# Timing of steering actions in locomotor interception of targets following curving trajectories

**DOI:** 10.1167/jov.23.3.11

**Published:** 2023-03-23

**Authors:** Albertha A. M. van Opstal, Remy Casanova, Frank T. J. M. Zaal, Reinoud J. Bootsma

**Affiliations:** 1 Aix Marseille Univ, CNRS, Inst Sci Mouvement, Marseille, France; 2 University Medical Center Groningen, Department of Human Movement Sciences, Groningen, The Netherlands

**Keywords:** visual guidance, interception, steering, locomotion, dynamics, bearing angle, CBA, visual information, acceleration cancelation, fractional order, QuID method

## Abstract

Recent work on the visual guidance of locomotor interception of nonuniformly moving targets argued for an early reliance on first-order (velocity-based) changes in the target's bearing angle that was complemented approximately 1 second later with reliance on second-order (acceleration-based) changes. Here we provide further support for this hypothesis in a virtual driving task, in which 19 participants steered a vehicle to intercept targets moving along receding circular trajectories. Adopting a set of carefully designed target trajectories, we tested discriminating predictions with respect to the timing and direction of the first steering action. Analyses of temporal and directional characteristics of first steering events revealed a pattern of results that was fully compatible with our predictions. Moreover, application of the recently developed QuID method, focusing on the temporal co-evolution of steering behavior and the potential information sources driving it, confirmed the operative progression from early reliance on first-order changes to subsequent (after approximately 1 second) reliance on a combination of first- and second-order changes in the target's bearing angle over the course of action at the individual-trial level. The finding of an evolution over time toward higher-order informational variables, potentially captured by a fractional-order time derivative, may have consequences for other locomotor interception tasks such as running to catch a fly ball.

## Introduction

**Figure 1.**
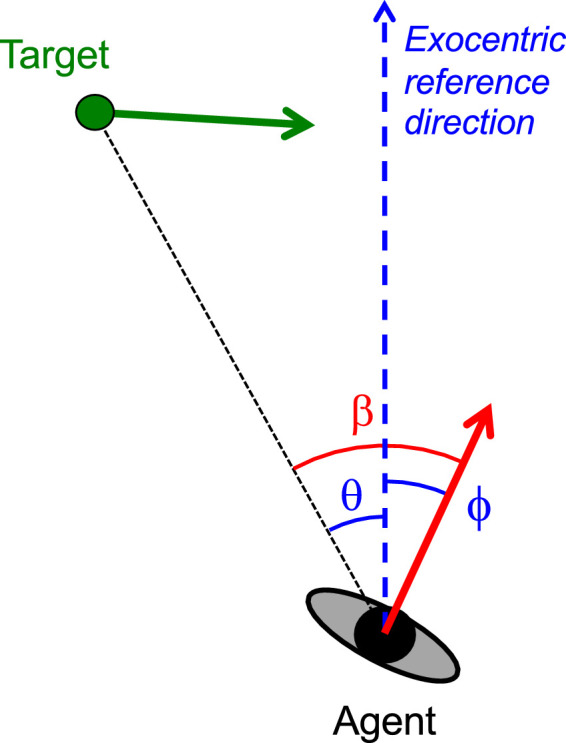
Definition of variables in a plane view of an agent moving through an environment containing a target moving in the same plane. Instantaneous velocity vectors are represented by arrows (red for agent, green for target). Agent heading ϕ and target bearing θ are defined with respect to an exocentric reference direction (dashed blue line). Target-heading angle β is defined by the eccentricity of the target with respect to the agent's direction of locomotion so that β = ϕ  − θ.

For targets moving in the horizontal plane, research into the visual guidance of human locomotor interception has concentrated typically on three candidate strategies. The first, a pure pursuit strategy in which the agent continuously seeks to move in the current direction of the target (Fajen & Warren, 2003; Rushton, Harris, Lloyd, & Wann, 1998), has been systematically ruled out when behavior unfolds within a structured environment (Casanova, Ceyte, & Bootsma, 2022; Ceyte, Casanova, & Bootsma, 2021; Fajen & Warren, 2004; Zhao et al., 2019, 2021). The two remaining strategies are based on the principle that maintaining constant either the target-heading angle β or the target's bearing angle θ (see Figure 1 for definitions) will guarantee future contact between agent and target (Berthelon & Mestre, 1993; Bootsma et al., 2016; Chapman, 1968; Chardenon et al., 2004; Cutting et al., 1995; Fajen & Warren, 2004, 2007; Kaiser & Mowafy, 1993; Lenoir et al., 1999; Pollack, 1995). These strategies, known as CTHA (for constant target-heading angle) and CBA (for constant bearing angle), are conventionally taken to be instantiated by first-order rate-of-change nulling, that is by dβ/dt-nulling for CTHA and dθ/dt-nulling for CBA.

While behavioral assessments, by means of measures of variability in β and θ observed over (later parts of) the unfolding action, have indicated that β may reveal less variability than θ (Zhao, Straub, & Rothkopf, 2019, 2021), such results do not directly speak to—and thereby do not suitably evaluate—the operational control principles at work. To address these principles, two methodological routes have been developed. First, model-based evaluations of the behavior resulting from a dβ/dt-nulling dynamics led Fajen and Warren (2007) to identify a qualitative difference between observed and expected behavior for intercepting uniformly moving targets under the specific condition of an agent initially lagging the target or, in other words, an agent confronted with an initially opening target-heading angle. Because a dβ/dt-nulling dynamics would only be able to reduce the lag, it cannot explain the observed result of a rapid and persistent sign switch in β, giving rise to the agent leading the target (Fajen & Warren, 2004, Center conditions). Replicated by Ceyte et al. (2021) and Casanova et al. (2022), this unequivocal result rules out unique reliance on dβ/dt information in the control of locomotor interception. However, even though this lag/lead issue did not arise in Fajen and Warren (2007) model-based evaluations of the behavior of a dθ/dt-nulling dynamics, this does not necessarily imply that locomotor interception would then, by ricochet, rely on dθ/dt information. Indeed, the second evaluation route, adopting what we have termed the Qualitative Inconsistency Detection (QuID) method (Van Opstal, Casanova, Zaal, & Bootsma, 2022; also see Bootsma, Ledouit, Casanova, & Zaal, 2016), has not only again demonstrated the insufficiency of unique reliance on dβ/dt information, but also of the insufficiency of unique reliance on dθ/dt information when to-be-intercepted targets follow curving trajectories. Briefly, this method evaluates the potential of any given informational candidate to have driven observed behavior at the level of the individual trial by searching for inconsistency in two distinct ways (see Van Opstal et al., 2022, for details). The first is action event anchored, examining whether, at a visuomotor delay before an observed steering event (defined as the onset of leftward or rightward turning), a candidate informational variable was indeed providing a drive in the required direction. The second is drive related, examining whether any substantial information-based drive in a direction opposite to that of current (leftward or rightward) steering indeed resulted in an observable new steering event. Application of these criteria to the curving target trajectory conditions in both Bootsma et al. (2016) and Van Opstal et al. (2022) studies revealed that (first-order) d^1^θ/dt^1^ information could adequately account for the behavior observed over approximately the first second of action but needed to be complemented with (second-order) d^2^θ/dt^2^ information from thereon. Here, we explore this intriguing finding further, now relating it in more detail to the advent of steering events.

The present contribution builds on the observation by Van Opstal et al. (2022; also see Warren & Fajen, 2008) that locomotor interception of targets moving along receding circular trajectories was generally characterized by locomotor paths with two inflection points. In other words, over the course of action, participants tended to change steering direction twice, with an initial leftward turn followed by a later rightward turn or an initial rightward turn followed by a later leftward turn. Although Van Opstal et al. (2022) noted that the first turn was typically initiated within the first second after target appearance, their study did not further scrutinize the timing of steering events for differences between experimental conditions. Yet, inspection of their data revealed that the timing of the first steering event varied as a function of the target's motion characteristics.^1^ Targets that initially moved outward generally elicited early first steering events, with their frequency peaks located within the first 0.5 seconds after the target had appeared, whereas for targets that initially moved inwards and started from the most eccentric position, such first steering events came a little later, with first-event frequency peaks now occurring between 0.5 and 1.0 seconds after target appearance.

**Figure 2.**
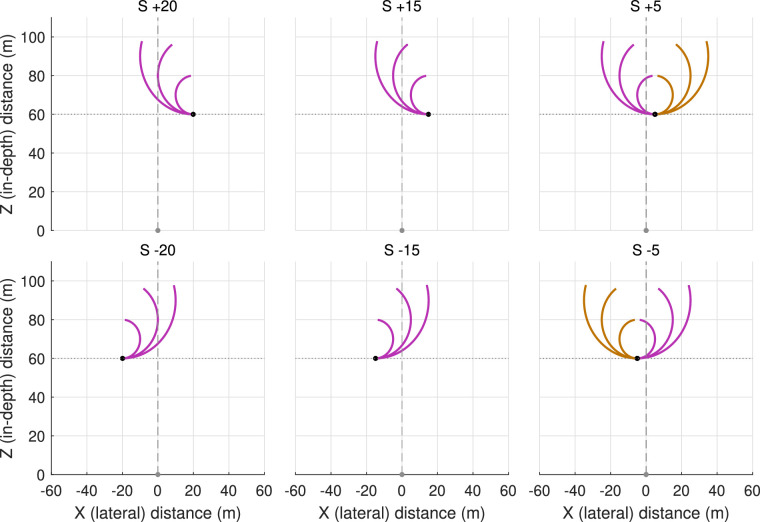
Initial conditions and target trajectories used in the present experiment. The participant's position at the onset of an experimental trial, marked by a gray dot, corresponded with the X–Z coordinate system's (0,0) origin. At that moment in time, a target (represented by a black dot) appeared at a lateral position S of +20, +15, +5, –5, –15, or –20 m, at a constant in-depth distance of 60 m. Targets could move along three different circular trajectories (10-m, 20-m, and 30-m radii), initially always moving leftward for S +20 and S +15 conditions, initially always moving rightward for S –15 and S –20 conditions, and initially moving either leftward or rightward for S +5 and S –5 conditions. For global analysis purposes, the 24 experimental target conditions were subsequently mirror-collapsed over negative left and positive right S conditions into S20, S15, and S5, recoding target motion direction to inward or outward. On inward trajectories (pink curves) the target initially moved toward the X = 0 axis corresponding to the participant's initial movement direction, whereas on outward trajectories (brown curves) the target initially moved away from this axis.

In the present contribution we tested whether these differences in the timing of the steering events when intercepting targets moving along different trajectories were related to the different temporal evolution of the informational variable(s) that participants rely on for the control of action. To this end, we purposefully manipulated the initial magnitudes and early evolutions of the dθ/dt and d^2^θ/dt^2^ variables by varying the characteristics of the target's circular trajectory, in terms of both initial lateral offset and radius. Figure 2 presents the initial conditions and spatial target trajectories selected.

**Figure 3.**
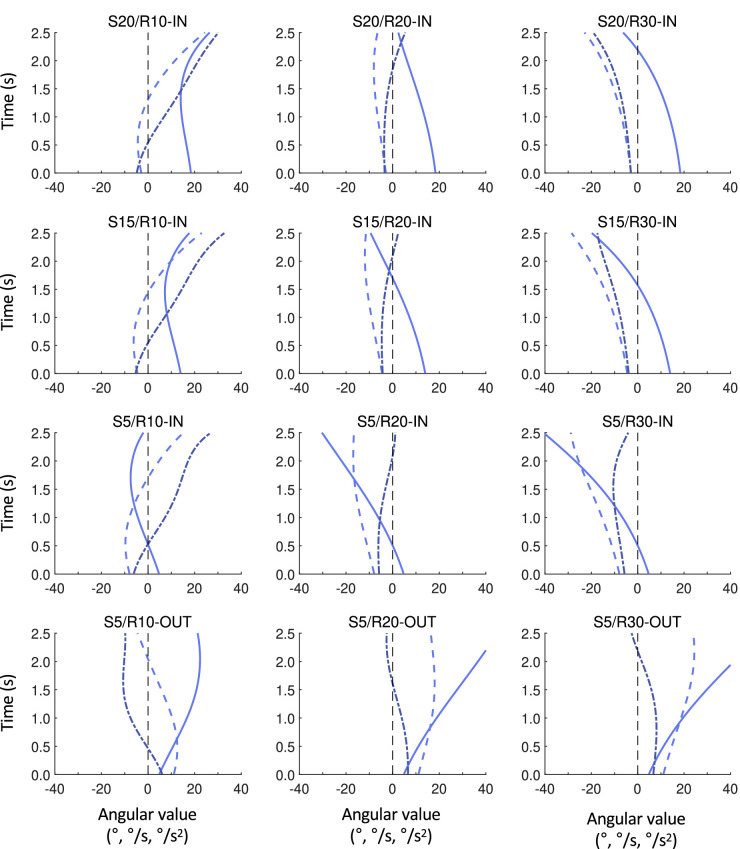
Simulation results, for each experimental condition separately, of the evolution over the first 2.5 seconds after the onset of a trial of bearing angle-based variables θ (full line), dθ/dt (dashed line), and d^2^θ/dt^2^ (dash–dotted line) as obtained for a nonsteering participant. These simulations allow evaluating the drives signaled by each informational candidate from the onset of a trial onward, up to the appearance of a first steering event. The simulations no longer apply after the first steering event, as they are calculated for continuous straight-ahead participant motion.

Figure 3 presents, for the rightward S20, S15, and S5 initial offsets and R10, R20, and R30 trajectory radii, the associated evolutions of θ, dθ/dt and d^2^θ/dt^2^ over the first 2.5 seconds of a trial, as obtained by numerical simulation for a nonsteering participant (i.e., a participant that continues to move straight ahead from the onset of a trial onward). Inclusion of the S20/R20-IN target trajectory, also used in Van Opstal et al. (2022) study, allowed using this condition as a reference. We recall that in this reference condition the first steering event was found to occur predominantly between 0.5 and 1.0 seconds after target appearance, with only few initiations before 0.5 seconds (see Supplementary Figure S1 for details). As can be seen from Figure 3, the S20/R20-IN trajectory (top row, middle panel) gives rise to initially relatively small negative dθ/dt (and d^2^θ/dt^2^) magnitudes. All three S5-OUT trajectories, in contrast, give rise to comparatively large positive initial values of dθ/dt (as well as positive initial values of d^2^θ/dt^2^), leading us to predict that these latter trajectories should be characterized by an early (i.e., predominantly within 0.5 seconds after target appearance) rightward-directed first steering action. By the same token, all three S5-IN trajectories give rise to comparatively large negative initial values of dθ/dt (as well as negative initial d^2^θ/dt^2^ values) and should therefore be characterized by an early (i.e., predominantly within 0.5 seconds after target appearance) leftward-directed first steering action. Compared with the S5-IN trajectories, both the S15/R20-IN and S15/R30-IN as well as the S20/R20-IN and S20/R30-IN trajectories are characterized by initially smaller but gradually increasing negative dθ/dt values (accompanied by negative d^2^θ/dt^2^ values), leading us to predict that for these trajectories initiation of the first steering action should be somewhat later (i.e., predominantly occur between 0.5 and 1.0 seconds) and leftward directed. Finally, the S15/R10-IN and S20/R10-IN trajectories are both characterized by small negative initial dθ/dt (and d^2^θ/dt^2^) values, leading us to predict that instances of an early initiation of a first steering action should be rare. Moreover, as the magnitude of dθ/dt decreases over time, switching from negative to positive at approximately 1.30 seconds for S20/R10-IN and at approximately 1.45 seconds for S15/R10-IN, and d^2^θ/dt^2^ rapidly becomes positive at approximately 0.55 seconds for both these trajectories, we predicted that the first steering action should tend to occur even later, between 1.0 and 1.5 seconds after target appearance for these two specific trajectories, with steering now rightward directed. This effect should moreover be somewhat more prominent for the S20/R10-IN trajectory. We stress that the predictions of belated (between 1.0 and 1.5 seconds after target appearance) first steering events for the S20/R10-IN and S15/R10-IN conditions are based on the coexistence of an initially weak and subsequently further decreasing dθ/dt-based drive and a coming to the fore of a considerable d^2^θ/dt^2^-based drive, resulting in a predominately late rightward-directed (rather than an earlier leftward-directed) first steering action.

Overall, based on early reliance on dθ/dt information and later (after approximately 1 second) reliance on a combination of dθ/dt and d^2^θ/dt^2^ information, these considerations allowed us to predict a distinct pattern of results with respect to when and in which direction the first steering event was to be expected to occur, as summarized in Table 1.

**Table 1.** Expected results in terms of predominant timing (0.5-s time bins) and direction (Right or Left) of first steering events for mirror-collapsed (positive S) conditions for each of the three trajectory radii

	S20-IN		S15-IN		S5-IN		S5-0UT	
	Timing (s)	Direction	Timing (s)	Direction	Timing (s)	Direction	Timing (s)	Direction
R10	1.0 < t ≤ 1.5	R	1.0 < t ≤ 1.5	R	t ≤ 0.5	L	t ≤ 0.5	R
R20	0.5 < t ≤ 1.0	L	0.5 < t ≤ 1.0	L	t ≤ 0.5	L	t ≤ 0.5	R
R30	0.5 < t ≤ 1.0	L	0.5 < t ≤ 1.0	L	t ≤ 0.5	L	t ≤ 0.5	R

## Methods

### Participants

Nineteen (post)graduate students from Aix-Marseille University (13 men and 6 women, aged 20.9 ± 2.8 years, *M* ± *SD*) participated in this experiment. They all reported normal or corrected-to-normal vision. All participants provided written informed consent before participating in the study. The study was approved by the French National Ethics Committee for Research in Sports Sciences (CERSTAPS) and conducted according to university regulations and the Declaration of Helsinki.

### Experimental set-up

The experiment took place in a large virtual reality facility (https://www.crvm.eu). The setting consisted of four projection surfaces: a 3 × 3-m floor surface and three 4-m high x 3-m wide walls. The two sidewalls were set at 90° angles with respect to the front wall. The basic driving simulator, consisting of a seat, a set of nonoperative pedals, and a steering wheel, was positioned in the middle of the floor surface, with the steering wheel at a distance of 1.10 m from the front wall. Stereopsis was ensured with Volfoni EDGE VR 3D Active glasses (120 Hz, 60 Hz per eye). These glasses were equipped with a configuration of reflective markers, allowing real-time motion capture of the head by an eight-camera Advanced Realtime Tracking (ART, Weilheim, Germany) opto-electronic system. The visual scene was refreshed at 60 Hz, taking into account the position and orientation of the participant's head relative to the virtual environment.

### Task and procedure

Using in-house developed software, we simulated a virtual environment consisting of a large grass-like flat plain, containing both fine and gross texture, bordered by distant mountains (see Supplementary Figure S2 for a depiction of apparatus and environment). The seated participant was instructed that on each trial the goal was to steer the car so as to intercept (i.e., drive into) a yellow cylinder (2-m radius, 3-m height) moving horizontally over the ground surface. Steering immediately affected heading direction, with the current steering wheel angle being proportionally mapped onto the current car turning rate. Before trial onset, participants moving at 20 m/s were to align locomotor direction with a yellow line by bringing the center of the seat within a maximal lateral distance of 3 cm from the middle of the line, while moving in a direction that deviated less than 0.1° from the line orientation. After successful alignment, the yellow line disappeared, and a red portal appeared 40 m ahead. Participants were instructed to keep their steering wheel centered when moving toward the portal, thus steering straight toward it. During that period, the steering wheel was deactivated with wheel orientation recalibrated to zero, so that when the participant crossed the portal and the target appeared they were moving straight ahead with ϕ = 0° and dϕ/dt = 0°/s. A trial ended when the participant drove into the target's circumference (successful interception), or when the car's in-depth (Z-axis) position exceeded the target's in-depth position by 20 m.

Targets moved at 10 m/s along receding circular trajectories of a 10-m, 20-m, or 30-m radius, starting at a constant in-depth (Z-axis) distance of 60 m, from six possible lateral (X-axis) departure positions at −20, −15, −5, +5, +15, or +20 m. Targets only moved inward for the ±20-m and ±15-m departure positions and either inward or outward for the ±5-m departure position, giving rise to a total of 24 different target trajectories (see Figure 2). The full set of 24 target trajectories was presented in a random order in each block of trials. Participants completed 5 blocks, for a total of 120 trials. To familiarize them with the environment and steering equipment, participants completed a block of 12 training trials in which they were to intercept targets moving along straight trajectories before the start of the experimental trials.

For global analysis purposes, for each of the three target trajectory radii (coded R10, R20, and R30), the experimental conditions were mirror collapsed for initial positions (coded S20, S15, and S5) with target movement direction recoded to IN and OUT, giving rise to 12 collapsed conditions.

### Data analysis

Participant position (x, z) and heading direction (ϕ) were sampled at 100 Hz and filtered using a fourth-order Butterworth filter with a 4-Hz cut-off frequency. For each individual trial, the presence of any salient steering action was determined following a two-criterion inclusion protocol (cf. Van Opstal et al., 2022). The first criterion was that the agent's heading angle (ϕ) changed by at least 10° over the course of the trial. The second criterion was that at some point, after a minimal 100-ms duration into the trial, a change in heading angle (dϕ/dt) exceeded an absolute value of 4°/s. Both criteria were met in 2271 of the total of 2280 trials; 9 trials were thus excluded from the analyses. For each included trial, the time of onset of a steering action was determined by searching backward in time from the moment of occurrence of dϕ/dt of greater than 4°/s to the moment that dϕ/dt first exceeded 1°/s for the first identified event or 0°/s for later events within the same trial, adding the criterion that the steering direction on a subsequent event must be opposite to the direction of the previous steering event. Finally, to ensure that the observed change in steering direction was substantial enough, we verified whether the heading angle changed by at least 4° after a change in steering direction. If not, the event was not taken into account. To avoid extreme values in the final part of the interception action, we limited the timeframe for our search to end 200 ms before the moment of interception.

## Results

Although the success rate was consistently greater than 90% for all R20 and R30 trajectories, intercepting the R10 trajectories proved to be more difficult, with success rates varying between 62% and 74% in these conditions. Because we presume that the principles of steering control do not vary over trials or over target trajectory conditions, all 2271 trials revealing one or more steering events were included in the analyses. The time from trial onset, marked by the appearance of the target, until the moment of contact with or closest distance to the target (i.e., action duration) varied over target trajectory conditions (means between 3.91 and 5.01 seconds). Within each target trajectory condition, however, action duration was remarkably stable over participants and trials (standard deviations of ≤0.08 seconds; see Table 2 for details).

**Table 2.** Participants’ overall action duration (AD, *M* ± *SD* over all trials per condition) and success rate (SR, *M* over participants per condition) for the mirror-collapsed experimental target trajectory conditions

	S20-IN		S15-IN		S5-IN		S5-0UT	
	AD (s)	SR (%)	AD (s)	SR (%)	AD (s)	SR (%)	AD (s)	SR (%)
R10	4.28 ± 0.07	62.1	4.16 ± 0.07	63.7	4.00 ± 0.06	62.1	3.91 ± 0.06	73.7
R20	4.69 ± 0.06	97.9	4.67 ± 0.05	96.3	4.74 ± 0.06	94.7	4.89 ± 0.08	93.2
R30	4.21 ± 0.03	100.0	4.30 ± 0.03	100.0	4.60 ± 0.05	100.0	5.01 ± 0.05	98.4

### Exemplary trials

**Figure 4.**
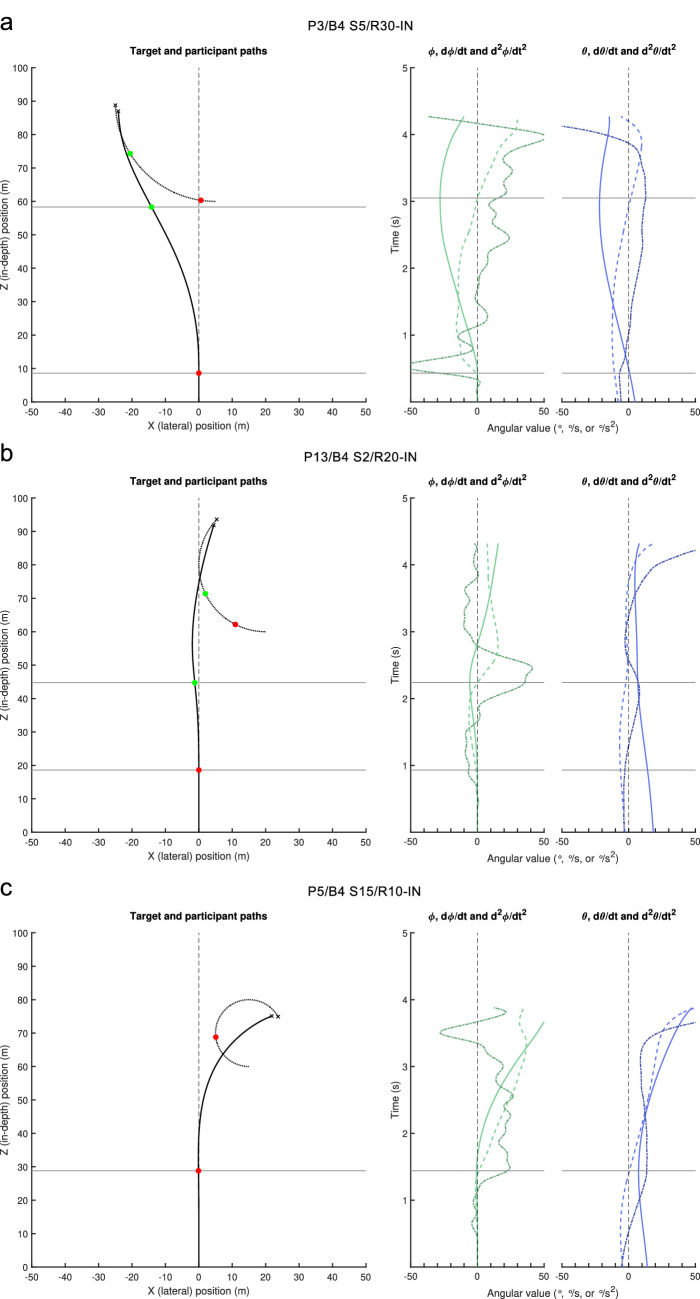
QuID plots for three exemplary trials. (a) P3, block 4, S5/R30-IN (leftward) condition, (b) P13, block 4, S20/R20-IN (leftward) condition, and (c) P5, block 4, S15/R10-IN (leftward) condition. The left graph in each panel presents the spatial paths followed by the target (dotted gray line) and the participant (black line). Steering events are marked by color-coded dots. Small crosses mark target and participant position at the moment of contact. Right graphs in each panel: time evolution (bottom to top) over the course of the trial of the participant's heading direction ϕ (in green) and the target's bearing angle θ (in blue) together with their first-order (dashed) and second-order (dash-dotted) time derivatives. Horizontal gray lines situate the steering events spatially (left graph) and temporally (right graphs). For each panel, an enlarged figure version is available as Supplementary Figure S3.

Before comprehensively addressing the specific hypotheses underlying the present study, we first examine a few exemplary trials (Figure 4; the full set of 2280 trials is available as Supplementary Material QuId Plots), focusing on the temporal coevolution of steering behavior and the potential information sources driving it. For each trial (i.e., each panel in Figure 4), the left graph presents the spatial paths followed by the target and the participant. Red and green dots mark target and participant positions at the moment of advent of the first and second steering events, respectively. Spatially situating the participants' turns in the left graph by horizontal gray lines, these turns are temporally situated by the corresponding horizontal lines in right graphs, presenting the time evolution (bottom to top) of the participant's heading direction ϕ (in green), and the target's bearing angle θ (in blue) together with their first-order and second-order time derivatives.

Figure 4a presents an exemplary S5/R30-IN trial. With the target initially moving IN (here leftward) and bearing angle θ closing, both dθ/dt and d^2^θ/dt^2^ are negative initially. Because their already considerable magnitudes gradually increase from the onset of the trial onward, these two informational variables immediately signal a substantial leftward drive. The first, indeed leftward-directed, steering action effectively begins early (within the first 0.5-second time bin). After the initiation of this first turn, the magnitude of dθ/dt initially continues to increase before subsequently decreasing, becoming positive shortly before the advent of a second steering event. At the same time, d^2^θ/dt^2^ rapidly evolves from negative to positive values. Both informational variables can therefore, in principle, account for the rightward-directed second steering action occurring after 3 seconds into the trial. A slightly different picture emerges for the exemplary S20/R20-IN trial presented in Figure 4b. Although both dθ/dt and d^2^θ/dt^2^ are again negative at the onset of the trial, their initial magnitudes are comparatively small. With magnitudes, notably for dθ/dt, gradually increasing over time, both variables signal a leftward drive. The first, indeed leftward-directed, steering action logically occurs somewhat later (shortly before 1 second, that is, within the second 0.5-second time bin) than for the above-described S5/R30-IN trial. After the initiation of this first steering action, dθ/dt remains negative, although decreasing in magnitude up to the initiation of a second steering action, occurring at approximately 2.2 seconds into the trial. The onset of this rightward-directed steering action, therefore, cannot be adequately accounted for by the (still leftward-oriented) drive signaled by dθ/dt. However, because the first steering action has led d^2^θ/dt^2^ to switch from negative to positive, its considerable positive magnitude before the second steering action is compatible with it driving the observed rightward-directed steering action. Finally, Figure 4c presents an exemplary S15/R10-IN trial, for which both dθ/dt and d^2^θ/dt^2^ are initially negative with relatively small magnitudes. Moreover, both are decreasing, with d^2^θ/dt^2^ becoming positive at approximately 0.5 seconds into the trial. The first steering action occurs after some 1.4 seconds (i.e., in the third 0.5-second time bin) and is rightward directed. With dθ/dt reaching zero at the moment of initiation, it cannot by itself adequately account for the onset of the rightward steering action. This can, however, be adequately accounted for by the concurrent large-magnitude drive signaled by positive d^2^θ/dt^2^ values. It is noteworthy that, in this S15/R10-IN trial, the target is intercepted without requiring a second steering action.

Overall, we note that, as hypothesized, participants’ behavior in these exemplary trials can be understood satisfactorily as resulting from participants’ early reliance on dθ/dt information and subsequent (from approximately 1 second into the trial) reliance on a combination of dθ/dt and d^2^θ/dt^2^ information, with a steering action being initiated when the informational drive has developed to a certain magnitude. To verify whether this principle held over the full dataset, we concentrated on hypotheses-specific global analyses in the remainder of this section. To this end, we report event-anchored results for the 12 mirror-collapsed target trajectory conditions.

### Timing of steering events

**Figure 5.**
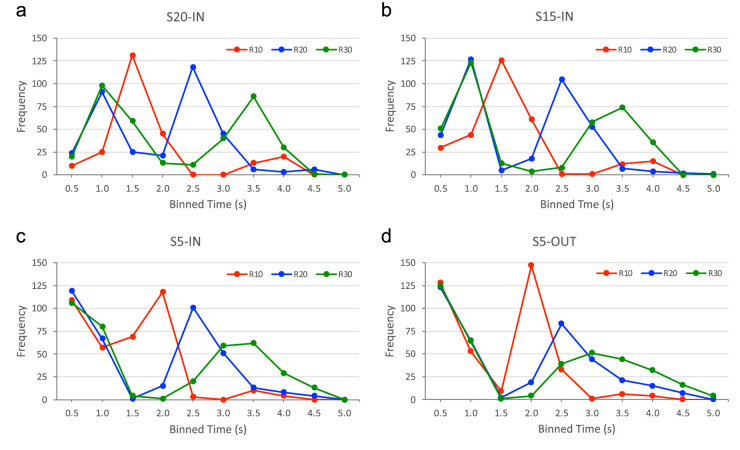
Frequency distributions over binned time (0.5-second steps) of occurrence of a steering event for the 12 mirror-collapsed target trajectory conditions. (a) S20-IN trajectories for R10, R20, and R30. (b) S15-IN trajectories for R10, R20, and R30. (c) S5-IN trajectories for R10, R20, and R30. (d) S5-OUT trajectories for R10, R20, and R30.

Figure 5 presents the frequency distributions (as a function of binned time) of the observed timing of steering events over the full duration of all trials for each experimental condition separately. In line with the observation that participants tended to change steering direction twice, the frequency distributions typically show two peaks (with the notable exceptions of the S15/R10-IN and S20/R10-IN trajectories, both revealing considerably fewer second steering events). Concentrating on first steering events, that is, first peaks in the frequency distributions, inspection of Figure 4 revealed a pattern of results fully compatible with our hypotheses (cf. Table 1). Indeed, all three S5-OUT (Figure 5d) and all three S5-IN (Figure 5c) target trajectories gave rise to a first frequency peak located in the first time bin, that is, within 0.5 second after trial onset. Both S15/R20-IN, S15/R30-IN (Figure 5b) and S20/R20-IN and S20/R30-IN (Figure 5a) target trajectories gave rise to a first frequency peak located in the second time-bin, that is between 0.5 and 1.0 seconds into the trial, whereas S15/R10 and S20/R10 trajectories gave rise to a first frequency peak located in the third time bin, that is, between 1.0 and 1.5 seconds into the trial. A separate assessment of the direction of first steering actions demonstrated that they were 99.9% compatible with our prediction that for inward-moving targets earlier (i.e., up to 1 seconds after target appearance) first steering actions would be leftward and later first steering actions would be rightward directed (as assessed for the mirror-collapsed conditions). All predicted effects as summarized in Table 1 were thus observed. As a side note, we highlight the striking similarity in frequency distributions for the S20/R20-IN condition between the present study and Van Opstal et al. (2022) study (see Supplementary Figure S1).

Inspection of the generally two-peaked frequency distributions also indicated that the timing of second steering events also systematically varied with experimental conditions, occurring relatively soon for the R10 (S5-IN and S5-OUT) trajectories (second peaks in the fourth time bin, that is between 1.5 and 2.0 seconds) (Figures 5c and d), somewhat later for the R20 trajectories (second peaks in fifth time bin, that is between 2.0 and 2.5 seconds) (Figure 5 all panels) and latest for the R30 trajectories (second peaks in sixth and seventh time bins, that is, between 2.5 and 3.5 seconds) (Figure 5 all panels). These results can be understood readily as resulting from the rapid changes in informational variables induced by targets moving along small radius trajectories and the slower changes induced by targets moving along larger radius trajectories.

### Roles of individual informational variables

**Figure 6.**
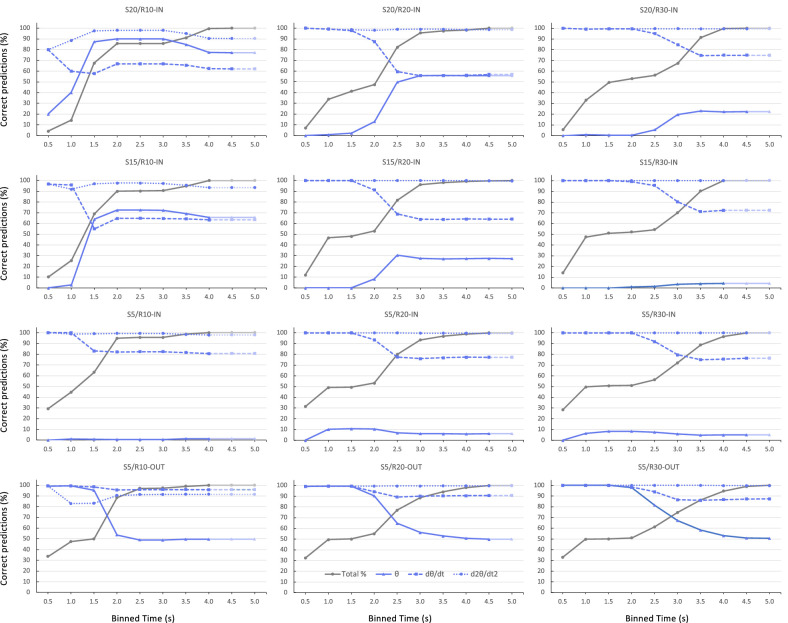
Cumulative percentage correct predictions over (0.5-second binned) time of upcoming steering direction signaled a visuomotor delay Δt = 0.1 second earlier by θ (full blue line), dθ/dt (dashed blue line), or d^2^θ /dt^2^ (dotted blue line) for each of the 12 experimental target trajectory conditions. The dark gray line presents the cumulative percentage over (binned) time of the total number of observed steering actions. Attenuated colors mark (empty) time bins extending beyond the condition's observed action duration. Qualitatively similar results were obtained for Δt = 0.2 second.

To substantiate our initial analysis, by means of inspection of exemplary trials, of the potential roles of informational variables in driving the observed steering behavior, we determined whether at a 100-ms visuomotor delay before appearance of any identified steering event the potential informational variables θ, dθ/dt, and d^2^θ/dt^2^ correctly signaled this upcoming event. Thus, upcoming negative values of turning rate dϕ/dt (indicating leftward steering) were considered to be correctly signaled by a negative value of the informational variable examined and upcoming positive values of dϕ/dt (rightward steering) were considered to be correctly signaled by a positive value of the informational variable examined. Applying this procedure to all steering events allowed an evaluation of the overall capacity of each individual informational variable to drive the particular identified steering events correctly.^2^ Figure 6 presents, for each target trajectory condition separately, the cumulative percentage over binned time of steering events that could be correctly explained by unique reliance on θ, dθ/dt, or d^2^θ/dt^2^. Although distortion effects owing to comparisons between bins with many steering events and bins with few steering actions were eliminated quite effectively by using cumulative rather than absolute percentages, we note that such effects persisted, notably for the first bin in the S20/R10-IN and S15/R10-IN conditions.

As was to be expected, the overall results clearly ruled out unique reliance on θ, because it quite systematically signaled steering in the wrong (i.e., contrary to observed) direction early on for all inward-moving target trajectories. Cumulated over all time bins, it rarely exceeded 60% of correct predictions. In line with our hypotheses, unique reliance on dθ/dt, in contrast, predicted the observed direction of steering correctly early on, with cumulative percentage correct predictions seen to begin declining from (close to) 100% after 1.0 second (i.e., in the third time bin) for the S15/R10-IN and S5/R10-IN trajectories, after 1.5 seconds (i.e., in the fourth time bin) for the R20 trajectories and after 2.0 seconds (i.e., in the fifth time bin) for the R30 trajectories. Because the number of steering events plateaued between 1 and 2 seconds for the R20 target trajectories and between 1.0 and 2.5 seconds for the R30 conditions, we cannot but conclude that our data only support potential unique reliance on dθ/dt over the first second of the interceptive actions. Indeed, for steering events occurring after 1 second into the trial, only 59.4% were correctly accounted for by reliance on dθ/dt (67.7% for R10 trials, 50.6% for R20 trials, 59.7% for R30 trials). Finally, unique reliance on d^2^θ/dt^2^ overall correctly predicted upcoming direction of steering for all target trajectories over the full lengths of the times series.

## Discussion

Hypotheses for the present study were derived following a two-step deduction process. First, based on earlier studies of locomotor interception of targets following curved trajectories (Bootsma et al., 2016; Van Opstal et al., 2022), we hypothesized that steering behavior would be driven by early reliance on dθ/dt information and later reliance on a combination of dθ/dt and d^2^θ/dt^2^ information, with the latter coming to the fore after approximately 1 second. Second, based on differences over target trajectory conditions in the timing of the first steering event identified in Van Opstal et al. (2022) data, we hypothesized that these differential timing effects could be understood as resulting from differences over such conditions in initial magnitudes and subsequent time evolutions of dθ/dt and d^2^θ/dt^2^. This logic led us to design the present experiment in which target trajectory conditions were selected to not only reproduce but also to enhance such differences. For the set of target trajectory conditions designed for these purposes, we predicted that, as a function of target trajectory conditions, initiation of the first steering action would predominantly occur either early, slightly later or even later or, more precisely, in the first, second or third 0.5-second time bin after target appearance. These predictions with respect to timing of the first steering event were moreover logically coupled to the expected (rightward or leftward) direction of the ensuing steering action, based on the (positive or negative) signs of the operative dθ/dt and d^2^θ/dt^2^ values.

In line with our earlier work, evidence in favor of our starting hypotheses of early reliance on dθ/dt information together with the gradual coming to the fore of reliance on d^2^θ/dt^2^-related information after approximately 1 second was obtained using the QuID method (see Van Opstal et al., 2022). For each observed steering event, we examined whether informational candidates (here θ, dθ/dt and d^2^θ/dt^2^) could have provided the drive required for that event, by comparing the direction of drive that each one of the informational variables was signaling a visuomotor delay earlier with the direction of steering actually observed. Unique reliance on θ information, that we included for the sake of completeness, could be discarded rapidly, because it only rarely correctly signaled the upcoming steering direction for initially inward moving targets. Although dθ/dt was found to signal the upcoming direction of steering over the first second into a trial correctly, unique reliance on dθ/dt information was no longer consistent with observed steering behavior later in the action, as revealed by the quite systematic decrease in the cumulative percentage that correctly explained steering events (see Figure 6).

In this event-anchored analysis, unique reliance on d^2^θ/dt^2^ information was found to be plausible over the full duration of trials, with cumulated percentages correct remaining close to 100 for all conditions. Yet, rather than taking this latter result as sufficient evidence for the idea that steering control uniquely relies on d^2^θ/dt^2^ information, we stress that the QuID method in fact searches for qualitative inconsistencies in two ways. Qualitative inconsistencies in explaining an upcoming steering event allows discarding reliance on a particular informational variable, as we have done above for θ information and for later dθ/dt information. However, to admit reliance on any informational variable we also need to consider the QuID method's second requirement, namely, that, if a steering action gives rise to a substantial change in the direction of drive provided by a given informational variable without provoking a fitting action, this also constitutes a qualitative inconsistency for this informational variable (cf. Van Opstal et al., 2022). An example of the latter can be observed in the exemplary trial presented in Figure 4a, where the first steering action leads d^2^θ/dt^2^ to become positive rapidly. Yet, notwithstanding the considerable drive signaled by d^2^θ/dt^2^ after this first steering action, the second steering action does not occur until much later. In line with our earlier work, this regularly observed pattern of prolonged episodes with dθ/dt and d^2^θ/dt^2^ signaling drives in opposite direction without any steering action intervening once again leads us to argue that steering control relies on an informational combination of dθ/dt and d^2^θ/dt^2^, perhaps in the form of a fractional time derivative order between 1 and 2 (see Bootsma et al., 2016 and Van Opstal et al., 2022 for further developments of this idea).

The concept of a reliance on information contained in some form of combination of dθ/dt and d^2^θ/dt^2^ may have important repercussions, not only on the debate on the informational variable(s) guiding the locomotor behavior that we considered in the present experiment, but also on the informational basis of other locomotor behavior, such as in fly-ball catching. Indeed, the proposition that forward-backward displacement in running to catch fly balls would be guided by nulling the acceleration of the ball's (environment-centered) elevation angle ε, that is by d^2^ε/dt^2^-nulling (Chapman, 1968; Fink, Foo, & Warren, 2009; McLeod, Reed, & Dienes, 2001; Michaels & Oudejans, 1992; Todd, 1981; Zaal & Michaels, 2003) has been questioned, claiming that human sensitivity to optical acceleration would be insufficient (Brouwer, Brenner, & Smeets, 2002; but see Babler & Dannemiller, 1993 and Zaal, Bongers, Pepping, & Bootsma, 2012). Furthermore, it has been argued that in an account of running to catch fly balls, the interceptability of the ball should also be factored in and that this would be incompatible with relying on nulling d^2^ε/dt^2^ (Fajen, 2007; Fajen, Diaz, & Cramer, 2011; Oudejans, Michaels, Bakker, & Dolné, 1996; Postma, Smith, Pepping, van Andel, & Zaal, 2017; Potsma, Lemmink, & Zaal, 2018). Application of the QuID method to fly-ball catching trials under a well-chosen variety of ball flight conditions, considering the potential use of informational variables on trial-to-trial basis might very well provide the means to uncover the informational basis for such a task as well.

The originality of the present contribution resides in the demonstration of a quantitative link between the magnitudes of coevolving dθ/dt and d^2^θ/dt^2^ time series and the onset of a first steering action. Close scrutiny of Van Opstal et al. (2022) conditions and results allowed us to select target trajectories that, relative to the S20/R20-IN reference condition with first steering events predominantly occurring in the second 0.5-second time bin, allowed us to predict earlier (first time bin) and later (third time bin) first steering events. With these predictions based on the early reliance on dθ/dt information and later reliance on an informational dθ/dt and d^2^θ/dt^2^ combination, as described elsewhere in this article, the finding of close correspondence between our predicted and observed results clearly reinforces our informational proposition. This is especially the case for the—not earlier observed—relatively late first steering action initiations observed when dθ/dt and d^2^θ/dt^2^ signals initially have small magnitudes and subsequently evolve differently, as in the S20/R10-IN and S15/R10-IN trajectory conditions.

The predictions made in the present contribution with respect to expected pattern of timing of the first steering events were based on differences between the S20/R20-IN reference condition (that was also tested in Van Opstal et al., 2022) and a set of other target trajectory conditions, purposefully designed on the basis of qualitative aspects of their associated time evolutions of dθ/dt and d^2^θ/dt^2^ signals (as presented in Figure 3). In so doing, we opted for a comparative approach, allowing us, for the time being, to stay away from considerations with respect to the absolute magnitudes of informational variables (reported, for these reasons, only as Supplementary Information in Table S1). By the same token, for now we refrain from speculating on the nature of the underlying control law, relying only on the single hypothesis required for application of the QuID method (cf. Van Opstal et al., 2022) that control is based on online nulling of pertinent informational variable(s). While we do hope to ultimately be able to provide a full-fledged model of steering behavior, allowing quantitative predictions to be made for both uniformly and nonuniformly moving targets (see Bootsma et al., 2016), we suggest that—as done in the present contribution—the principled scrutinizing of informational variables potentially relied upon is both a useful and necessary intermediate step.

Finally, we would like to point out that the fact that the full set of empirical findings of the present experiment accurately corresponded to the effects predicted for each individual target trajectory examined has two other implications by itself. First, differences between (admittedly a priori capable) individual participants do not seem to play a significant role in the way locomotor interception behavior is controlled (also see Bootsma et al., 2016; Casanova et al., 2022; Ceyte et al., 2021; Van Opstal et al., 2022). Participants acquainted with the use of a steering wheel readily performed our interception-by-steering task after a dozen of practice trials, with little variation in their modus operandi observed over blocks of trials (Casanova et al., 2022; Ceyte et al., 2021; Van Opstal et al., 2022). We note that, notwithstanding such swiftly established control principles, some kind of calibration may take place over practice, as suggested by the observed increase in success rate over the five blocks of trials (from 74.8% to 93.9%). Second, the identification of operational control principles is best served by analysis at the level of individual trials. Designed to filter out supposedly irrelevant variations between individual trials, the common practice of focusing on ensemble averages (e.g., Fajen & Warren, 2004; Zhao et al., 2019, 2021) may sometimes obscure the very nature of the operative control principles. For example, ensemble averaging over trials would definitely camouflage this study's pertinent finding for the S20/R10-IN target trajectory that the first steering action was typically rightward directed when it occurred between 1.0 and 1.5 seconds after trial onset (as it predominantly did), but was in fact typically leftward directed when it occurred earlier (as it more rarely did). We note that both these observations fit with the control principle of early reliance on dθ/dt information and a 1-second later reliance on (a combination of dθ/dt and) d^2^θ/dt^2^ information.

## Supplementary Material

Supplement 1

Supplement 2

Supplement 3

Supplement 4

Supplement 5

## Footnotes

1 In Van Opstal et al. (2022) study targets could appear straight ahead of the participant or at (left or right) lateral offsets of 10 or 20 m (offsets coded S0, S10 and S20, respectively) and followed receding circular trajectories of 20 or 40 m radius (coded R20 and R40). While targets following S0 trajectories necessarily always initially moved outward, targets following S10 and S20 trajectories could initially move either inward or outward (coded IN and OUT). Frequency distributions of the moments of initiation of steering actions under the different experimental conditions, reported by Van Opstal et al. (2022) in their Table 2, can be found in graphical form in the present contribution's Supplementary Figure S1.

2 Average observed values of θ, dθ/dt, and d^2^θ/dt^2^ at 100 ms before a steering event are reported per time bin for each target trajectory condition in Supplementary Table S1.
