# Supplementary material for: Timing of steering actions in locomotor interception of targets following curving trajectories
Source: J Vis. 2023 Mar 23;23(3):11. doi: 10.1167/jov.23.3.11 (PMC10050912; doi:10.1167/jov.23.3.11)

Supplementary Figure S1: Frequency distributions over time bins (0.5-s steps) of occurrence of a steering event as reported by Van Opstal et al. (2022, Table 1) for their 10 (mirror-collapsed) target trajectory conditions (For R20 in blue and R40 in green: S20-IN, S10-IN, S20-OUT, S10-OUT, S0-OUT).

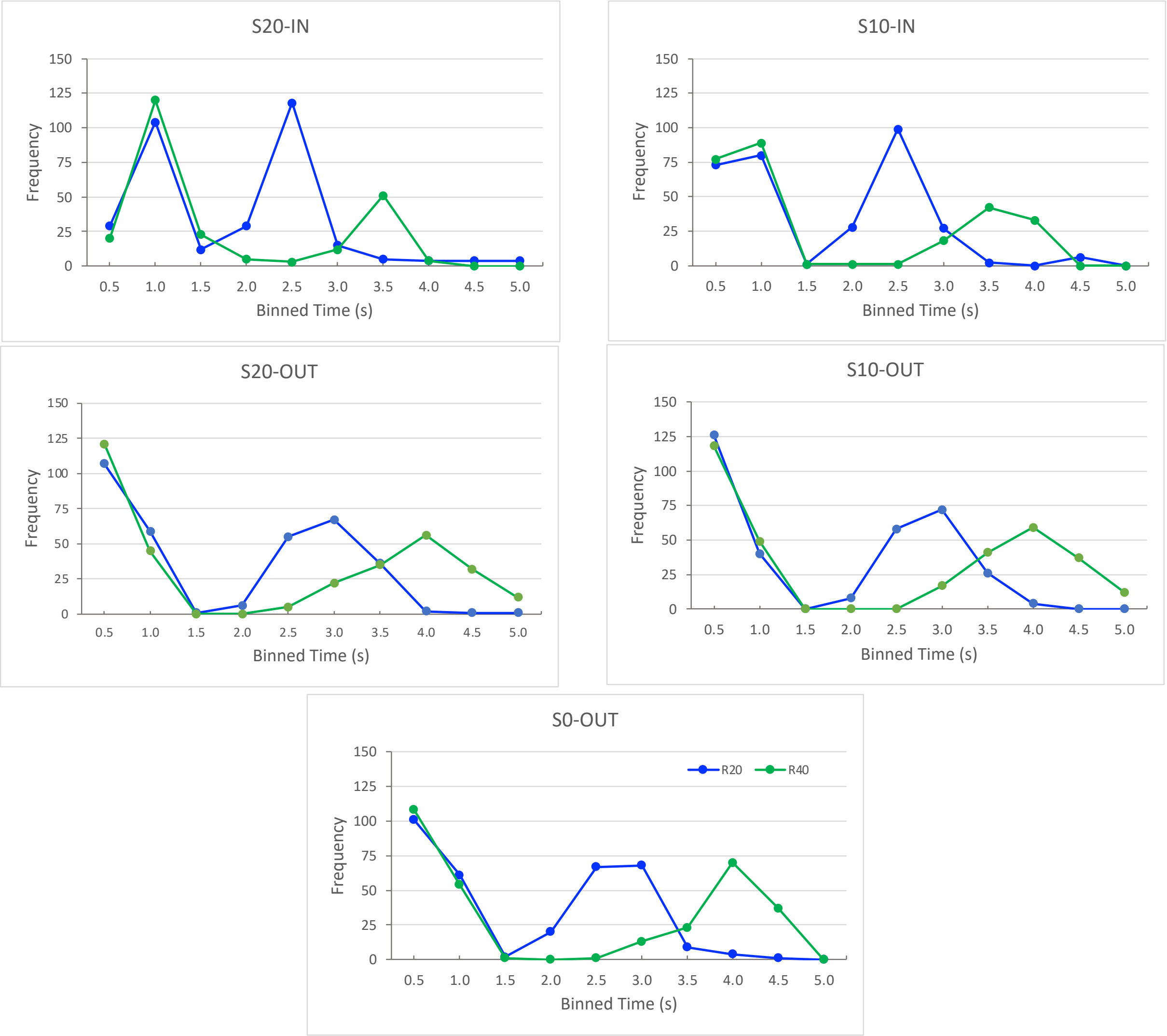

Supplement: Supplement 1 [file jovi-23-3-11_s001.pdf]
