# Supplementary material for: Timing of steering actions in locomotor interception of targets following curving trajectories
Source: J Vis. 2023 Mar 23;23(3):11. doi: 10.1167/jov.23.3.11 (PMC10050912; doi:10.1167/jov.23.3.11)

Supplementary Figure 2: Photo of the experimental set-up depicting a participant seated in the driving simulator placed inside the virtual reality system. The simulated environment was projected on the three (3-m wide by 4-m high) walls and the (3 m by 3 m) ground surface. Participant head position and orientation was tracked via markers on the stereo-glasses. From the participant's point of view the environment consisted of a large grass-like flat plain, containing both fine and gross texture, bordered by distant mountains.

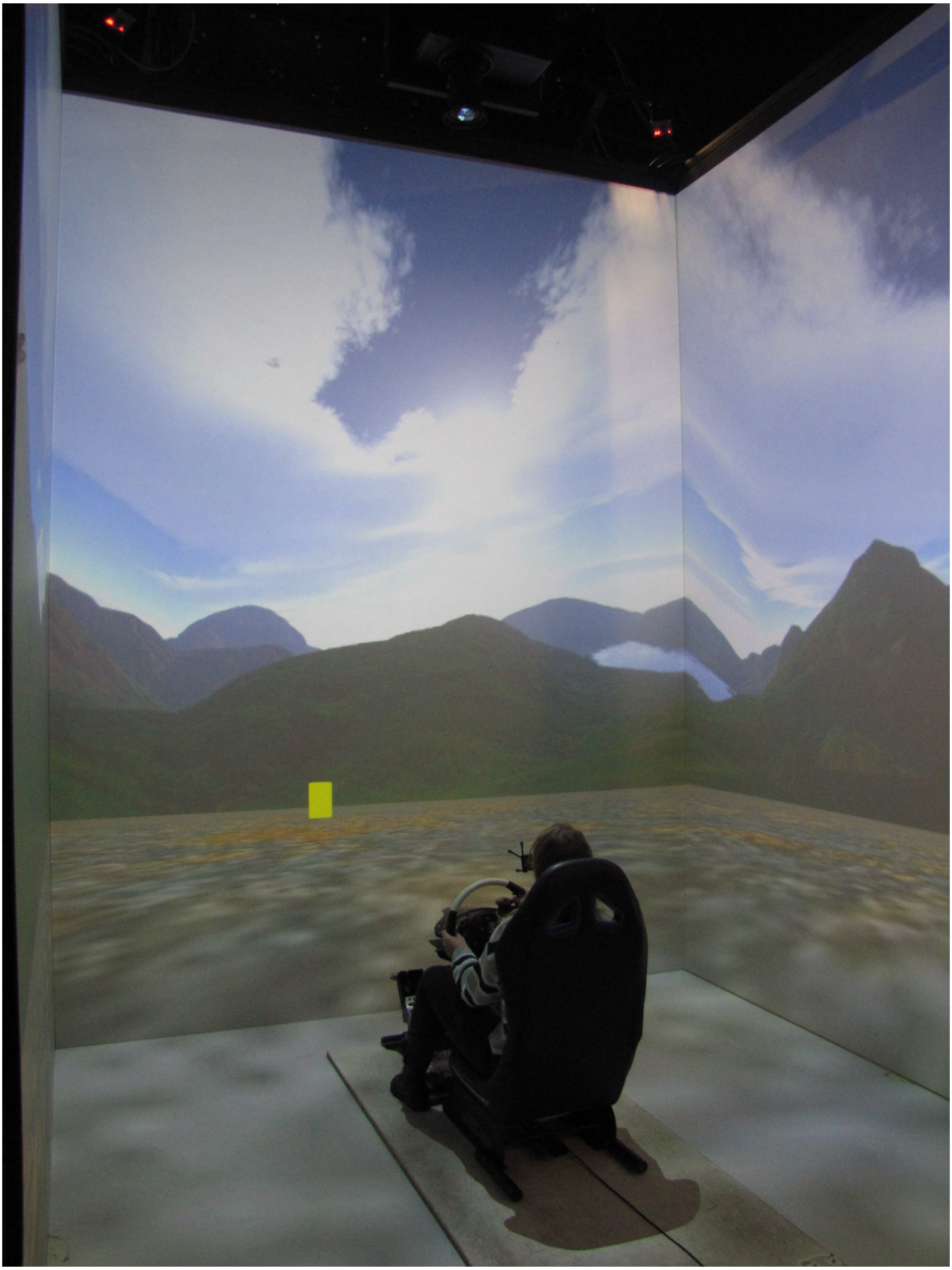

Supplement: Supplement 2 [file jovi-23-3-11_s002.pdf]
