# Supplementary material for: Timing of steering actions in locomotor interception of targets following curving trajectories
Source: J Vis. 2023 Mar 23;23(3):11. doi: 10.1167/jov.23.3.11 (PMC10050912; doi:10.1167/jov.23.3.11)

Supplementary Figure 3a: Page-sized versions of each panel of Figure 4.
(**a**) P3, Block 4, S5/R30-IN (leftward) condition

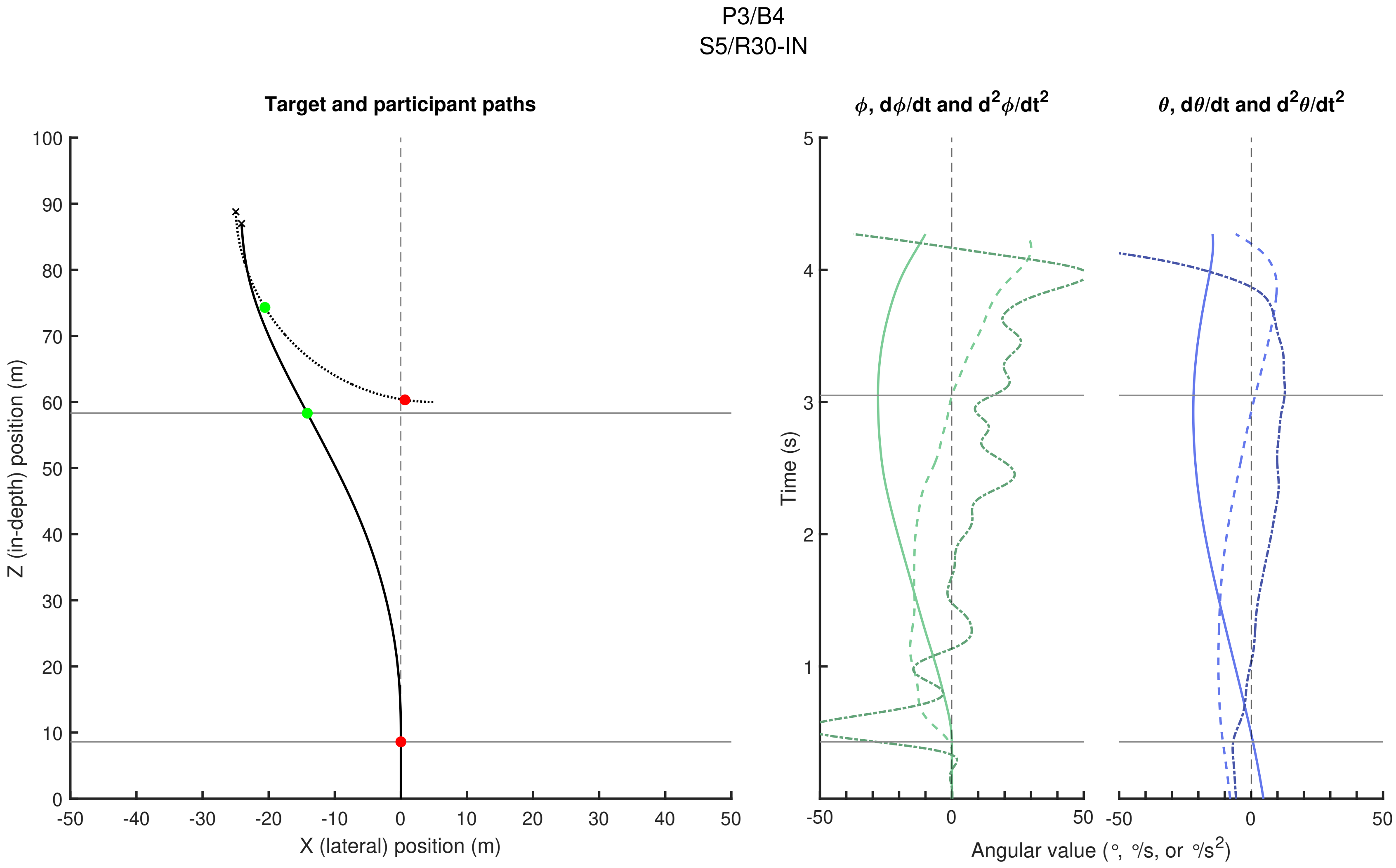

Supplementary Figure S3b: Page-sized versions of each panel of Figure 4.
(**b**) P13, Block 4, S20/R20-IN (leftward) condition

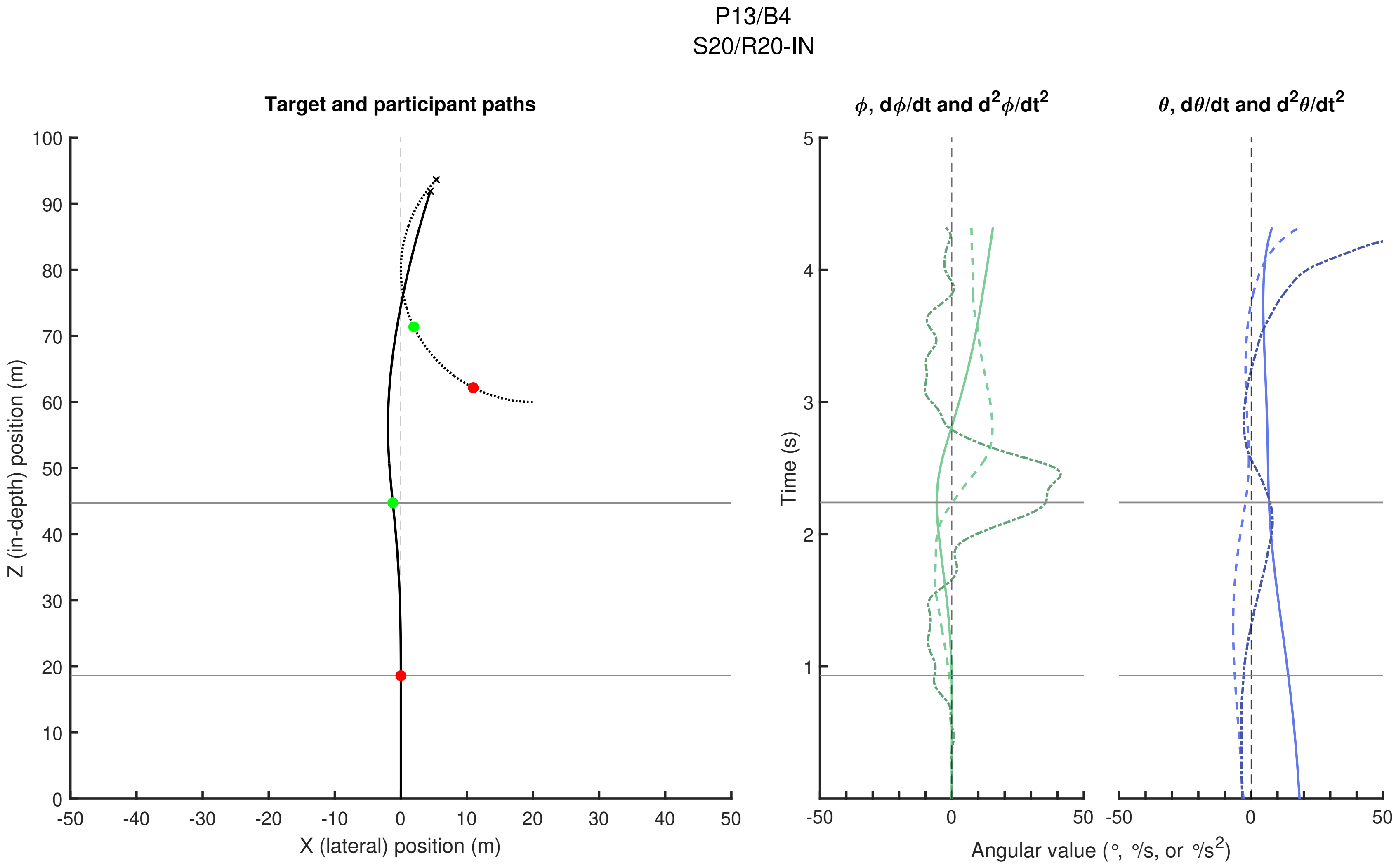

Supplementary Figure 3c: Page-sized versions of each panel of Figure 4.
(**c**) P5, Block 4, S15/R10-IN (leftward) condition.

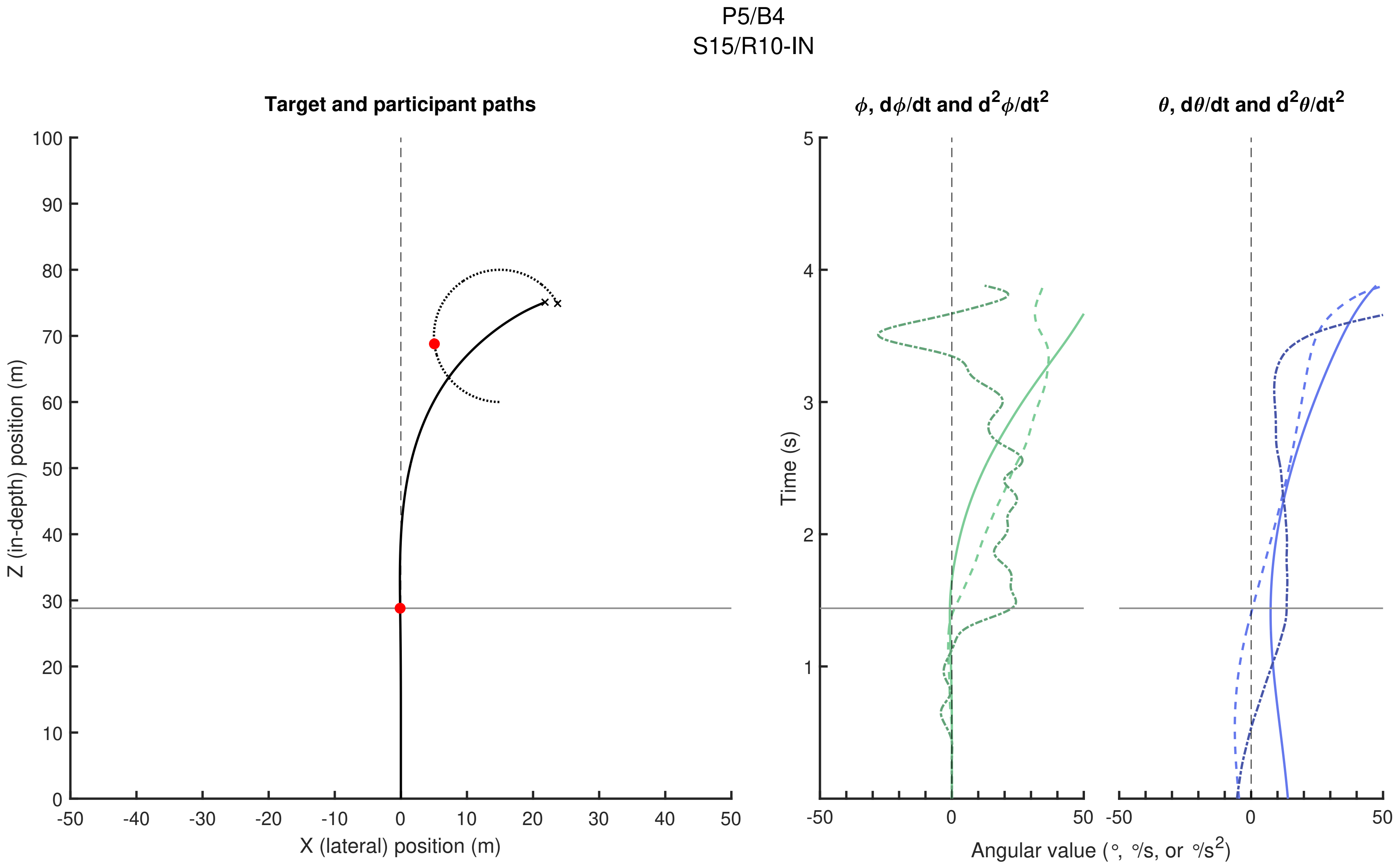

Supplement: Supplement 3 [file jovi-23-3-11_s003.pdf]
