# Supplementary material for: Timing of steering actions in locomotor interception of targets following curving trajectories
Source: J Vis. 2023 Mar 23;23(3):11. doi: 10.1167/jov.23.3.11 (PMC10050912; doi:10.1167/jov.23.3.11)
Supplement: Supplement 4 [file jovi-23-3-11_s004.pdf]

Supplementary Table S1: Means and Standard Deviations (between parentheses), per 0.5-s time bin, of actually observed values of θ, dθ/dt and $d^2θ/dt^2$ at a 100-ms visuomotor delay before a steering event, for all 4227 identified steering events, for each of the 12 experimental conditions. Cells with less than 5 values are marked NaN.

θ (°)	Bin 1	Bin 2	Bin 3	Bin 4	Bin 5	Bin 6	Bin 7	Bin 8	Bin 9	Bin 10
**Upper bin limit**	**0.5 s**	**1.0 s**	**1.5 s**	**2.0 s**	**2.5 s**	**3.0 s**	**3.5 s**	**4.0 s**	**4.5 s**	**5.0 s**
S20/R10-IN	17.4 (0.5)	15.9 (0.7)	14.4 (0.3)	14.6 (0.4)	NaN	NaN	26.5 (11.6)	33.4 (14.3)	NaN	NaN
S20/R20-IN	17.3 (0.3)	16.1 (0.7)	12.8 (1.0)	10.1 (1.3)	8 (1.8)	4.7 (2.5)	-0.1 (3.5)	NaN	4.5 (8.0)	NaN
S20/R30-IN	17.4 50.4)	15.9 (0.7)	12.6 (1.1)	9.4 (1.1)	6.7 (2.7)	4.0 (2.9)	-1.7 (3.5)	-6.7 (4.3)	NaN	NaN
S15/R10-IN	12.5 (0.5)	11.3 (0.5)	8.1 (0.5)	8.3 (0.8)	NaN	NaN	16.4 (11.2)	24.7 (13.4)	NaN	NaN
S15/R20-IN	12.4 (0.5)	11 (0.8)	6.5 (1.7)	2.7 (1.6)	1.0 (1.9)	-2.6 (2.0)	-7.6 (3.6)	NaN	NaN	NaN)
S15/R30-IN	12.4 (0.5)	11 (0.8)	5.5 (1.6)	NaN	-0.4 (1.1)	-3.8 (2.6)	-10 (4.5)	-15.1 (7.2)	NaN	NaN
S5/R10-IN	1.9 (0.6)	0.8 (0.4)	-4.7 (0.7)	-5 (1.2)	NaN	NaN	5.2 (11.9)	NaN	NaN	NaN
S5/R20-IN	1.8 (0.6)	0.2 (1.1)	NaN	-10 (2.0)	-13.3 (2.1)	-17.6 (3.1)	-23.6 1.9)	-28.6 (2.2)	NaN	NaN
S5/R30-IN	2.0 (0.8)	0.4 (0.9)	NaN	NaN	-13.9 (1.9)	-19.7 (3.0)	-24.9 (4.1)	-34.6 (7.1)	-45.7 (7.3)	NaN
S5/R10-OUT	8.6 (0.7)	11 (1.4)	16.1 (1.5)	17.2 (1.6)	19.1 (1.8)	NaN	13.6 (6.3)	NaN	NaN	NaN
S5/R20-OUT	8.6 (0.8)	11.2 (1.5)	NaN	21.5 (1.4)	25.7 (2.4)	30 (2.4)	37.4 (3.0)	42.8 (2.9)	45.9 (2.7)	NaN
S5/R30-OUT	8.5 (0.9)	11.3 (1.7)	NaN	NaN	27.5 (2.3)	32.5 (3.1)	38.5 (4.0)	48 (7.3)	55.7 (5.3)	NaN

dθ/dt (°/s)	Bin 1	Bin 2	Bin 3	Bin 4	Bin 5	Bin 6	Bin 7	Bin 8	Bin 9	Bin 10
**Upper bin limit**	**0.5 s**	**1.0 s**	**1.5 s**	**2.0 s**	**2.5 s**	**3.0 s**	**3.5 s**	**4.0 s**	**4.5 s**	**5.0 s**
S20/R10-IN	-3.9 (0.4)	-4.1 (0.4)	0.0 (1.5)	3.4 (1.2)	NaN	NaN	12 (19.6)	18 (23.6)	NaN	NaN
S20/R20-IN	-4 (0.2)	-5 (0.5)	-6.8 (1.3)	-3.9 (3.0)	-1.8 (2.4)	-1.4 (2.7)	0.2 (2.9)	NaN	-7.0 (16.8)	NaN
S20/R30-IN	-3.8 (0.4)	-5.2 (0.7)	-7.9 (1.0)	-10.4 (1.4)	-3.3 (4.5)	-1.3 (2.3)	-1.4 (4.4)	1.3 (7.2)	NaN	NaN
S15/R10-IN	-5.7 (0.4)	-6.1 (0.1)	-0.8 (1.7)	3.3 (2.4)	NaN	NaN	4.6 (14.6)	15.1 (28.7)	NaN	NaN
S15/R20-IN	-5.8 0.4)	-6.9 (0.6)	-9.4 (0.8)	-4.2 (2.6)	-1.5 (2.3)	-0.9 (2.3)	1.3 (3.7)	NaN	NaN	NaN
S15/R30-IN	-5.8 (0.4)	-7.0 (0.7)	-11.1 (1.2)	NaN	-3.3 (2.2)	-1.6 2.8)	-1.0 (3.6)	1.7 (6.9)	NaN	NaN
S5/R10-IN	-9.4 (0.3)	-9.7 (0.1)	-0.4 (2.0)	2.6 (3.1)	NaN	NaN	7.3 (20.8)	NaN	NaN	NaN
S5/R20-IN	-9.9 (0.4)	-10.8 (0.6)	NaN	-3.1 (4.4)	-0.4 (2.0)	0.7 (2.7)	2.3 (1.5)	6.0 (4.1)	NaN	NaN
S5/R30-IN	-9.8 0.5)	-10.9 (0.6)	NaN	NaN	-1.5 (1.9)	-0.5 (2.0)	0.4 (2.4)	2.7 (4.5)	8.4 (6.9)	NaN
S5/R10-OUT	12.4 (0.2)	12.5 (0.3)	-1.1 (5.5)	-4.4 (3.5)	-6 (3.0)	NaN	4.4 (2.9)	NaN	NaN	NaN
S5/R20-OUT	13.1 (0.4)	14.3 (0.6)	NaN	0.3 (2.5)	-1.7 (2.7)	-3.1 (2.3)	-4.2 (3.0)	-4.5 (2.9)	-7.4 (4.5)	NaN
S5/R30-OUT	13.3 (0.5)	14.8 (0.9)	NaN	NaN	-0.8 (2.2)	-0.5 (2.5)	-1.9 (1.7)	-3.4 (5.0)	-6.5 (4.0)	NaN

$d^2θ/dt^2$ (°/s^2)	Bin 1	Bin 2	Bin 3	Bin 4	Bin 5	Bin 6	Bin 7	Bin 8	Bin 9	Bin 10
**Upper bin limit**	**0.5 s**	**1.0 s**	**1.5 s**	**2.0 s**	**2.5 s**	**3.0 s**	**3.5 s**	**4.0 s**	**4.5 s**	**5.0 s**
S20/R10-IN	-2.7 (1.0)	1.2 (2.5)	11.5 (2.3)	15.4 (1.2)	NaN	NaN	16.5 (28.0)	37.2 (64.9)	NaN	NaN
S20/R20-IN	-3.6 (0.2)	-3.7 (0.4)	-2.8 (1.6)	3.7 (3.7)	8.5 (2.7)	11.7 (3.6)	17.4 (4.4)	NaN)	15.8 (71.8)	NaN
S20/R30-IN	-3.5 (0.3)	-4.4 (0.6)	-6.3 (0.9)	-8.4 (1.9)	4.7 (6.1)	10 (4.7)	14.9 (12.8)	33.0 (30.2)	NaN	NaN
S15/R10-IN	-3.1 (0.9)	-0.8 (1.2)	12.7 (2.1)	16.8 (1.8)	NaN	NaN	4.4 (23.9)	34.3 (87.1)	NaN	NaN
S15/R20-IN	-4.5 (0.2)	-4.7 (0.4)	-4.0 (0.3)	5.6 (2.5)	9.7 (2.2)	12.8 (2.8)	18.6 (5.8)	NaN	NaN	60.6
S15/R30-IN	-4.7 (0.3)	-5.5 (0.5)	-8.0 (0.8)	NaN	4.9 (6.2)	9.7 (5.2)	15.8 (9.5)	29.9 (28.4)	NaN	NaN
S5/R10-IN	-2.9 (0.8)	-1.4 (0.8)	15.6 (1.9)	17.4 (2.4)	NaN	NaN	11.4 (50.9)	NaN)	NaN	NaN
S5/R20-IN	-6.1 (0.2)	-6.1 (0.2)	NaN	7.9 (4.0)	11.9 (2.1)	13.7 (2.8)	17 (2.7)	28.1 (9.6)	NaN	NaN
S5/R30-IN	-6.8 (0.4)	-7.4 (0.4)	NaN	NaN	8.4 (2.4)	12.1 (3.1)	15.2 (4.0)	22.8 (11.7)	44.9 (29.1)	NaN
S5/R10-OUT	2.1 (0.8)	-0.8 (1.9)	-17.2 (4.7)	-17.9 (2.8)	-16.4 (3.0)	NaN	28.9 (12.6)	NaN	NaN	NaN
S5/R20-OUT	6.4 (0.2)	6.0 (0.4)	NaN	-10.7 (1.9)	-12.4 (2.2)	-13.9 (2.1)	-15.9 (3.9)	-16.4 (5.8)	-24.6 (12.5)	NaN
S5/R30-OUT	7.5 (0.3)	7.9 (0.4)	NaN	NaN	-9.7 (2.2)	-10.1 (3.0)	-13.4 (2.7)	-17.8 (5.8)	-26.2 (8.1)	NaN
