# Supplementary material for: Timing of steering actions in locomotor interception of targets following curving trajectories
Source: J Vis. 2023 Mar 23;23(3):11. doi: 10.1167/jov.23.3.11 (PMC10050912; doi:10.1167/jov.23.3.11)

P1/B1
S20/R10-IN

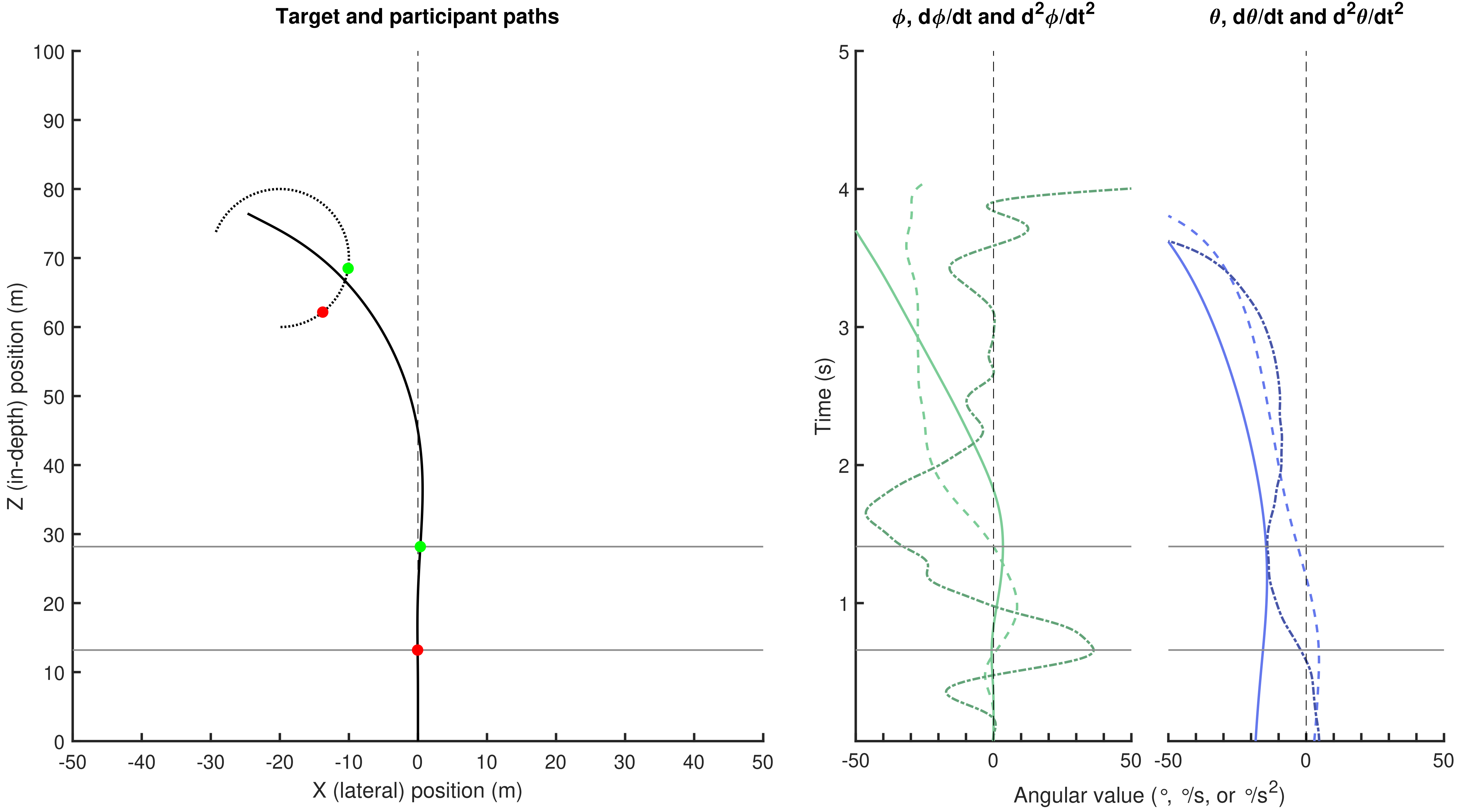

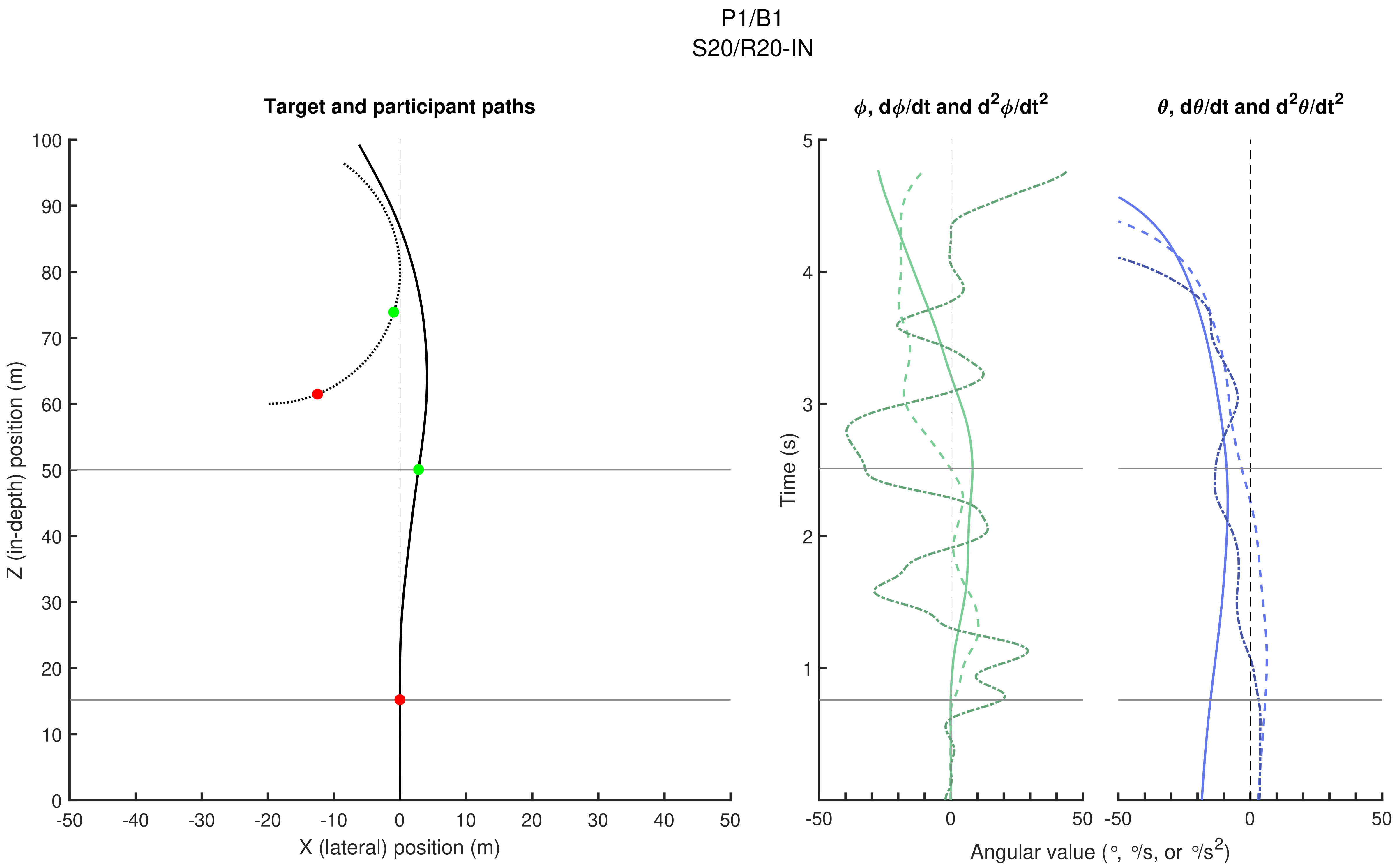
P1/B1
S20/R20-IN
Target and participant paths
φ, dφ/dt and d²φ/dt²
θ, dθ/dt and d²θ/dt²
Z (in-depth) position (m)
X (lateral) position (m)
Time (s)
Angular value (°, °/s, or °/s²)
100
90
80
70
60
50
40
30
20
10
0
-50
-40
-30
-20
-10
0
10
20
30
40
50
5
4
3
2
1
-50
0
50
-50
0
50

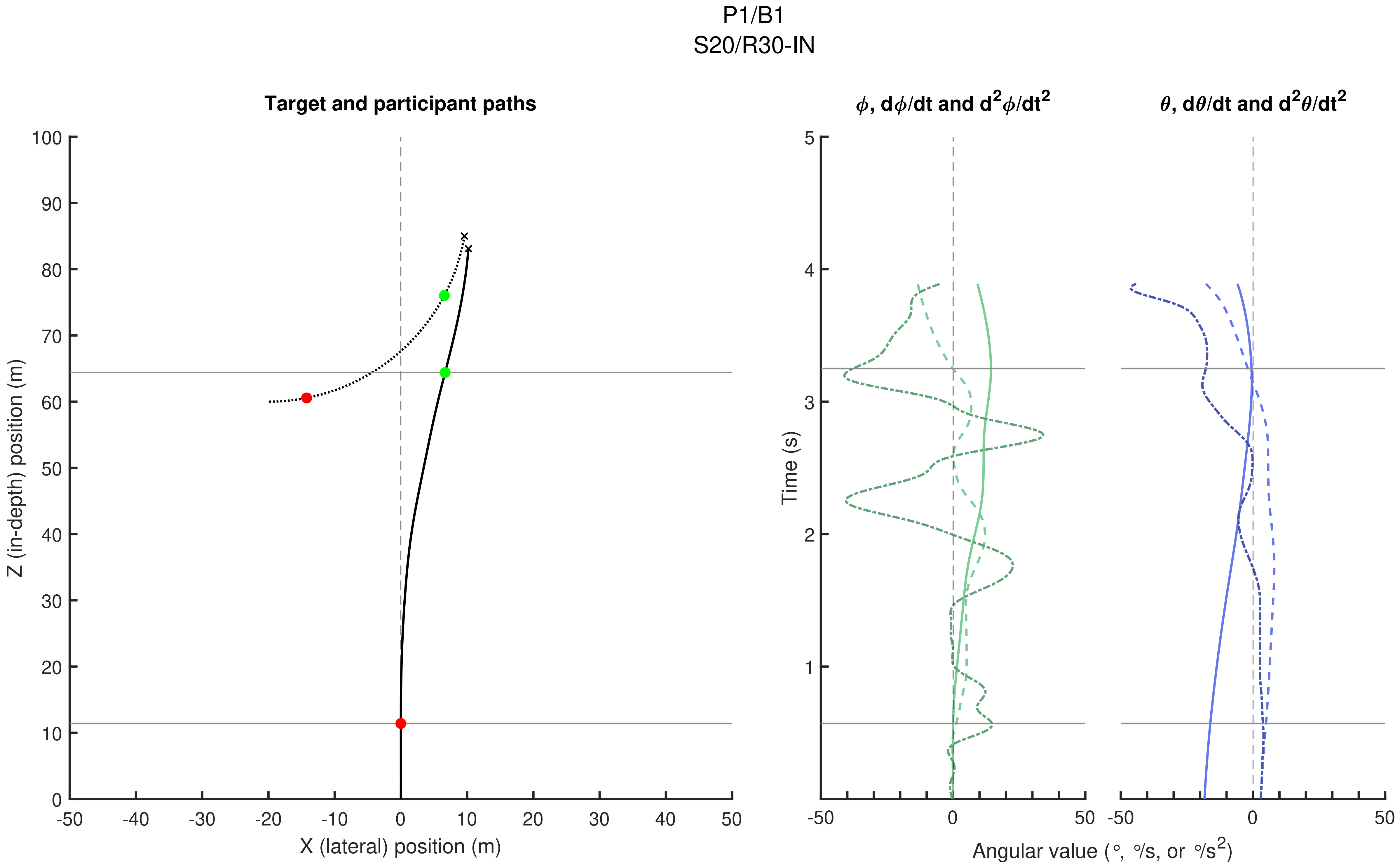
P1/B1
S20/R30-IN
Target and participant paths
Z (in-depth) position (m)
X (lateral) position (m)
ϕ, dϕ/dt and d$^2\phi$/dt^2
θ, dθ/dt and d$^2\theta$/dt^2
Time (s)
Angular value (°, °/s, or °/s^2)

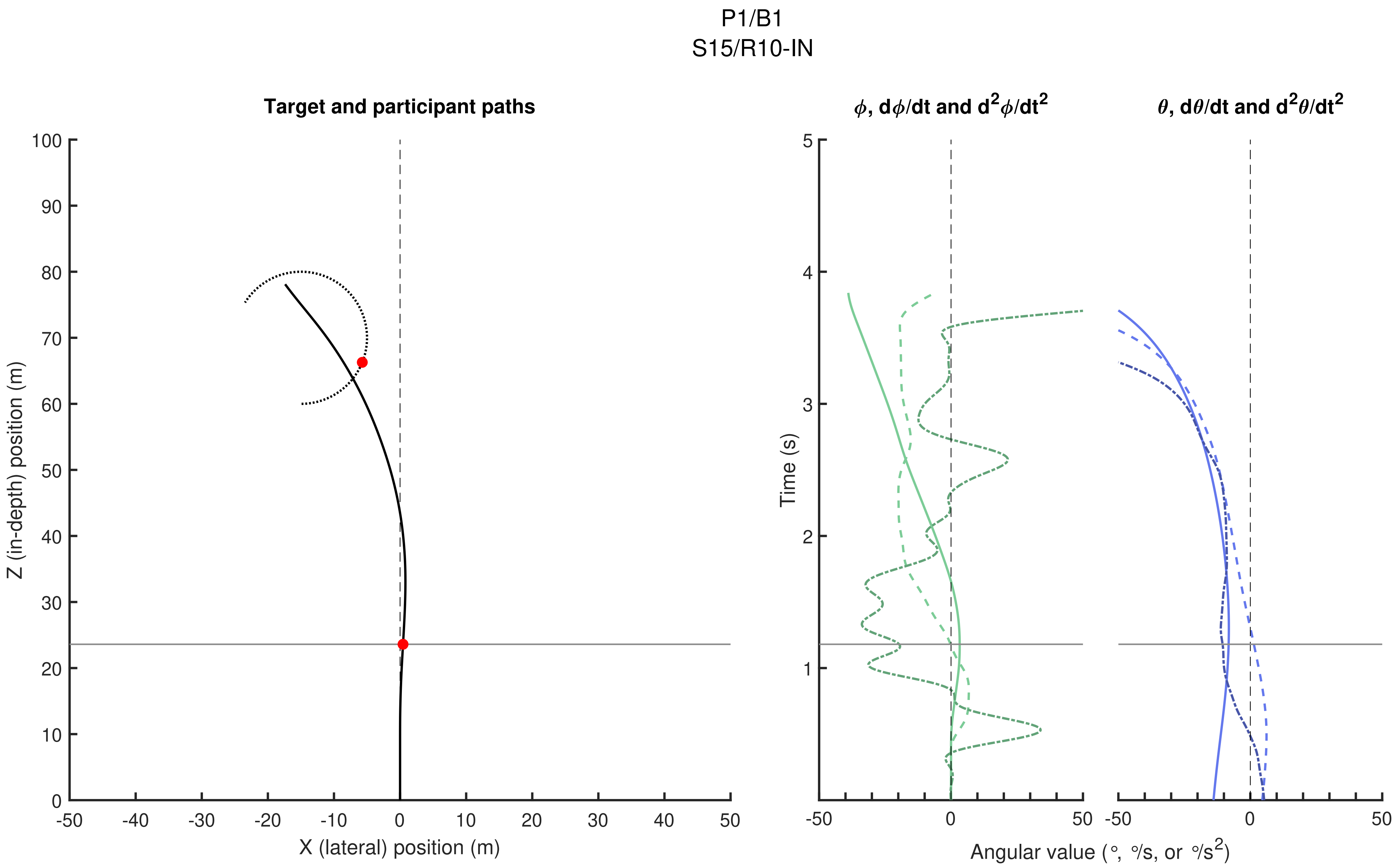
P1/B1
S15/R10-IN
Target and participant paths
Z (in-depth) position (m)
X (lateral) position (m)
0
10
20
30
40
50
60
70
80
90
100
-50
-40
-30
-20
-10
0
10
20
30
40
50
ϕ, dϕ/dt and d²ϕ/dt²
θ, dθ/dt and d²θ/dt²
Time (s)
1
2
3
4
5
-50
0
50
-50
0
50
Angular value (°, °/s, or °/s²)

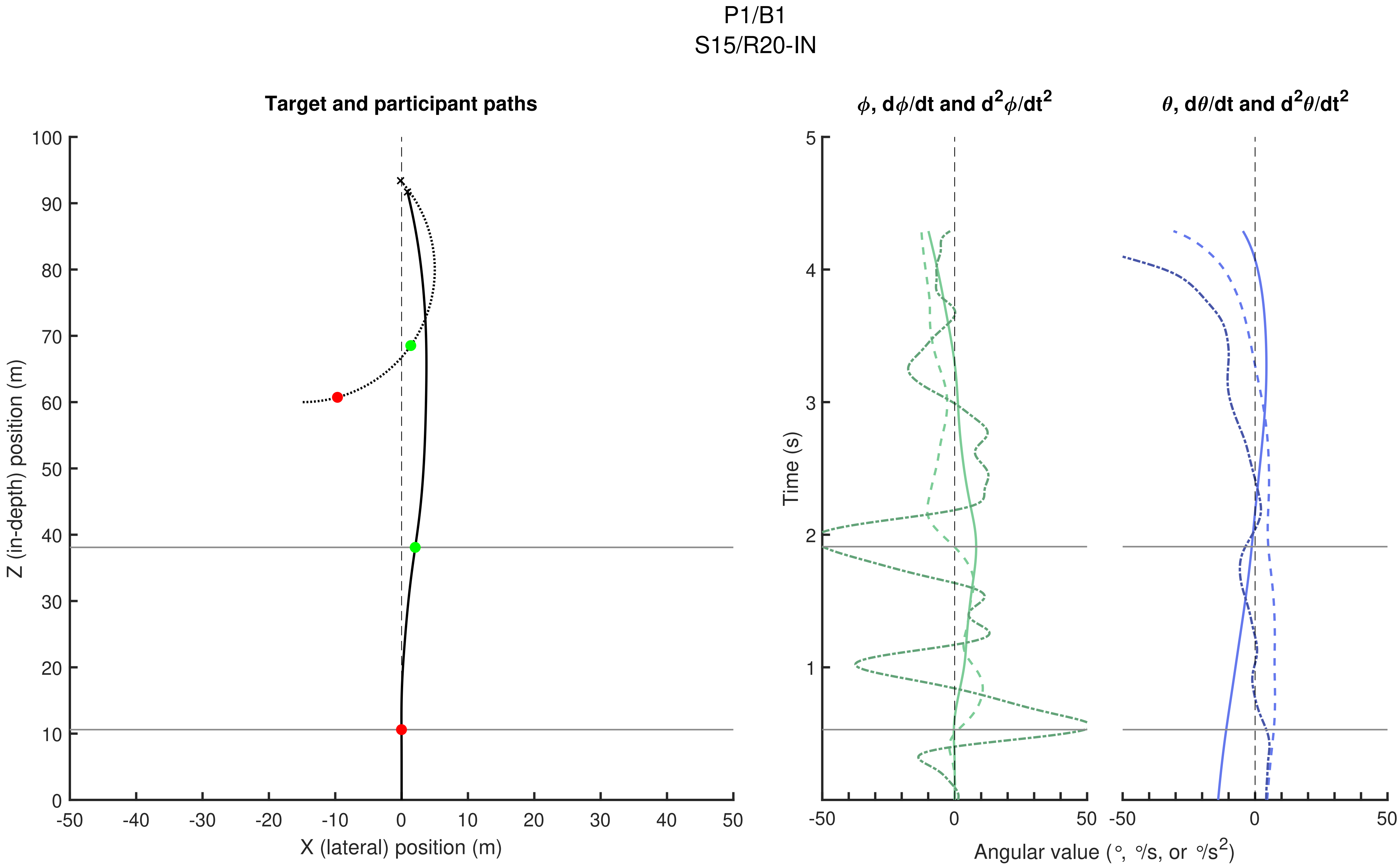
P1/B1
S15/R20-IN
Target and participant paths
Z (in-depth) position (m)
X (lateral) position (m)
ϕ, dϕ/dt and d²ϕ/dt²
θ, dθ/dt and d²θ/dt²
Time (s)
Angular value (°, °/s, or °/s²)

P1/B1
S15/R30-IN

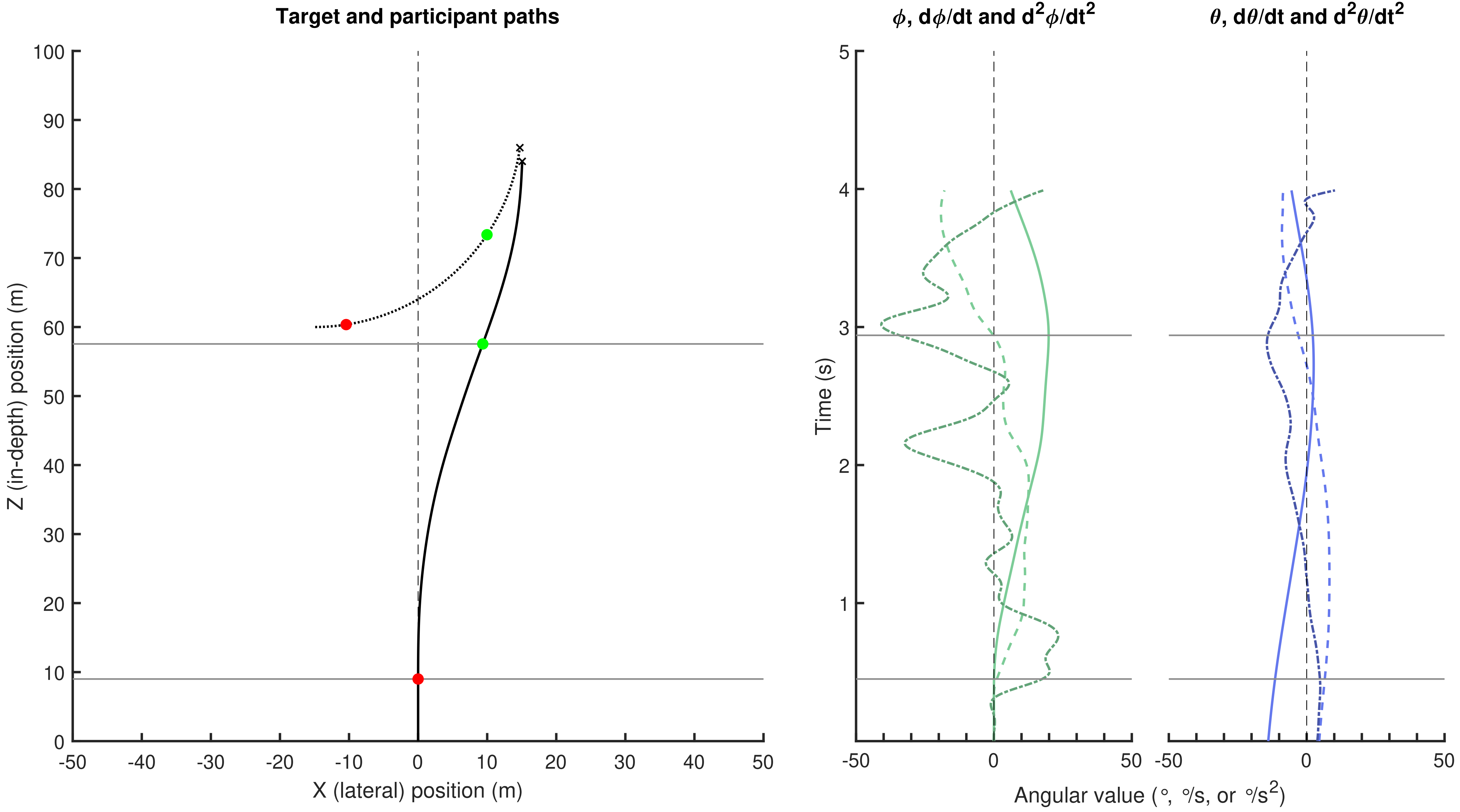

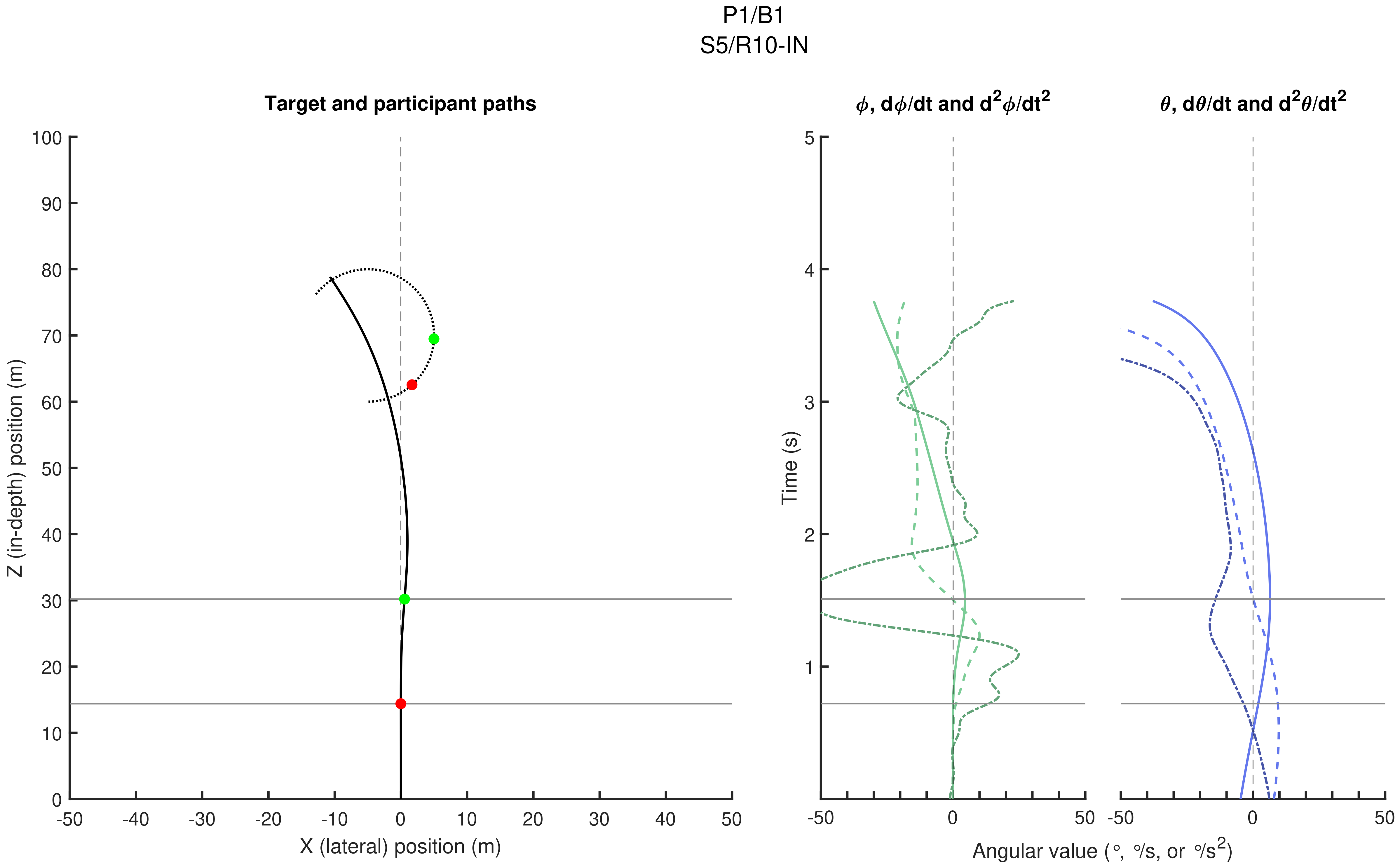
P1/B1
S5/R10-IN
Target and participant paths
Z (in-depth) position (m)
X (lateral) position (m)
100
90
80
70
60
50
40
30
20
10
0
-50
-40
-30
-20
-10
0
10
20
30
40
50
ϕ, dϕ/dt and d²ϕ/dt²
θ, dθ/dt and d²θ/dt²
Time (s)
5
4
3
2
1
-50
0
50
Angular value (°, °/s, or °/s²)

P1/B1
S5/R10-OUT

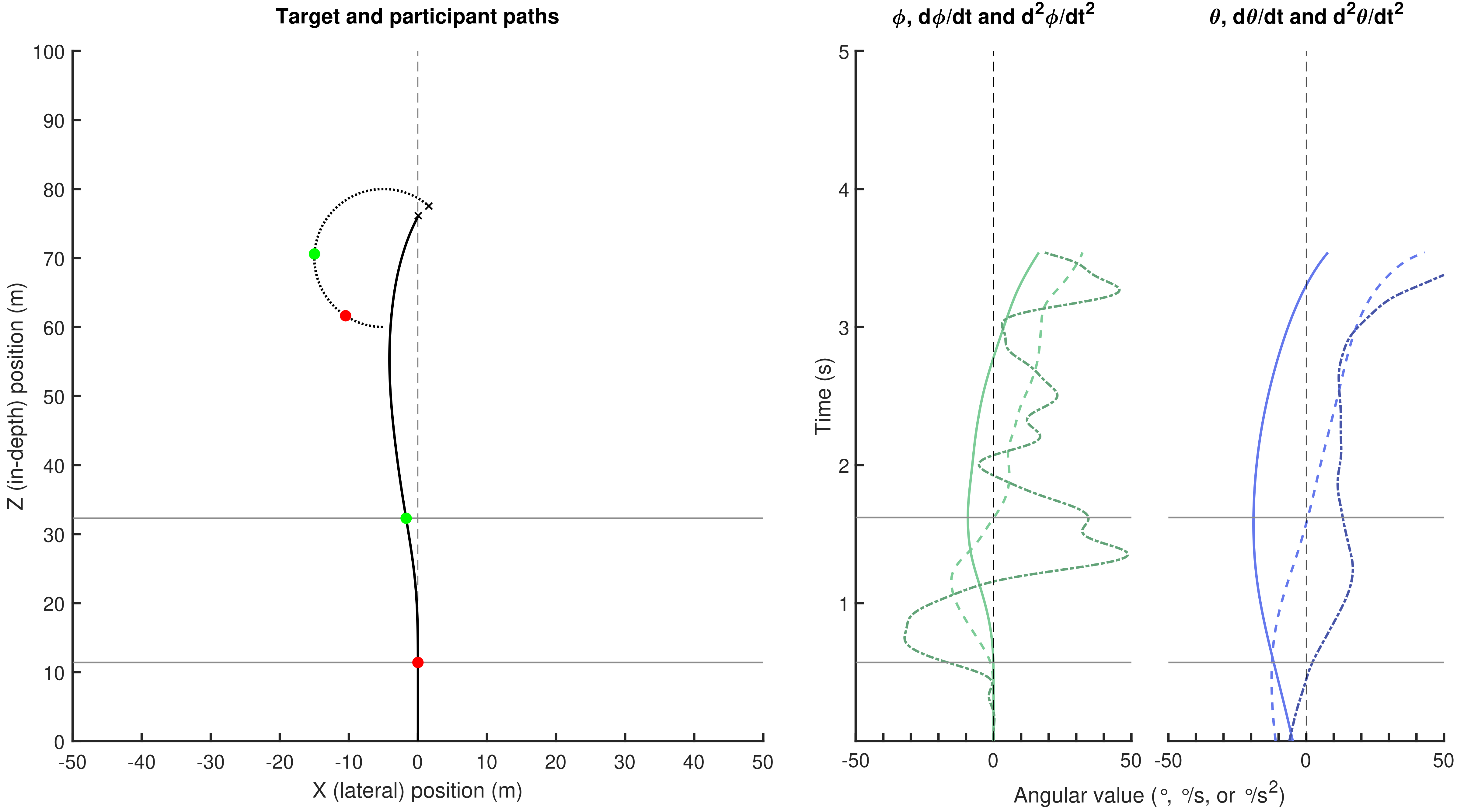

P1/B1
S5/R20-IN

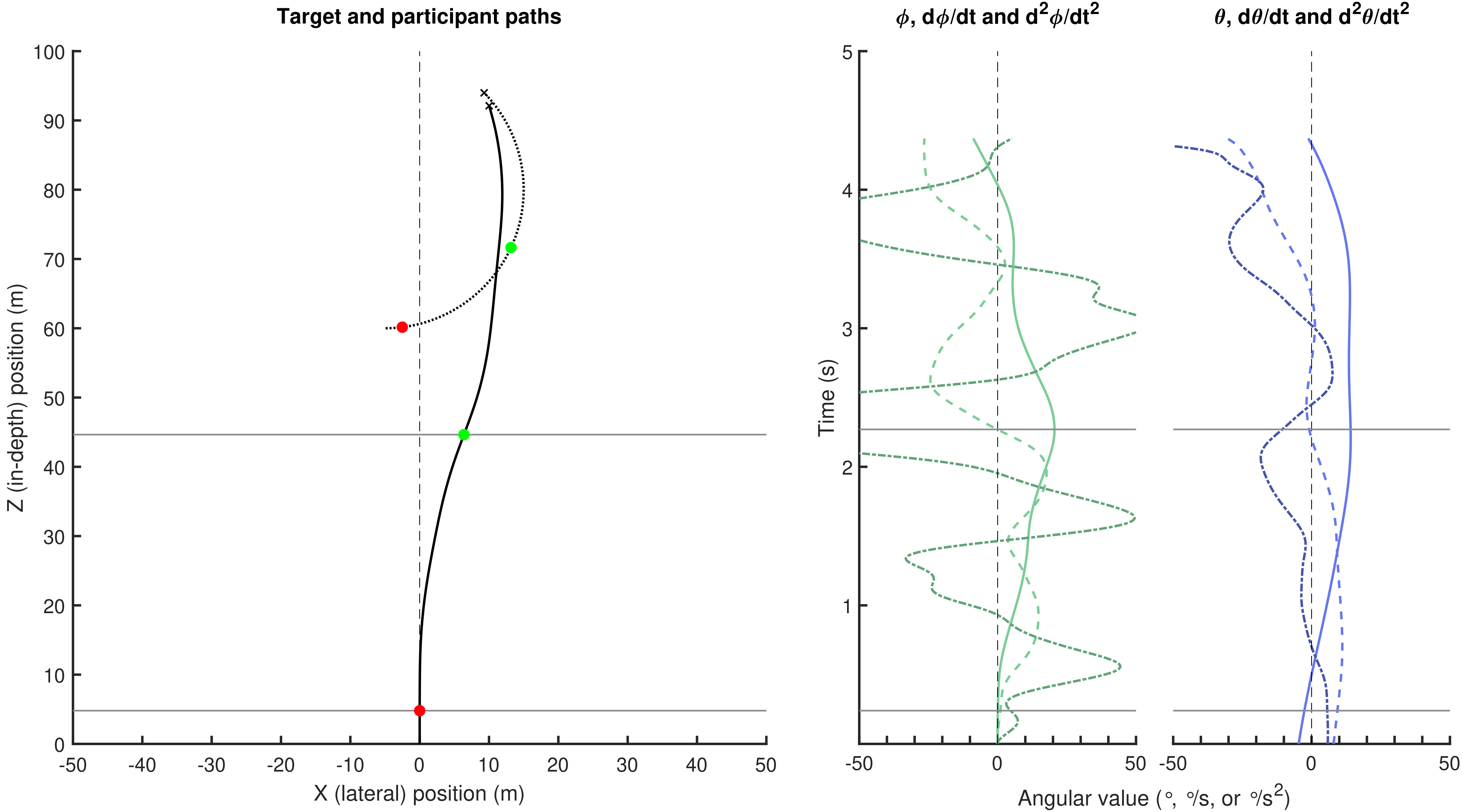

P1/B1
S5/R20-OUT

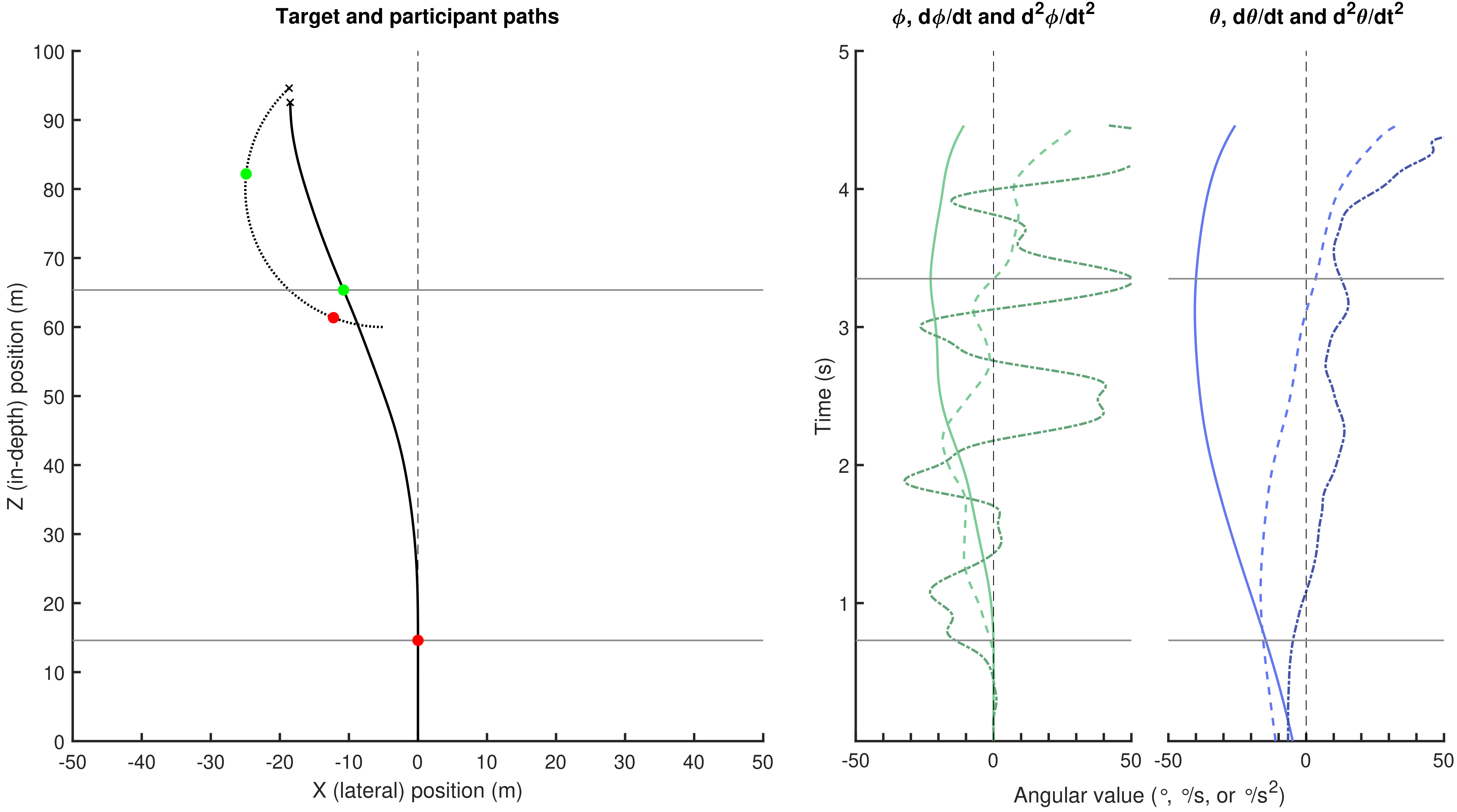

P1/B1
S5/R30-IN

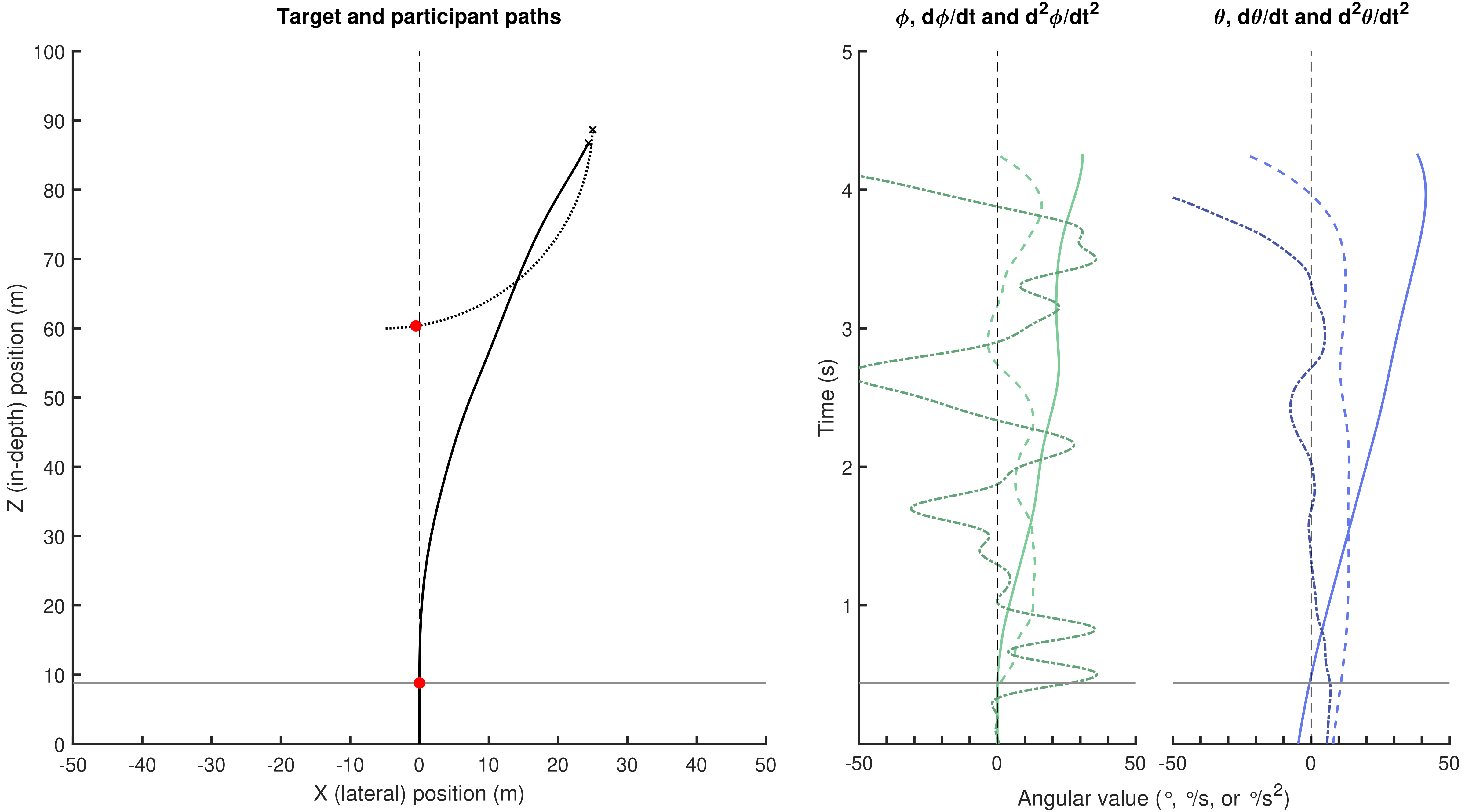

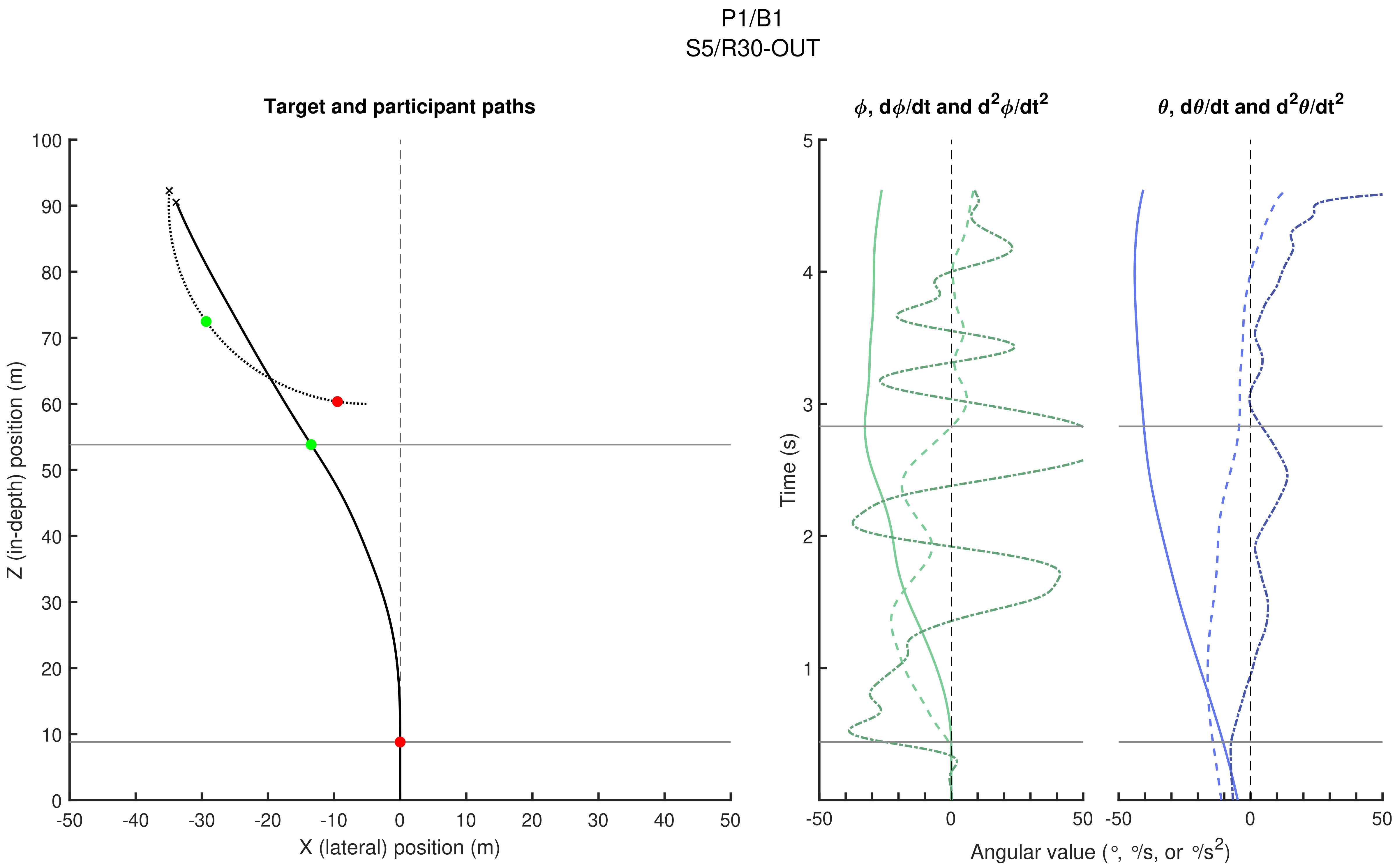

P1/B1
S5/R30-OUT
Target and participant paths
Z (in-depth) position (m)
X (lateral) position (m)
ϕ, dϕ/dt and d$^2\phi$/dt^2
θ, dθ/dt and d$^2\theta$/dt^2
Time (s)
Angular value (°, °/s, or °/s^2)

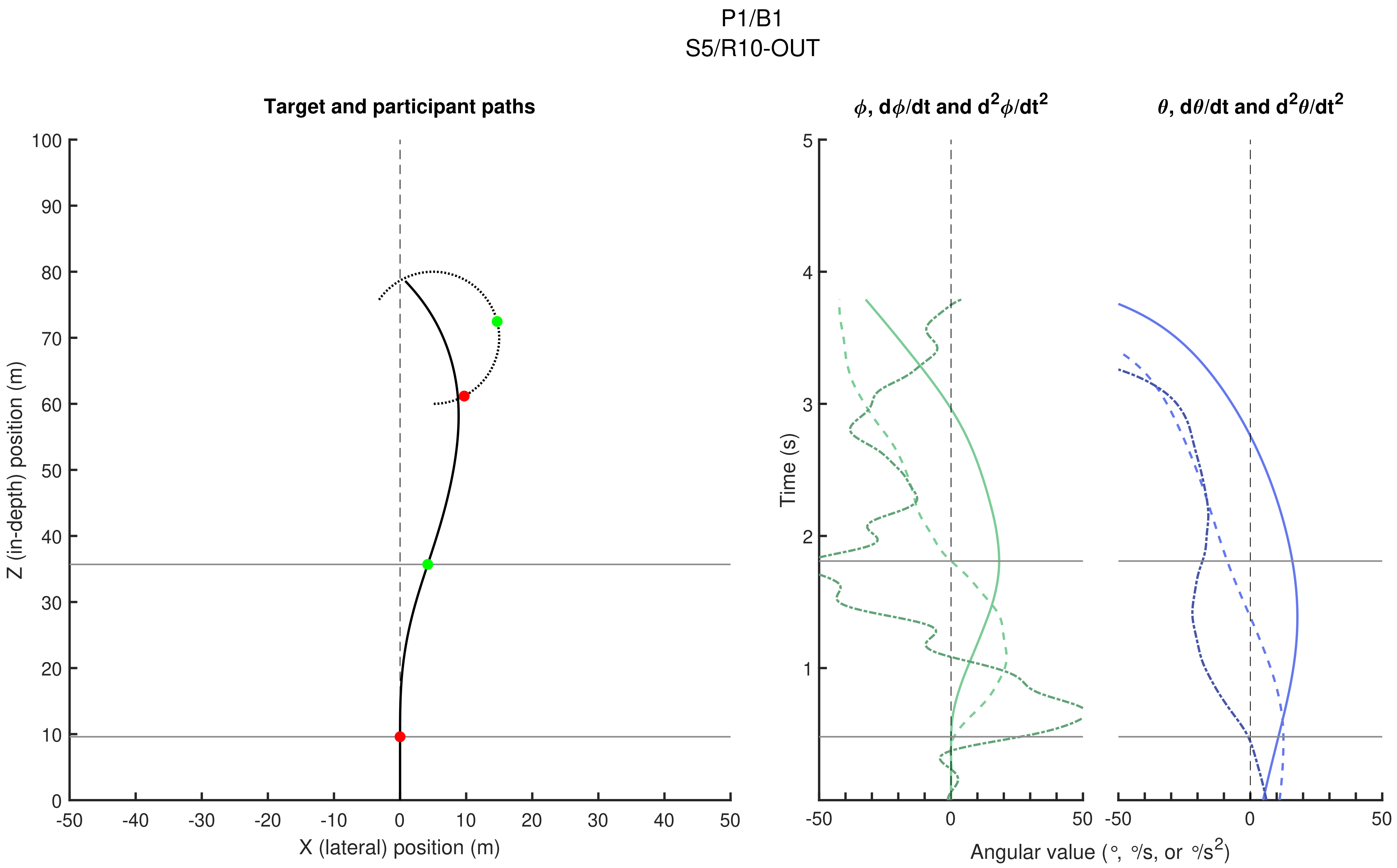
P1/B1
S5/R10-OUT
Target and participant paths
Z (in-depth) position (m)
X (lateral) position (m)
ϕ, dϕ/dt and d²ϕ/dt²
θ, dθ/dt and d²θ/dt²
Time (s)
Angular value (°, °/s, or °/s²)

P1/B1
S5/R10-IN

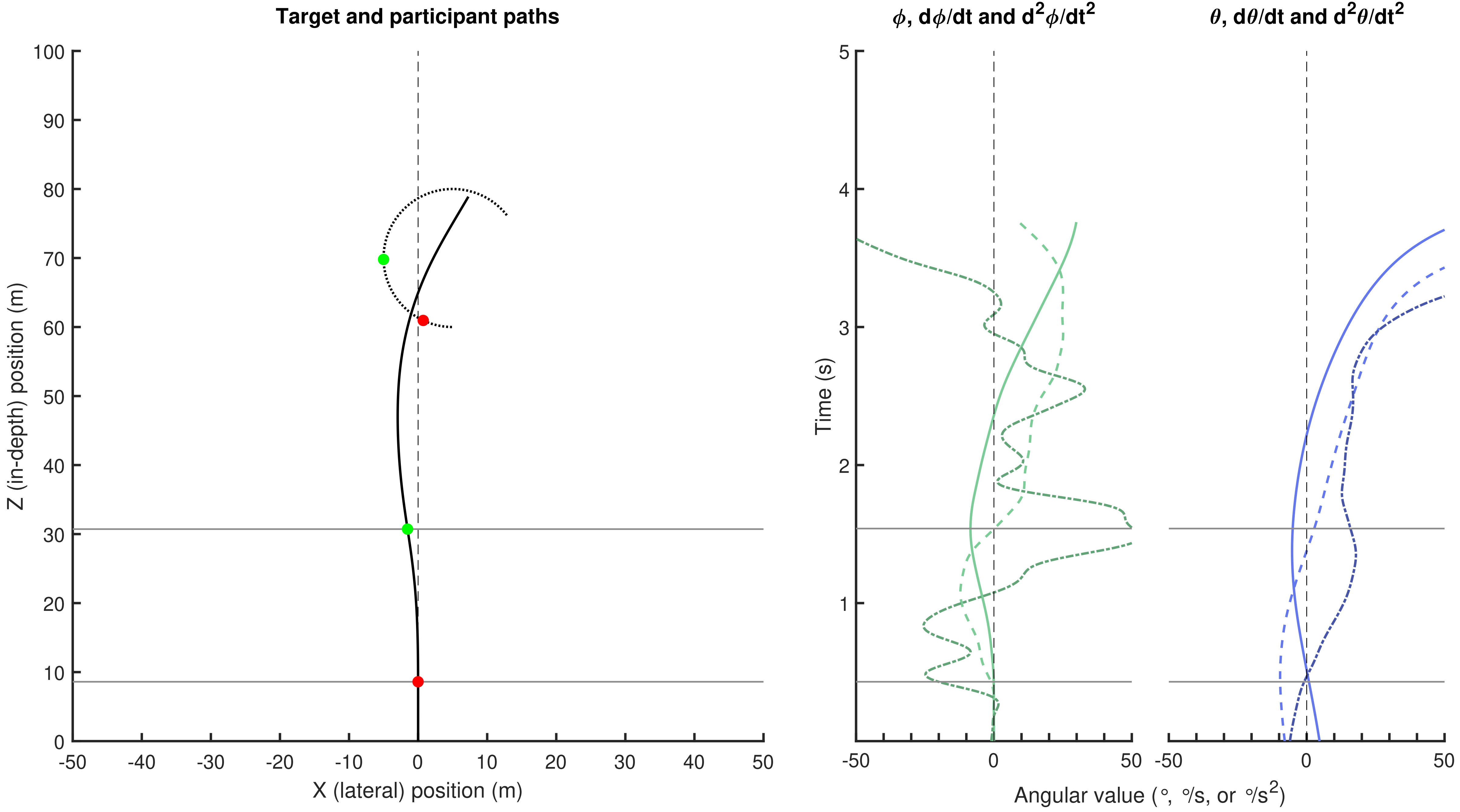

P1/B1
S5/R20-OUT

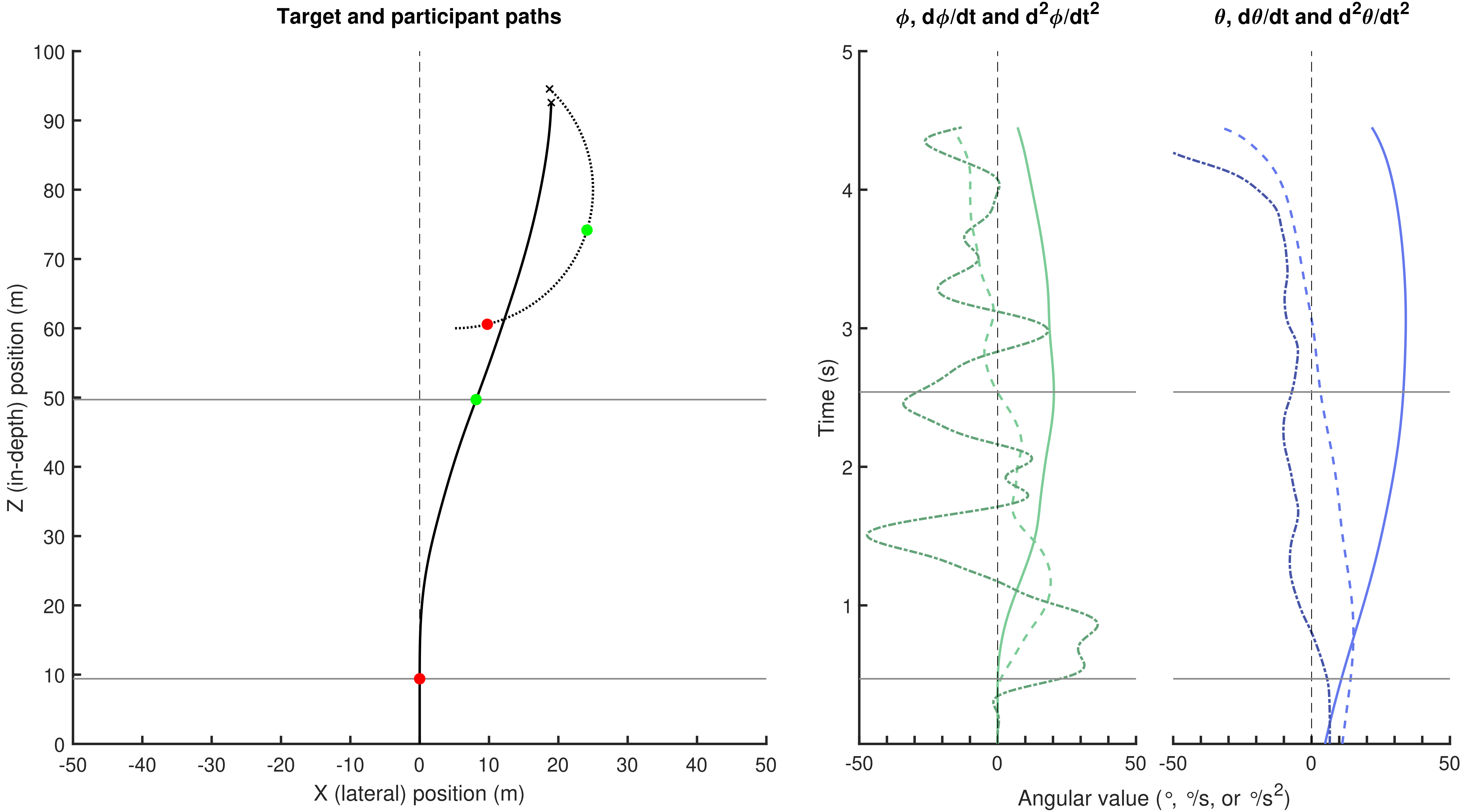

P1/B1
S5/R20-IN

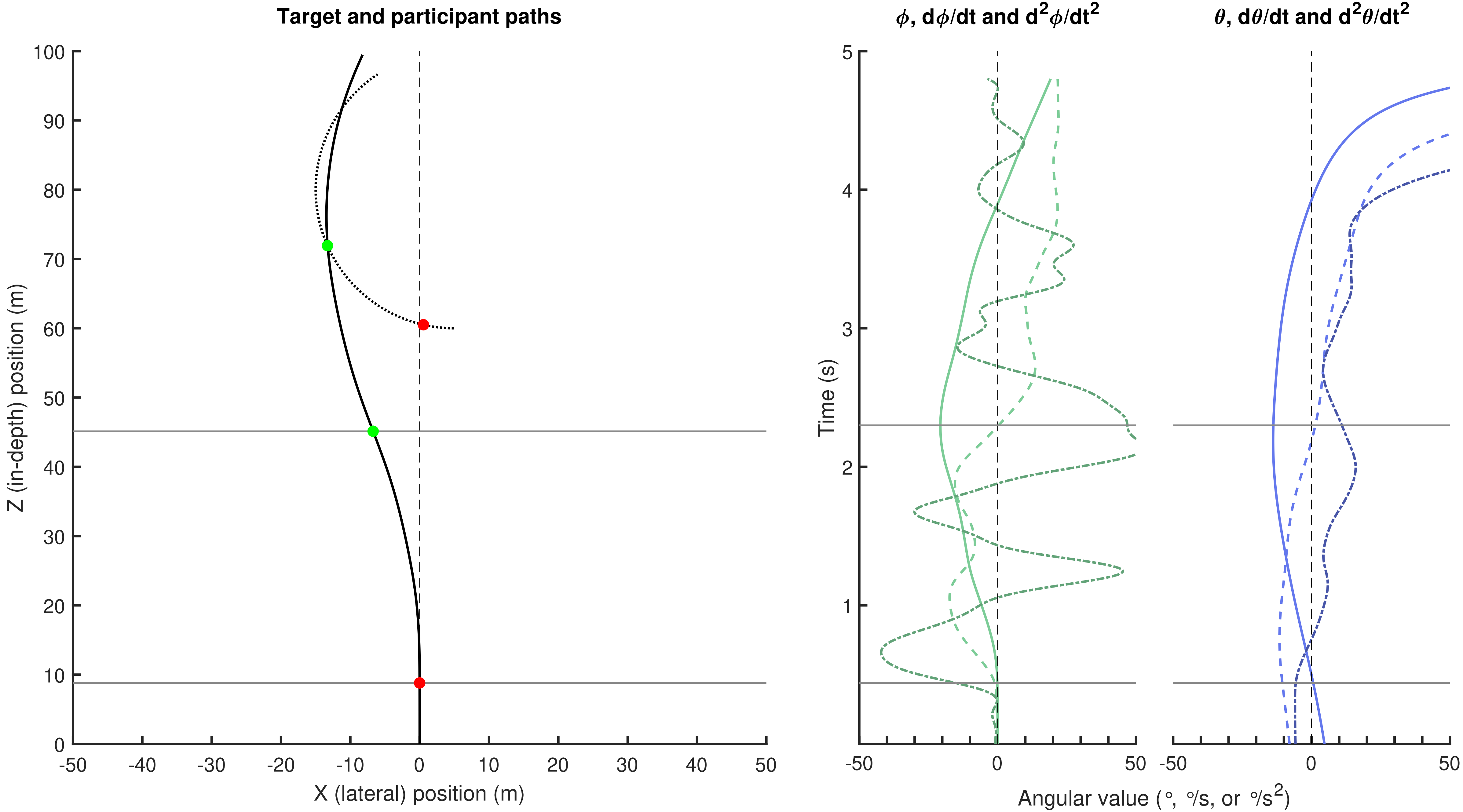

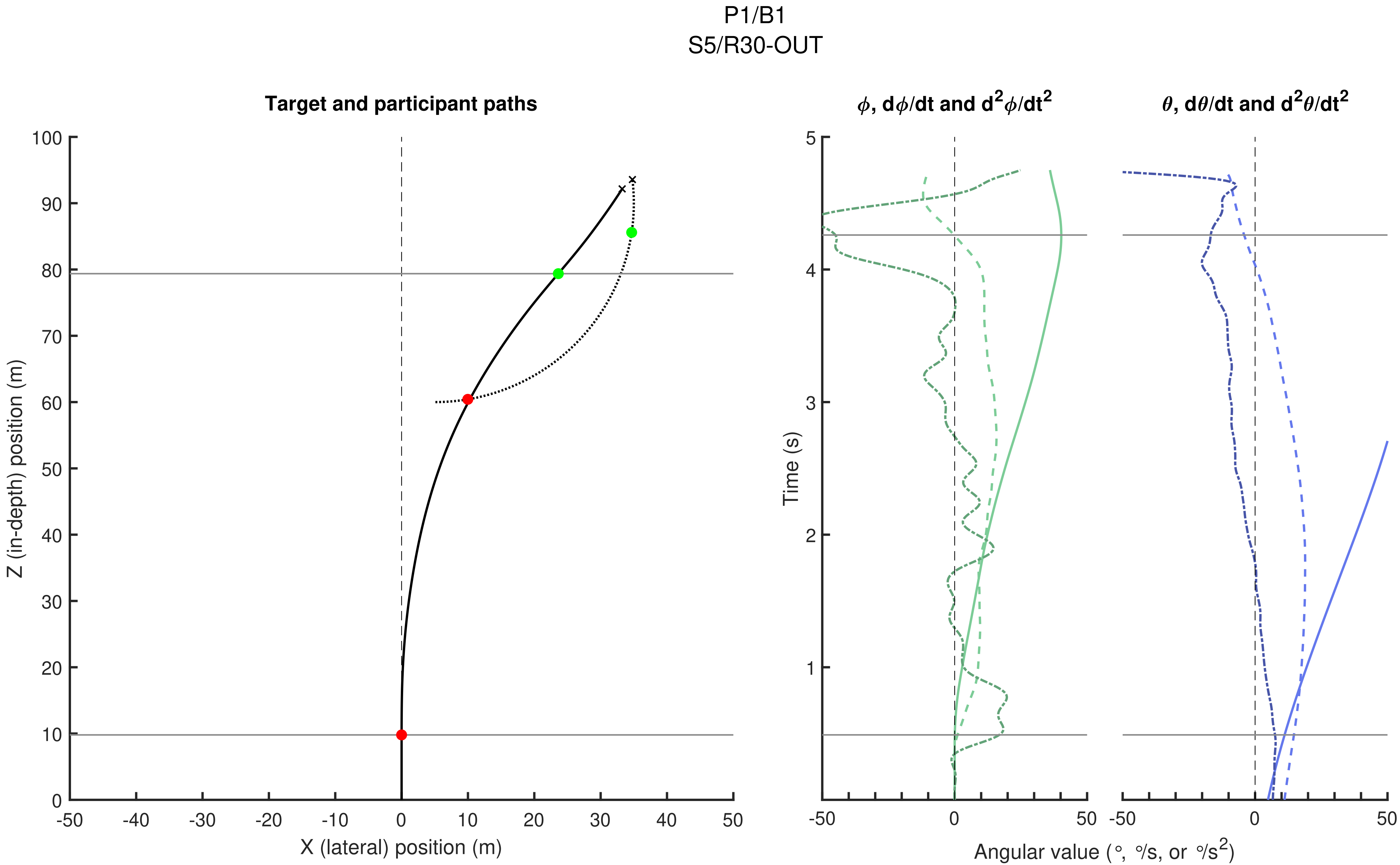
P1/B1
S5/R30-OUT
Target and participant paths
Z (in-depth) position (m)
X (lateral) position (m)
ϕ, dϕ/dt and d²ϕ/dt²
θ, dθ/dt and d²θ/dt²
Time (s)
Angular value (°, °/s, or °/s²)

P1/B1
S5/R30-IN

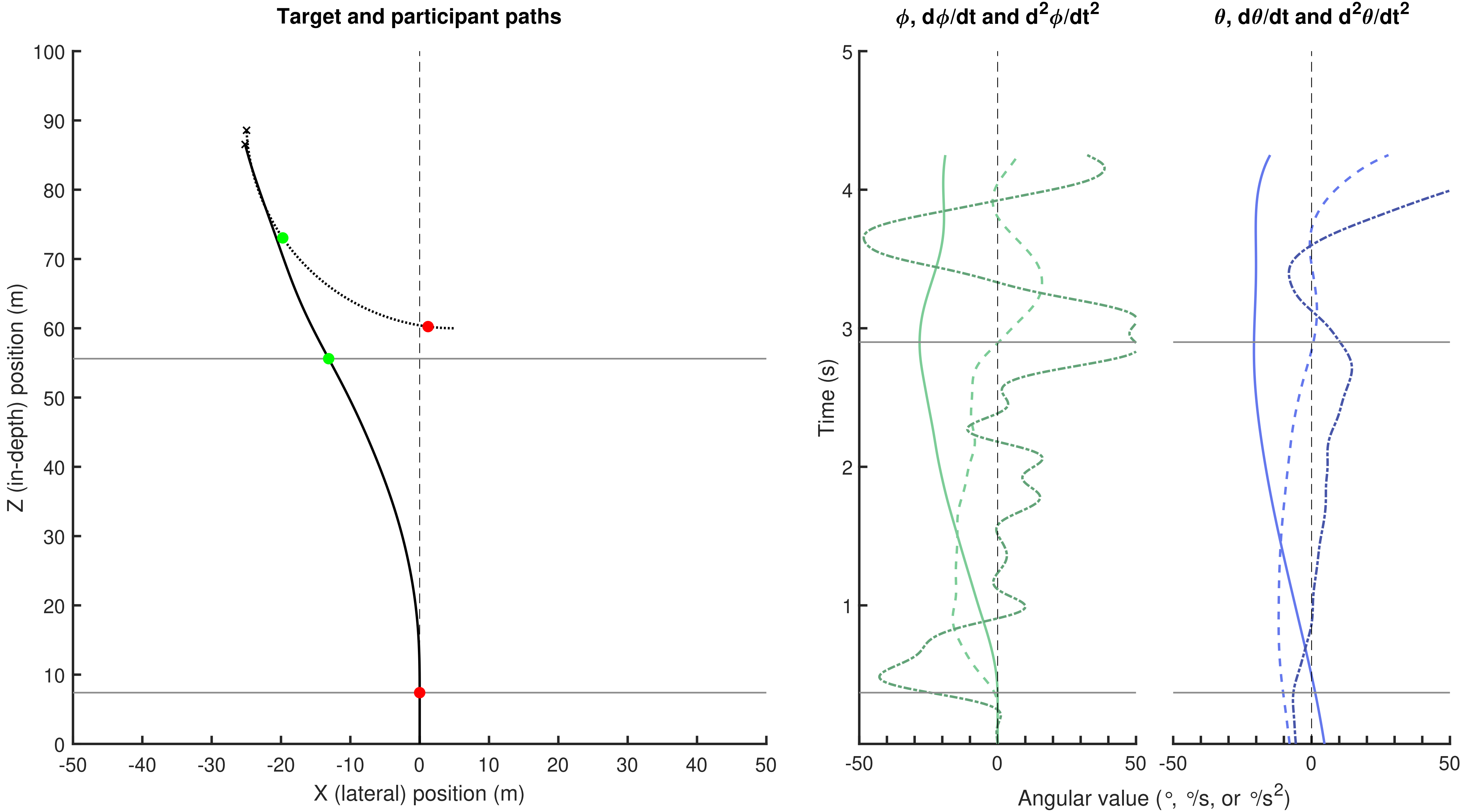

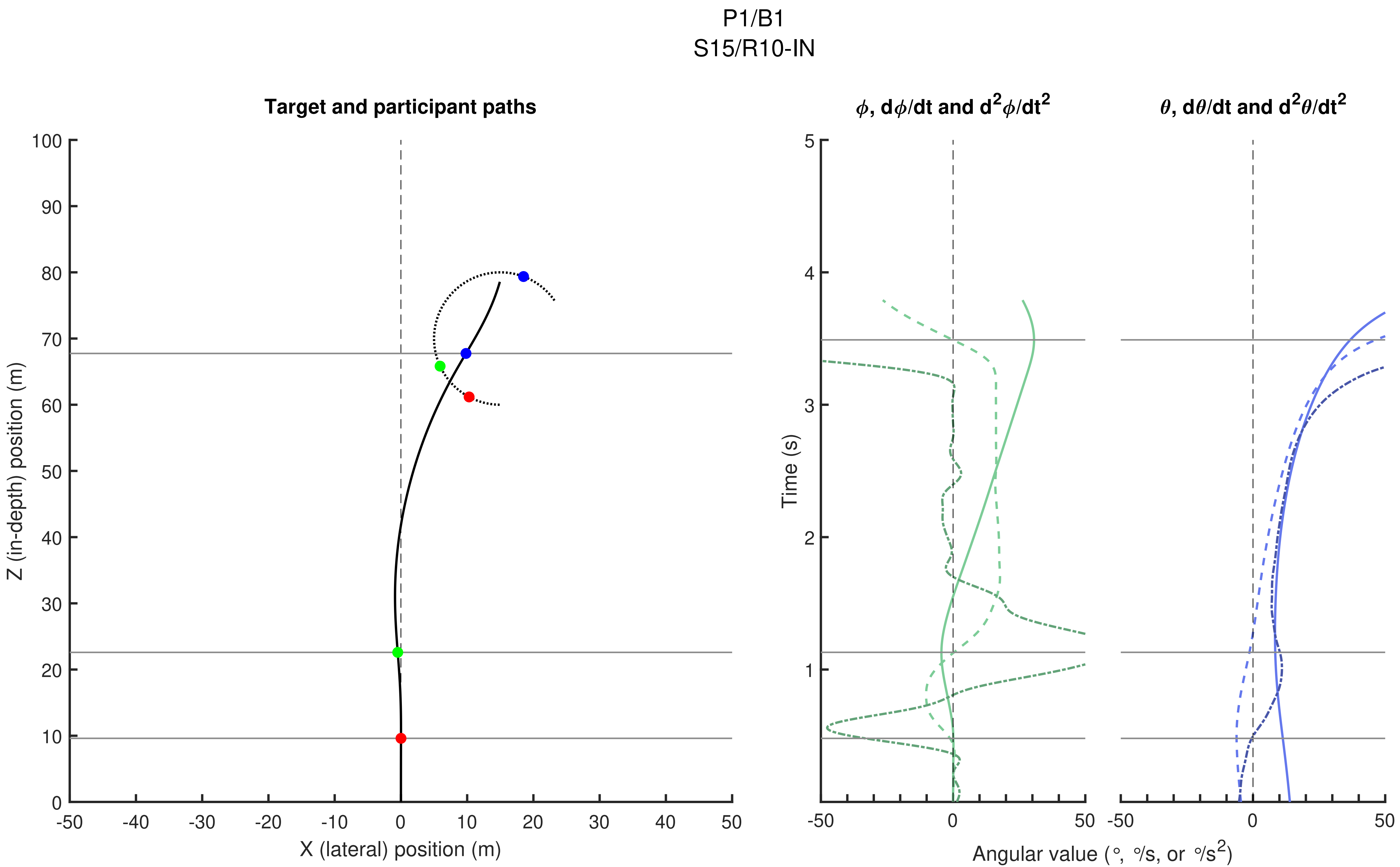

P1/B1
S15/R10-IN
Target and participant paths
Z (in-depth) position (m)
X (lateral) position (m)
φ, dφ/dt and d²φ/dt²
θ, dθ/dt and d²θ/dt²
Time (s)
Angular value (°, °/s, or °/s²)

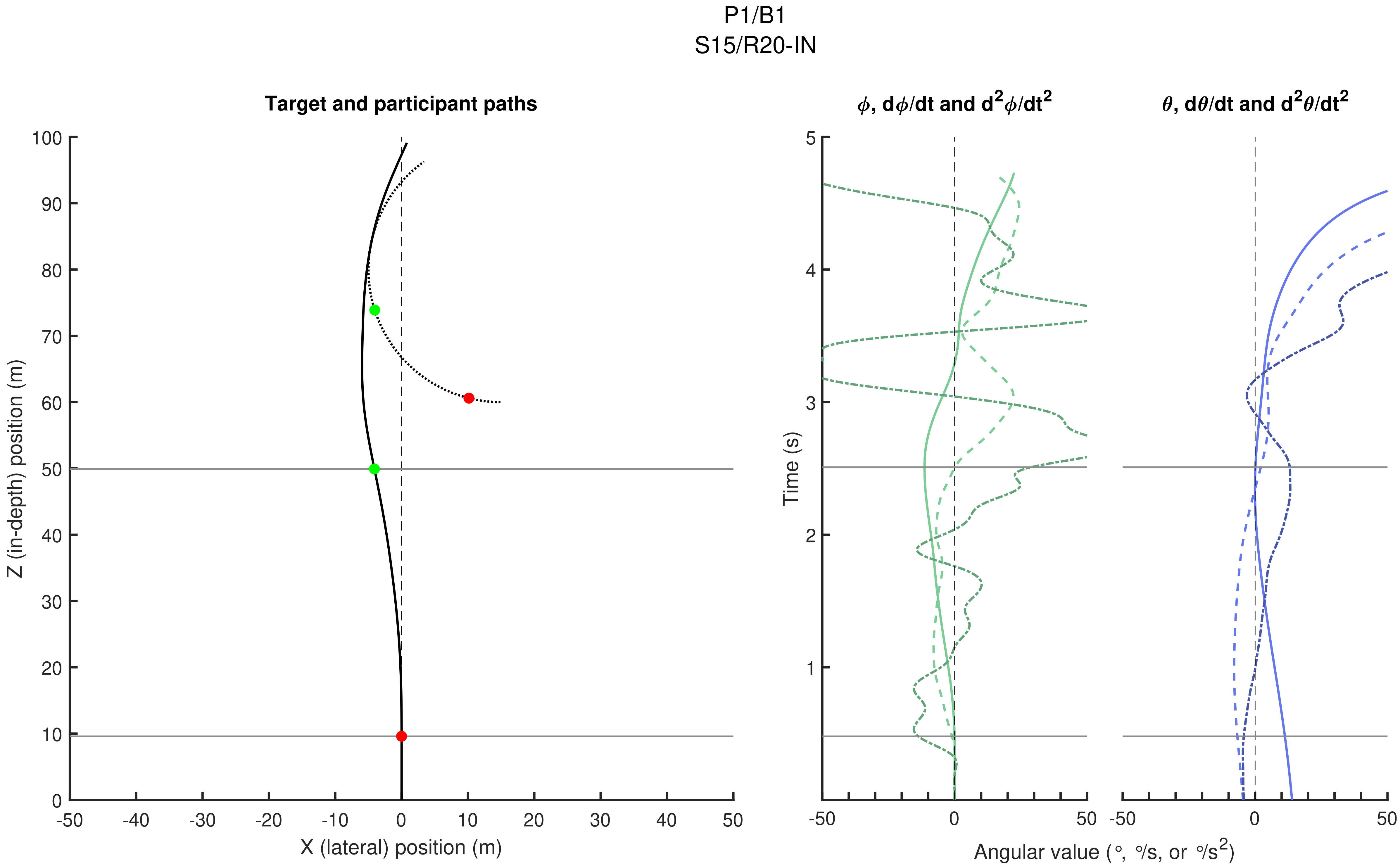

P1/B1
S15/R20-IN
Target and participant paths
Z (in-depth) position (m)
X (lateral) position (m)
100
90
80
70
60
50
40
30
20
10
0
-50
-40
-30
-20
-10
0
10
20
30
40
50
ϕ, dϕ/dt and d²ϕ/dt²
θ, dθ/dt and d²θ/dt²
Time (s)
5
4
3
2
1
-50
0
50
-50
0
50
Angular value (°, °/s, or °/s²)

P1/B1
S15/R30-IN

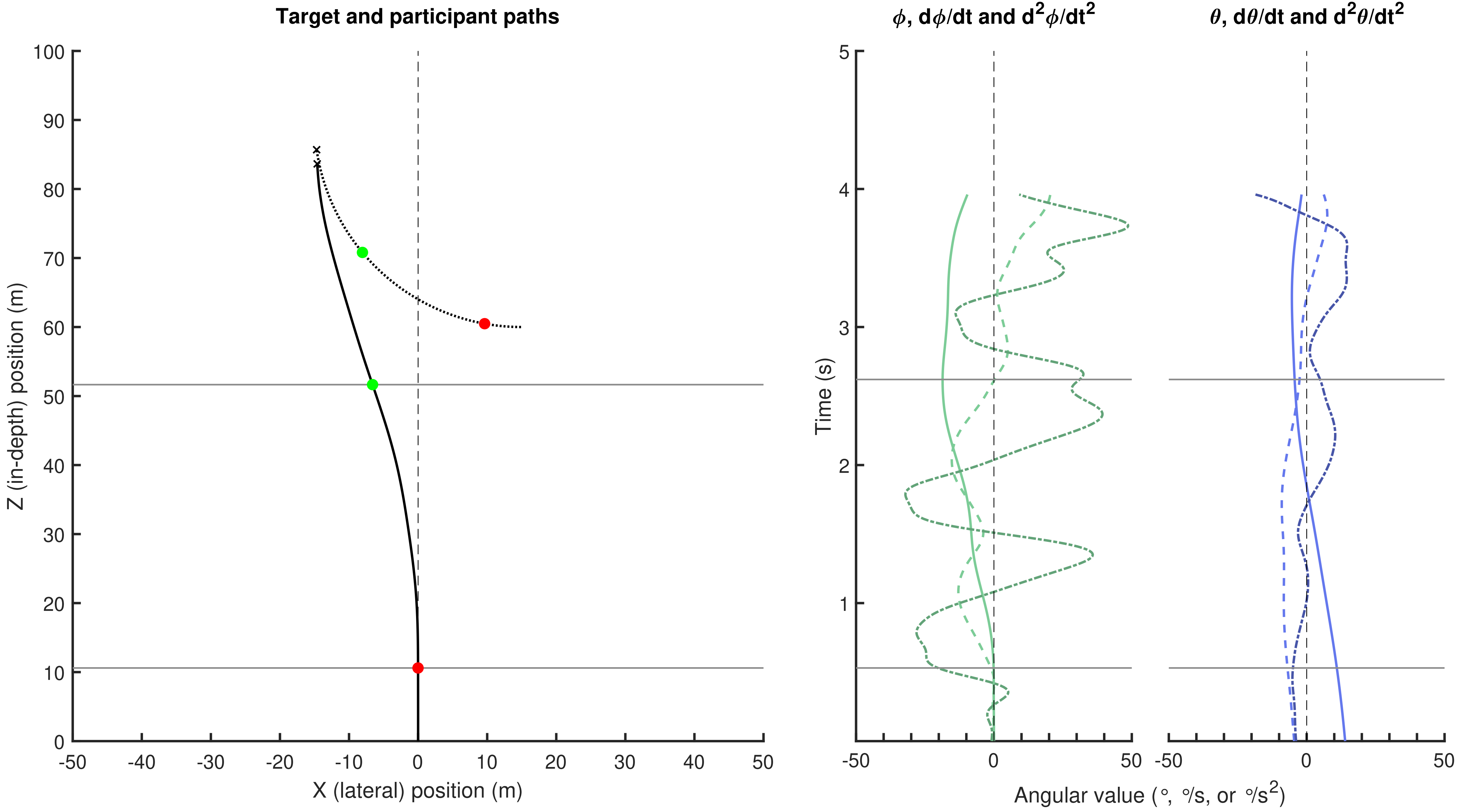

P1/B1
S20/R10-IN

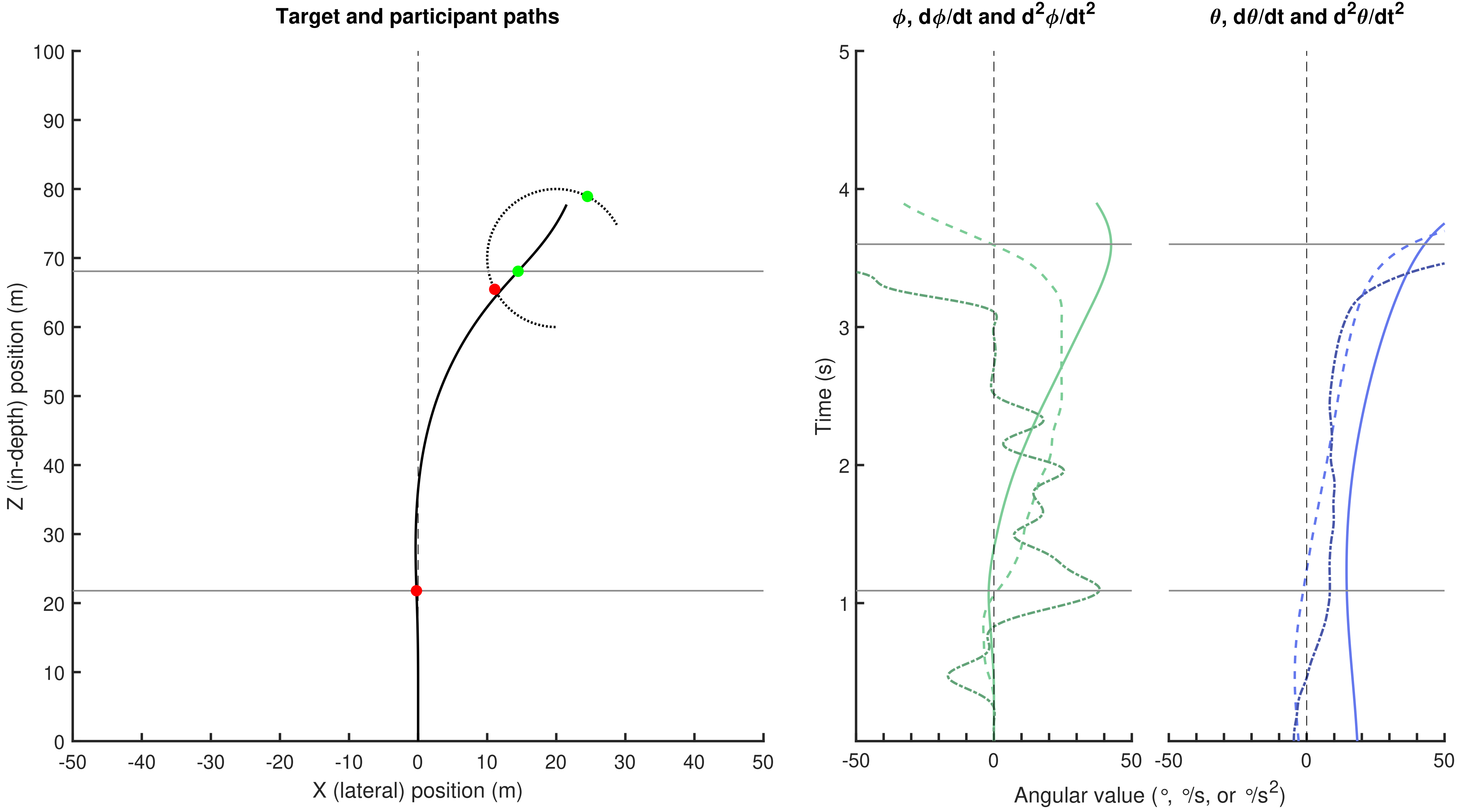

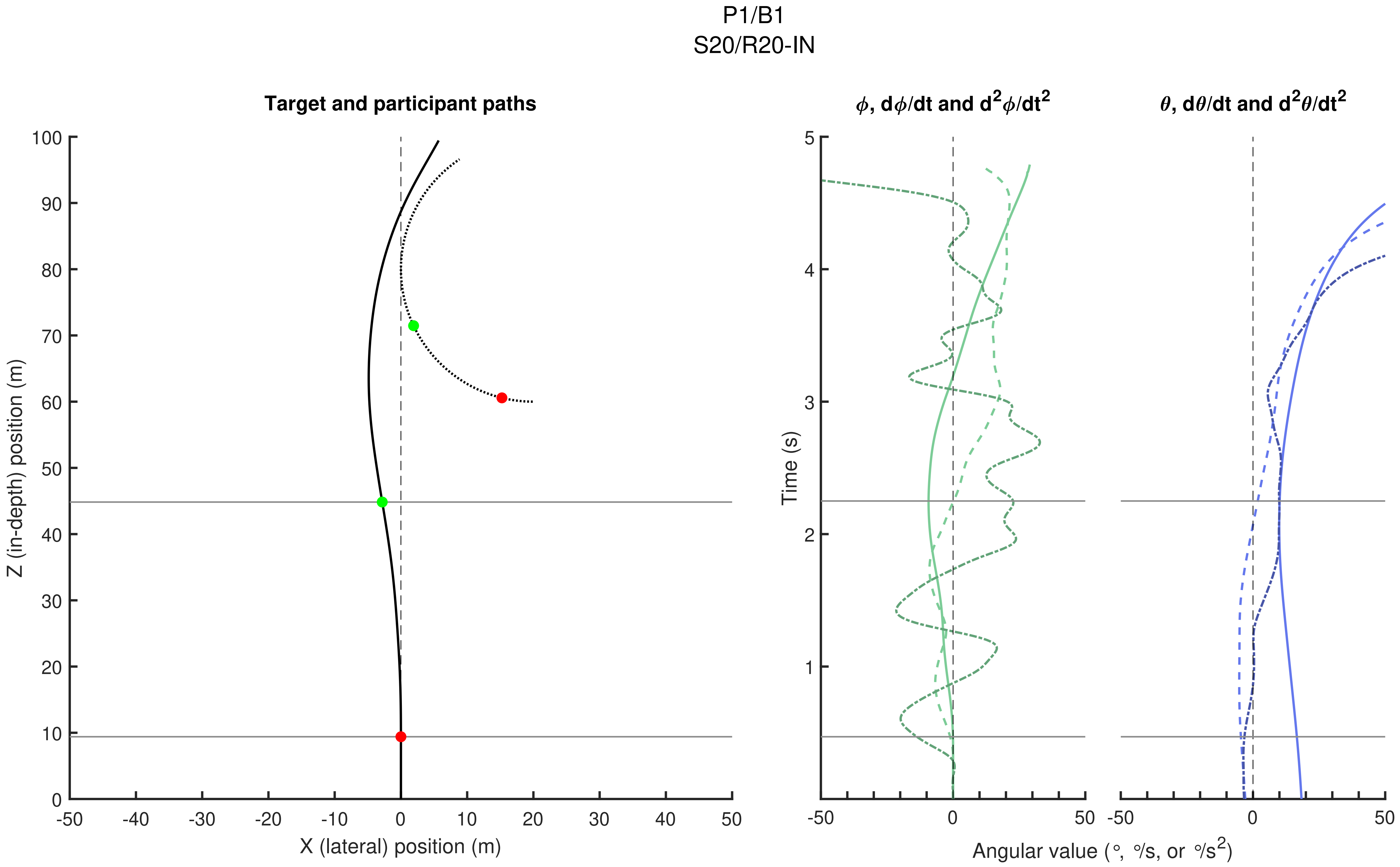

P1/B1
S20/R20-IN
Target and participant paths
Z (in-depth) position (m)
X (lateral) position (m)
100
90
80
70
60
50
40
30
20
10
0
-50
-40
-30
-20
-10
0
10
20
30
40
50
ϕ, dϕ/dt and d²ϕ/dt²
θ, dθ/dt and d²θ/dt²
Time (s)
5
4
3
2
1
-50
0
50
-50
0
50
Angular value (°, °/s, or °/s²)

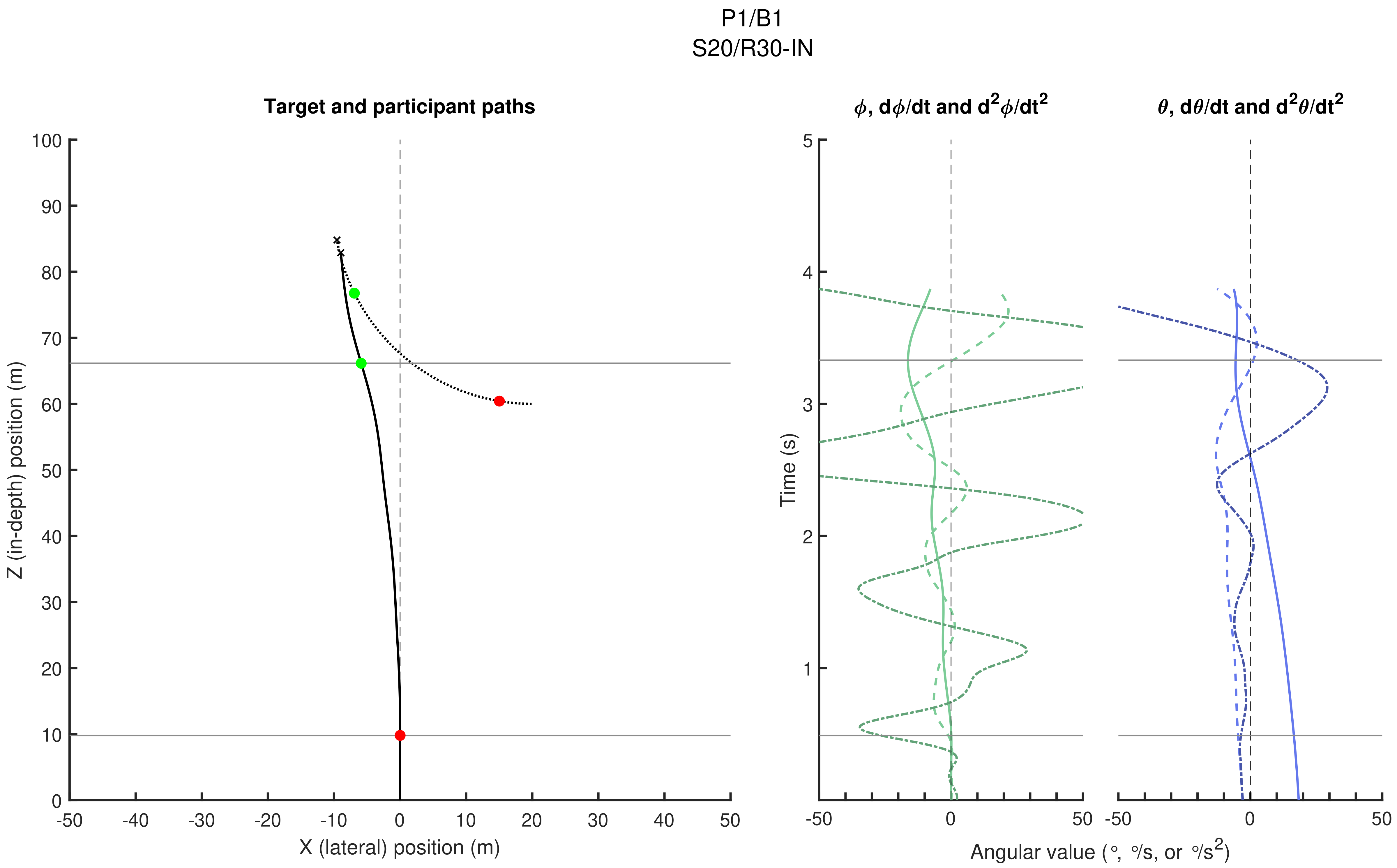
P1/B1
S20/R30-IN
Target and participant paths
Z (in-depth) position (m)
X (lateral) position (m)
ϕ, dϕ/dt and d$^2\phi$/dt^2
θ, dθ/dt and d$^2\theta$/dt^2
Time (s)
Angular value (°, °/s, or °/s^2)

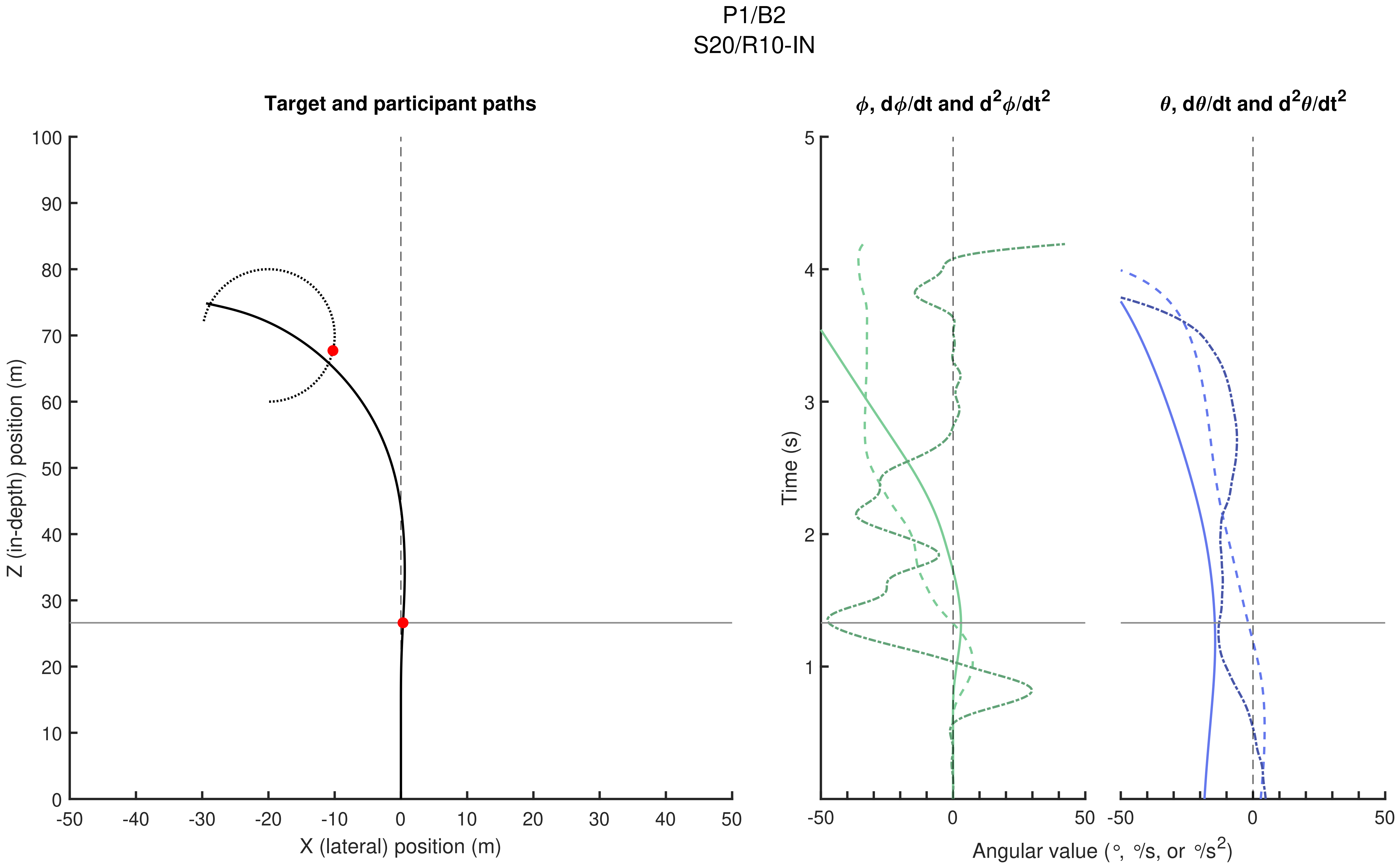

P1/B2
S20/R10-IN
Target and participant paths
Z (in-depth) position (m)
X (lateral) position (m)
100
90
80
70
60
50
40
30
20
10
0
-50
-40
-30
-20
-10
0
10
20
30
40
50
ϕ, dϕ/dt and d2ϕ/dt2
θ, dθ/dt and d2θ/dt2
Time (s)
5
4
3
2
1
-50
0
50
Angular value (°, °/s, or °/s2)

P1/B2
S20/R20-IN

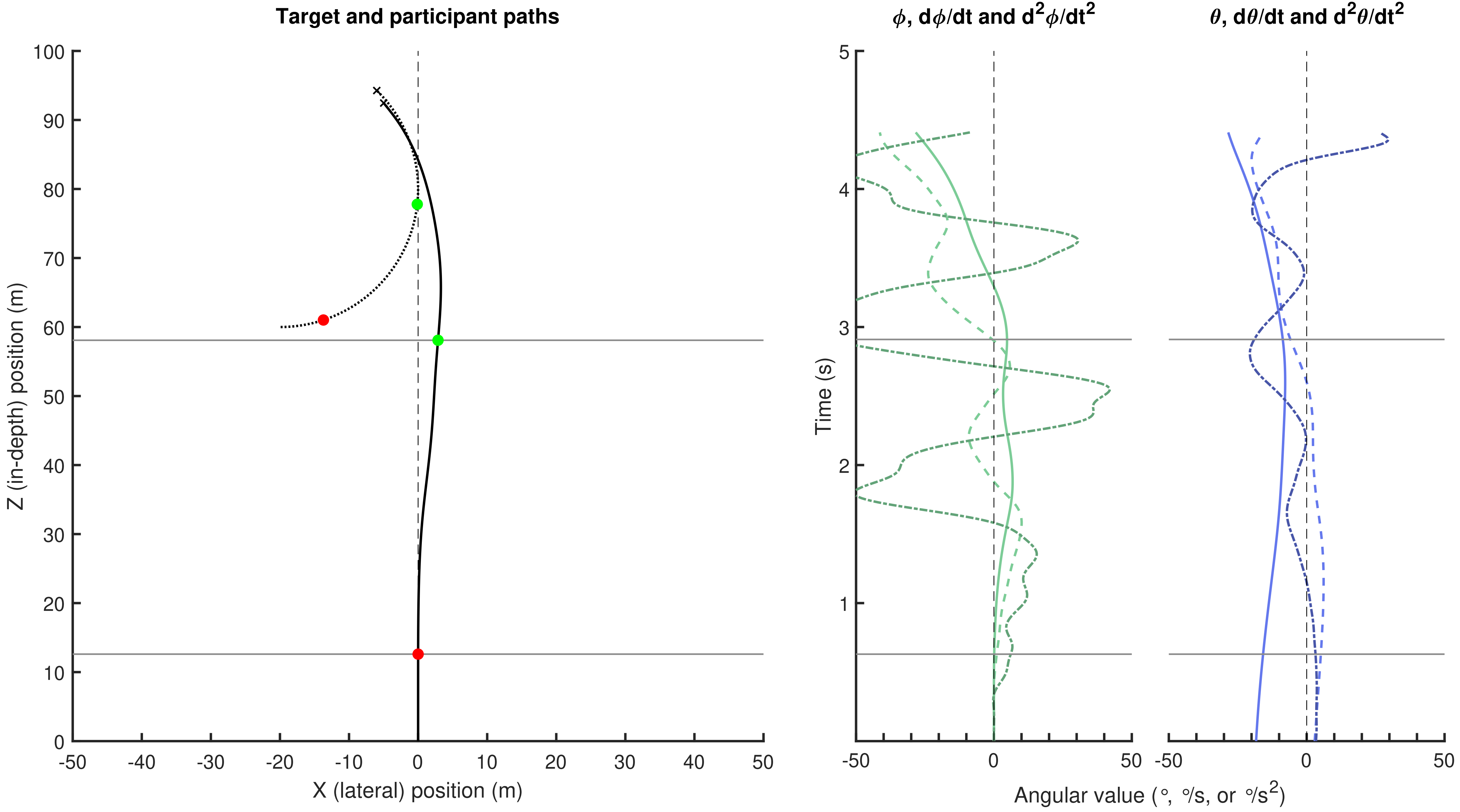

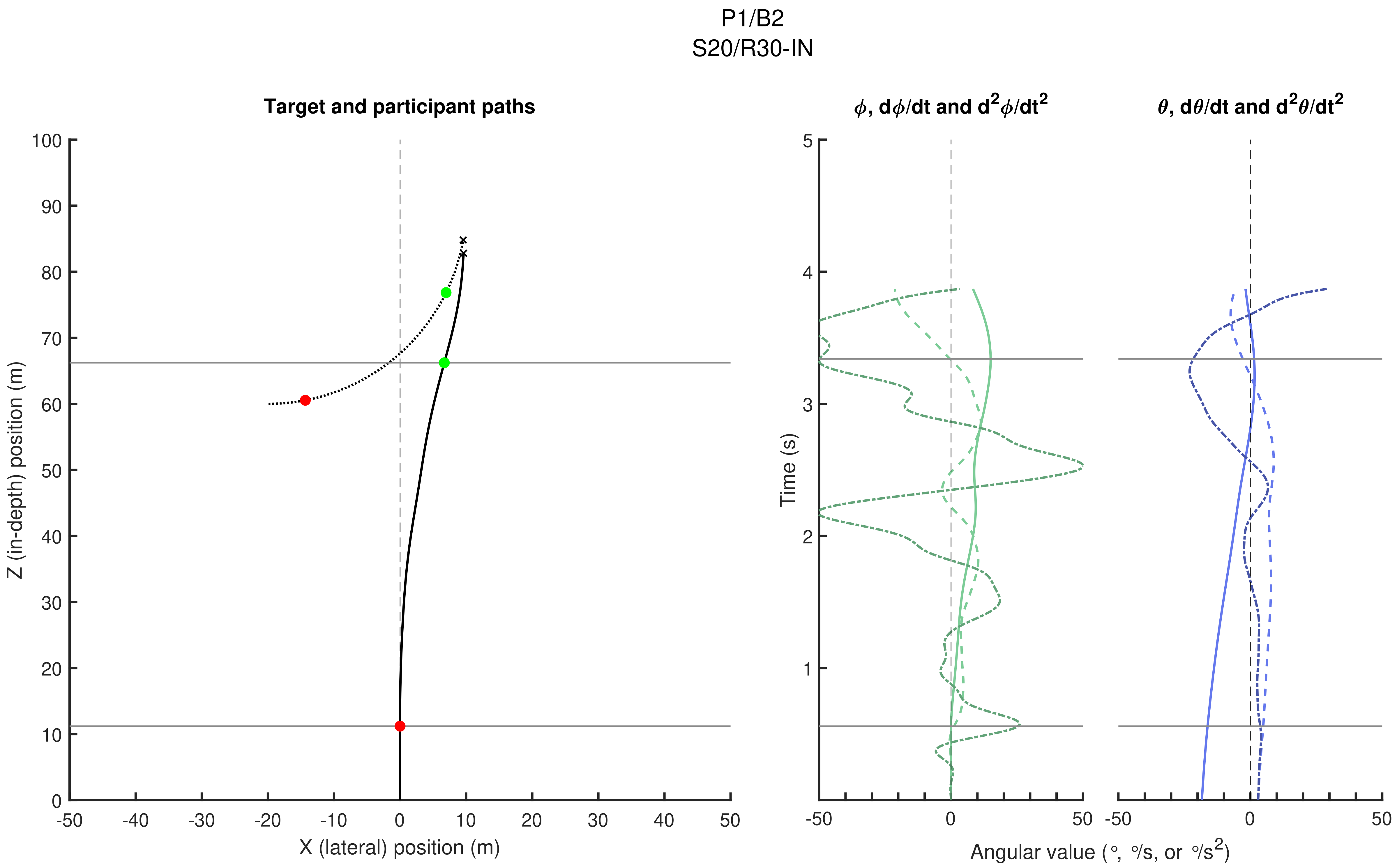

P1/B2
S20/R30-IN
Target and participant paths
Z (in-depth) position (m)
X (lateral) position (m)
ϕ, dϕ/dt and d$^2\phi$/dt^2
θ, dθ/dt and d$^2\theta$/dt^2
Time (s)
Angular value (°, °/s, or °/s^2)

P1/B2
S15/R10-IN

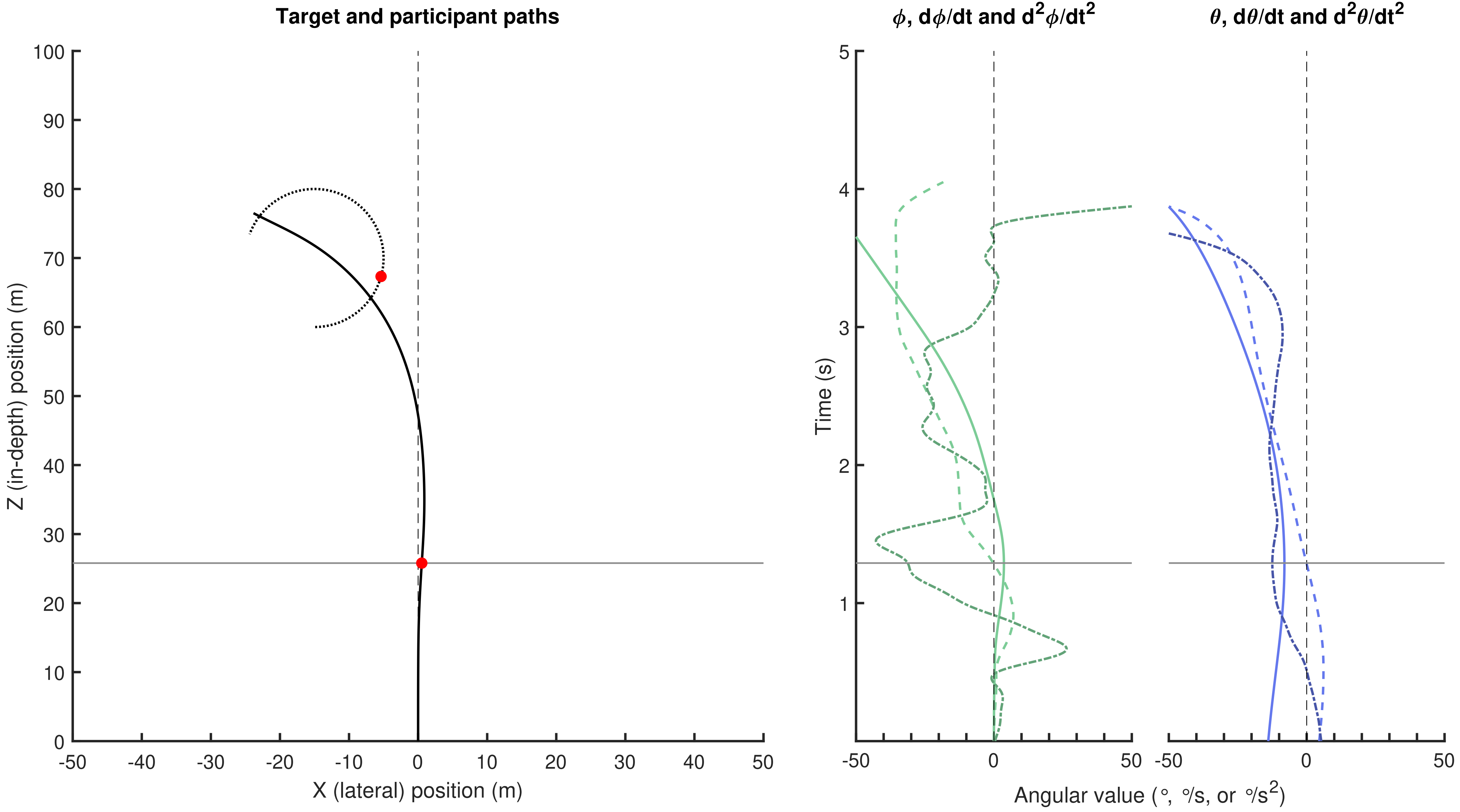

P1/B2
S15/R20-IN

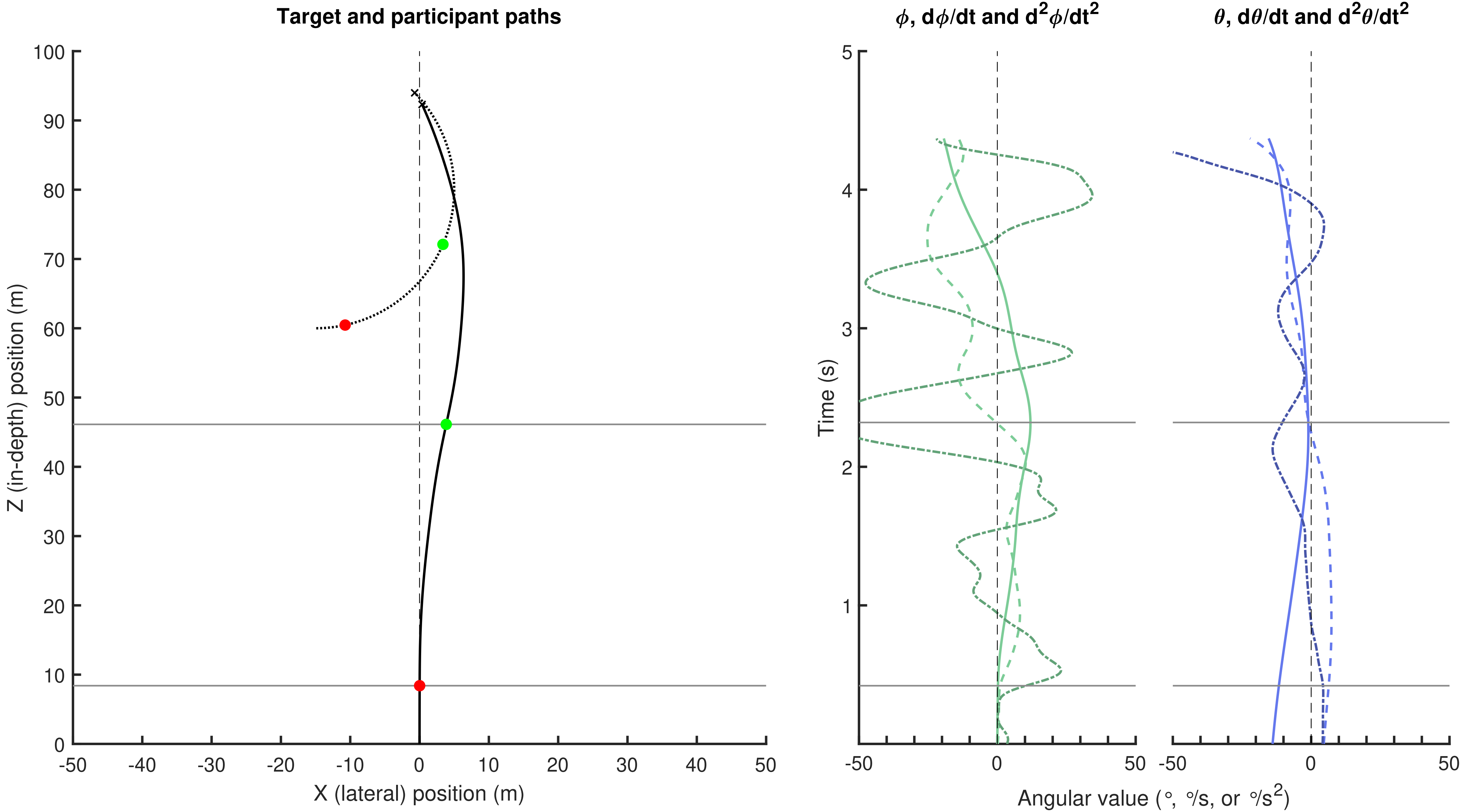

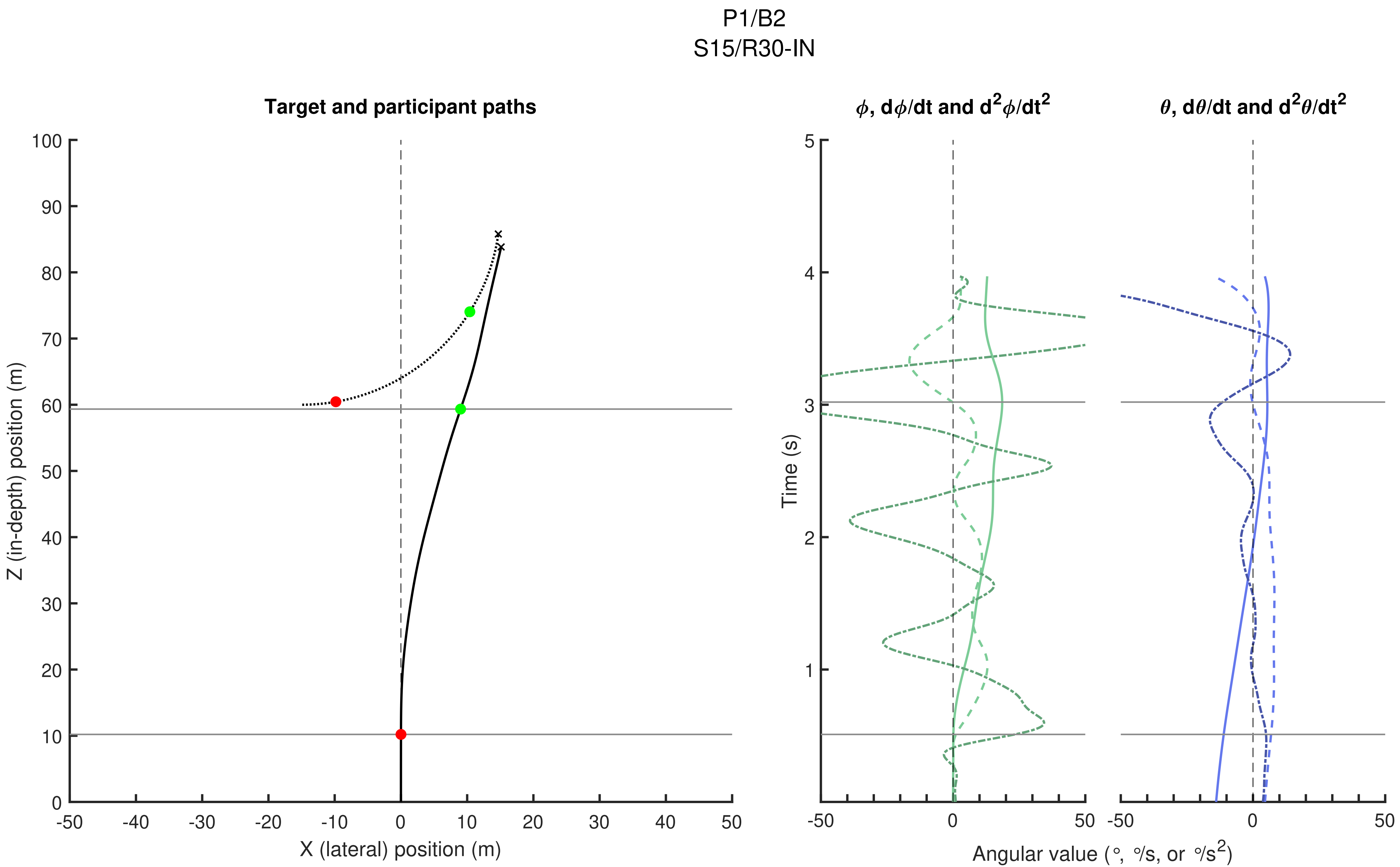

P1/B2
S15/R30-IN
Target and participant paths
Z (in-depth) position (m)
X (lateral) position (m)
φ, dφ/dt and d²φ/dt²
θ, dθ/dt and d²θ/dt²
Time (s)
Angular value (°, °/s, or °/s²)

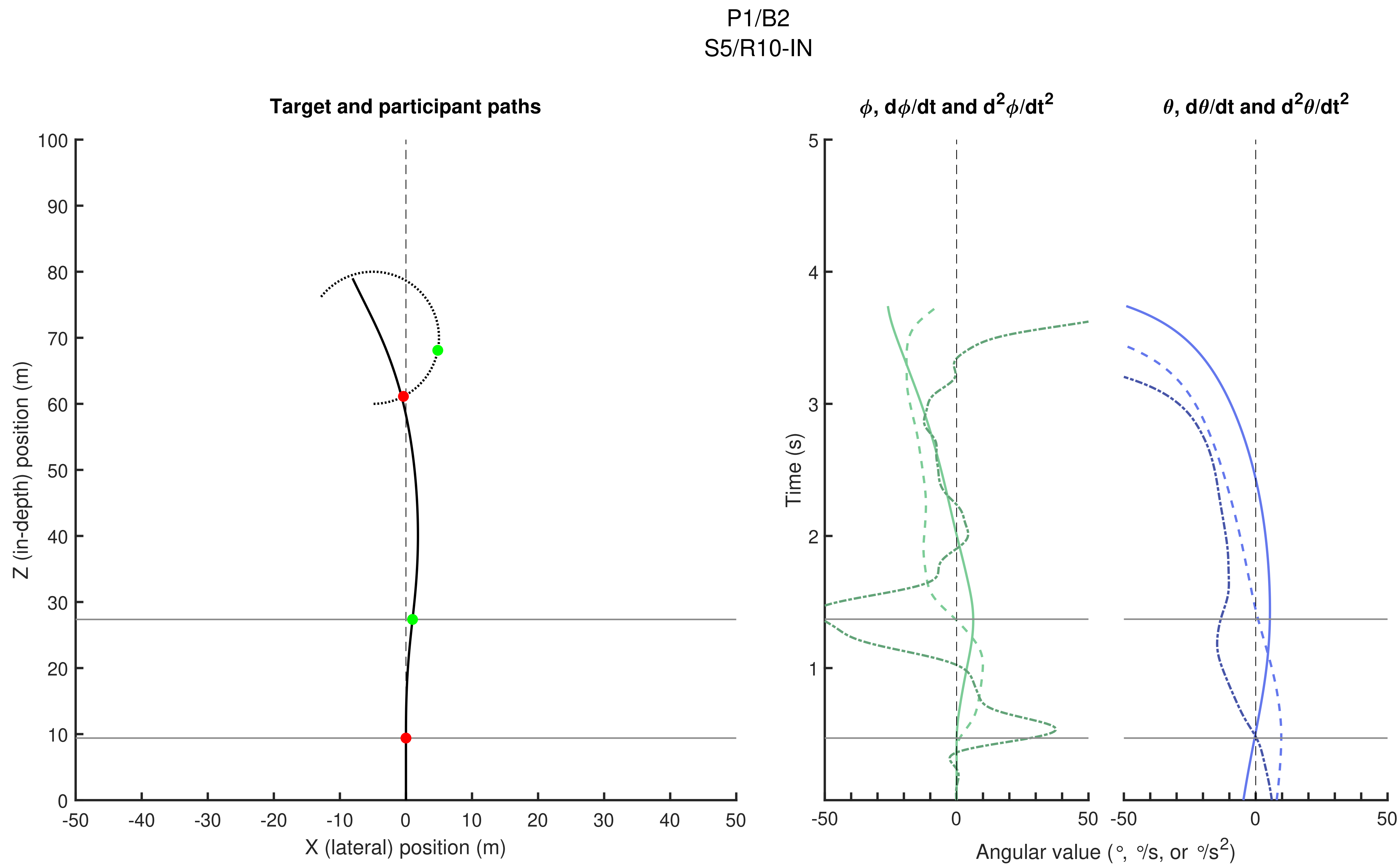
P1/B2
S5/R10-IN
Target and participant paths
ϕ, dϕ/dt and d²ϕ/dt²
θ, dθ/dt and d²θ/dt²
Z (in-depth) position (m)
X (lateral) position (m)
Time (s)
Angular value (°, °/s, or °/s²)
100
90
80
70
60
50
40
30
20
10
0
-50
-40
-30
-20
-10
0
10
20
30
40
50
5
4
3
2
1
-50
0
50
-50
0
50

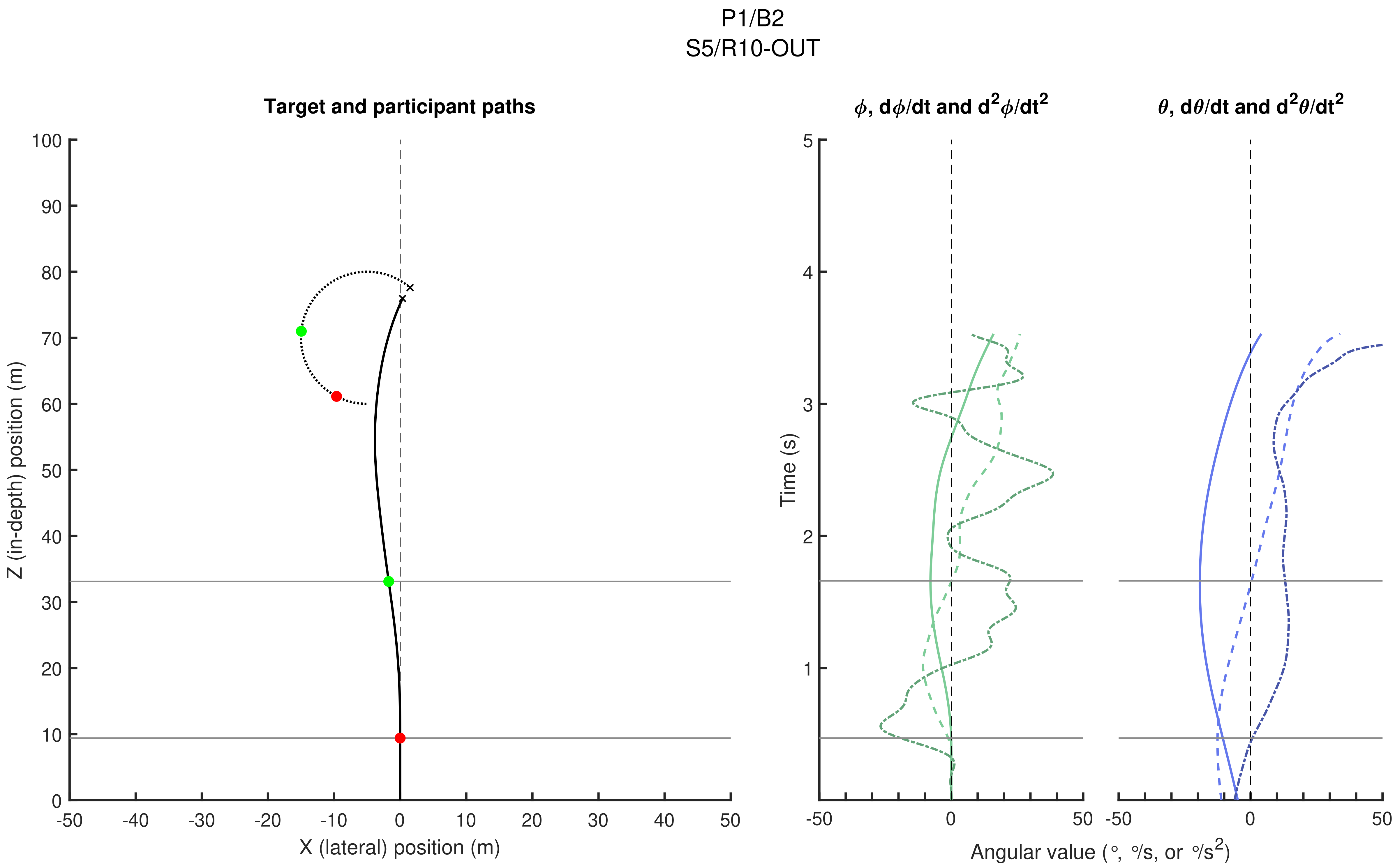
P1/B2
S5/R10-OUT
Target and participant paths
Z (in-depth) position (m)
X (lateral) position (m)
φ, dφ/dt and d²φ/dt²
θ, dθ/dt and d²θ/dt²
Time (s)
Angular value (°, °/s, or °/s²)

P1/B2
S5/R20-IN

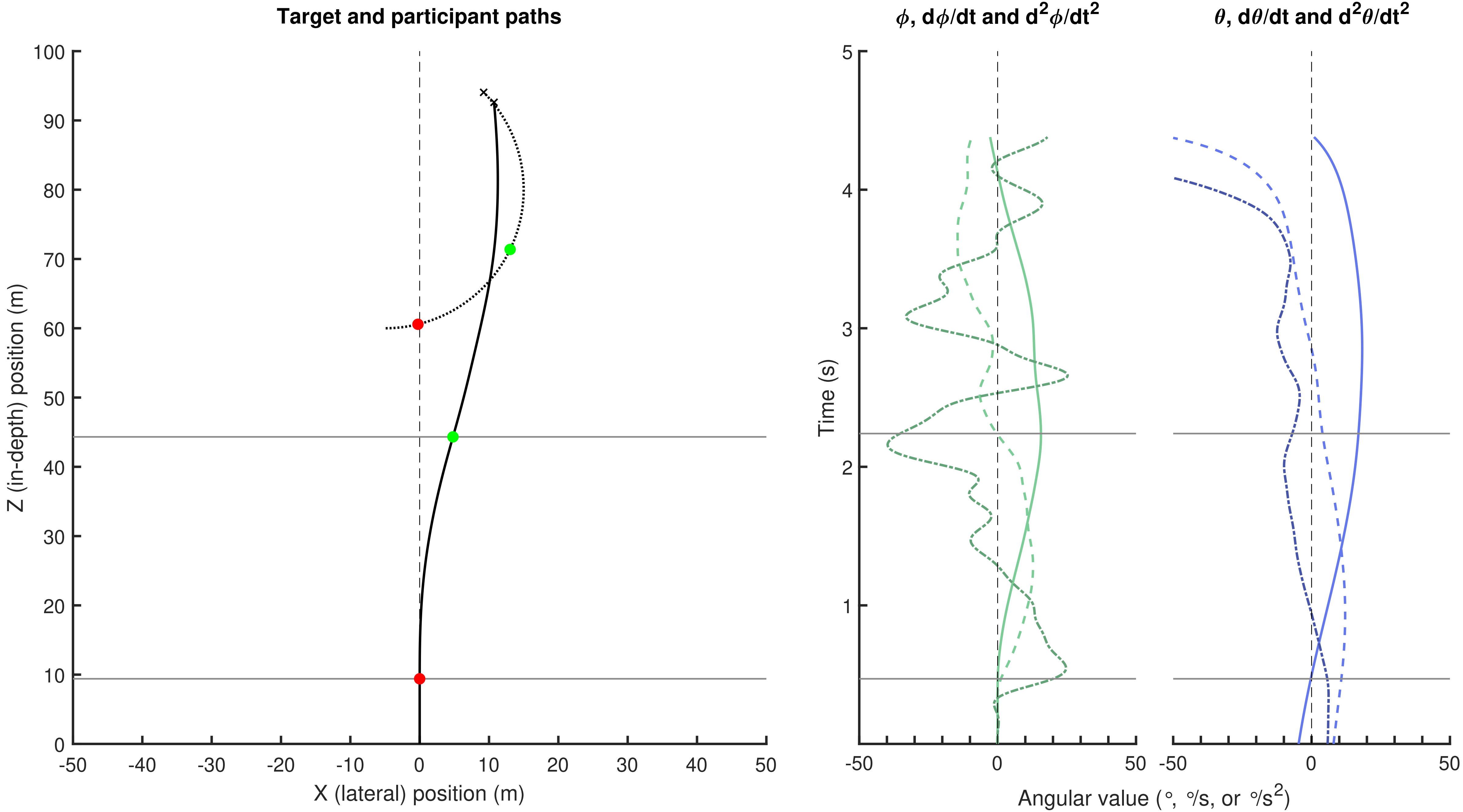

P1/B2
S5/R20-OUT

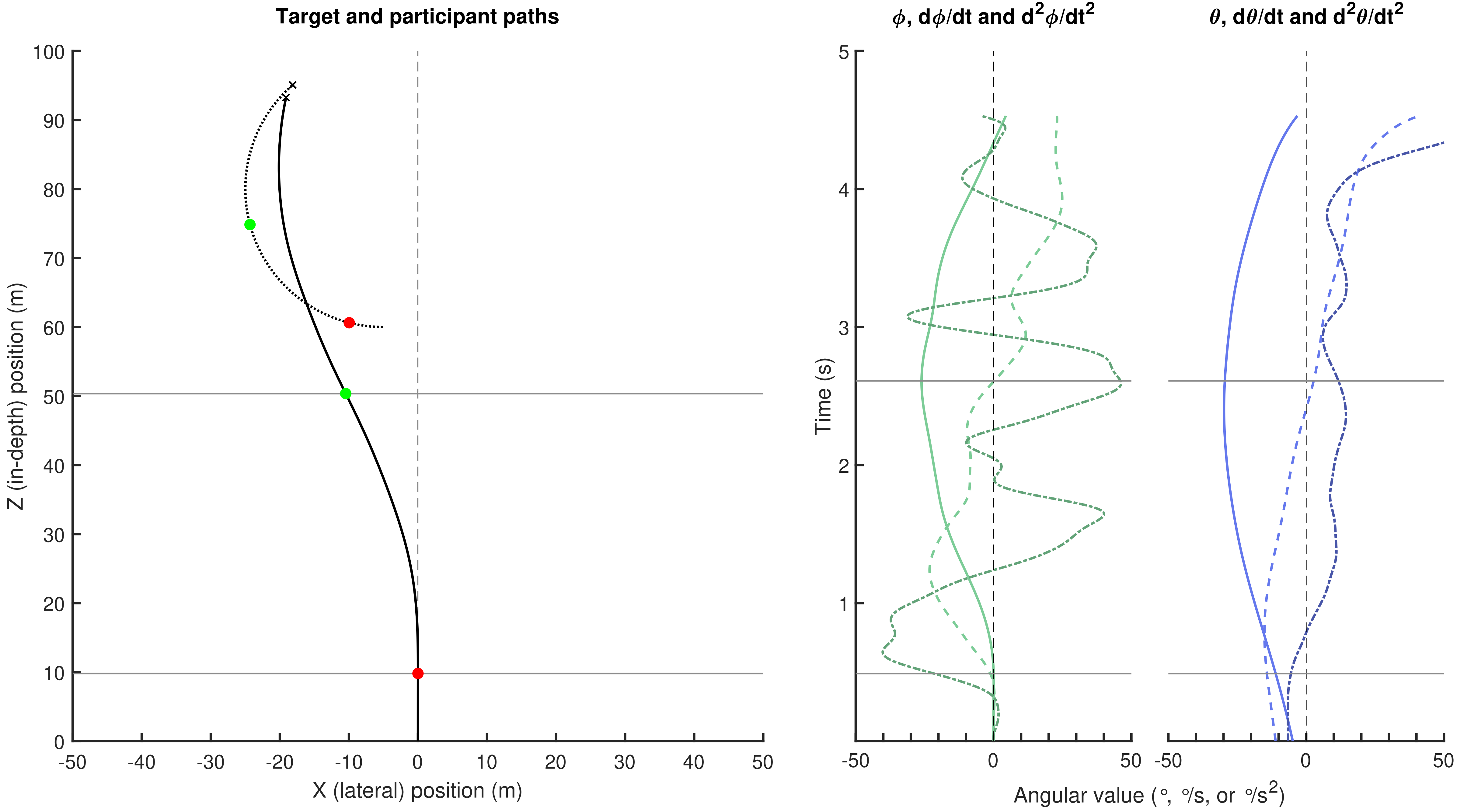

P1/B2
S5/R30-IN

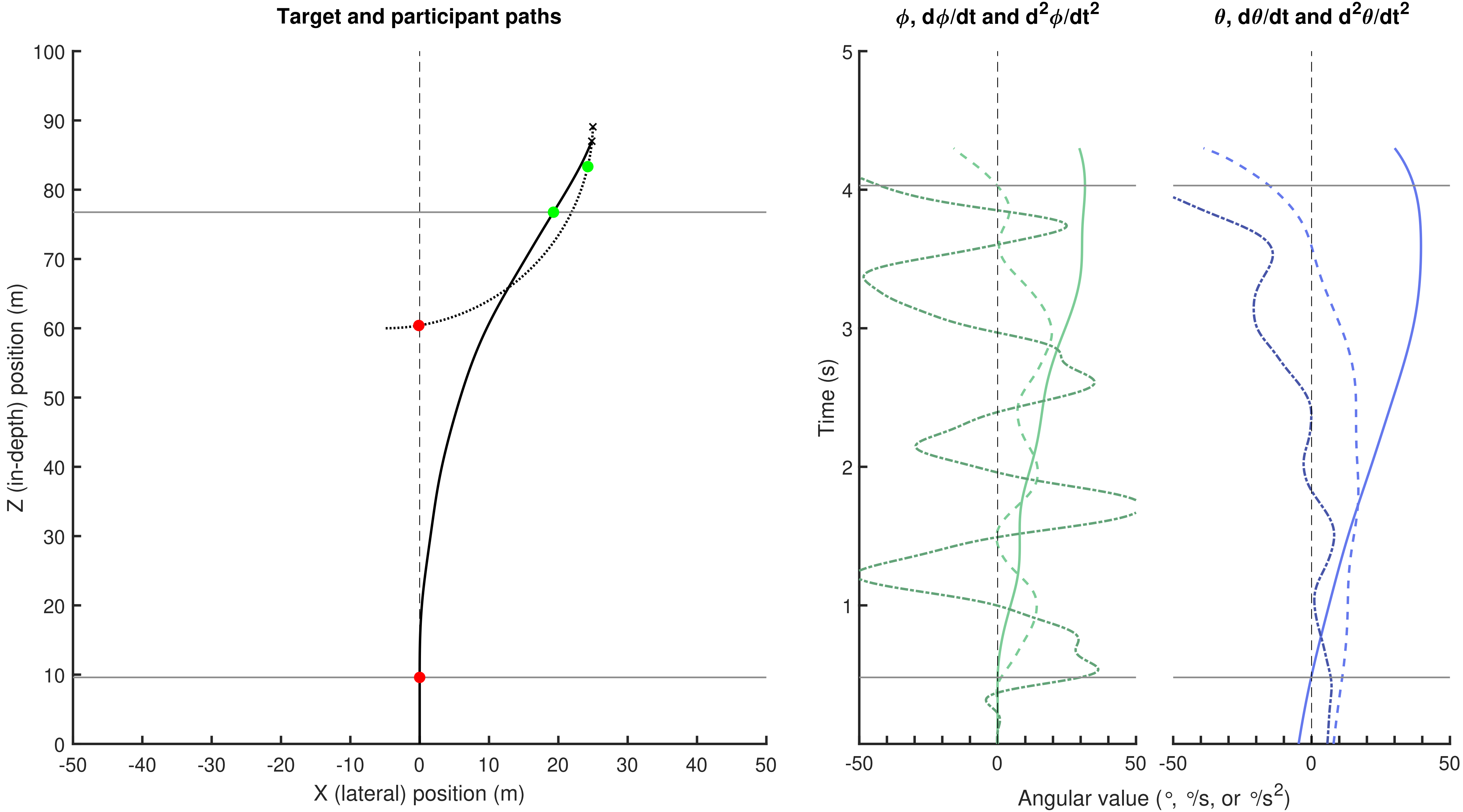

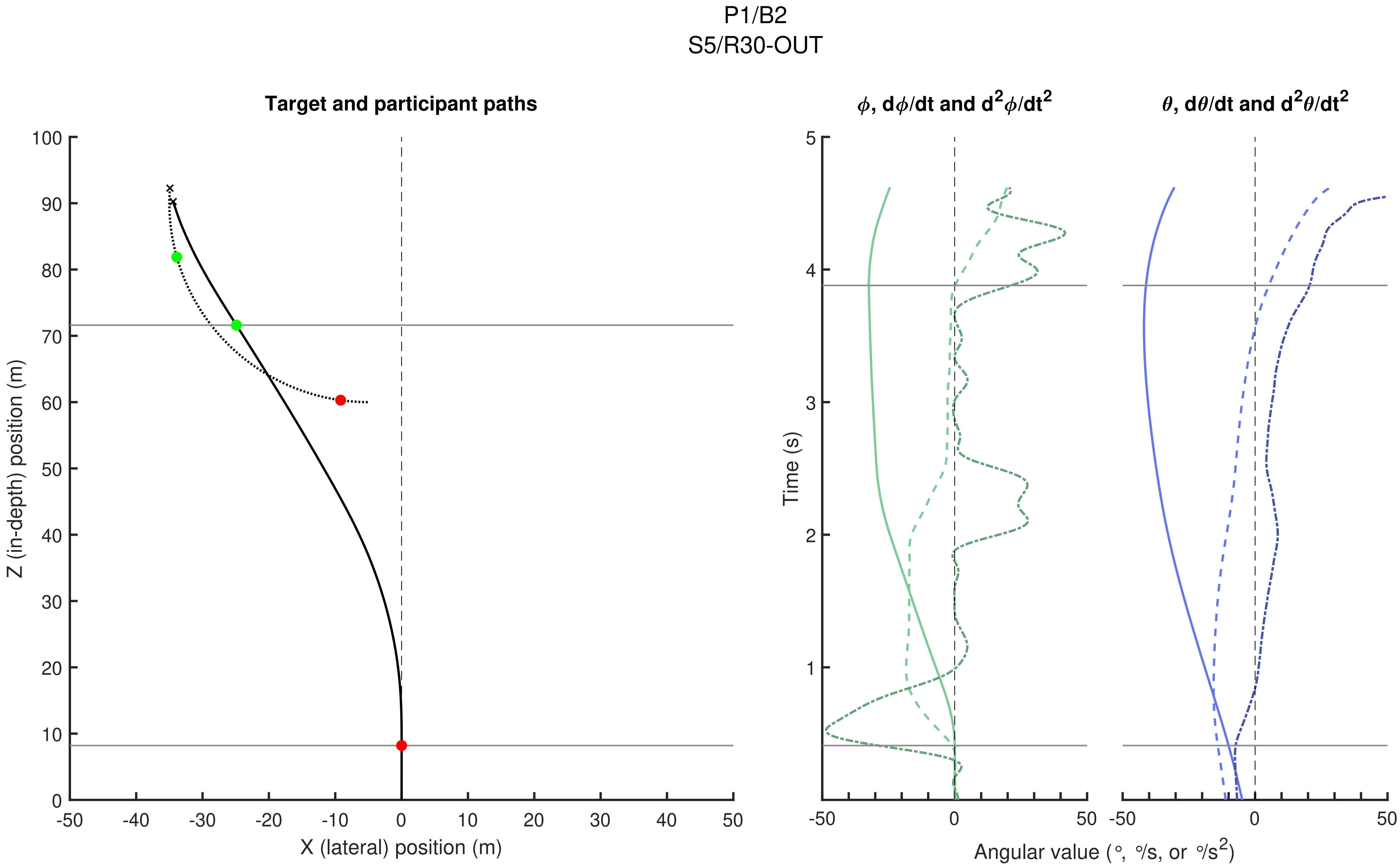
P1/B2
S5/R30-OUT
Target and participant paths
Z (in-depth) position (m)
X (lateral) position (m)
ϕ, dϕ/dt and d²ϕ/dt²
θ, dθ/dt and d²θ/dt²
Time (s)
Angular value (°, °/s, or °/s²)

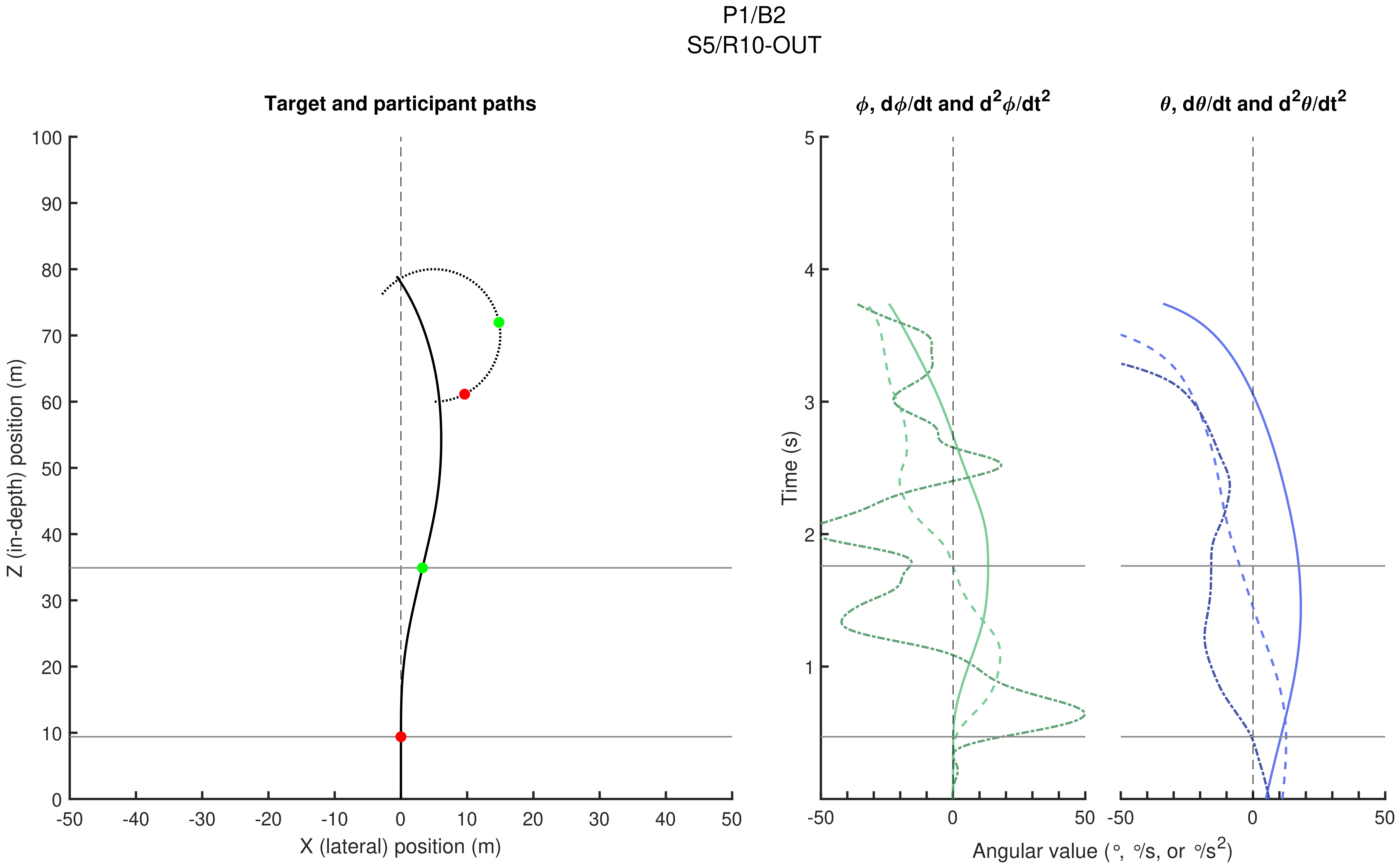
P1/B2
S5/R10-OUT
Target and participant paths
Z (in-depth) position (m)
X (lateral) position (m)
100
90
80
70
60
50
40
30
20
10
0
-50
-40
-30
-20
-10
0
10
20
30
40
50
ϕ, dϕ/dt and d$^2\phi$/dt^2
θ, dθ/dt and d$^2\theta$/dt^2
Time (s)
5
4
3
2
1
-50
0
50
-50
0
50
Angular value (°, °/s, or °/s^2)

P1/B2
S5/R10-IN

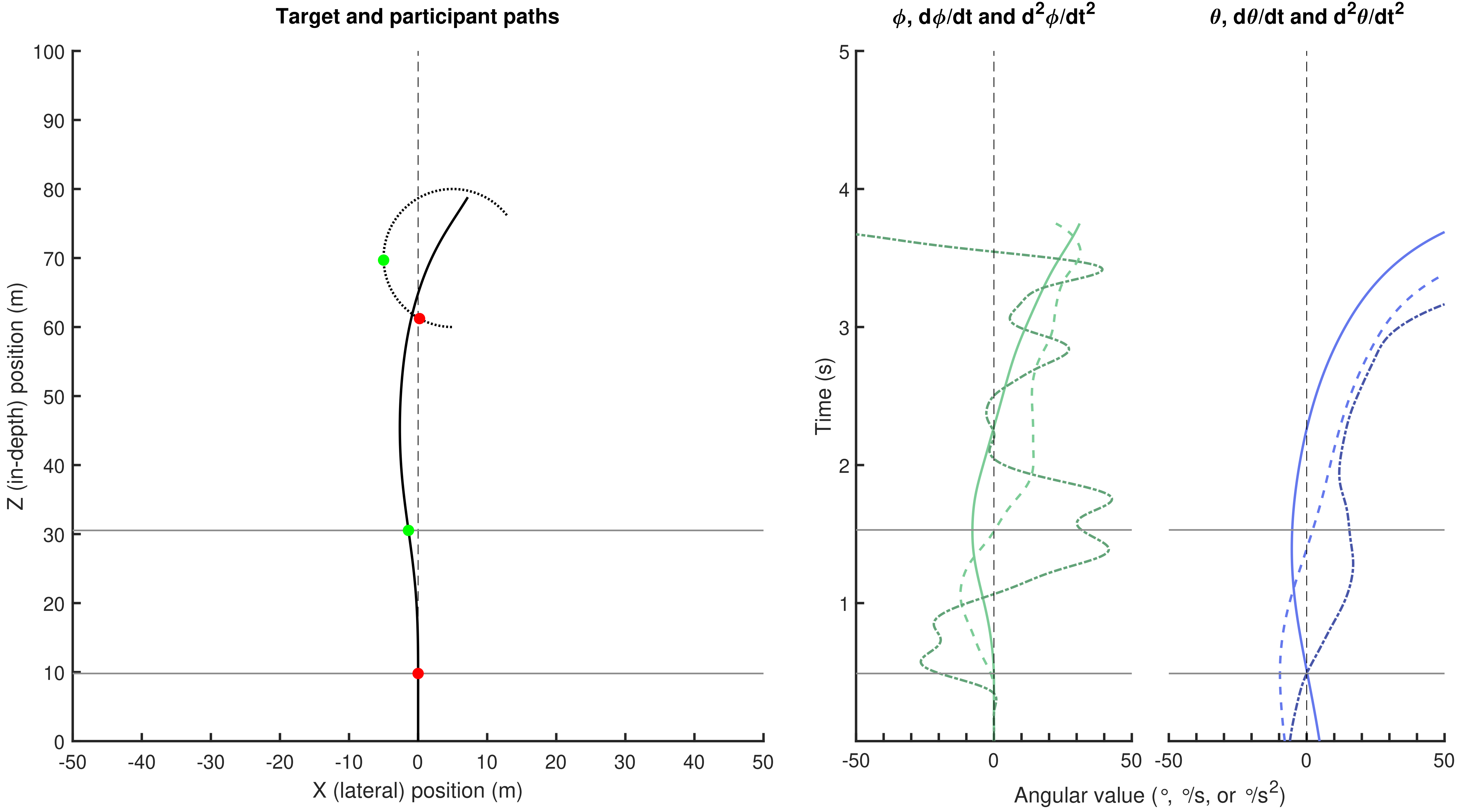

P1/B2
S5/R20-OUT

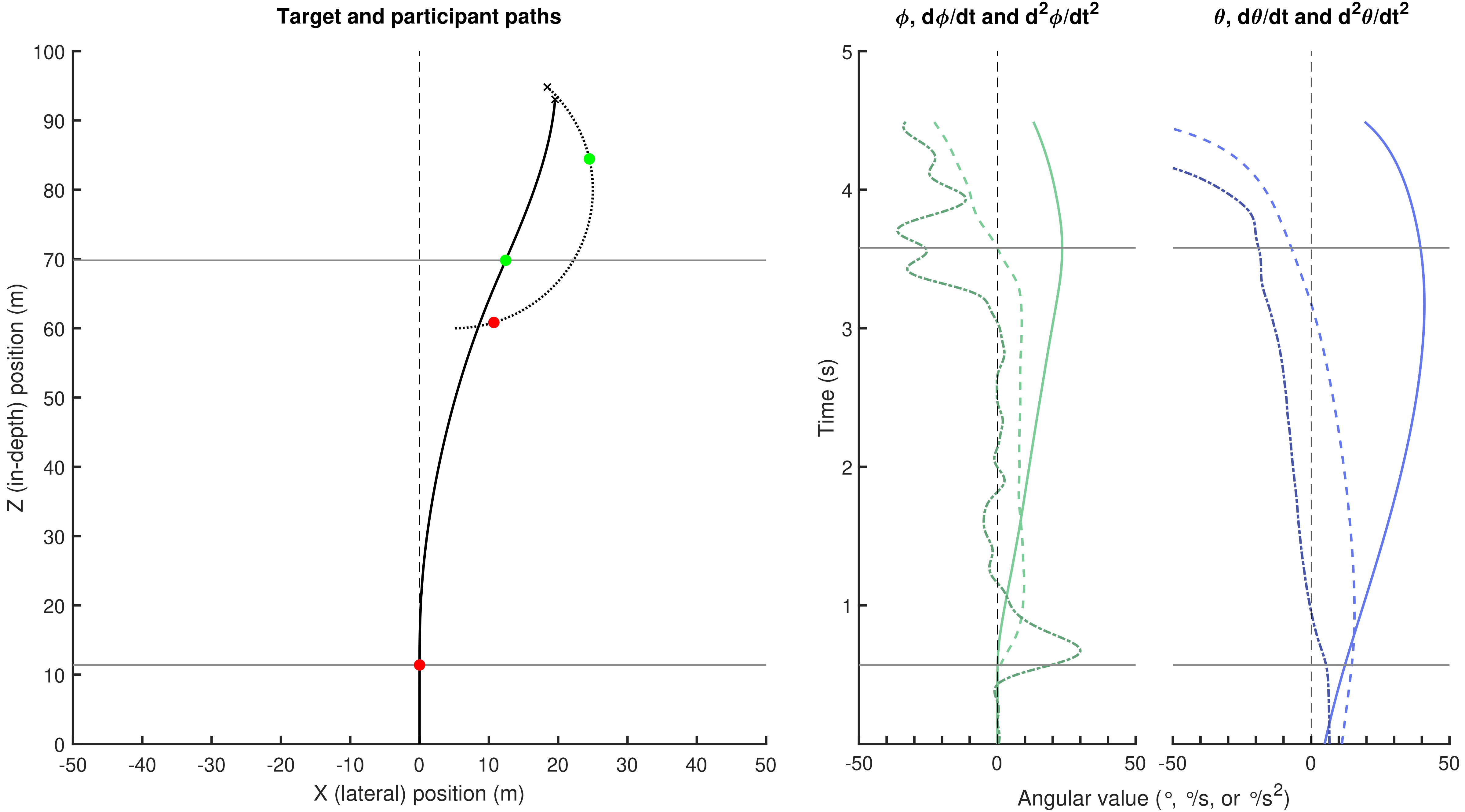

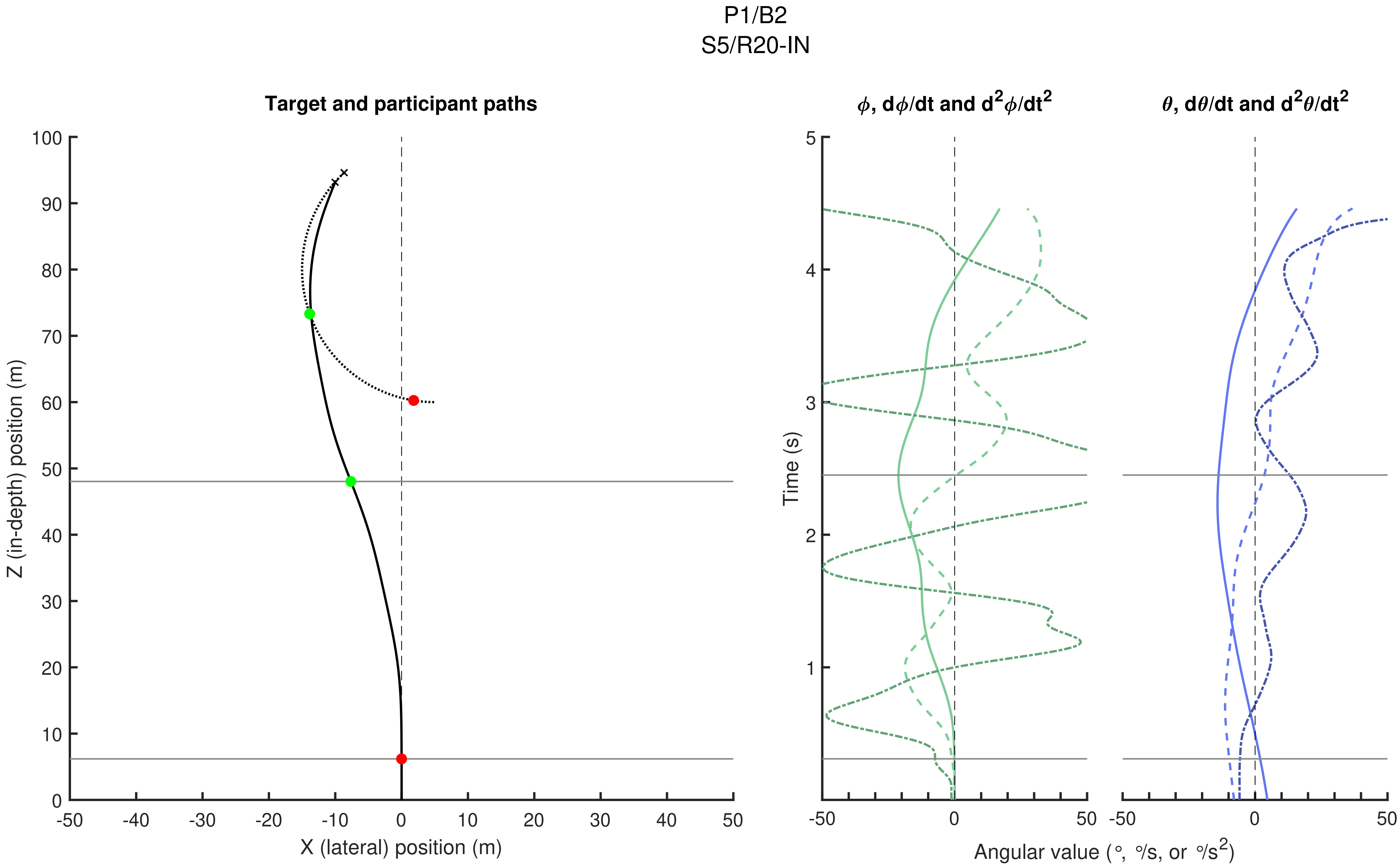

P1/B2
S5/R20-IN
Target and participant paths
ϕ, dϕ/dt and d$^2\phi$/dt^2
θ, dθ/dt and d$^2\theta$/dt^2
Z (in-depth) position (m)
X (lateral) position (m)
Time (s)
Angular value (°, °/s, or °/s^2)
100
90
80
70
60
50
40
30
20
10
0
-50
-40
-30
-20
-10
0
10
20
30
40
50
5
4
3
2
1
-50
0
50
-50
0
50

P1/B2
S5/R30-OUT

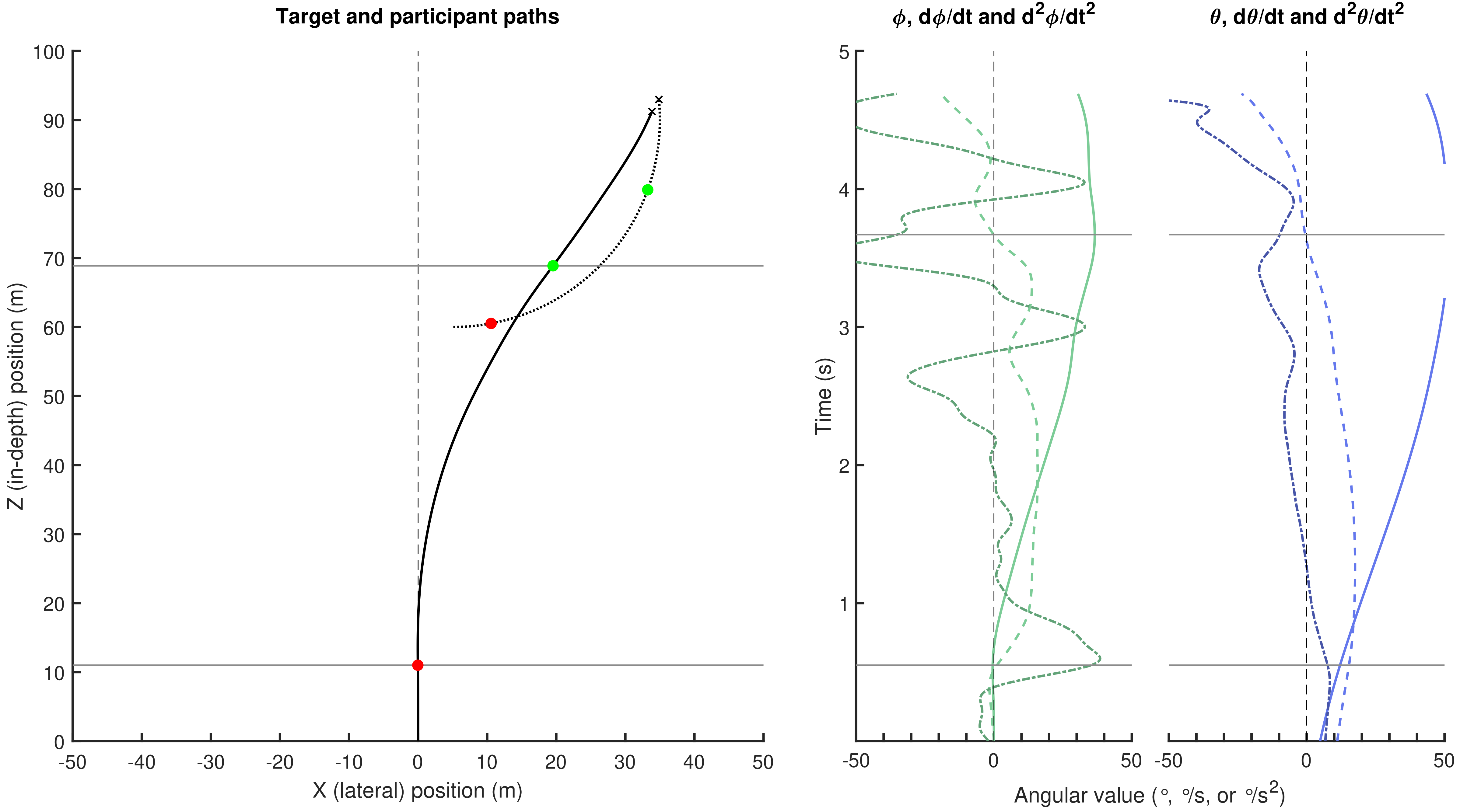

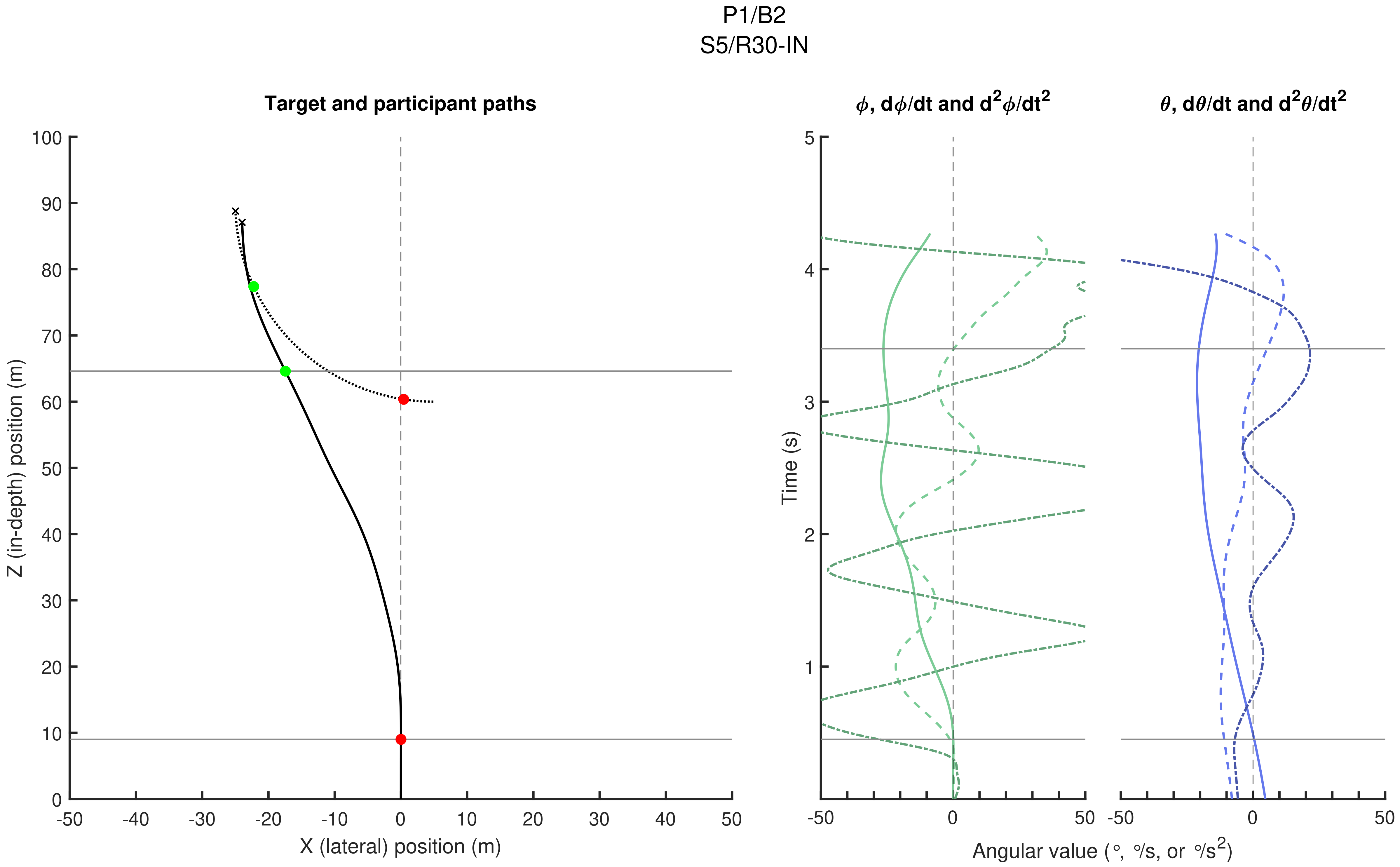

P1/B2
S5/R30-IN
Target and participant paths
Z (in-depth) position (m)
X (lateral) position (m)
100
90
80
70
60
50
40
30
20
10
0
-50
-40
-30
-20
-10
0
10
20
30
40
50
φ, dφ/dt and d²φ/dt²
θ, dθ/dt and d²θ/dt²
Time (s)
5
4
3
2
1
Angular value (°, °/s, or °/s²)

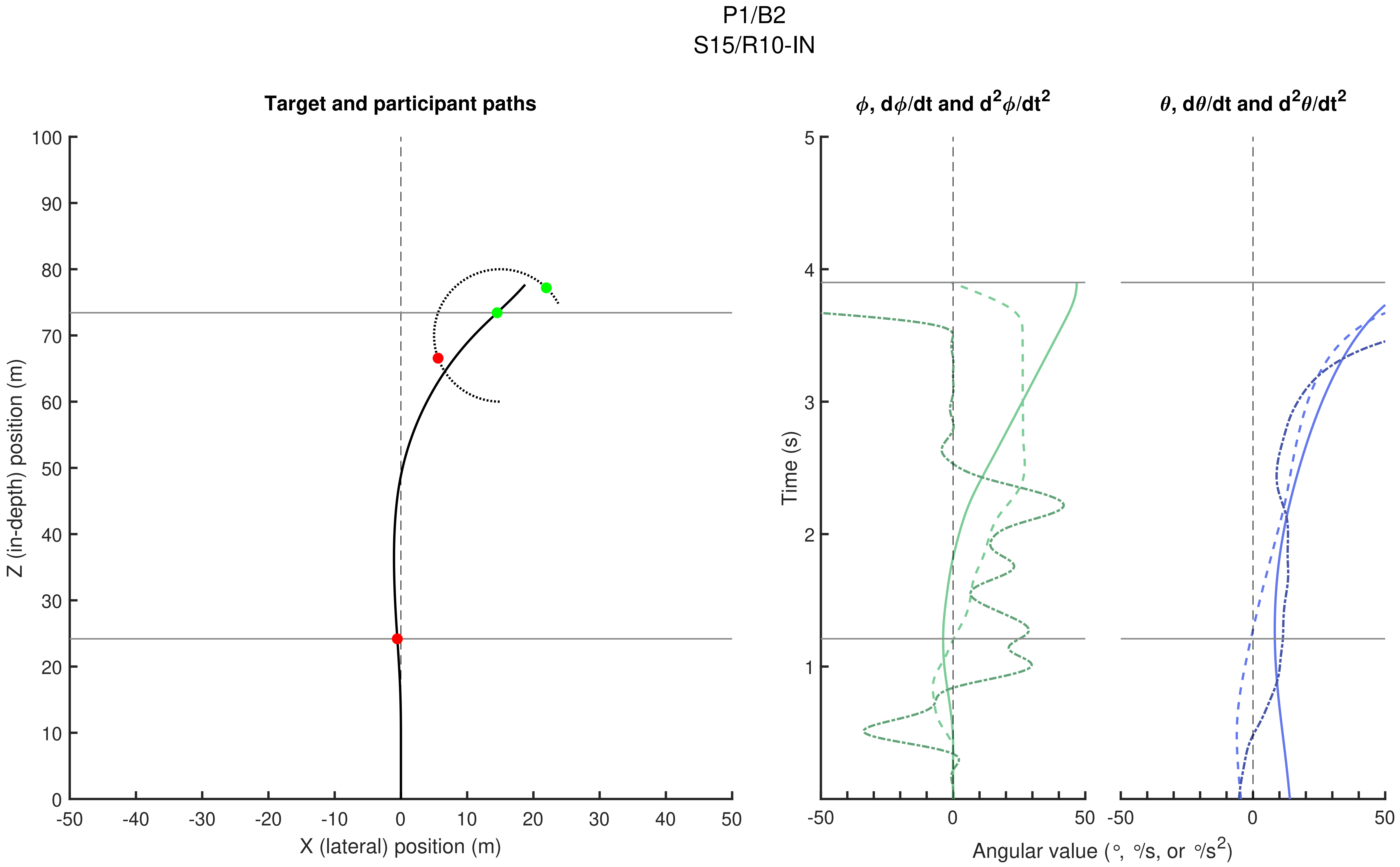

P1/B2
S15/R10-IN
Target and participant paths
Z (in-depth) position (m)
X (lateral) position (m)
ϕ, dϕ/dt and d²ϕ/dt²
θ, dθ/dt and d²θ/dt²
Time (s)
Angular value (°, °/s, or °/s²)

P1/B2
S15/R20-IN

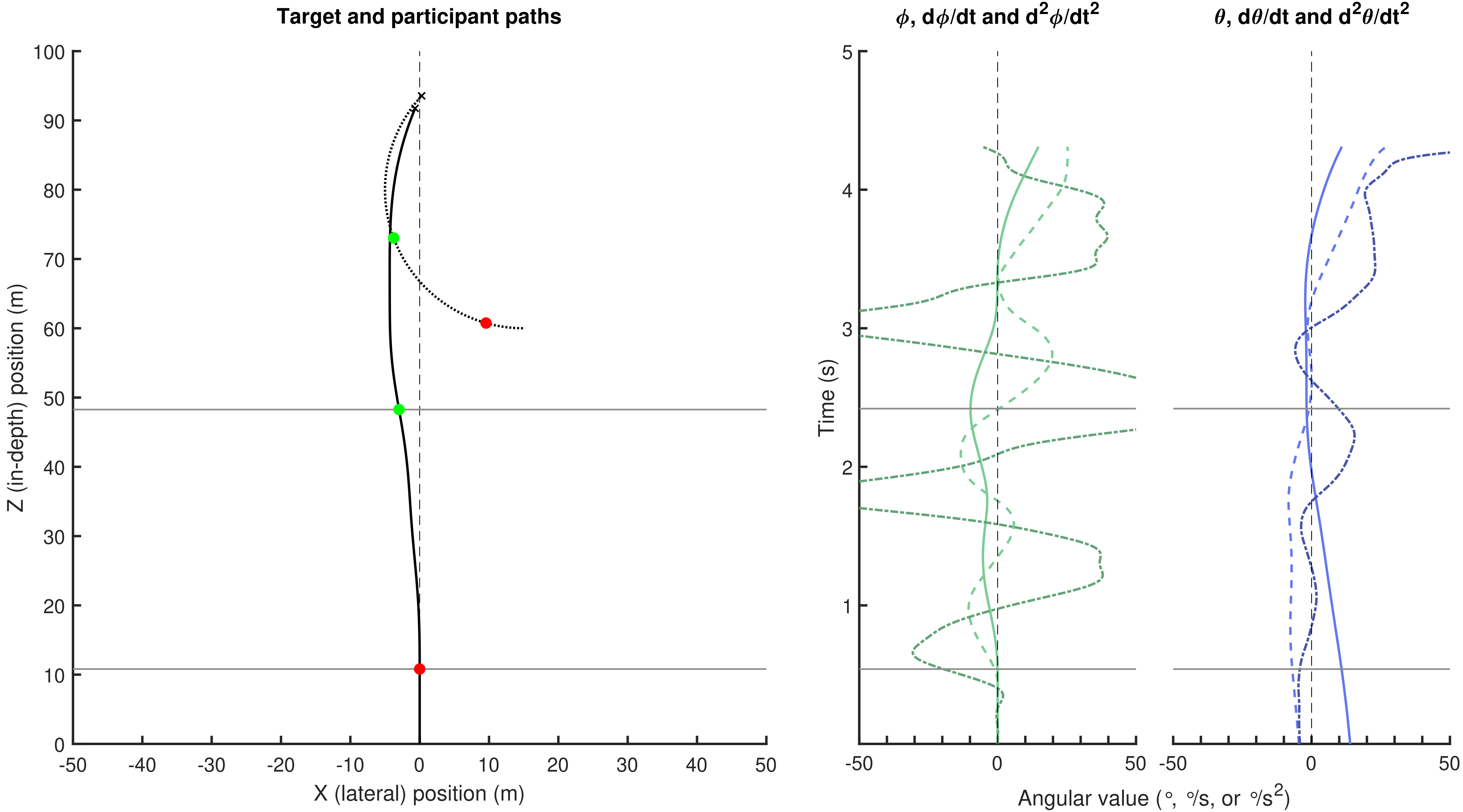

P1/B2
S15/R30-IN

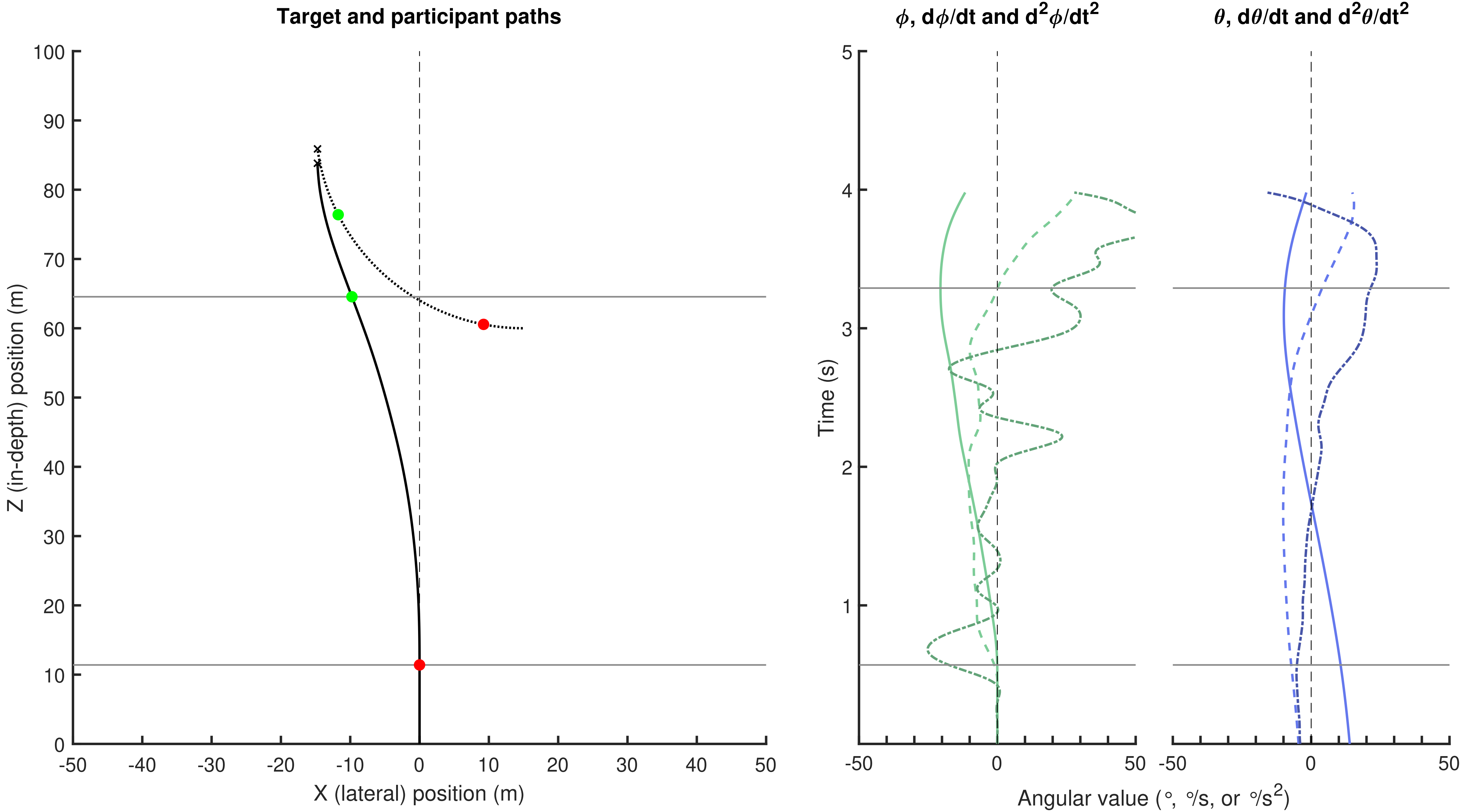

P1/B2
S20/R10-IN

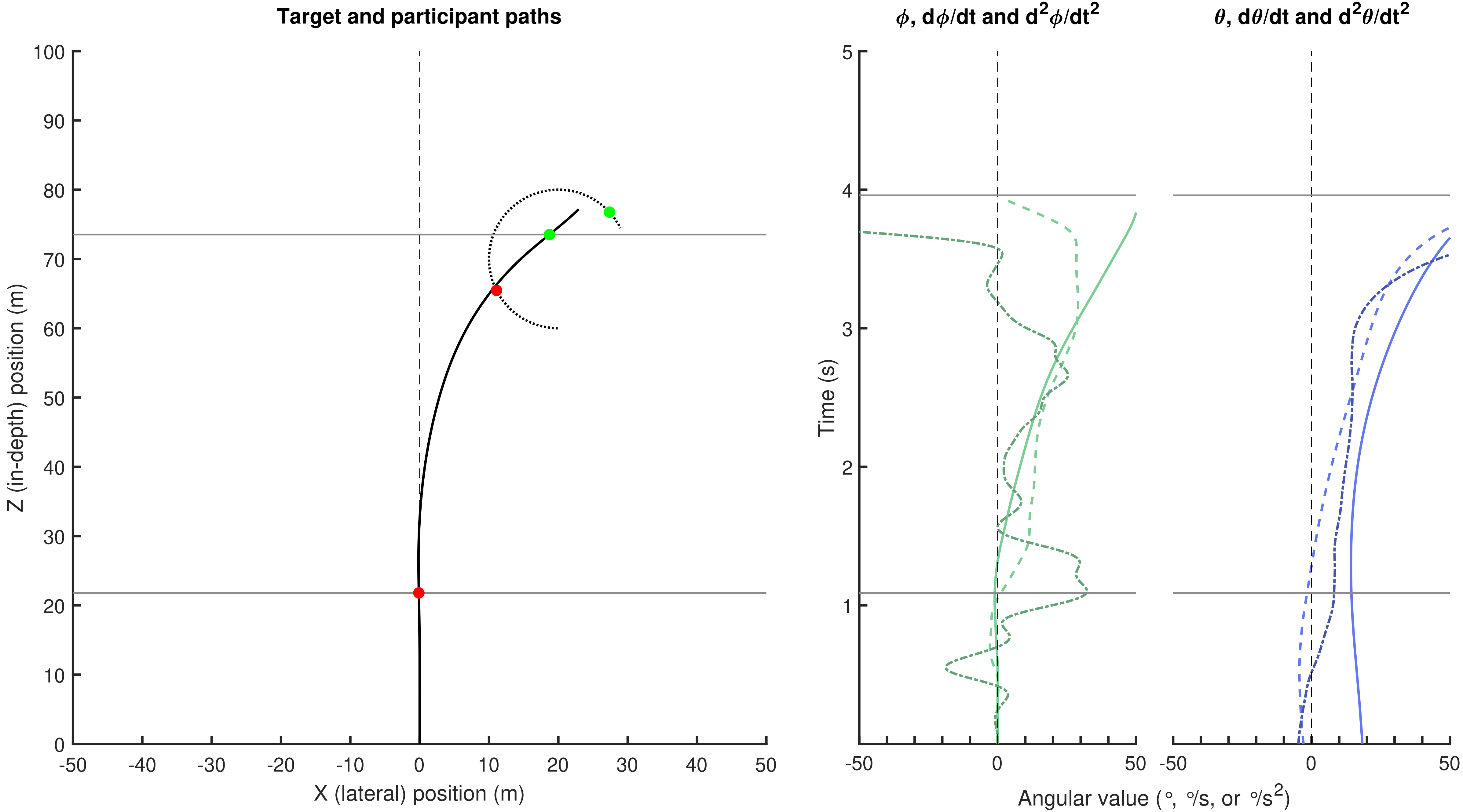

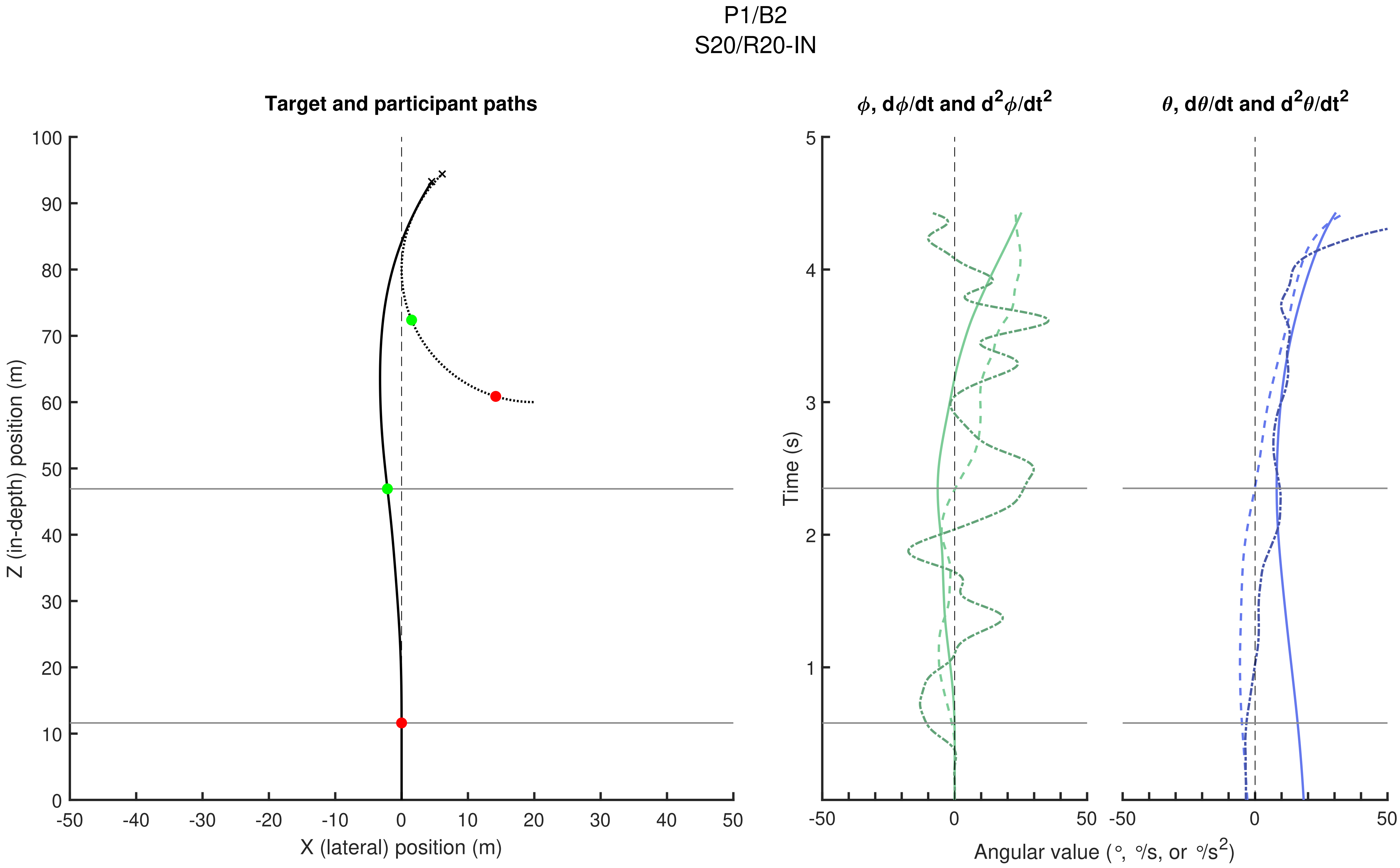

P1/B2
S20/R20-IN
Target and participant paths
Z (in-depth) position (m)
X (lateral) position (m)
φ, dφ/dt and d²φ/dt²
θ, dθ/dt and d²θ/dt²
Time (s)
Angular value (°, °/s, or °/s²)

P1/B2
S20/R30-IN

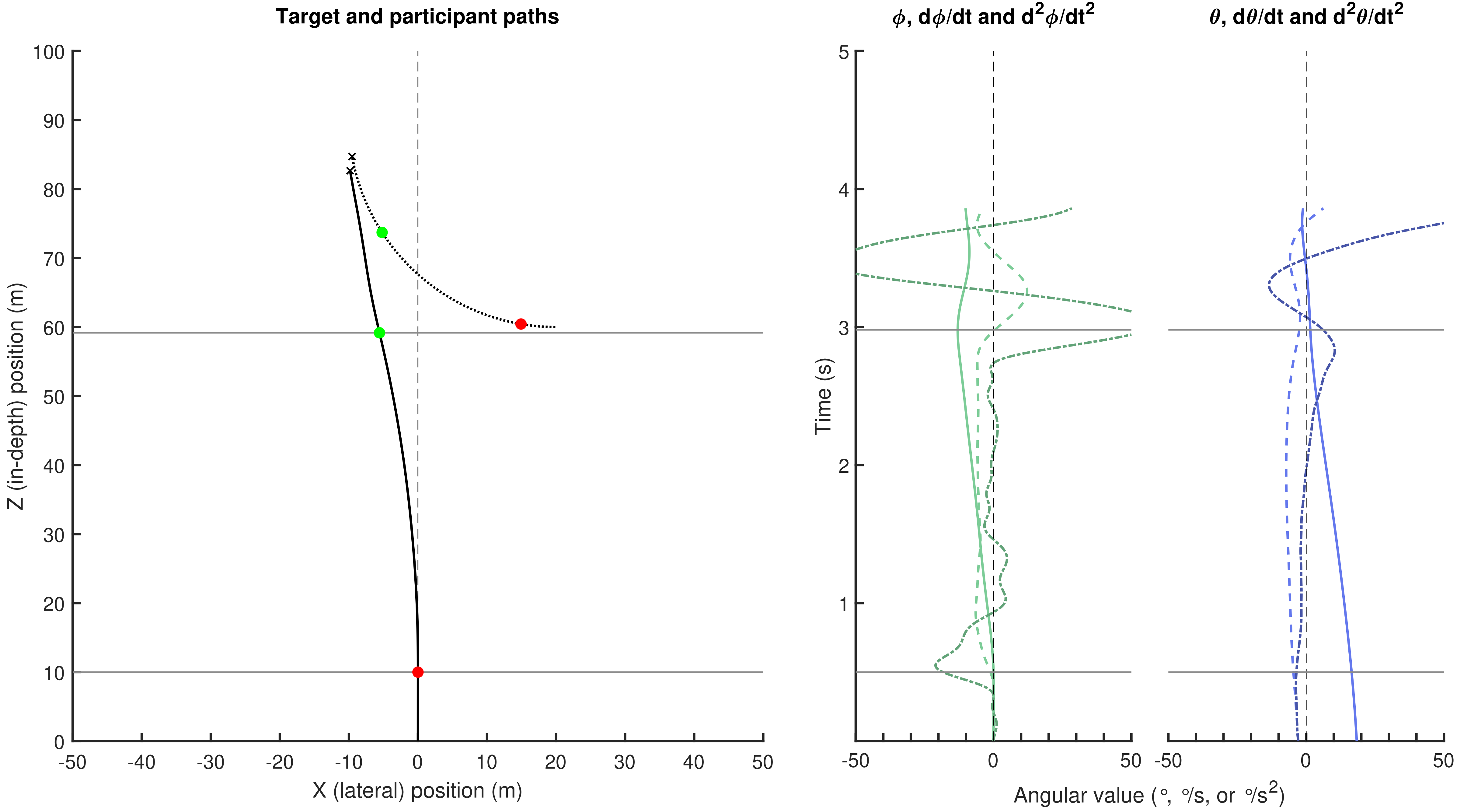

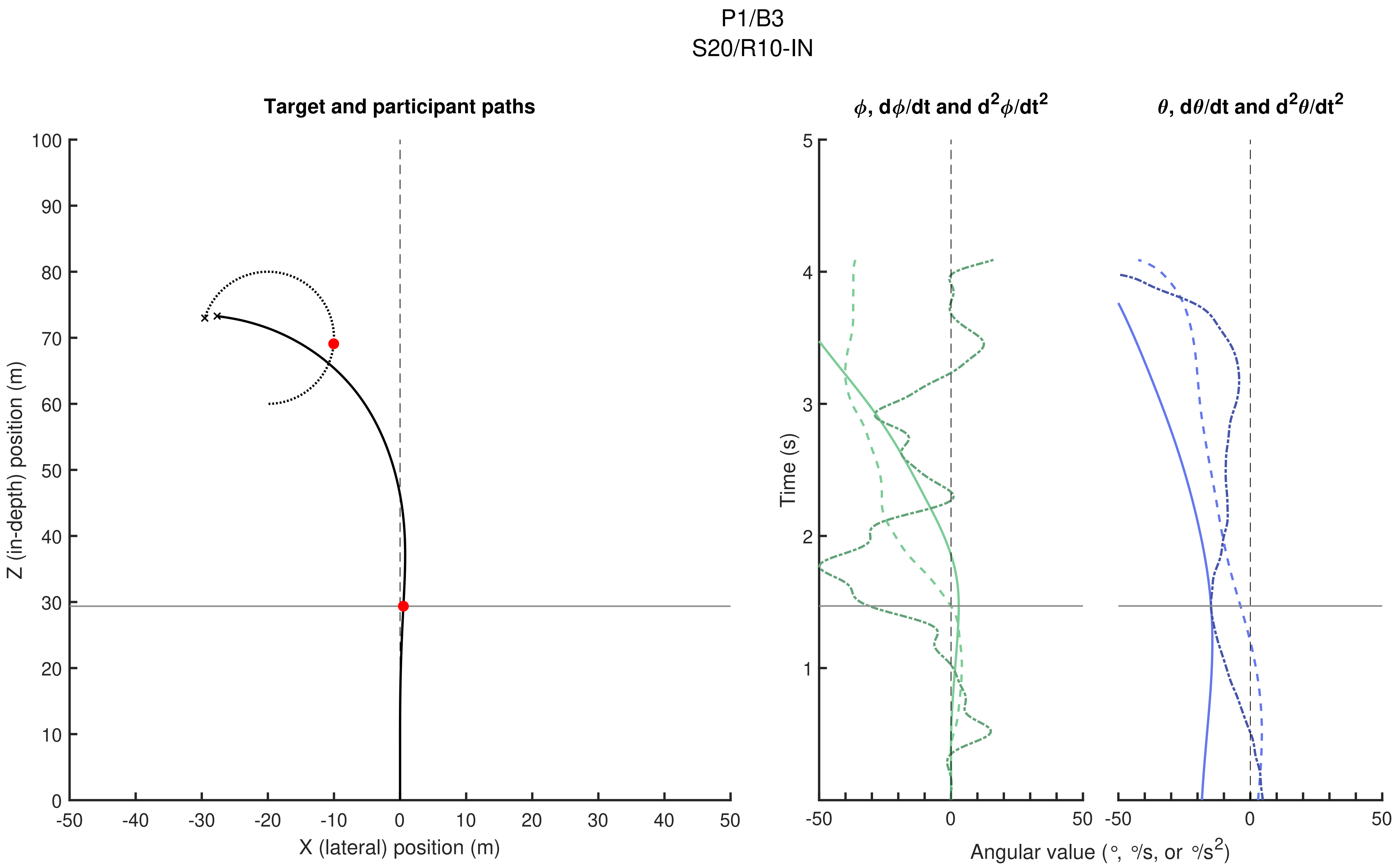
P1/B3
S20/R10-IN
Target and participant paths
Z (in-depth) position (m)
X (lateral) position (m)
ϕ, dϕ/dt and d$^2\phi$/dt^2
θ, dθ/dt and d$^2\theta$/dt^2
Time (s)
Angular value (°, °/s, or °/s^2)

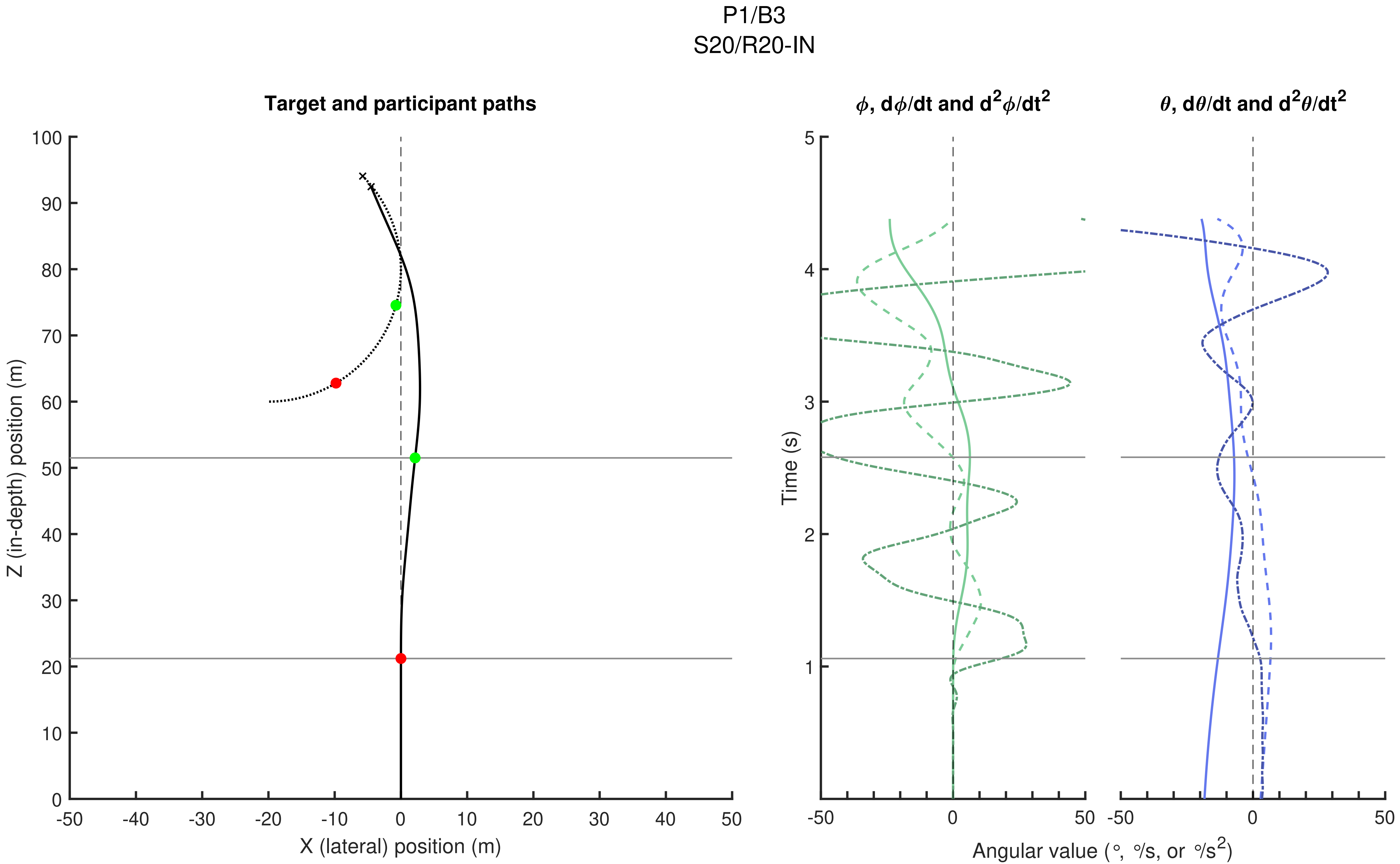
P1/B3
S20/R20-IN
Target and participant paths
Z (in-depth) position (m)
X (lateral) position (m)
ϕ, dϕ/dt and d$^2\phi$/dt^2
θ, dθ/dt and d$^2\theta$/dt^2
Time (s)
Angular value (°, °/s, or °/s^2)

P1/B3
S20/R30-IN

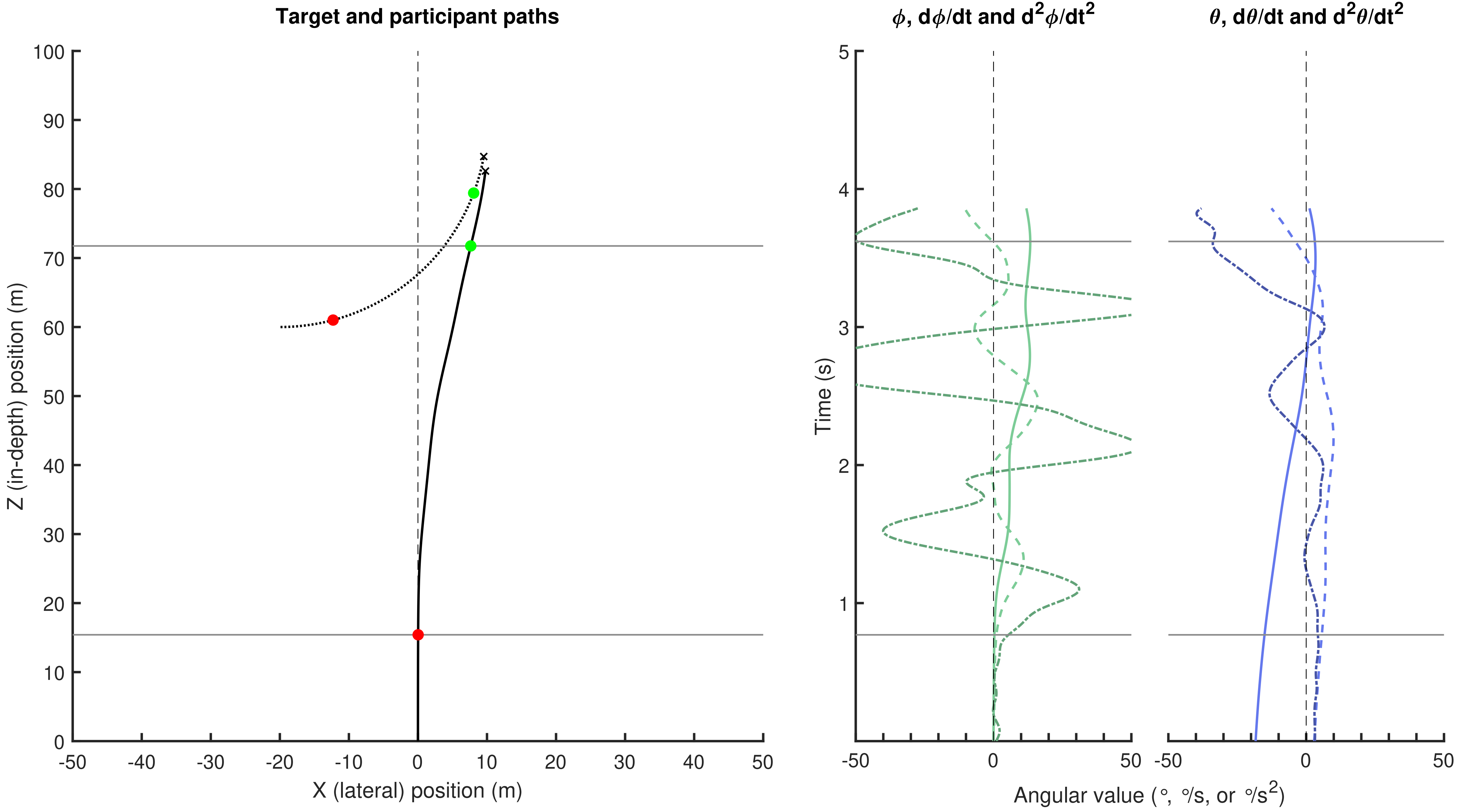

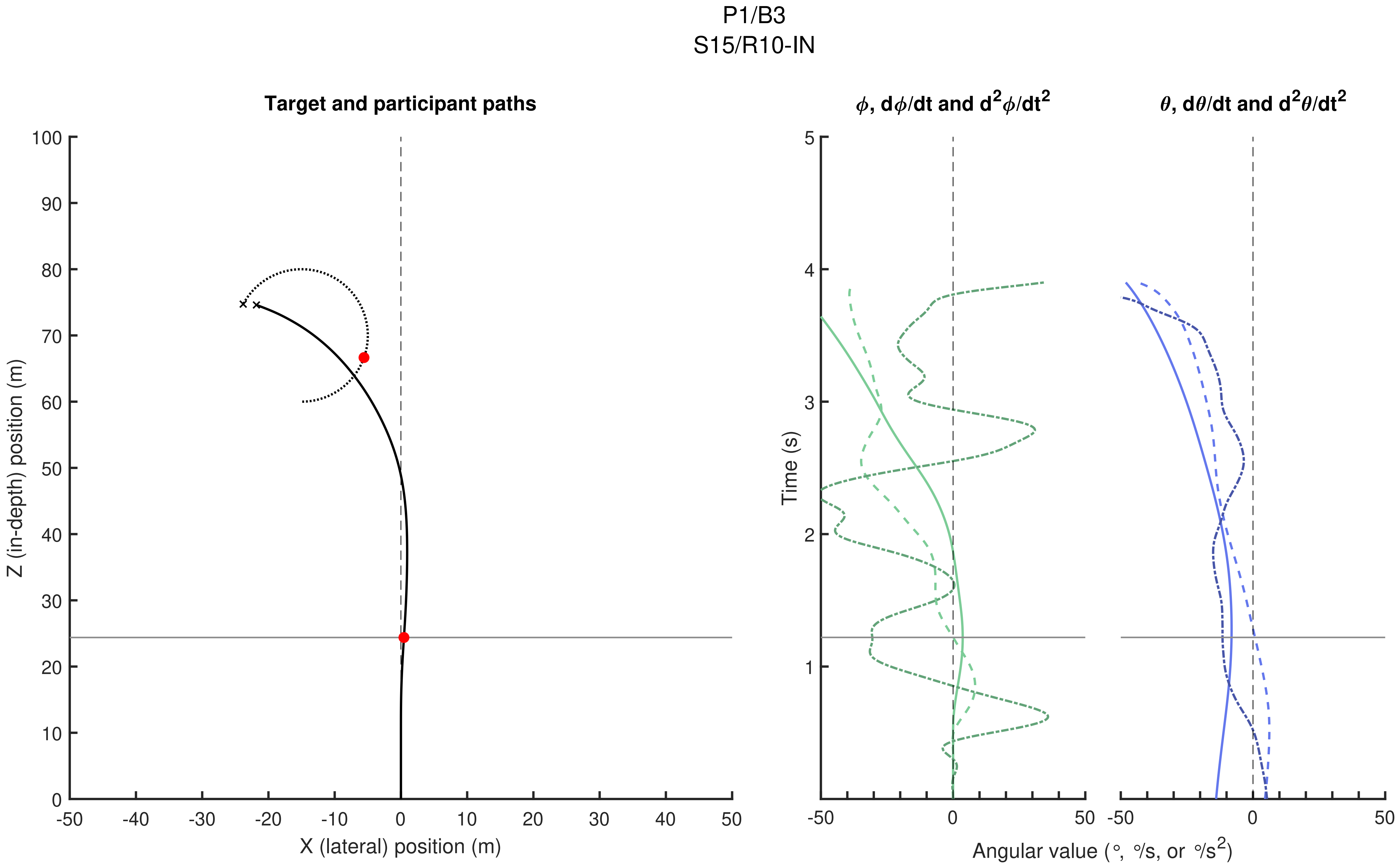
P1/B3
S15/R10-IN
Target and participant paths
Z (in-depth) position (m)
X (lateral) position (m)
φ, dφ/dt and d²φ/dt²
θ, dθ/dt and d²θ/dt²
Time (s)
Angular value (°, °/s, or °/s²)

P1/B3
S15/R20-IN

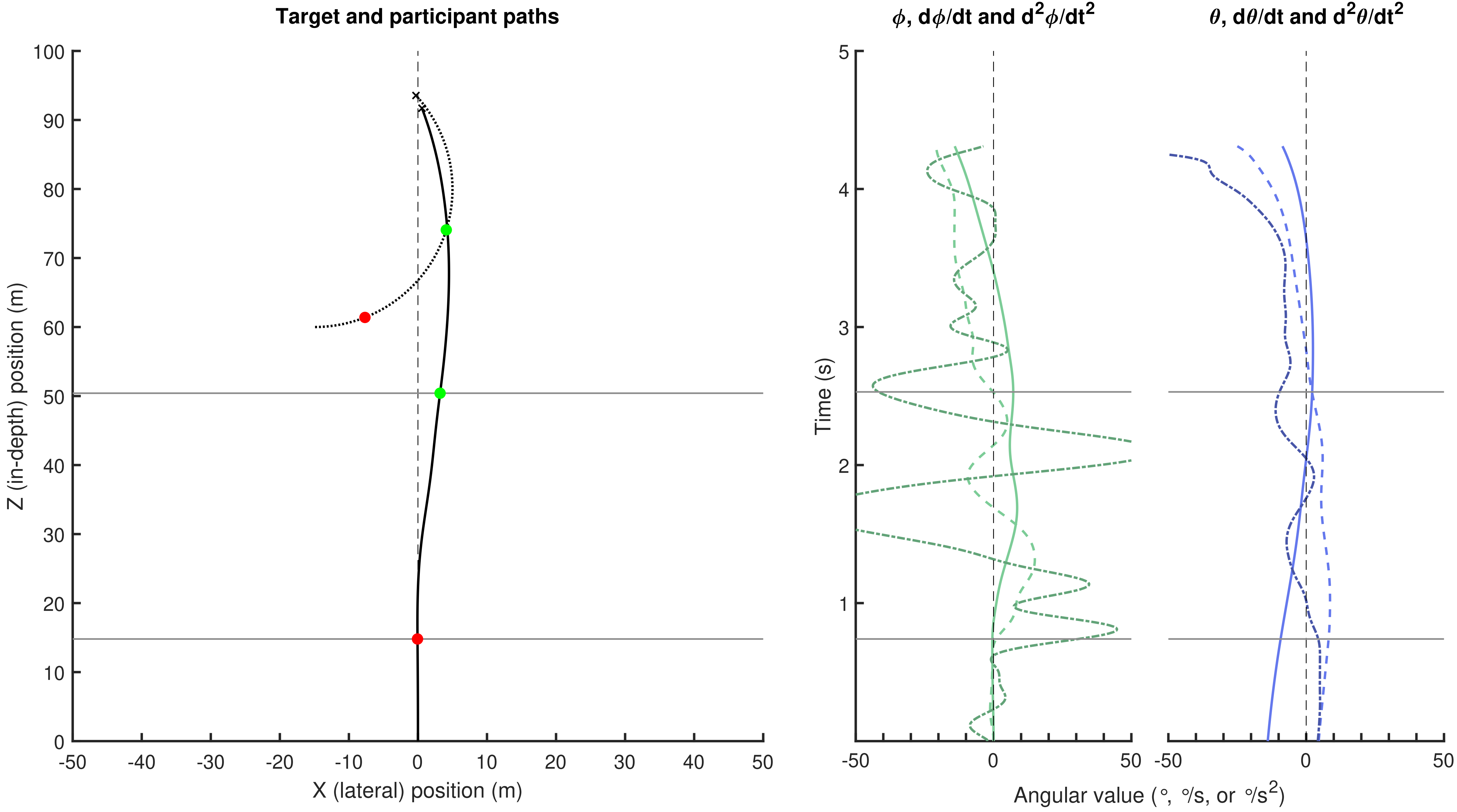

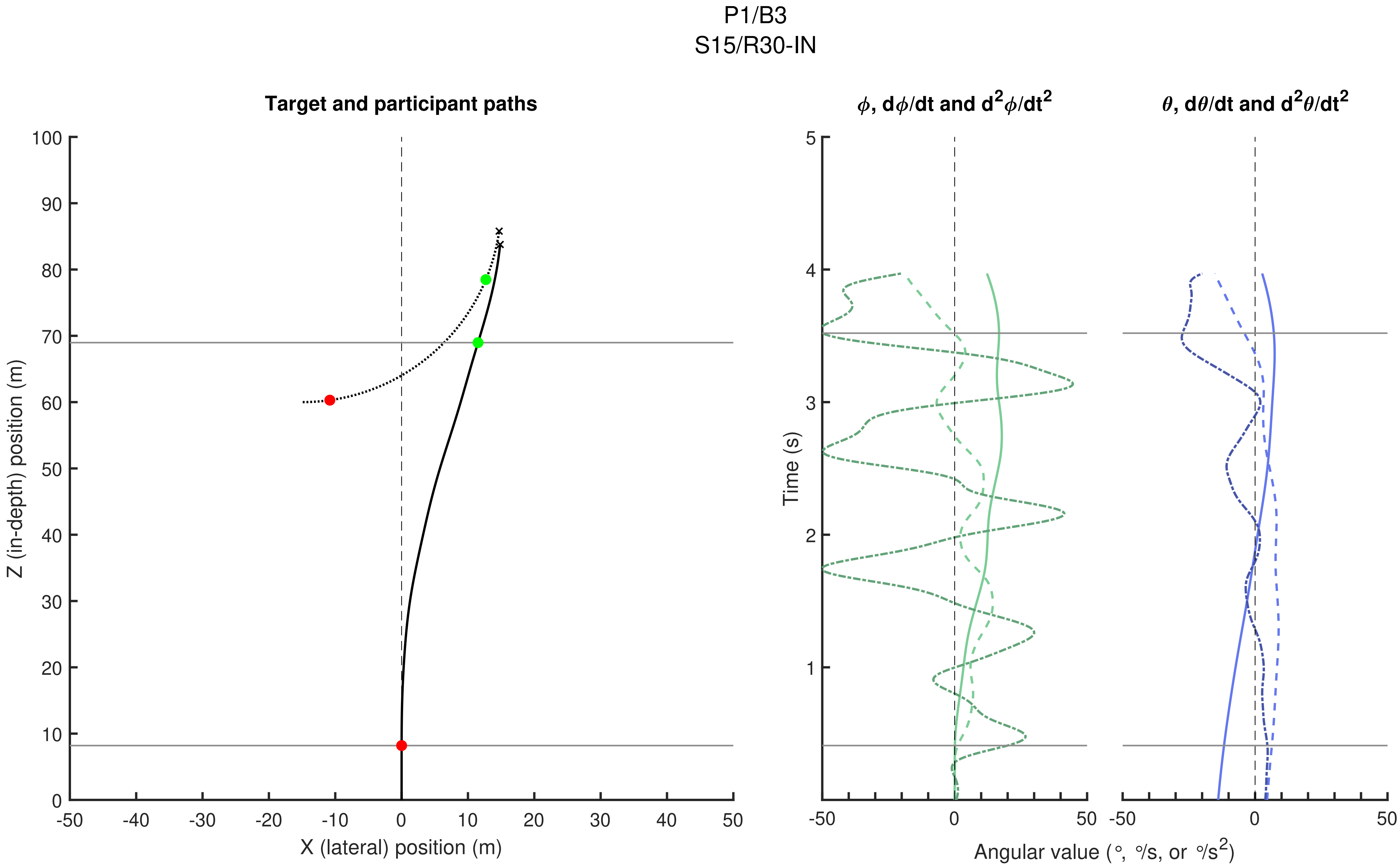

P1/B3
S15/R30-IN
Target and participant paths
Z (in-depth) position (m)
X (lateral) position (m)
φ, dφ/dt and d²φ/dt²
θ, dθ/dt and d²θ/dt²
Time (s)
Angular value (°, °/s, or °/s²)

P1/B3
S5/R10-IN

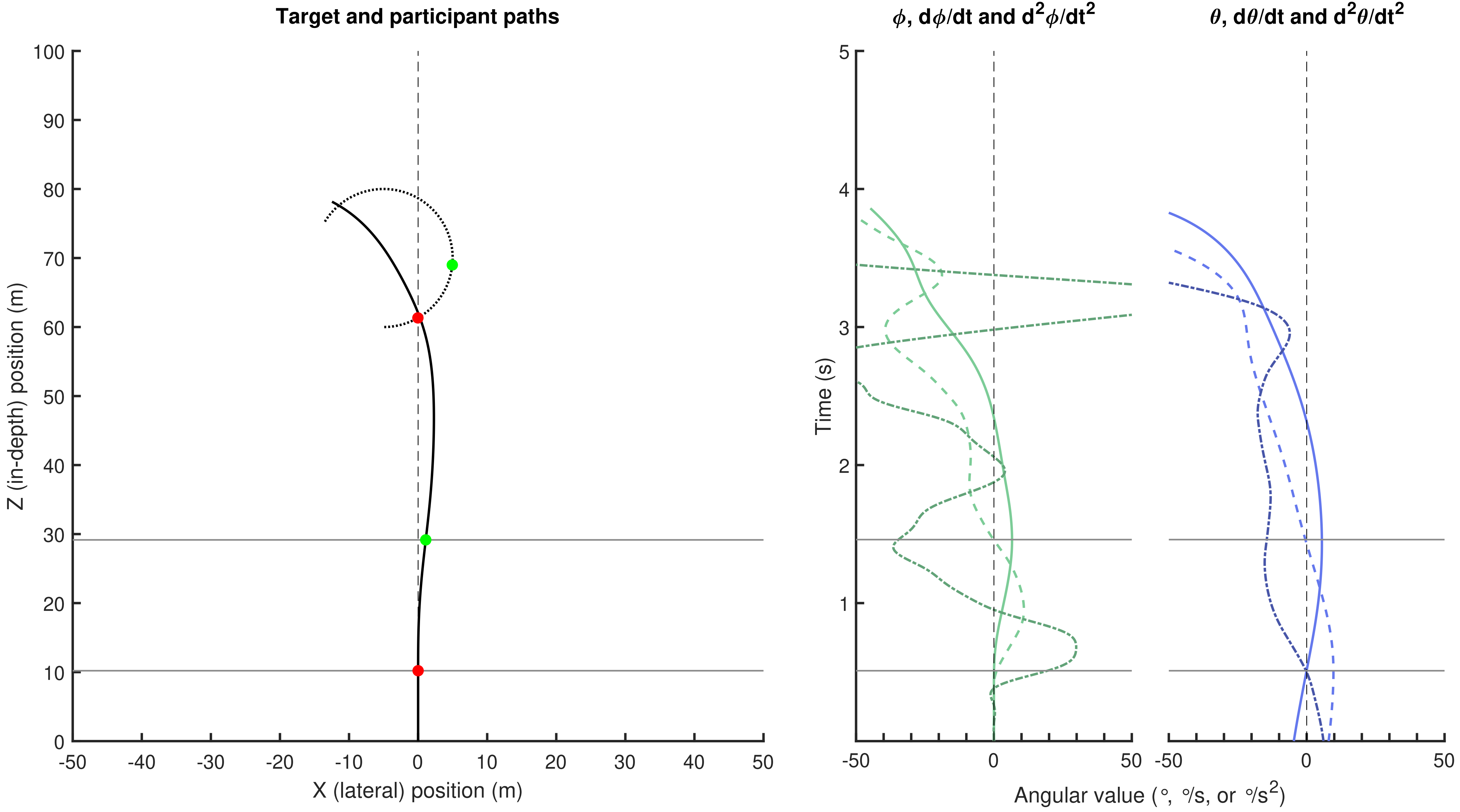

P1/B3
S5/R10-OUT

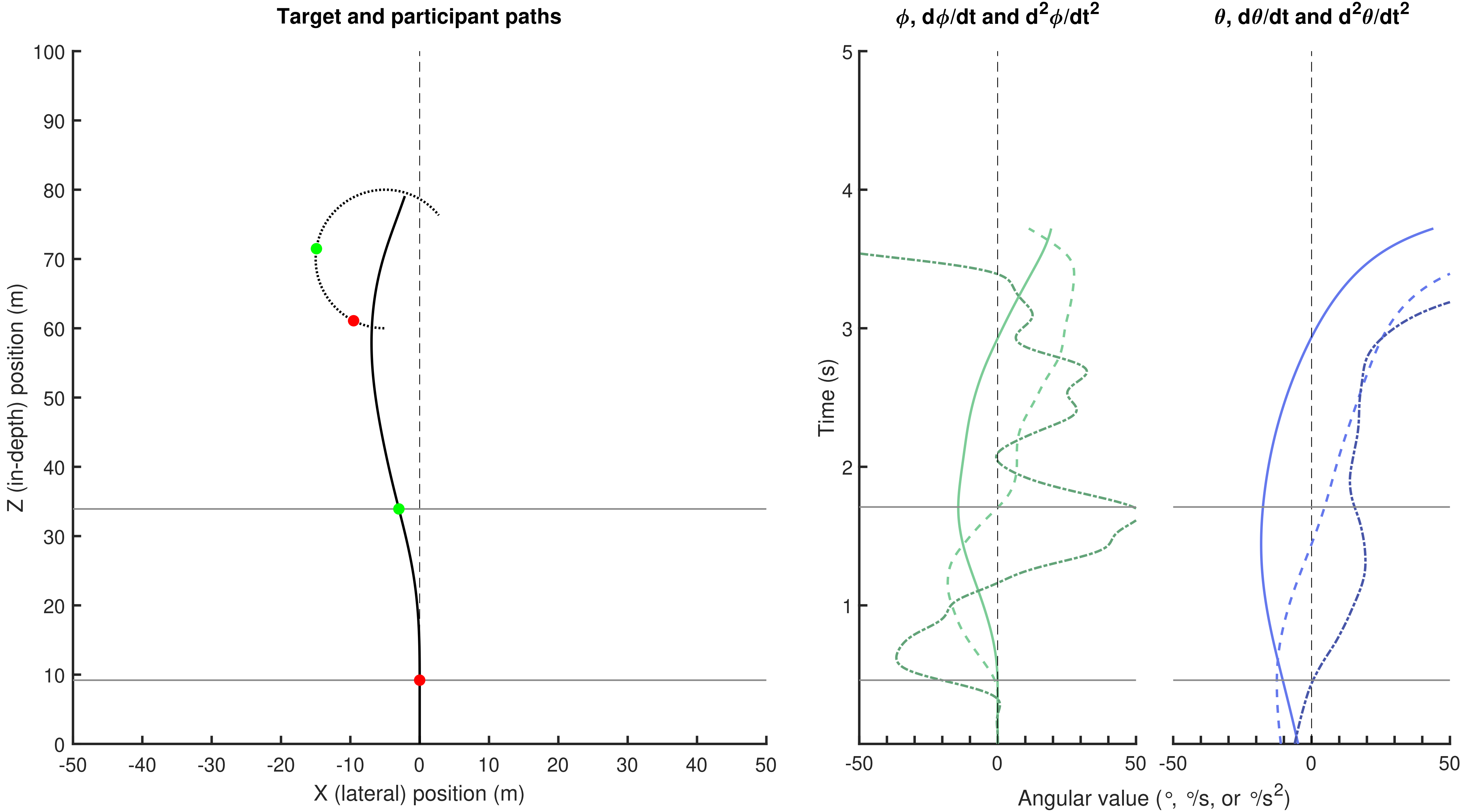

P1/B3
S5/R20-IN

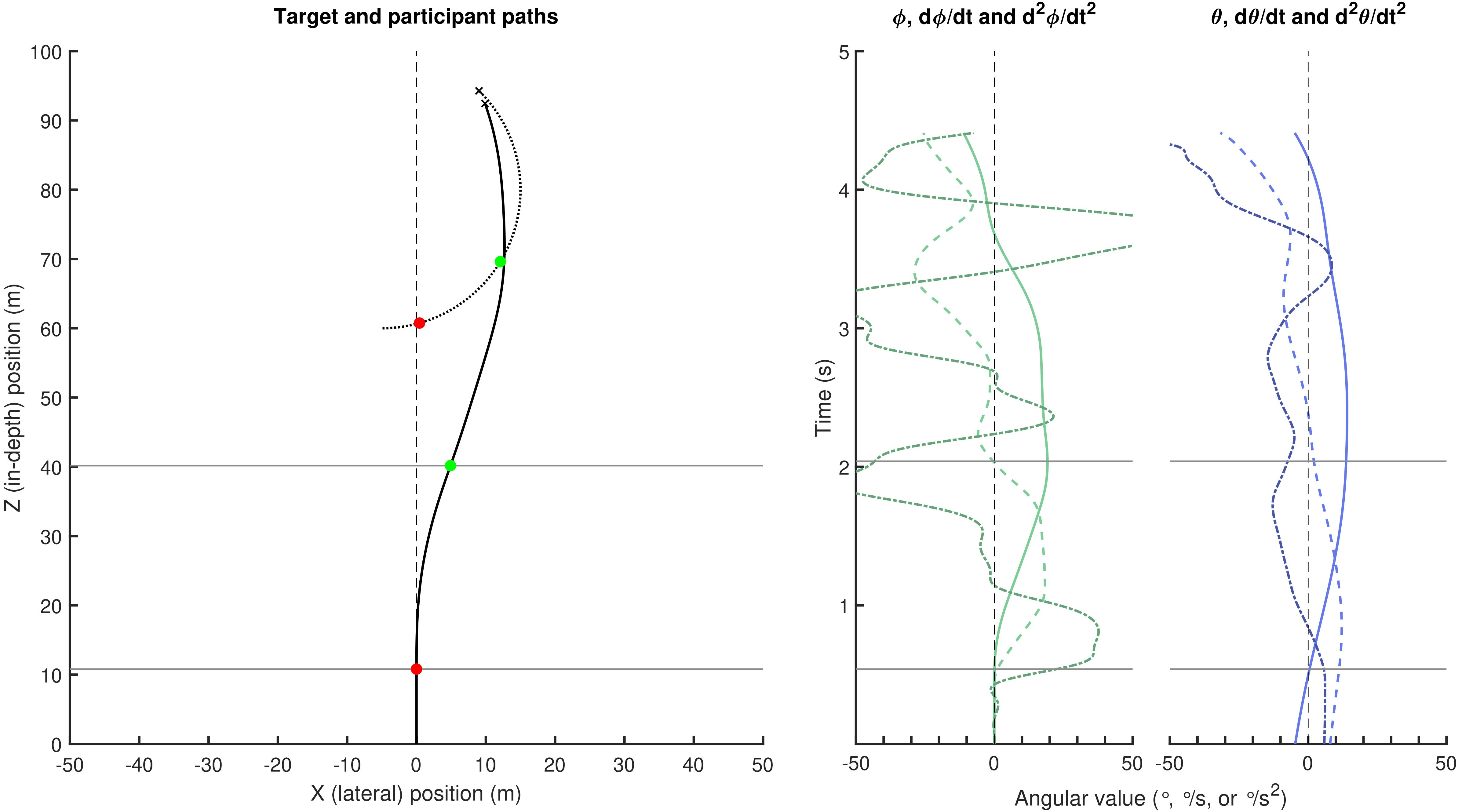

P1/B3
S5/R20-OUT

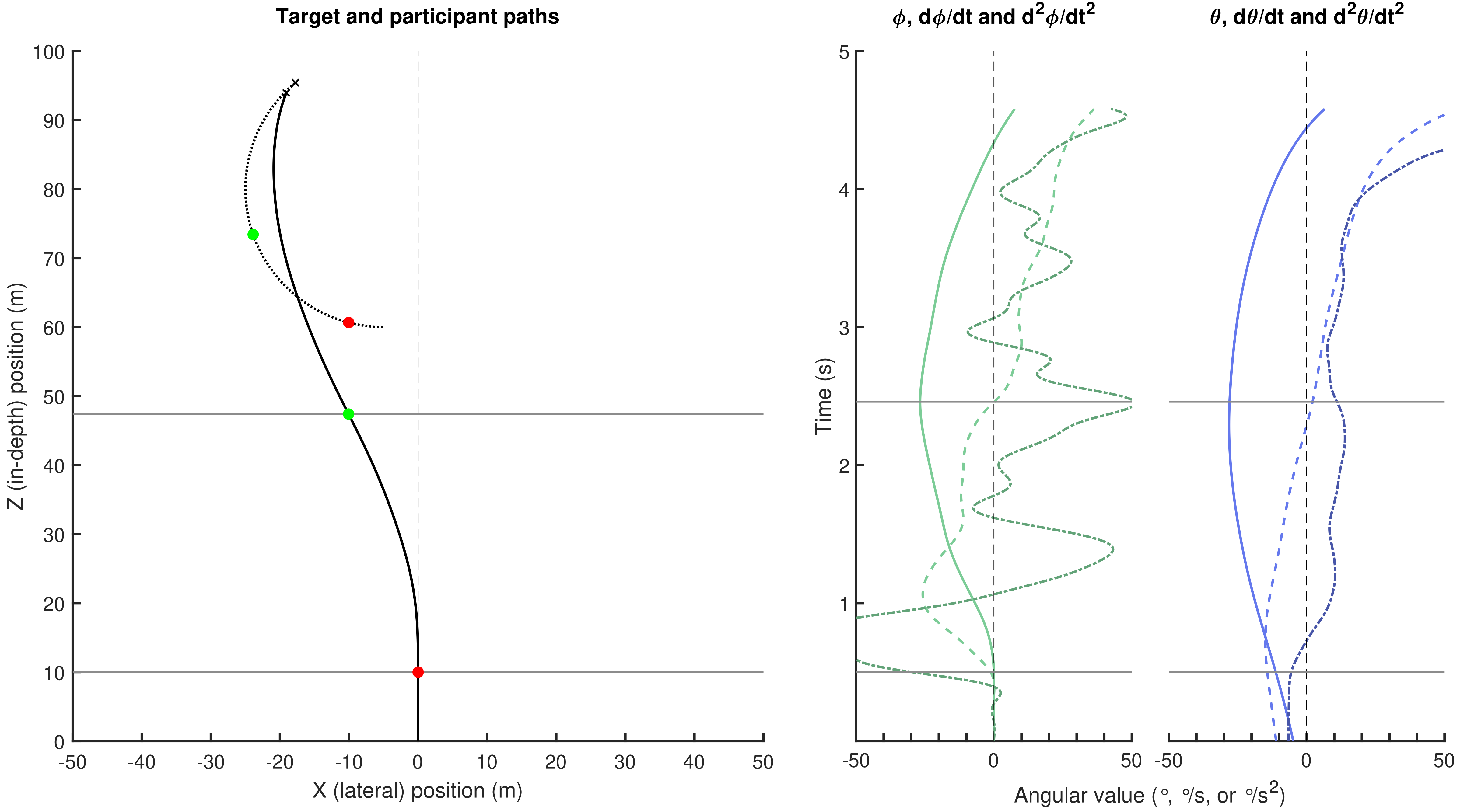

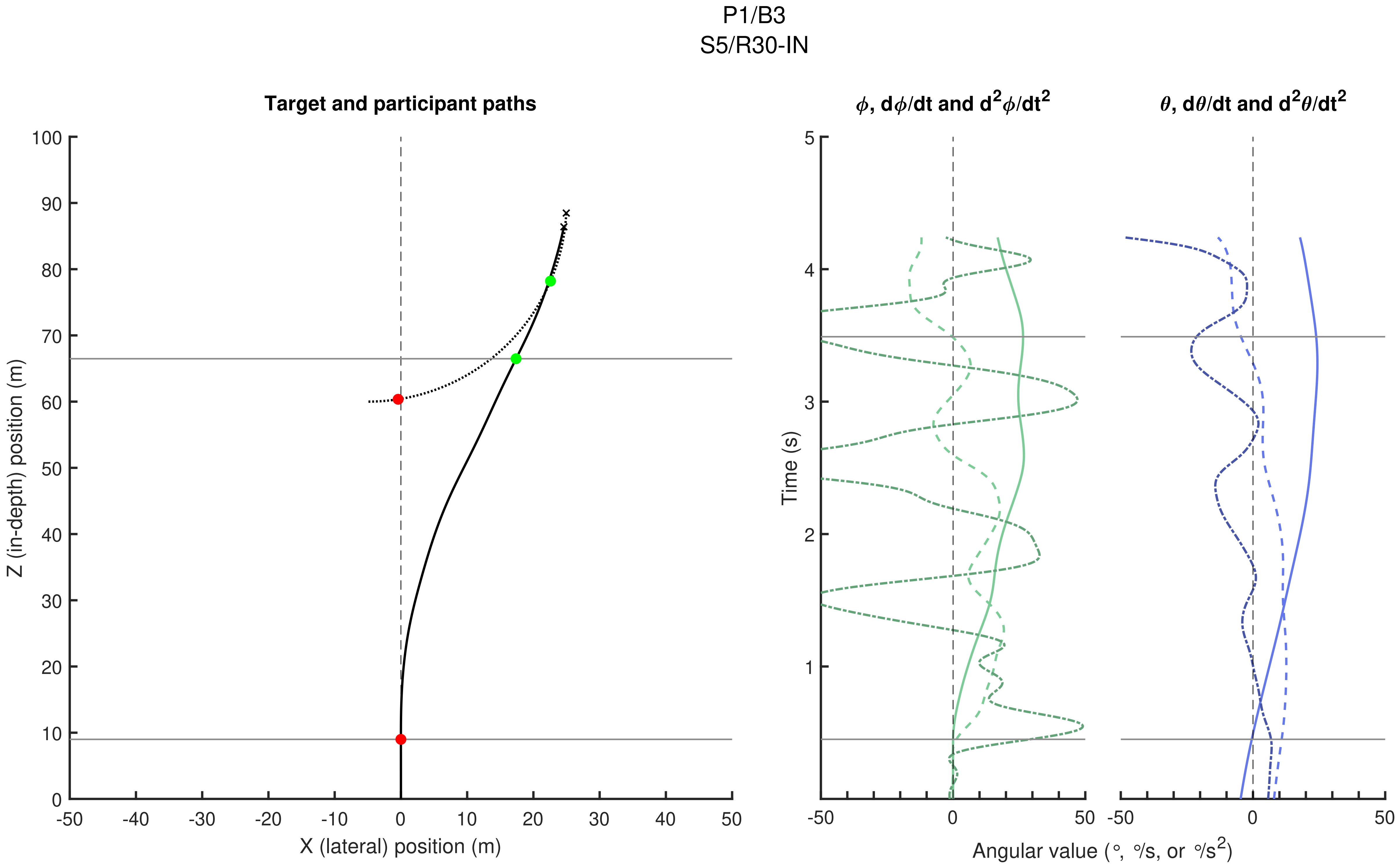
P1/B3
S5/R30-IN
Target and participant paths
Z (in-depth) position (m)
X (lateral) position (m)
ϕ, dϕ/dt and d$^2\phi$/dt^2
θ, dθ/dt and d$^2\theta$/dt^2
Time (s)
Angular value (°, °/s, or °/s^2)

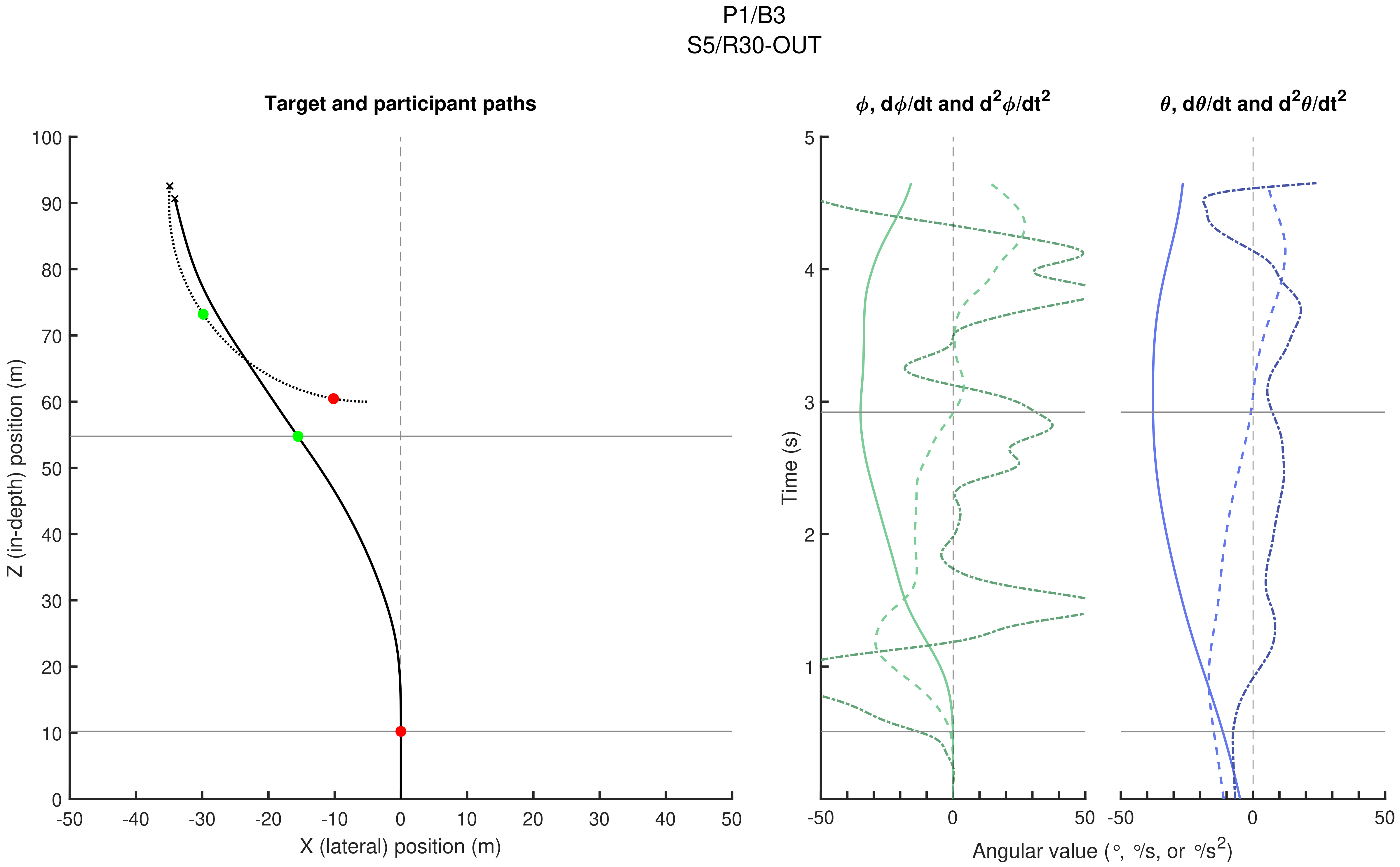
P1/B3
S5/R30-OUT
Target and participant paths
Z (in-depth) position (m)
X (lateral) position (m)
100
90
80
70
60
50
40
30
20
10
0
-50
-40
-30
-20
-10
0
10
20
30
40
50
ϕ, dϕ/dt and d$^2\phi$/dt^2
θ, dθ/dt and d$^2\theta$/dt^2
Time (s)
5
4
3
2
1
-50
0
50
-50
0
50
Angular value (°, °/s, or °/s^2)

P1/B3
S5/R10-OUT

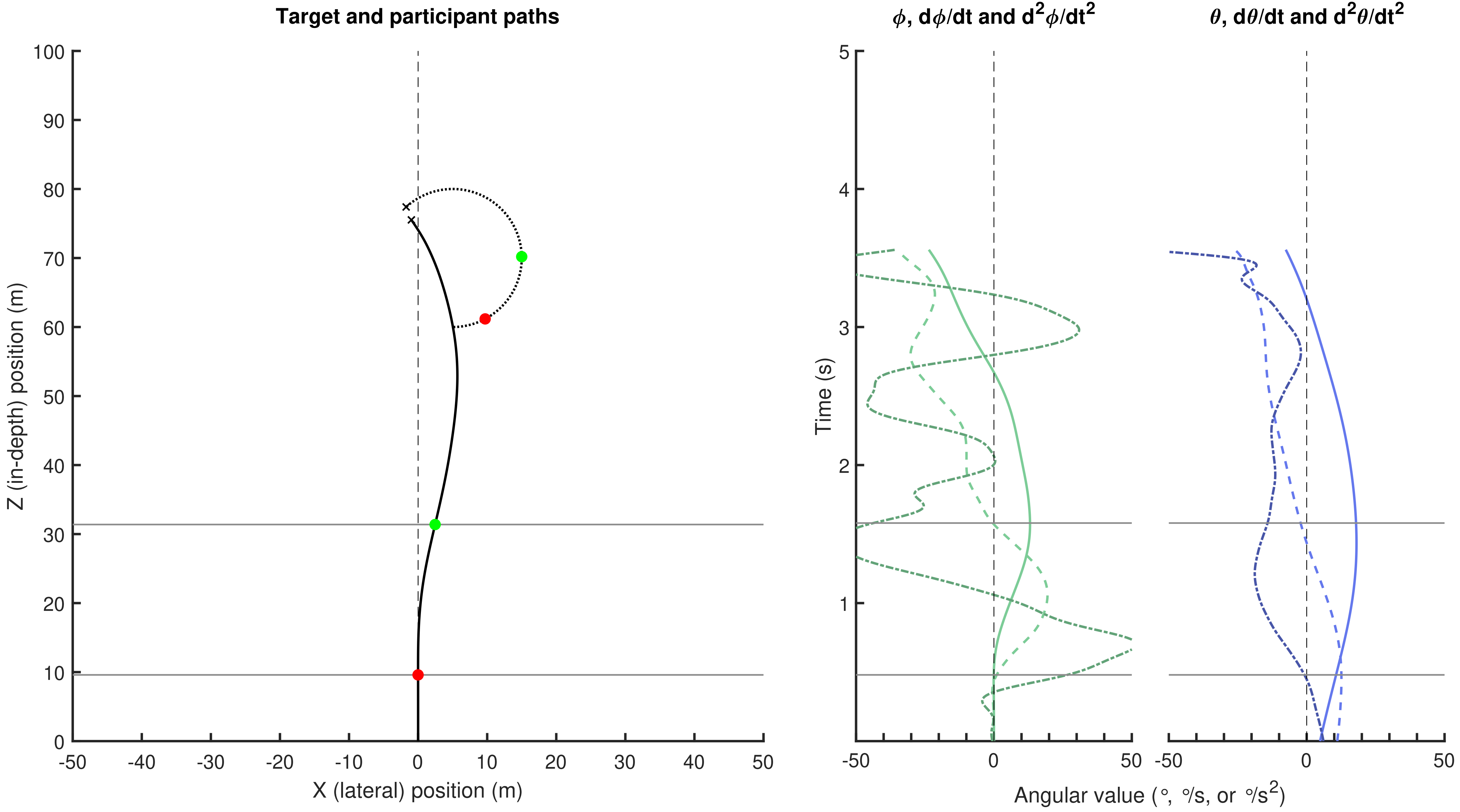

P1/B3
S5/R10-IN

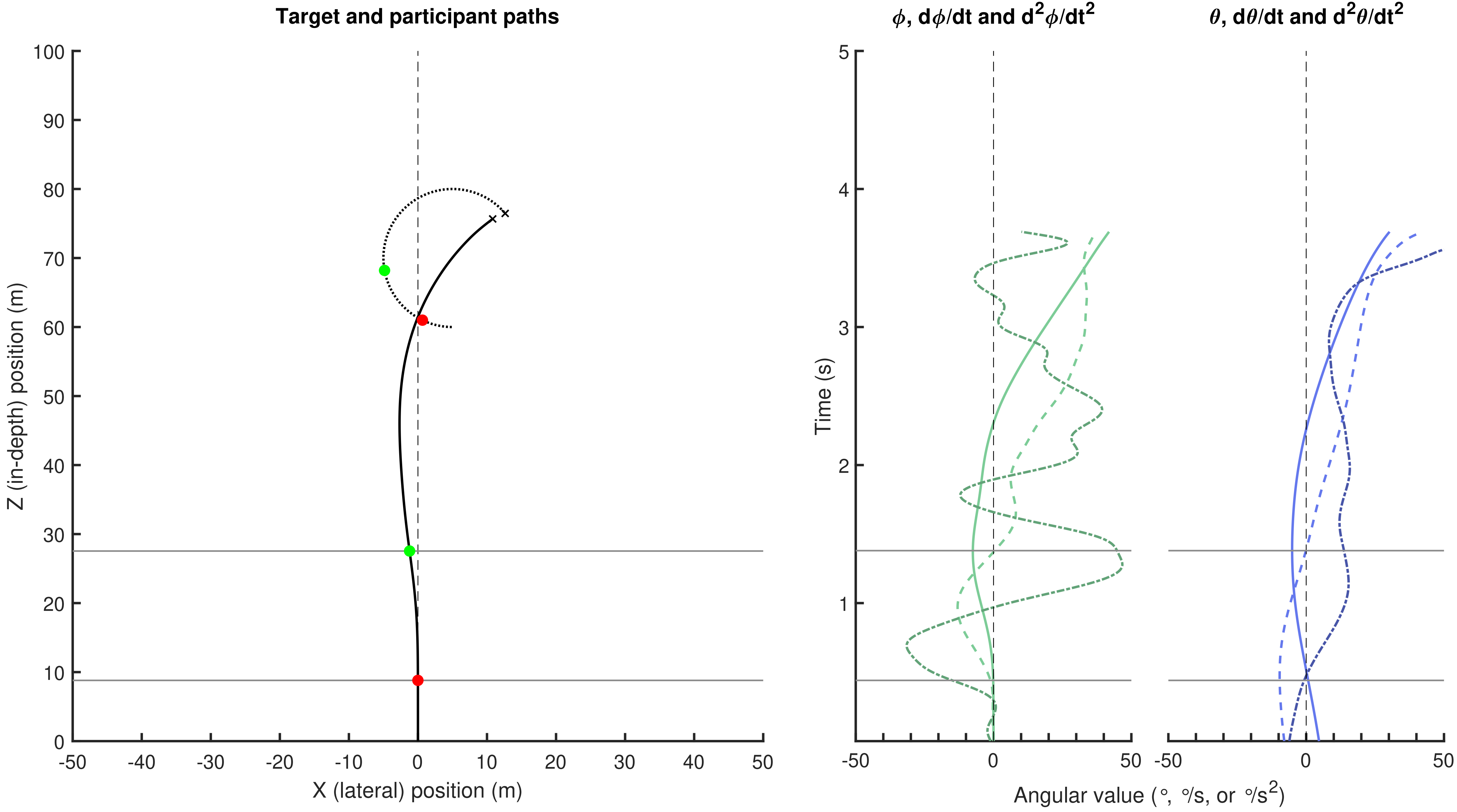

P1/B3
S5/R20-OUT

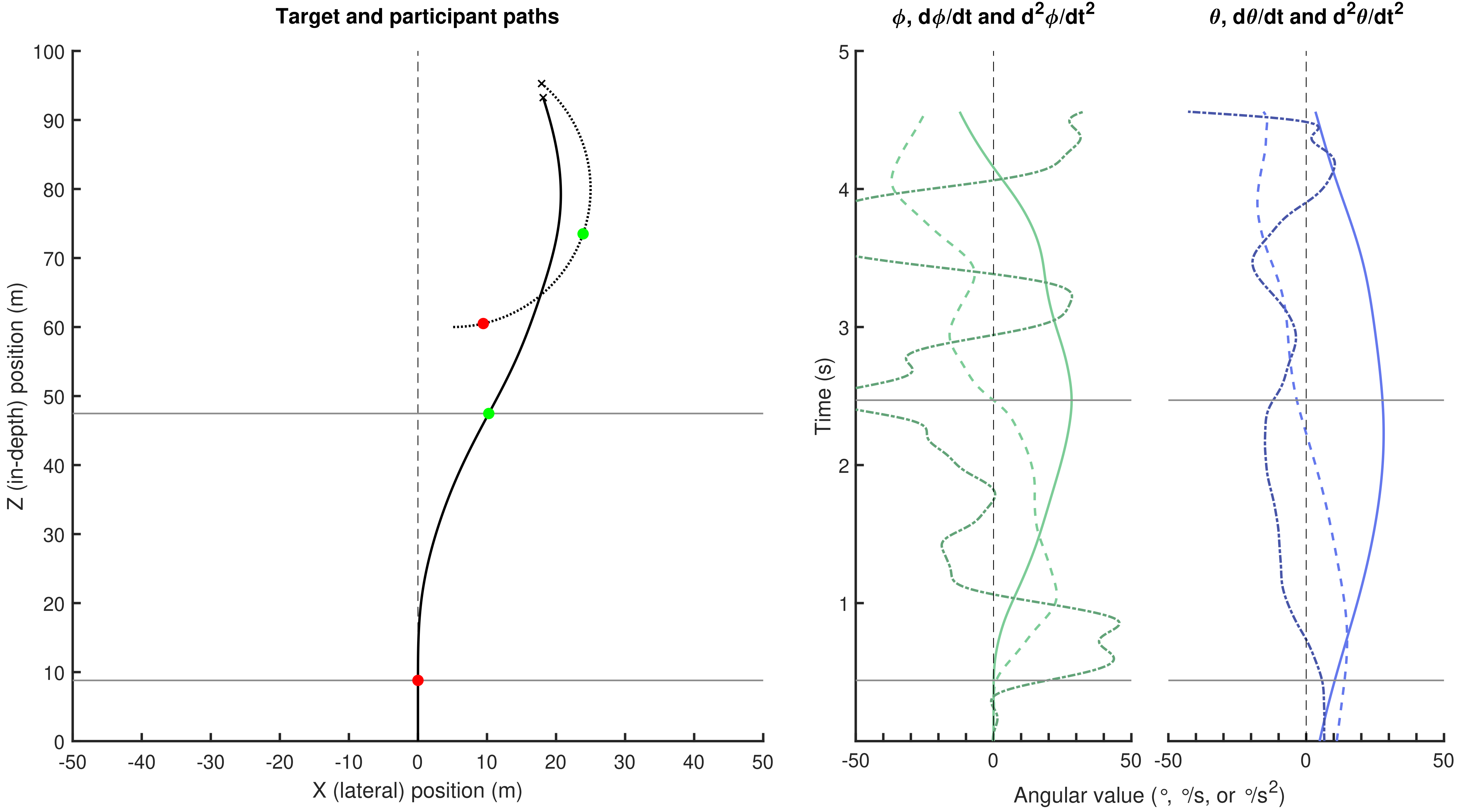

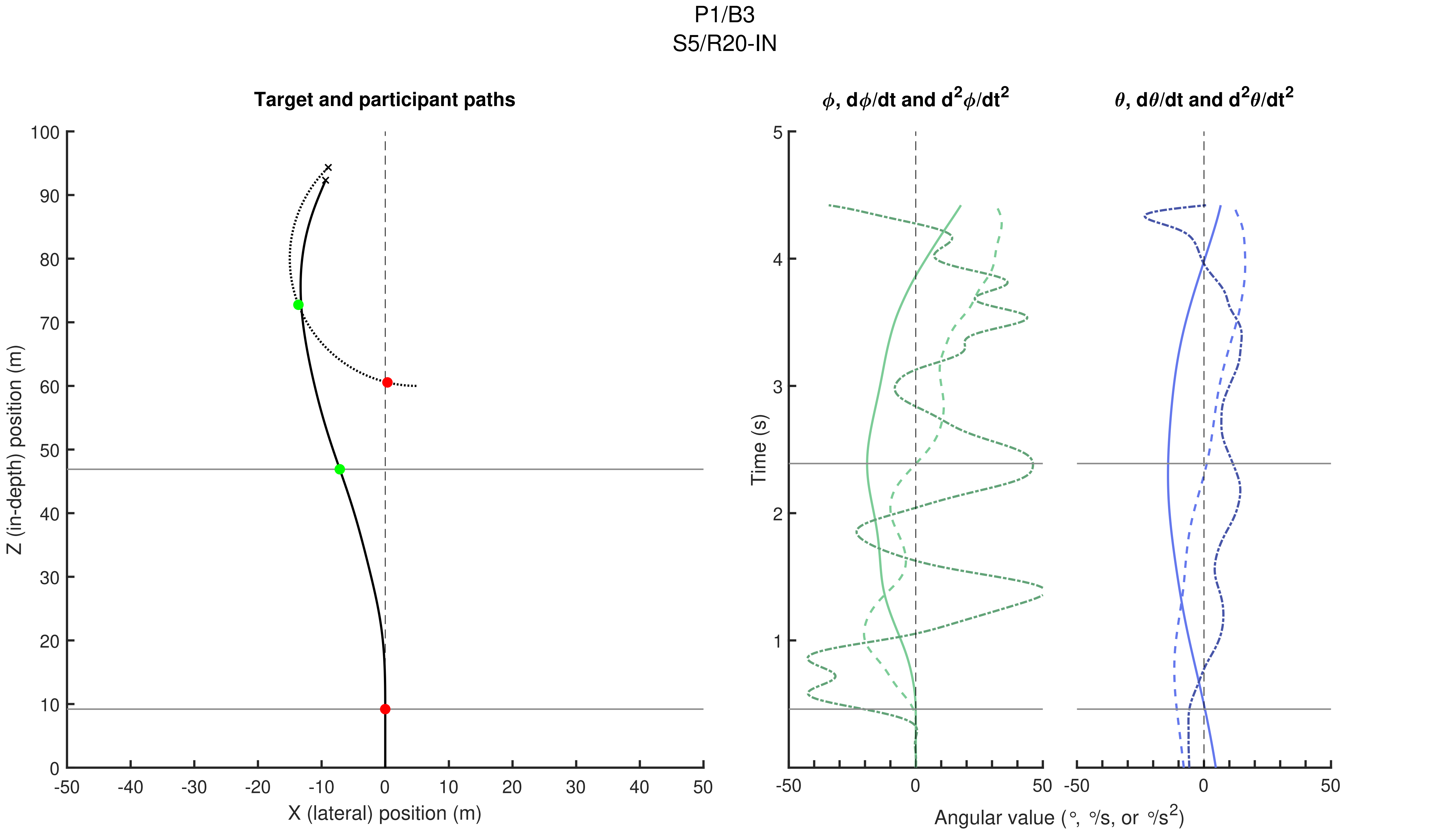
P1/B3
S5/R20-IN
Target and participant paths
Z (in-depth) position (m)
X (lateral) position (m)
ϕ, dϕ/dt and d²ϕ/dt²
θ, dθ/dt and d²θ/dt²
Time (s)
Angular value (°, °/s, or °/s²)

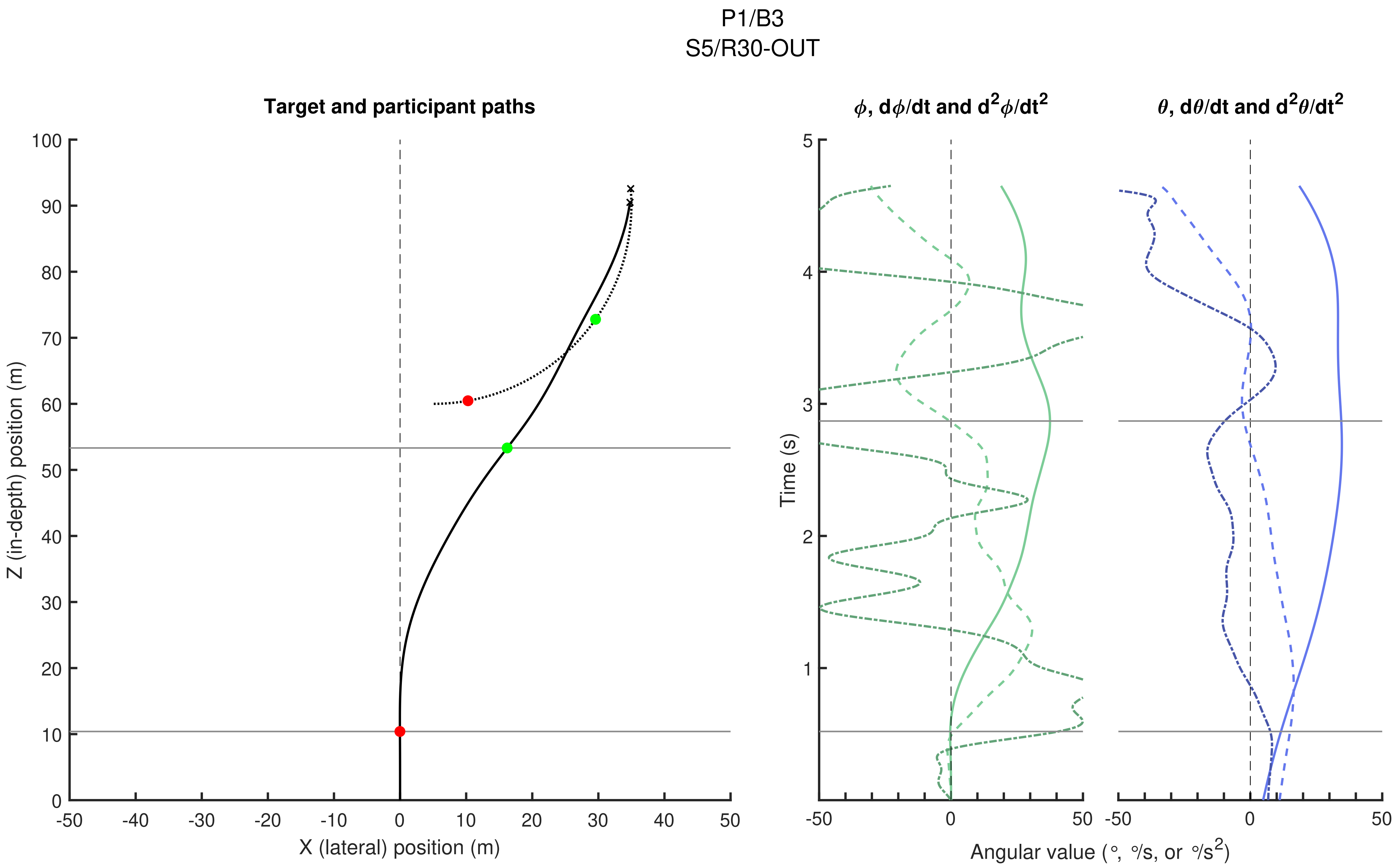

P1/B3
S5/R30-OUT
Target and participant paths
Z (in-depth) position (m)
X (lateral) position (m)
φ, dφ/dt and d²φ/dt²
θ, dθ/dt and d²θ/dt²
Time (s)
Angular value (°, °/s, or °/s²)

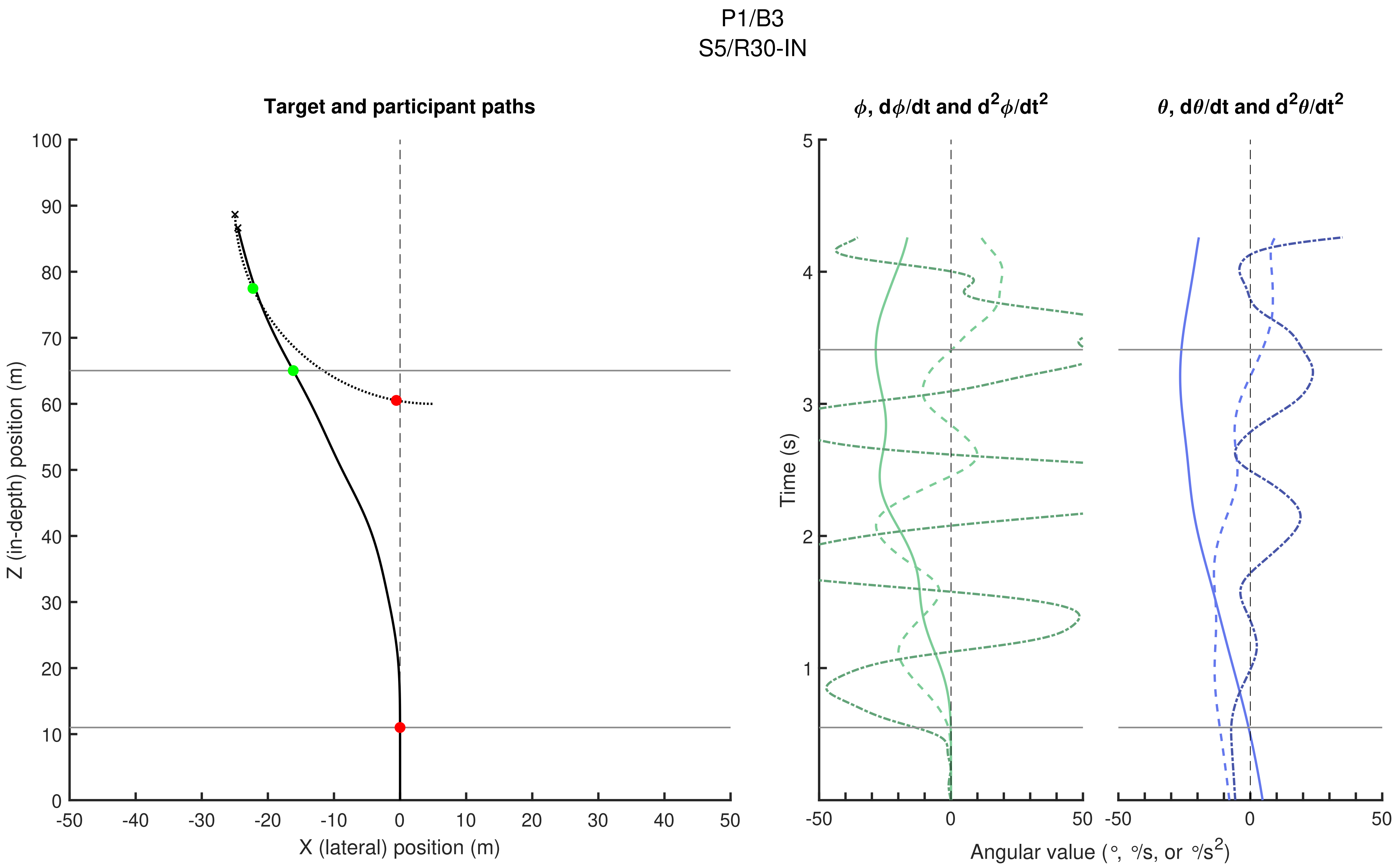
P1/B3
S5/R30-IN
Target and participant paths
Z (in-depth) position (m)
X (lateral) position (m)
100
90
80
70
60
50
40
30
20
10
0
-50
-40
-30
-20
-10
0
10
20
30
40
50
ϕ, dϕ/dt and d$^2\phi$/dt^2
θ, dθ/dt and d$^2\theta$/dt^2
Time (s)
5
4
3
2
1
-50
0
50
-50
0
50
Angular value (°, °/s, or °/s^2)

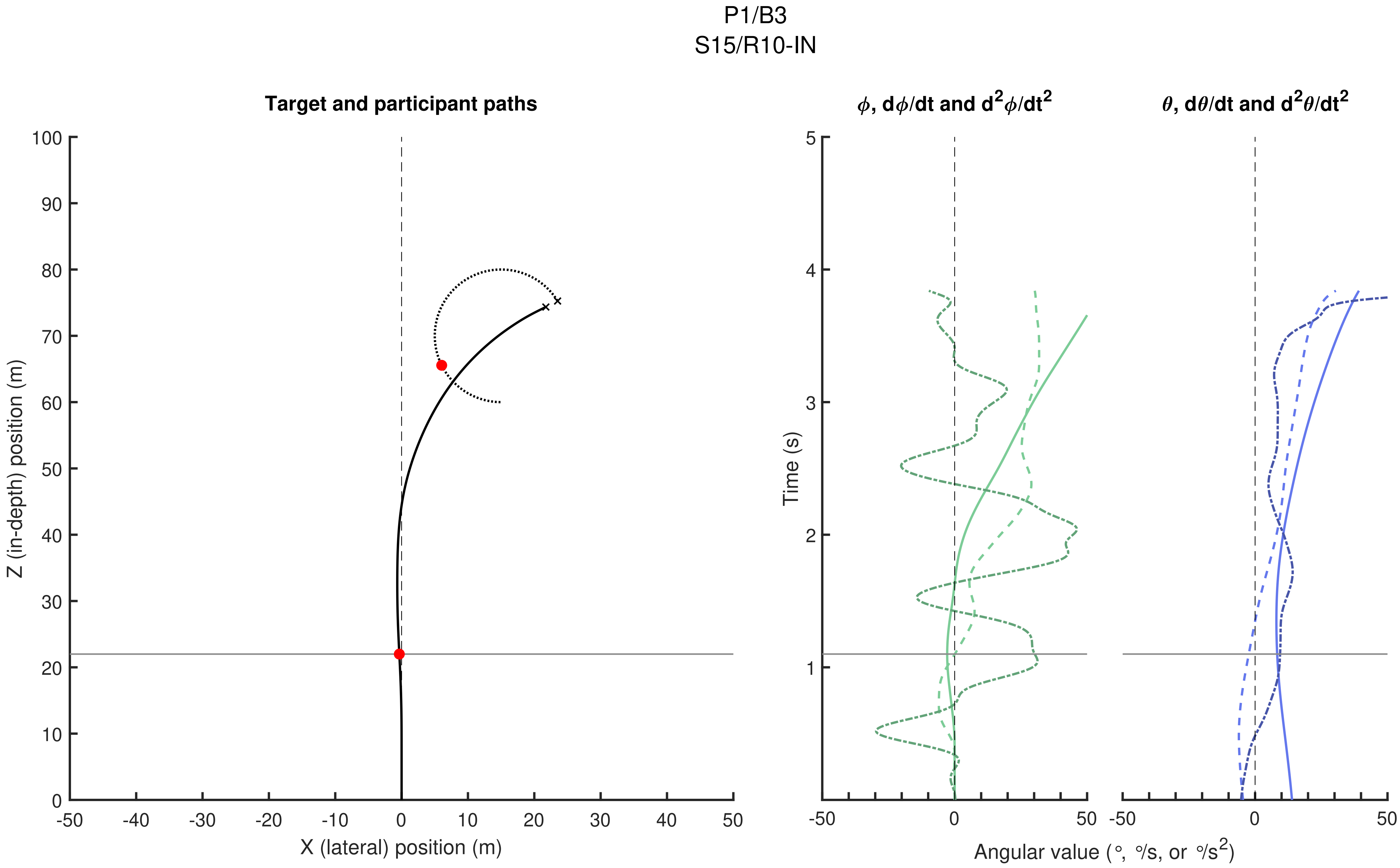

P1/B3
S15/R10-IN
Target and participant paths
Z (in-depth) position (m)
X (lateral) position (m)
φ, dφ/dt and d²φ/dt²
θ, dθ/dt and d²θ/dt²
Time (s)
Angular value (°, °/s, or °/s²)

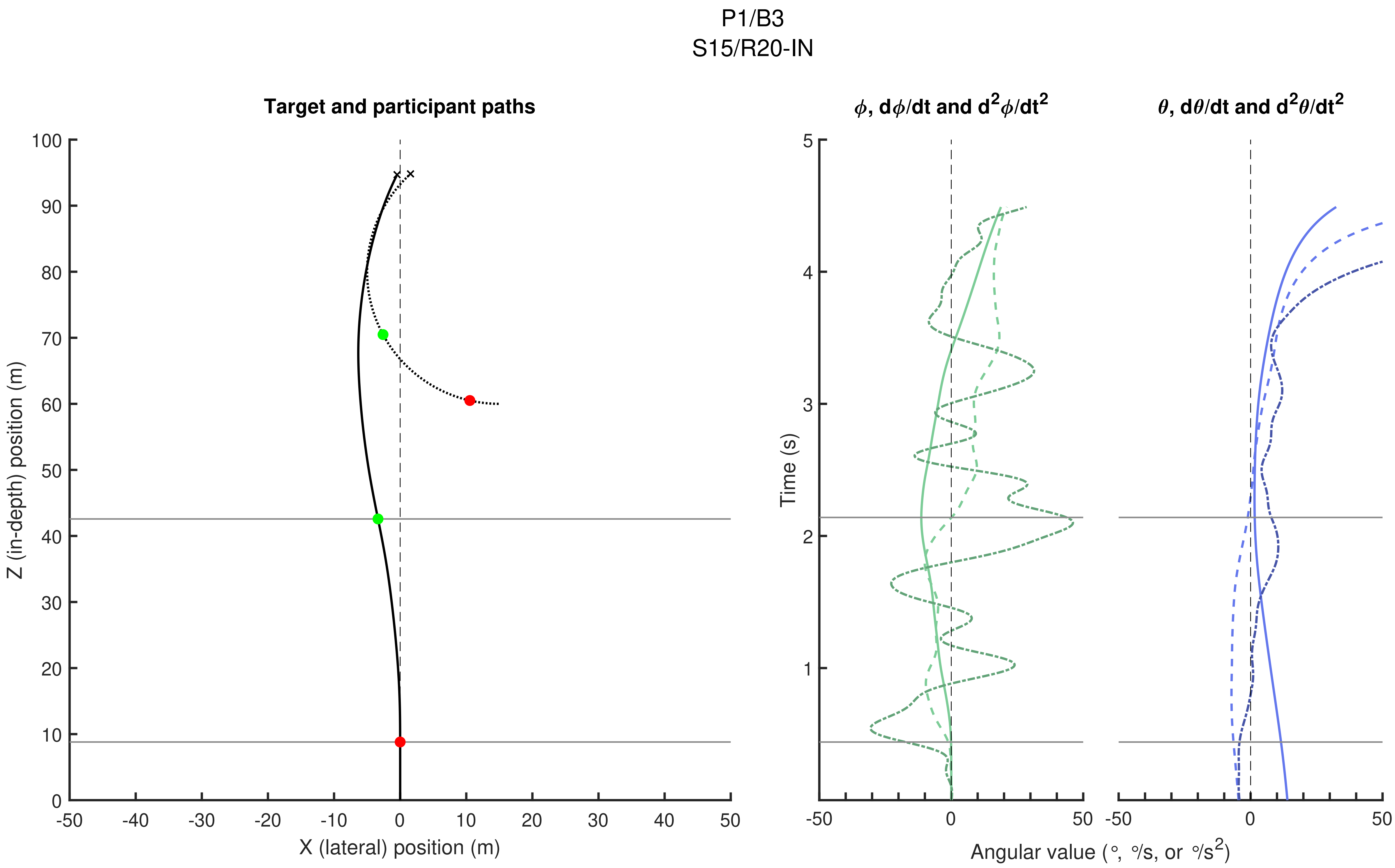

P1/B3
S15/R20-IN
Target and participant paths
Z (in-depth) position (m)
X (lateral) position (m)
ϕ, dϕ/dt and d²ϕ/dt²
θ, dθ/dt and d²θ/dt²
Time (s)
Angular value (°, °/s, or °/s²)
100
90
80
70
60
50
40
30
20
10
0
-50
-40
-30
-20
-10
0
10
20
30
40
50
5
4
3
2
1
-50
0
50
-50
0
50

P1/B3
S15/R30-IN

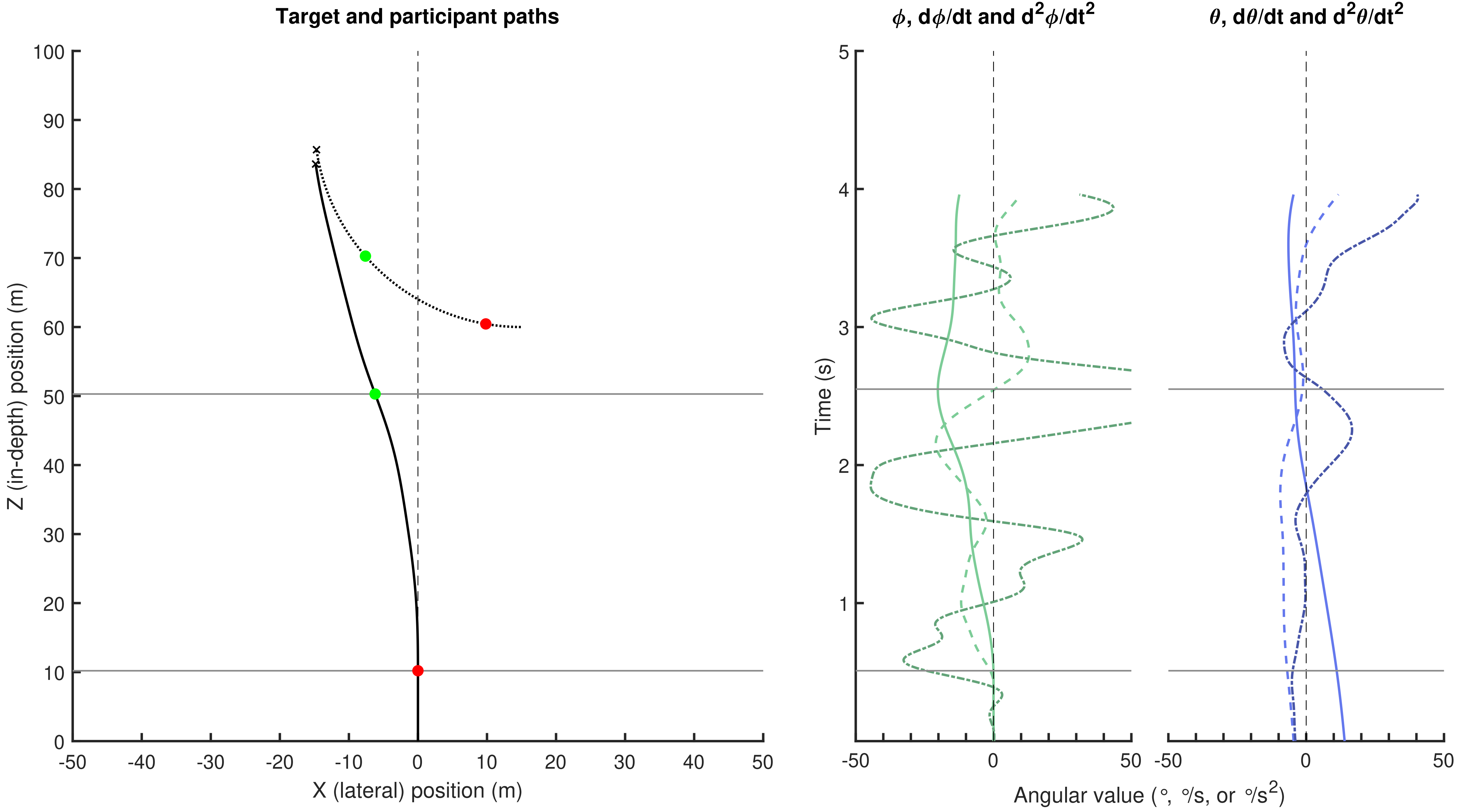

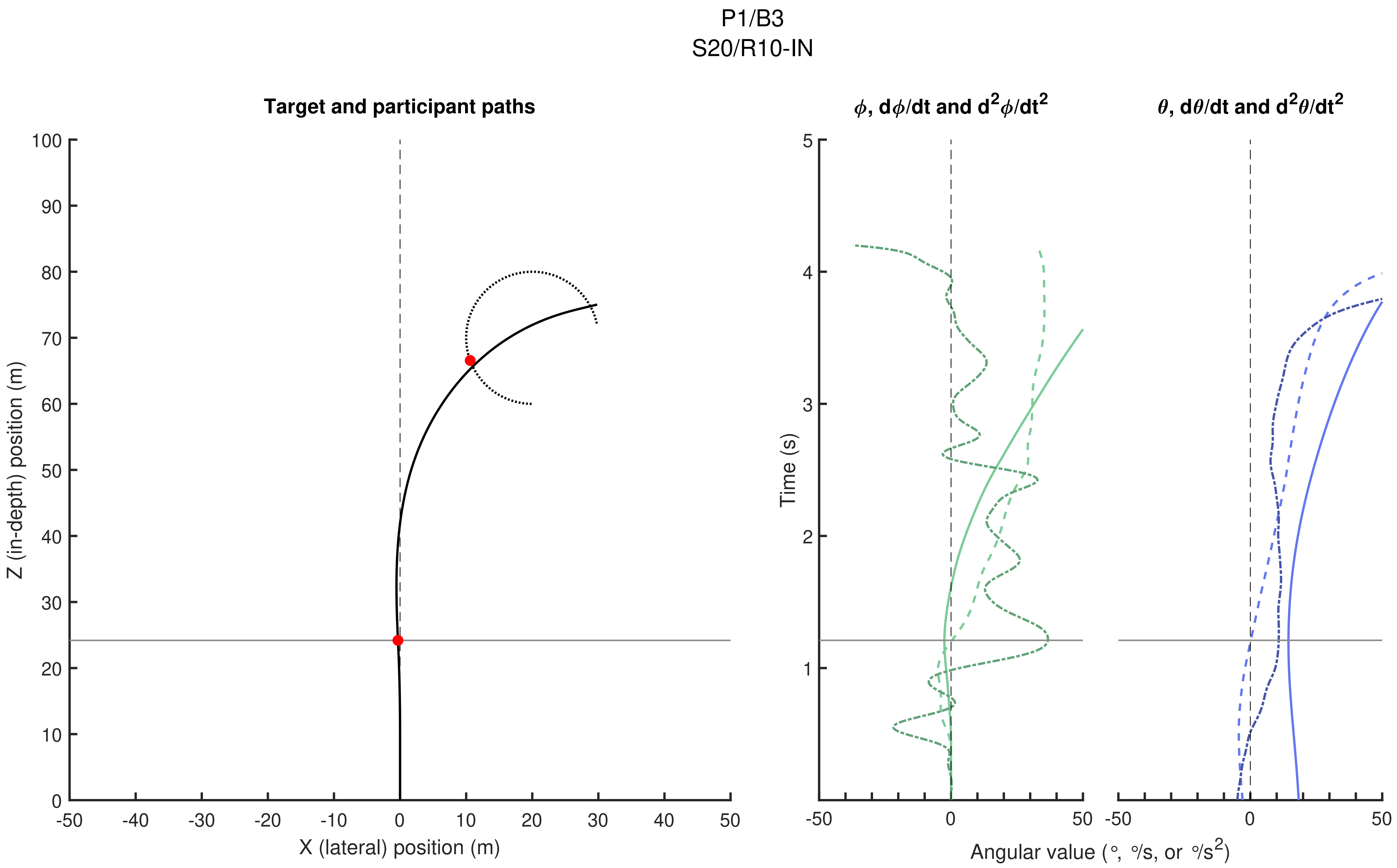

P1/B3
S20/R10-IN
Target and participant paths
Z (in-depth) position (m)
X (lateral) position (m)
ϕ, dϕ/dt and d$^2\phi$/dt^2
θ, dθ/dt and d$^2\theta$/dt^2
Time (s)
Angular value (°, °/s, or °/s^2)

P1/B3
S20/R20-IN

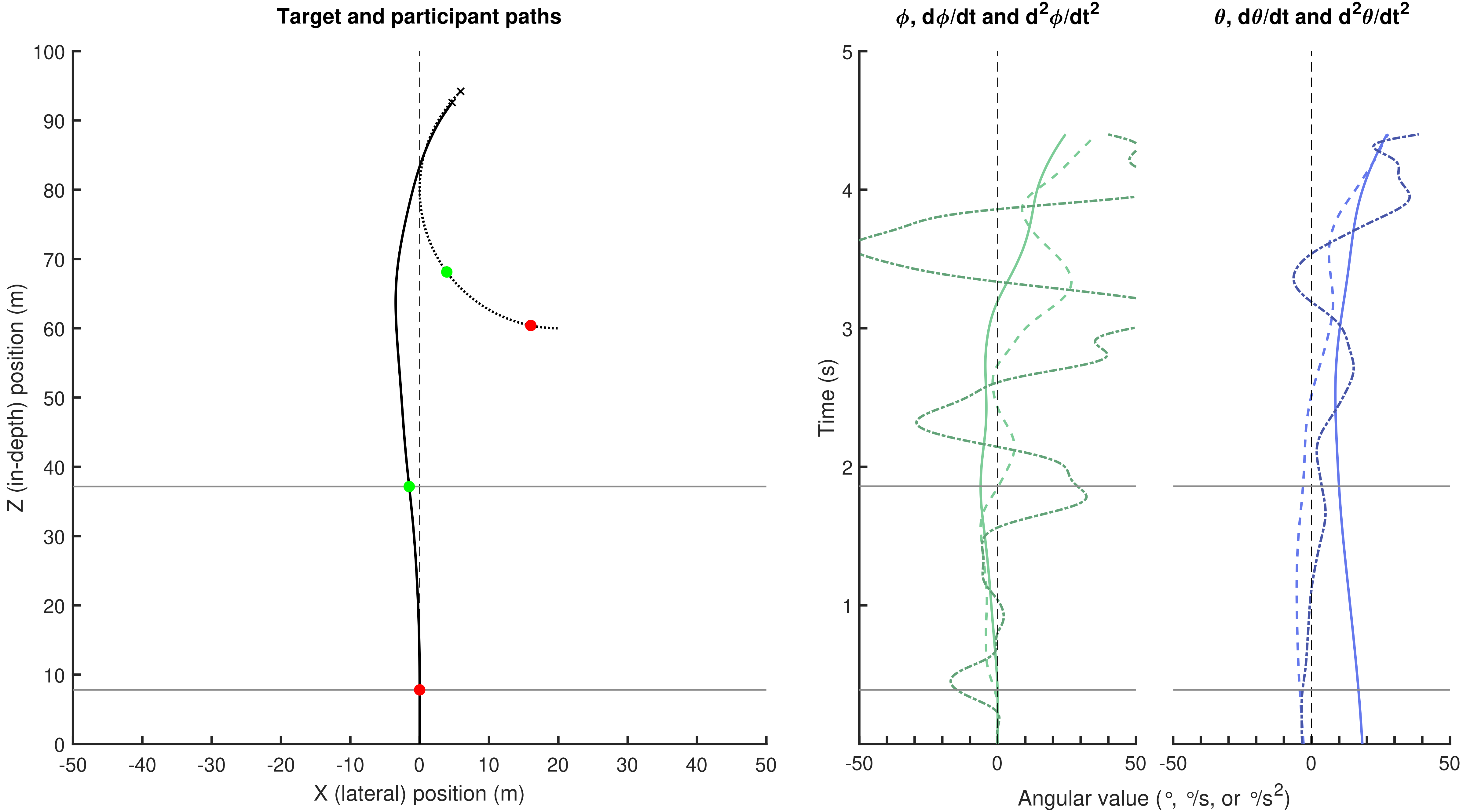

P1/B3
S20/R30-IN

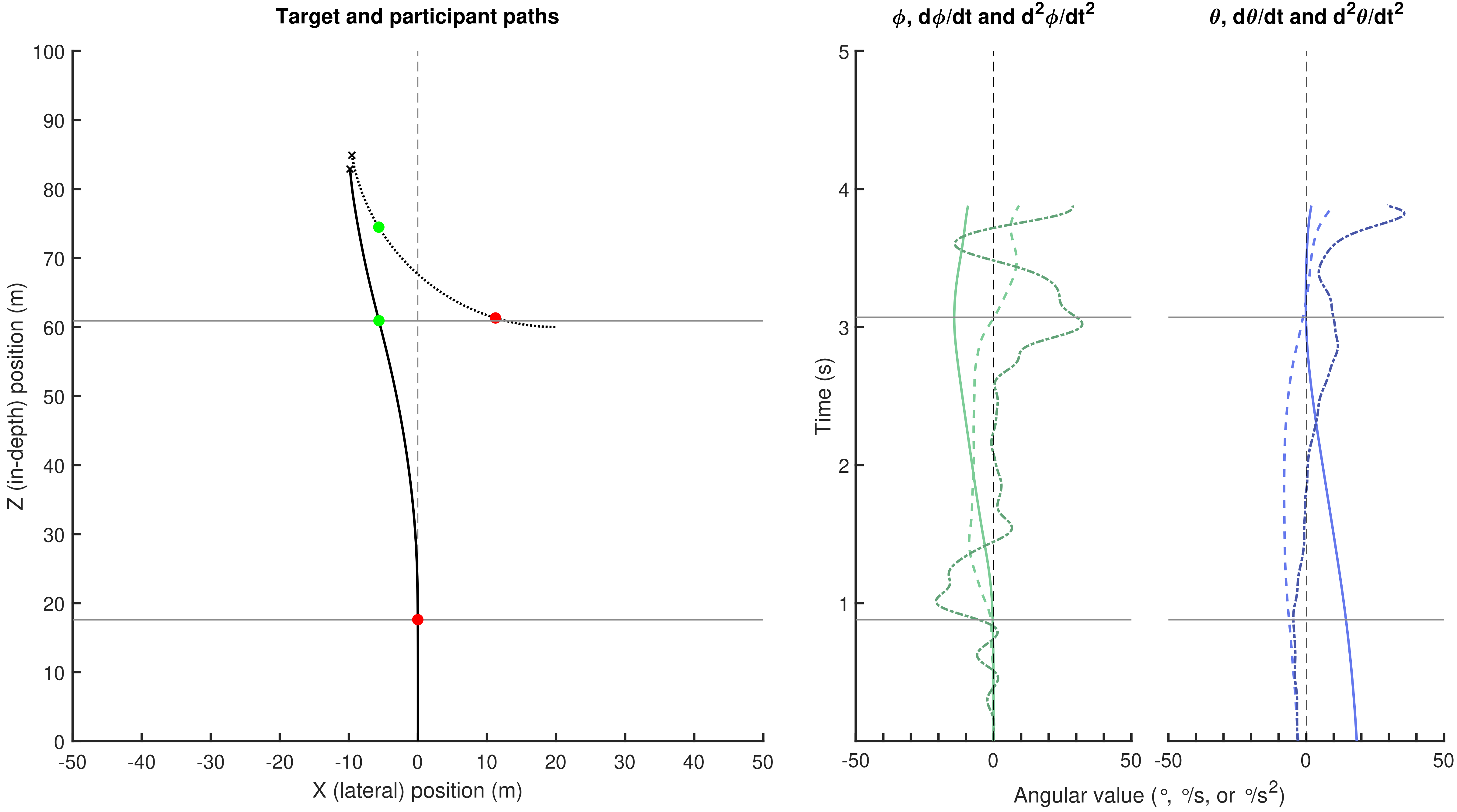

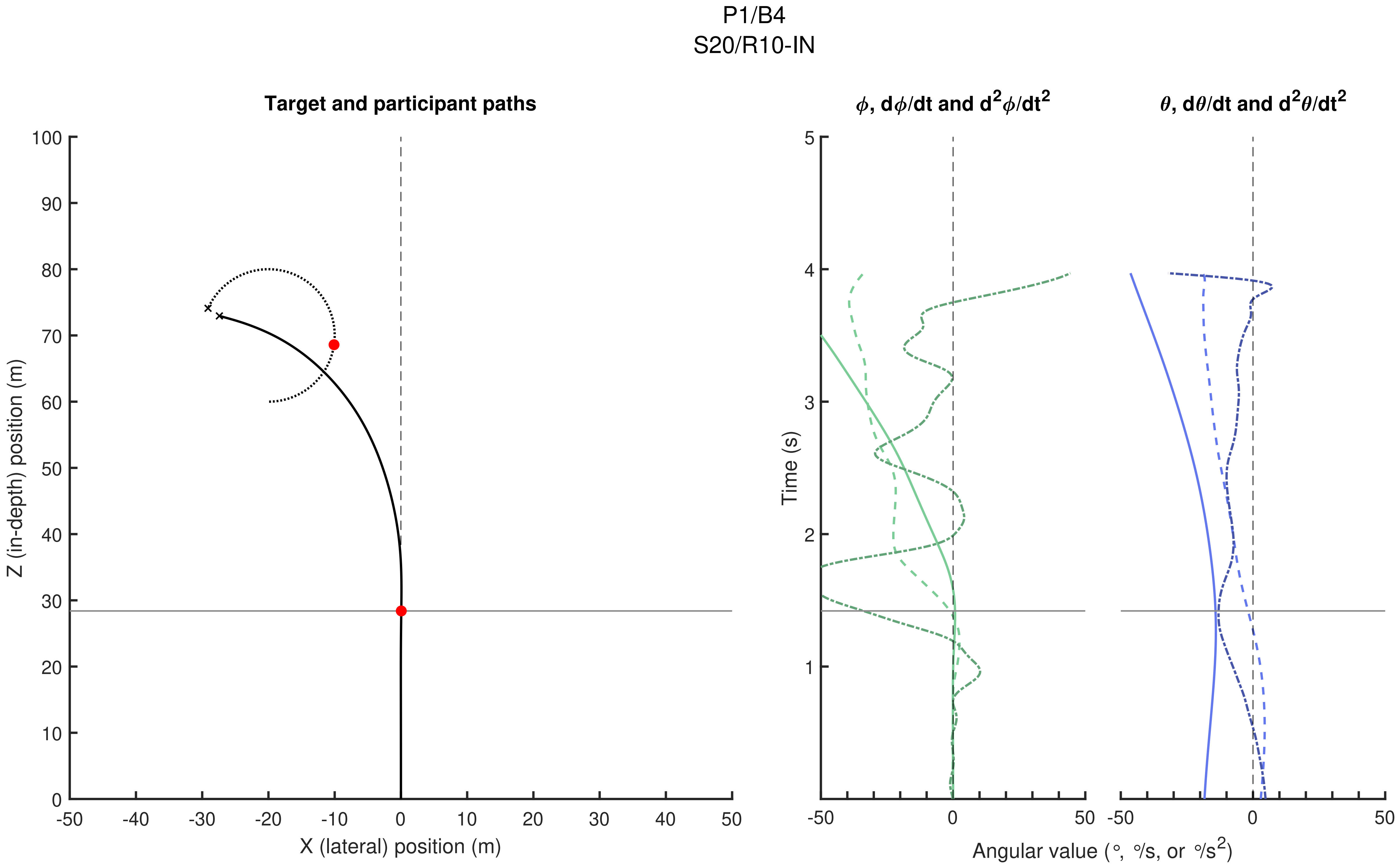

P1/B4
S20/R10-IN
Target and participant paths
Z (in-depth) position (m)
X (lateral) position (m)
ϕ, dϕ/dt and d$^2\phi$/dt^2
θ, dθ/dt and d$^2\theta$/dt^2
Time (s)
Angular value (°, °/s, or °/s^2)

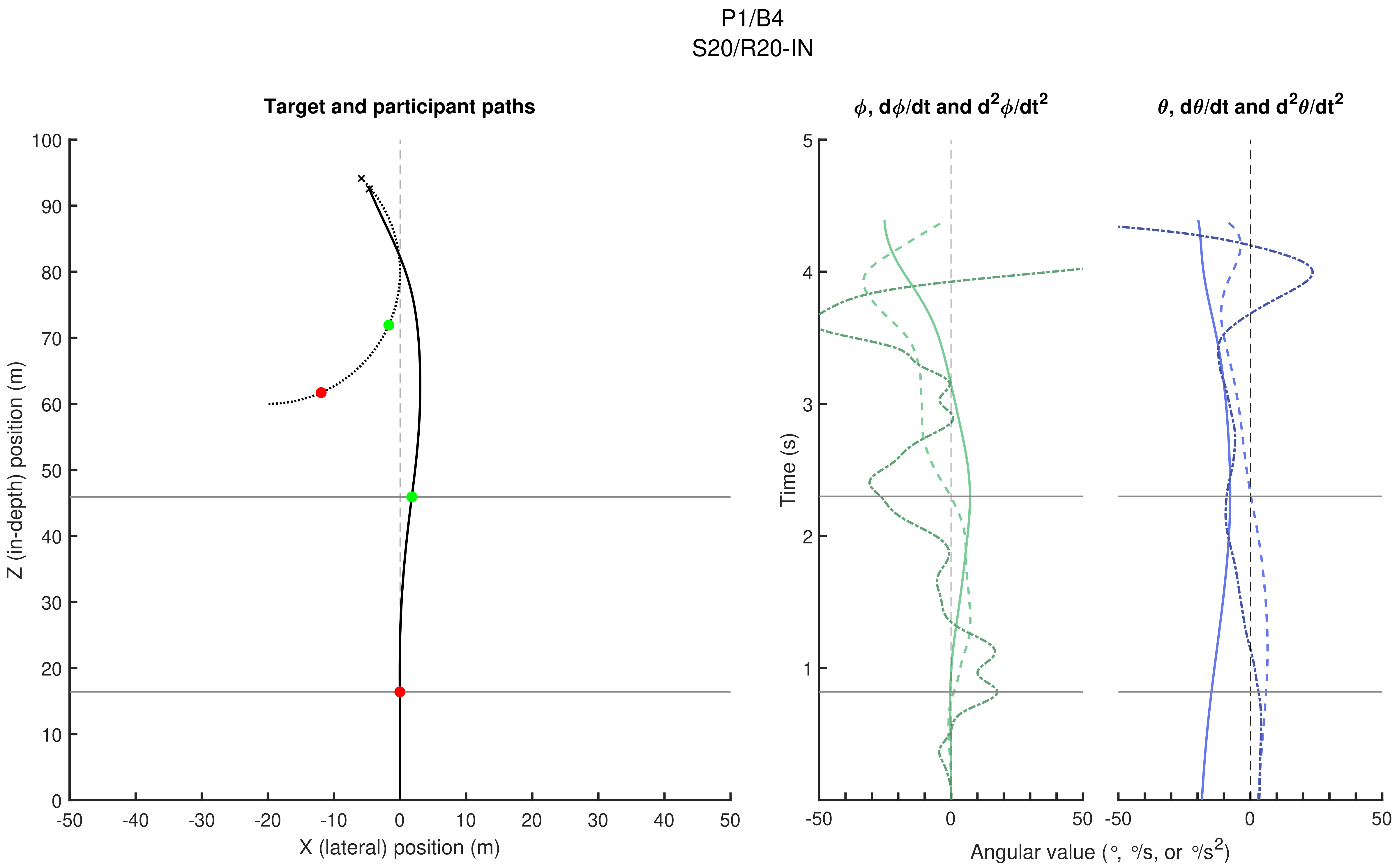
P1/B4
S20/R20-IN
Target and participant paths
ϕ, dϕ/dt and d$^2\phi$/dt^2
θ, dθ/dt and d$^2\theta$/dt^2
Z (in-depth) position (m)
X (lateral) position (m)
Time (s)
Angular value (°, °/s, or °/s^2)
100
90
80
70
60
50
40
30
20
10
0
-50
-40
-30
-20
-10
0
10
20
30
40
50
5
4
3
2
1
-50
0
50
-50
0
50

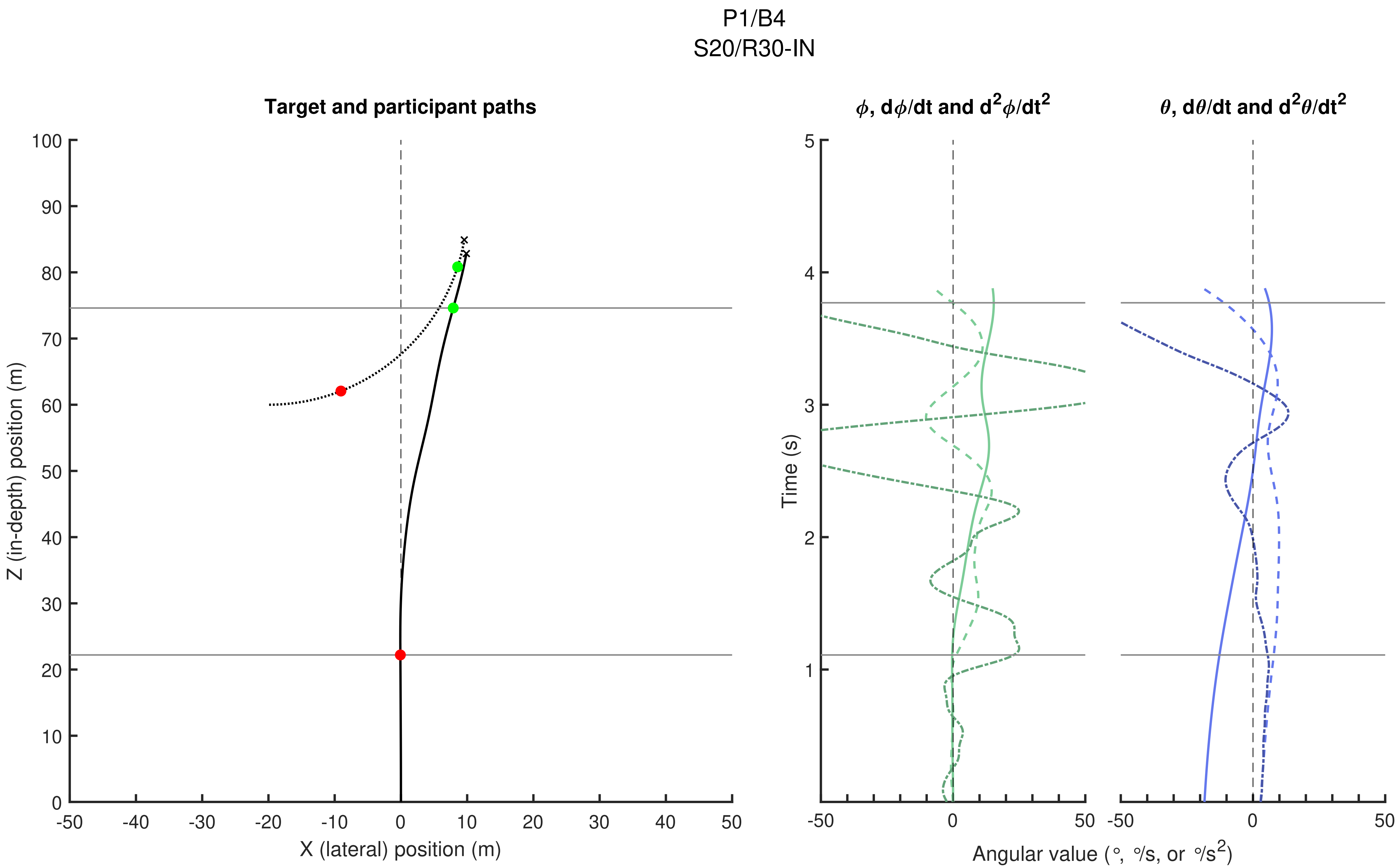

P1/B4
S20/R30-IN
Target and participant paths
Z (in-depth) position (m)
X (lateral) position (m)
ϕ, dϕ/dt and d$^2\phi$/dt^2
θ, dθ/dt and d$^2\theta$/dt^2
Time (s)
Angular value (°, °/s, or °/s^2)

P1/B4
S15/R10-IN

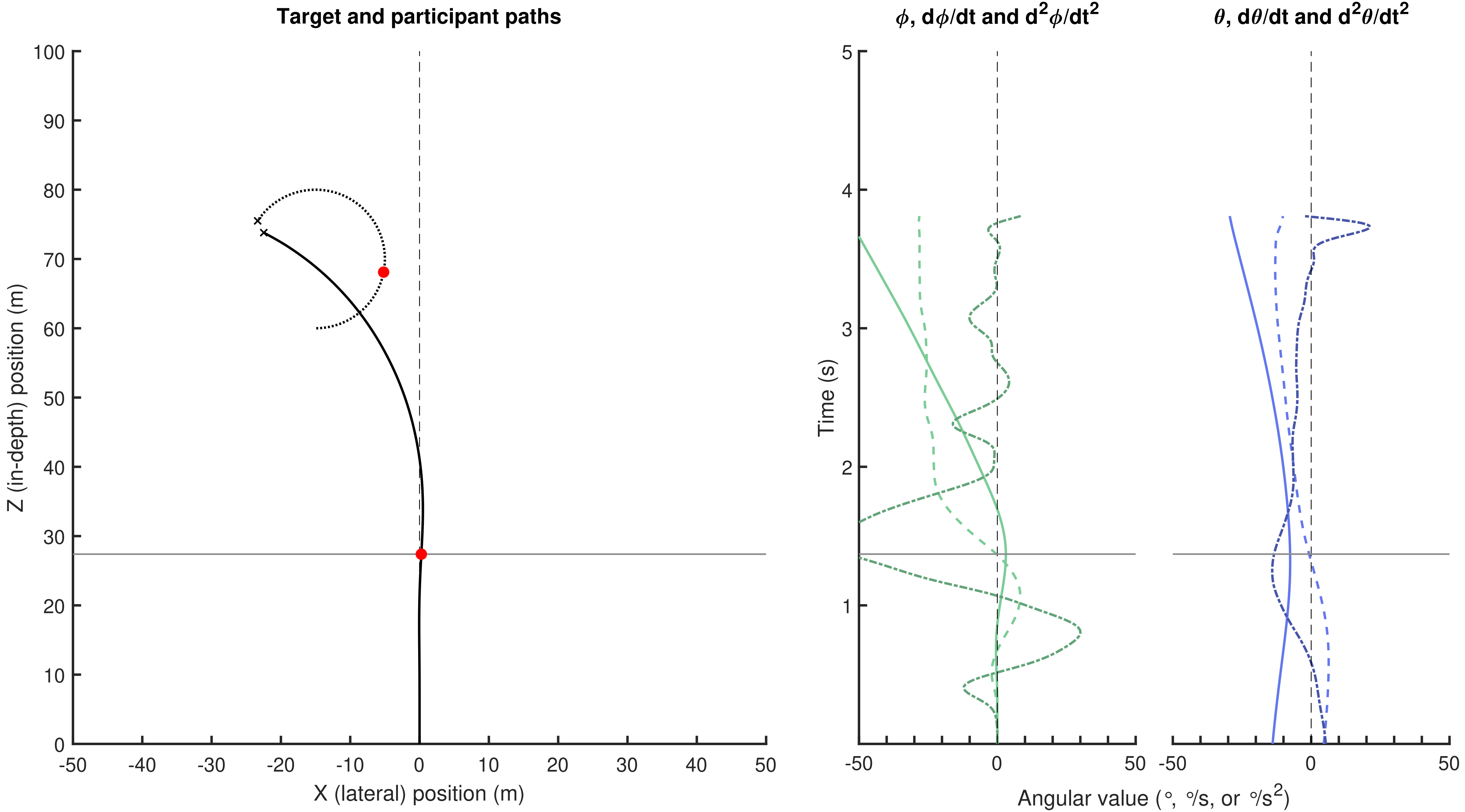

P1/B4
S15/R20-IN

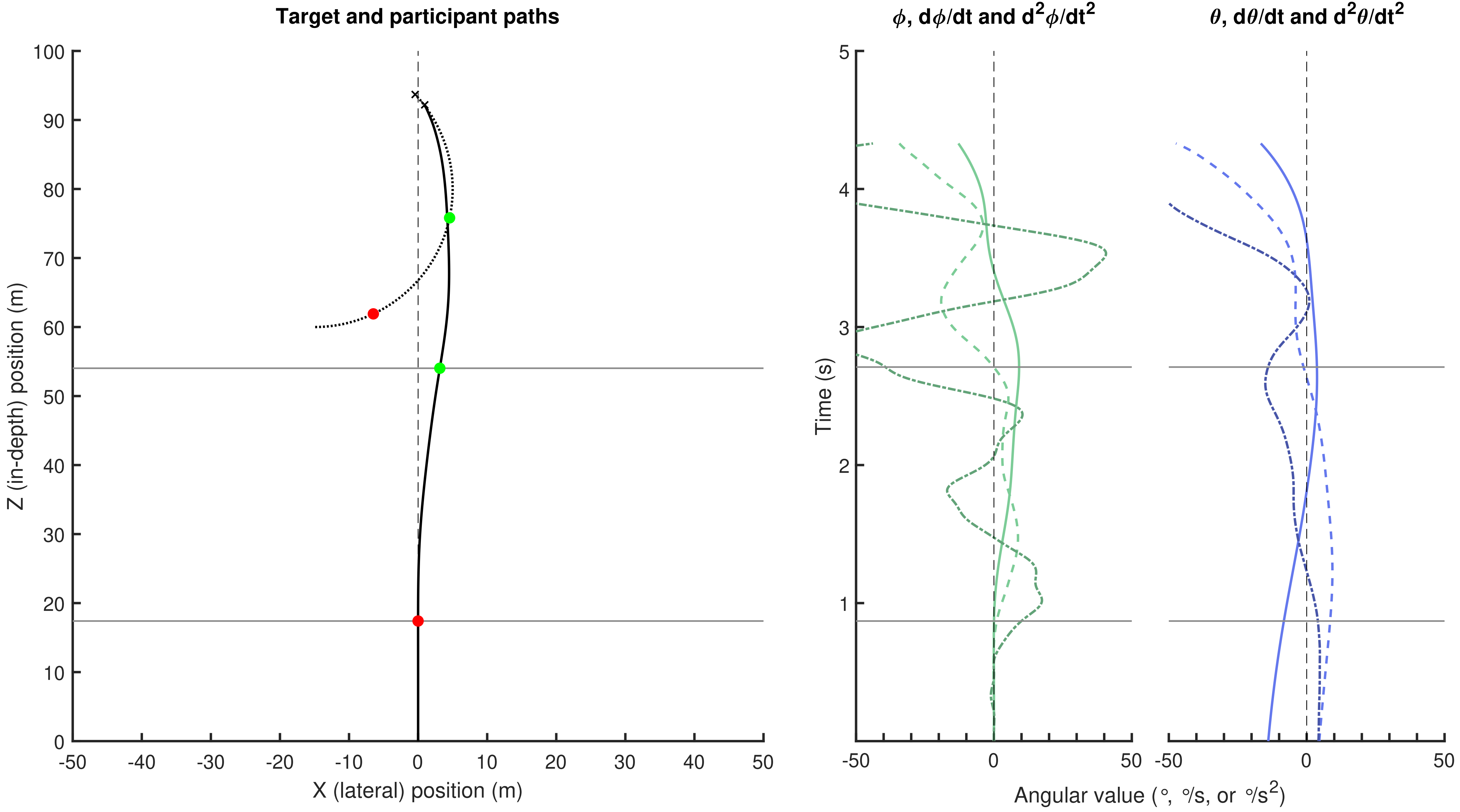

P1/B4
S15/R30-IN

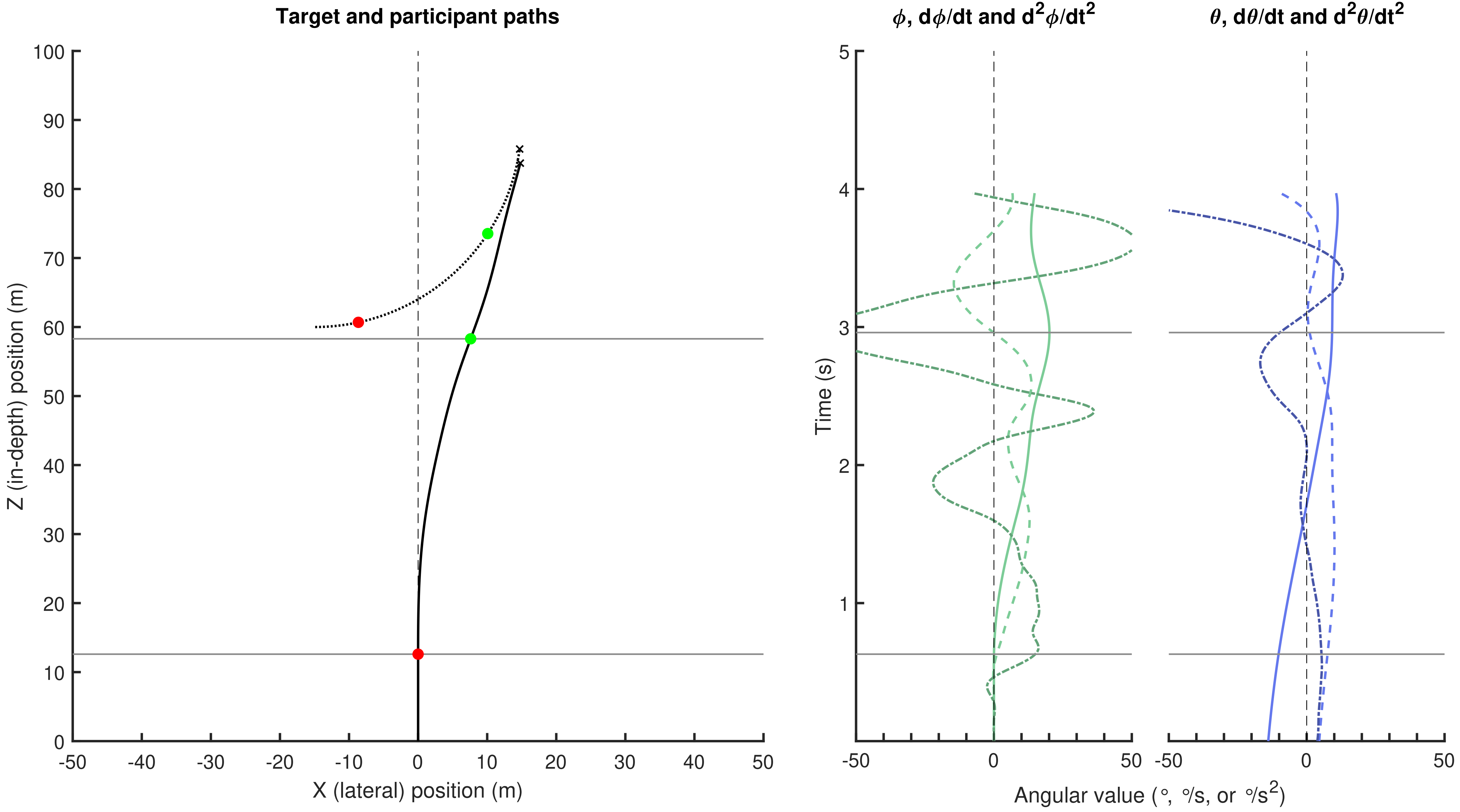

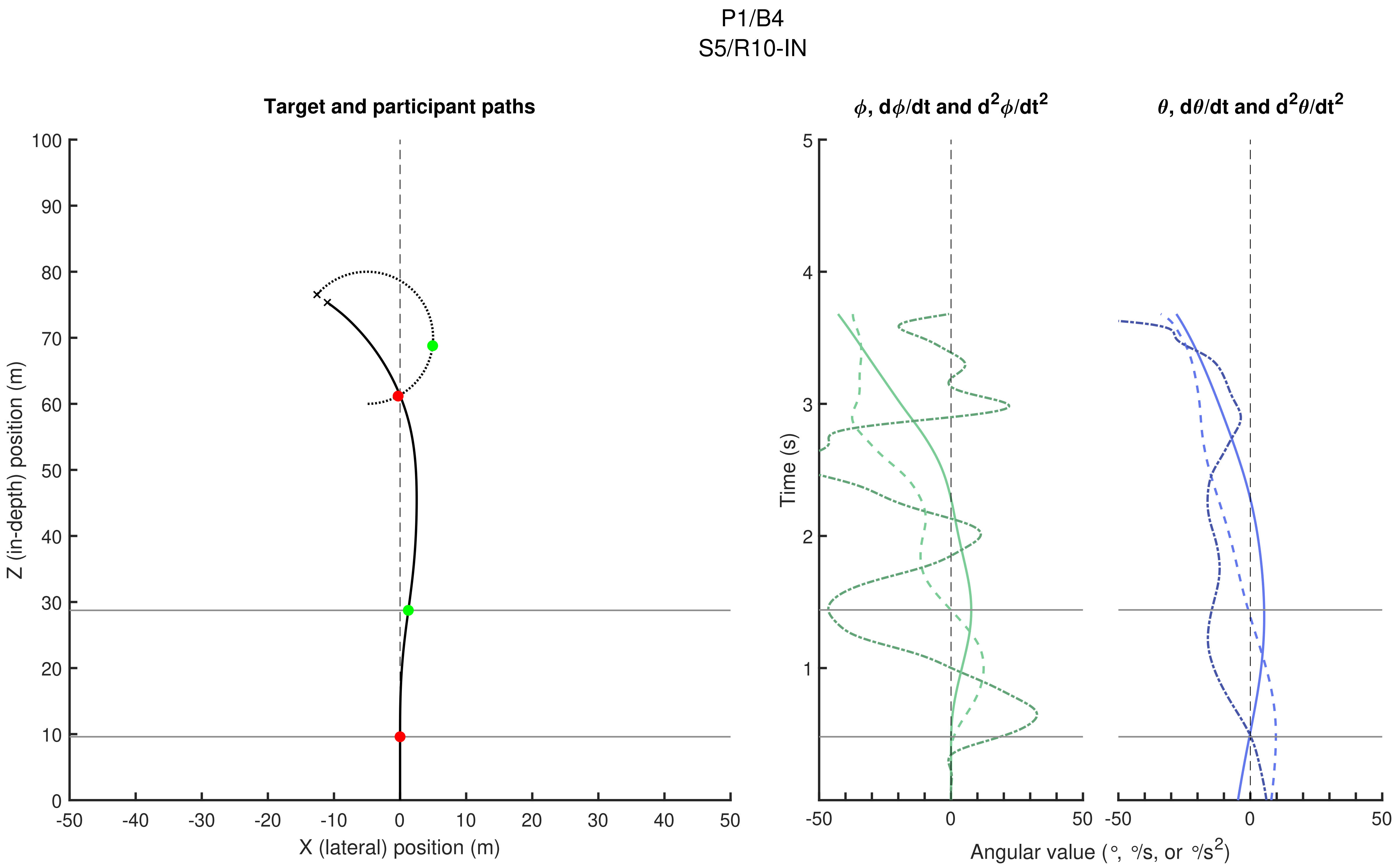
P1/B4
S5/R10-IN
Target and participant paths
ϕ, dϕ/dt and d$^2\phi$/dt^2
θ, dθ/dt and d$^2\theta$/dt^2
Z (in-depth) position (m)
X (lateral) position (m)
Time (s)
Angular value (°, °/s, or °/s^2)
100
90
80
70
60
50
40
30
20
10
0
-50
-40
-30
-20
-10
0
10
20
30
40
50
5
4
3
2
1

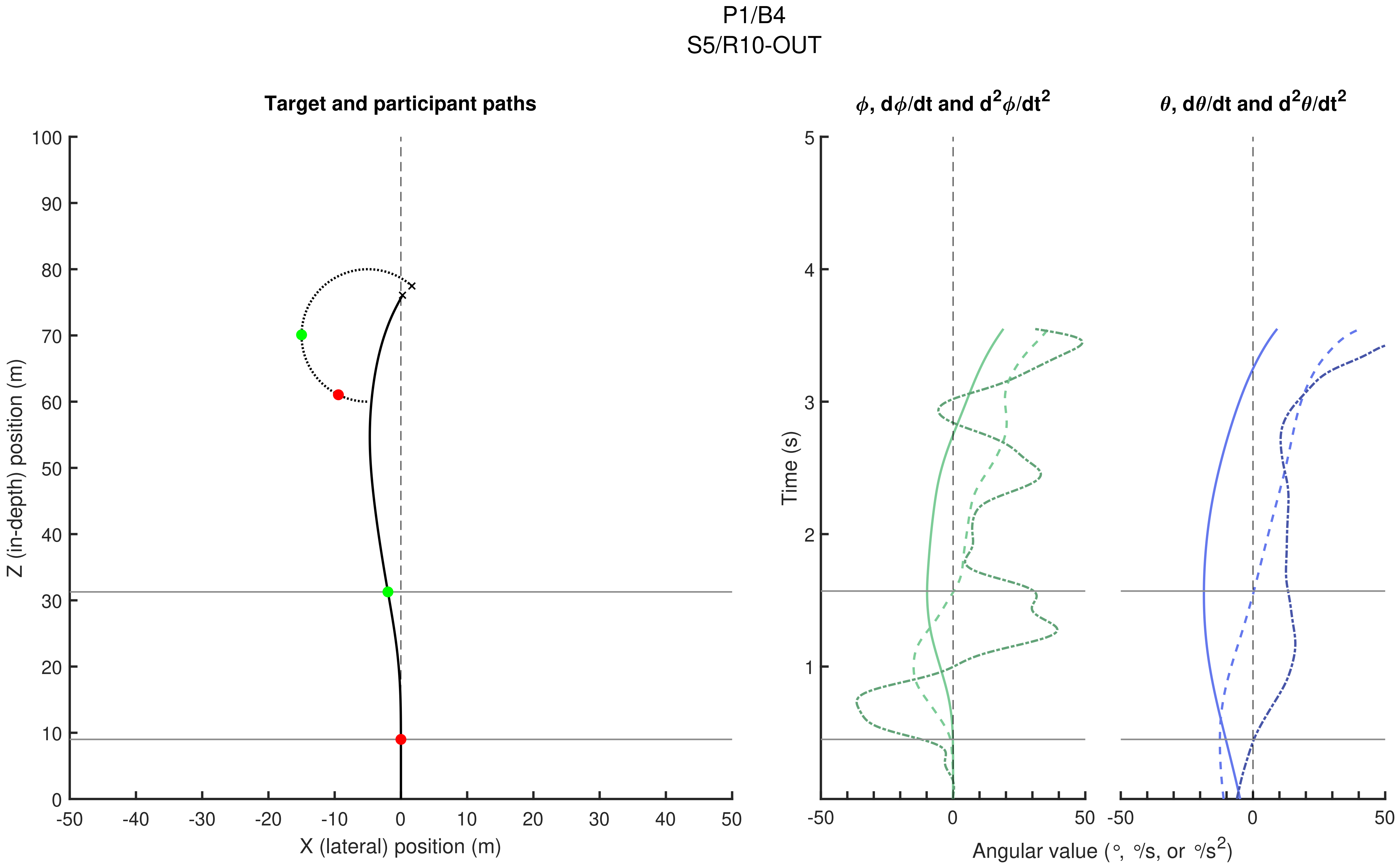
P1/B4
S5/R10-OUT
Target and participant paths
Z (in-depth) position (m)
X (lateral) position (m)
φ, dφ/dt and d²φ/dt²
θ, dθ/dt and d²θ/dt²
Time (s)
Angular value (°, °/s, or °/s²)

P1/B4
S5/R20-IN

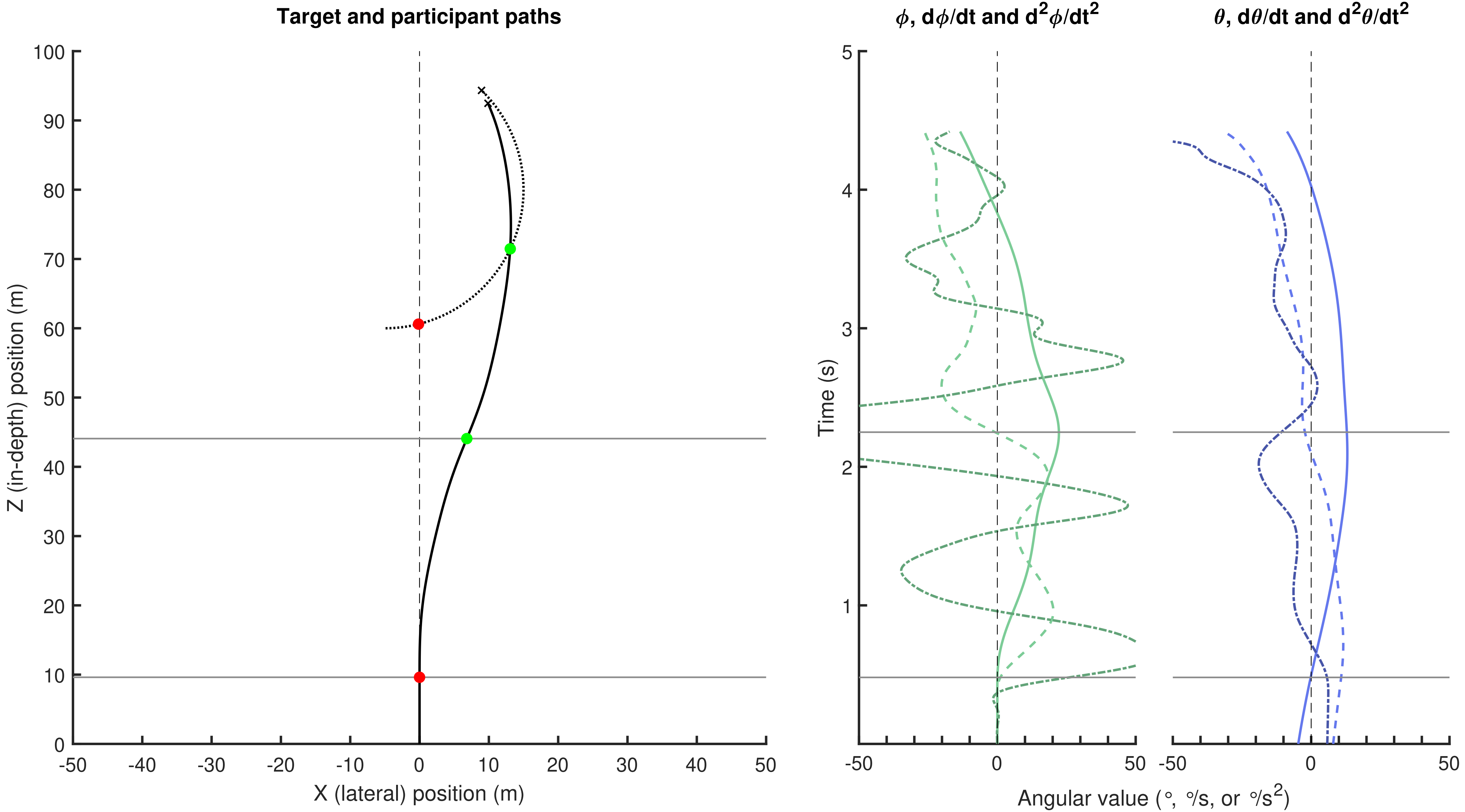

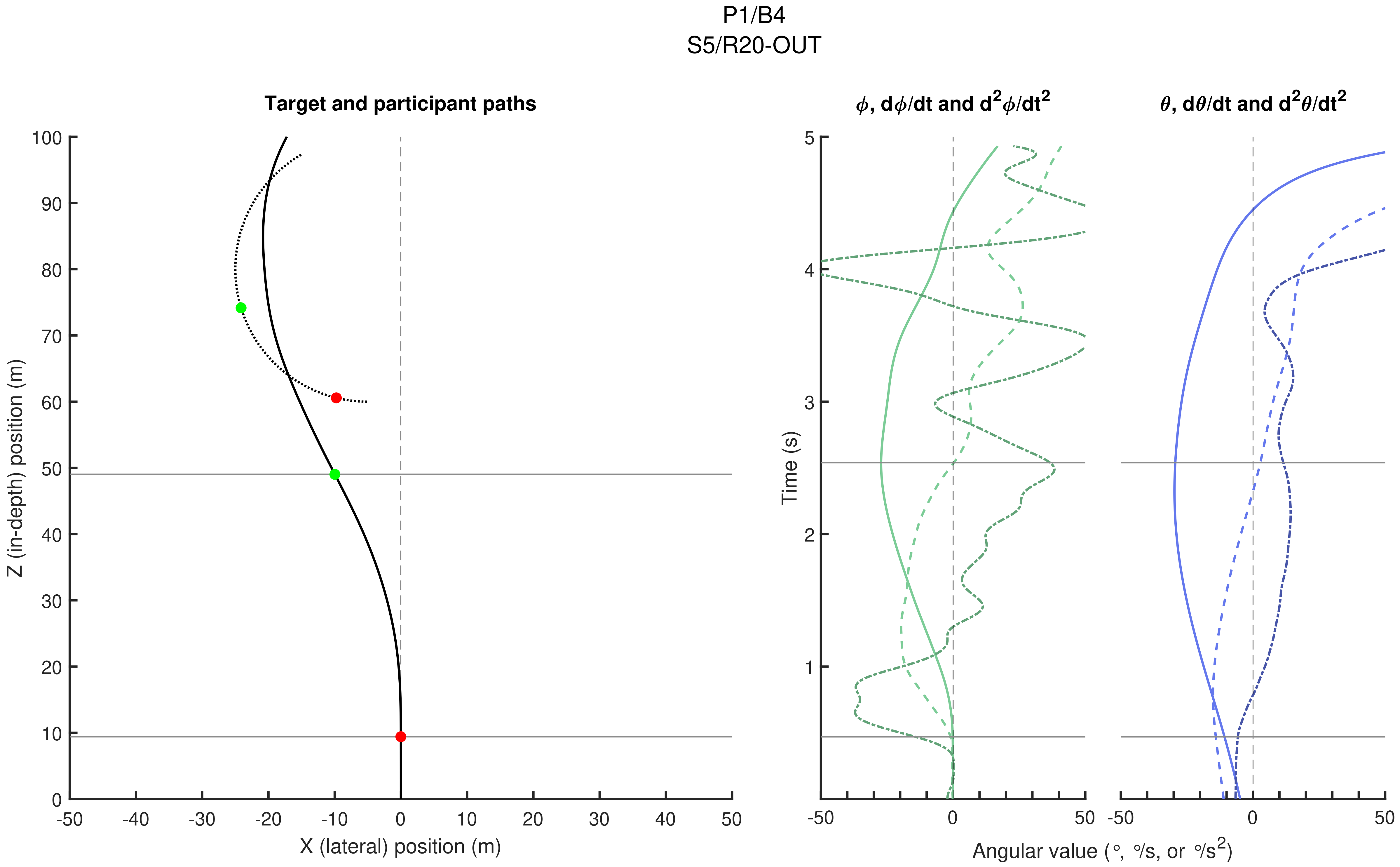

P1/B4
S5/R20-OUT
Target and participant paths
Z (in-depth) position (m)
X (lateral) position (m)
ϕ, dϕ/dt and d$^2\phi$/dt^2
θ, dθ/dt and d$^2\theta$/dt^2
Time (s)
Angular value (°, °/s, or °/s^2)

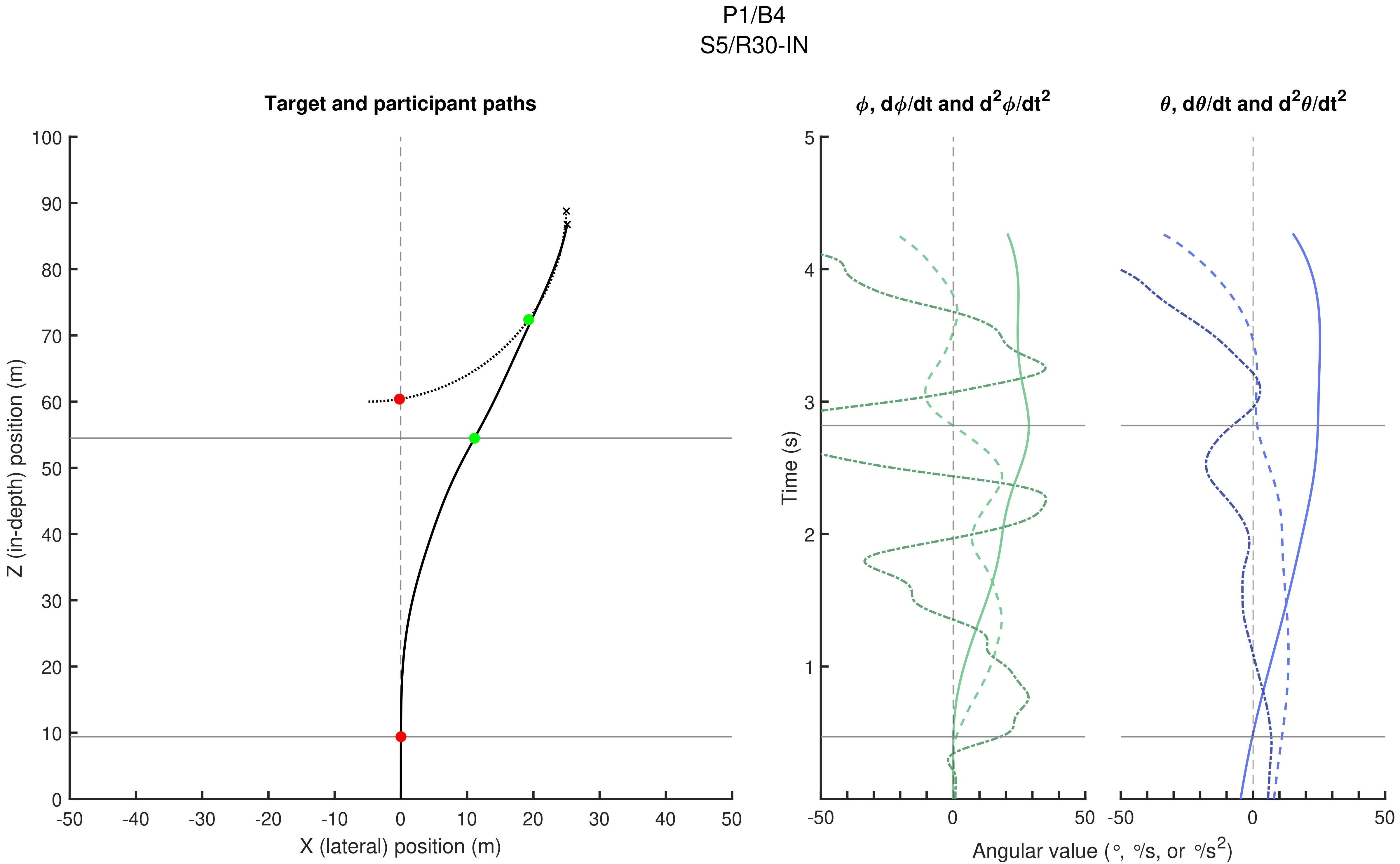
P1/B4
S5/R30-IN
Target and participant paths
Z (in-depth) position (m)
X (lateral) position (m)
ϕ, dϕ/dt and d²ϕ/dt²
θ, dθ/dt and d²θ/dt²
Time (s)
Angular value (°, °/s, or °/s²)

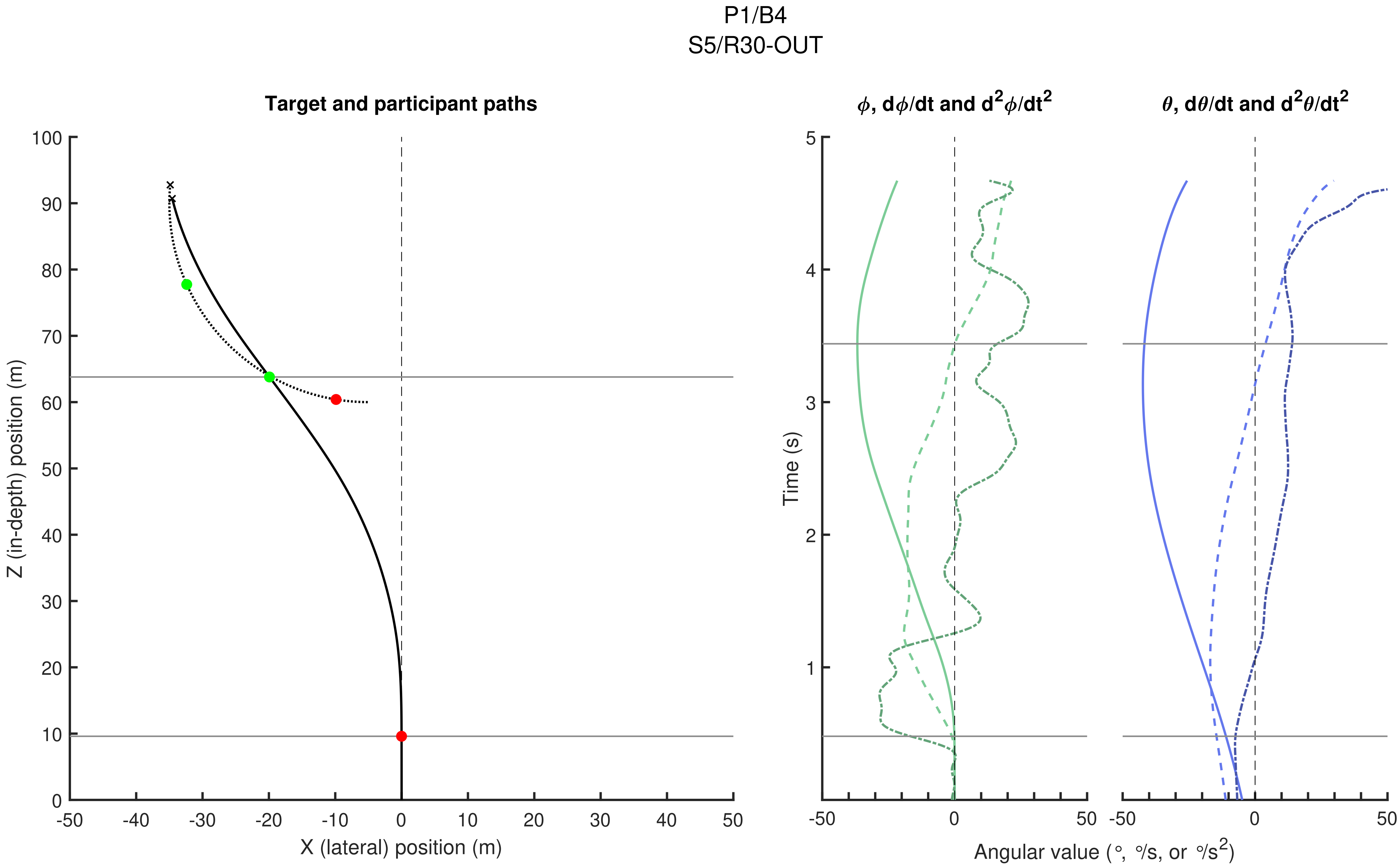
P1/B4
S5/R30-OUT
Target and participant paths
ϕ, dϕ/dt and d$^2\phi$/dt^2
θ, dθ/dt and d$^2\theta$/dt^2
Z (in-depth) position (m)
X (lateral) position (m)
Time (s)
Angular value (°, °/s, or °/s^2)
100
90
80
70
60
50
40
30
20
10
0
-50
-40
-30
-20
-10
0
10
20
30
40
50
5
4
3
2
1
-50
0
50
-50
0
50

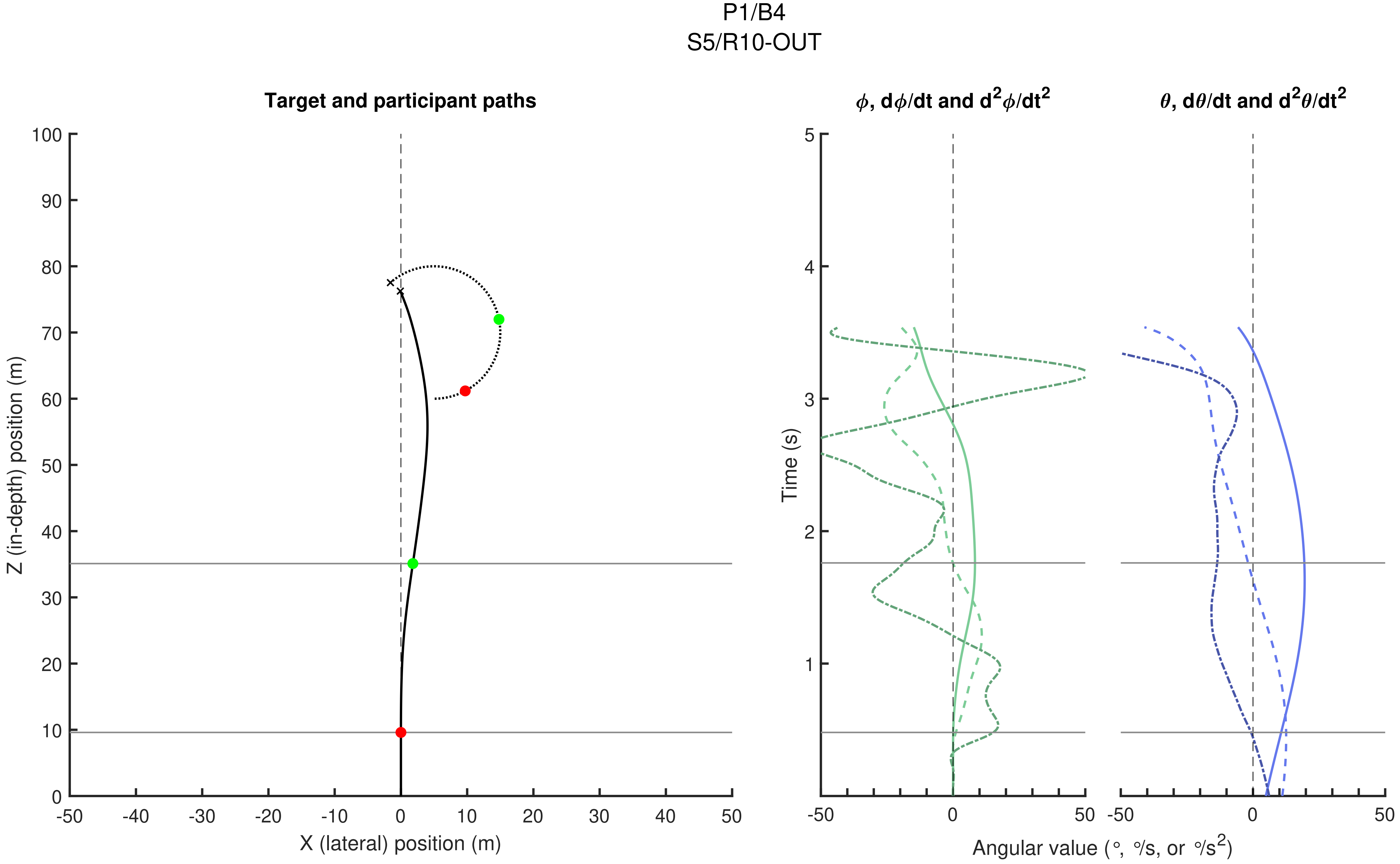
P1/B4
S5/R10-OUT
Target and participant paths
Z (in-depth) position (m)
X (lateral) position (m)
φ, dφ/dt and d²φ/dt²
θ, dθ/dt and d²θ/dt²
Time (s)
Angular value (°, °/s, or °/s²)

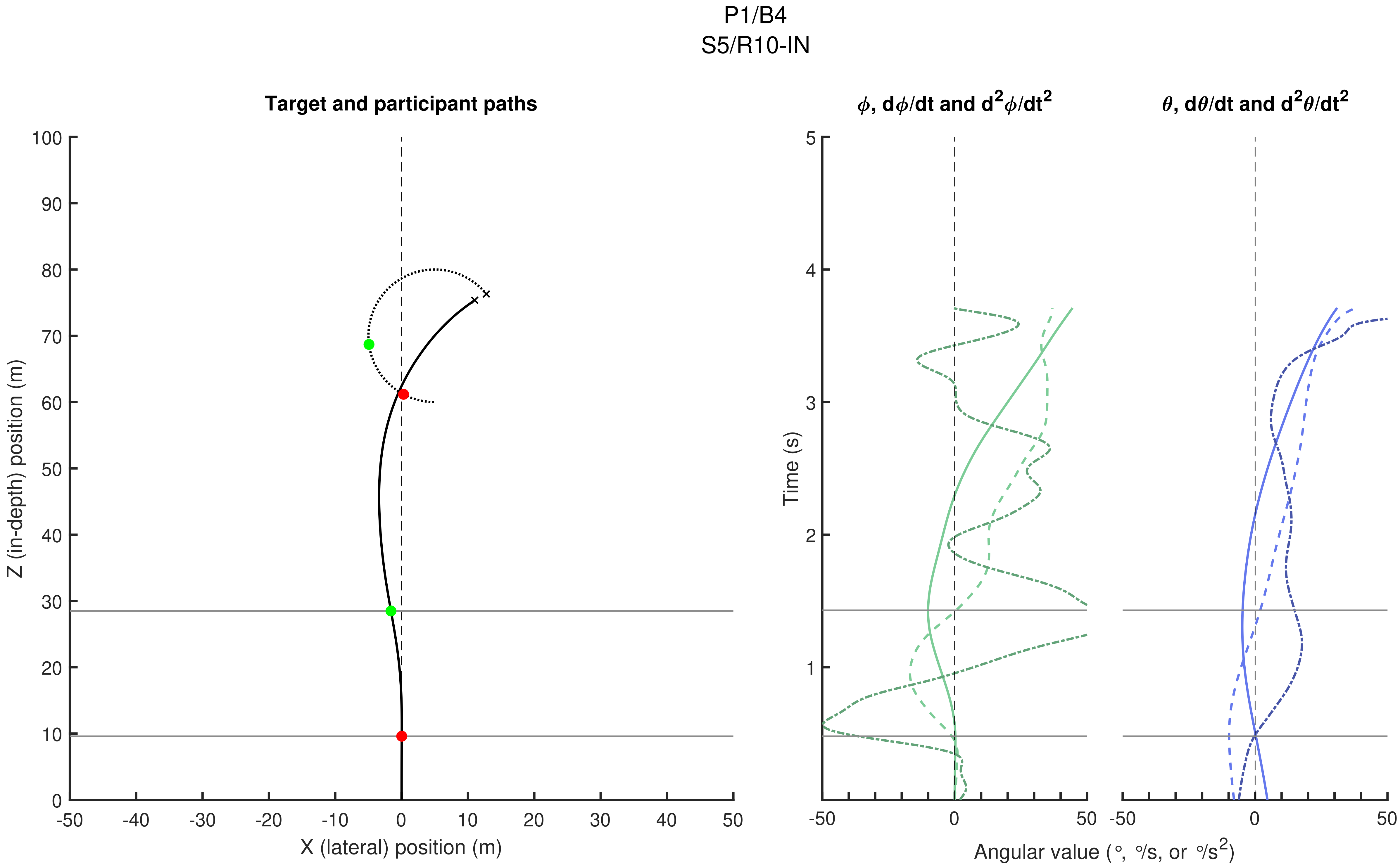

P1/B4
S5/R10-IN
Target and participant paths
Z (in-depth) position (m)
X (lateral) position (m)
φ, dφ/dt and d²φ/dt²
θ, dθ/dt and d²θ/dt²
Time (s)
Angular value (°, °/s, or °/s²)

P1/B4
S5/R20-OUT

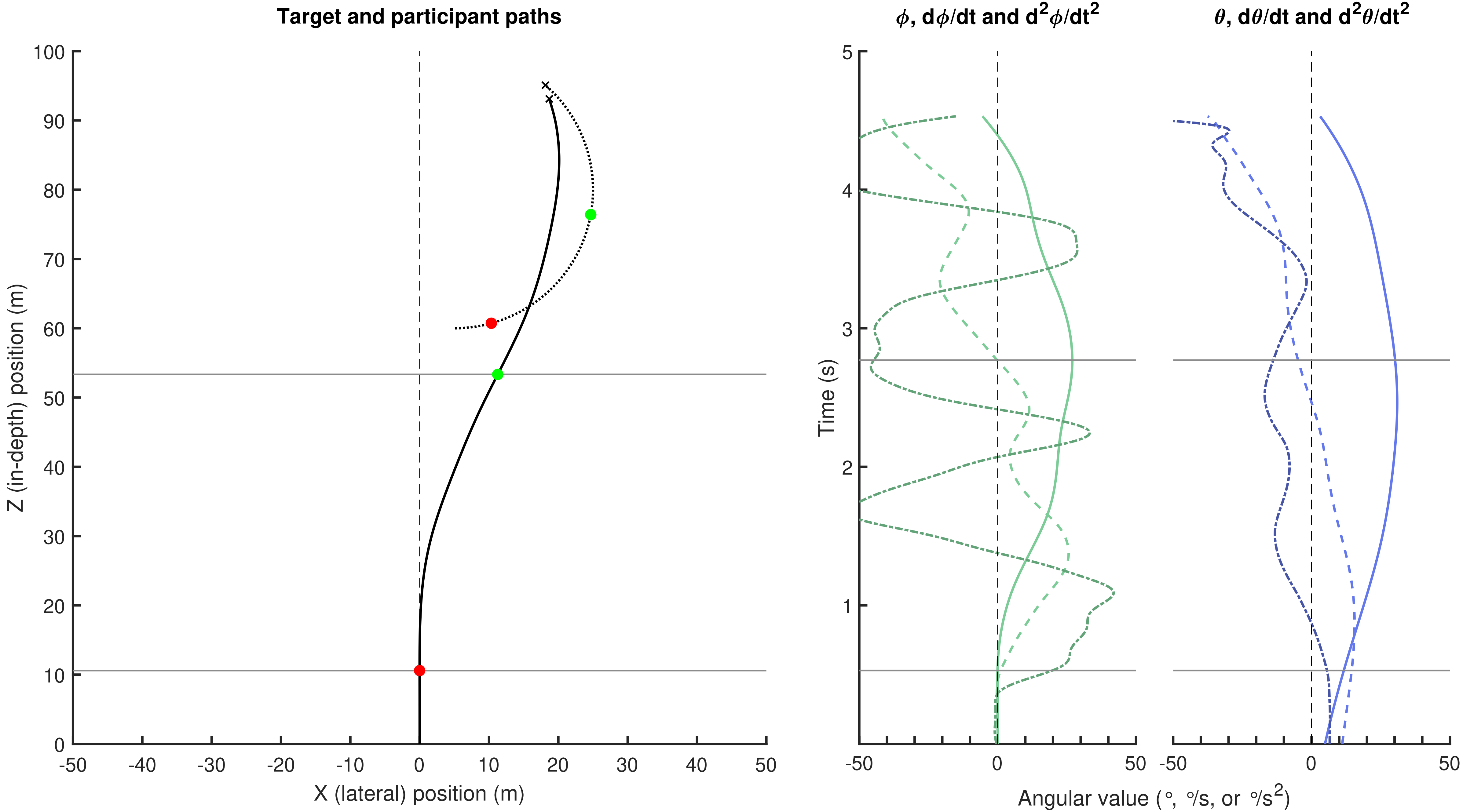

P1/B4
S5/R20-IN

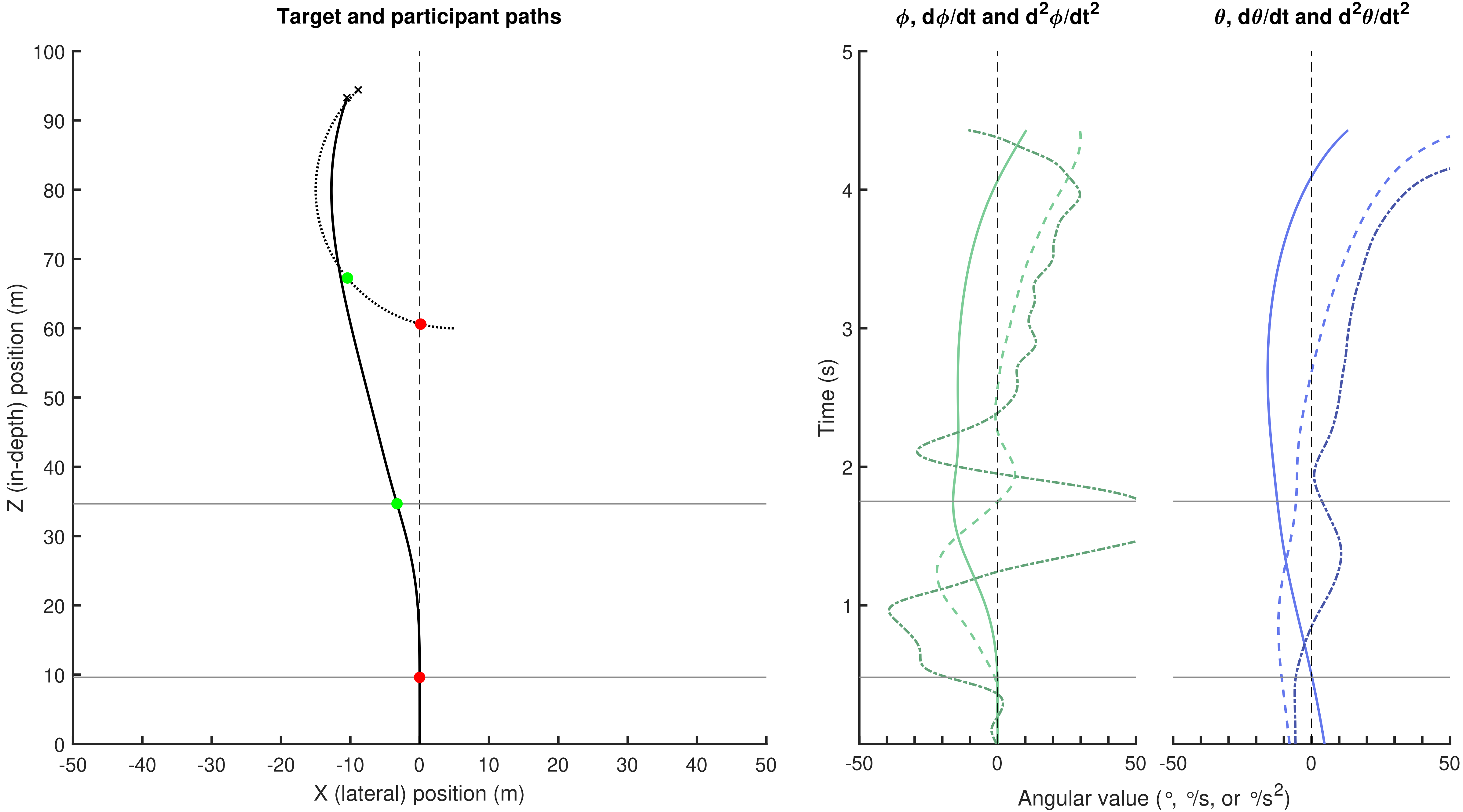

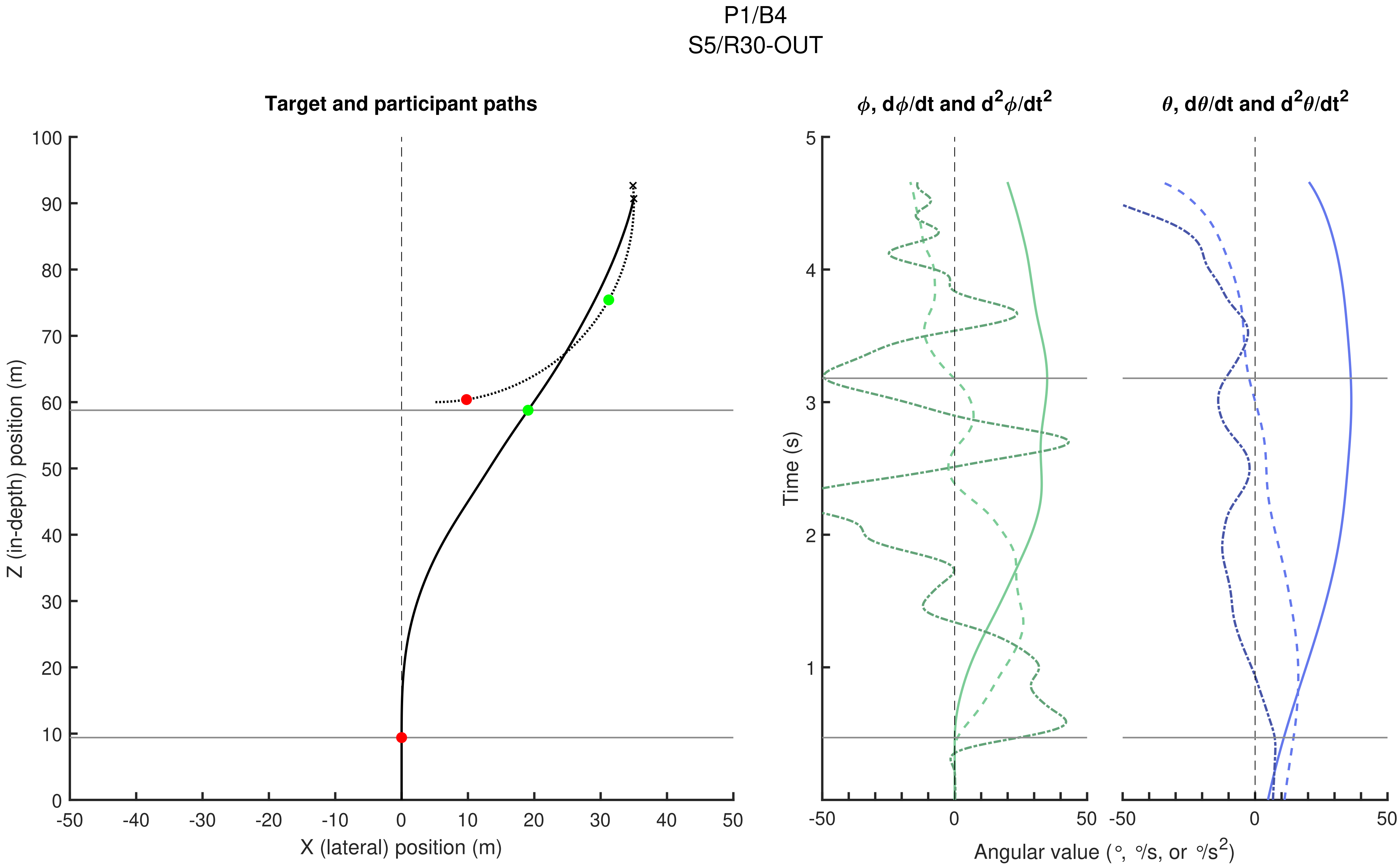

P1/B4
S5/R30-OUT
Target and participant paths
Z (in-depth) position (m)
X (lateral) position (m)
100
90
80
70
60
50
40
30
20
10
0
-50
-40
-30
-20
-10
0
10
20
30
40
50
ϕ, dϕ/dt and d$^2\phi$/dt^2
θ, dθ/dt and d$^2\theta$/dt^2
Time (s)
5
4
3
2
1
-50
0
50
-50
0
50
Angular value (°, °/s, or °/s^2)

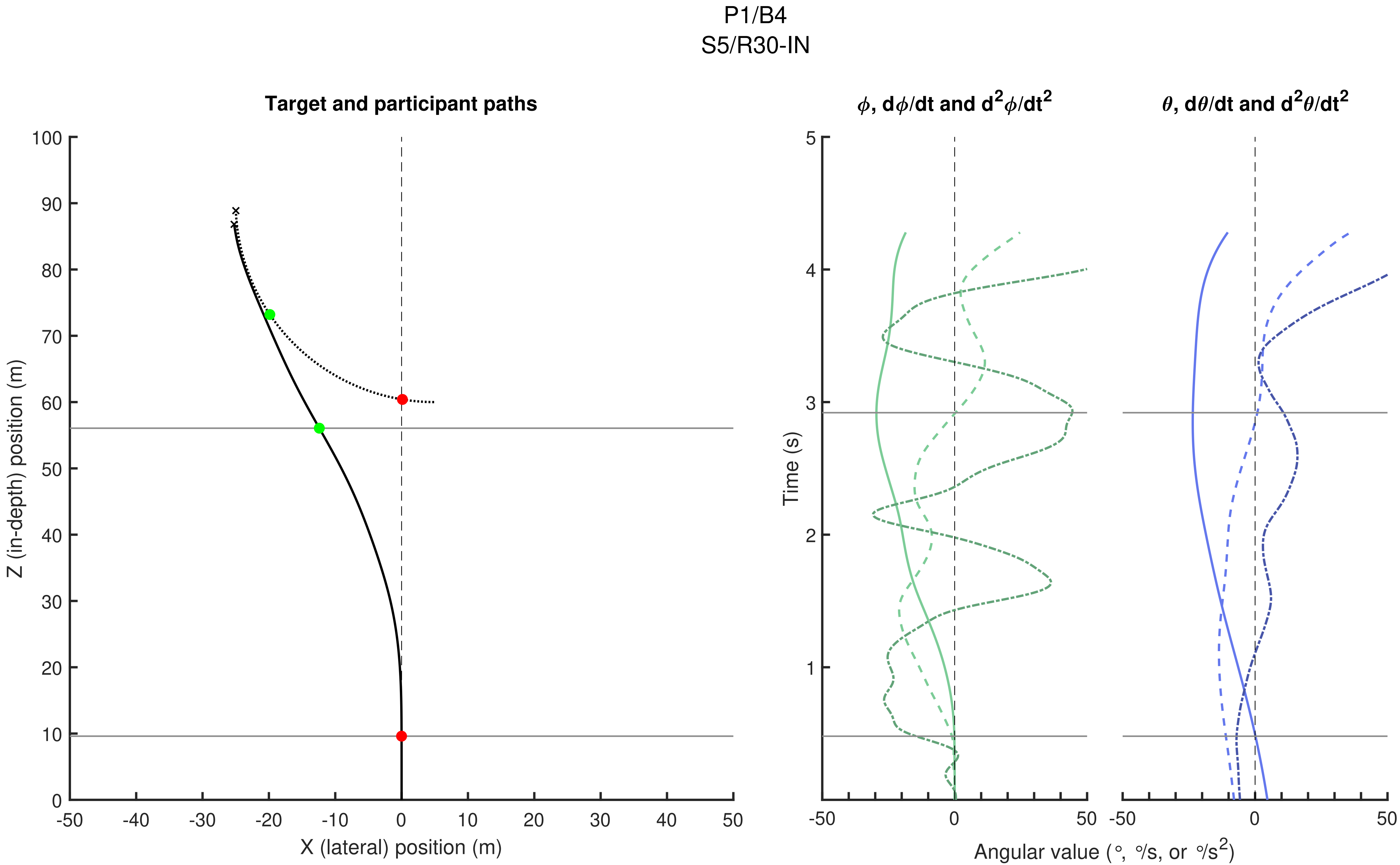
P1/B4
S5/R30-IN
Target and participant paths
Z (in-depth) position (m)
X (lateral) position (m)
100
90
80
70
60
50
40
30
20
10
0
-50
-40
-30
-20
-10
0
10
20
30
40
50
ϕ, dϕ/dt and d$^2\phi$/dt^2
θ, dθ/dt and d$^2\theta$/dt^2
Time (s)
5
4
3
2
1
-50
0
50
Angular value (°, °/s, or °/s^2)

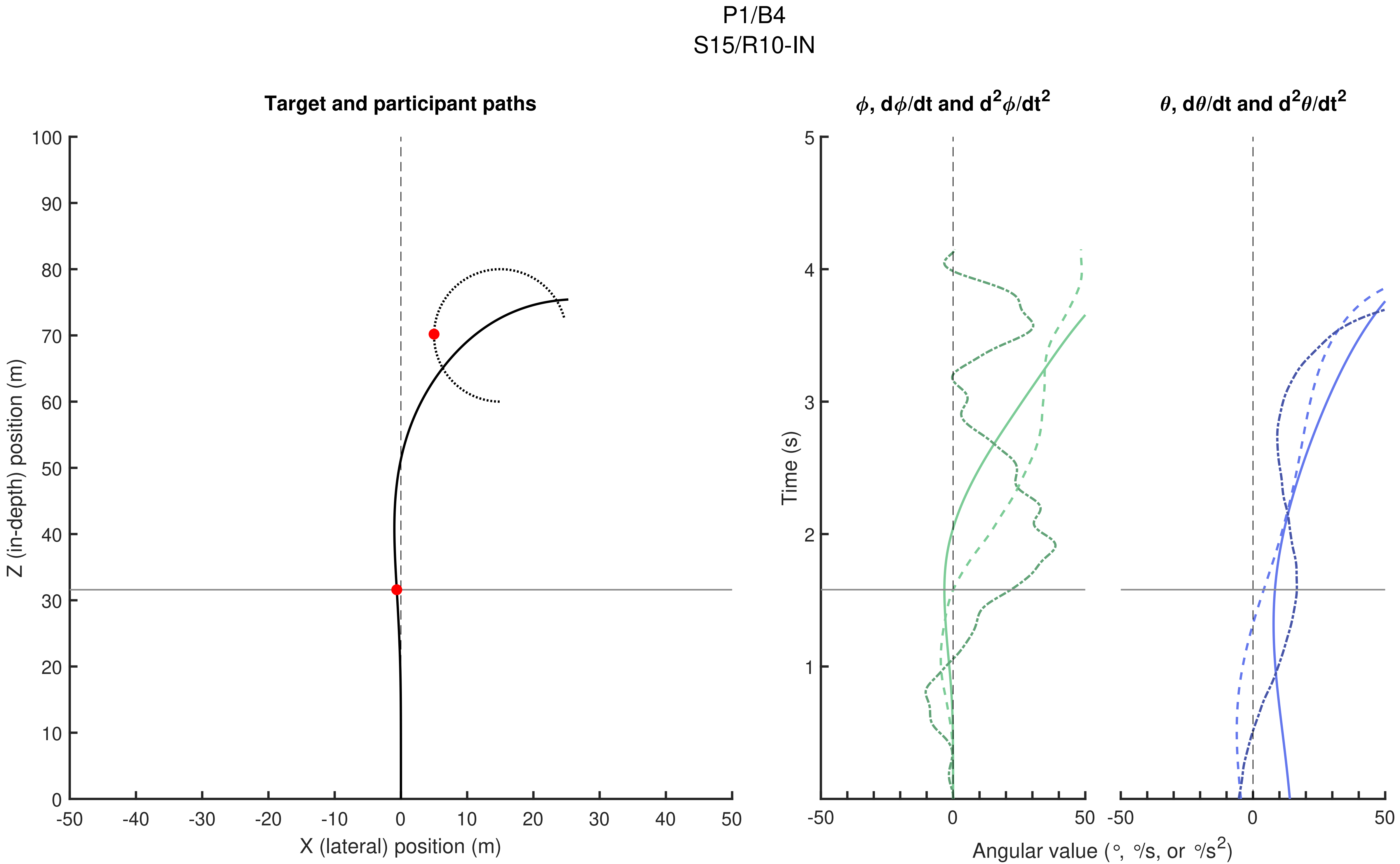

P1/B4
S15/R10-IN
Target and participant paths
ϕ, dϕ/dt and d$^2\phi$/dt^2
θ, dθ/dt and d$^2\theta$/dt^2
Z (in-depth) position (m)
X (lateral) position (m)
Time (s)
Angular value (°, °/s, or °/s^2)
100
90
80
70
60
50
40
30
20
10
0
-50
-40
-30
-20
-10
0
10
20
30
40
50
5
4
3
2
1
-50
0
50
-50
0
50

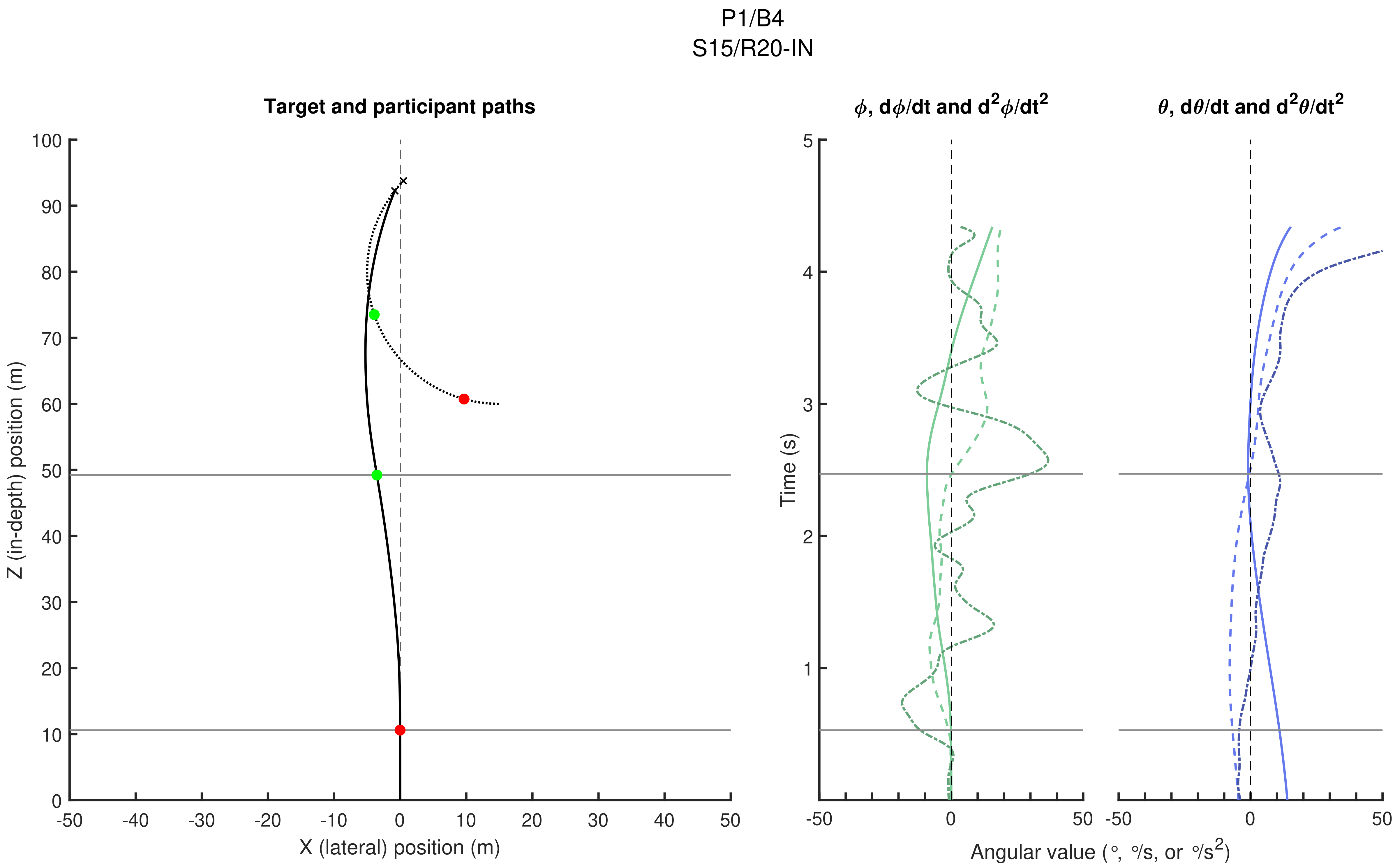
P1/B4
S15/R20-IN
Target and participant paths
Z (in-depth) position (m)
X (lateral) position (m)
100
90
80
70
60
50
40
30
20
10
0
-50
-40
-30
-20
-10
0
10
20
30
40
50
ϕ, dϕ/dt and d$^2\phi$/dt^2
θ, dθ/dt and d$^2\theta$/dt^2
Time (s)
5
4
3
2
1
-50
0
50
-50
0
50
Angular value (°, °/s, or °/s^2)

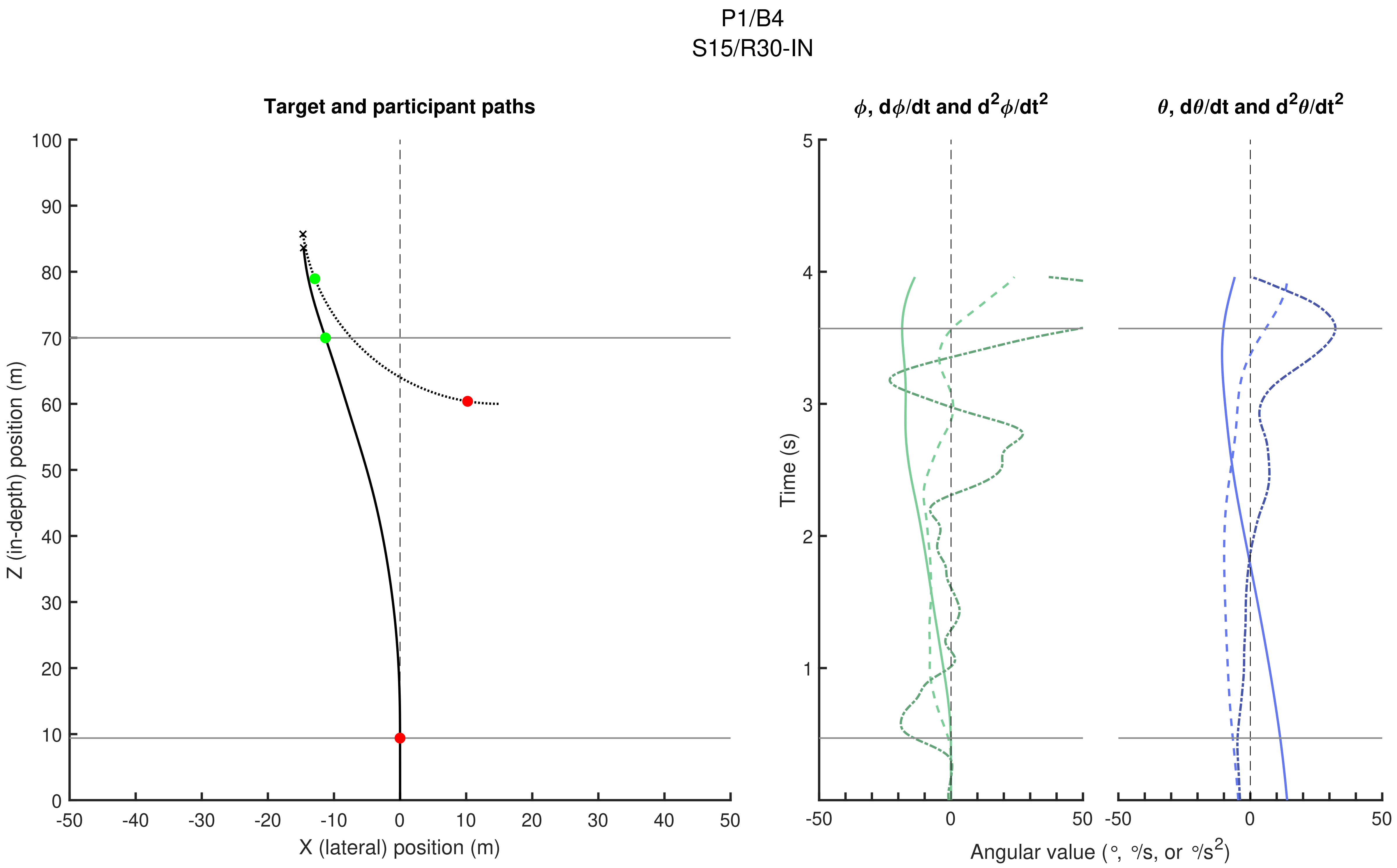
P1/B4
S15/R30-IN
Target and participant paths
Z (in-depth) position (m)
X (lateral) position (m)
100
90
80
70
60
50
40
30
20
10
0
-50
-40
-30
-20
-10
0
10
20
30
40
50
ϕ, dϕ/dt and d$^2\phi$/dt^2
θ, dθ/dt and d$^2\theta$/dt^2
Time (s)
5
4
3
2
1
-50
0
50
-50
0
50
Angular value (°, °/s, or °/s^2)

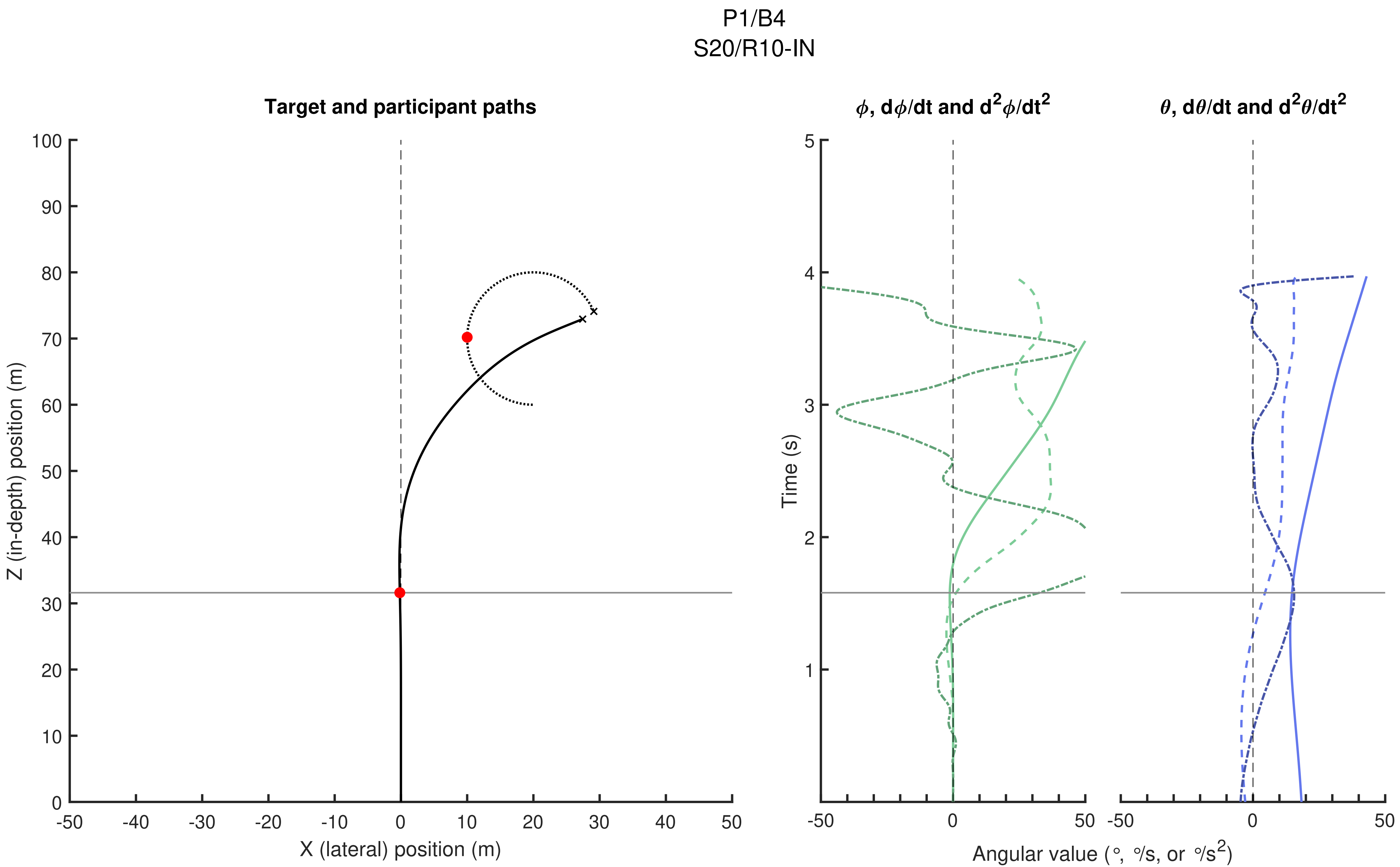

P1/B4
S20/R10-IN
Target and participant paths
Z (in-depth) position (m)
X (lateral) position (m)
ϕ, dϕ/dt and d$^2\phi$/dt^2
θ, dθ/dt and d$^2\theta$/dt^2
Time (s)
Angular value (°, °/s, or °/s^2)

P1/B4
S20/R20-IN

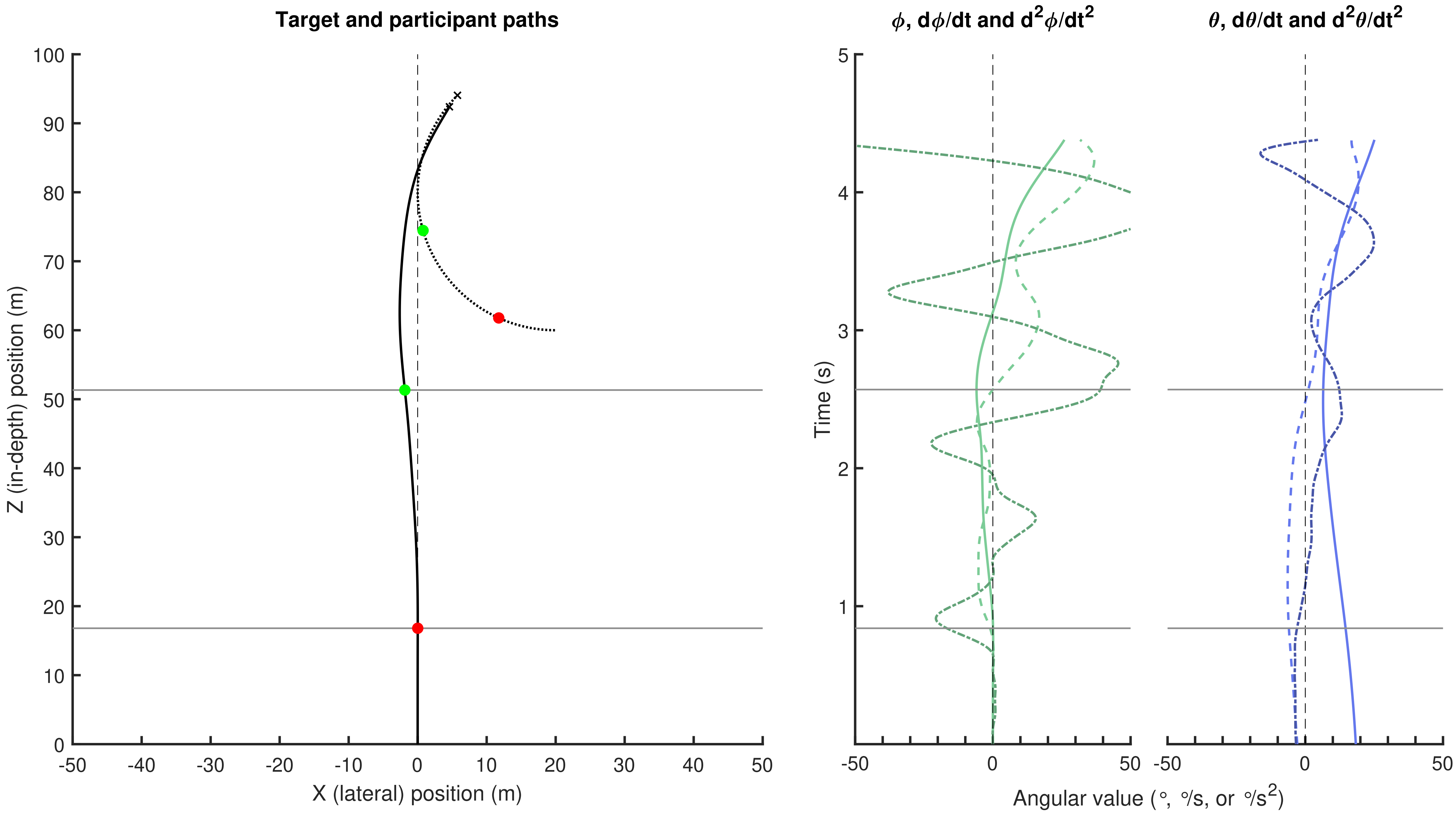

P1/B4
S20/R30-IN

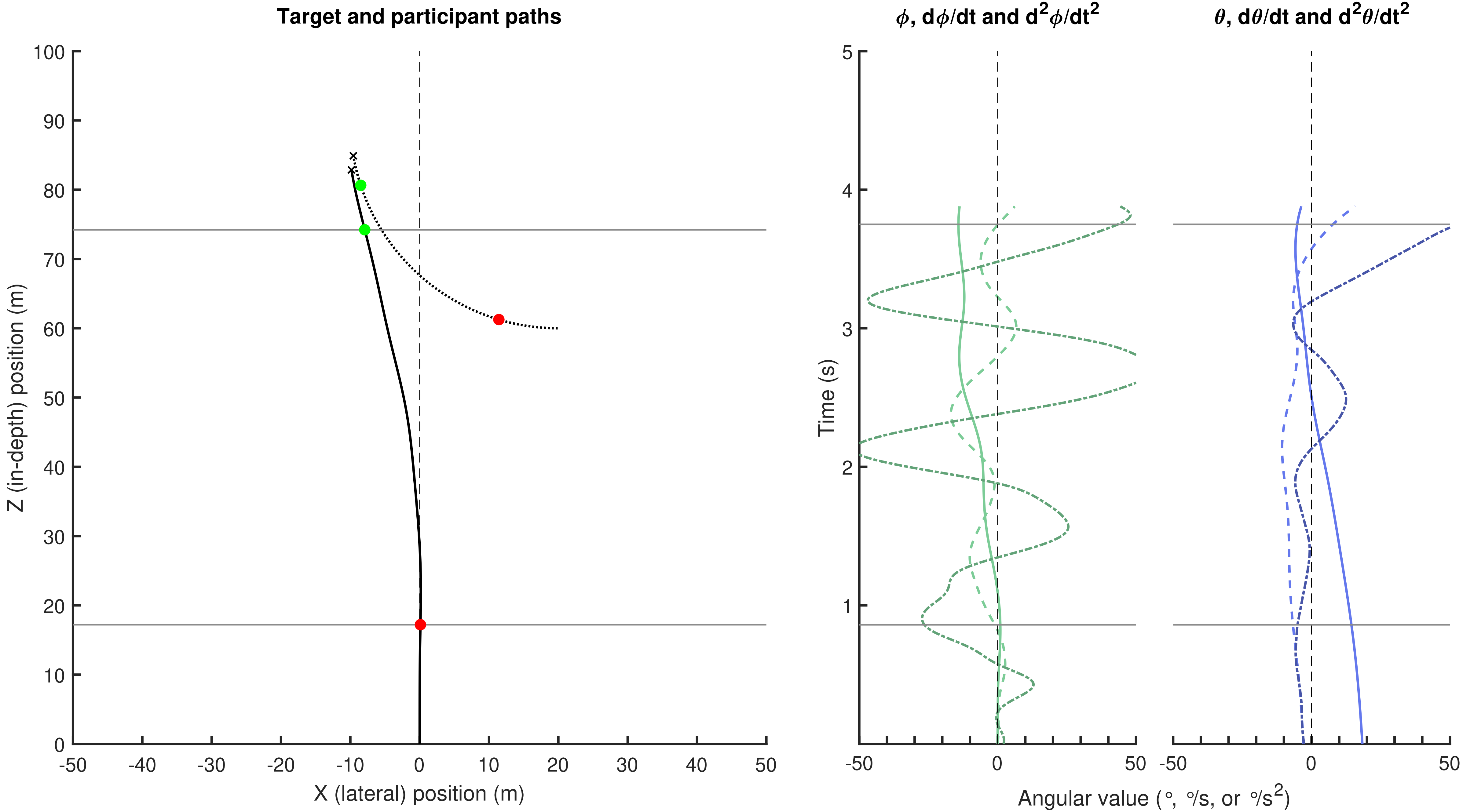

P1/B5
S20/R10-IN

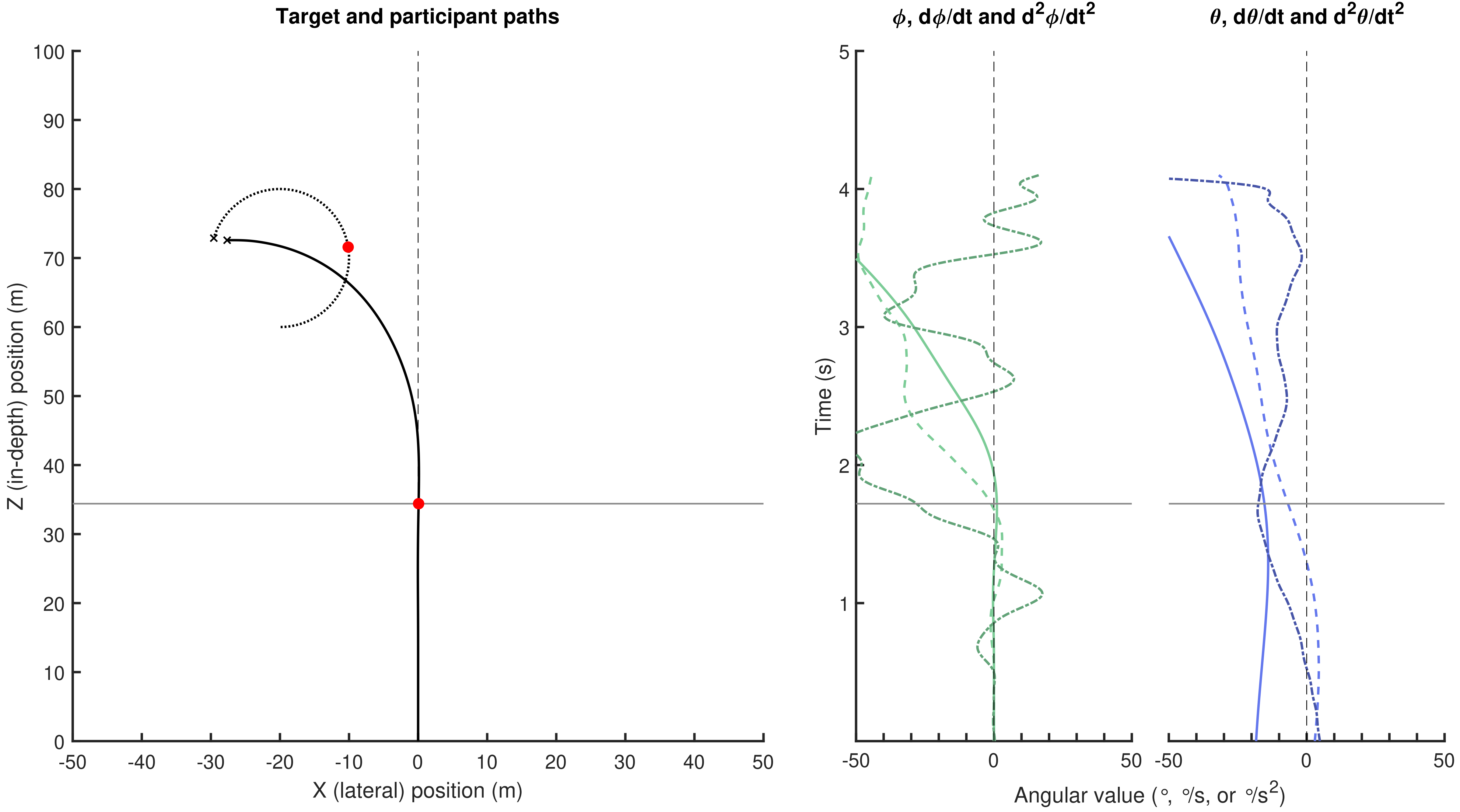

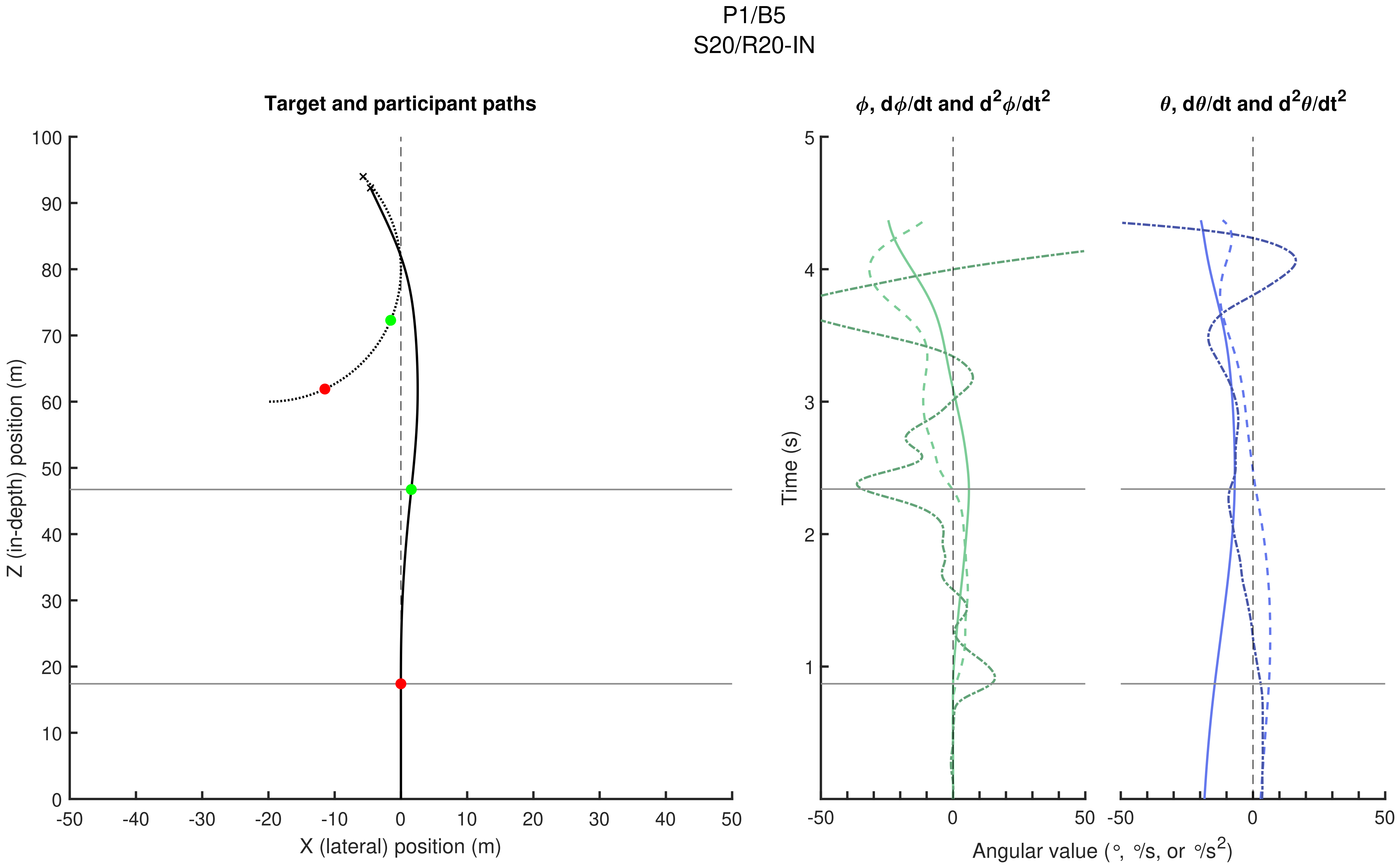
P1/B5
S20/R20-IN
Target and participant paths
Z (in-depth) position (m)
X (lateral) position (m)
ϕ, dϕ/dt and d$^2\phi$/dt^2
θ, dθ/dt and d$^2\theta$/dt^2
Time (s)
Angular value (°, °/s, or °/s^2)

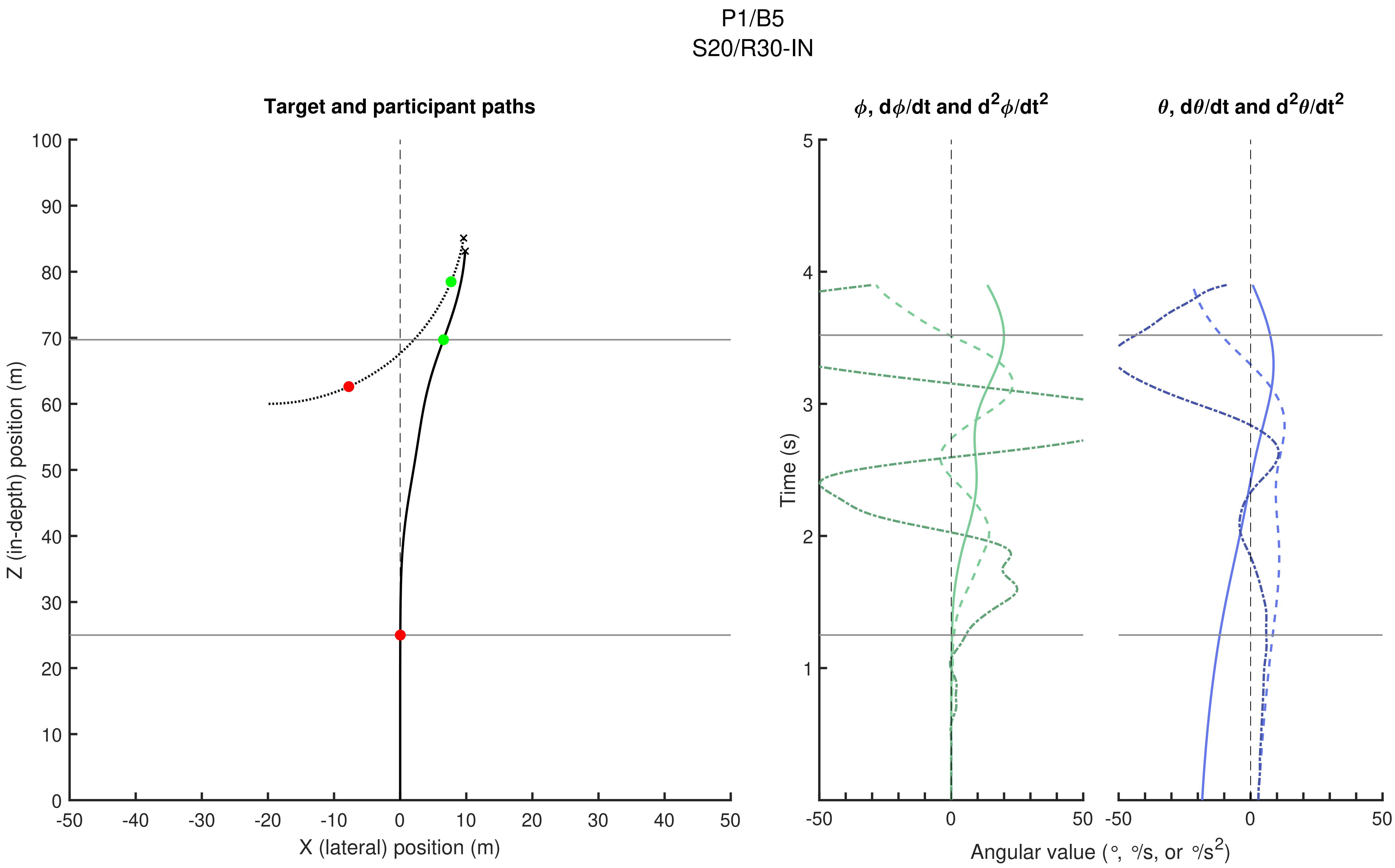

P1/B5
S20/R30-IN
Target and participant paths
ϕ, dϕ/dt and d$^2\phi$/dt^2
θ, dθ/dt and d$^2\theta$/dt^2
Z (in-depth) position (m)
X (lateral) position (m)
Time (s)
Angular value (°, °/s, or °/s^2)
100
90
80
70
60
50
40
30
20
10
0
-50
-40
-30
-20
-10
0
10
20
30
40
50
5
4
3
2
1
-50
0
50
-50
0
50

P1/B5
S15/R10-IN

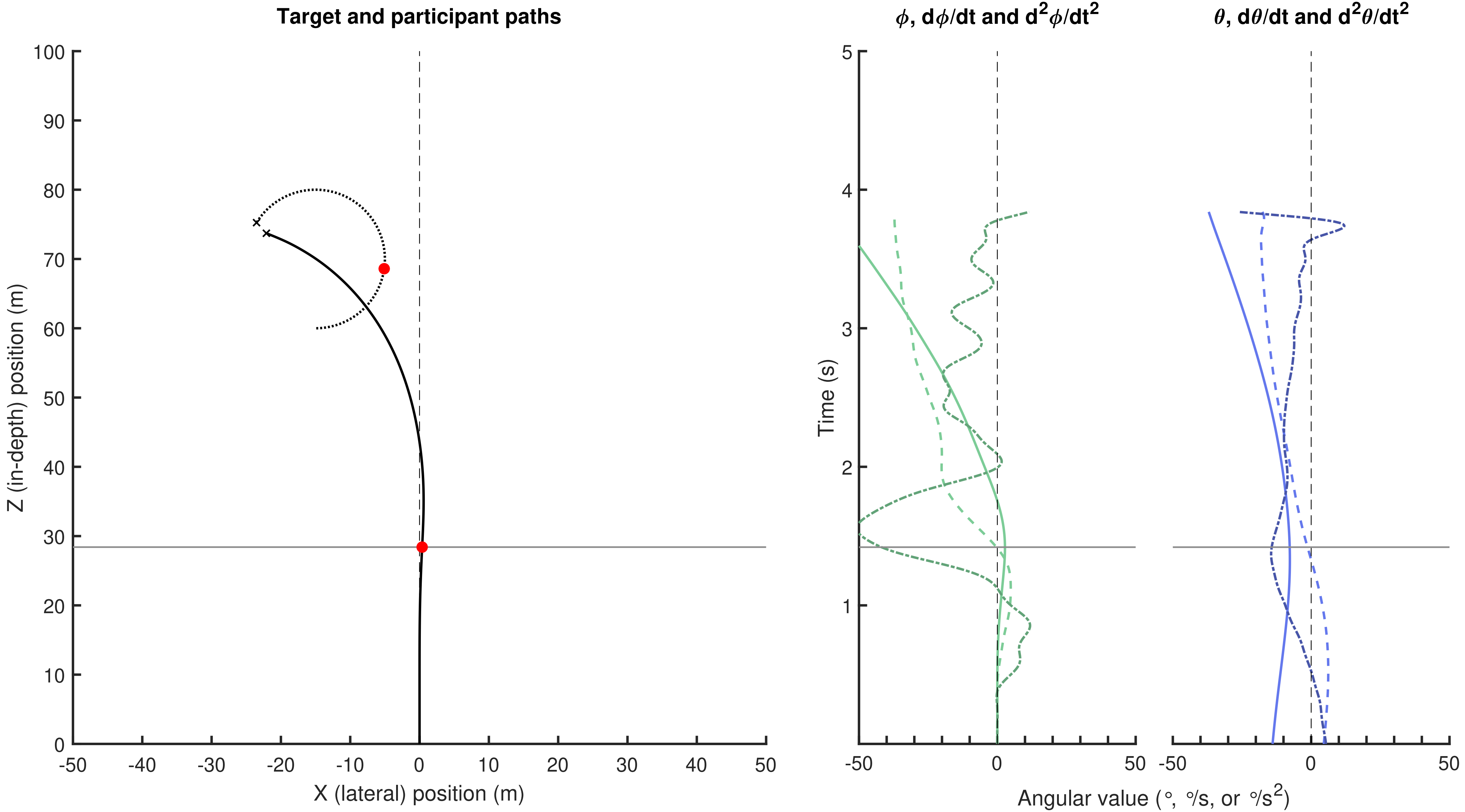

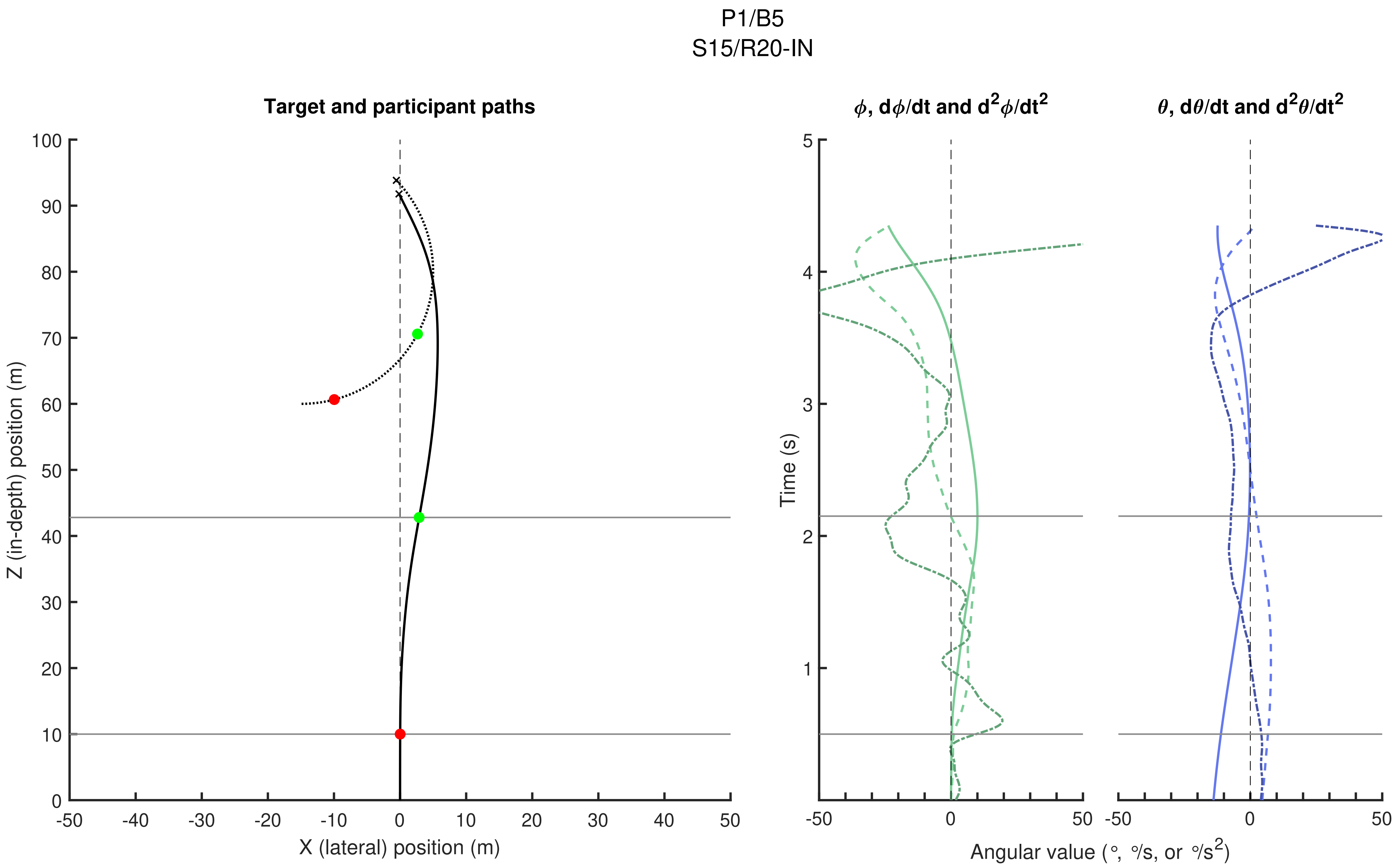
P1/B5
S15/R20-IN
Target and participant paths
Z (in-depth) position (m)
X (lateral) position (m)
φ, dφ/dt and d²φ/dt²
θ, dθ/dt and d²θ/dt²
Time (s)
Angular value (°, °/s, or °/s²)

P1/B5
S15/R30-IN

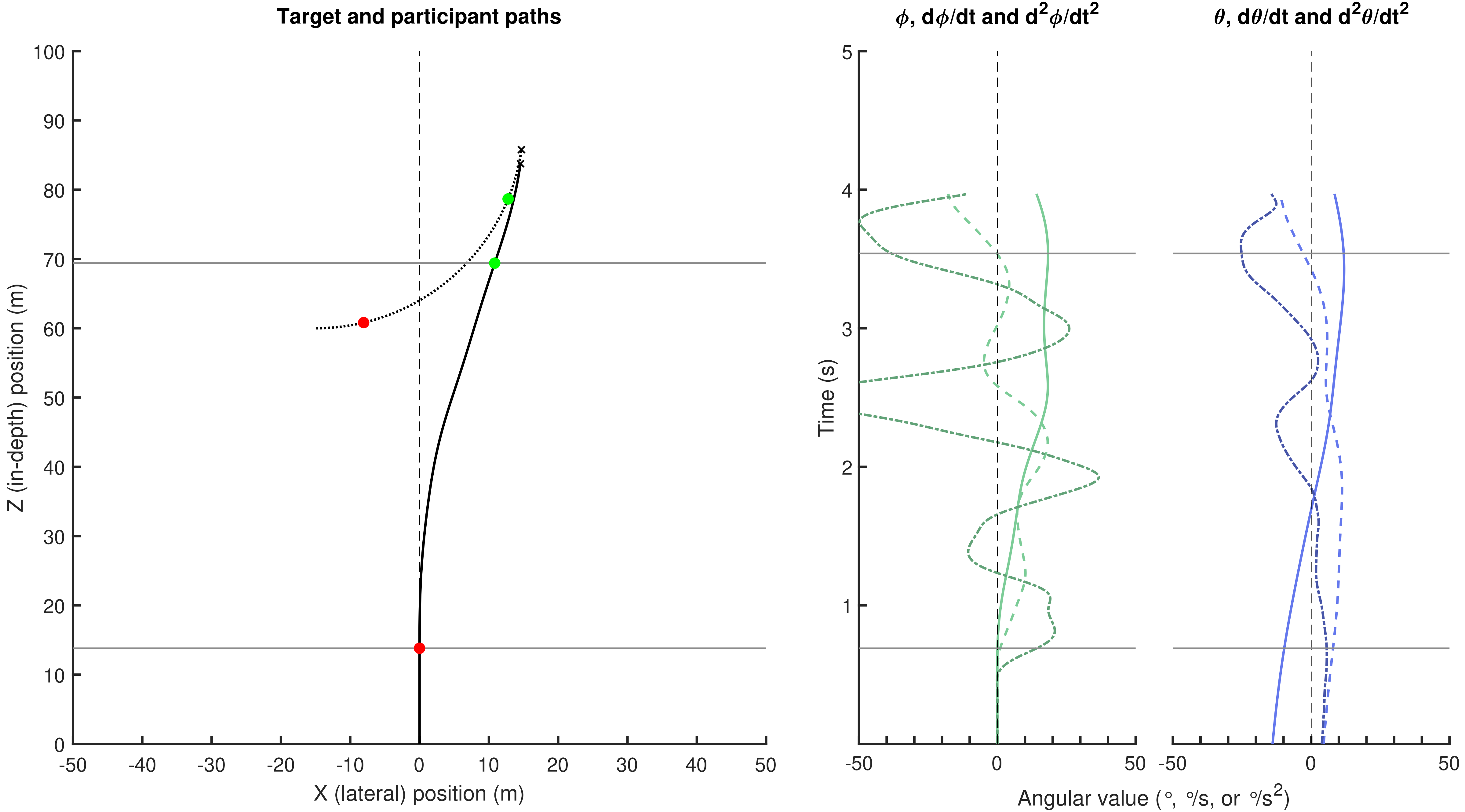

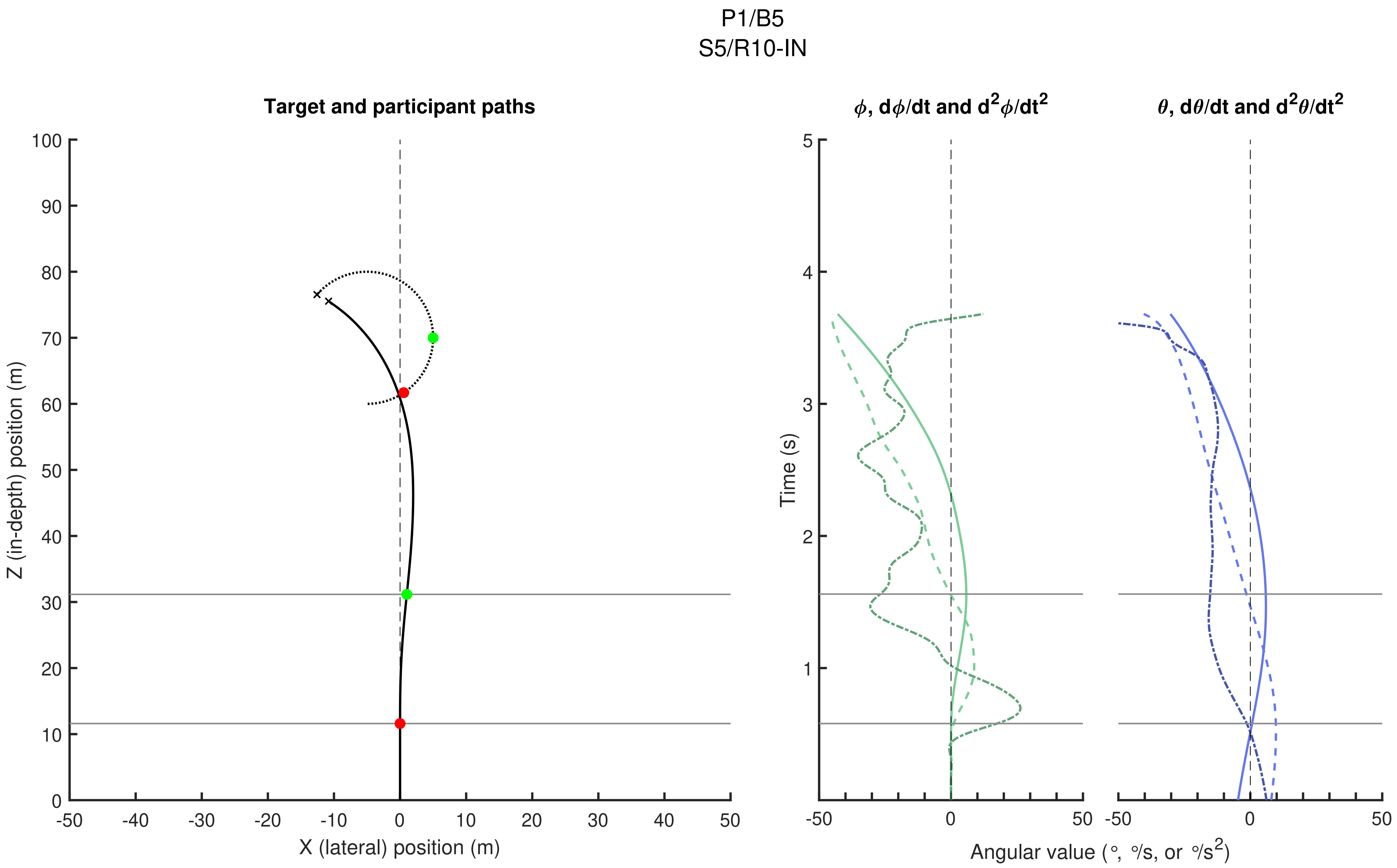
P1/B5
S5/R10-IN
Target and participant paths
Z (in-depth) position (m)
X (lateral) position (m)
ϕ, dϕ/dt and d2ϕ/dt2
θ, dθ/dt and d2θ/dt2
Time (s)
Angular value (°, °/s, or °/s2)

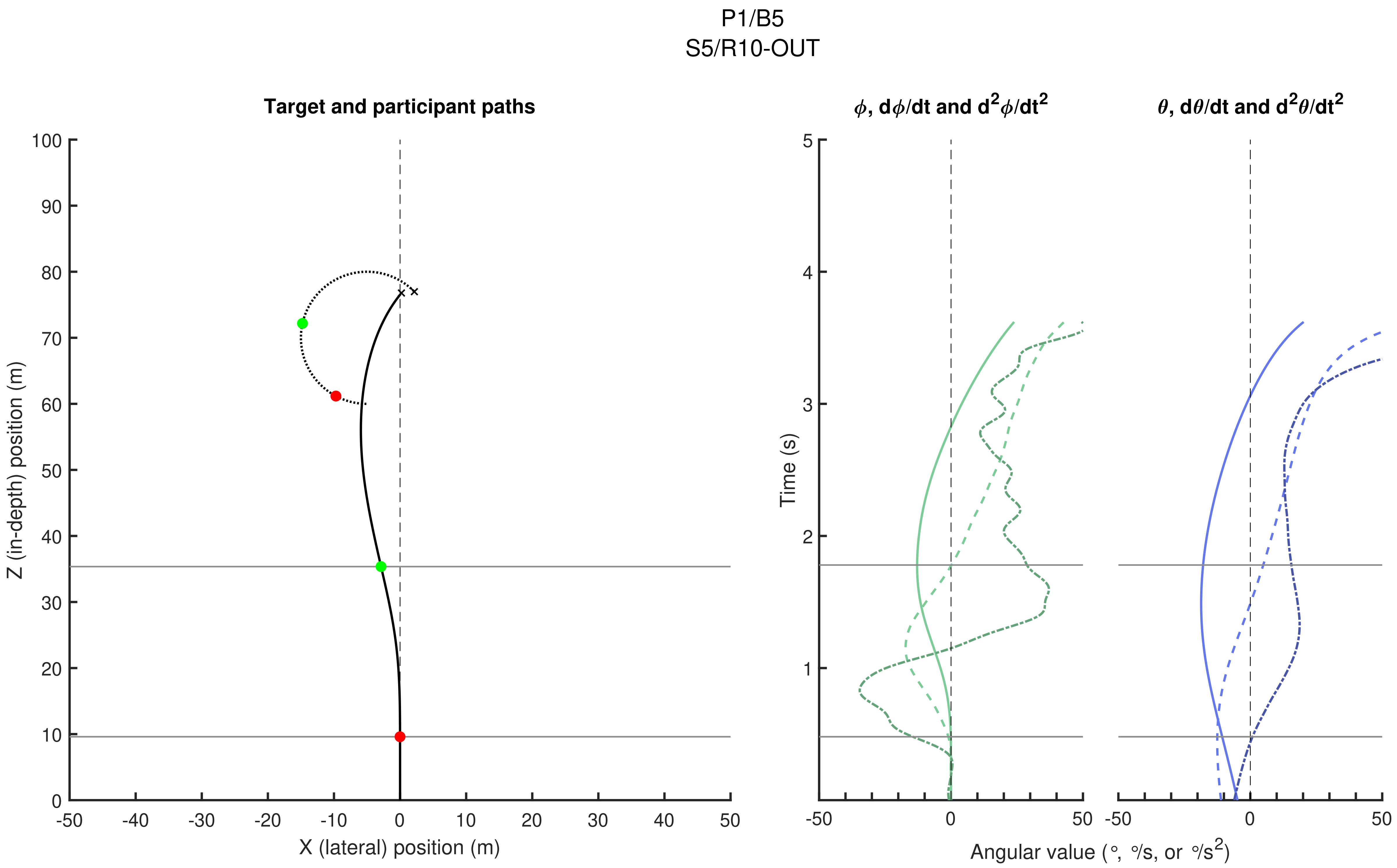

P1/B5
S5/R10-OUT
Target and participant paths
Z (in-depth) position (m)
X (lateral) position (m)
ϕ, dϕ/dt and d$^2\phi$/dt^2
θ, dθ/dt and d$^2\theta$/dt^2
Time (s)
Angular value (°, °/s, or °/s^2)

P1/B5
S5/R20-IN

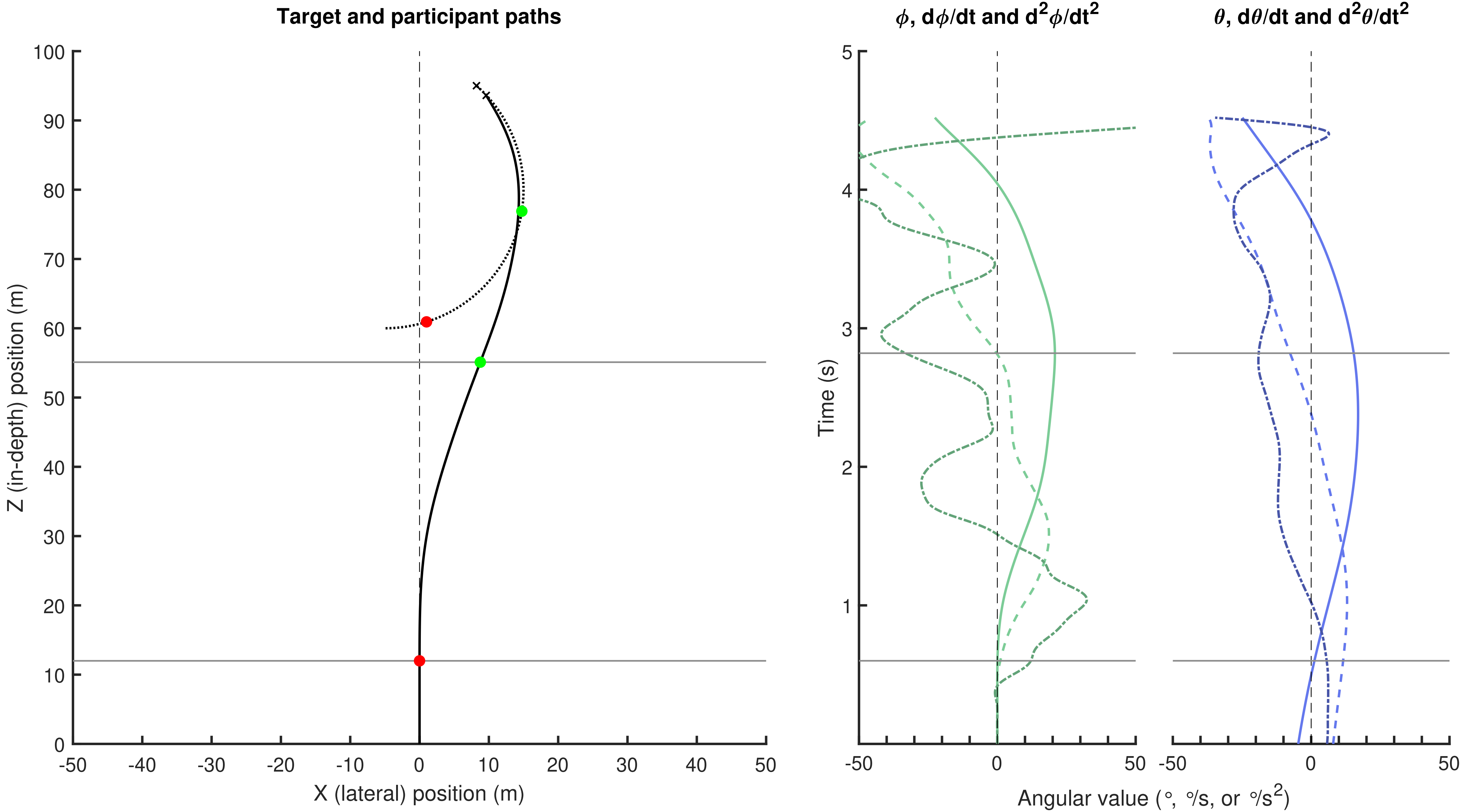

P1/B5
S5/R20-OUT

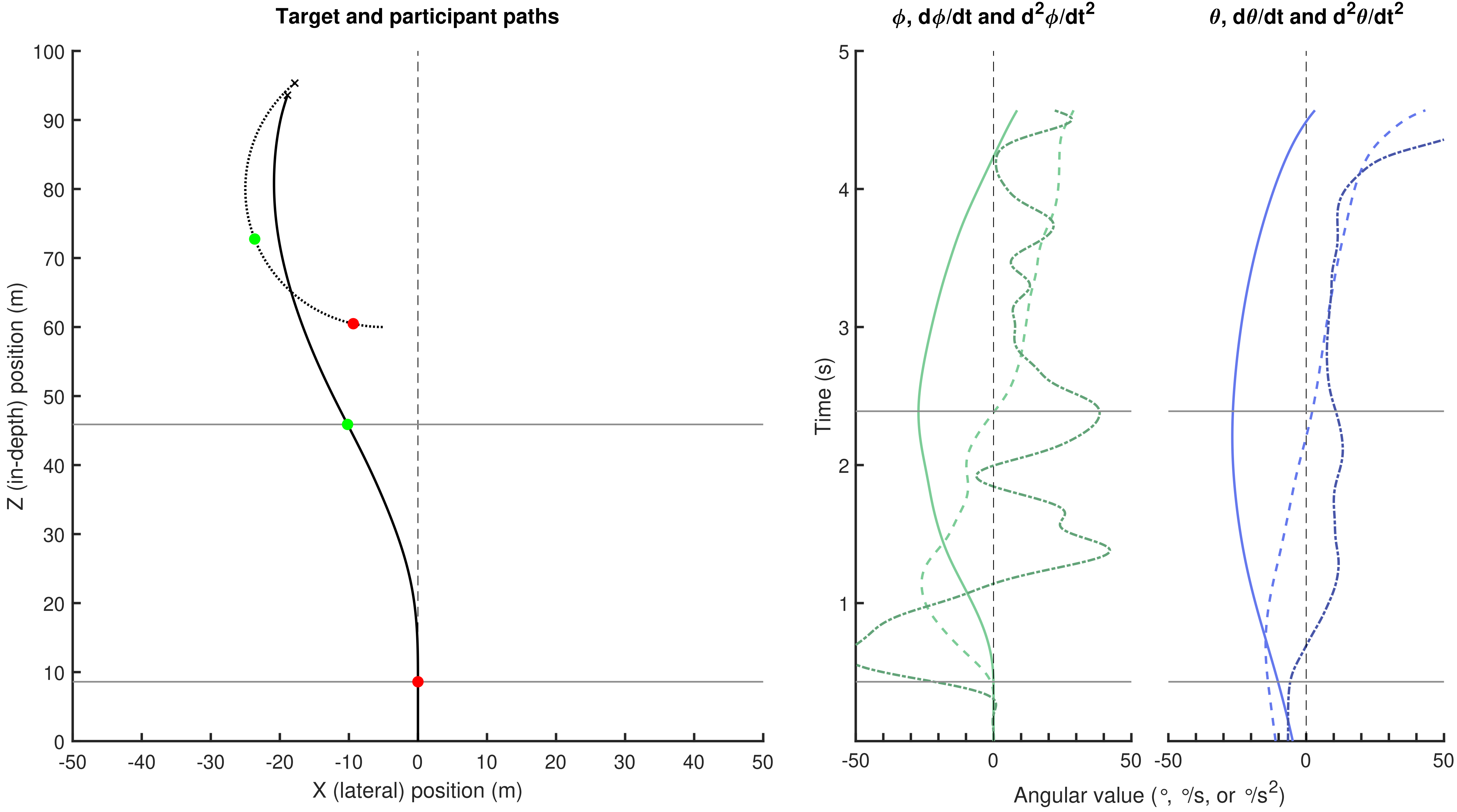

P1/B5
S5/R30-IN

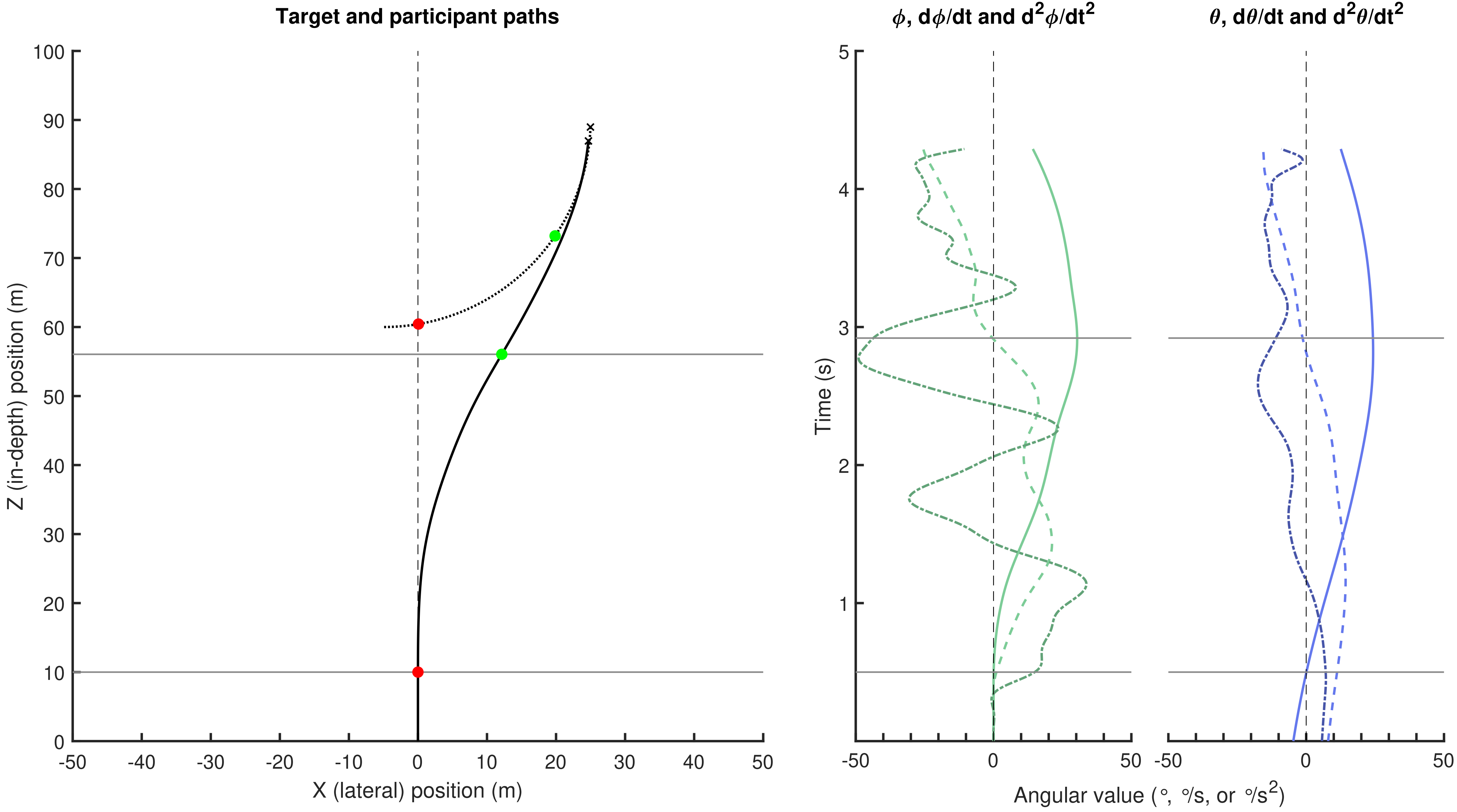

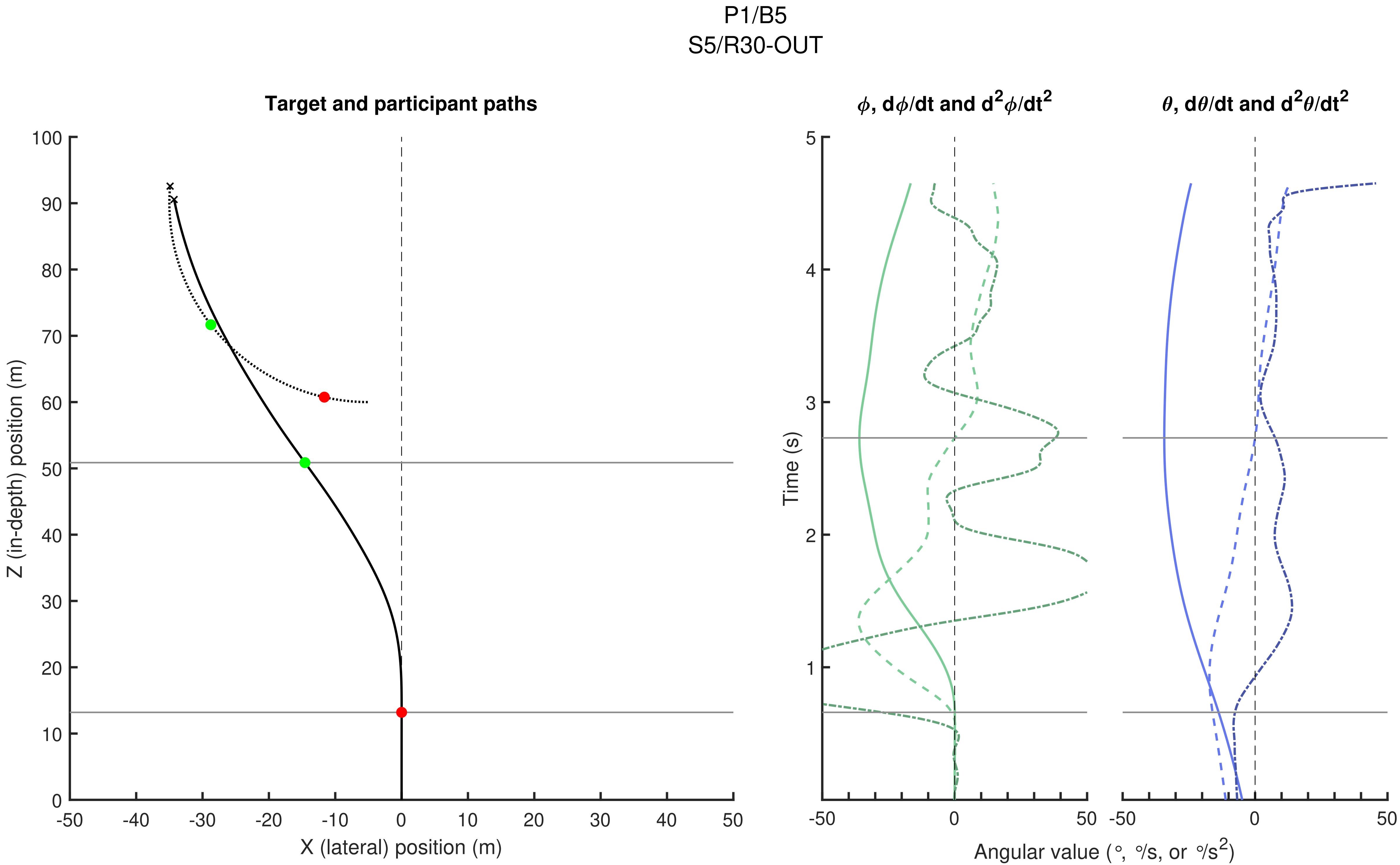

P1/B5
S5/R30-OUT
Target and participant paths
Z (in-depth) position (m)
X (lateral) position (m)
ϕ, dϕ/dt and d²ϕ/dt²
θ, dθ/dt and d²θ/dt²
Time (s)
Angular value (°, °/s, or °/s²)

P1/B5
S5/R10-OUT

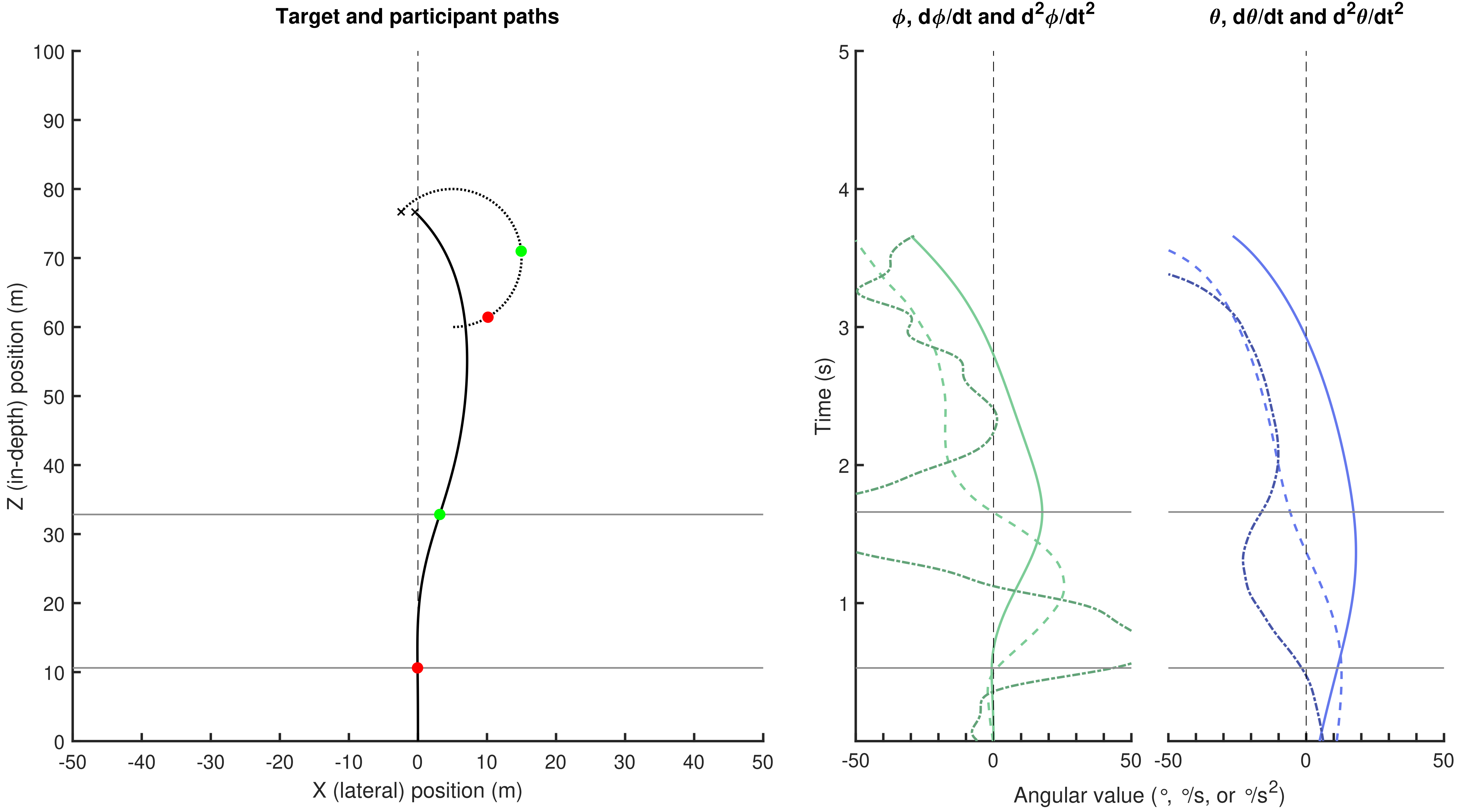

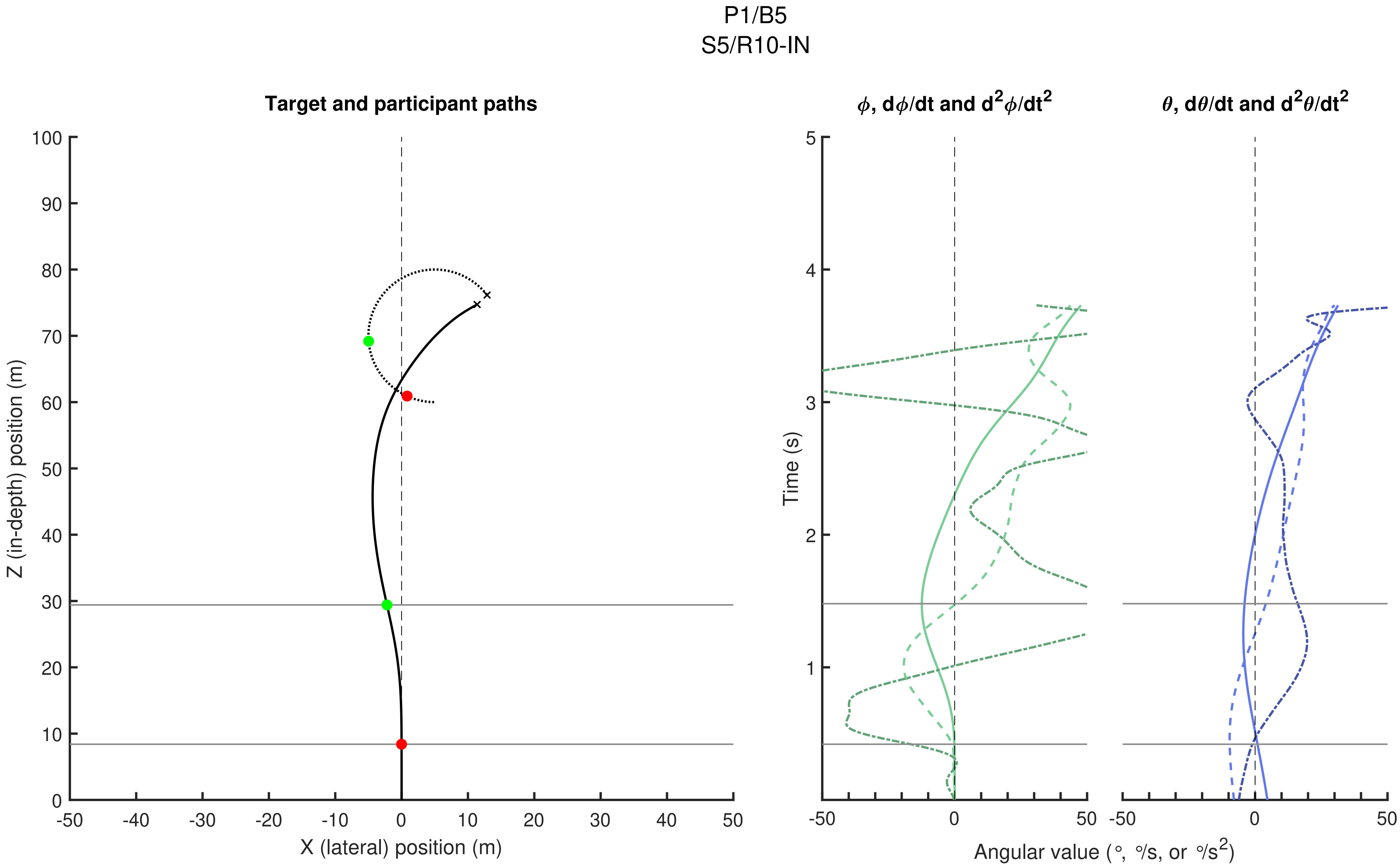

P1/B5
S5/R10-IN
Target and participant paths
Z (in-depth) position (m)
X (lateral) position (m)
φ, dφ/dt and d²φ/dt²
θ, dθ/dt and d²θ/dt²
Time (s)
Angular value (°, °/s, or °/s²)

P1/B5
S5/R20-OUT

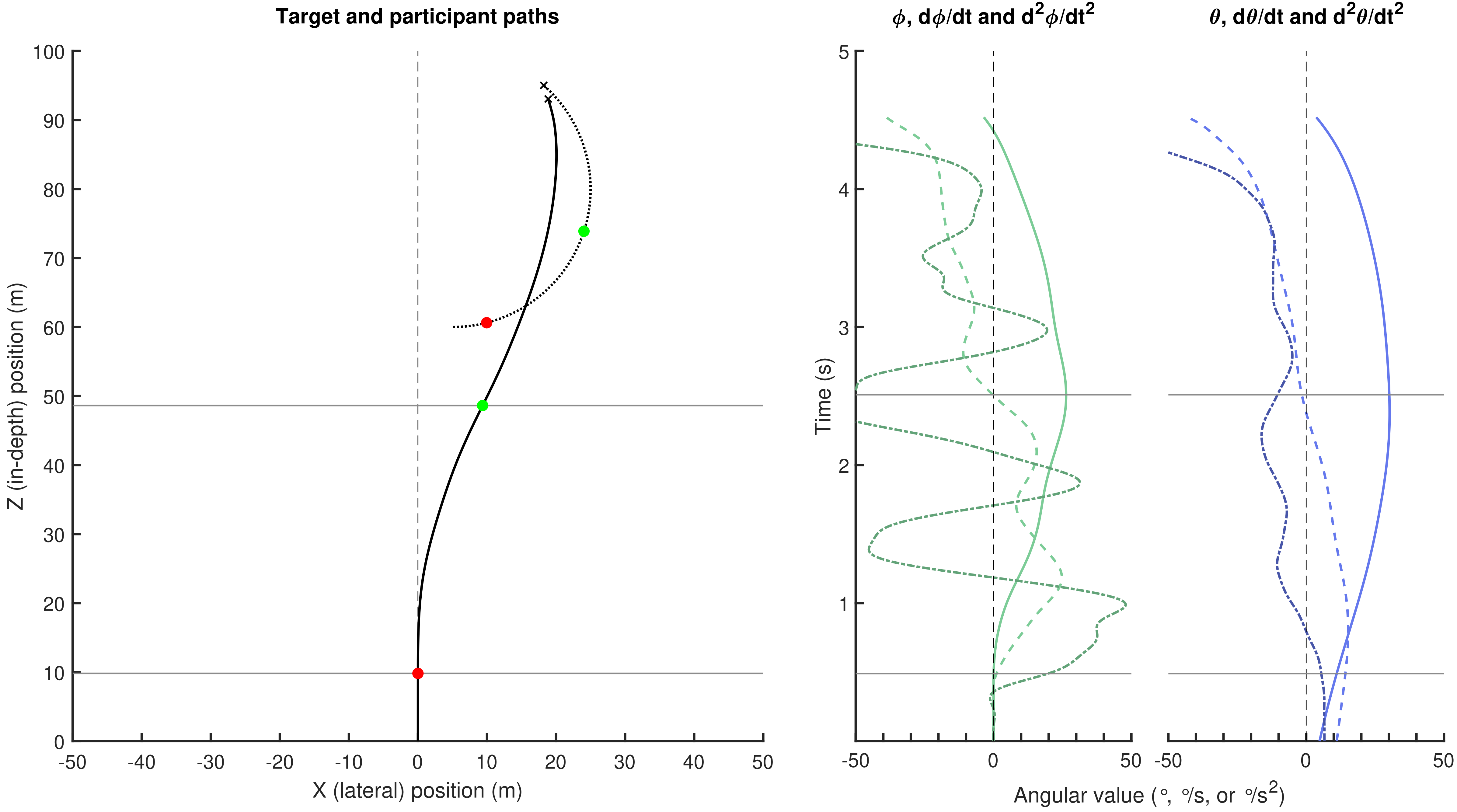

P1/B5
S5/R20-IN

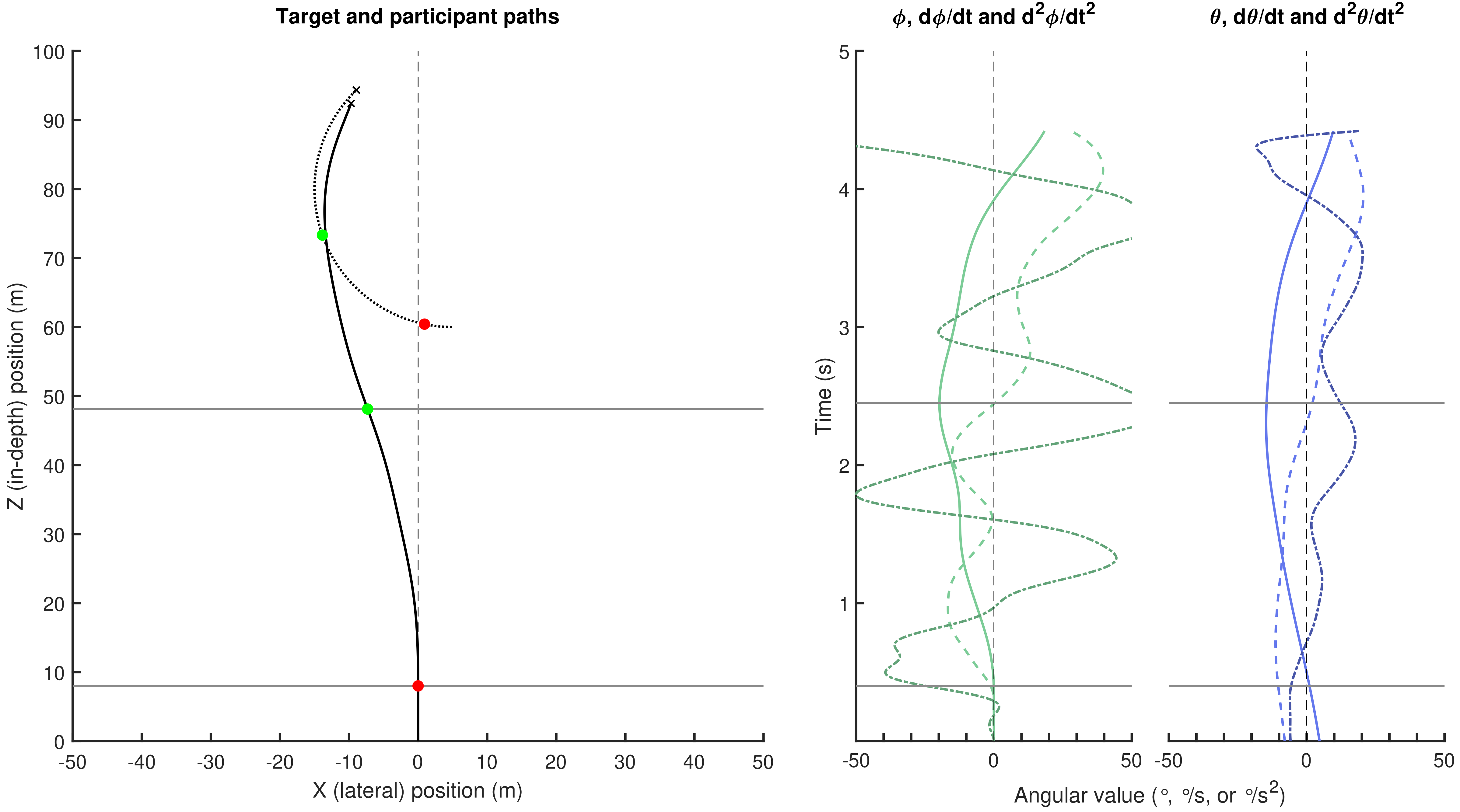

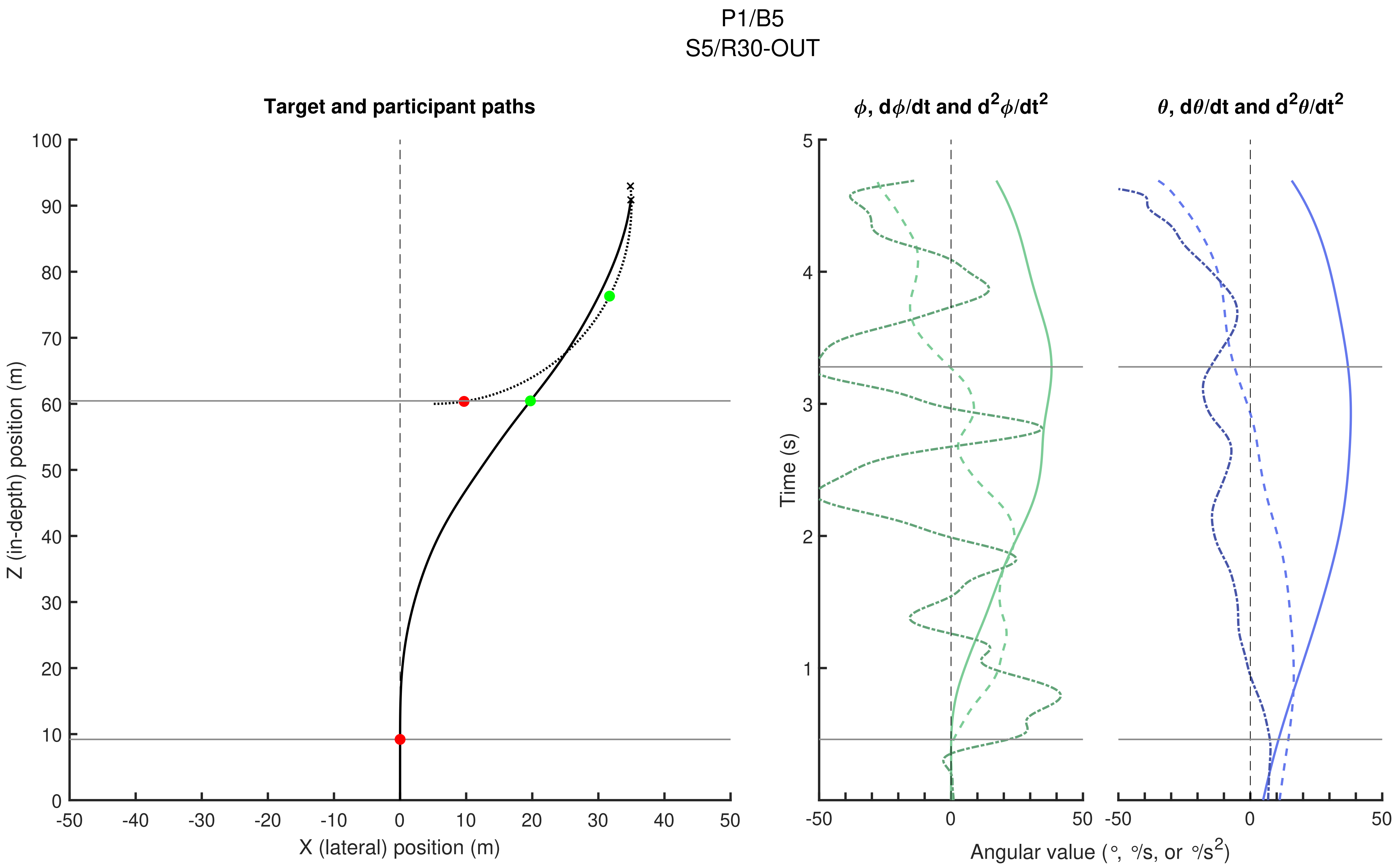
P1/B5
S5/R30-OUT
Target and participant paths
Z (in-depth) position (m)
X (lateral) position (m)
ϕ, dϕ/dt and d²ϕ/dt²
θ, dθ/dt and d²θ/dt²
Time (s)
Angular value (°, °/s, or °/s²)

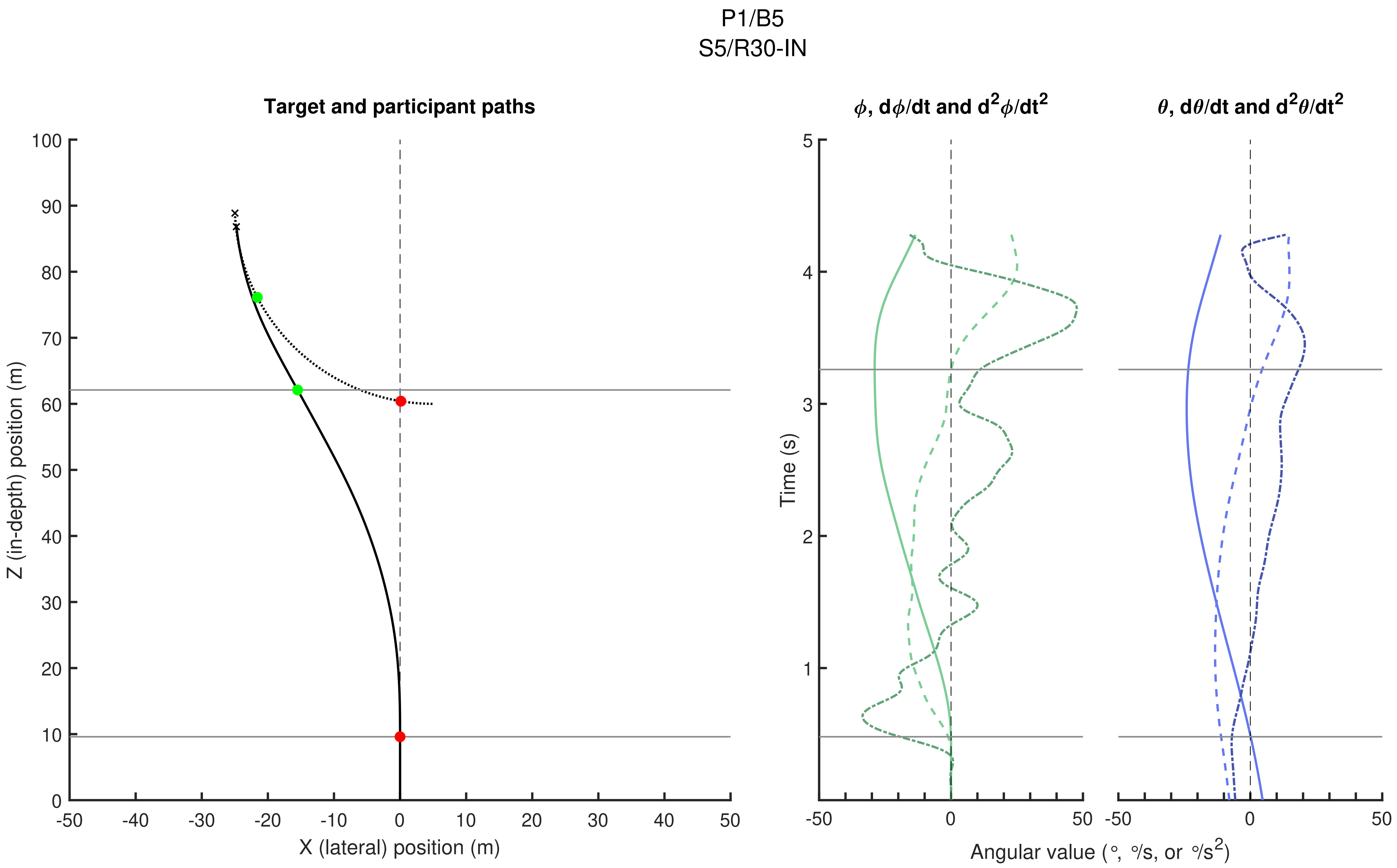
P1/B5
S5/R30-IN
Target and participant paths
Z (in-depth) position (m)
X (lateral) position (m)
ϕ, dϕ/dt and d$^2\phi$/dt^2
θ, dθ/dt and d$^2\theta$/dt^2
Time (s)
Angular value (°, °/s, or °/s^2)

P1/B5
S15/R10-IN

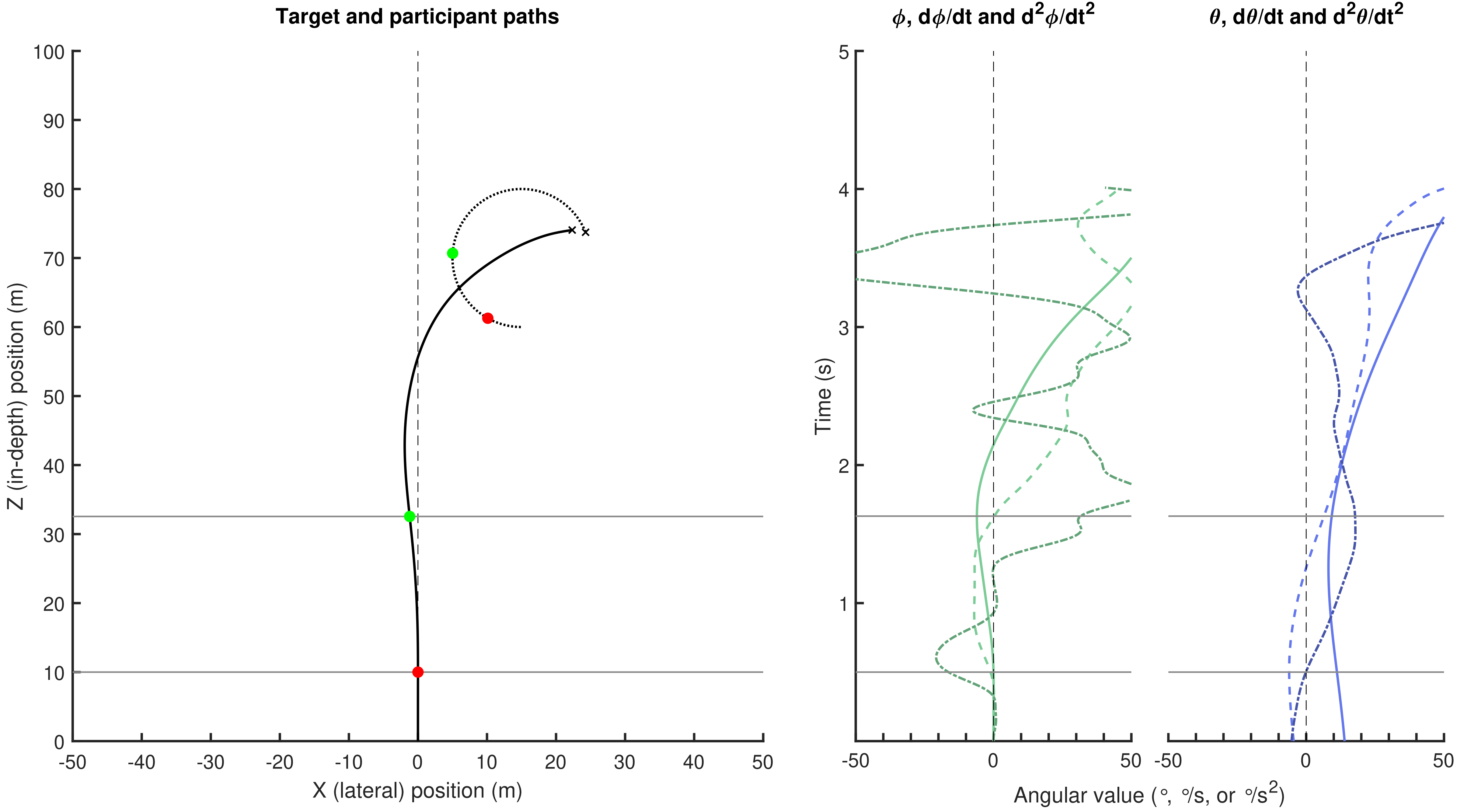

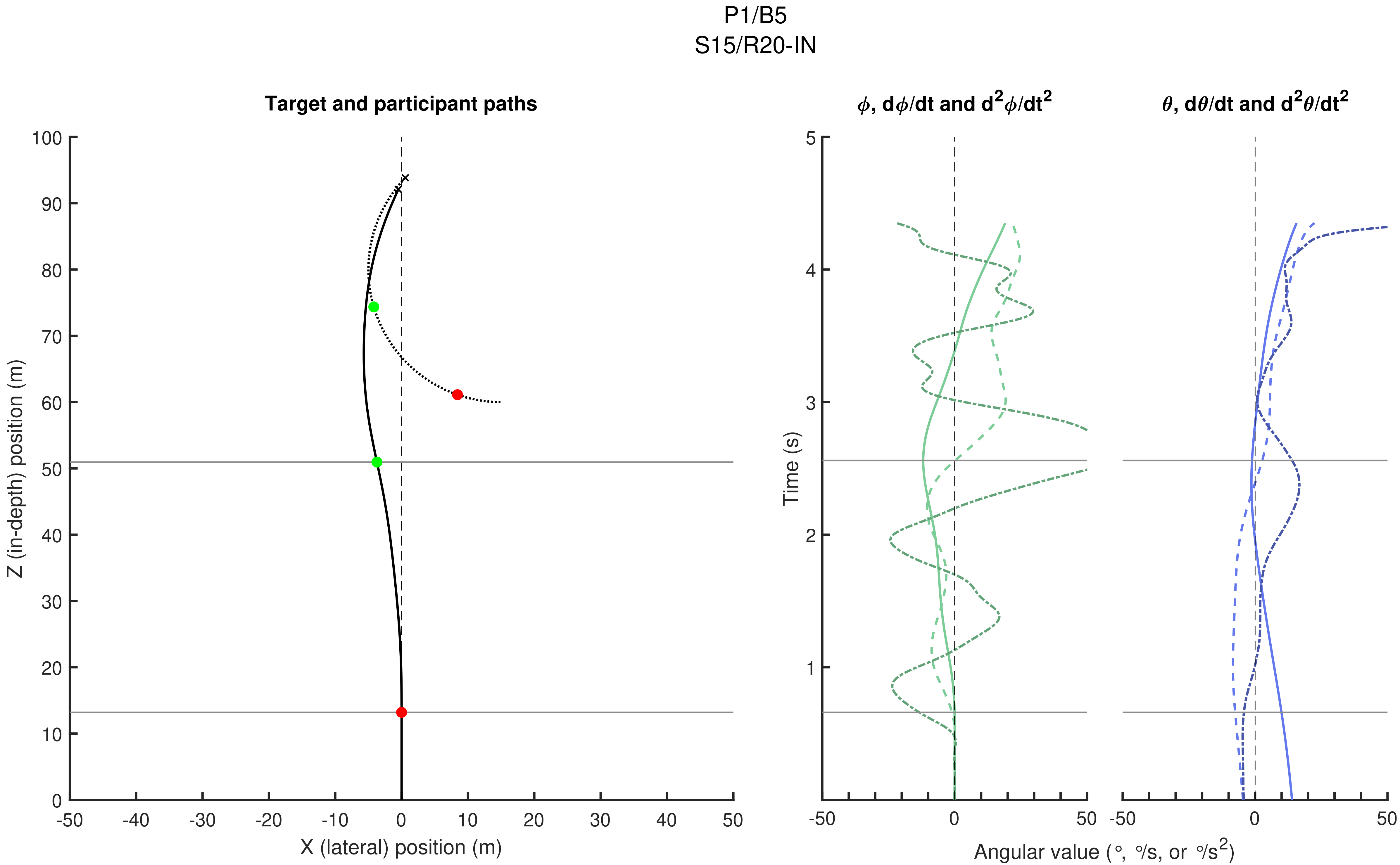
P1/B5
S15/R20-IN
Target and participant paths
Z (in-depth) position (m)
X (lateral) position (m)
100
90
80
70
60
50
40
30
20
10
0
-50
-40
-30
-20
-10
0
10
20
30
40
50
ϕ, dϕ/dt and d²ϕ/dt²
θ, dθ/dt and d²θ/dt²
Time (s)
5
4
3
2
1
-50
0
50
-50
0
50
Angular value (°, °/s, or °/s²)

P1/B5
S15/R30-IN

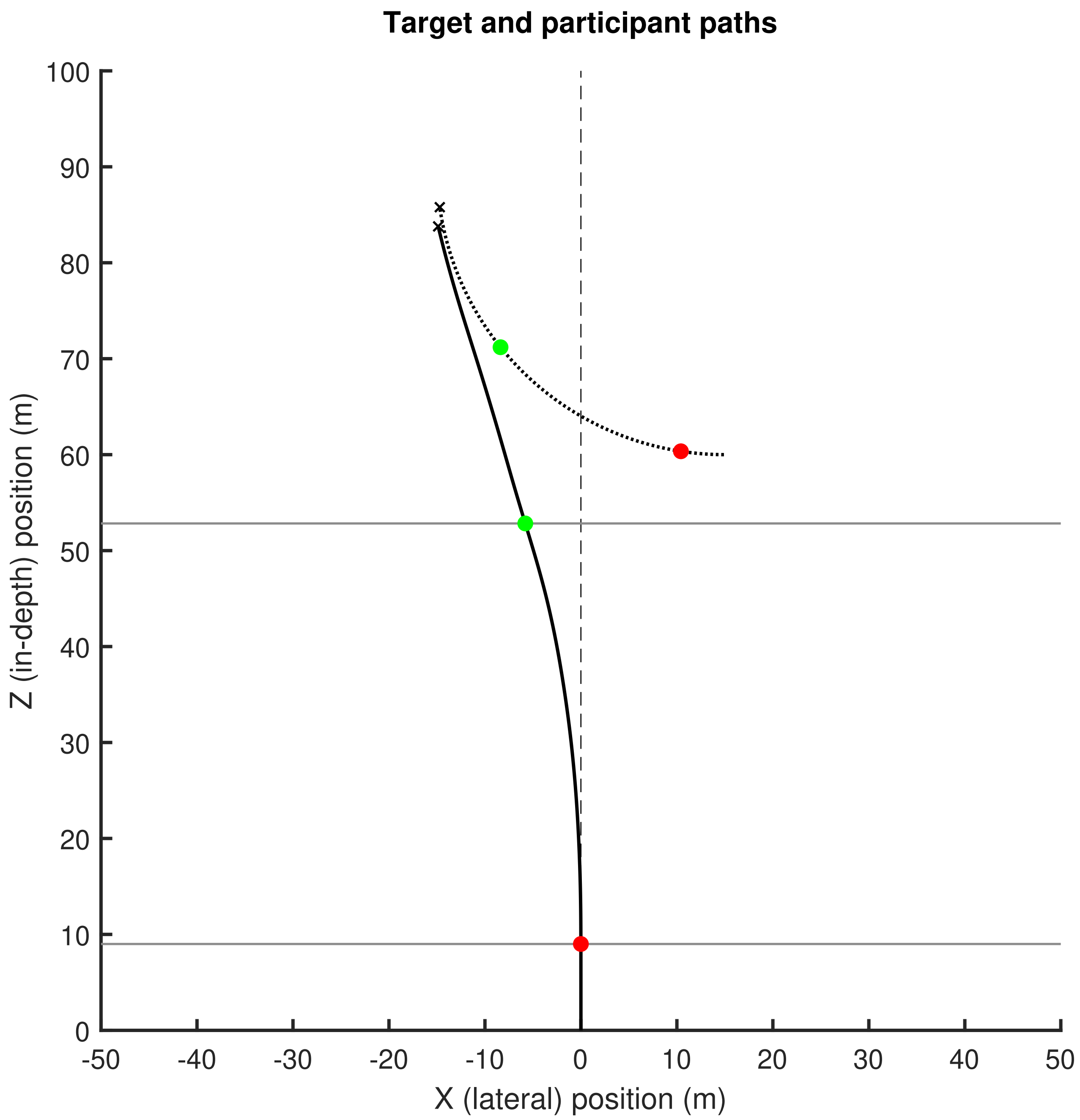

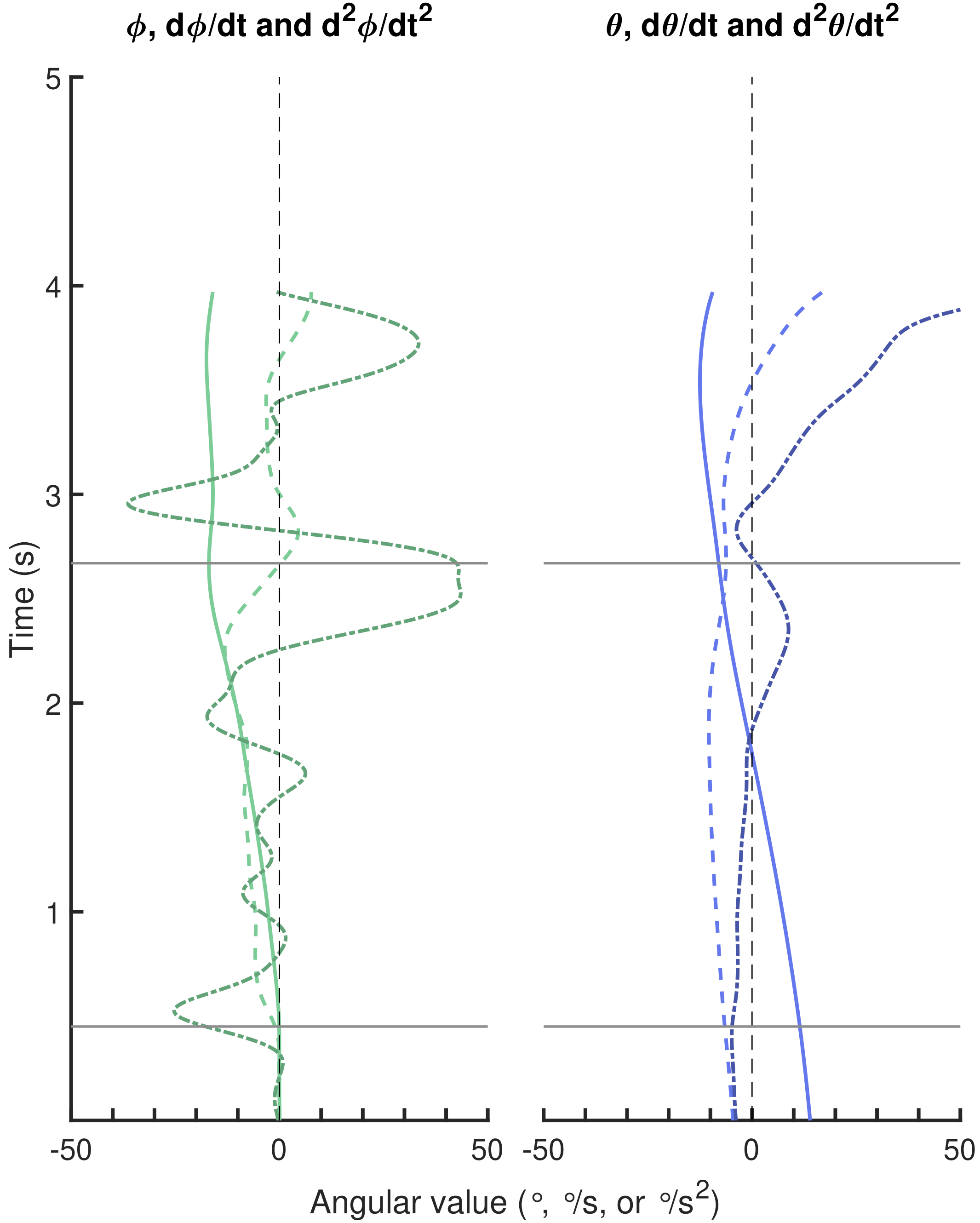

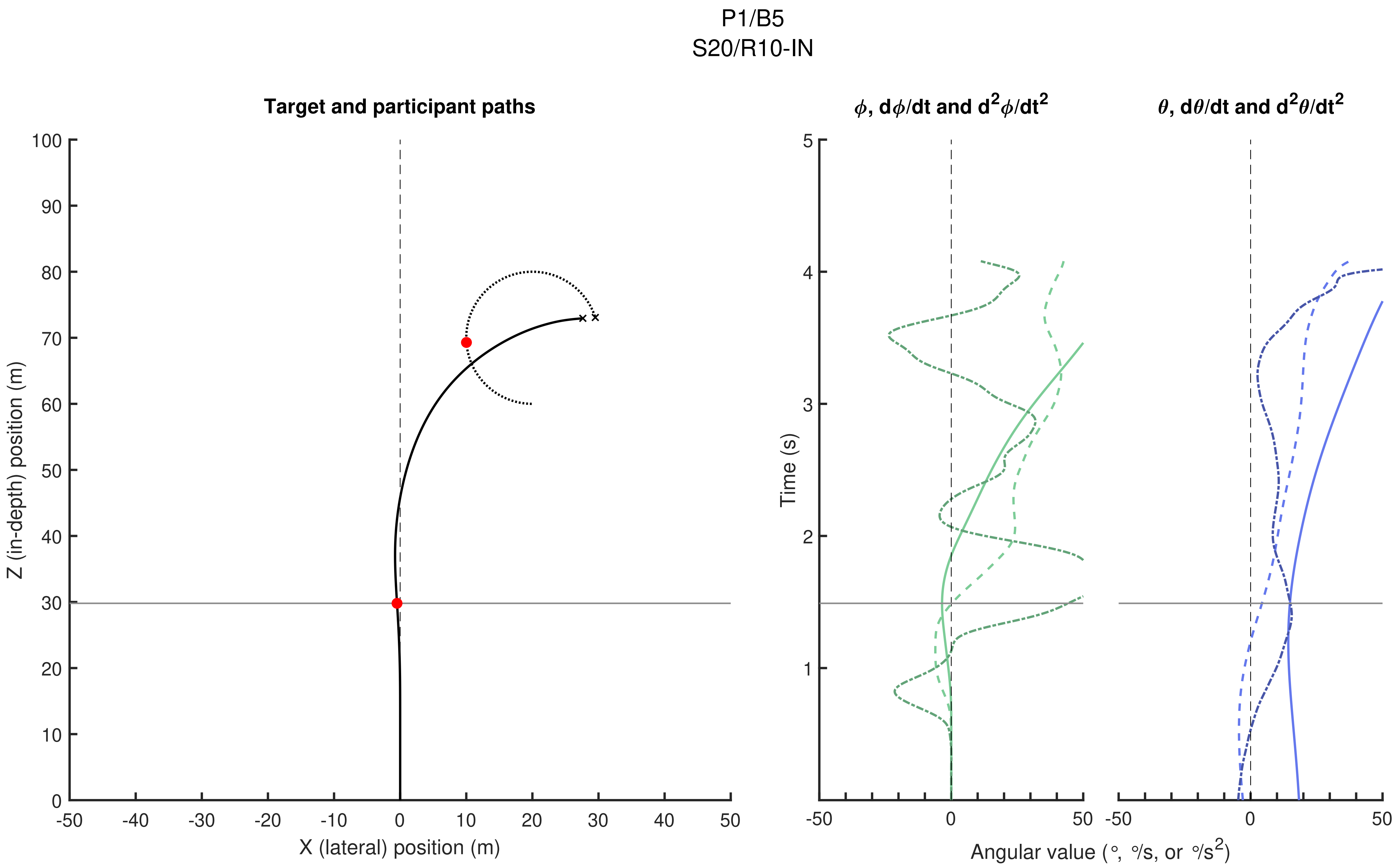

P1/B5
S20/R10-IN
Target and participant paths
Z (in-depth) position (m)
X (lateral) position (m)
ϕ, dϕ/dt and d$^2\phi$/dt^2
θ, dθ/dt and d$^2\theta$/dt^2
Time (s)
Angular value (°, °/s, or °/s^2)

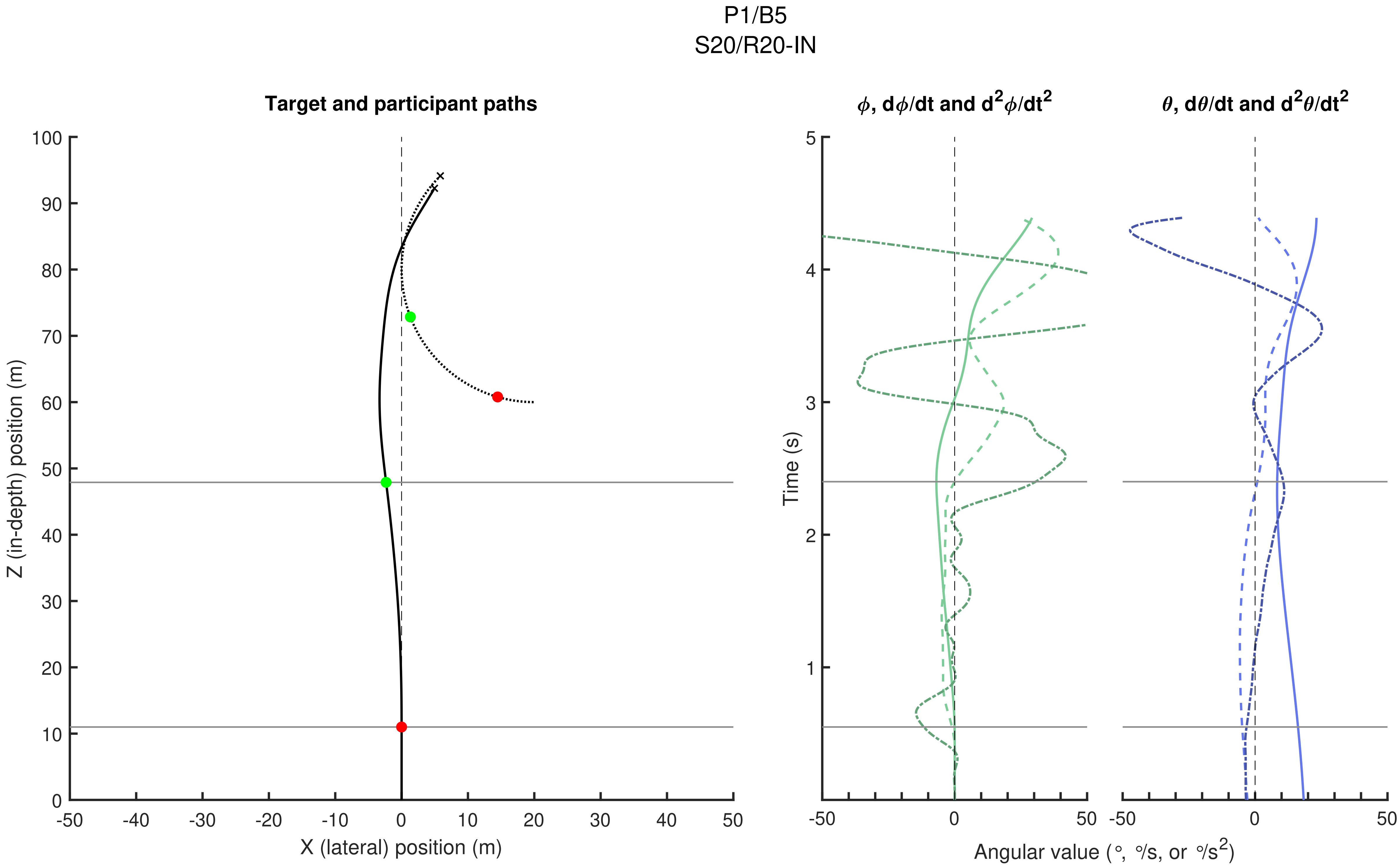

P1/B5
S20/R20-IN
Target and participant paths
ϕ, dϕ/dt and d²ϕ/dt²
θ, dθ/dt and d²θ/dt²
Z (in-depth) position (m)
X (lateral) position (m)
Time (s)
Angular value (°, °/s, or °/s²)

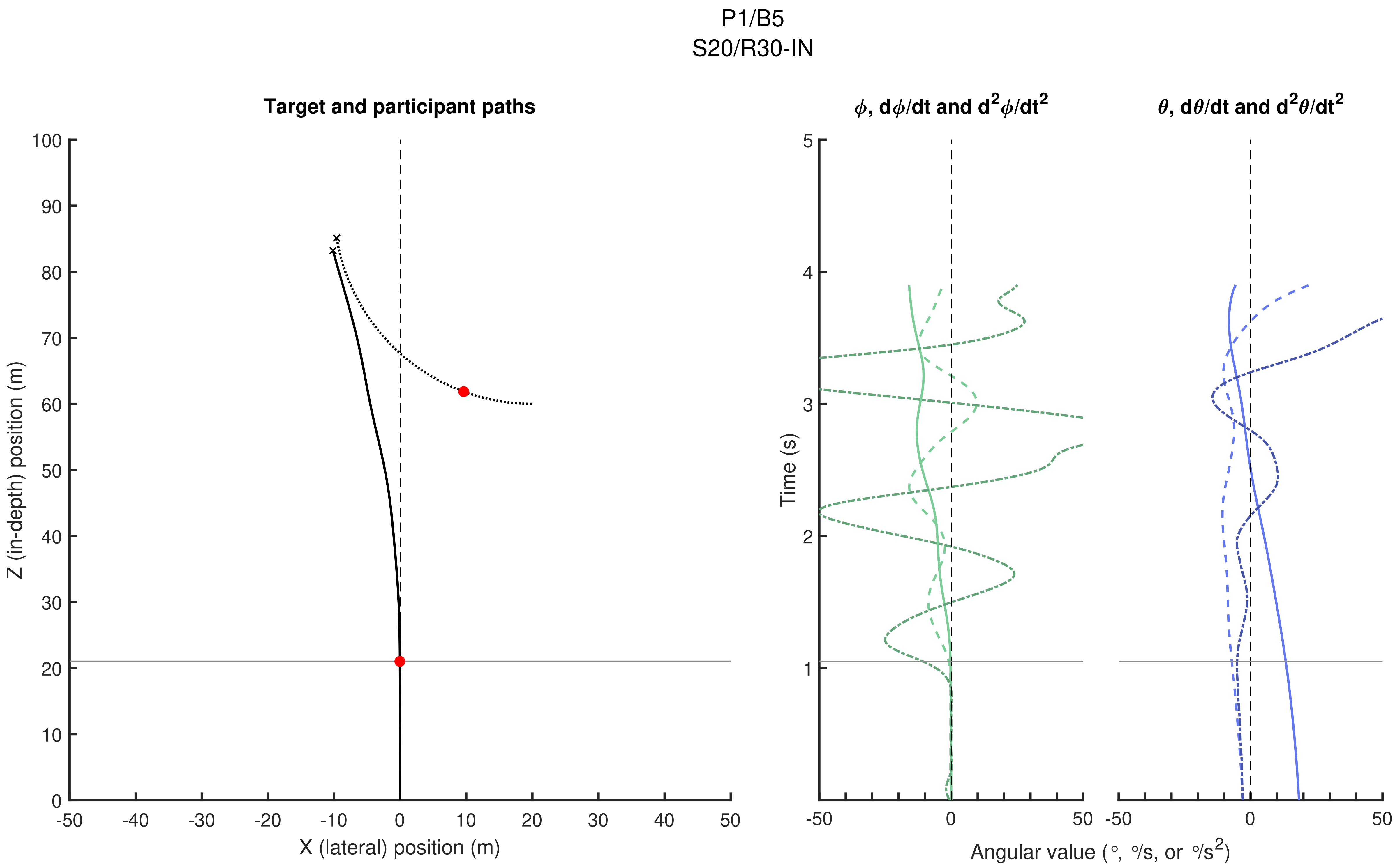
P1/B5
S20/R30-IN
Target and participant paths
Z (in-depth) position (m)
X (lateral) position (m)
ϕ, dϕ/dt and d$^2\phi$/dt^2
θ, dθ/dt and d$^2\theta$/dt^2
Time (s)
Angular value (°, °/s, or °/s^2)

P2/B1
S20/R10-IN

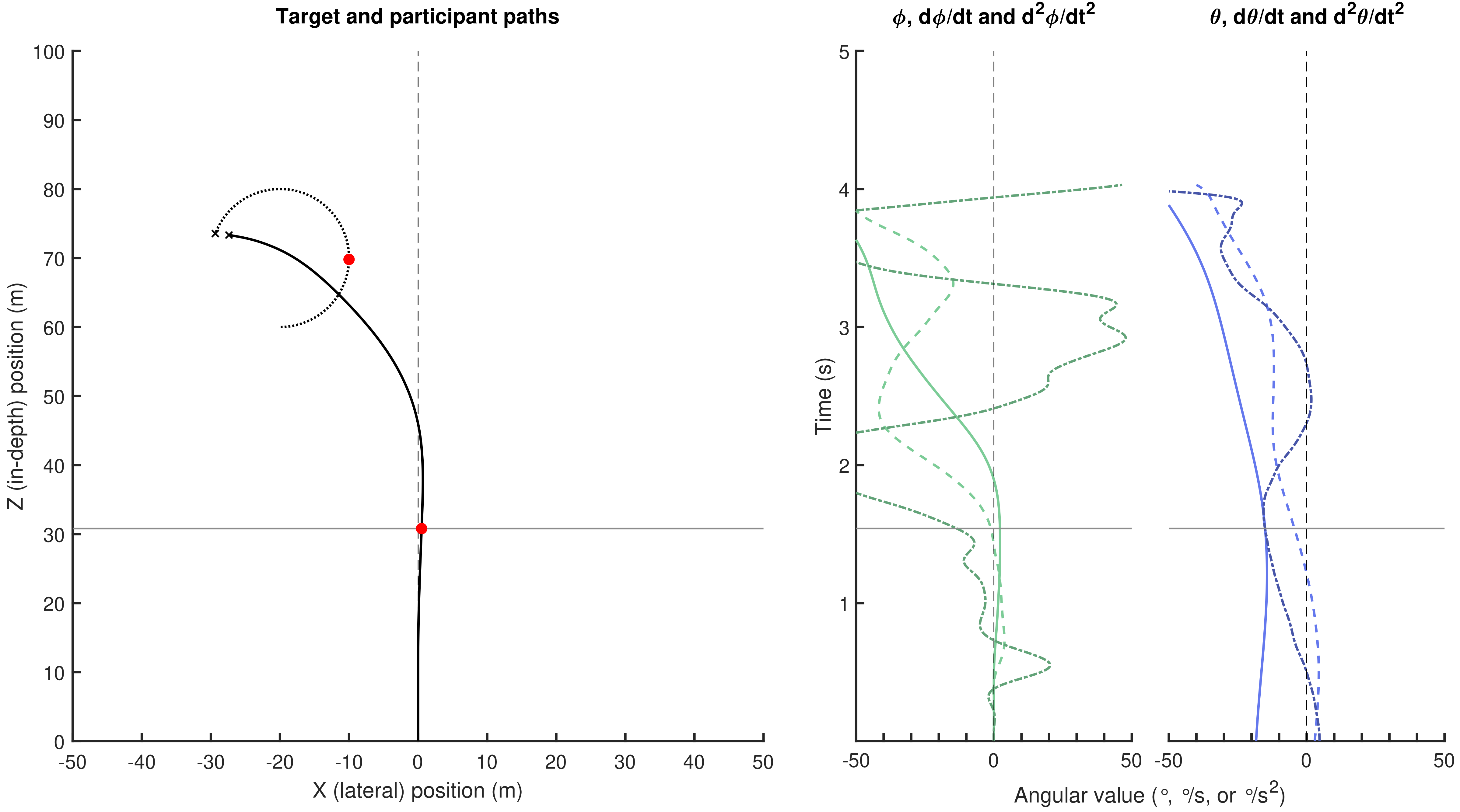

P2/B1
S20/R20-IN

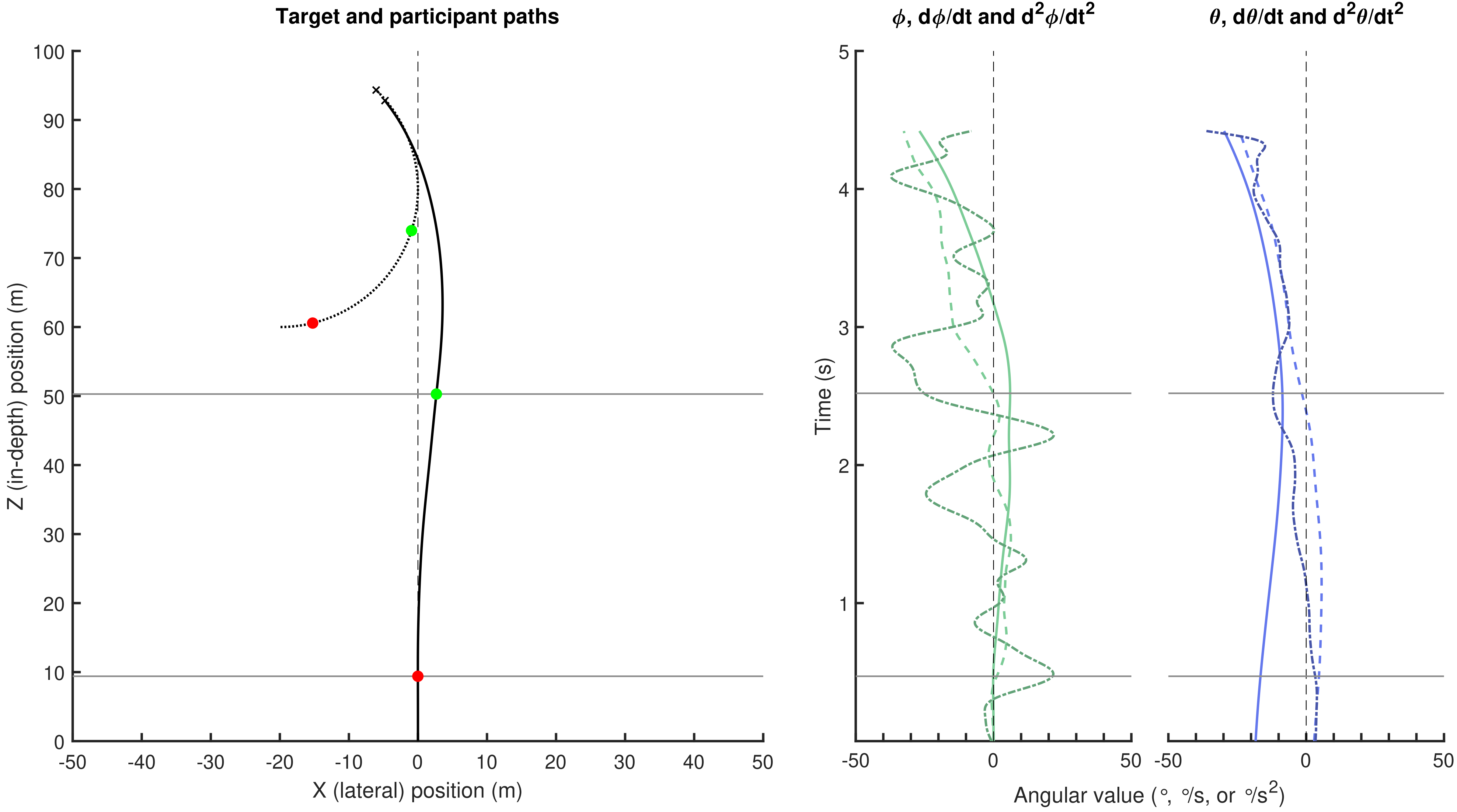

P2/B1
S20/R30-IN

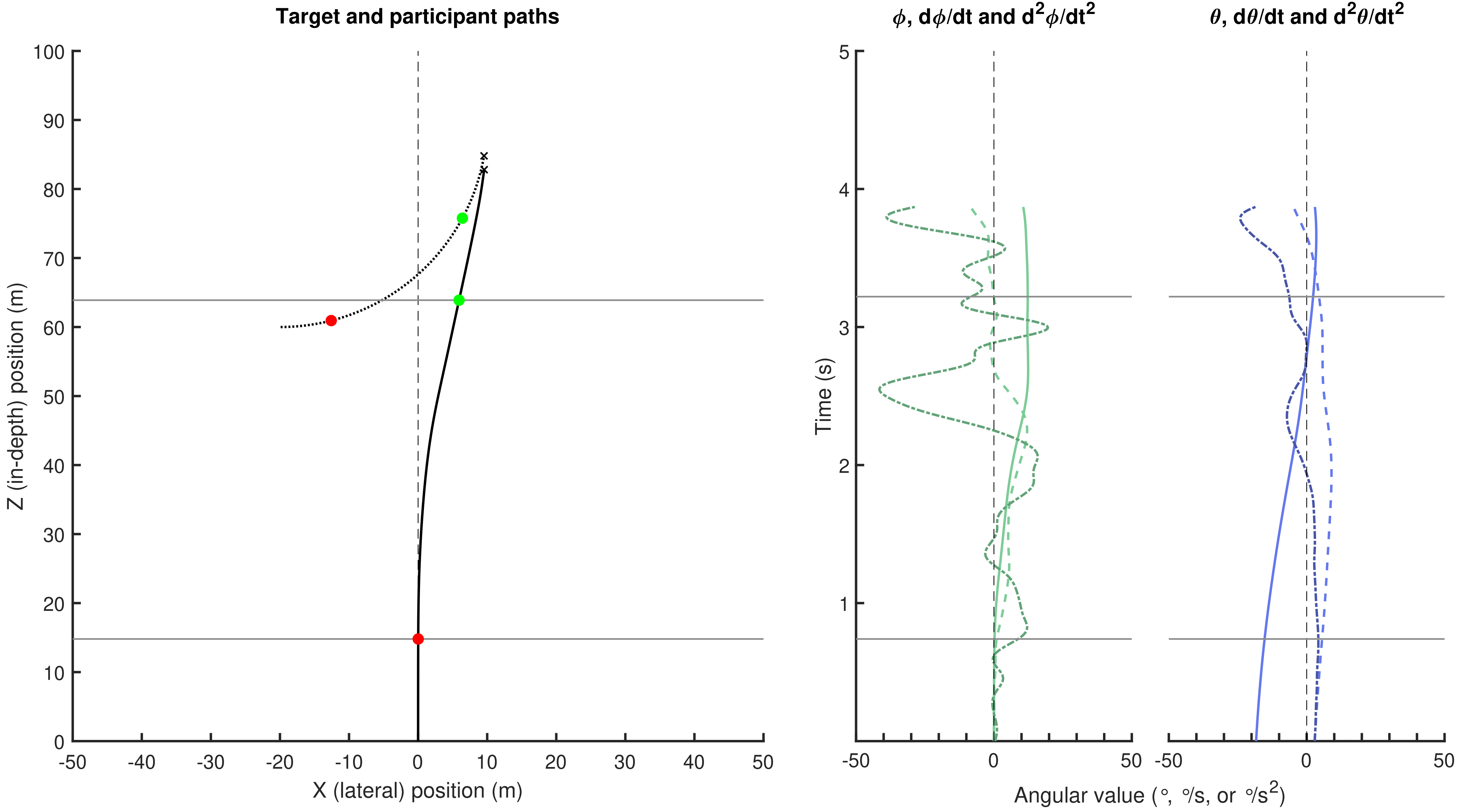

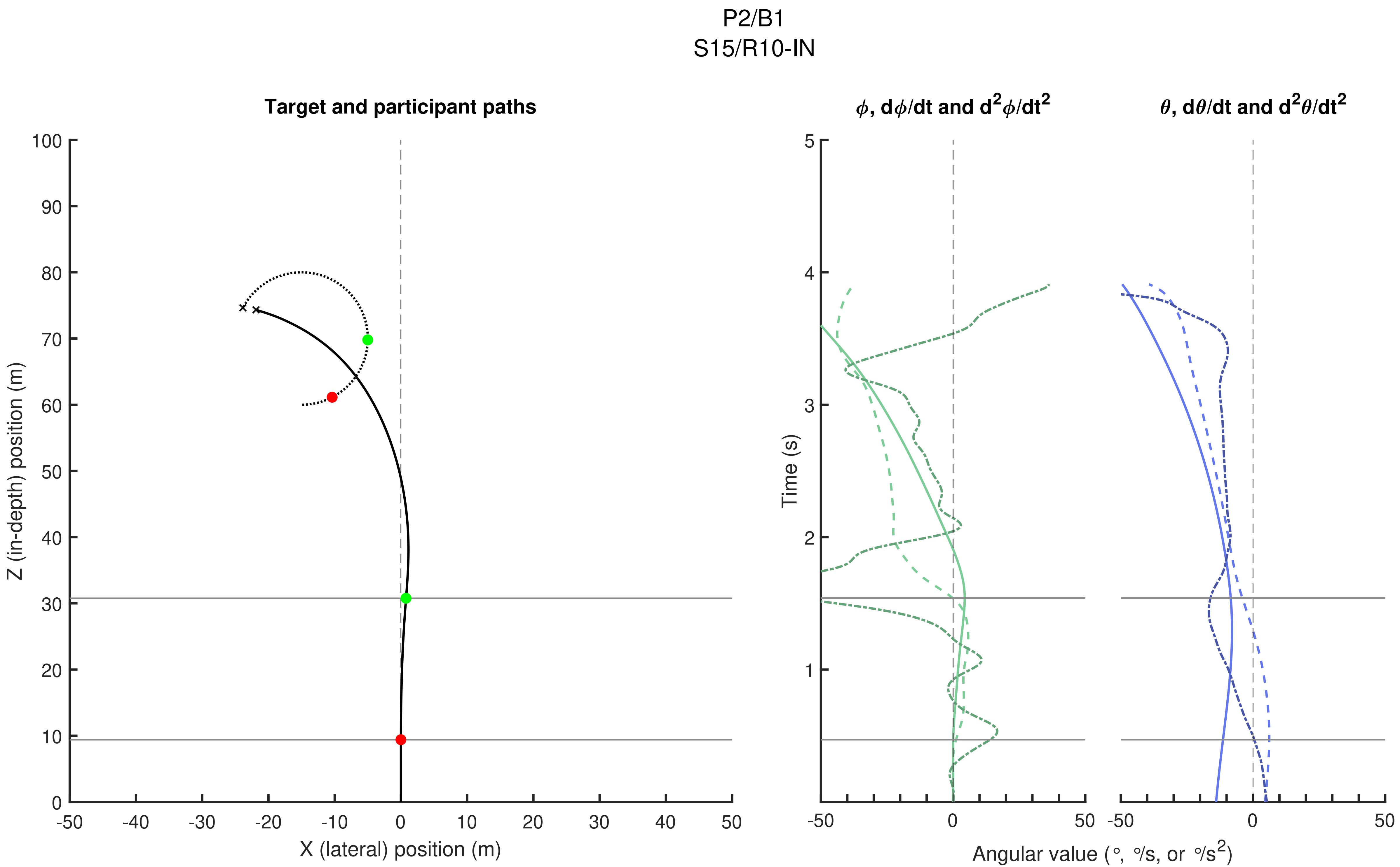

P2/B1
S15/R10-IN
Target and participant paths
Z (in-depth) position (m)
X (lateral) position (m)
100
90
80
70
60
50
40
30
20
10
0
-50
-40
-30
-20
-10
0
10
20
30
40
50
φ, dφ/dt and d²φ/dt²
θ, dθ/dt and d²θ/dt²
Time (s)
5
4
3
2
1
Angular value (°, °/s, or °/s²)

P2/B1
S15/R20-IN

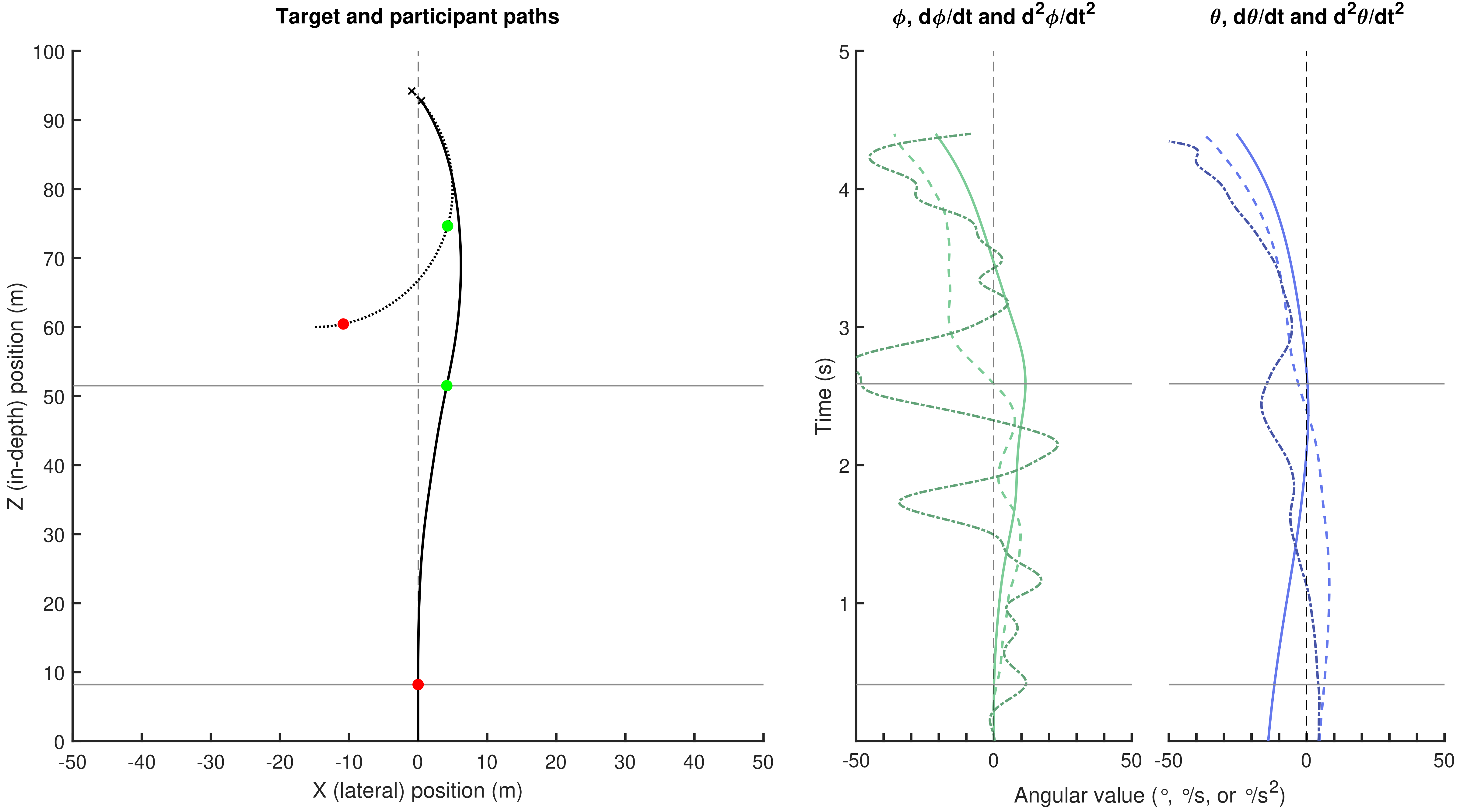

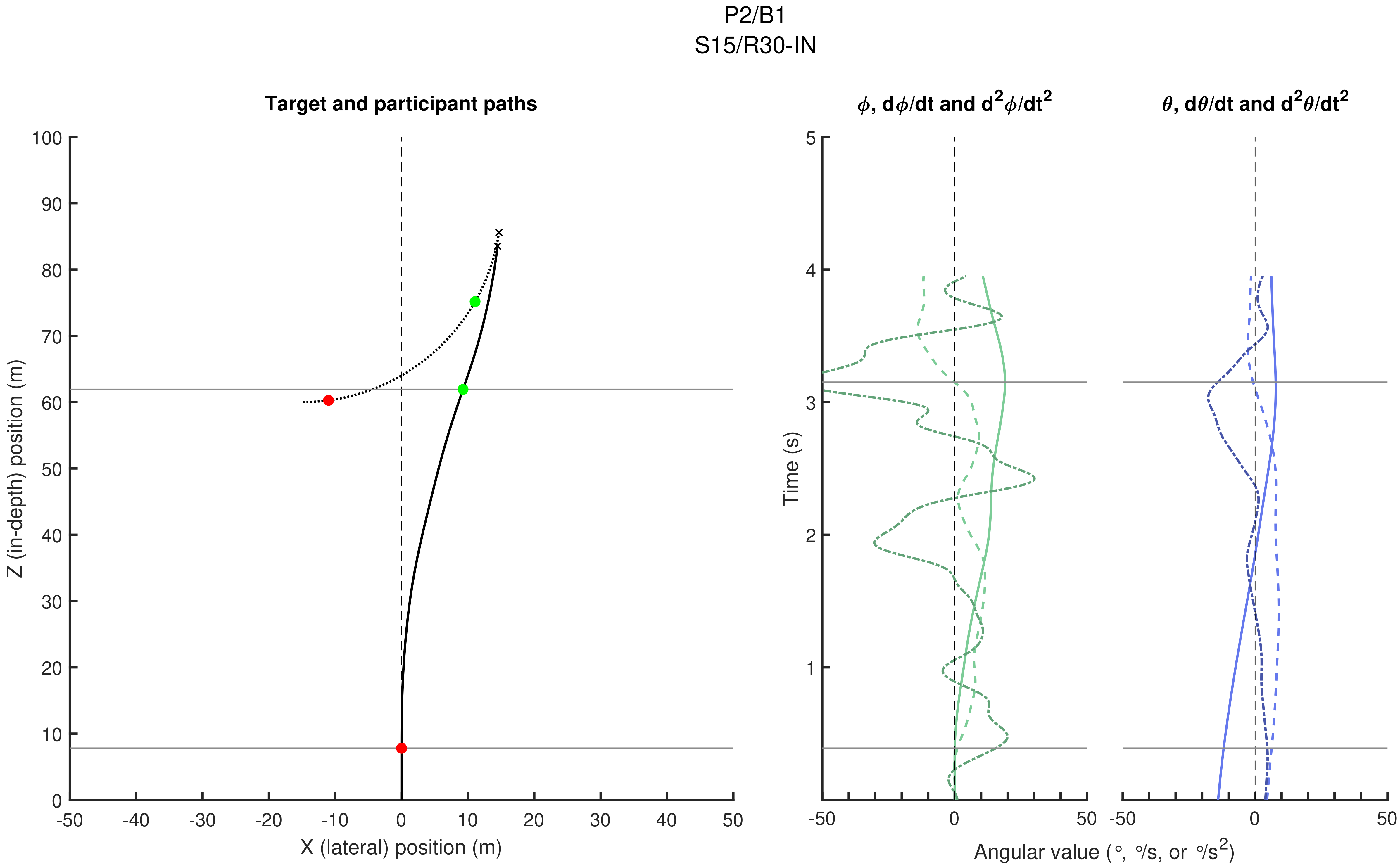

P2/B1
S15/R30-IN
Target and participant paths
φ, dφ/dt and d²φ/dt²
θ, dθ/dt and d²θ/dt²
Z (in-depth) position (m)
X (lateral) position (m)
Time (s)
Angular value (°, °/s, or °/s²)

P2/B1
S5/R10-IN

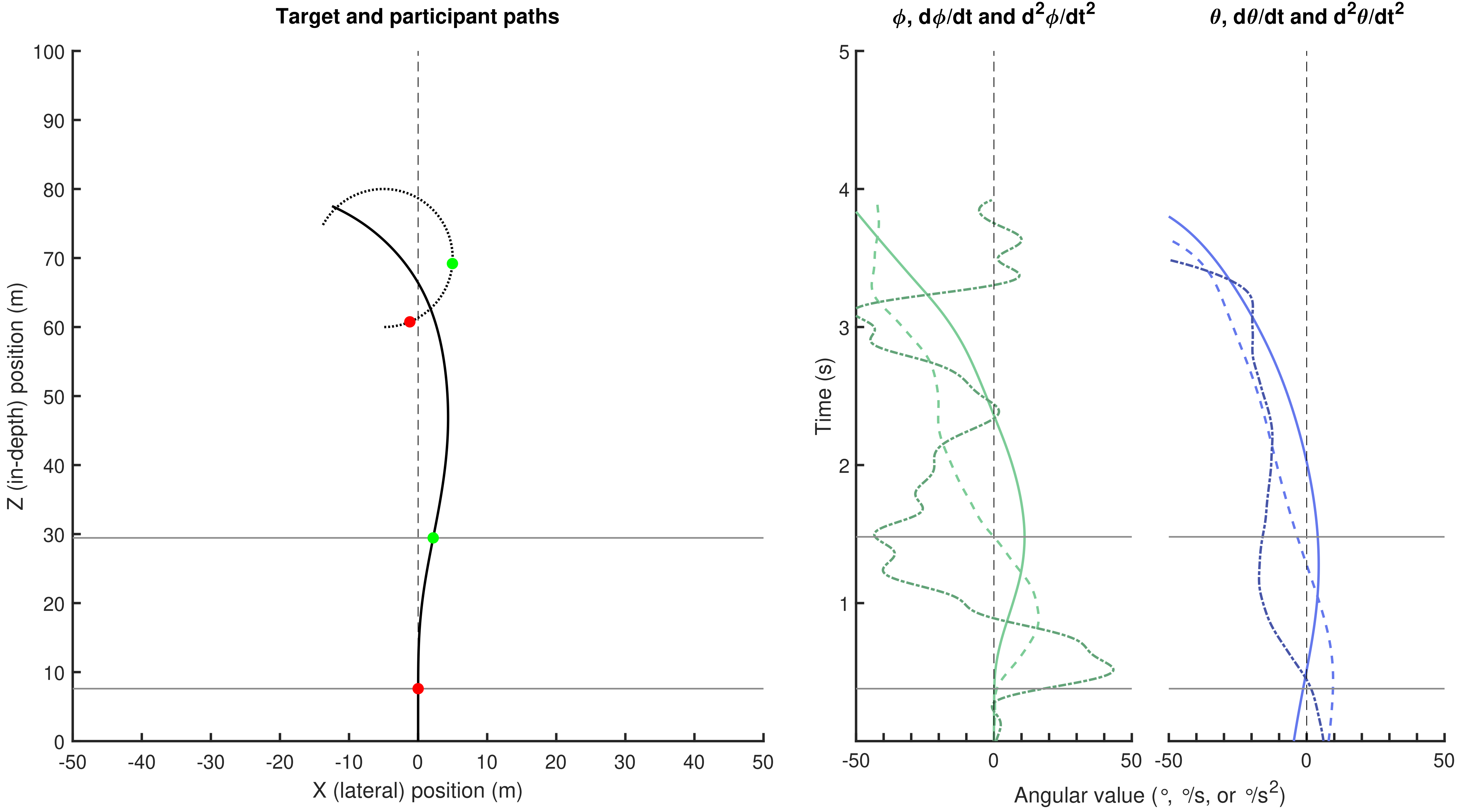

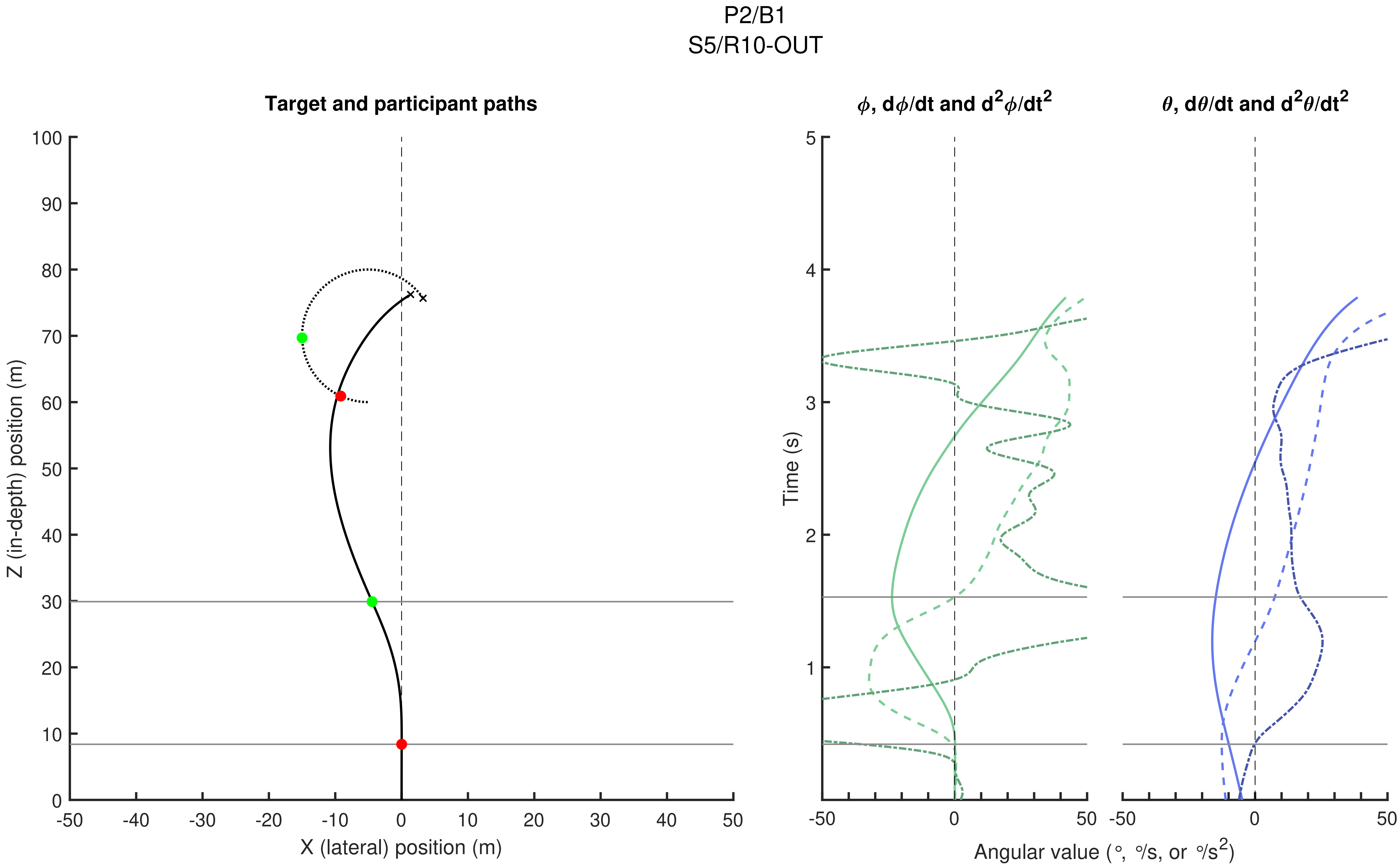

P2/B1
S5/R10-OUT
Target and participant paths
Z (in-depth) position (m)
X (lateral) position (m)
ϕ, dϕ/dt and d$^2\phi$/dt^2
θ, dθ/dt and d$^2\theta$/dt^2
Time (s)
Angular value (°, °/s, or °/s^2)
100
90
80
70
60
50
40
30
20
10
0
-50
-40
-30
-20
-10
0
10
20
30
40
50
5
4
3
2
1
-50
0
50
-50
0
50

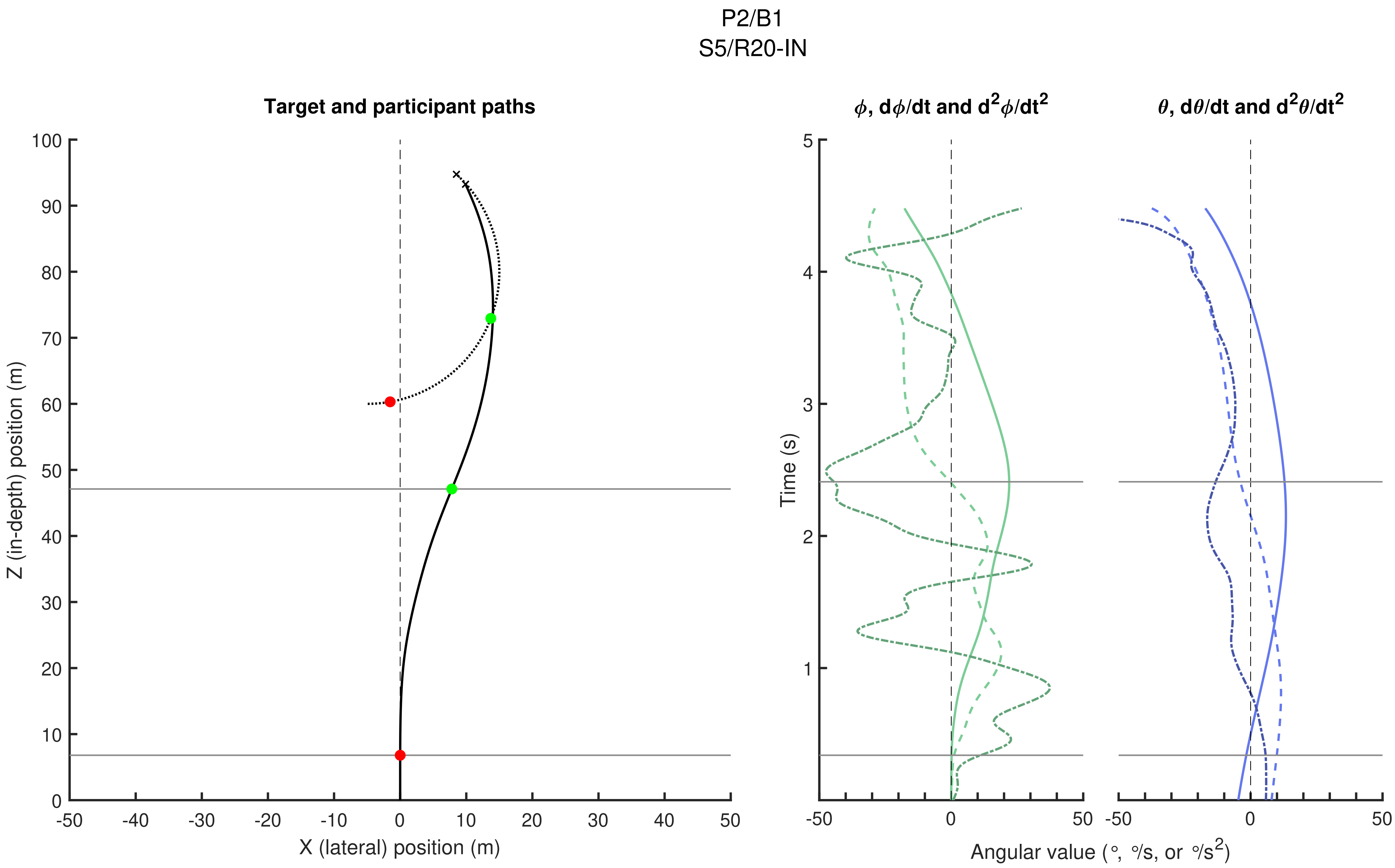

P2/B1
S5/R20-IN
Target and participant paths
Z (in-depth) position (m)
X (lateral) position (m)
ϕ, dϕ/dt and d$^2\phi$/dt^2
θ, dθ/dt and d$^2\theta$/dt^2
Time (s)
Angular value (°, °/s, or °/s^2)

P2/B1
S5/R20-OUT

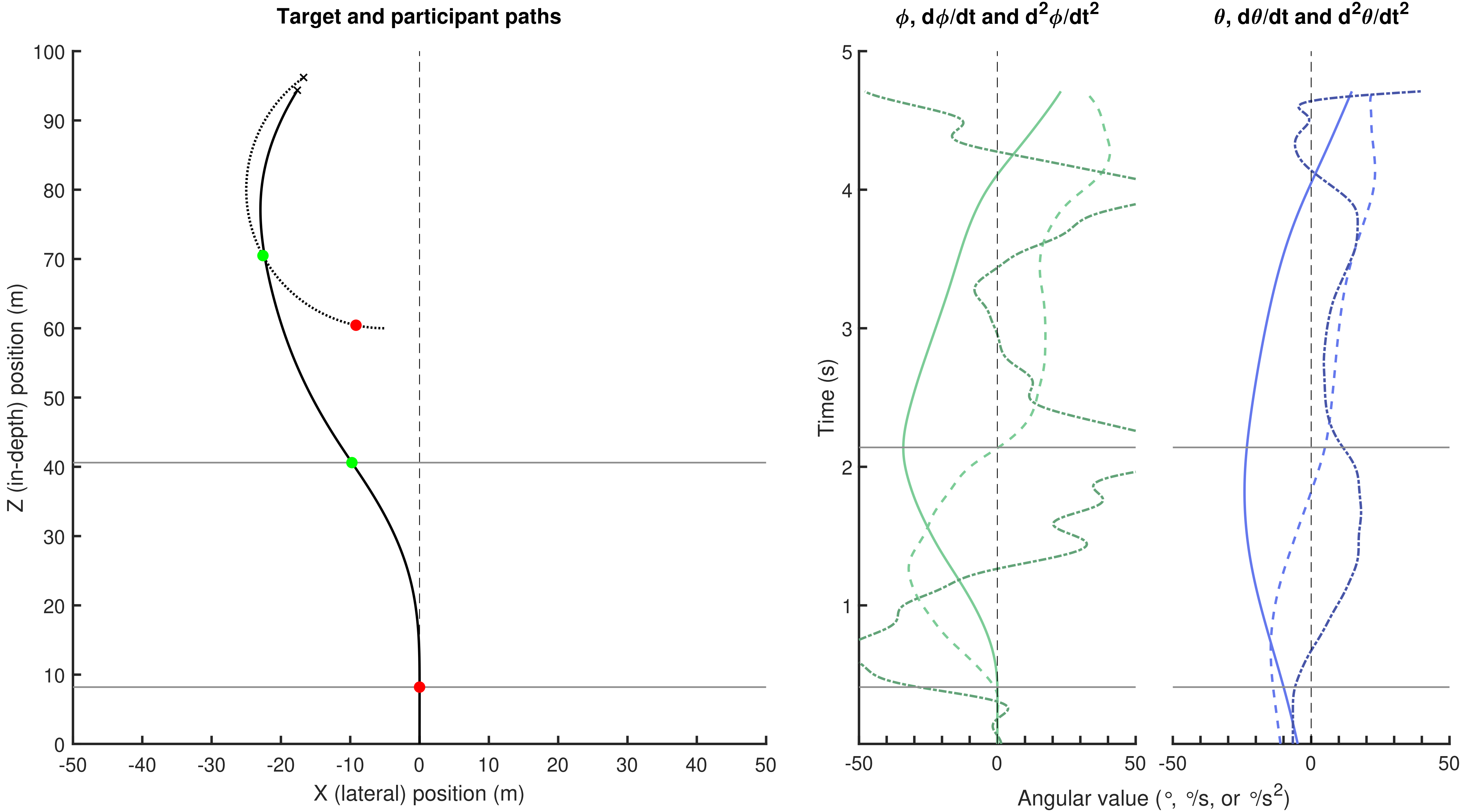

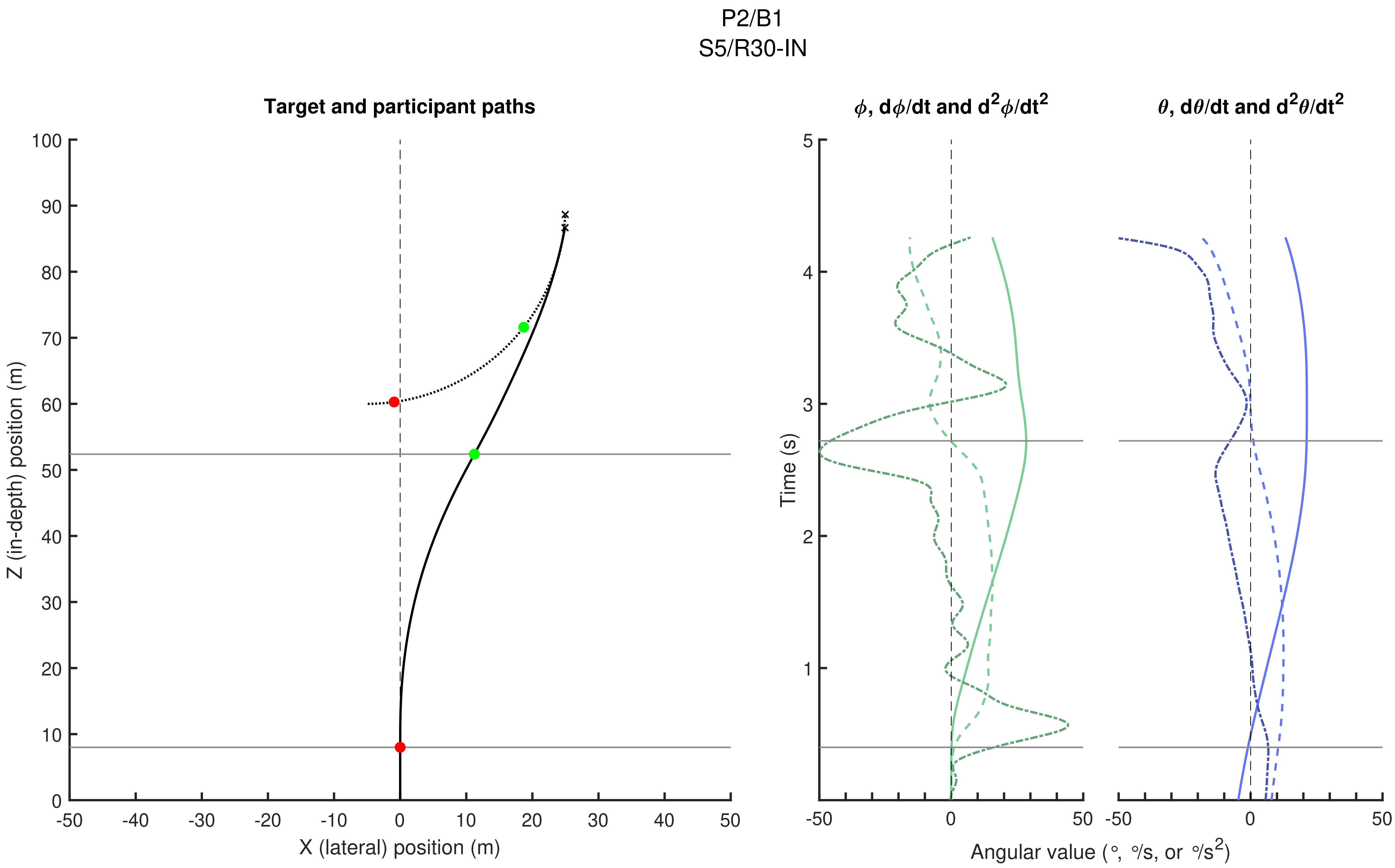

P2/B1
S5/R30-IN
Target and participant paths
Z (in-depth) position (m)
X (lateral) position (m)
ϕ, dϕ/dt and d$^2\phi$/dt^2
θ, dθ/dt and d$^2\theta$/dt^2
Time (s)
Angular value (°, °/s, or °/s^2)

P2/B1
S5/R30-OUT

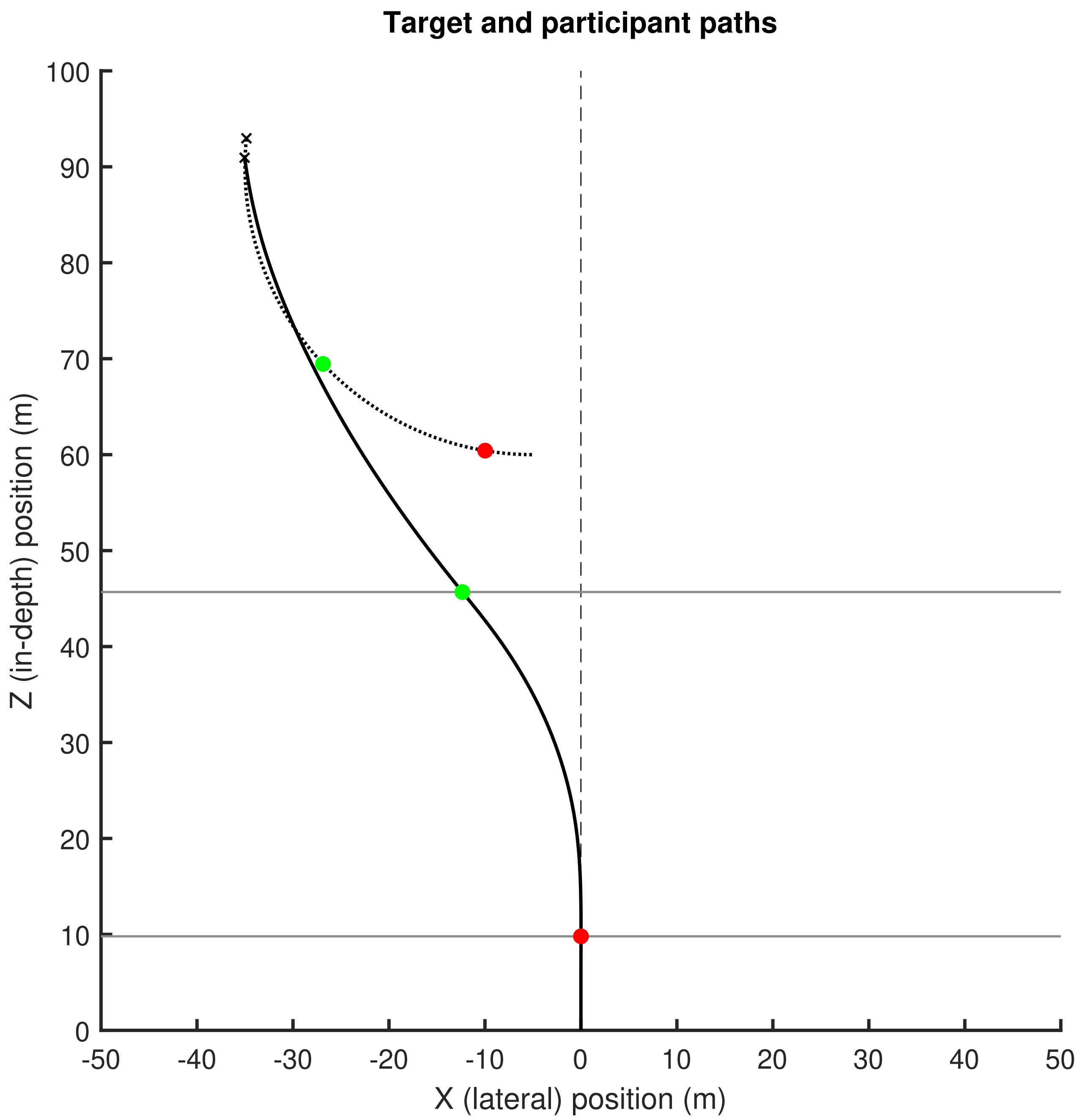

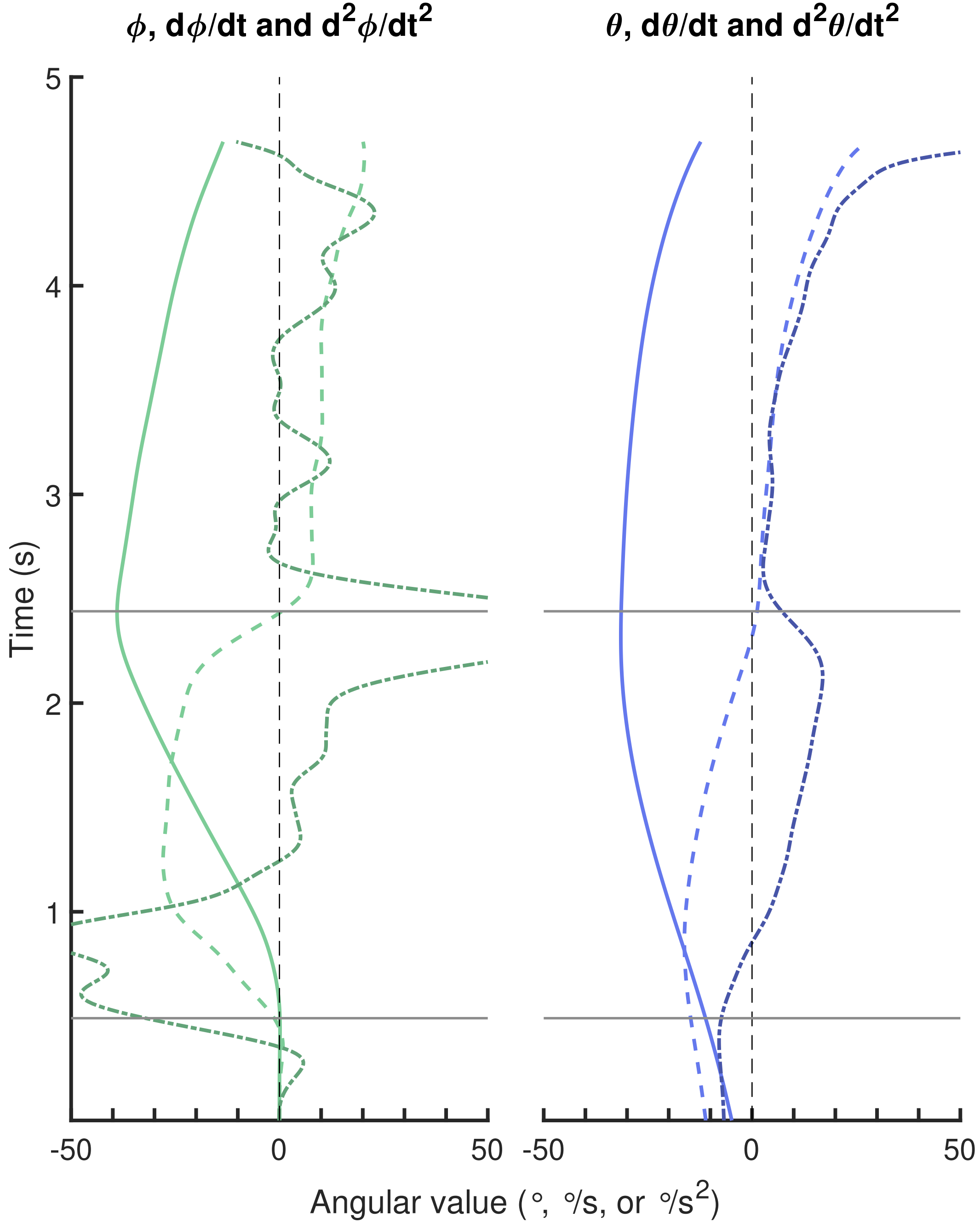

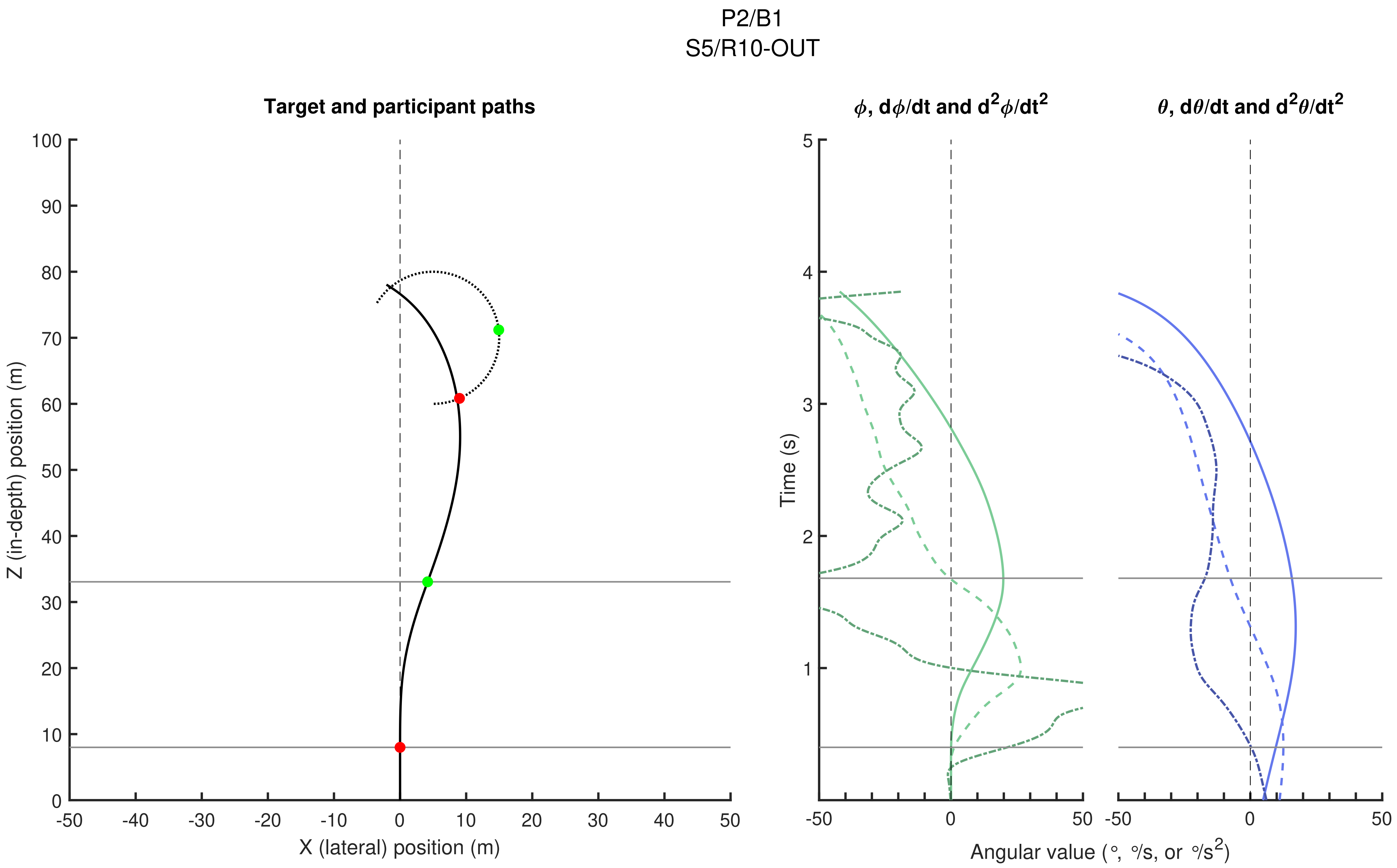

P2/B1
S5/R10-OUT
Target and participant paths
ϕ, dϕ/dt and d$^2\phi$/dt^2
θ, dθ/dt and d$^2\theta$/dt^2
Z (in-depth) position (m)
X (lateral) position (m)
Time (s)
Angular value (°, °/s, or °/s^2)

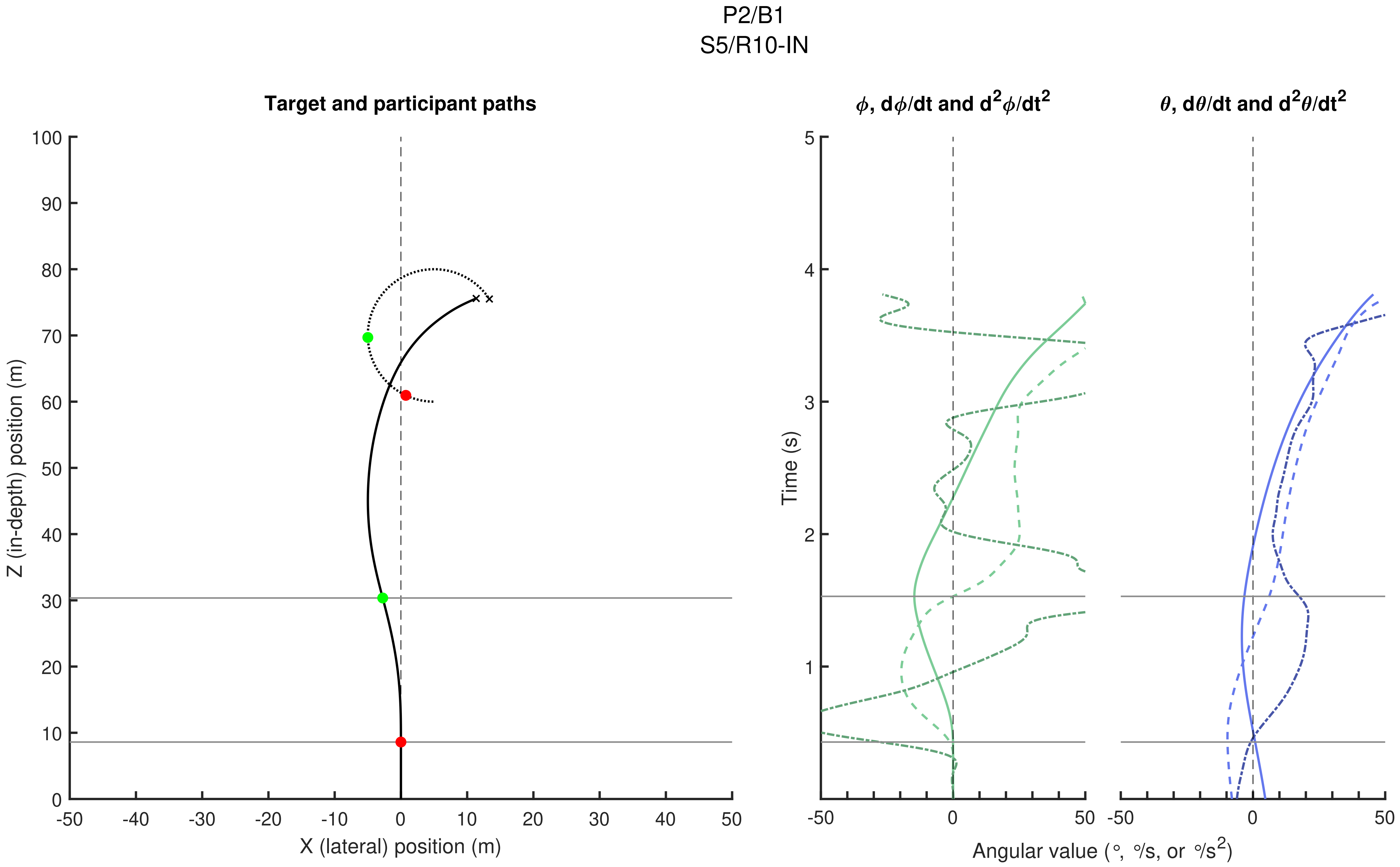
P2/B1
S5/R10-IN
Target and participant paths
Z (in-depth) position (m)
X (lateral) position (m)
ϕ, dϕ/dt and d²ϕ/dt²
θ, dθ/dt and d²θ/dt²
Time (s)
Angular value (°, °/s, or °/s²)

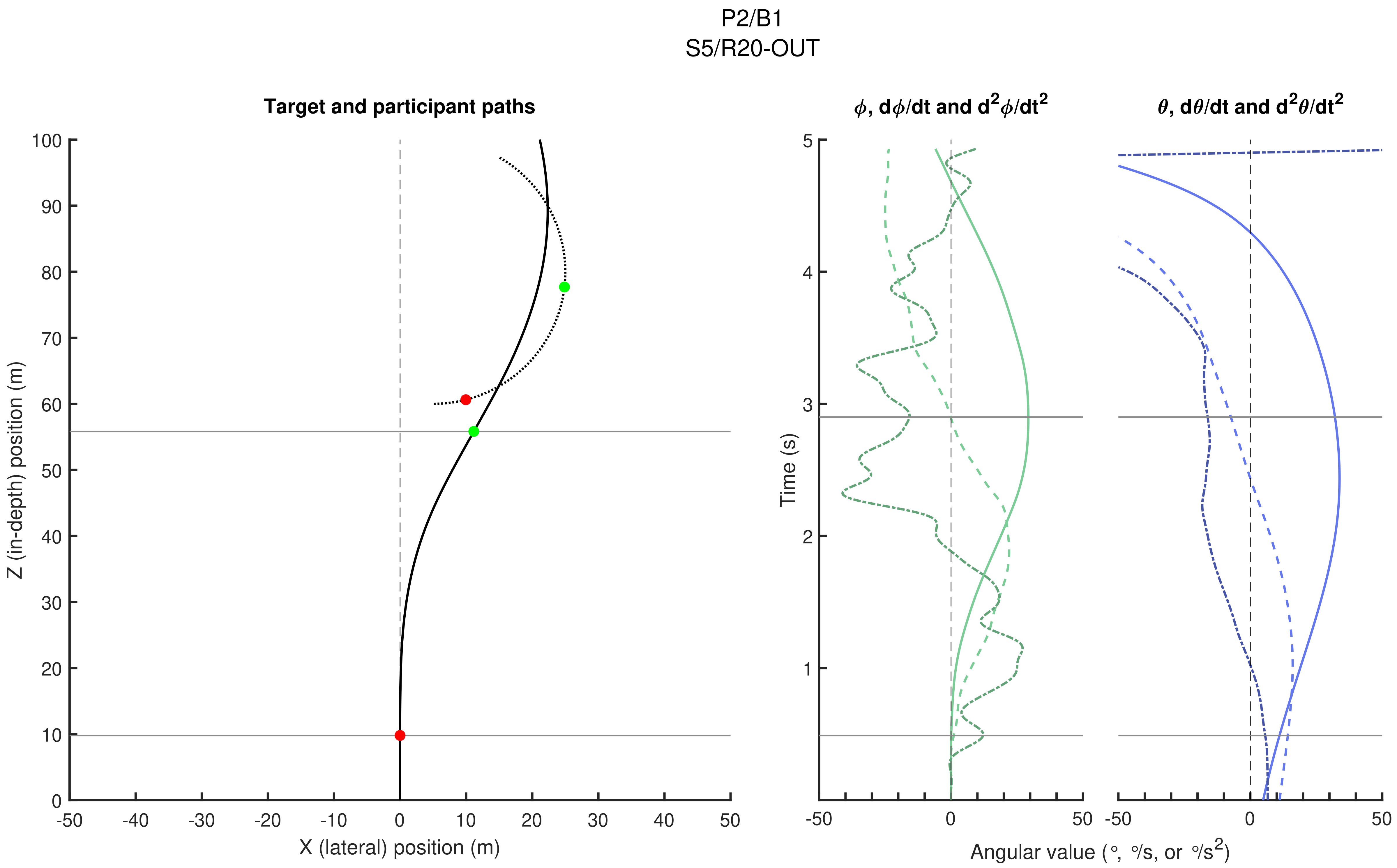
P2/B1
S5/R20-OUT
Target and participant paths
ϕ, dϕ/dt and d$^2\phi$/dt^2
θ, dθ/dt and d$^2\theta$/dt^2
Z (in-depth) position (m)
X (lateral) position (m)
Time (s)
Angular value (°, °/s, or °/s^2)
100
90
80
70
60
50
40
30
20
10
0
-50
-40
-30
-20
-10
0
10
20
30
40
50
5
4
3
2
1
-50
0
50
-50
0
50

P2/B1
S5/R20-IN

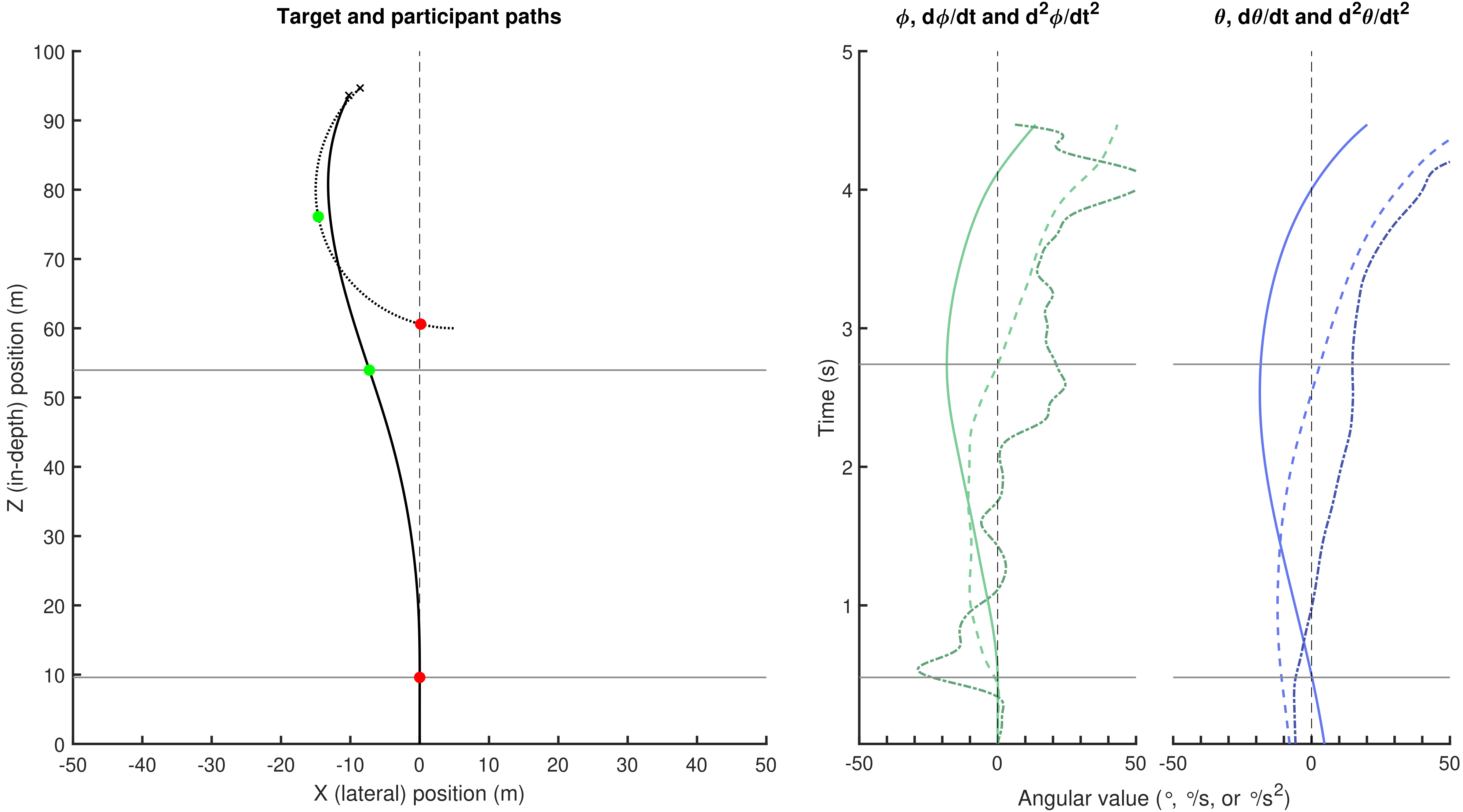

P2/B1
S5/R30-OUT

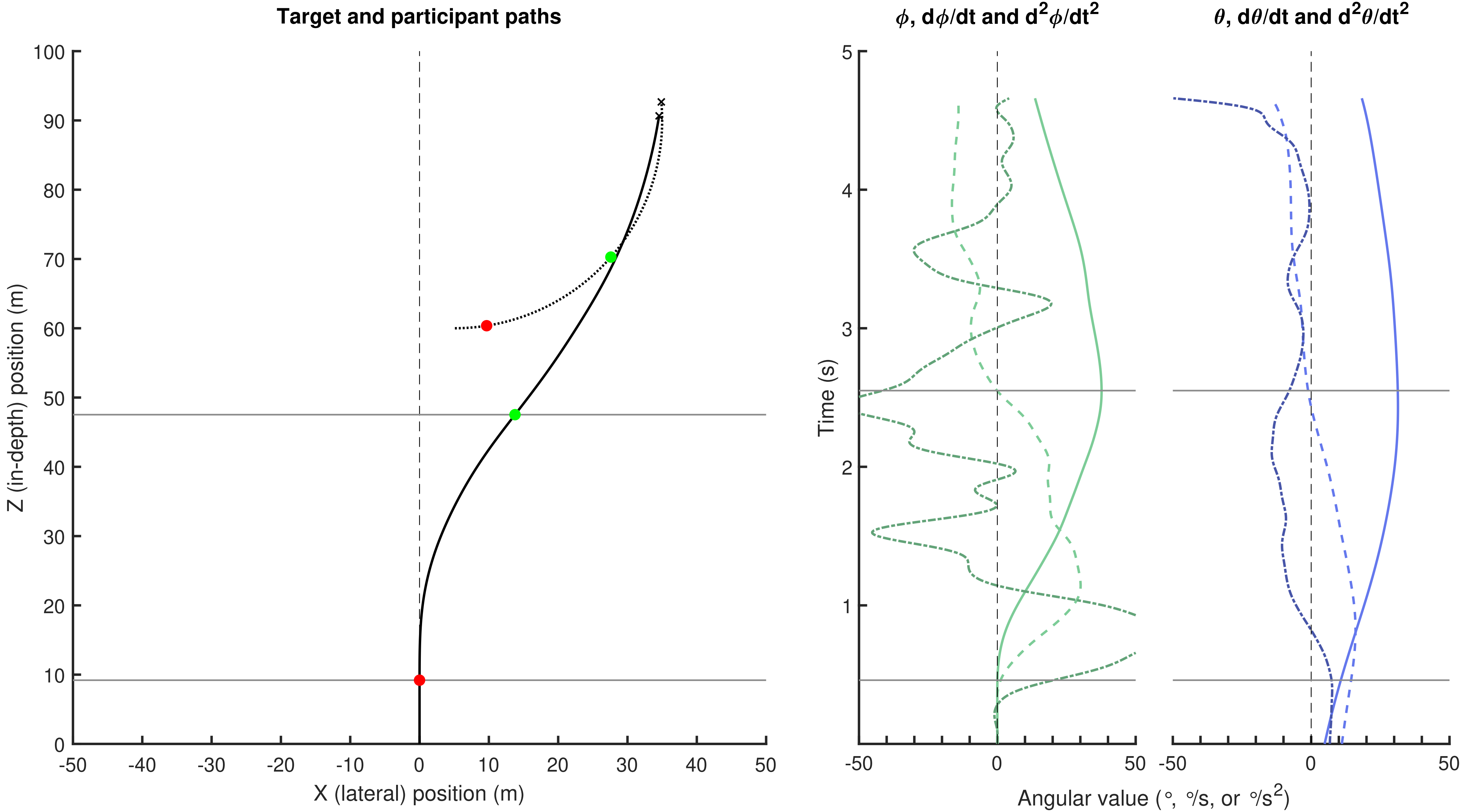

P2/B1
S5/R30-IN

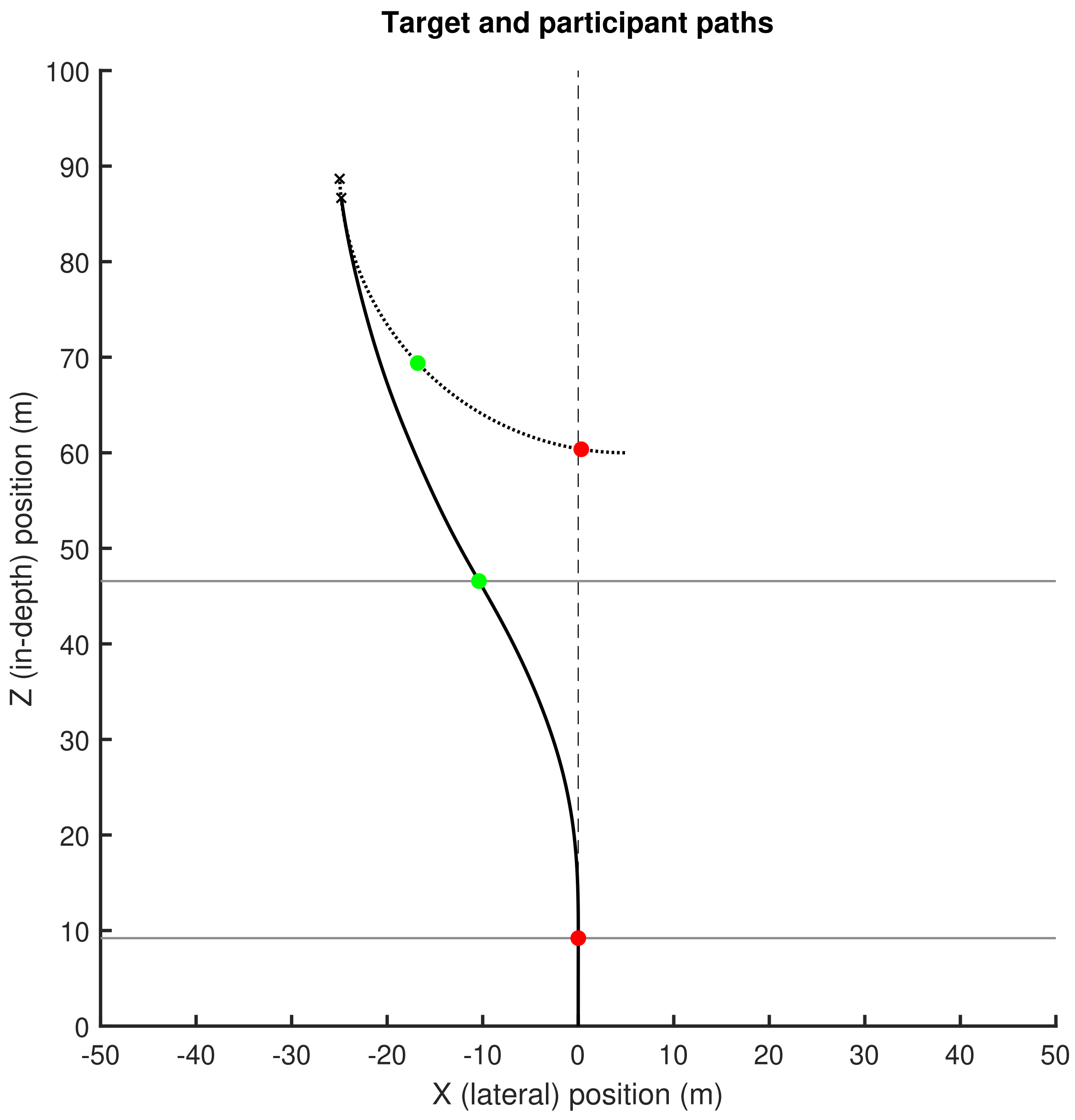

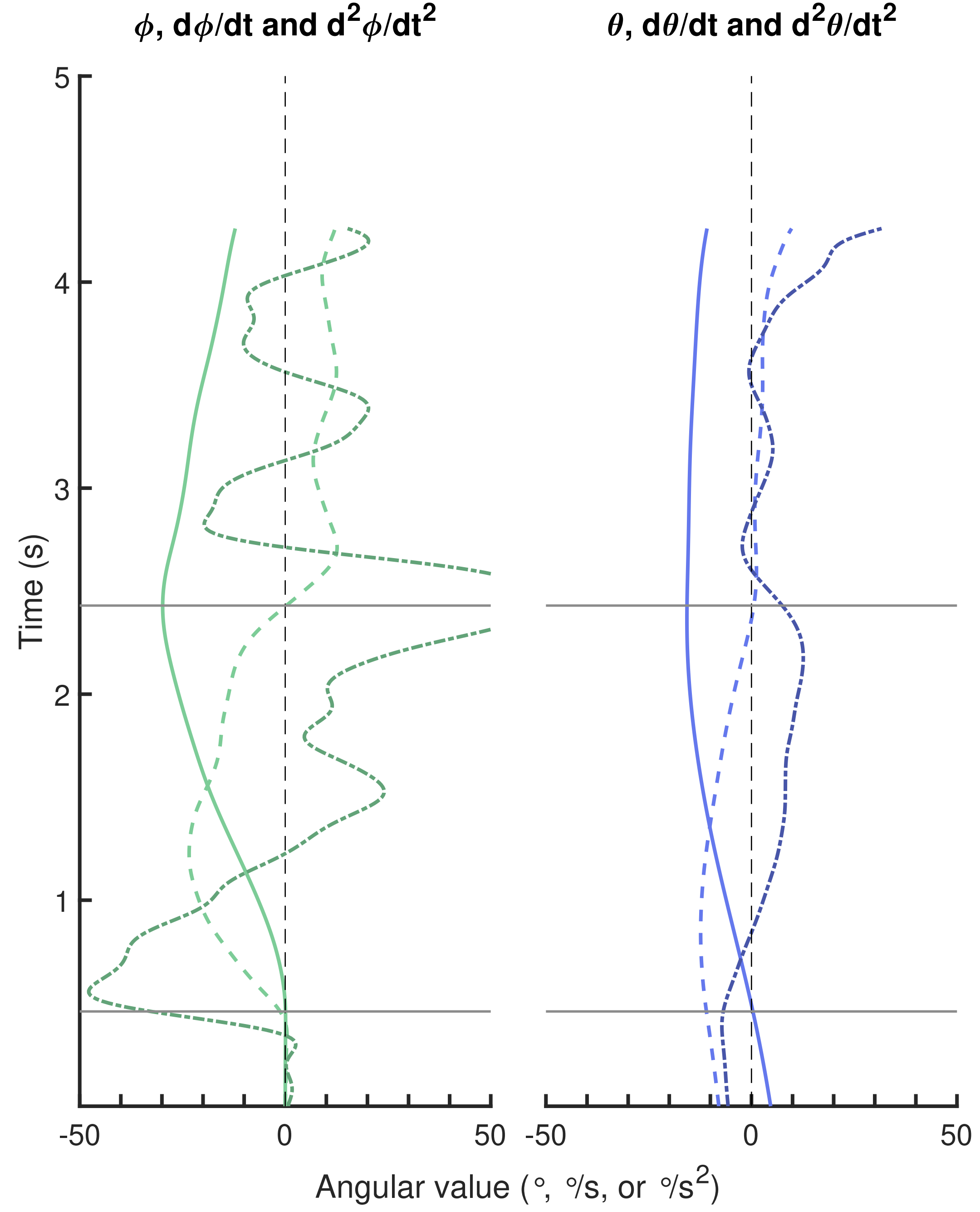

P2/B1
S15/R10-IN

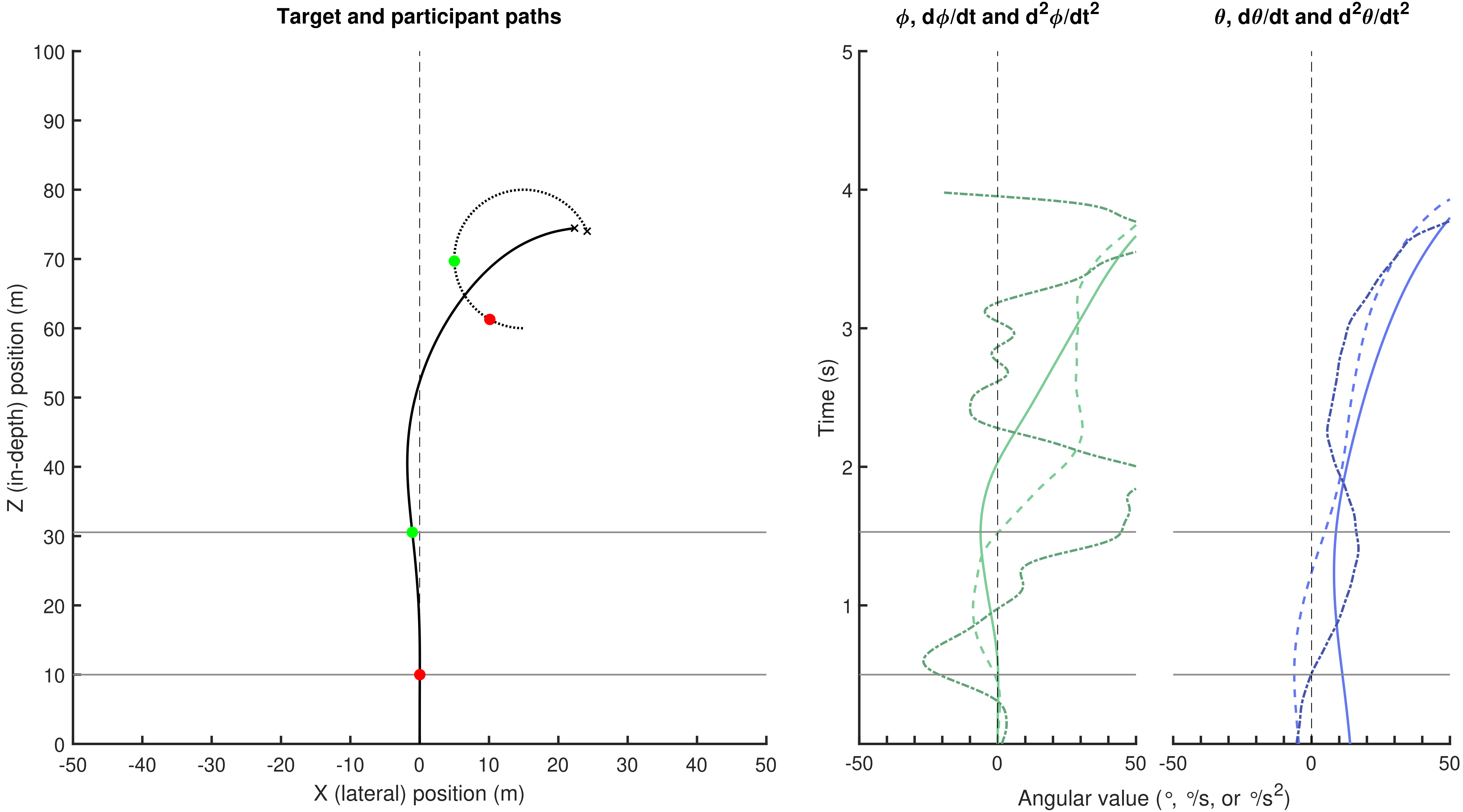

P2/B1
S15/R20-IN

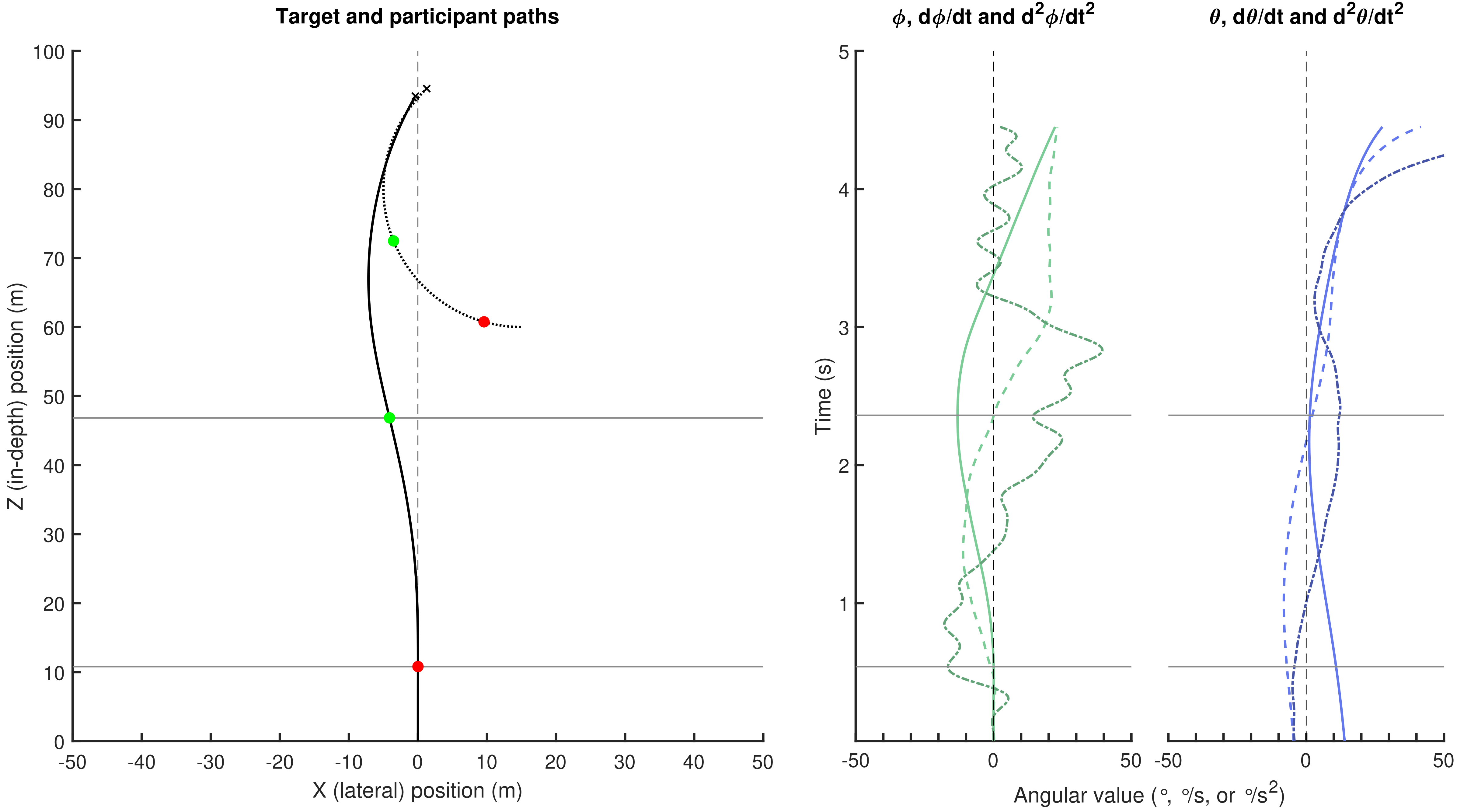

P2/B1
S15/R30-IN

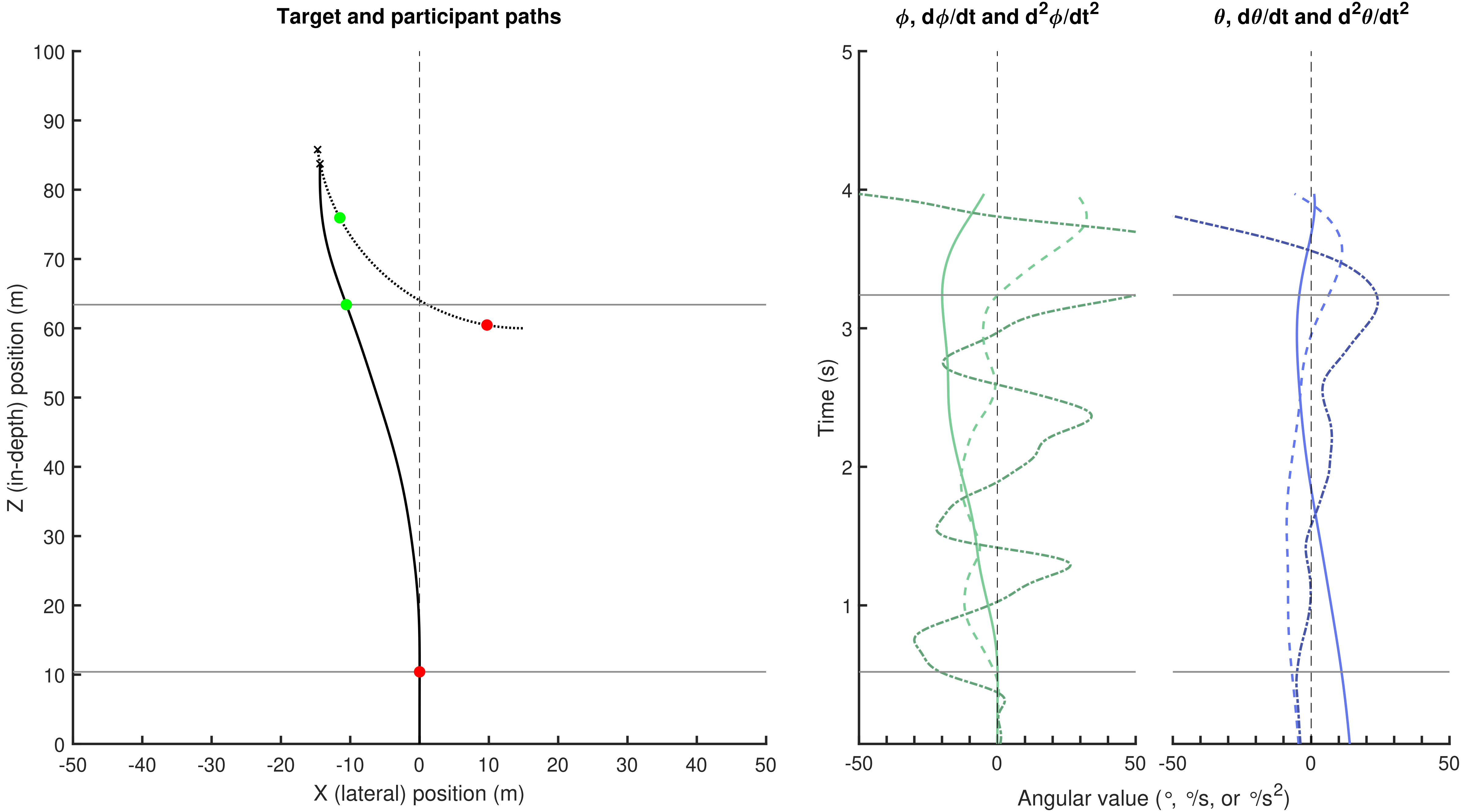

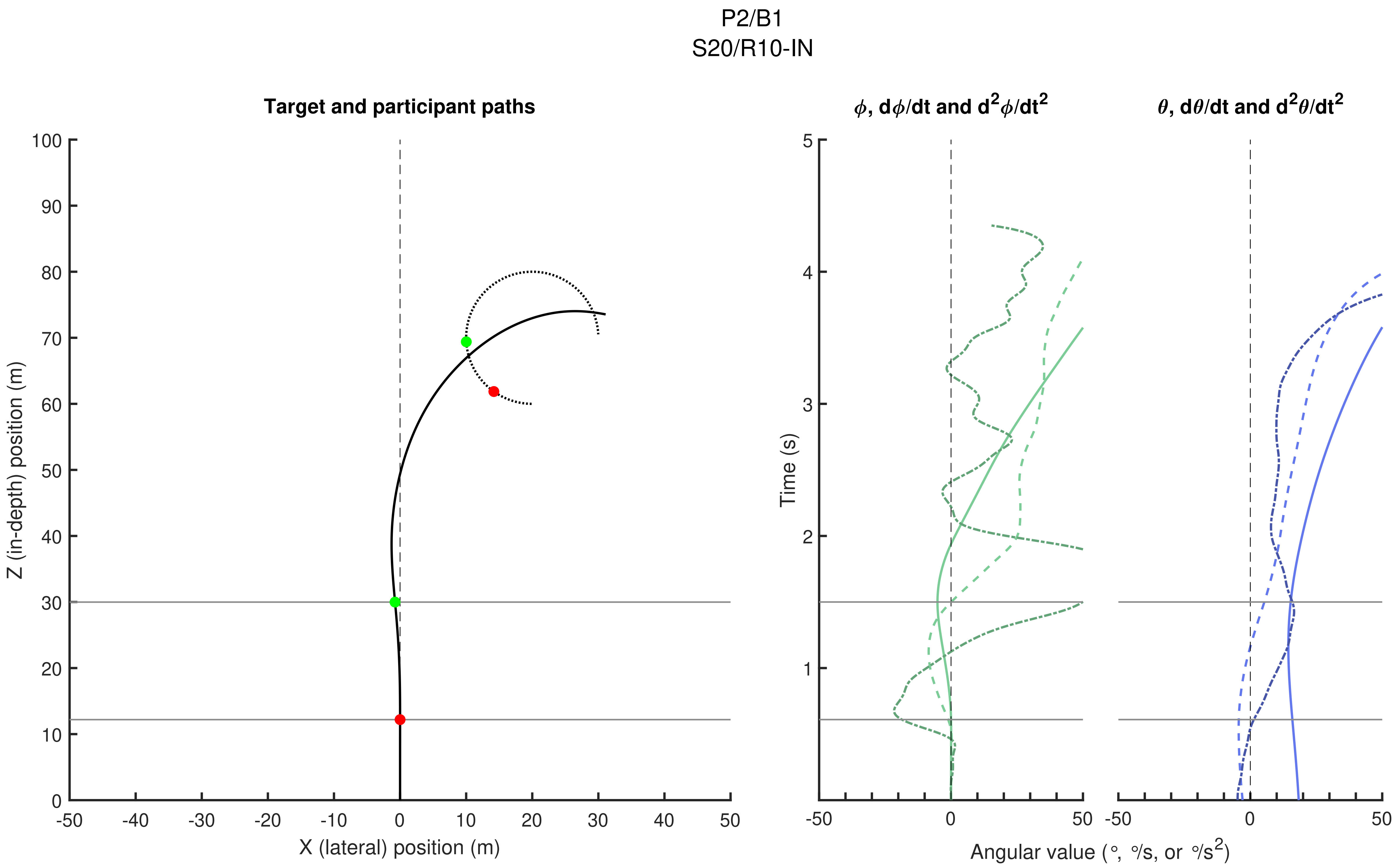

P2/B1
S20/R10-IN
Target and participant paths
Z (in-depth) position (m)
X (lateral) position (m)
100
90
80
70
60
50
40
30
20
10
0
-50
-40
-30
-20
-10
0
10
20
30
40
50
ϕ, dϕ/dt and d$^2\phi$/dt^2
θ, dθ/dt and d$^2\theta$/dt^2
Time (s)
5
4
3
2
1
-50
0
50
-50
0
50
Angular value (°, °/s, or °/s^2)

P2/B1
S20/R20-IN

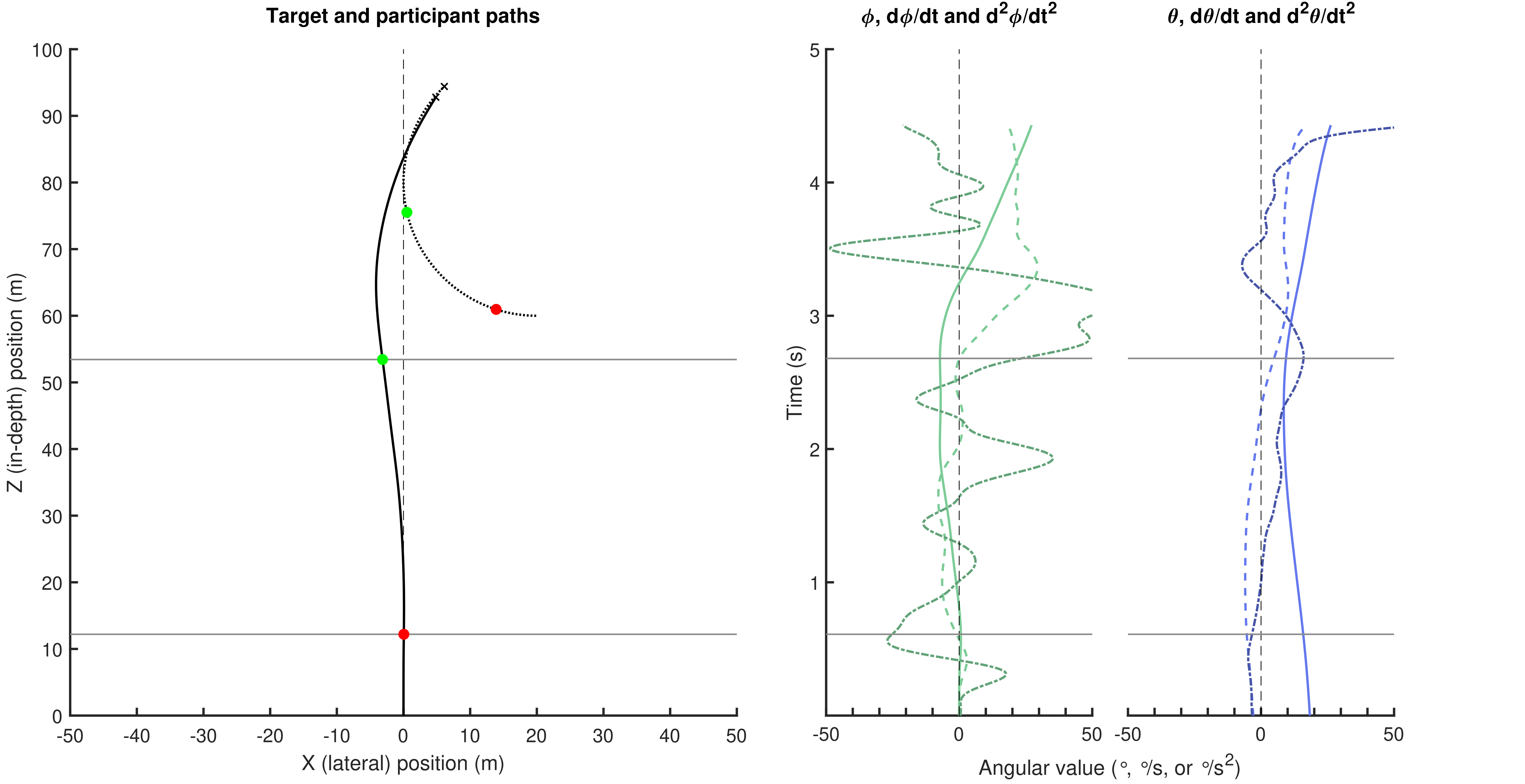

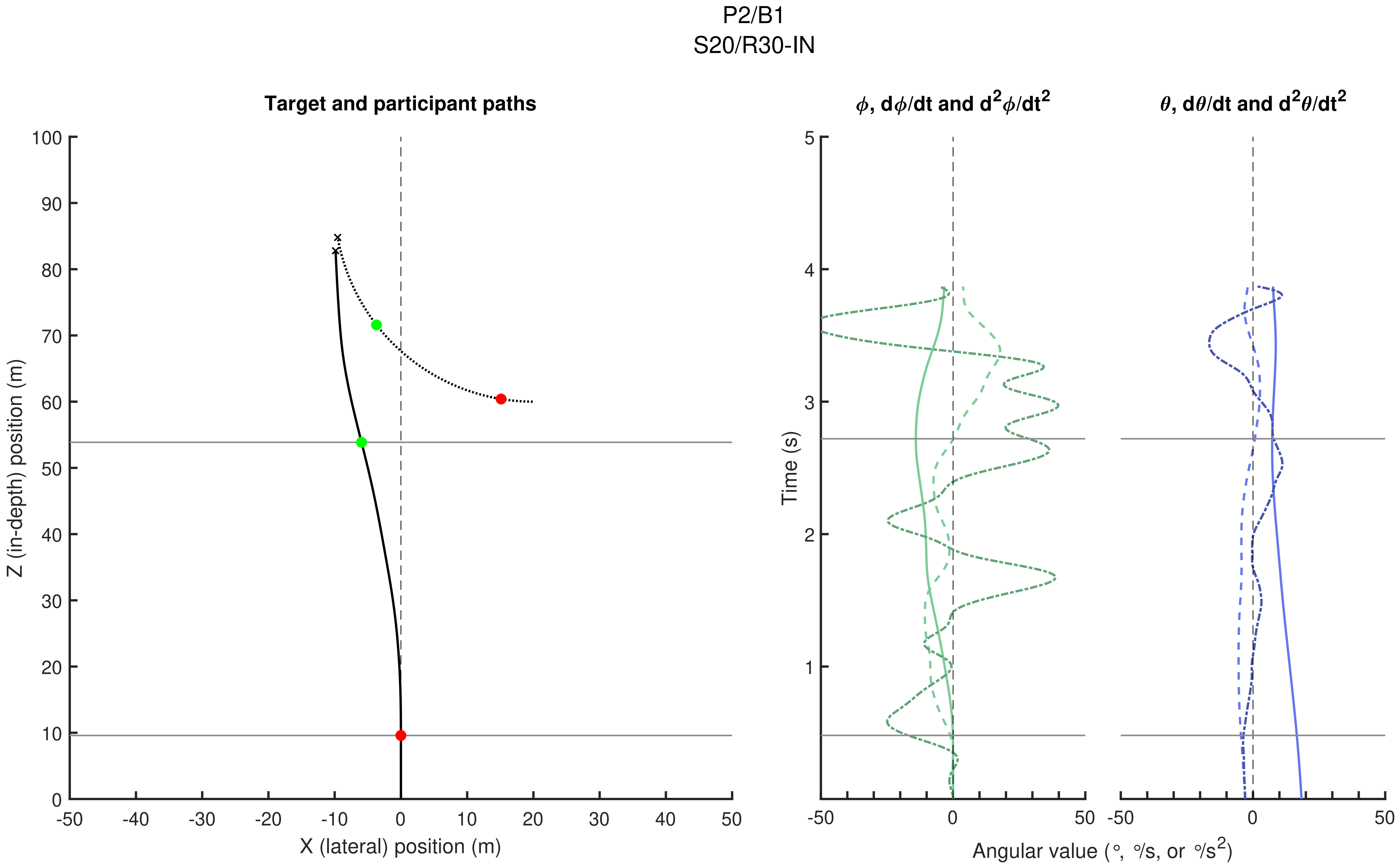

P2/B1
S20/R30-IN
Target and participant paths
Z (in-depth) position (m)
X (lateral) position (m)
-50
-40
-30
-20
-10
0
10
20
30
40
50
100
90
80
70
60
50
40
30
20
10
0
ϕ, dϕ/dt and d$^2\phi$/dt^2
θ, dθ/dt and d$^2\theta$/dt^2
Time (s)
5
4
3
2
1
-50
0
50
-50
0
50
Angular value (°, °/s, or °/s^2)

P2/B2
S20/R10-IN

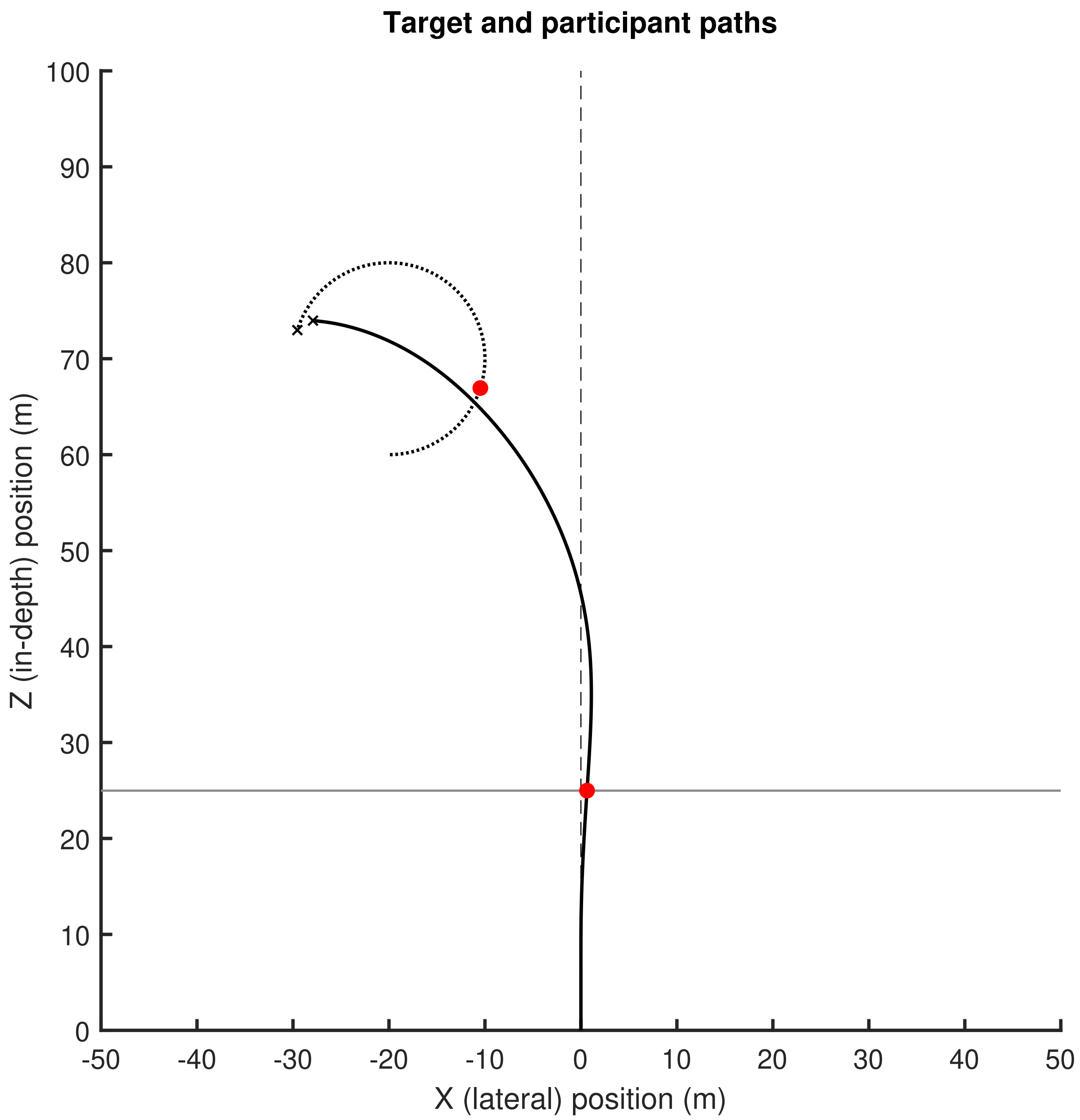

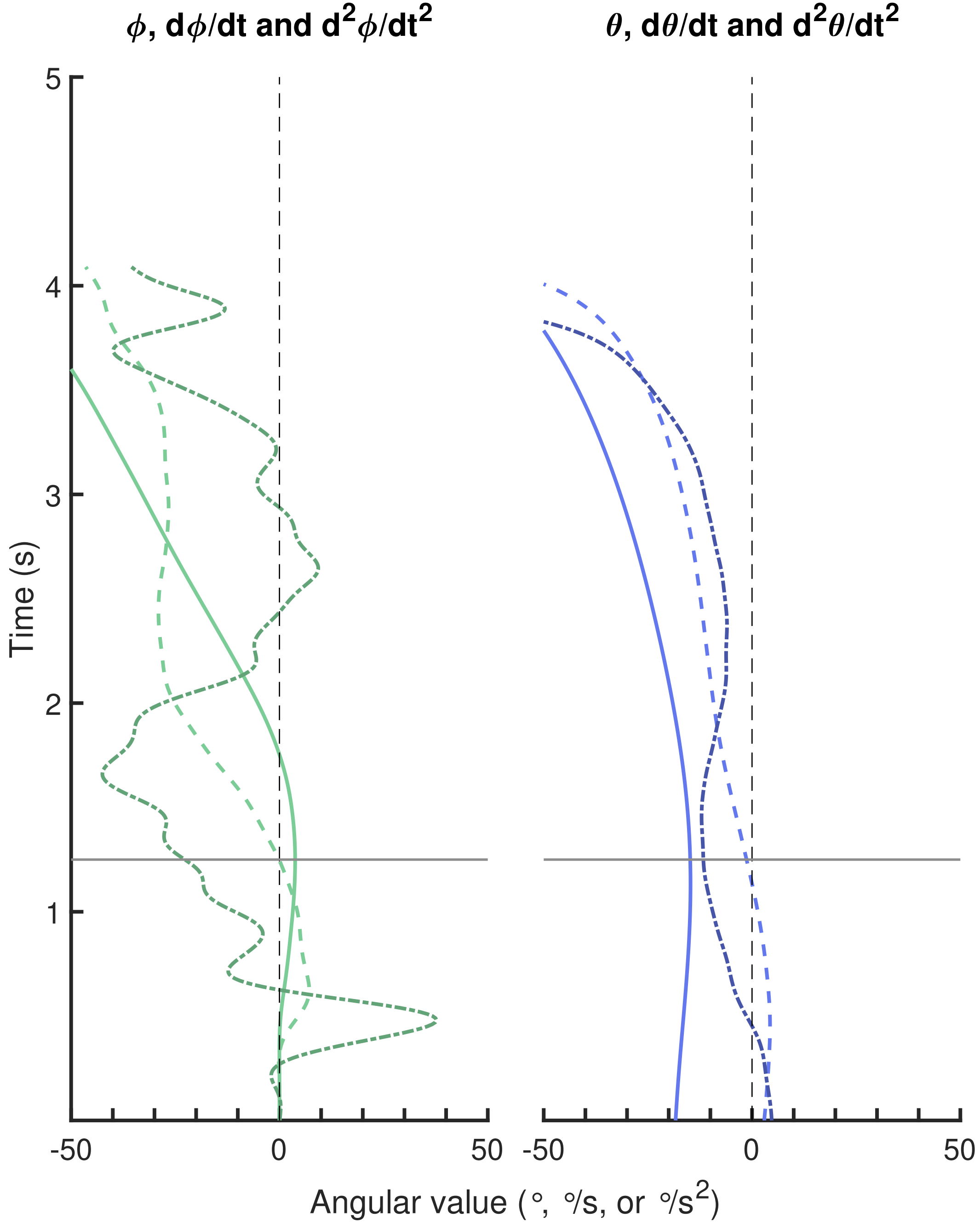

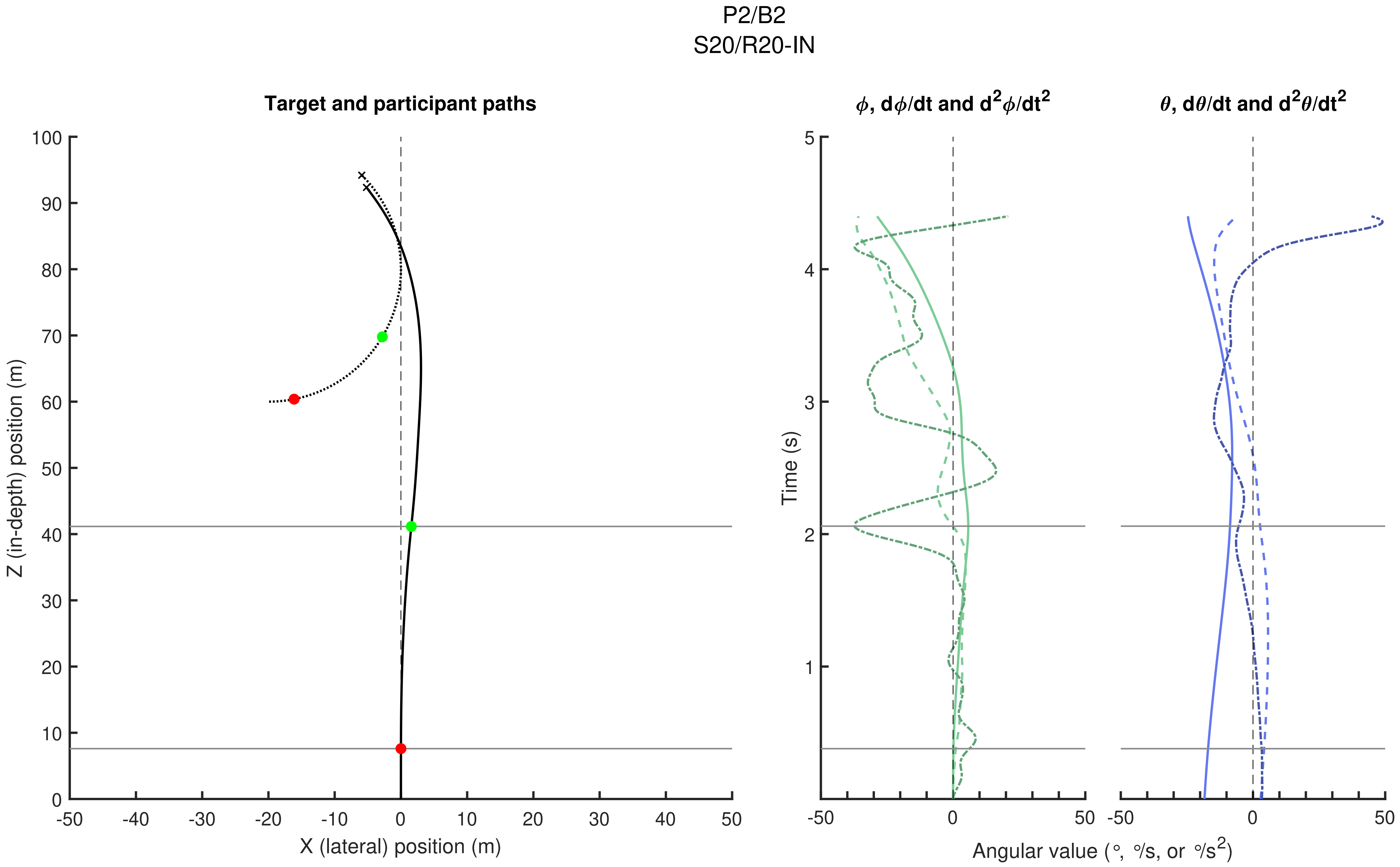
P2/B2
S20/R20-IN
Target and participant paths
Z (in-depth) position (m)
X (lateral) position (m)
ϕ, dϕ/dt and d$^2\phi$/dt^2
θ, dθ/dt and d$^2\theta$/dt^2
Time (s)
Angular value (°, °/s, or °/s^2)

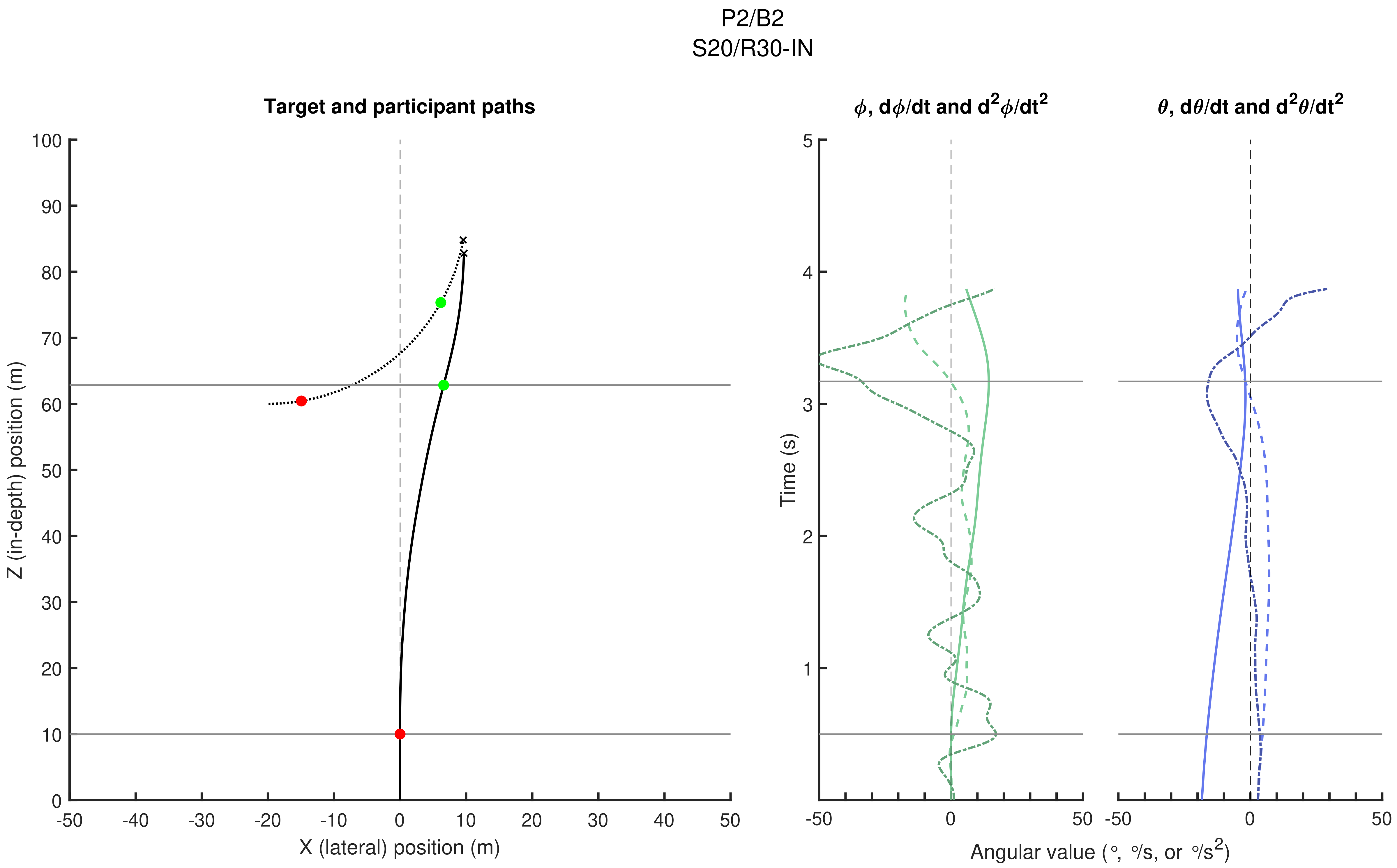
P2/B2
S20/R30-IN
Target and participant paths
Z (in-depth) position (m)
X (lateral) position (m)
φ, dφ/dt and d²φ/dt²
θ, dθ/dt and d²θ/dt²
Time (s)
Angular value (°, °/s, or °/s²)

P2/B2
S15/R10-IN

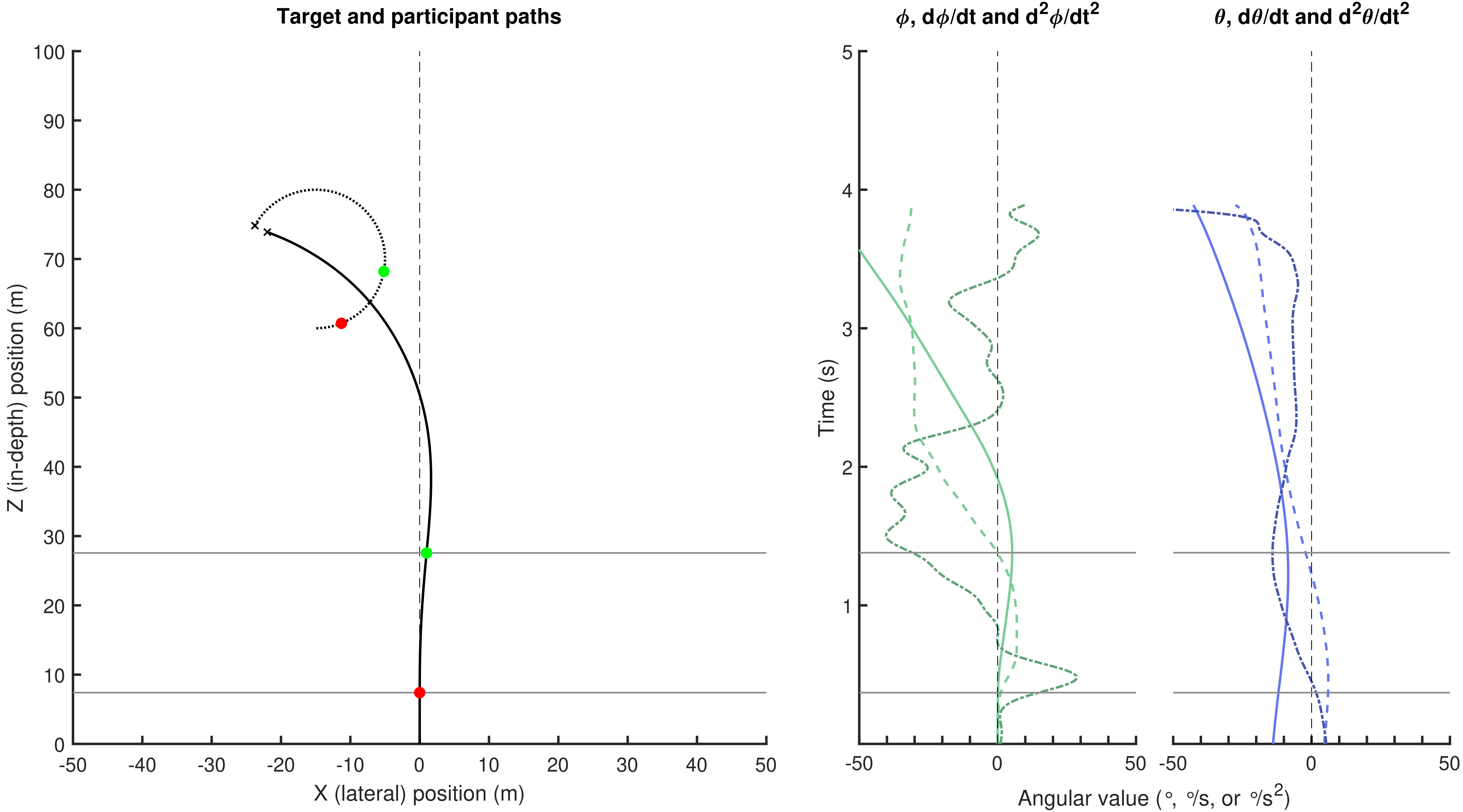

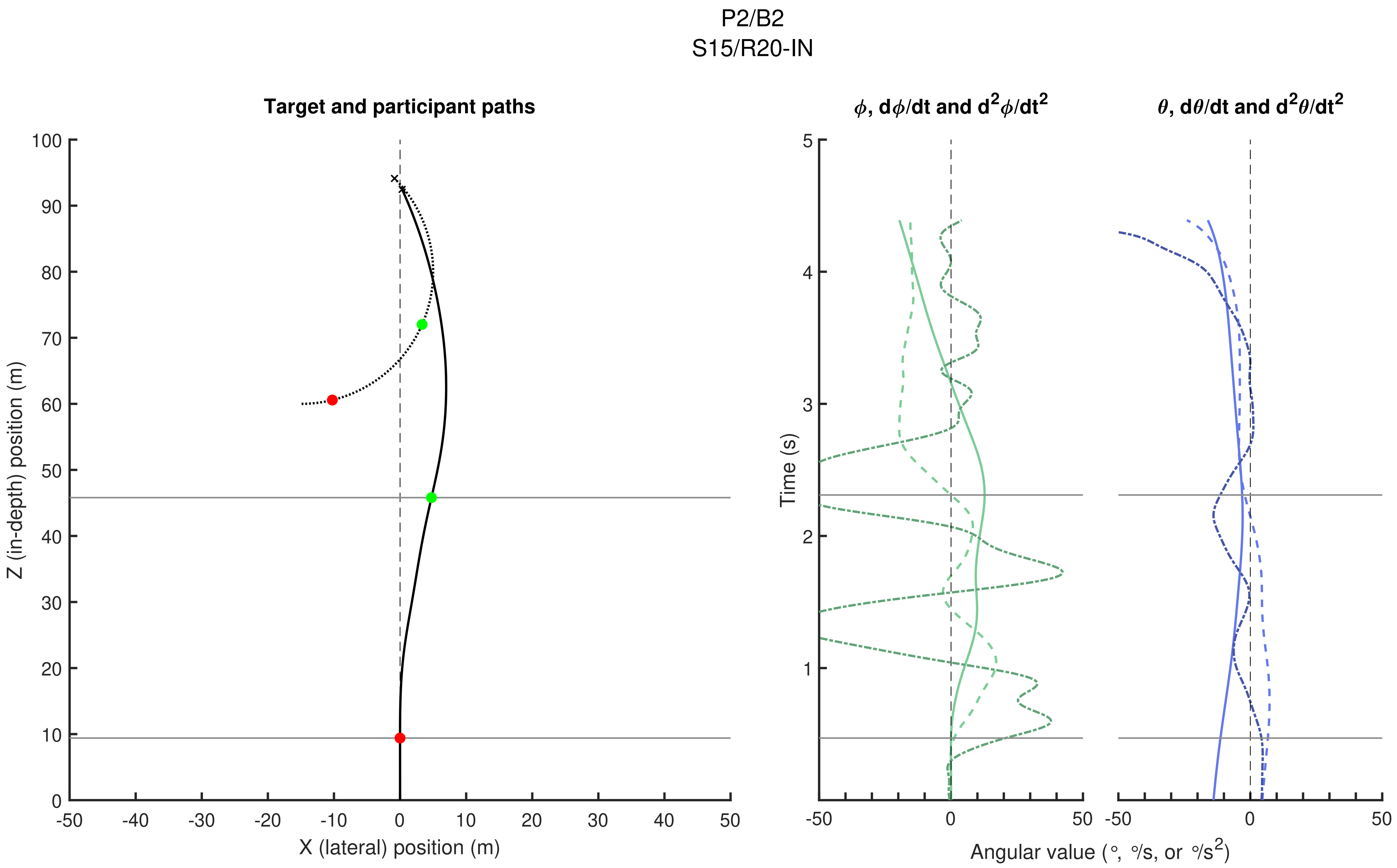
P2/B2
S15/R20-IN
Target and participant paths
Z (in-depth) position (m)
X (lateral) position (m)
100
90
80
70
60
50
40
30
20
10
0
-50
-40
-30
-20
-10
0
10
20
30
40
50
ϕ, dϕ/dt and d$^2\phi$/dt^2
θ, dθ/dt and d$^2\theta$/dt^2
Time (s)
5
4
3
2
1
-50
0
50
-50
0
50
Angular value (°, °/s, or °/s^2)

P2/B2
S15/R30-IN

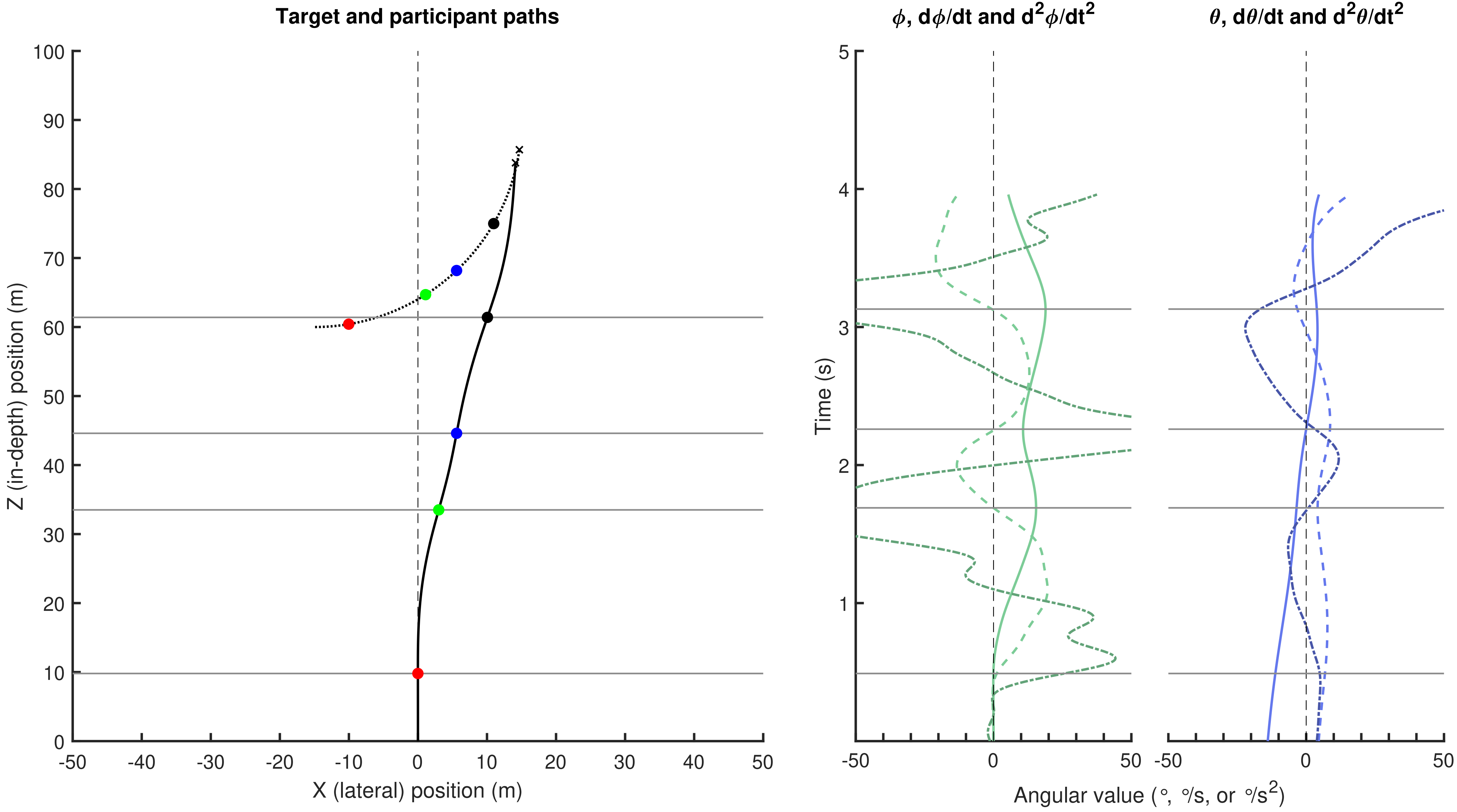

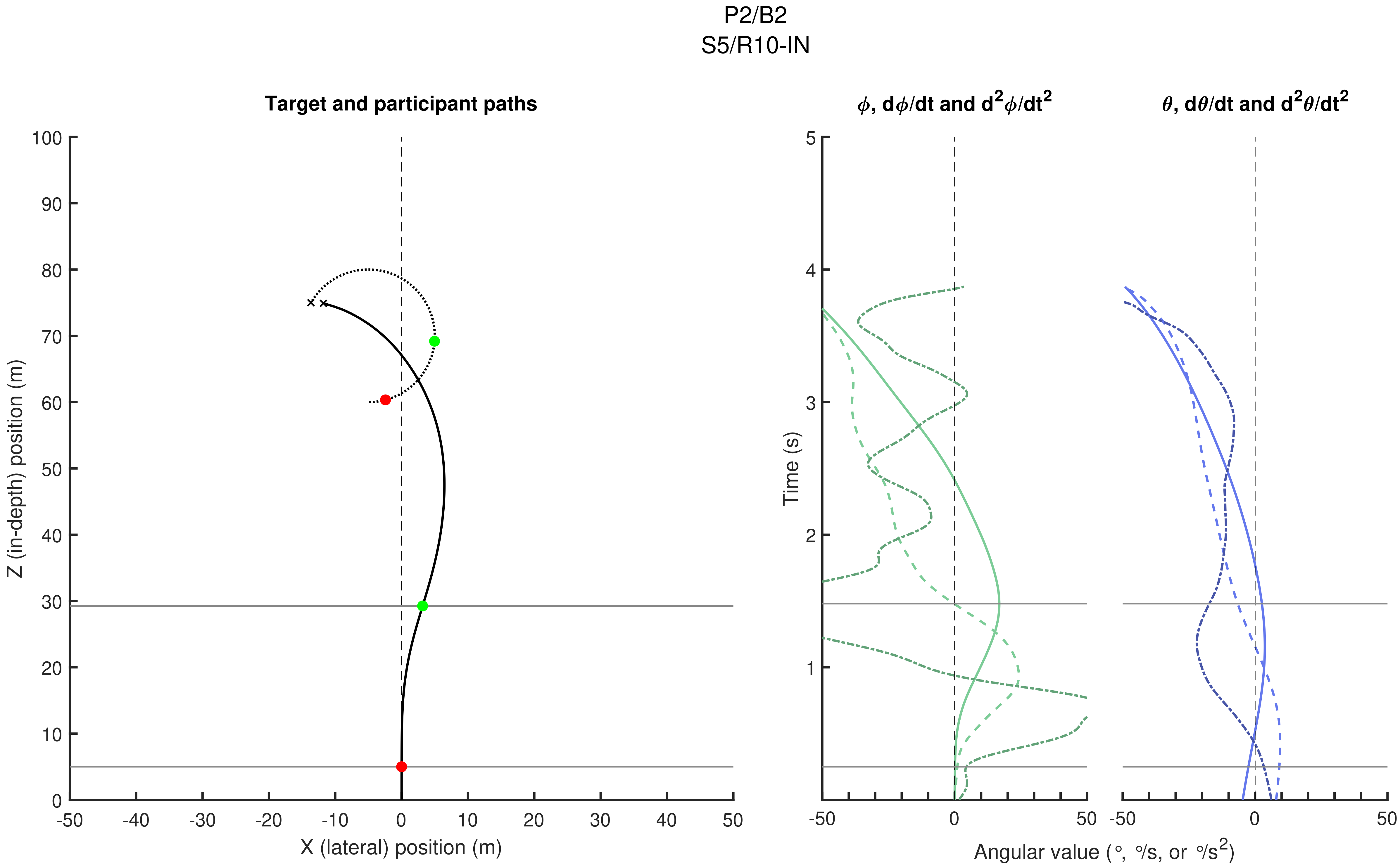
P2/B2
S5/R10-IN
Target and participant paths
ϕ, dϕ/dt and d$^2\phi$/dt^2
θ, dθ/dt and d$^2\theta$/dt^2
Z (in-depth) position (m)
X (lateral) position (m)
Time (s)
Angular value (°, °/s, or °/s^2)
100
90
80
70
60
50
40
30
20
10
0
-50
-40
-30
-20
-10
0
10
20
30
40
50
5
4
3
2
1
-50
0
50
-50
0
50

P2/B2
S5/R10-OUT

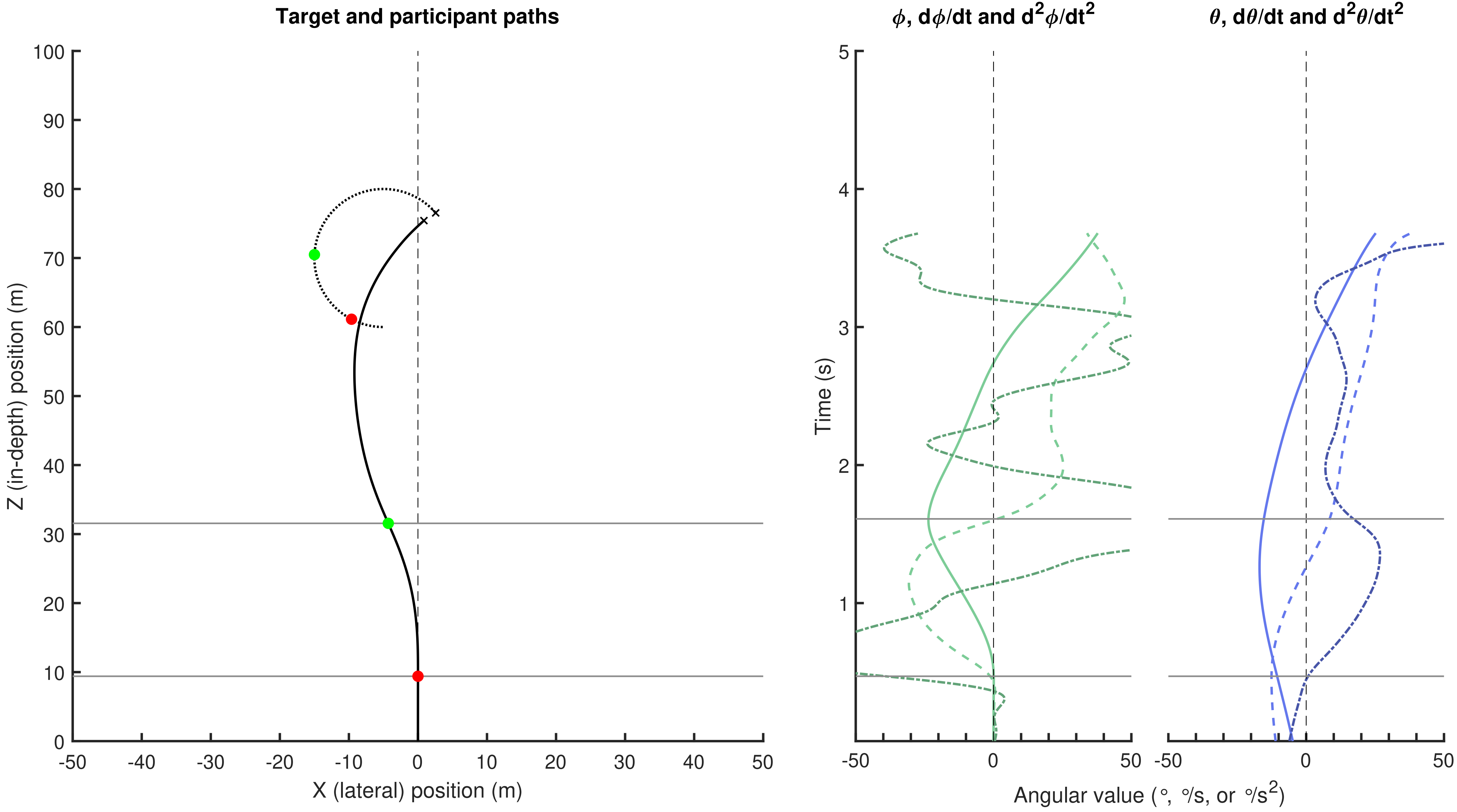

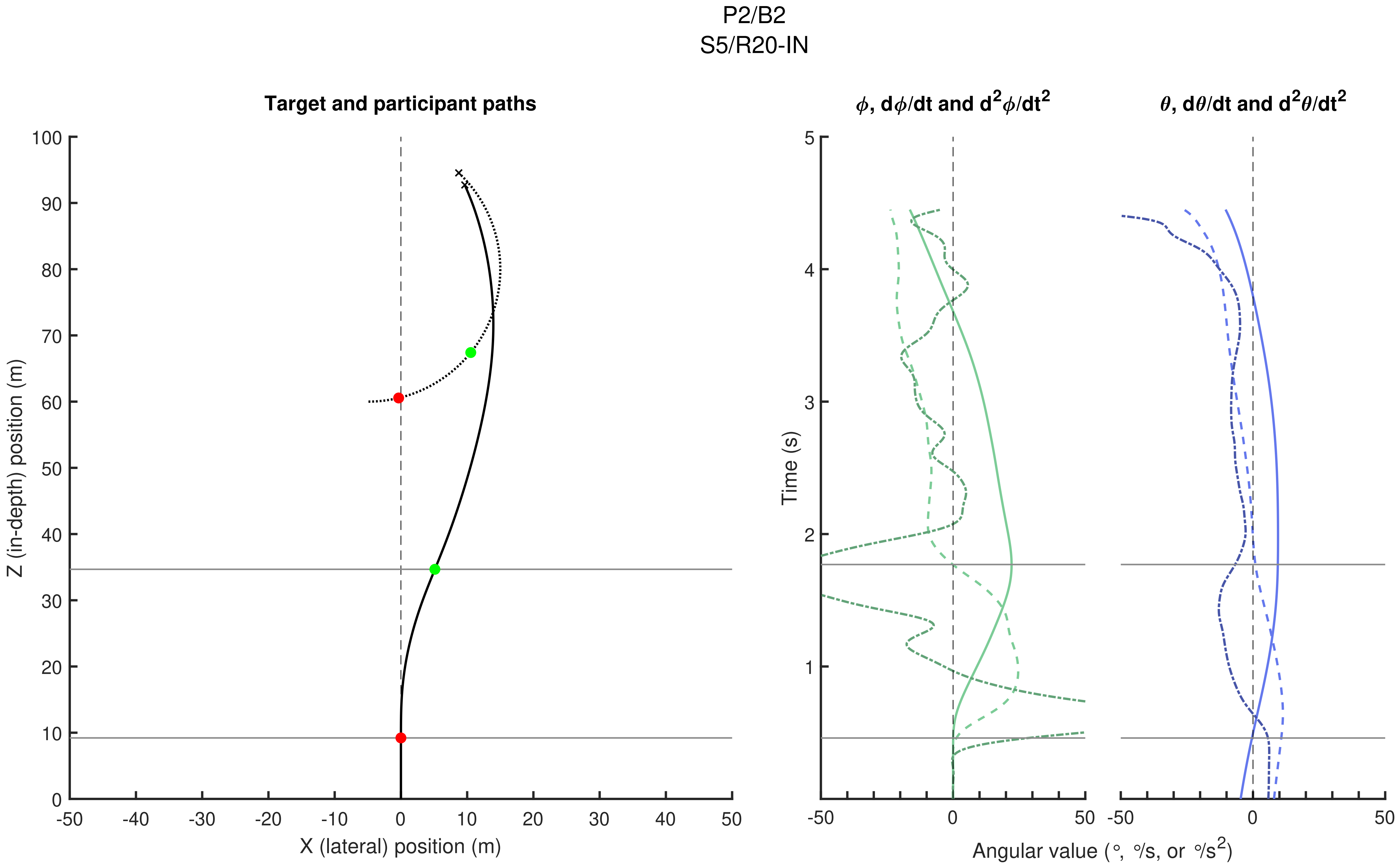

P2/B2
S5/R20-IN
Target and participant paths
Z (in-depth) position (m)
X (lateral) position (m)
100
90
80
70
60
50
40
30
20
10
0
-50
-40
-30
-20
-10
0
10
20
30
40
50
ϕ, dϕ/dt and d$^2\phi$/dt^2
θ, dθ/dt and d$^2\theta$/dt^2
Time (s)
5
4
3
2
1
-50
0
50
-50
0
50
Angular value (°, °/s, or °/s^2)

P2/B2
S5/R20-OUT

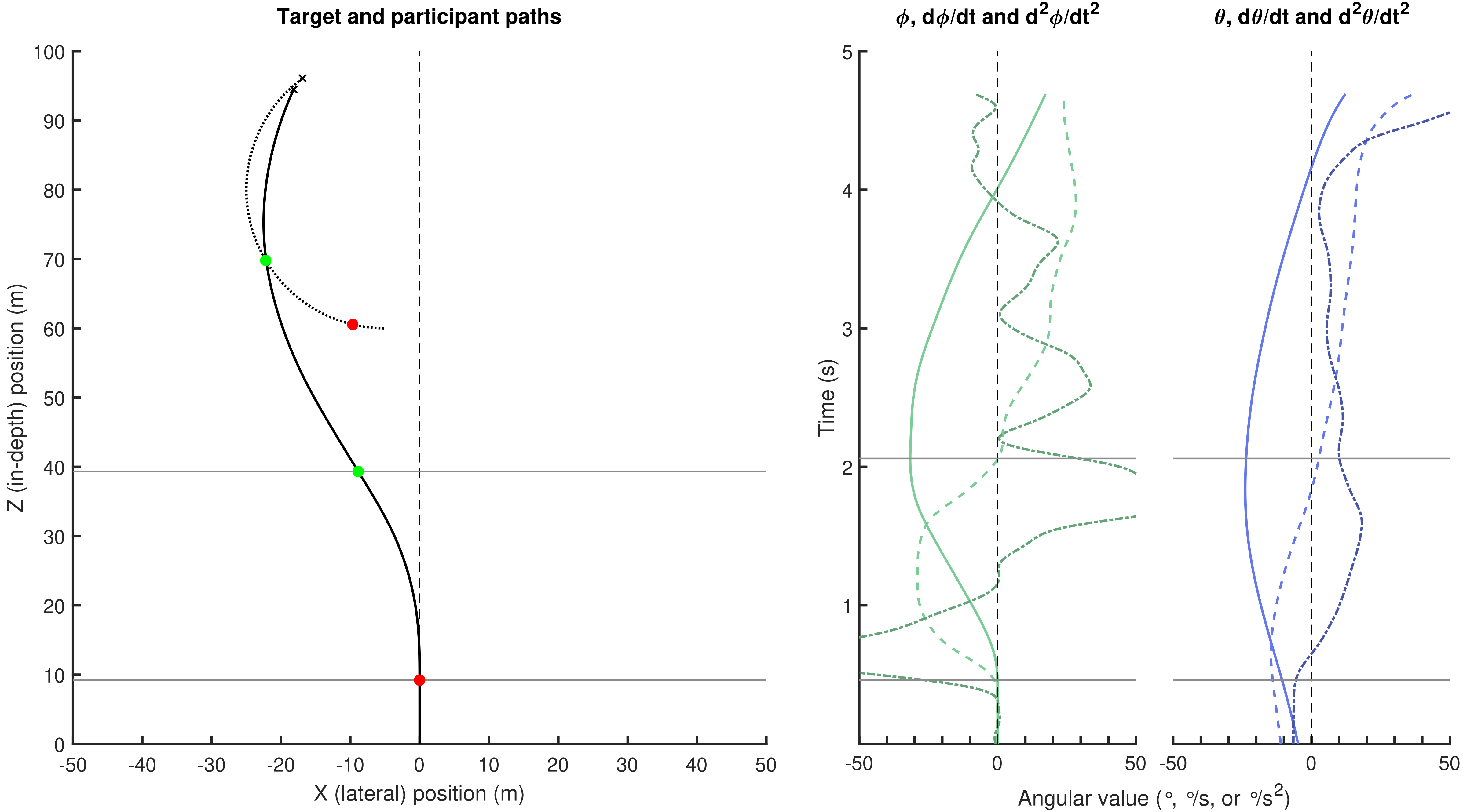

P2/B2
S5/R30-IN

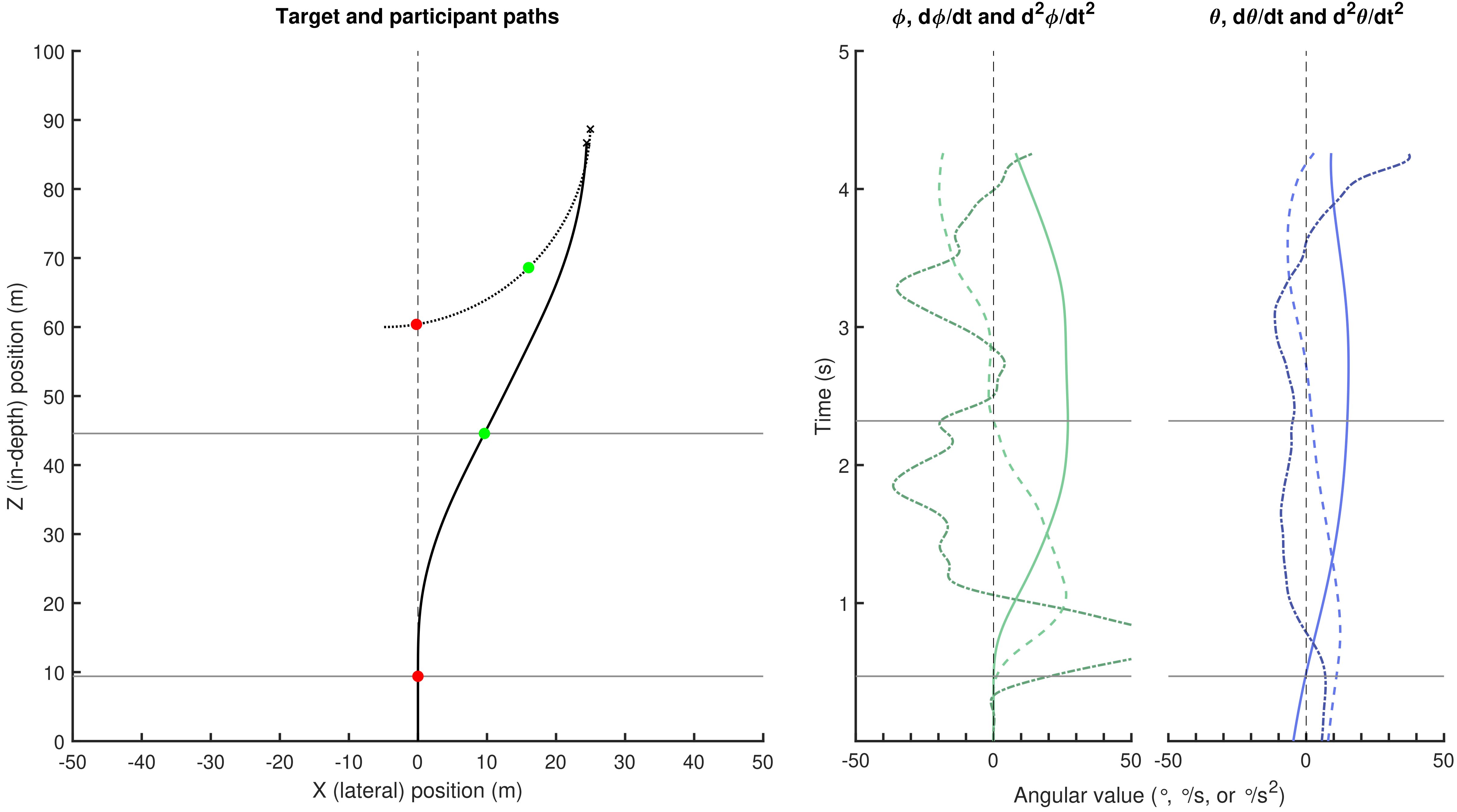

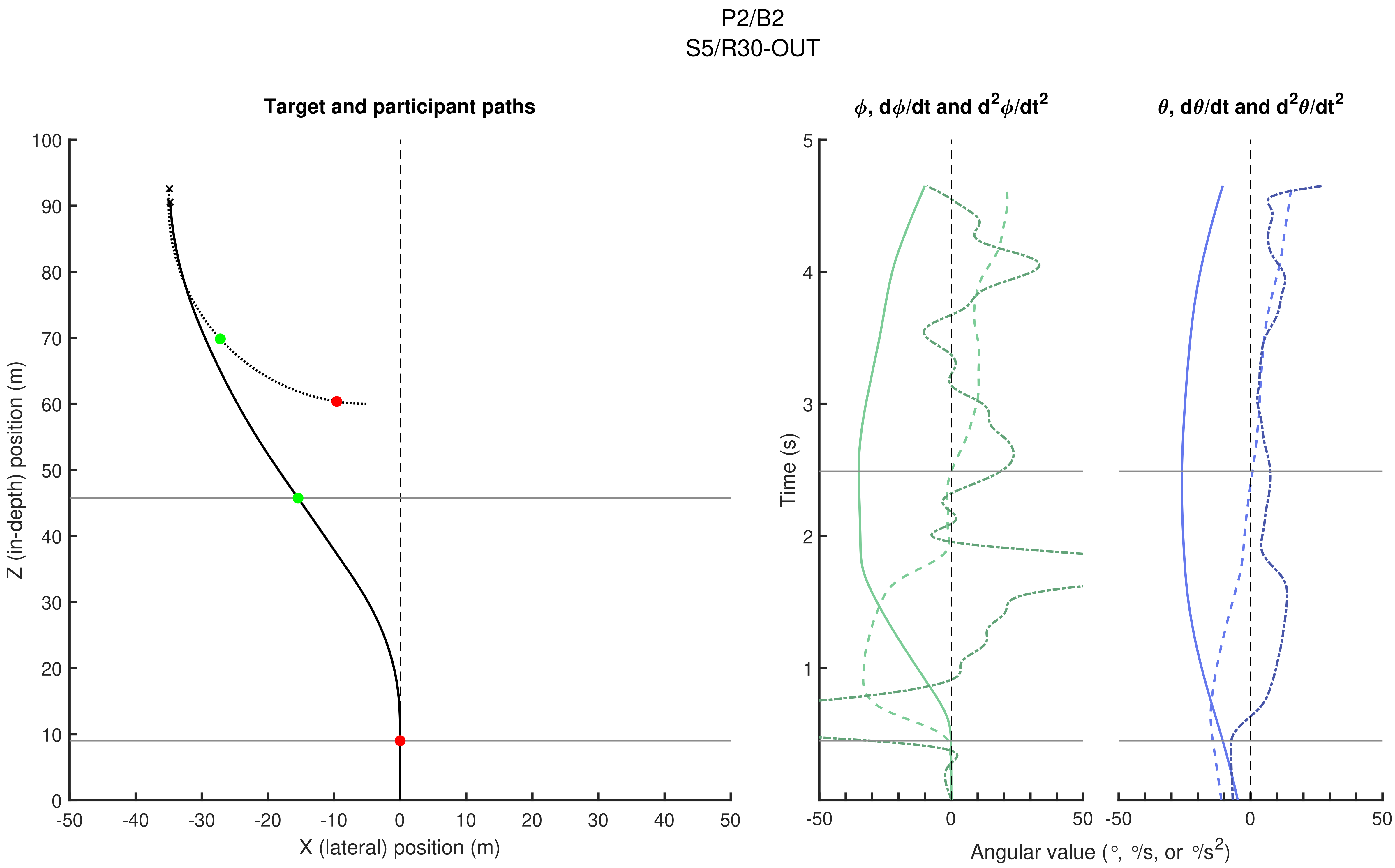

P2/B2
S5/R30-OUT
Target and participant paths
Z (in-depth) position (m)
X (lateral) position (m)
ϕ, dϕ/dt and d$^2\phi$/dt^2
θ, dθ/dt and d$^2\theta$/dt^2
Time (s)
Angular value (°, °/s, or °/s^2)

P2/B2
S5/R10-OUT

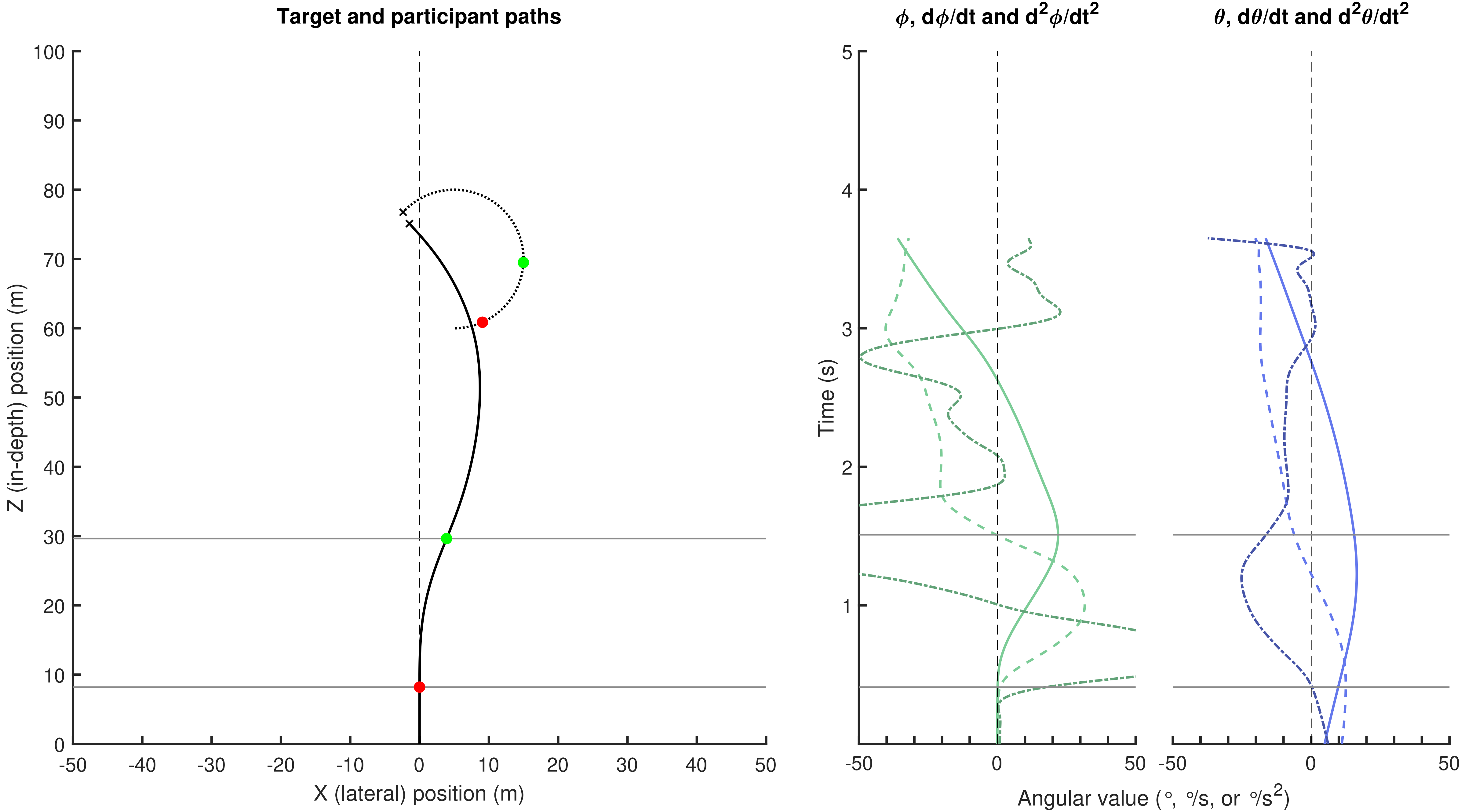

P2/B2
S5/R10-IN

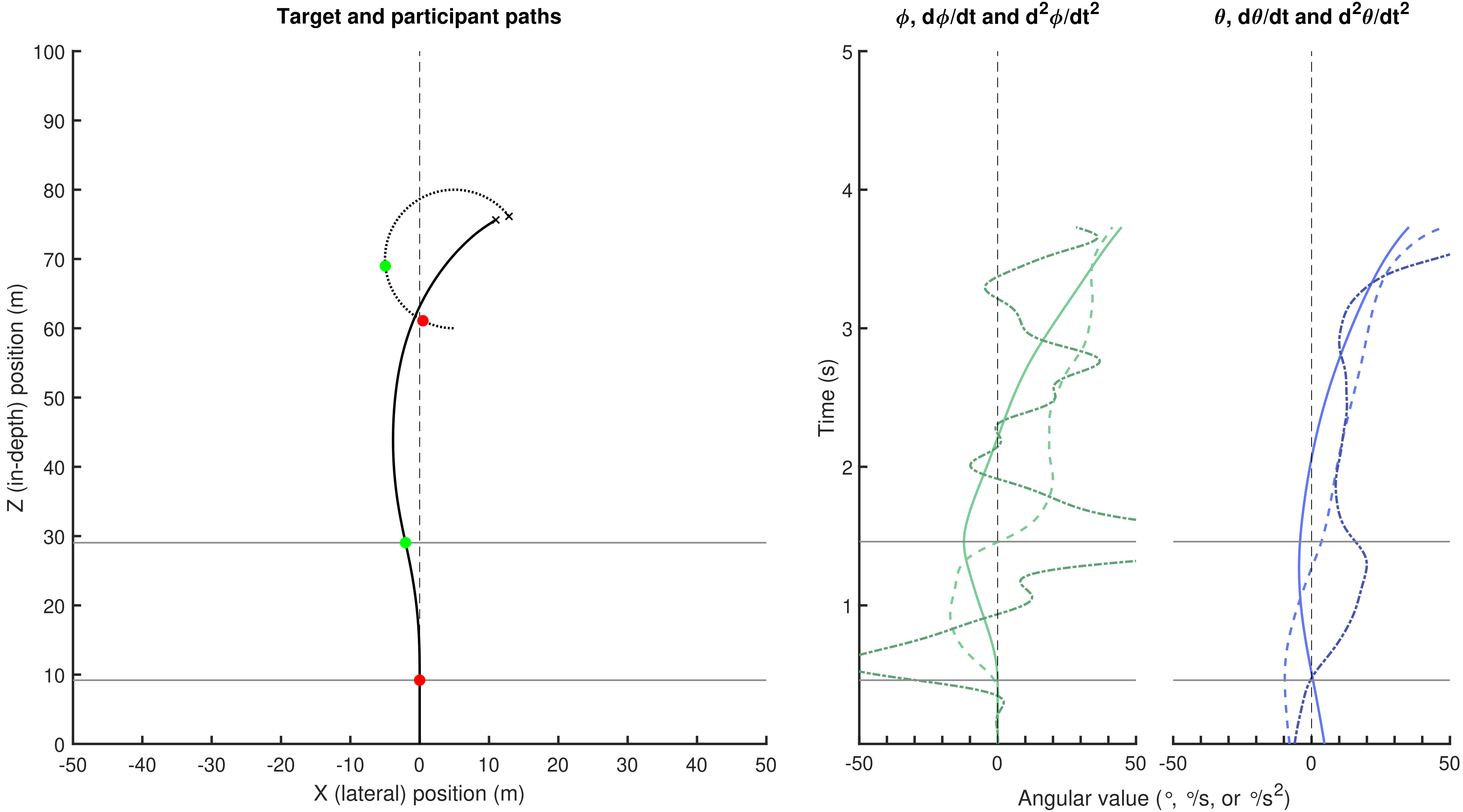

P2/B2
S5/R20-OUT

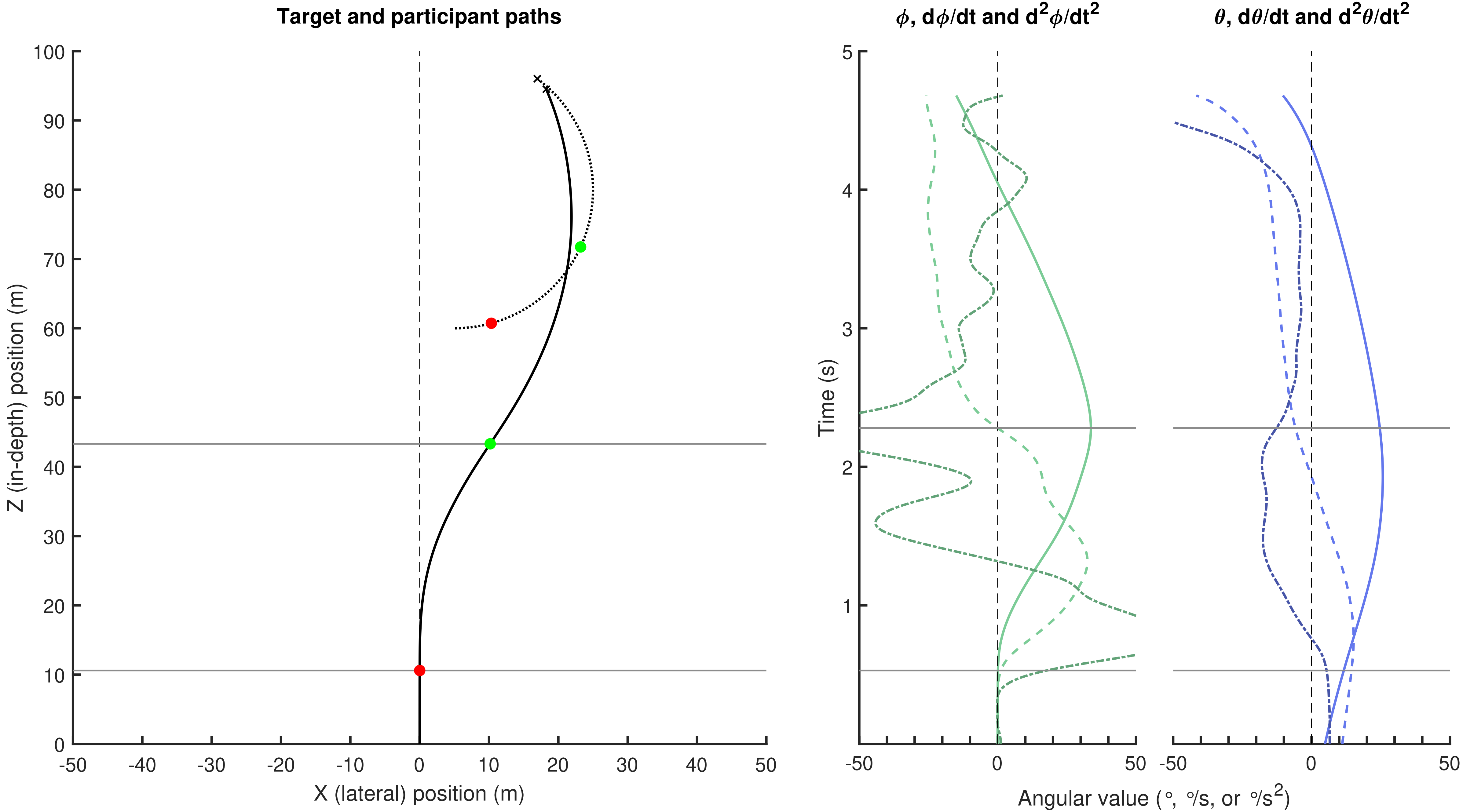

P2/B2
S5/R20-IN

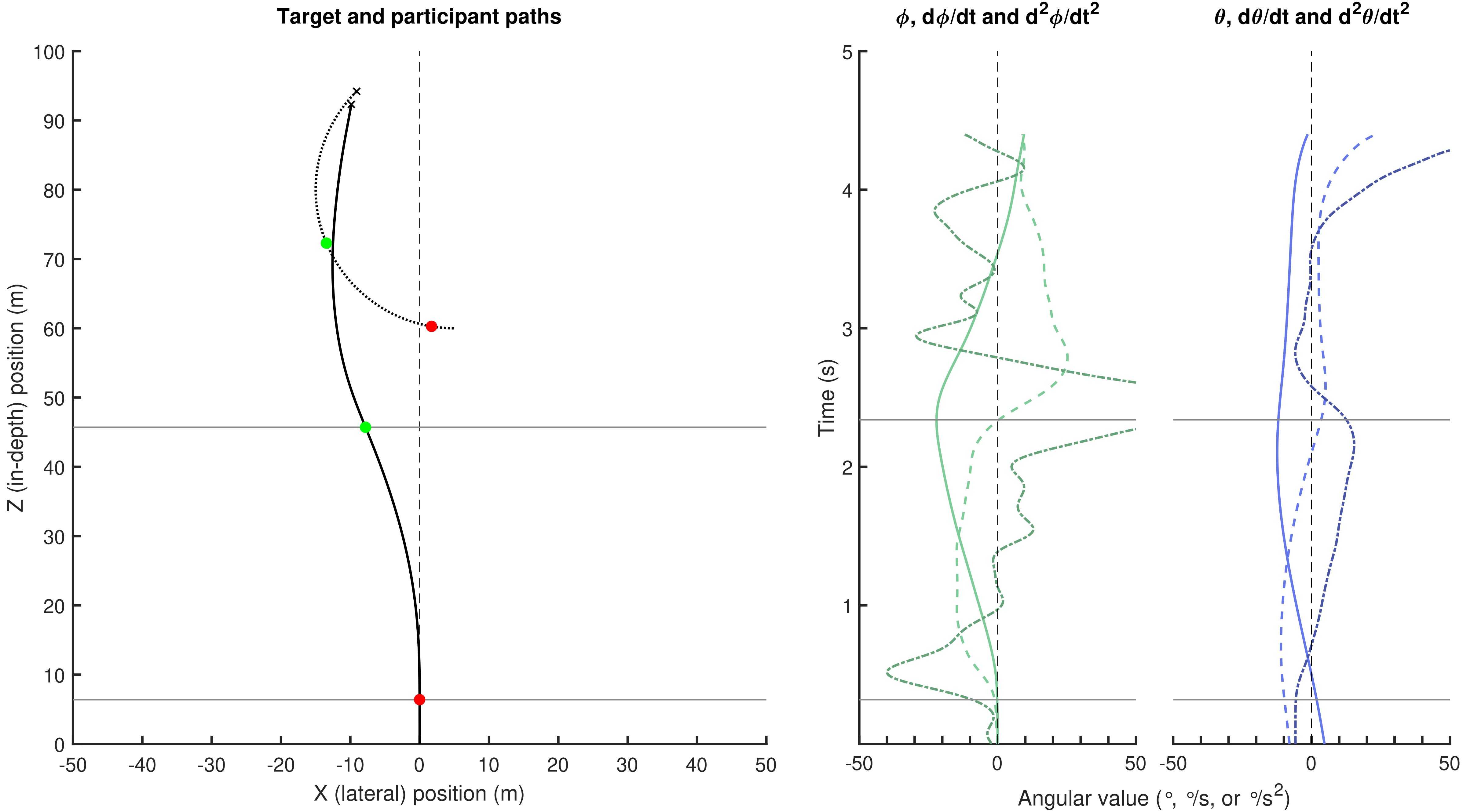

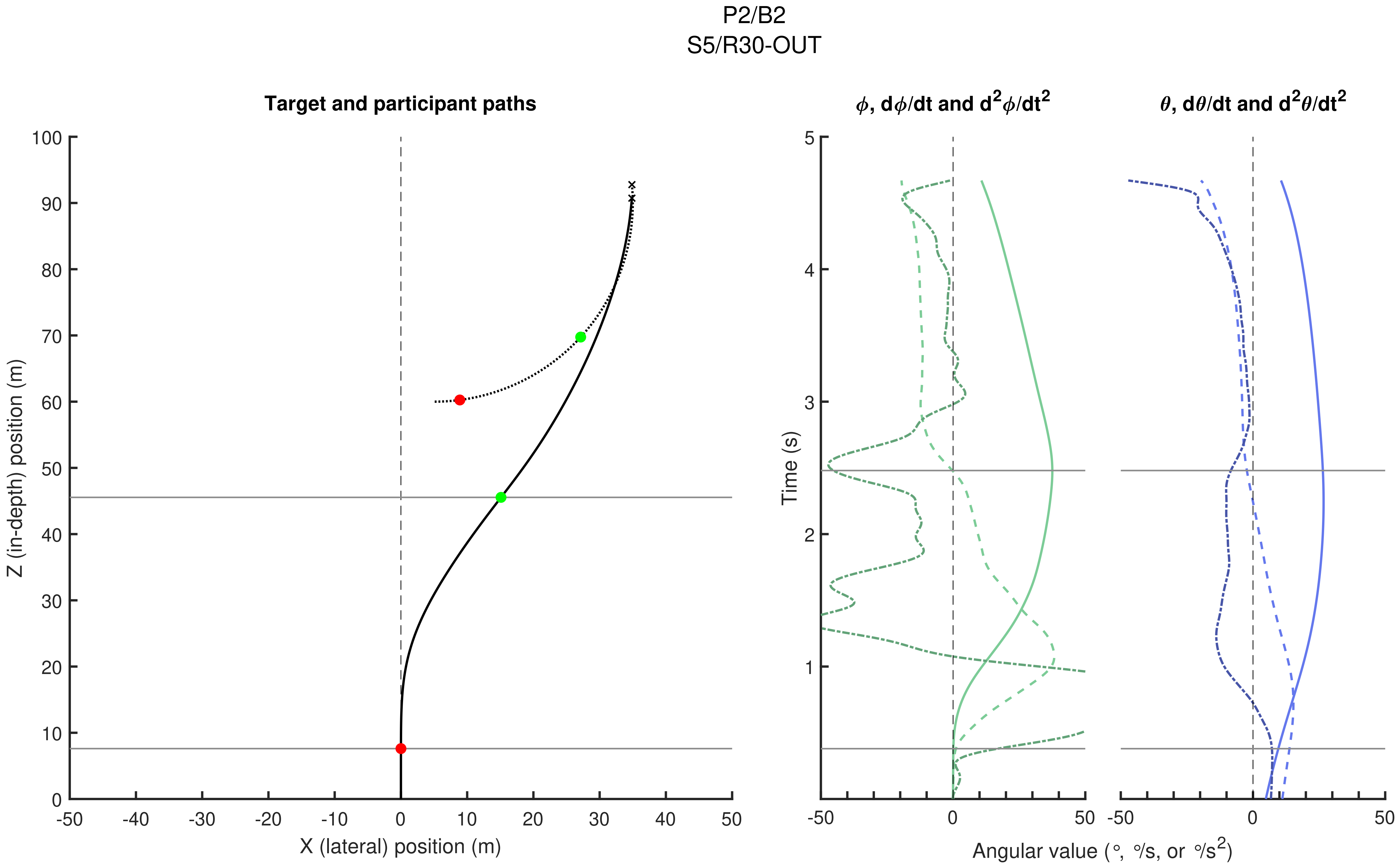

P2/B2
S5/R30-OUT
Target and participant paths
Z (in-depth) position (m)
X (lateral) position (m)
100
90
80
70
60
50
40
30
20
10
0
-50
-40
-30
-20
-10
0
10
20
30
40
50
ϕ, dϕ/dt and d$^2\phi$/dt^2
θ, dθ/dt and d$^2\theta$/dt^2
Time (s)
5
4
3
2
1
-50
0
50
-50
0
50
Angular value (°, °/s, or °/s^2)

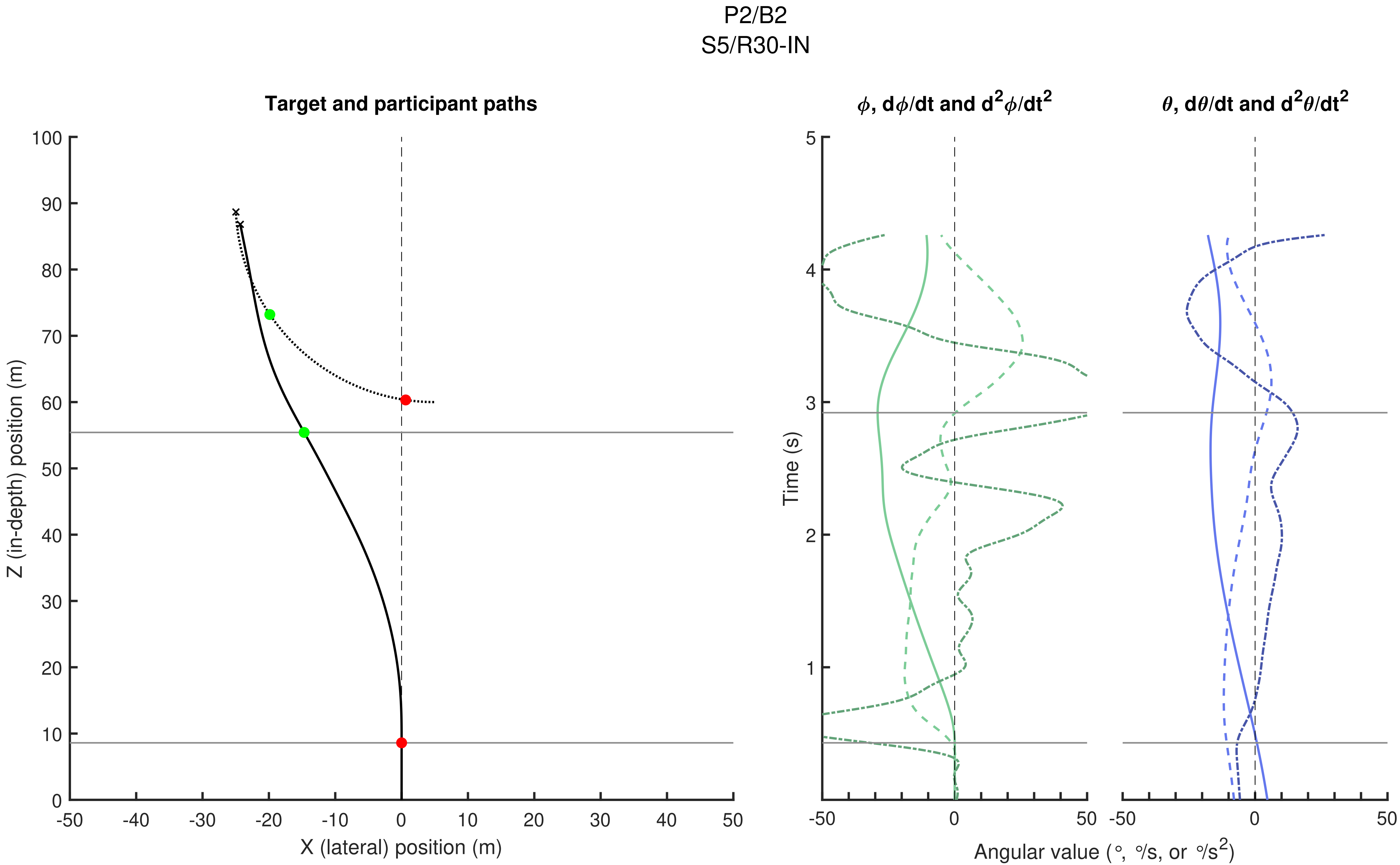
P2/B2
S5/R30-IN
Target and participant paths
Z (in-depth) position (m)
X (lateral) position (m)
ϕ, dϕ/dt and d$^2\phi$/dt^2
θ, dθ/dt and d$^2\theta$/dt^2
Time (s)
Angular value (°, °/s, or °/s^2)

P2/B2
S15/R10-IN

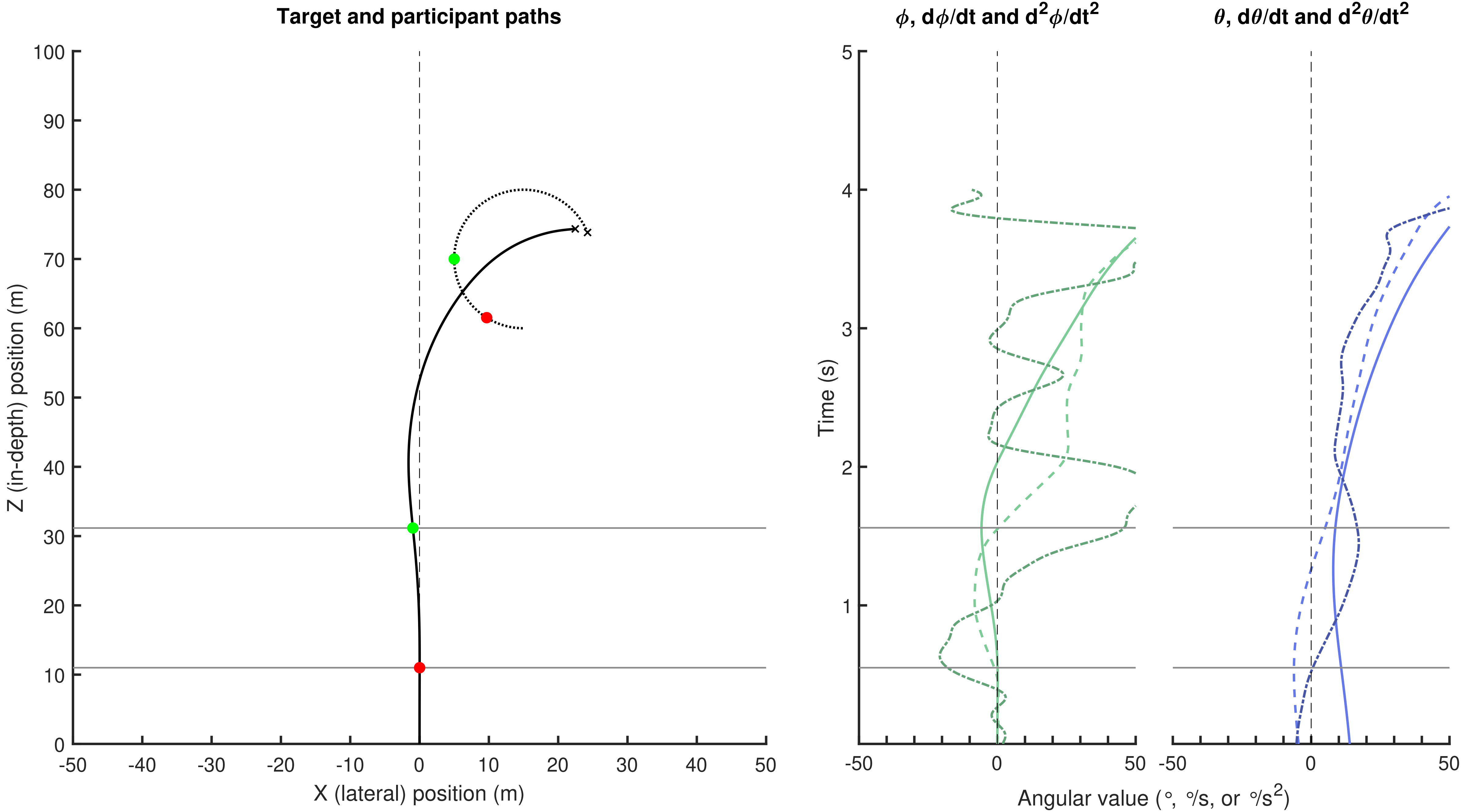

P2/B2
S15/R20-IN

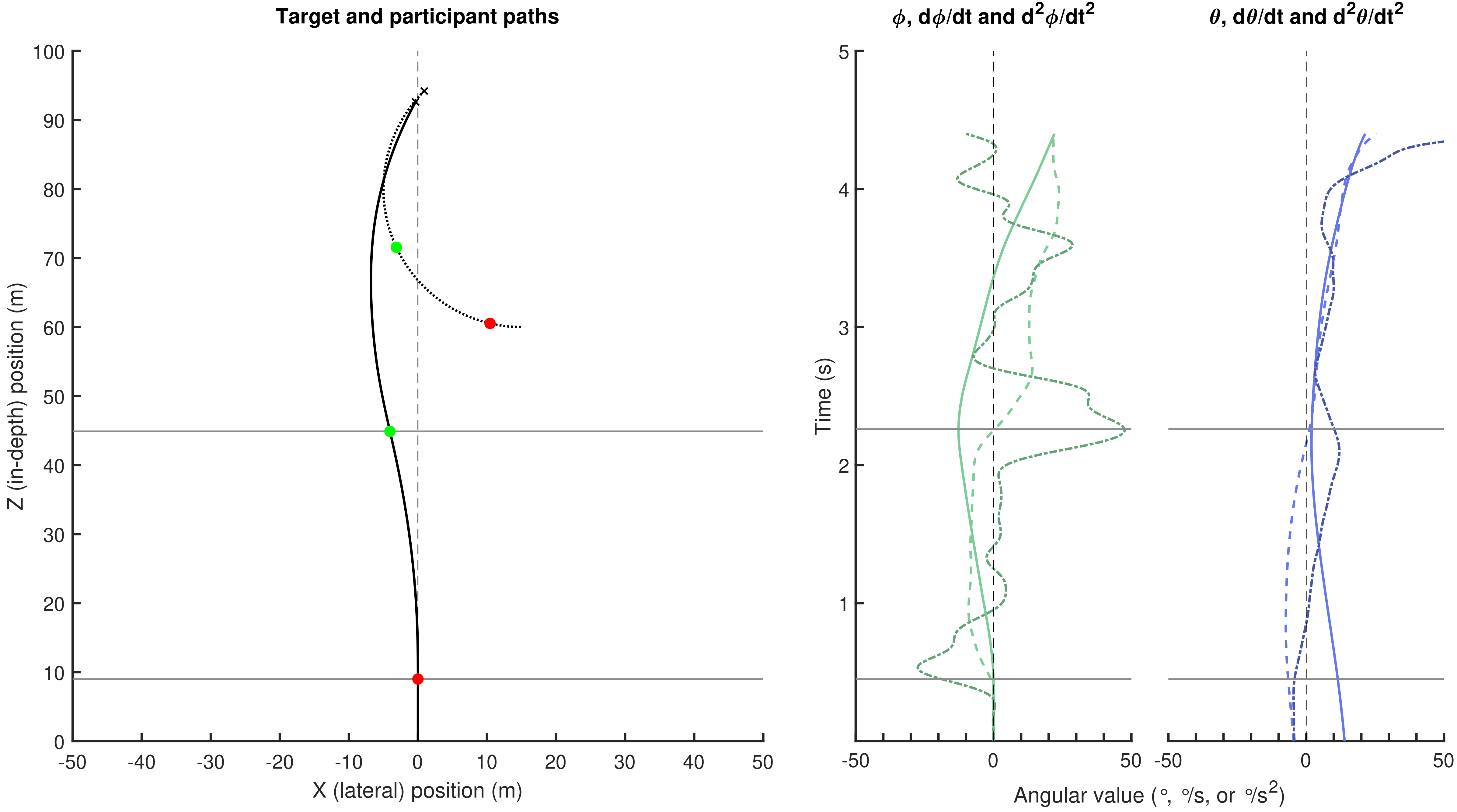

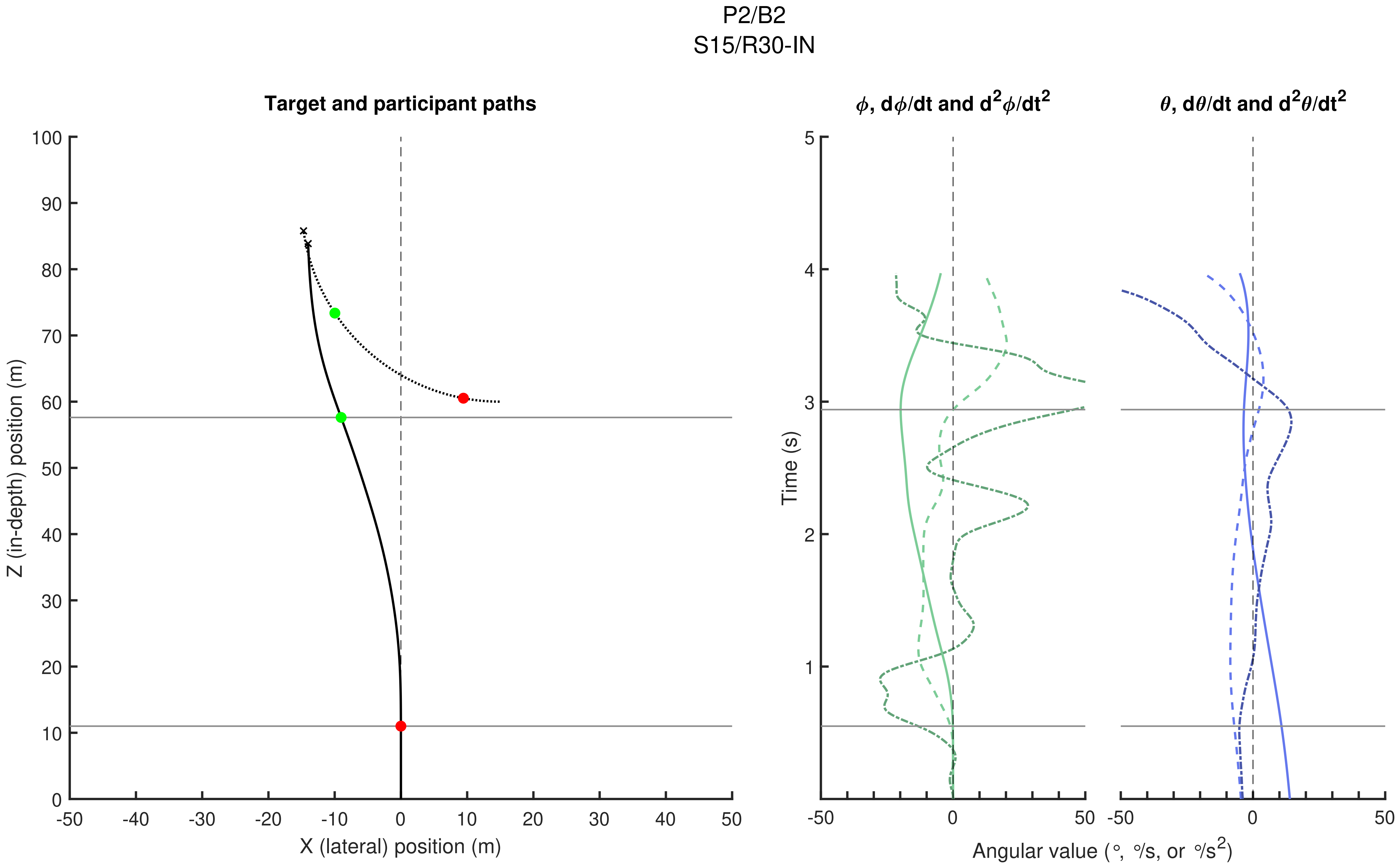
P2/B2
S15/R30-IN
Target and participant paths
Z (in-depth) position (m)
X (lateral) position (m)
ϕ, dϕ/dt and d$^2\phi$/dt^2
θ, dθ/dt and d$^2\theta$/dt^2
Time (s)
Angular value (°, °/s, or °/s^2)

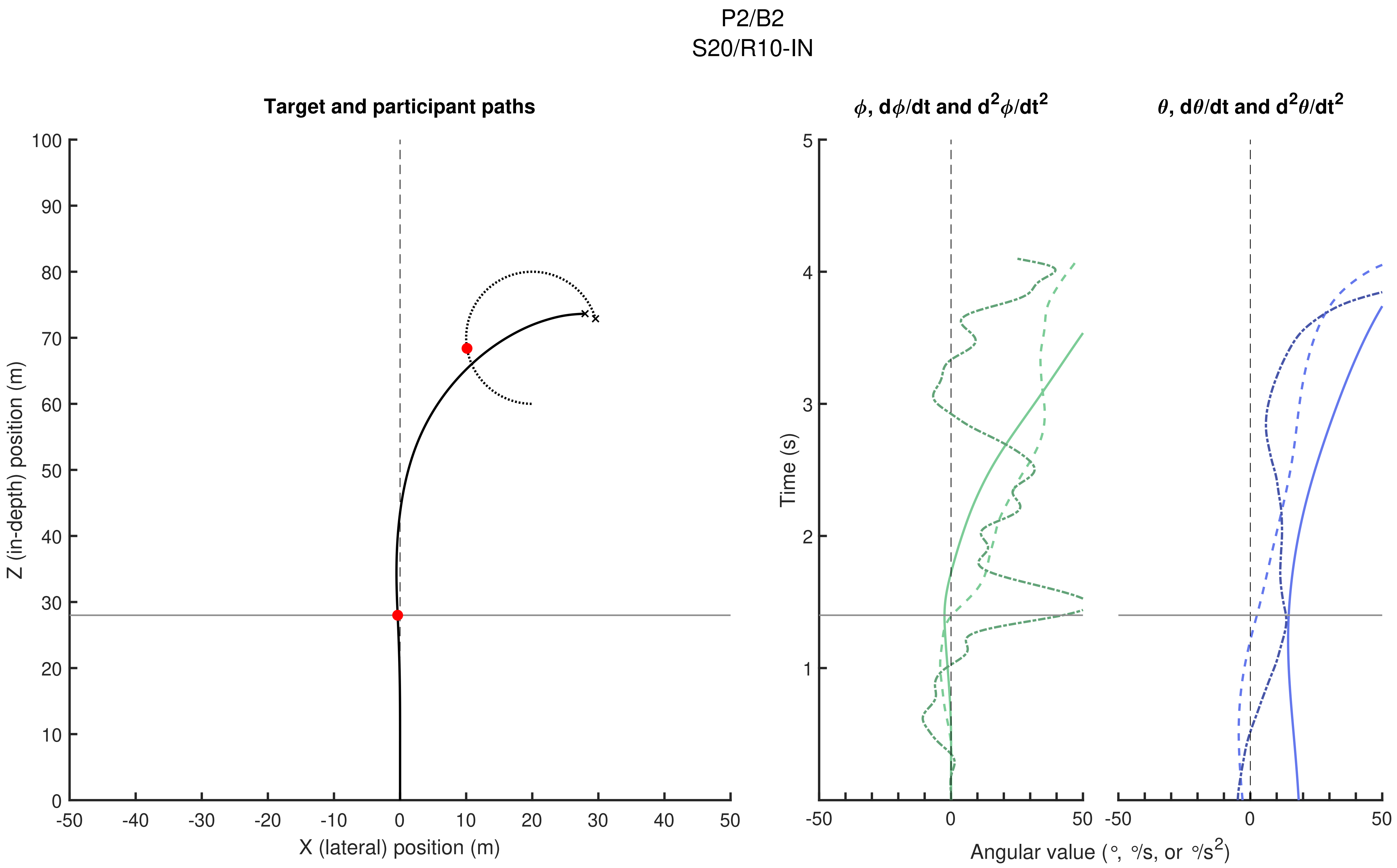
P2/B2
S20/R10-IN
Target and participant paths
Z (in-depth) position (m)
X (lateral) position (m)
100
90
80
70
60
50
40
30
20
10
0
-50
-40
-30
-20
-10
0
10
20
30
40
50
φ, dφ/dt and d²φ/dt²
θ, dθ/dt and d²θ/dt²
Time (s)
5
4
3
2
1
-50
0
50
-50
0
50
Angular value (°, °/s, or °/s²)

P2/B2
S20/R20-IN

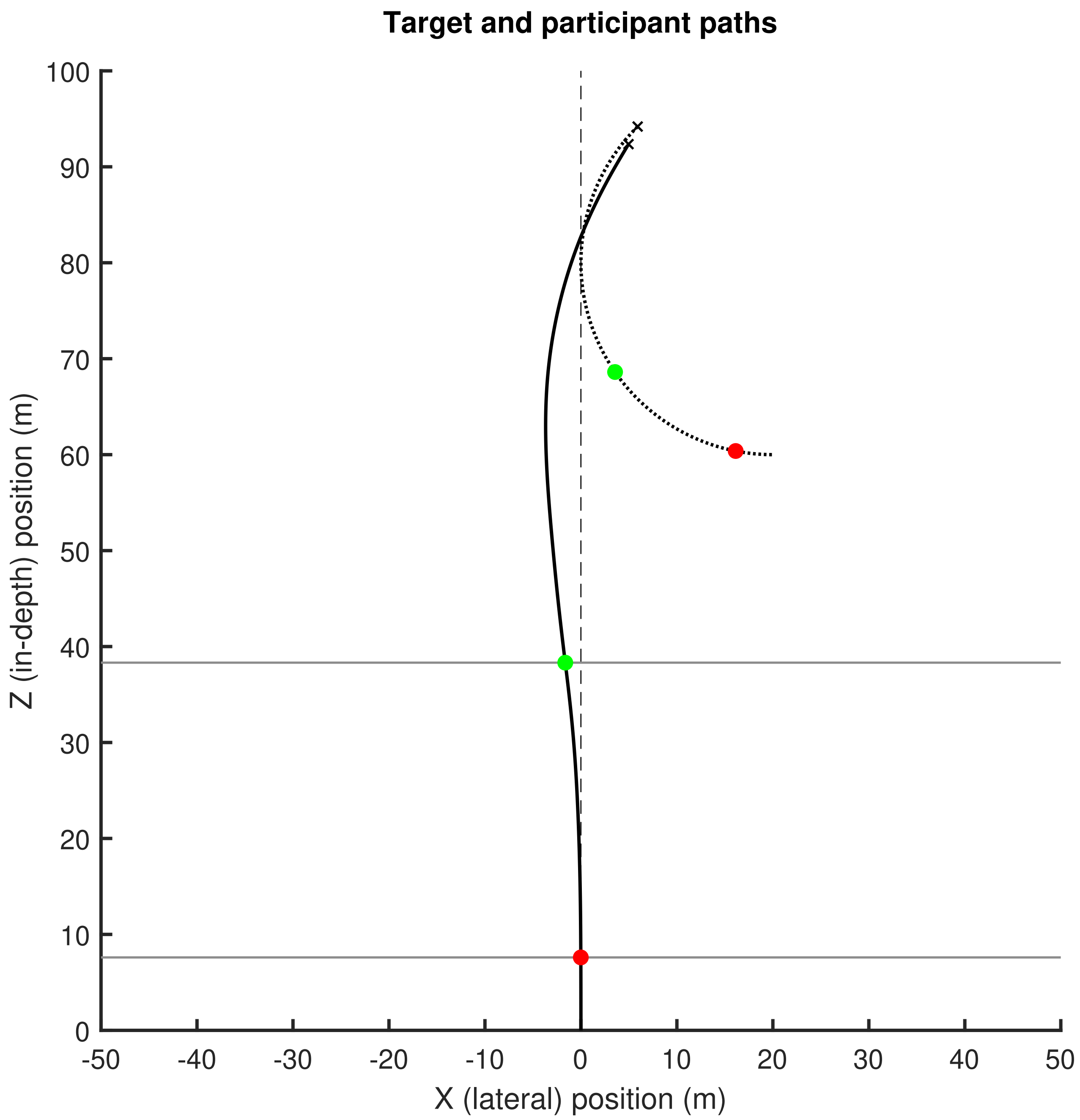

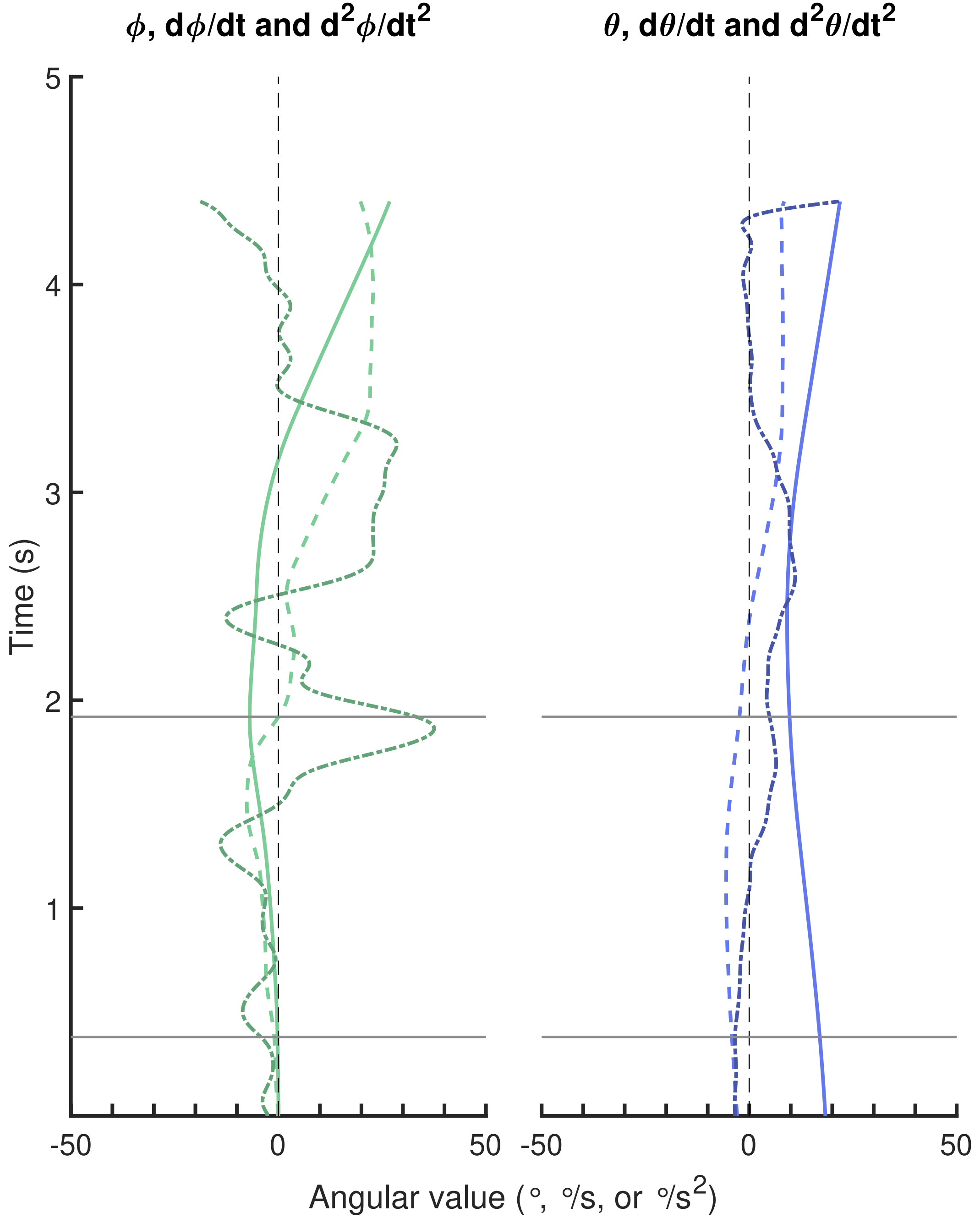

P2/B2
S20/R30-IN

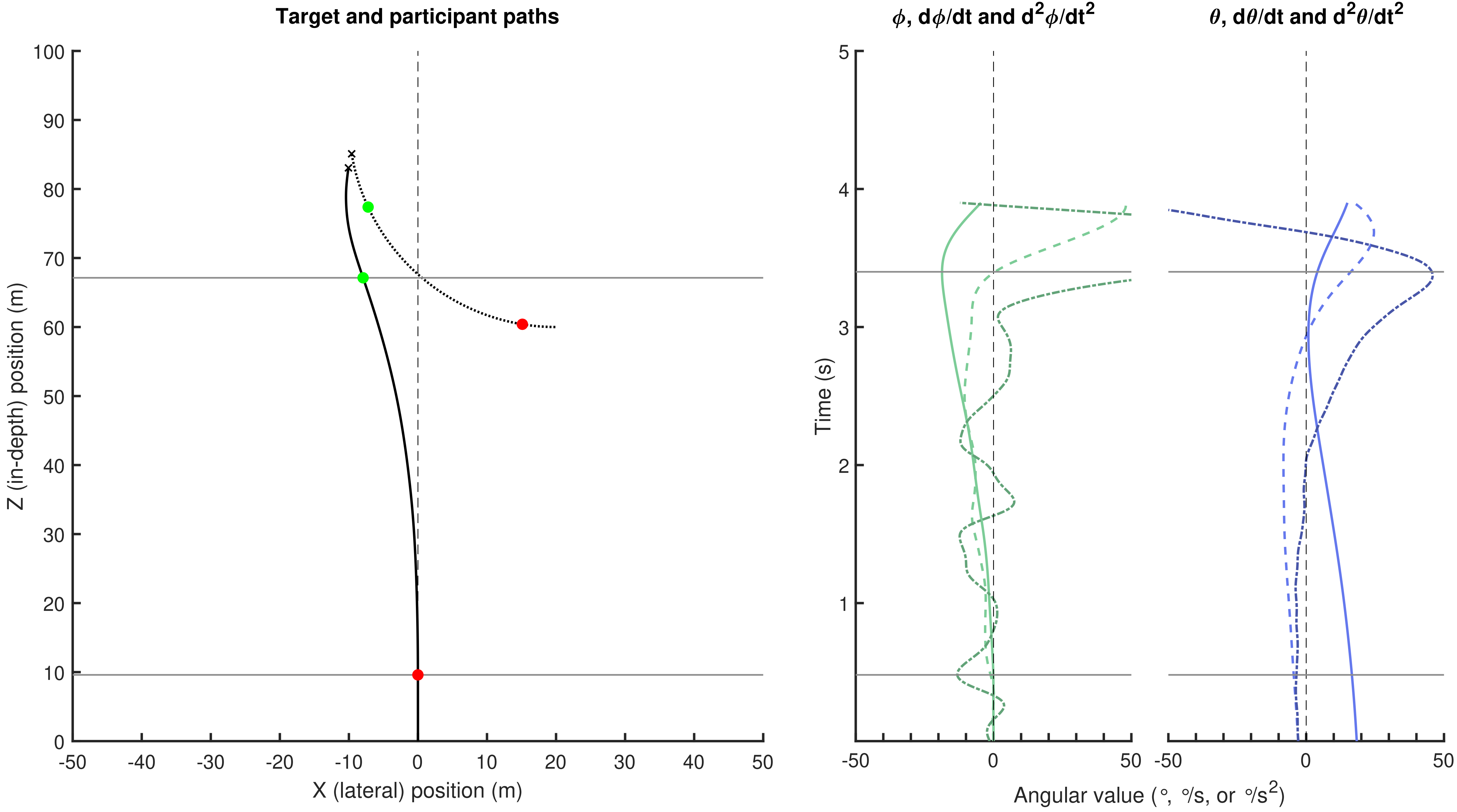

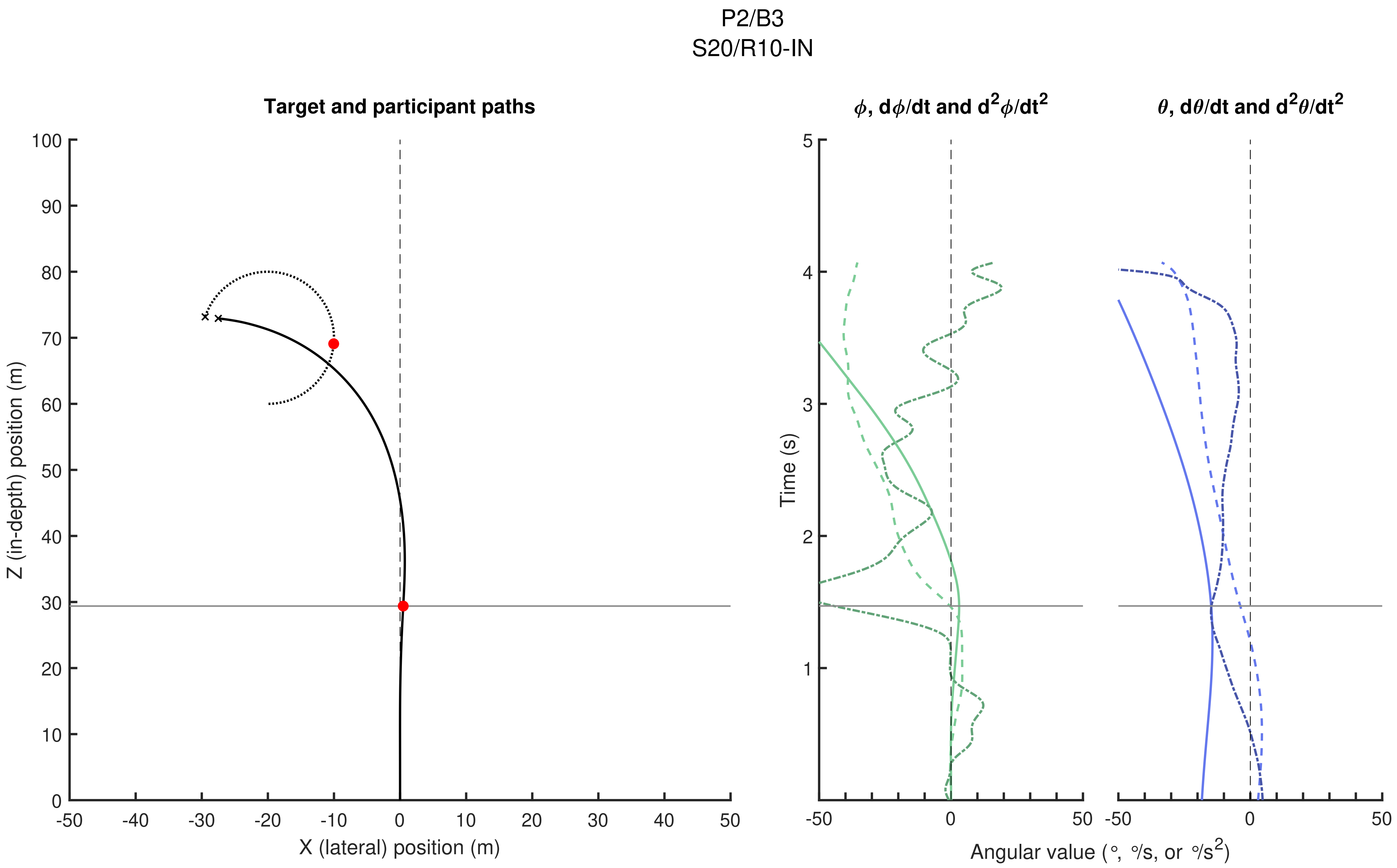

P2/B3
S20/R10-IN
Target and participant paths
Z (in-depth) position (m)
X (lateral) position (m)
ϕ, dϕ/dt and d$^2\phi$/dt^2
θ, dθ/dt and d$^2\theta$/dt^2
Time (s)
Angular value (°, °/s, or °/s^2)

P2/B3
S20/R20-IN

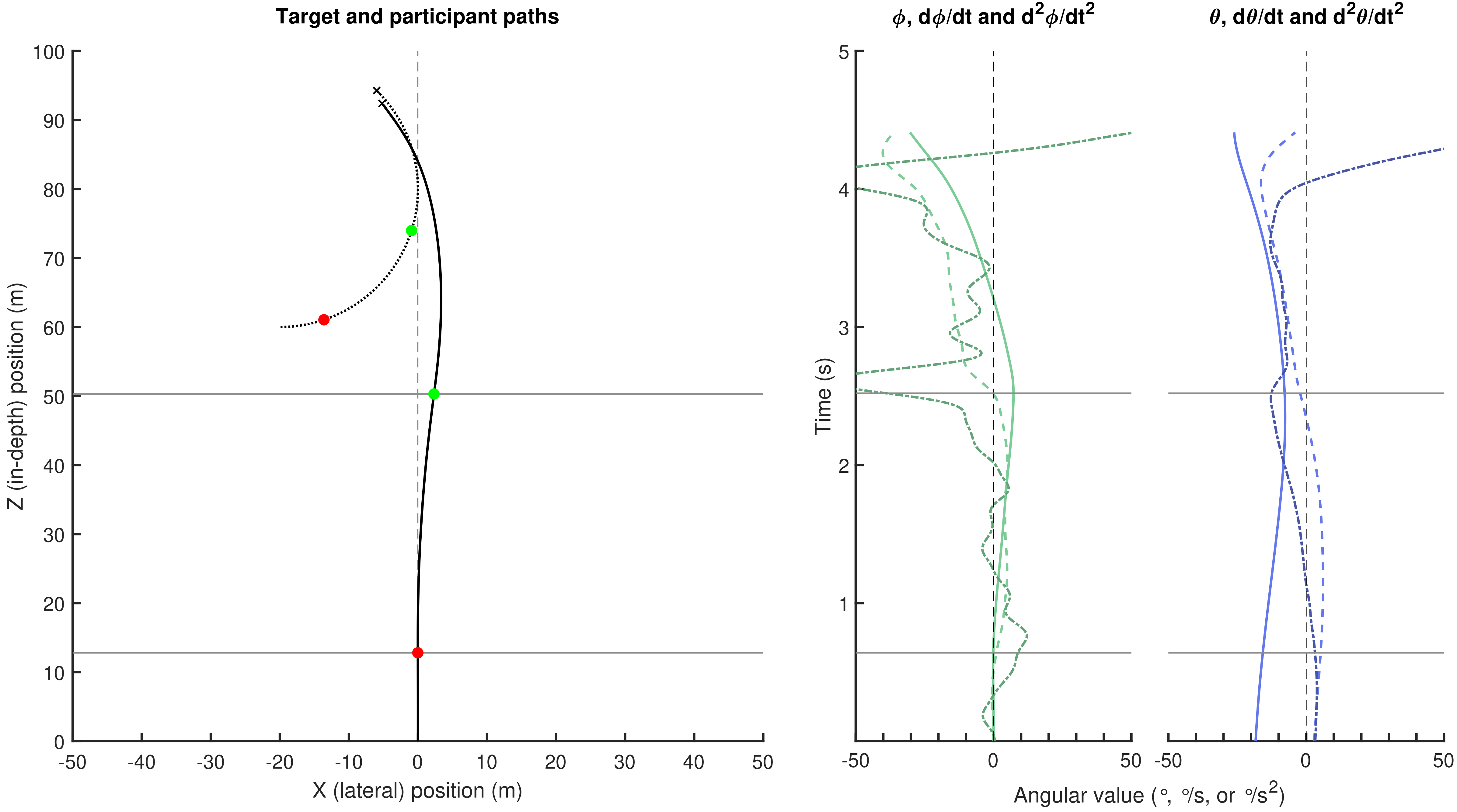

P2/B3
S20/R30-IN

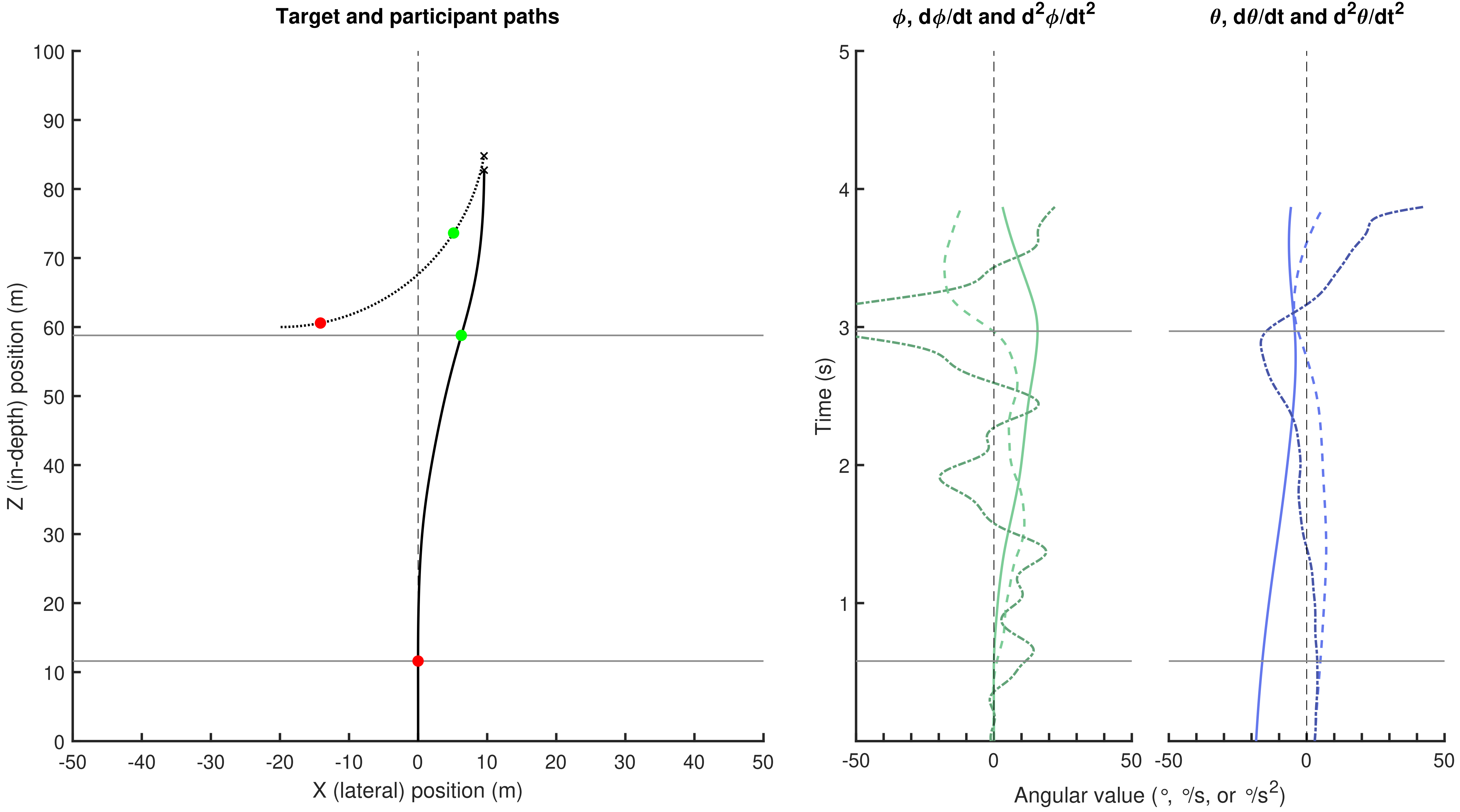

P2/B3
S15/R10-IN

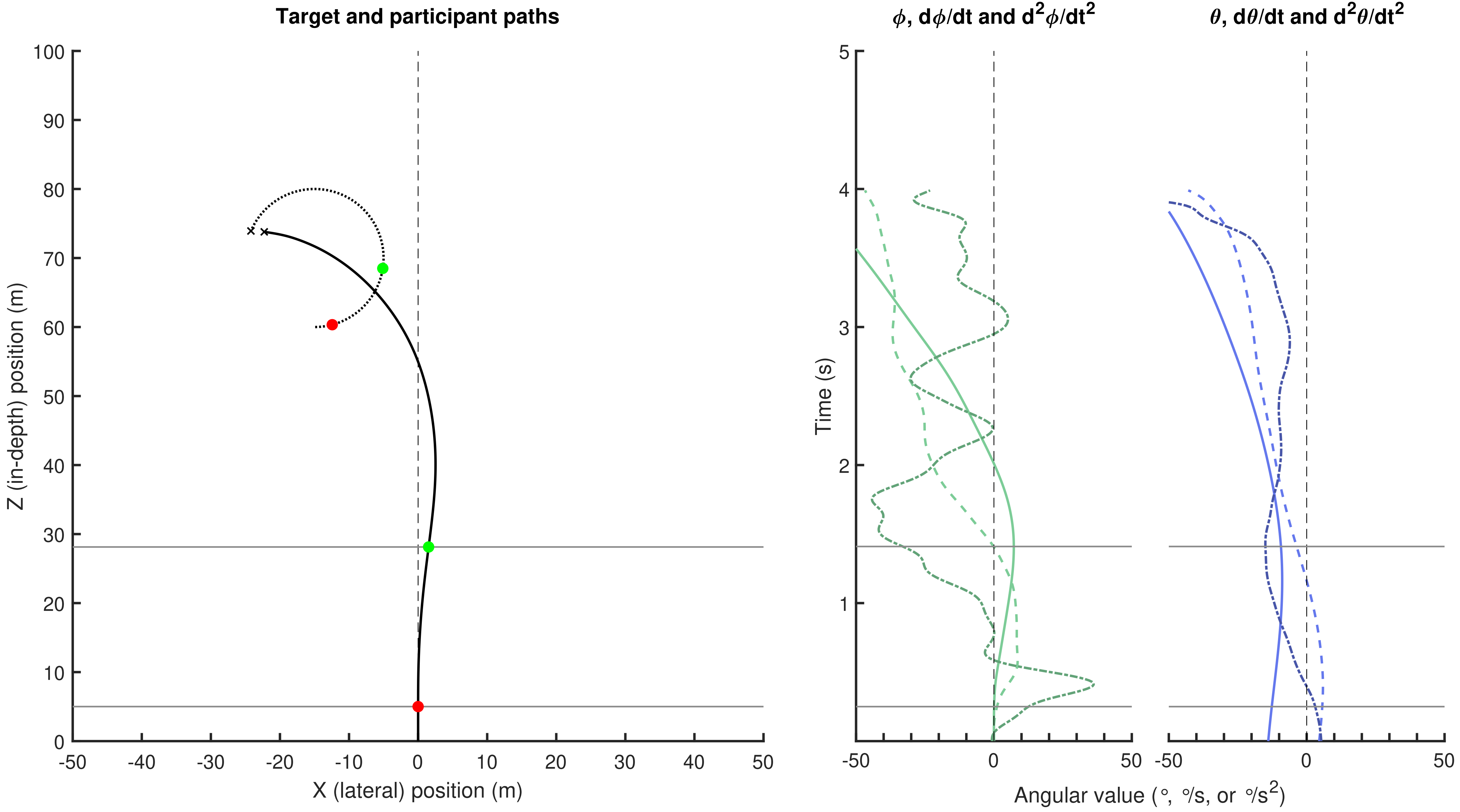

P2/B3
S15/R20-IN

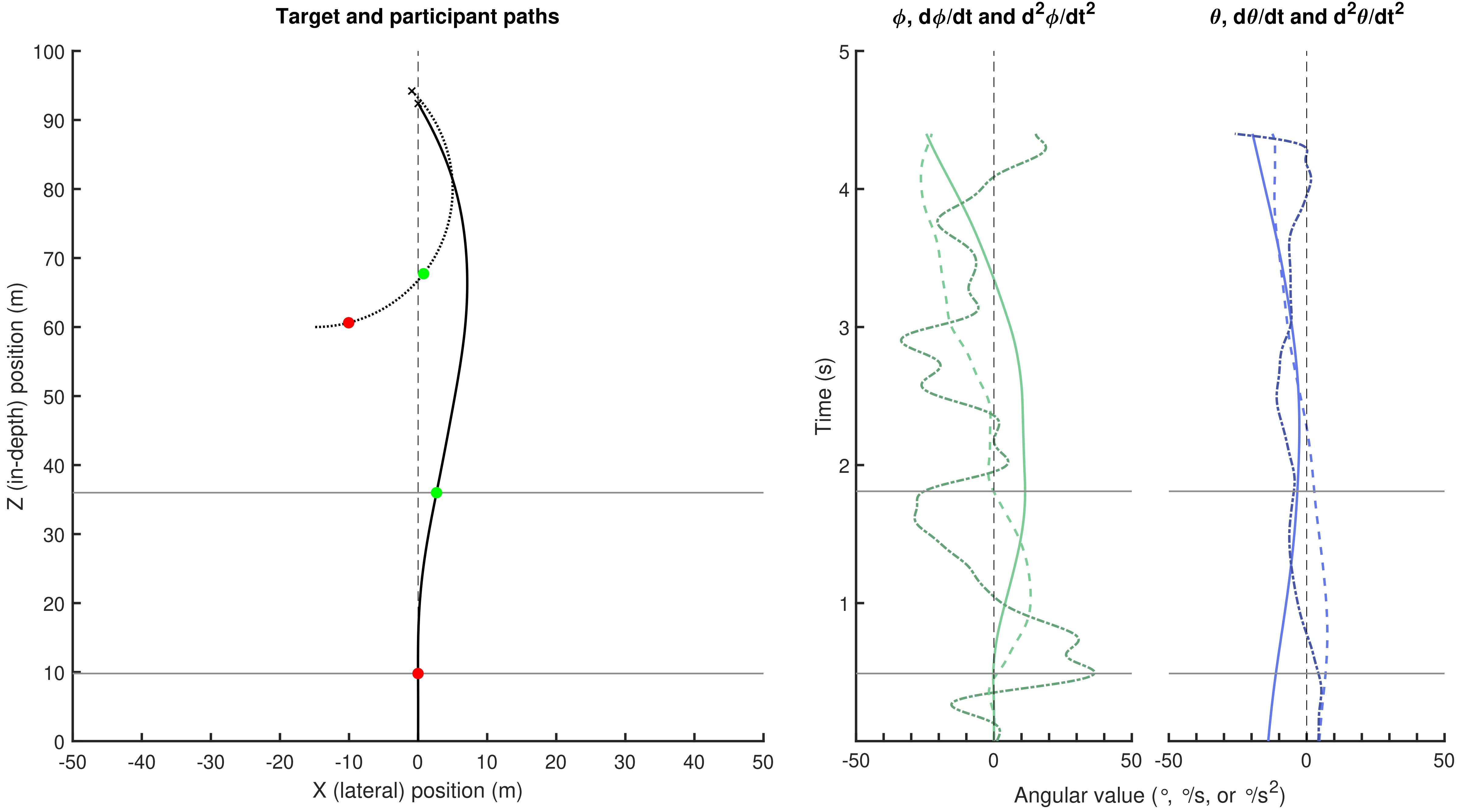

P2/B3
S15/R30-IN

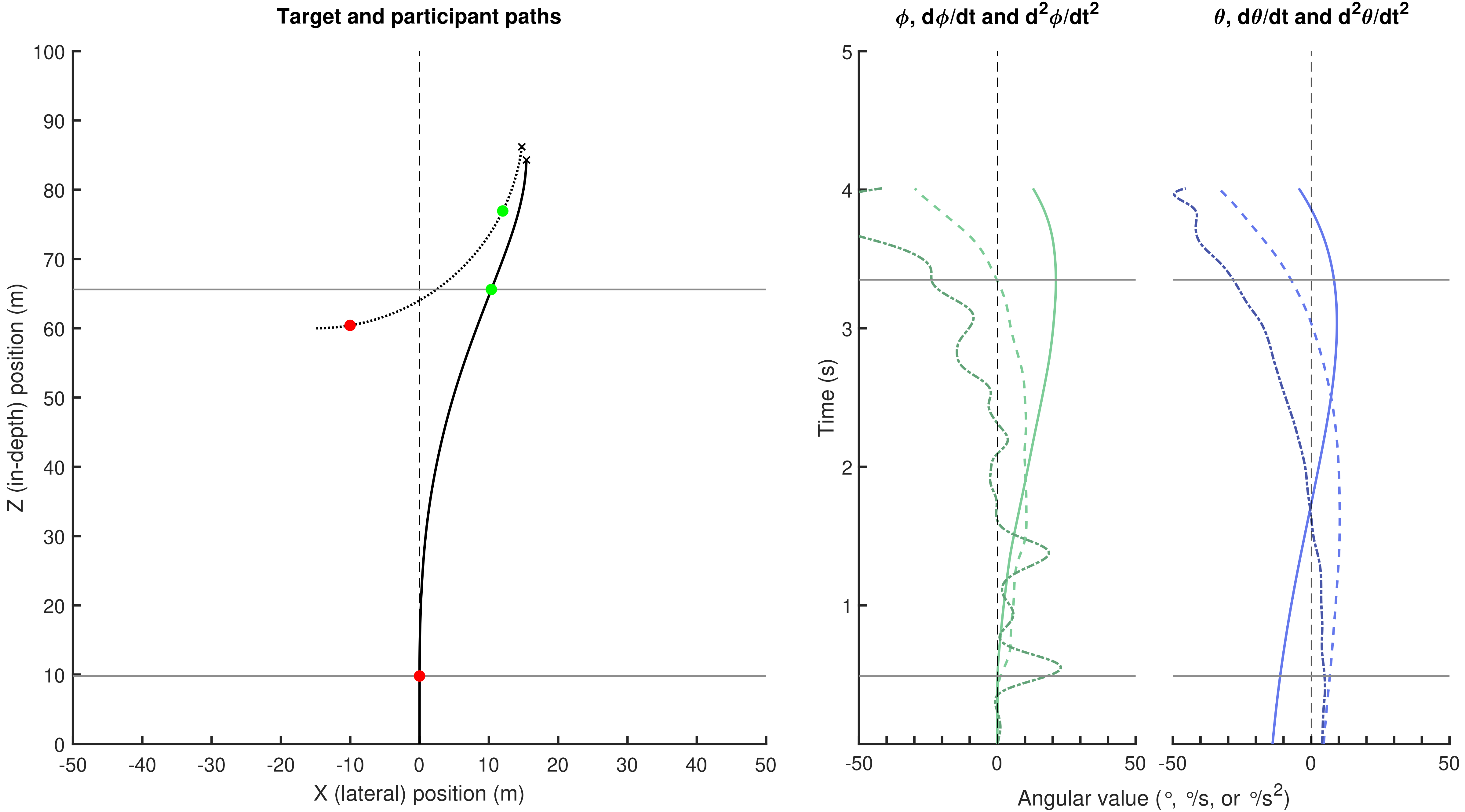

P2/B3
S5/R10-IN

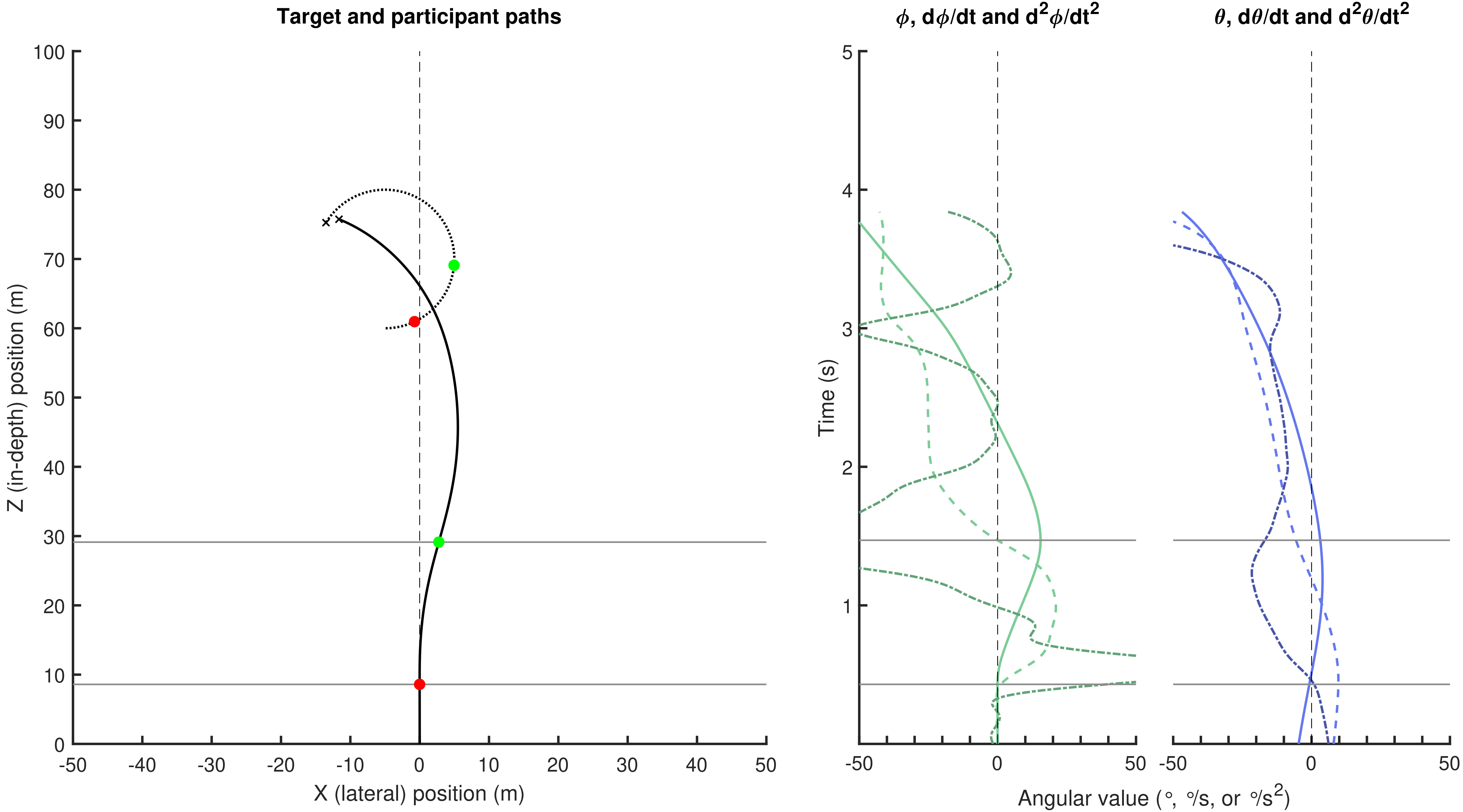

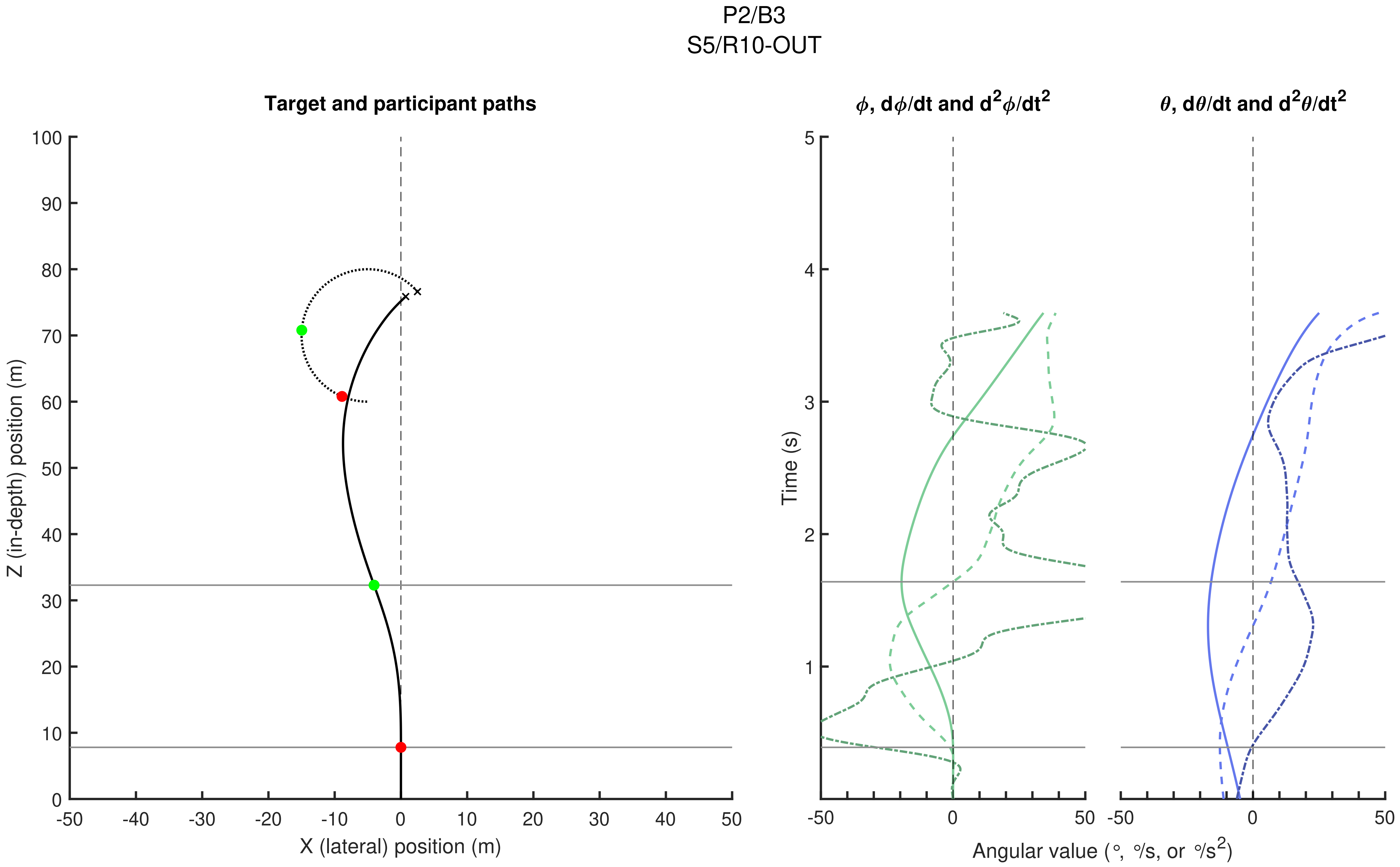
P2/B3
S5/R10-OUT
Target and participant paths
Z (in-depth) position (m)
X (lateral) position (m)
ϕ, dϕ/dt and d$^2\phi$/dt^2
θ, dθ/dt and d$^2\theta$/dt^2
Time (s)
Angular value (°, °/s, or °/s^2)

P2/B3
S5/R20-IN

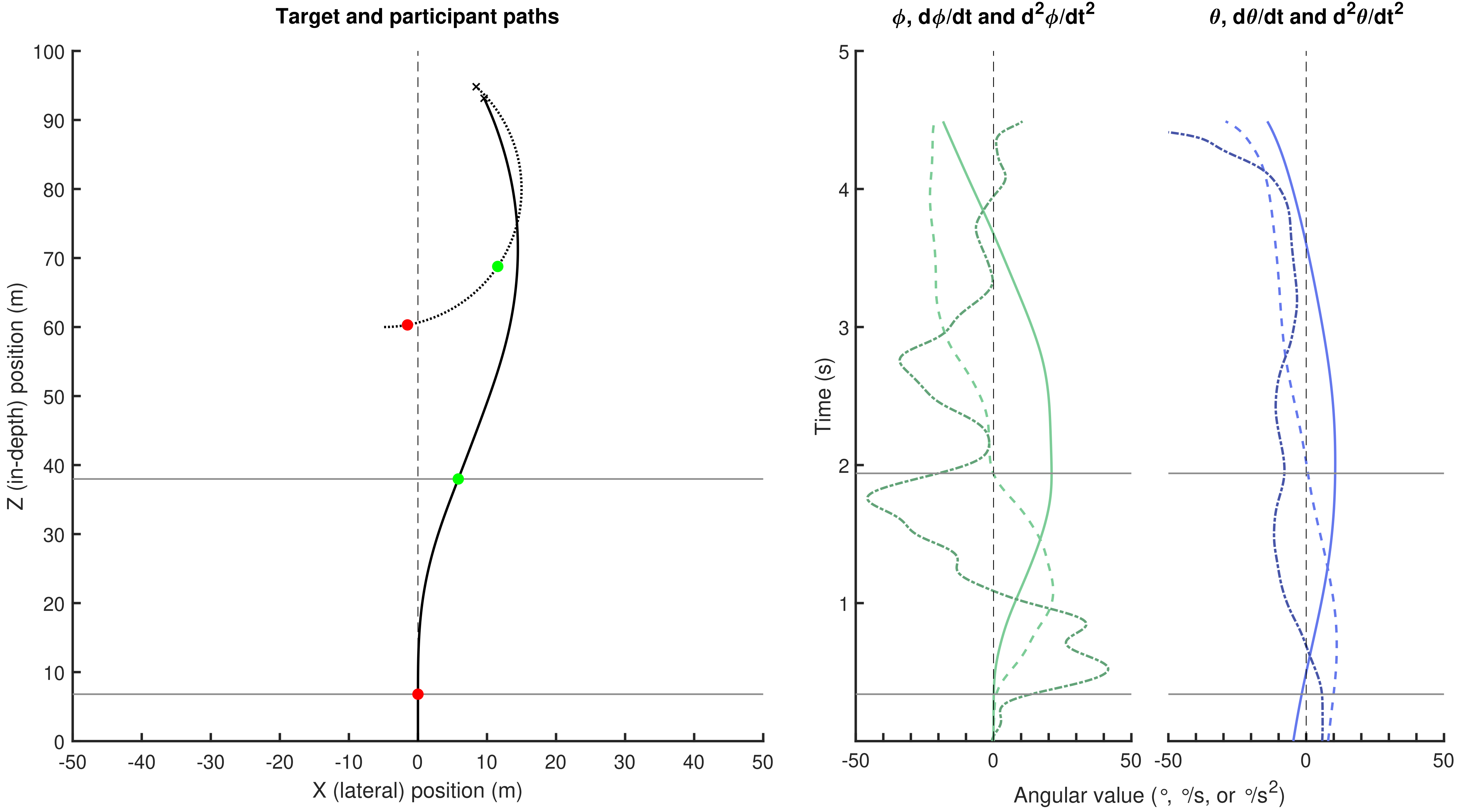

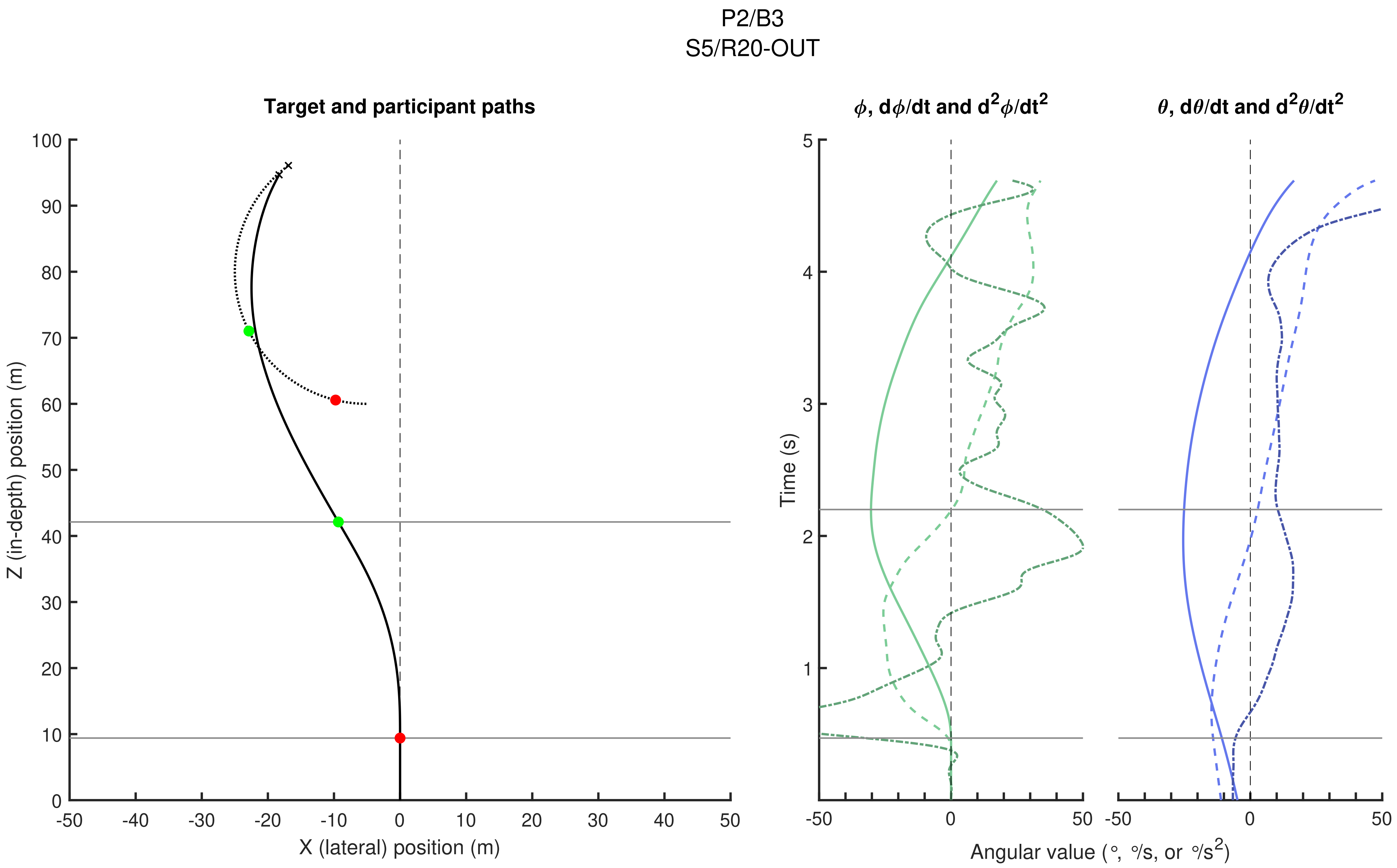

P2/B3
S5/R20-OUT
Target and participant paths
Z (in-depth) position (m)
X (lateral) position (m)
φ, dφ/dt and d²φ/dt²
θ, dθ/dt and d²θ/dt²
Time (s)
Angular value (°, °/s, or °/s²)

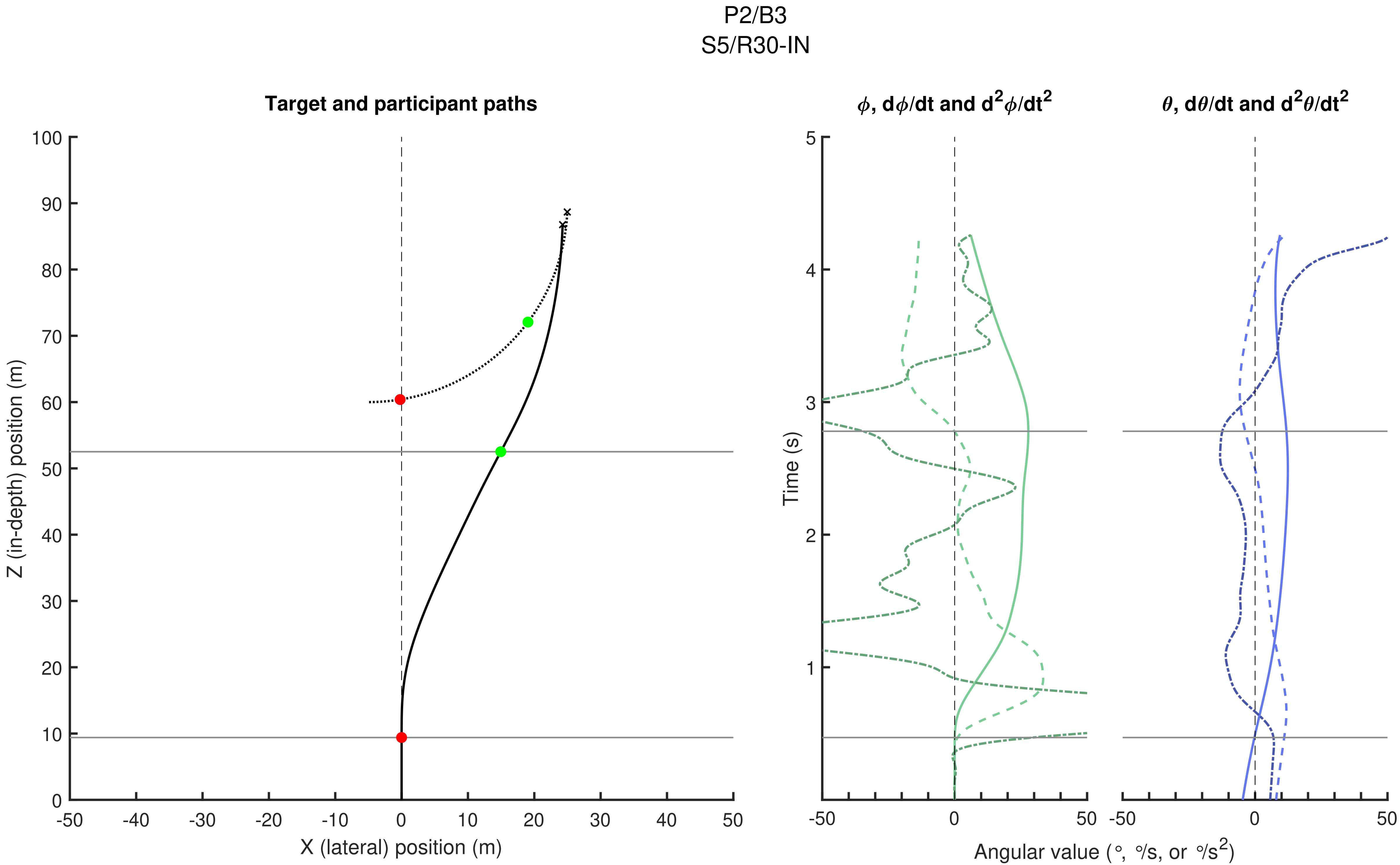

P2/B3
S5/R30-IN
Target and participant paths
Z (in-depth) position (m)
X (lateral) position (m)
0
10
20
30
40
50
60
70
80
90
100
-50
-40
-30
-20
-10
0
10
20
30
40
50
φ, dφ/dt and d²φ/dt²
θ, dθ/dt and d²θ/dt²
Time (s)
1
2
3
4
5
-50
0
50
-50
0
50
Angular value (°, °/s, or °/s²)

P2/B3
S5/R30-OUT

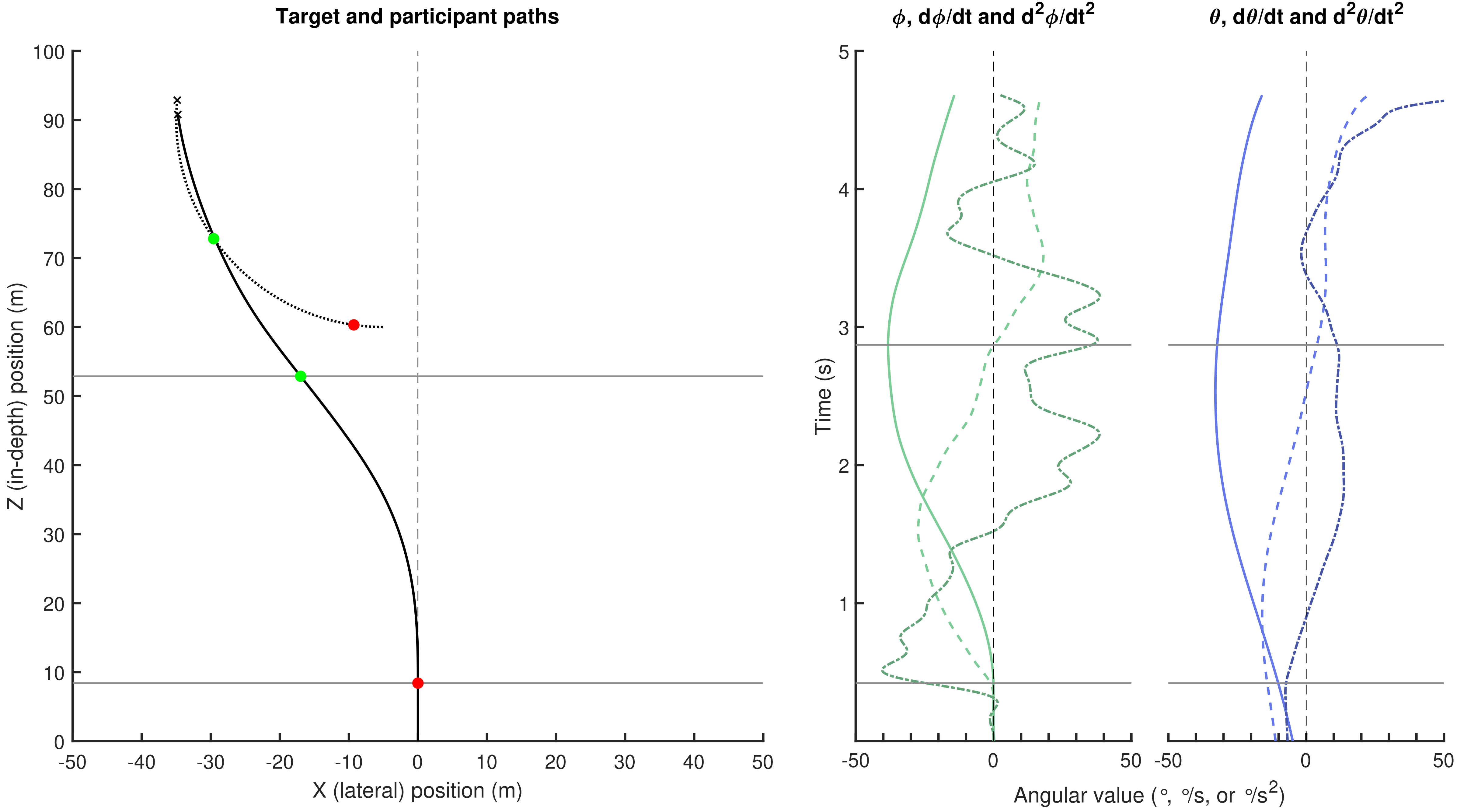

P2/B3
S5/R10-OUT

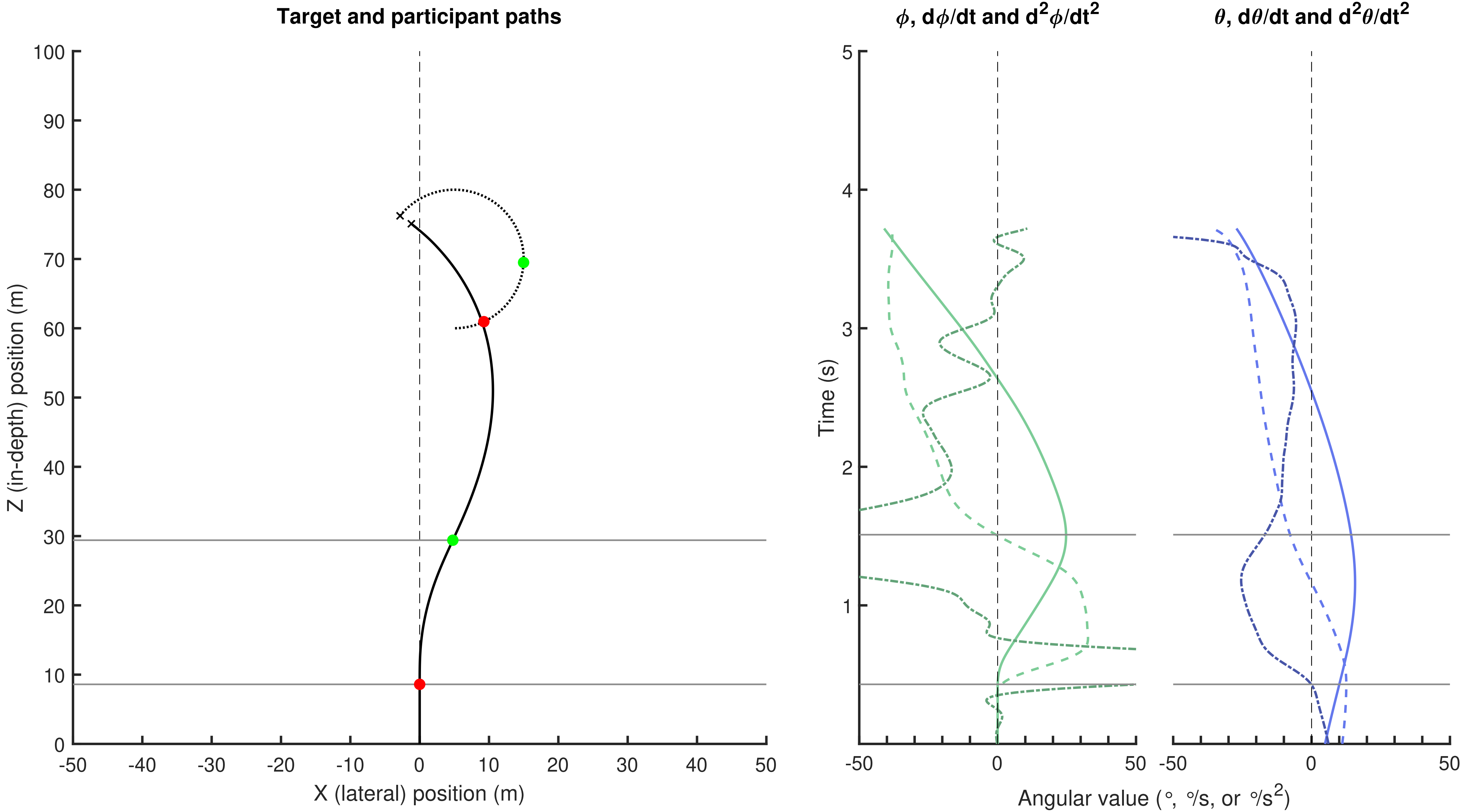

P2/B3
S5/R10-IN

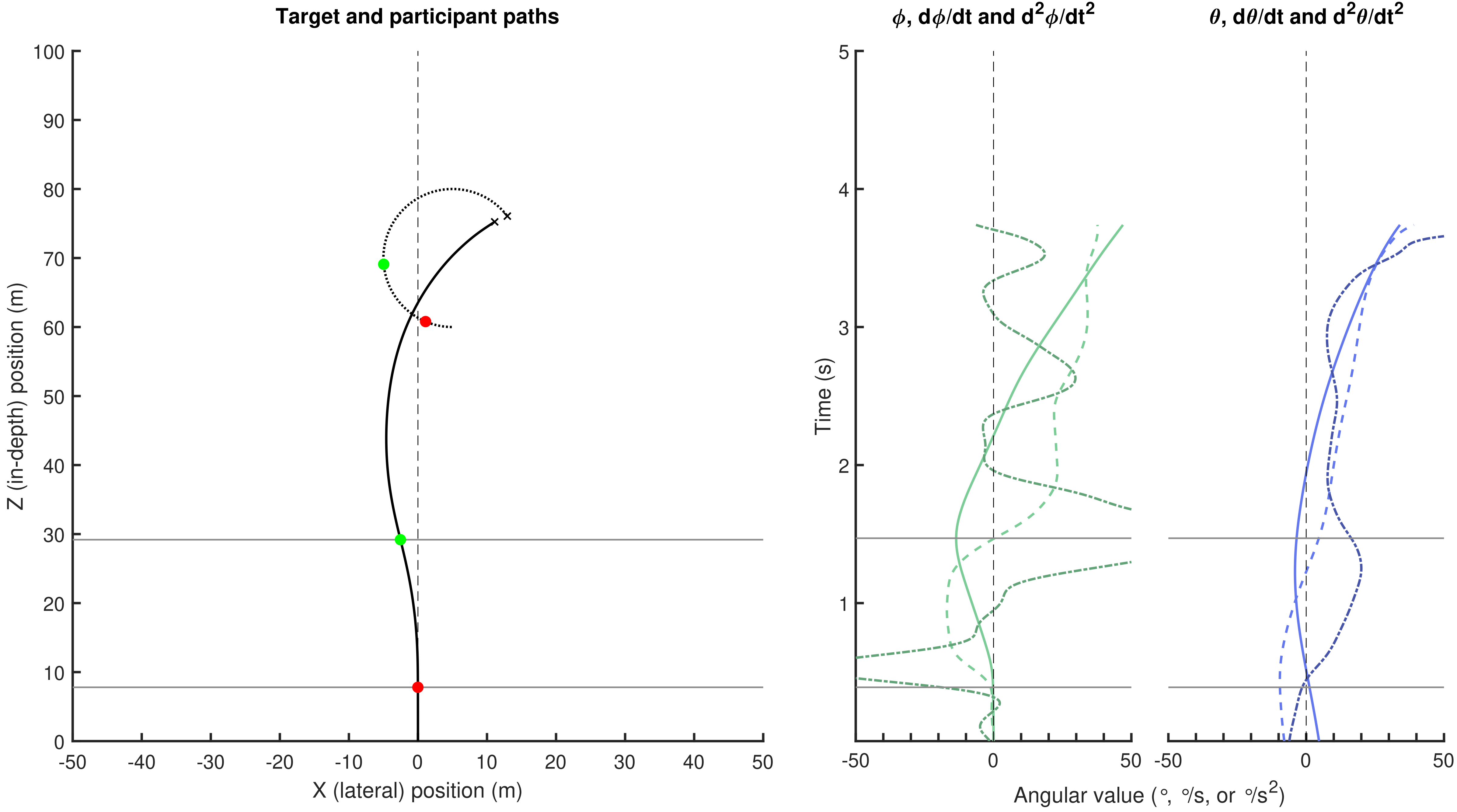

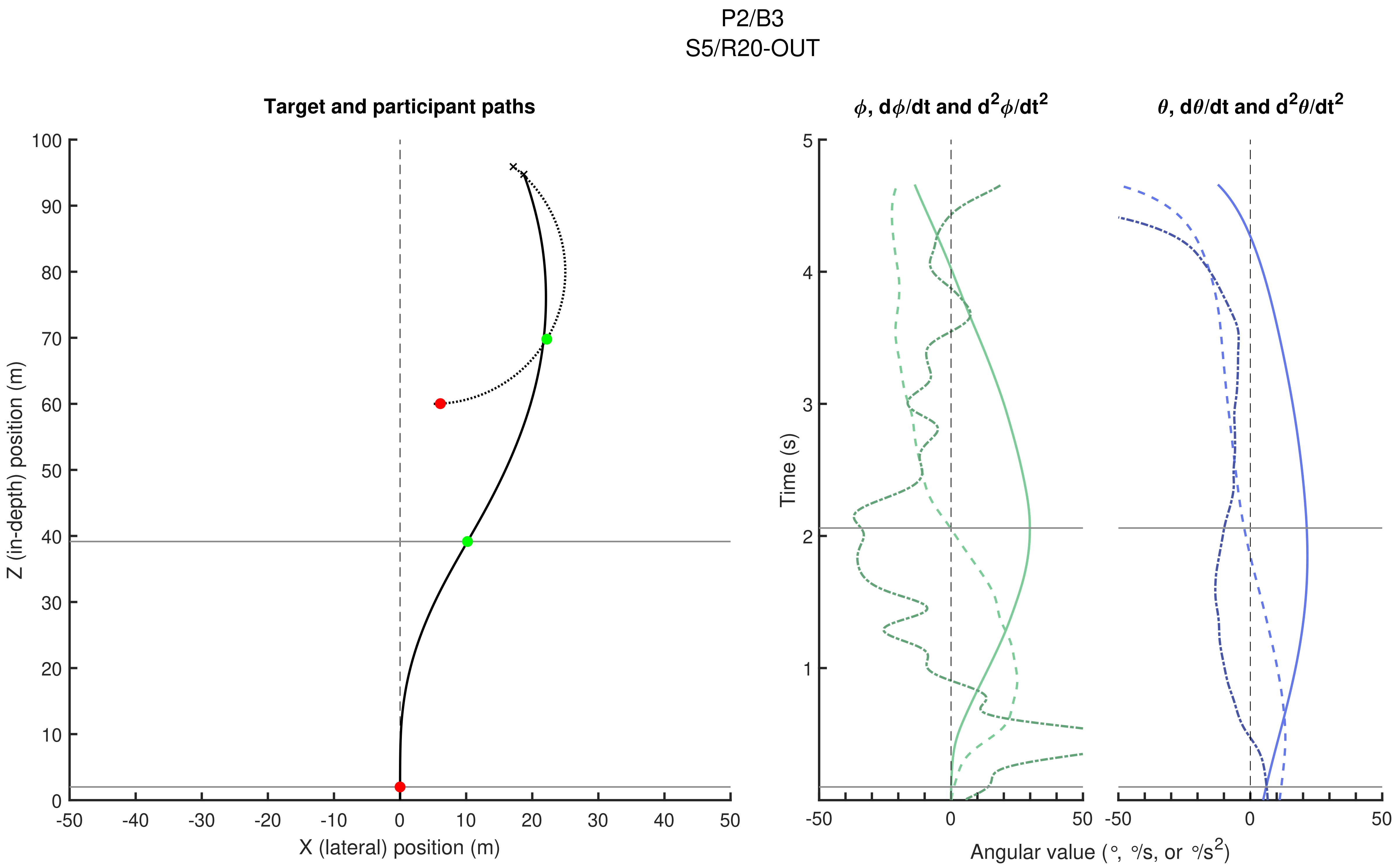

P2/B3
S5/R20-OUT
Target and participant paths
ϕ, dϕ/dt and d$^2\phi$/dt^2
θ, dθ/dt and d$^2\theta$/dt^2
Z (in-depth) position (m)
X (lateral) position (m)
Time (s)
Angular value (°, °/s, or °/s^2)

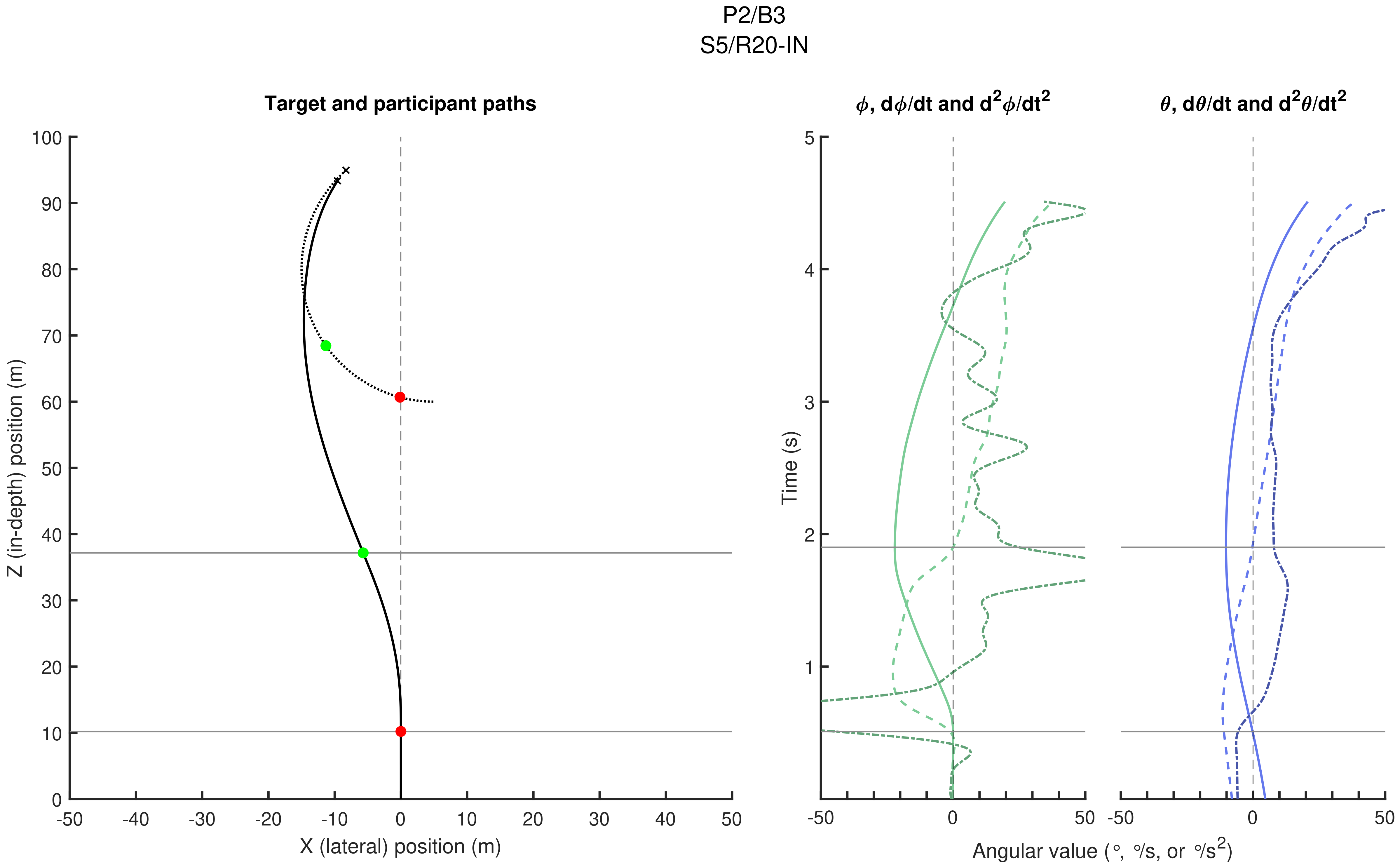
P2/B3
S5/R20-IN
Target and participant paths
Z (in-depth) position (m)
X (lateral) position (m)
100
90
80
70
60
50
40
30
20
10
0
-50
-40
-30
-20
-10
0
10
20
30
40
50
ϕ, dϕ/dt and d$^2\phi$/dt^2
θ, dθ/dt and d$^2\theta$/dt^2
Time (s)
5
4
3
2
1
-50
0
50
-50
0
50
Angular value (°, °/s, or °/s^2)

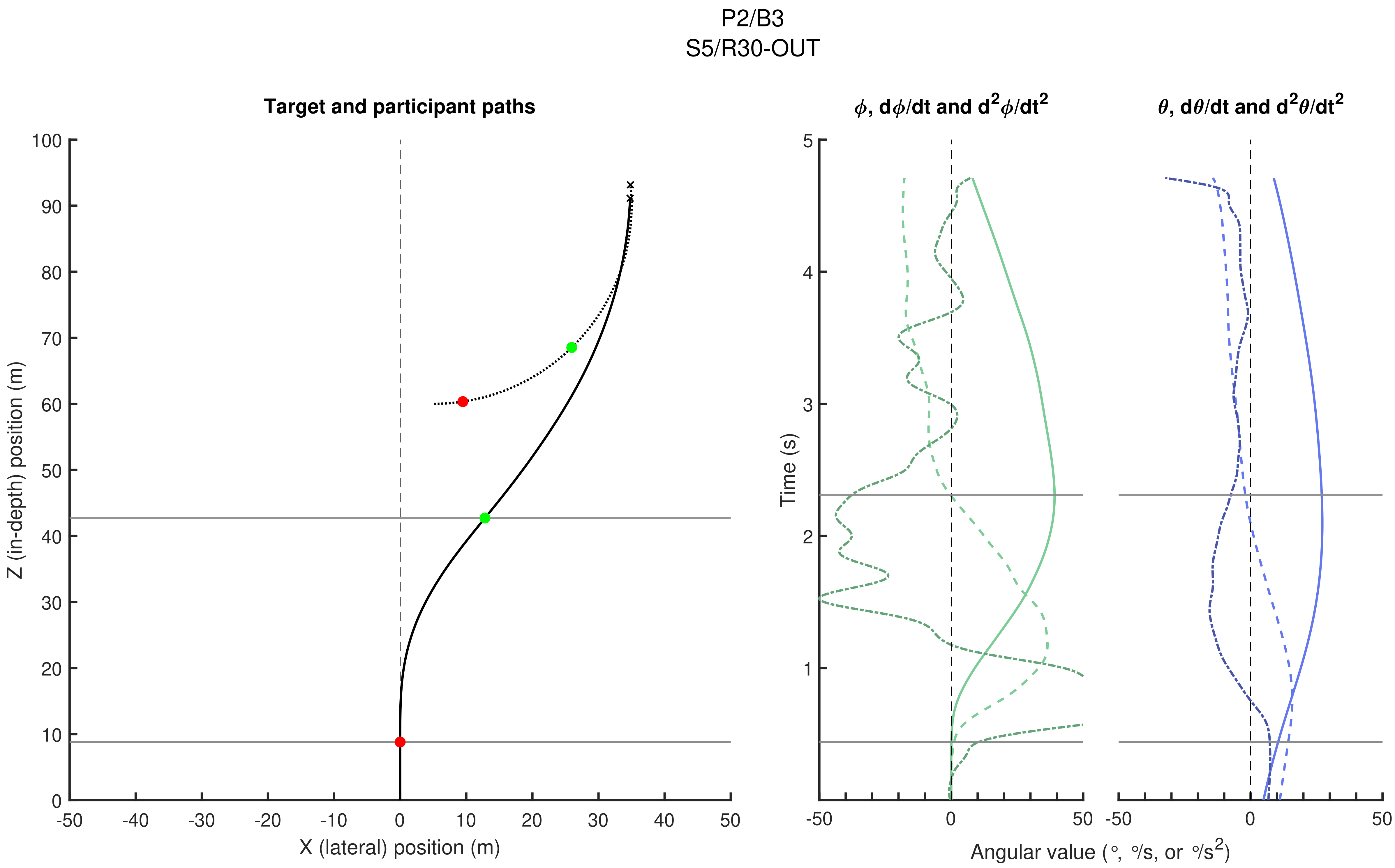

P2/B3
S5/R30-OUT
Target and participant paths
Z (in-depth) position (m)
X (lateral) position (m)
ϕ, dϕ/dt and d²ϕ/dt²
θ, dθ/dt and d²θ/dt²
Time (s)
Angular value (°, °/s, or °/s²)

P2/B3
S5/R30-IN

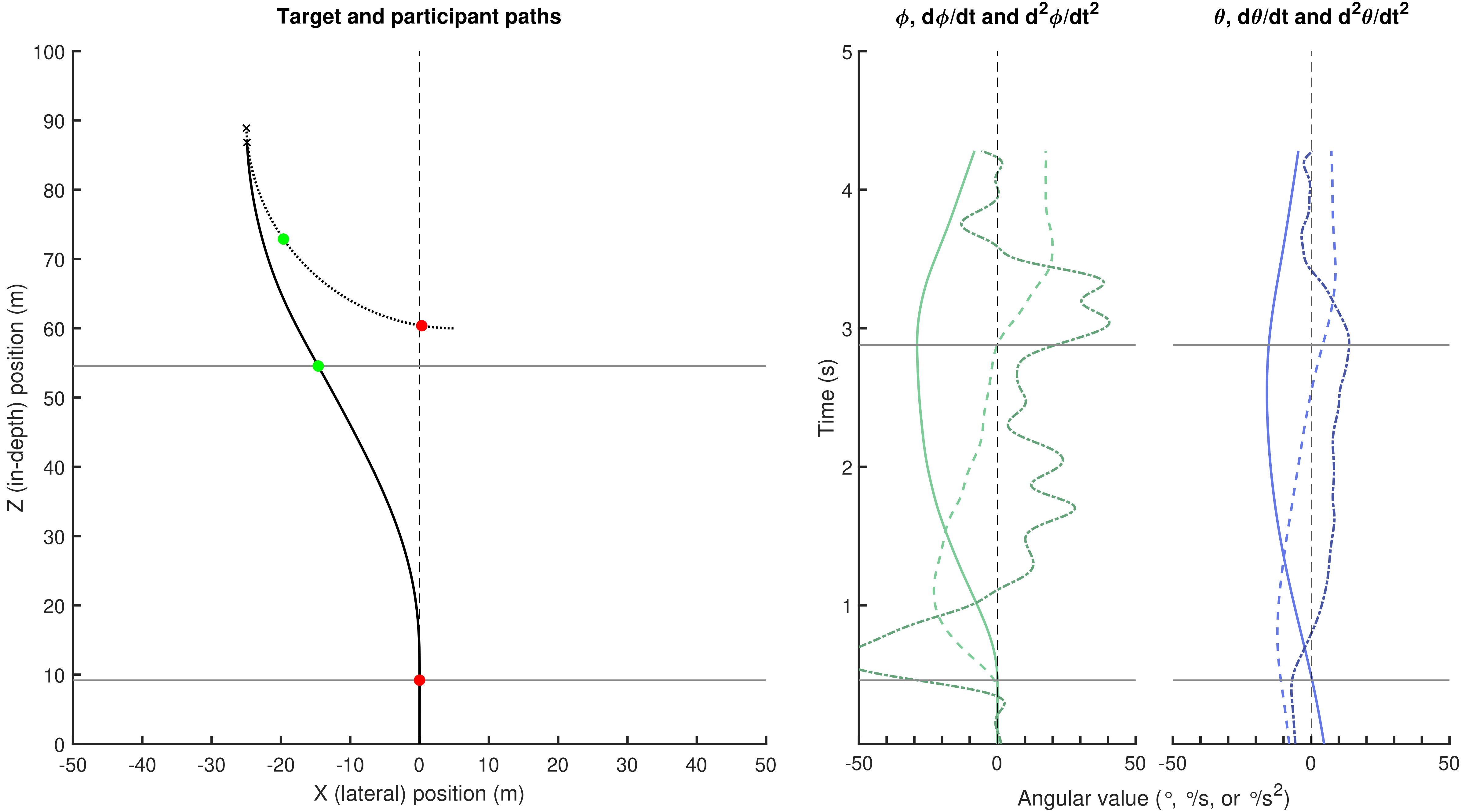

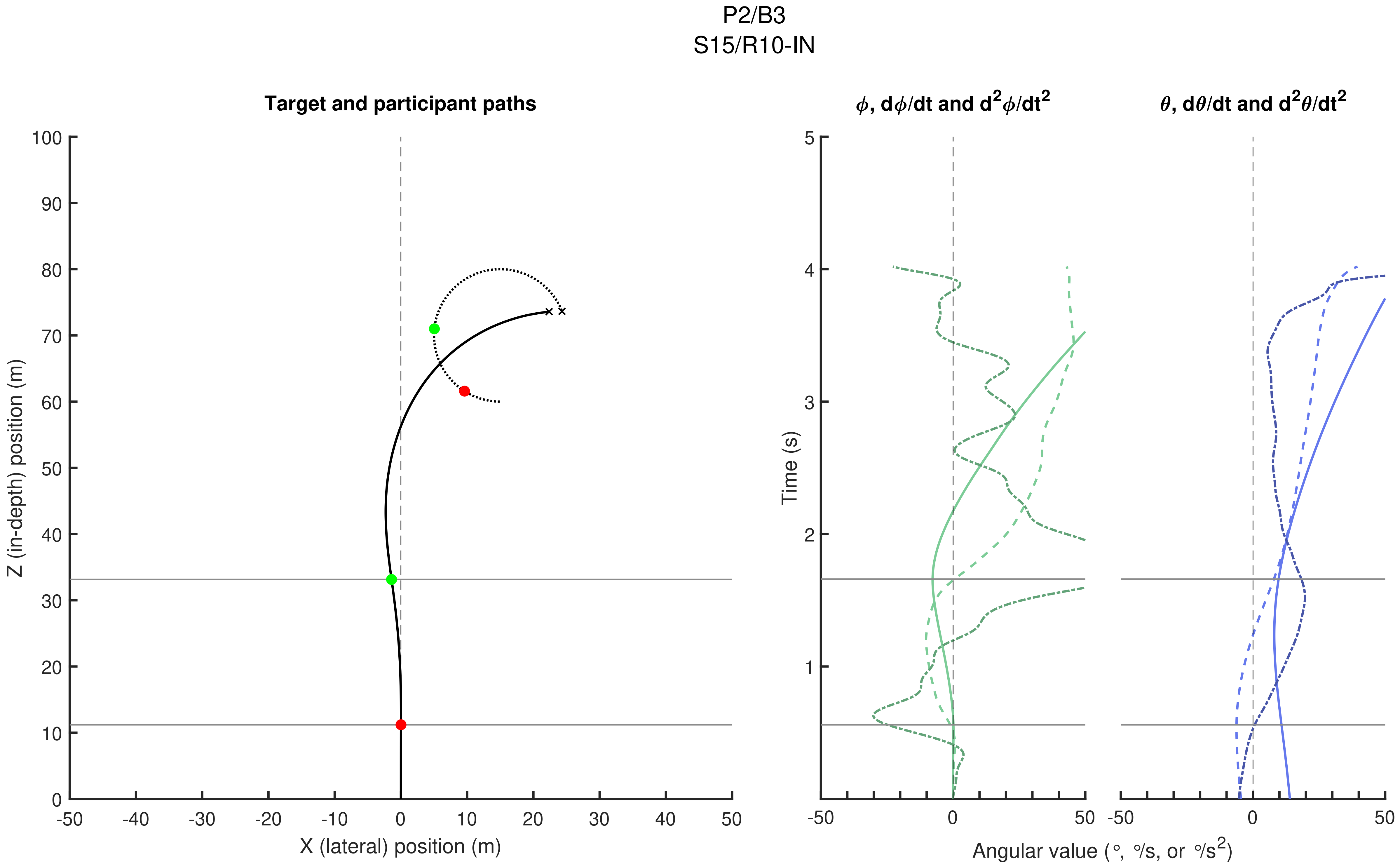

P2/B3
S15/R10-IN
Target and participant paths
Z (in-depth) position (m)
X (lateral) position (m)
ϕ, dϕ/dt and d$^2\phi$/dt^2
θ, dθ/dt and d$^2\theta$/dt^2
Time (s)
Angular value (°, °/s, or °/s^2)

P2/B3
S15/R20-IN

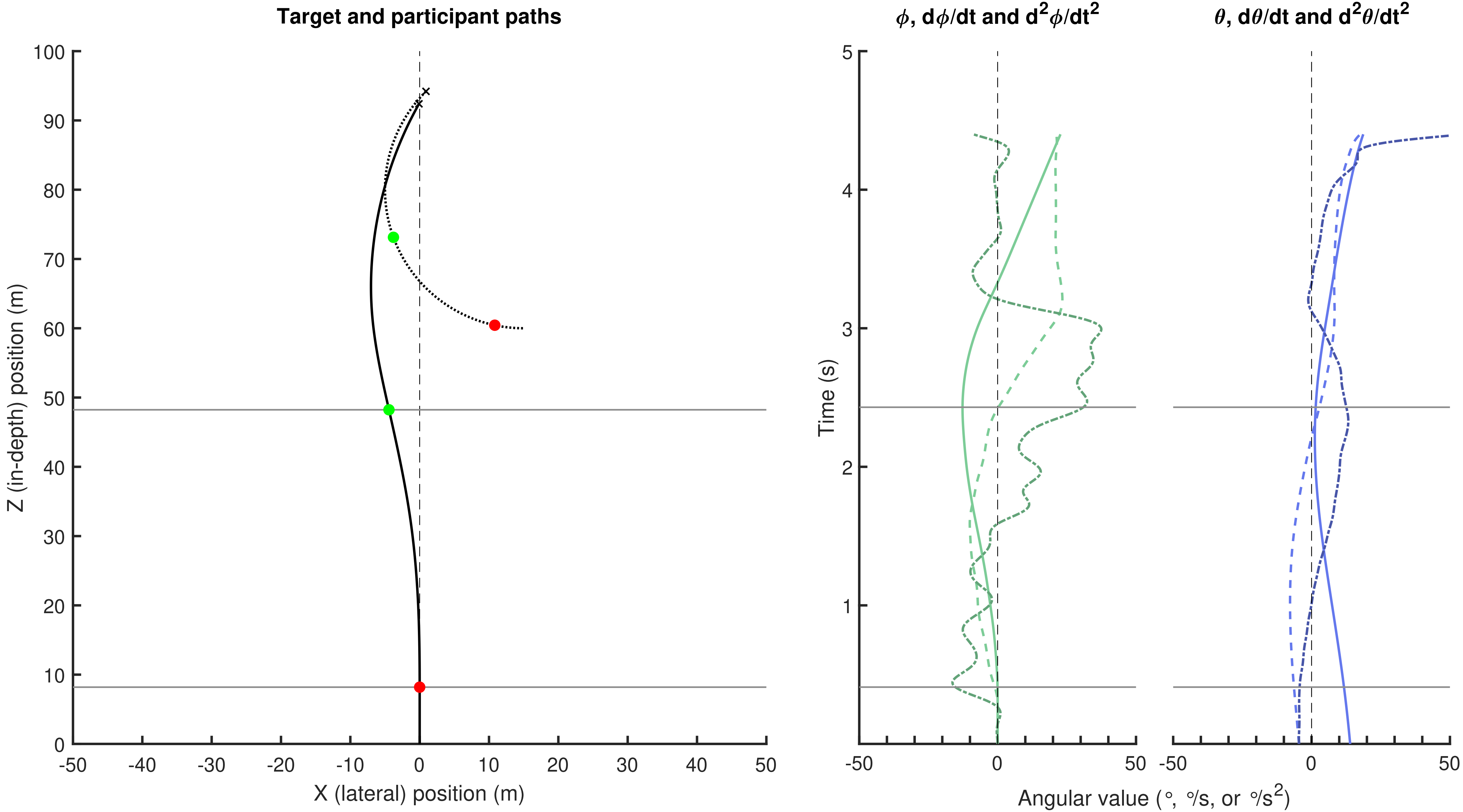

P2/B3
S15/R30-IN

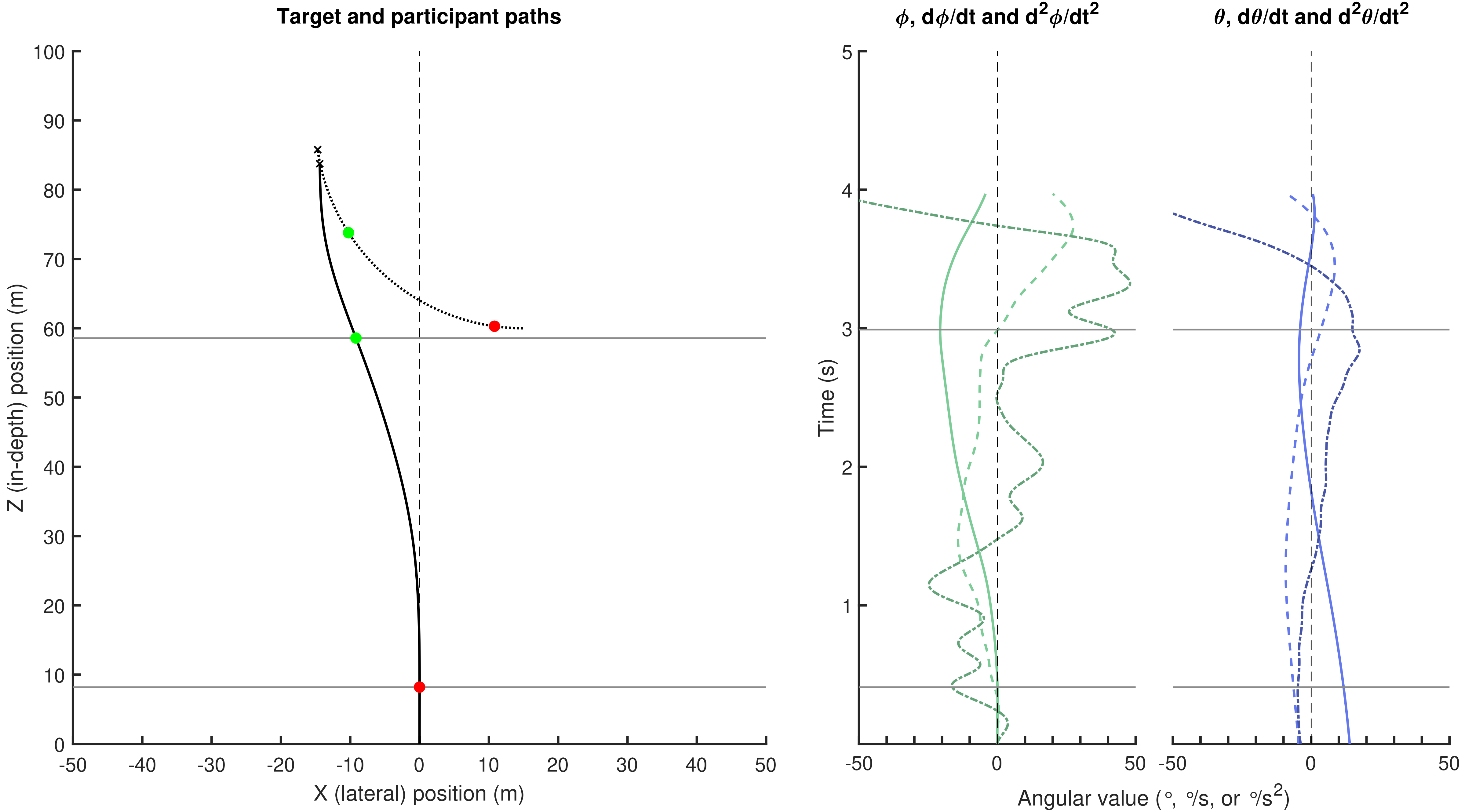

P2/B3
S20/R10-IN

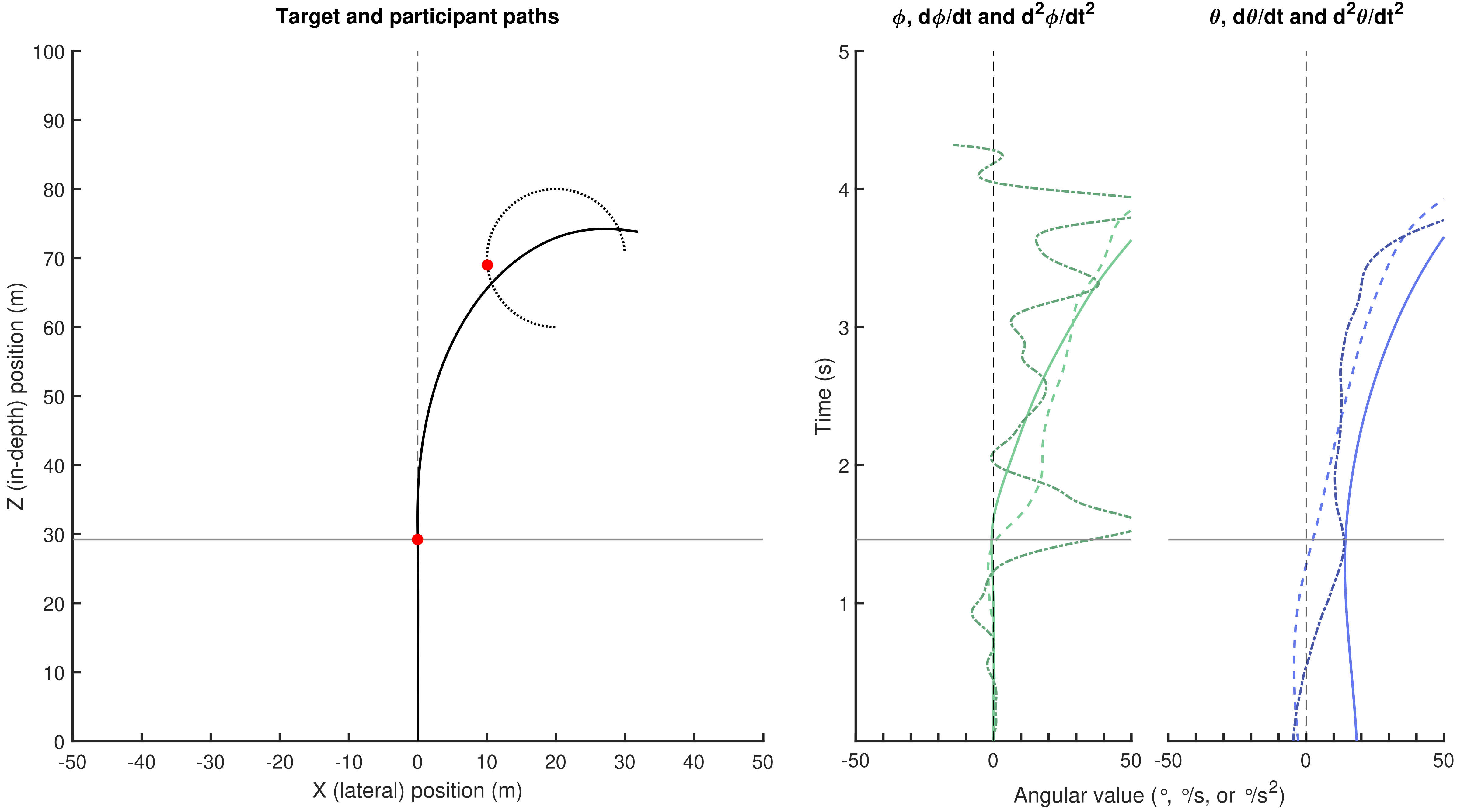

P2/B3
S20/R20-IN

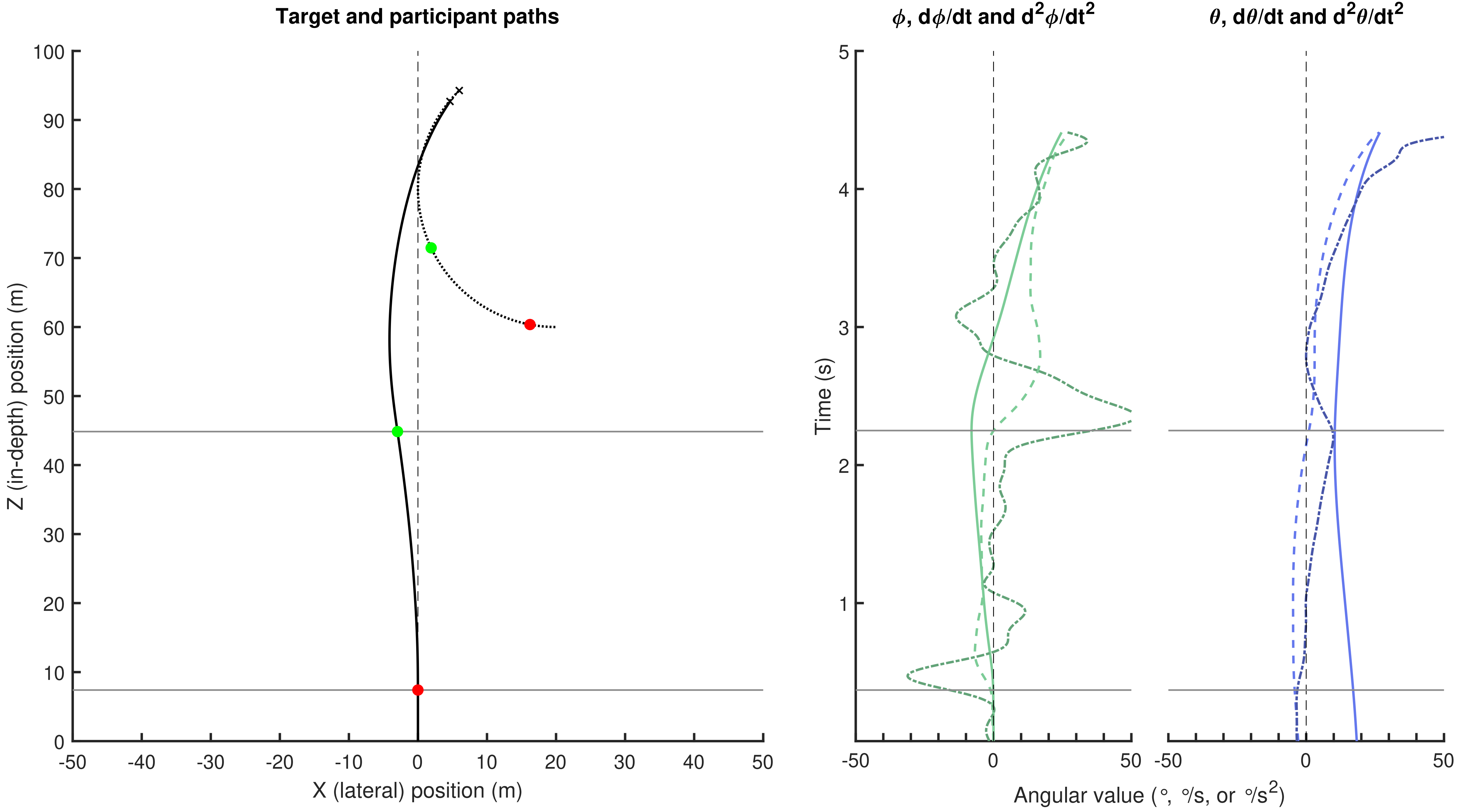

P2/B3
S20/R30-IN

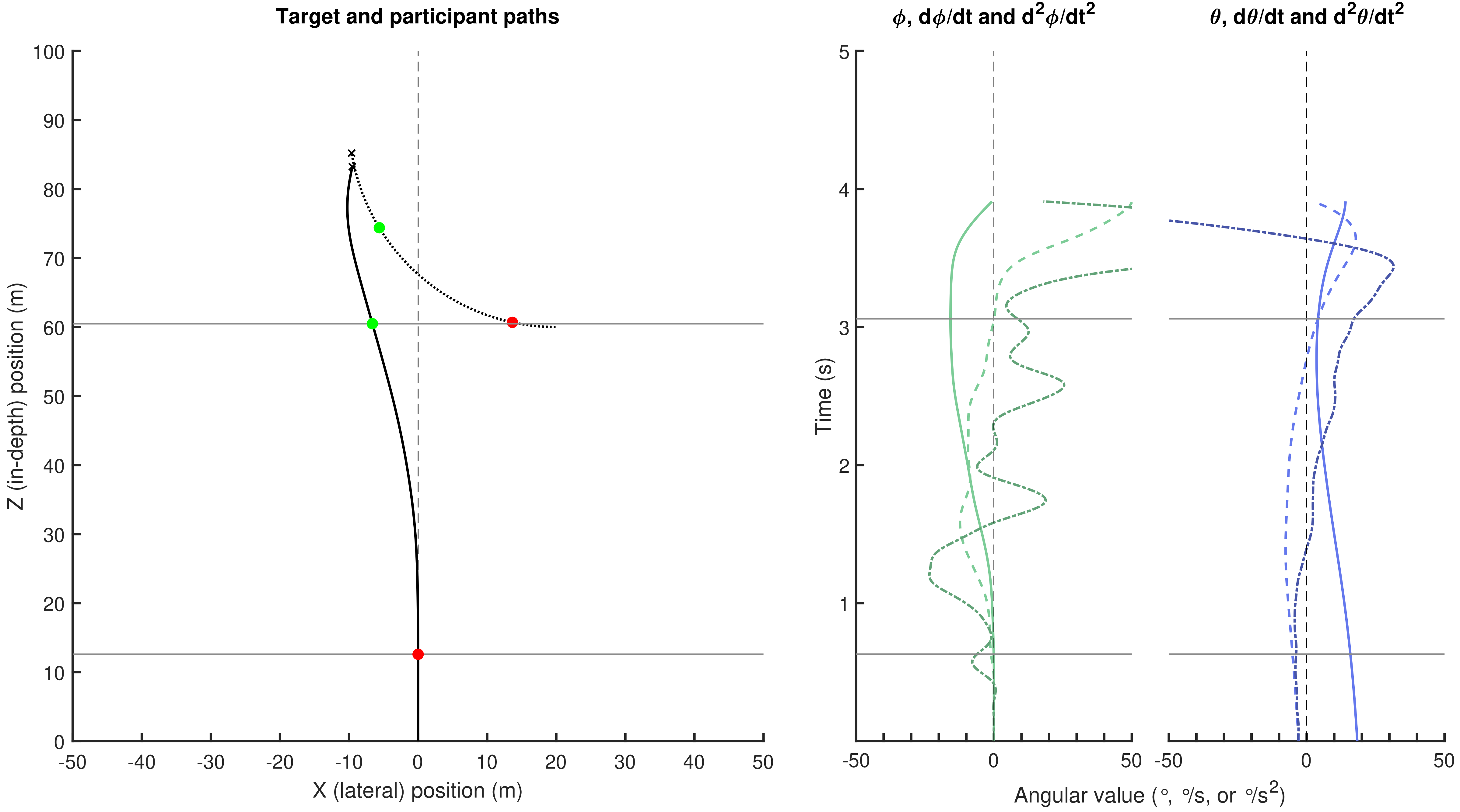

P2/B4
S20/R10-IN

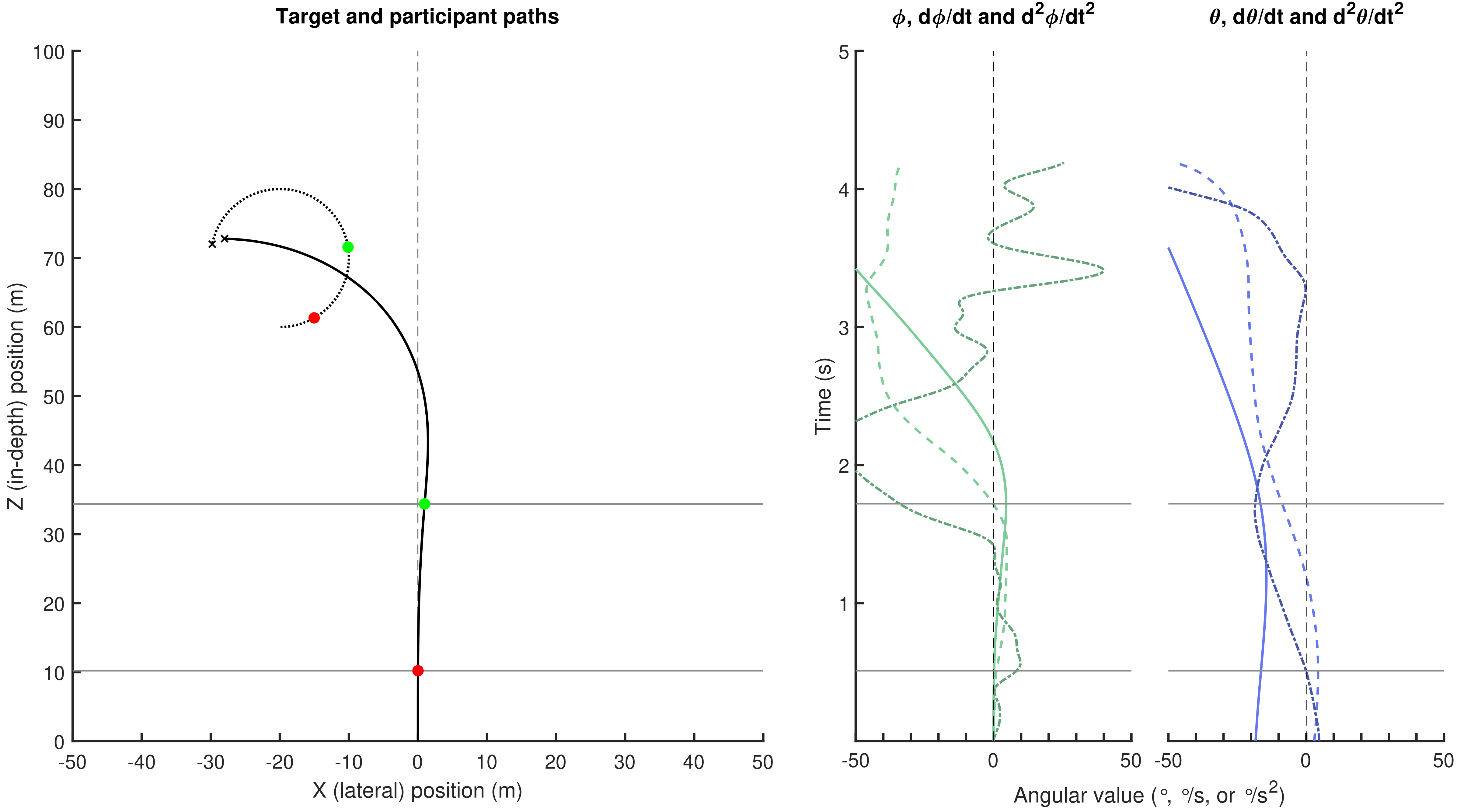

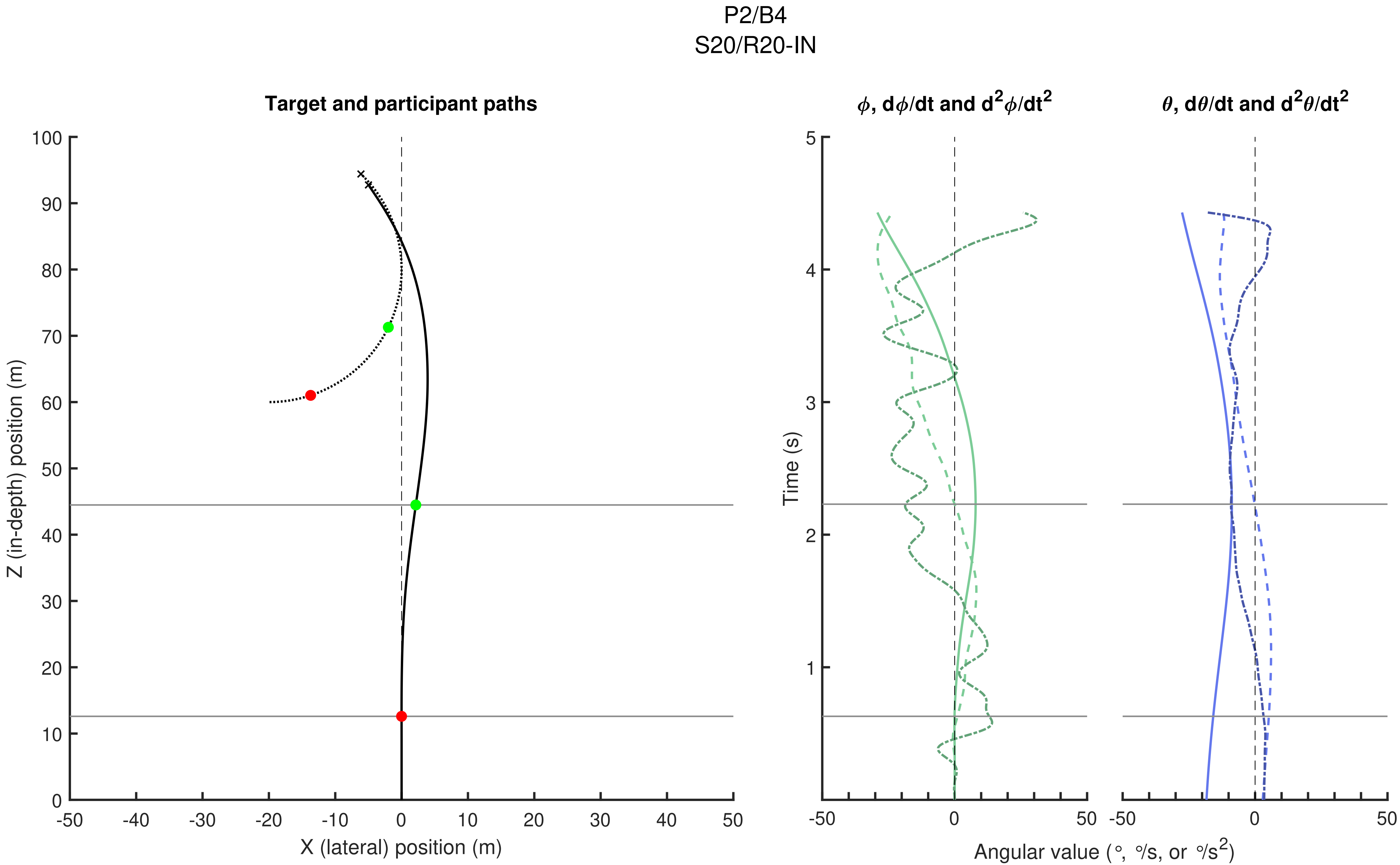

P2/B4
S20/R20-IN
Target and participant paths
Z (in-depth) position (m)
X (lateral) position (m)
φ, dφ/dt and d²φ/dt²
θ, dθ/dt and d²θ/dt²
Time (s)
Angular value (°, °/s, or °/s²)

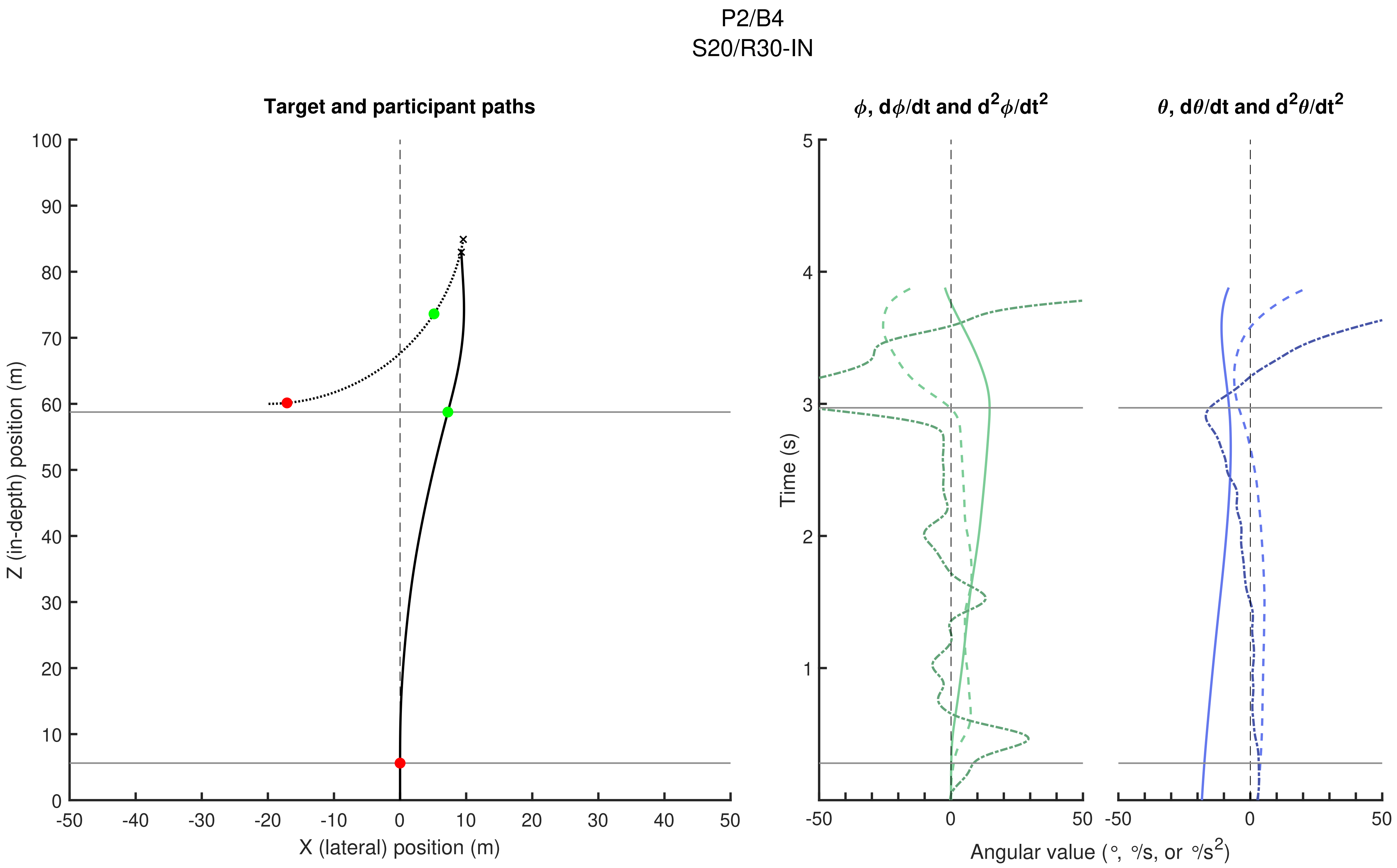

P2/B4
S20/R30-IN
Target and participant paths
Z (in-depth) position (m)
X (lateral) position (m)
ϕ, dϕ/dt and d²ϕ/dt²
θ, dθ/dt and d²θ/dt²
Time (s)
Angular value (°, °/s, or °/s²)

P2/B4
S15/R10-IN

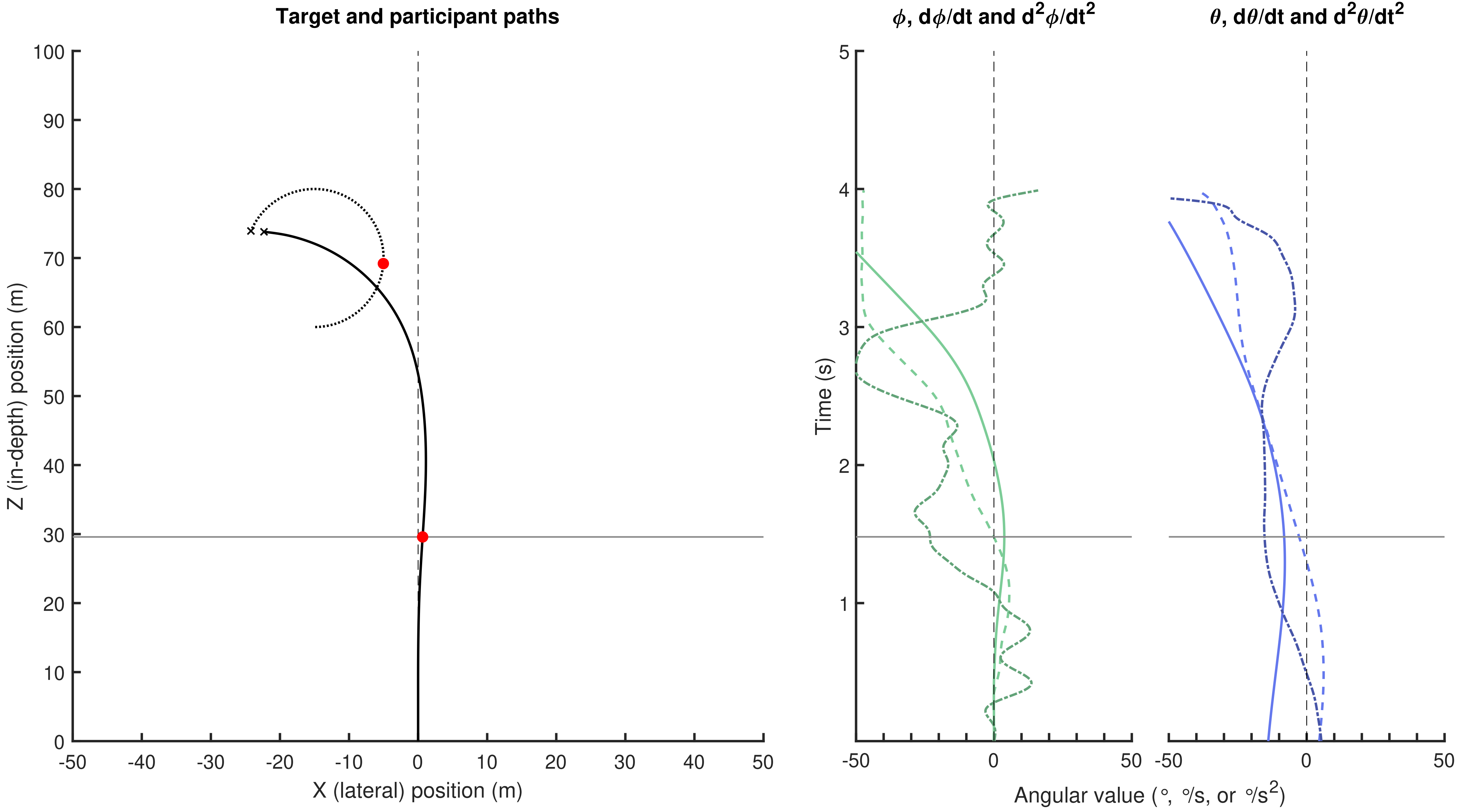

P2/B4
S15/R20-IN

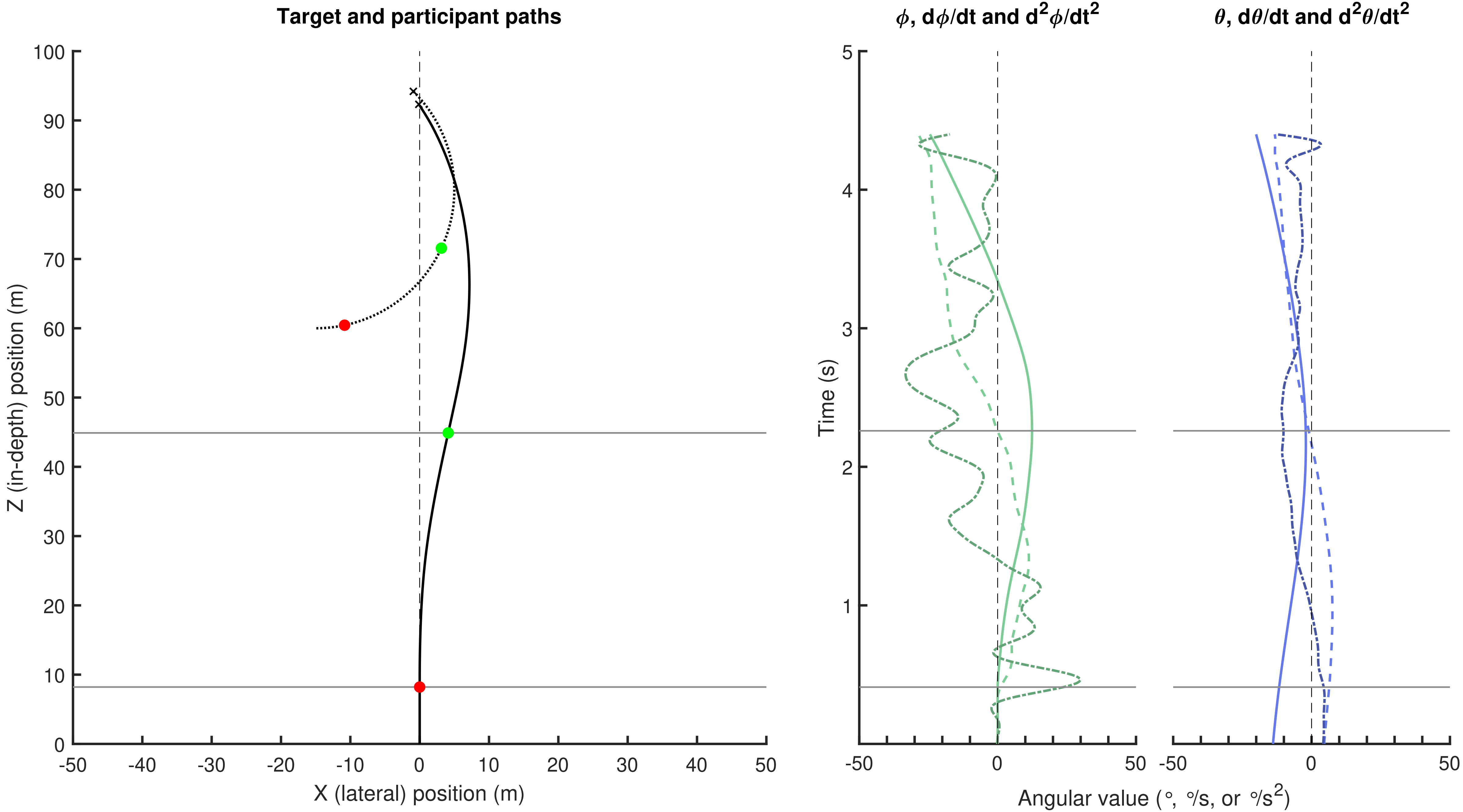

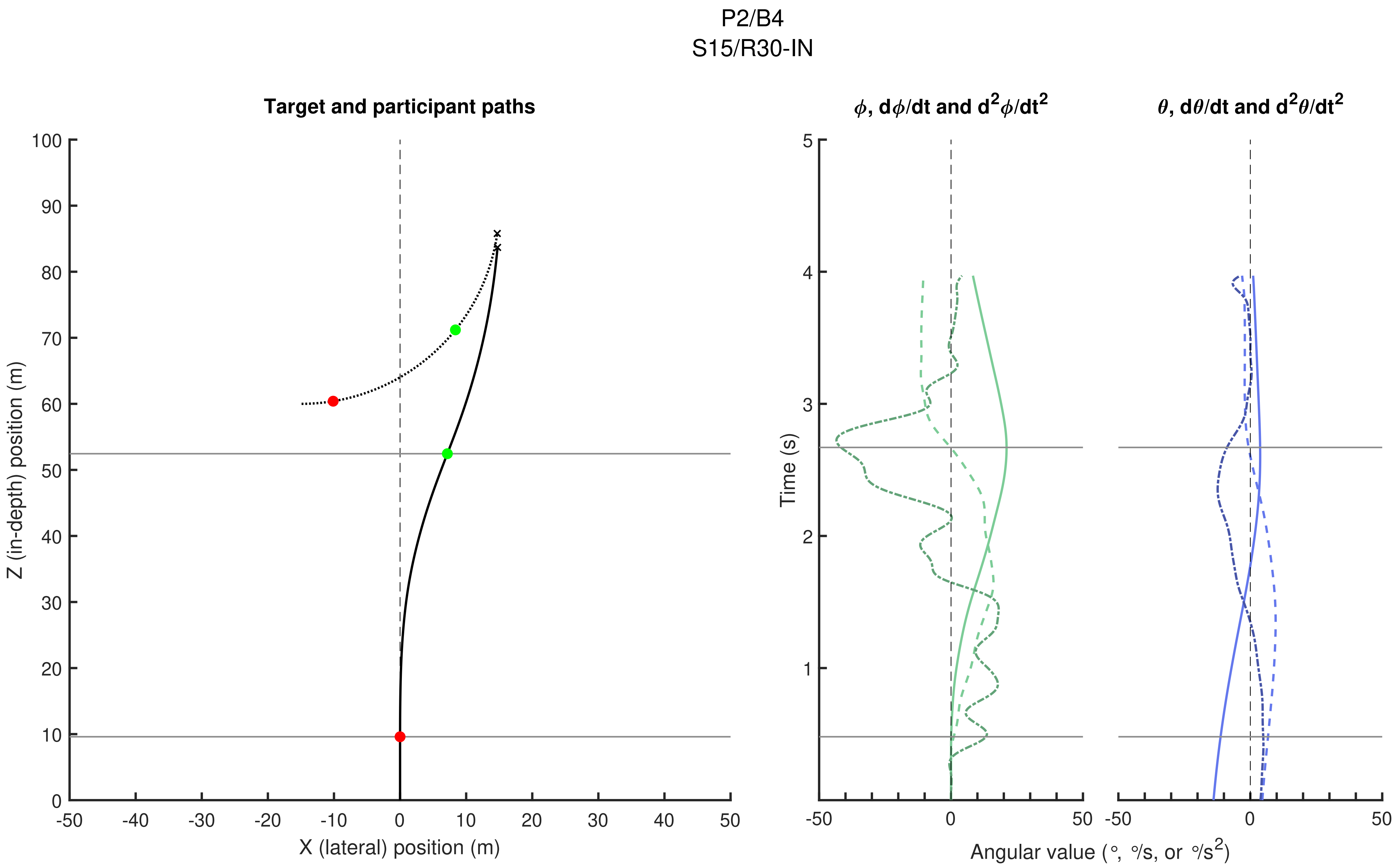
P2/B4
S15/R30-IN
Target and participant paths
Z (in-depth) position (m)
X (lateral) position (m)
100
90
80
70
60
50
40
30
20
10
0
-50
-40
-30
-20
-10
0
10
20
30
40
50
ϕ, dϕ/dt and d$^2\phi$/dt^2
θ, dθ/dt and d$^2\theta$/dt^2
Time (s)
5
4
3
2
1
-50
0
50
-50
0
50
Angular value (°, °/s, or °/s^2)

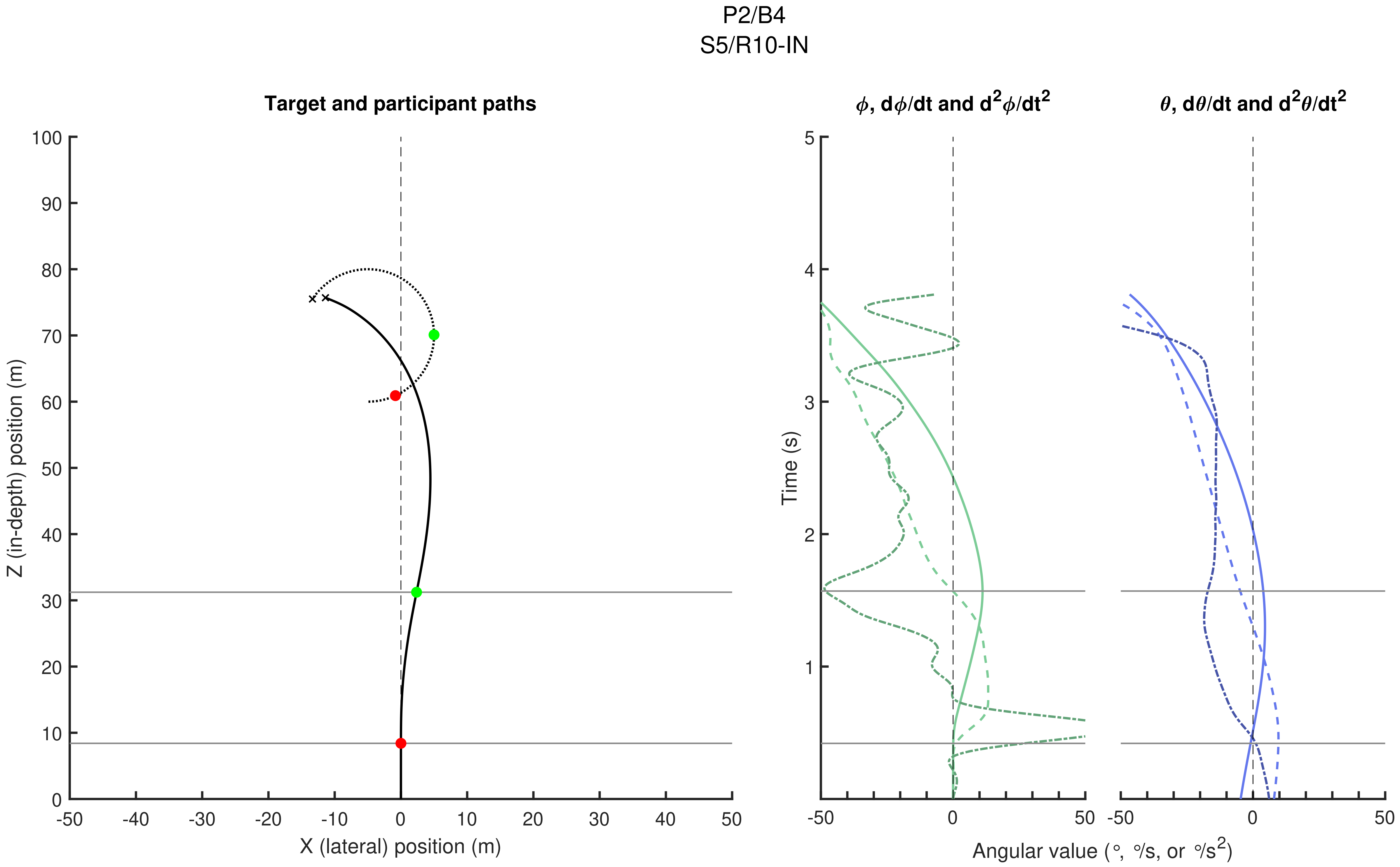
P2/B4
S5/R10-IN
Target and participant paths
ϕ, dϕ/dt and d$^2\phi$/dt^2
θ, dθ/dt and d$^2\theta$/dt^2
Z (in-depth) position (m)
X (lateral) position (m)
Time (s)
Angular value (°, °/s, or °/s^2)
100
90
80
70
60
50
40
30
20
10
0
-50
-40
-30
-20
-10
0
10
20
30
40
50
5
4
3
2
1
-50
0
50
-50
0
50

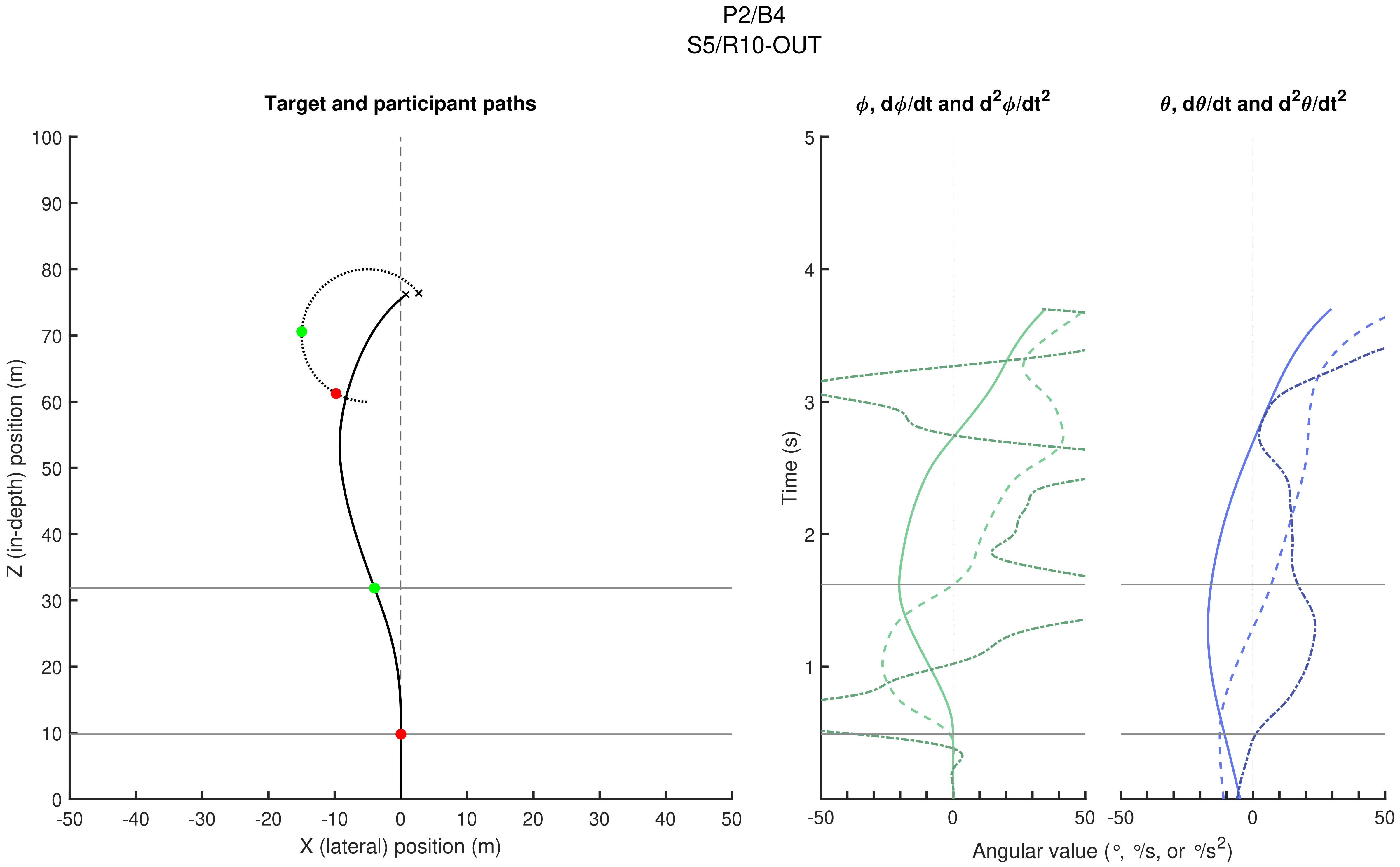

P2/B4
S5/R10-OUT
Target and participant paths
Z (in-depth) position (m)
X (lateral) position (m)
ϕ, dϕ/dt and d$^2\phi$/dt^2
θ, dθ/dt and d$^2\theta$/dt^2
Time (s)
Angular value (°, °/s, or °/s^2)

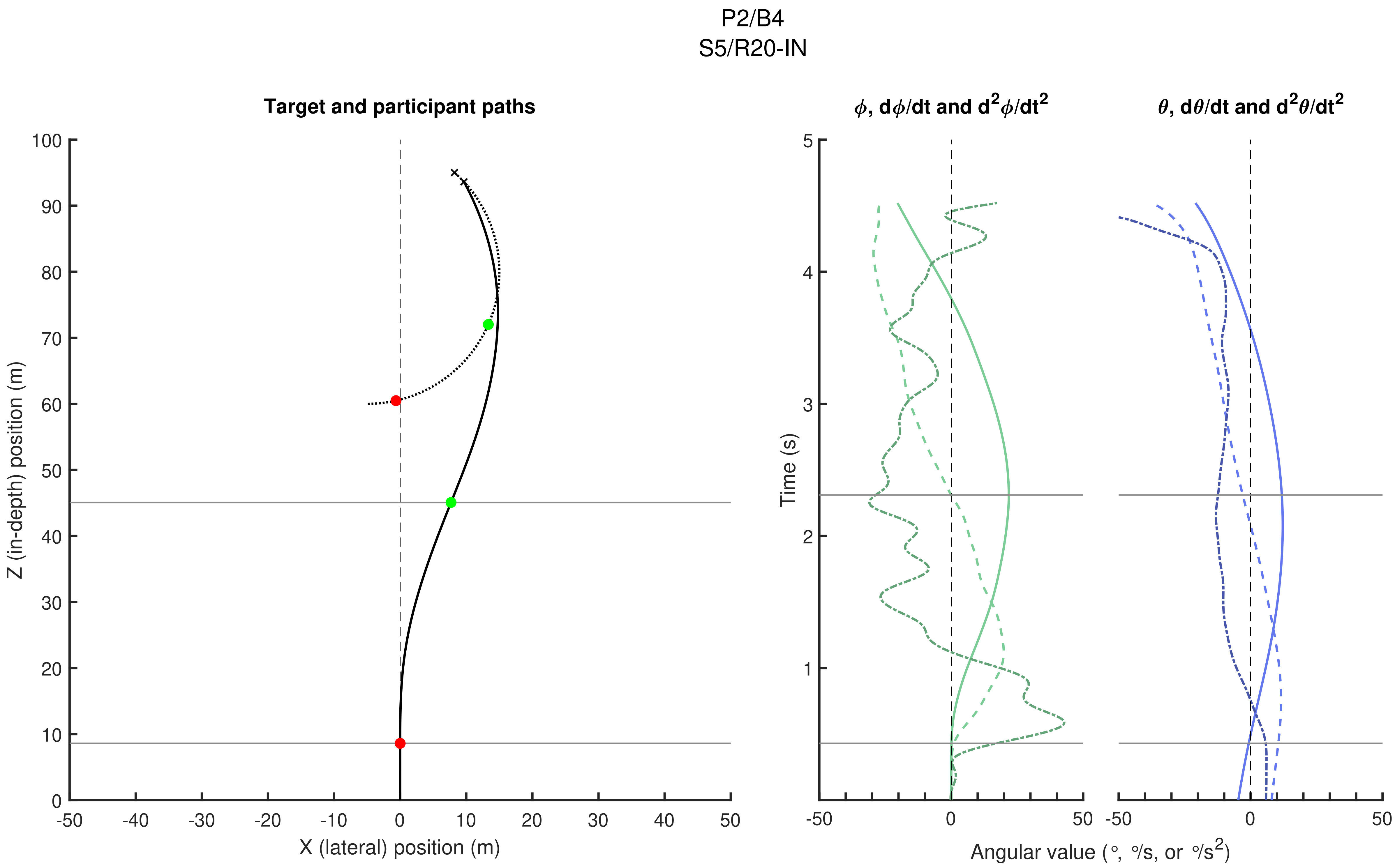
P2/B4
S5/R20-IN
Target and participant paths
Z (in-depth) position (m)
X (lateral) position (m)
ϕ, dϕ/dt and d$^2\phi$/dt^2
θ, dθ/dt and d$^2\theta$/dt^2
Time (s)
Angular value (°, °/s, or °/s^2)

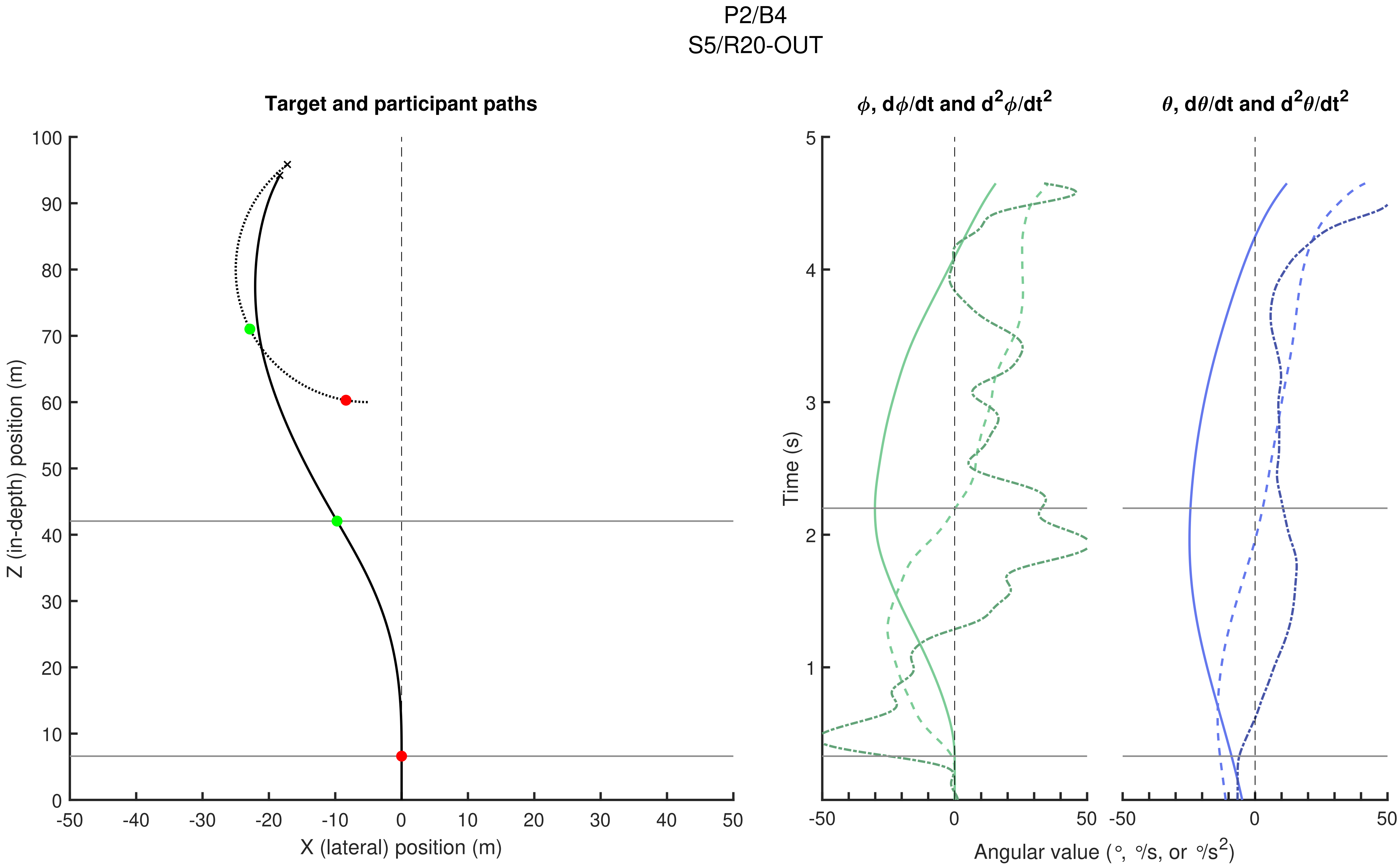
P2/B4
S5/R20-OUT
Target and participant paths
Z (in-depth) position (m)
X (lateral) position (m)
-50
-40
-30
-20
-10
0
10
20
30
40
50
0
10
20
30
40
50
60
70
80
90
100
ϕ, dϕ/dt and d$^2\phi$/dt^2
θ, dθ/dt and d$^2\theta$/dt^2
Time (s)
1
2
3
4
5
Angular value (°, °/s, or °/s^2)

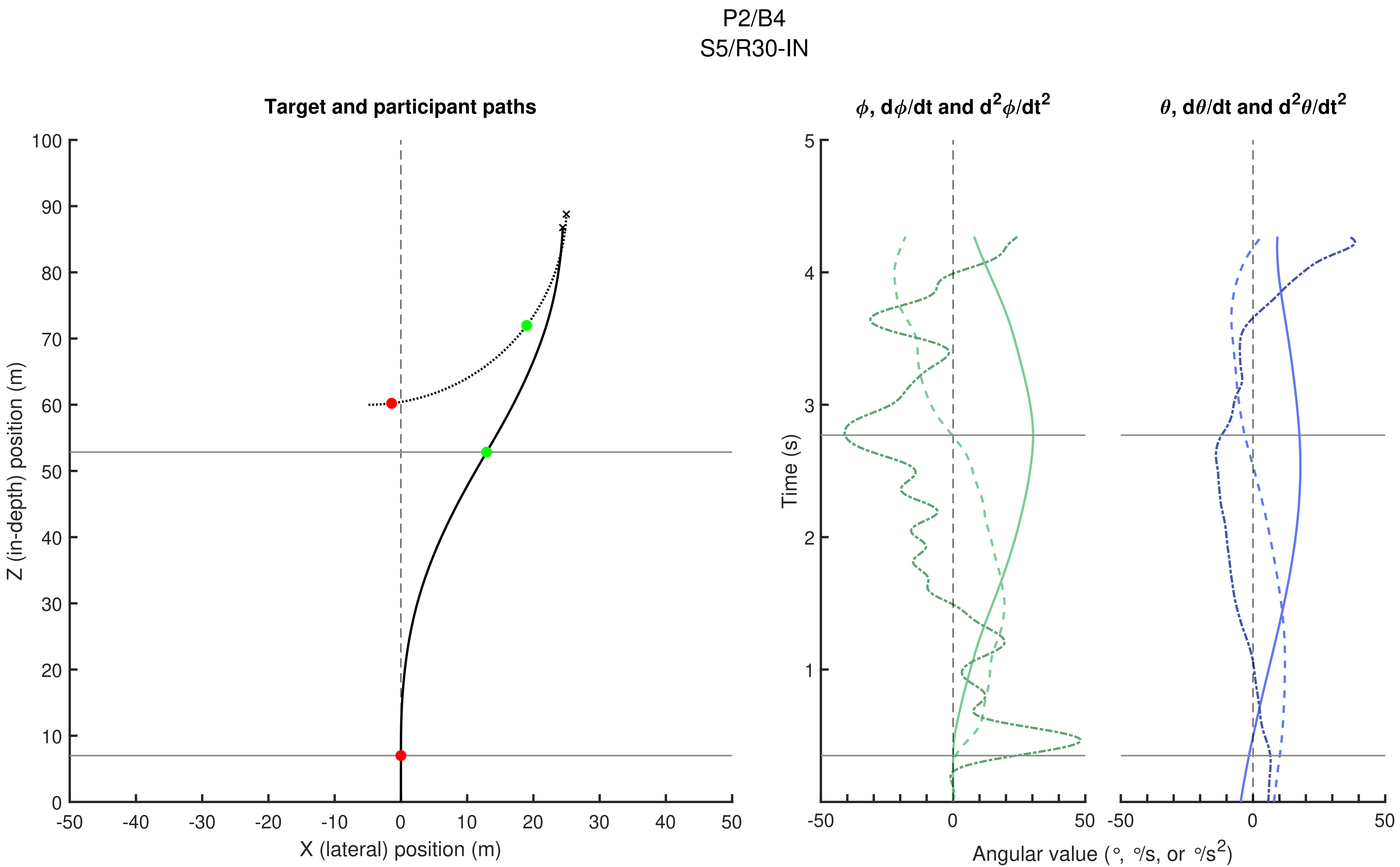

P2/B4
S5/R30-IN
Target and participant paths
Z (in-depth) position (m)
X (lateral) position (m)
φ, dφ/dt and d²φ/dt²
θ, dθ/dt and d²θ/dt²
Time (s)
Angular value (°, °/s, or °/s²)

P2/B4
S5/R30-OUT

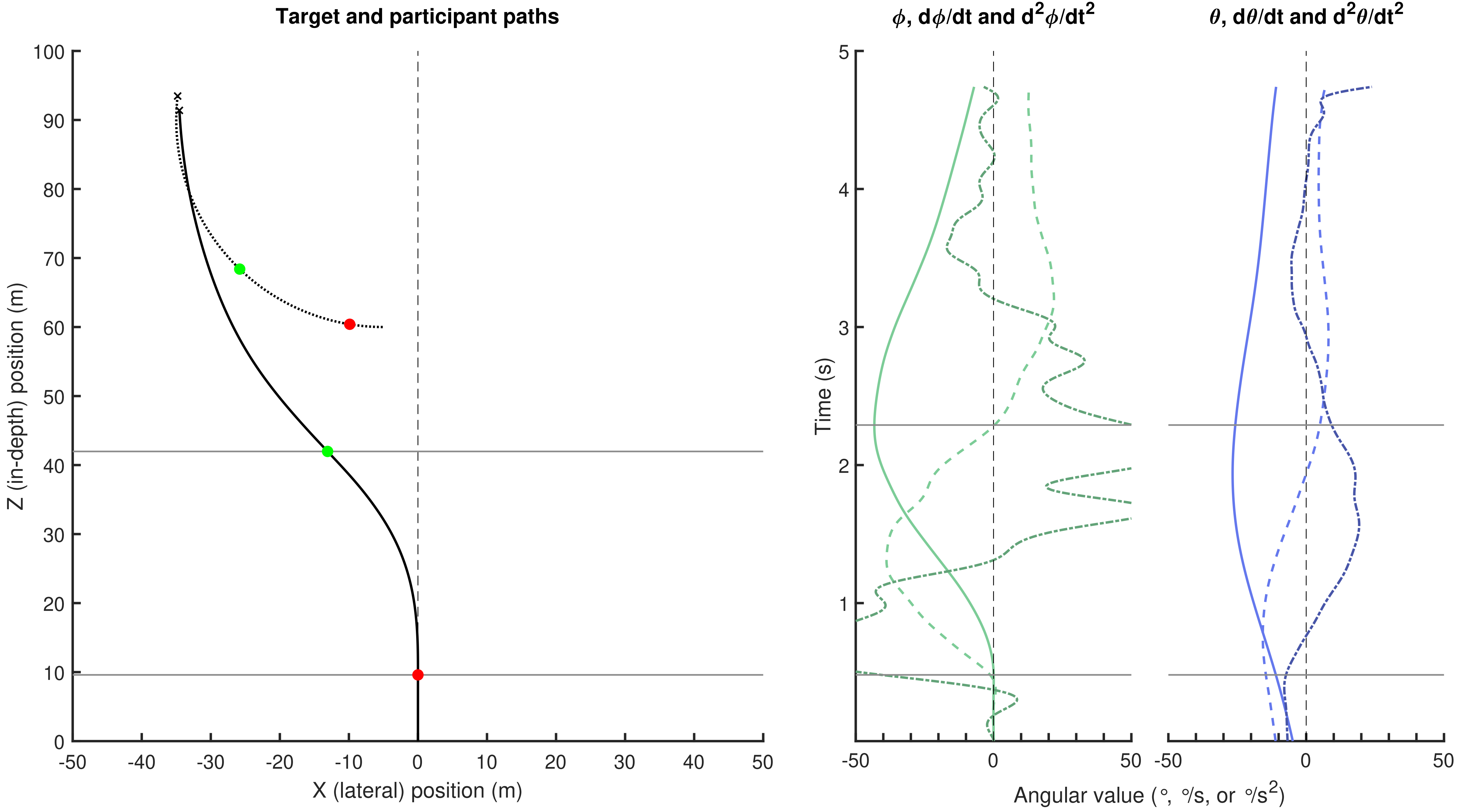

P2/B4
S5/R10-OUT

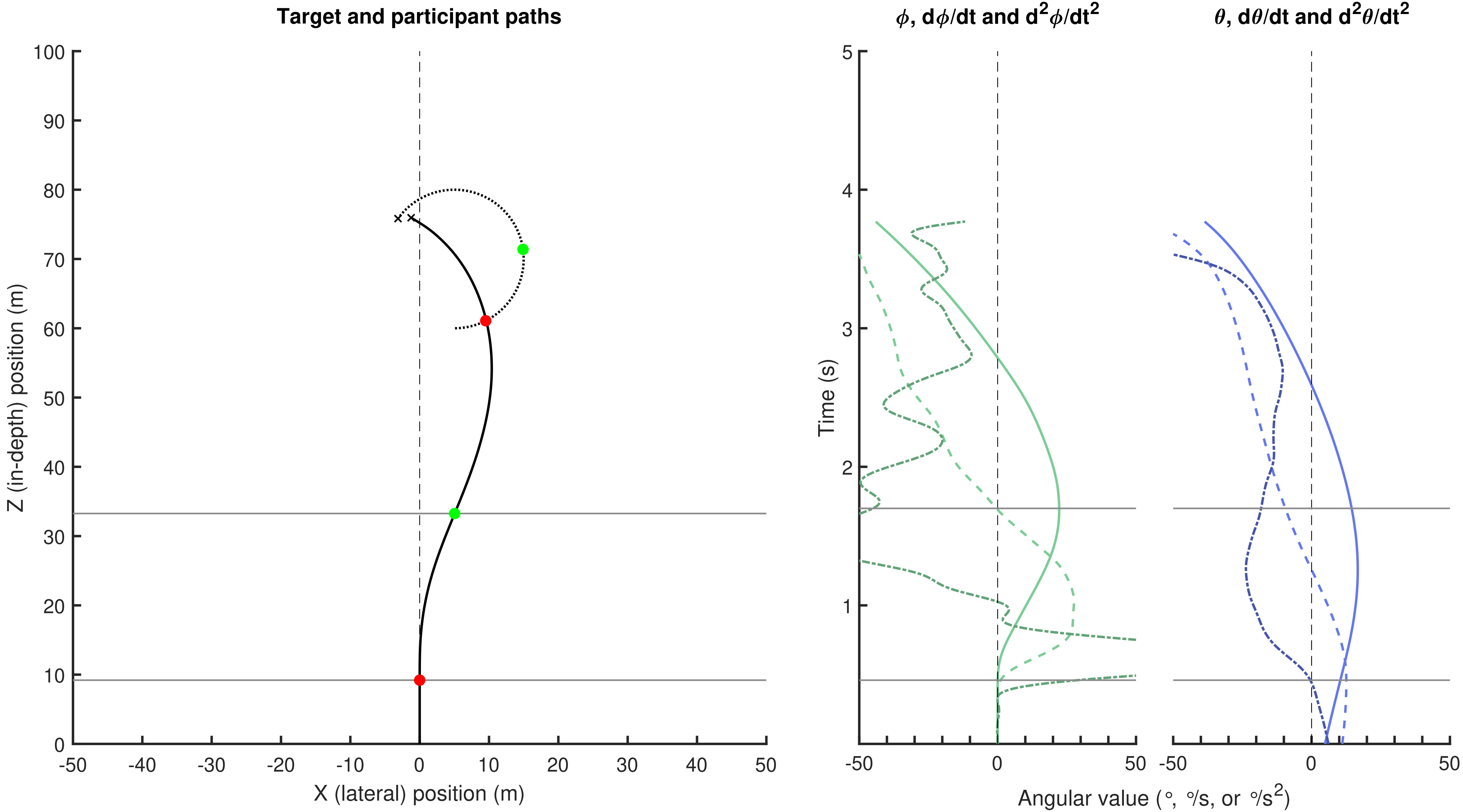

P2/B4
S5/R10-IN

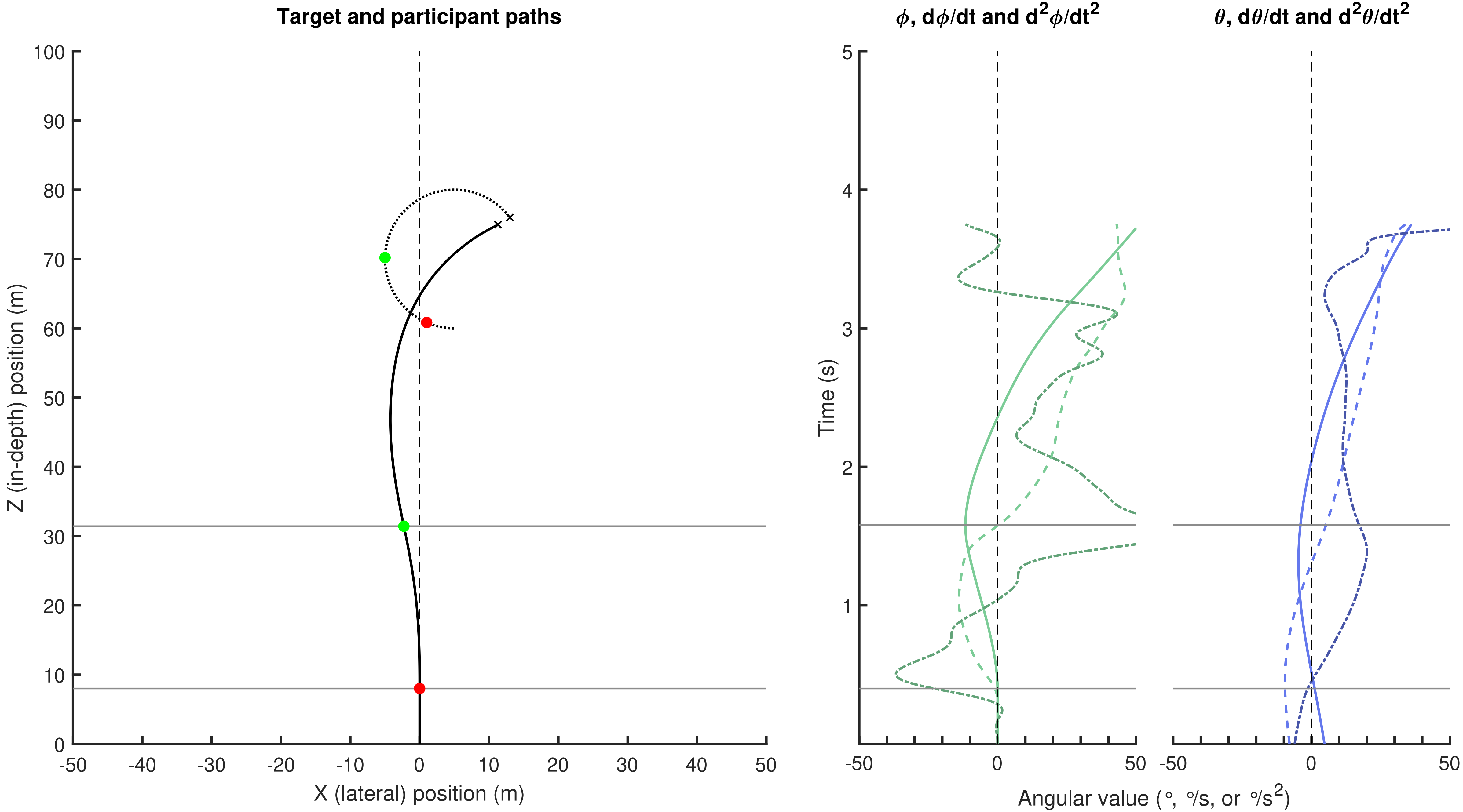

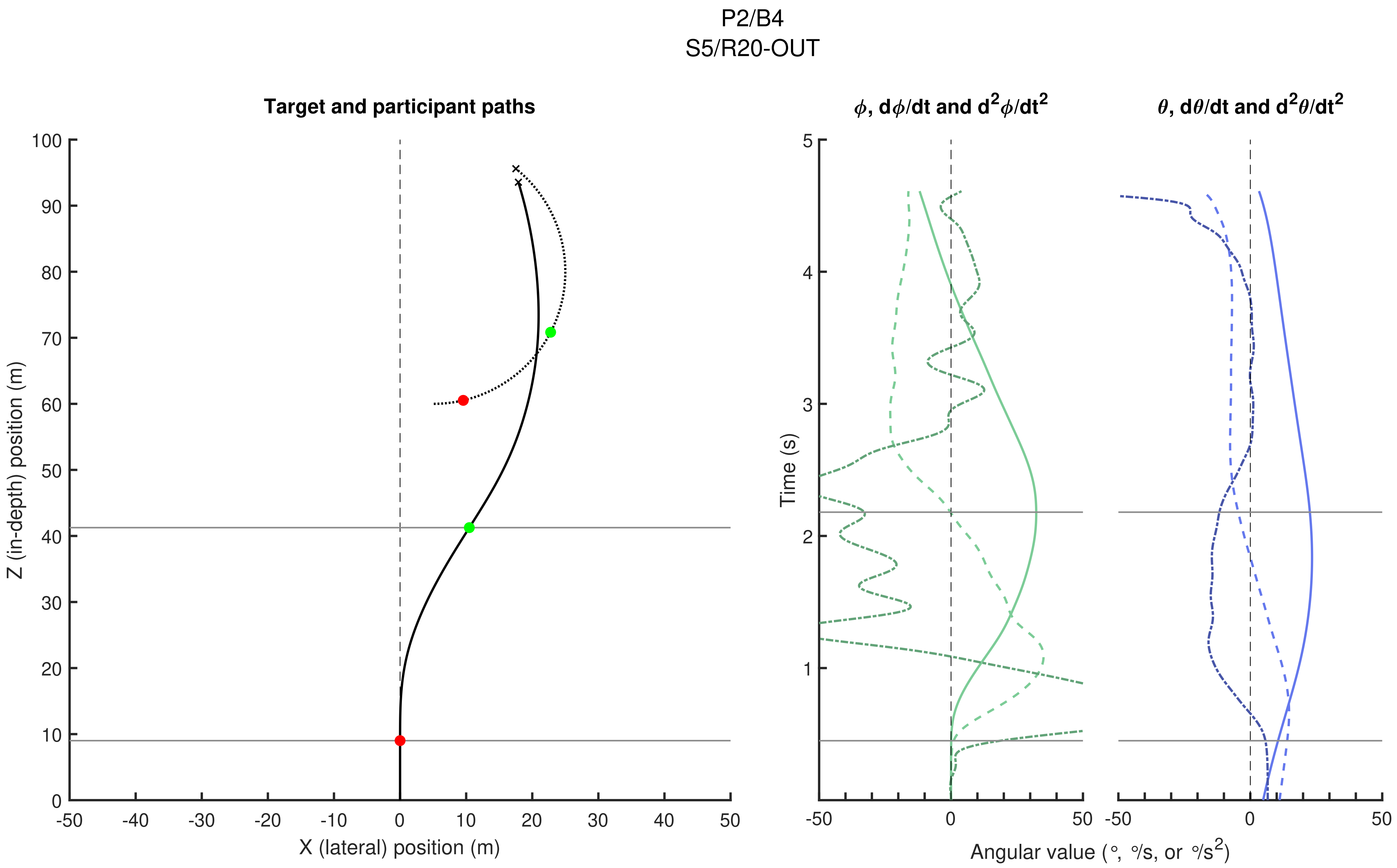

P2/B4
S5/R20-OUT
Target and participant paths
Z (in-depth) position (m)
X (lateral) position (m)
φ, dφ/dt and d²φ/dt²
θ, dθ/dt and d²θ/dt²
Time (s)
Angular value (°, °/s, or °/s²)

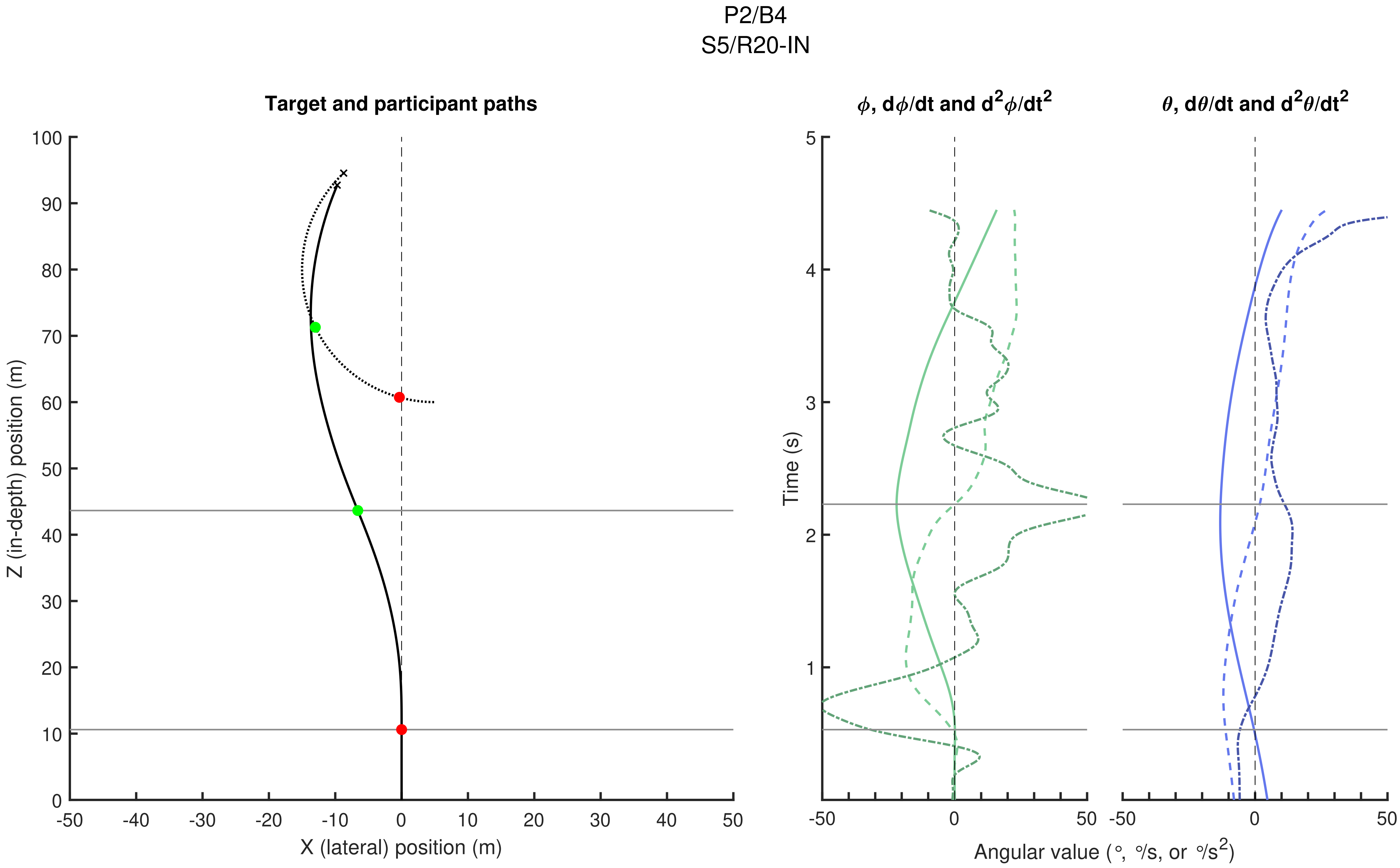

P2/B4
S5/R20-IN
Target and participant paths
Z (in-depth) position (m)
X (lateral) position (m)
ϕ, dϕ/dt and d²ϕ/dt²
θ, dθ/dt and d²θ/dt²
Time (s)
Angular value (°, °/s, or °/s²)

P2/B4
S5/R30-OUT

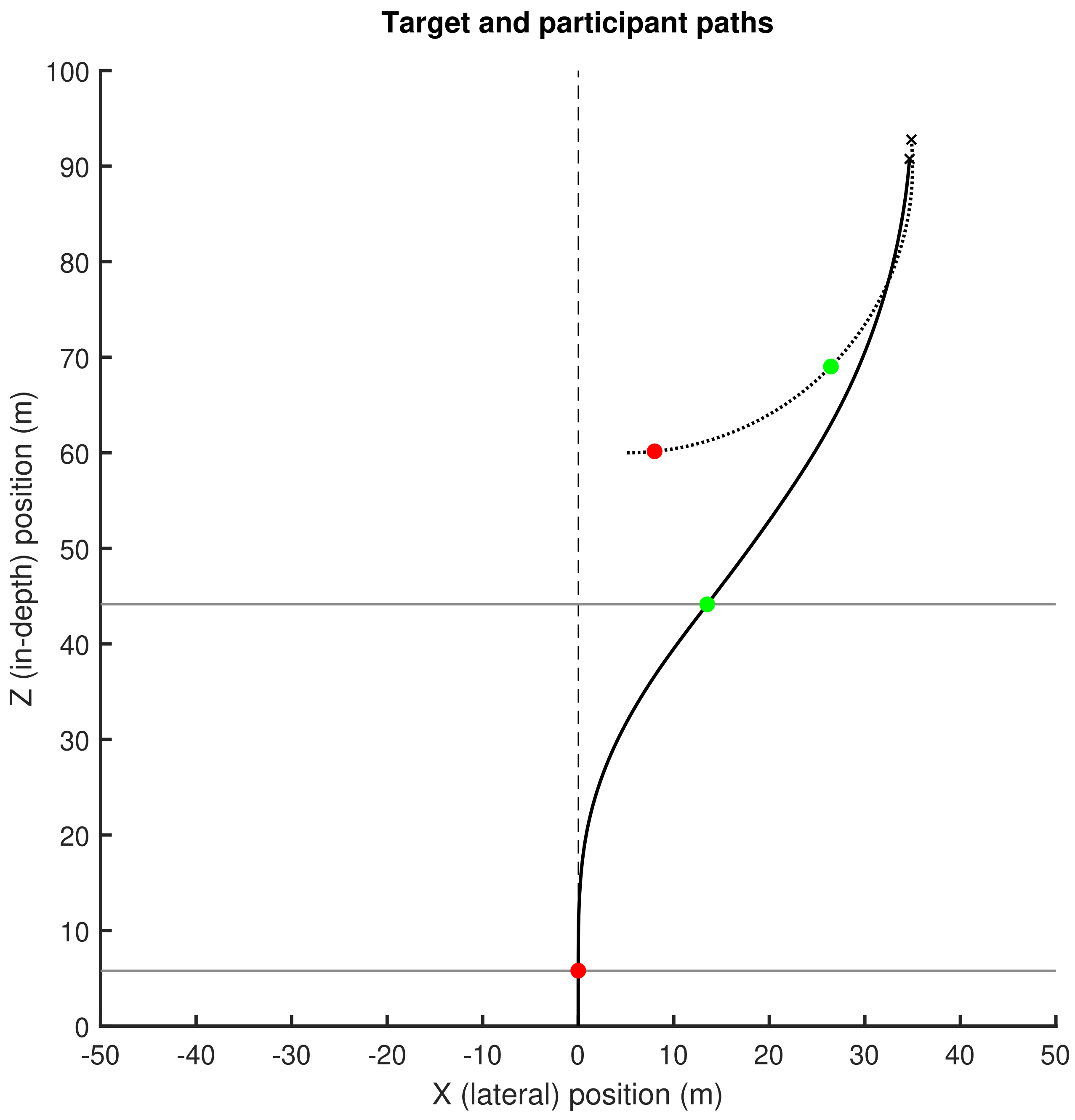

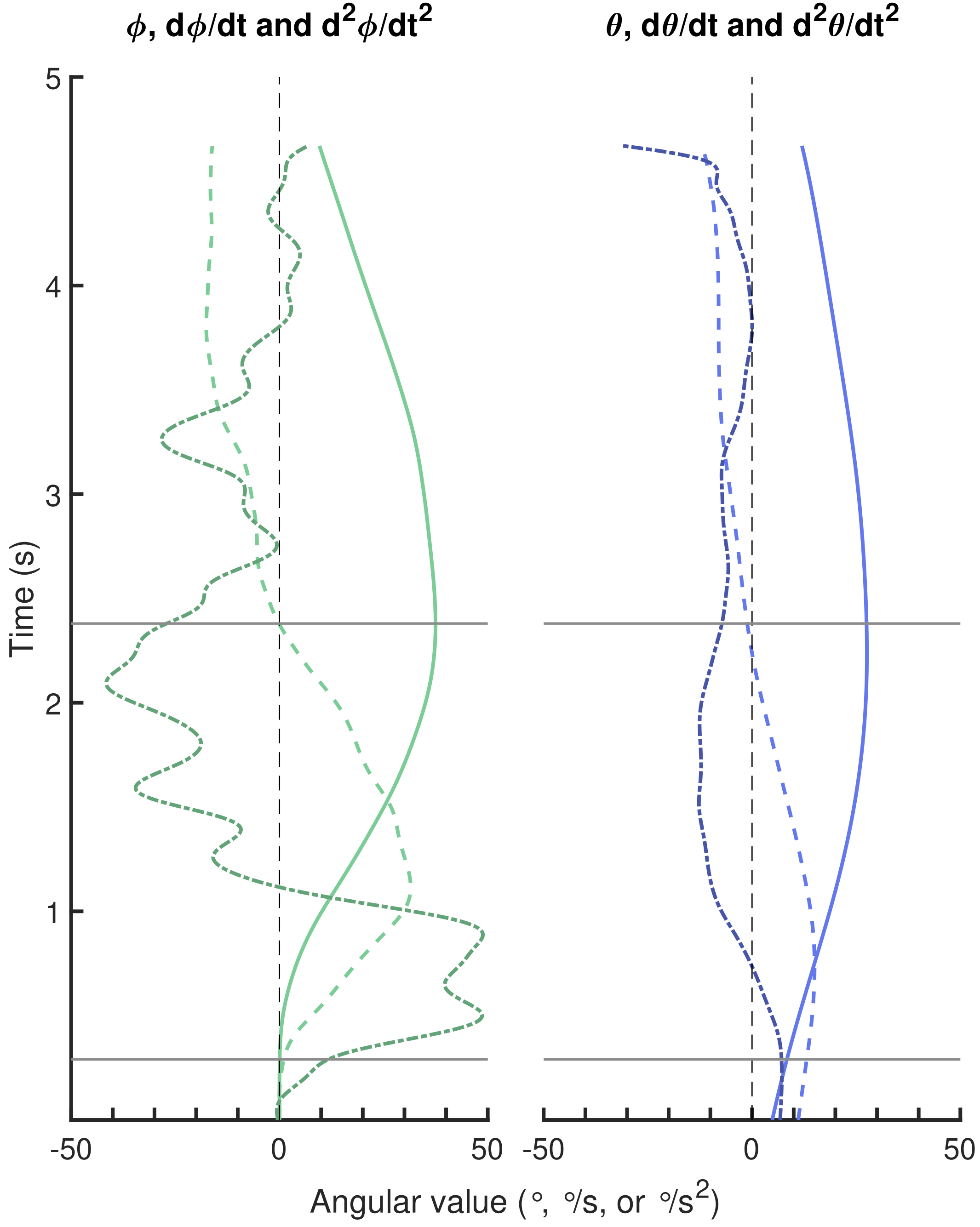

P2/B4
S5/R30-IN

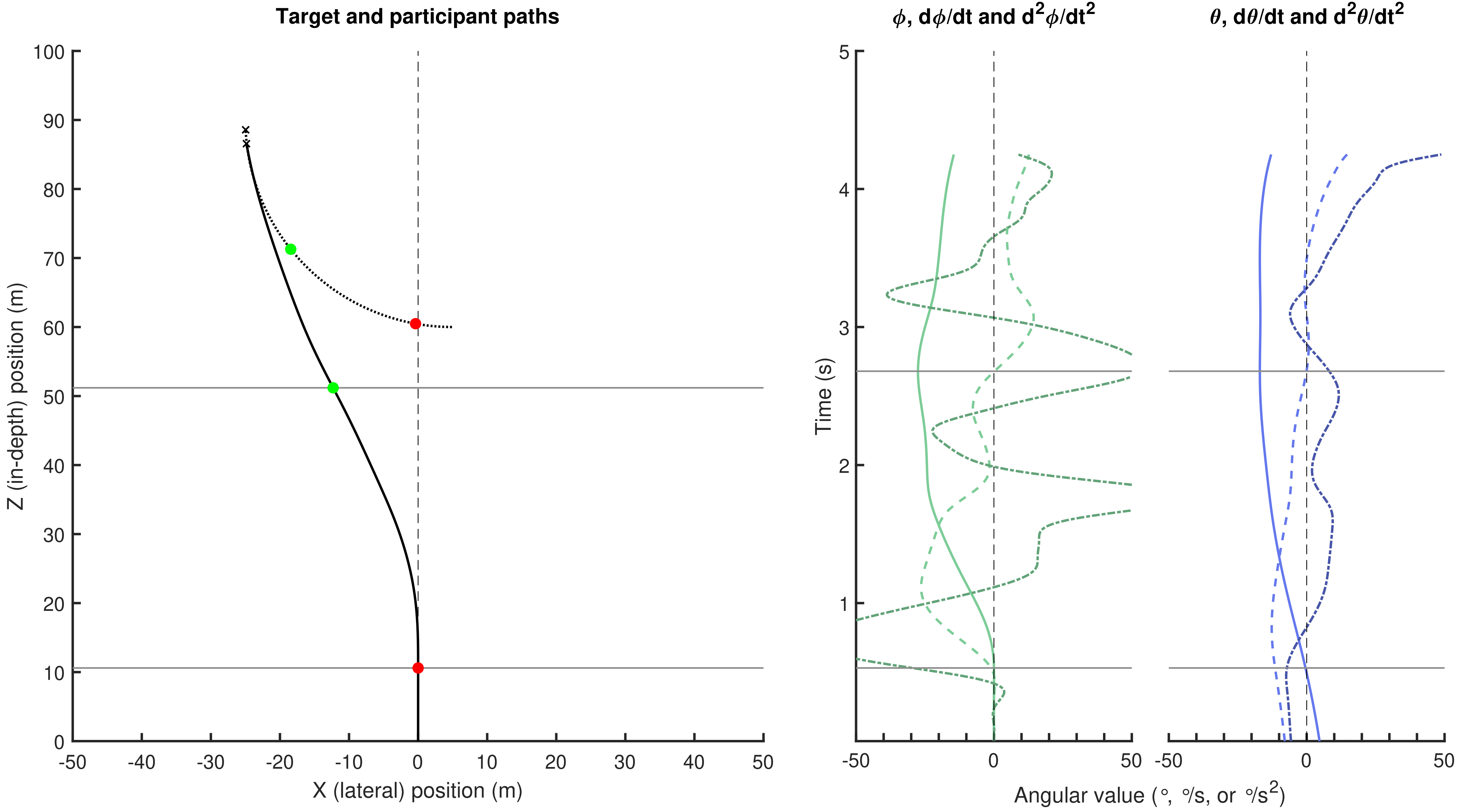

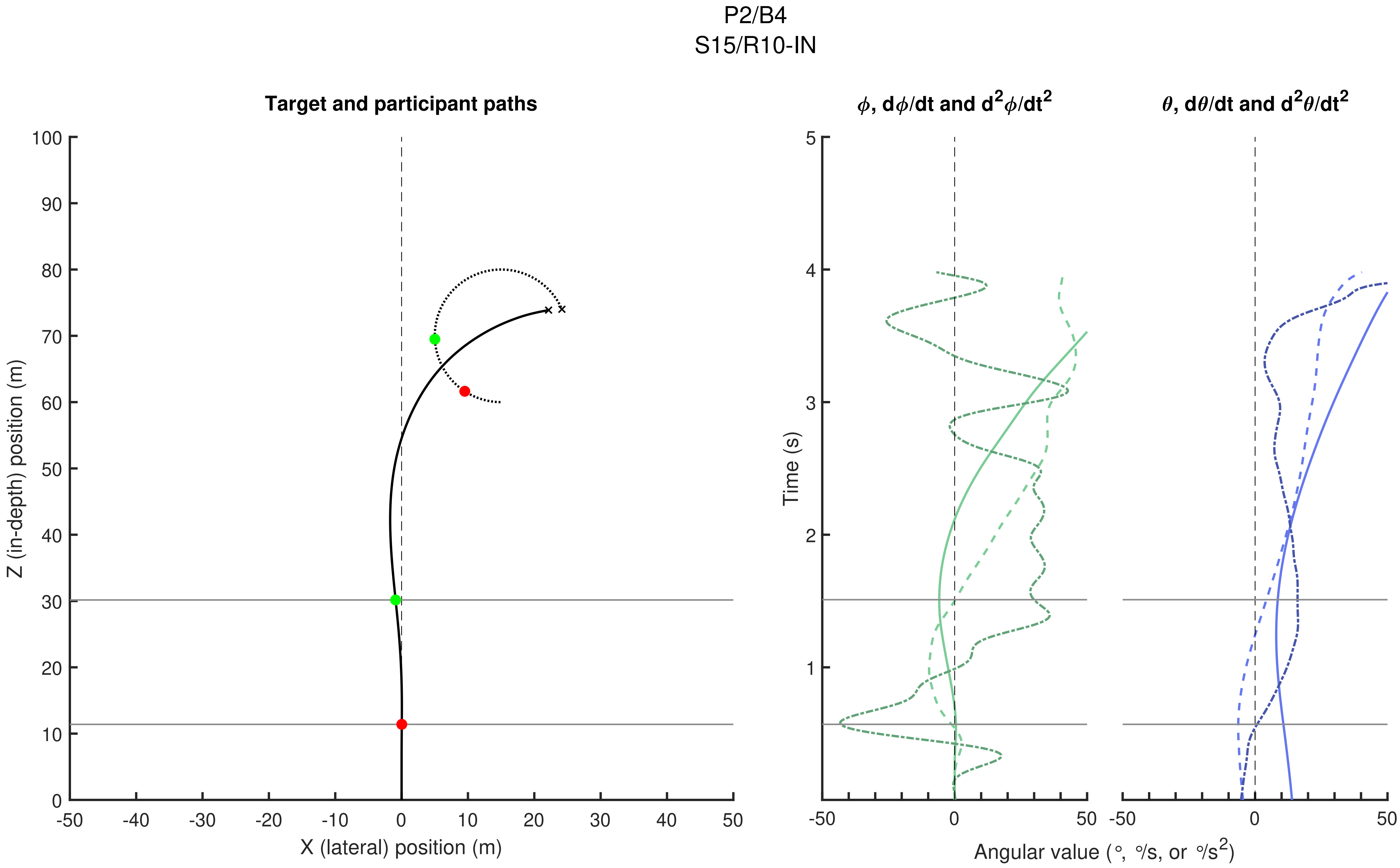

P2/B4
S15/R10-IN
Target and participant paths
Z (in-depth) position (m)
X (lateral) position (m)
ϕ, dϕ/dt and d$^2\phi$/dt^2
θ, dθ/dt and d$^2\theta$/dt^2
Time (s)
Angular value (°, °/s, or °/s^2)

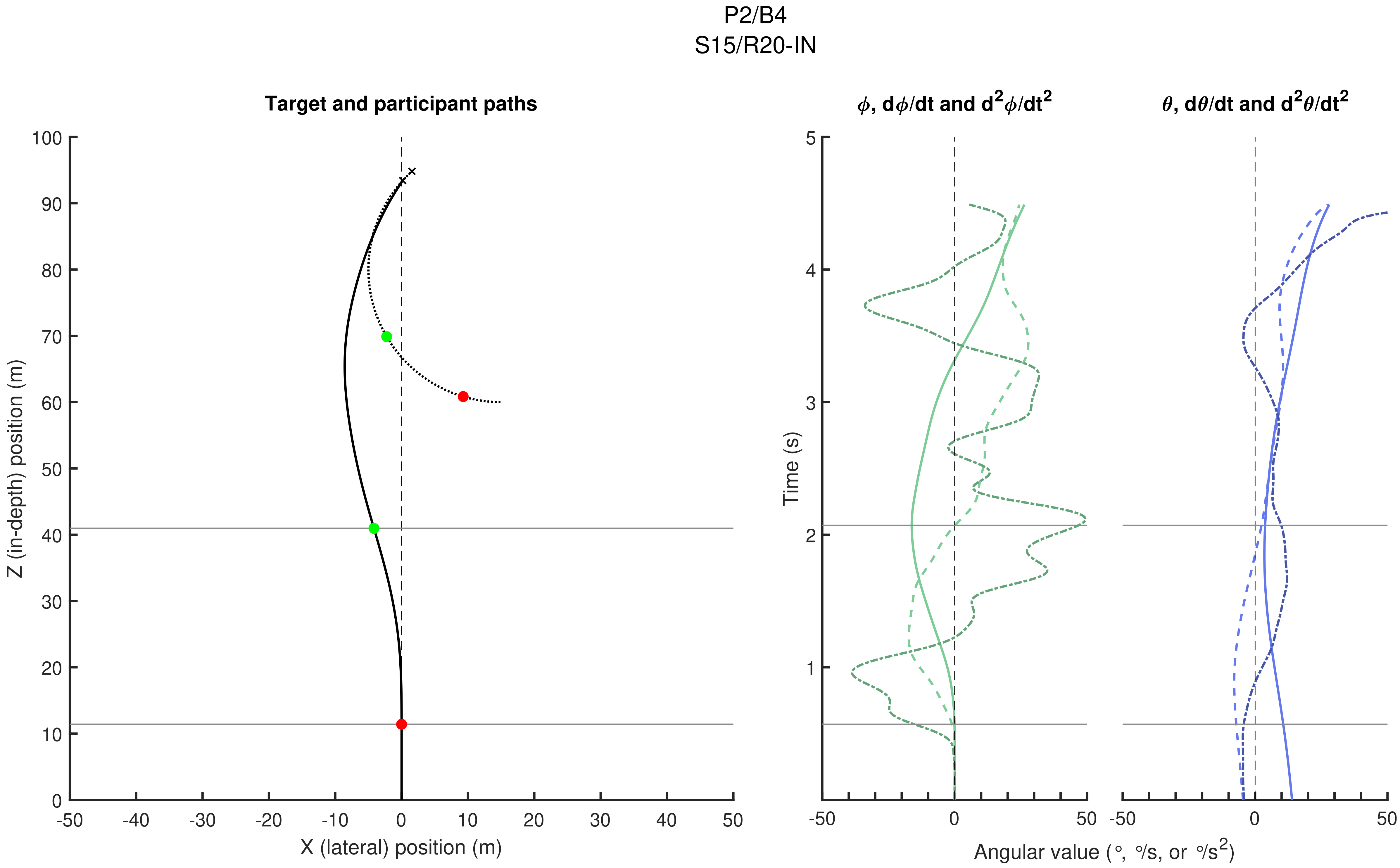
P2/B4
S15/R20-IN
Target and participant paths
Z (in-depth) position (m)
X (lateral) position (m)
ϕ, dϕ/dt and d²ϕ/dt²
θ, dθ/dt and d²θ/dt²
Time (s)
Angular value (°, °/s, or °/s²)

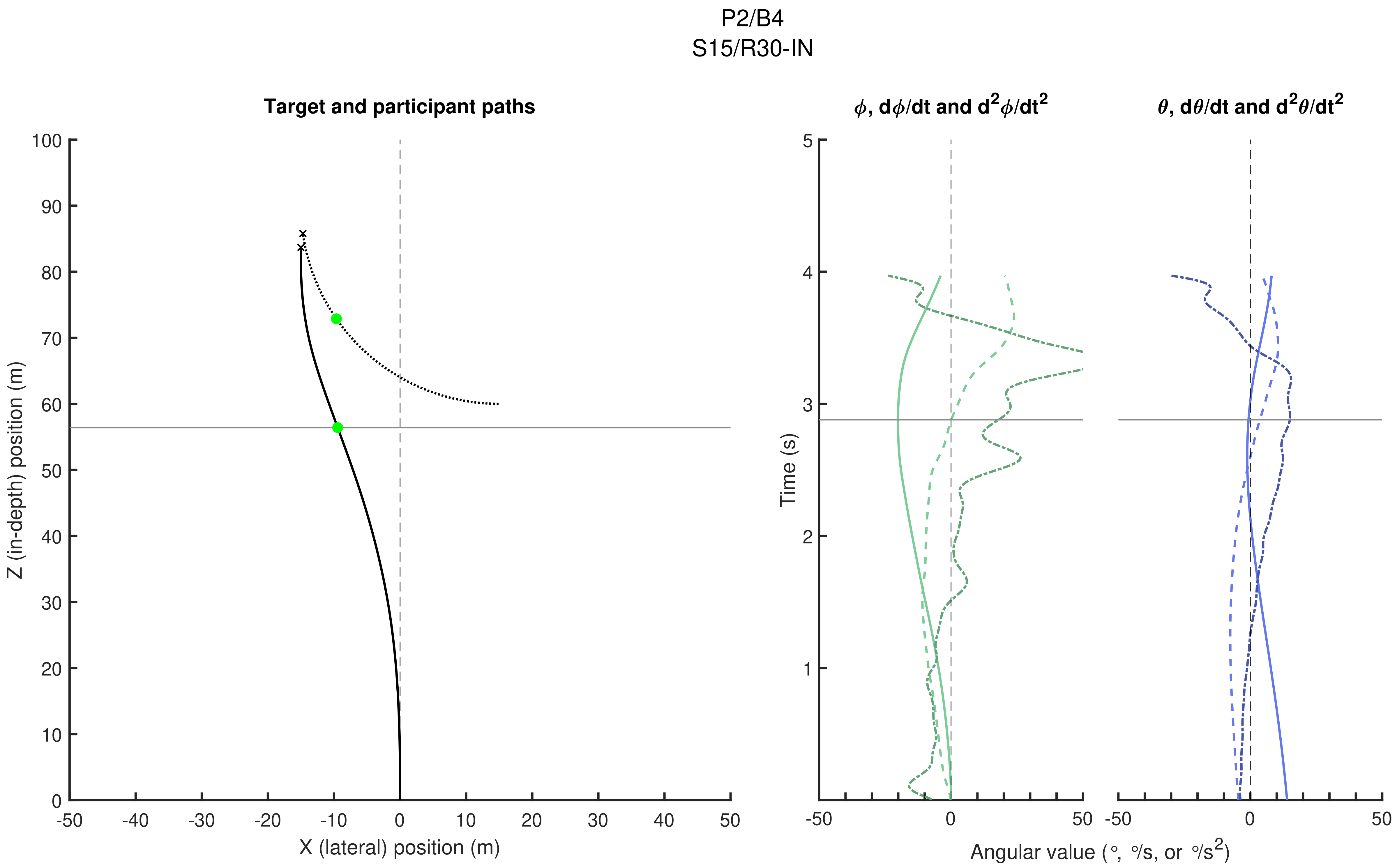

P2/B4
S15/R30-IN
Target and participant paths
Z (in-depth) position (m)
X (lateral) position (m)
ϕ, dϕ/dt and d$^2\phi$/dt^2
θ, dθ/dt and d$^2\theta$/dt^2
Time (s)
Angular value (°, °/s, or °/s^2)

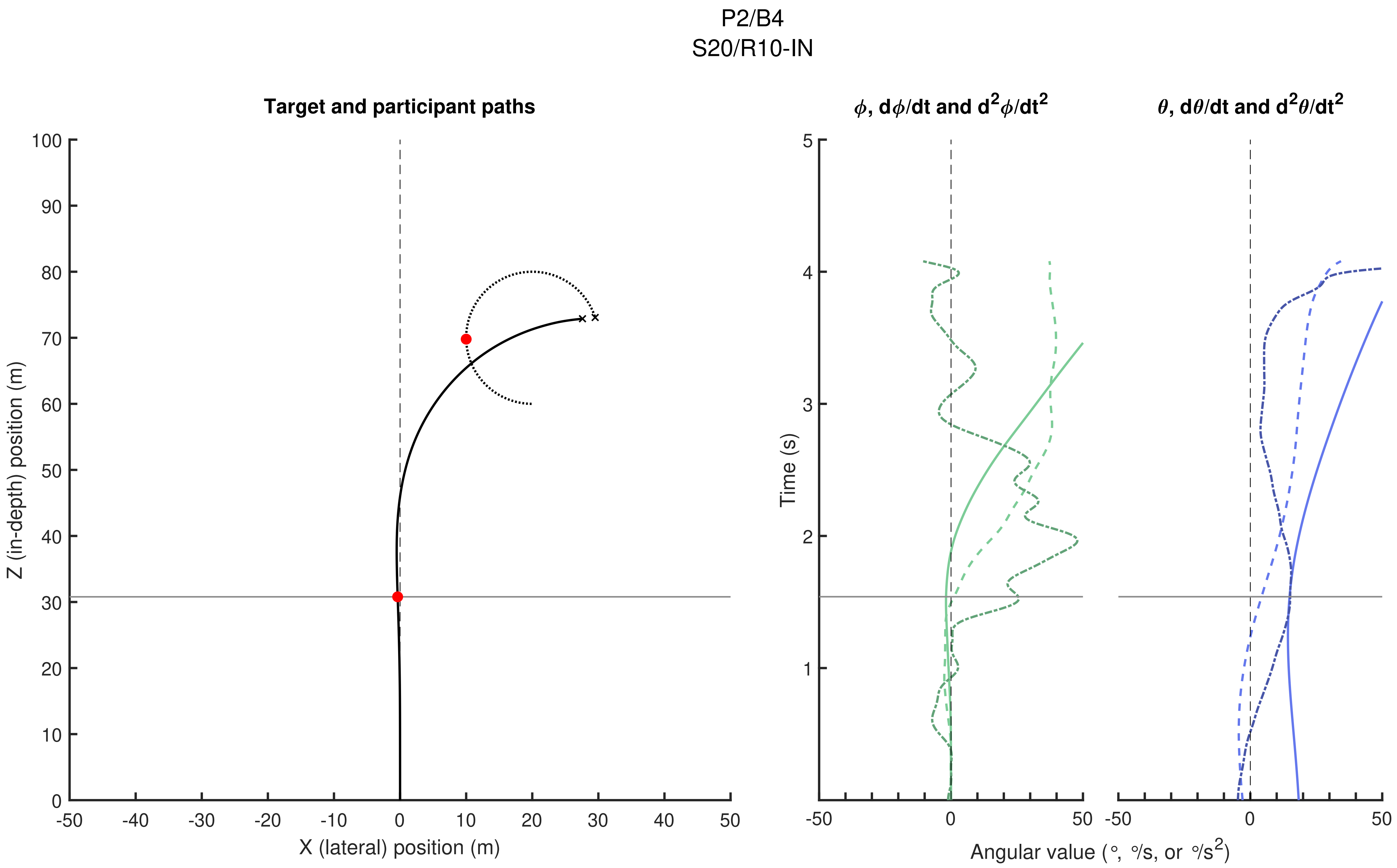

P2/B4
S20/R10-IN
Target and participant paths
ϕ, dϕ/dt and d$^2\phi$/dt^2
θ, dθ/dt and d$^2\theta$/dt^2
Z (in-depth) position (m)
X (lateral) position (m)
Time (s)
Angular value (°, °/s, or °/s^2)

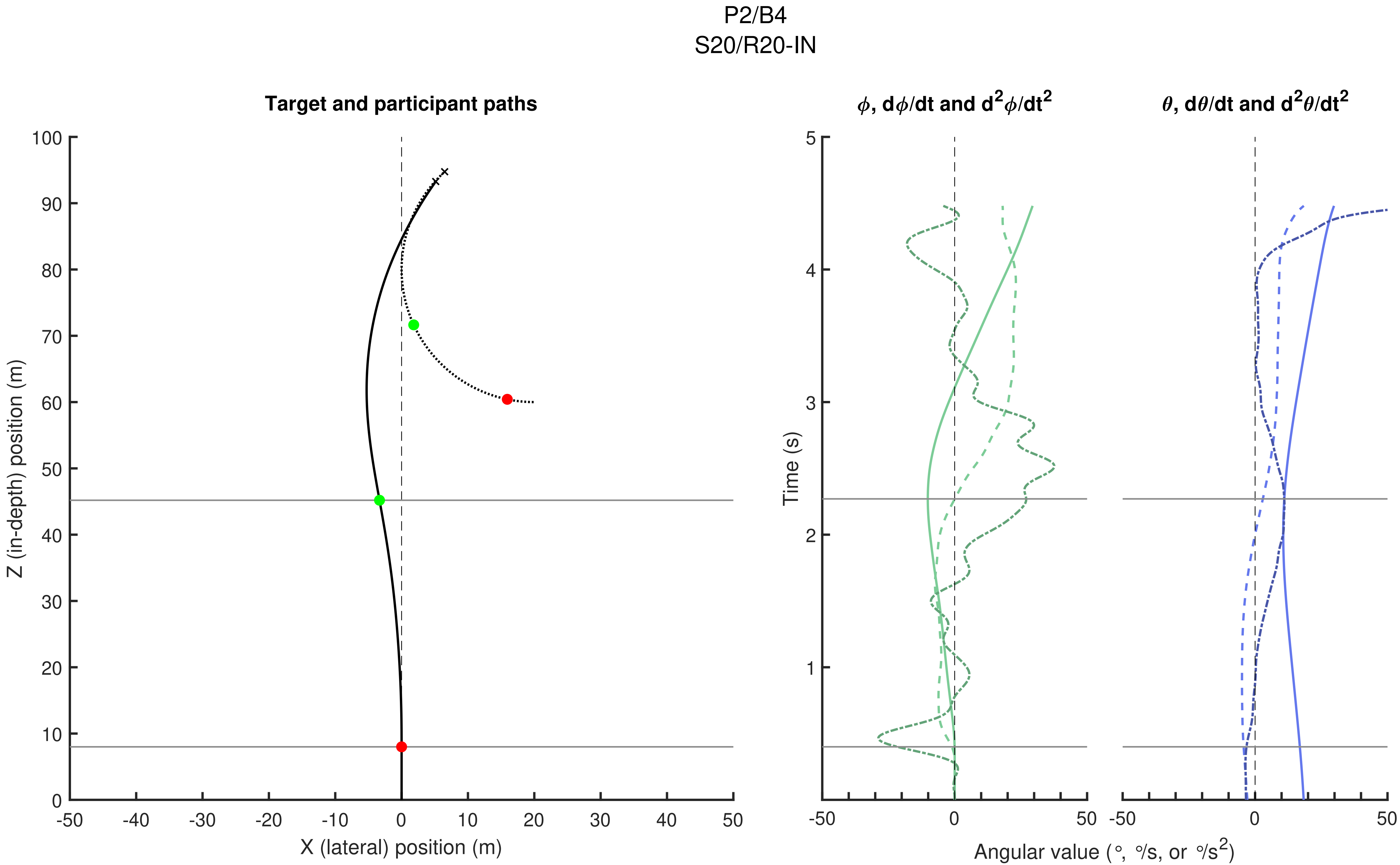

P2/B4
S20/R20-IN
Target and participant paths
Z (in-depth) position (m)
X (lateral) position (m)
100
90
80
70
60
50
40
30
20
10
0
-50
-40
-30
-20
-10
0
10
20
30
40
50
ϕ, dϕ/dt and d²ϕ/dt²
θ, dθ/dt and d²θ/dt²
Time (s)
5
4
3
2
1
-50
0
50
-50
0
50
Angular value (°, °/s, or °/s²)

P2/B4
S20/R30-IN

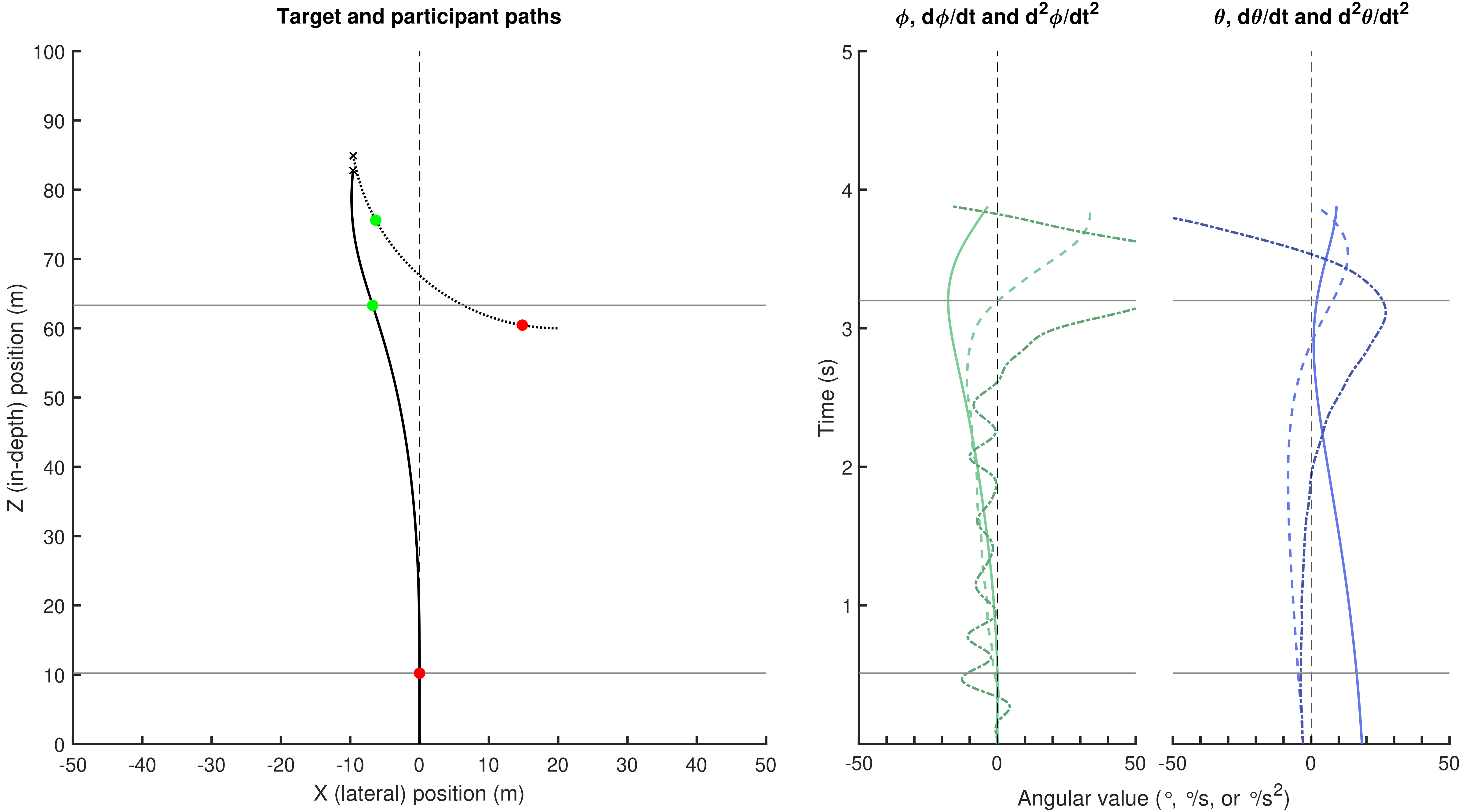

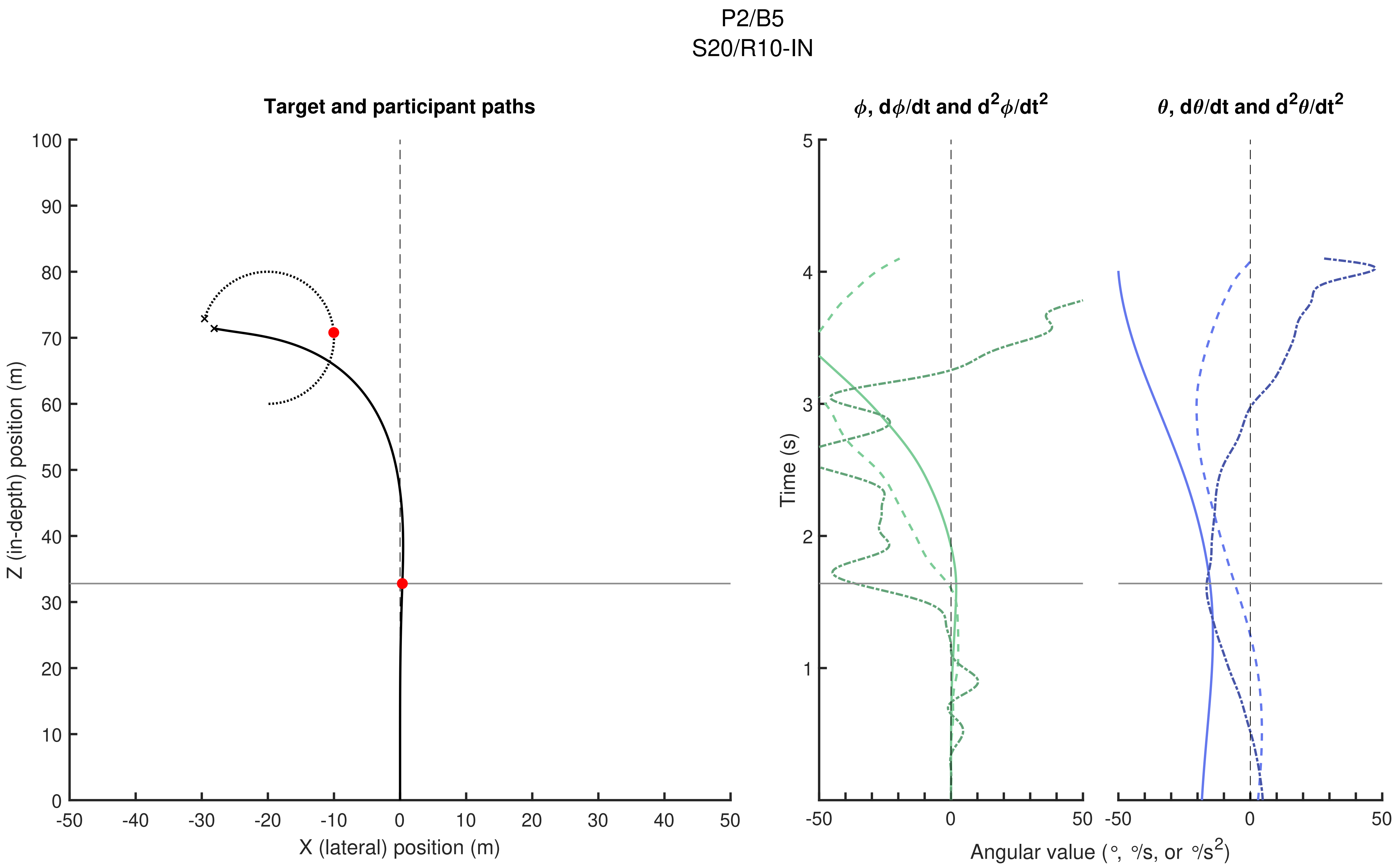
P2/B5
S20/R10-IN
Target and participant paths
Z (in-depth) position (m)
X (lateral) position (m)
ϕ, dϕ/dt and d$^2\phi$/dt^2
θ, dθ/dt and d$^2\theta$/dt^2
Time (s)
Angular value (°, °/s, or °/s^2)

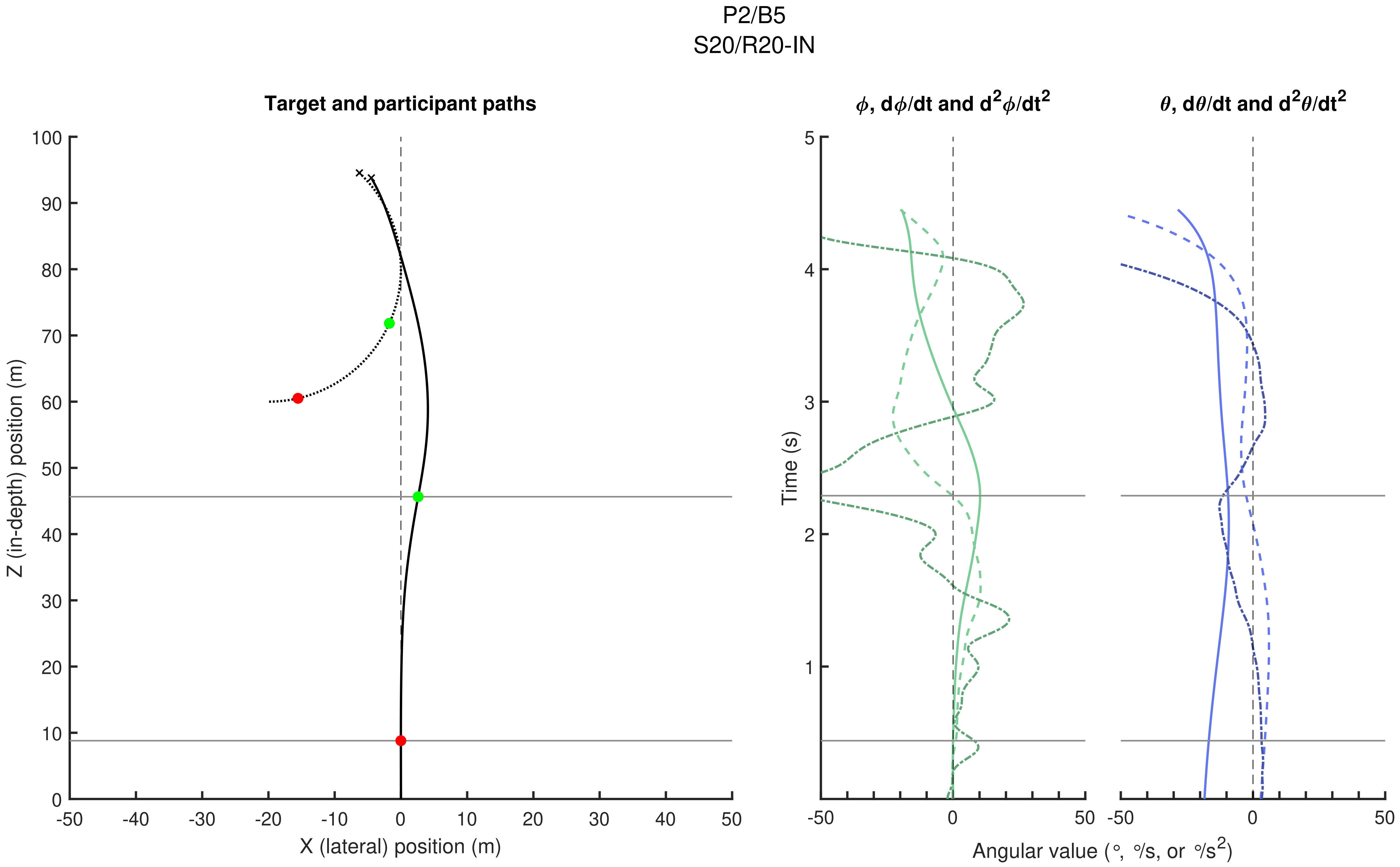
P2/B5
S20/R20-IN
Target and participant paths
Z (in-depth) position (m)
X (lateral) position (m)
φ, dφ/dt and d²φ/dt²
θ, dθ/dt and d²θ/dt²
Time (s)
Angular value (°, °/s, or °/s²)

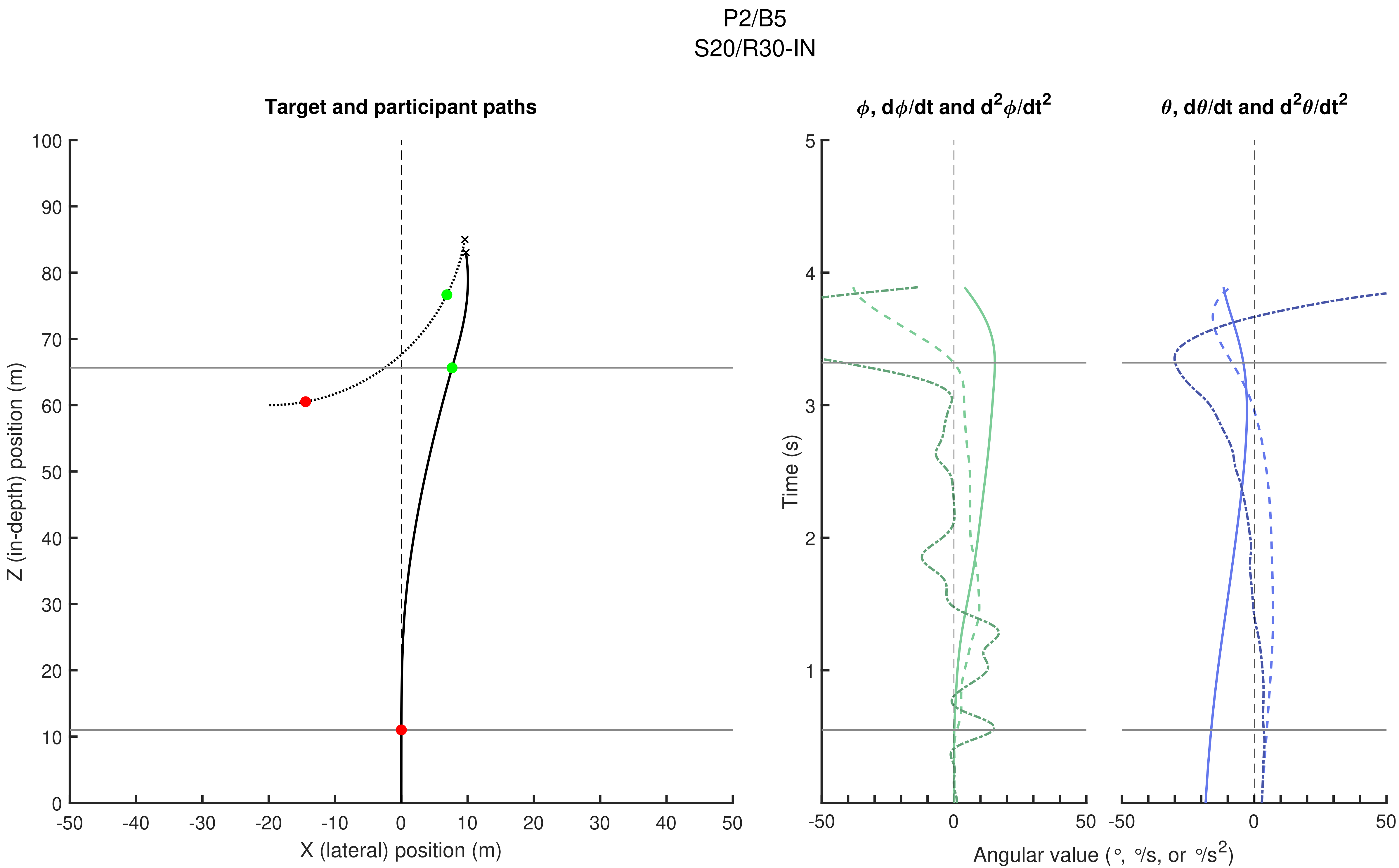

P2/B5
S20/R30-IN
Target and participant paths
Z (in-depth) position (m)
X (lateral) position (m)
φ, dφ/dt and d²φ/dt²
θ, dθ/dt and d²θ/dt²
Time (s)
Angular value (°, °/s, or °/s²)

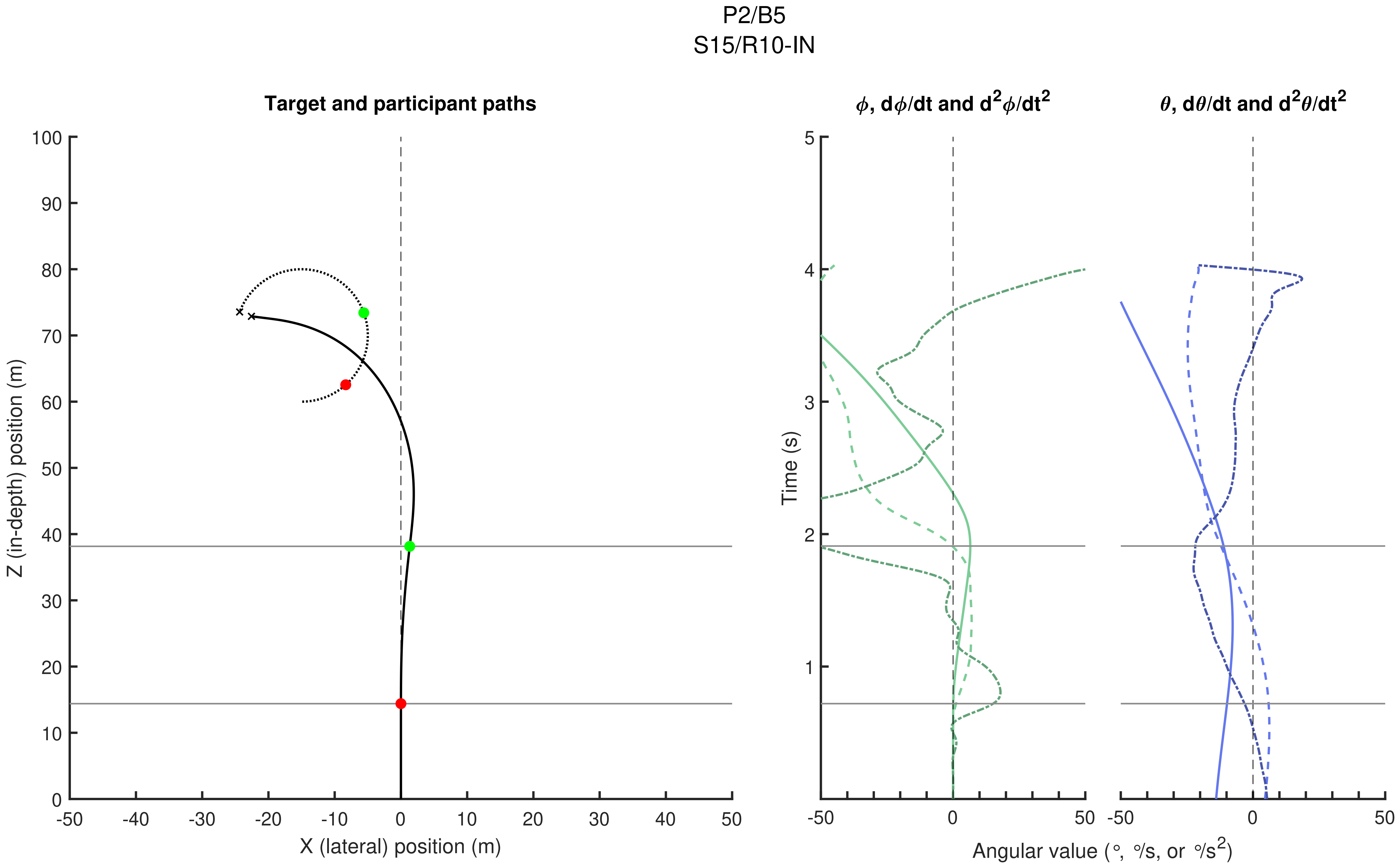
P2/B5
S15/R10-IN
Target and participant paths
Z (in-depth) position (m)
X (lateral) position (m)
100
90
80
70
60
50
40
30
20
10
0
-50
-40
-30
-20
-10
0
10
20
30
40
50
ϕ, dϕ/dt and d²ϕ/dt²
θ, dθ/dt and d²θ/dt²
Time (s)
5
4
3
2
1
-50
0
50
-50
0
50
Angular value (°, °/s, or °/s²)

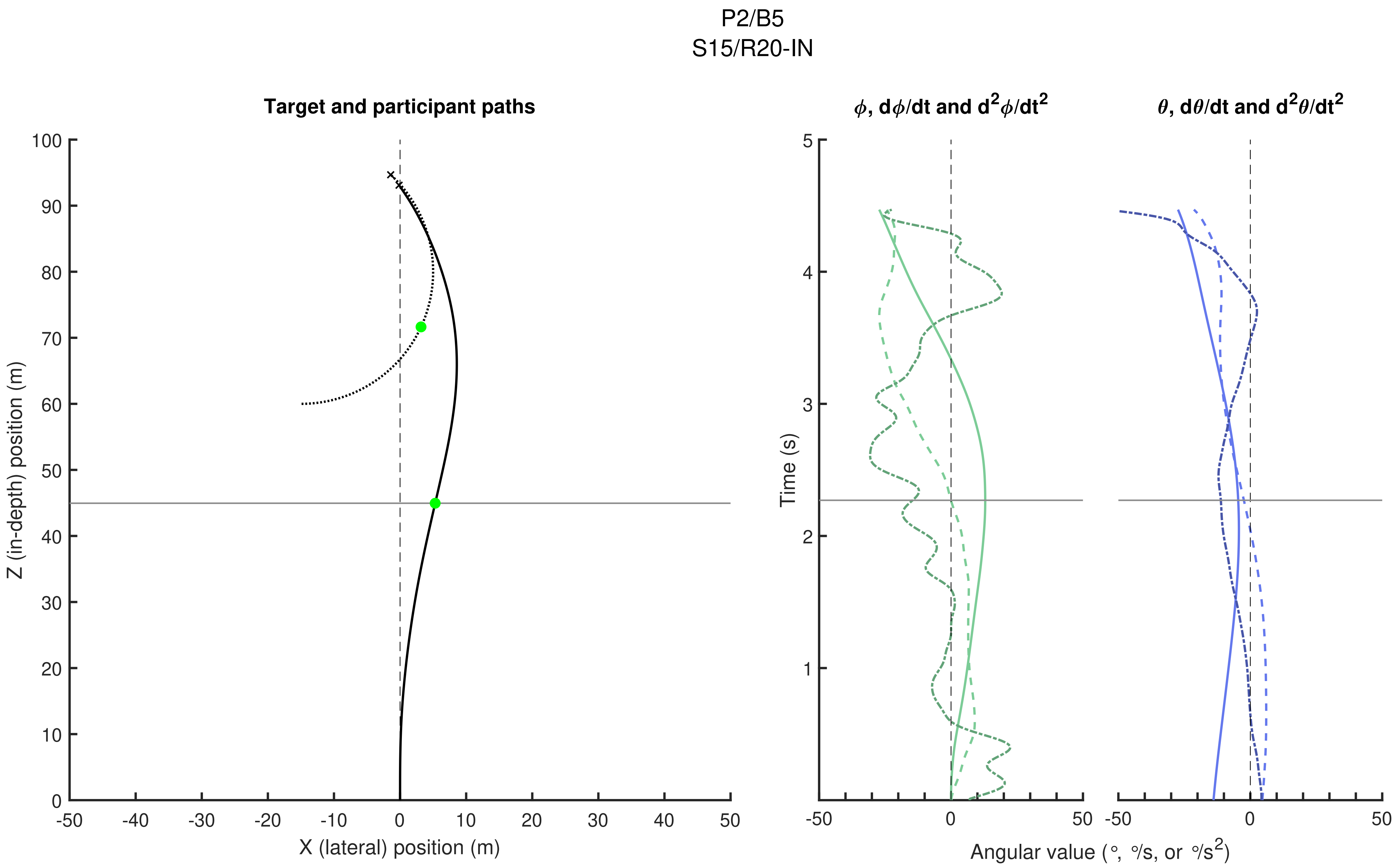
P2/B5
S15/R20-IN
Target and participant paths
φ, dφ/dt and d²φ/dt²
θ, dθ/dt and d²θ/dt²
Z (in-depth) position (m)
X (lateral) position (m)
Time (s)
Angular value (°, °/s, or °/s²)

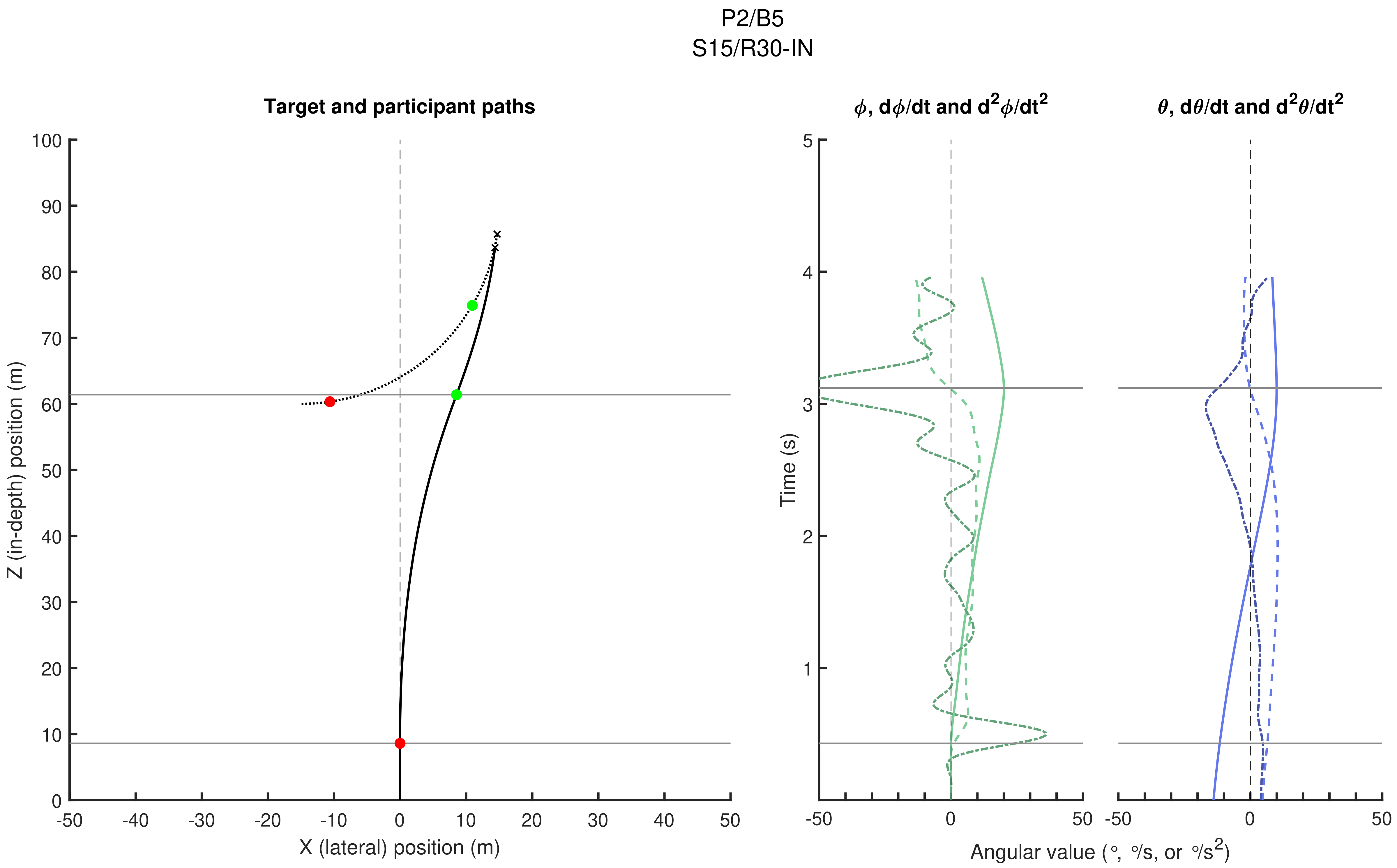

P2/B5
S15/R30-IN
Target and participant paths
Z (in-depth) position (m)
X (lateral) position (m)
0
10
20
30
40
50
60
70
80
90
100
-50
-40
-30
-20
-10
0
10
20
30
40
50
ϕ, dϕ/dt and d$^2\phi$/dt^2
θ, dθ/dt and d$^2\theta$/dt^2
Time (s)
1
2
3
4
5
-50
0
50
-50
0
50
Angular value (°, °/s, or °/s^2)

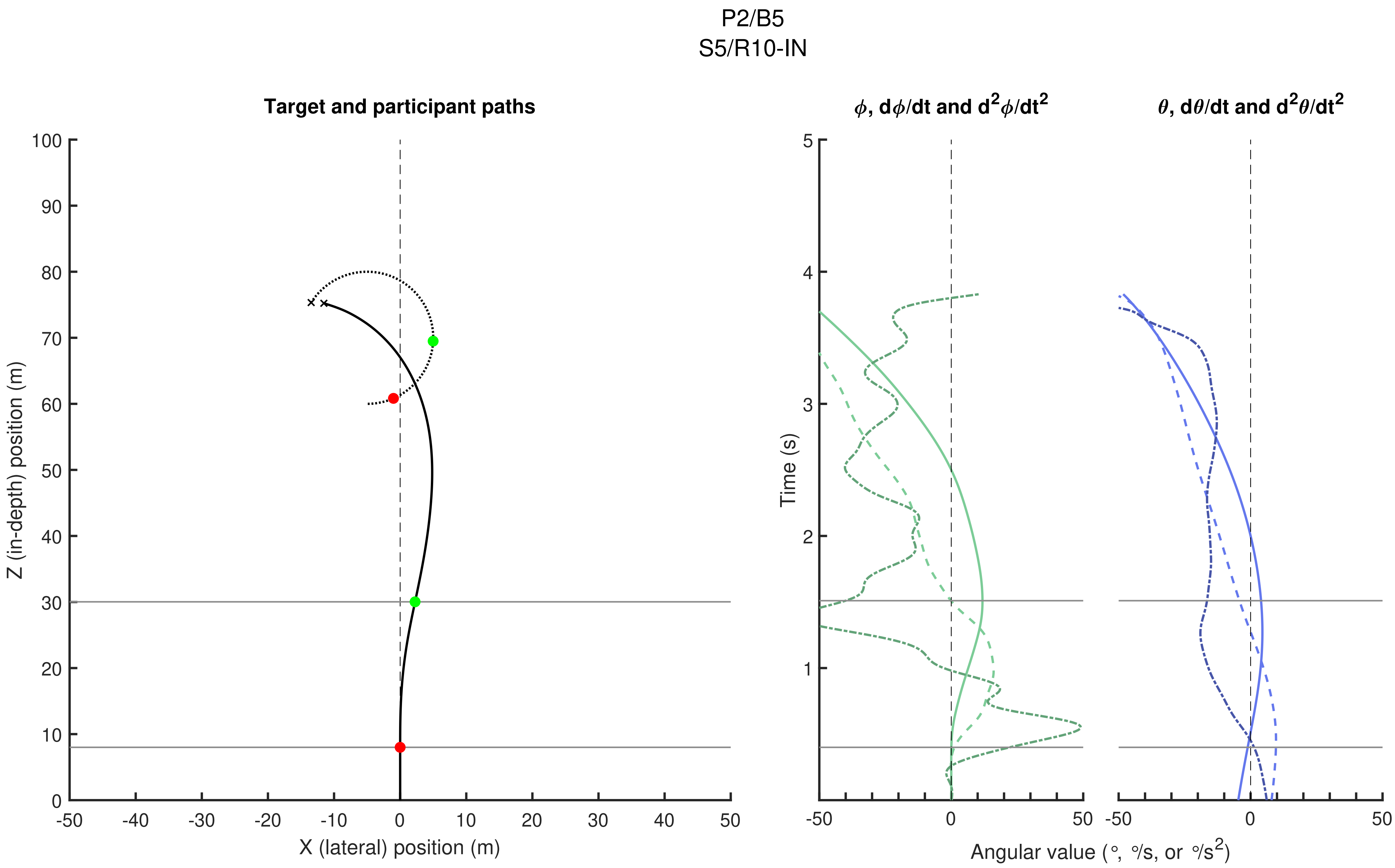

P2/B5
S5/R10-IN
Target and participant paths
Z (in-depth) position (m)
X (lateral) position (m)
ϕ, dϕ/dt and d²ϕ/dt²
θ, dθ/dt and d²θ/dt²
Time (s)
Angular value (°, °/s, or °/s²)

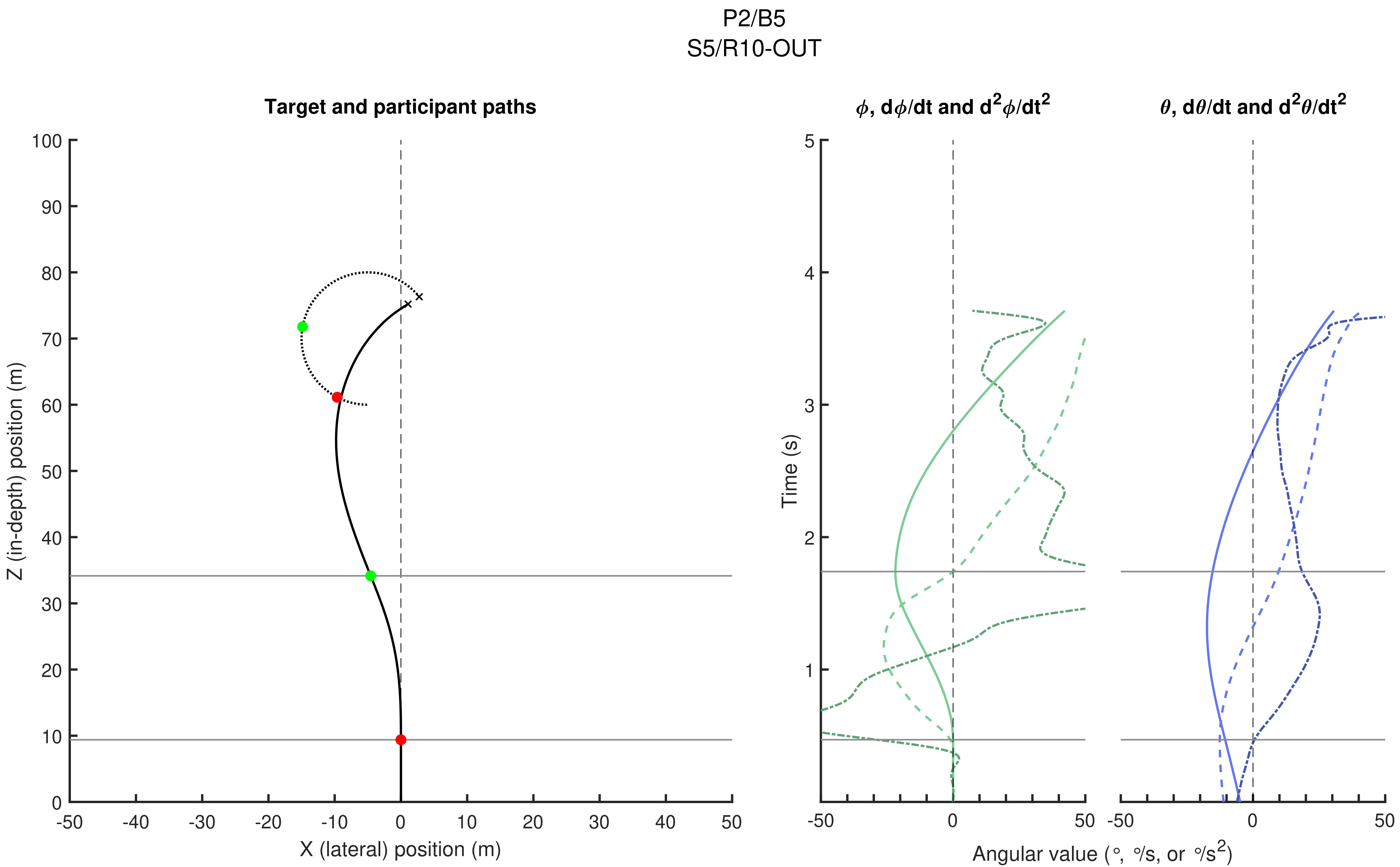

P2/B5
S5/R10-OUT
Target and participant paths
Z (in-depth) position (m)
X (lateral) position (m)
100
90
80
70
60
50
40
30
20
10
0
-50
-40
-30
-20
-10
0
10
20
30
40
50
φ, dφ/dt and d²φ/dt²
θ, dθ/dt and d²θ/dt²
Time (s)
5
4
3
2
1
-50
0
50
-50
0
50
Angular value (°, °/s, or °/s²)

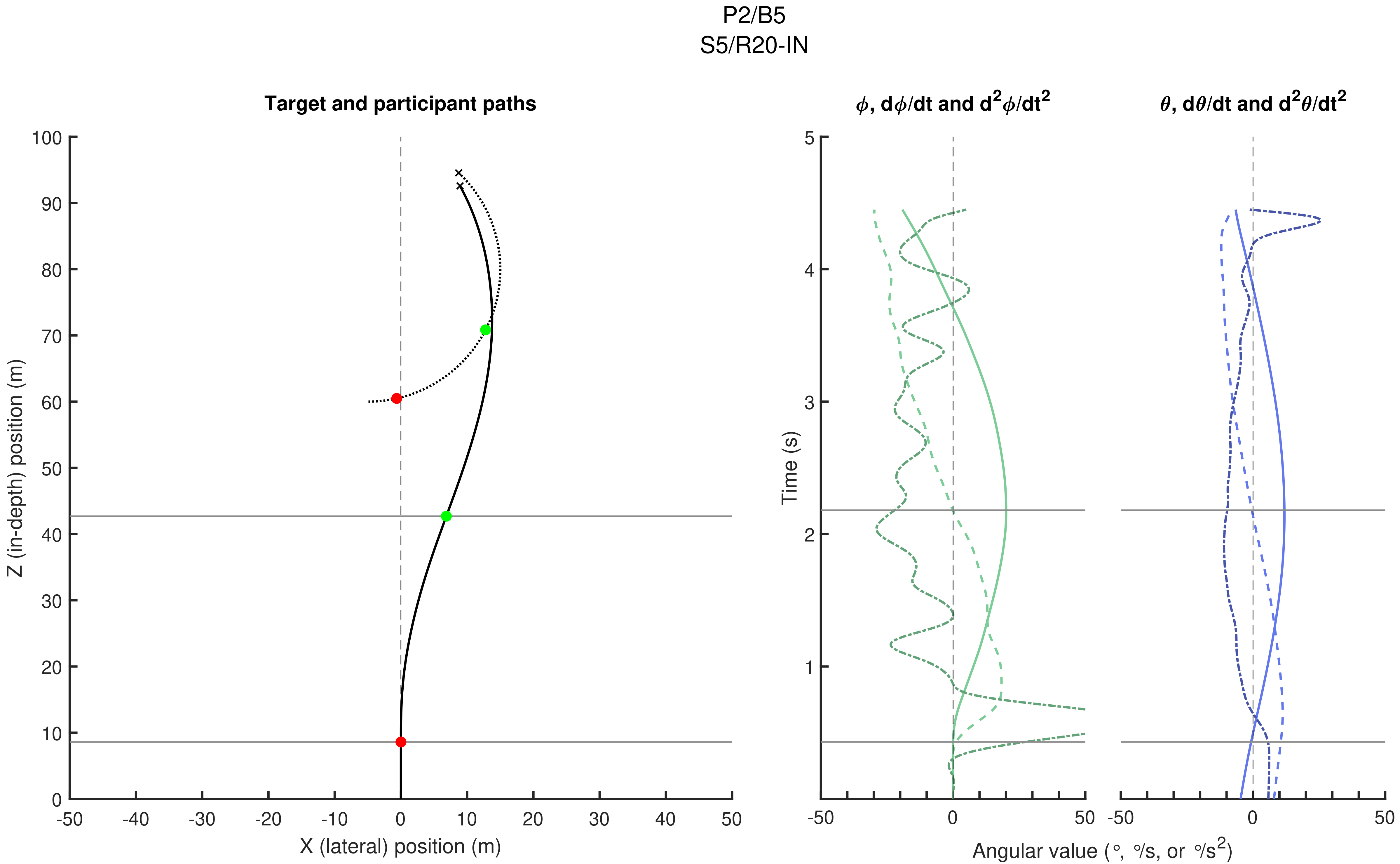

P2/B5
S5/R20-IN
Target and participant paths
Z (in-depth) position (m)
X (lateral) position (m)
ϕ, dϕ/dt and d²ϕ/dt²
θ, dθ/dt and d²θ/dt²
Time (s)
Angular value (°, °/s, or °/s²)

P2/B5
S5/R20-OUT

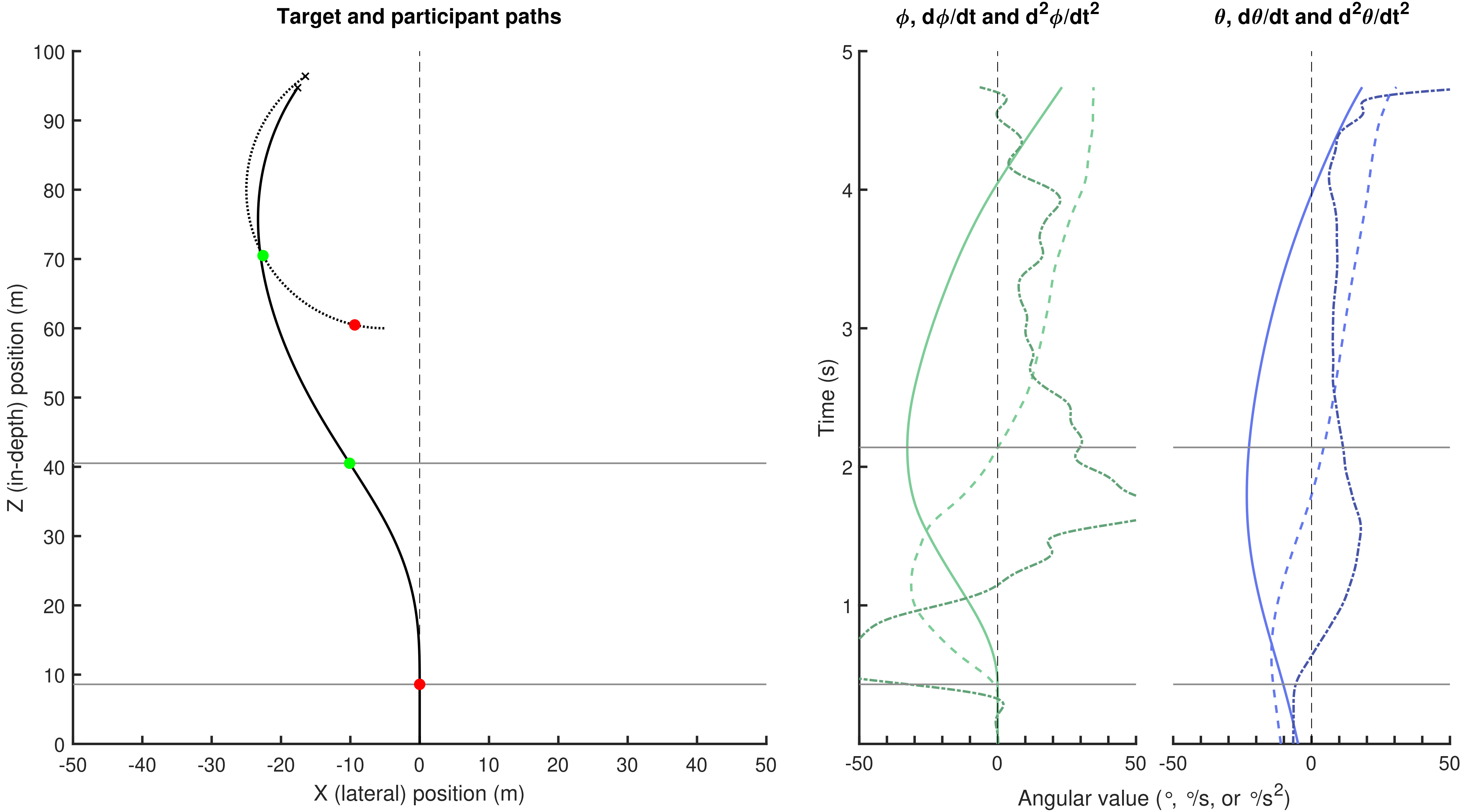

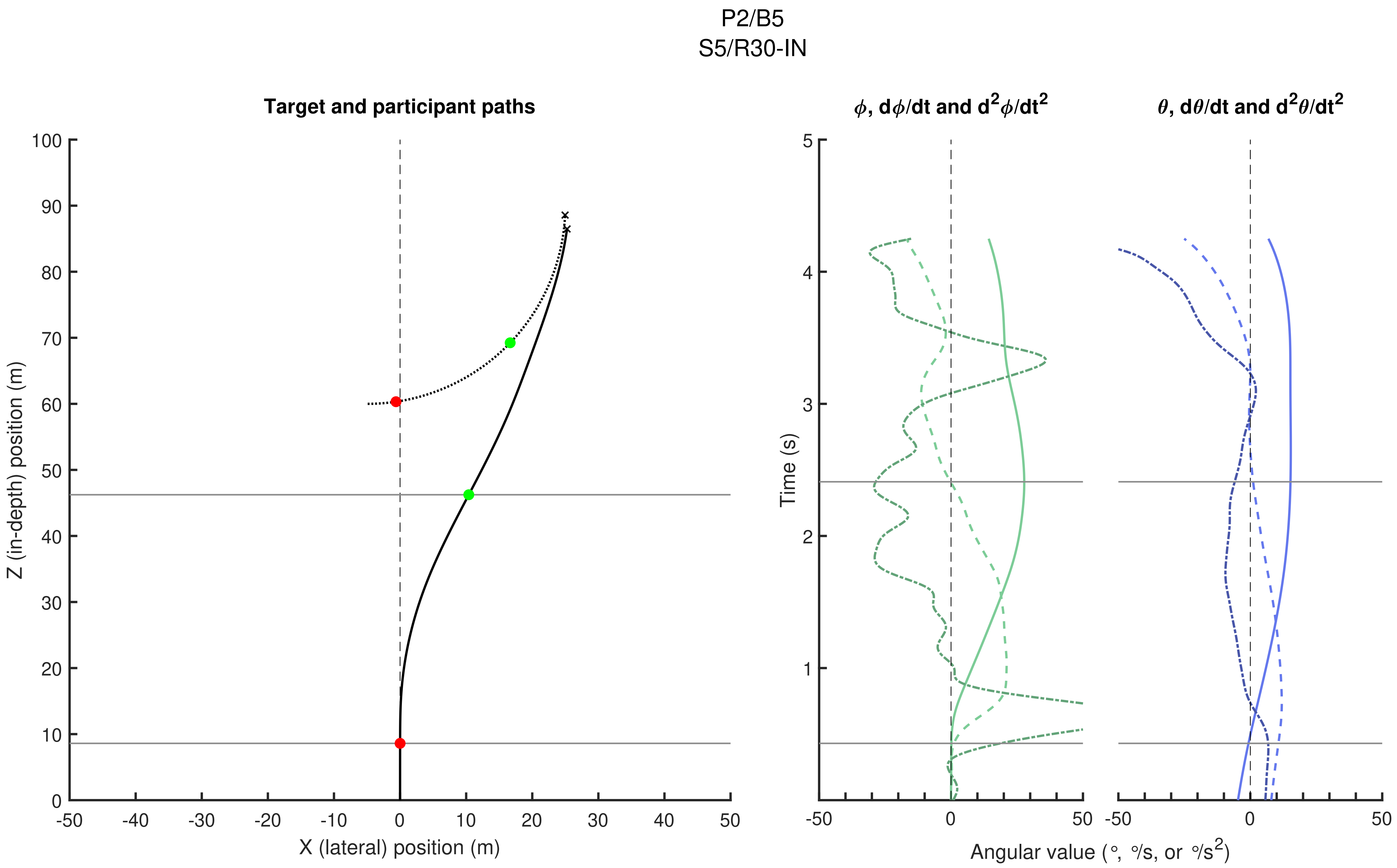
P2/B5
S5/R30-IN
Target and participant paths
Z (in-depth) position (m)
X (lateral) position (m)
ϕ, dϕ/dt and d²ϕ/dt²
θ, dθ/dt and d²θ/dt²
Time (s)
Angular value (°, °/s, or °/s²)

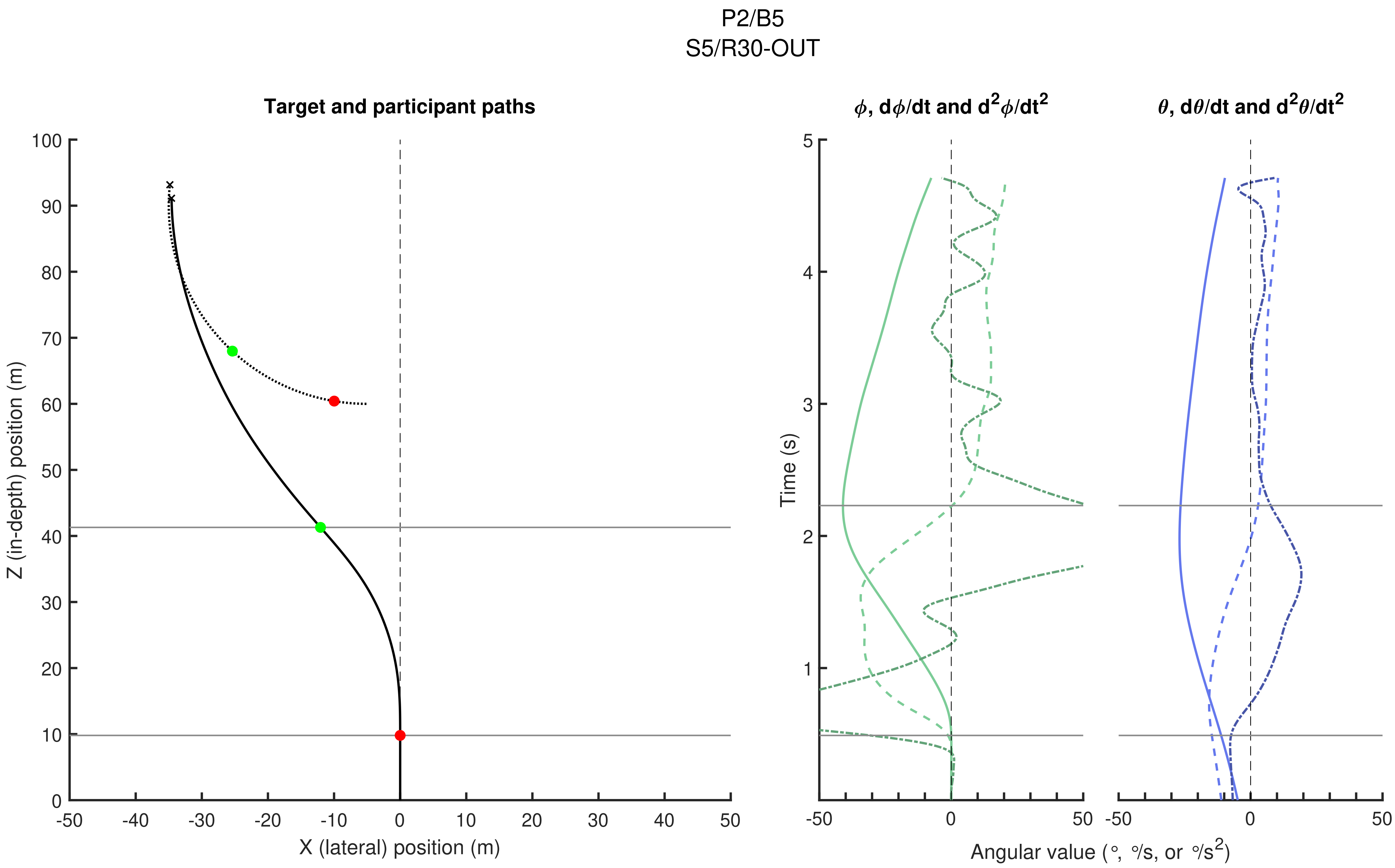
P2/B5
S5/R30-OUT
Target and participant paths
Z (in-depth) position (m)
X (lateral) position (m)
ϕ, dϕ/dt and d$^2\phi$/dt^2
θ, dθ/dt and d$^2\theta$/dt^2
Time (s)
Angular value (°, °/s, or °/s^2)

P2/B5
S5/R10-OUT

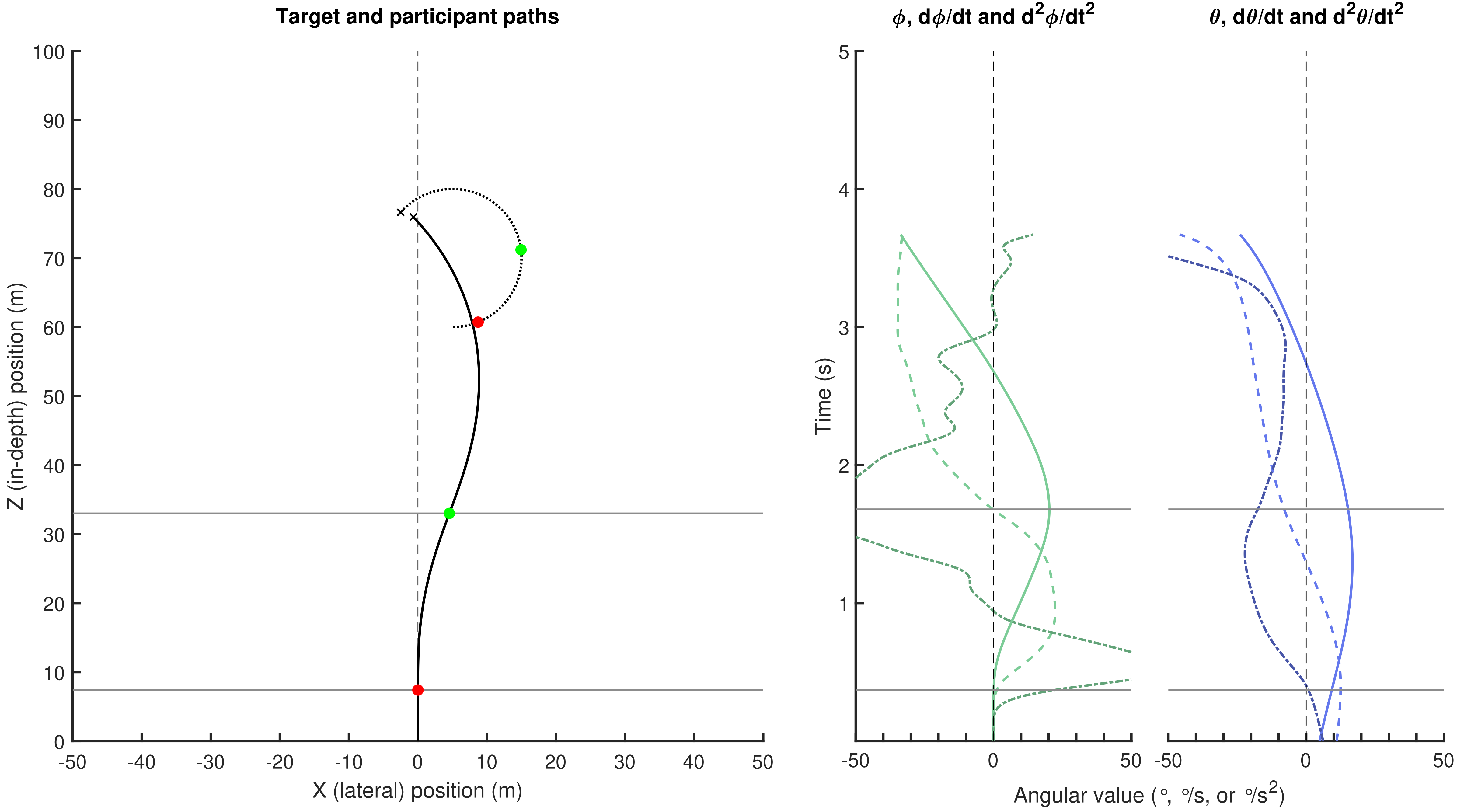

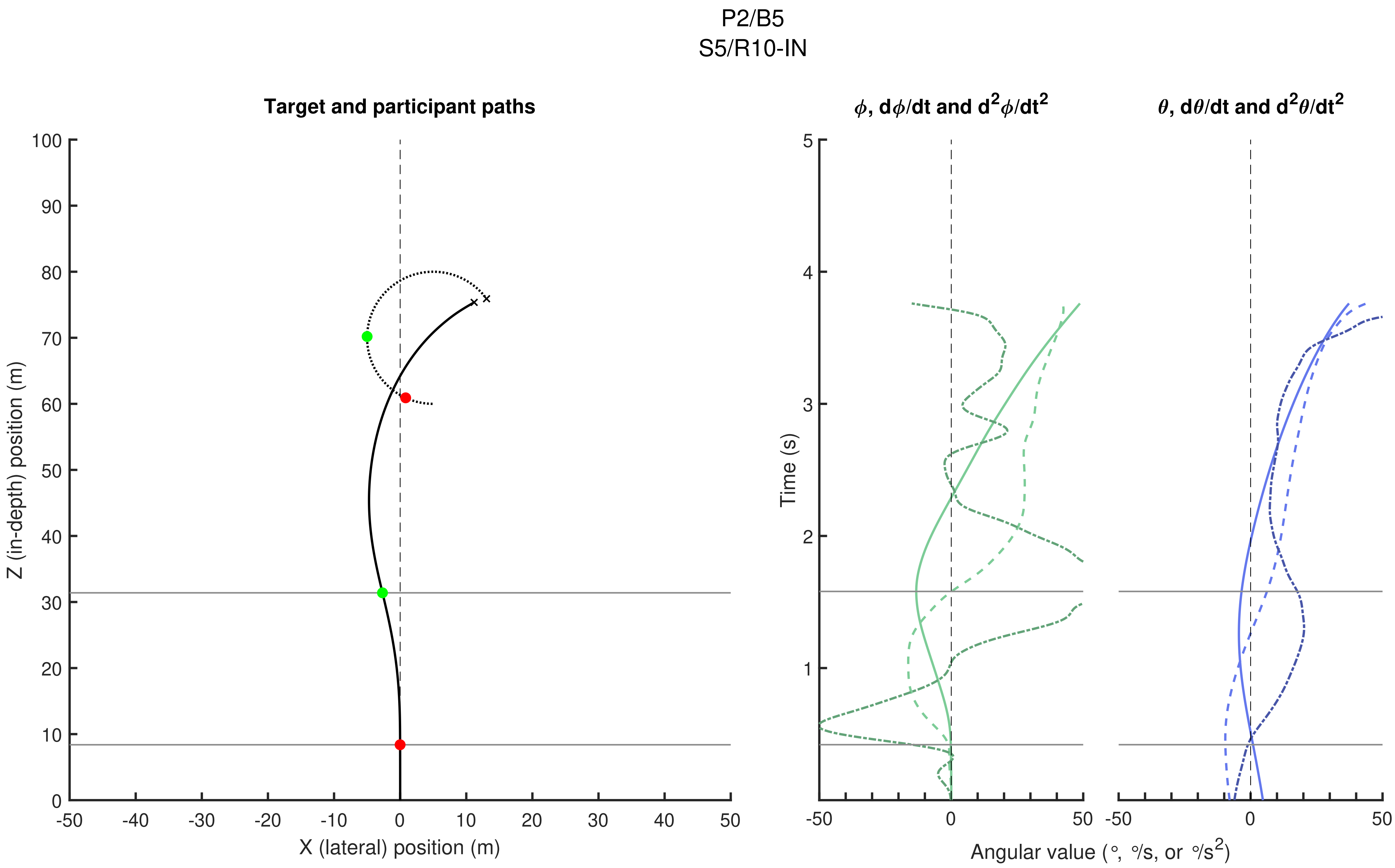
P2/B5
S5/R10-IN
Target and participant paths
Z (in-depth) position (m)
X (lateral) position (m)
ϕ, dϕ/dt and d$^2\phi$/dt^2
θ, dθ/dt and d$^2\theta$/dt^2
Time (s)
Angular value (°, °/s, or °/s^2)

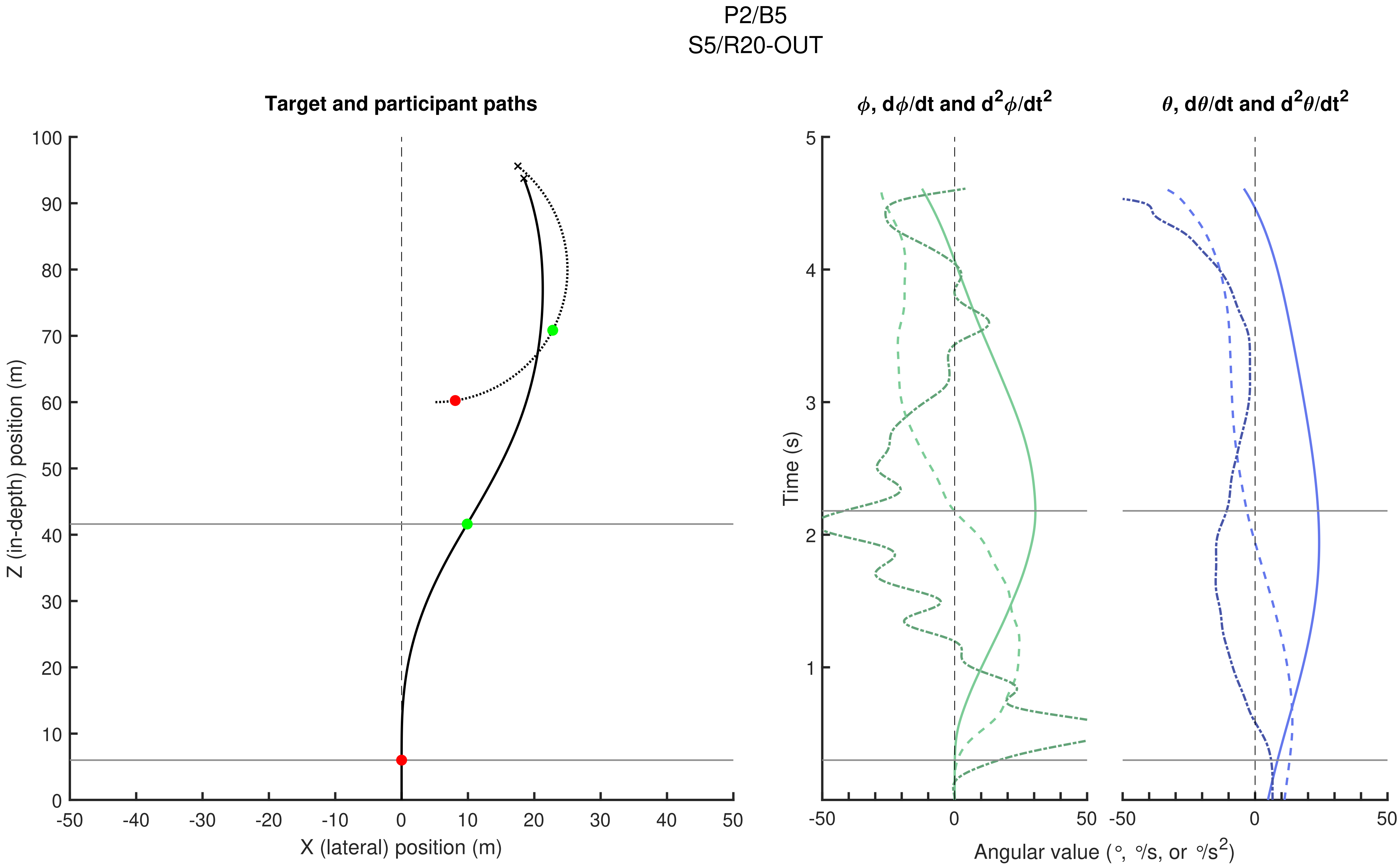

P2/B5
S5/R20-OUT
Target and participant paths
Z (in-depth) position (m)
X (lateral) position (m)
ϕ, dϕ/dt and d$^2\phi$/dt^2
θ, dθ/dt and d$^2\theta$/dt^2
Time (s)
Angular value (°, °/s, or °/s^2)

P2/B5
S5/R20-IN

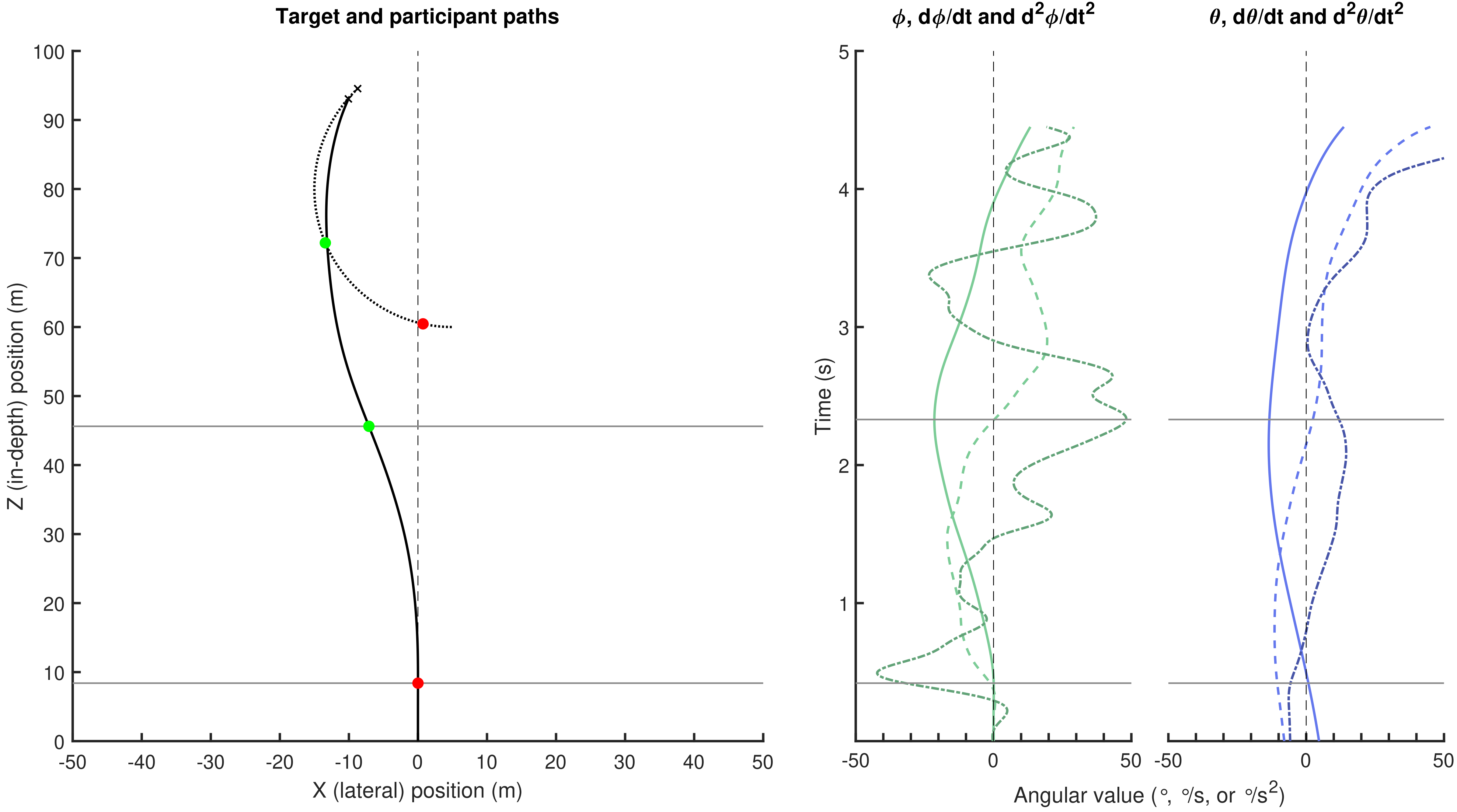

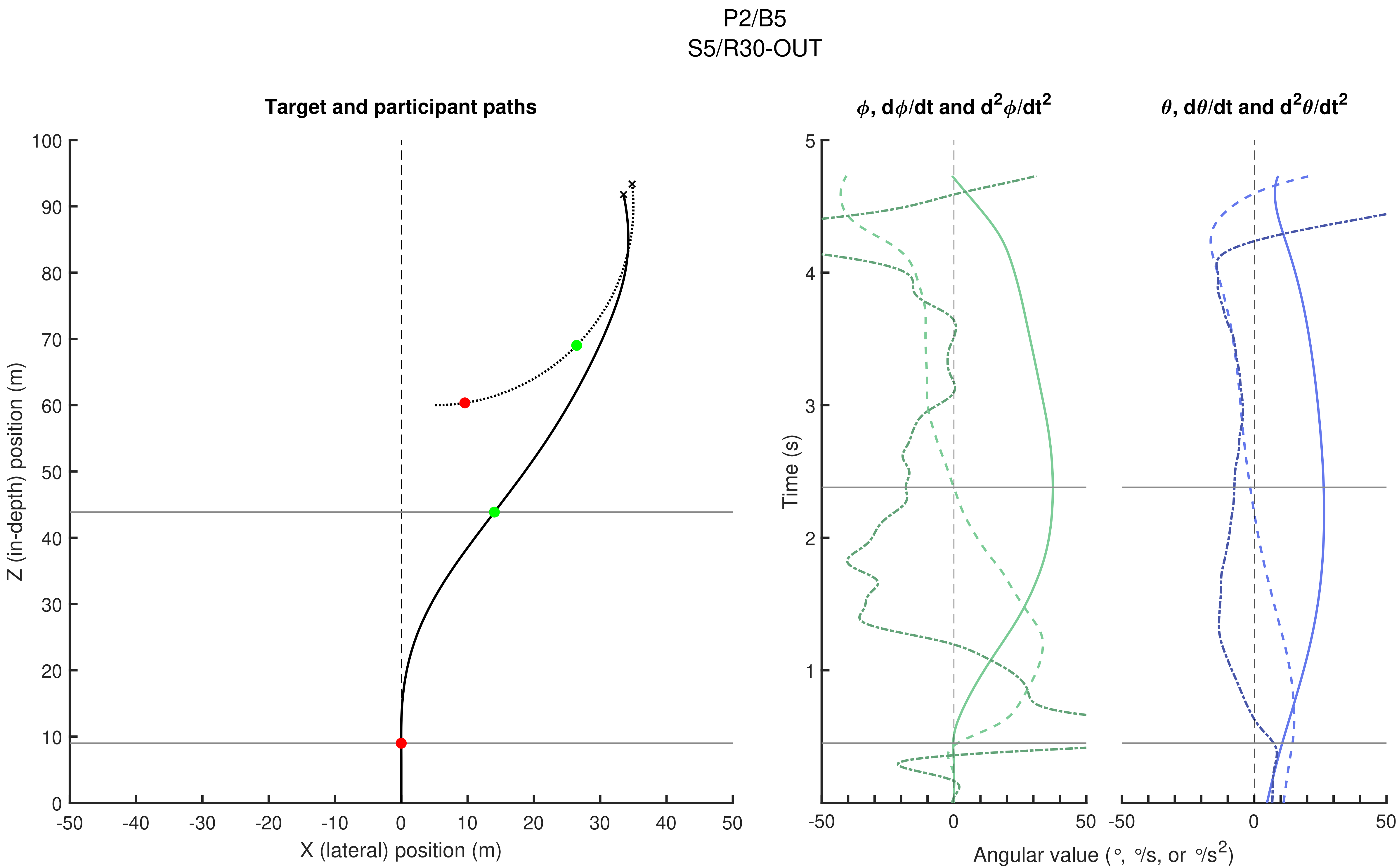
P2/B5
S5/R30-OUT
Target and participant paths
Z (in-depth) position (m)
X (lateral) position (m)
φ, dφ/dt and d²φ/dt²
θ, dθ/dt and d²θ/dt²
Time (s)
Angular value (°, °/s, or °/s²)

P2/B5
S5/R30-IN

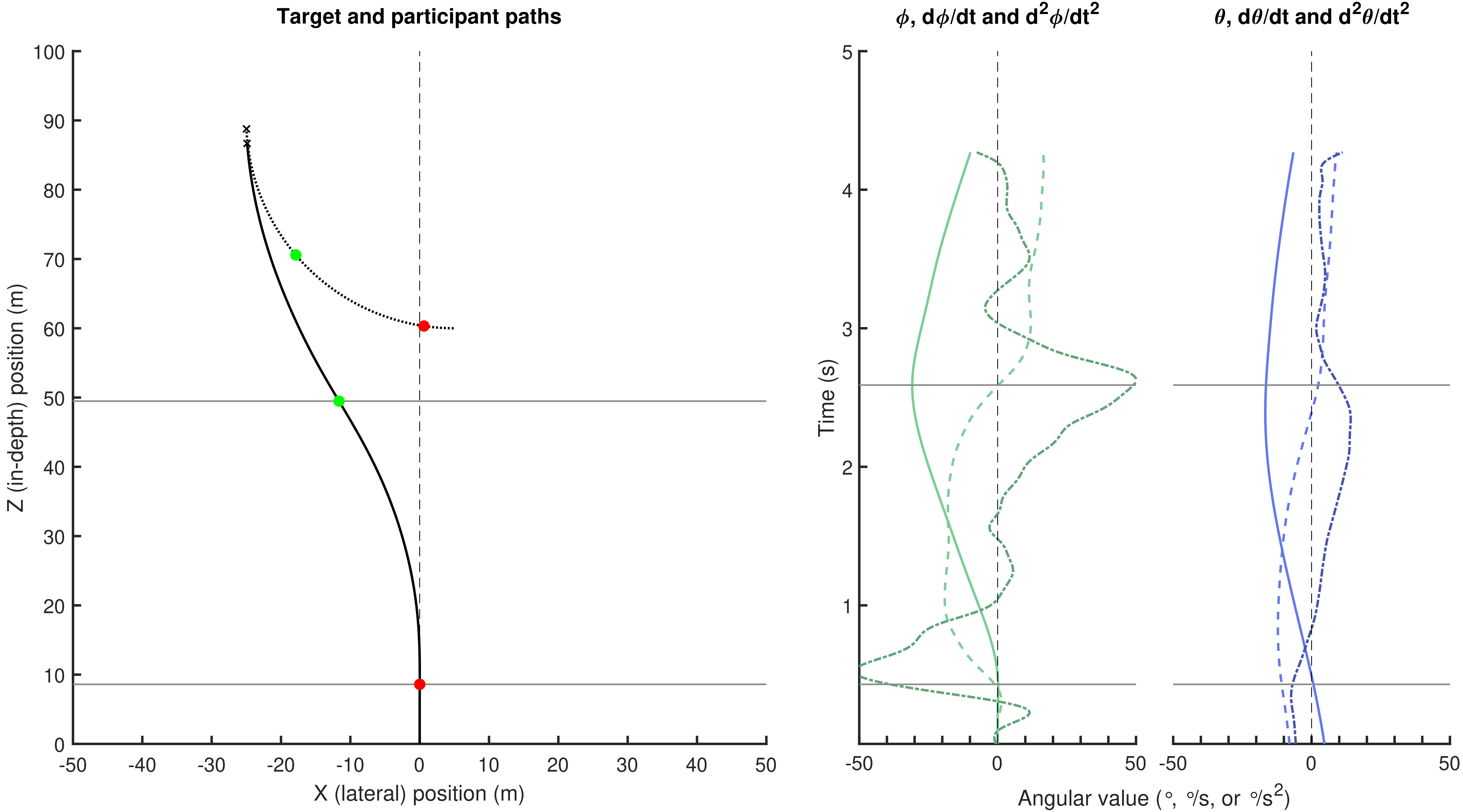

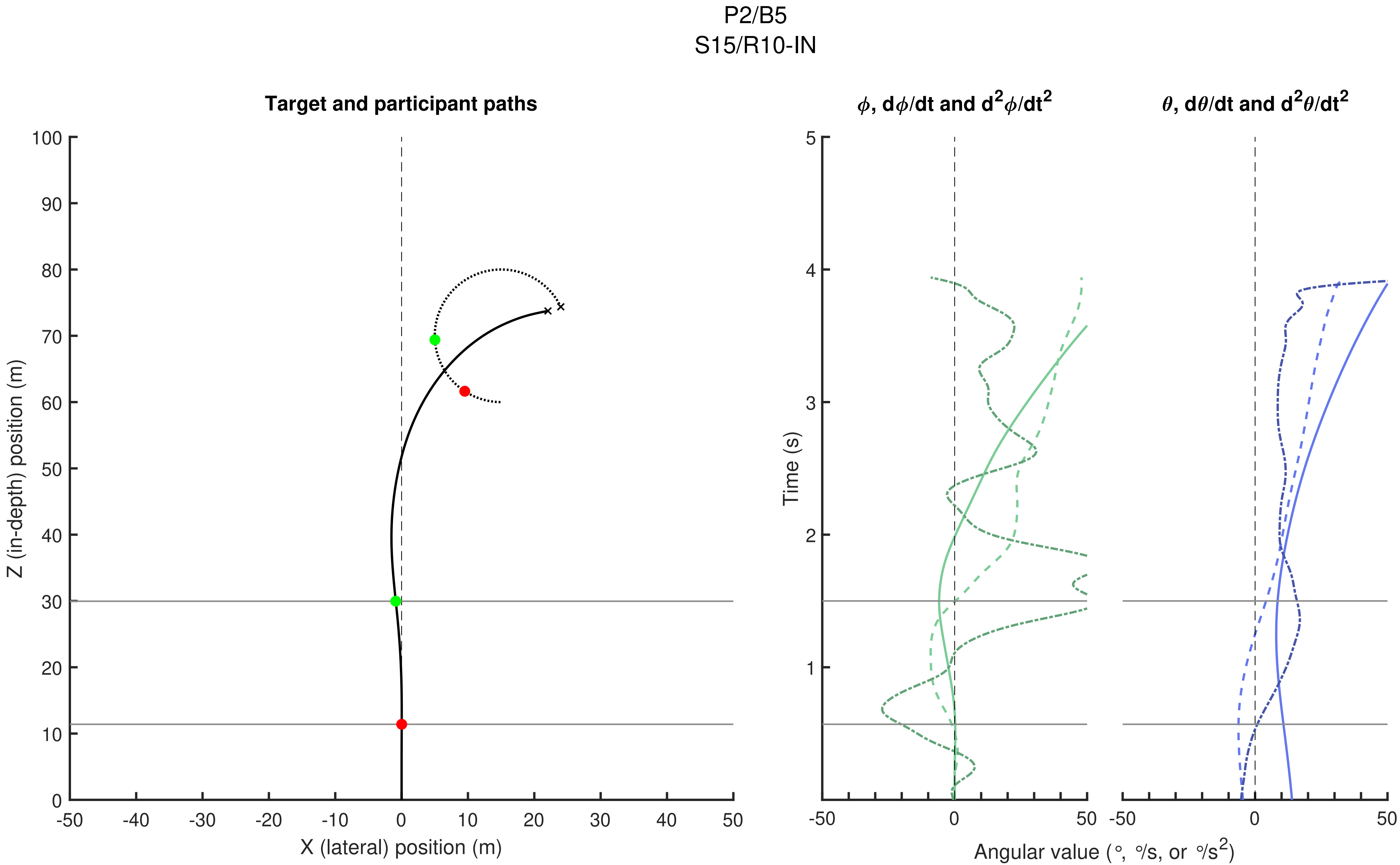

P2/B5
S15/R10-IN
Target and participant paths
Z (in-depth) position (m)
X (lateral) position (m)
ϕ, dϕ/dt and d²ϕ/dt²
θ, dθ/dt and d²θ/dt²
Time (s)
Angular value (°, °/s, or °/s²)

P2/B5
S15/R20-IN

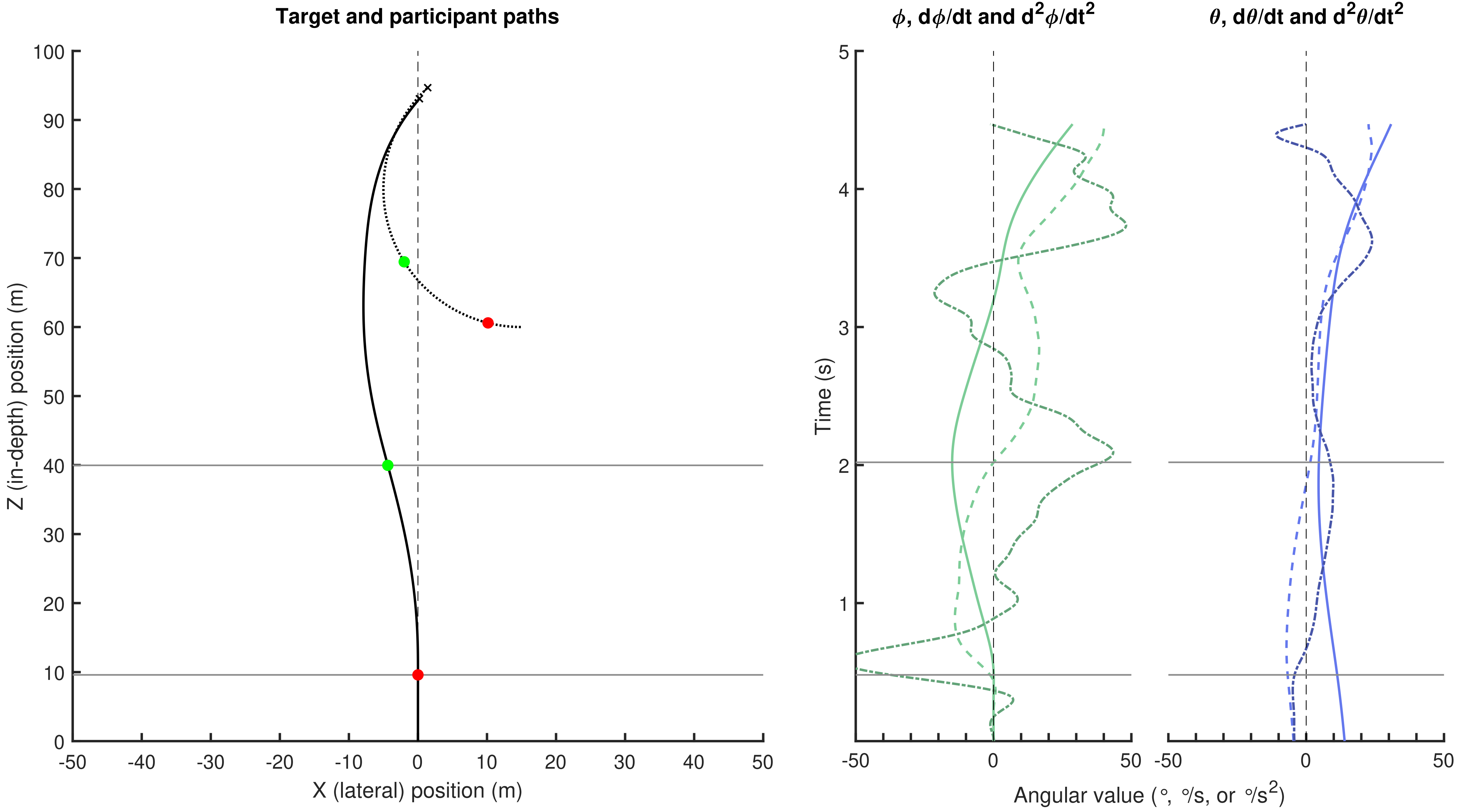

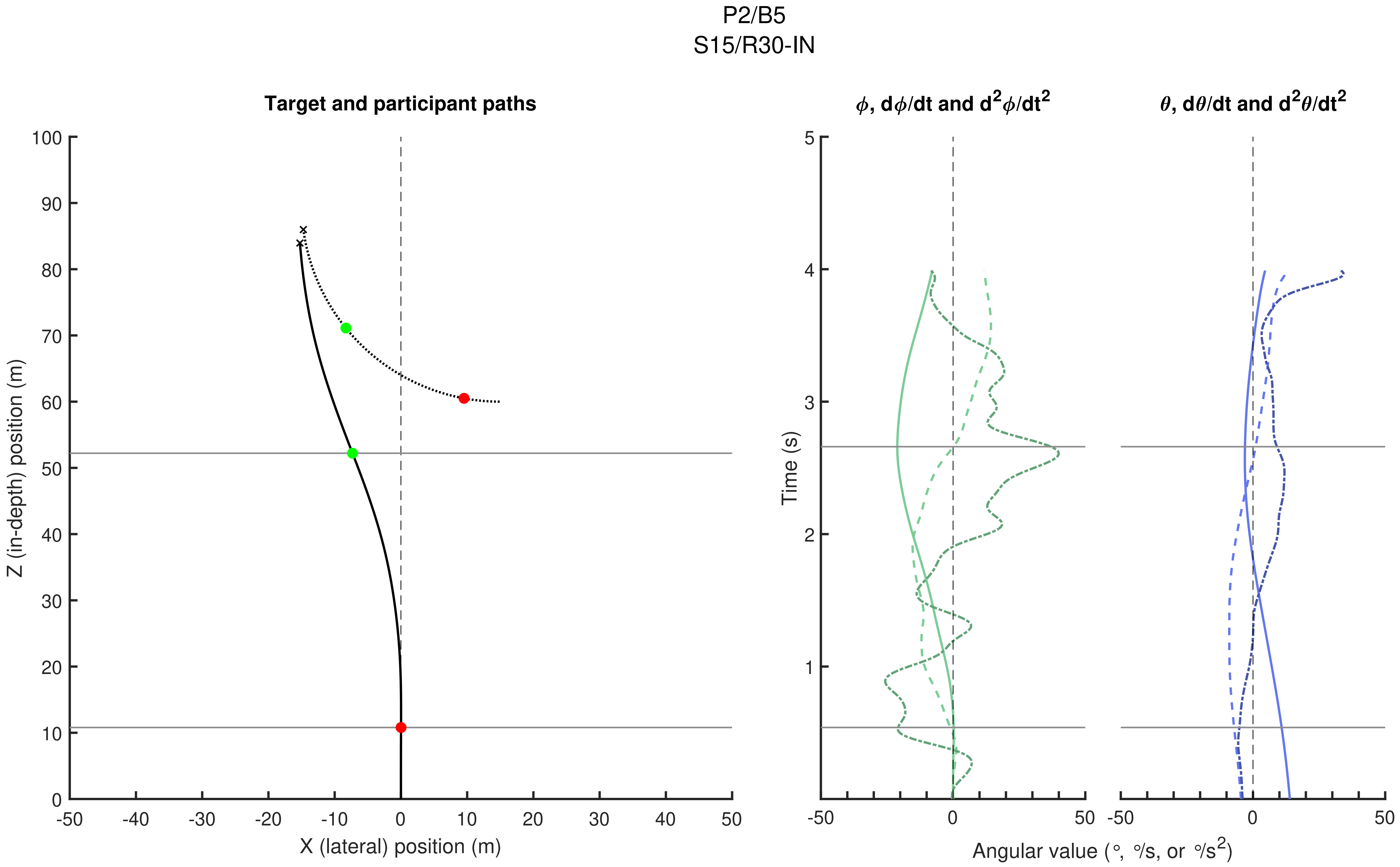

P2/B5
S15/R30-IN
Target and participant paths
ϕ, dϕ/dt and d$^2\phi$/dt^2
θ, dθ/dt and d$^2\theta$/dt^2
100
90
80
70
60
50
40
30
20
10
0
-50
-40
-30
-20
-10
0
10
20
30
40
50
Z (in-depth) position (m)
X (lateral) position (m)
5
4
3
2
1
Time (s)
-50
0
50
-50
0
50
Angular value (°, °/s, or °/s^2)

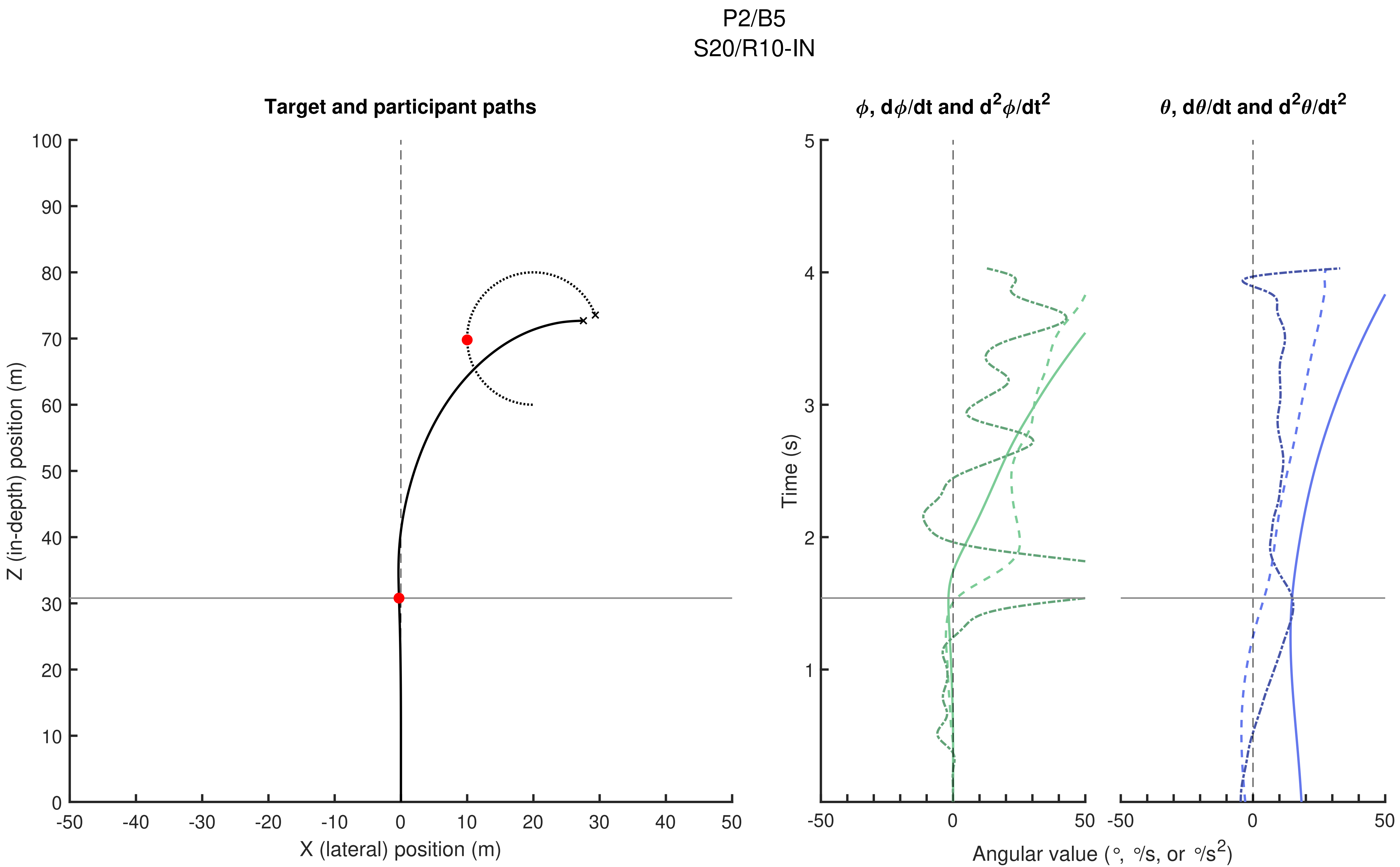
P2/B5
S20/R10-IN
Target and participant paths
Z (in-depth) position (m)
X (lateral) position (m)
φ, dφ/dt and d²φ/dt²
θ, dθ/dt and d²θ/dt²
Time (s)
Angular value (°, °/s, or °/s²)

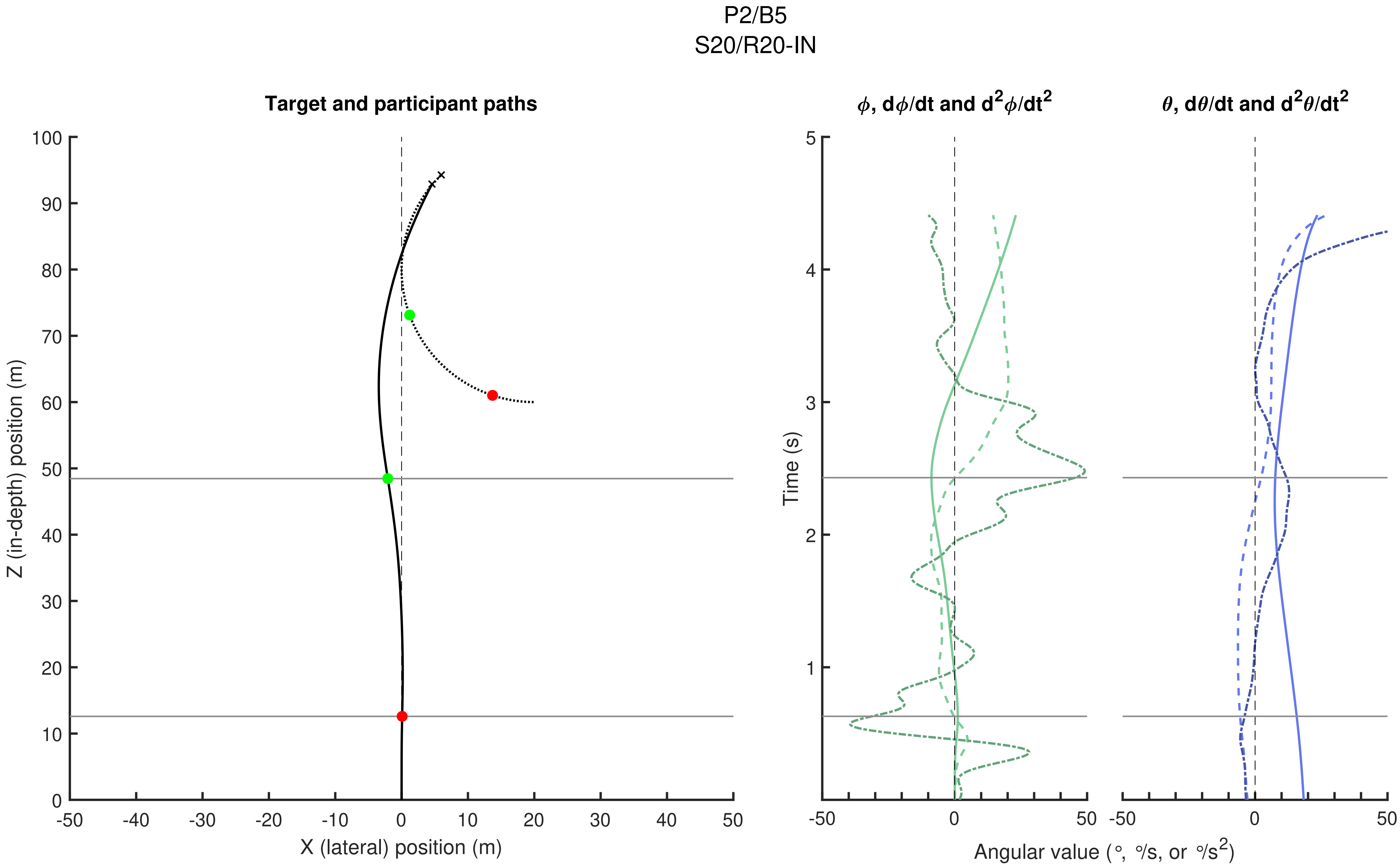
P2/B5
S20/R20-IN
Target and participant paths
Z (in-depth) position (m)
X (lateral) position (m)
ϕ, dϕ/dt and d$^2\phi$/dt^2
θ, dθ/dt and d$^2\theta$/dt^2
Time (s)
Angular value (°, °/s, or °/s^2)

P2/B5
S20/R30-IN

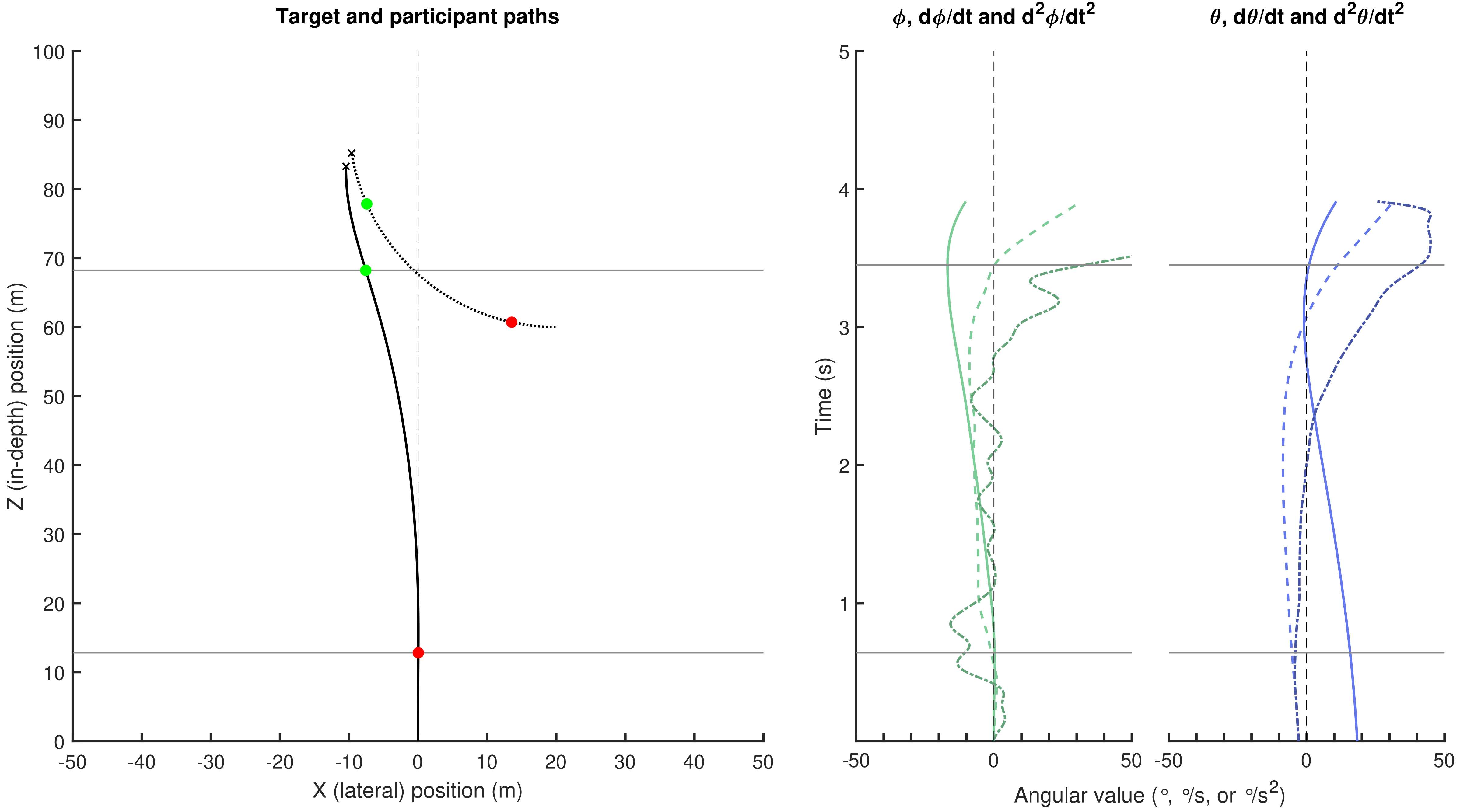

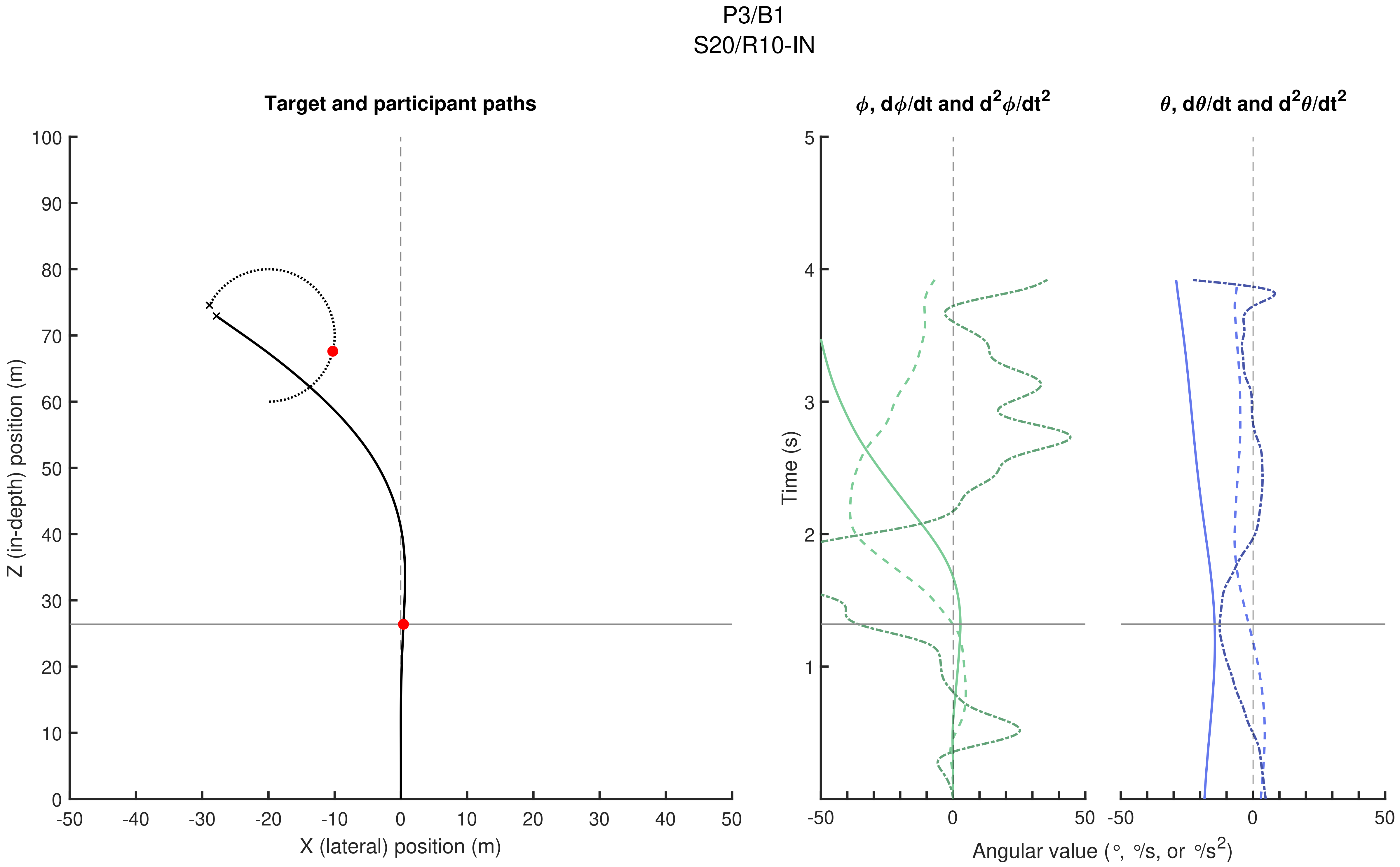
P3/B1
S20/R10-IN
Target and participant paths
Z (in-depth) position (m)
X (lateral) position (m)
φ, dφ/dt and d²φ/dt²
θ, dθ/dt and d²θ/dt²
Time (s)
Angular value (°, °/s, or °/s²)

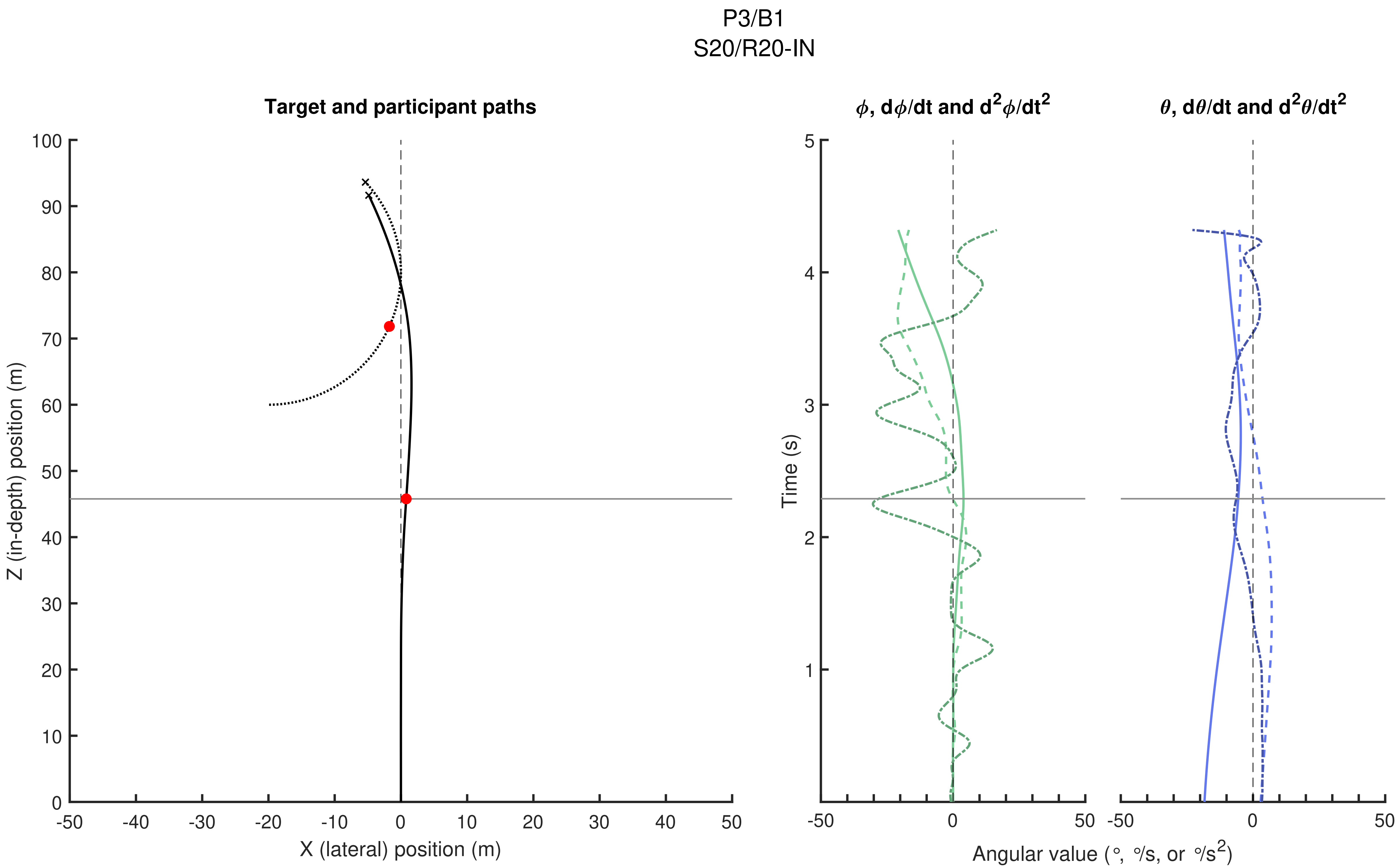
P3/B1
S20/R20-IN
Target and participant paths
ϕ, dϕ/dt and d$^2\phi$/dt^2
θ, dθ/dt and d$^2\theta$/dt^2
Z (in-depth) position (m)
X (lateral) position (m)
Time (s)
Angular value (°, °/s, or °/s^2)

P3/B1
S20/R30-IN

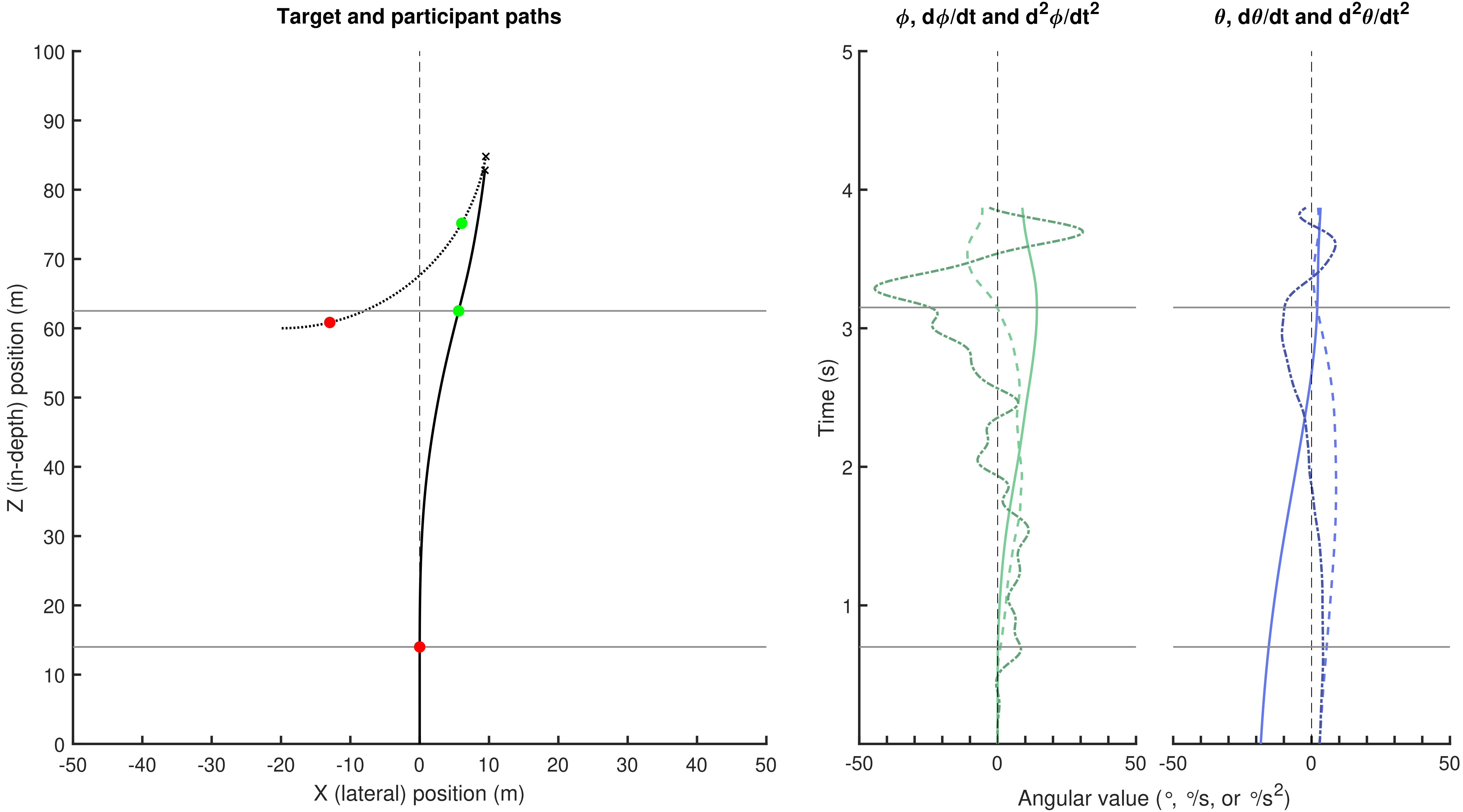

P3/B1
S15/R10-IN

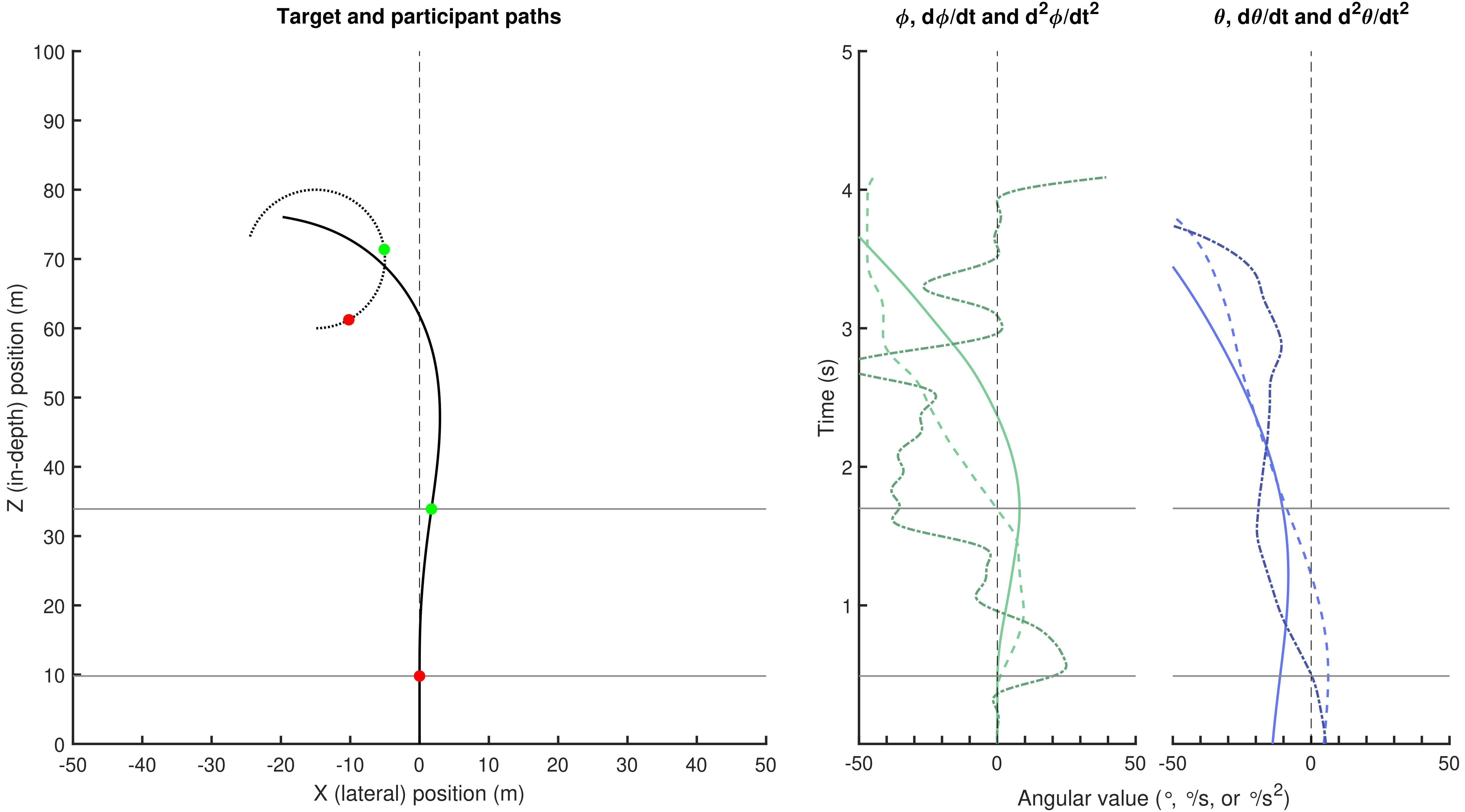

P3/B1
S15/R20-IN

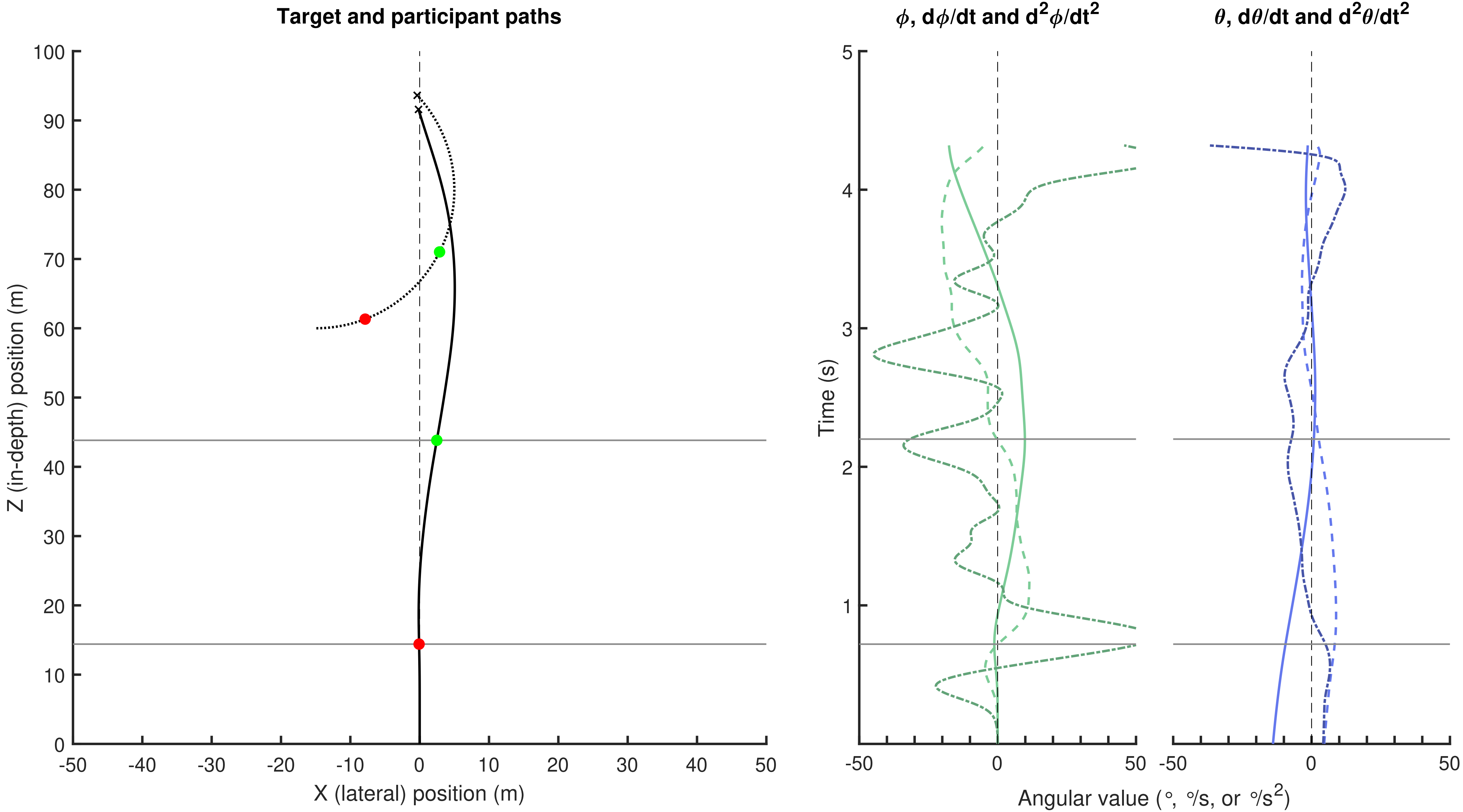

P3/B1
S15/R30-IN

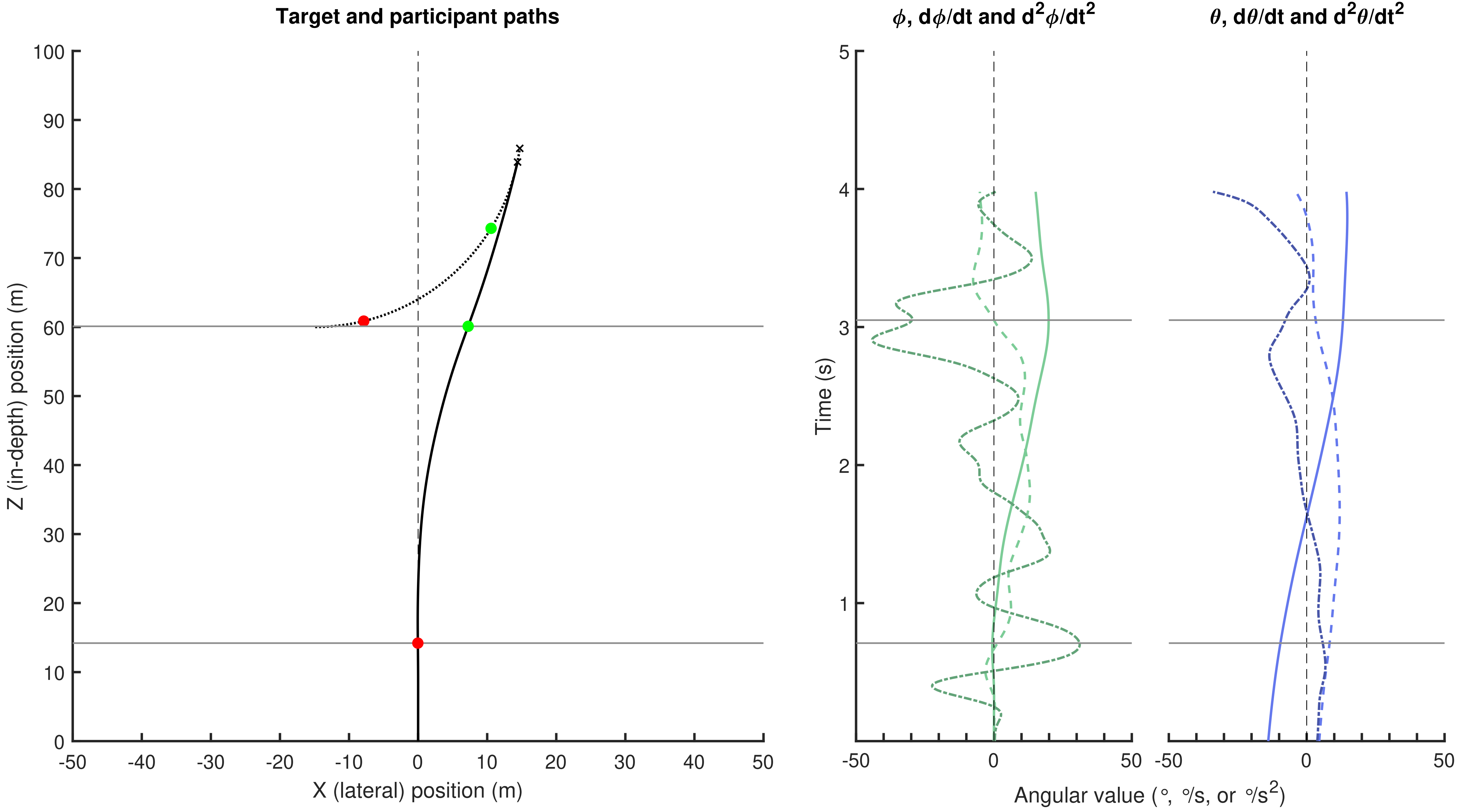

P3/B1
S5/R10-IN

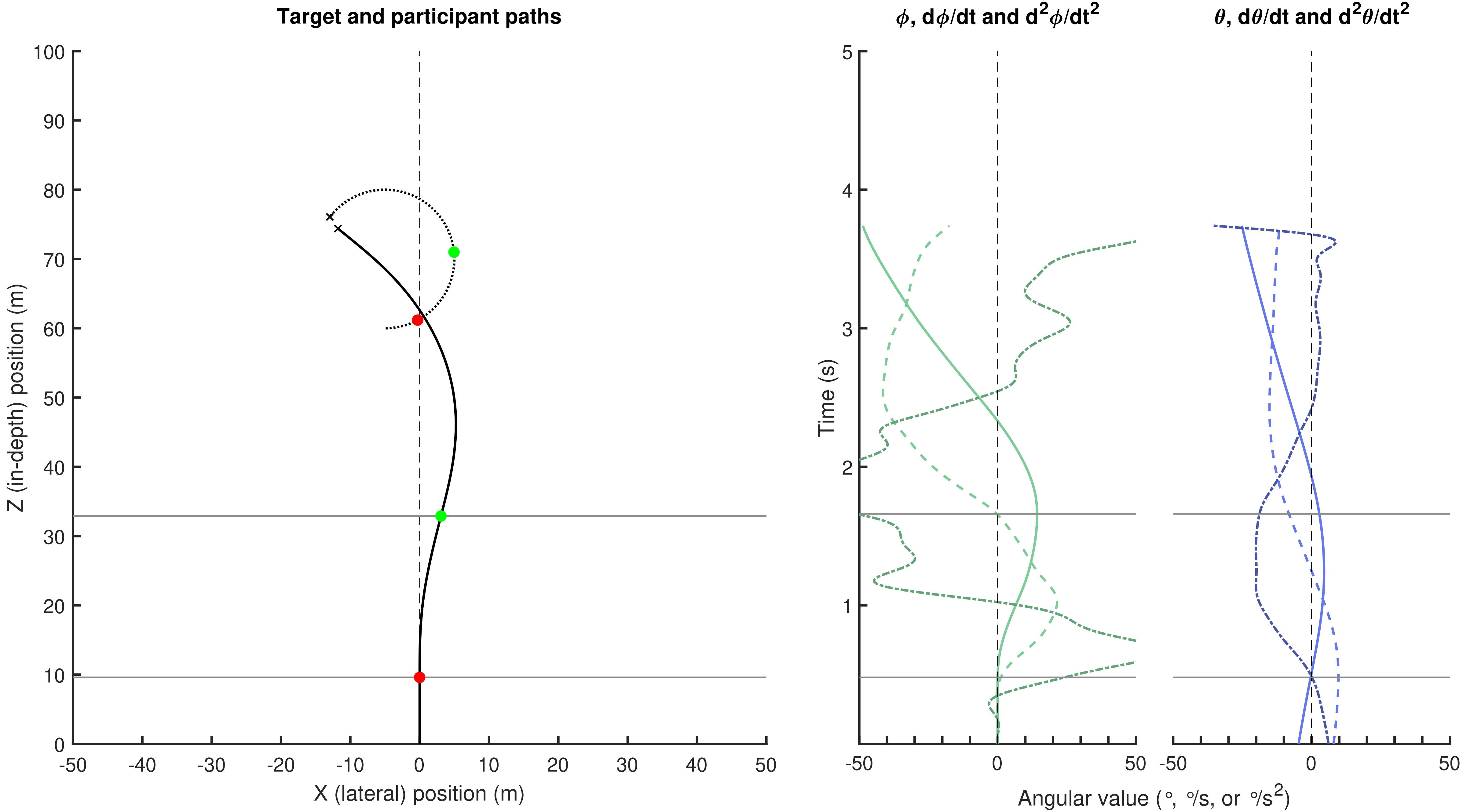

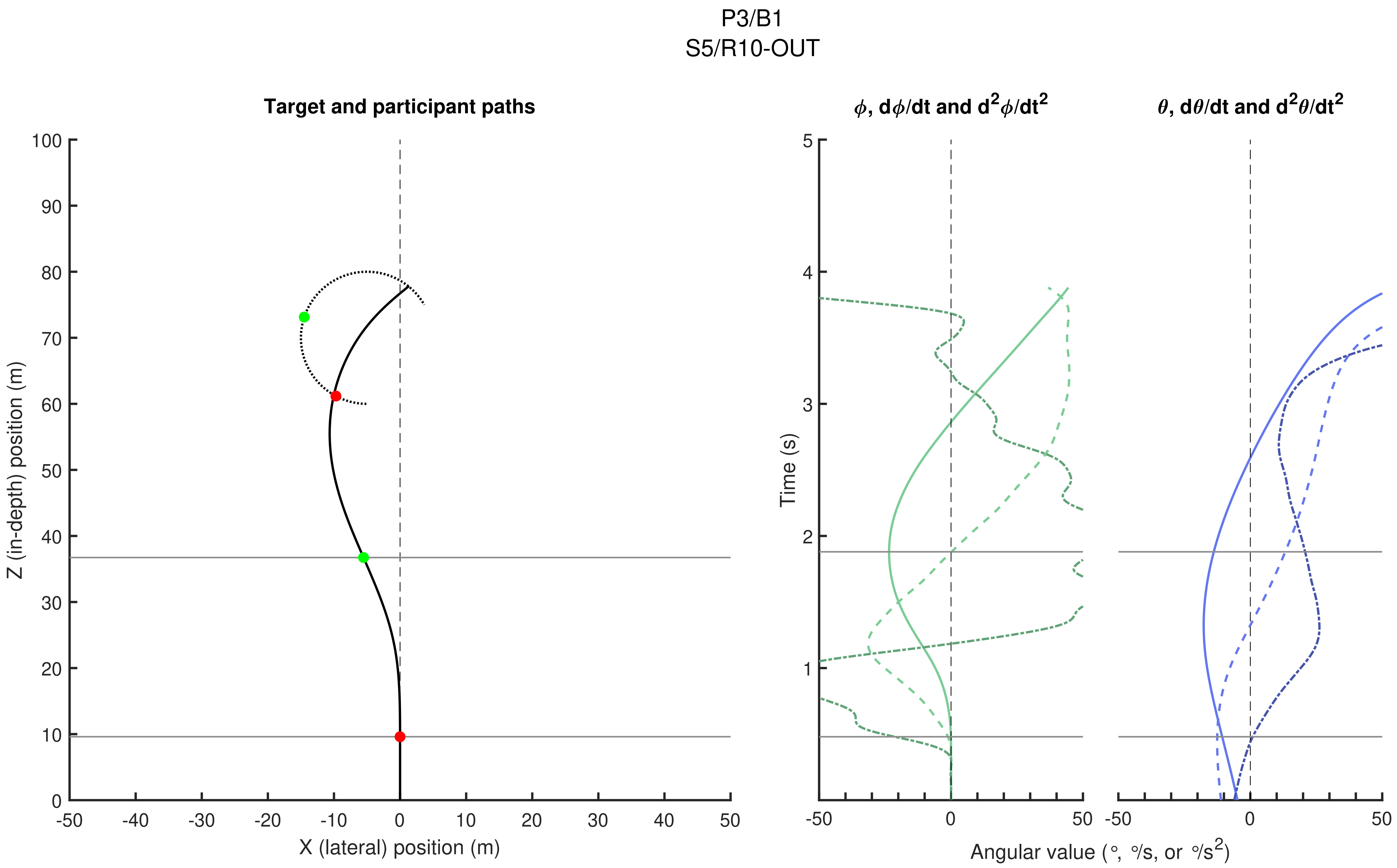
P3/B1
S5/R10-OUT
Target and participant paths
Z (in-depth) position (m)
X (lateral) position (m)
ϕ, dϕ/dt and d$^2\phi$/dt^2
θ, dθ/dt and d$^2\theta$/dt^2
Time (s)
Angular value (°, °/s, or °/s^2)

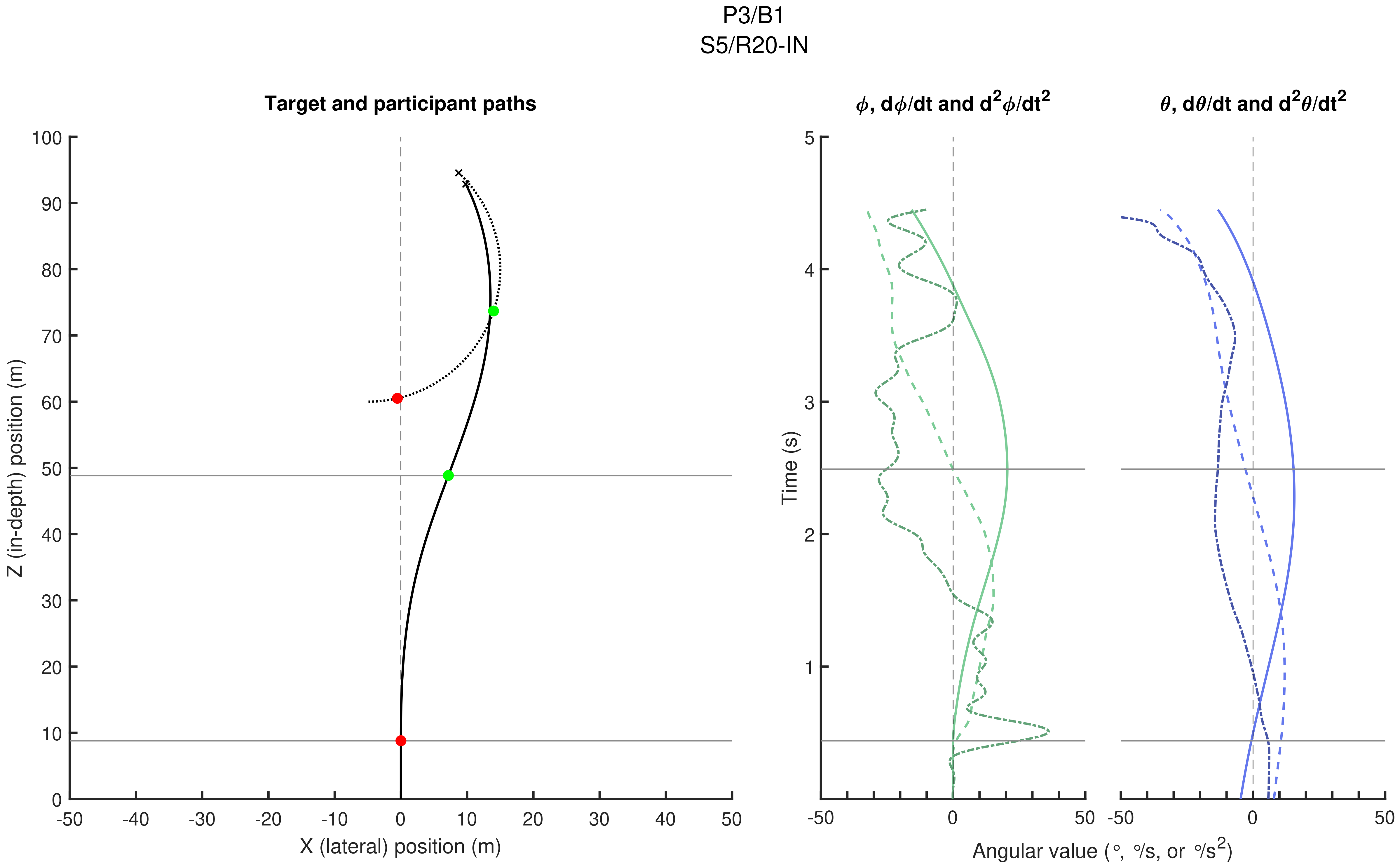

P3/B1
S5/R20-IN
Target and participant paths
Z (in-depth) position (m)
X (lateral) position (m)
0
10
20
30
40
50
60
70
80
90
100
-50
-40
-30
-20
-10
0
10
20
30
40
50
ϕ, dϕ/dt and d²ϕ/dt²
θ, dθ/dt and d²θ/dt²
Time (s)
1
2
3
4
5
-50
0
50
-50
0
50
Angular value (°, °/s, or °/s²)

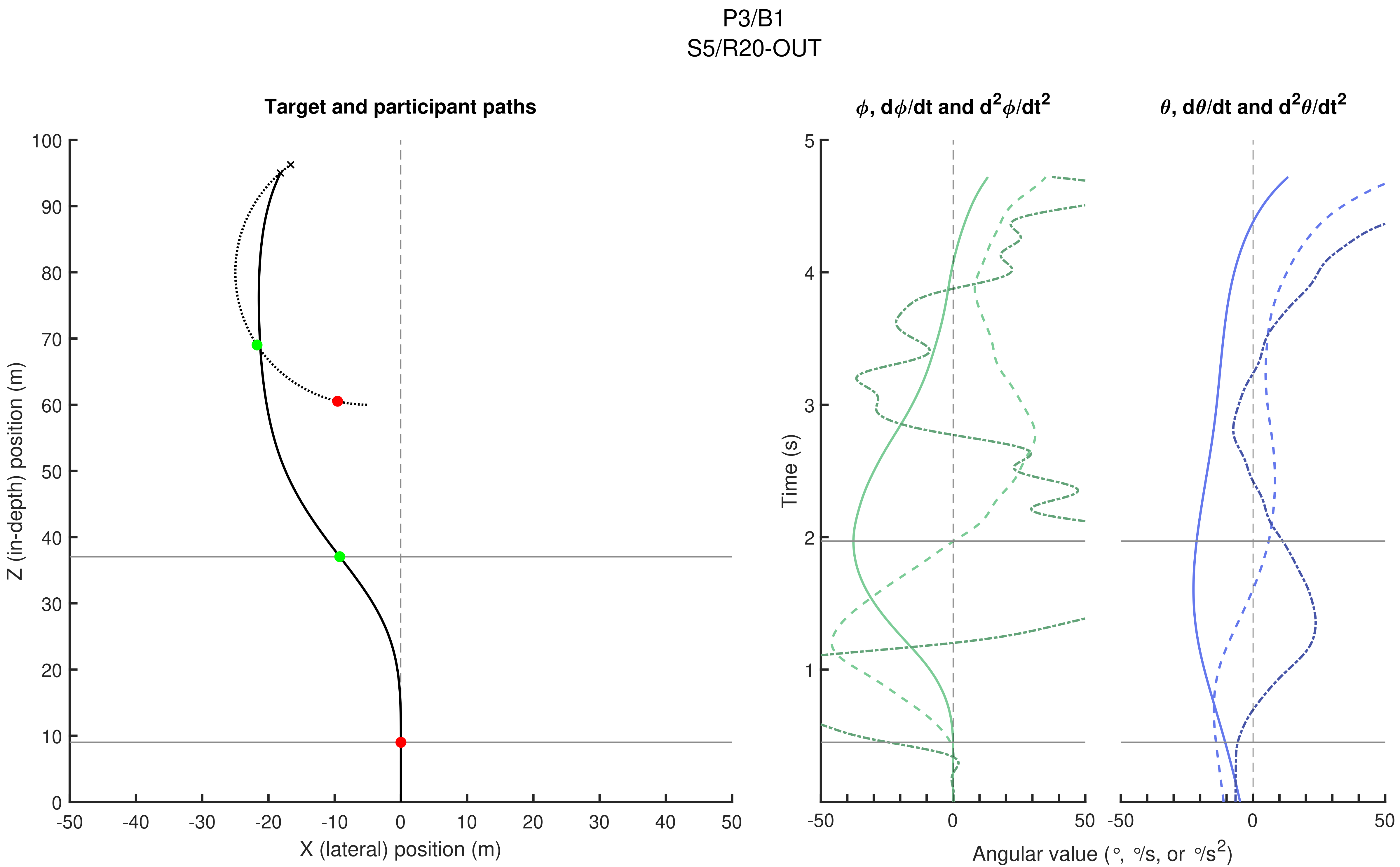
P3/B1
S5/R20-OUT
Target and participant paths
Z (in-depth) position (m)
X (lateral) position (m)
ϕ, dϕ/dt and d²ϕ/dt²
θ, dθ/dt and d²θ/dt²
Time (s)
Angular value (°, °/s, or °/s²)

P3/B1
S5/R30-IN

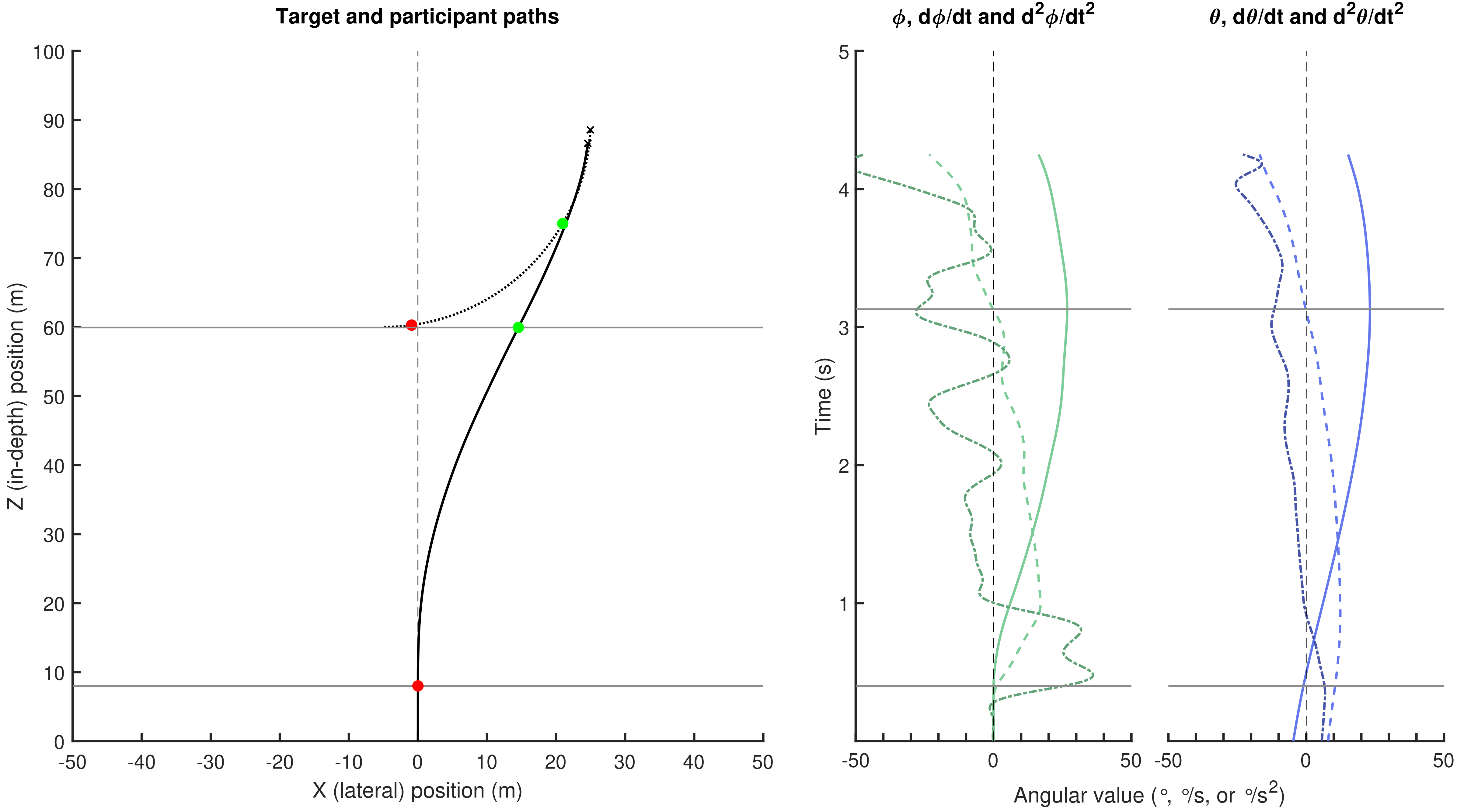

P3/B1
S5/R30-OUT

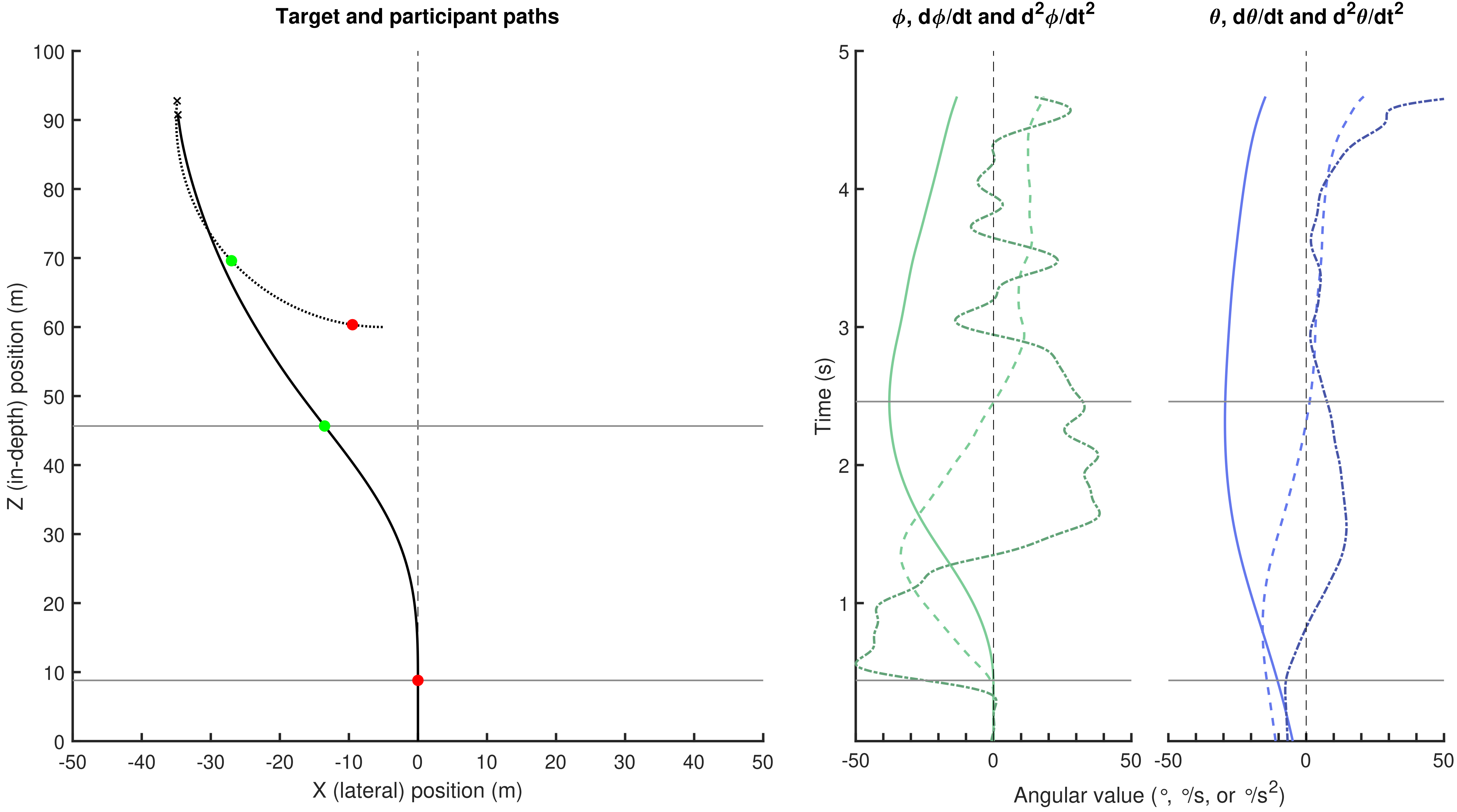

P3/B1
S5/R10-OUT

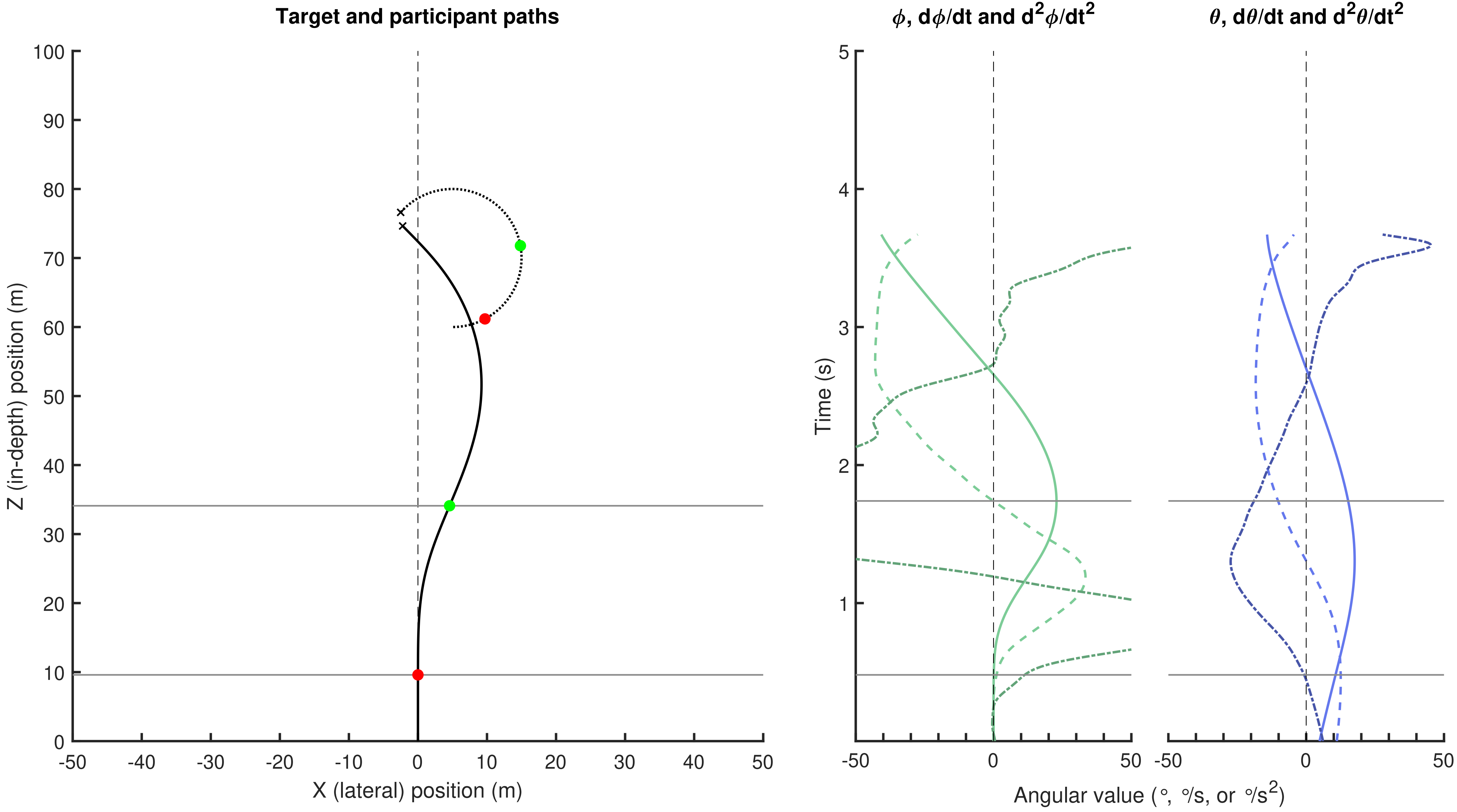

P3/B1
S5/R10-IN

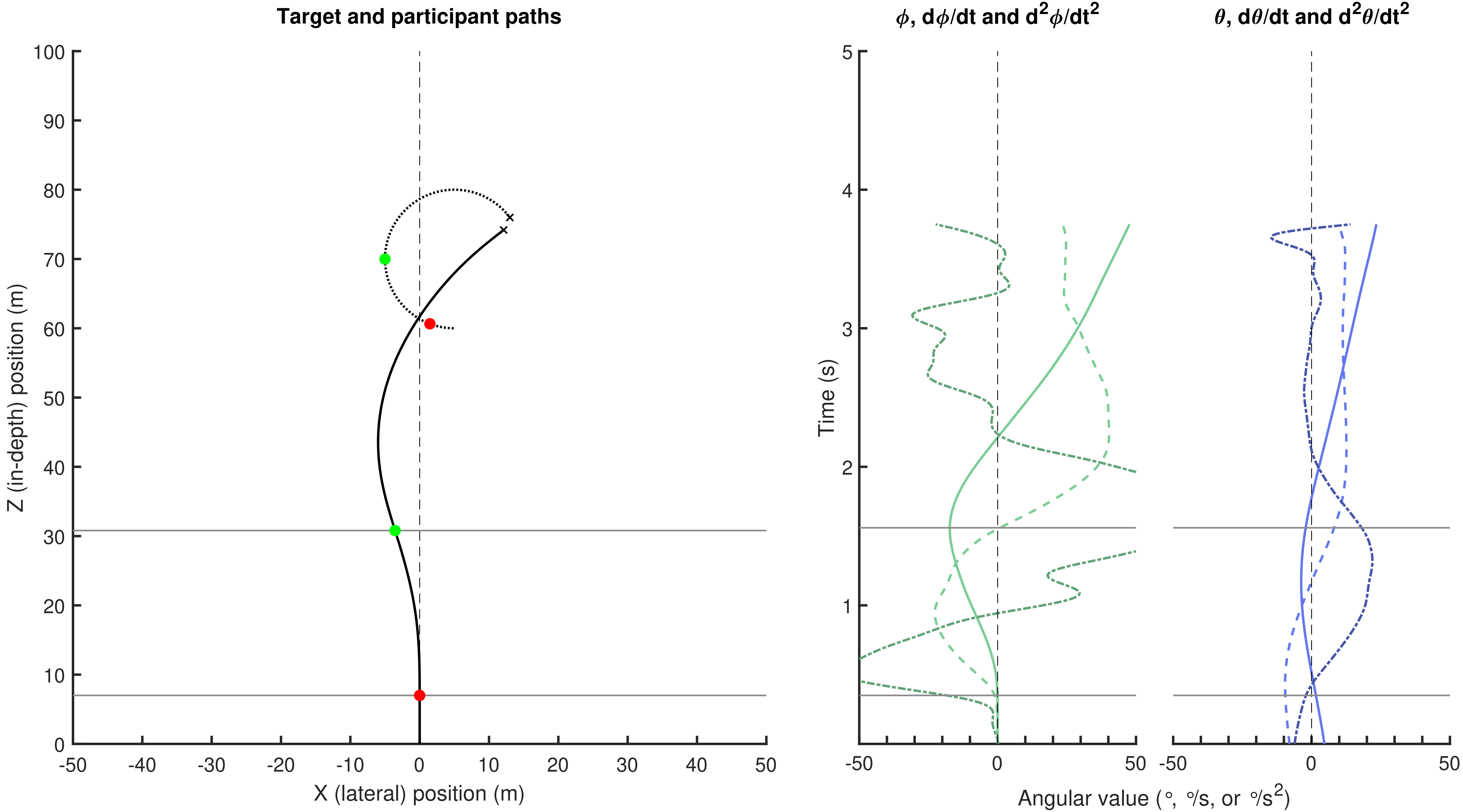

P3/B1
S5/R20-OUT

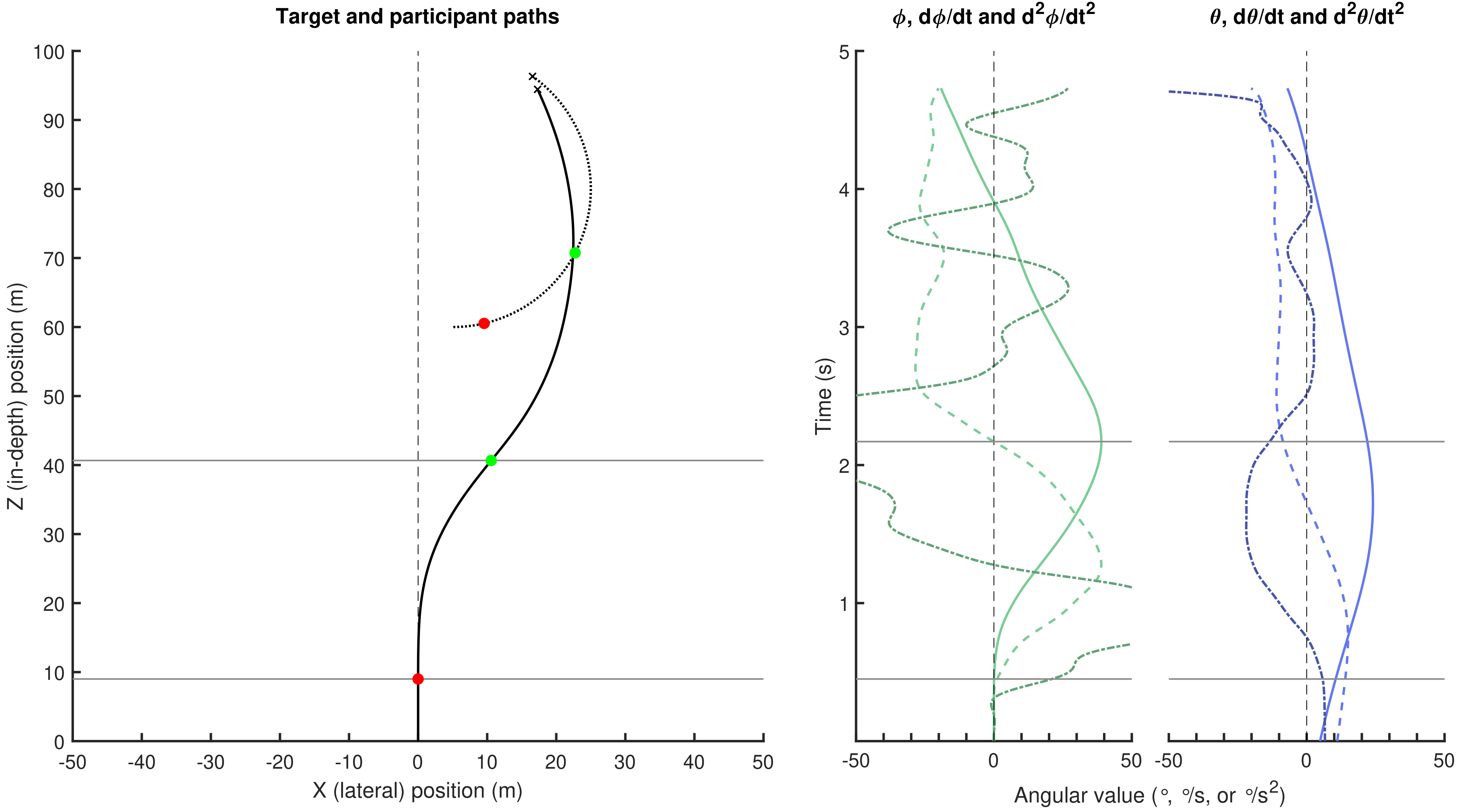

P3/B1
S5/R20-IN

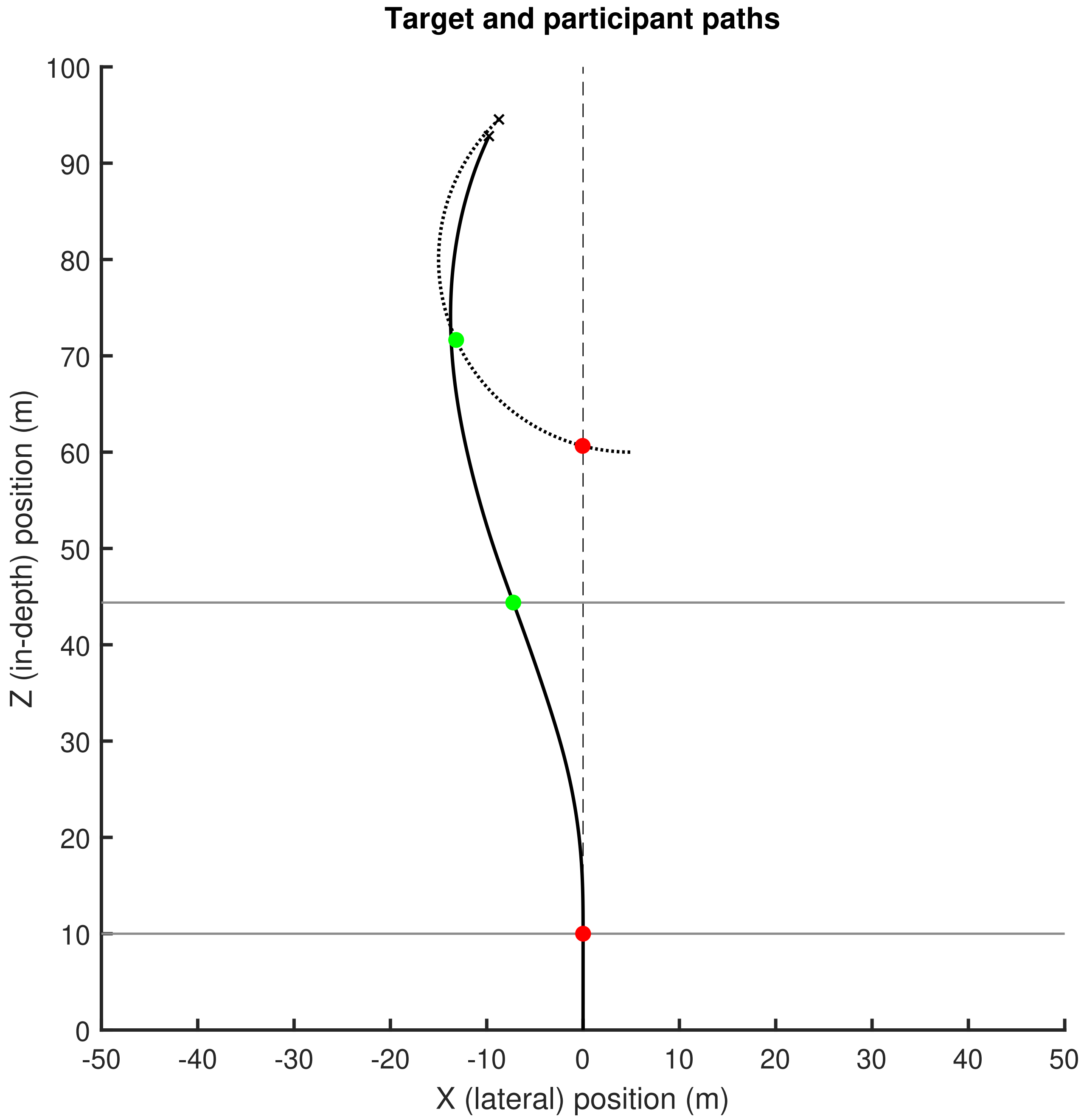

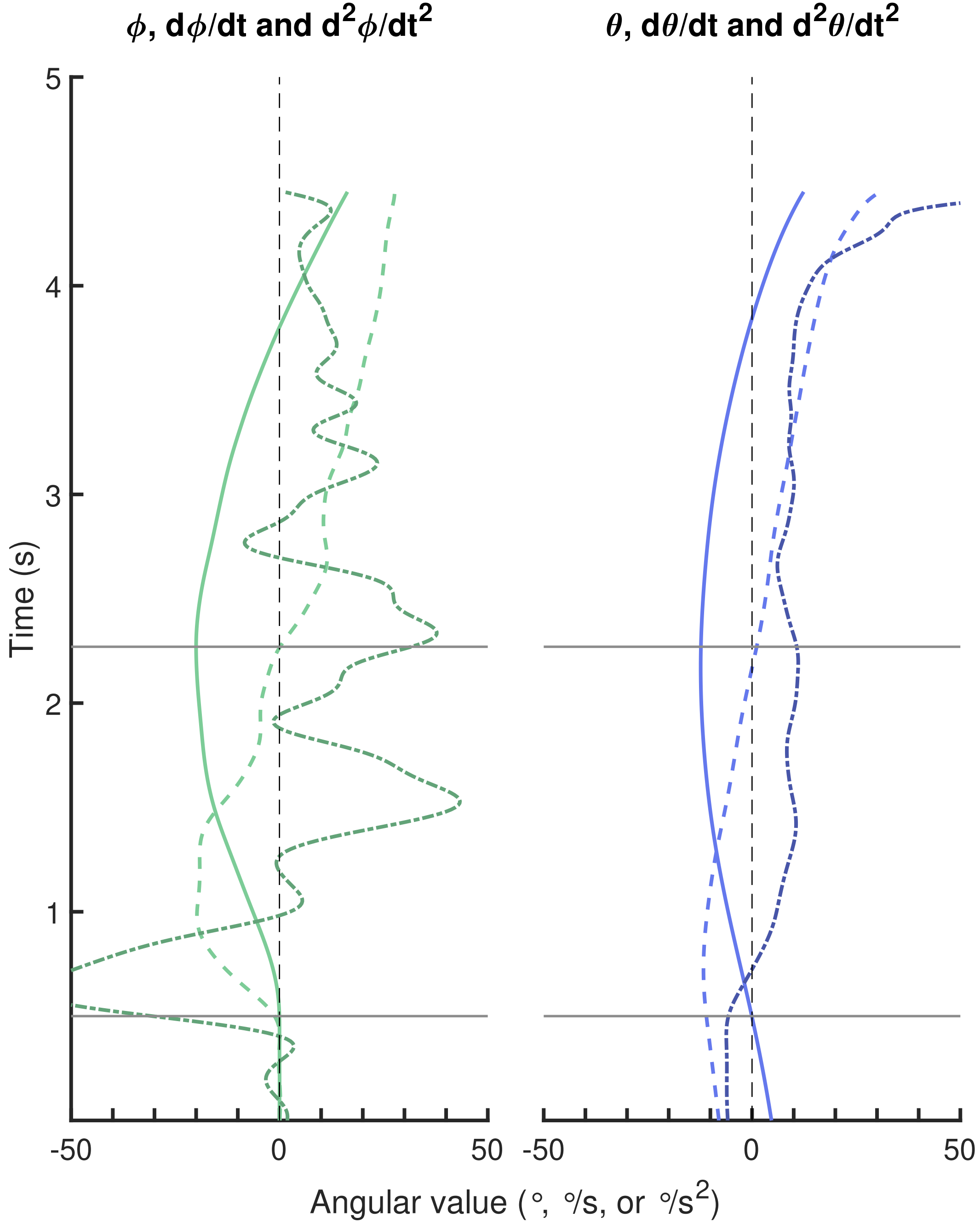

P3/B1
S5/R30-OUT

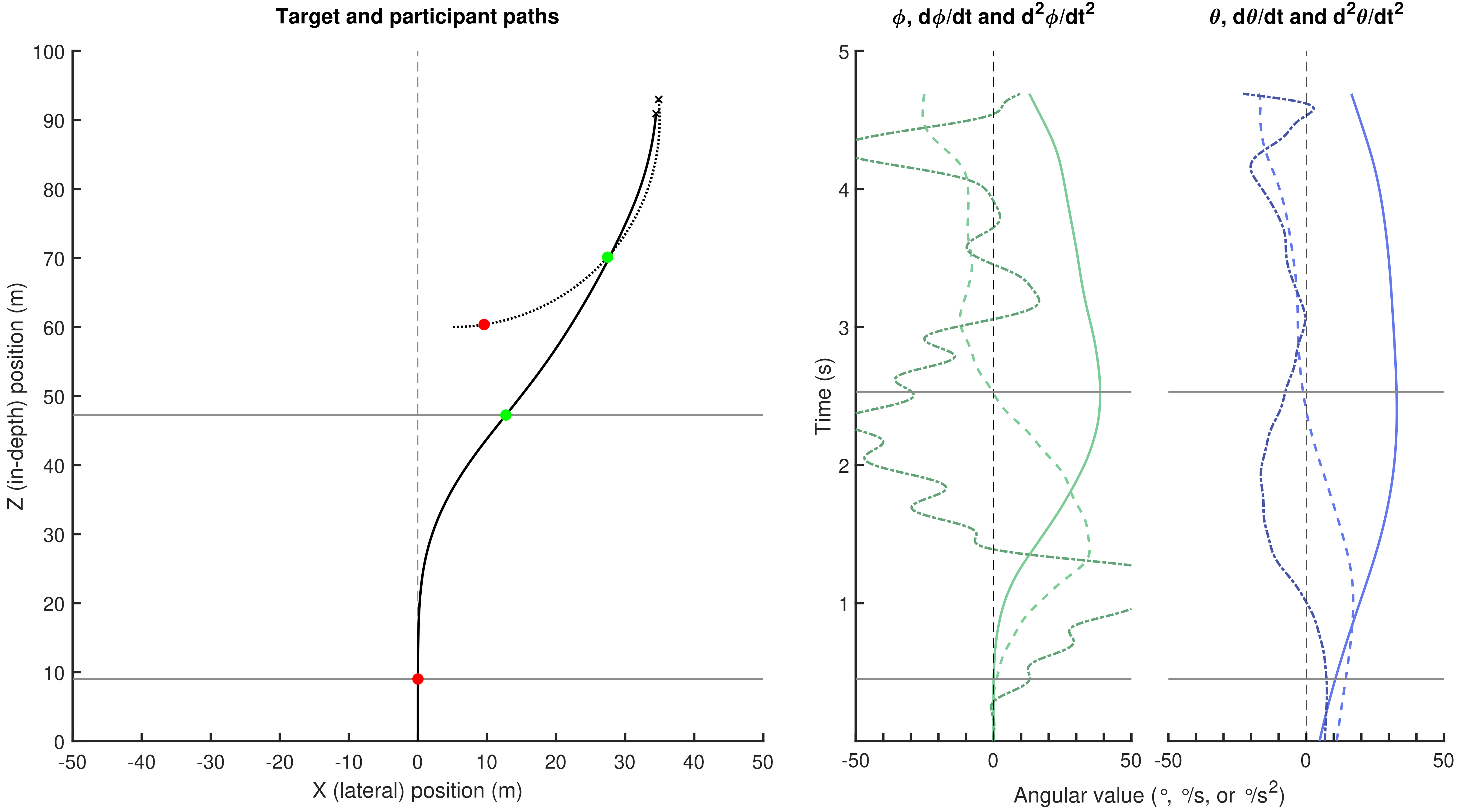

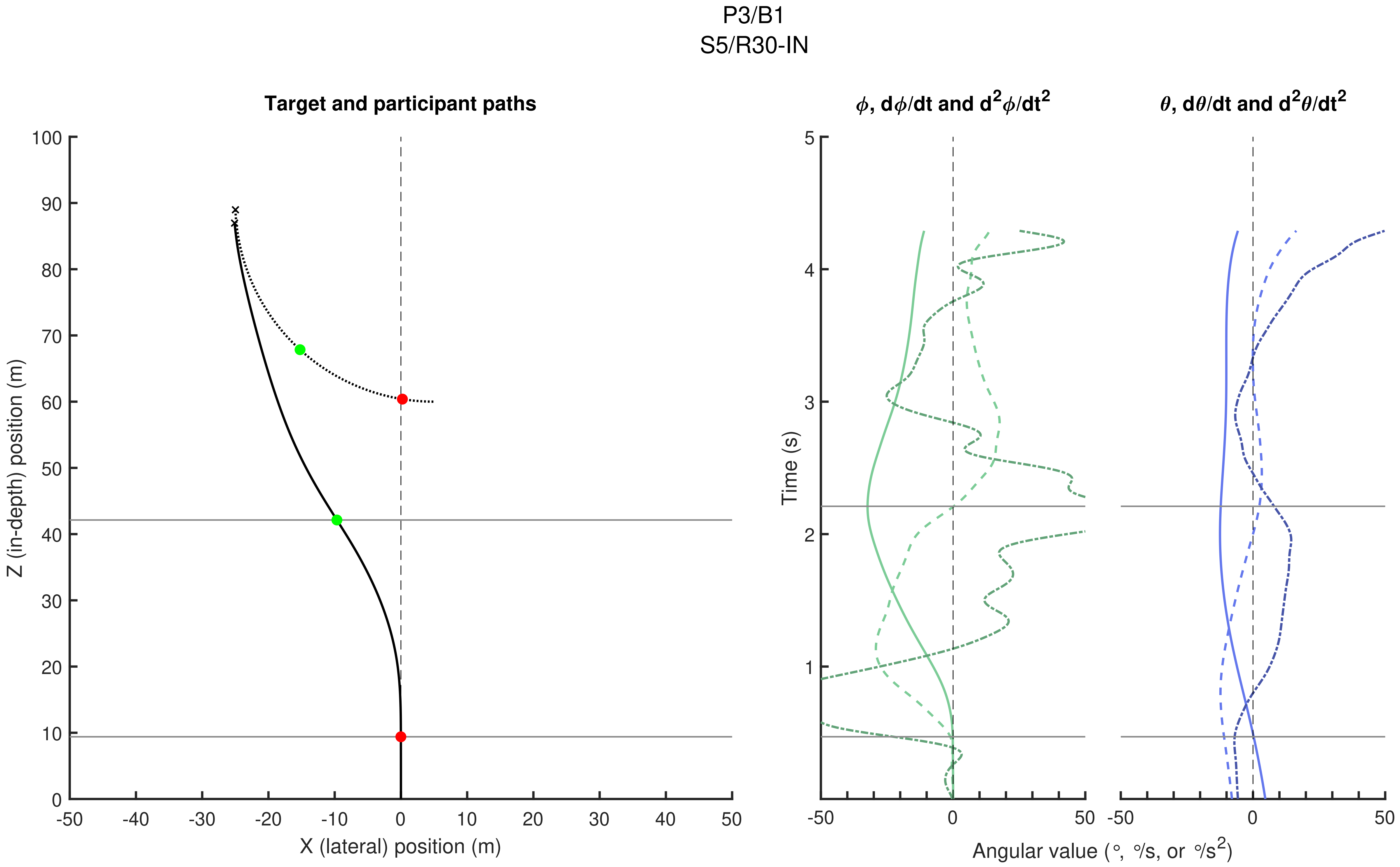
P3/B1
S5/R30-IN
Target and participant paths
Z (in-depth) position (m)
X (lateral) position (m)
φ, dφ/dt and d²φ/dt²
θ, dθ/dt and d²θ/dt²
Time (s)
Angular value (°, °/s, or °/s²)

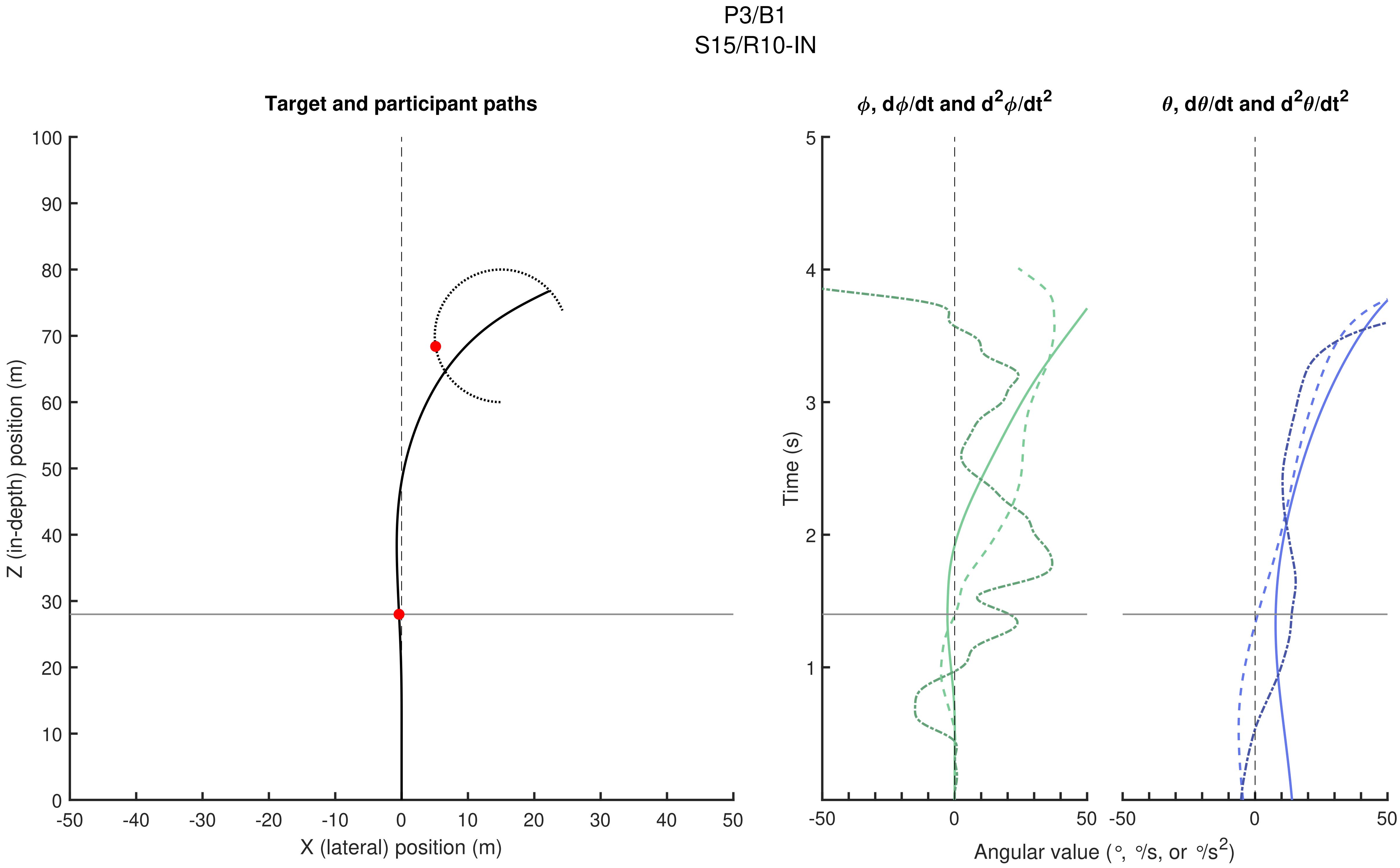
P3/B1
S15/R10-IN
Target and participant paths
ϕ, dϕ/dt and d$^2\phi$/dt^2
θ, dθ/dt and d$^2\theta$/dt^2
Z (in-depth) position (m)
X (lateral) position (m)
Time (s)
Angular value (°, °/s, or °/s^2)
100
90
80
70
60
50
40
30
20
10
0
-50
-40
-30
-20
-10
0
10
20
30
40
50
5
4
3
2
1
-50
0
50
-50
0
50

P3/B1
S15/R20-IN

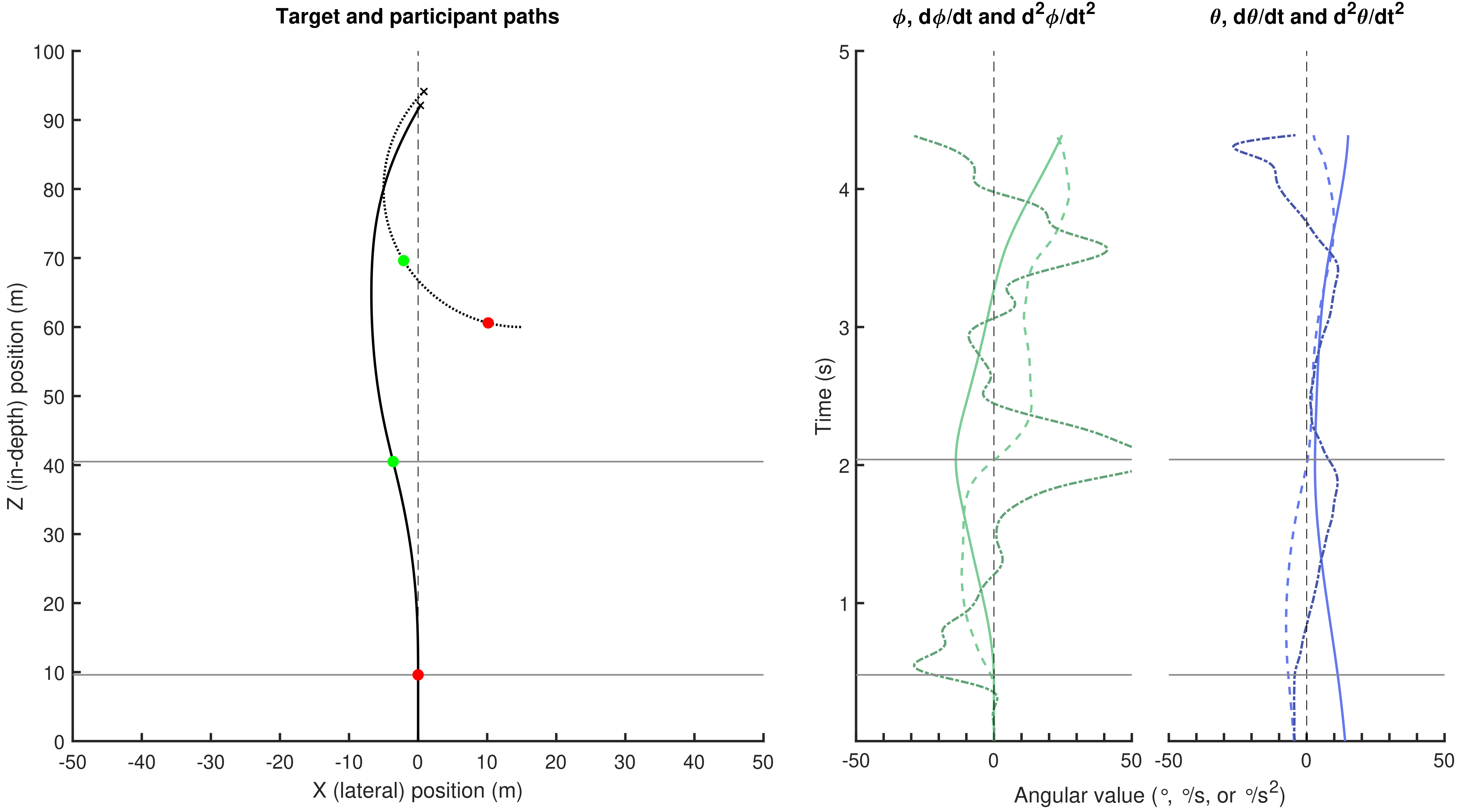

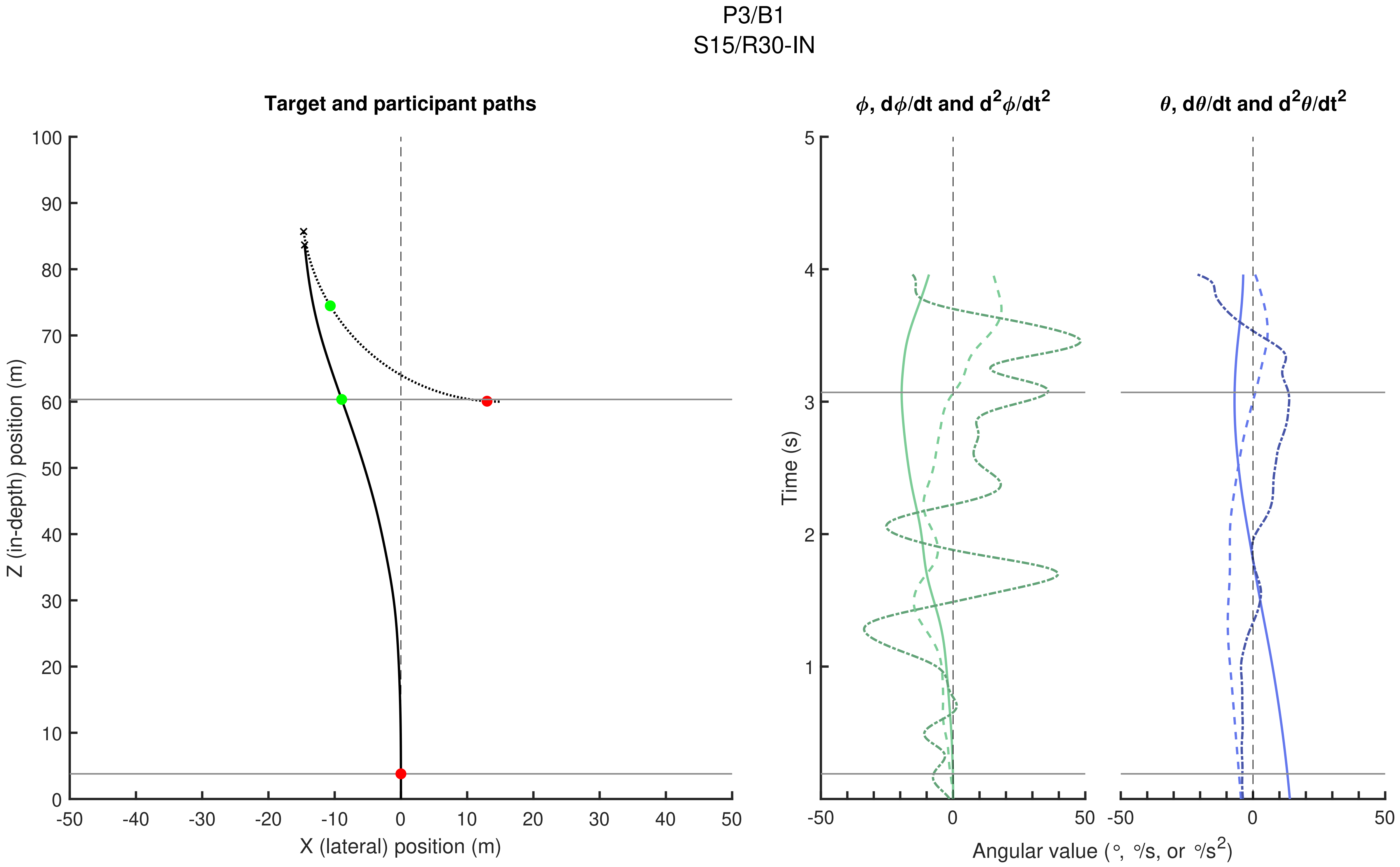

P3/B1
S15/R30-IN
Target and participant paths
Z (in-depth) position (m)
X (lateral) position (m)
ϕ, dϕ/dt and d$^2\phi$/dt^2
θ, dθ/dt and d$^2\theta$/dt^2
Time (s)
Angular value (°, °/s, or °/s^2)

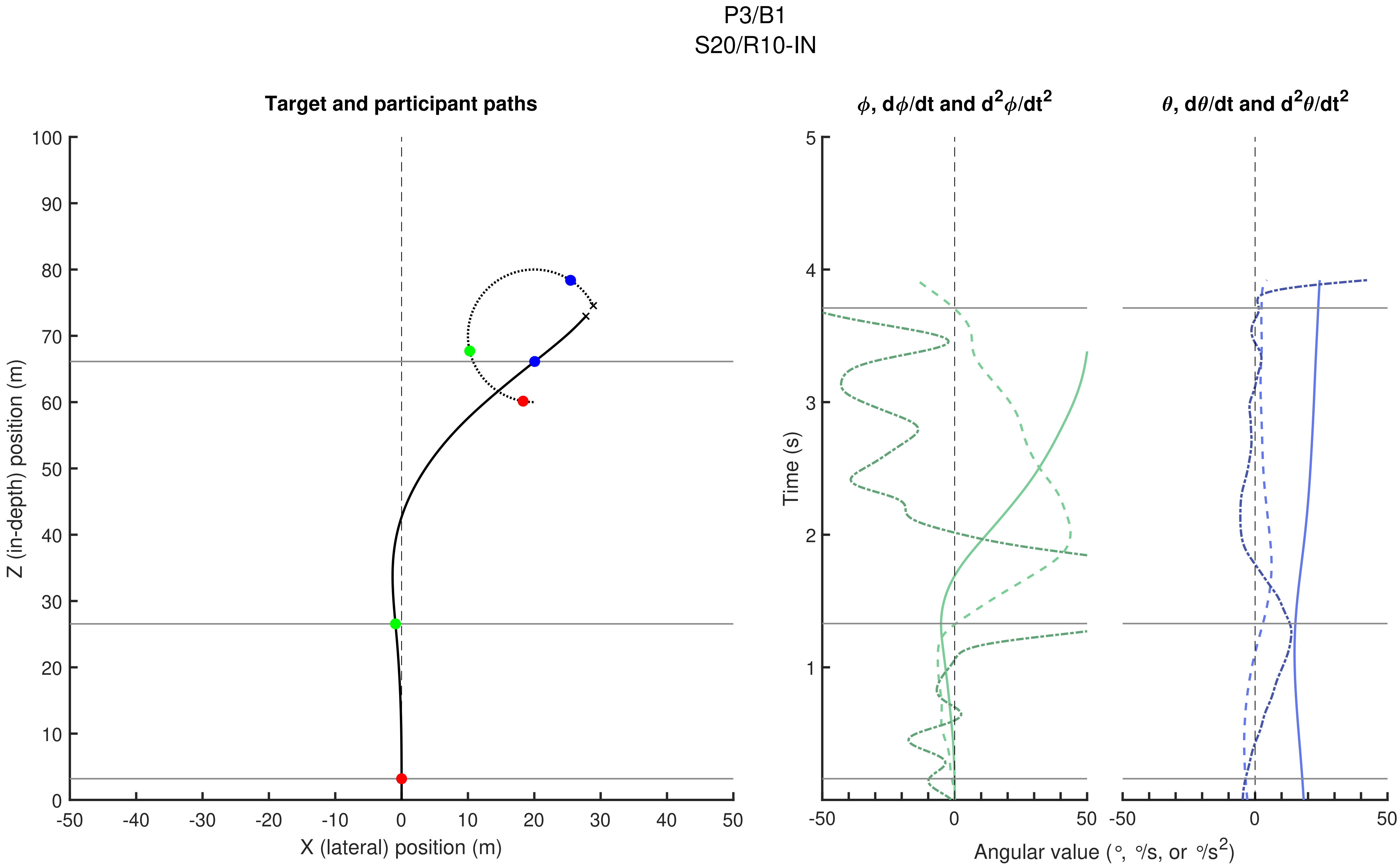

P3/B1
S20/R10-IN
Target and participant paths
Z (in-depth) position (m)
X (lateral) position (m)
100
90
80
70
60
50
40
30
20
10
0
-50
-40
-30
-20
-10
0
10
20
30
40
50
ϕ, dϕ/dt and d$^2\phi$/dt^2
θ, dθ/dt and d$^2\theta$/dt^2
Time (s)
5
4
3
2
1
-50
0
50
-50
0
50
Angular value (°, °/s, or °/s^2)

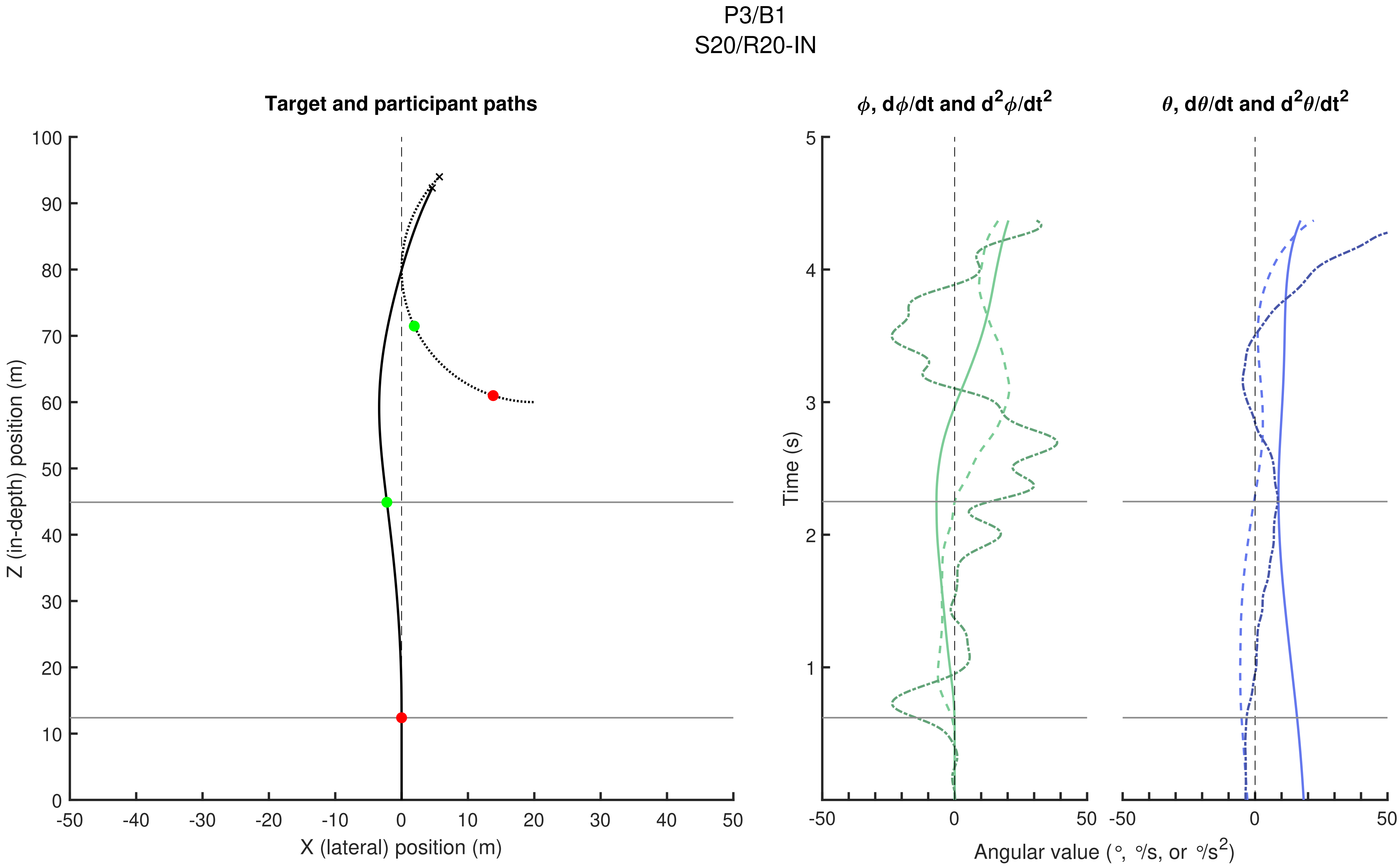
P3/B1
S20/R20-IN
Target and participant paths
Z (in-depth) position (m)
X (lateral) position (m)
100
90
80
70
60
50
40
30
20
10
0
-50
-40
-30
-20
-10
0
10
20
30
40
50
ϕ, dϕ/dt and d²ϕ/dt²
θ, dθ/dt and d²θ/dt²
Time (s)
5
4
3
2
1
-50
0
50
Angular value (°, °/s, or °/s²)

P3/B1
S20/R30-IN

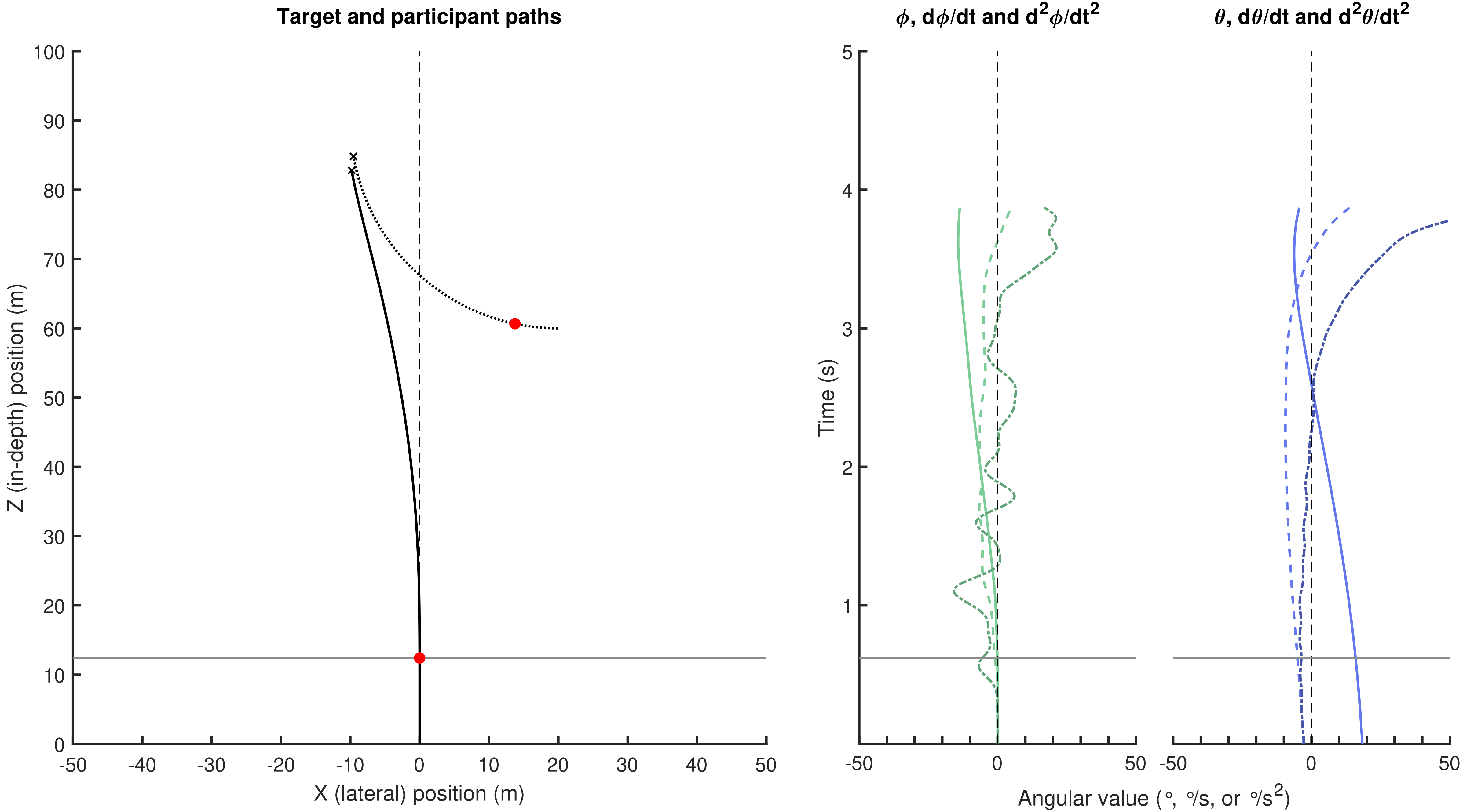

P3/B2
S20/R10-IN

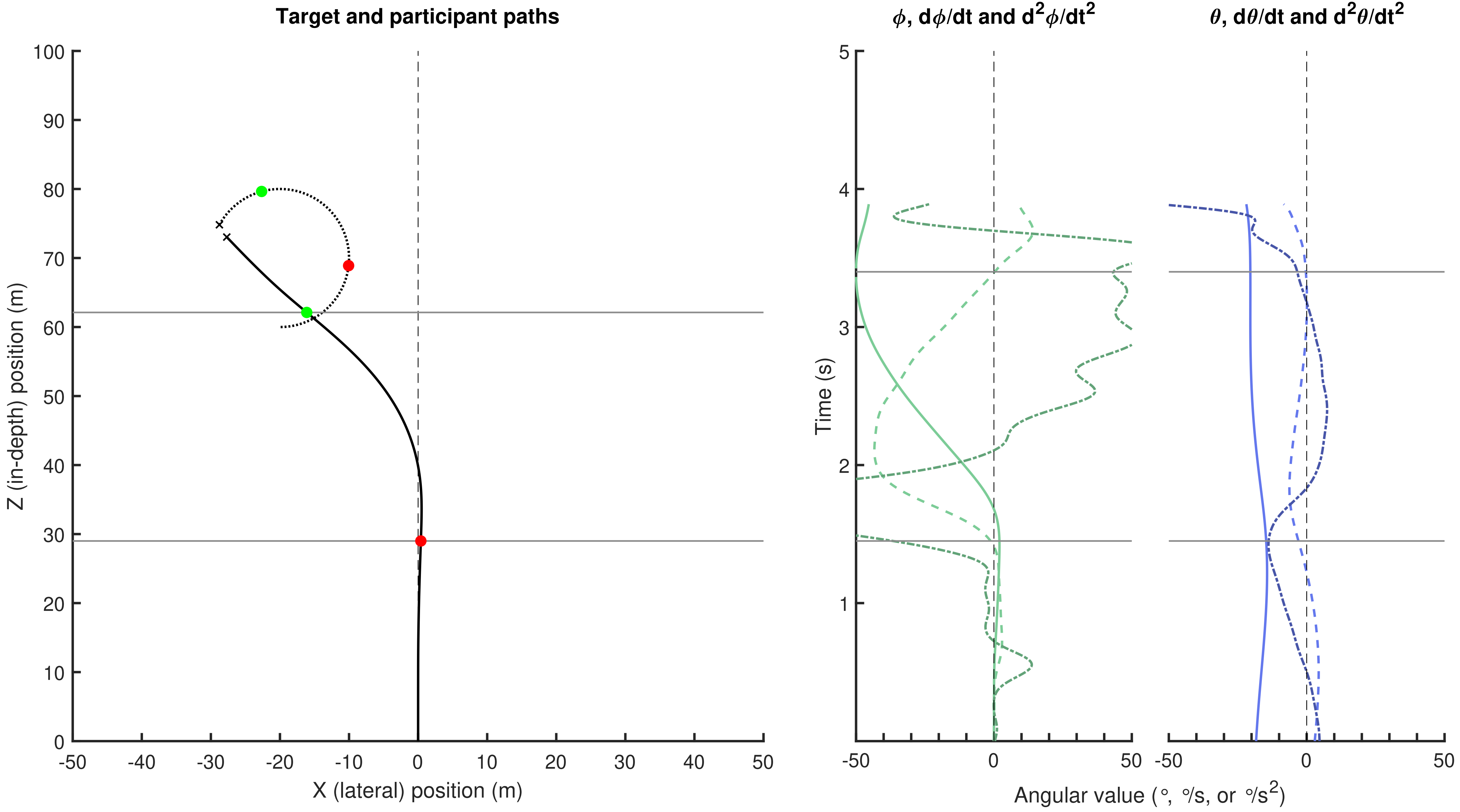

P3/B2
S20/R20-IN

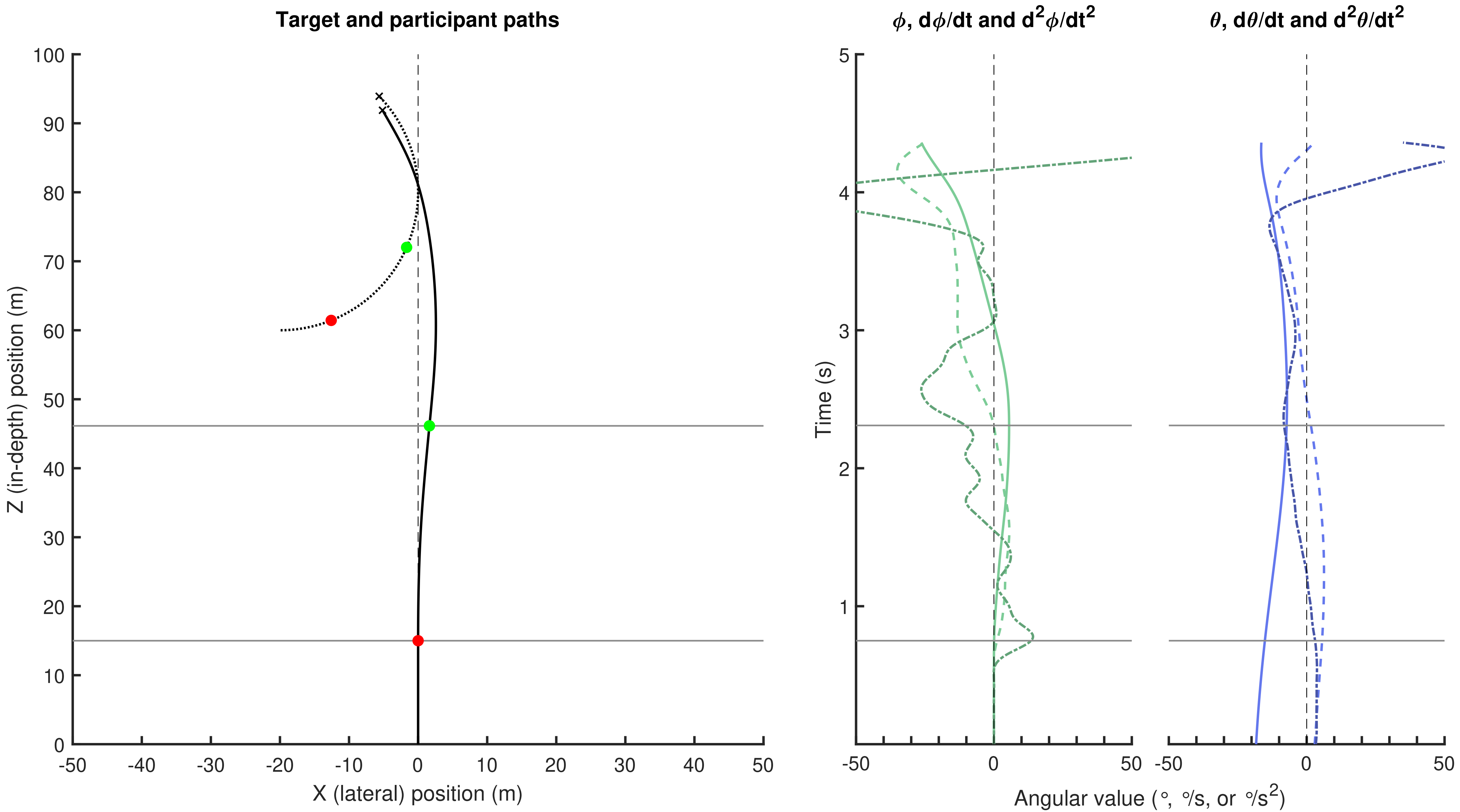

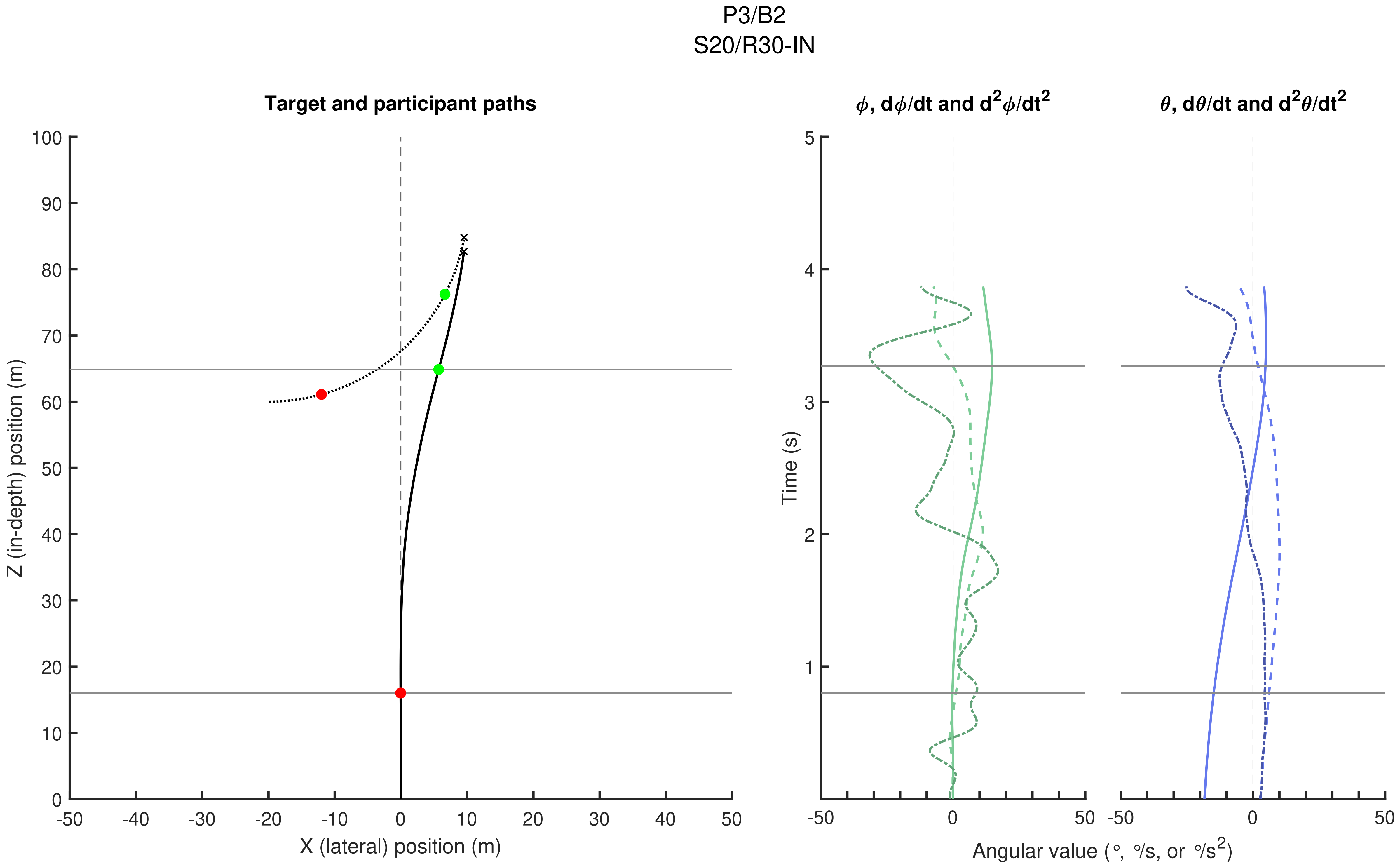
P3/B2
S20/R30-IN
Target and participant paths
Z (in-depth) position (m)
X (lateral) position (m)
ϕ, dϕ/dt and d$^2\phi$/dt^2
θ, dθ/dt and d$^2\theta$/dt^2
Time (s)
Angular value (°, °/s, or °/s^2)

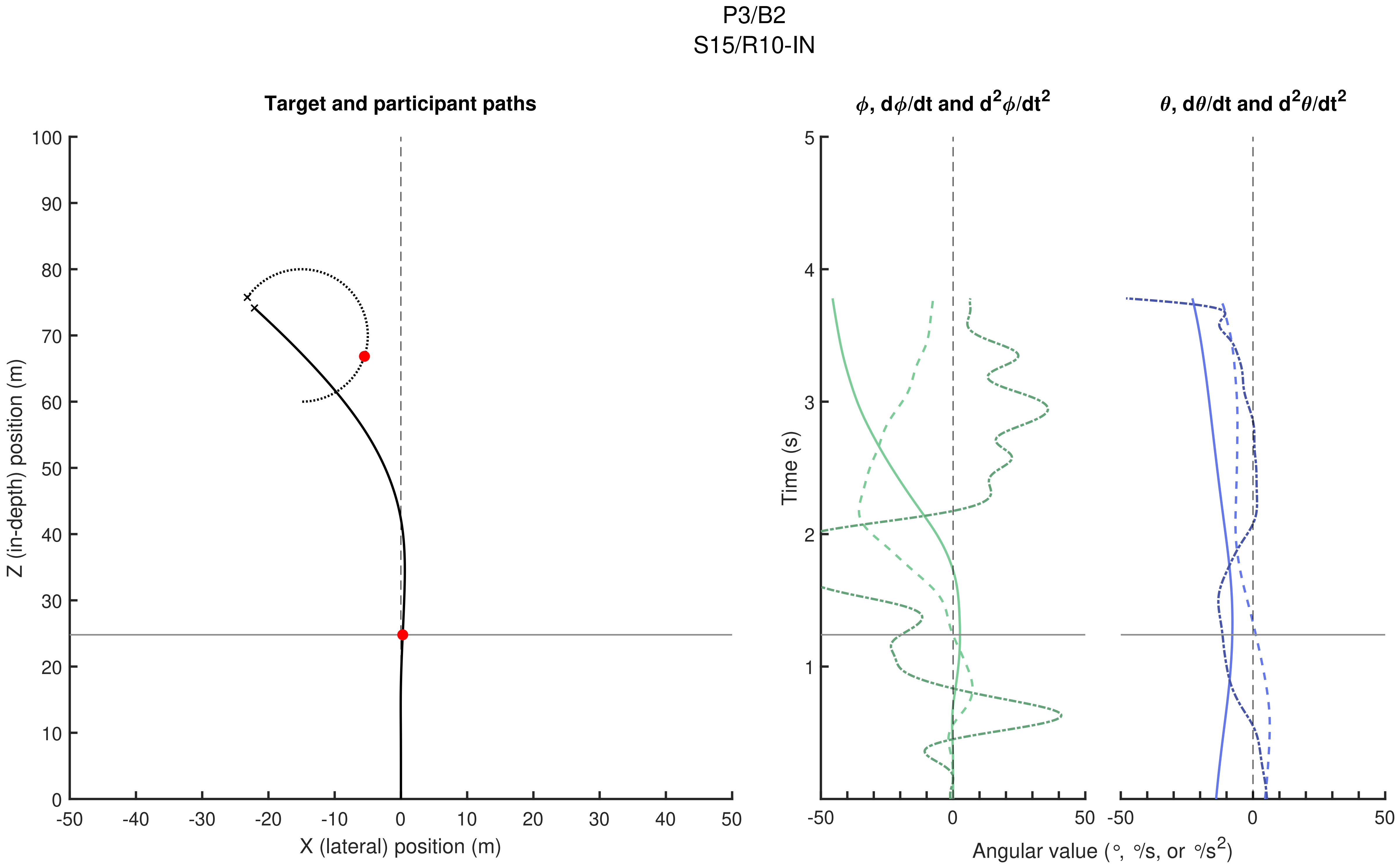

P3/B2
S15/R10-IN
Target and participant paths
Z (in-depth) position (m)
X (lateral) position (m)
ϕ, dϕ/dt and d²ϕ/dt²
θ, dθ/dt and d²θ/dt²
Time (s)
Angular value (°, °/s, or °/s²)

P3/B2
S15/R20-IN

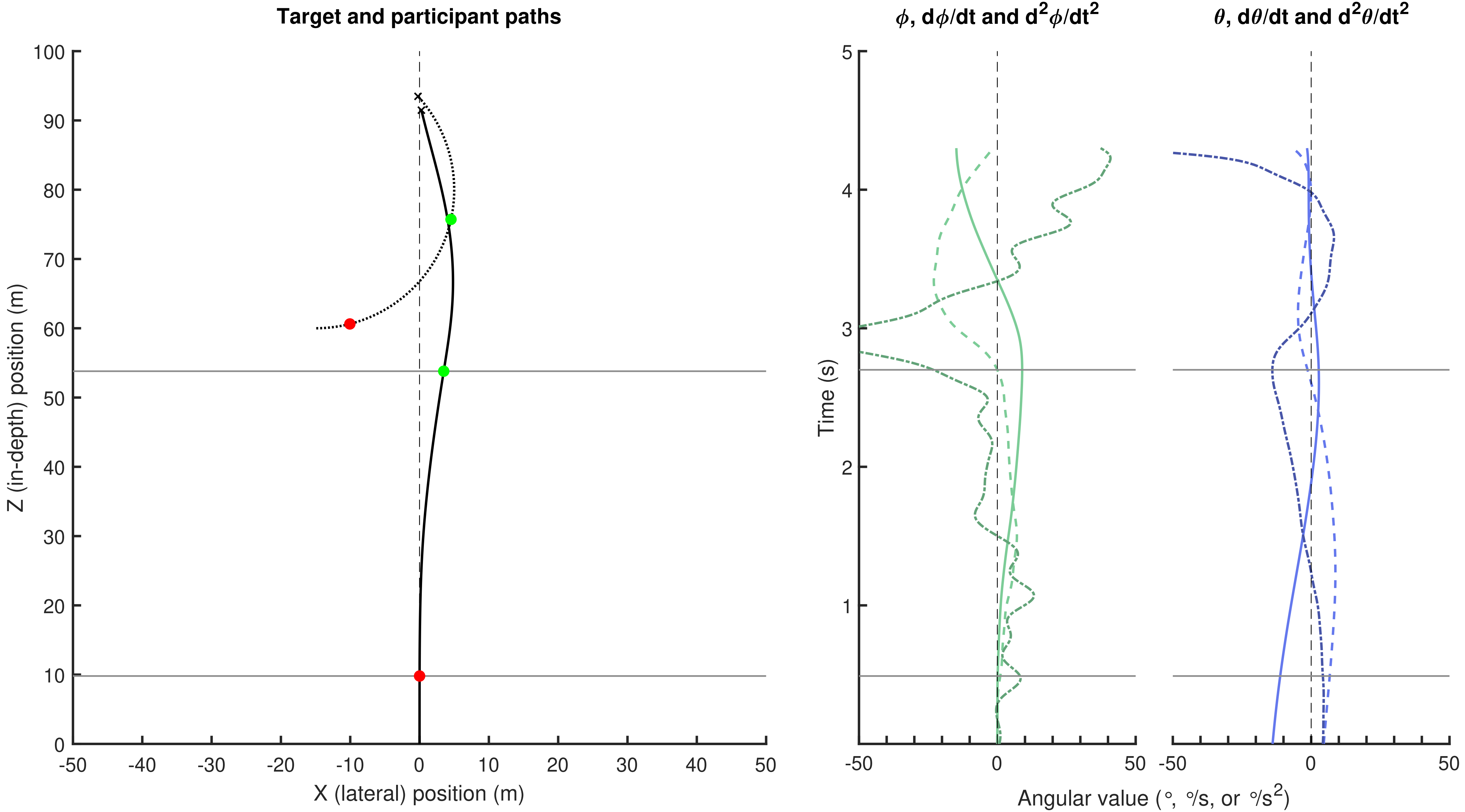

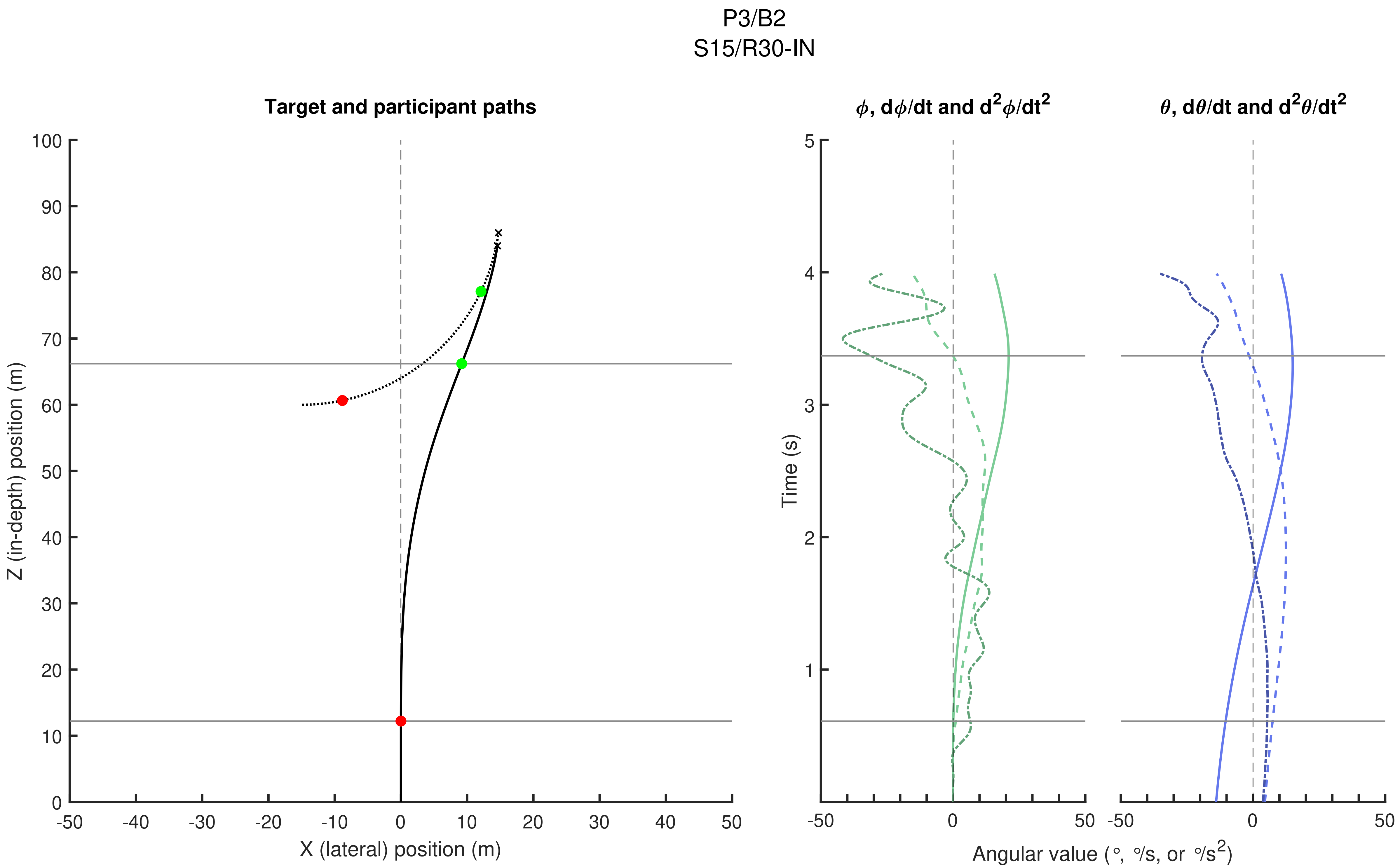
P3/B2
S15/R30-IN
Target and participant paths
Z (in-depth) position (m)
X (lateral) position (m)
ϕ, dϕ/dt and d²ϕ/dt²
θ, dθ/dt and d²θ/dt²
Time (s)
Angular value (°, °/s, or °/s²)

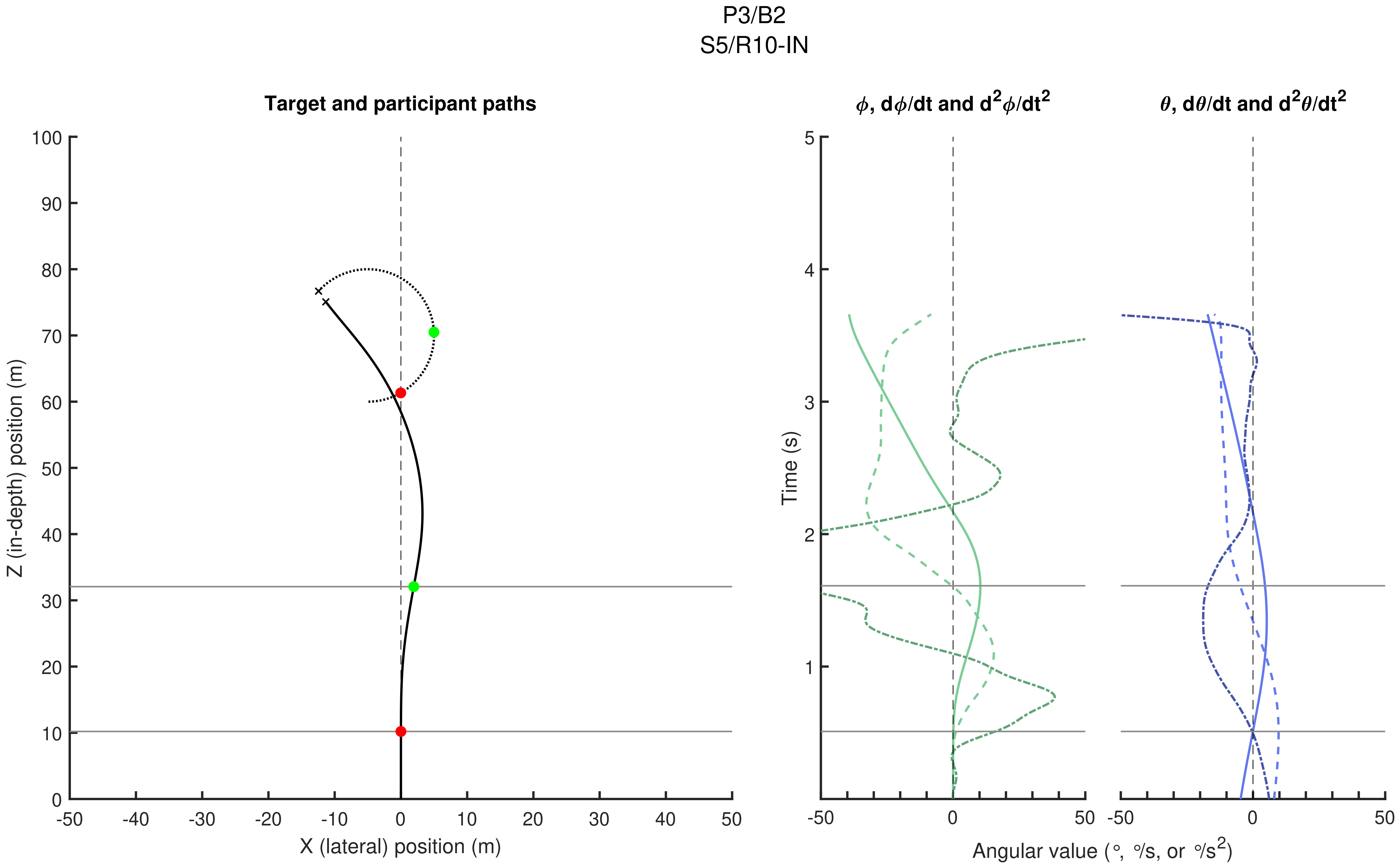
P3/B2
S5/R10-IN
Target and participant paths
Z (in-depth) position (m)
X (lateral) position (m)
0
10
20
30
40
50
60
70
80
90
100
-50
-40
-30
-20
-10
0
10
20
30
40
50
ϕ, dϕ/dt and d$^2\phi$/dt^2
θ, dθ/dt and d$^2\theta$/dt^2
Time (s)
1
2
3
4
5
-50
0
50
-50
0
50
Angular value (°, °/s, or °/s^2)

P3/B2
S5/R10-OUT

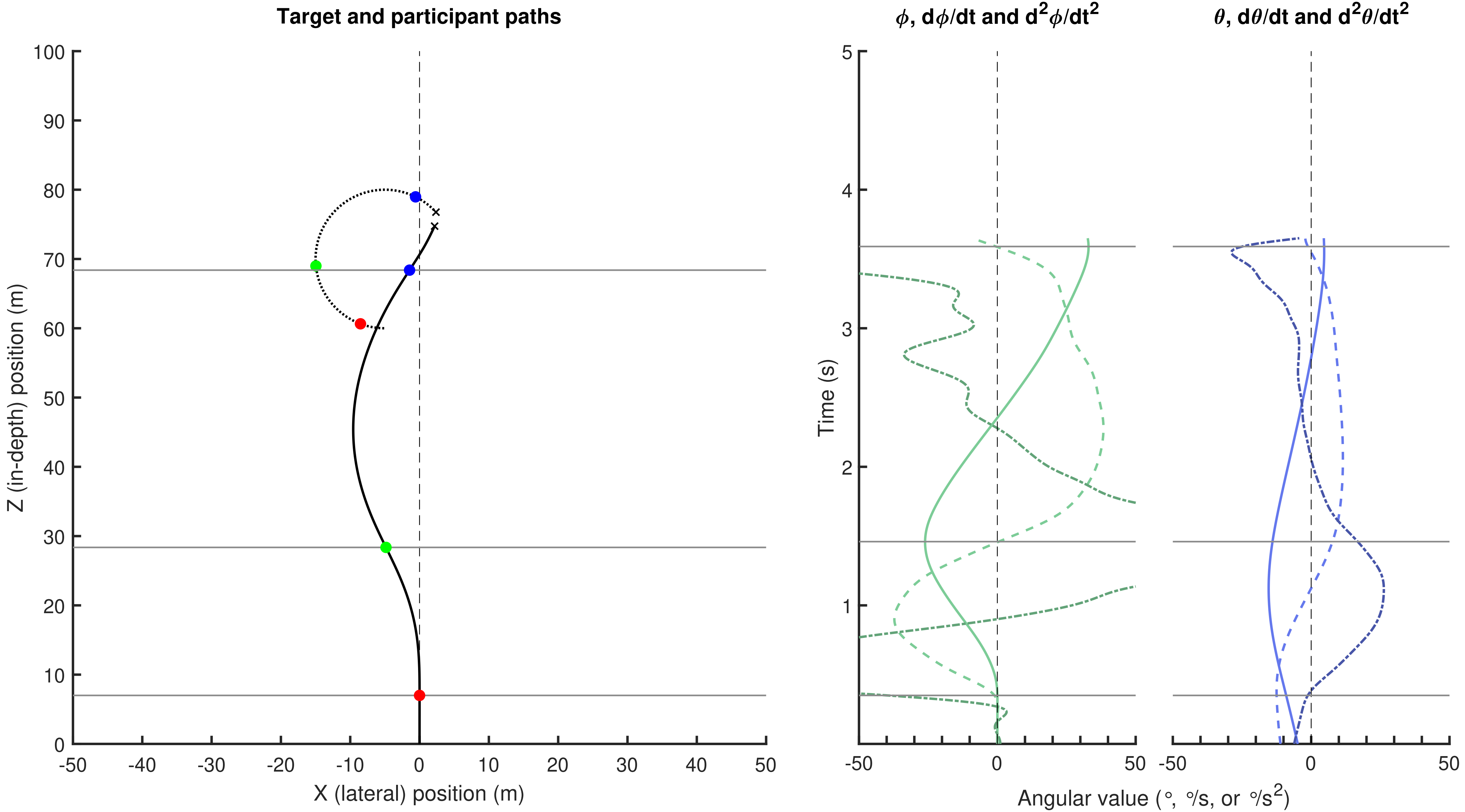

P3/B2
S5/R20-IN

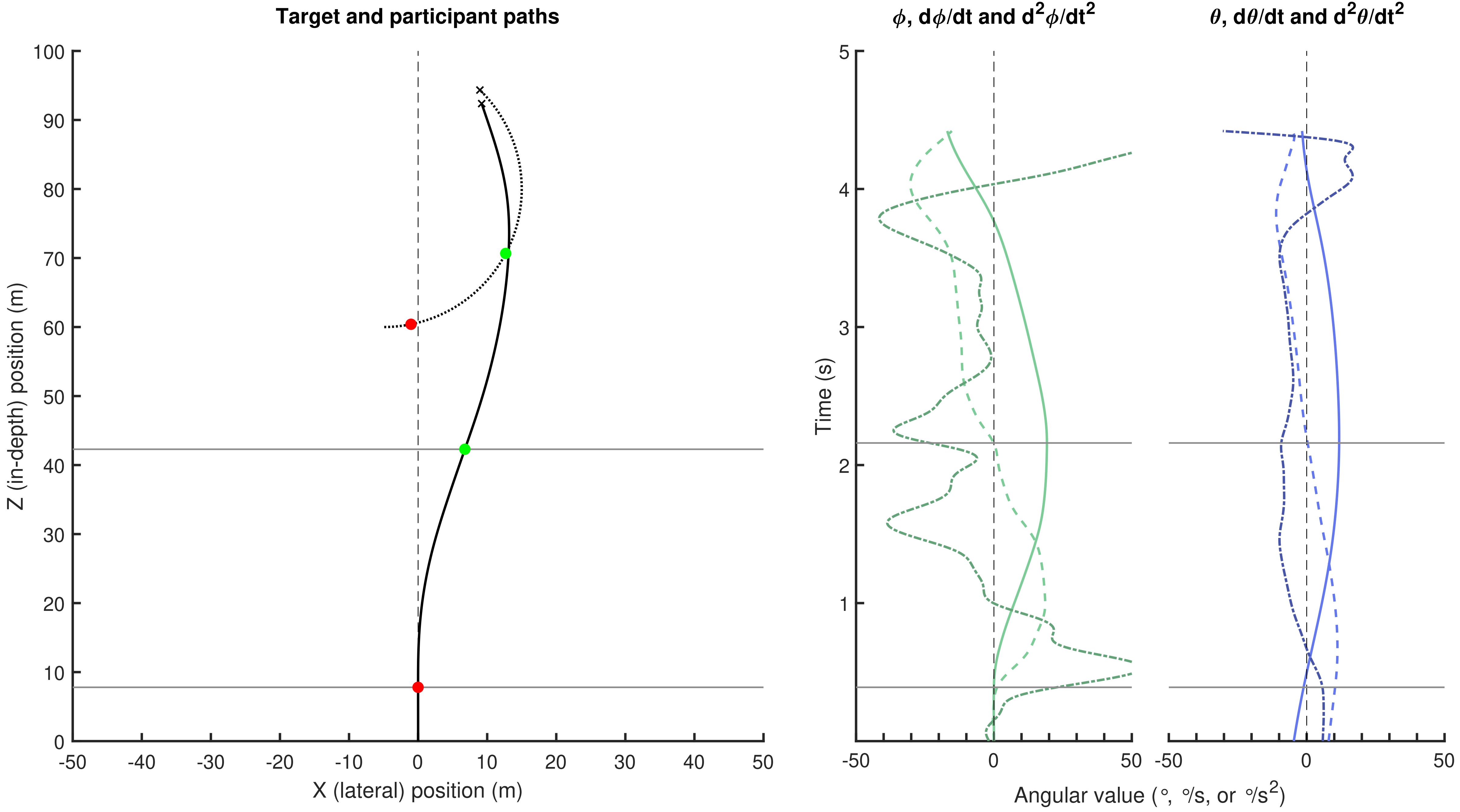

P3/B2
S5/R20-OUT

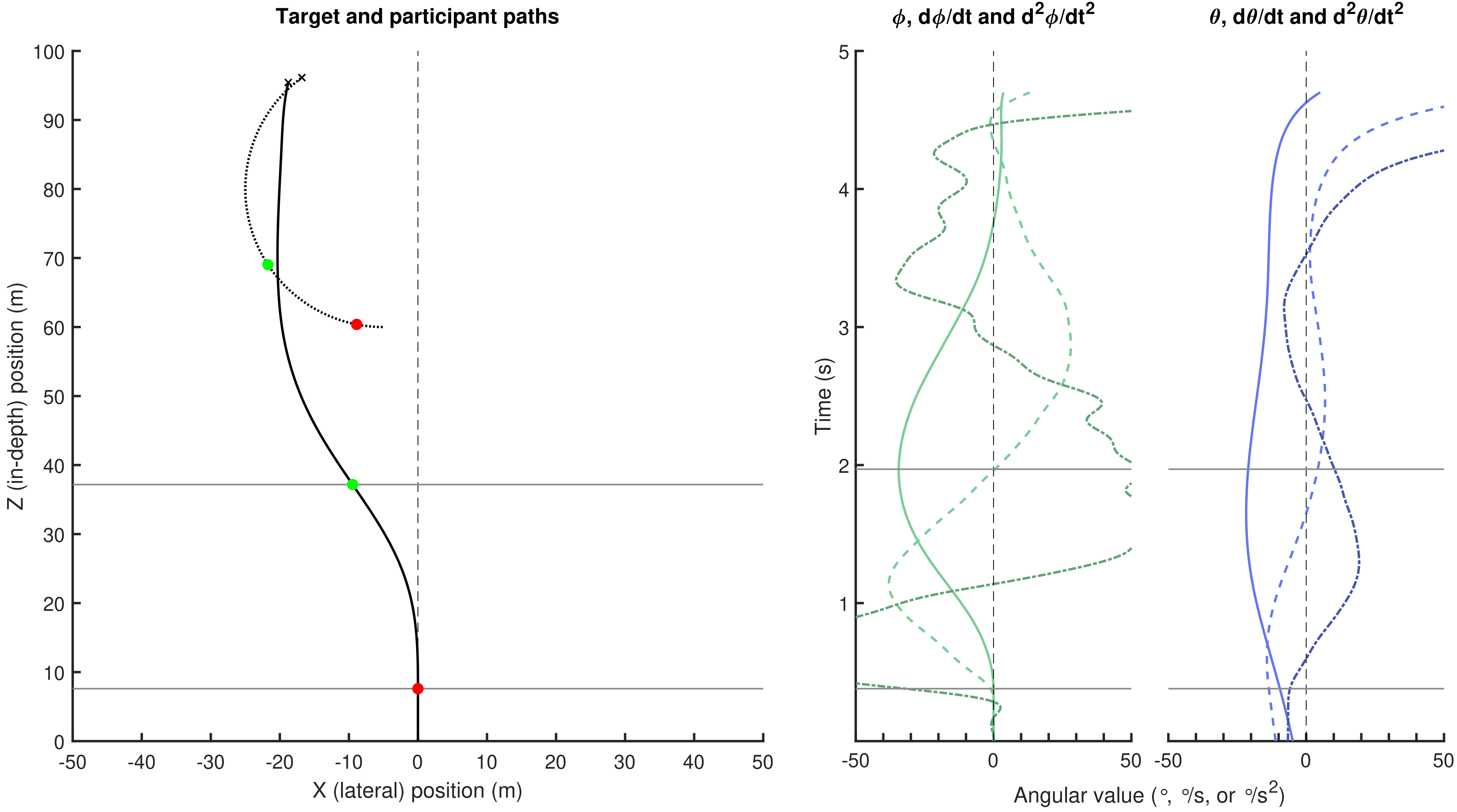

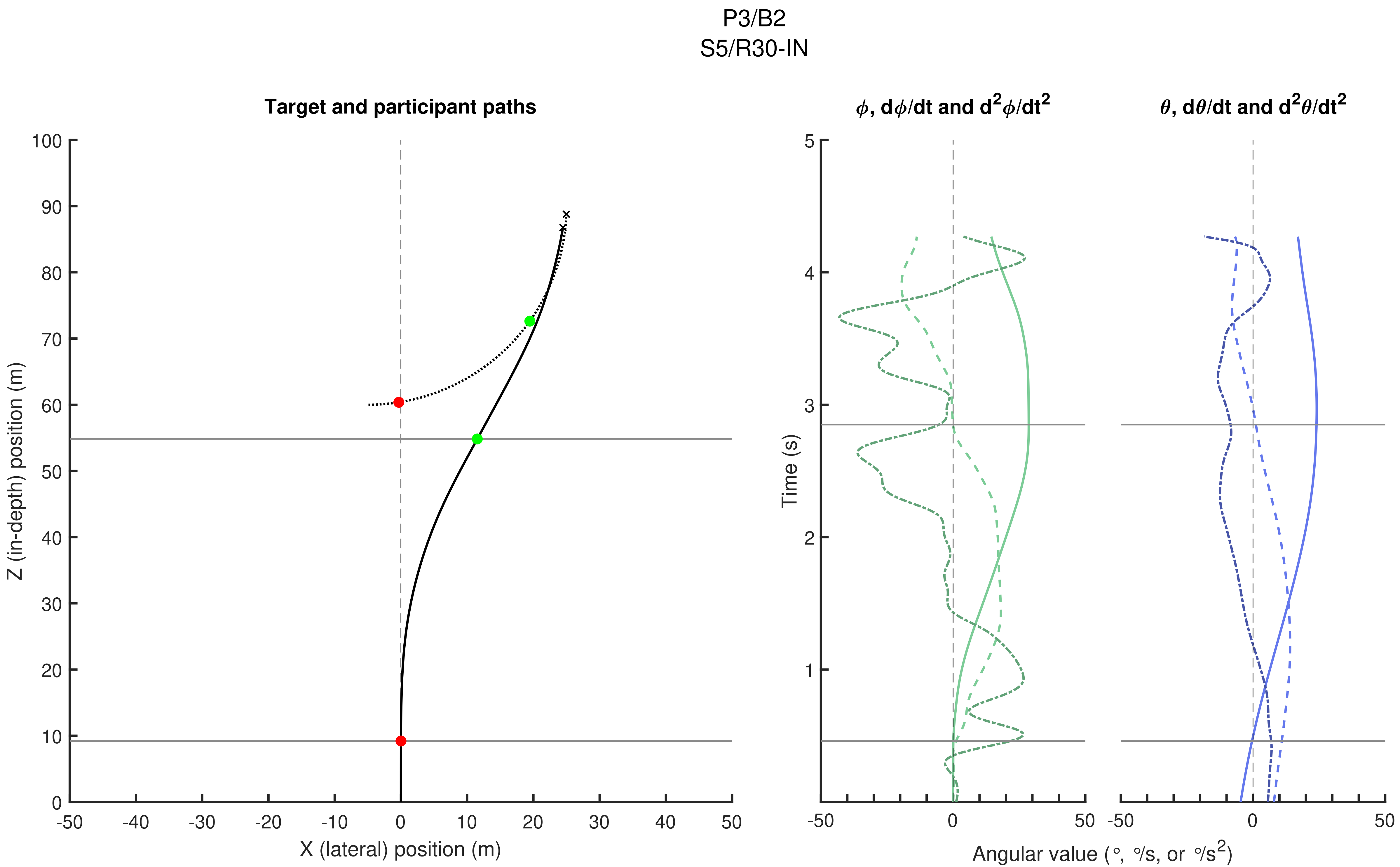

P3/B2
S5/R30-IN
Target and participant paths
Z (in-depth) position (m)
X (lateral) position (m)
ϕ, dϕ/dt and d$^2\phi$/dt^2
θ, dθ/dt and d$^2\theta$/dt^2
Time (s)
Angular value (°, °/s, or °/s^2)

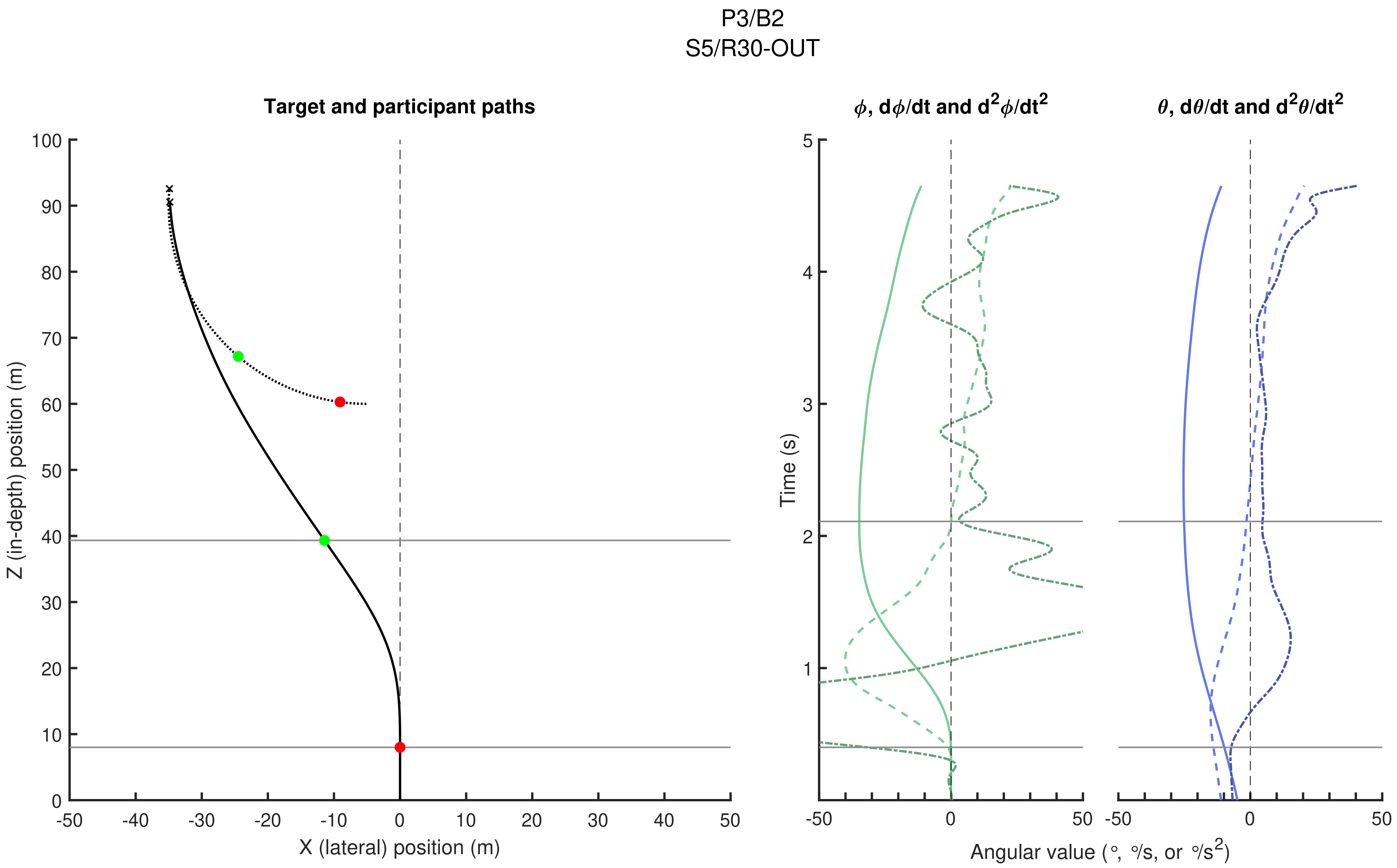

P3/B2
S5/R30-OUT
Target and participant paths
Z (in-depth) position (m)
X (lateral) position (m)
ϕ, dϕ/dt and d²ϕ/dt²
θ, dθ/dt and d²θ/dt²
Time (s)
Angular value (°, °/s, or °/s²)

P3/B2
S5/R10-OUT

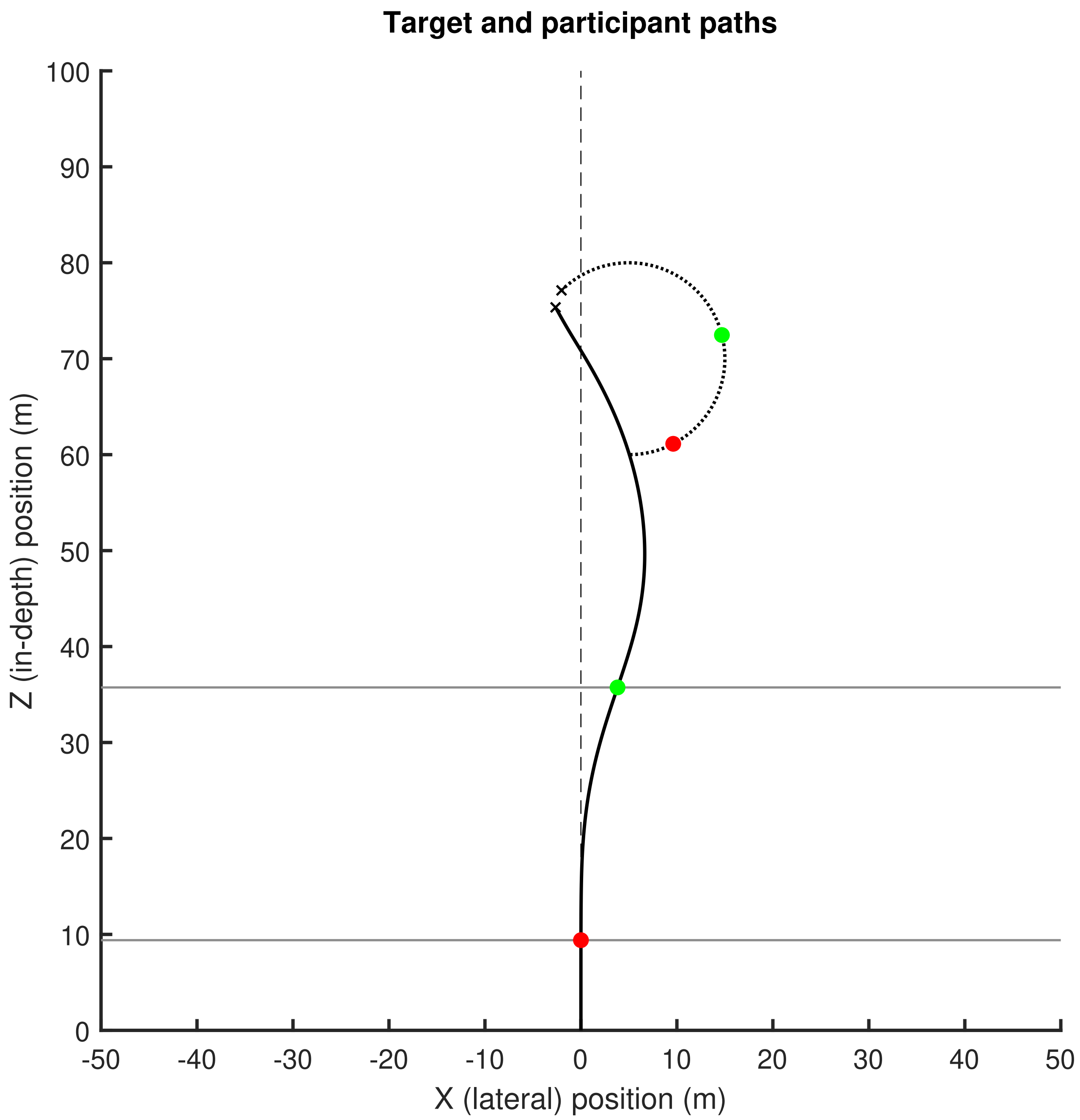

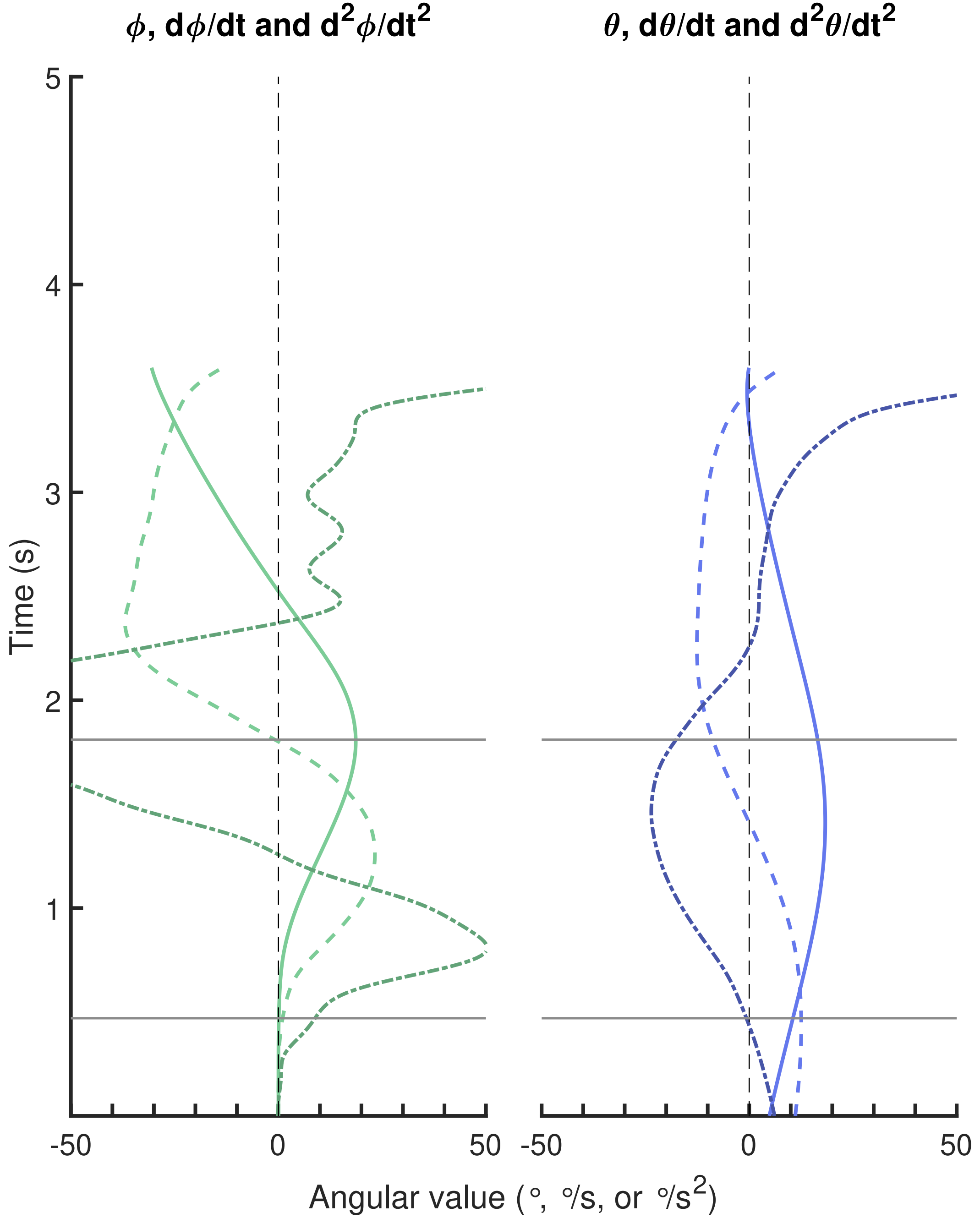

P3/B2
S5/R10-IN

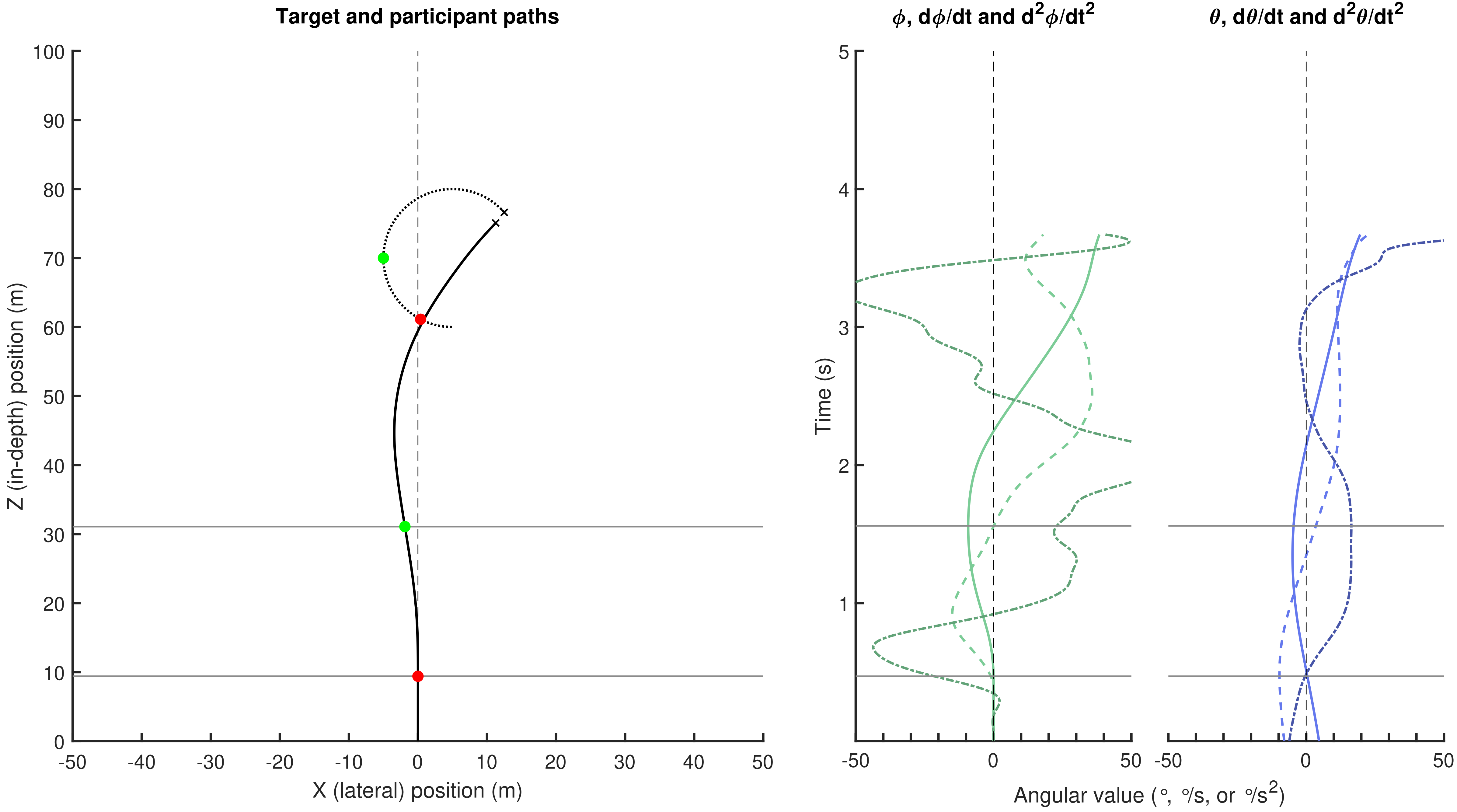

P3/B2
S5/R20-OUT

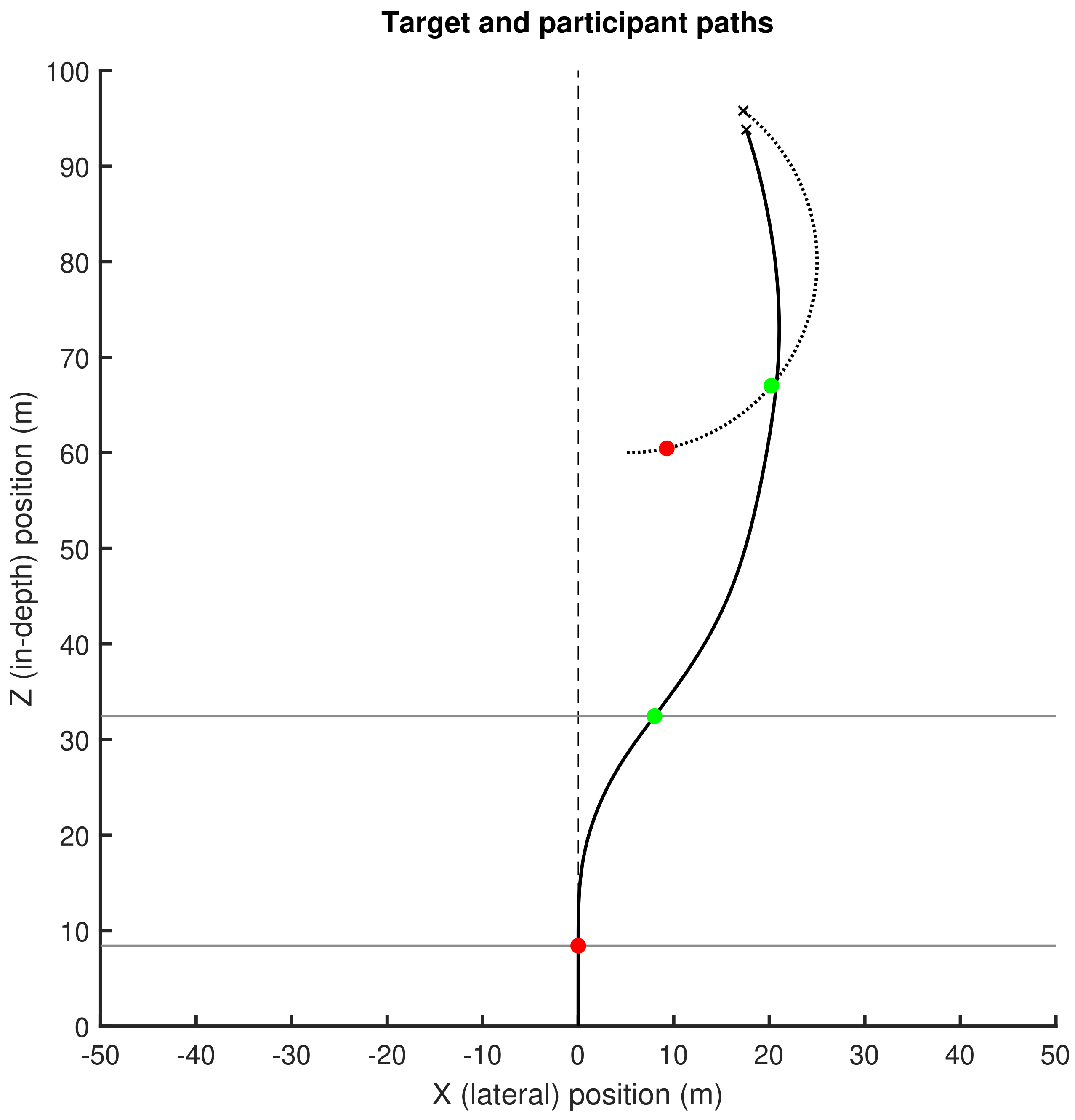

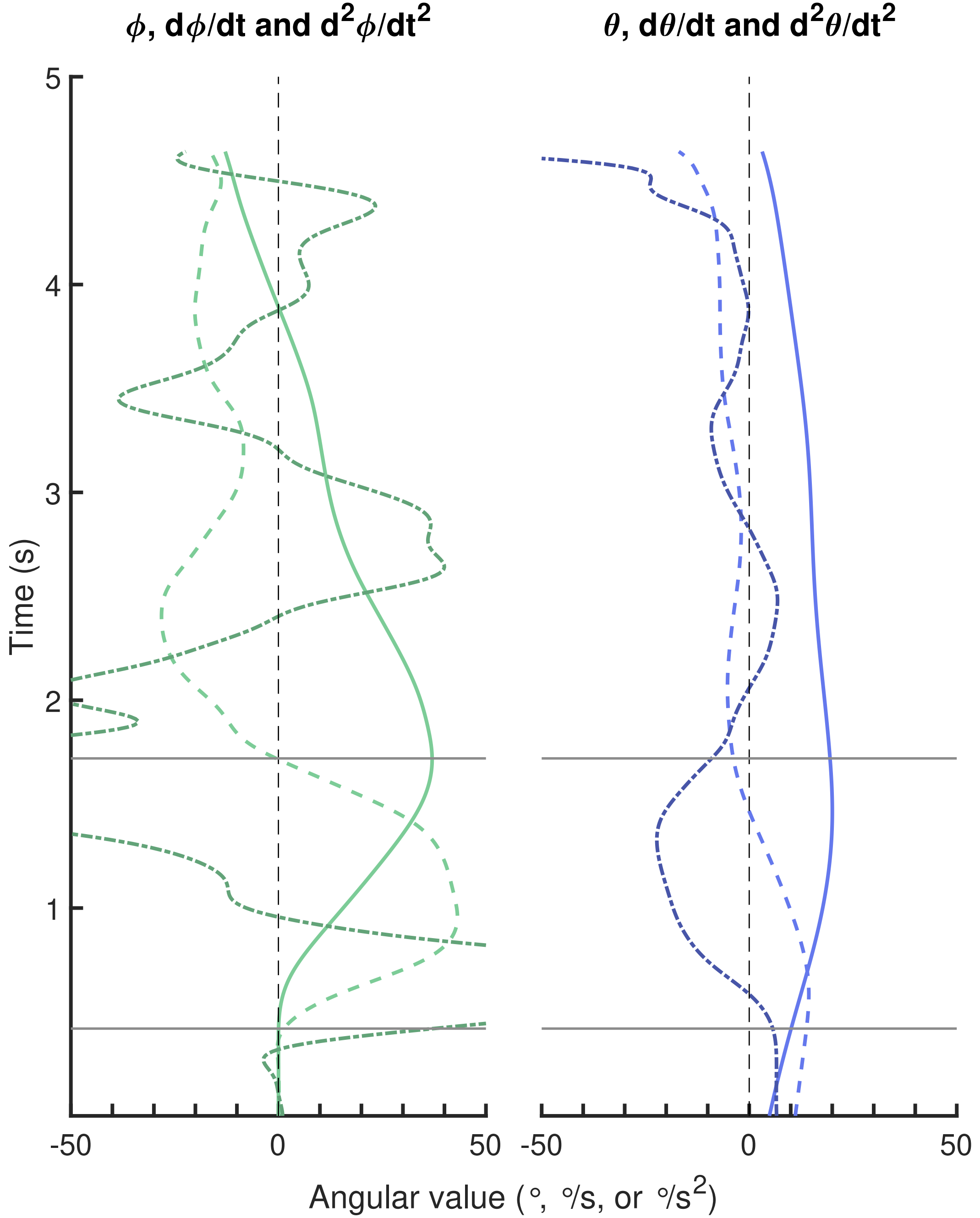

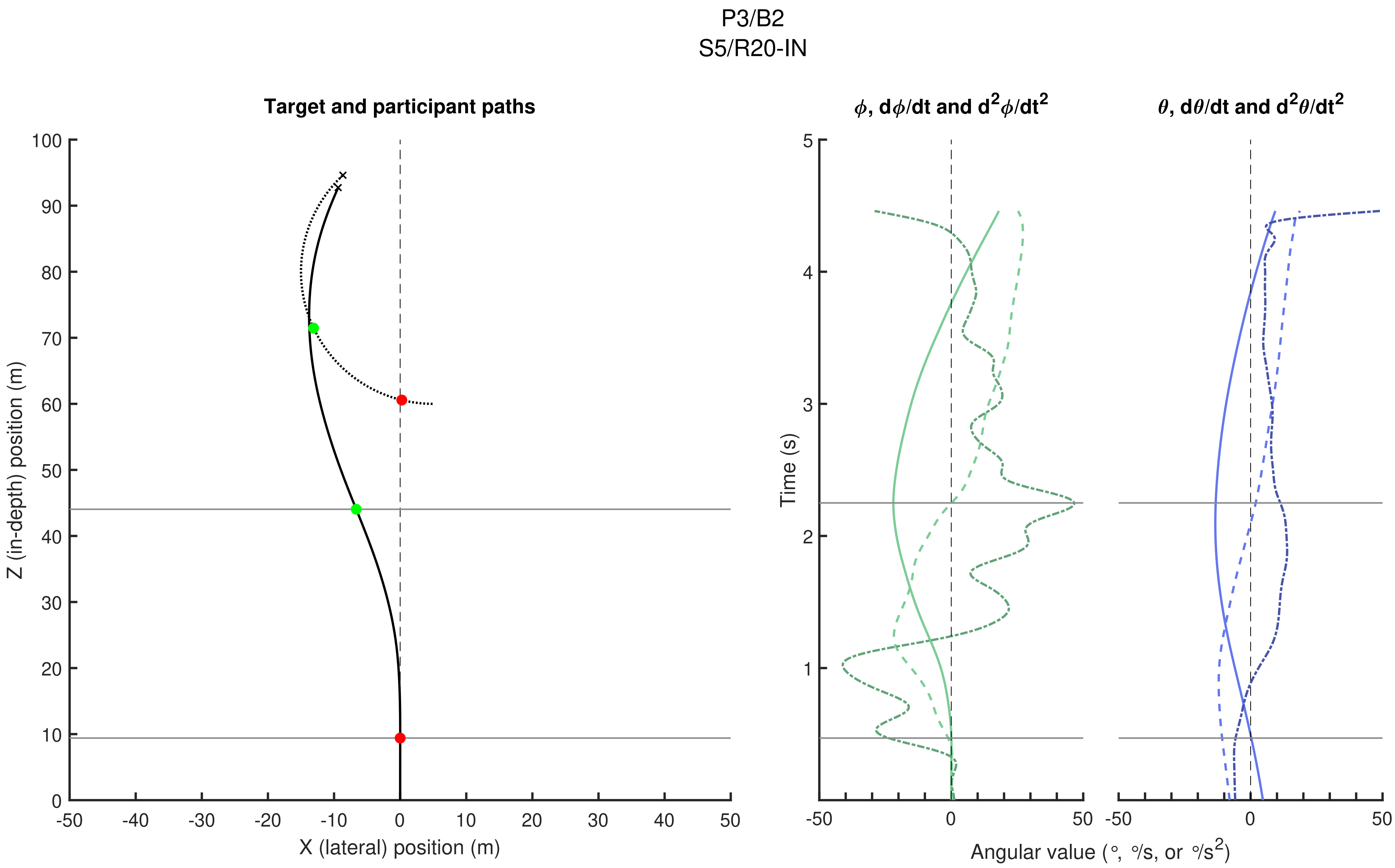
P3/B2
S5/R20-IN
Target and participant paths
Z (in-depth) position (m)
X (lateral) position (m)
ϕ, dϕ/dt and d$^2\phi$/dt^2
θ, dθ/dt and d$^2\theta$/dt^2
Time (s)
Angular value (°, °/s, or °/s^2)

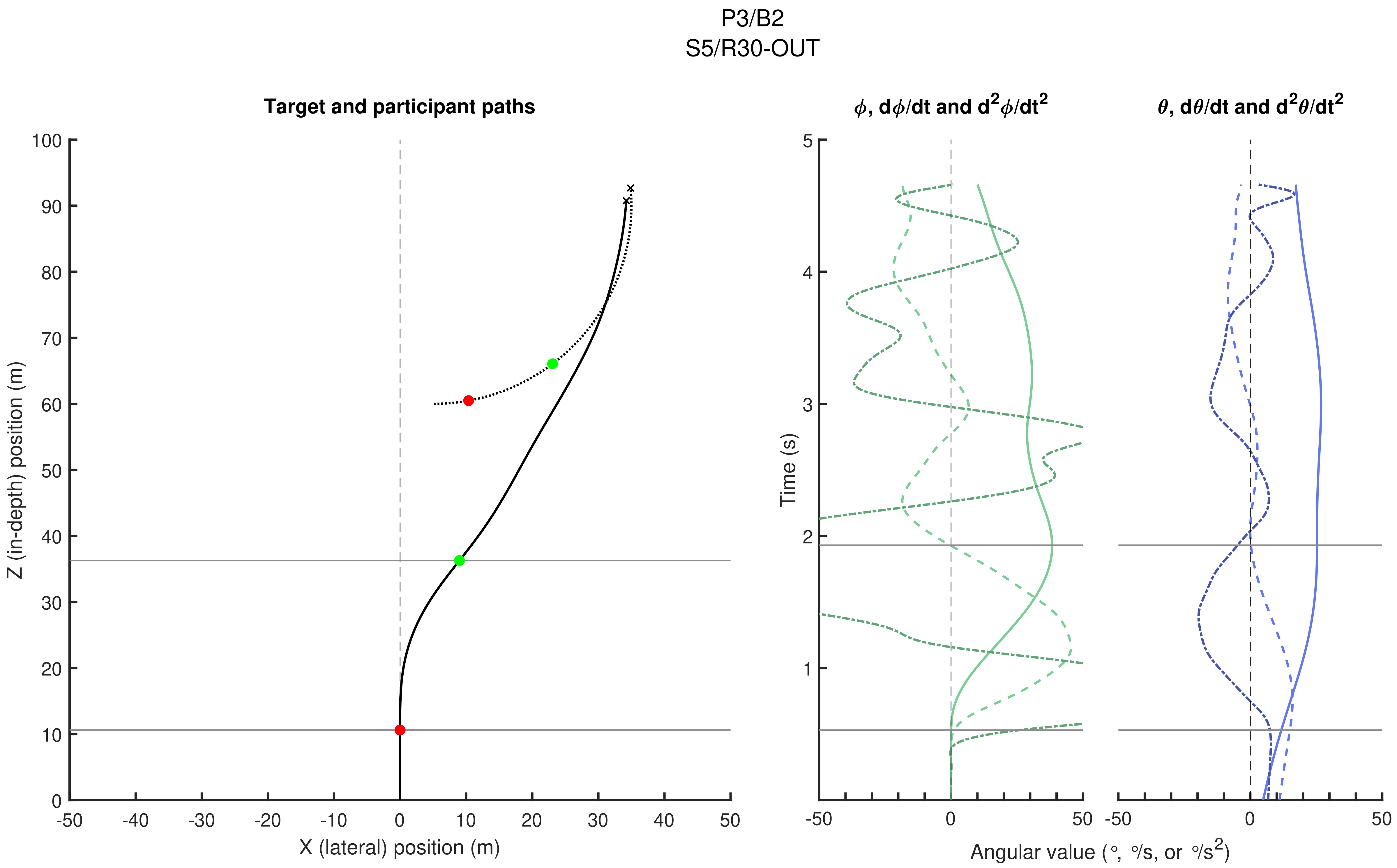
P3/B2
S5/R30-OUT
Target and participant paths
ϕ, dϕ/dt and d²ϕ/dt²
θ, dθ/dt and d²θ/dt²
Z (in-depth) position (m)
X (lateral) position (m)
Time (s)
Angular value (°, °/s, or °/s²)
100
90
80
70
60
50
40
30
20
10
0
-50
-40
-30
-20
-10
0
10
20
30
40
50
5
4
3
2
1
-50
0
50
-50
0
50

P3/B2
S5/R30-IN

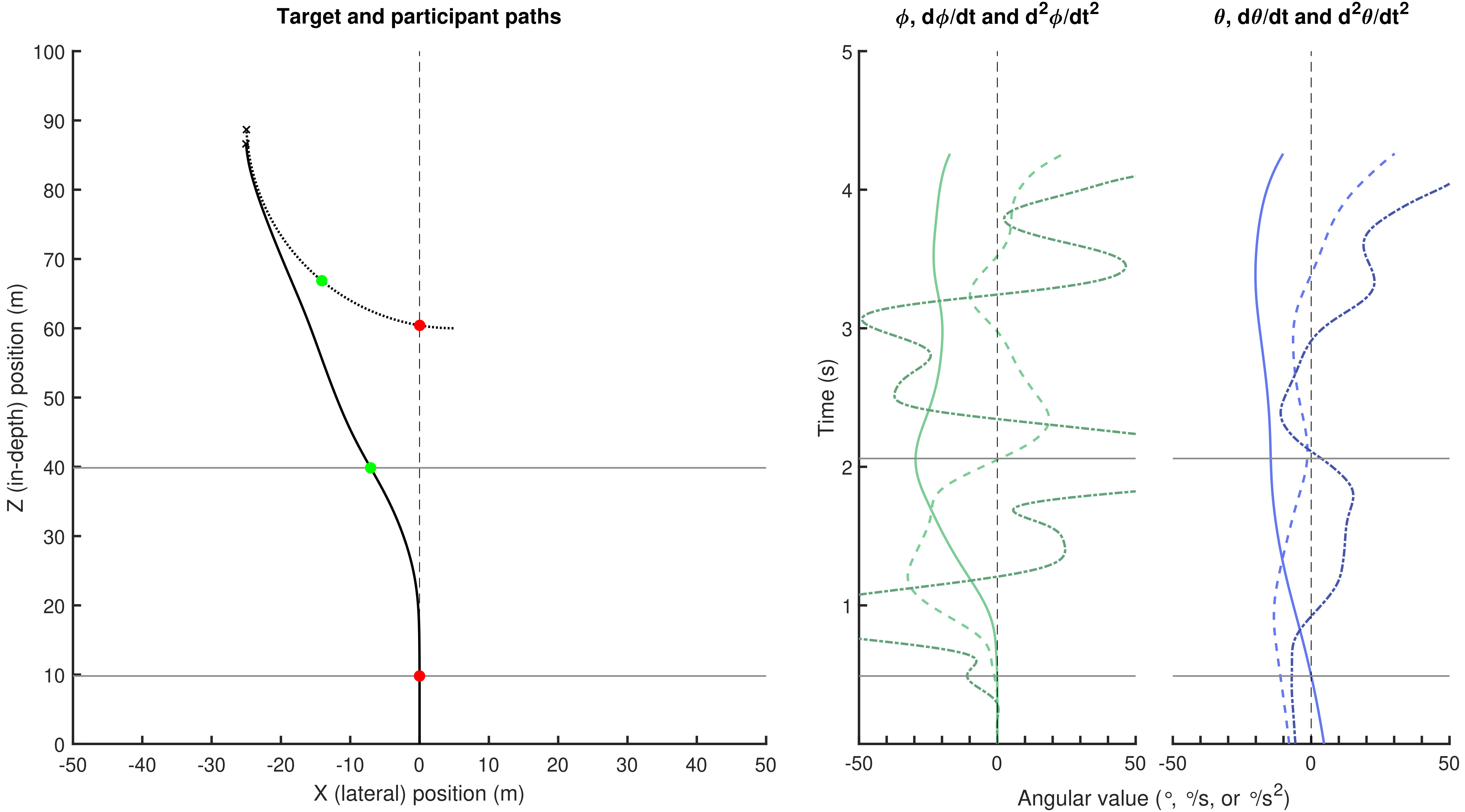

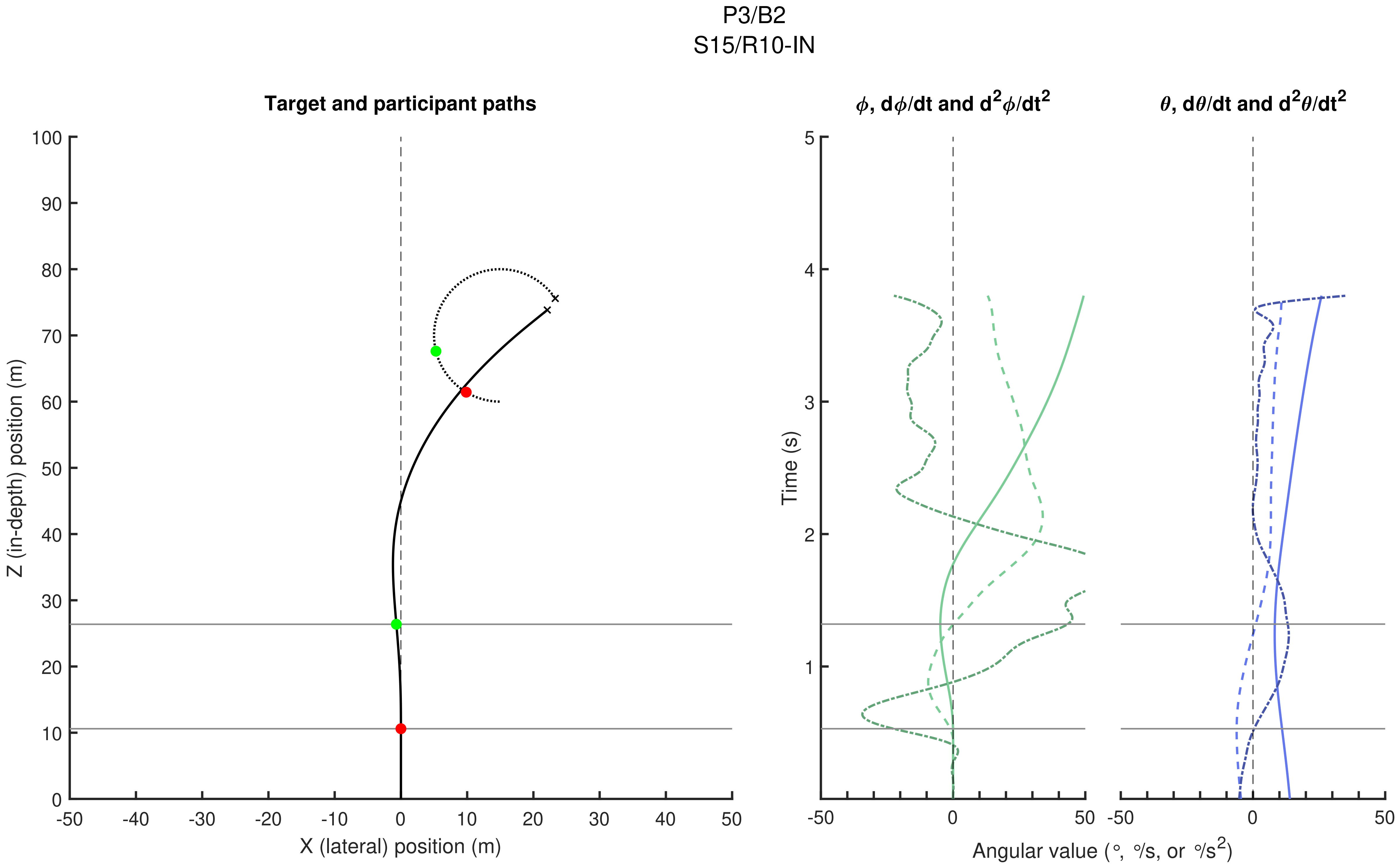

P3/B2
S15/R10-IN
Target and participant paths
ϕ, dϕ/dt and d$^2\phi$/dt^2
θ, dθ/dt and d$^2\theta$/dt^2
Z (in-depth) position (m)
X (lateral) position (m)
Time (s)
Angular value (°, °/s, or °/s^2)

P3/B2
S15/R20-IN

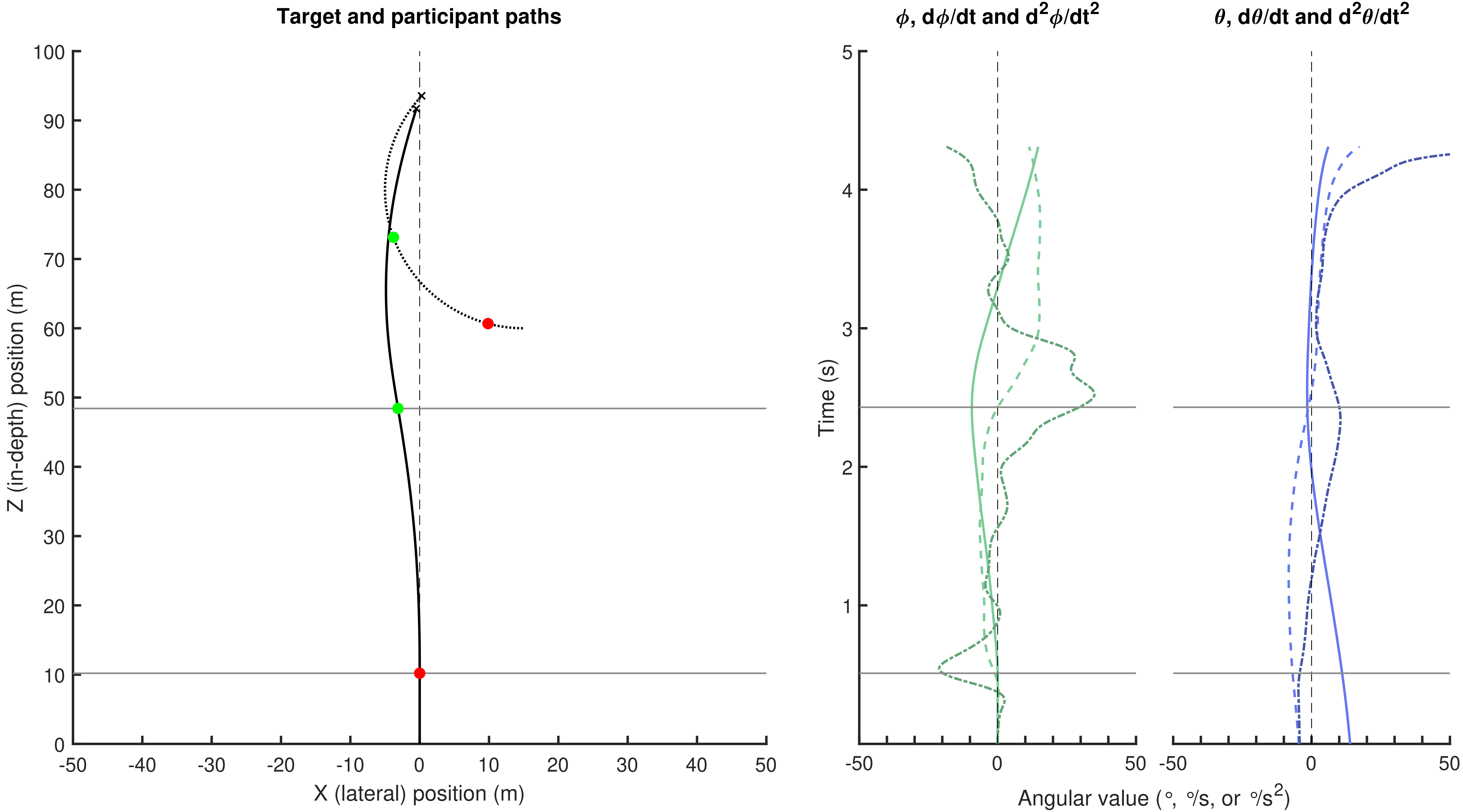

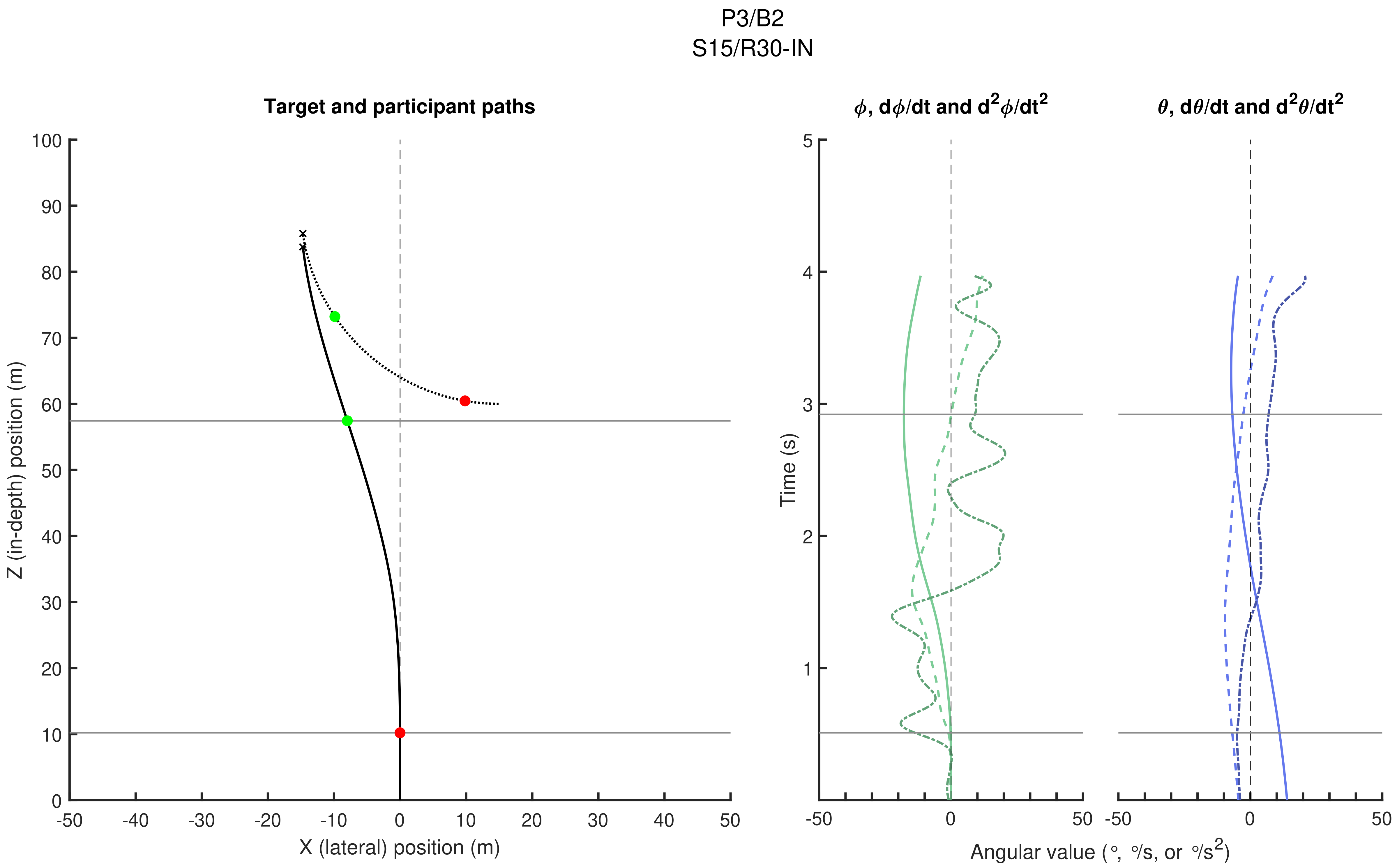

P3/B2
S15/R30-IN
Target and participant paths
ϕ, dϕ/dt and d$^2\phi$/dt^2
θ, dθ/dt and d$^2\theta$/dt^2
Z (in-depth) position (m)
X (lateral) position (m)
Time (s)
Angular value (°, °/s, or °/s^2)
100
90
80
70
60
50
40
30
20
10
0
-50
-40
-30
-20
-10
0
10
20
30
40
50
5
4
3
2
1

P3/B2
S20/R10-IN

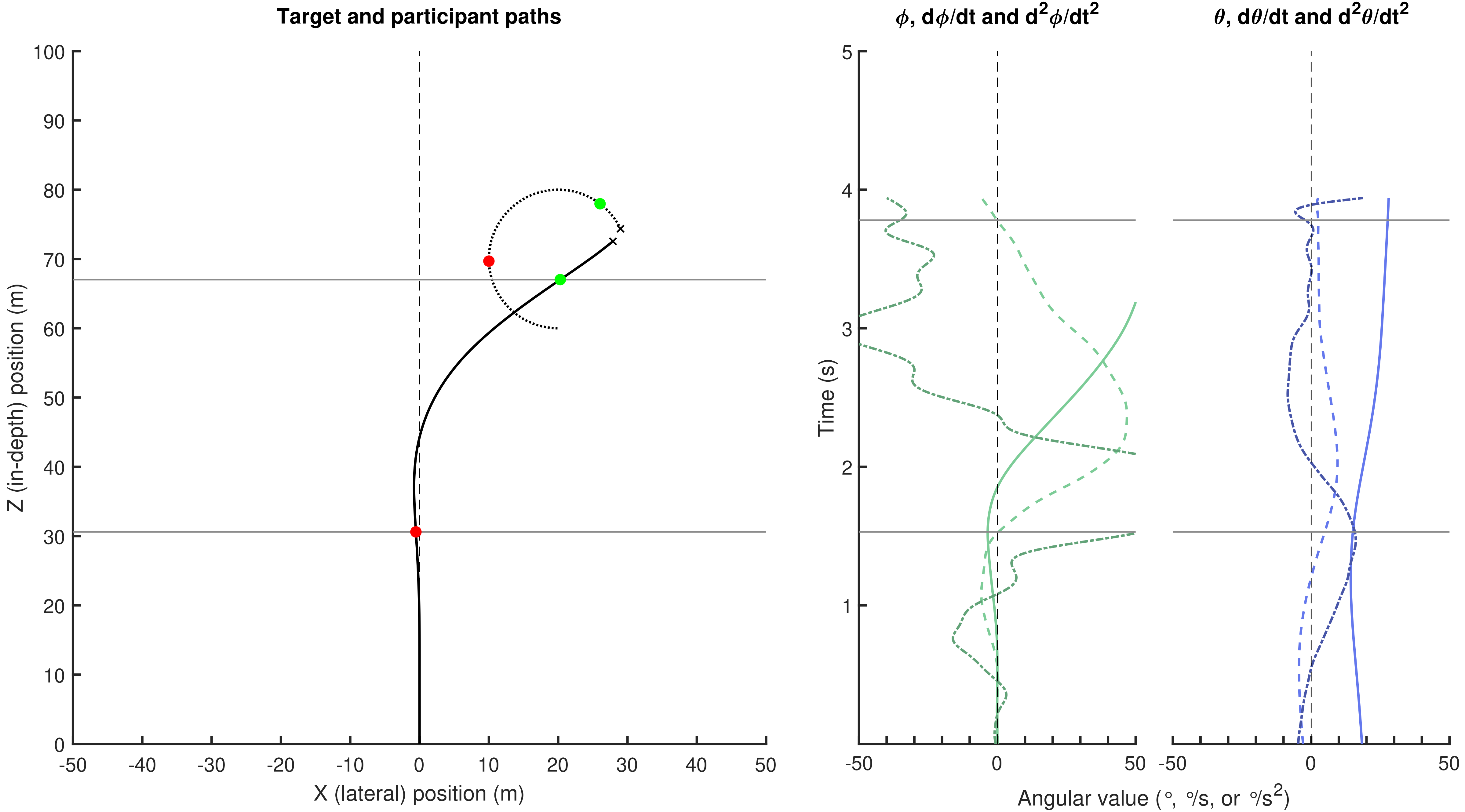

P3/B2
S20/R20-IN

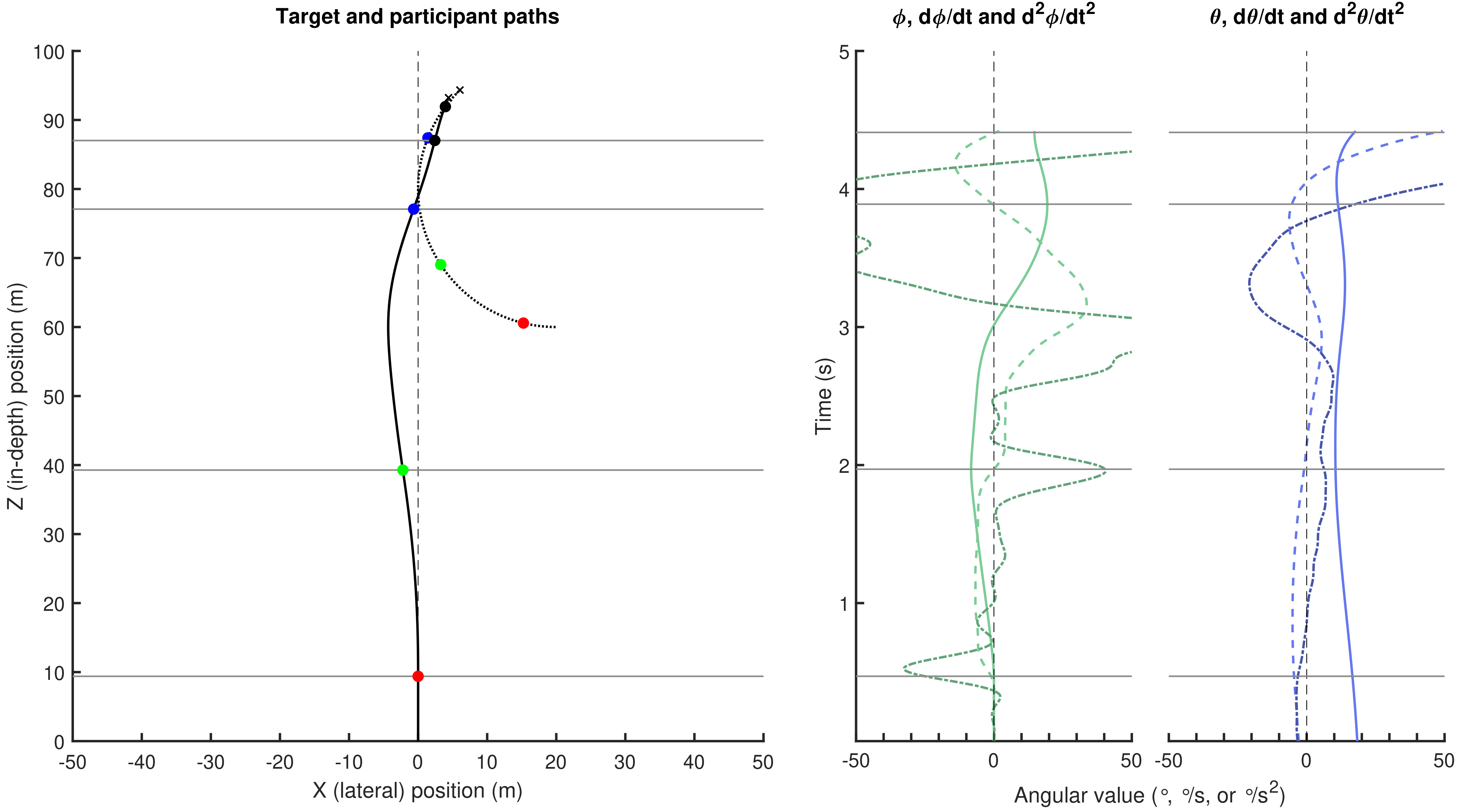

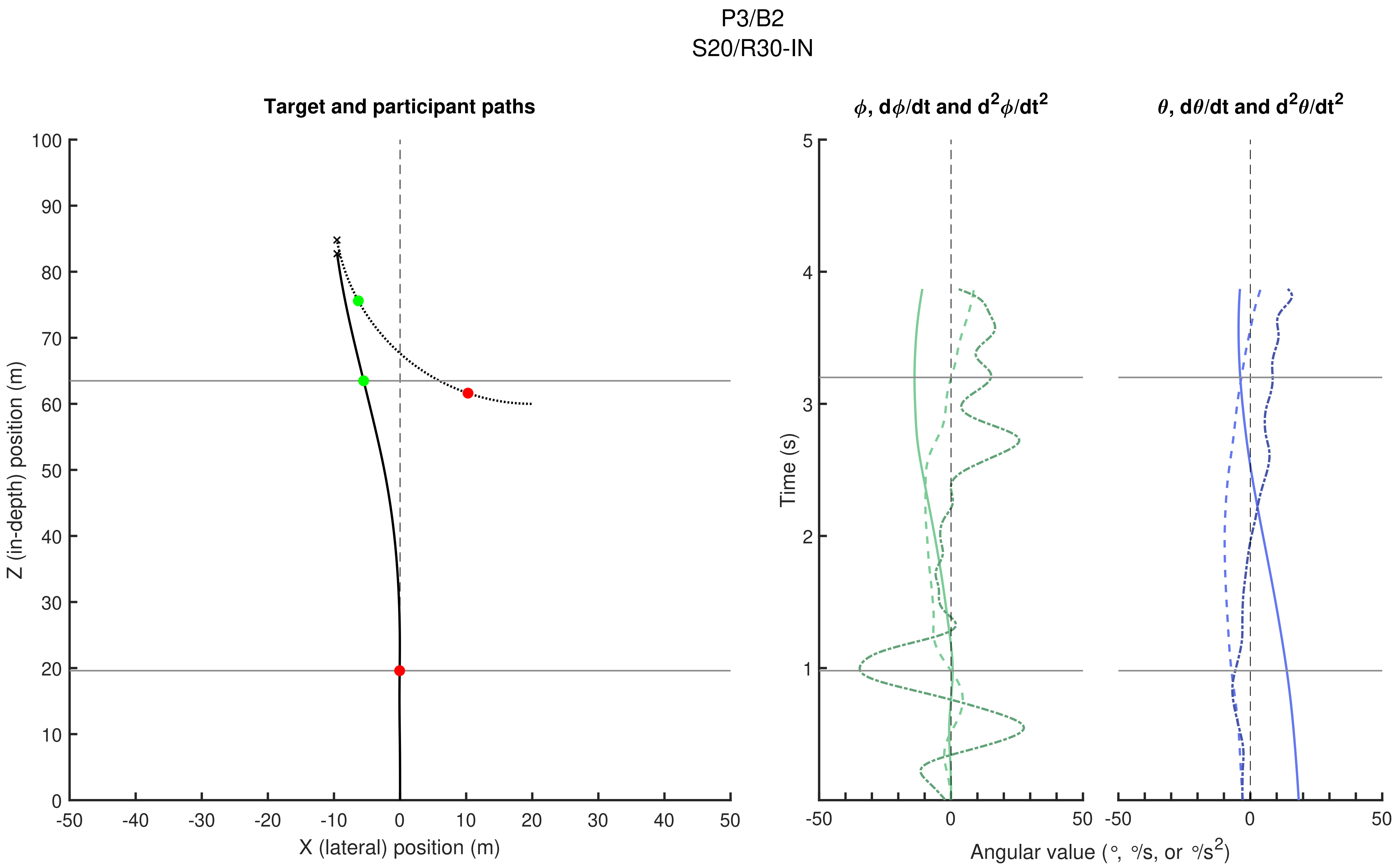

P3/B2
S20/R30-IN
Target and participant paths
Z (in-depth) position (m)
X (lateral) position (m)
φ, dφ/dt and d²φ/dt²
θ, dθ/dt and d²θ/dt²
Time (s)
Angular value (°, °/s, or °/s²)

P3/B3
S20/R10-IN

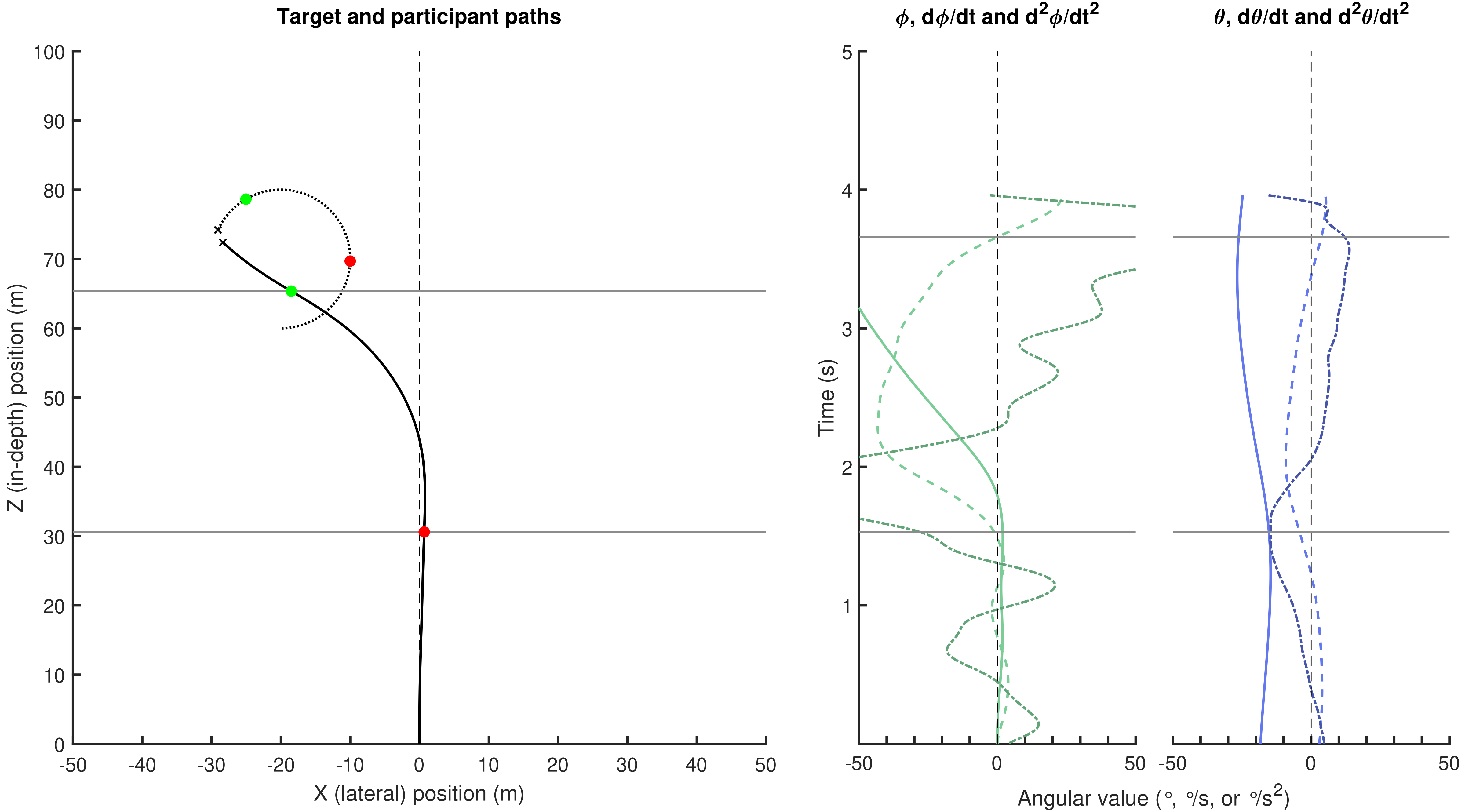

P3/B3
S20/R20-IN

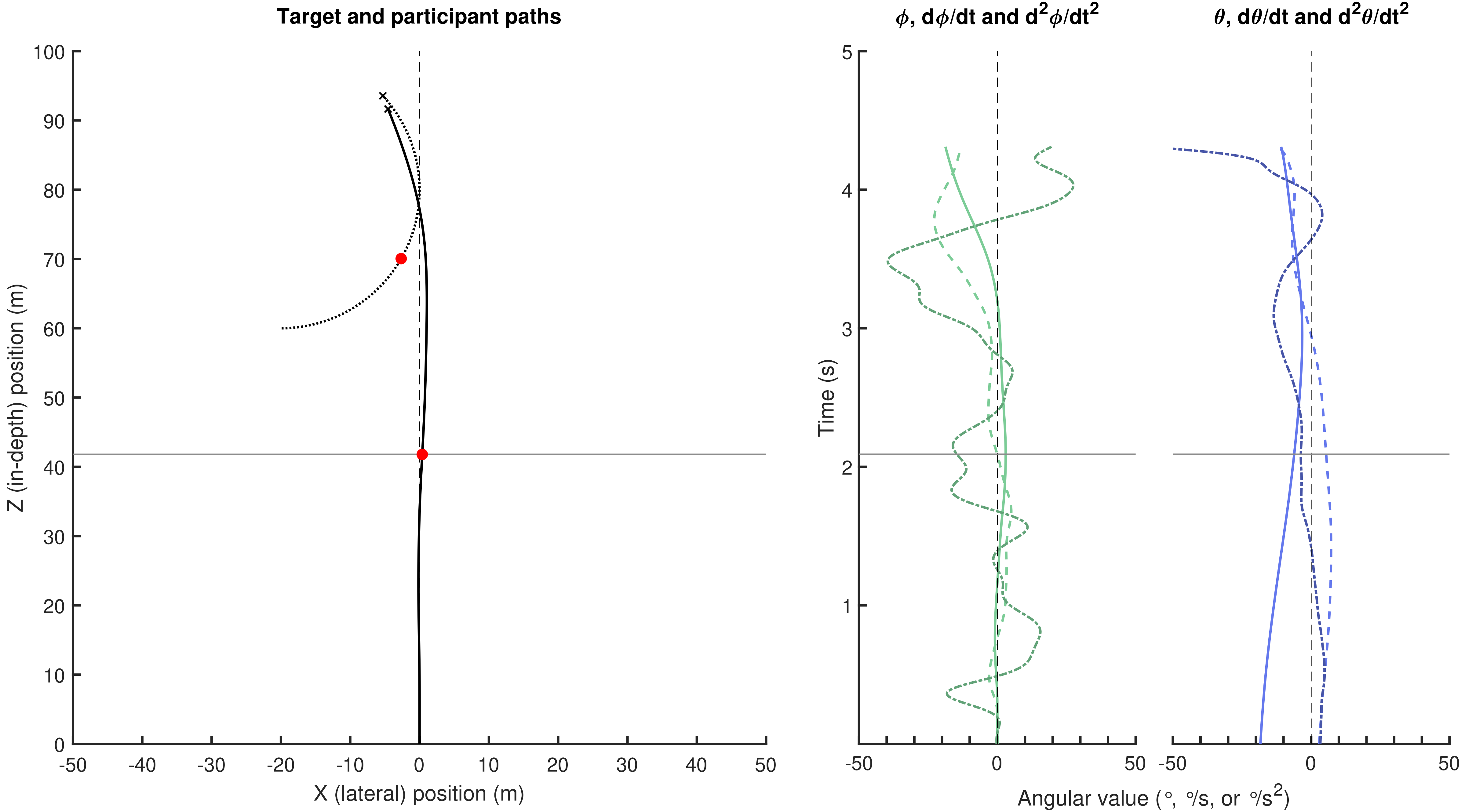

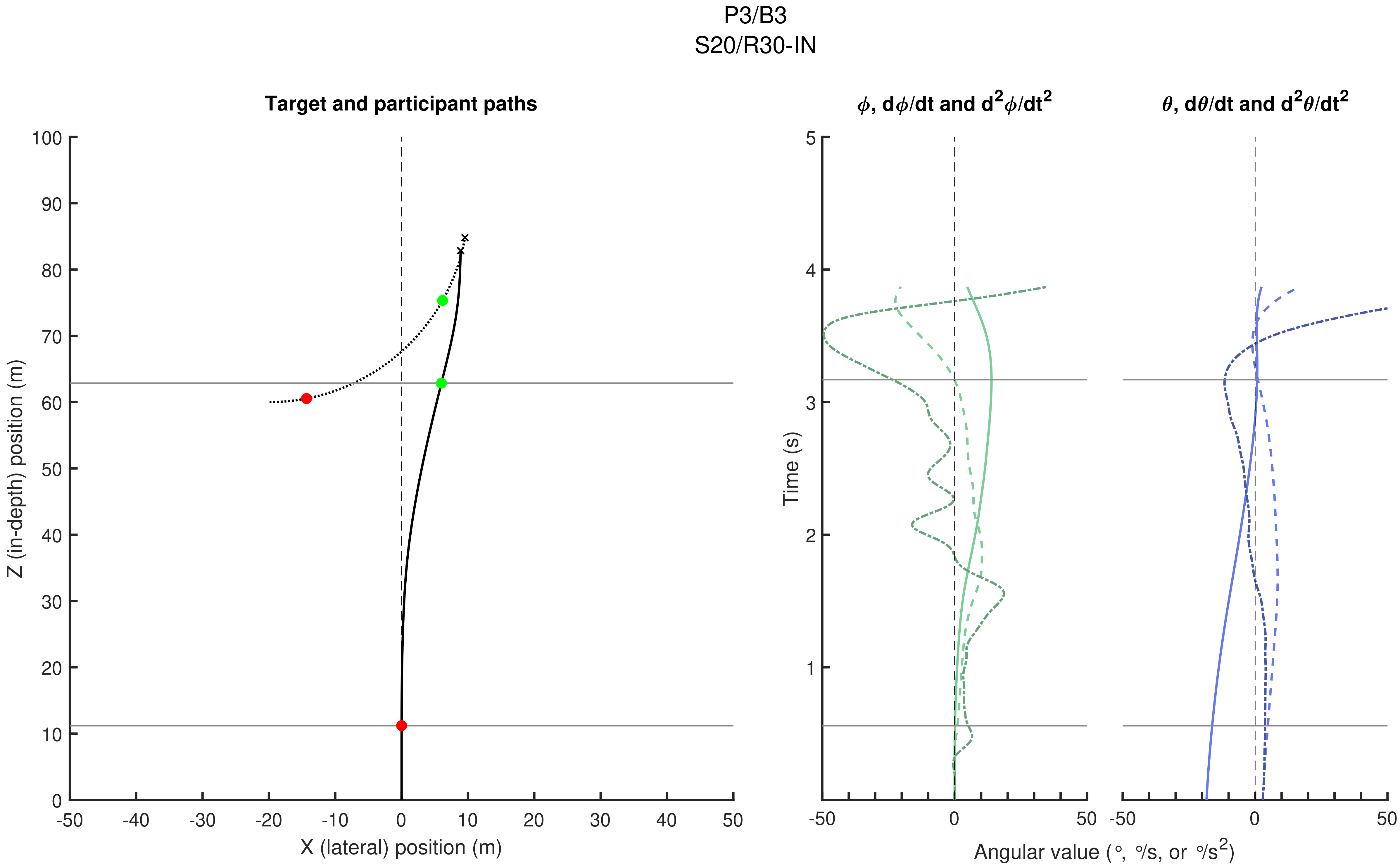
P3/B3
S20/R30-IN
Target and participant paths
Z (in-depth) position (m)
X (lateral) position (m)
0
10
20
30
40
50
60
70
80
90
100
-50
-40
-30
-20
-10
0
10
20
30
40
50
ϕ, dϕ/dt and d$^2\phi$/dt^2
θ, dθ/dt and d$^2\theta$/dt^2
Time (s)
1
2
3
4
5
-50
0
50
-50
0
50
Angular value (°, °/s, or °/s^2)

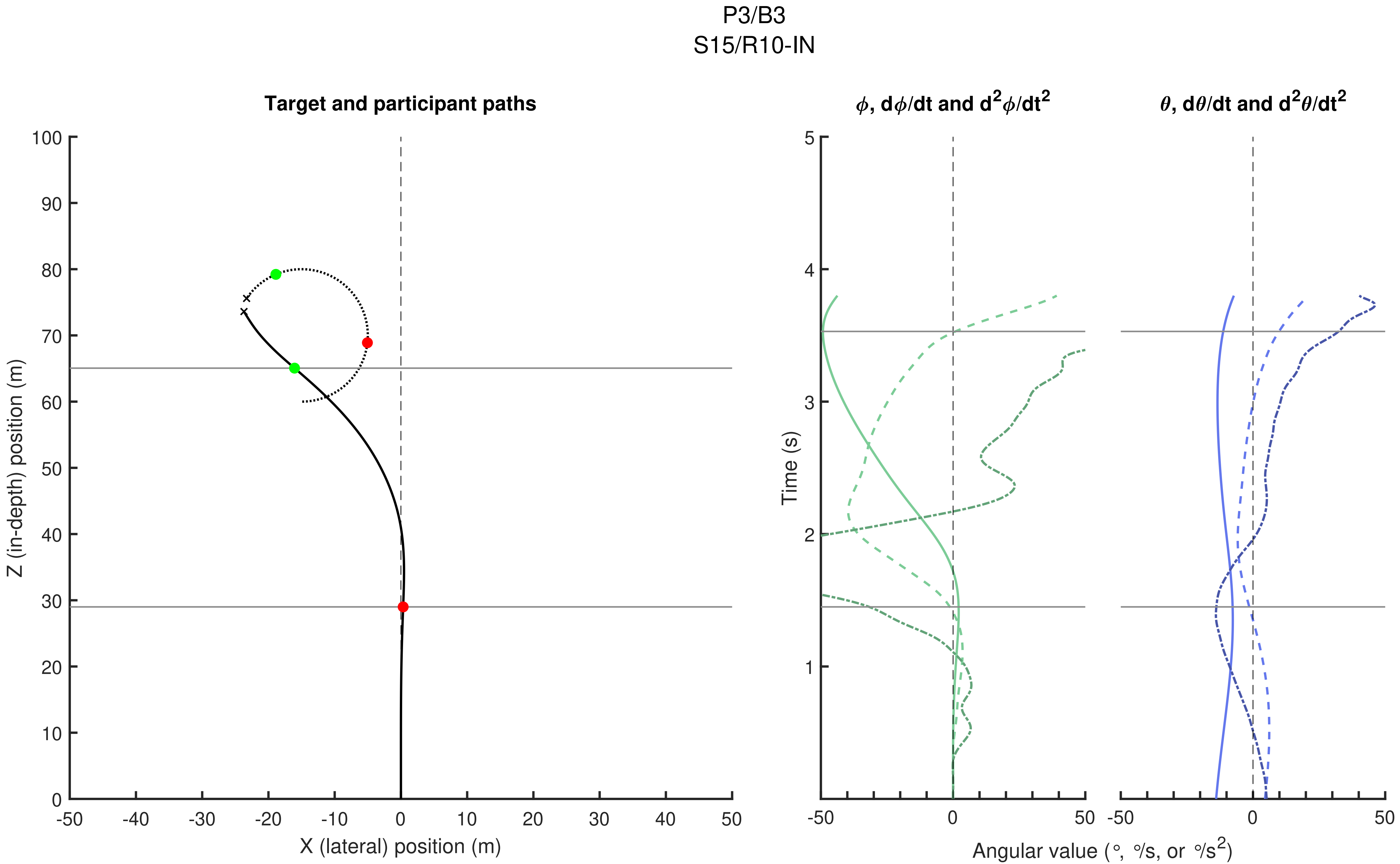
P3/B3
S15/R10-IN
Target and participant paths
Z (in-depth) position (m)
X (lateral) position (m)
ϕ, dϕ/dt and d²ϕ/dt²
θ, dθ/dt and d²θ/dt²
Time (s)
Angular value (°, °/s, or °/s²)

P3/B3
S15/R20-IN

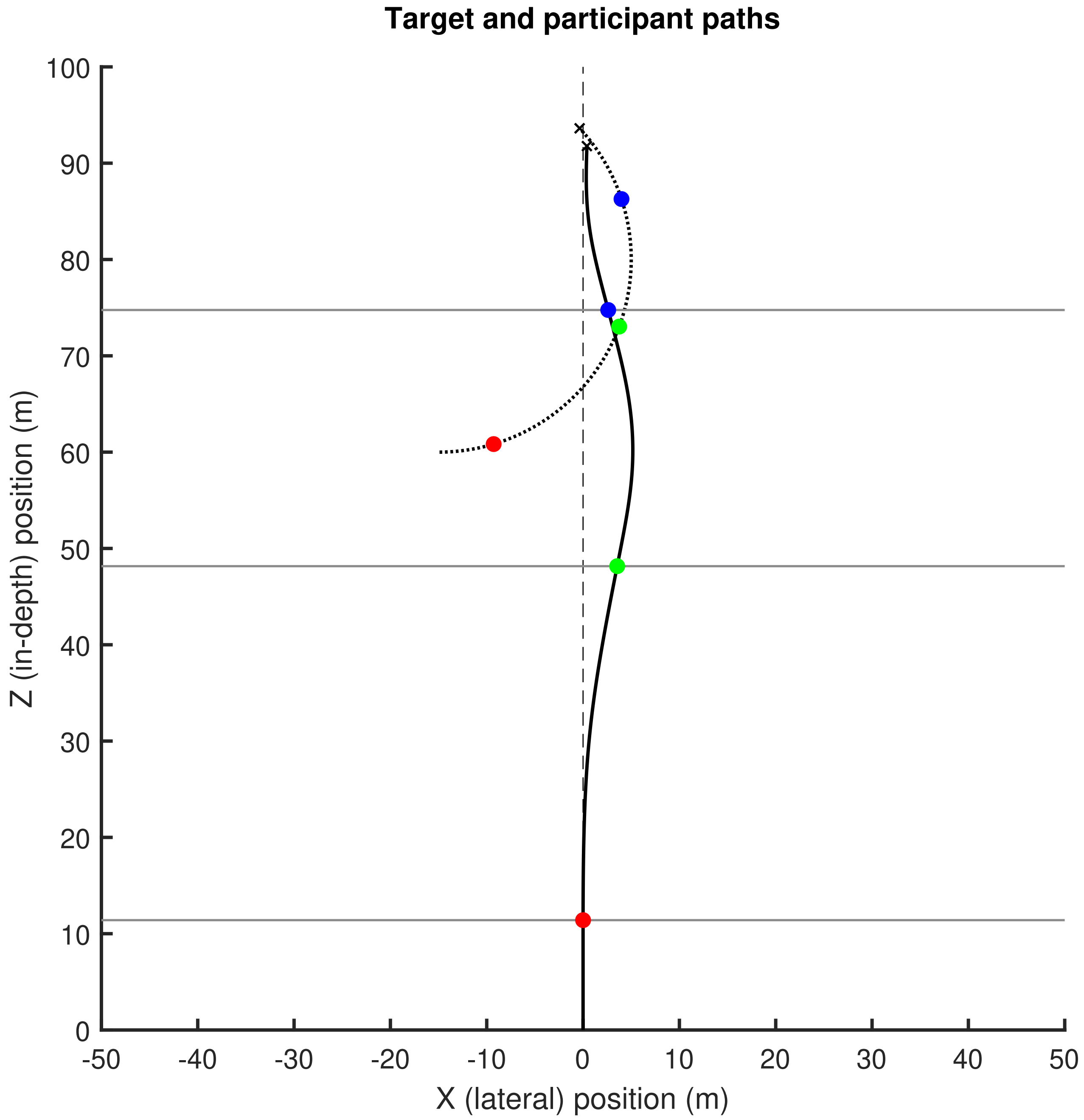

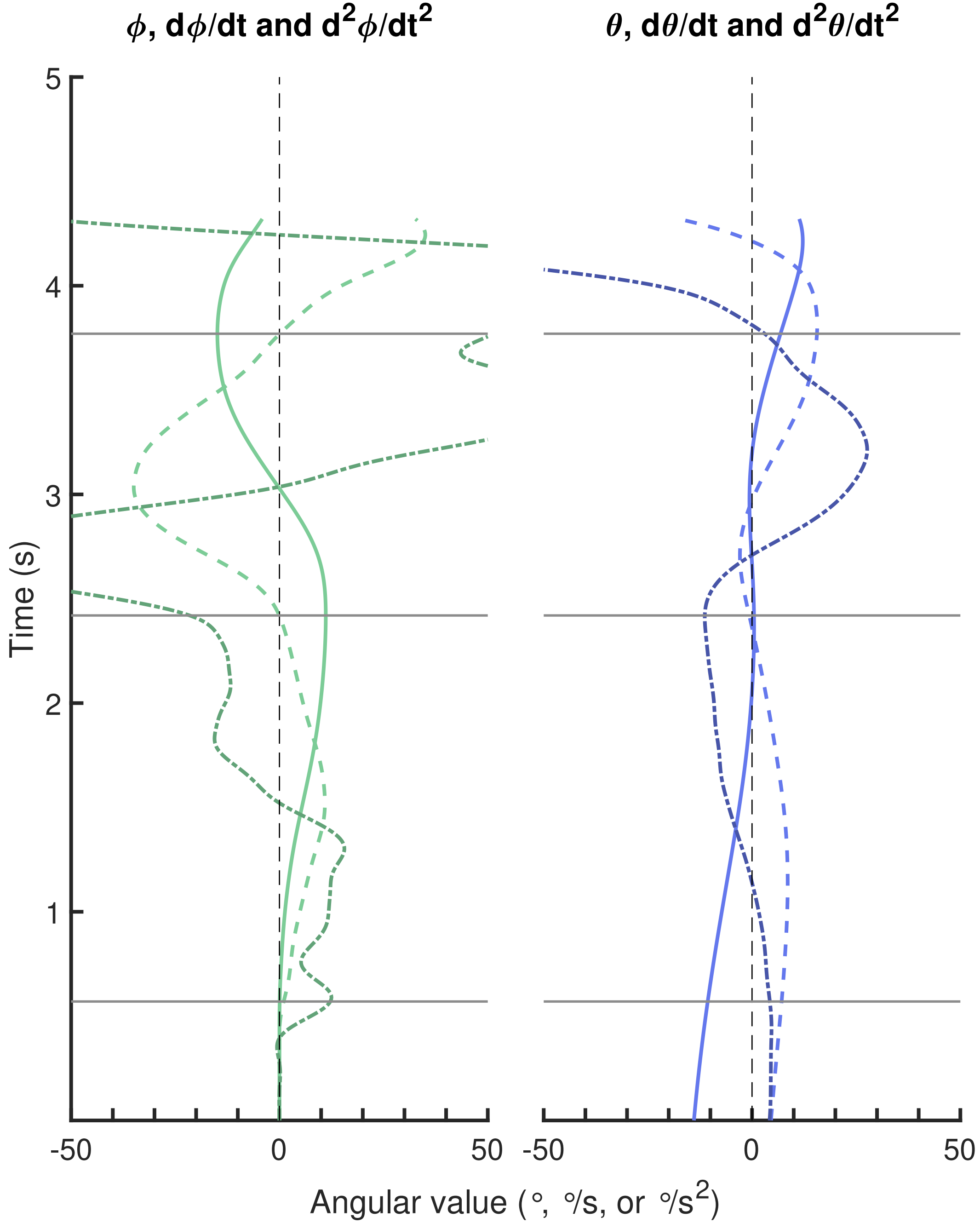

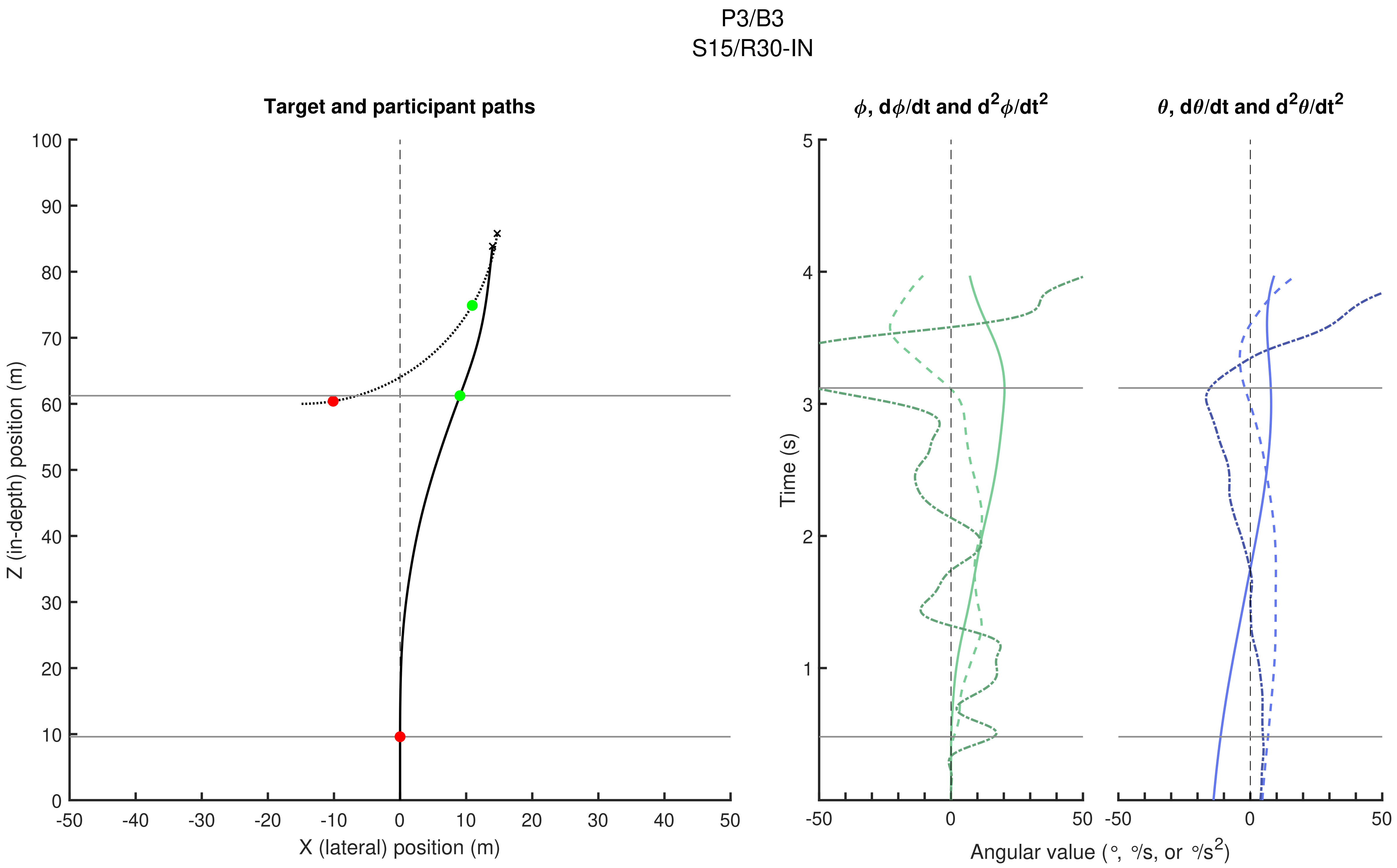
P3/B3
S15/R30-IN
Target and participant paths
Z (in-depth) position (m)
X (lateral) position (m)
φ, dφ/dt and d²φ/dt²
θ, dθ/dt and d²θ/dt²
Time (s)
Angular value (°, °/s, or °/s²)

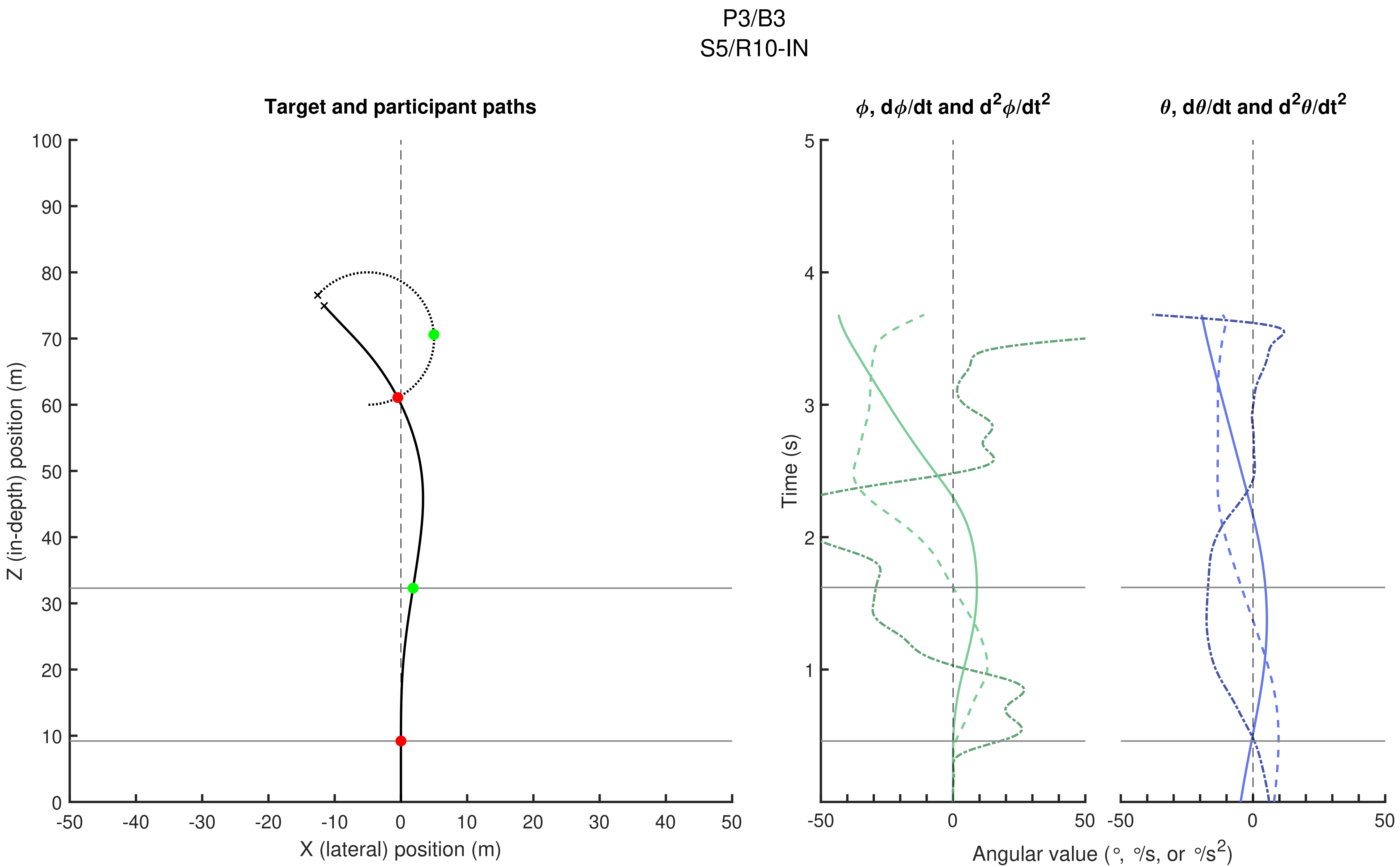
P3/B3
S5/R10-IN
Target and participant paths
ϕ, dϕ/dt and d$^2\phi$/dt^2
θ, dθ/dt and d$^2\theta$/dt^2
Z (in-depth) position (m)
X (lateral) position (m)
Time (s)
Angular value (°, °/s, or °/s^2)

P3/B3
S5/R10-OUT

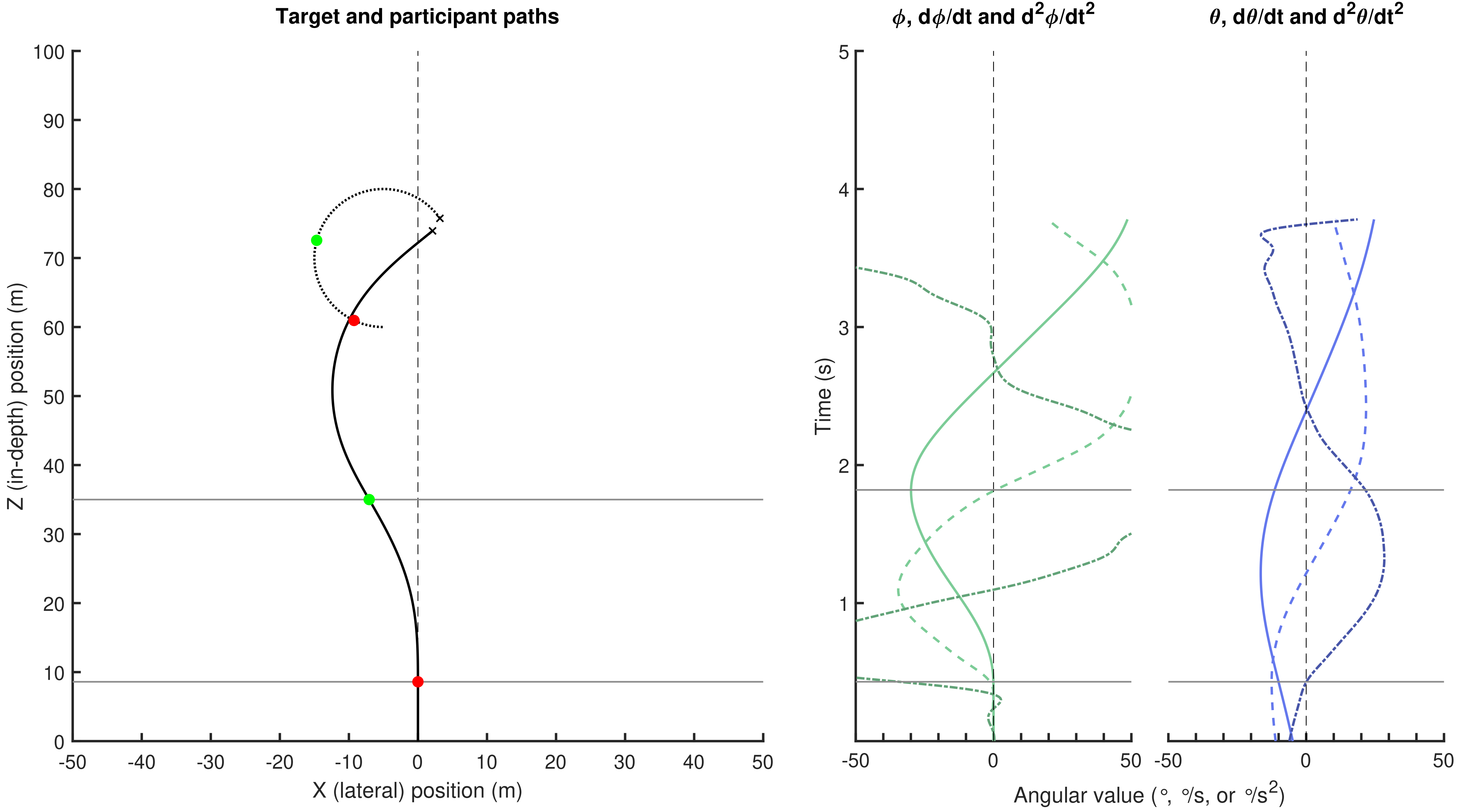

P3/B3
S5/R20-IN

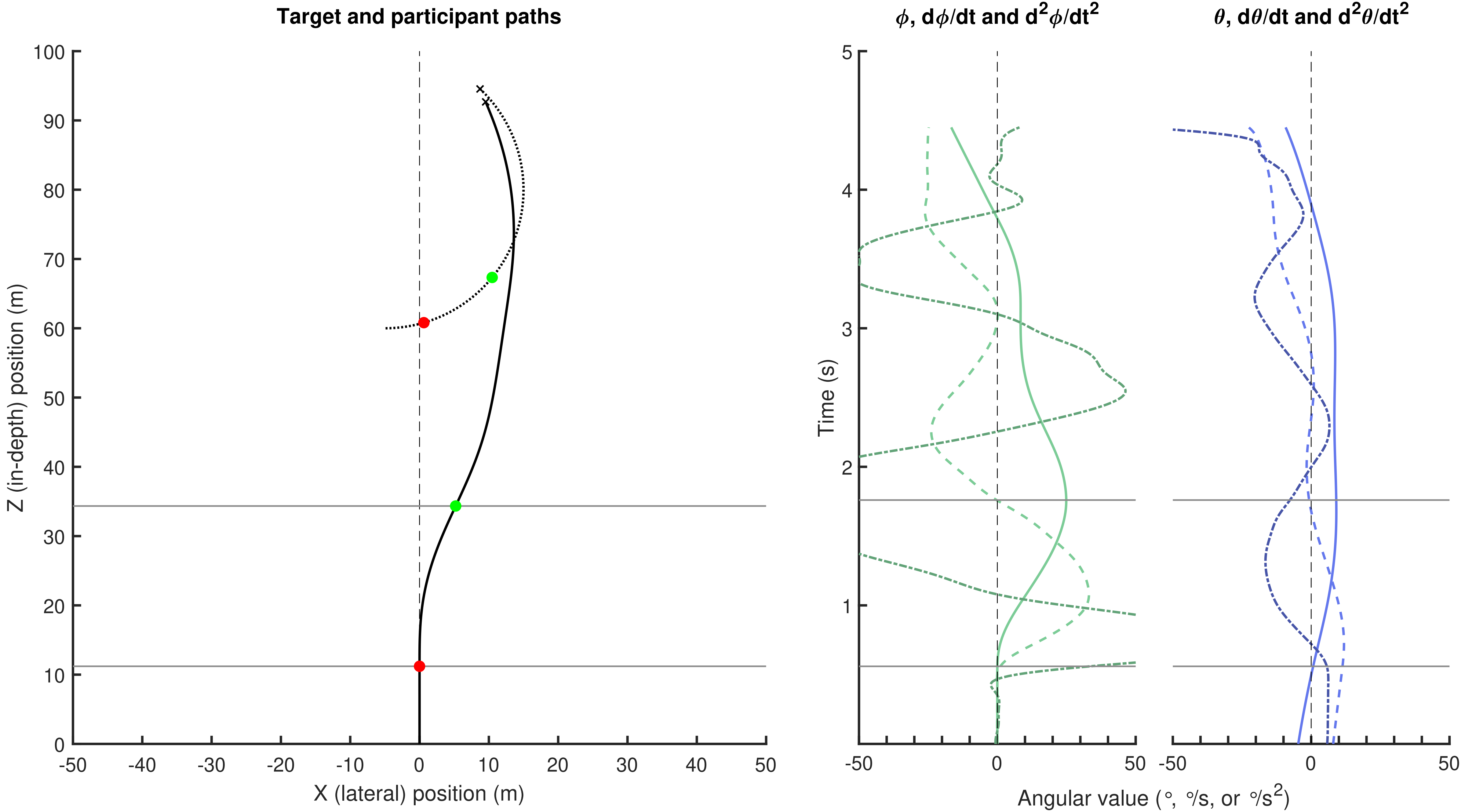

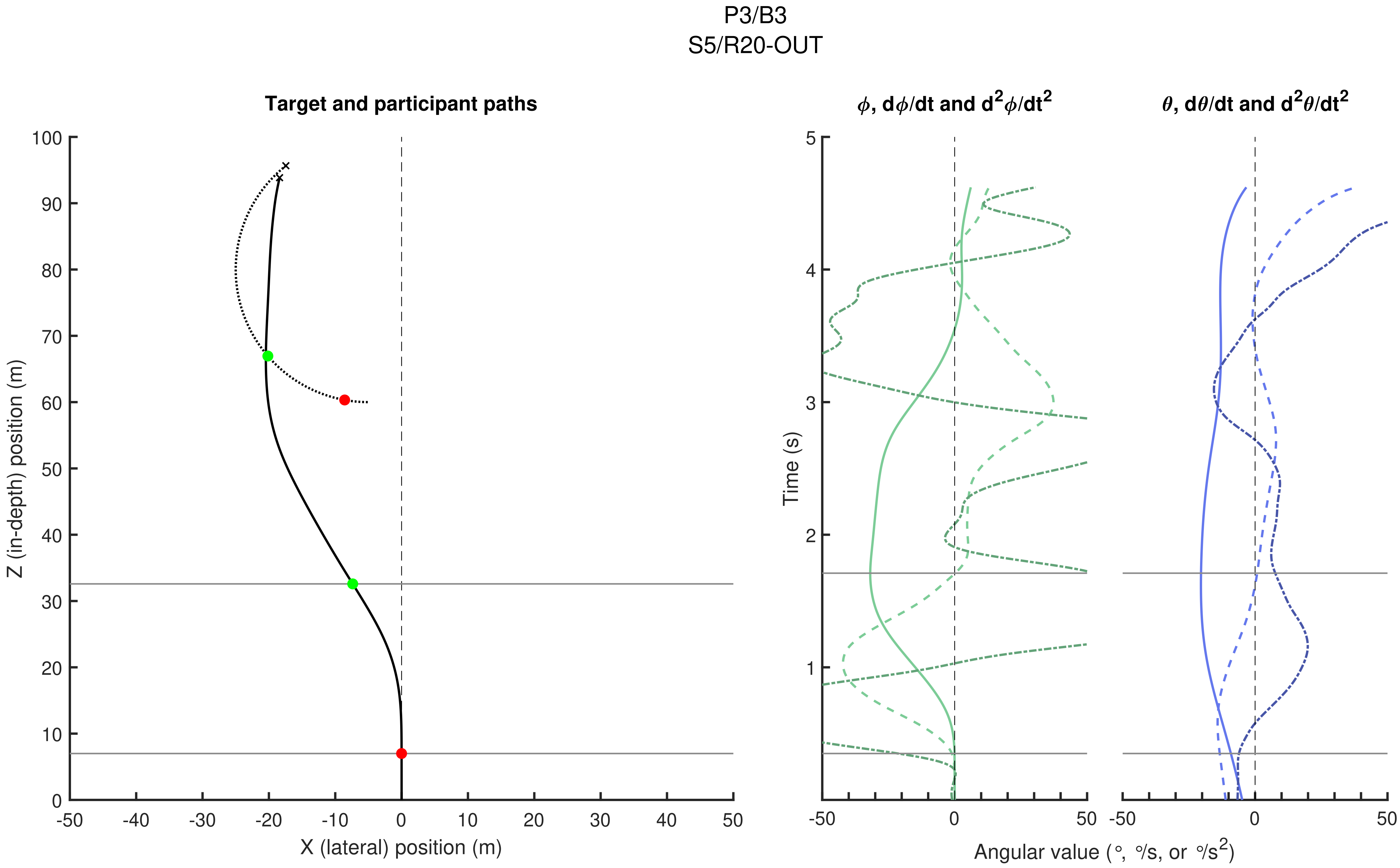

P3/B3
S5/R20-OUT
Target and participant paths
Z (in-depth) position (m)
X (lateral) position (m)
ϕ, dϕ/dt and d$^2\phi$/dt^2
θ, dθ/dt and d$^2\theta$/dt^2
Time (s)
Angular value (°, °/s, or °/s^2)

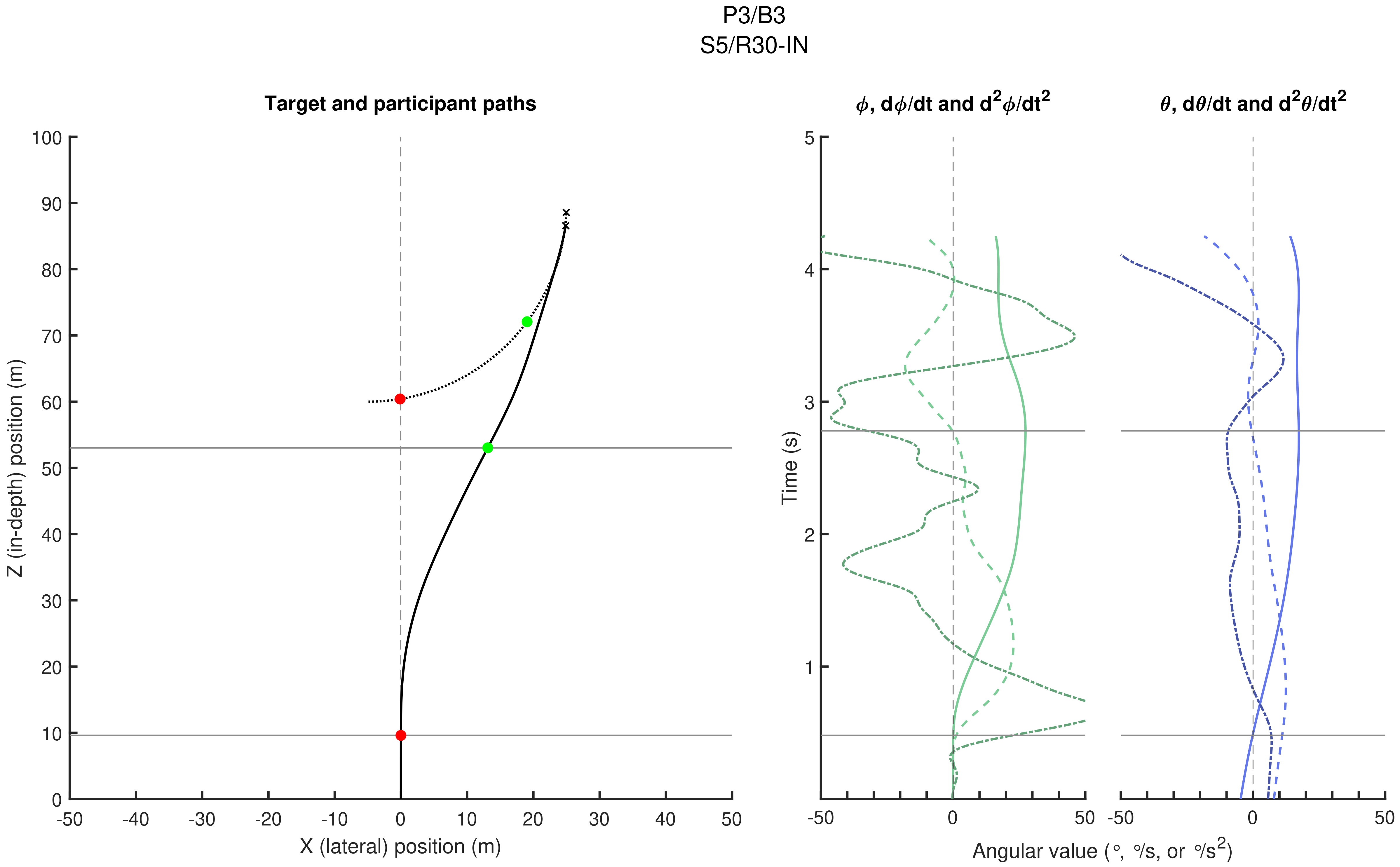
P3/B3
S5/R30-IN
Target and participant paths
Z (in-depth) position (m)
X (lateral) position (m)
ϕ, dϕ/dt and d²ϕ/dt²
θ, dθ/dt and d²θ/dt²
Time (s)
Angular value (°, °/s, or °/s²)
100
90
80
70
60
50
40
30
20
10
0
-50
-40
-30
-20
-10
0
10
20
30
40
50
5
4
3
2
1
-50
0
50
-50
0
50

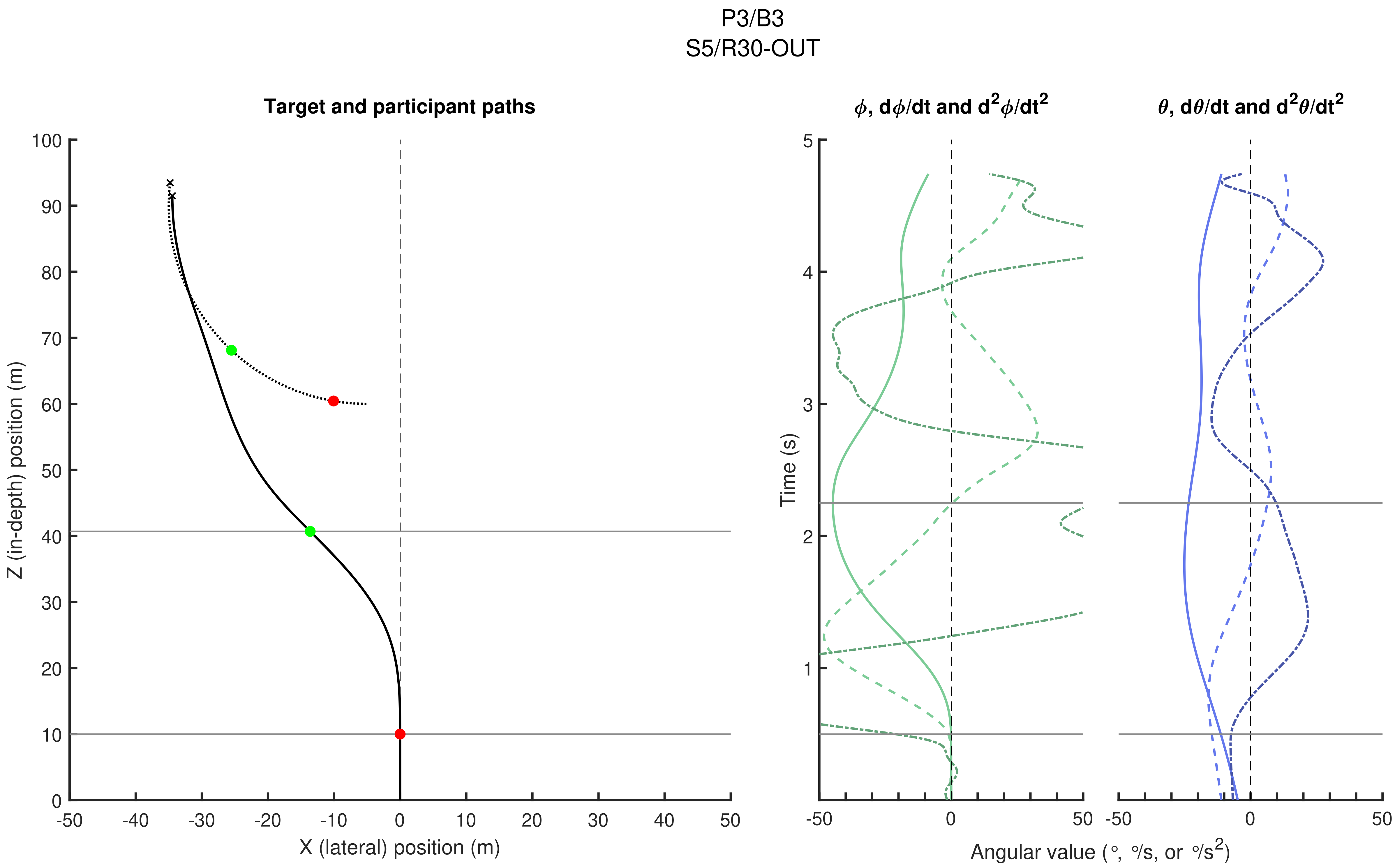

P3/B3
S5/R30-OUT
Target and participant paths
Z (in-depth) position (m)
X (lateral) position (m)
ϕ, dϕ/dt and d²ϕ/dt²
θ, dθ/dt and d²θ/dt²
Time (s)
Angular value (°, °/s, or °/s²)

P3/B3
S5/R10-OUT

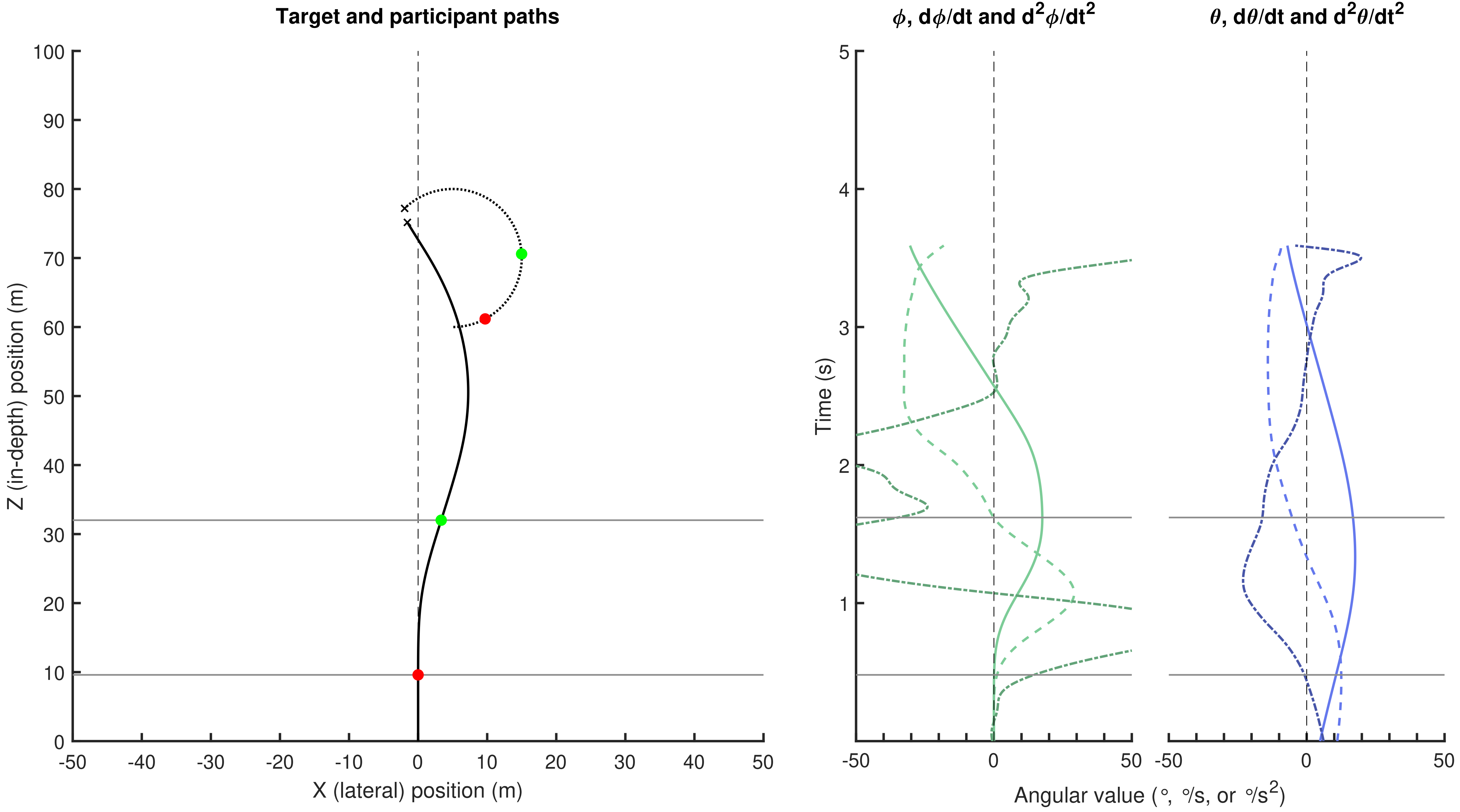

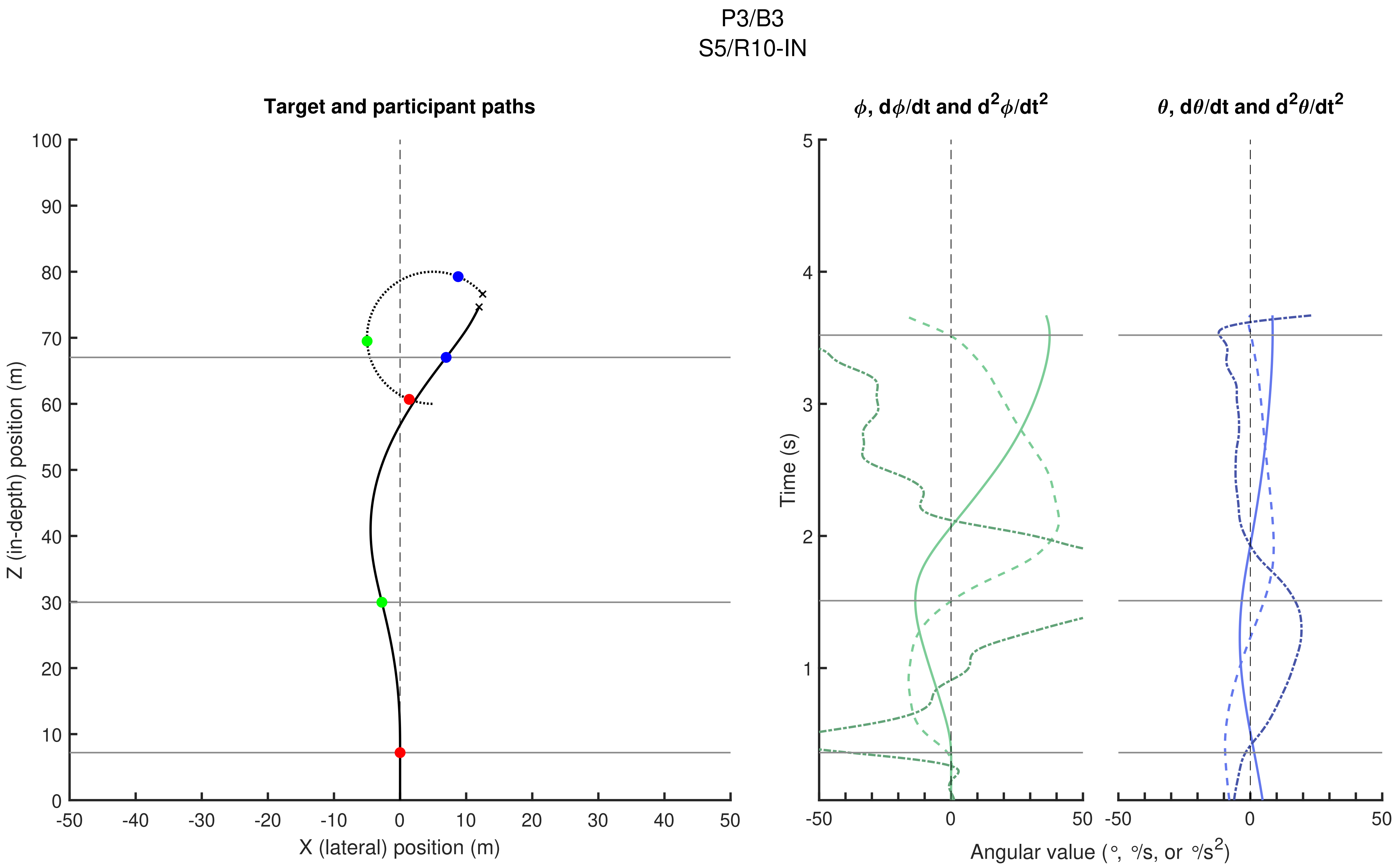
P3/B3
S5/R10-IN
Target and participant paths
Z (in-depth) position (m)
X (lateral) position (m)
ϕ, dϕ/dt and d$^2\phi$/dt^2
θ, dθ/dt and d$^2\theta$/dt^2
Time (s)
Angular value (°, °/s, or °/s^2)

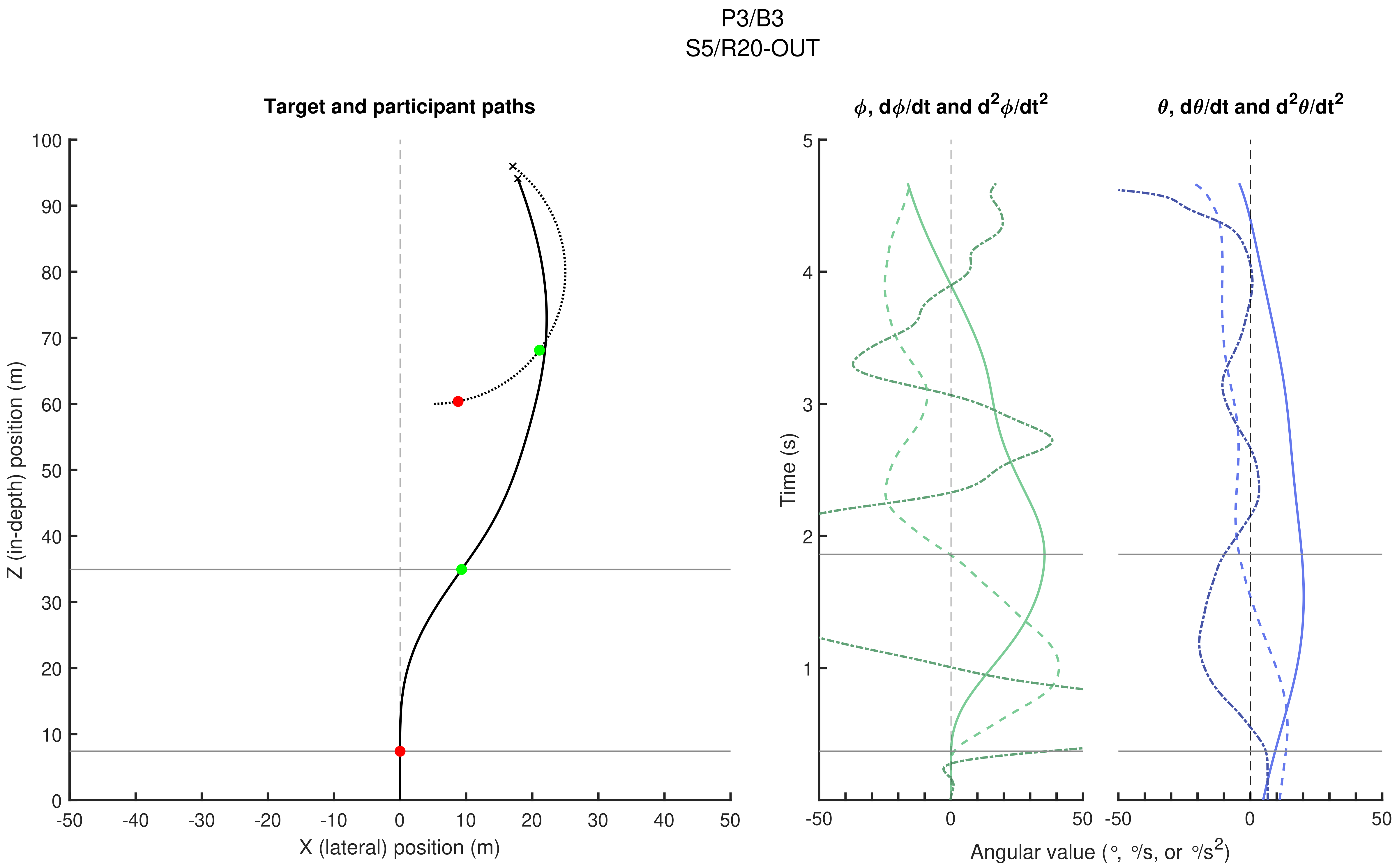

P3/B3
S5/R20-OUT
Target and participant paths
Z (in-depth) position (m)
X (lateral) position (m)
φ, dφ/dt and d²φ/dt²
θ, dθ/dt and d²θ/dt²
Time (s)
Angular value (°, °/s, or °/s²)

P3/B3
S5/R20-IN

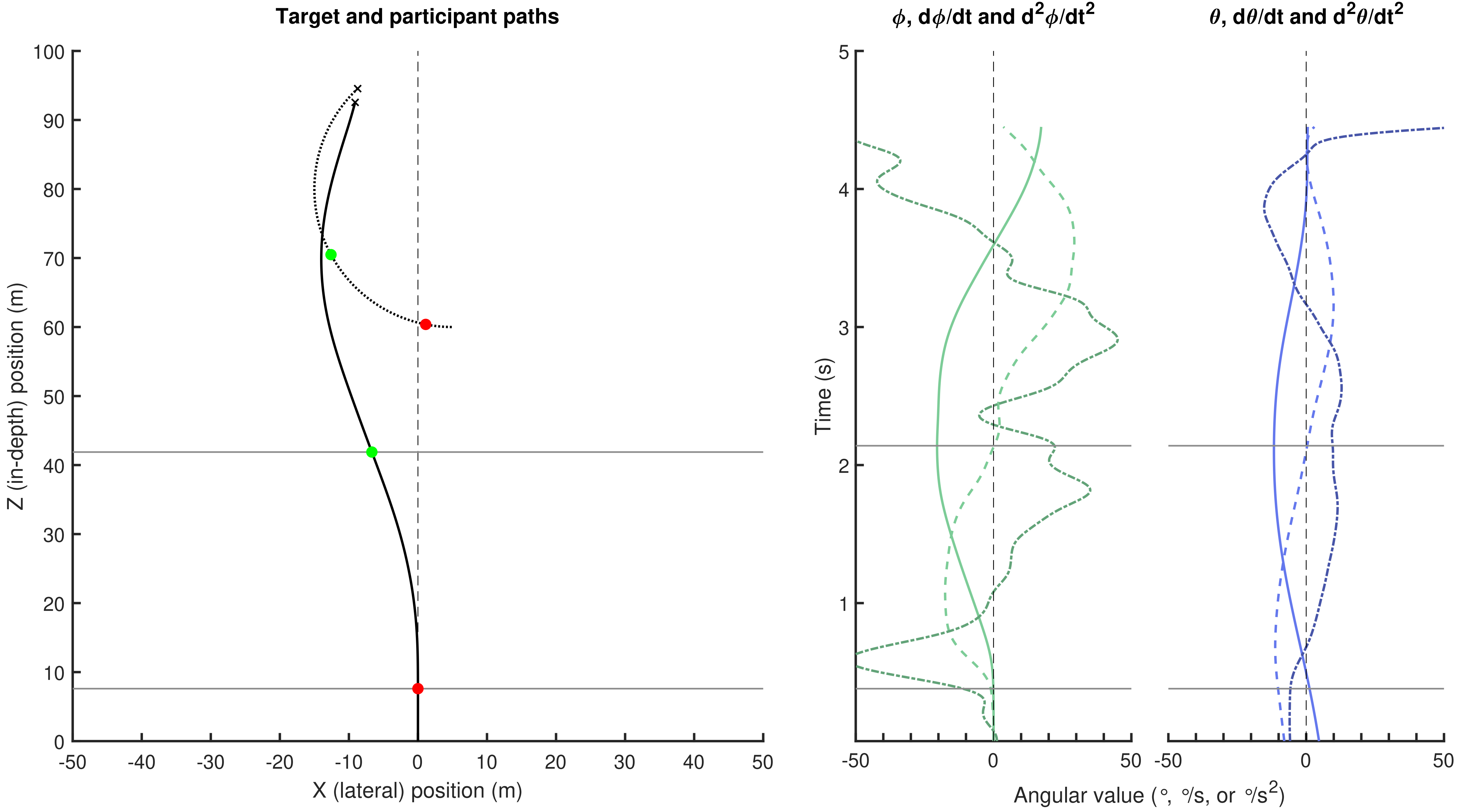

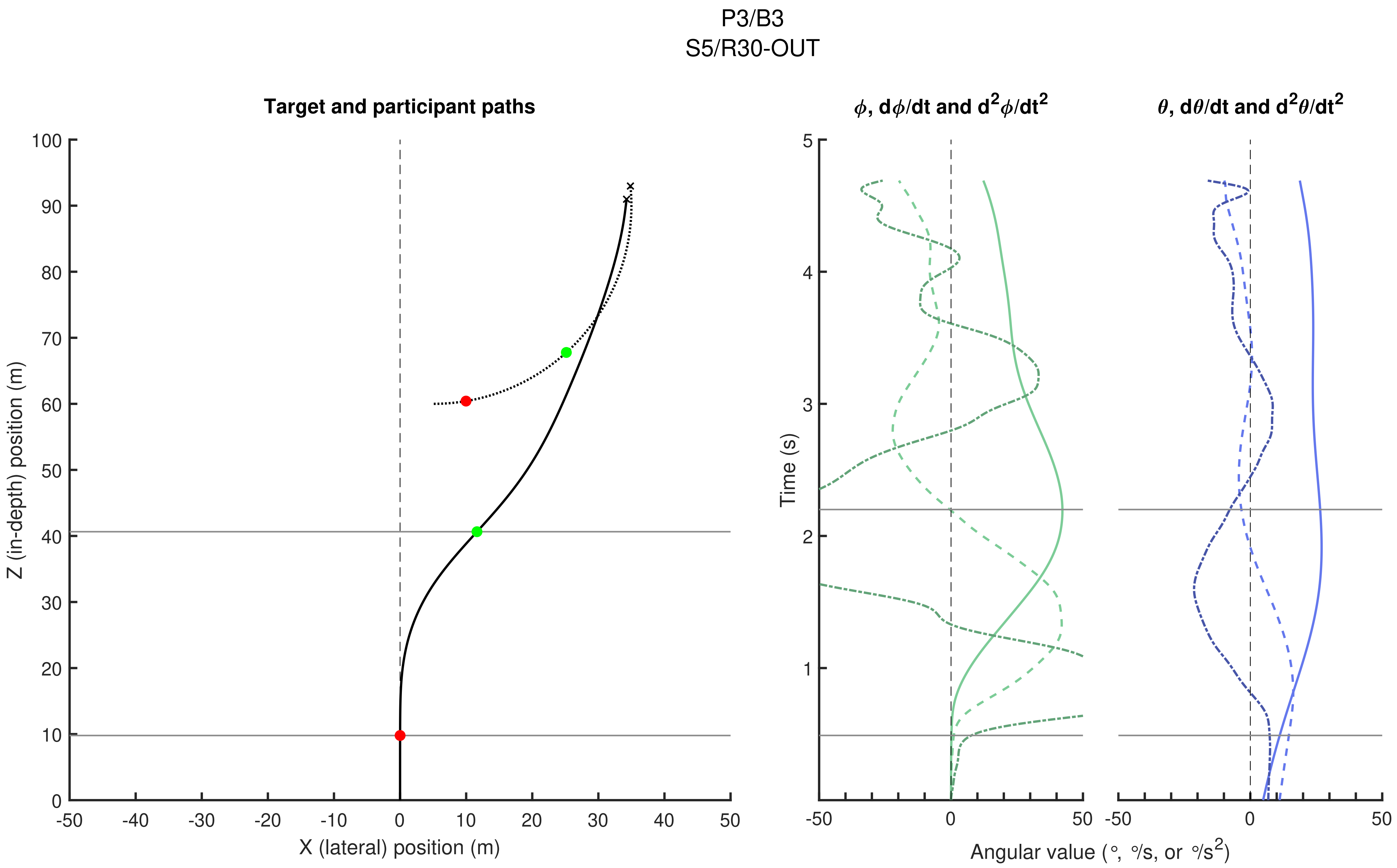

P3/B3
S5/R30-OUT
Target and participant paths
ϕ, dϕ/dt and d²ϕ/dt²
θ, dθ/dt and d²θ/dt²
Z (in-depth) position (m)
X (lateral) position (m)
Time (s)
Angular value (°, °/s, or °/s²)
0
10
20
30
40
50
60
70
80
90
100
-50
-40
-30
-20
-10
1
2
3
4
5

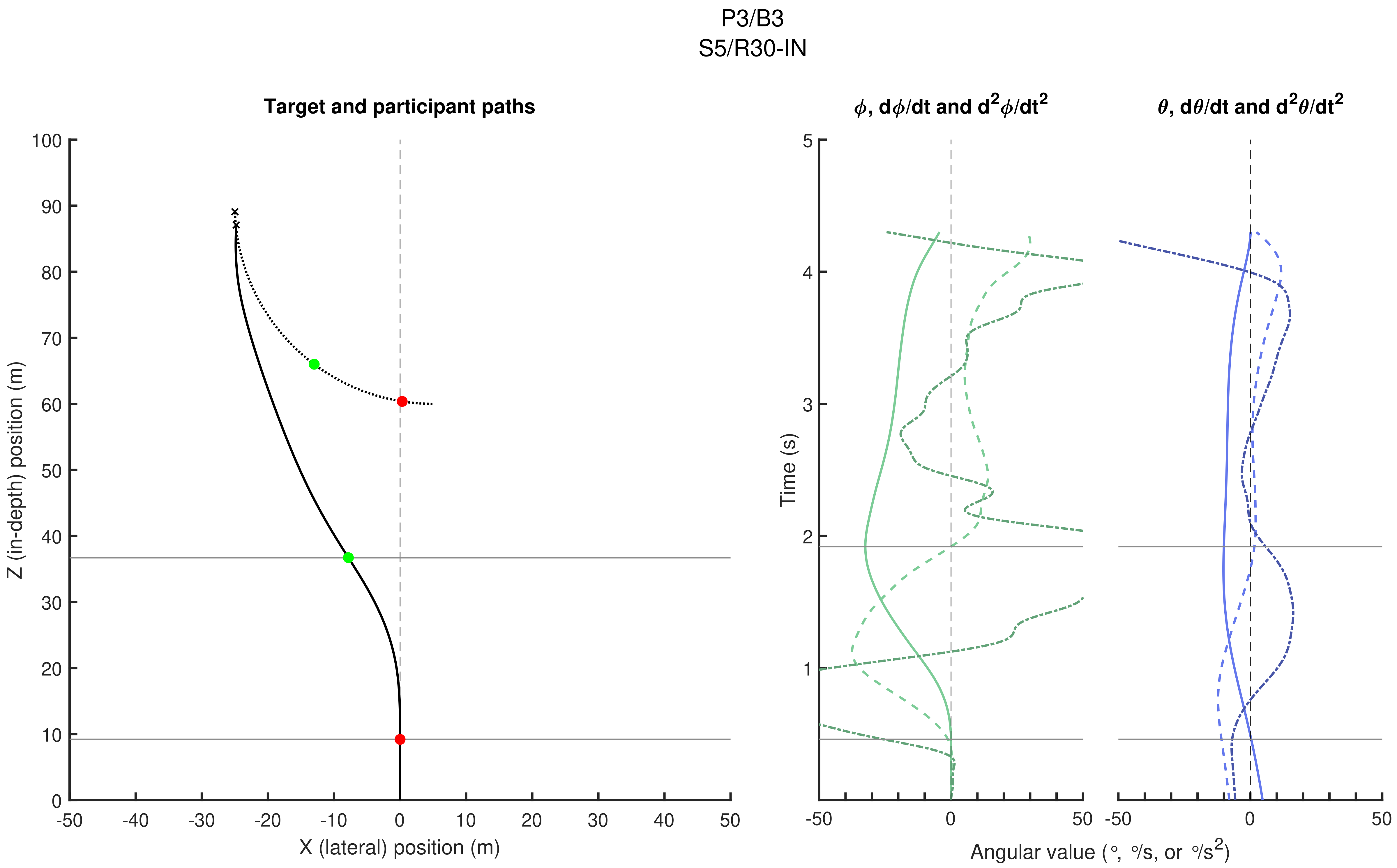

P3/B3
S5/R30-IN
Target and participant paths
Z (in-depth) position (m)
X (lateral) position (m)
ϕ, dϕ/dt and d²ϕ/dt²
θ, dθ/dt and d²θ/dt²
Time (s)
Angular value (°, °/s, or °/s²)

P3/B3
S15/R10-IN

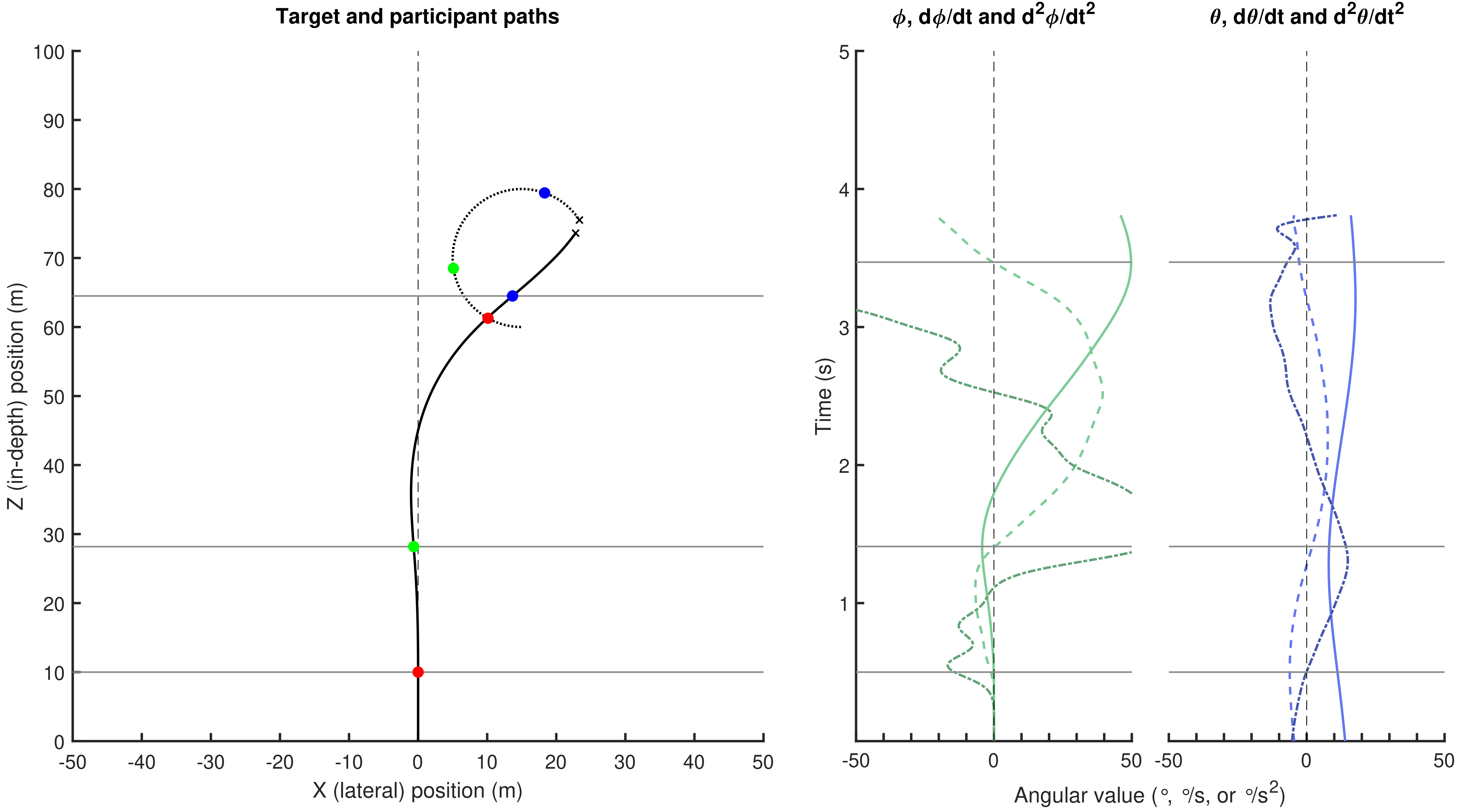

P3/B3
S15/R20-IN

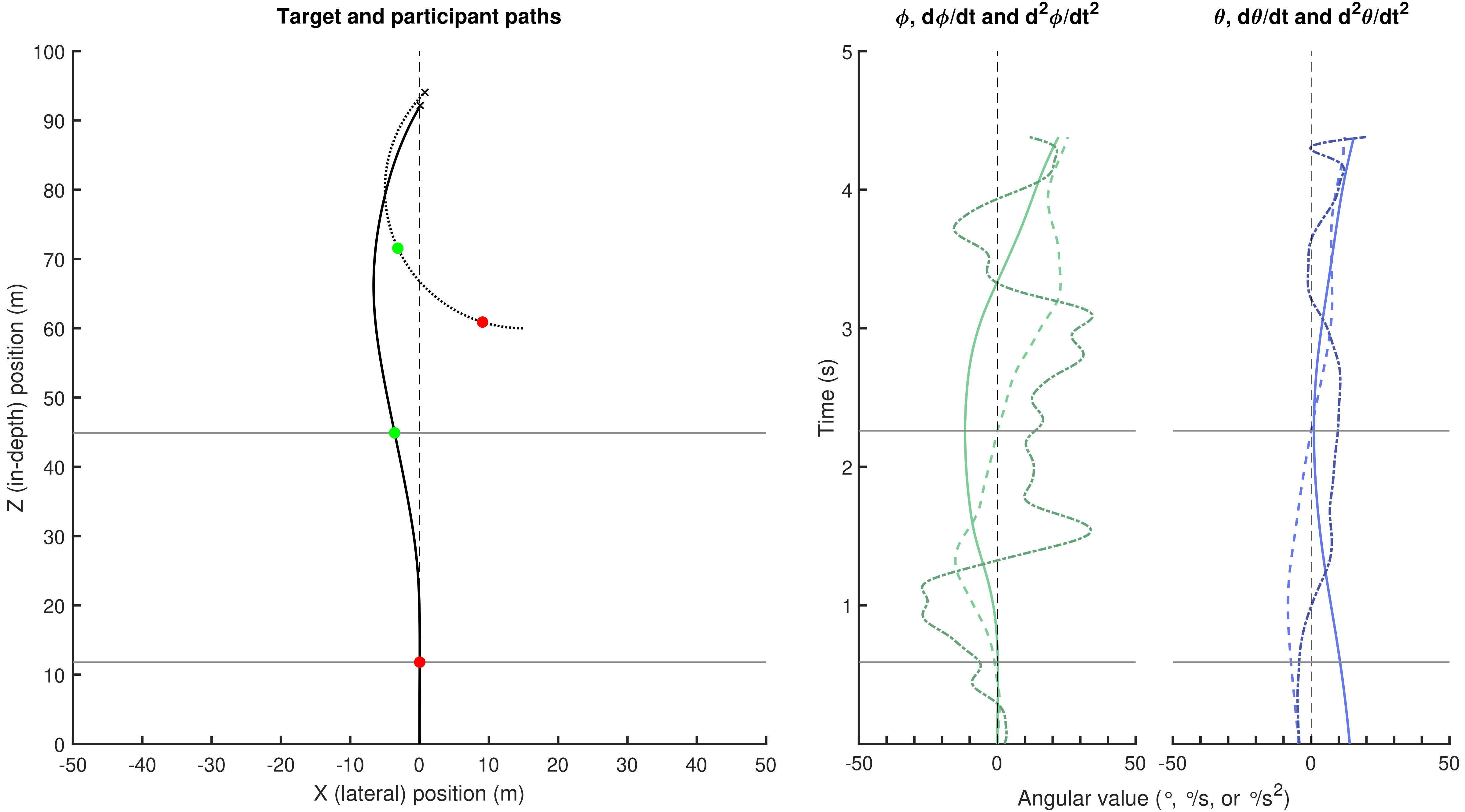

P3/B3
S15/R30-IN

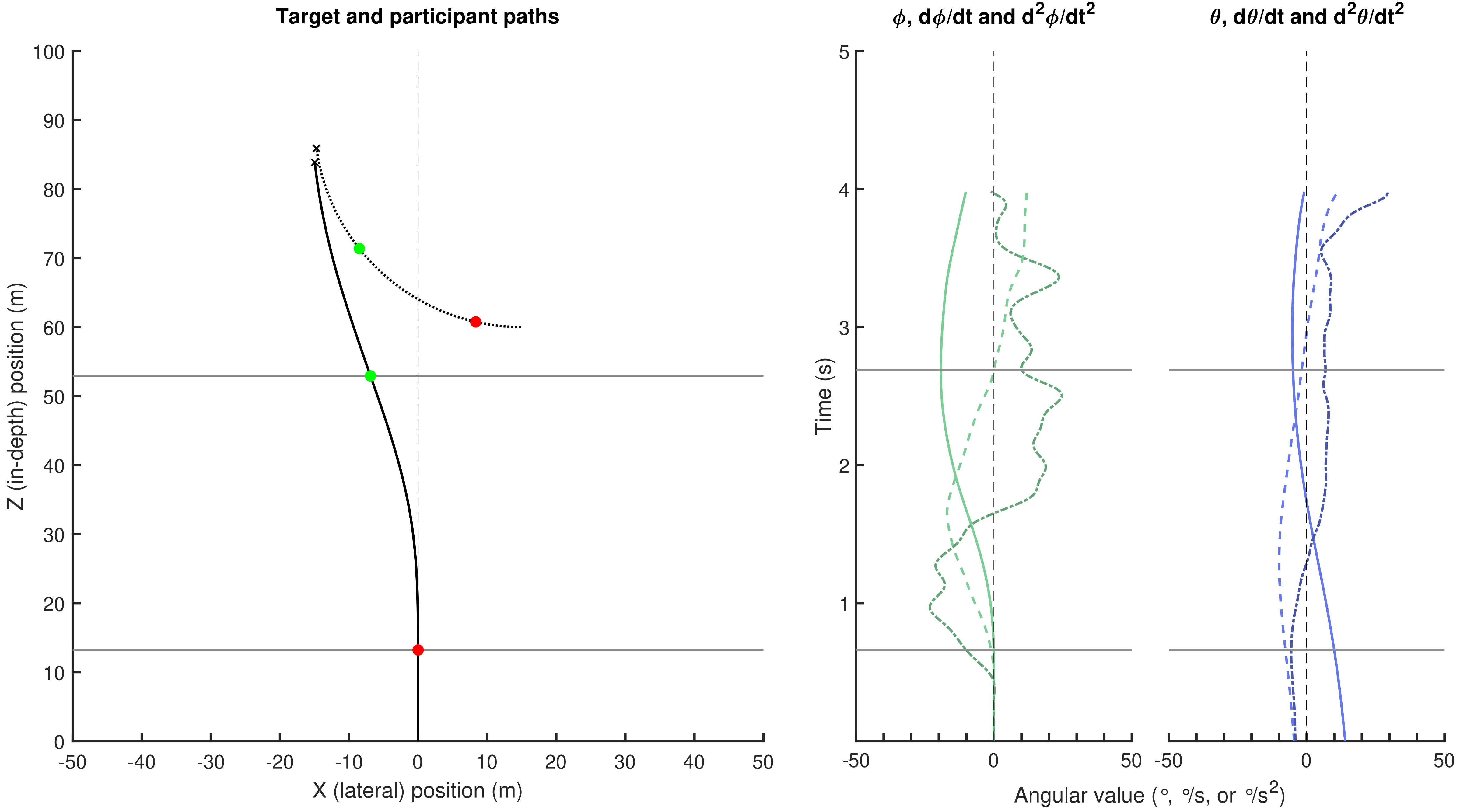

P3/B3
S20/R10-IN

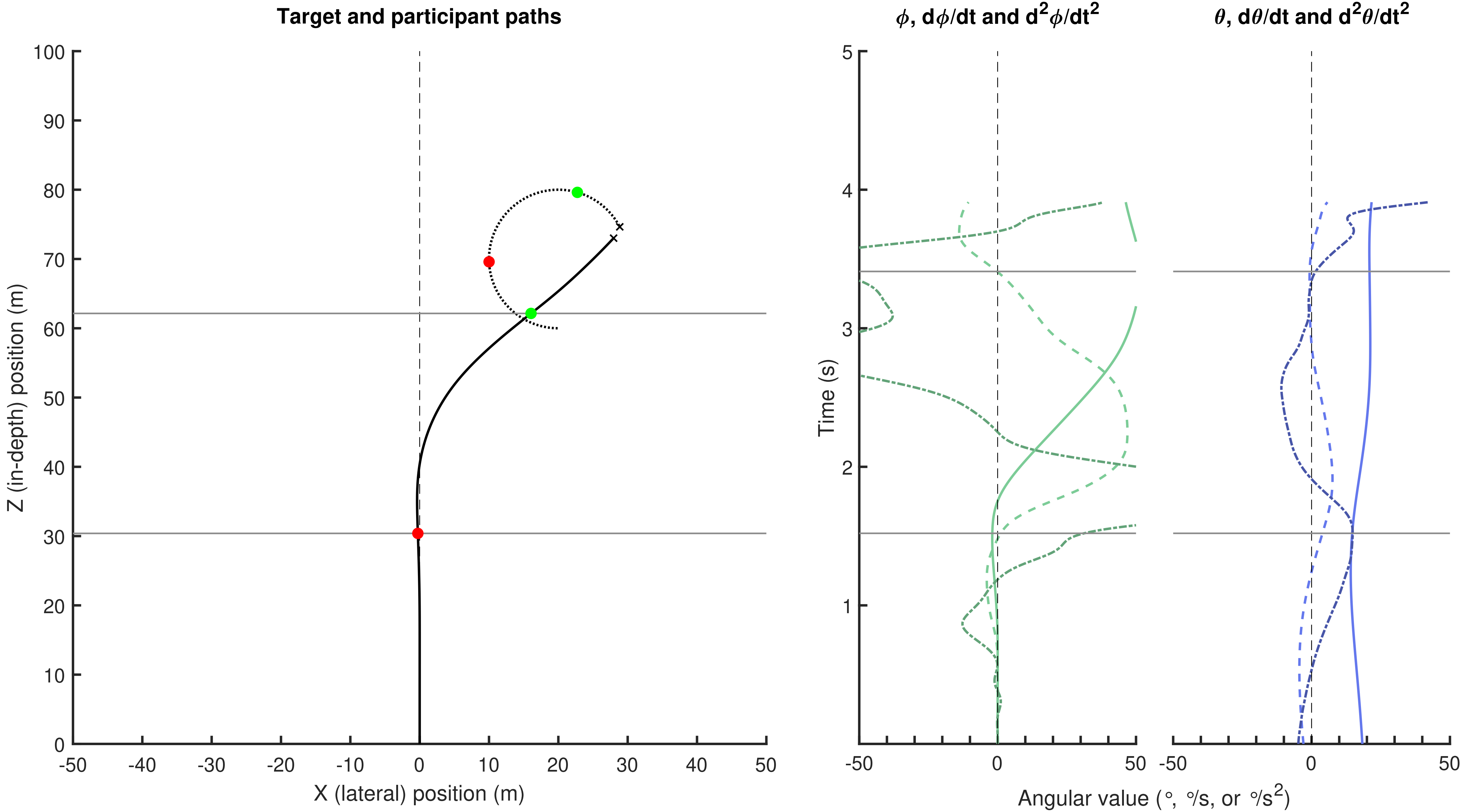

P3/B3
S20/R20-IN

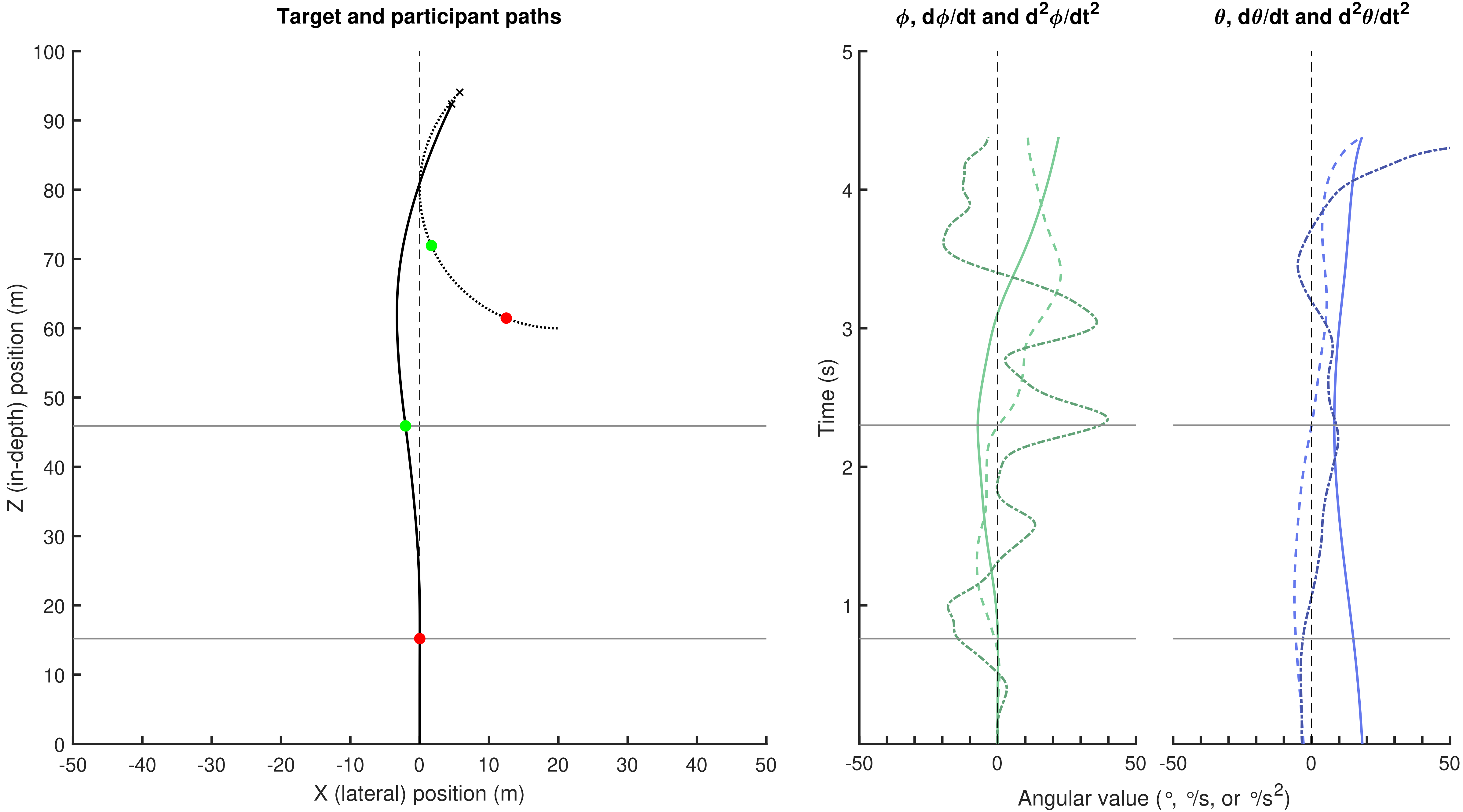

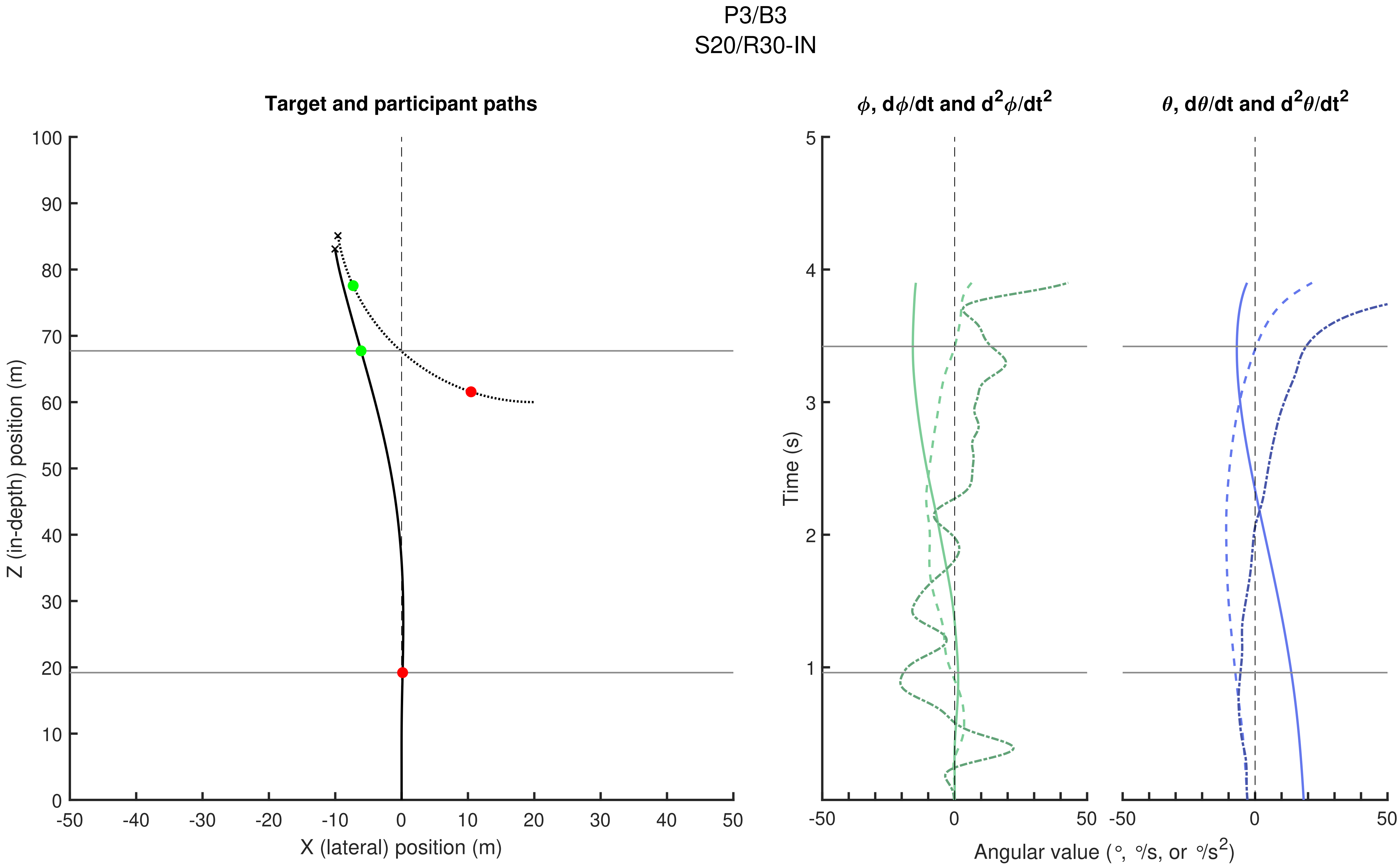

P3/B3
S20/R30-IN
Target and participant paths
Z (in-depth) position (m)
X (lateral) position (m)
ϕ, dϕ/dt and d$^2\phi$/dt^2
θ, dθ/dt and d$^2\theta$/dt^2
Time (s)
Angular value (°, °/s, or °/s^2)

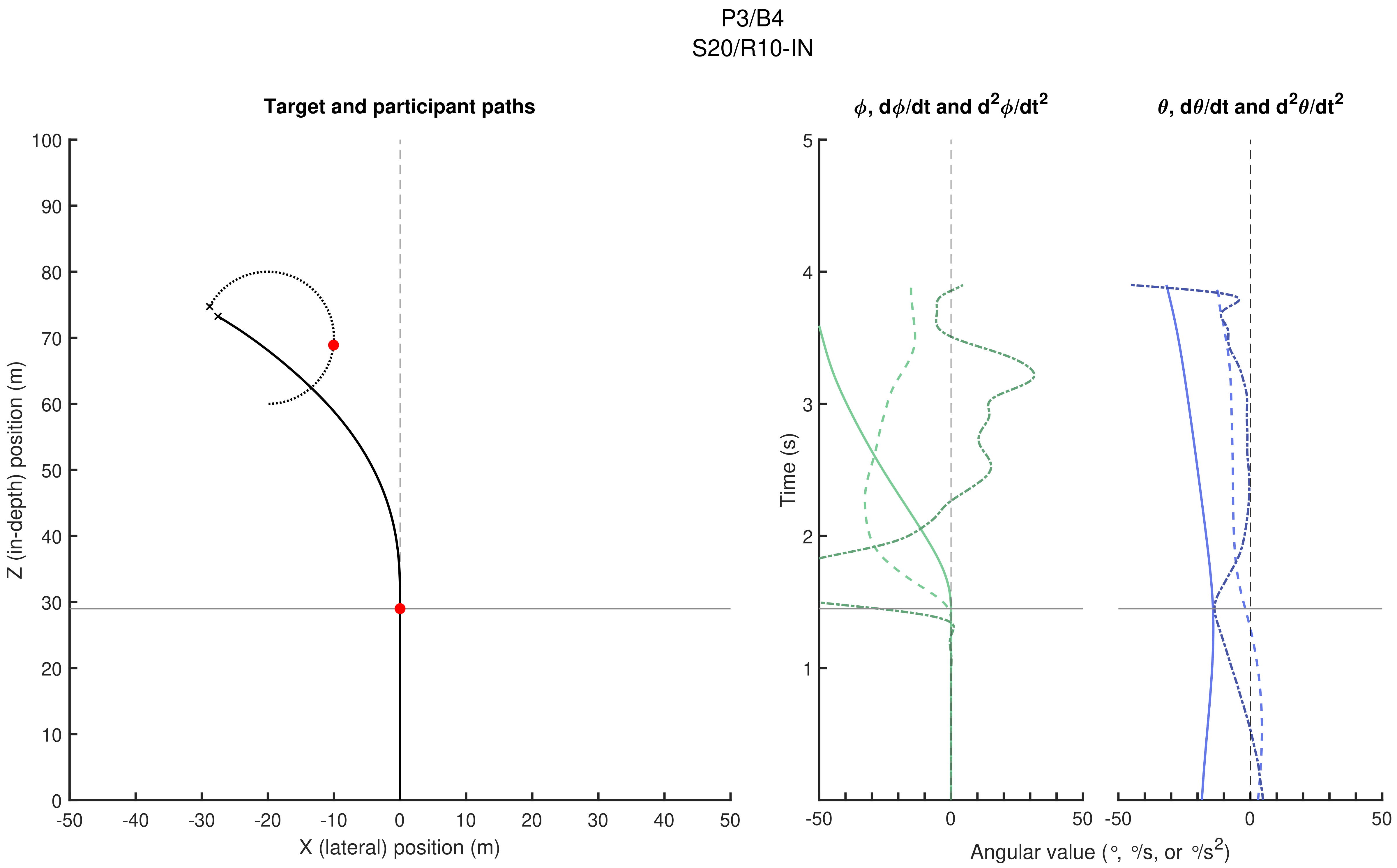

P3/B4
S20/R10-IN
Target and participant paths
Z (in-depth) position (m)
X (lateral) position (m)
ϕ, dϕ/dt and d$^2\phi$/dt^2
θ, dθ/dt and d$^2\theta$/dt^2
Time (s)
Angular value (°, °/s, or °/s^2)

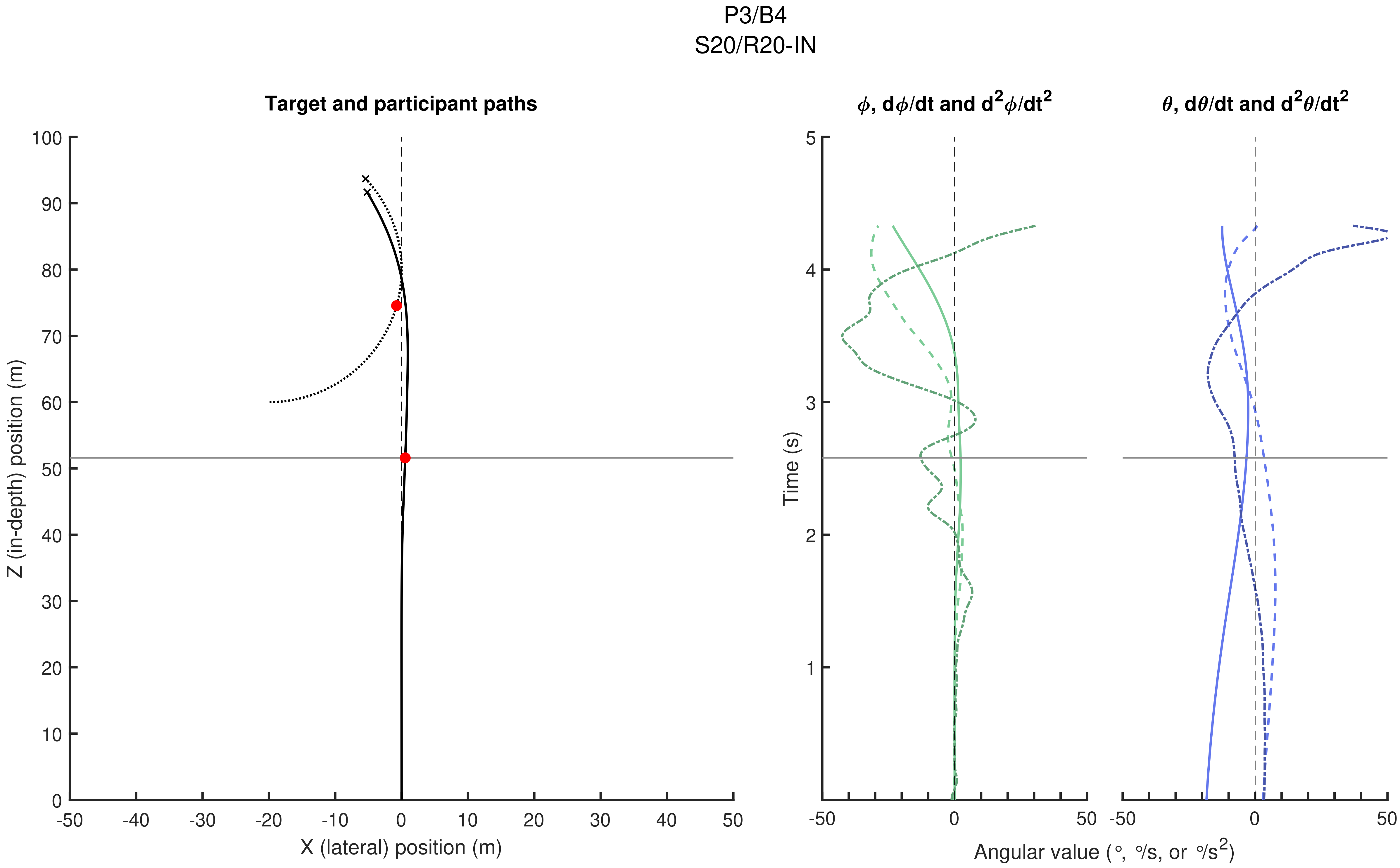
P3/B4
S20/R20-IN
Target and participant paths
Z (in-depth) position (m)
X (lateral) position (m)
100
90
80
70
60
50
40
30
20
10
0
-50
-40
-30
-20
-10
0
10
20
30
40
50
ϕ, dϕ/dt and d²ϕ/dt²
θ, dθ/dt and d²θ/dt²
Time (s)
5
4
3
2
1
-50
0
50
-50
0
50
Angular value (°, °/s, or °/s²)

P3/B4
S20/R30-IN

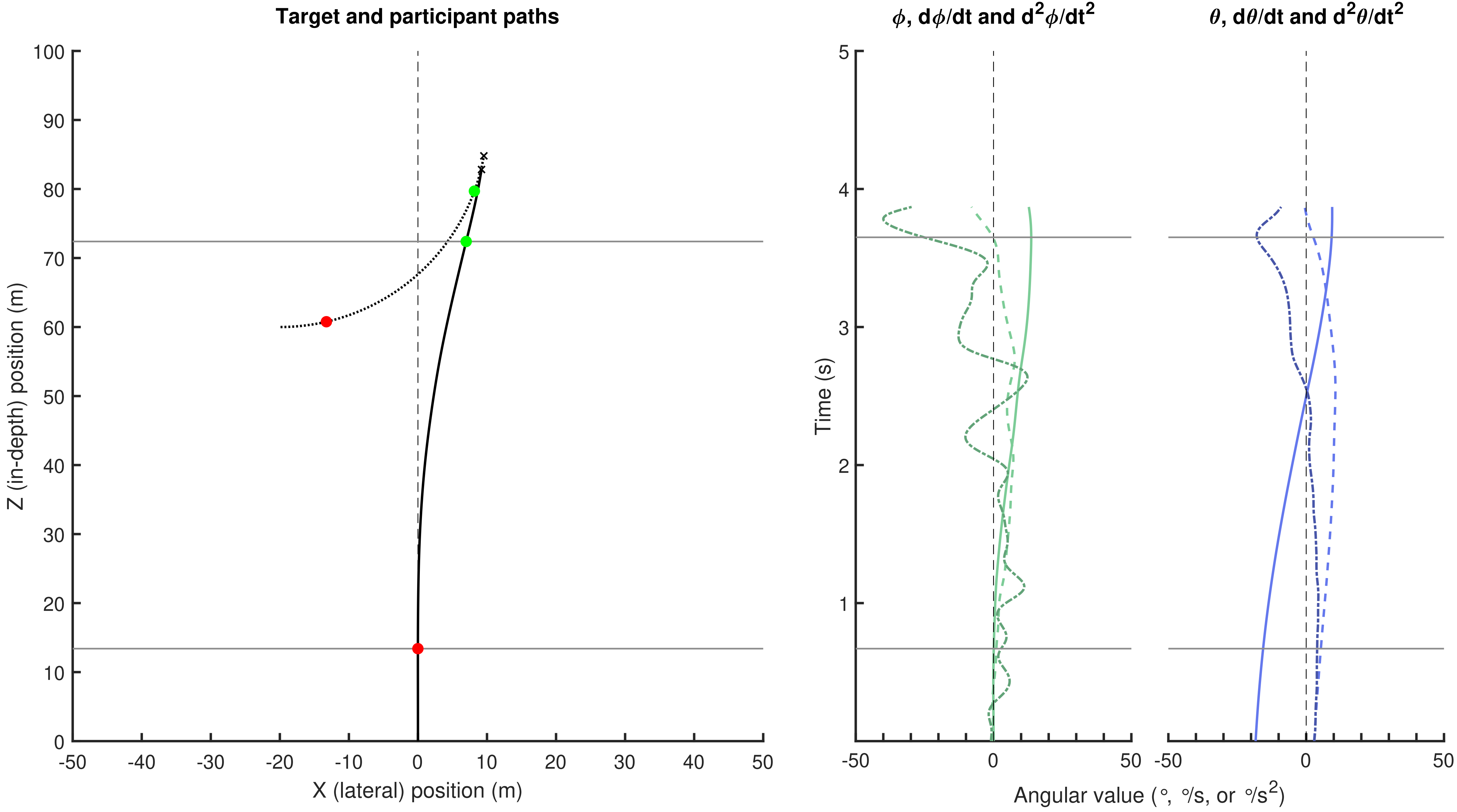

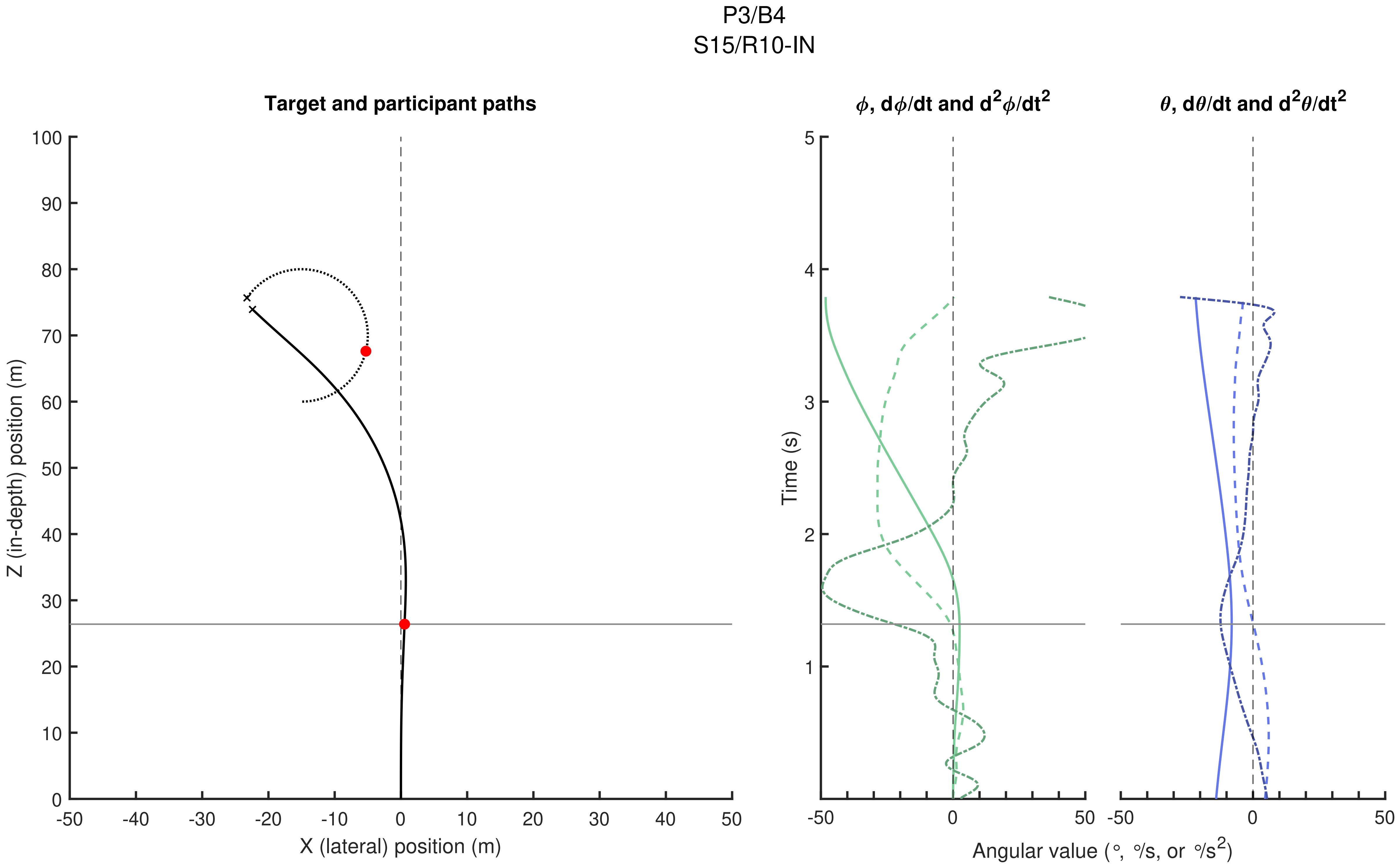
P3/B4
S15/R10-IN
Target and participant paths
Z (in-depth) position (m)
X (lateral) position (m)
φ, dφ/dt and d²φ/dt²
θ, dθ/dt and d²θ/dt²
Time (s)
Angular value (°, °/s, or °/s²)

P3/B4
S15/R20-IN

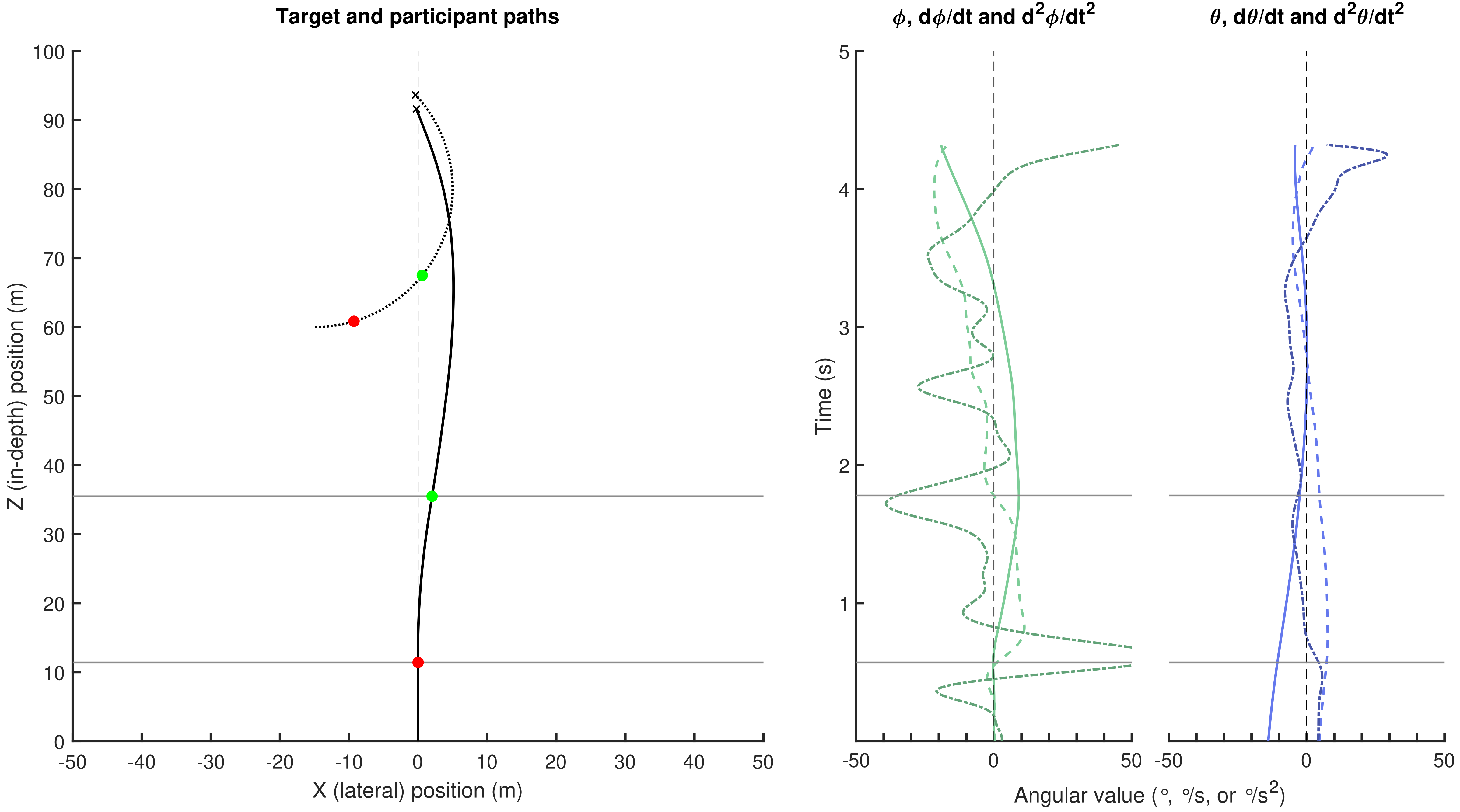

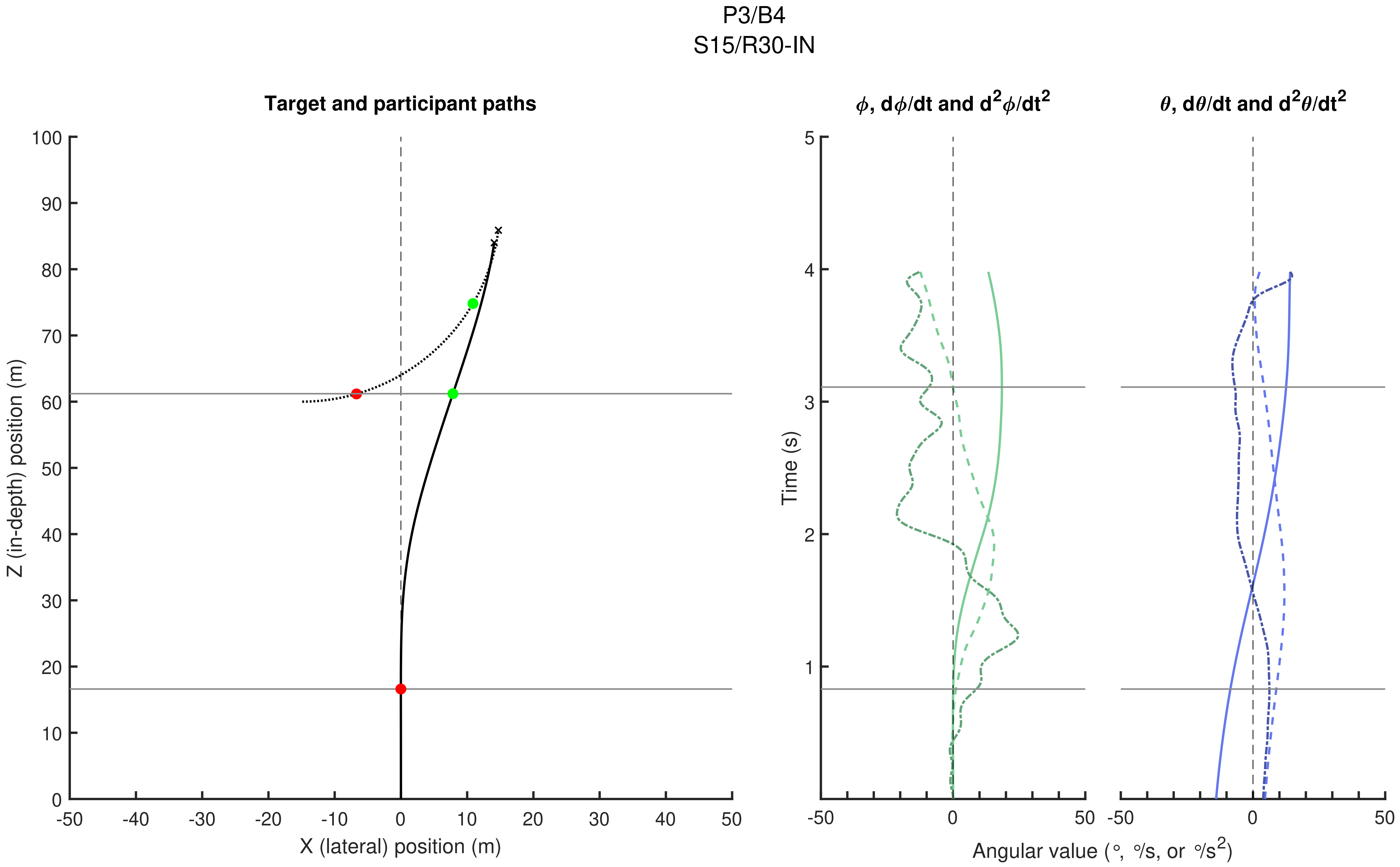
P3/B4
S15/R30-IN
Target and participant paths
Z (in-depth) position (m)
X (lateral) position (m)
100
90
80
70
60
50
40
30
20
10
0
-50
-40
-30
-20
-10
0
10
20
30
40
50
ϕ, dϕ/dt and d$^2\phi$/dt^2
θ, dθ/dt and d$^2\theta$/dt^2
Time (s)
5
4
3
2
1
-50
0
50
-50
0
50
Angular value (°, °/s, or °/s^2)

P3/B4
S5/R10-IN

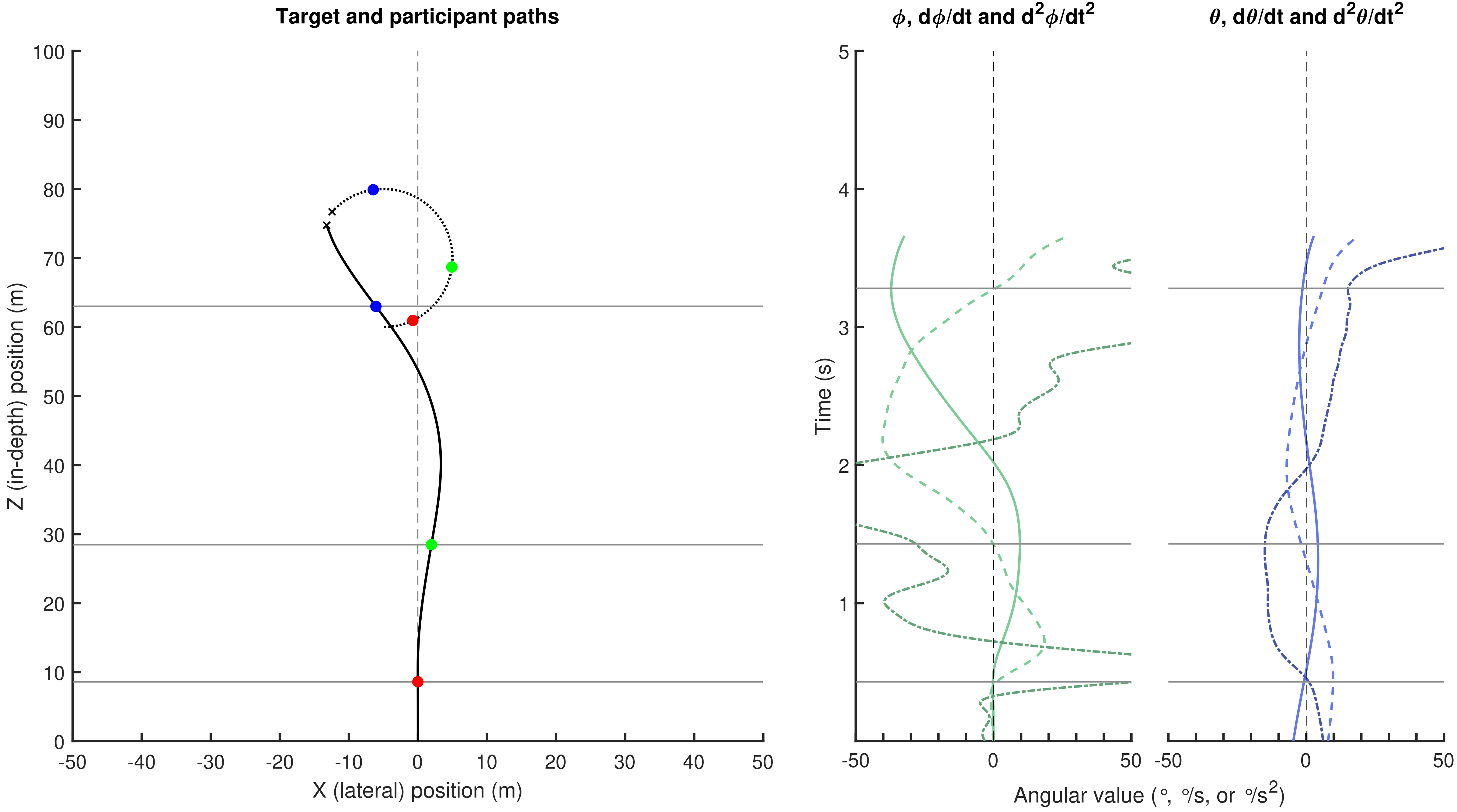

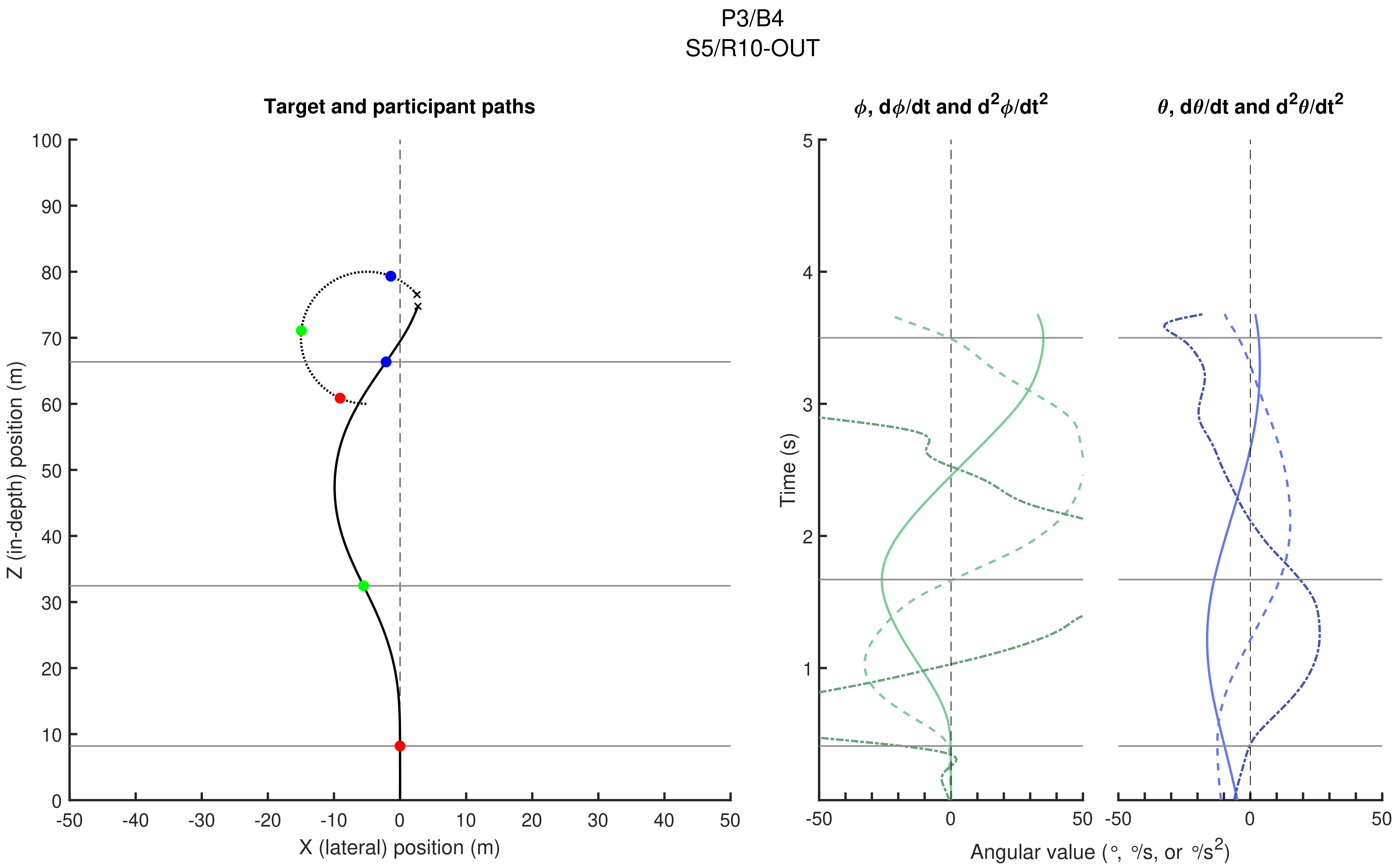

P3/B4
S5/R10-OUT
Target and participant paths
Z (in-depth) position (m)
X (lateral) position (m)
ϕ, dϕ/dt and d$^2\phi$/dt^2
θ, dθ/dt and d$^2\theta$/dt^2
Time (s)
Angular value (°, °/s, or °/s^2)

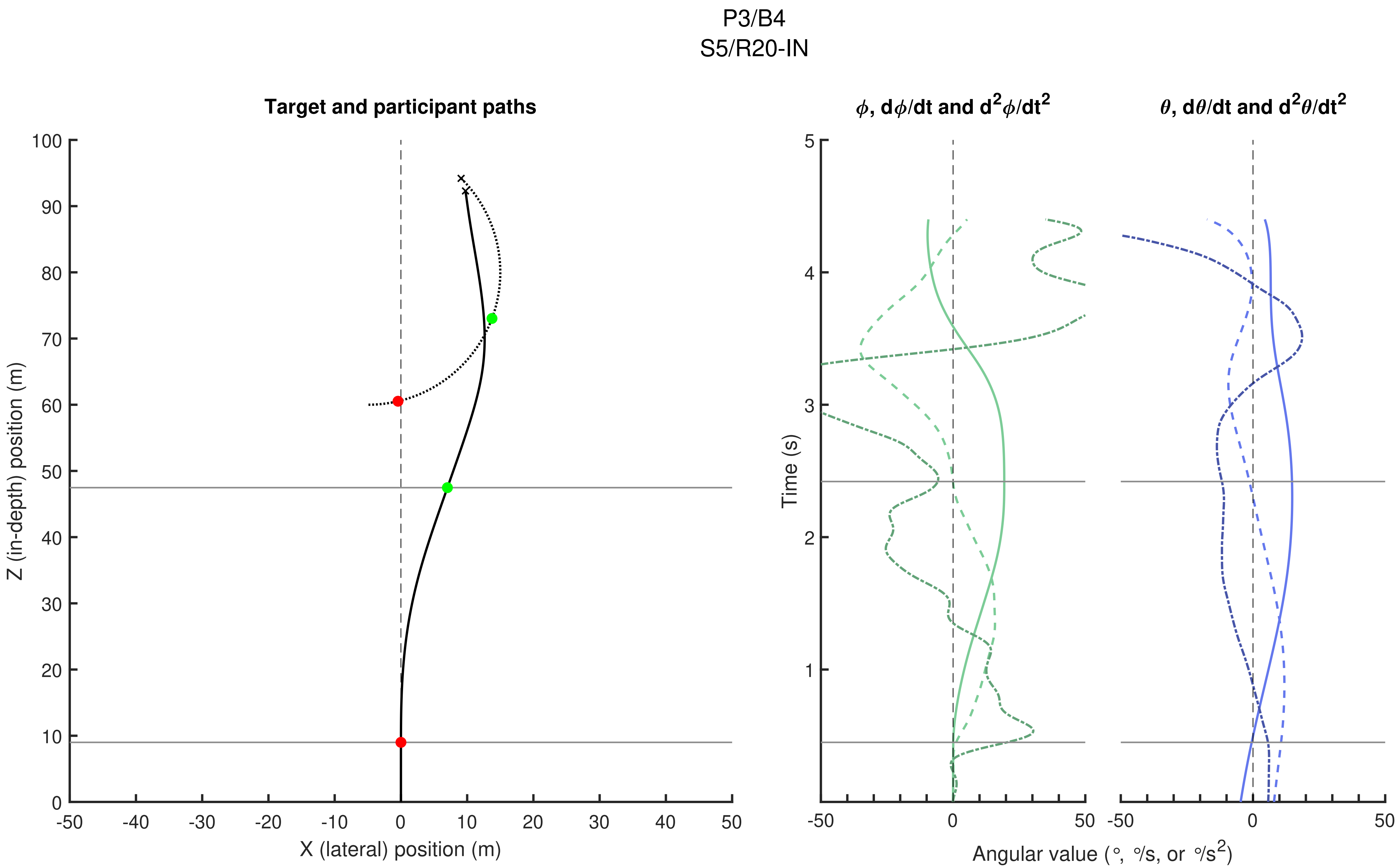

P3/B4
S5/R20-IN
Target and participant paths
Z (in-depth) position (m)
X (lateral) position (m)
ϕ, dϕ/dt and d$^2\phi$/dt^2
θ, dθ/dt and d$^2\theta$/dt^2
Time (s)
Angular value (°, °/s, or °/s^2)

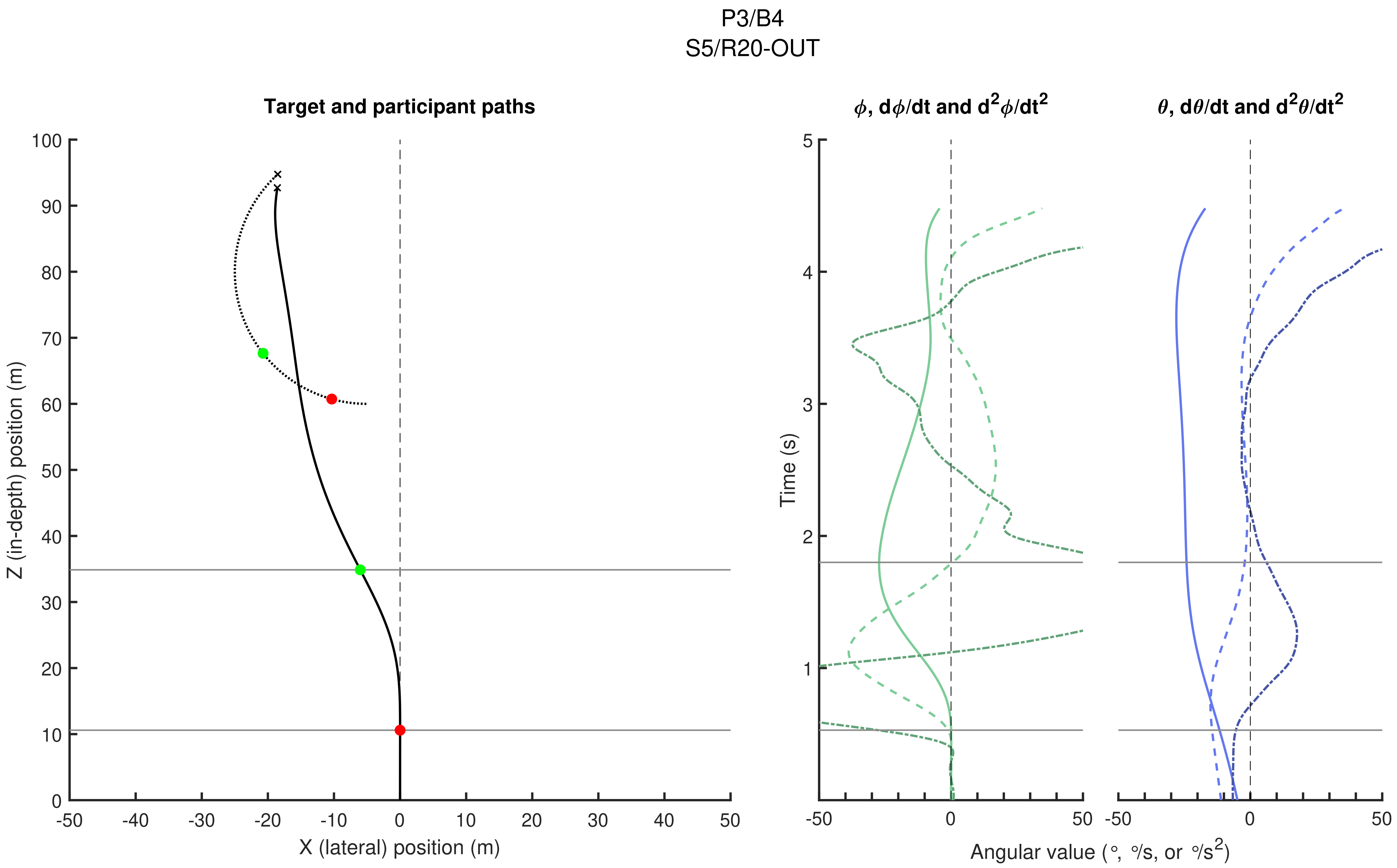

P3/B4
S5/R20-OUT
Target and participant paths
Z (in-depth) position (m)
X (lateral) position (m)
ϕ, dϕ/dt and d²ϕ/dt²
θ, dθ/dt and d²θ/dt²
Time (s)
Angular value (°, °/s, or °/s²)

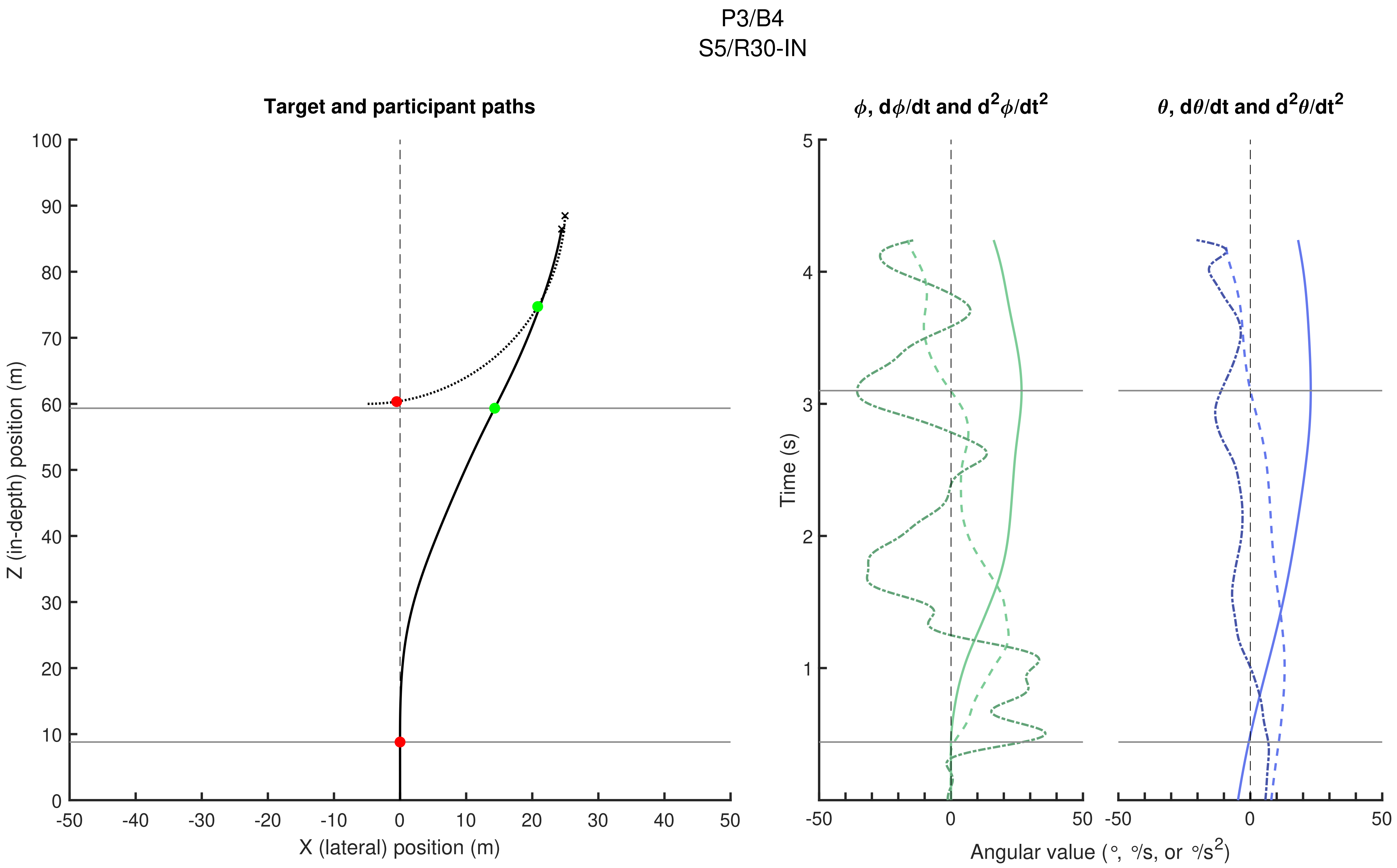
P3/B4
S5/R30-IN
Target and participant paths
ϕ, dϕ/dt and d$^2\phi$/dt^2
θ, dθ/dt and d$^2\theta$/dt^2
Z (in-depth) position (m)
X (lateral) position (m)
Time (s)
Angular value (°, °/s, or °/s^2)
0
10
20
30
40
50
60
70
80
90
100
-50
-40
-30
-20
-10
0
10
20
30
40
50
1
2
3
4
5
-50
0
50
-50
0
50

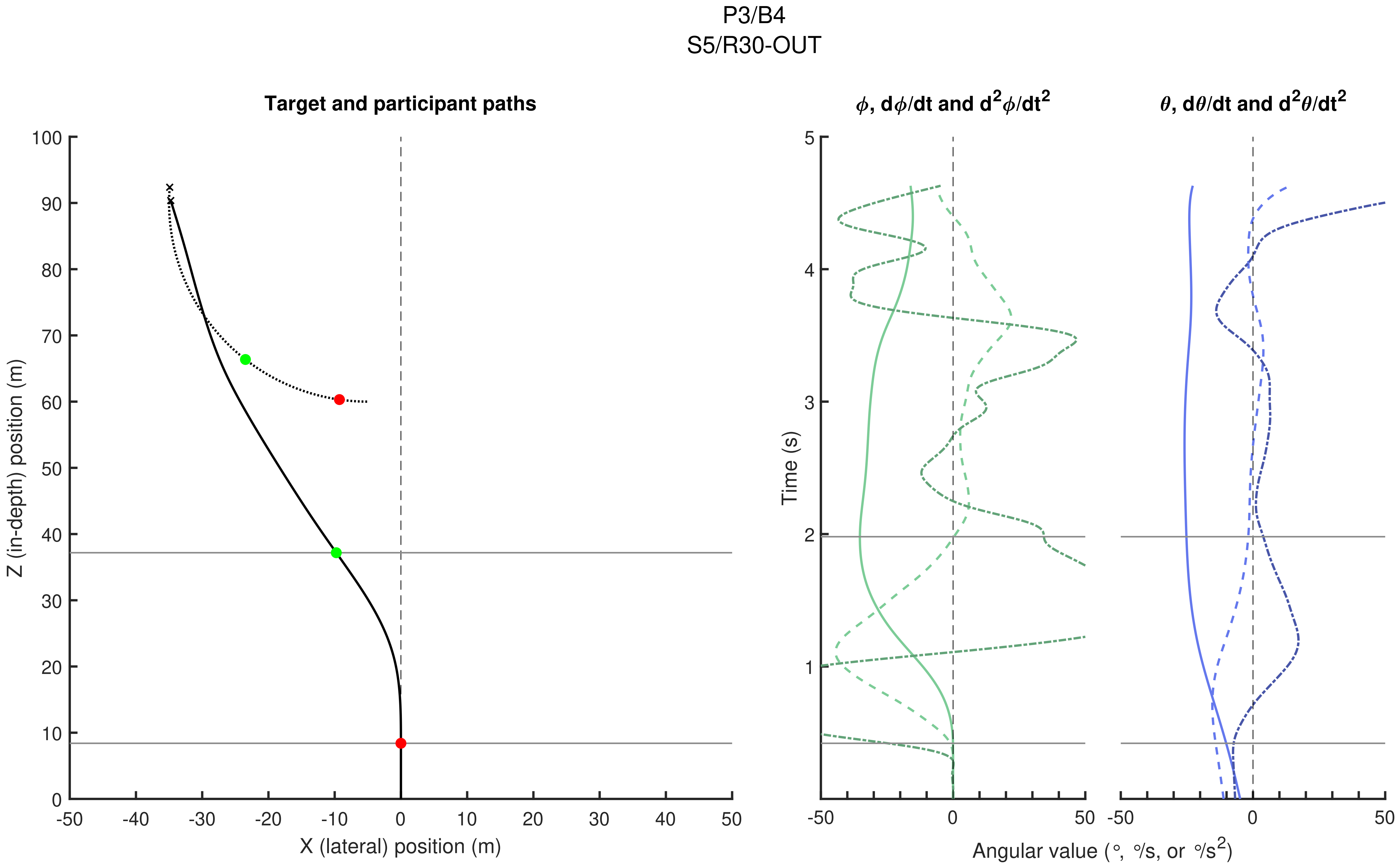

P3/B4
S5/R30-OUT
Target and participant paths
Z (in-depth) position (m)
X (lateral) position (m)
ϕ, dϕ/dt and d²ϕ/dt²
θ, dθ/dt and d²θ/dt²
Time (s)
Angular value (°, °/s, or °/s²)

P3/B4
S5/R10-OUT

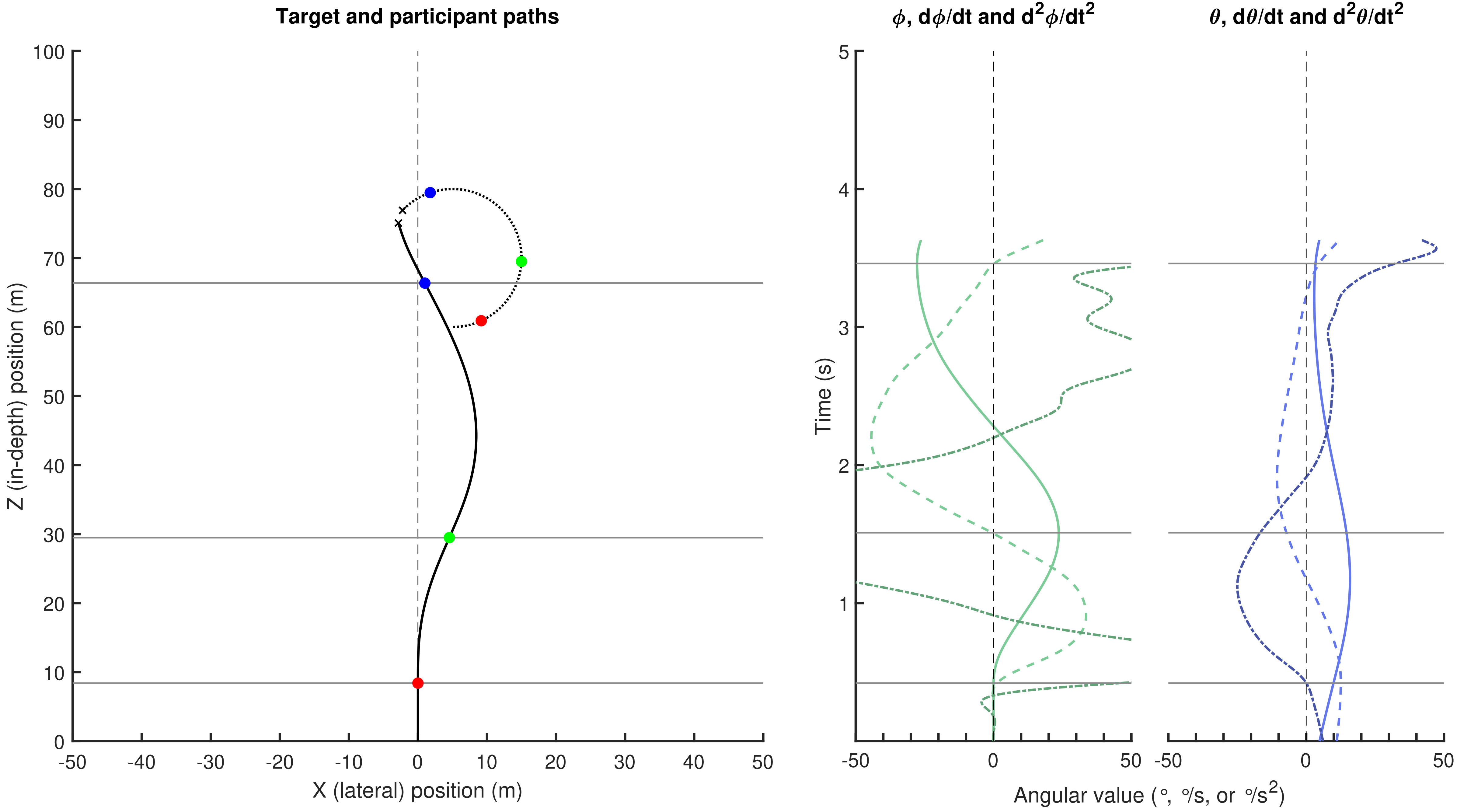

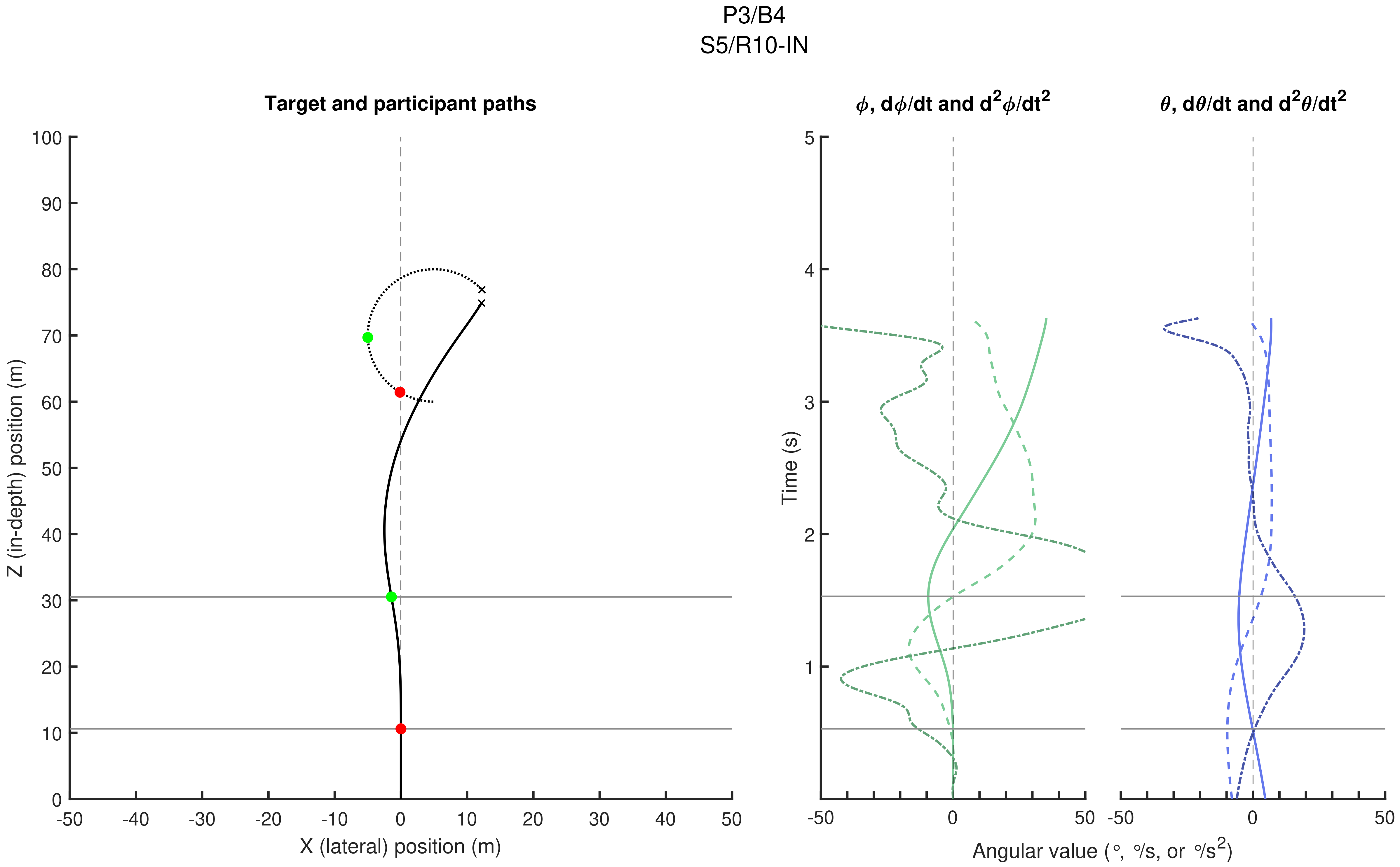
P3/B4
S5/R10-IN
Target and participant paths
Z (in-depth) position (m)
X (lateral) position (m)
φ, dφ/dt and d²φ/dt²
θ, dθ/dt and d²θ/dt²
Time (s)
Angular value (°, °/s, or °/s²)

P3/B4
S5/R20-OUT

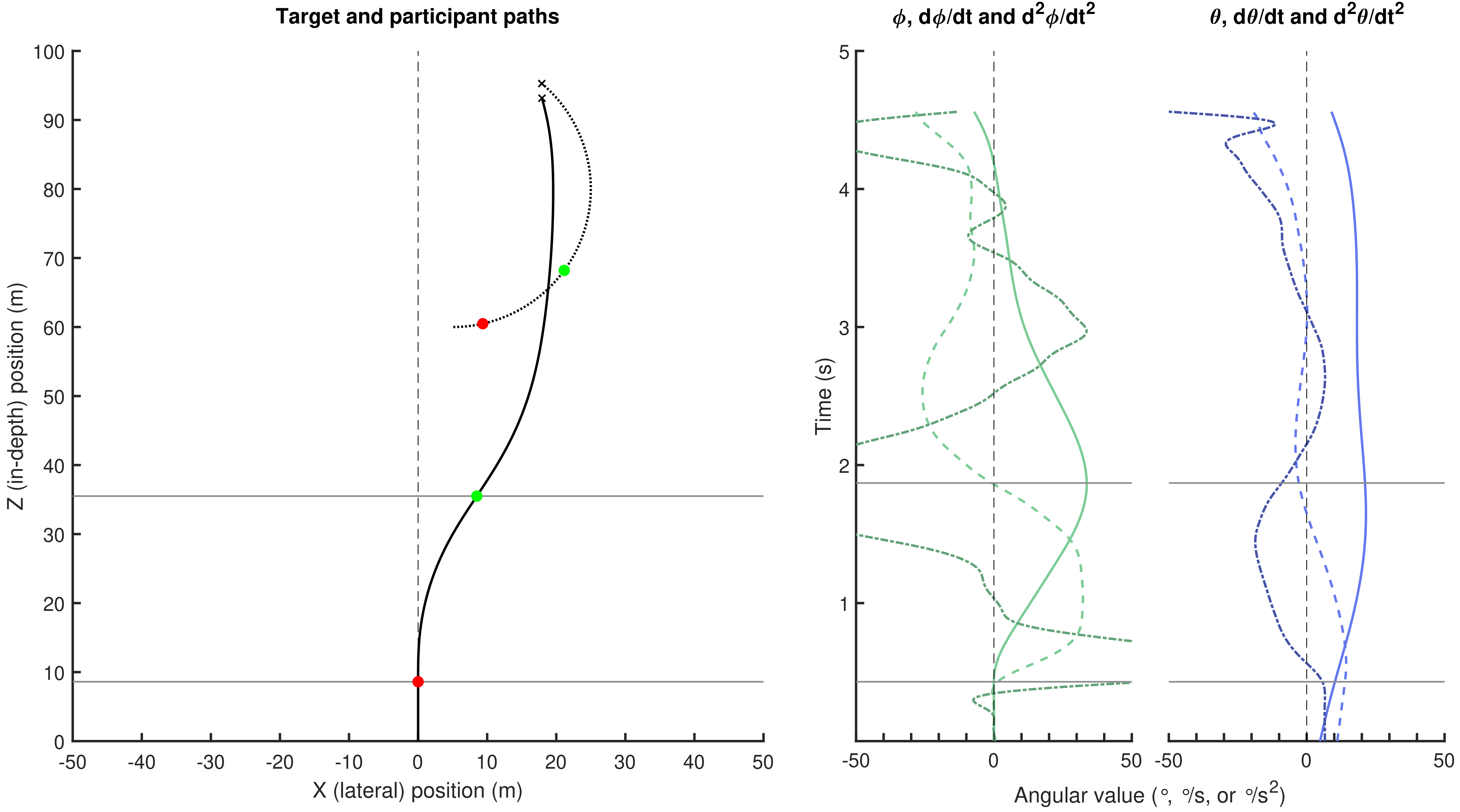

P3/B4
S5/R20-IN

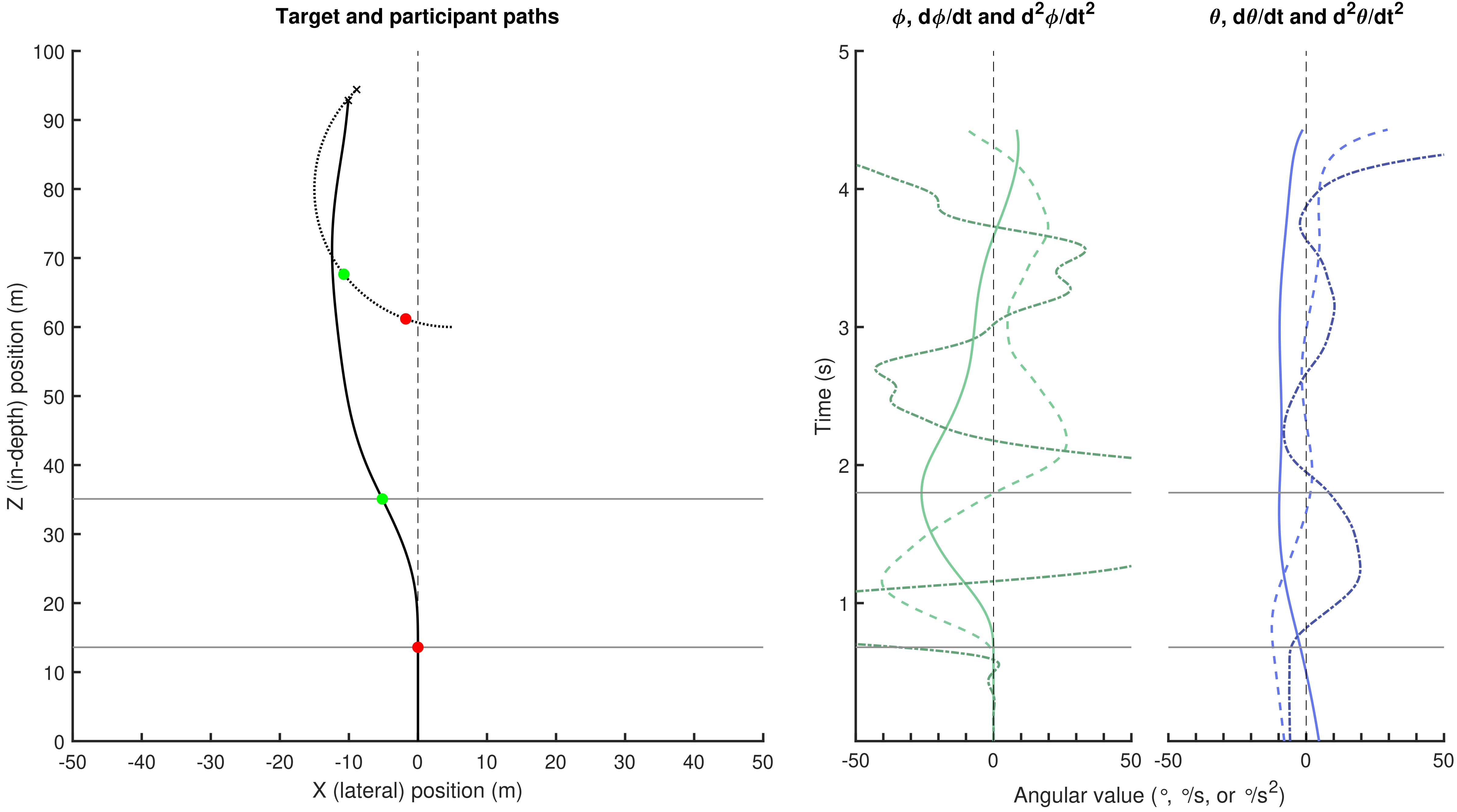

P3/B4
S5/R30-OUT

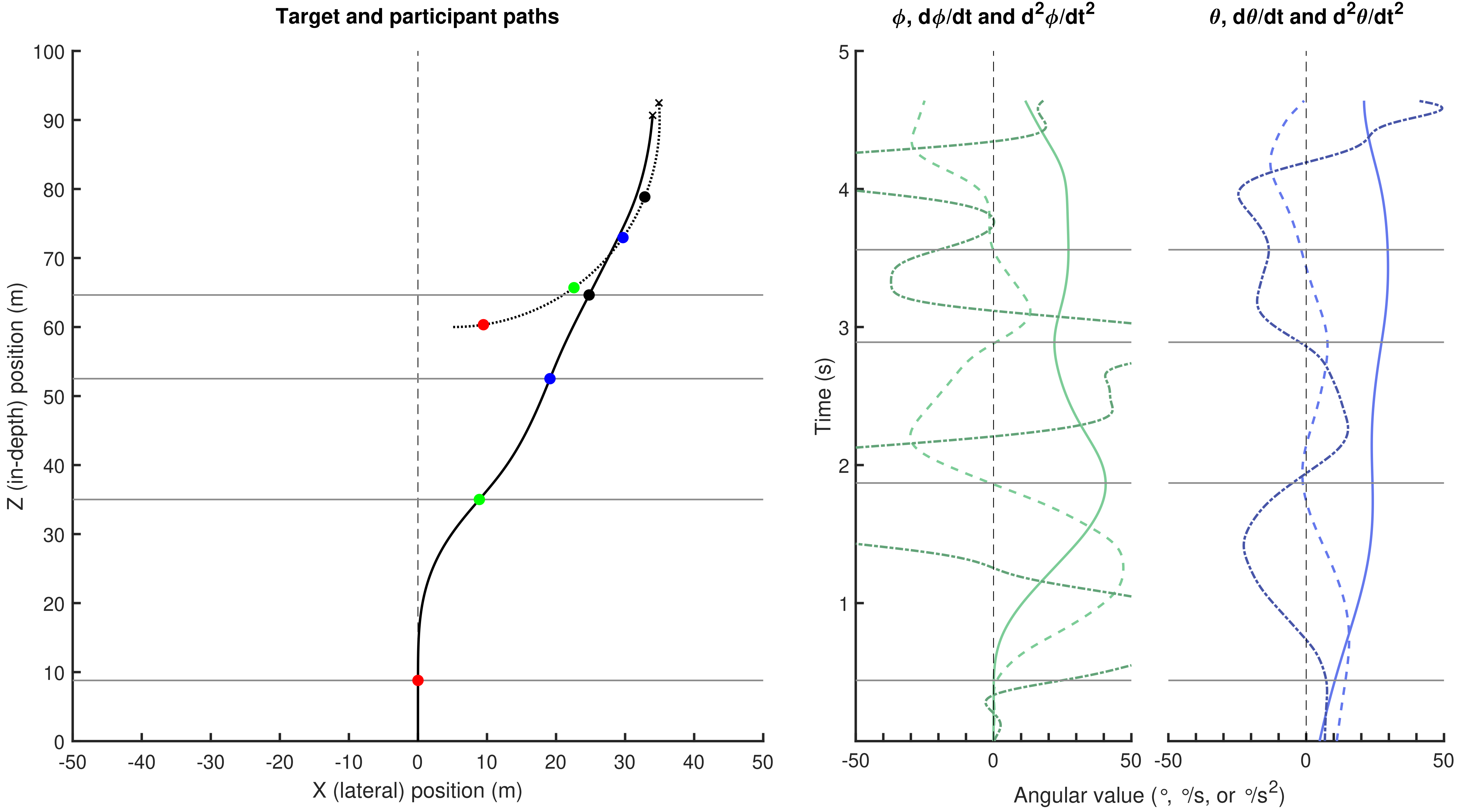

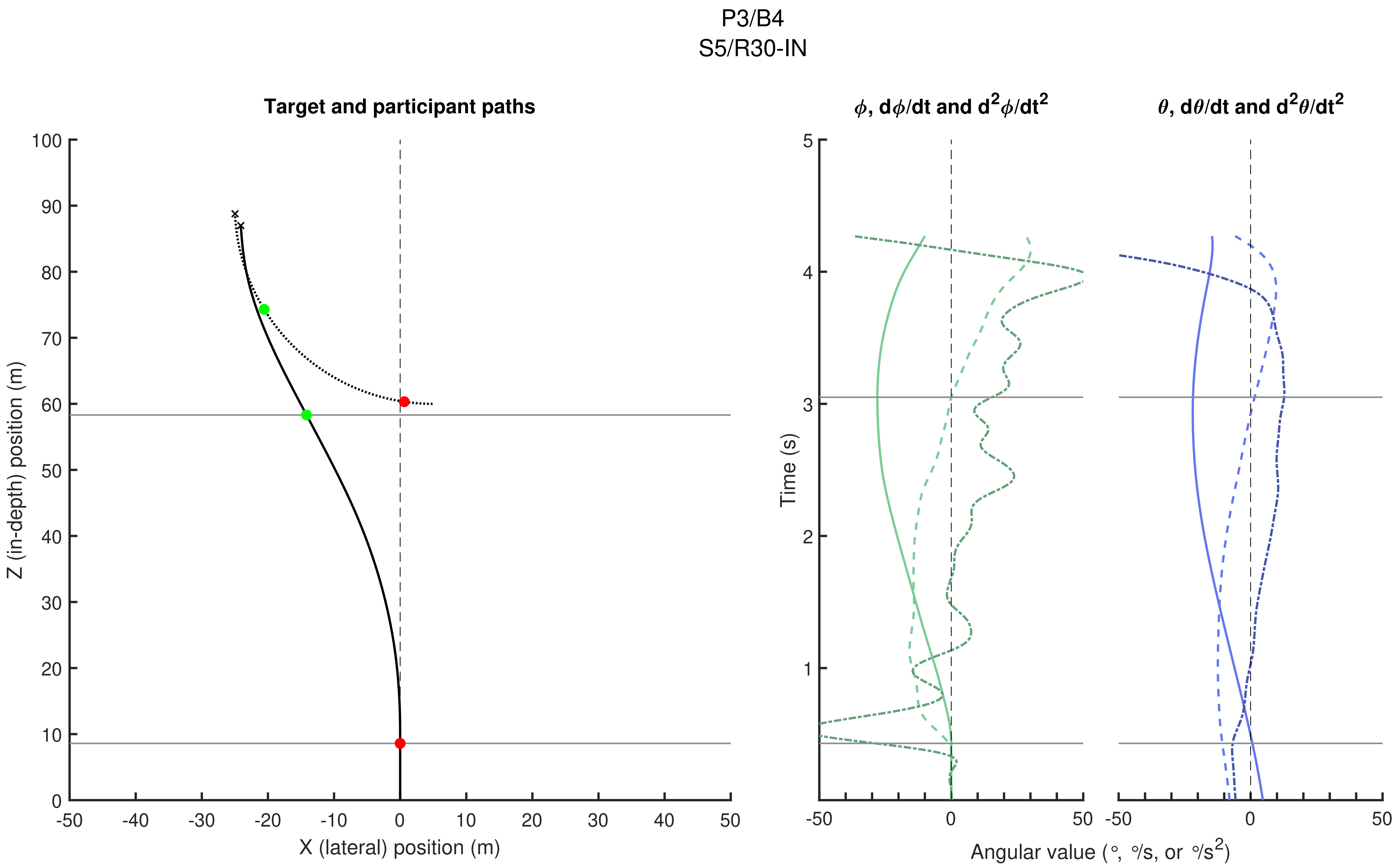
P3/B4
S5/R30-IN
Target and participant paths
Z (in-depth) position (m)
X (lateral) position (m)
ϕ, dϕ/dt and d²ϕ/dt²
θ, dθ/dt and d²θ/dt²
Time (s)
Angular value (°, °/s, or °/s²)

P3/B4
S15/R10-IN

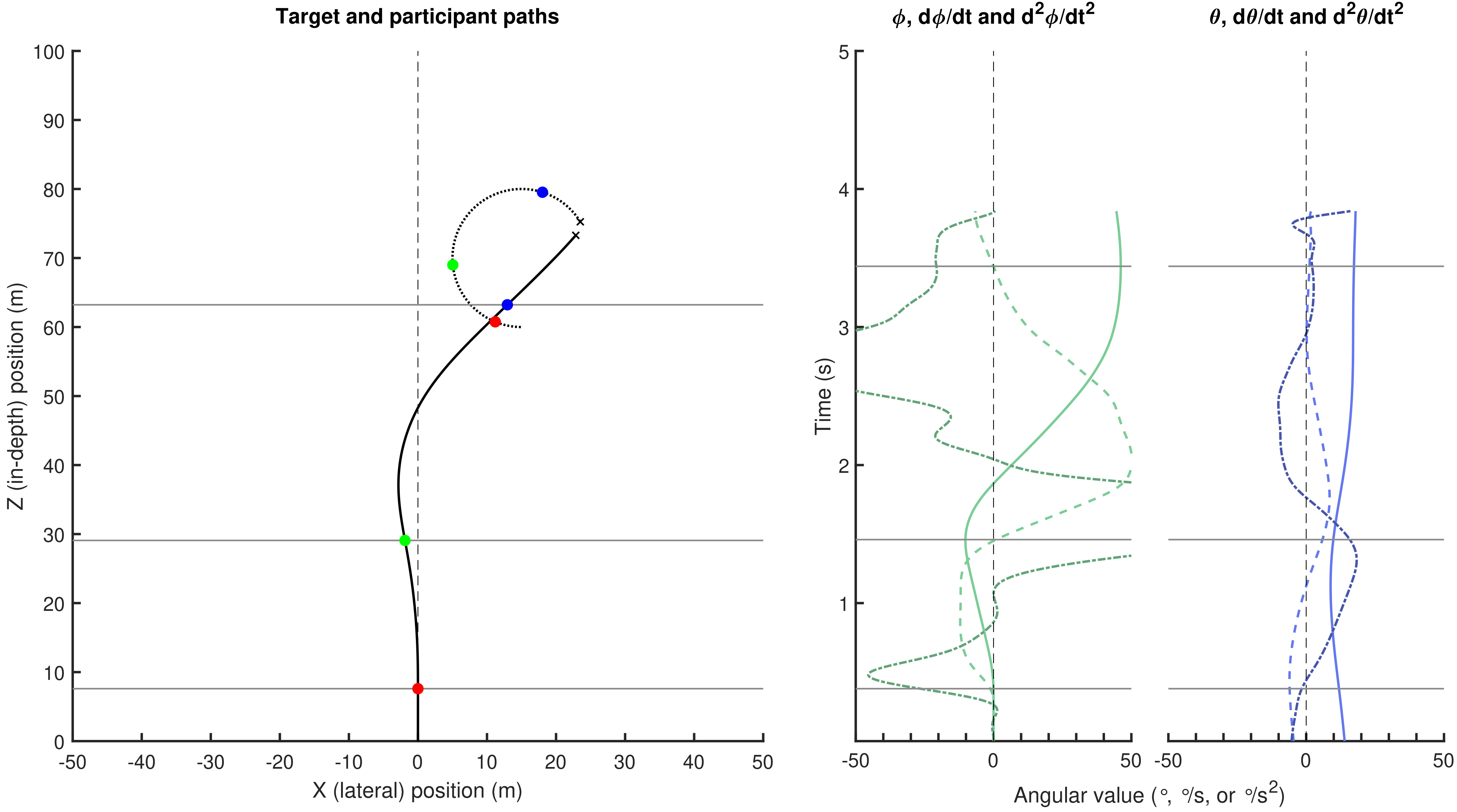

P3/B4
S15/R20-IN

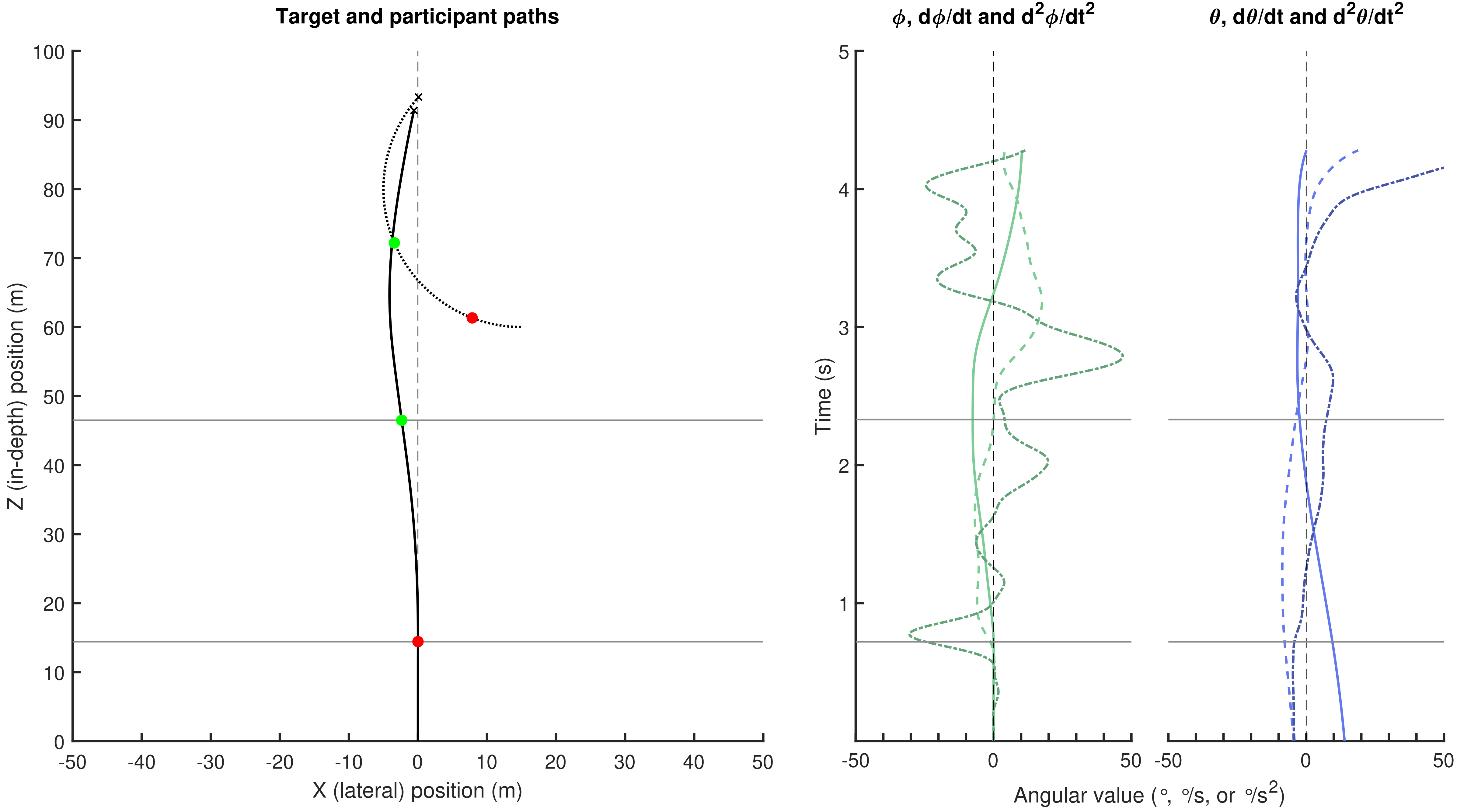

P3/B4
S15/R30-IN

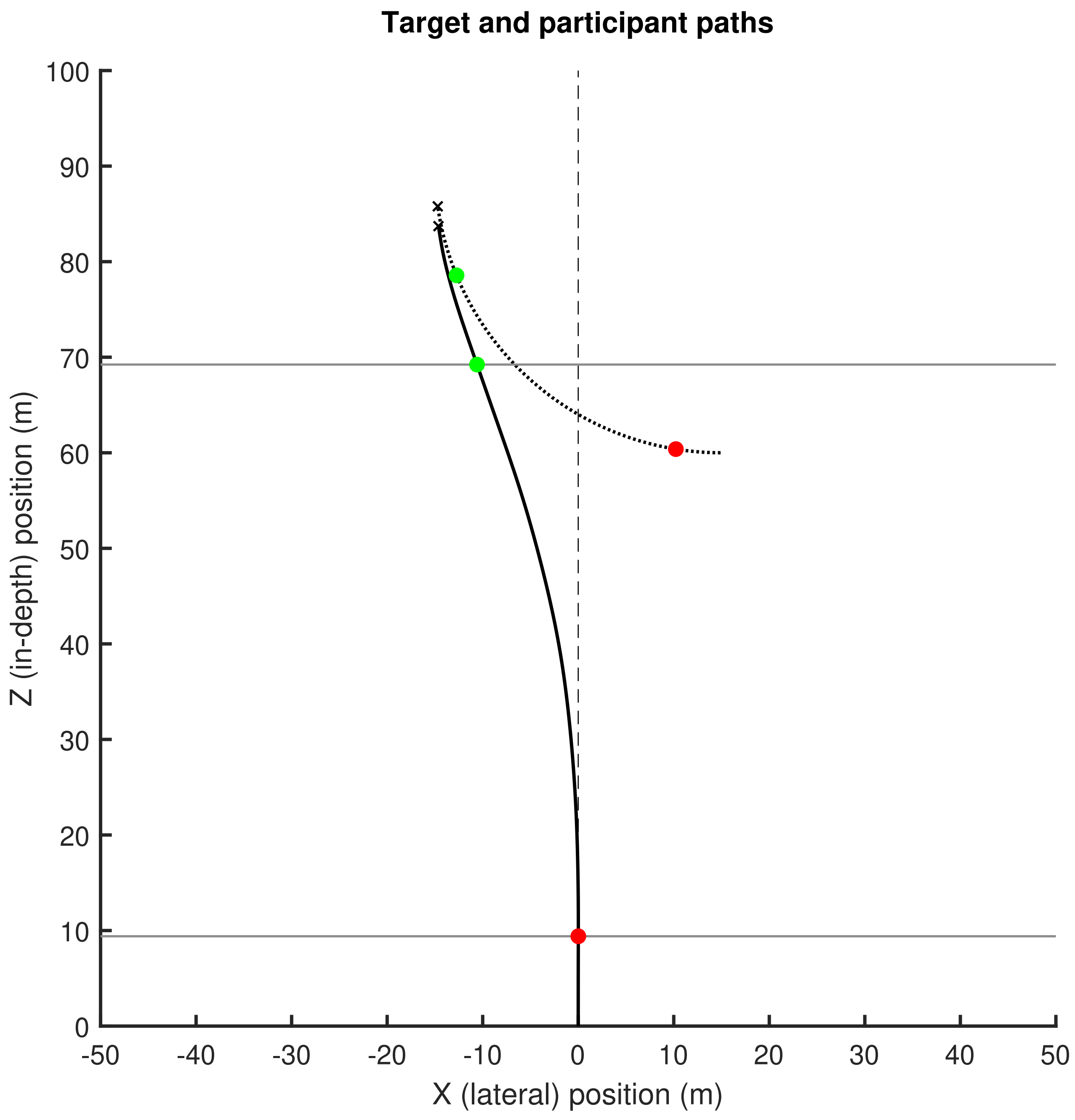

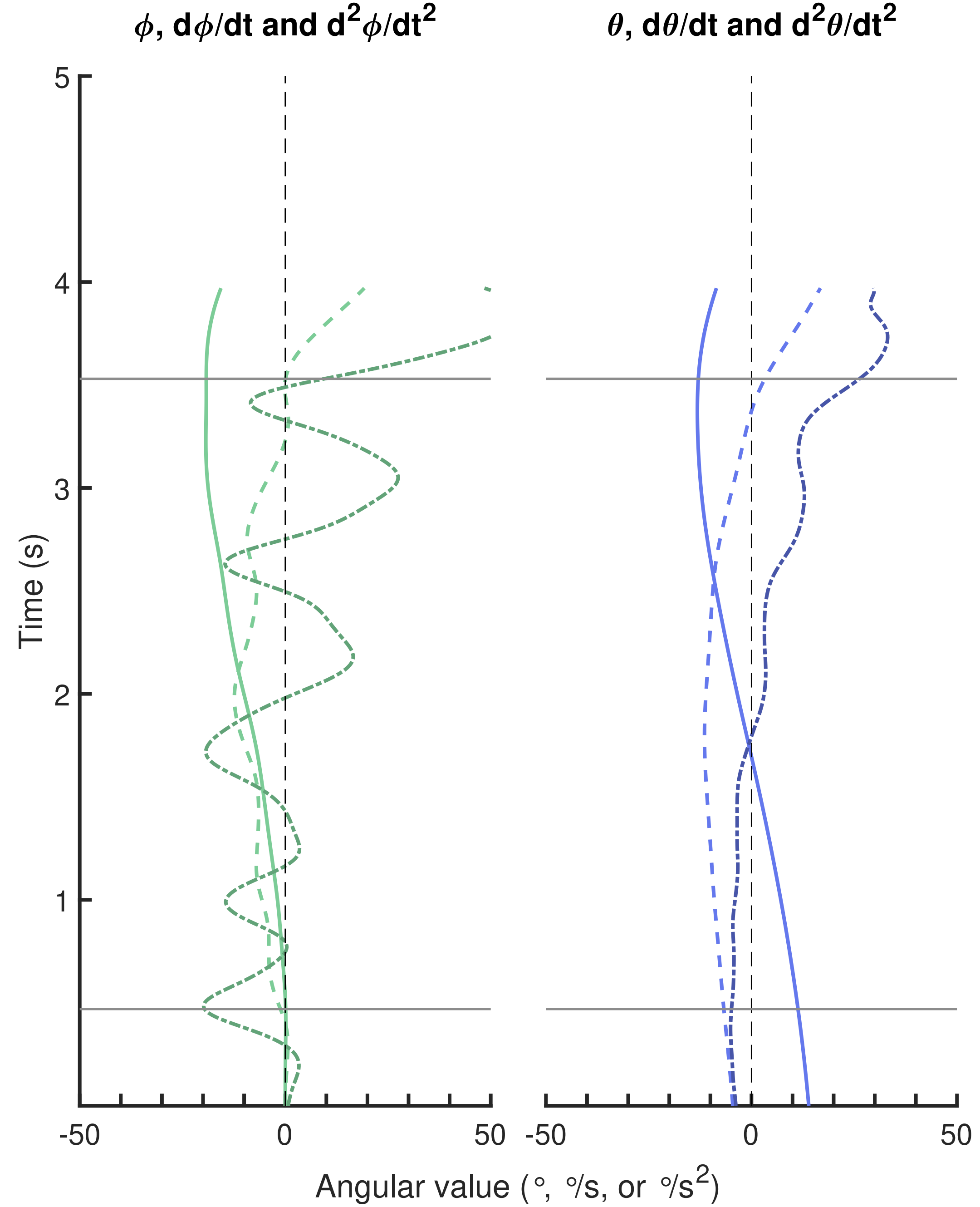

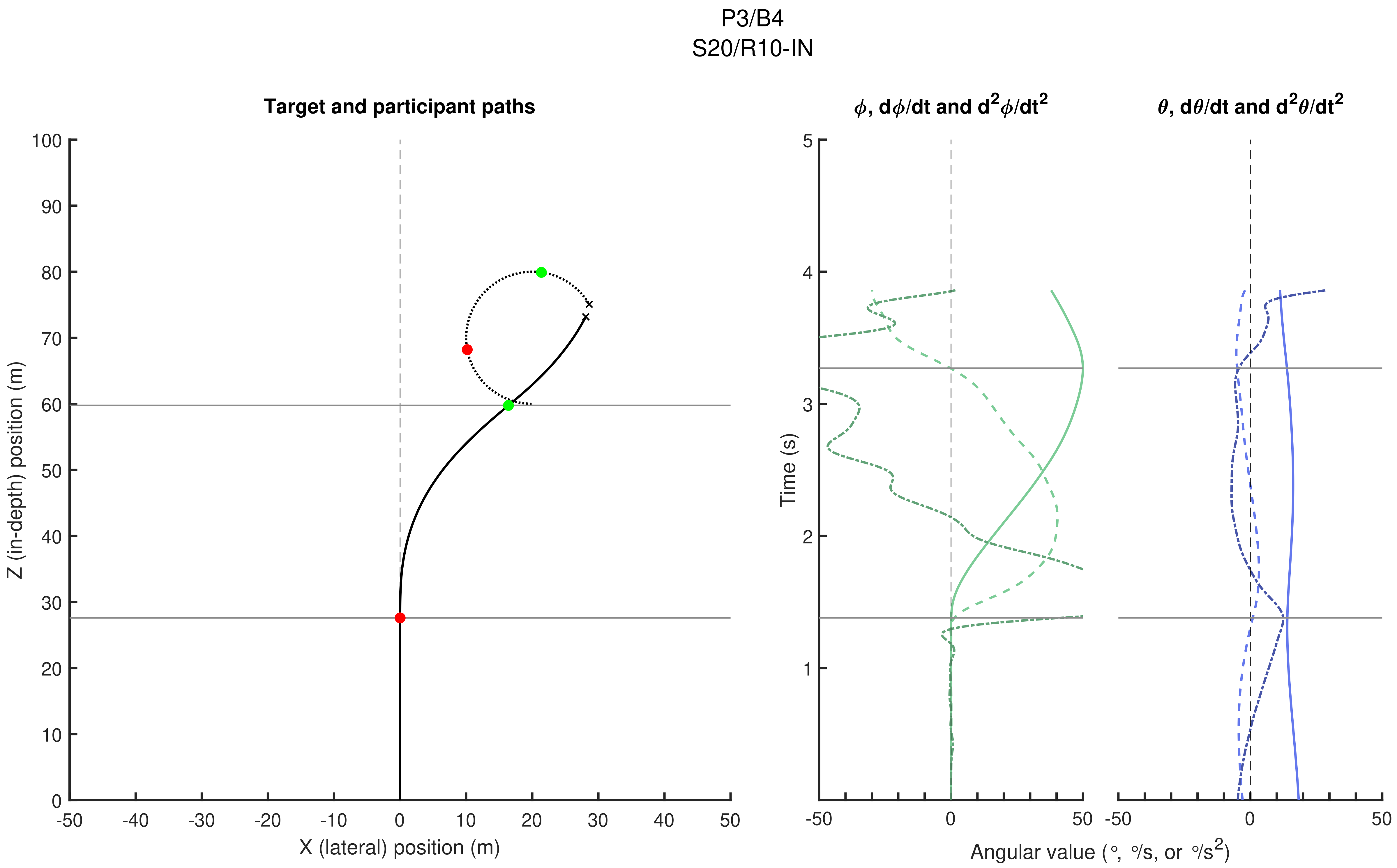
P3/B4
S20/R10-IN
Target and participant paths
Z (in-depth) position (m)
X (lateral) position (m)
ϕ, dϕ/dt and d$^2\phi$/dt^2
θ, dθ/dt and d$^2\theta$/dt^2
Time (s)
Angular value (°, °/s, or °/s^2)

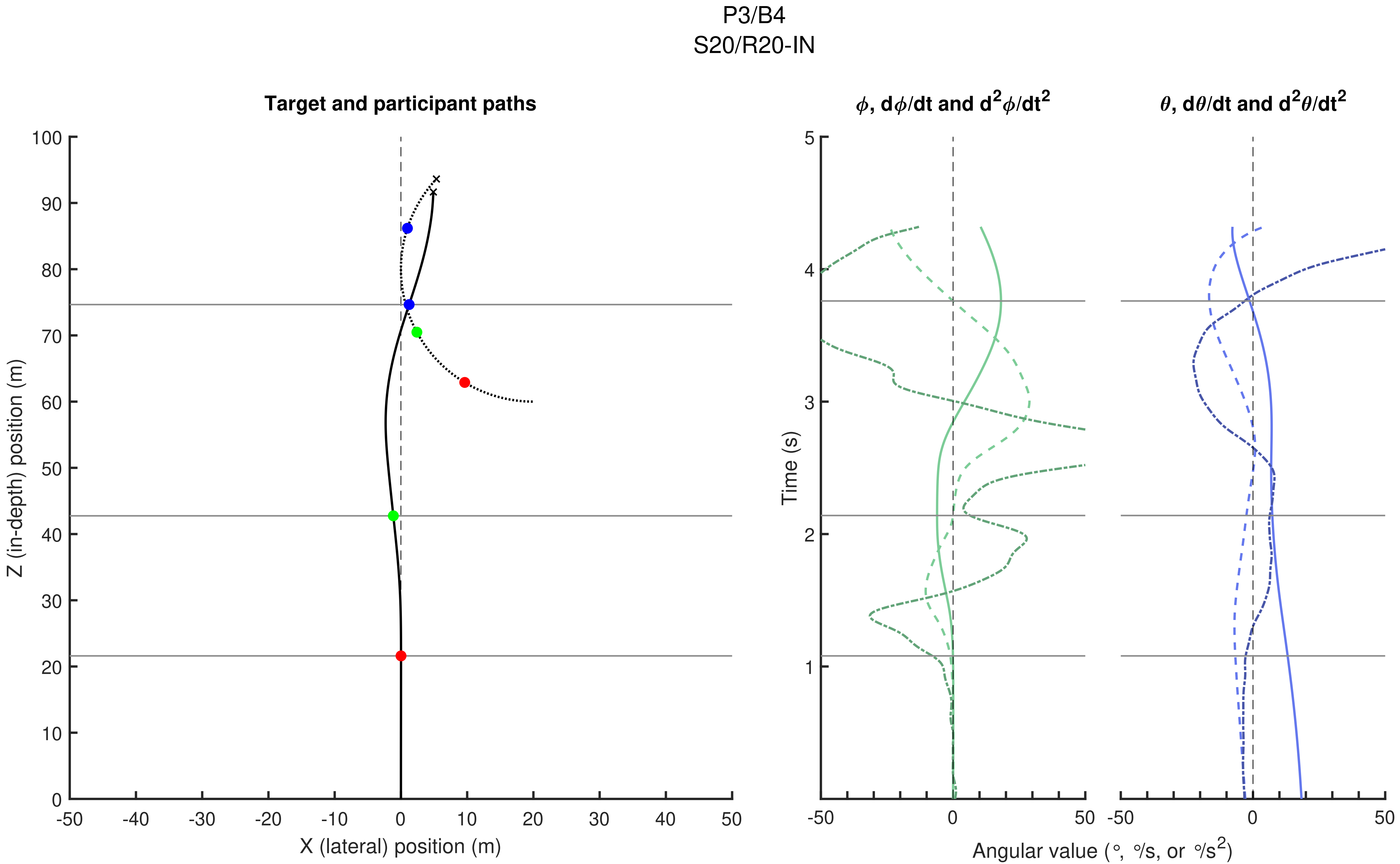

P3/B4
S20/R20-IN
Target and participant paths
Z (in-depth) position (m)
X (lateral) position (m)
ϕ, dϕ/dt and d²ϕ/dt²
θ, dθ/dt and d²θ/dt²
Time (s)
Angular value (°, °/s, or °/s²)

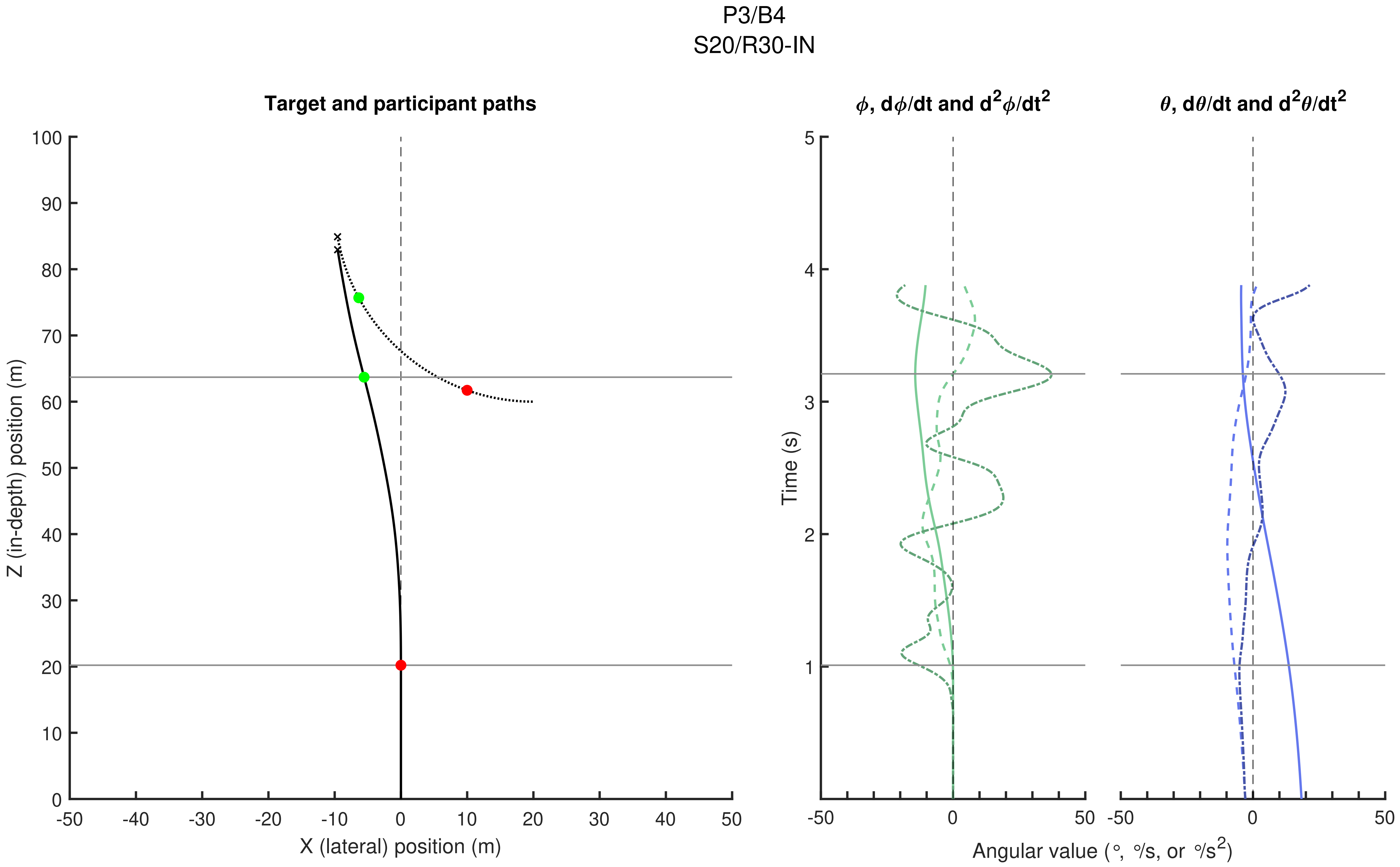
P3/B4
S20/R30-IN
Target and participant paths
Z (in-depth) position (m)
X (lateral) position (m)
φ, dφ/dt and d²φ/dt²
θ, dθ/dt and d²θ/dt²
Time (s)
Angular value (°, °/s, or °/s²)

P3/B5
S20/R10-IN

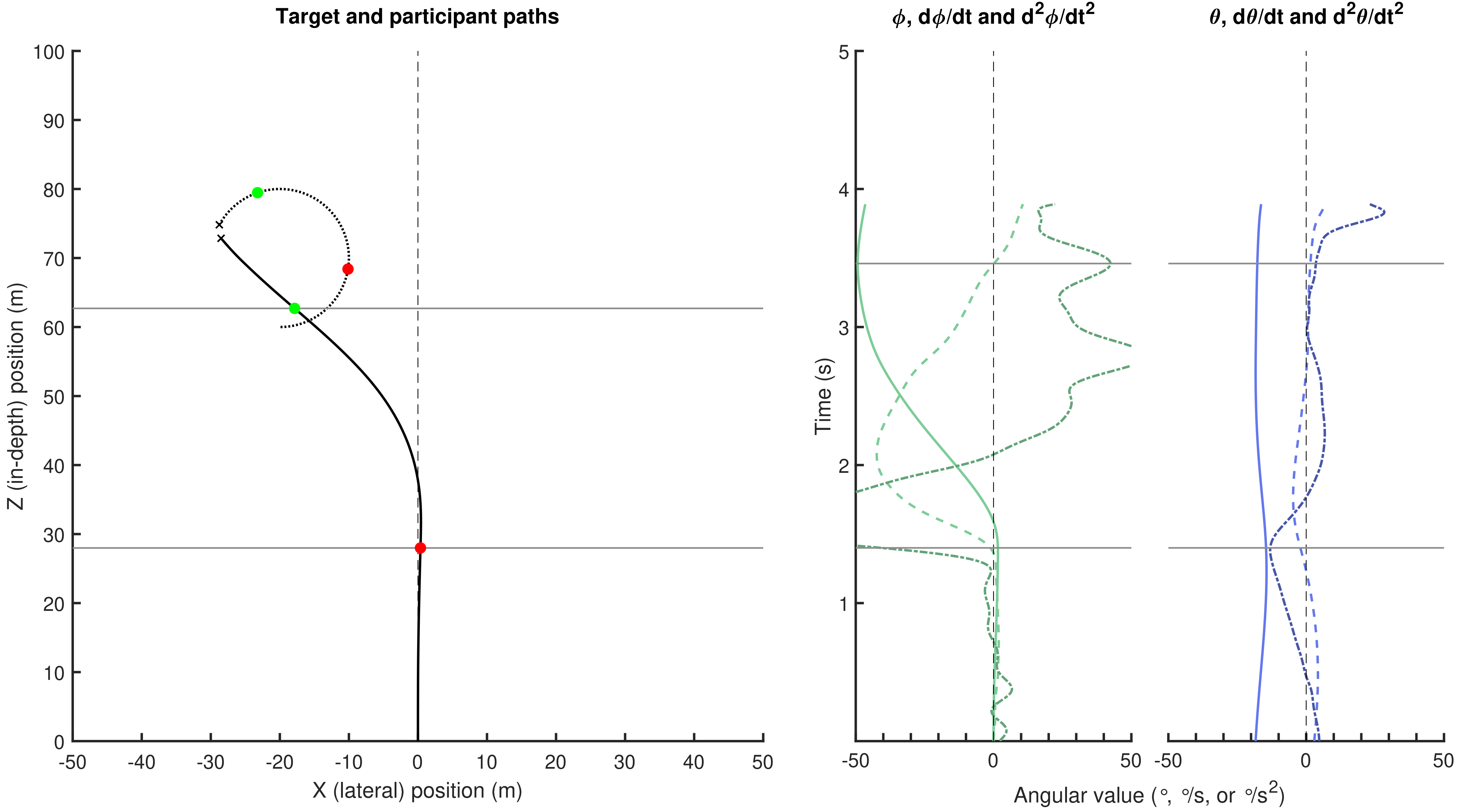

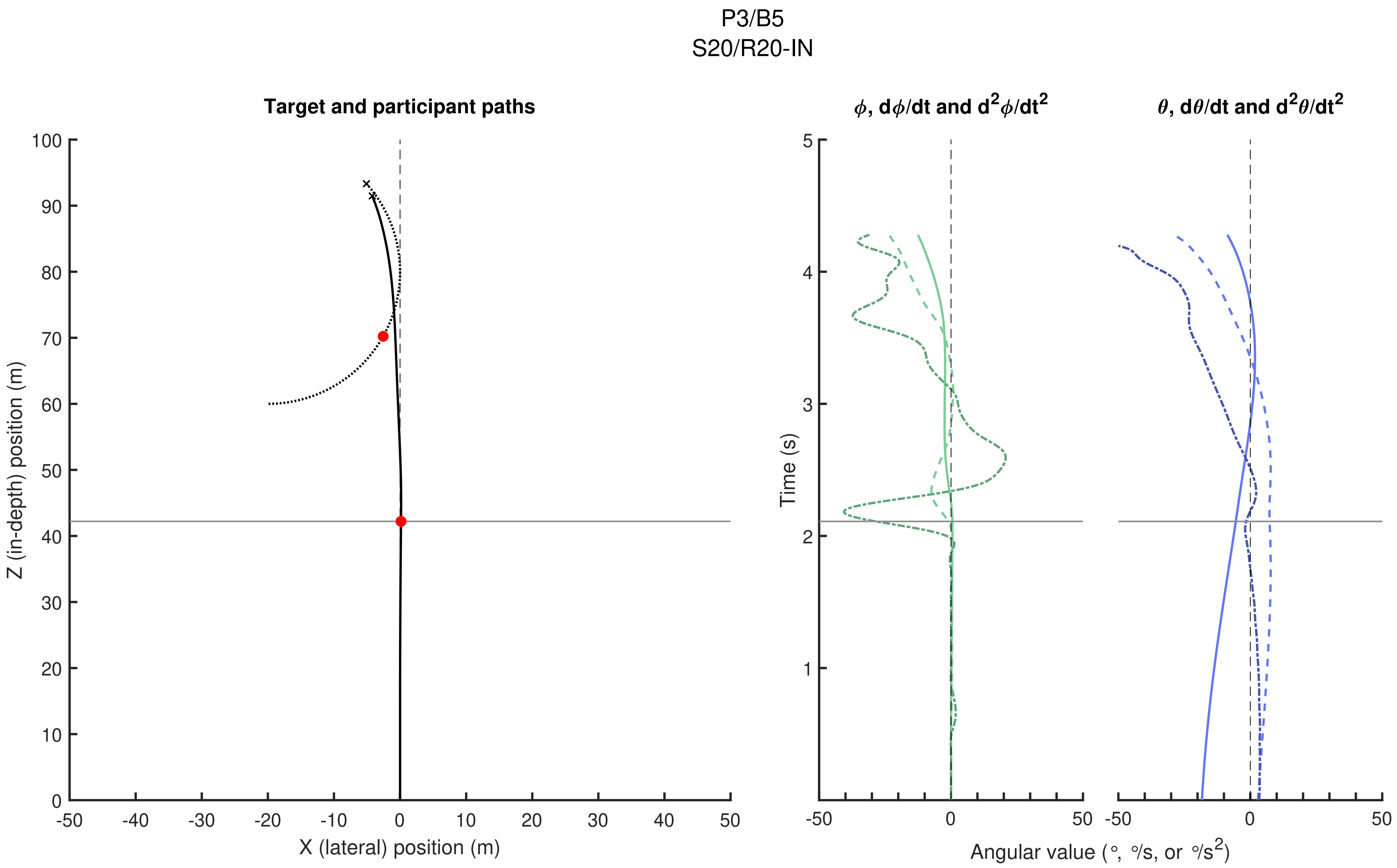
P3/B5
S20/R20-IN
Target and participant paths
ϕ, dϕ/dt and d²ϕ/dt²
θ, dθ/dt and d²θ/dt²
Z (in-depth) position (m)
X (lateral) position (m)
Time (s)
Angular value (°, °/s, or °/s²)
100
90
80
70
60
50
40
30
20
10
0
-50
-40
-30
-20
-10
0
10
20
30
40
50
5
4
3
2
1

P3/B5
S20/R30-IN

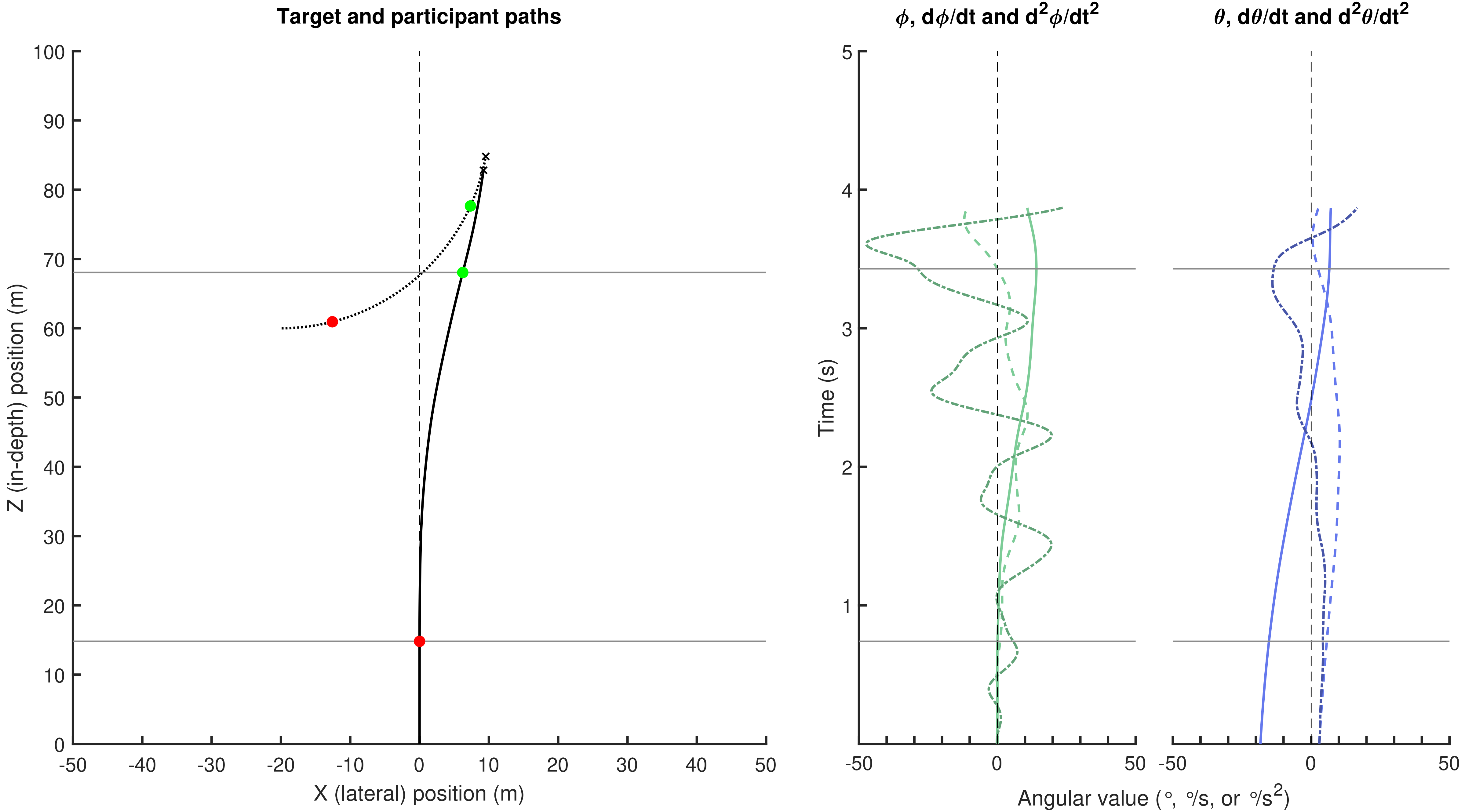

P3/B5
S15/R10-IN

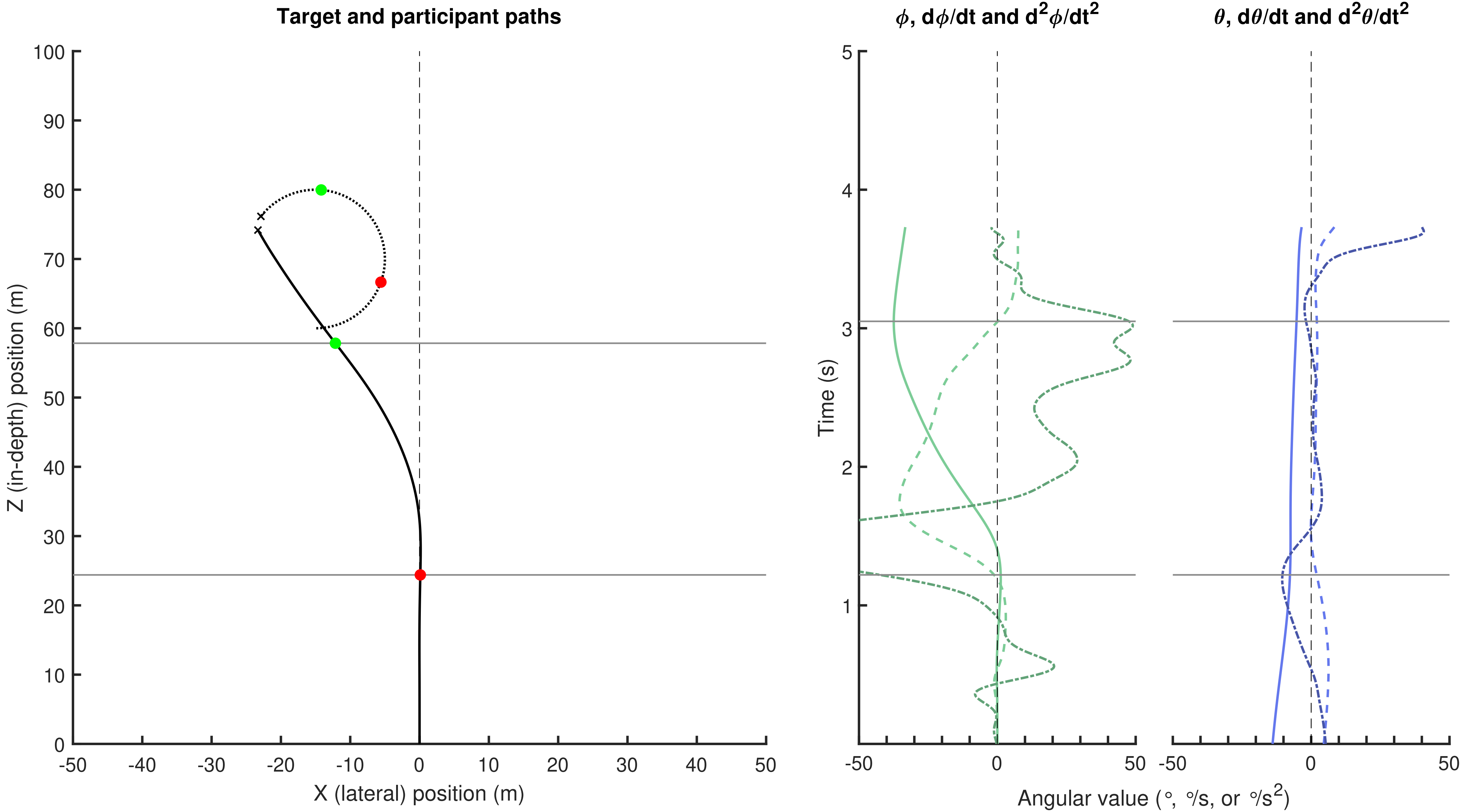

P3/B5
S15/R20-IN

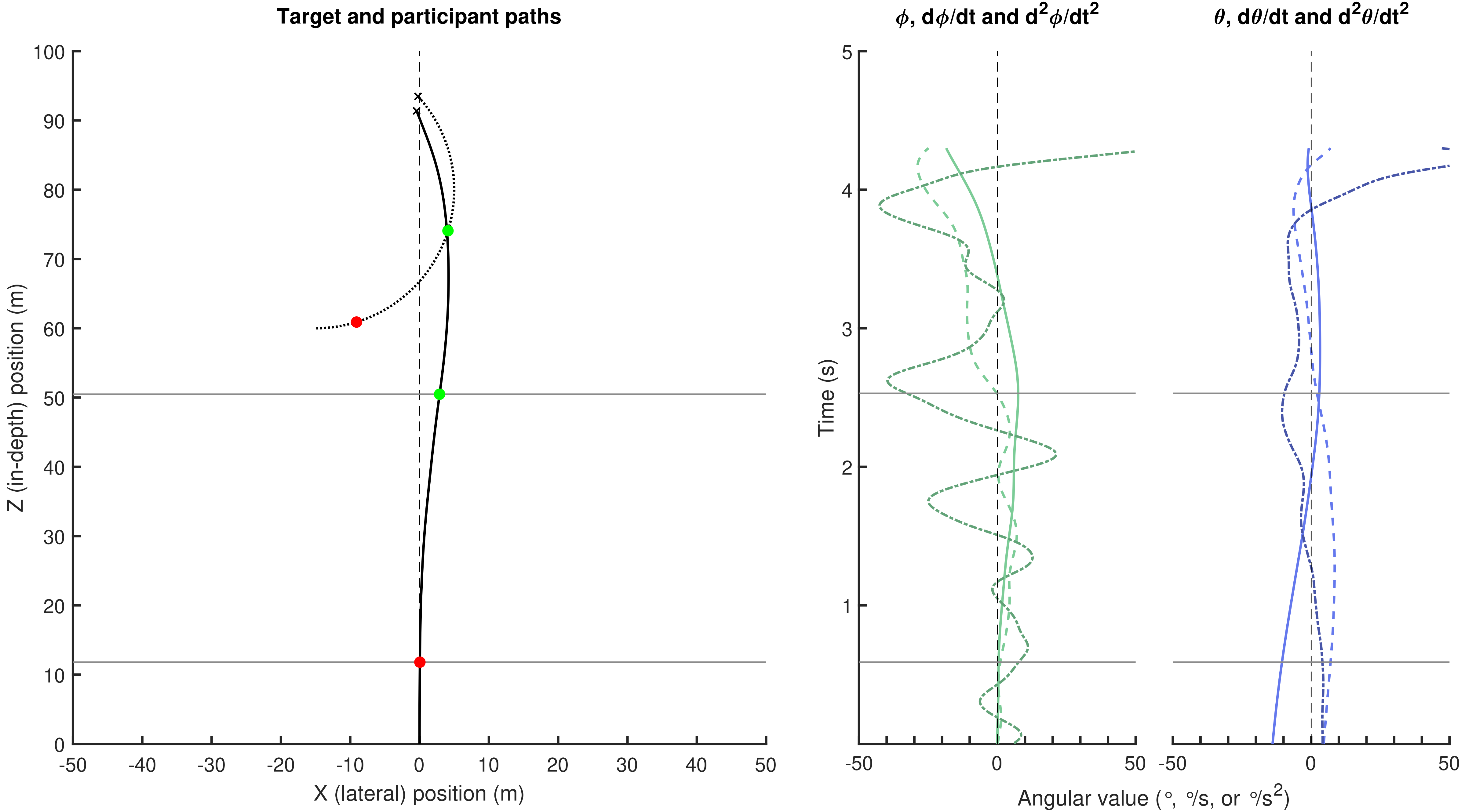

P3/B5
S15/R30-IN

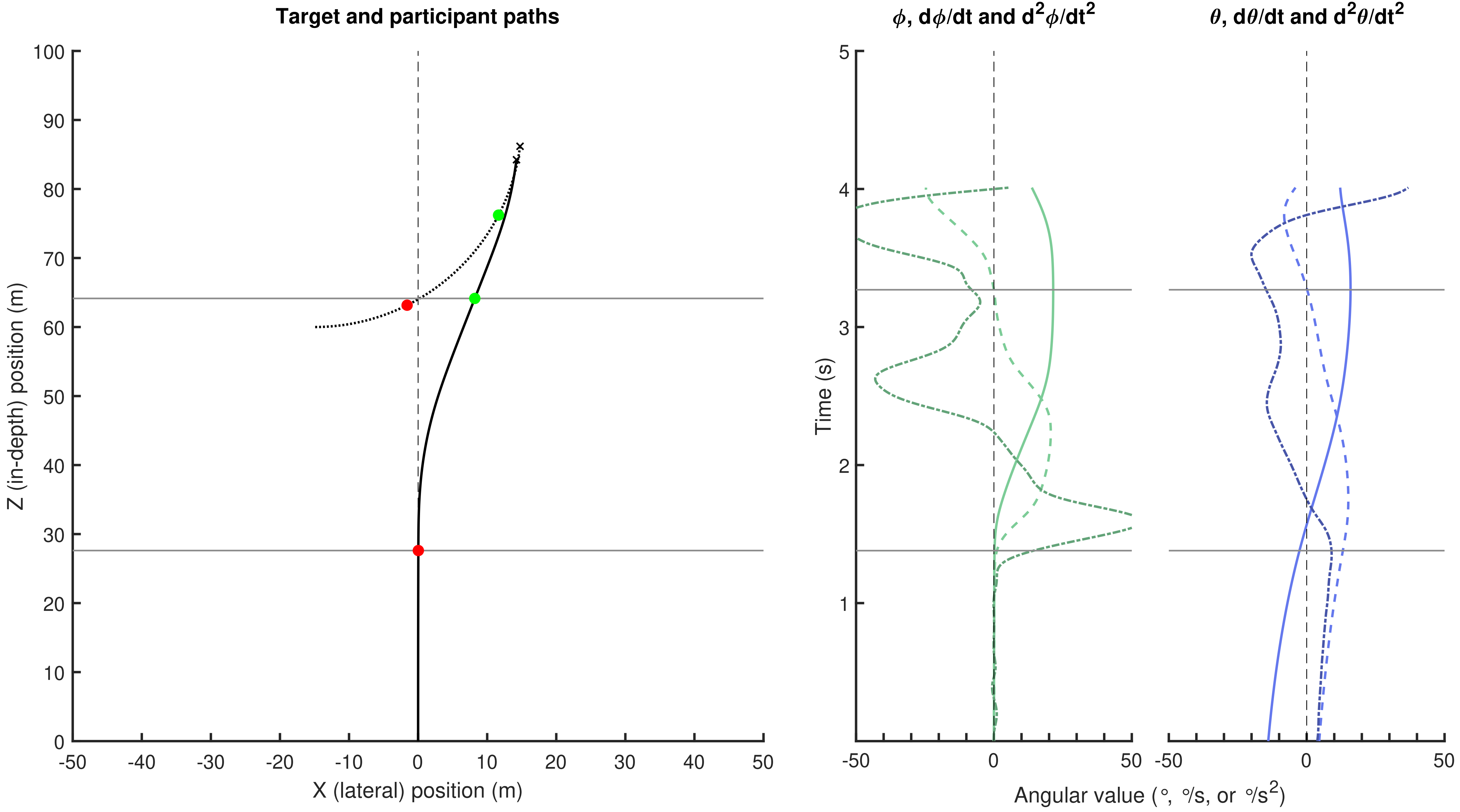

P3/B5
S5/R10-IN

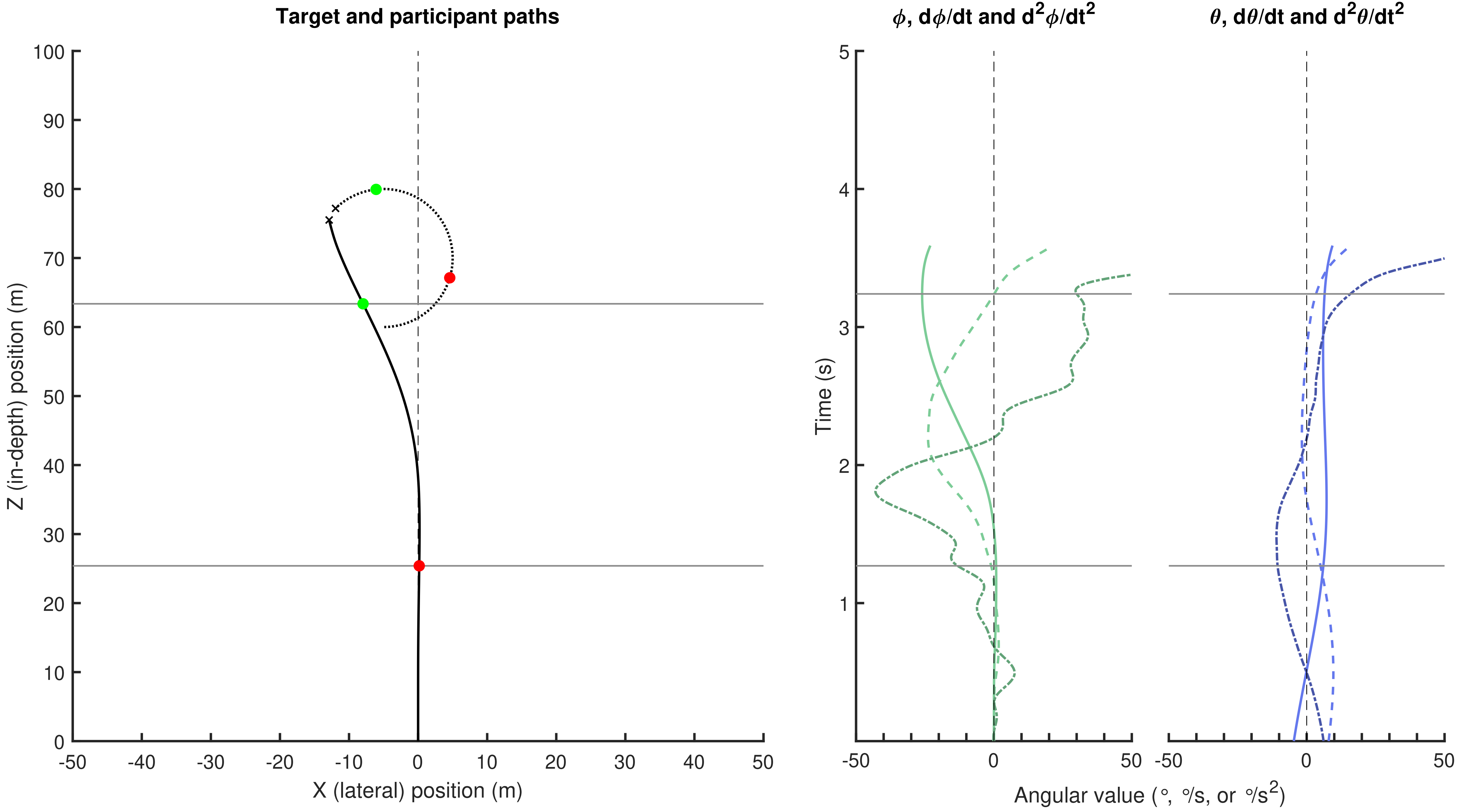

P3/B5
S5/R10-OUT

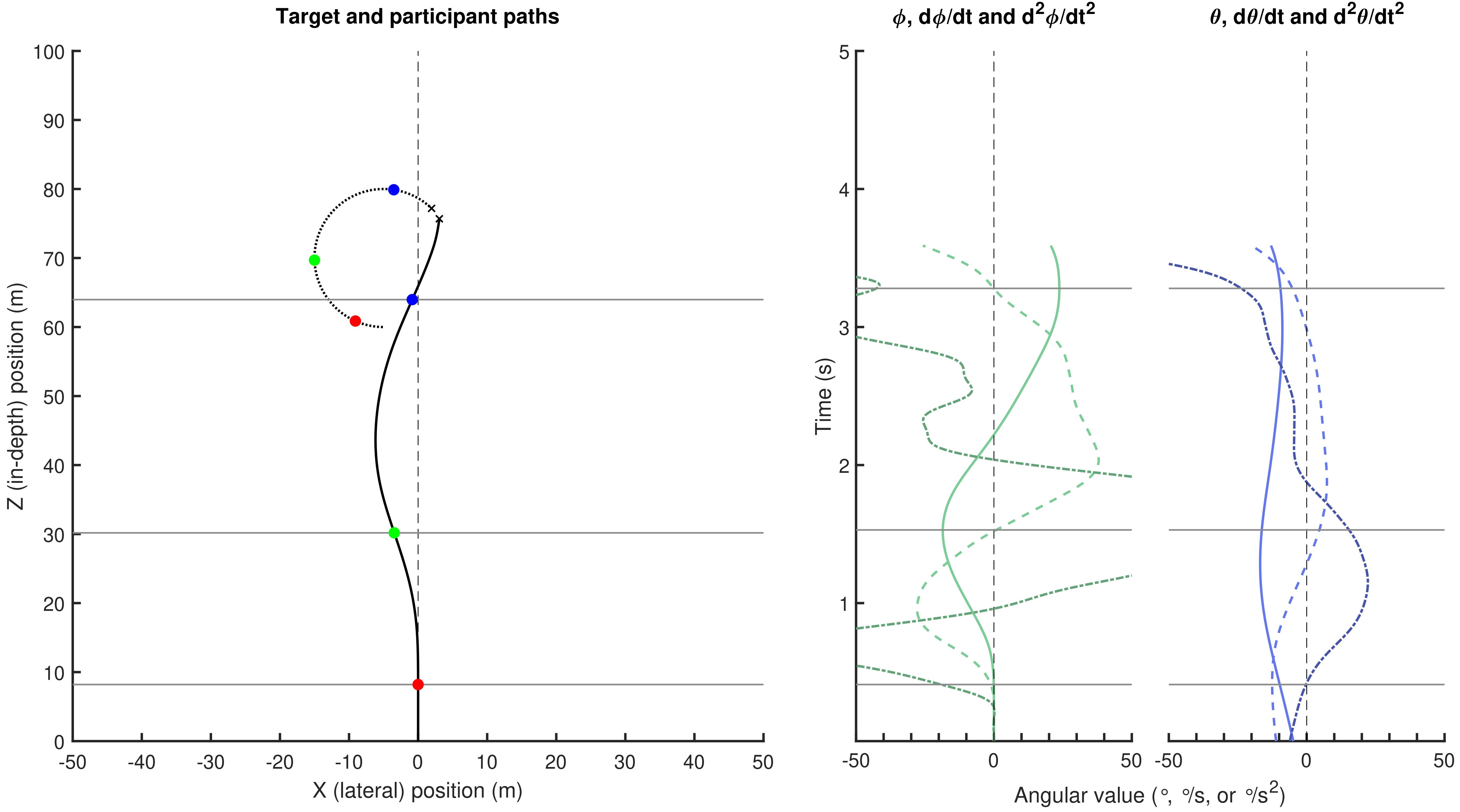

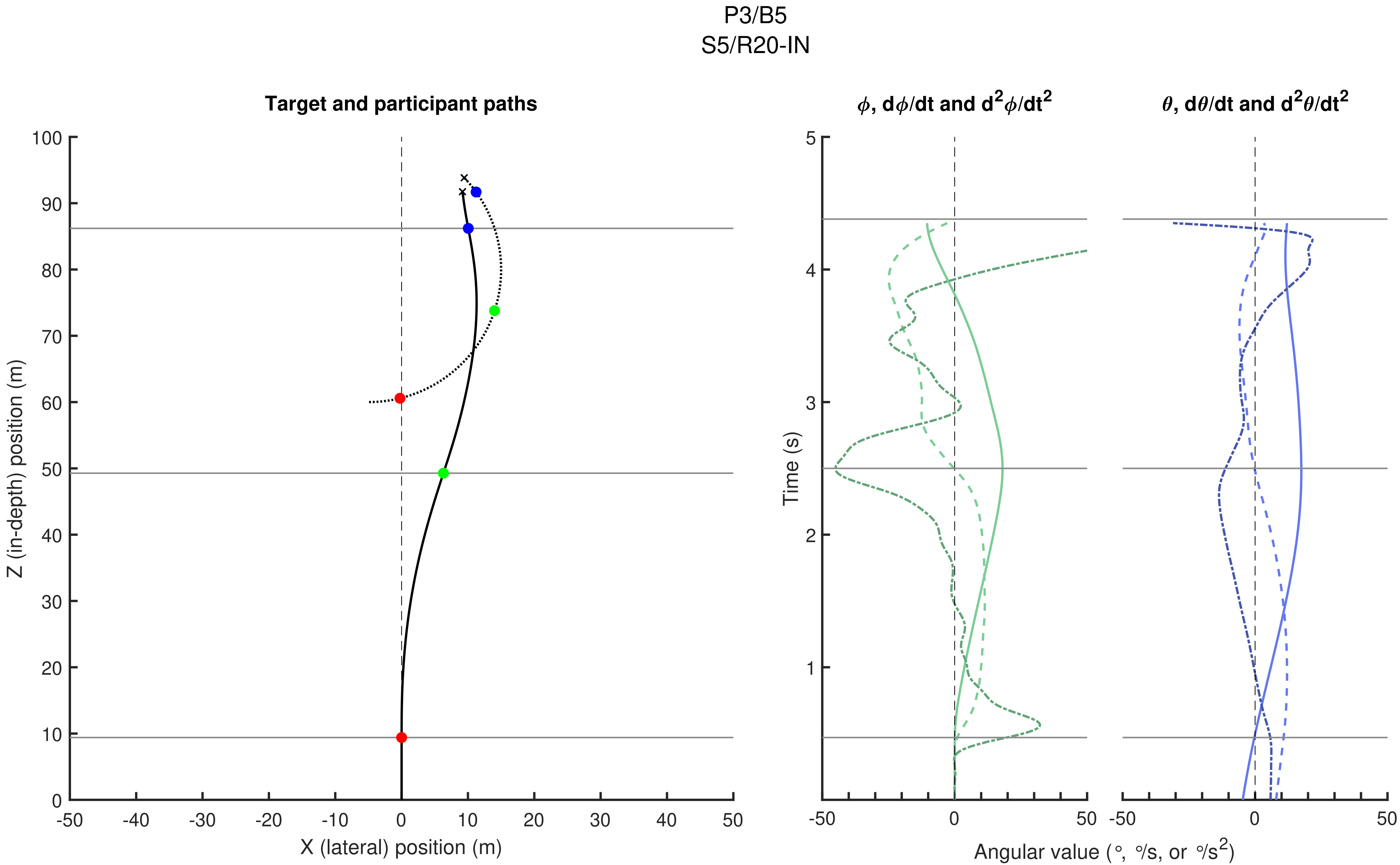

P3/B5
S5/R20-IN
Target and participant paths
Z (in-depth) position (m)
X (lateral) position (m)
-50
-40
-30
-20
-10
0
10
20
30
40
50
0
10
20
30
40
50
60
70
80
90
100
ϕ, dϕ/dt and d²ϕ/dt²
θ, dθ/dt and d²θ/dt²
Time (s)
1
2
3
4
5
-50
0
50
Angular value (°, °/s, or °/s²)

P3/B5
S5/R20-OUT

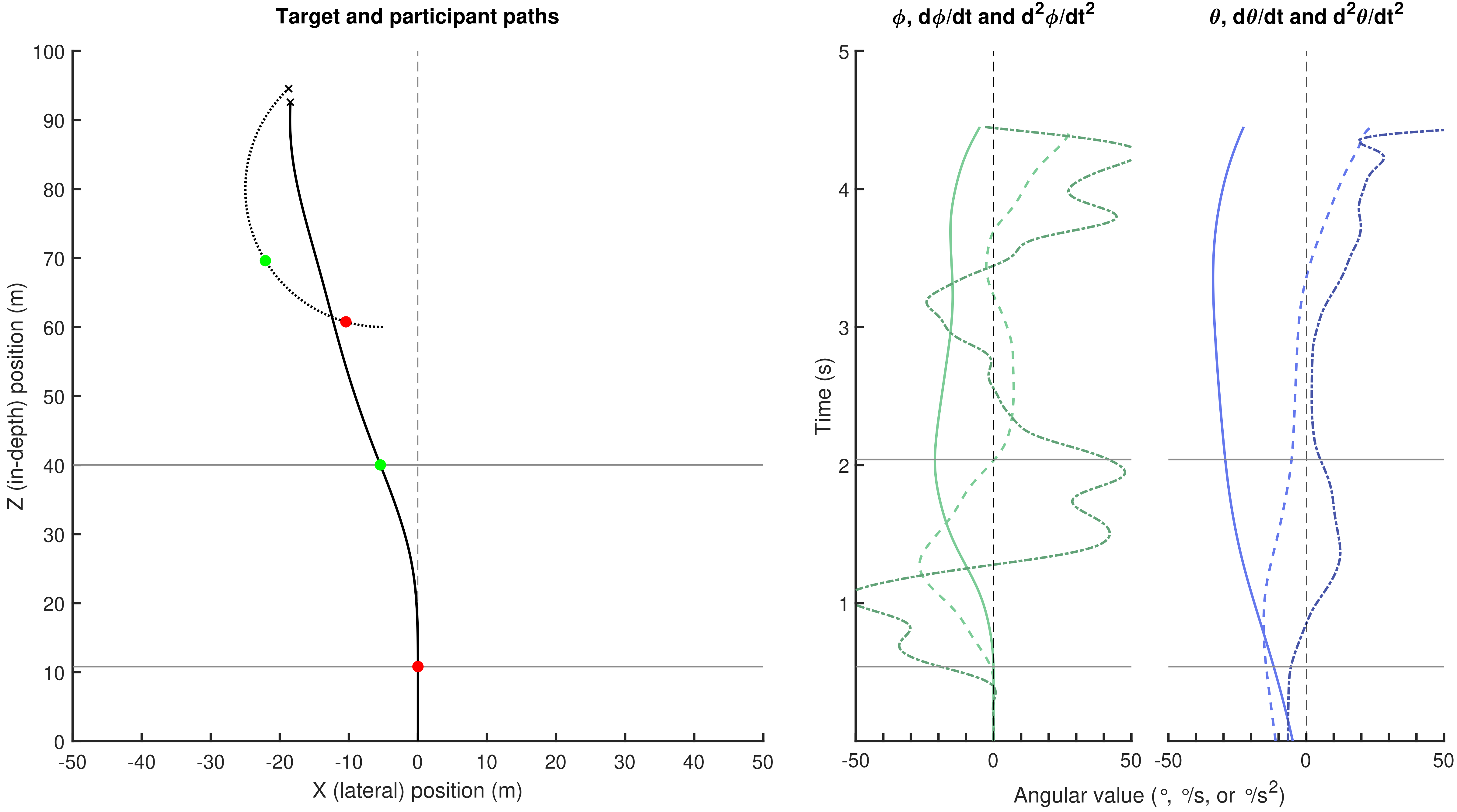

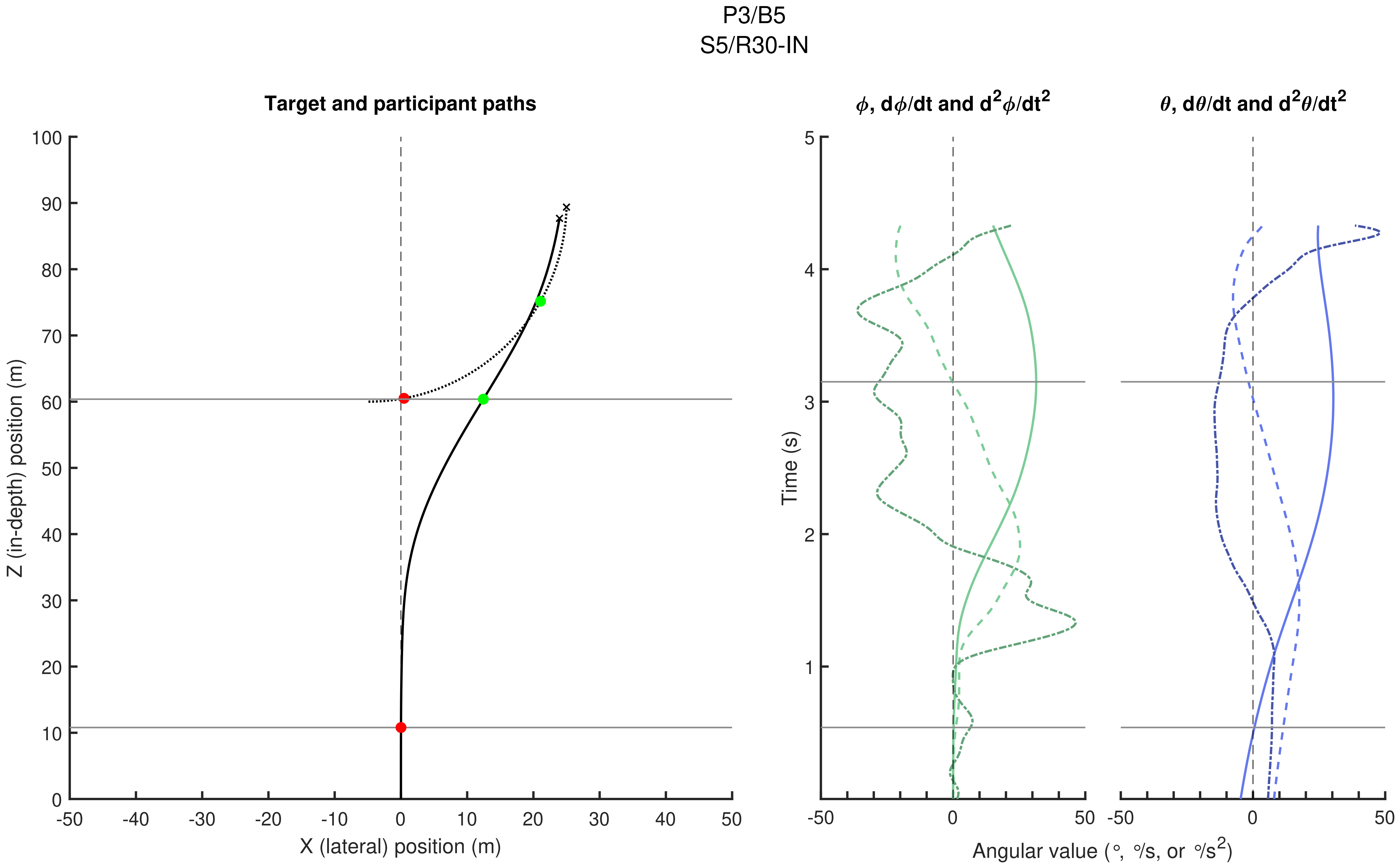

P3/B5
S5/R30-IN
Target and participant paths
Z (in-depth) position (m)
X (lateral) position (m)
ϕ, dϕ/dt and d$^2\phi$/dt^2
θ, dθ/dt and d$^2\theta$/dt^2
Time (s)
Angular value (°, °/s, or °/s^2)

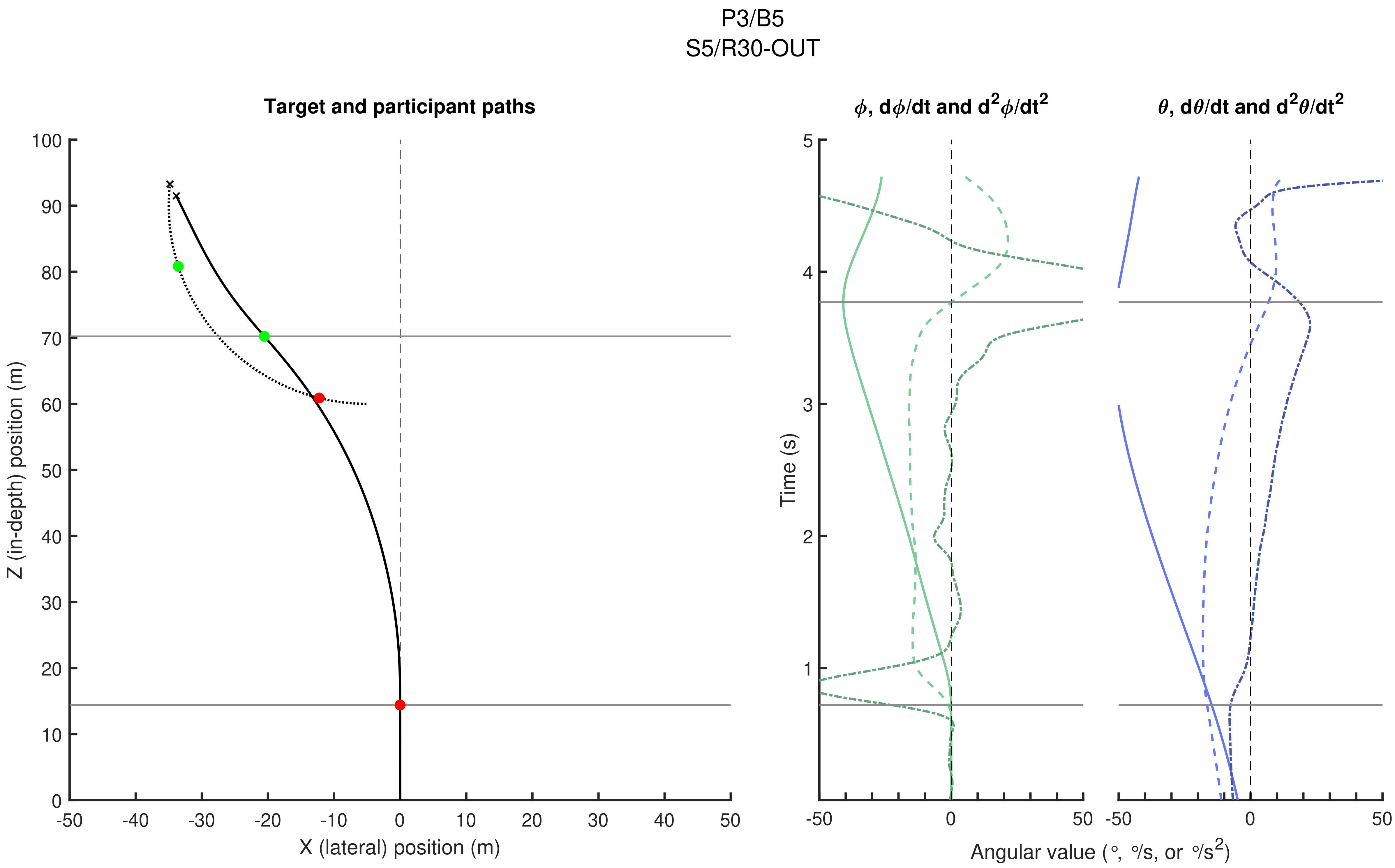
P3/B5
S5/R30-OUT
Target and participant paths
Z (in-depth) position (m)
X (lateral) position (m)
ϕ, dϕ/dt and d²ϕ/dt²
θ, dθ/dt and d²θ/dt²
Time (s)
Angular value (°, °/s, or °/s²)

P3/B5
S5/R10-OUT

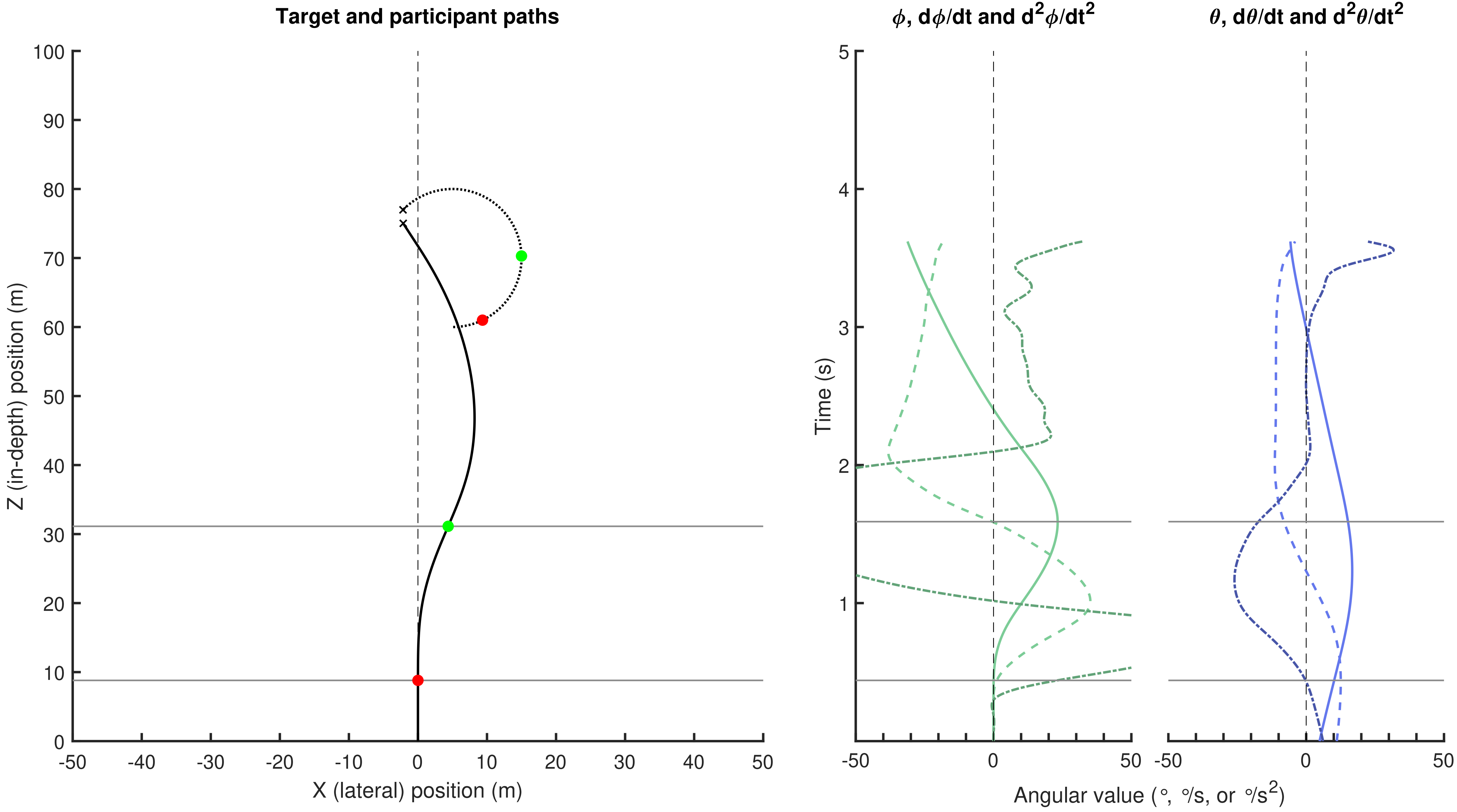

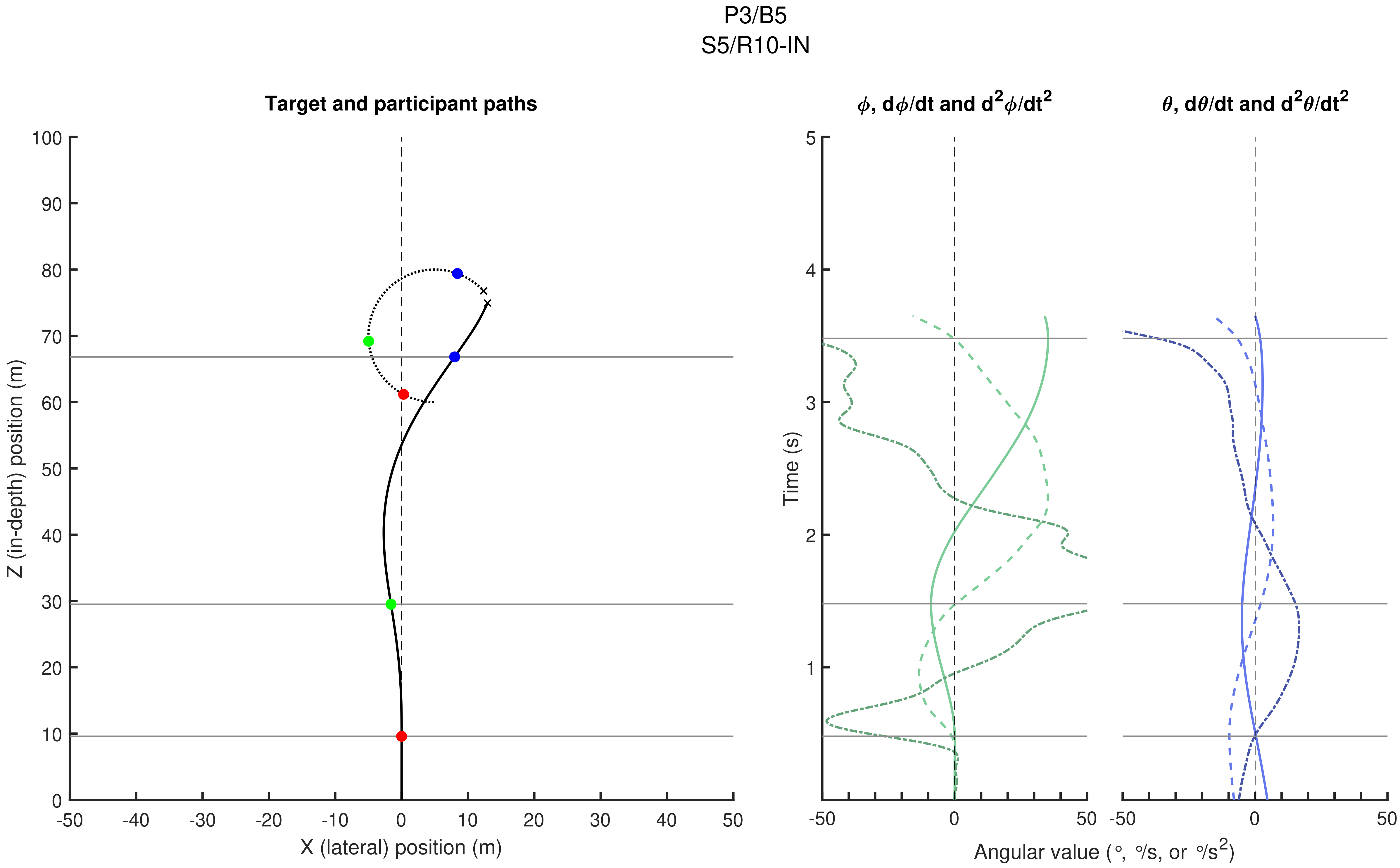
P3/B5
S5/R10-IN
Target and participant paths
Z (in-depth) position (m)
X (lateral) position (m)
100
90
80
70
60
50
40
30
20
10
0
-50
-40
-30
-20
-10
0
10
20
30
40
50
ϕ, dϕ/dt and d²ϕ/dt²
θ, dθ/dt and d²θ/dt²
Time (s)
5
4
3
2
1
-50
0
50
-50
0
50
Angular value (°, °/s, or °/s²)

P3/B5
S5/R20-OUT

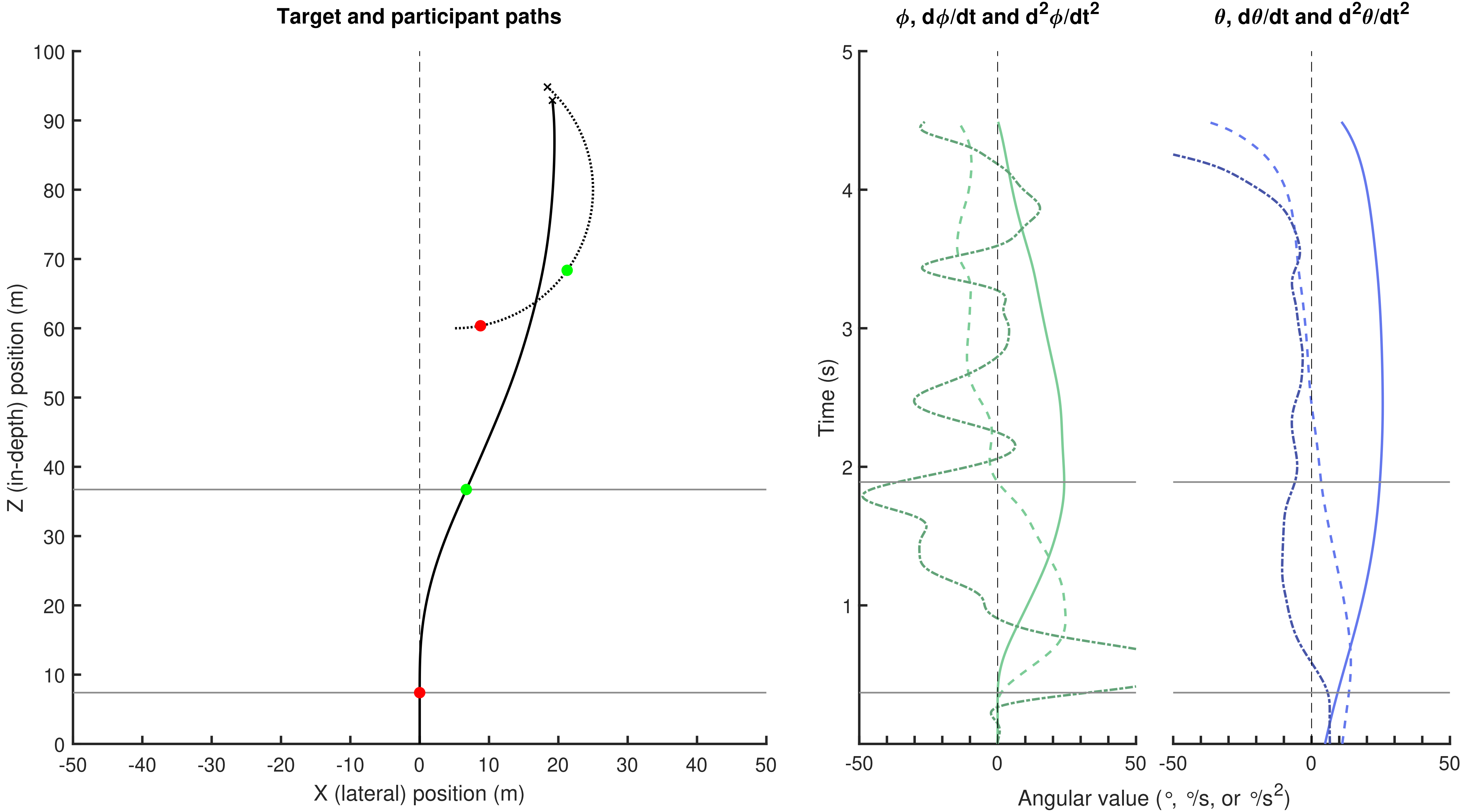

P3/B5
S5/R20-IN

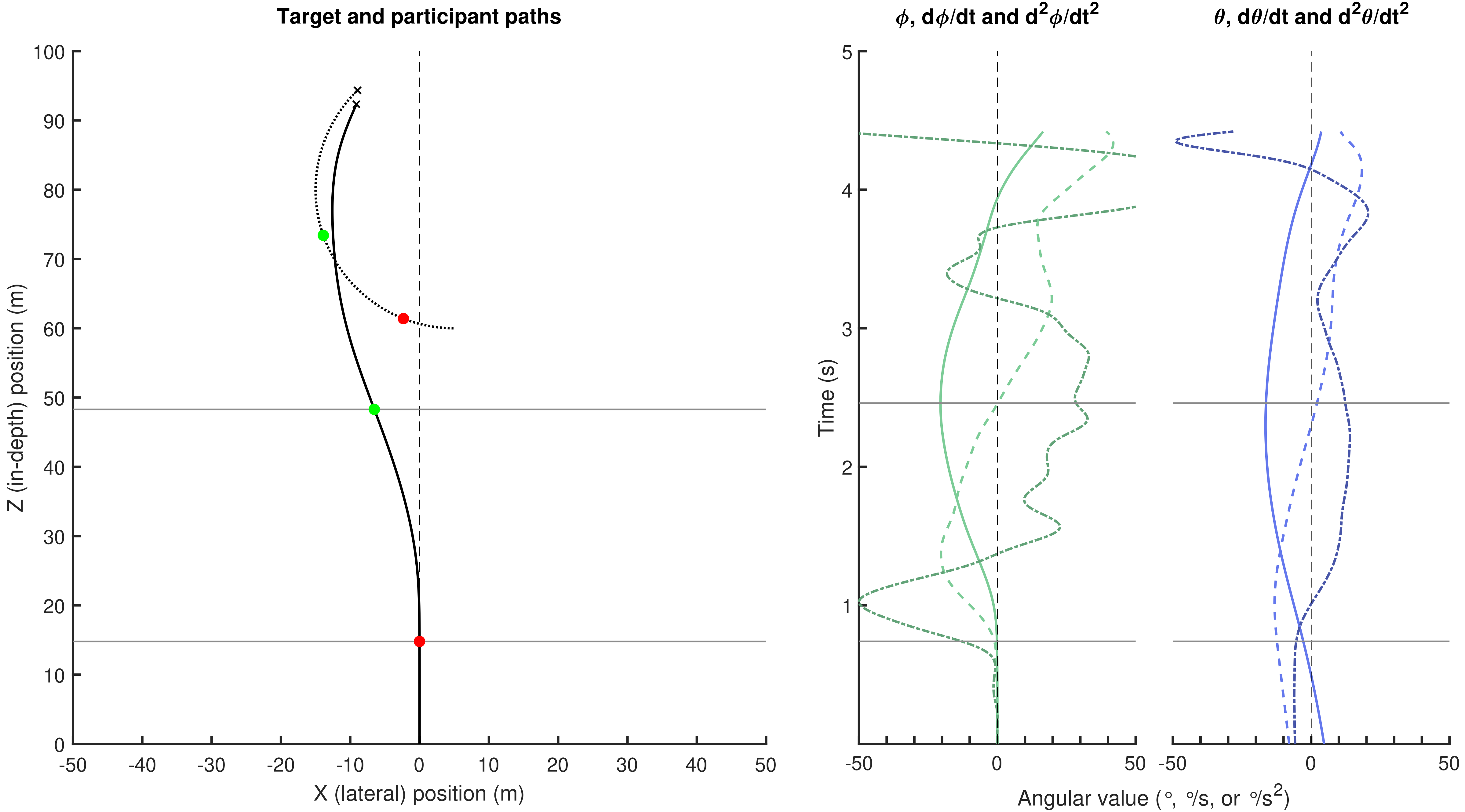

P3/B5
S5/R30-OUT

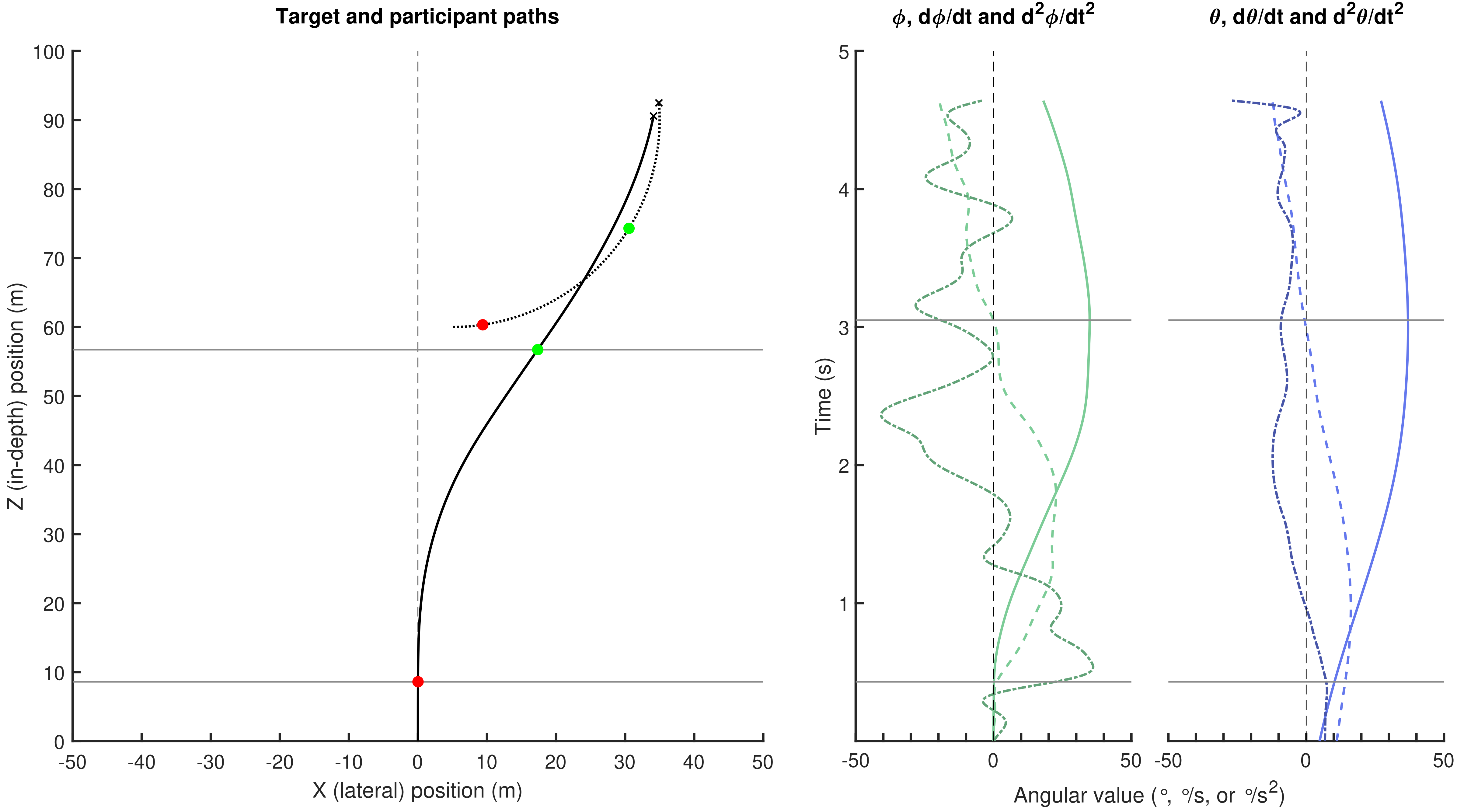

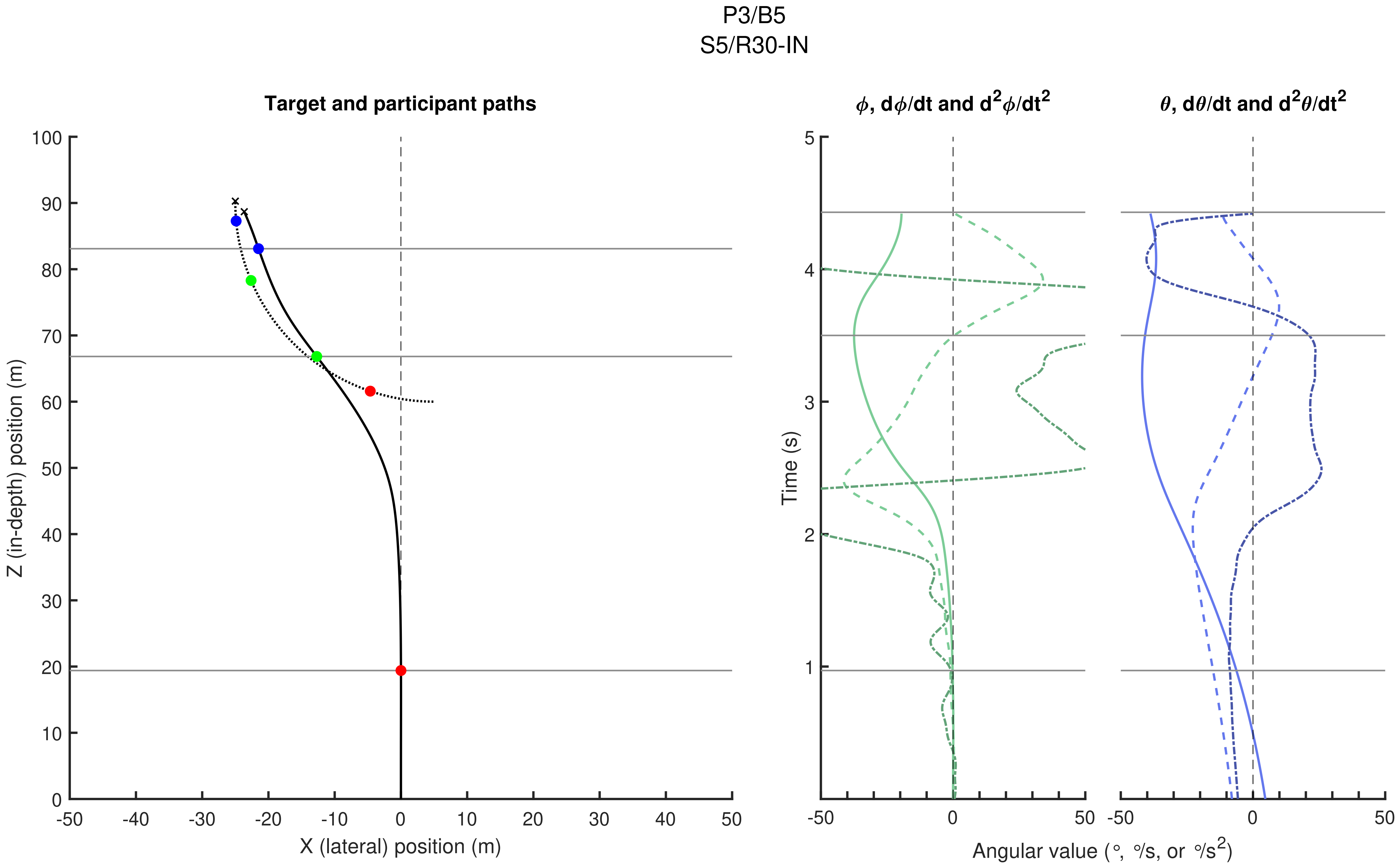
P3/B5
S5/R30-IN
Target and participant paths
Z (in-depth) position (m)
X (lateral) position (m)
ϕ, dϕ/dt and d$^2\phi$/dt^2
θ, dθ/dt and d$^2\theta$/dt^2
Time (s)
Angular value (°, °/s, or °/s^2)

P3/B5
S15/R10-IN

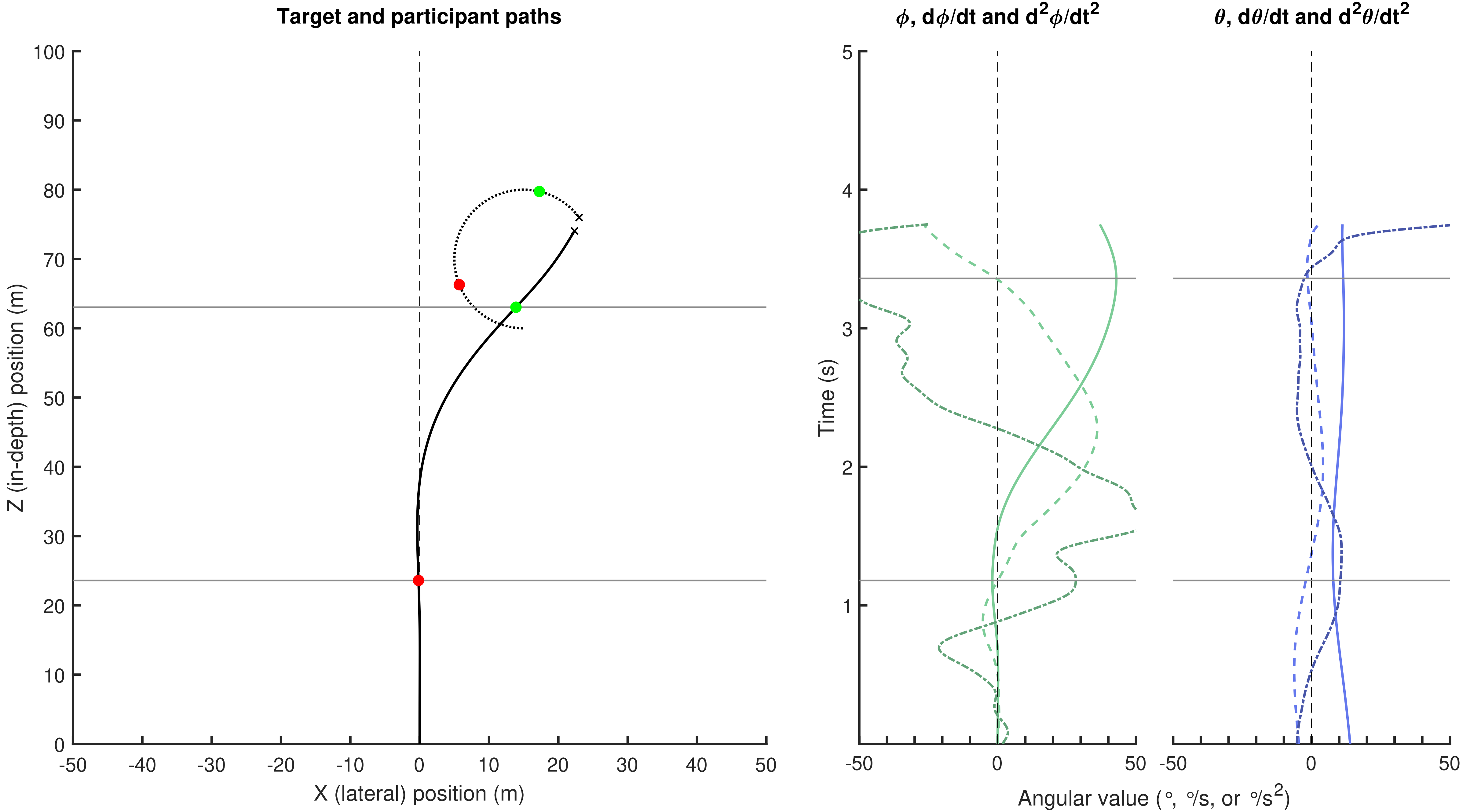

P3/B5
S15/R20-IN

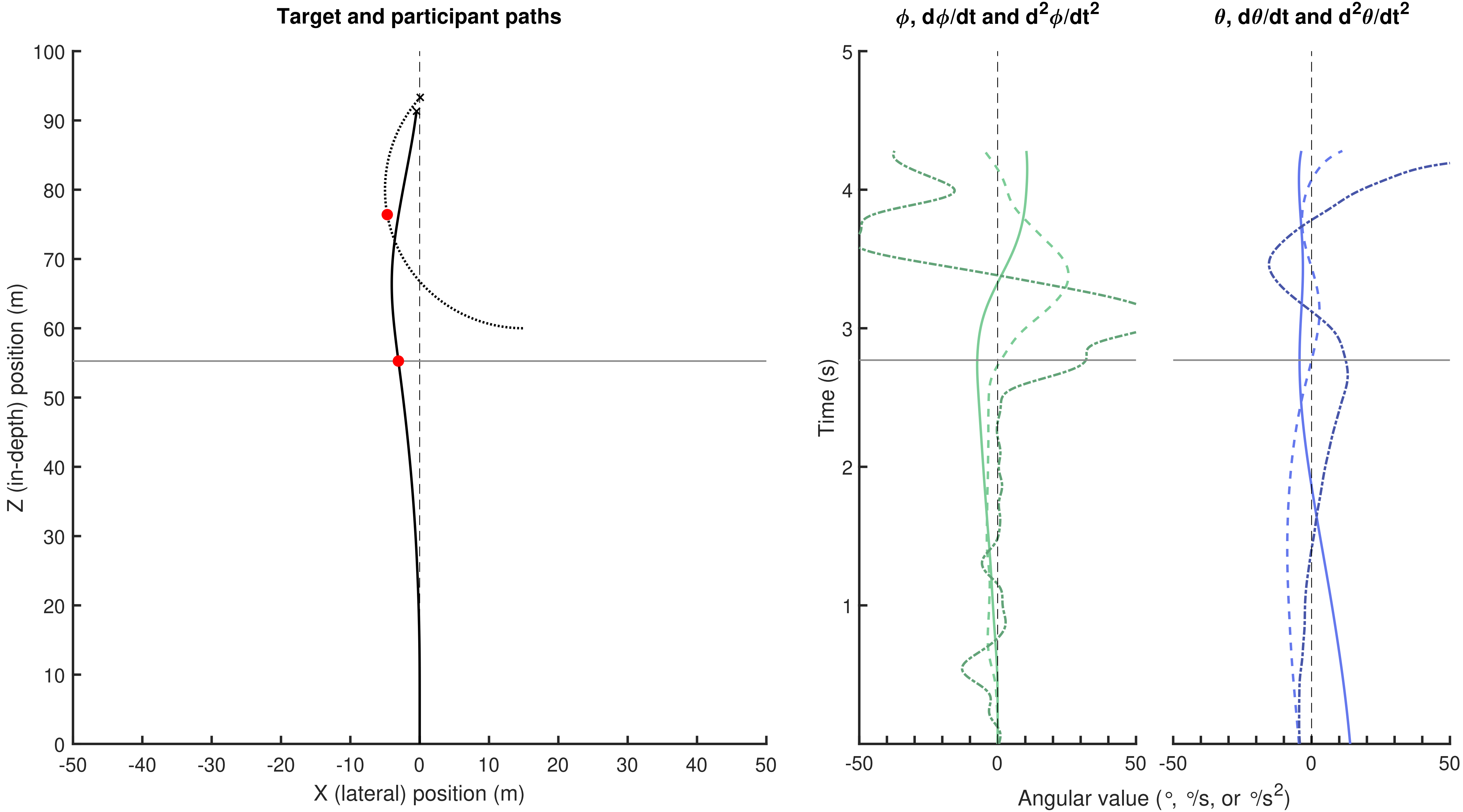

P3/B5
S15/R30-IN

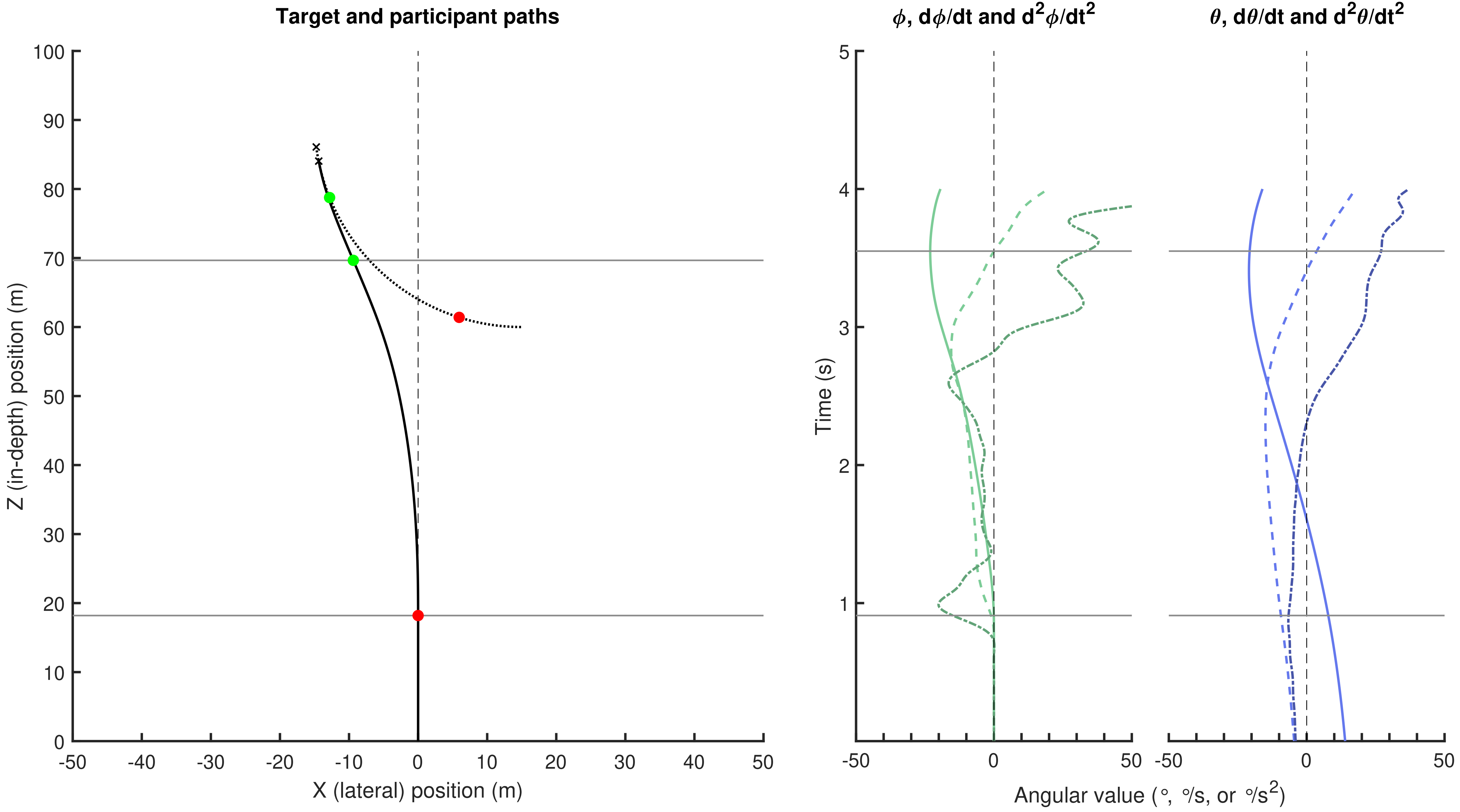

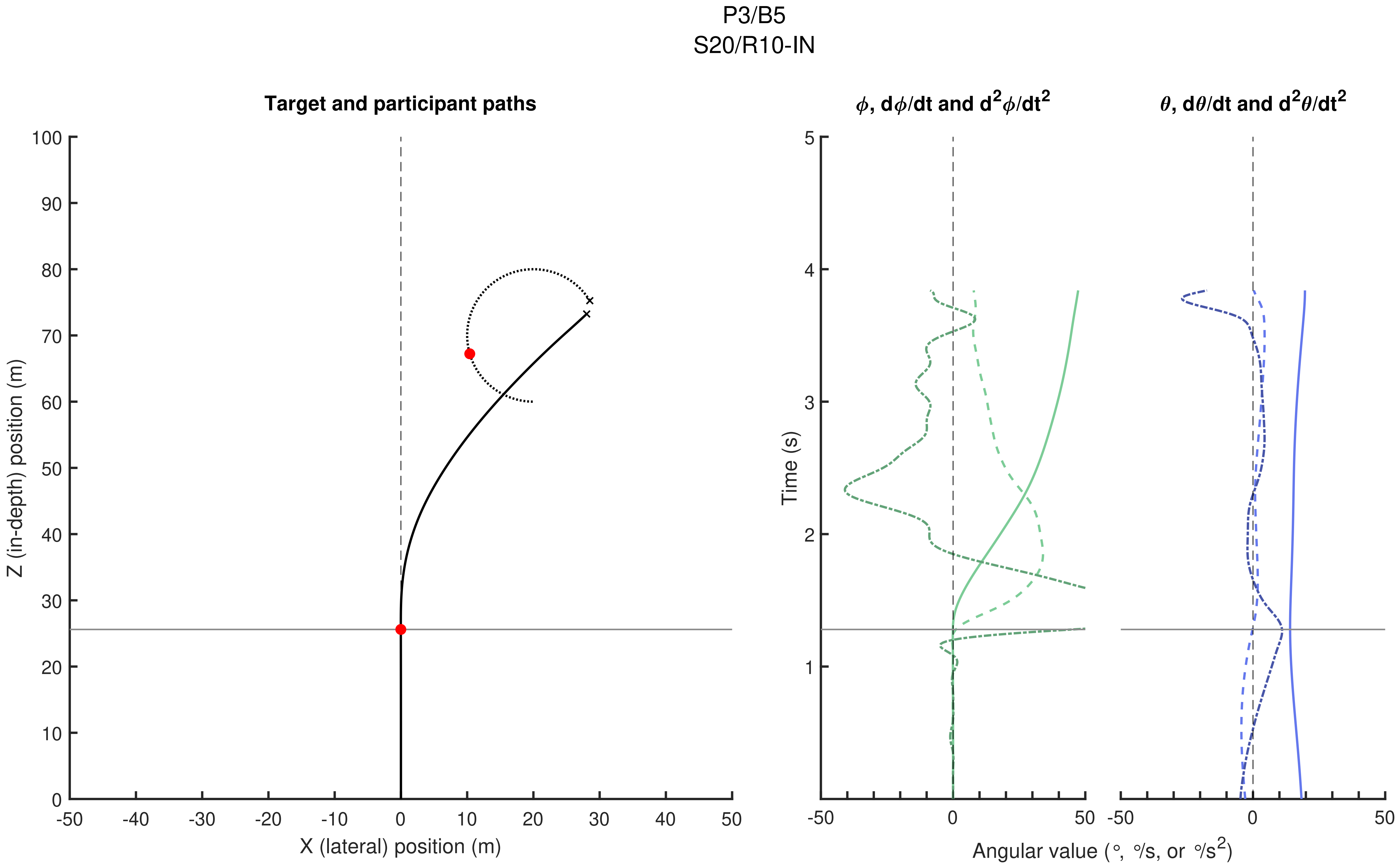
P3/B5
S20/R10-IN
Target and participant paths
Z (in-depth) position (m)
X (lateral) position (m)
100
90
80
70
60
50
40
30
20
10
0
-50
-40
-30
-20
-10
0
10
20
30
40
50
ϕ, dϕ/dt and d$^2\phi$/dt^2
θ, dθ/dt and d$^2\theta$/dt^2
Time (s)
5
4
3
2
1
-50
0
50
-50
0
50
Angular value (°, °/s, or °/s^2)

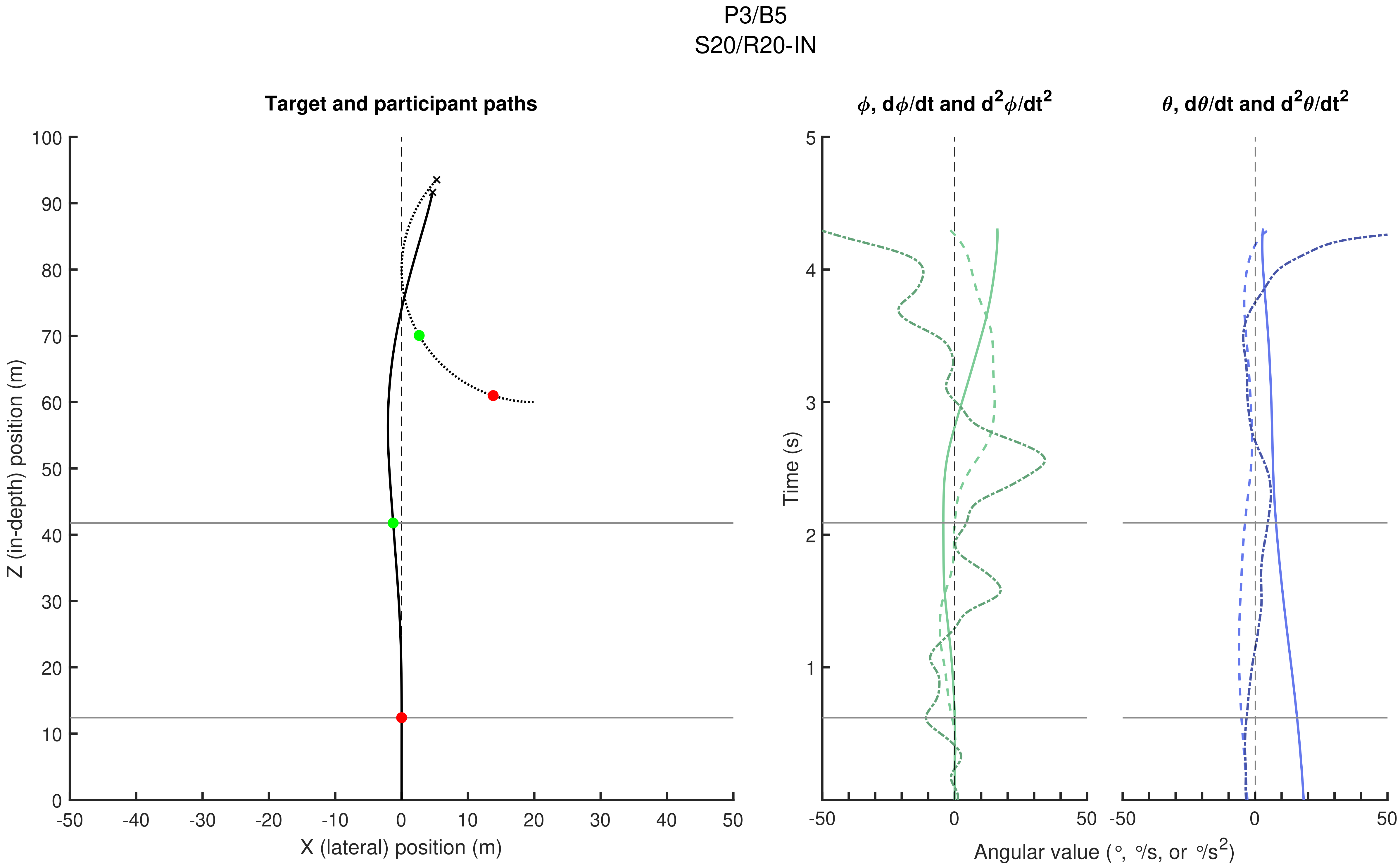

P3/B5
S20/R20-IN
Target and participant paths
Z (in-depth) position (m)
X (lateral) position (m)
ϕ, dϕ/dt and d²ϕ/dt²
θ, dθ/dt and d²θ/dt²
Time (s)
Angular value (°, °/s, or °/s²)

P3/B5
S20/R30-IN

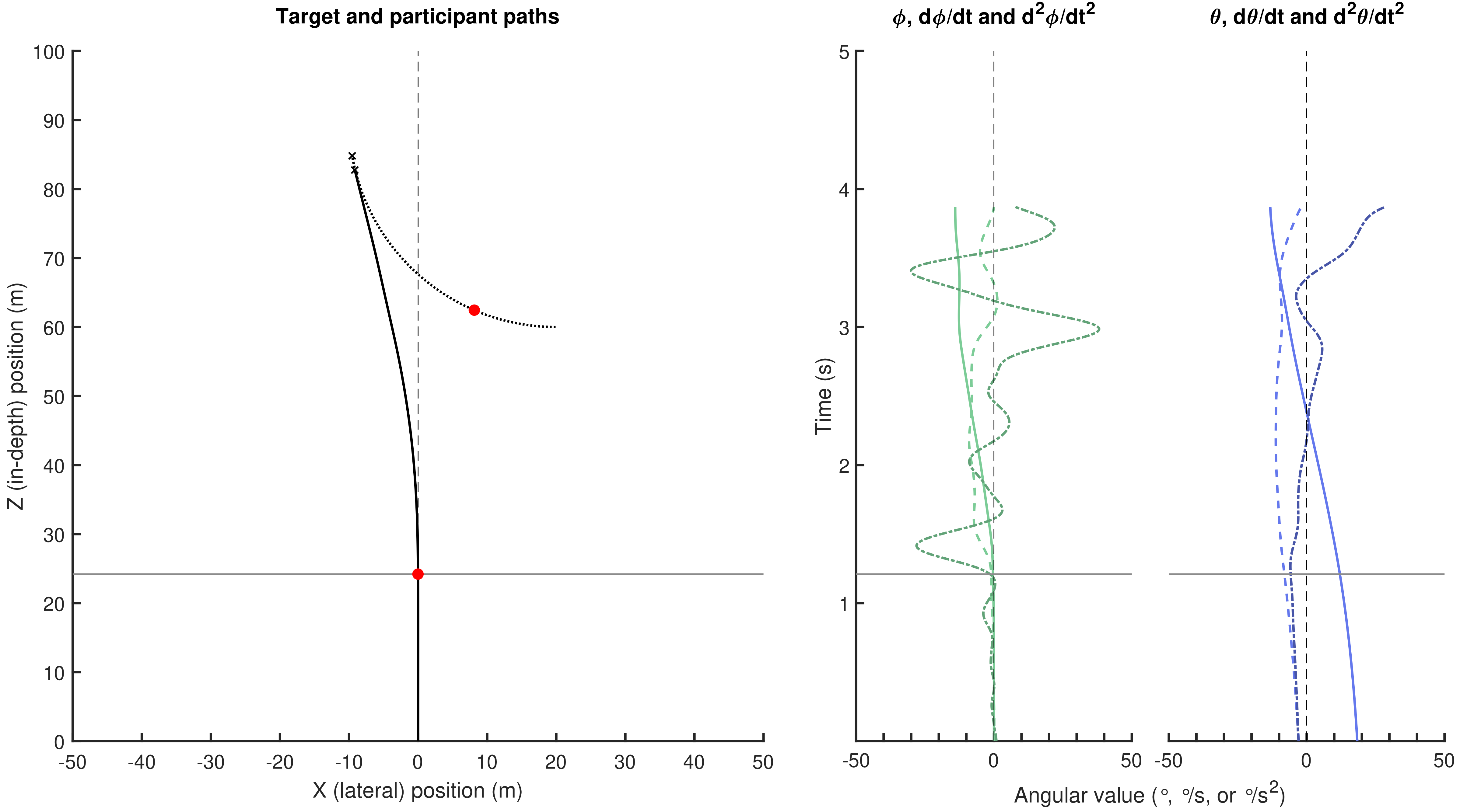

P4/B1
S20/R10-IN

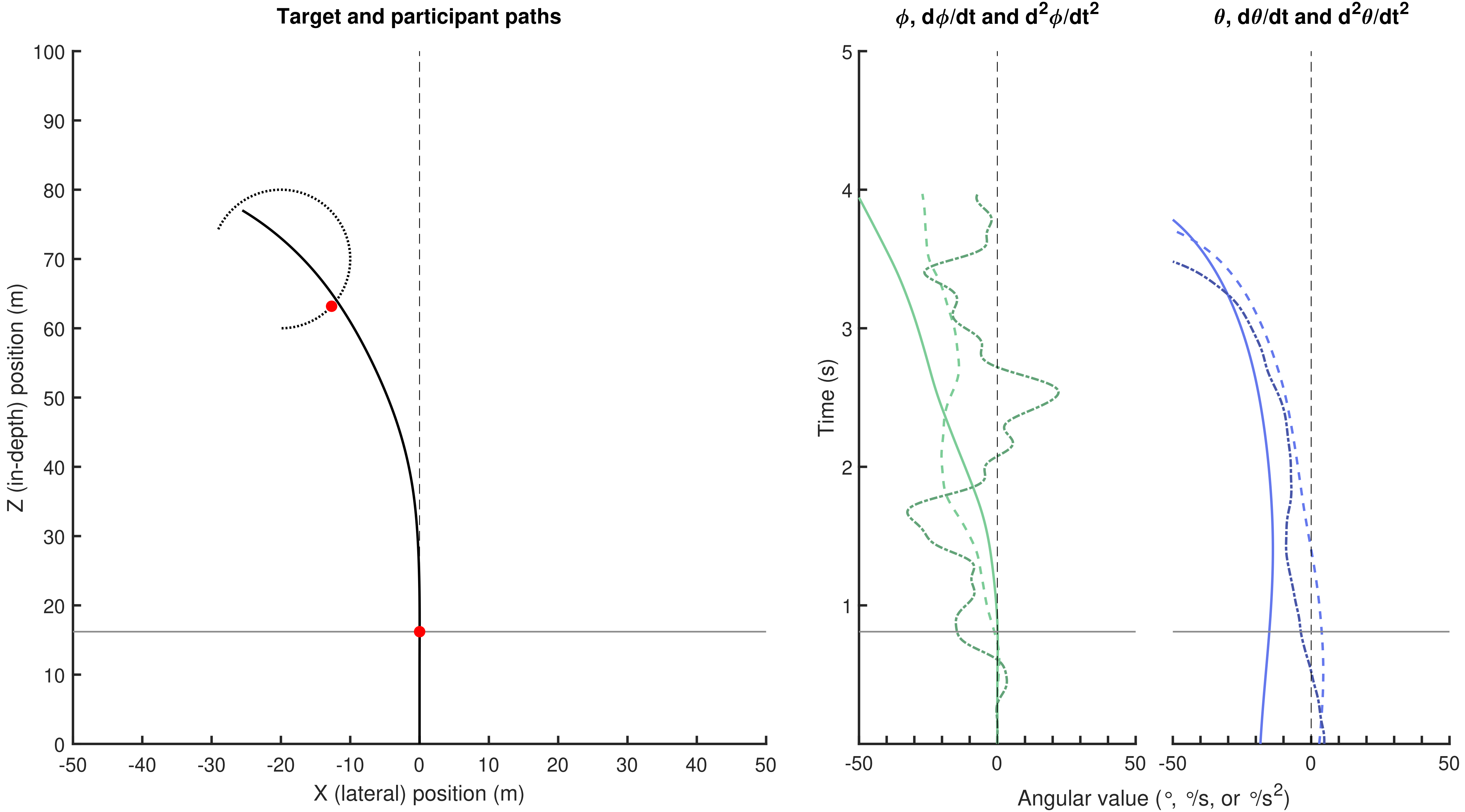

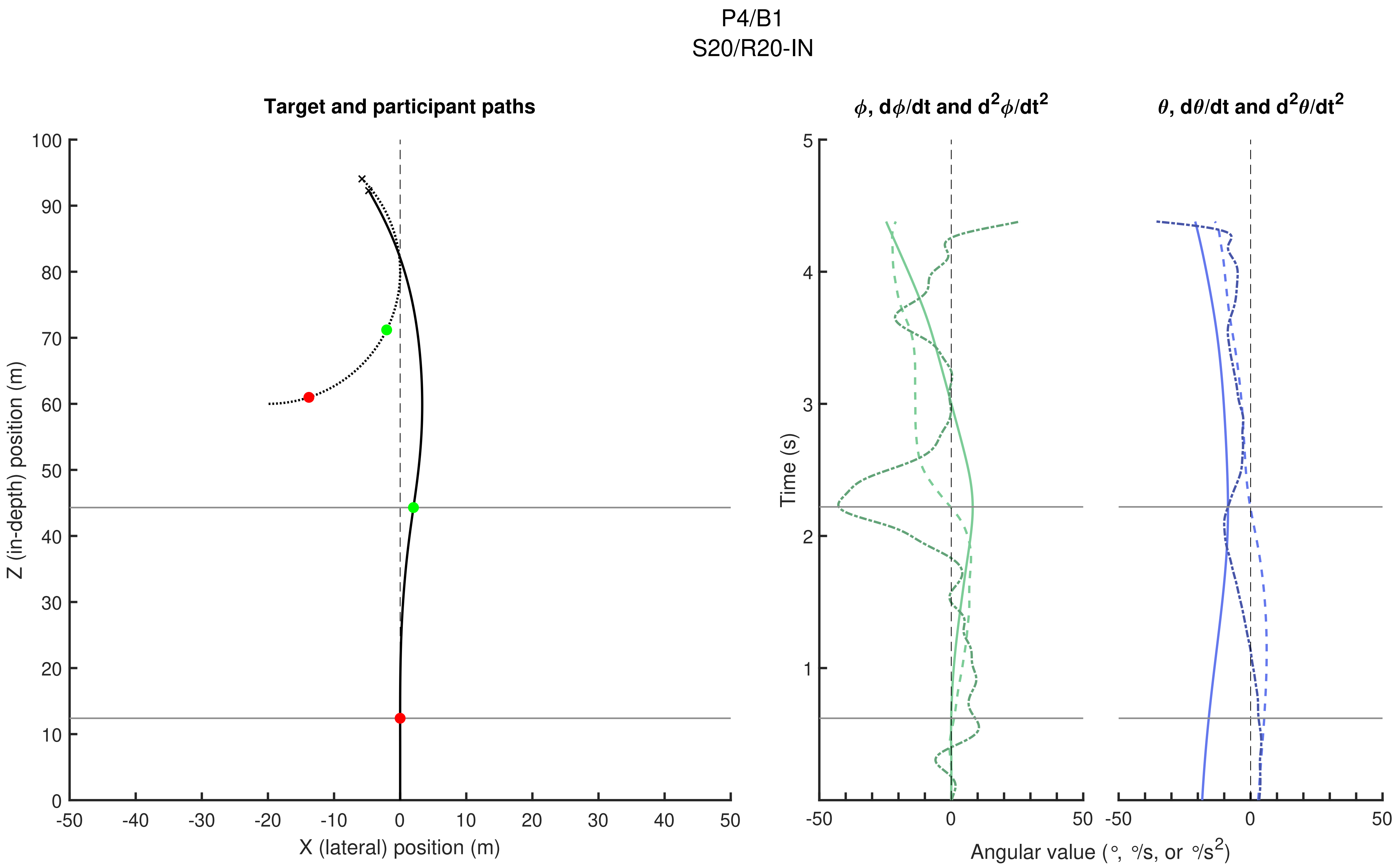
P4/B1
S20/R20-IN
Target and participant paths
Z (in-depth) position (m)
X (lateral) position (m)
0
10
20
30
40
50
60
70
80
90
100
-50
-40
-30
-20
-10
0
10
20
30
40
50
ϕ, dϕ/dt and d$^2\phi$/dt^2
θ, dθ/dt and d$^2\theta$/dt^2
Time (s)
1
2
3
4
5
-50
0
50
-50
0
50
Angular value (°, °/s, or °/s^2)

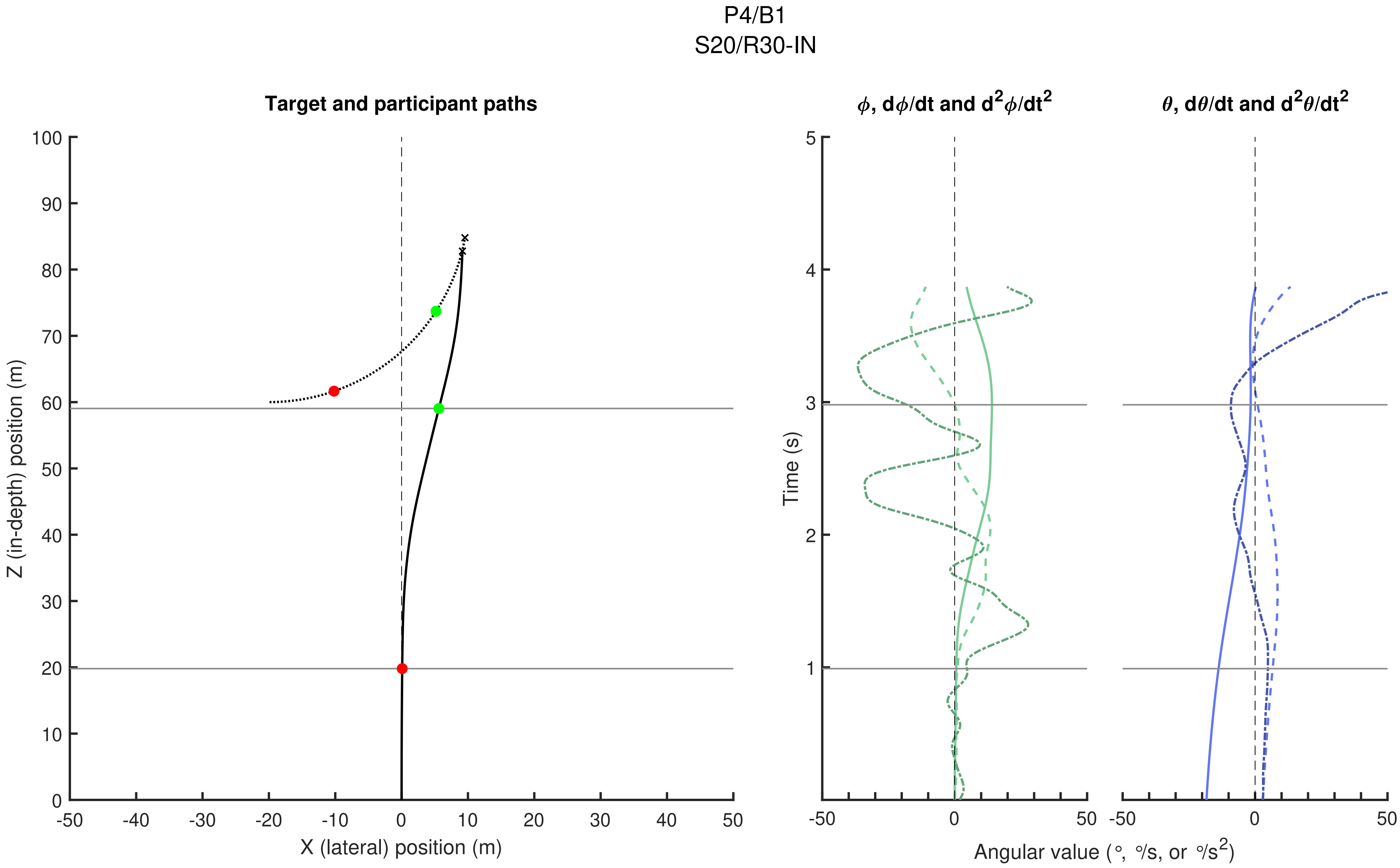
P4/B1
S20/R30-IN
Target and participant paths
Z (in-depth) position (m)
X (lateral) position (m)
100
90
80
70
60
50
40
30
20
10
0
-50
-40
-30
-20
-10
0
10
20
30
40
50
ϕ, dϕ/dt and d²ϕ/dt²
θ, dθ/dt and d²θ/dt²
Time (s)
5
4
3
2
1
-50
0
50
-50
0
50
Angular value (°, °/s, or °/s²)

P4/B1
S15/R10-IN

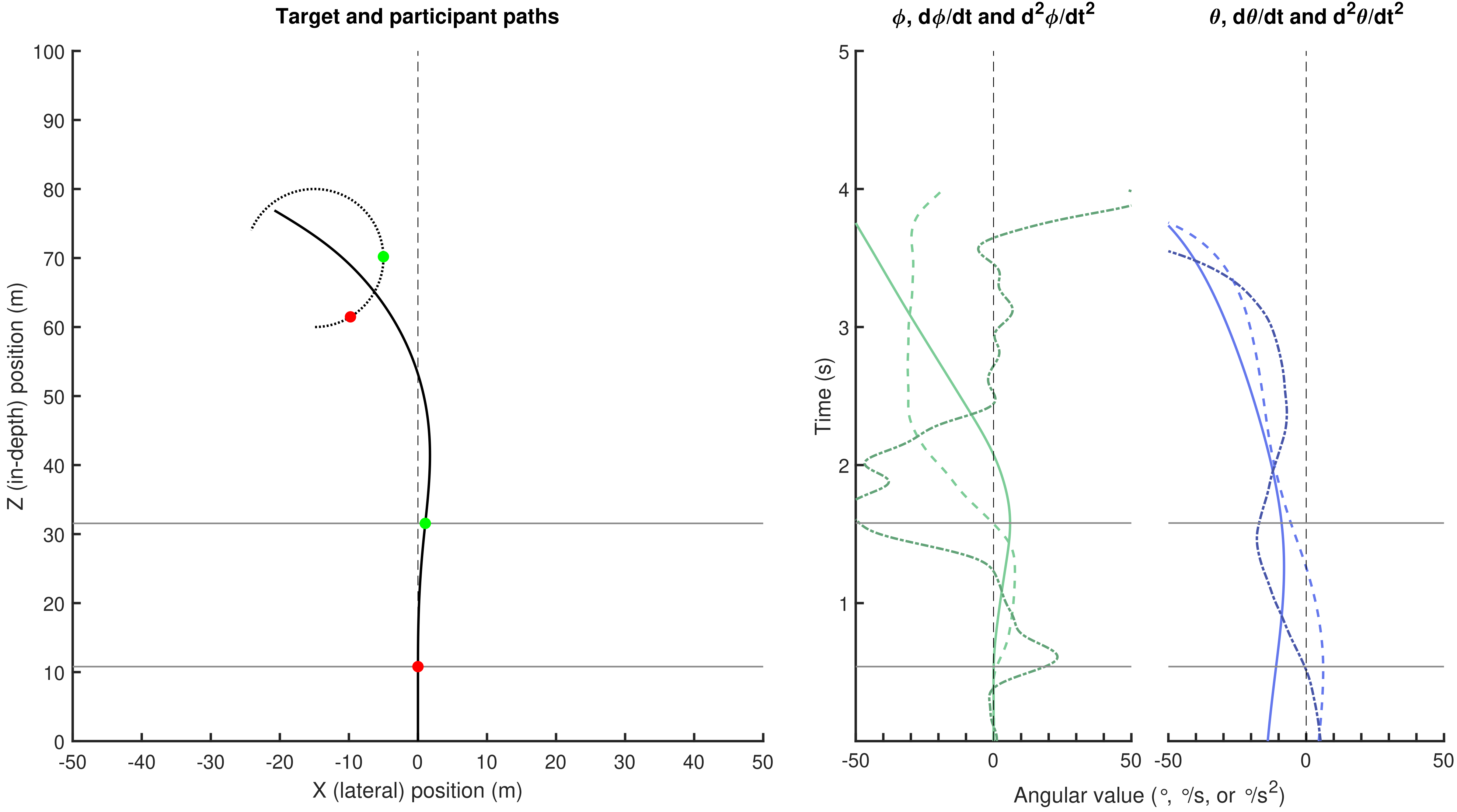

P4/B1
S15/R20-IN

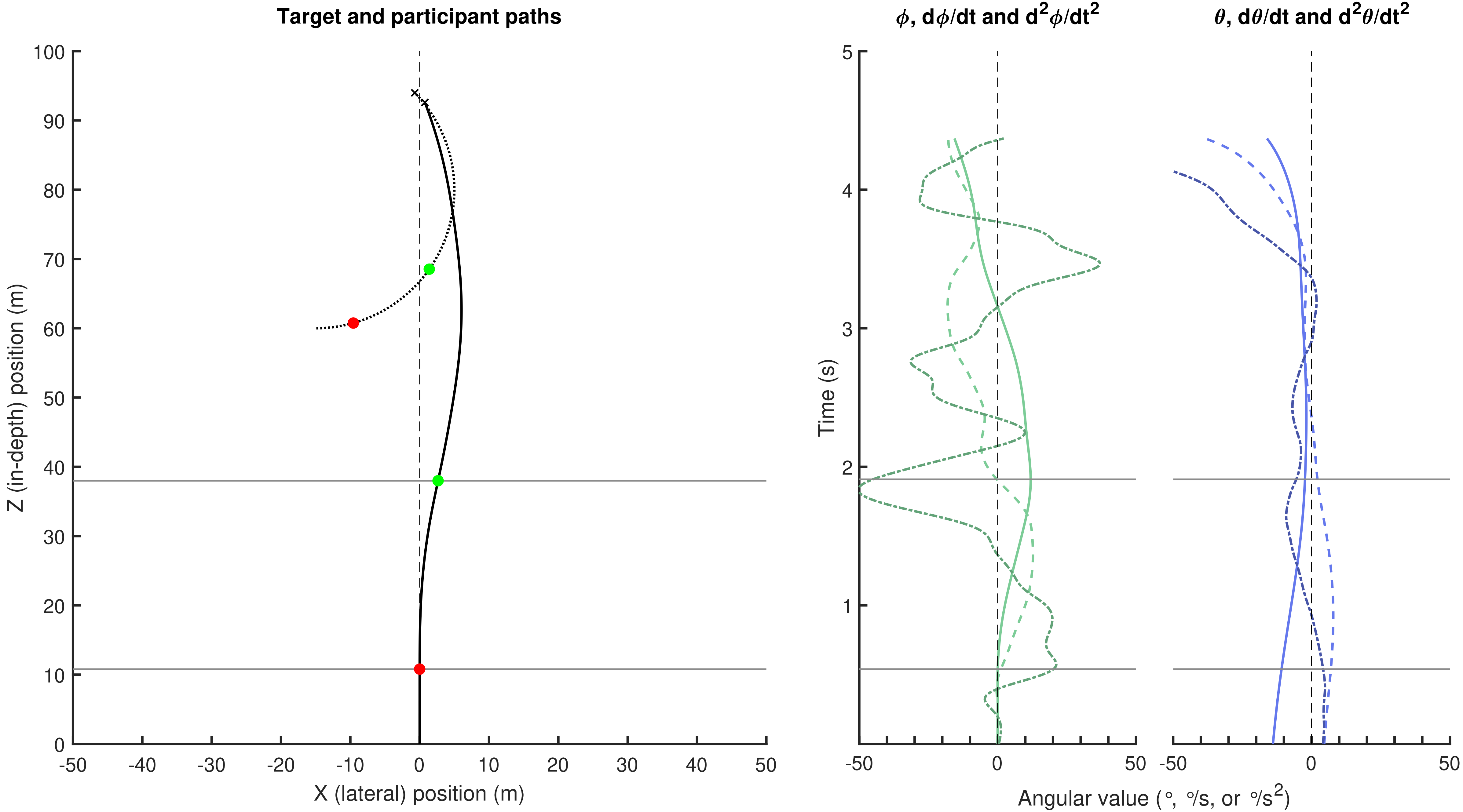

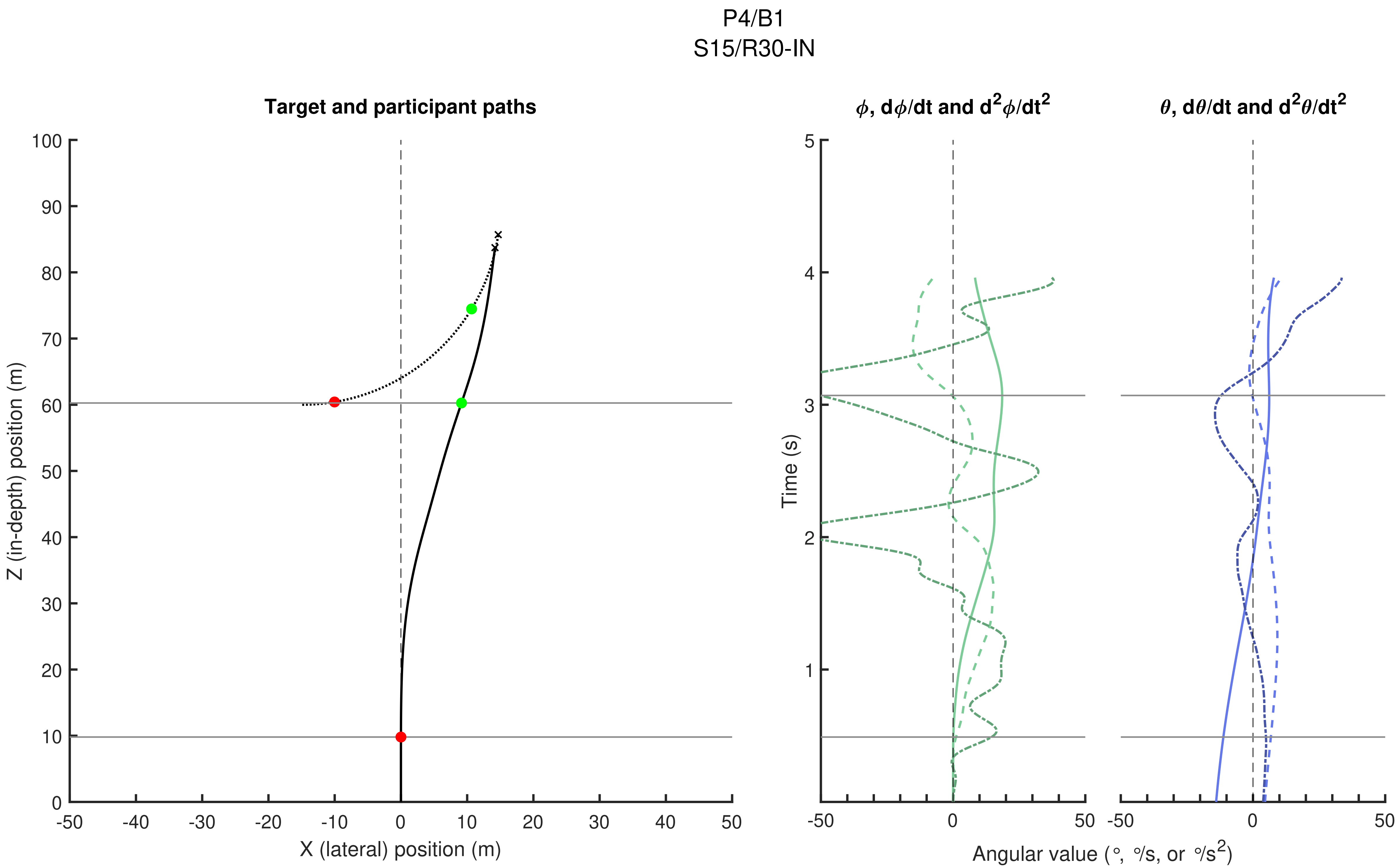
P4/B1
S15/R30-IN
Target and participant paths
Z (in-depth) position (m)
X (lateral) position (m)
ϕ, dϕ/dt and d$^2\phi$/dt^2
θ, dθ/dt and d$^2\theta$/dt^2
Time (s)
Angular value (°, °/s, or °/s^2)

P4/B1
S5/R10-IN

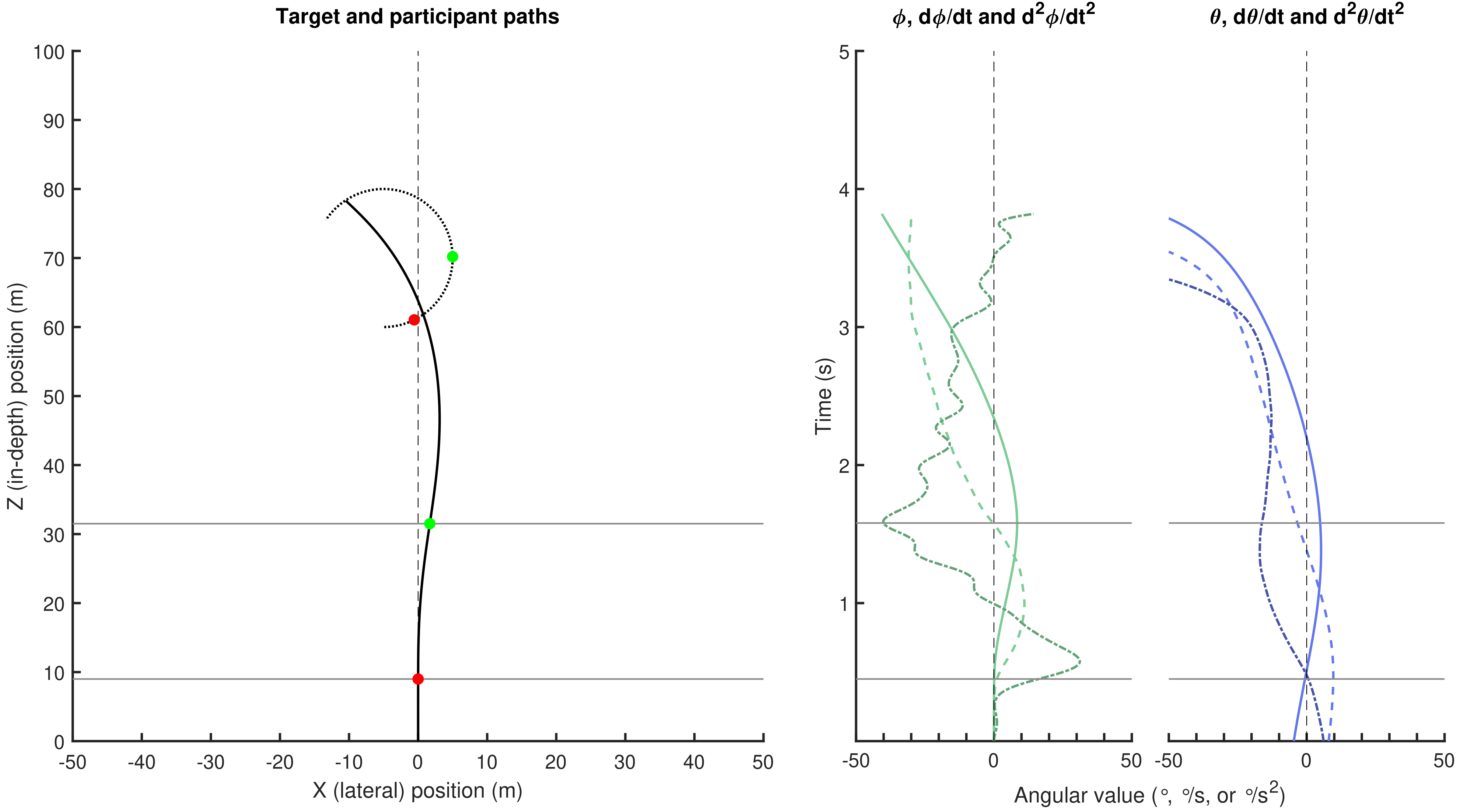

P4/B1
S5/R10-OUT

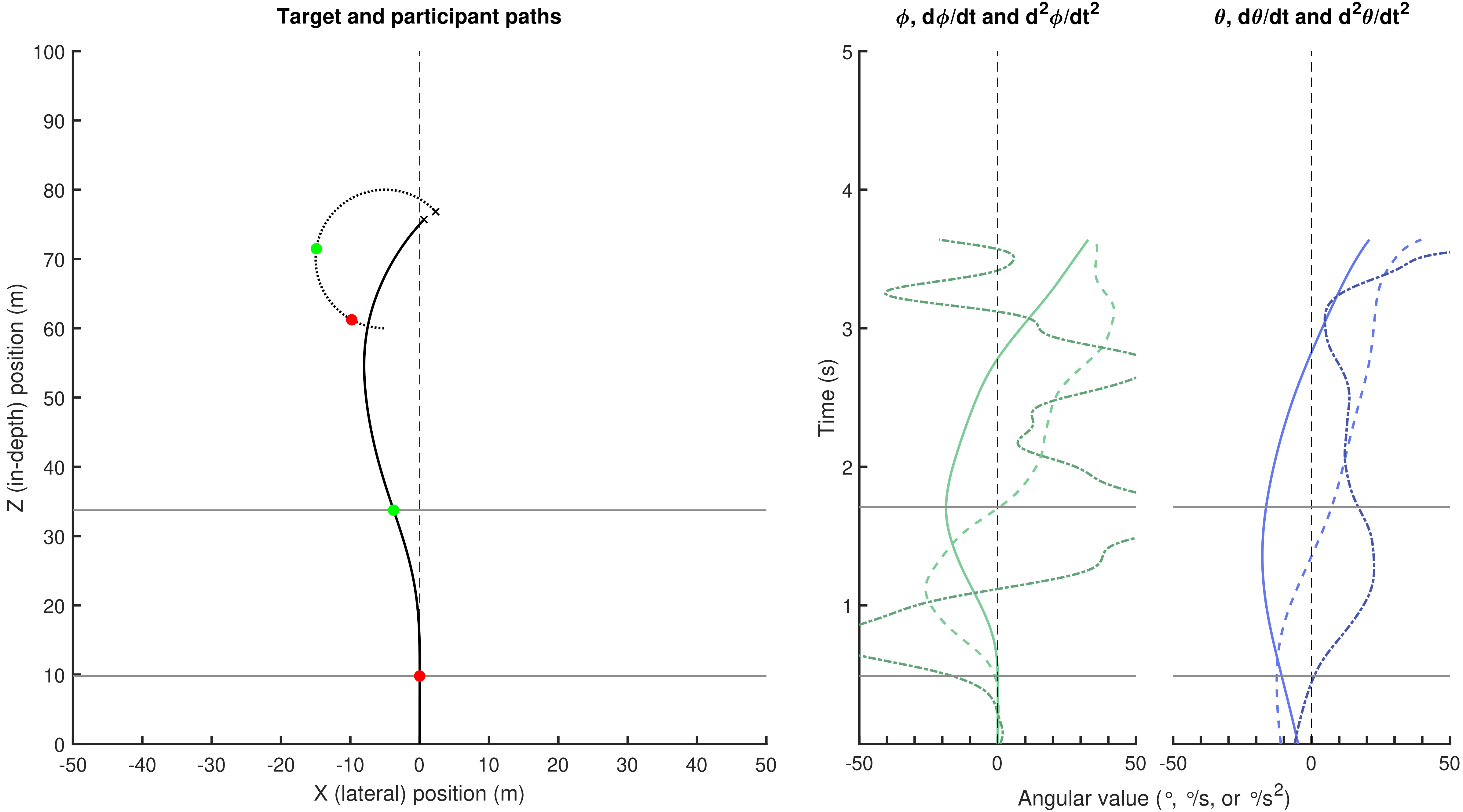

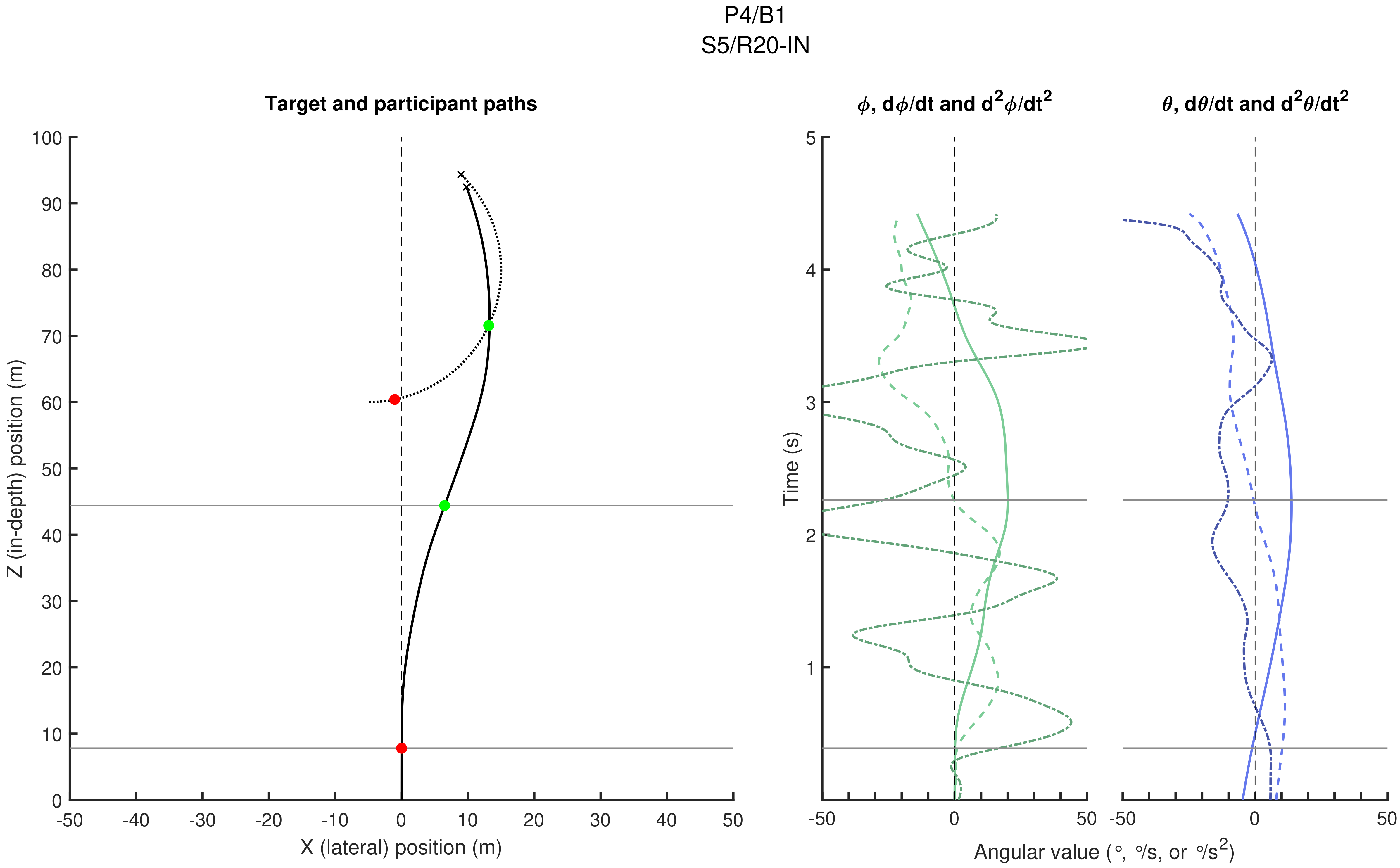
P4/B1
S5/R20-IN
Target and participant paths
Z (in-depth) position (m)
X (lateral) position (m)
100
90
80
70
60
50
40
30
20
10
0
-50
-40
-30
-20
-10
0
10
20
30
40
50
ϕ, dϕ/dt and d$^2\phi$/dt^2
θ, dθ/dt and d$^2\theta$/dt^2
Time (s)
5
4
3
2
1
-50
0
50
-50
0
50
Angular value (°, °/s, or °/s^2)

P4/B1
S5/R20-OUT

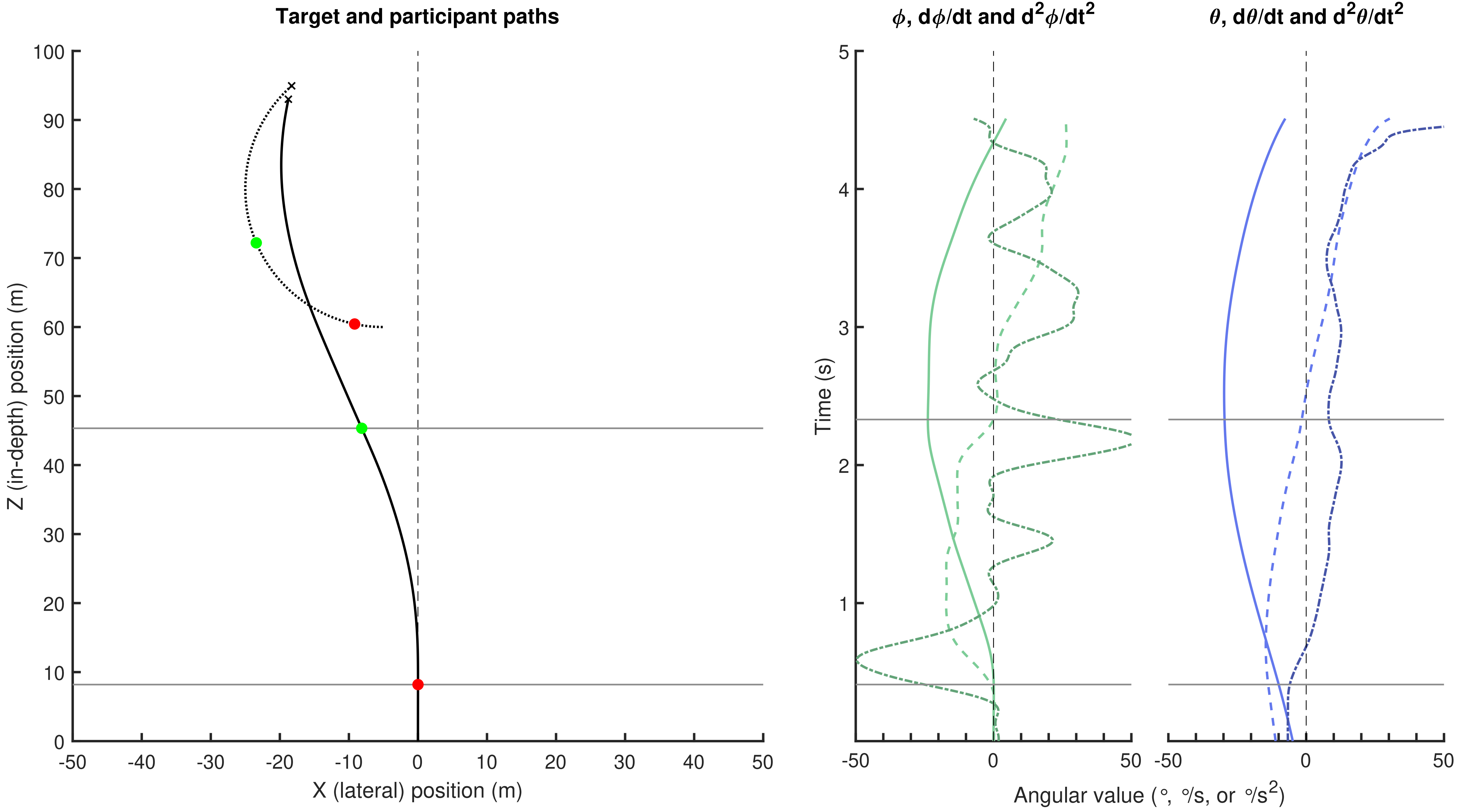

P4/B1
S5/R30-IN

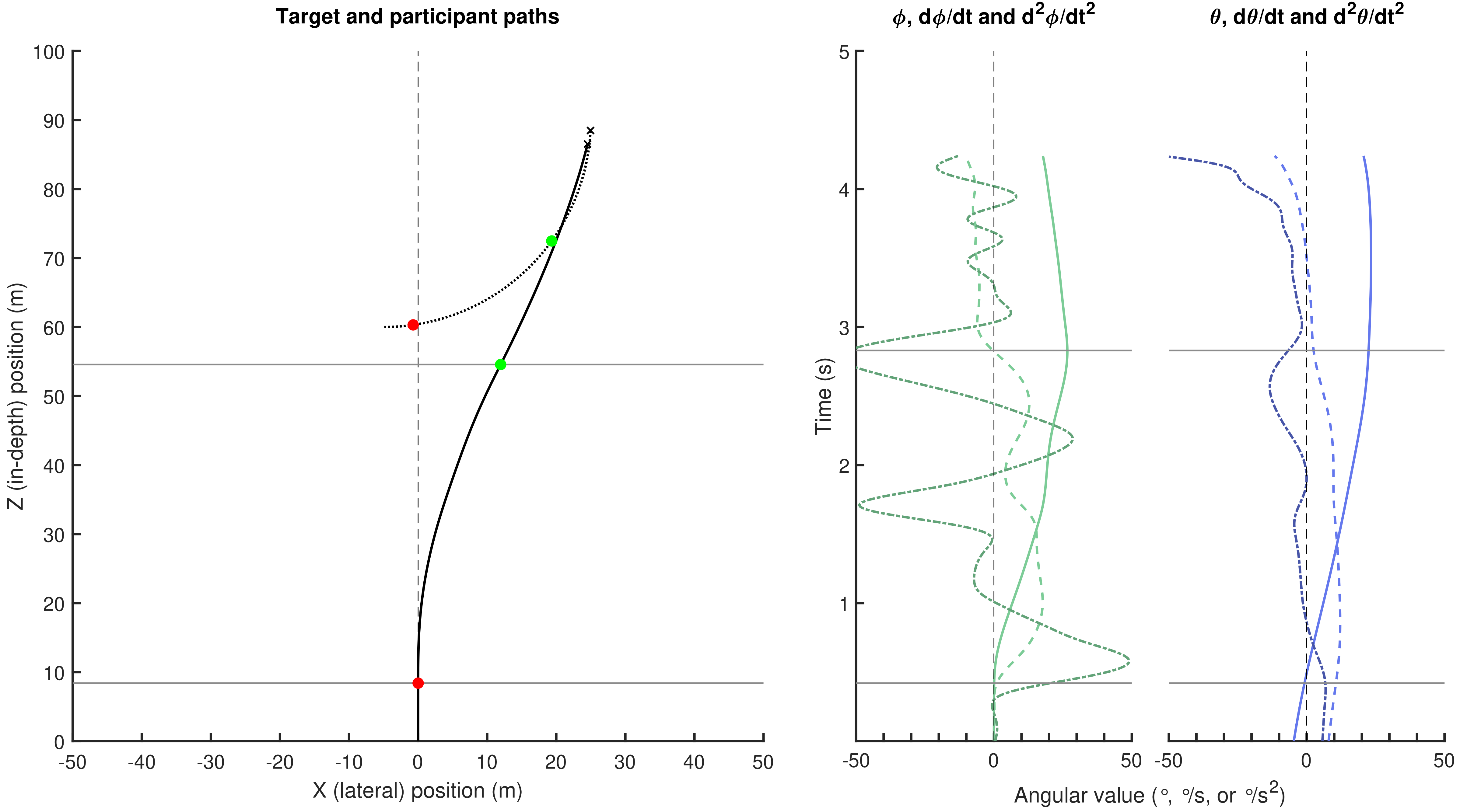

P4/B1
S5/R30-OUT

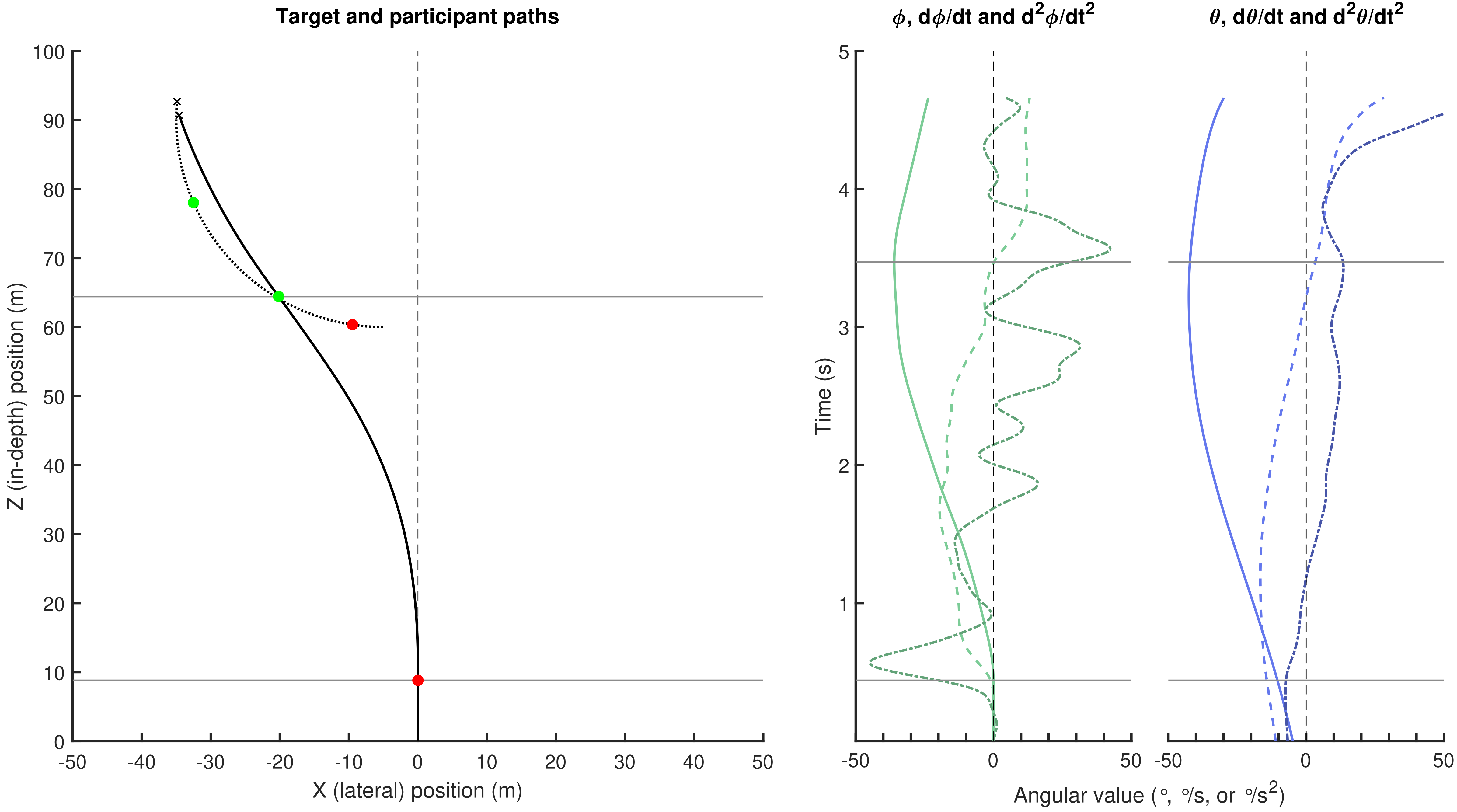

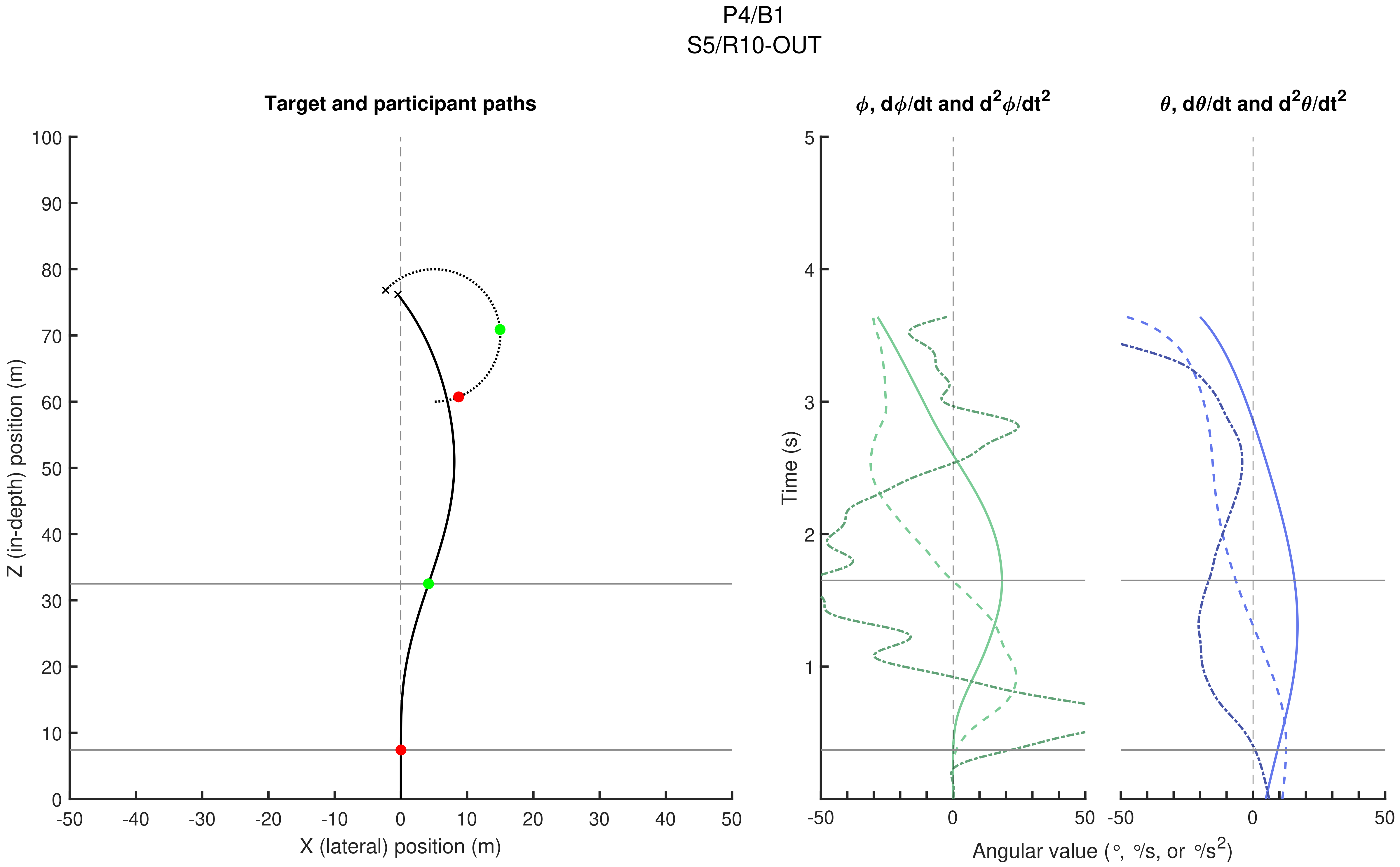
P4/B1
S5/R10-OUT
Target and participant paths
Z (in-depth) position (m)
X (lateral) position (m)
φ, dφ/dt and d²φ/dt²
θ, dθ/dt and d²θ/dt²
Time (s)
Angular value (°, °/s, or °/s²)

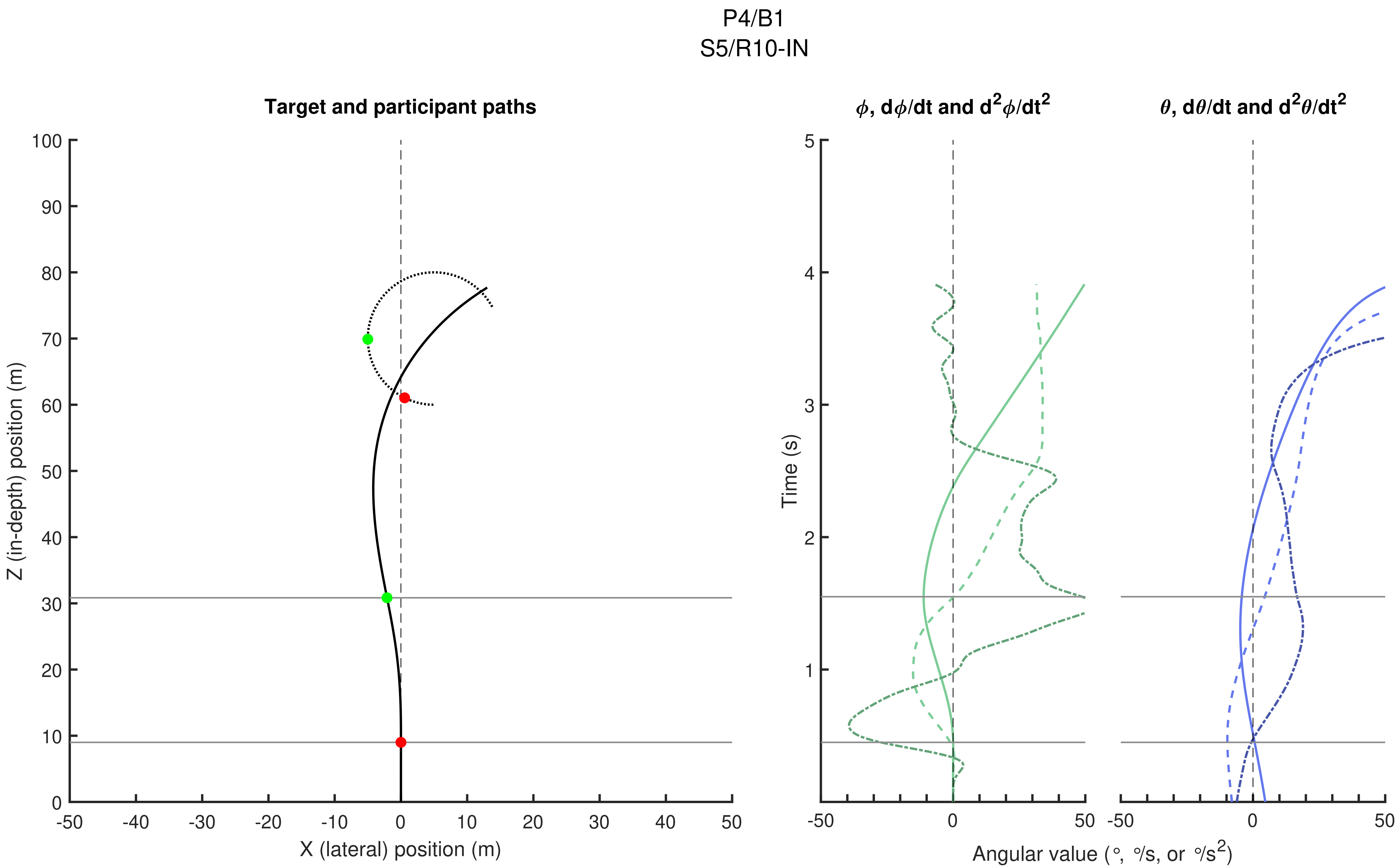

P4/B1
S5/R10-IN
Target and participant paths
Z (in-depth) position (m)
X (lateral) position (m)
ϕ, dϕ/dt and d$^2\phi$/dt^2
θ, dθ/dt and d$^2\theta$/dt^2
Time (s)
Angular value (°, °/s, or °/s^2)

P4/B1
S5/R20-OUT

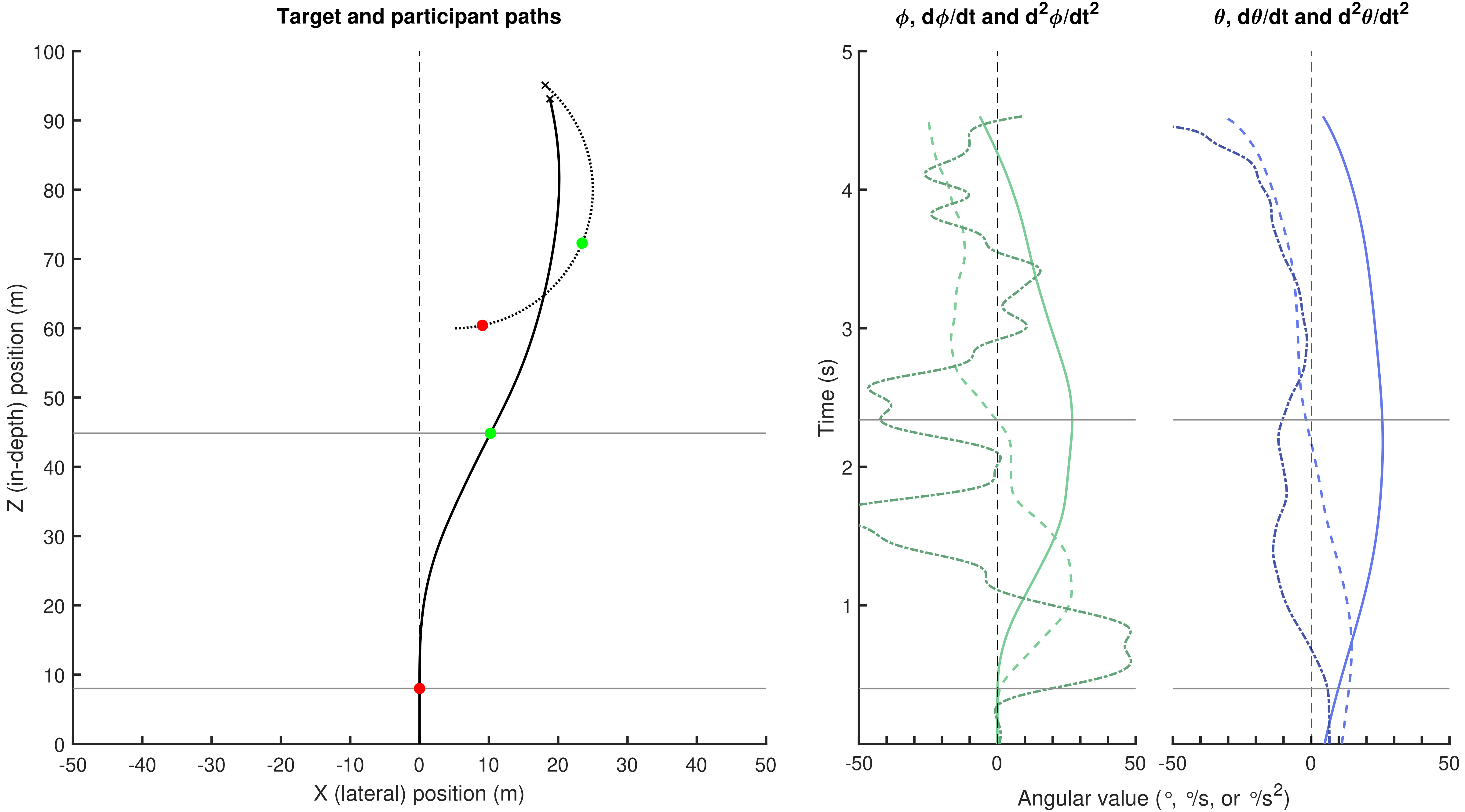

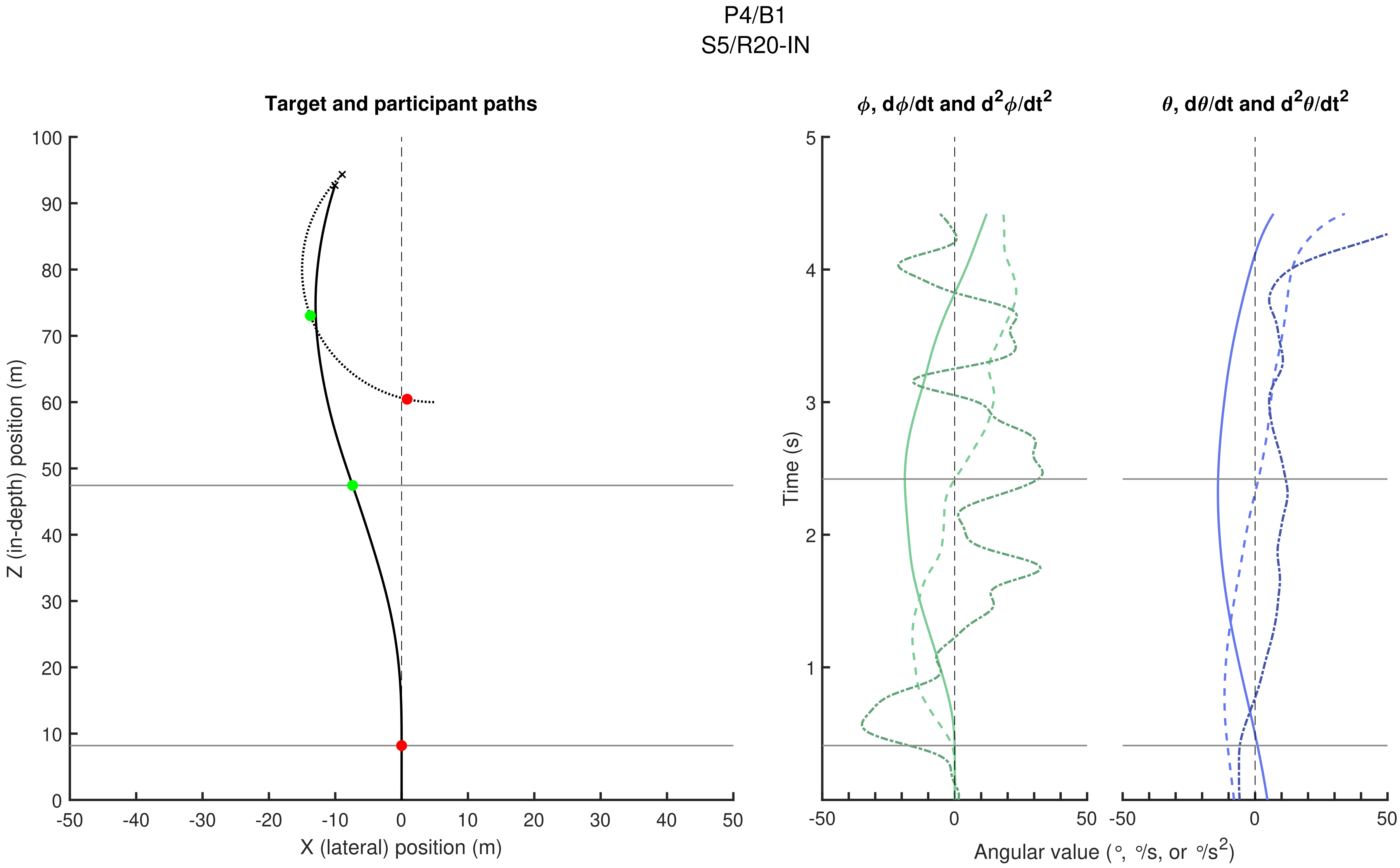
P4/B1
S5/R20-IN
Target and participant paths
Z (in-depth) position (m)
X (lateral) position (m)
100
90
80
70
60
50
40
30
20
10
0
-50
-40
-30
-20
-10
0
10
20
30
40
50
ϕ, dϕ/dt and d²ϕ/dt²
θ, dθ/dt and d²θ/dt²
Time (s)
5
4
3
2
1
-50
0
50
-50
0
50
Angular value (°, °/s, or °/s²)

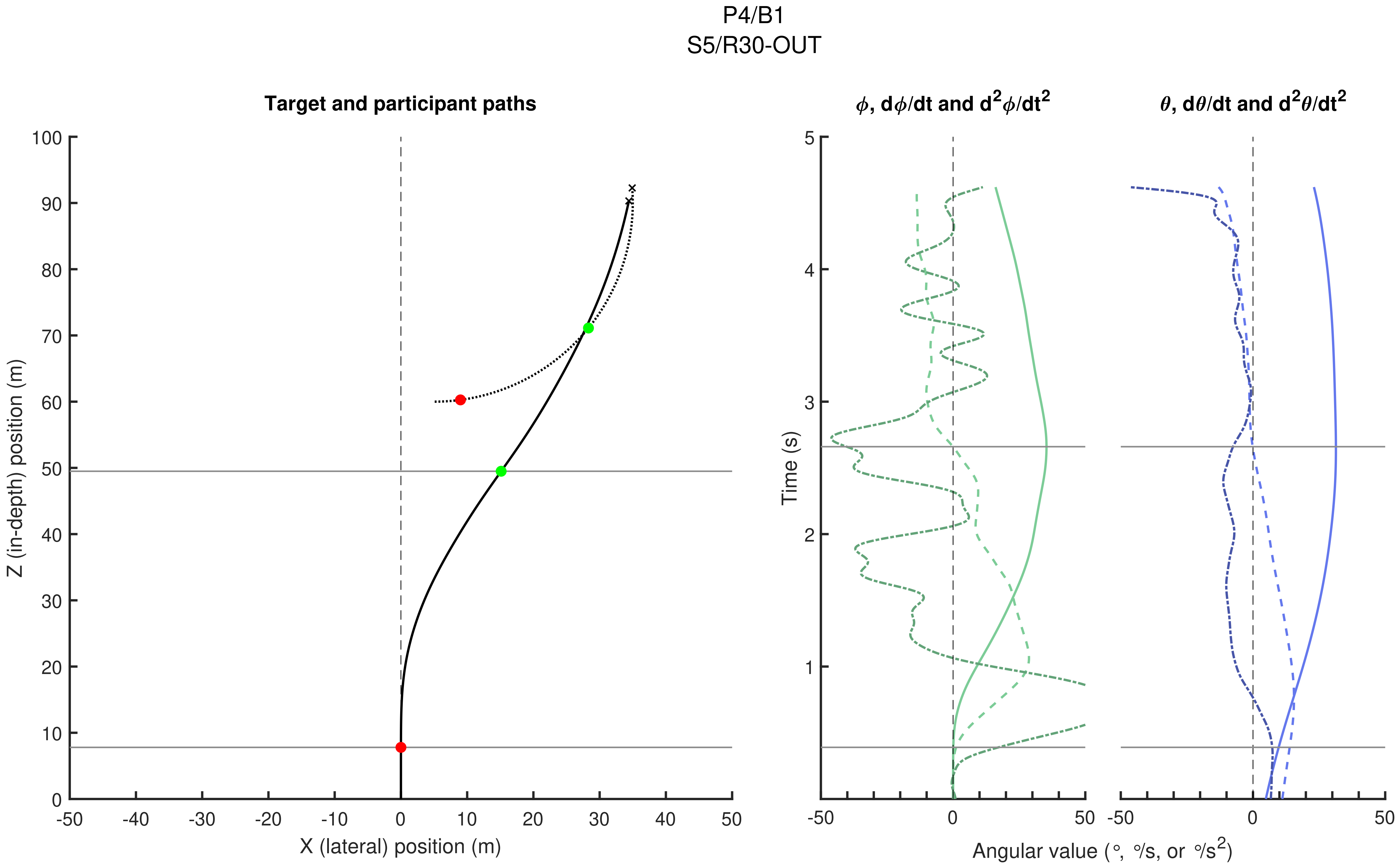
P4/B1
S5/R30-OUT
Target and participant paths
Z (in-depth) position (m)
X (lateral) position (m)
100
90
80
70
60
50
40
30
20
10
0
-50
-40
-30
-20
-10
0
10
20
30
40
50
ϕ, dϕ/dt and d²ϕ/dt²
θ, dθ/dt and d²θ/dt²
Time (s)
5
4
3
2
1
-50
0
50
-50
0
50
Angular value (°, °/s, or °/s²)

P4/B1
S5/R30-IN

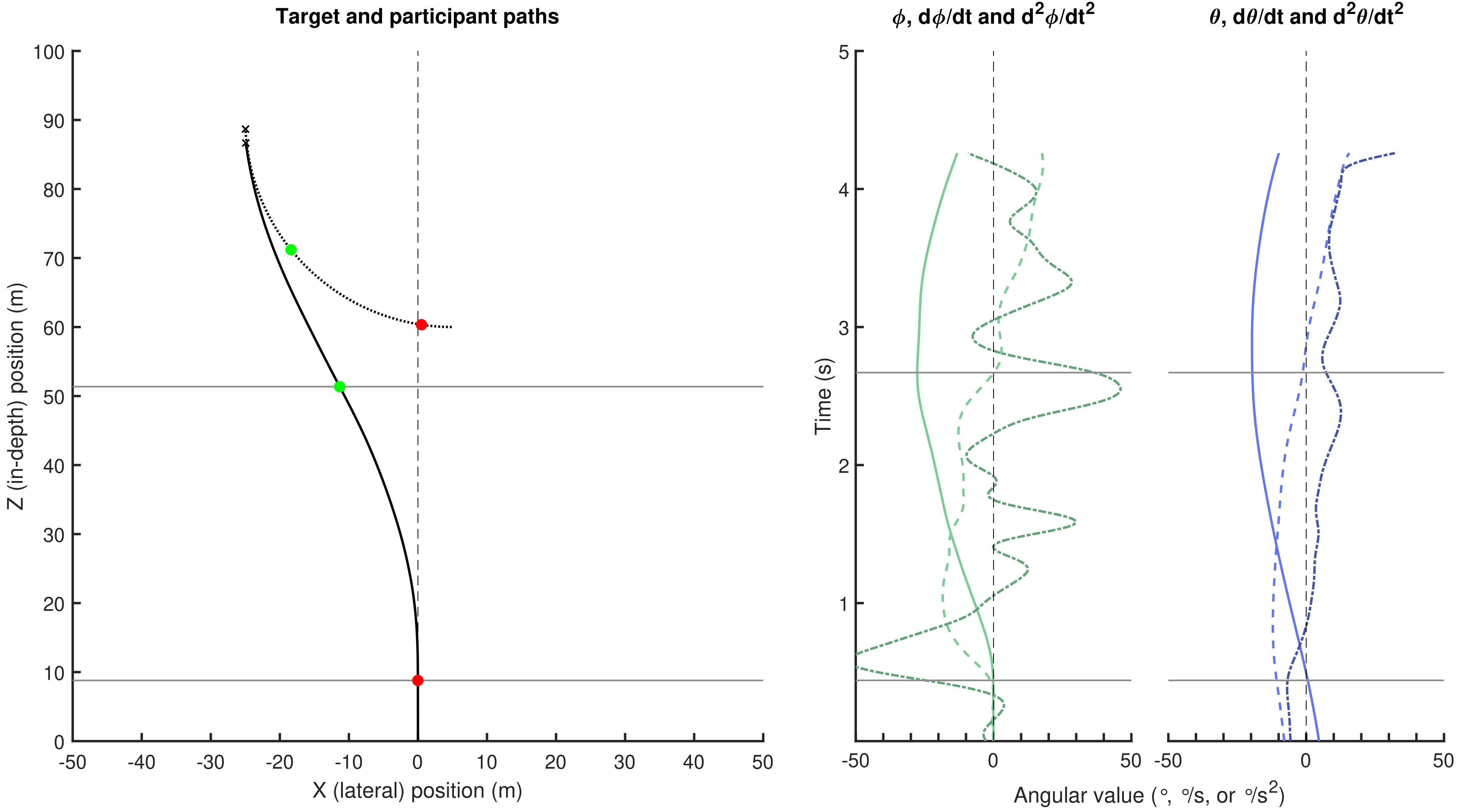

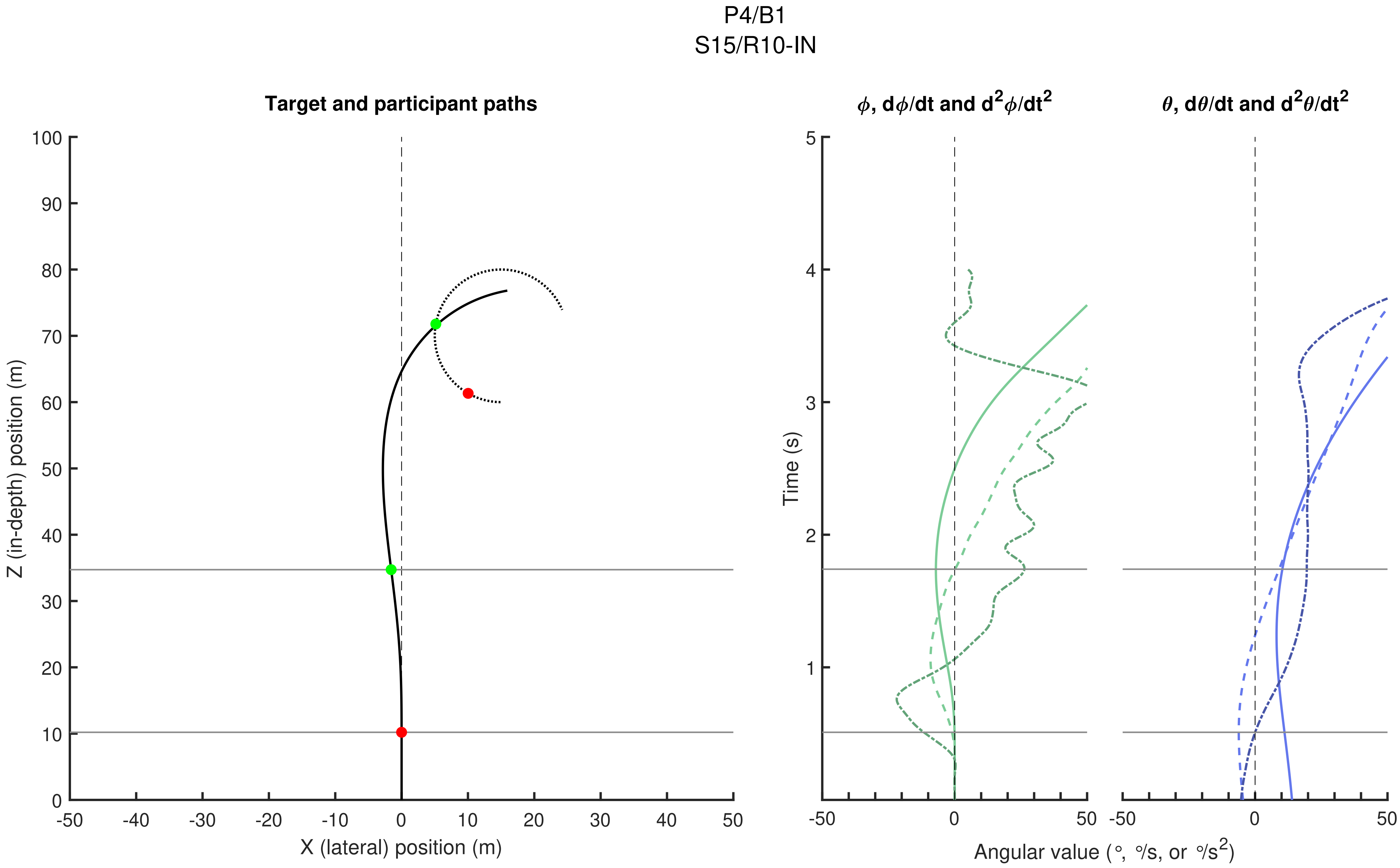

P4/B1
S15/R10-IN
Target and participant paths
Z (in-depth) position (m)
X (lateral) position (m)
φ, dφ/dt and d²φ/dt²
θ, dθ/dt and d²θ/dt²
Time (s)
Angular value (°, °/s, or °/s²)

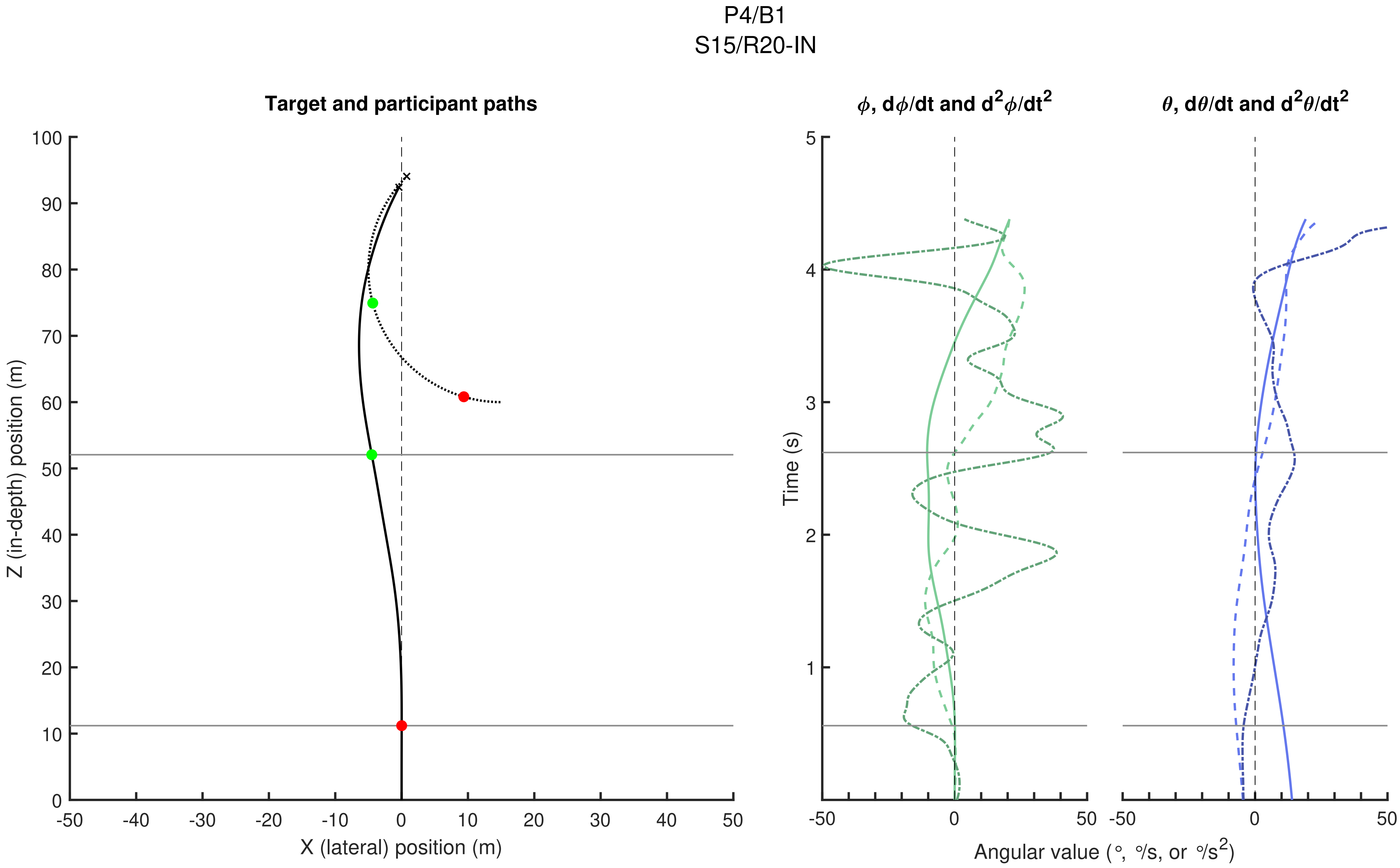

P4/B1
S15/R20-IN
Target and participant paths
Z (in-depth) position (m)
X (lateral) position (m)
ϕ, dϕ/dt and d$^2\phi$/dt^2
θ, dθ/dt and d$^2\theta$/dt^2
Time (s)
Angular value (°, °/s, or °/s^2)

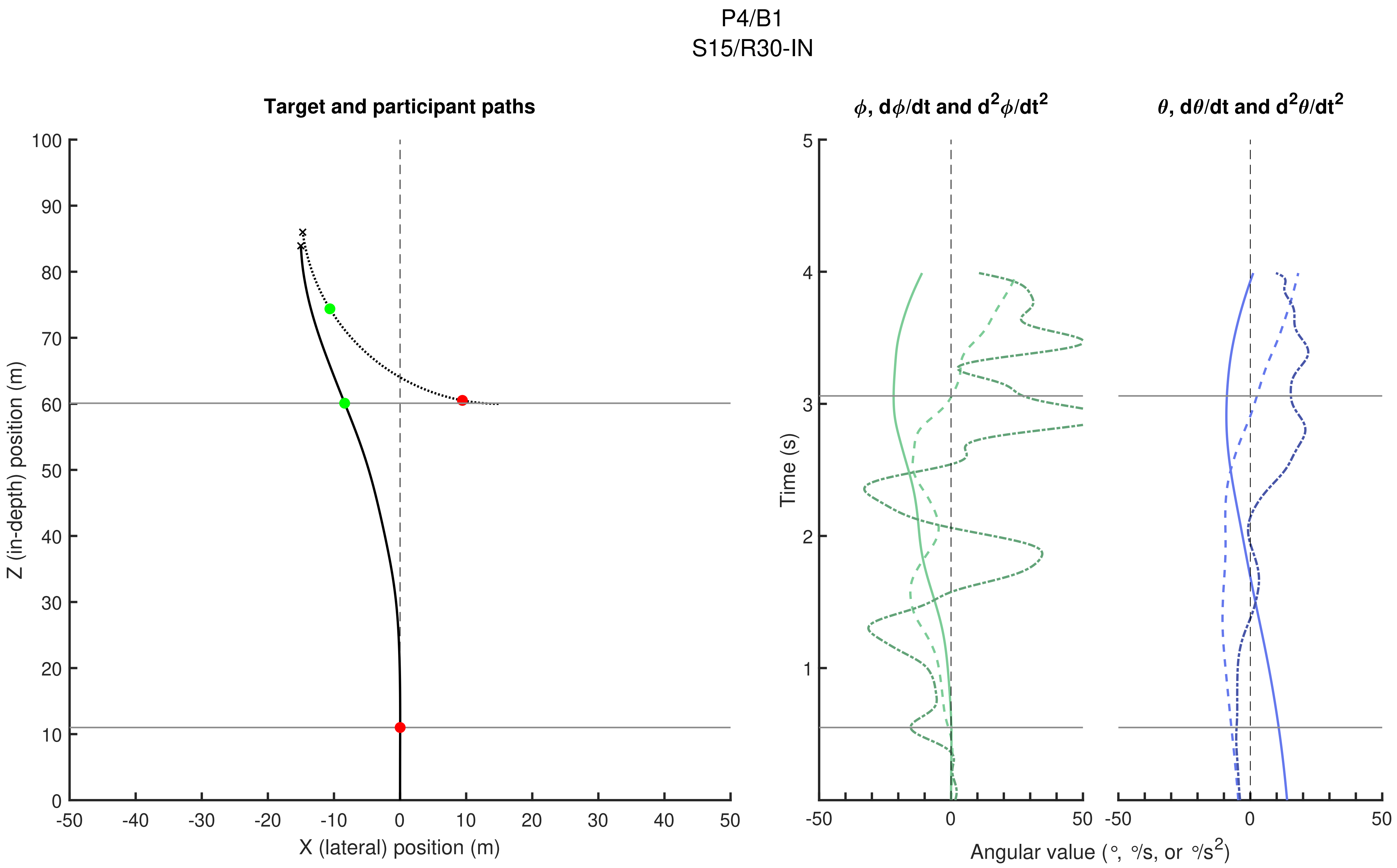
P4/B1
S15/R30-IN
Target and participant paths
Z (in-depth) position (m)
X (lateral) position (m)
100
90
80
70
60
50
40
30
20
10
0
-50
-40
-30
-20
-10
0
10
20
30
40
50
ϕ, dϕ/dt and d²ϕ/dt²
θ, dθ/dt and d²θ/dt²
Time (s)
5
4
3
2
1
-50
0
50
-50
0
50
Angular value (°, °/s, or °/s²)

P4/B1
S20/R10-IN

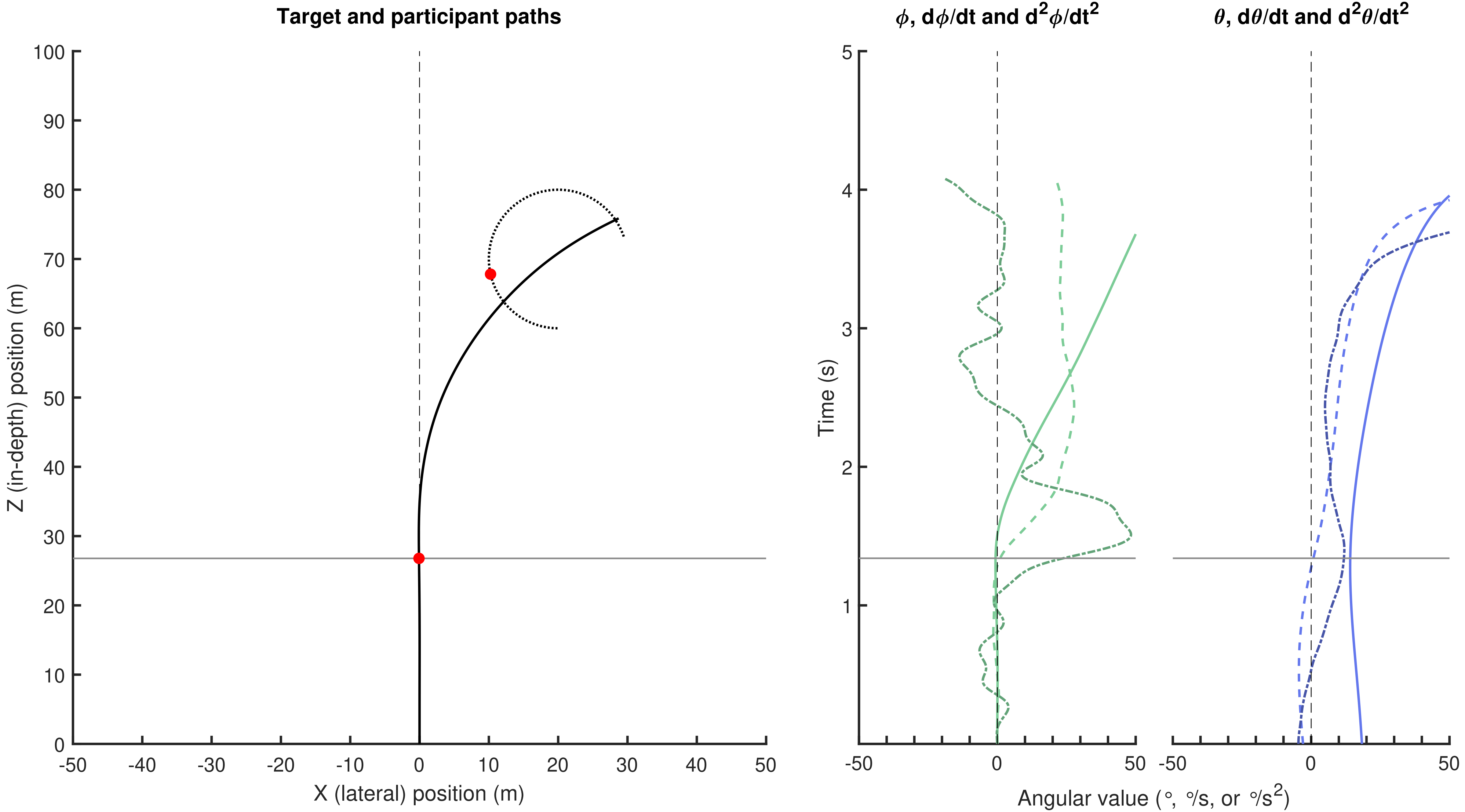

P4/B1
S20/R20-IN

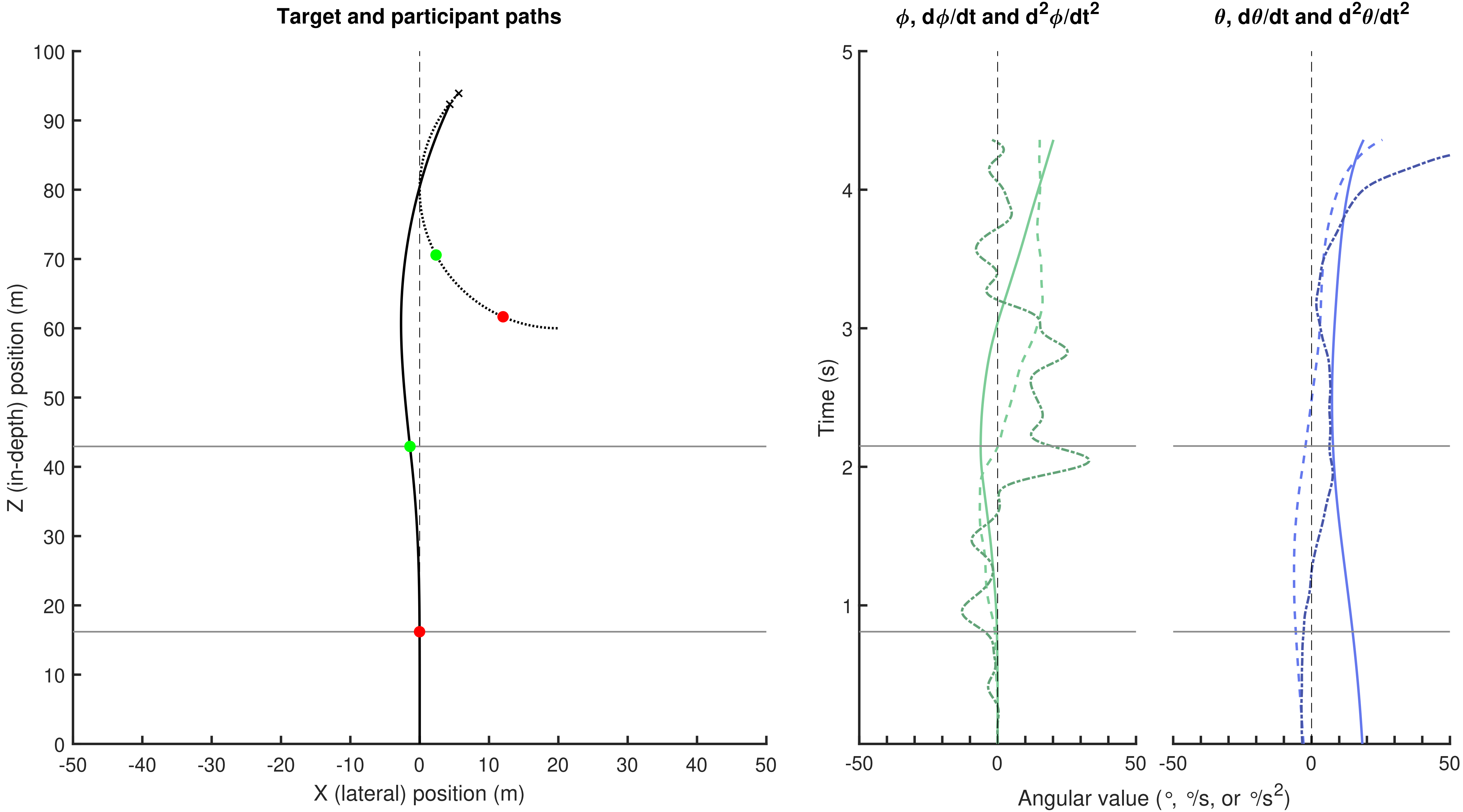

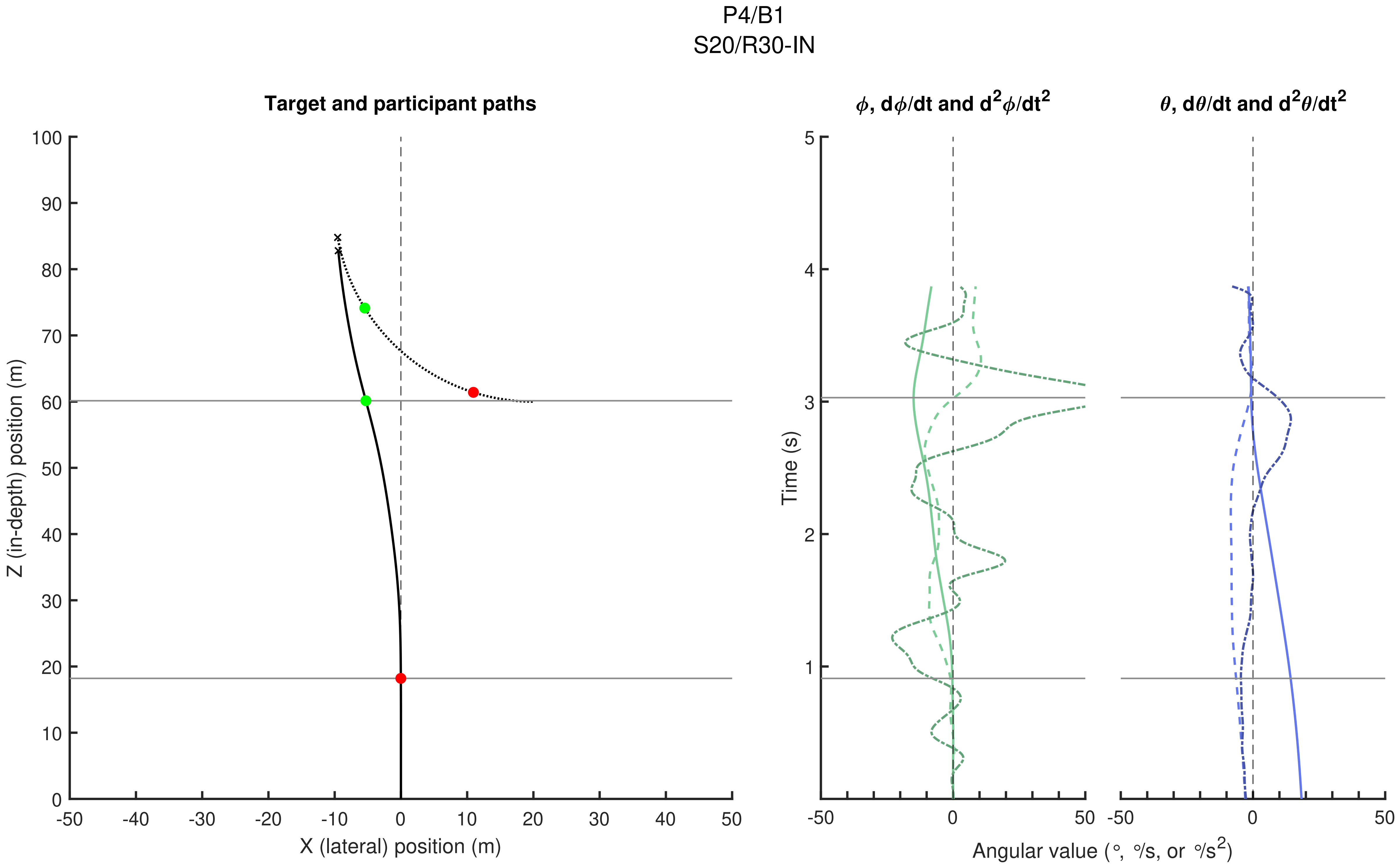
P4/B1
S20/R30-IN
Target and participant paths
Z (in-depth) position (m)
X (lateral) position (m)
ϕ, dϕ/dt and d$^2\phi$/dt^2
θ, dθ/dt and d$^2\theta$/dt^2
Time (s)
Angular value (°, °/s, or °/s^2)

P4/B2
S20/R10-IN

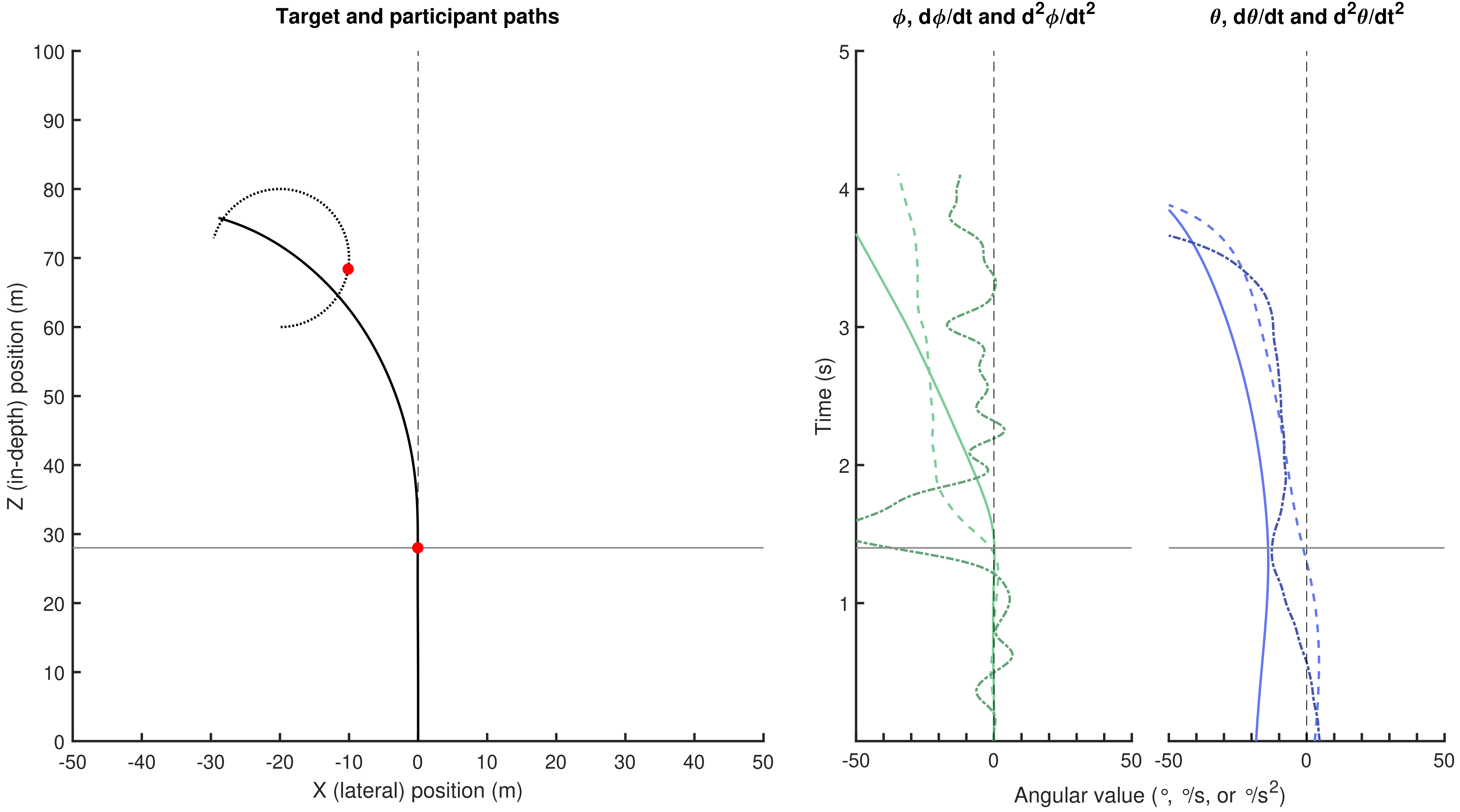

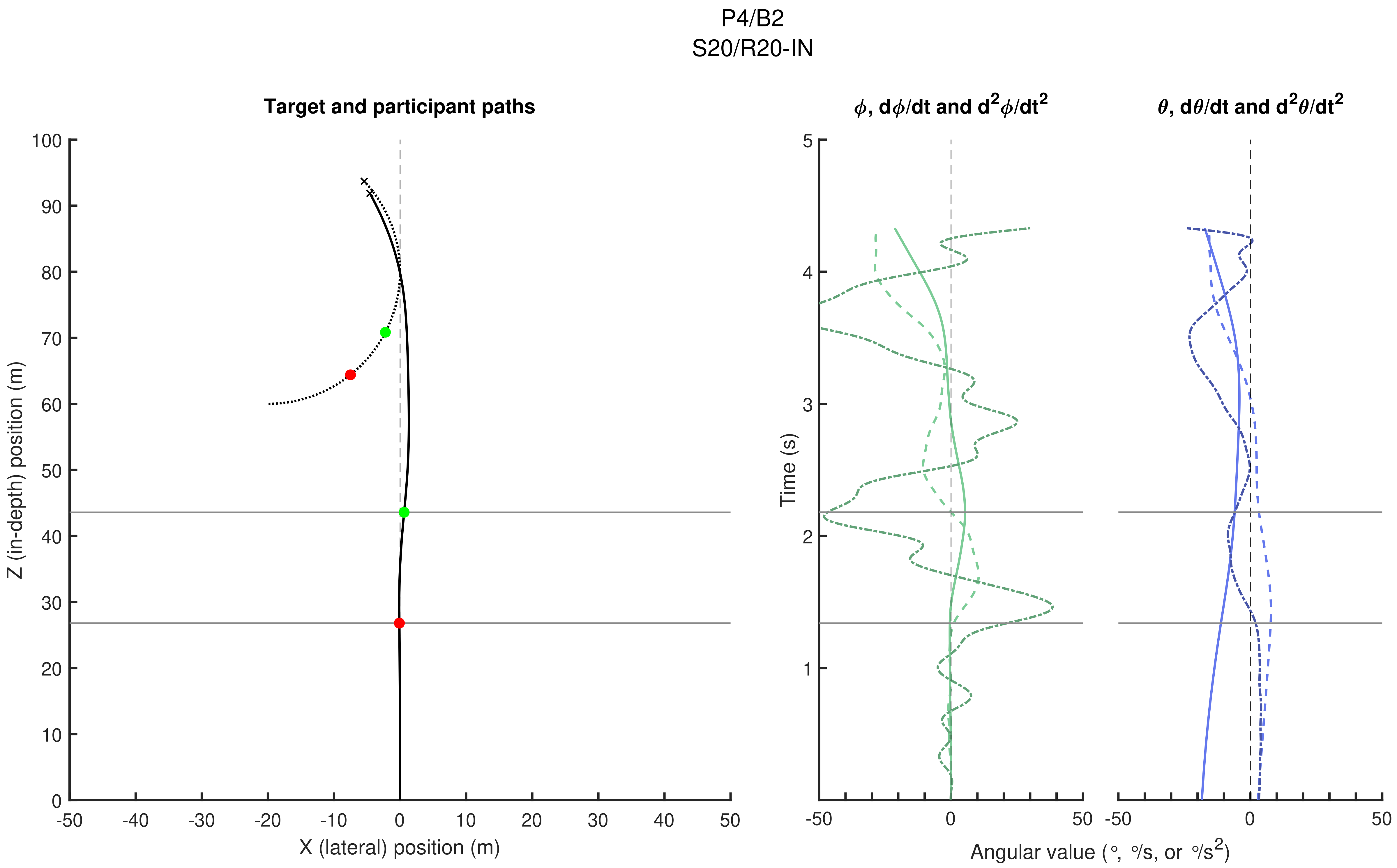
P4/B2
S20/R20-IN
Target and participant paths
Z (in-depth) position (m)
X (lateral) position (m)
100
90
80
70
60
50
40
30
20
10
0
-50
-40
-30
-20
-10
0
10
20
30
40
50
ϕ, dϕ/dt and d²ϕ/dt²
θ, dθ/dt and d²θ/dt²
Time (s)
5
4
3
2
1
-50
0
50
Angular value (°, °/s, or °/s²)

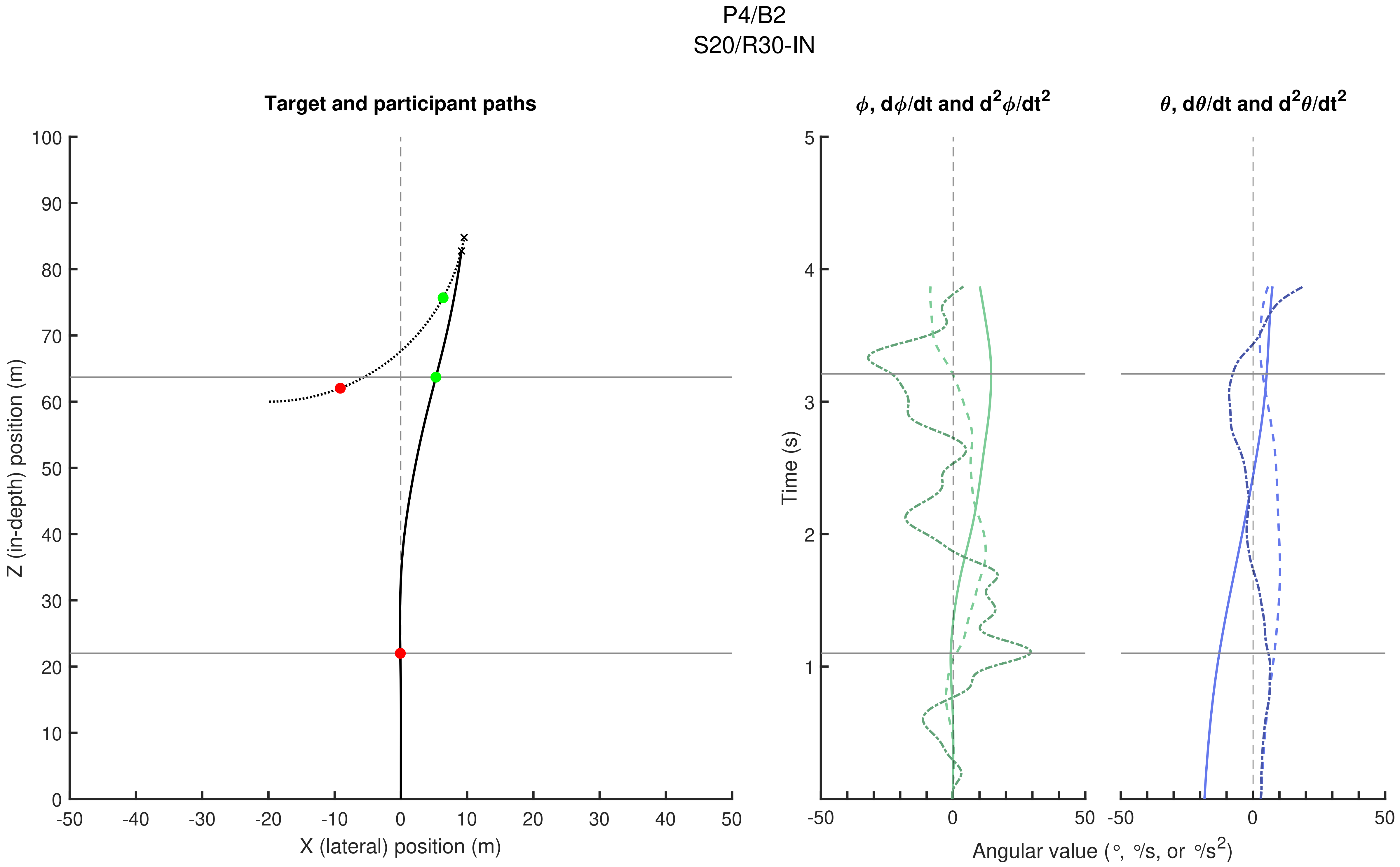

P4/B2
S20/R30-IN
Target and participant paths
ϕ, dϕ/dt and d$^2\phi$/dt^2
θ, dθ/dt and d$^2\theta$/dt^2
Z (in-depth) position (m)
X (lateral) position (m)
Time (s)
Angular value (°, °/s, or °/s^2)
100
90
80
70
60
50
40
30
20
10
0
-50
-40
-30
-20
-10
0
10
20
30
40
50
5
4
3
2
1
-50
0
50
-50
0
50

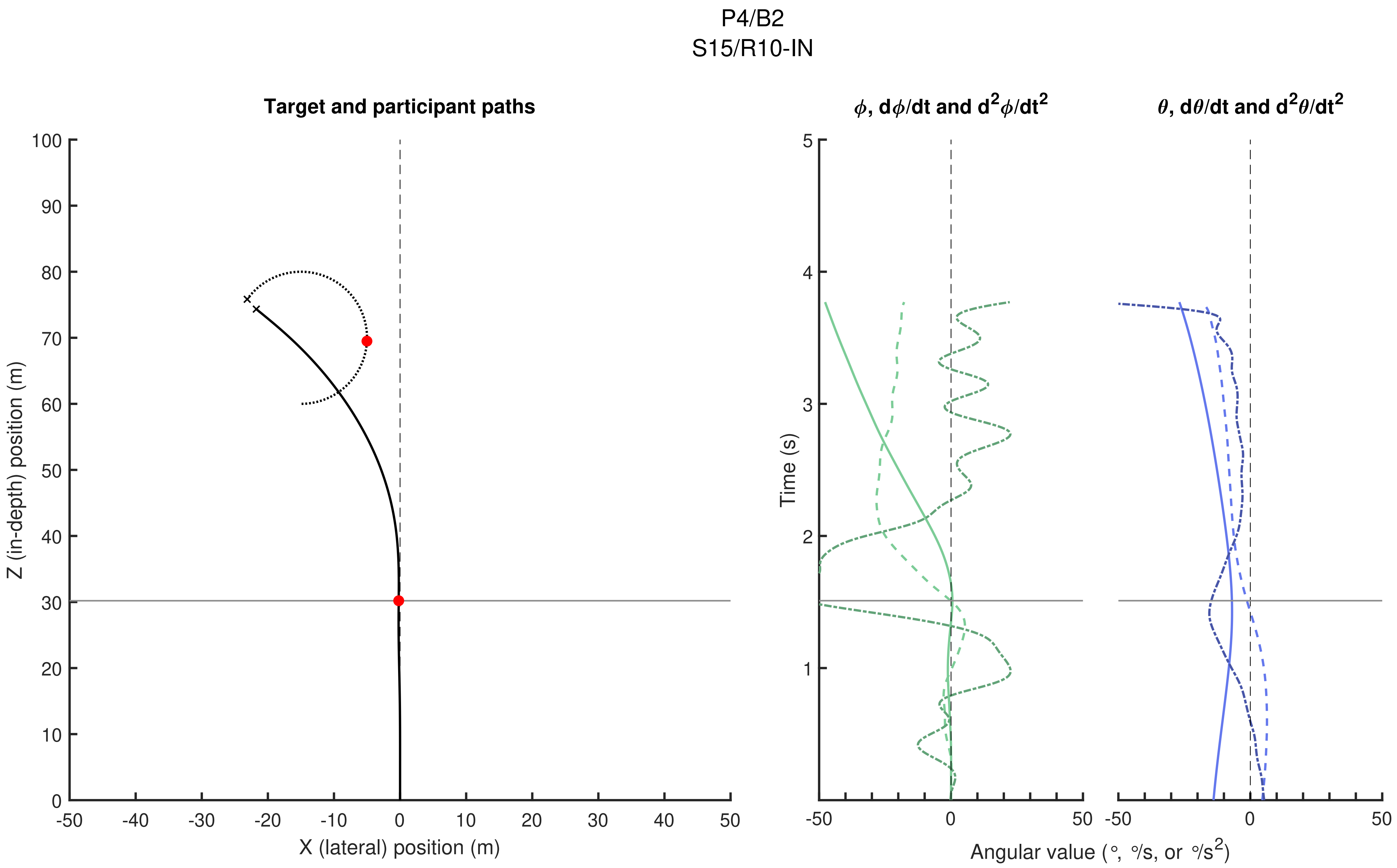
P4/B2
S15/R10-IN
Target and participant paths
Z (in-depth) position (m)
X (lateral) position (m)
ϕ, dϕ/dt and d²ϕ/dt²
θ, dθ/dt and d²θ/dt²
Time (s)
Angular value (°, °/s, or °/s²)
100
90
80
70
60
50
40
30
20
10
0
-50
-40
-30
-20
-10
0
10
20
30
40
50
5
4
3
2
1
-50
0
50
-50
0
50

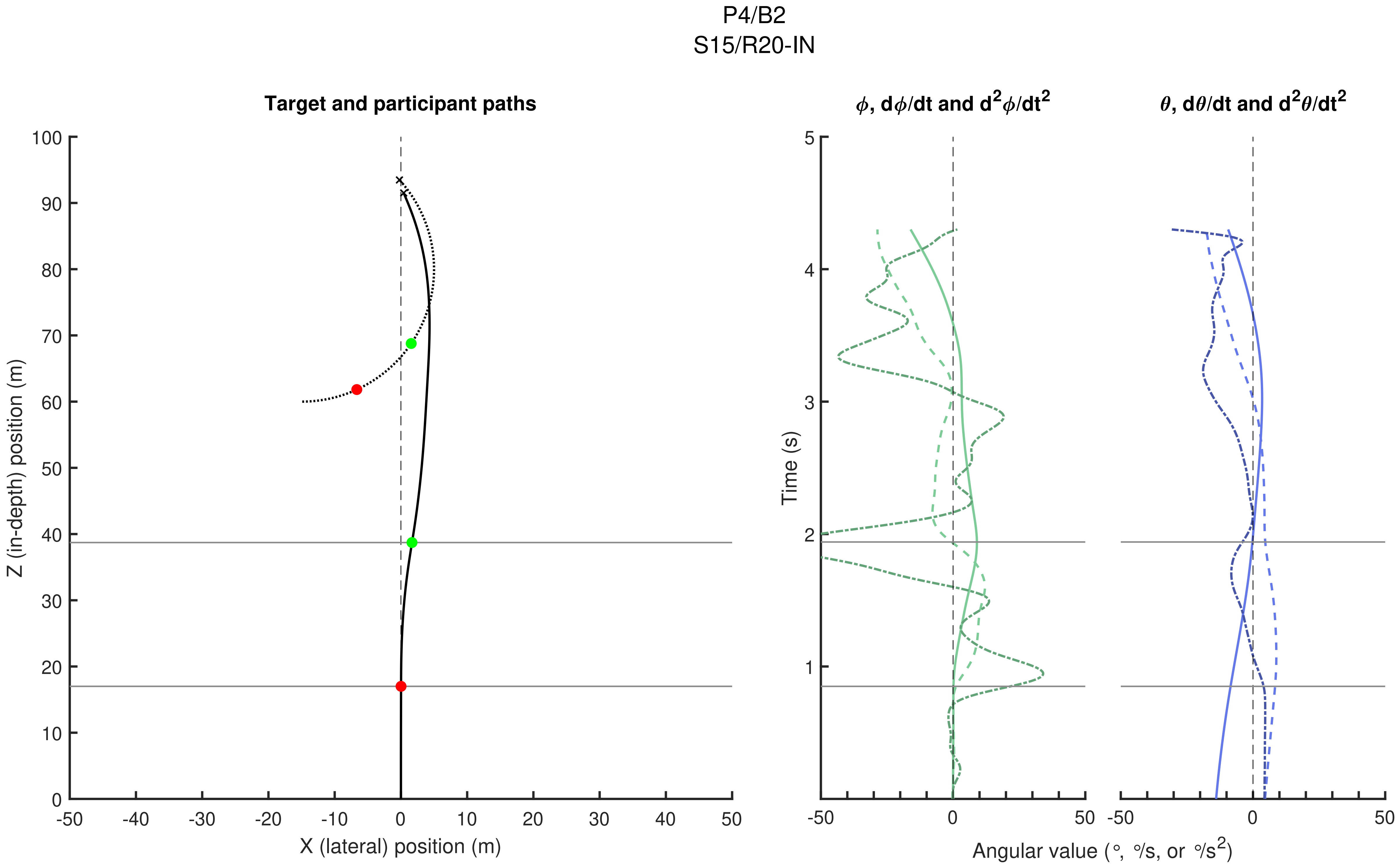
P4/B2
S15/R20-IN
Target and participant paths
Z (in-depth) position (m)
X (lateral) position (m)
ϕ, dϕ/dt and d$^2\phi$/dt^2
θ, dθ/dt and d$^2\theta$/dt^2
Time (s)
Angular value (°, °/s, or °/s^2)

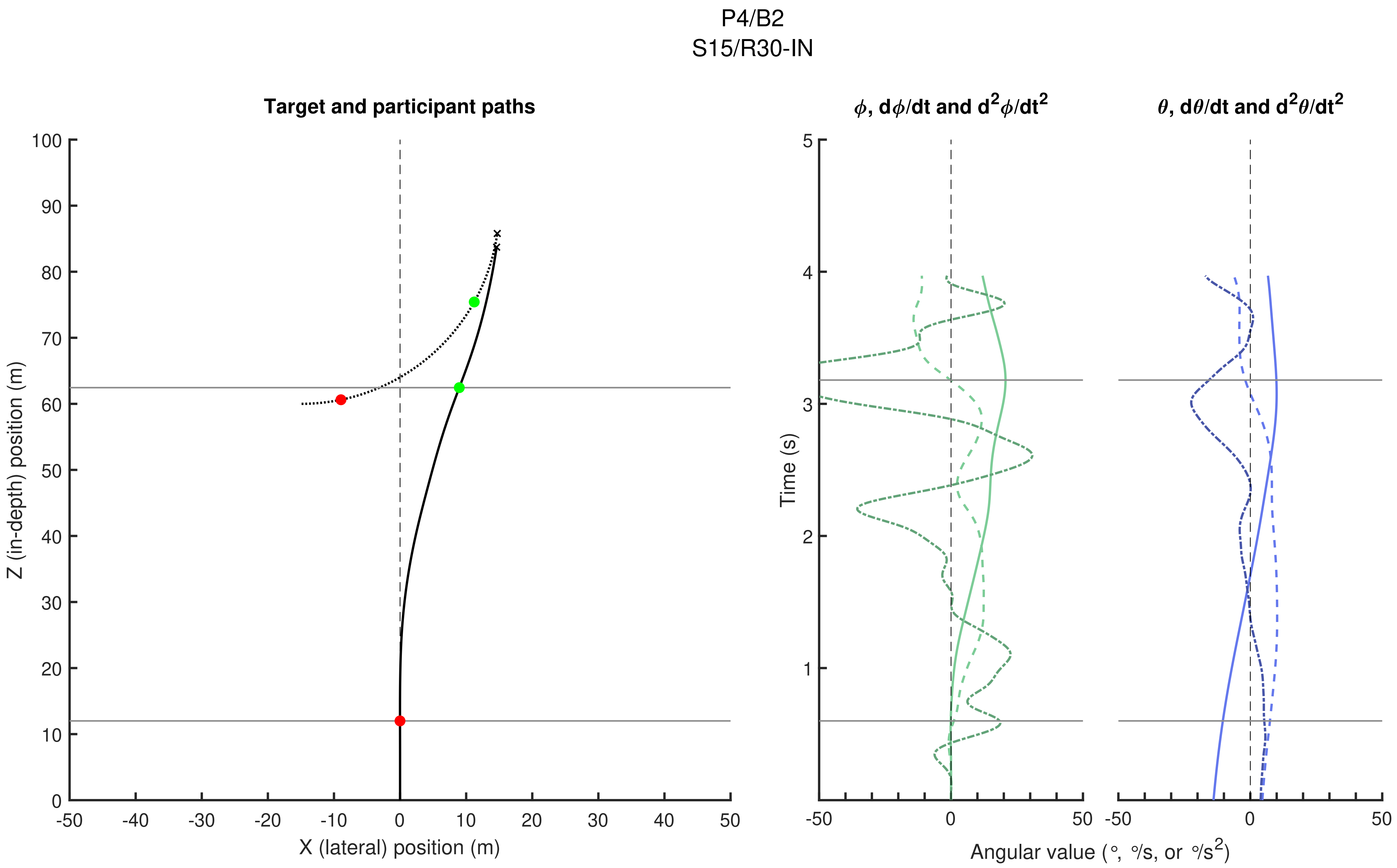

P4/B2
S15/R30-IN
Target and participant paths
Z (in-depth) position (m)
X (lateral) position (m)
ϕ, dϕ/dt and d$^2\phi$/dt^2
θ, dθ/dt and d$^2\theta$/dt^2
Time (s)
Angular value (°, °/s, or °/s^2)

P4/B2
S5/R10-IN

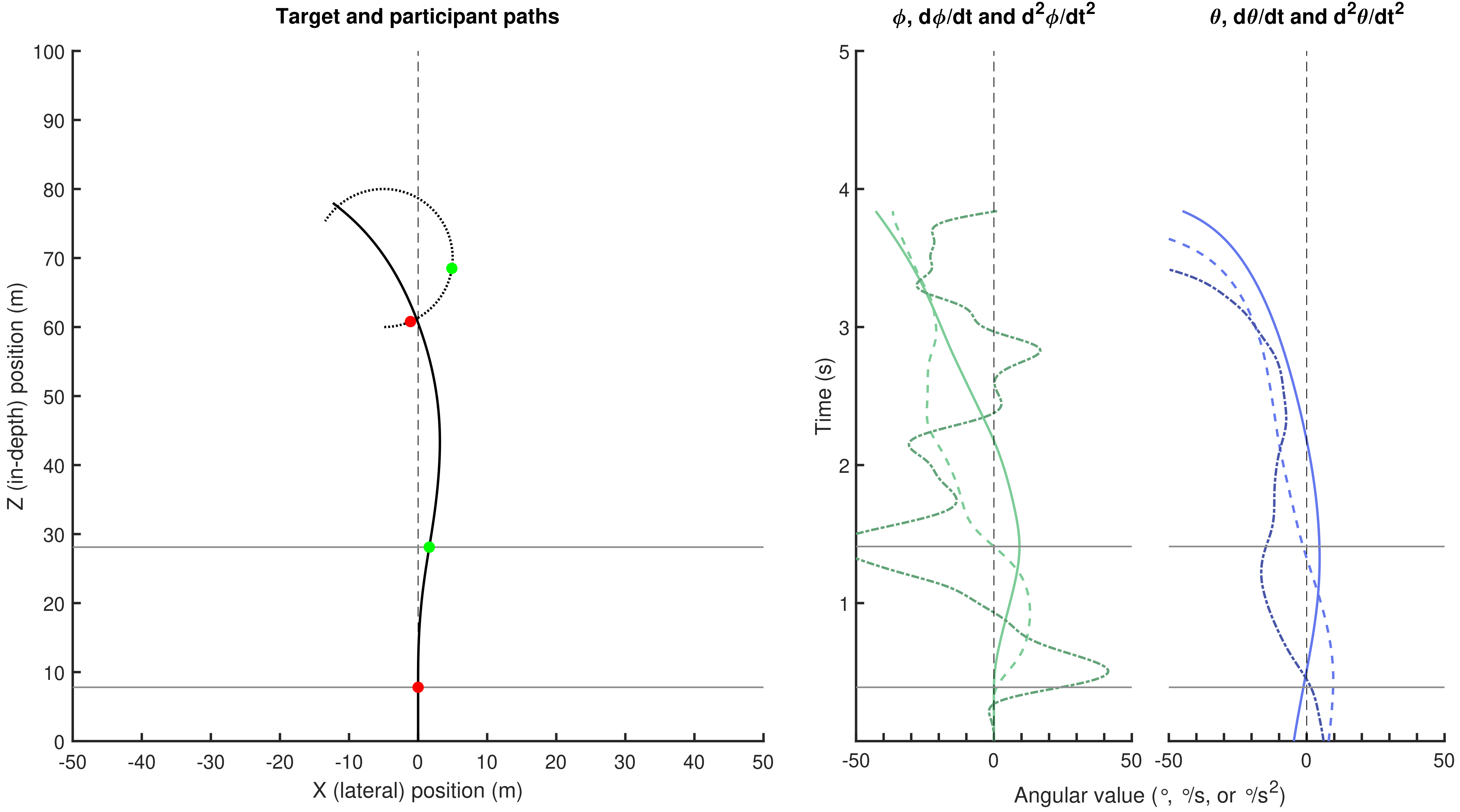

P4/B2
S5/R10-OUT

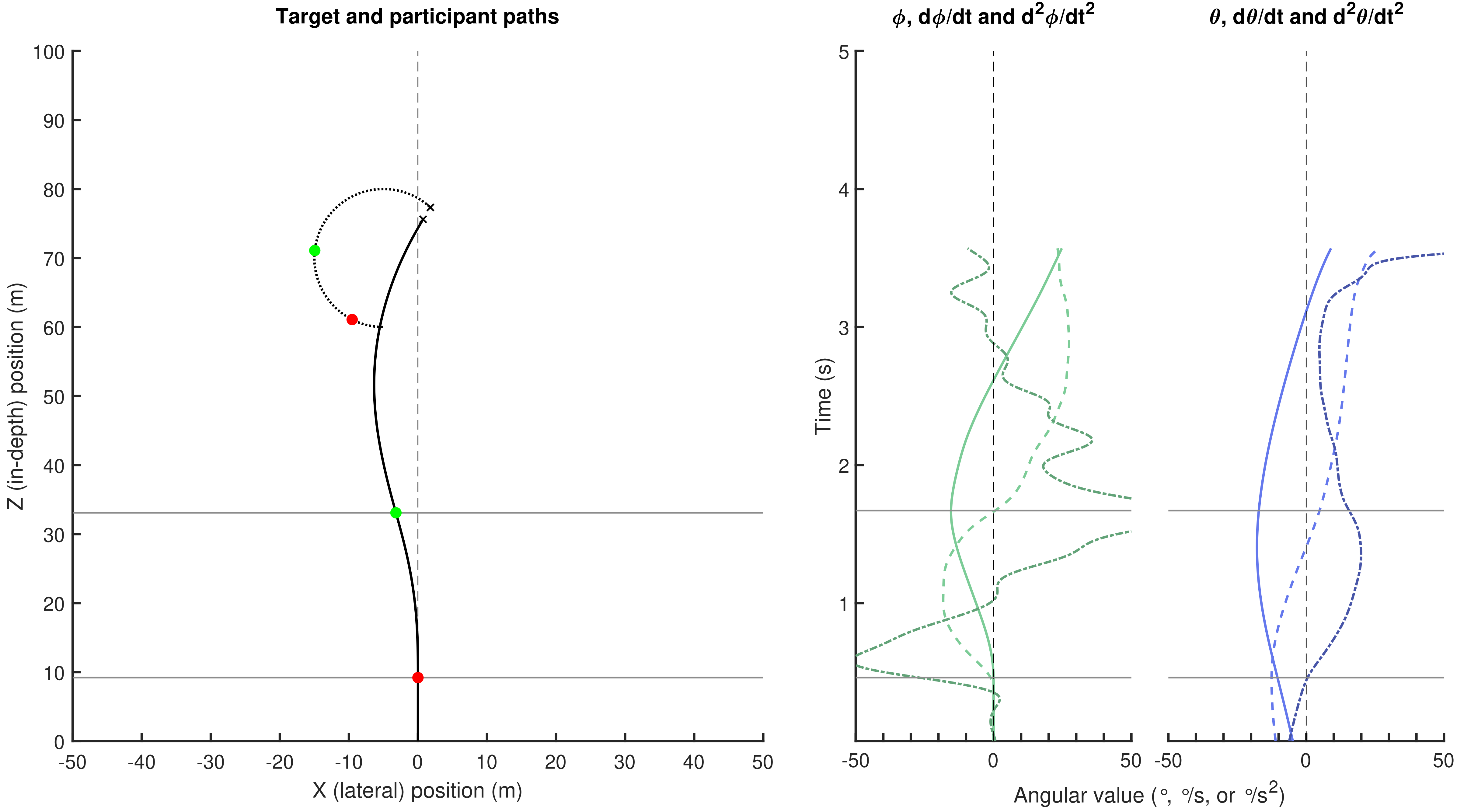

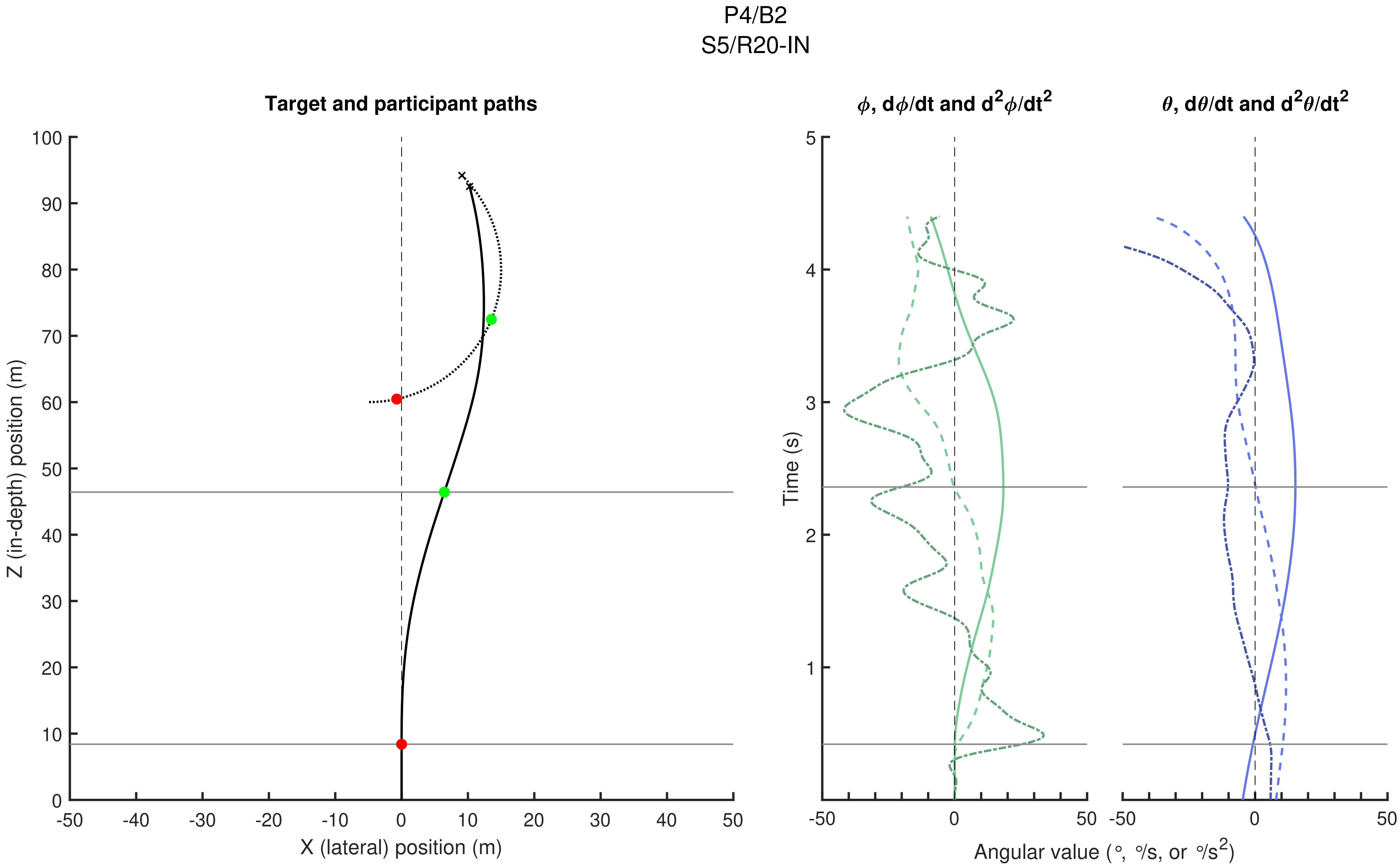
P4/B2
S5/R20-IN
Target and participant paths
Z (in-depth) position (m)
X (lateral) position (m)
φ, dφ/dt and d²φ/dt²
θ, dθ/dt and d²θ/dt²
Time (s)
Angular value (°, °/s, or °/s²)

P4/B2
S5/R20-OUT

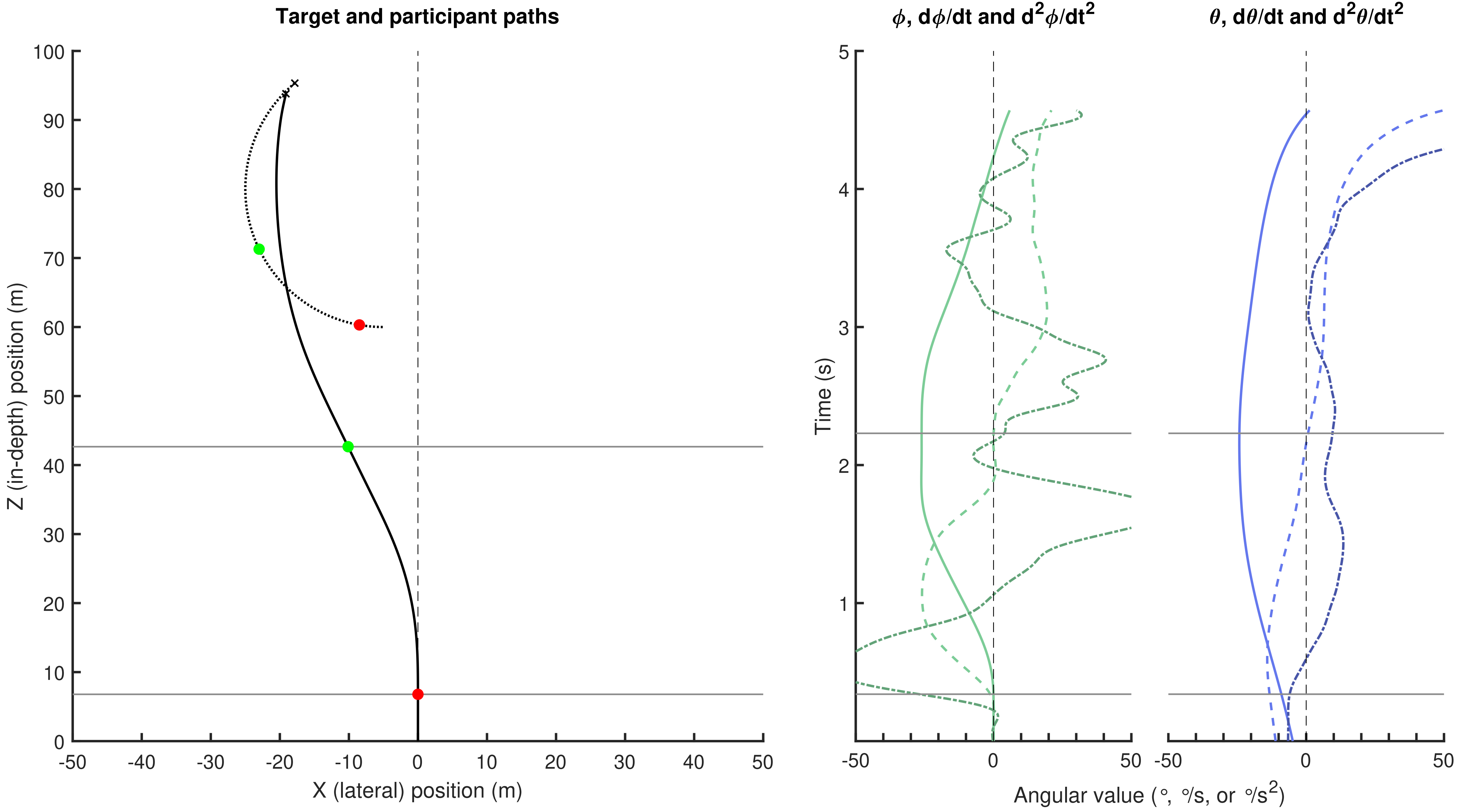

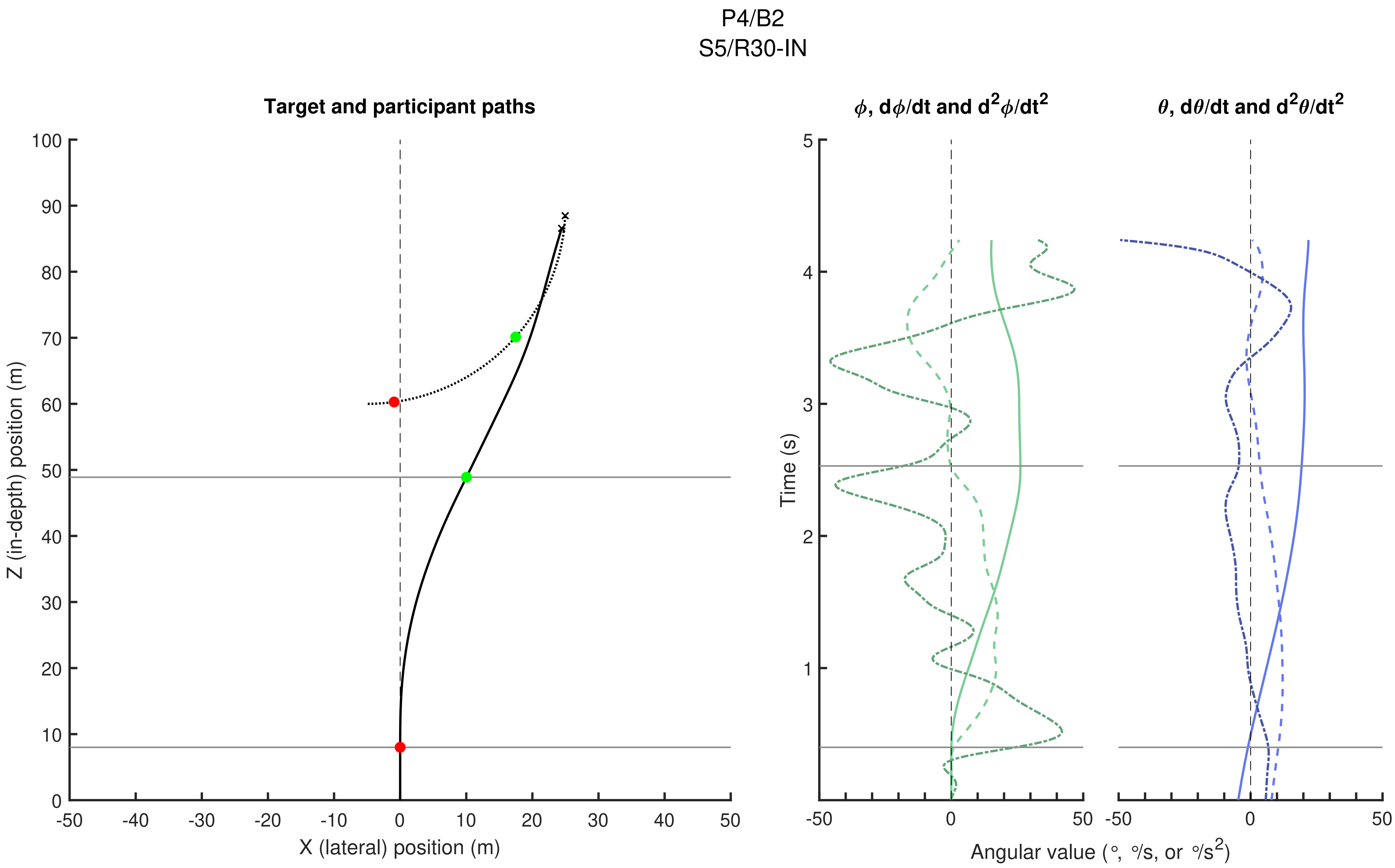
P4/B2
S5/R30-IN
Target and participant paths
Z (in-depth) position (m)
X (lateral) position (m)
φ, dφ/dt and d²φ/dt²
θ, dθ/dt and d²θ/dt²
Time (s)
Angular value (°, °/s, or °/s²)

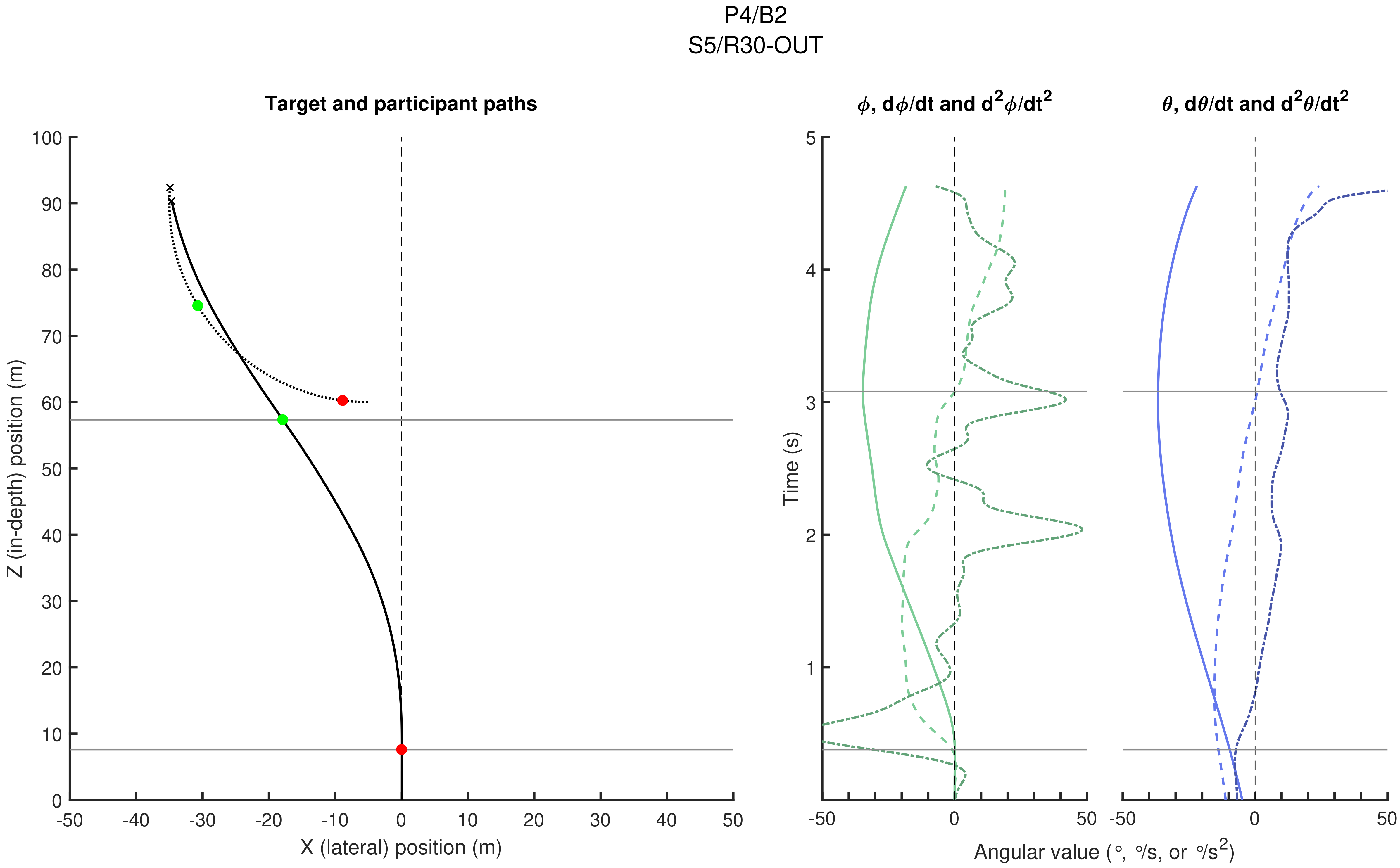
P4/B2
S5/R30-OUT
Target and participant paths
Z (in-depth) position (m)
X (lateral) position (m)
ϕ, dϕ/dt and d$^2\phi$/dt^2
θ, dθ/dt and d$^2\theta$/dt^2
Time (s)
Angular value (°, °/s, or °/s^2)

P4/B2
S5/R10-OUT

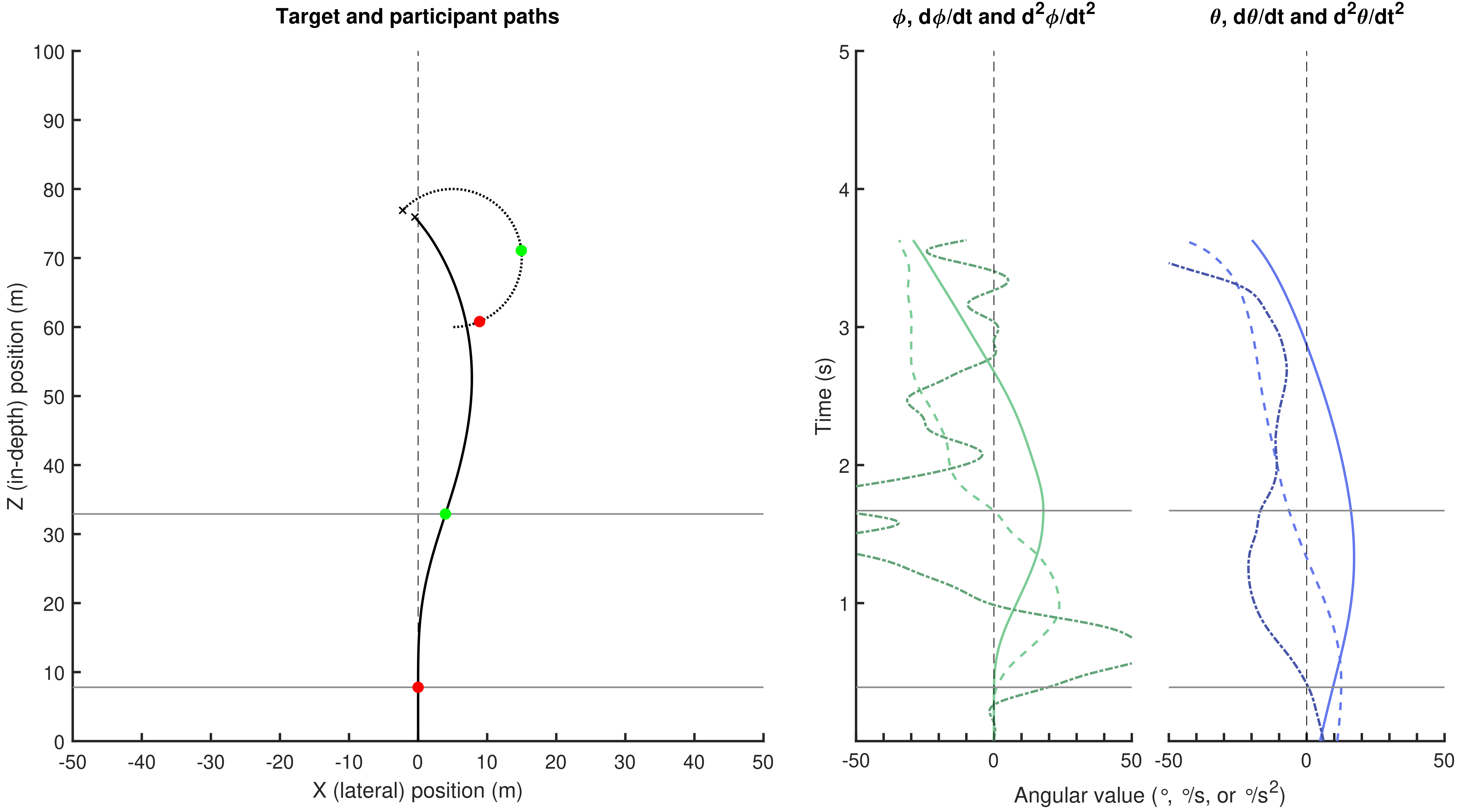

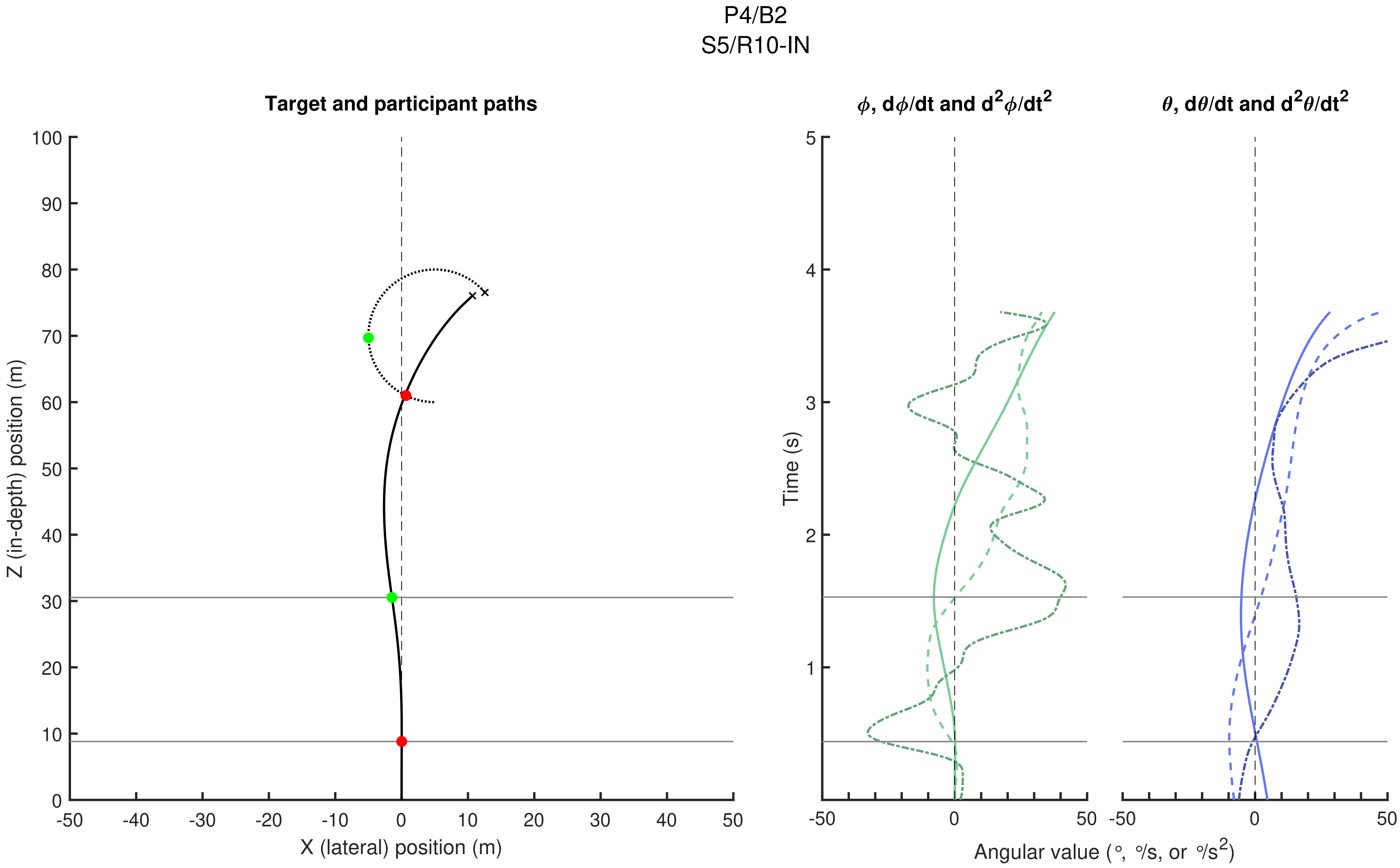
P4/B2
S5/R10-IN
Target and participant paths
Z (in-depth) position (m)
X (lateral) position (m)
100
90
80
70
60
50
40
30
20
10
0
-50
-40
-30
-20
-10
0
10
20
30
40
50
φ, dφ/dt and d²φ/dt²
θ, dθ/dt and d²θ/dt²
Time (s)
5
4
3
2
1
-50
0
50
-50
0
50
Angular value (°, °/s, or °/s²)

P4/B2
S5/R20-OUT

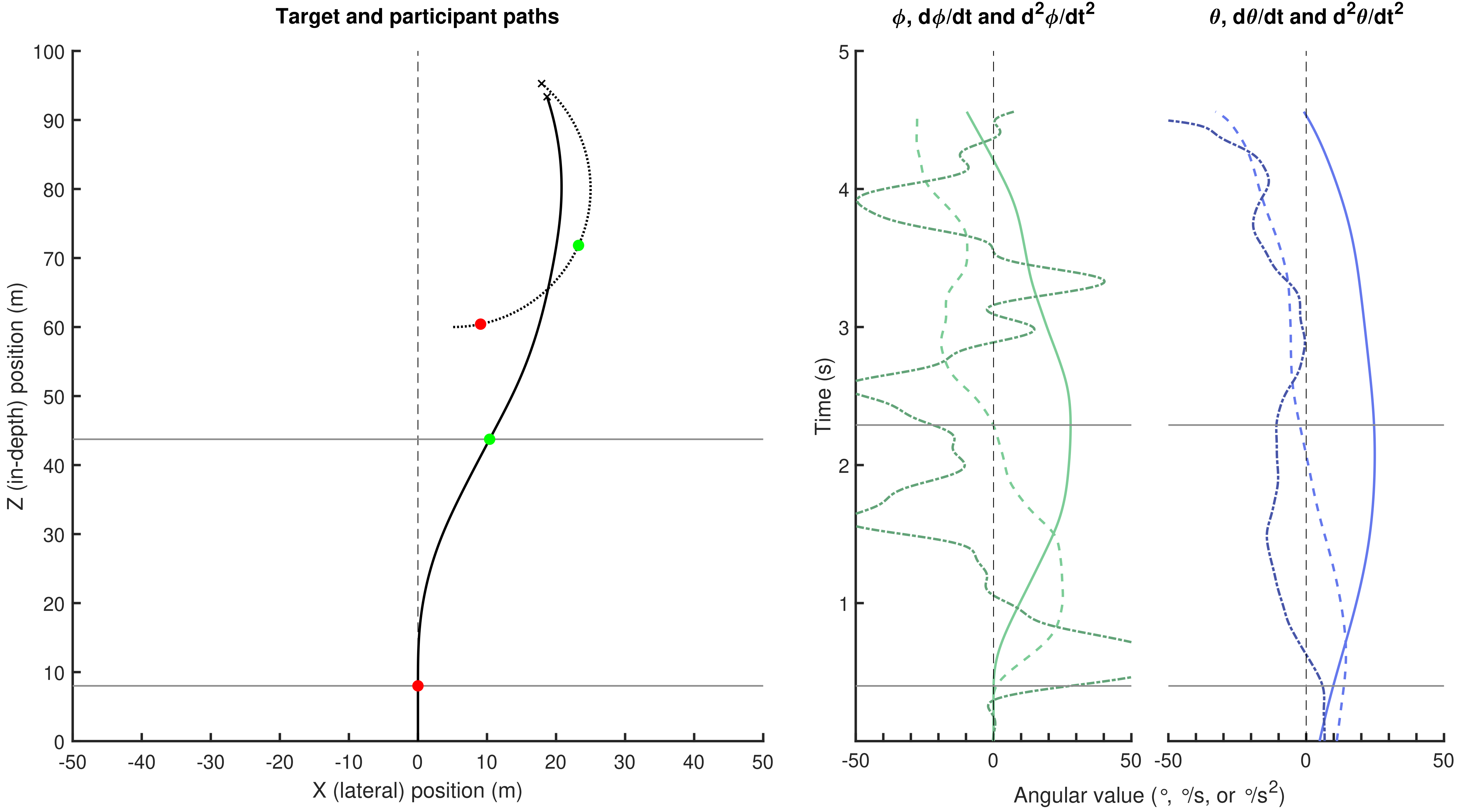

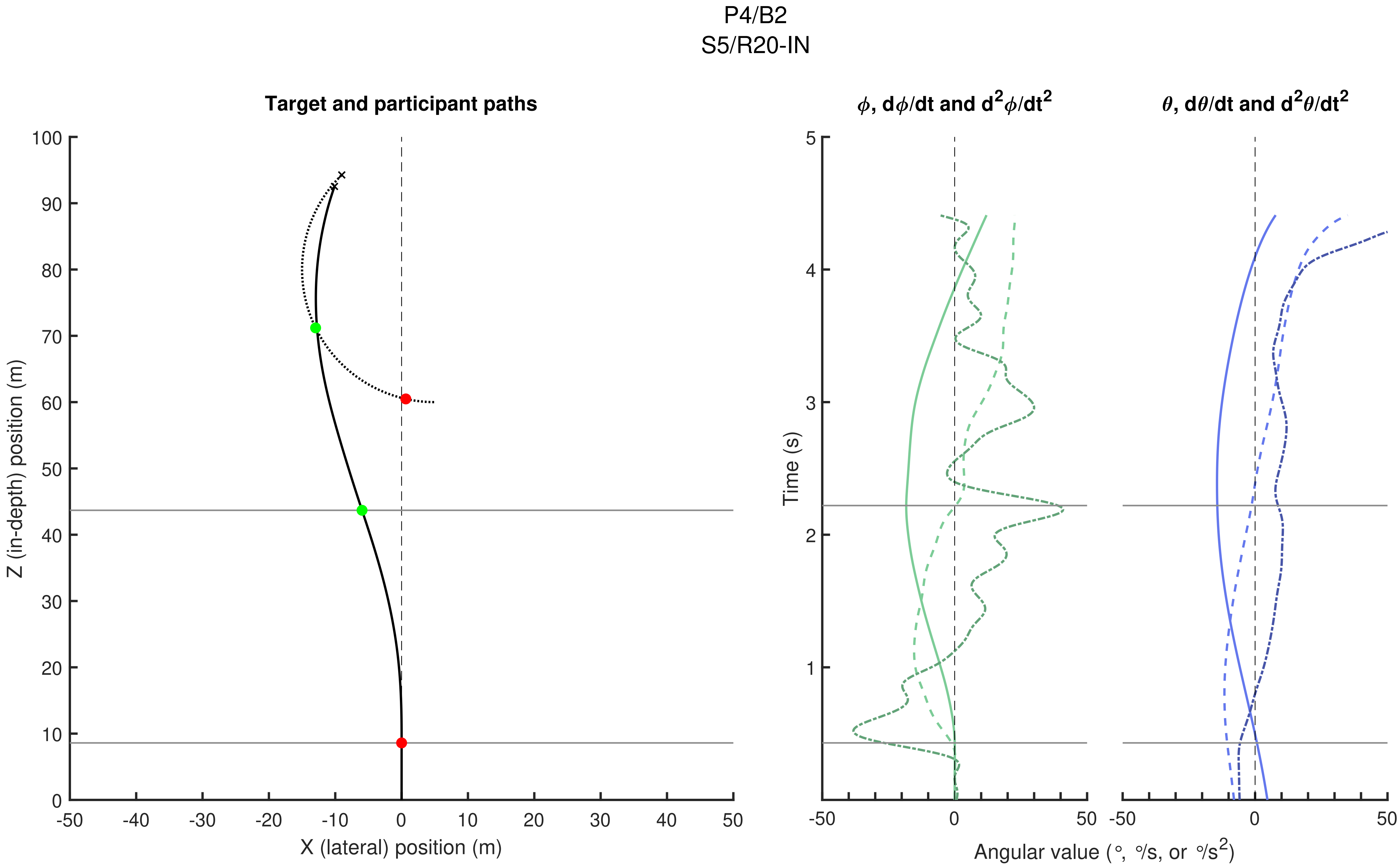

P4/B2
S5/R20-IN
Target and participant paths
Z (in-depth) position (m)
X (lateral) position (m)
ϕ, dϕ/dt and d²ϕ/dt²
θ, dθ/dt and d²θ/dt²
Time (s)
Angular value (°, °/s, or °/s²)

P4/B2
S5/R30-OUT

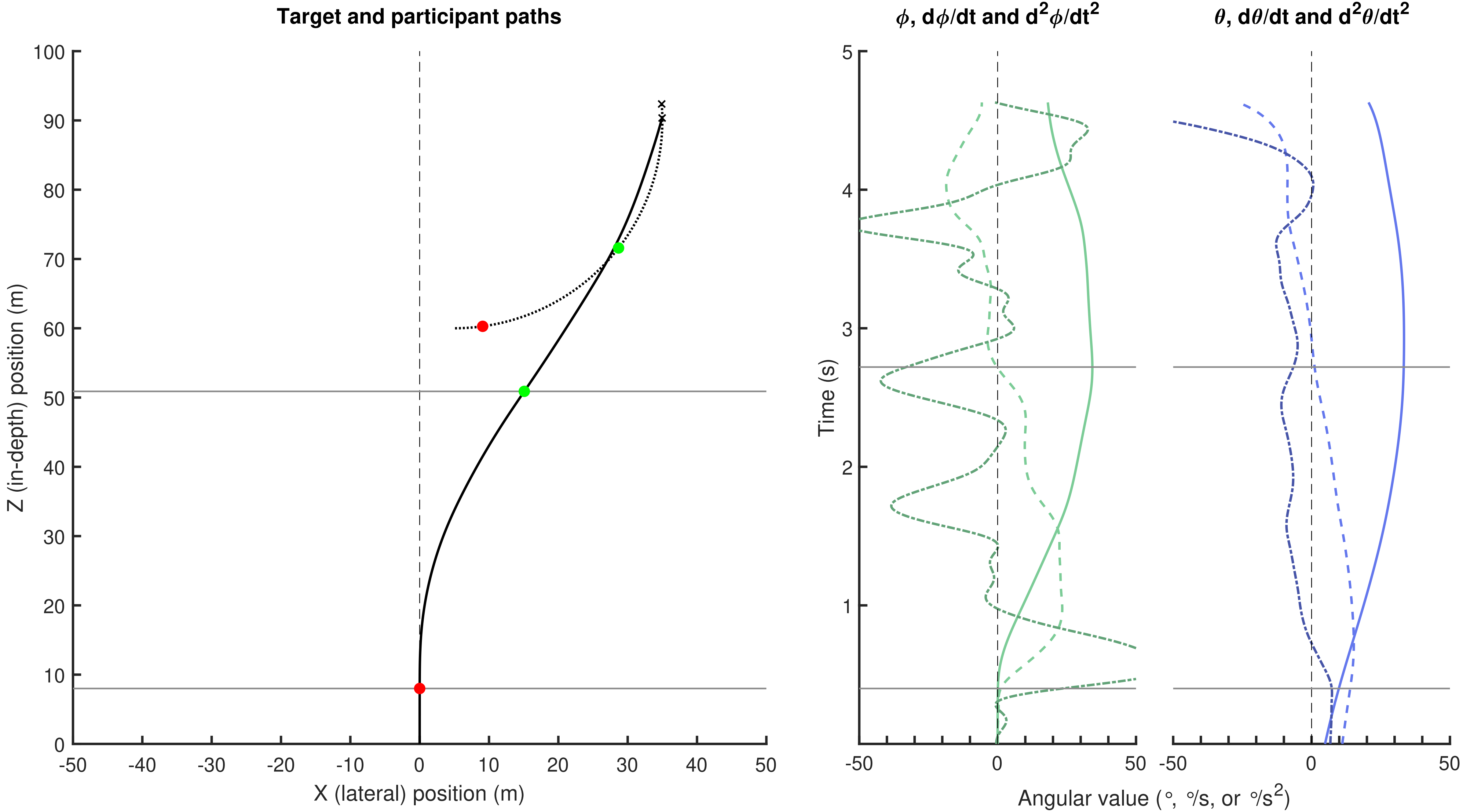

P4/B2
S5/R30-IN

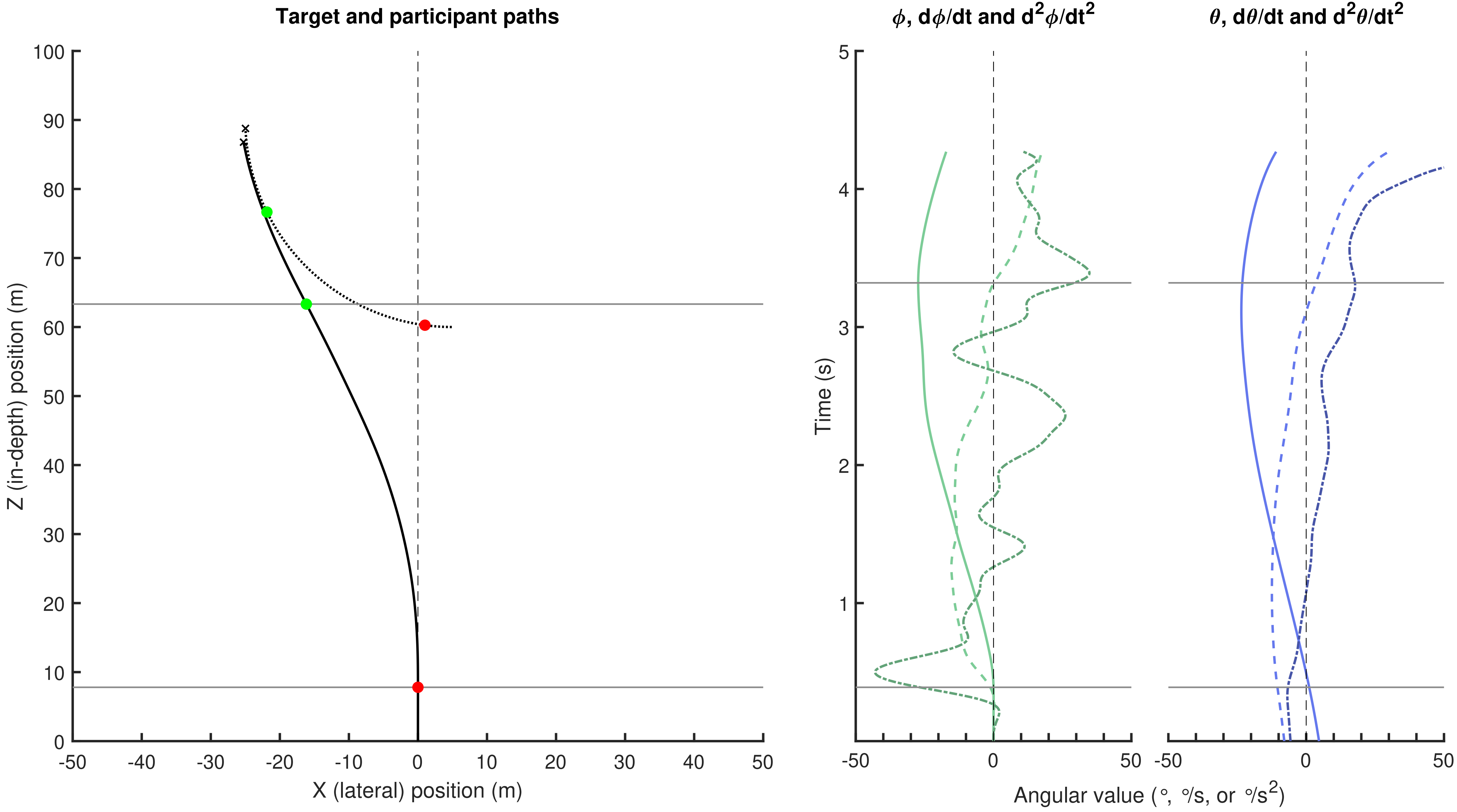

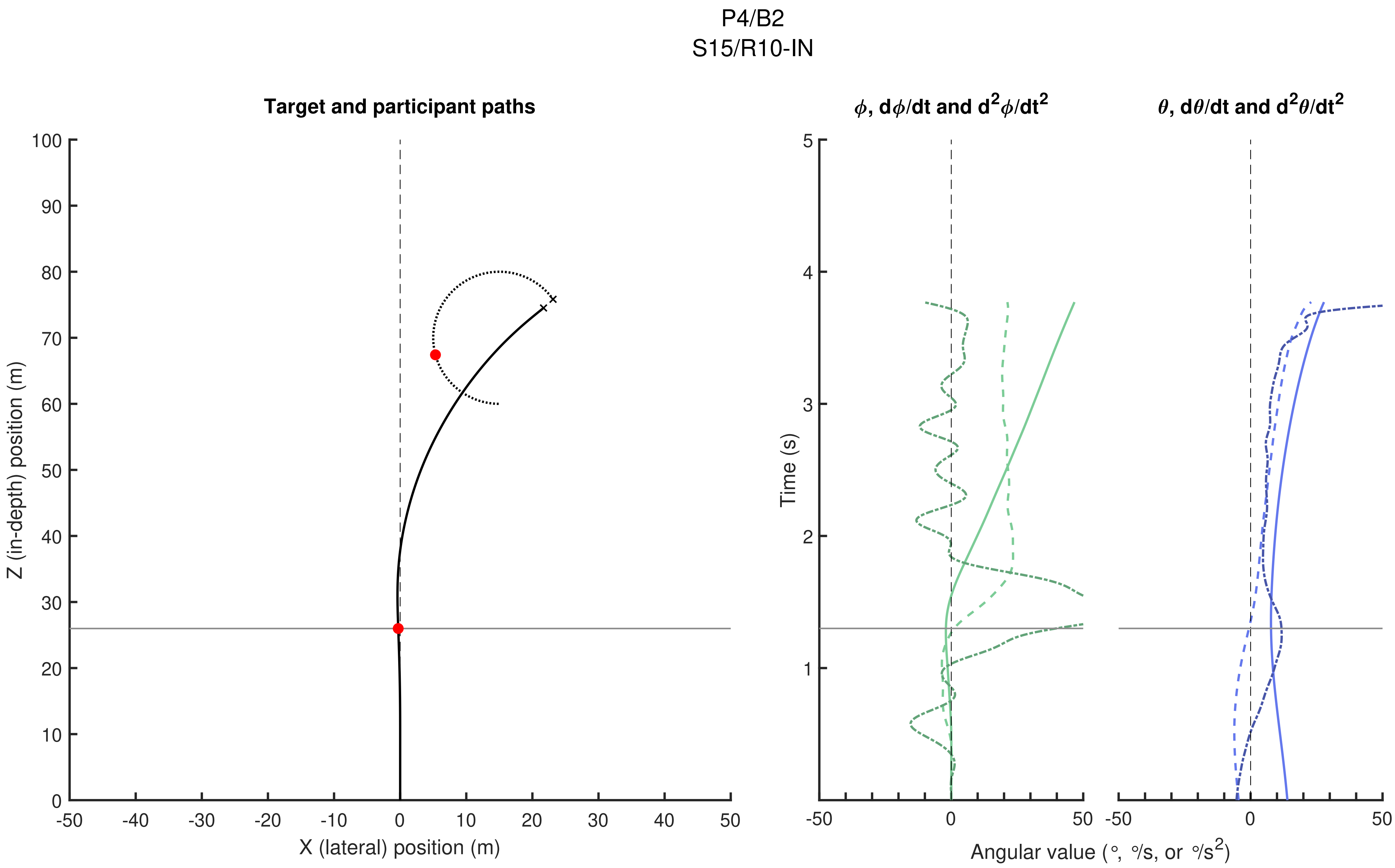
P4/B2
S15/R10-IN
Target and participant paths
Z (in-depth) position (m)
X (lateral) position (m)
ϕ, dϕ/dt and d²ϕ/dt²
θ, dθ/dt and d²θ/dt²
Time (s)
Angular value (°, °/s, or °/s²)

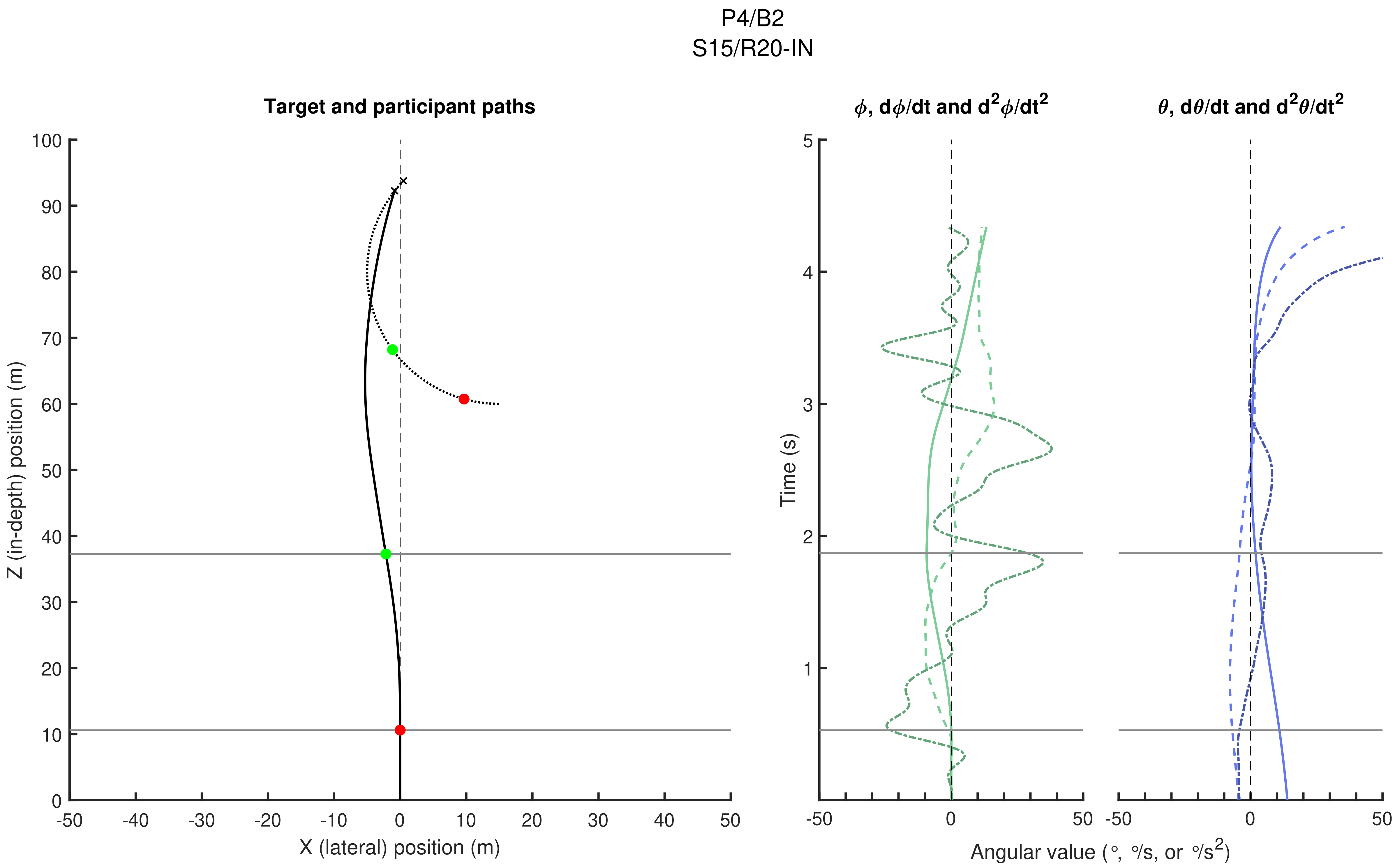

P4/B2
S15/R20-IN
Target and participant paths
Z (in-depth) position (m)
X (lateral) position (m)
ϕ, dϕ/dt and d$^2\phi$/dt^2
θ, dθ/dt and d$^2\theta$/dt^2
Time (s)
Angular value (°, °/s, or °/s^2)

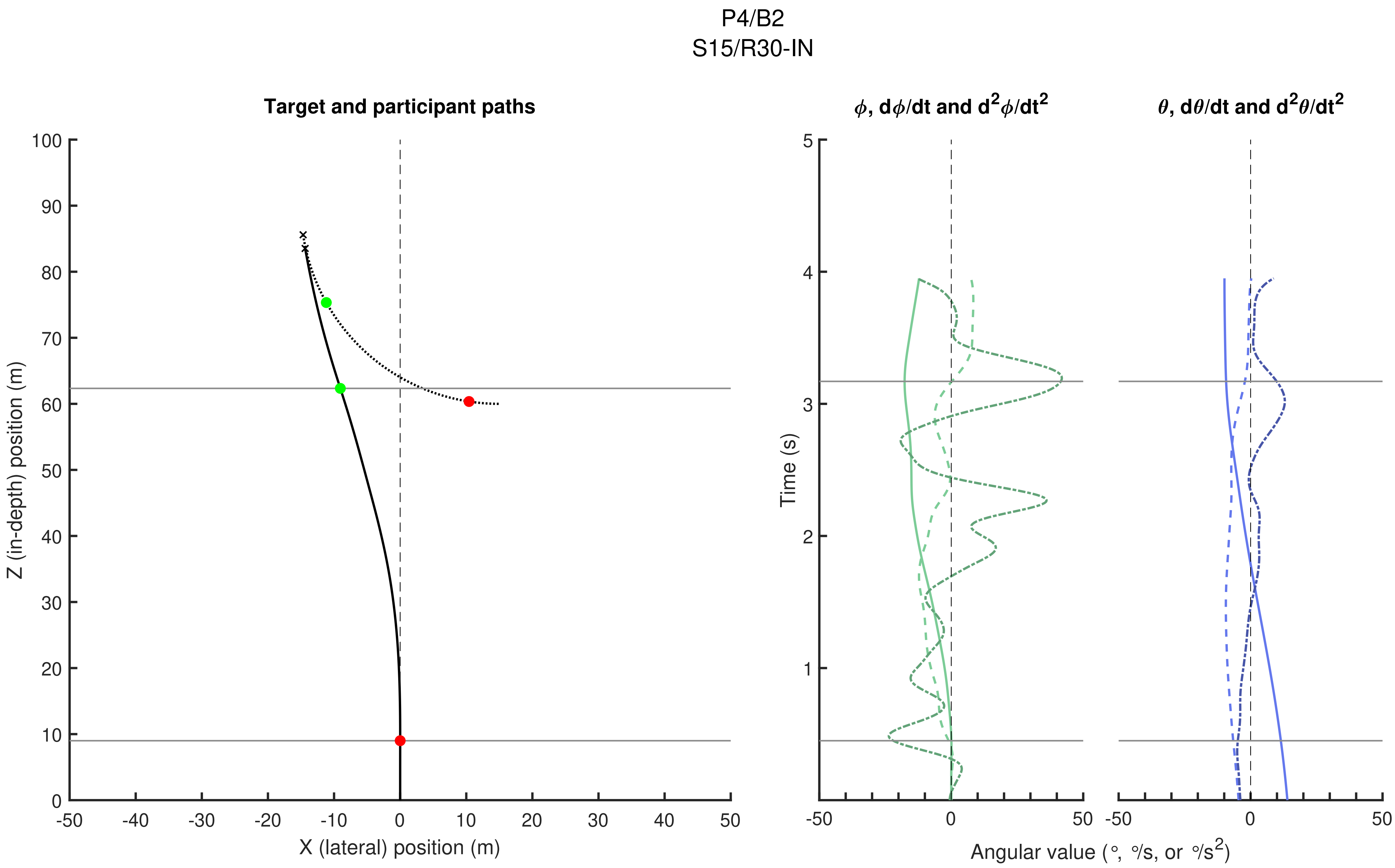
P4/B2
S15/R30-IN
Target and participant paths
Z (in-depth) position (m)
X (lateral) position (m)
φ, dφ/dt and d²φ/dt²
θ, dθ/dt and d²θ/dt²
Time (s)
Angular value (°, °/s, or °/s²)

P4/B2
S20/R10-IN

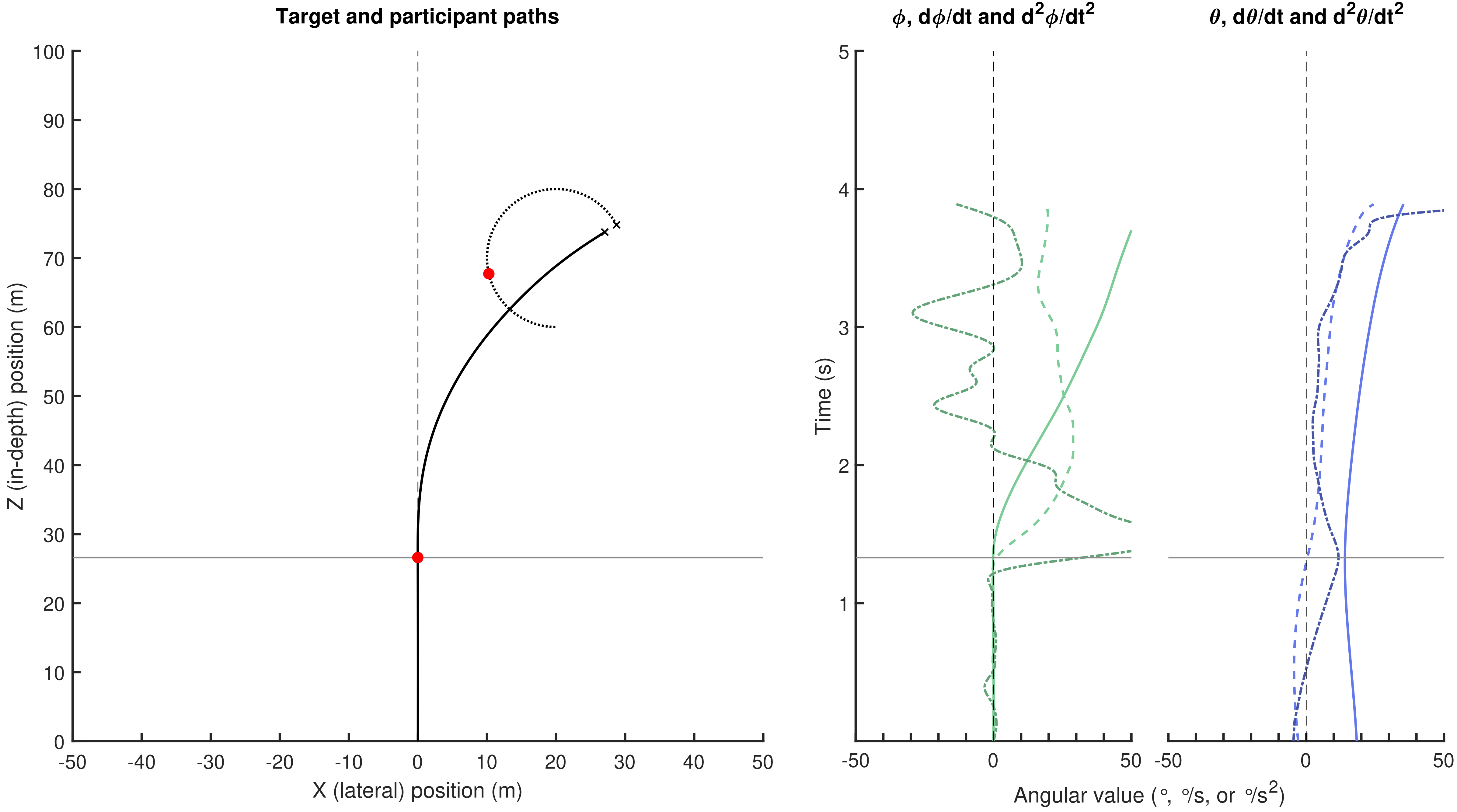

P4/B2
S20/R20-IN

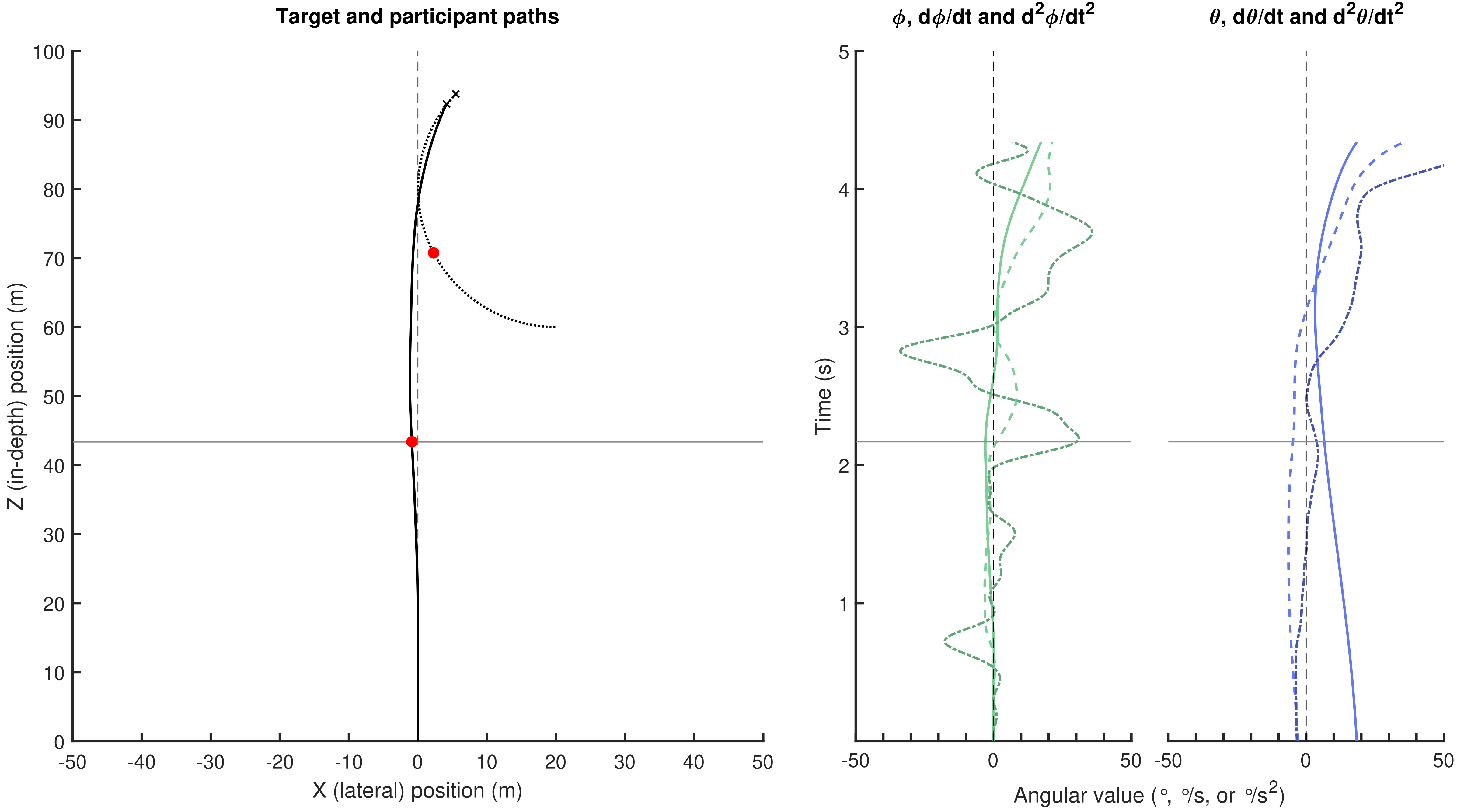

P4/B2
S20/R30-IN

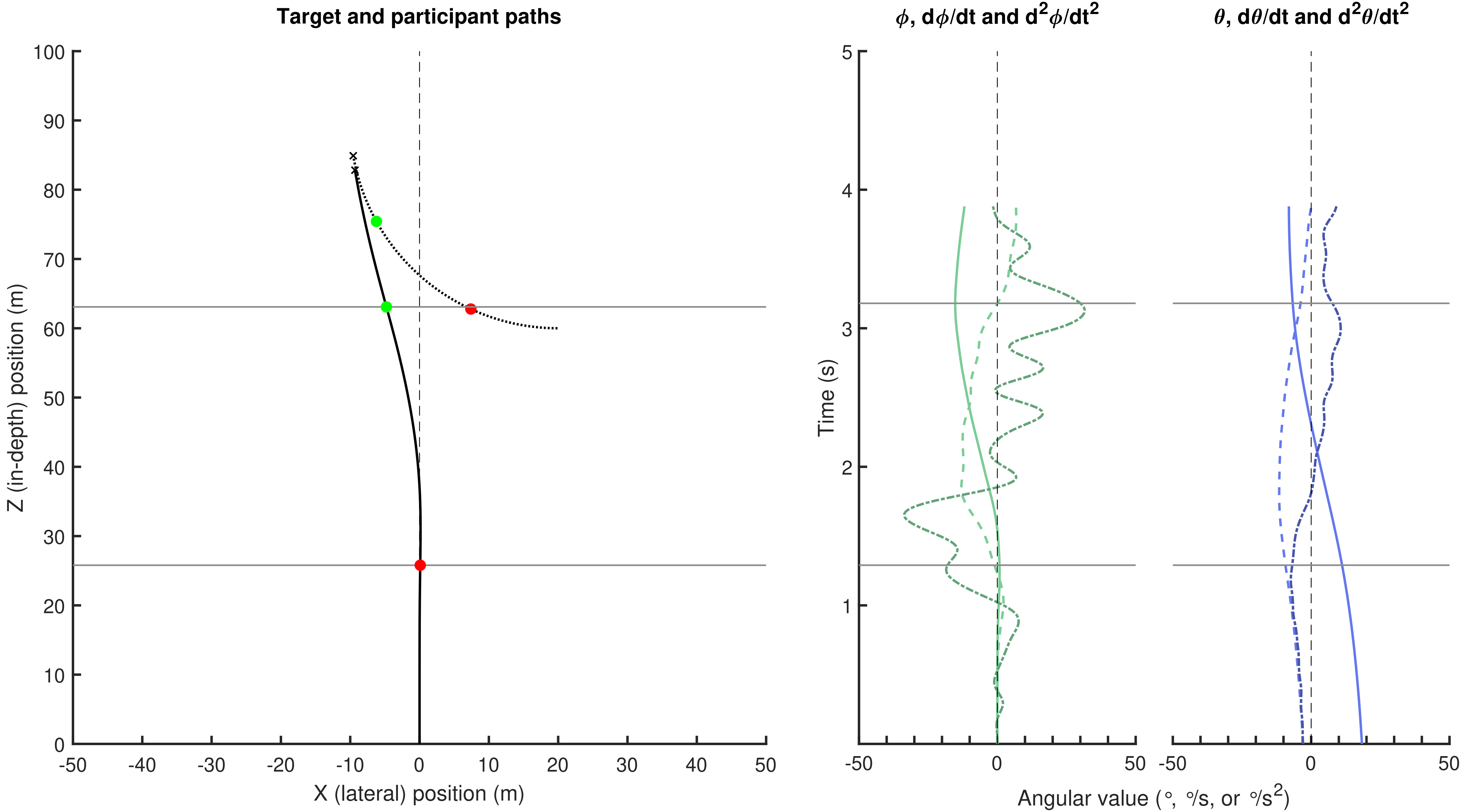

P4/B3
S20/R10-IN

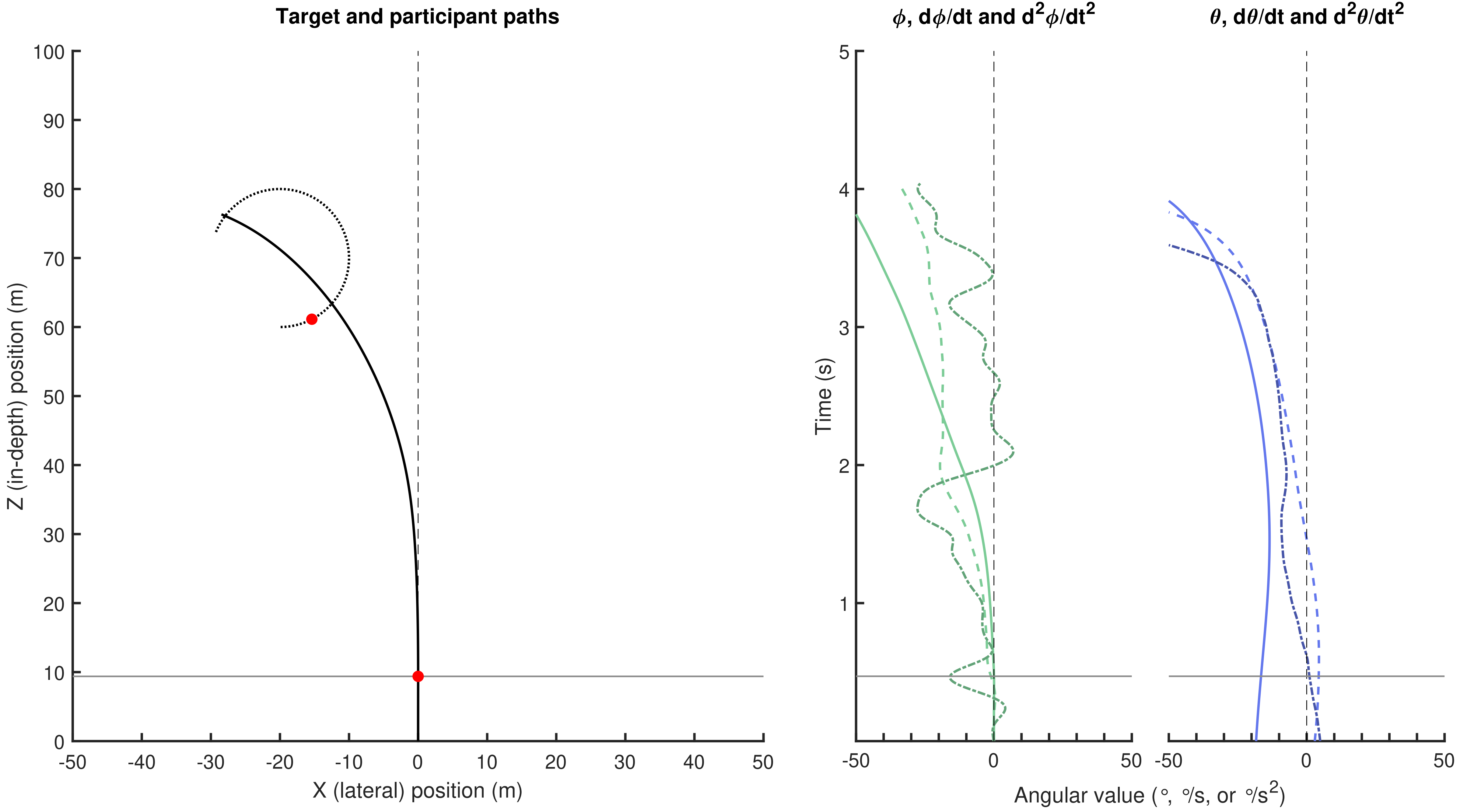

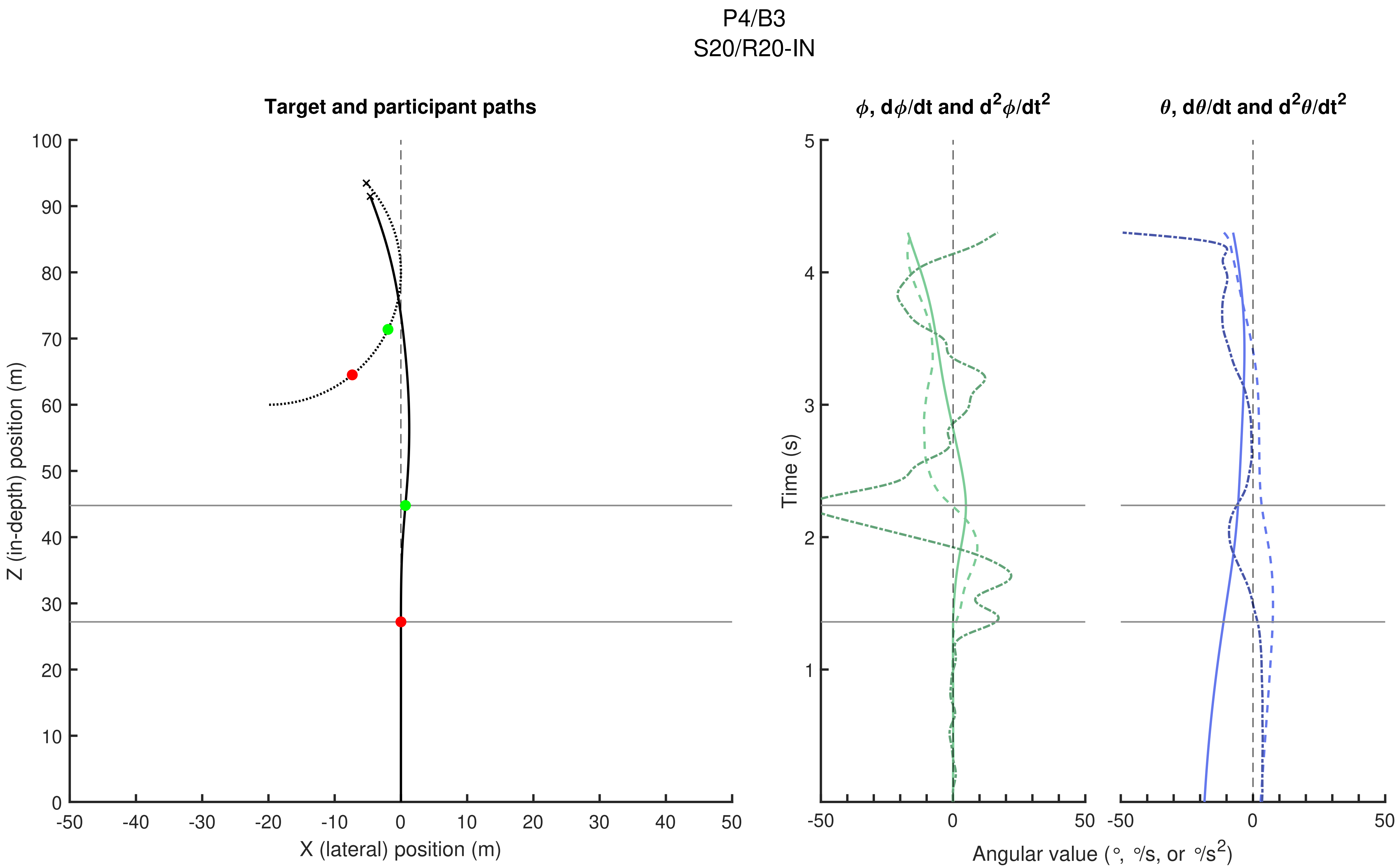
P4/B3
S20/R20-IN
Target and participant paths
Z (in-depth) position (m)
X (lateral) position (m)
ϕ, dϕ/dt and d²ϕ/dt²
θ, dθ/dt and d²θ/dt²
Time (s)
Angular value (°, °/s, or °/s²)

P4/B3
S20/R30-IN

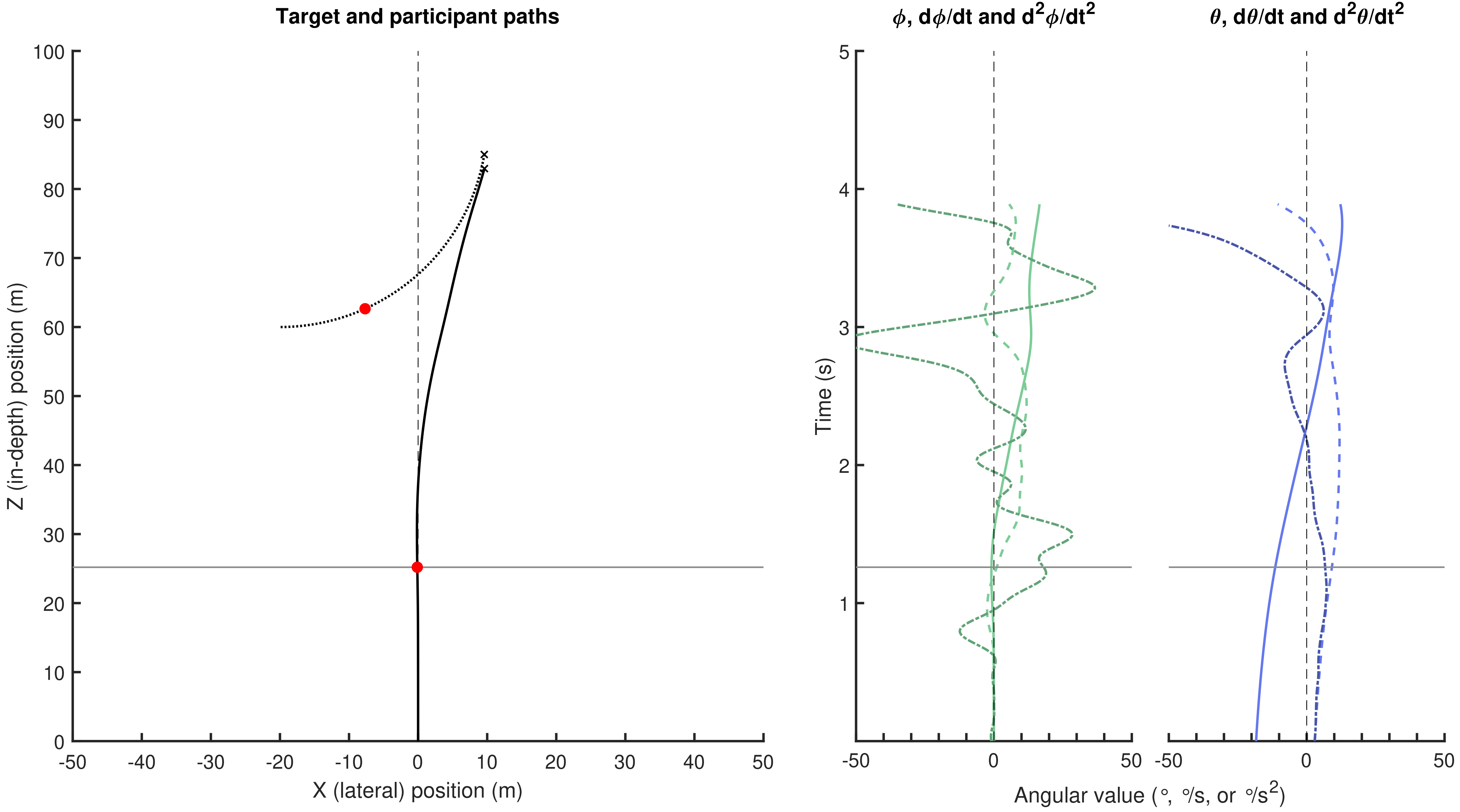

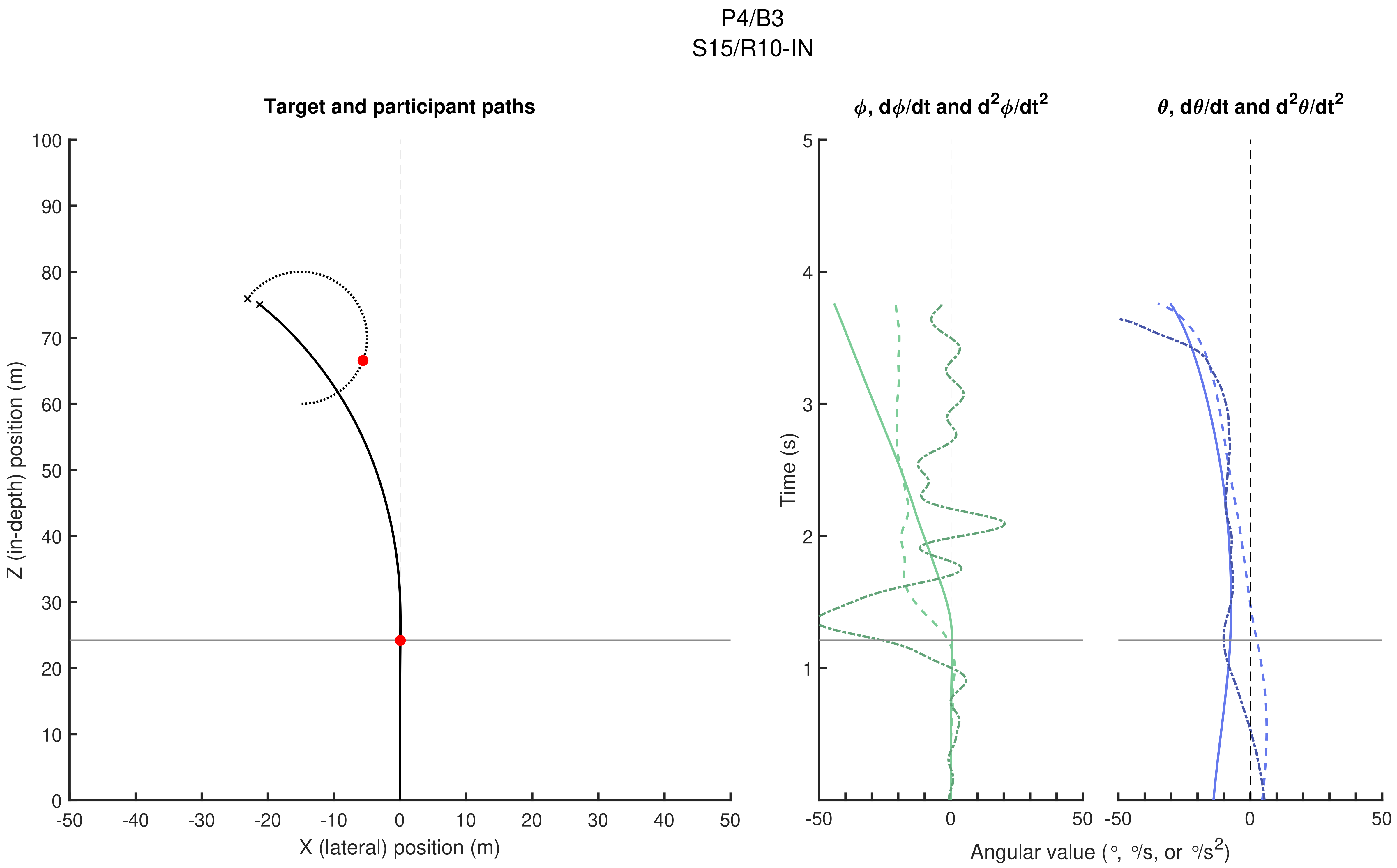
P4/B3
S15/R10-IN
Target and participant paths
Z (in-depth) position (m)
X (lateral) position (m)
ϕ, dϕ/dt and d²ϕ/dt²
θ, dθ/dt and d²θ/dt²
Time (s)
Angular value (°, °/s, or °/s²)

P4/B3
S15/R20-IN

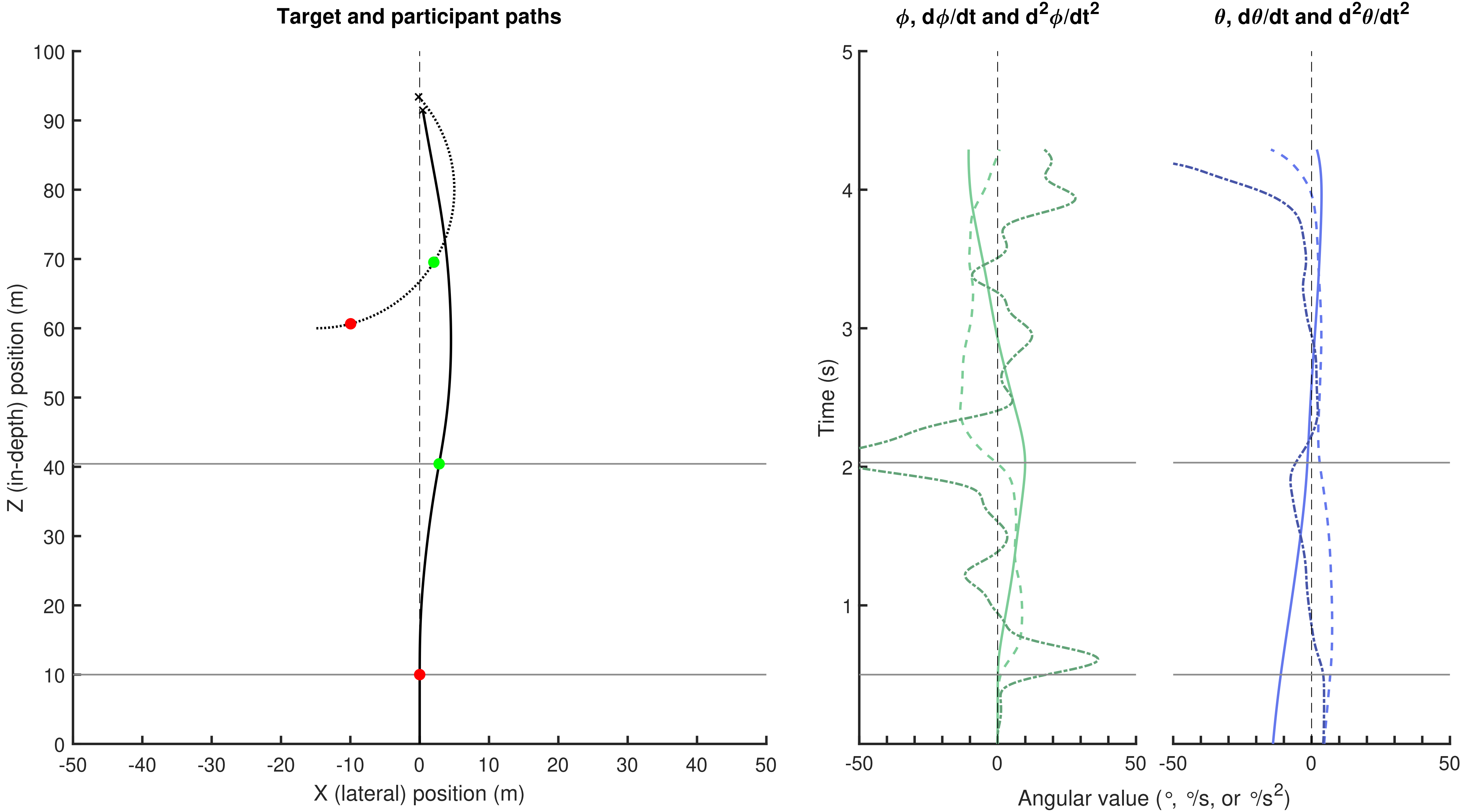

P4/B3
S15/R30-IN

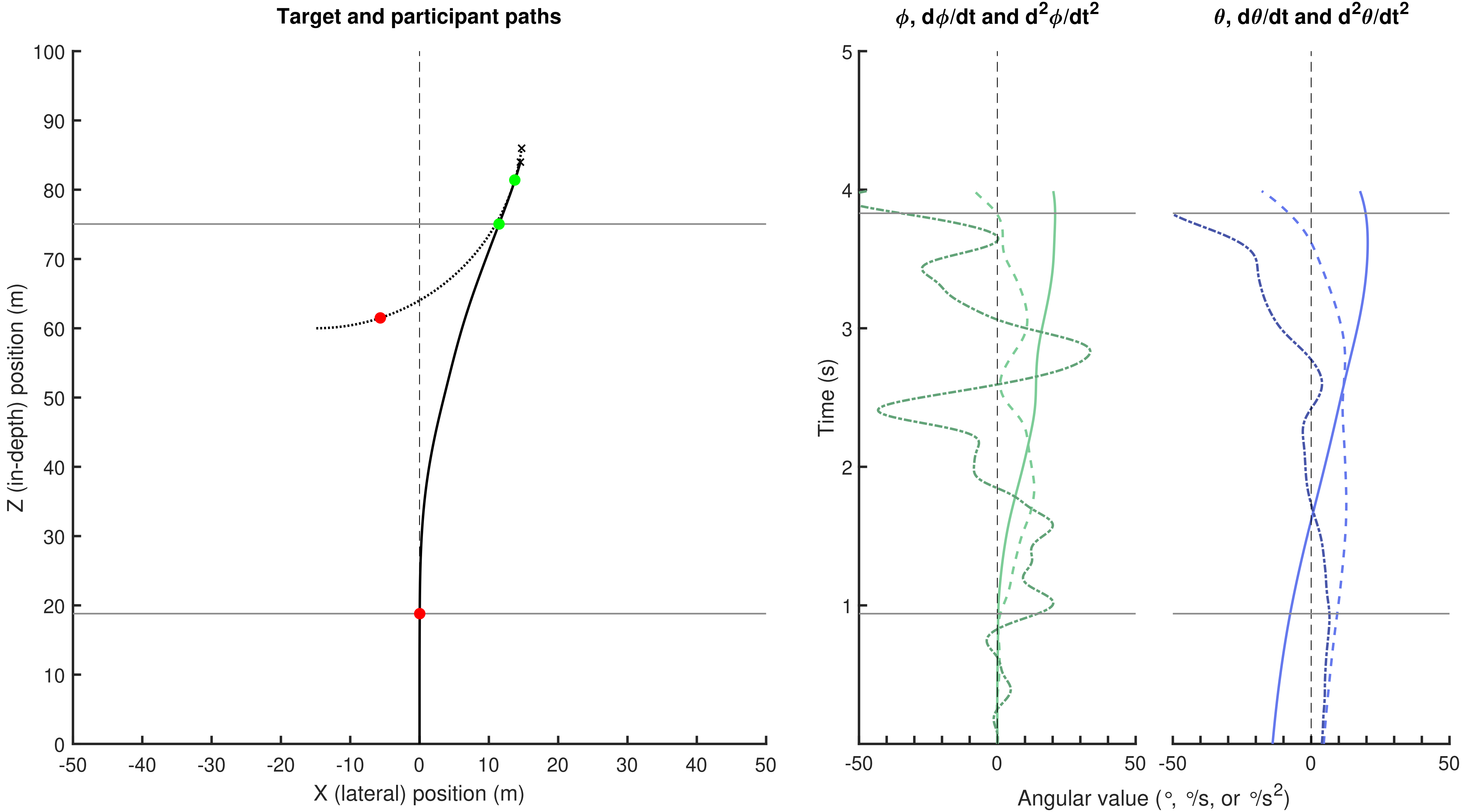

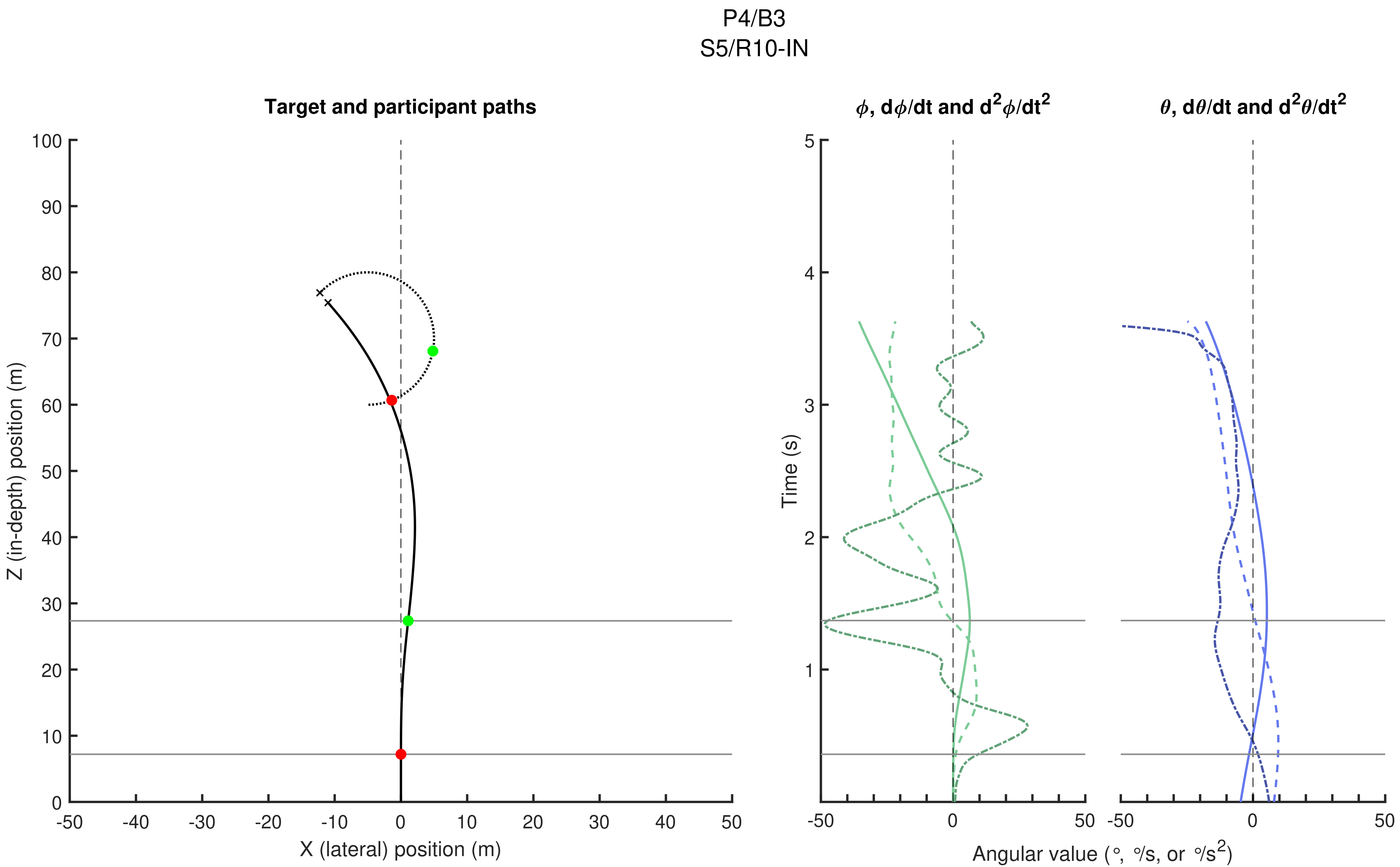
P4/B3
S5/R10-IN
Target and participant paths
Z (in-depth) position (m)
X (lateral) position (m)
-50
-40
-30
-20
-10
0
10
20
30
40
50
100
90
80
70
60
ϕ, dϕ/dt and d$^2\phi$/dt^2
θ, dθ/dt and d$^2\theta$/dt^2
Time (s)
1
2
3
4
5
Angular value (°, °/s, or °/s^2)

P4/B3
S5/R10-OUT

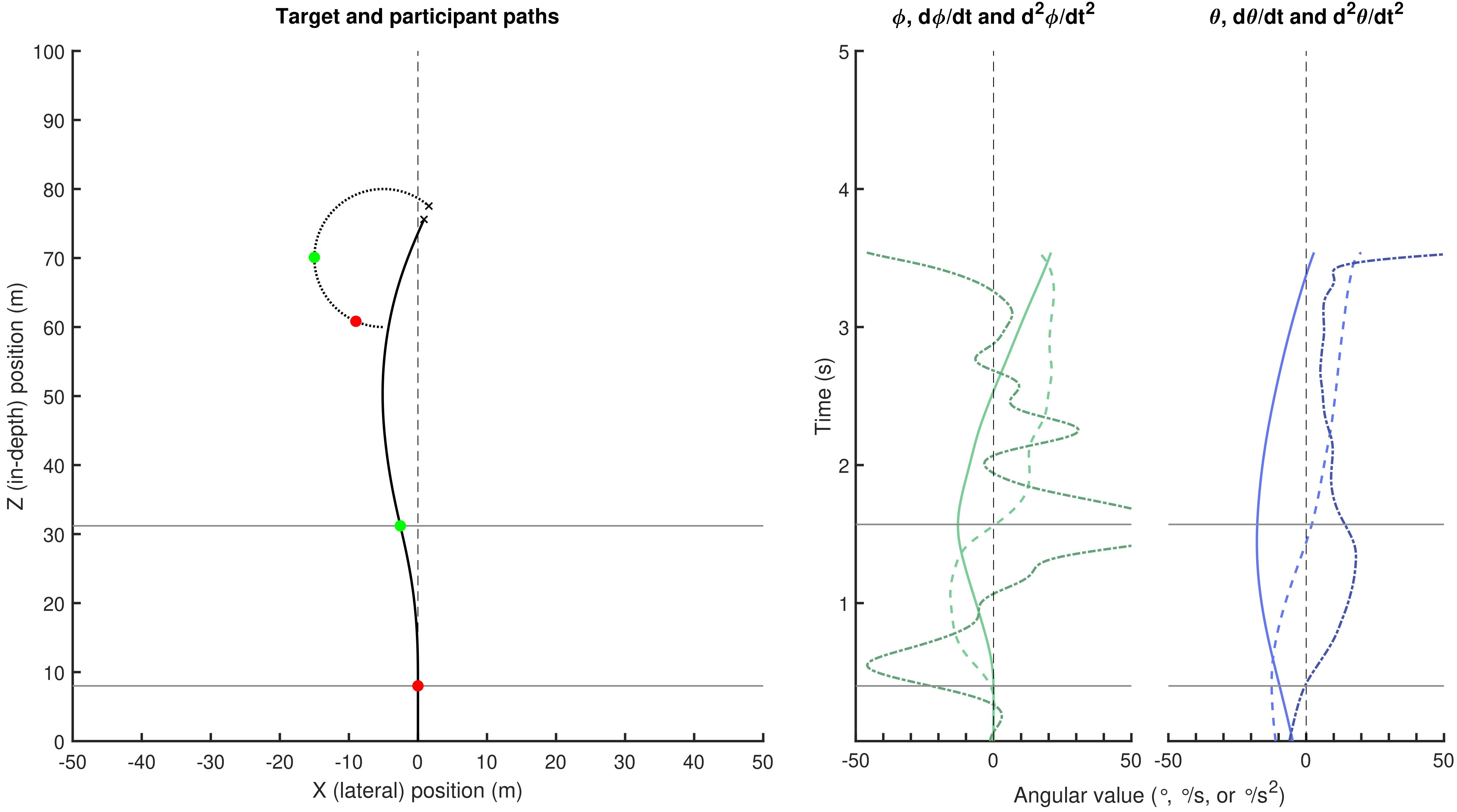

P4/B3
S5/R20-IN

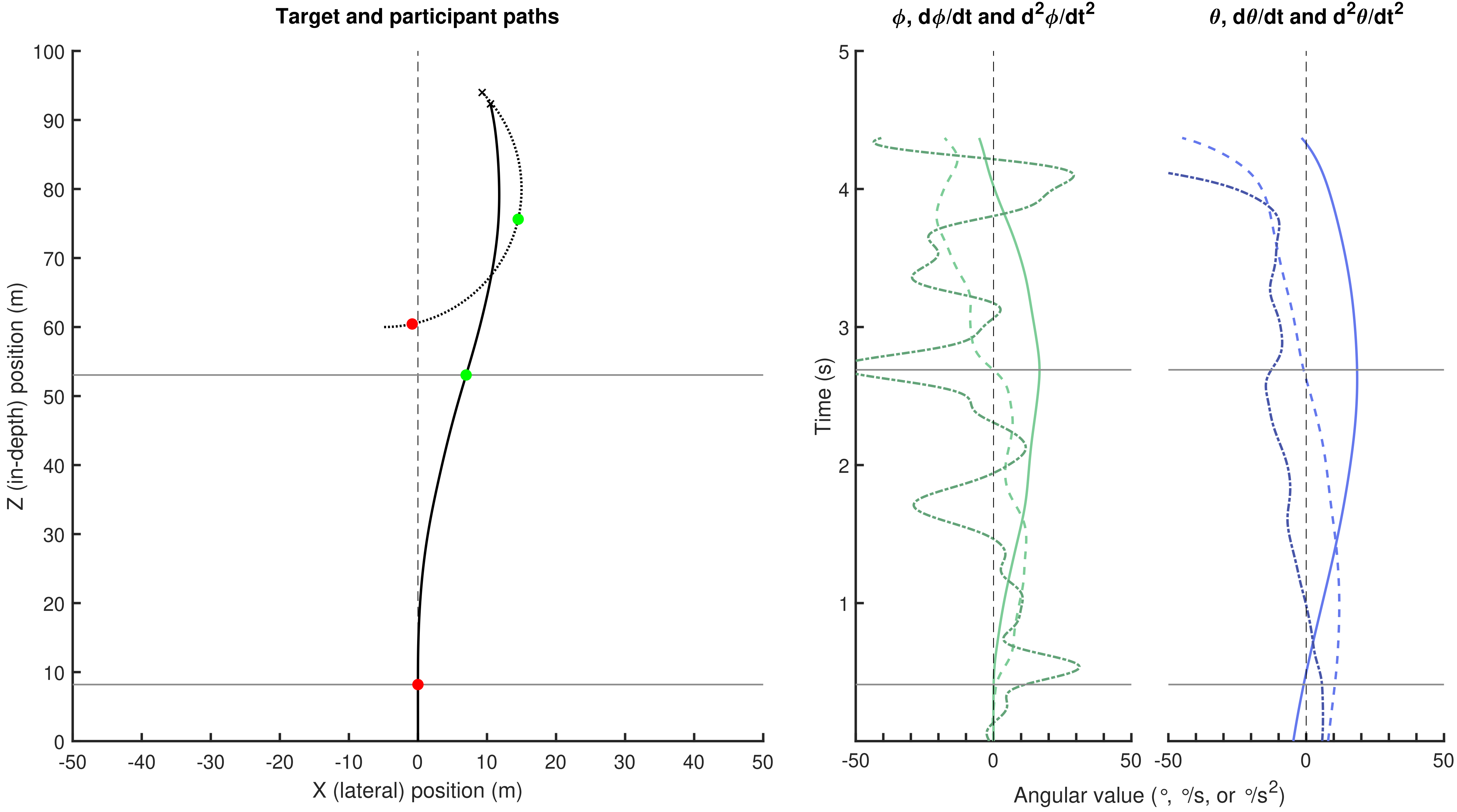

P4/B3
S5/R20-OUT

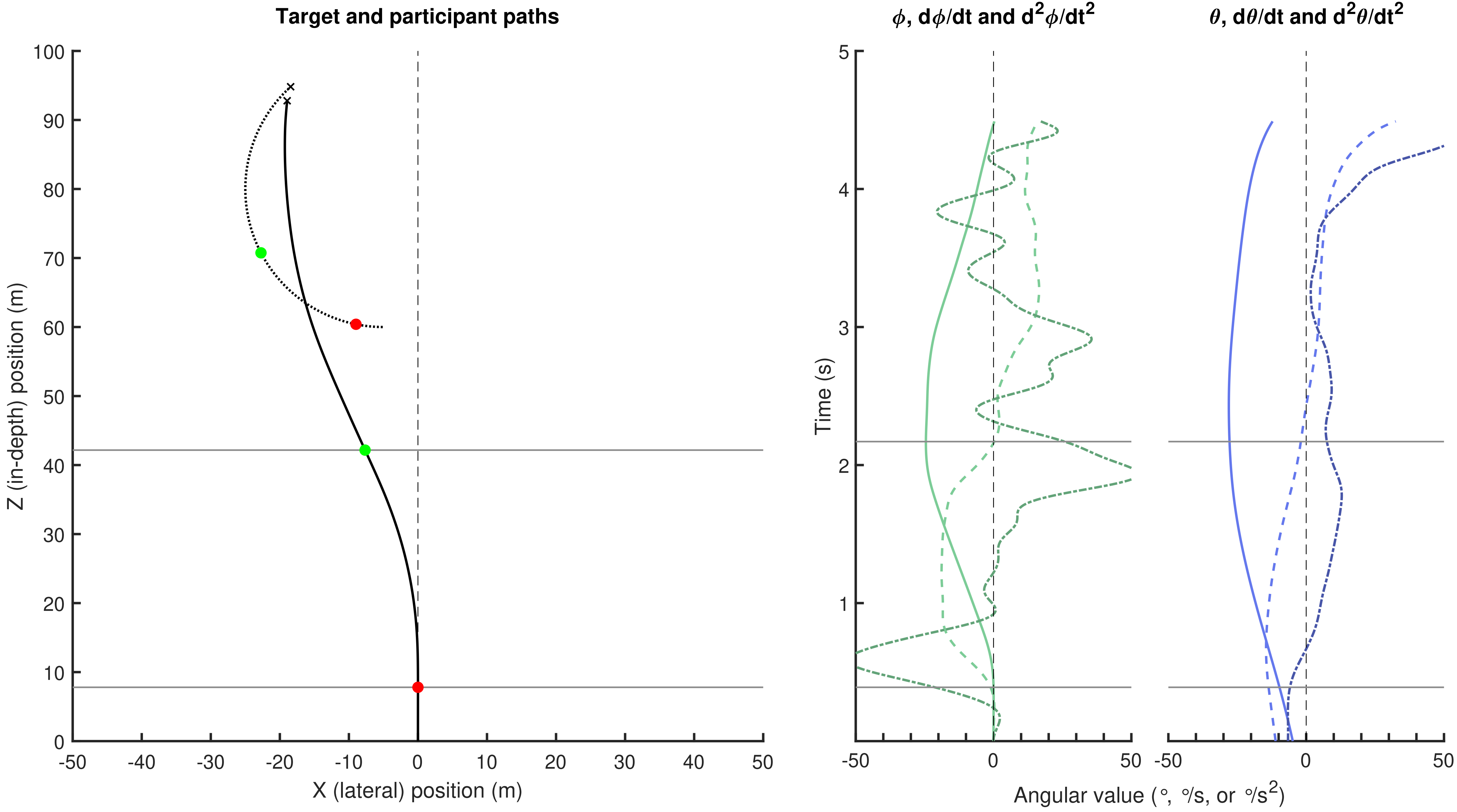

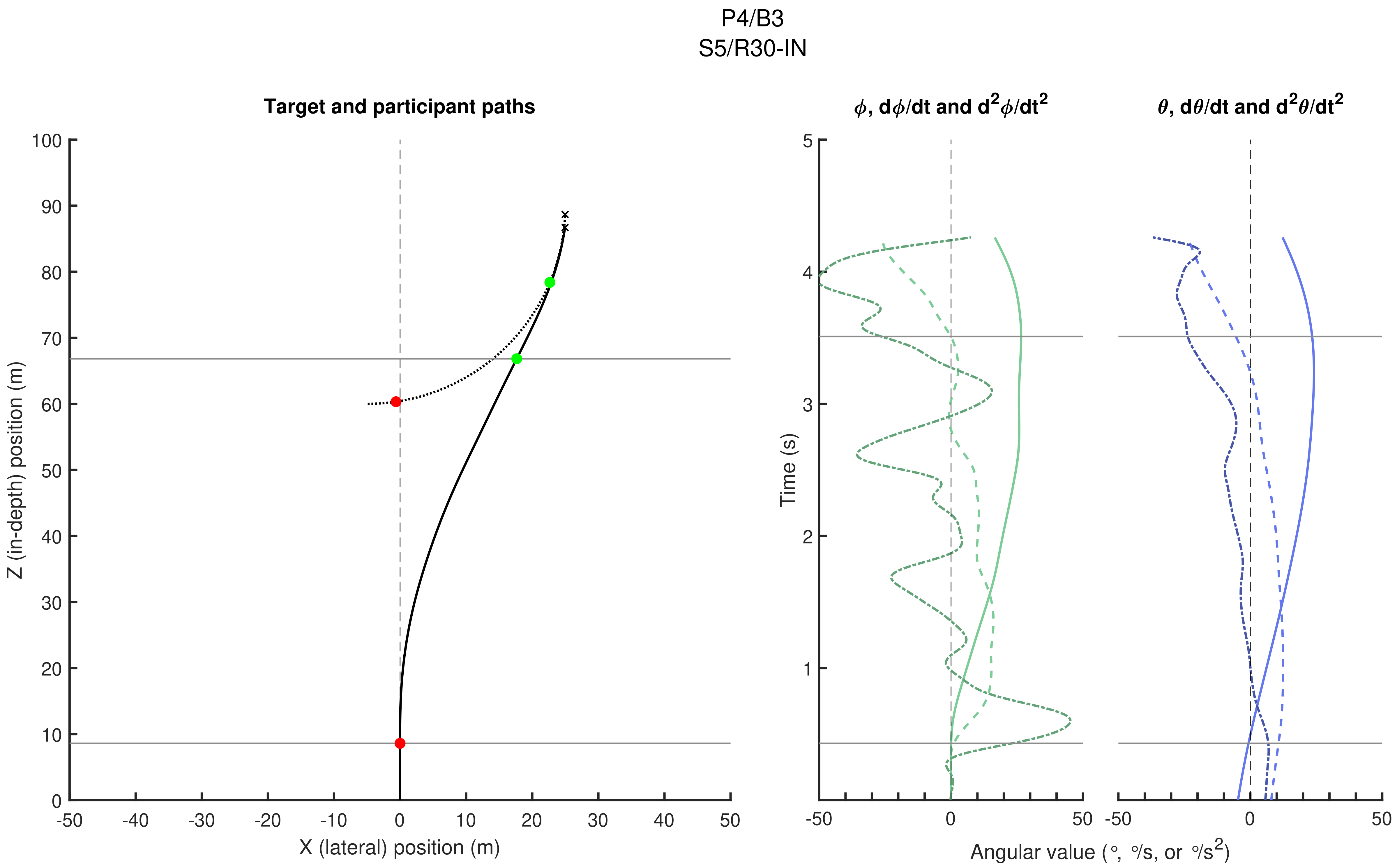
P4/B3
S5/R30-IN
Target and participant paths
Z (in-depth) position (m)
X (lateral) position (m)
100
90
80
70
60
50
40
30
20
10
0
-50
-40
-30
-20
-10
0
10
20
30
40
50
ϕ, dϕ/dt and d$^2\phi$/dt^2
θ, dθ/dt and d$^2\theta$/dt^2
Time (s)
5
4
3
2
1
-50
0
50
-50
0
50
Angular value (°, °/s, or °/s^2)

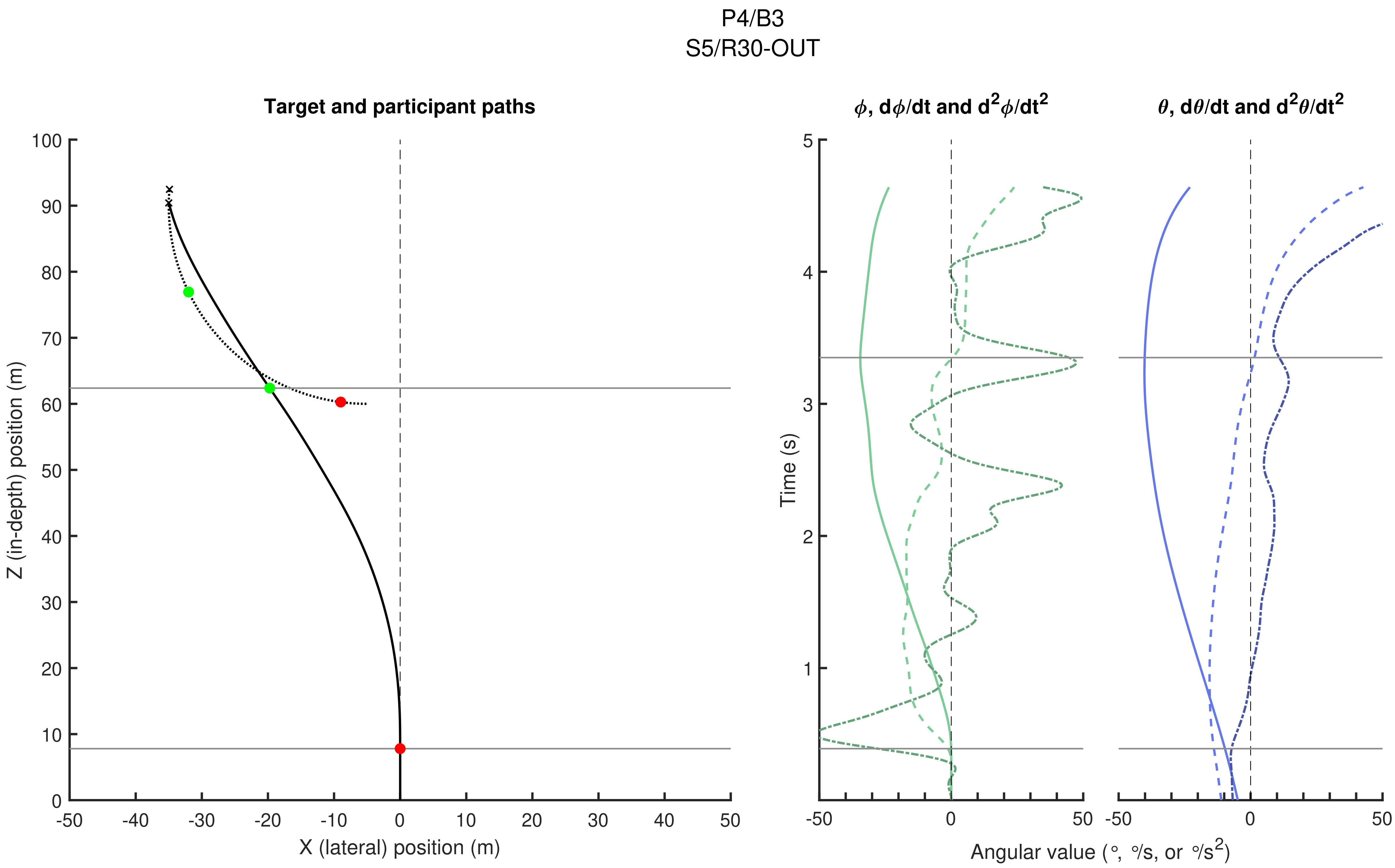

P4/B3
S5/R30-OUT
Target and participant paths
Z (in-depth) position (m)
X (lateral) position (m)
ϕ, dϕ/dt and d$^2\phi$/dt^2
θ, dθ/dt and d$^2\theta$/dt^2
Time (s)
Angular value (°, °/s, or °/s^2)

P4/B3
S5/R10-OUT

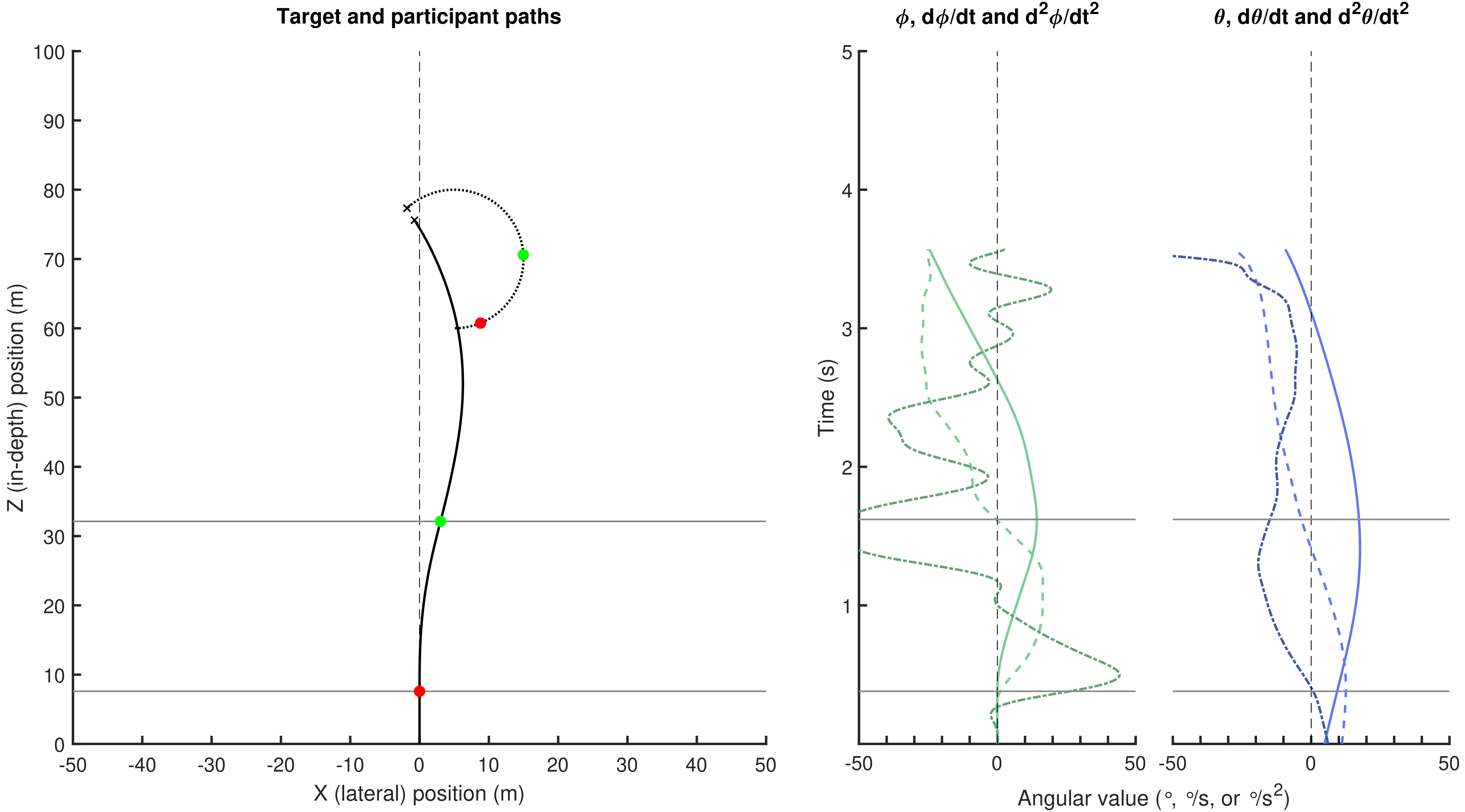

P4/B3
S5/R10-IN

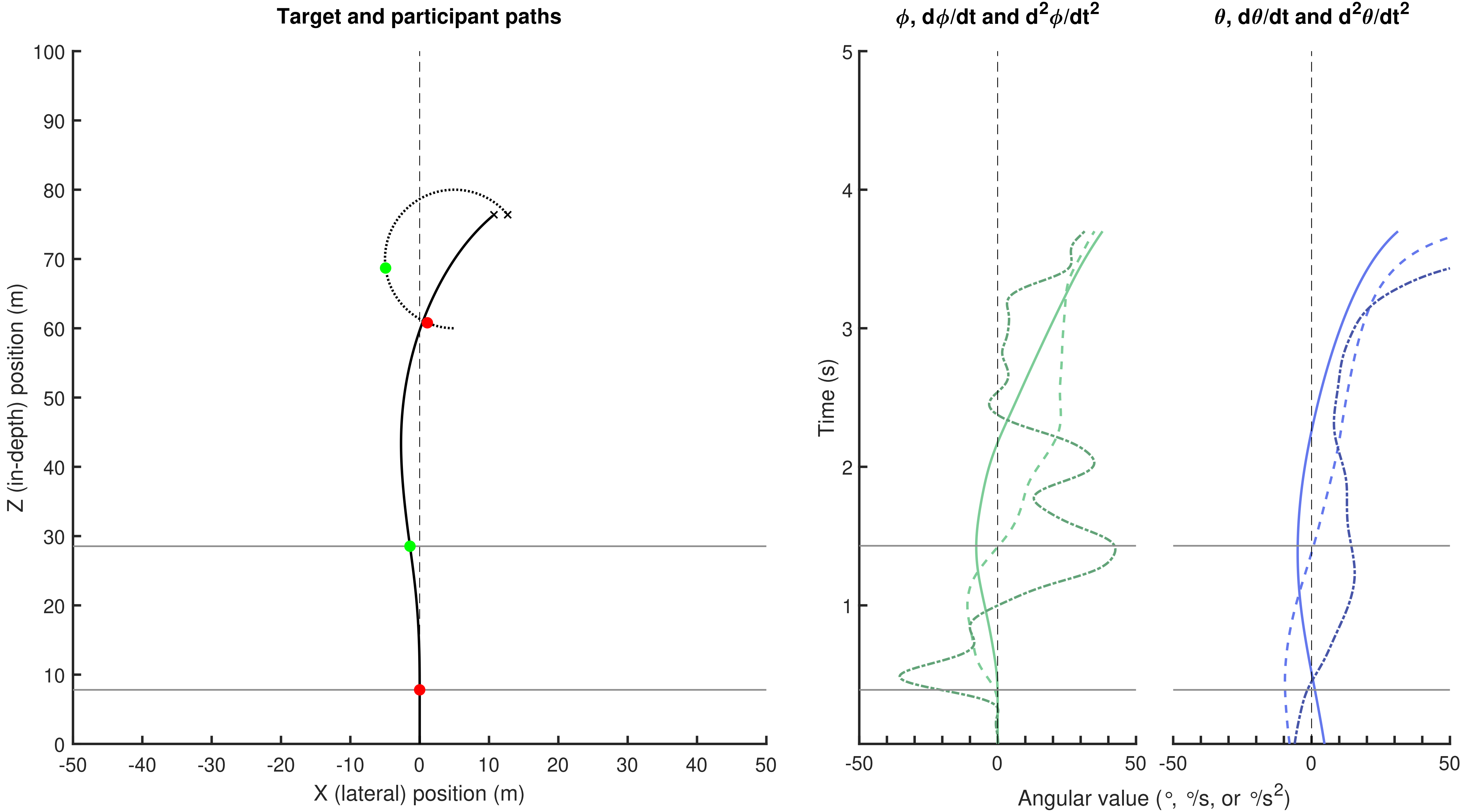

P4/B3
S5/R20-OUT

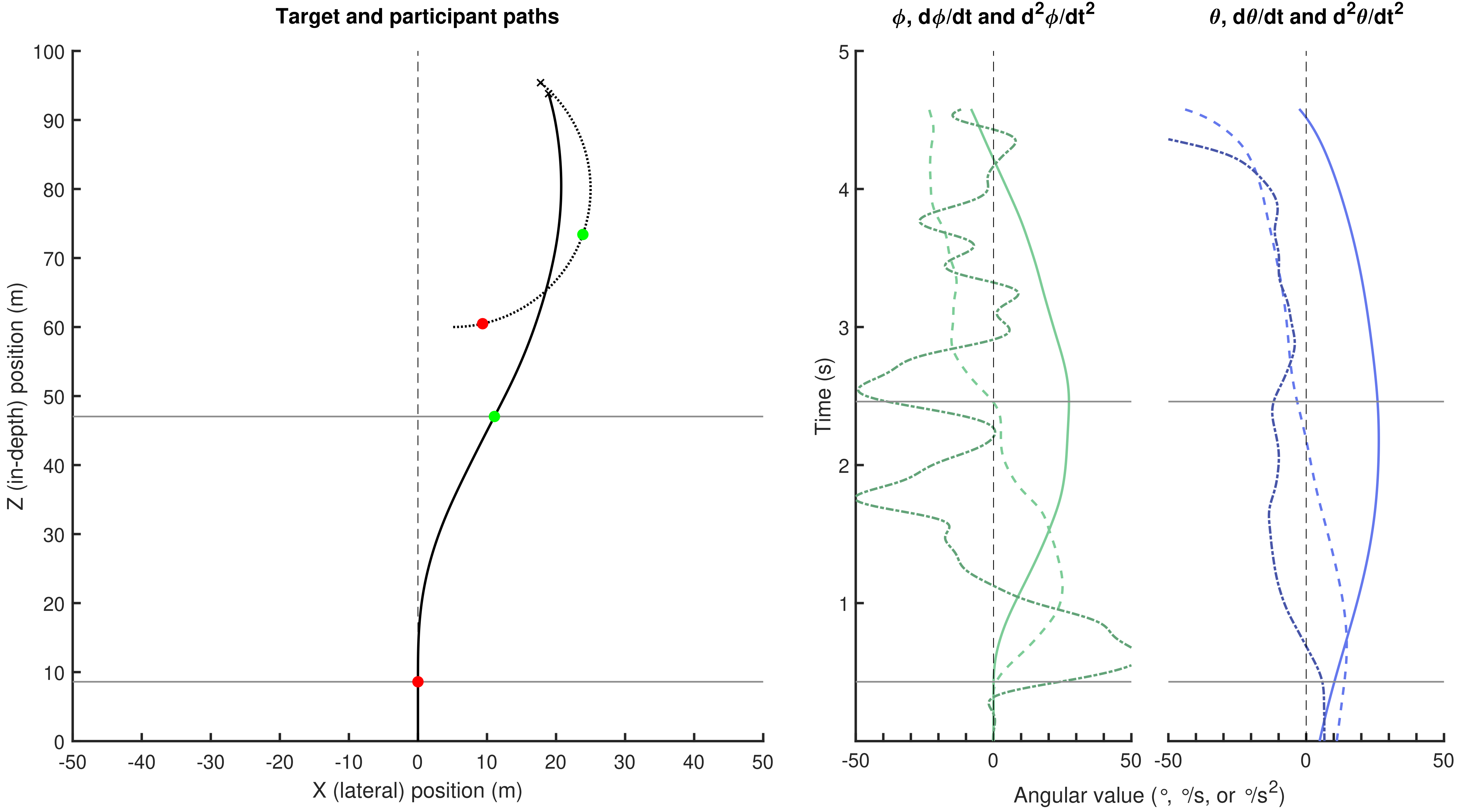

P4/B3
S5/R20-IN

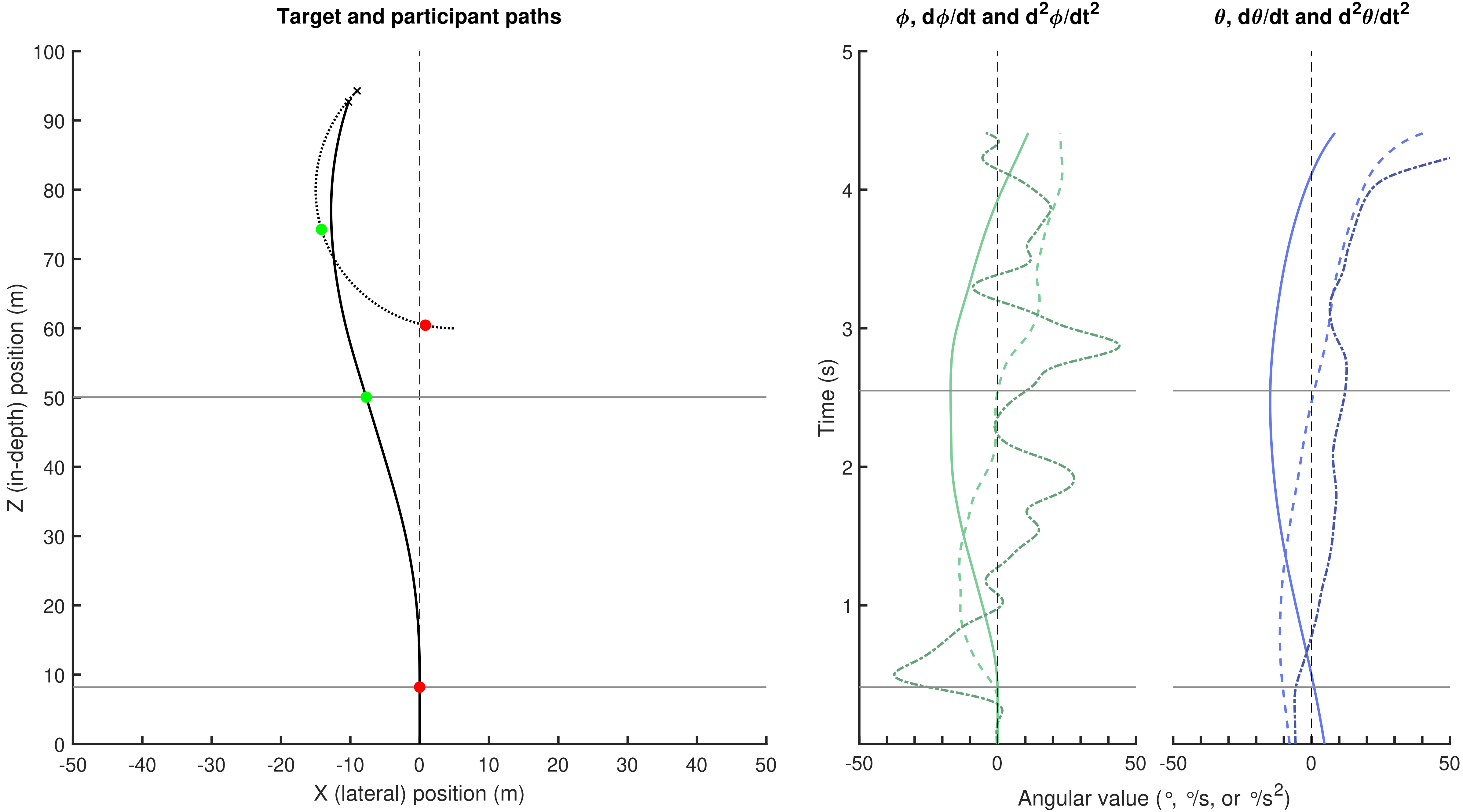

P4/B3
S5/R30-OUT

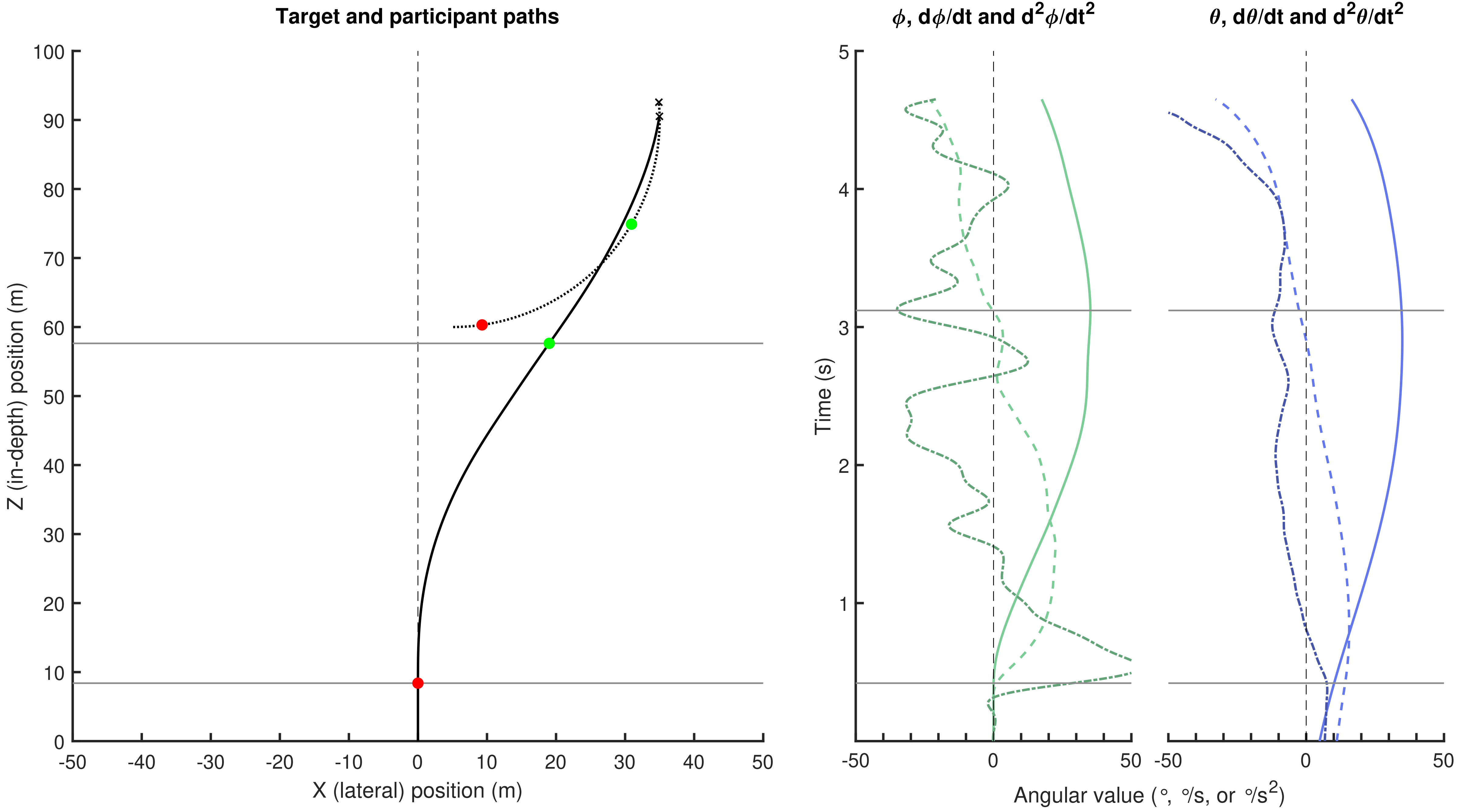

P4/B3
S5/R30-IN

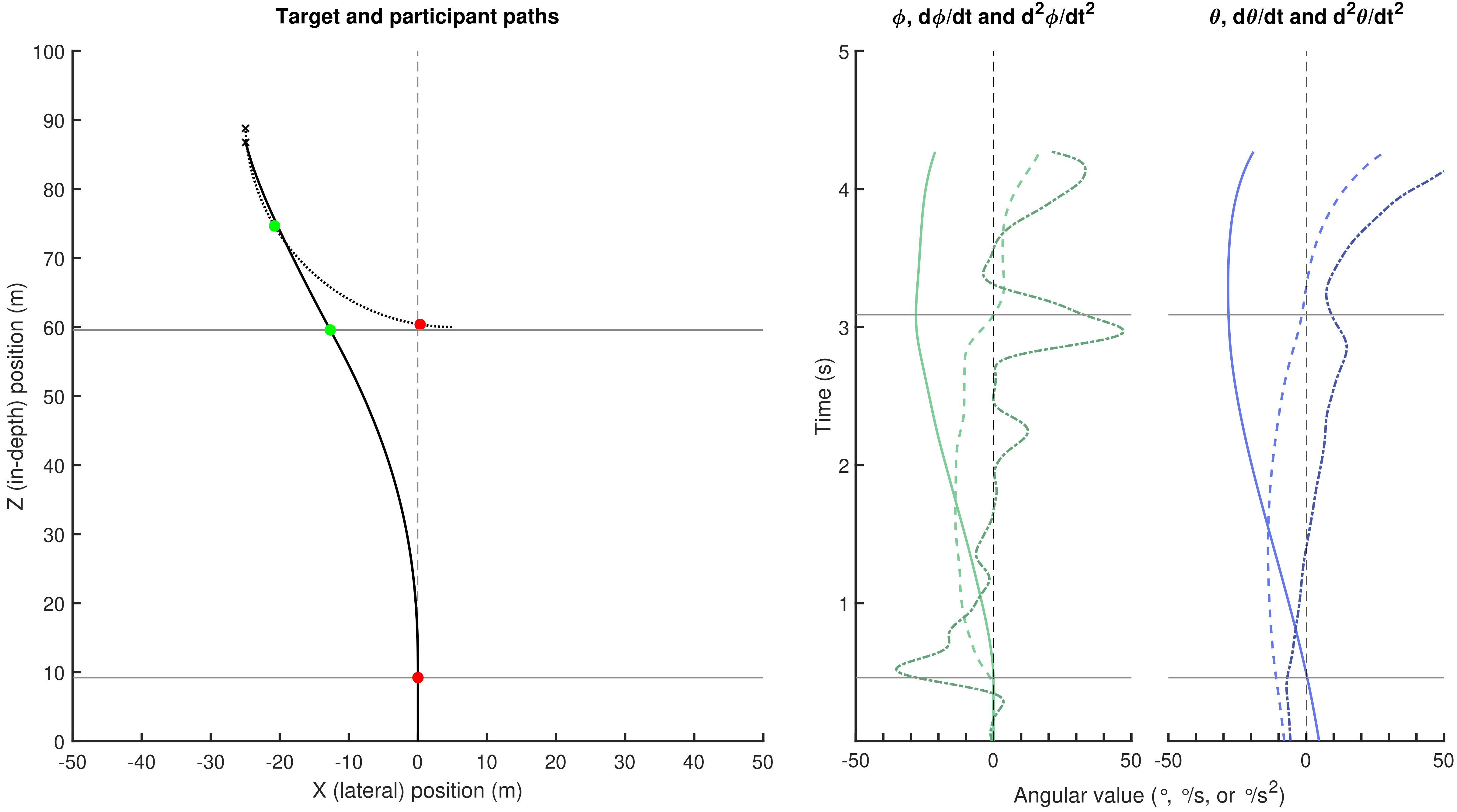

P4/B3
S15/R10-IN

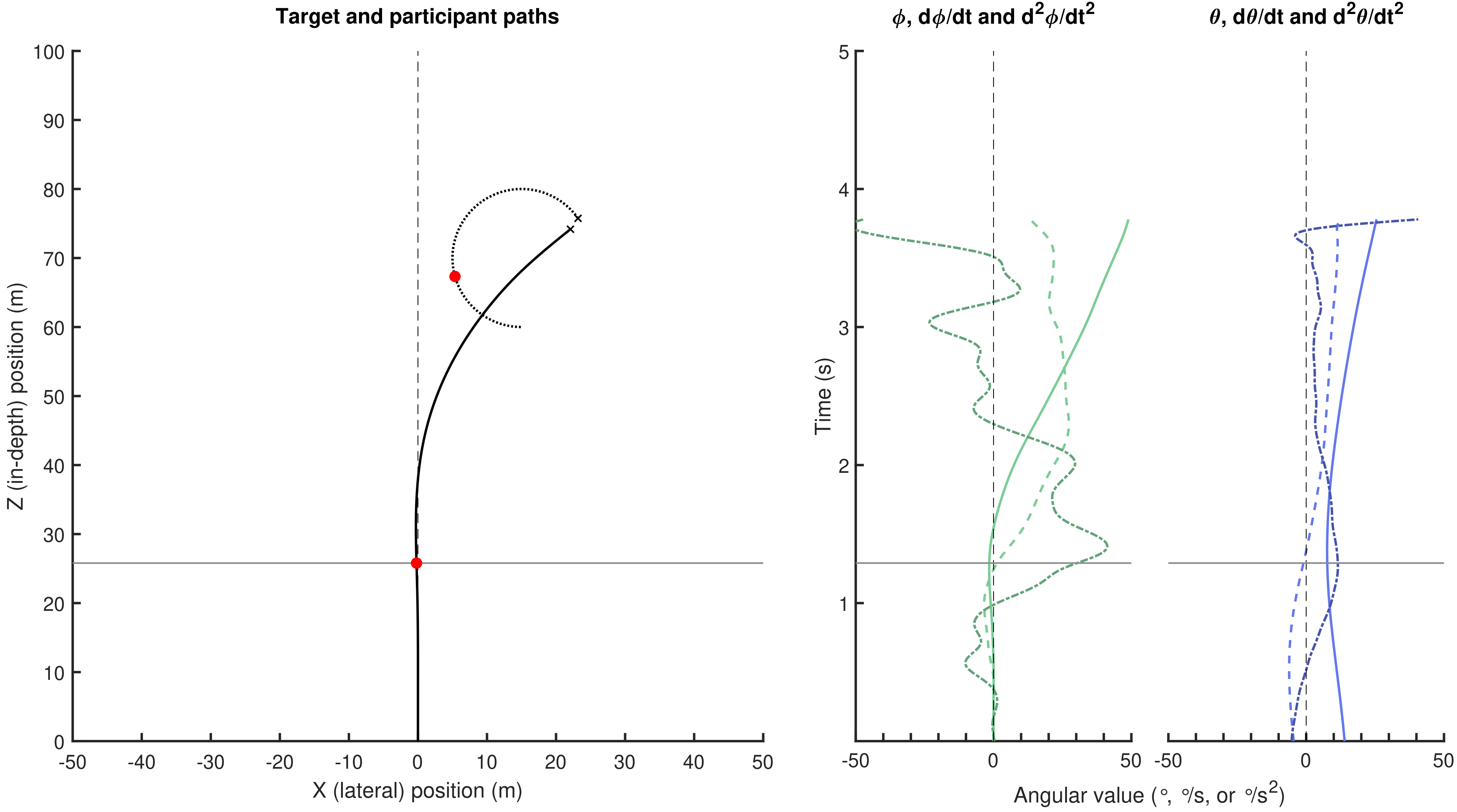

P4/B3
S15/R20-IN

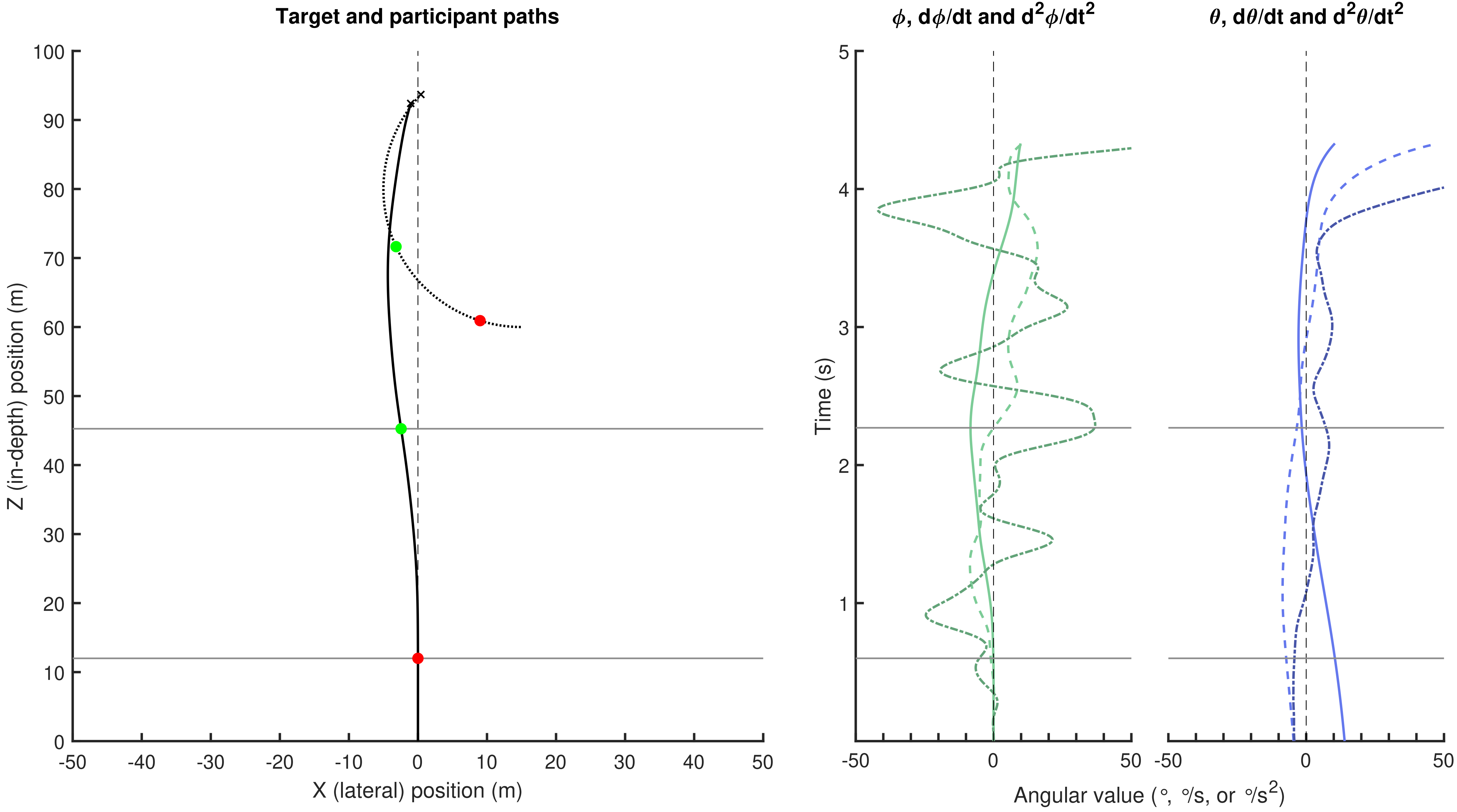

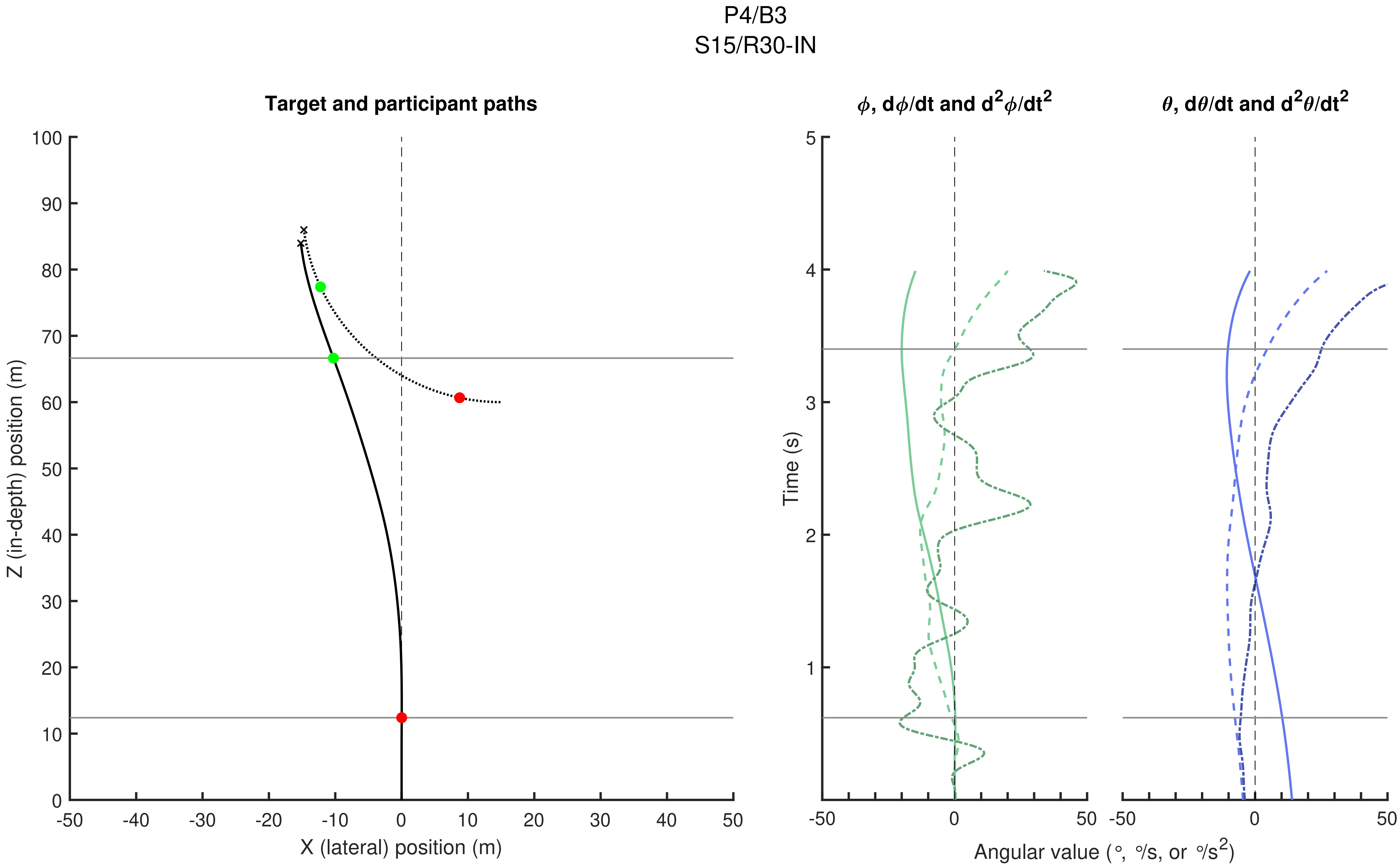
P4/B3
S15/R30-IN
Target and participant paths
Z (in-depth) position (m)
X (lateral) position (m)
φ, dφ/dt and d²φ/dt²
θ, dθ/dt and d²θ/dt²
Time (s)
Angular value (°, °/s, or °/s²)

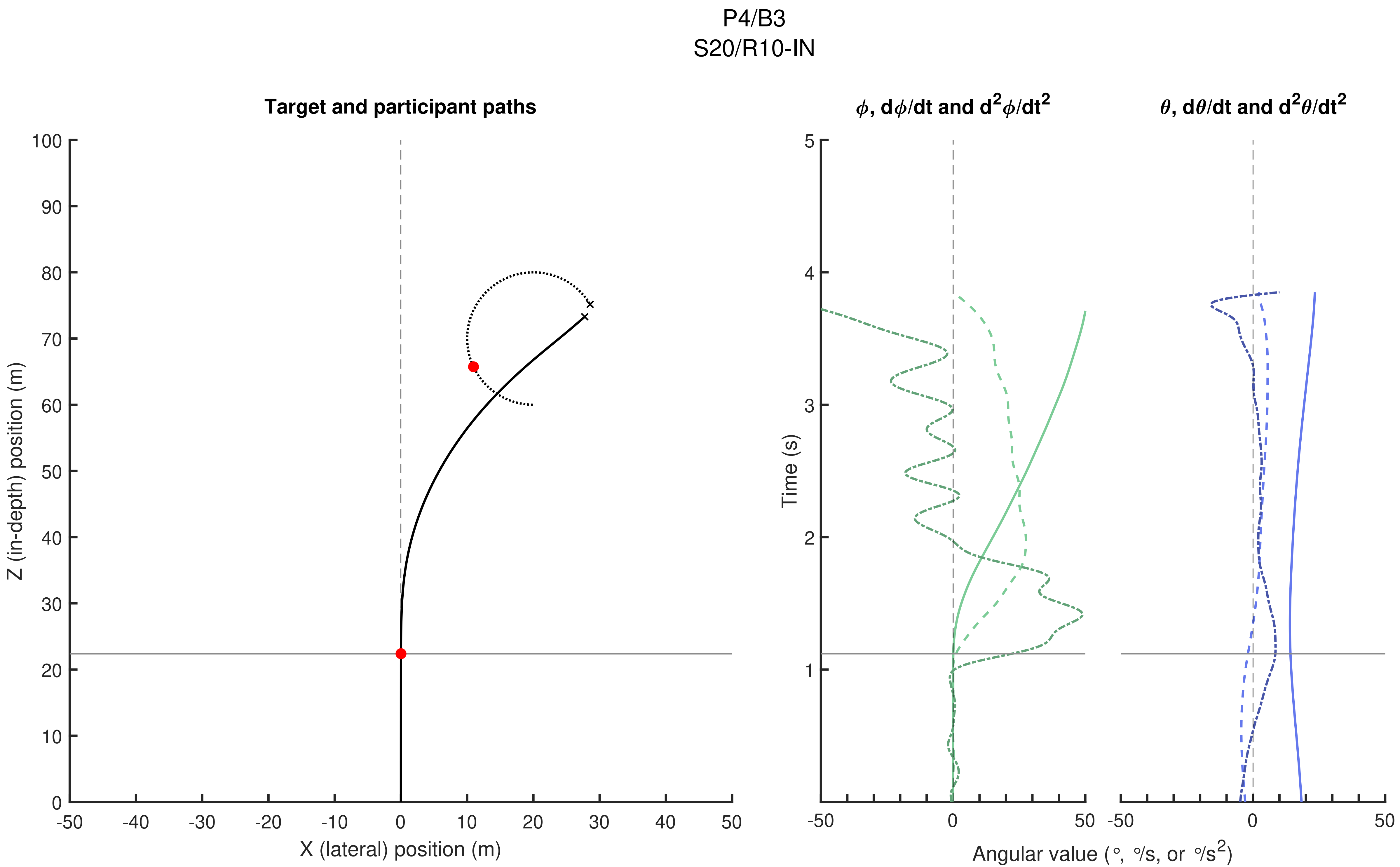
P4/B3
S20/R10-IN
Target and participant paths
Z (in-depth) position (m)
X (lateral) position (m)
ϕ, dϕ/dt and d$^2\phi$/dt^2
θ, dθ/dt and d$^2\theta$/dt^2
Time (s)
Angular value (°, °/s, or °/s^2)

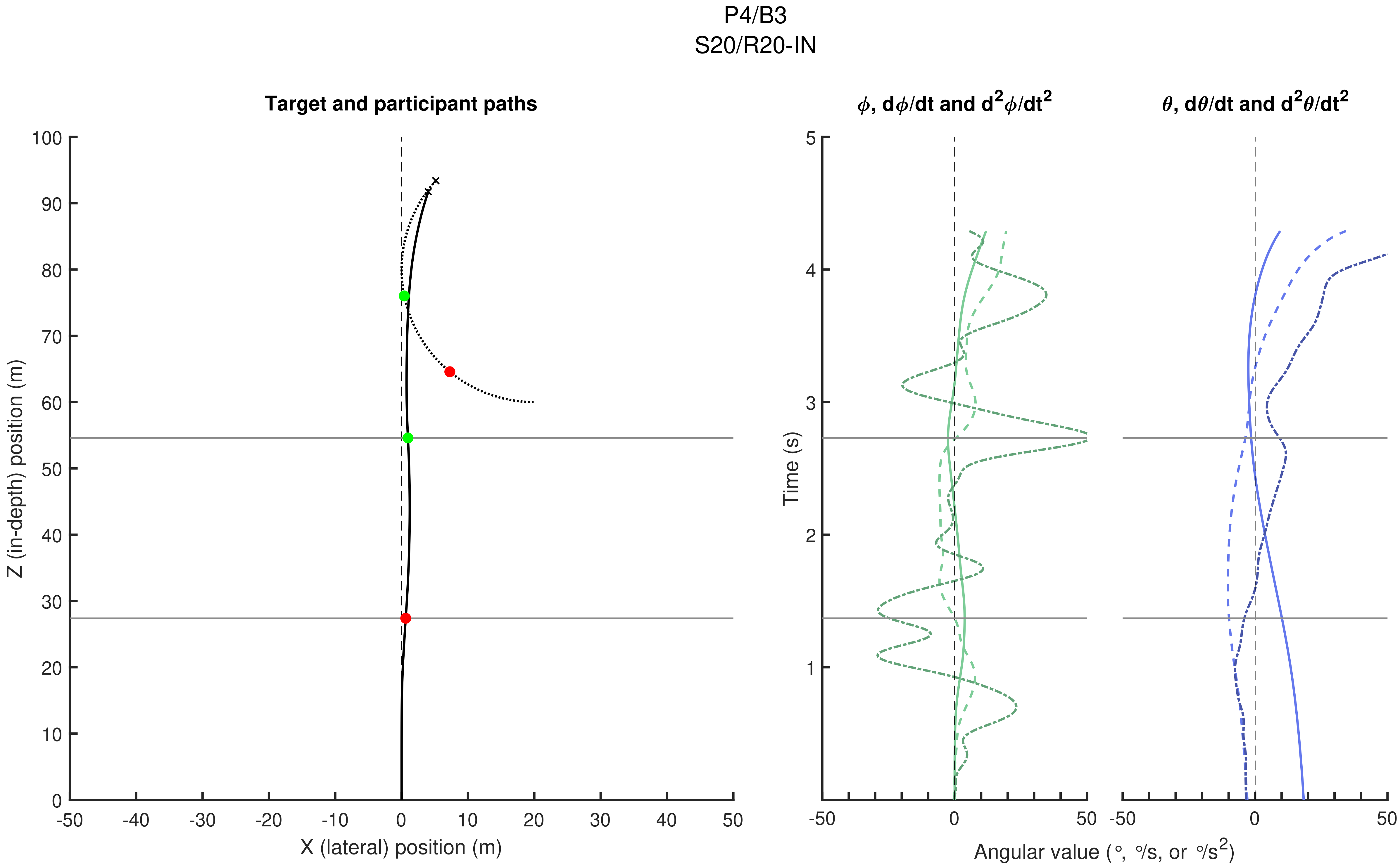
P4/B3
S20/R20-IN
Target and participant paths
Z (in-depth) position (m)
X (lateral) position (m)
φ, dφ/dt and d²φ/dt²
θ, dθ/dt and d²θ/dt²
Time (s)
Angular value (°, °/s, or °/s²)

P4/B3
S20/R30-IN

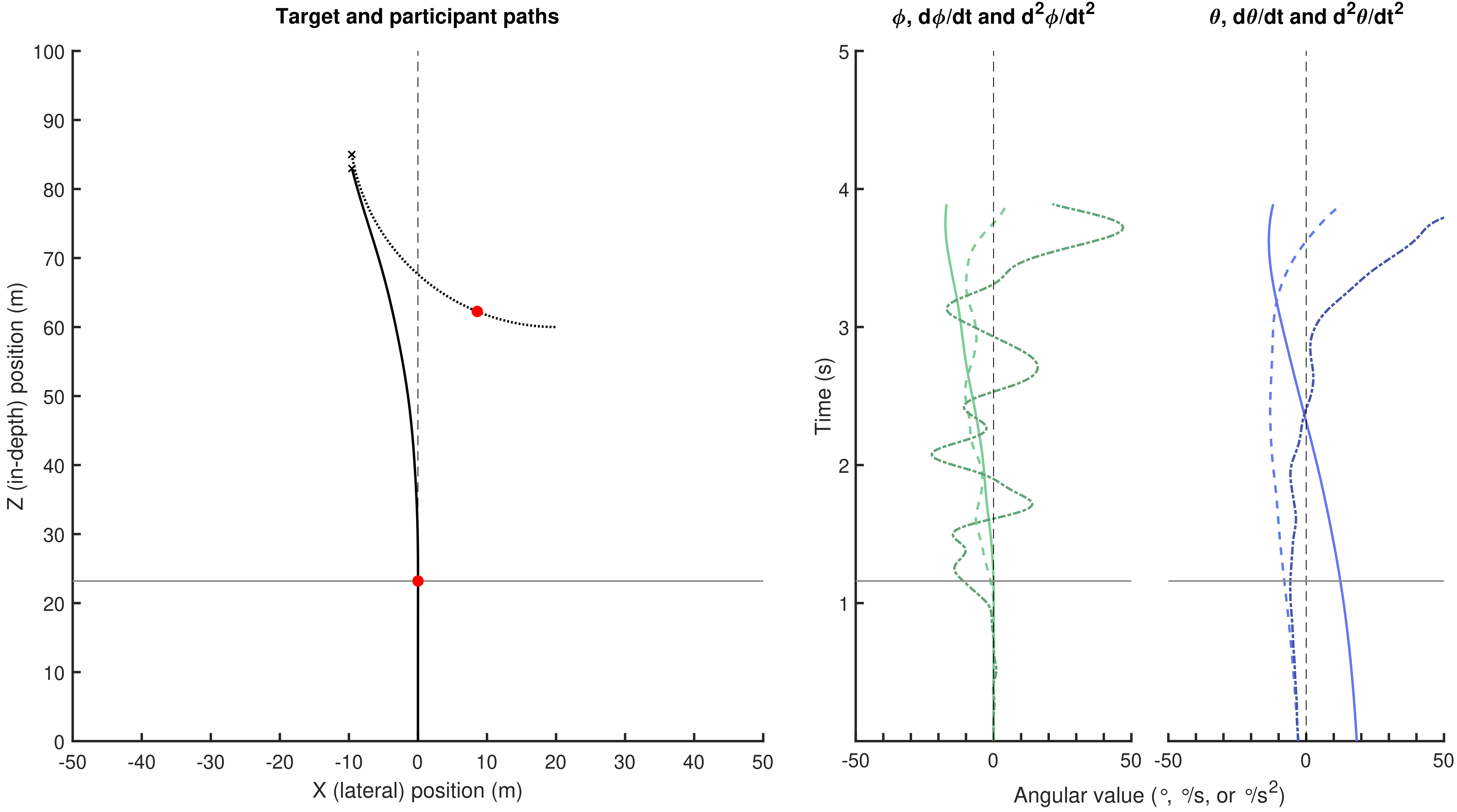

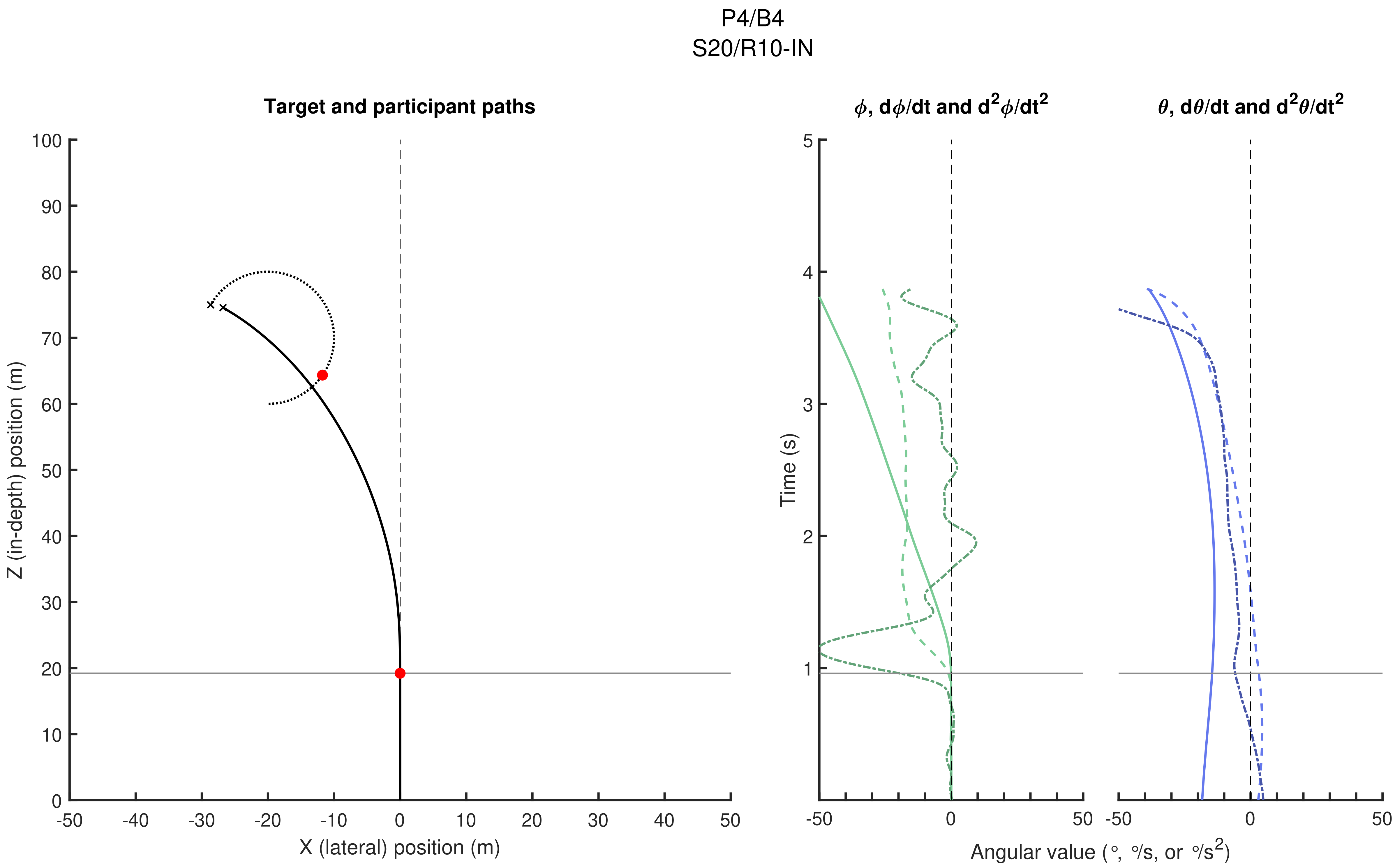

P4/B4
S20/R10-IN
Target and participant paths
ϕ, dϕ/dt and d$^2\phi$/dt^2
θ, dθ/dt and d$^2\theta$/dt^2
Z (in-depth) position (m)
X (lateral) position (m)
Time (s)
Angular value (°, °/s, or °/s^2)

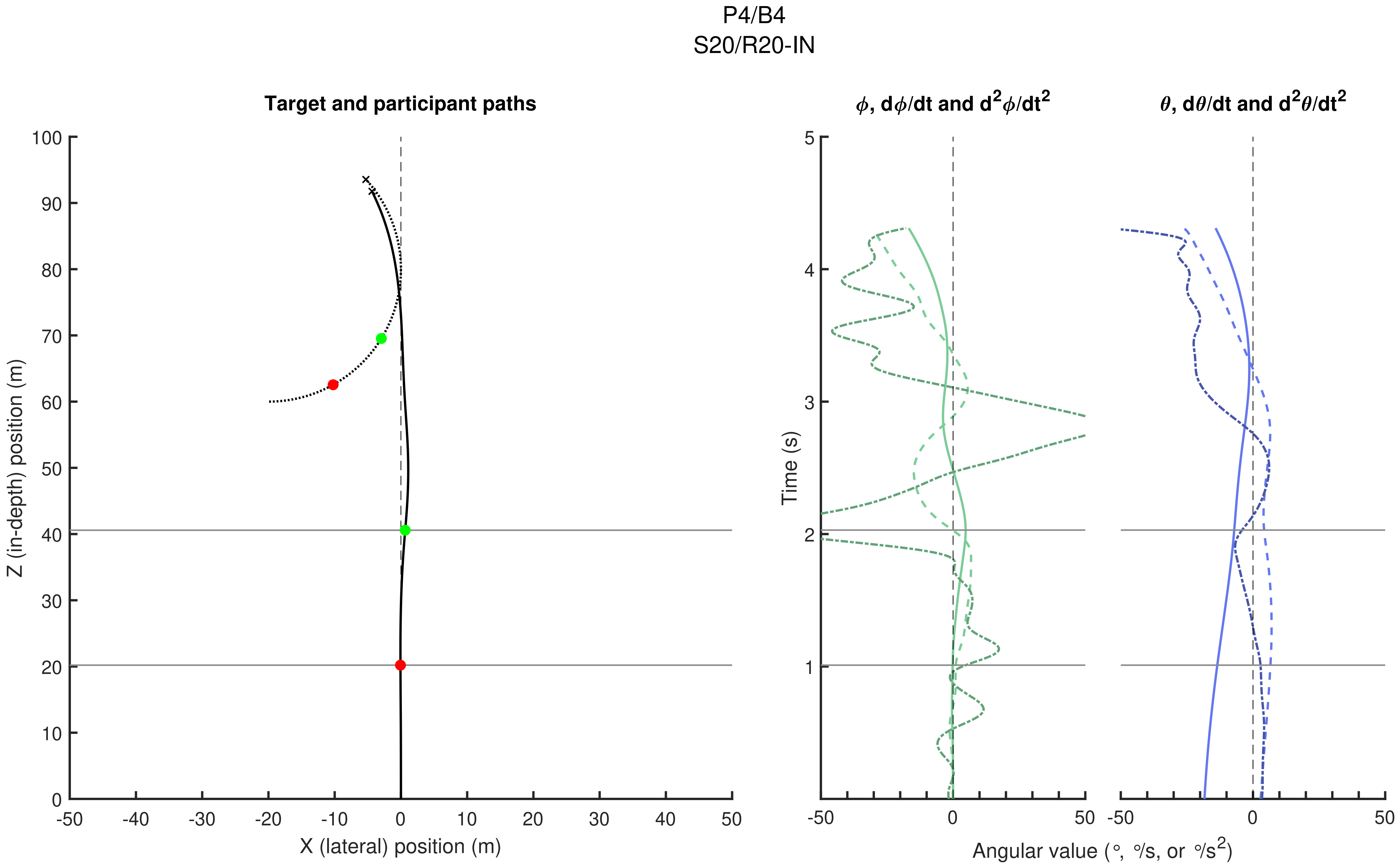
P4/B4
S20/R20-IN
Target and participant paths
Z (in-depth) position (m)
X (lateral) position (m)
ϕ, dϕ/dt and d²ϕ/dt²
θ, dθ/dt and d²θ/dt²
Time (s)
Angular value (°, °/s, or °/s²)

P4/B4
S20/R30-IN

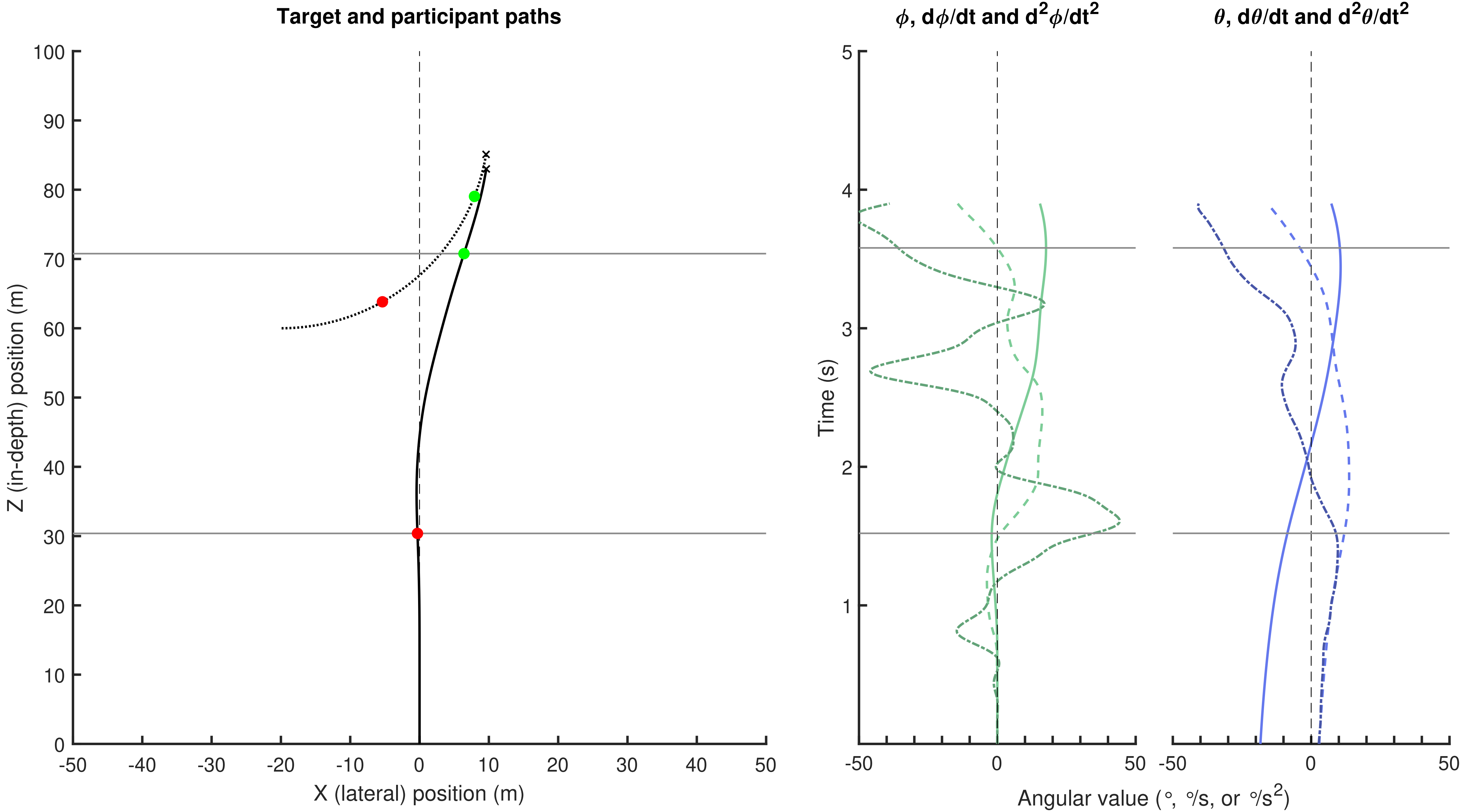

P4/B4
S15/R10-IN

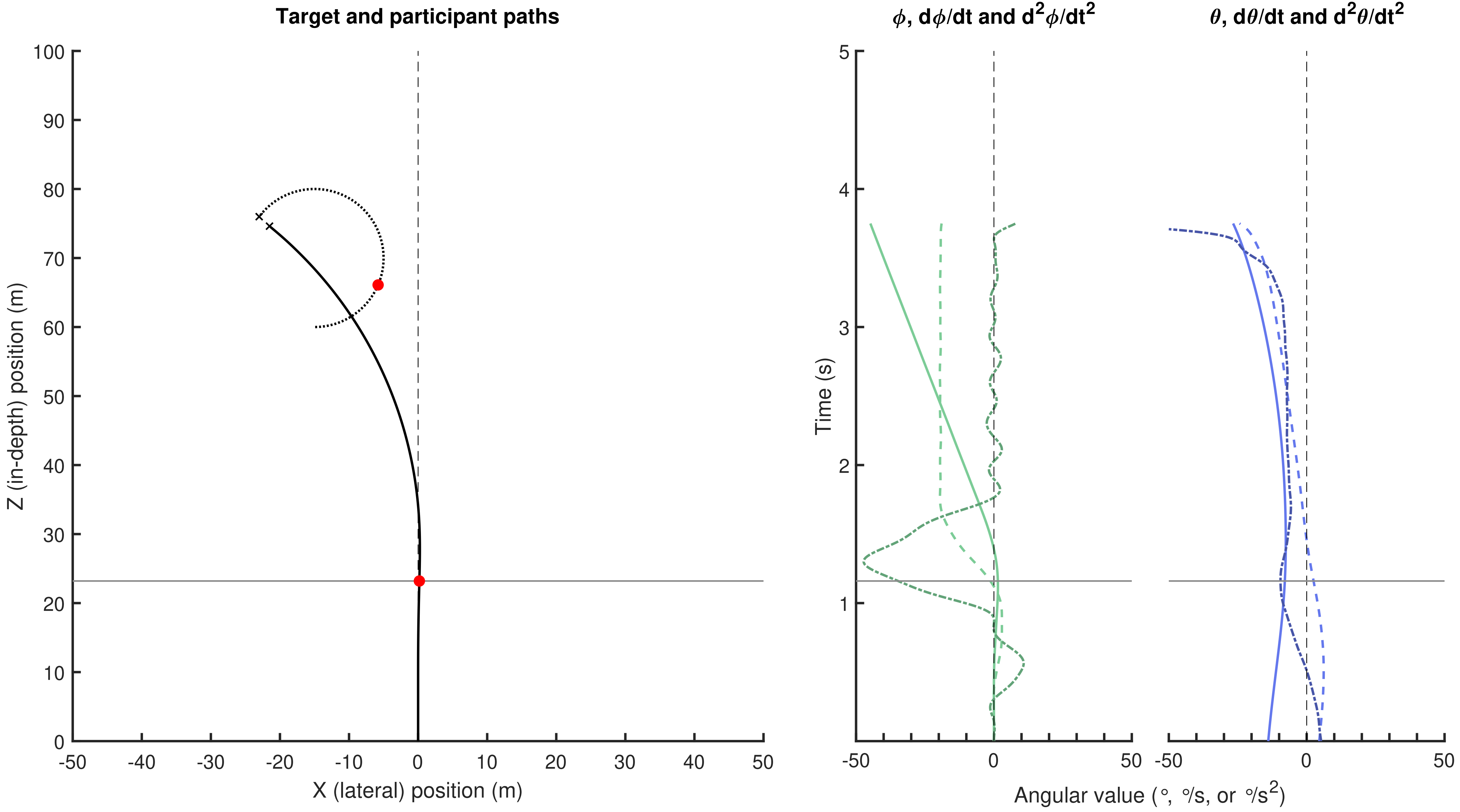

P4/B4
S15/R20-IN

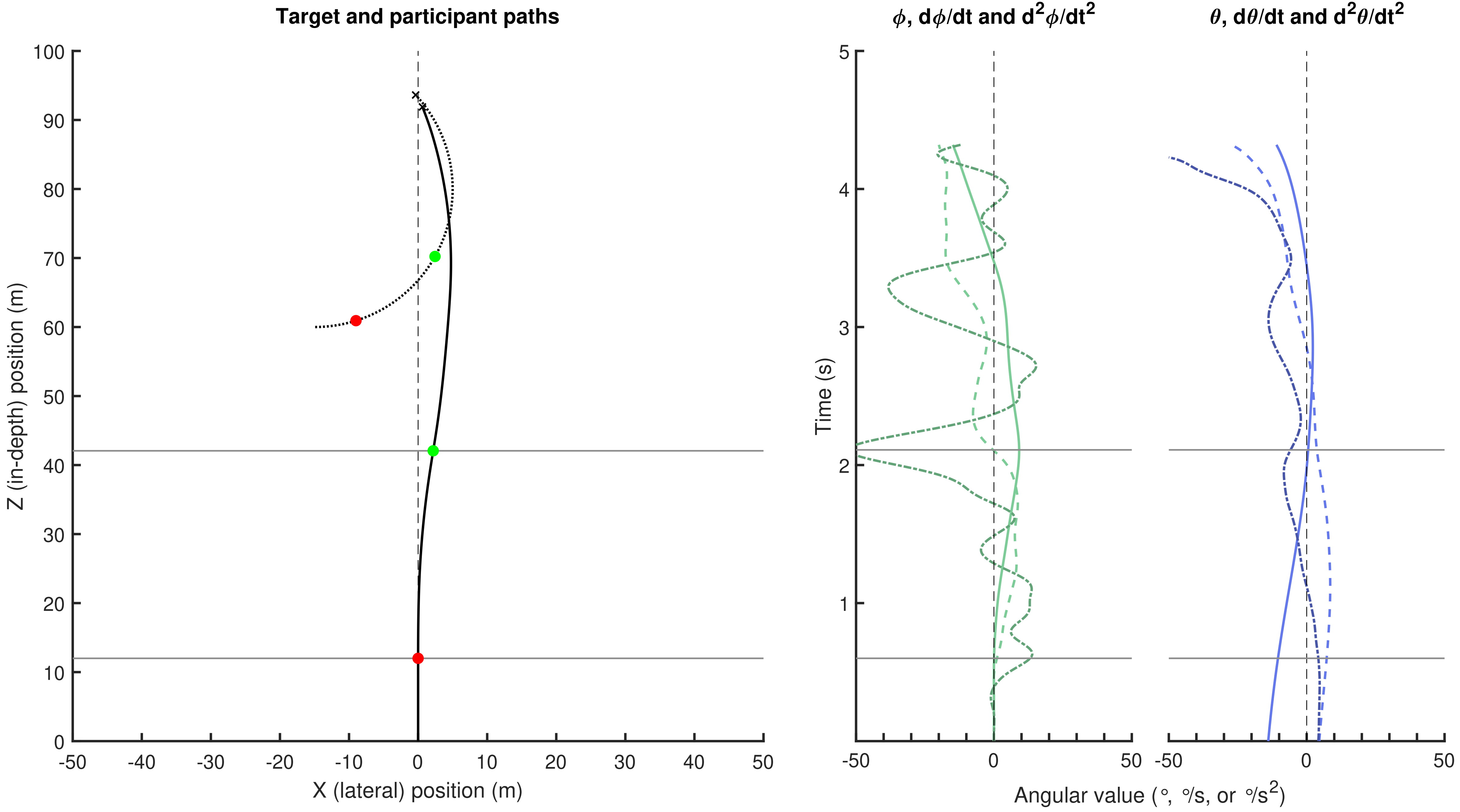

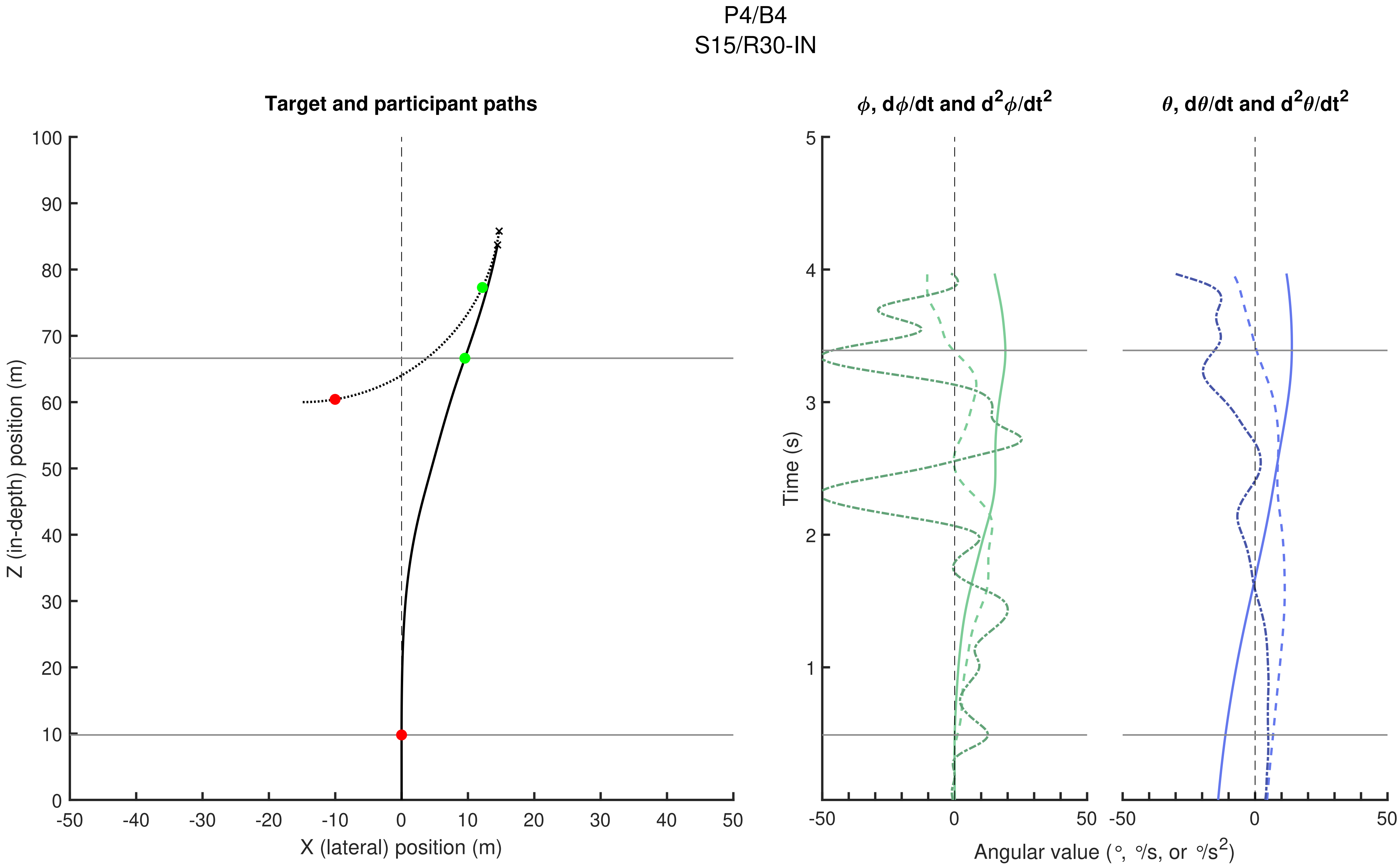

P4/B4
S15/R30-IN
Target and participant paths
ϕ, dϕ/dt and d$^2\phi$/dt^2
θ, dθ/dt and d$^2\theta$/dt^2
Z (in-depth) position (m)
X (lateral) position (m)
Time (s)
Angular value (°, °/s, or °/s^2)

P4/B4
S5/R10-IN

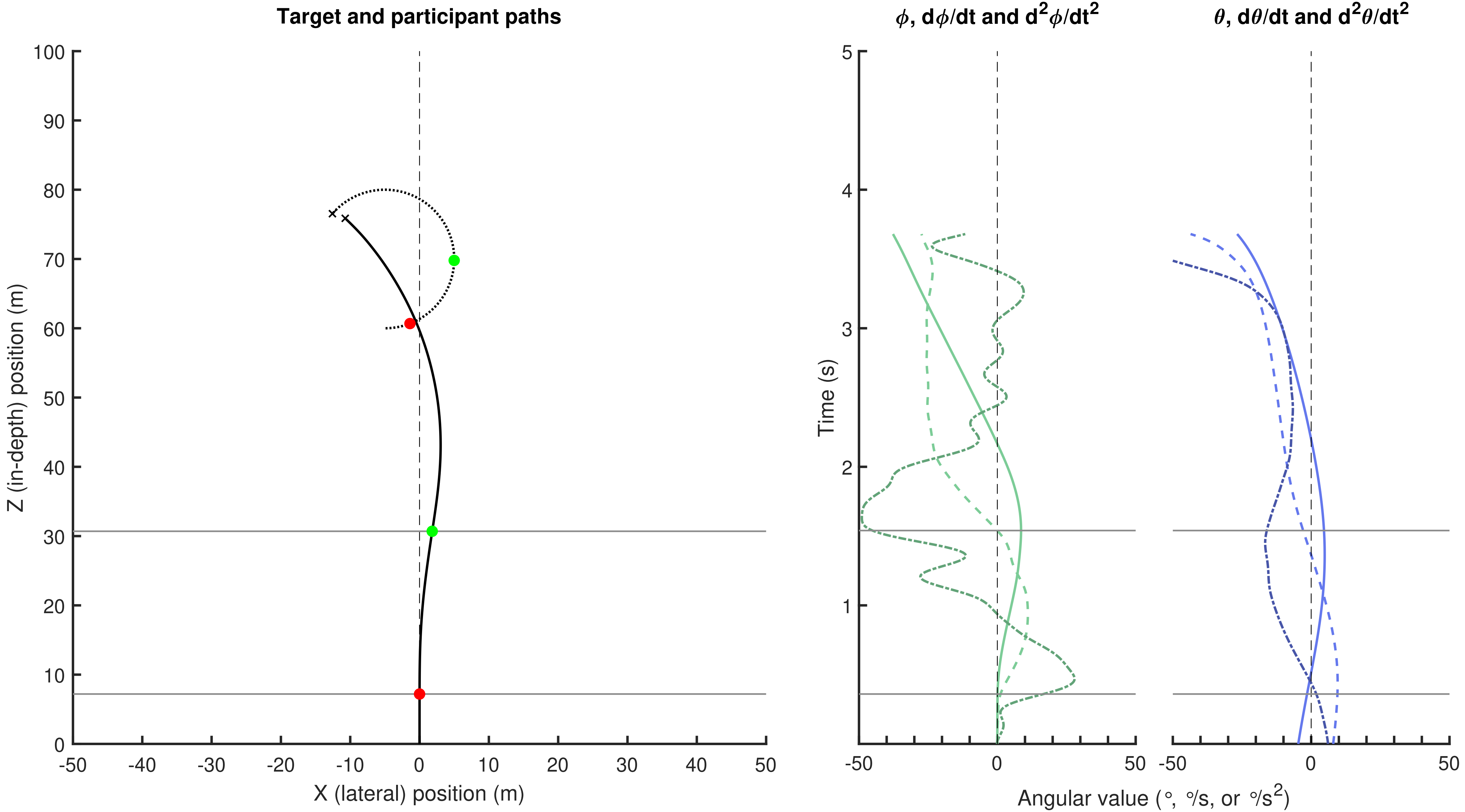

P4/B4
S5/R10-OUT

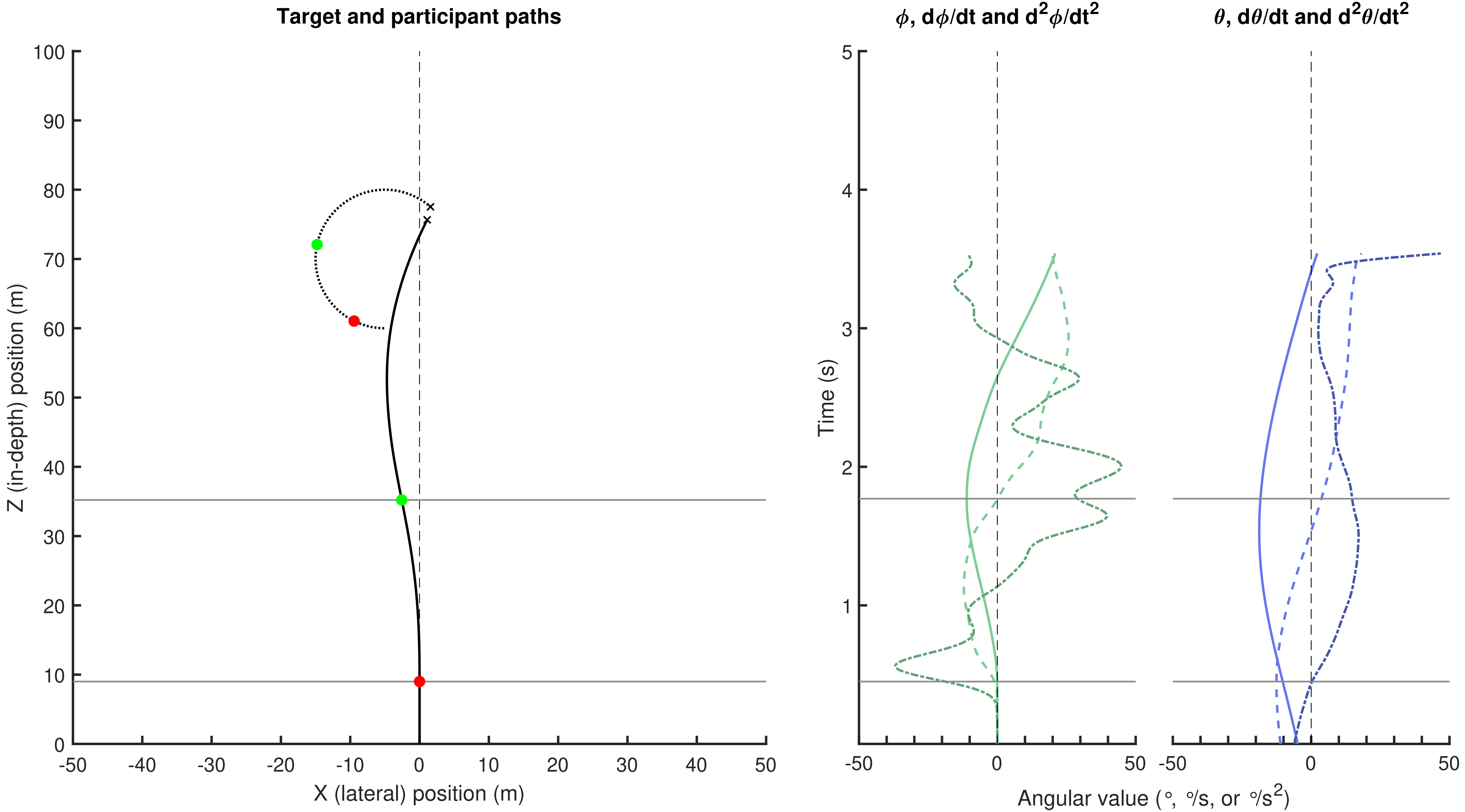

P4/B4
S5/R20-IN

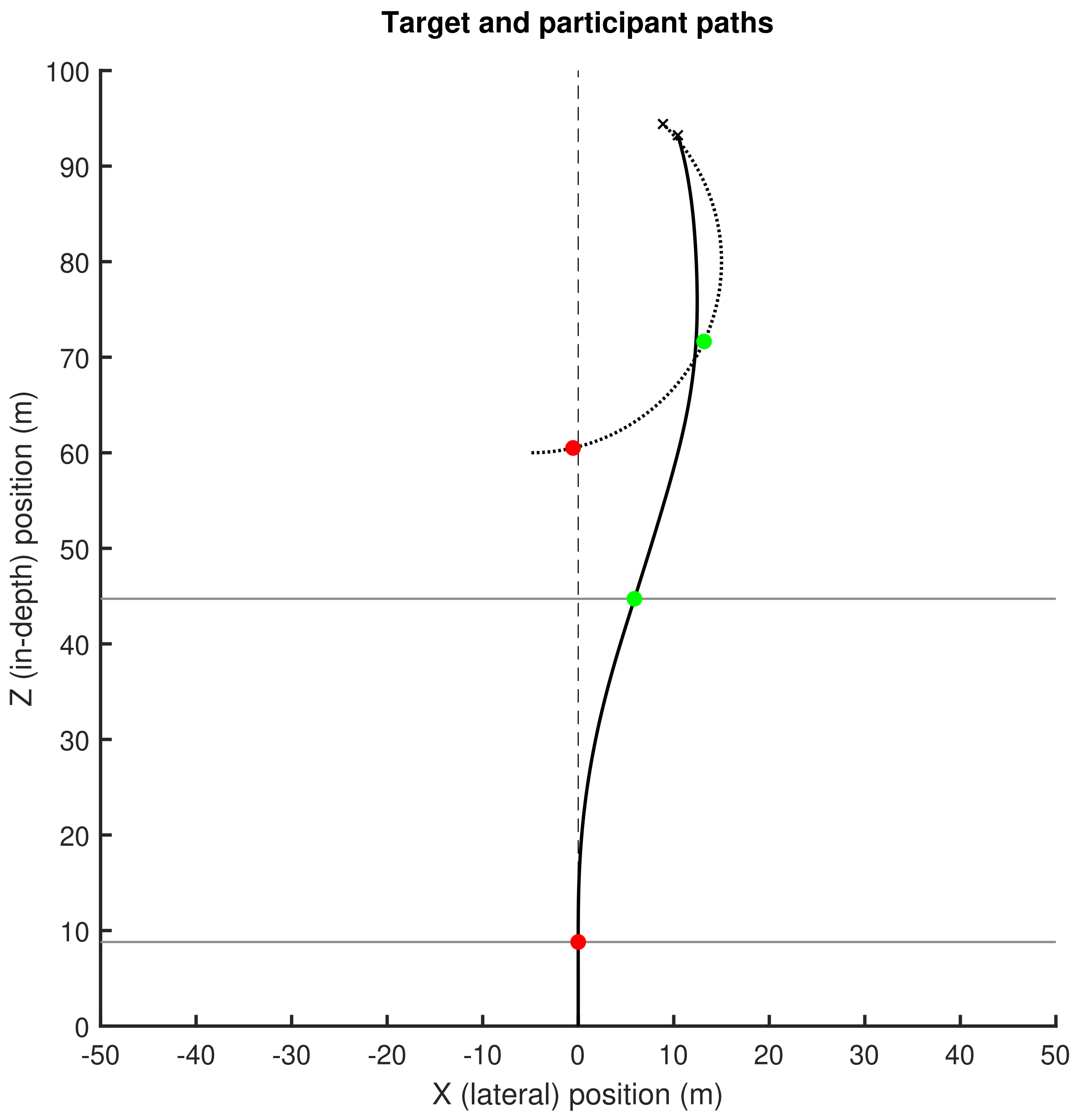

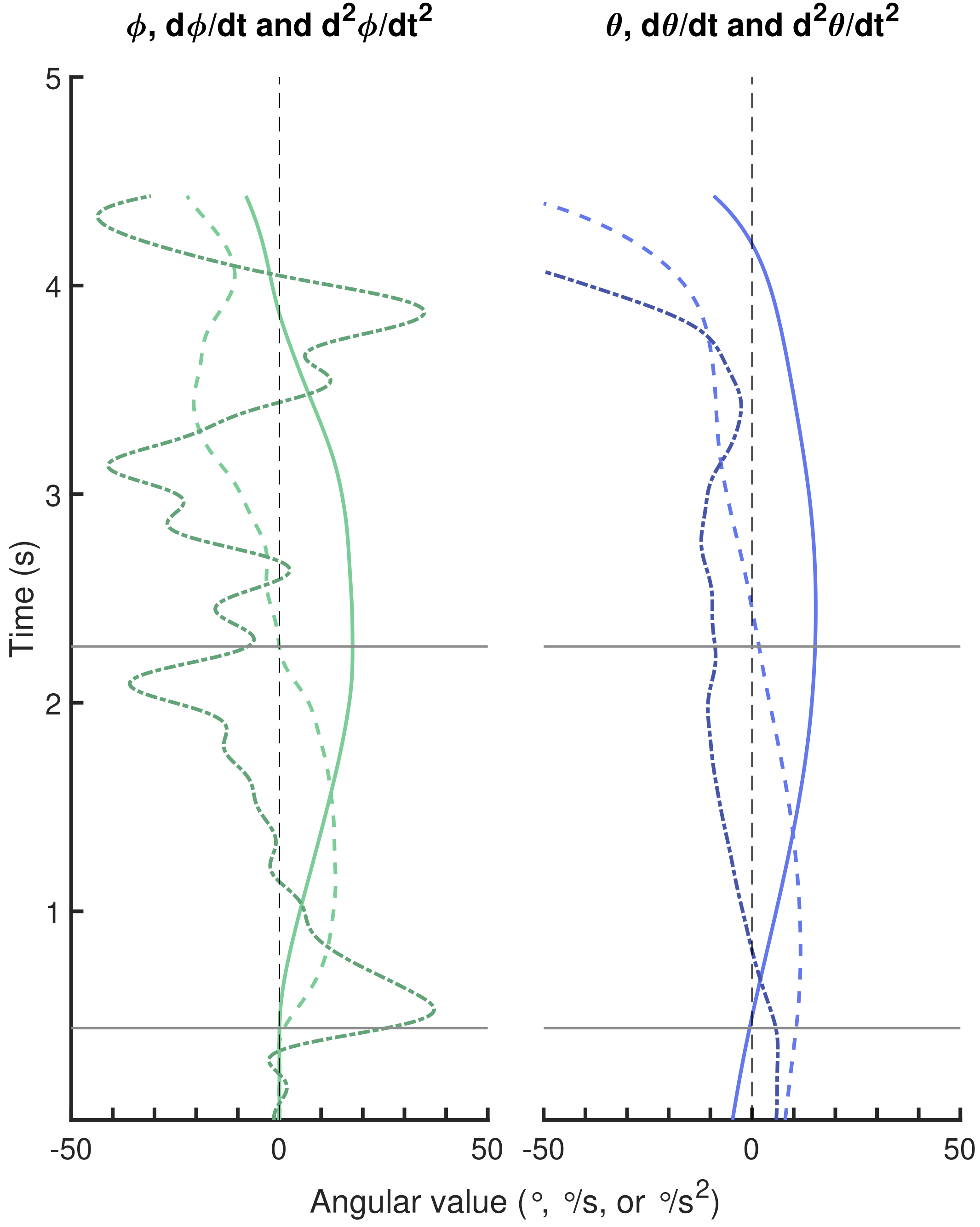

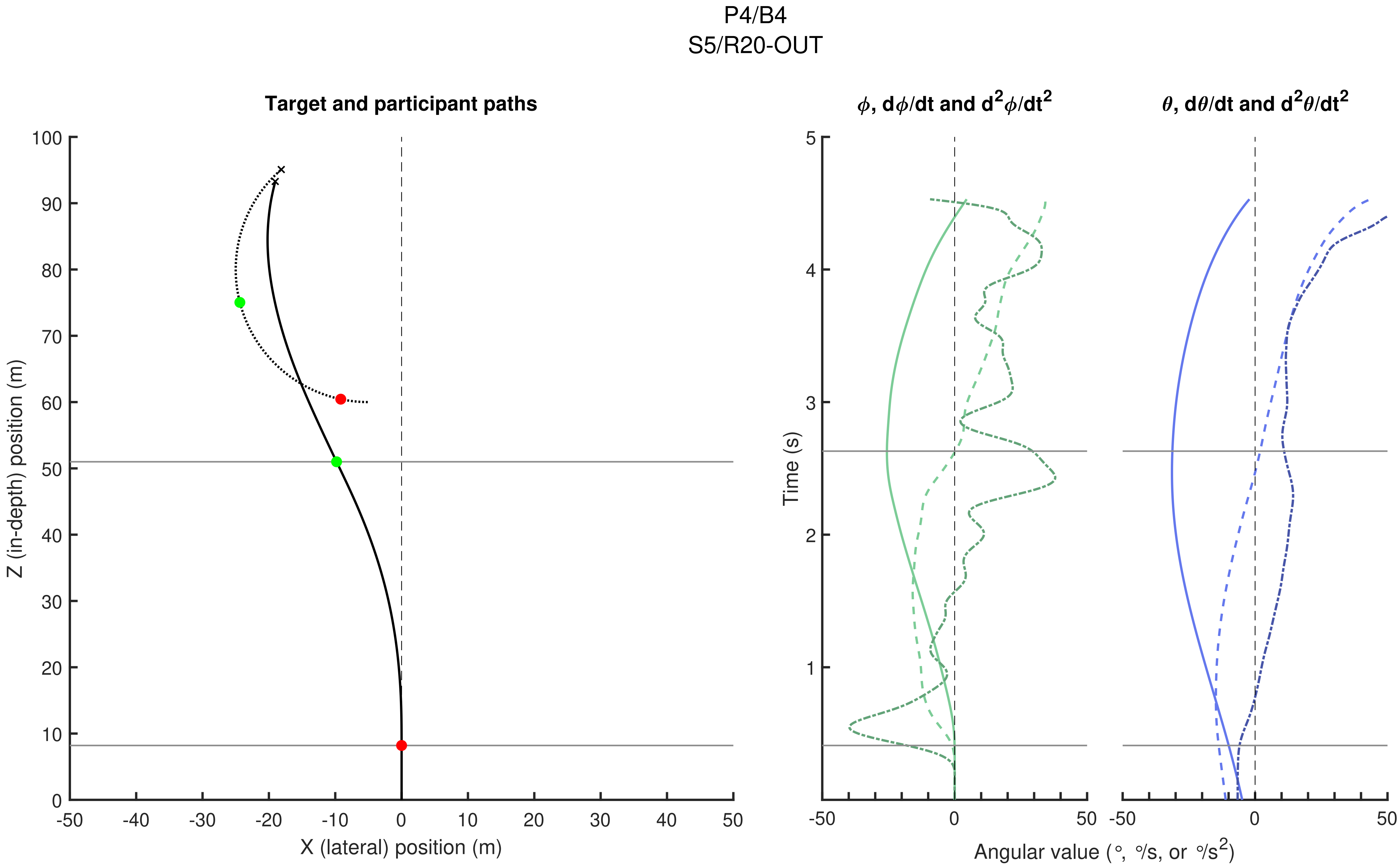

P4/B4
S5/R20-OUT
Target and participant paths
Z (in-depth) position (m)
X (lateral) position (m)
ϕ, dϕ/dt and d$^2\phi$/dt^2
θ, dθ/dt and d$^2\theta$/dt^2
Time (s)
Angular value (°, °/s, or °/s^2)

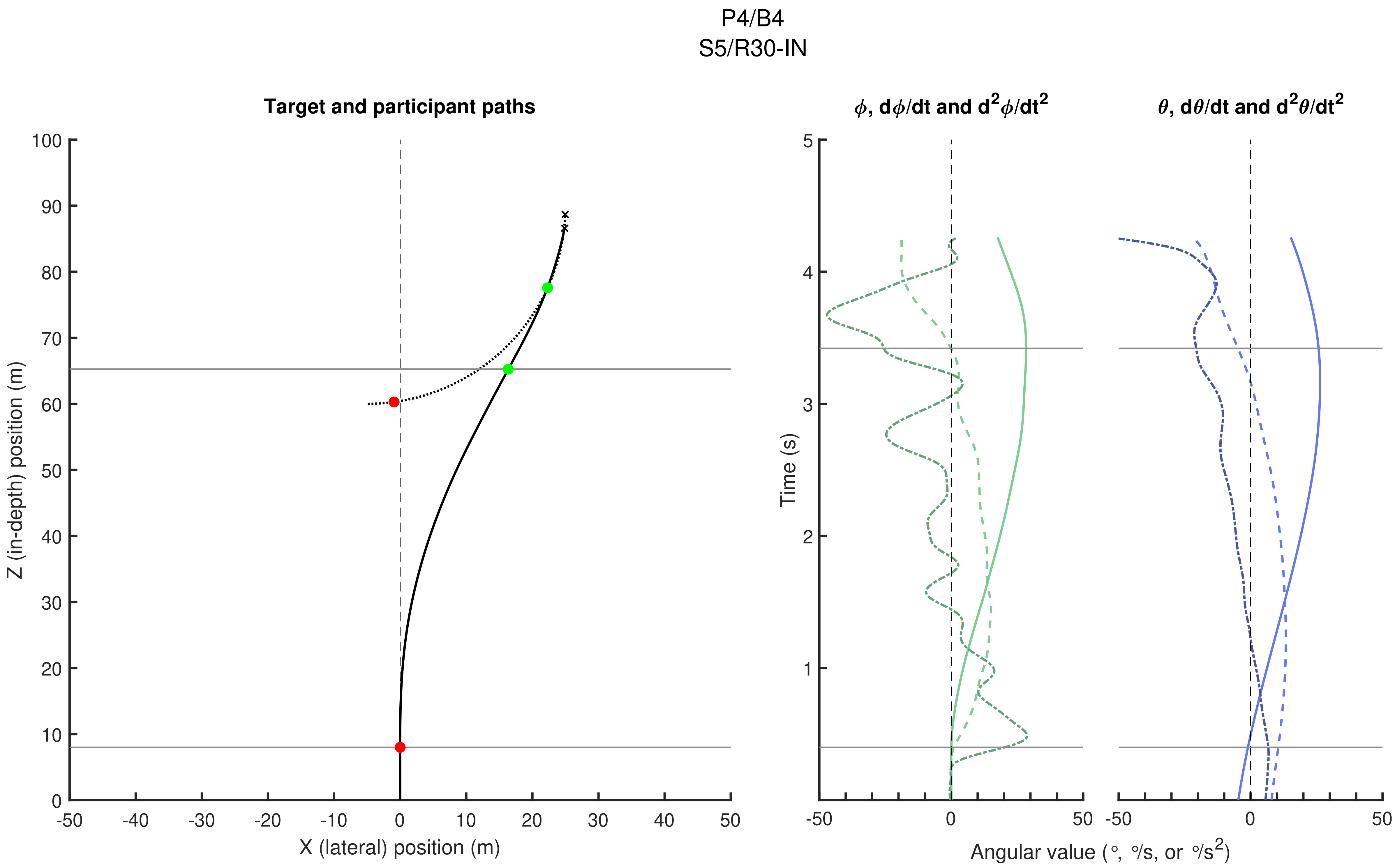
P4/B4
S5/R30-IN
Target and participant paths
ϕ, dϕ/dt and d$^2\phi$/dt^2
θ, dθ/dt and d$^2\theta$/dt^2
Z (in-depth) position (m)
X (lateral) position (m)
Time (s)
Angular value (°, °/s, or °/s^2)
100
90
80
70
60
50
40
30
20
10
0
-50
-40
-30
-20
-10
0
10
20
30
40
50
5
4
3
2
1
-50
0
50
-50
0
50

P4/B4
S5/R30-OUT

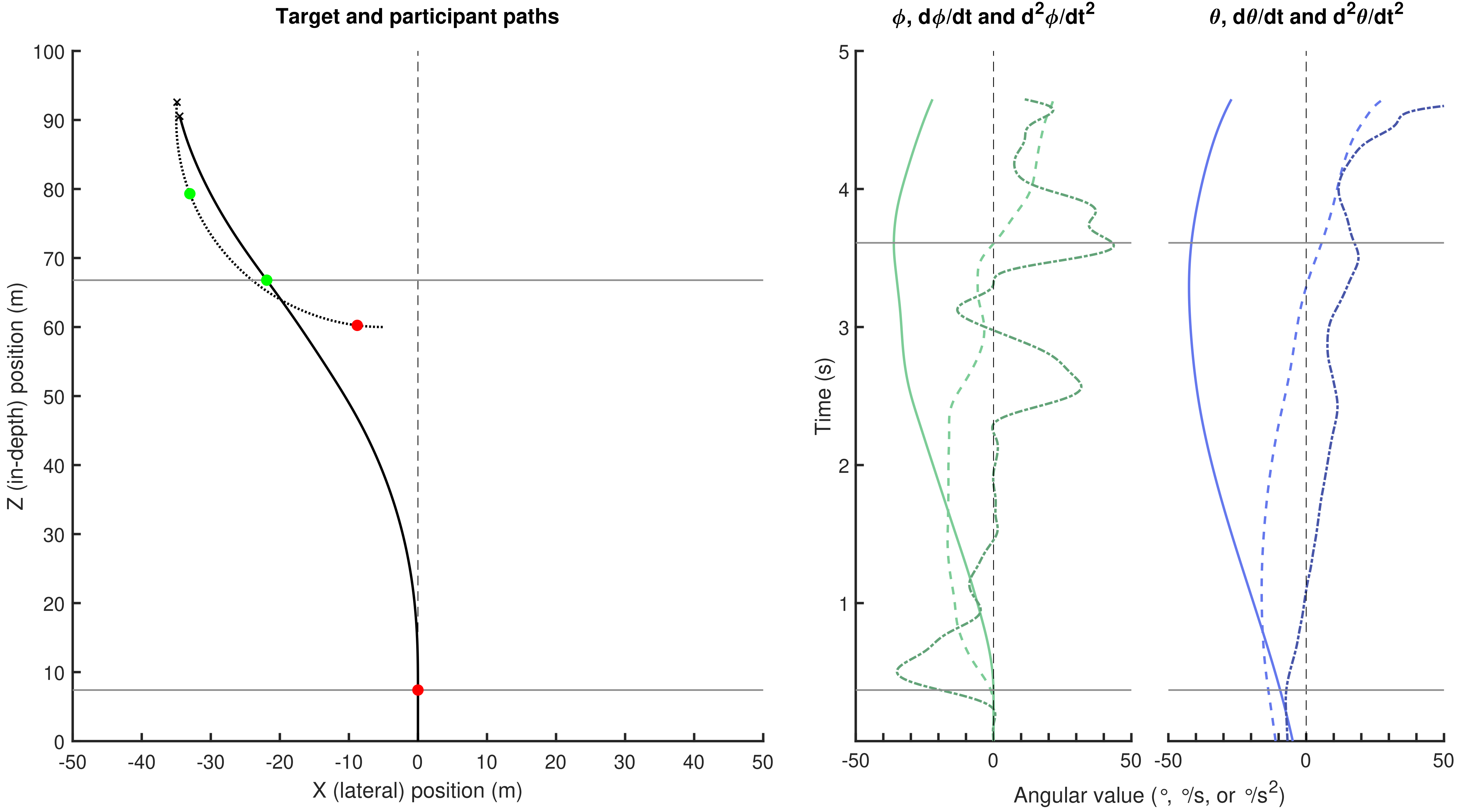

P4/B4
S5/R10-OUT

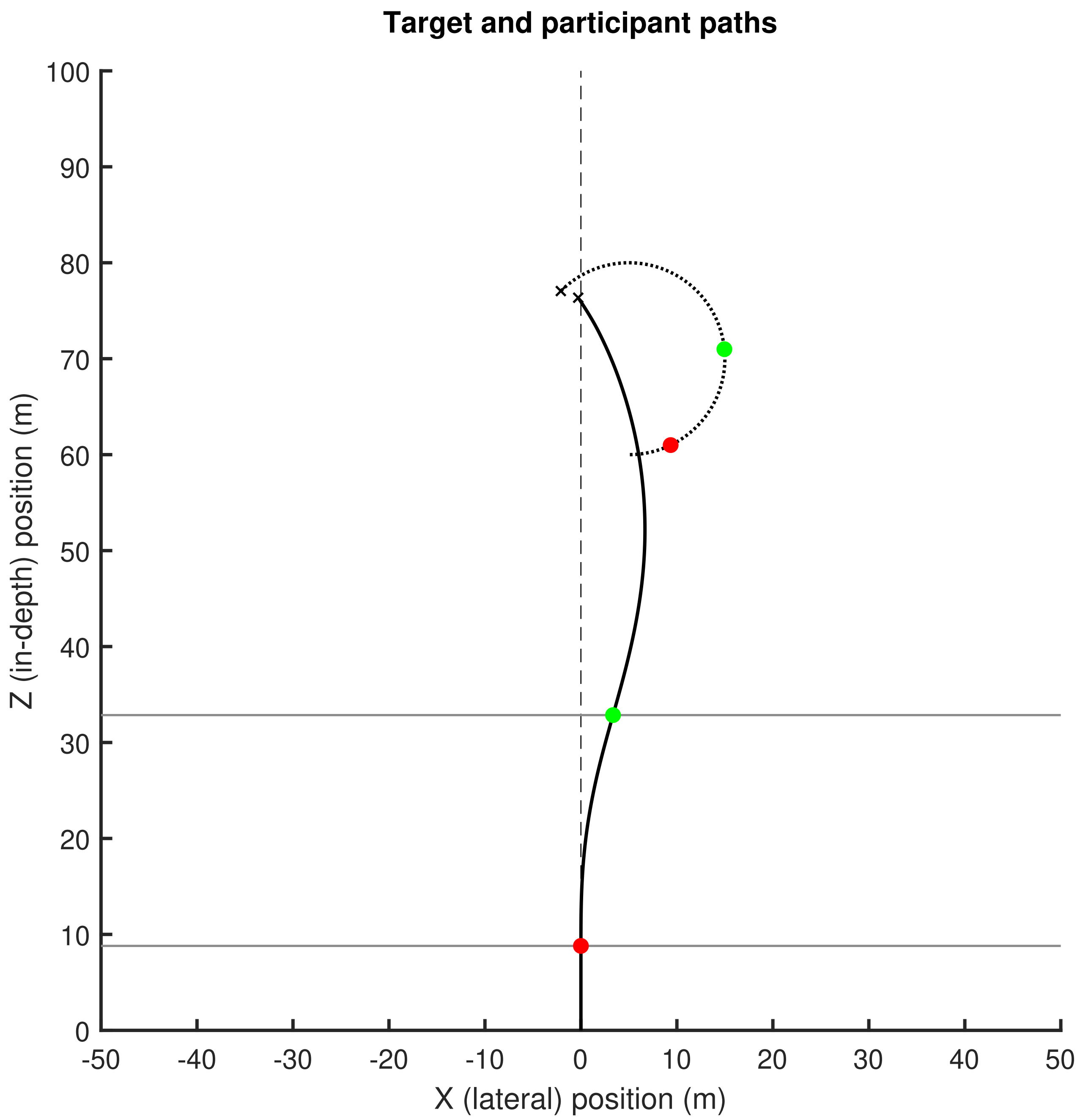

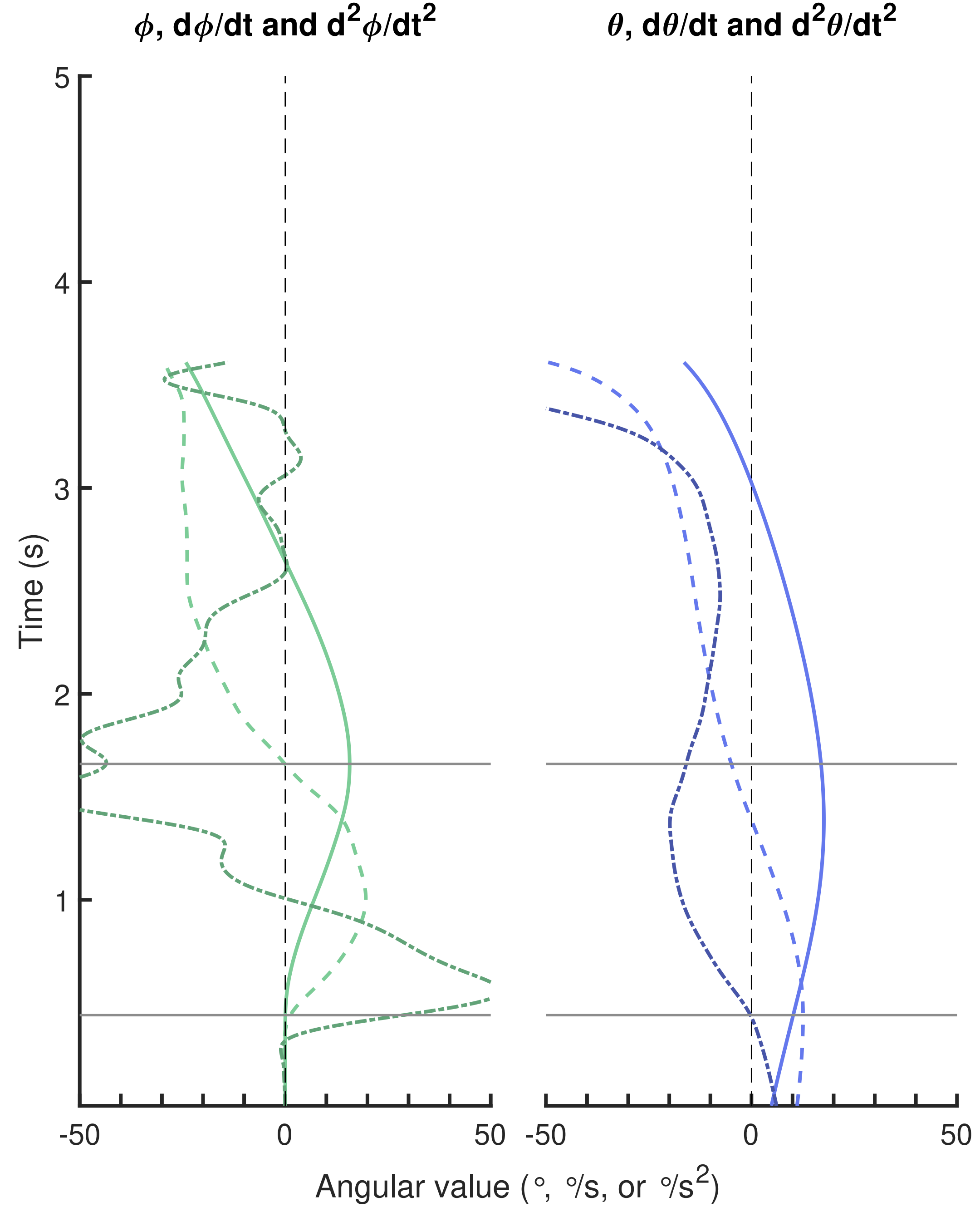

P4/B4
S5/R10-IN

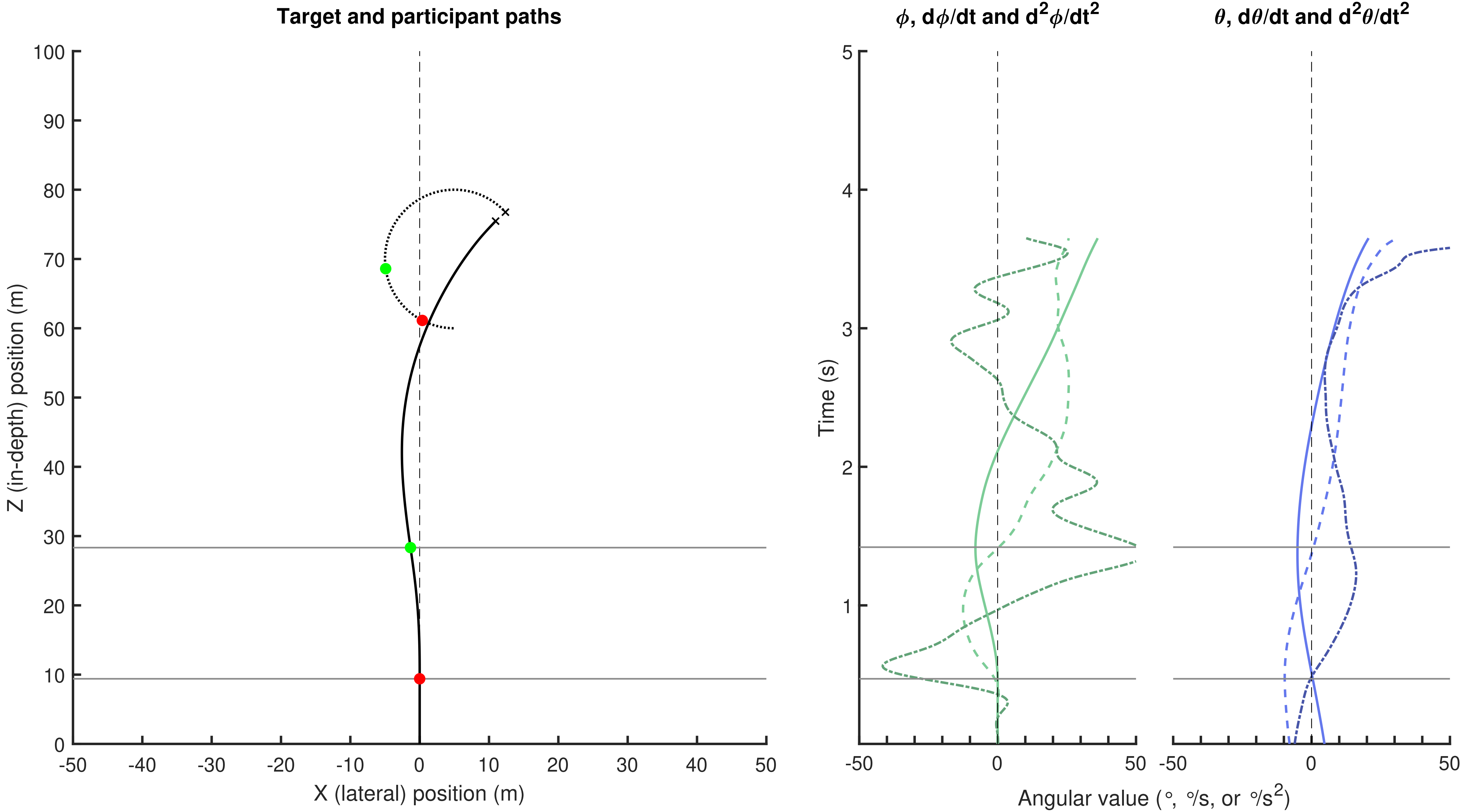

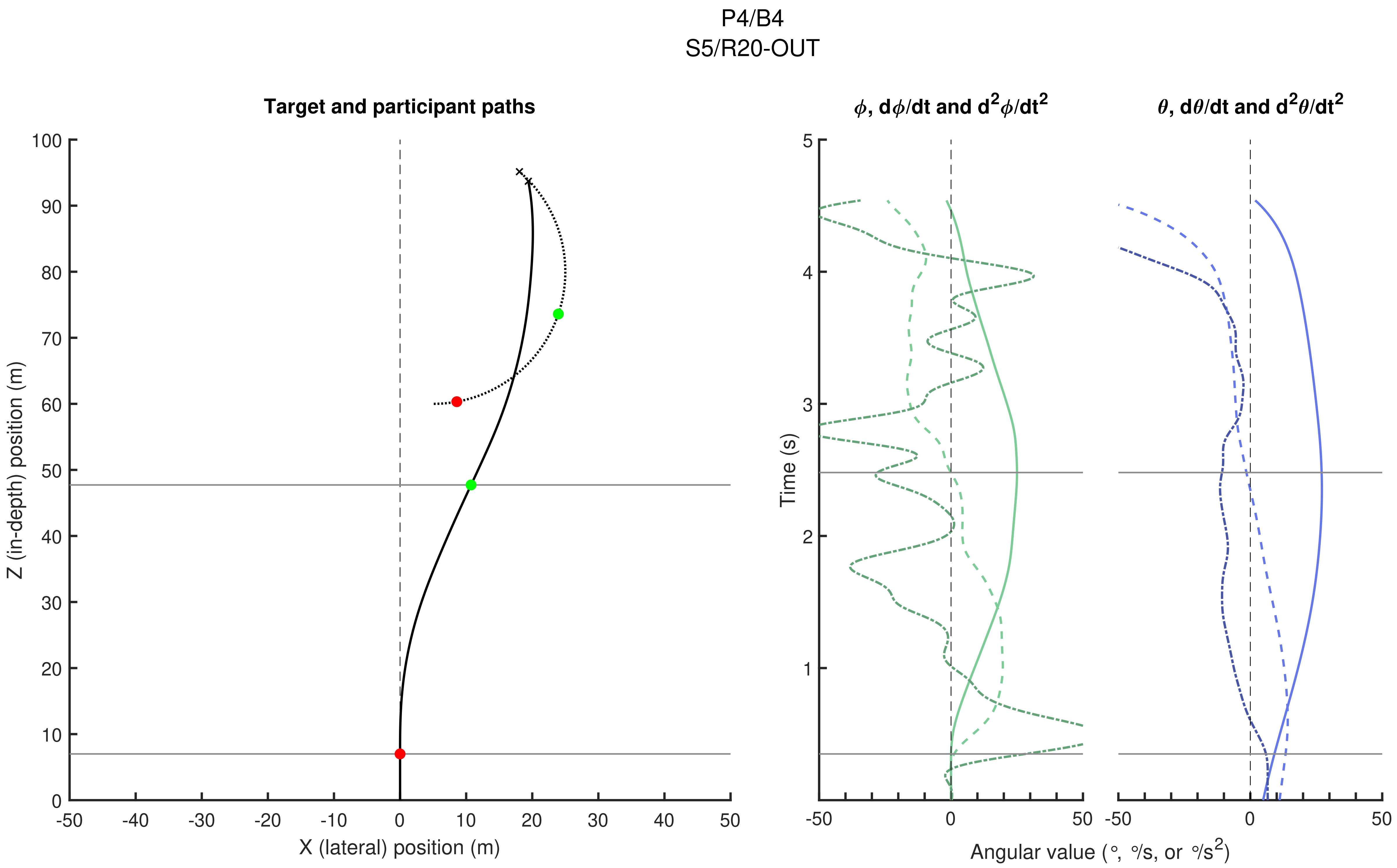
P4/B4
S5/R20-OUT
Target and participant paths
Z (in-depth) position (m)
X (lateral) position (m)
-50 -40 -30 -20 -10 0 10 20 30 40 50
0 10 20 30 40 50 60 70 80 90 100
ϕ, dϕ/dt and d$^2\phi$/dt^2
θ, dθ/dt and d$^2\theta$/dt^2
Time (s)
1 2 3 4 5
-50 0 50
Angular value (°, °/s, or °/s^2)

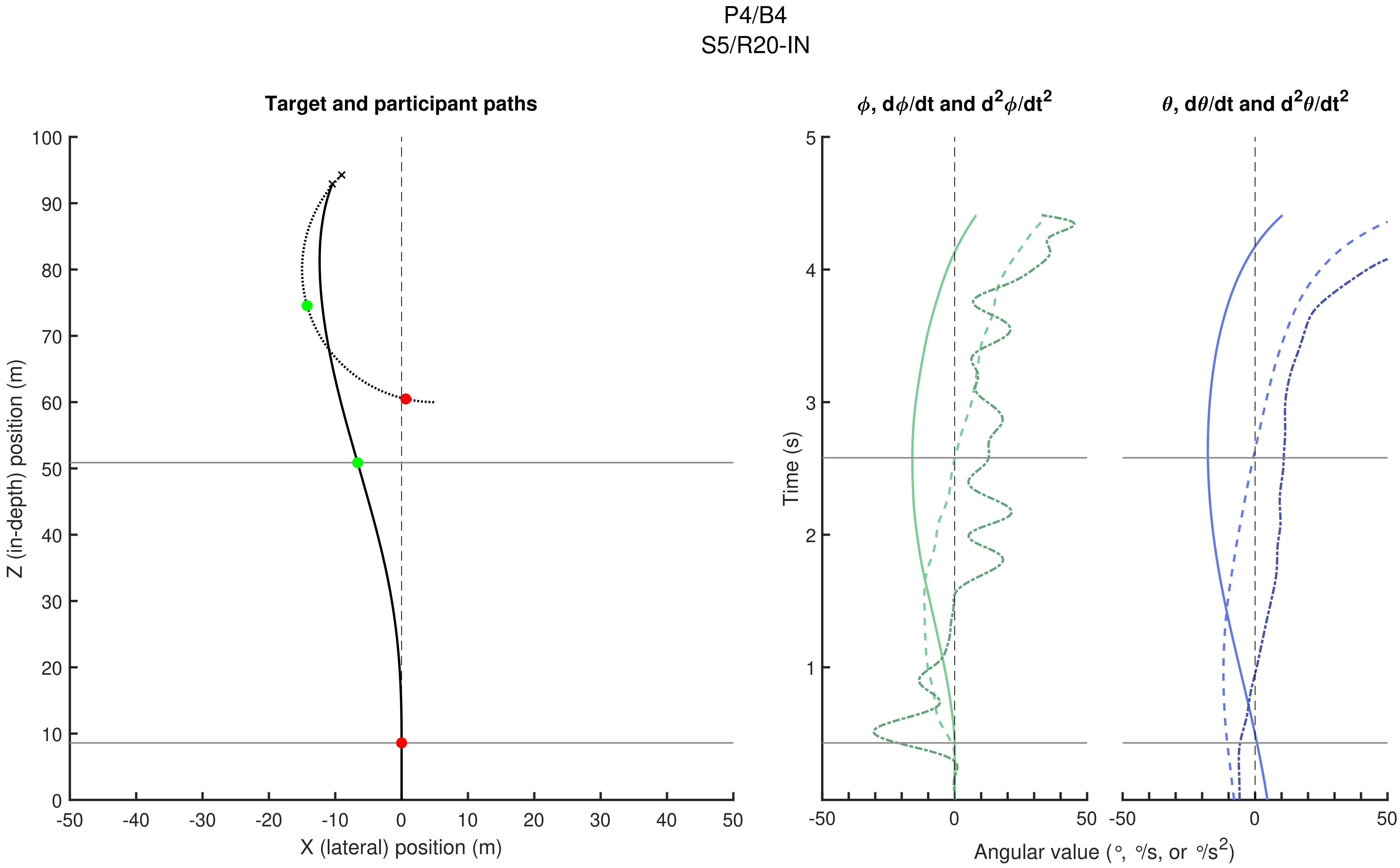

P4/B4
S5/R20-IN
Target and participant paths
Z (in-depth) position (m)
X (lateral) position (m)
ϕ, dϕ/dt and d$^2\phi$/dt^2
θ, dθ/dt and d$^2\theta$/dt^2
Time (s)
Angular value (°, °/s, or °/s^2)

P4/B4
S5/R30-OUT

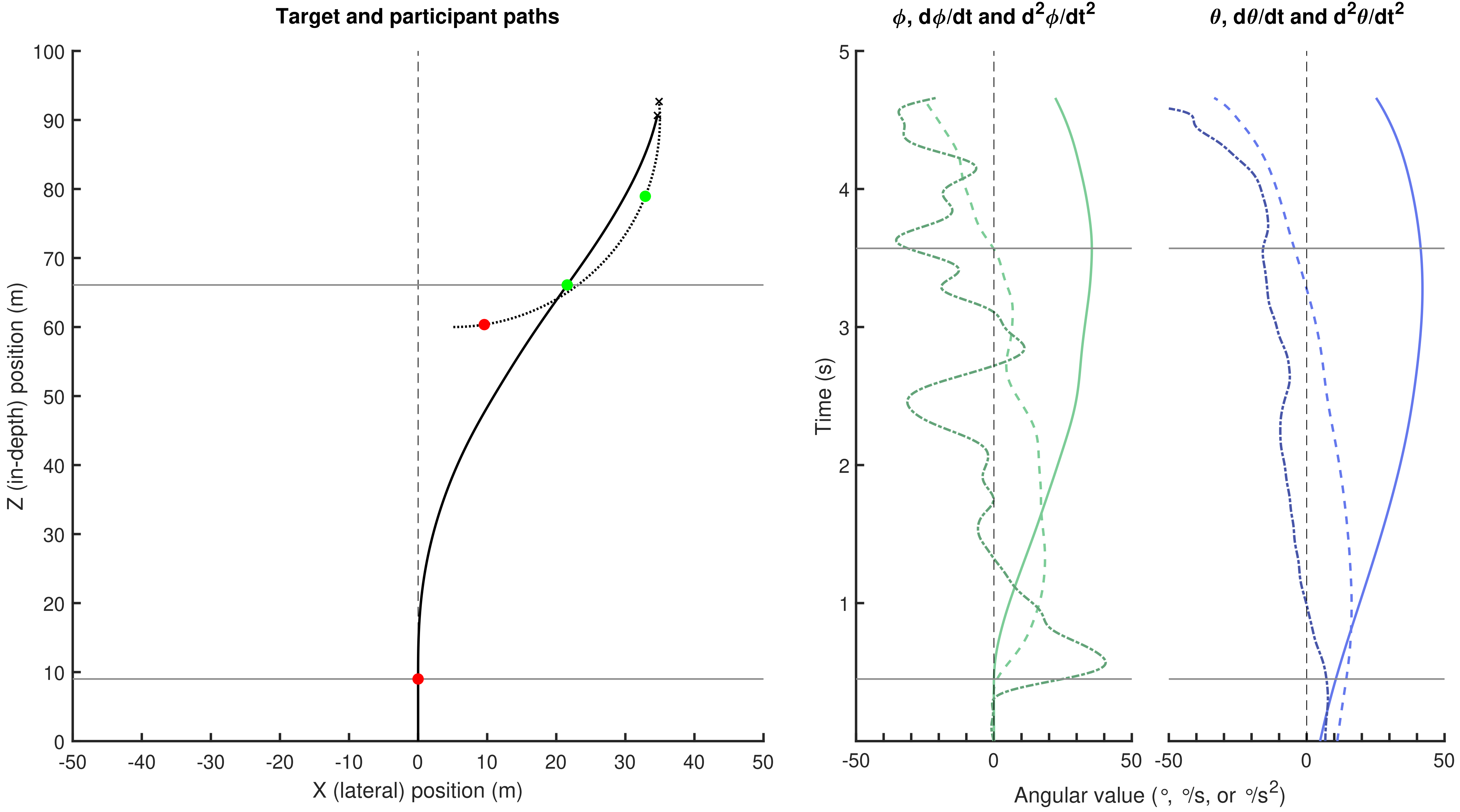

P4/B4
S5/R30-IN

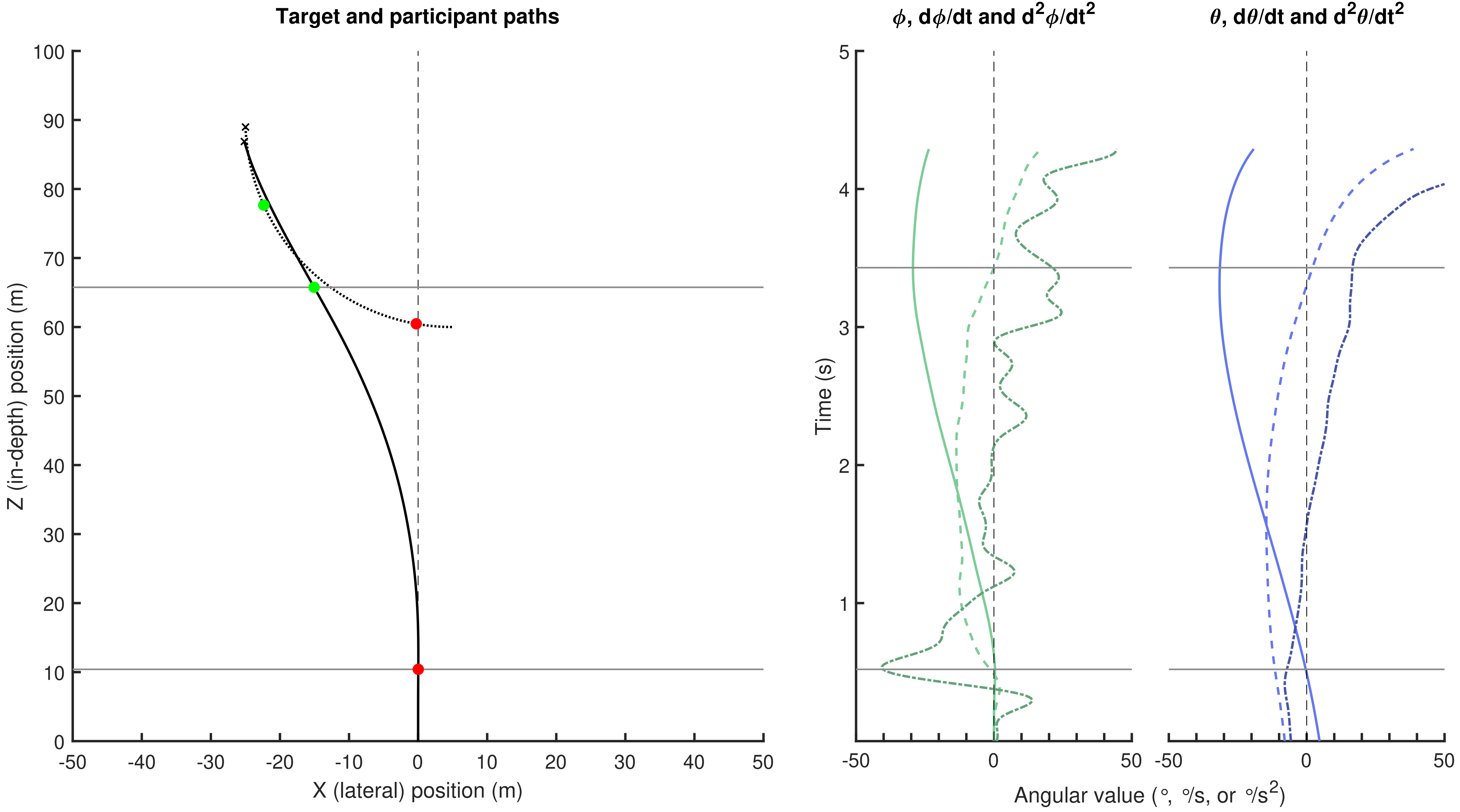

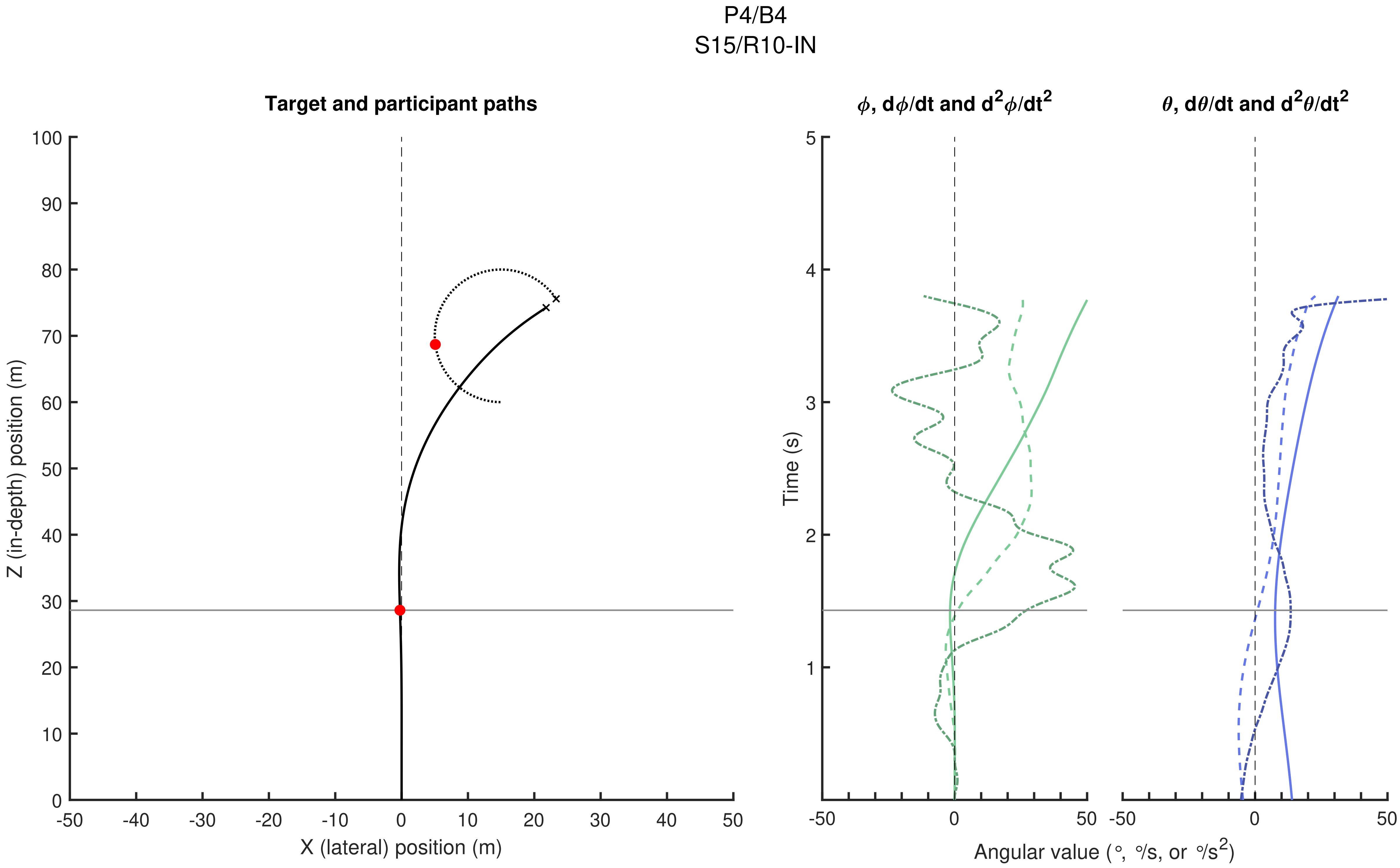

P4/B4
S15/R10-IN
Target and participant paths
ϕ, dϕ/dt and d$^2\phi$/dt^2
θ, dθ/dt and d$^2\theta$/dt^2
100
90
80
70
60
50
40
30
20
10
0
-50
-40
-30
-20
-10
0
10
20
30
40
50
Z (in-depth) position (m)
X (lateral) position (m)
5
4
3
2
1
Time (s)
-50
0
50
-50
0
50
Angular value (°, °/s, or °/s^2)

P4/B4
S15/R20-IN

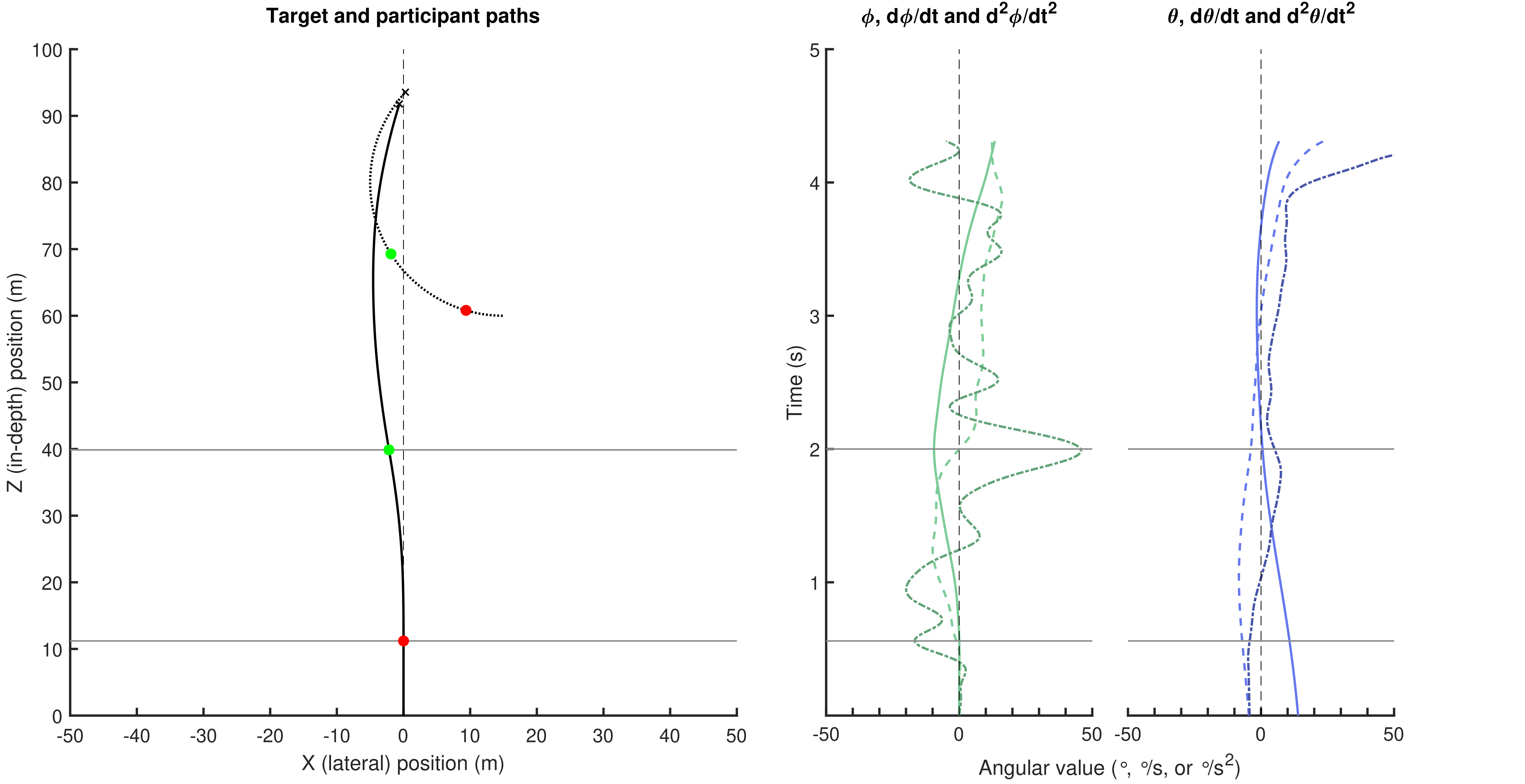

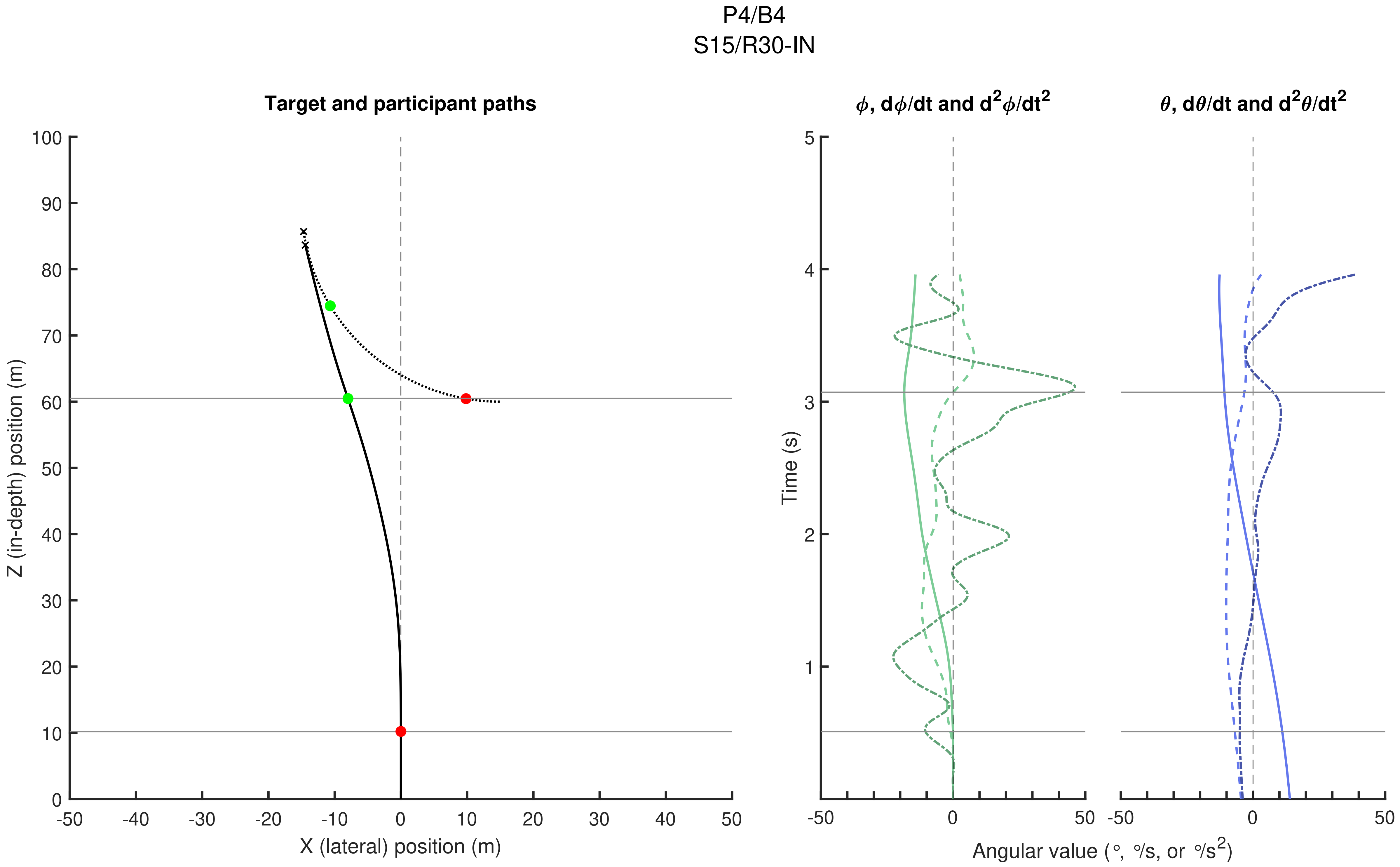
P4/B4
S15/R30-IN
Target and participant paths
Z (in-depth) position (m)
X (lateral) position (m)
0
10
20
30
40
50
60
70
80
90
100
-50
-40
-30
-20
-10
0
10
20
30
40
50
ϕ, dϕ/dt and d²ϕ/dt²
θ, dθ/dt and d²θ/dt²
Time (s)
1
2
3
4
5
-50
0
50
-50
0
50
Angular value (°, °/s, or °/s²)

P4/B4
S20/R10-IN

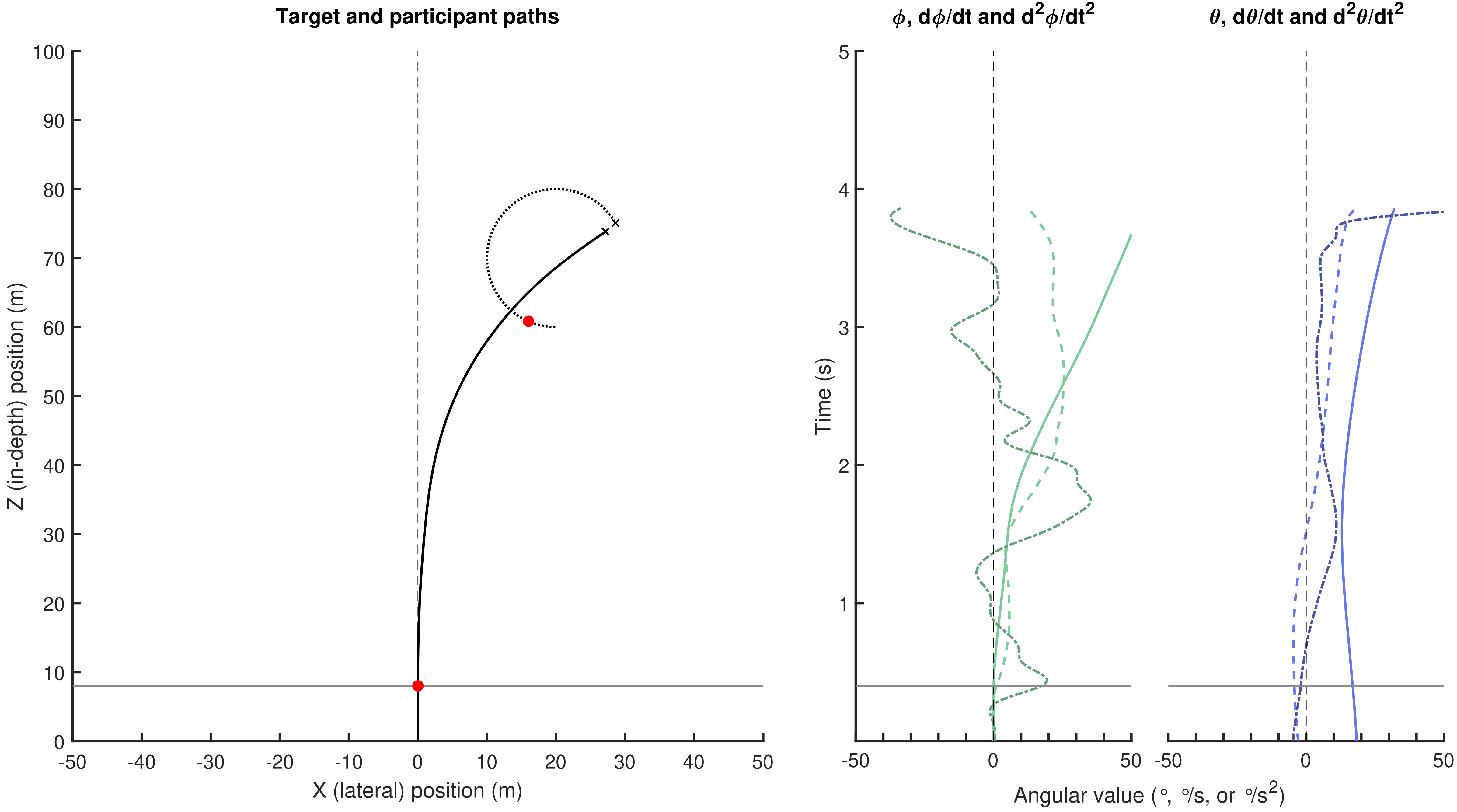

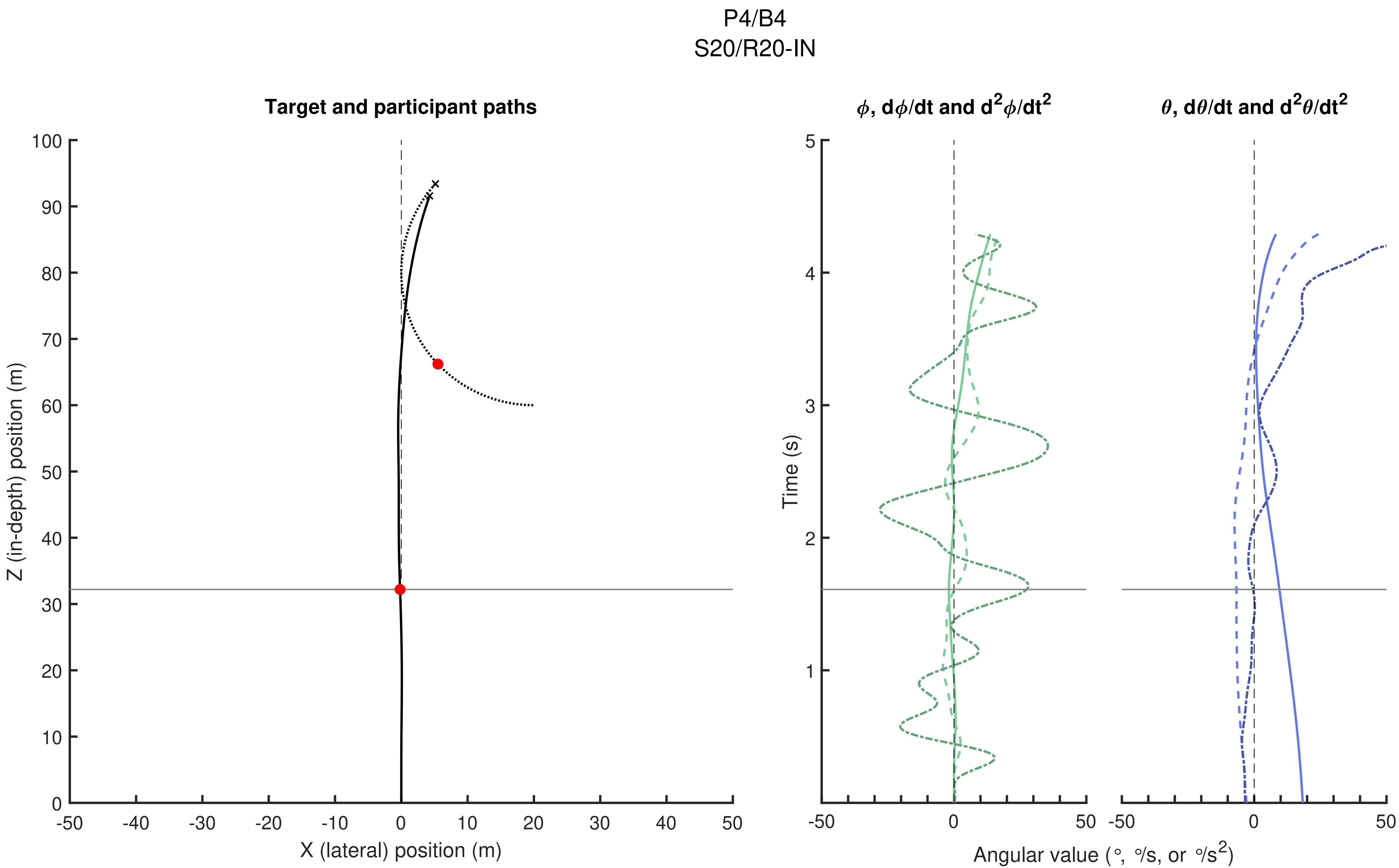

P4/B4
S20/R20-IN
Target and participant paths
Z (in-depth) position (m)
X (lateral) position (m)
φ, dφ/dt and d²φ/dt²
θ, dθ/dt and d²θ/dt²
Time (s)
Angular value (°, °/s, or °/s²)

P4/B4
S20/R30-IN

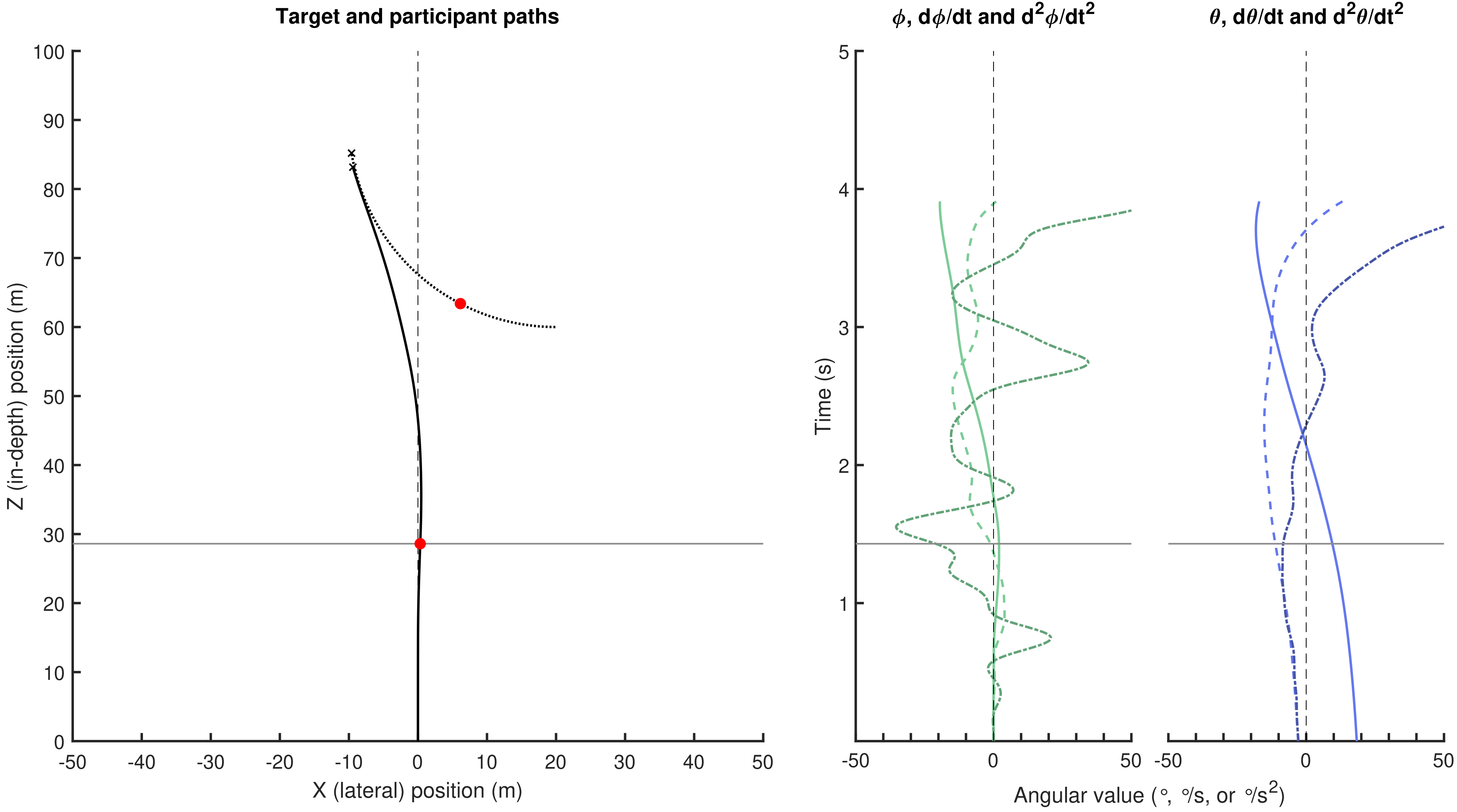

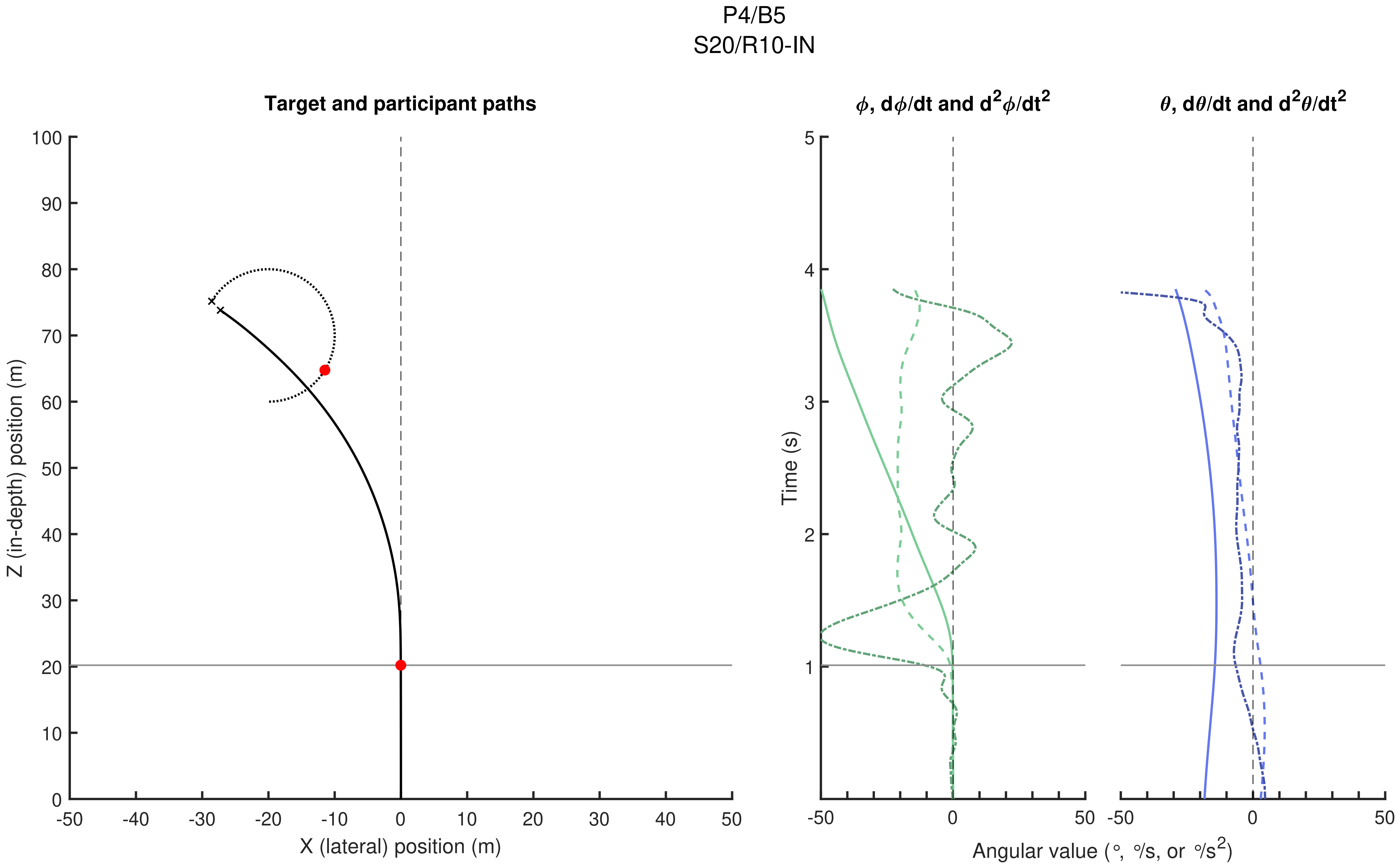
P4/B5
S20/R10-IN
Target and participant paths
Z (in-depth) position (m)
X (lateral) position (m)
100
90
80
70
60
50
40
30
20
10
0
-50
-40
-30
-20
-10
0
10
20
30
40
50
ϕ, dϕ/dt and d²ϕ/dt²
θ, dθ/dt and d²θ/dt²
Time (s)
5
4
3
2
1
-50
0
50
Angular value (°, °/s, or °/s²)

P4/B5
S20/R20-IN

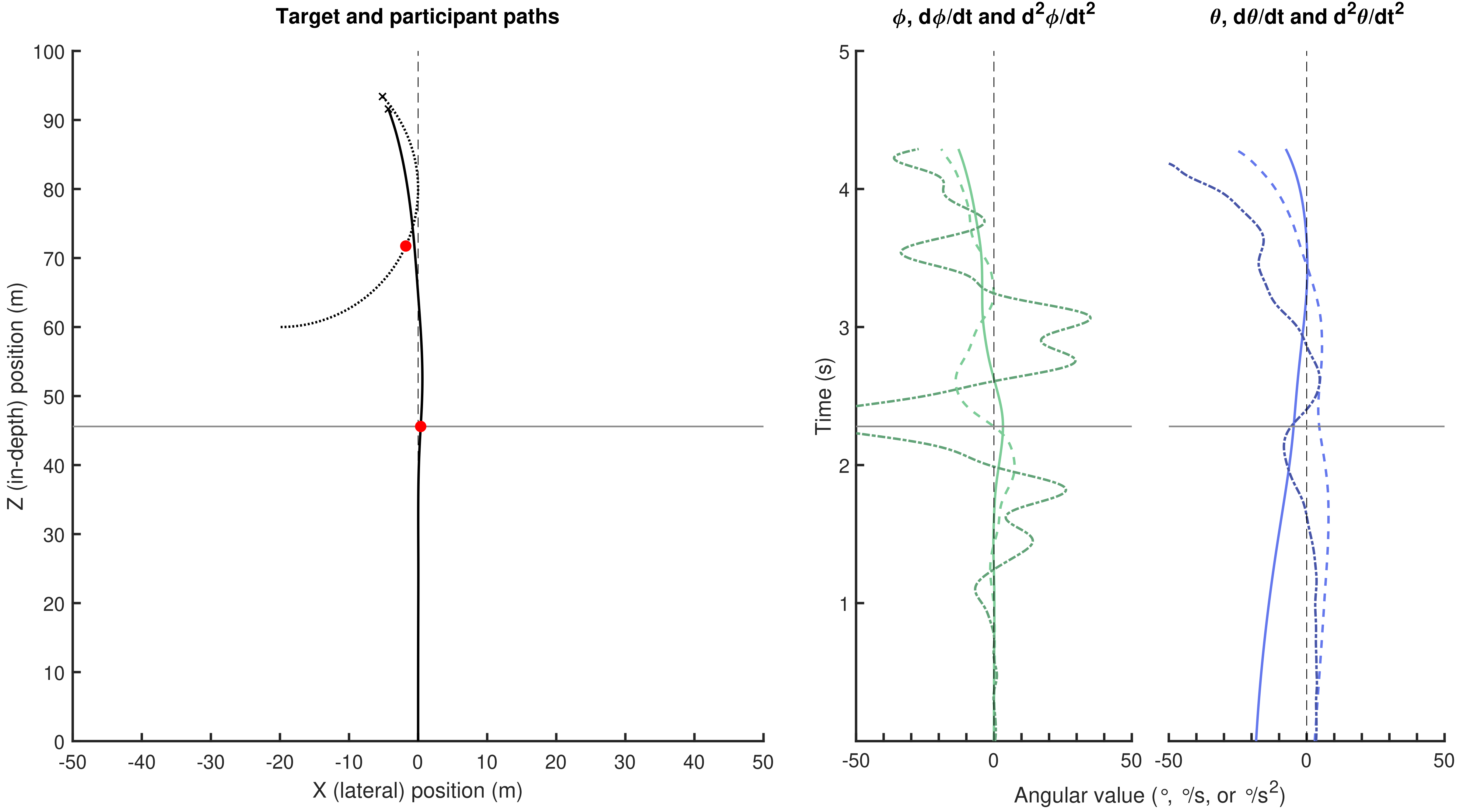

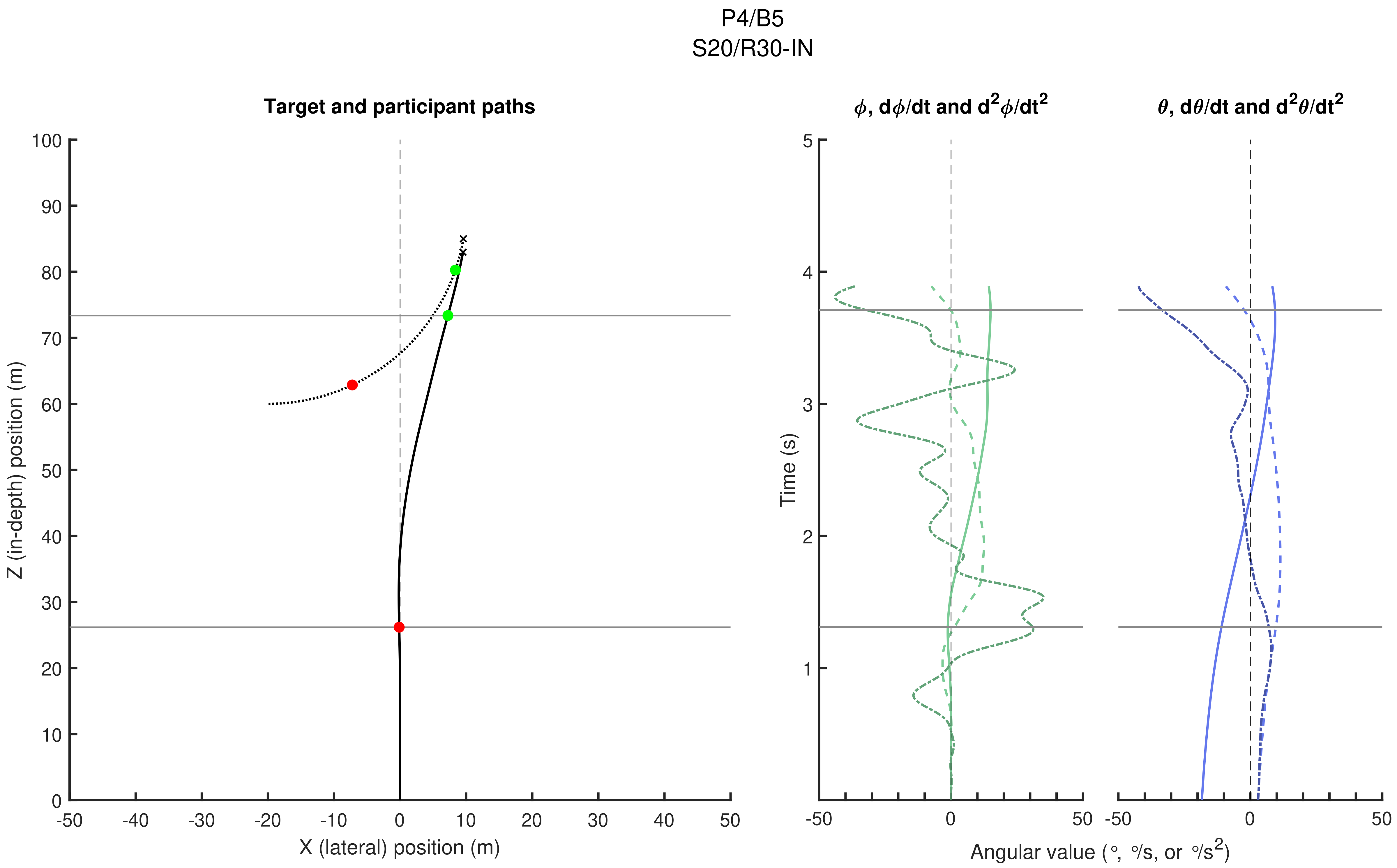
P4/B5
S20/R30-IN
Target and participant paths
Z (in-depth) position (m)
X (lateral) position (m)
ϕ, dϕ/dt and d$^2\phi$/dt^2
θ, dθ/dt and d$^2\theta$/dt^2
Time (s)
Angular value (°, °/s, or °/s^2)

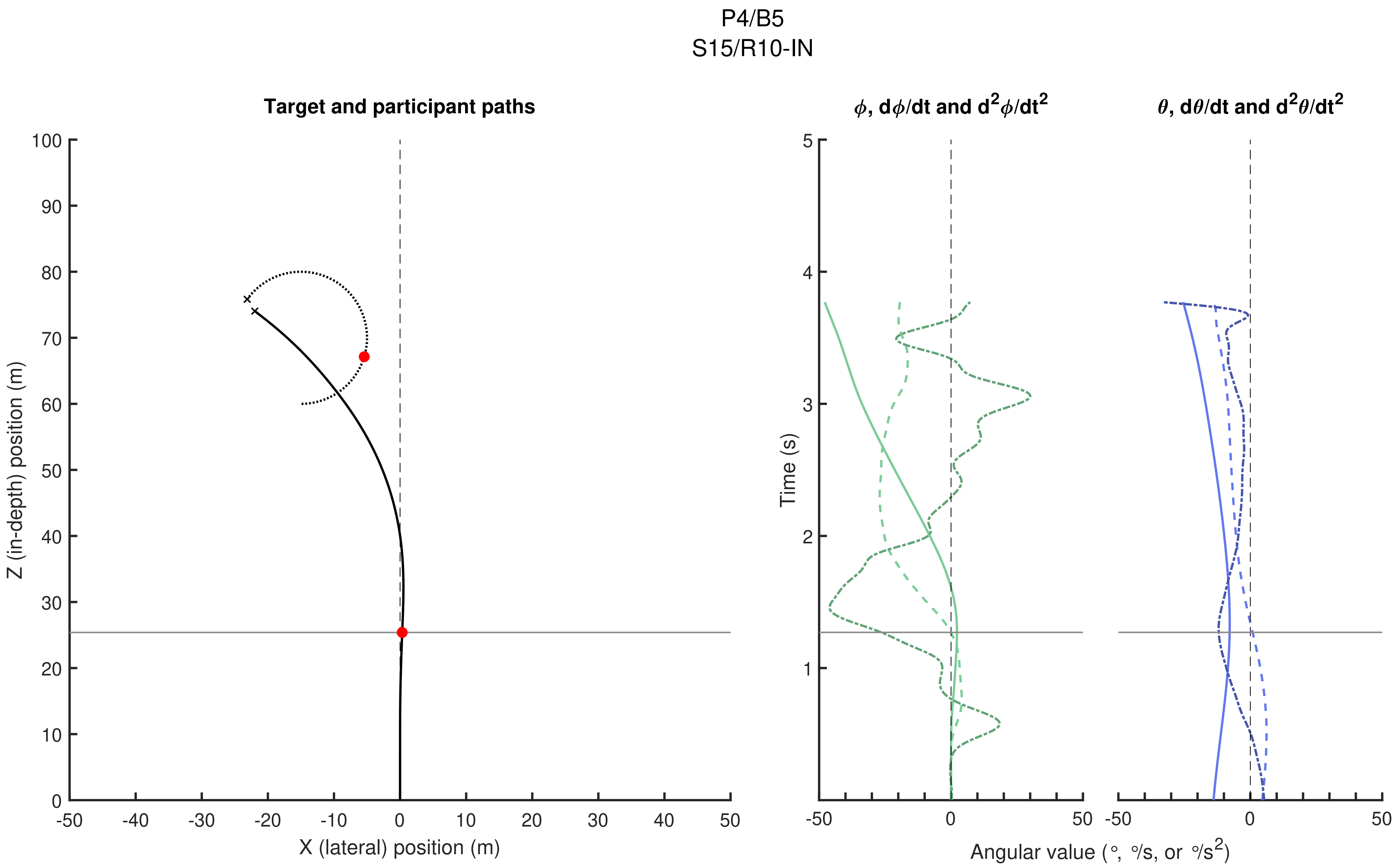

P4/B5
S15/R10-IN
Target and participant paths
Z (in-depth) position (m)
X (lateral) position (m)
ϕ, dϕ/dt and d²ϕ/dt²
θ, dθ/dt and d²θ/dt²
Time (s)
Angular value (°, °/s, or °/s²)

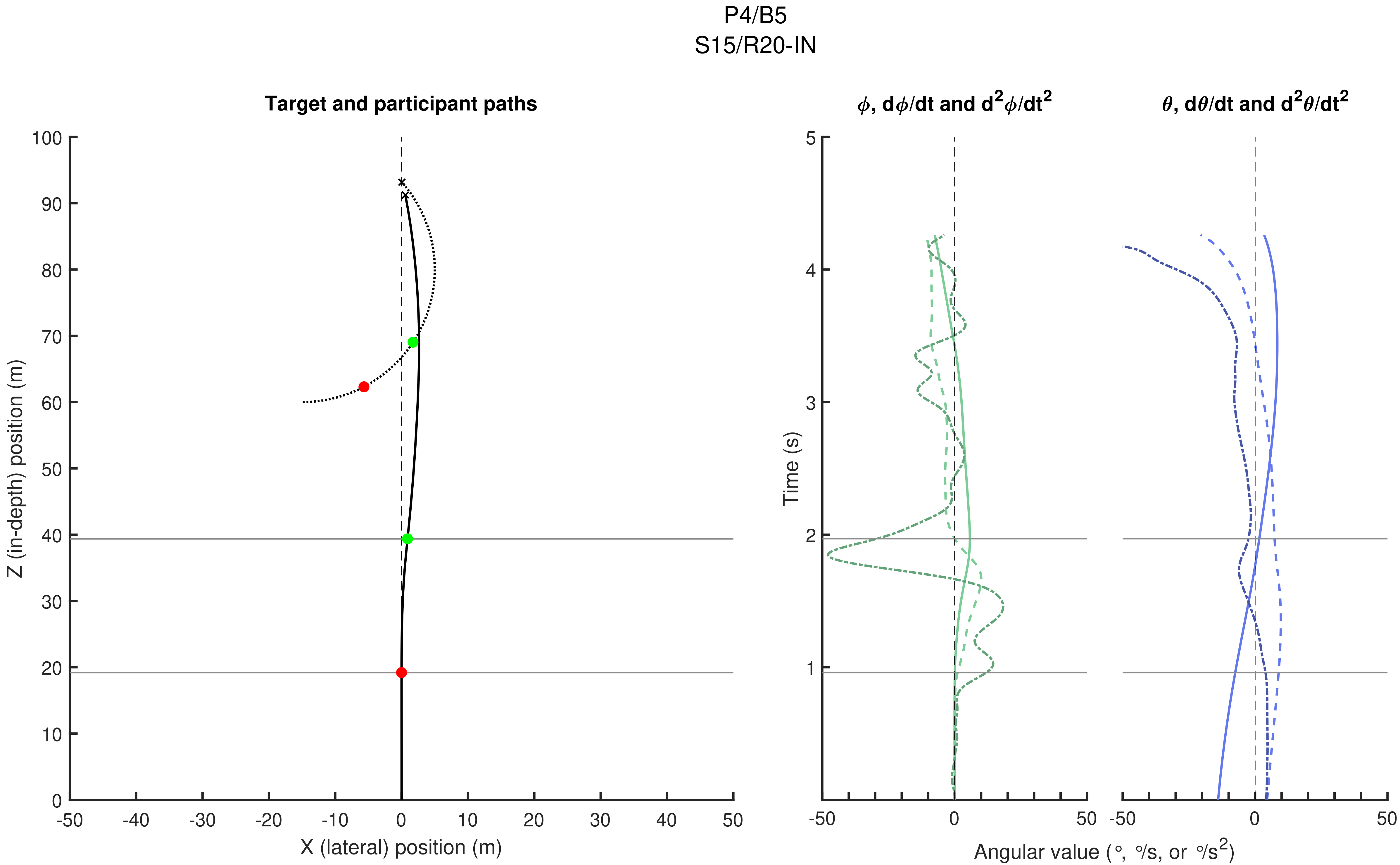
P4/B5
S15/R20-IN
Target and participant paths
Z (in-depth) position (m)
X (lateral) position (m)
ϕ, dϕ/dt and d²ϕ/dt²
θ, dθ/dt and d²θ/dt²
Time (s)
Angular value (°, °/s, or °/s²)
100
90
80
70
60
50
40
30
20
10
0
-50
-40
-30
-20
-10
0
10
20
30
40
50
5
4
3
2
1
-50
0
50
-50
0
50

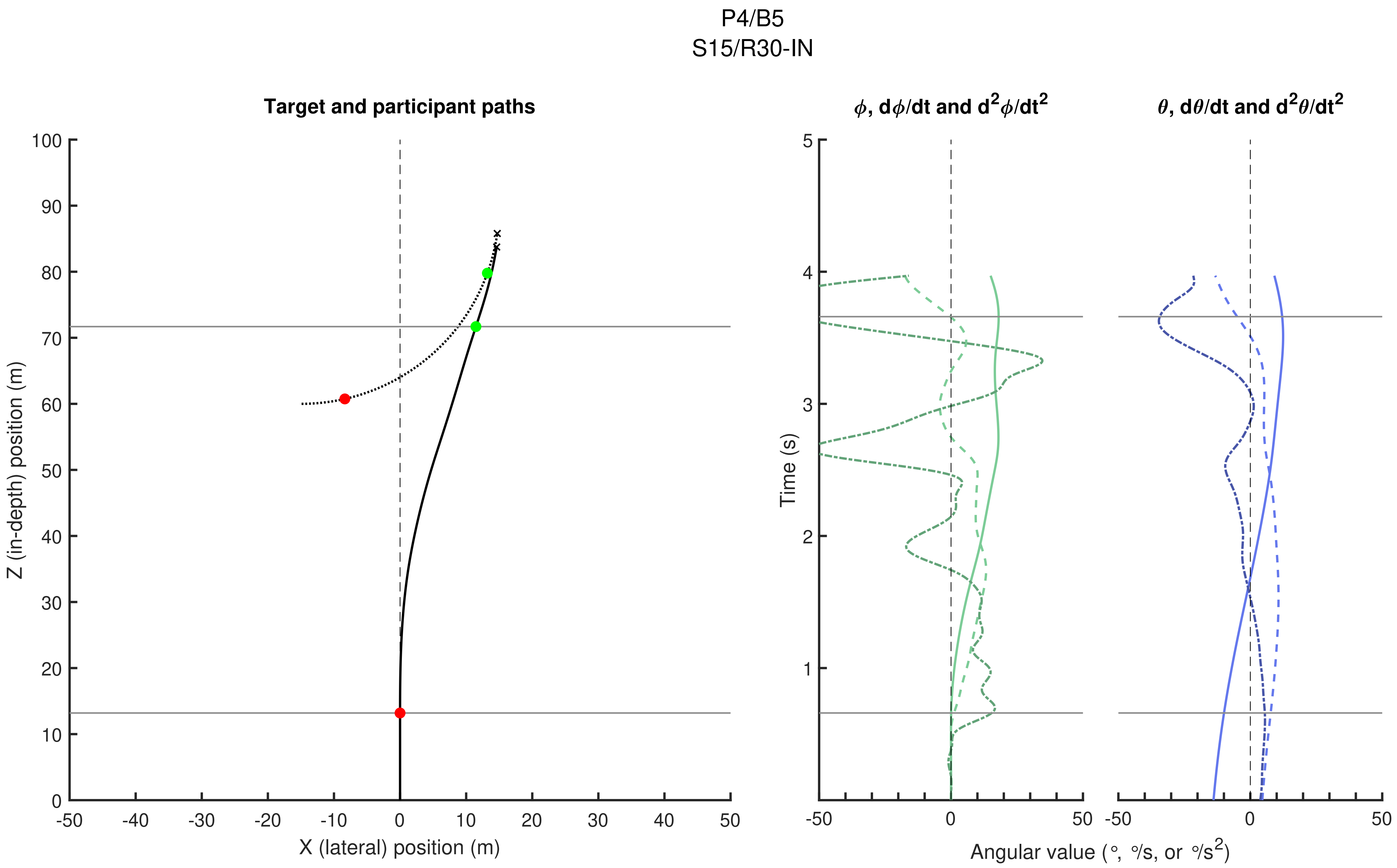

P4/B5
S15/R30-IN
Target and participant paths
Z (in-depth) position (m)
X (lateral) position (m)
ϕ, dϕ/dt and d$^2\phi$/dt^2
θ, dθ/dt and d$^2\theta$/dt^2
Time (s)
Angular value (°, °/s, or °/s^2)

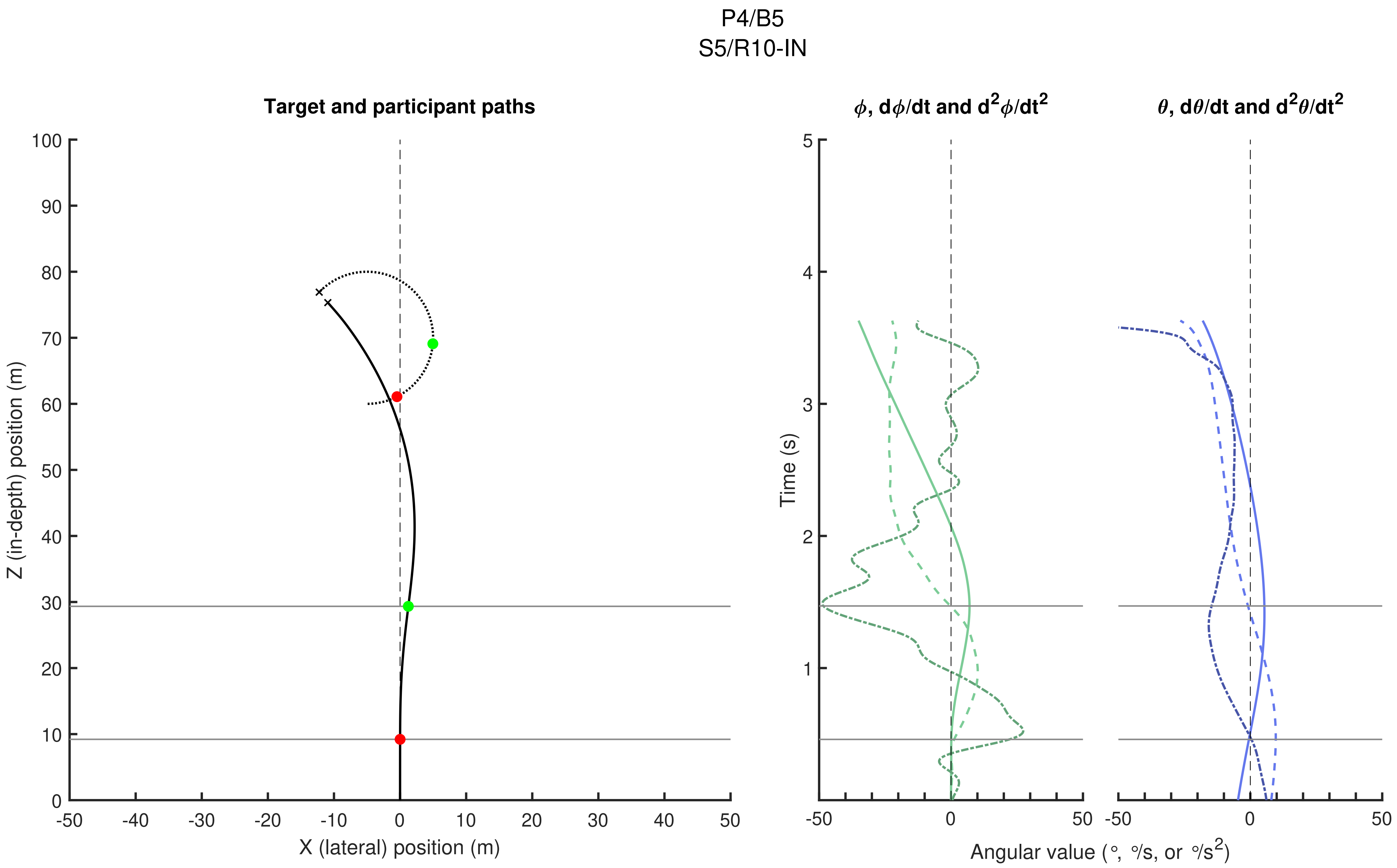
P4/B5
S5/R10-IN
Target and participant paths
ϕ, dϕ/dt and d²ϕ/dt²
θ, dθ/dt and d²θ/dt²
Z (in-depth) position (m)
X (lateral) position (m)
Time (s)
Angular value (°, °/s, or °/s²)

P4/B5
S5/R10-OUT

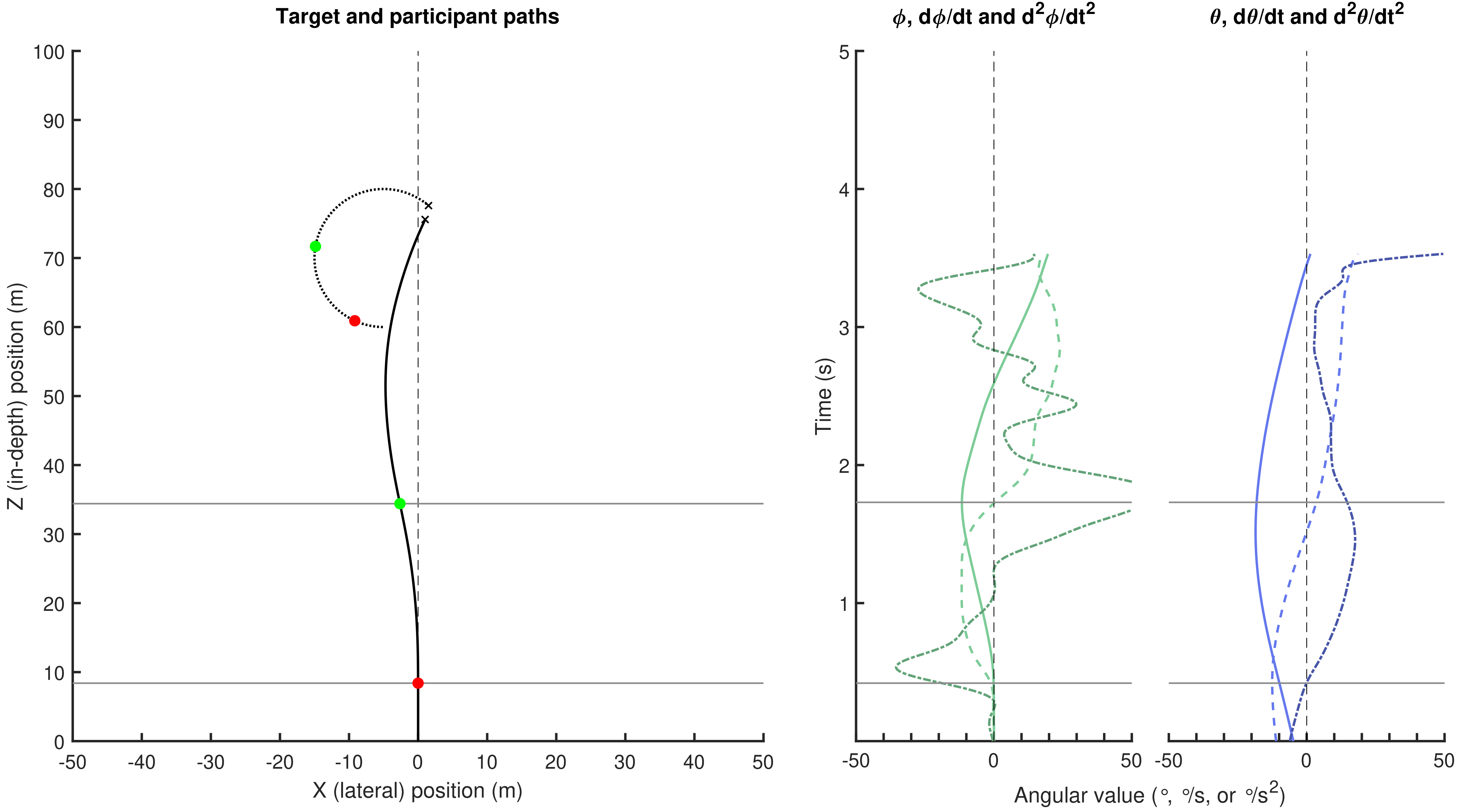

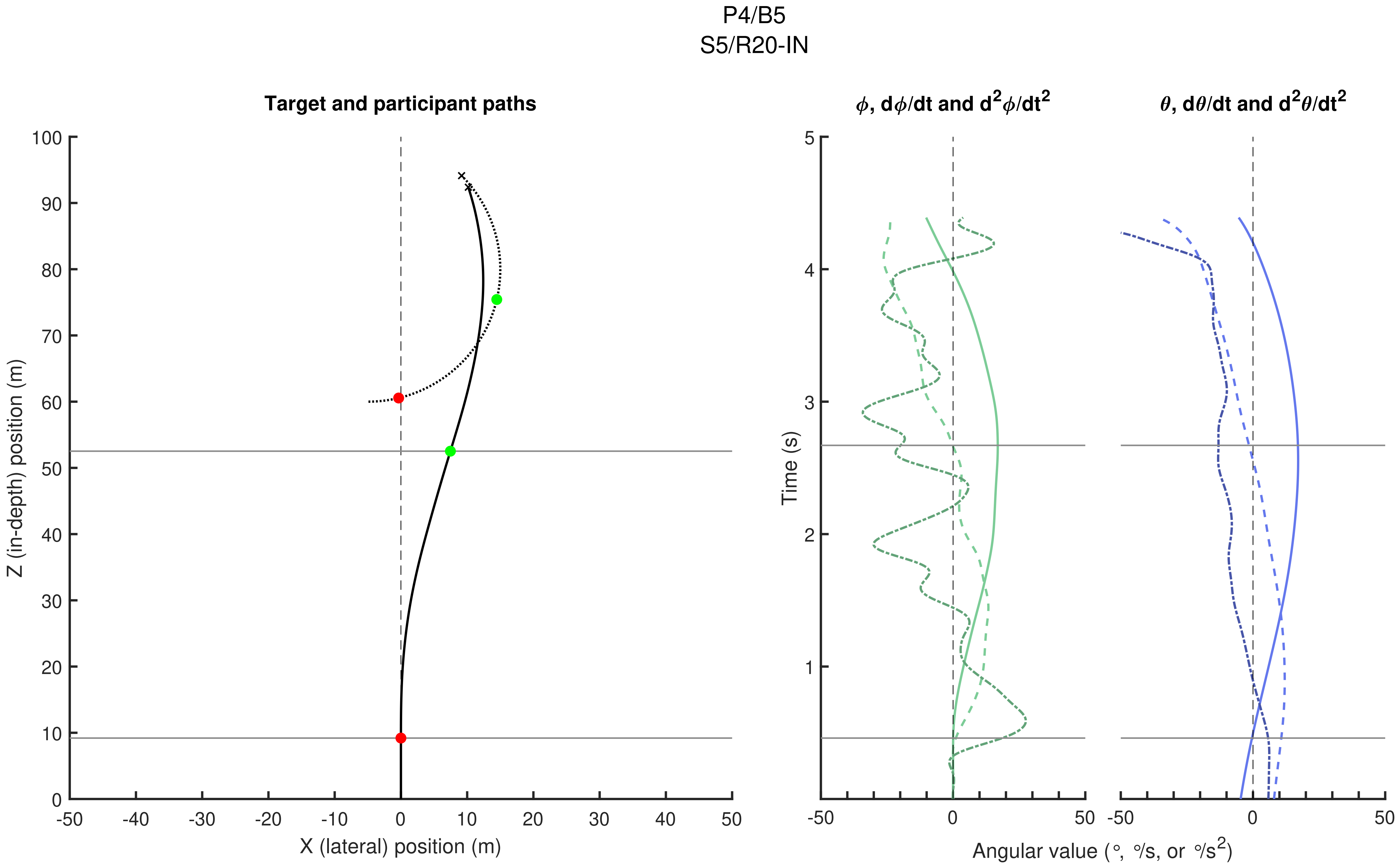
P4/B5
S5/R20-IN
Target and participant paths
Z (in-depth) position (m)
X (lateral) position (m)
ϕ, dϕ/dt and d²ϕ/dt²
θ, dθ/dt and d²θ/dt²
Time (s)
Angular value (°, °/s, or °/s²)

P4/B5
S5/R20-OUT

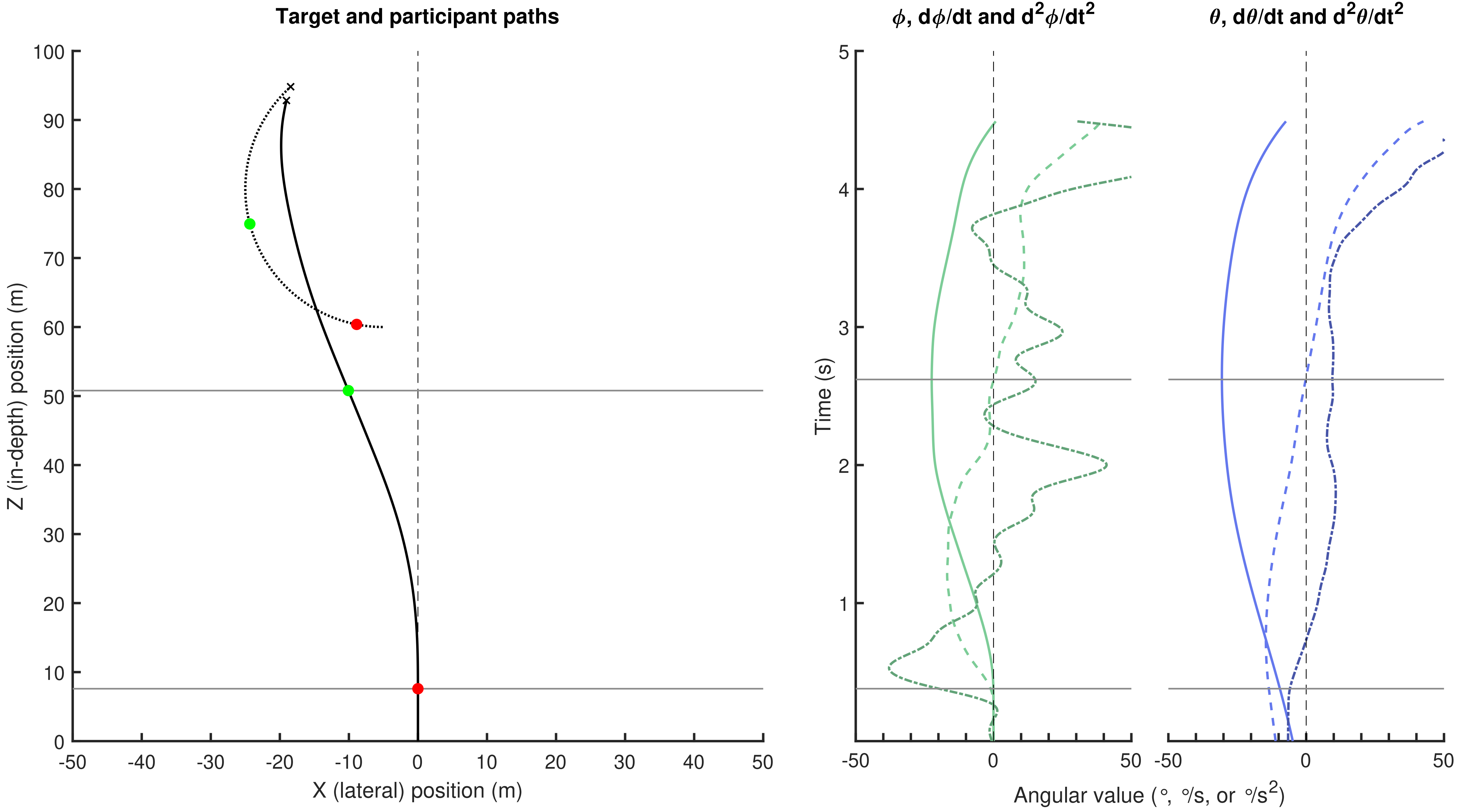

P4/B5
S5/R30-IN

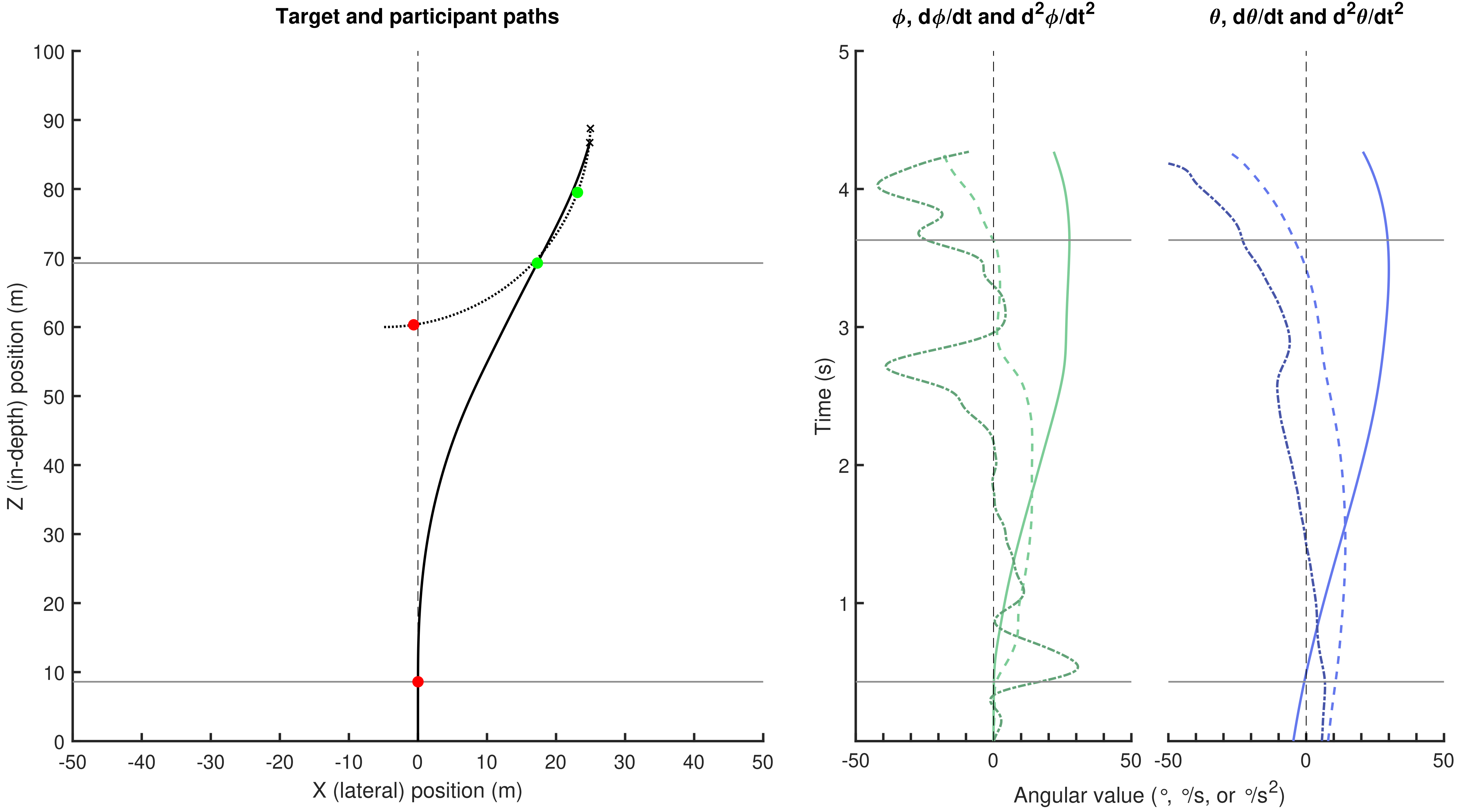

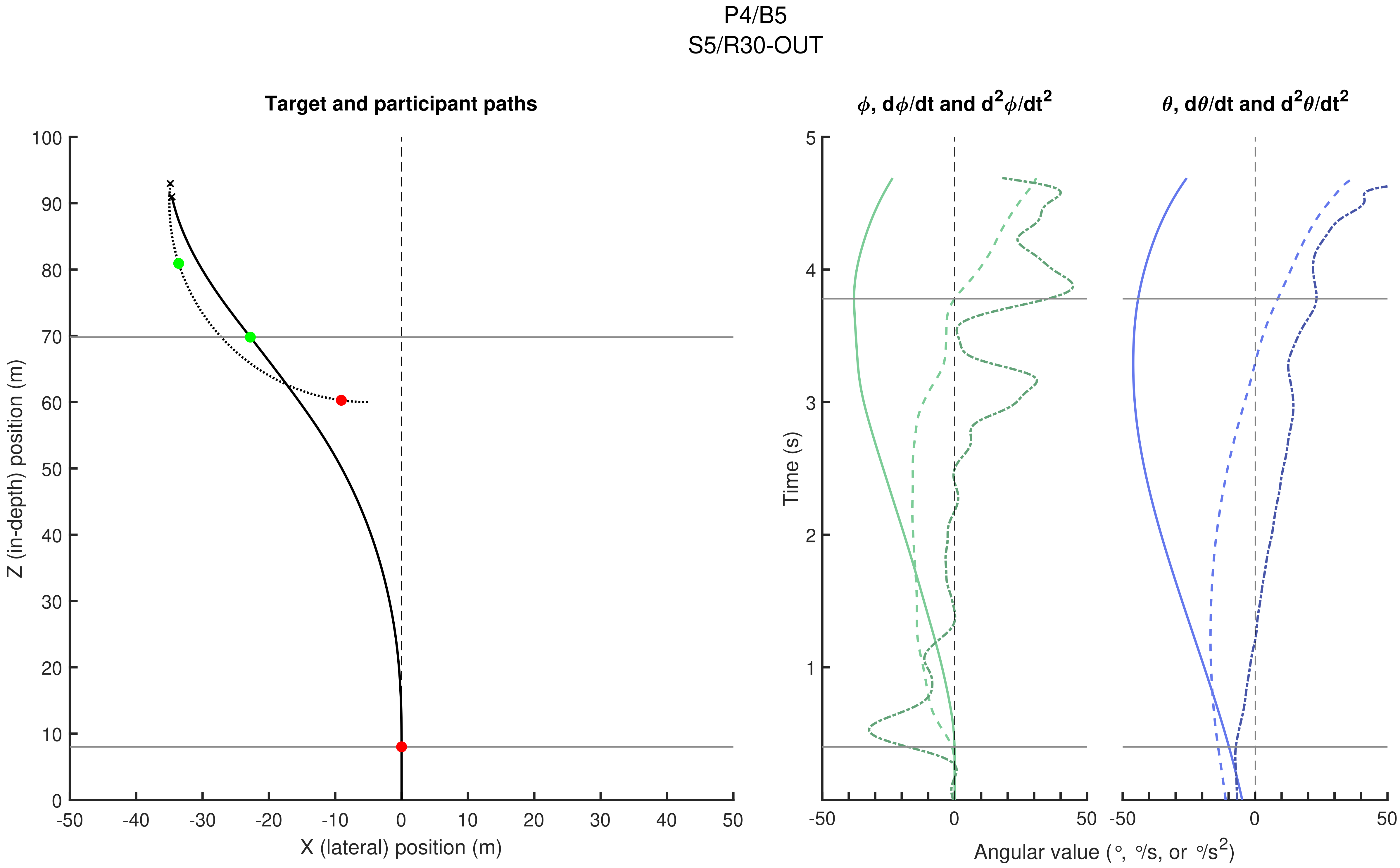
P4/B5
S5/R30-OUT
Target and participant paths
Z (in-depth) position (m)
X (lateral) position (m)
ϕ, dϕ/dt and d$^2\phi$/dt^2
θ, dθ/dt and d$^2\theta$/dt^2
Time (s)
Angular value (°, °/s, or °/s^2)

P4/B5
S5/R10-OUT

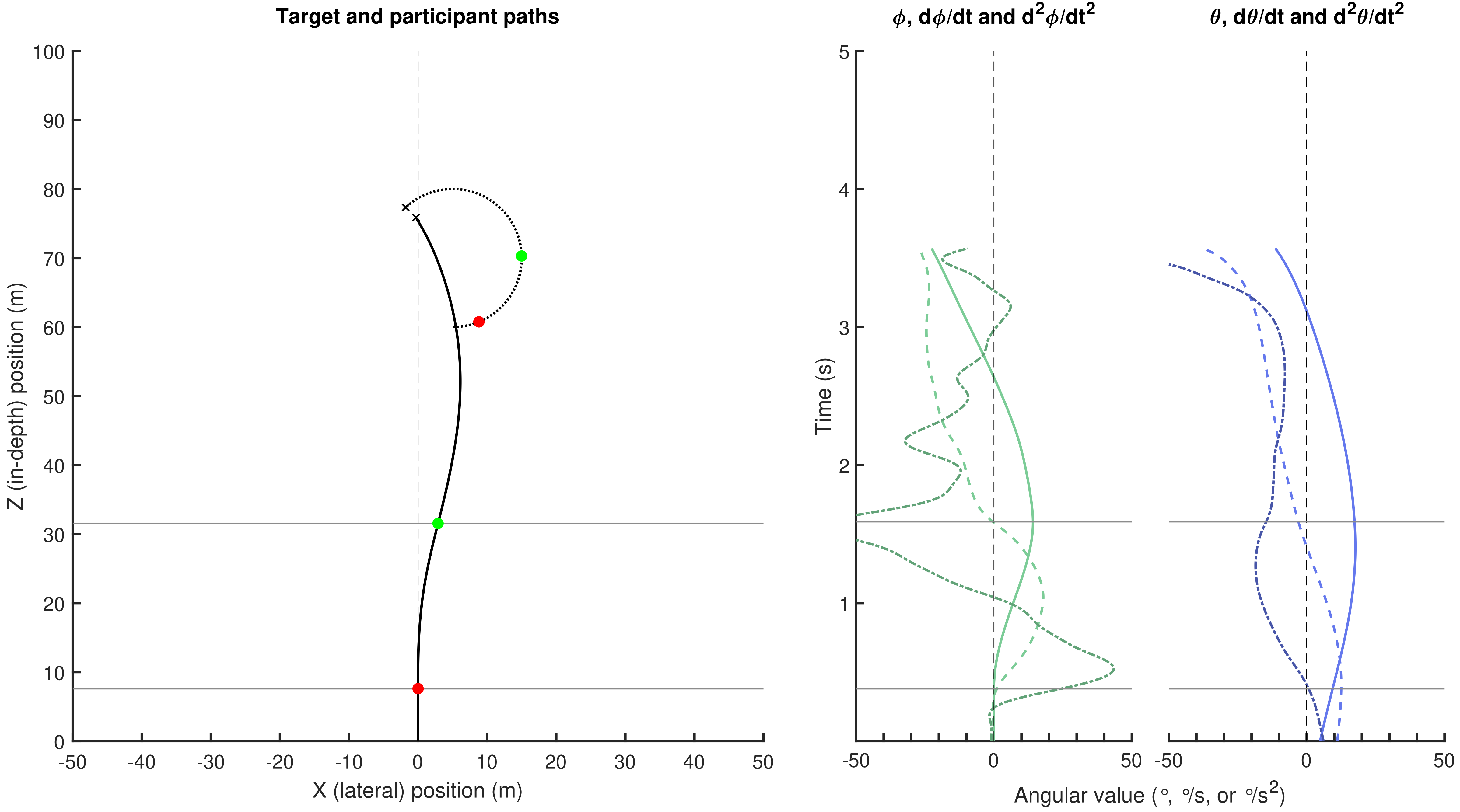

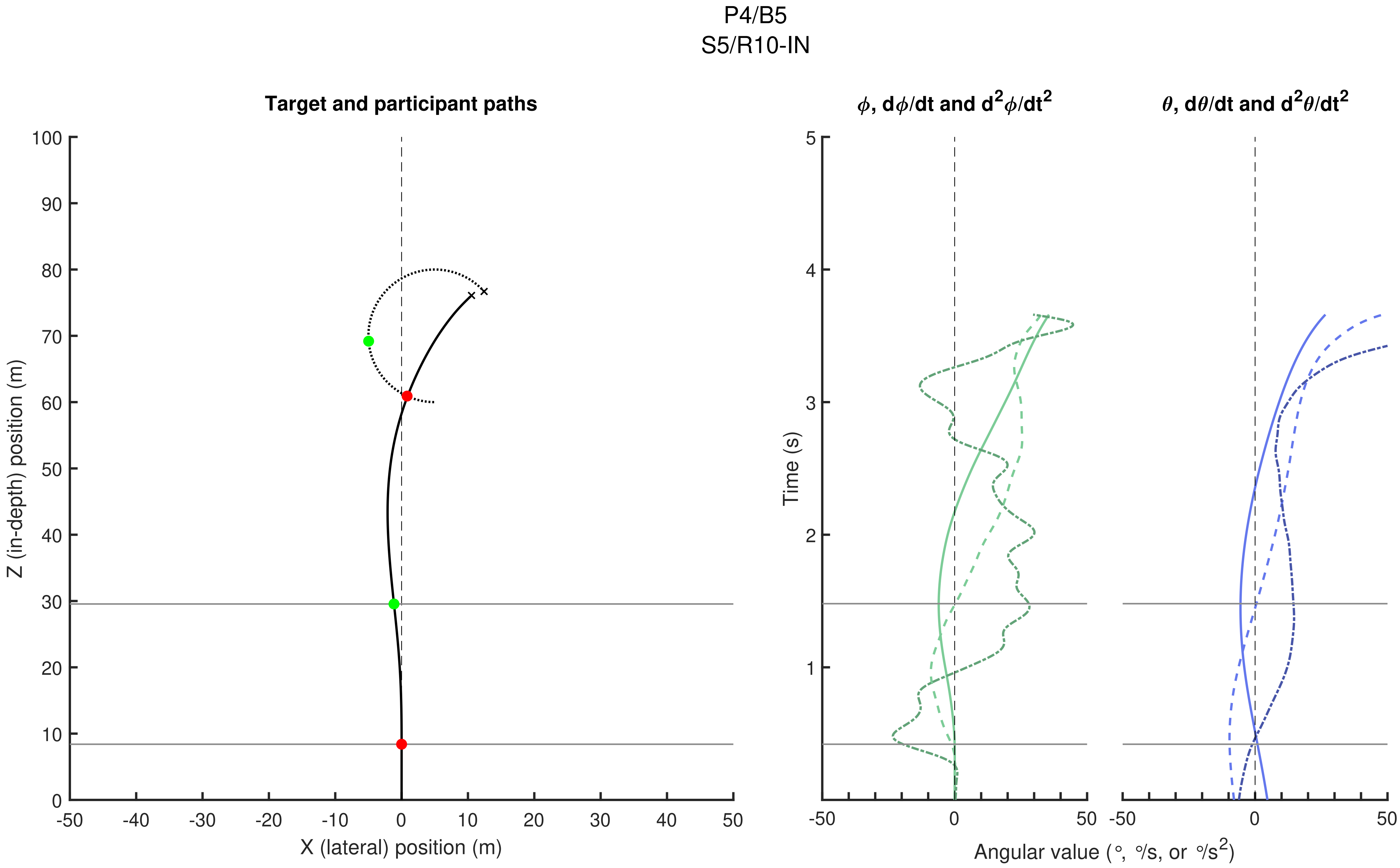
P4/B5
S5/R10-IN
Target and participant paths
Z (in-depth) position (m)
X (lateral) position (m)
100
90
80
70
60
50
40
30
20
10
0
-50
-40
-30
-20
-10
0
10
20
30
40
50
ϕ, dϕ/dt and d²ϕ/dt²
θ, dθ/dt and d²θ/dt²
Time (s)
5
4
3
2
1
-50
0
50
-50
0
50
Angular value (°, °/s, or °/s²)

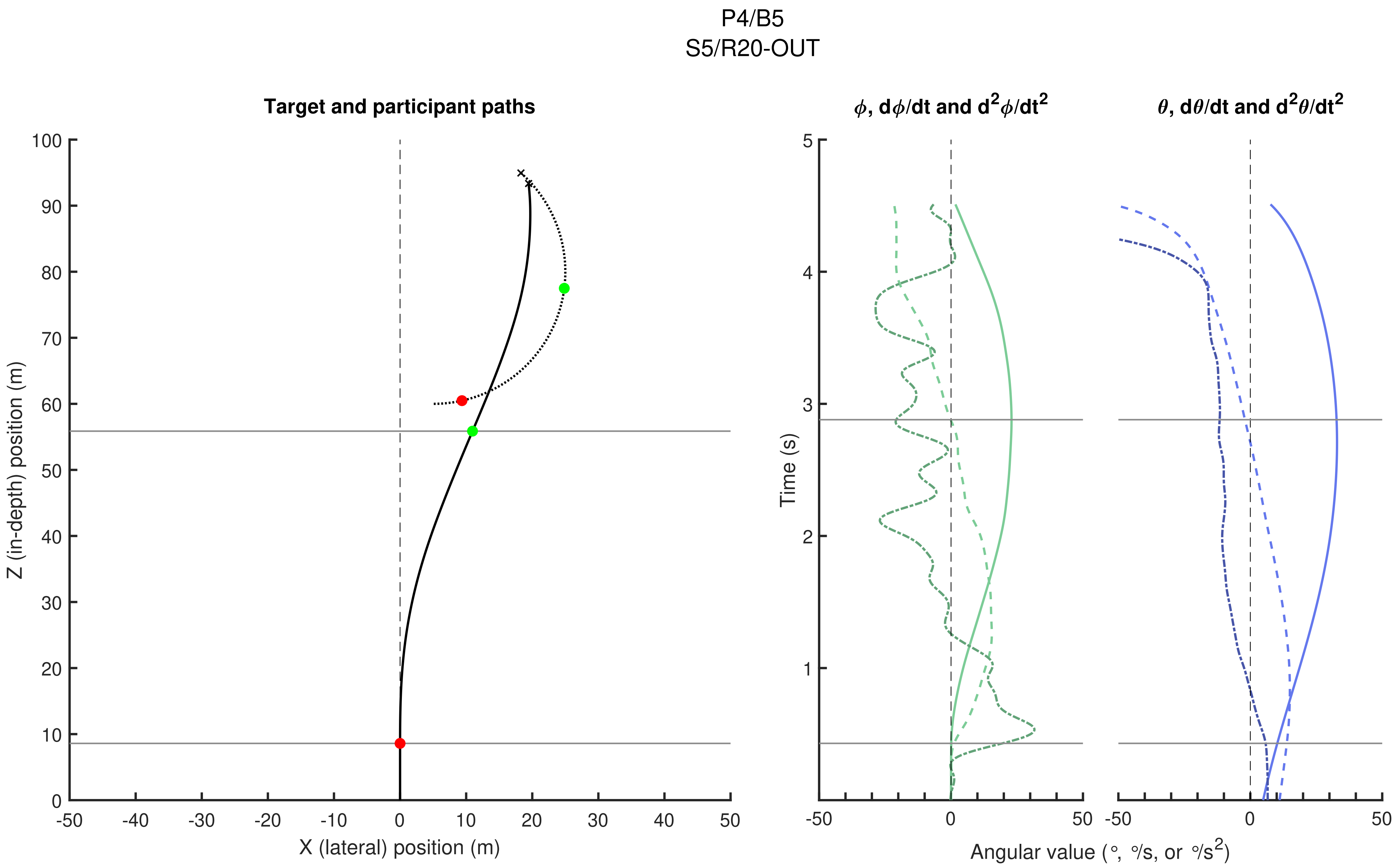
P4/B5
S5/R20-OUT
Target and participant paths
Z (in-depth) position (m)
X (lateral) position (m)
φ, dφ/dt and d²φ/dt²
θ, dθ/dt and d²θ/dt²
Time (s)
Angular value (°, °/s, or °/s²)

P4/B5
S5/R20-IN

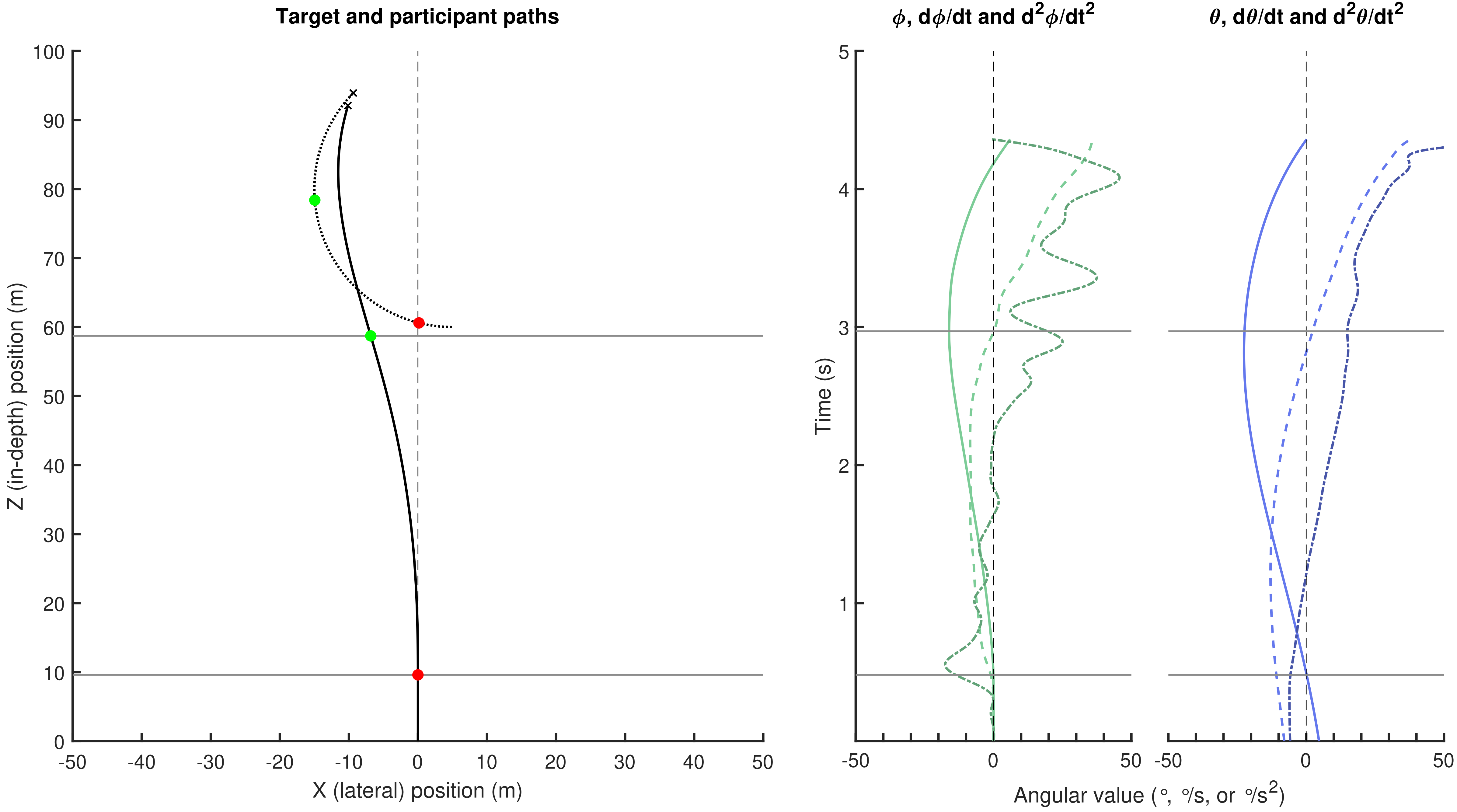

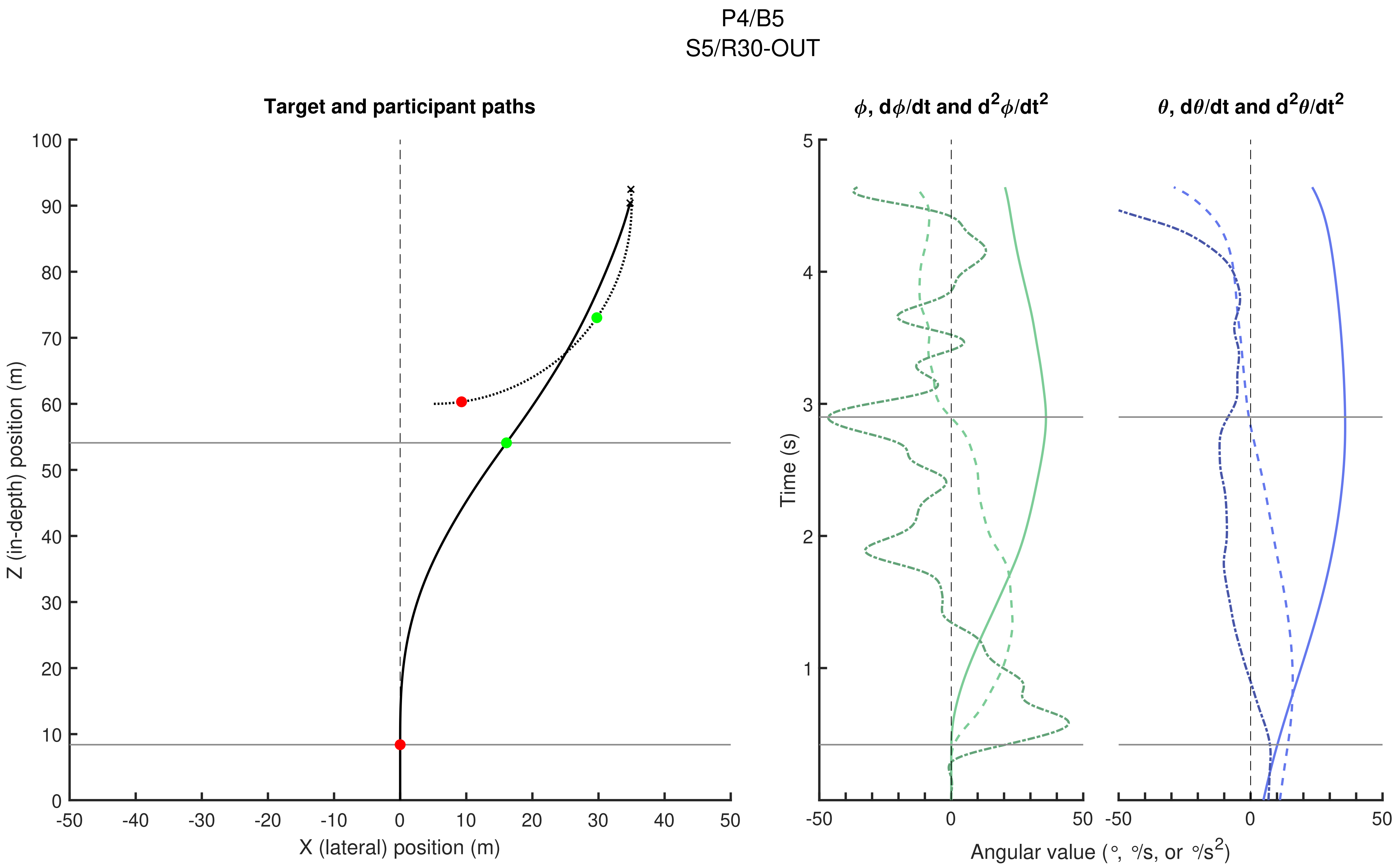

P4/B5
S5/R30-OUT
Target and participant paths
Z (in-depth) position (m)
X (lateral) position (m)
ϕ, dϕ/dt and d$^2\phi$/dt^2
θ, dθ/dt and d$^2\theta$/dt^2
Time (s)
Angular value (°, °/s, or °/s^2)

P4/B5
S5/R30-IN

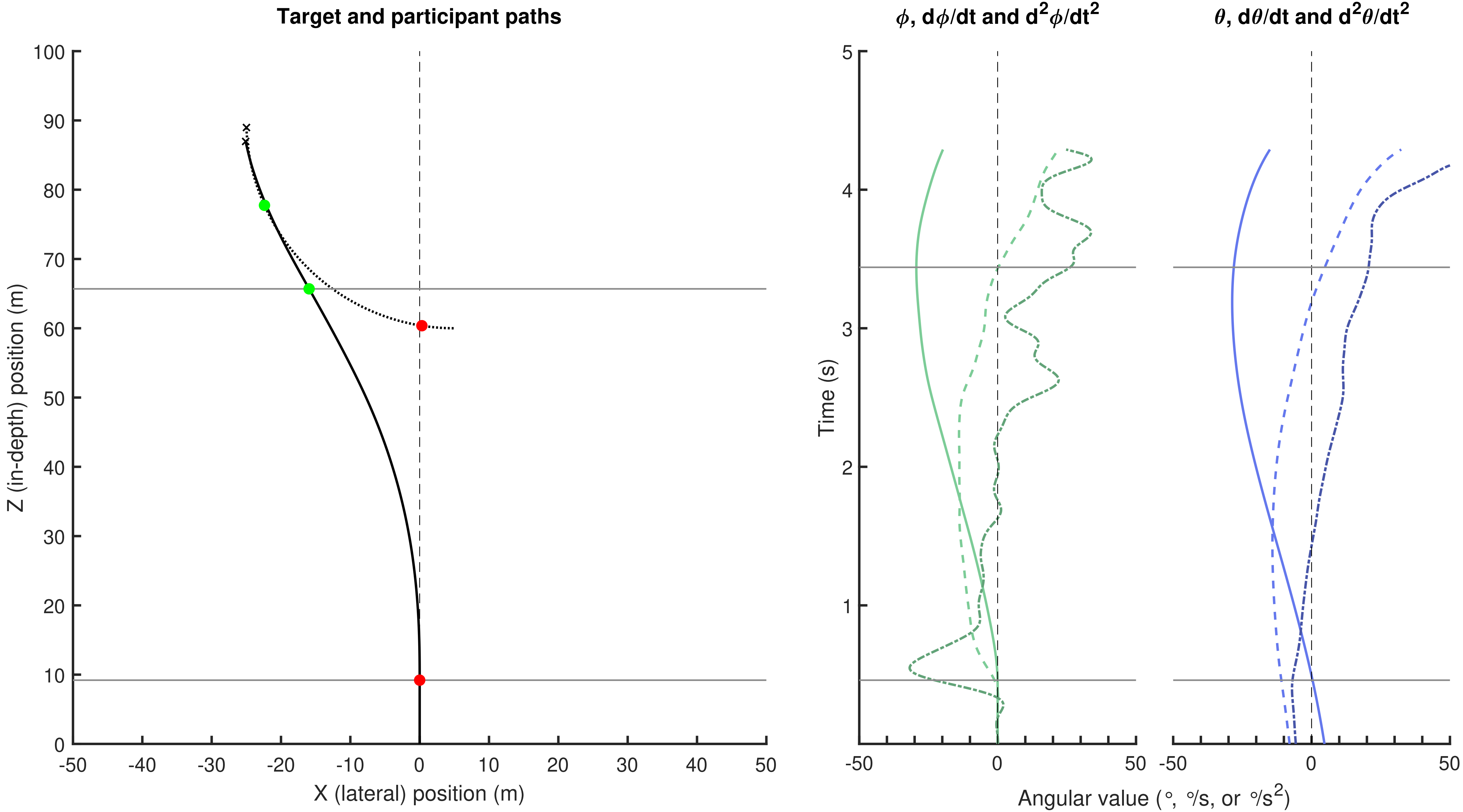

P4/B5
S15/R10-IN

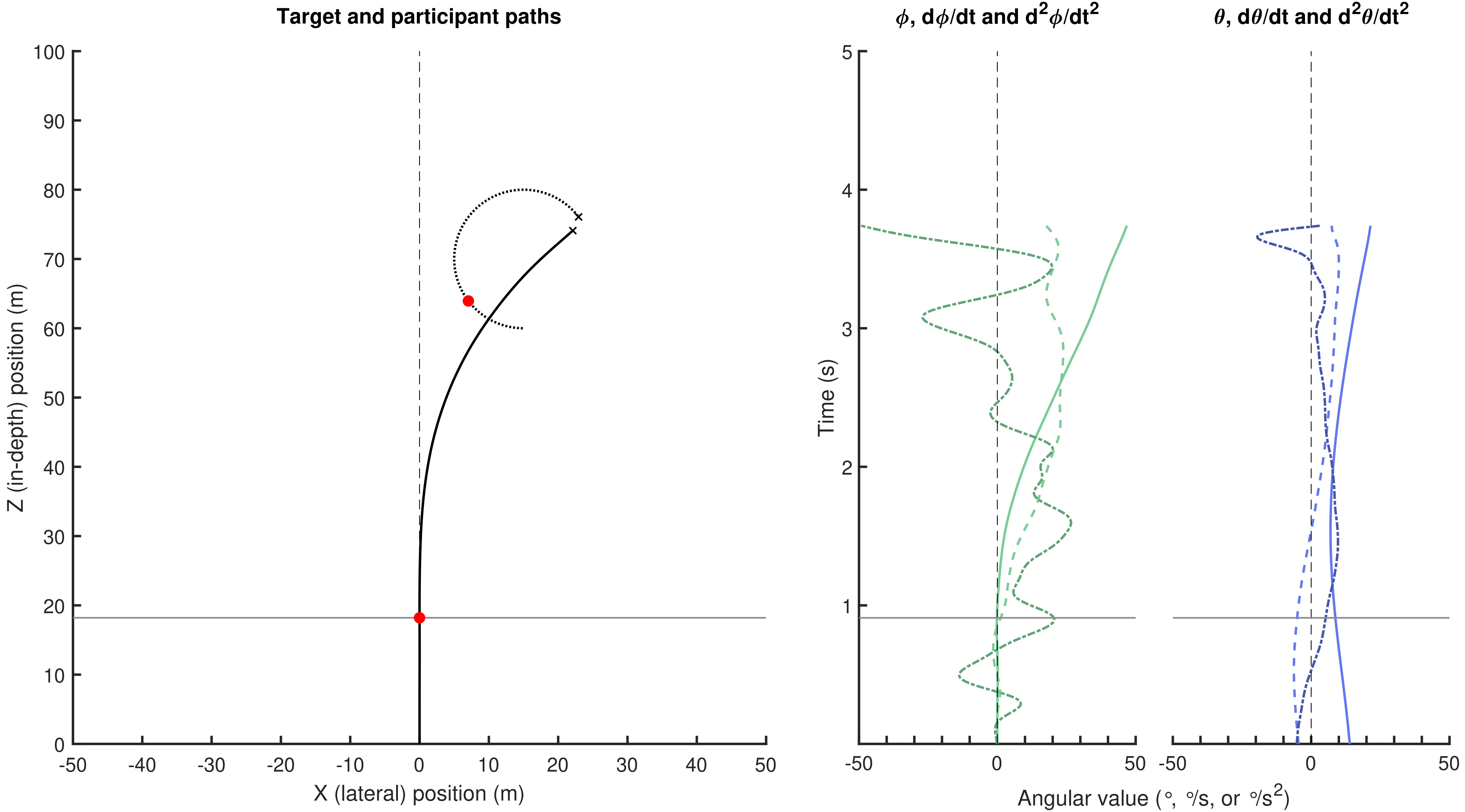

P4/B5
S15/R20-IN

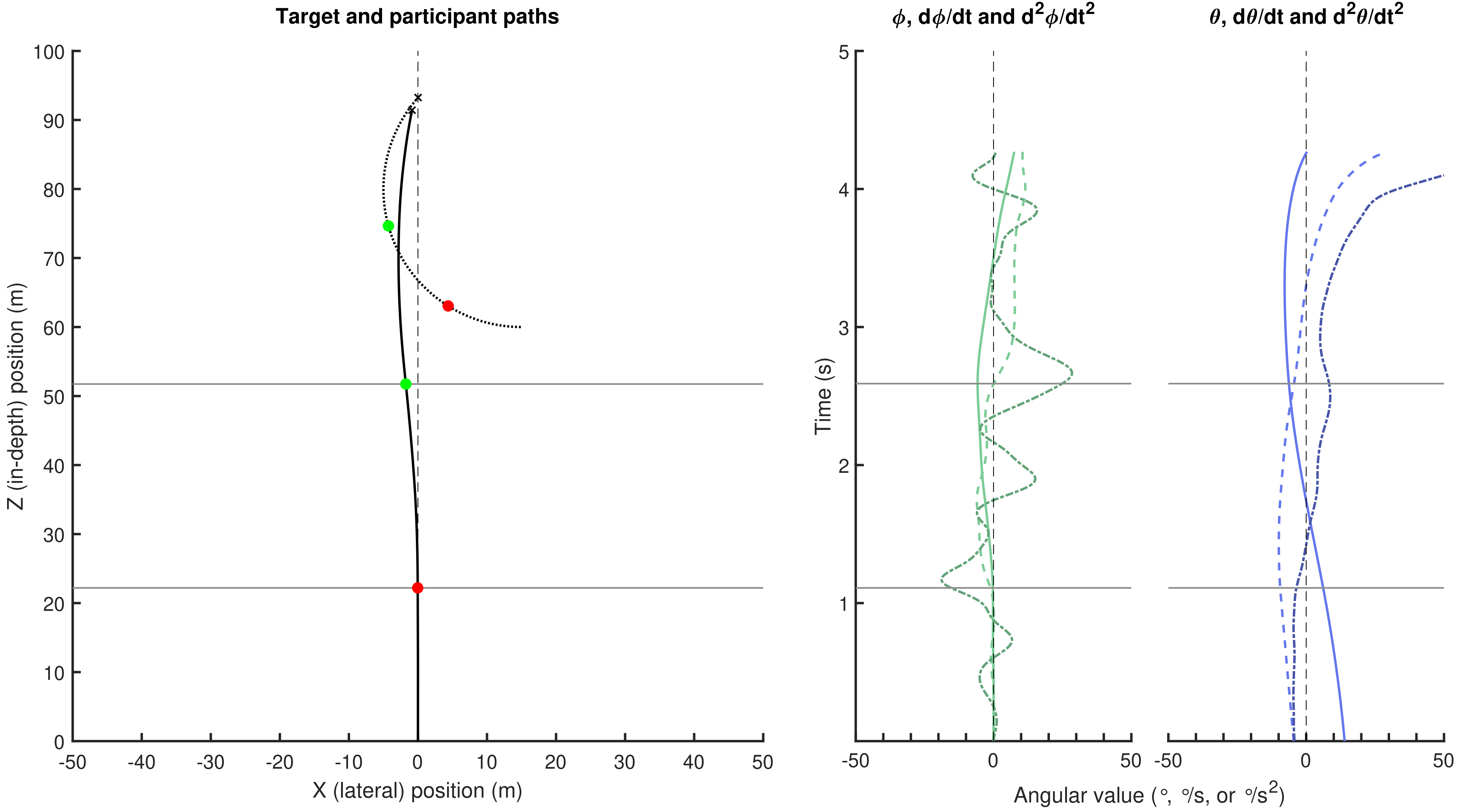

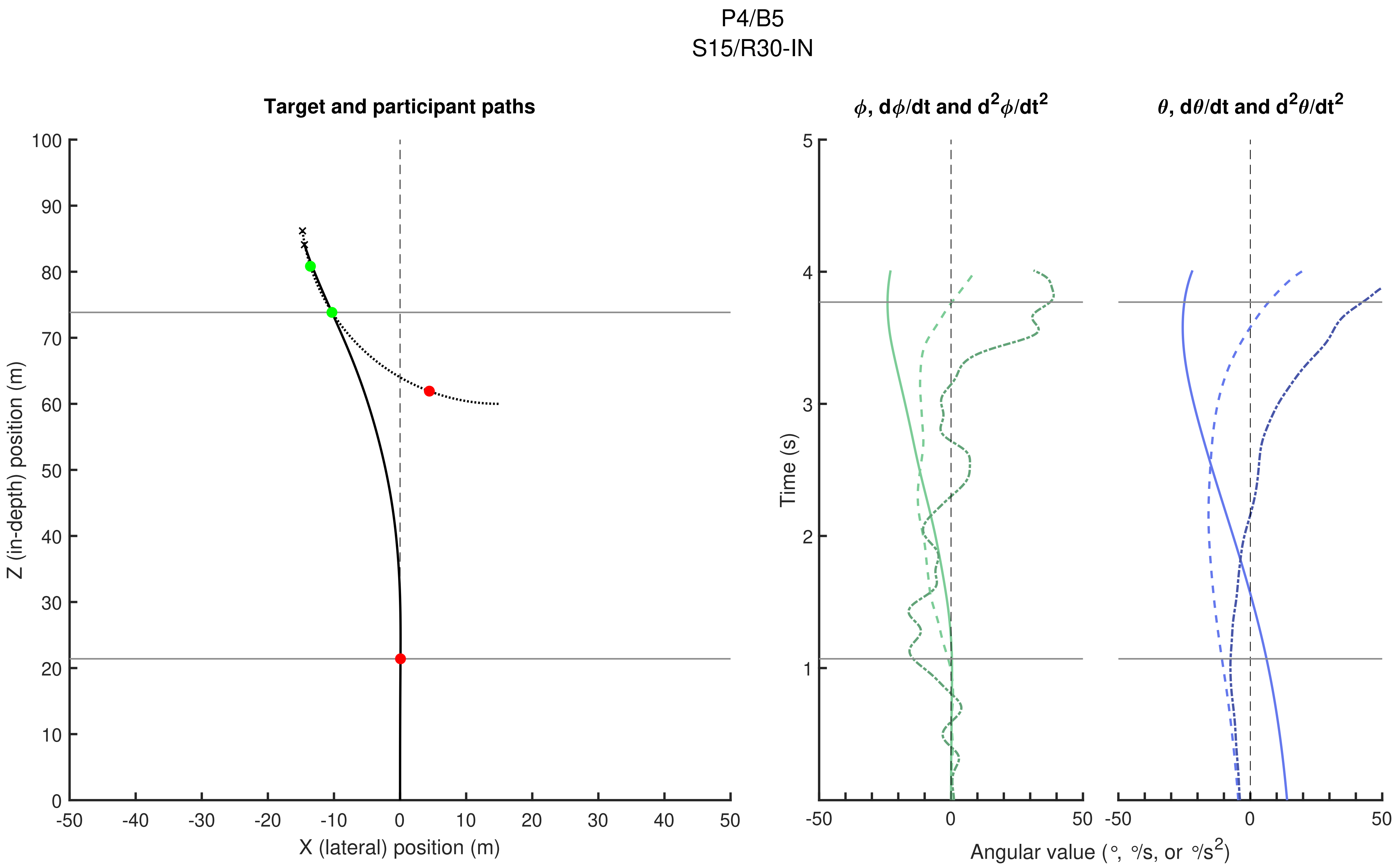

P4/B5
S15/R30-IN
Target and participant paths
Z (in-depth) position (m)
X (lateral) position (m)
ϕ, dϕ/dt and d$^2\phi$/dt^2
θ, dθ/dt and d$^2\theta$/dt^2
Time (s)
Angular value (°, °/s, or °/s^2)

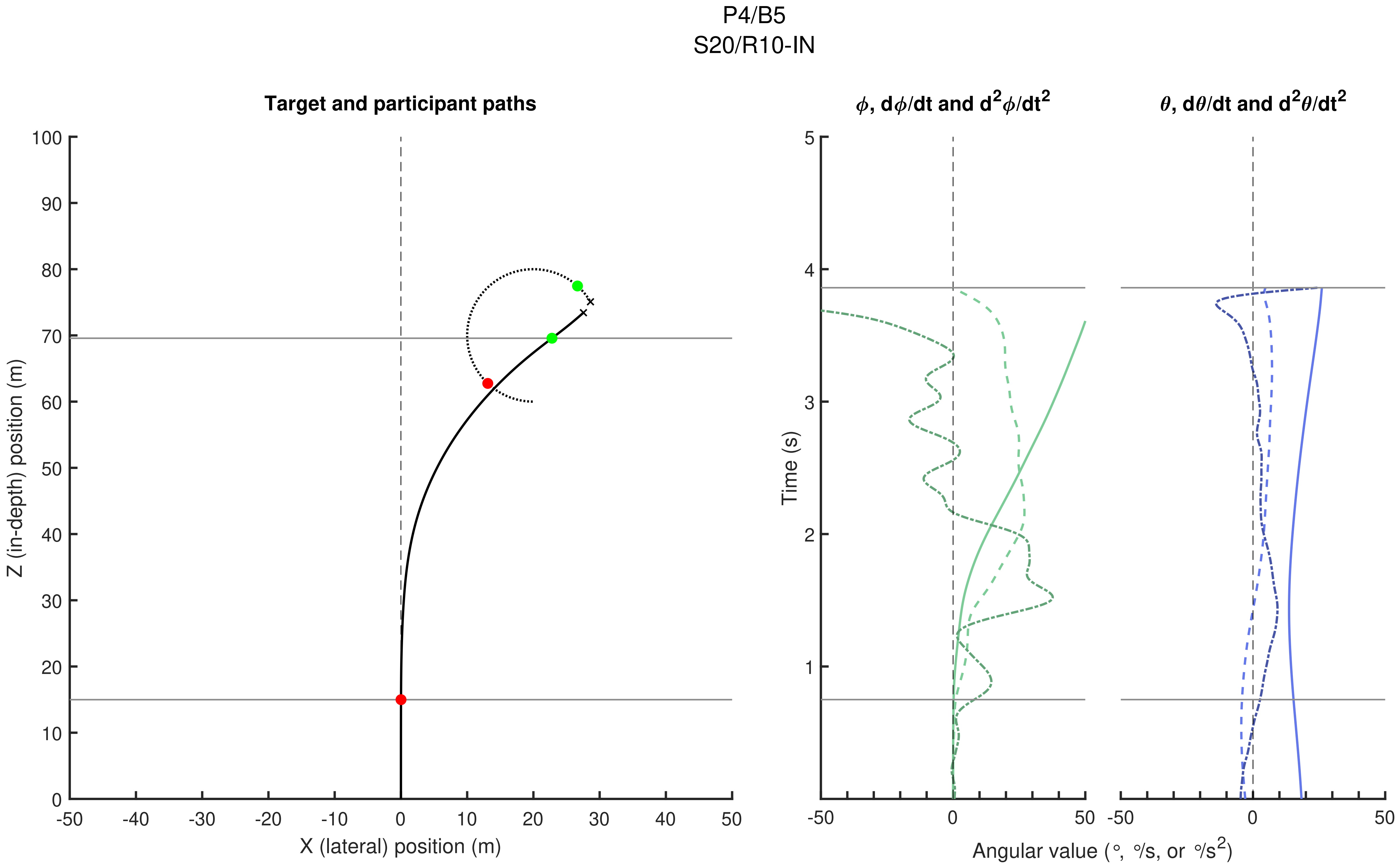
P4/B5
S20/R10-IN
Target and participant paths
Z (in-depth) position (m)
X (lateral) position (m)
φ, dφ/dt and d²φ/dt²
θ, dθ/dt and d²θ/dt²
Time (s)
Angular value (°, °/s, or °/s²)

P4/B5
S20/R20-IN

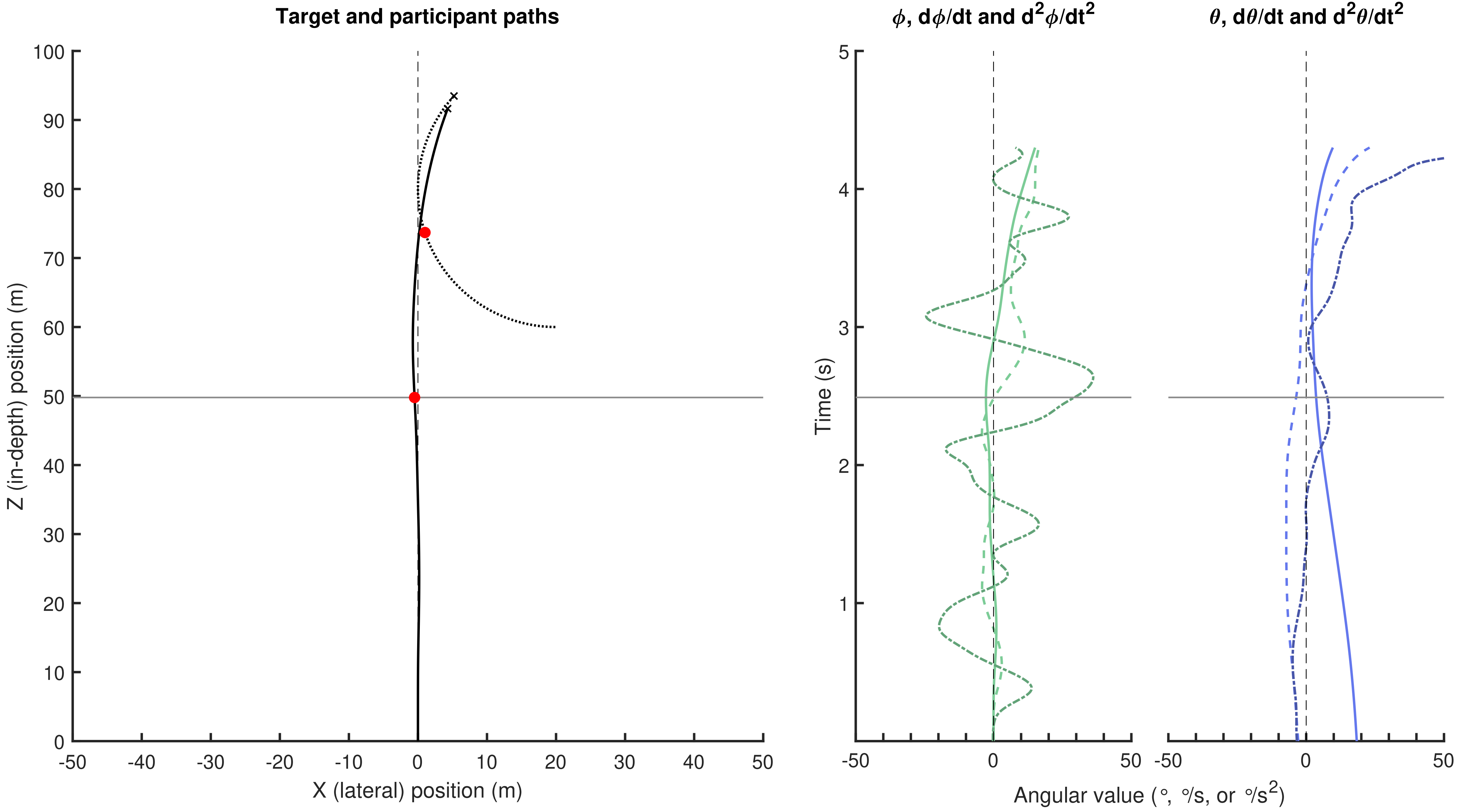

P4/B5
S20/R30-IN

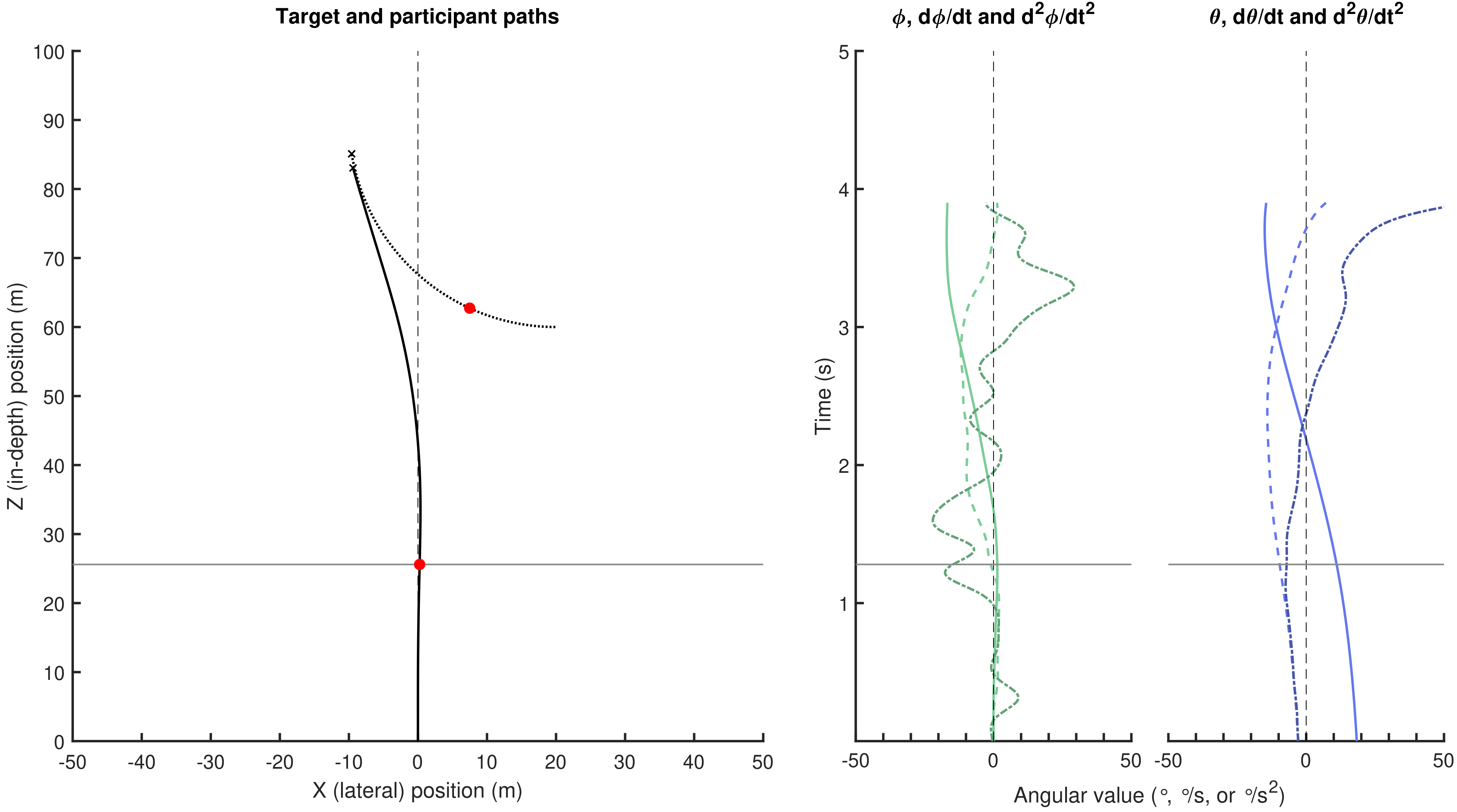

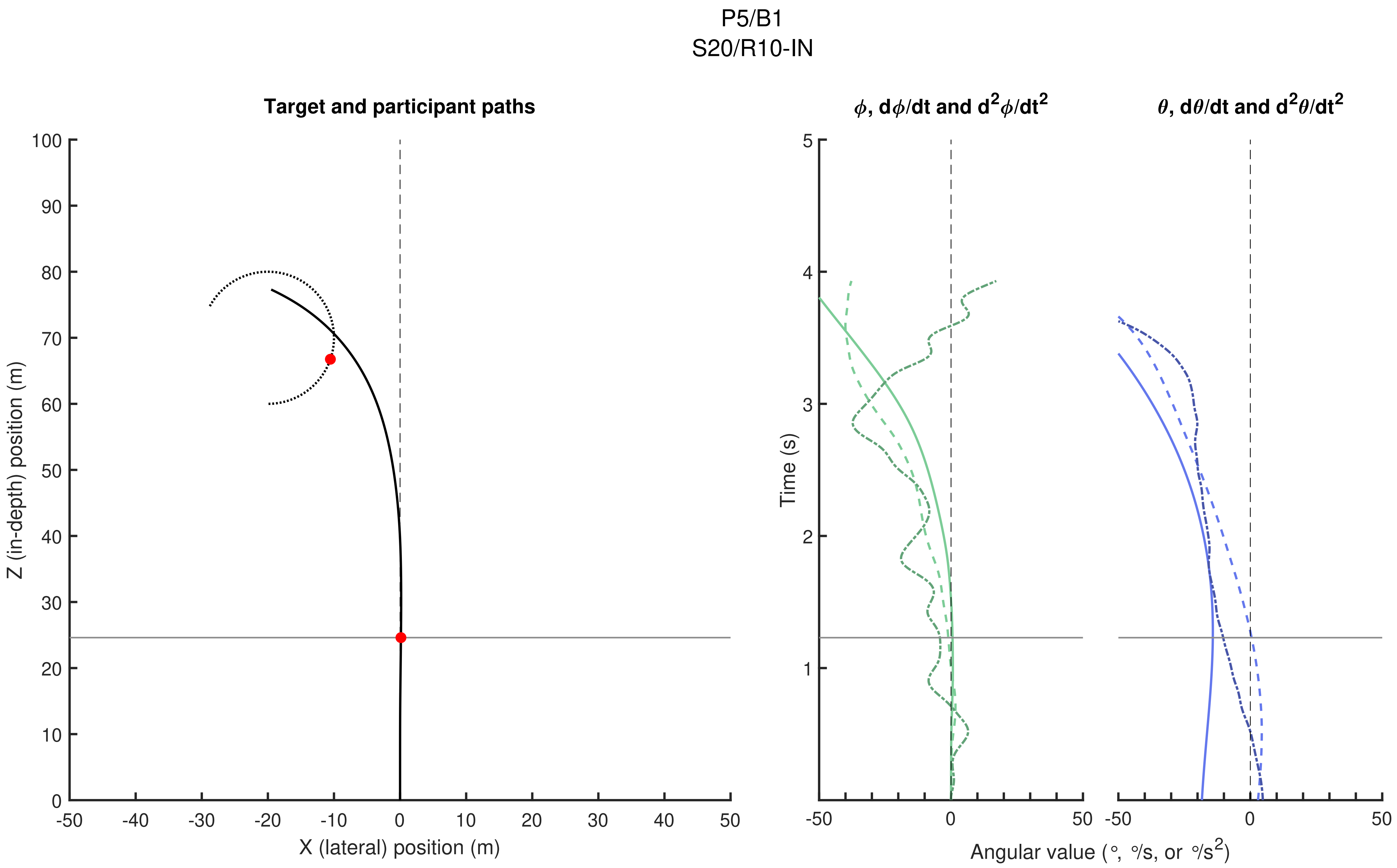
P5/B1
S20/R10-IN
Target and participant paths
Z (in-depth) position (m)
X (lateral) position (m)
ϕ, dϕ/dt and d$^2\phi$/dt^2
θ, dθ/dt and d$^2\theta$/dt^2
Time (s)
Angular value (°, °/s, or °/s^2)

P5/B1
S20/R20-IN

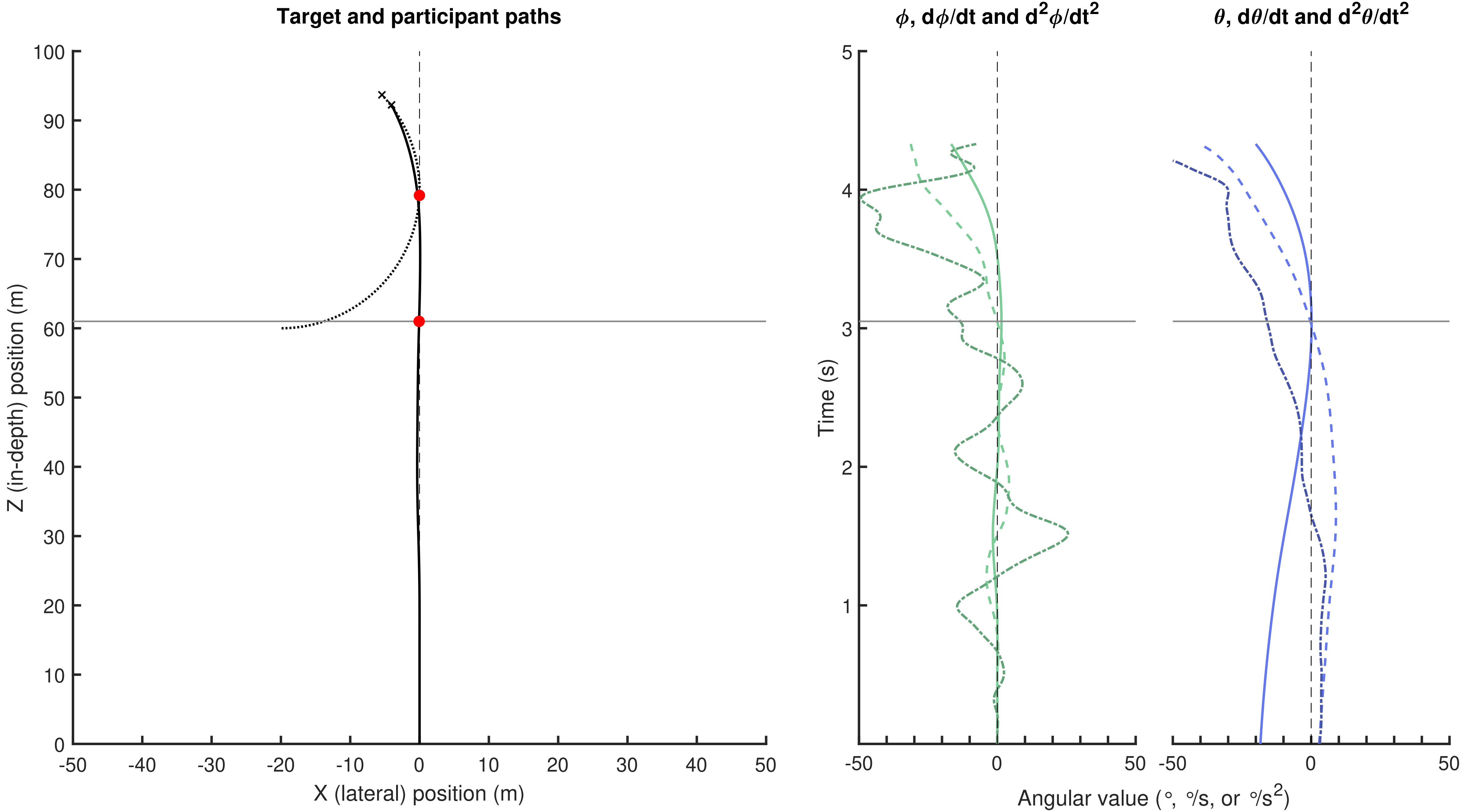

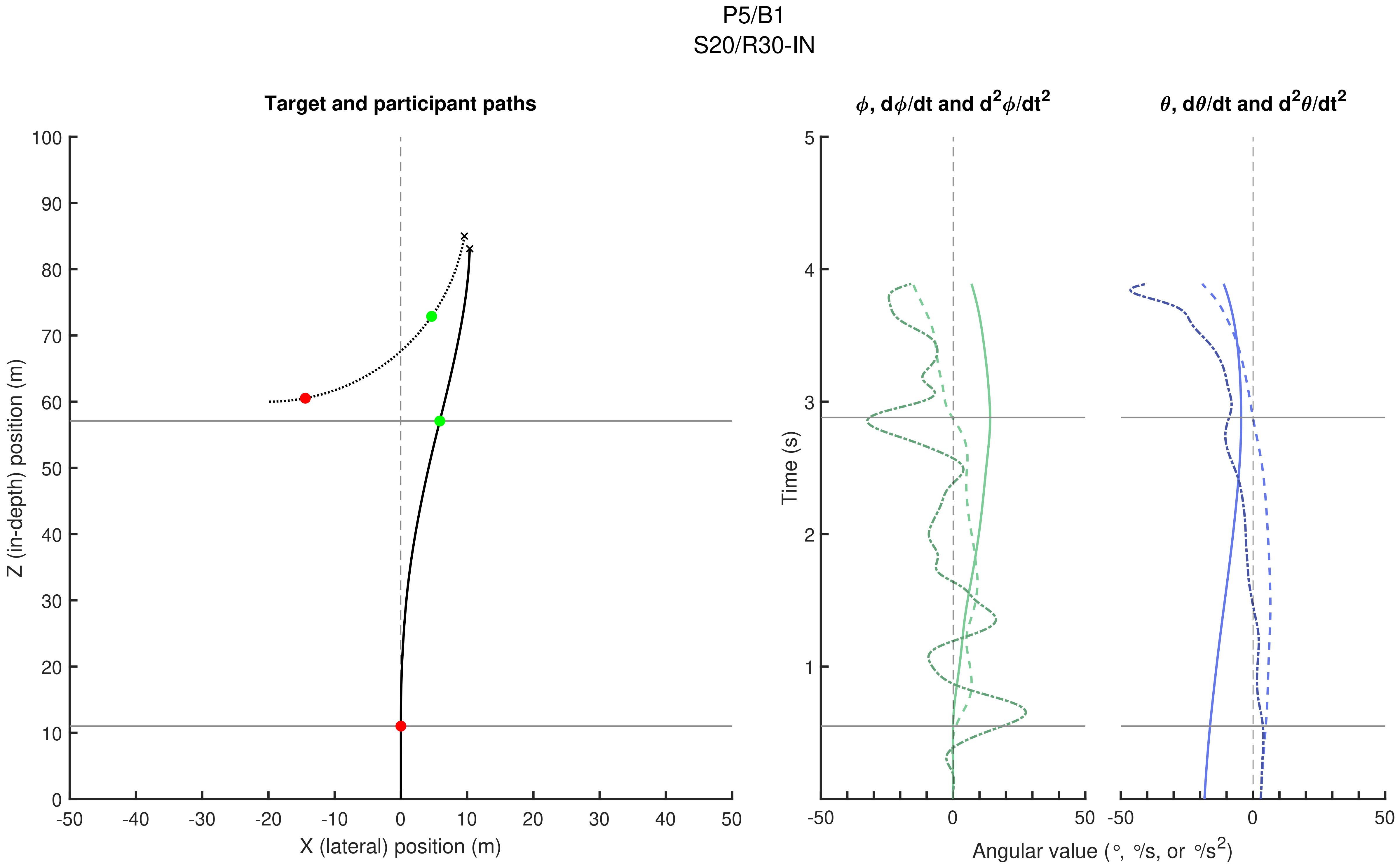
P5/B1
S20/R30-IN
Target and participant paths
ϕ, dϕ/dt and d$^2\phi$/dt^2
θ, dθ/dt and d$^2\theta$/dt^2
Z (in-depth) position (m)
X (lateral) position (m)
Time (s)
Angular value (°, °/s, or °/s^2)
100
90
80
70
60
50
40
30
20
10
0
-50
-40
-30
-20
-10
0
10
20
30
40
50
5
4
3
2
1
-50
0
50
-50
0
50

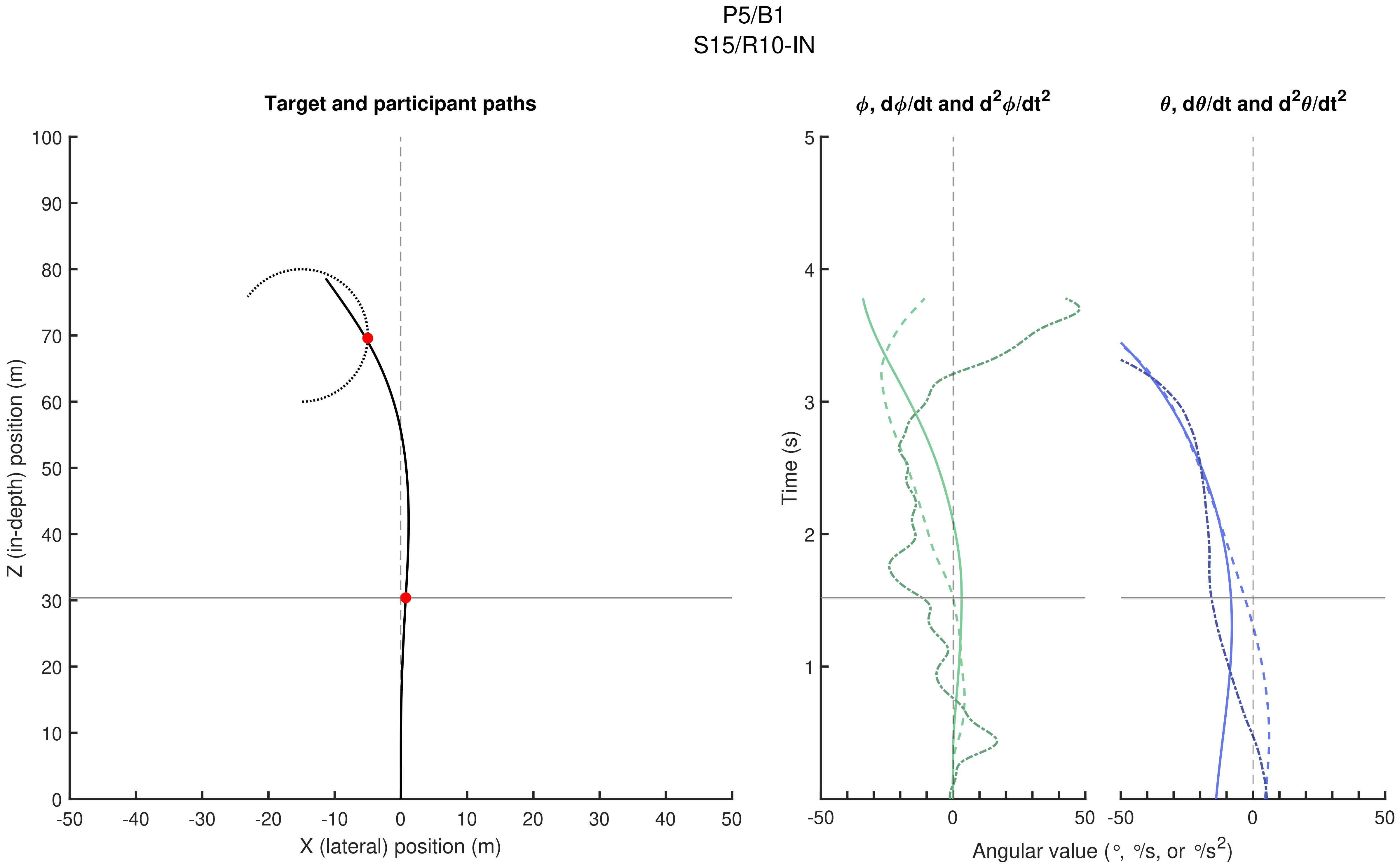

P5/B1
S15/R10-IN
Target and participant paths
Z (in-depth) position (m)
X (lateral) position (m)
100
90
80
70
60
50
40
30
20
10
0
-50
-40
-30
-20
-10
0
10
20
30
40
50
ϕ, dϕ/dt and d$^2\phi$/dt^2
θ, dθ/dt and d$^2\theta$/dt^2
Time (s)
5
4
3
2
1
-50
0
50
-50
0
50
Angular value (°, °/s, or °/s^2)

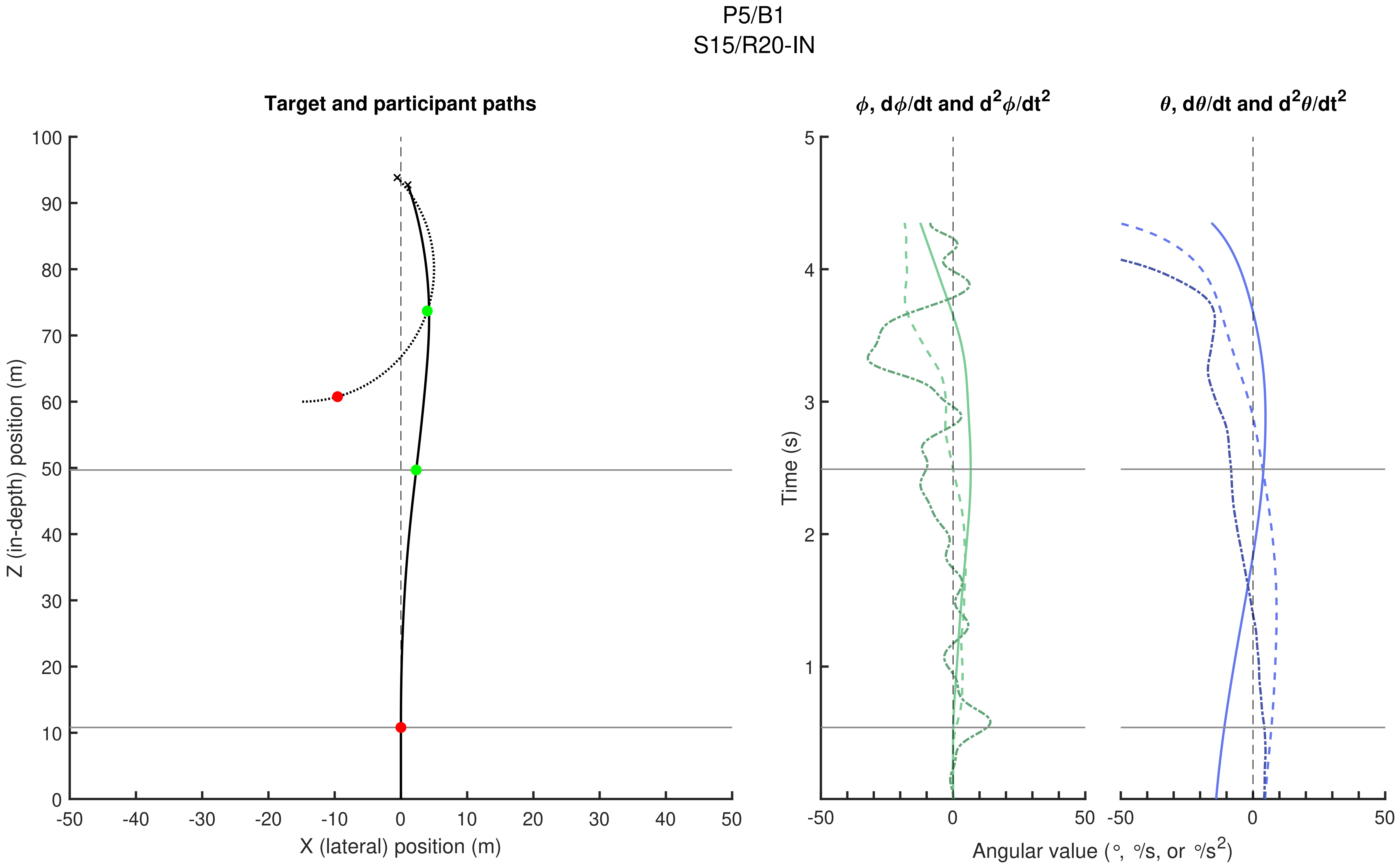

P5/B1
S15/R20-IN
Target and participant paths
Z (in-depth) position (m)
X (lateral) position (m)
100
90
80
70
60
50
40
30
20
10
0
-50
-40
-30
-20
-10
0
10
20
30
40
50
ϕ, dϕ/dt and d$^2\phi$/dt^2
θ, dθ/dt and d$^2\theta$/dt^2
Time (s)
5
4
3
2
1
-50
0
50
-50
0
50
Angular value (°, °/s, or °/s^2)

P5/B1
S15/R30-IN

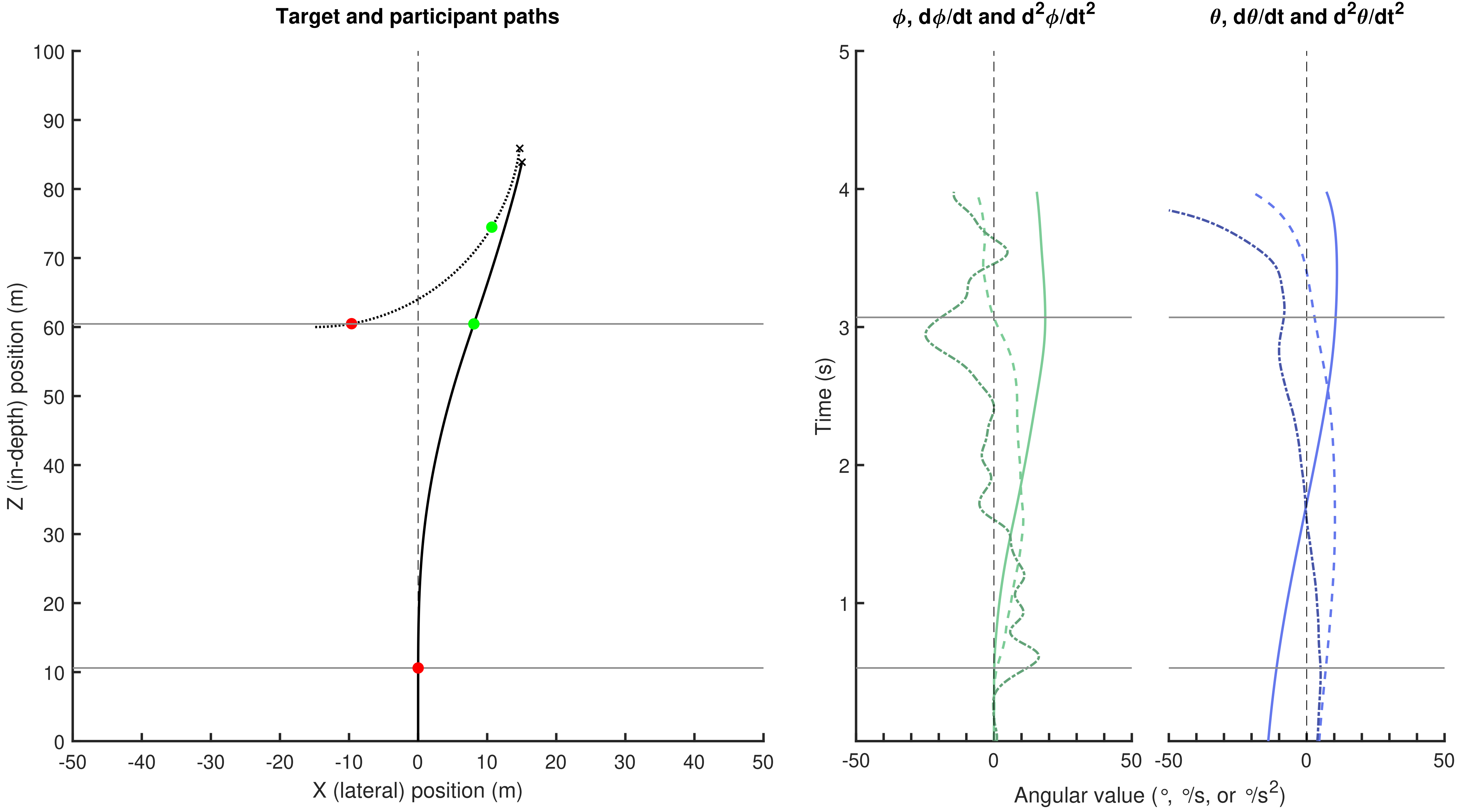

P5/B1
S5/R10-IN

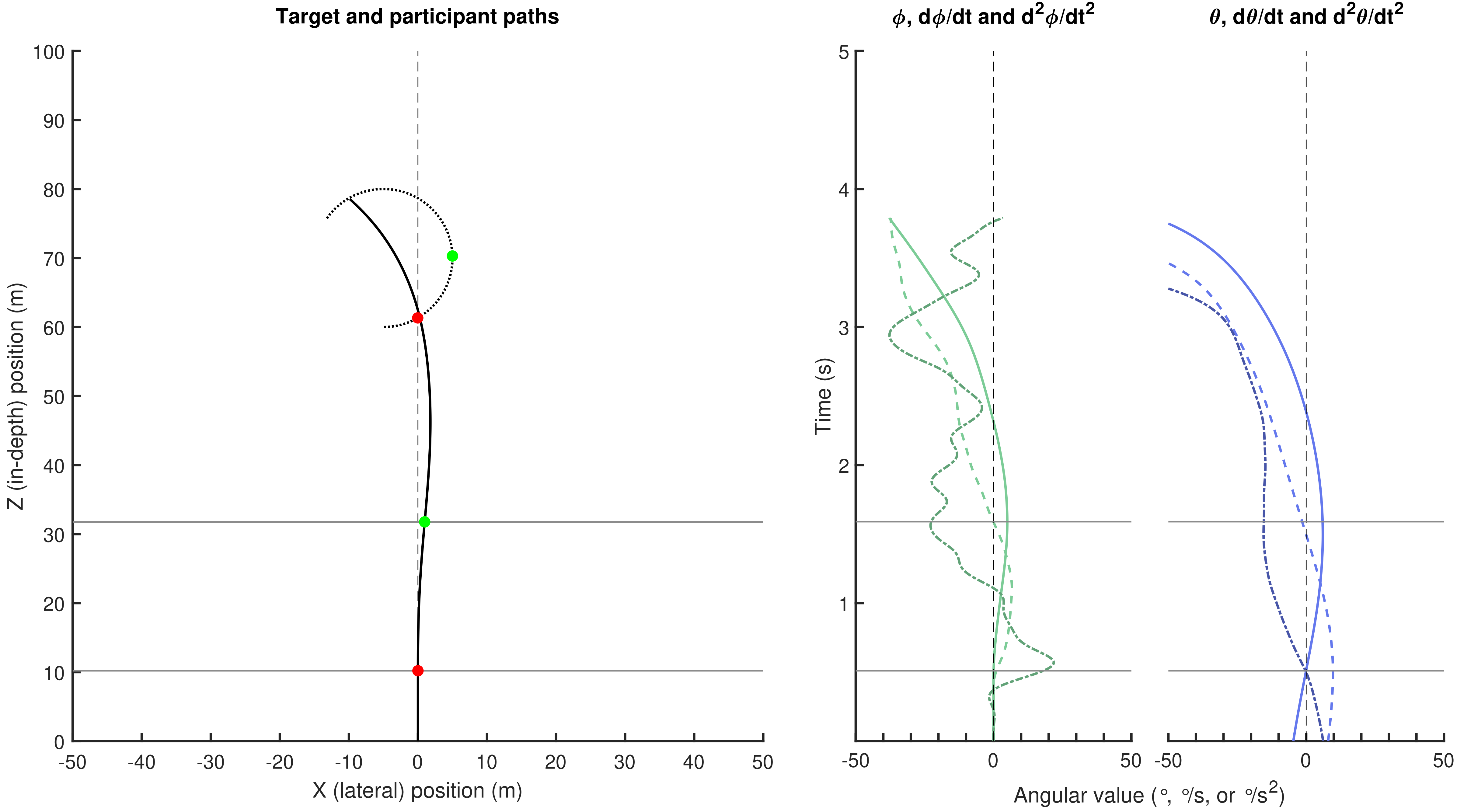

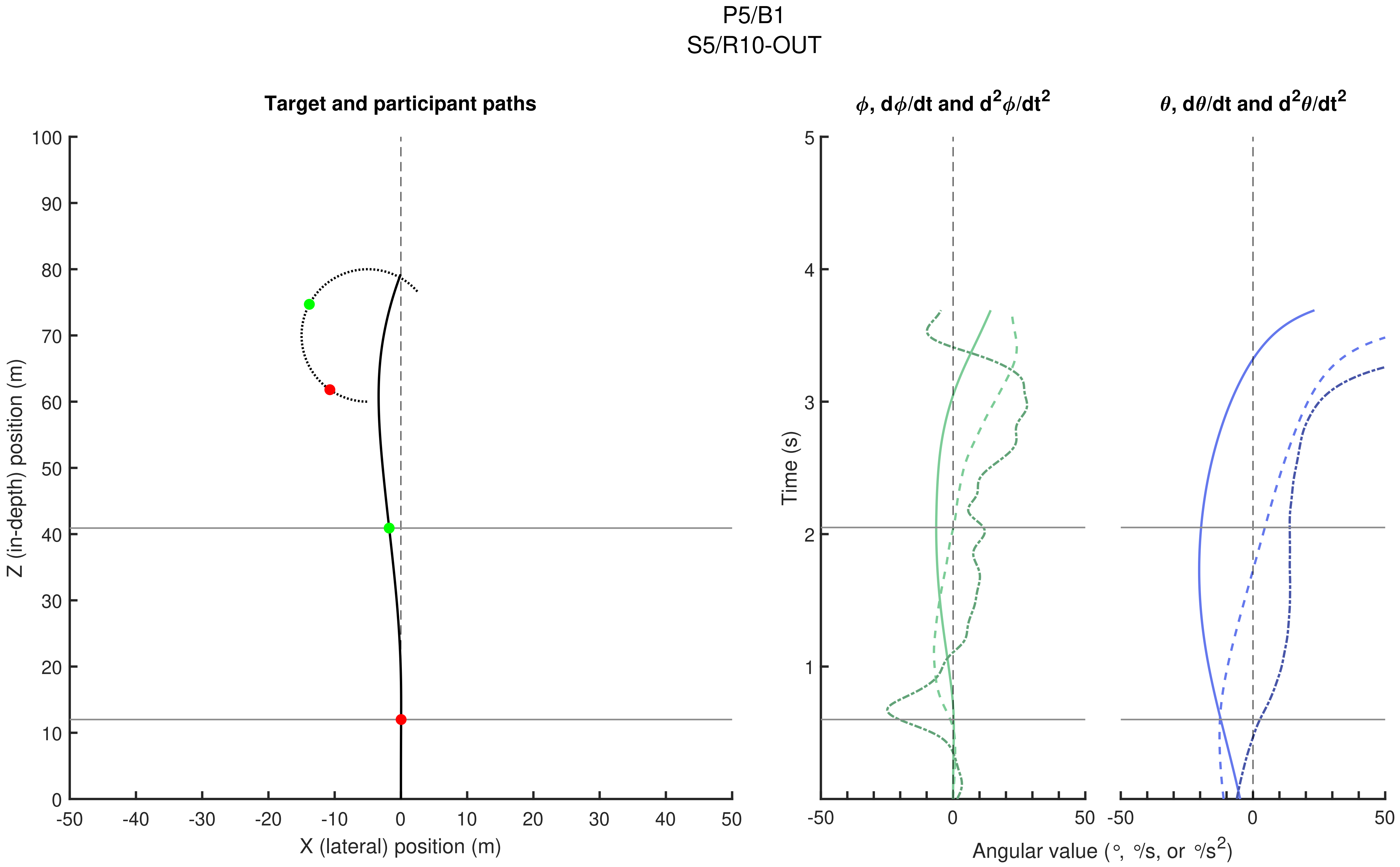

P5/B1
S5/R10-OUT
Target and participant paths
ϕ, dϕ/dt and d$^2\phi$/dt^2
θ, dθ/dt and d$^2\theta$/dt^2
Z (in-depth) position (m)
X (lateral) position (m)
Time (s)
Angular value (°, °/s, or °/s^2)
100
90
80
70
60
50
40
30
20
10
0
-50
-40
-30
-20
-10
0
10
20
30
40
50
5
4
3
2
1
-50
0
50
-50
0
50

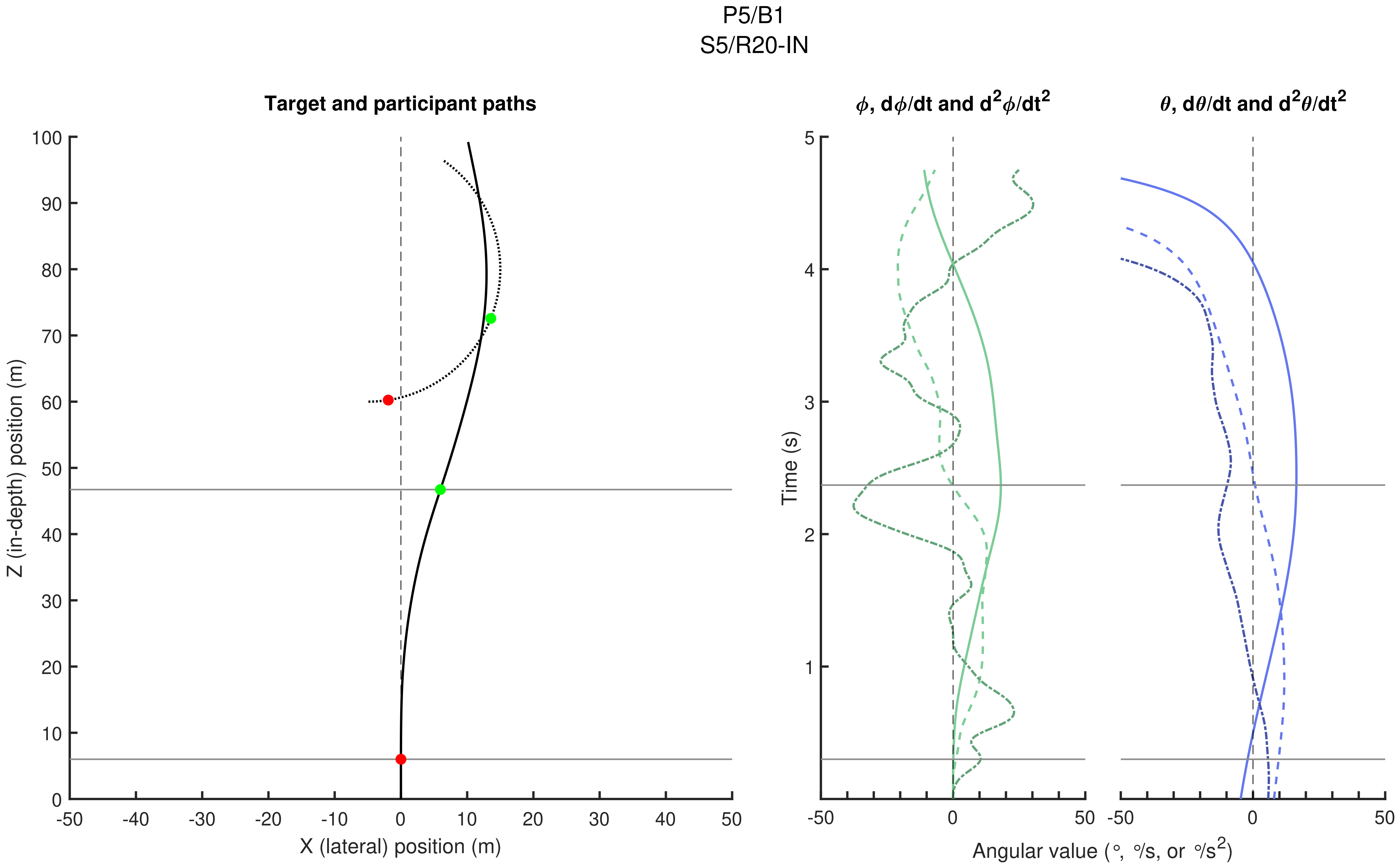
P5/B1
S5/R20-IN
Target and participant paths
Z (in-depth) position (m)
X (lateral) position (m)
ϕ, dϕ/dt and d$^2\phi$/dt^2
θ, dθ/dt and d$^2\theta$/dt^2
Time (s)
Angular value (°, °/s, or °/s^2)

P5/B1
S5/R20-OUT

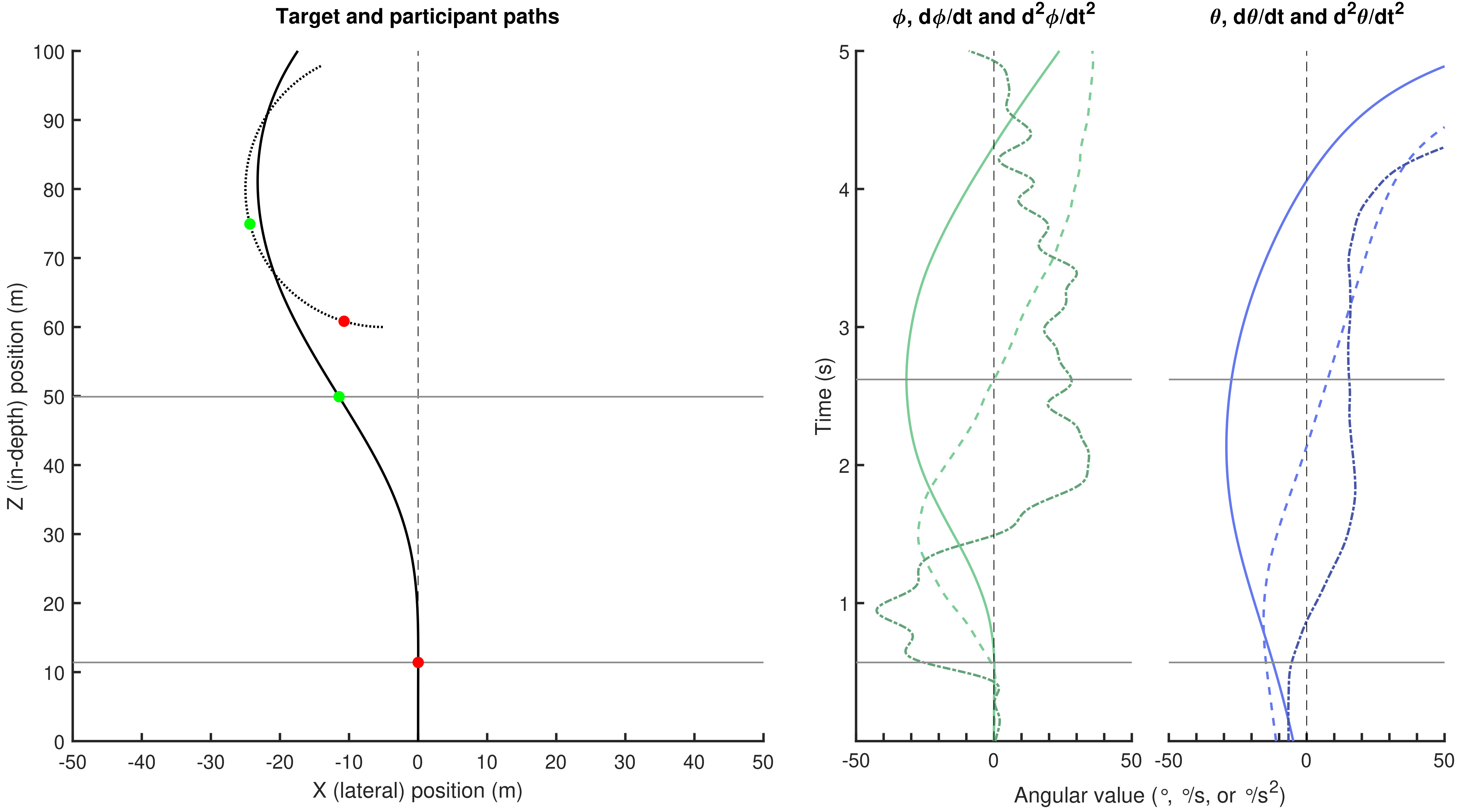

P5/B1
S5/R30-IN

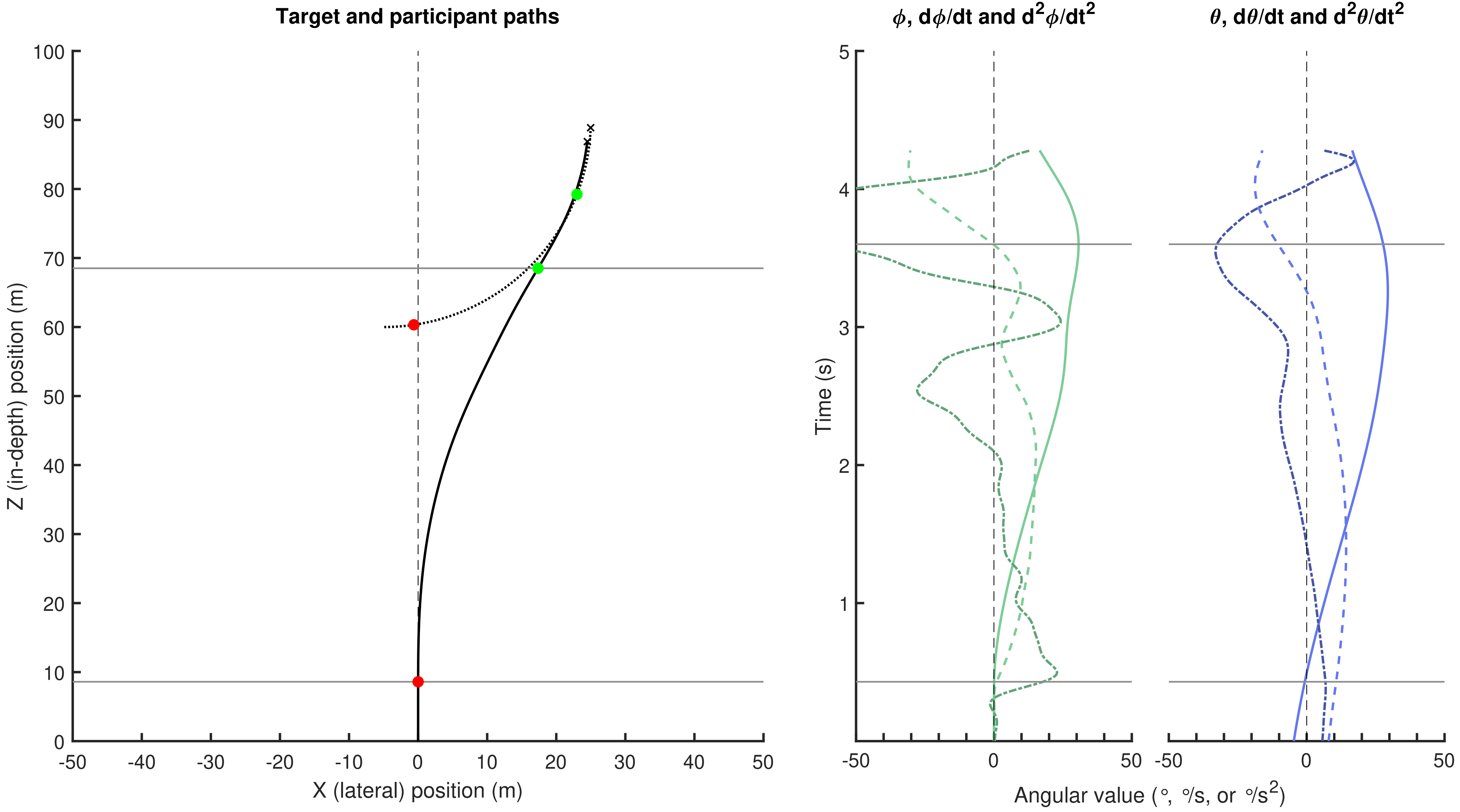

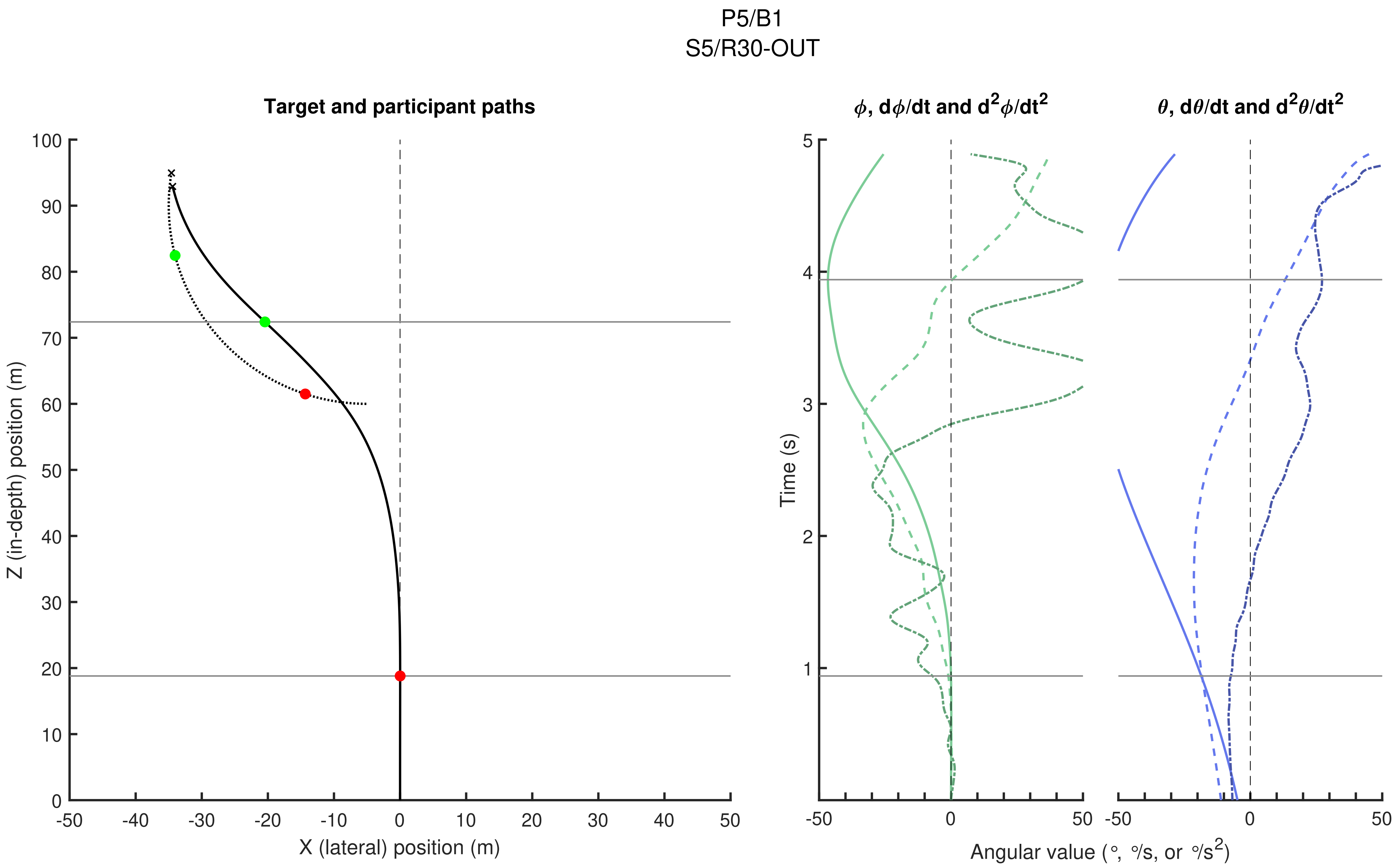

P5/B1
S5/R30-OUT
Target and participant paths
Z (in-depth) position (m)
X (lateral) position (m)
100
90
80
70
60
50
40
30
20
10
0
-50
-40
-30
-20
-10
0
10
20
30
40
50
ϕ, dϕ/dt and d$^2\phi$/dt^2
θ, dθ/dt and d$^2\theta$/dt^2
Time (s)
5
4
3
2
1
-50
0
50
-50
0
50
Angular value (°, °/s, or °/s^2)

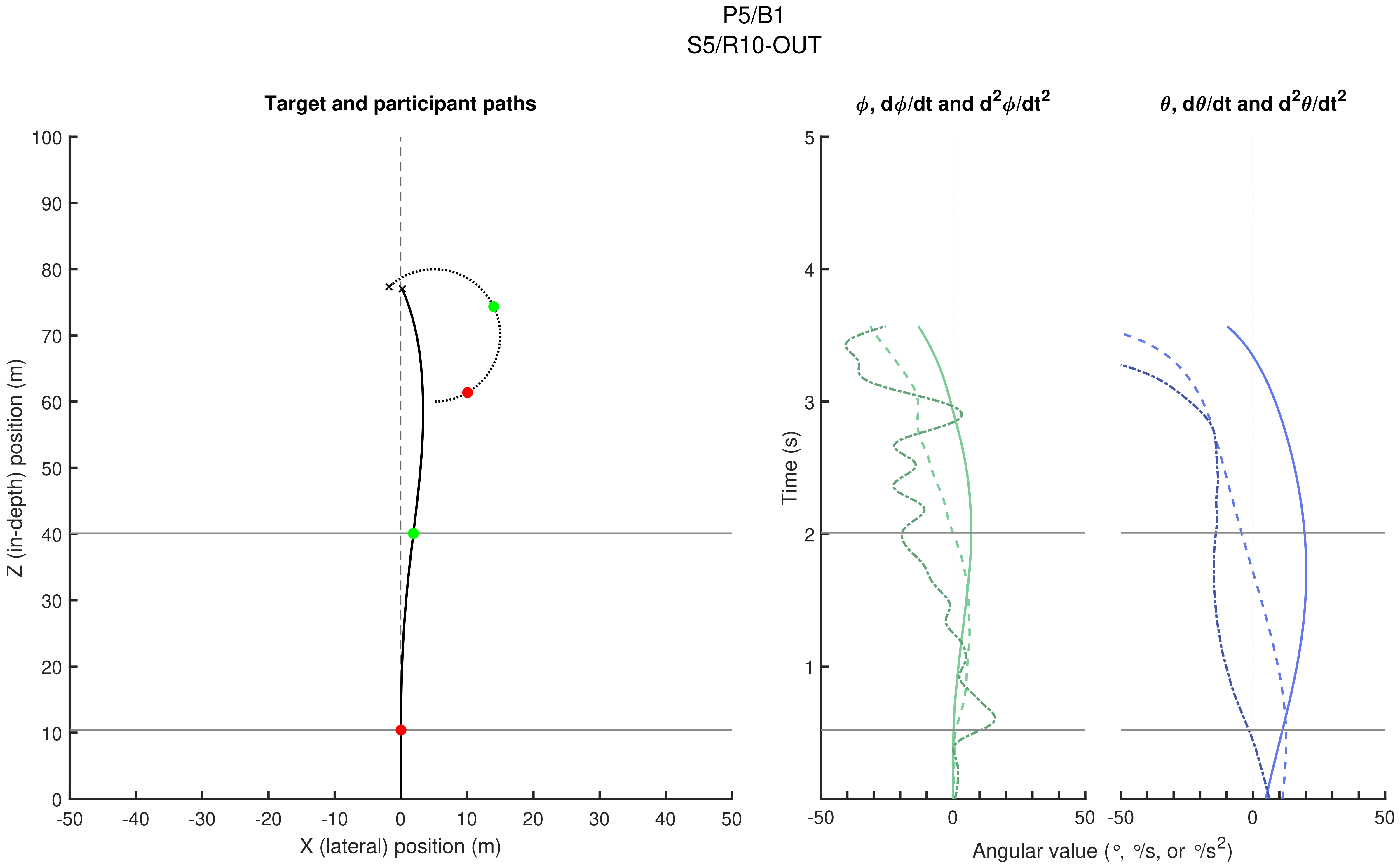
P5/B1
S5/R10-OUT
Target and participant paths
Z (in-depth) position (m)
X (lateral) position (m)
-50
-40
-30
-20
-10
0
10
20
30
40
50
60
70
80
90
100
ϕ, dϕ/dt and d$^2\phi$/dt^2
θ, dθ/dt and d$^2\theta$/dt^2
Time (s)
1
2
3
4
5
Angular value (°, °/s, or °/s^2)

P5/B1
S5/R10-IN

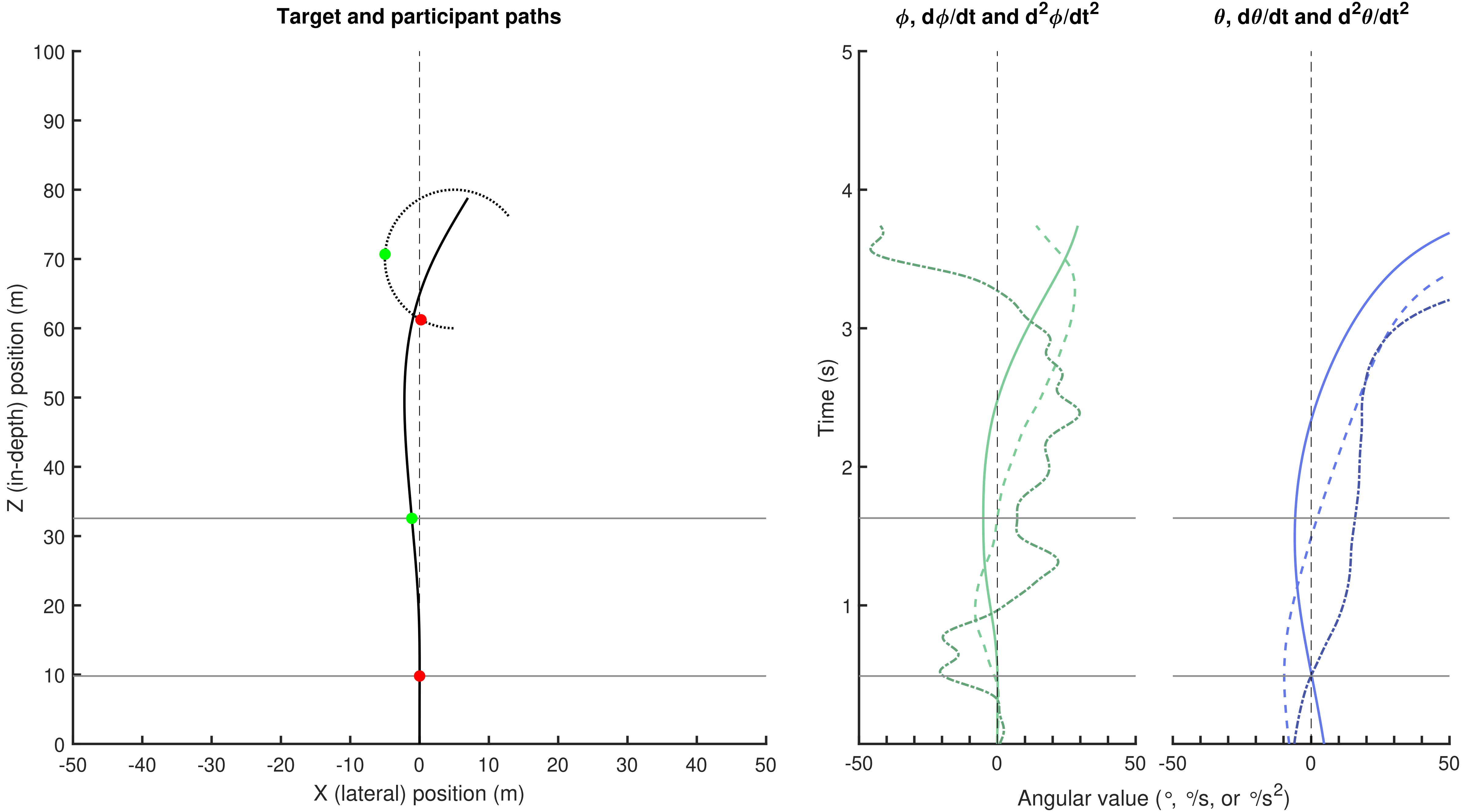

P5/B1
S5/R20-OUT

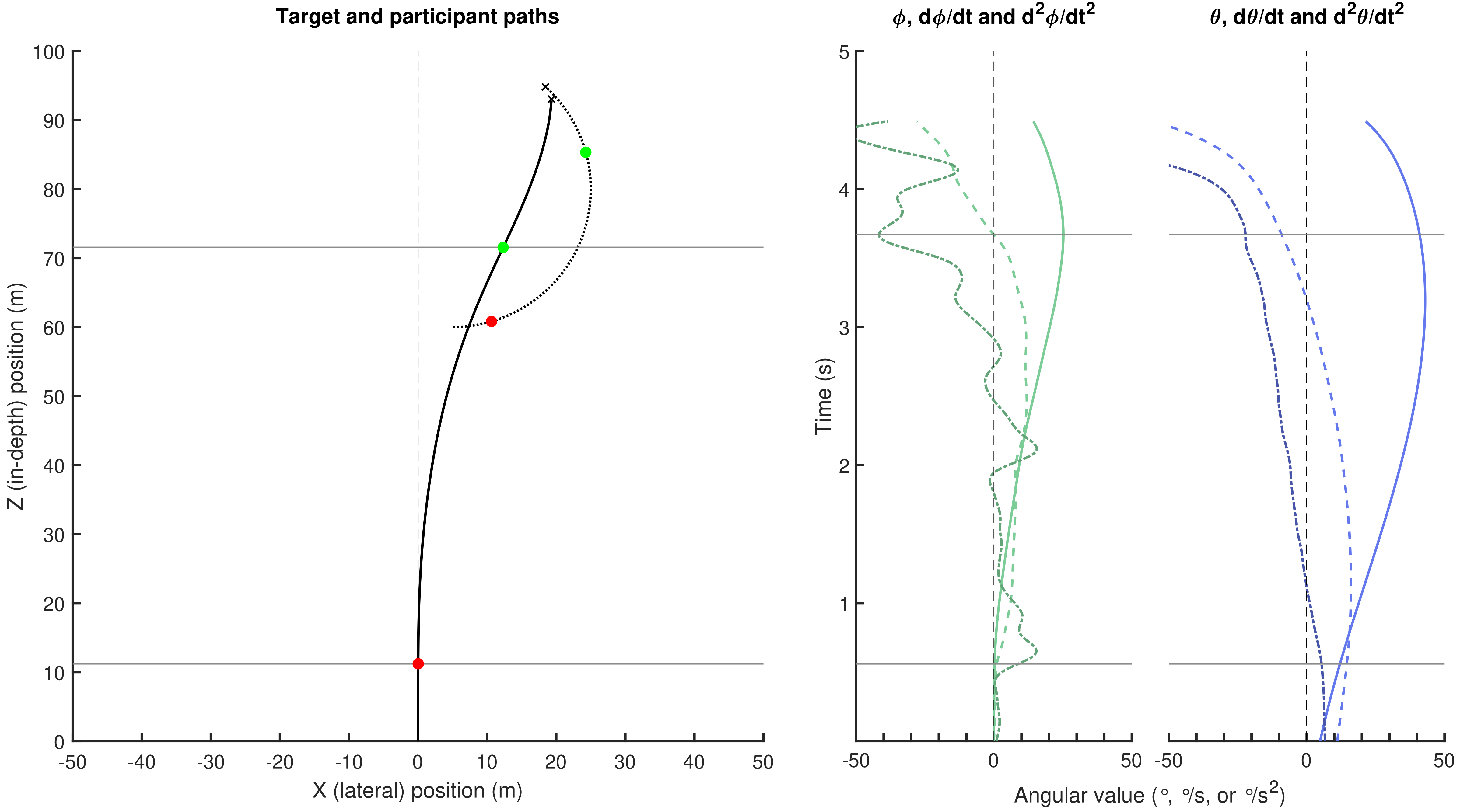

P5/B1
S5/R20-IN

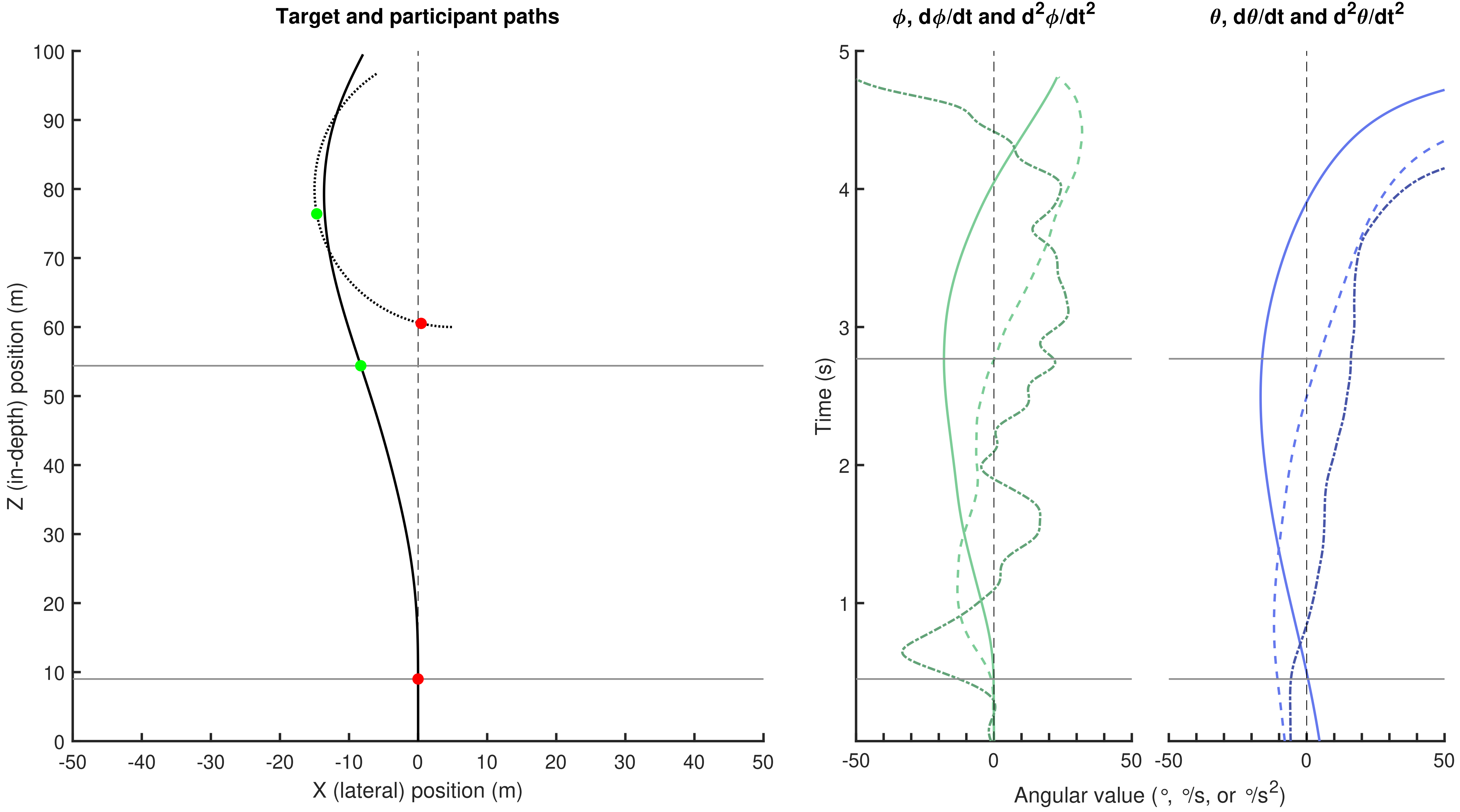

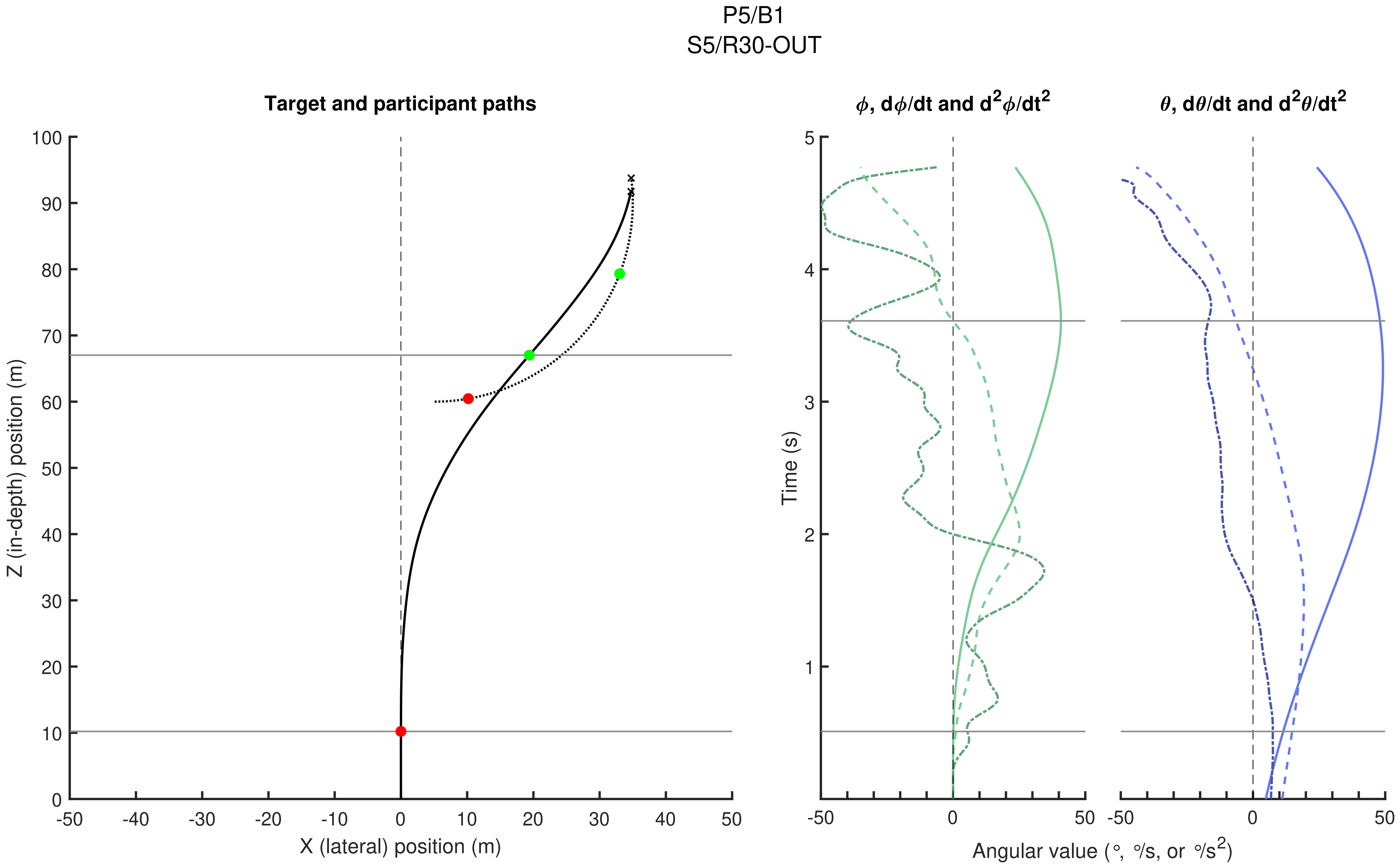
P5/B1
S5/R30-OUT
Target and participant paths
Z (in-depth) position (m)
X (lateral) position (m)
100
90
80
70
60
50
40
30
20
10
0
-50
-40
-30
-20
-10
0
10
20
30
40
50
ϕ, dϕ/dt and d$^2\phi$/dt^2
θ, dθ/dt and d$^2\theta$/dt^2
Time (s)
5
4
3
2
1
-50
0
50
Angular value (°, °/s, or °/s^2)

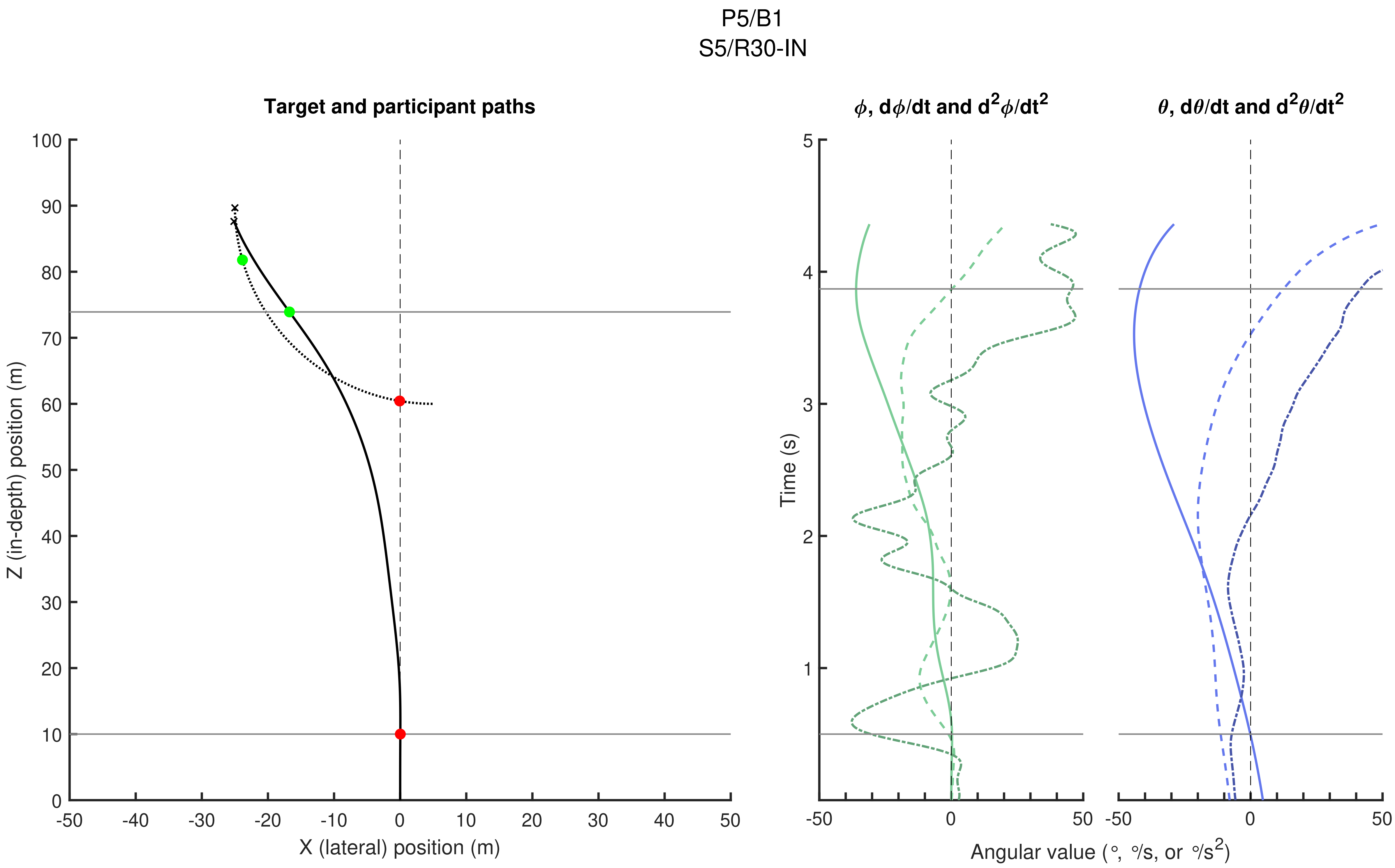
P5/B1
S5/R30-IN
Target and participant paths
Z (in-depth) position (m)
X (lateral) position (m)
ϕ, dϕ/dt and d$^2\phi$/dt^2
θ, dθ/dt and d$^2\theta$/dt^2
Time (s)
Angular value (°, °/s, or °/s^2)

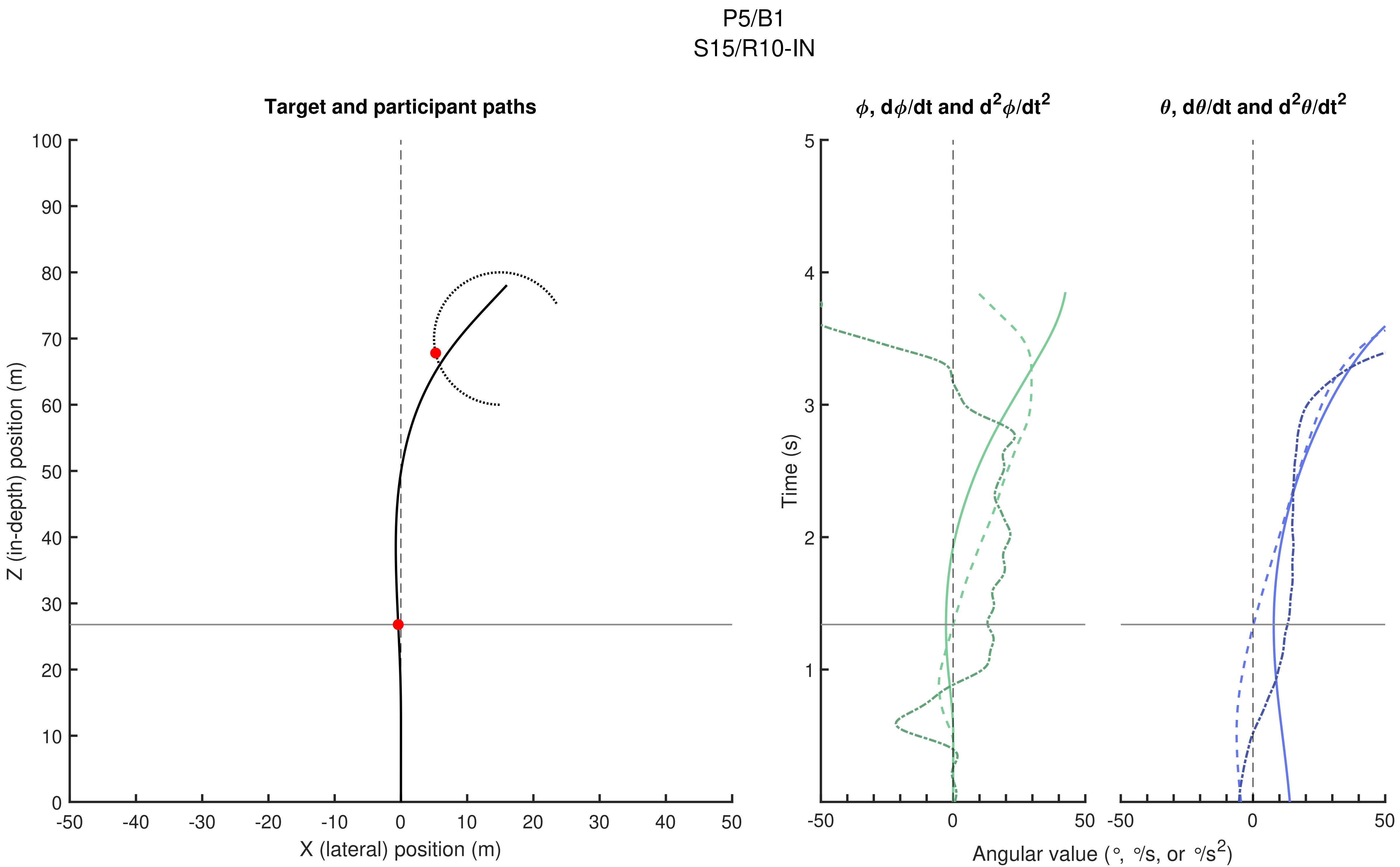
P5/B1
S15/R10-IN
Target and participant paths
Z (in-depth) position (m)
X (lateral) position (m)
100
90
80
70
60
50
40
30
20
10
0
-50
-40
-30
-20
-10
0
10
20
30
40
50
ϕ, dϕ/dt and d$^2\phi$/dt^2
θ, dθ/dt and d$^2\theta$/dt^2
Time (s)
5
4
3
2
1
-50
0
50
-50
0
50
Angular value (°, °/s, or °/s^2)

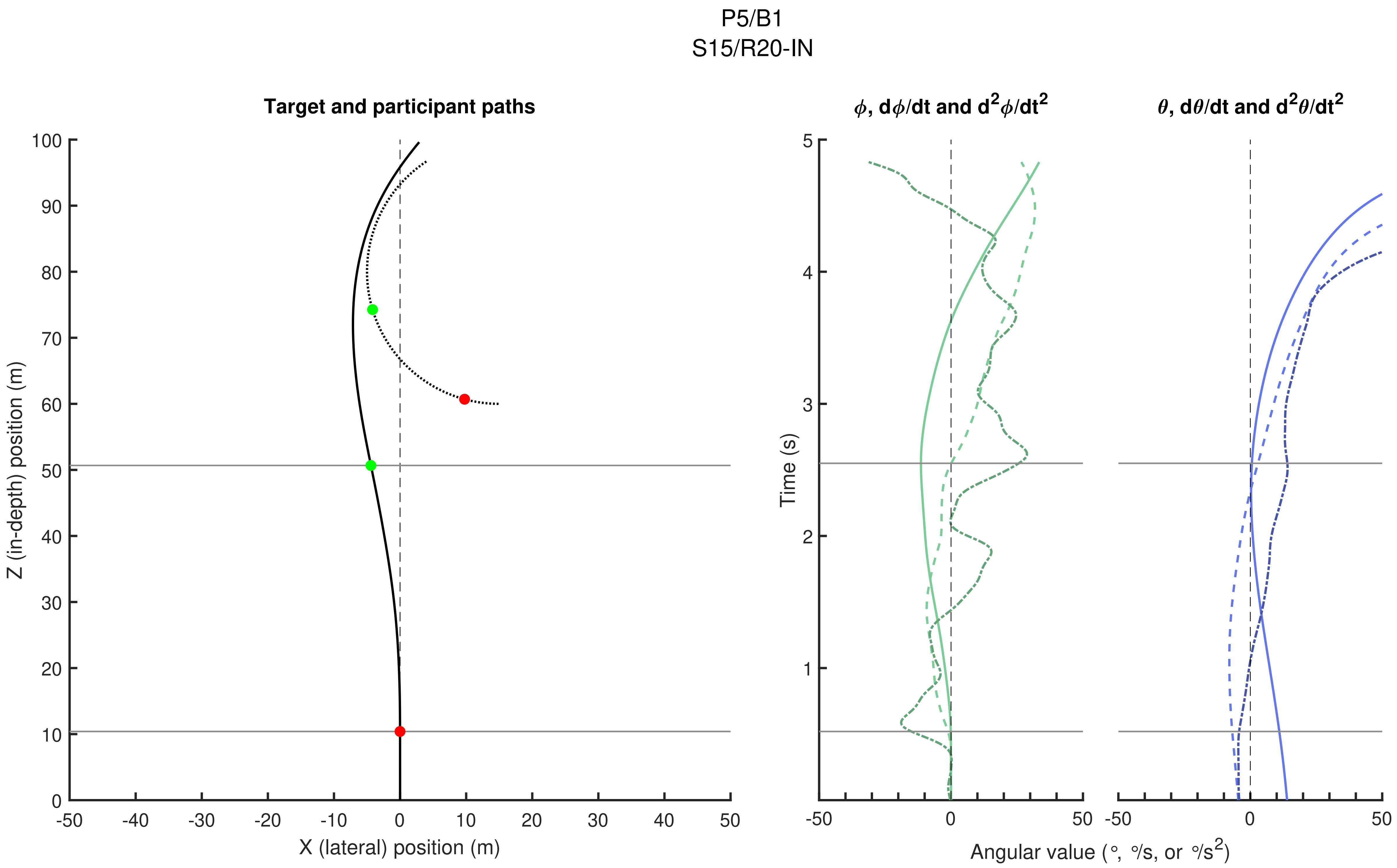

P5/B1
S15/R20-IN
Target and participant paths
Z (in-depth) position (m)
X (lateral) position (m)
ϕ, dϕ/dt and d²ϕ/dt²
θ, dθ/dt and d²θ/dt²
Time (s)
Angular value (°, °/s, or °/s²)

P5/B1
S15/R30-IN

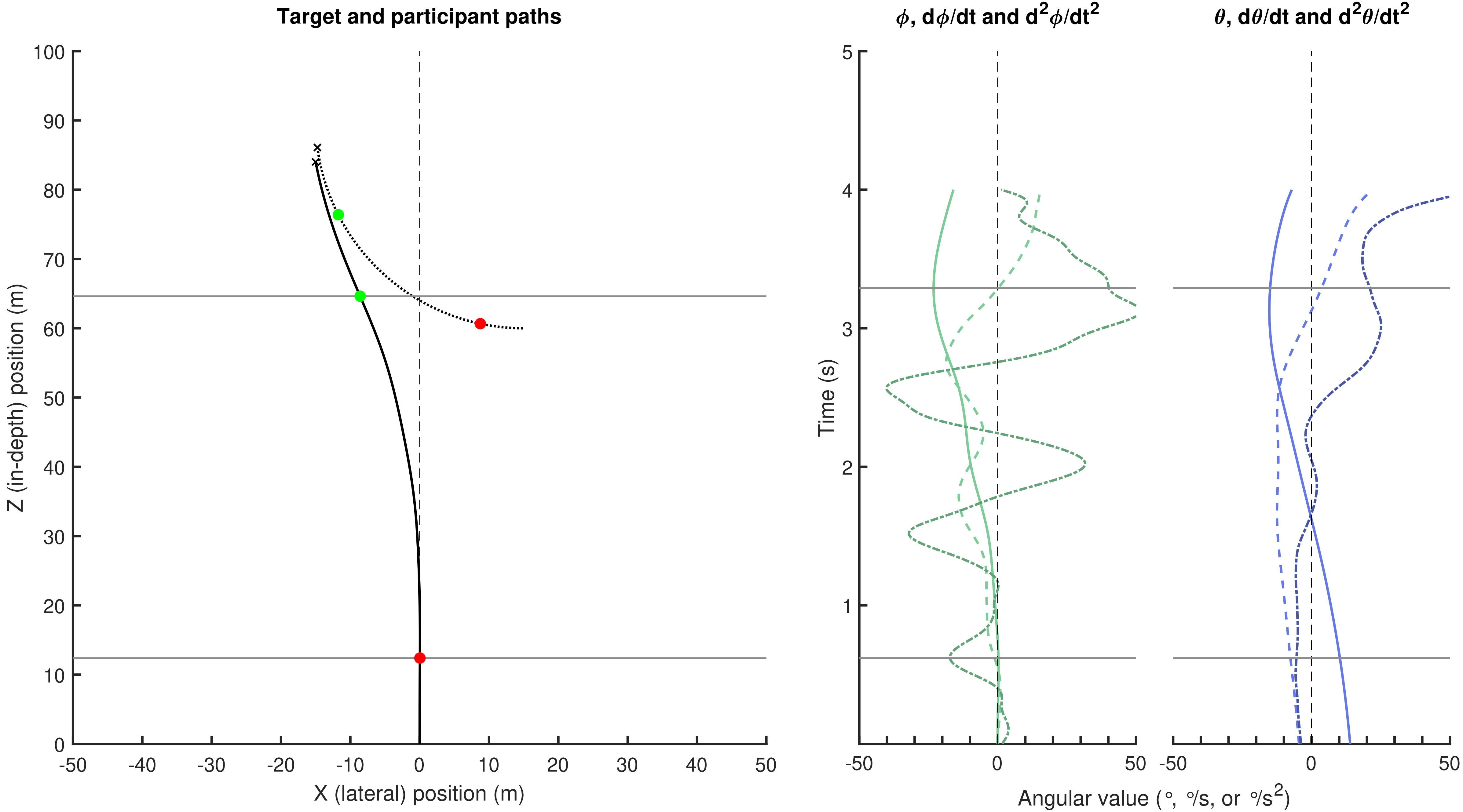

P5/B1
S20/R10-IN

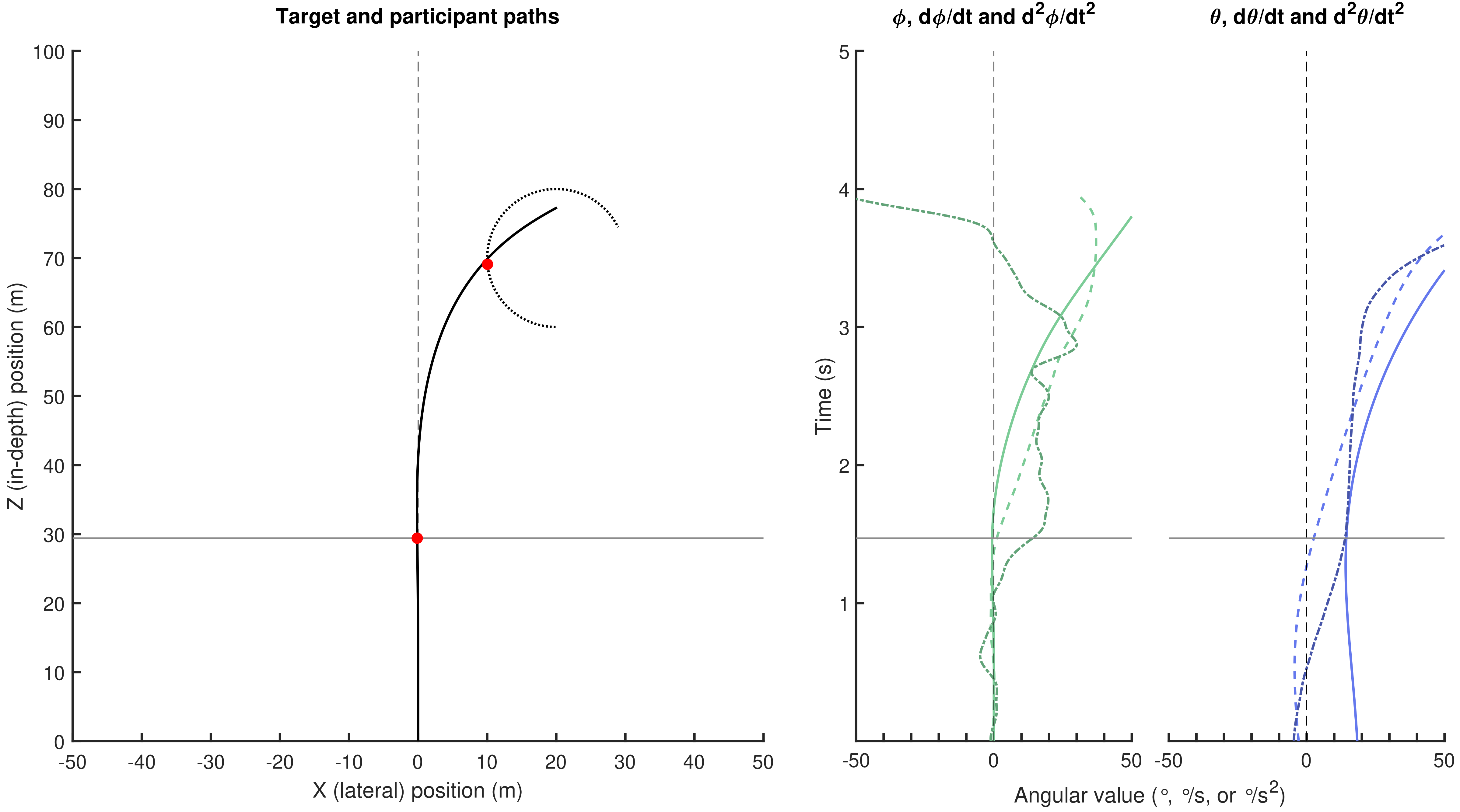

P5/B1
S20/R20-IN

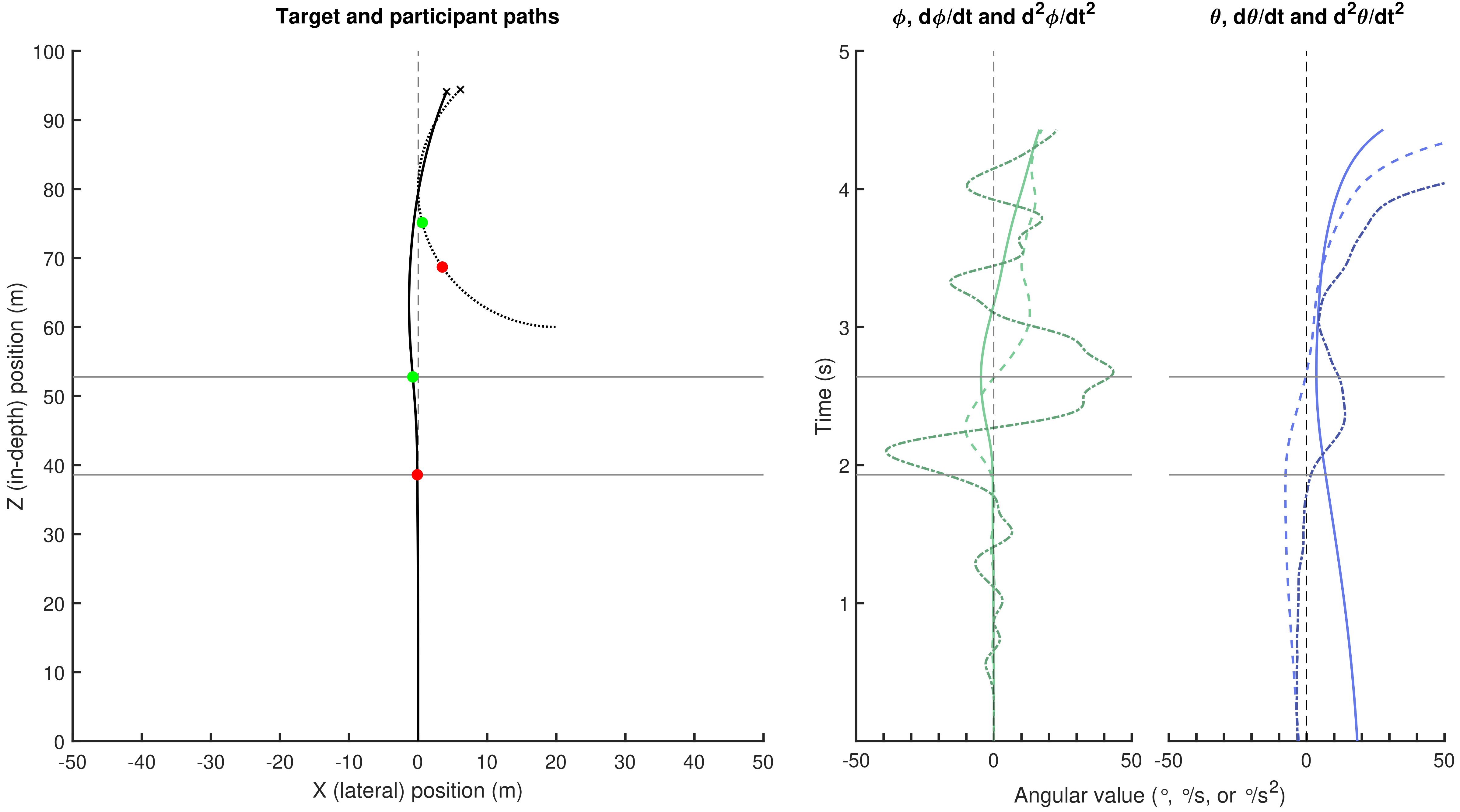

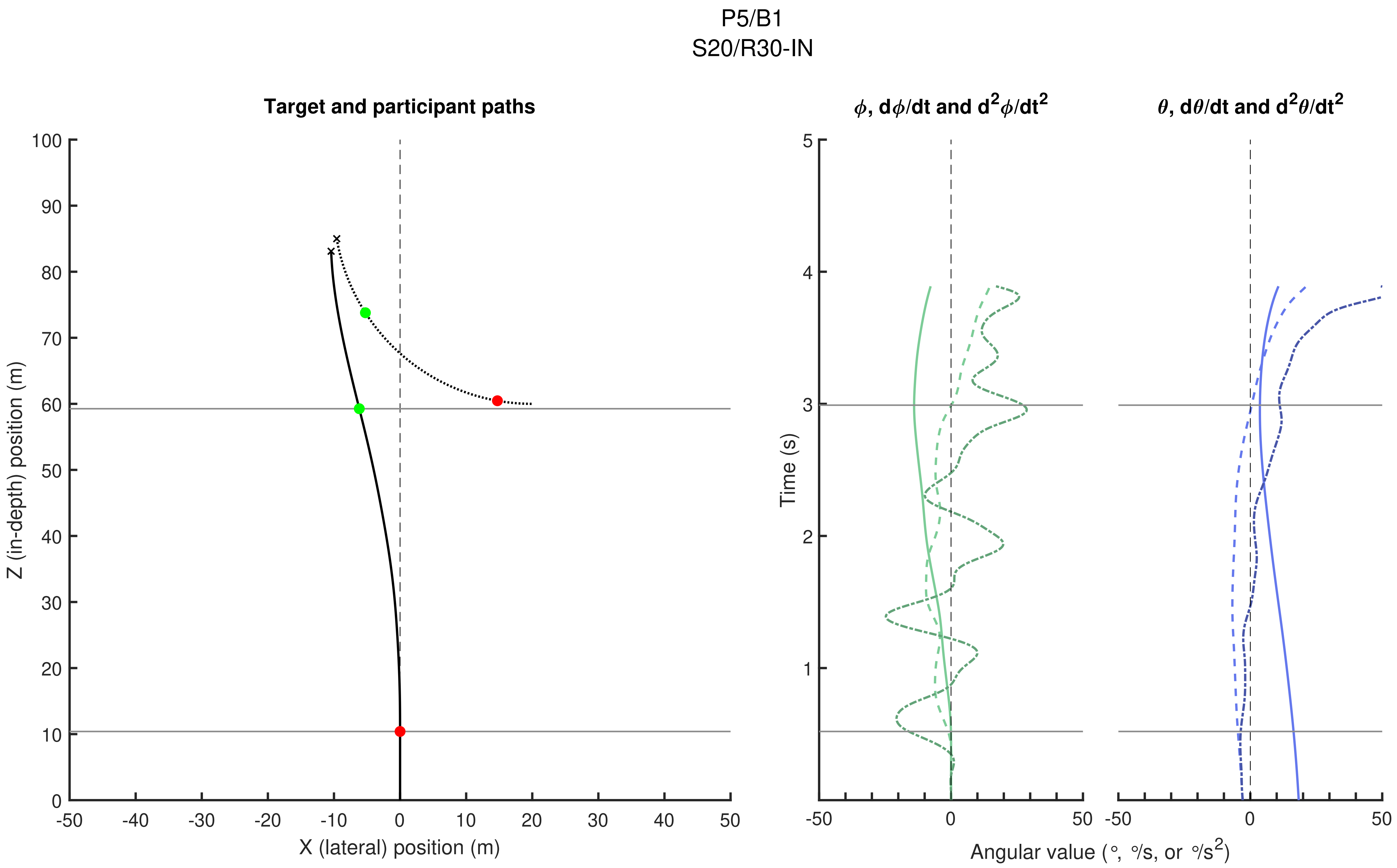

P5/B1
S20/R30-IN
Target and participant paths
ϕ, dϕ/dt and d$^2\phi$/dt^2
θ, dθ/dt and d$^2\theta$/dt^2
Z (in-depth) position (m)
X (lateral) position (m)
Time (s)
Angular value (°, °/s, or °/s^2)

P5/B2
S20/R10-IN

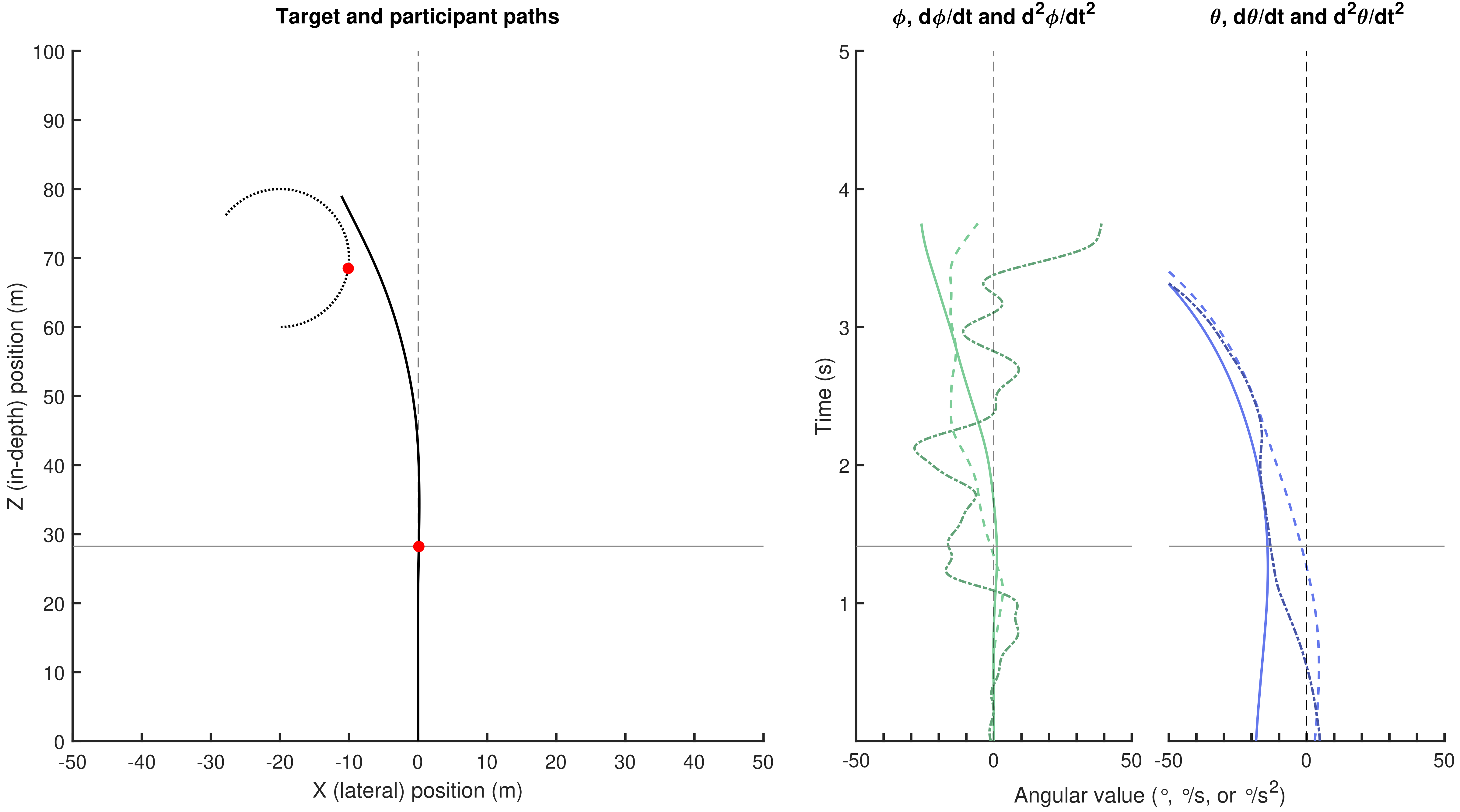

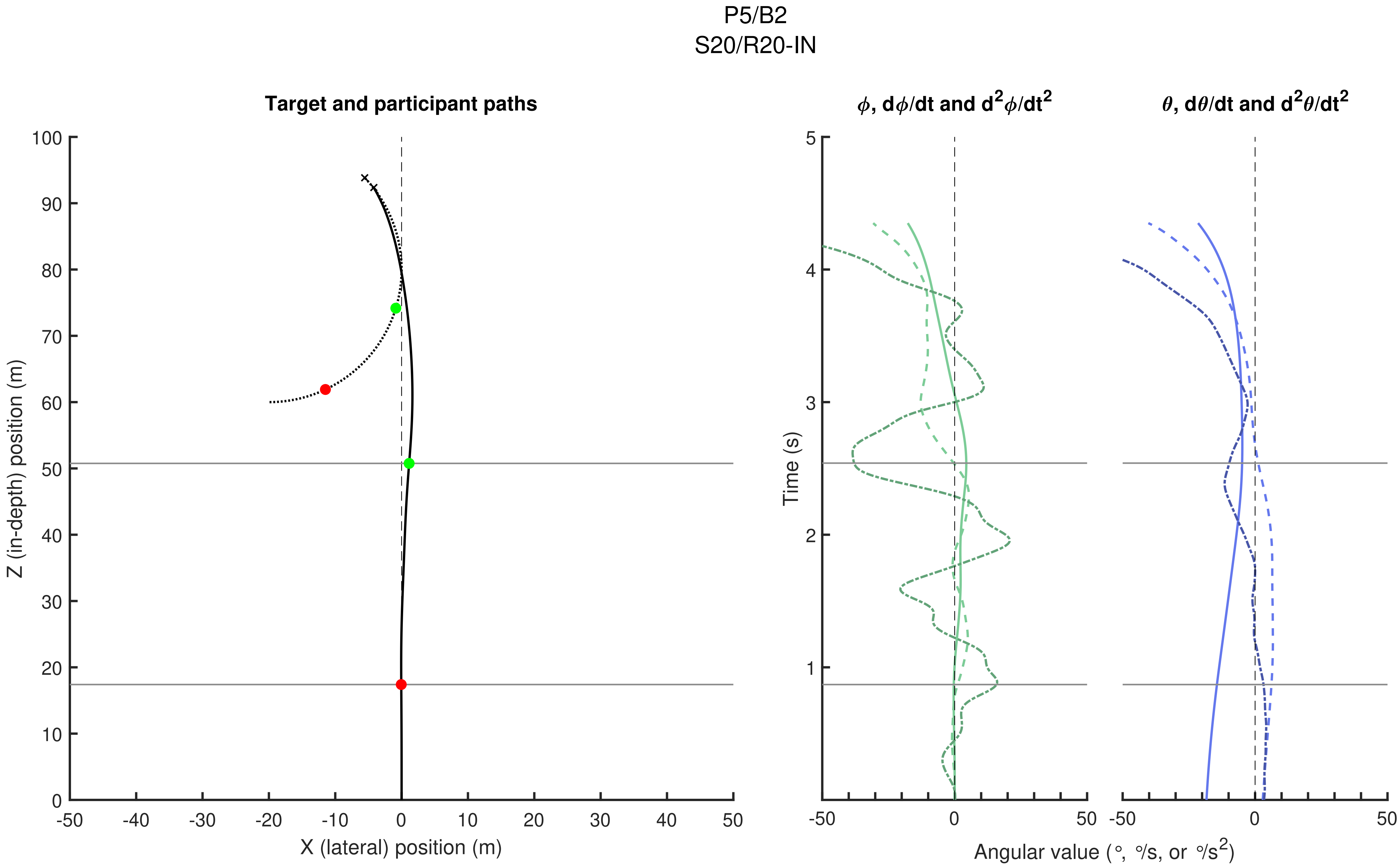
P5/B2
S20/R20-IN
Target and participant paths
Z (in-depth) position (m)
X (lateral) position (m)
100
90
80
70
60
50
40
30
20
10
0
-50
-40
-30
-20
-10
0
10
20
30
40
50
ϕ, dϕ/dt and d$^2\phi$/dt^2
θ, dθ/dt and d$^2\theta$/dt^2
Time (s)
5
4
3
2
1
-50
0
50
-50
0
50
Angular value (°, °/s, or °/s^2)

P5/B2
S20/R30-IN

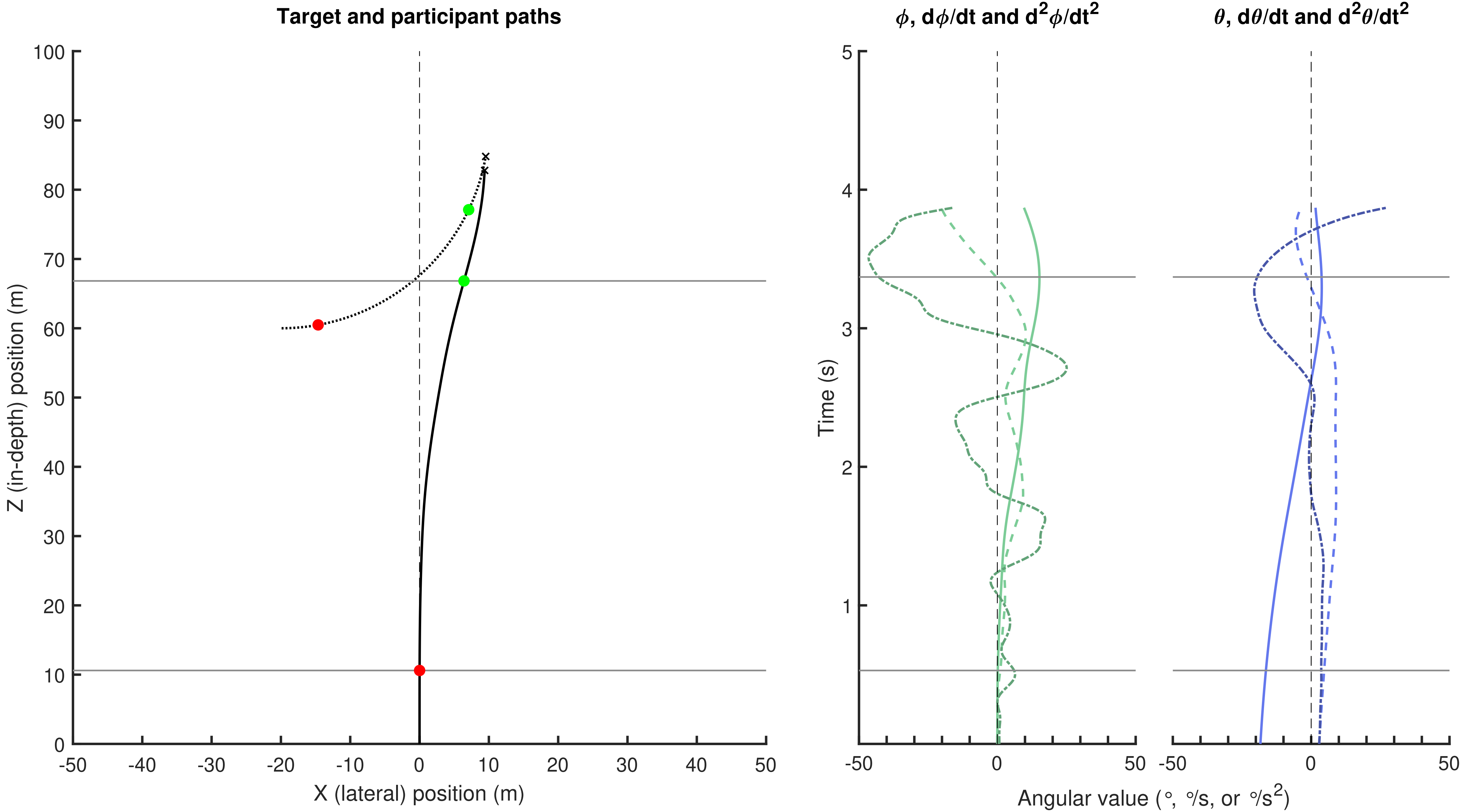

P5/B2
S15/R10-IN

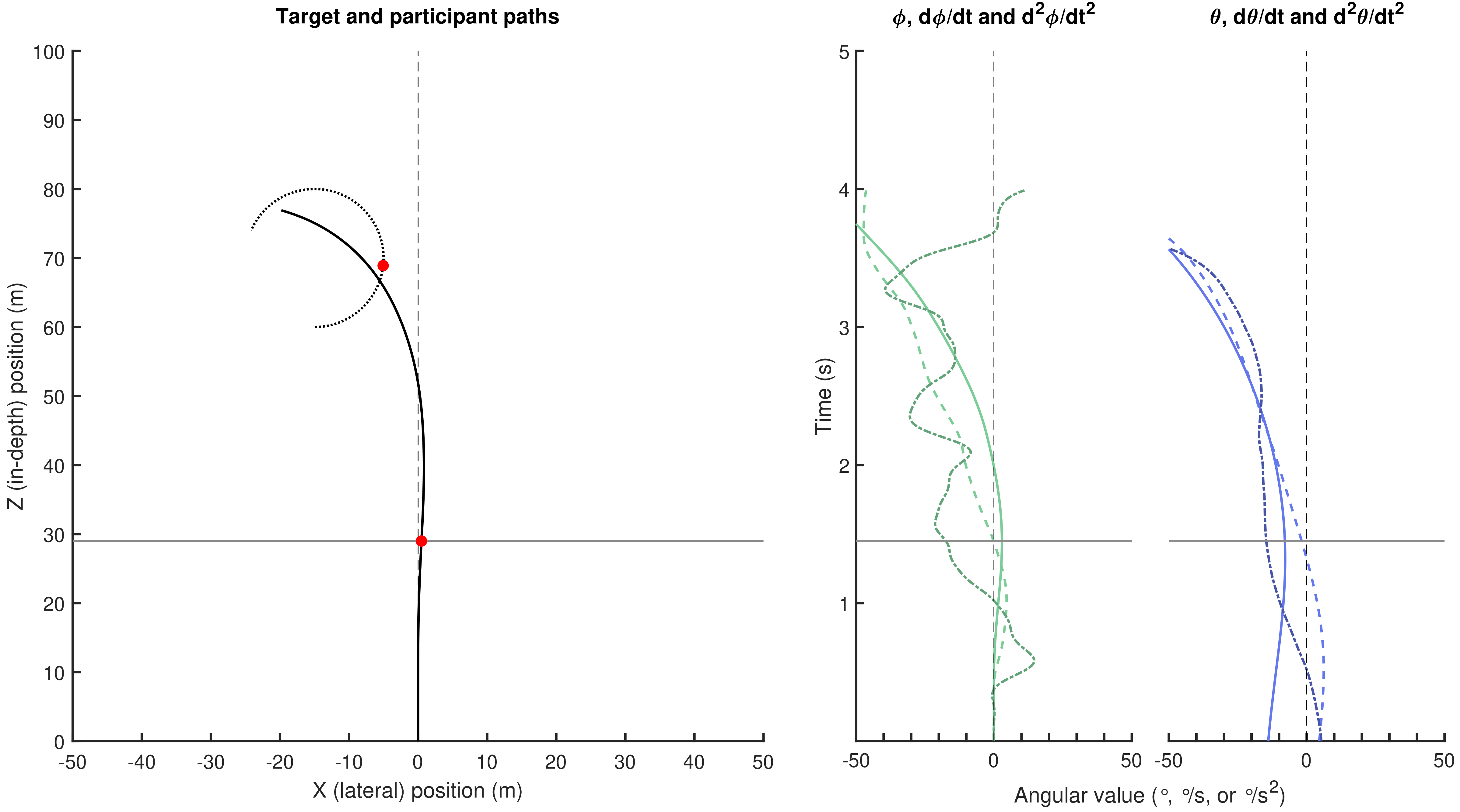

P5/B2
S15/R20-IN

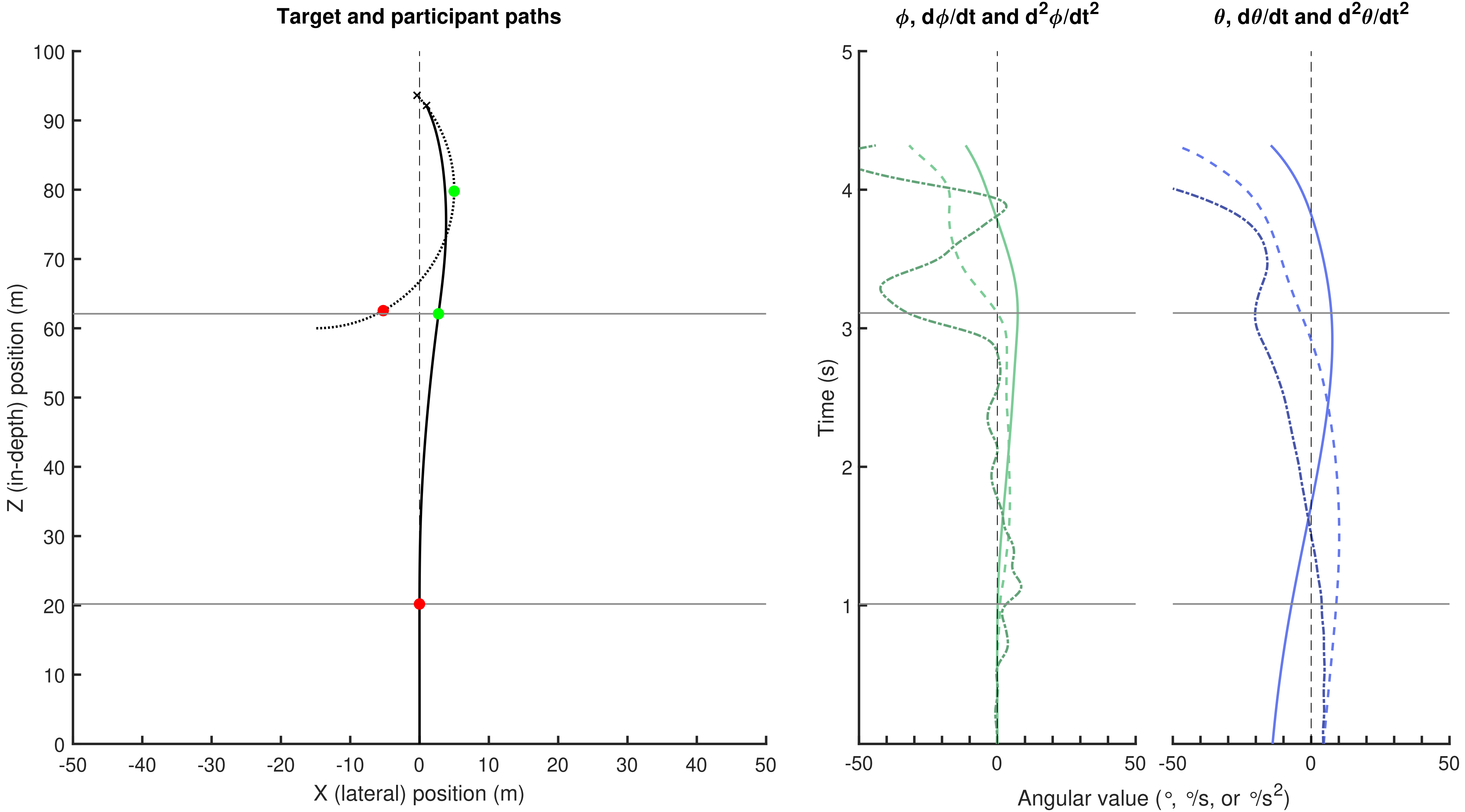

P5/B2
S15/R30-IN

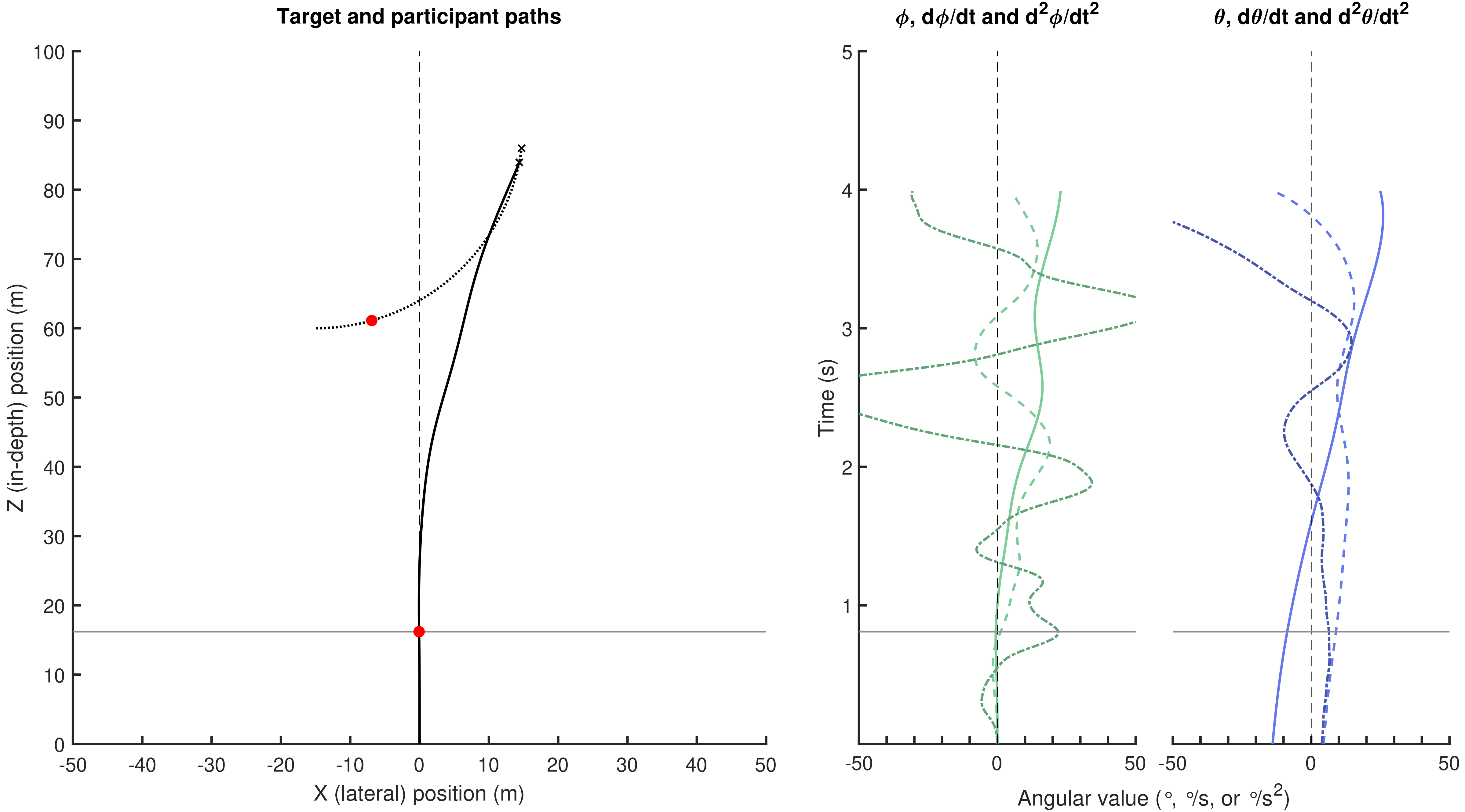

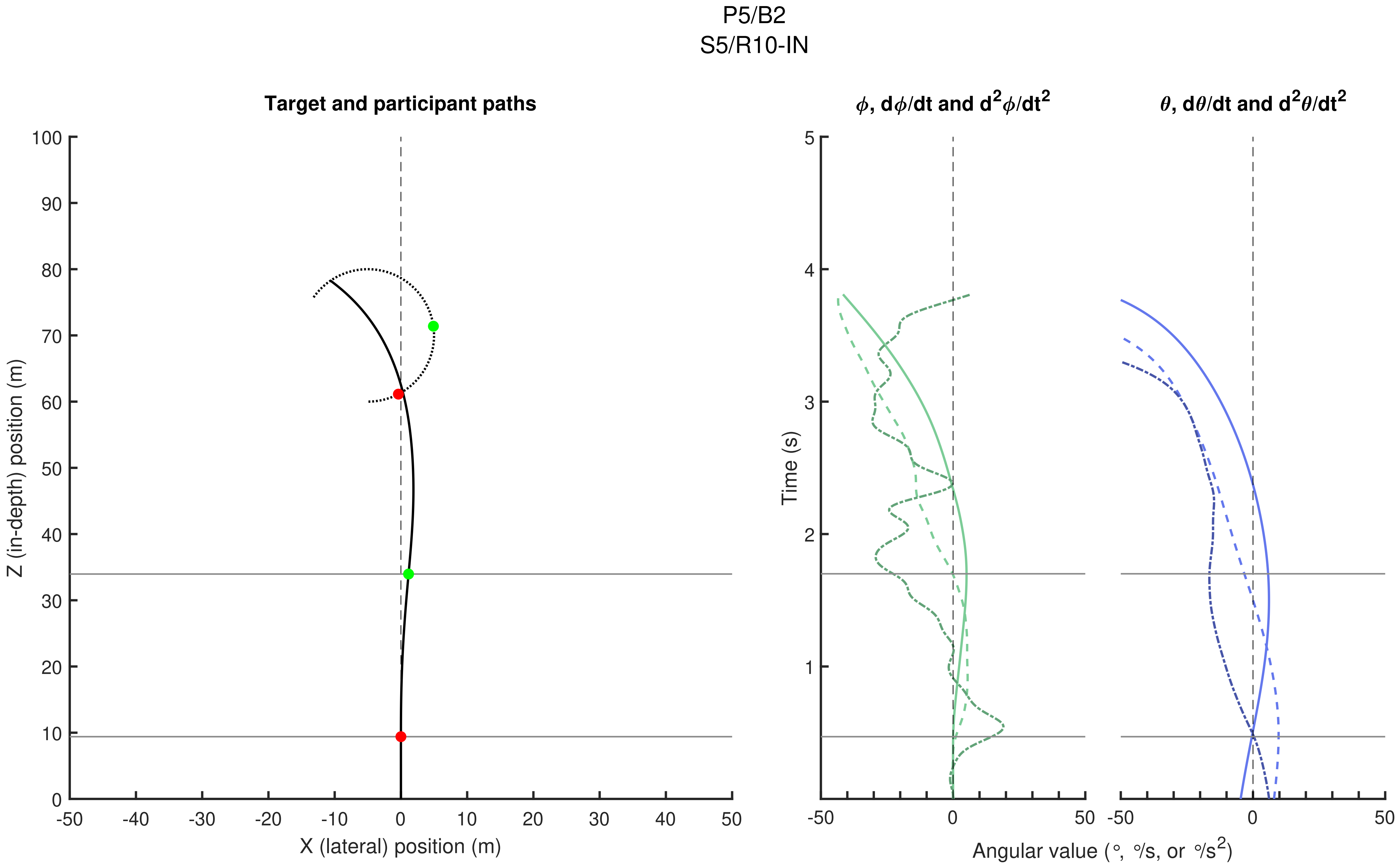
P5/B2
S5/R10-IN
Target and participant paths
Z (in-depth) position (m)
X (lateral) position (m)
100
90
80
70
60
50
40
30
20
10
0
-50
-40
-30
-20
-10
0
10
20
30
40
50
ϕ, dϕ/dt and d²ϕ/dt²
θ, dθ/dt and d²θ/dt²
Time (s)
5
4
3
2
1
-50
0
50
-50
0
50
Angular value (°, °/s, or °/s²)

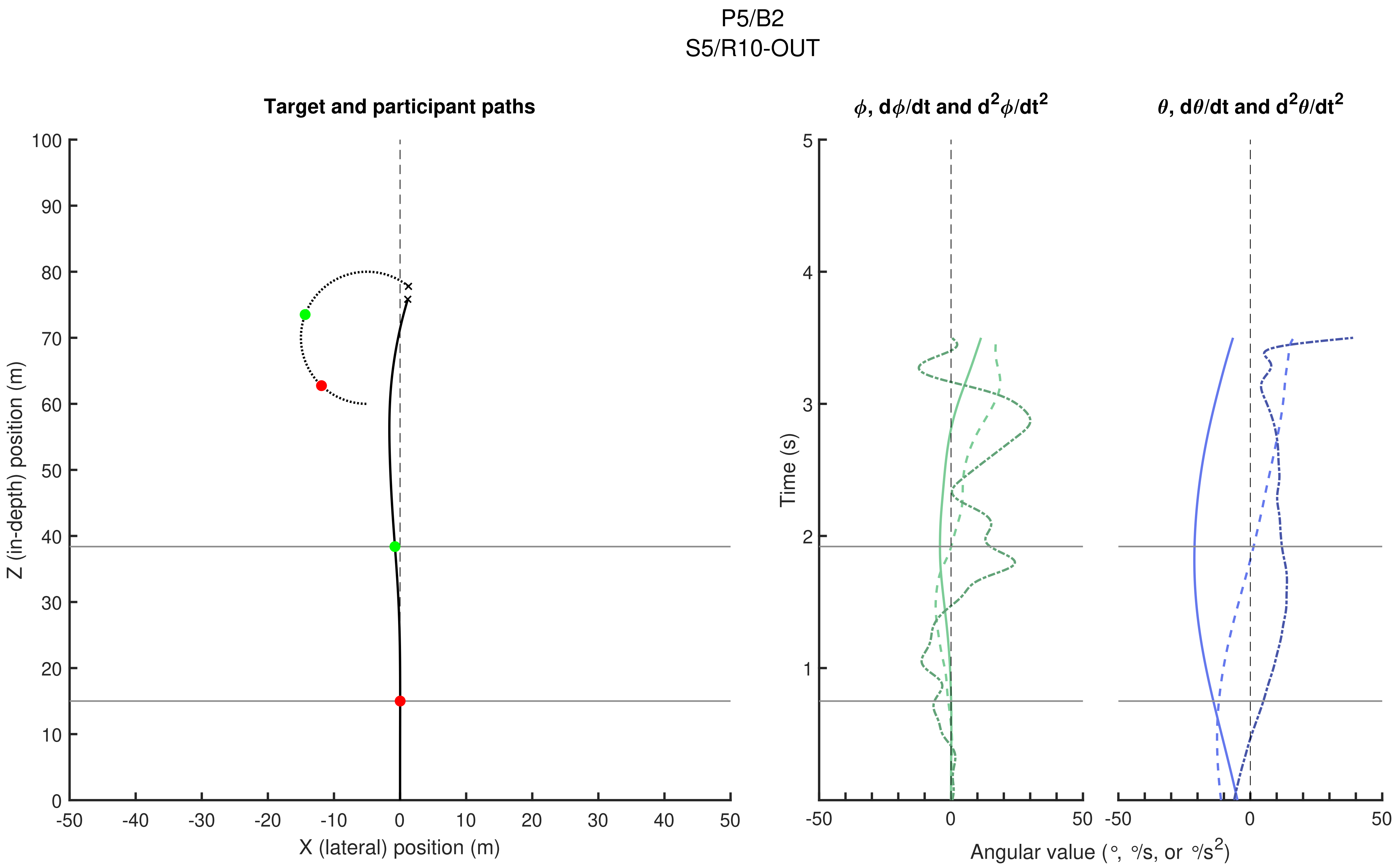
P5/B2
S5/R10-OUT
Target and participant paths
Z (in-depth) position (m)
X (lateral) position (m)
100
90
80
70
60
50
40
30
20
10
0
-50
-40
-30
-20
-10
0
10
20
30
40
50
ϕ, dϕ/dt and d²ϕ/dt²
θ, dθ/dt and d²θ/dt²
Time (s)
5
4
3
2
1
-50
0
50
-50
0
50
Angular value (°, °/s, or °/s²)

P5/B2
S5/R20-IN

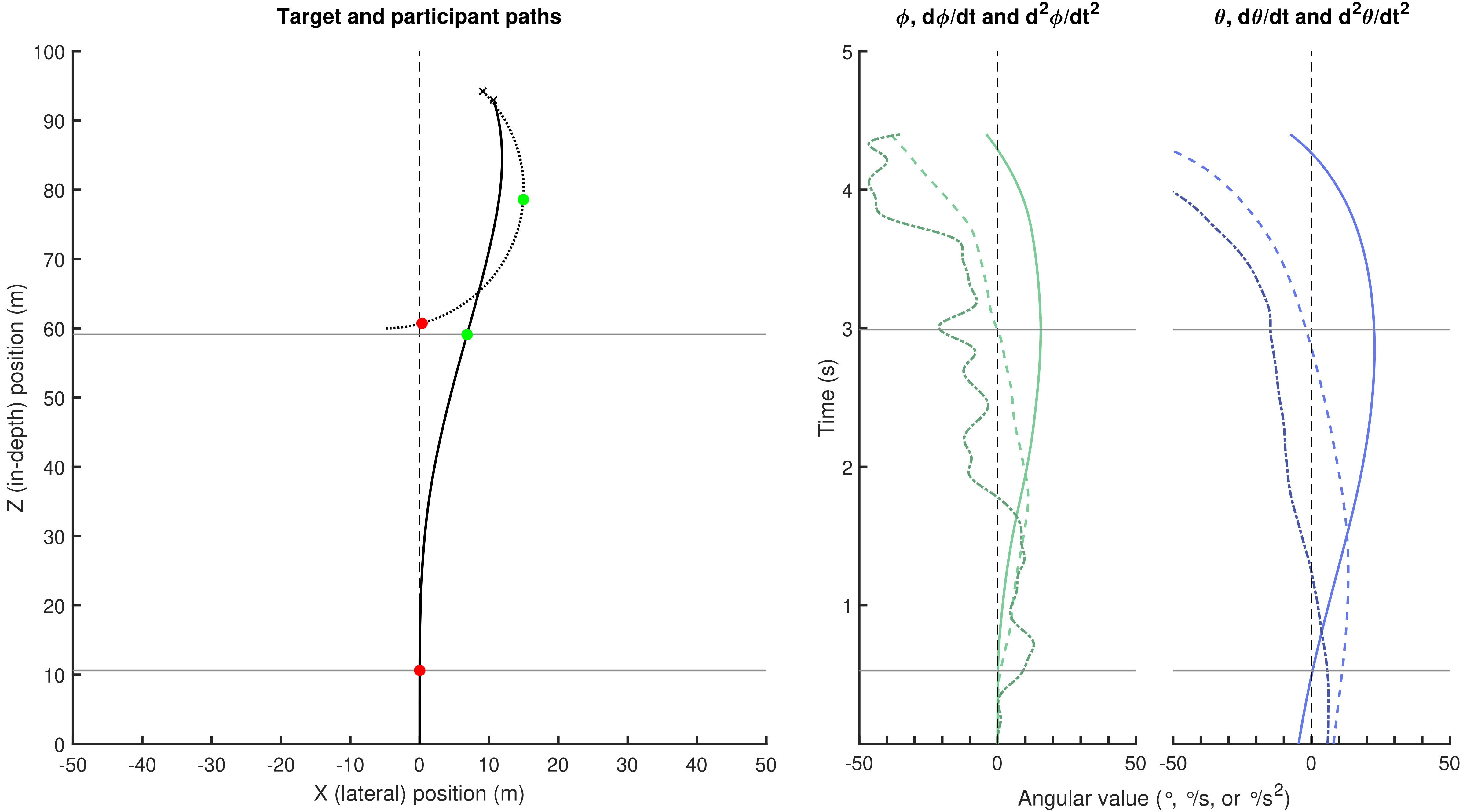

P5/B2
S5/R20-OUT

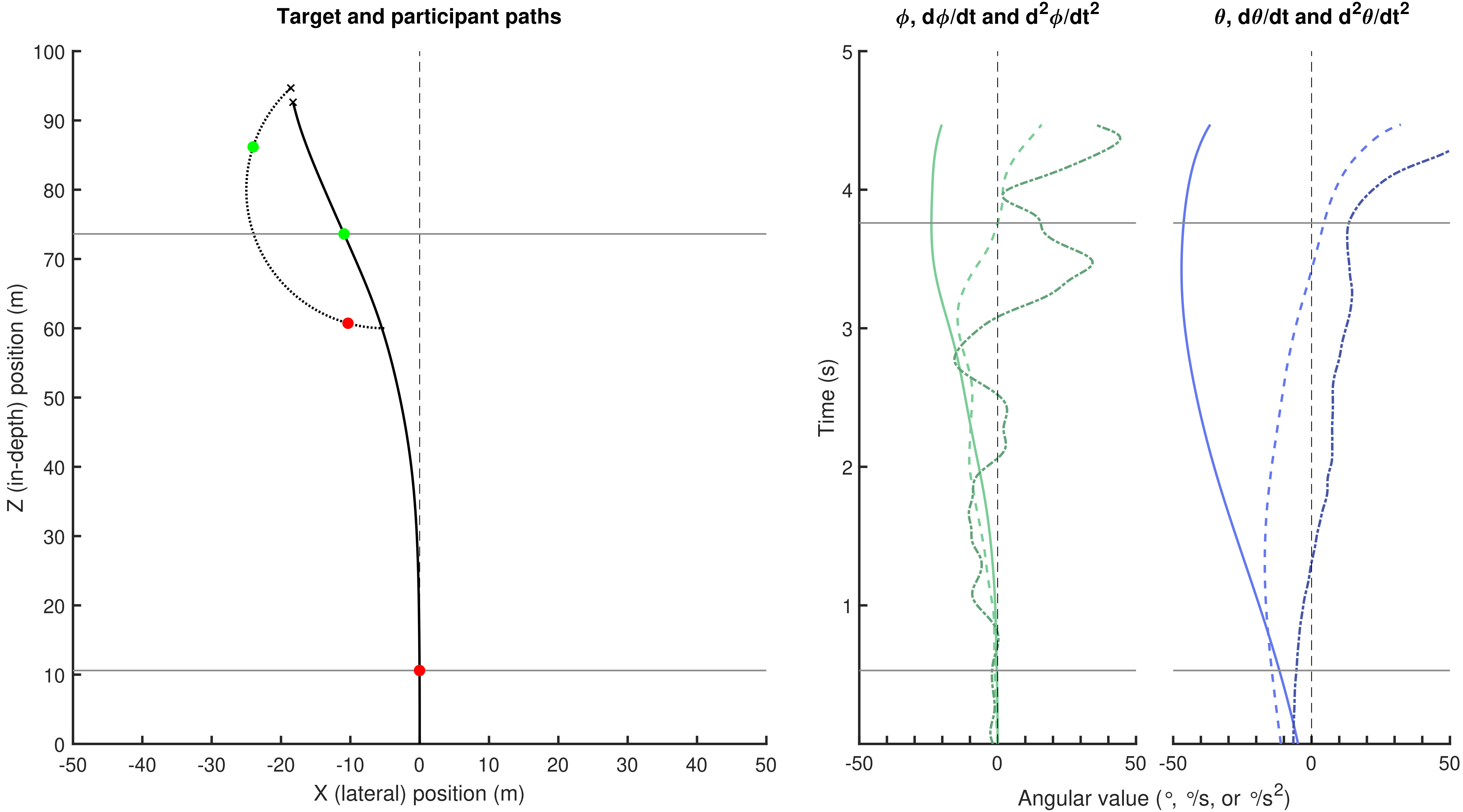

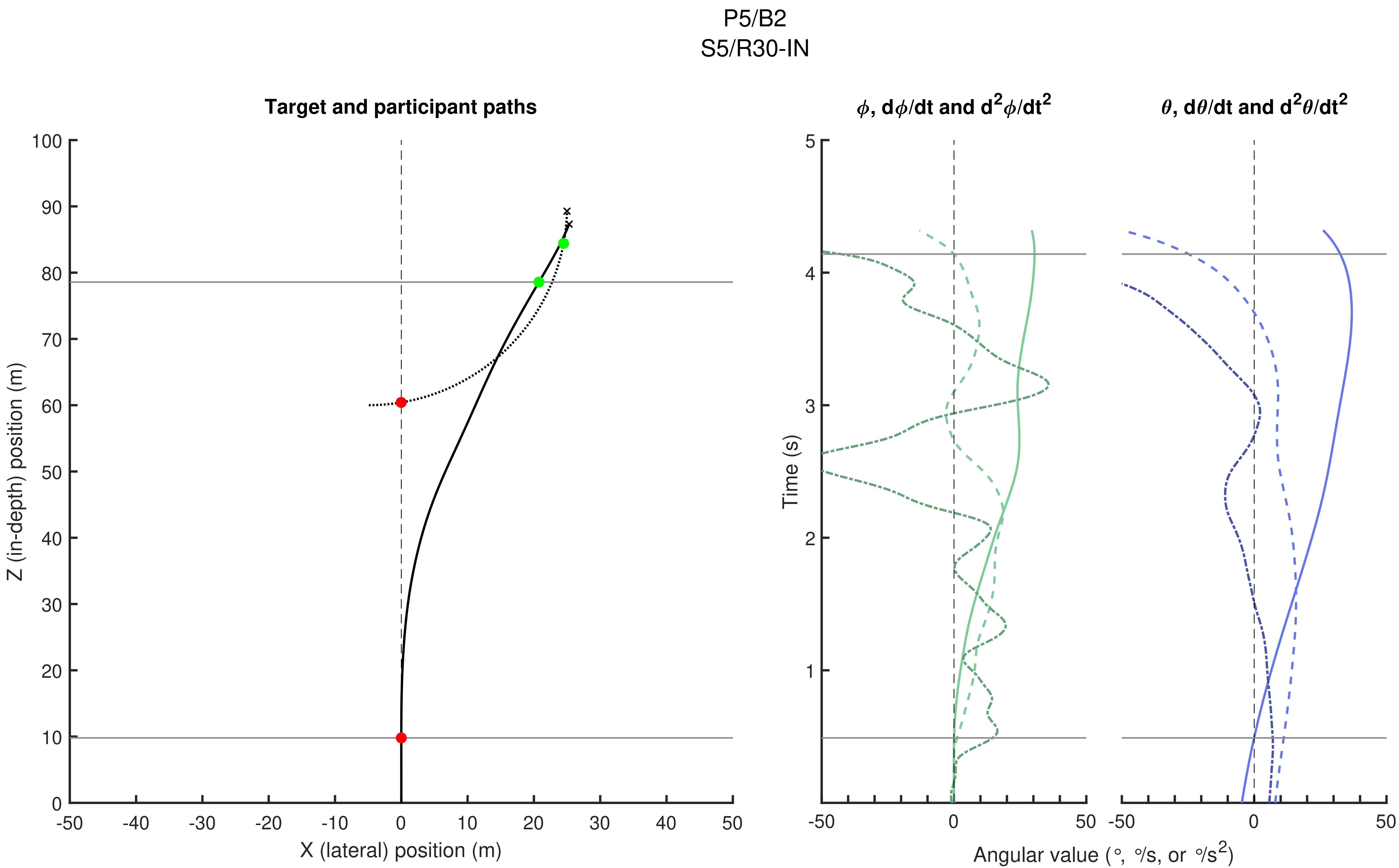
P5/B2
S5/R30-IN
Target and participant paths
Z (in-depth) position (m)
X (lateral) position (m)
ϕ, dϕ/dt and d$^2\phi$/dt^2
θ, dθ/dt and d$^2\theta$/dt^2
Time (s)
Angular value (°, °/s, or °/s^2)

P5/B2
S5/R30-OUT

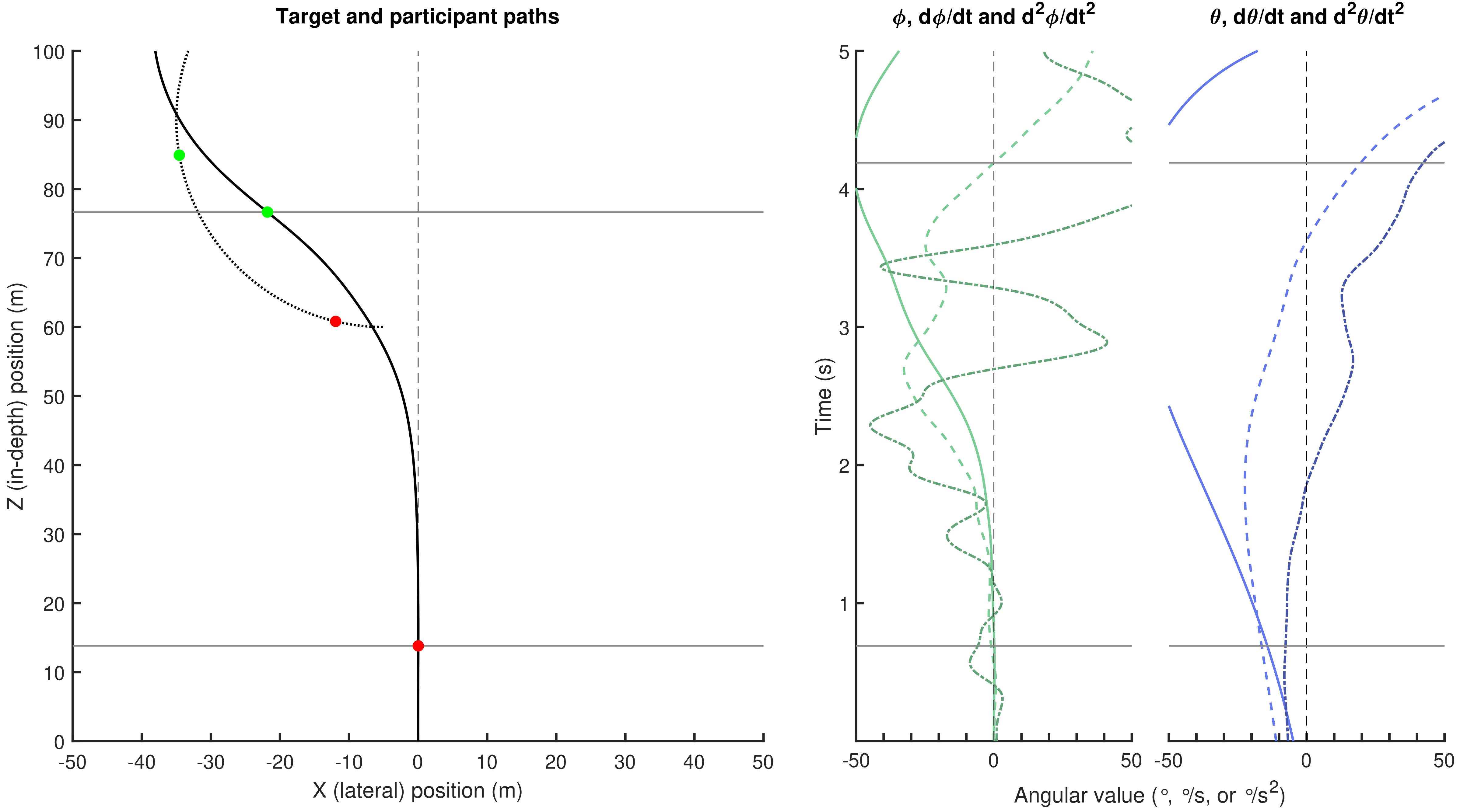

P5/B2
S5/R10-OUT

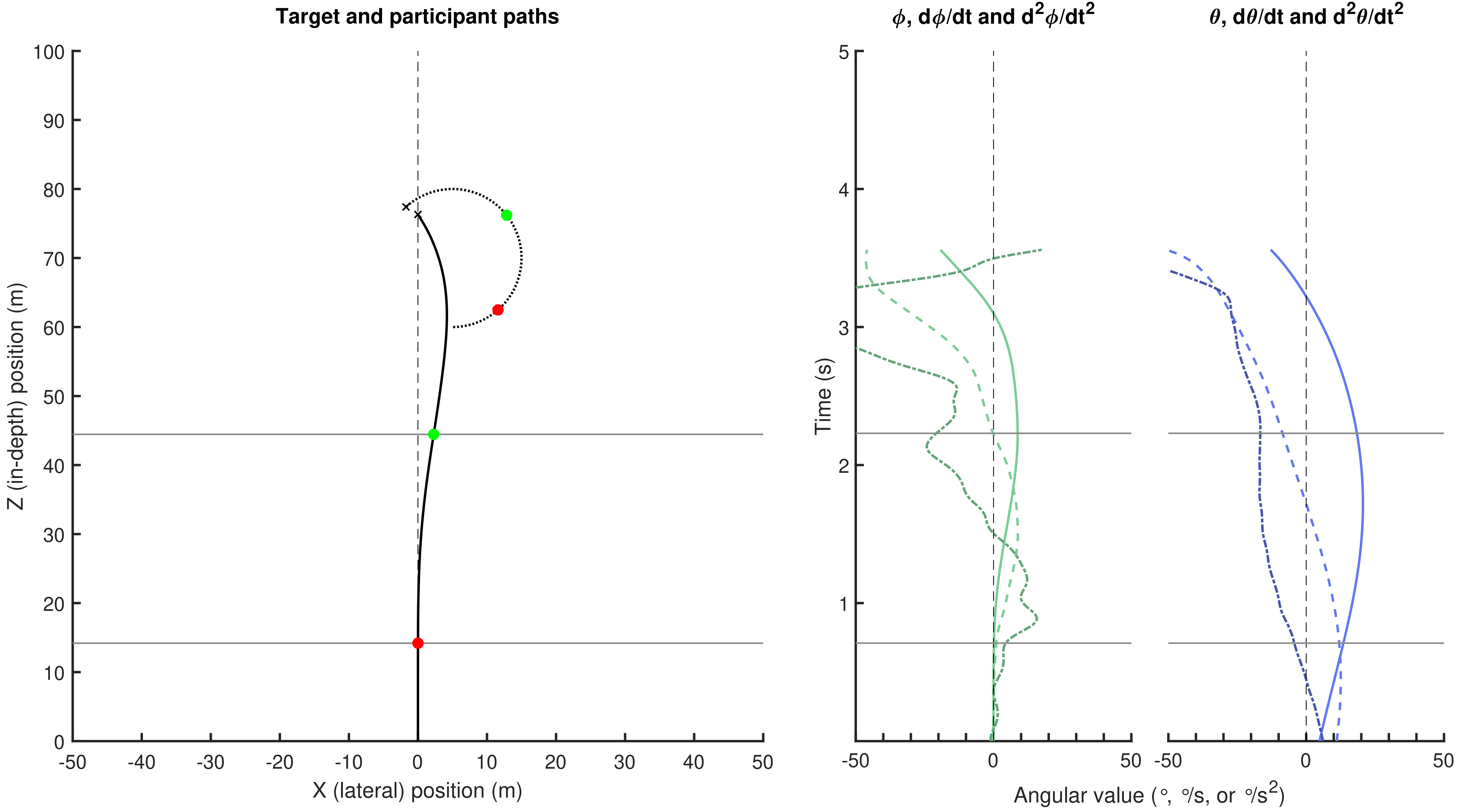

P5/B2
S5/R10-IN

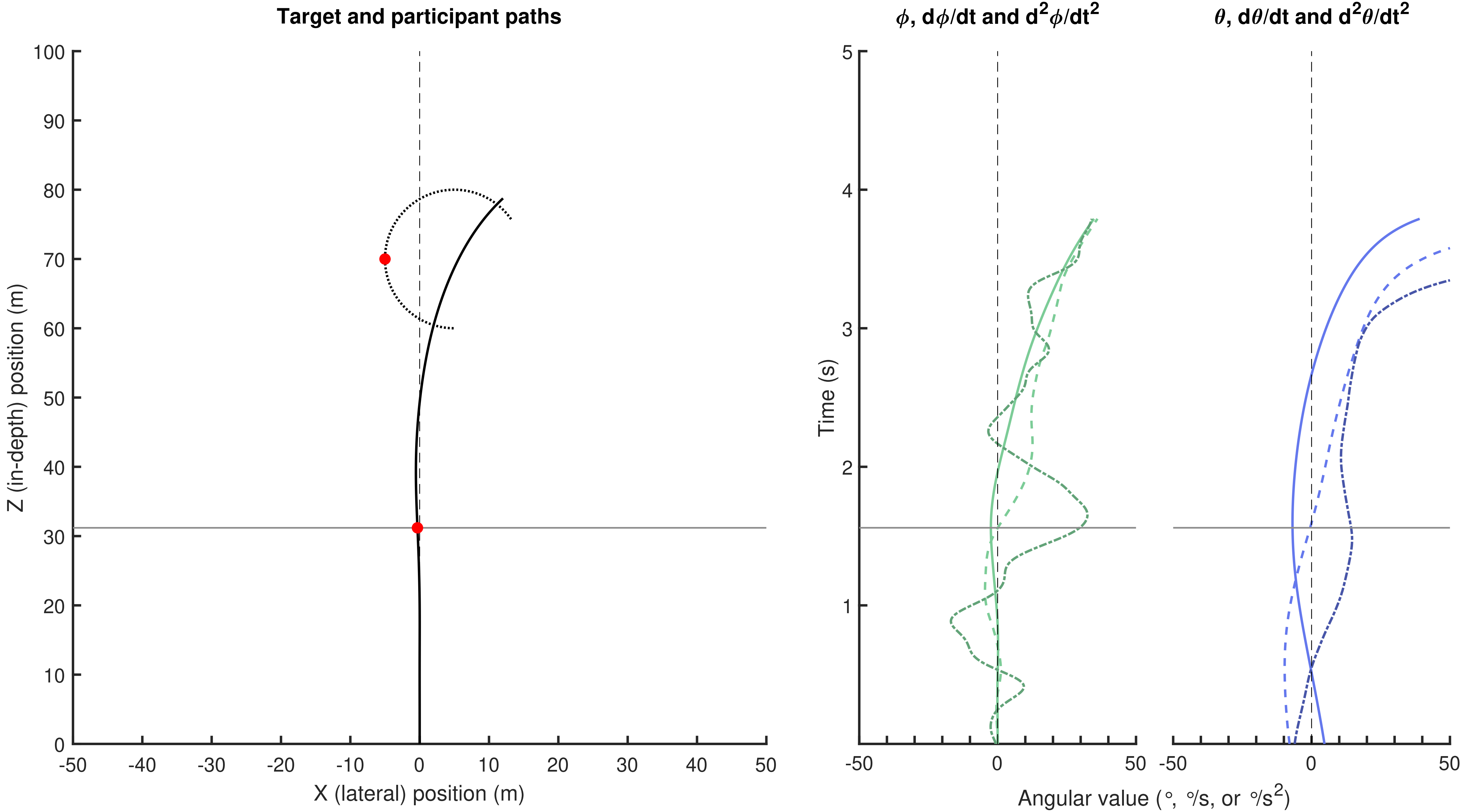

P5/B2
S5/R20-OUT

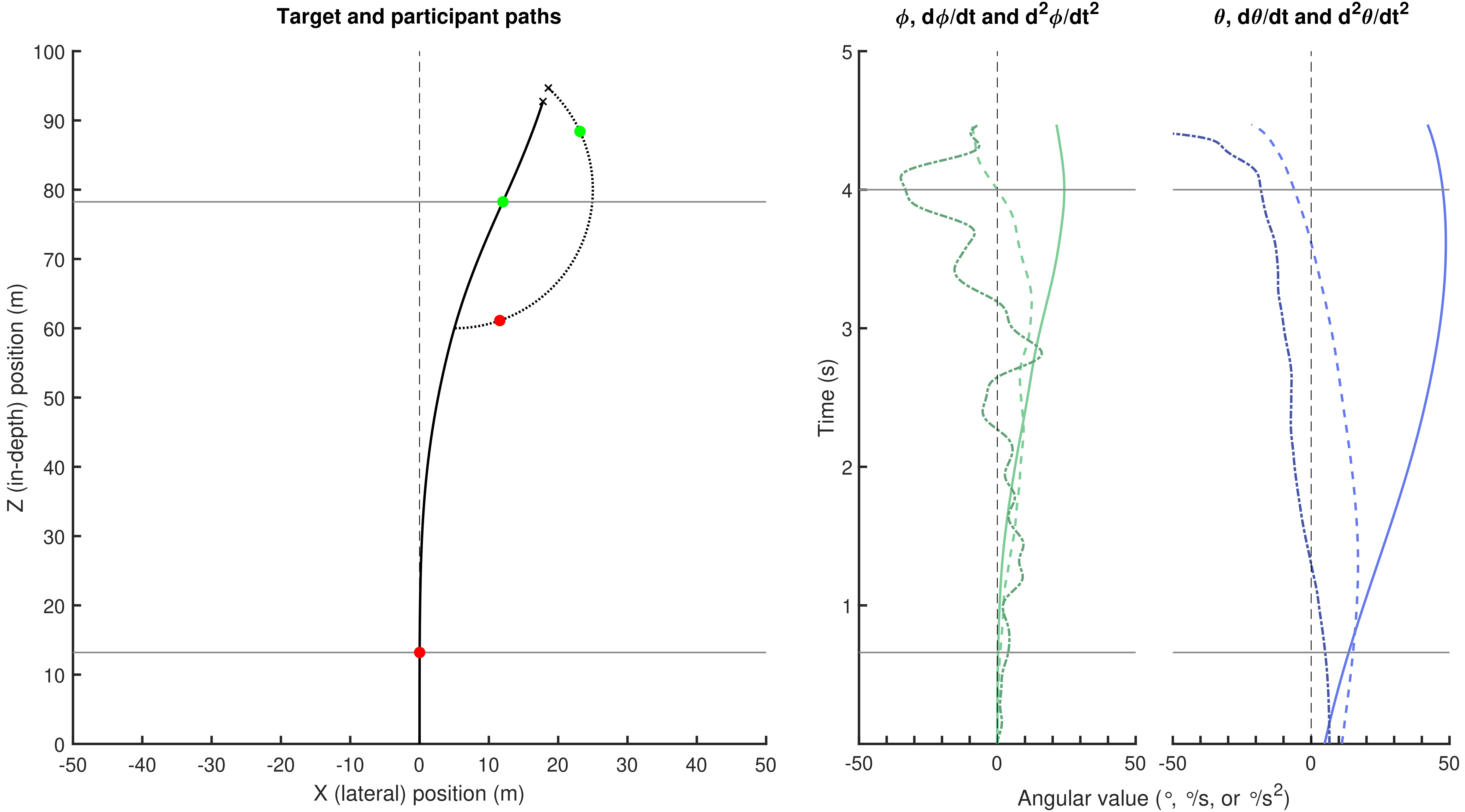

P5/B2
S5/R20-IN

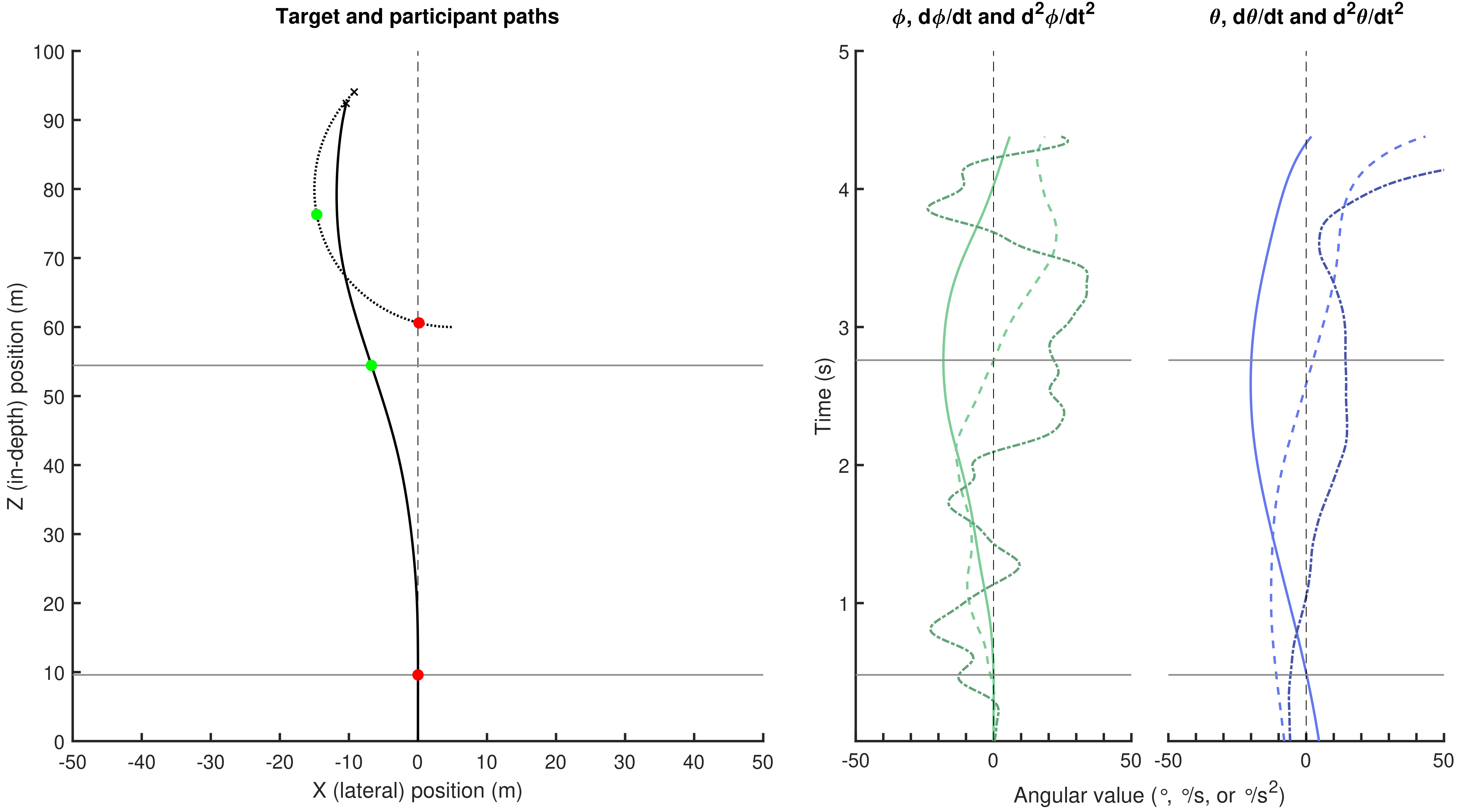

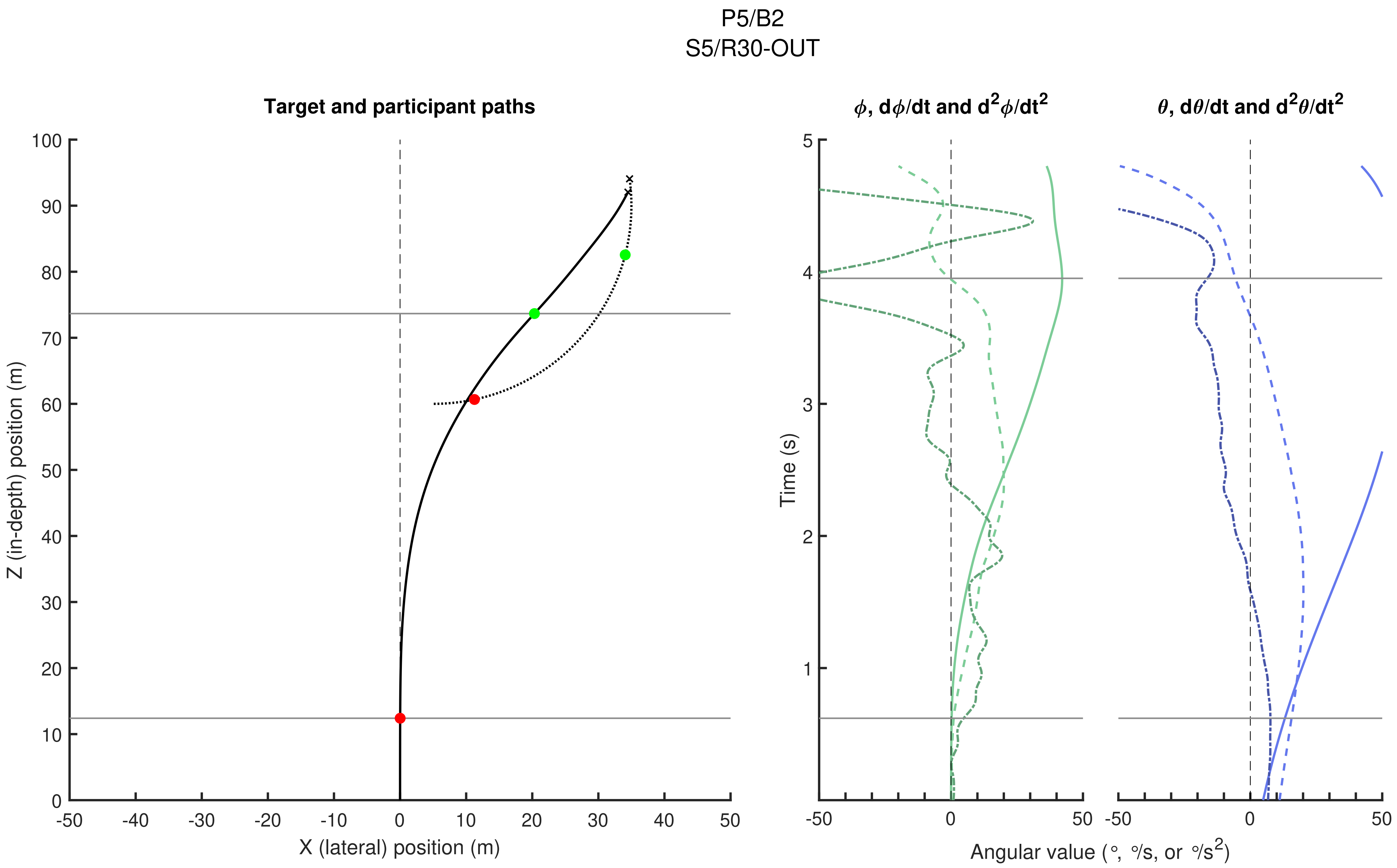

P5/B2
S5/R30-OUT
Target and participant paths
Z (in-depth) position (m)
X (lateral) position (m)
ϕ, dϕ/dt and d²ϕ/dt²
θ, dθ/dt and d²θ/dt²
Time (s)
Angular value (°, °/s, or °/s²)

P5/B2
S5/R30-IN

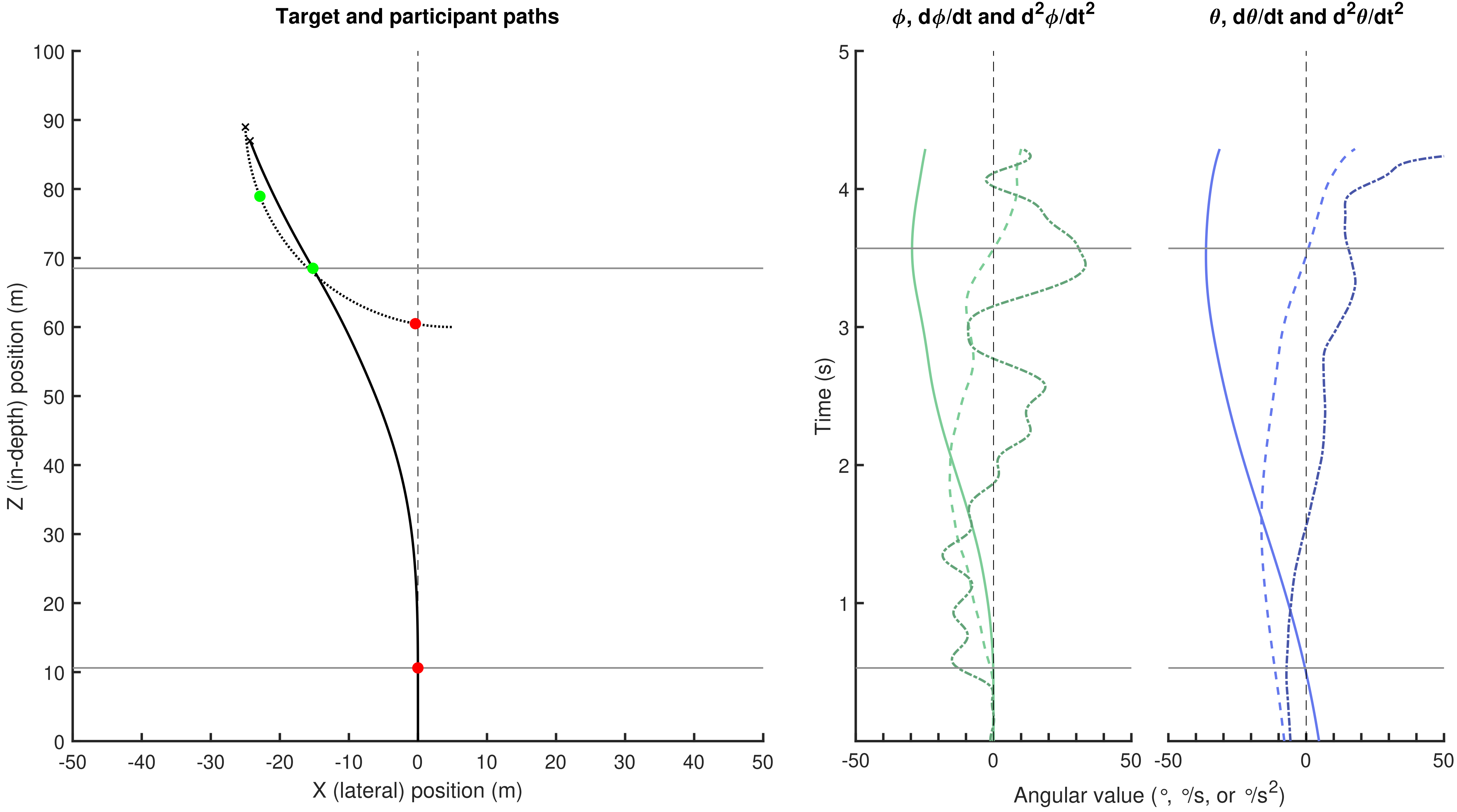

P5/B2
S15/R10-IN

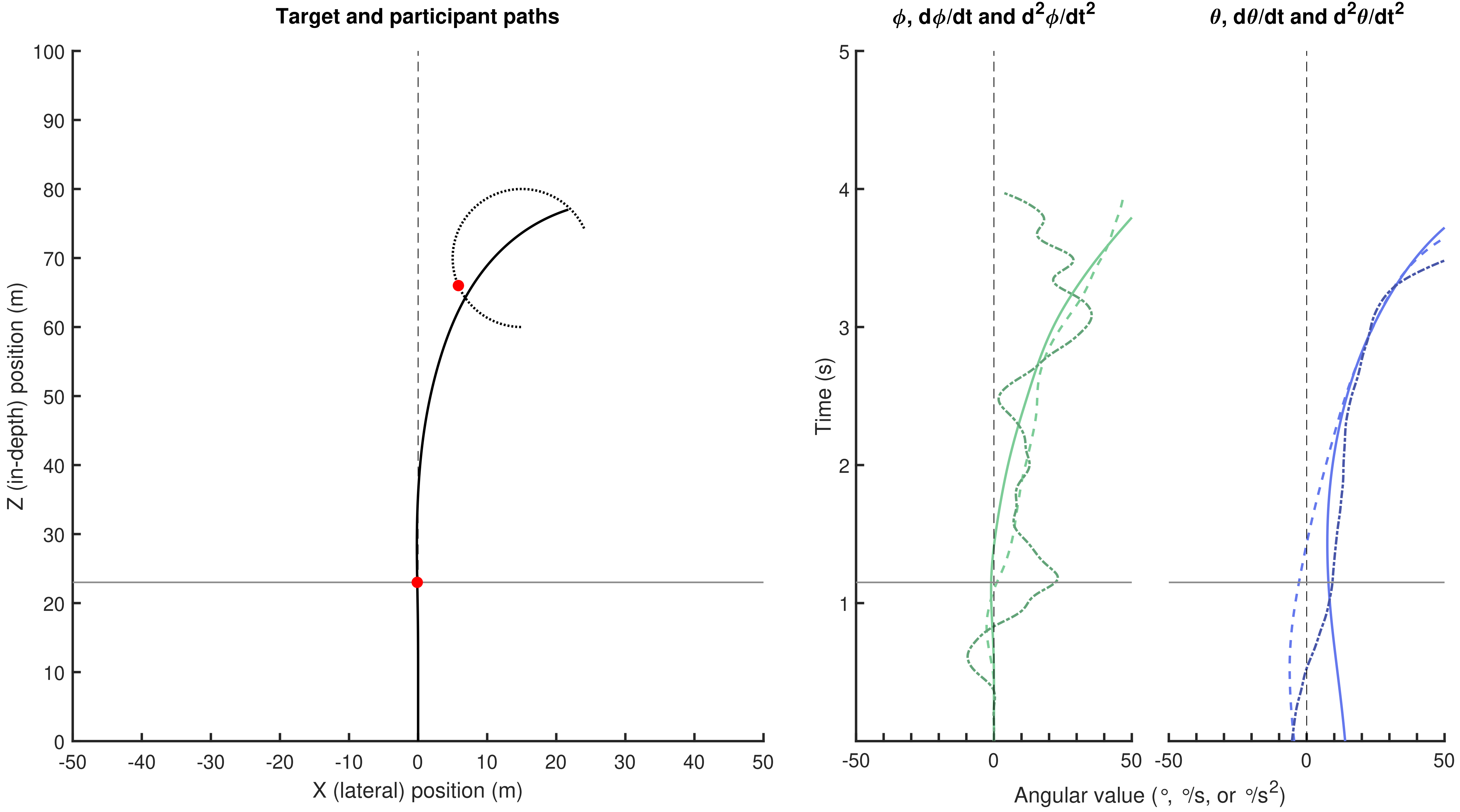

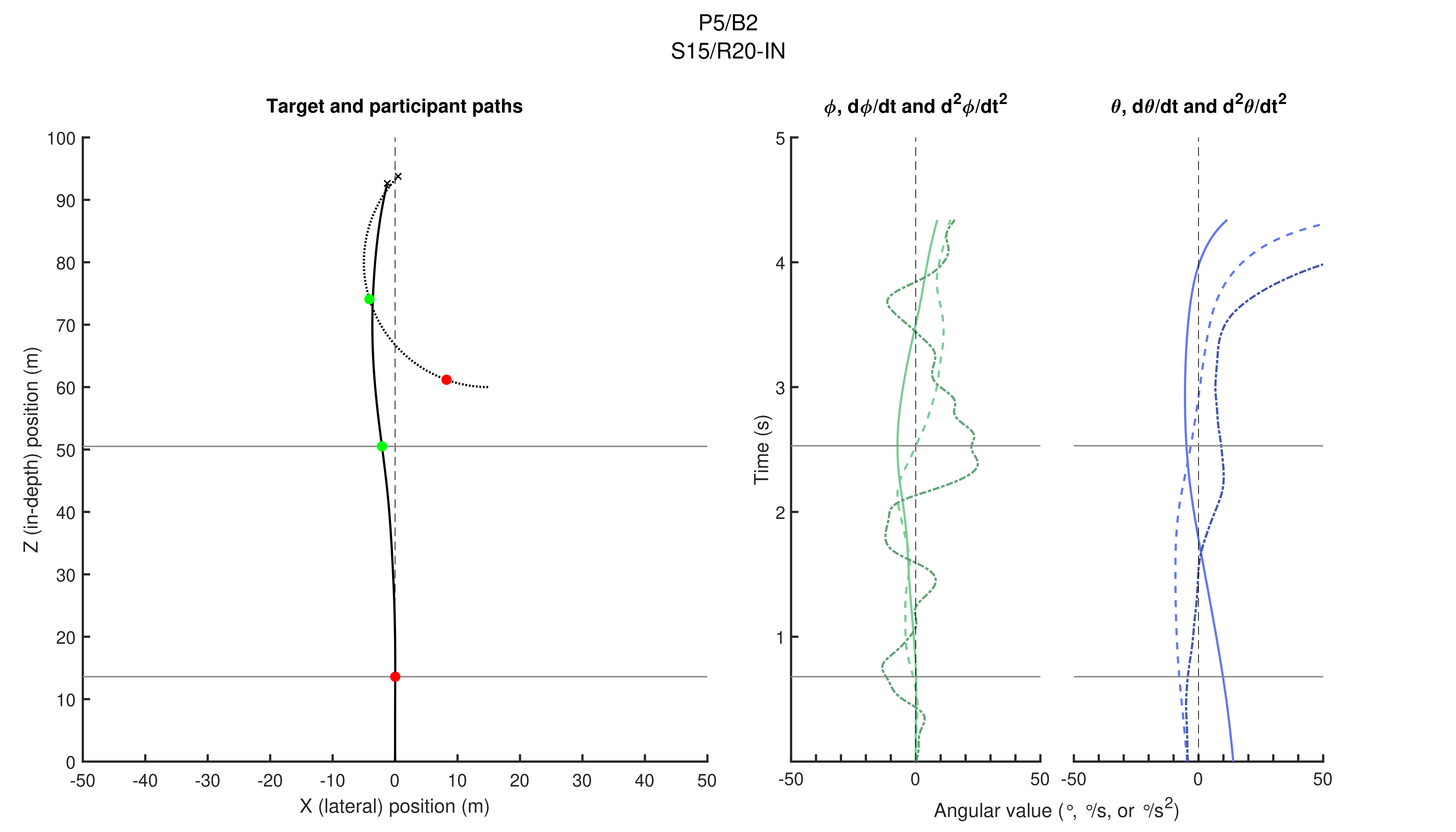

P5/B2
S15/R20-IN
Target and participant paths
Z (in-depth) position (m)
X (lateral) position (m)
ϕ, dϕ/dt and d$^2\phi$/dt^2
θ, dθ/dt and d$^2\theta$/dt^2
Time (s)
Angular value (°, °/s, or °/s^2)

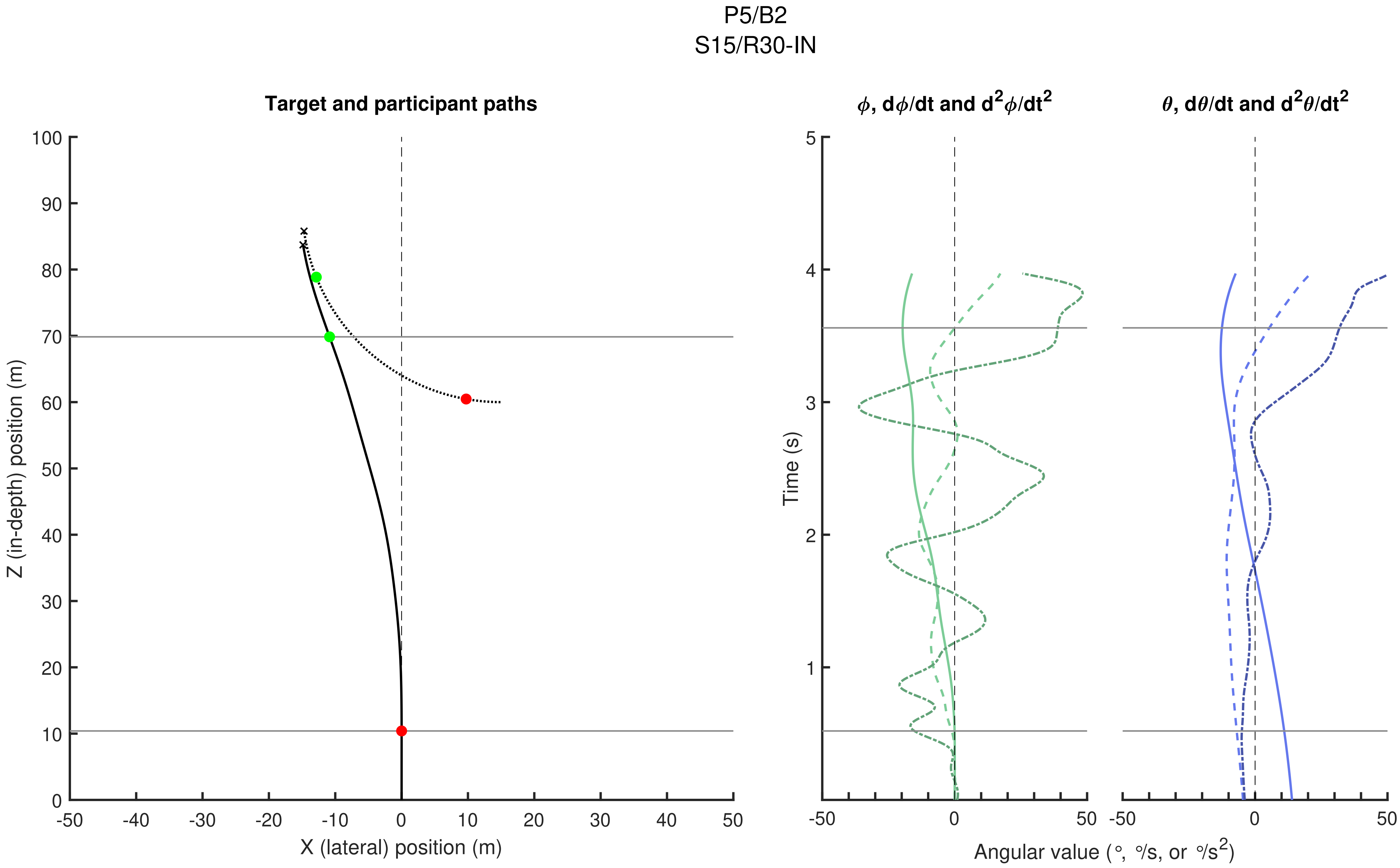
P5/B2
S15/R30-IN
Target and participant paths
Z (in-depth) position (m)
X (lateral) position (m)
ϕ, dϕ/dt and d$^2\phi$/dt^2
θ, dθ/dt and d$^2\theta$/dt^2
Time (s)
Angular value (°, °/s, or °/s^2)

P5/B2
S20/R10-IN

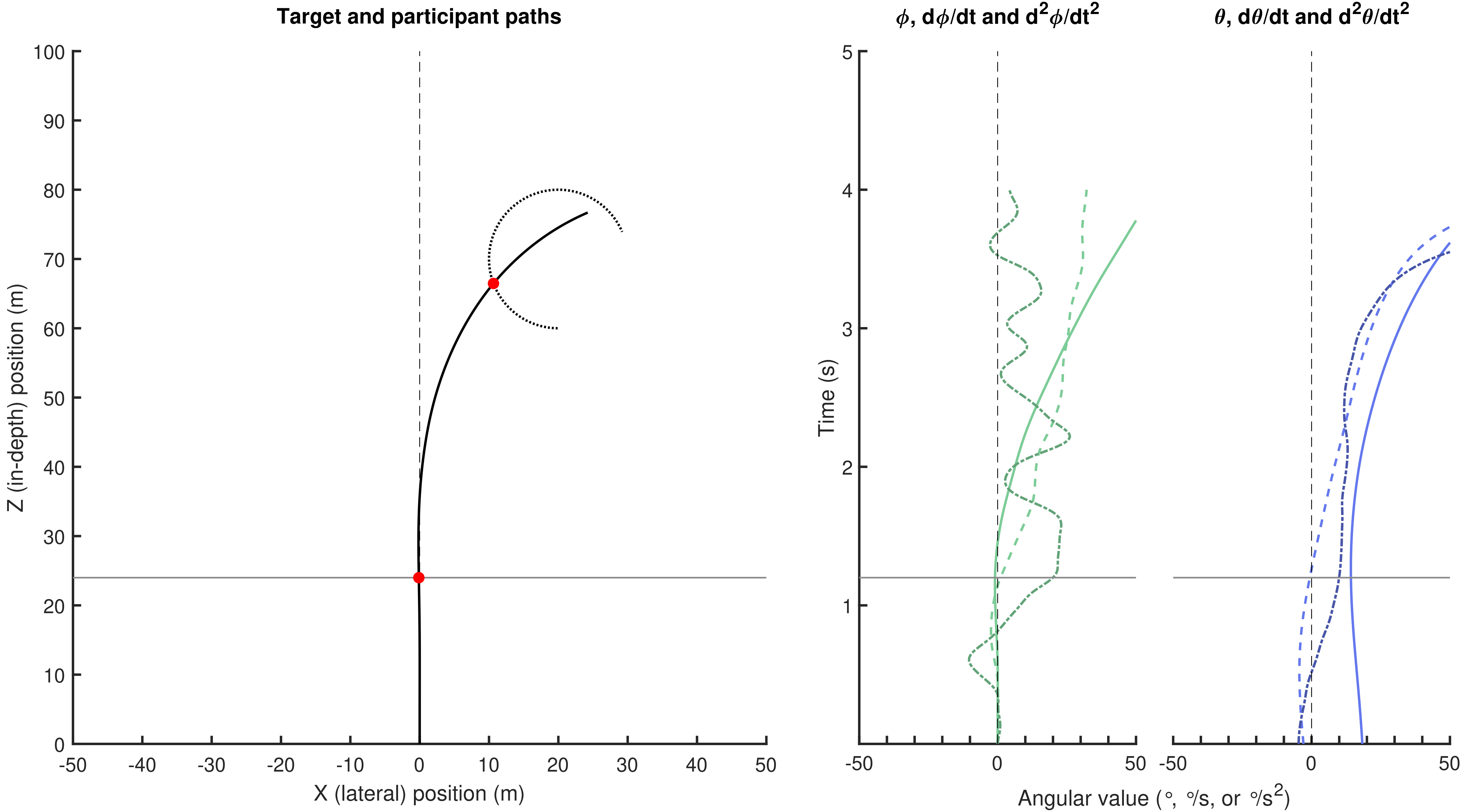

P5/B2
S20/R20-IN

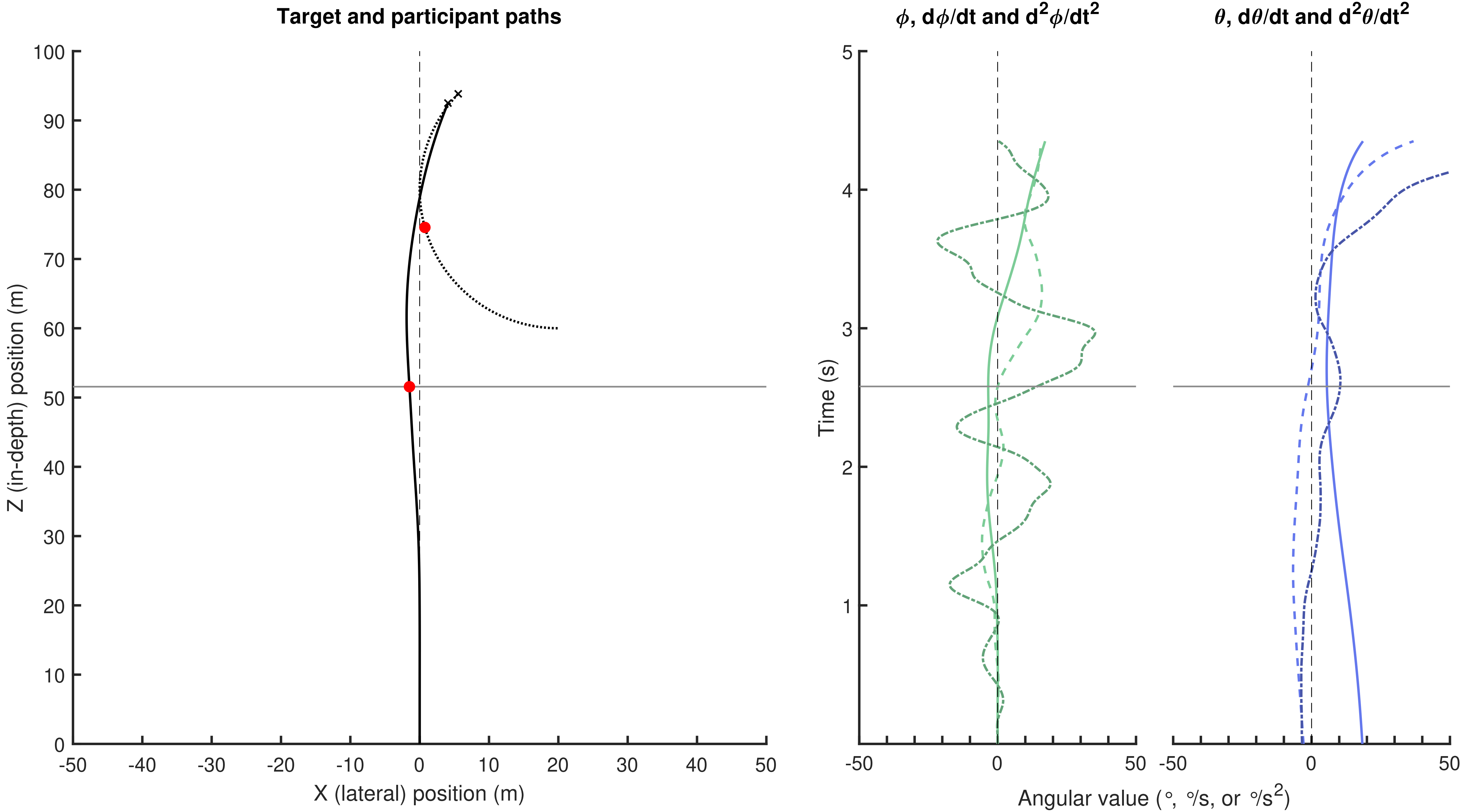

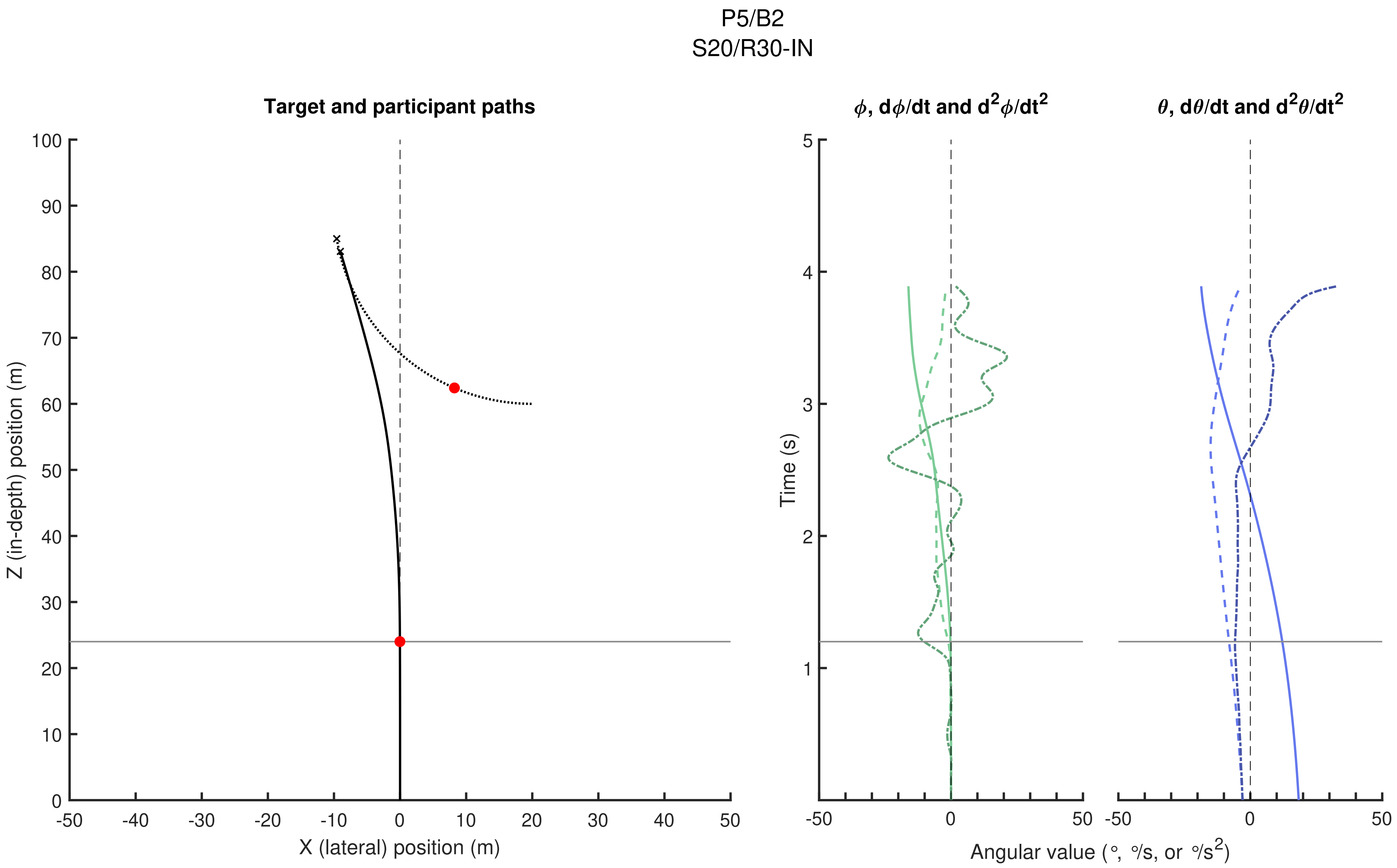
P5/B2
S20/R30-IN
Target and participant paths
Z (in-depth) position (m)
X (lateral) position (m)
100
90
80
70
60
50
40
30
20
10
0
-50
-40
-30
-20
-10
0
10
20
30
40
50
ϕ, dϕ/dt and d$^2\phi$/dt^2
θ, dθ/dt and d$^2\theta$/dt^2
Time (s)
5
4
3
2
1
-50
0
50
-50
0
50
Angular value (°, °/s, or °/s^2)

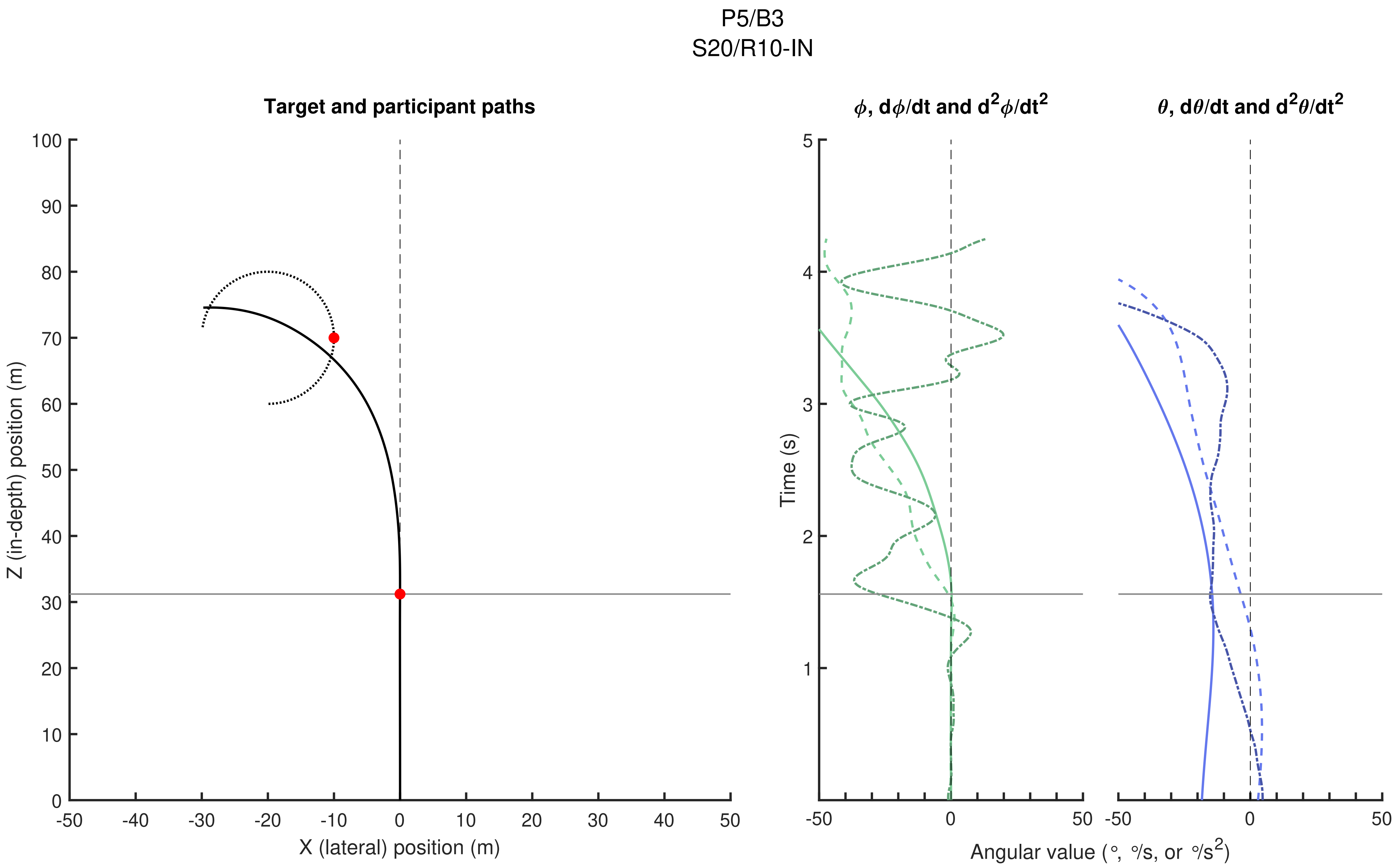
P5/B3
S20/R10-IN
Target and participant paths
Z (in-depth) position (m)
X (lateral) position (m)
ϕ, dϕ/dt and d$^2\phi$/dt^2
θ, dθ/dt and d$^2\theta$/dt^2
Time (s)
Angular value (°, °/s, or °/s^2)

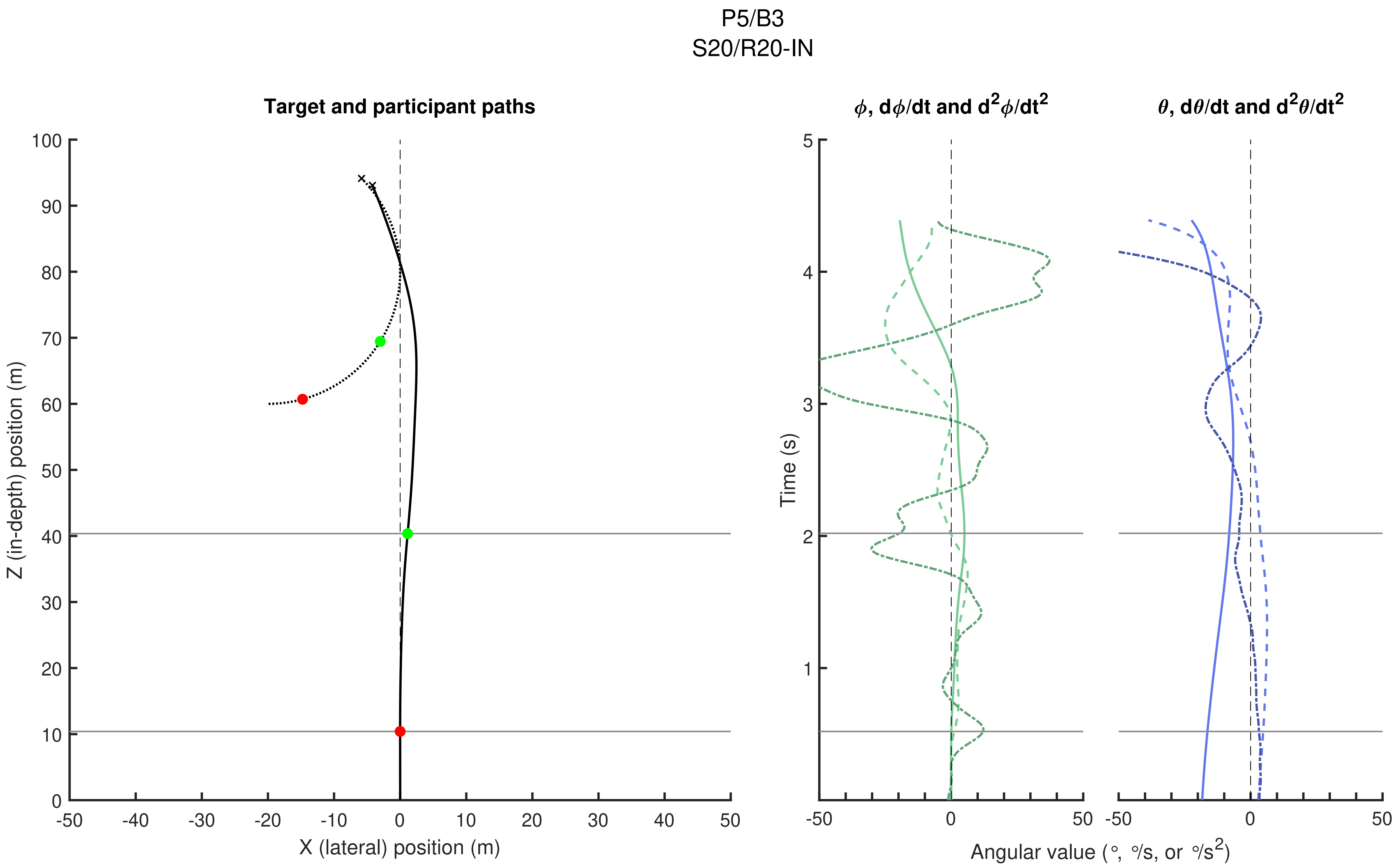
P5/B3
S20/R20-IN
Target and participant paths
Z (in-depth) position (m)
X (lateral) position (m)
φ, dφ/dt and d²φ/dt²
θ, dθ/dt and d²θ/dt²
Time (s)
Angular value (°, °/s, or °/s²)

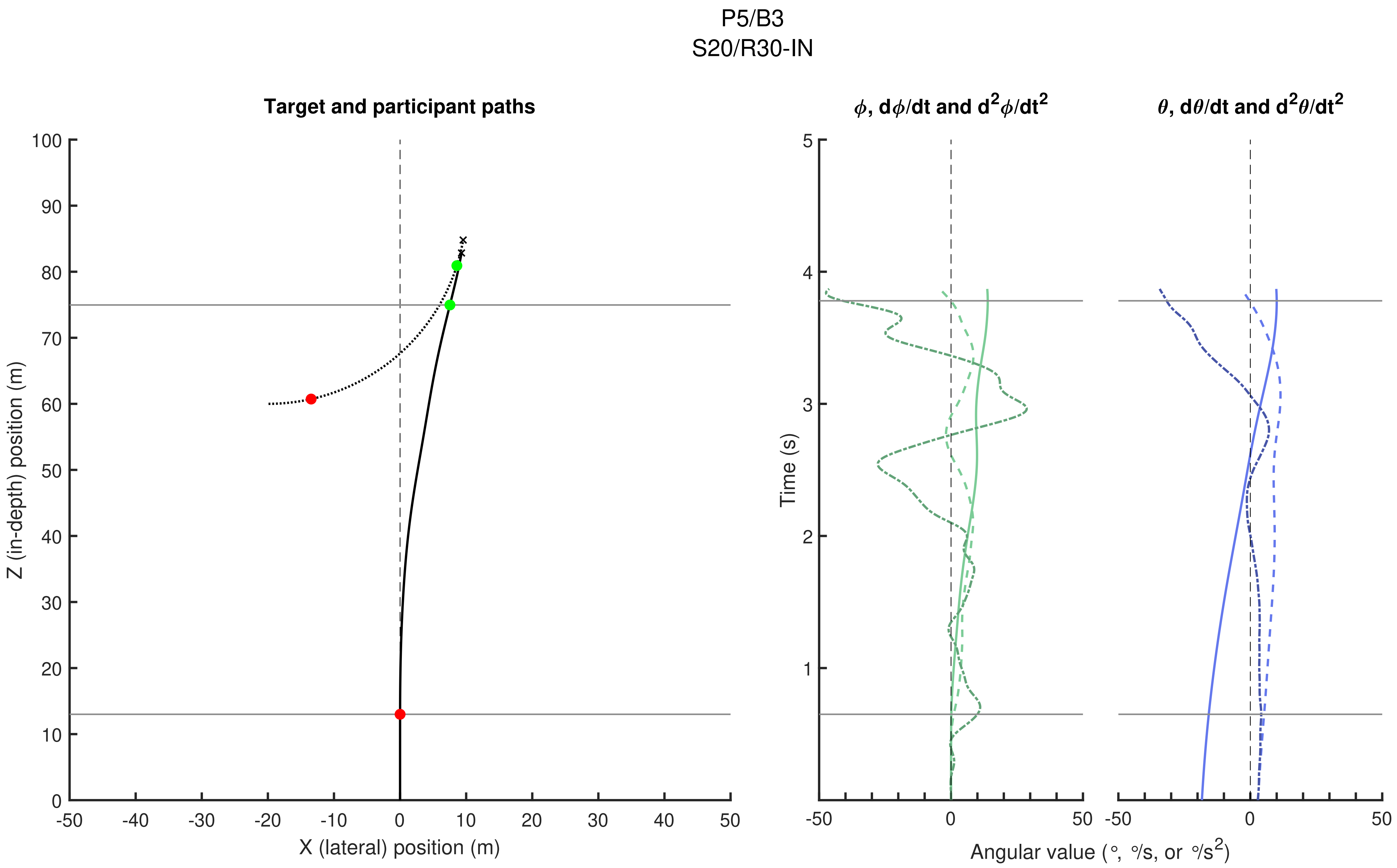
P5/B3
S20/R30-IN
Target and participant paths
Z (in-depth) position (m)
X (lateral) position (m)
ϕ, dϕ/dt and d$^2\phi$/dt^2
θ, dθ/dt and d$^2\theta$/dt^2
Time (s)
Angular value (°, °/s, or °/s^2)

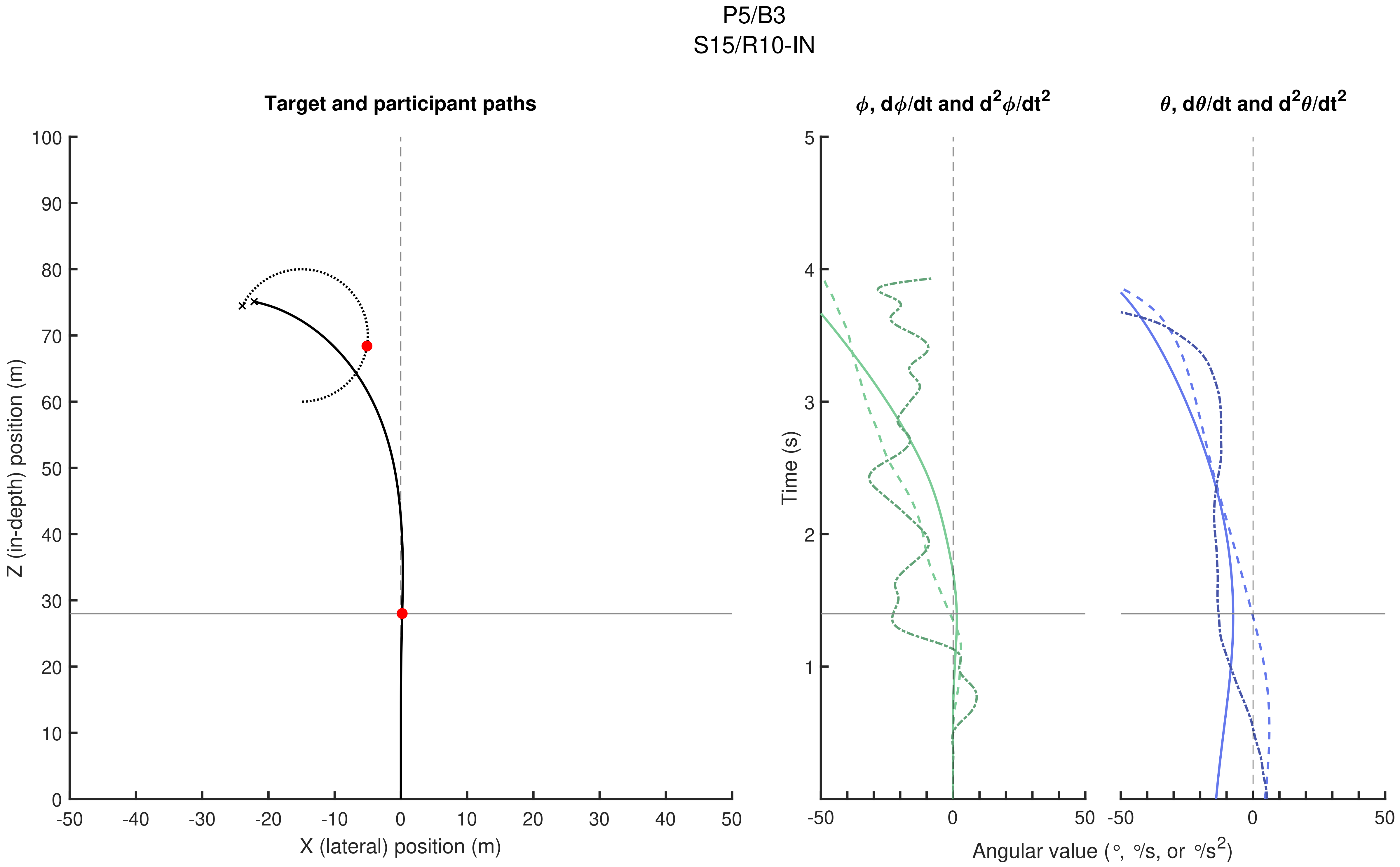
P5/B3
S15/R10-IN
Target and participant paths
ϕ, dϕ/dt and d$^2\phi$/dt^2
θ, dθ/dt and d$^2\theta$/dt^2
Z (in-depth) position (m)
X (lateral) position (m)
Time (s)
Angular value (°, °/s, or °/s^2)
100
90
80
70
60
50
40
30
20
10
0
-50
-40
-30
-20
-10
0
10
20
30
40
50
5
4
3
2
1
-50
0
50
-50
0
50

P5/B3
S15/R20-IN

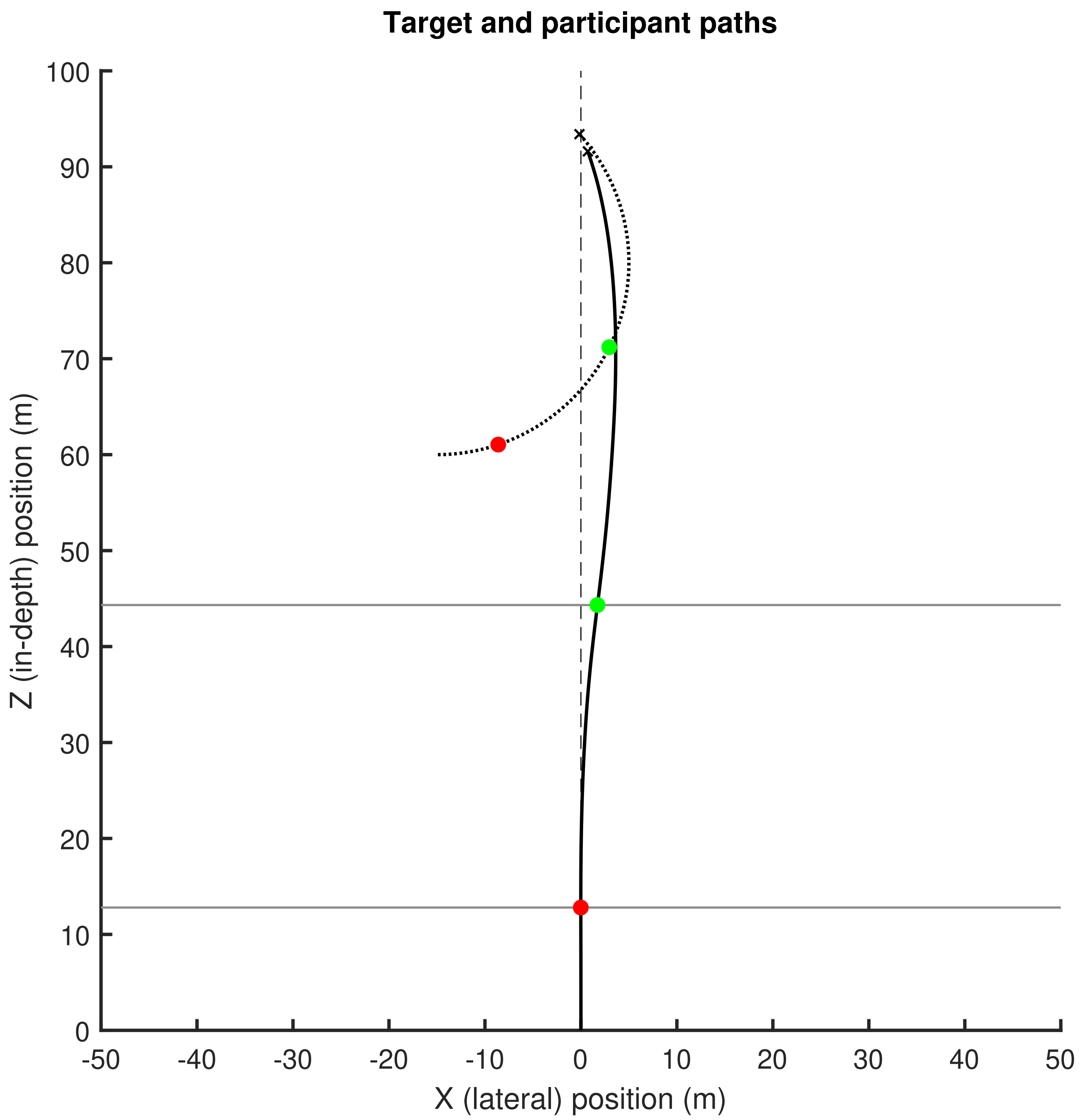

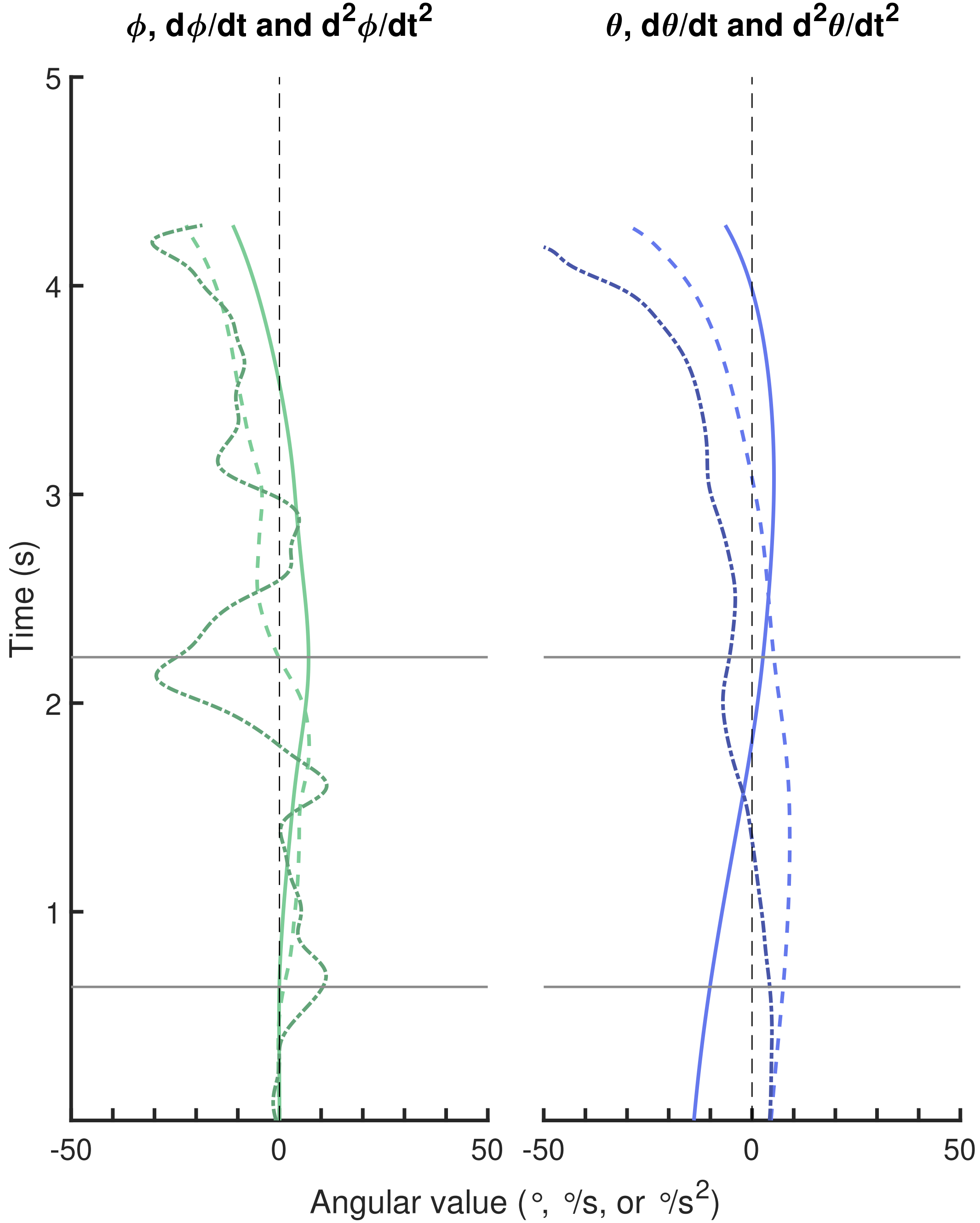

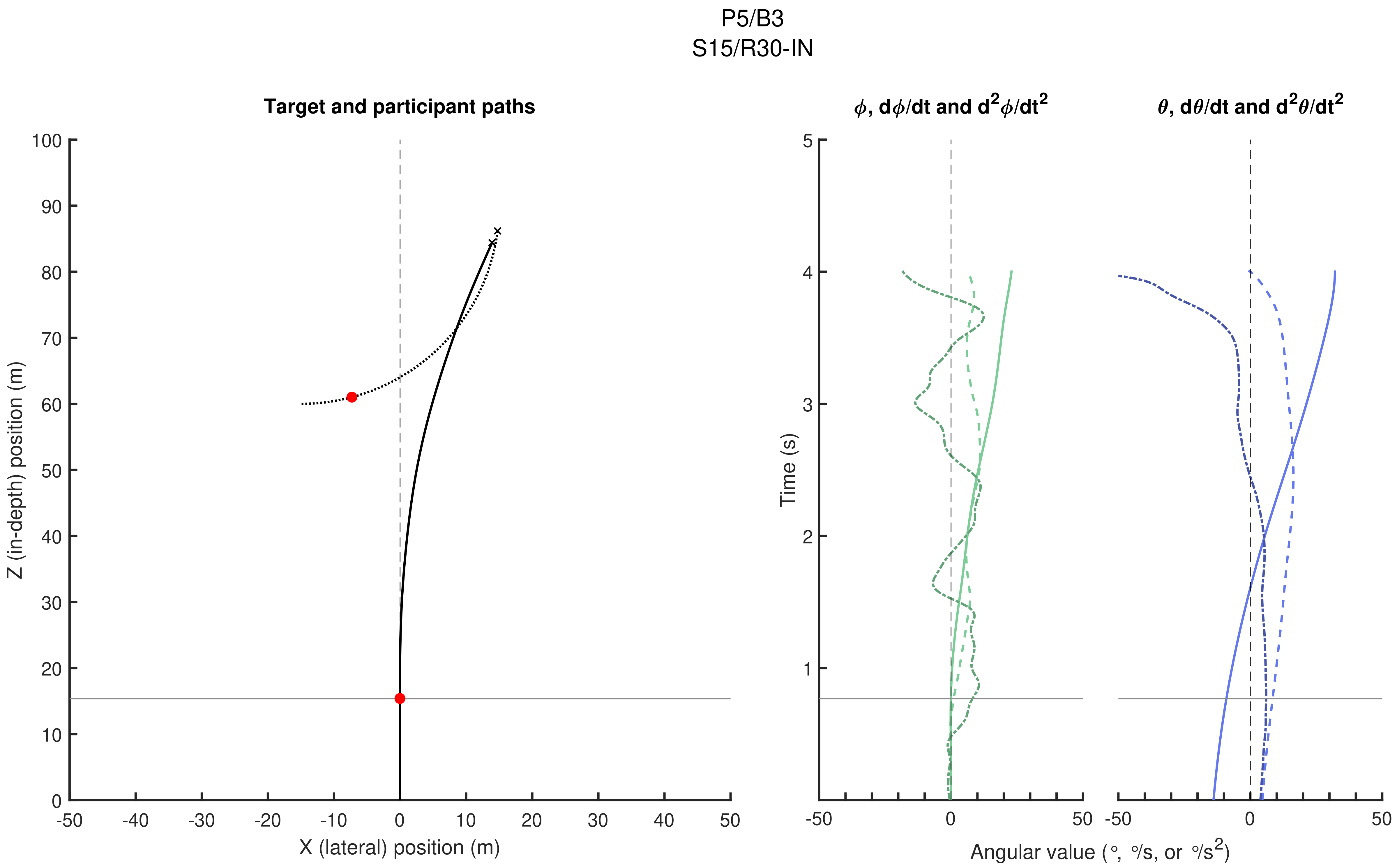
P5/B3
S15/R30-IN
Target and participant paths
ϕ, dϕ/dt and d$^2\phi$/dt^2
θ, dθ/dt and d$^2\theta$/dt^2
Z (in-depth) position (m)
X (lateral) position (m)
Time (s)
Angular value (°, °/s, or °/s^2)
100
90
80
70
60
50
40
30
20
10
0
-50
-40
-30
-20
-10
0
10
20
30
40
50
5
4
3
2
1
-50
0
50
-50
0
50

P5/B3
S5/R10-IN

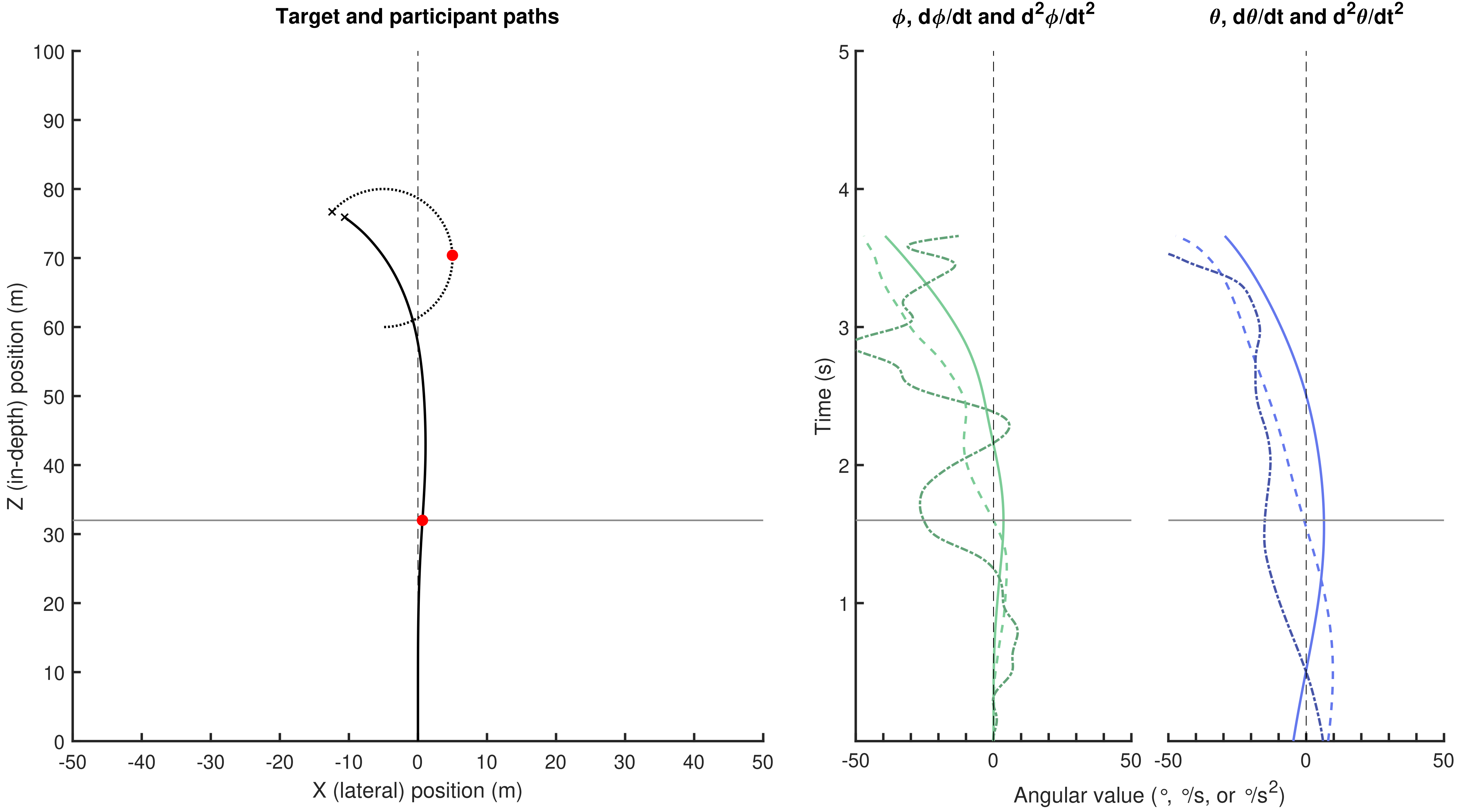

P5/B3
S5/R10-OUT

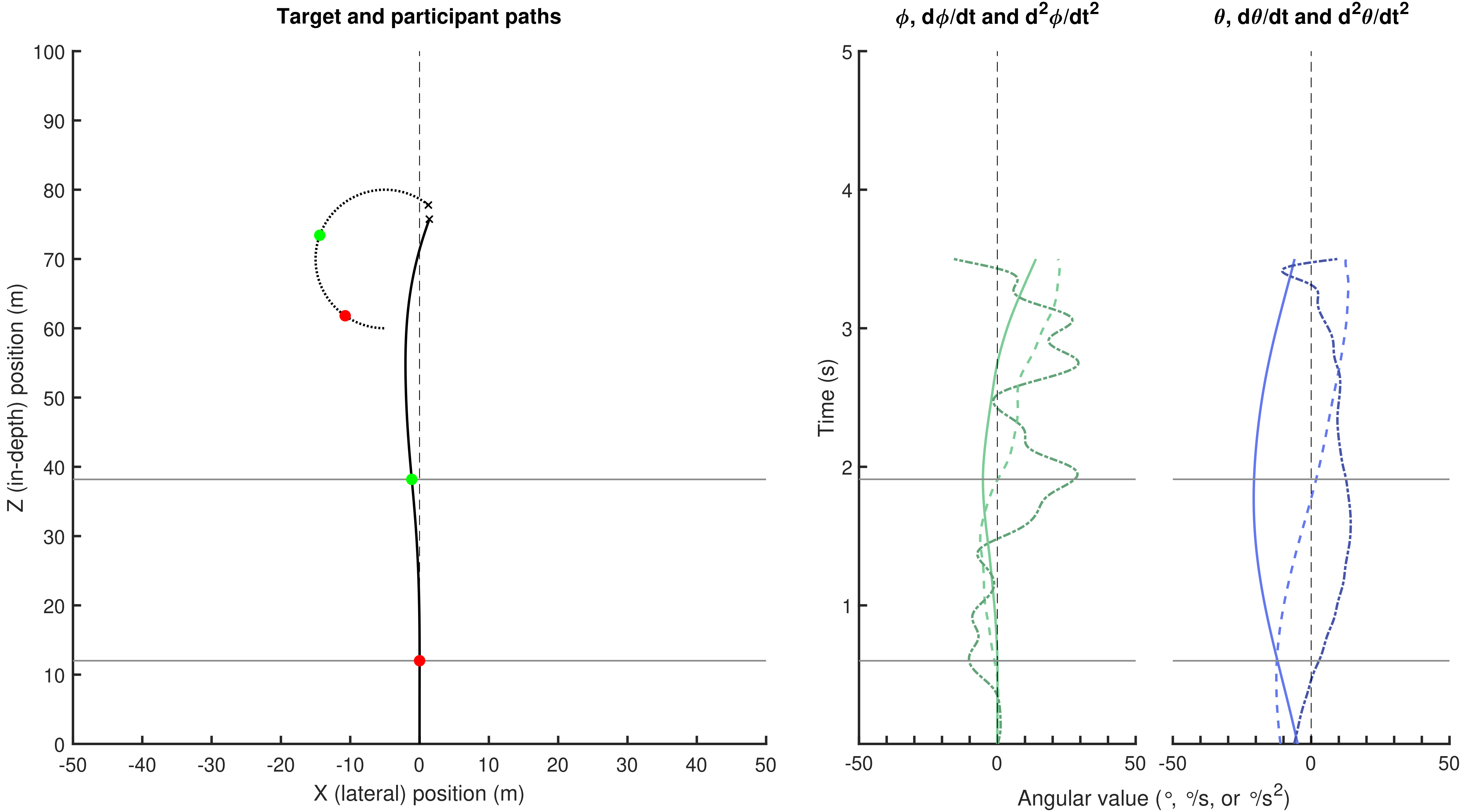

P5/B3
S5/R20-IN

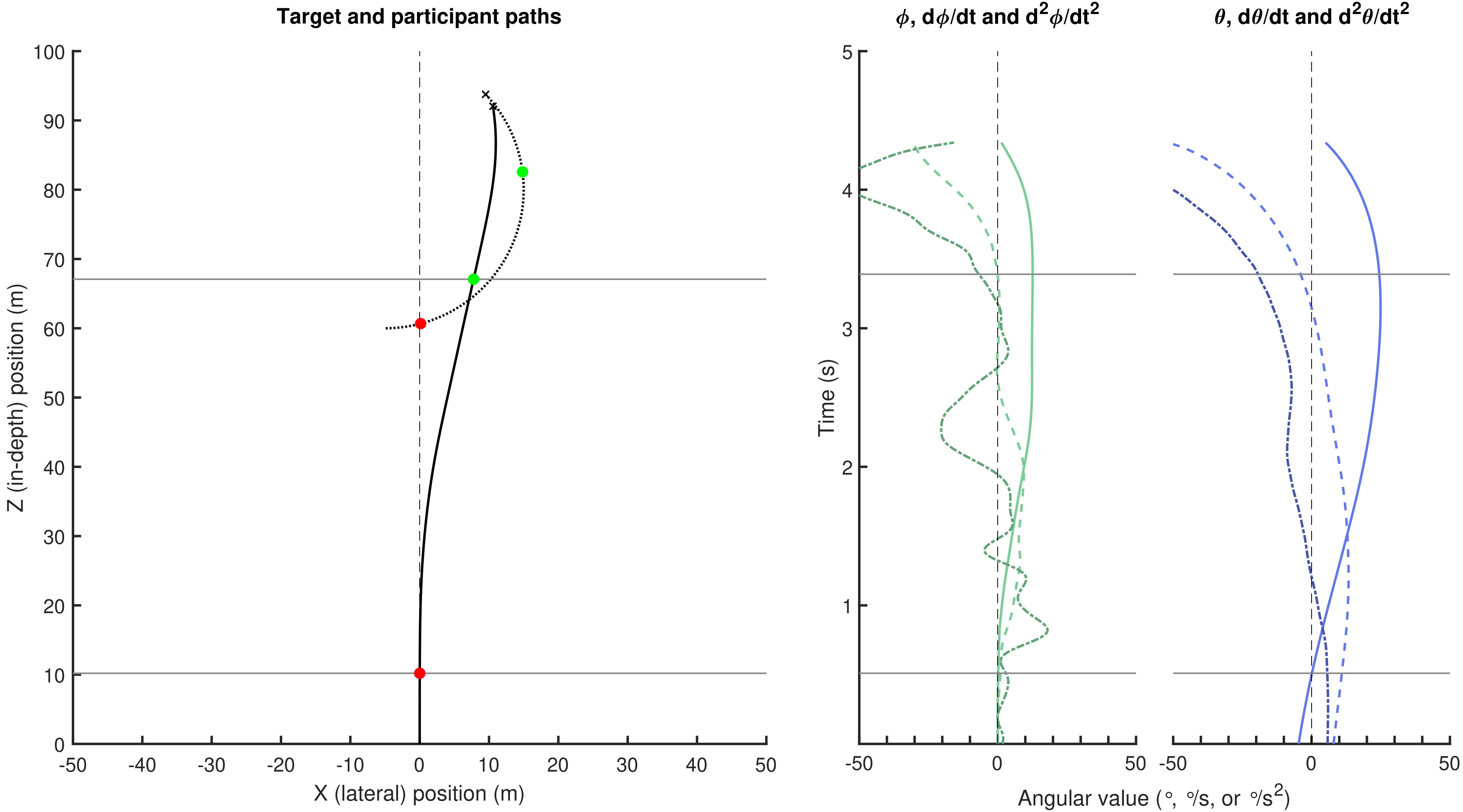

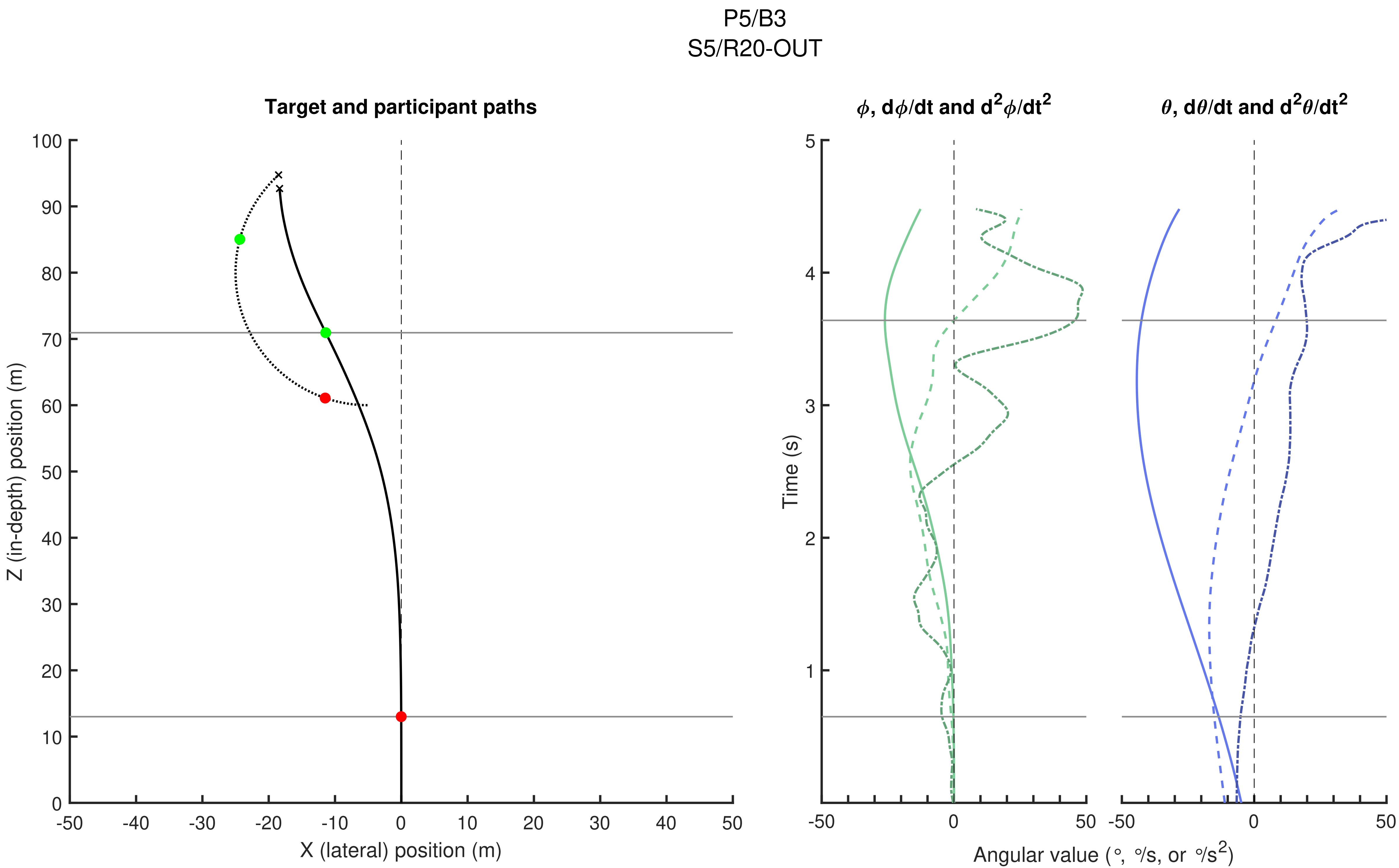

P5/B3
S5/R20-OUT
Target and participant paths
Z (in-depth) position (m)
X (lateral) position (m)
0
10
20
30
40
50
60
70
80
90
100
-50
-40
-30
-20
-10
0
10
20
30
40
50
φ, dφ/dt and d²φ/dt²
θ, dθ/dt and d²θ/dt²
Time (s)
1
2
3
4
5
-50
0
50
-50
0
50
Angular value (°, °/s, or °/s²)

P5/B3
S5/R30-IN

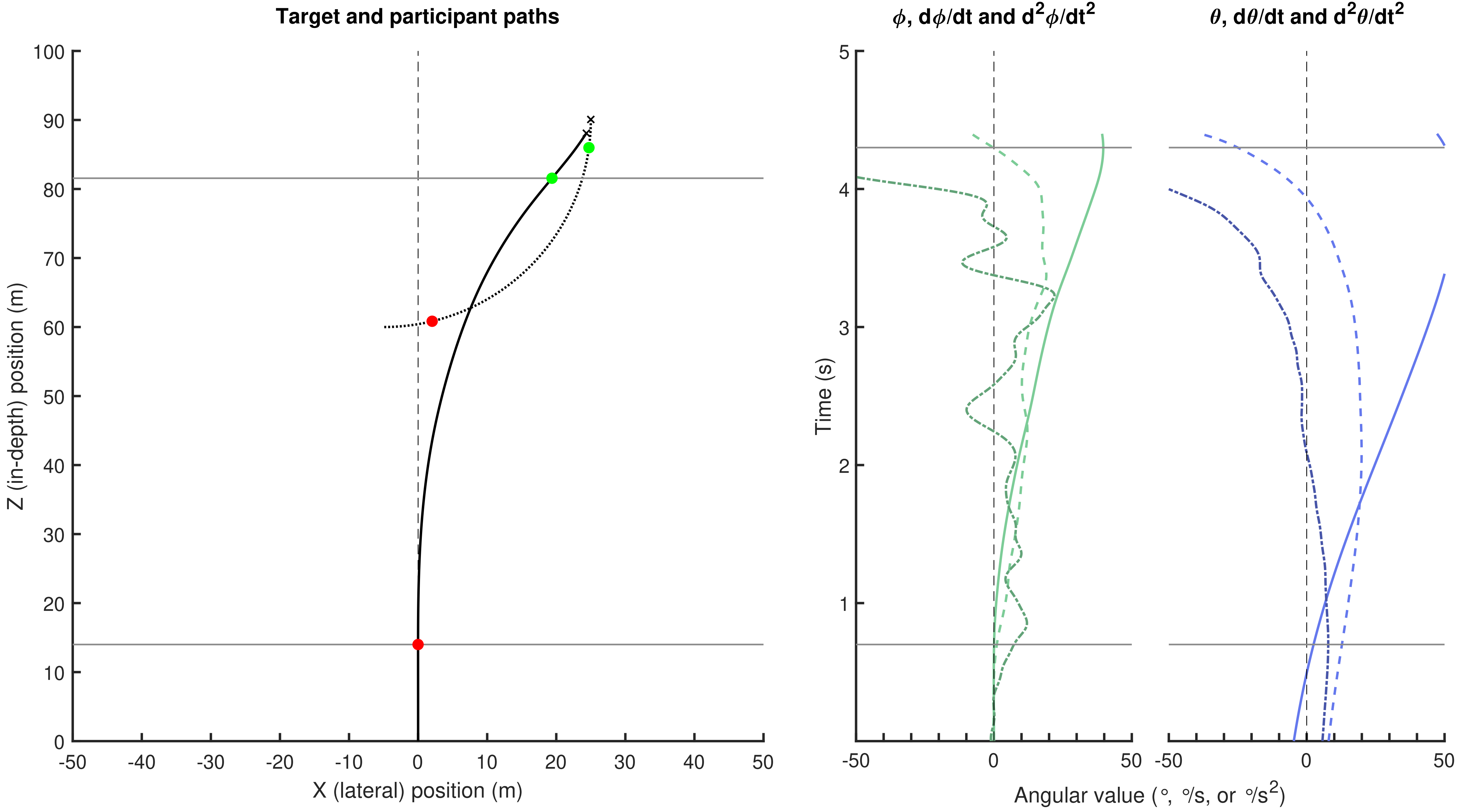

P5/B3
S5/R30-OUT

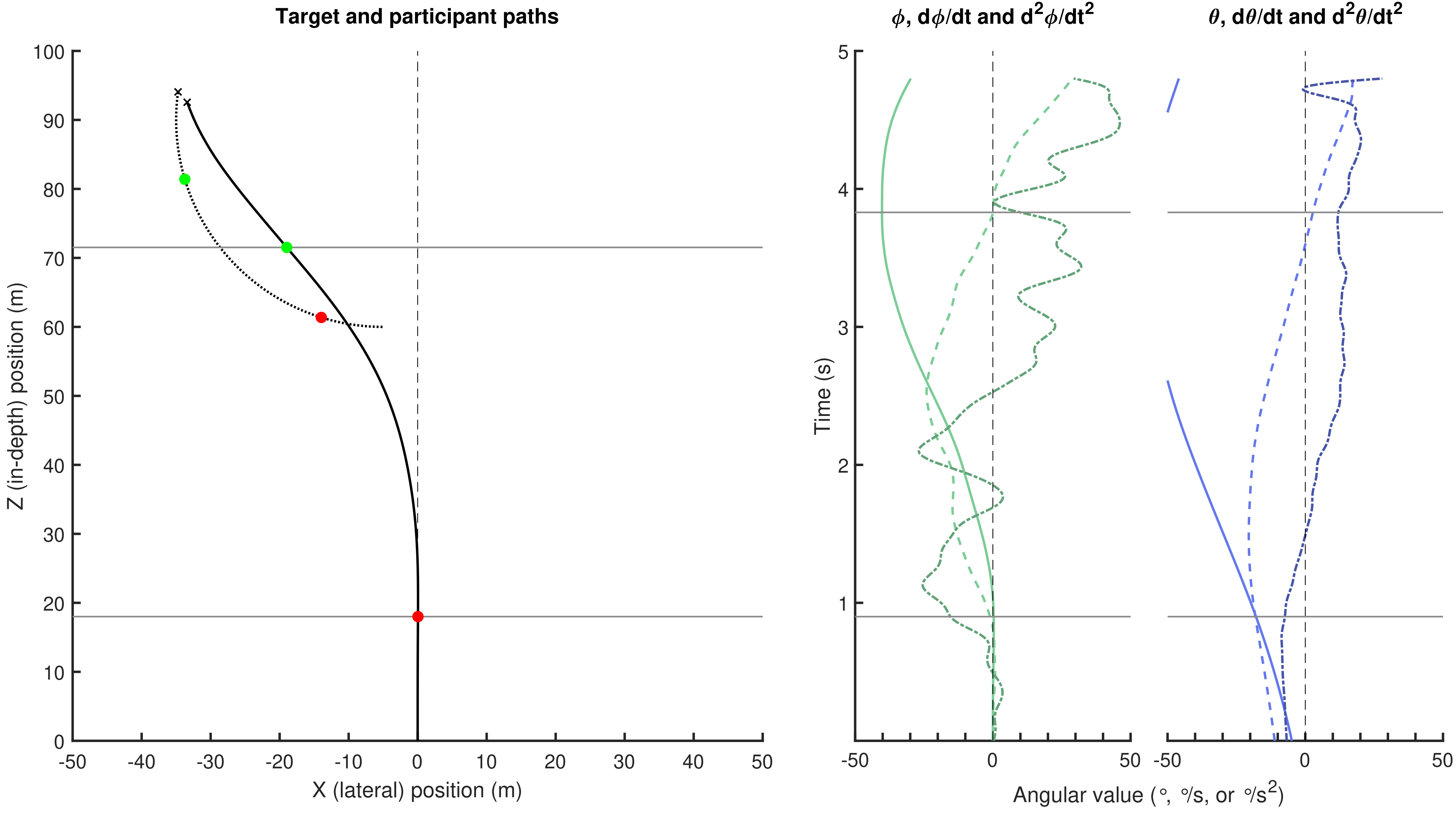

P5/B3
S5/R10-OUT

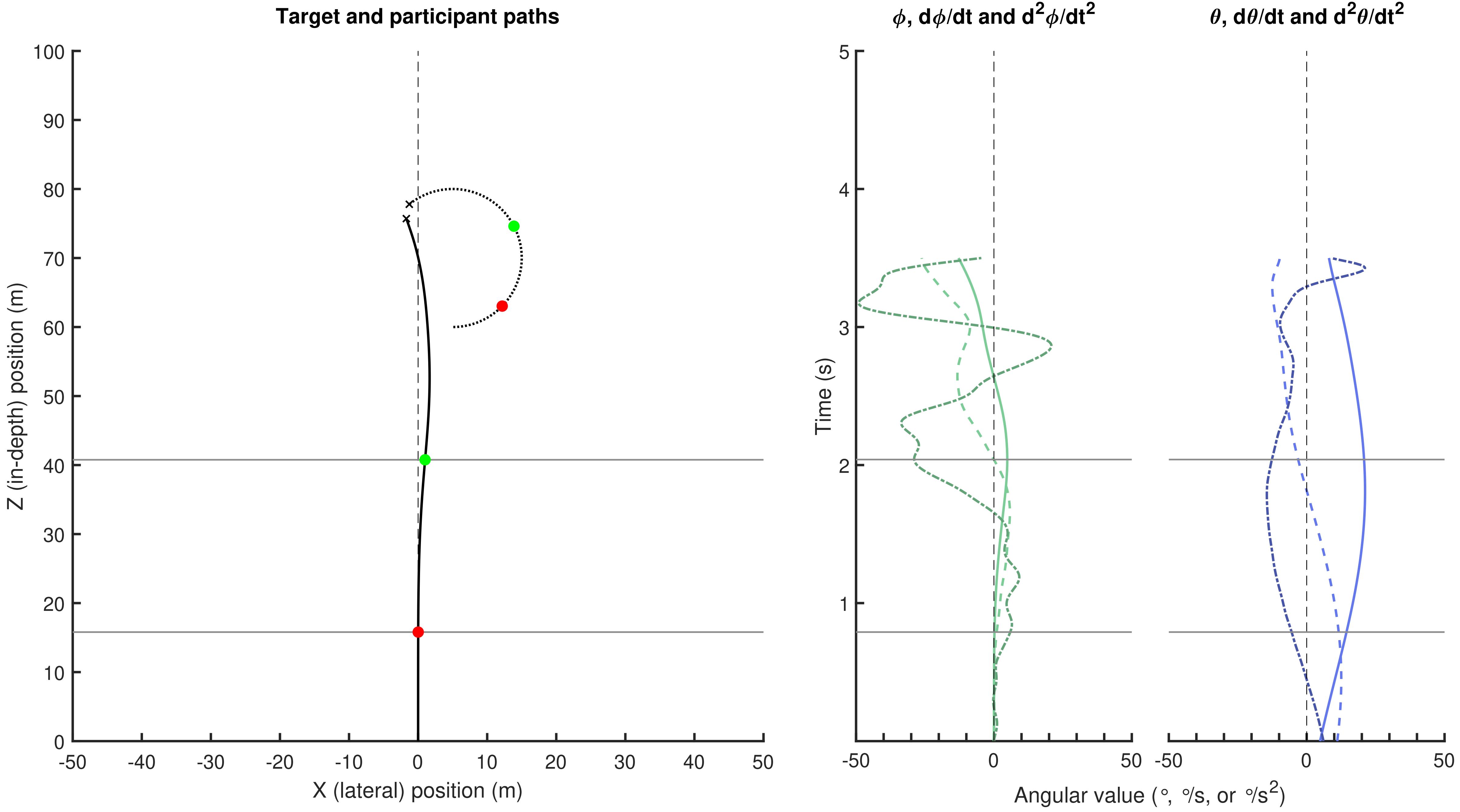

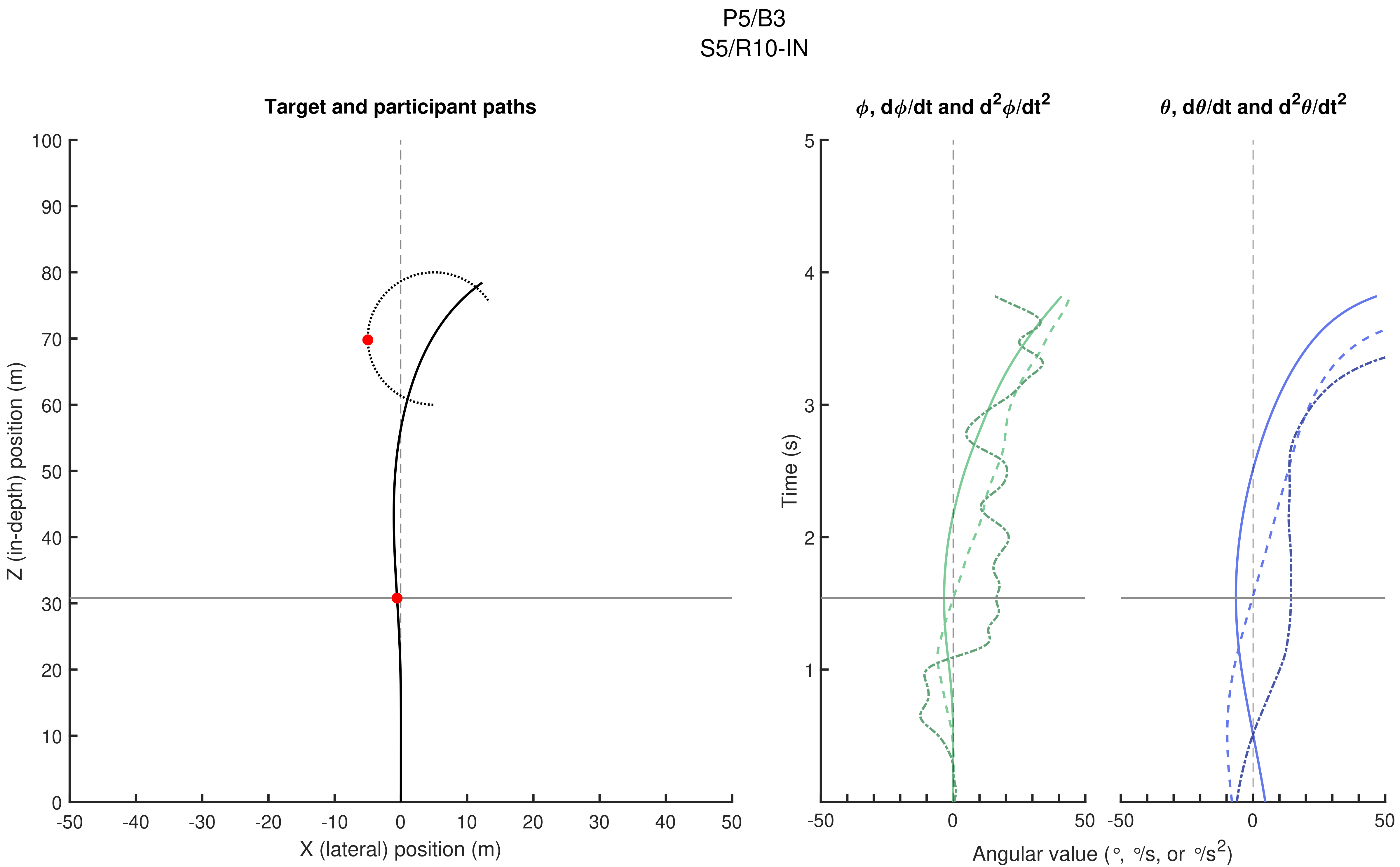
P5/B3
S5/R10-IN
Target and participant paths
Z (in-depth) position (m)
X (lateral) position (m)
100
90
80
70
60
50
40
30
20
10
0
-50
-40
-30
-20
-10
0
10
20
30
40
50
ϕ, dϕ/dt and d²ϕ/dt²
θ, dθ/dt and d²θ/dt²
Time (s)
5
4
3
2
1
-50
0
50
-50
0
50
Angular value (°, °/s, or °/s²)

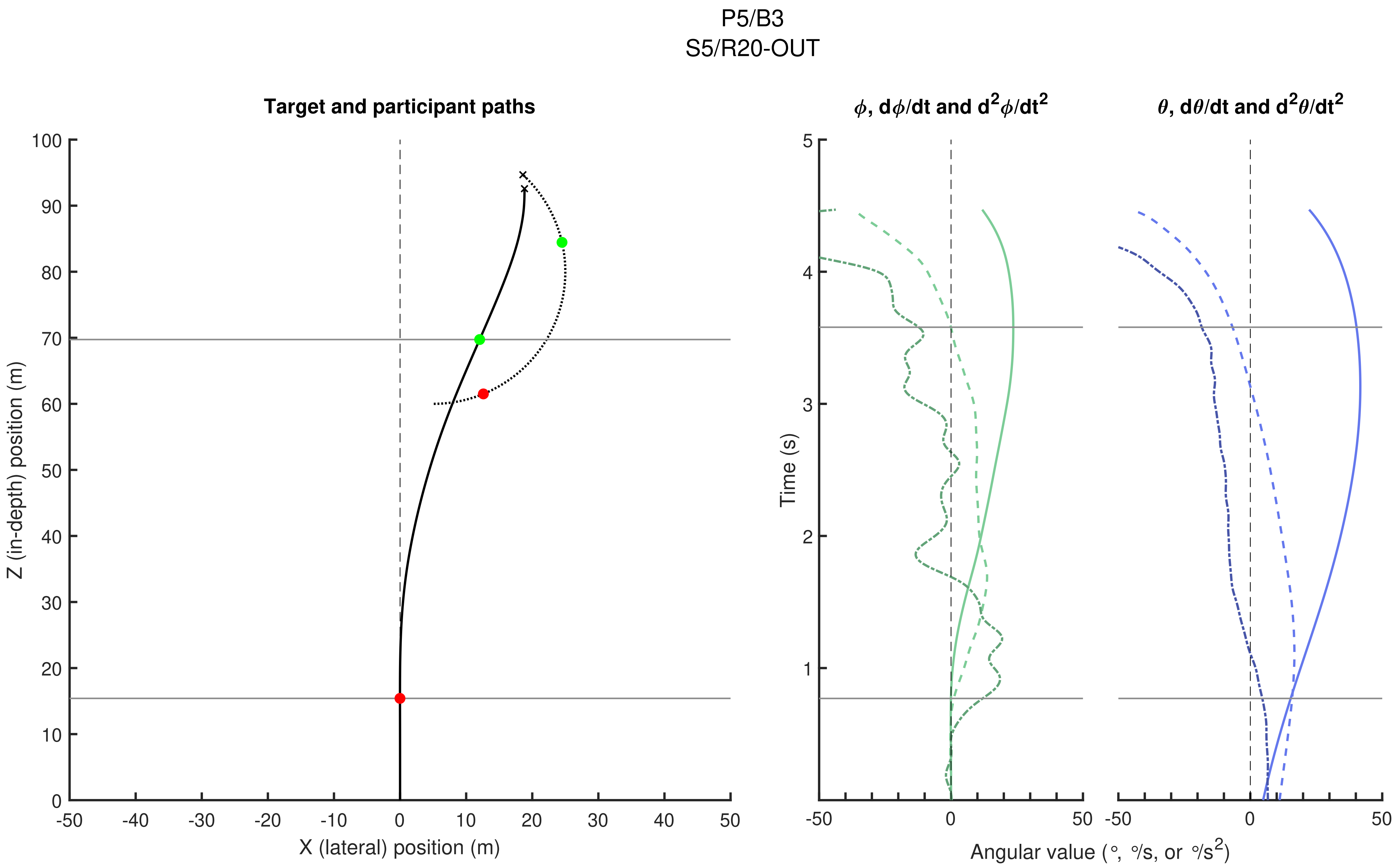
P5/B3
S5/R20-OUT
Target and participant paths
Z (in-depth) position (m)
X (lateral) position (m)
φ, dφ/dt and d²φ/dt²
θ, dθ/dt and d²θ/dt²
Time (s)
Angular value (°, °/s, or °/s²)

P5/B3
S5/R20-IN

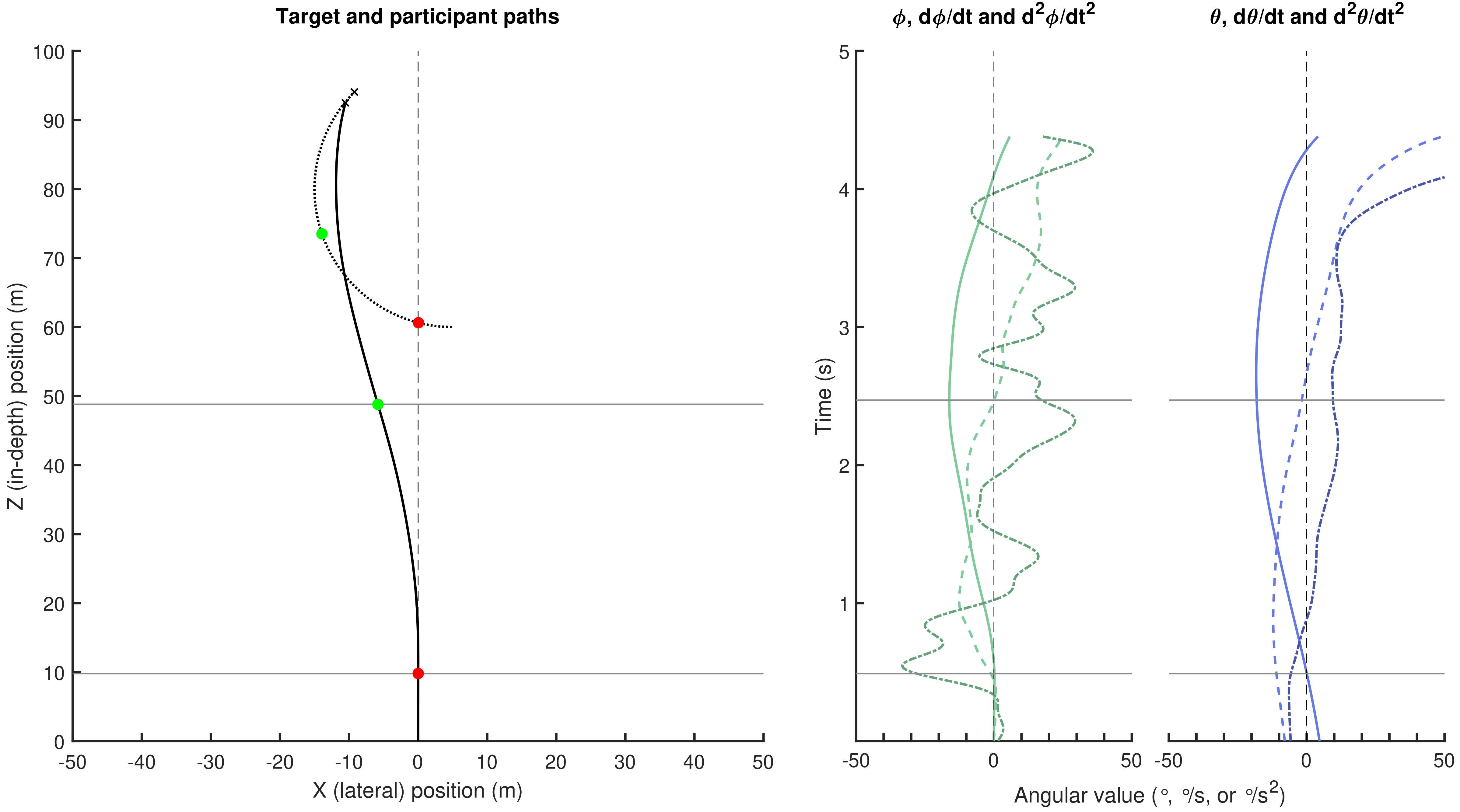

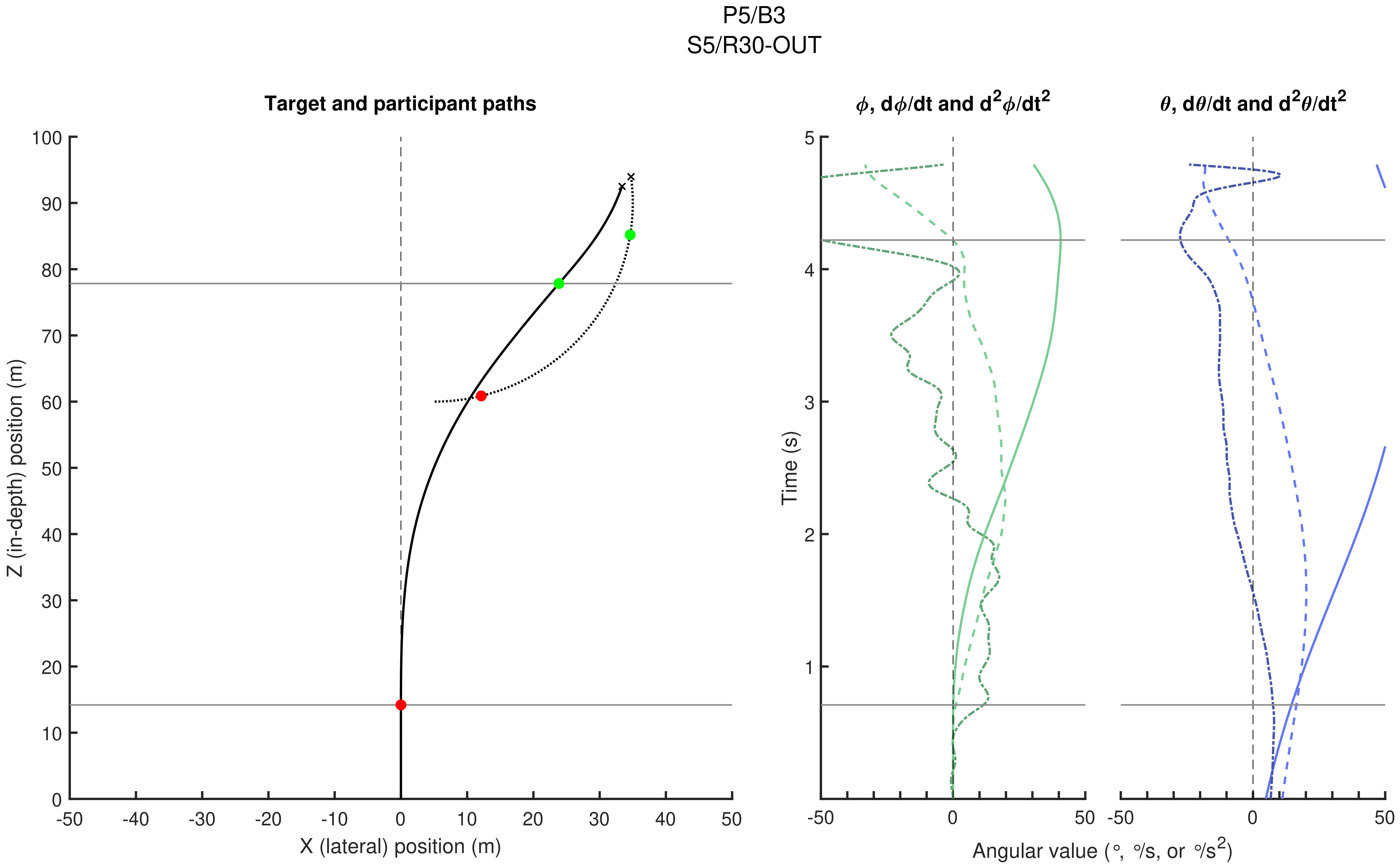

P5/B3
S5/R30-OUT
Target and participant paths
Z (in-depth) position (m)
X (lateral) position (m)
100
90
80
70
60
50
40
30
20
10
0
-50
-40
-30
-20
-10
0
10
20
30
40
50
ϕ, dϕ/dt and d$^2\phi$/dt^2
θ, dθ/dt and d$^2\theta$/dt^2
Time (s)
5
4
3
2
1
-50
0
50
-50
0
50
Angular value (°, °/s, or °/s^2)

P5/B3
S5/R30-IN

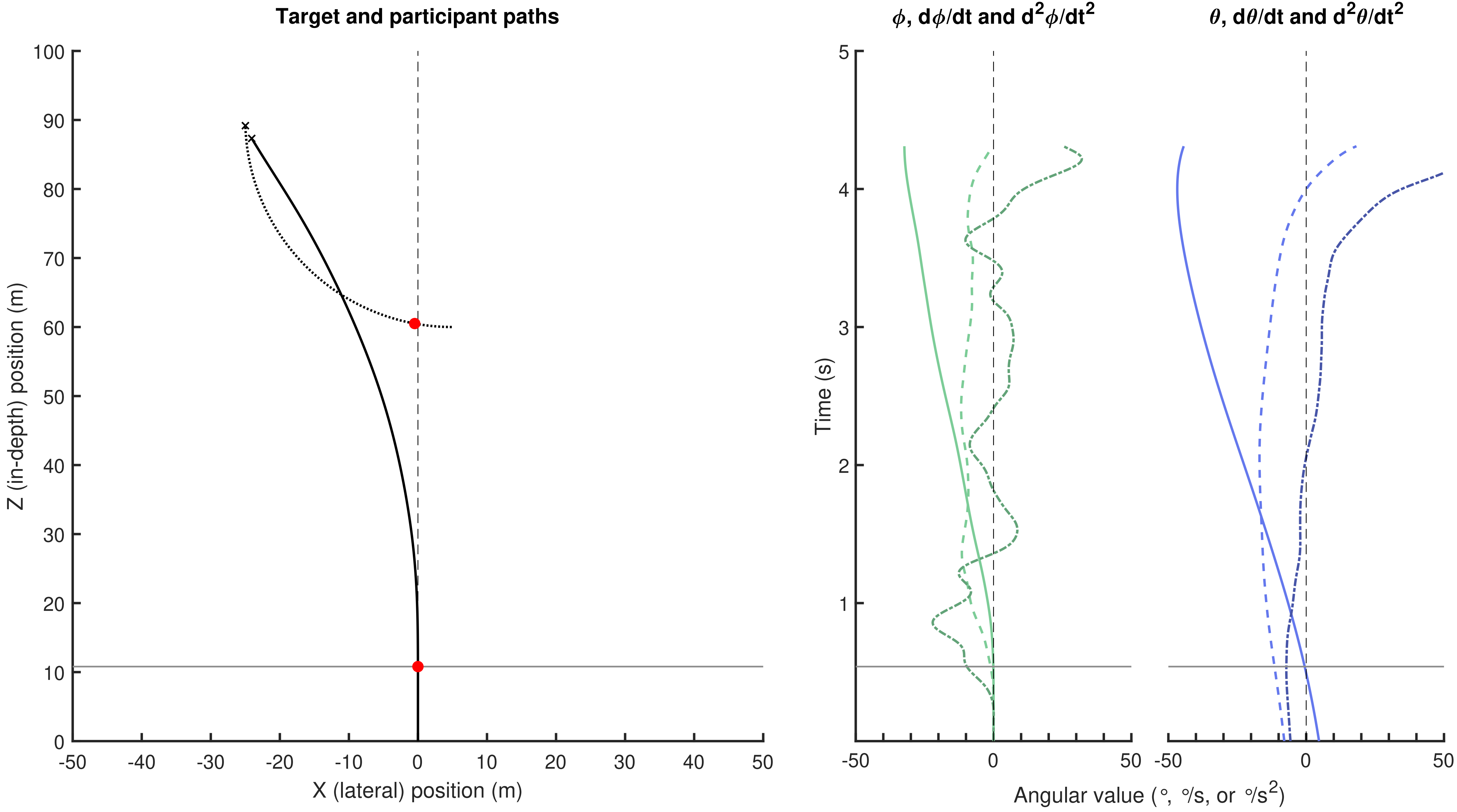

P5/B3
S15/R10-IN

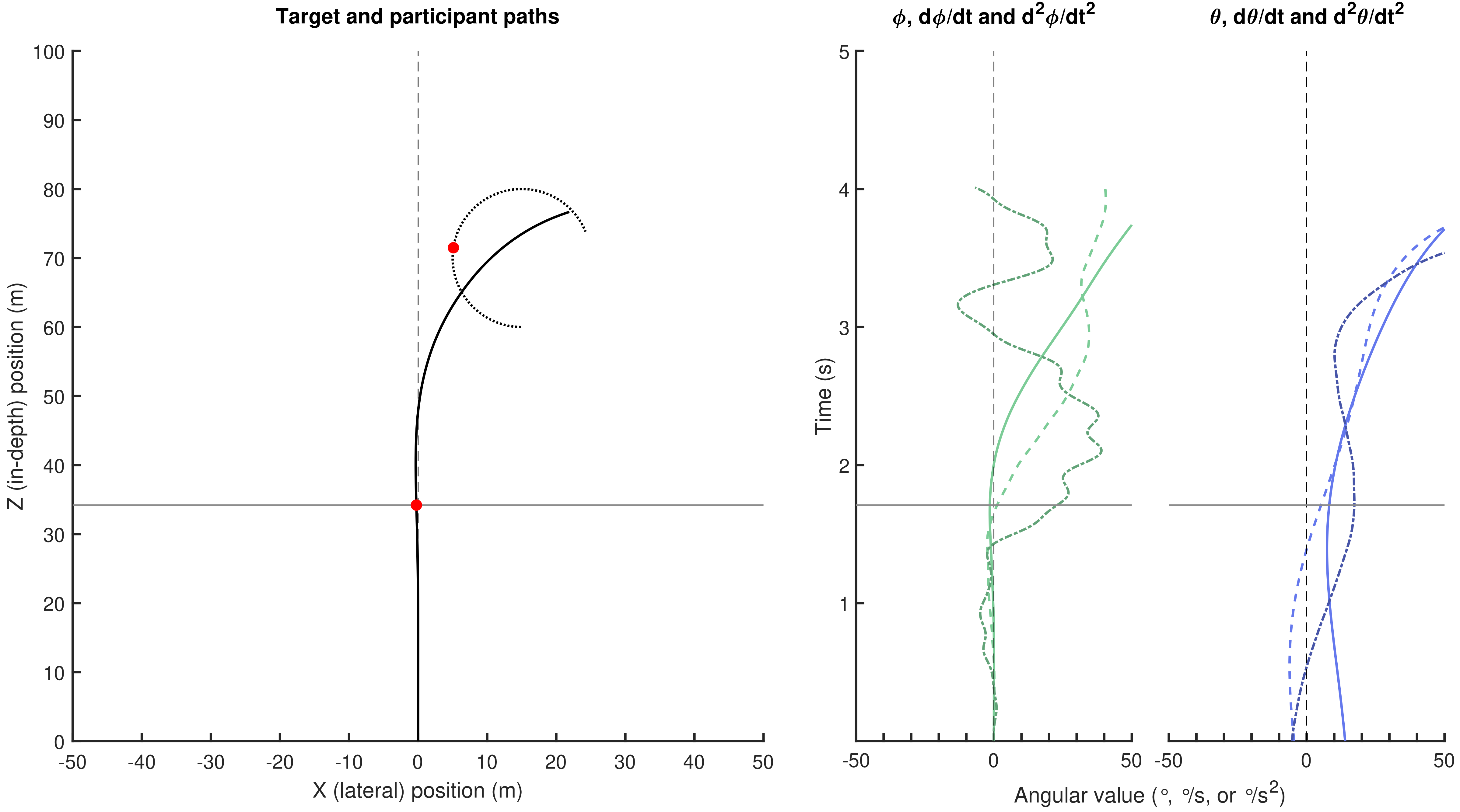

P5/B3
S15/R20-IN

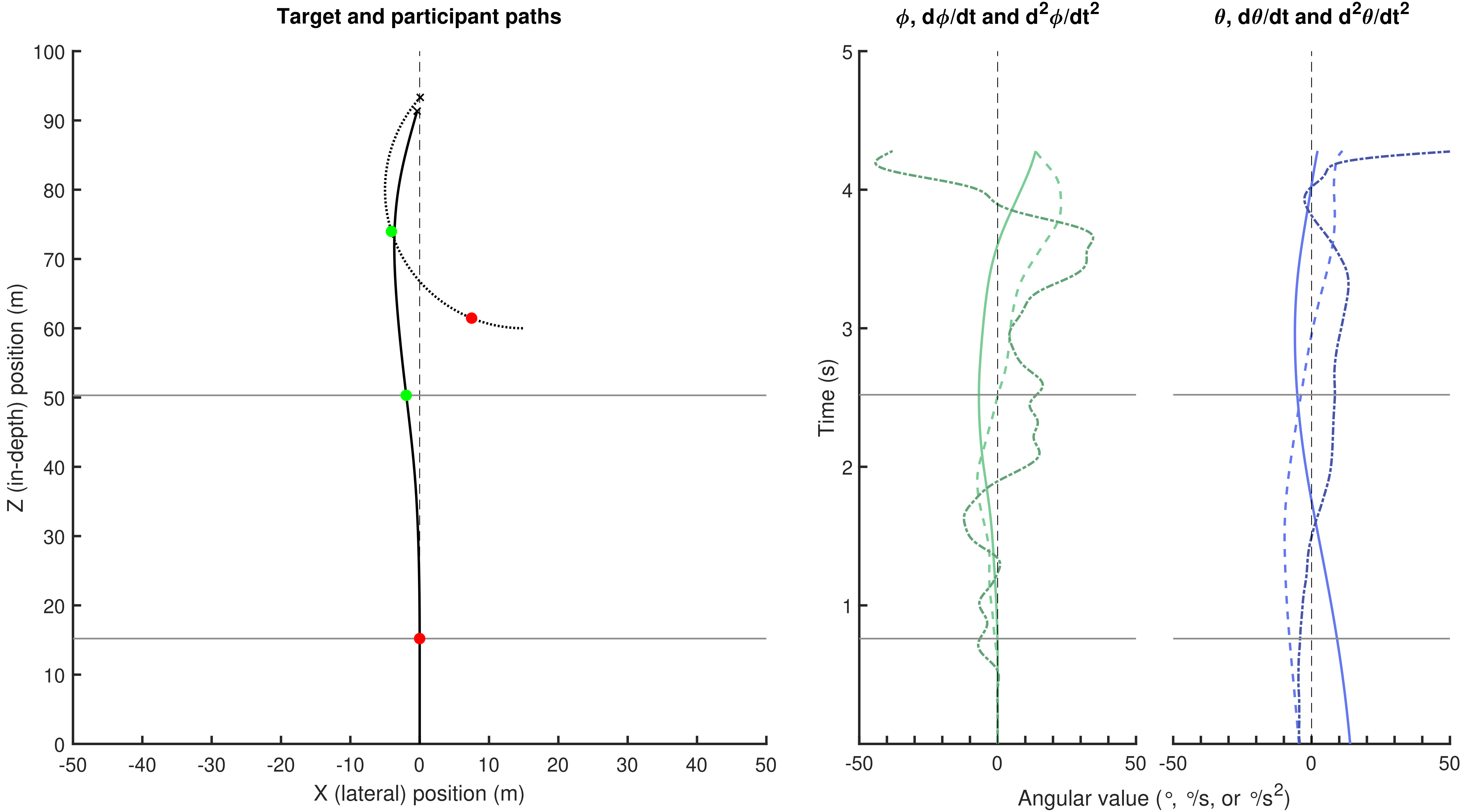

P5/B3
S15/R30-IN

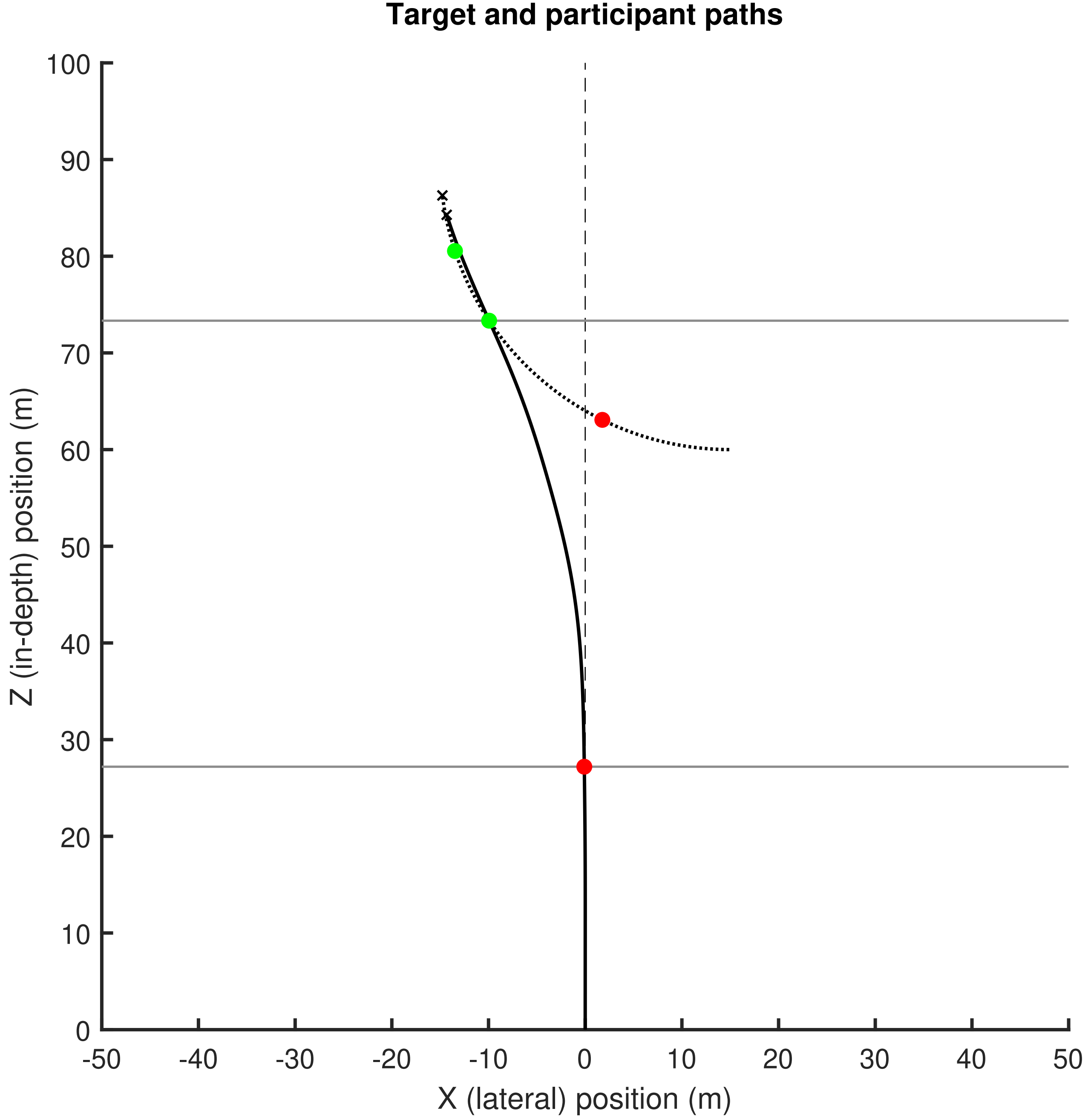

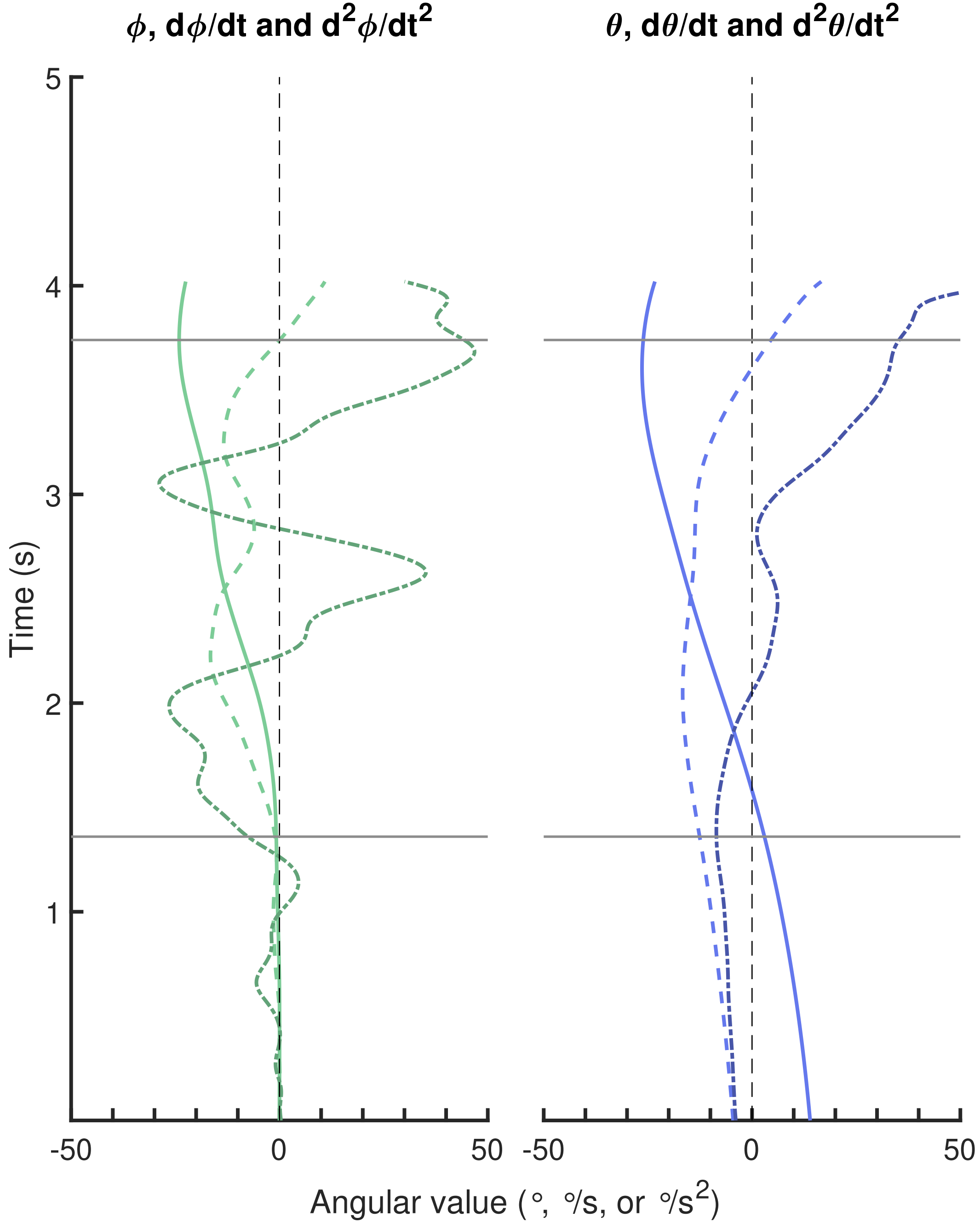

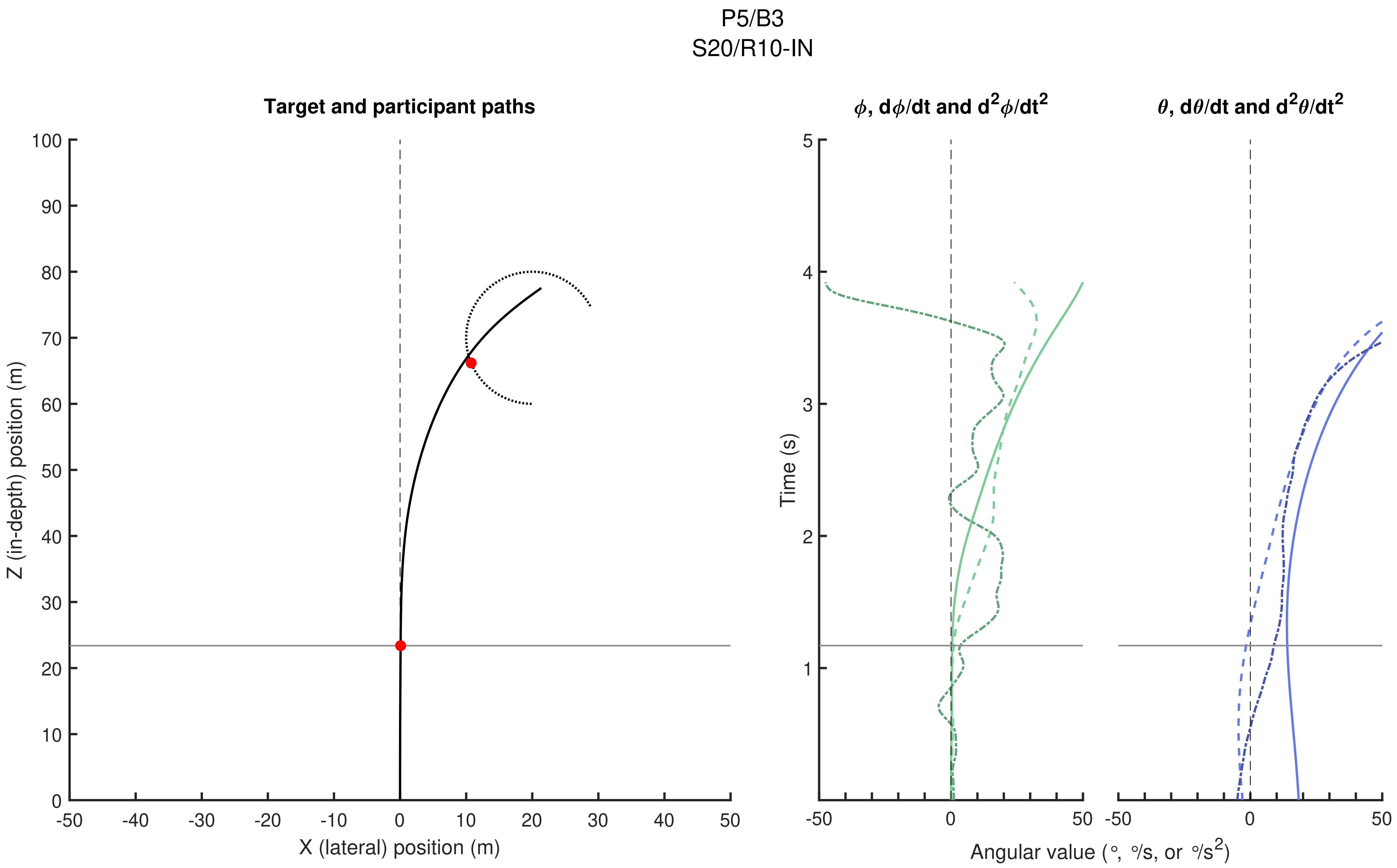
P5/B3
S20/R10-IN
Target and participant paths
Z (in-depth) position (m)
X (lateral) position (m)
100
90
80
70
60
50
40
30
20
10
0
-50
-40
-30
-20
-10
0
10
20
30
40
50
ϕ, dϕ/dt and d$^2\phi$/dt^2
θ, dθ/dt and d$^2\theta$/dt^2
Time (s)
5
4
3
2
1
-50
0
50
-50
0
50
Angular value (°, °/s, or °/s^2)

P5/B3
S20/R20-IN

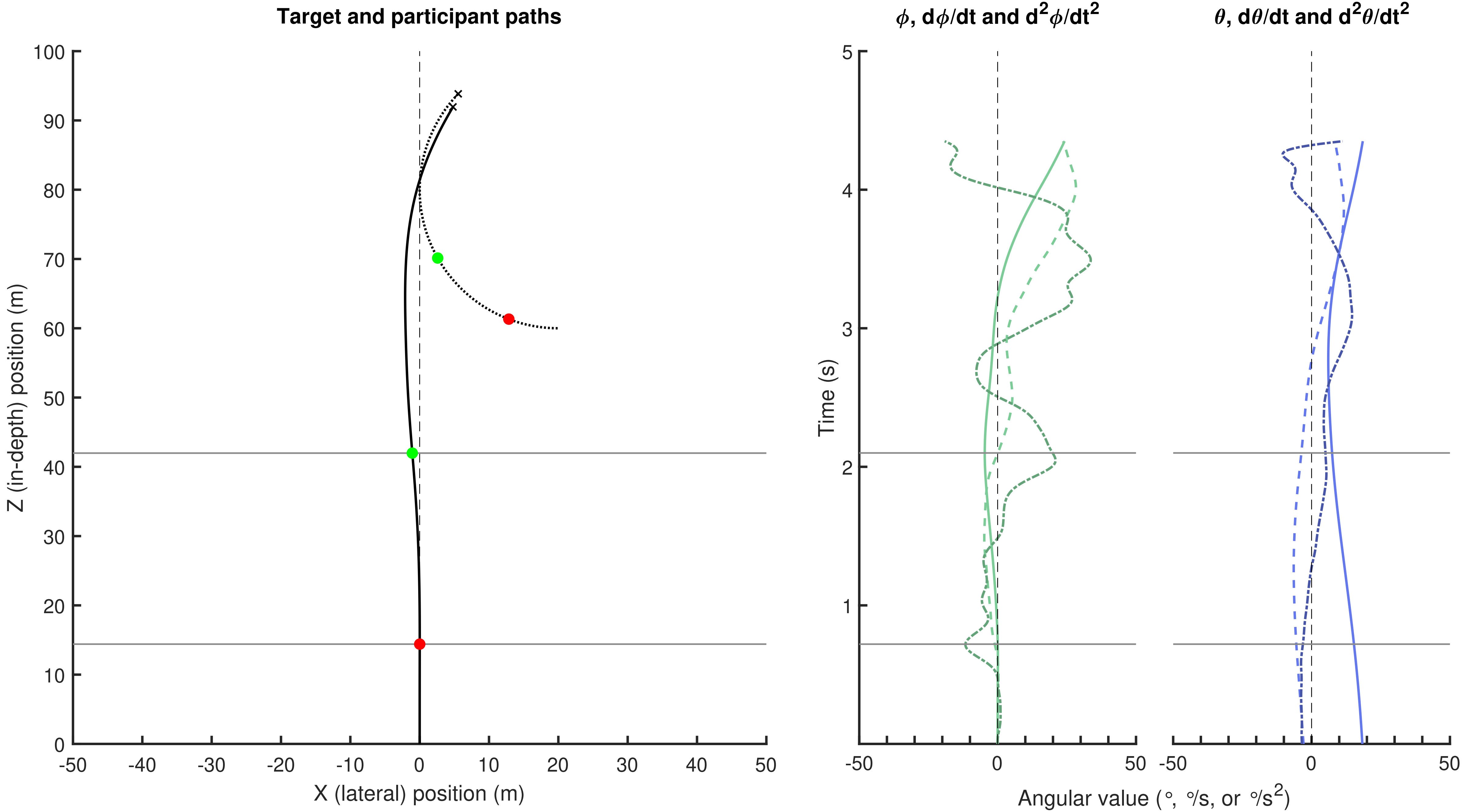

P5/B3
S20/R30-IN

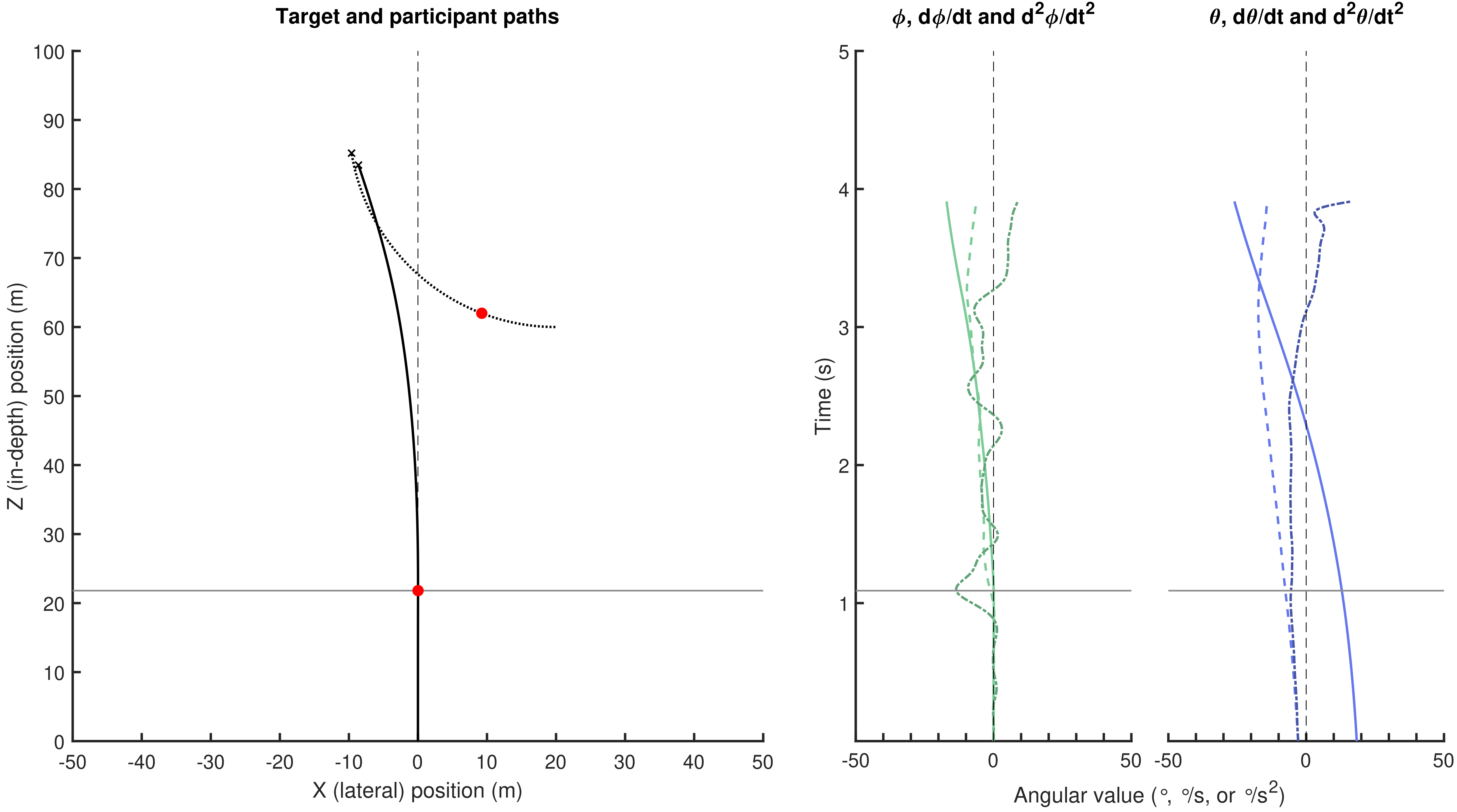

P5/B4
S20/R10-IN

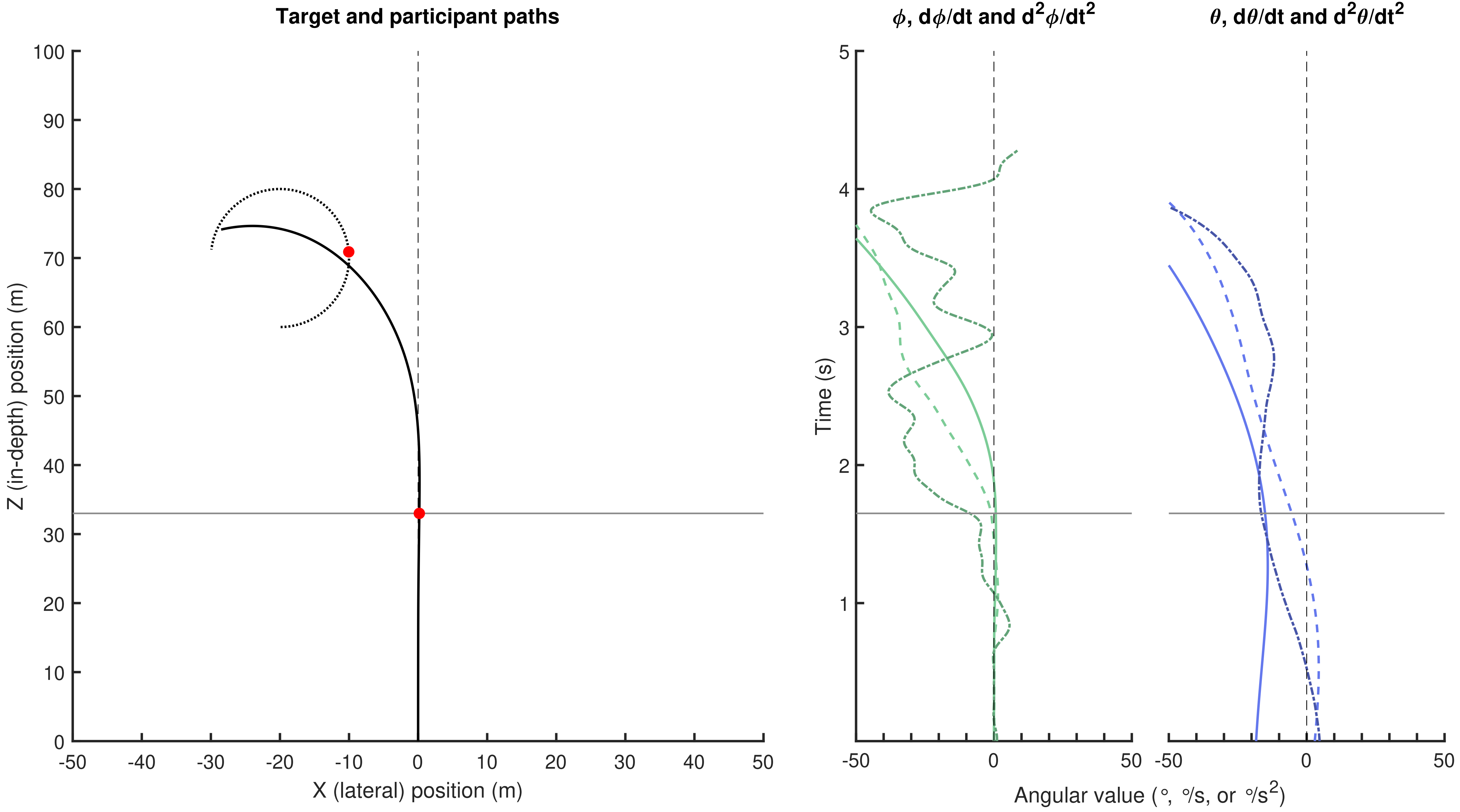

P5/B4
S20/R20-IN

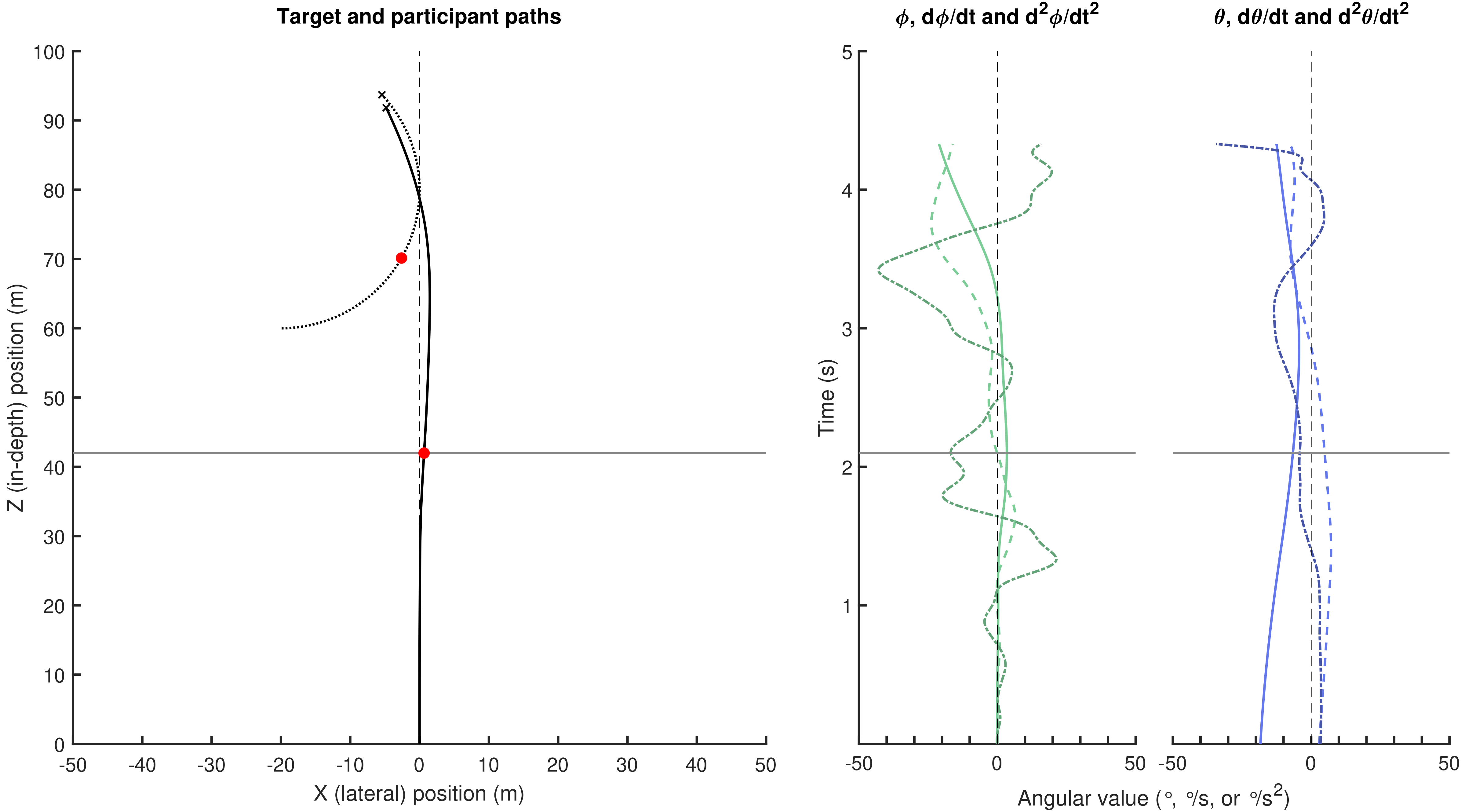

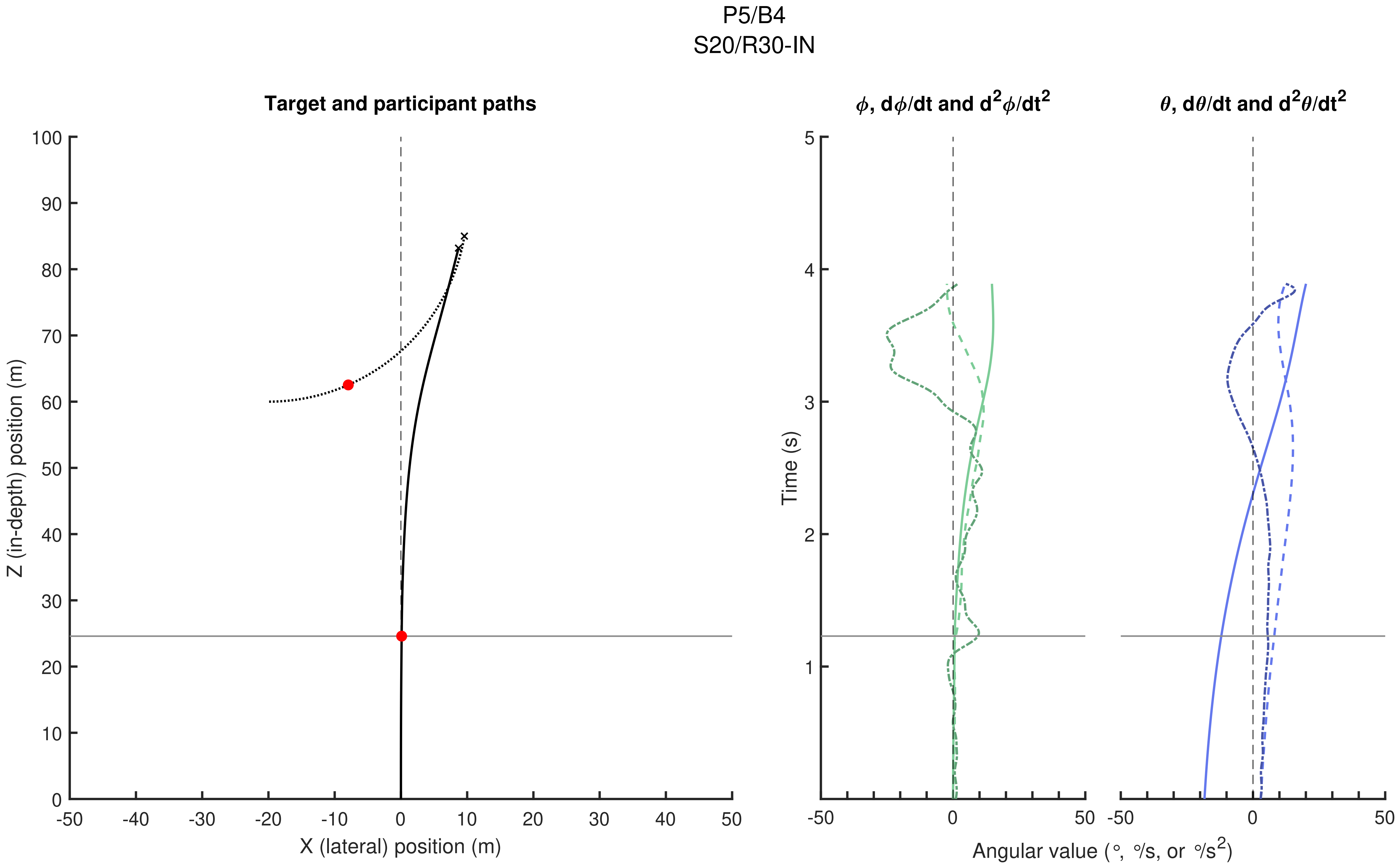
P5/B4
S20/R30-IN
Target and participant paths
Z (in-depth) position (m)
X (lateral) position (m)
ϕ, dϕ/dt and d²ϕ/dt²
θ, dθ/dt and d²θ/dt²
Time (s)
Angular value (°, °/s, or °/s²)

P5/B4
S15/R10-IN

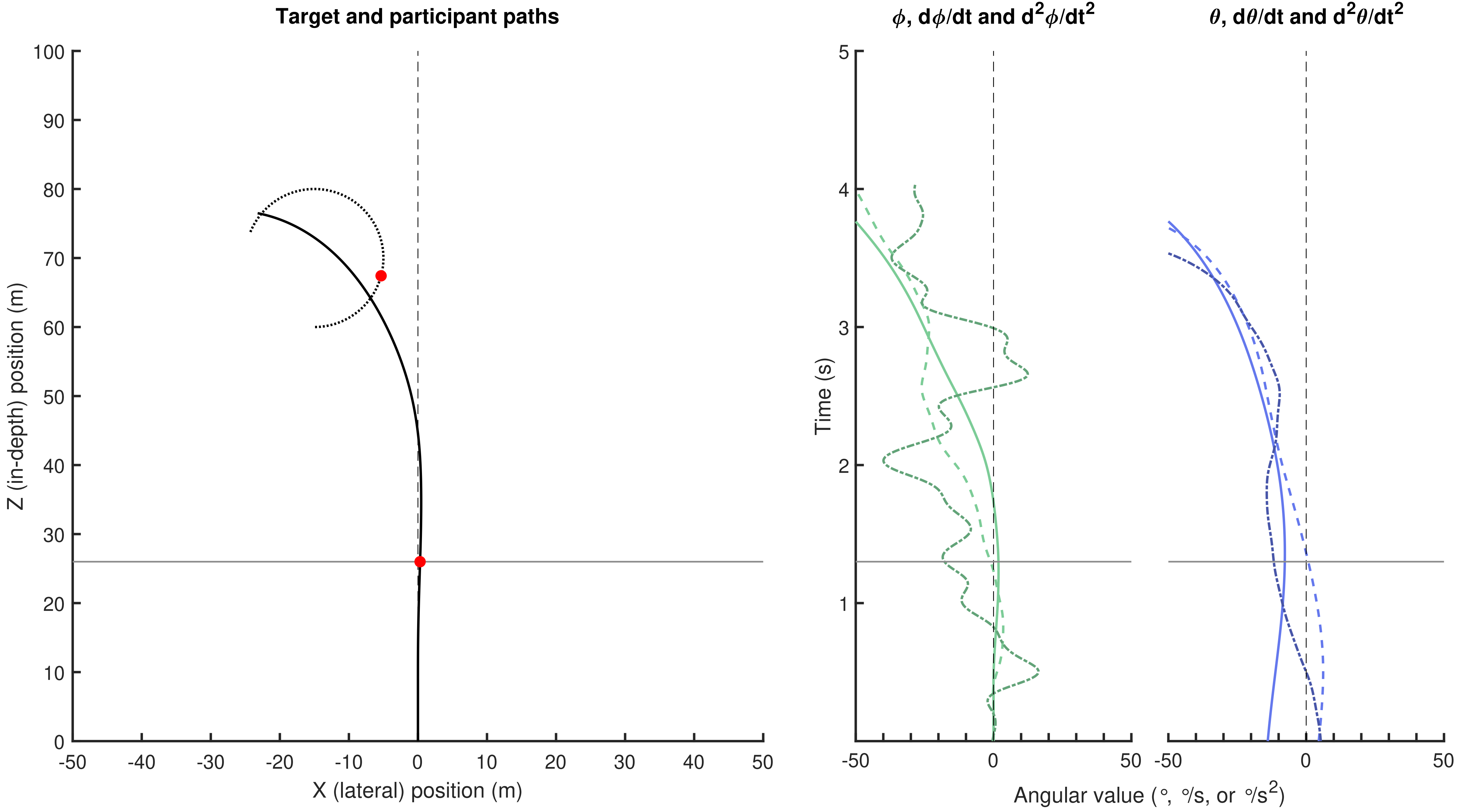

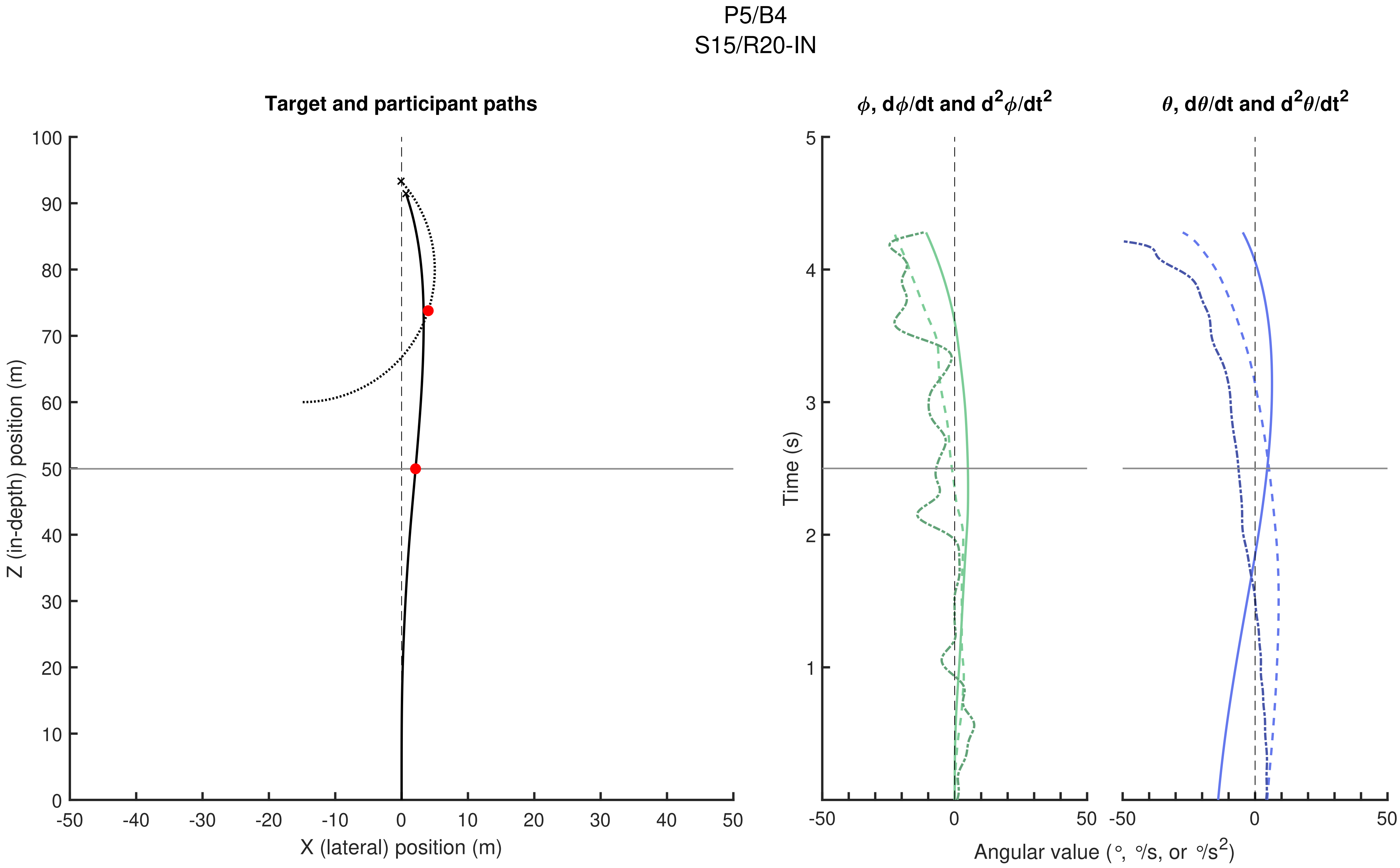

P5/B4
S15/R20-IN
Target and participant paths
ϕ, dϕ/dt and d$^2\phi$/dt^2
θ, dθ/dt and d$^2\theta$/dt^2
Z (in-depth) position (m)
X (lateral) position (m)
Time (s)
Angular value (°, °/s, or °/s^2)
100
90
80
70
60
50
40
30
20
10
0
-50
-40
-30
-20
-10
0
10
20
30
40
50
5
4
3
2
1

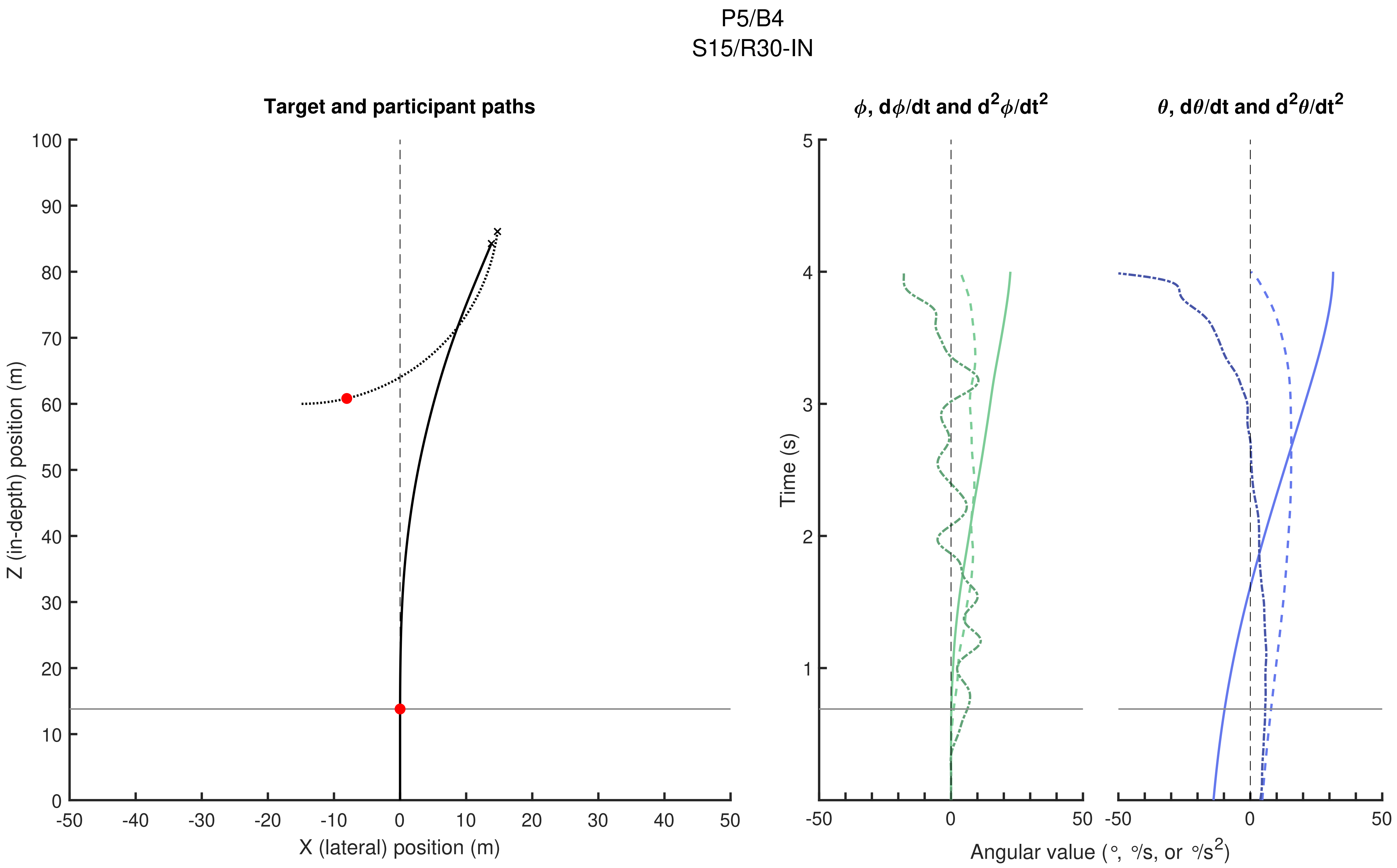
P5/B4
S15/R30-IN
Target and participant paths
Z (in-depth) position (m)
X (lateral) position (m)
100
90
80
70
60
50
40
30
20
10
0
-50
-40
-30
-20
-10
0
10
20
30
40
50
ϕ, dϕ/dt and d$^2\phi$/dt^2
θ, dθ/dt and d$^2\theta$/dt^2
Time (s)
5
4
3
2
1
-50
0
50
-50
0
50
Angular value (°, °/s, or °/s^2)

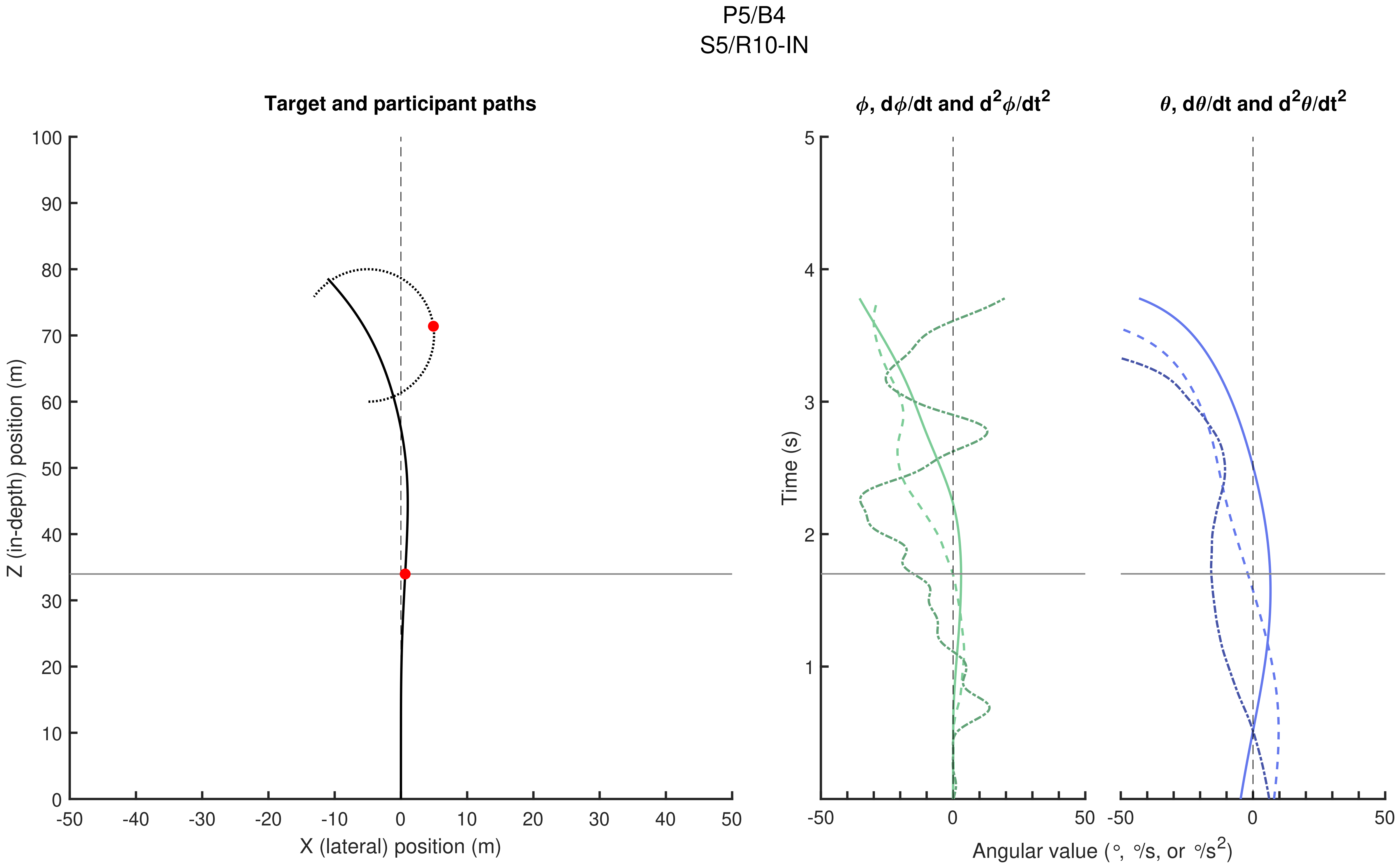
P5/B4
S5/R10-IN
Target and participant paths
Z (in-depth) position (m)
X (lateral) position (m)
φ, dφ/dt and d²φ/dt²
θ, dθ/dt and d²θ/dt²
Time (s)
Angular value (°, °/s, or °/s²)

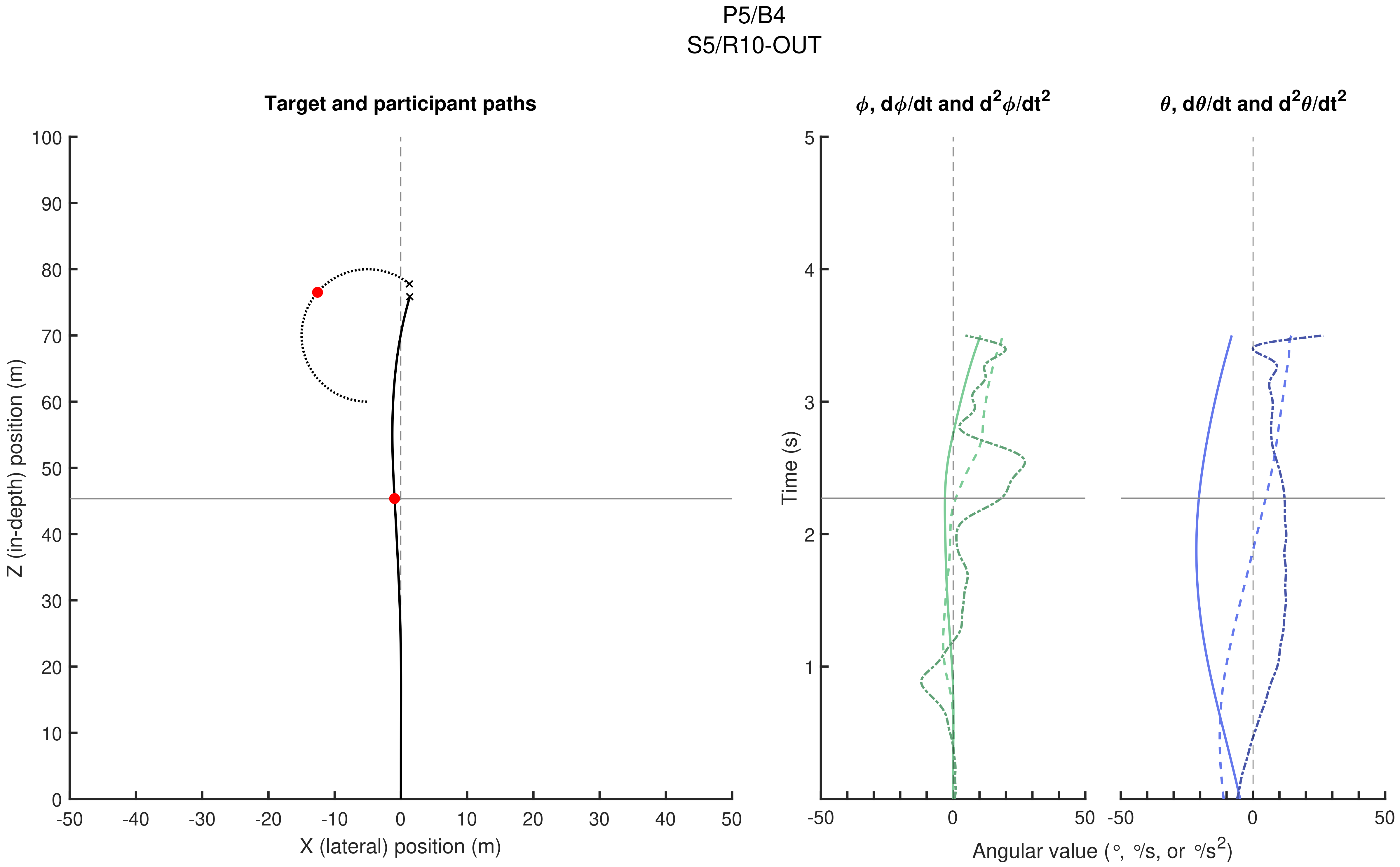
P5/B4
S5/R10-OUT
Target and participant paths
Z (in-depth) position (m)
X (lateral) position (m)
ϕ, dϕ/dt and d$^2\phi$/dt^2
θ, dθ/dt and d$^2\theta$/dt^2
Time (s)
Angular value (°, °/s, or °/s^2)
100
90
80
70
60
50
40
30
20
10
0
-50
-40
-30
-20
-10
0
10
20
30
40
50
5
4
3
2
1

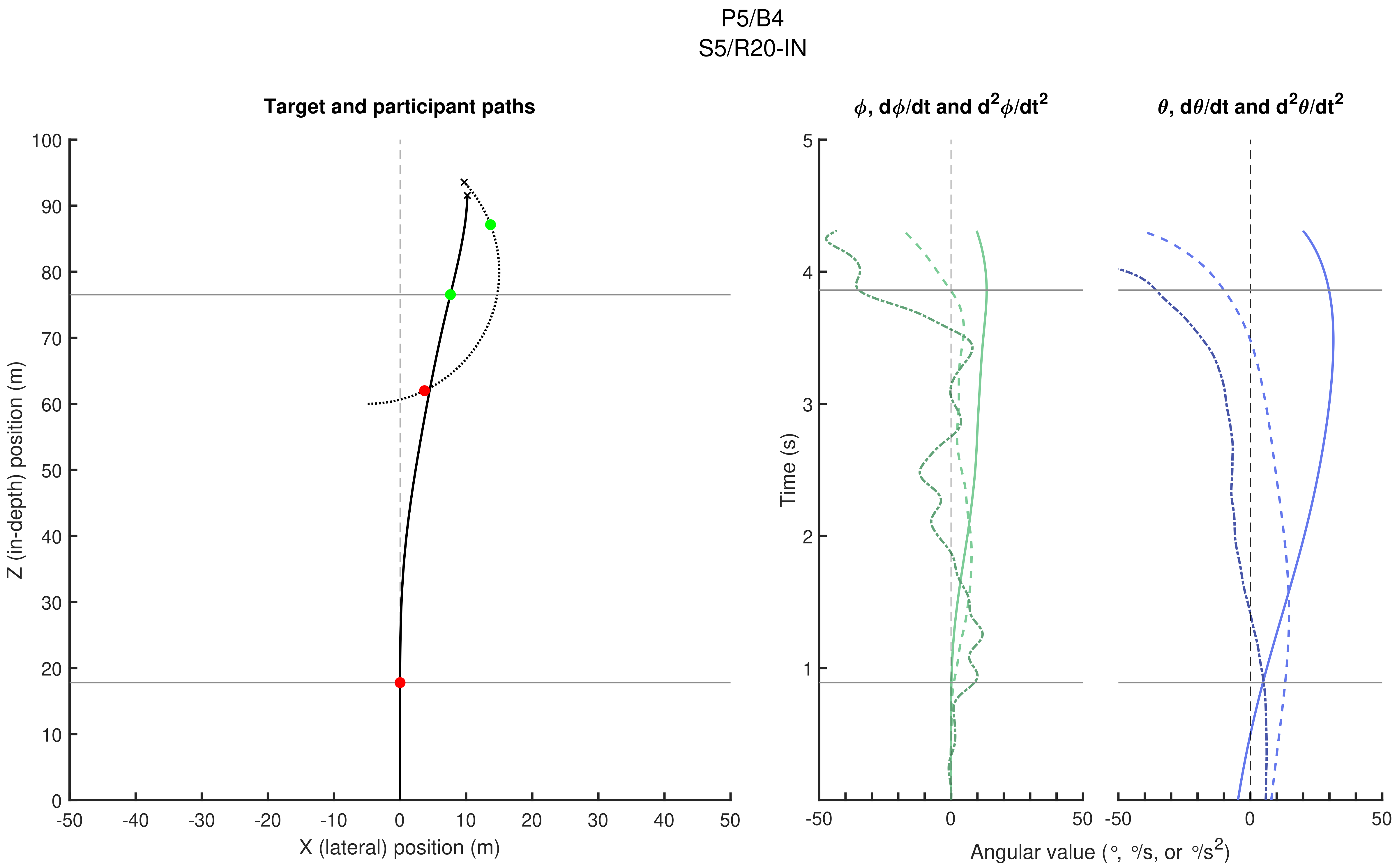
P5/B4
S5/R20-IN
Target and participant paths
Z (in-depth) position (m)
X (lateral) position (m)
ϕ, dϕ/dt and d²ϕ/dt²
θ, dθ/dt and d²θ/dt²
Time (s)
Angular value (°, °/s, or °/s²)

P5/B4
S5/R20-OUT

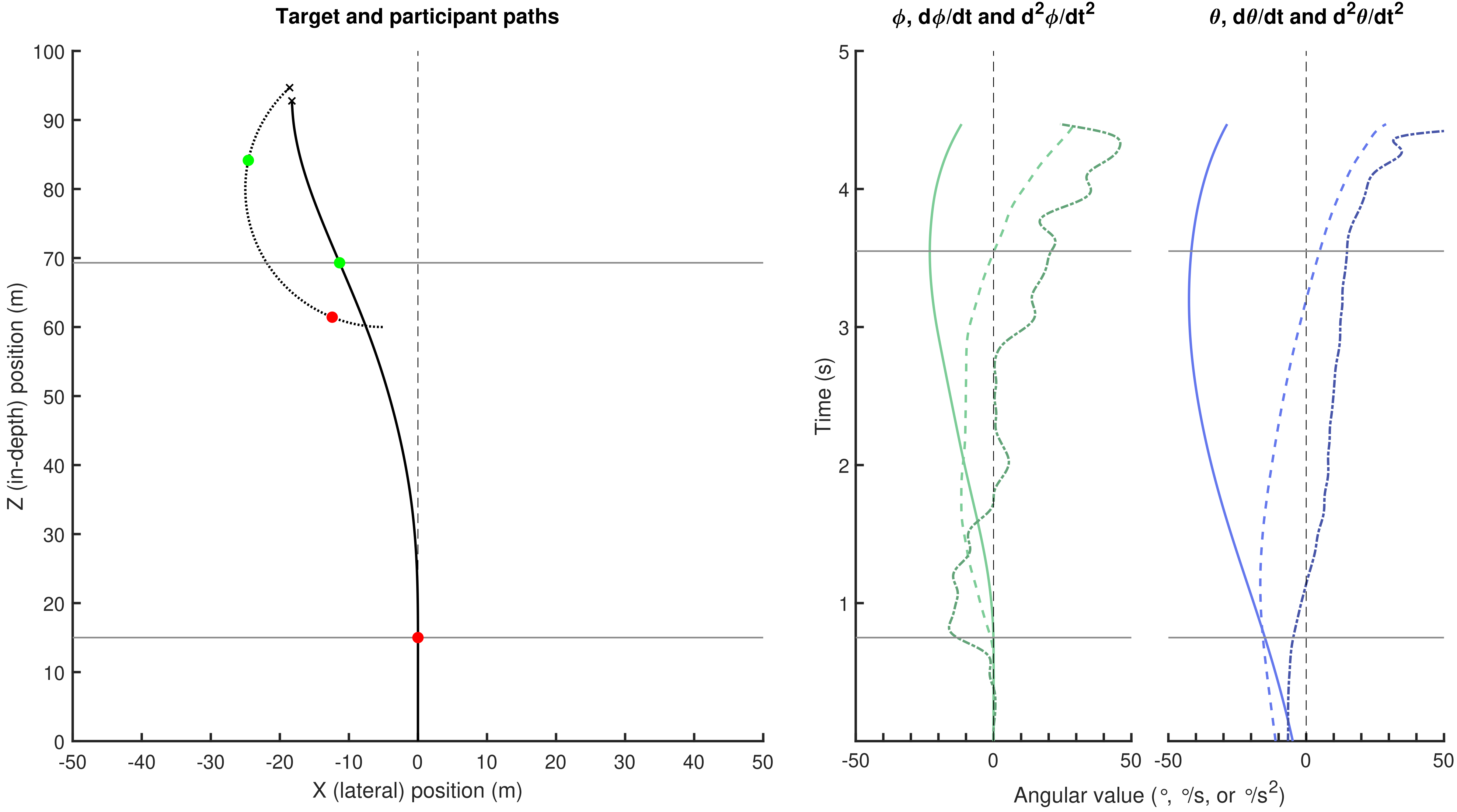

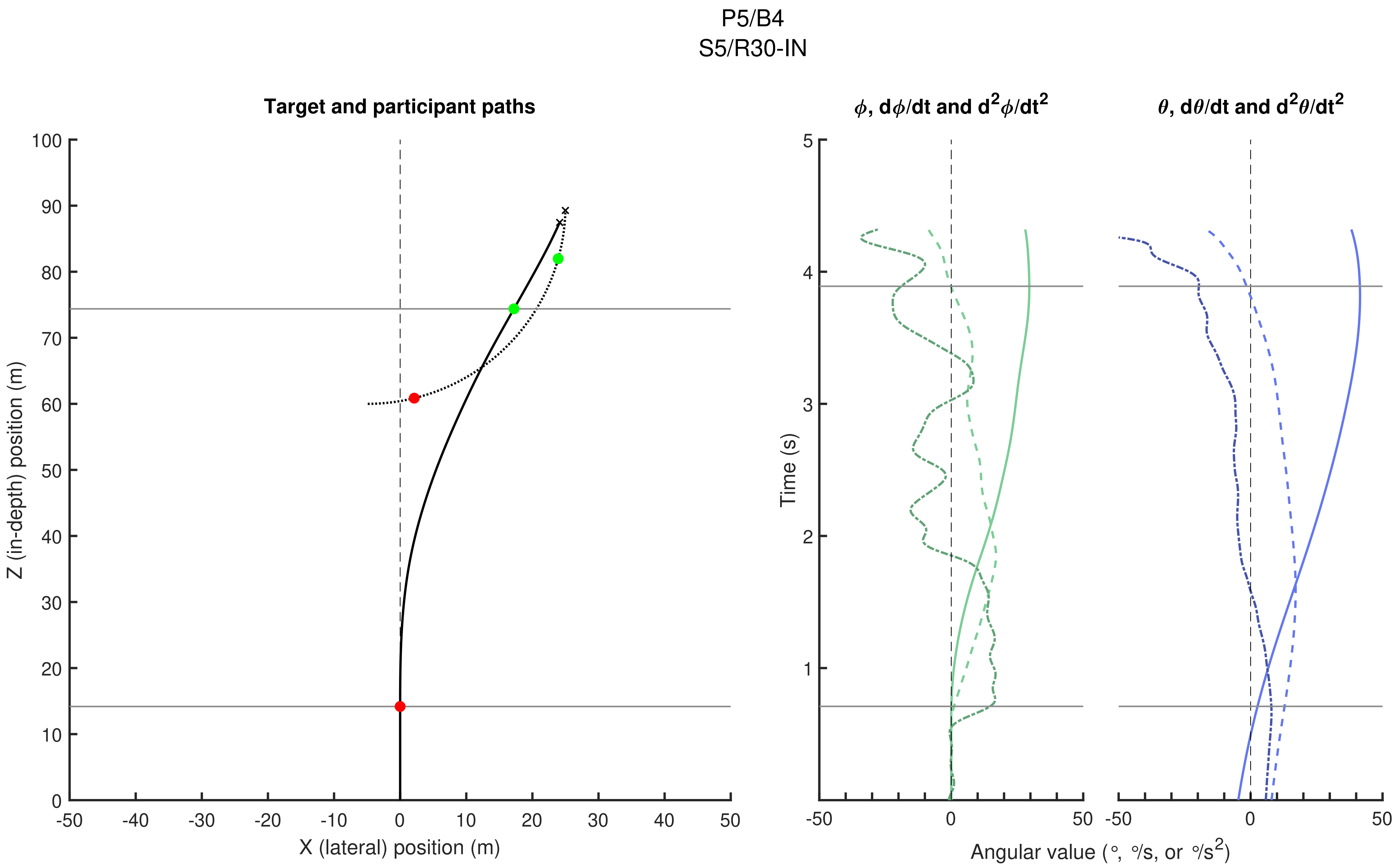

P5/B4
S5/R30-IN
Target and participant paths
Z (in-depth) position (m)
X (lateral) position (m)
100
90
80
70
60
50
40
30
20
10
0
-50
-40
-30
-20
-10
0
10
20
30
40
50
ϕ, dϕ/dt and d$^2\phi$/dt^2
θ, dθ/dt and d$^2\theta$/dt^2
Time (s)
5
4
3
2
1
-50
0
50
-50
0
50
Angular value (°, °/s, or °/s^2)

P5/B4
S5/R30-OUT

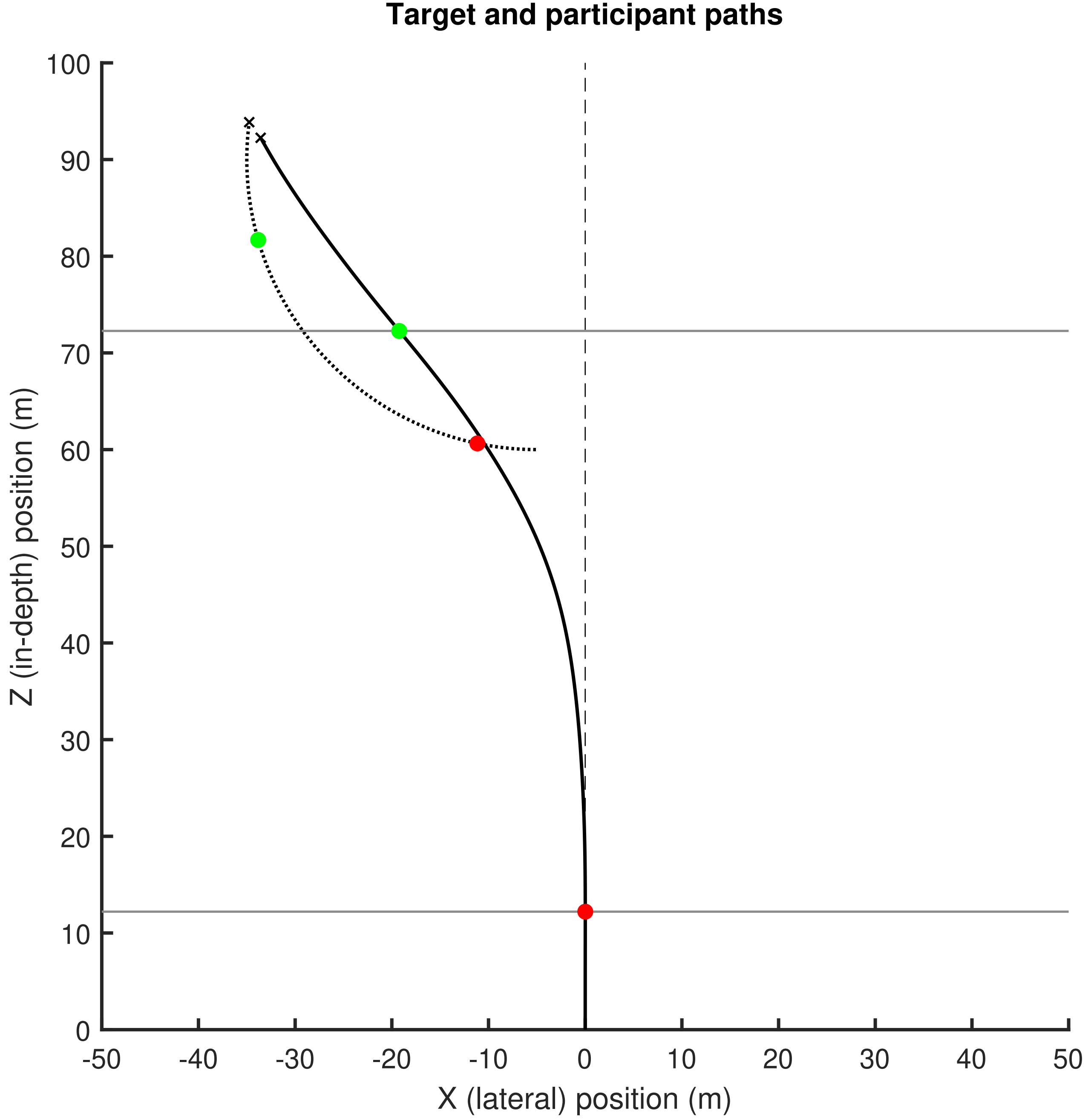

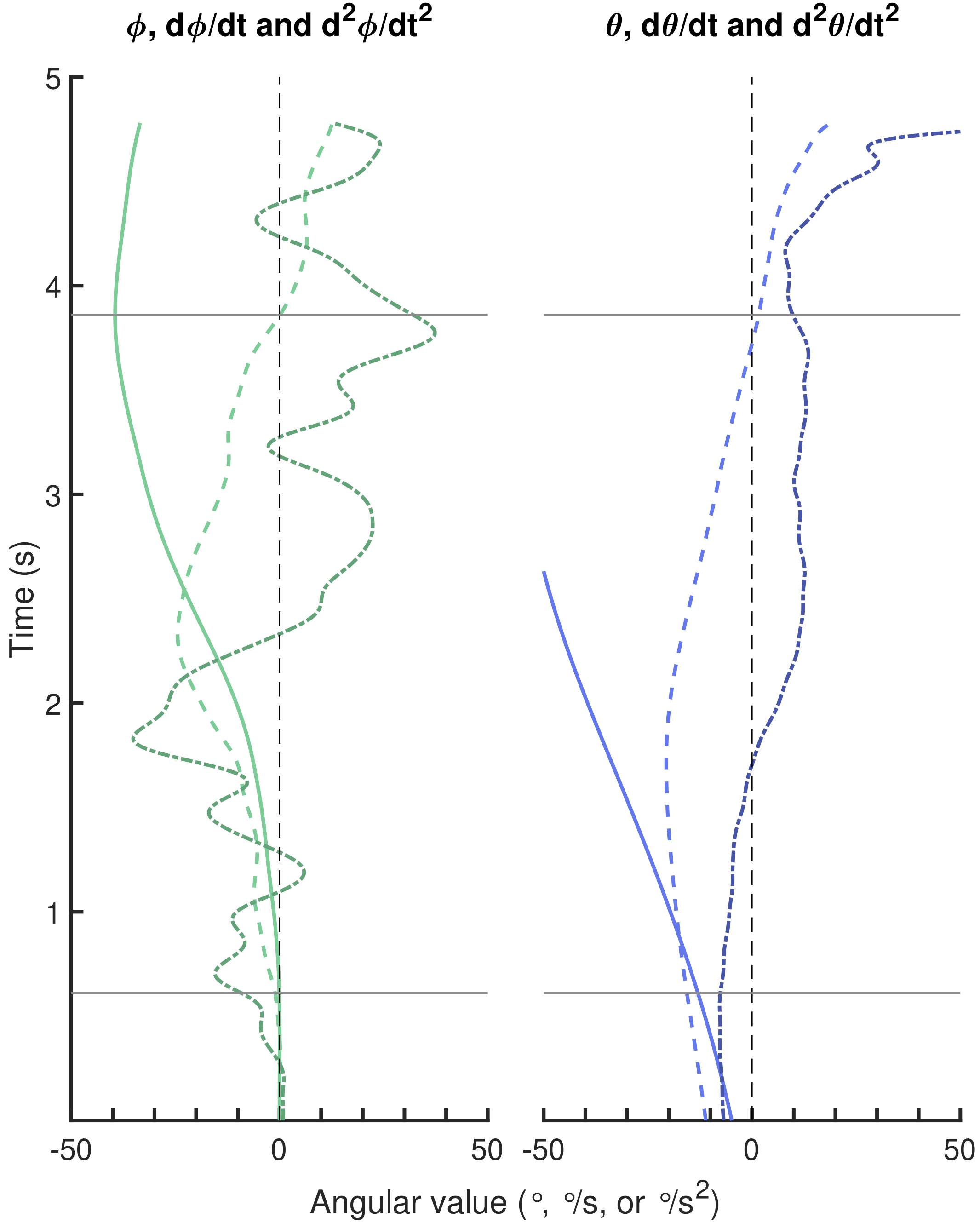

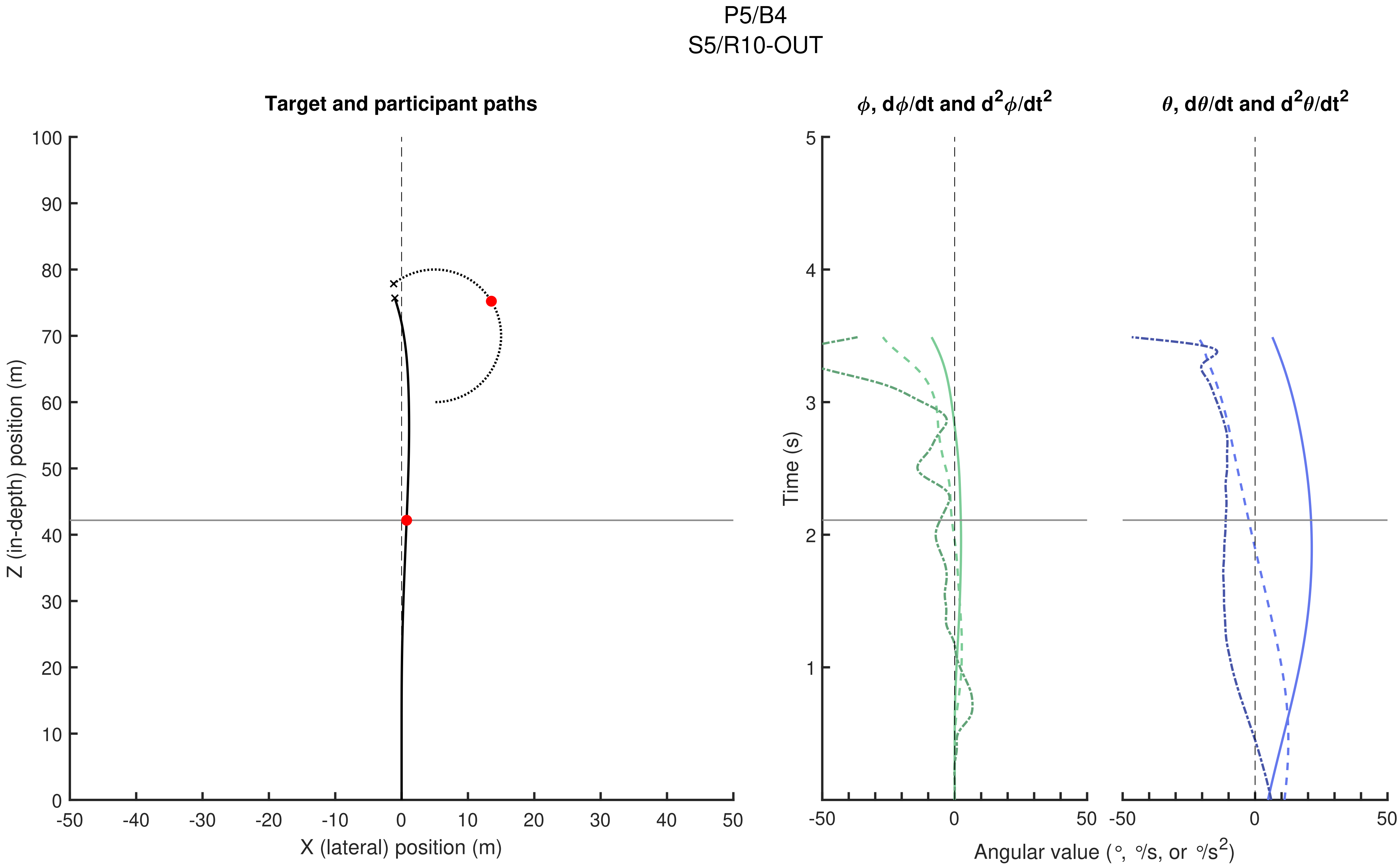
P5/B4
S5/R10-OUT
Target and participant paths
Z (in-depth) position (m)
X (lateral) position (m)
100
90
80
70
60
50
40
30
20
10
0
-50
-40
-30
-20
-10
0
10
20
30
40
50
ϕ, dϕ/dt and d$^2\phi$/dt^2
θ, dθ/dt and d$^2\theta$/dt^2
Time (s)
5
4
3
2
1
-50
0
50
-50
0
50
Angular value (°, °/s, or °/s^2)

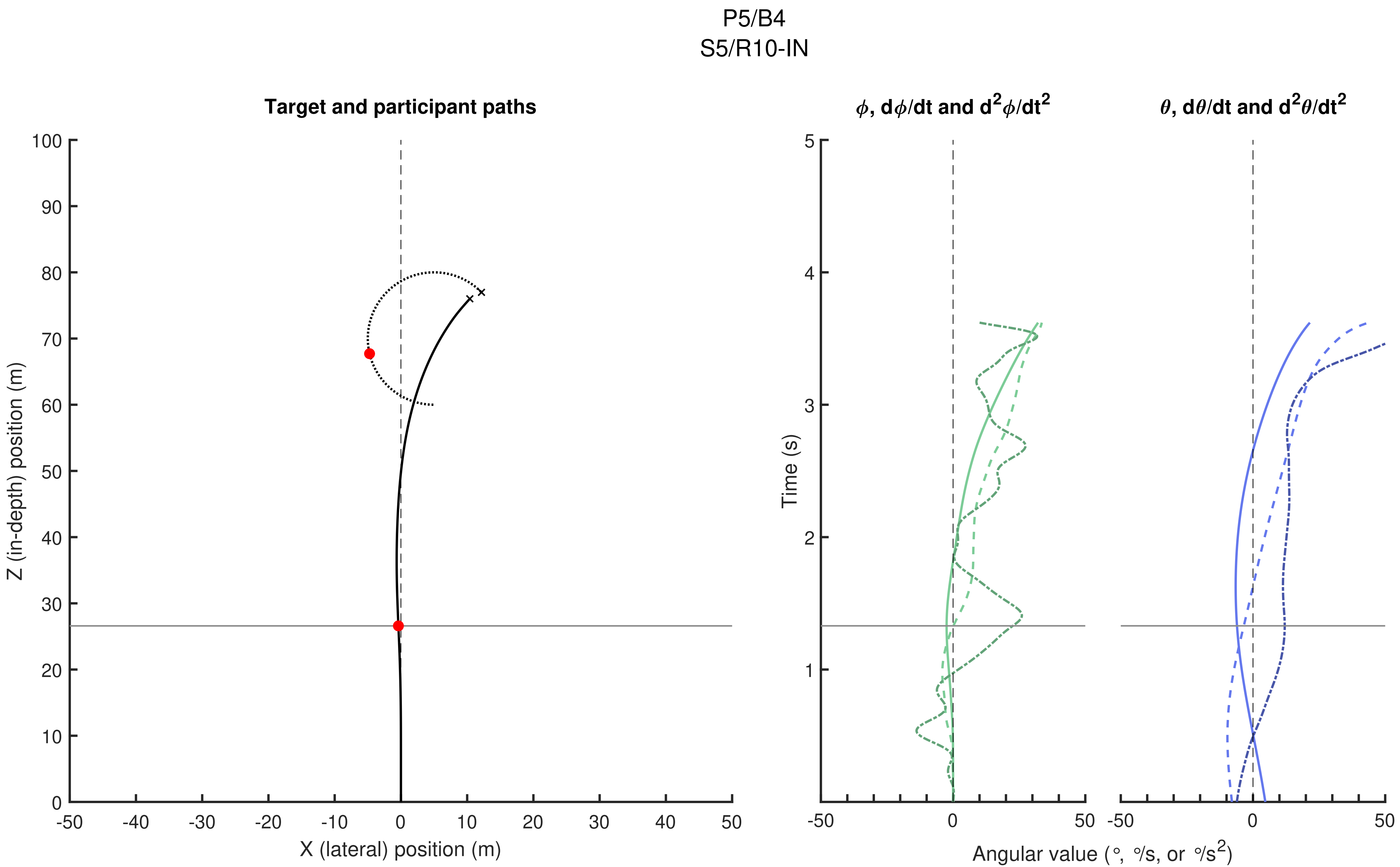

P5/B4
S5/R10-IN
Target and participant paths
Z (in-depth) position (m)
X (lateral) position (m)
φ, dφ/dt and d²φ/dt²
θ, dθ/dt and d²θ/dt²
Time (s)
Angular value (°, °/s, or °/s²)

P5/B4
S5/R20-OUT

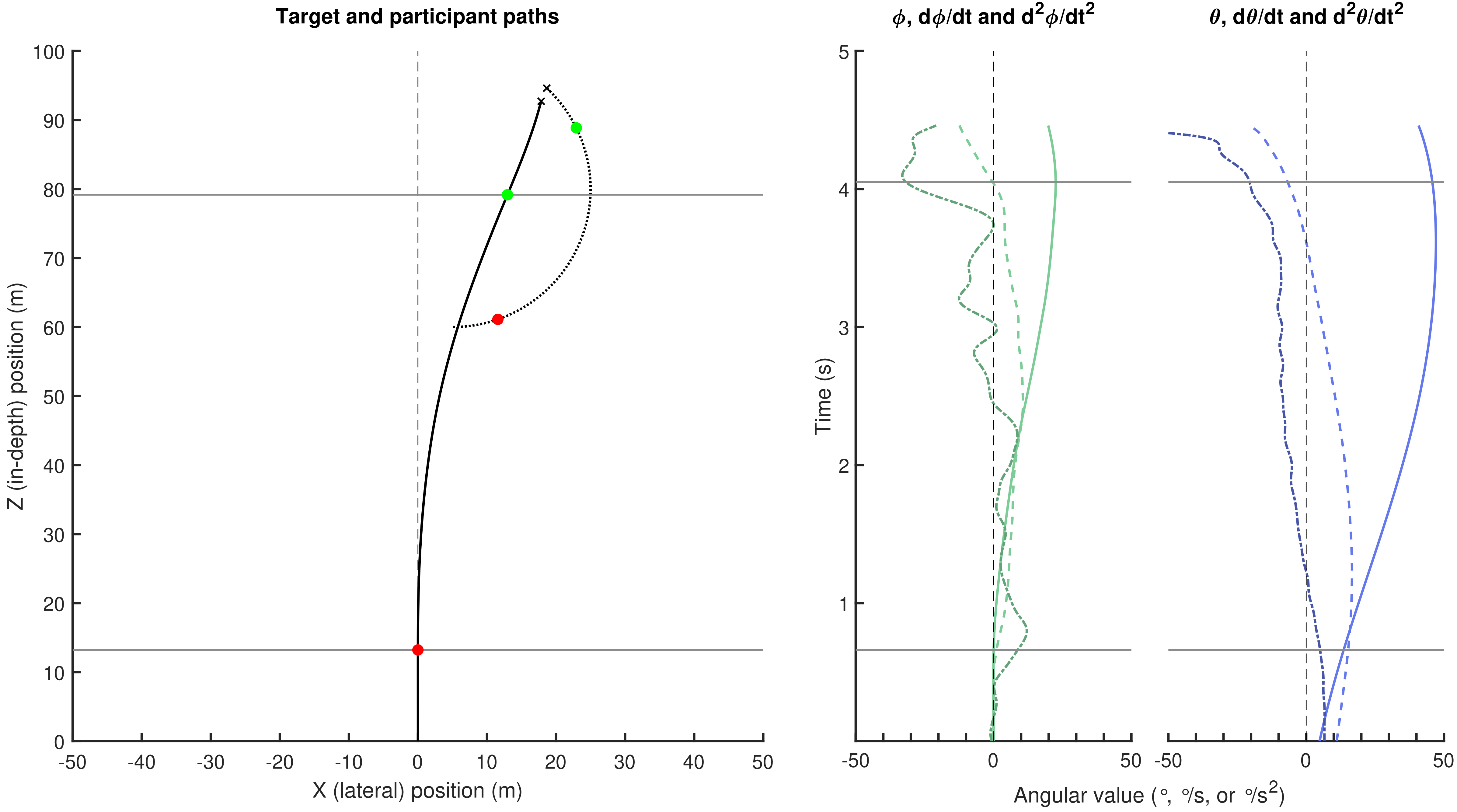

P5/B4
S5/R20-IN

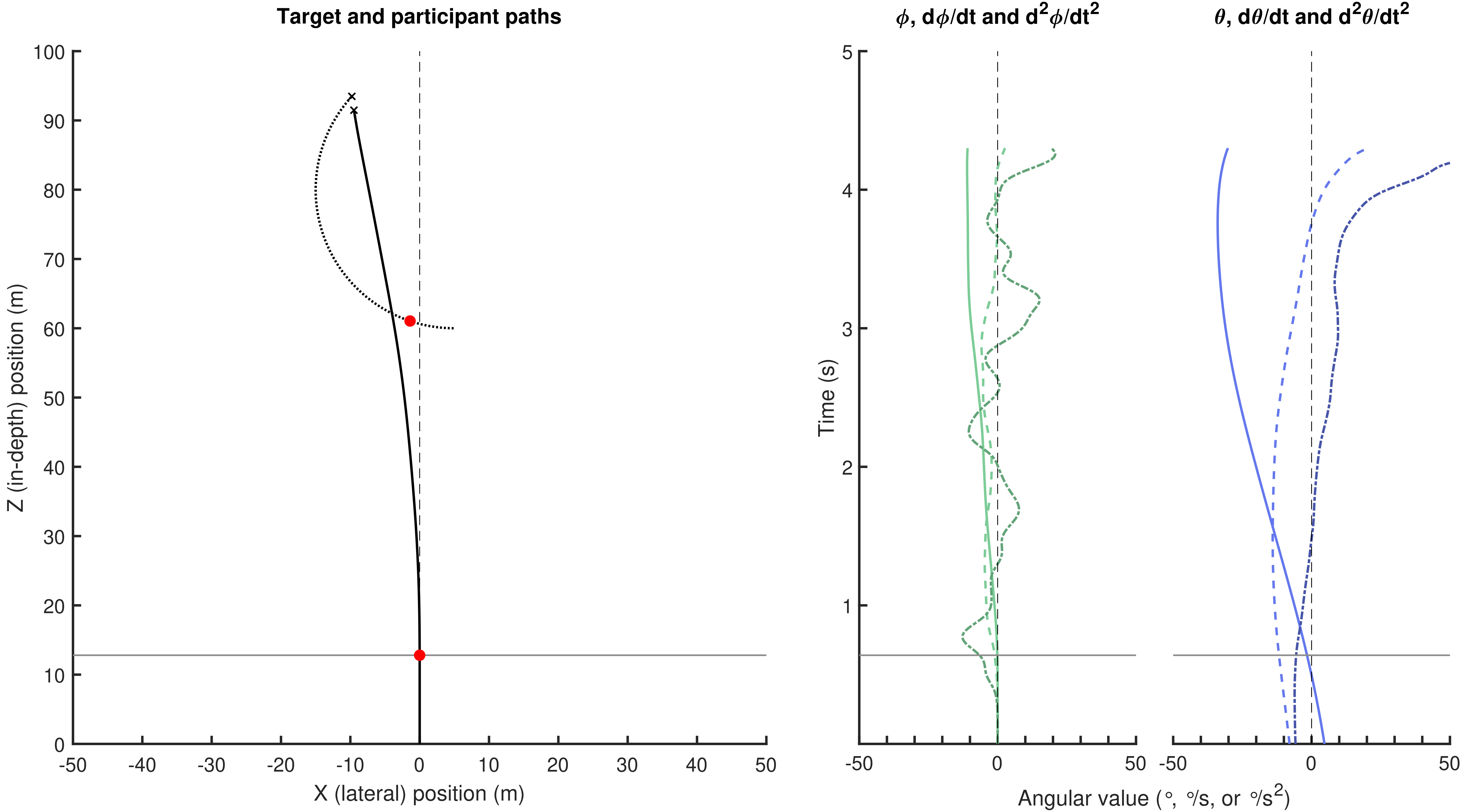

P5/B4
S5/R30-OUT

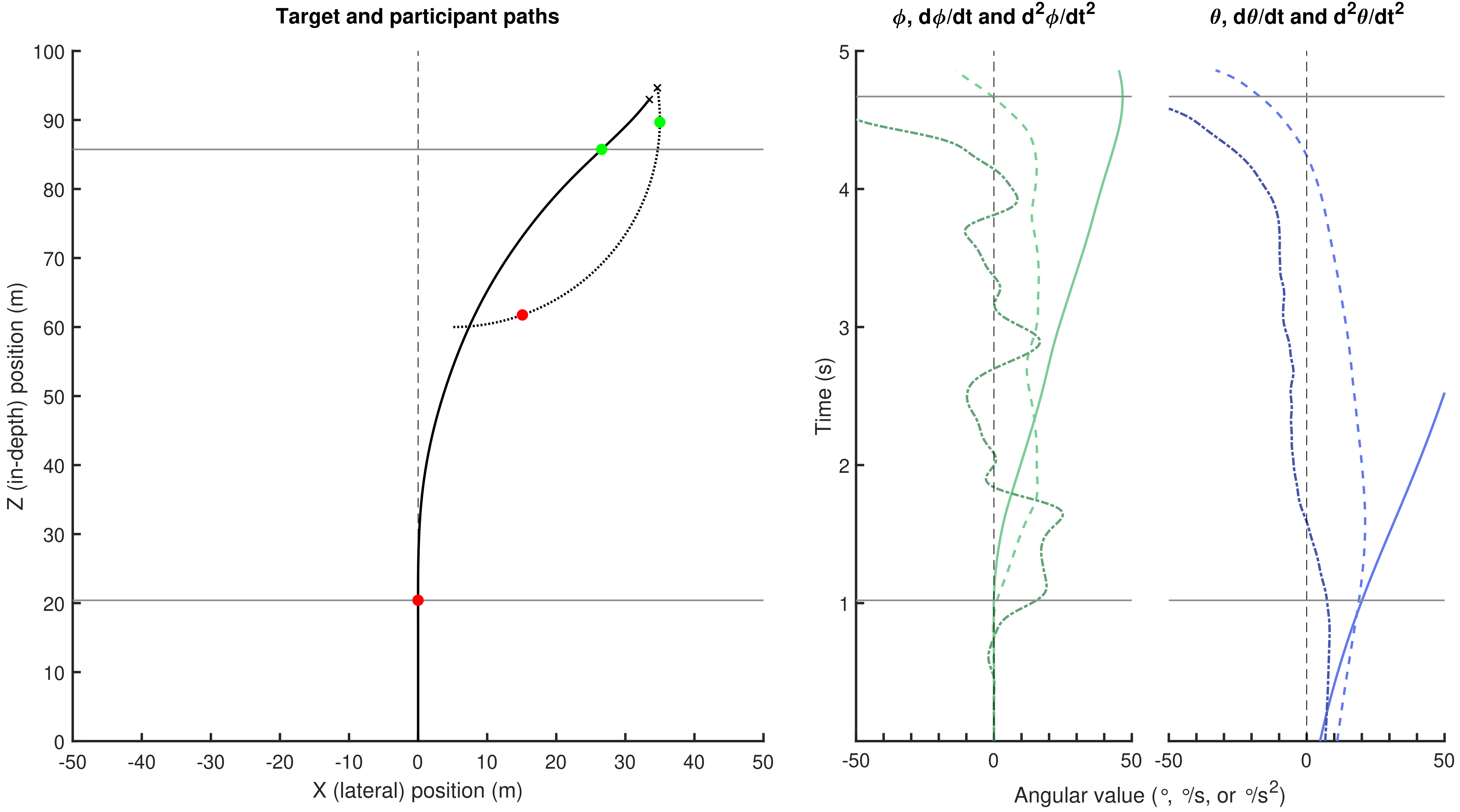

P5/B4
S5/R30-IN

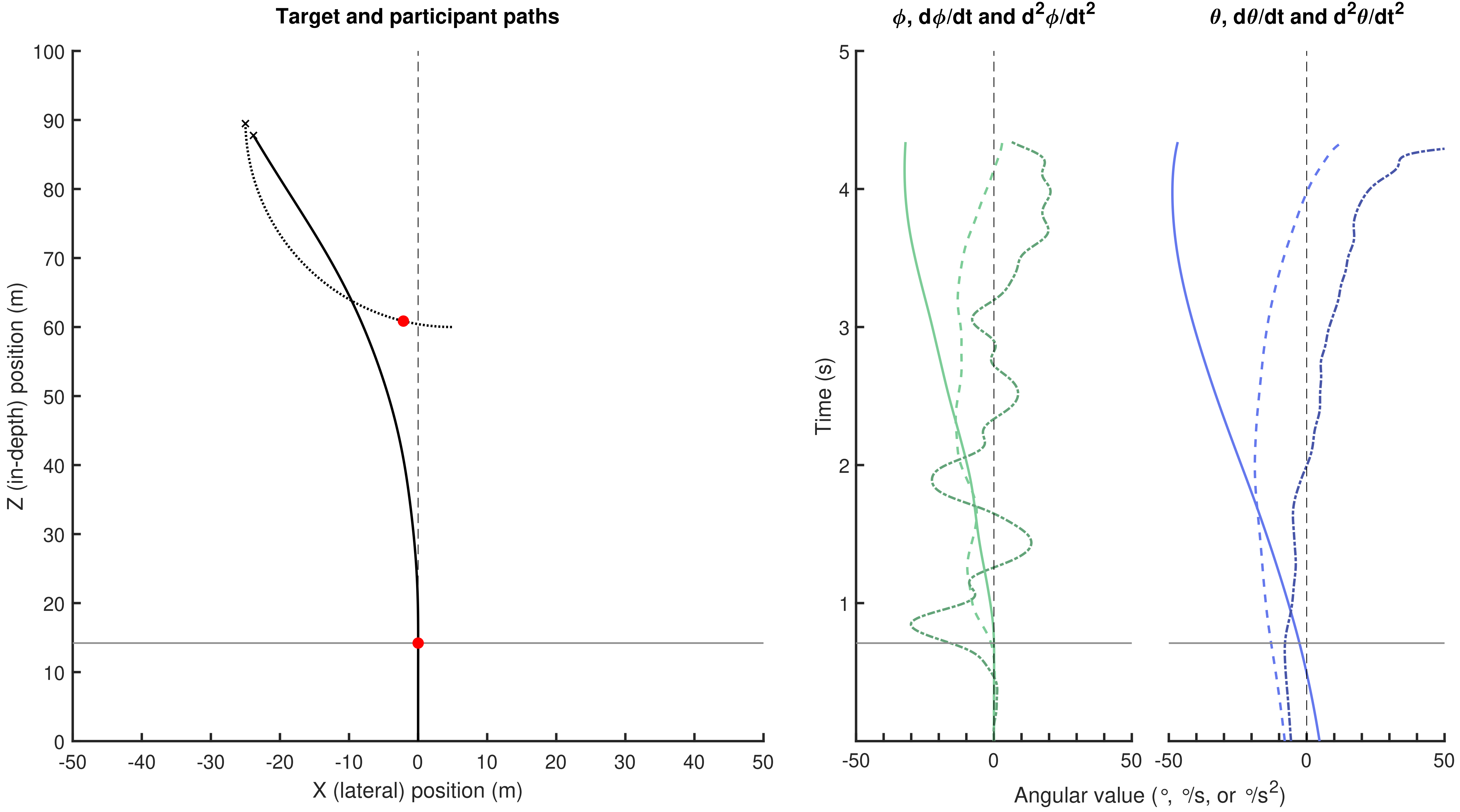

P5/B4
S15/R10-IN

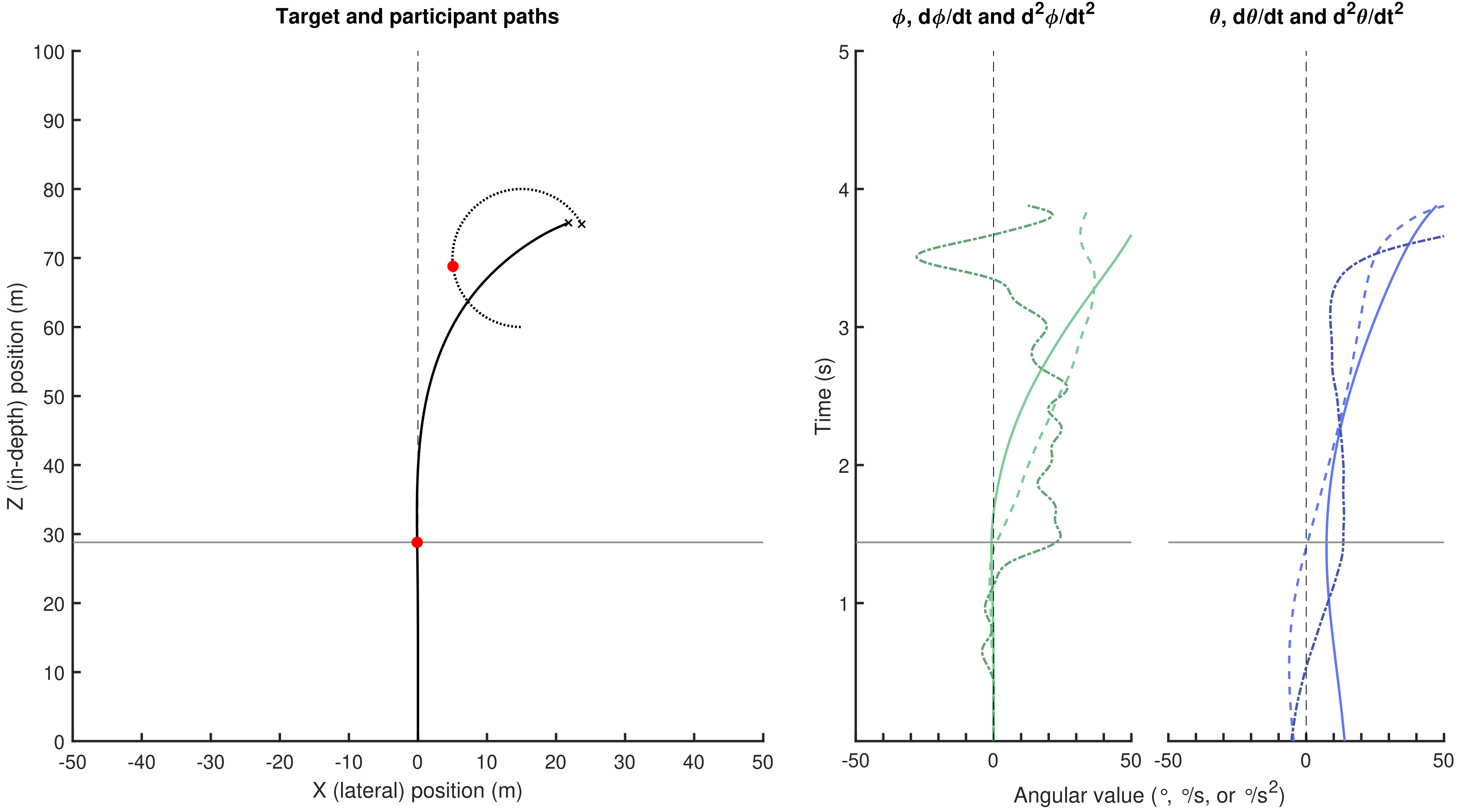

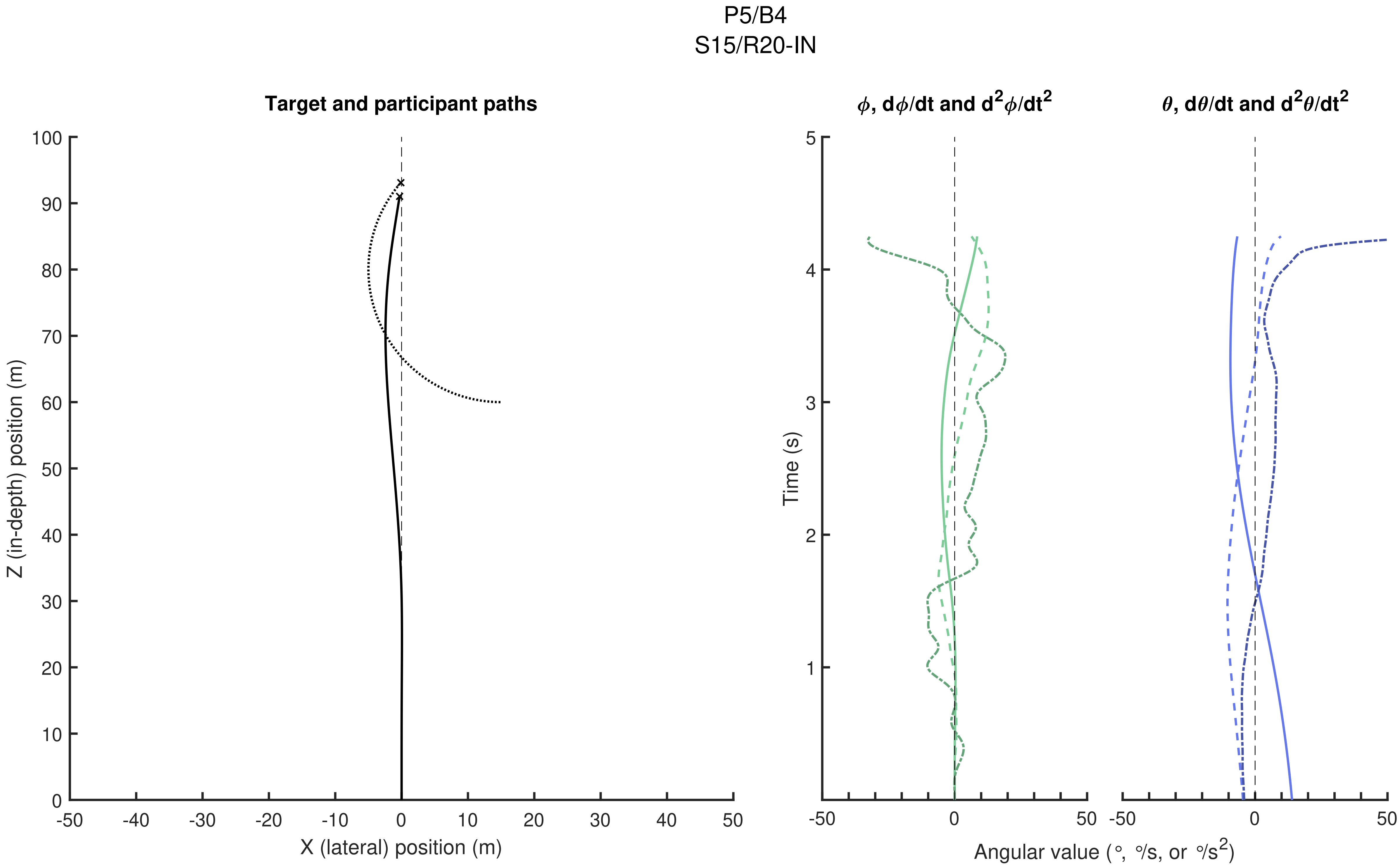

P5/B4
S15/R20-IN
Target and participant paths
Z (in-depth) position (m)
X (lateral) position (m)
100
90
80
70
60
50
40
30
20
10
0
-50
-40
-30
-20
-10
0
10
20
30
40
50
φ, dφ/dt and d²φ/dt²
θ, dθ/dt and d²θ/dt²
Time (s)
5
4
3
2
1
-50
0
50
-50
0
50
Angular value (°, °/s, or °/s²)

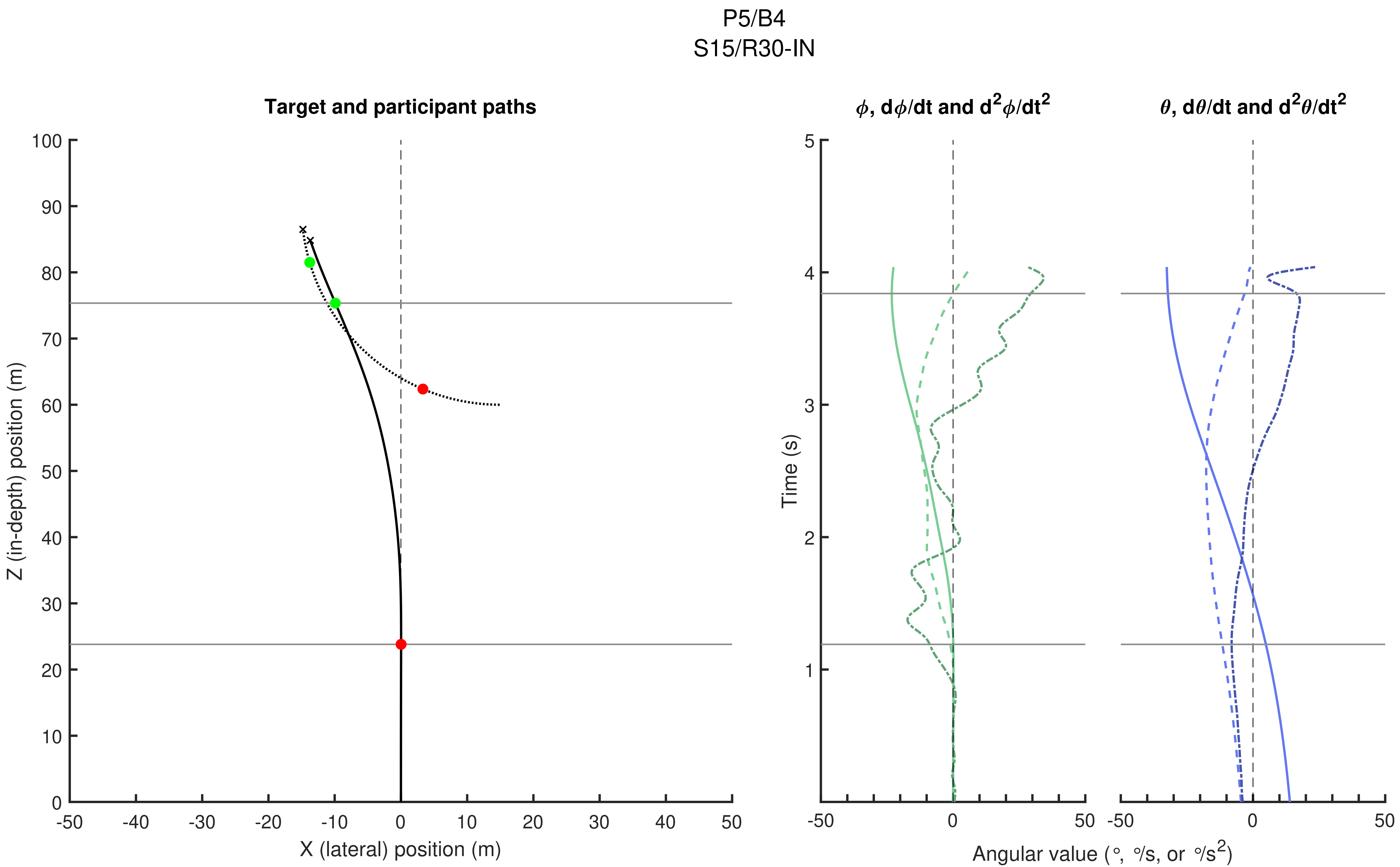
P5/B4
S15/R30-IN
Target and participant paths
Z (in-depth) position (m)
X (lateral) position (m)
ϕ, dϕ/dt and d$^2\phi$/dt^2
θ, dθ/dt and d$^2\theta$/dt^2
Time (s)
Angular value (°, °/s, or °/s^2)

P5/B4
S20/R10-IN

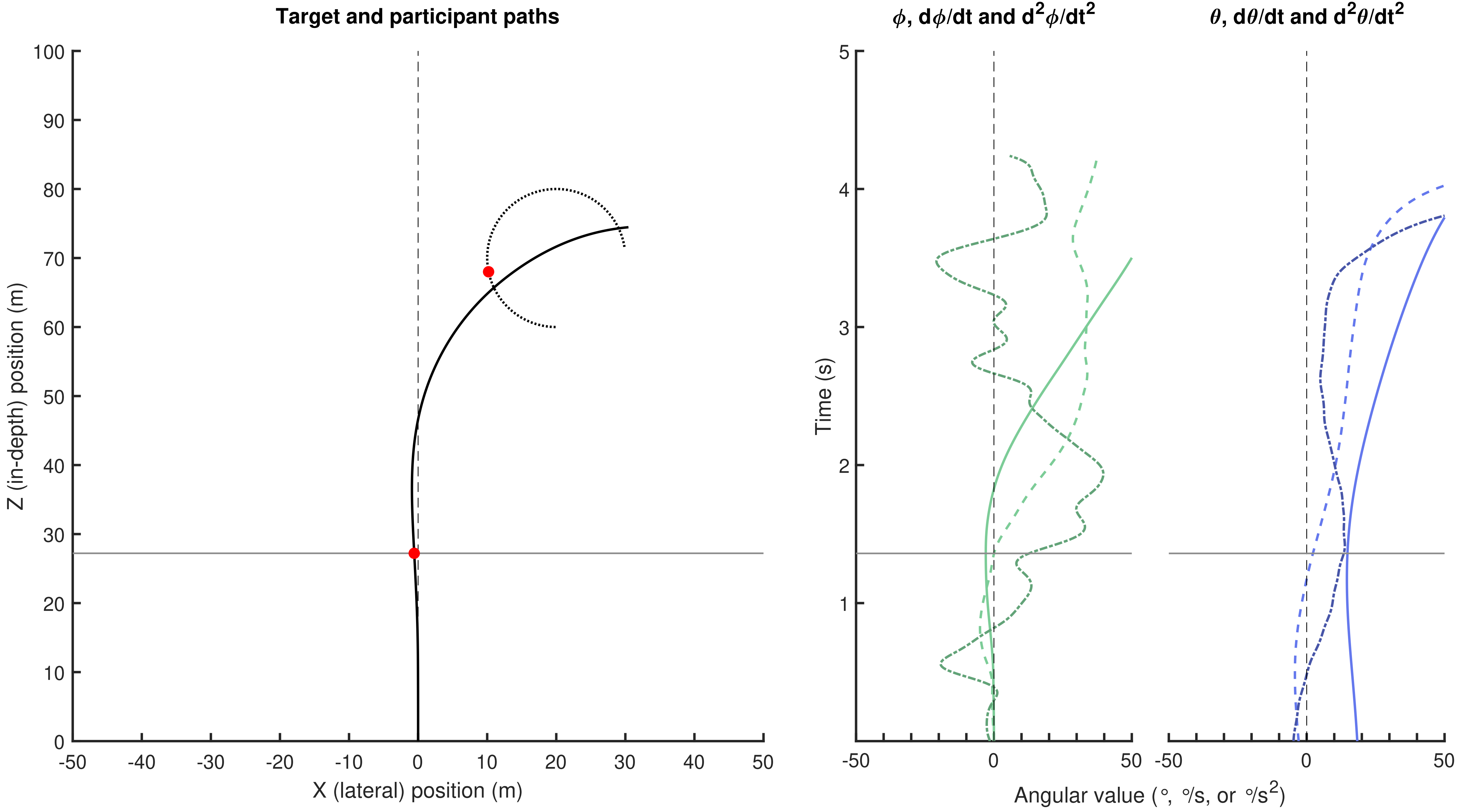

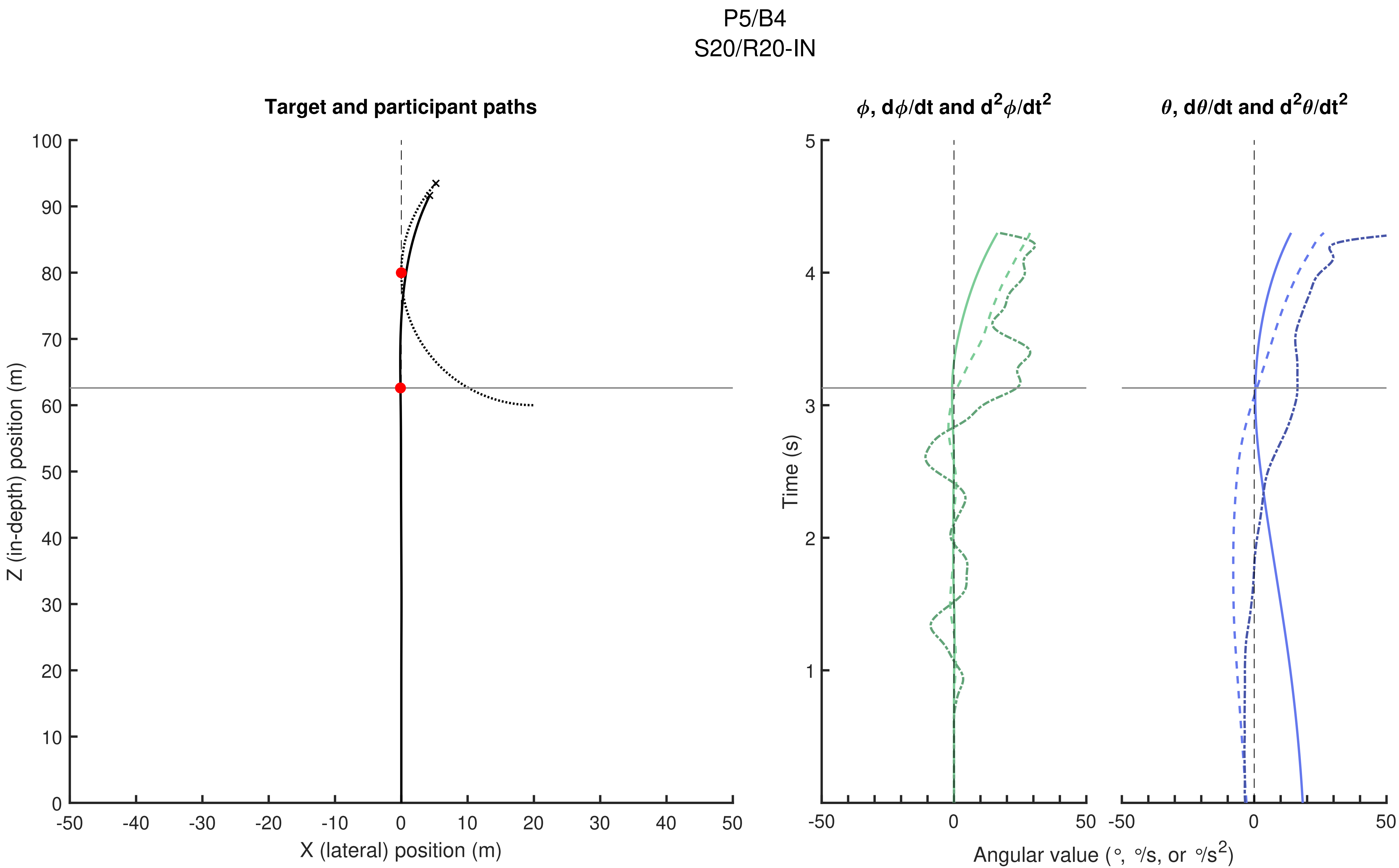
P5/B4
S20/R20-IN
Target and participant paths
ϕ, dϕ/dt and d$^2\phi$/dt^2
θ, dθ/dt and d$^2\theta$/dt^2
Z (in-depth) position (m)
X (lateral) position (m)
Time (s)
Angular value (°, °/s, or °/s^2)
100
90
80
70
60
50
40
30
20
10
0
-50
-40
-30
-20
-10
0
10
20
30
40
50
5
4
3
2
1
-50
0
50
-50
0
50

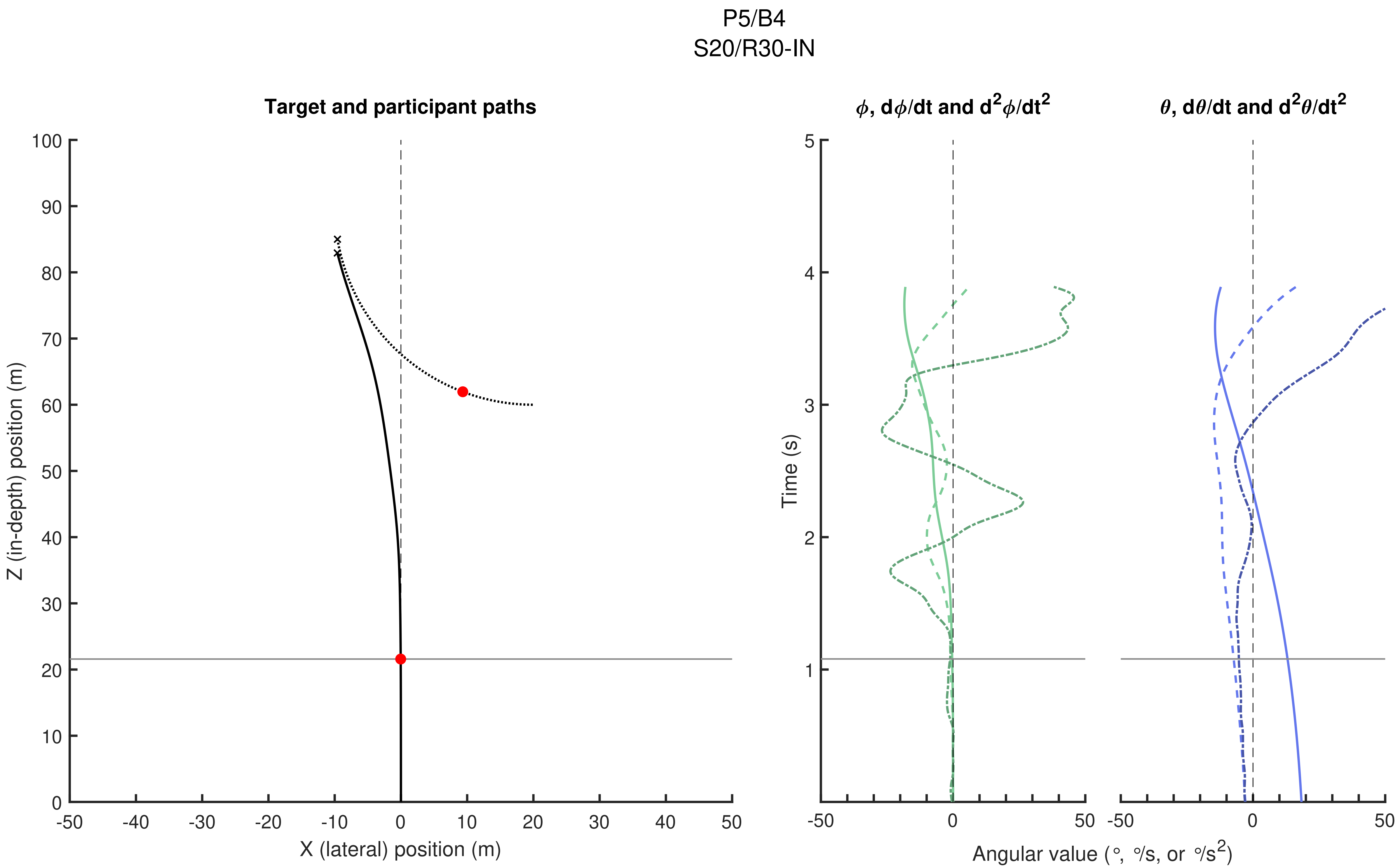

P5/B4
S20/R30-IN
Target and participant paths
ϕ, dϕ/dt and d$^2\phi$/dt^2
θ, dθ/dt and d$^2\theta$/dt^2
Z (in-depth) position (m)
X (lateral) position (m)
Time (s)
Angular value (°, °/s, or °/s^2)

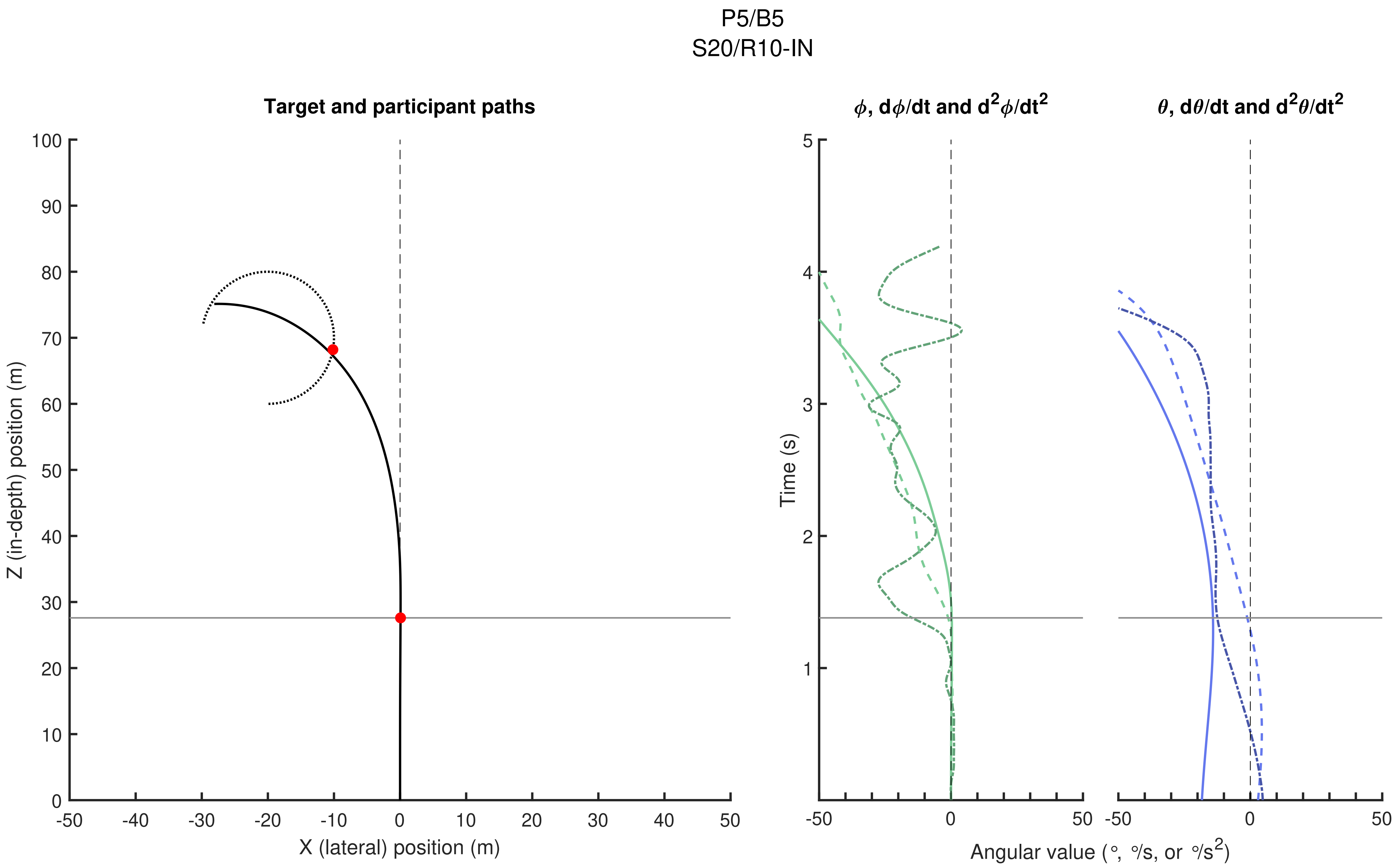
P5/B5
S20/R10-IN
Target and participant paths
Z (in-depth) position (m)
X (lateral) position (m)
100
90
80
70
60
50
40
30
20
10
0
-50
-40
-30
-20
-10
0
10
20
30
40
50
ϕ, dϕ/dt and d²ϕ/dt²
θ, dθ/dt and d²θ/dt²
Time (s)
5
4
3
2
1
-50
0
50
-50
0
50
Angular value (°, °/s, or °/s²)

P5/B5
S20/R20-IN

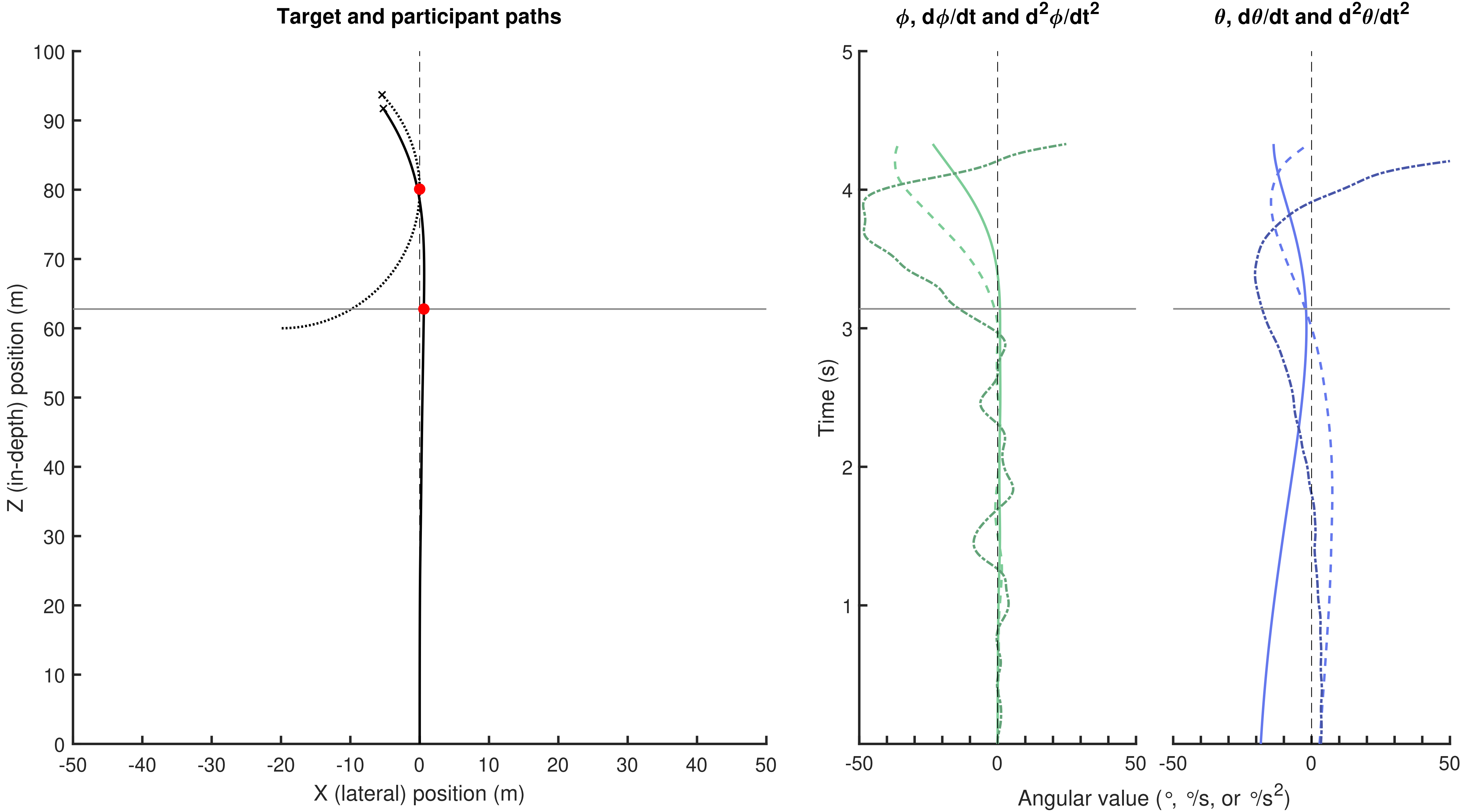

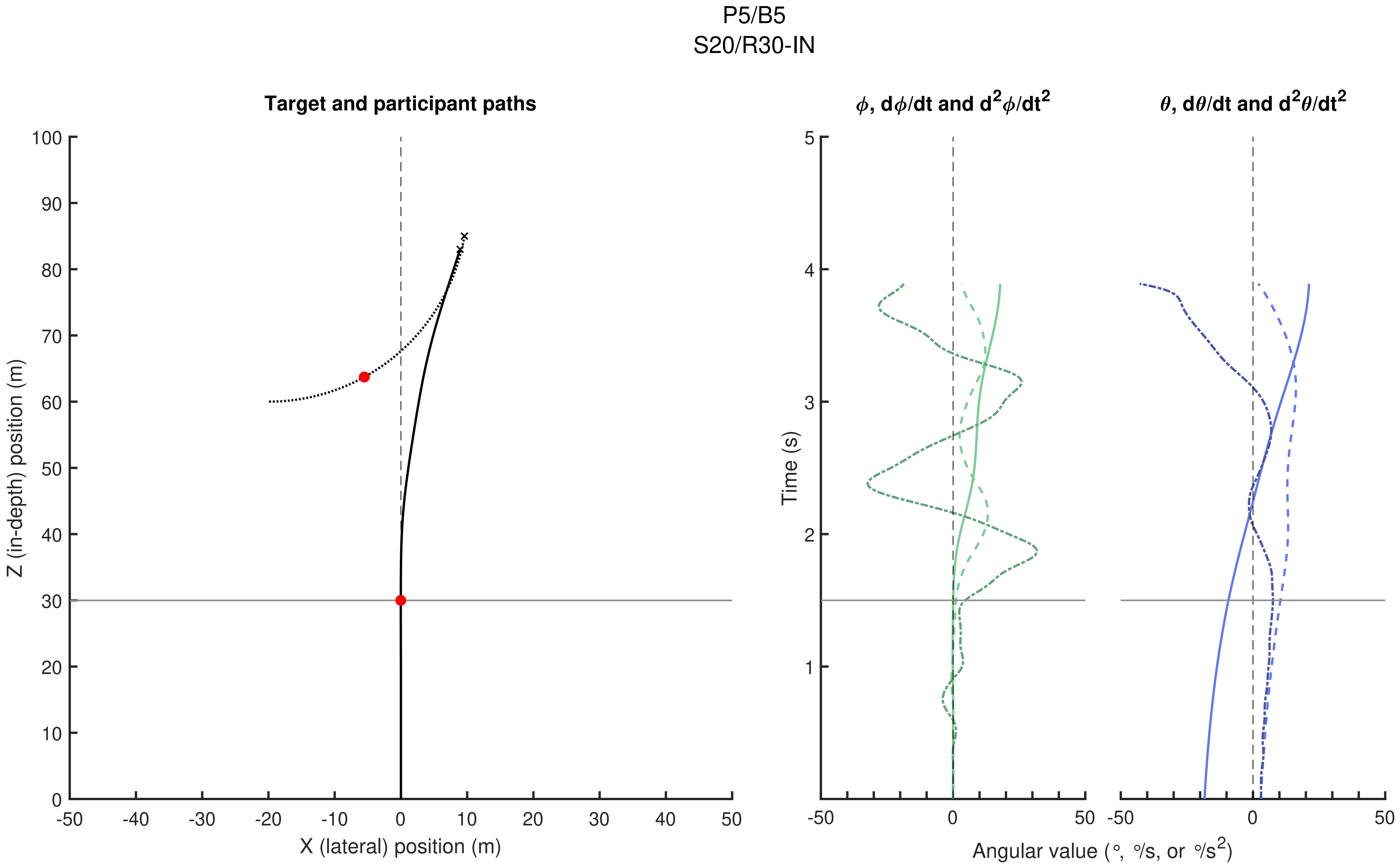

P5/B5
S20/R30-IN
Target and participant paths
Z (in-depth) position (m)
X (lateral) position (m)
ϕ, dϕ/dt and d$^2\phi$/dt^2
θ, dθ/dt and d$^2\theta$/dt^2
Time (s)
Angular value (°, °/s, or °/s^2)

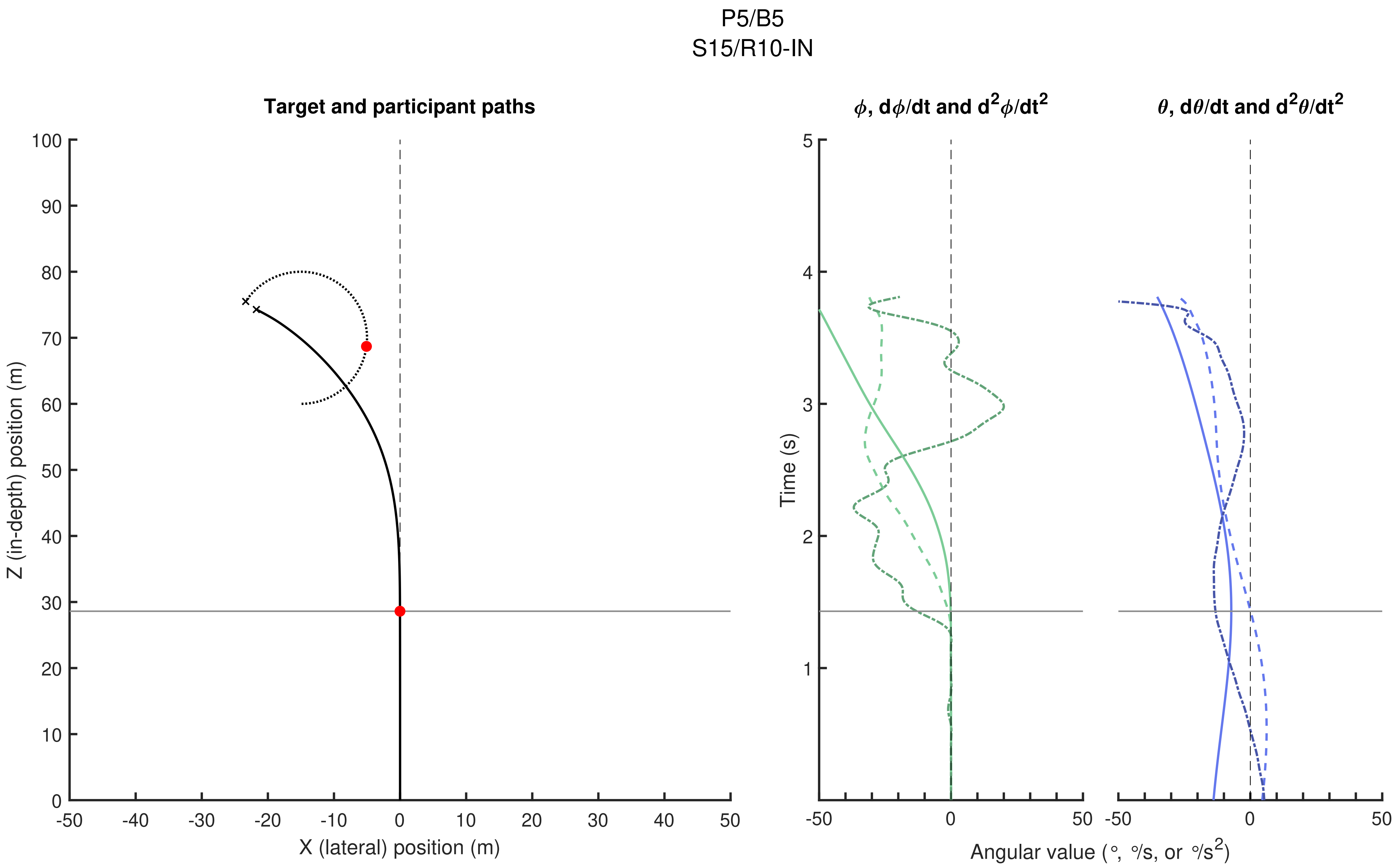
P5/B5
S15/R10-IN
Target and participant paths
Z (in-depth) position (m)
X (lateral) position (m)
ϕ, dϕ/dt and d$^2\phi$/dt^2
θ, dθ/dt and d$^2\theta$/dt^2
Time (s)
Angular value (°, °/s, or °/s^2)

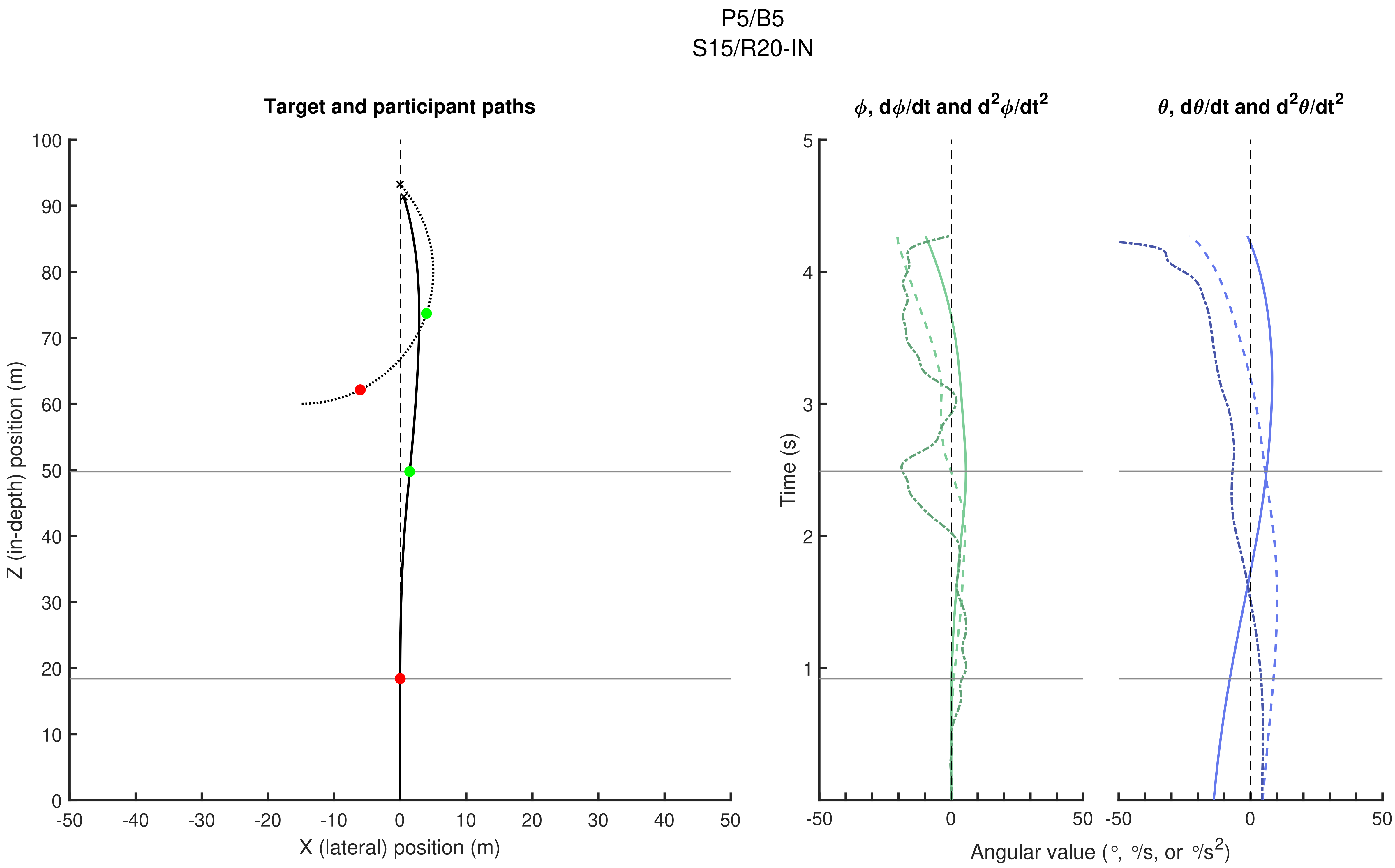
P5/B5
S15/R20-IN
Target and participant paths
Z (in-depth) position (m)
X (lateral) position (m)
φ, dφ/dt and d²φ/dt²
θ, dθ/dt and d²θ/dt²
Time (s)
Angular value (°, °/s, or °/s²)

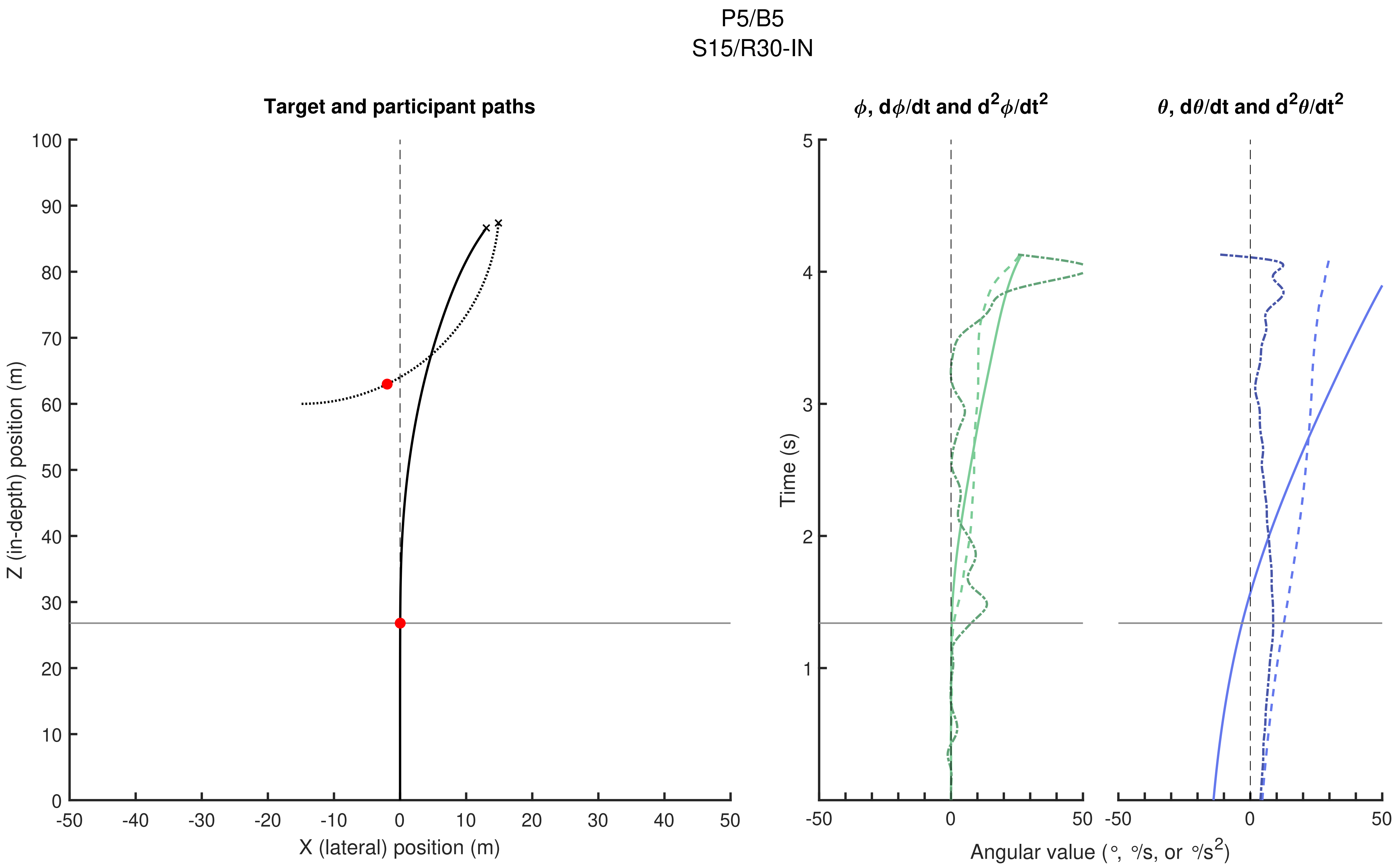
P5/B5
S15/R30-IN
Target and participant paths
ϕ, dϕ/dt and d$^2\phi$/dt^2
θ, dθ/dt and d$^2\theta$/dt^2
Z (in-depth) position (m)
X (lateral) position (m)
Time (s)
Angular value (°, °/s, or °/s^2)

P5/B5
S5/R10-IN

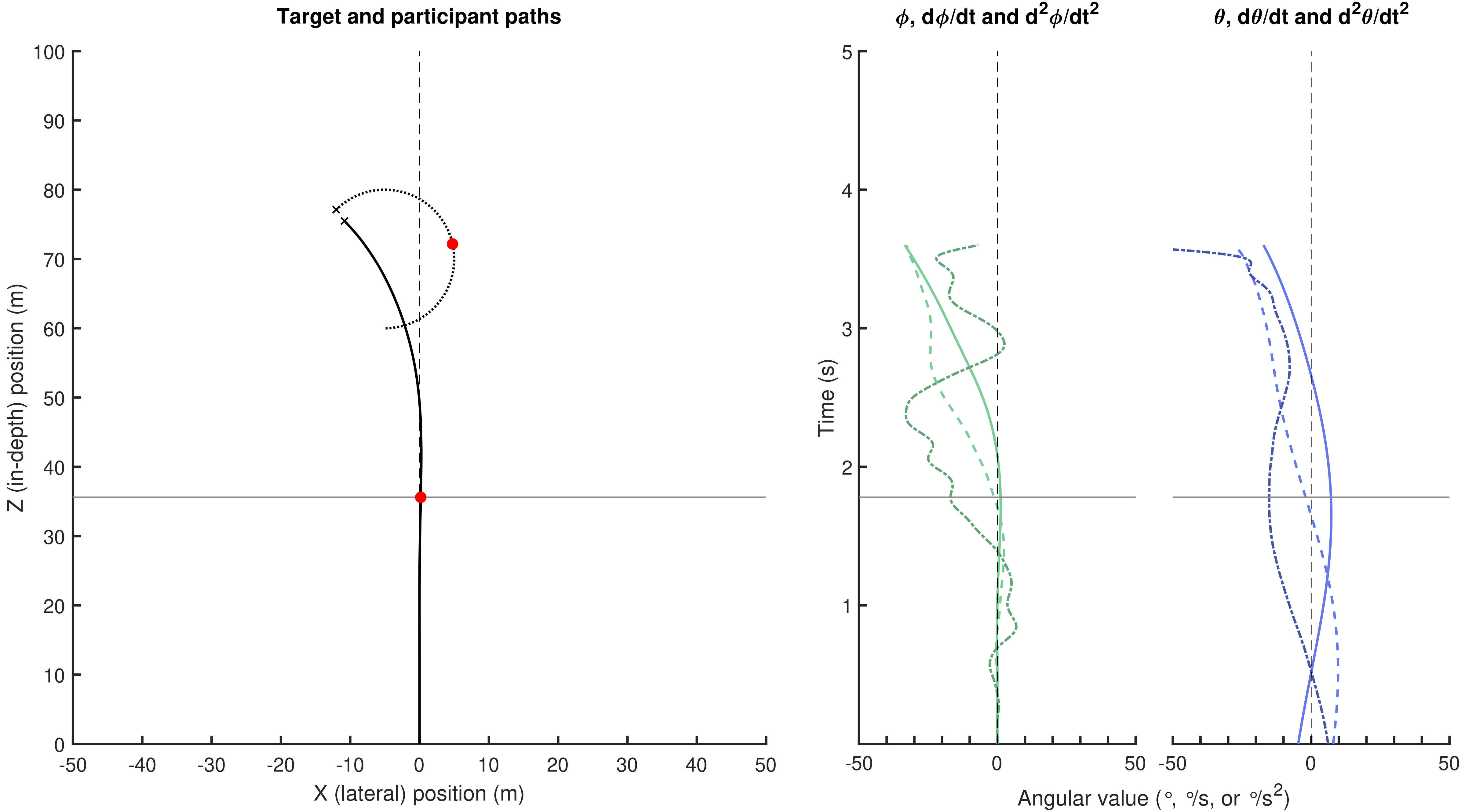

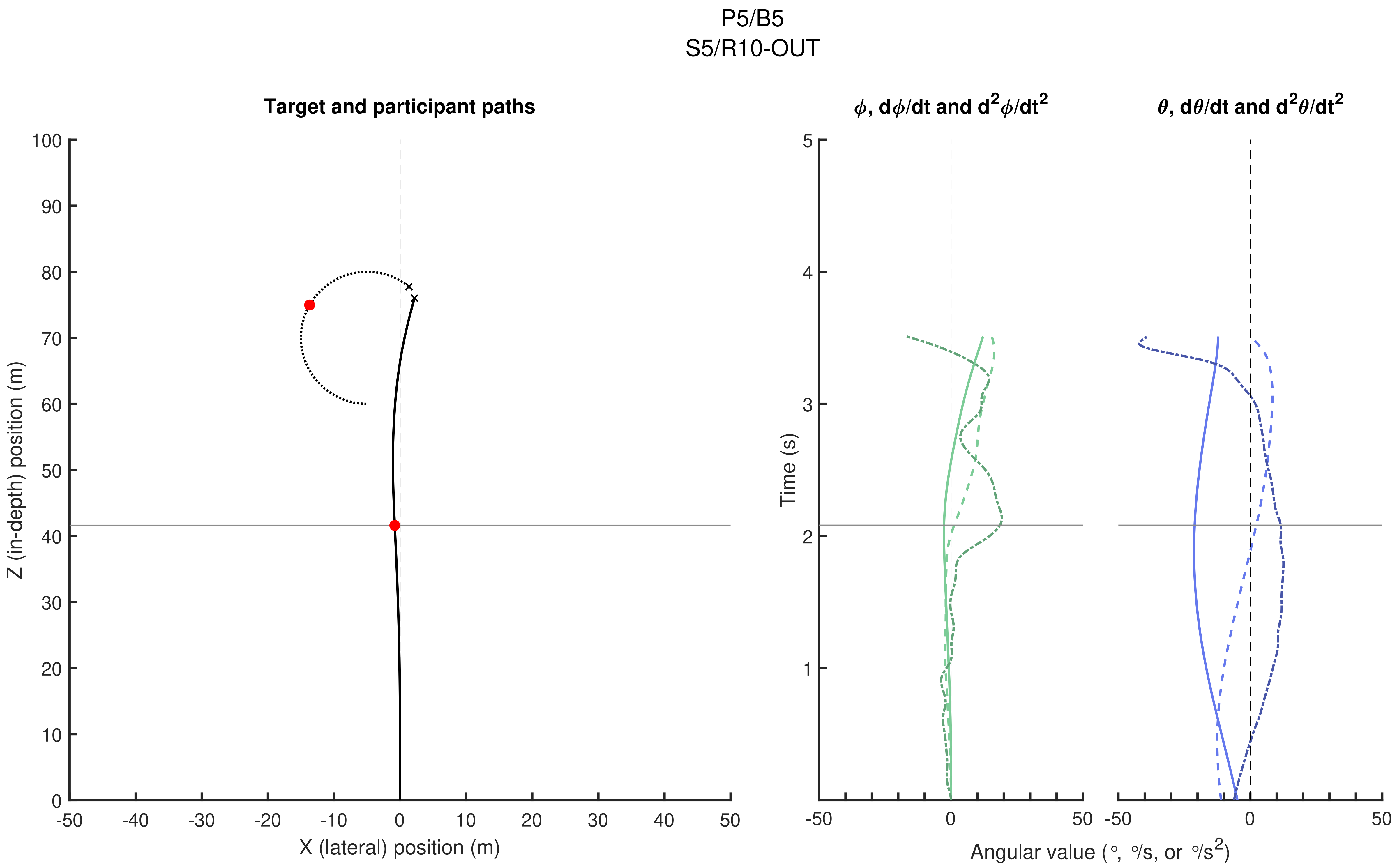
P5/B5
S5/R10-OUT
Target and participant paths
Z (in-depth) position (m)
X (lateral) position (m)
ϕ, dϕ/dt and d$^2\phi$/dt^2
θ, dθ/dt and d$^2\theta$/dt^2
Time (s)
Angular value (°, °/s, or °/s^2)

P5/B5
S5/R20-IN

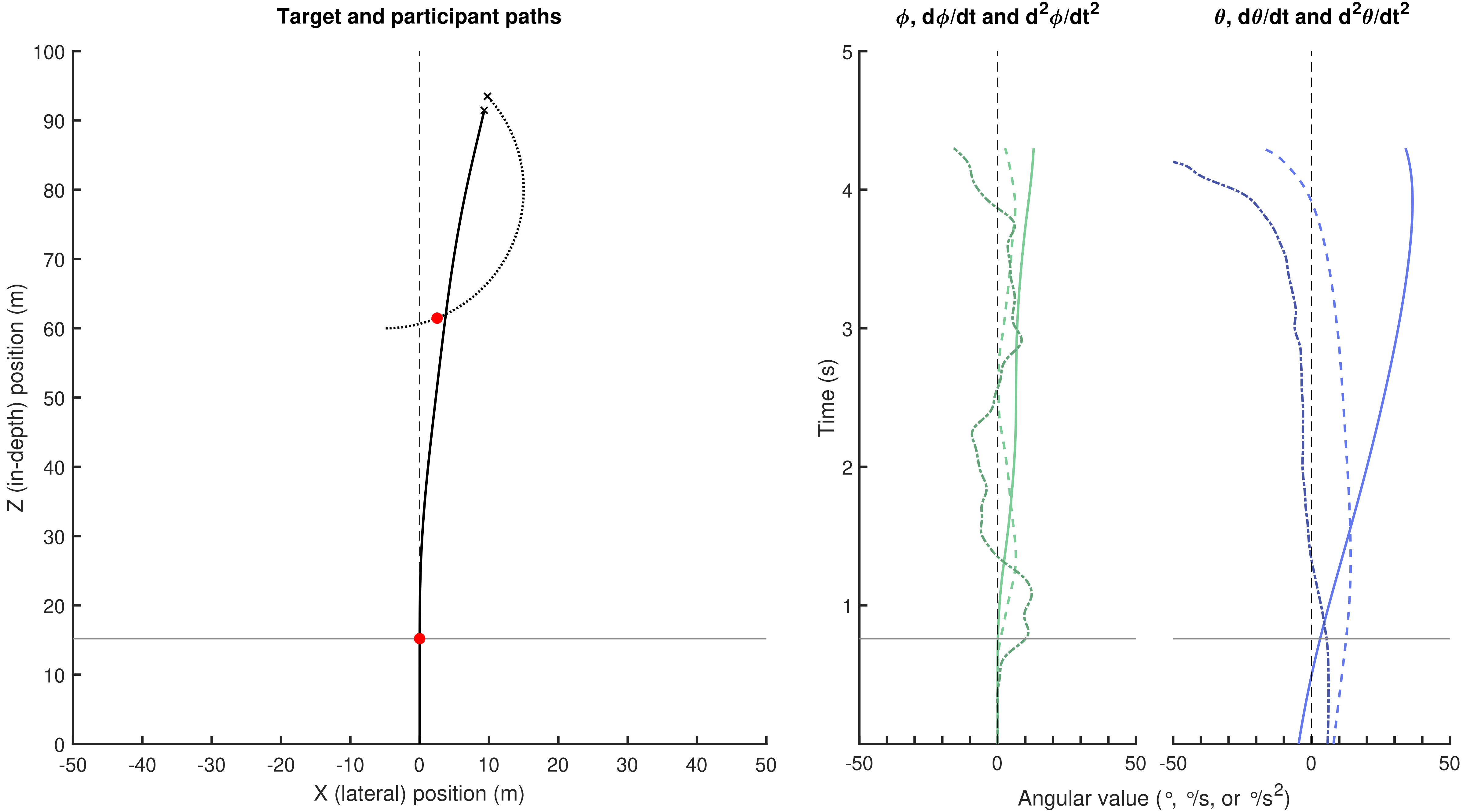

P5/B5
S5/R20-OUT

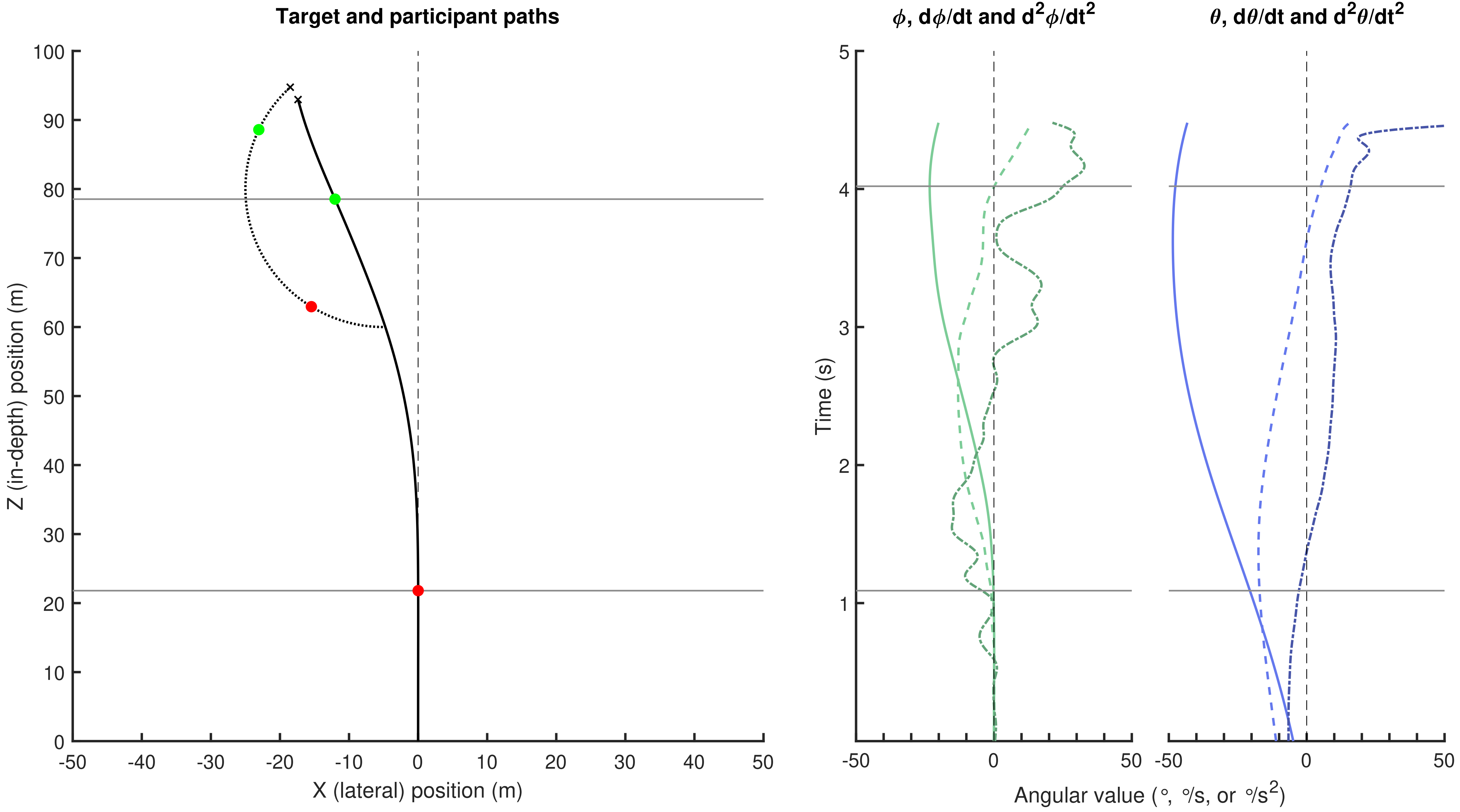

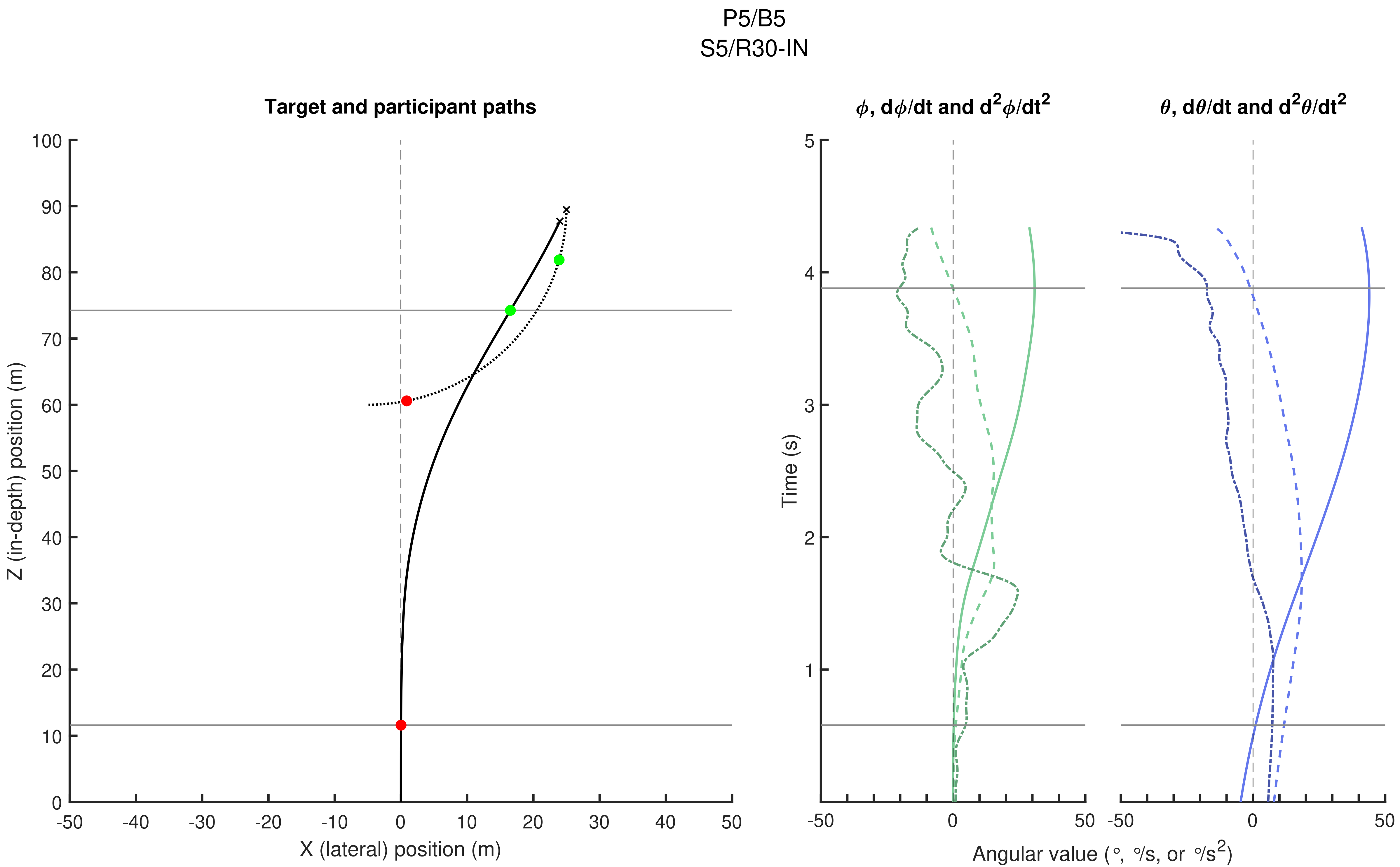

P5/B5
S5/R30-IN
Target and participant paths
Z (in-depth) position (m)
X (lateral) position (m)
ϕ, dϕ/dt and d$^2\phi$/dt^2
θ, dθ/dt and d$^2\theta$/dt^2
Time (s)
Angular value (°, °/s, or °/s^2)

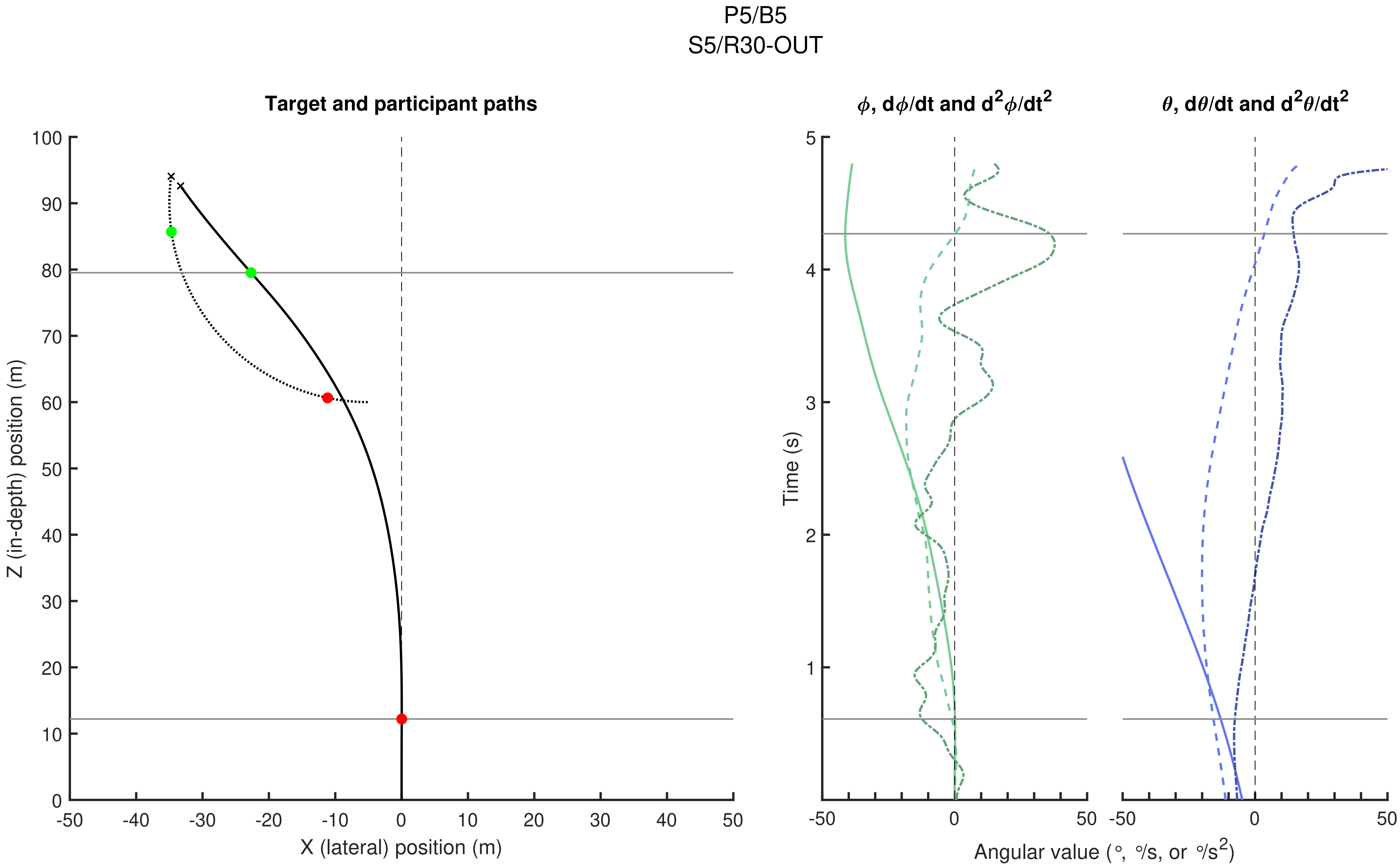
P5/B5
S5/R30-OUT
Target and participant paths
Z (in-depth) position (m)
X (lateral) position (m)
ϕ, dϕ/dt and d$^2\phi$/dt^2
θ, dθ/dt and d$^2\theta$/dt^2
Time (s)
Angular value (°, °/s, or °/s^2)

P5/B5
S5/R10-OUT

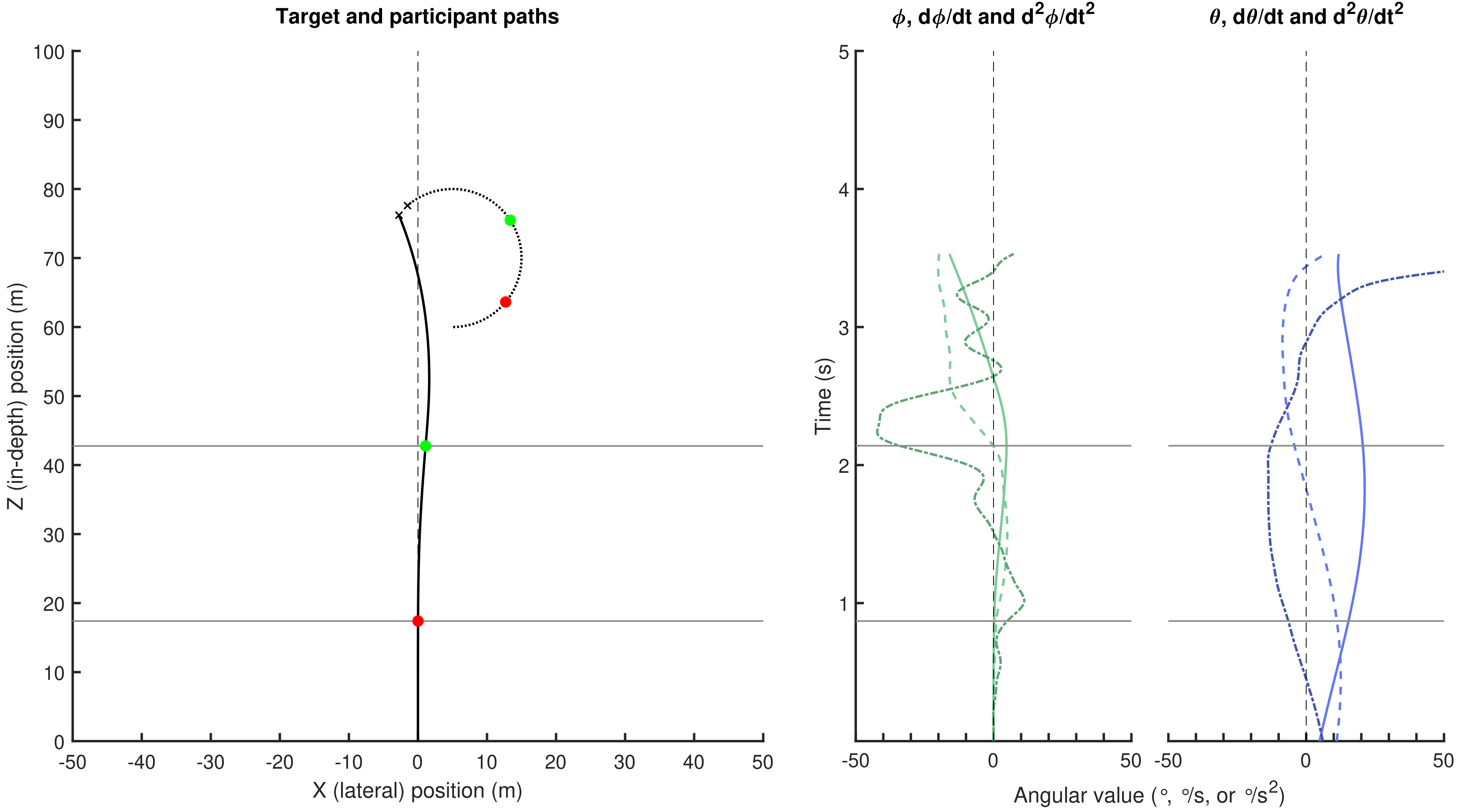

P5/B5
S5/R10-IN

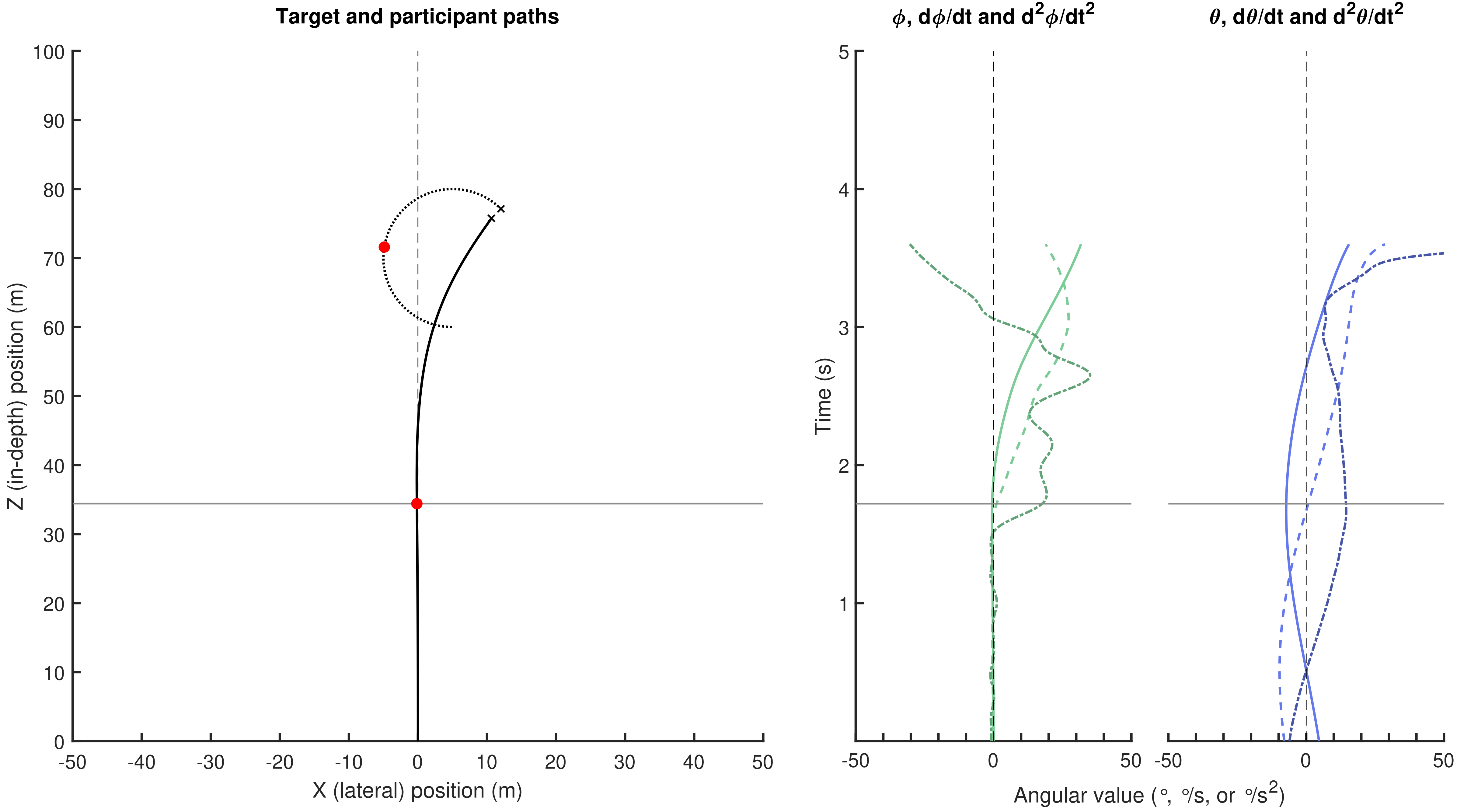

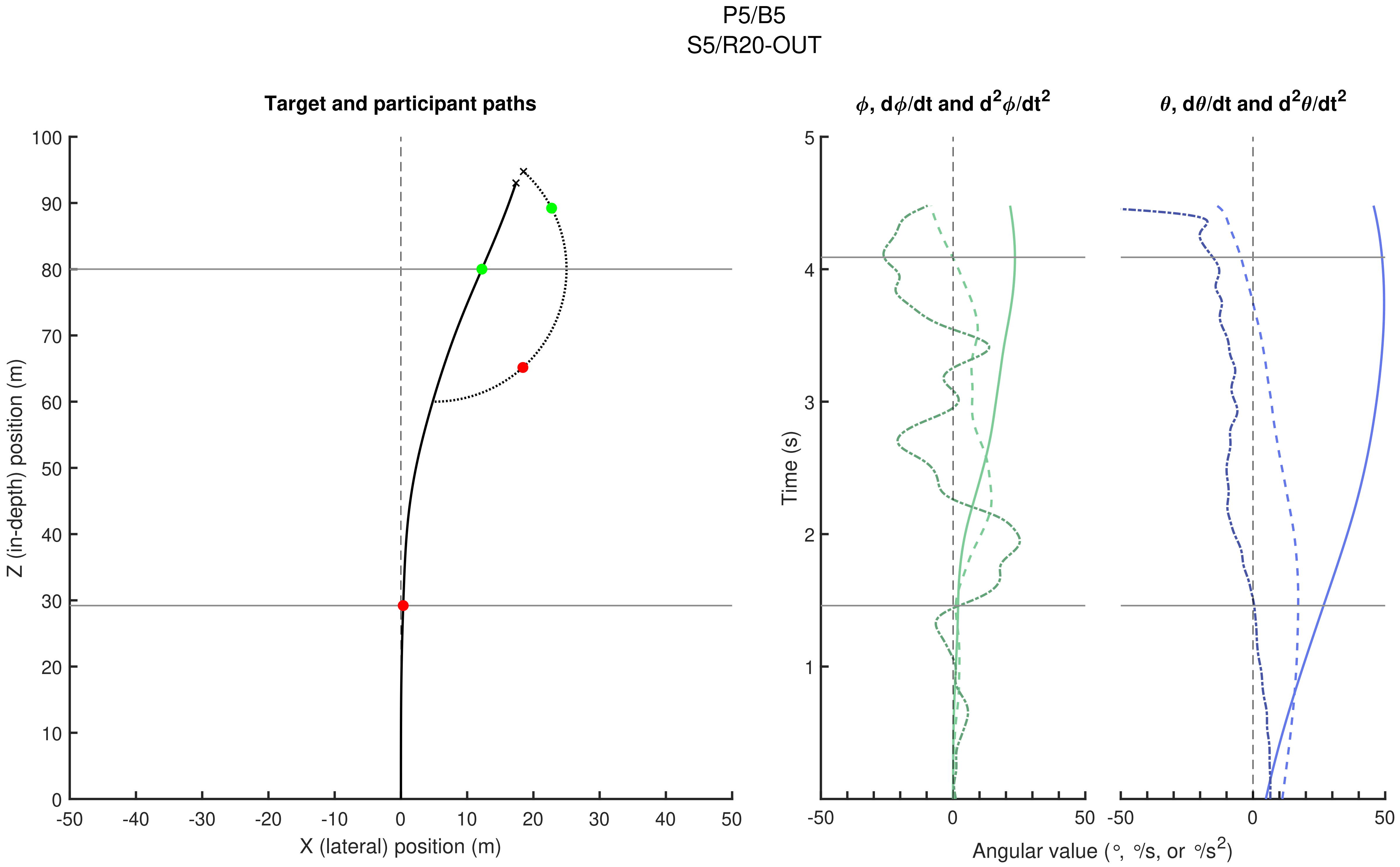
P5/B5
S5/R20-OUT
Target and participant paths
φ, dφ/dt and d²φ/dt²
θ, dθ/dt and d²θ/dt²
Z (in-depth) position (m)
X (lateral) position (m)
Time (s)
Angular value (°, °/s, or °/s²)
100
90
80
70
60
50
40
30
20
10
0
-50
-40
-30
-20
-10
0
10
20
30
40
50
5
4
3
2
1
-50
0
50
-50
0
50

P5/B5
S5/R20-IN

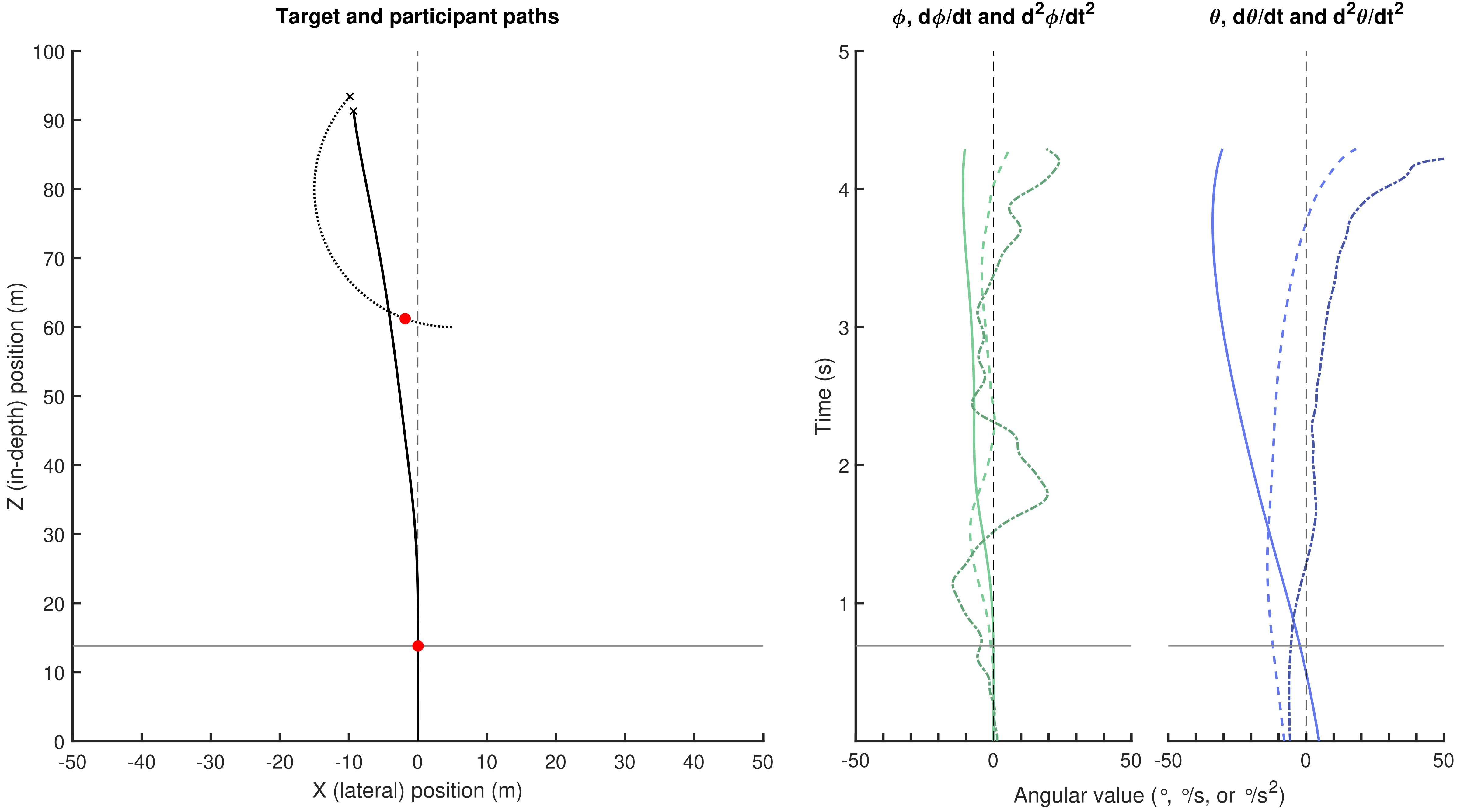

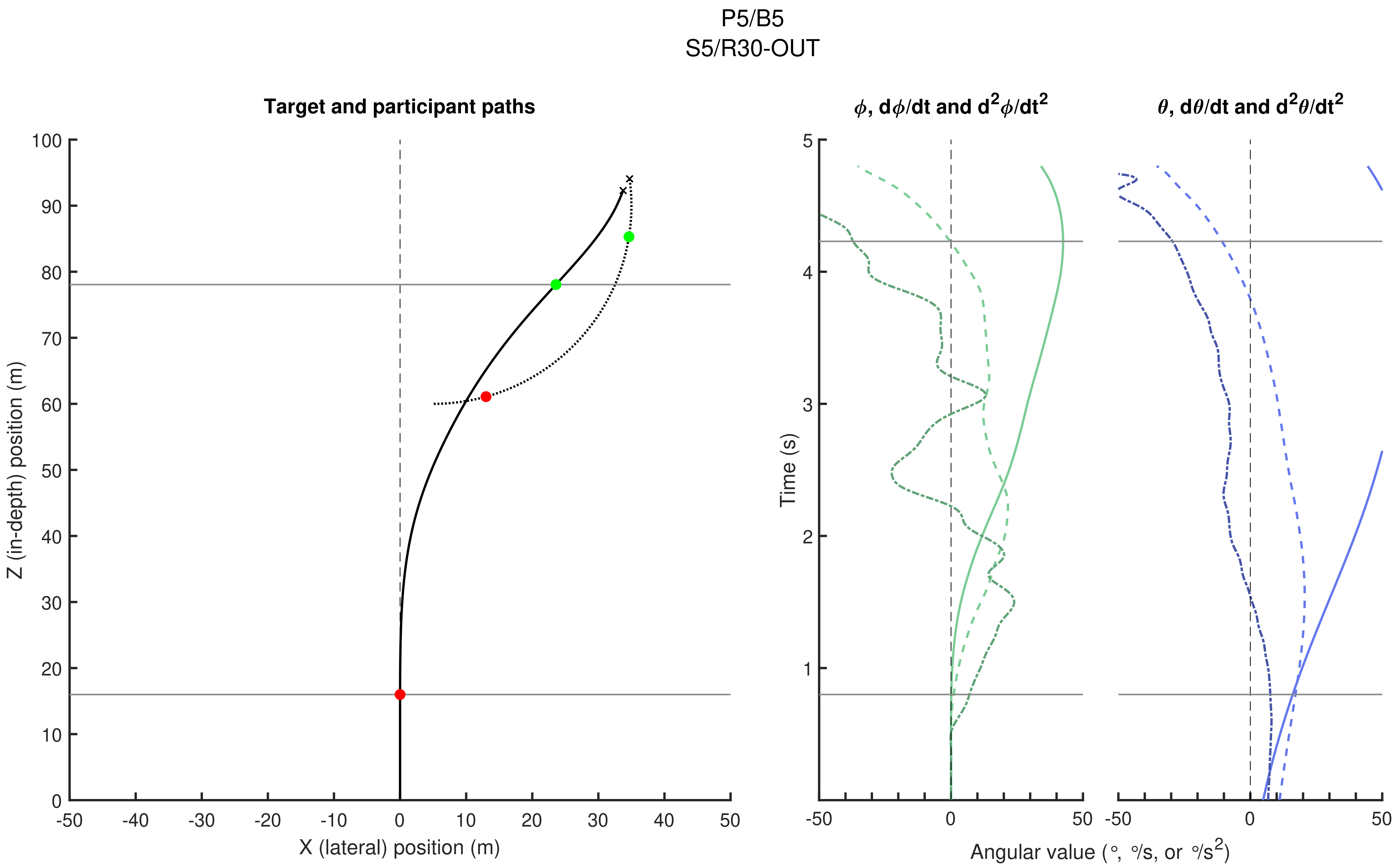
P5/B5
S5/R30-OUT
Target and participant paths
Z (in-depth) position (m)
X (lateral) position (m)
ϕ, dϕ/dt and d²ϕ/dt²
θ, dθ/dt and d²θ/dt²
Time (s)
Angular value (°, °/s, or °/s²)

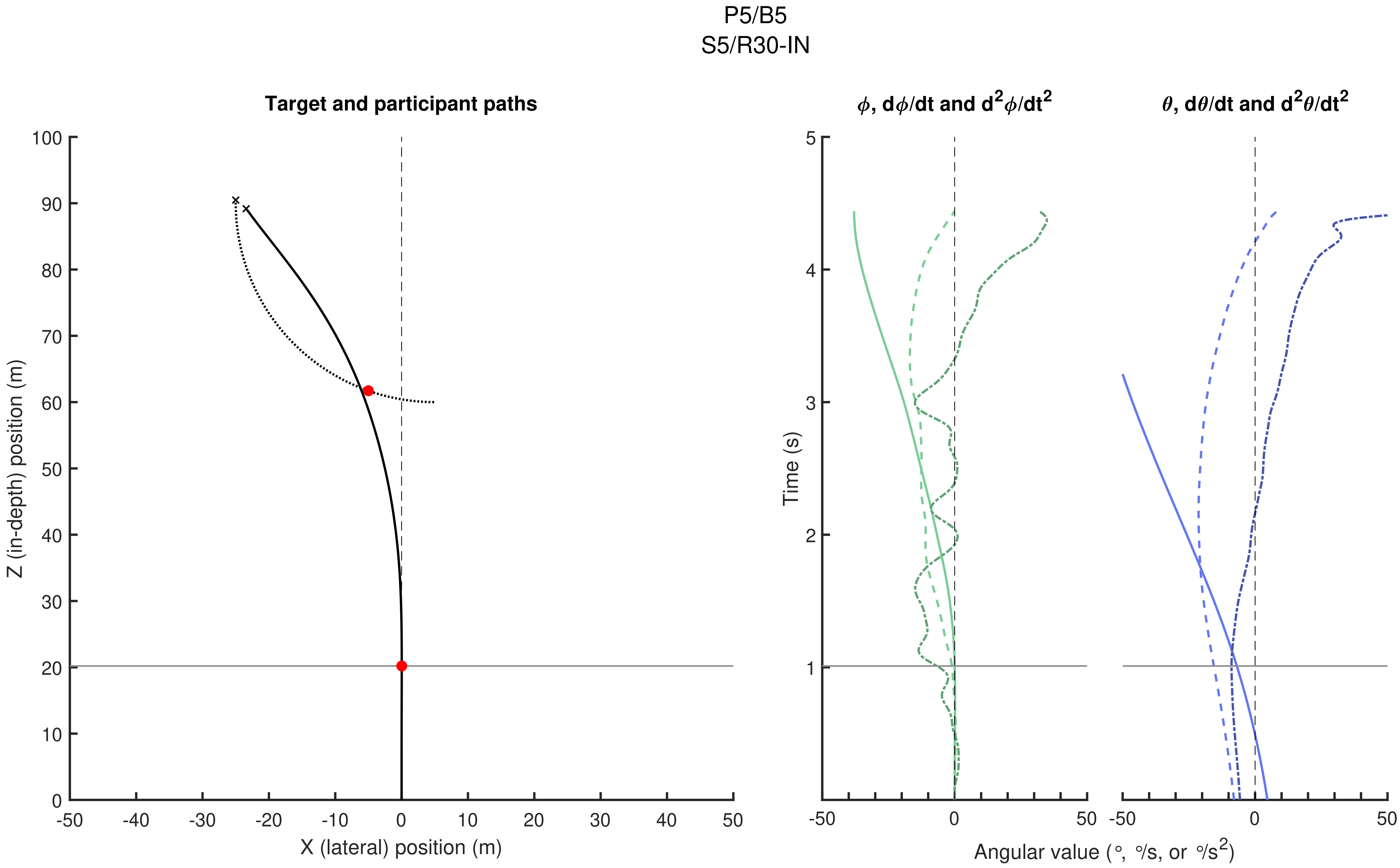
P5/B5
S5/R30-IN
Target and participant paths
ϕ, dϕ/dt and d$^2\phi$/dt^2
θ, dθ/dt and d$^2\theta$/dt^2
Z (in-depth) position (m)
X (lateral) position (m)
Time (s)
Angular value (°, °/s, or °/s^2)
100
90
80
70
60
50
40
30
20
10
0
-50
-40
-30
-20
-10
0
10
20
30
40
50
5
4
3
2
1

P5/B5
S15/R10-IN

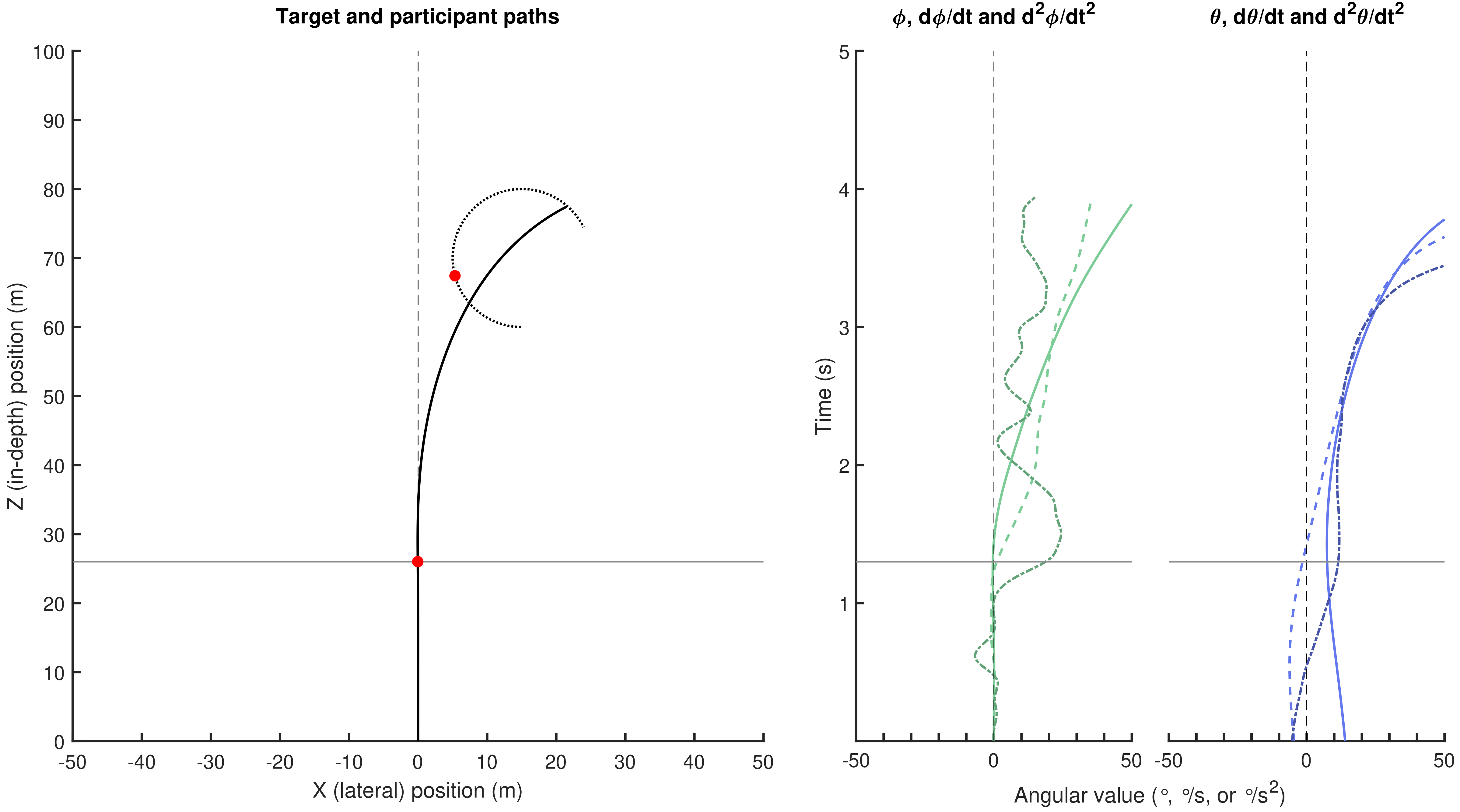

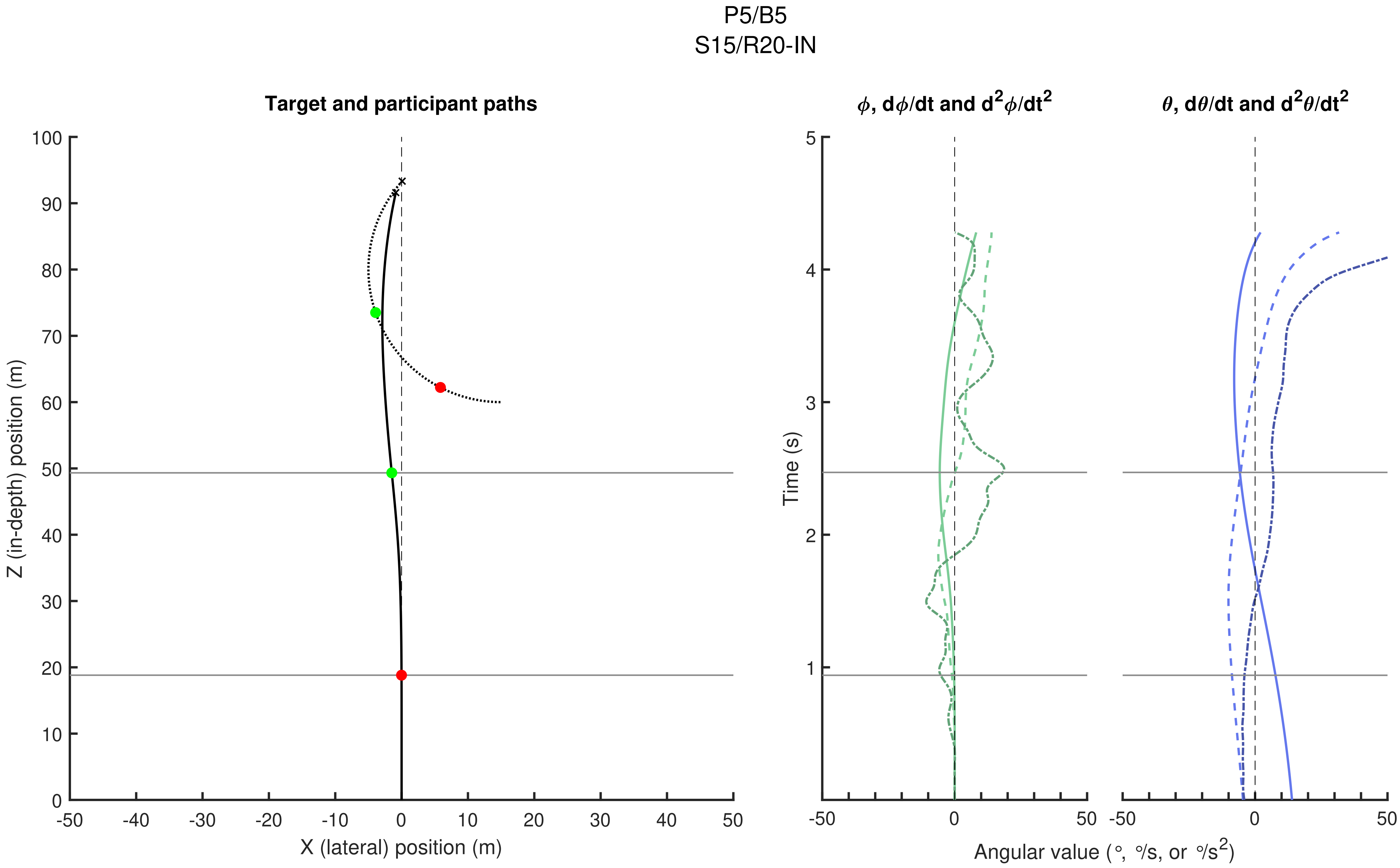
P5/B5
S15/R20-IN
Target and participant paths
Z (in-depth) position (m)
X (lateral) position (m)
φ, dφ/dt and d²φ/dt²
θ, dθ/dt and d²θ/dt²
Time (s)
Angular value (°, °/s, or °/s²)
100
90
80
70
60
50
40
30
20
10
0
-50
-40
-30
-20
-10
0
10
20
30
40
50
5
4
3
2
1
-50
0
50
-50
0
50

P5/B5
S15/R30-IN

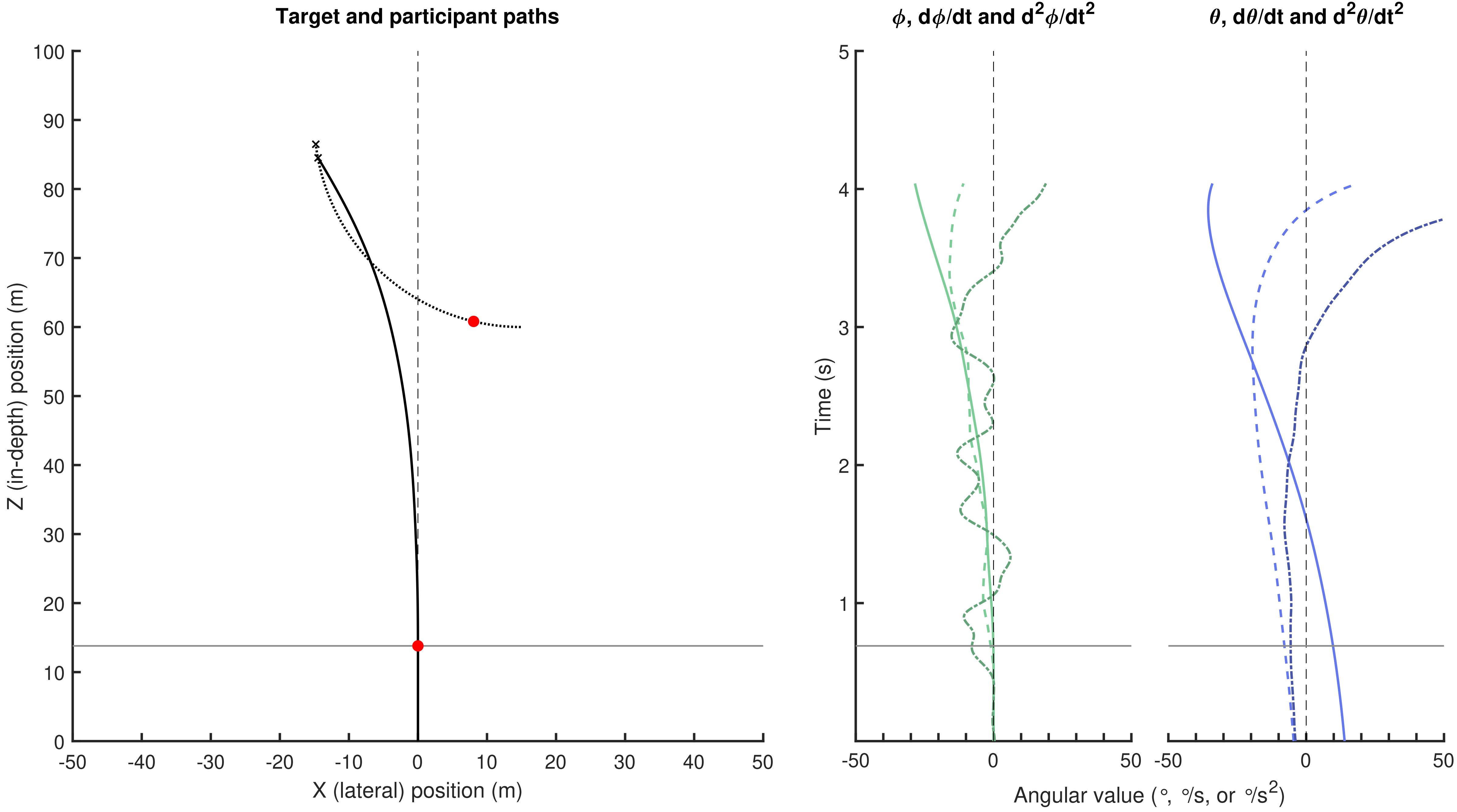

P5/B5
S20/R10-IN

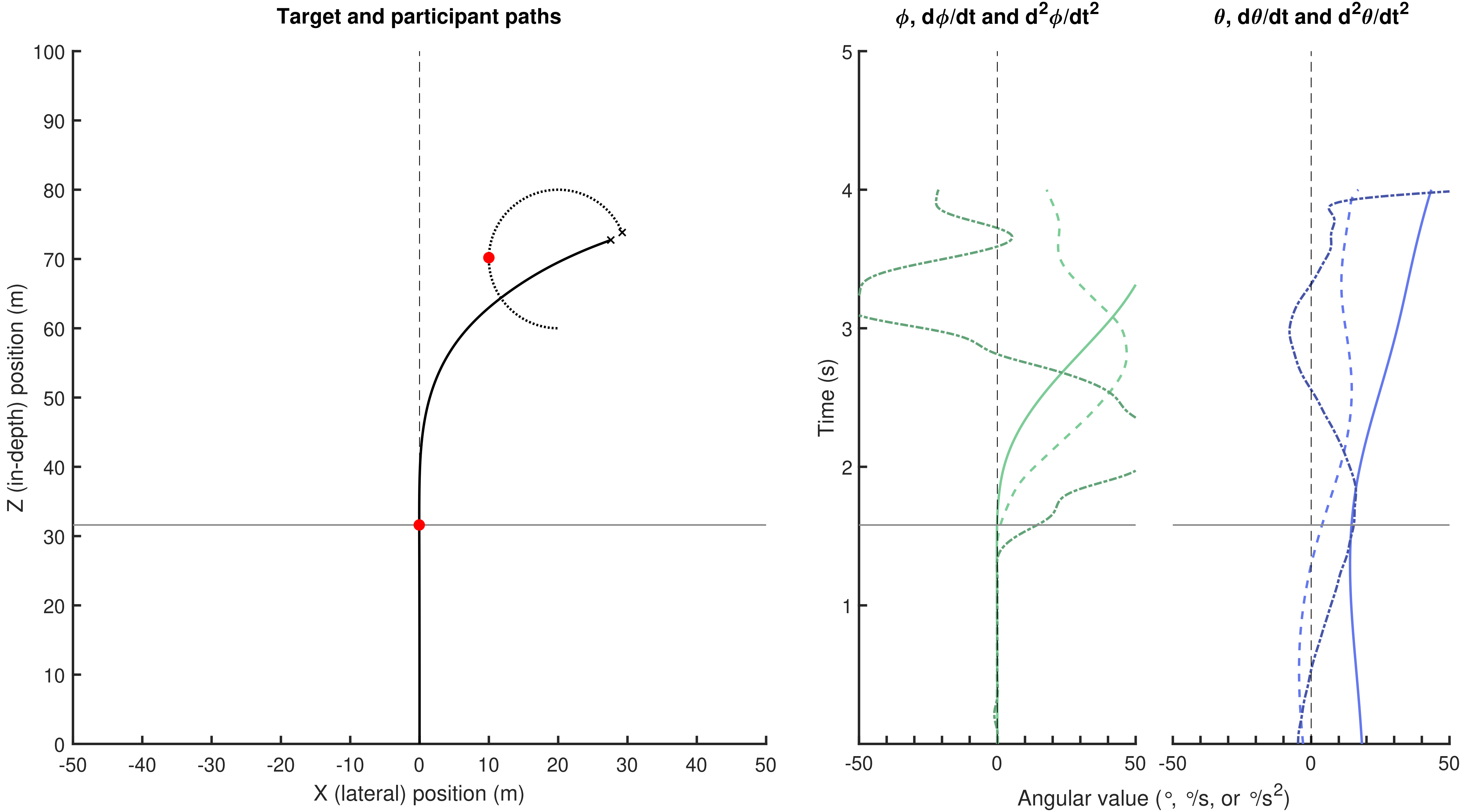

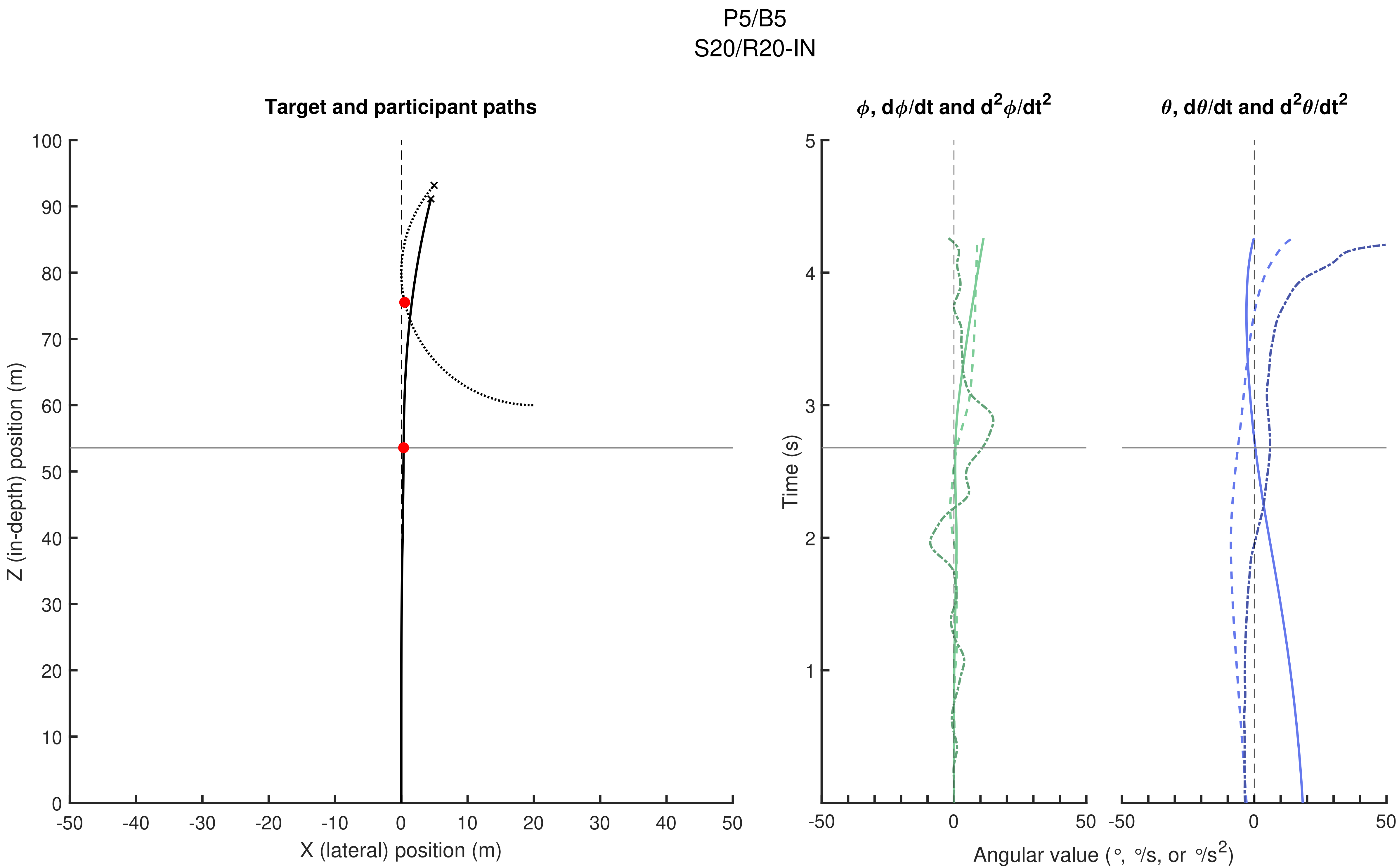
P5/B5
S20/R20-IN
Target and participant paths
ϕ, dϕ/dt and d²ϕ/dt²
θ, dθ/dt and d²θ/dt²
Z (in-depth) position (m)
X (lateral) position (m)
Time (s)
Angular value (°, °/s, or °/s²)
100
90
80
70
60
50
40
30
20
10
0
-50
-40
-30
-20
-10
0
10
20
30
40
50
5
4
3
2
1
-50
0
50
-50
0
50

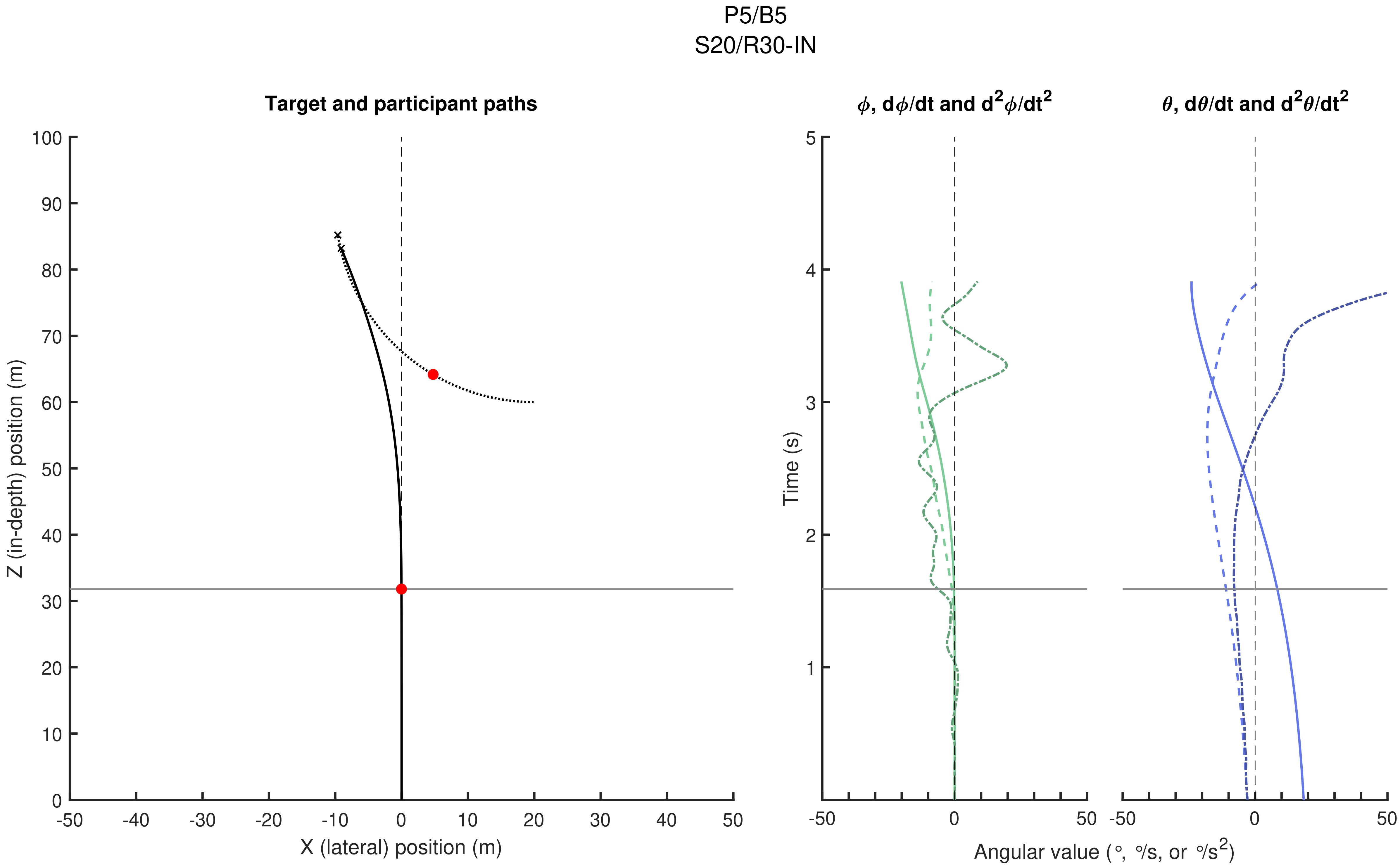

P5/B5
S20/R30-IN
Target and participant paths
Z (in-depth) position (m)
X (lateral) position (m)
100
90
80
70
60
50
40
30
20
10
0
-50
-40
-30
-20
-10
0
10
20
30
40
50
ϕ, dϕ/dt and d²ϕ/dt²
θ, dθ/dt and d²θ/dt²
Time (s)
5
4
3
2
1
-50
0
50
-50
0
50
Angular value (°, °/s, or °/s²)

P6/B1
S20/R10-IN

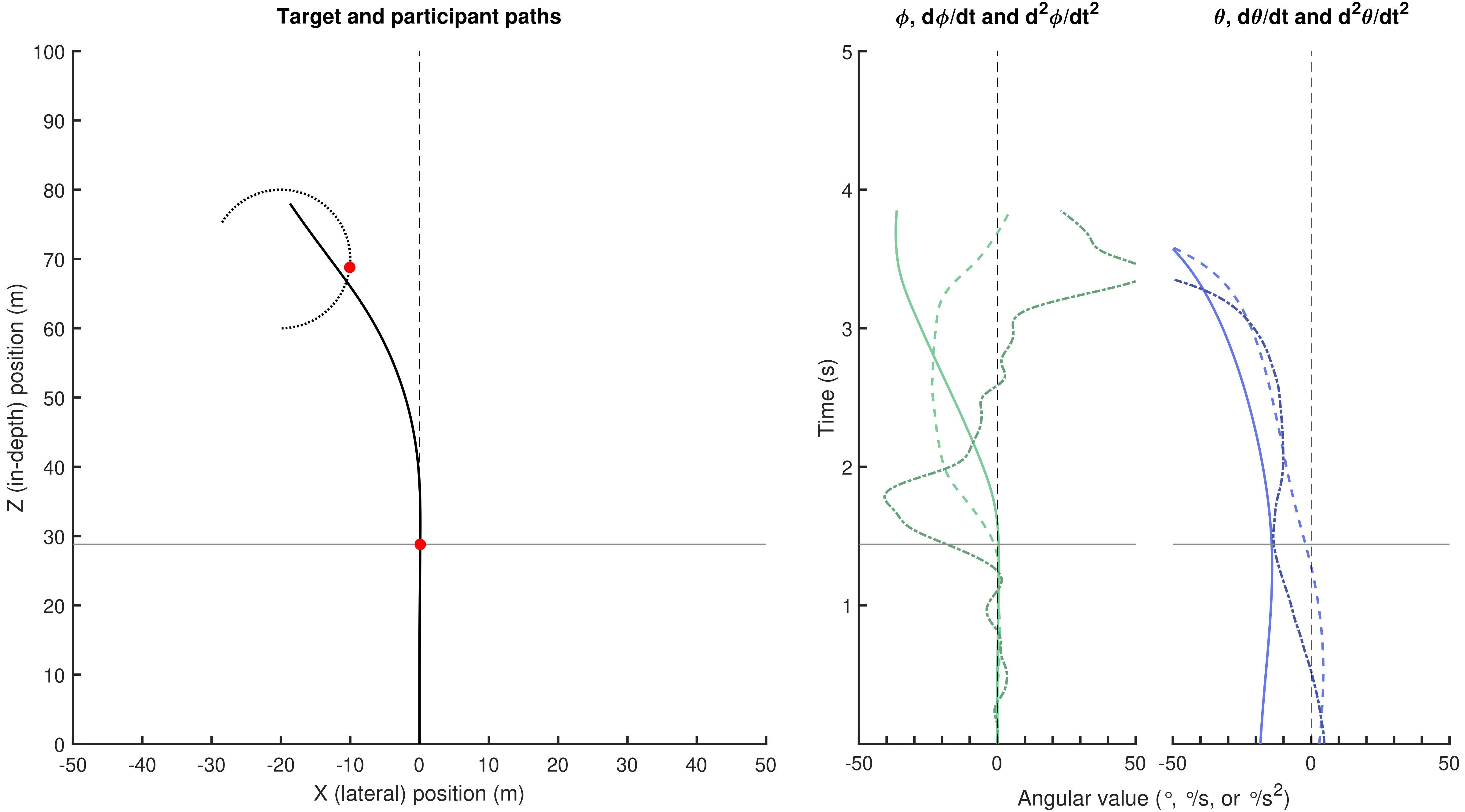

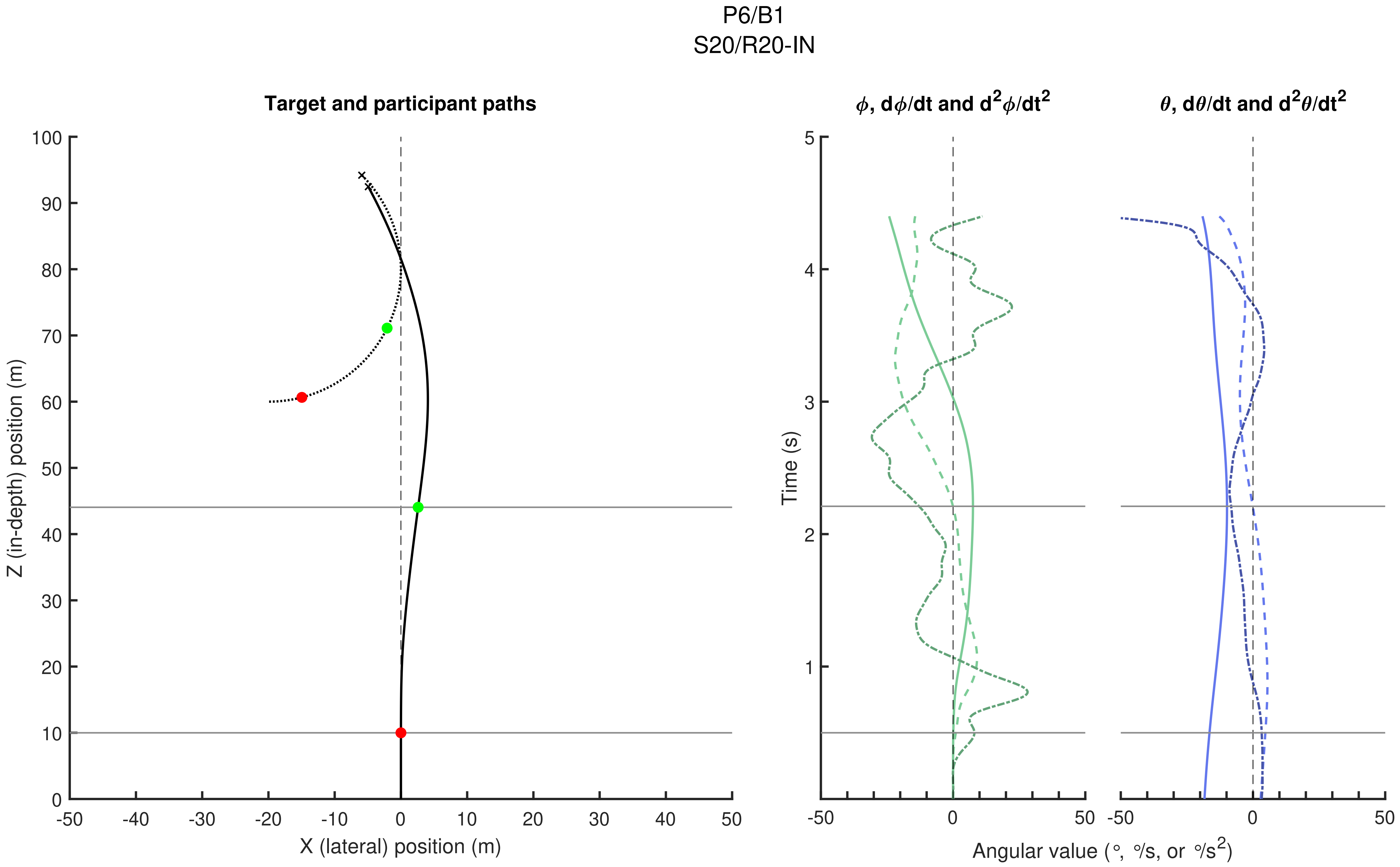
P6/B1
S20/R20-IN
Target and participant paths
Z (in-depth) position (m)
X (lateral) position (m)
ϕ, dϕ/dt and d$^2\phi$/dt^2
θ, dθ/dt and d$^2\theta$/dt^2
Time (s)
Angular value (°, °/s, or °/s^2)

P6/B1
S20/R30-IN

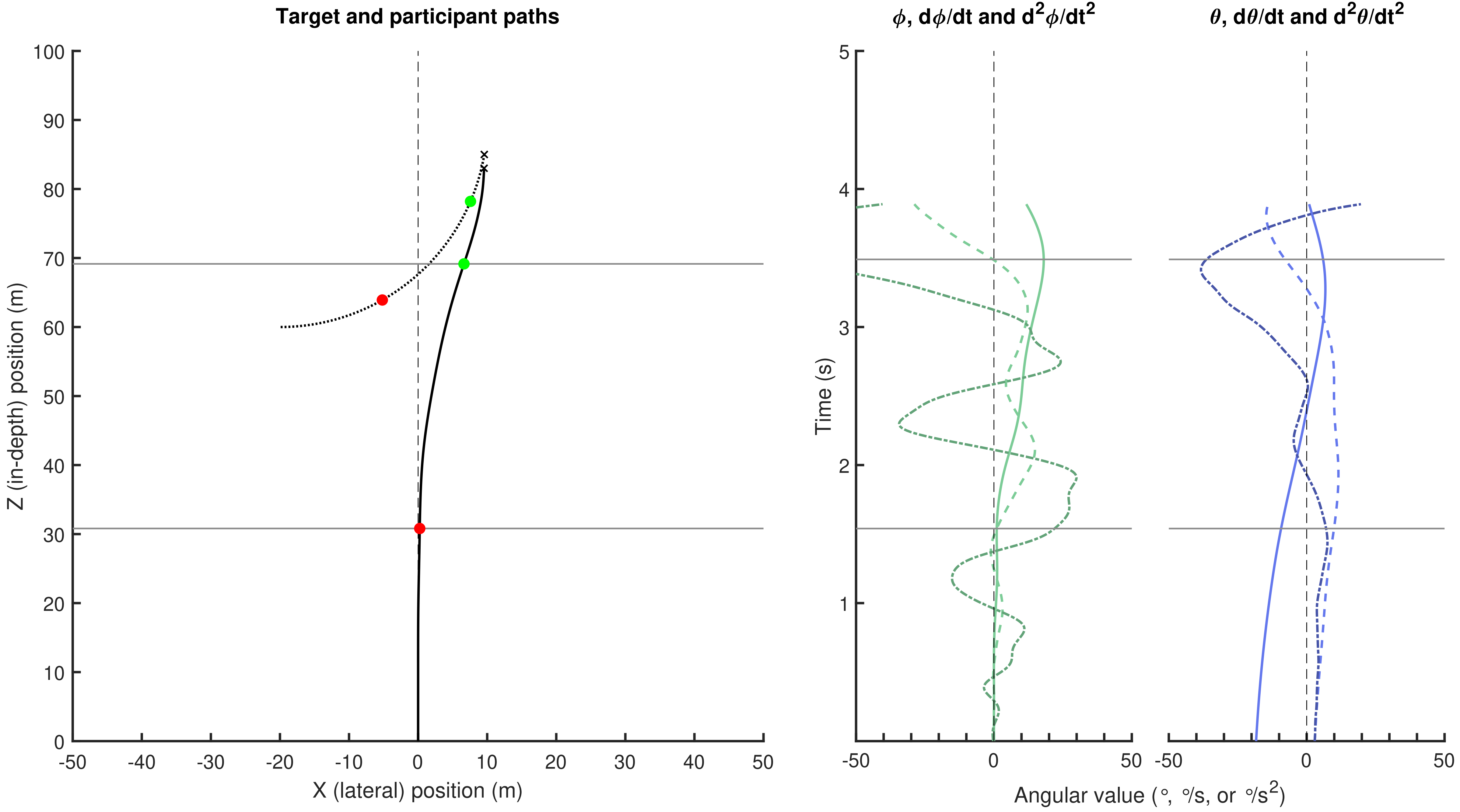

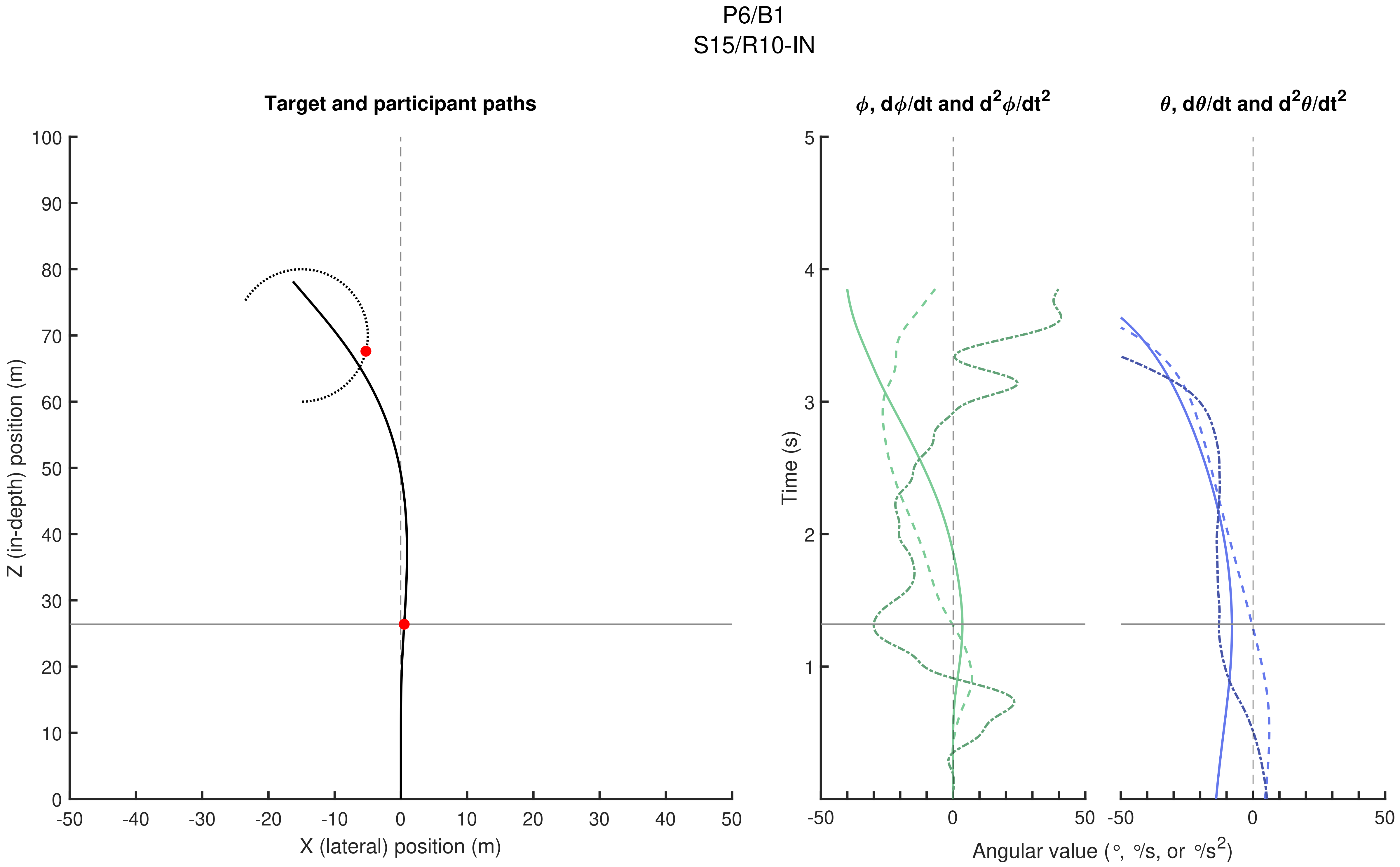

P6/B1
S15/R10-IN
Target and participant paths
Z (in-depth) position (m)
X (lateral) position (m)
φ, dφ/dt and d²φ/dt²
θ, dθ/dt and d²θ/dt²
Time (s)
Angular value (°, °/s, or °/s²)

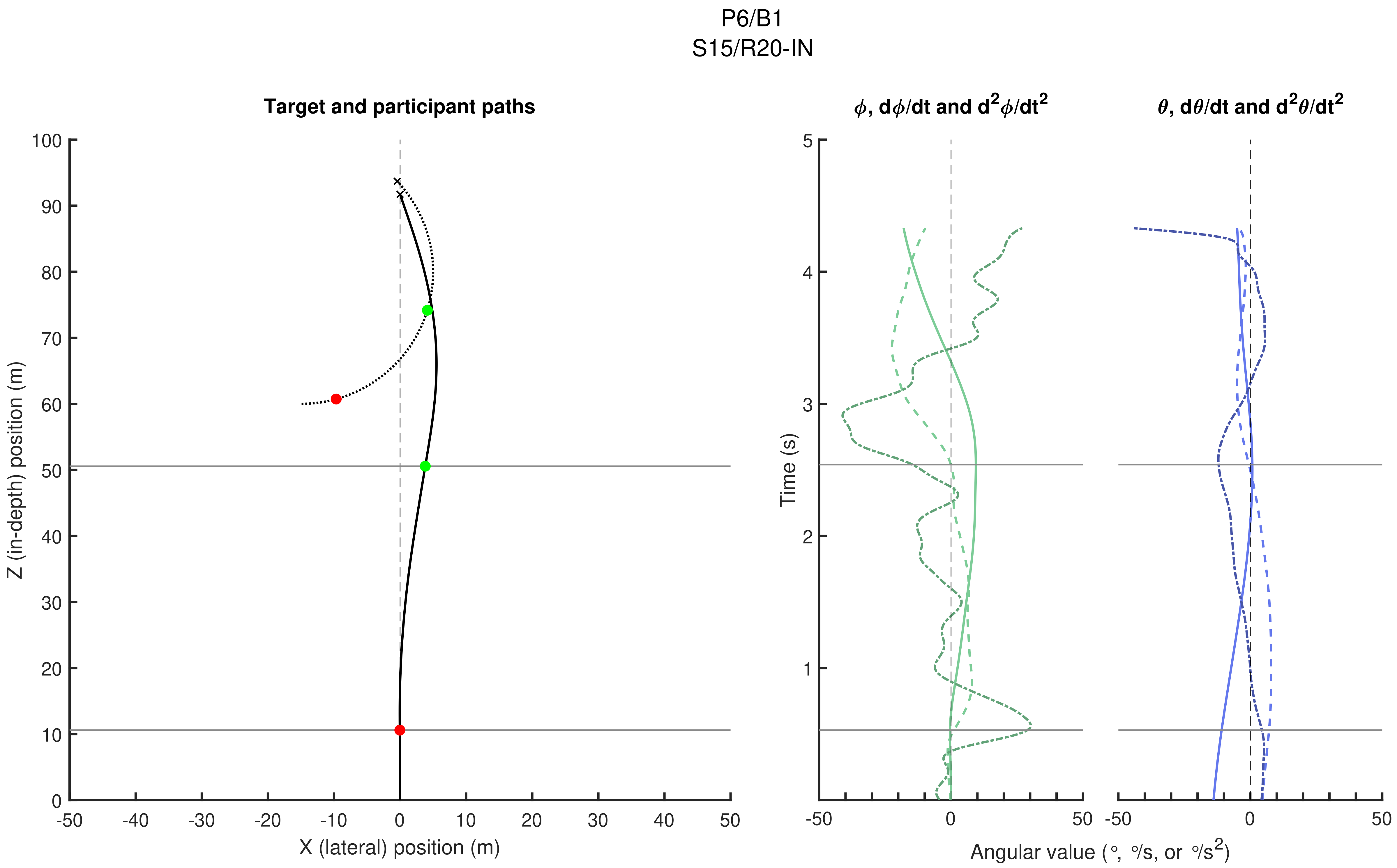
P6/B1
S15/R20-IN
Target and participant paths
Z (in-depth) position (m)
X (lateral) position (m)
ϕ, dϕ/dt and d$^2\phi$/dt^2
θ, dθ/dt and d$^2\theta$/dt^2
Time (s)
Angular value (°, °/s, or °/s^2)

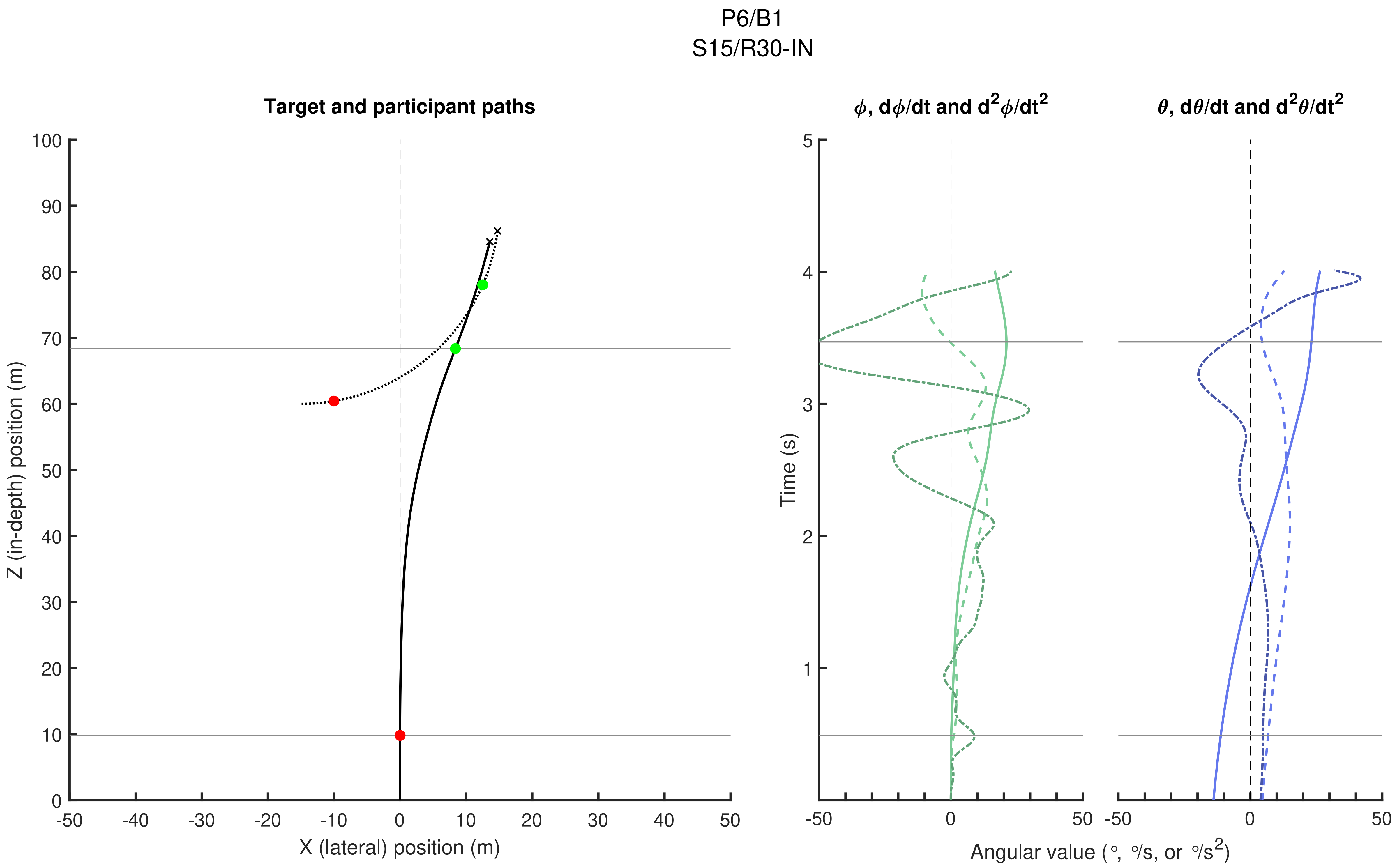
P6/B1
S15/R30-IN
Target and participant paths
Z (in-depth) position (m)
X (lateral) position (m)
ϕ, dϕ/dt and d$^2\phi$/dt^2
θ, dθ/dt and d$^2\theta$/dt^2
Time (s)
Angular value (°, °/s, or °/s^2)

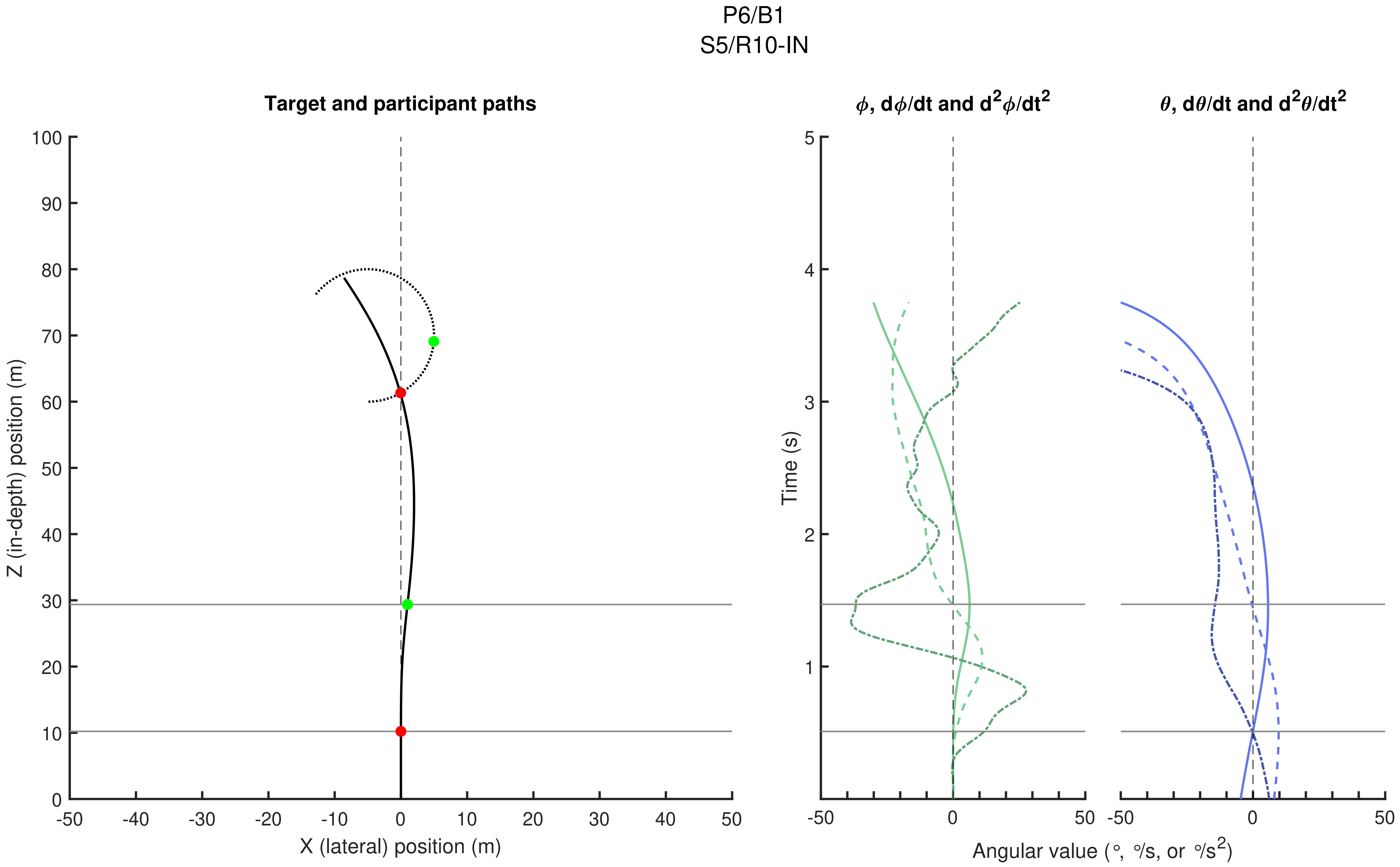

P6/B1
S5/R10-IN
Target and participant paths
Z (in-depth) position (m)
X (lateral) position (m)
ϕ, dϕ/dt and d²ϕ/dt²
θ, dθ/dt and d²θ/dt²
Time (s)
Angular value (°, °/s, or °/s²)

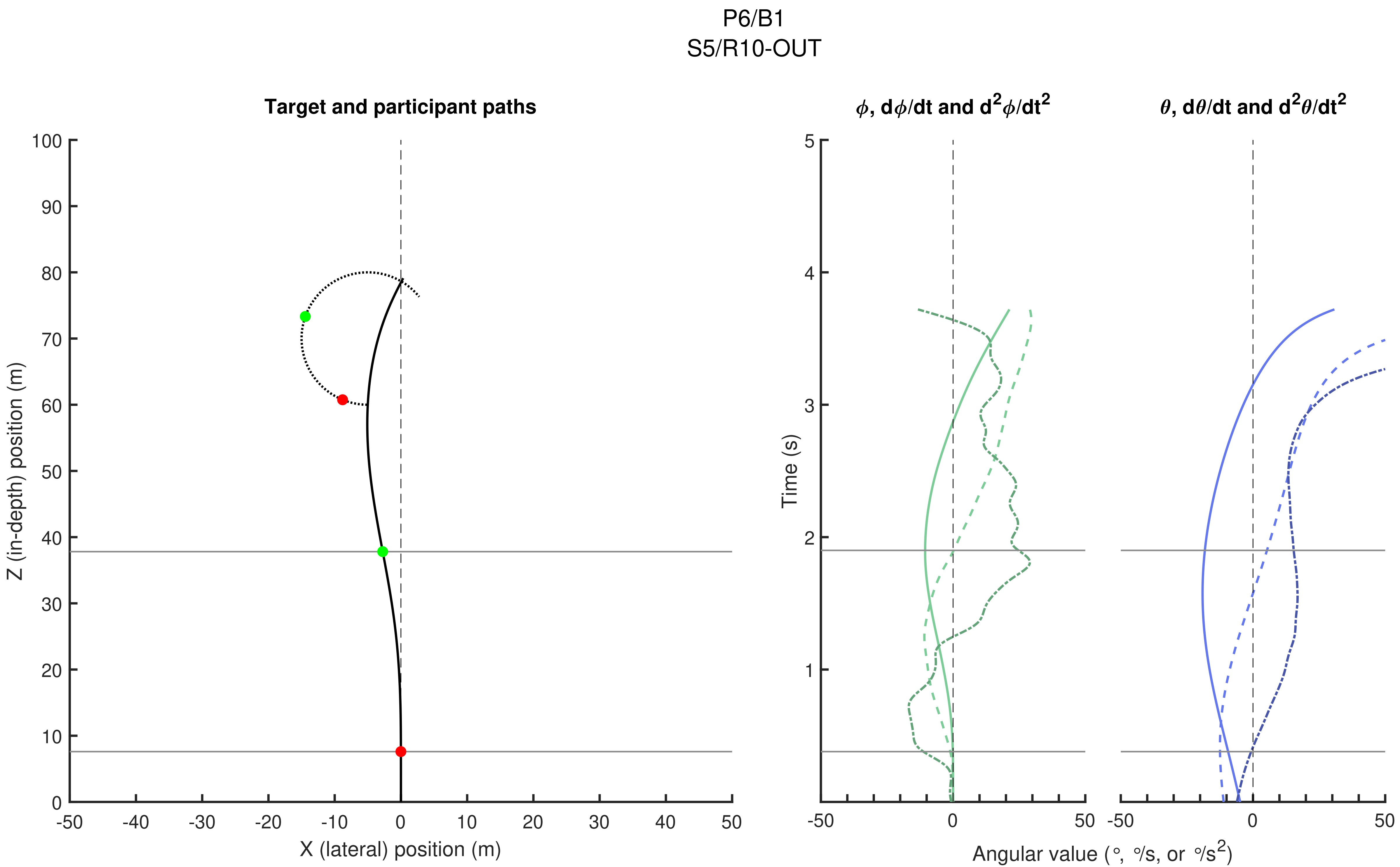

P6/B1
S5/R10-OUT
Target and participant paths
Z (in-depth) position (m)
X (lateral) position (m)
100
90
80
70
60
50
40
30
20
10
0
-50
-40
-30
-20
-10
0
10
20
30
40
50
ϕ, dϕ/dt and d²ϕ/dt²
θ, dθ/dt and d²θ/dt²
Time (s)
5
4
3
2
1
-50
0
50
Angular value (°, °/s, or °/s²)

P6/B1
S5/R20-IN

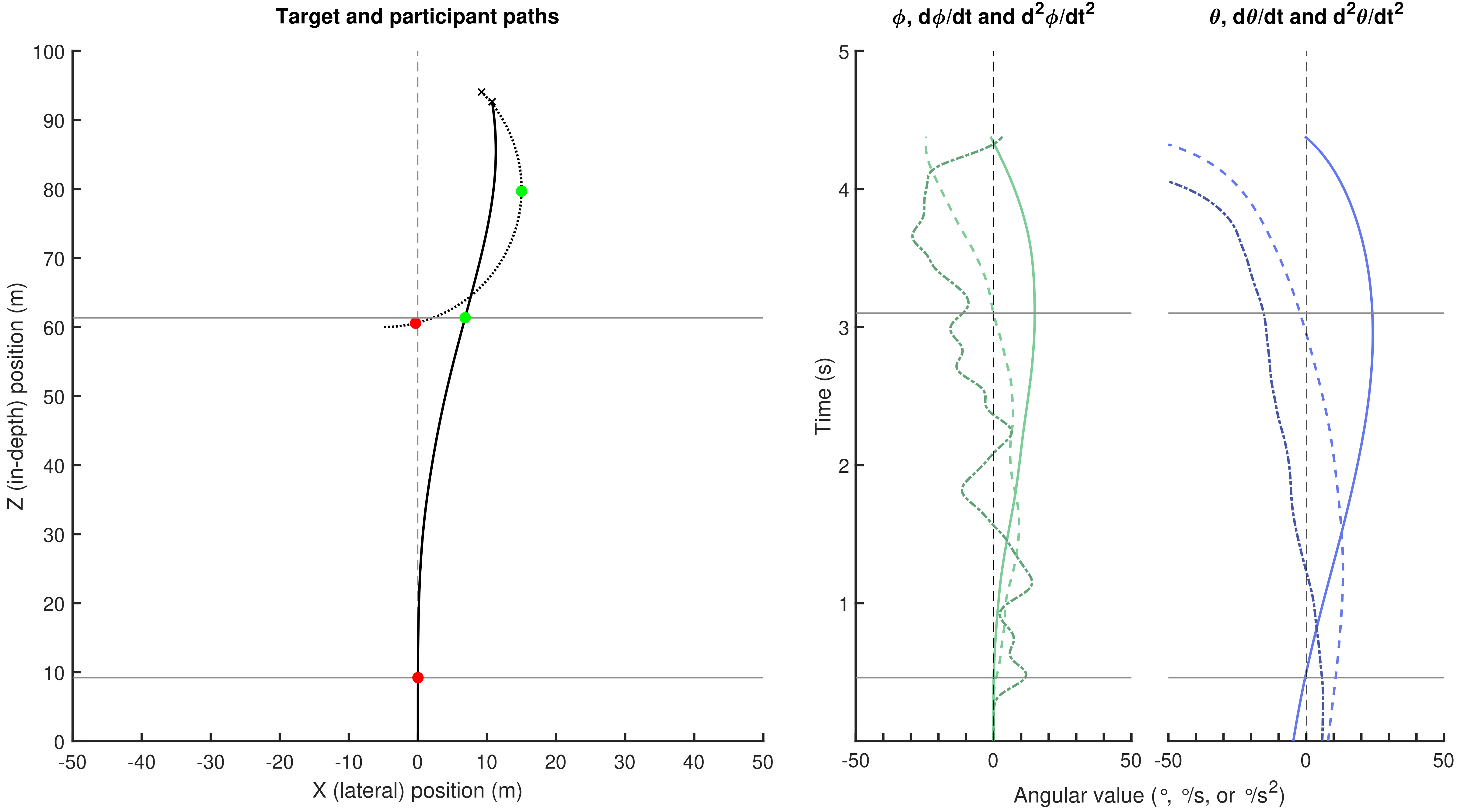

P6/B1
S5/R20-OUT

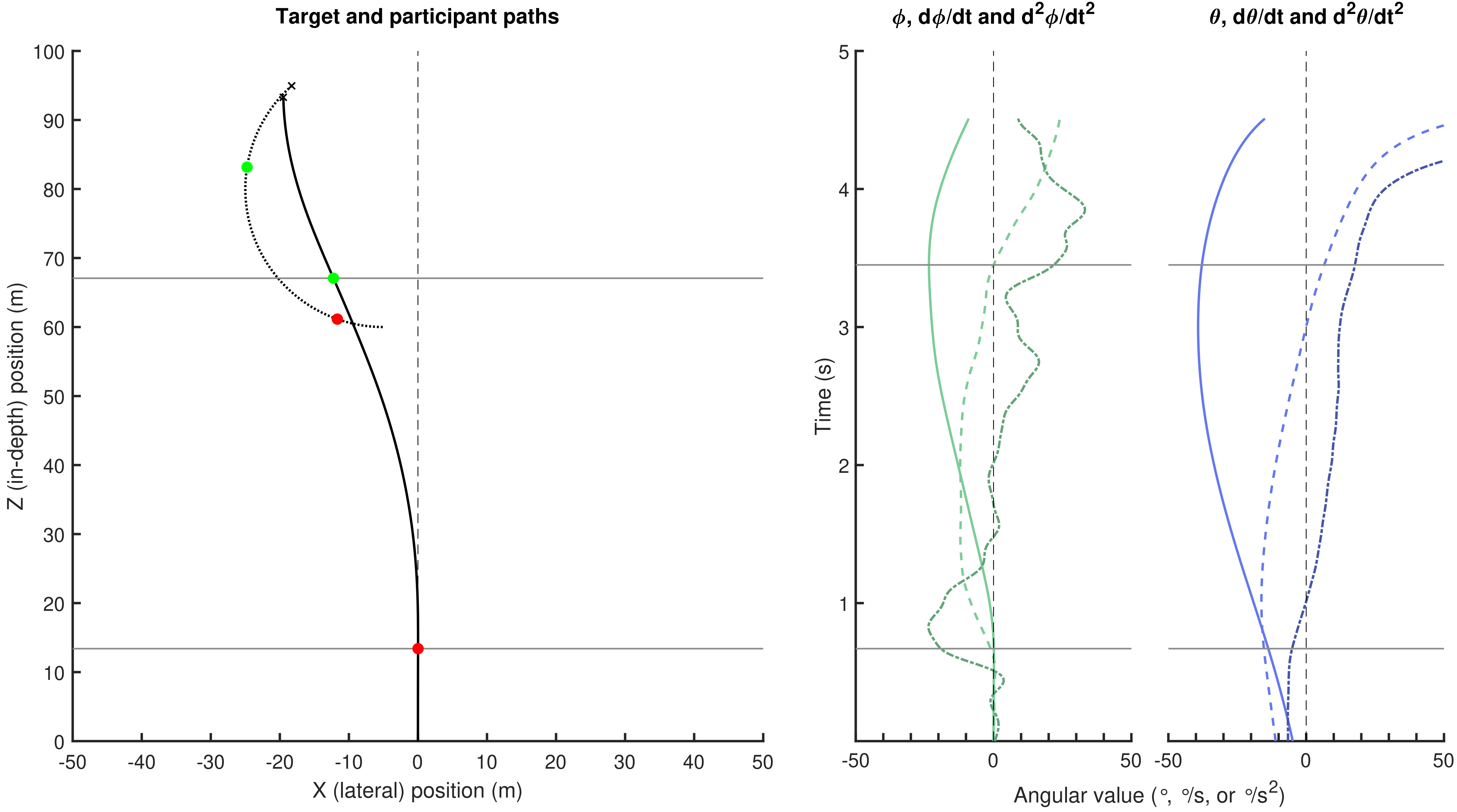

P6/B1
S5/R30-IN

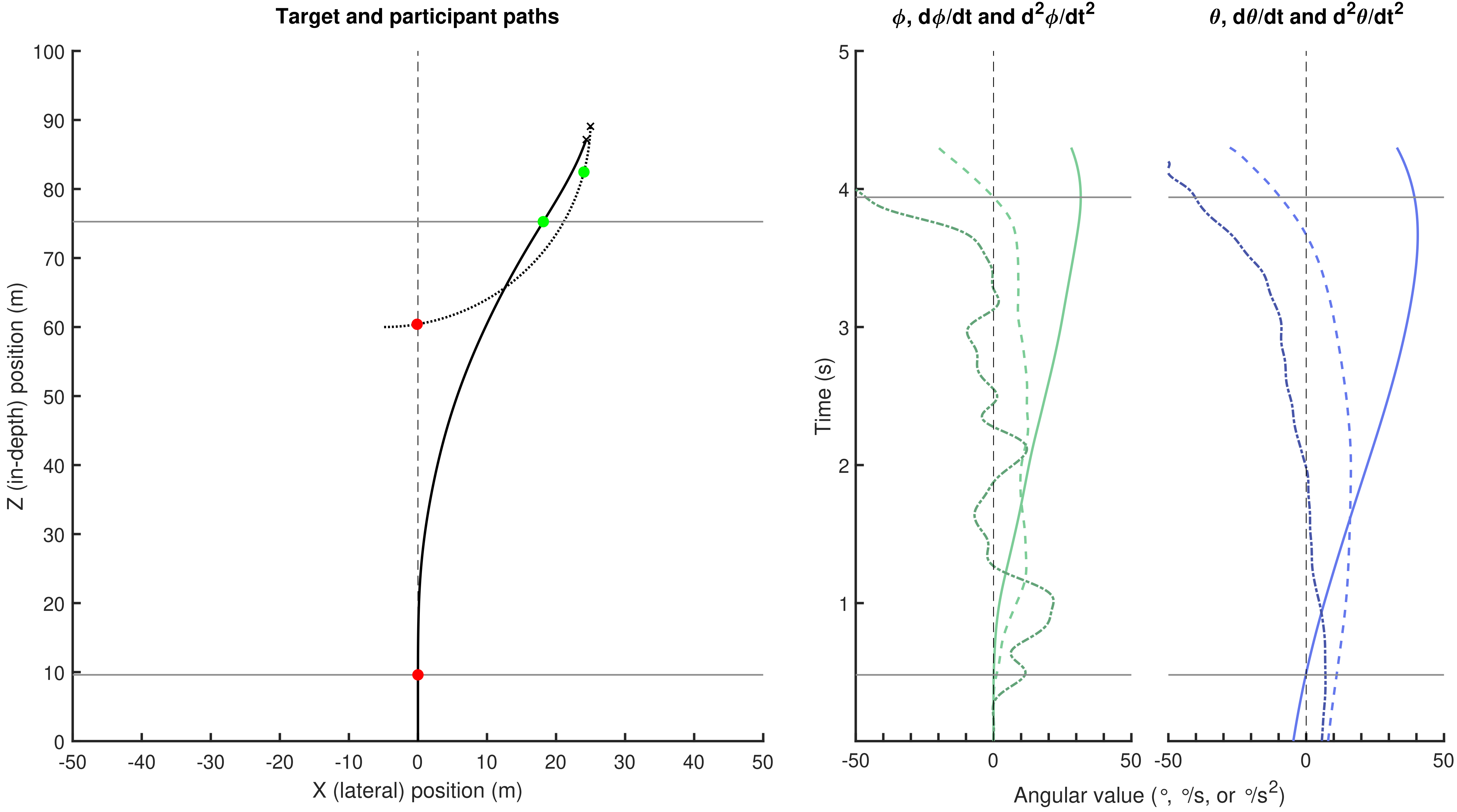

P6/B1
S5/R30-OUT

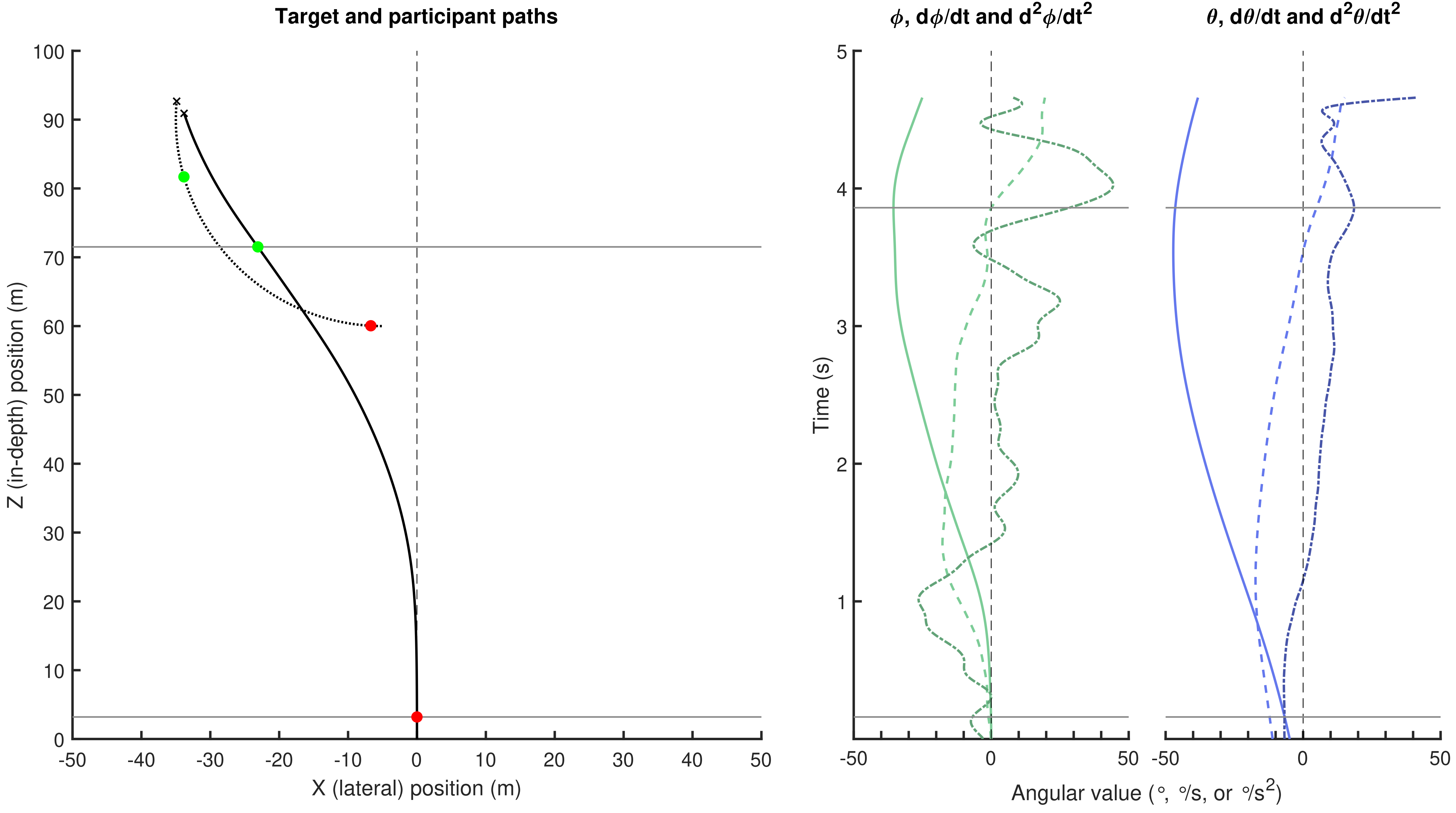

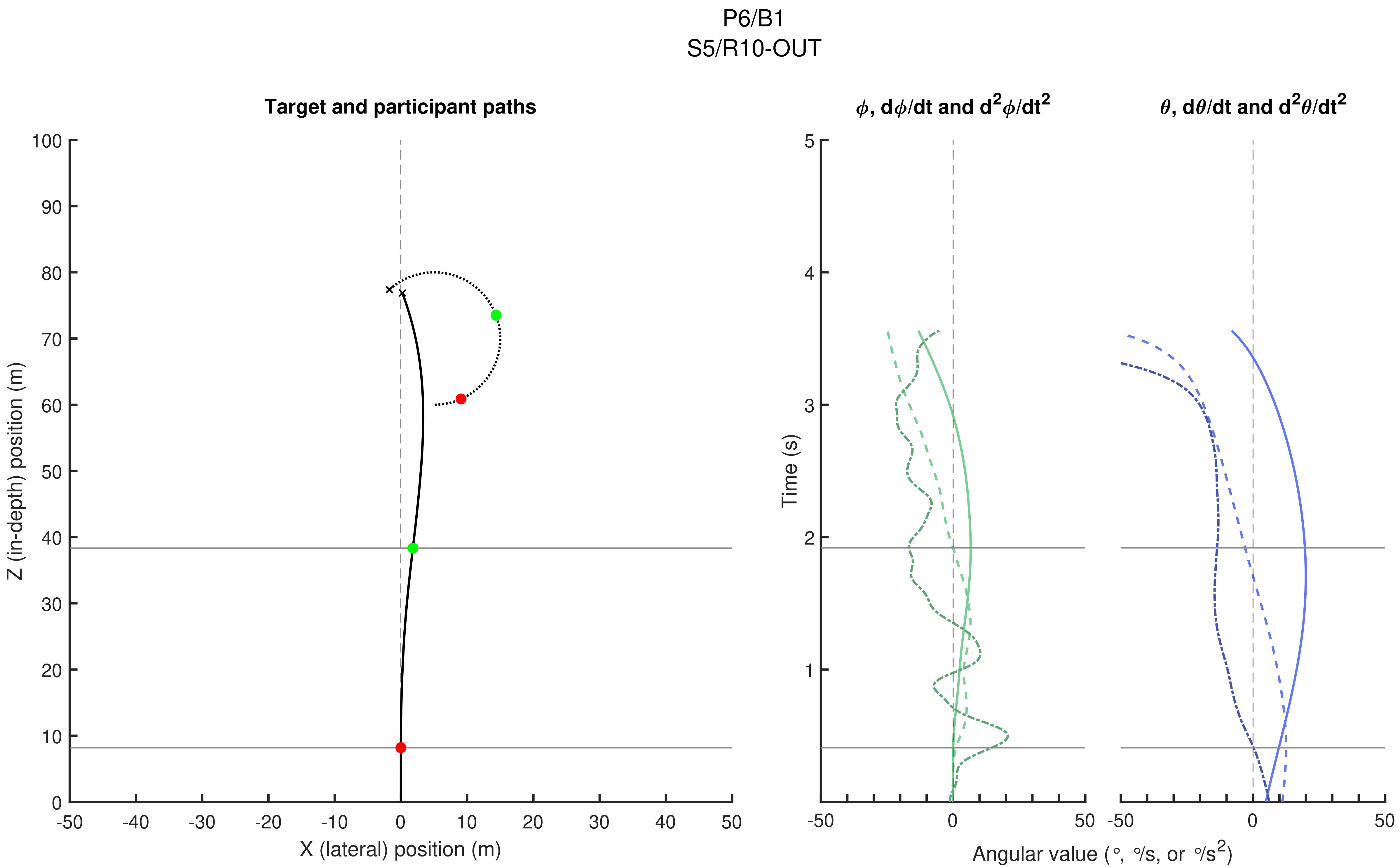

P6/B1
S5/R10-OUT
Target and participant paths
Z (in-depth) position (m)
X (lateral) position (m)
0
10
20
30
40
50
60
70
80
90
100
-50
-40
-30
-20
-10
0
10
20
30
40
50
φ, dφ/dt and d²φ/dt²
θ, dθ/dt and d²θ/dt²
Time (s)
0
1
2
3
4
5
-50
0
50
-50
0
50
Angular value (°, °/s, or °/s²)

P6/B1
S5/R10-IN

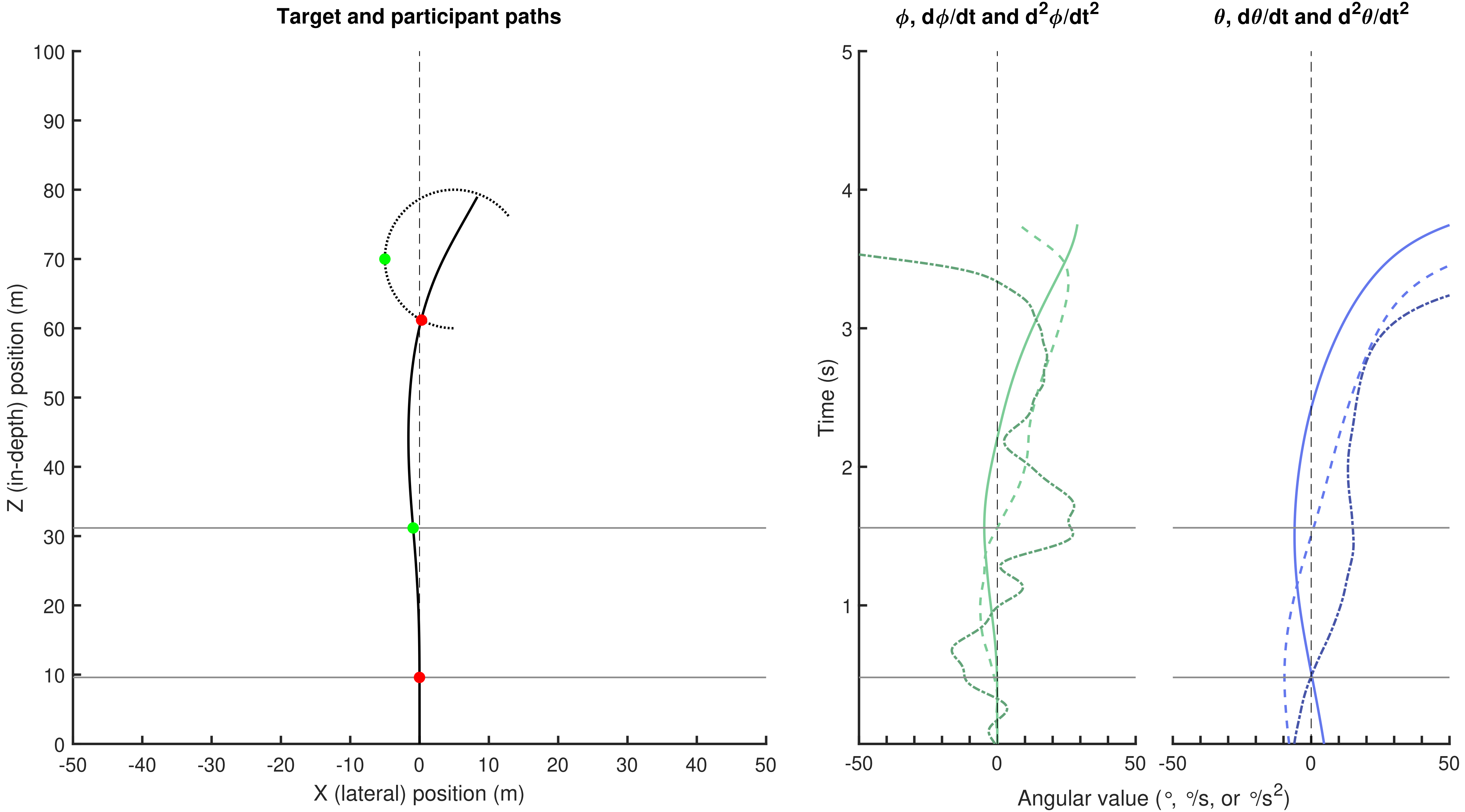

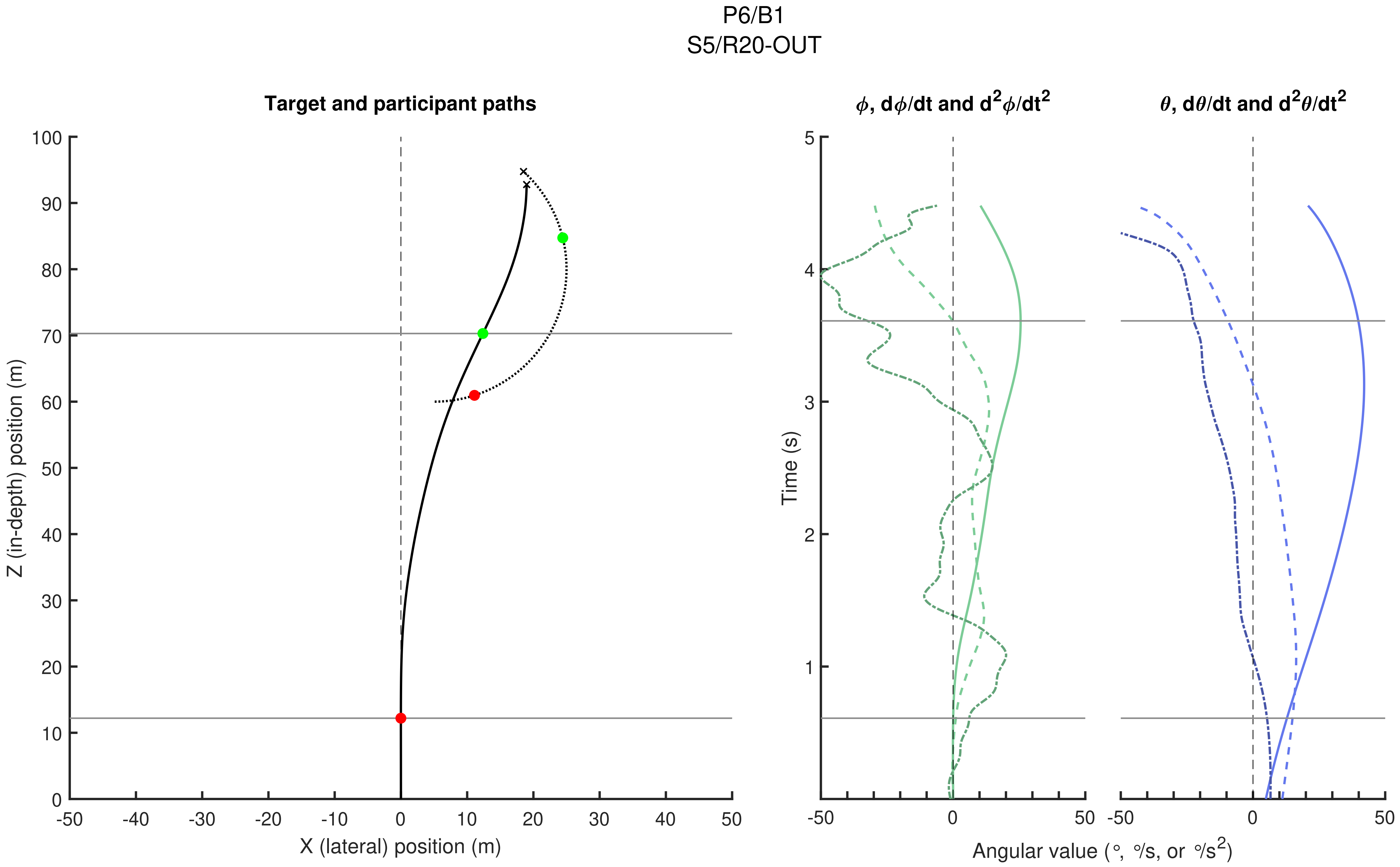

P6/B1
S5/R20-OUT
Target and participant paths
Z (in-depth) position (m)
X (lateral) position (m)
ϕ, dϕ/dt and d$^2\phi$/dt^2
θ, dθ/dt and d$^2\theta$/dt^2
Time (s)
Angular value (°, °/s, or °/s^2)

P6/B1
S5/R20-IN

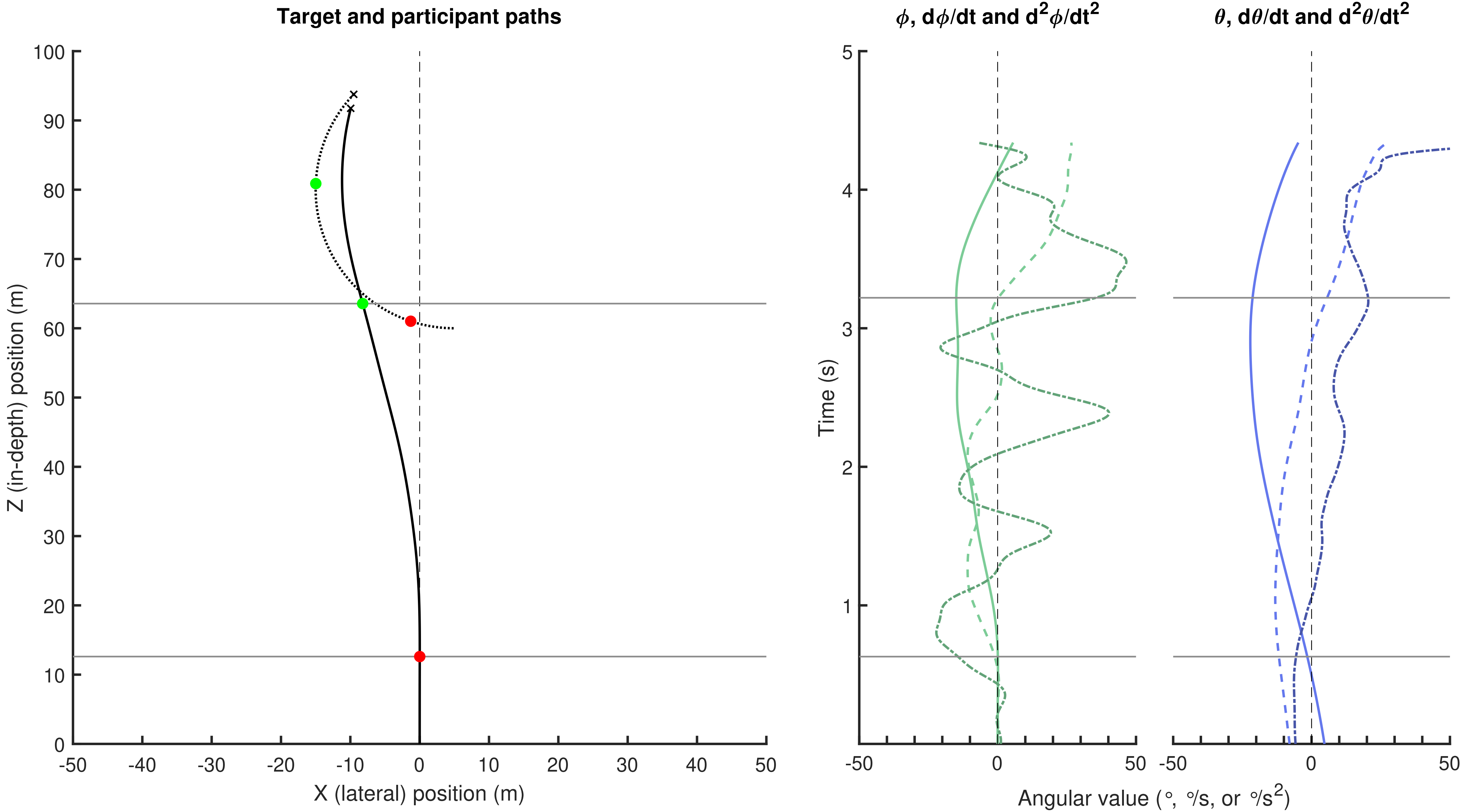

P6/B1
S5/R30-OUT

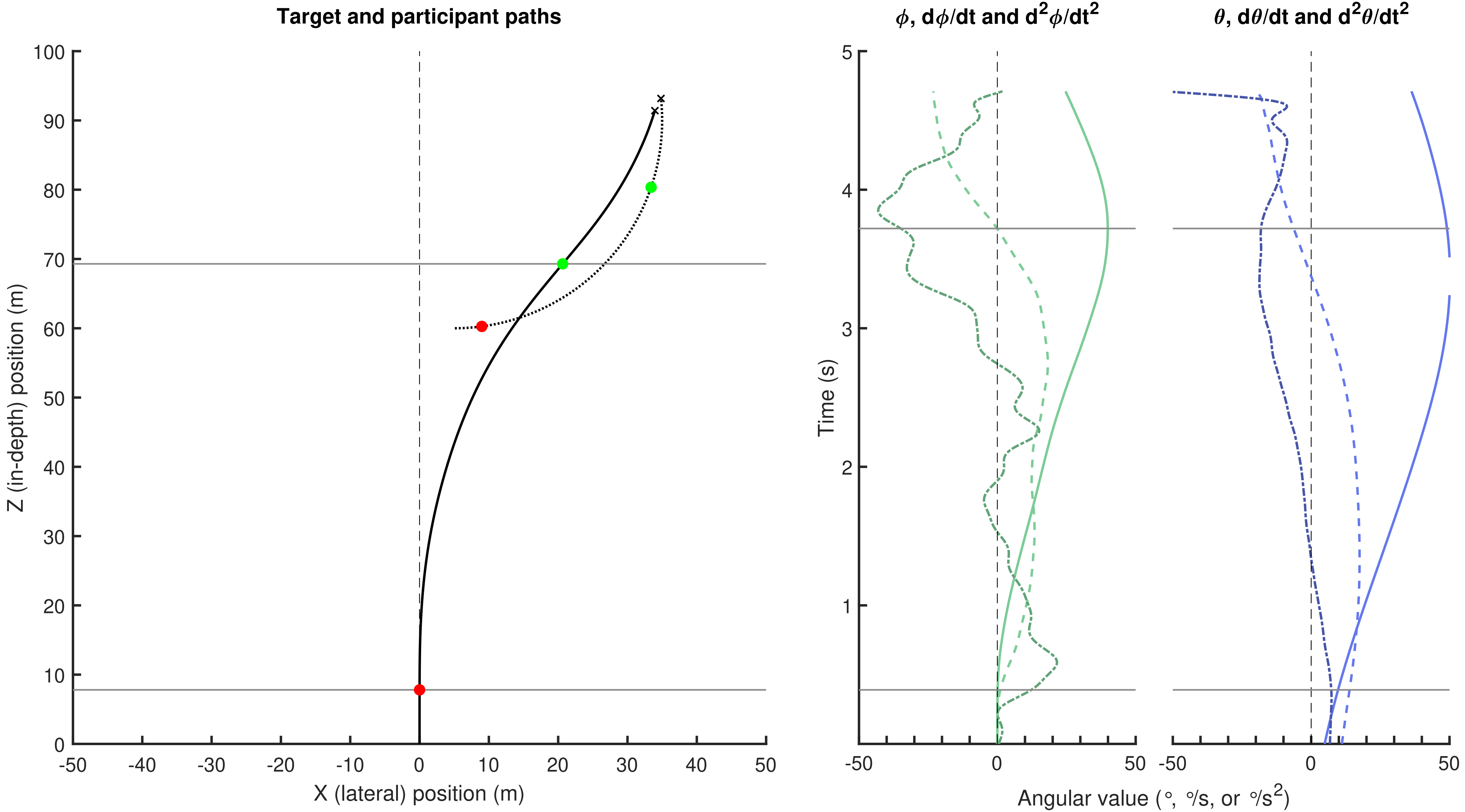

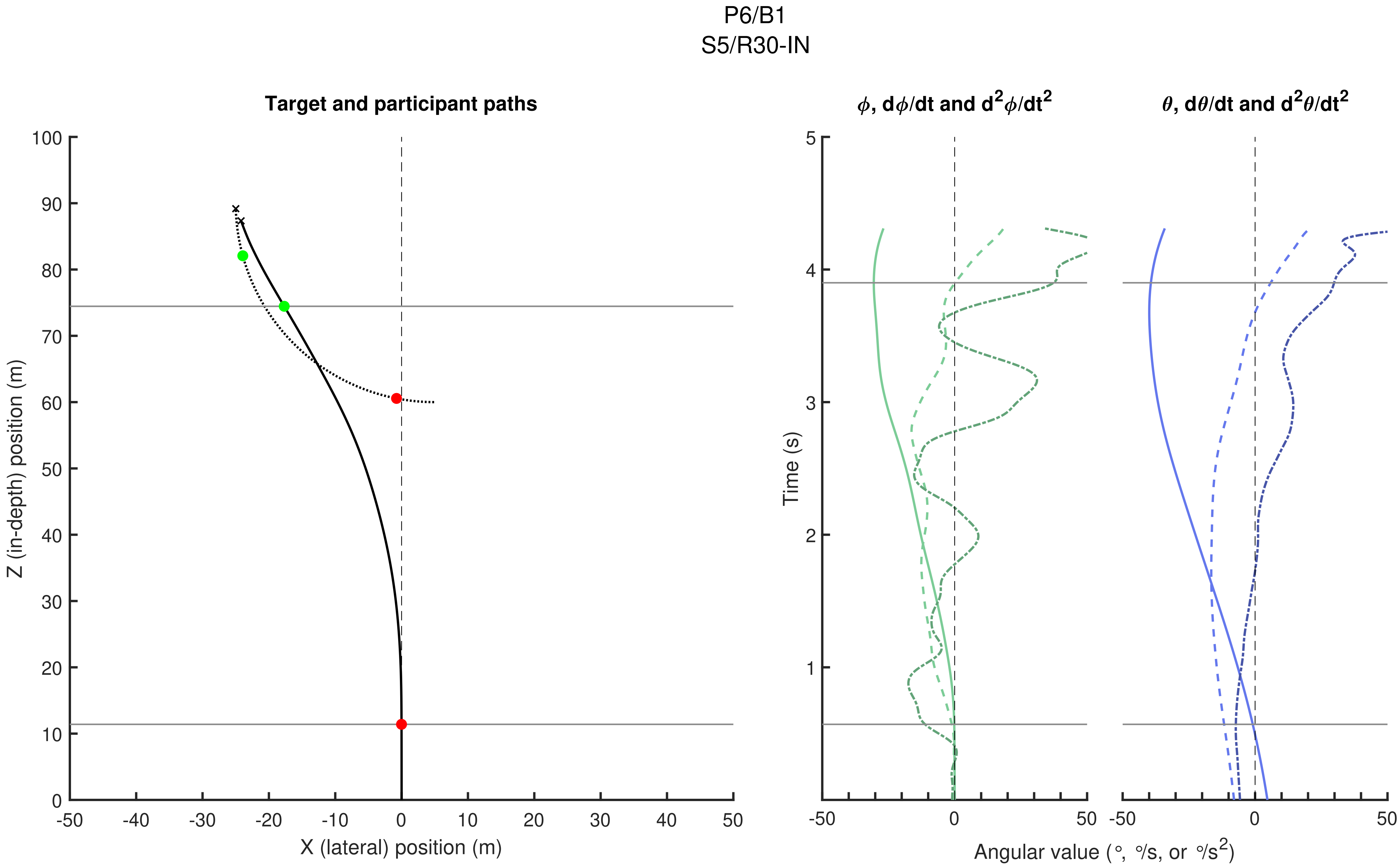

P6/B1
S5/R30-IN
Target and participant paths
Z (in-depth) position (m)
X (lateral) position (m)
100
90
80
70
60
50
40
30
20
10
0
-50
-40
-30
-20
-10
0
10
20
30
40
50
ϕ, dϕ/dt and d²ϕ/dt²
θ, dθ/dt and d²θ/dt²
Time (s)
5
4
3
2
1
-50
0
50
-50
0
50
Angular value (°, °/s, or °/s²)

P6/B1
S15/R10-IN

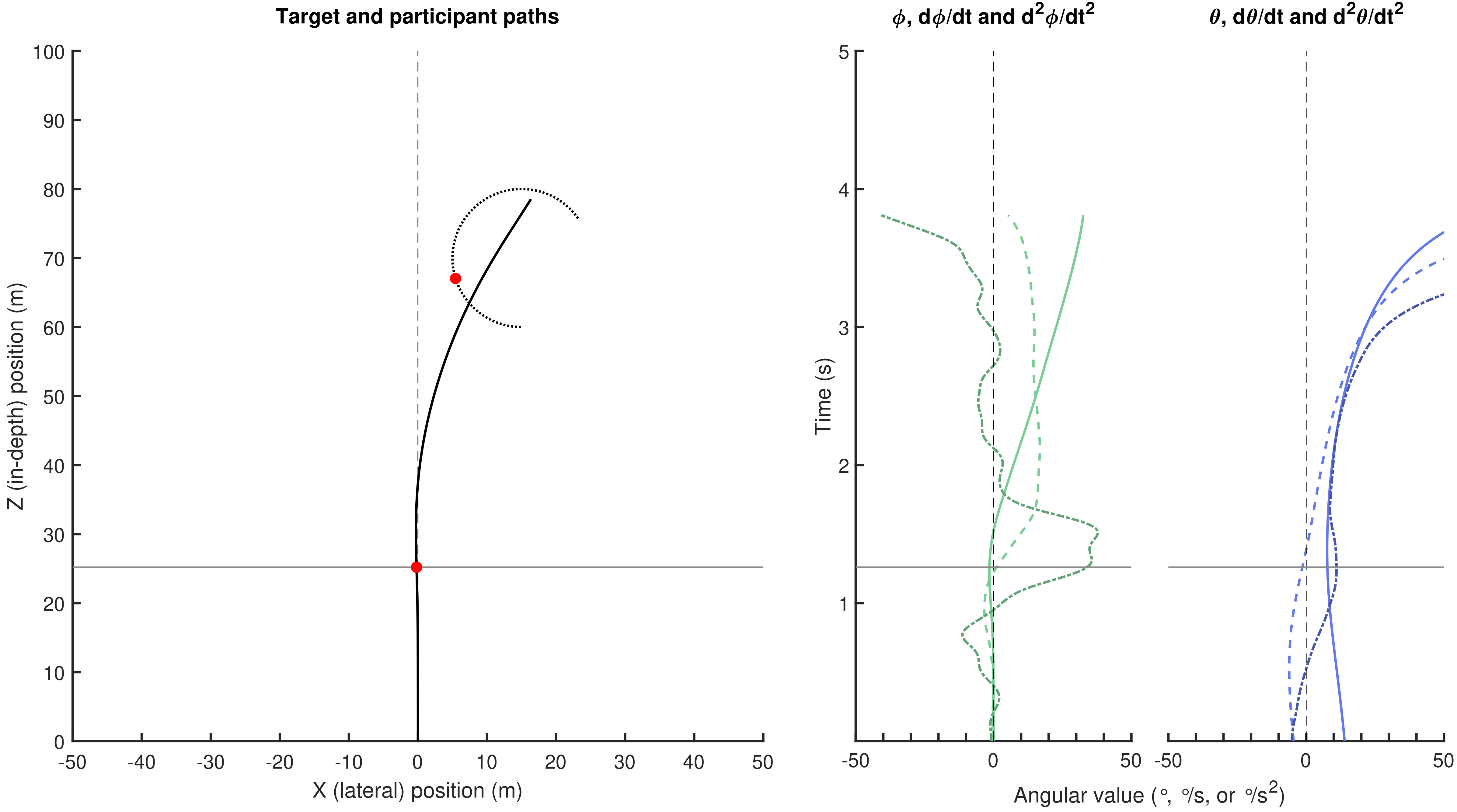

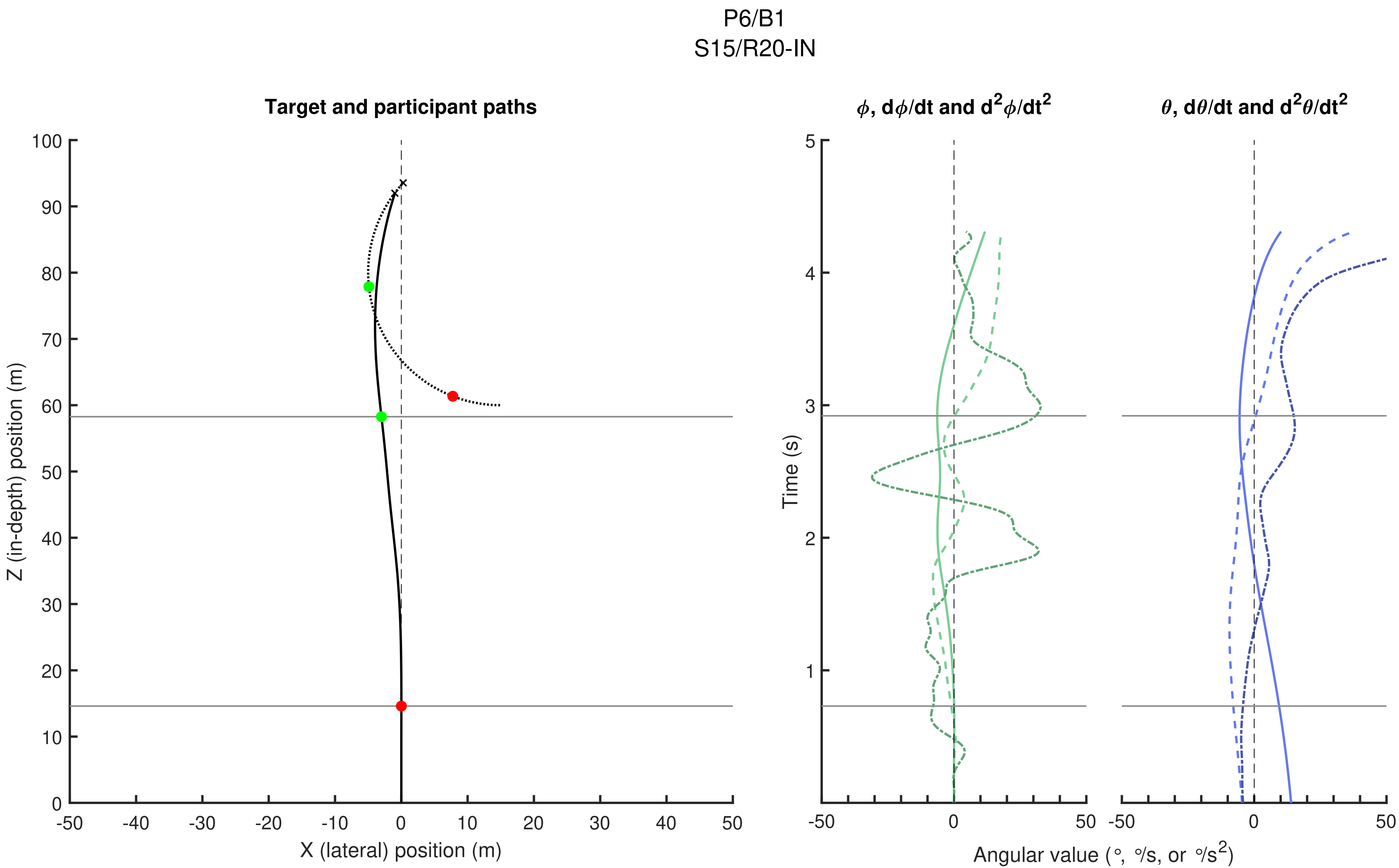

P6/B1
S15/R20-IN
Target and participant paths
Z (in-depth) position (m)
X (lateral) position (m)
ϕ, dϕ/dt and d$^2\phi$/dt^2
θ, dθ/dt and d$^2\theta$/dt^2
Time (s)
Angular value (°, °/s, or °/s^2)

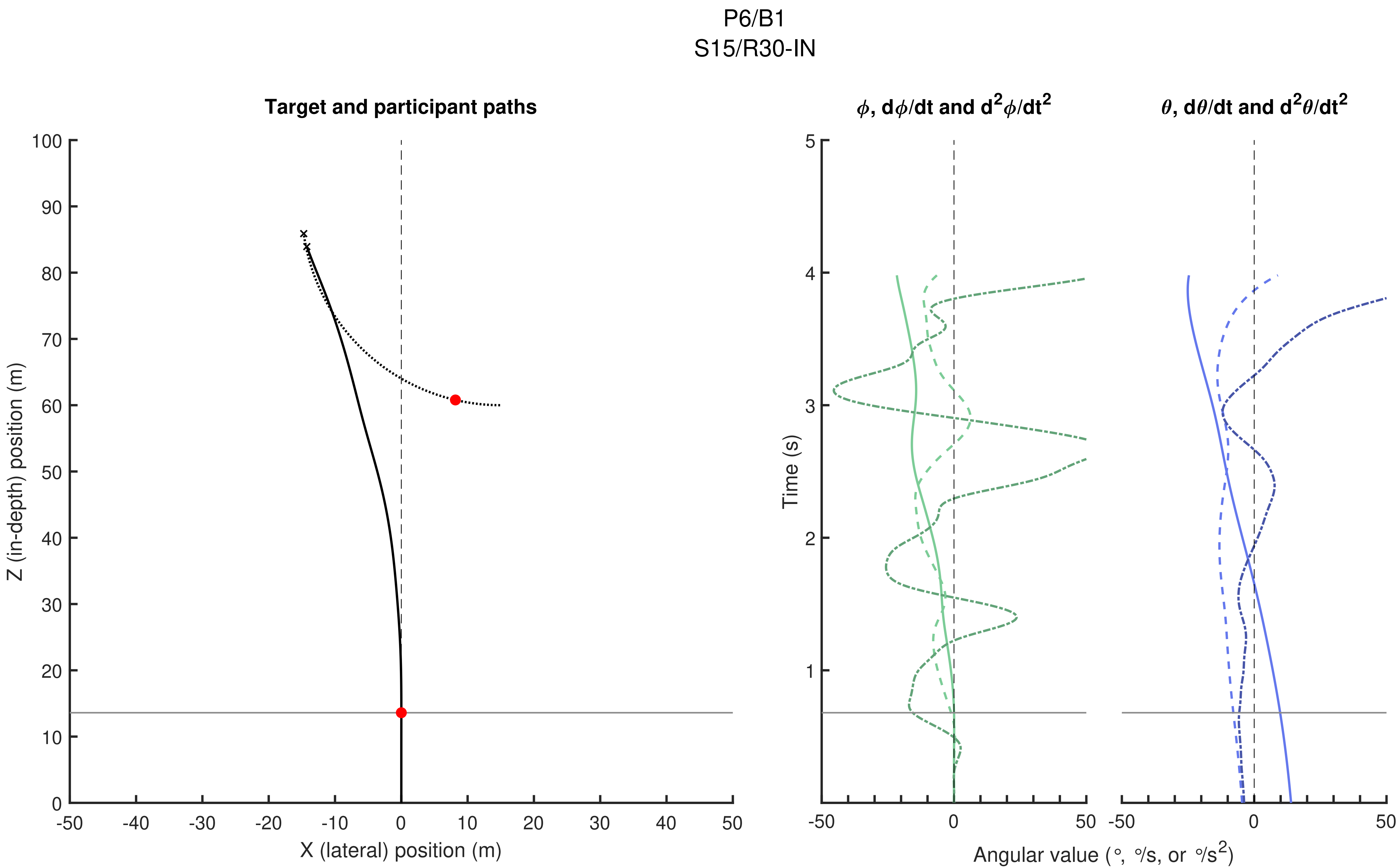

P6/B1
S15/R30-IN
Target and participant paths
Z (in-depth) position (m)
X (lateral) position (m)
ϕ, dϕ/dt and d$^2\phi$/dt^2
θ, dθ/dt and d$^2\theta$/dt^2
Time (s)
Angular value (°, °/s, or °/s^2)

P6/B1
S20/R10-IN

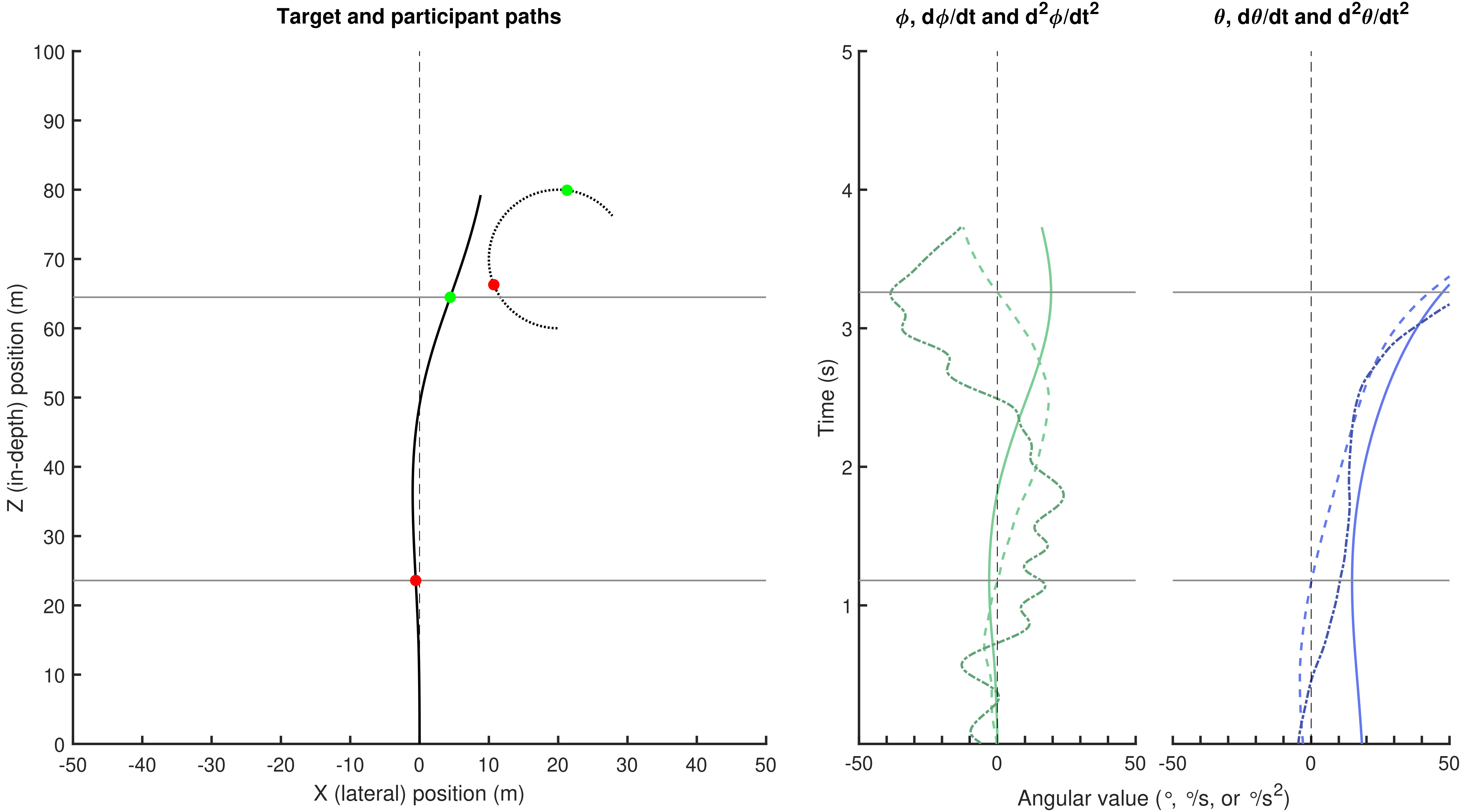

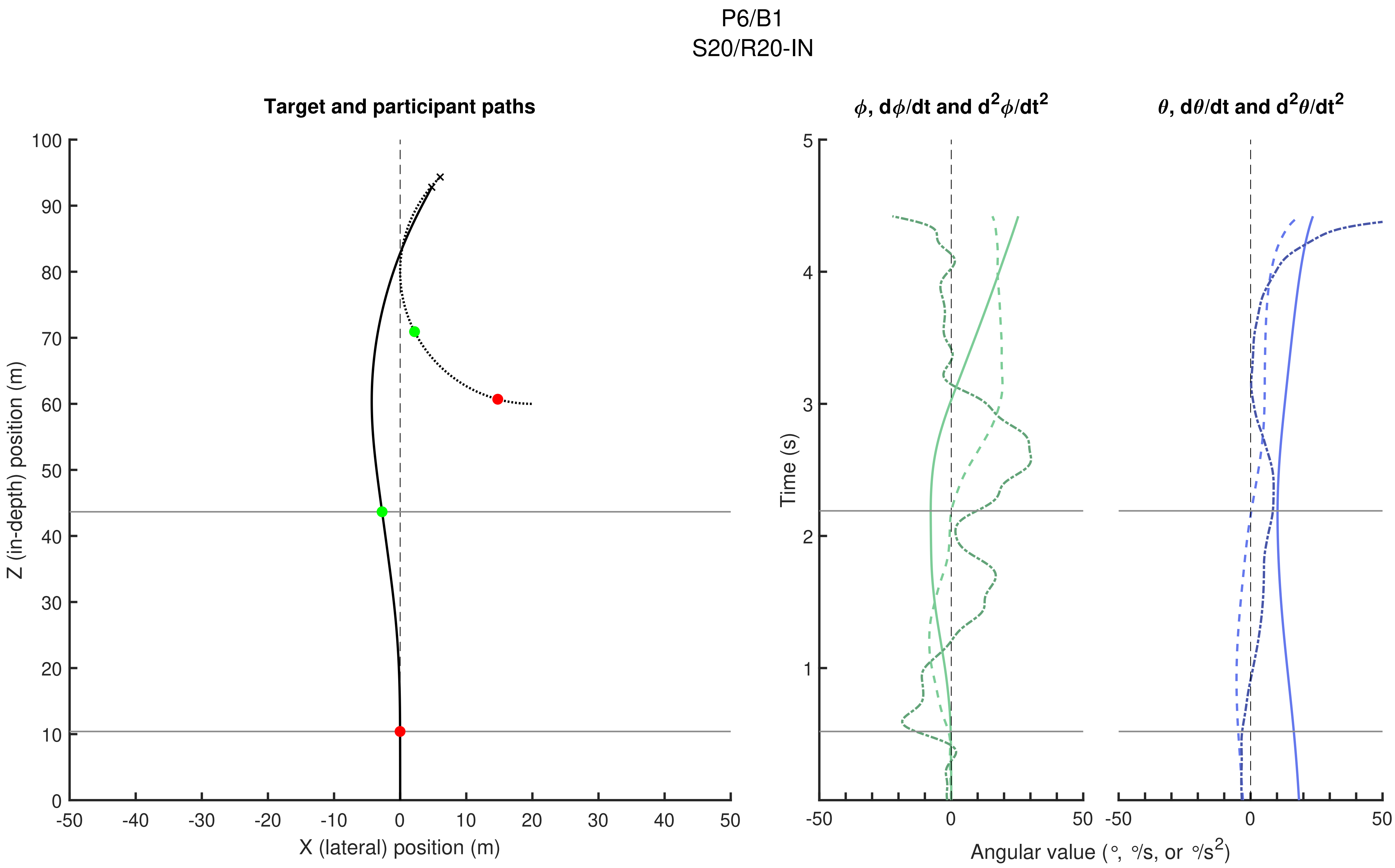

P6/B1
S20/R20-IN
Target and participant paths
Z (in-depth) position (m)
X (lateral) position (m)
ϕ, dϕ/dt and d²ϕ/dt²
θ, dθ/dt and d²θ/dt²
Time (s)
Angular value (°, °/s, or °/s²)

P6/B1
S20/R30-IN

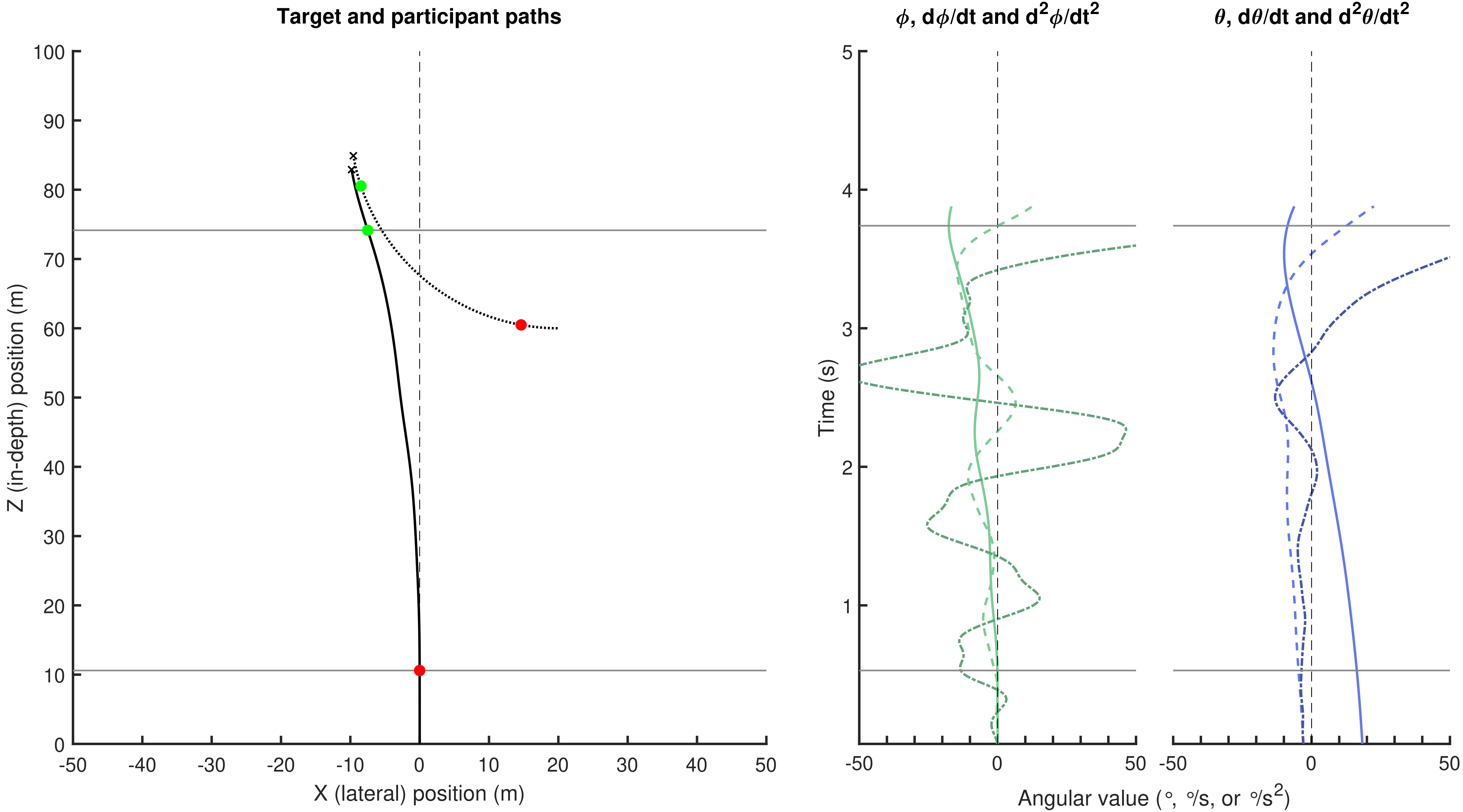

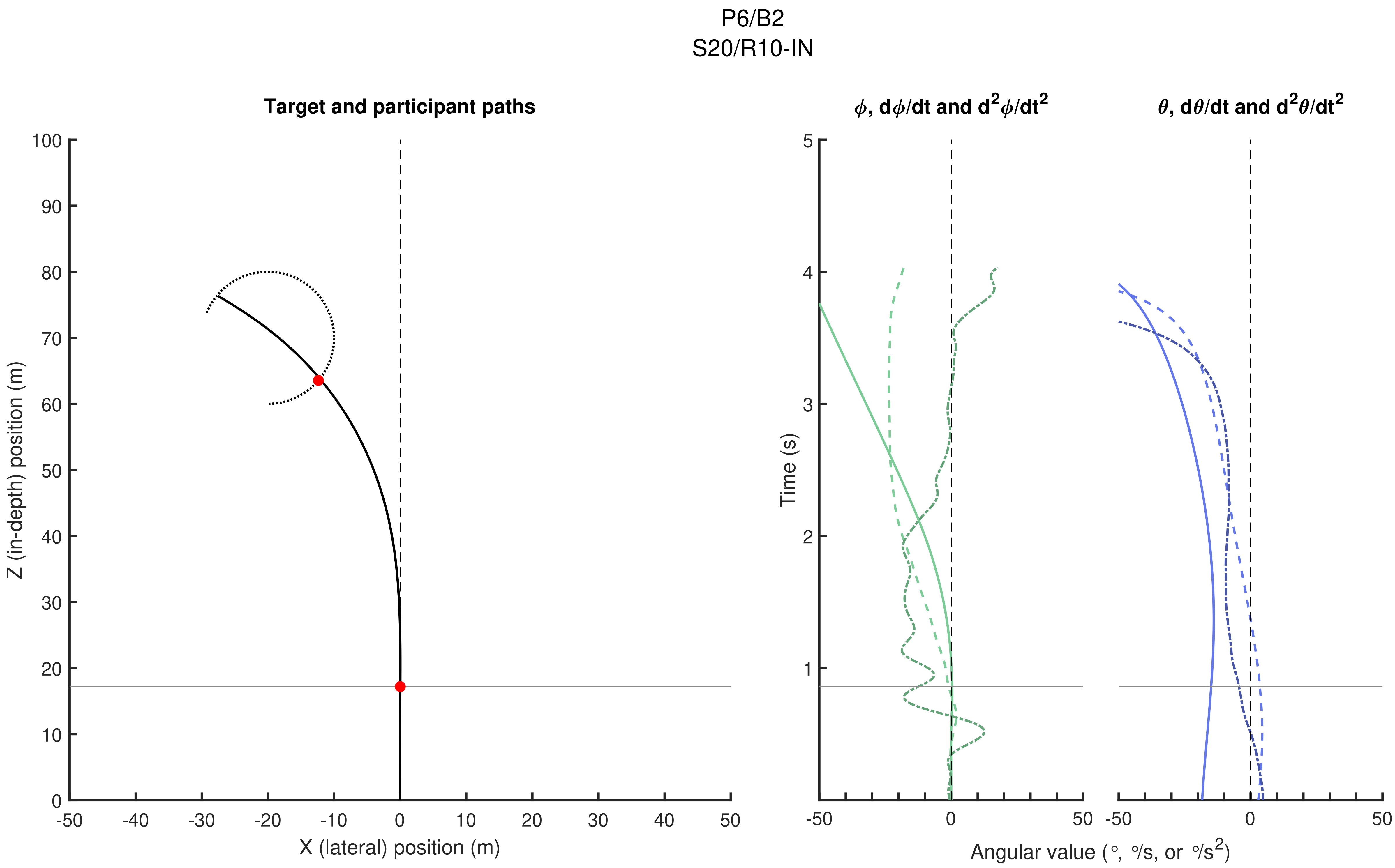
P6/B2
S20/R10-IN
Target and participant paths
Z (in-depth) position (m)
X (lateral) position (m)
100
90
80
70
60
50
40
30
20
10
0
-50
-40
-30
-20
-10
0
10
20
30
40
50
ϕ, dϕ/dt and d$^2\phi$/dt^2
θ, dθ/dt and d$^2\theta$/dt^2
Time (s)
5
4
3
2
1
-50
0
50
-50
0
50
Angular value (°, °/s, or °/s^2)

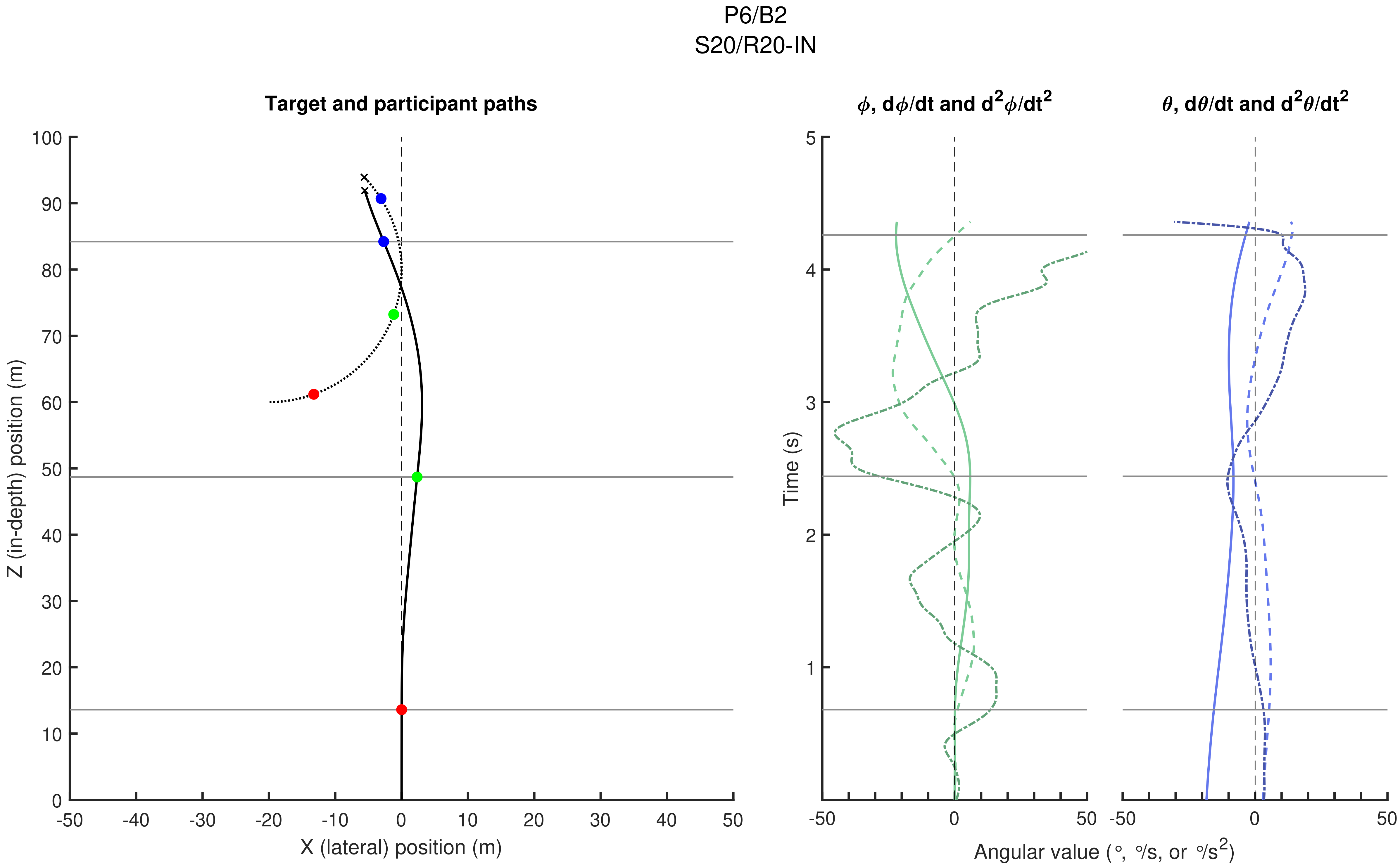
P6/B2
S20/R20-IN
Target and participant paths
Z (in-depth) position (m)
X (lateral) position (m)
φ, dφ/dt and d²φ/dt²
θ, dθ/dt and d²θ/dt²
Time (s)
Angular value (°, °/s, or °/s²)

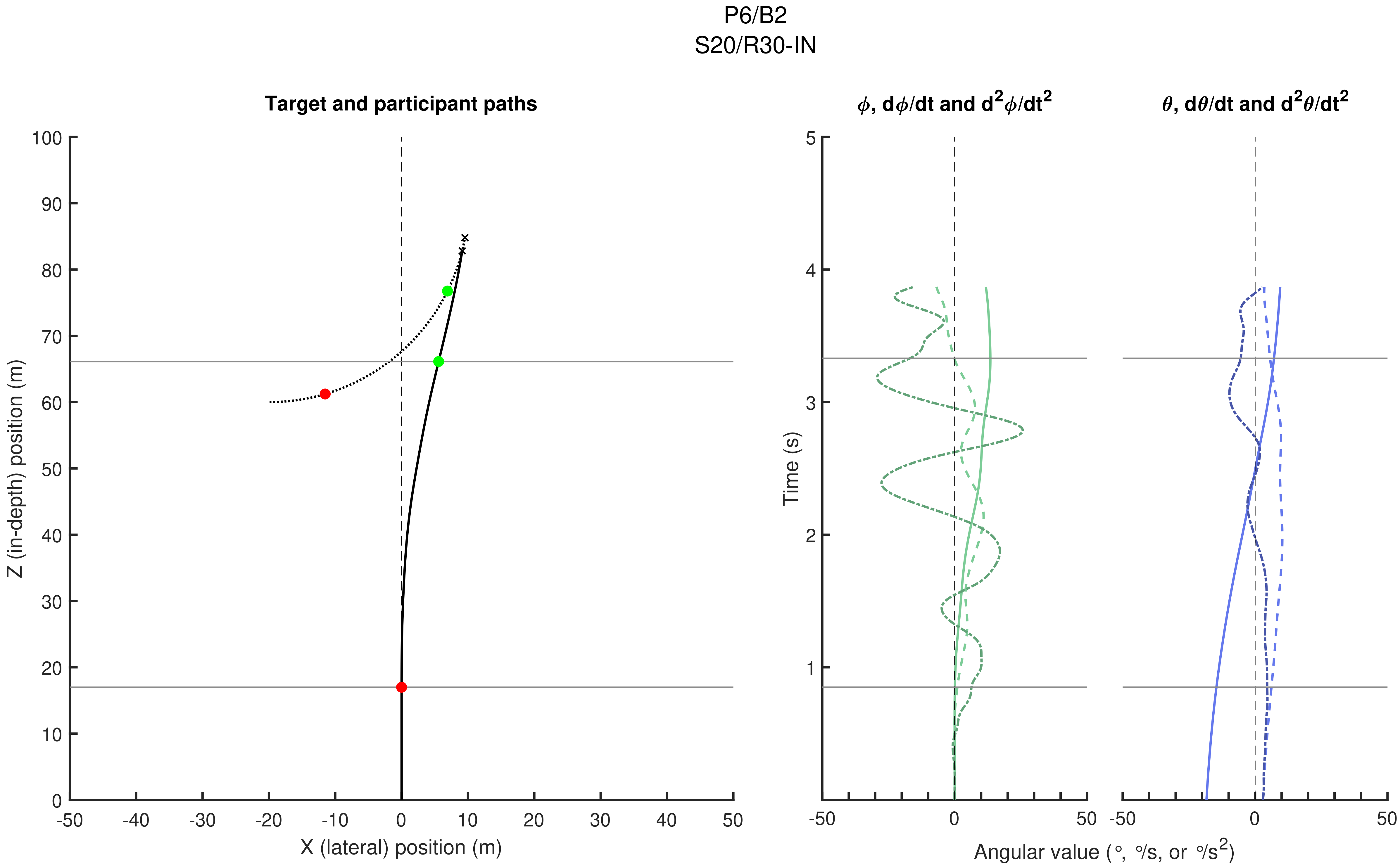
P6/B2
S20/R30-IN
Target and participant paths
ϕ, dϕ/dt and d$^2\phi$/dt^2
θ, dθ/dt and d$^2\theta$/dt^2
Z (in-depth) position (m)
X (lateral) position (m)
Time (s)
Angular value (°, °/s, or °/s^2)
100
90
80
70
60
50
40
30
20
10
0
-50
-40
-30
-20
-10
0
10
20
30
40
50
5
4
3
2
1
-50
0
50
-50
0
50

P6/B2
S15/R10-IN

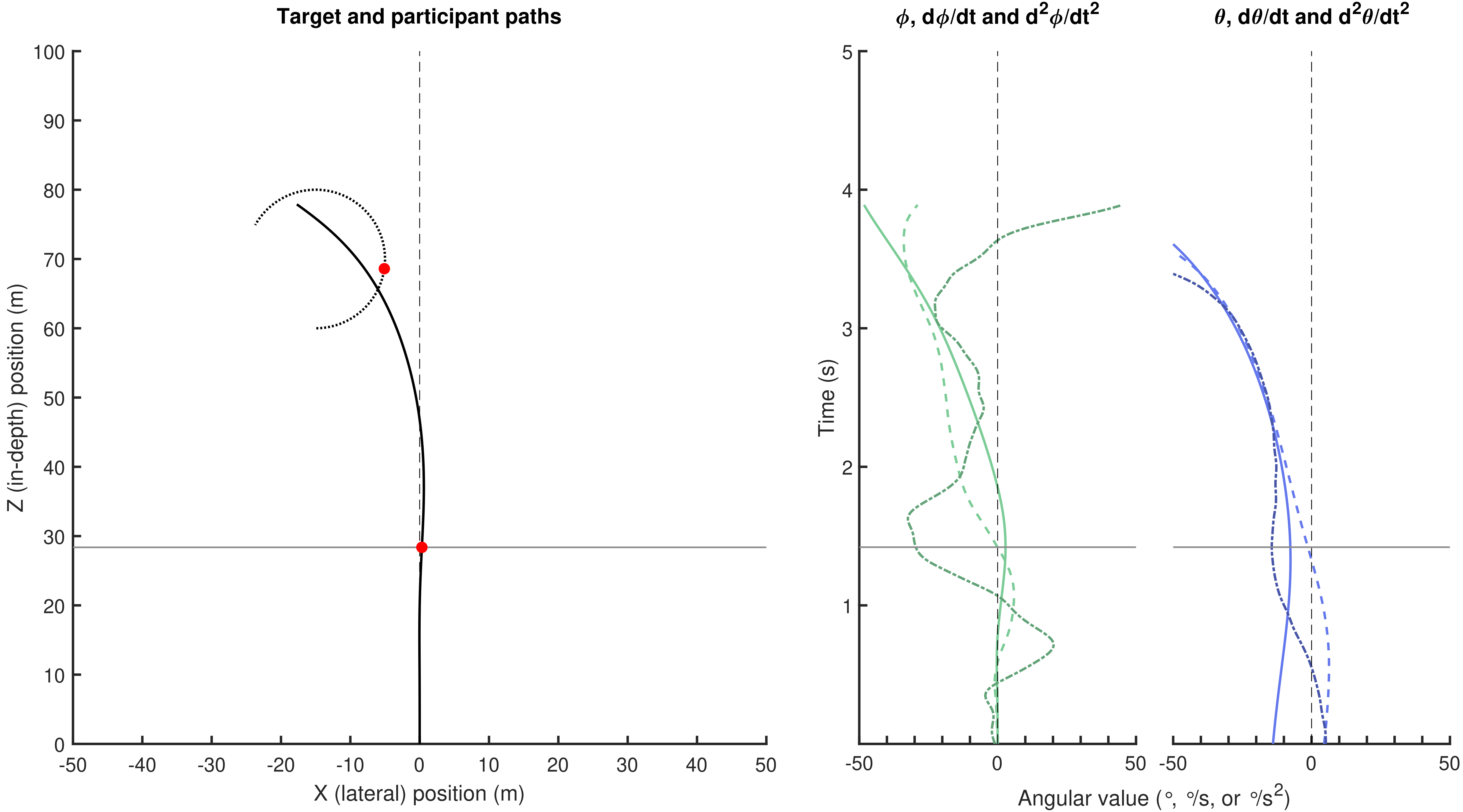

P6/B2
S15/R20-IN

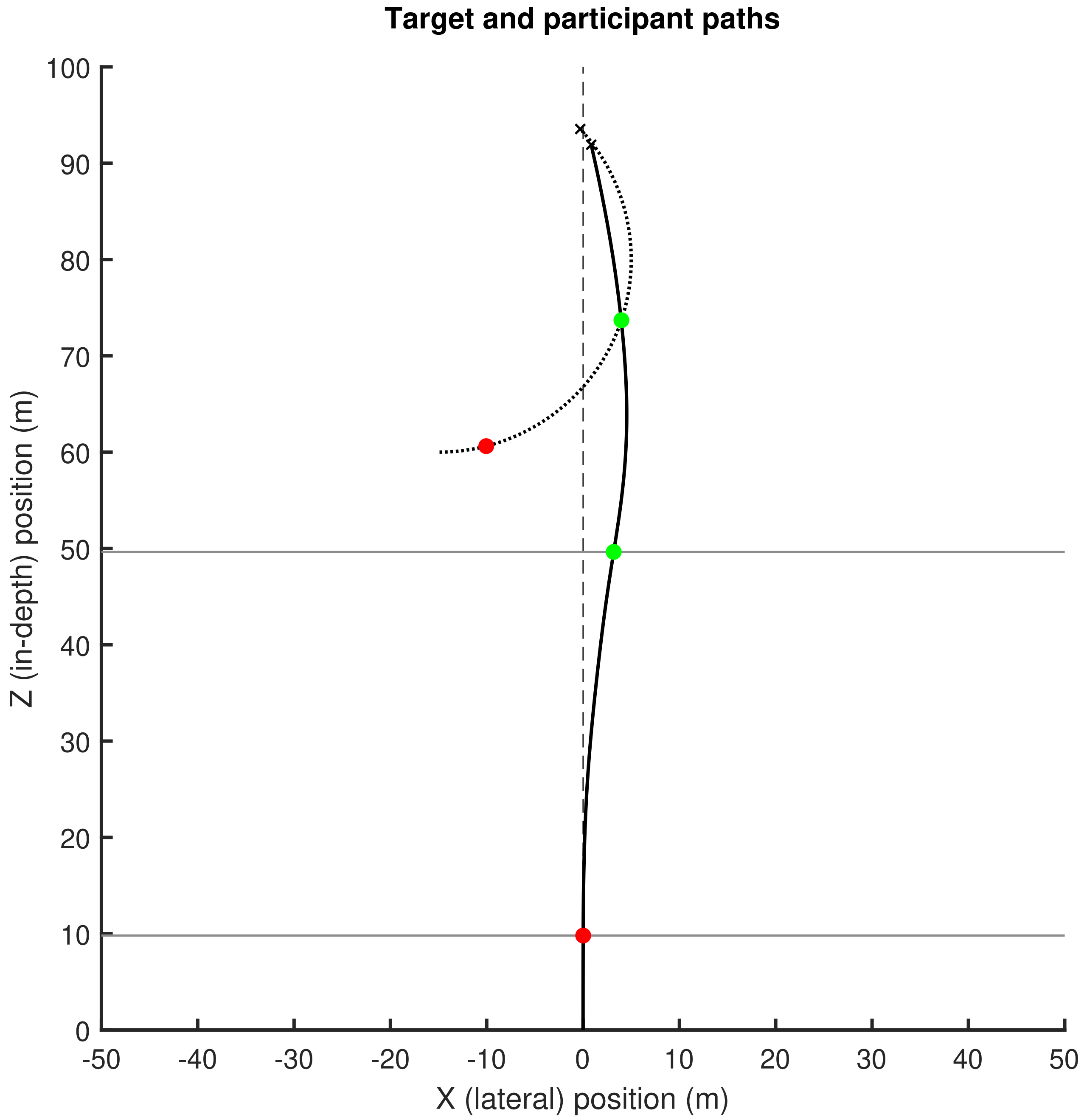

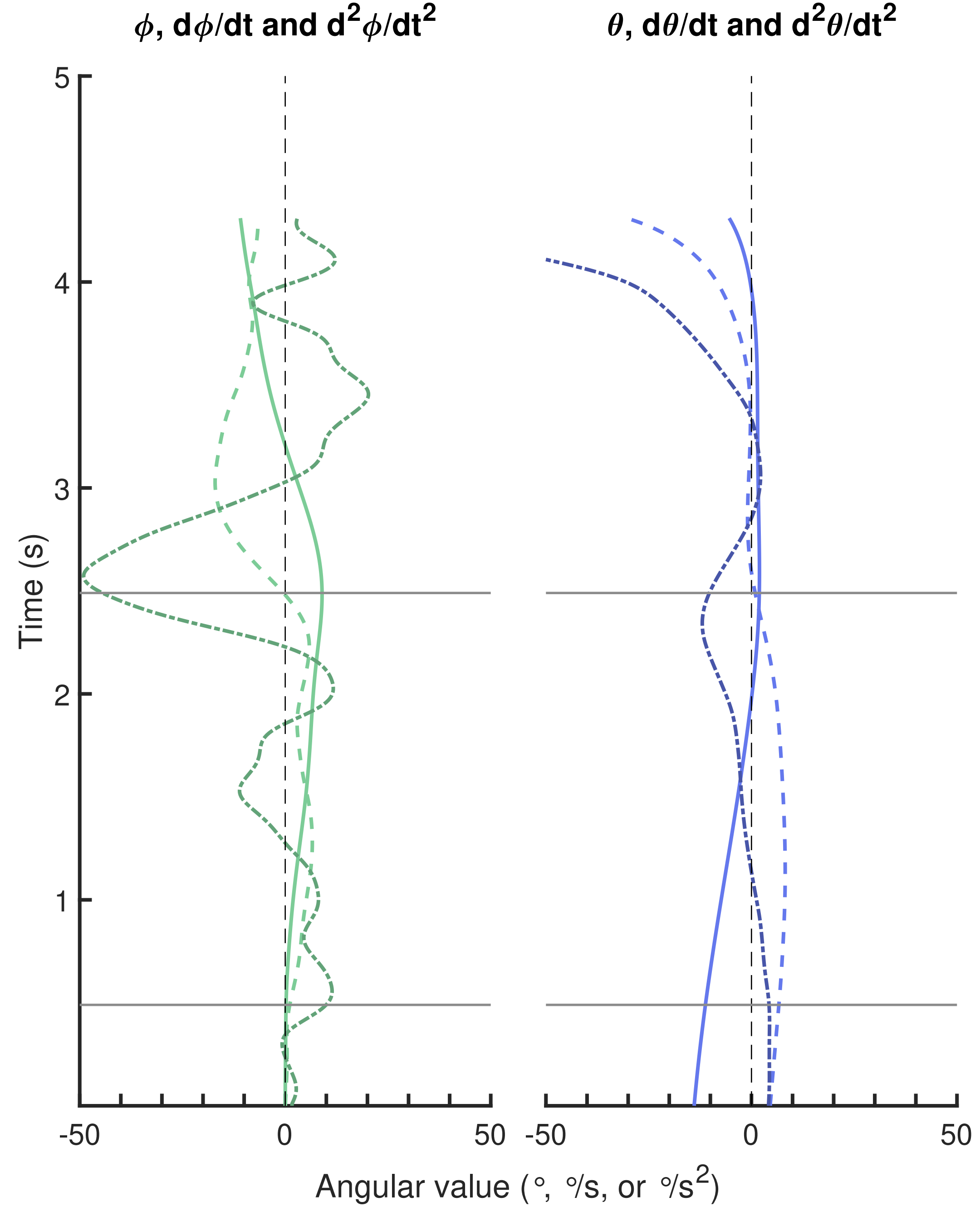

P6/B2
S15/R30-IN

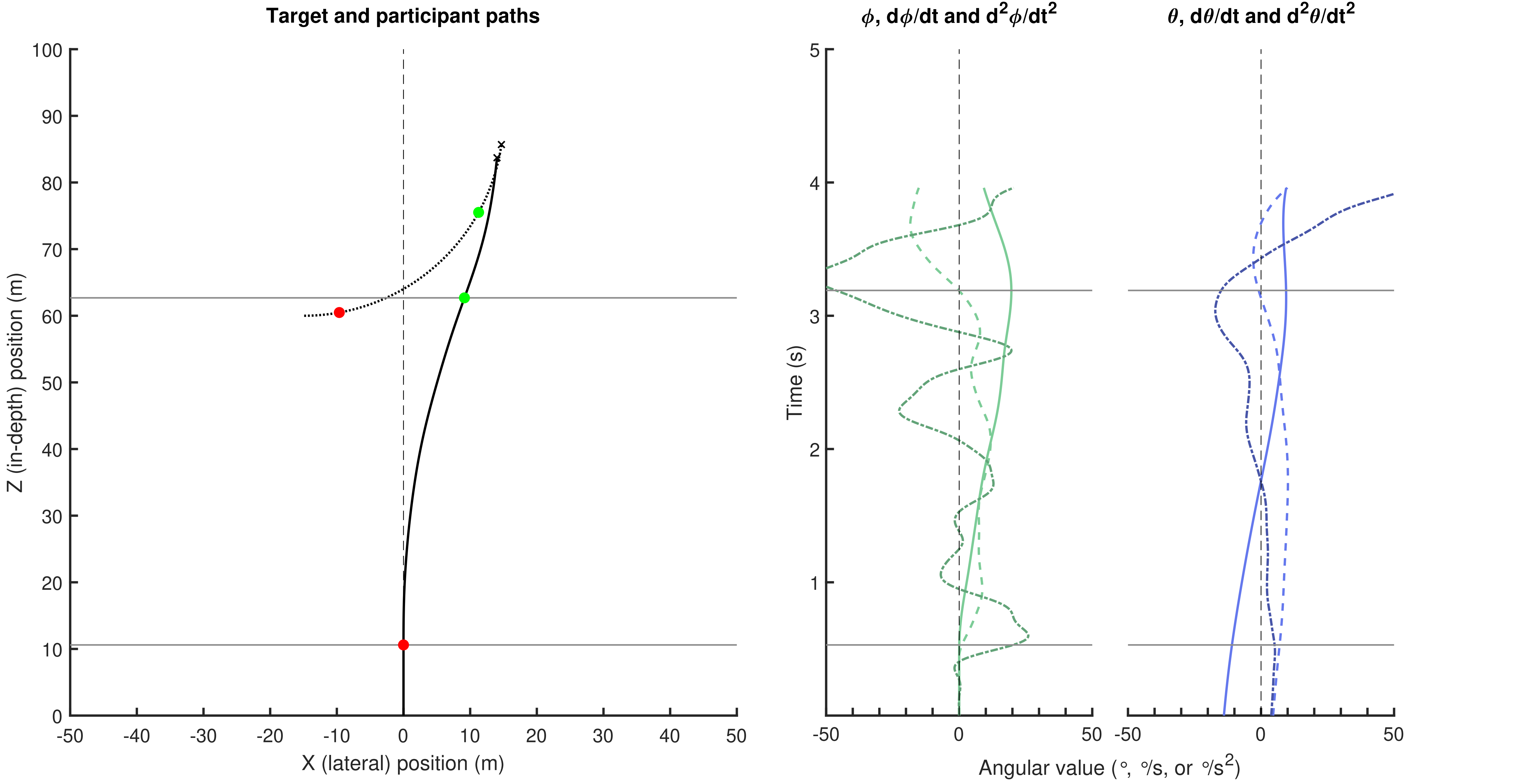

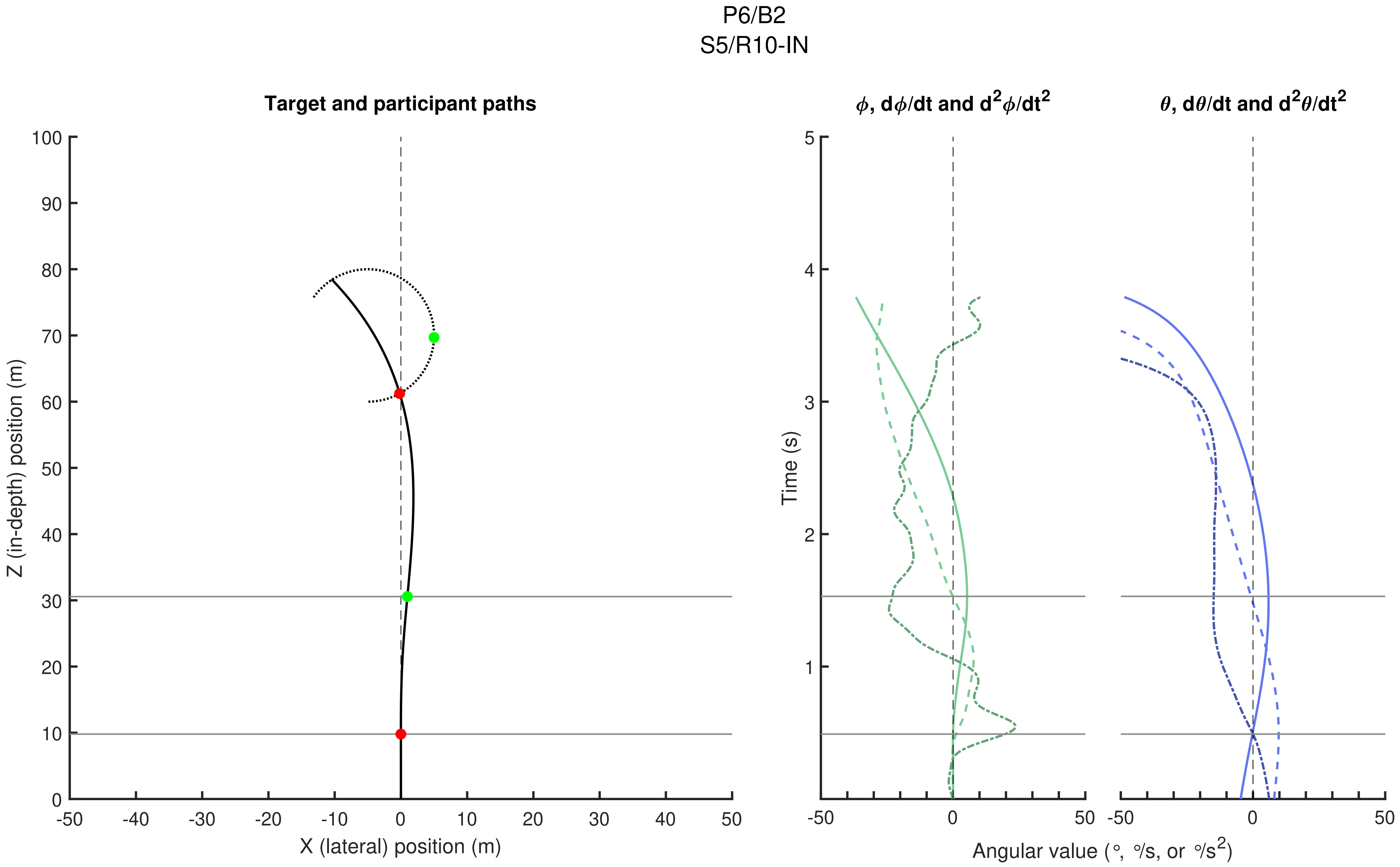
P6/B2
S5/R10-IN
Target and participant paths
Z (in-depth) position (m)
X (lateral) position (m)
0
10
20
30
40
50
60
70
80
90
100
-50
-40
-30
-20
-10
0
10
20
30
40
50
φ, dφ/dt and d²φ/dt²
θ, dθ/dt and d²θ/dt²
Time (s)
1
2
3
4
5
-50
0
50
-50
0
50
Angular value (°, °/s, or °/s²)

P6/B2
S5/R10-OUT

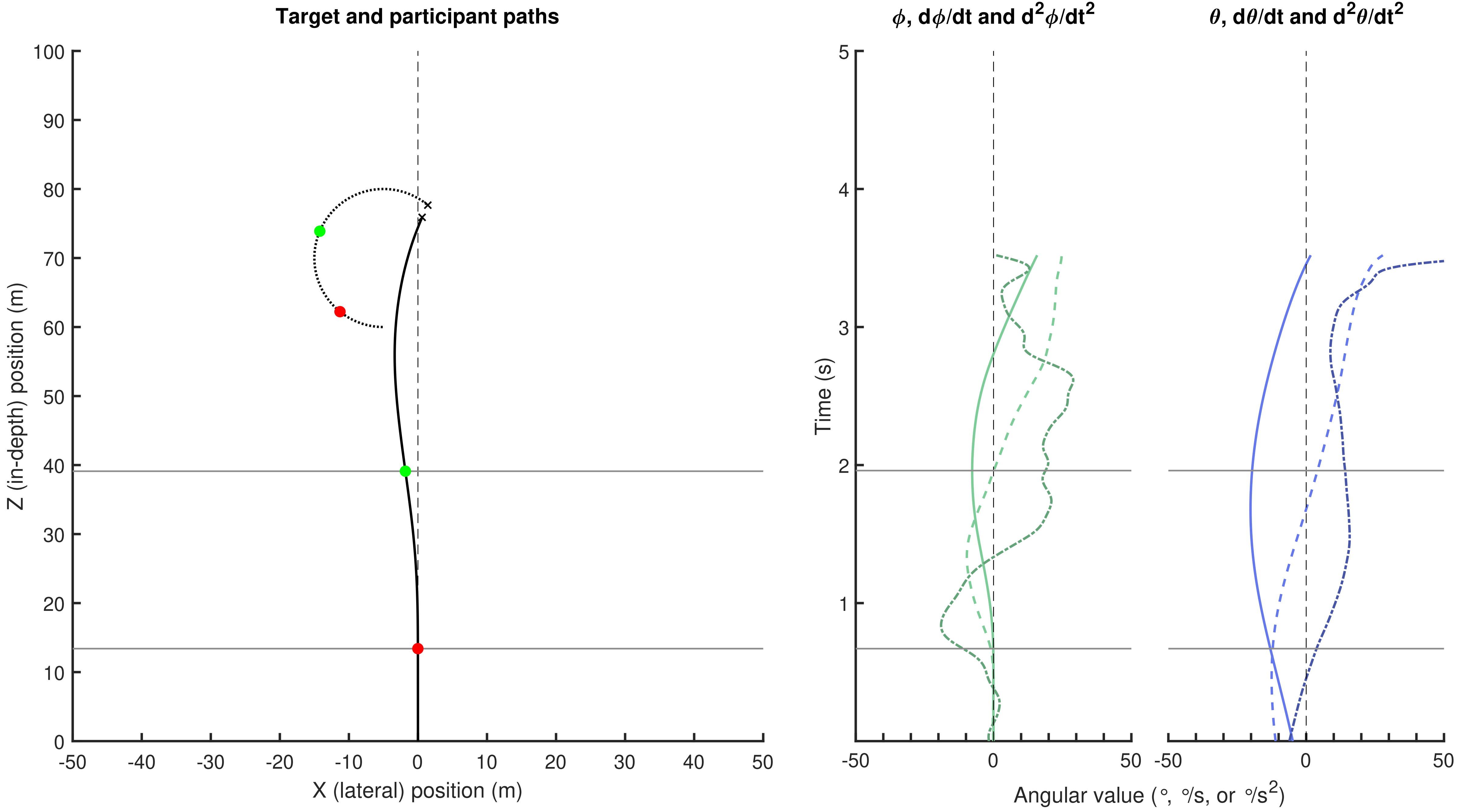

P6/B2
S5/R20-IN

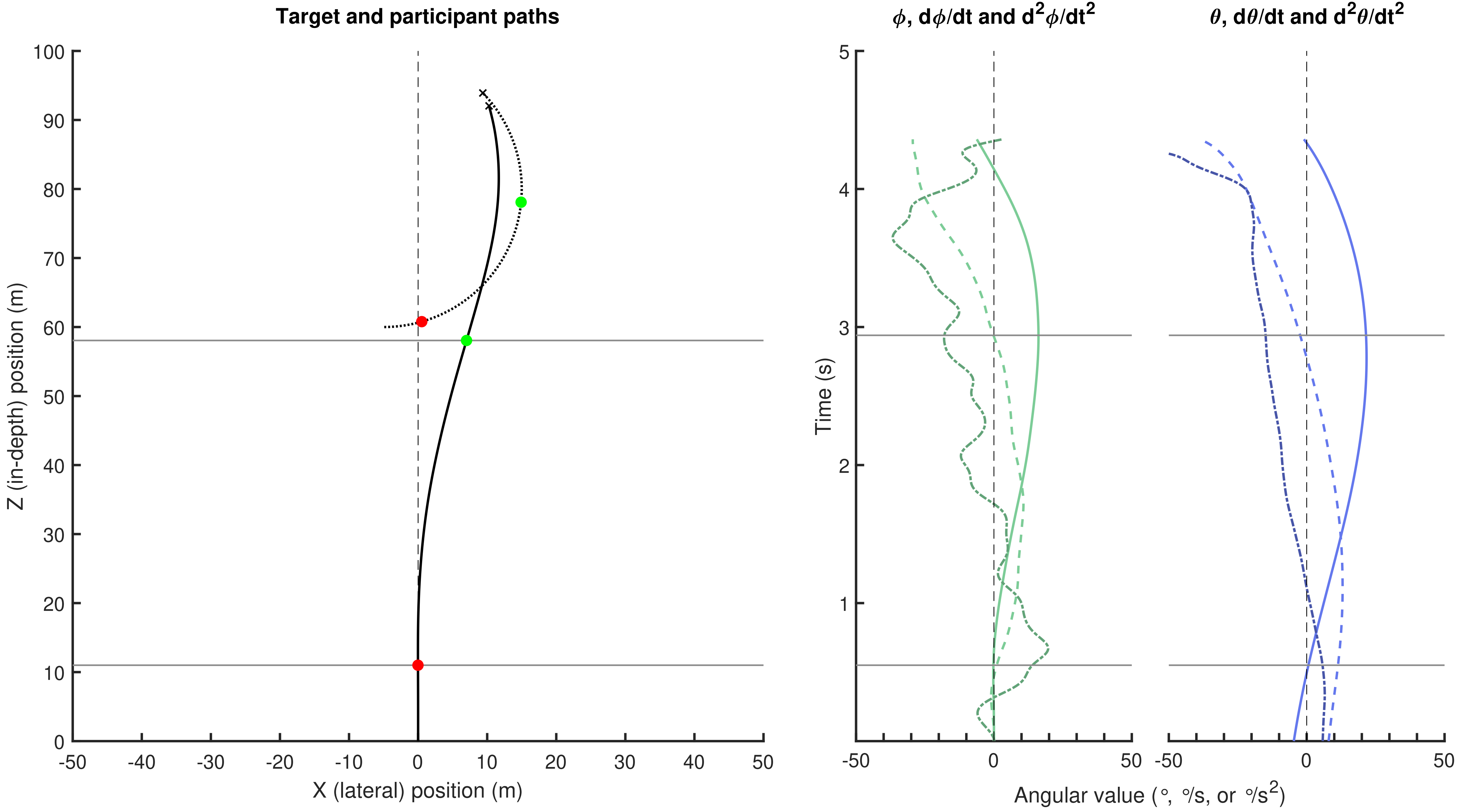

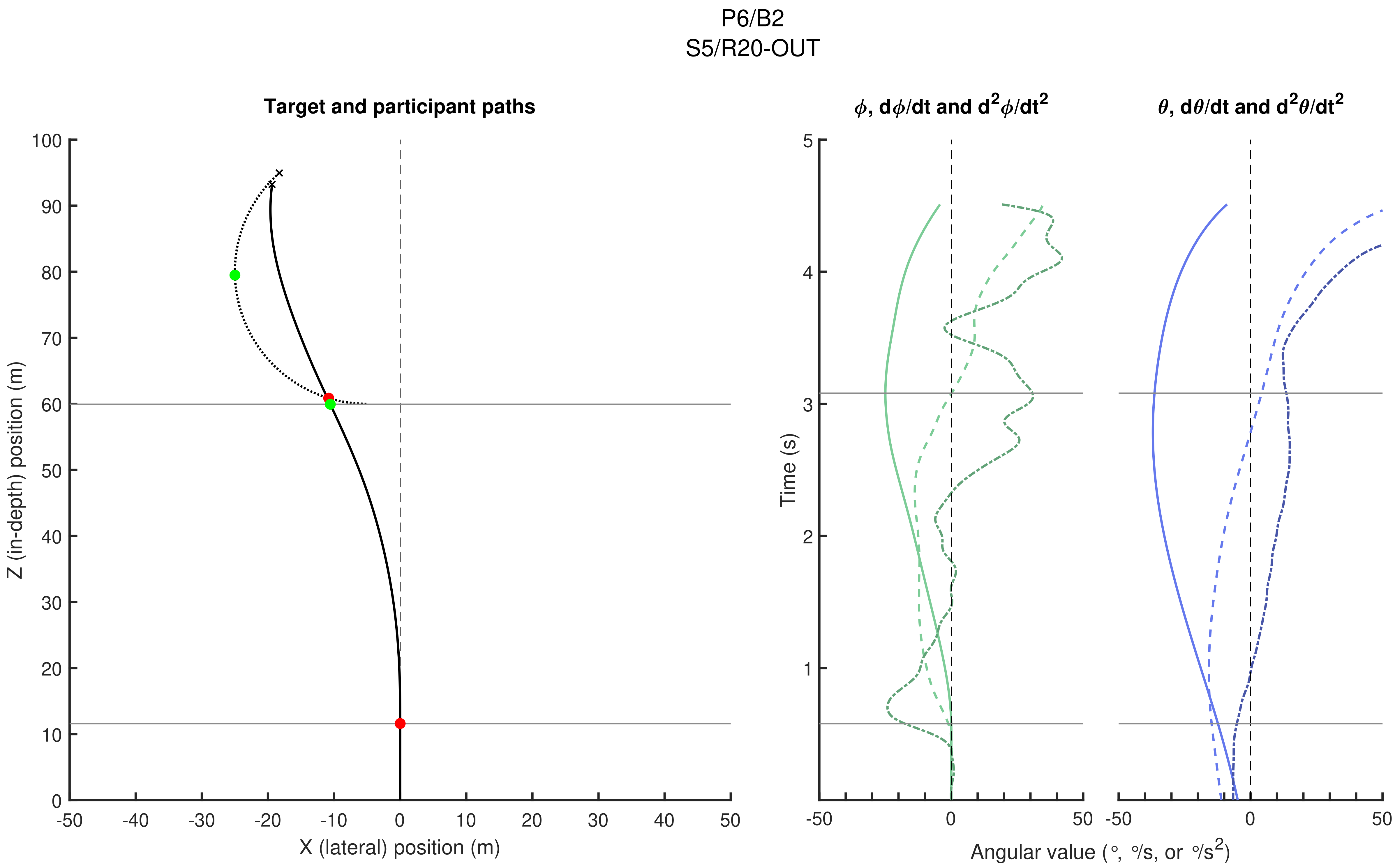

P6/B2
S5/R20-OUT
Target and participant paths
Z (in-depth) position (m)
X (lateral) position (m)
100
90
80
70
60
50
40
30
20
10
0
-50
-40
-30
-20
-10
0
10
20
30
40
50
ϕ, dϕ/dt and d²ϕ/dt²
θ, dθ/dt and d²θ/dt²
Time (s)
5
4
3
2
1
-50
0
50
-50
0
50
Angular value (°, °/s, or °/s²)

P6/B2
S5/R30-IN

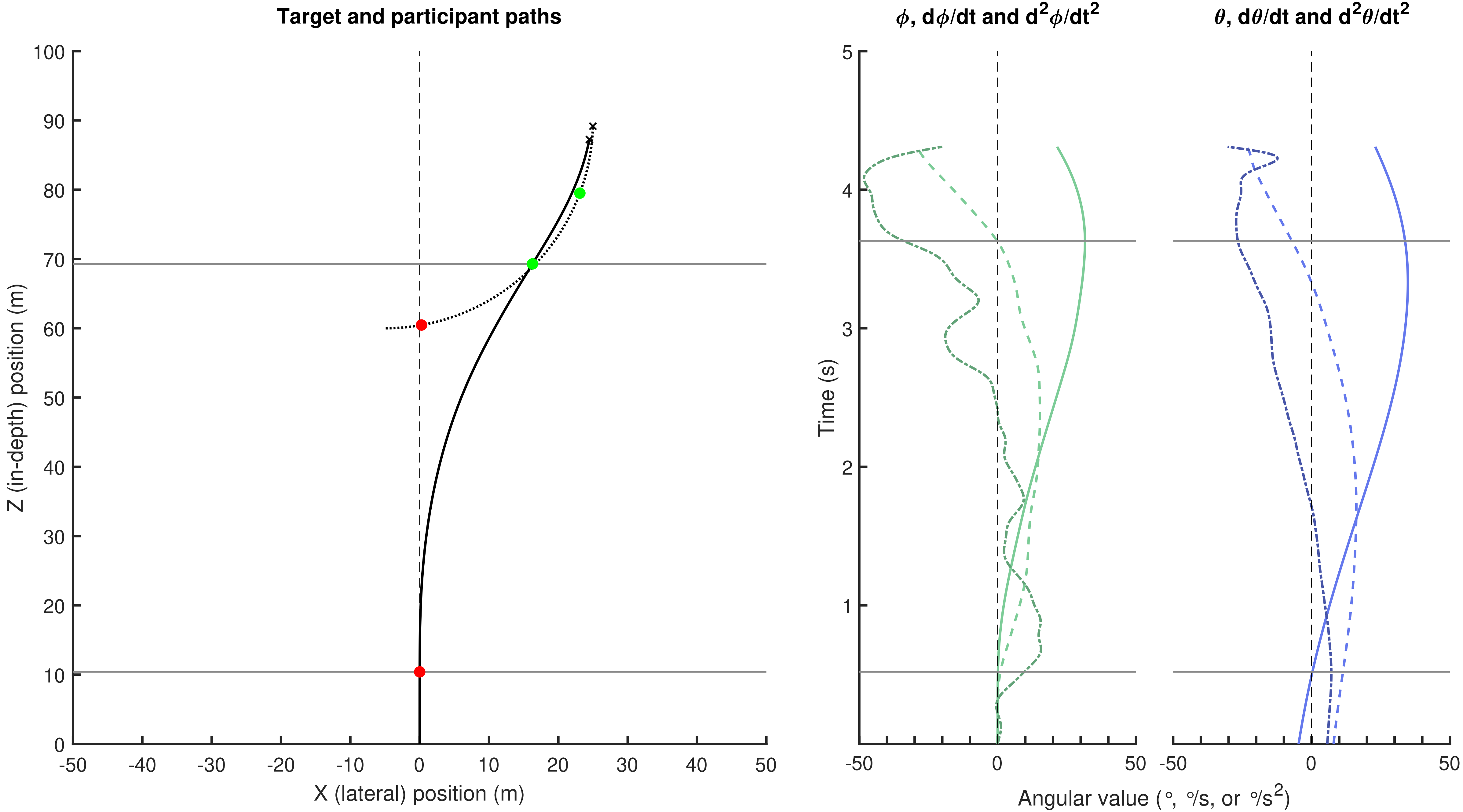

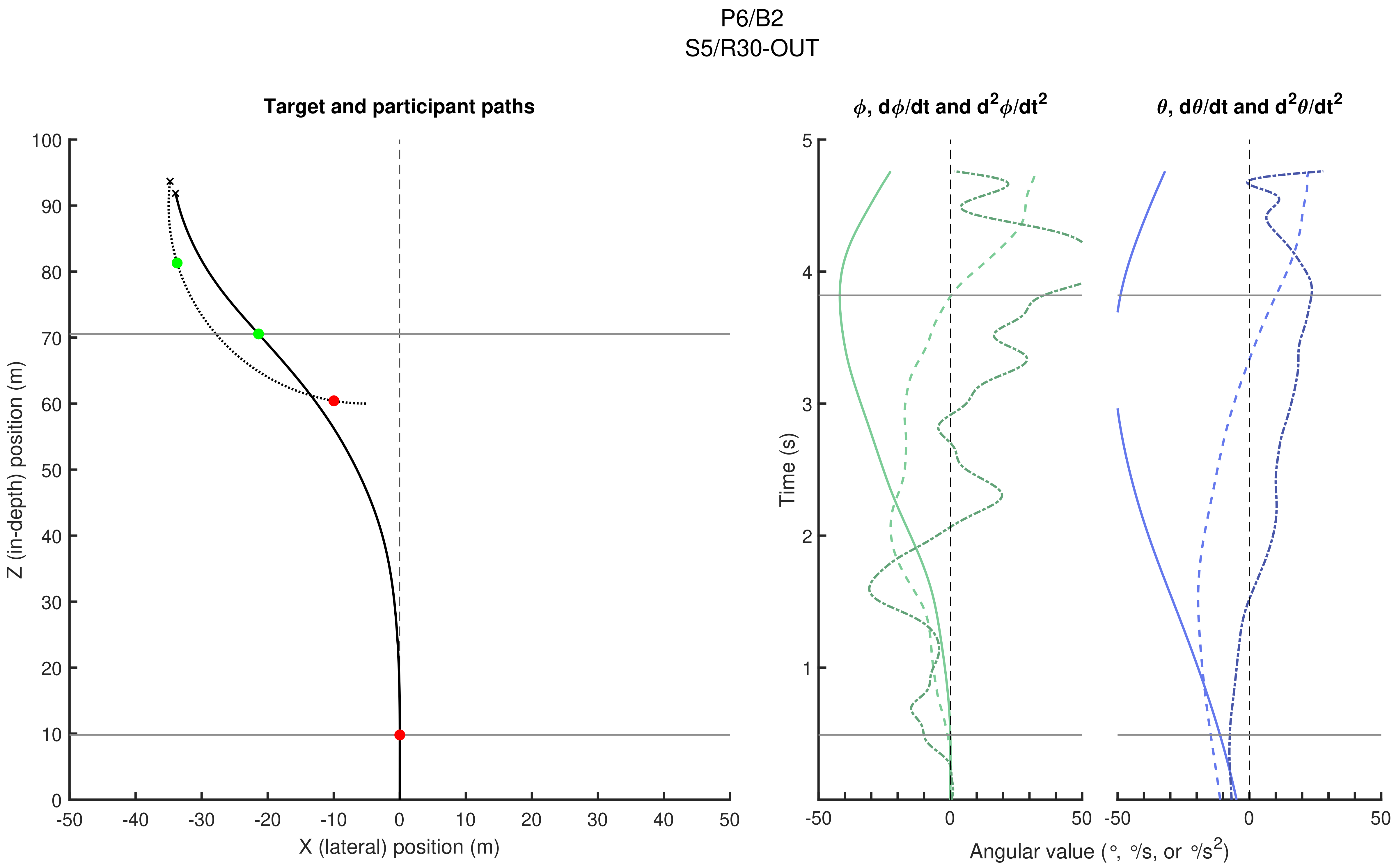

P6/B2
S5/R30-OUT
Target and participant paths
Z (in-depth) position (m)
X (lateral) position (m)
ϕ, dϕ/dt and d$^2\phi$/dt^2
θ, dθ/dt and d$^2\theta$/dt^2
Time (s)
Angular value (°, °/s, or °/s^2)

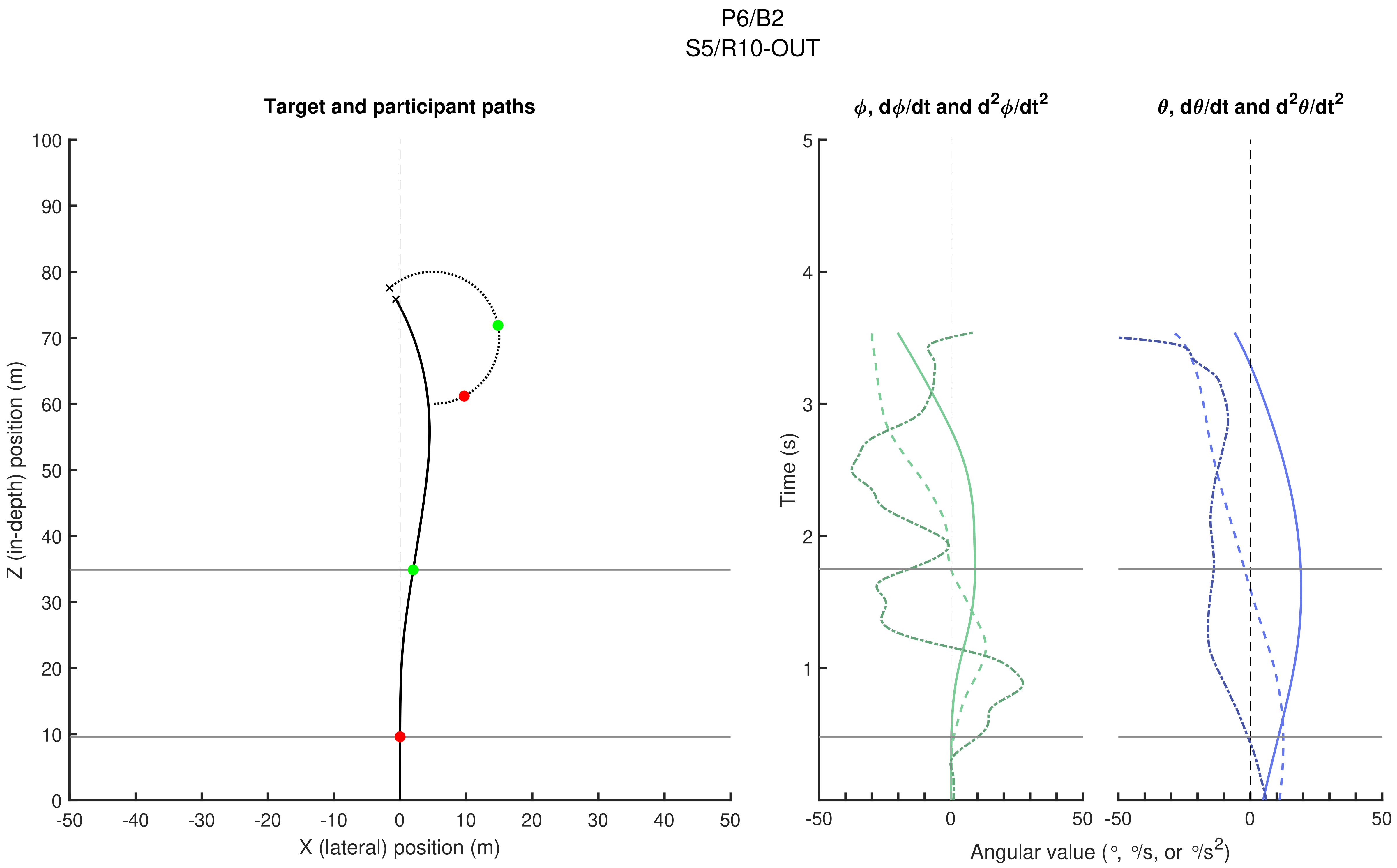
P6/B2
S5/R10-OUT
Target and participant paths
Z (in-depth) position (m)
X (lateral) position (m)
100
90
80
70
60
50
40
30
20
10
0
-50
-40
-30
-20
-10
0
10
20
30
40
50
φ, dφ/dt and d²φ/dt²
θ, dθ/dt and d²θ/dt²
Time (s)
5
4
3
2
1
-50
0
50
-50
0
50
Angular value (°, °/s, or °/s²)

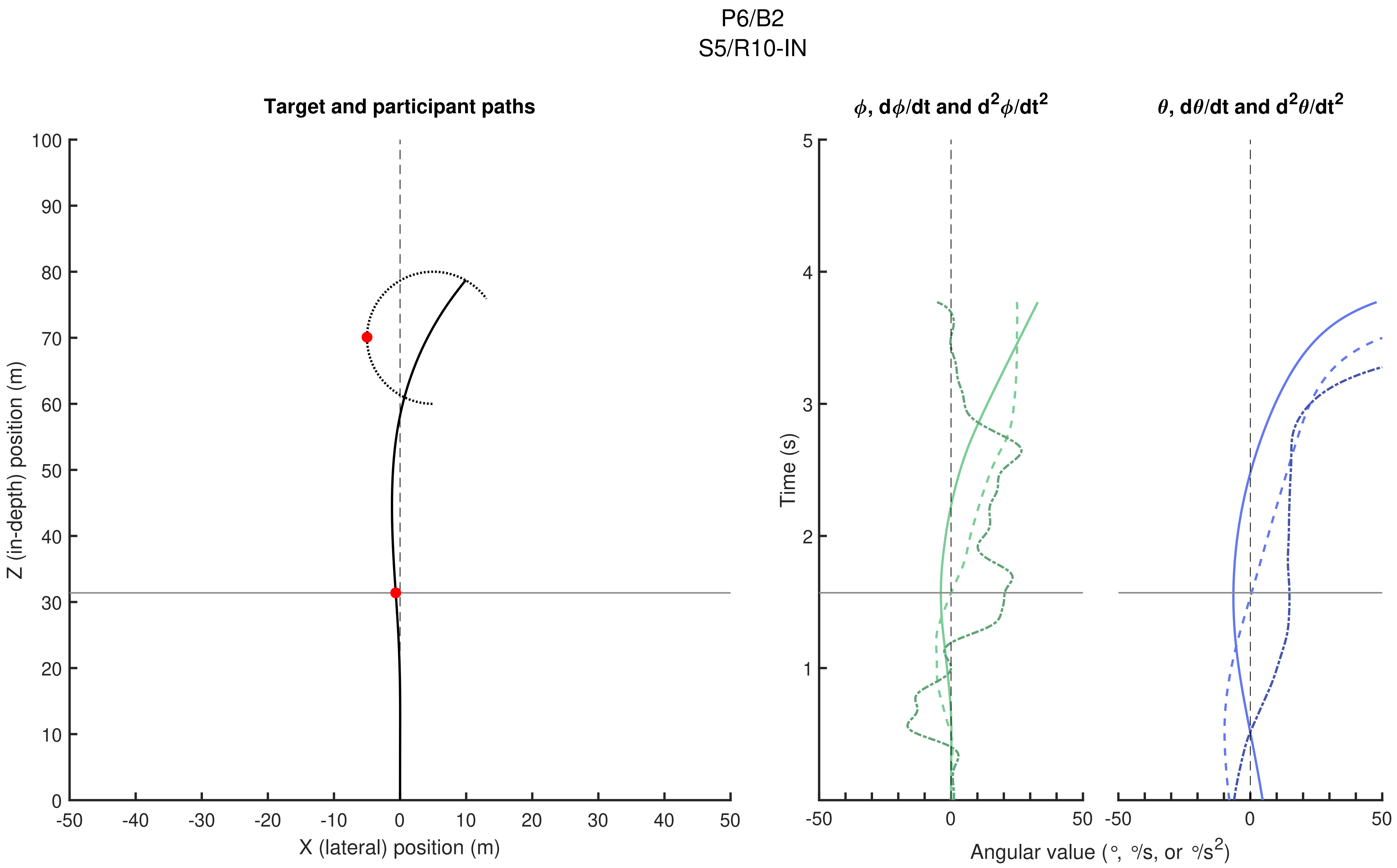

P6/B2
S5/R10-IN
Target and participant paths
ϕ, dϕ/dt and d$^2\phi$/dt^2
θ, dθ/dt and d$^2\theta$/dt^2
Z (in-depth) position (m)
X (lateral) position (m)
Time (s)
Angular value (°, °/s, or °/s^2)
100
90
80
70
60
50
40
30
20
10
0
-50
-40
-30
-20
-10
0
10
20
30
40
50
5
4
3
2
1

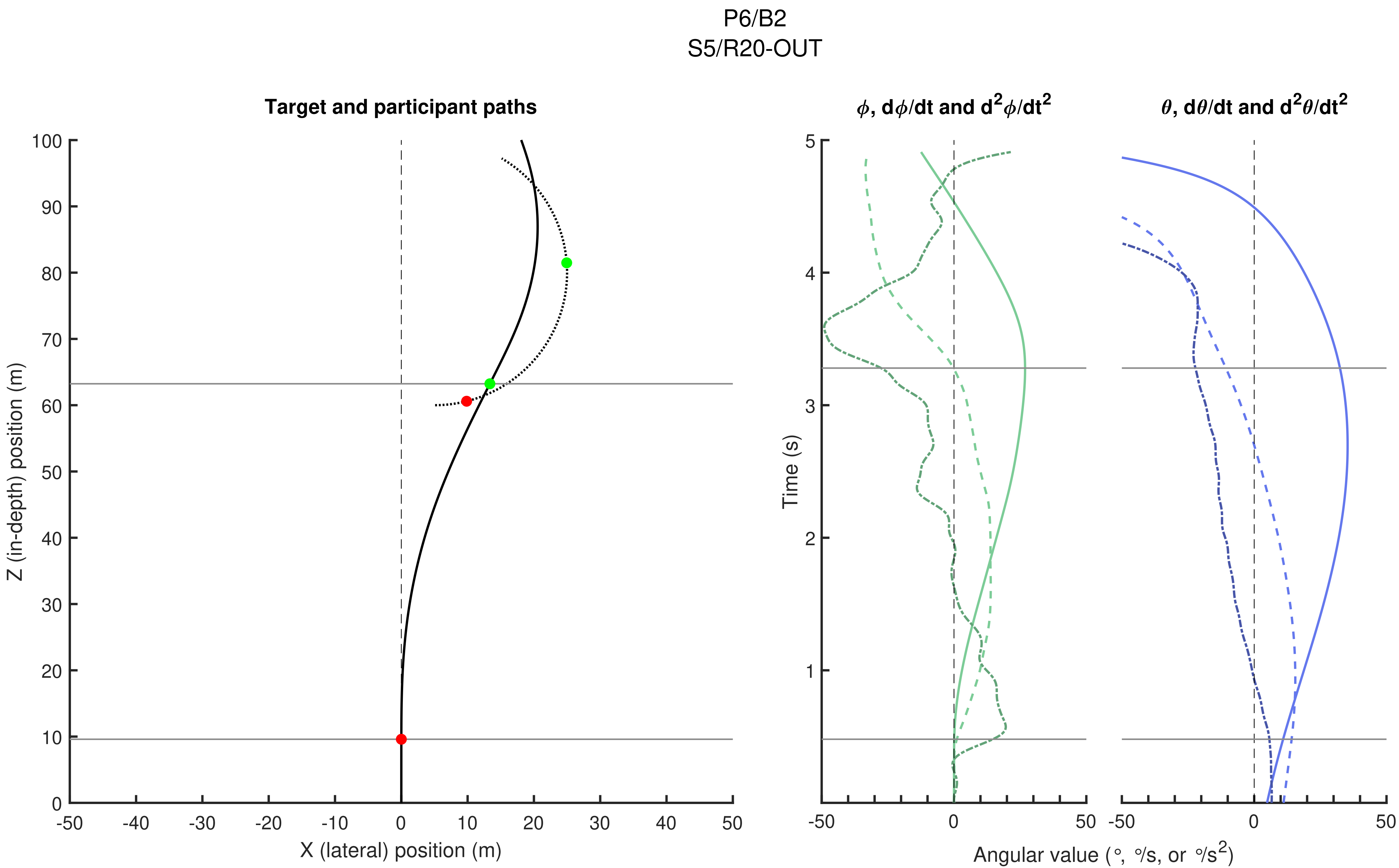
P6/B2
S5/R20-OUT
Target and participant paths
Z (in-depth) position (m)
X (lateral) position (m)
100
90
80
70
60
50
40
30
20
10
0
-50
-40
-30
-20
-10
0
10
20
30
40
50
ϕ, dϕ/dt and d²ϕ/dt²
θ, dθ/dt and d²θ/dt²
Time (s)
5
4
3
2
1
-50
0
50
-50
0
50
Angular value (°, °/s, or °/s²)

P6/B2
S5/R20-IN

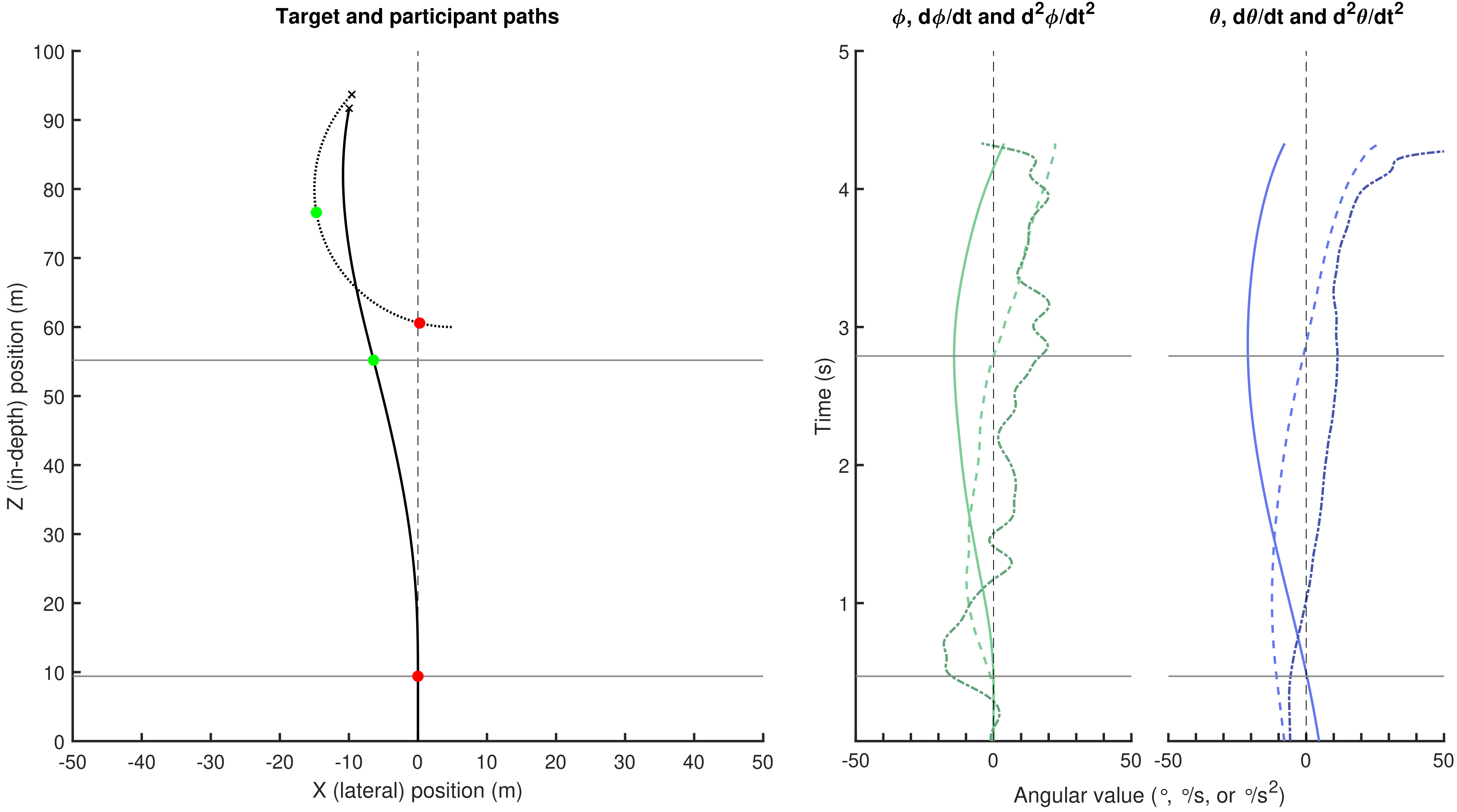

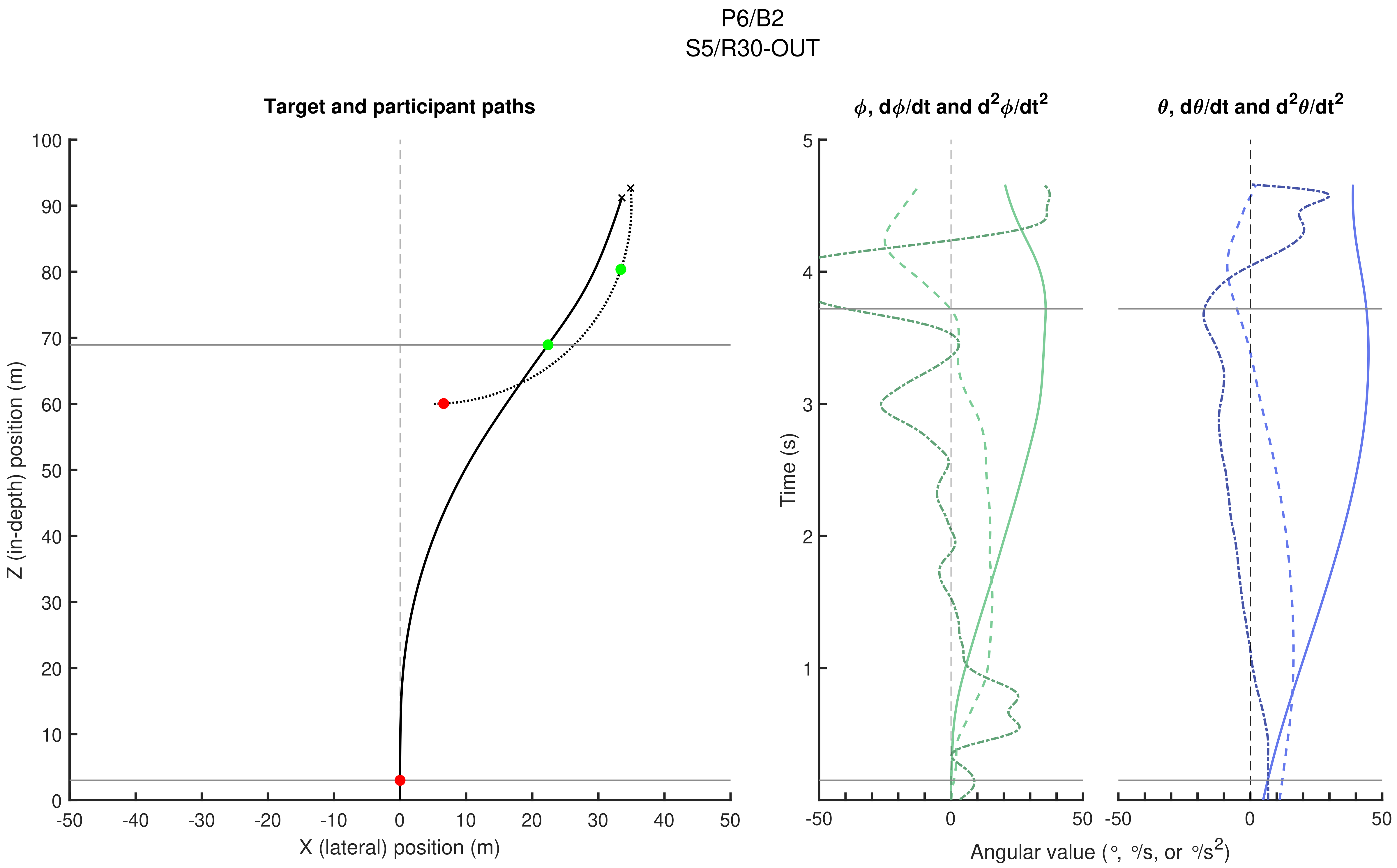
P6/B2
S5/R30-OUT
Target and participant paths
Z (in-depth) position (m)
X (lateral) position (m)
φ, dφ/dt and d²φ/dt²
θ, dθ/dt and d²θ/dt²
Time (s)
Angular value (°, °/s, or °/s²)

P6/B2
S5/R30-IN

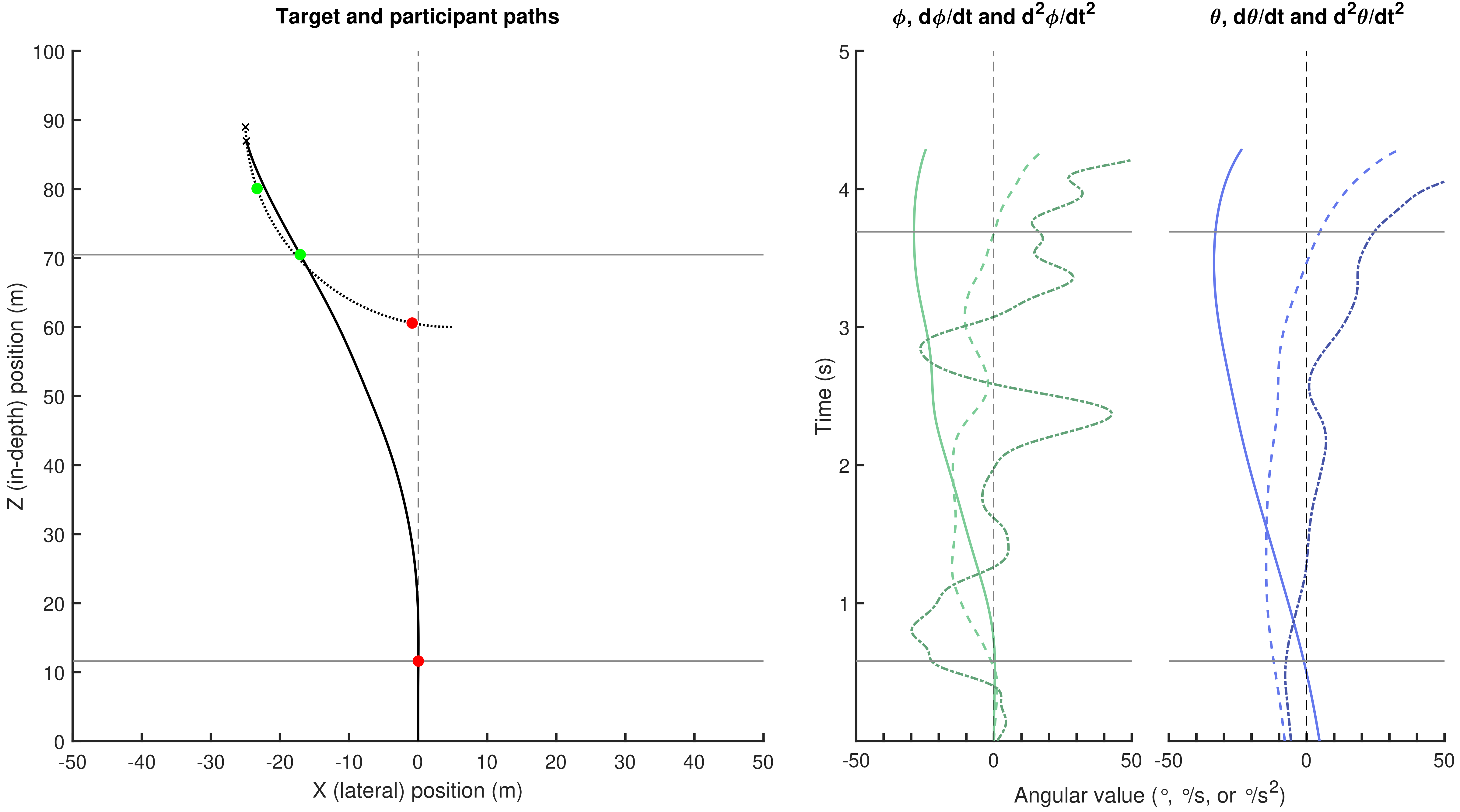

P6/B2
S15/R10-IN

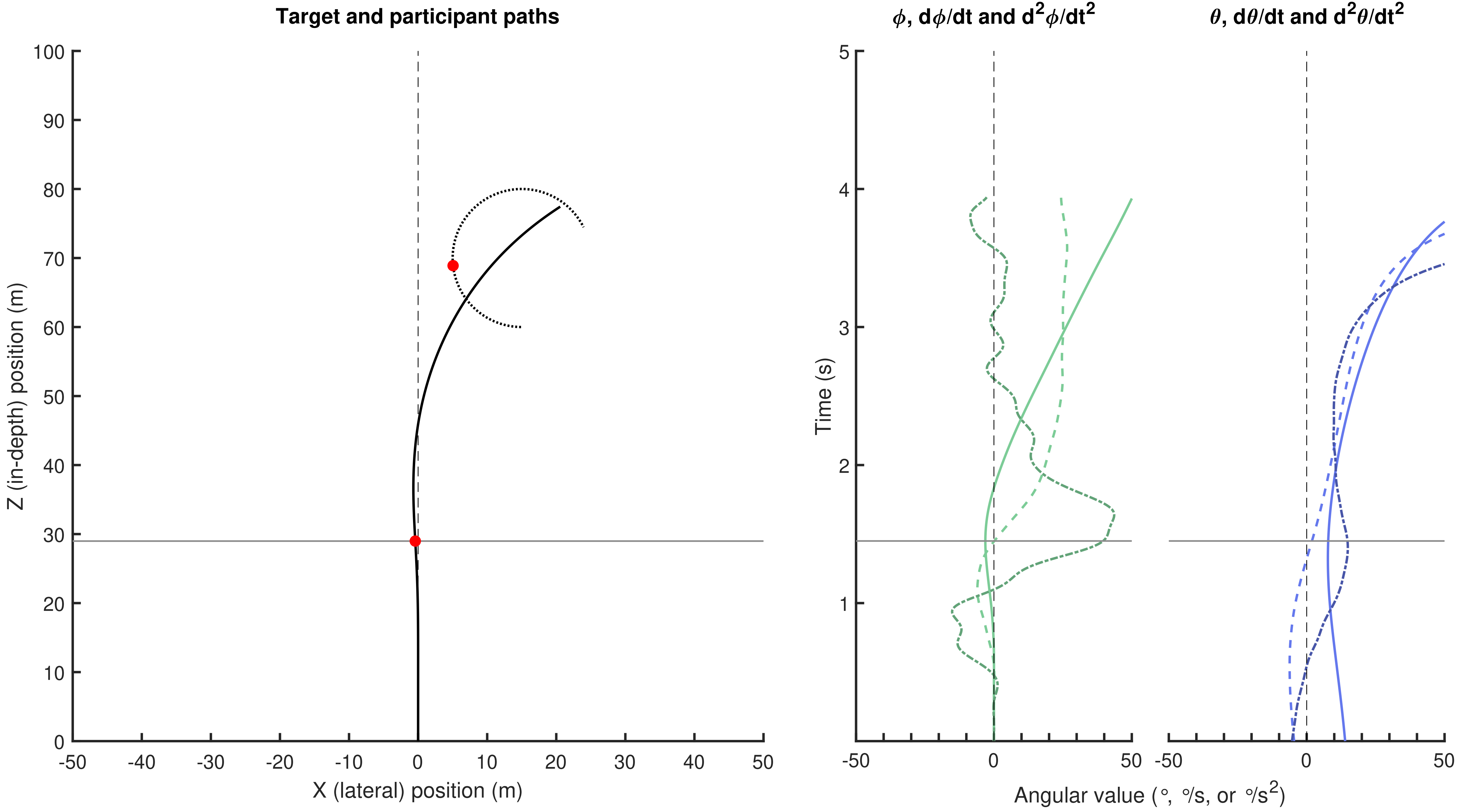

P6/B2
S15/R20-IN

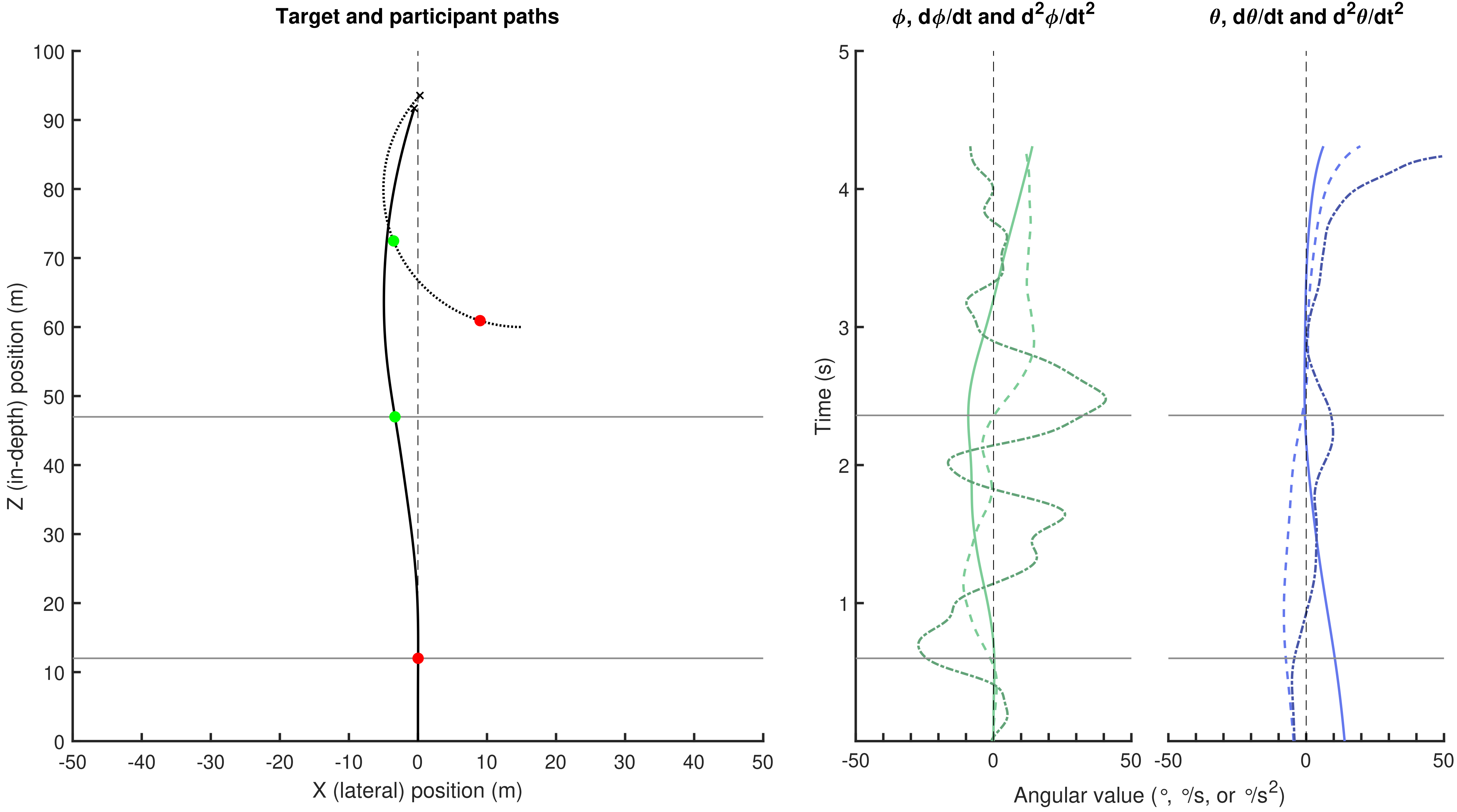

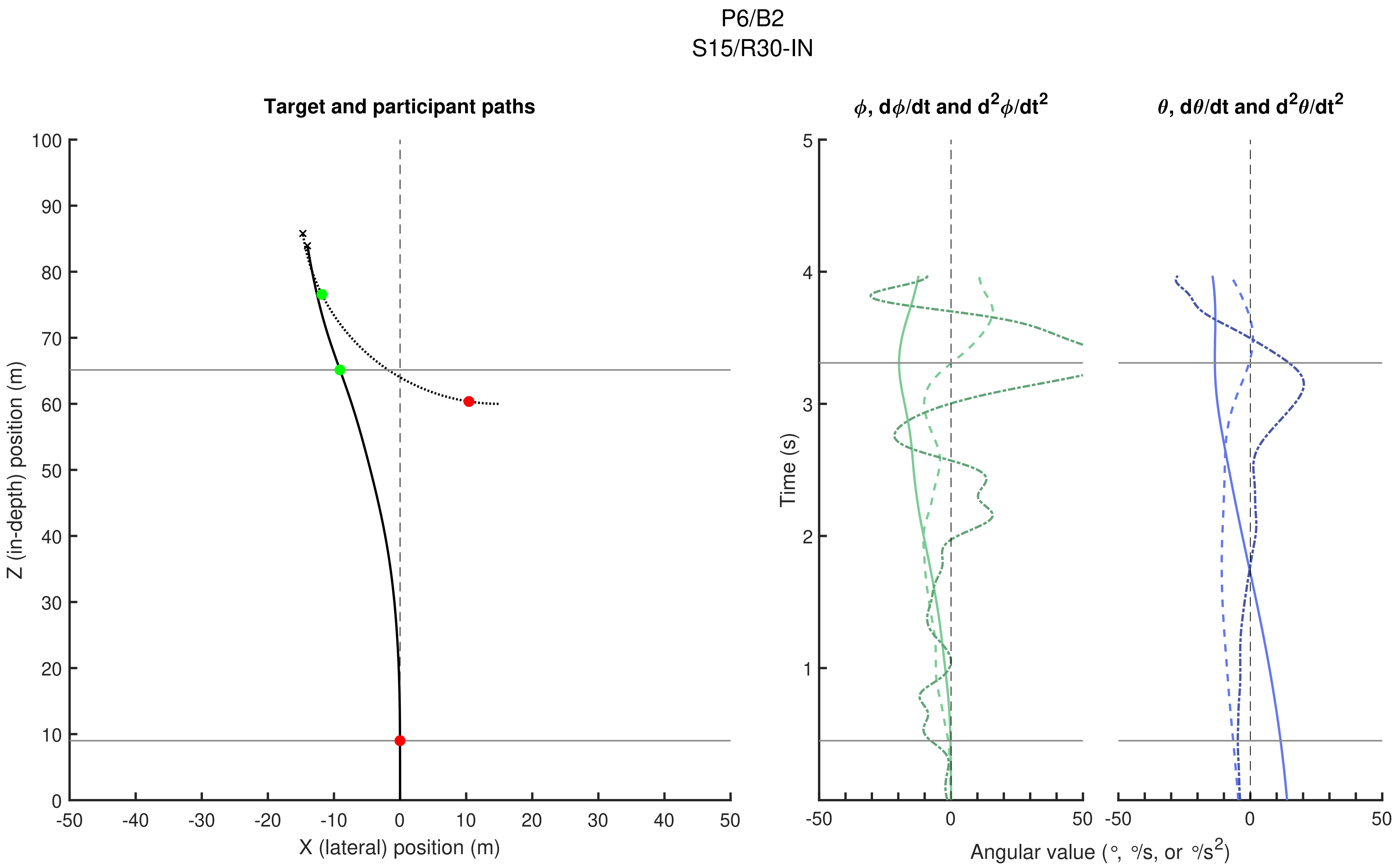

P6/B2
S15/R30-IN
Target and participant paths
ϕ, dϕ/dt and d$^2\phi$/dt^2
θ, dθ/dt and d$^2\theta$/dt^2
Z (in-depth) position (m)
X (lateral) position (m)
Time (s)
Angular value (°, °/s, or °/s^2)

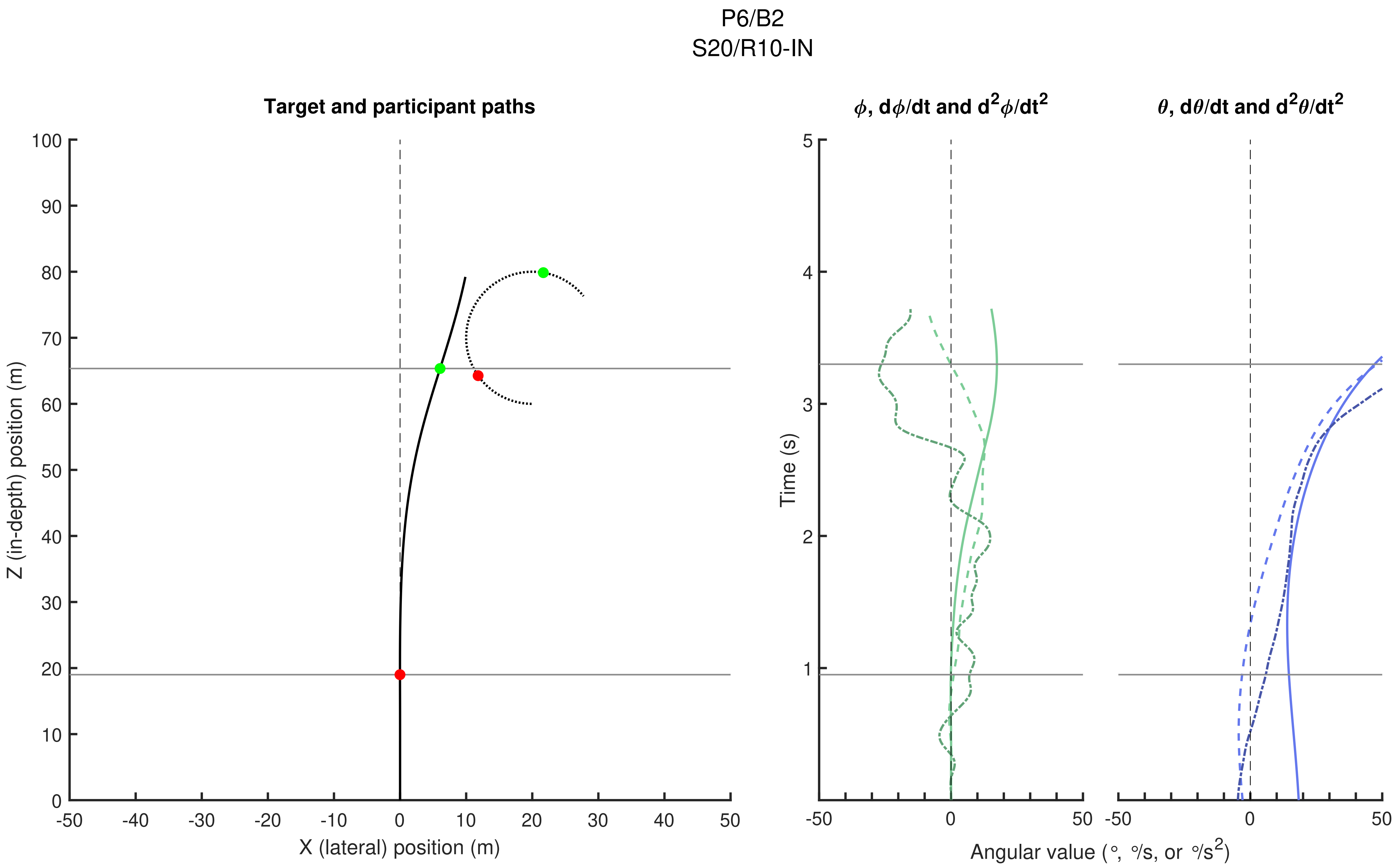

P6/B2
S20/R10-IN
Target and participant paths
Z (in-depth) position (m)
X (lateral) position (m)
ϕ, dϕ/dt and d$^2\phi$/dt^2
θ, dθ/dt and d$^2\theta$/dt^2
Time (s)
Angular value (°, °/s, or °/s^2)

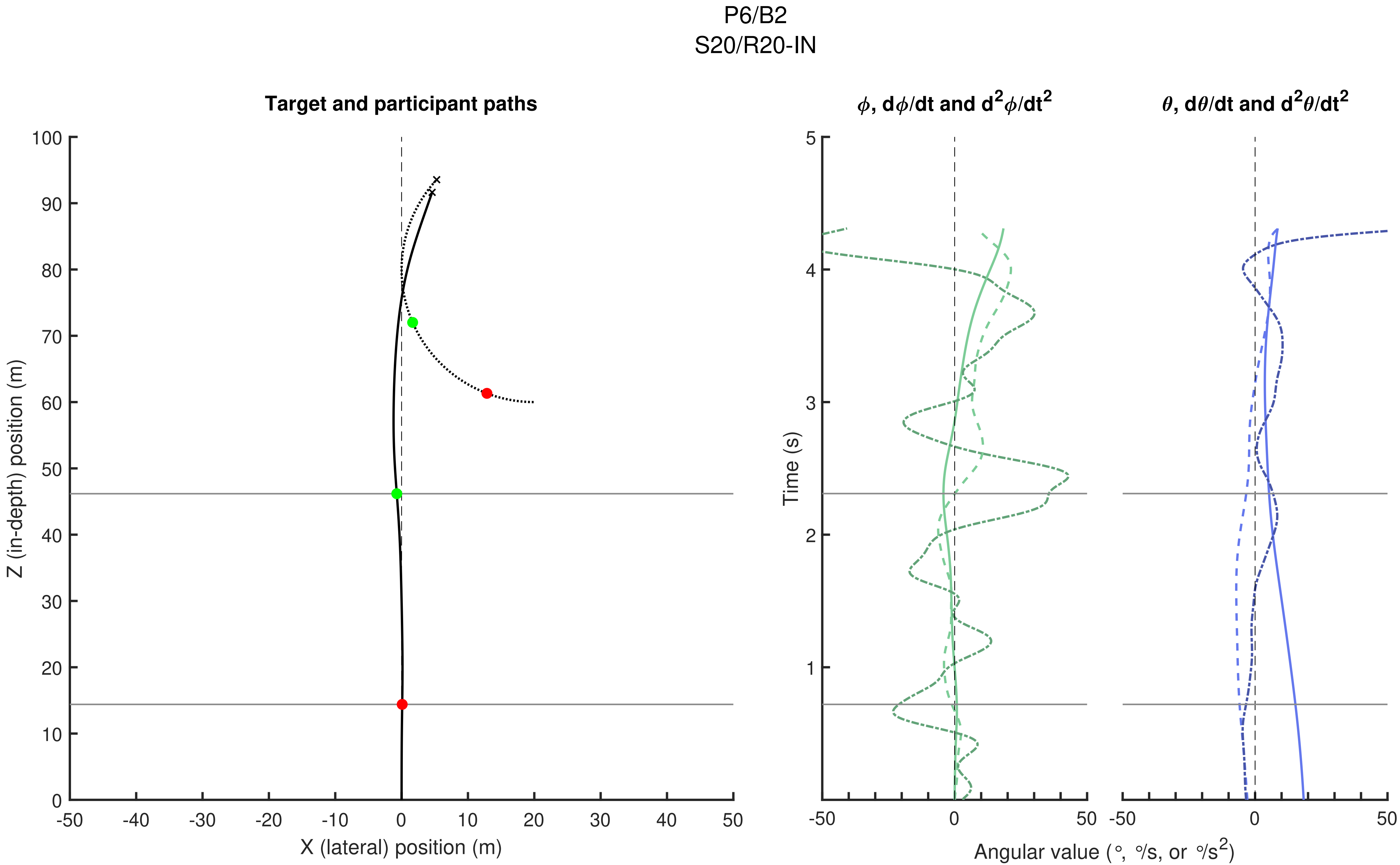
P6/B2
S20/R20-IN
Target and participant paths
ϕ, dϕ/dt and d$^2\phi$/dt^2
θ, dθ/dt and d$^2\theta$/dt^2
Z (in-depth) position (m)
X (lateral) position (m)
Time (s)
Angular value (°, °/s, or °/s^2)

P6/B2
S20/R30-IN

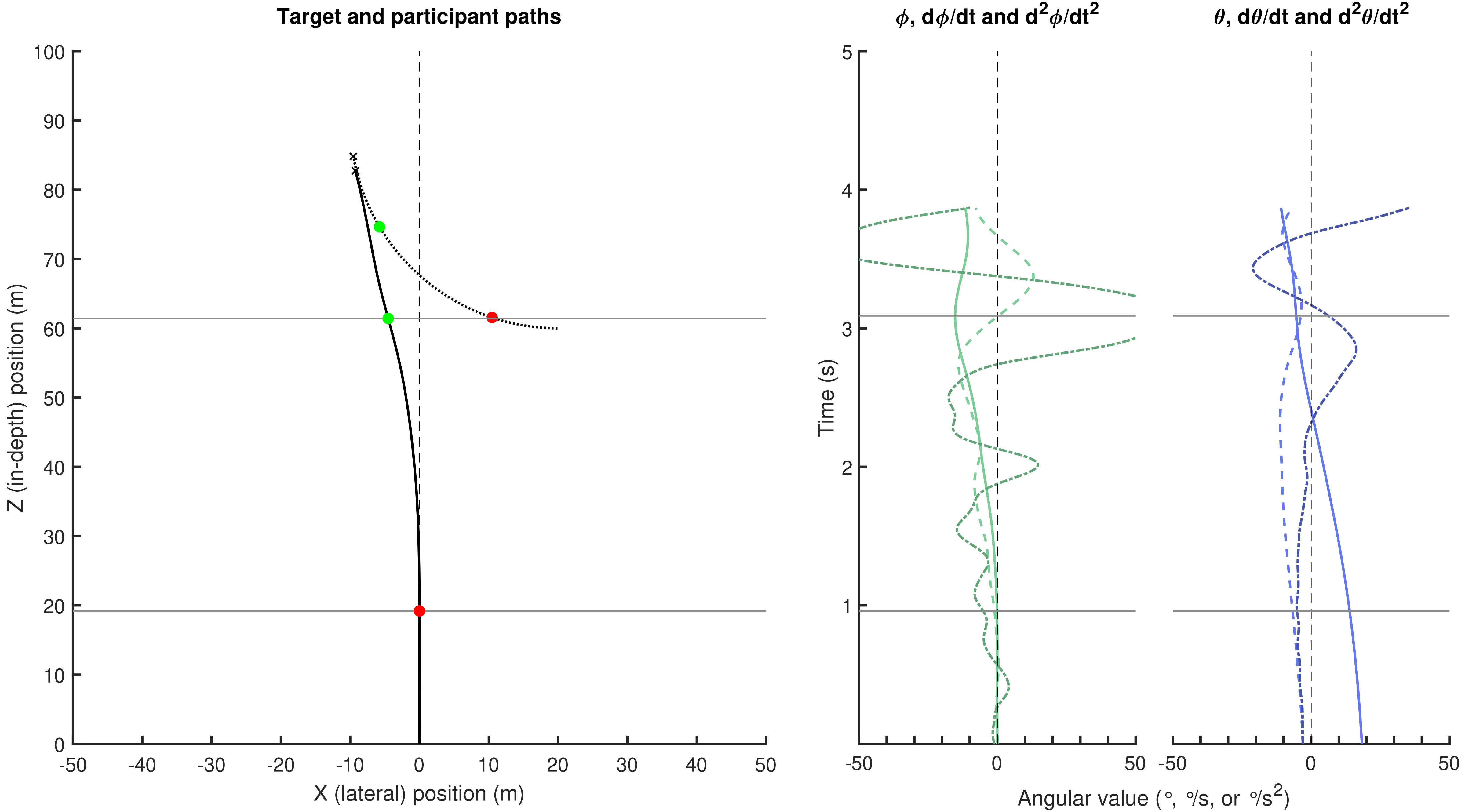

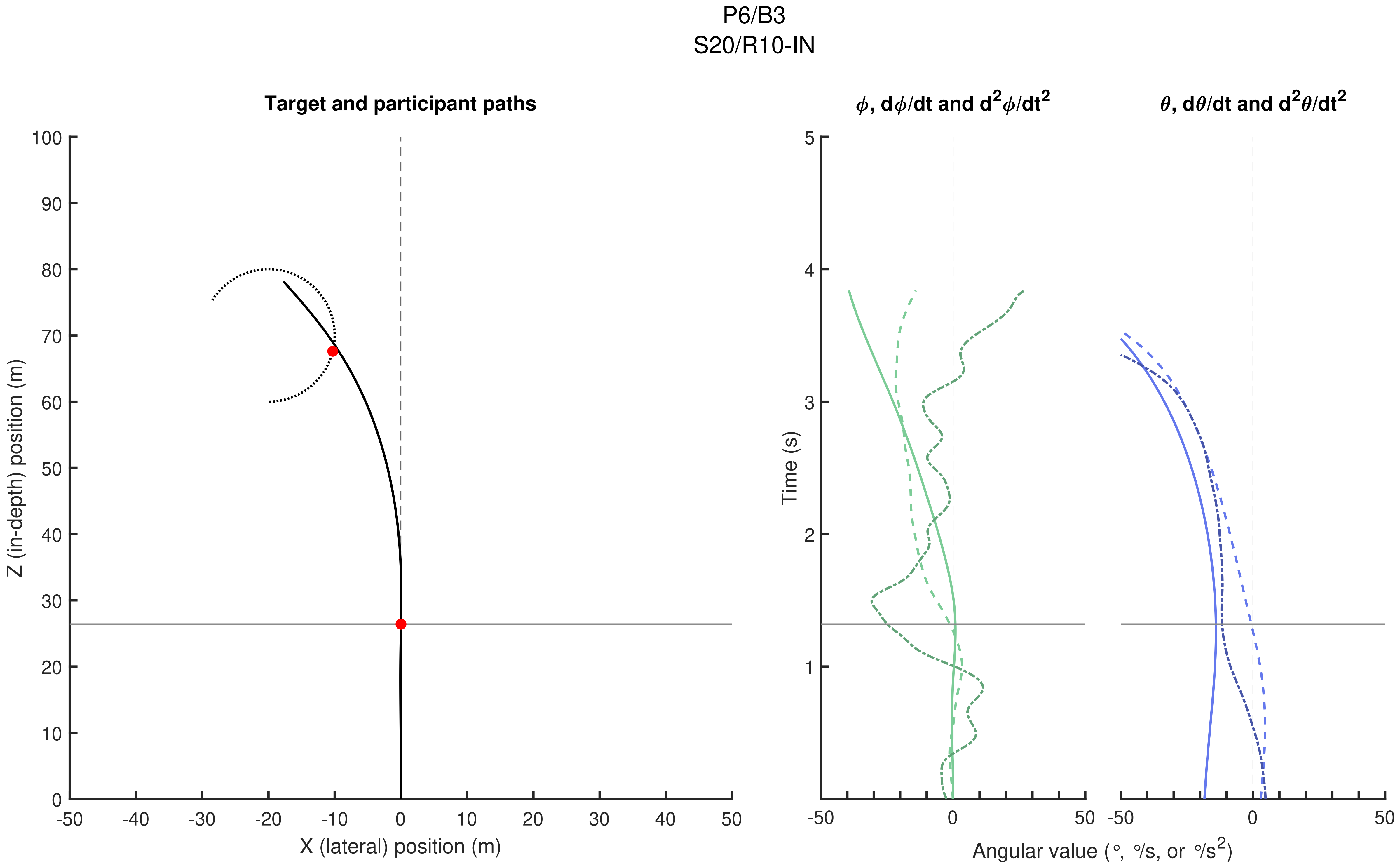

P6/B3
S20/R10-IN
Target and participant paths
Z (in-depth) position (m)
X (lateral) position (m)
ϕ, dϕ/dt and d$^2\phi$/dt^2
θ, dθ/dt and d$^2\theta$/dt^2
Time (s)
Angular value (°, °/s, or °/s^2)

P6/B3
S20/R20-IN

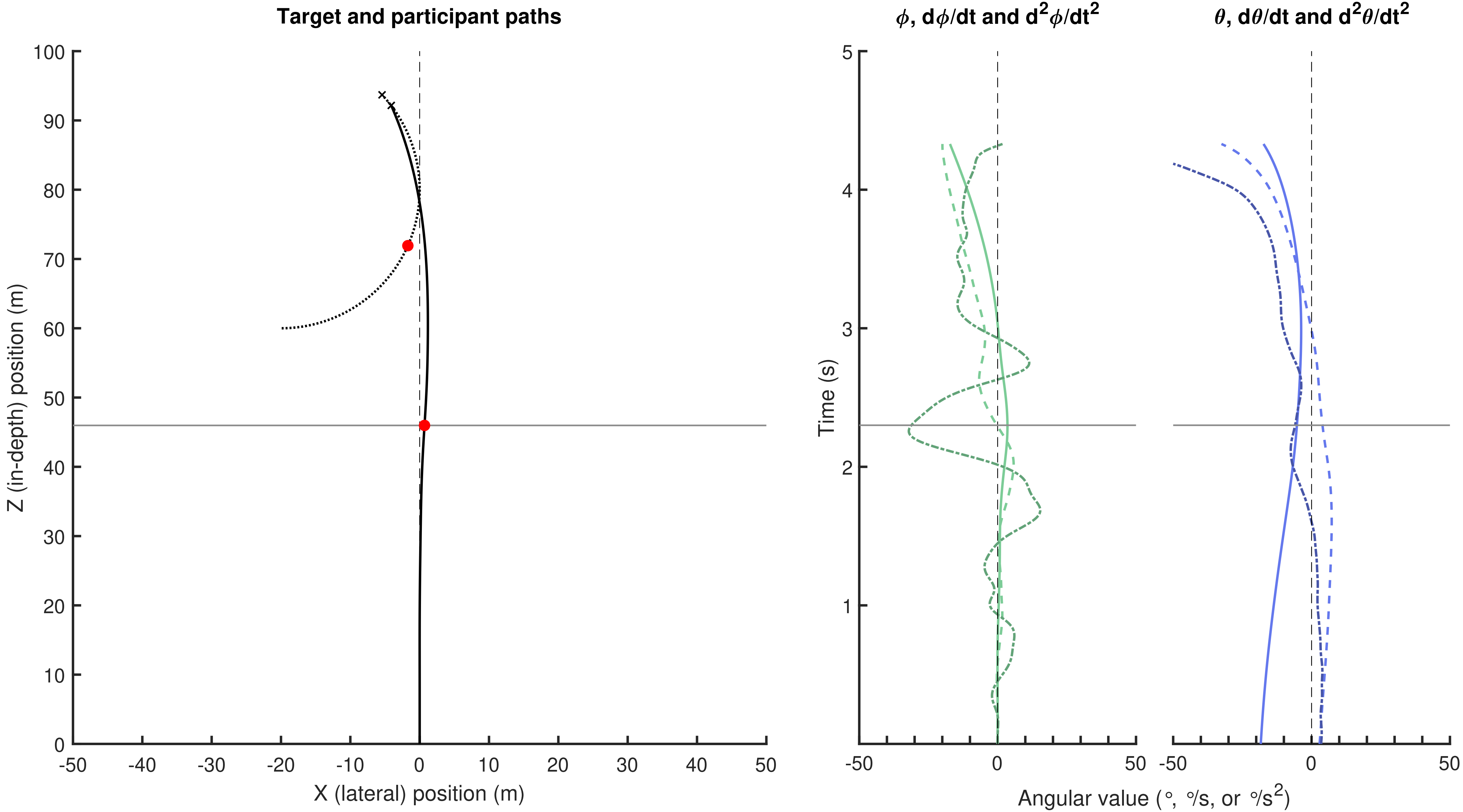

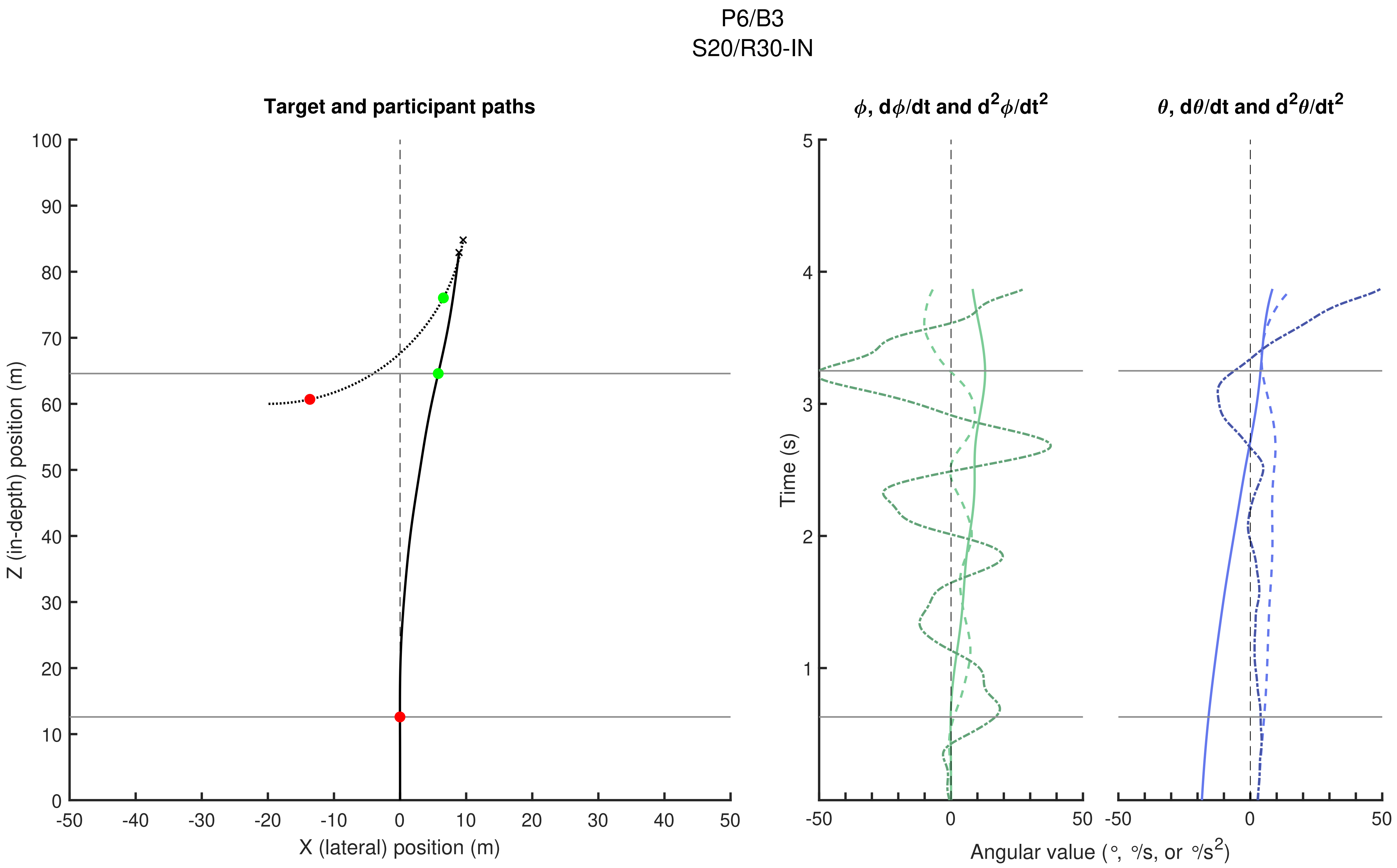
P6/B3
S20/R30-IN
Target and participant paths
Z (in-depth) position (m)
X (lateral) position (m)
ϕ, dϕ/dt and d$^2\phi$/dt^2
θ, dθ/dt and d$^2\theta$/dt^2
Time (s)
Angular value (°, °/s, or °/s^2)

P6/B3
S15/R10-IN

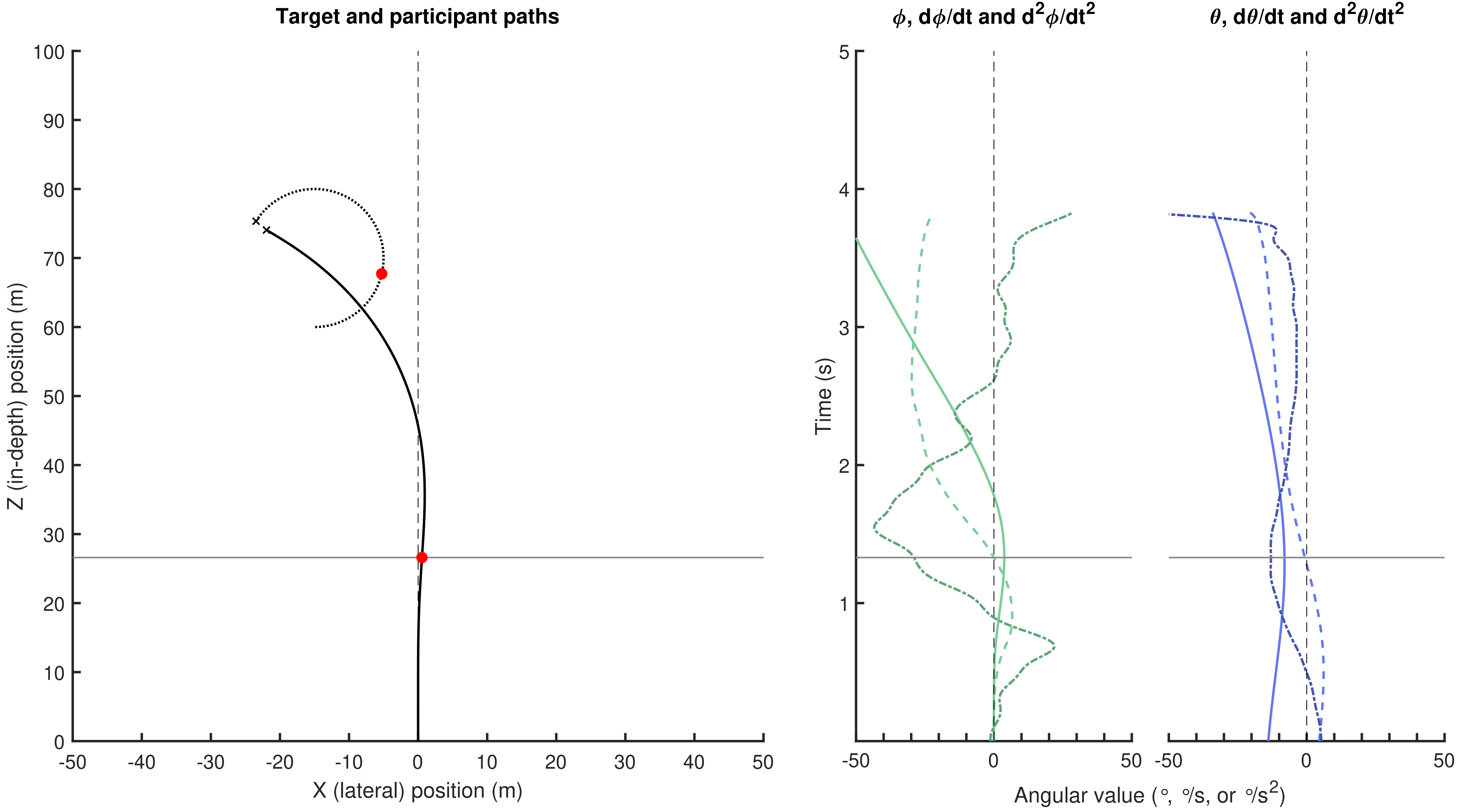

P6/B3
S15/R20-IN

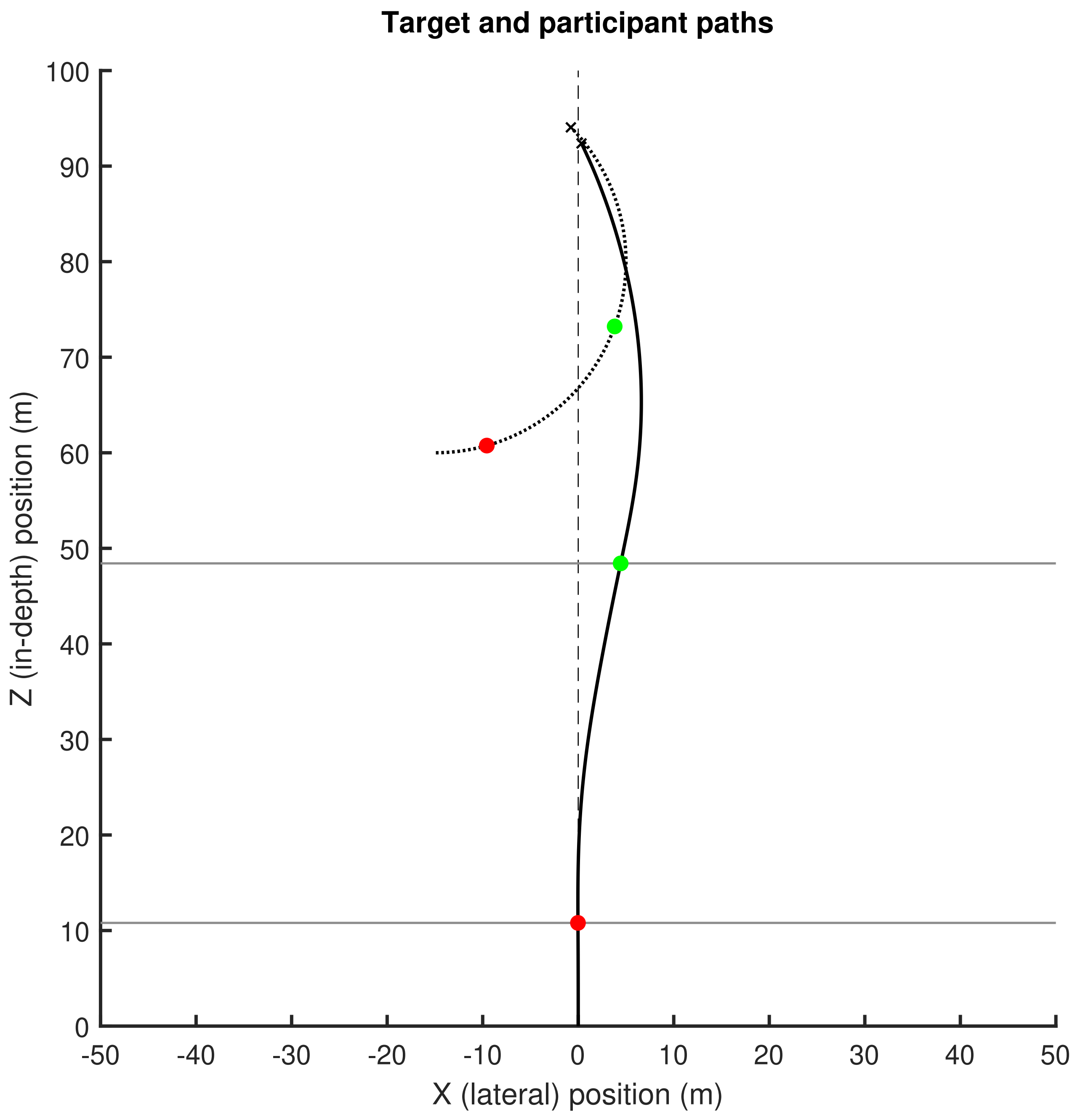

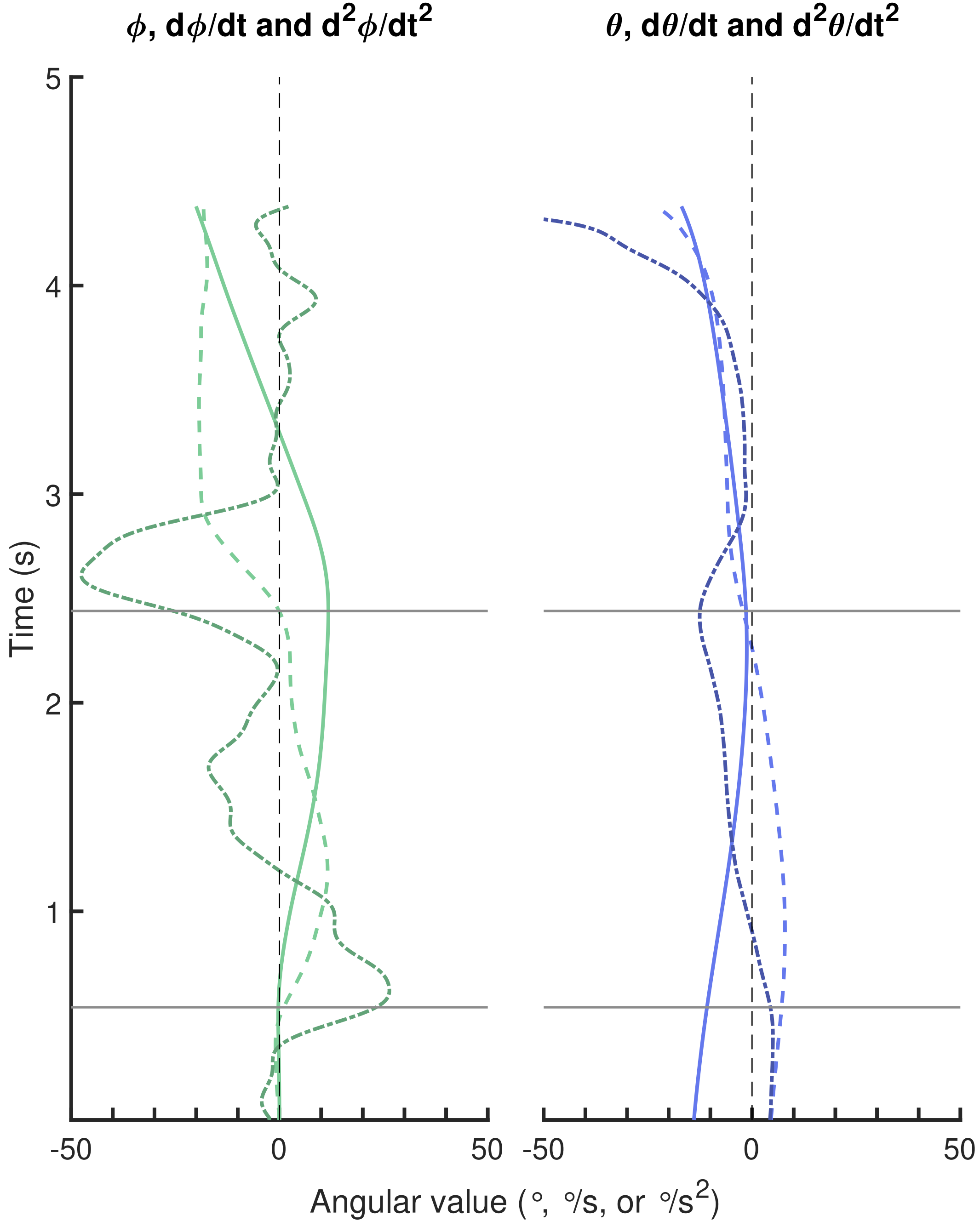

P6/B3
S15/R30-IN

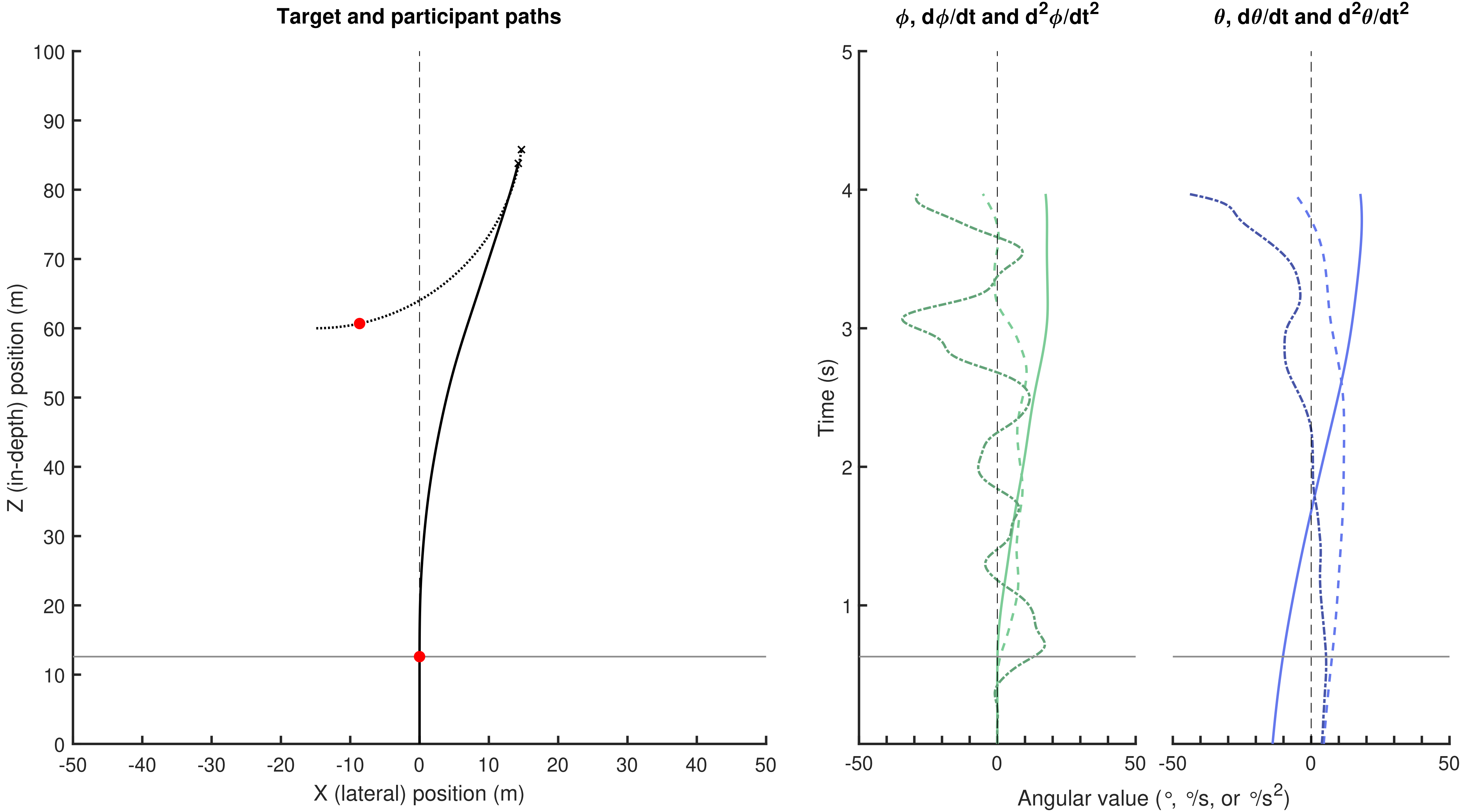

P6/B3
S5/R10-IN

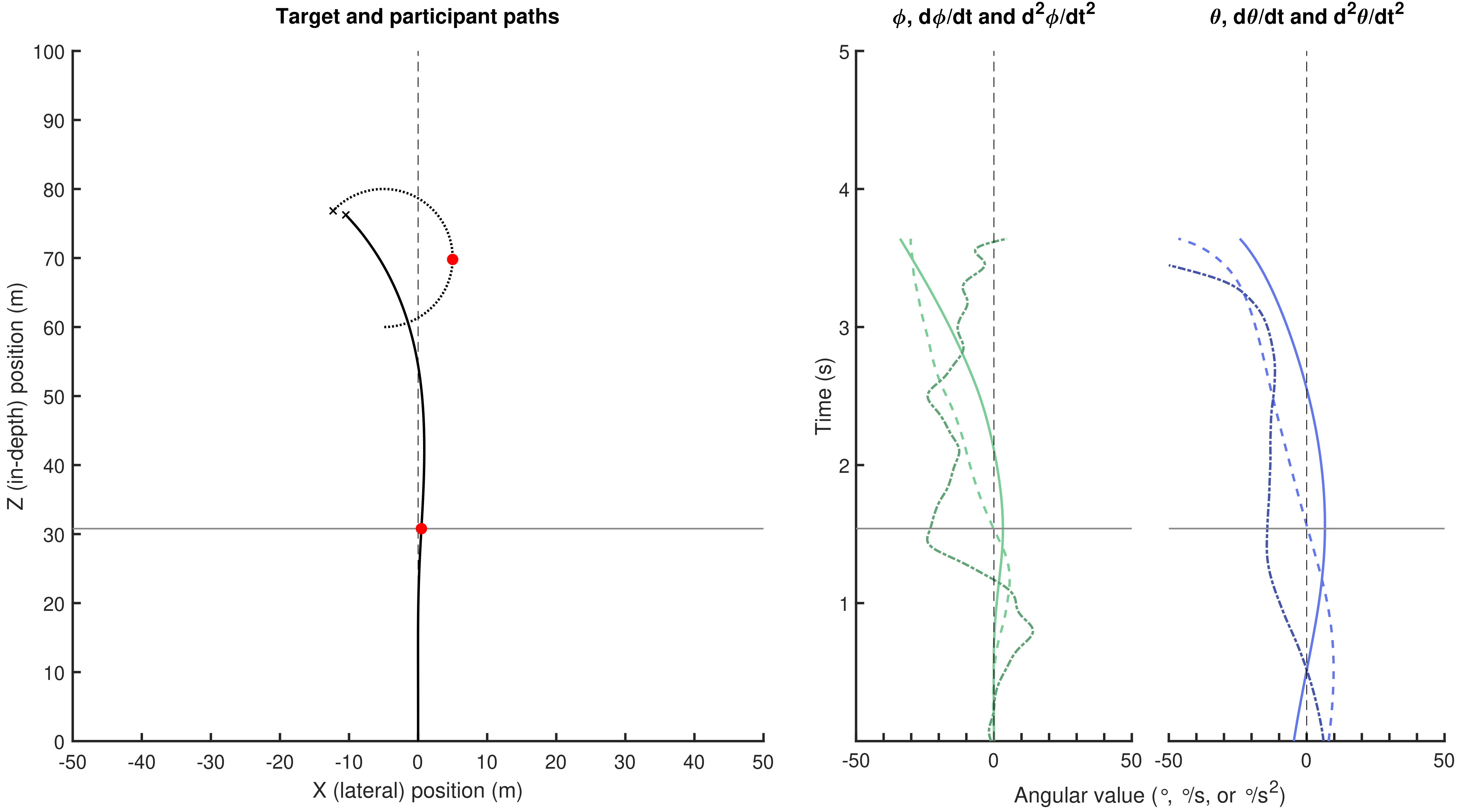

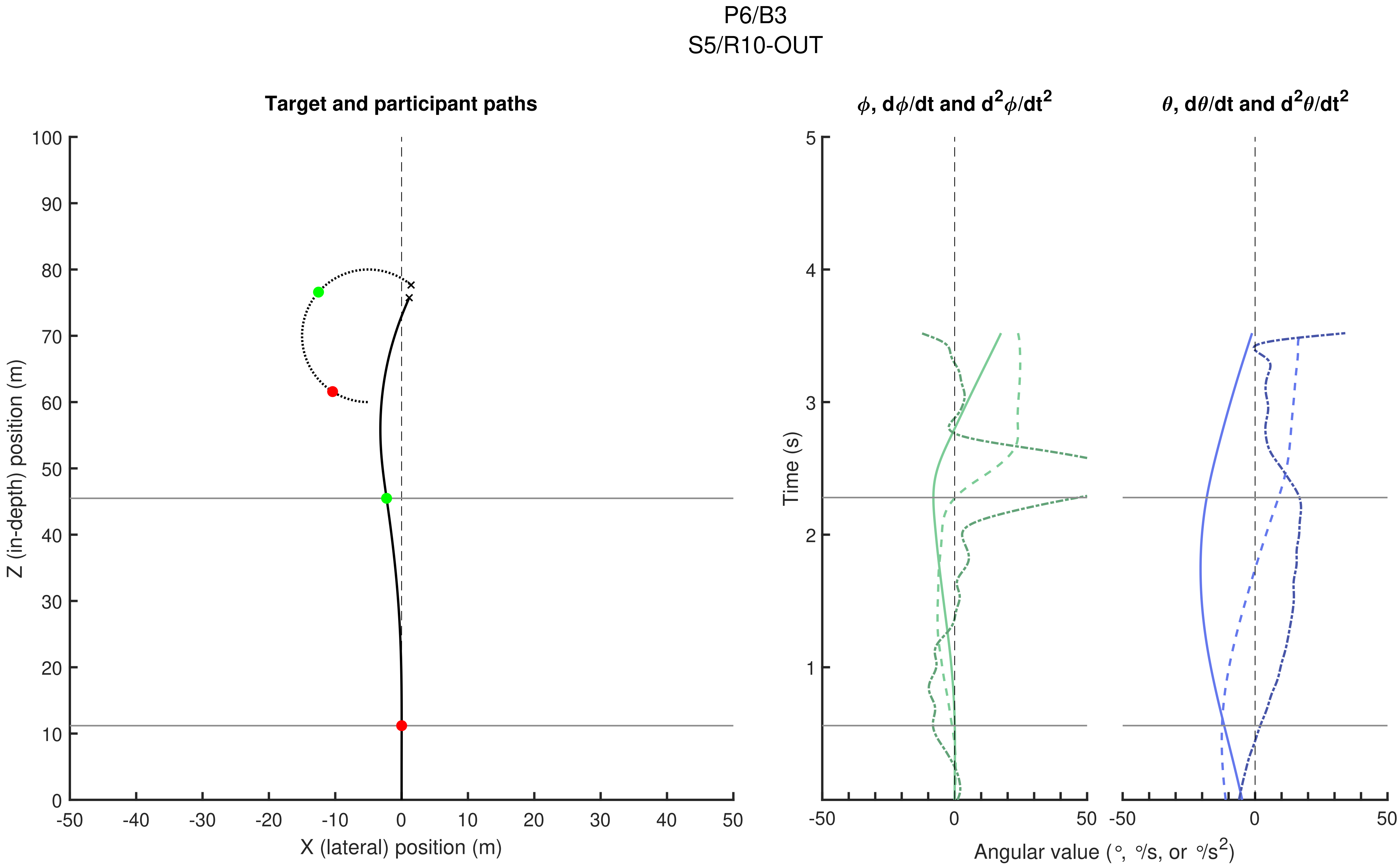
P6/B3
S5/R10-OUT
Target and participant paths
Z (in-depth) position (m)
X (lateral) position (m)
ϕ, dϕ/dt and d$^2\phi$/dt^2
θ, dθ/dt and d$^2\theta$/dt^2
Time (s)
Angular value (°, °/s, or °/s^2)

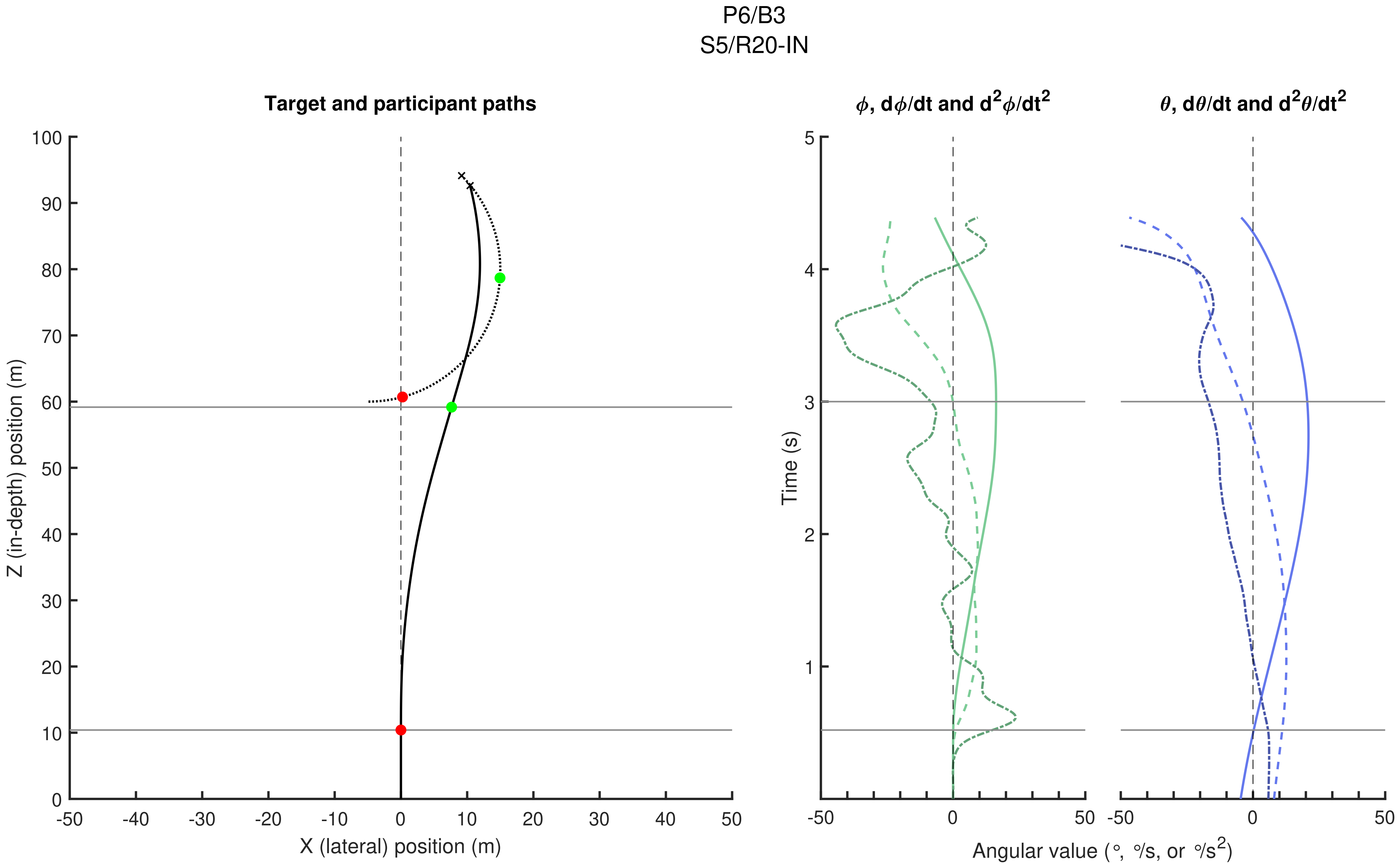
P6/B3
S5/R20-IN
Target and participant paths
Z (in-depth) position (m)
X (lateral) position (m)
0
10
20
30
40
50
60
70
80
90
100
-50
-40
-30
-20
-10
0
10
20
30
40
50
ϕ, dϕ/dt and d$^2\phi$/dt^2
θ, dθ/dt and d$^2\theta$/dt^2
Time (s)
1
2
3
4
5
-50
0
50
-50
0
50
Angular value (°, °/s, or °/s^2)

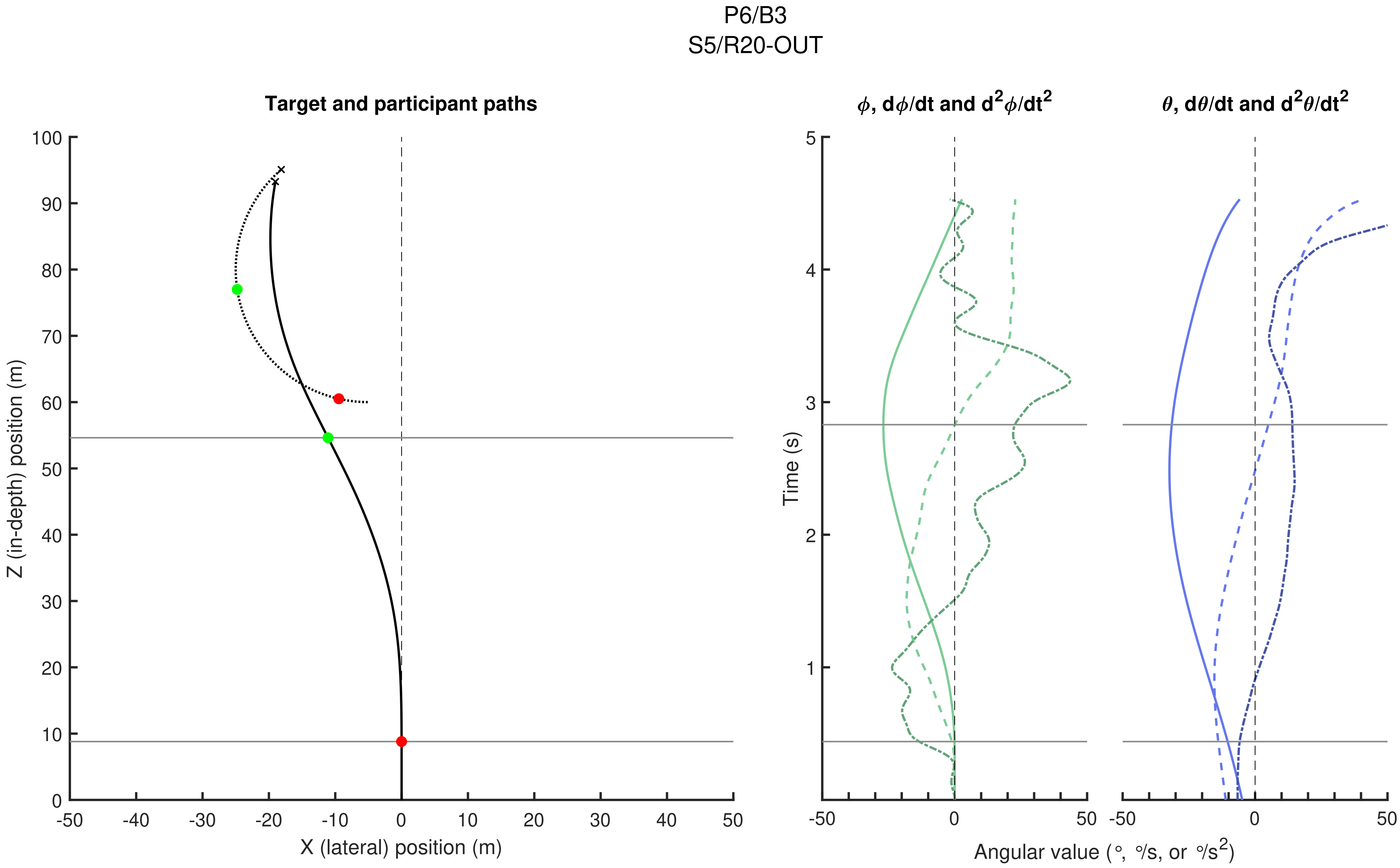
P6/B3
S5/R20-OUT
Target and participant paths
Z (in-depth) position (m)
X (lateral) position (m)
ϕ, dϕ/dt and d²ϕ/dt²
θ, dθ/dt and d²θ/dt²
Time (s)
Angular value (°, °/s, or °/s²)

P6/B3
S5/R30-IN

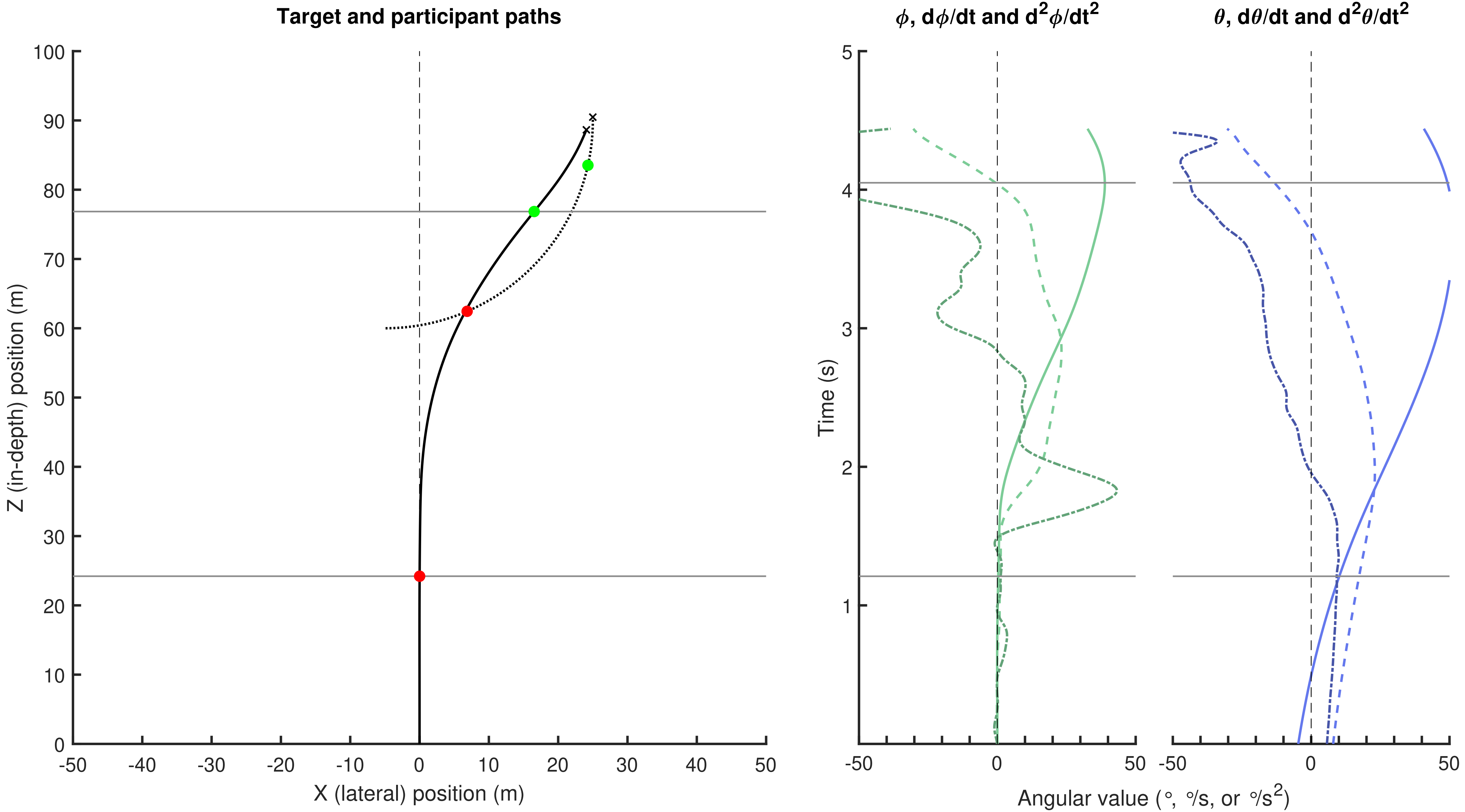

P6/B3
S5/R30-OUT

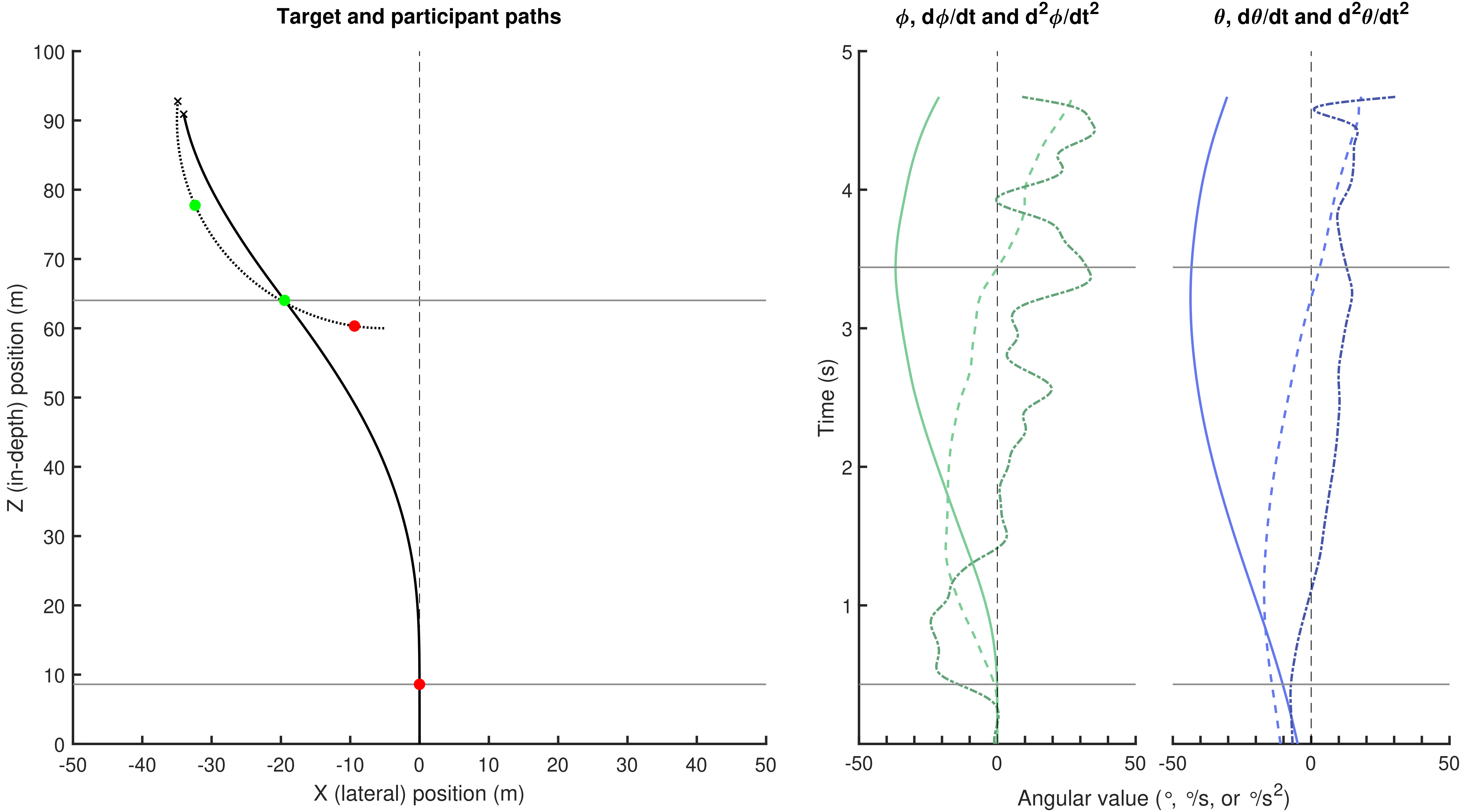

P6/B3
S5/R10-OUT

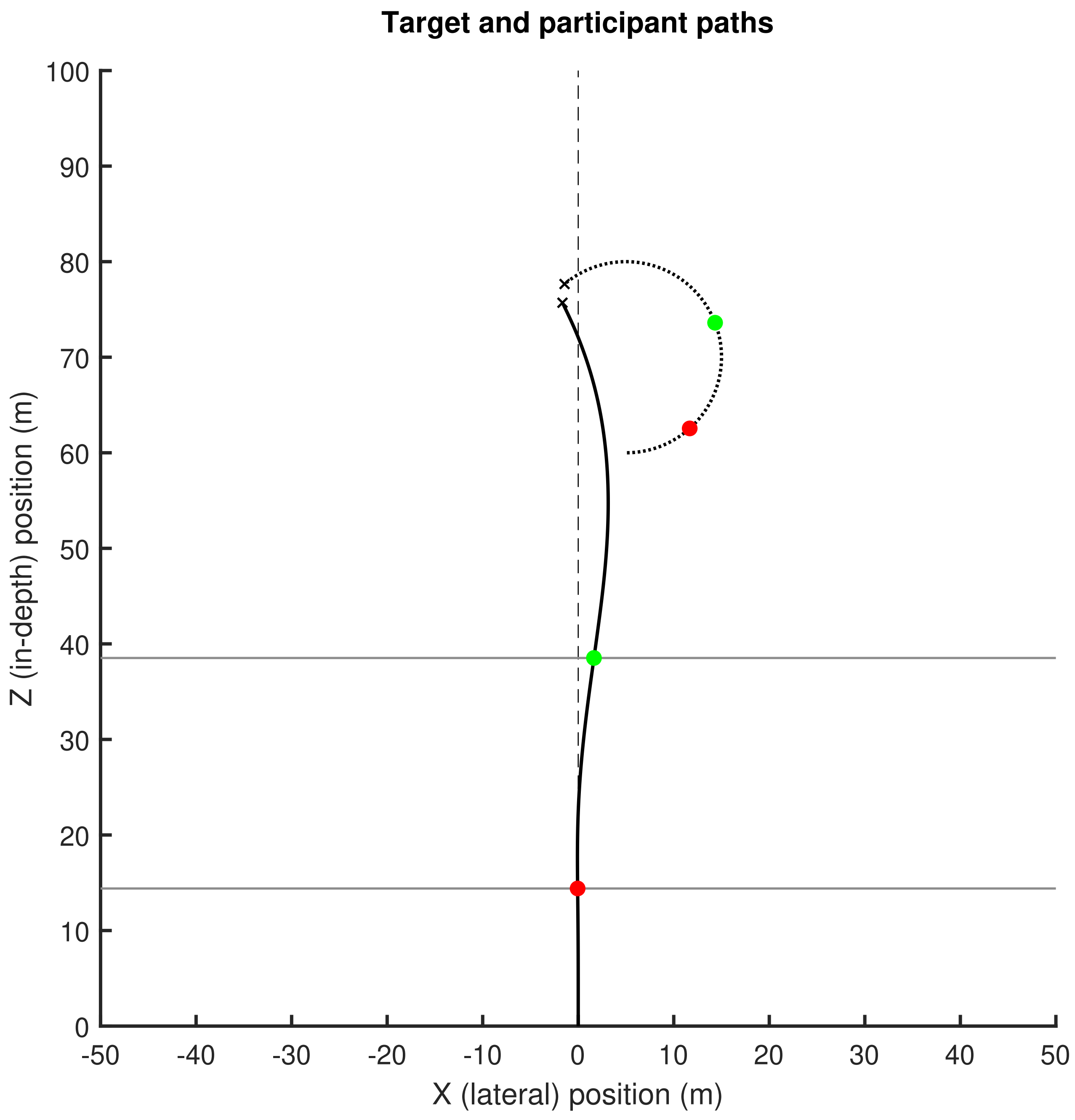

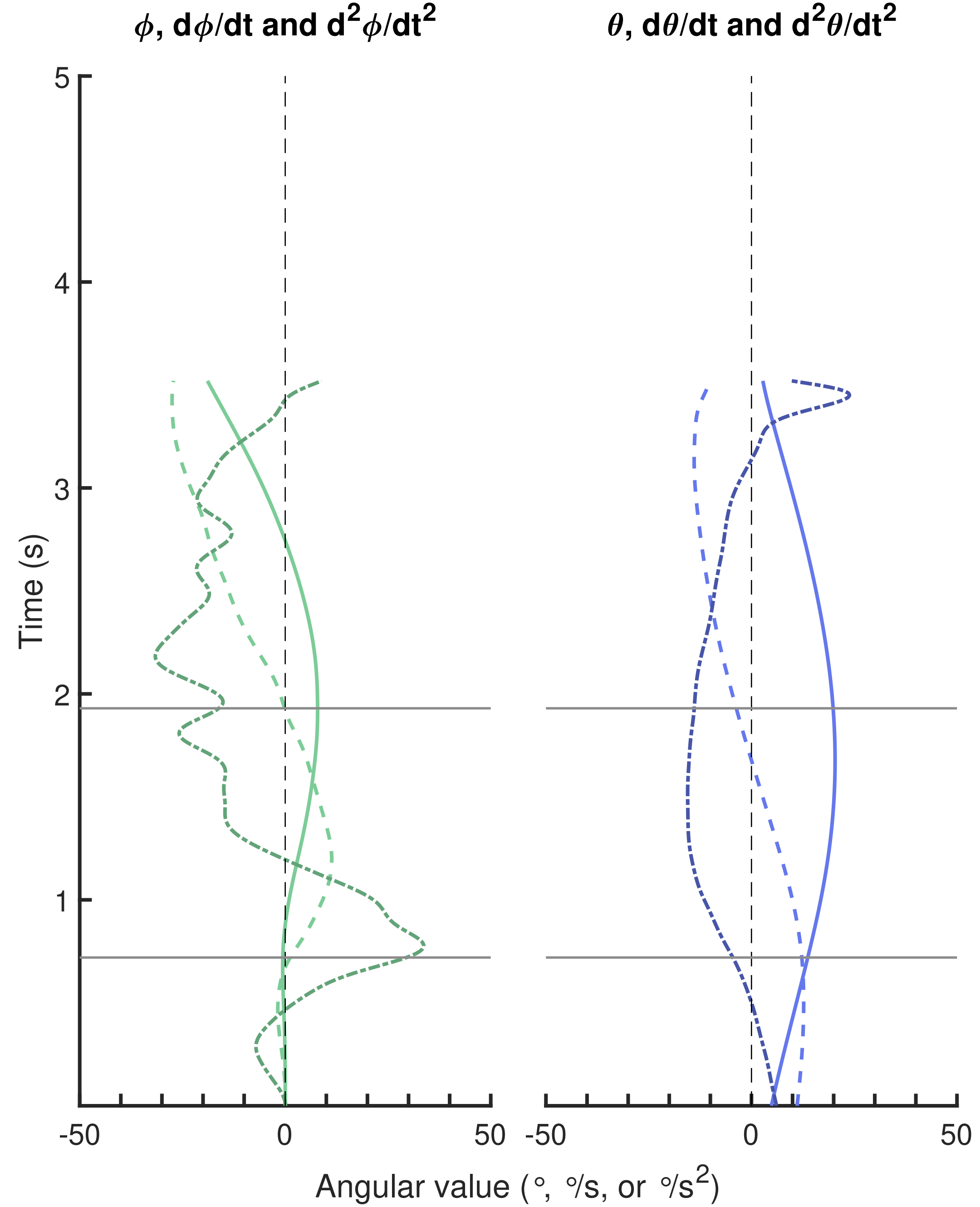

P6/B3
S5/R10-IN

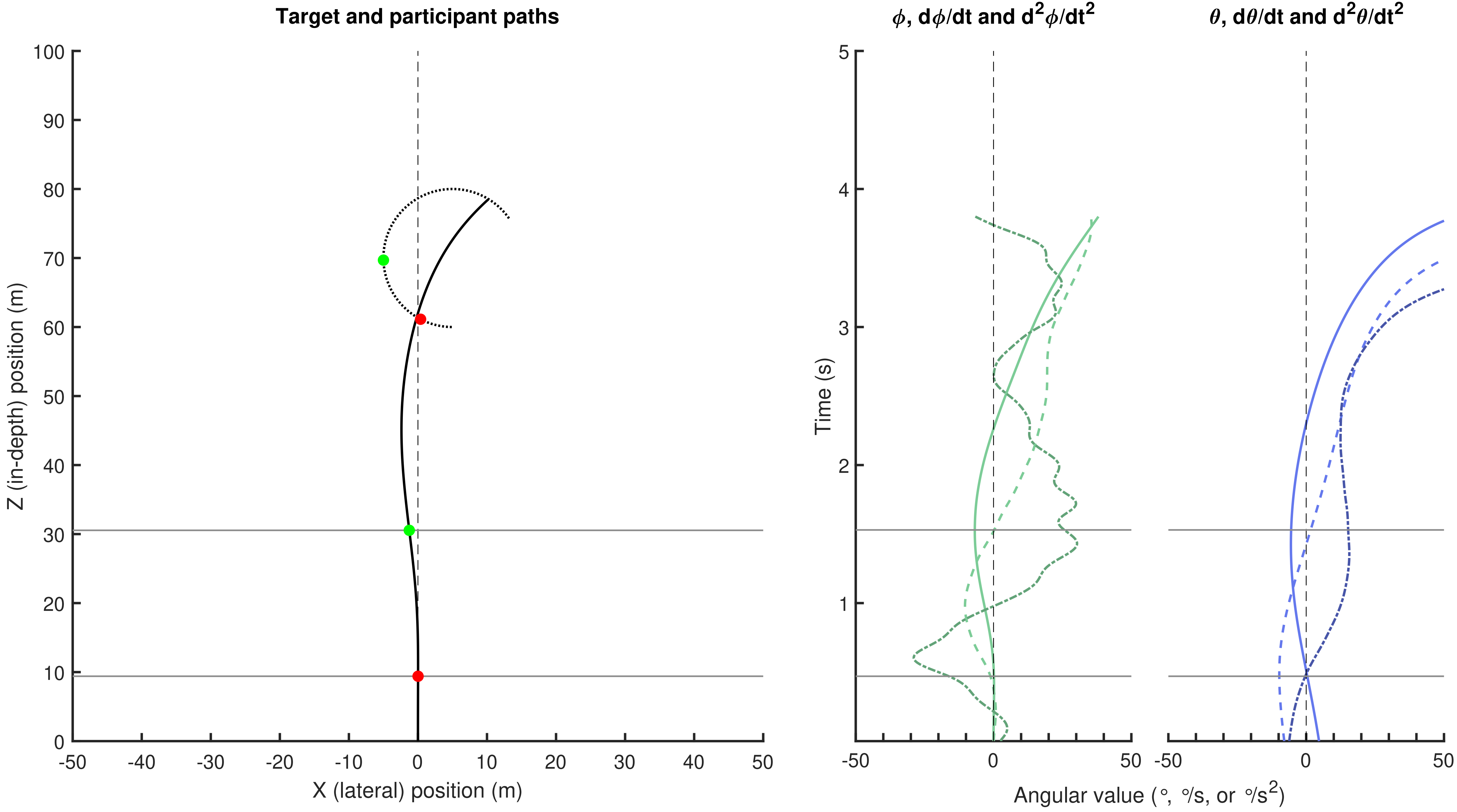

P6/B3
S5/R20-OUT

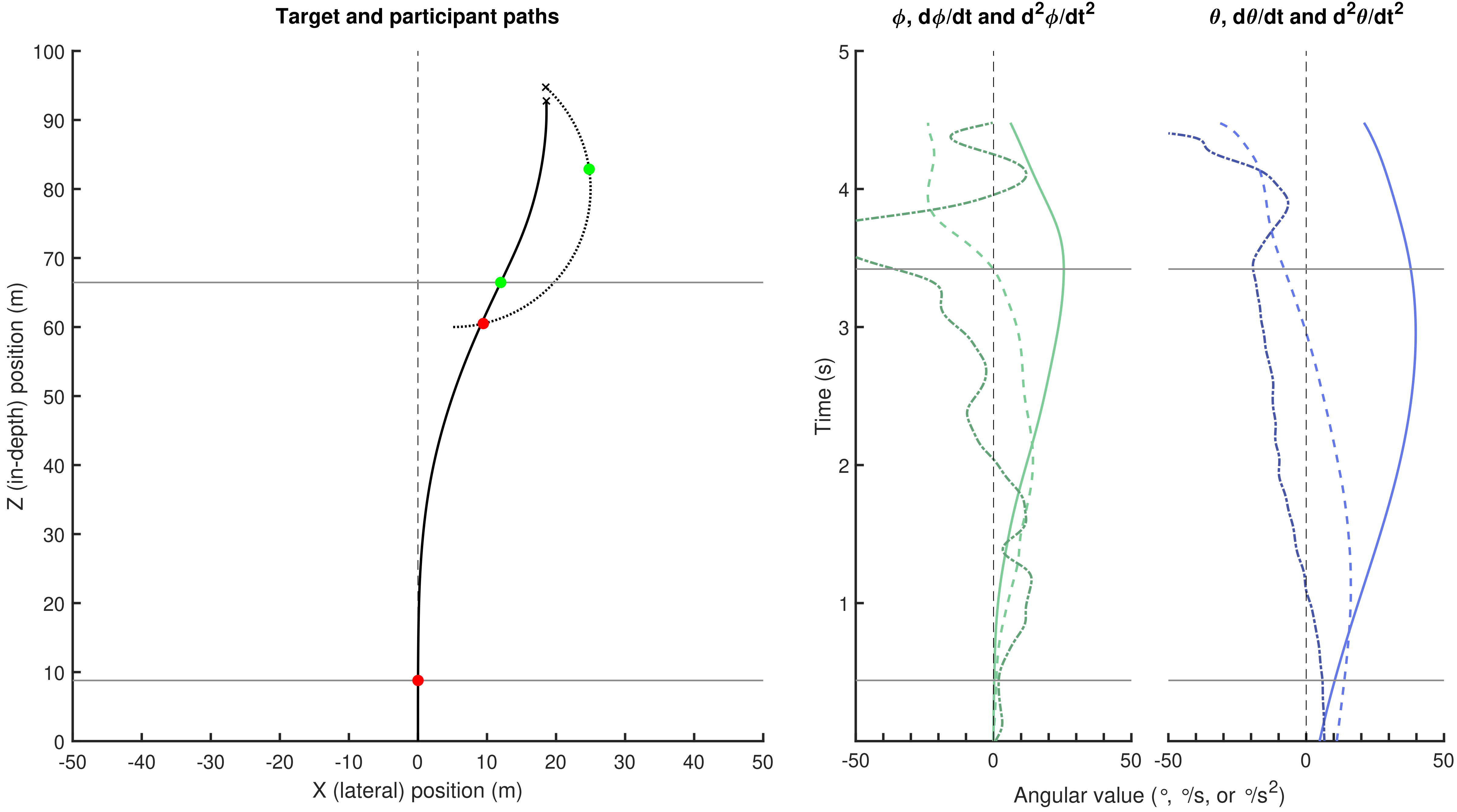

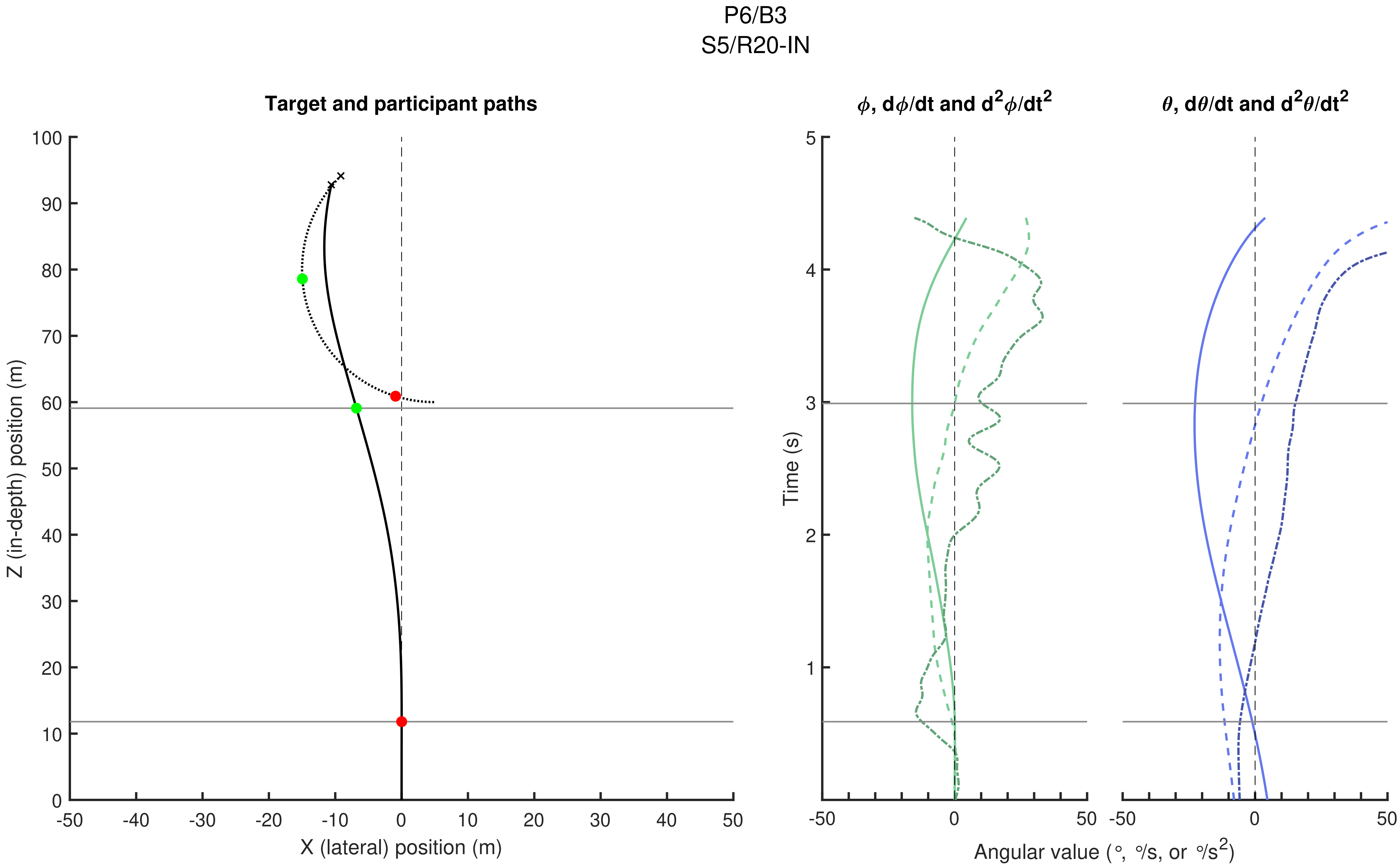

P6/B3
S5/R20-IN
Target and participant paths
Z (in-depth) position (m)
X (lateral) position (m)
ϕ, dϕ/dt and d²ϕ/dt²
θ, dθ/dt and d²θ/dt²
Time (s)
Angular value (°, °/s, or °/s²)

P6/B3
S5/R30-OUT

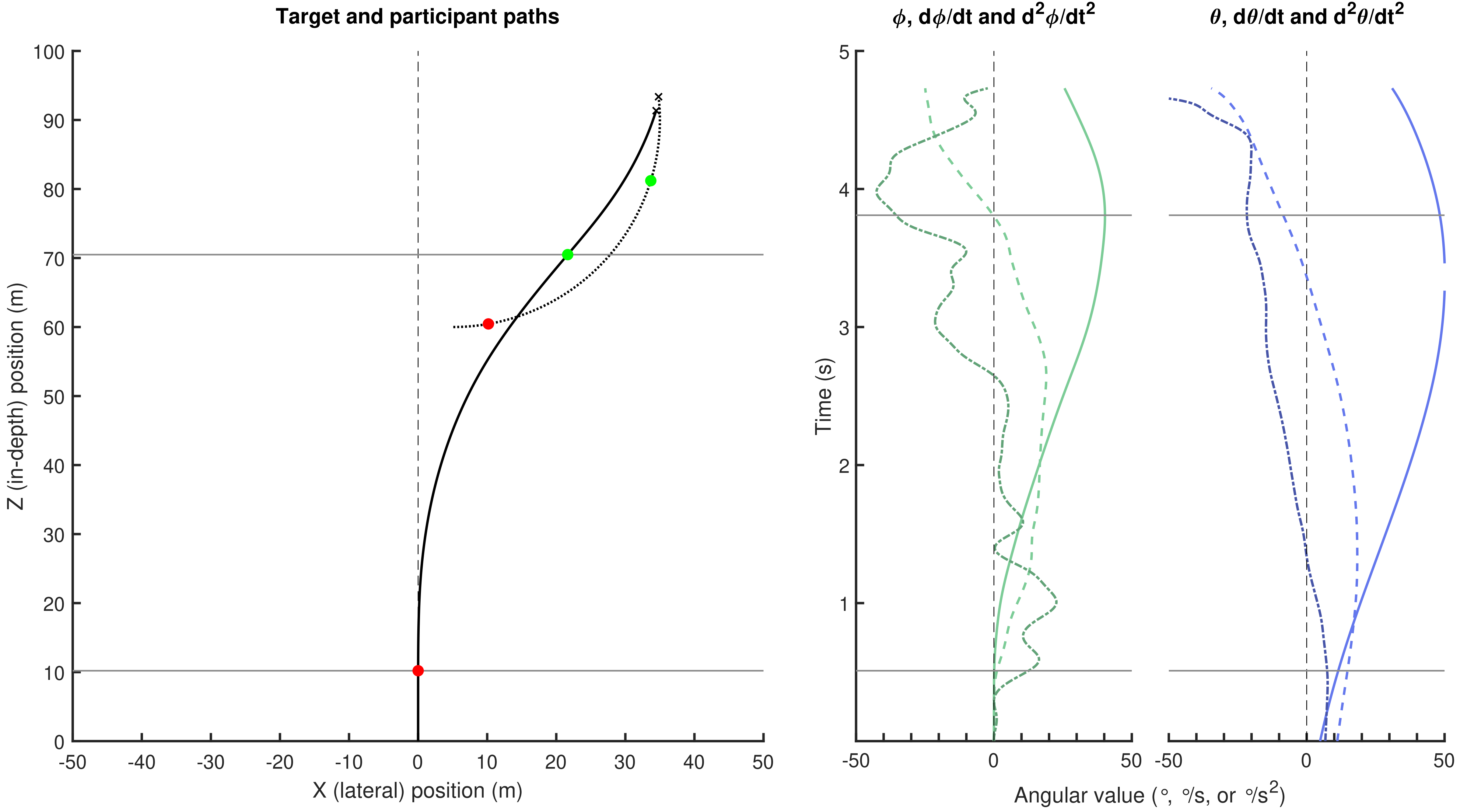

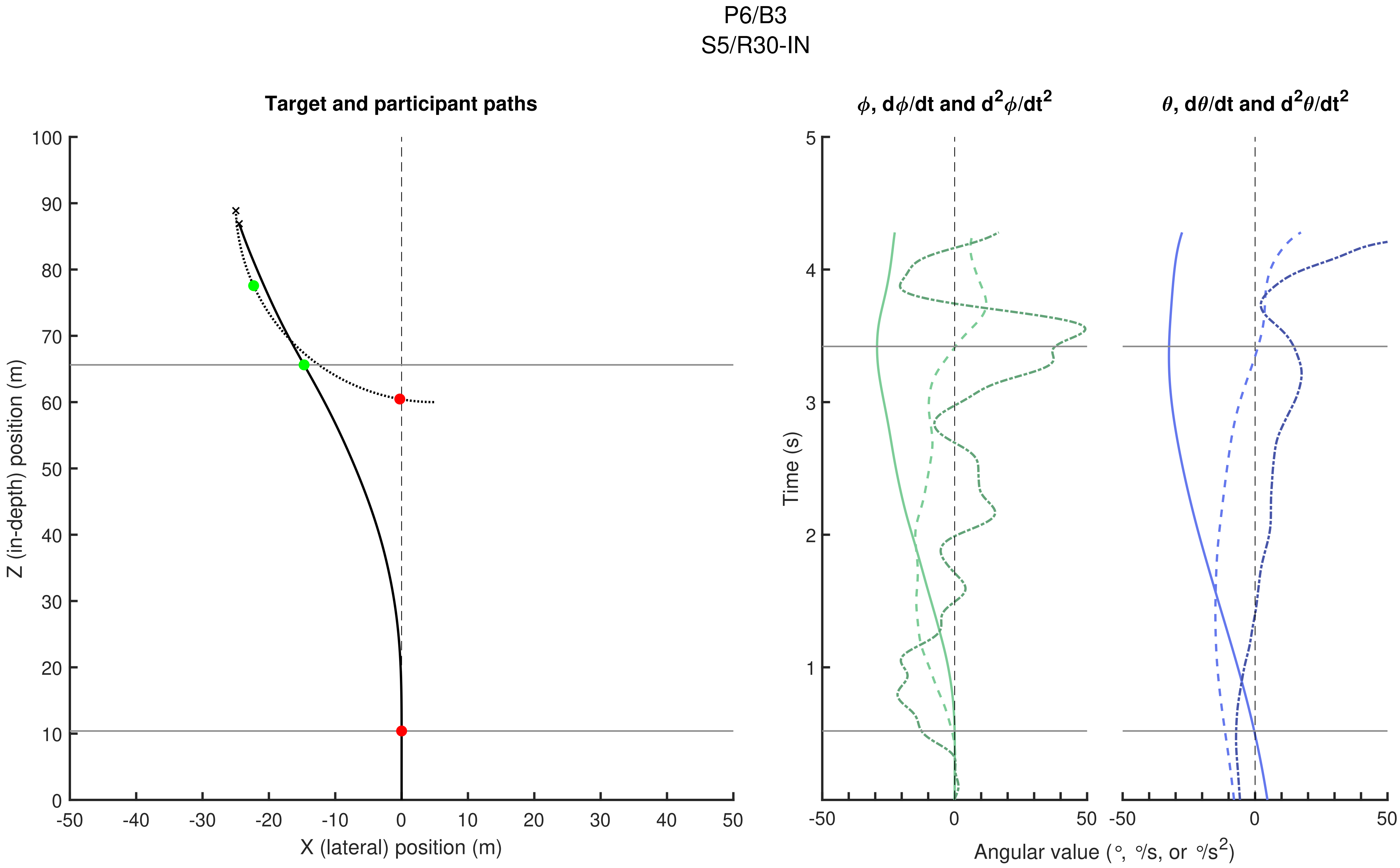
P6/B3
S5/R30-IN
Target and participant paths
Z (in-depth) position (m)
X (lateral) position (m)
100
90
80
70
60
50
40
30
20
10
0
-50
-40
-30
-20
-10
0
10
20
30
40
50
ϕ, dϕ/dt and d²ϕ/dt²
θ, dθ/dt and d²θ/dt²
Time (s)
5
4
3
2
1
-50
0
50
-50
0
50
Angular value (°, °/s, or °/s²)

P6/B3
S15/R10-IN

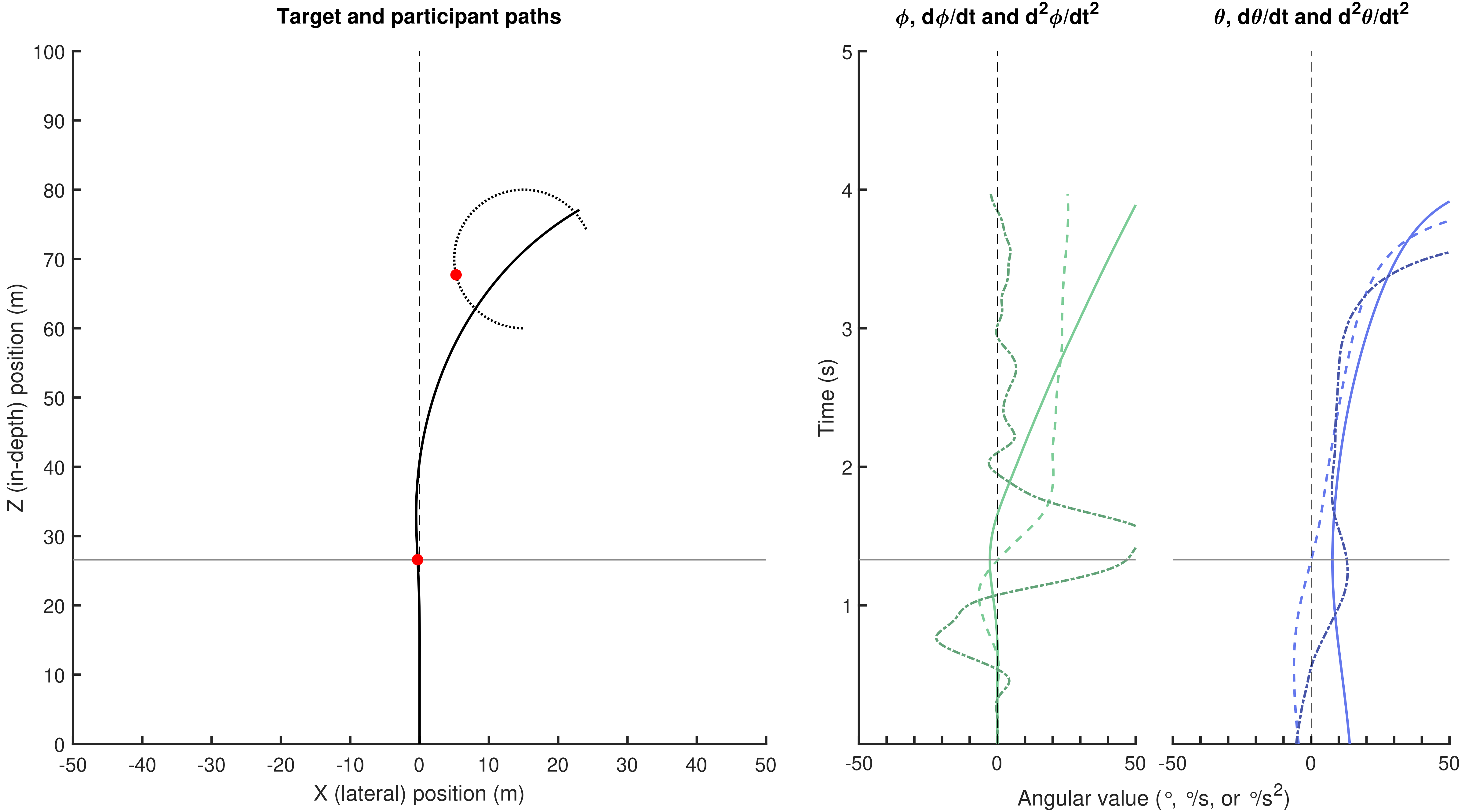

P6/B3
S15/R20-IN

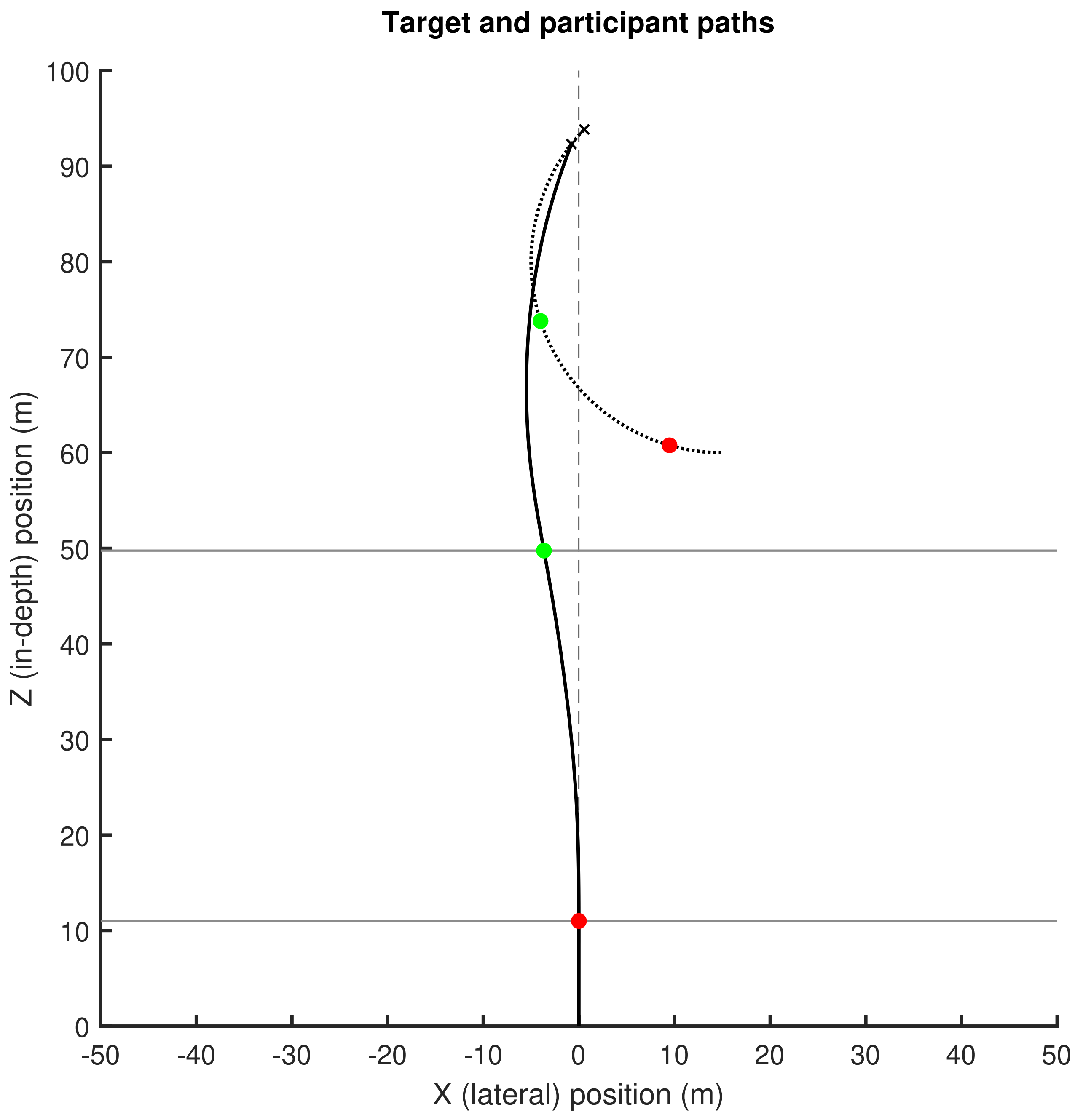

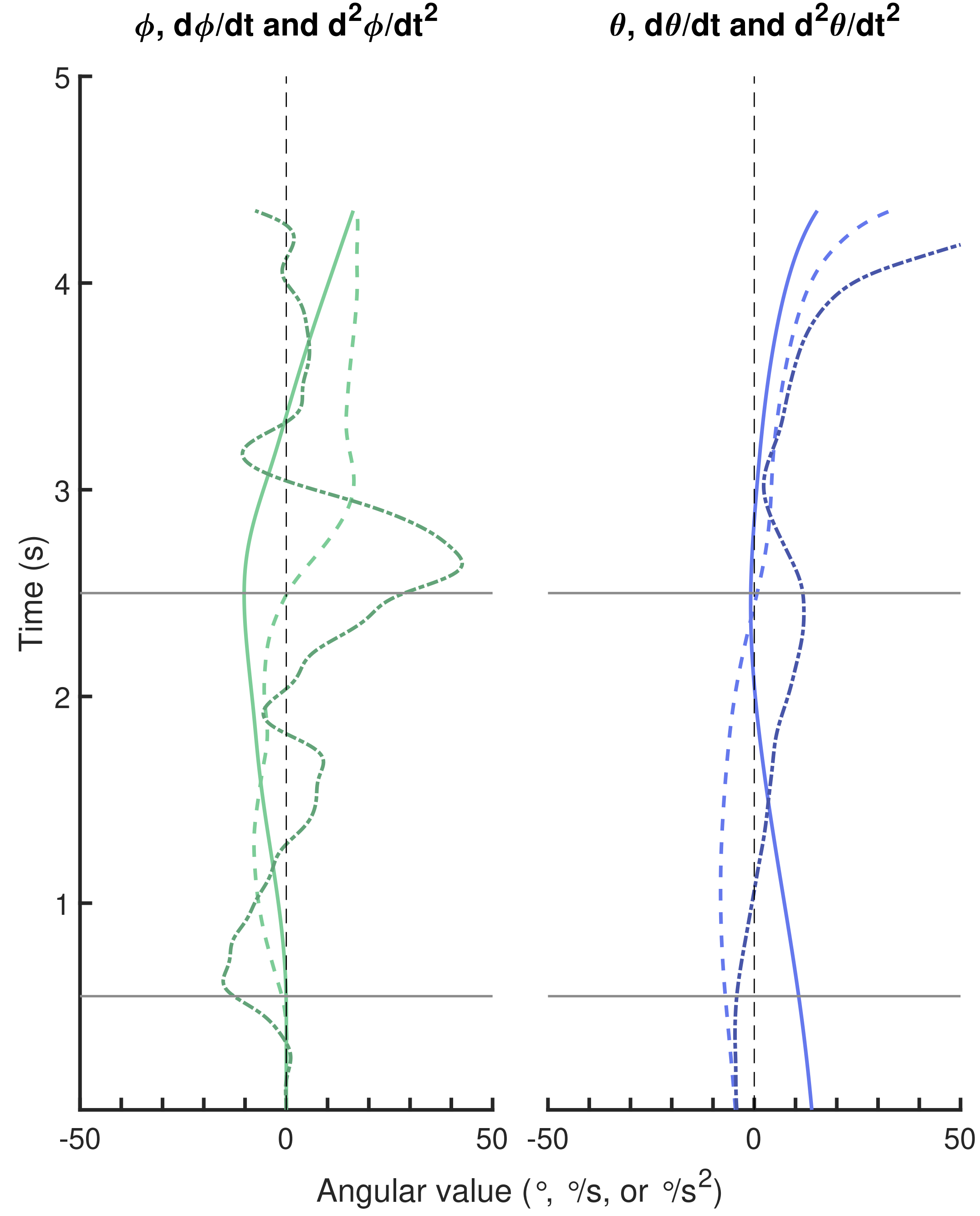

P6/B3
S15/R30-IN

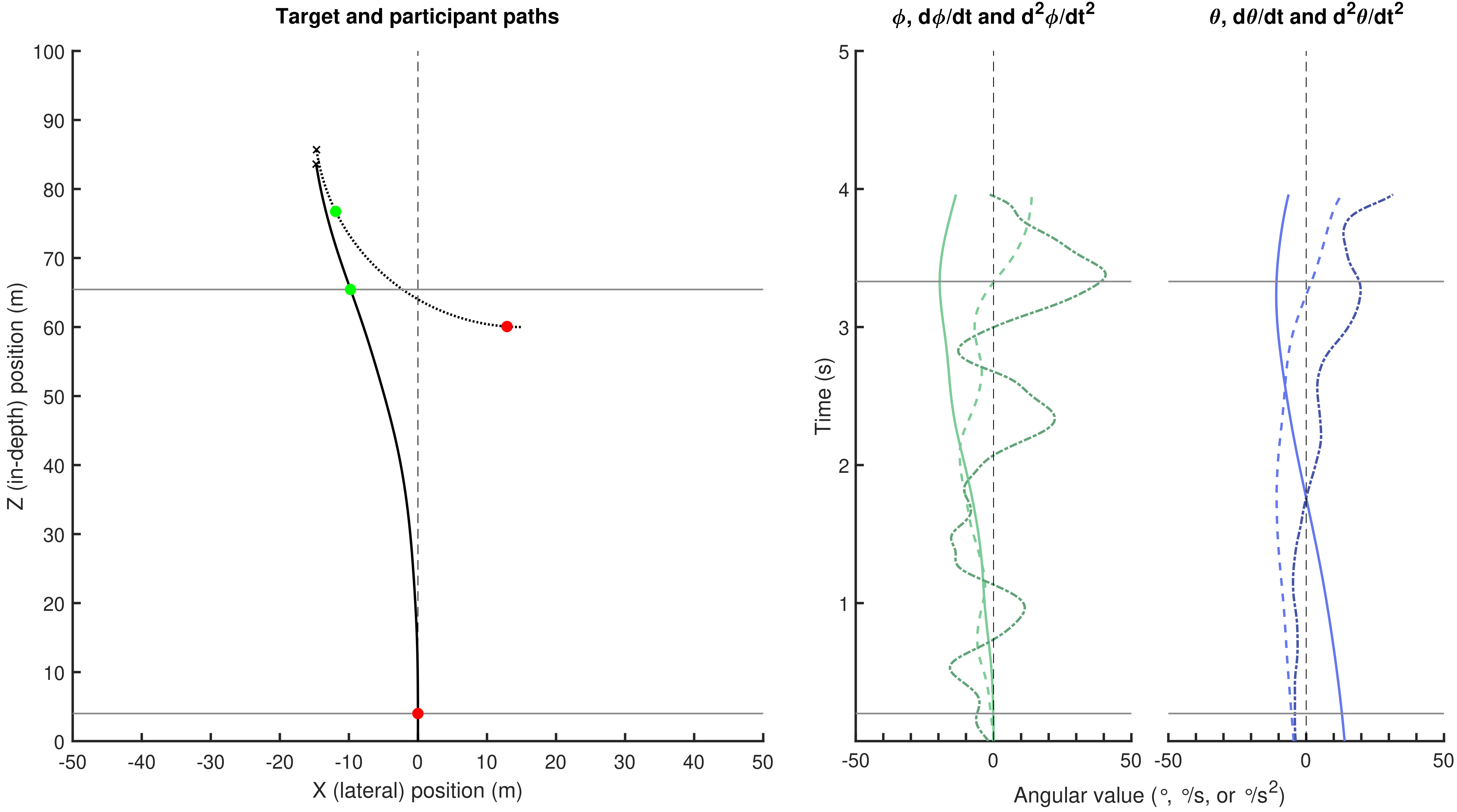

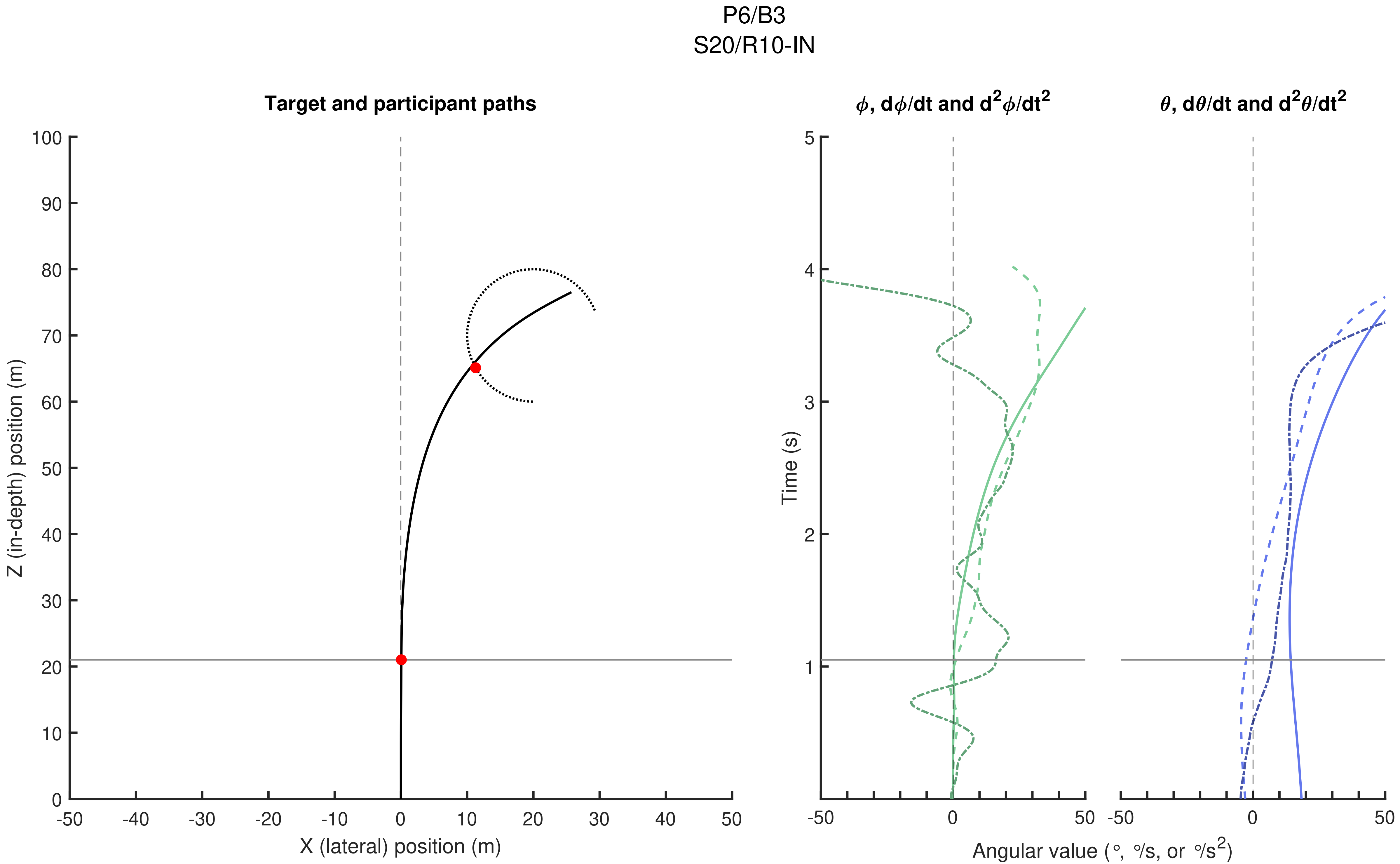
P6/B3
S20/R10-IN
Target and participant paths
φ, dφ/dt and d²φ/dt²
θ, dθ/dt and d²θ/dt²
Z (in-depth) position (m)
X (lateral) position (m)
Time (s)
Angular value (°, °/s, or °/s²)
100
90
80
70
60
50
40
30
20
10
0
-50
-40
-30
-20
-10
0
10
20
30
40
50
5
4
3
2
1
-50
0
50
-50
0
50

P6/B3
S20/R20-IN

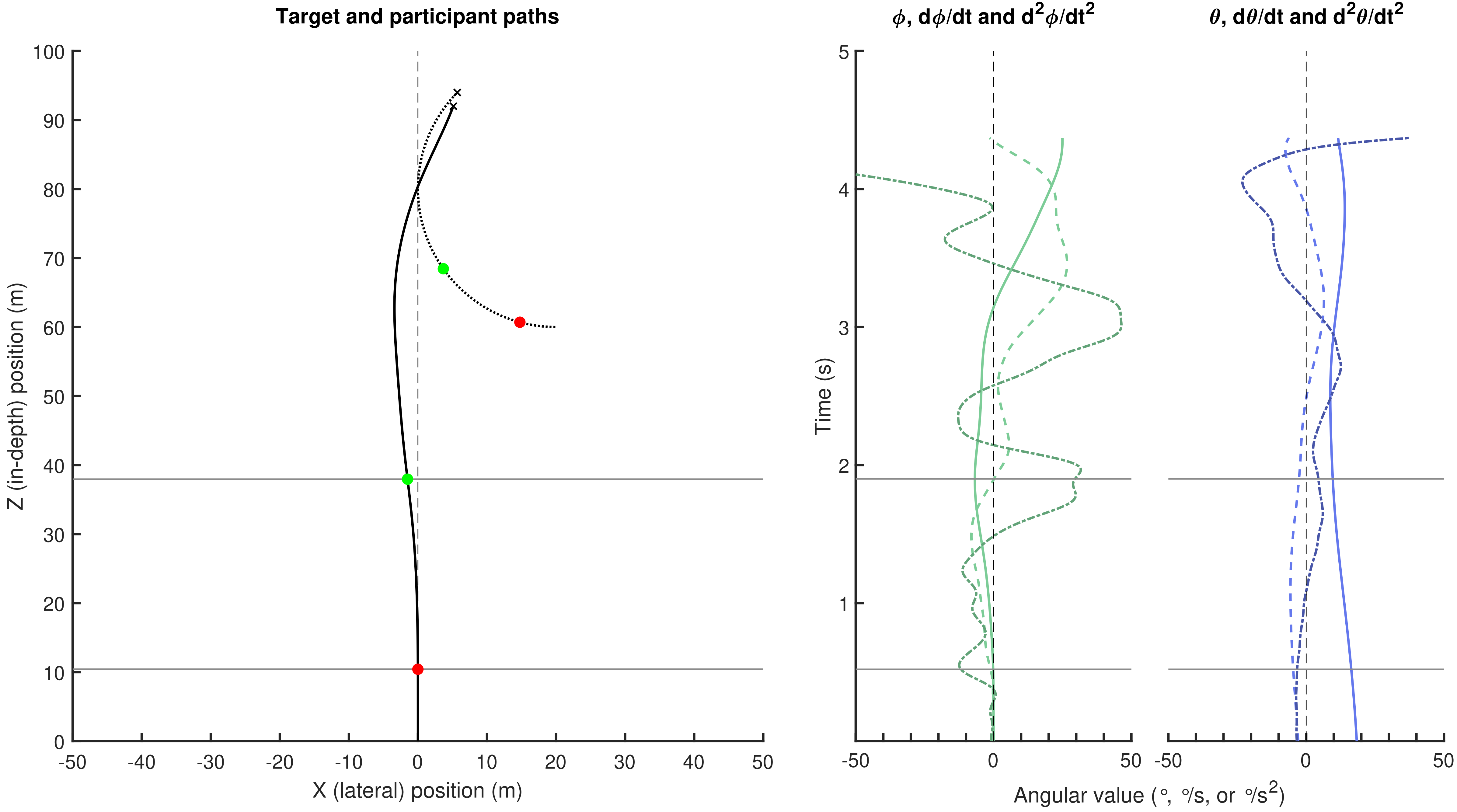

P6/B3
S20/R30-IN

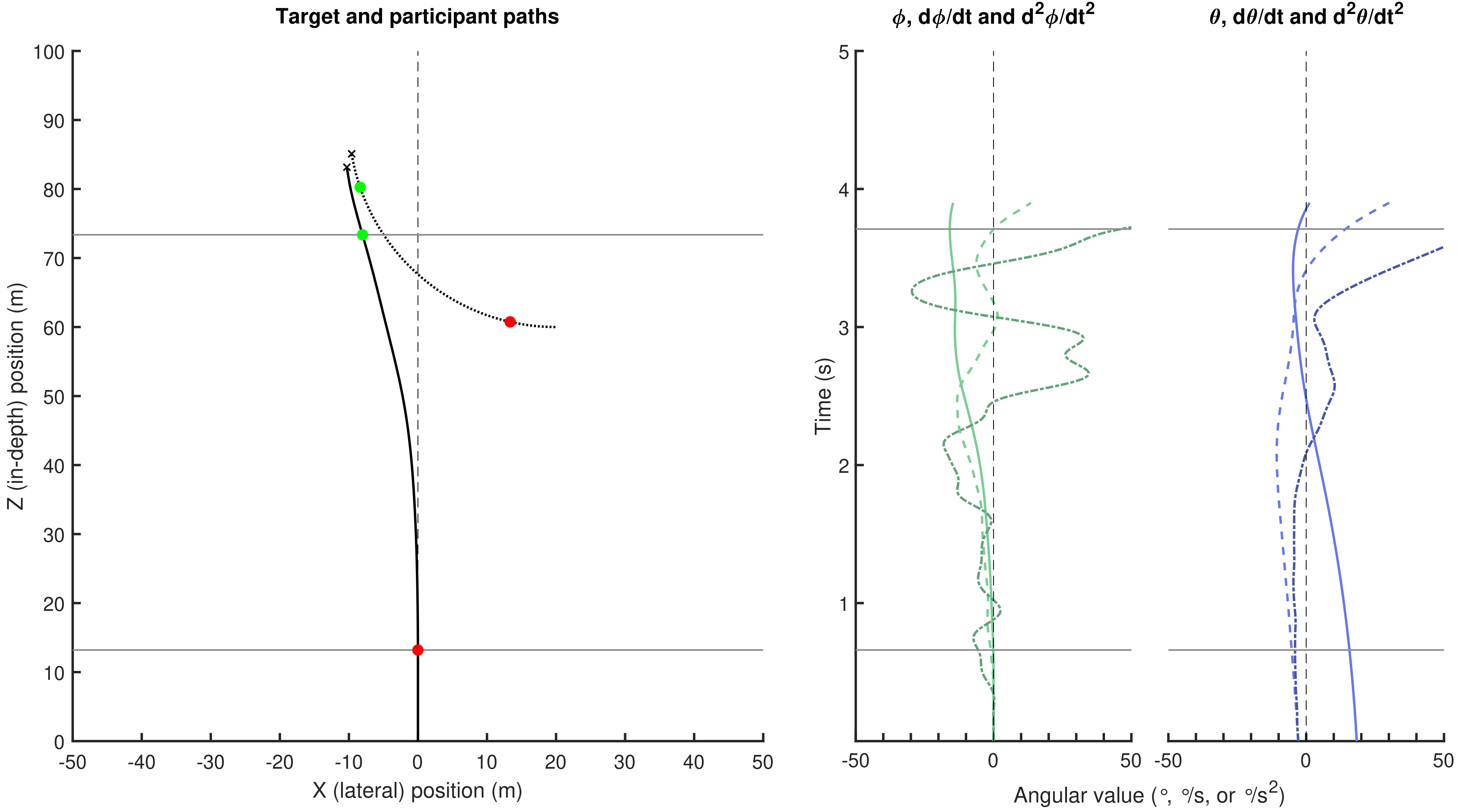

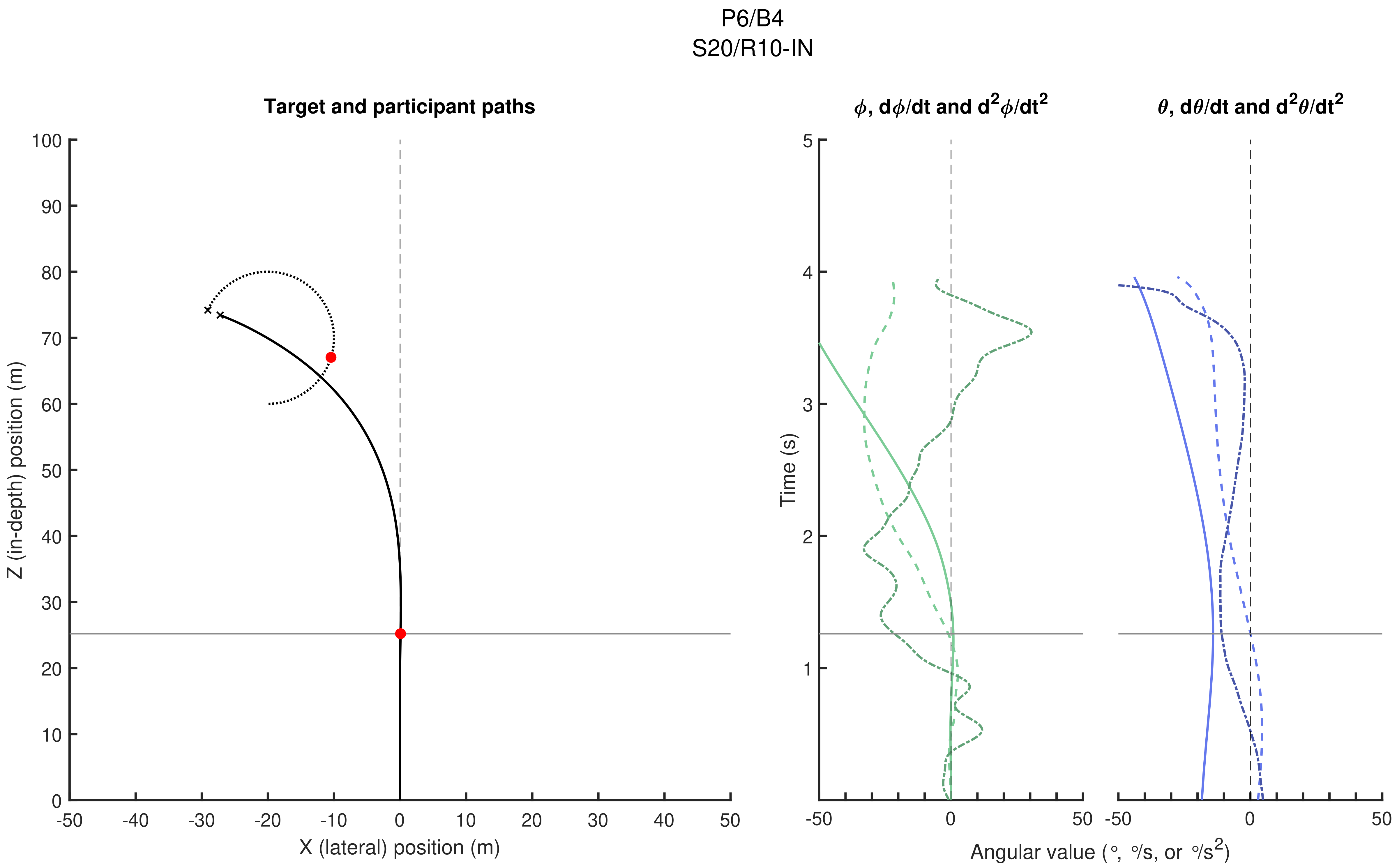

P6/B4
S20/R10-IN
Target and participant paths
Z (in-depth) position (m)
X (lateral) position (m)
100
90
80
70
60
50
40
30
20
10
0
-50
-40
-30
-20
-10
0
10
20
30
40
50
ϕ, dϕ/dt and d$^2\phi$/dt^2
θ, dθ/dt and d$^2\theta$/dt^2
Time (s)
5
4
3
2
1
-50
0
50
-50
0
50
Angular value (°, °/s, or °/s^2)

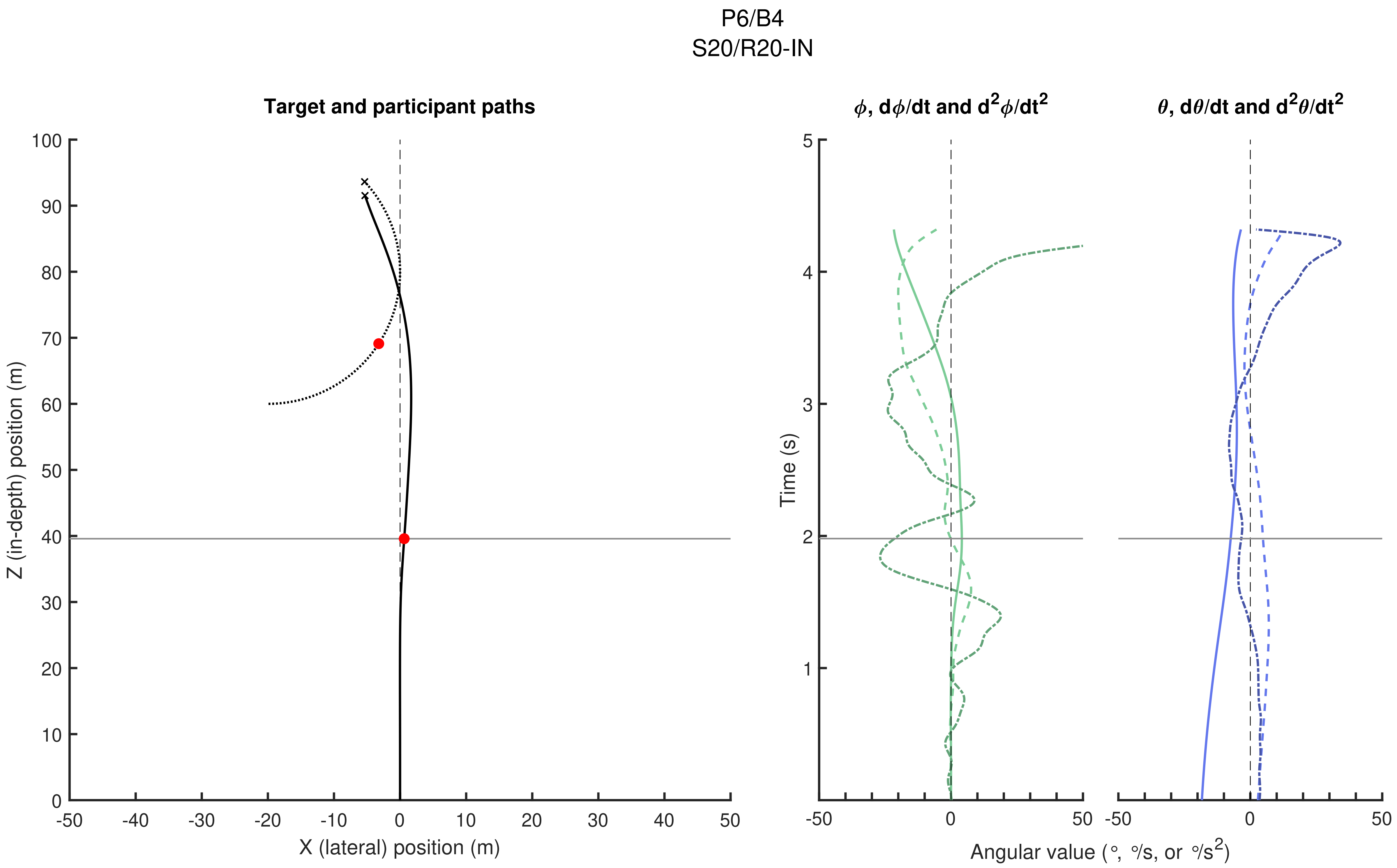
P6/B4
S20/R20-IN
Target and participant paths
Z (in-depth) position (m)
X (lateral) position (m)
100
90
80
70
60
50
40
30
20
10
0
-50
-40
-30
-20
-10
0
10
20
30
40
50
ϕ, dϕ/dt and d2ϕ/dt2
θ, dθ/dt and d2θ/dt2
Time (s)
5
4
3
2
1
-50
0
50
-50
0
50
Angular value (°, °/s, or °/s2)

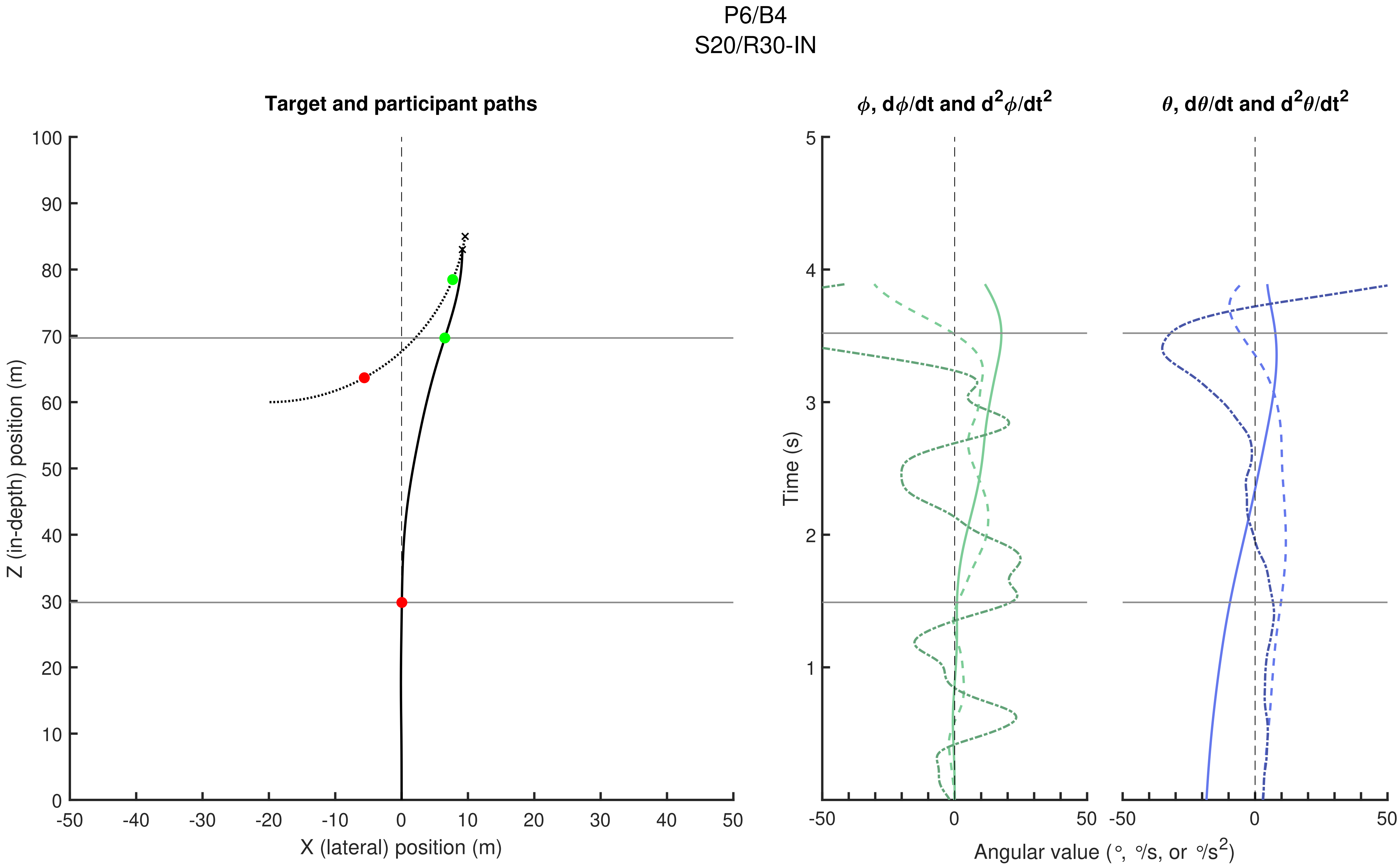

P6/B4
S20/R30-IN
Target and participant paths
Z (in-depth) position (m)
X (lateral) position (m)
100
90
80
70
60
50
40
30
20
10
0
-50
-40
-30
-20
-10
0
10
20
30
40
50
ϕ, dϕ/dt and d²ϕ/dt²
θ, dθ/dt and d²θ/dt²
Time (s)
5
4
3
2
1
-50
0
50
-50
0
50
Angular value (°, °/s, or °/s²)

P6/B4
S15/R10-IN

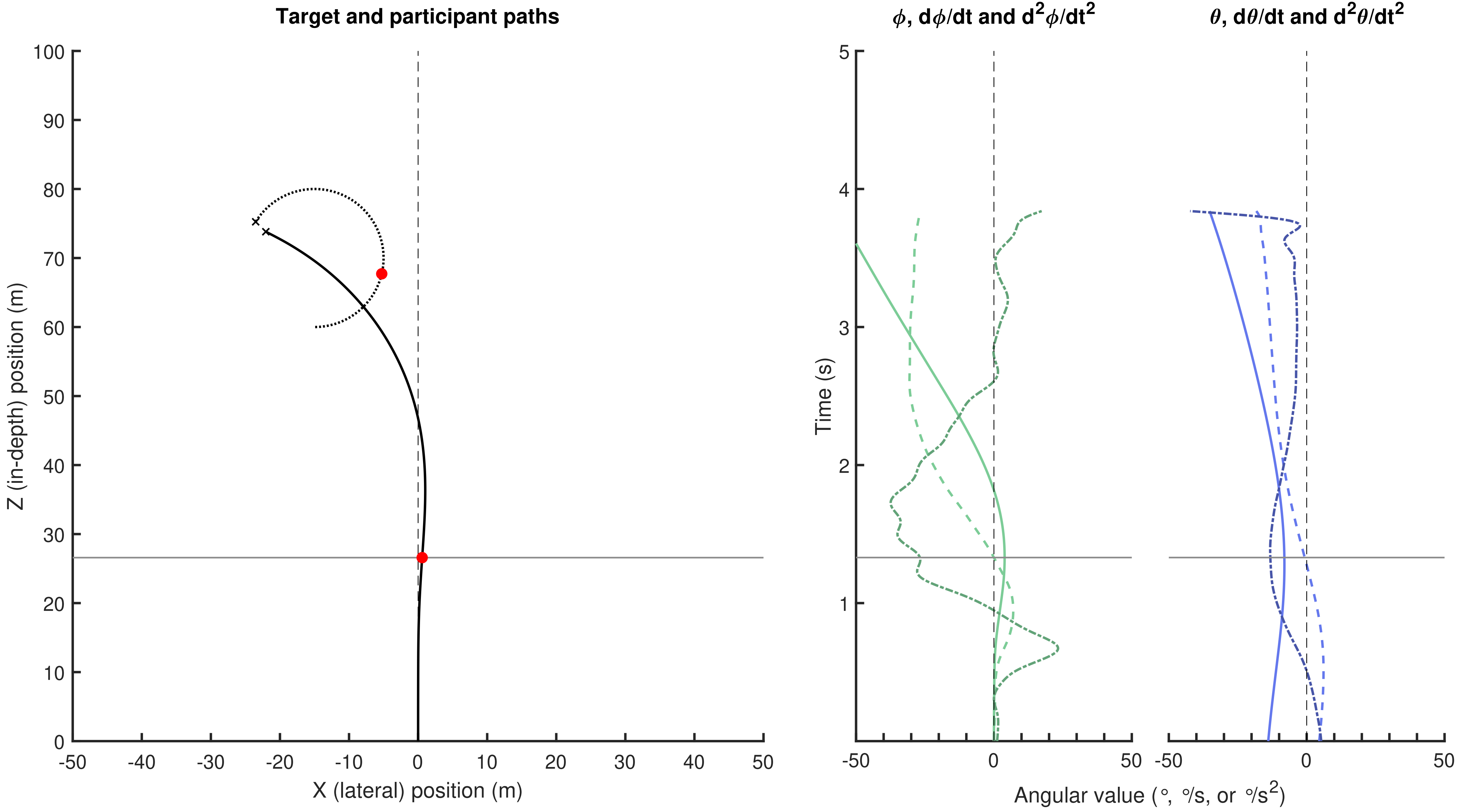

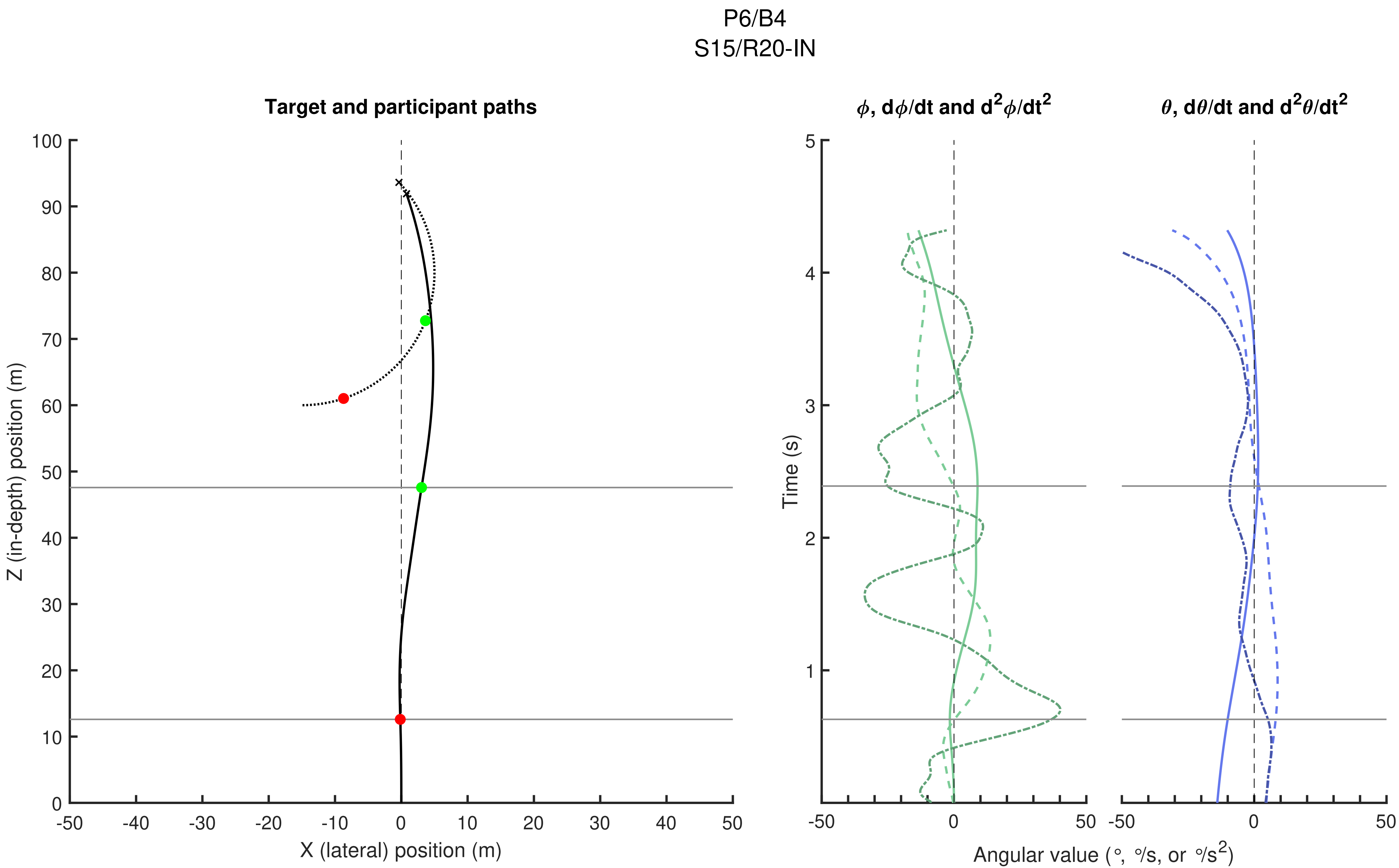
P6/B4
S15/R20-IN
Target and participant paths
Z (in-depth) position (m)
X (lateral) position (m)
0
10
20
30
40
50
60
70
80
90
100
-50
-40
-30
-20
-10
0
10
20
30
40
50
ϕ, dϕ/dt and d²ϕ/dt²
θ, dθ/dt and d²θ/dt²
Time (s)
0
1
2
3
4
5
-50
0
50
-50
0
50
Angular value (°, °/s, or °/s²)

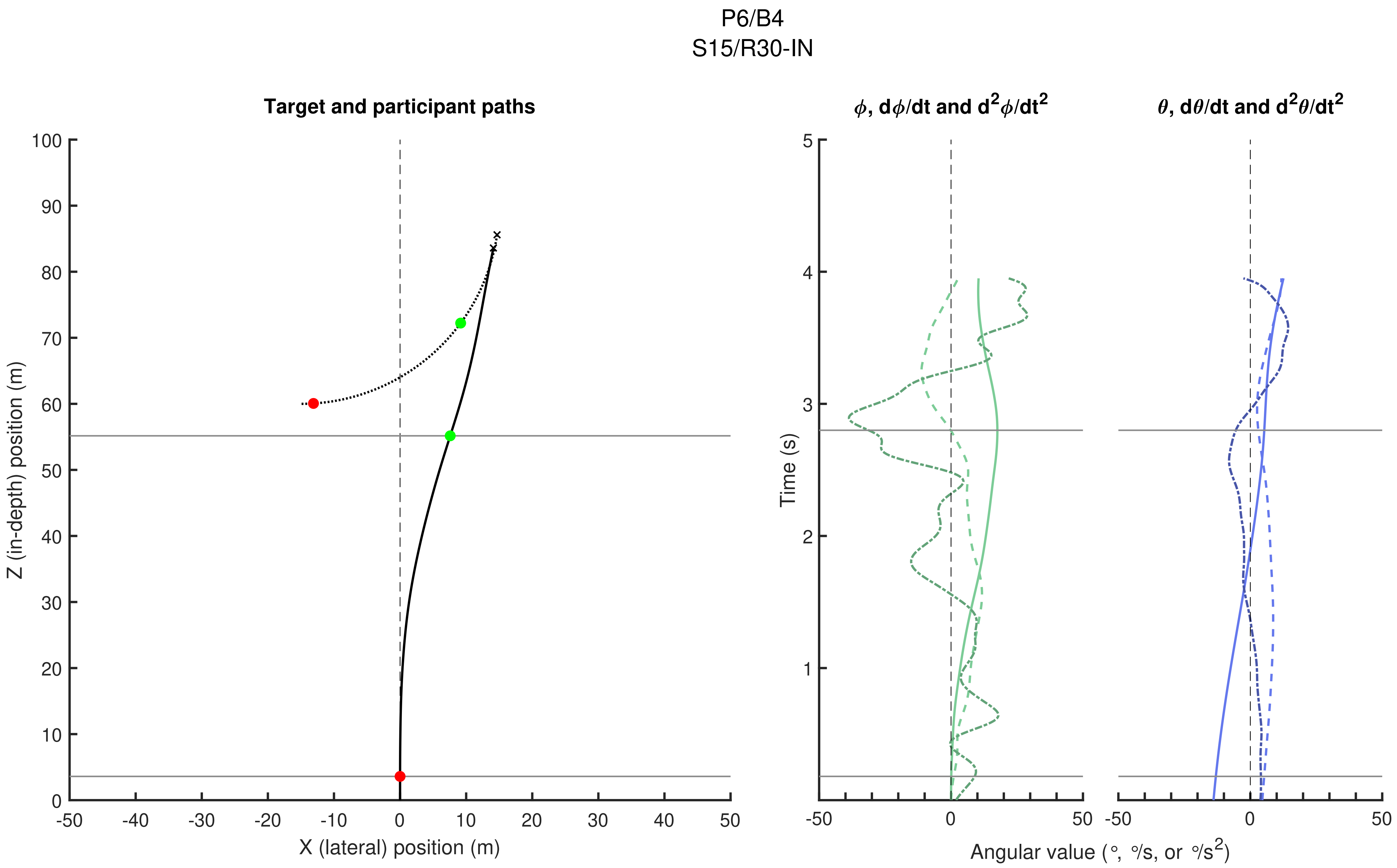

P6/B4
S15/R30-IN
Target and participant paths
Z (in-depth) position (m)
X (lateral) position (m)
100
90
80
70
60
50
40
30
20
10
0
-50
-40
-30
-20
-10
0
10
20
30
40
50
ϕ, dϕ/dt and d$^2\phi$/dt^2
θ, dθ/dt and d$^2\theta$/dt^2
Time (s)
5
4
3
2
1
-50
0
50
-50
0
50
Angular value (°, °/s, or °/s^2)

P6/B4
S5/R10-IN

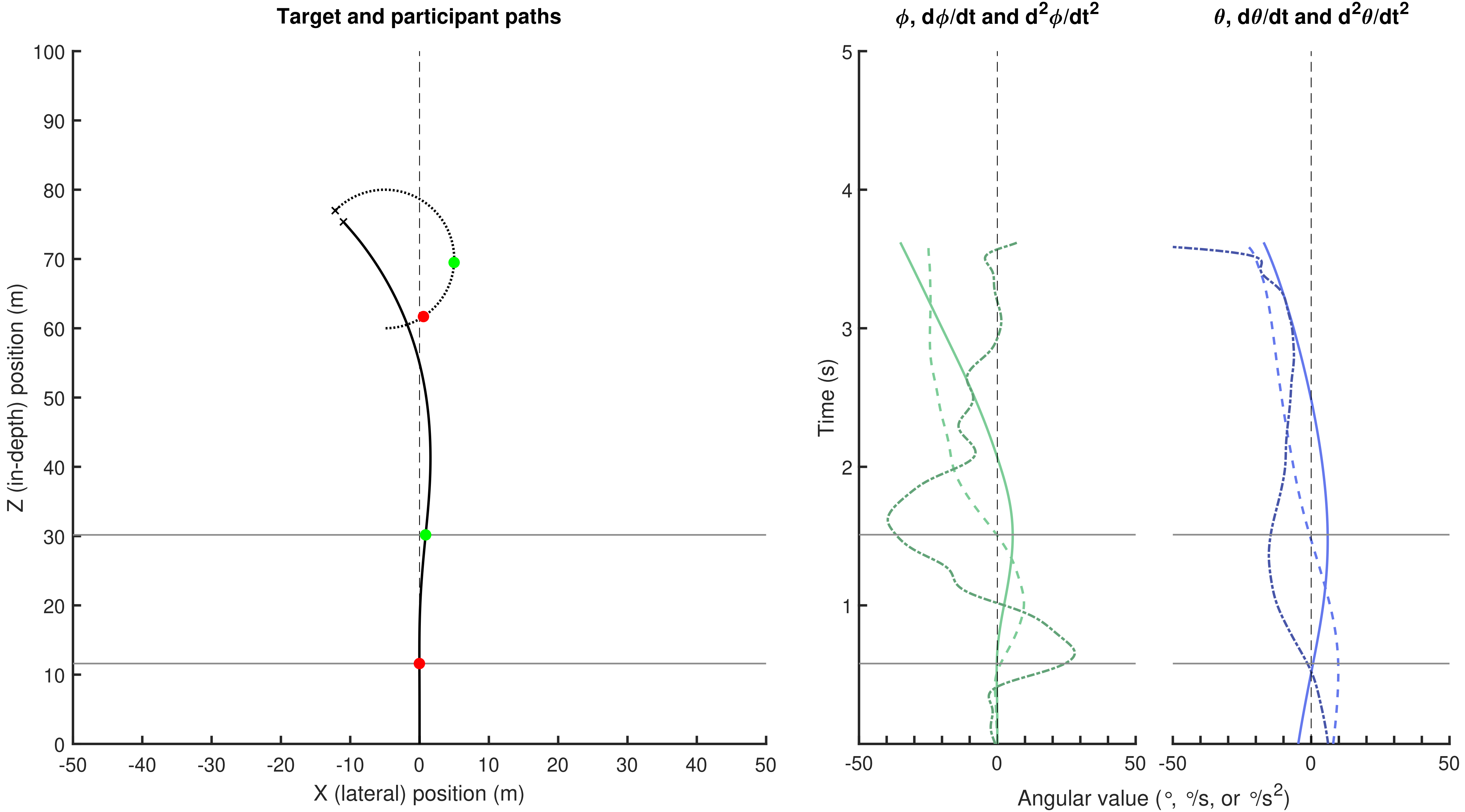

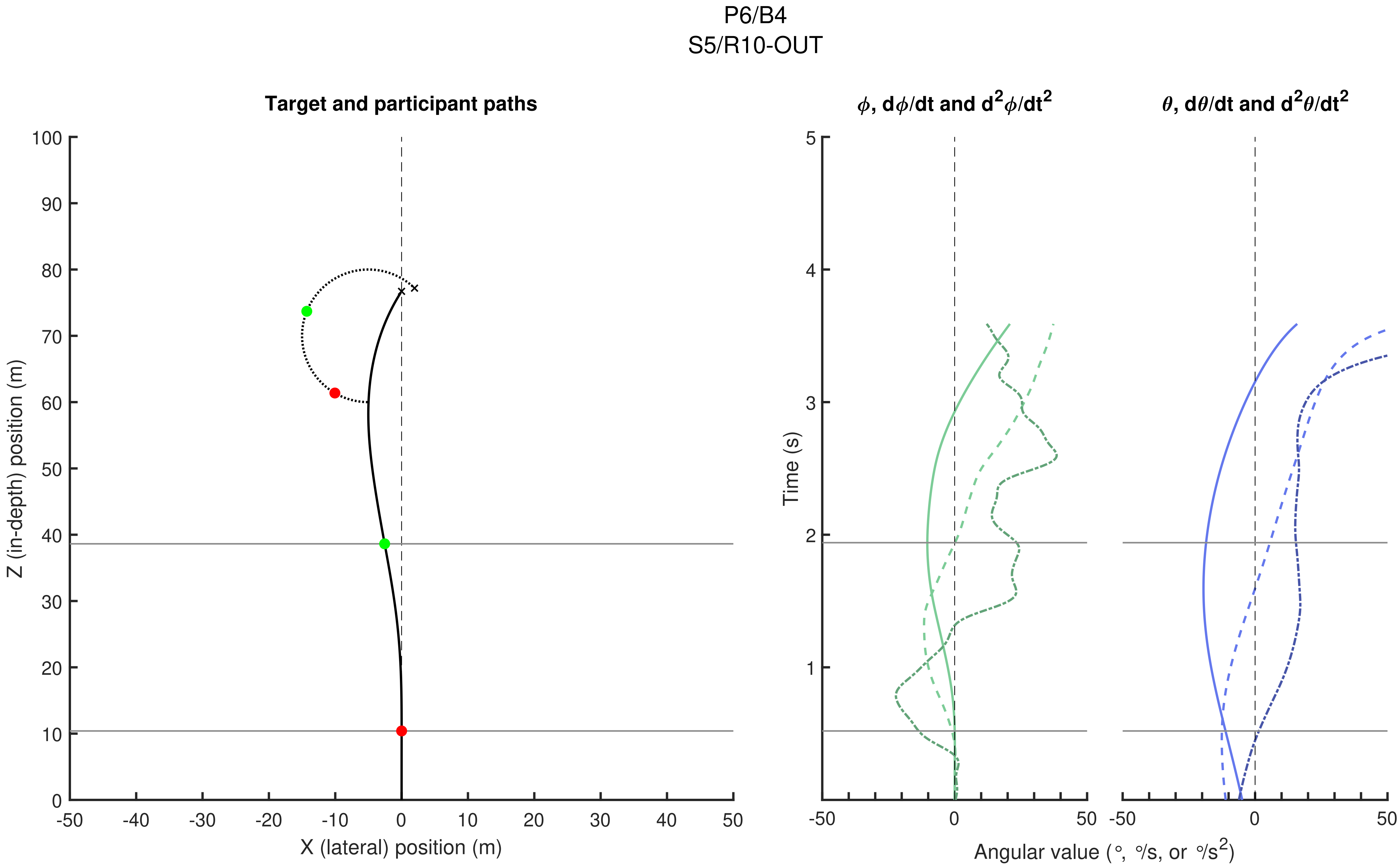
P6/B4
S5/R10-OUT
Target and participant paths
Z (in-depth) position (m)
X (lateral) position (m)
-50
-40
-30
-20
-10
0
10
20
30
40
50
100
90
80
70
60
50
40
30
20
10
0
ϕ, dϕ/dt and d$^2\phi$/dt^2
θ, dθ/dt and d$^2\theta$/dt^2
Time (s)
5
4
3
2
1
-50
0
50
Angular value (°, °/s, or °/s^2)

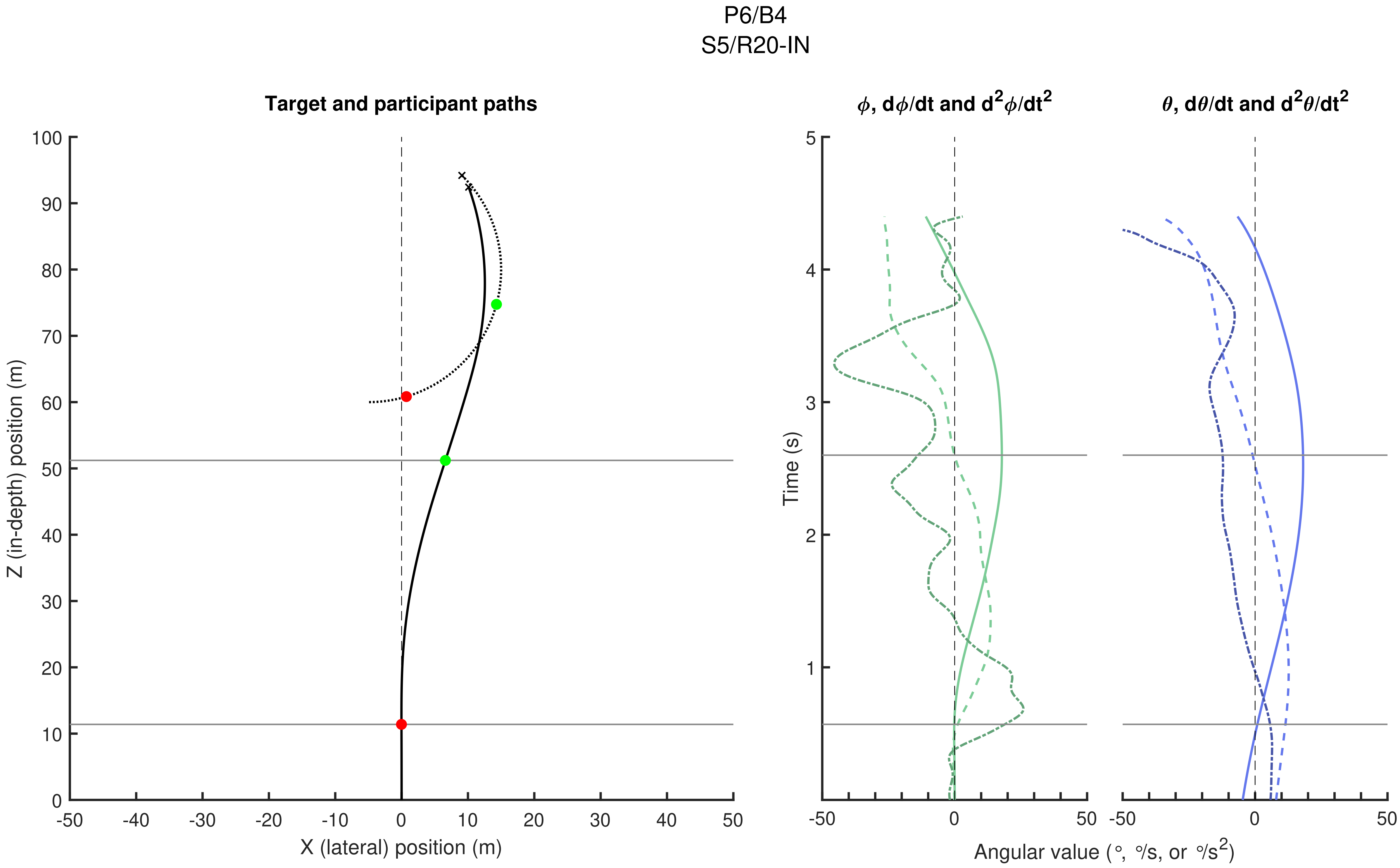

P6/B4
S5/R20-IN
Target and participant paths
Z (in-depth) position (m)
X (lateral) position (m)
ϕ, dϕ/dt and d$^2\phi$/dt^2
θ, dθ/dt and d$^2\theta$/dt^2
Time (s)
Angular value (°, °/s, or °/s^2)

P6/B4
S5/R20-OUT

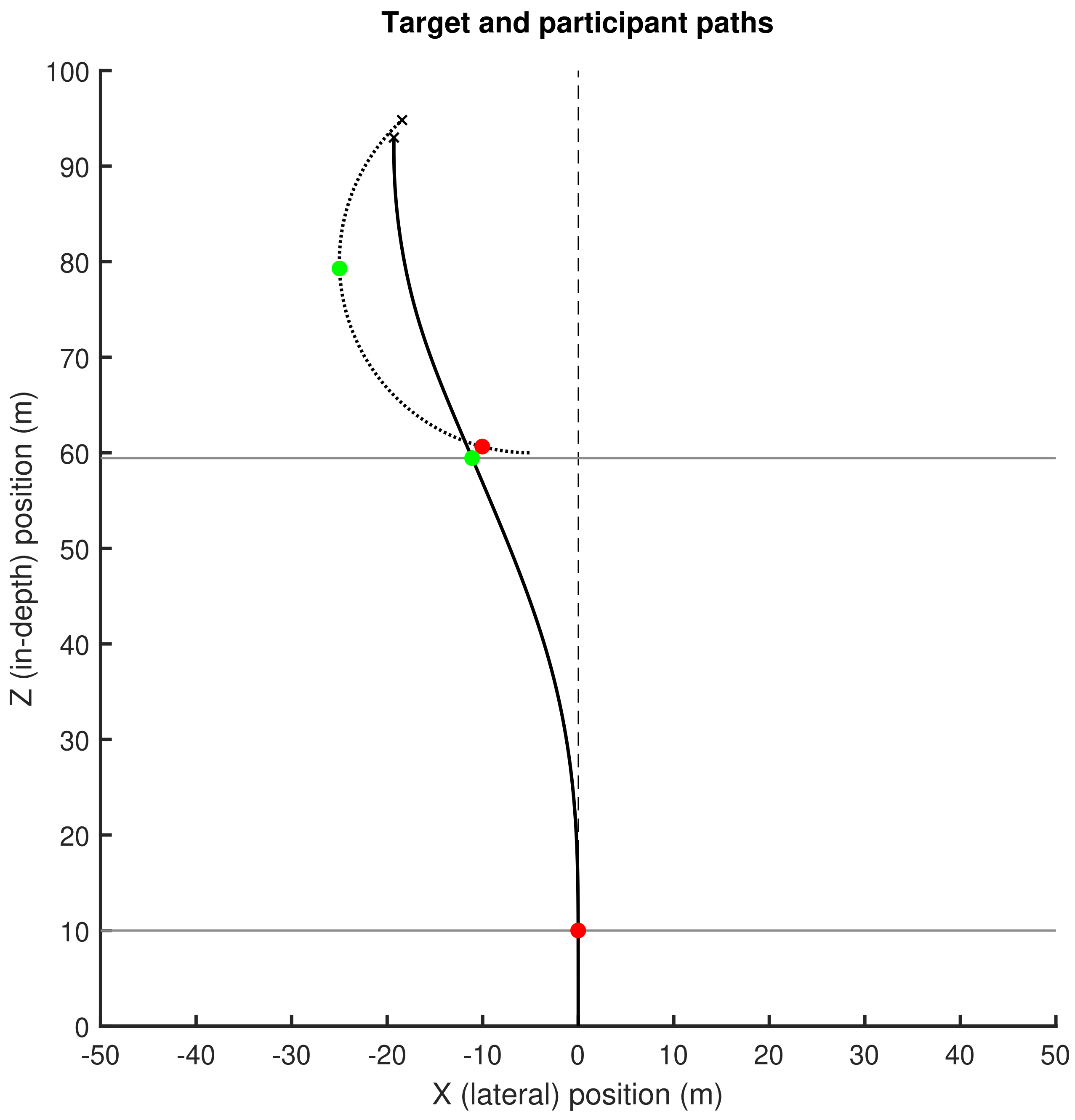

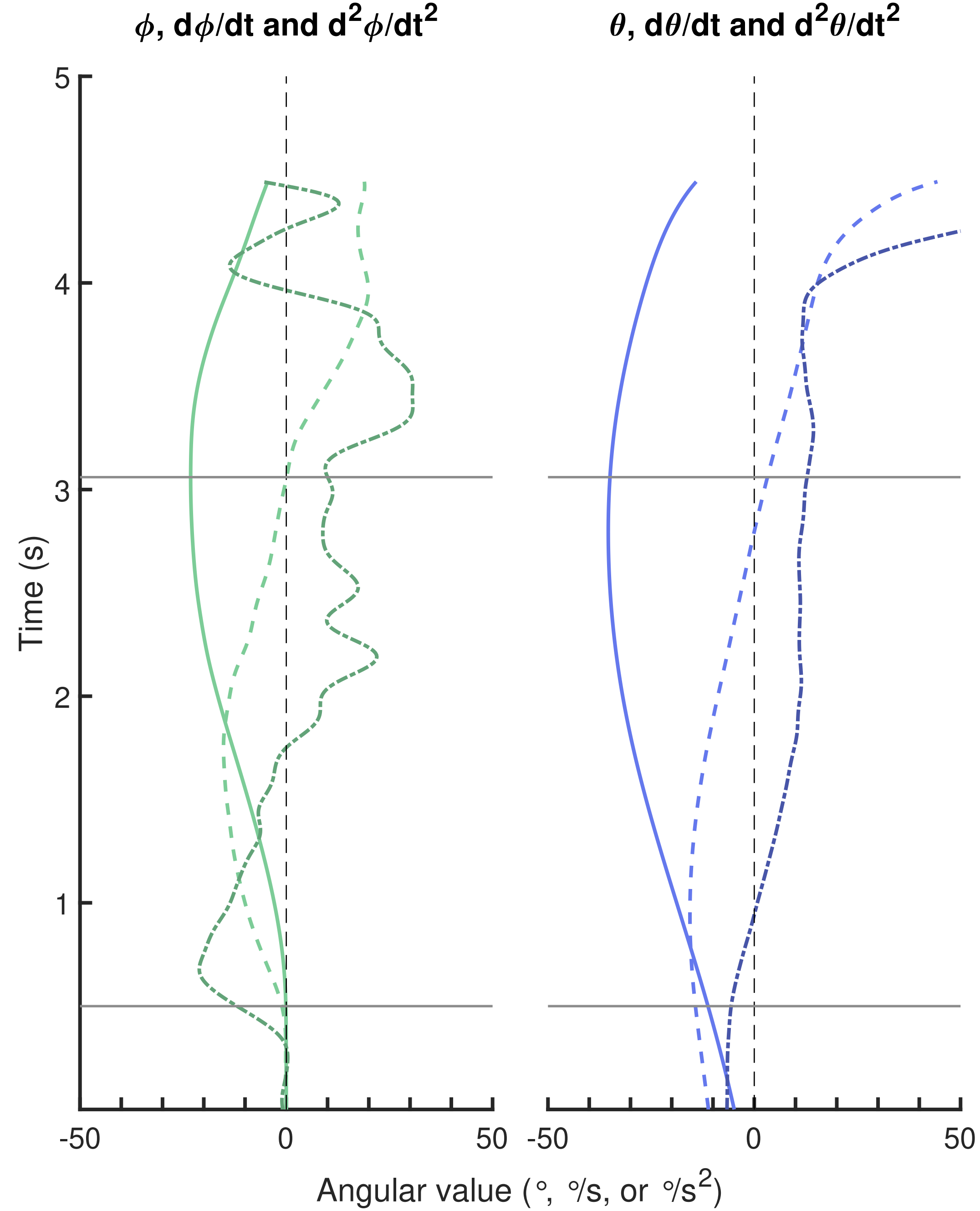

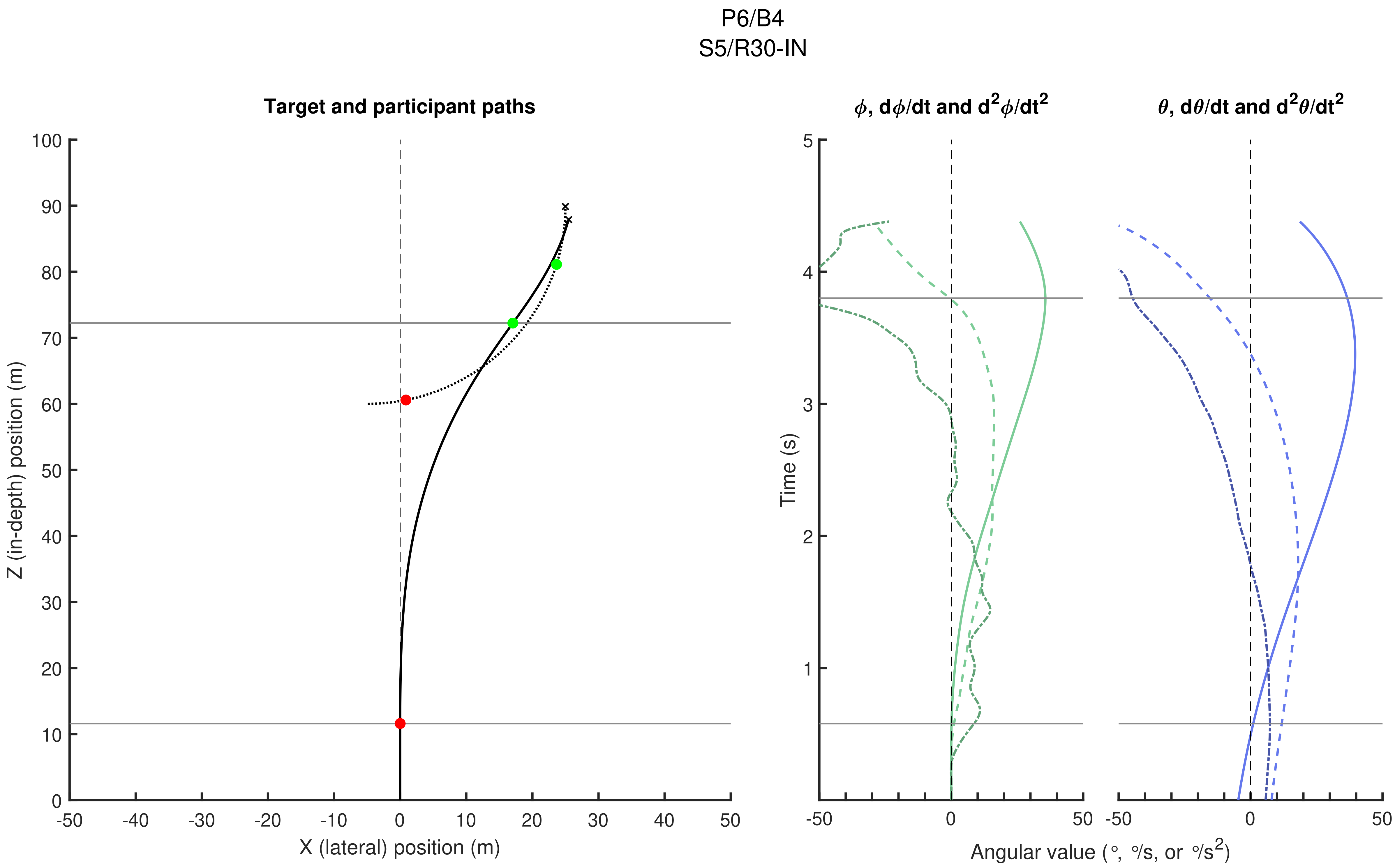

P6/B4
S5/R30-IN
Target and participant paths
Z (in-depth) position (m)
X (lateral) position (m)
φ, dφ/dt and d²φ/dt²
θ, dθ/dt and d²θ/dt²
Time (s)
Angular value (°, °/s, or °/s²)

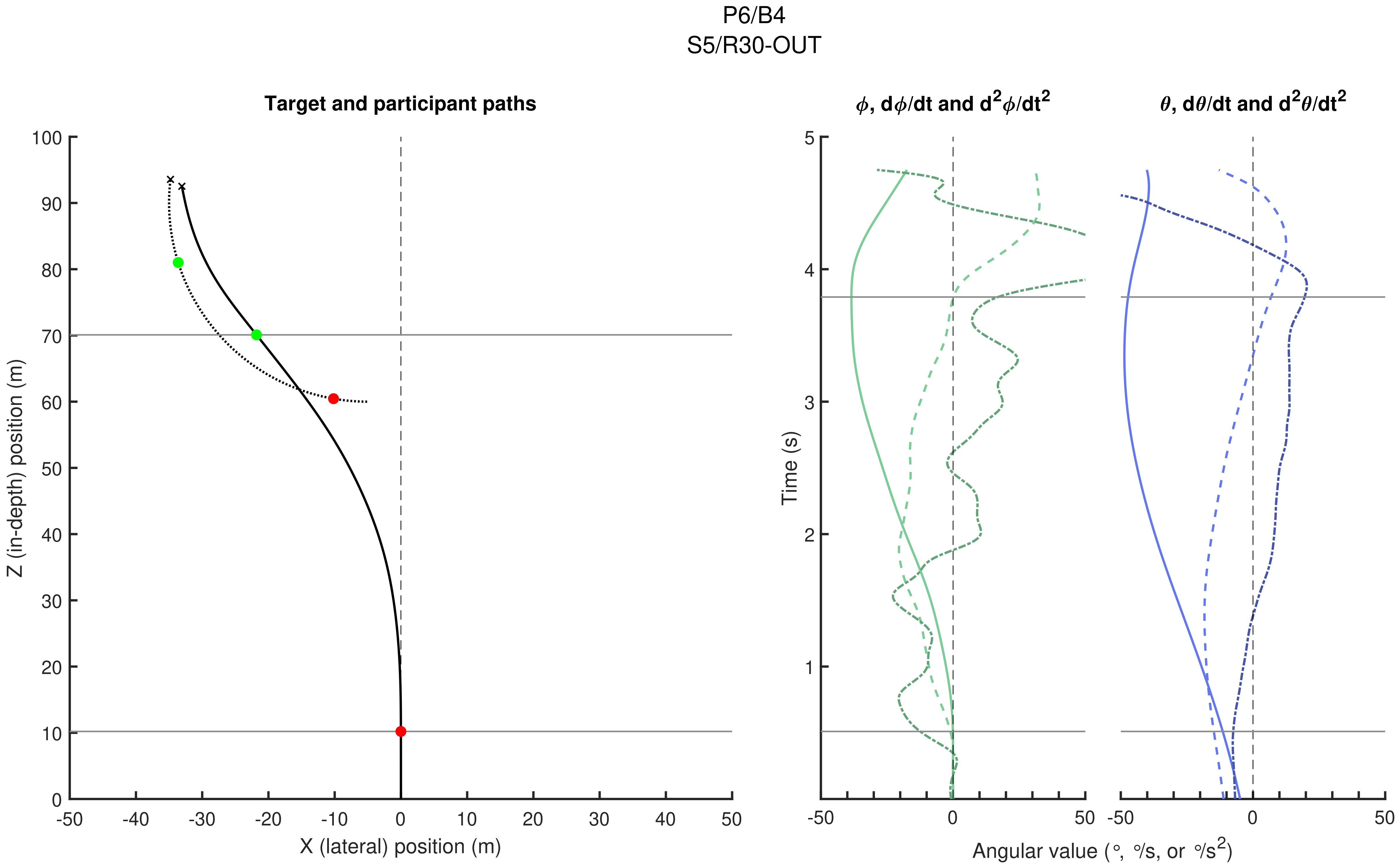
P6/B4
S5/R30-OUT
Target and participant paths
ϕ, dϕ/dt and d$^2\phi$/dt^2
θ, dθ/dt and d$^2\theta$/dt^2
Z (in-depth) position (m)
X (lateral) position (m)
Time (s)
Angular value (°, °/s, or °/s^2)

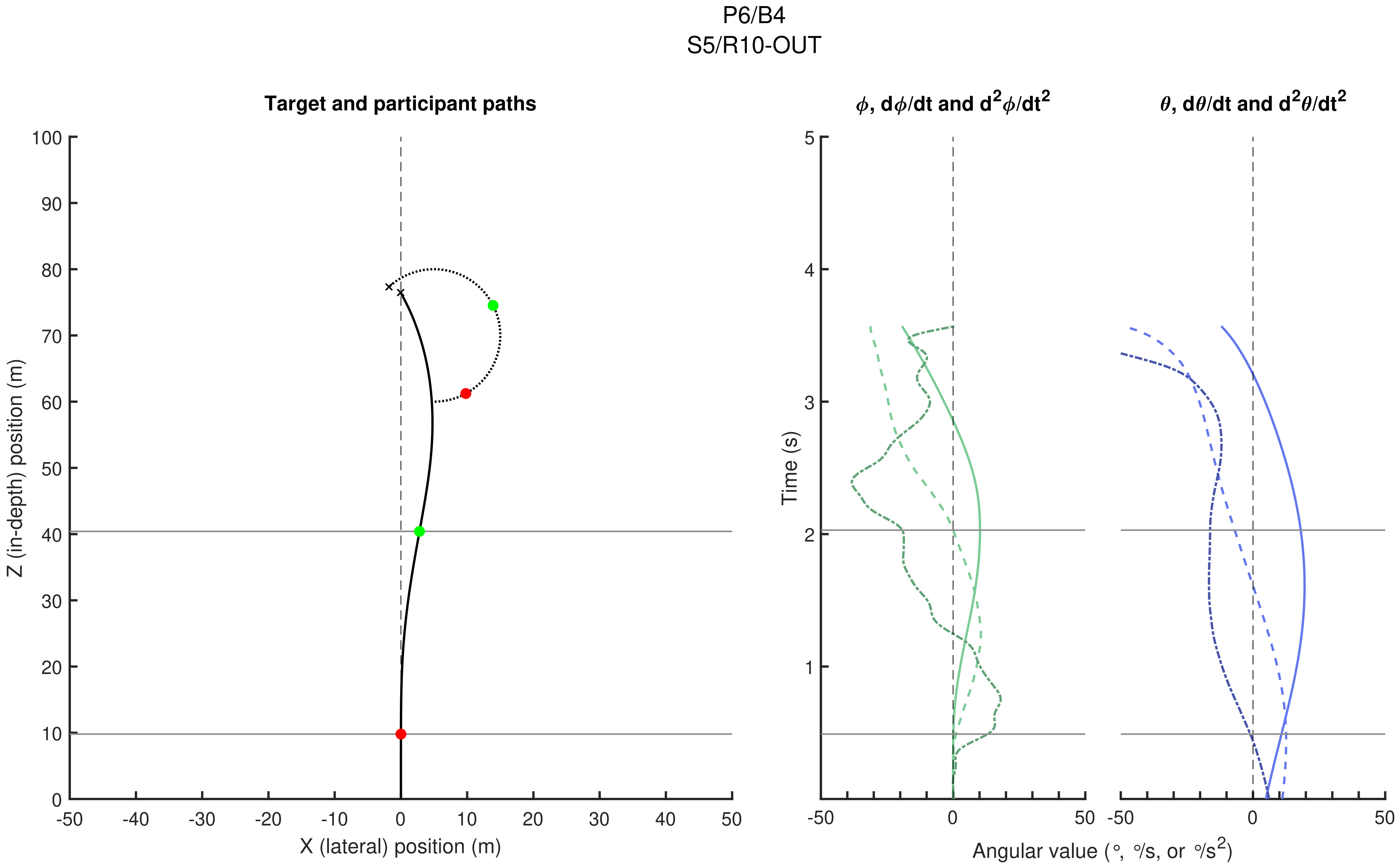

P6/B4
S5/R10-OUT
Target and participant paths
Z (in-depth) position (m)
X (lateral) position (m)
ϕ, dϕ/dt and d$^2\phi$/dt^2
θ, dθ/dt and d$^2\theta$/dt^2
Time (s)
Angular value (°, °/s, or °/s^2)

P6/B4
S5/R10-IN

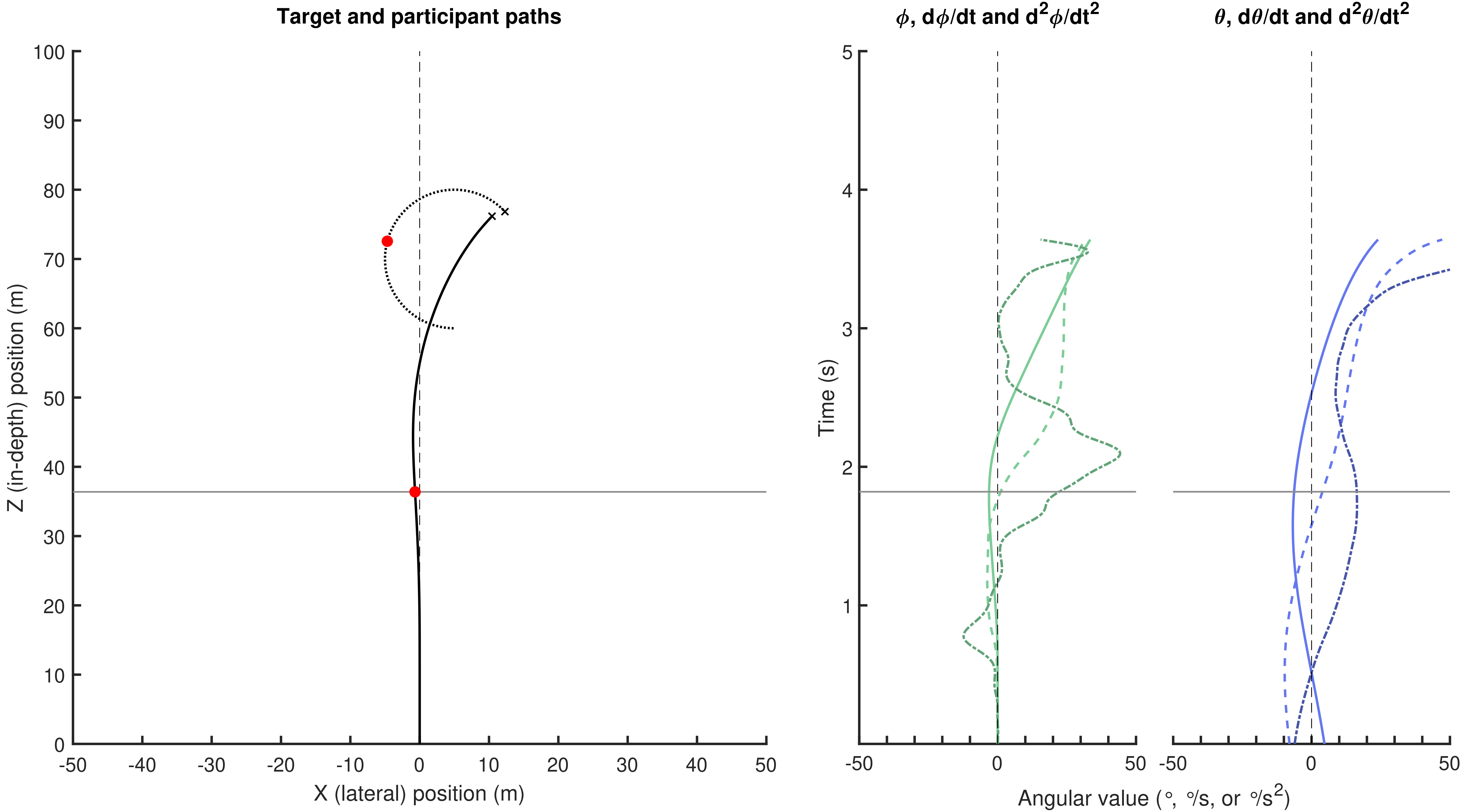

P6/B4
S5/R20-OUT

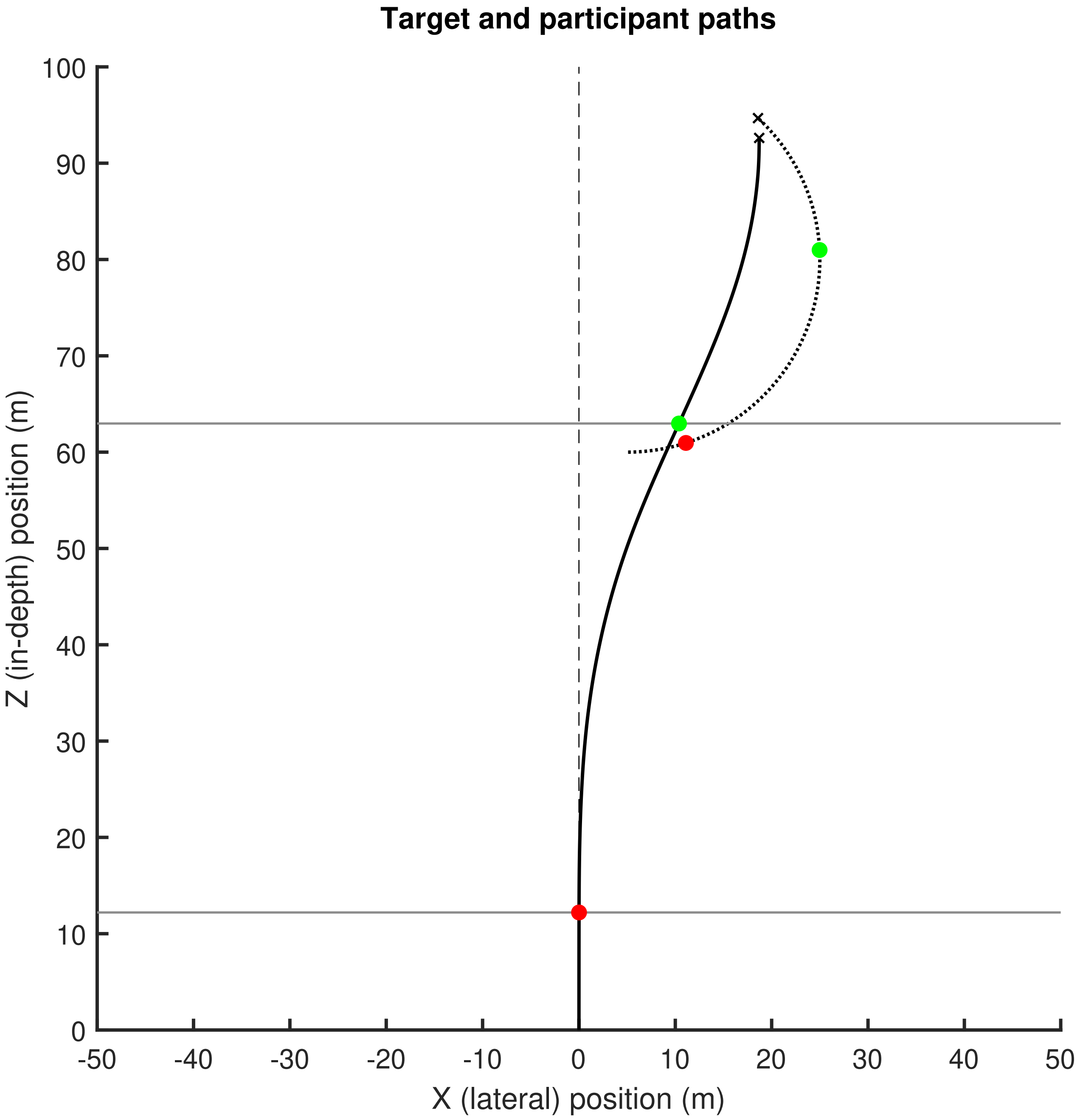

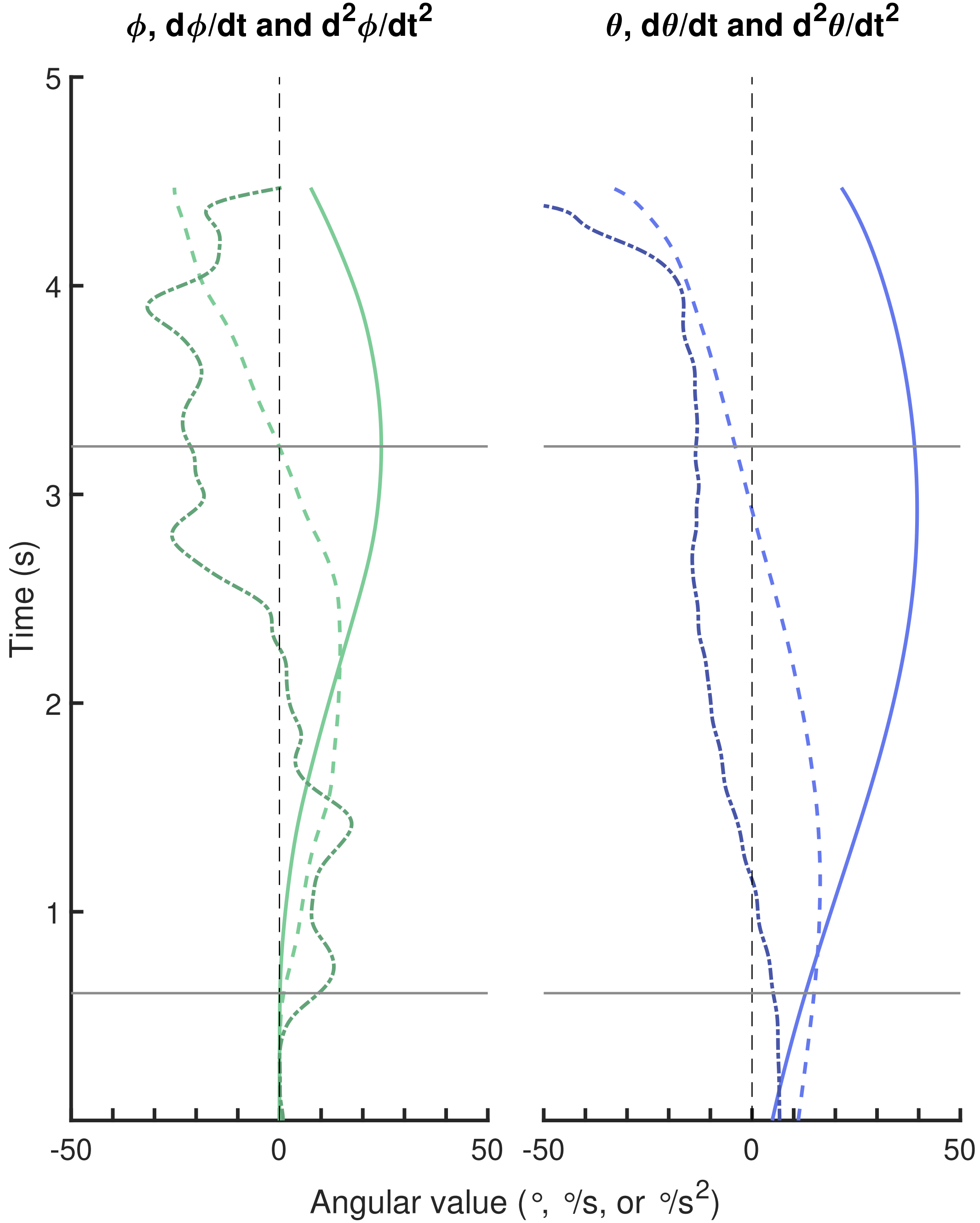

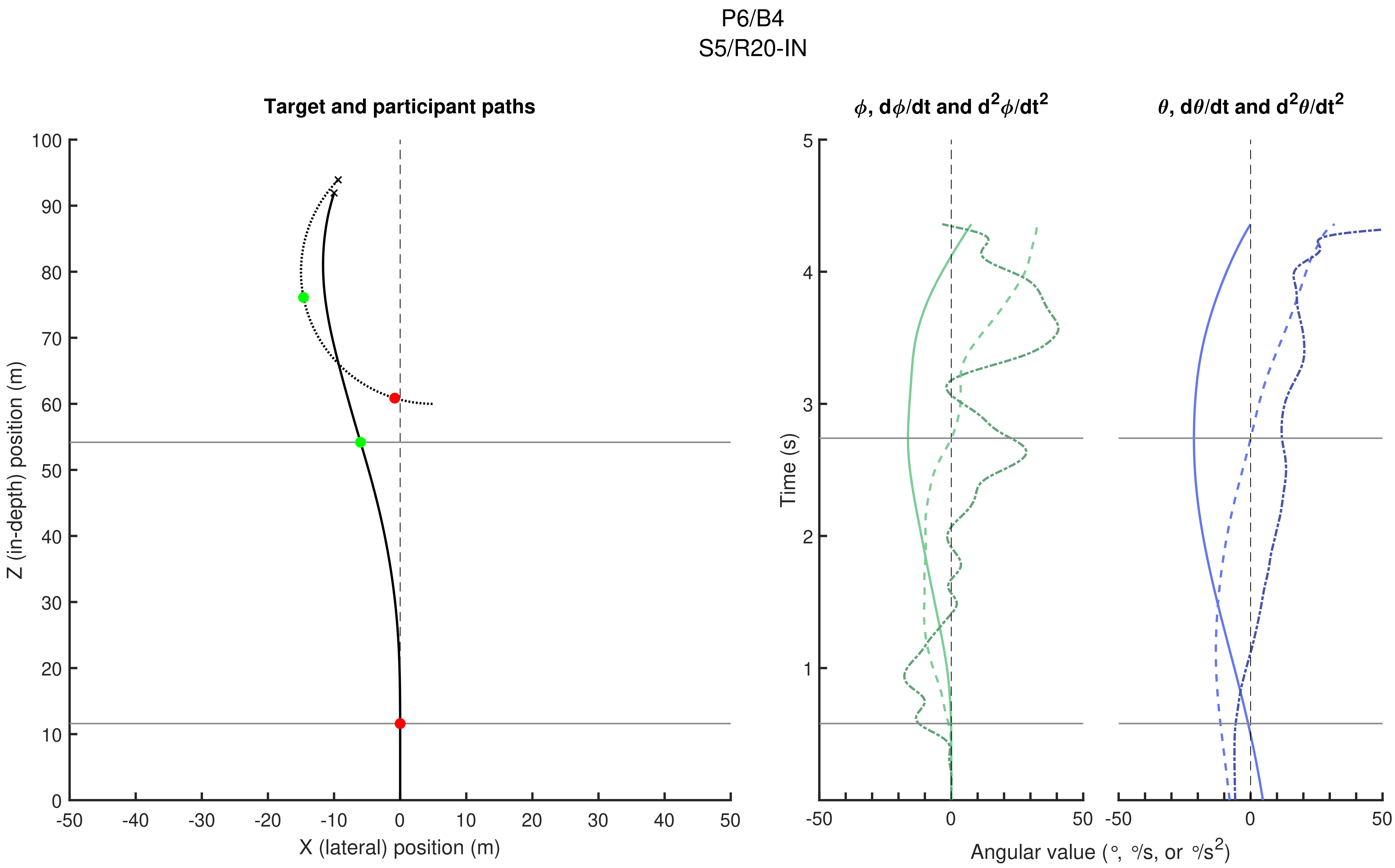
P6/B4
S5/R20-IN
Target and participant paths
Z (in-depth) position (m)
X (lateral) position (m)
100
90
80
70
60
50
40
30
20
10
0
-50
-40
-30
-20
-10
0
10
20
30
40
50
ϕ, dϕ/dt and d$^2\phi$/dt^2
θ, dθ/dt and d$^2\theta$/dt^2
Time (s)
5
4
3
2
1
-50
0
50
-50
0
50
Angular value (°, °/s, or °/s^2)

P6/B4
S5/R30-OUT

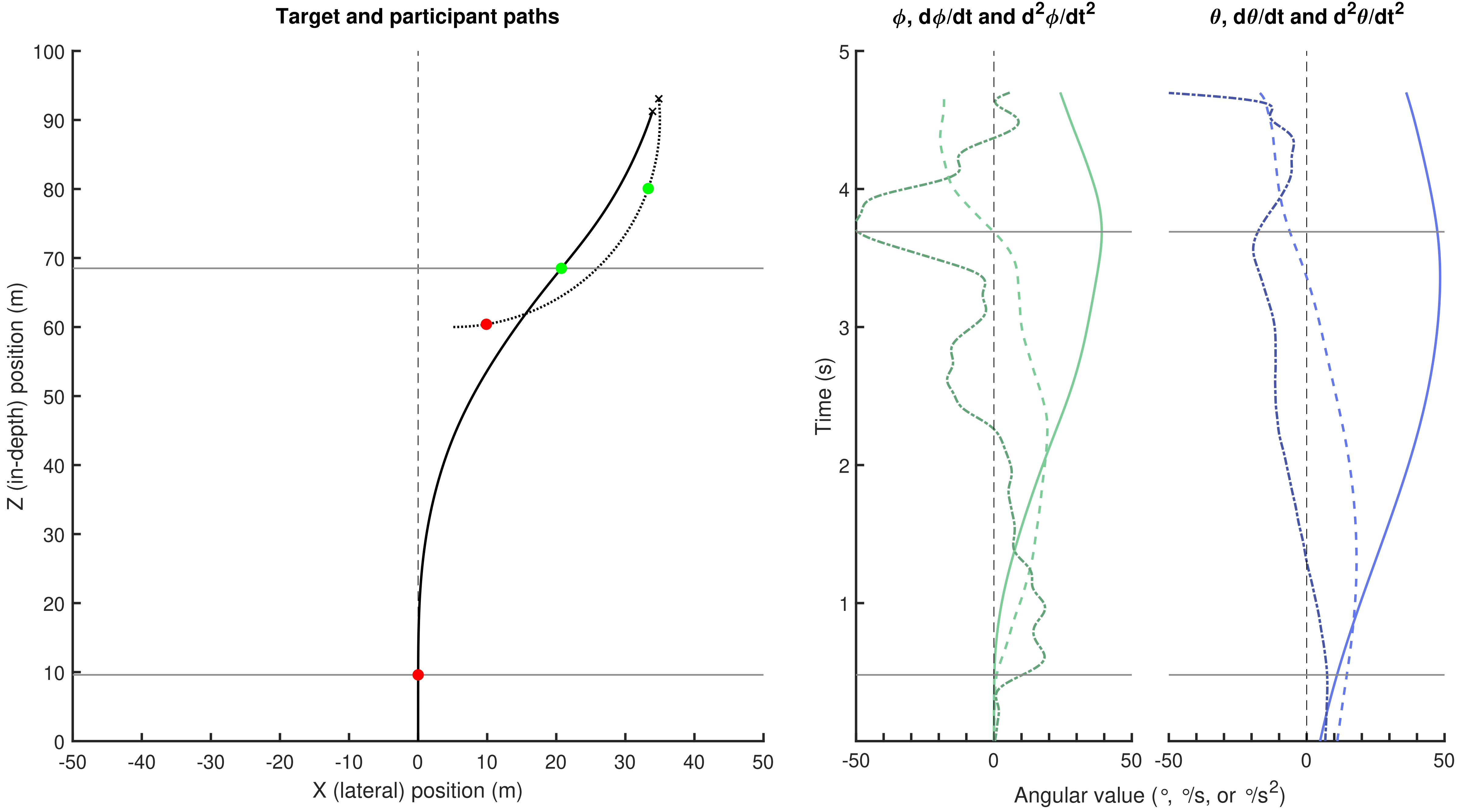

P6/B4
S5/R30-IN

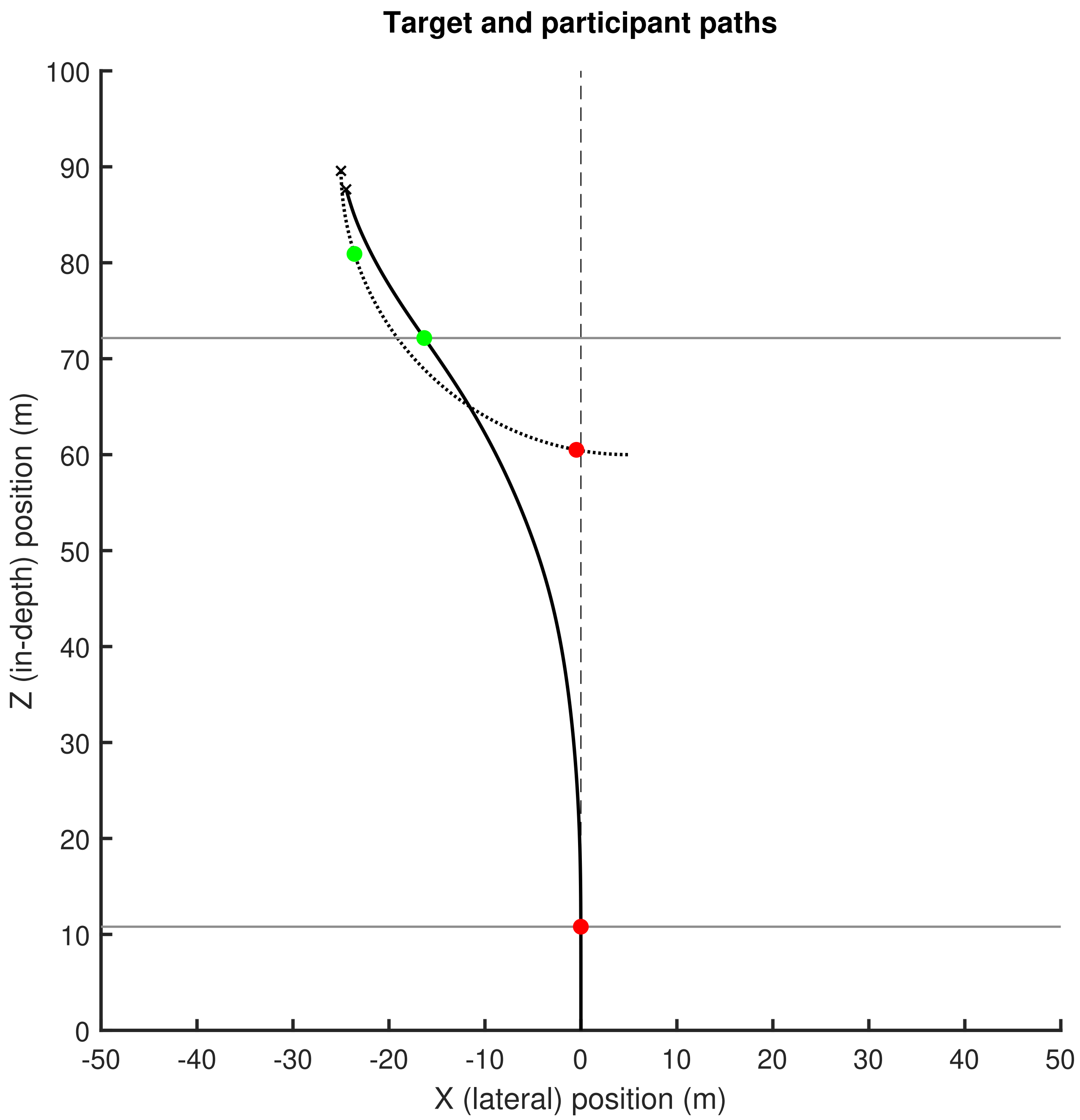

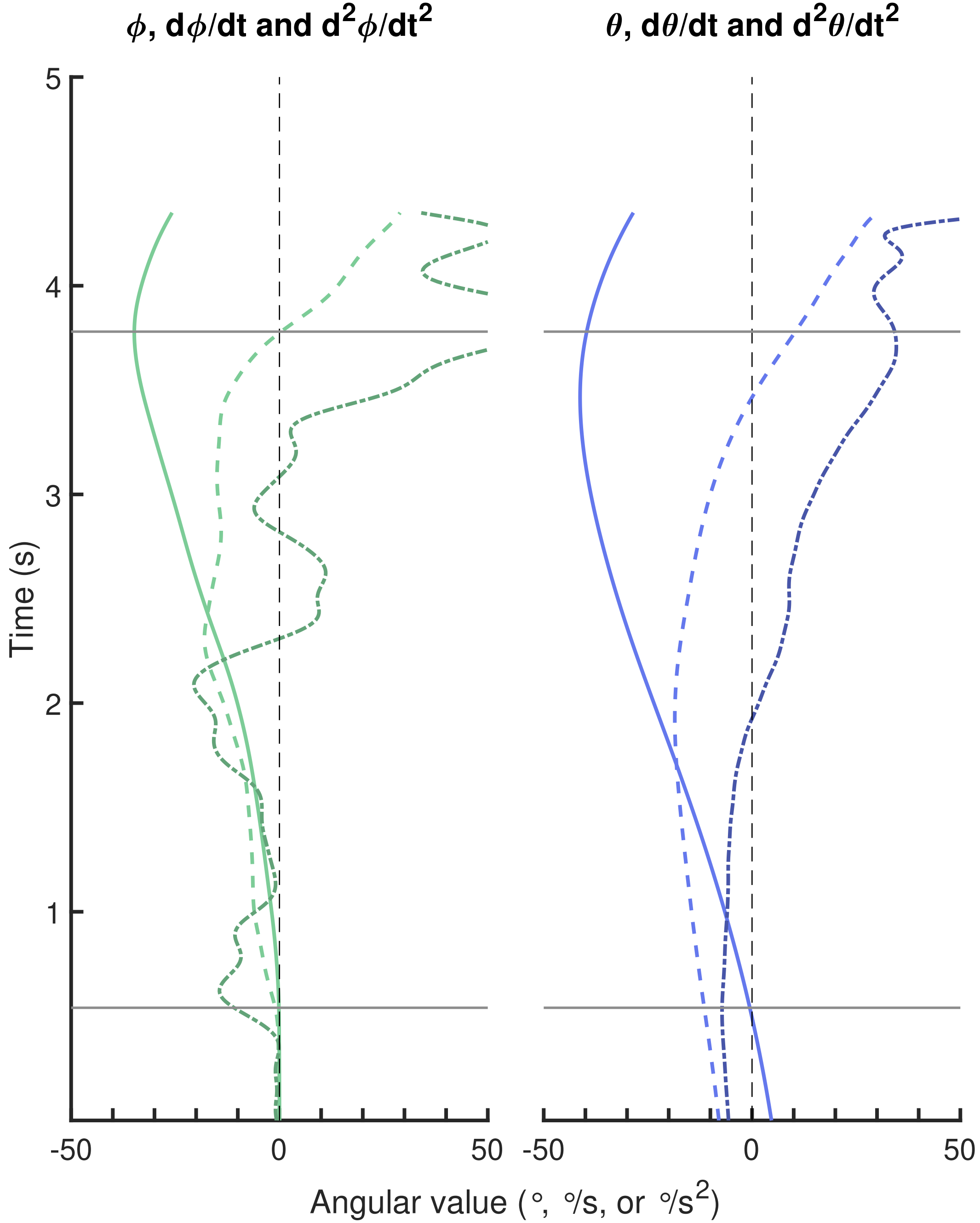

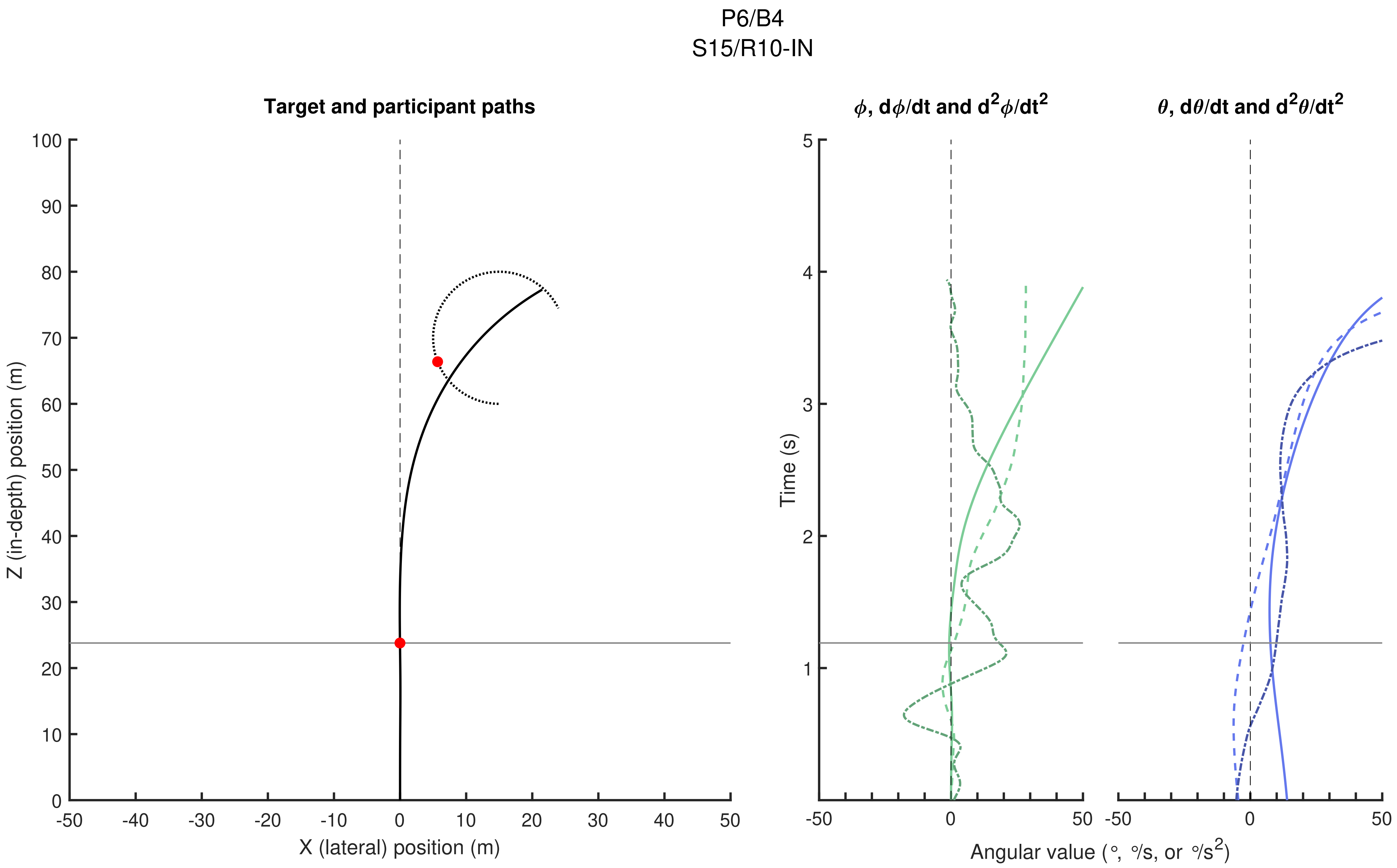
P6/B4
S15/R10-IN
Target and participant paths
Z (in-depth) position (m)
X (lateral) position (m)
ϕ, dϕ/dt and d²ϕ/dt²
θ, dθ/dt and d²θ/dt²
Time (s)
Angular value (°, °/s, or °/s²)

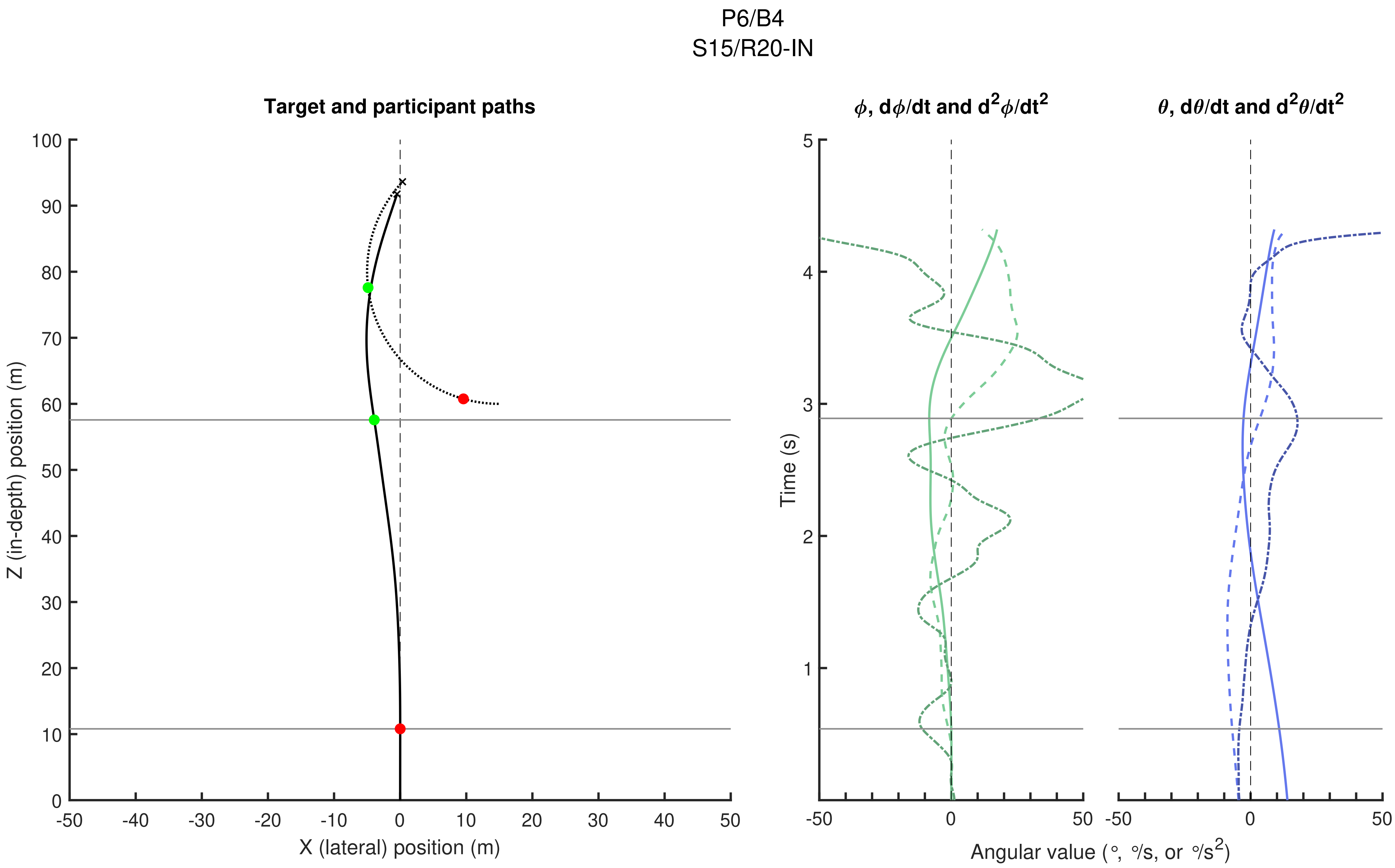
P6/B4
S15/R20-IN
Target and participant paths
Z (in-depth) position (m)
X (lateral) position (m)
100
90
80
70
60
50
40
30
20
10
0
-50
-40
-30
-20
-10
0
10
20
30
40
50
ϕ, dϕ/dt and d²ϕ/dt²
θ, dθ/dt and d²θ/dt²
Time (s)
5
4
3
2
1
-50
0
50
-50
0
50
Angular value (°, °/s, or °/s²)

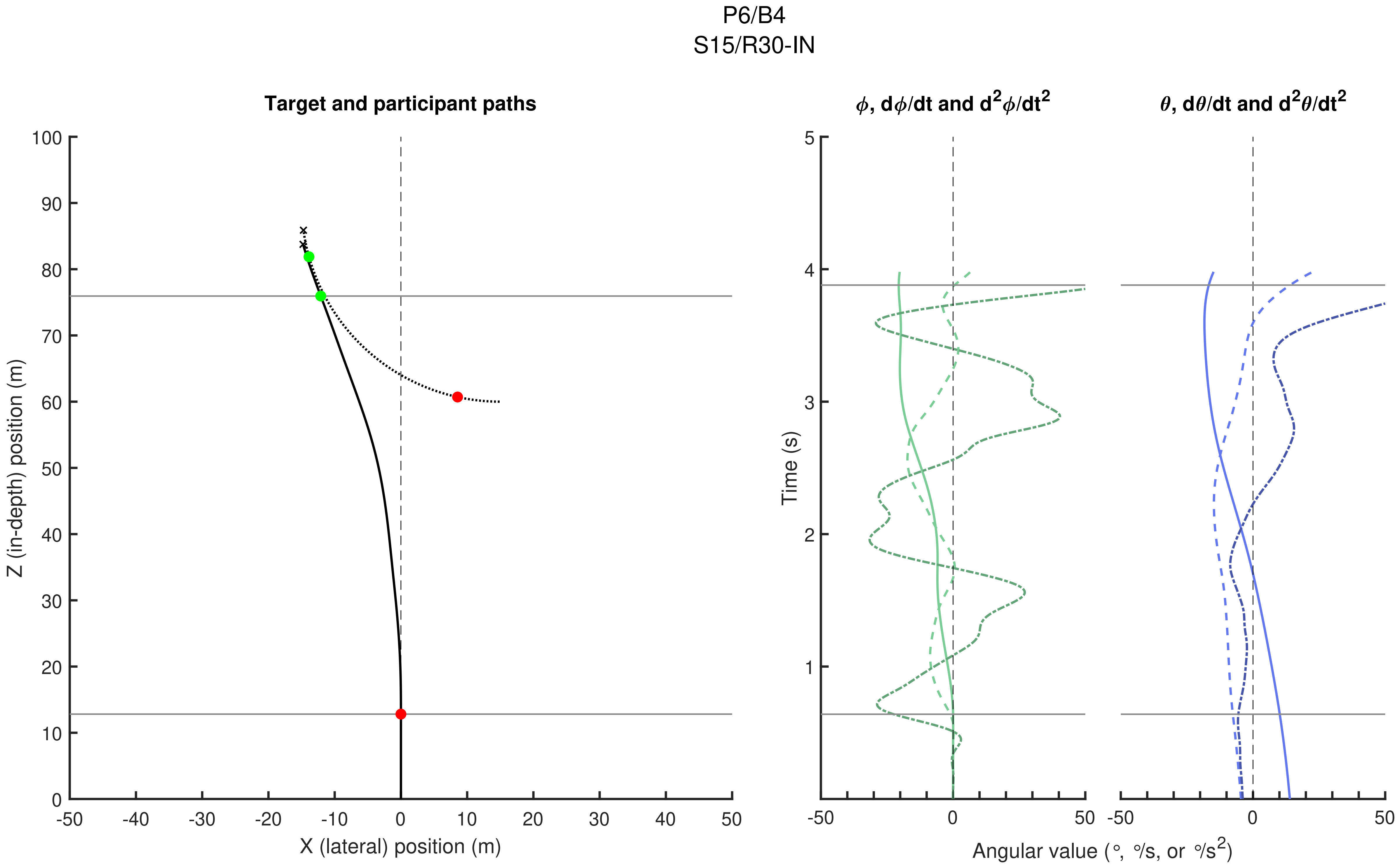

P6/B4
S15/R30-IN
Target and participant paths
Z (in-depth) position (m)
X (lateral) position (m)
ϕ, dϕ/dt and d$^2\phi$/dt^2
θ, dθ/dt and d$^2\theta$/dt^2
Time (s)
Angular value (°, °/s, or °/s^2)

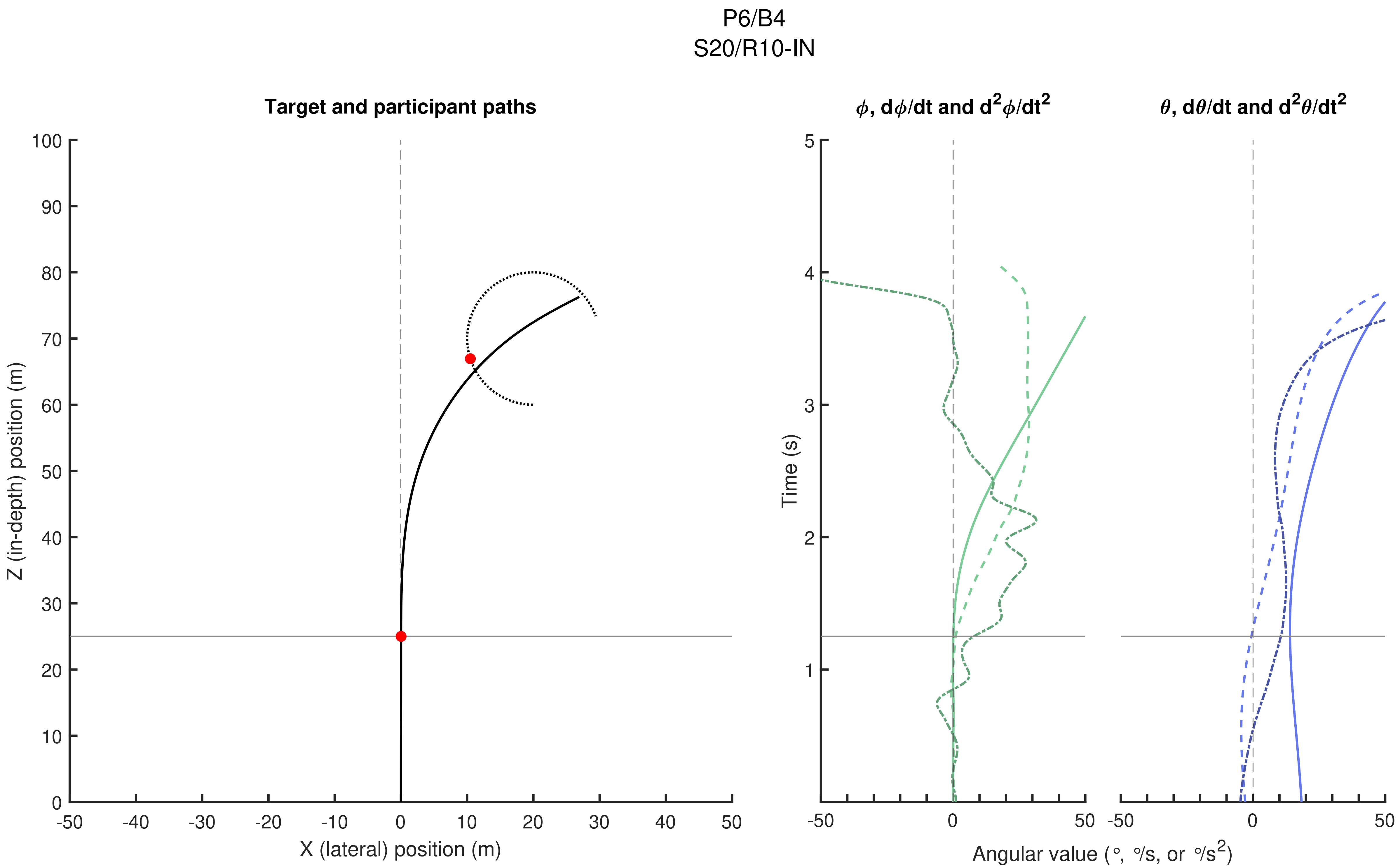

P6/B4
S20/R10-IN
Target and participant paths
Z (in-depth) position (m)
X (lateral) position (m)
ϕ, dϕ/dt and d$^2\phi$/dt^2
θ, dθ/dt and d$^2\theta$/dt^2
Time (s)
Angular value (°, °/s, or °/s^2)

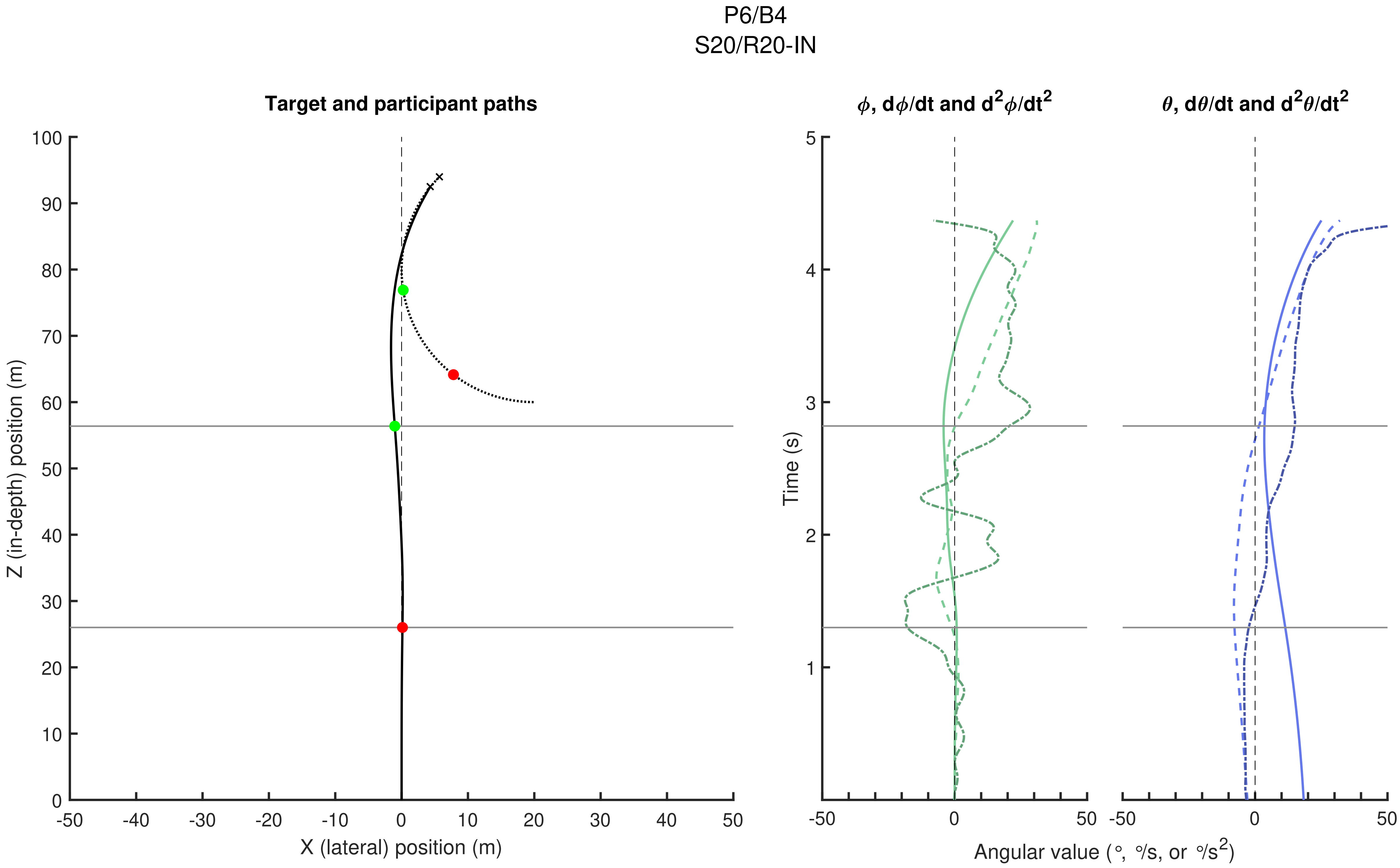

P6/B4
S20/R20-IN
Target and participant paths
Z (in-depth) position (m)
X (lateral) position (m)
ϕ, dϕ/dt and d$^2\phi$/dt^2
θ, dθ/dt and d$^2\theta$/dt^2
Time (s)
Angular value (°, °/s, or °/s^2)

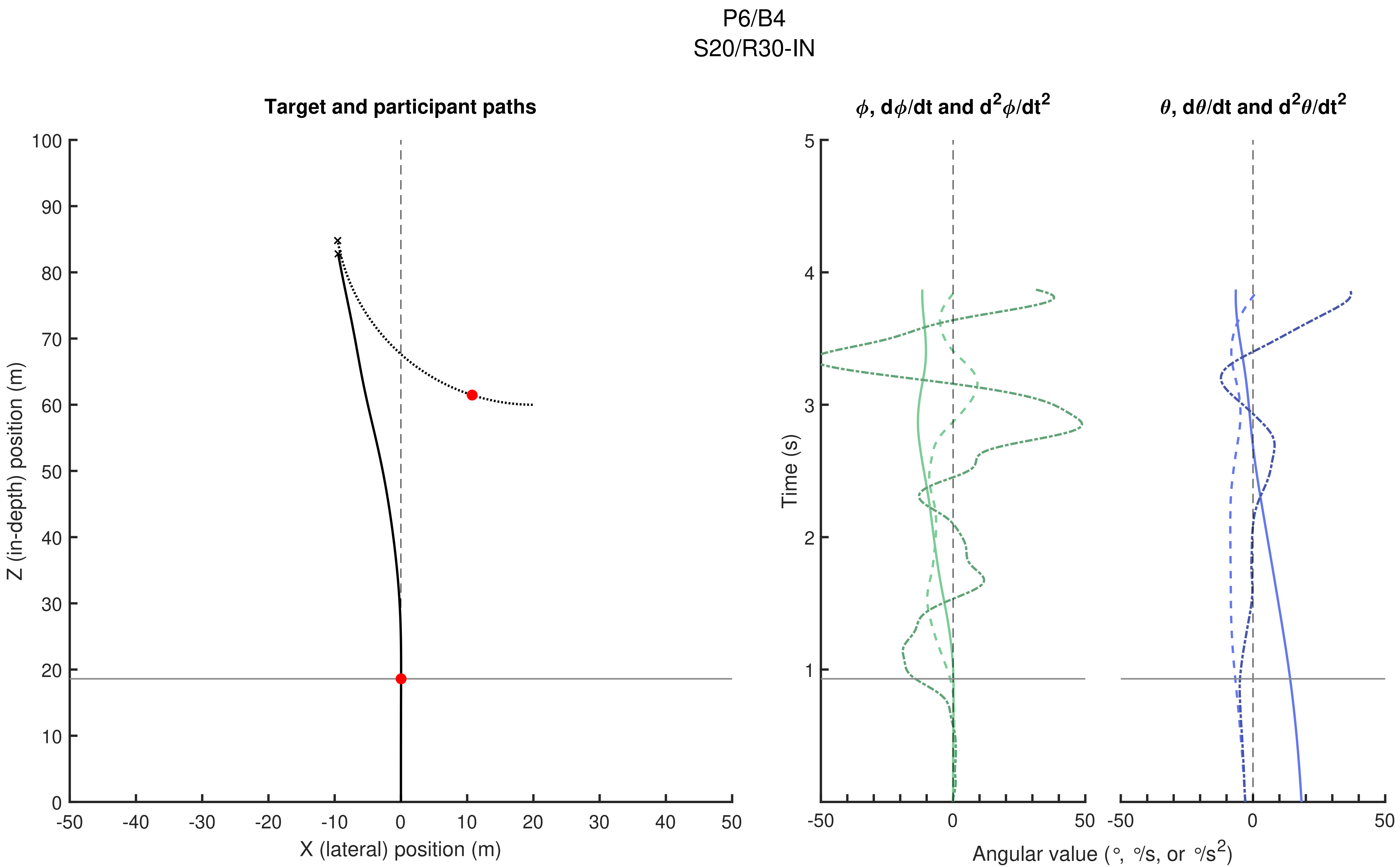
P6/B4
S20/R30-IN
Target and participant paths
Z (in-depth) position (m)
X (lateral) position (m)
100
90
80
70
60
50
40
30
20
10
0
-50
-40
-30
-20
-10
0
10
20
30
40
50
ϕ, dϕ/dt and d$^2\phi$/dt^2
θ, dθ/dt and d$^2\theta$/dt^2
Time (s)
5
4
3
2
1
-50
0
50
-50
0
50
Angular value (°, °/s, or °/s^2)

P6/B5
S20/R10-IN

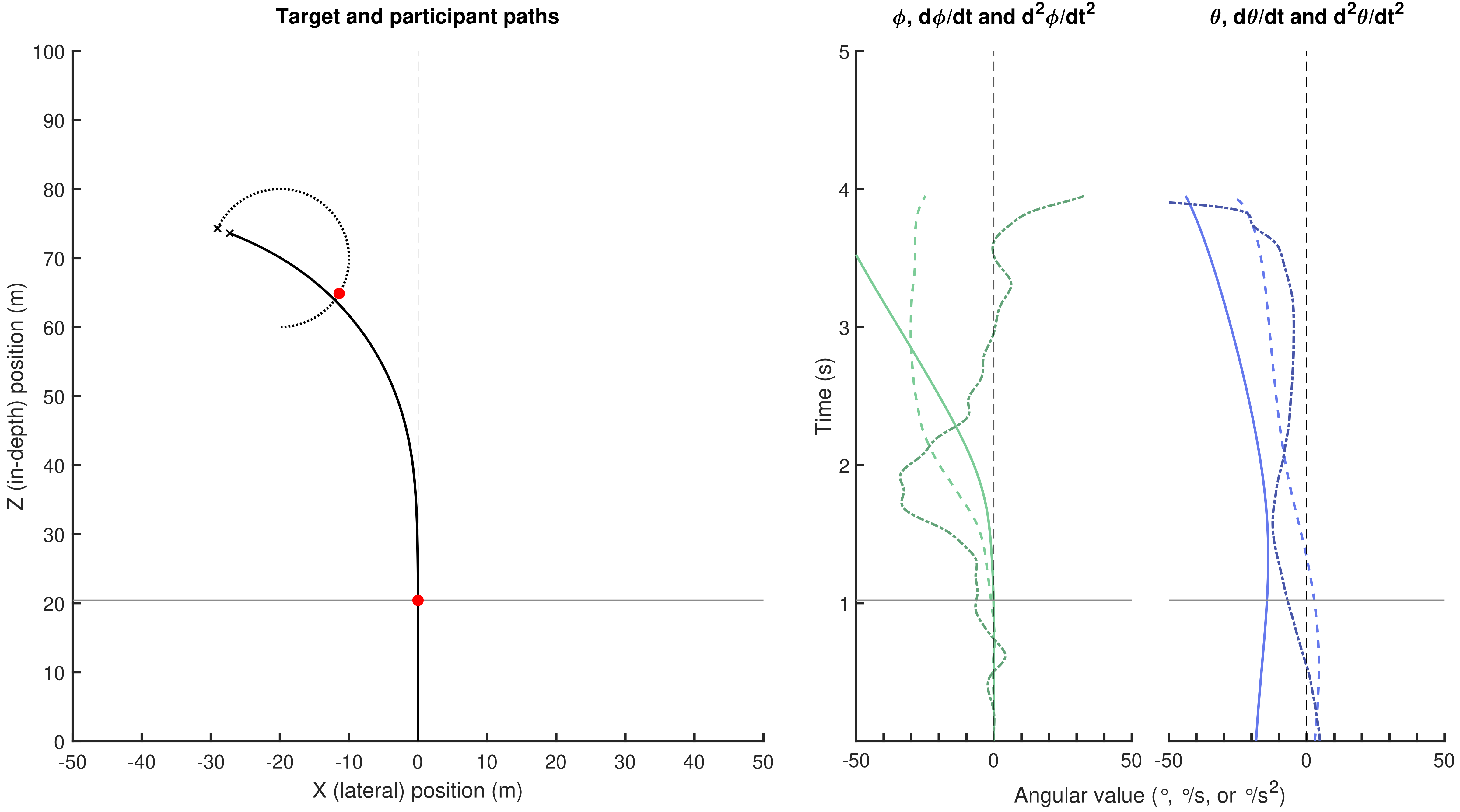

P6/B5
S20/R20-IN

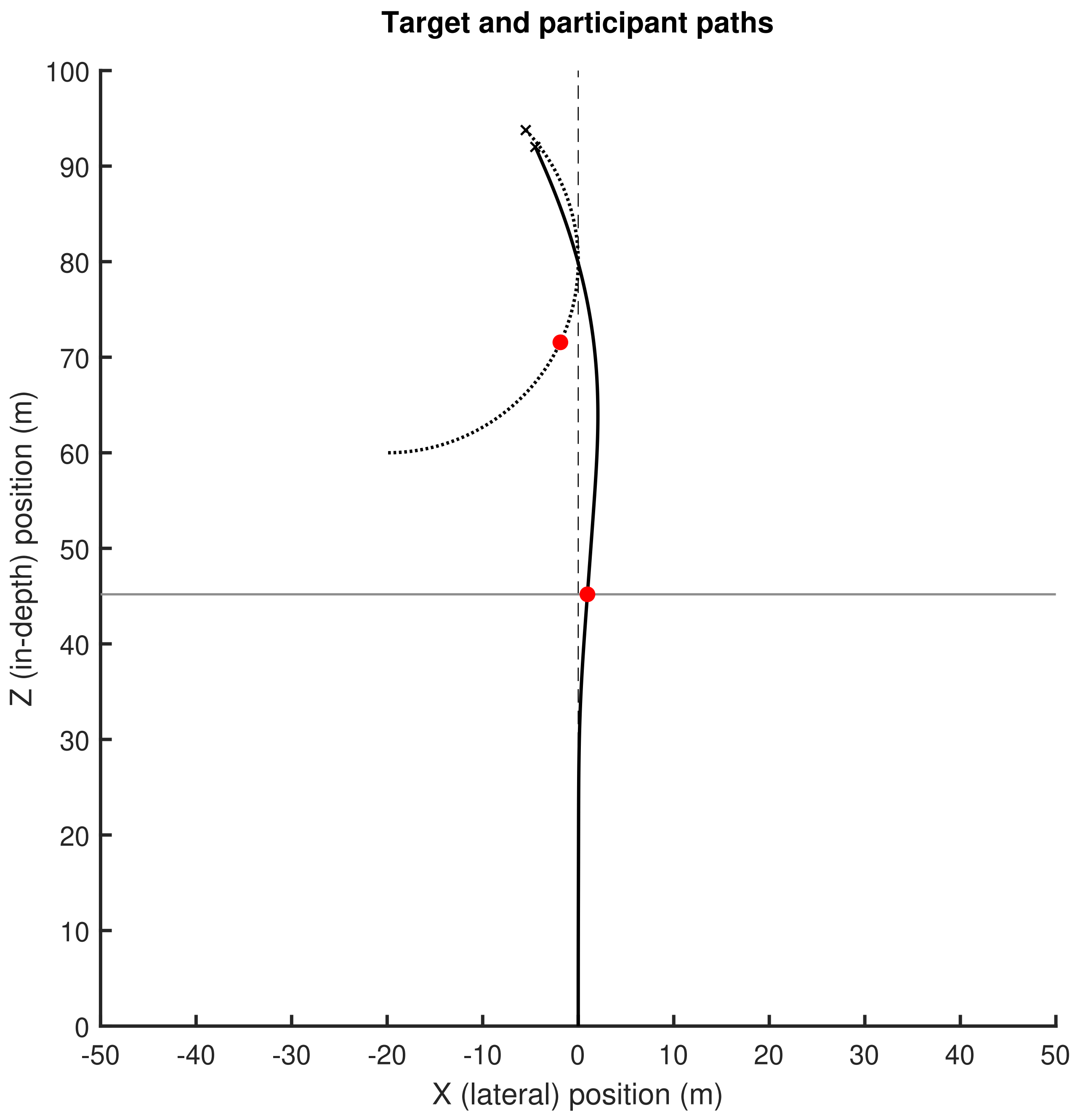

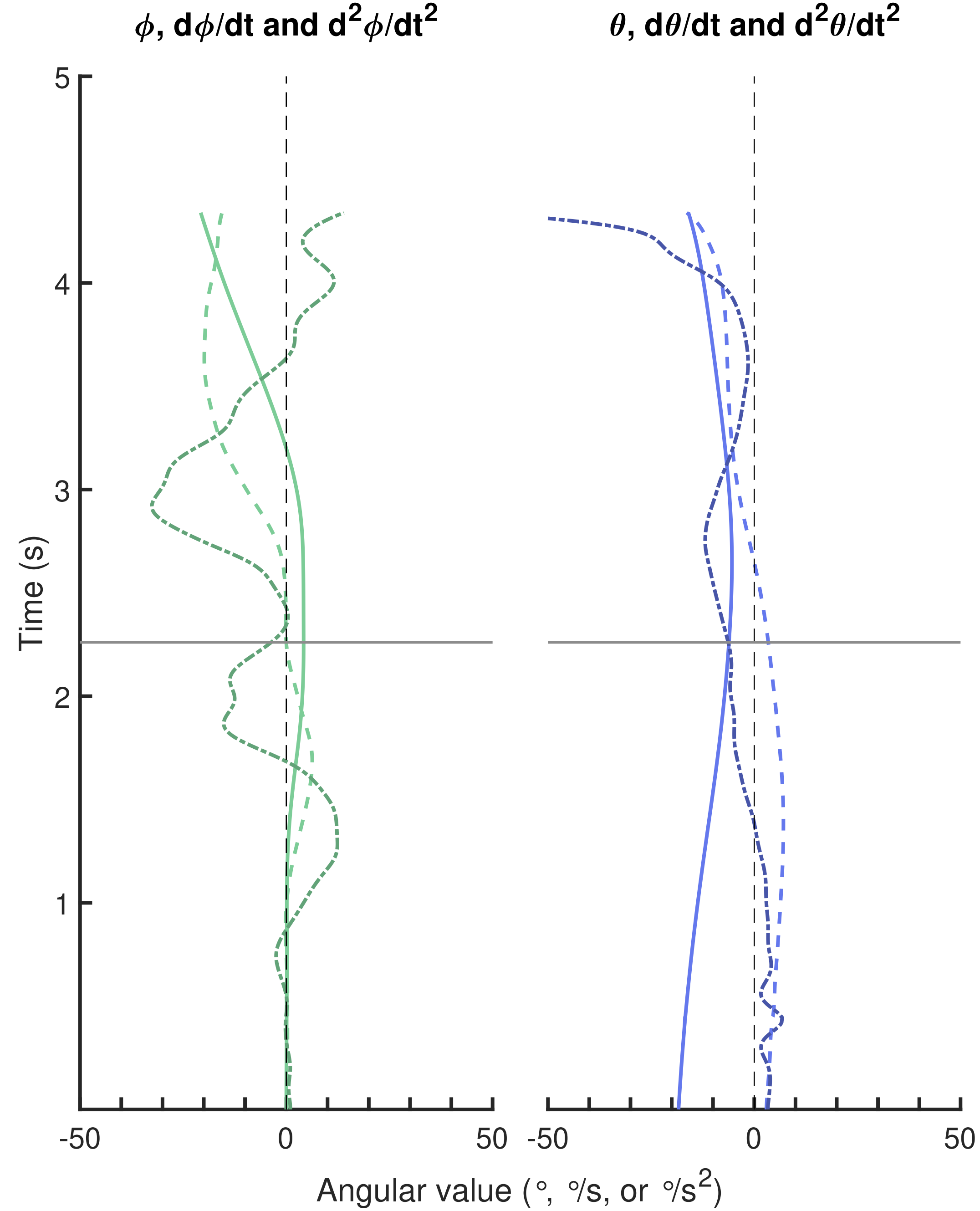

P6/B5
S20/R30-IN

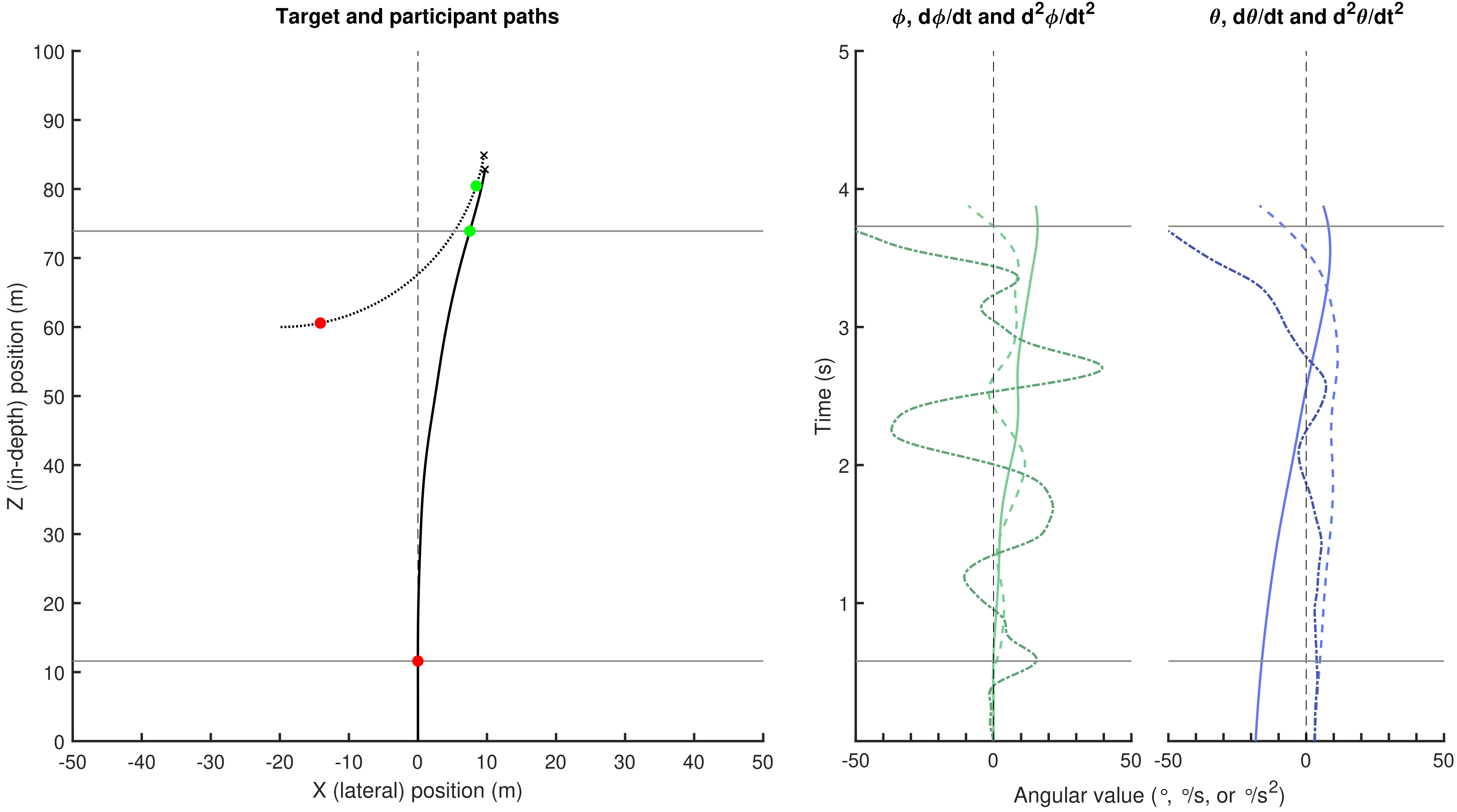

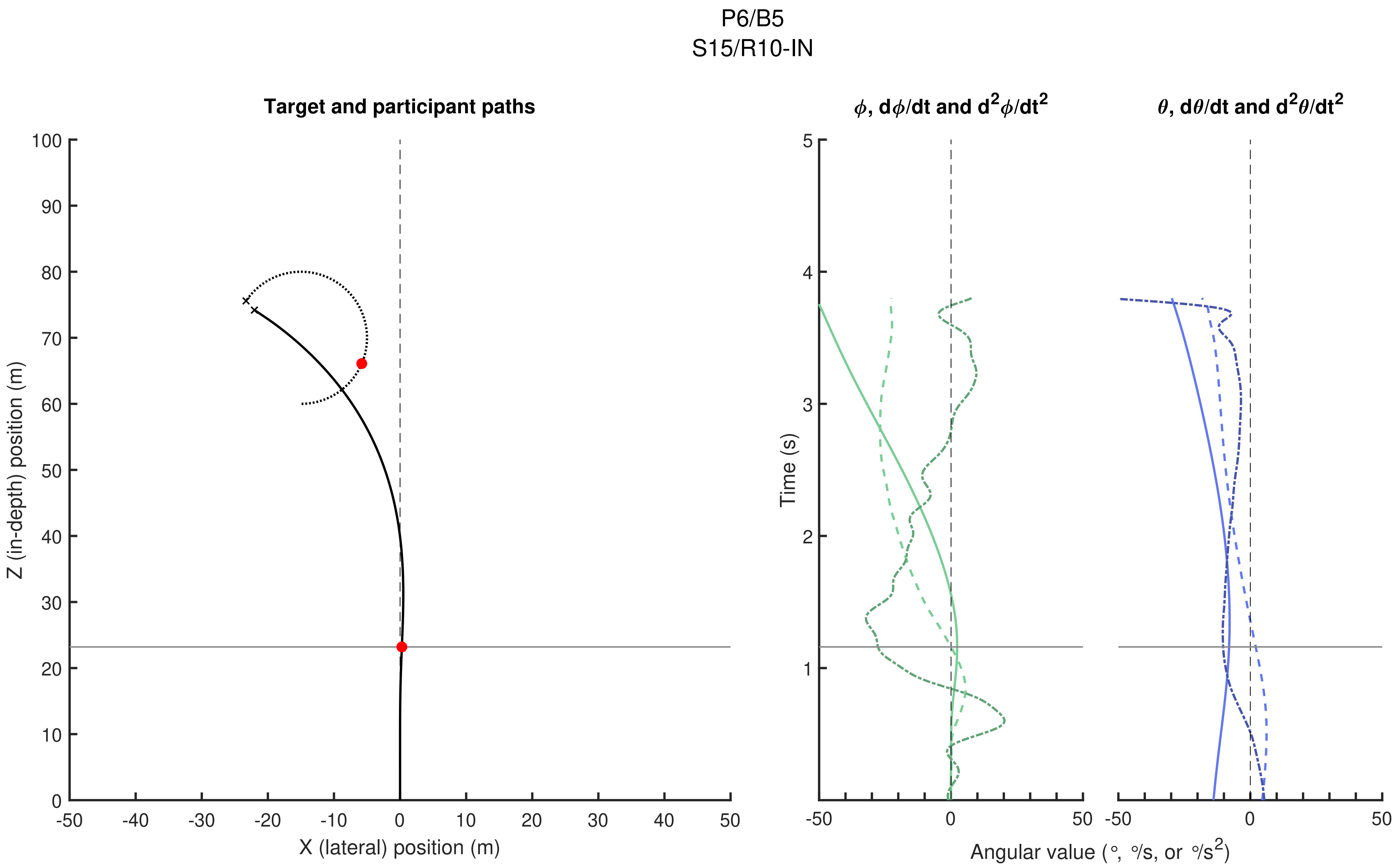

P6/B5
S15/R10-IN
Target and participant paths
Z (in-depth) position (m)
X (lateral) position (m)
100
90
80
70
60
50
40
30
20
10
0
-50
-40
-30
-20
-10
0
10
20
30
40
50
ϕ, dϕ/dt and d$^2\phi$/dt^2
θ, dθ/dt and d$^2\theta$/dt^2
Time (s)
5
4
3
2
1
-50
0
50
-50
0
50
Angular value (°, °/s, or °/s^2)

P6/B5
S15/R20-IN

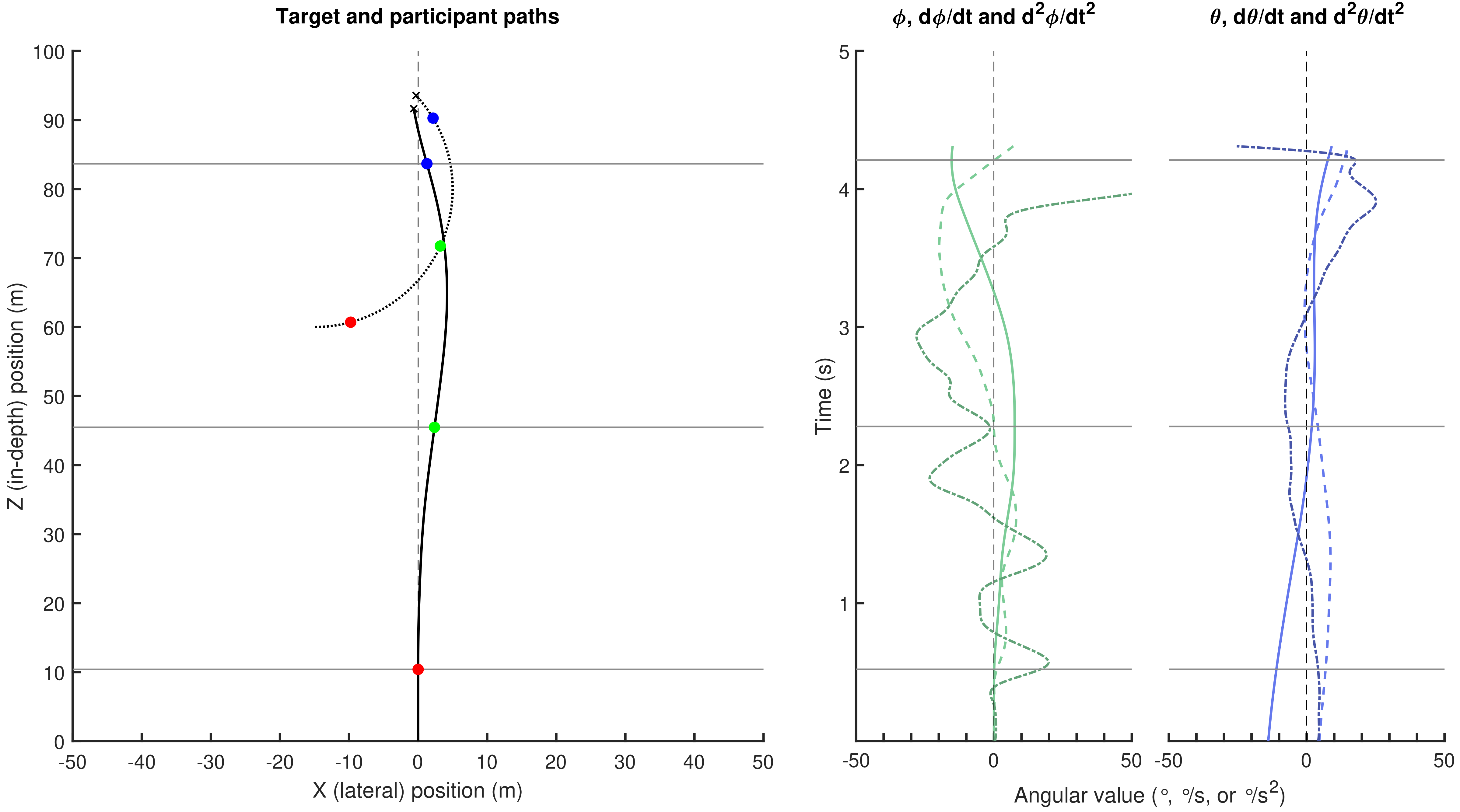

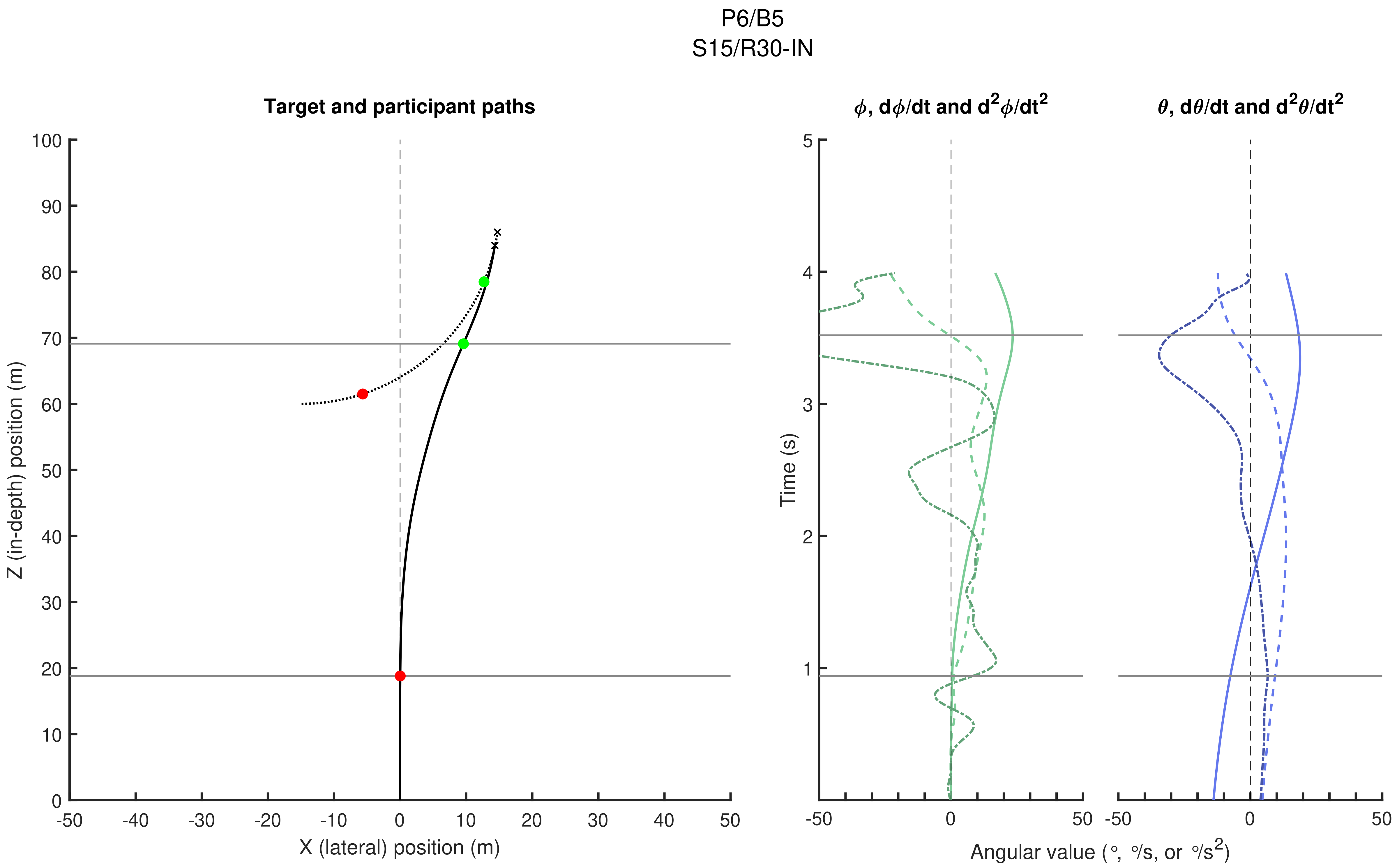

P6/B5
S15/R30-IN
Target and participant paths
Z (in-depth) position (m)
X (lateral) position (m)
100
90
80
70
60
50
40
30
20
10
0
-50
-40
-30
-20
-10
0
10
20
30
40
50
ϕ, dϕ/dt and d$^2\phi$/dt^2
θ, dθ/dt and d$^2\theta$/dt^2
Time (s)
5
4
3
2
1
-50
0
50
-50
0
50
Angular value (°, °/s, or °/s^2)

P6/B5
S5/R10-IN

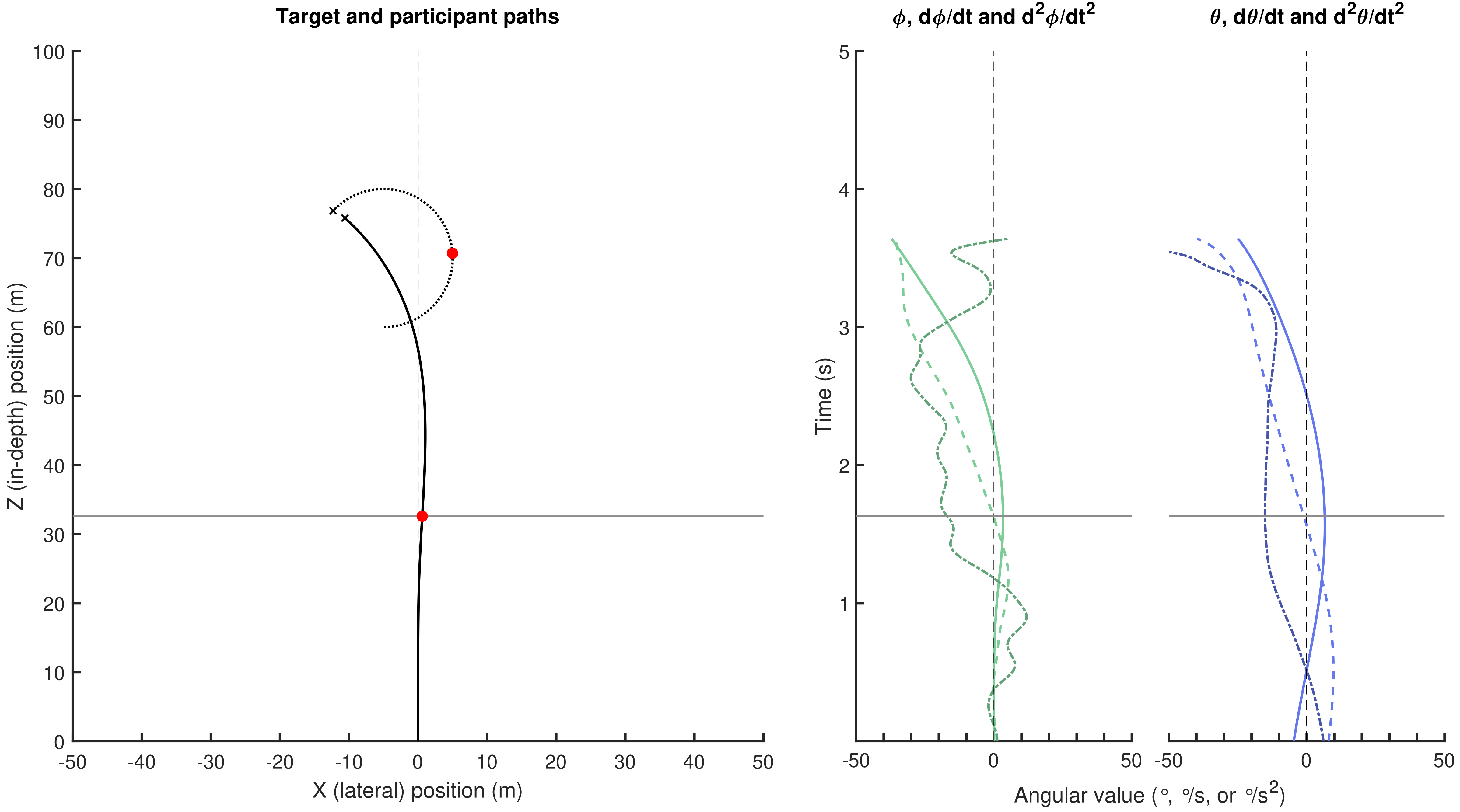

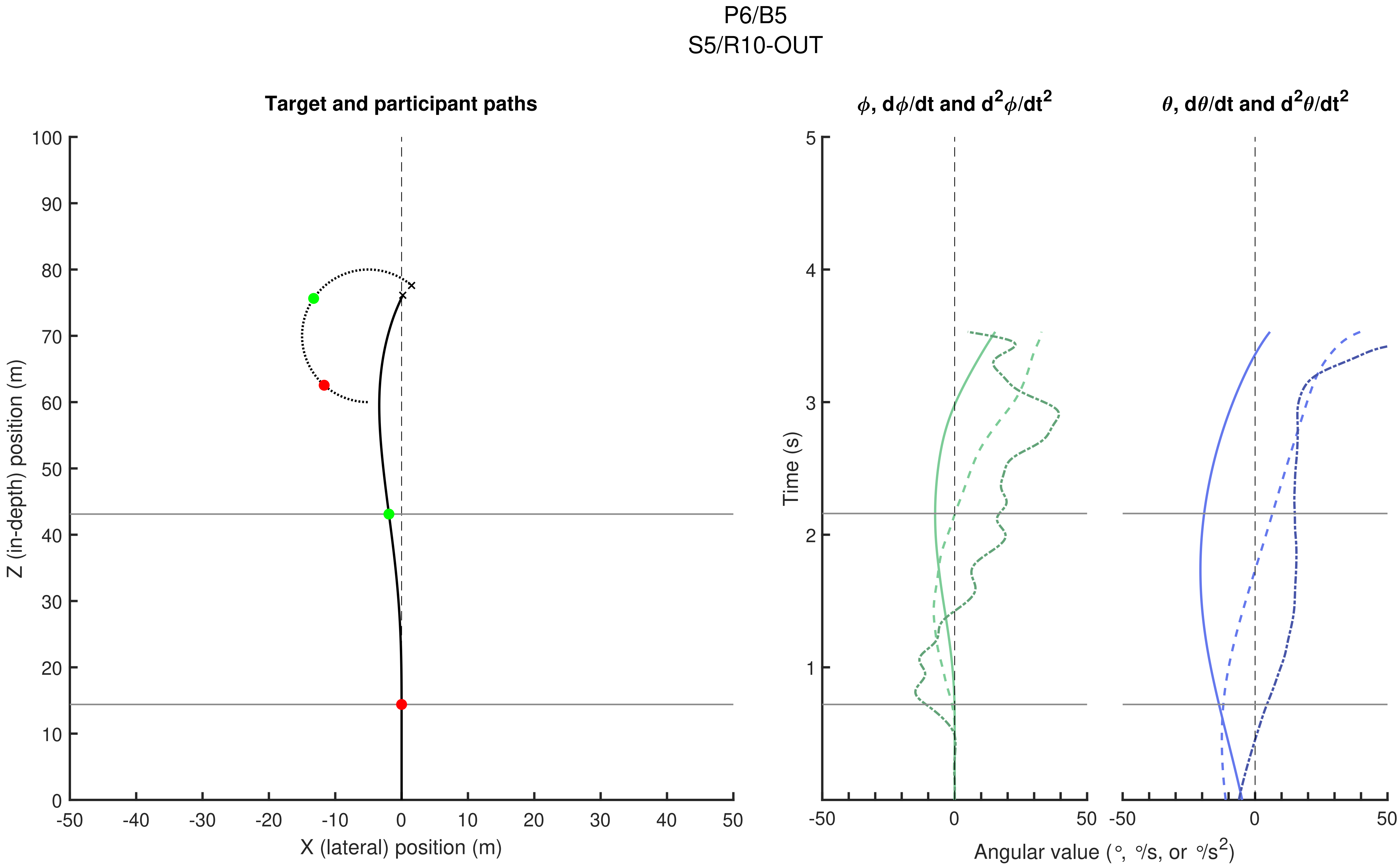

P6/B5
S5/R10-OUT
Target and participant paths
φ, dφ/dt and d²φ/dt²
θ, dθ/dt and d²θ/dt²
Z (in-depth) position (m)
X (lateral) position (m)
Time (s)
Angular value (°, °/s, or °/s²)

P6/B5
S5/R20-IN

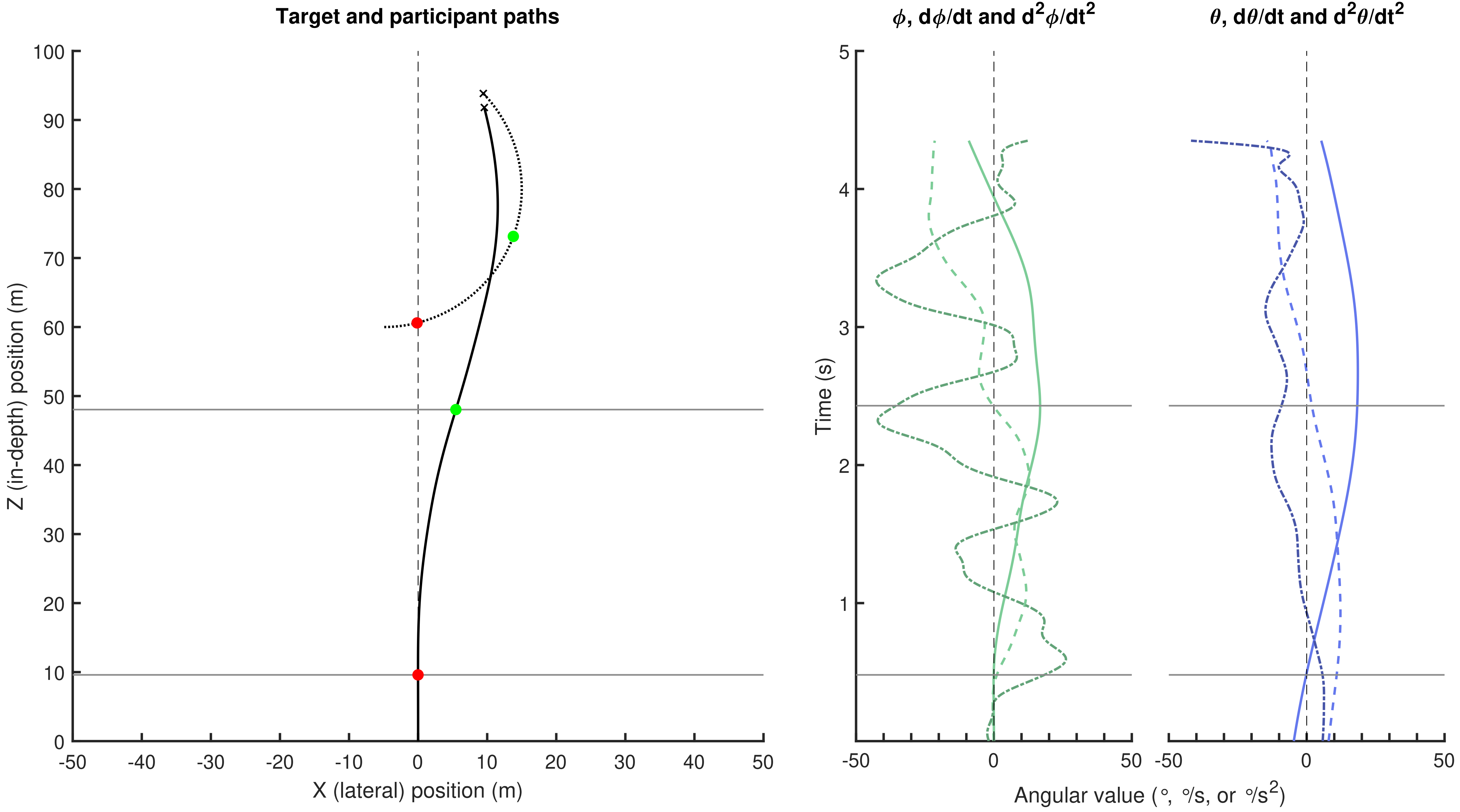

P6/B5
S5/R20-OUT

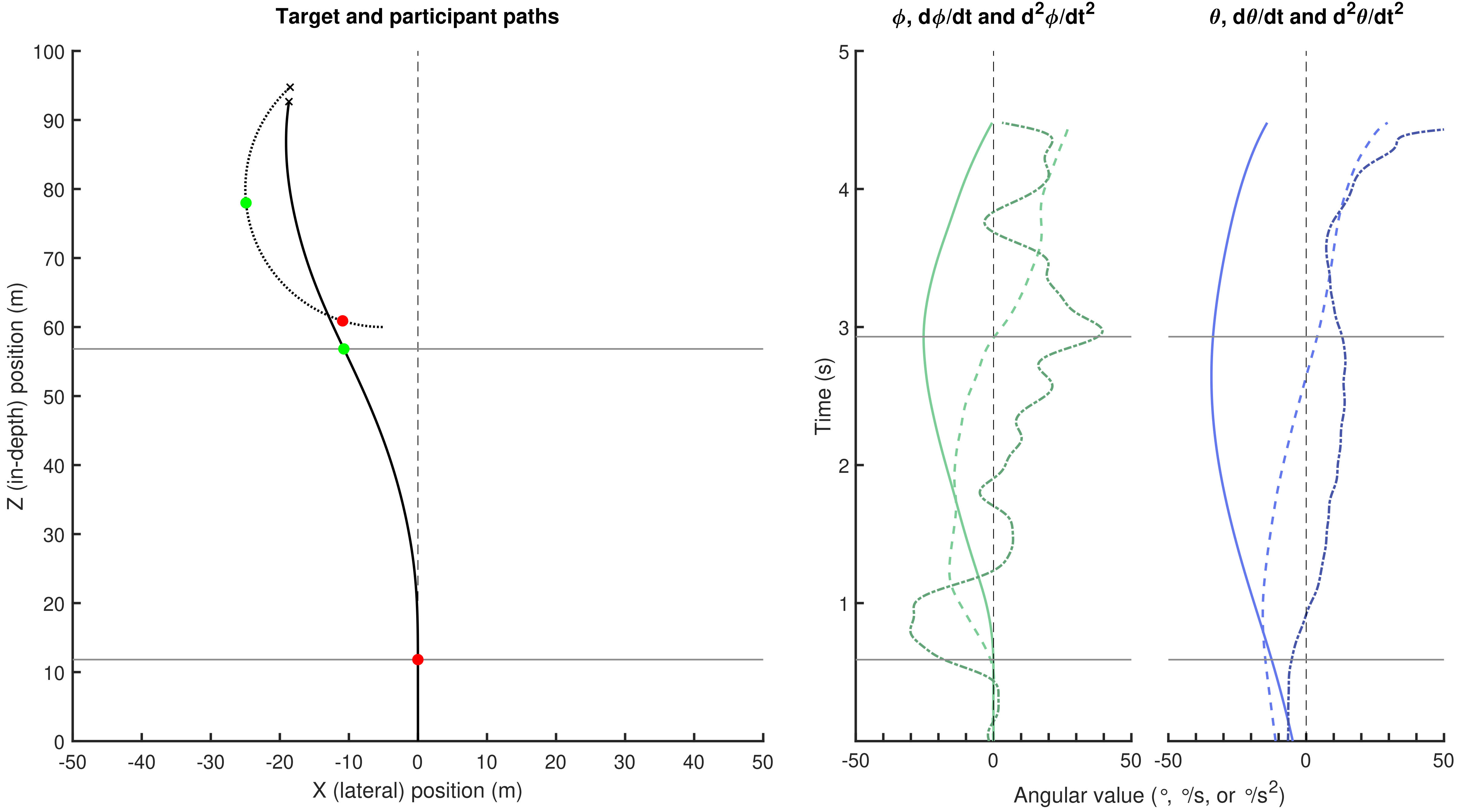

P6/B5
S5/R30-IN

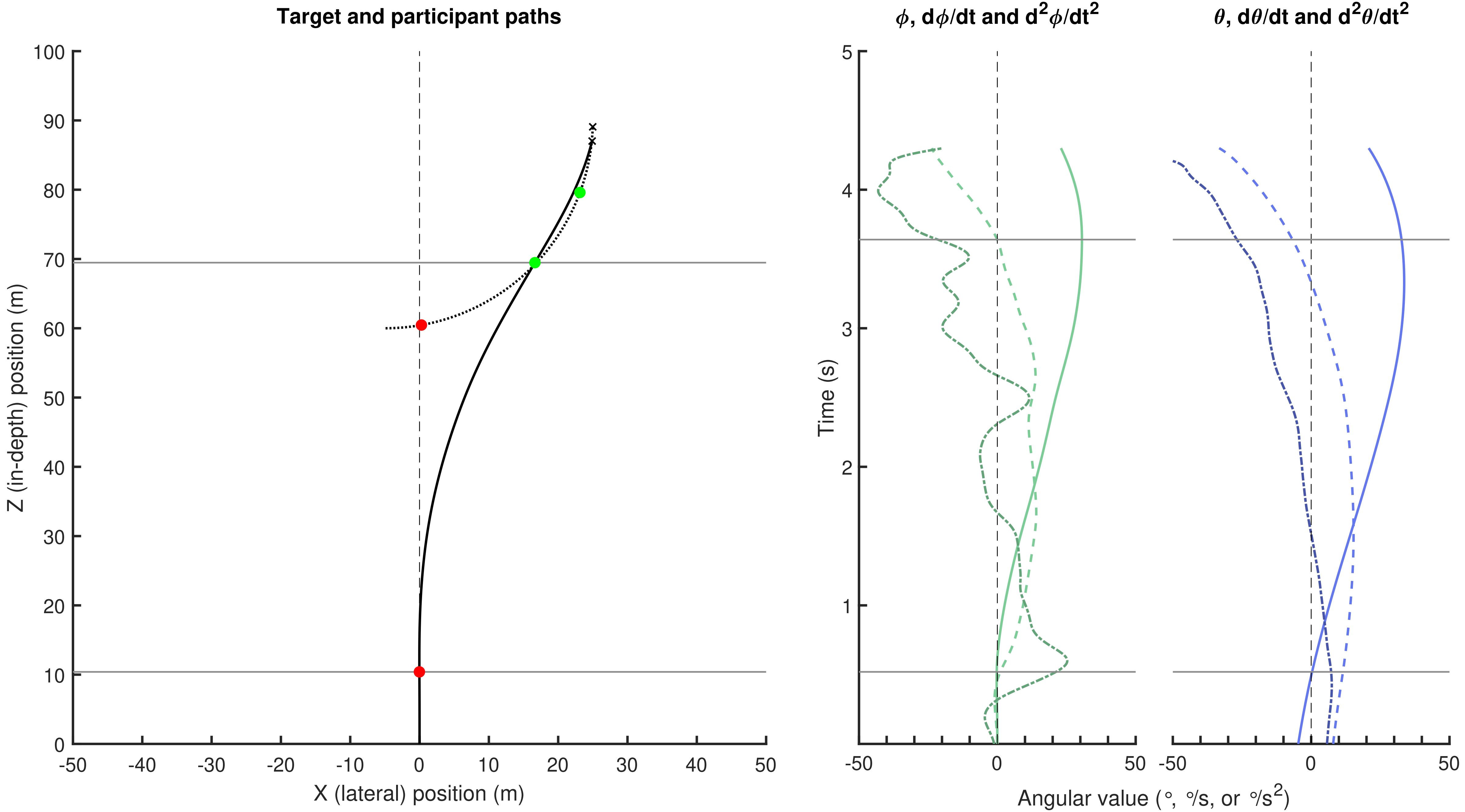

P6/B5
S5/R30-OUT

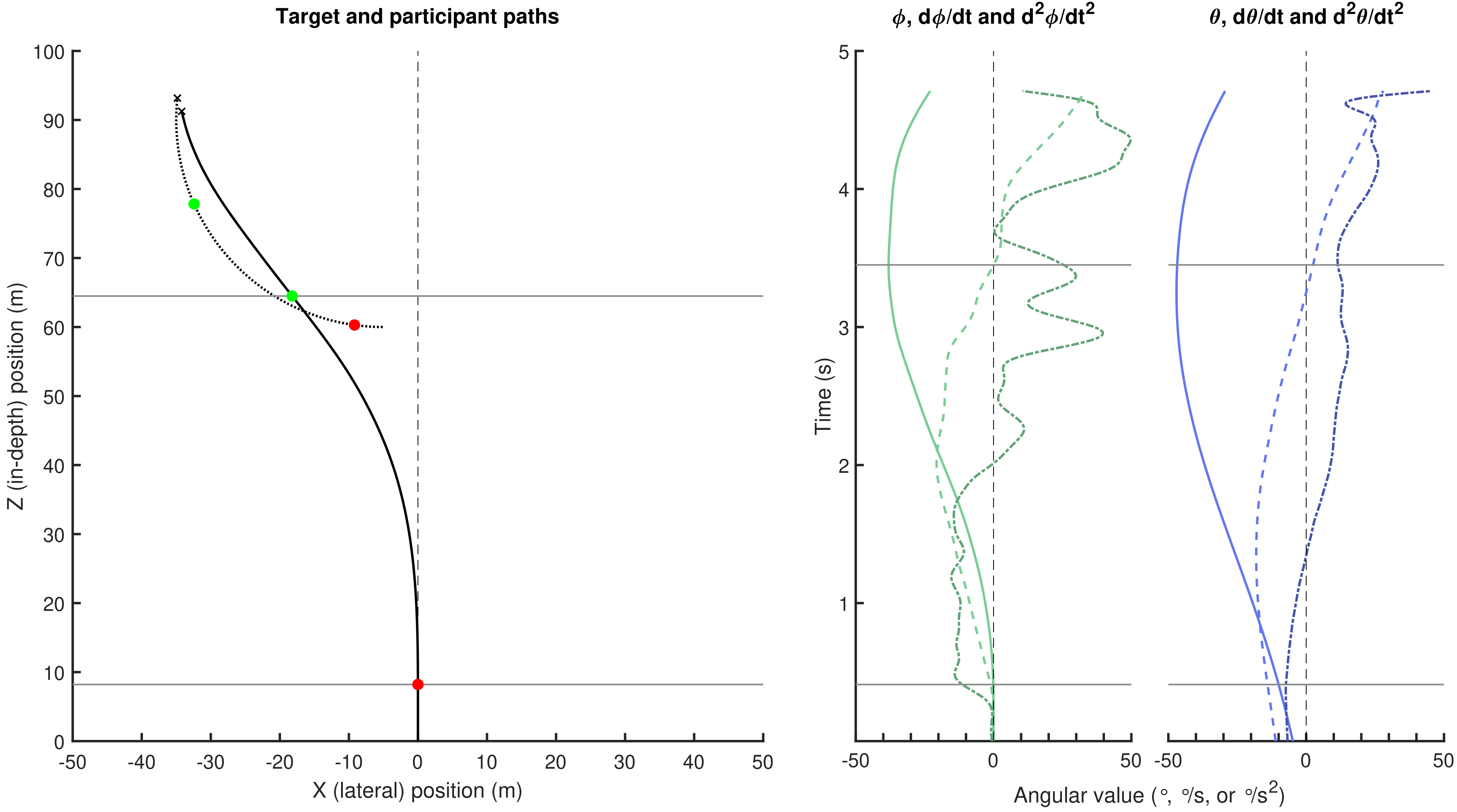

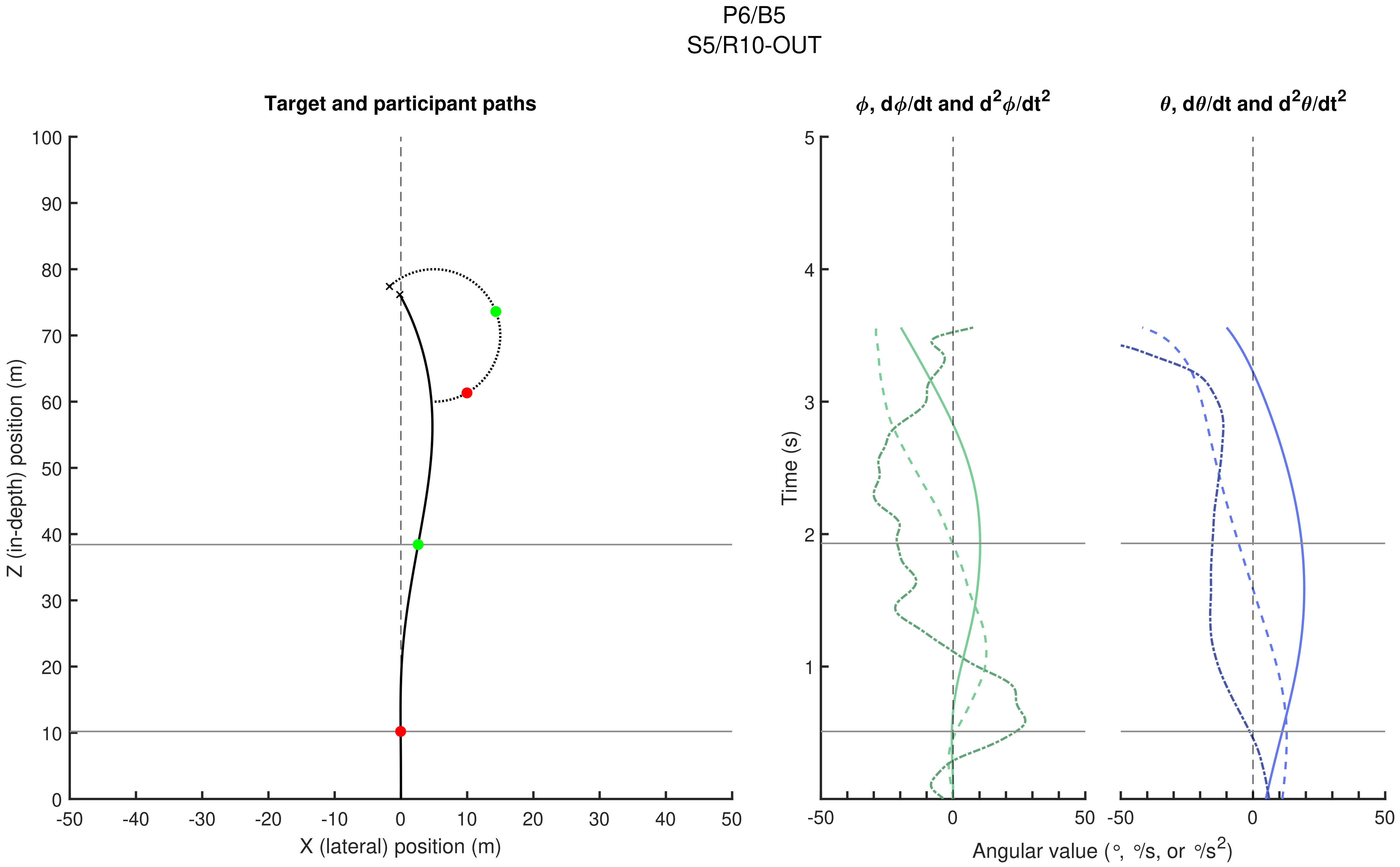

P6/B5
S5/R10-OUT
Target and participant paths
Z (in-depth) position (m)
X (lateral) position (m)
ϕ, dϕ/dt and d²ϕ/dt²
θ, dθ/dt and d²θ/dt²
Time (s)
Angular value (°, °/s, or °/s²)

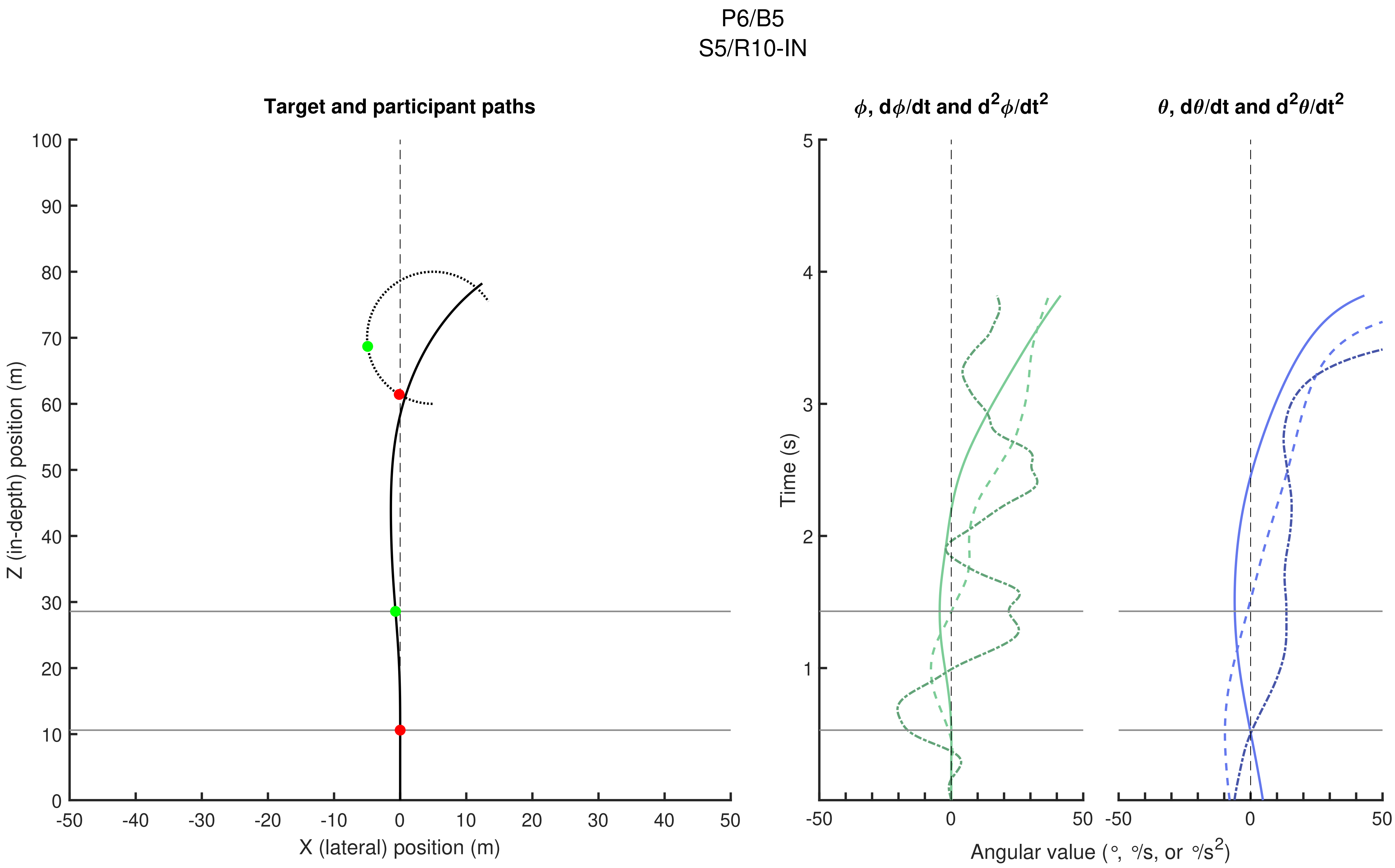

P6/B5
S5/R10-IN
Target and participant paths
Z (in-depth) position (m)
X (lateral) position (m)
ϕ, dϕ/dt and d$^2\phi$/dt^2
θ, dθ/dt and d$^2\theta$/dt^2
Time (s)
Angular value (°, °/s, or °/s^2)

P6/B5
S5/R20-OUT

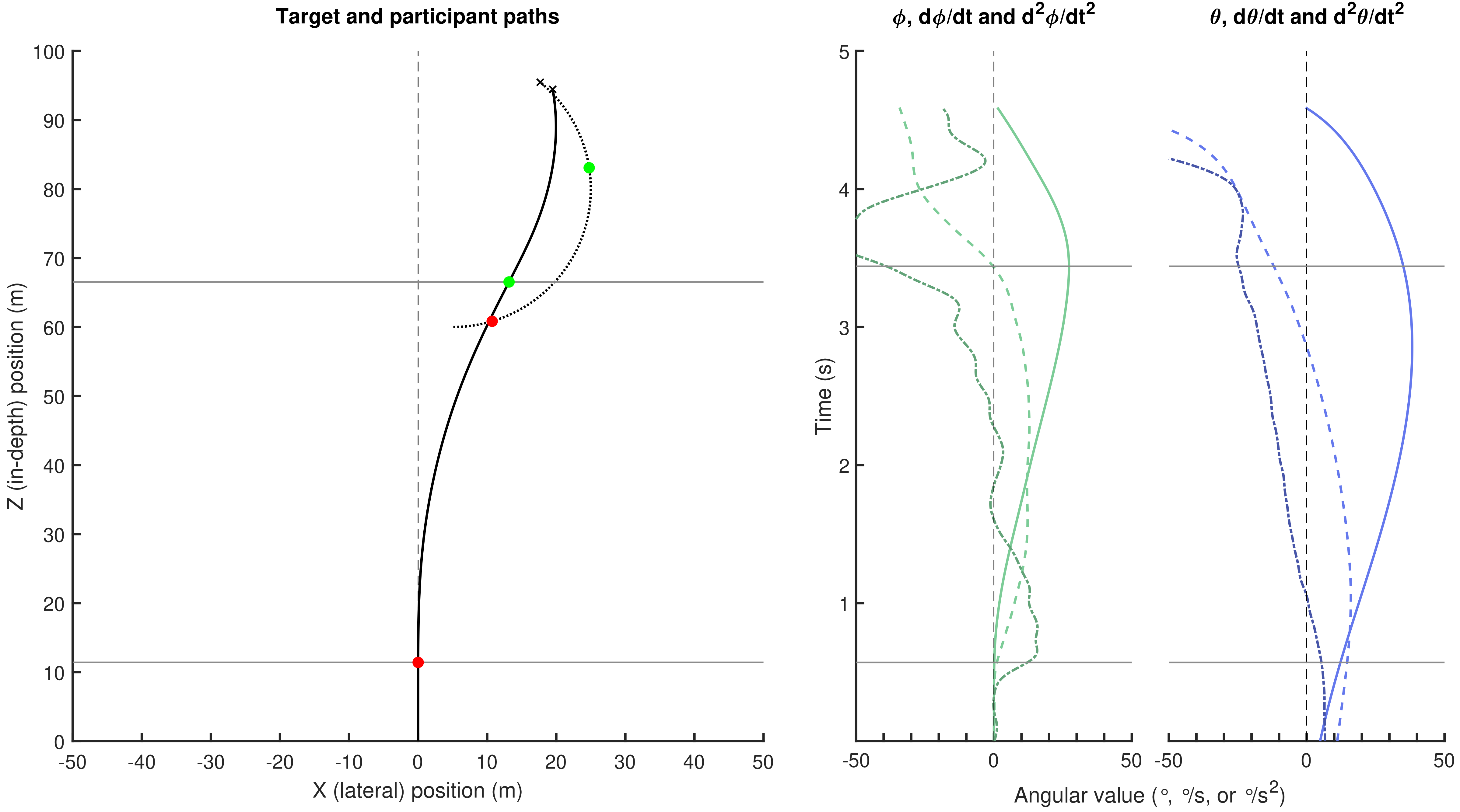

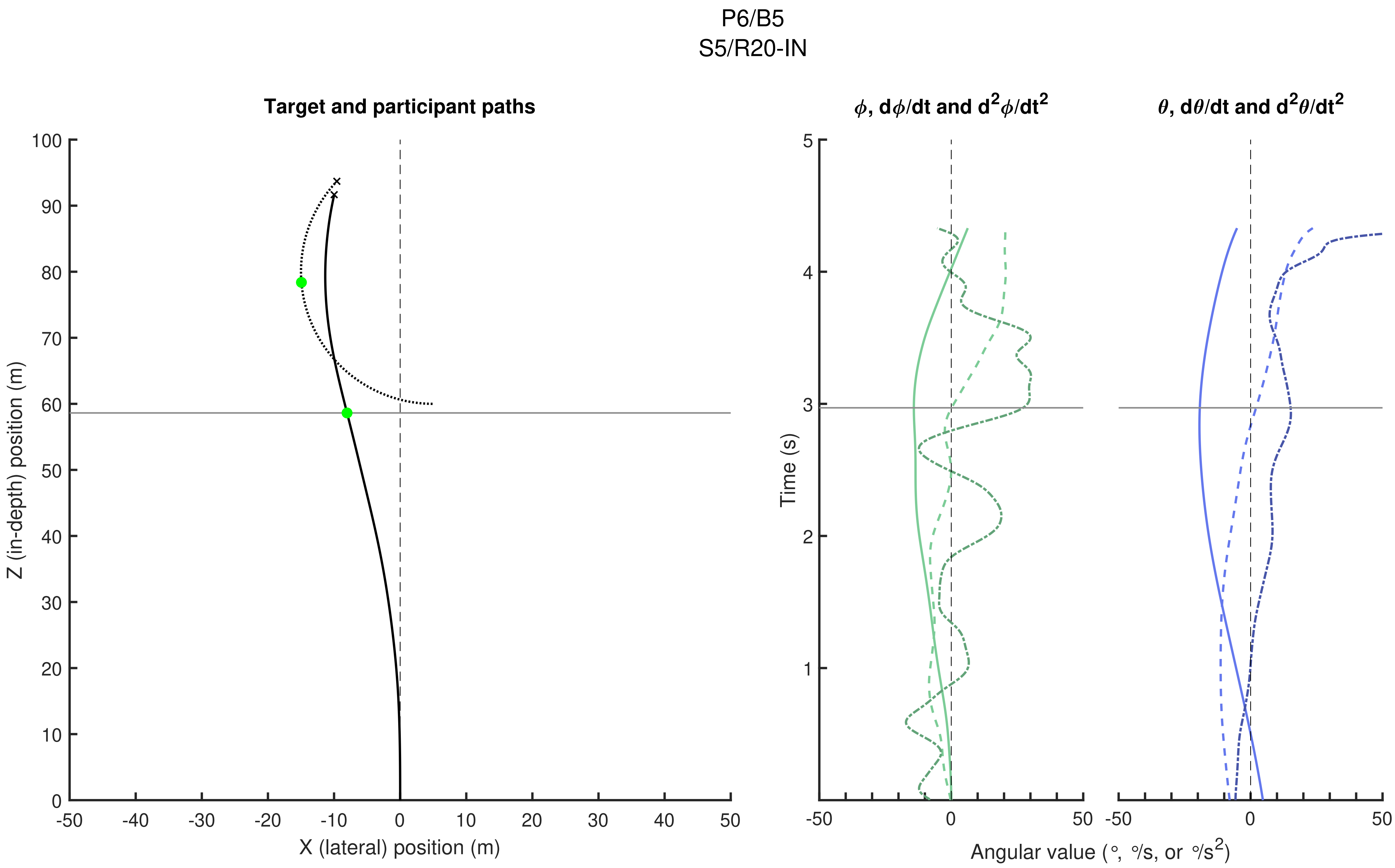

P6/B5
S5/R20-IN
Target and participant paths
Z (in-depth) position (m)
X (lateral) position (m)
ϕ, dϕ/dt and d$^2\phi$/dt^2
θ, dθ/dt and d$^2\theta$/dt^2
Time (s)
Angular value (°, °/s, or °/s^2)

P6/B5
S5/R30-OUT

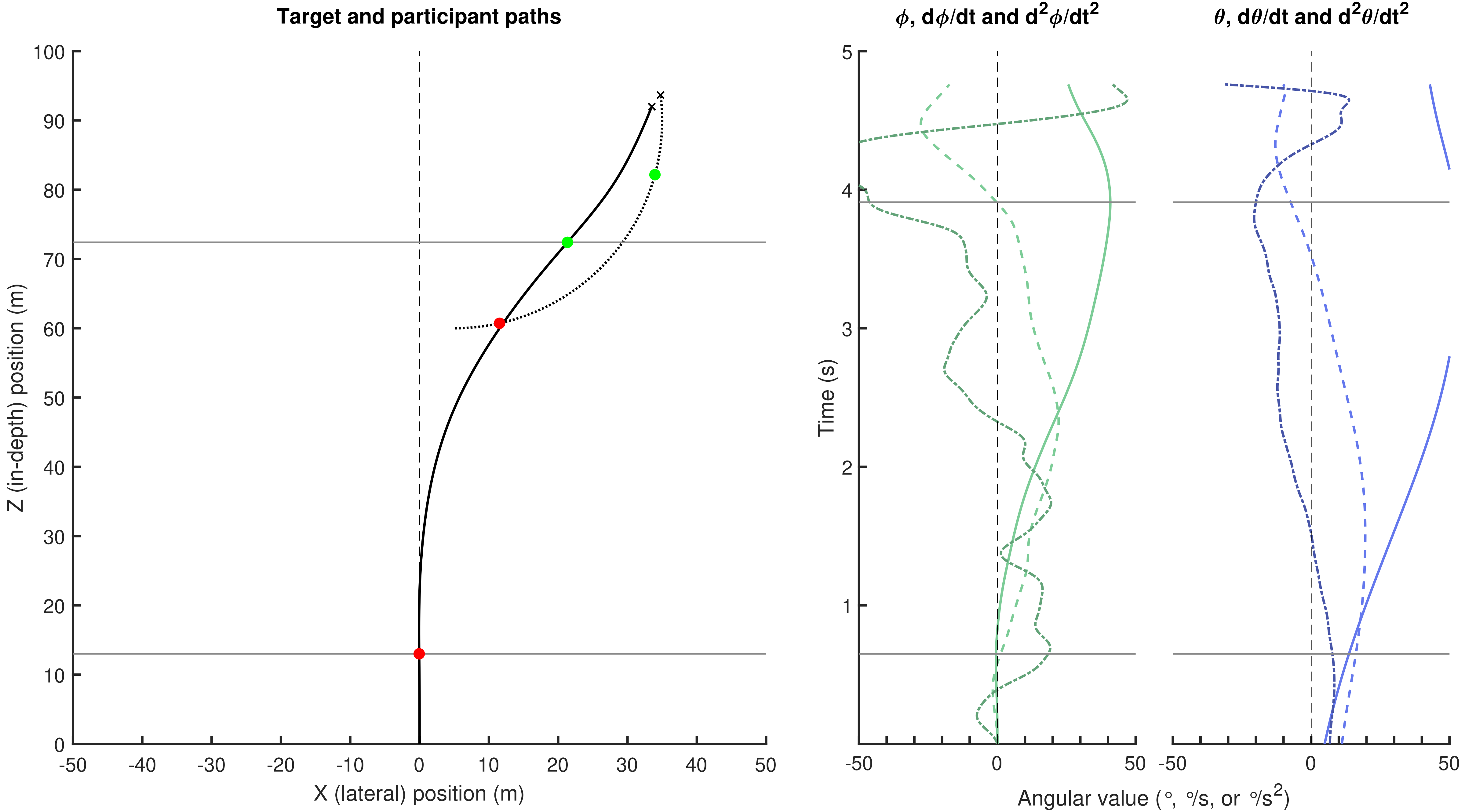

P6/B5
S5/R30-IN

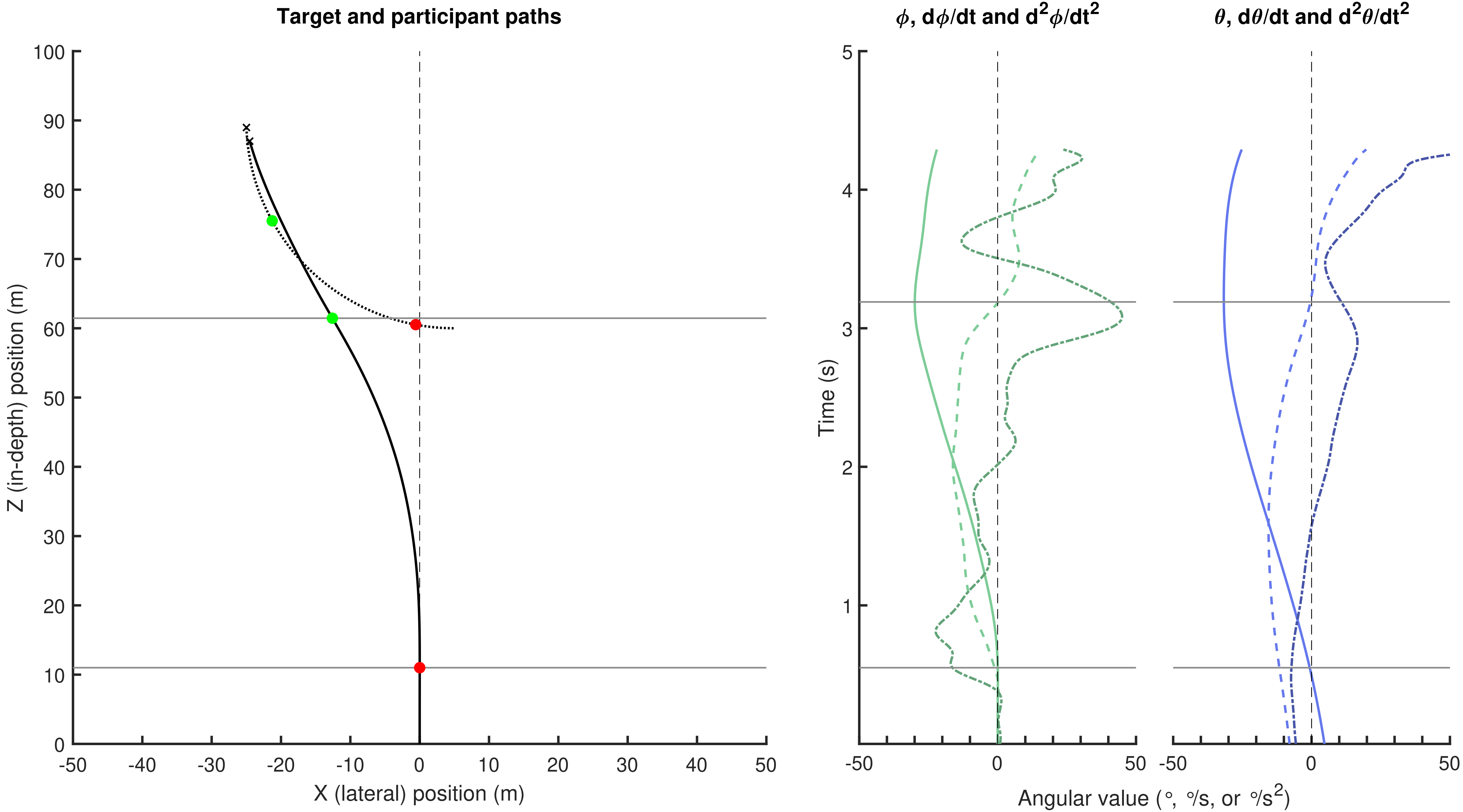

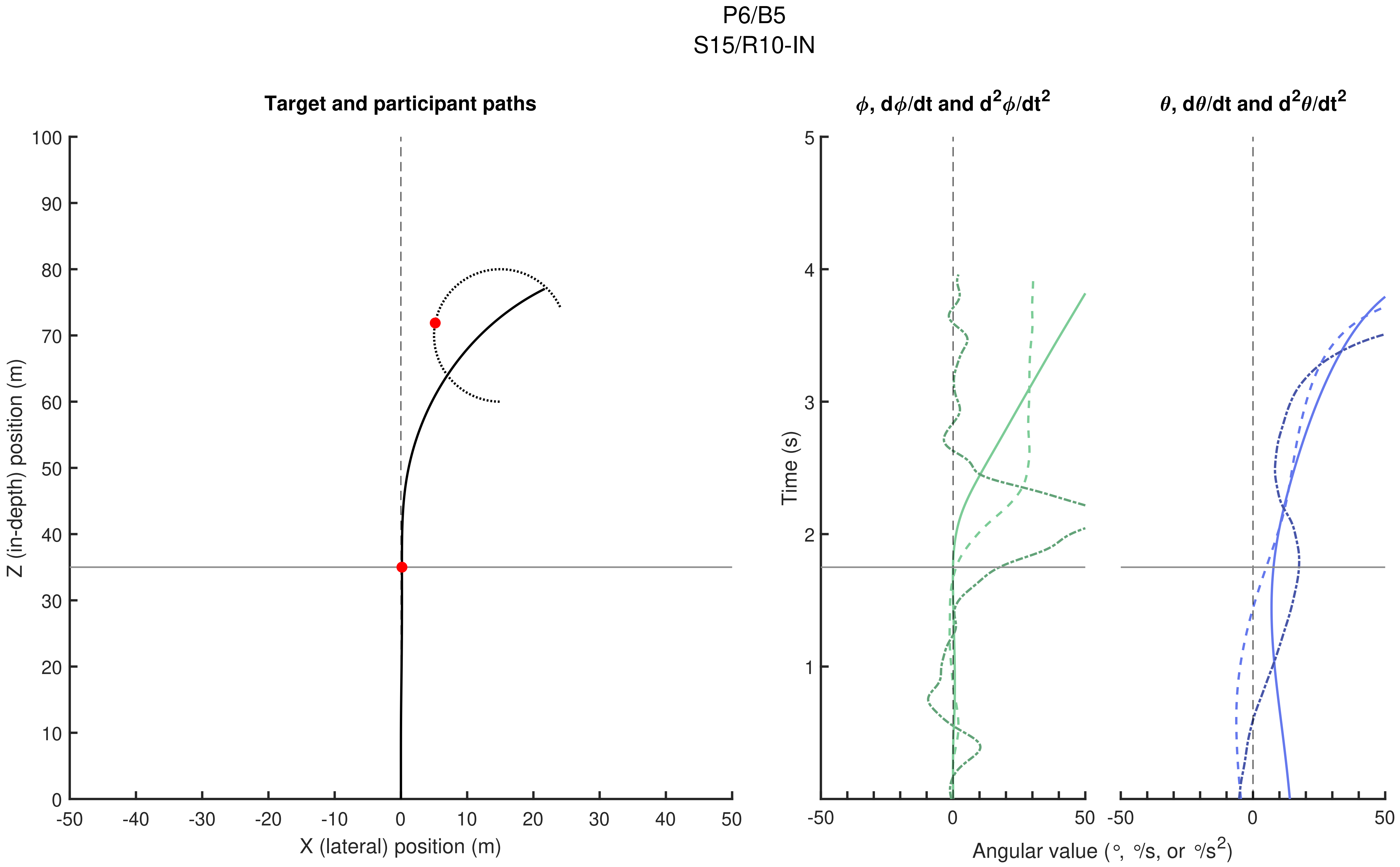
P6/B5
S15/R10-IN
Target and participant paths
Z (in-depth) position (m)
X (lateral) position (m)
ϕ, dϕ/dt and d$^2\phi$/dt^2
θ, dθ/dt and d$^2\theta$/dt^2
Time (s)
Angular value (°, °/s, or °/s^2)
100
90
80
70
60
50
40
30
20
10
0
-50
-40
-30
-20
-10
0
10
20
30
40
50
5
4
3
2
1
-50
0
50
-50
0
50

P6/B5
S15/R20-IN

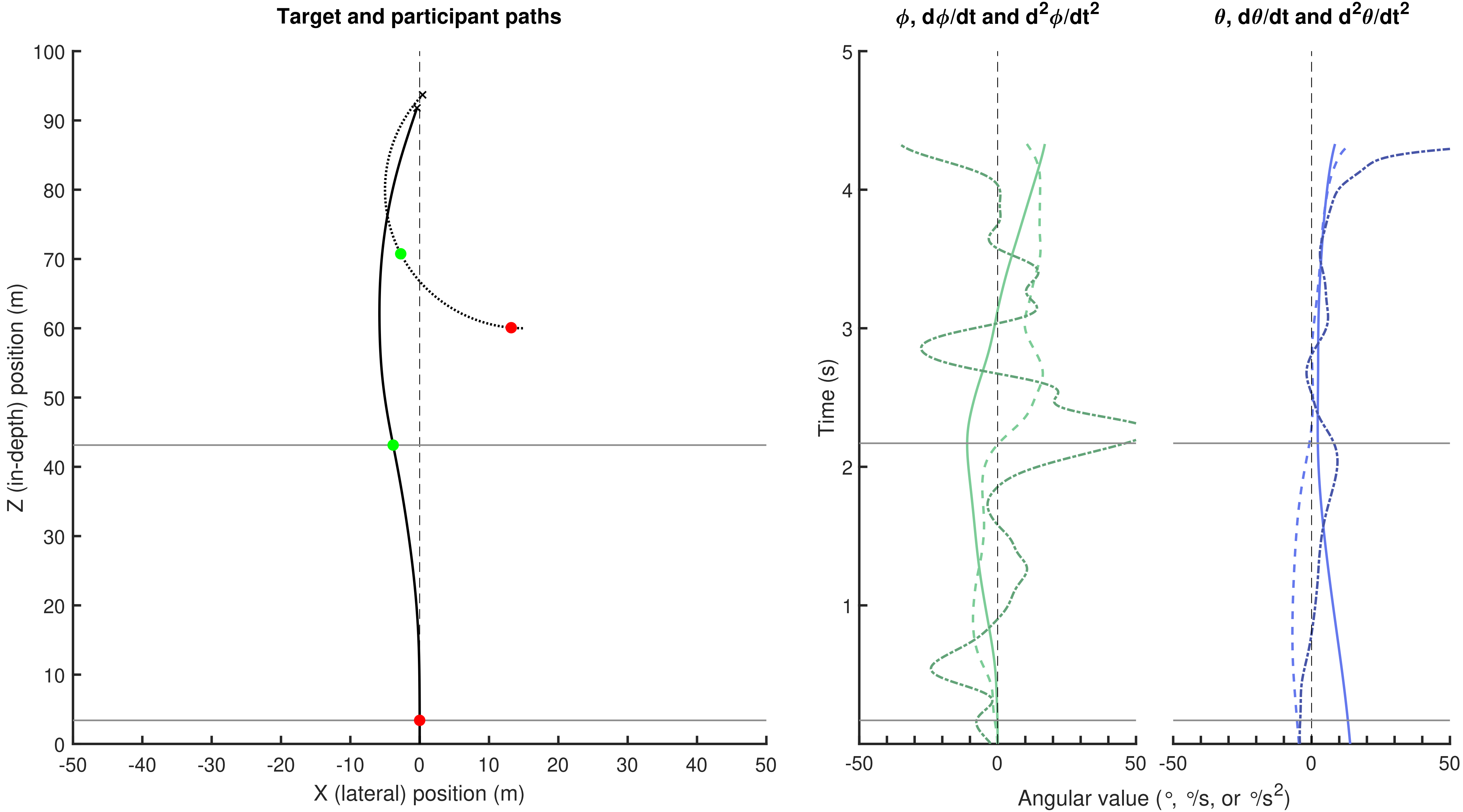

P6/B5
S15/R30-IN

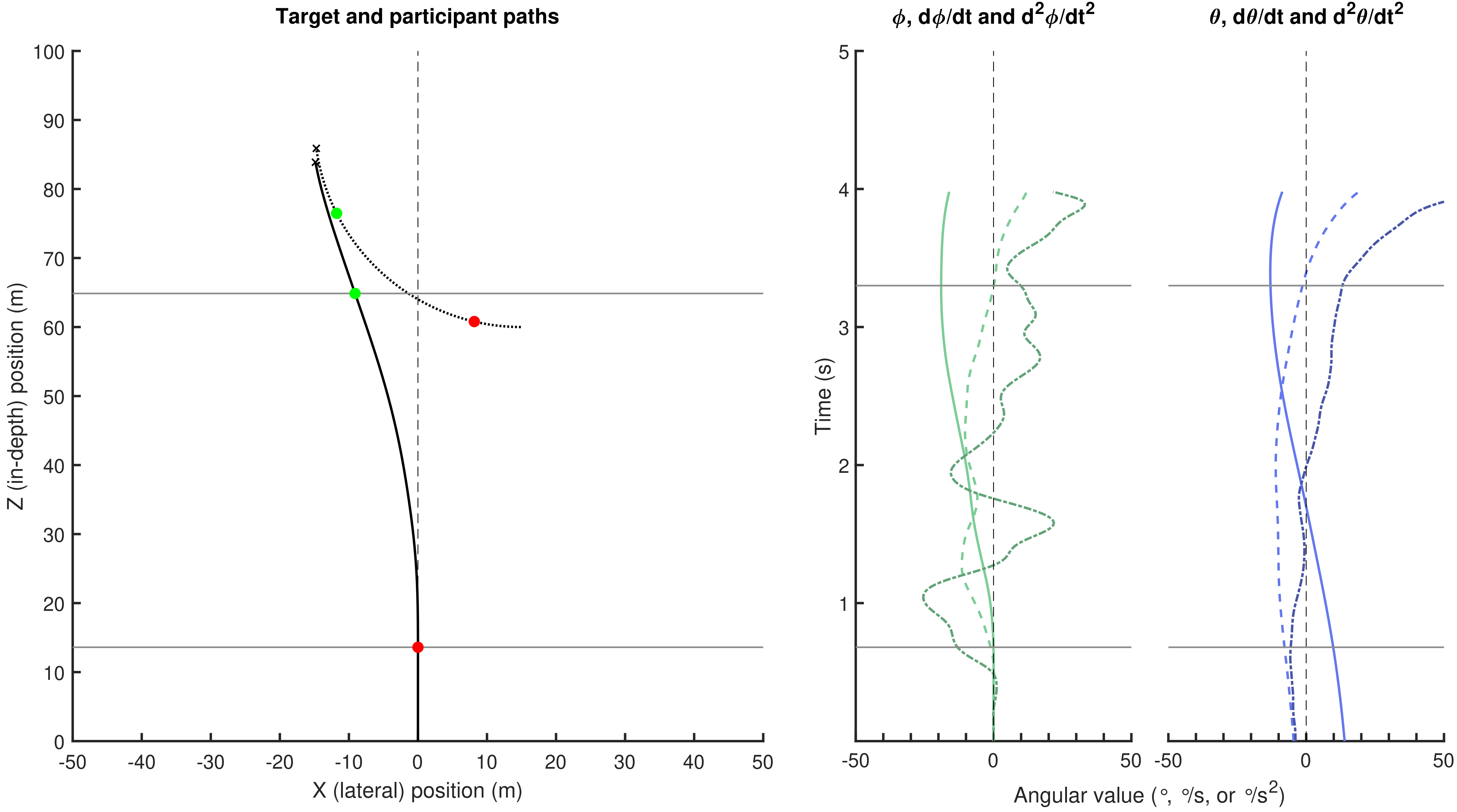

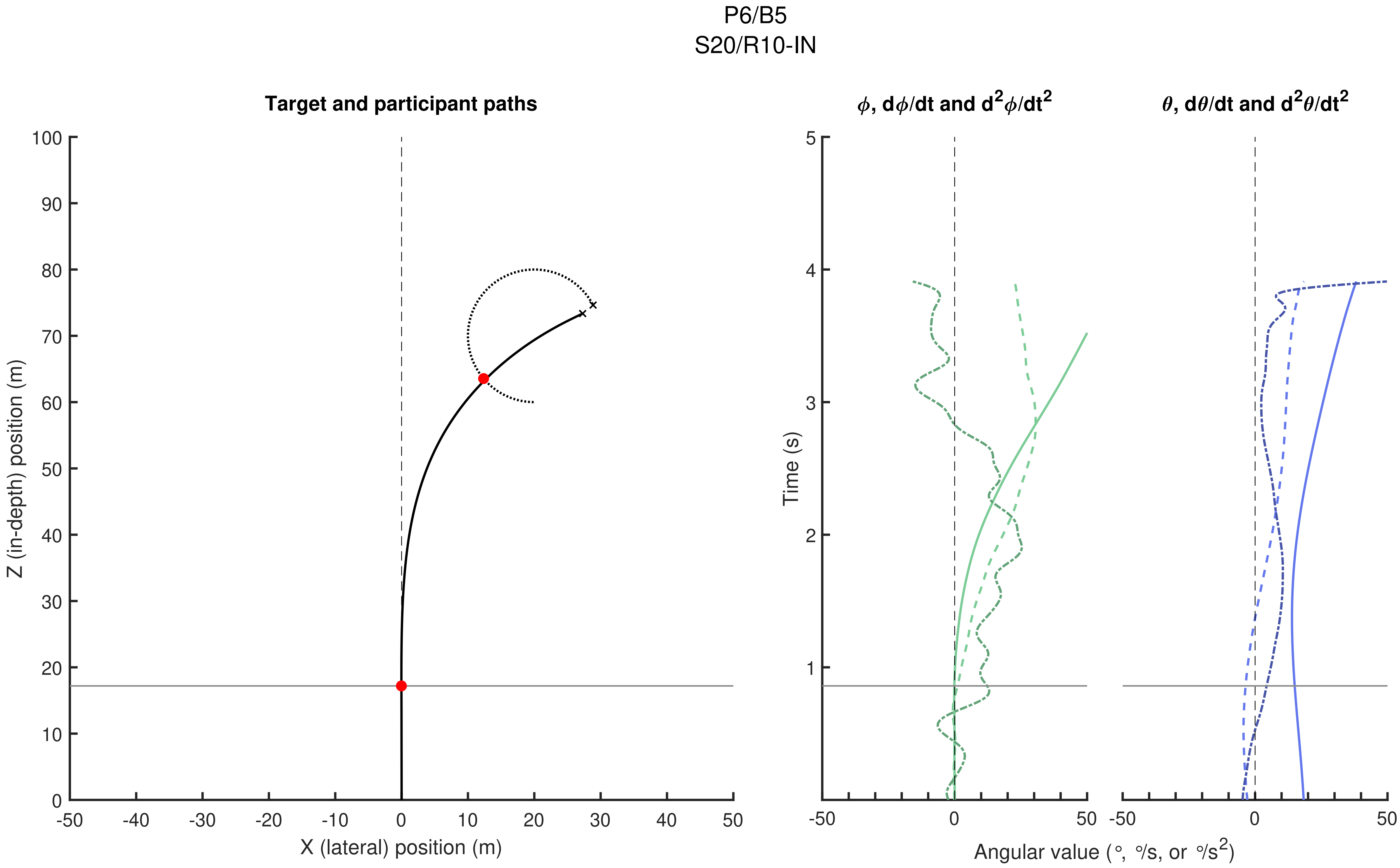

P6/B5
S20/R10-IN
Target and participant paths
Z (in-depth) position (m)
X (lateral) position (m)
100
90
80
70
60
50
40
30
20
10
0
-50
-40
-30
-20
-10
0
10
20
30
40
50
ϕ, dϕ/dt and d$^2\phi$/dt^2
θ, dθ/dt and d$^2\theta$/dt^2
Time (s)
5
4
3
2
1
-50
0
50
-50
0
50
Angular value (°, °/s, or °/s^2)

P6/B5
S20/R20-IN

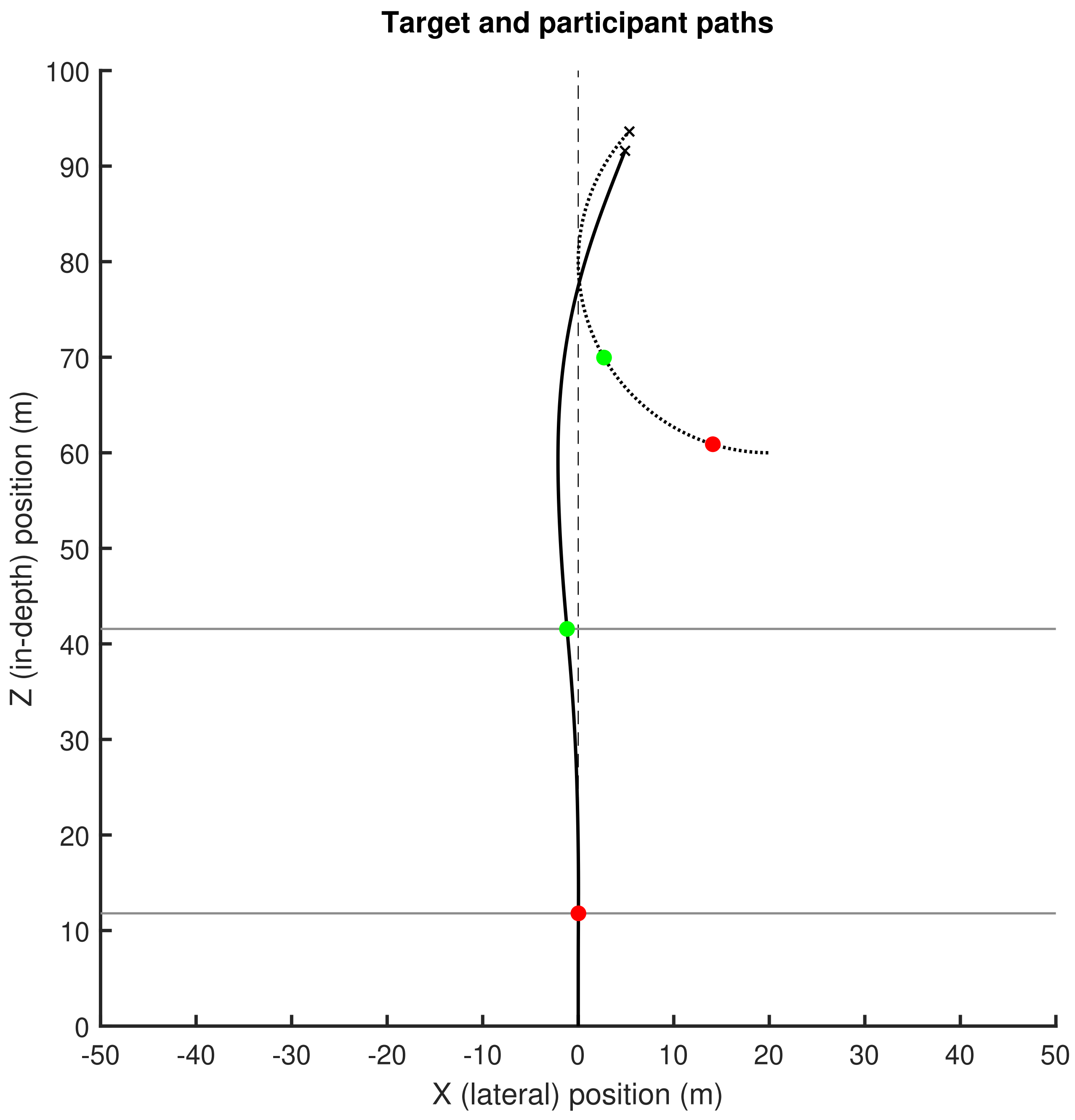

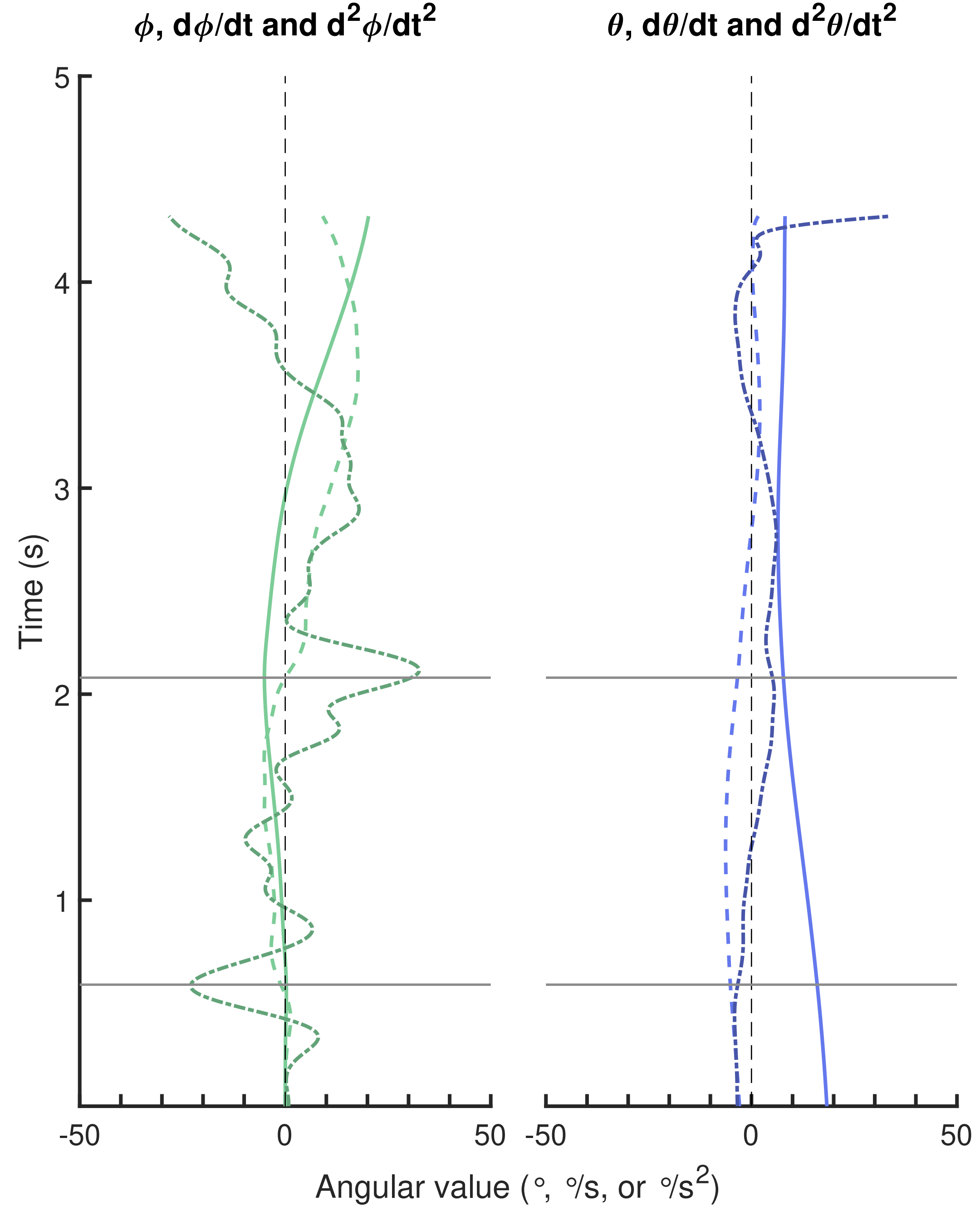

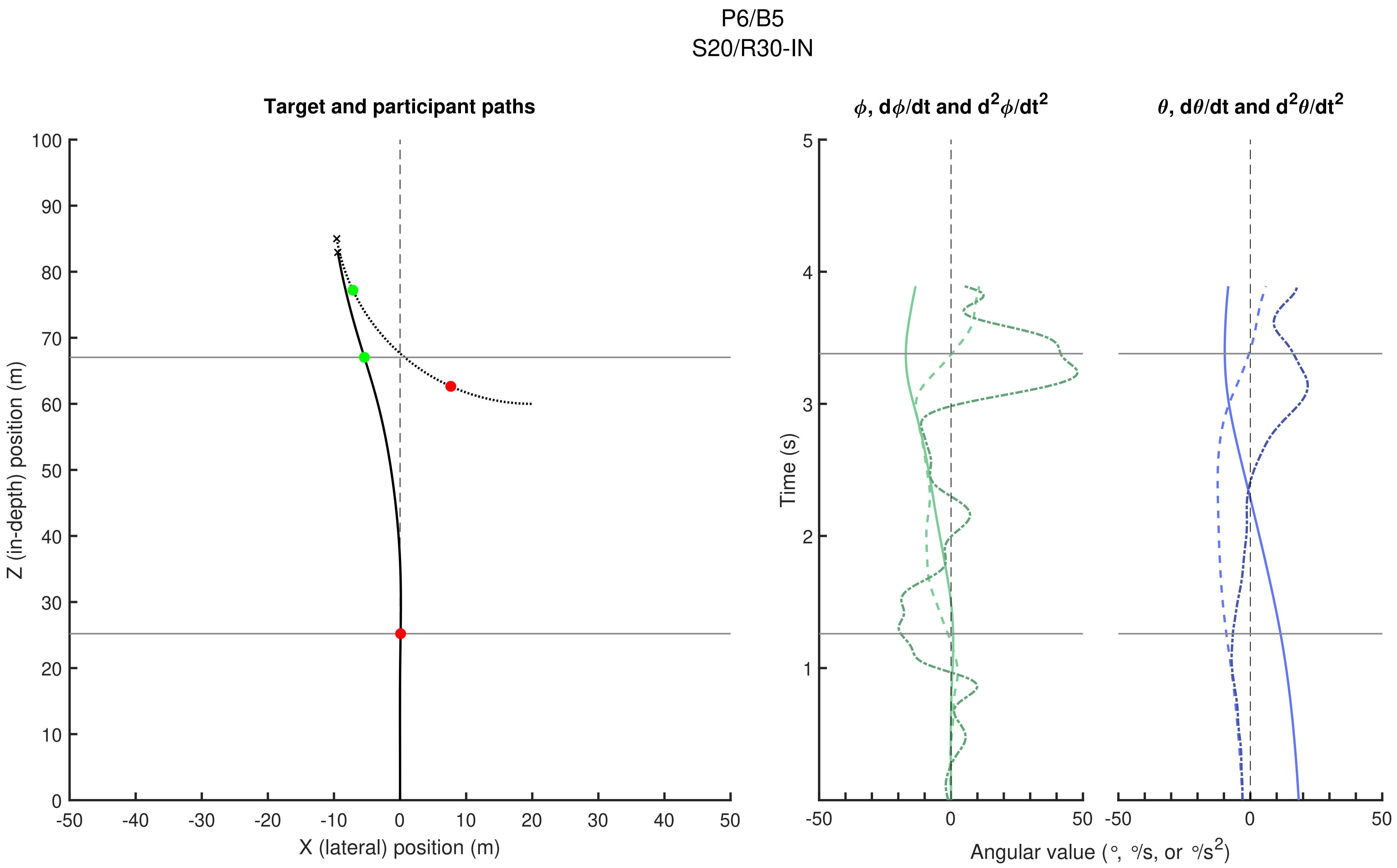

P6/B5
S20/R30-IN
Target and participant paths
Z (in-depth) position (m)
X (lateral) position (m)
100
90
80
70
60
50
40
30
20
10
0
-50
-40
-30
-20
-10
0
10
20
30
40
50
ϕ, dϕ/dt and d$^2\phi$/dt^2
θ, dθ/dt and d$^2\theta$/dt^2
Time (s)
5
4
3
2
1
-50
0
50
-50
0
50
Angular value (°, °/s, or °/s^2)

P7/B1
S20/R10-IN

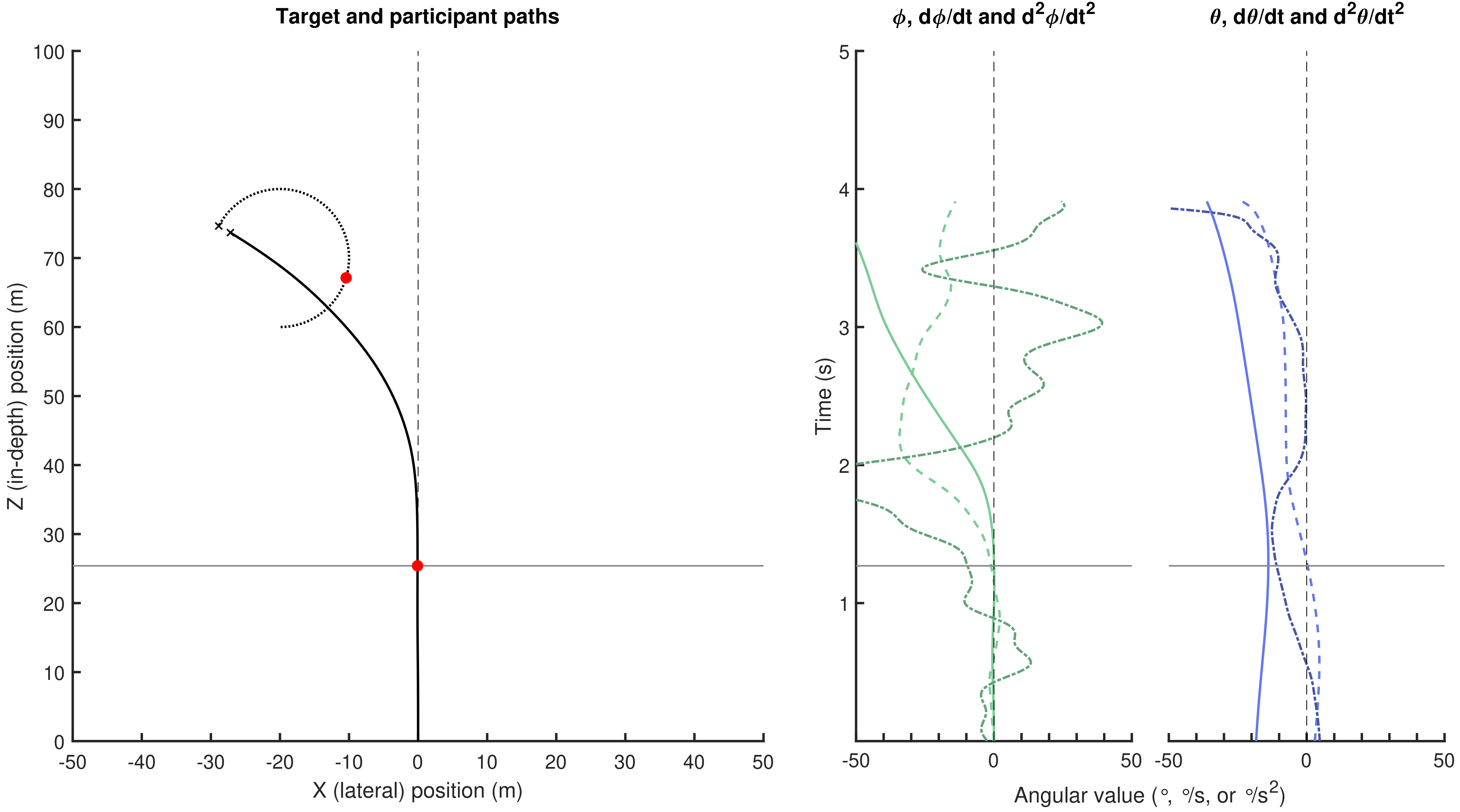

P7/B1
S20/R20-IN

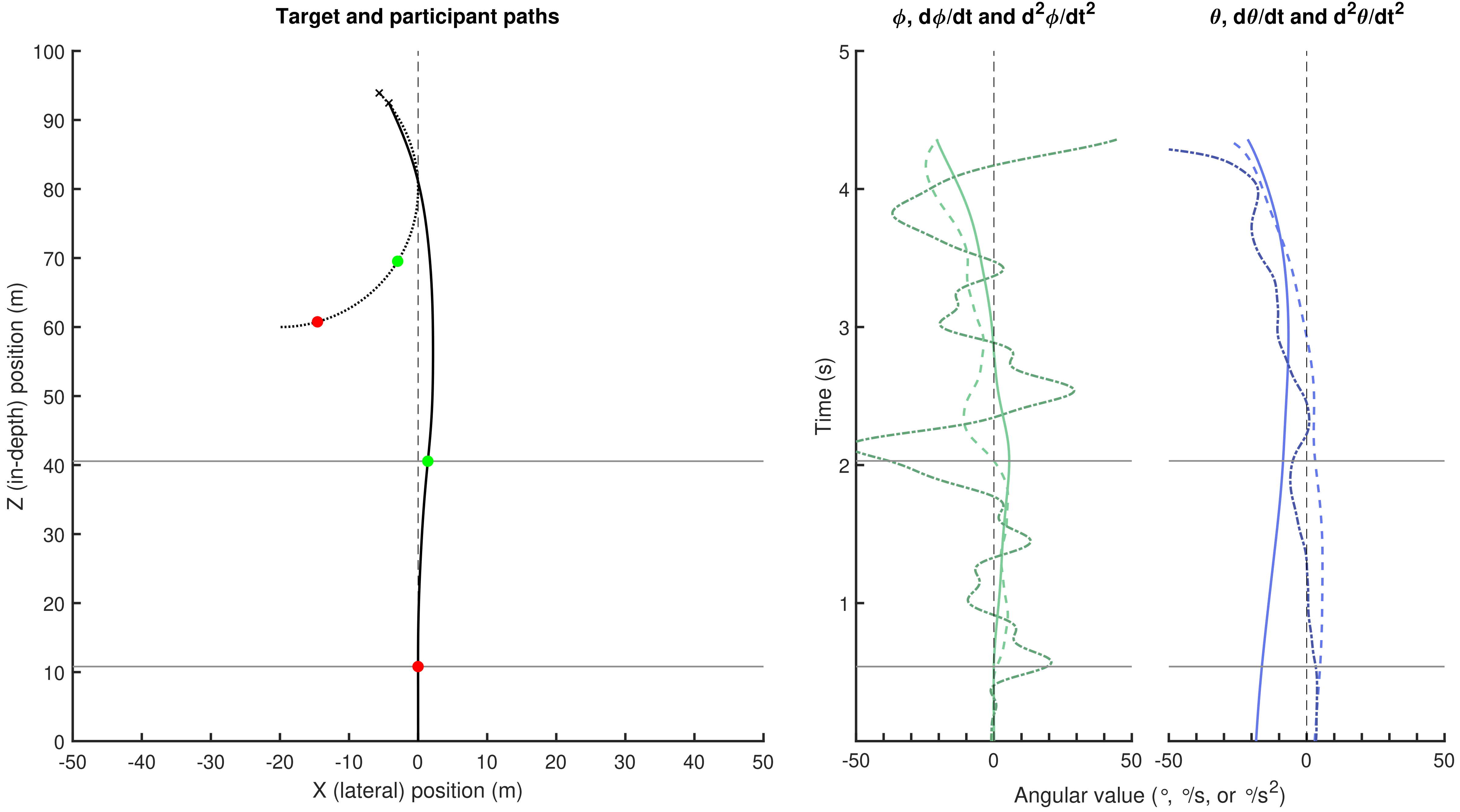

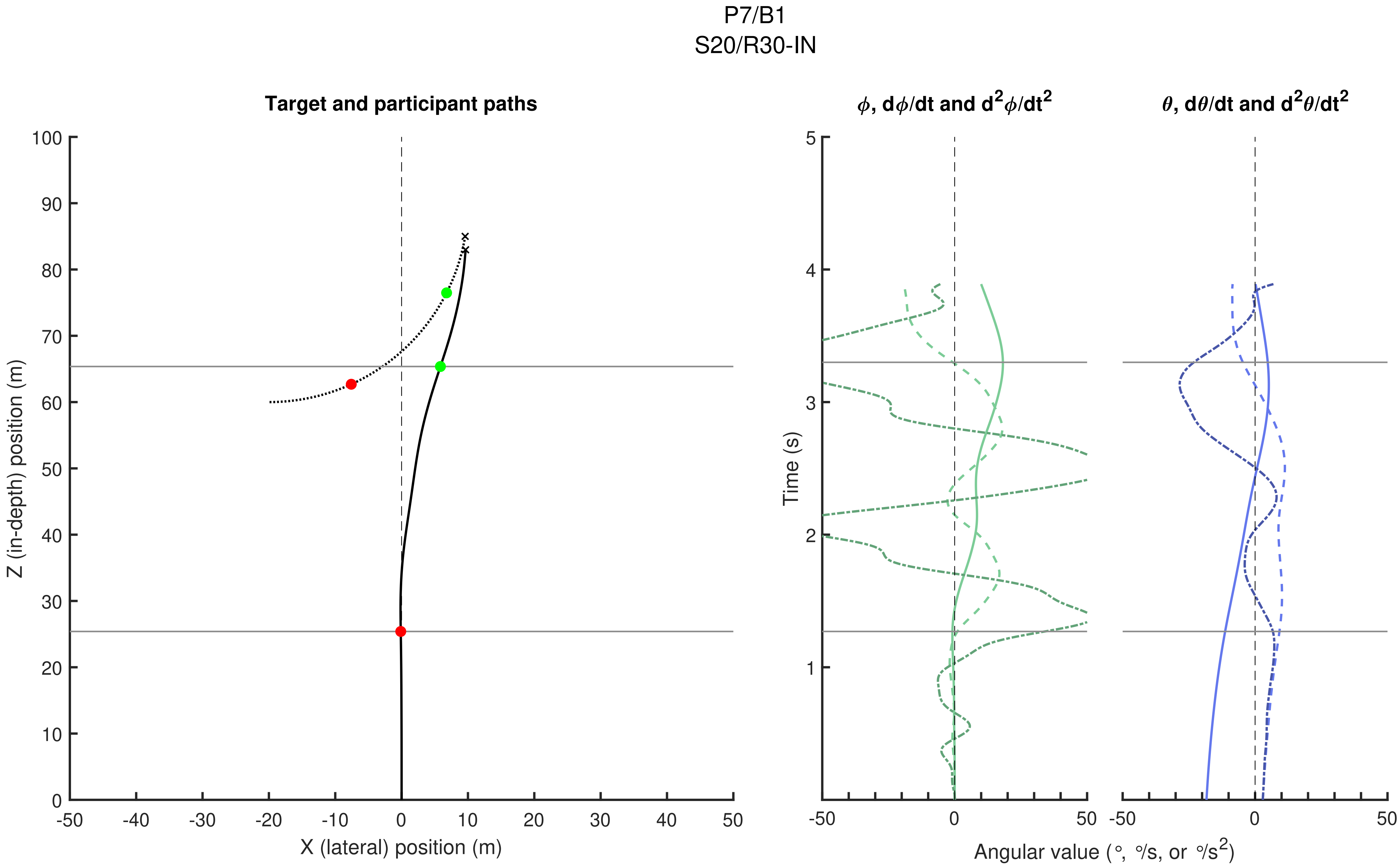

P7/B1
S20/R30-IN
Target and participant paths
Z (in-depth) position (m)
X (lateral) position (m)
ϕ, dϕ/dt and d$^2\phi$/dt^2
θ, dθ/dt and d$^2\theta$/dt^2
Time (s)
Angular value (°, °/s, or °/s^2)

P7/B1
S15/R10-IN

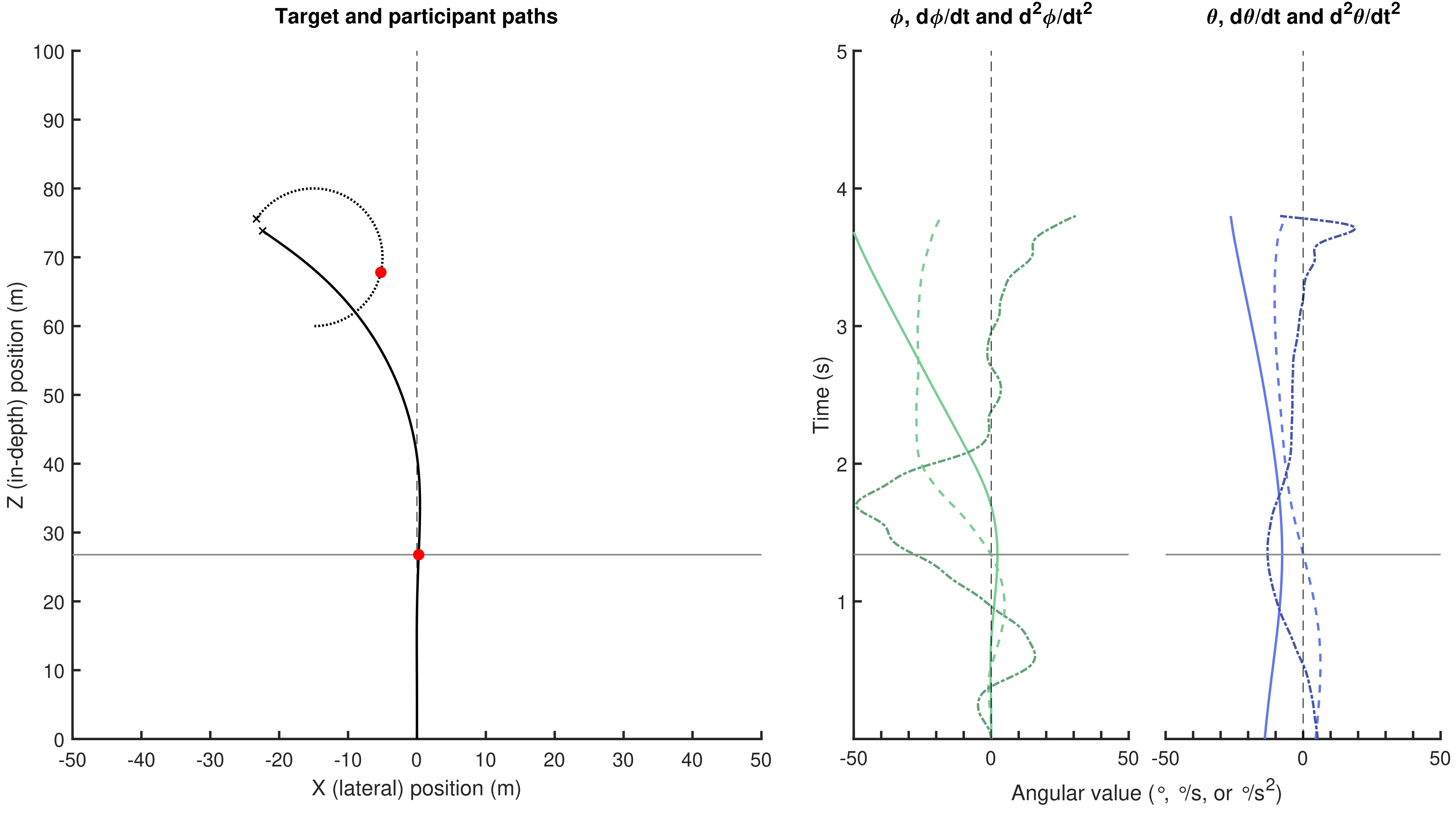

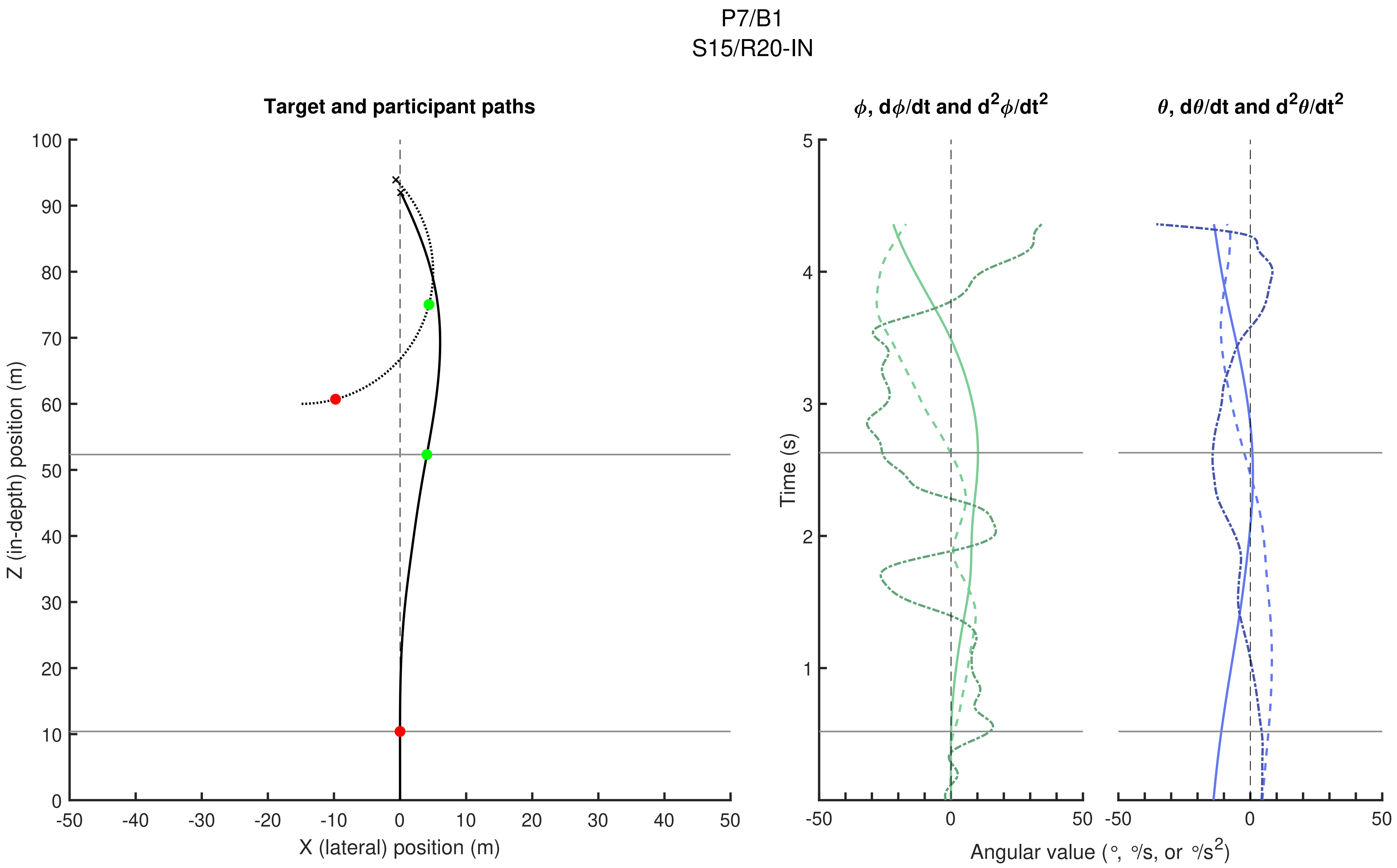

P7/B1
S15/R20-IN
Target and participant paths
ϕ, dϕ/dt and d$^2\phi$/dt^2
θ, dθ/dt and d$^2\theta$/dt^2
Z (in-depth) position (m)
X (lateral) position (m)
Time (s)
Angular value (°, °/s, or °/s^2)
100
90
80
70
60
50
40
30
20
10
0
-50
-40
-30
-20
-10
0
10
20
30
40
50
5
4
3
2
1
-50
0
50
-50
0
50

P7/B1
S15/R30-IN

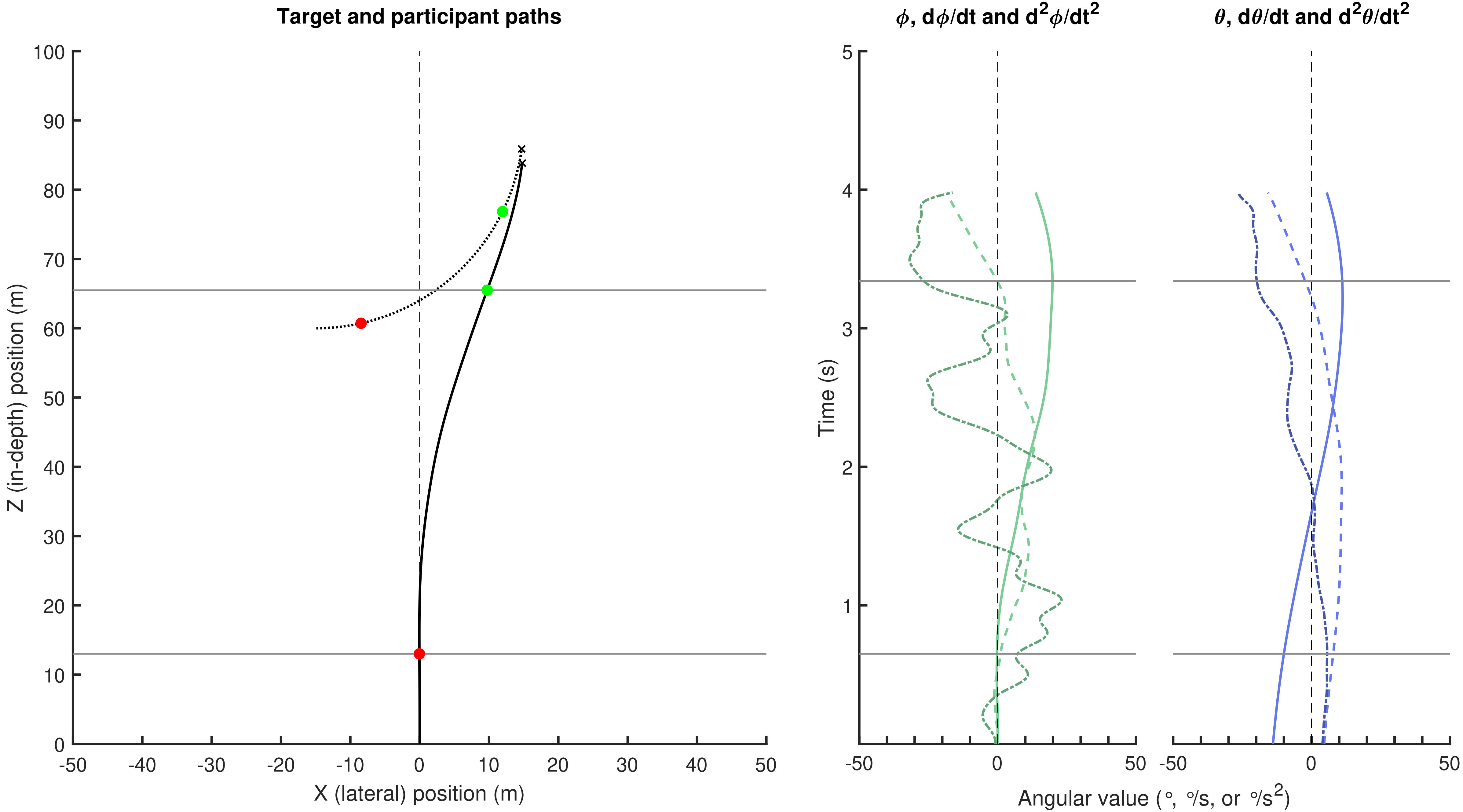

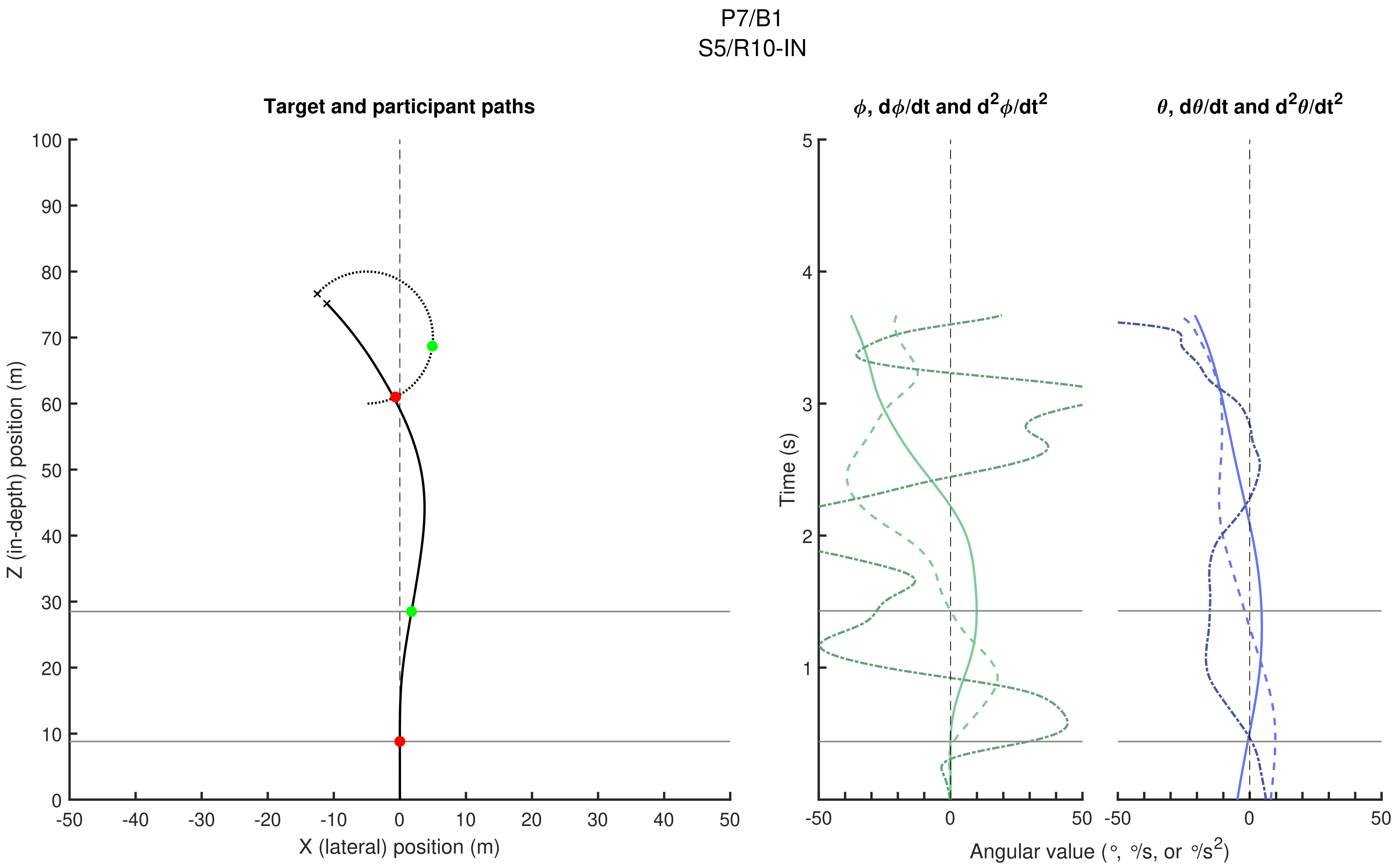

P7/B1
S5/R10-IN
Target and participant paths
Z (in-depth) position (m)
X (lateral) position (m)
100
90
80
70
60
50
40
30
20
10
0
-50
-40
-30
-20
-10
0
10
20
30
40
50
ϕ, dϕ/dt and d$^2\phi$/dt^2
θ, dθ/dt and d$^2\theta$/dt^2
Time (s)
5
4
3
2
1
-50
0
50
-50
0
50
Angular value (°, °/s, or °/s^2)

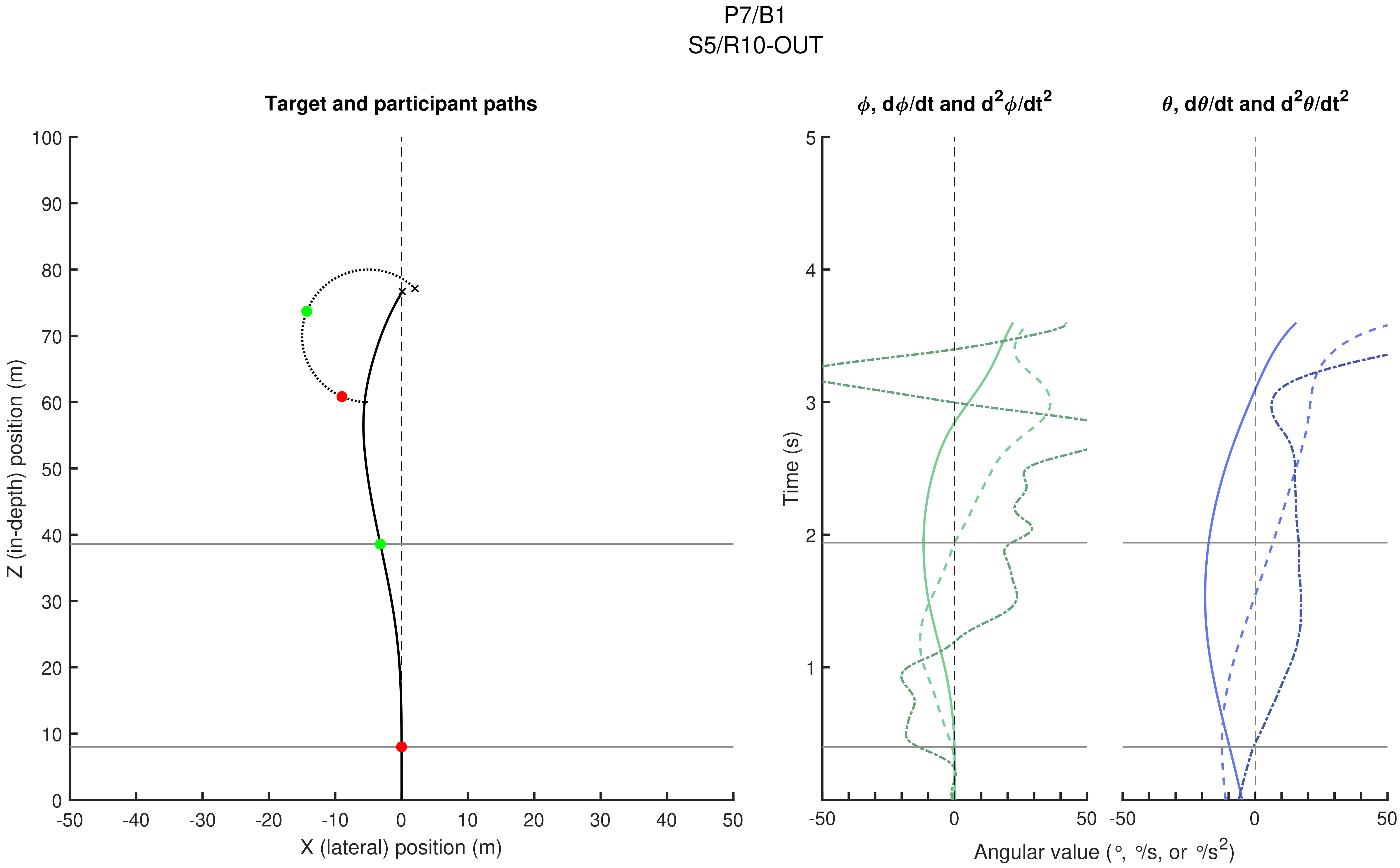
P7/B1
S5/R10-OUT
Target and participant paths
Z (in-depth) position (m)
X (lateral) position (m)
φ, dφ/dt and d²φ/dt²
θ, dθ/dt and d²θ/dt²
Time (s)
Angular value (°, °/s, or °/s²)

P7/B1
S5/R20-IN

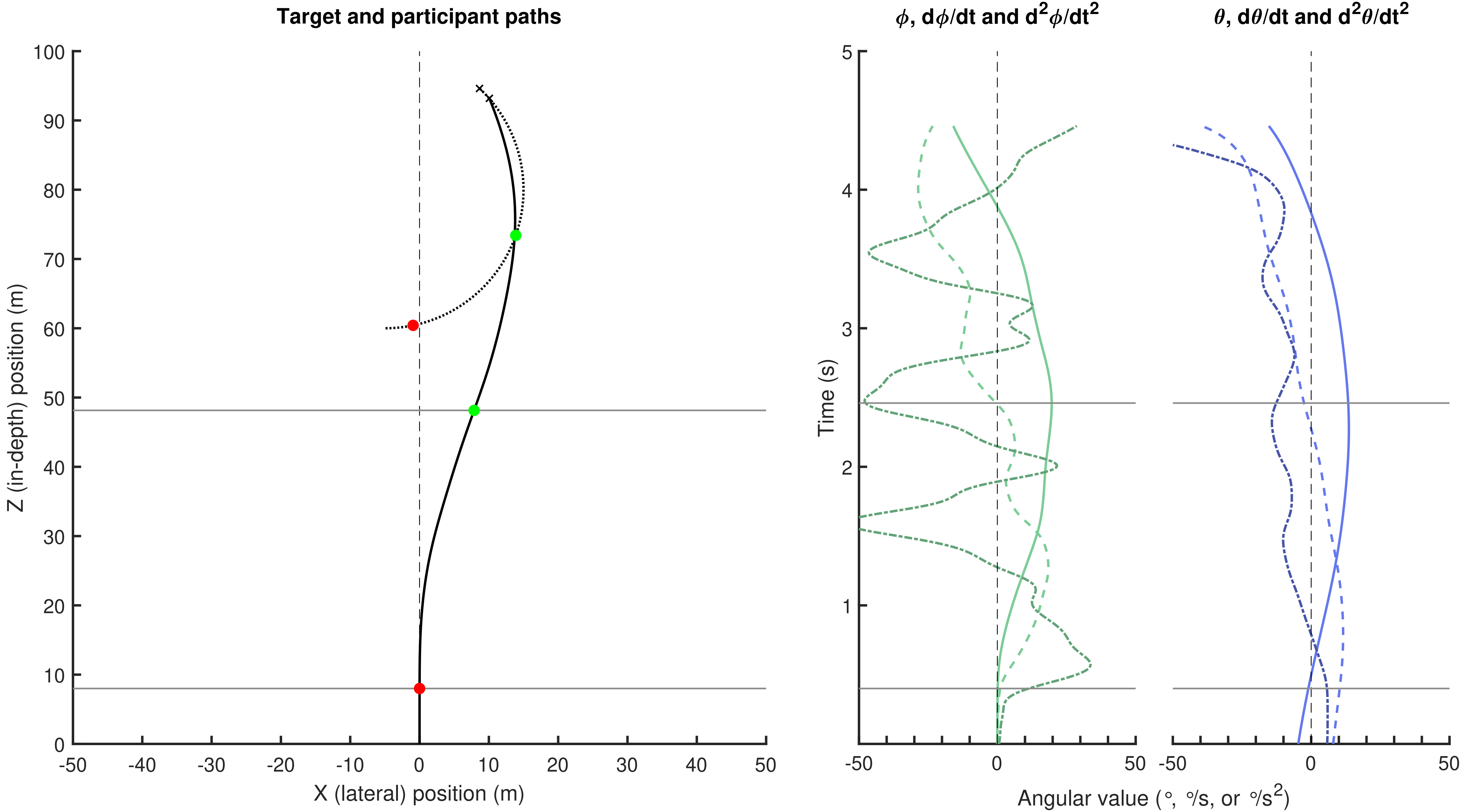

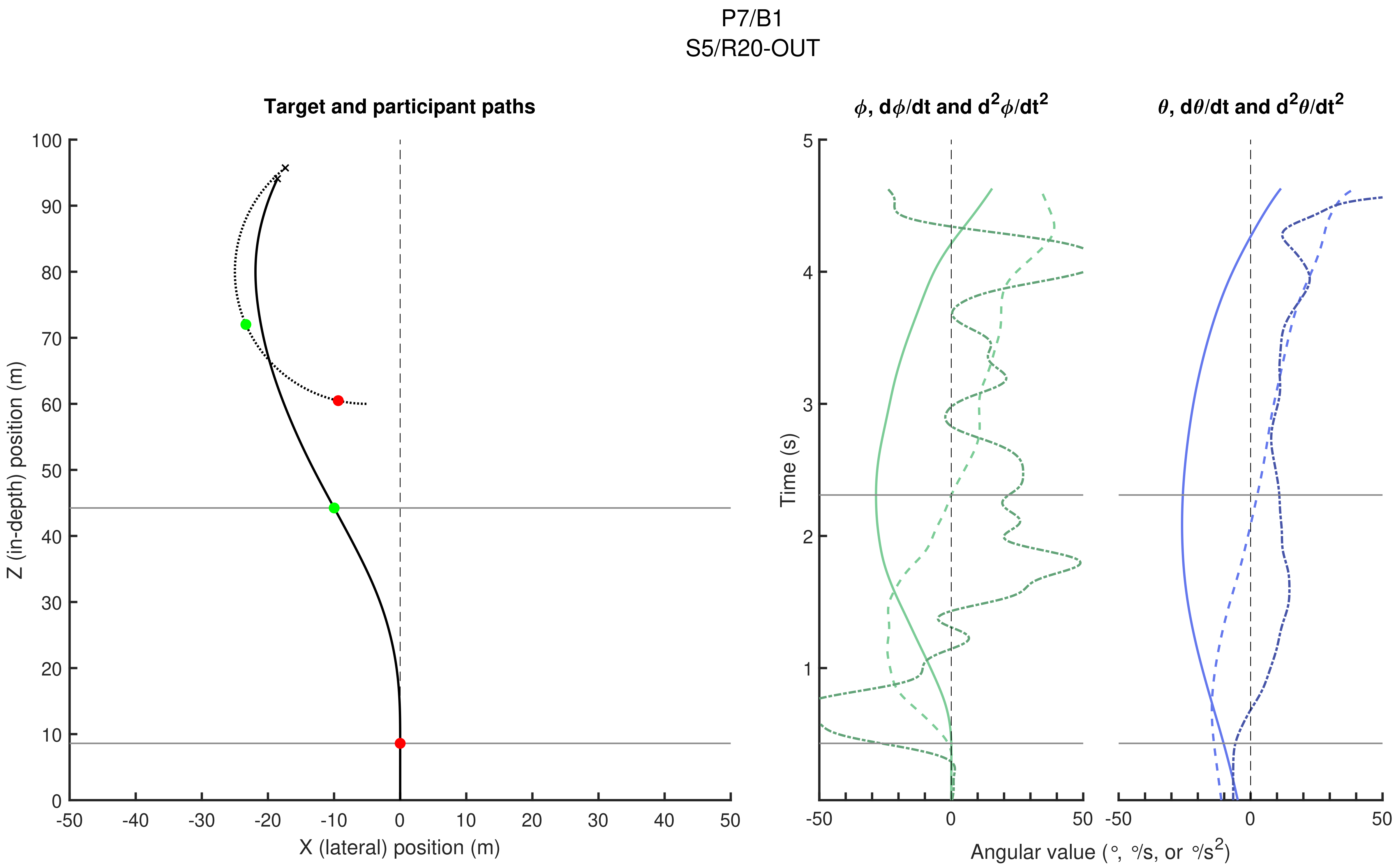

P7/B1
S5/R20-OUT
Target and participant paths
ϕ, dϕ/dt and d$^2\phi$/dt^2
θ, dθ/dt and d$^2\theta$/dt^2
Z (in-depth) position (m)
X (lateral) position (m)
Time (s)
Angular value (°, °/s, or °/s^2)

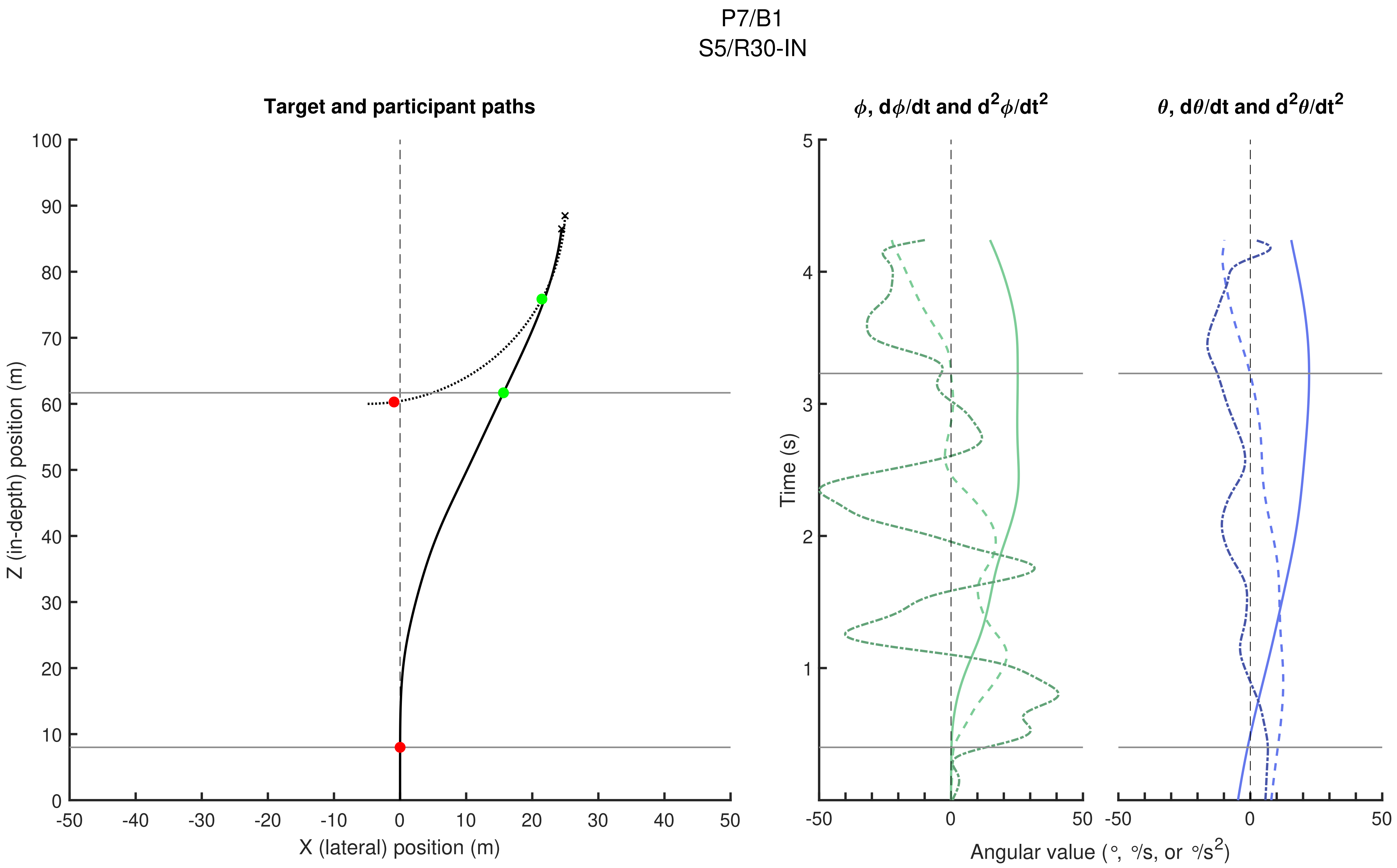
P7/B1
S5/R30-IN
Target and participant paths
Z (in-depth) position (m)
X (lateral) position (m)
ϕ, dϕ/dt and d²ϕ/dt²
θ, dθ/dt and d²θ/dt²
Time (s)
Angular value (°, °/s, or °/s²)

P7/B1
S5/R30-OUT

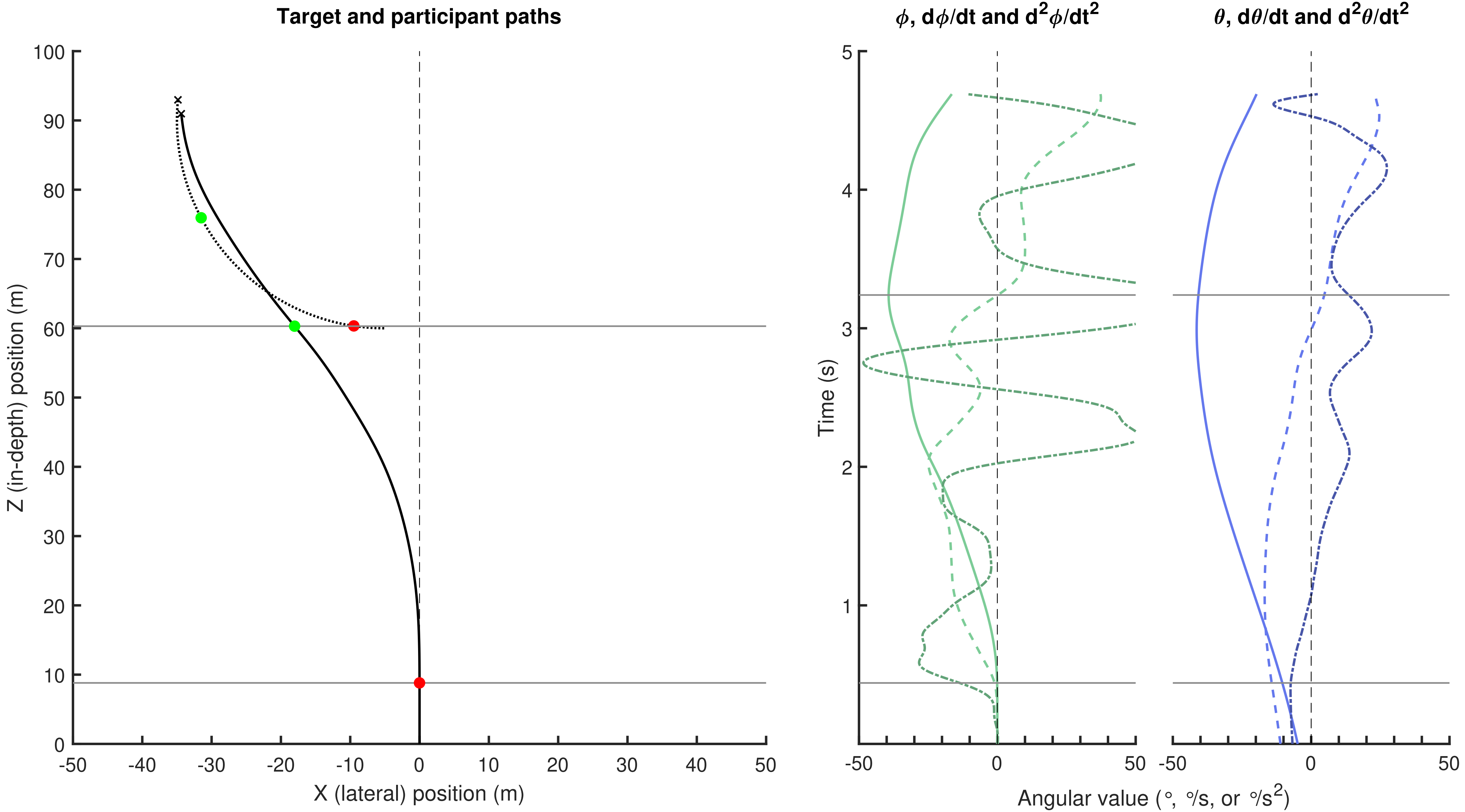

P7/B1
S5/R10-OUT

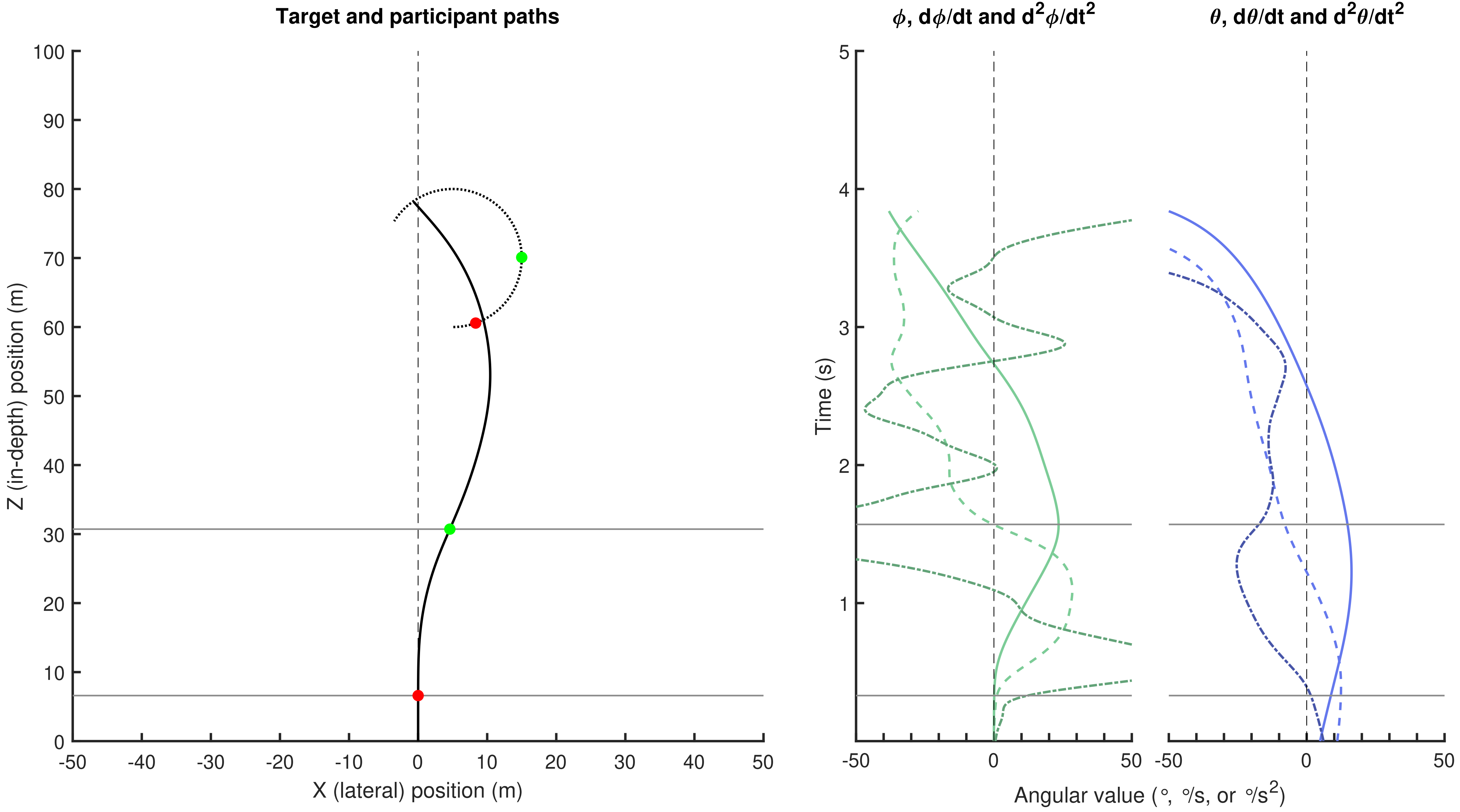

P7/B1
S5/R10-IN

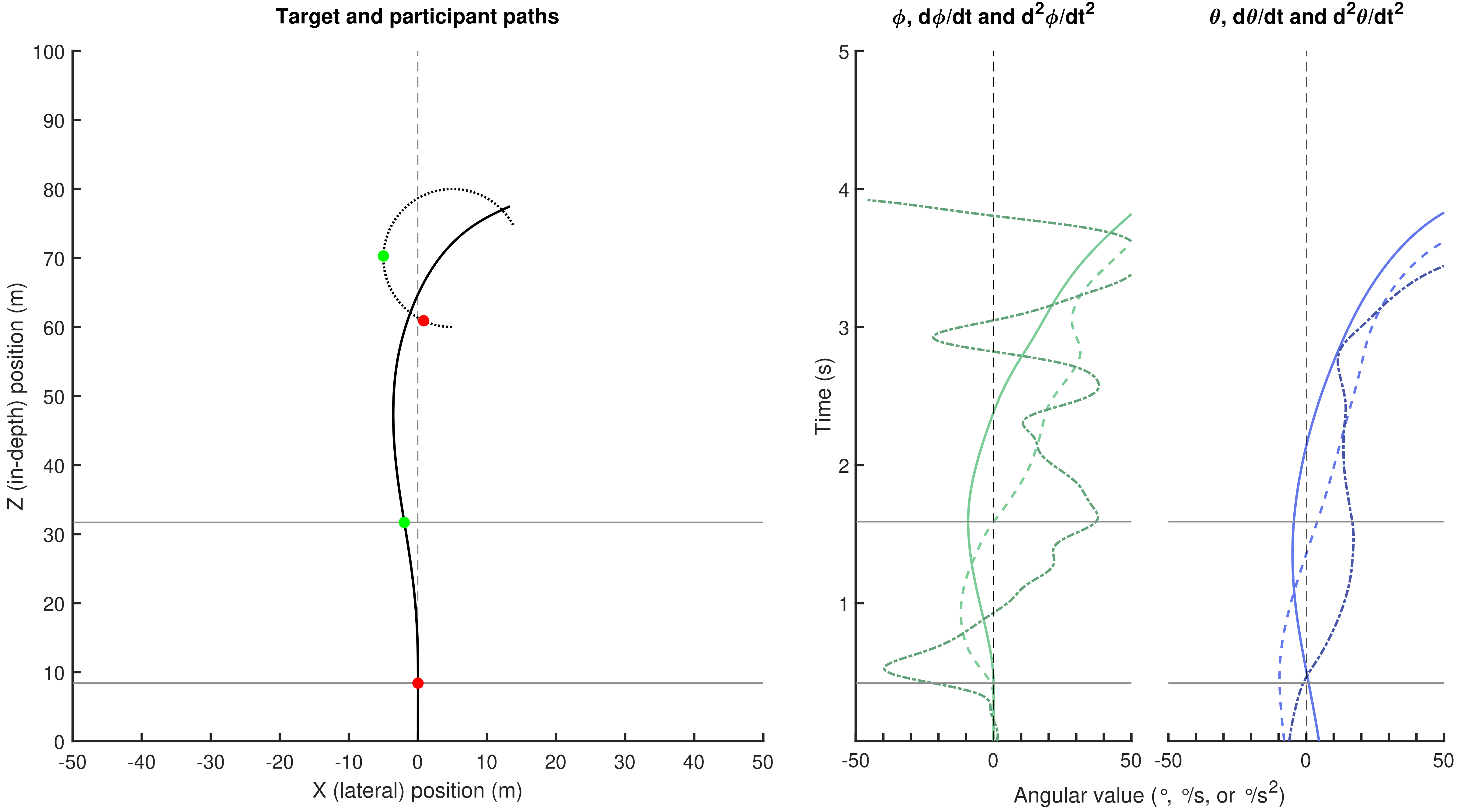

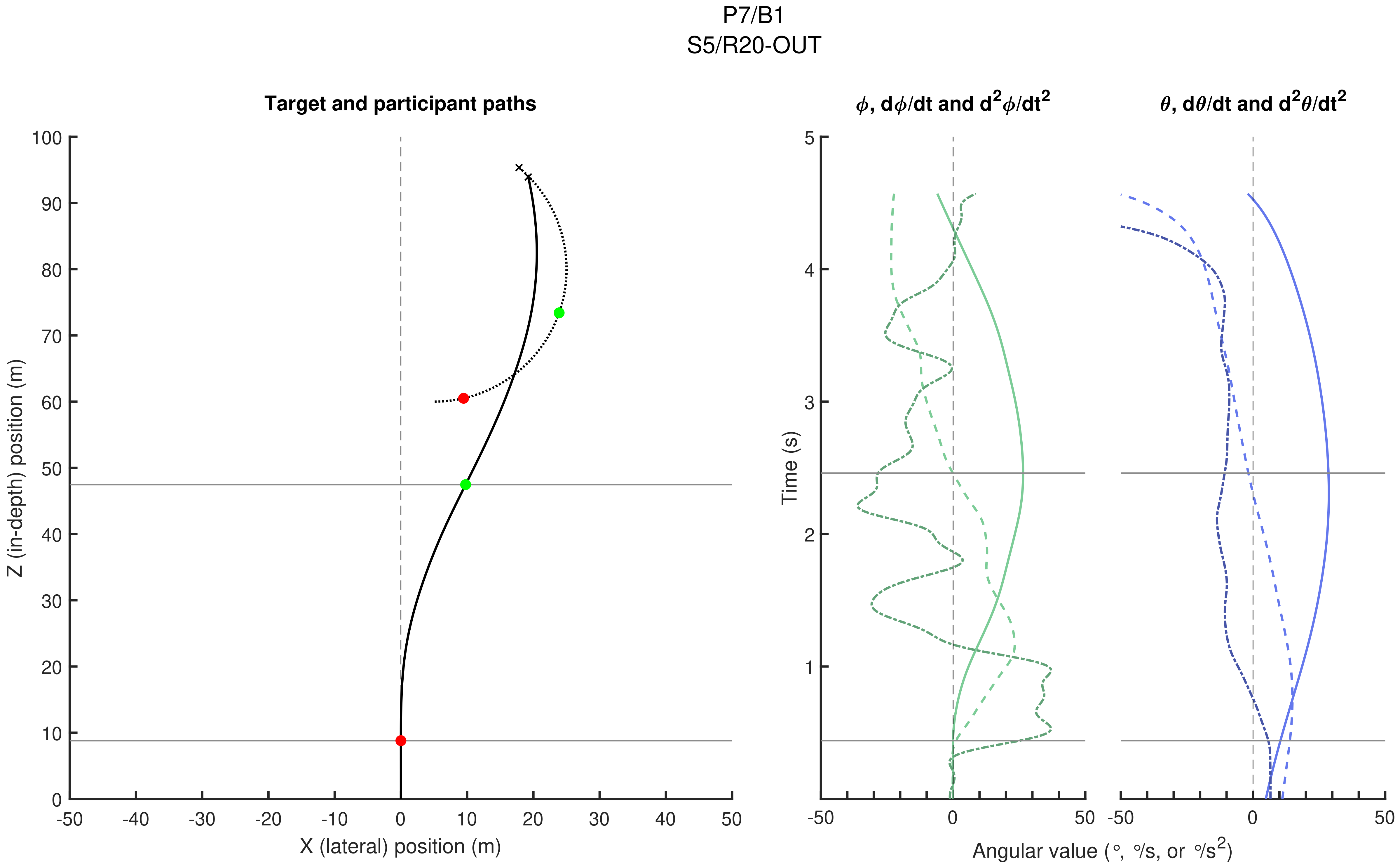

P7/B1
S5/R20-OUT
Target and participant paths
Z (in-depth) position (m)
X (lateral) position (m)
ϕ, dϕ/dt and d$^2\phi$/dt^2
θ, dθ/dt and d$^2\theta$/dt^2
Time (s)
Angular value (°, °/s, or °/s^2)

P7/B1
S5/R20-IN

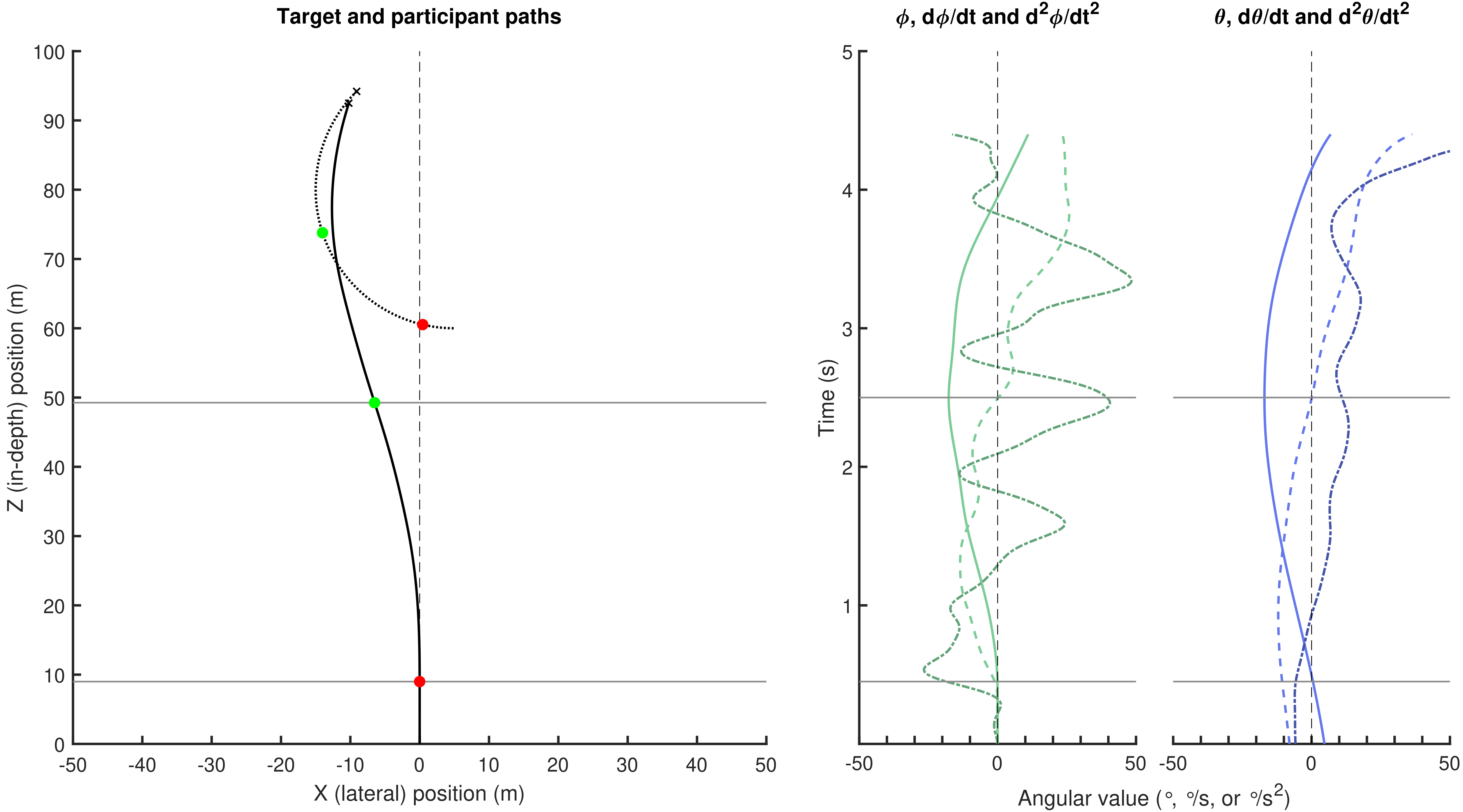

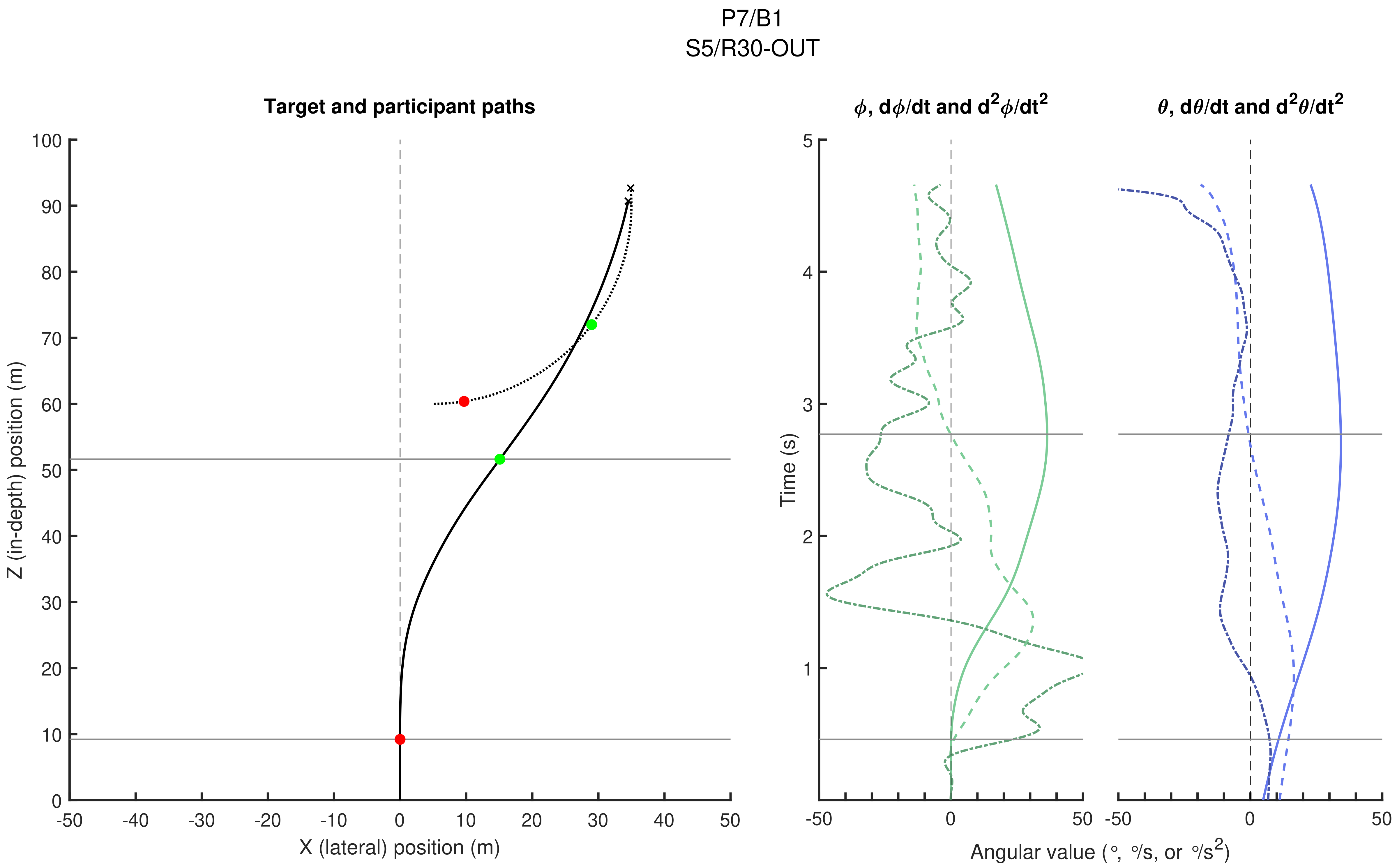

P7/B1
S5/R30-OUT
Target and participant paths
Z (in-depth) position (m)
X (lateral) position (m)
φ, dφ/dt and d²φ/dt²
θ, dθ/dt and d²θ/dt²
Time (s)
Angular value (°, °/s, or °/s²)

P7/B1
S5/R30-IN

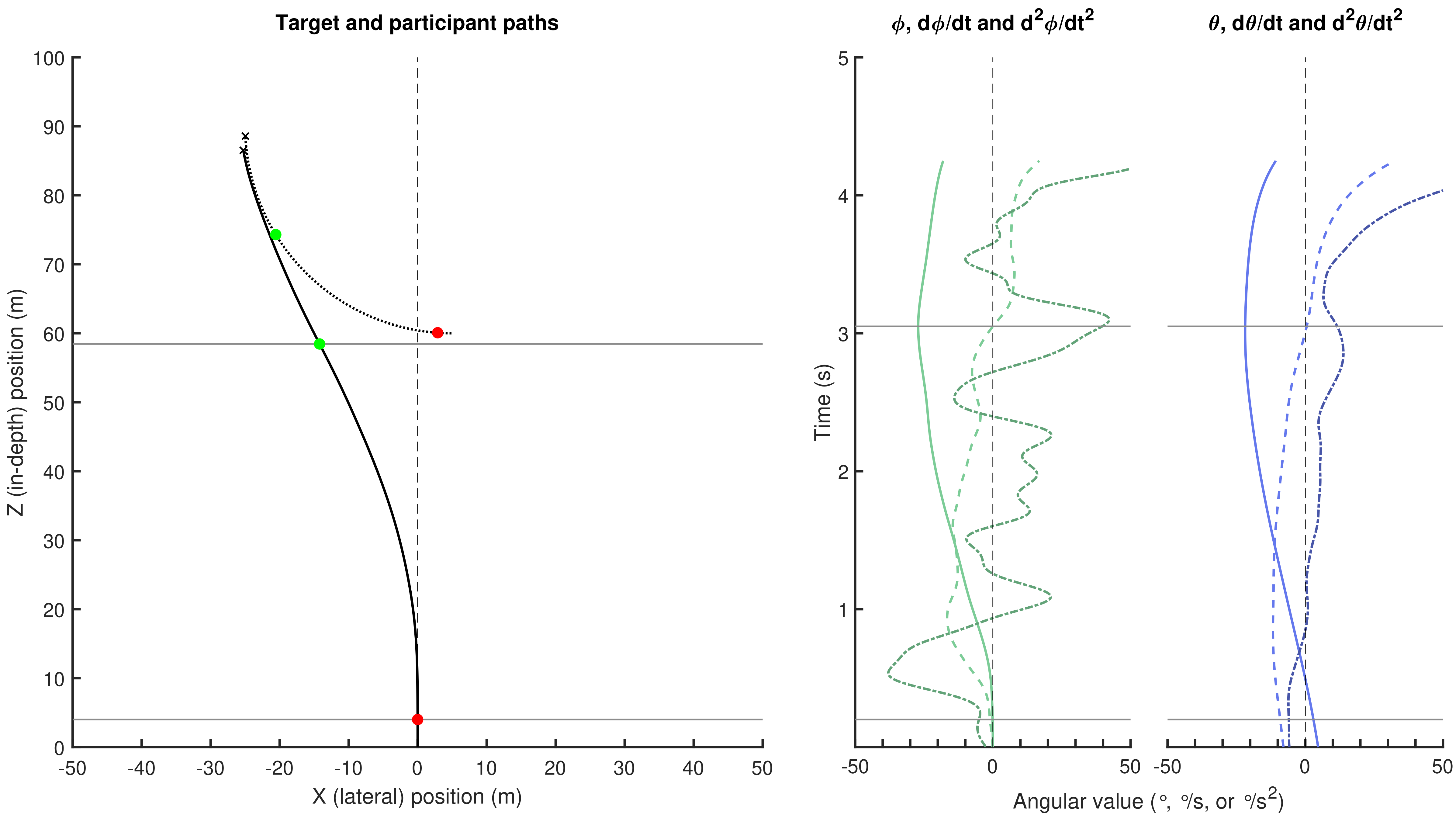

P7/B1
S15/R10-IN

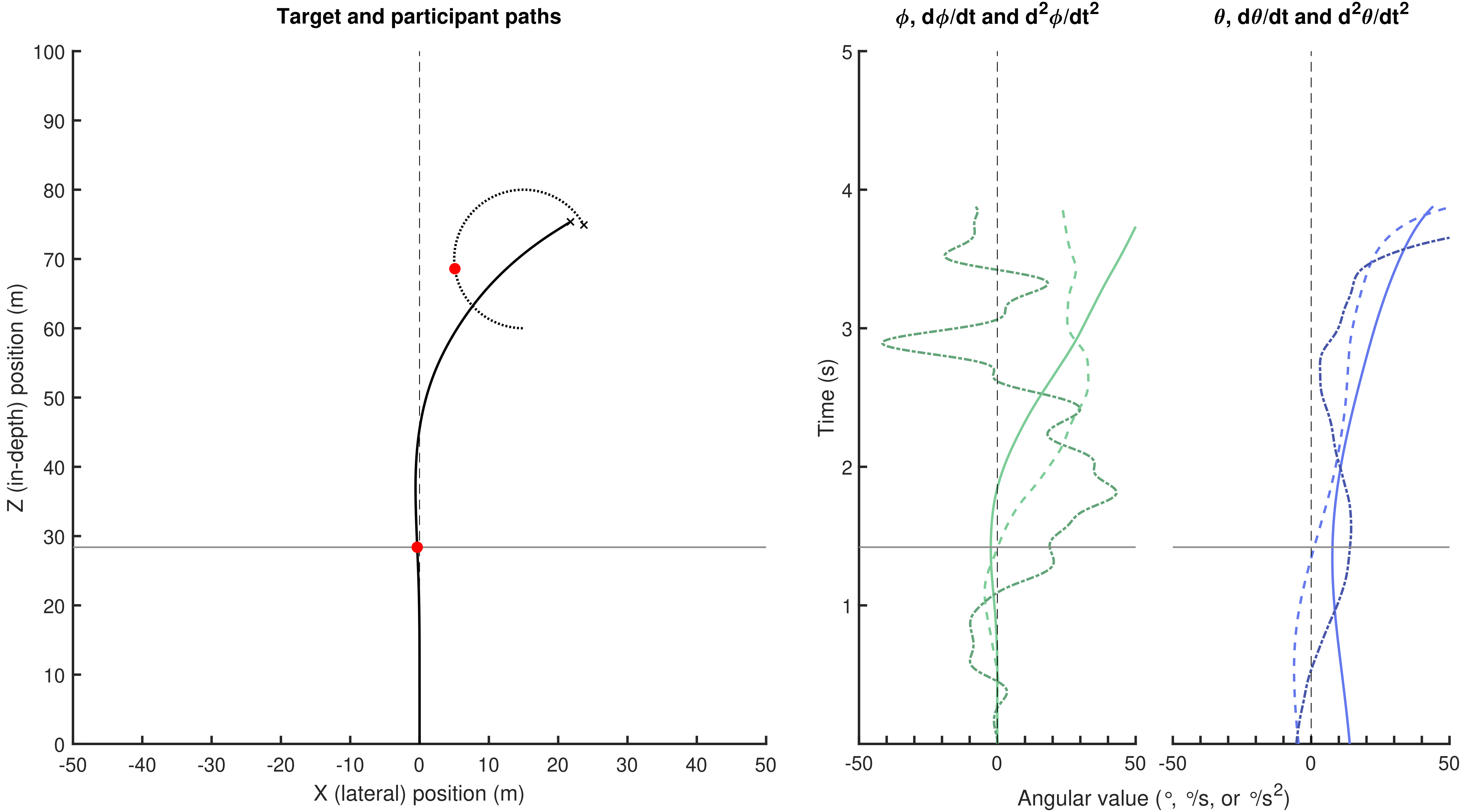

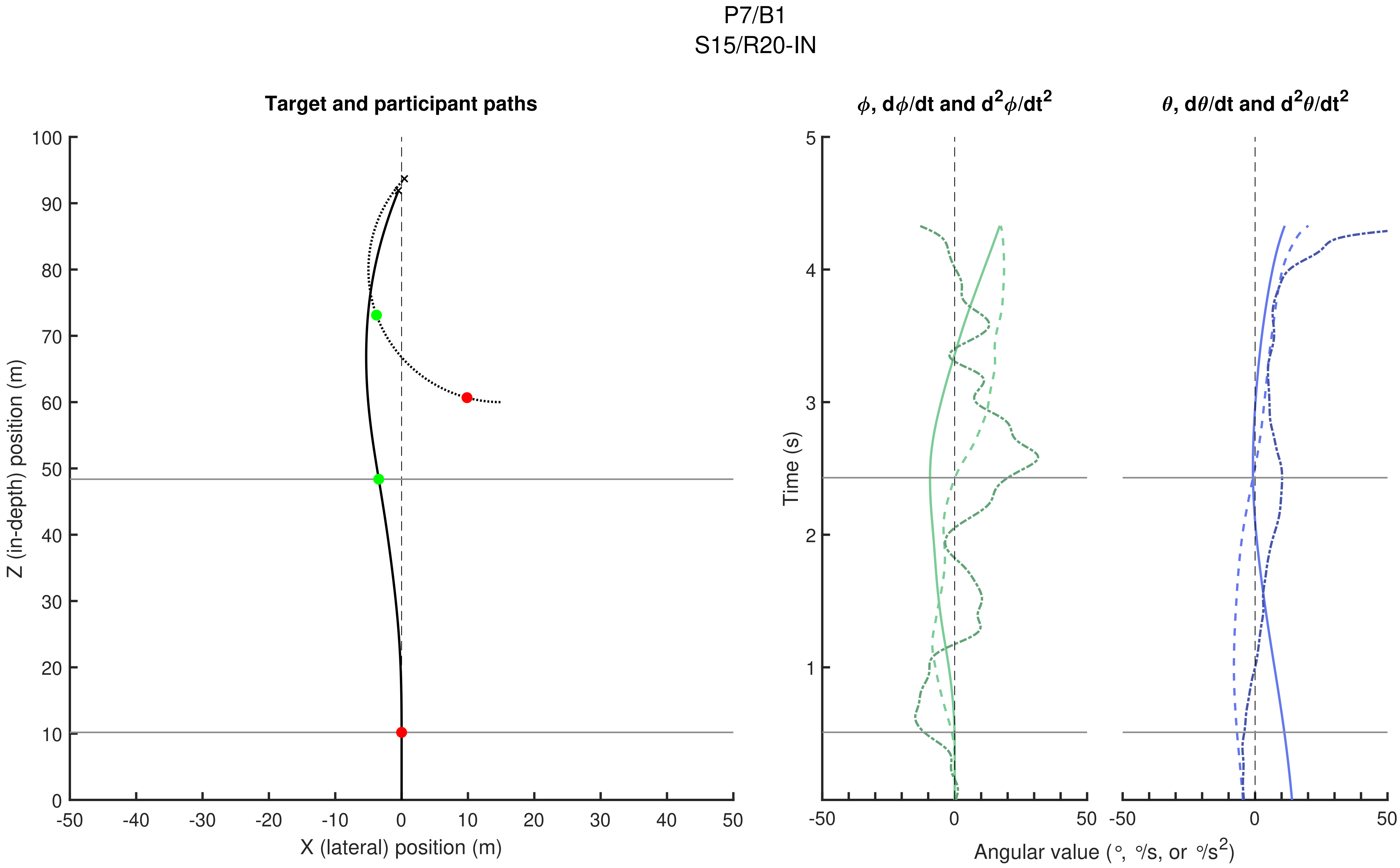
P7/B1
S15/R20-IN
Target and participant paths
Z (in-depth) position (m)
X (lateral) position (m)
φ, dφ/dt and d²φ/dt²
θ, dθ/dt and d²θ/dt²
Time (s)
Angular value (°, °/s, or °/s²)

P7/B1
S15/R30-IN

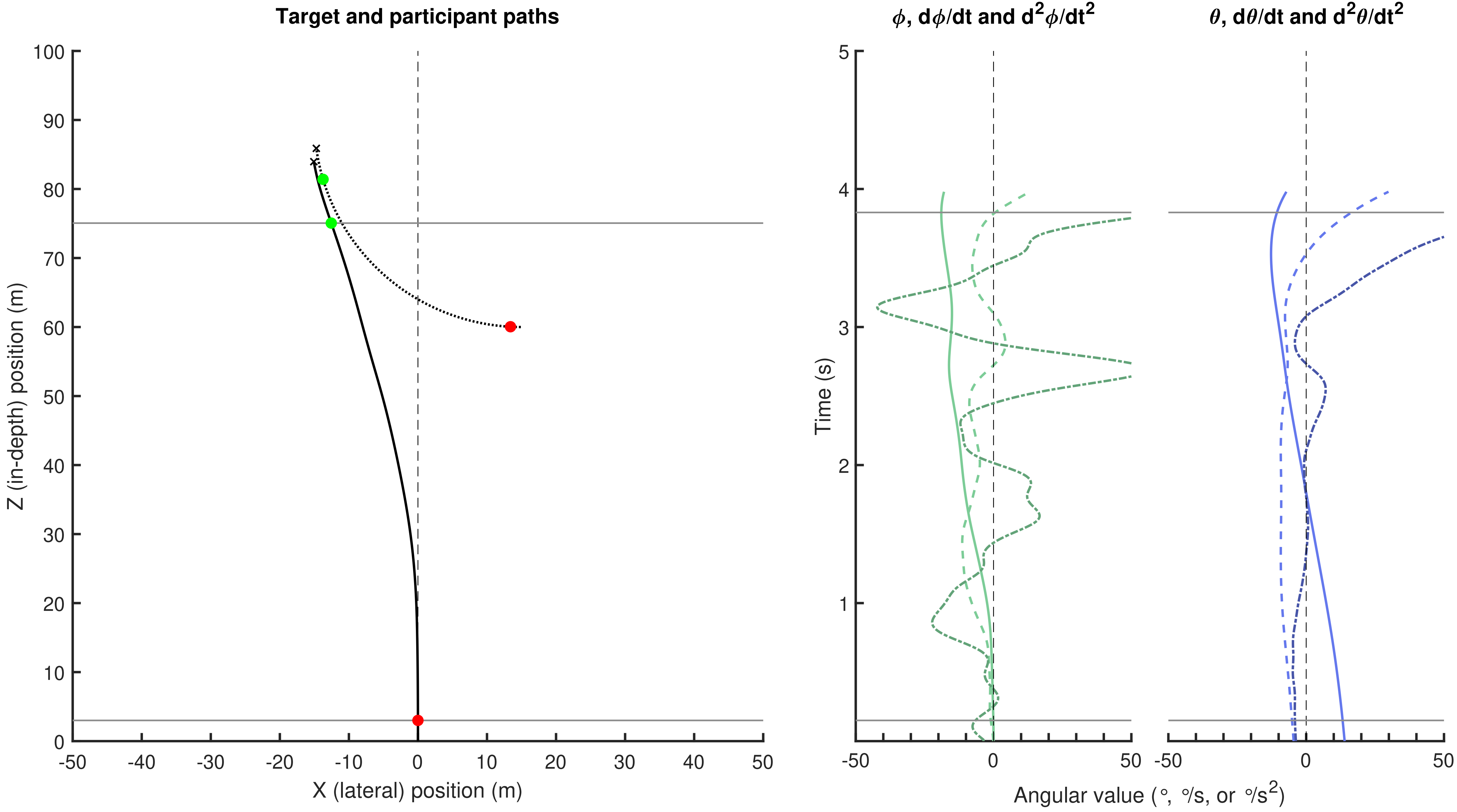

P7/B1
S20/R10-IN

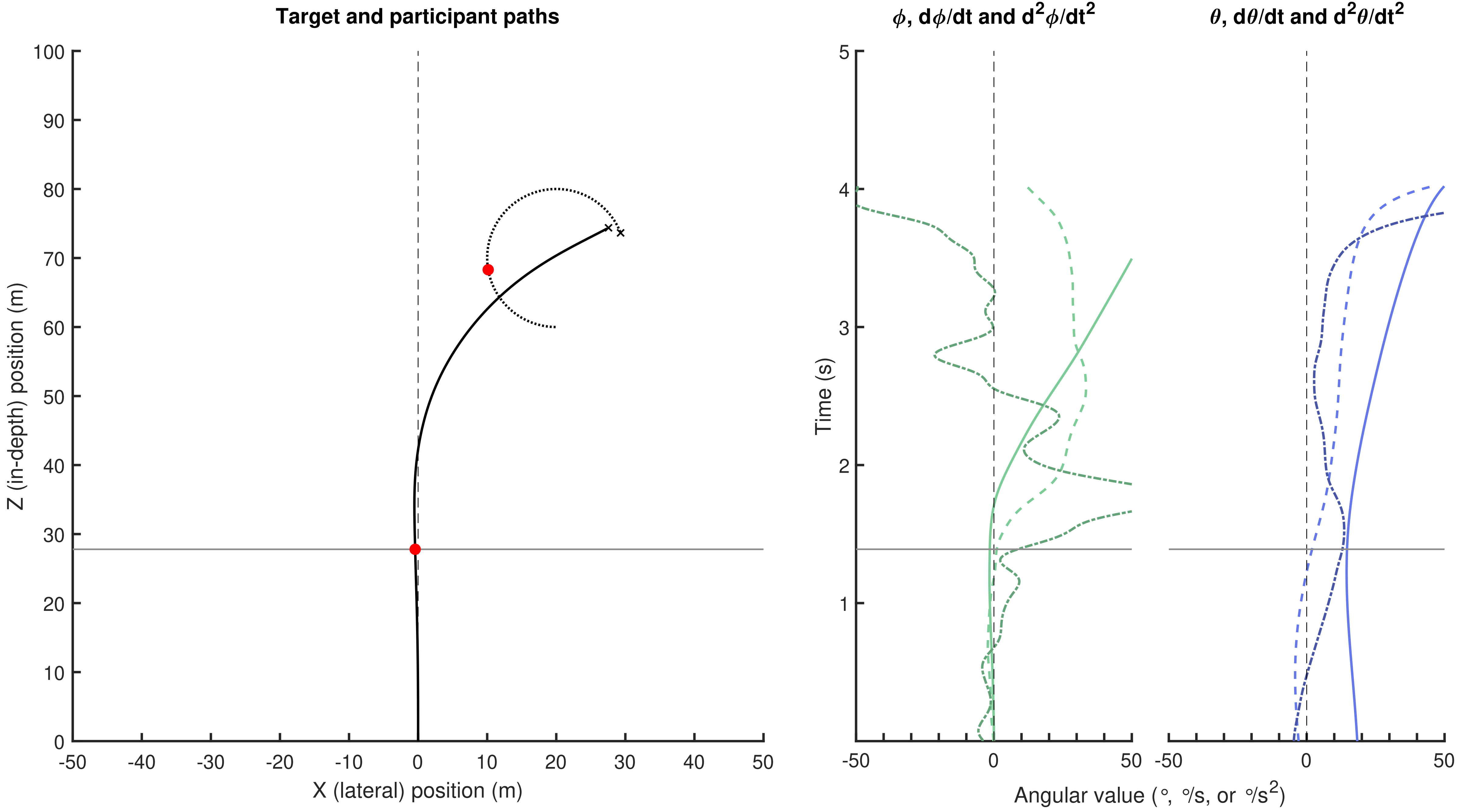

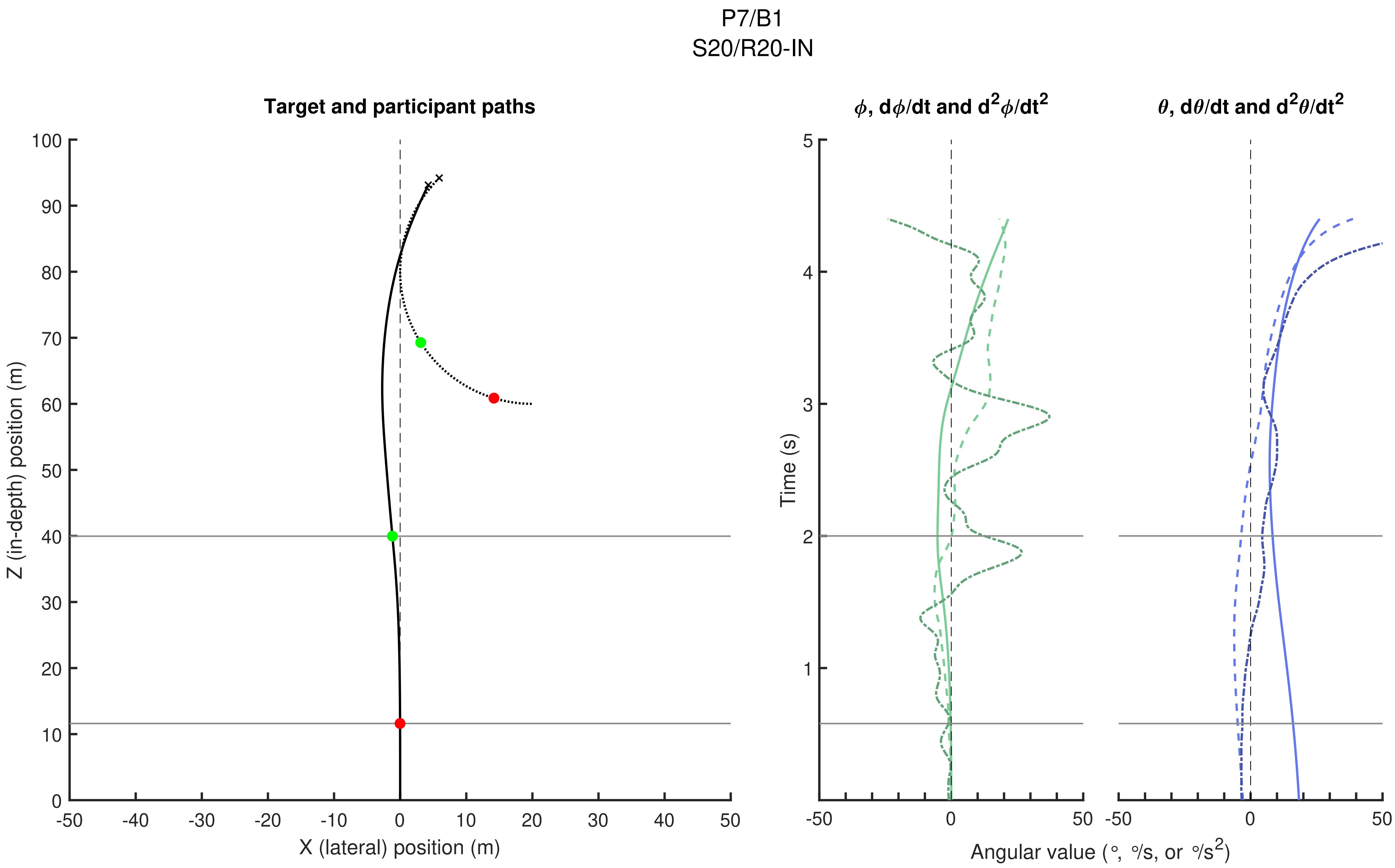
P7/B1
S20/R20-IN
Target and participant paths
Z (in-depth) position (m)
X (lateral) position (m)
ϕ, dϕ/dt and d$^2\phi$/dt^2
θ, dθ/dt and d$^2\theta$/dt^2
Time (s)
Angular value (°, °/s, or °/s^2)

P7/B1
S20/R30-IN

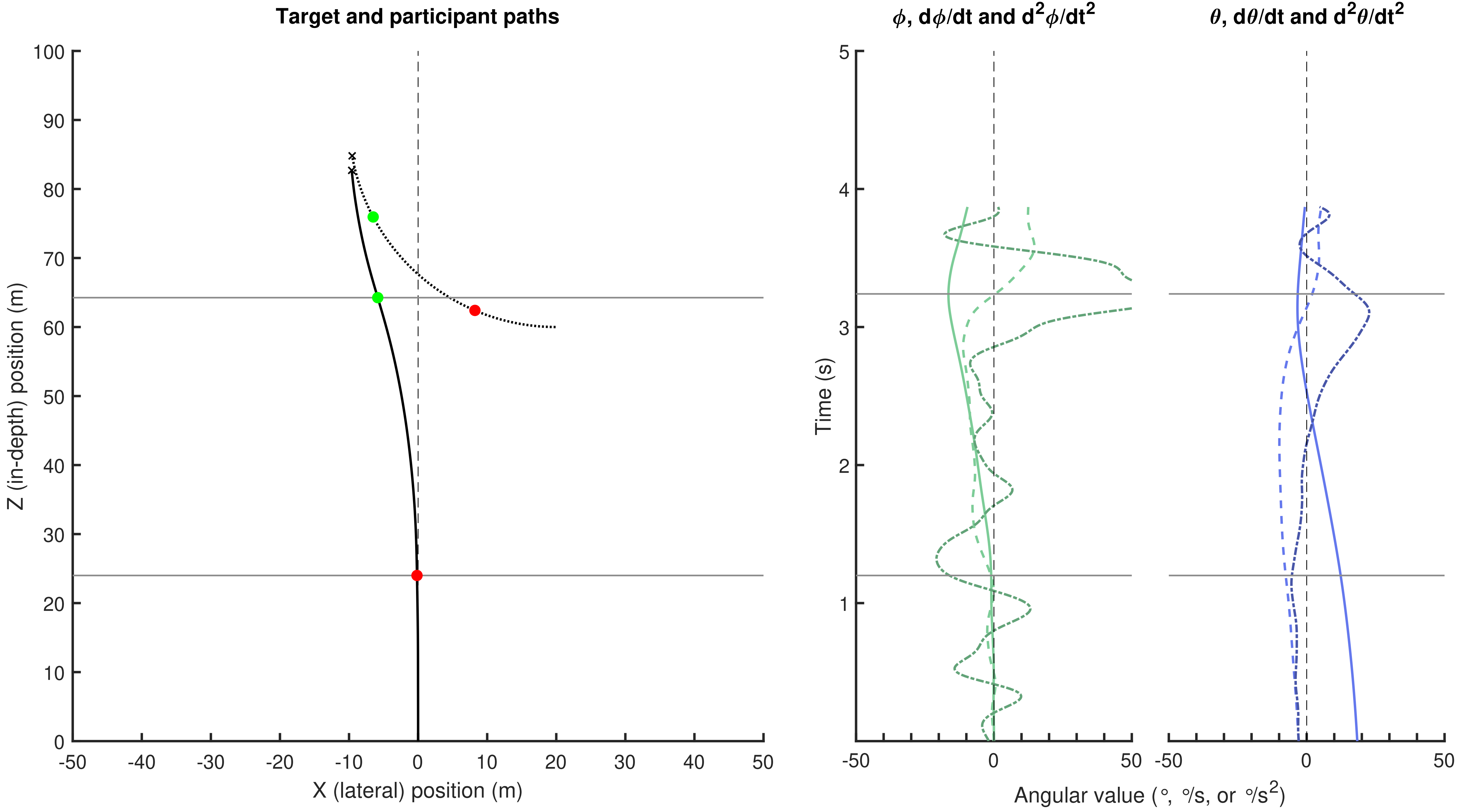

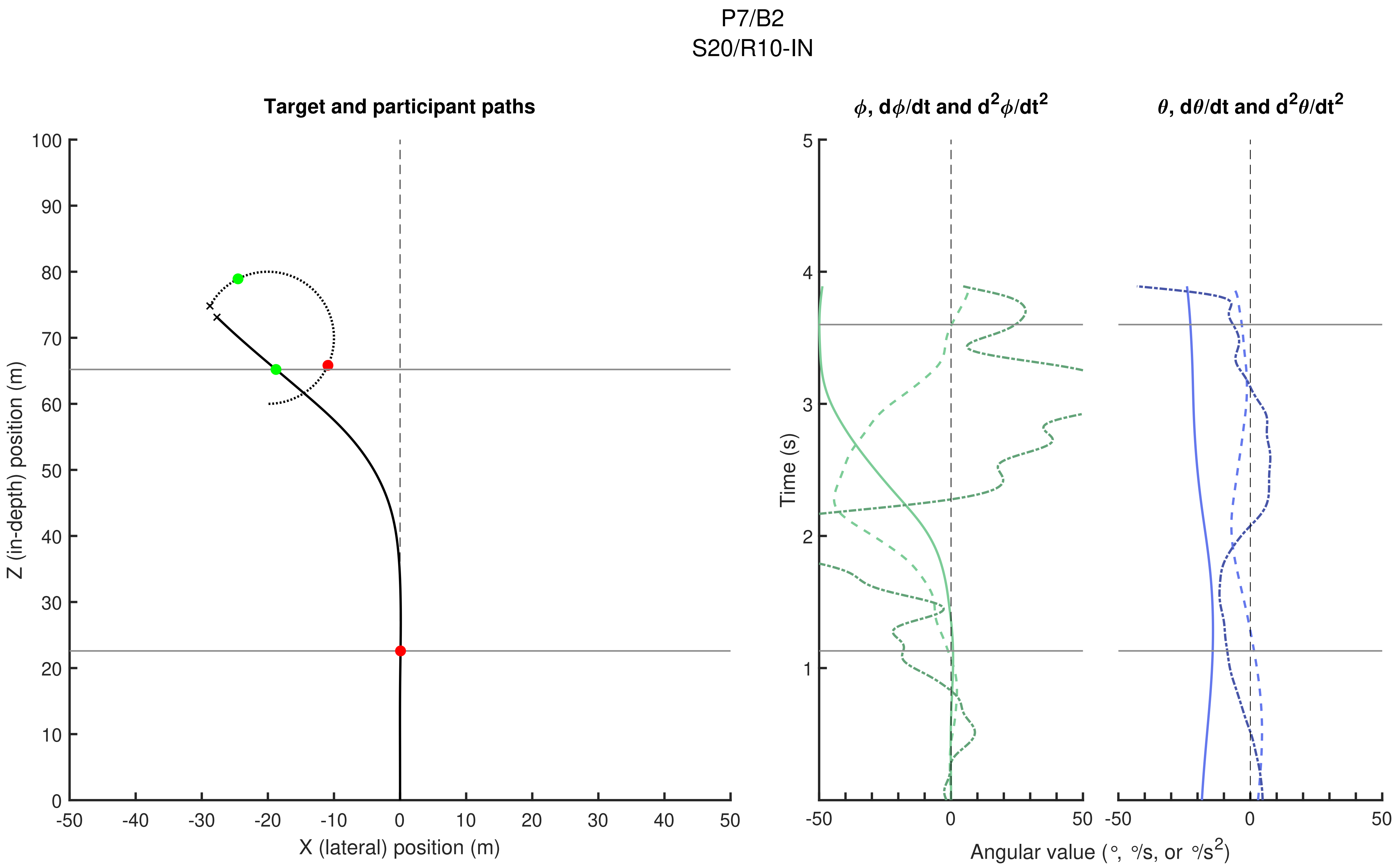

P7/B2
S20/R10-IN
Target and participant paths
Z (in-depth) position (m)
X (lateral) position (m)
ϕ, dϕ/dt and d$^2\phi$/dt^2
θ, dθ/dt and d$^2\theta$/dt^2
Time (s)
Angular value (°, °/s, or °/s^2)

P7/B2
S20/R20-IN

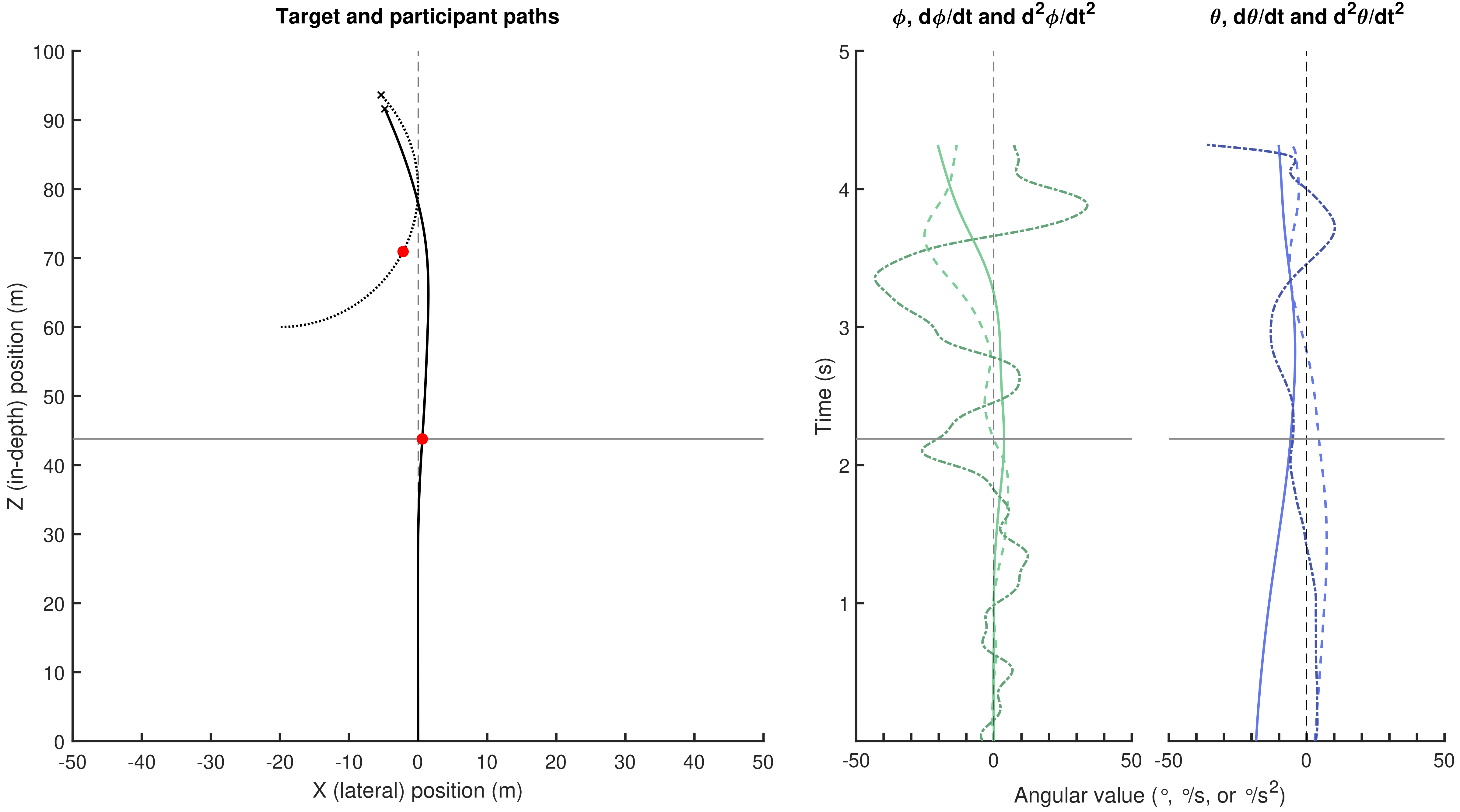

P7/B2
S20/R30-IN

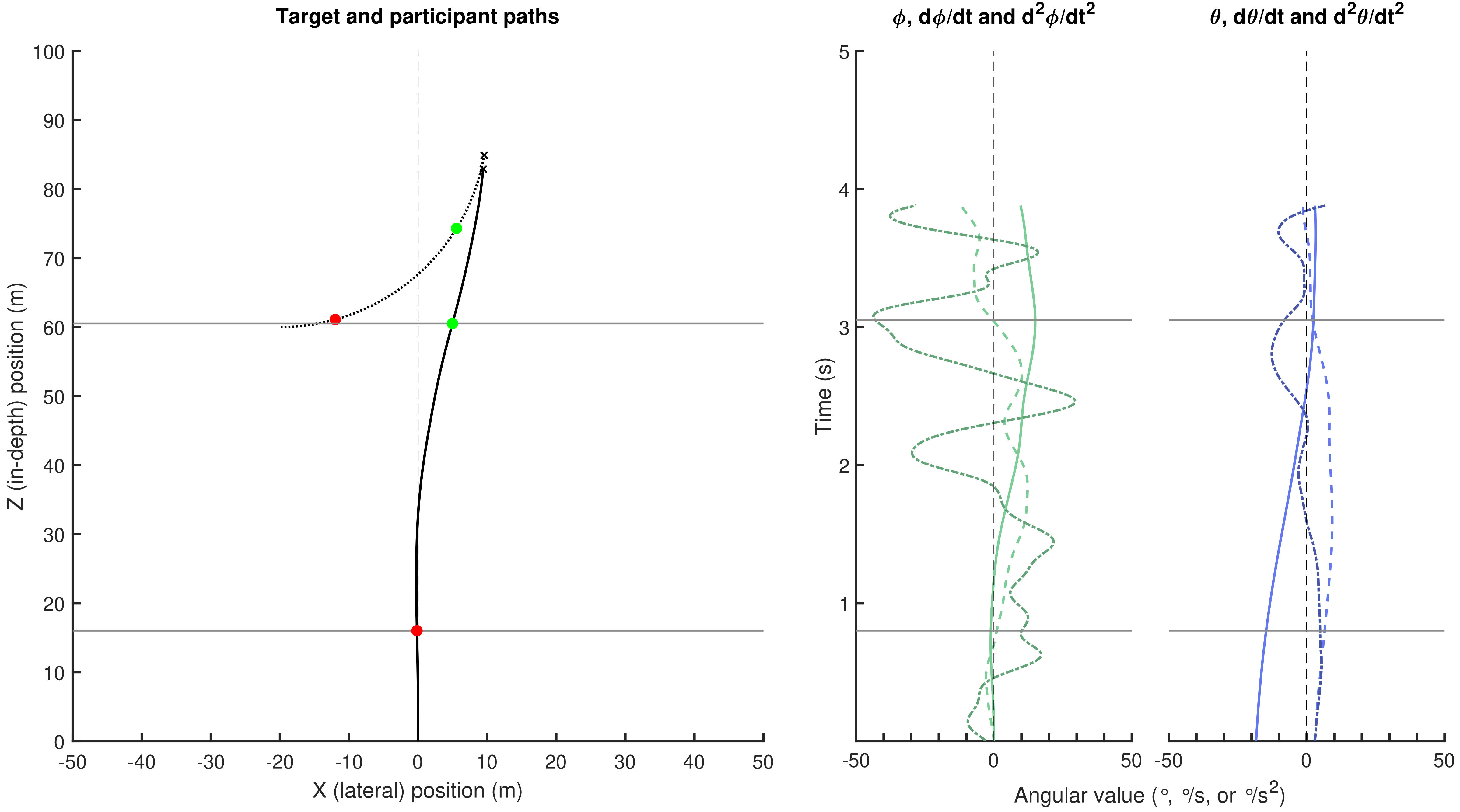

P7/B2
S15/R10-IN

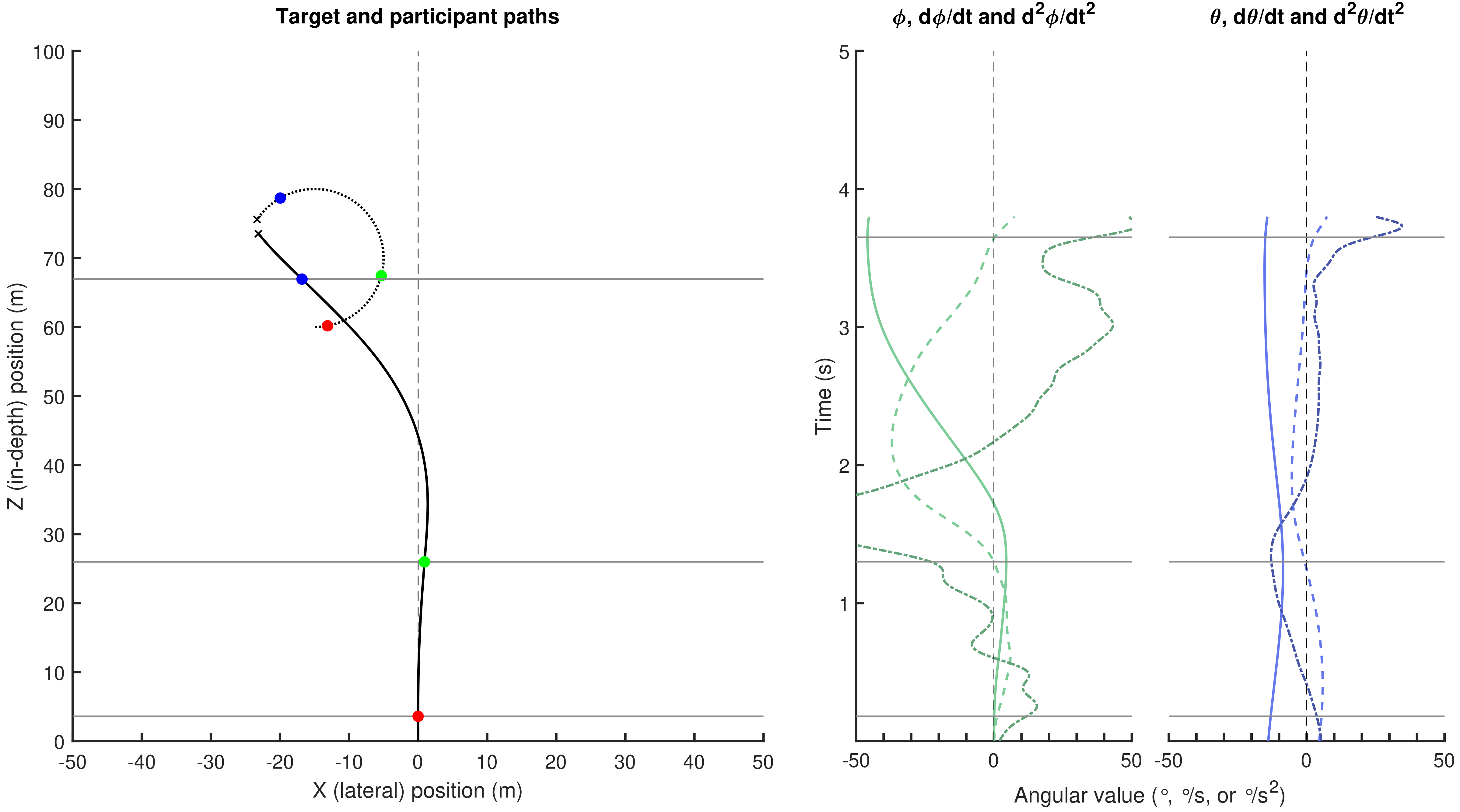

P7/B2
S15/R20-IN

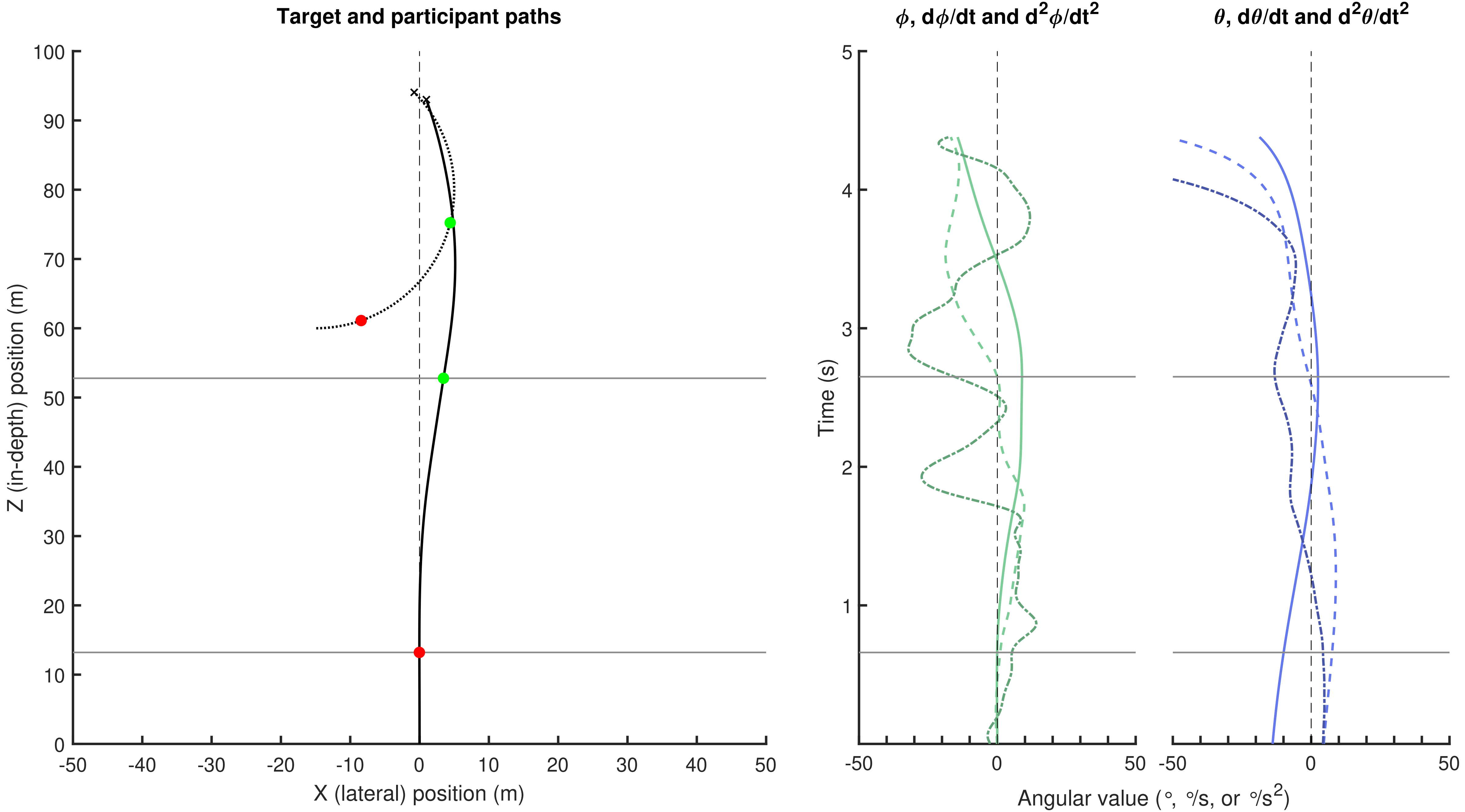

P7/B2
S15/R30-IN

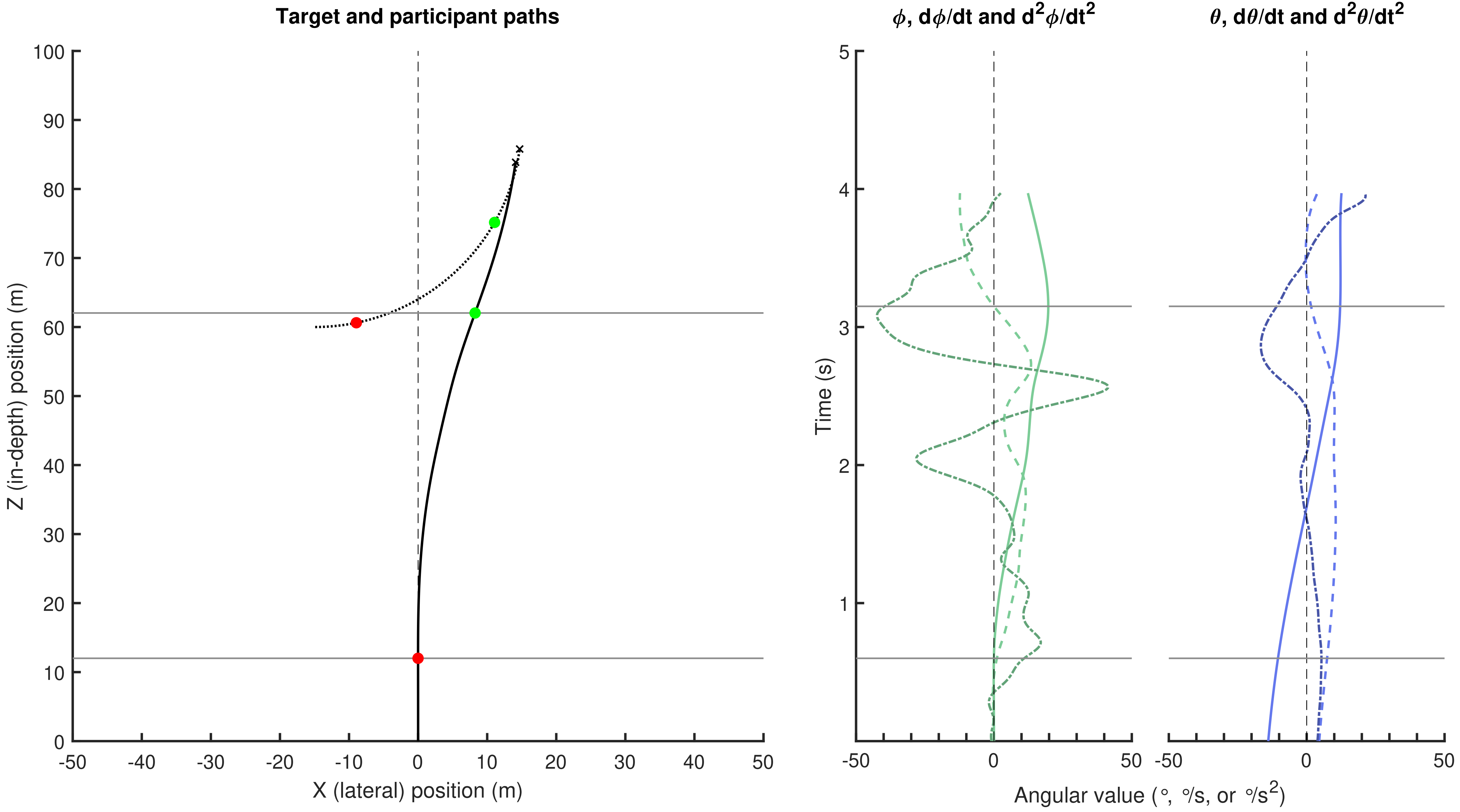

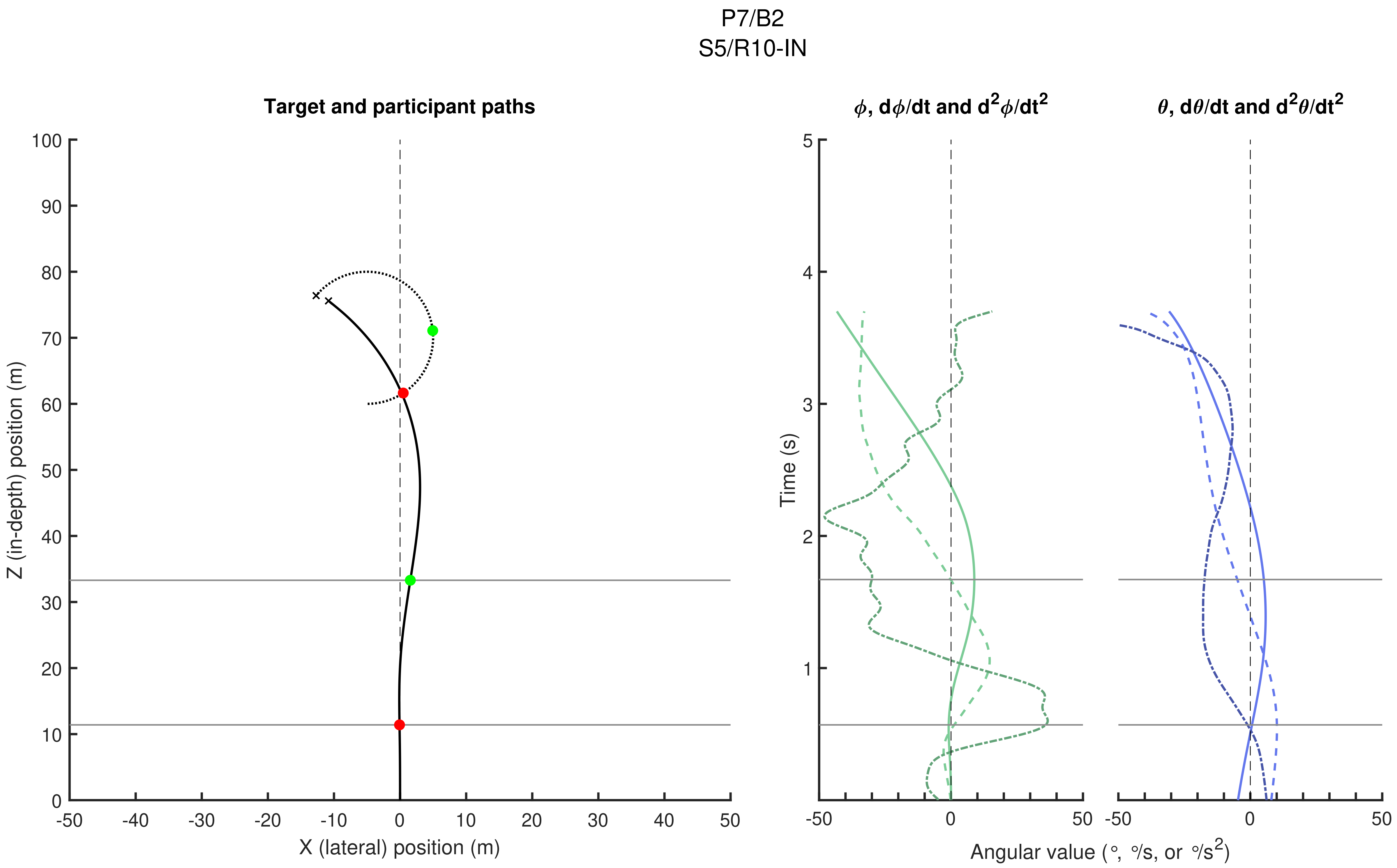

P7/B2
S5/R10-IN
Target and participant paths
Z (in-depth) position (m)
X (lateral) position (m)
ϕ, dϕ/dt and d²ϕ/dt²
θ, dθ/dt and d²θ/dt²
Time (s)
Angular value (°, °/s, or °/s²)

P7/B2
S5/R10-OUT

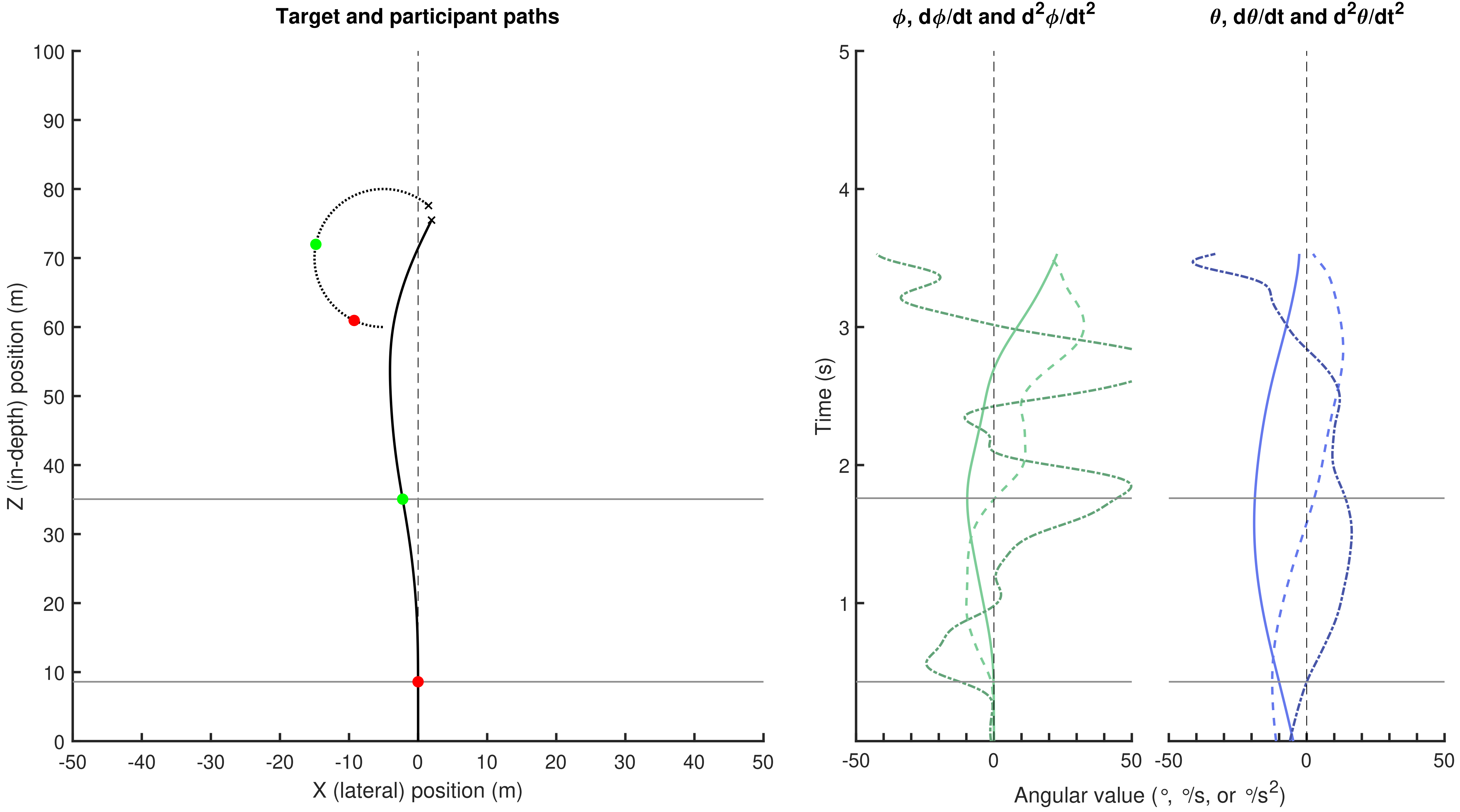

P7/B2
S5/R20-IN

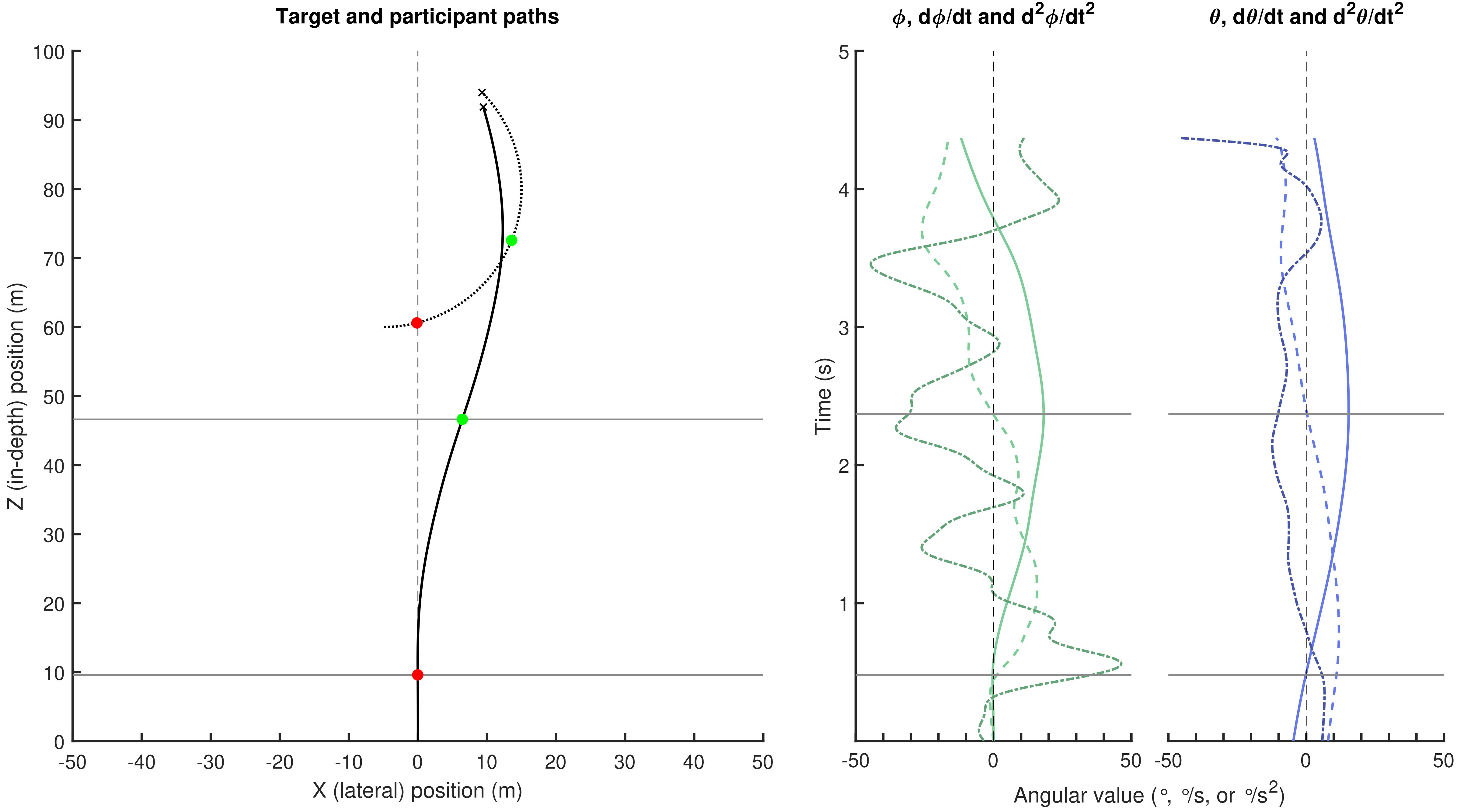

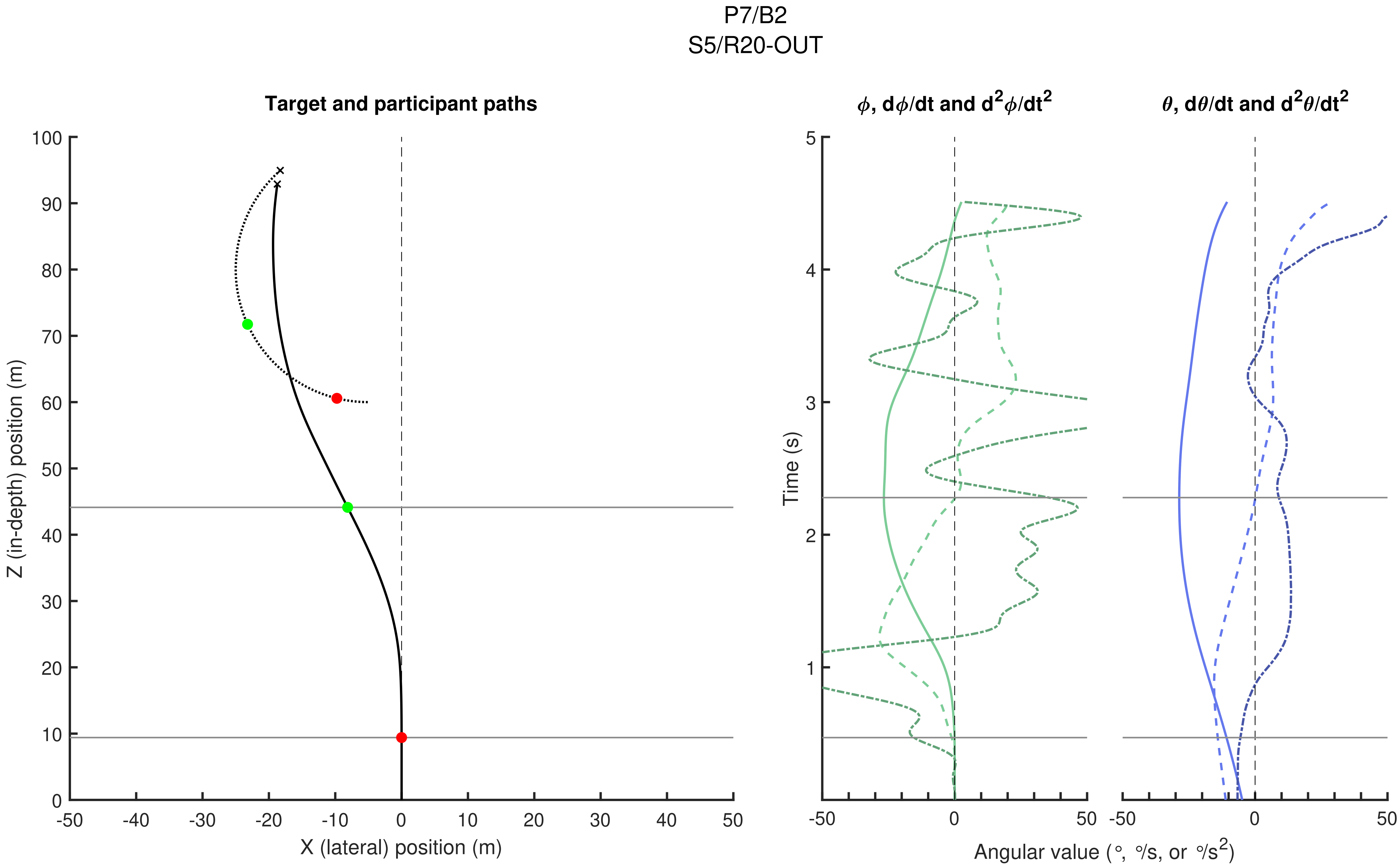

P7/B2
S5/R20-OUT
Target and participant paths
Z (in-depth) position (m)
X (lateral) position (m)
100
90
80
70
60
50
40
30
20
10
0
-50
-40
-30
-20
-10
0
10
20
30
40
50
ϕ, dϕ/dt and d$^2\phi$/dt^2
θ, dθ/dt and d$^2\theta$/dt^2
Time (s)
5
4
3
2
1
-50
0
50
-50
0
50
Angular value (°, °/s, or °/s^2)

P7/B2
S5/R30-IN

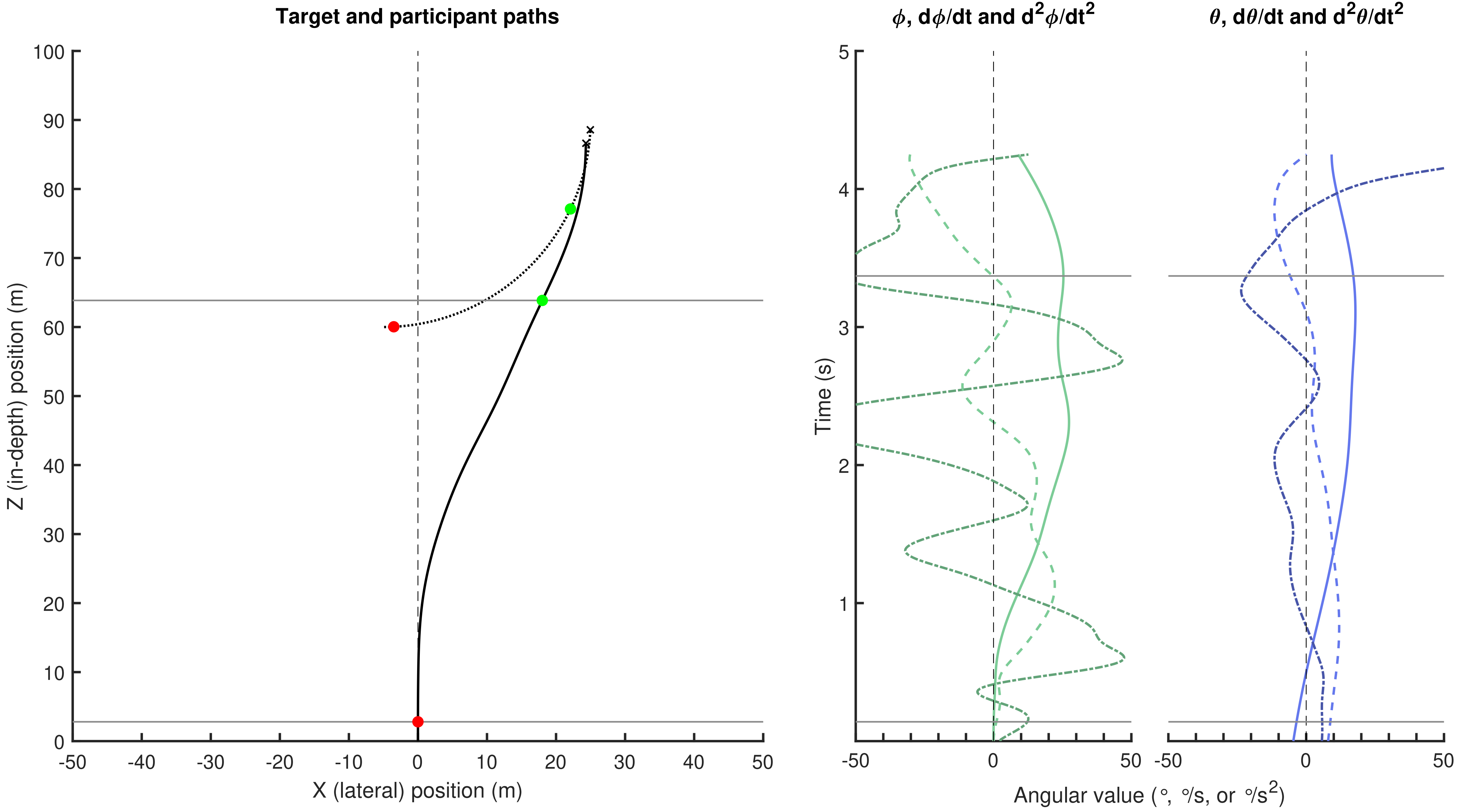

P7/B2
S5/R30-OUT

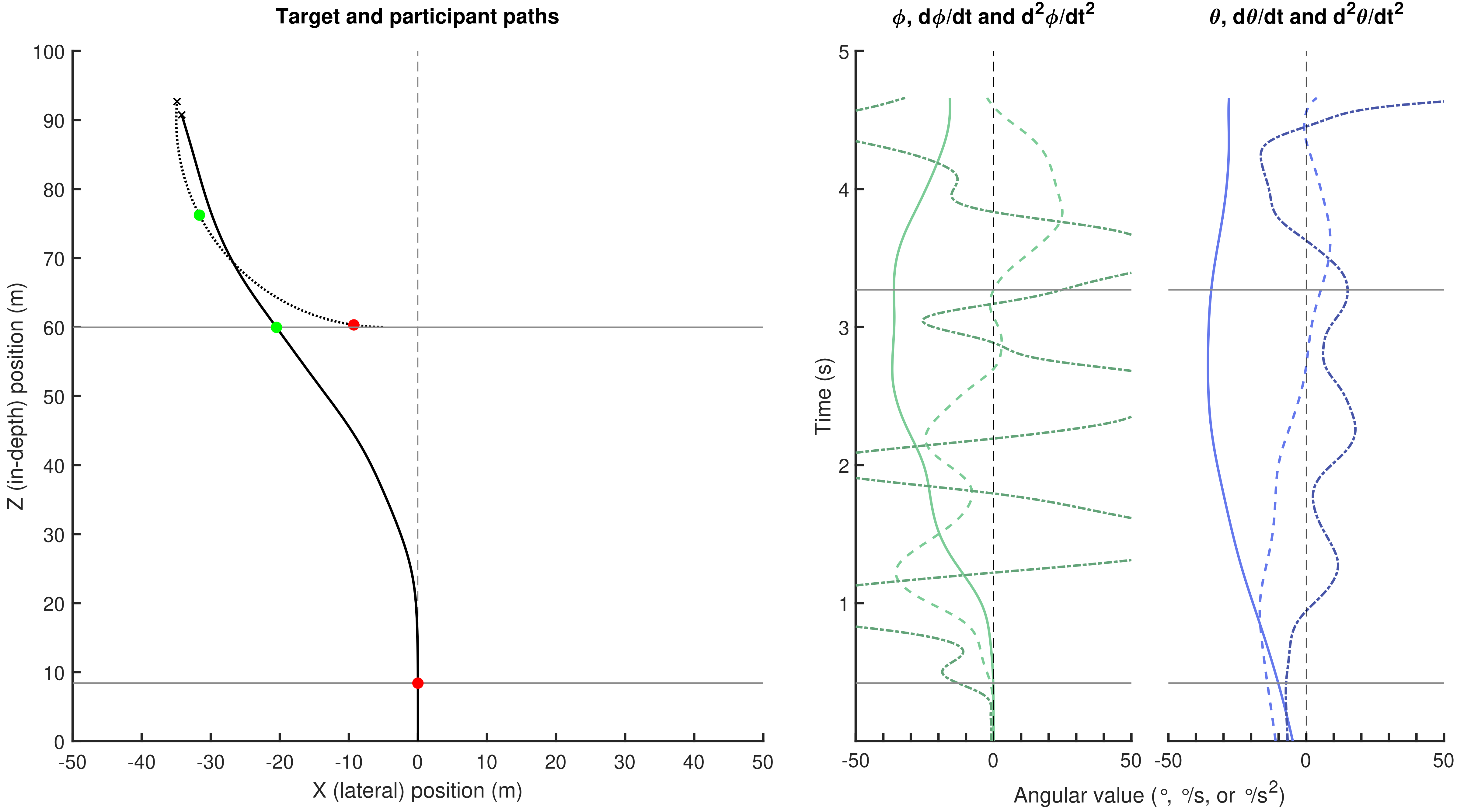

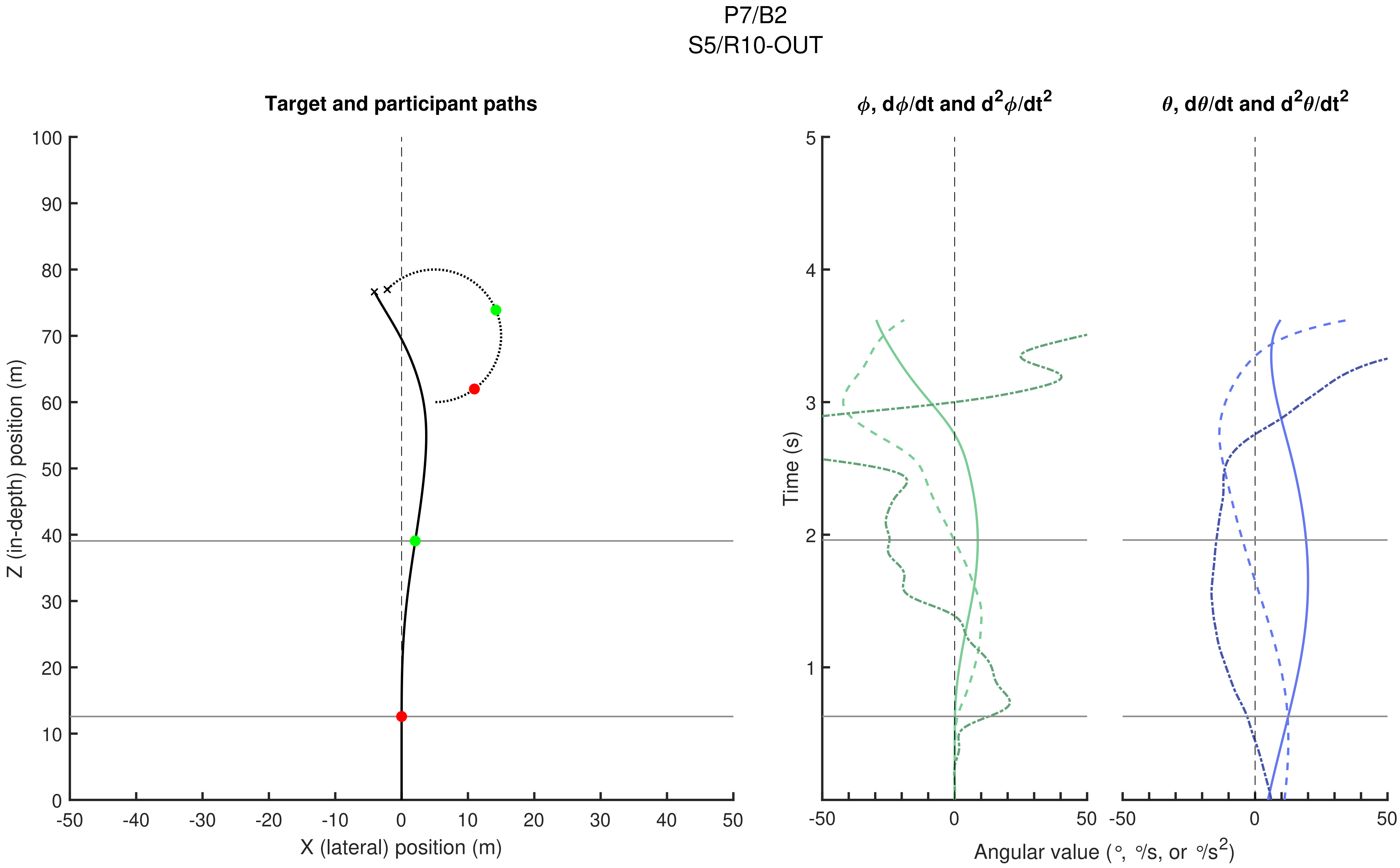
P7/B2
S5/R10-OUT
Target and participant paths
ϕ, dϕ/dt and d$^2\phi$/dt^2
θ, dθ/dt and d$^2\theta$/dt^2
Z (in-depth) position (m)
X (lateral) position (m)
Time (s)
Angular value (°, °/s, or °/s^2)
100
90
80
70
60
50
40
30
20
10
0
-50
-40
-30
-20
-10
0
10
20
30
40
50
5
4
3
2
1
-50
0
50
-50
0
50

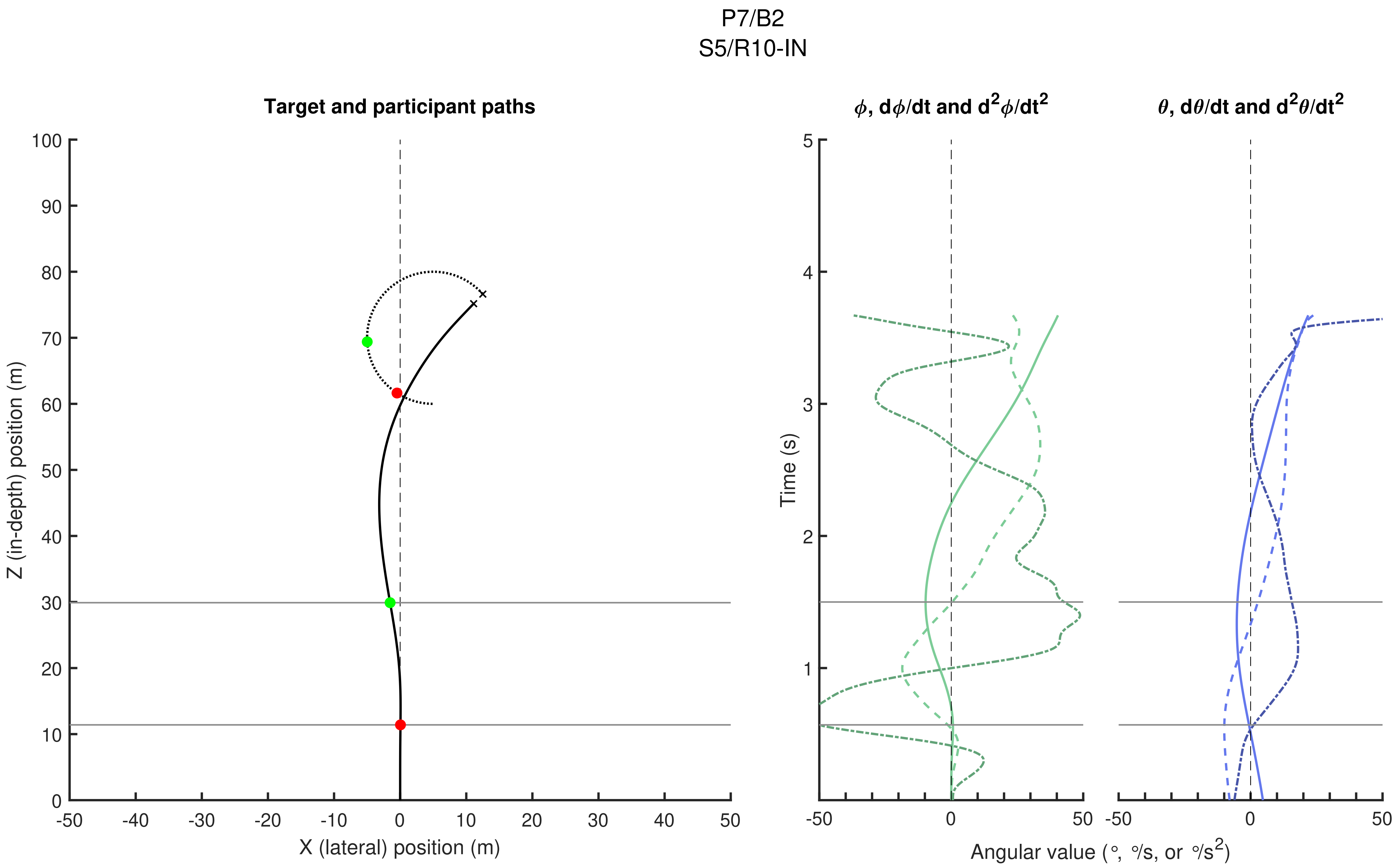

P7/B2
S5/R10-IN
Target and participant paths
Z (in-depth) position (m)
X (lateral) position (m)
ϕ, dϕ/dt and d²ϕ/dt²
θ, dθ/dt and d²θ/dt²
Time (s)
Angular value (°, °/s, or °/s²)

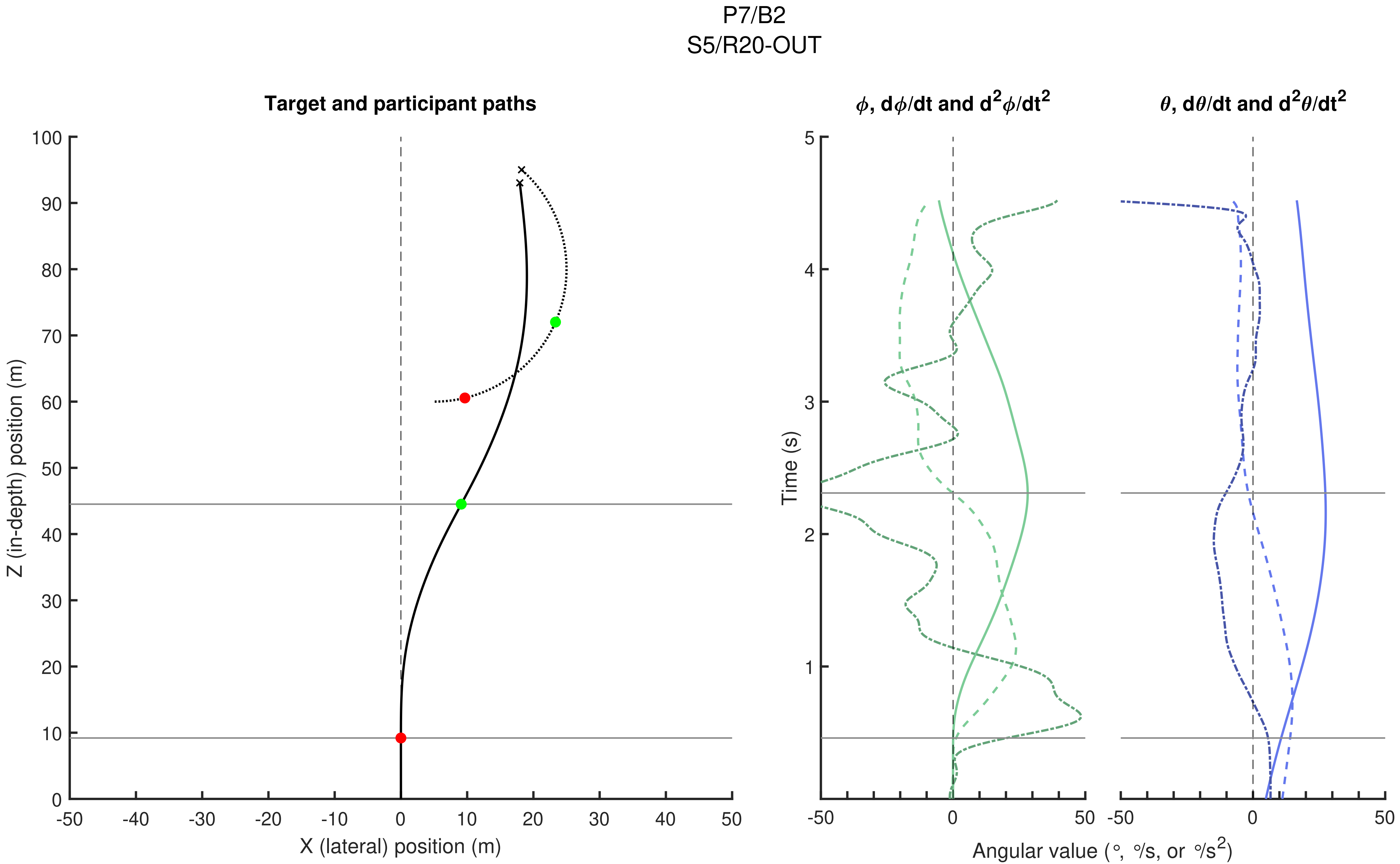

P7/B2
S5/R20-OUT
Target and participant paths
Z (in-depth) position (m)
X (lateral) position (m)
φ, dφ/dt and d²φ/dt²
θ, dθ/dt and d²θ/dt²
Time (s)
Angular value (°, °/s, or °/s²)

P7/B2
S5/R20-IN

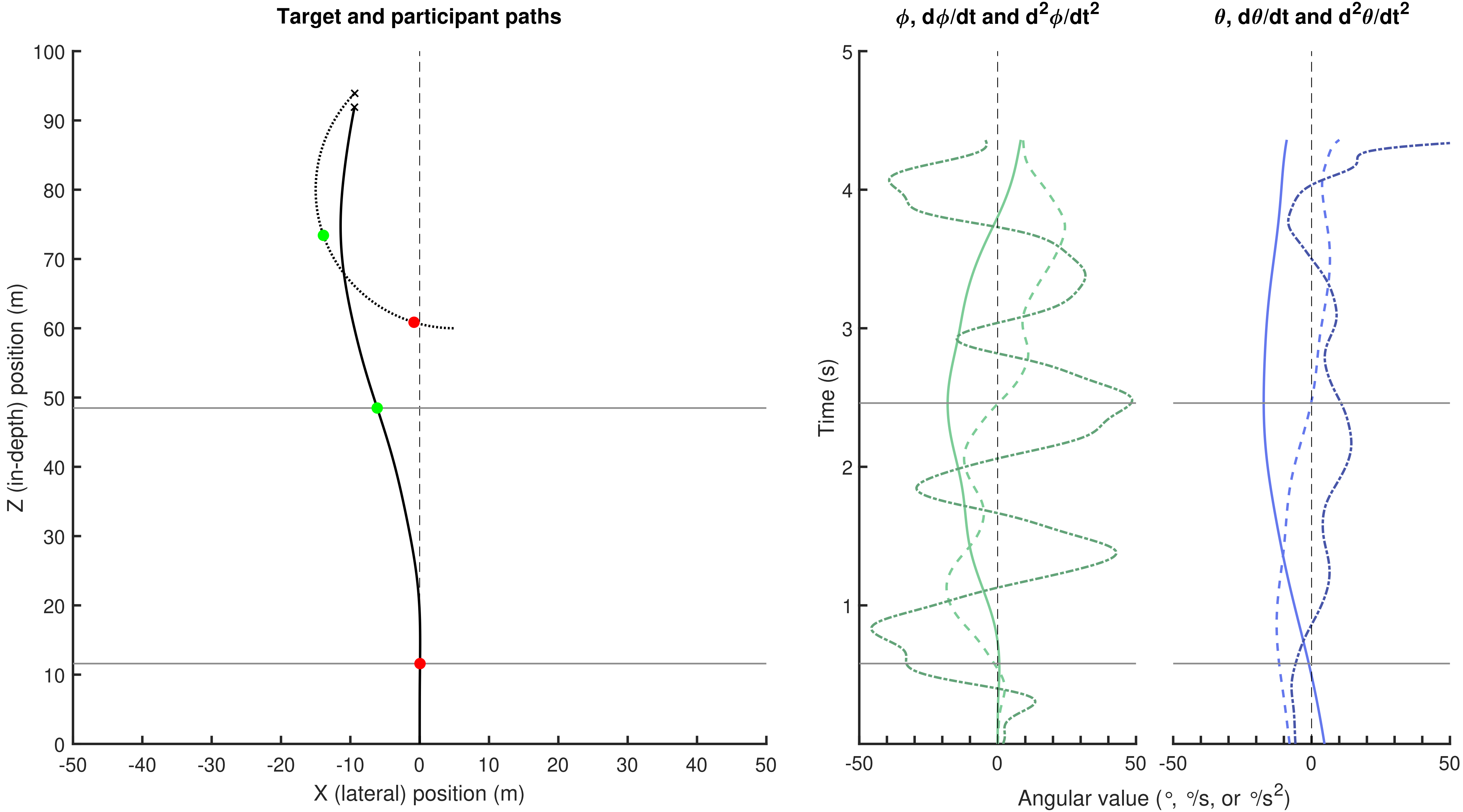

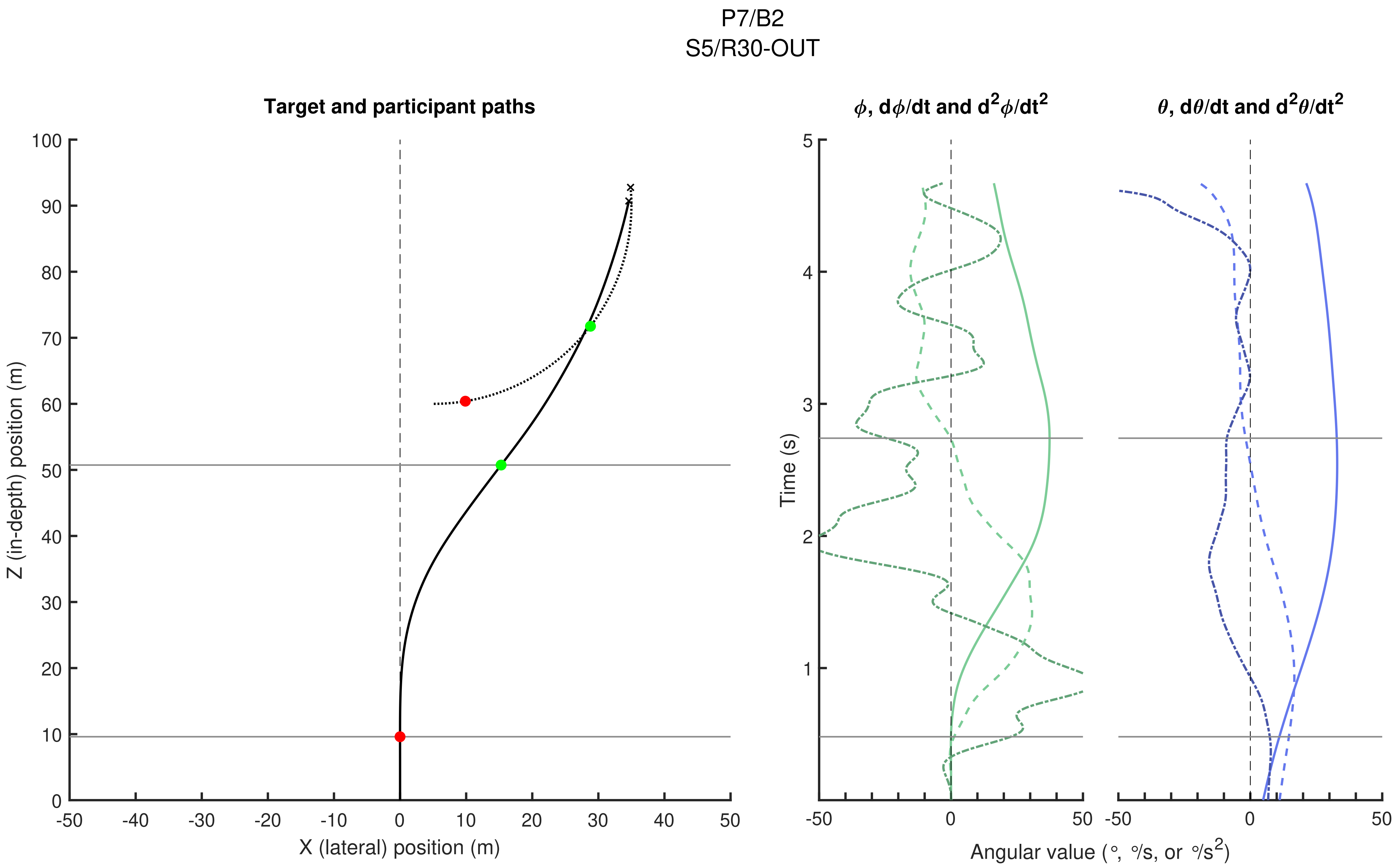
P7/B2
S5/R30-OUT
Target and participant paths
Z (in-depth) position (m)
X (lateral) position (m)
-50
-40
-30
-20
-10
0
10
20
30
40
50
60
70
80
90
100
φ, dφ/dt and d²φ/dt²
θ, dθ/dt and d²θ/dt²
Time (s)
1
2
3
4
5
Angular value (°, °/s, or °/s²)

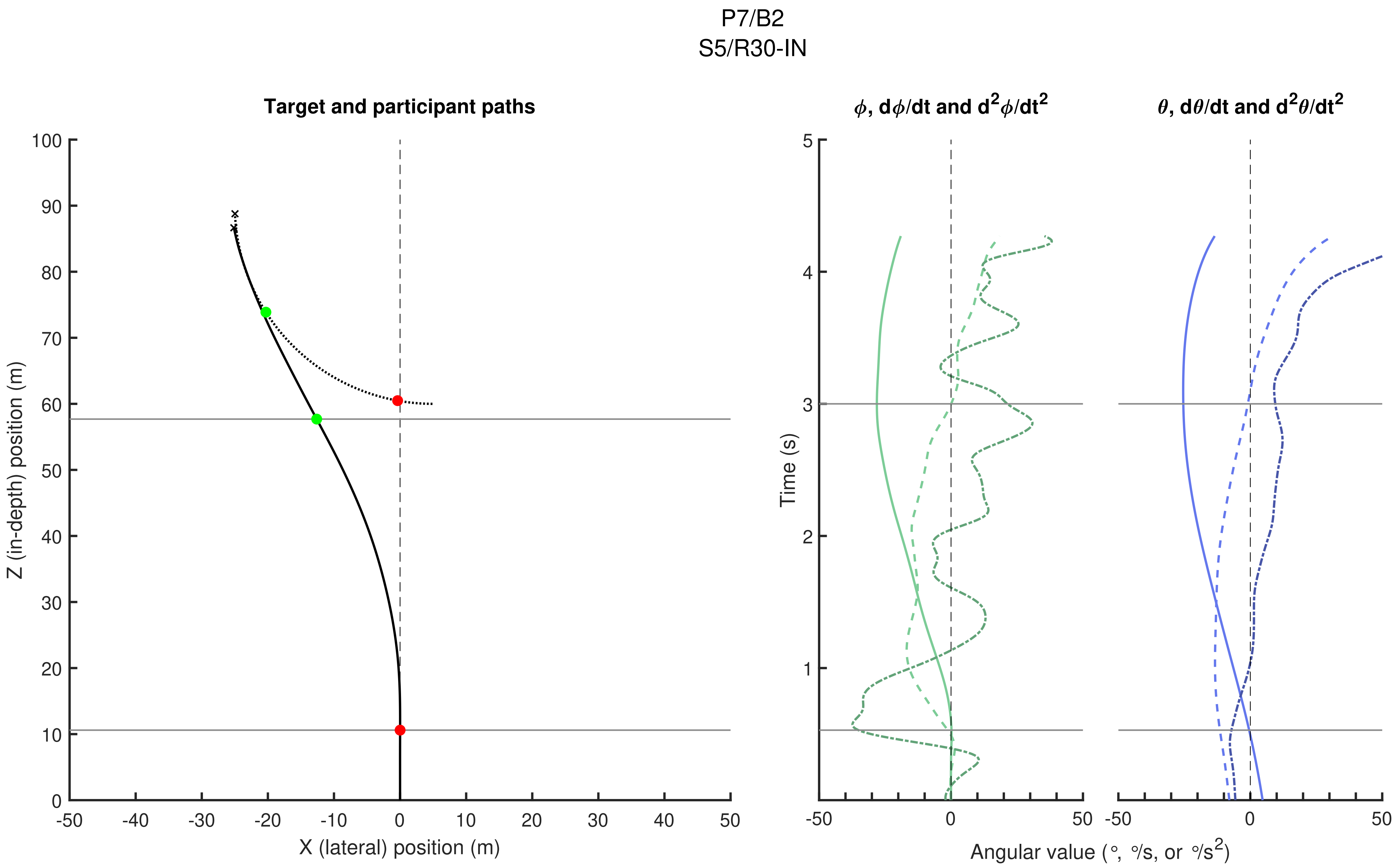
P7/B2
S5/R30-IN
Target and participant paths
Z (in-depth) position (m)
X (lateral) position (m)
φ, dφ/dt and d²φ/dt²
θ, dθ/dt and d²θ/dt²
Time (s)
Angular value (°, °/s, or °/s²)
100
90
80
70
60
50
40
30
20
10
0
-50
-40
-30
-20
-10
0
10
20
30
40
50
5
4
3
2
1
-50
0
50
-50
0
50

P7/B2
S15/R10-IN

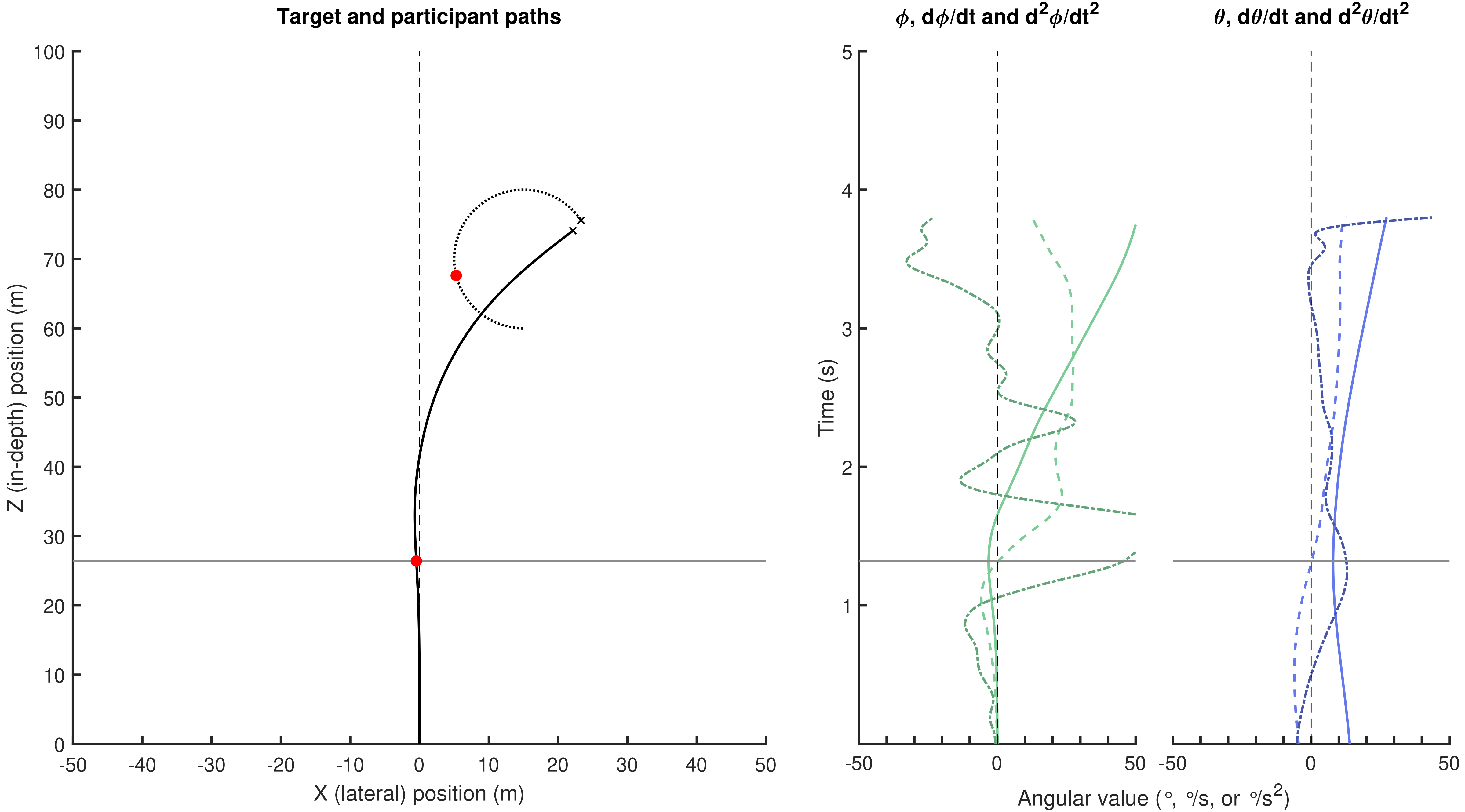

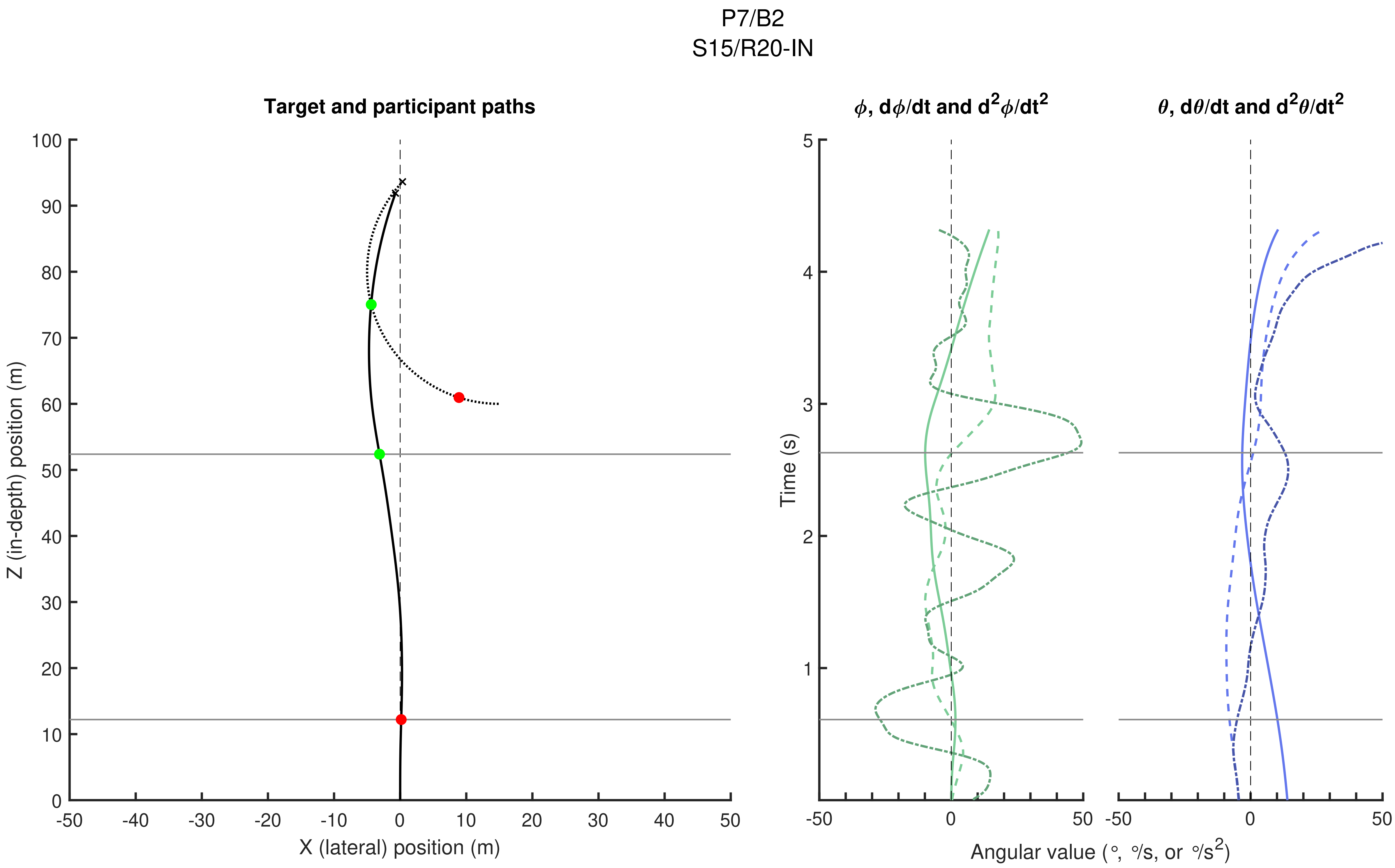

P7/B2
S15/R20-IN
Target and participant paths
ϕ, dϕ/dt and d$^2\phi$/dt^2
θ, dθ/dt and d$^2\theta$/dt^2
Z (in-depth) position (m)
X (lateral) position (m)
Time (s)
Angular value (°, °/s, or °/s^2)

P7/B2
S15/R30-IN

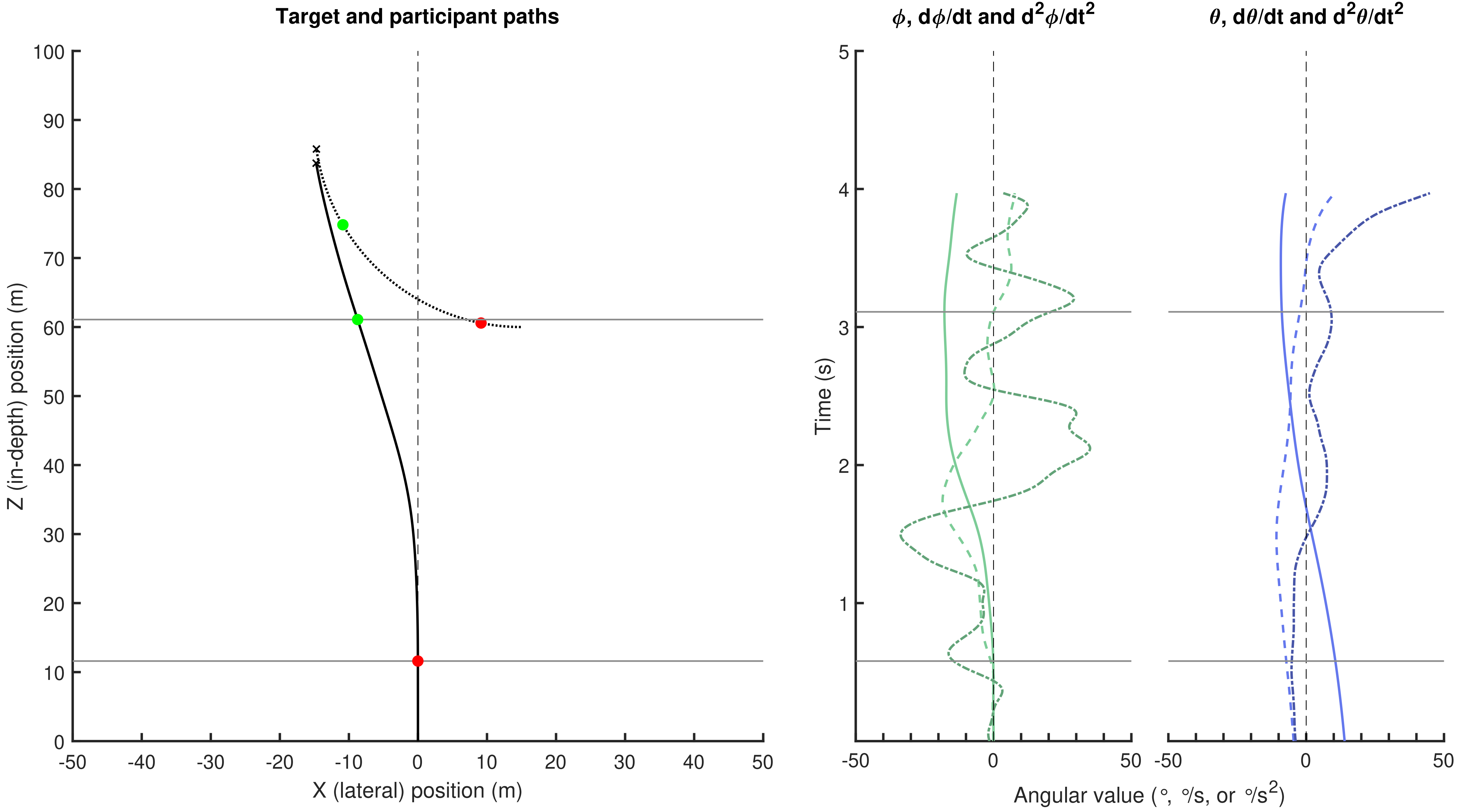

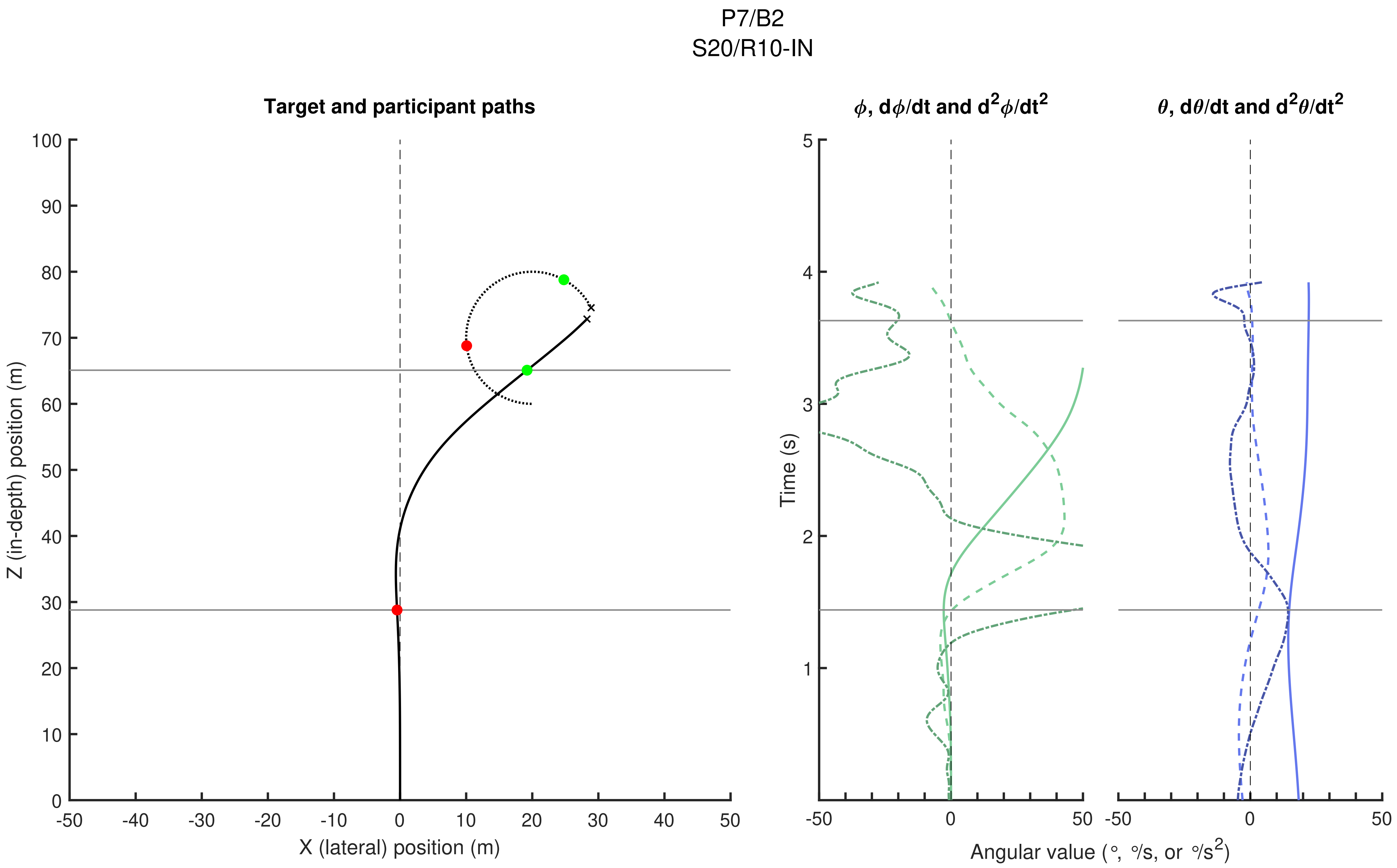
P7/B2
S20/R10-IN
Target and participant paths
Z (in-depth) position (m)
X (lateral) position (m)
ϕ, dϕ/dt and d²ϕ/dt²
θ, dθ/dt and d²θ/dt²
Time (s)
Angular value (°, °/s, or °/s²)

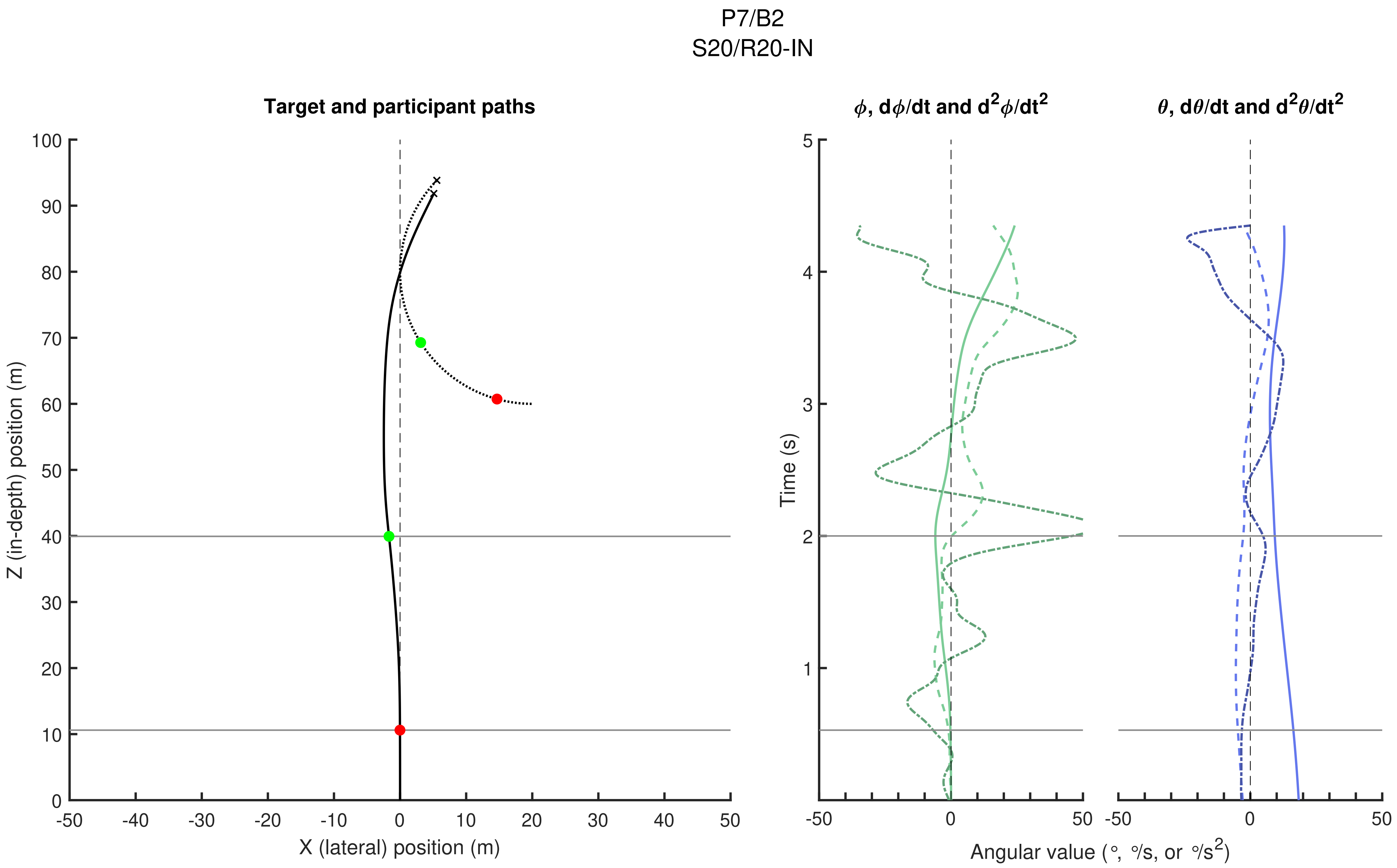

P7/B2
S20/R20-IN
Target and participant paths
ϕ, dϕ/dt and d$^2\phi$/dt^2
θ, dθ/dt and d$^2\theta$/dt^2
Z (in-depth) position (m)
X (lateral) position (m)
Time (s)
Angular value (°, °/s, or °/s^2)
100
90
80
70
60
50
40
30
20
10
0
-50
-40
-30
-20
-10
0
10
20
30
40
50
5
4
3
2
1
-50
0
50
-50
0
50

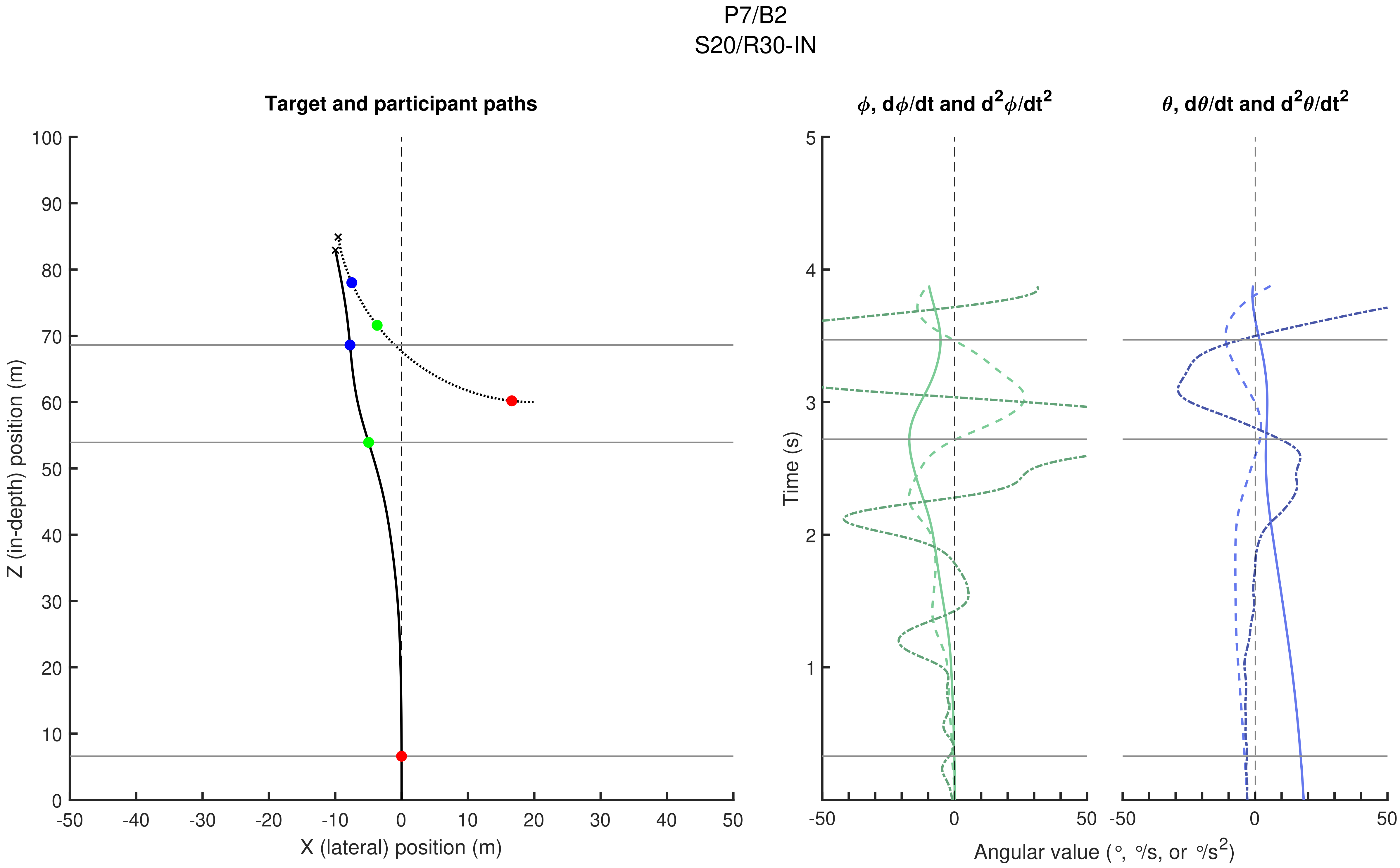

P7/B2
S20/R30-IN
Target and participant paths
Z (in-depth) position (m)
X (lateral) position (m)
ϕ, dϕ/dt and d$^2\phi$/dt^2
θ, dθ/dt and d$^2\theta$/dt^2
Time (s)
Angular value (°, °/s, or °/s^2)

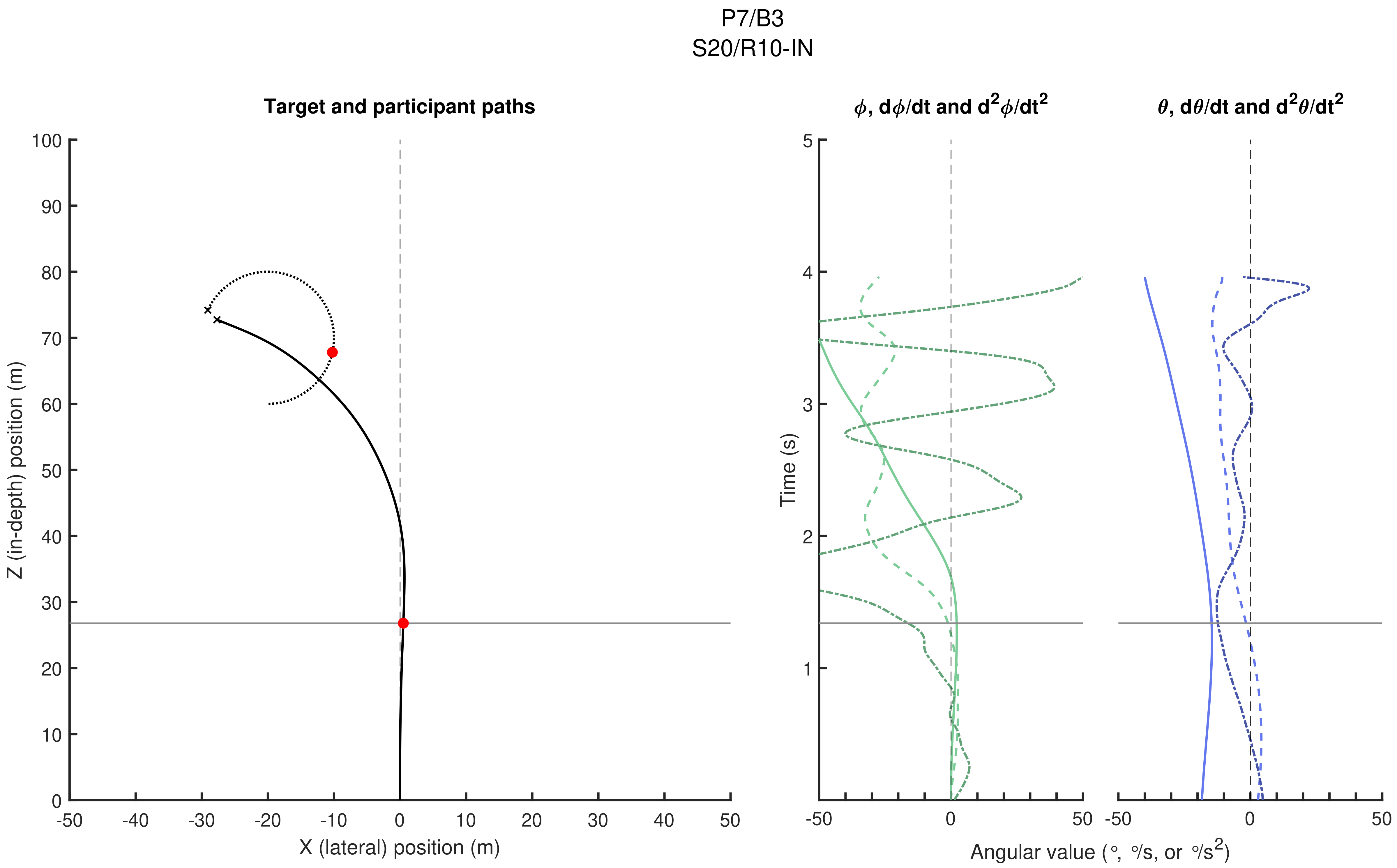
P7/B3
S20/R10-IN
Target and participant paths
Z (in-depth) position (m)
X (lateral) position (m)
φ, dφ/dt and d²φ/dt²
θ, dθ/dt and d²θ/dt²
Time (s)
Angular value (°, °/s, or °/s²)

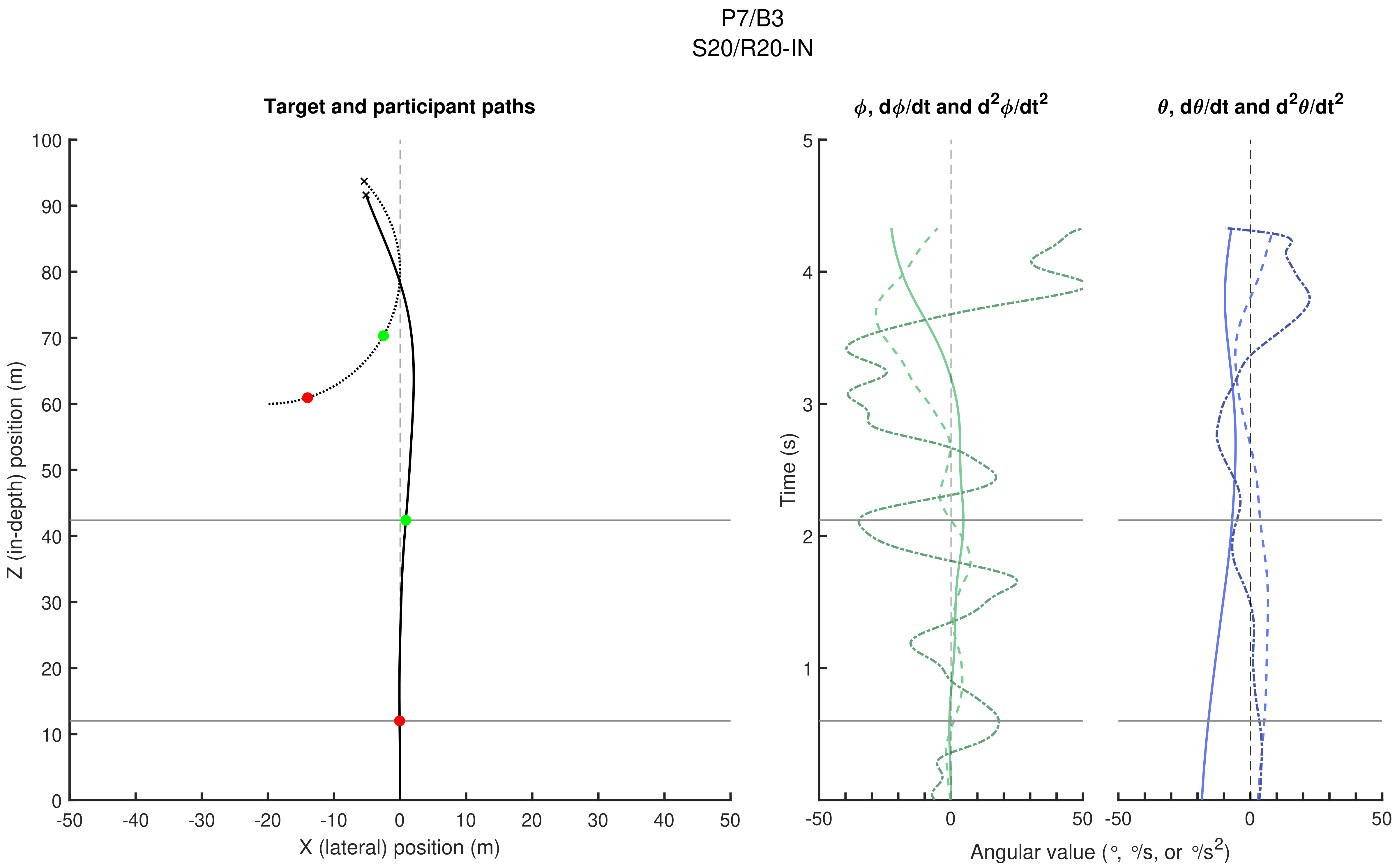

P7/B3
S20/R20-IN
Target and participant paths
Z (in-depth) position (m)
X (lateral) position (m)
ϕ, dϕ/dt and d$^2\phi$/dt^2
θ, dθ/dt and d$^2\theta$/dt^2
Time (s)
Angular value (°, °/s, or °/s^2)

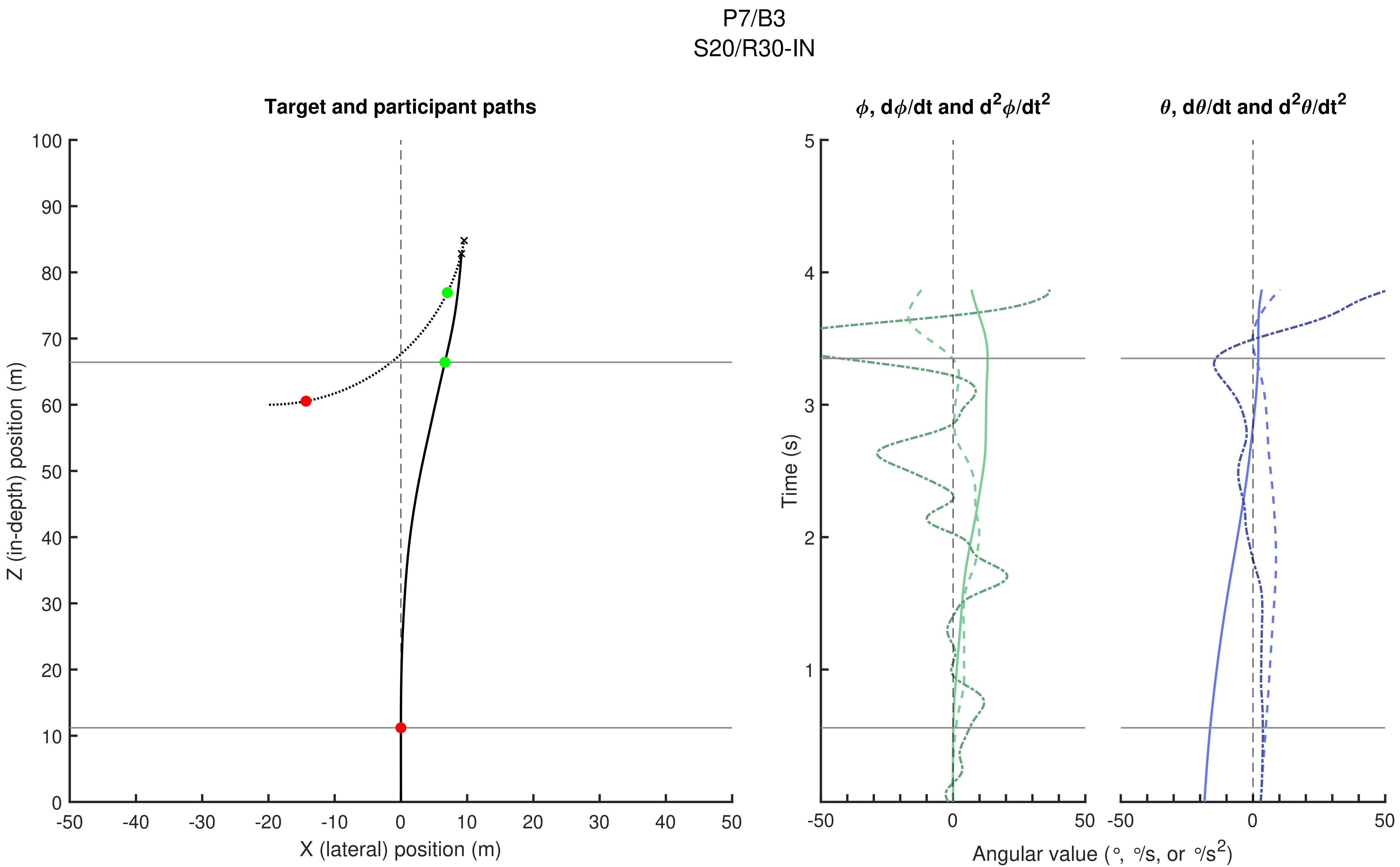
P7/B3
S20/R30-IN
Target and participant paths
ϕ, dϕ/dt and d²ϕ/dt²
θ, dθ/dt and d²θ/dt²
Z (in-depth) position (m)
X (lateral) position (m)
Time (s)
Angular value (°, °/s, or °/s²)
100
90
80
70
60
50
40
30
20
10
0
-50
-40
-30
-20
-10
0
10
20
30
40
50
5
4
3
2
1
-50
0
50
-50
0
50

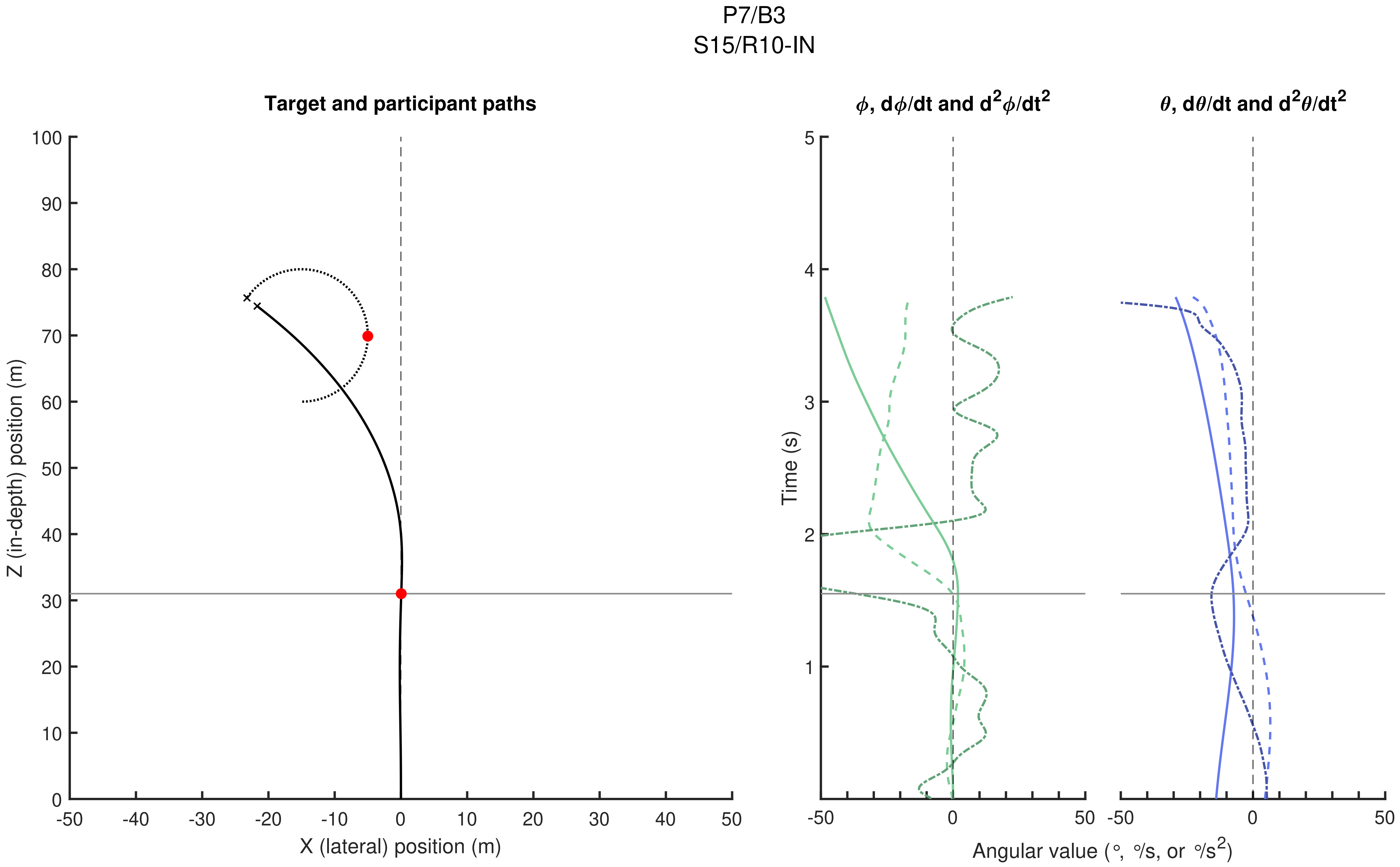
P7/B3
S15/R10-IN
Target and participant paths
Z (in-depth) position (m)
X (lateral) position (m)
φ, dφ/dt and d²φ/dt²
θ, dθ/dt and d²θ/dt²
Time (s)
Angular value (°, °/s, or °/s²)

P7/B3
S15/R20-IN

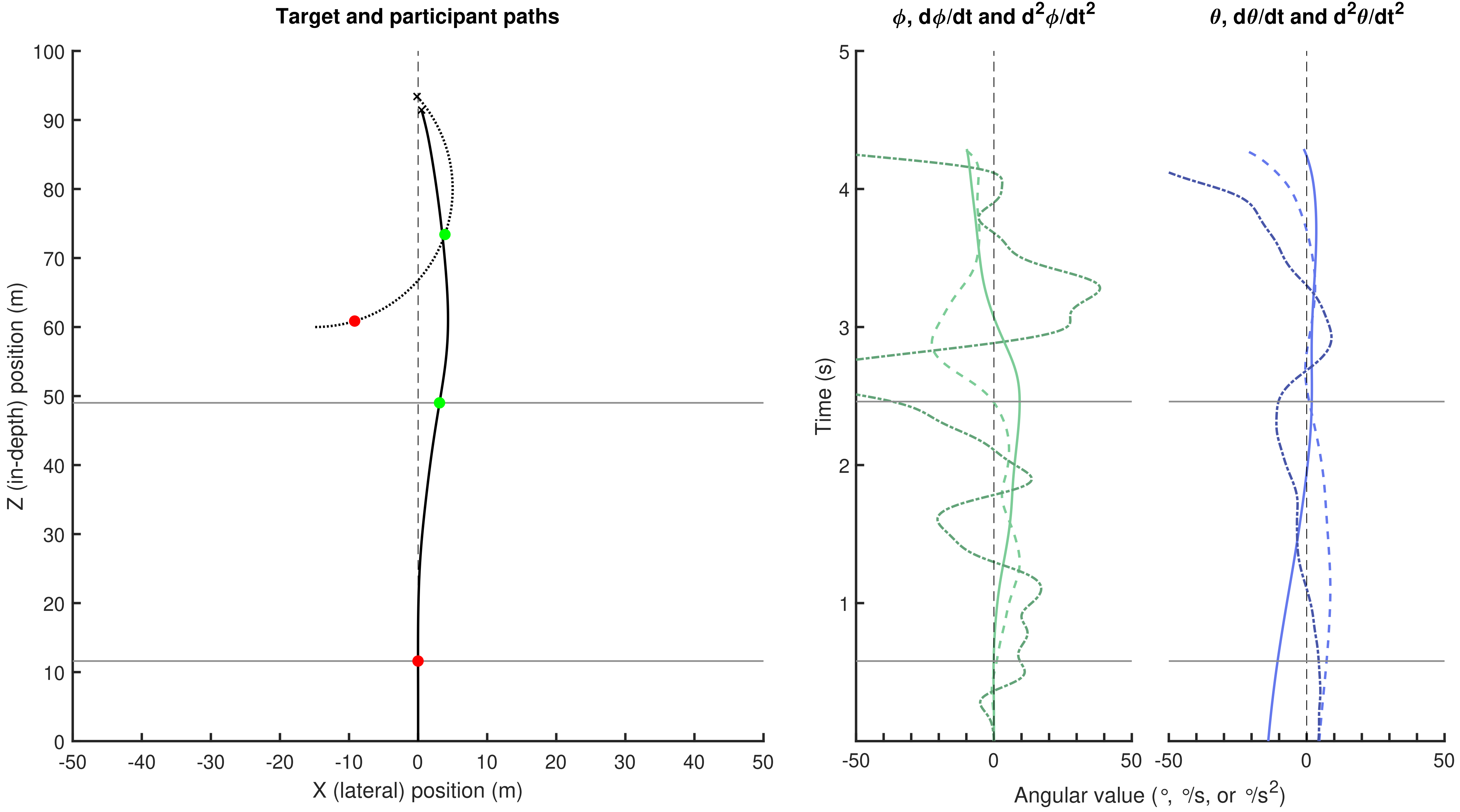

P7/B3
S15/R30-IN

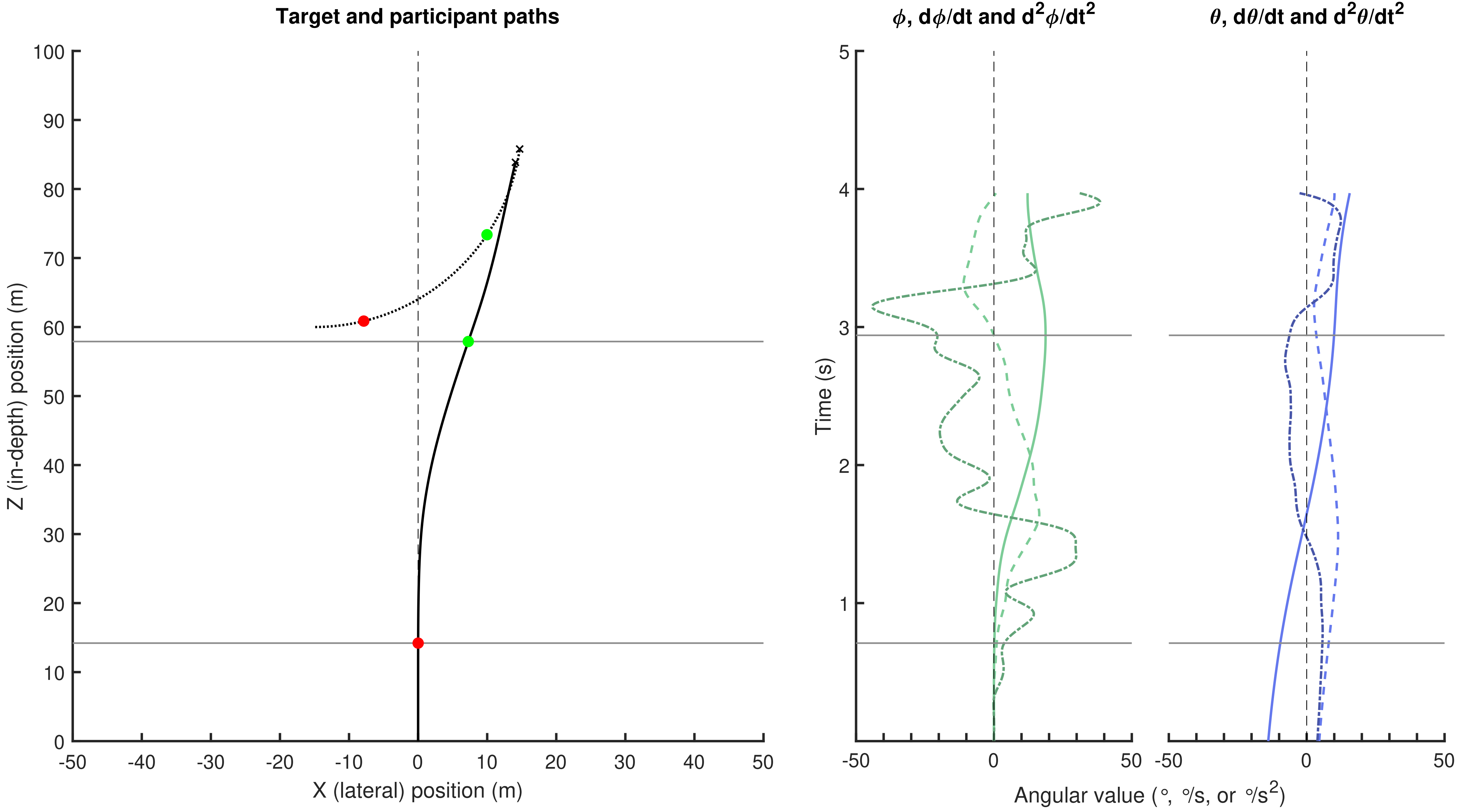

P7/B3
S5/R10-IN

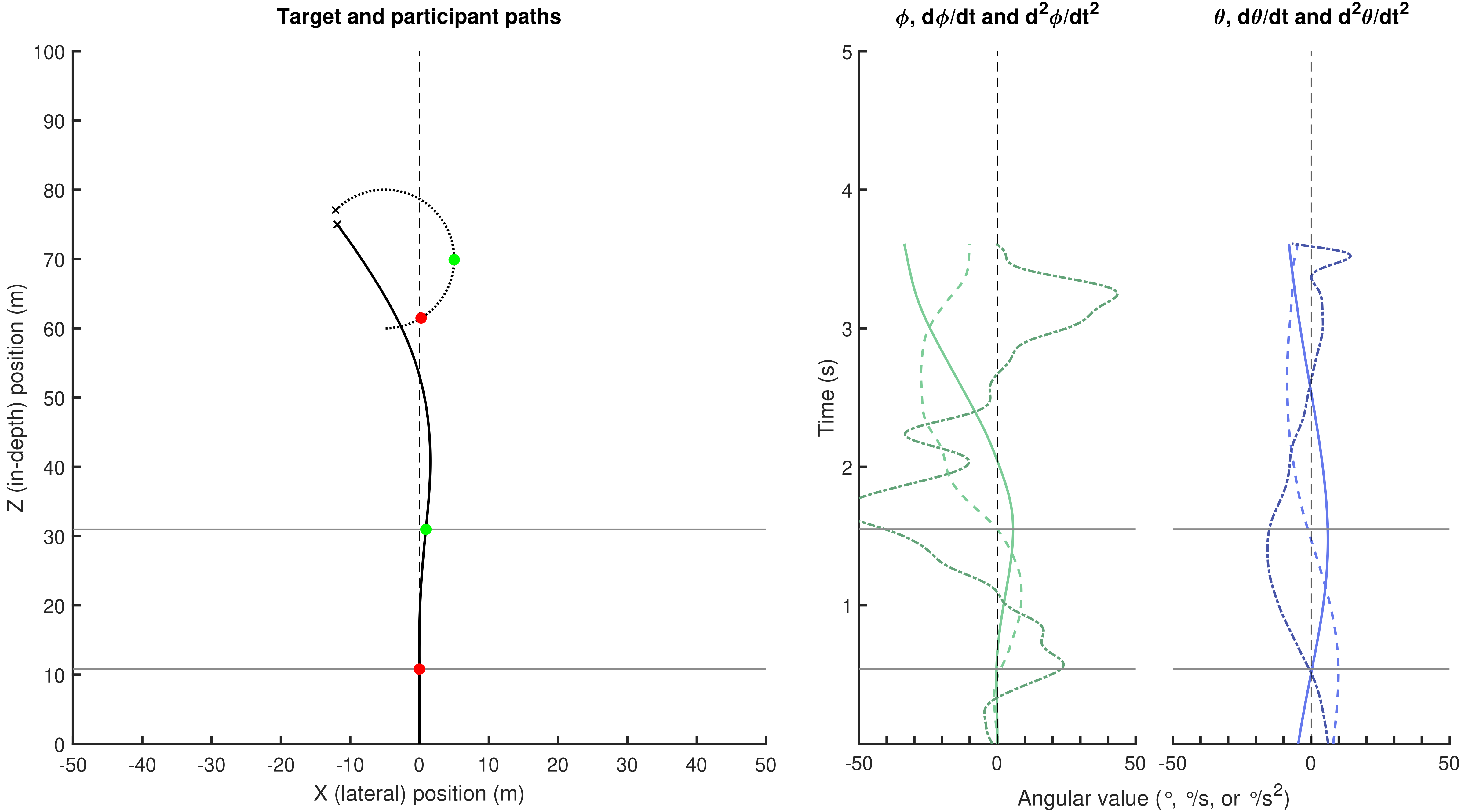

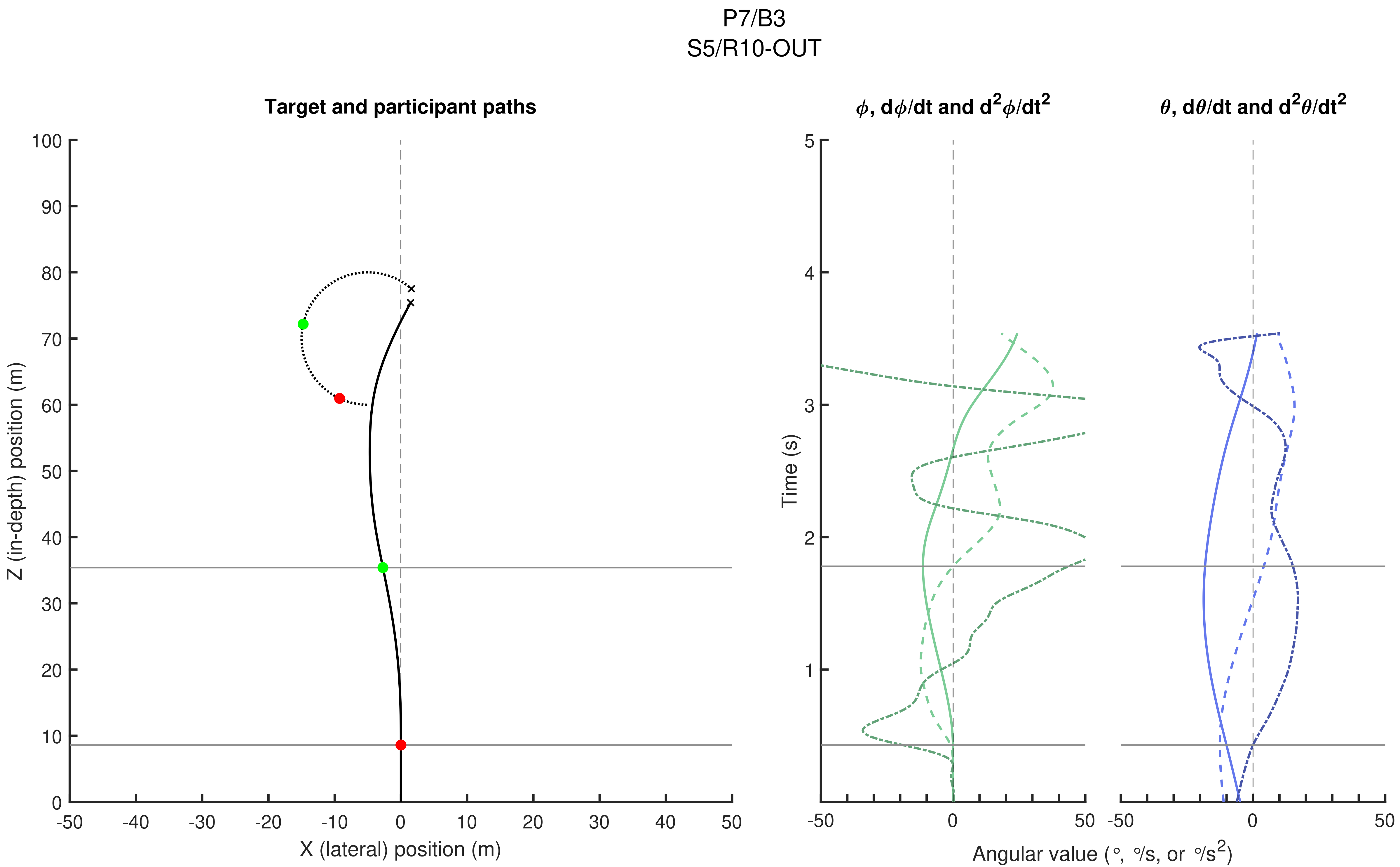

P7/B3
S5/R10-OUT
Target and participant paths
ϕ, dϕ/dt and d$^2\phi$/dt^2
θ, dθ/dt and d$^2\theta$/dt^2
Z (in-depth) position (m)
X (lateral) position (m)
Time (s)
Angular value (°, °/s, or °/s^2)
100
90
80
70
60
50
40
30
20
10
0
-50
-40
-30
-20
-10
0
10
20
30
40
50
5
4
3
2
1
-50
0
50
-50
0
50

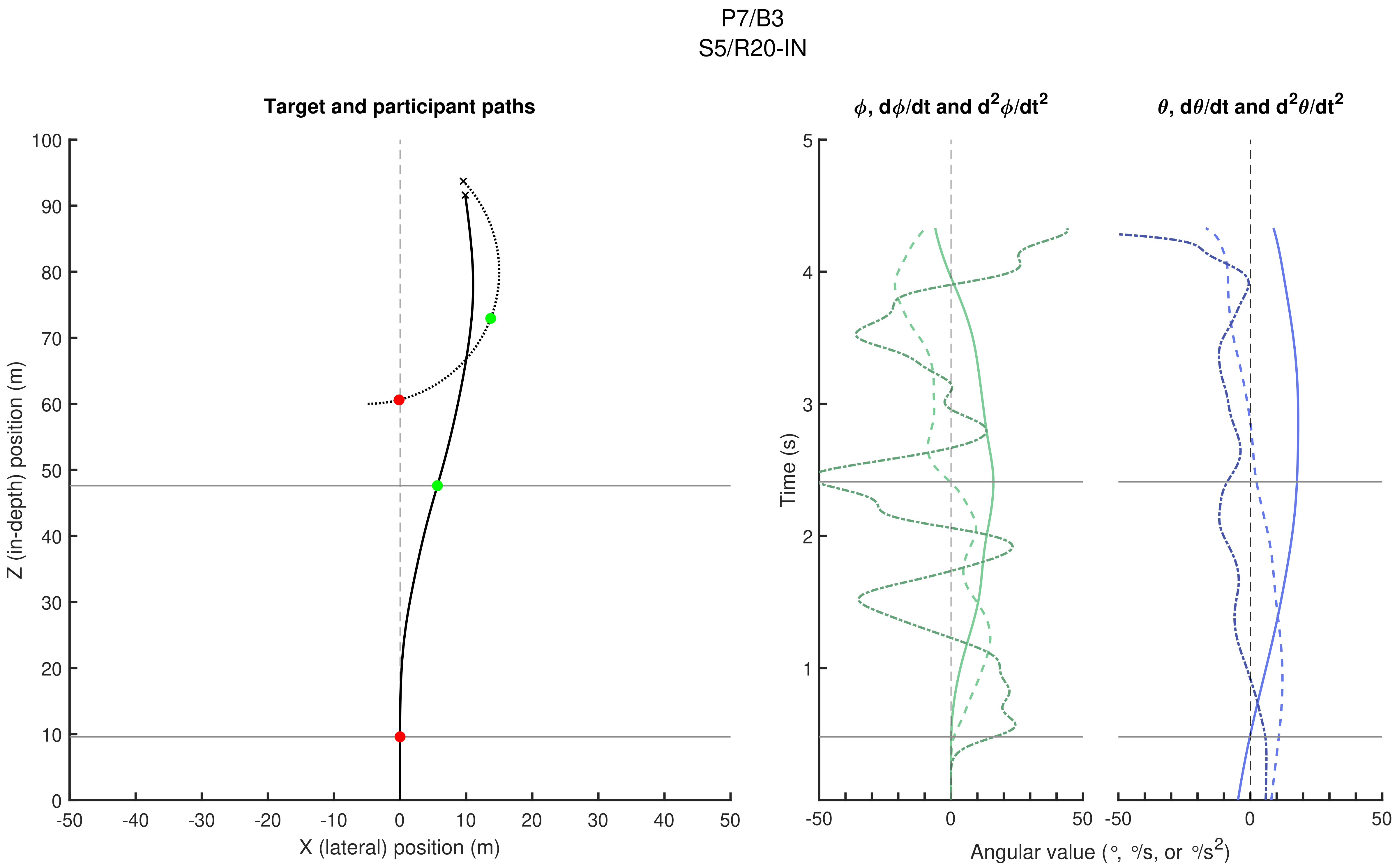
P7/B3
S5/R20-IN
Target and participant paths
Z (in-depth) position (m)
X (lateral) position (m)
ϕ, dϕ/dt and d$^2\phi$/dt^2
θ, dθ/dt and d$^2\theta$/dt^2
Time (s)
Angular value (°, °/s, or °/s^2)

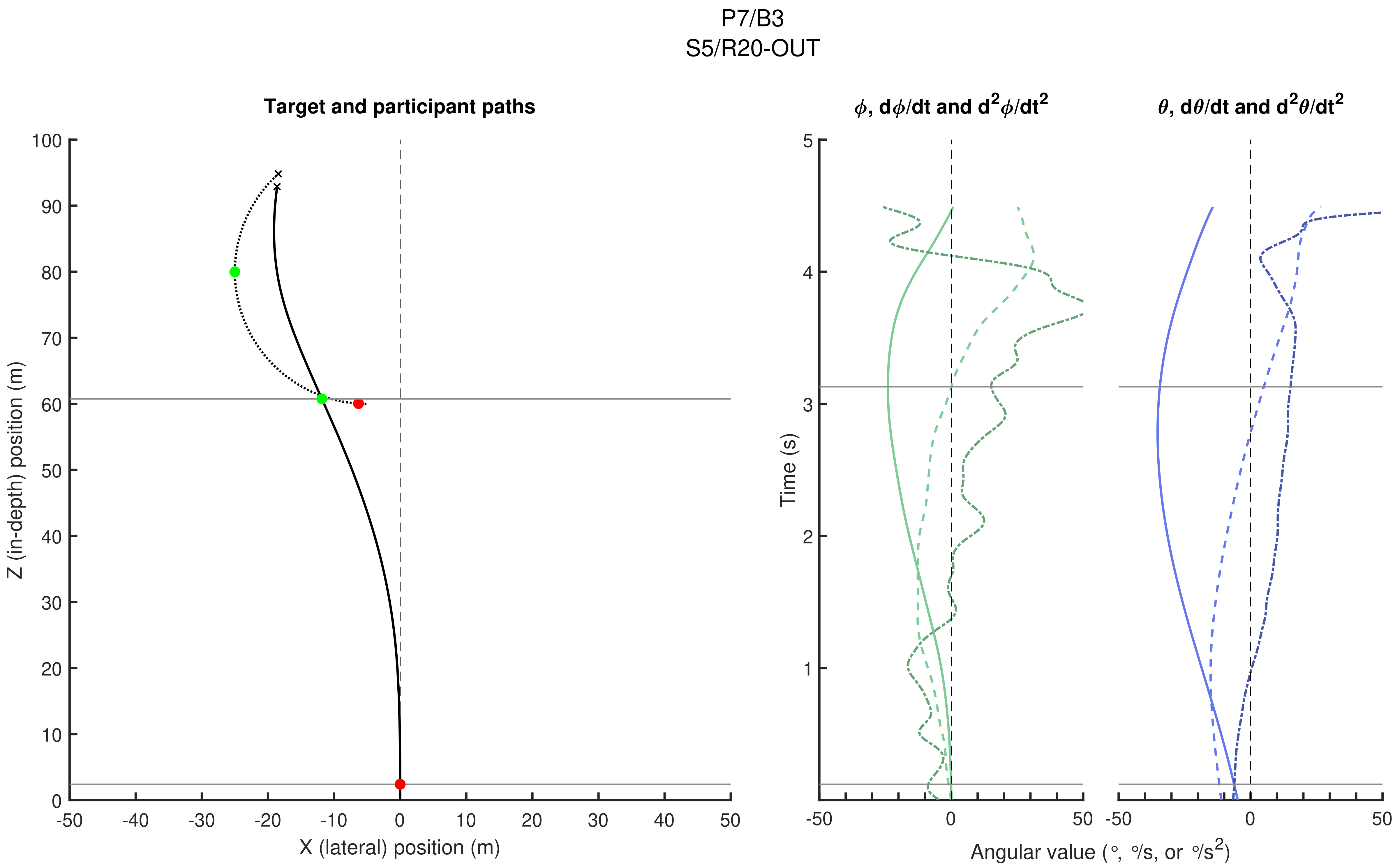
P7/B3
S5/R20-OUT
Target and participant paths
Z (in-depth) position (m)
X (lateral) position (m)
ϕ, dϕ/dt and d²ϕ/dt²
θ, dθ/dt and d²θ/dt²
Time (s)
Angular value (°, °/s, or °/s²)

P7/B3
S5/R30-IN

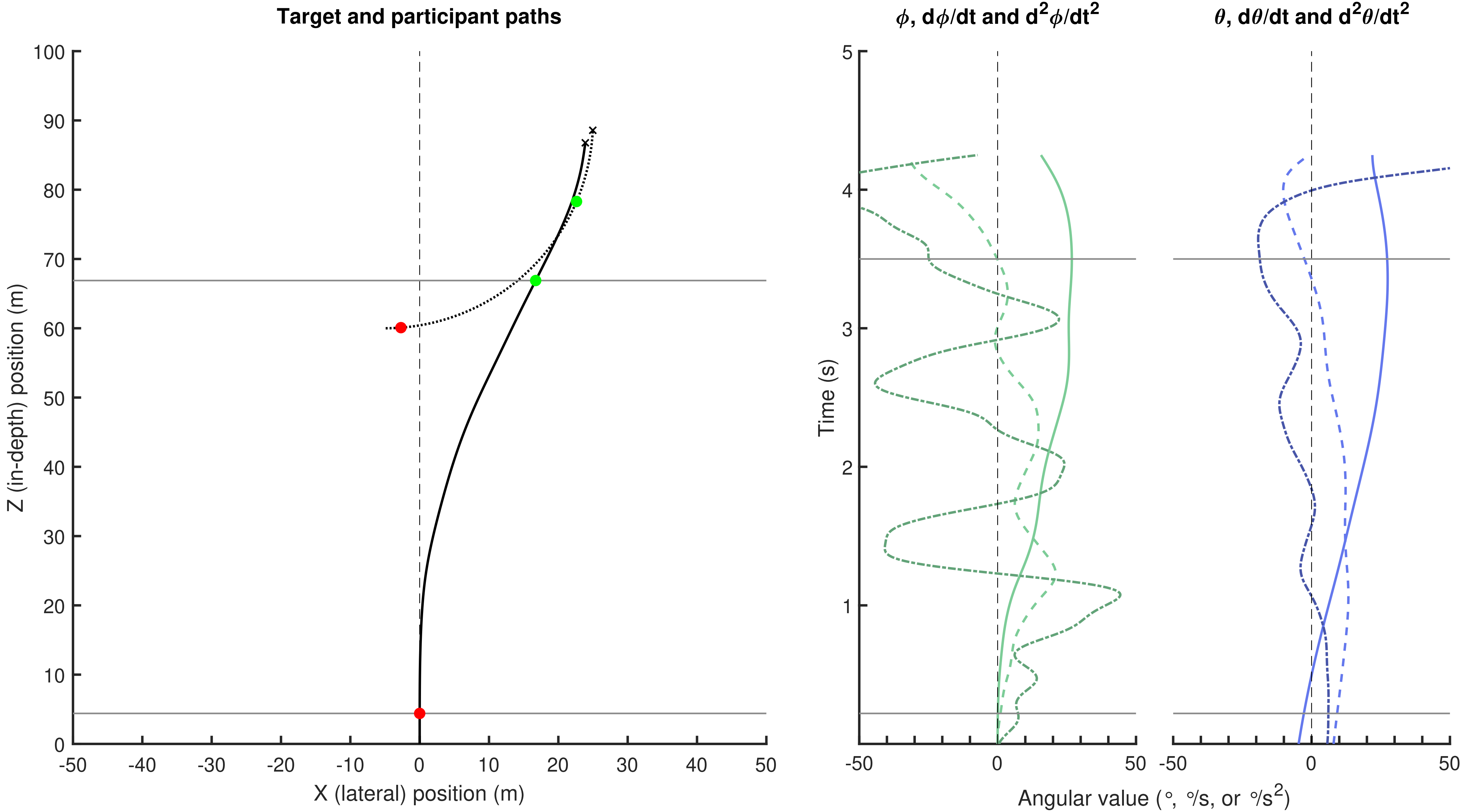

P7/B3
S5/R30-OUT

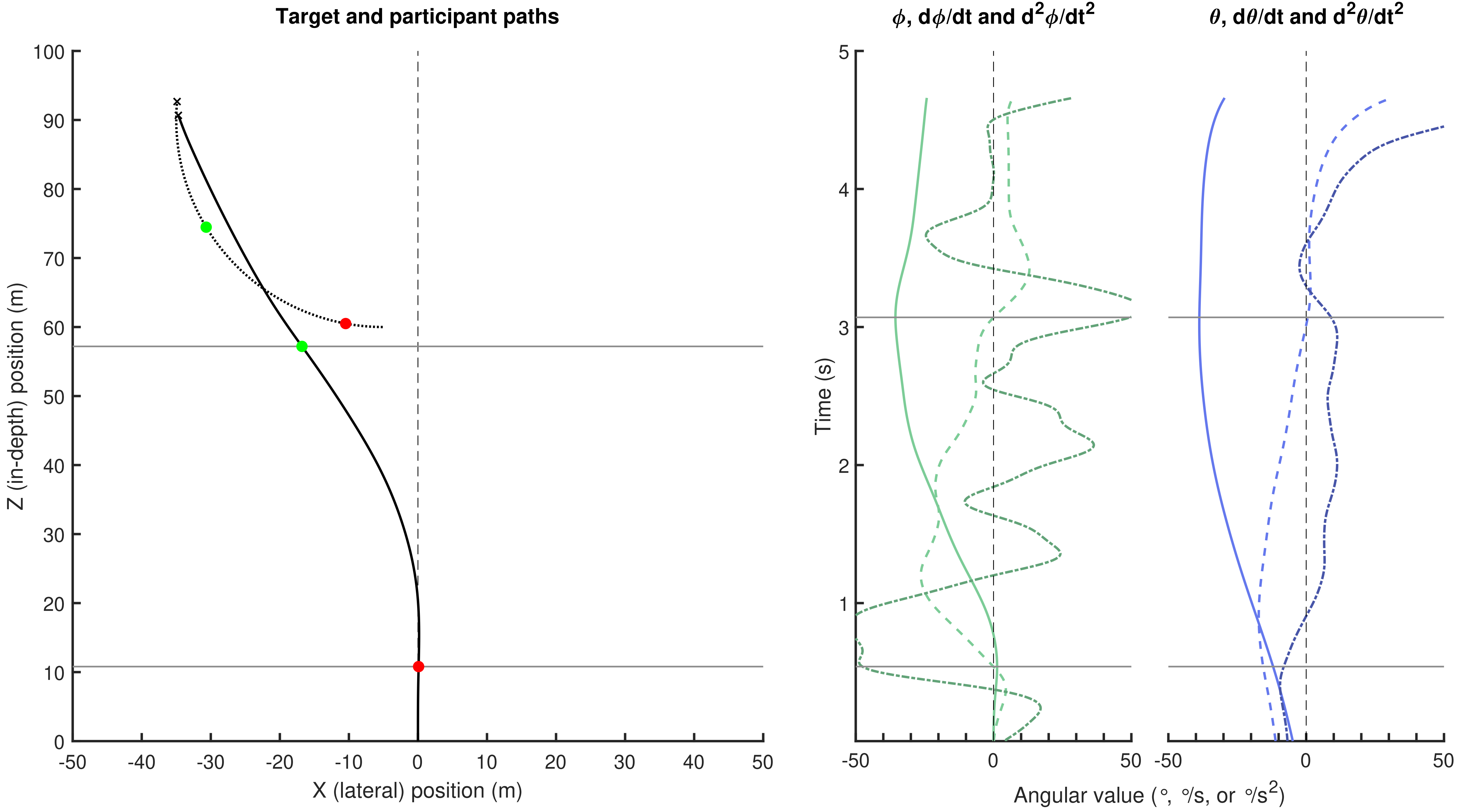

P7/B3
S5/R10-OUT

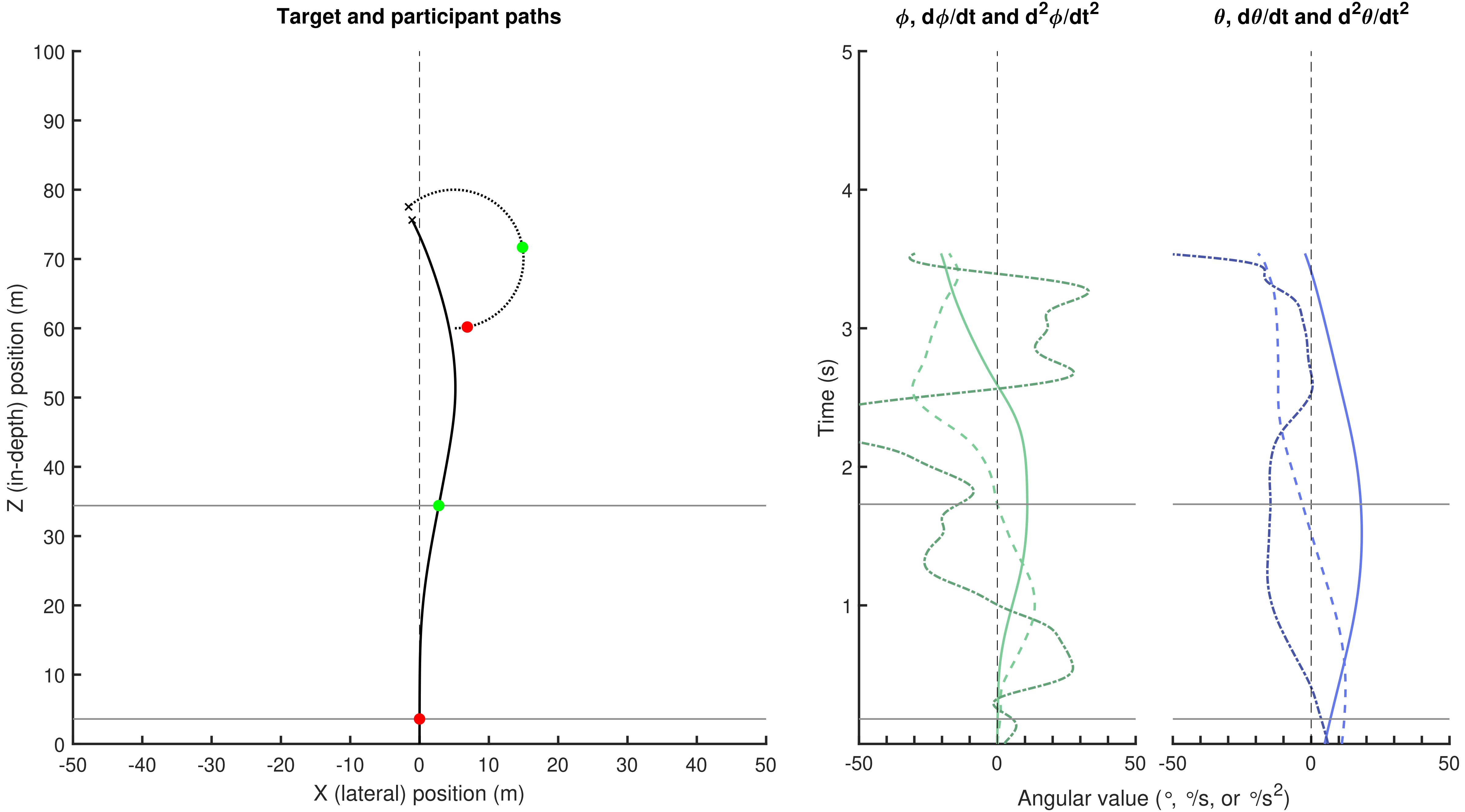

P7/B3
S5/R10-IN

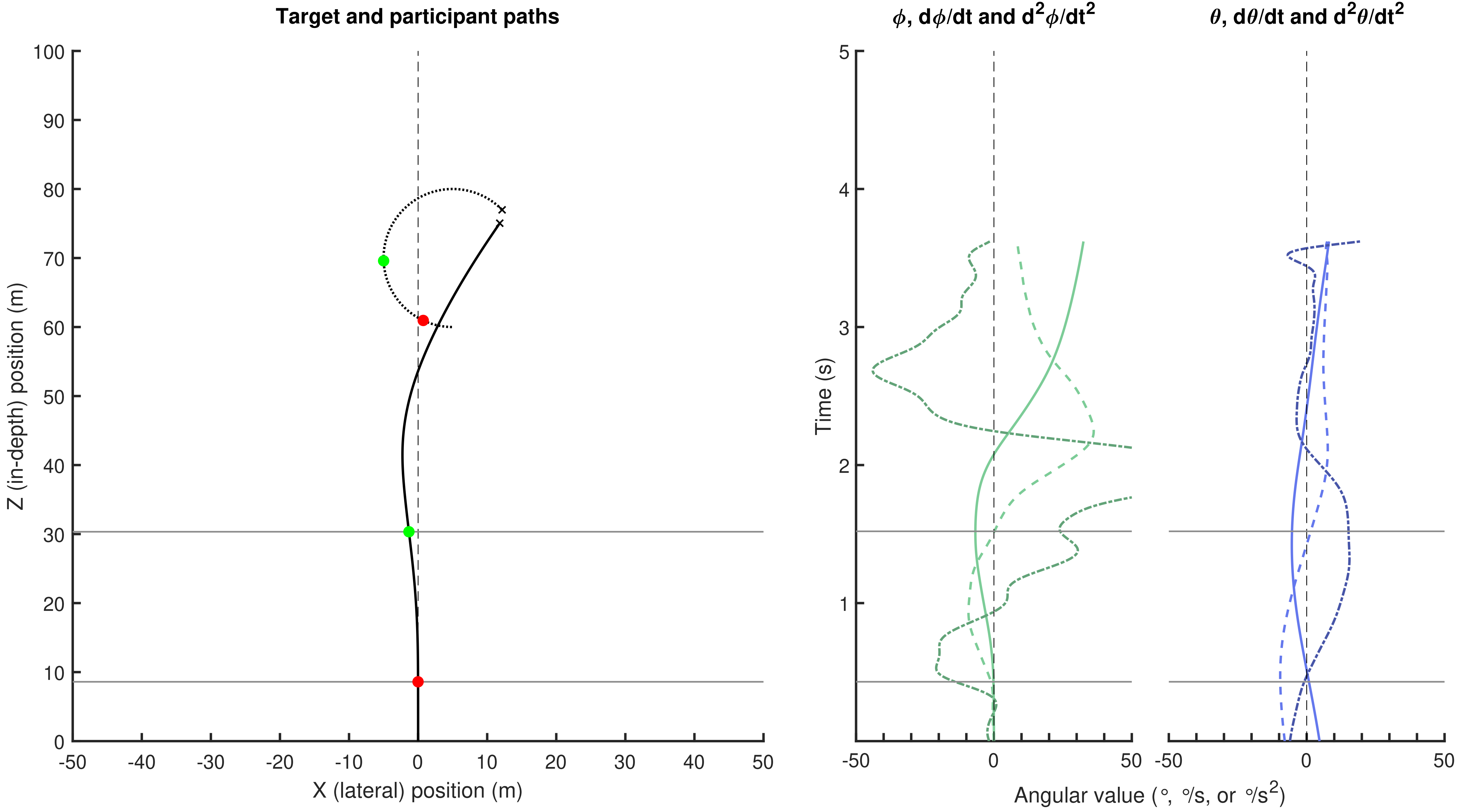

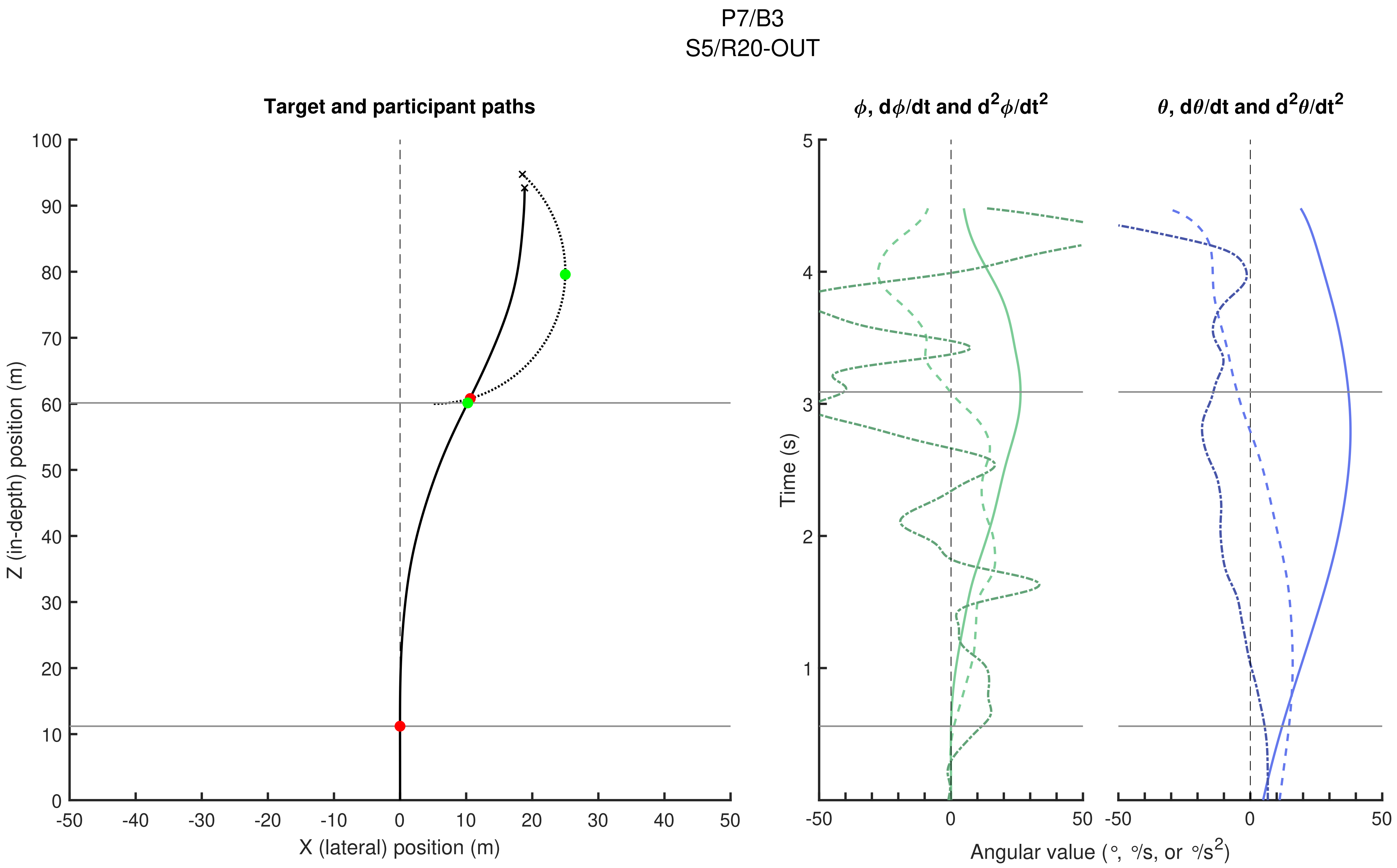
P7/B3
S5/R20-OUT
Target and participant paths
Z (in-depth) position (m)
X (lateral) position (m)
100
90
80
70
60
50
40
30
20
10
0
-50
-40
-30
-20
-10
0
10
20
30
40
50
ϕ, dϕ/dt and d$^2\phi$/dt^2
θ, dθ/dt and d$^2\theta$/dt^2
Time (s)
5
4
3
2
1
-50
0
50
-50
0
50
Angular value (°, °/s, or °/s^2)

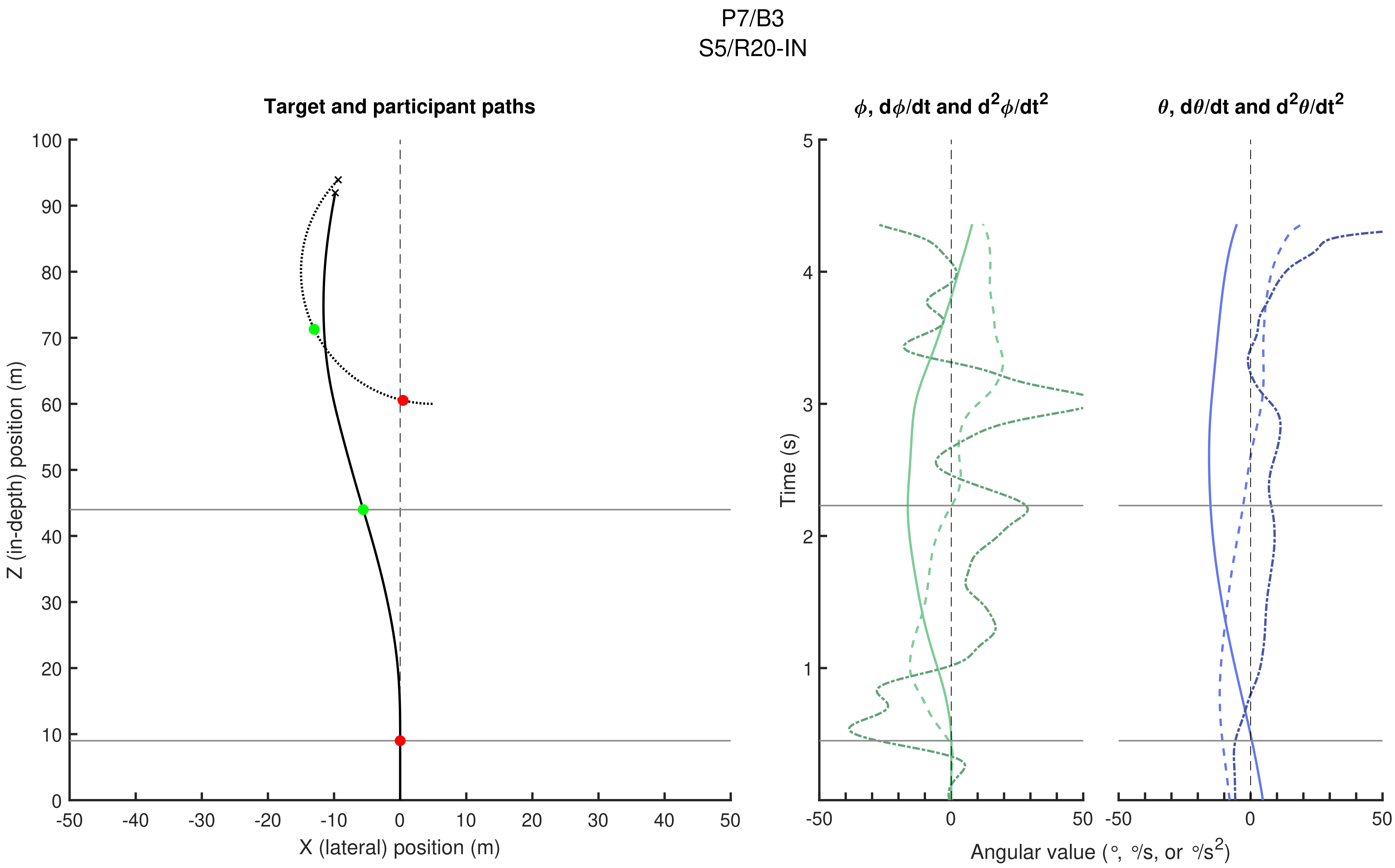
P7/B3
S5/R20-IN
Target and participant paths
Z (in-depth) position (m)
X (lateral) position (m)
ϕ, dϕ/dt and d²ϕ/dt²
θ, dθ/dt and d²θ/dt²
Time (s)
Angular value (°, °/s, or °/s²)

P7/B3
S5/R30-OUT

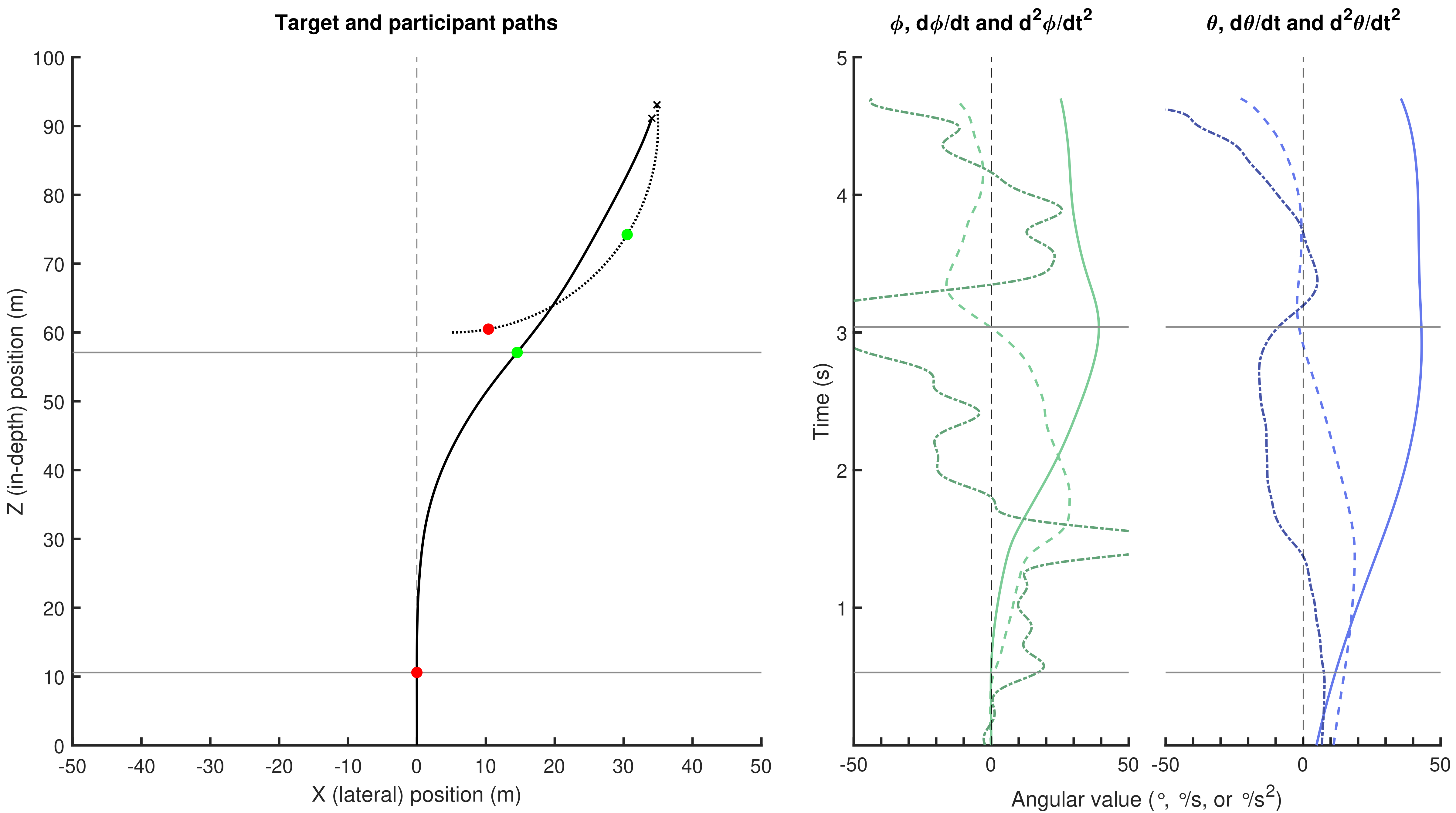

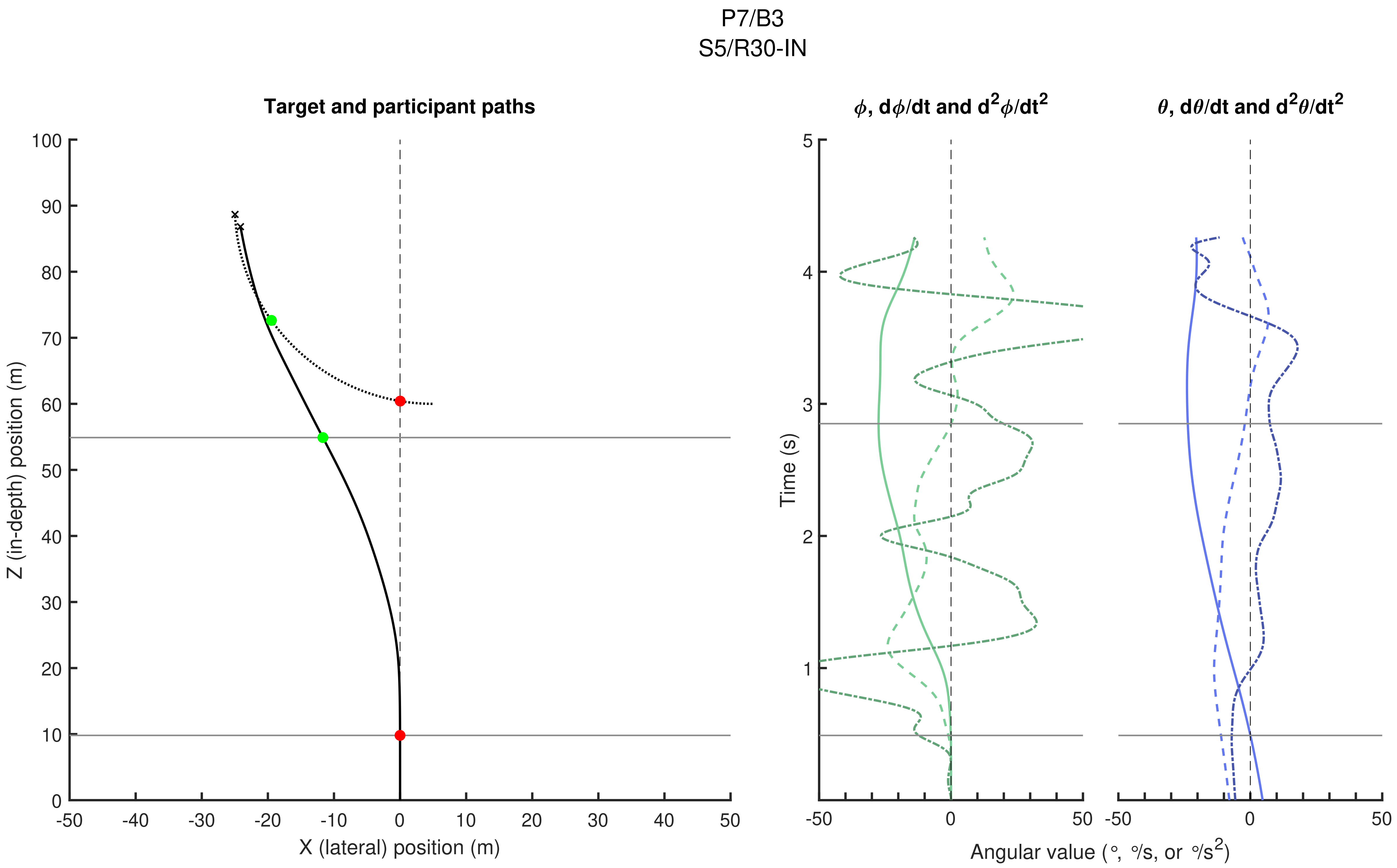

P7/B3
S5/R30-IN
Target and participant paths
Z (in-depth) position (m)
X (lateral) position (m)
ϕ, dϕ/dt and d$^2\phi$/dt^2
θ, dθ/dt and d$^2\theta$/dt^2
Time (s)
Angular value (°, °/s, or °/s^2)

P7/B3
S15/R10-IN

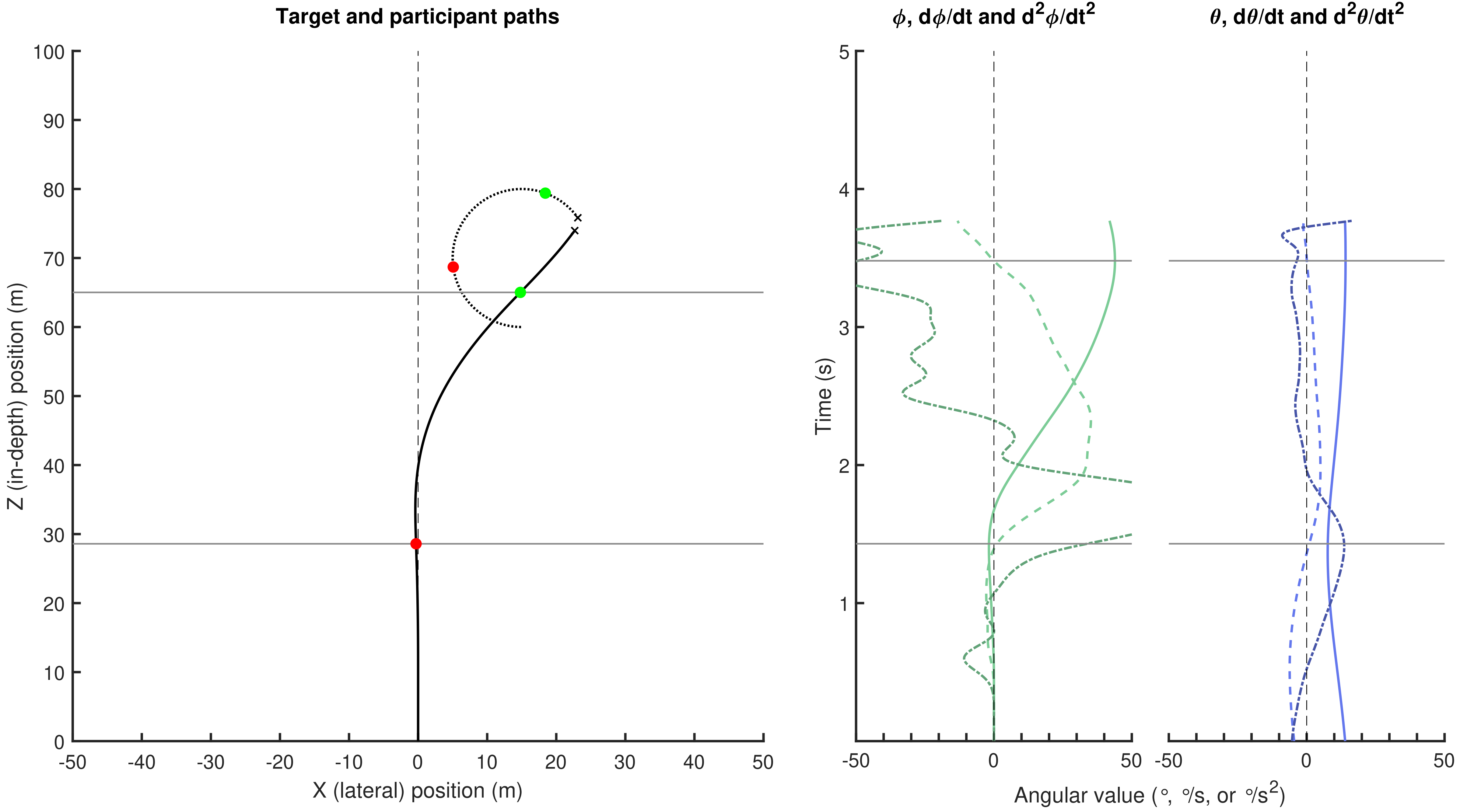

P7/B3
S15/R20-IN

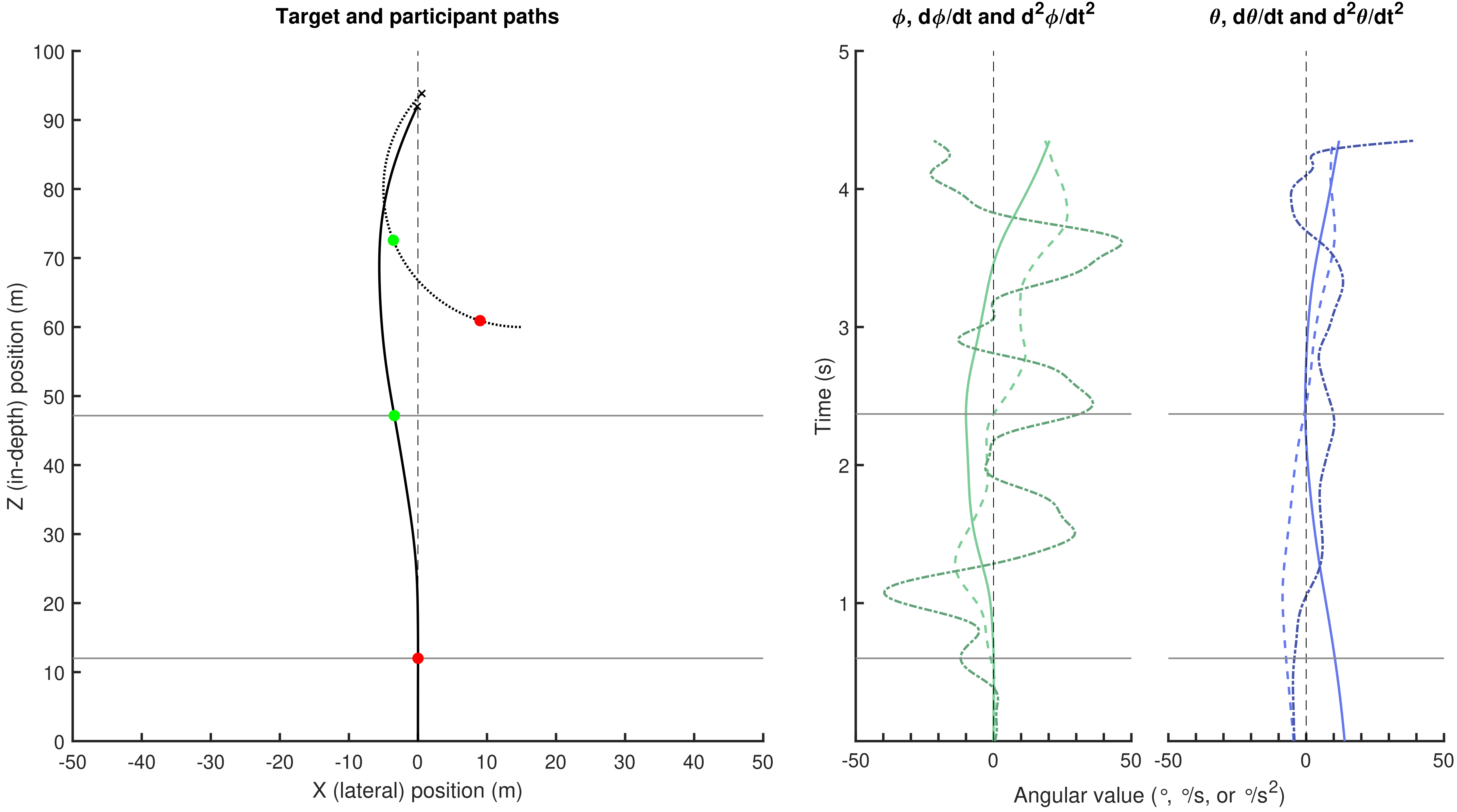

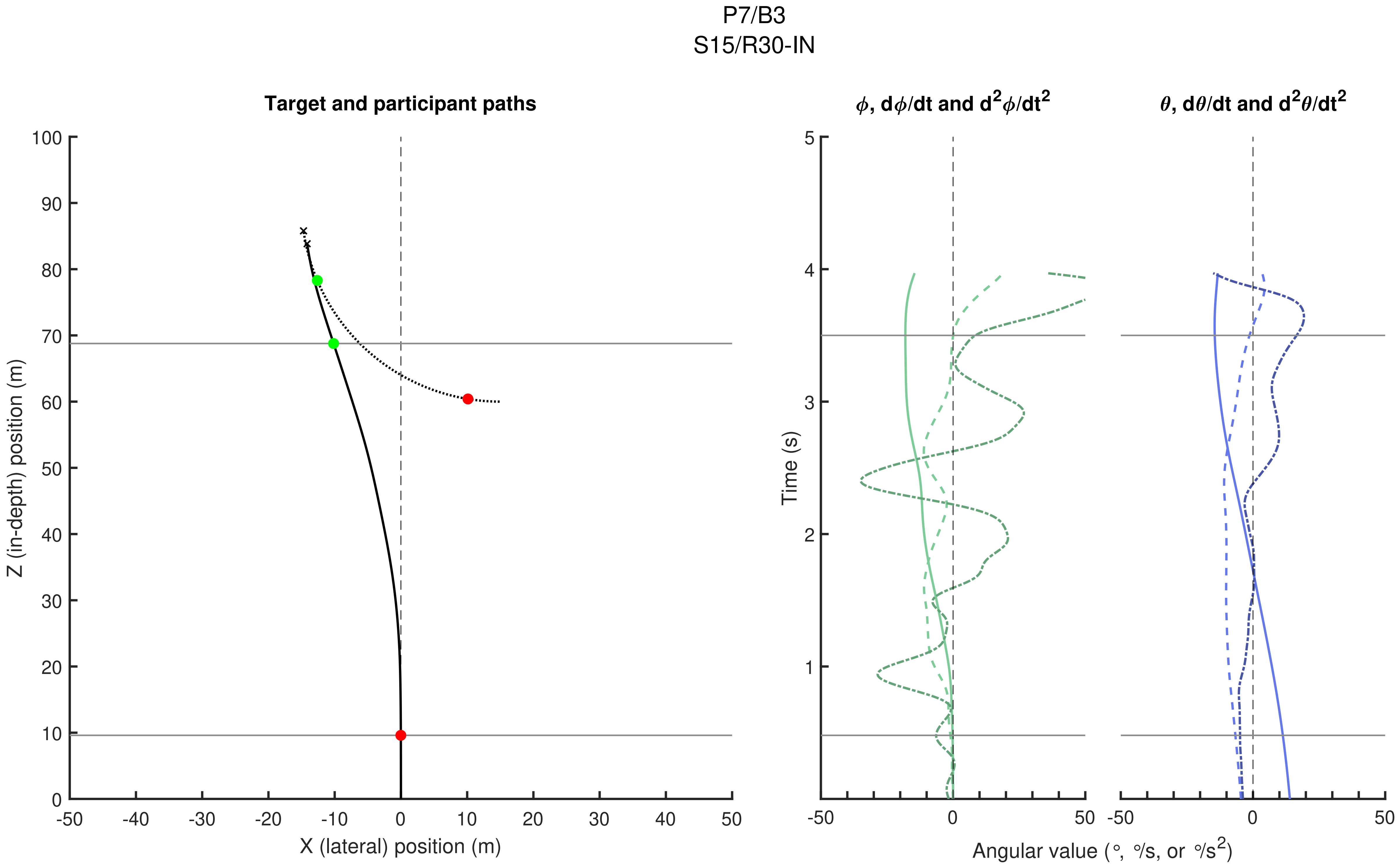

P7/B3
S15/R30-IN
Target and participant paths
Z (in-depth) position (m)
X (lateral) position (m)
ϕ, dϕ/dt and d$^2\phi$/dt^2
θ, dθ/dt and d$^2\theta$/dt^2
Time (s)
Angular value (°, °/s, or °/s^2)

P7/B3
S20/R10-IN

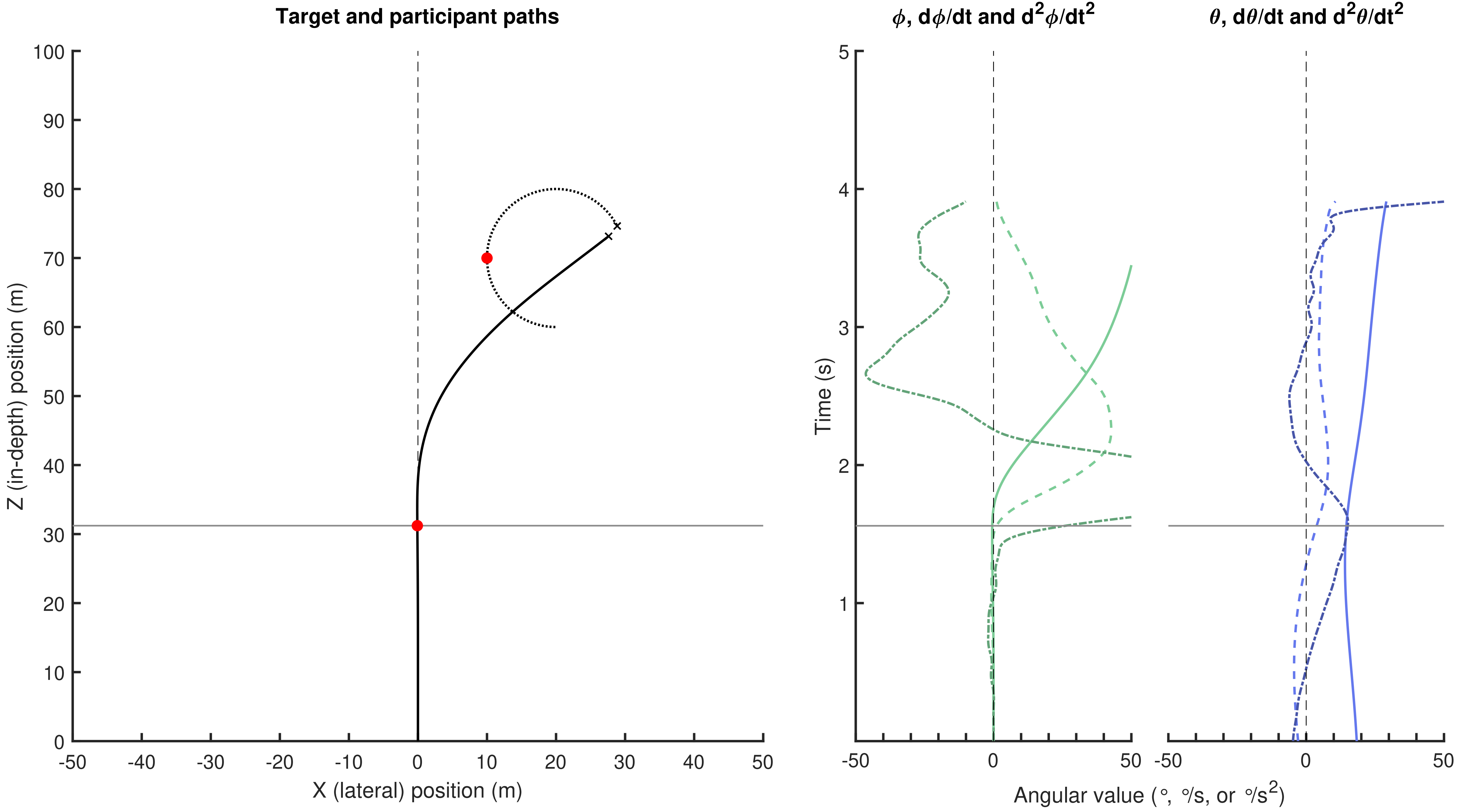

P7/B3
S20/R20-IN

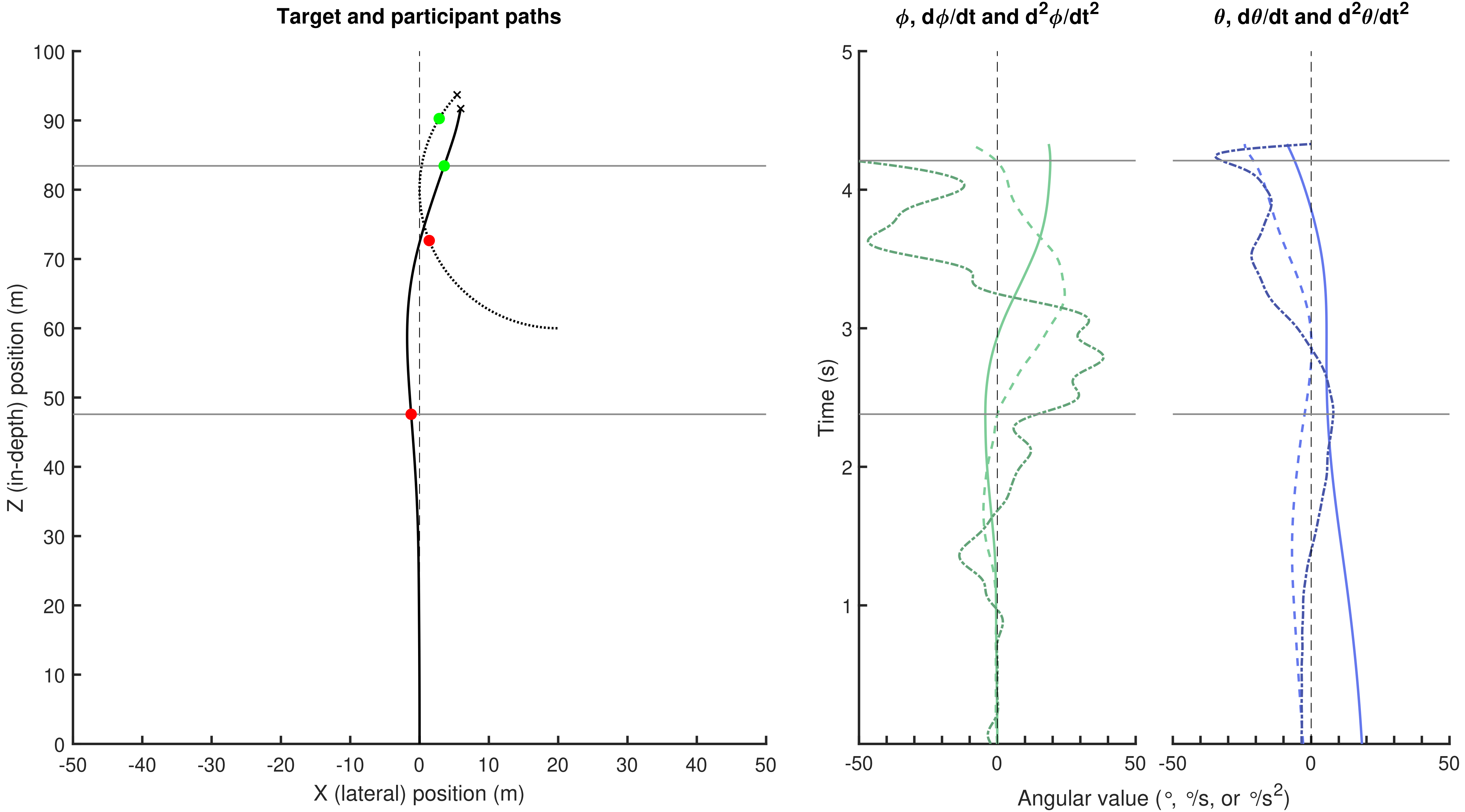

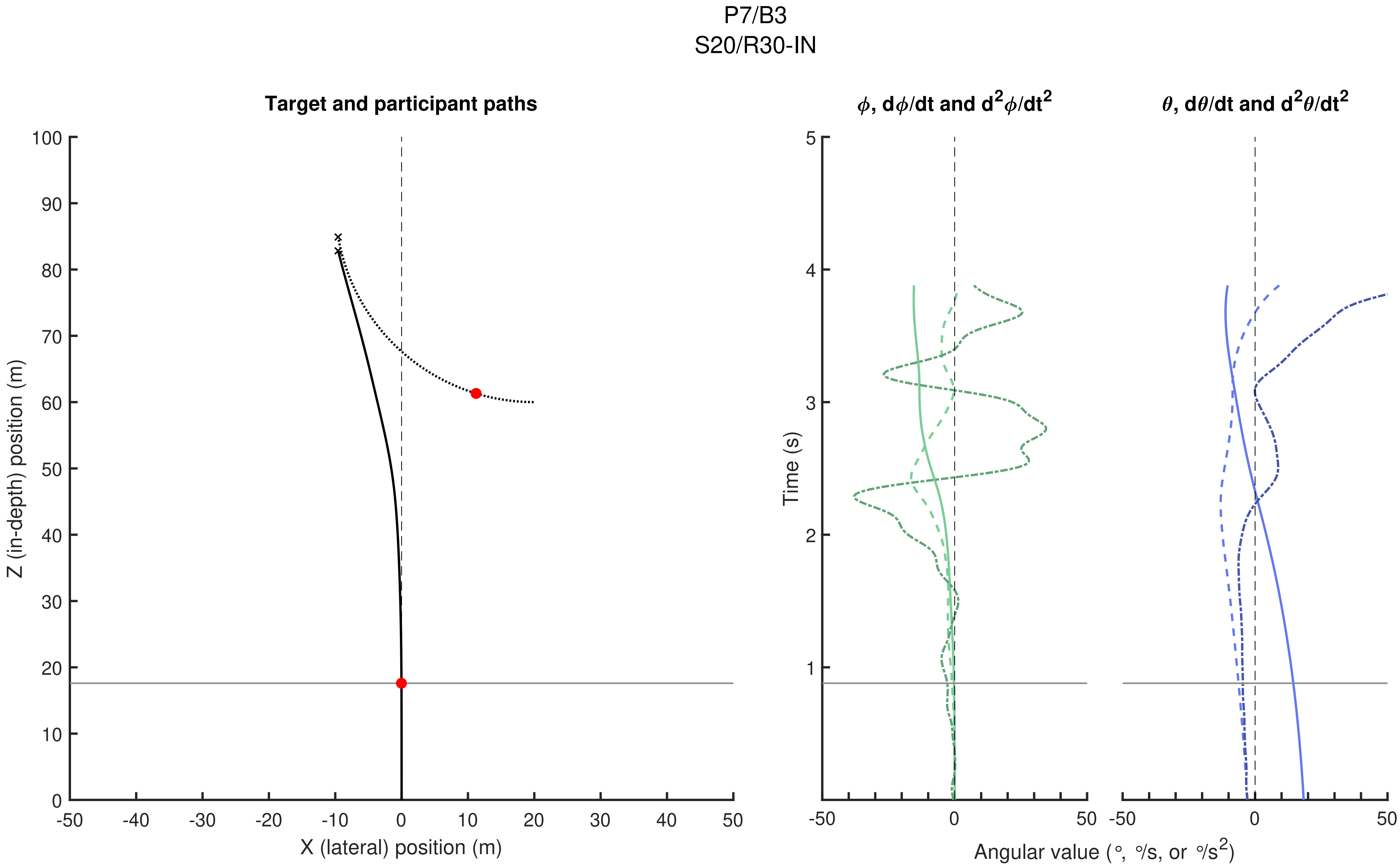
P7/B3
S20/R30-IN
Target and participant paths
Z (in-depth) position (m)
X (lateral) position (m)
φ, dφ/dt and d²φ/dt²
θ, dθ/dt and d²θ/dt²
Time (s)
Angular value (°, °/s, or °/s²)

P7/B4
S20/R10-IN

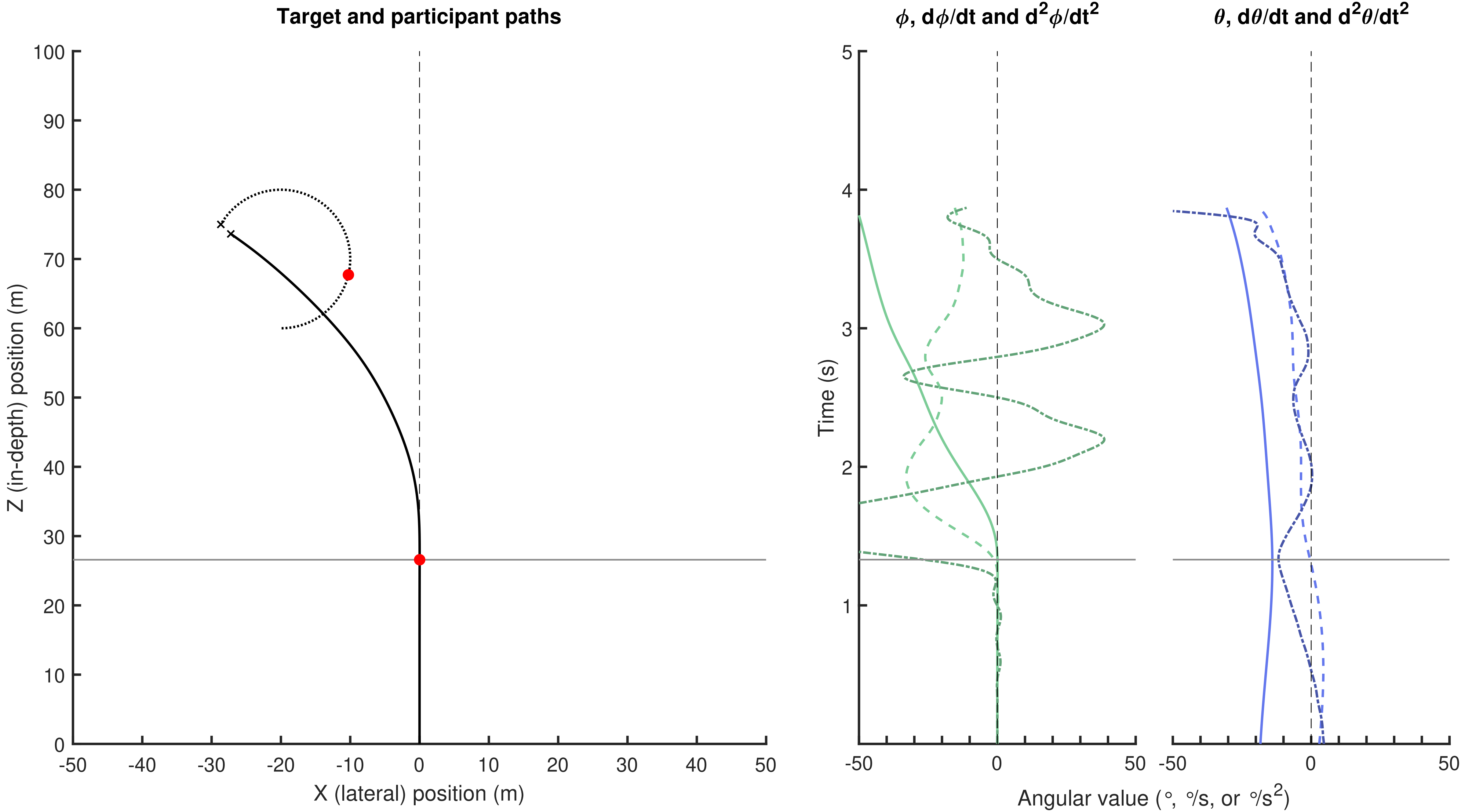

P7/B4
S20/R20-IN

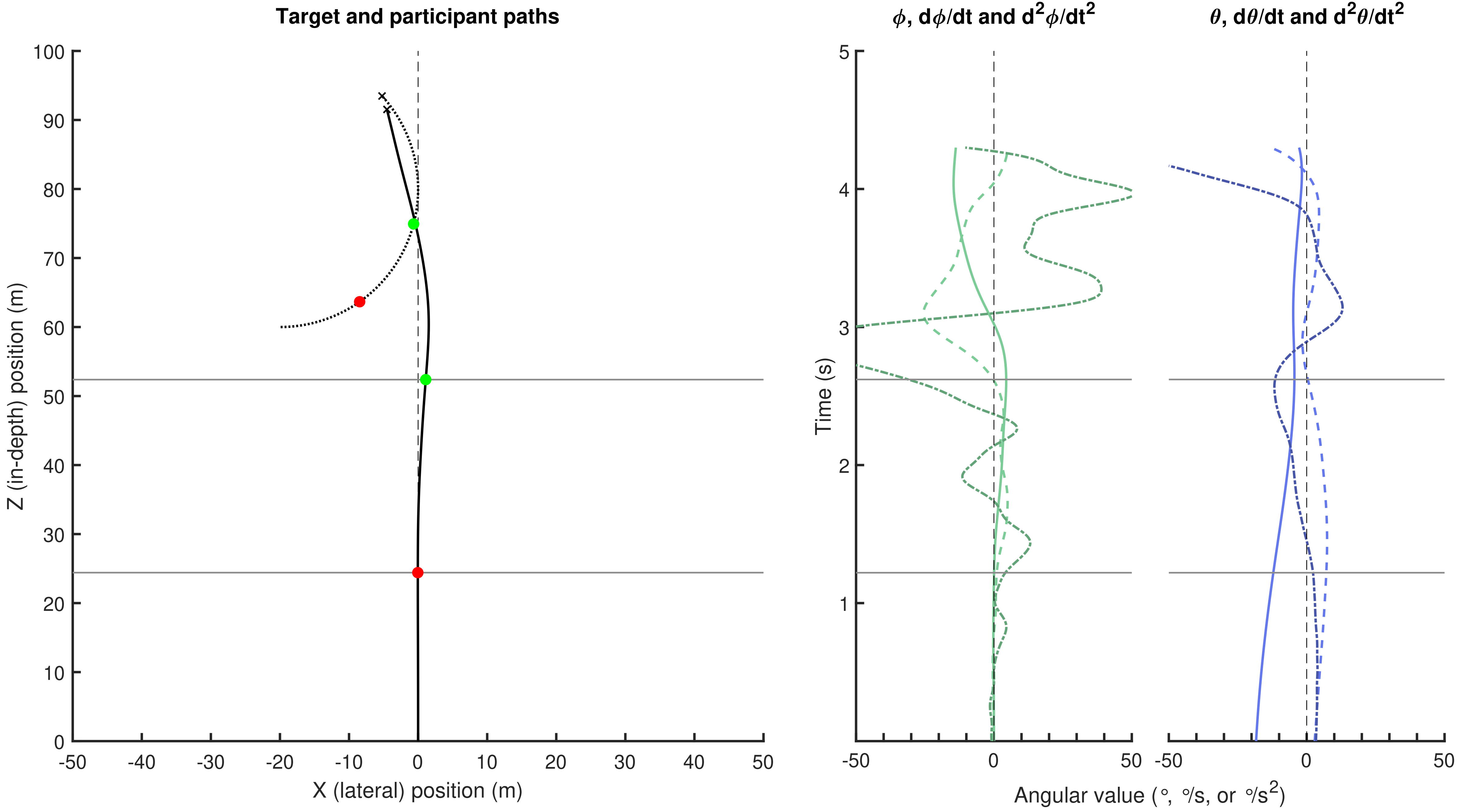

P7/B4
S20/R30-IN

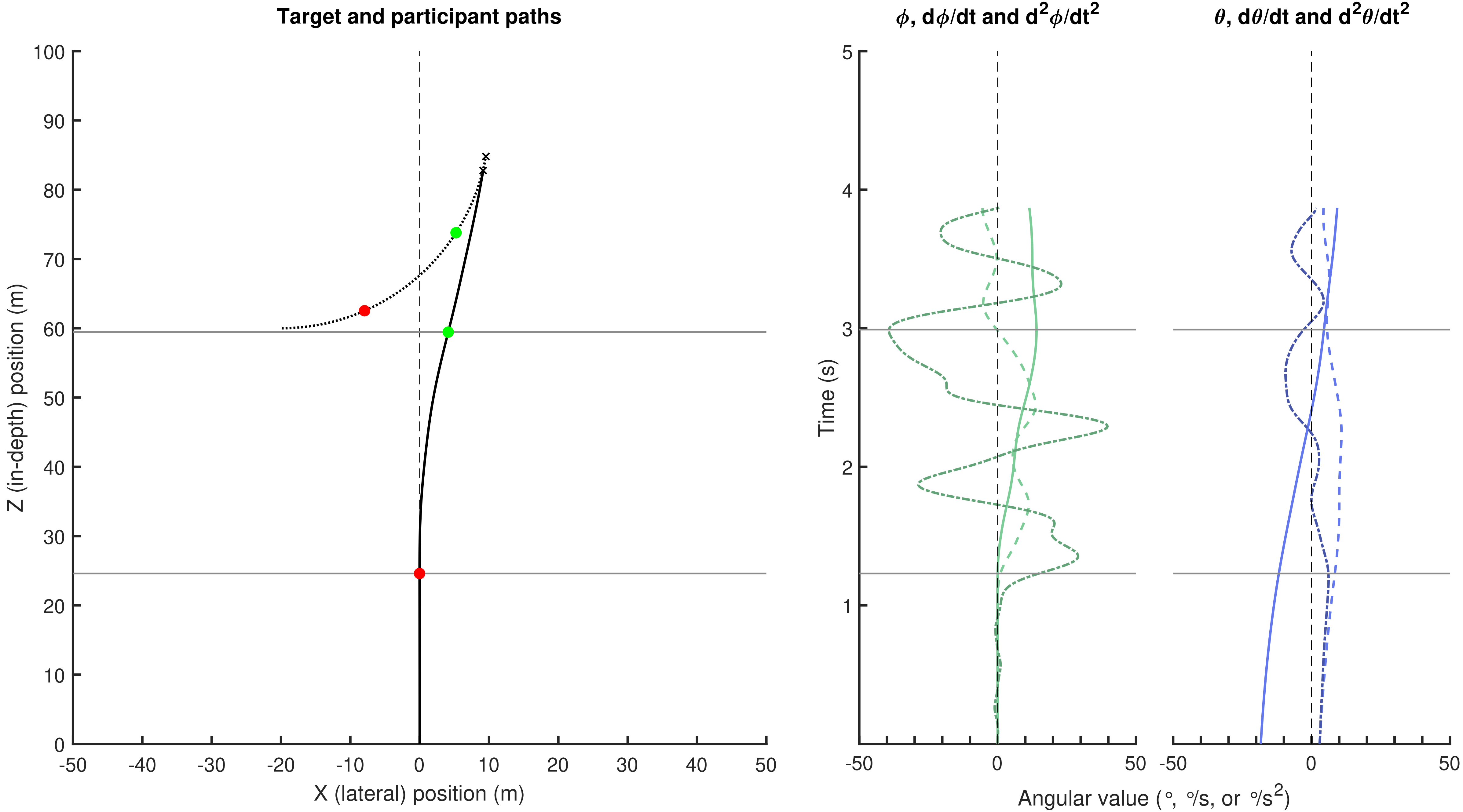

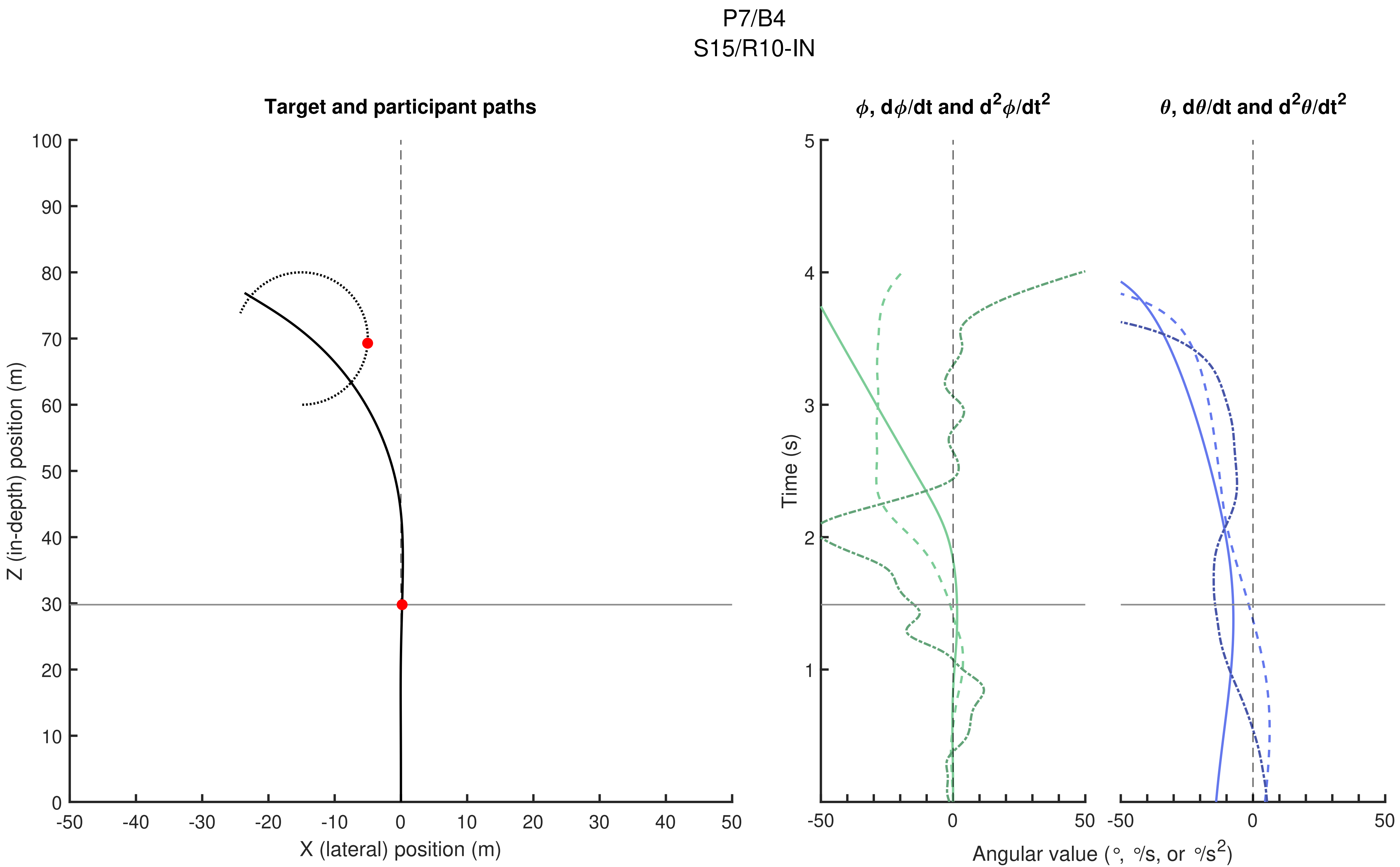

P7/B4
S15/R10-IN
Target and participant paths
Z (in-depth) position (m)
X (lateral) position (m)
ϕ, dϕ/dt and d$^2\phi$/dt^2
θ, dθ/dt and d$^2\theta$/dt^2
Time (s)
Angular value (°, °/s, or °/s^2)

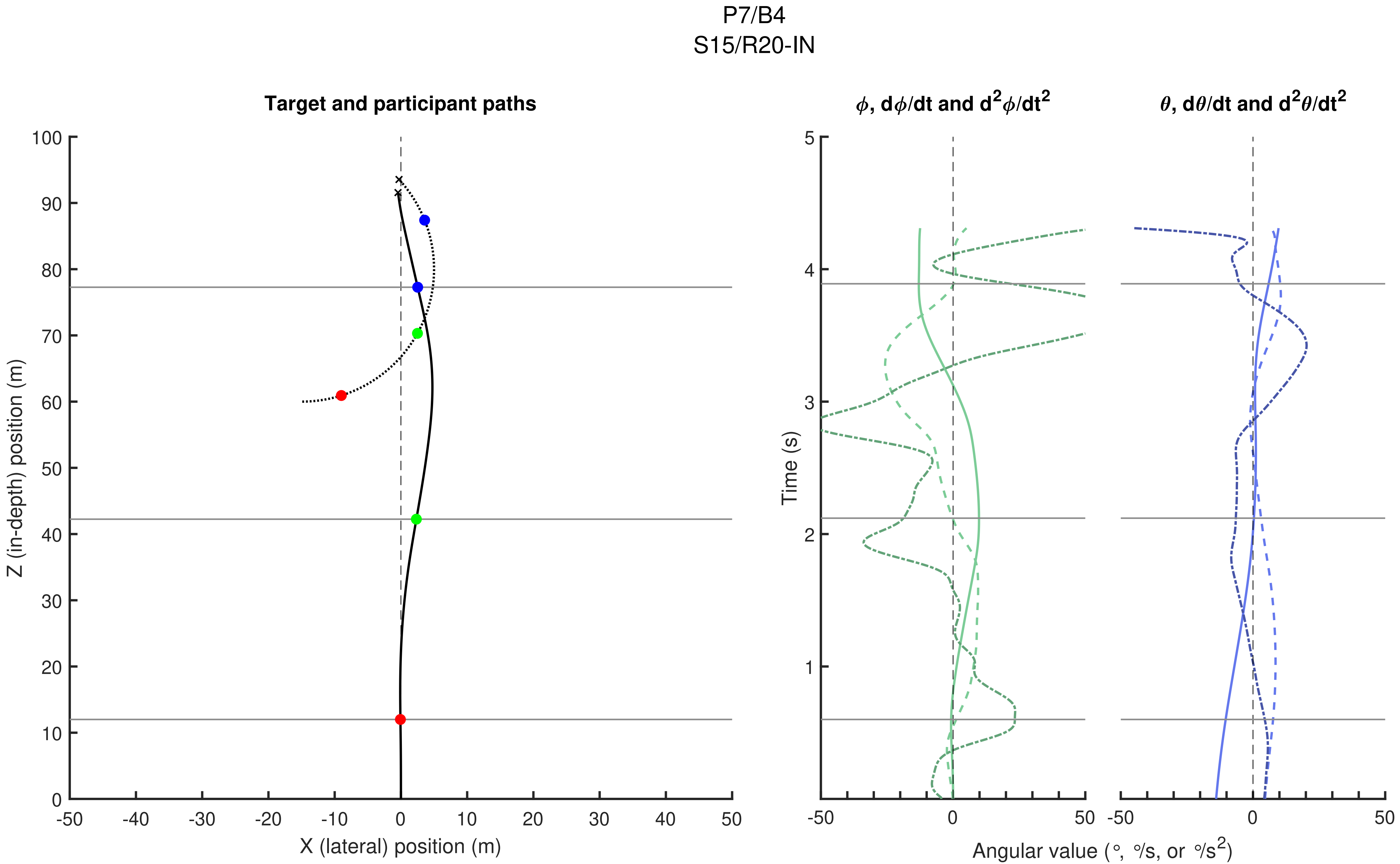

P7/B4
S15/R20-IN
Target and participant paths
φ, dφ/dt and d²φ/dt²
θ, dθ/dt and d²θ/dt²
Z (in-depth) position (m)
X (lateral) position (m)
Time (s)
Angular value (°, °/s, or °/s²)

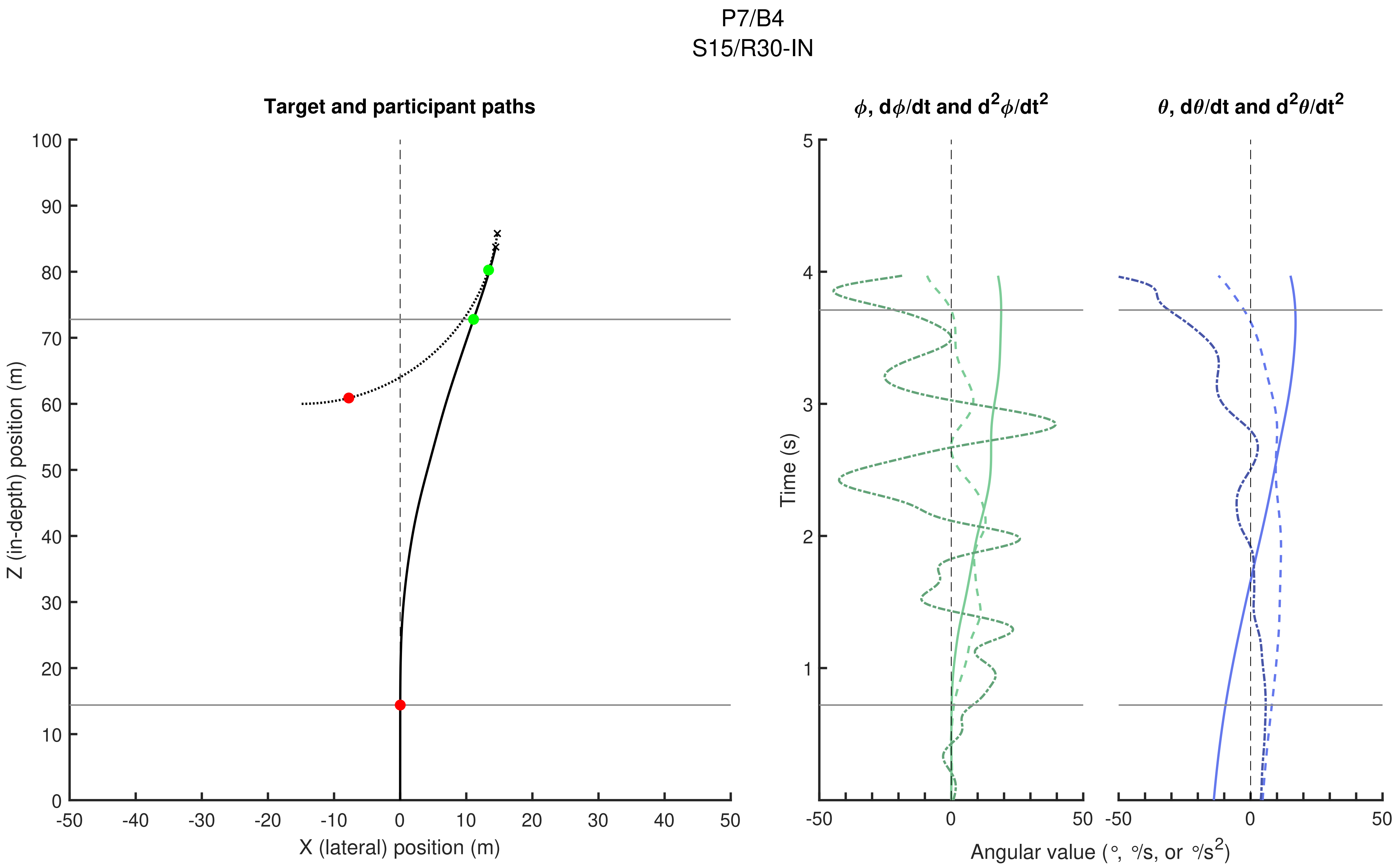

P7/B4
S15/R30-IN
Target and participant paths
Z (in-depth) position (m)
X (lateral) position (m)
100
90
80
70
60
50
40
30
20
10
0
-50
-40
-30
-20
-10
0
10
20
30
40
50
ϕ, dϕ/dt and d²ϕ/dt²
θ, dθ/dt and d²θ/dt²
Time (s)
5
4
3
2
1
-50
0
50
-50
0
50
Angular value (°, °/s, or °/s²)

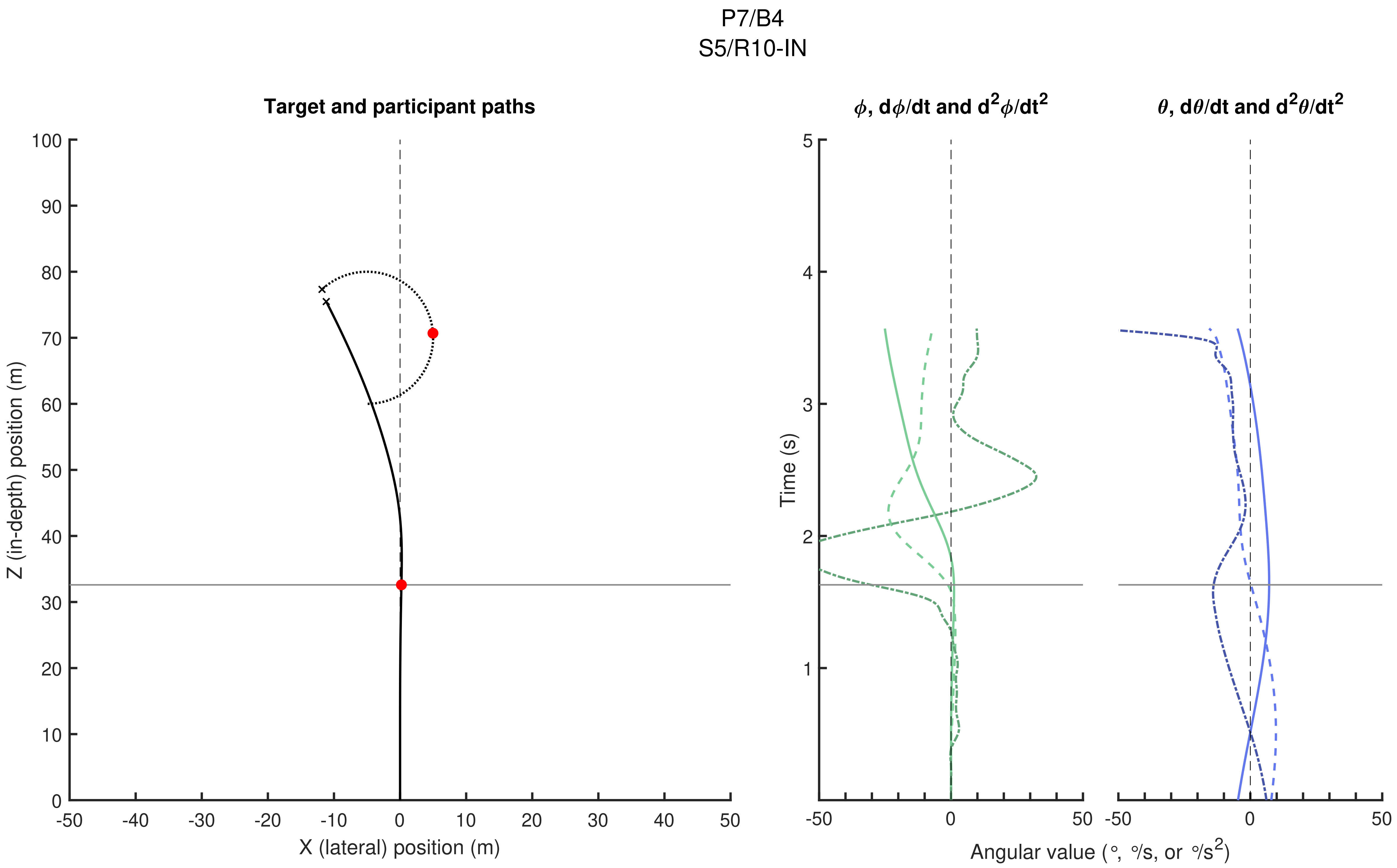

P7/B4
S5/R10-IN
Target and participant paths
Z (in-depth) position (m)
X (lateral) position (m)
100
90
80
70
60
50
40
30
20
10
0
-50
-40
-30
-20
-10
0
10
20
30
40
50
ϕ, dϕ/dt and d²ϕ/dt²
θ, dθ/dt and d²θ/dt²
Time (s)
5
4
3
2
1
-50
0
50
Angular value (°, °/s, or °/s²)

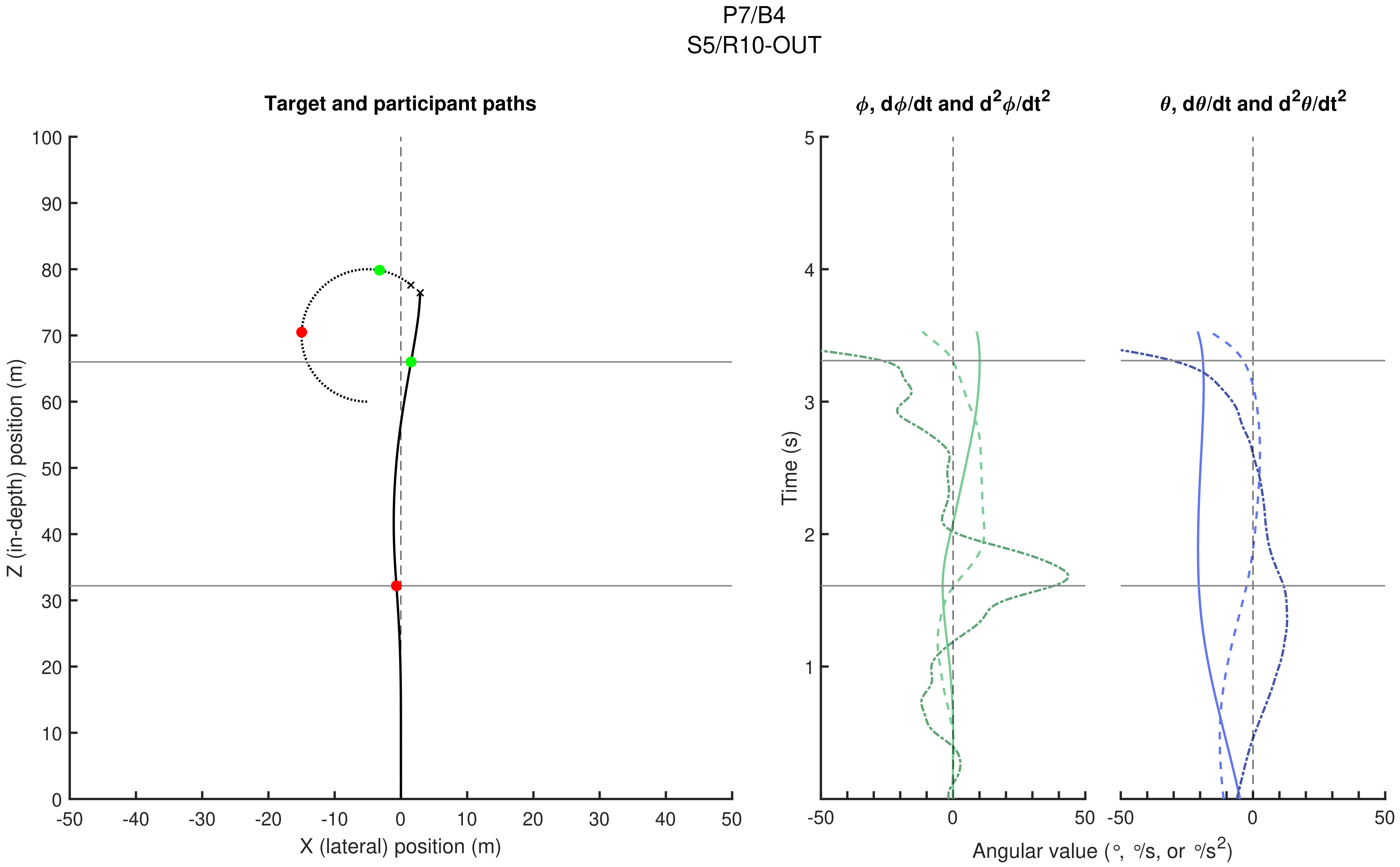

P7/B4
S5/R10-OUT
Target and participant paths
Z (in-depth) position (m)
X (lateral) position (m)
ϕ, dϕ/dt and d$^2\phi$/dt^2
θ, dθ/dt and d$^2\theta$/dt^2
Time (s)
Angular value (°, °/s, or °/s^2)

P7/B4
S5/R20-IN

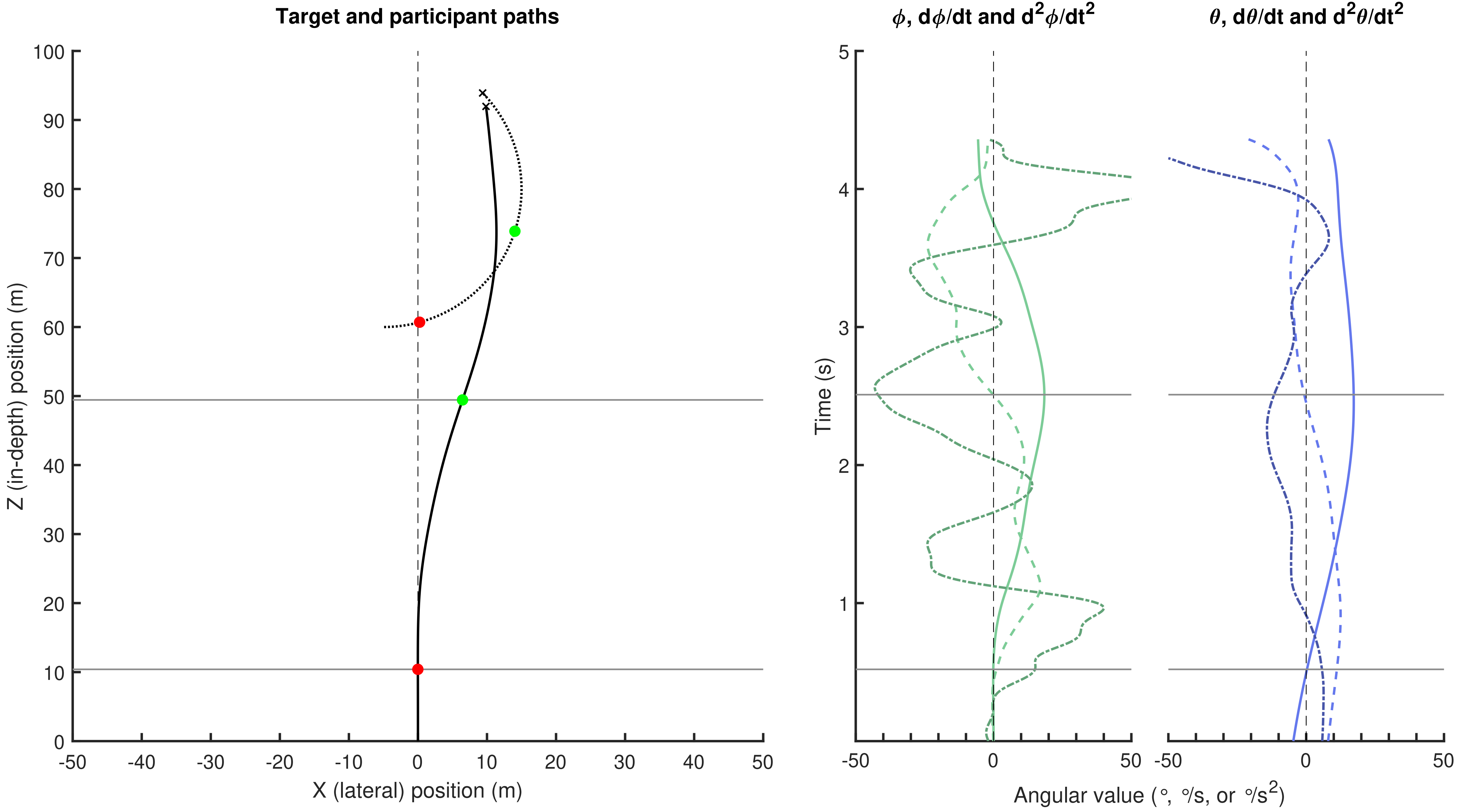

P7/B4
S5/R20-OUT

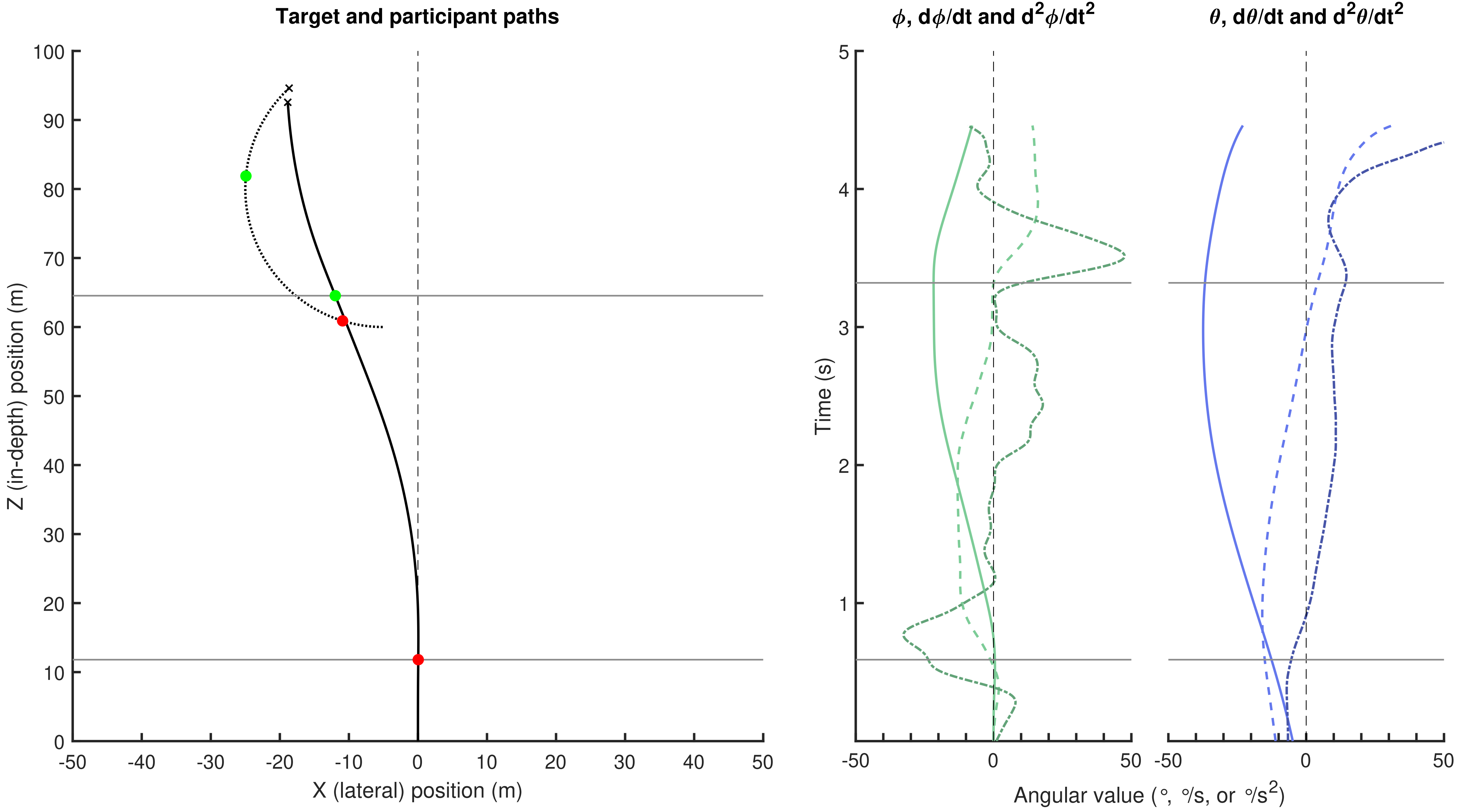

P7/B4
S5/R30-IN

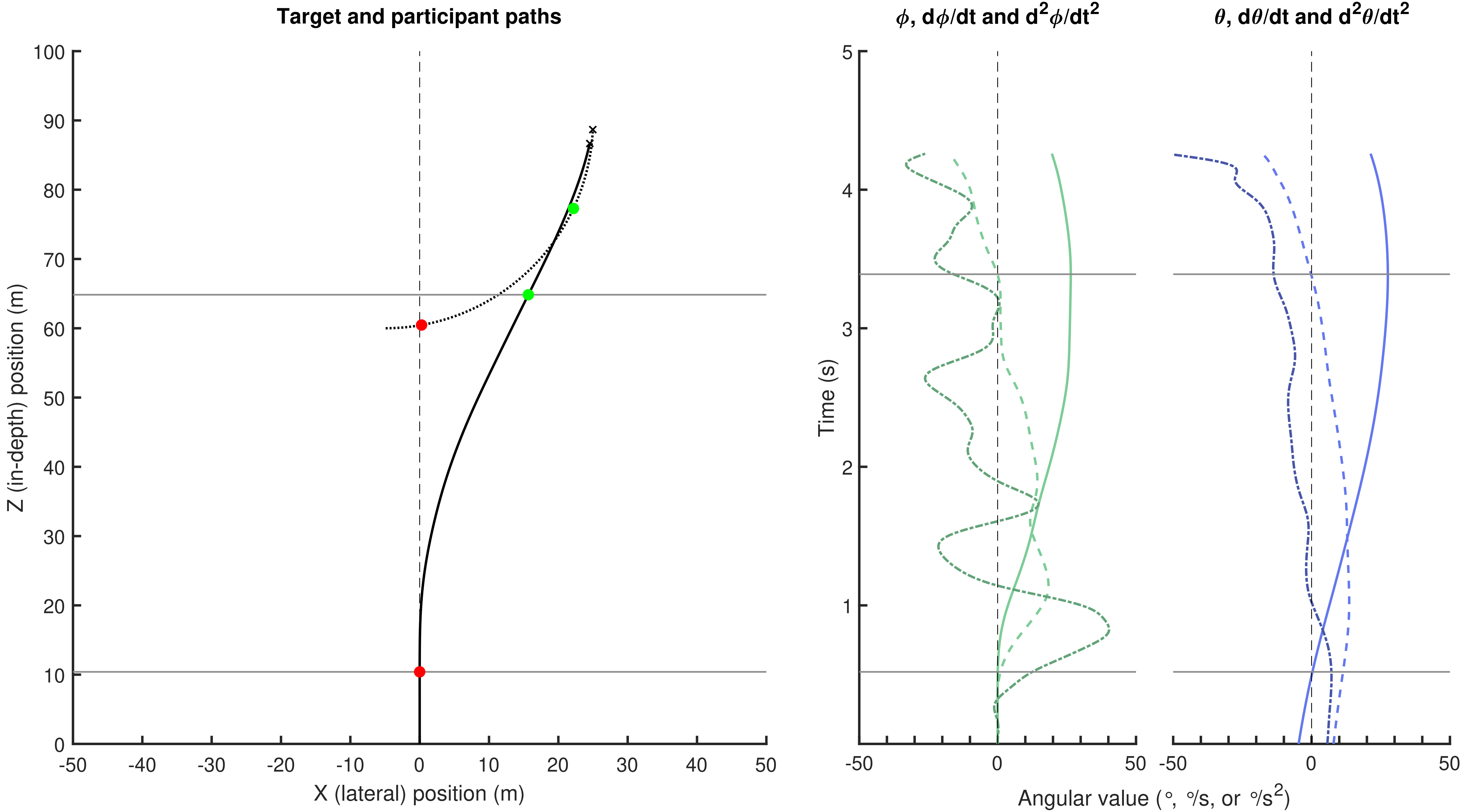

P7/B4
S5/R30-OUT

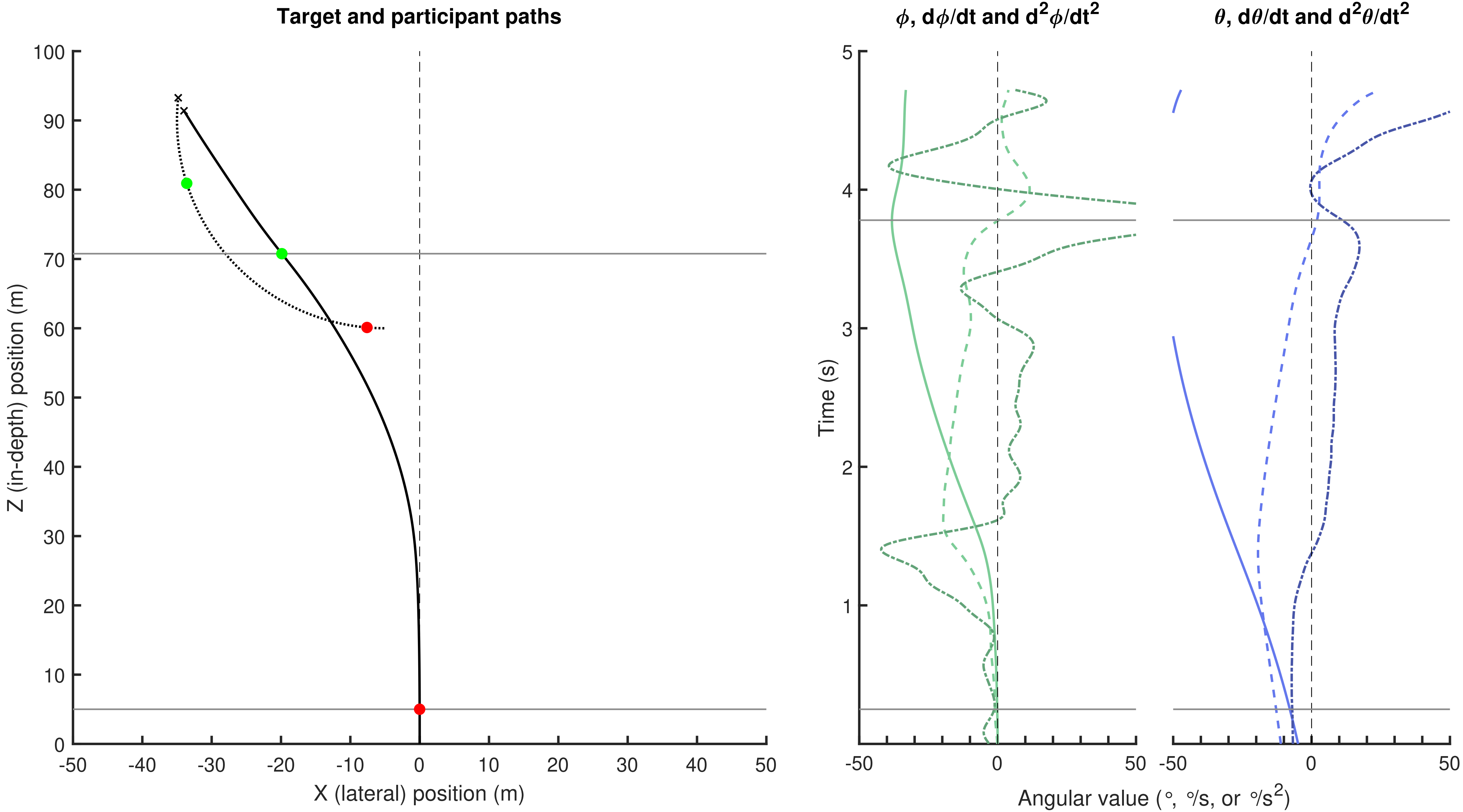

P7/B4
S5/R10-OUT

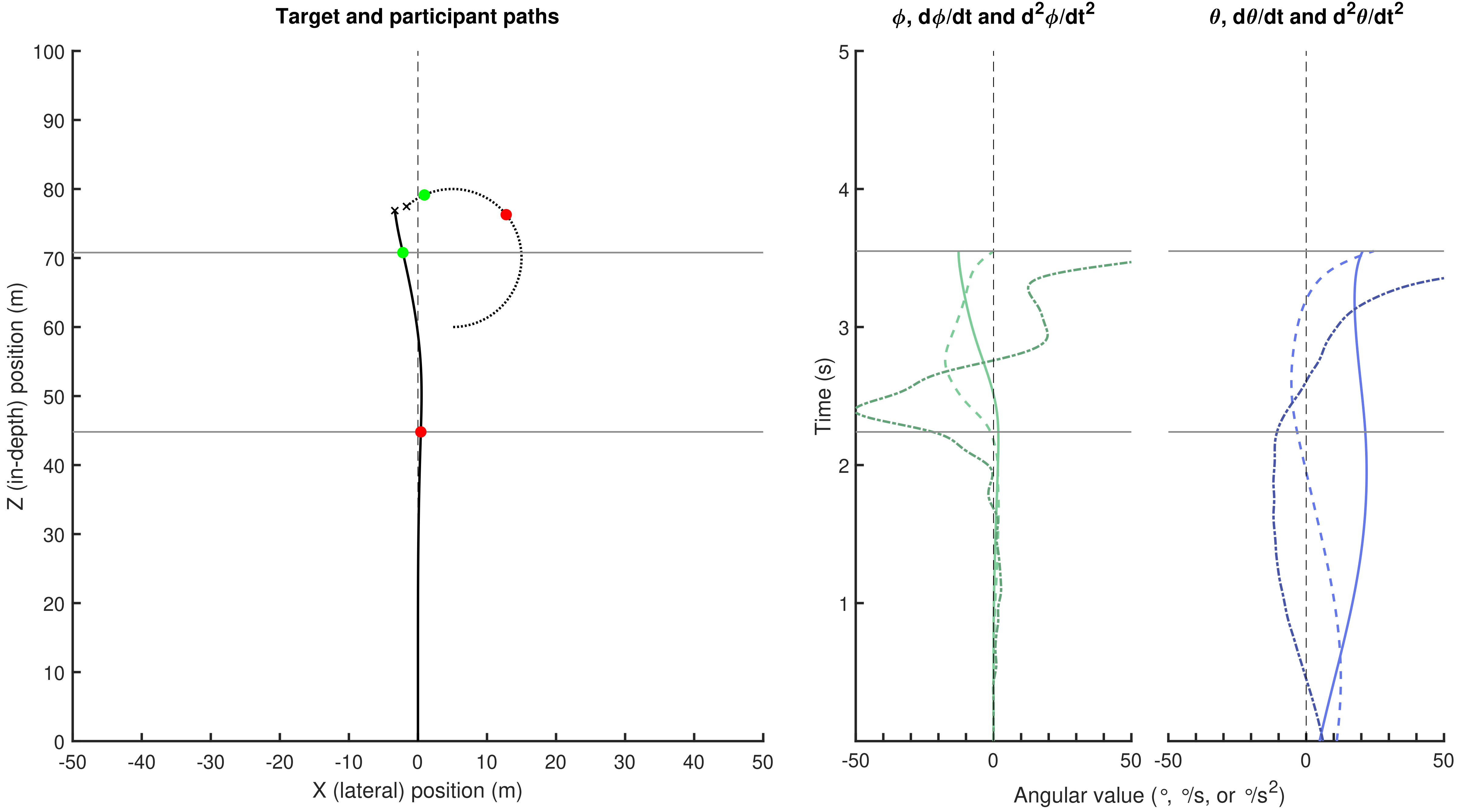

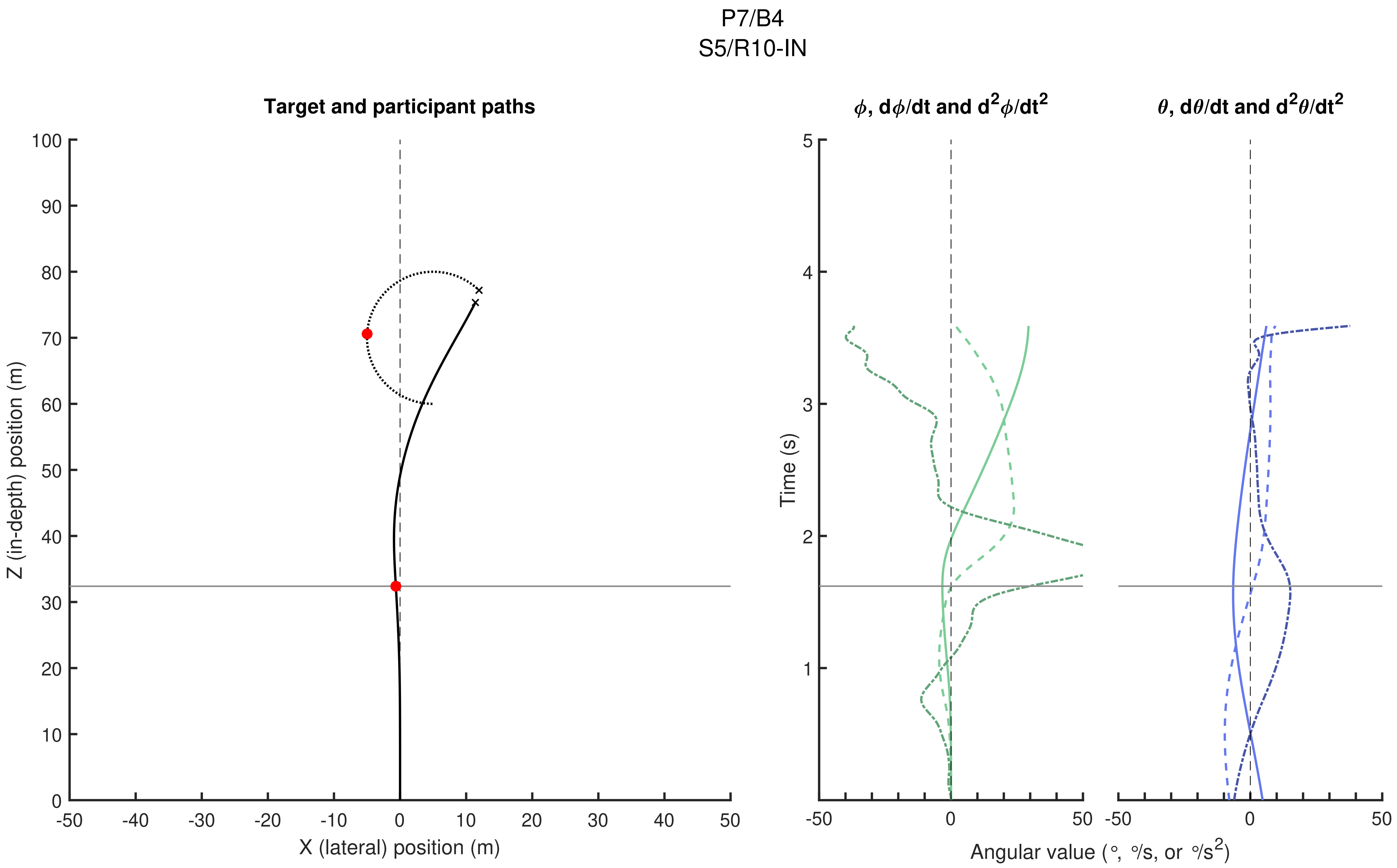

P7/B4
S5/R10-IN
Target and participant paths
Z (in-depth) position (m)
X (lateral) position (m)
ϕ, dϕ/dt and d$^2\phi$/dt^2
θ, dθ/dt and d$^2\theta$/dt^2
Time (s)
Angular value (°, °/s, or °/s^2)

P7/B4
S5/R20-OUT

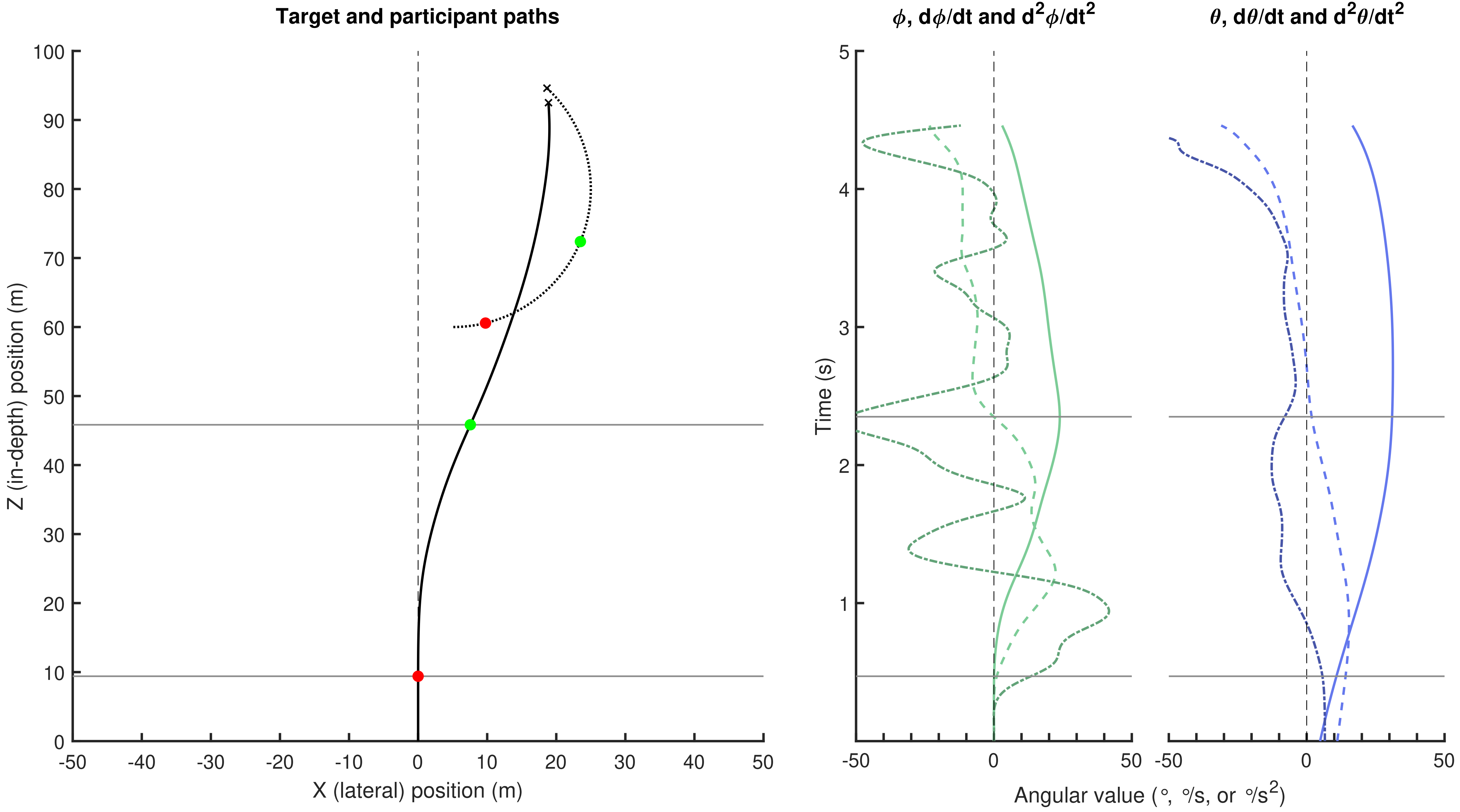

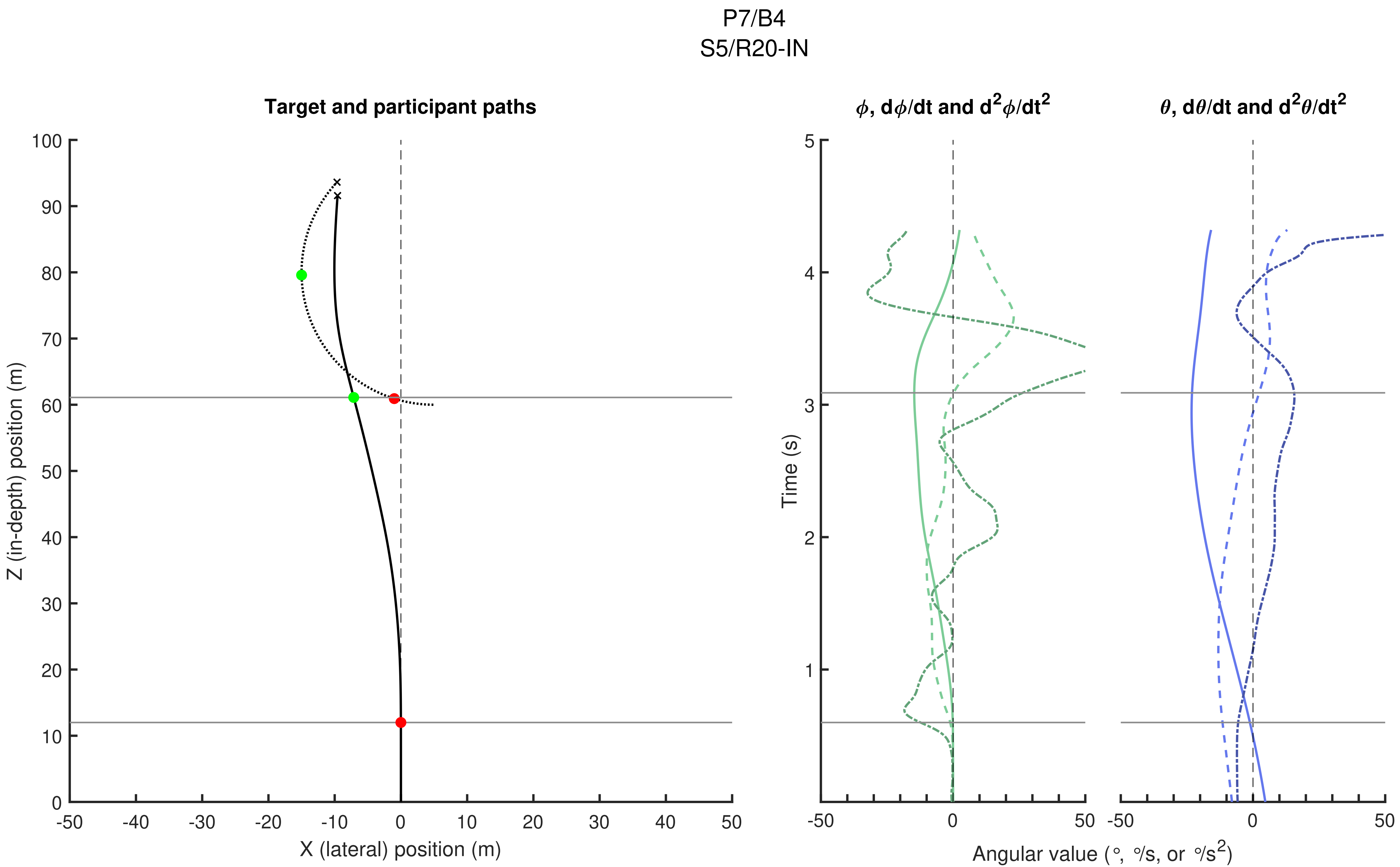

P7/B4
S5/R20-IN
Target and participant paths
Z (in-depth) position (m)
X (lateral) position (m)
100
90
80
70
60
50
40
30
20
10
0
-50
-40
-30
-20
-10
0
10
20
30
40
50
ϕ, dϕ/dt and d$^2\phi$/dt^2
θ, dθ/dt and d$^2\theta$/dt^2
Time (s)
5
4
3
2
1
-50
0
50
Angular value (°, °/s, or °/s^2)

P7/B4
S5/R30-OUT

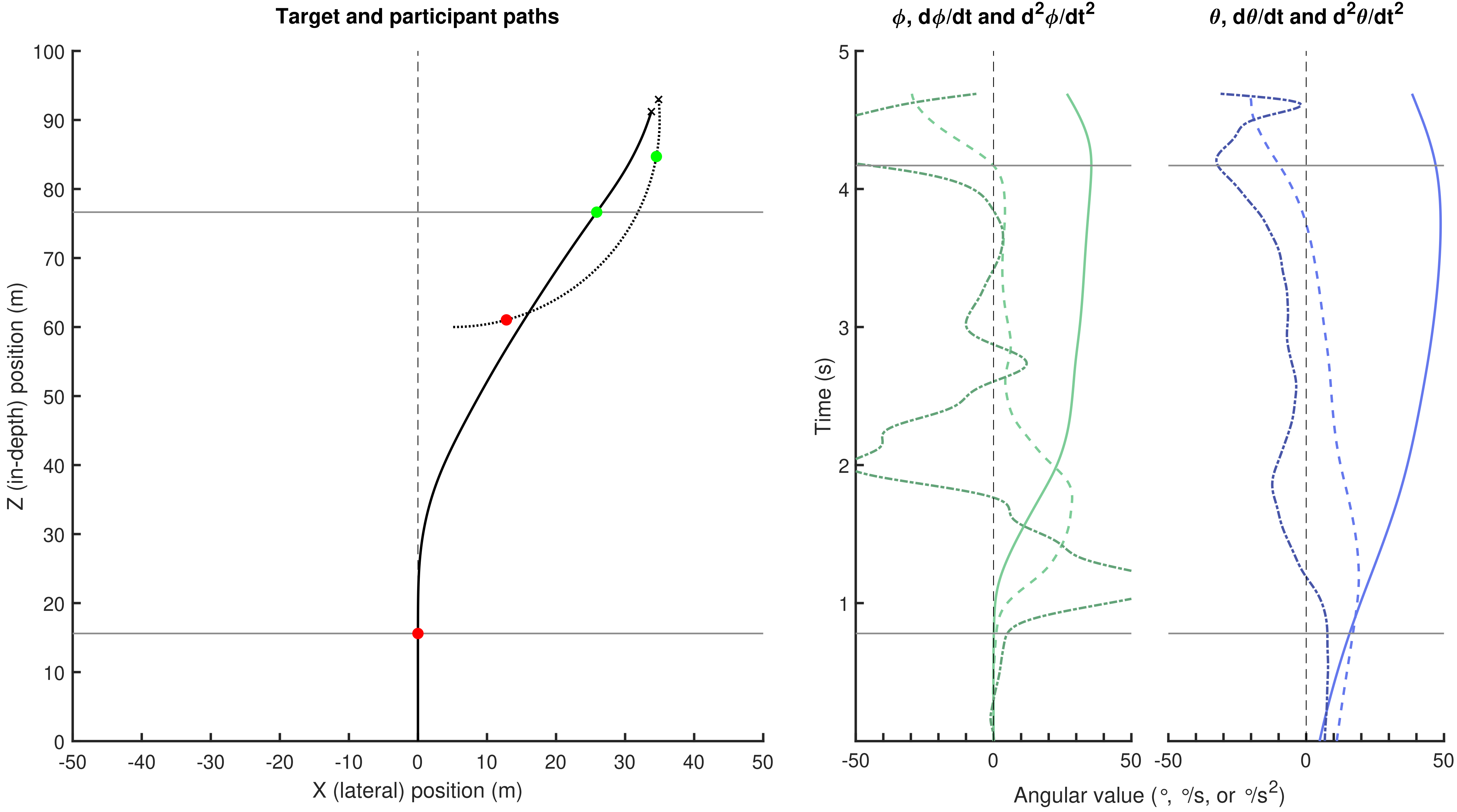

P7/B4
S5/R30-IN

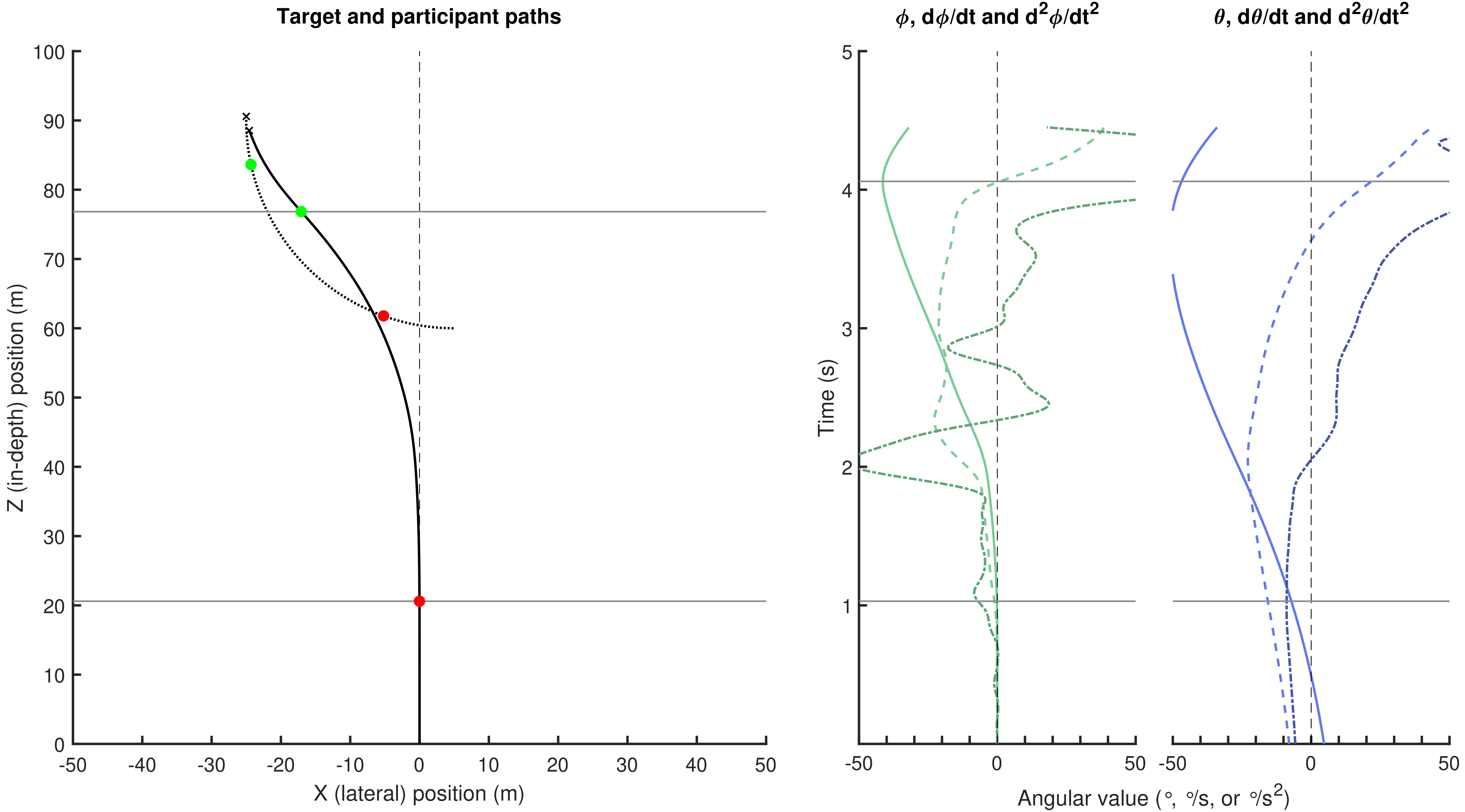

P7/B4
S15/R10-IN

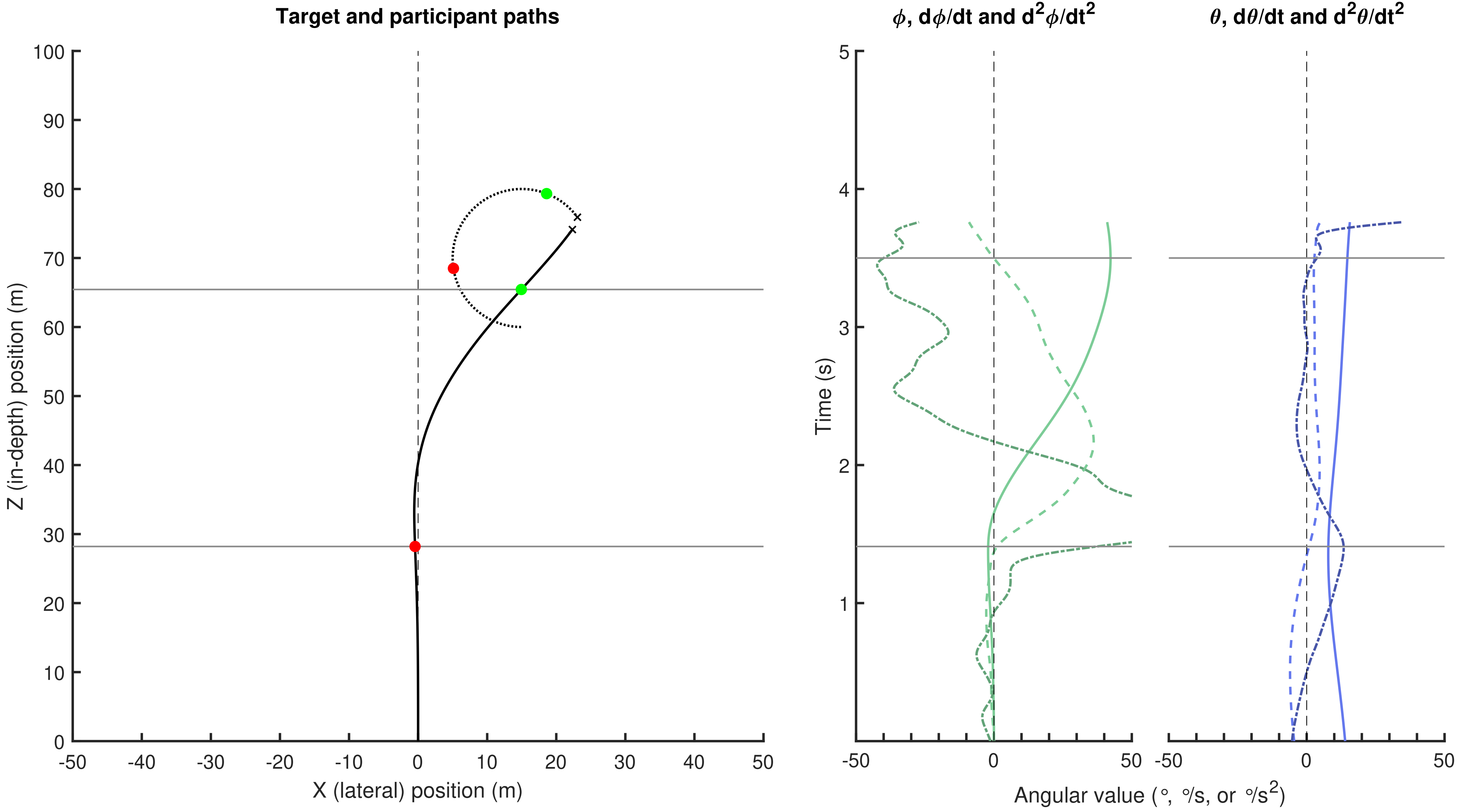

P7/B4
S15/R20-IN

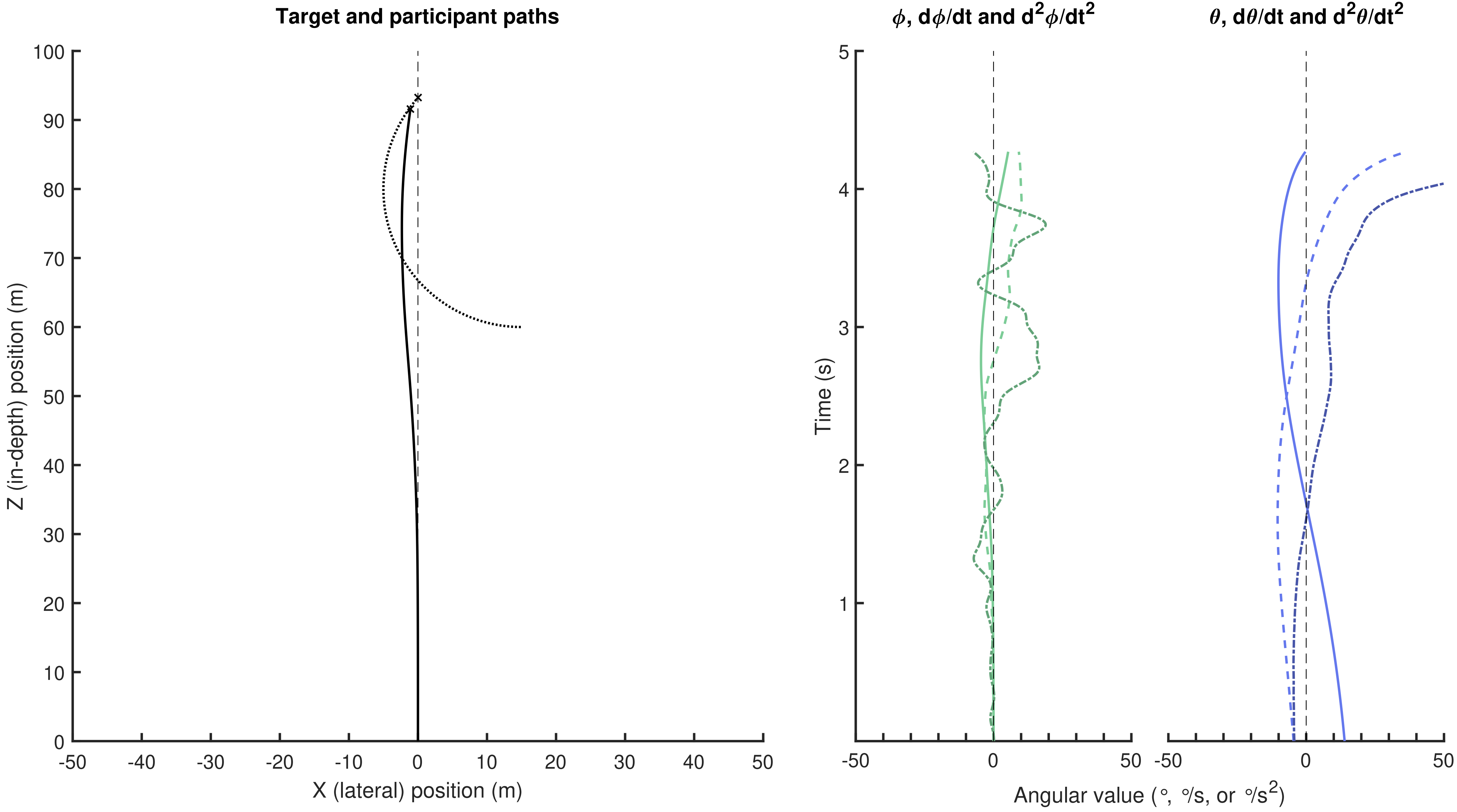

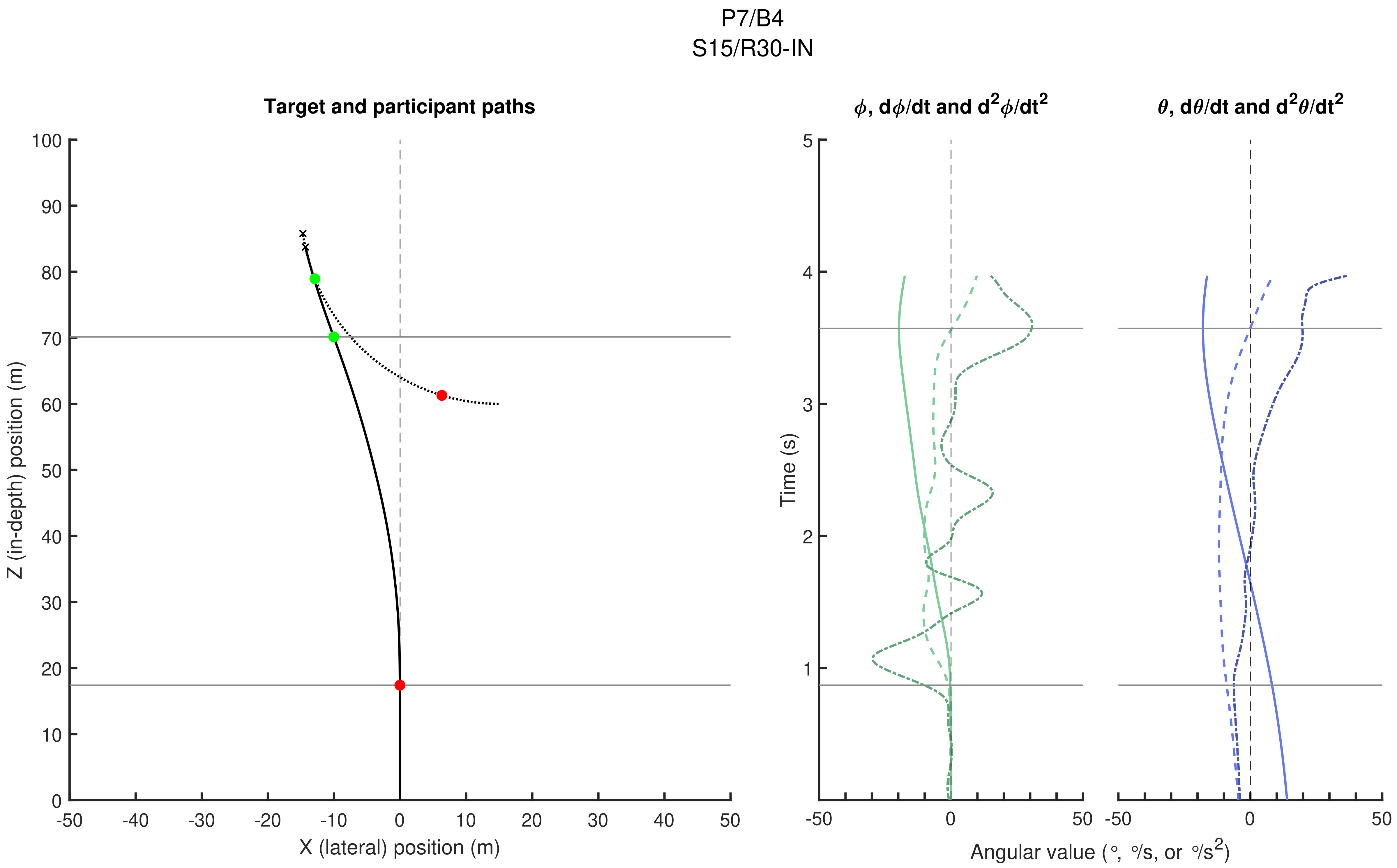
P7/B4
S15/R30-IN
Target and participant paths
Z (in-depth) position (m)
X (lateral) position (m)
ϕ, dϕ/dt and d$^2\phi$/dt^2
θ, dθ/dt and d$^2\theta$/dt^2
Time (s)
Angular value (°, °/s, or °/s^2)

P7/B4
S20/R10-IN

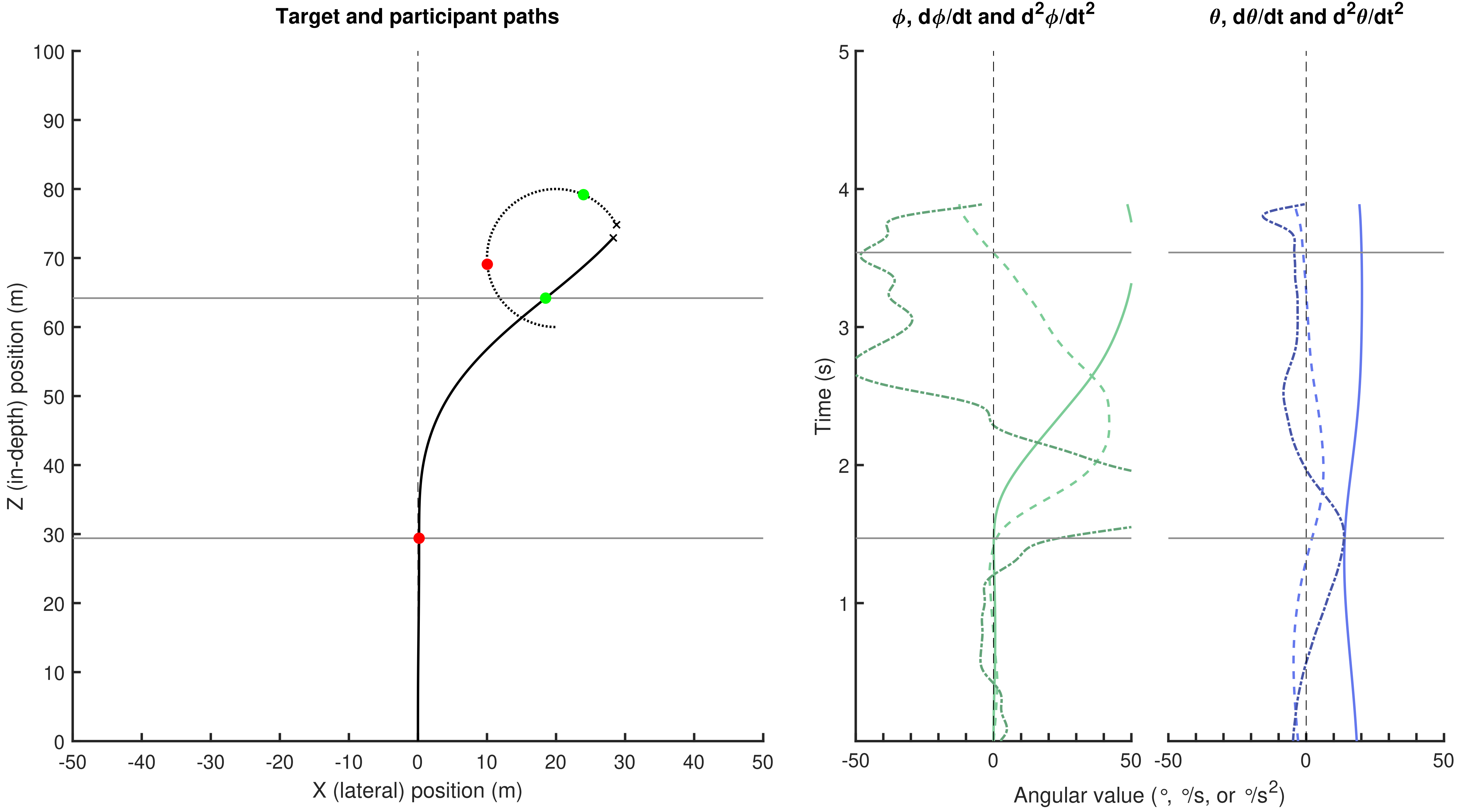

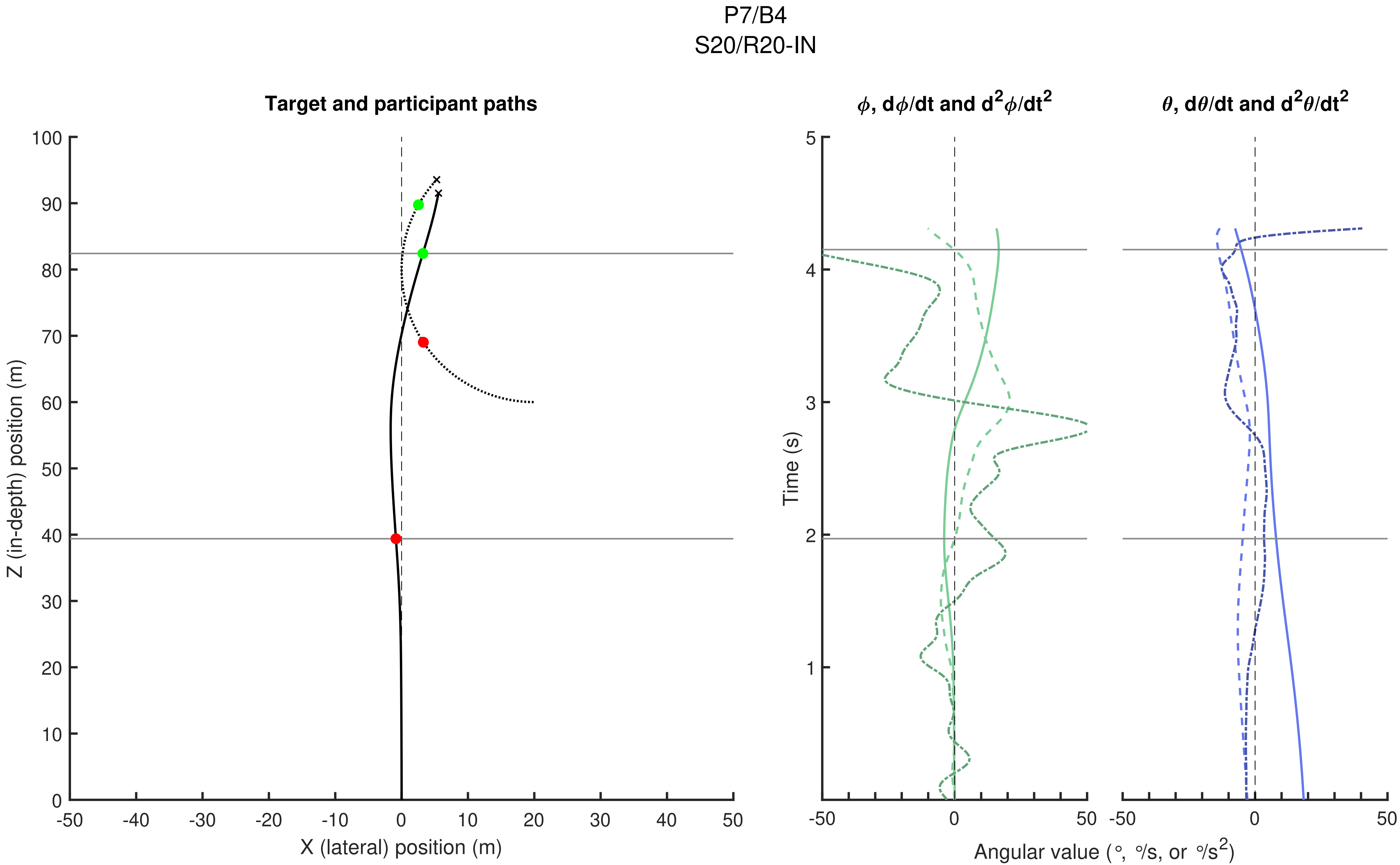

P7/B4
S20/R20-IN
Target and participant paths
Z (in-depth) position (m)
X (lateral) position (m)
100
90
80
70
60
50
40
30
20
10
0
-50
-40
-30
-20
-10
0
10
20
30
40
50
ϕ, dϕ/dt and d²ϕ/dt²
θ, dθ/dt and d²θ/dt²
Time (s)
5
4
3
2
1
-50
0
50
-50
0
50
Angular value (°, °/s, or °/s²)

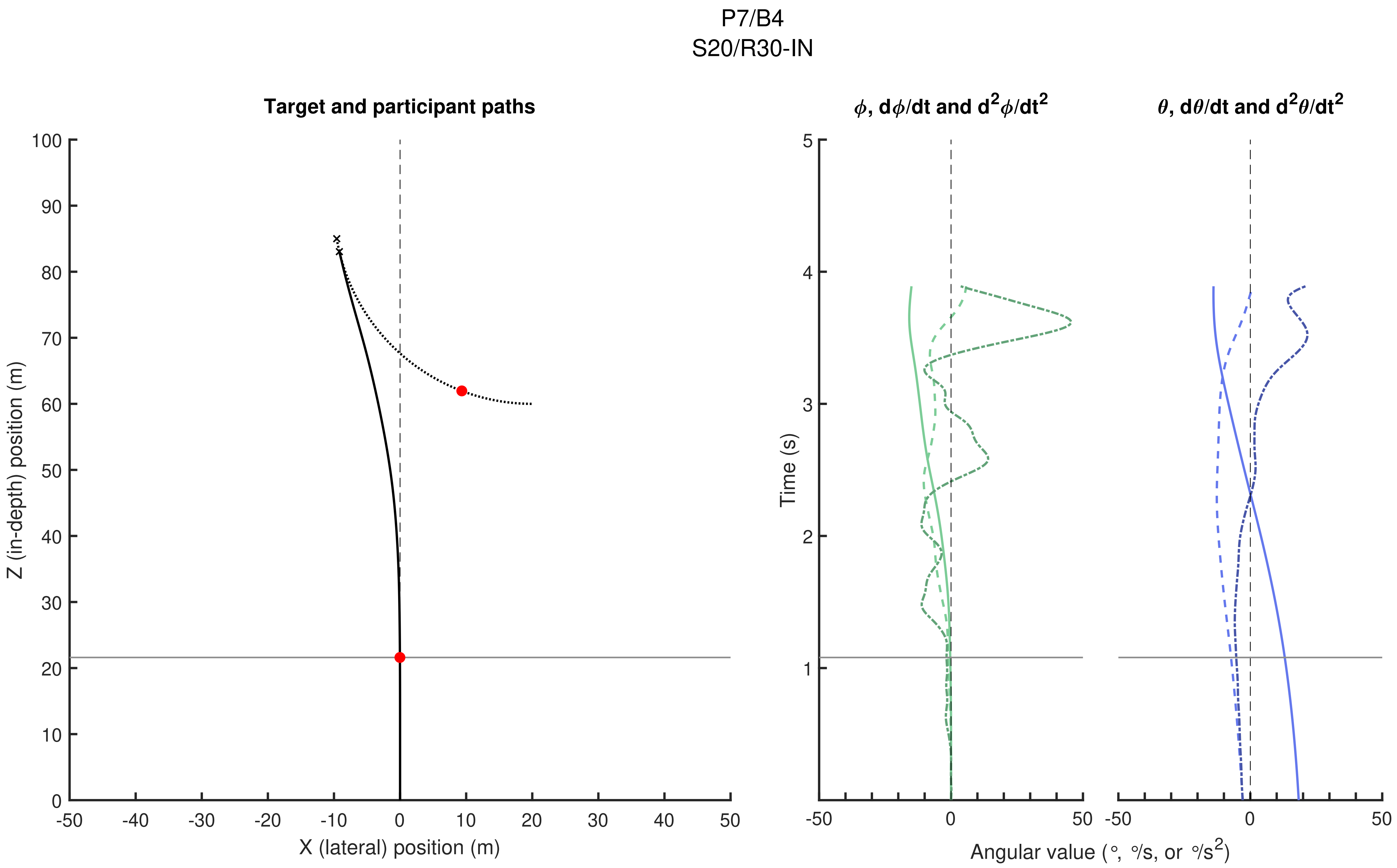
P7/B4
S20/R30-IN
Target and participant paths
Z (in-depth) position (m)
X (lateral) position (m)
ϕ, dϕ/dt and d$^2\phi$/dt^2
θ, dθ/dt and d$^2\theta$/dt^2
Time (s)
Angular value (°, °/s, or °/s^2)

P7/B5
S20/R10-IN

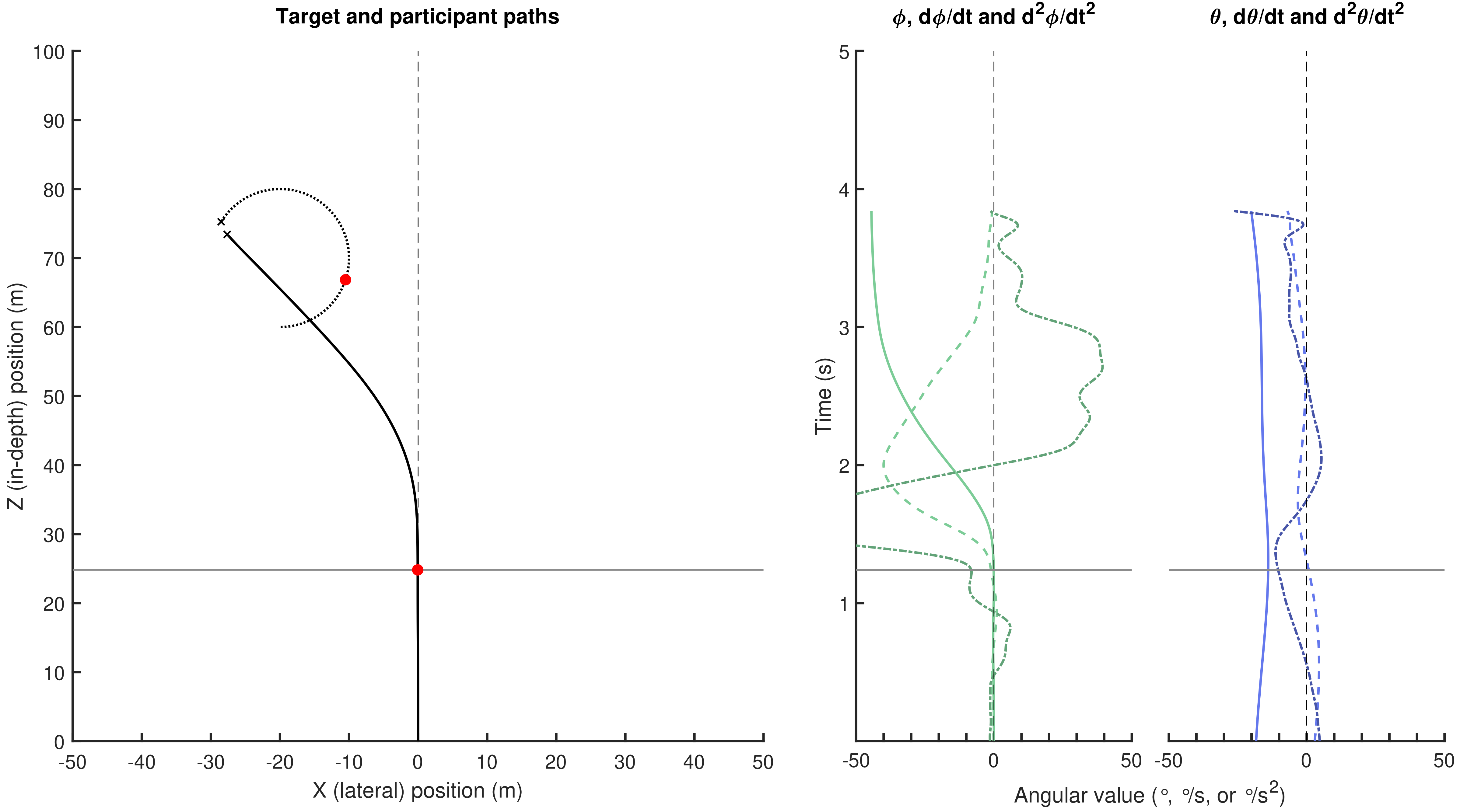

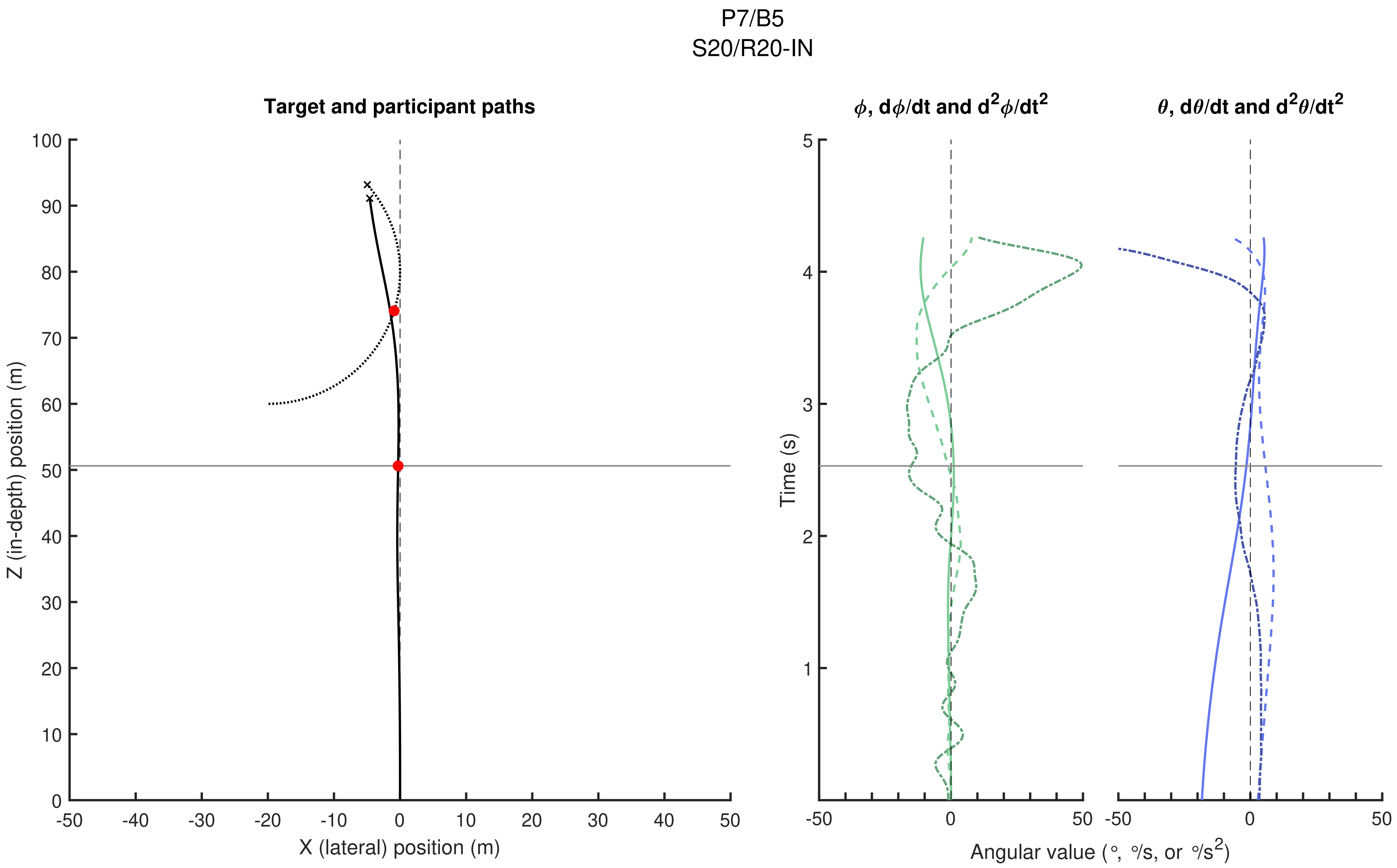

P7/B5
S20/R20-IN
Target and participant paths
Z (in-depth) position (m)
X (lateral) position (m)
ϕ, dϕ/dt and d²ϕ/dt²
θ, dθ/dt and d²θ/dt²
Time (s)
Angular value (°, °/s, or °/s²)

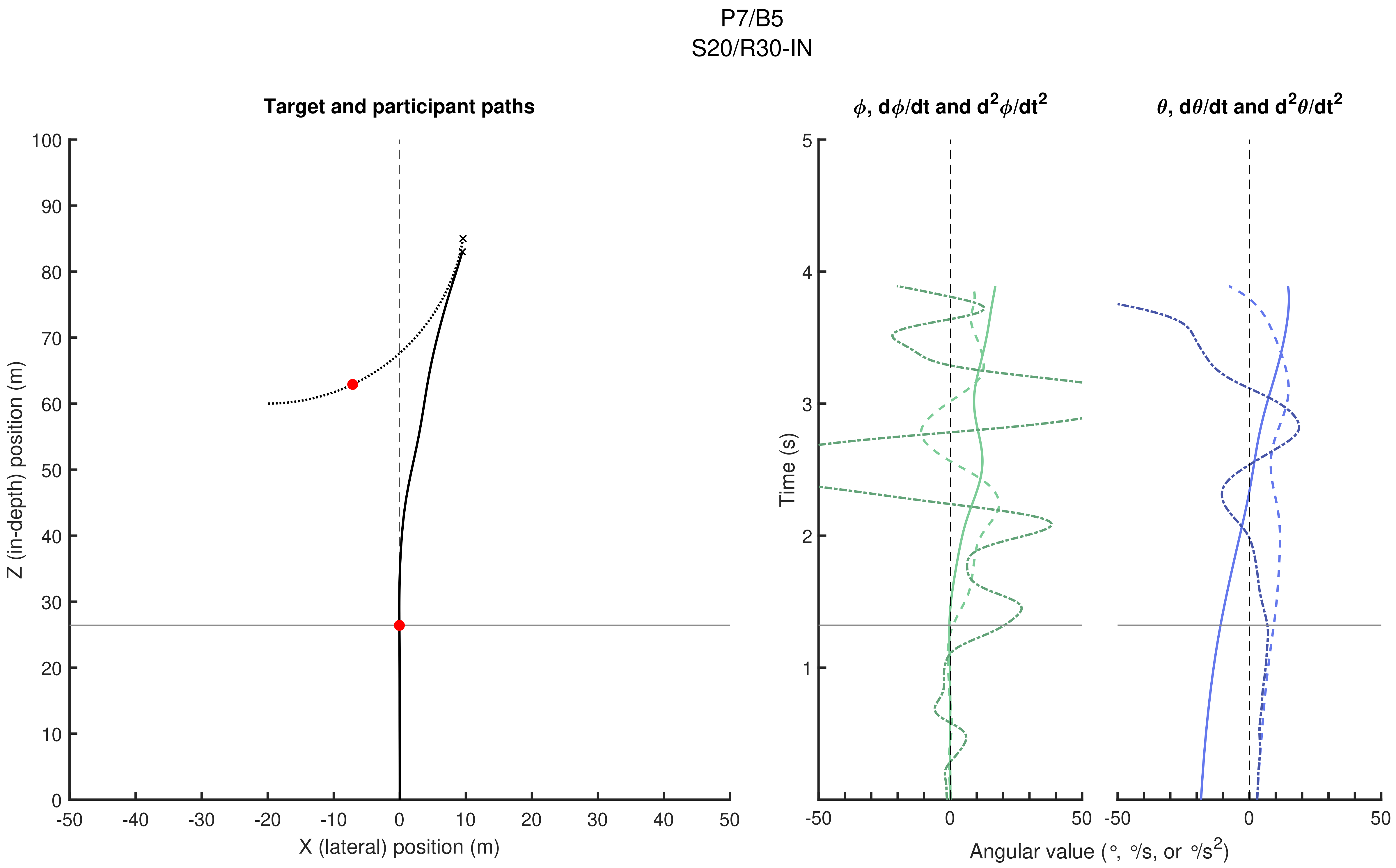

P7/B5
S20/R30-IN
Target and participant paths
Z (in-depth) position (m)
X (lateral) position (m)
ϕ, dϕ/dt and d$^2\phi$/dt^2
θ, dθ/dt and d$^2\theta$/dt^2
Time (s)
Angular value (°, °/s, or °/s^2)

P7/B5
S15/R10-IN

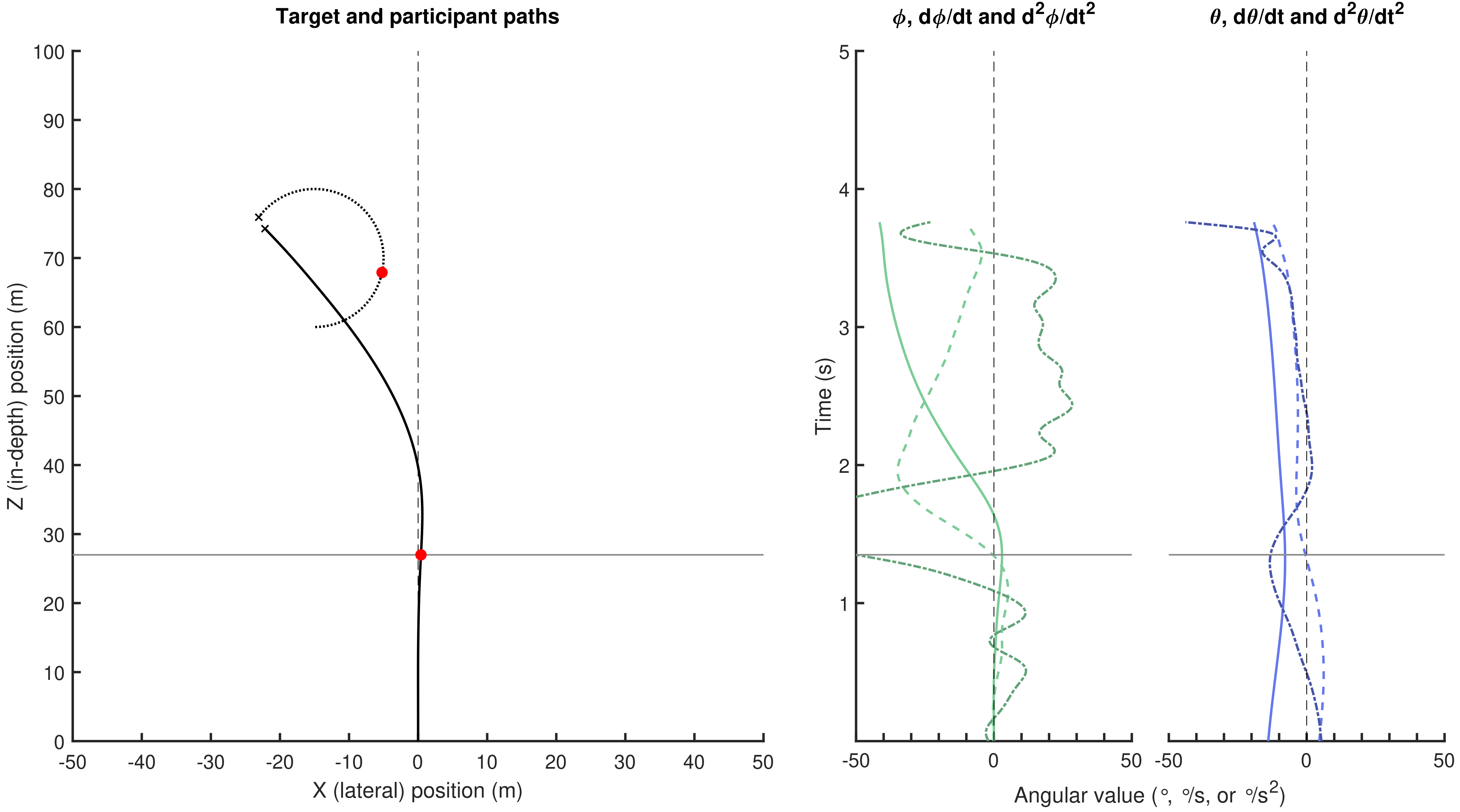

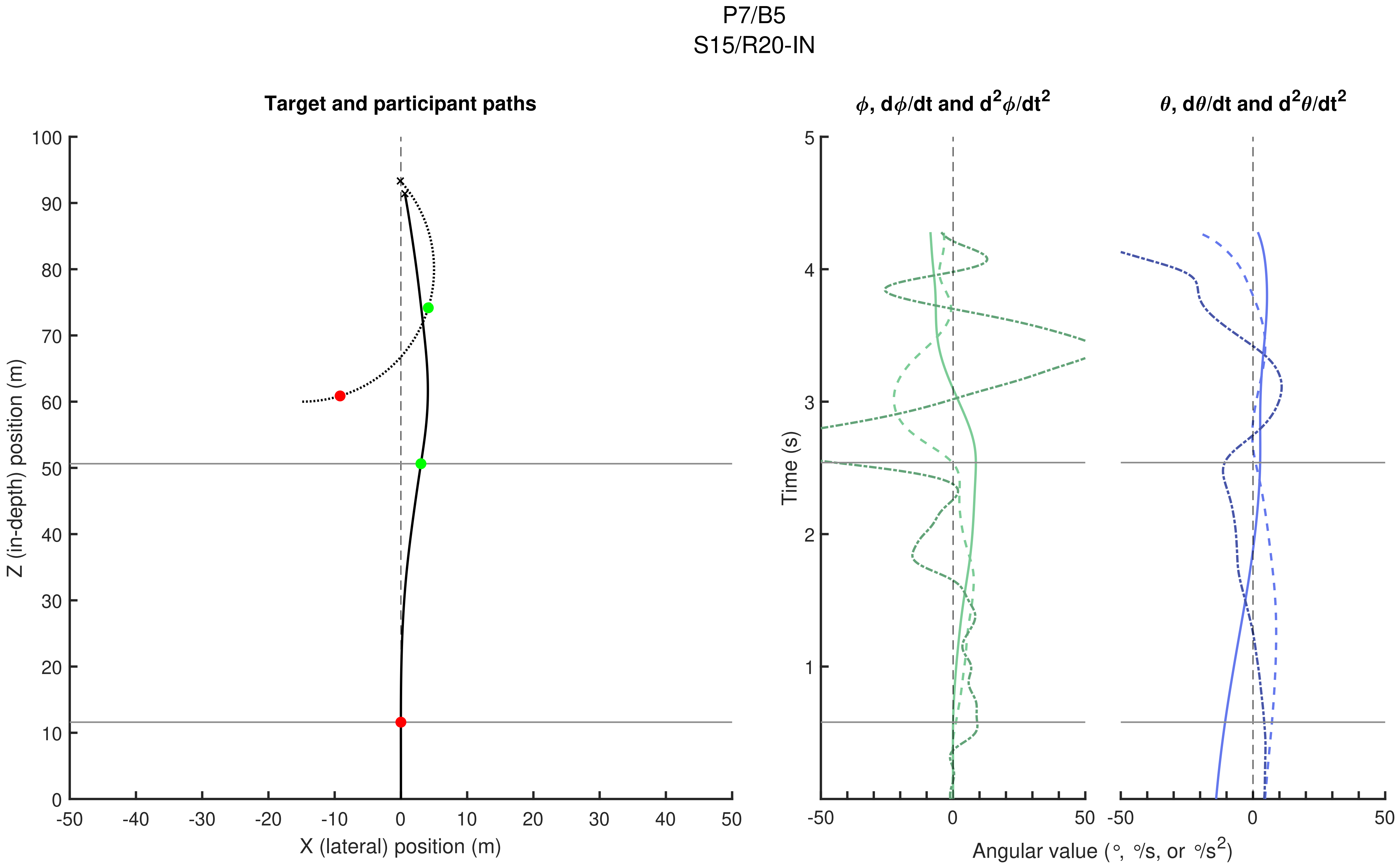
P7/B5
S15/R20-IN
Target and participant paths
Z (in-depth) position (m)
X (lateral) position (m)
ϕ, dϕ/dt and d$^2\phi$/dt^2
θ, dθ/dt and d$^2\theta$/dt^2
Time (s)
Angular value (°, °/s, or °/s^2)

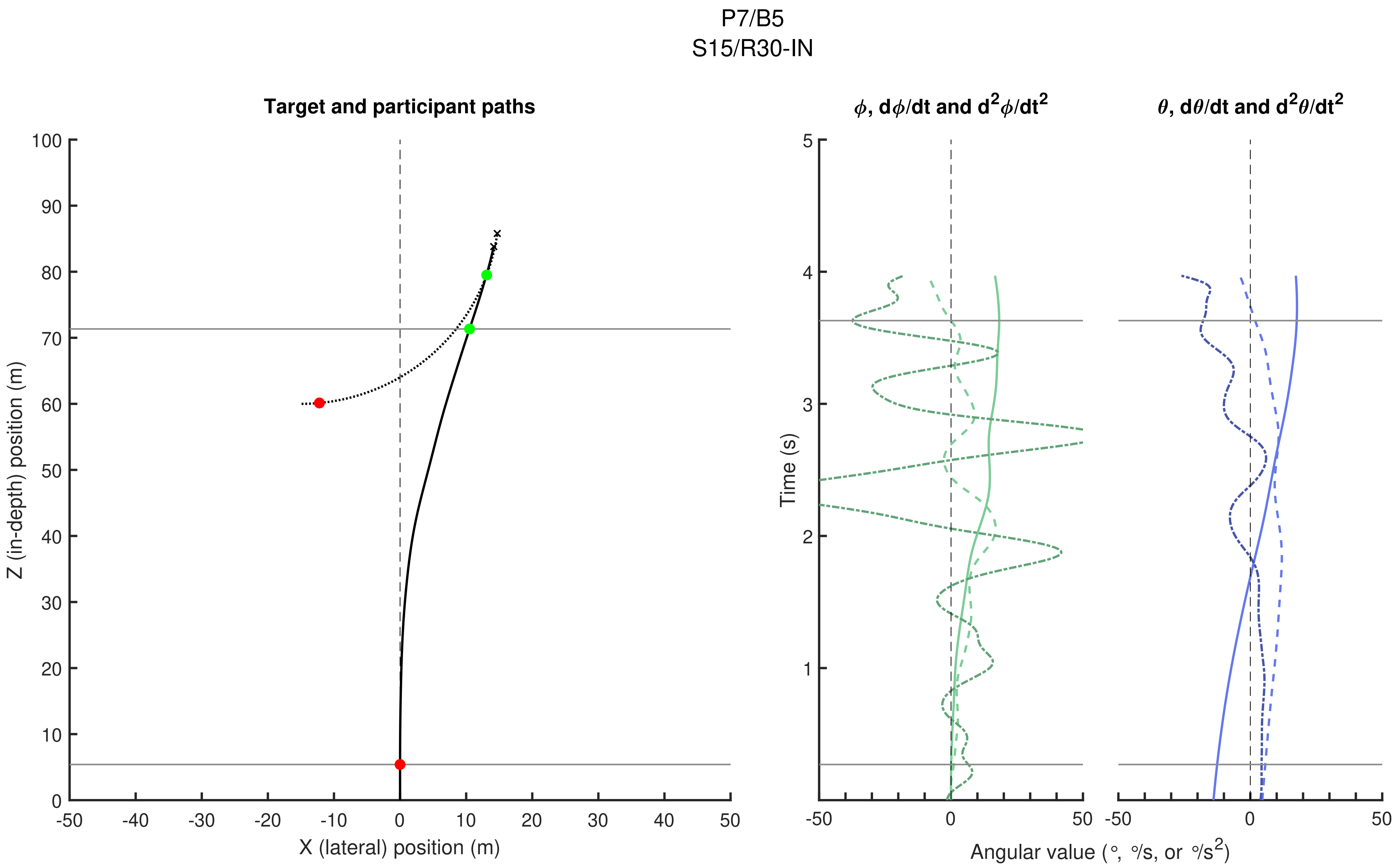

P7/B5
S15/R30-IN
Target and participant paths
Z (in-depth) position (m)
X (lateral) position (m)
φ, dφ/dt and d²φ/dt²
θ, dθ/dt and d²θ/dt²
Time (s)
Angular value (°, °/s, or °/s²)

P7/B5
S5/R10-IN

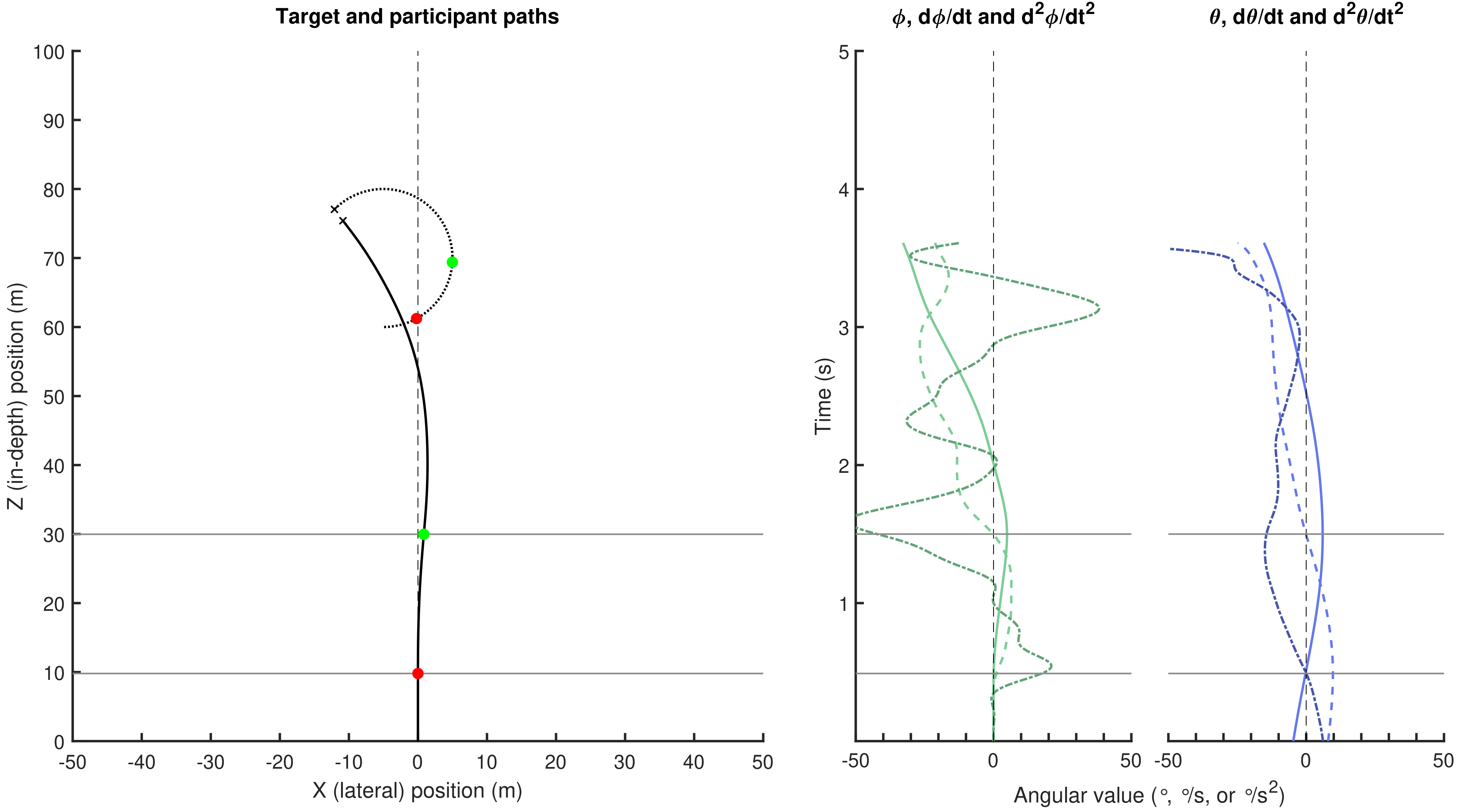

P7/B5
S5/R10-OUT

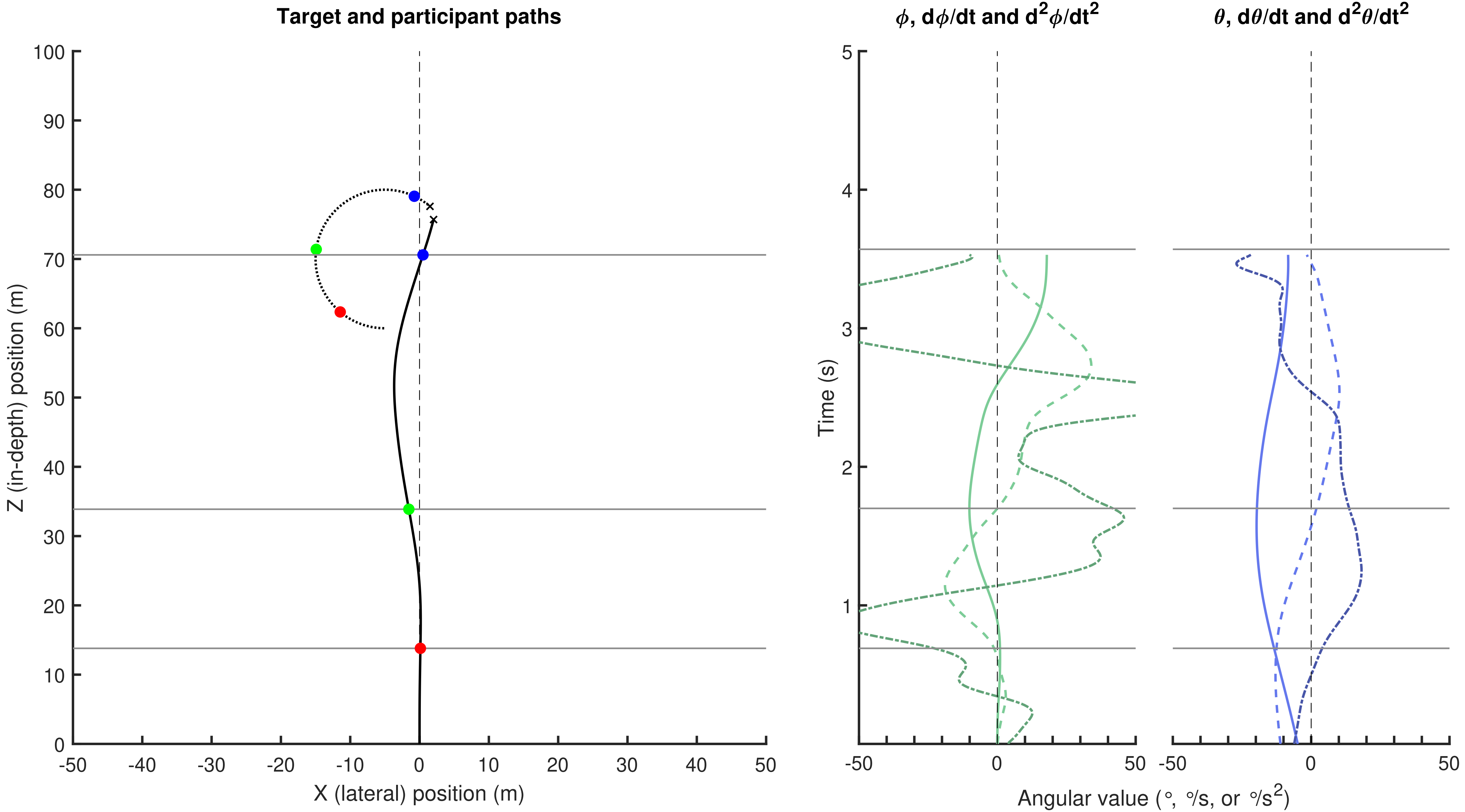

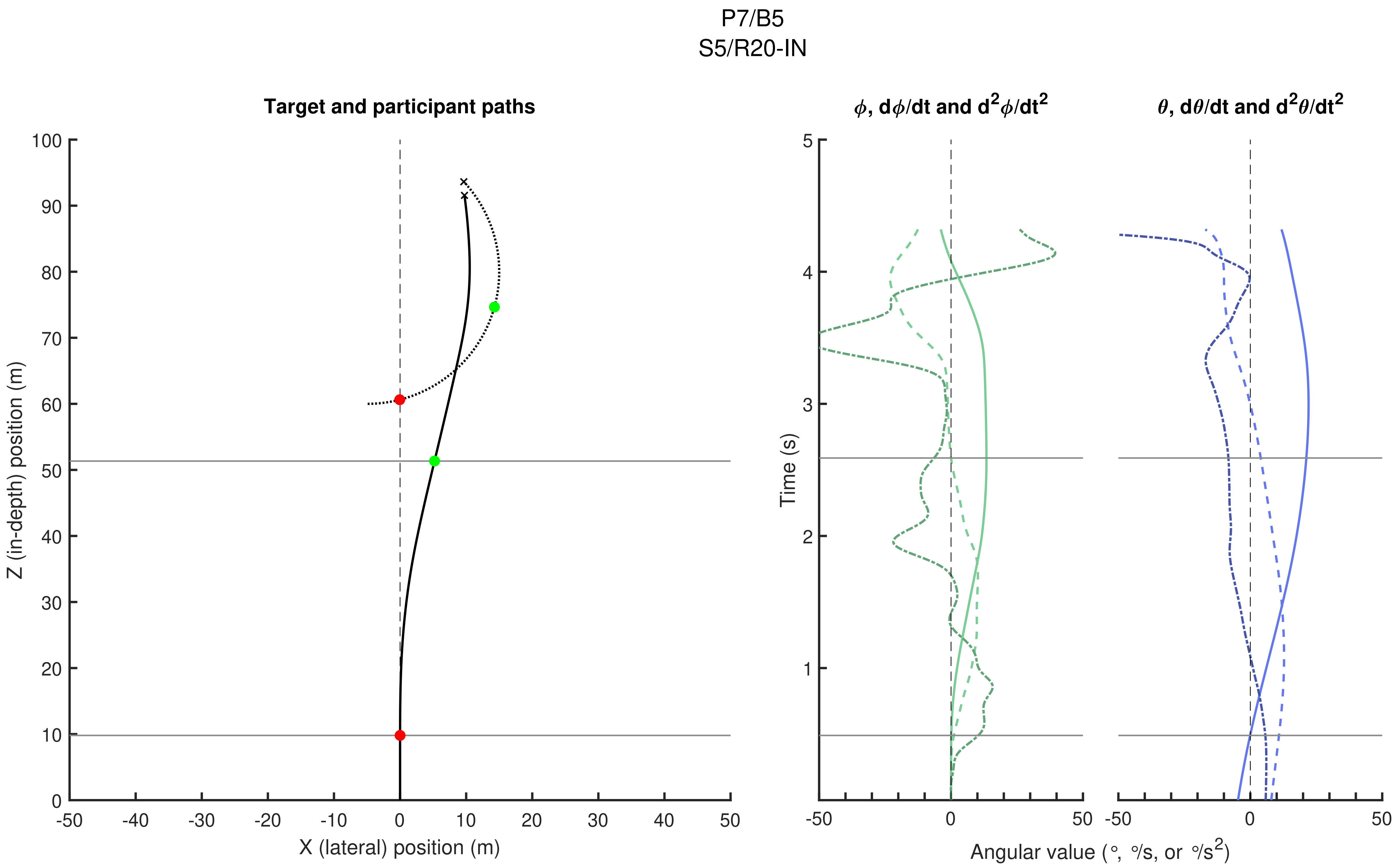
P7/B5
S5/R20-IN
Target and participant paths
Z (in-depth) position (m)
X (lateral) position (m)
0
10
20
30
40
50
60
70
80
90
100
-50
-40
-30
-20
-10
0
10
20
30
40
50
ϕ, dϕ/dt and d$^2\phi$/dt^2
θ, dθ/dt and d$^2\theta$/dt^2
Time (s)
1
2
3
4
5
-50
0
50
-50
0
50
Angular value (°, °/s, or °/s^2)

P7/B5
S5/R20-OUT

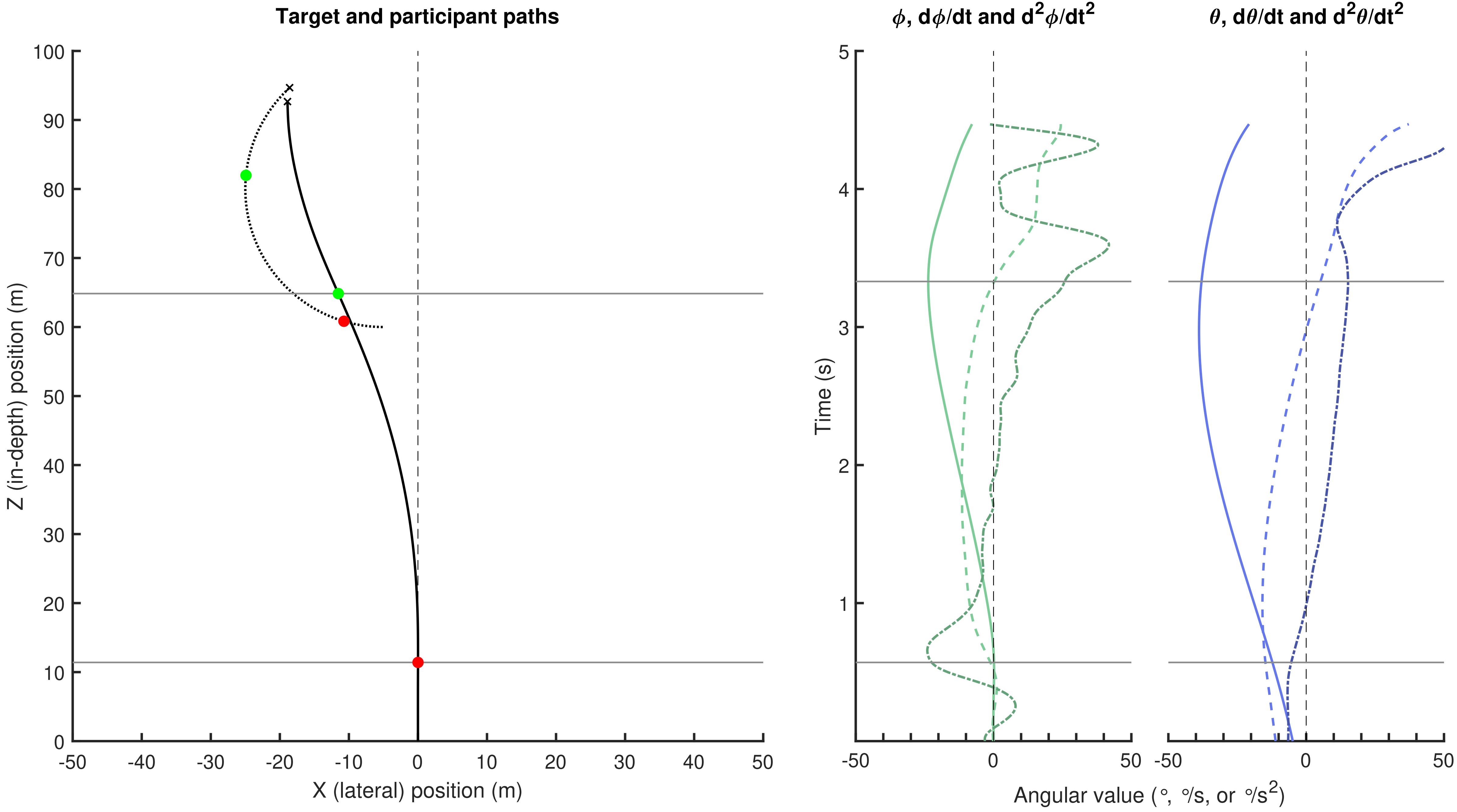

P7/B5
S5/R30-IN

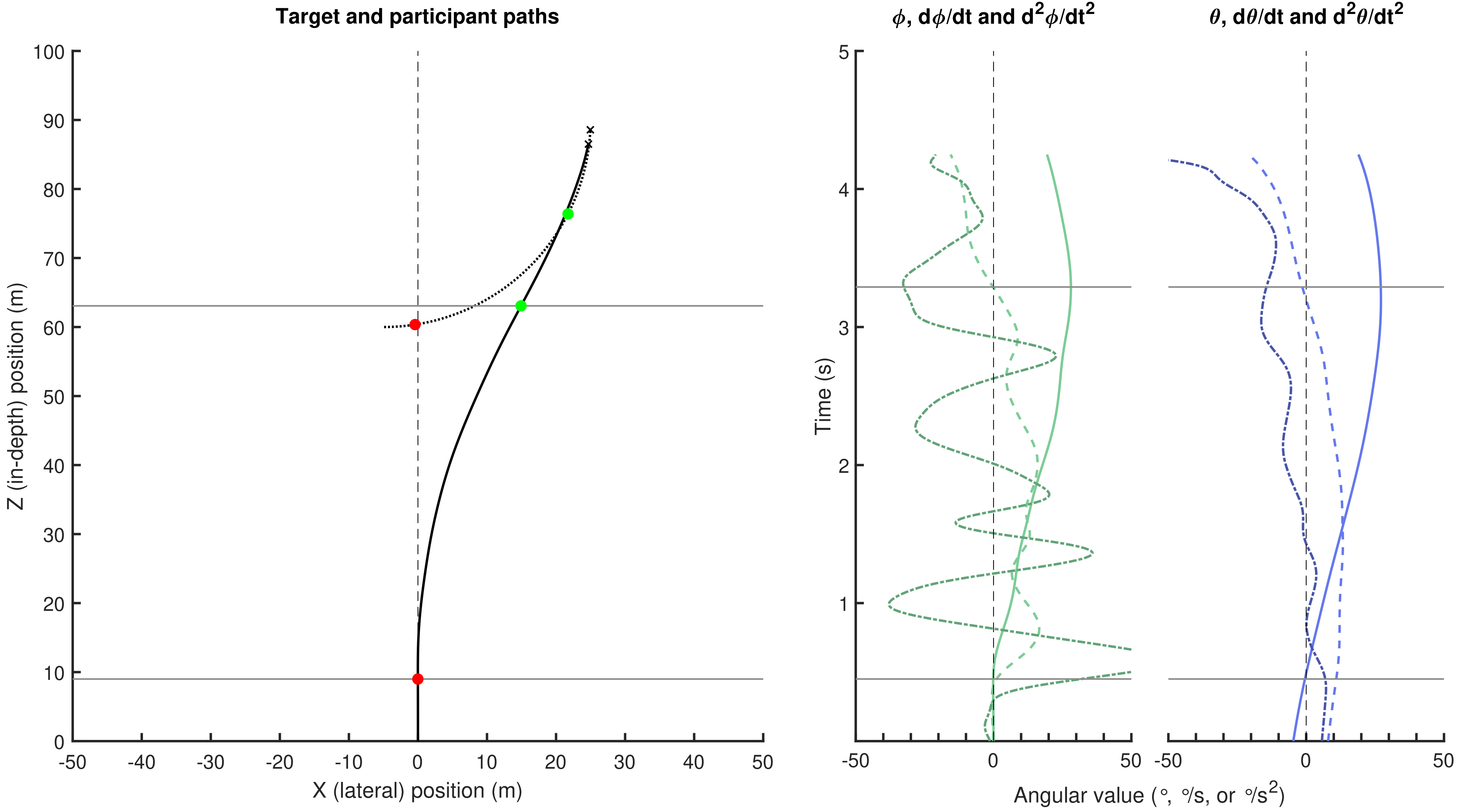

P7/B5
S5/R30-OUT

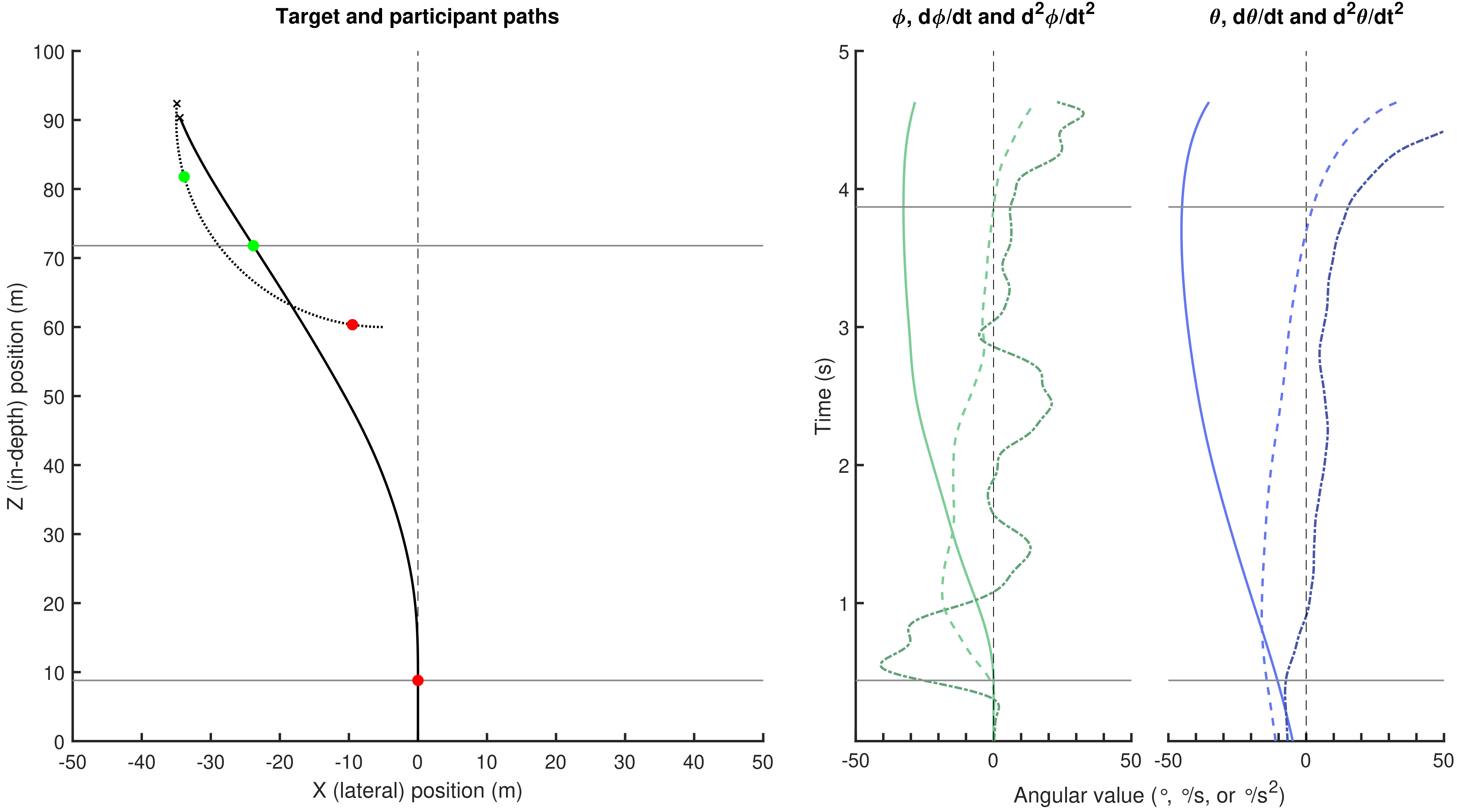

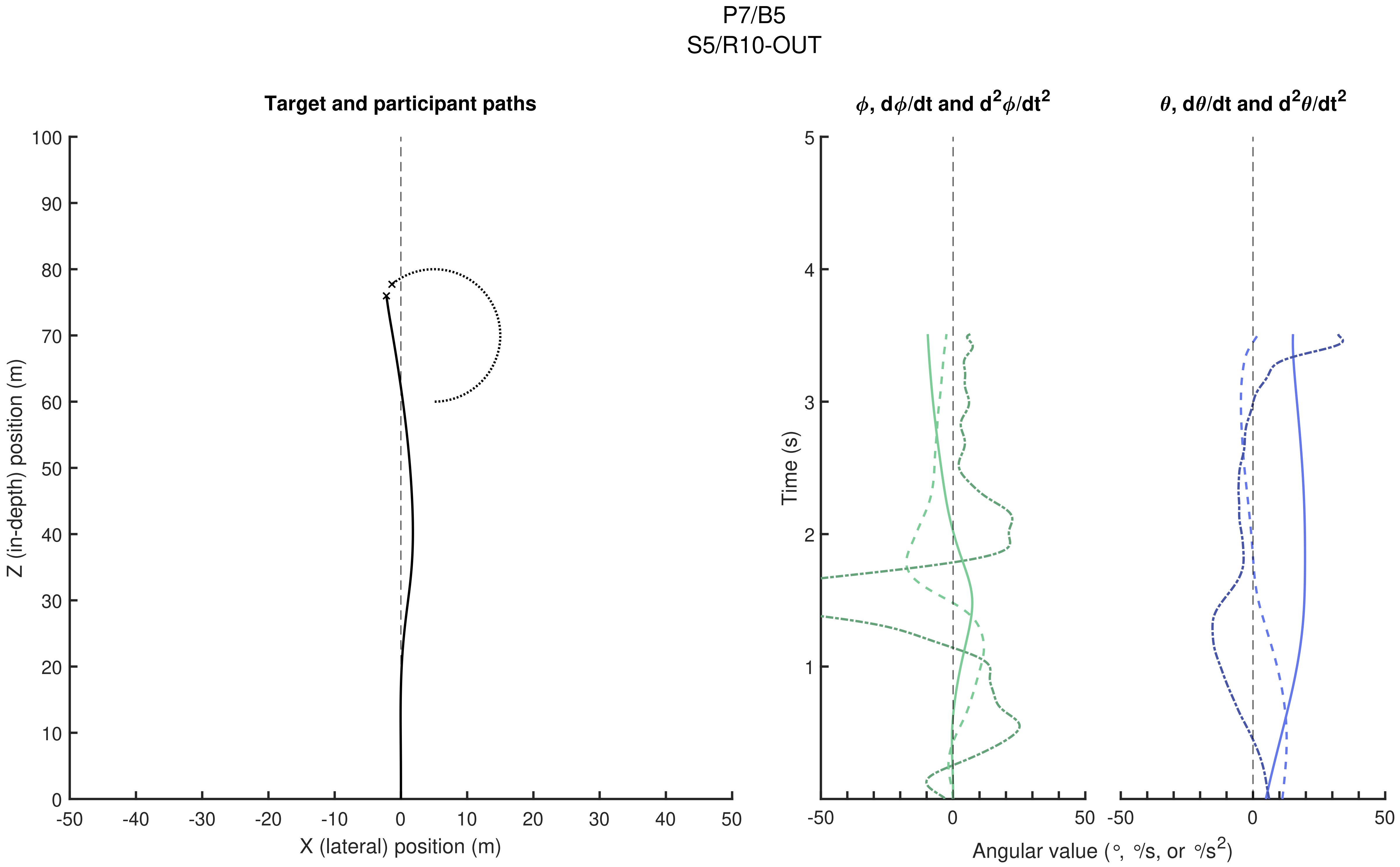
P7/B5
S5/R10-OUT
Target and participant paths
Z (in-depth) position (m)
X (lateral) position (m)
100
90
80
70
60
50
40
30
20
10
0
-50
-40
-30
-20
-10
0
10
20
30
40
50
ϕ, dϕ/dt and d$^2\phi$/dt^2
θ, dθ/dt and d$^2\theta$/dt^2
Time (s)
5
4
3
2
1
-50
0
50
-50
0
50
Angular value (°, °/s, or °/s^2)

P7/B5
S5/R10-IN

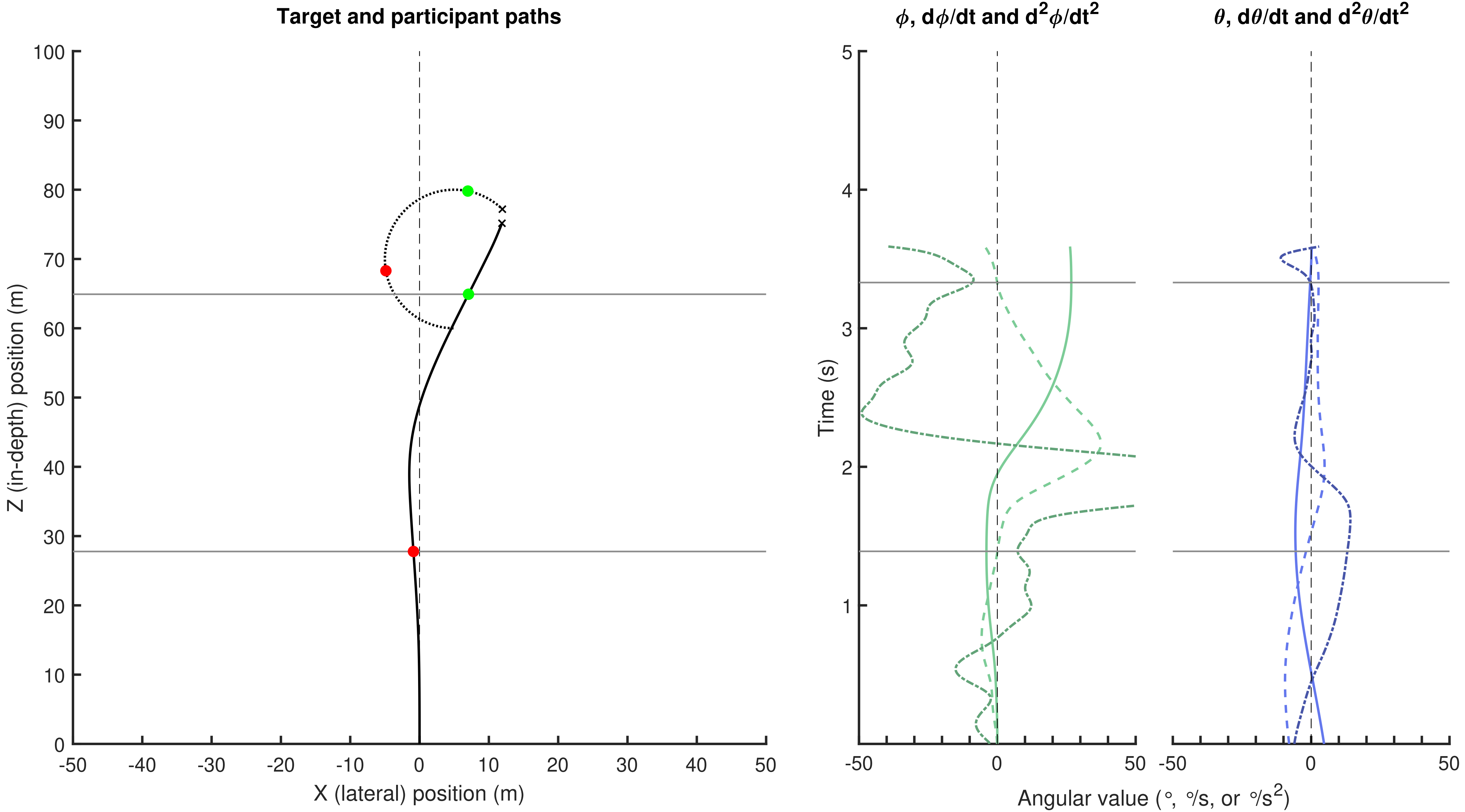

P7/B5
S5/R20-OUT

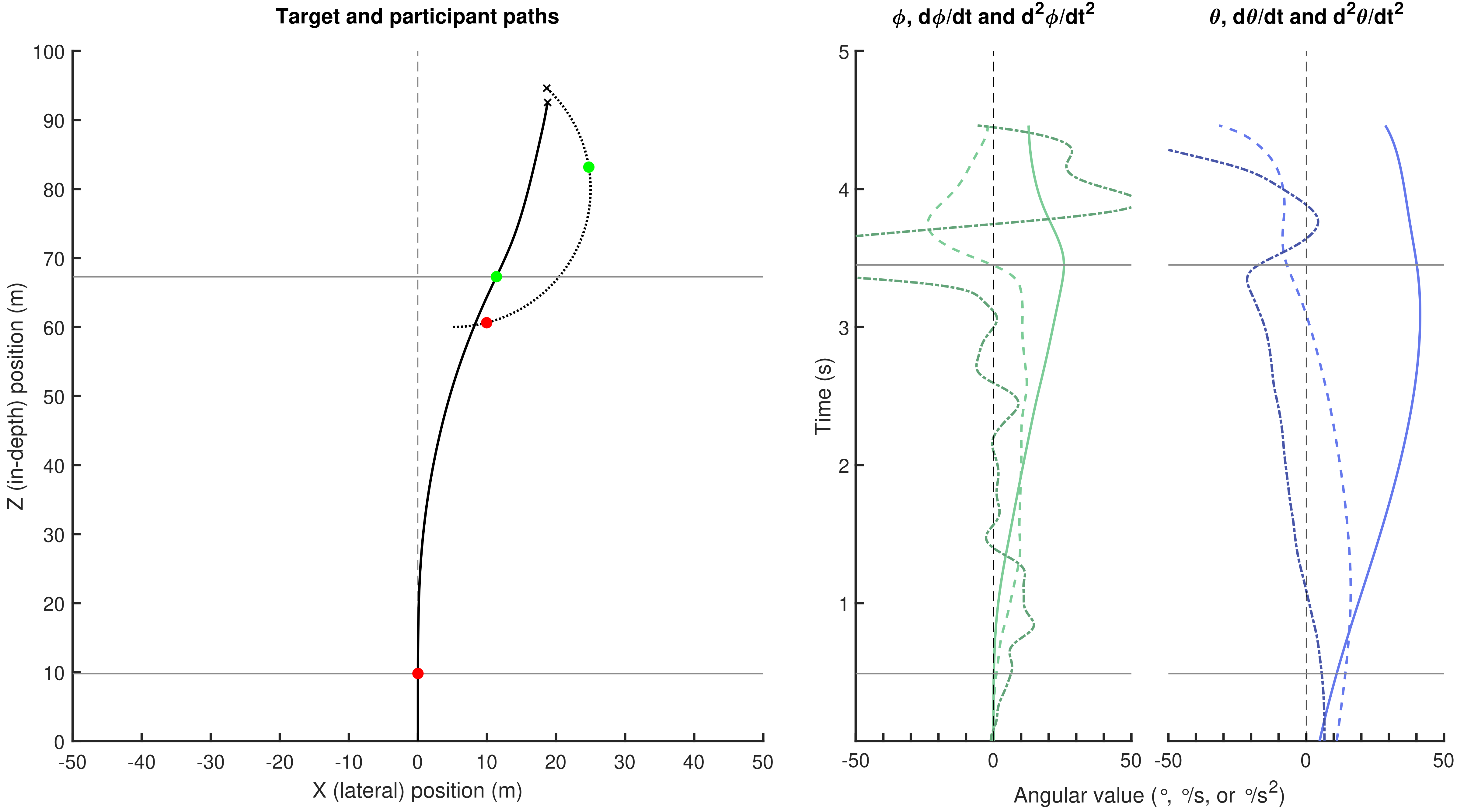

P7/B5
S5/R20-IN

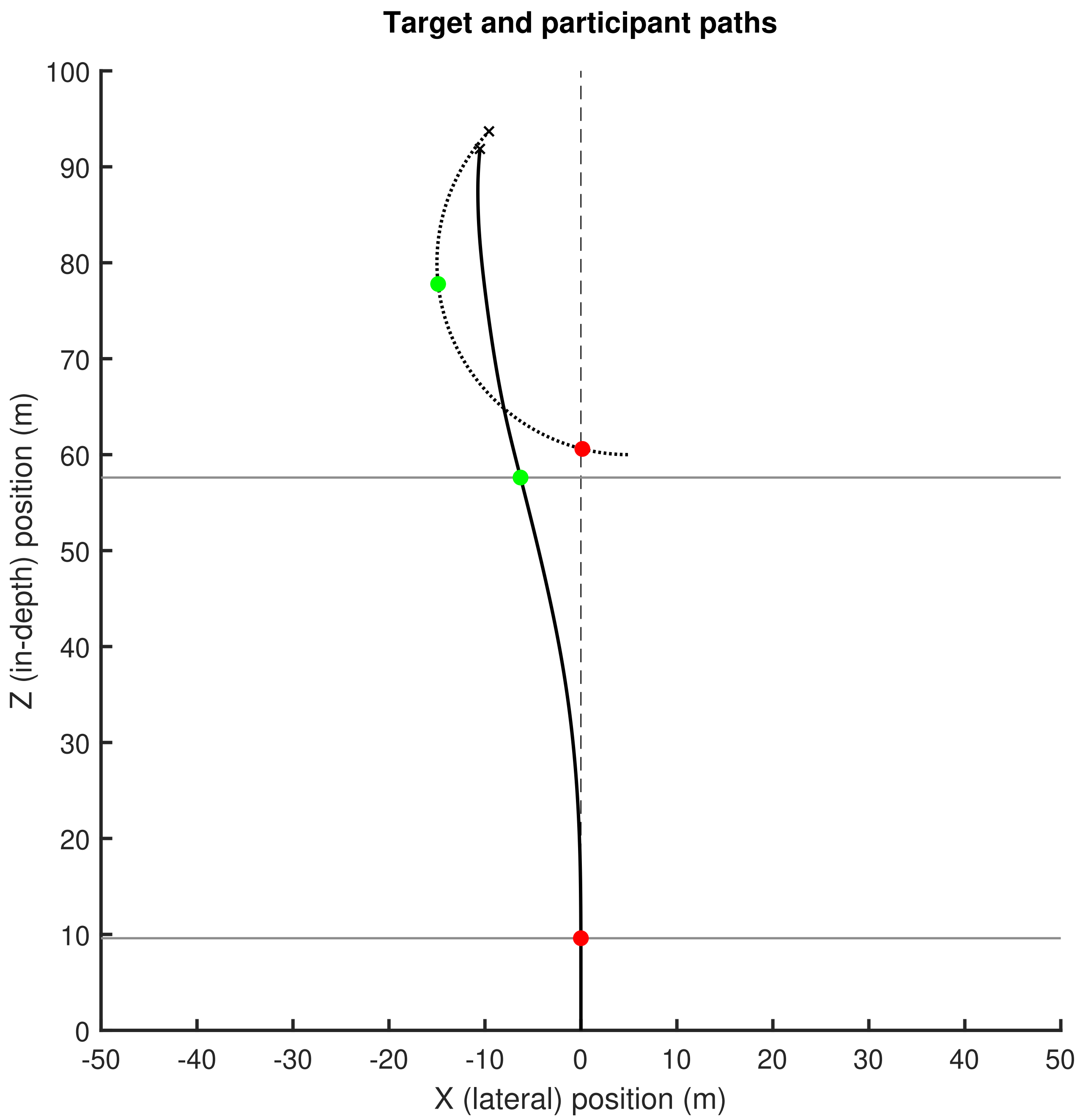

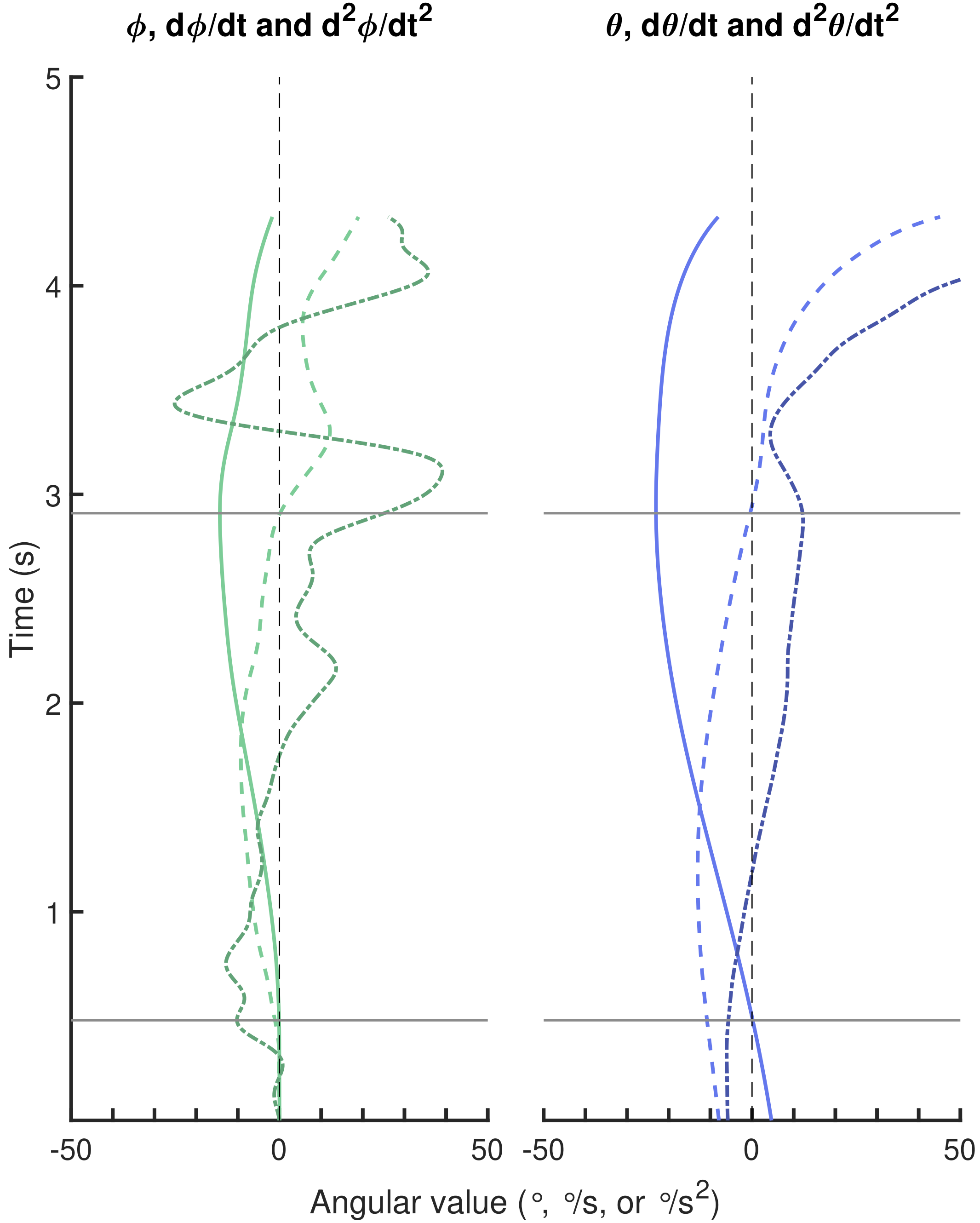

P7/B5
S5/R30-OUT

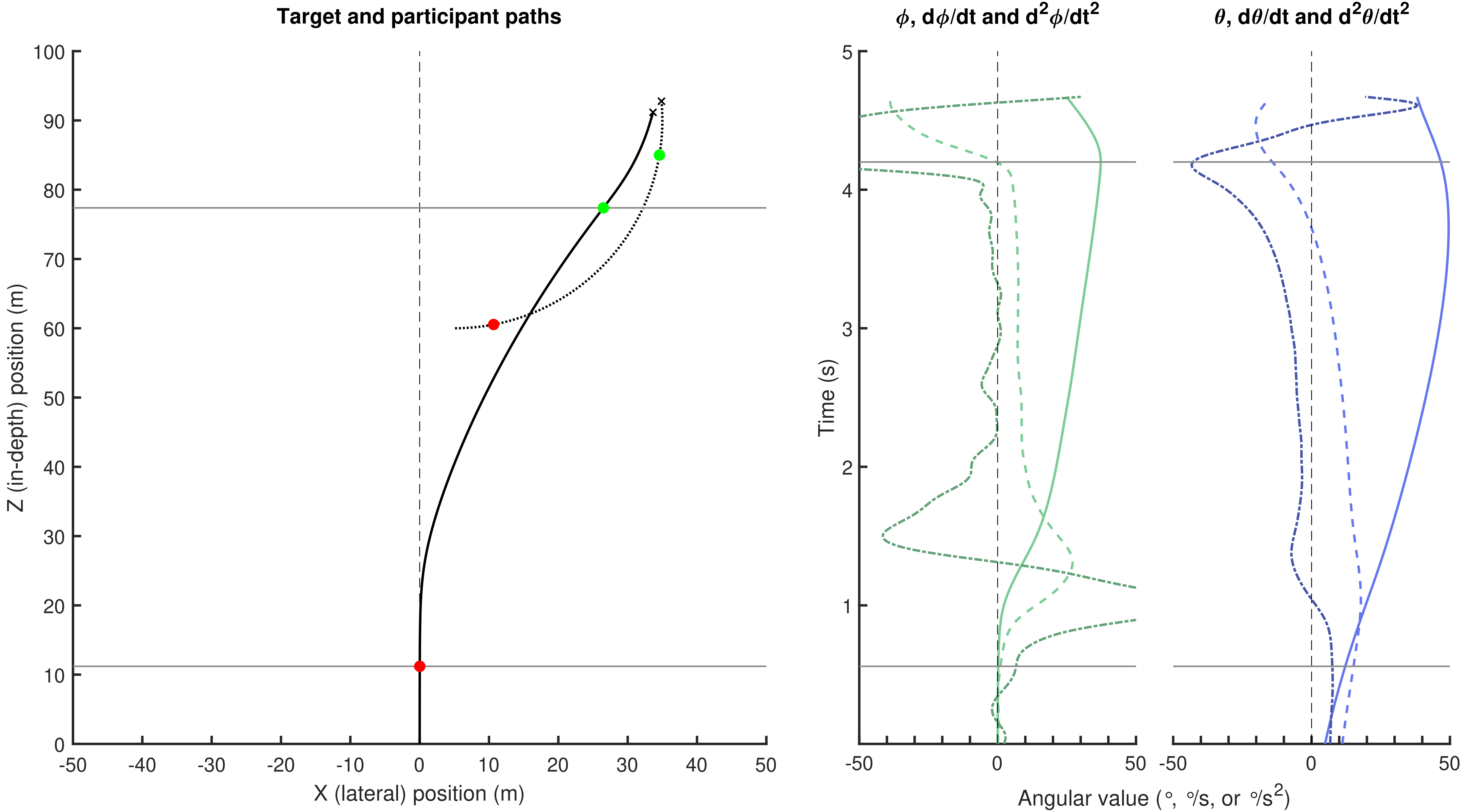

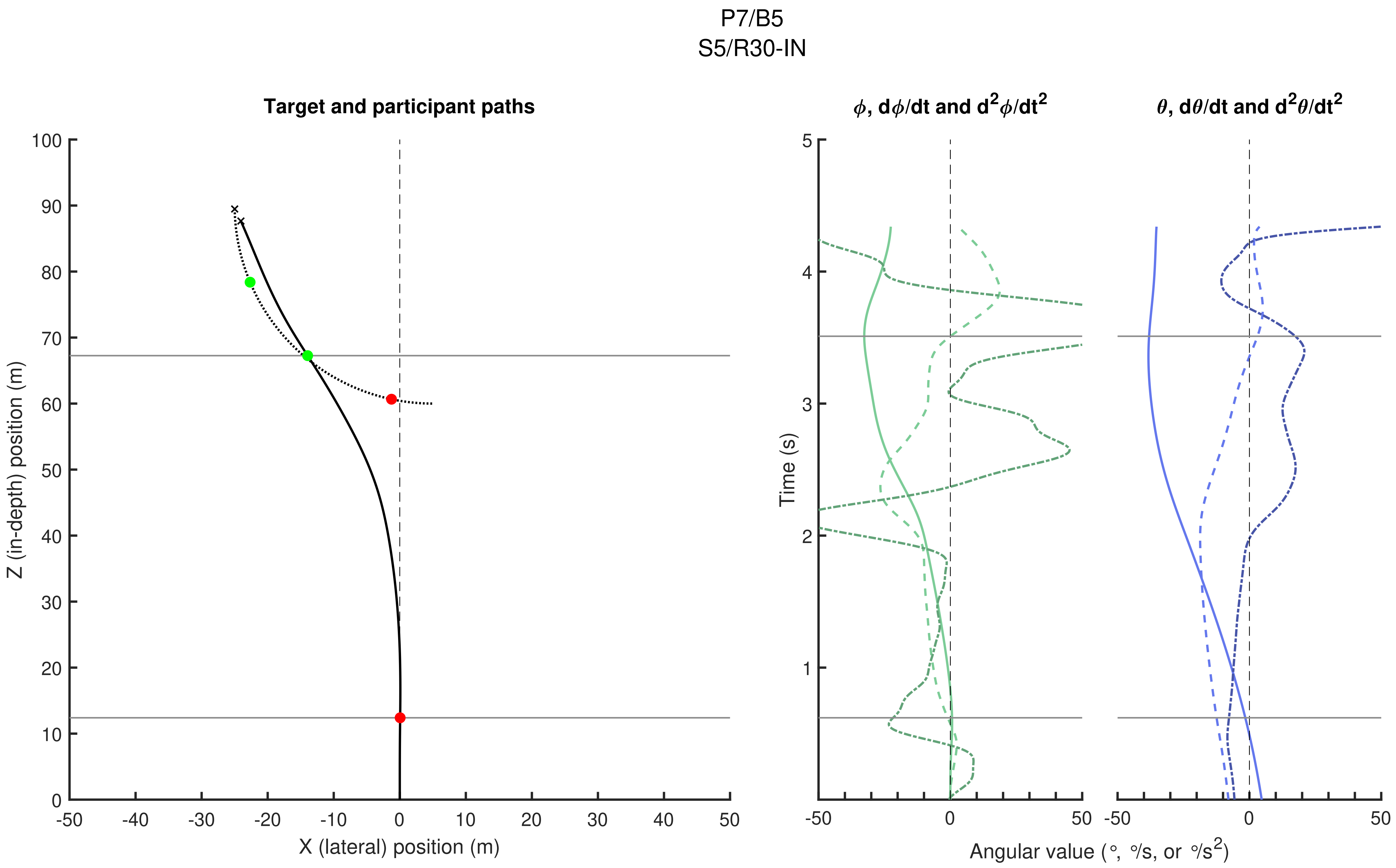
P7/B5
S5/R30-IN
Target and participant paths
Z (in-depth) position (m)
X (lateral) position (m)
100
90
80
70
60
50
40
30
20
10
0
-50
-40
-30
-20
-10
0
10
20
30
40
50
ϕ, dϕ/dt and d²ϕ/dt²
θ, dθ/dt and d²θ/dt²
Time (s)
5
4
3
2
1
-50
0
50
-50
0
50
Angular value (°, °/s, or °/s²)

P7/B5
S15/R10-IN

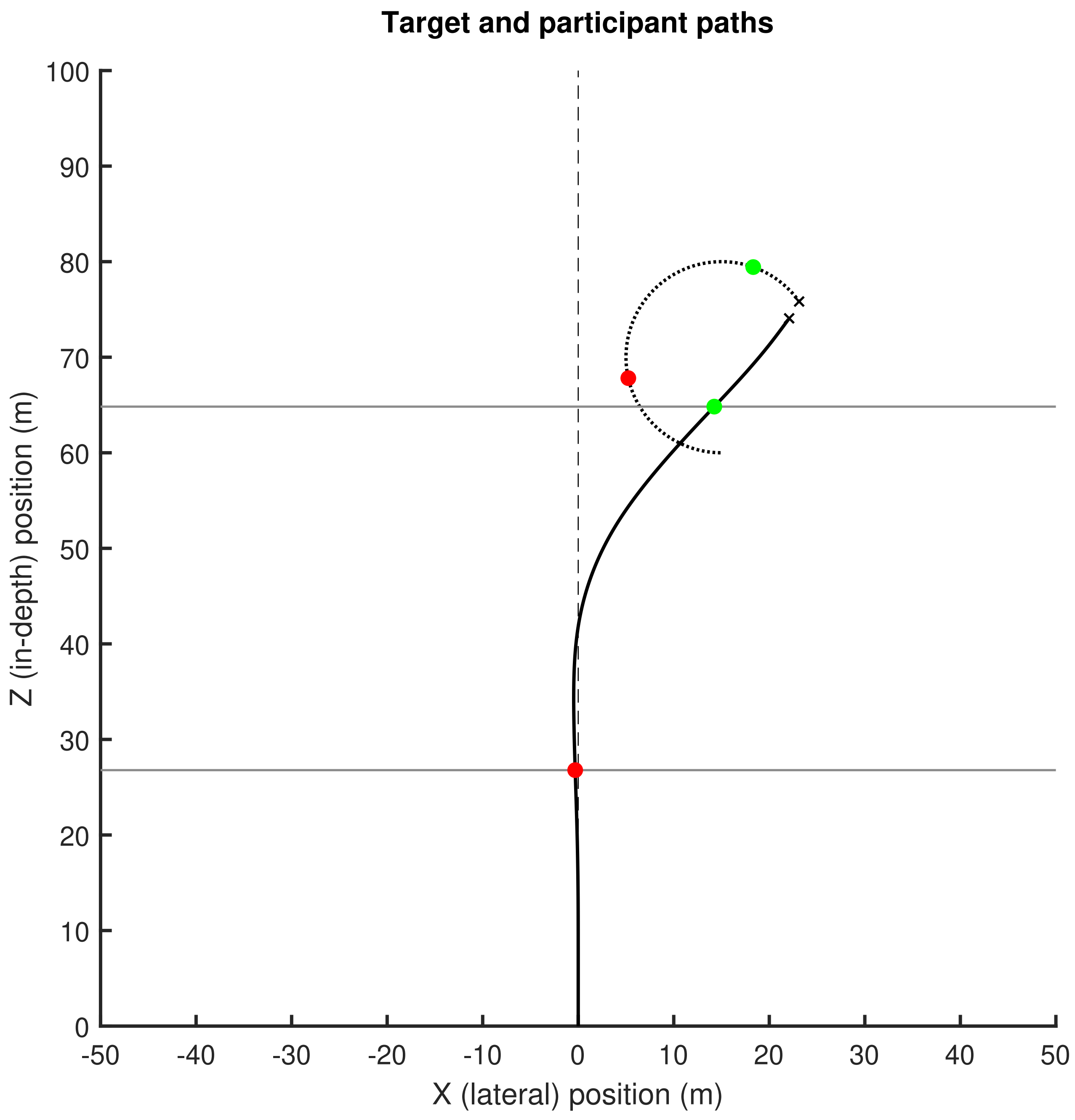

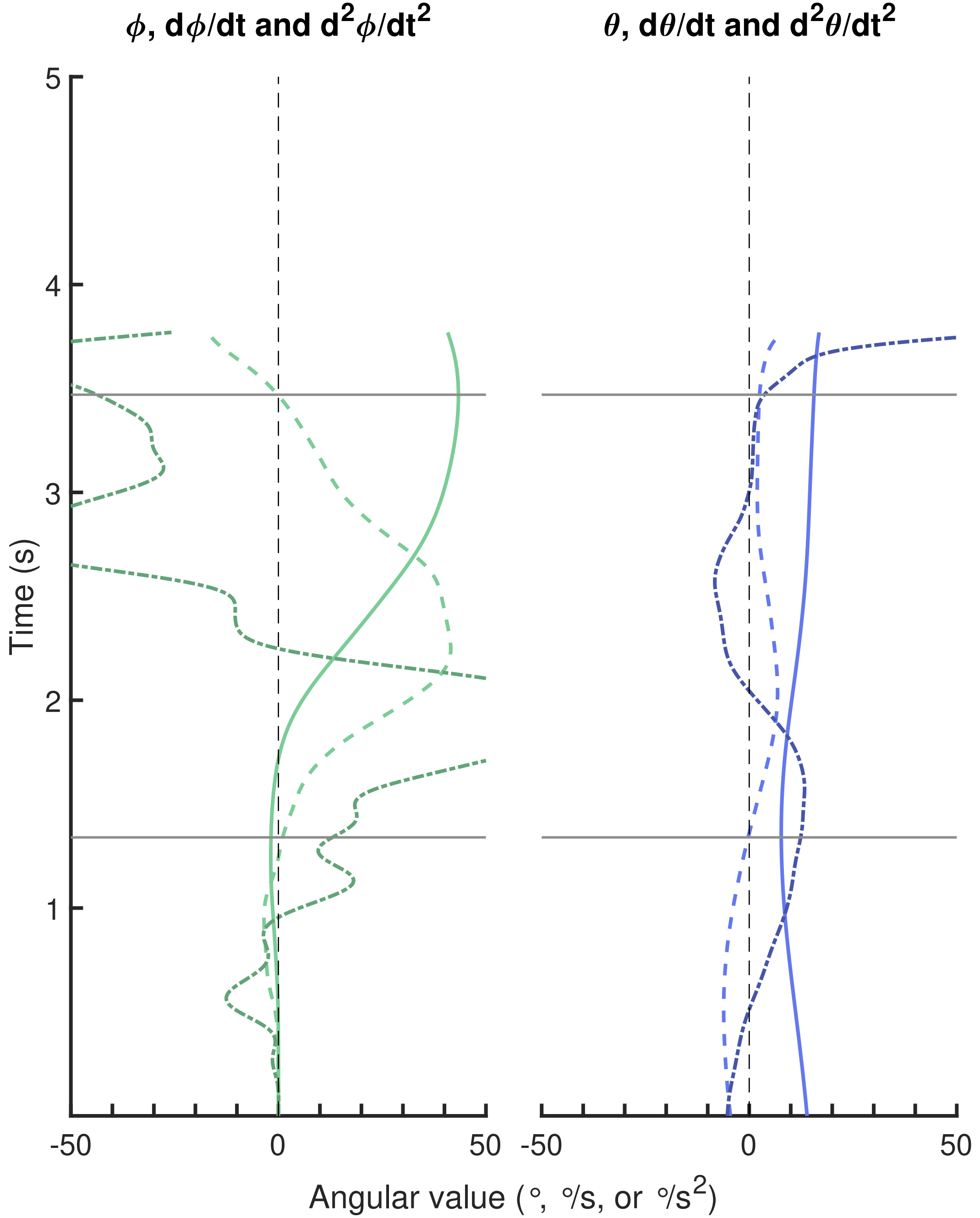

P7/B5
S15/R20-IN

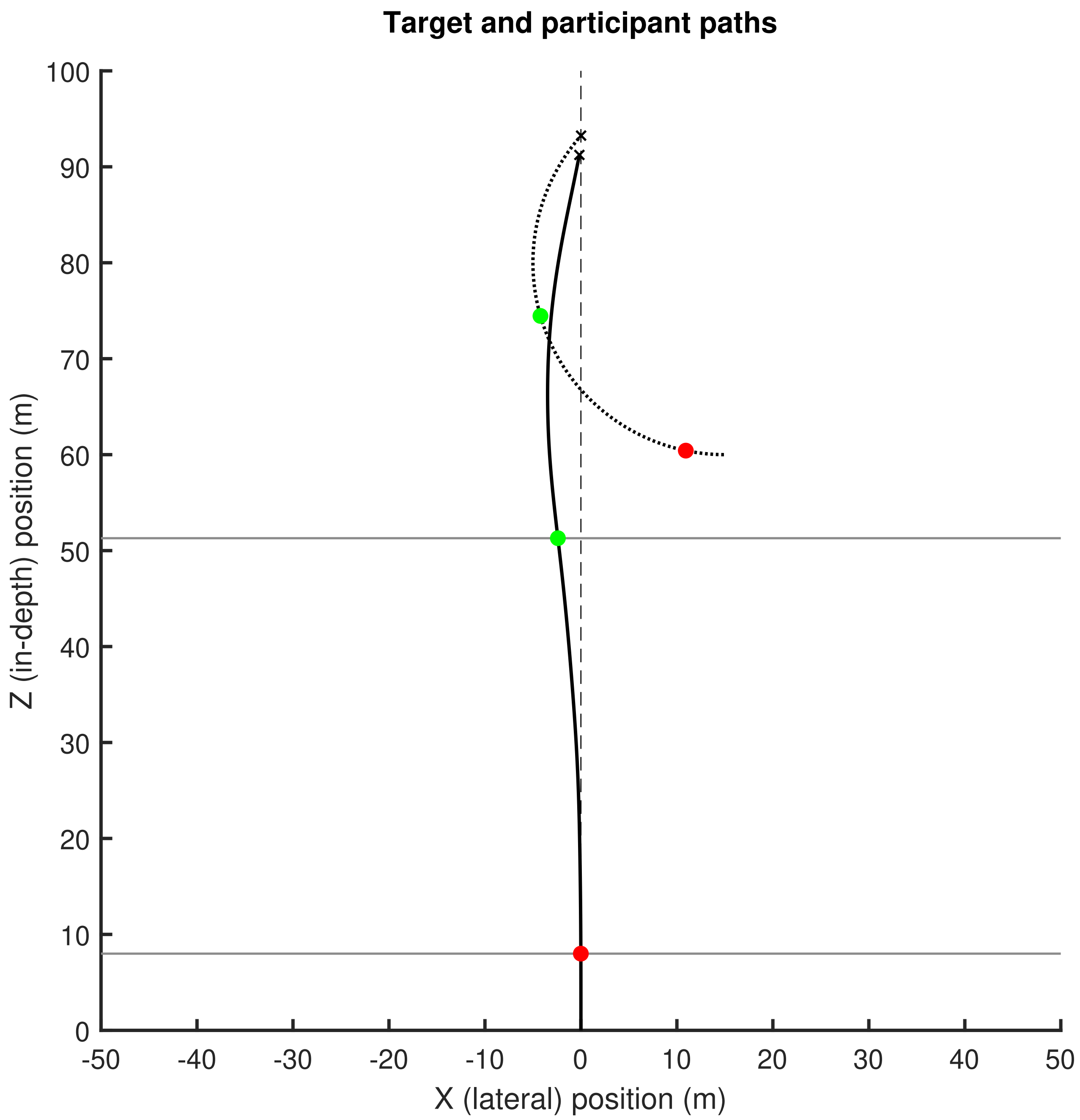

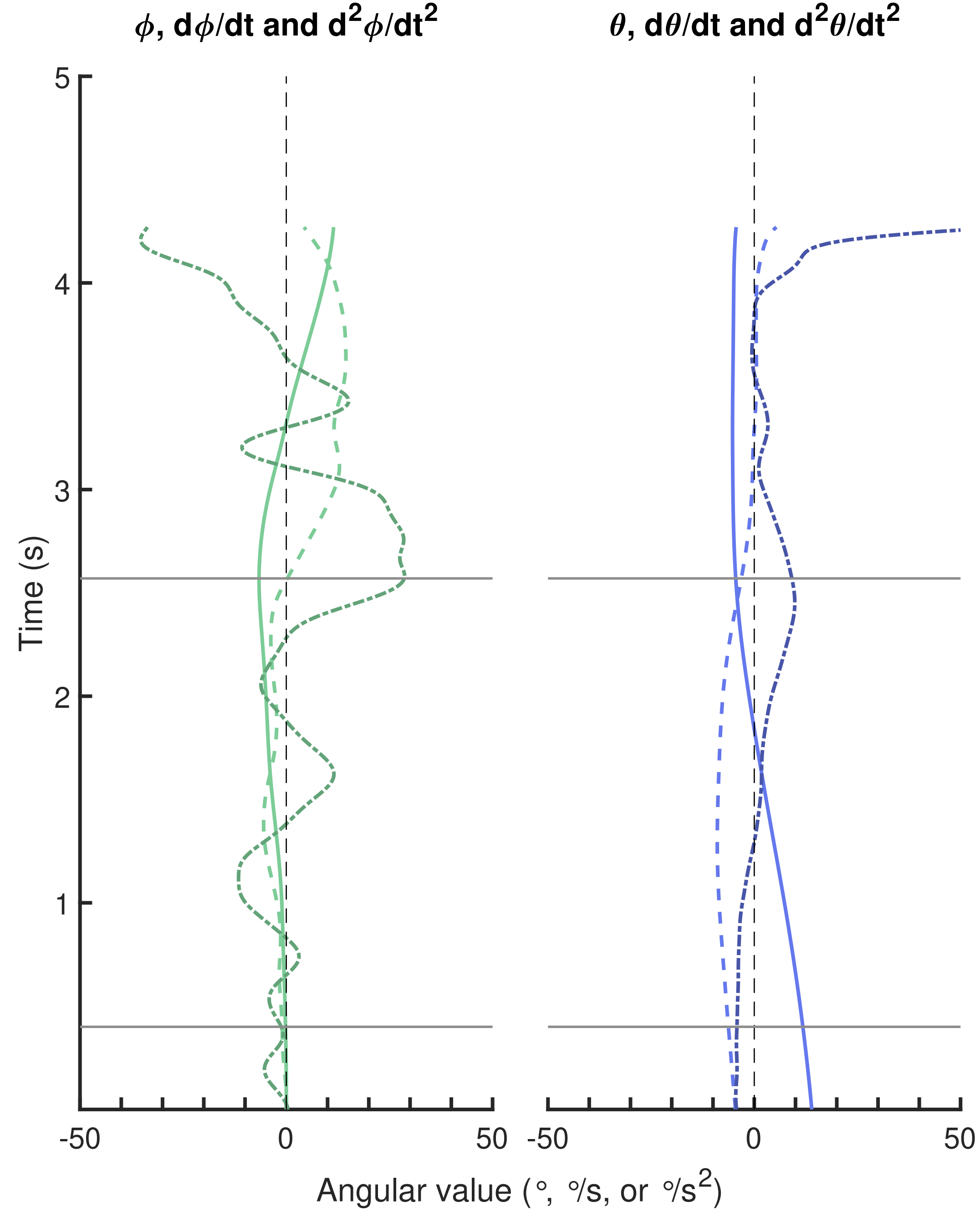

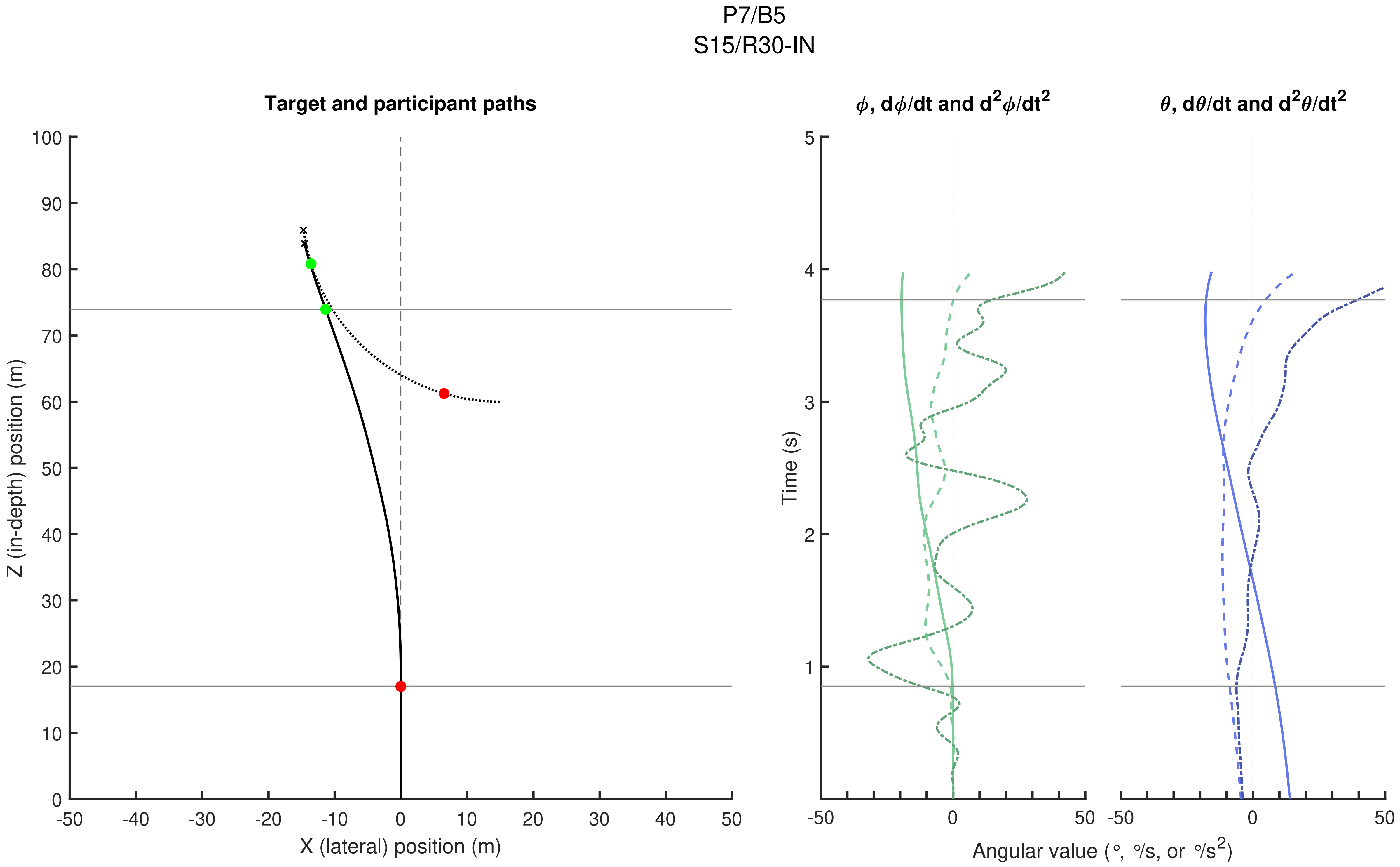
P7/B5
S15/R30-IN
Target and participant paths
Z (in-depth) position (m)
X (lateral) position (m)
φ, dφ/dt and d²φ/dt²
θ, dθ/dt and d²θ/dt²
Time (s)
Angular value (°, °/s, or °/s²)

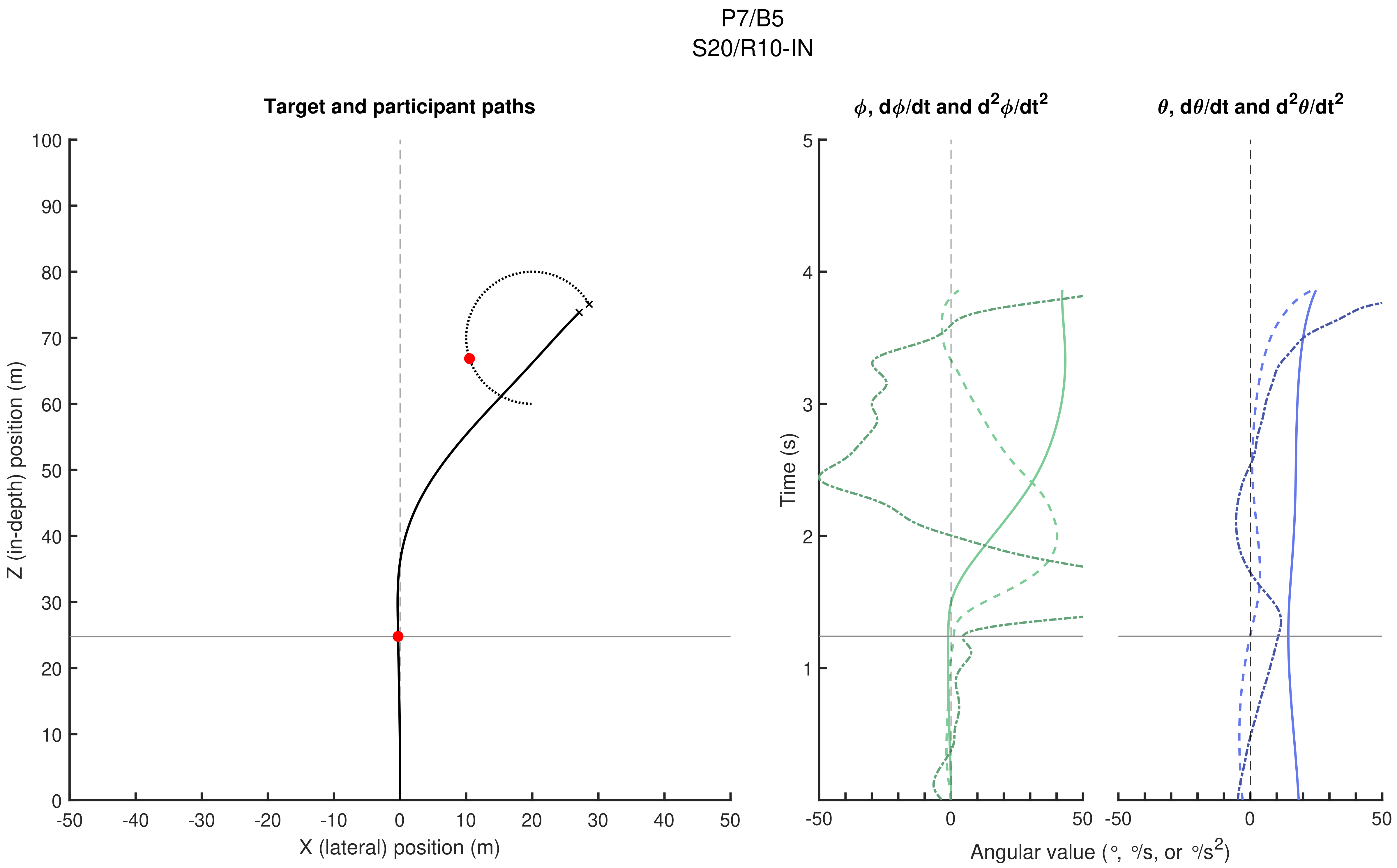
P7/B5
S20/R10-IN
Target and participant paths
Z (in-depth) position (m)
X (lateral) position (m)
0
10
20
30
40
50
60
70
80
90
100
-50
-40
-30
-20
-10
0
10
20
30
40
50
ϕ, dϕ/dt and d²ϕ/dt²
θ, dθ/dt and d²θ/dt²
Time (s)
1
2
3
4
5
-50
0
50
-50
0
50
Angular value (°, °/s, or °/s²)

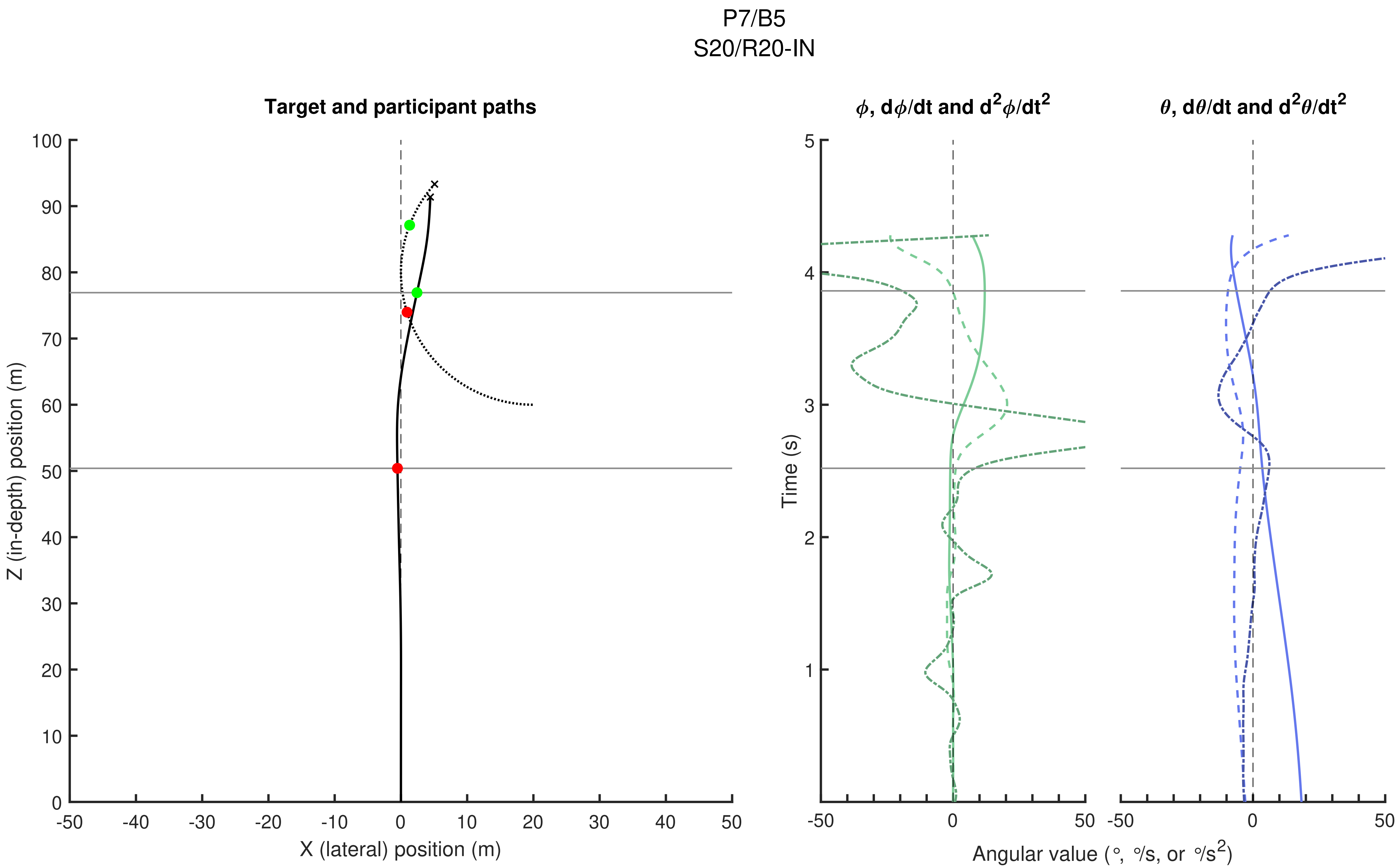
P7/B5
S20/R20-IN
Target and participant paths
Z (in-depth) position (m)
X (lateral) position (m)
100
90
80
70
60
50
40
30
20
10
0
-50
-40
-30
-20
-10
0
10
20
30
40
50
ϕ, dϕ/dt and d²ϕ/dt²
θ, dθ/dt and d²θ/dt²
Time (s)
5
4
3
2
1
-50
0
50
-50
0
50
Angular value (°, °/s, or °/s²)

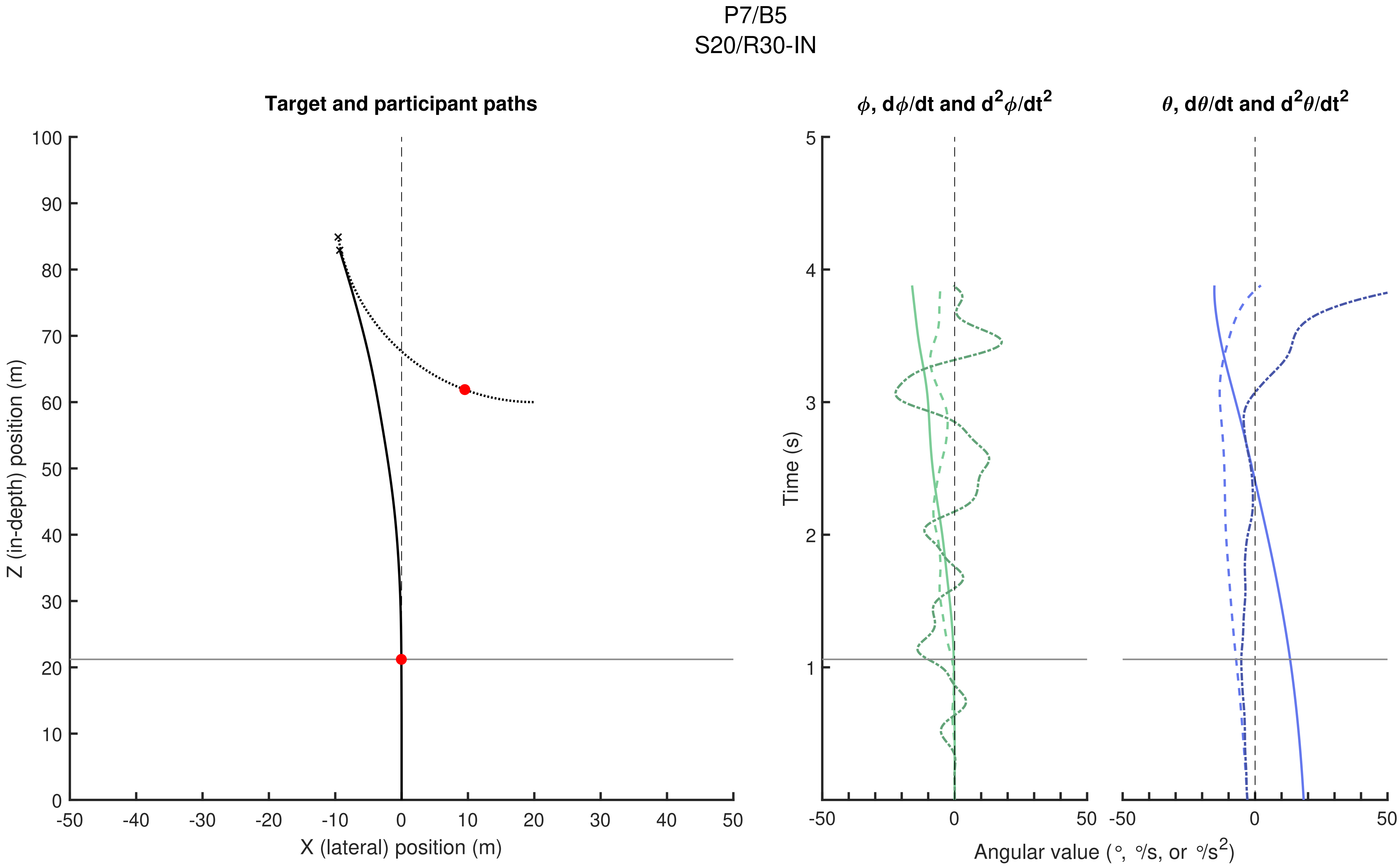

P7/B5
S20/R30-IN
Target and participant paths
Z (in-depth) position (m)
100
90
80
70
60
50
40
30
20
10
0
-50
-40
-30
-20
-10
0
10
20
30
40
50
X (lateral) position (m)
φ, dφ/dt and d²φ/dt²
θ, dθ/dt and d²θ/dt²
Time (s)
5
4
3
2
1
-50
0
50
-50
0
50
Angular value (°, °/s, or °/s²)

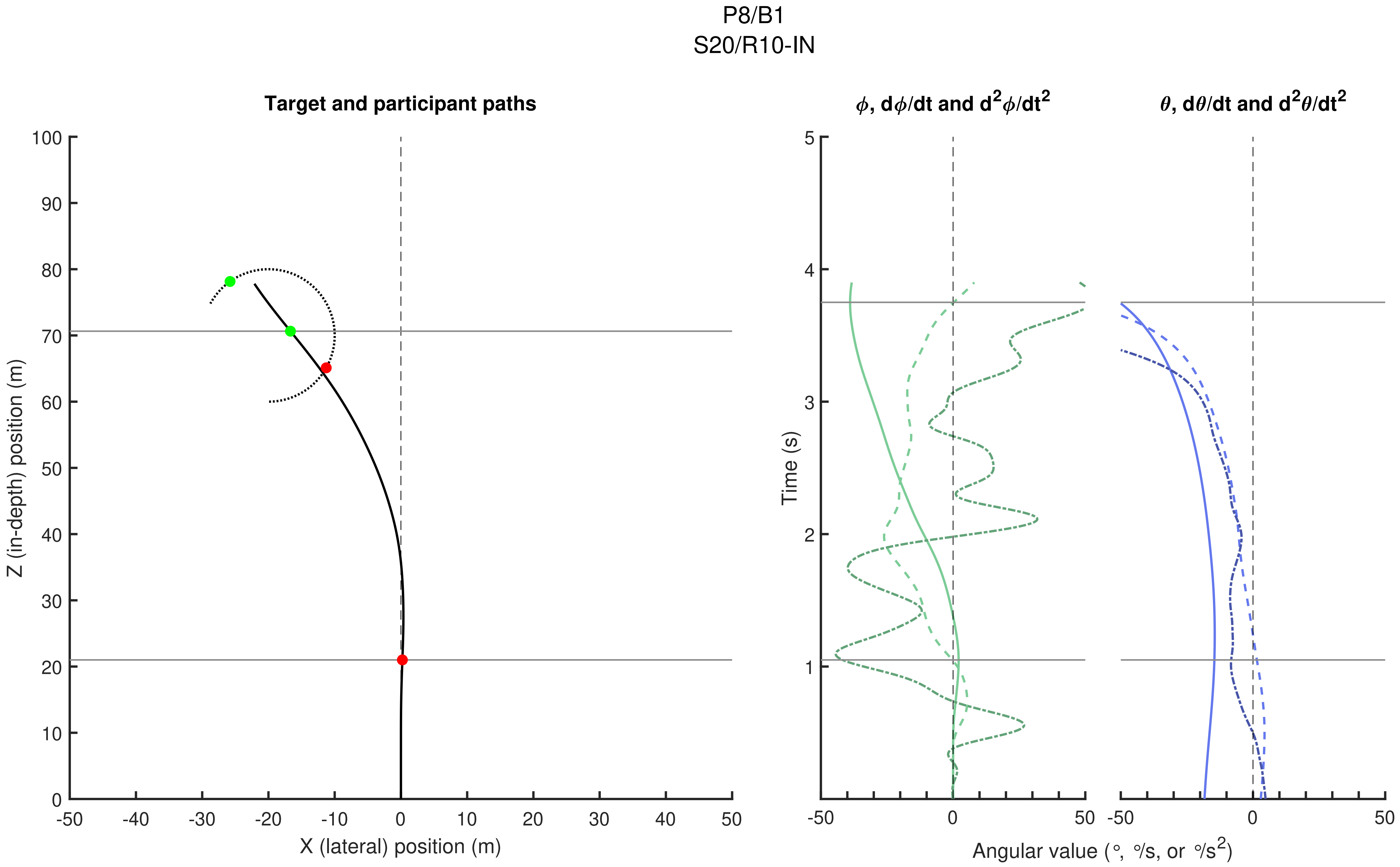

P8/B1
S20/R10-IN
Target and participant paths
Z (in-depth) position (m)
X (lateral) position (m)
ϕ, dϕ/dt and d$^2\phi$/dt^2
θ, dθ/dt and d$^2\theta$/dt^2
Time (s)
Angular value (°, °/s, or °/s^2)

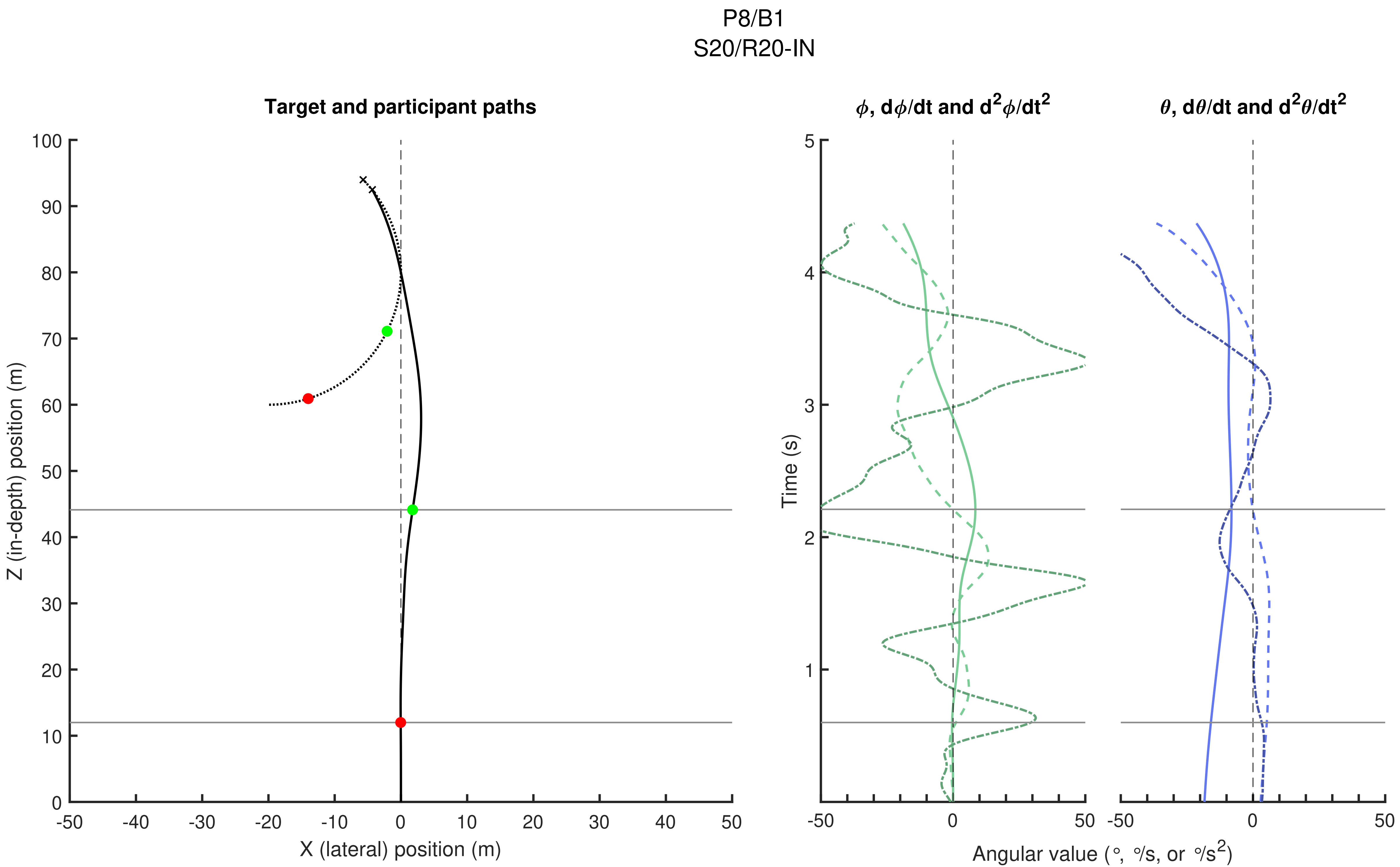
P8/B1
S20/R20-IN
Target and participant paths
ϕ, dϕ/dt and d$^2\phi$/dt^2
θ, dθ/dt and d$^2\theta$/dt^2
Z (in-depth) position (m)
X (lateral) position (m)
Time (s)
Angular value (°, °/s, or °/s^2)
100
90
80
70
60
50
40
30
20
10
0
-50
-40
-30
-20
-10
0
10
20
30
40
50
5
4
3
2
1

P8/B1
S20/R30-IN

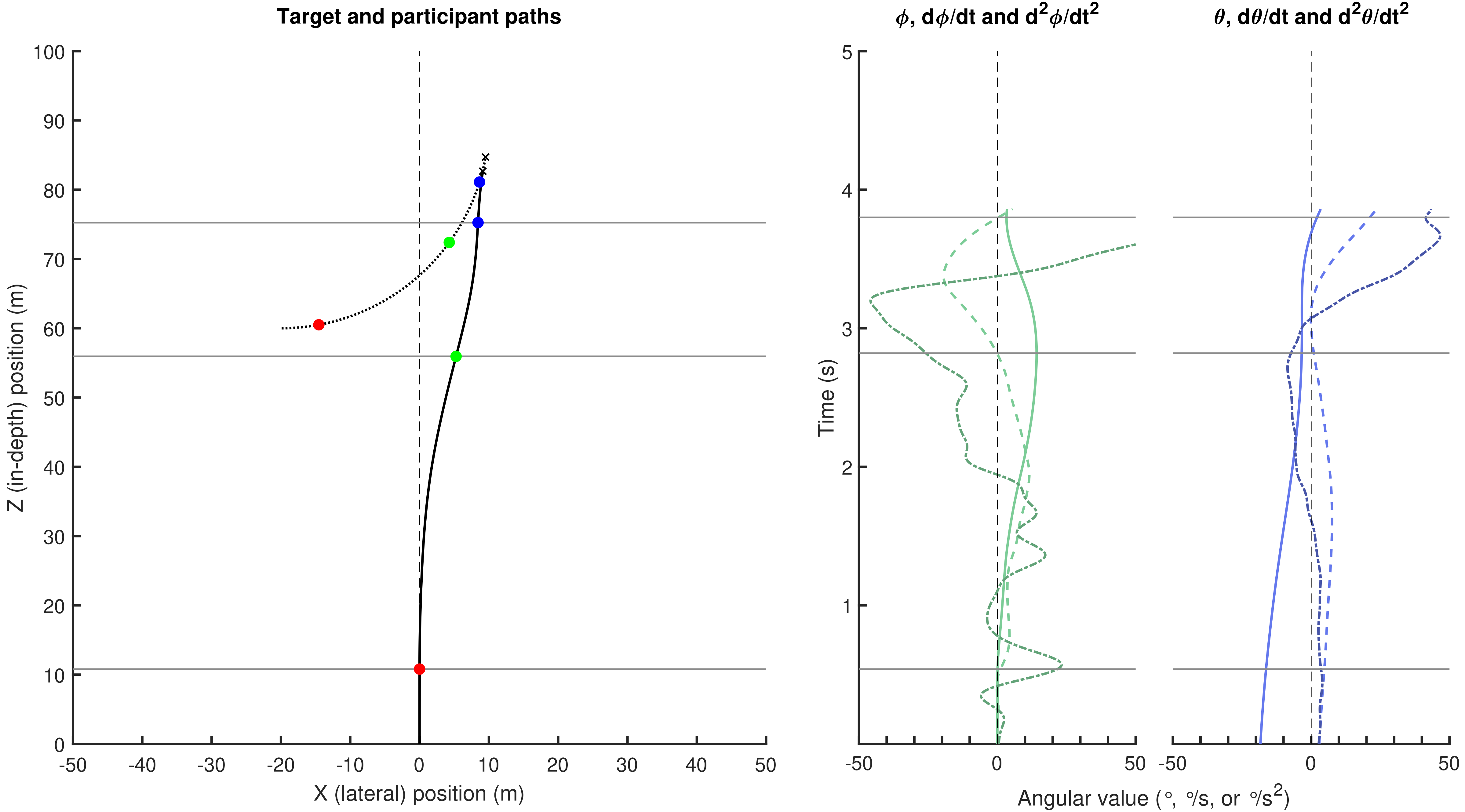

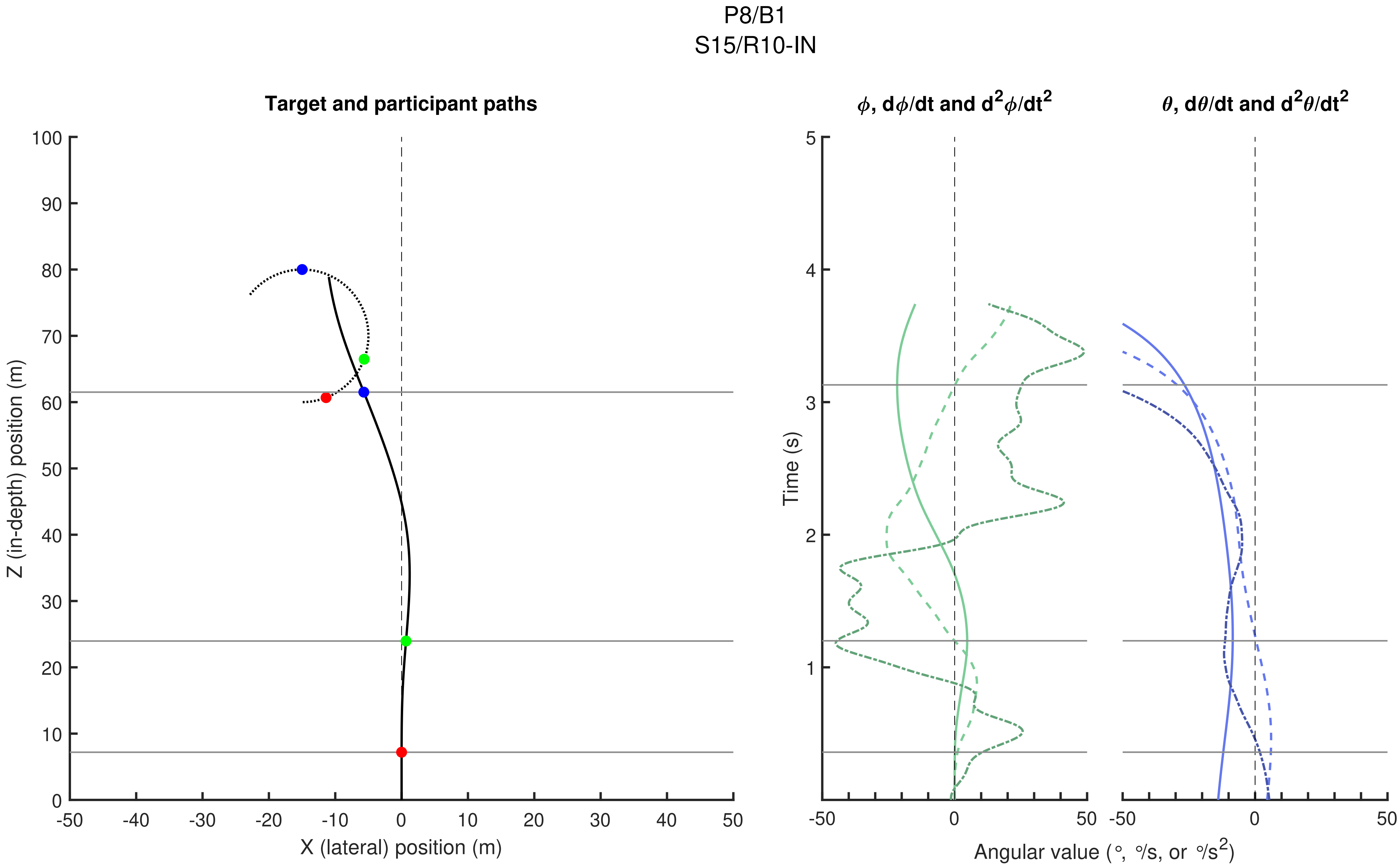

P8/B1
S15/R10-IN
Target and participant paths
Z (in-depth) position (m)
X (lateral) position (m)
ϕ, dϕ/dt and d²ϕ/dt²
θ, dθ/dt and d²θ/dt²
Time (s)
Angular value (°, °/s, or °/s²)

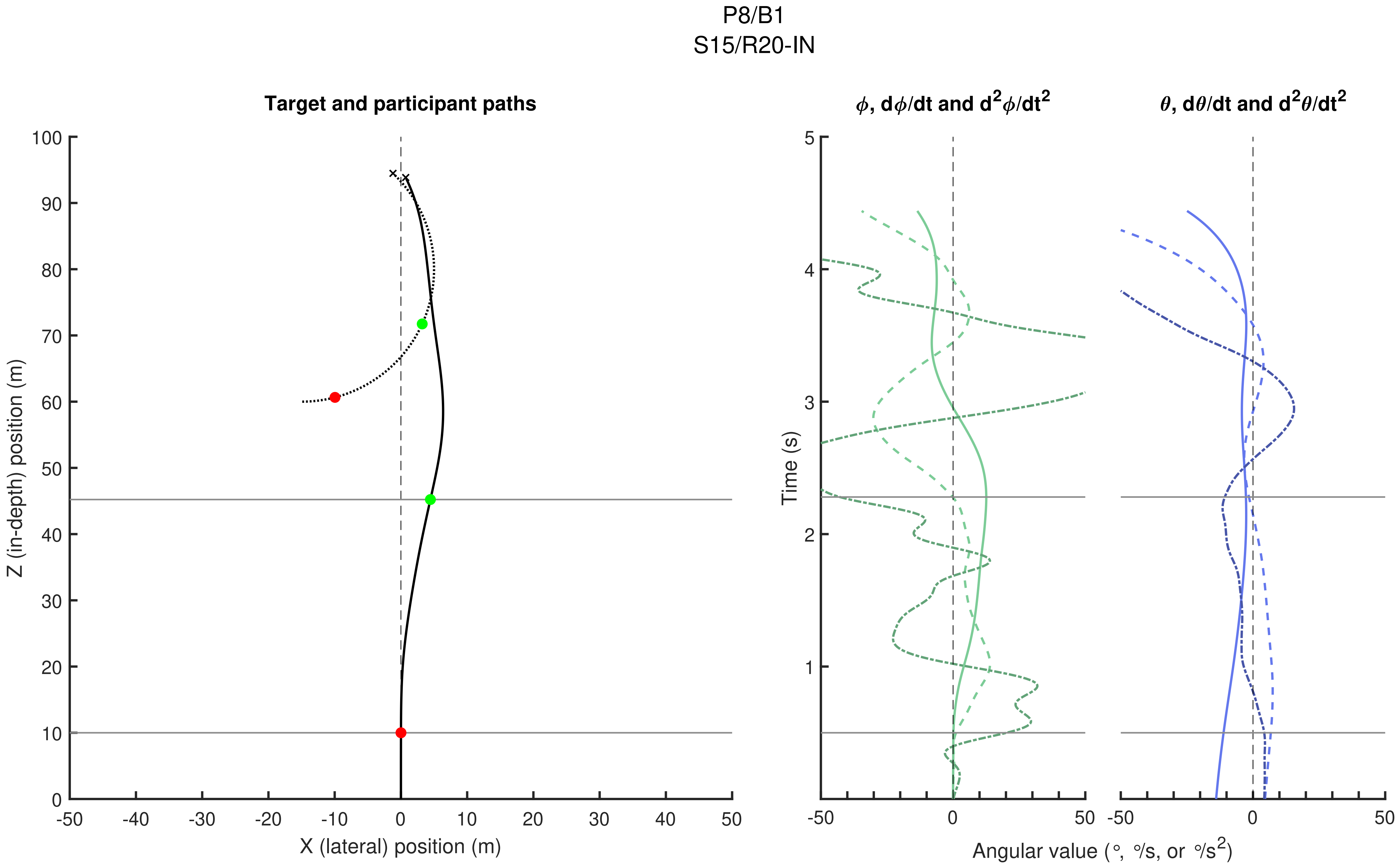
P8/B1
S15/R20-IN
Target and participant paths
Z (in-depth) position (m)
X (lateral) position (m)
ϕ, dϕ/dt and d²ϕ/dt²
θ, dθ/dt and d²θ/dt²
Time (s)
Angular value (°, °/s, or °/s²)

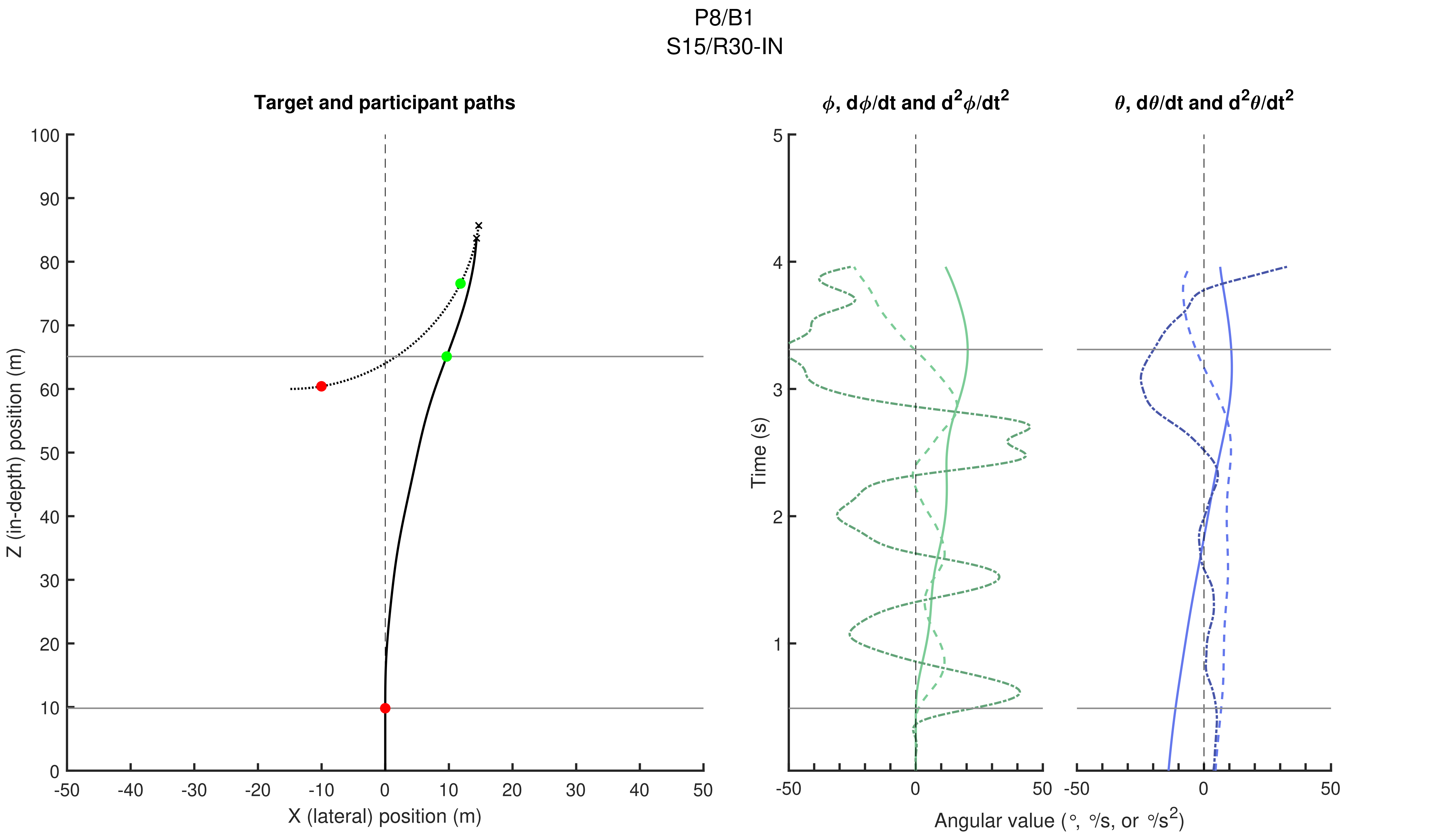
P8/B1
S15/R30-IN
Target and participant paths
Z (in-depth) position (m)
X (lateral) position (m)
φ, dφ/dt and d²φ/dt²
θ, dθ/dt and d²θ/dt²
Time (s)
Angular value (°, °/s, or °/s²)

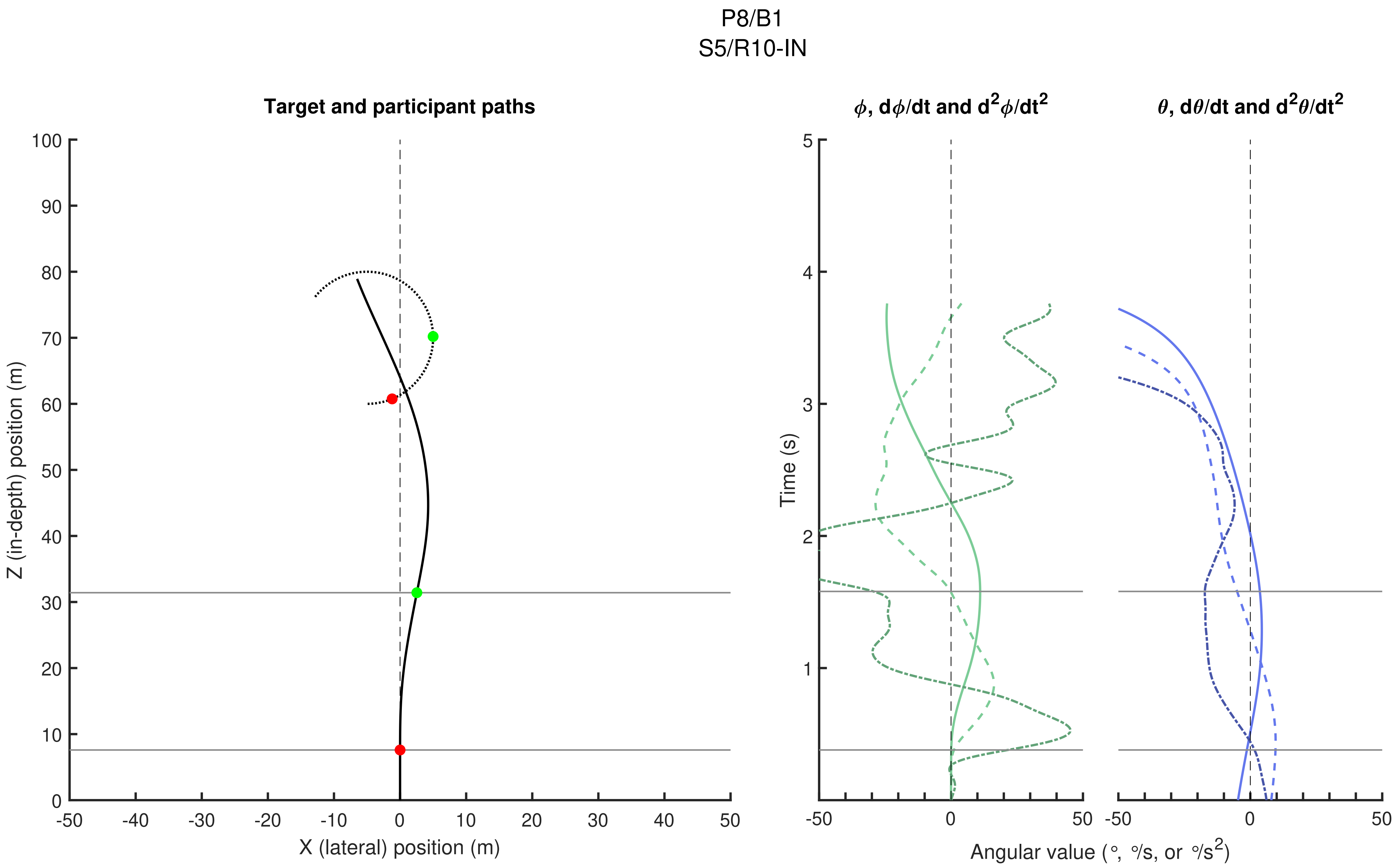
P8/B1
S5/R10-IN
Target and participant paths
Z (in-depth) position (m)
X (lateral) position (m)
φ, dφ/dt and d²φ/dt²
θ, dθ/dt and d²θ/dt²
Time (s)
Angular value (°, °/s, or °/s²)

P8/B1
S5/R10-OUT

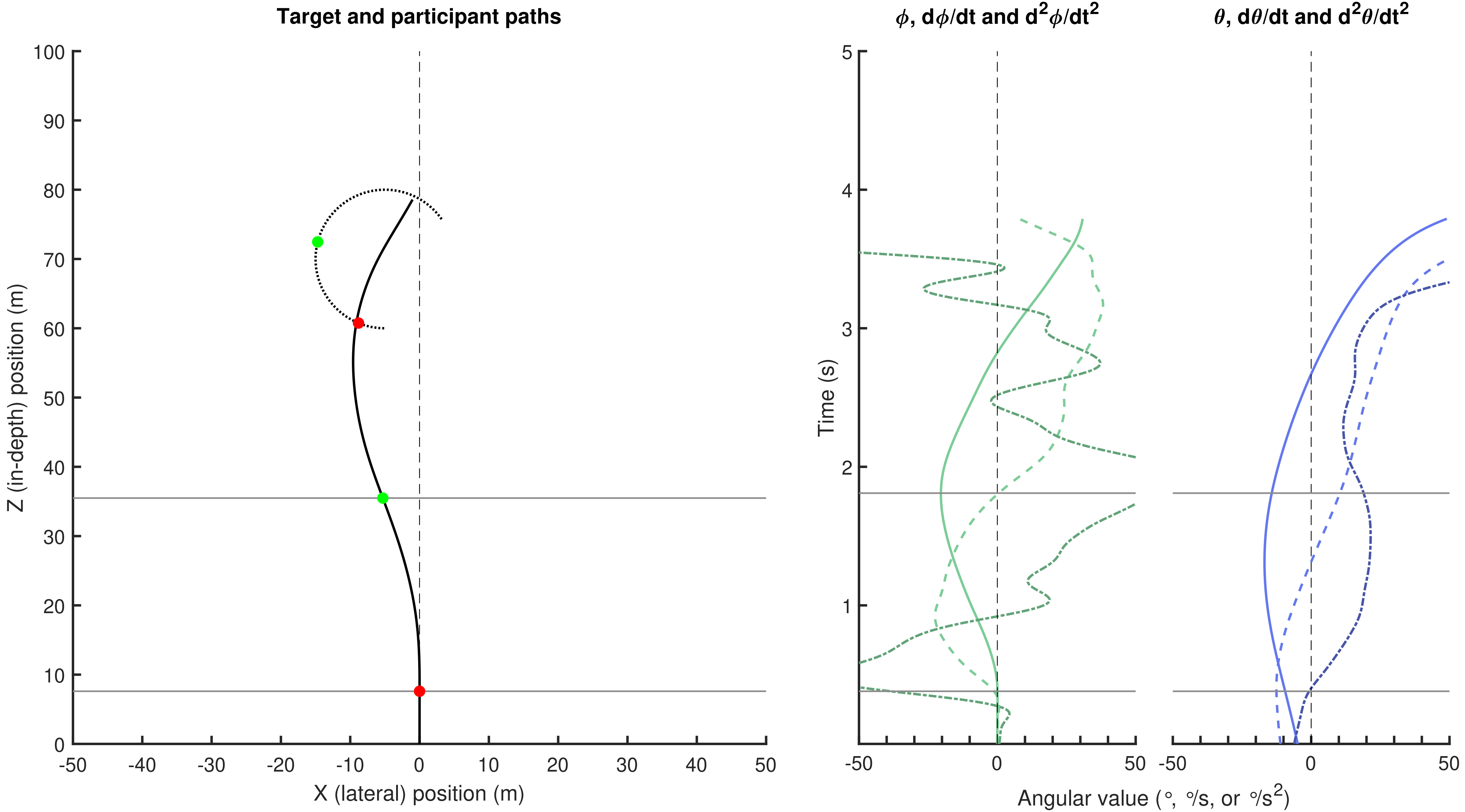

P8/B1
S5/R20-IN

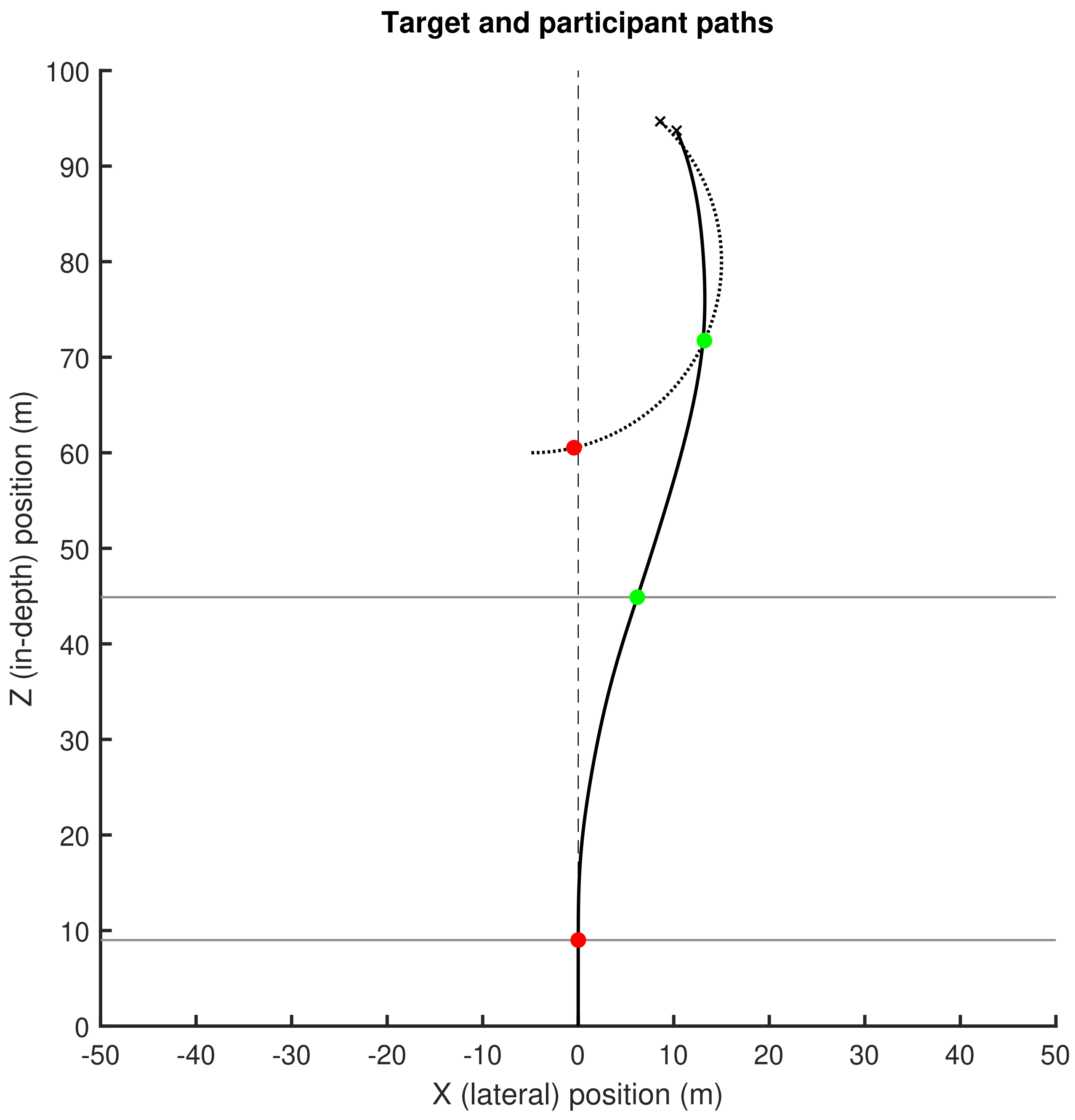

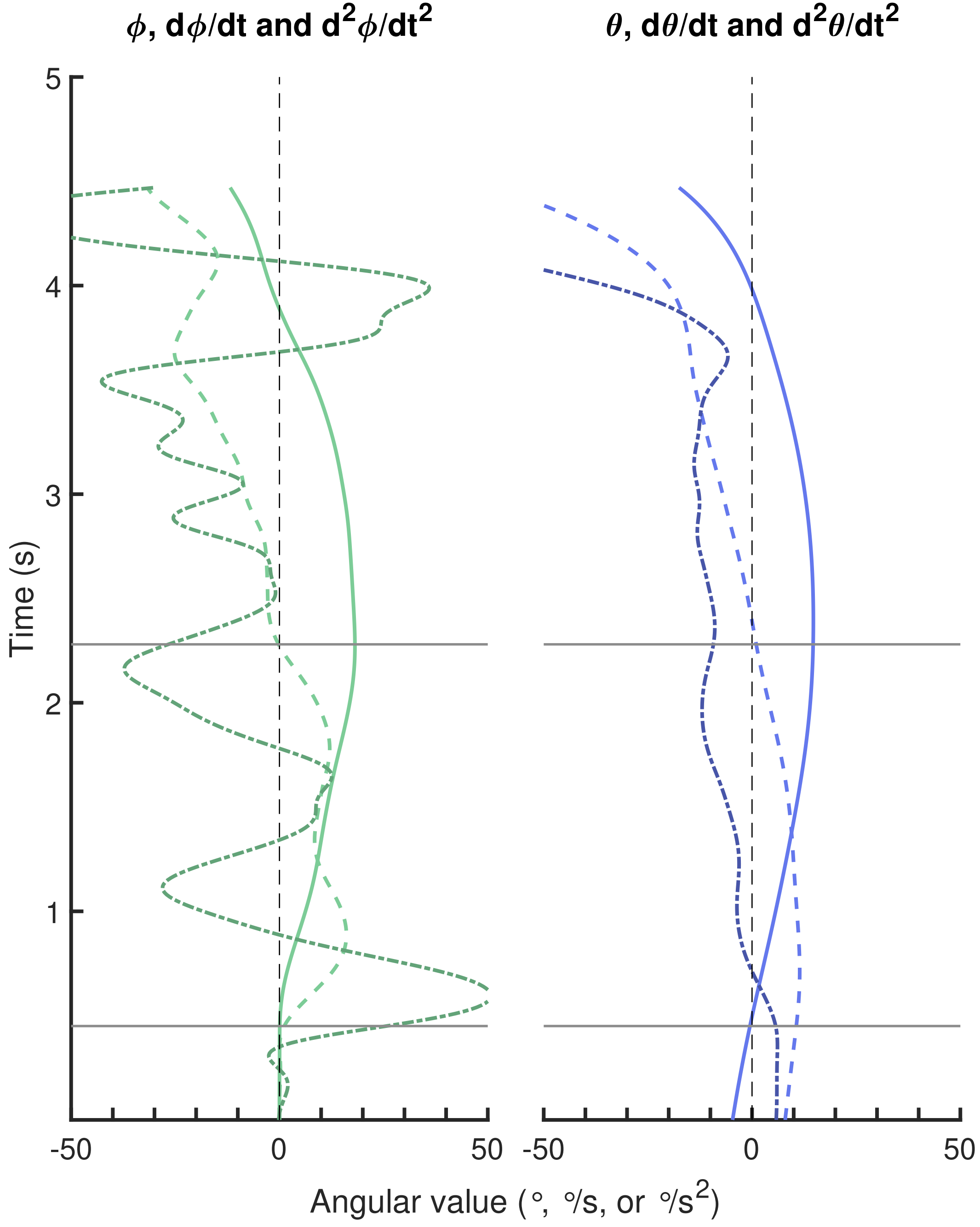

P8/B1
S5/R20-OUT

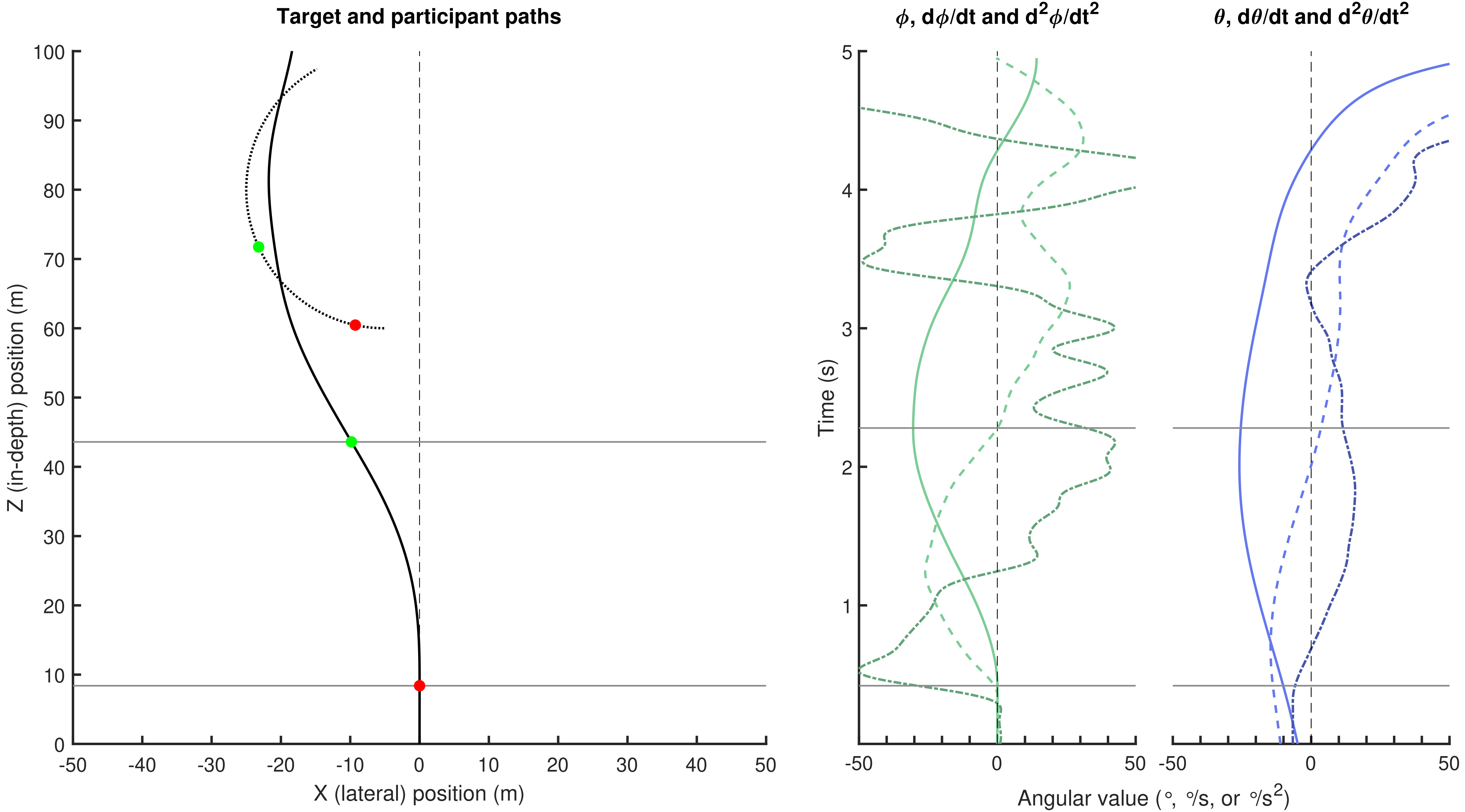

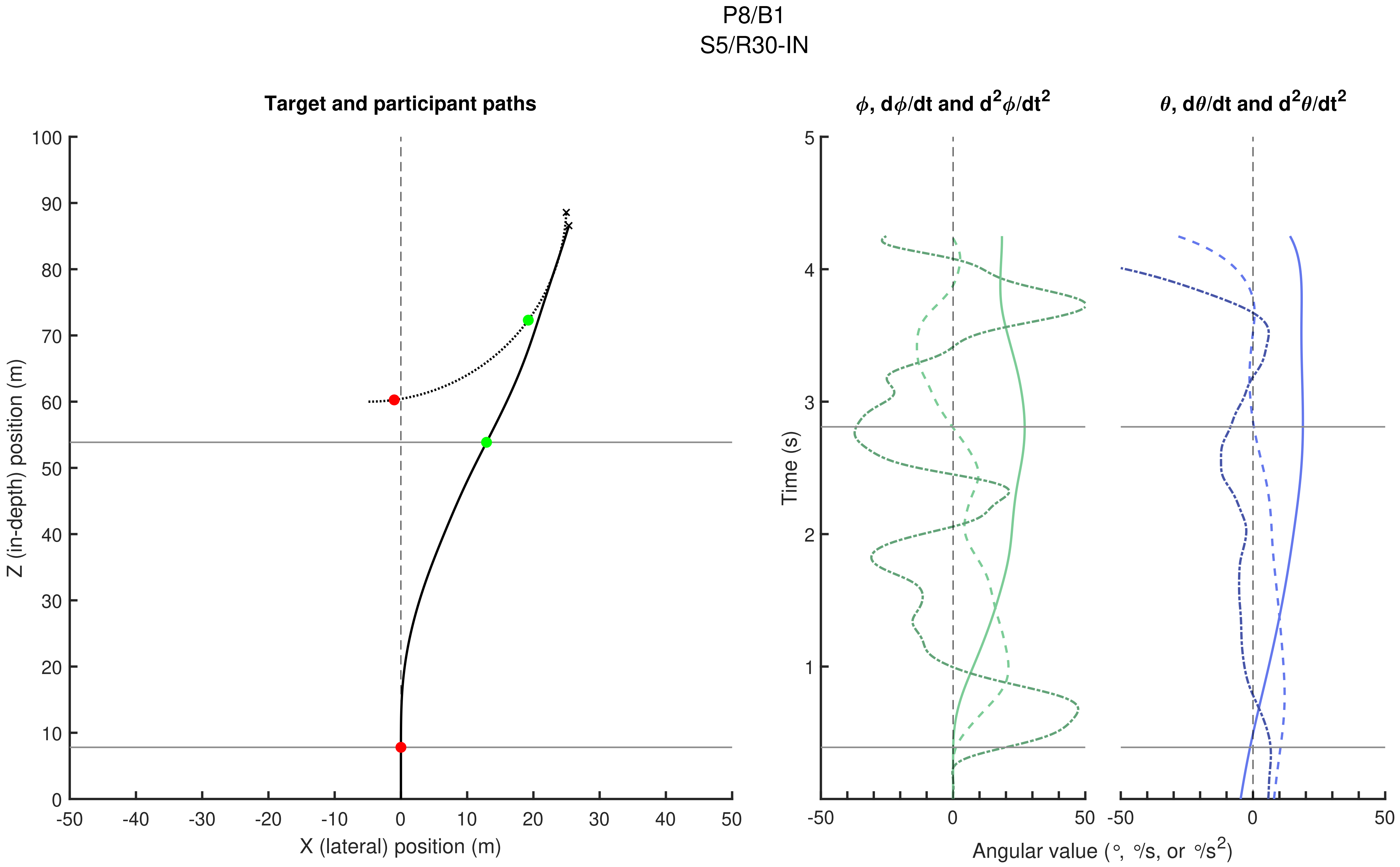

P8/B1
S5/R30-IN
Target and participant paths
Z (in-depth) position (m)
X (lateral) position (m)
ϕ, dϕ/dt and d²ϕ/dt²
θ, dθ/dt and d²θ/dt²
Time (s)
Angular value (°, °/s, or °/s²)

P8/B1
S5/R30-OUT

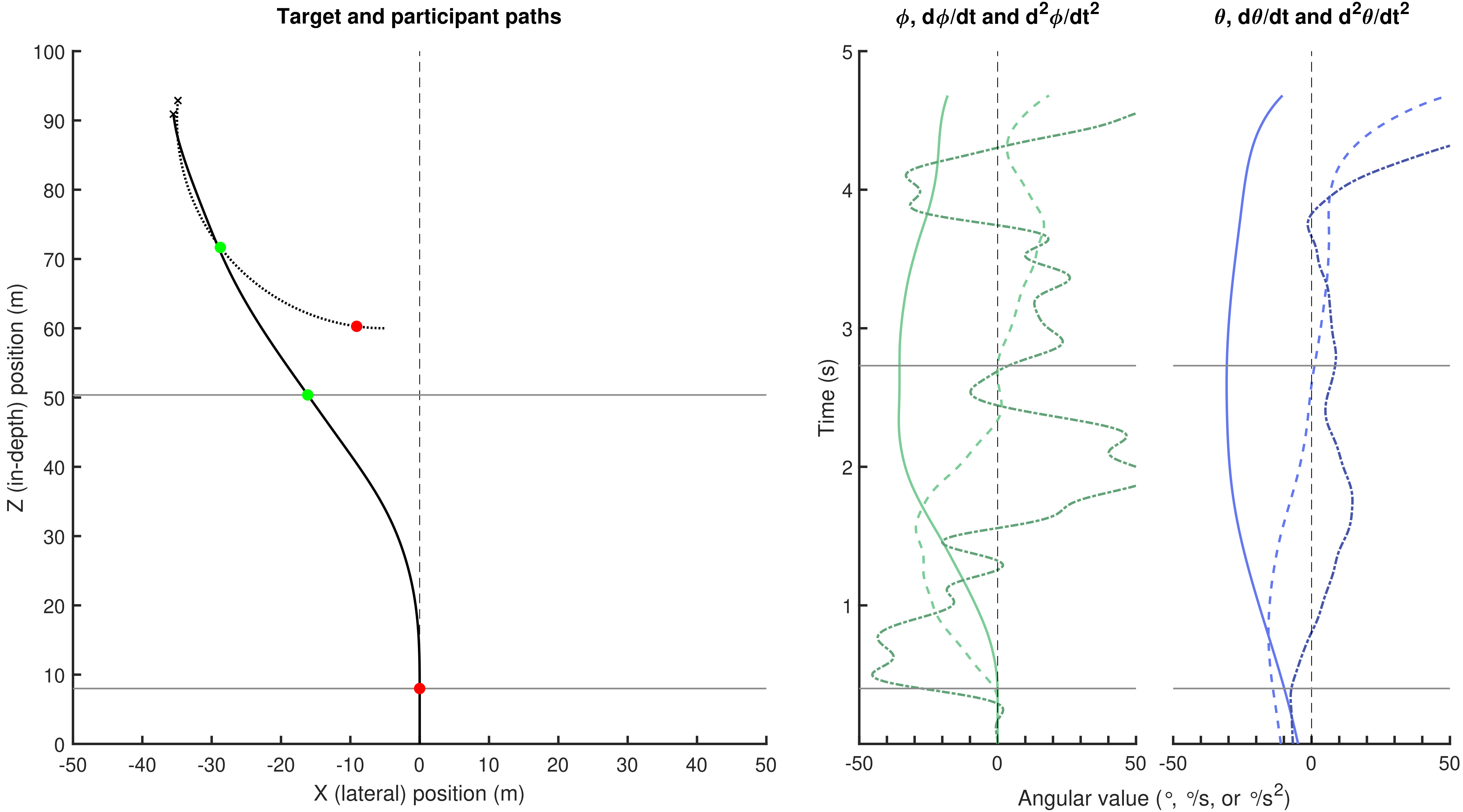

P8/B1
S5/R10-OUT

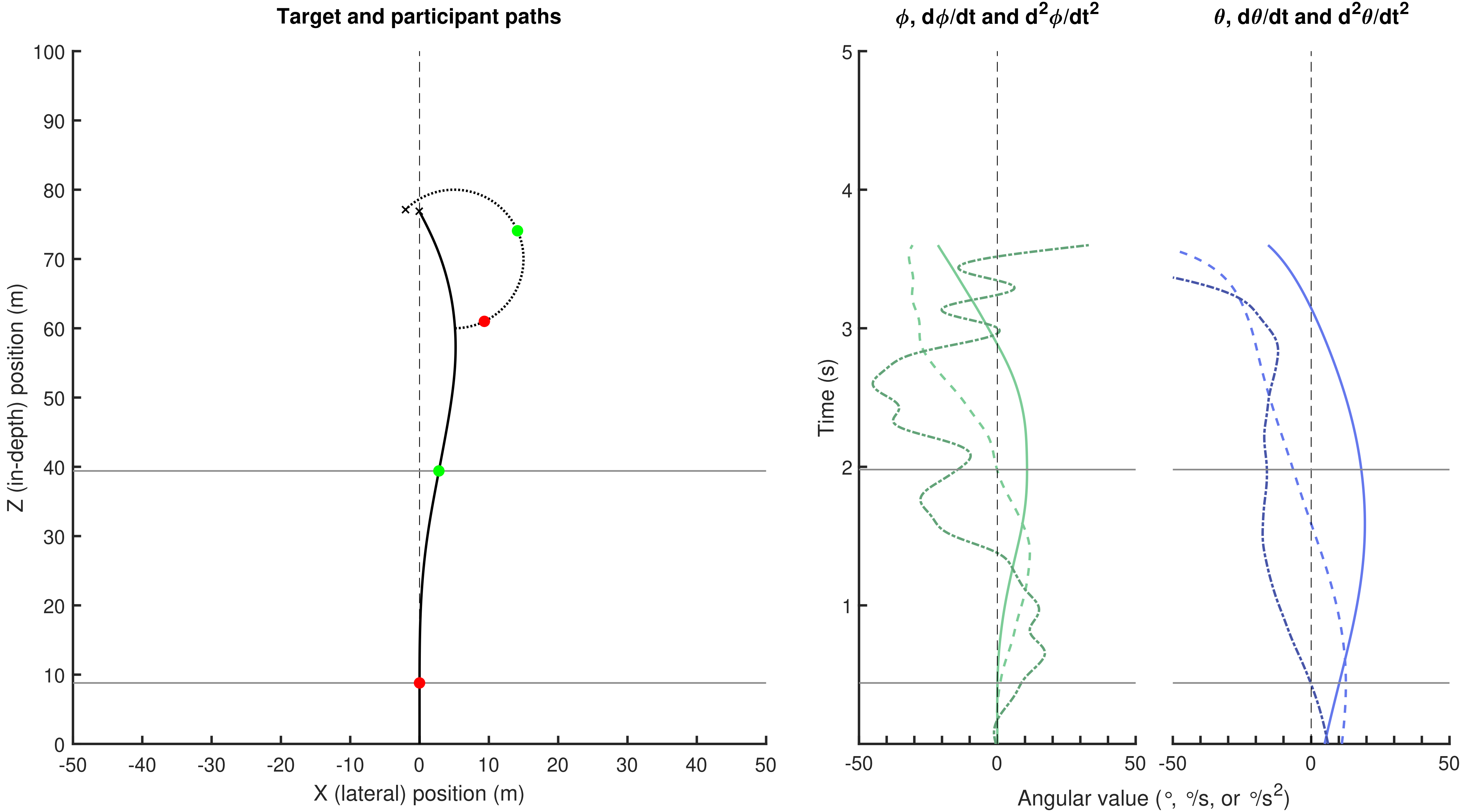

P8/B1
S5/R10-IN

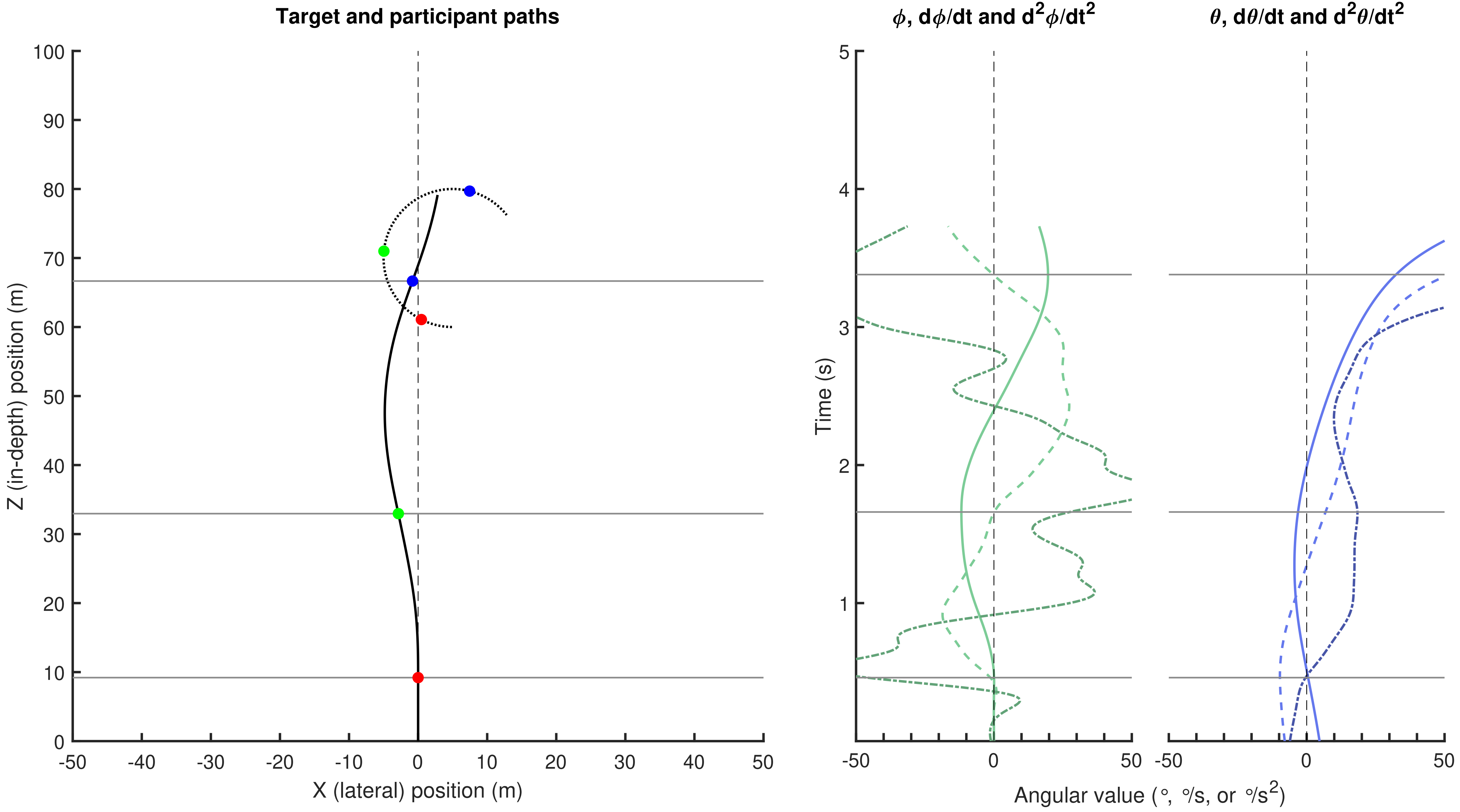

P8/B1
S5/R20-OUT

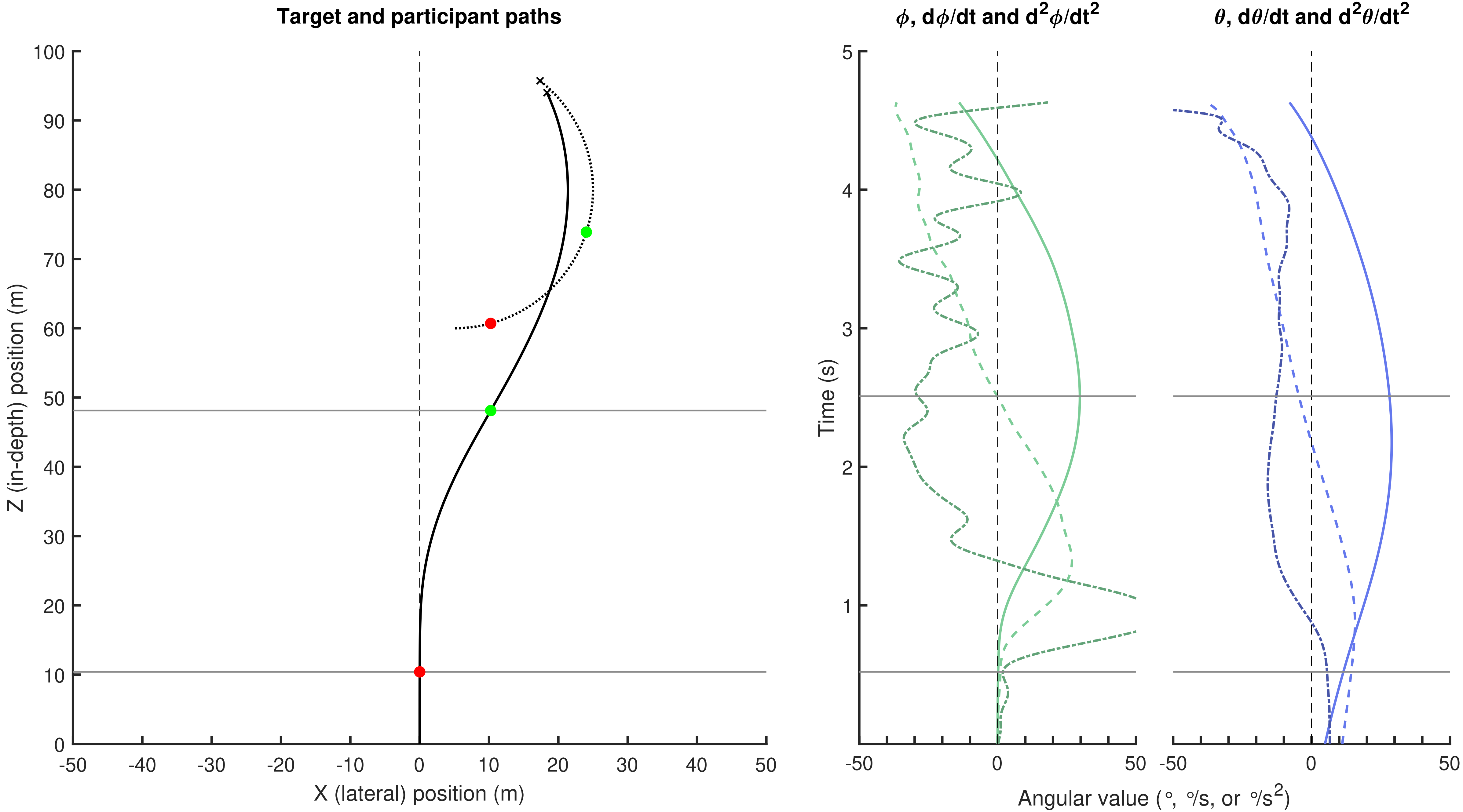

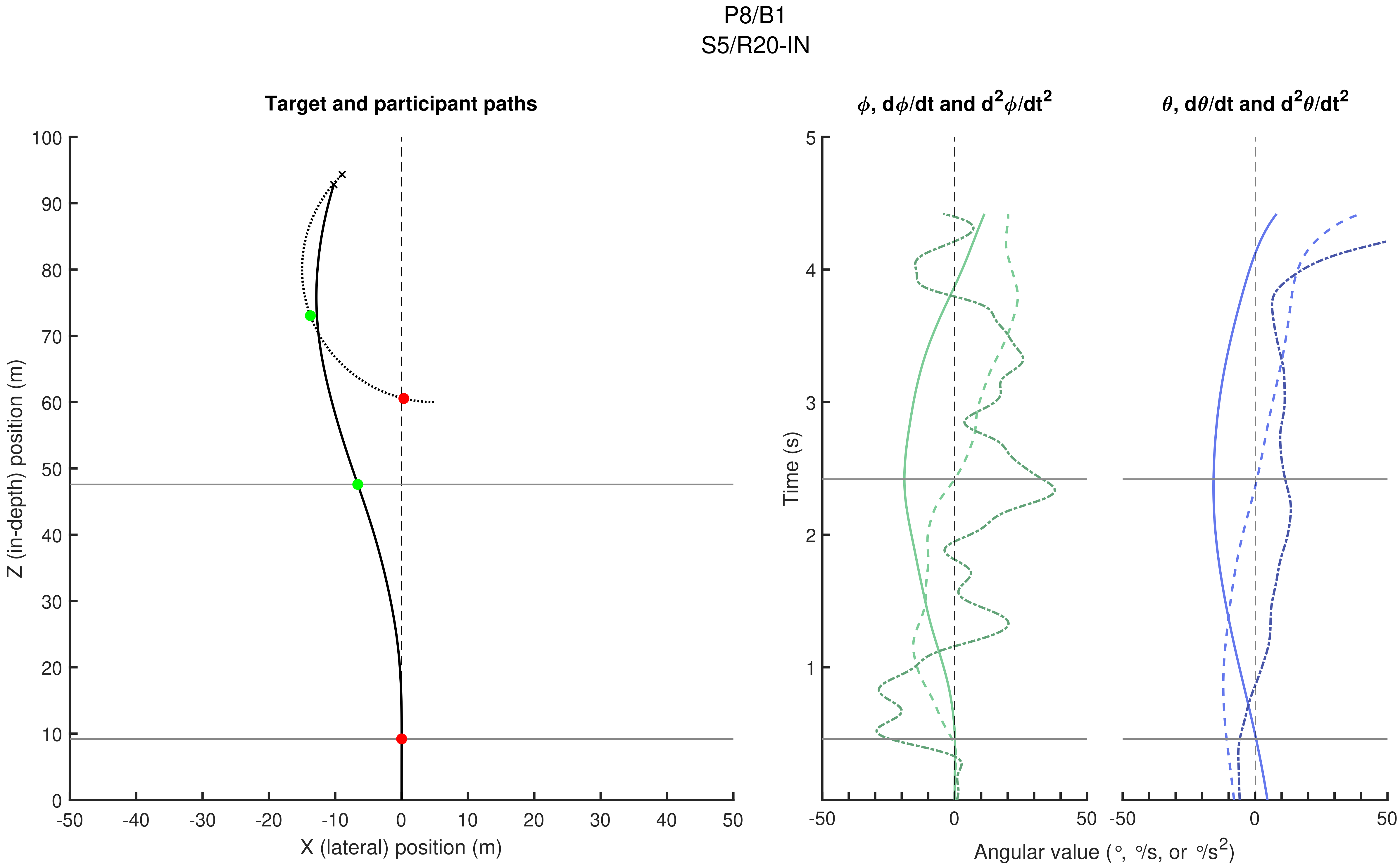
P8/B1
S5/R20-IN
Target and participant paths
Z (in-depth) position (m)
X (lateral) position (m)
ϕ, dϕ/dt and d$^2\phi$/dt^2
θ, dθ/dt and d$^2\theta$/dt^2
Time (s)
Angular value (°, °/s, or °/s^2)

P8/B1
S5/R30-OUT

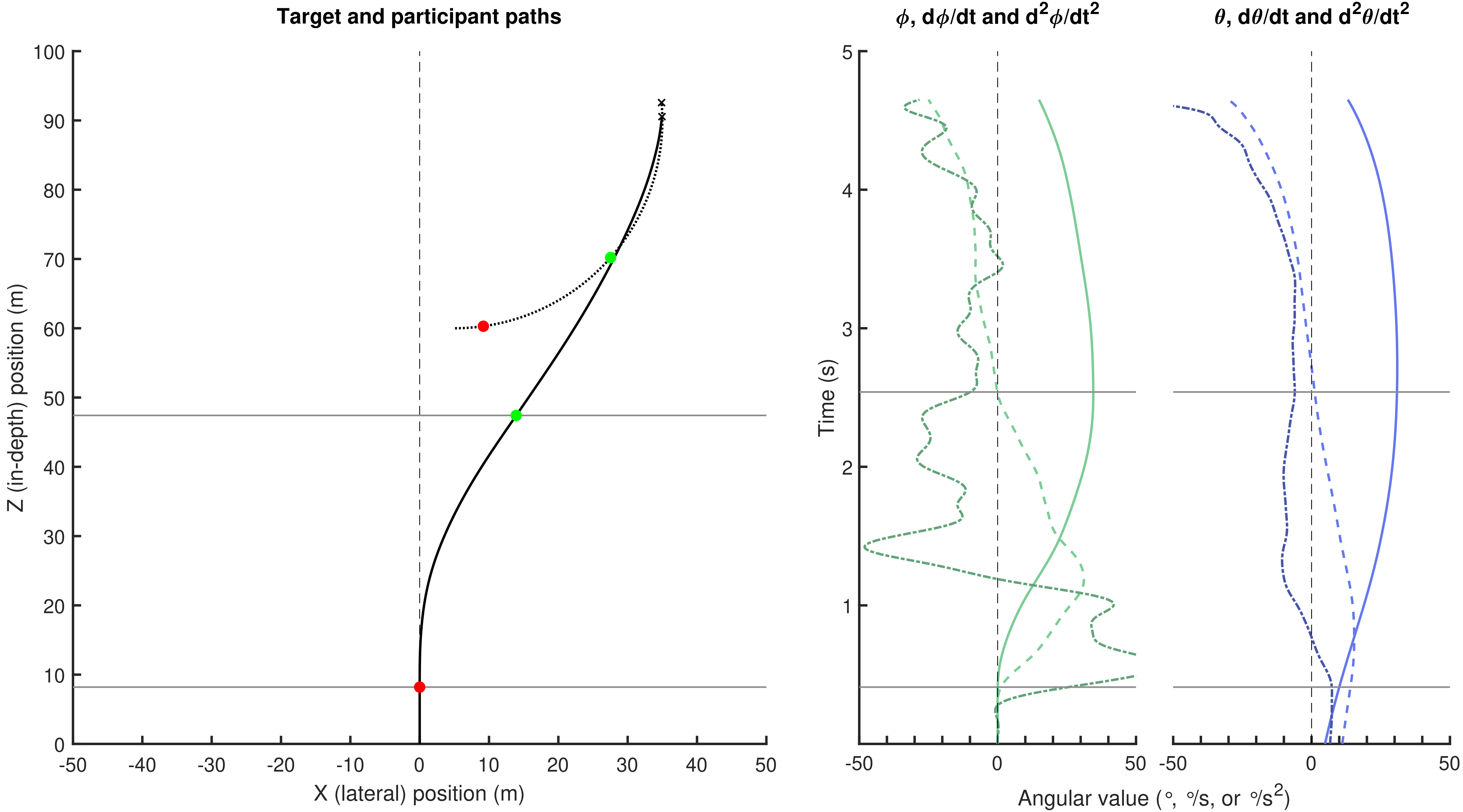

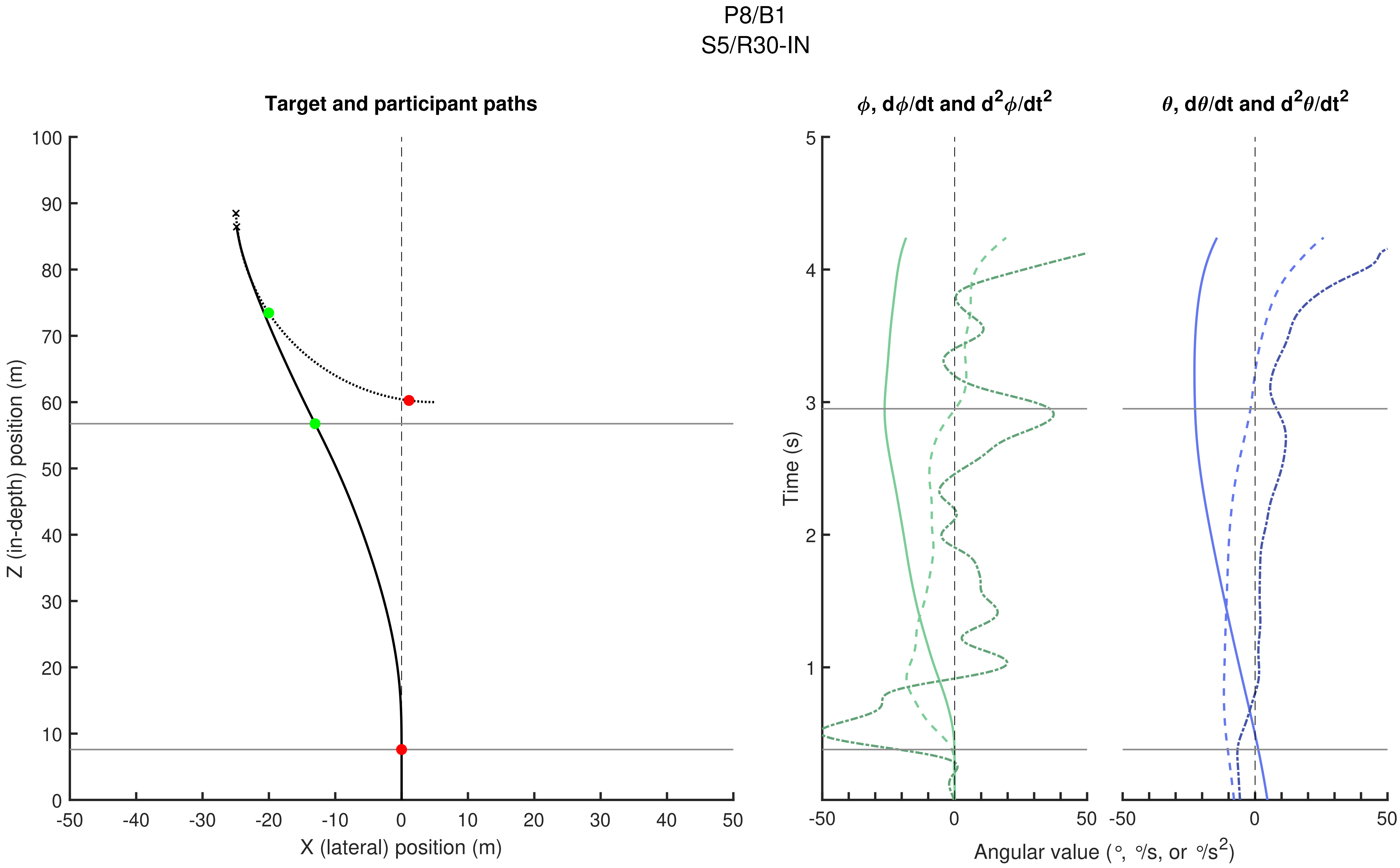

P8/B1
S5/R30-IN
Target and participant paths
Z (in-depth) position (m)
X (lateral) position (m)
100
90
80
70
60
50
40
30
20
10
0
-50
-40
-30
-20
-10
0
10
20
30
40
50
φ, dφ/dt and d²φ/dt²
θ, dθ/dt and d²θ/dt²
Time (s)
5
4
3
2
1
-50
0
50
-50
0
50
Angular value (°, °/s, or °/s²)

P8/B1
S15/R10-IN

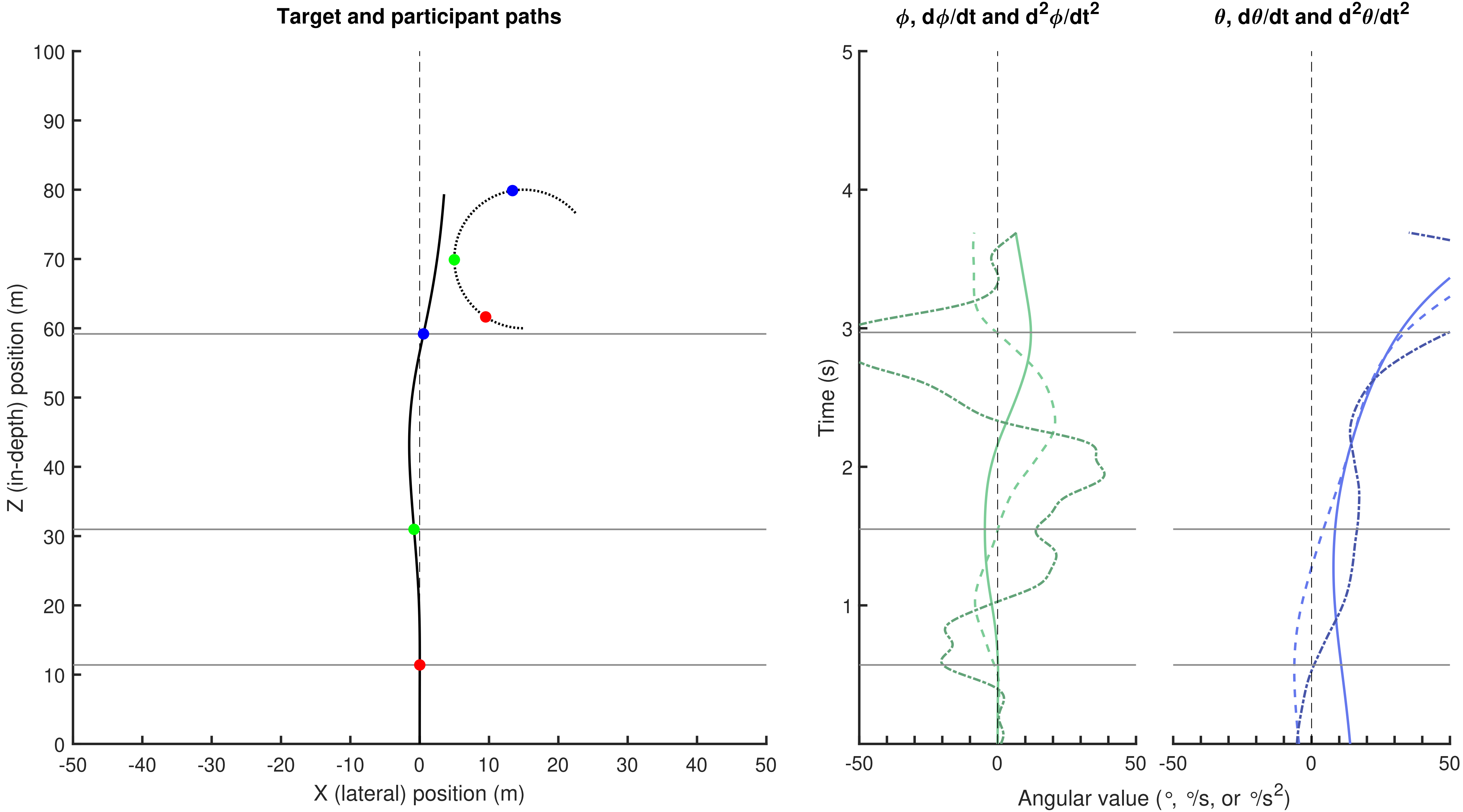

P8/B1
S15/R20-IN

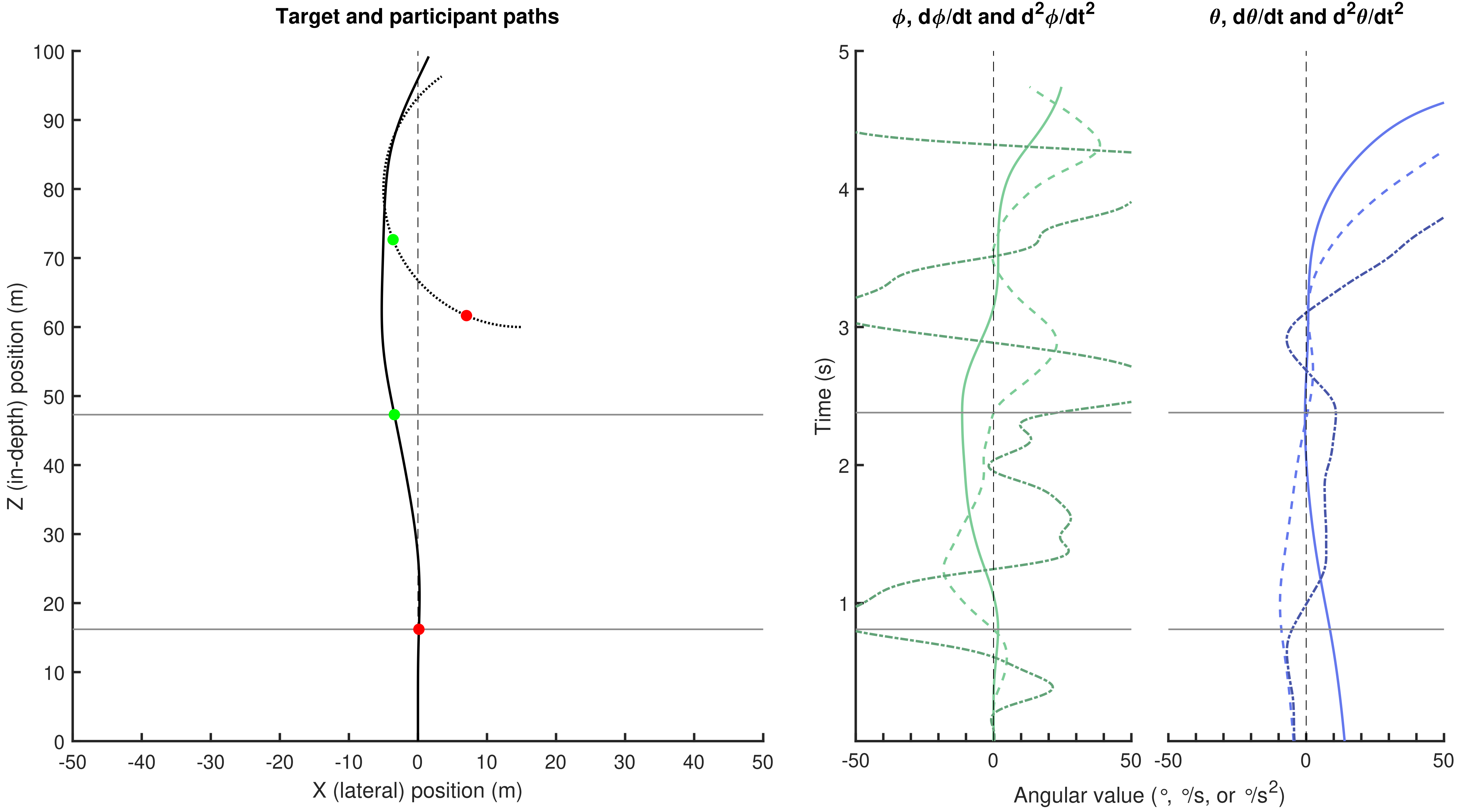

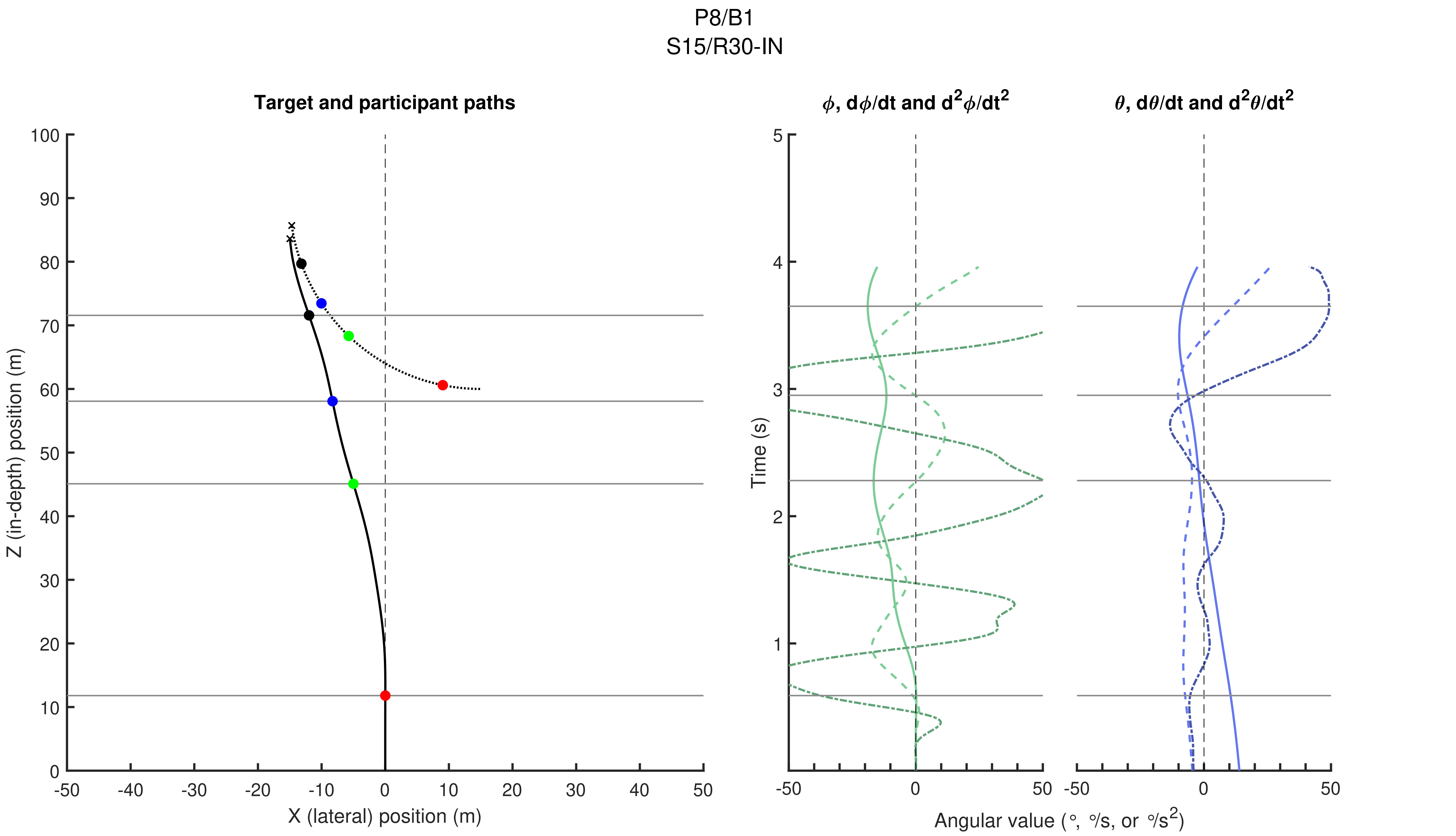

P8/B1
S15/R30-IN
Target and participant paths
Z (in-depth) position (m)
X (lateral) position (m)
ϕ, dϕ/dt and d$^2\phi$/dt^2
θ, dθ/dt and d$^2\theta$/dt^2
Time (s)
Angular value (°, °/s, or °/s^2)

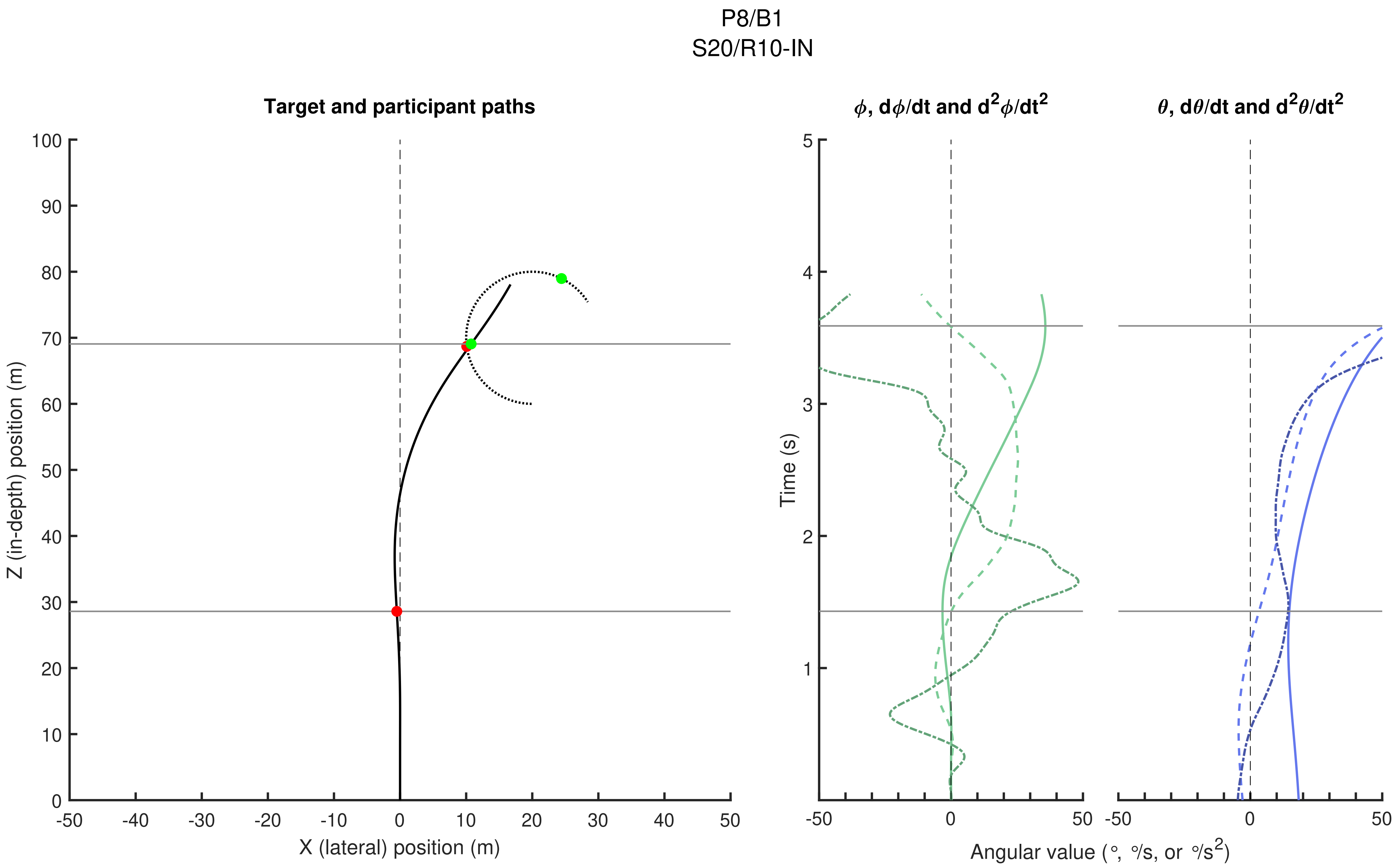
P8/B1
S20/R10-IN
Target and participant paths
Z (in-depth) position (m)
X (lateral) position (m)
100
90
80
70
60
50
40
30
20
10
0
-50
-40
-30
-20
-10
0
10
20
30
40
50
ϕ, dϕ/dt and d$^2\phi$/dt^2
θ, dθ/dt and d$^2\theta$/dt^2
Time (s)
5
4
3
2
1
-50
0
50
-50
0
50
Angular value (°, °/s, or °/s^2)

P8/B1
S20/R20-IN

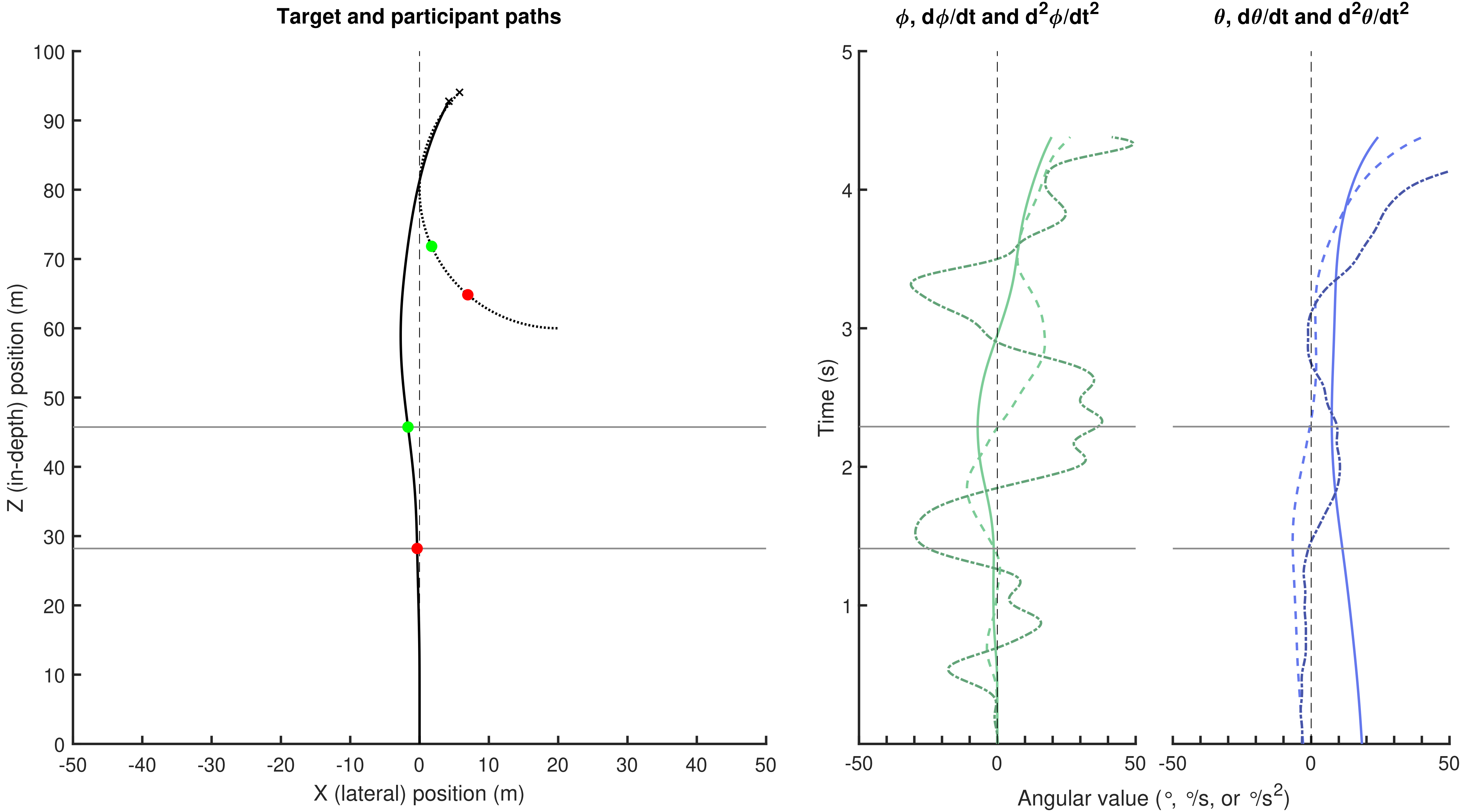

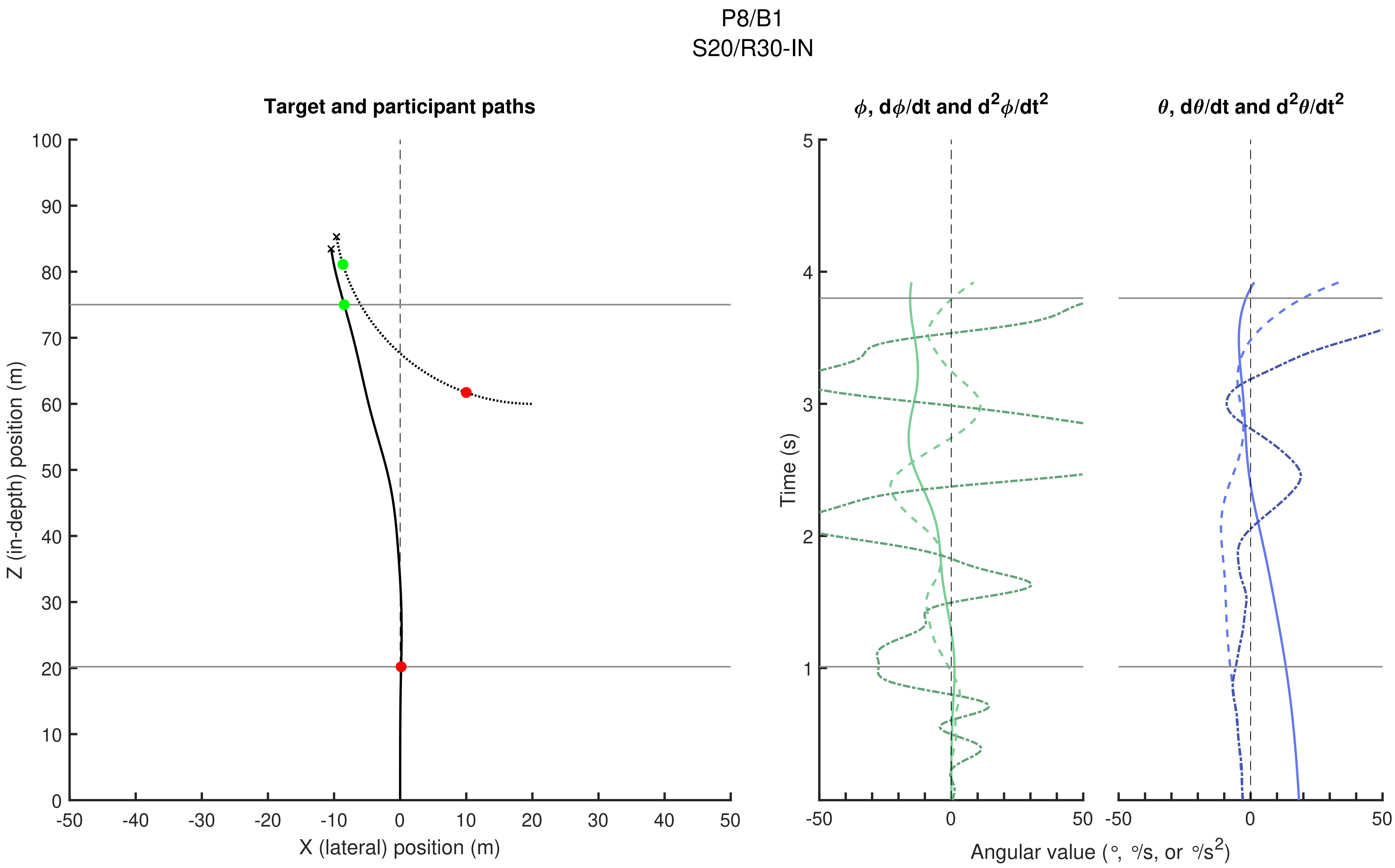

P8/B1
S20/R30-IN
Target and participant paths
Z (in-depth) position (m)
X (lateral) position (m)
100
90
80
70
60
50
40
30
20
10
0
-50
-40
-30
-20
-10
0
10
20
30
40
50
ϕ, dϕ/dt and d²ϕ/dt²
θ, dθ/dt and d²θ/dt²
Time (s)
5
4
3
2
1
-50
0
50
-50
0
50
Angular value (°, °/s, or °/s²)

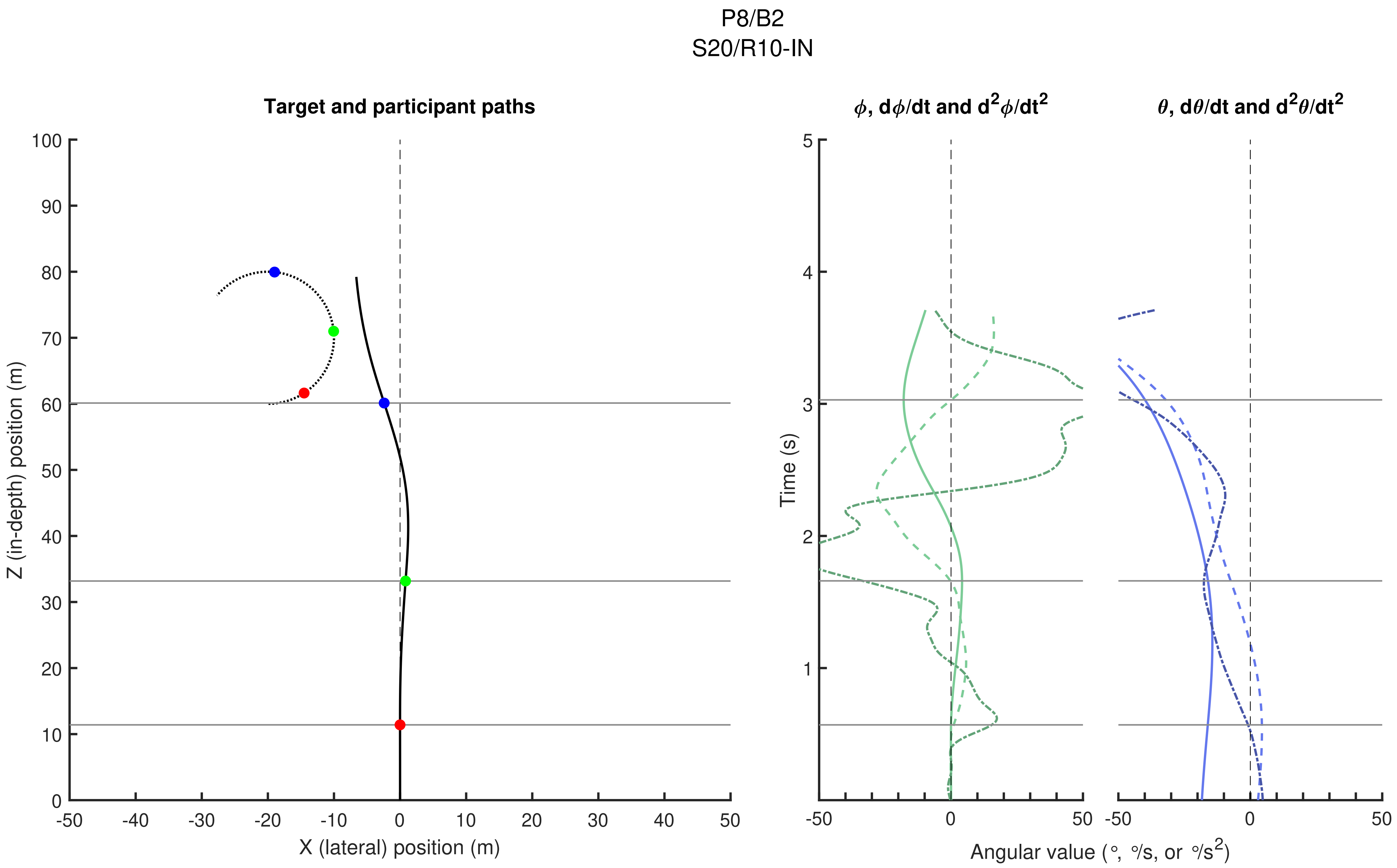

P8/B2
S20/R10-IN
Target and participant paths
ϕ, dϕ/dt and d²ϕ/dt²
θ, dθ/dt and d²θ/dt²
Z (in-depth) position (m)
X (lateral) position (m)
Time (s)
Angular value (°, °/s, or °/s²)

P8/B2
S20/R20-IN

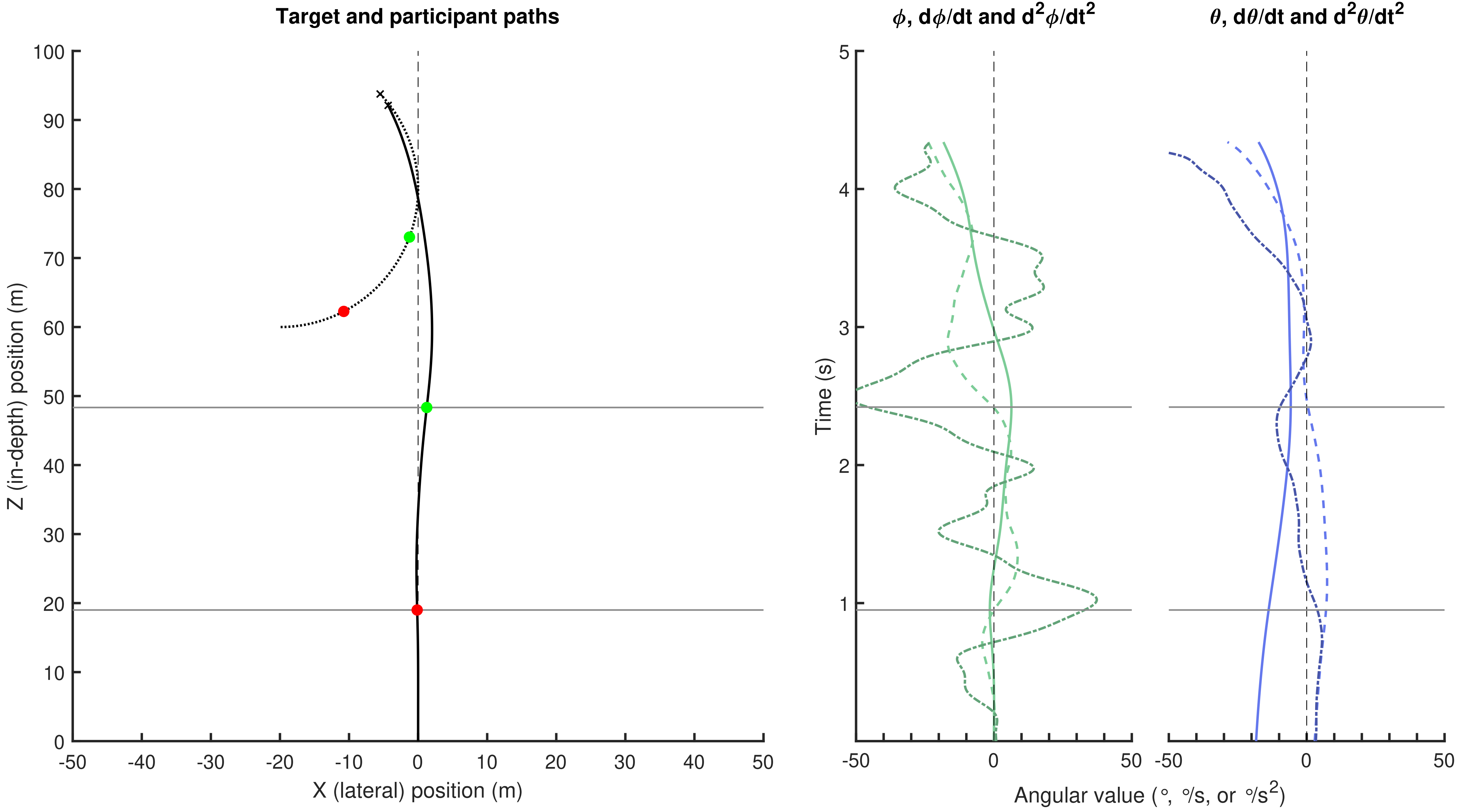

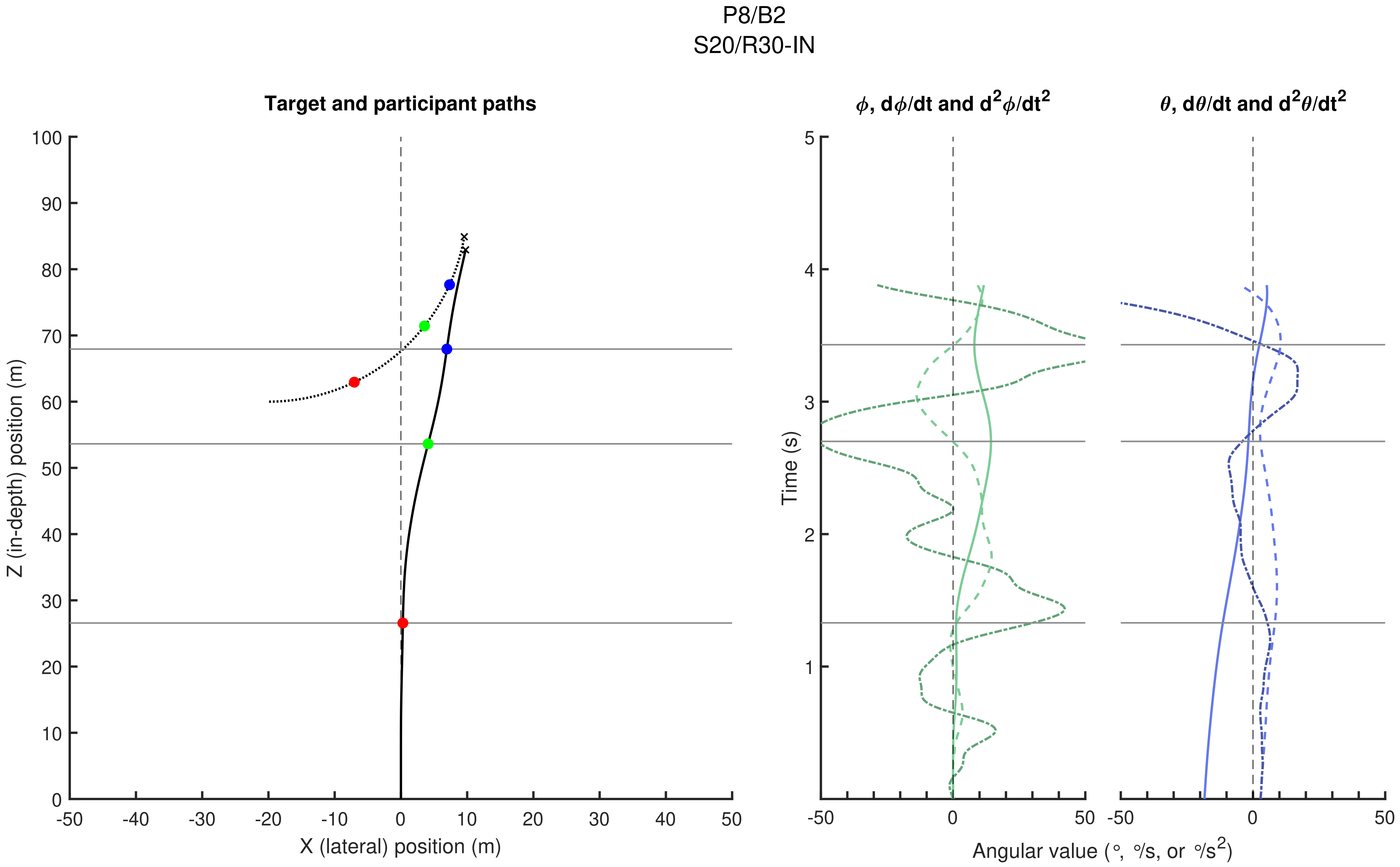
P8/B2
S20/R30-IN
Target and participant paths
Z (in-depth) position (m)
X (lateral) position (m)
ϕ, dϕ/dt and d²ϕ/dt²
θ, dθ/dt and d²θ/dt²
Time (s)
Angular value (°, °/s, or °/s²)

P8/B2
S15/R10-IN

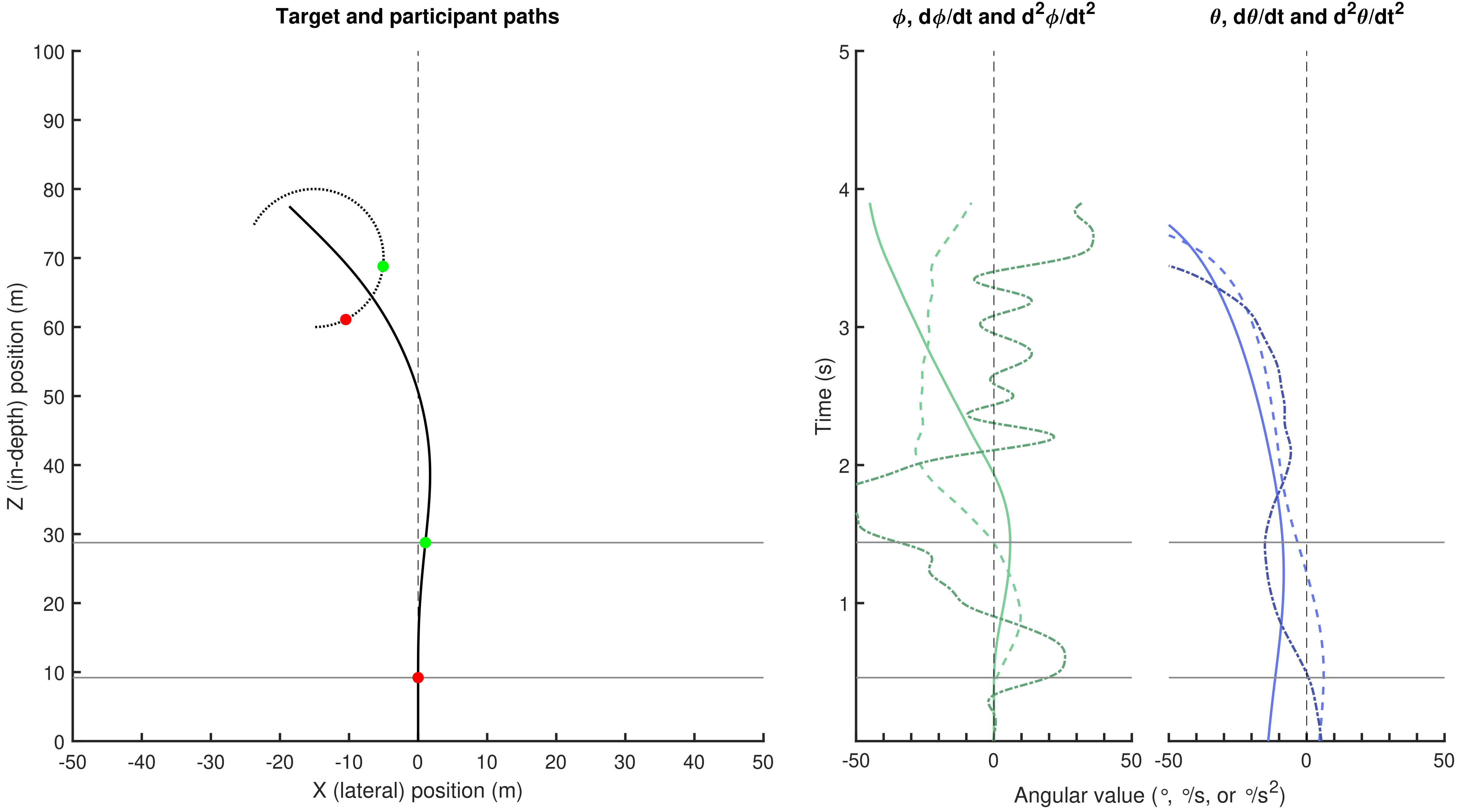

P8/B2
S15/R20-IN

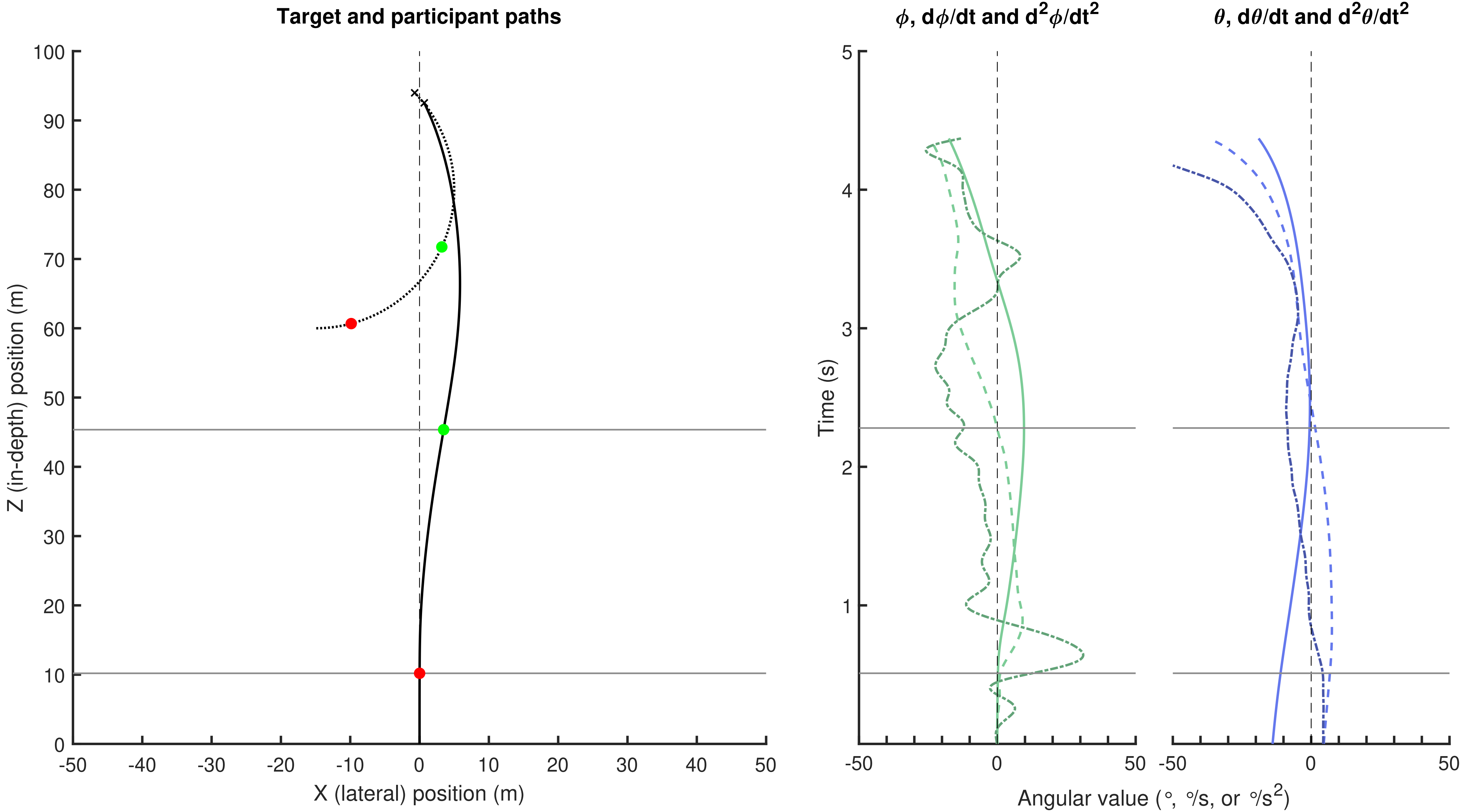

P8/B2
S15/R30-IN

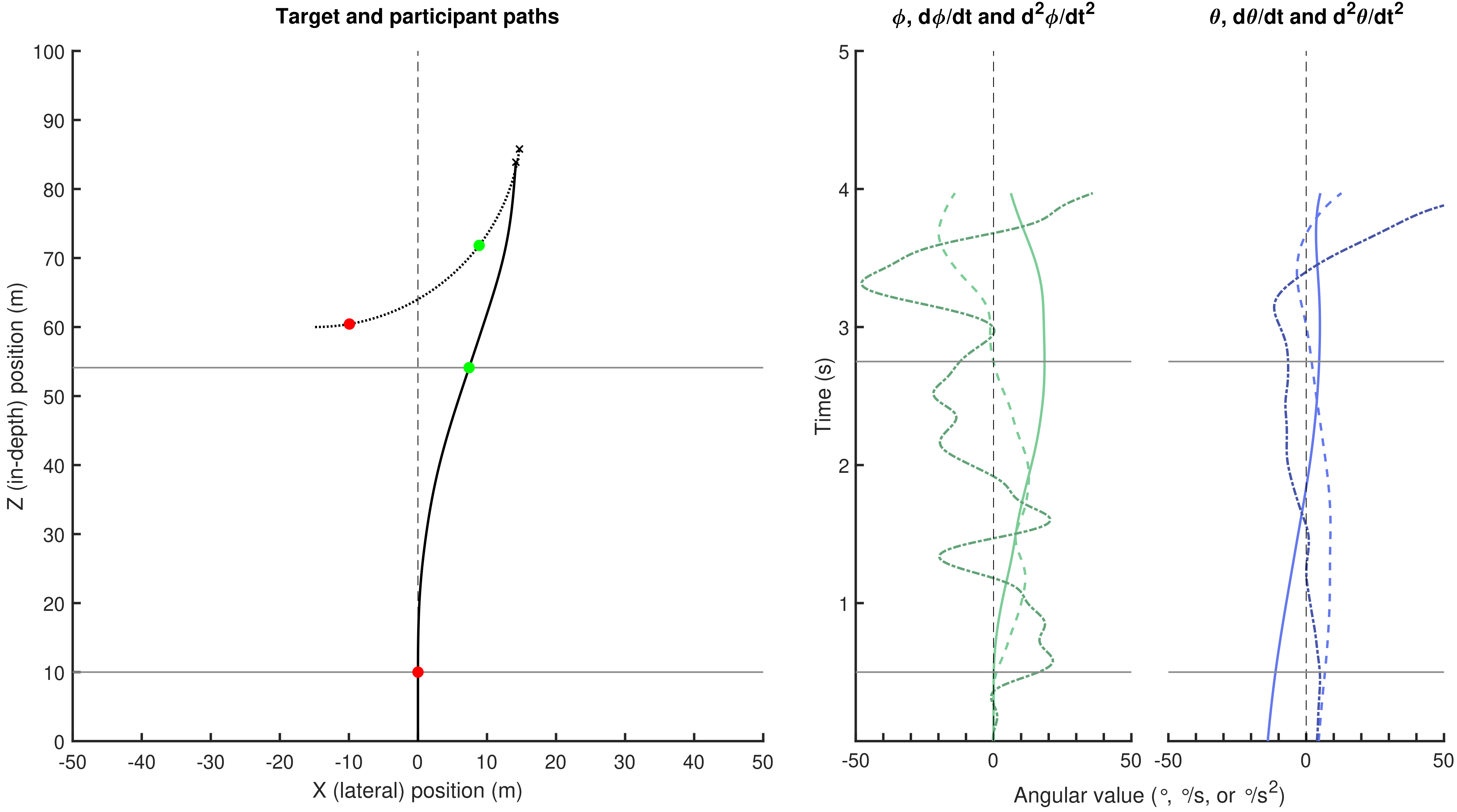

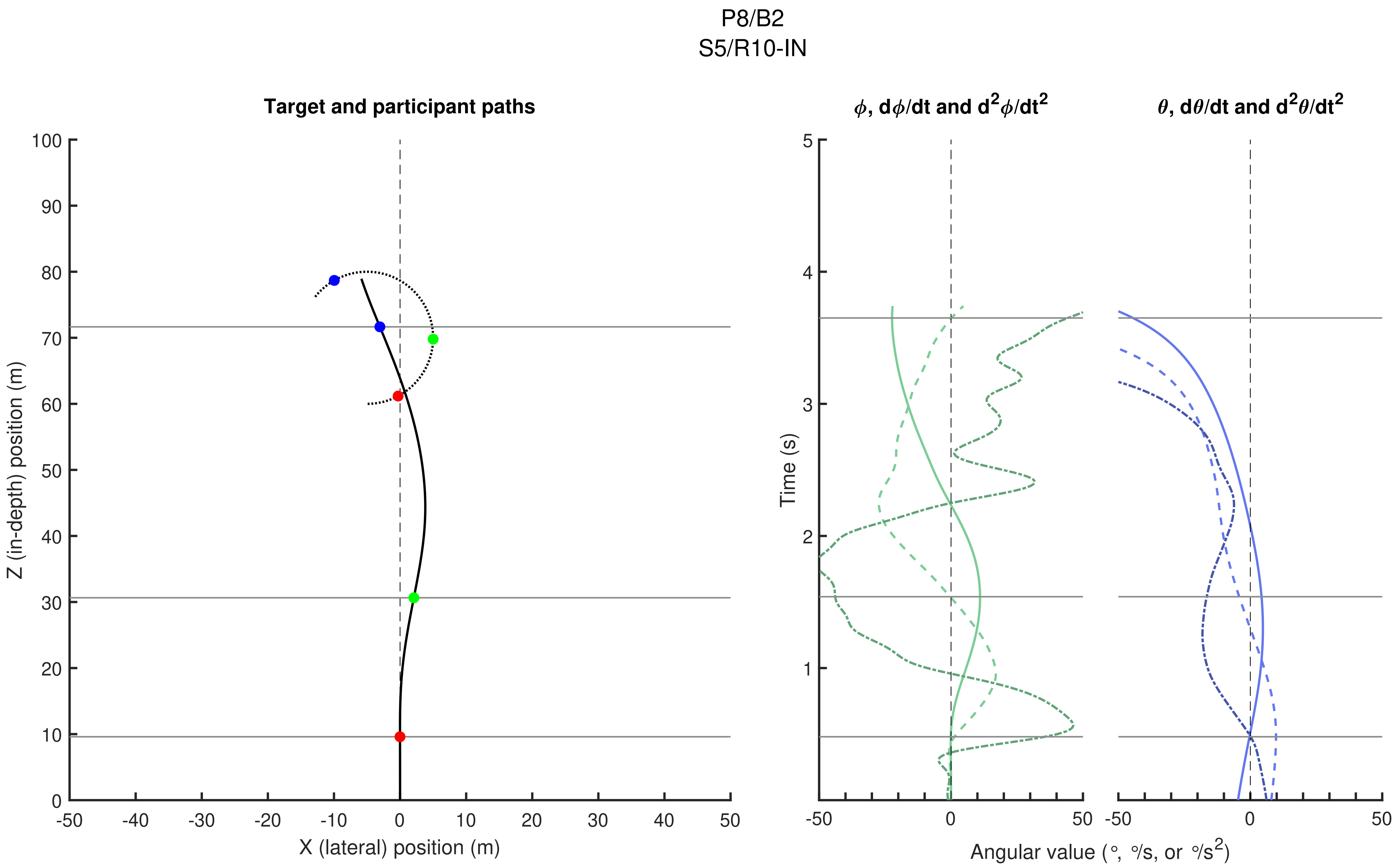

P8/B2
S5/R10-IN
Target and participant paths
φ, dφ/dt and d²φ/dt²
θ, dθ/dt and d²θ/dt²
Z (in-depth) position (m)
X (lateral) position (m)
Time (s)
Angular value (°, °/s, or °/s²)

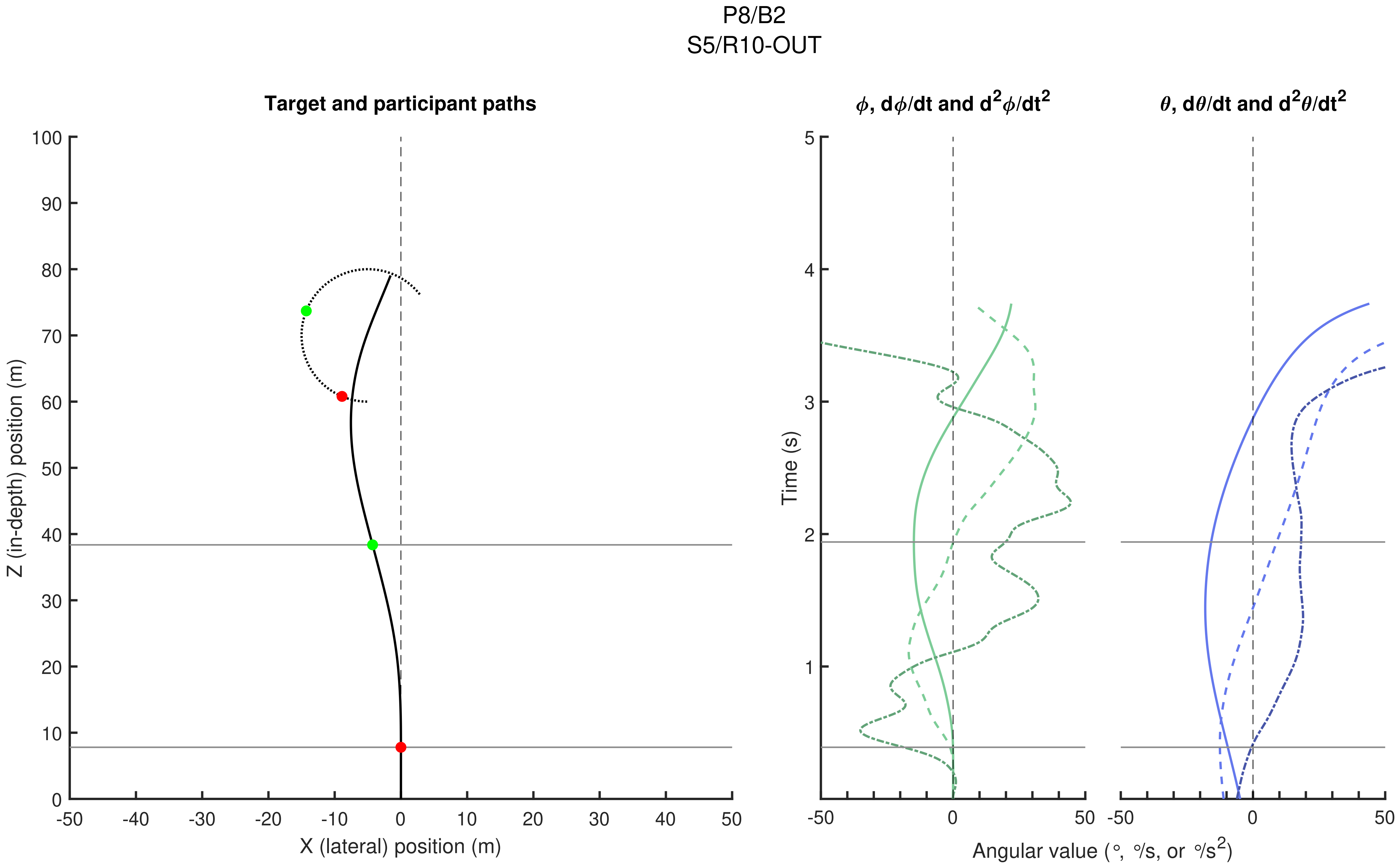
P8/B2
S5/R10-OUT
Target and participant paths
Z (in-depth) position (m)
X (lateral) position (m)
φ, dφ/dt and d²φ/dt²
θ, dθ/dt and d²θ/dt²
Time (s)
Angular value (°, °/s, or °/s²)

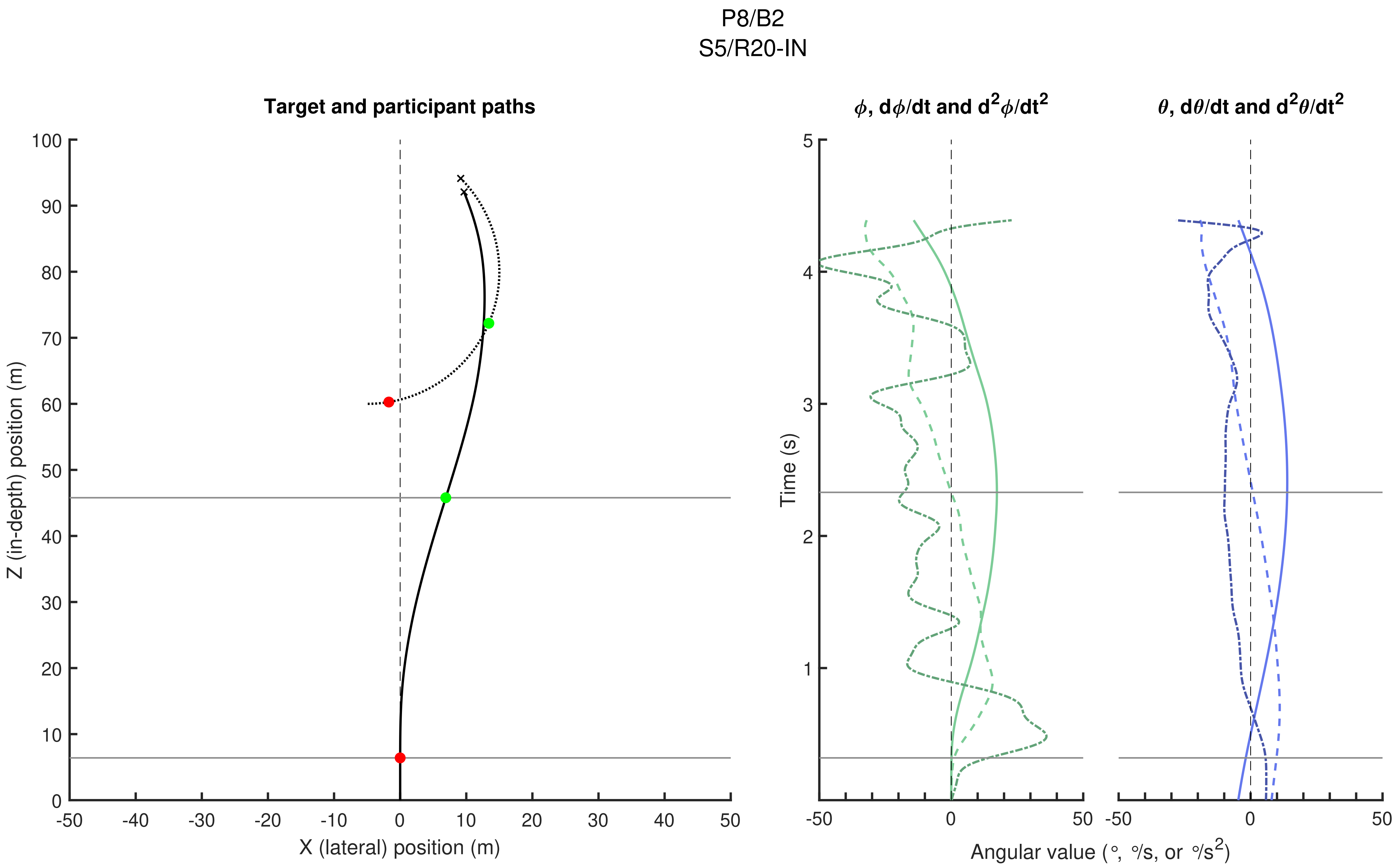
P8/B2
S5/R20-IN
Target and participant paths
Z (in-depth) position (m)
X (lateral) position (m)
ϕ, dϕ/dt and d$^2\phi$/dt^2
θ, dθ/dt and d$^2\theta$/dt^2
Time (s)
Angular value (°, °/s, or °/s^2)
100
90
80
70
60
50
40
30
20
10
0
-50
-40
-30
-20
-10
0
10
20
30
40
50
5
4
3
2
1
-50
0
50
-50
0
50

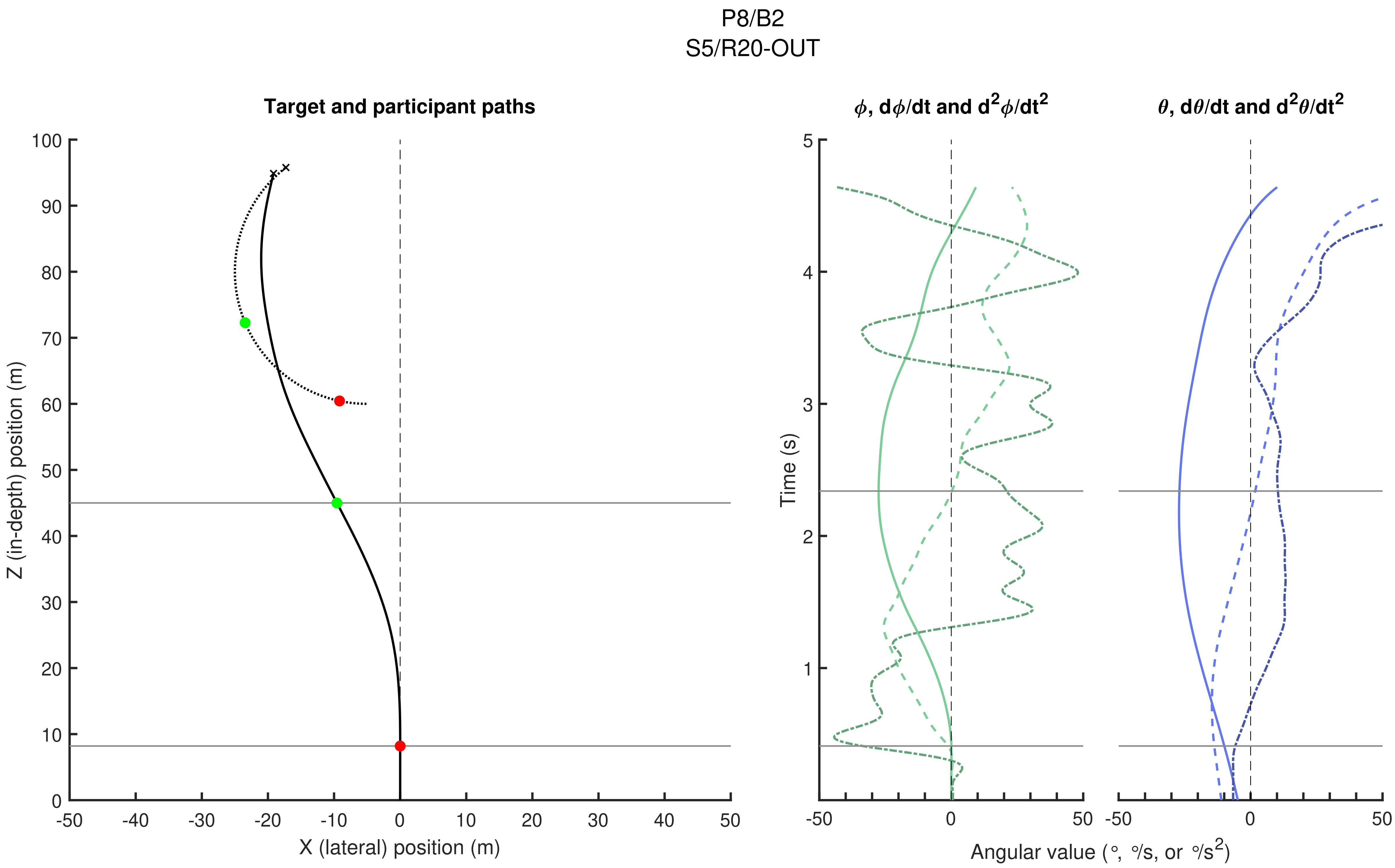

P8/B2
S5/R20-OUT
Target and participant paths
Z (in-depth) position (m)
X (lateral) position (m)
φ, dφ/dt and d²φ/dt²
θ, dθ/dt and d²θ/dt²
Time (s)
Angular value (°, °/s, or °/s²)

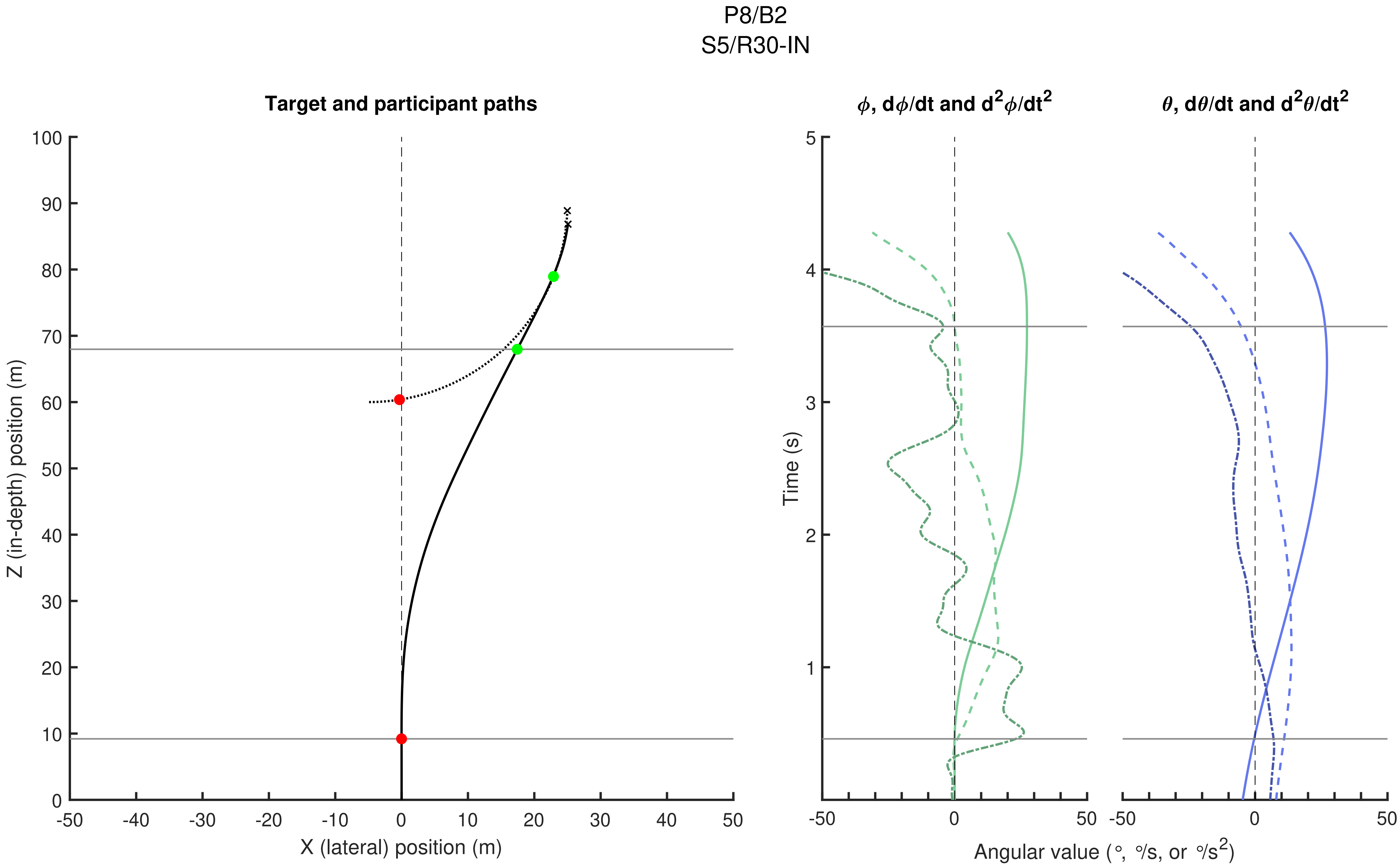

P8/B2
S5/R30-IN
Target and participant paths
Z (in-depth) position (m)
X (lateral) position (m)
ϕ, dϕ/dt and d$^2\phi$/dt^2
θ, dθ/dt and d$^2\theta$/dt^2
Time (s)
Angular value (°, °/s, or °/s^2)

P8/B2
S5/R30-OUT

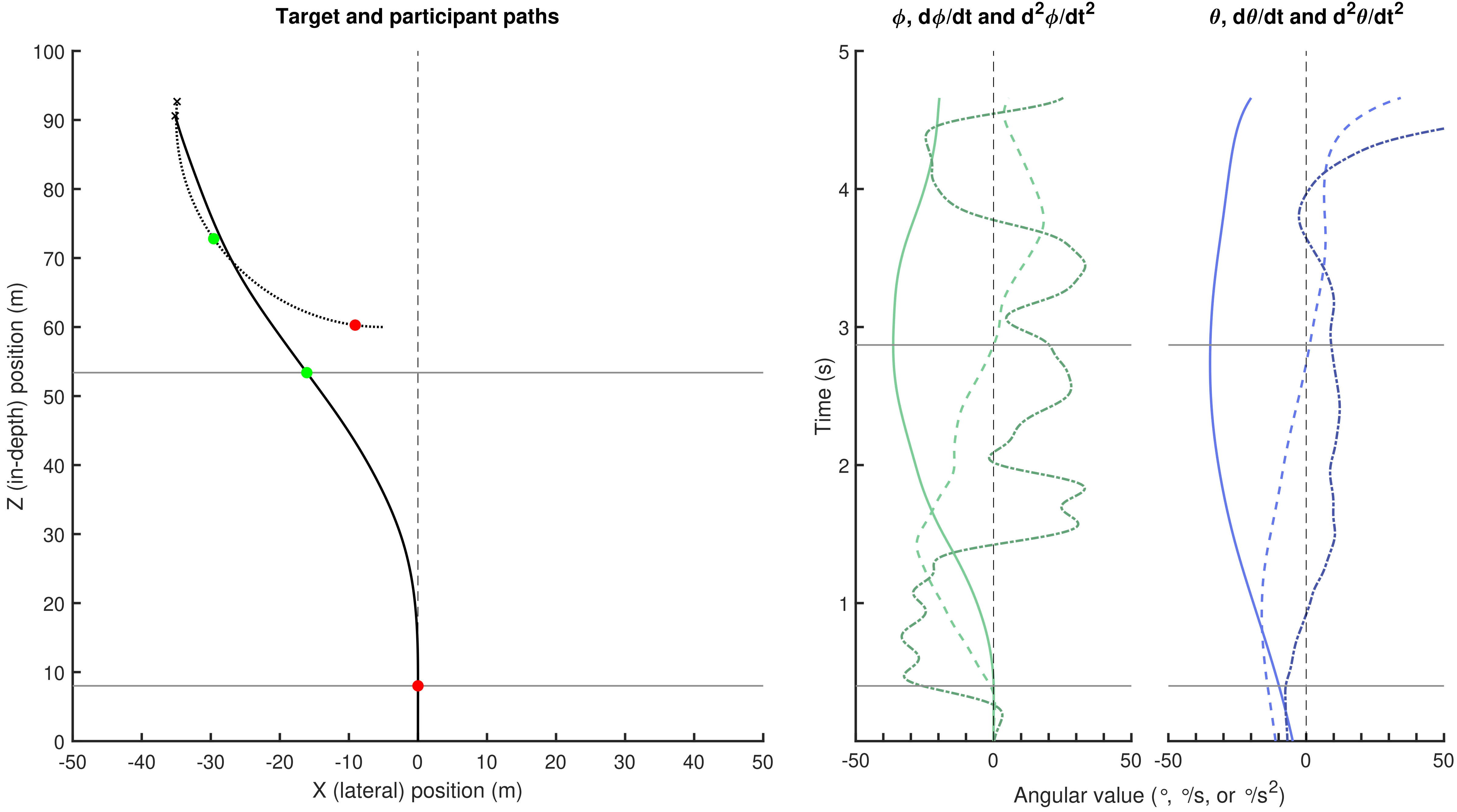

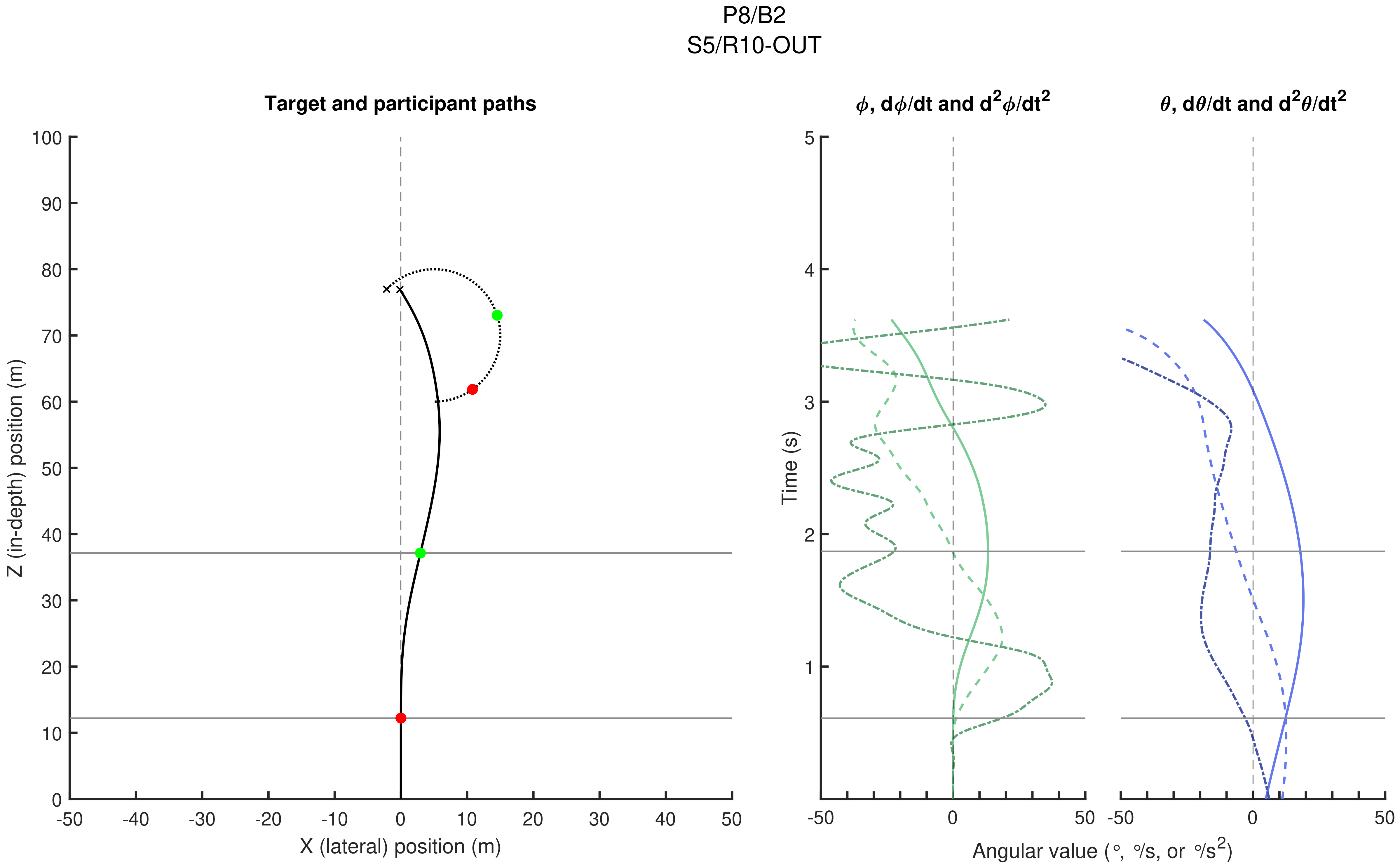

P8/B2
S5/R10-OUT
Target and participant paths
ϕ, dϕ/dt and d$^2\phi$/dt^2
θ, dθ/dt and d$^2\theta$/dt^2
Z (in-depth) position (m)
X (lateral) position (m)
Time (s)
Angular value (°, °/s, or °/s^2)

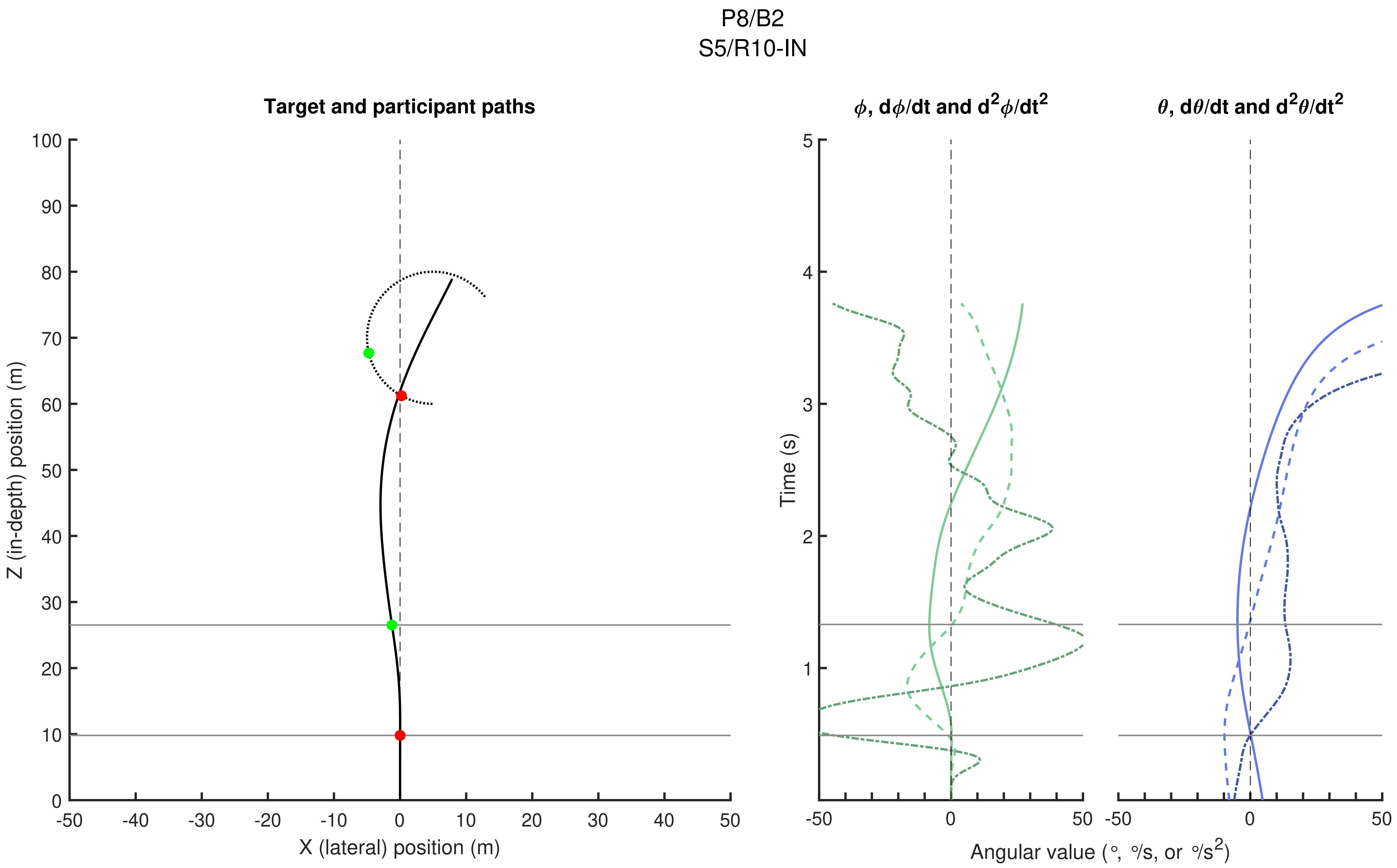

P8/B2
S5/R10-IN
Target and participant paths
Z (in-depth) position (m)
X (lateral) position (m)
φ, dφ/dt and d²φ/dt²
θ, dθ/dt and d²θ/dt²
Time (s)
Angular value (°, °/s, or °/s²)

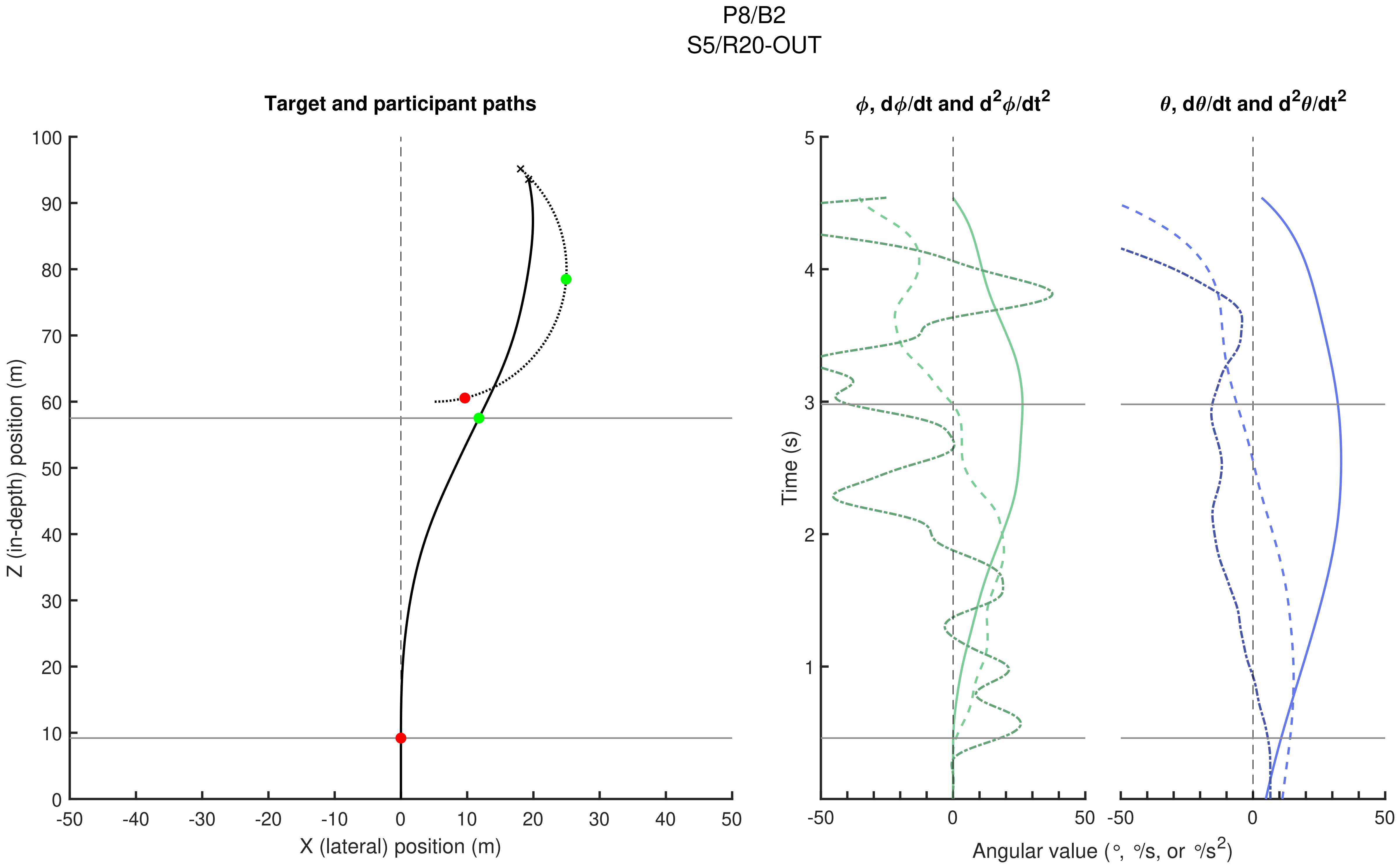

P8/B2
S5/R20-OUT
Target and participant paths
Z (in-depth) position (m)
X (lateral) position (m)
100
90
80
70
60
50
40
30
20
10
0
-50
-40
-30
-20
-10
0
10
20
30
40
50
φ, dφ/dt and d²φ/dt²
θ, dθ/dt and d²θ/dt²
Time (s)
5
4
3
2
1
-50
0
50
-50
0
50
Angular value (°, °/s, or °/s²)

P8/B2
S5/R20-IN

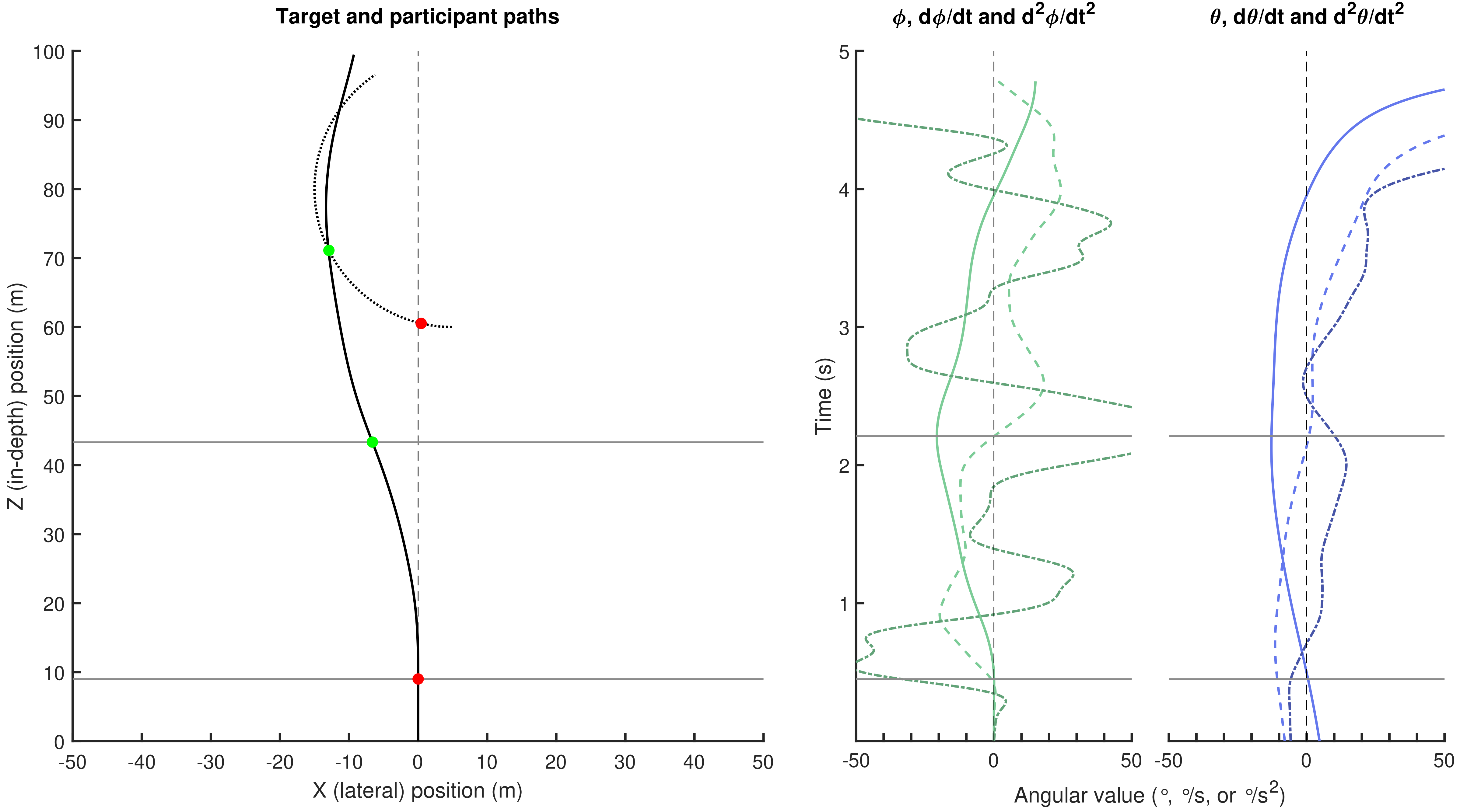

P8/B2
S5/R30-OUT

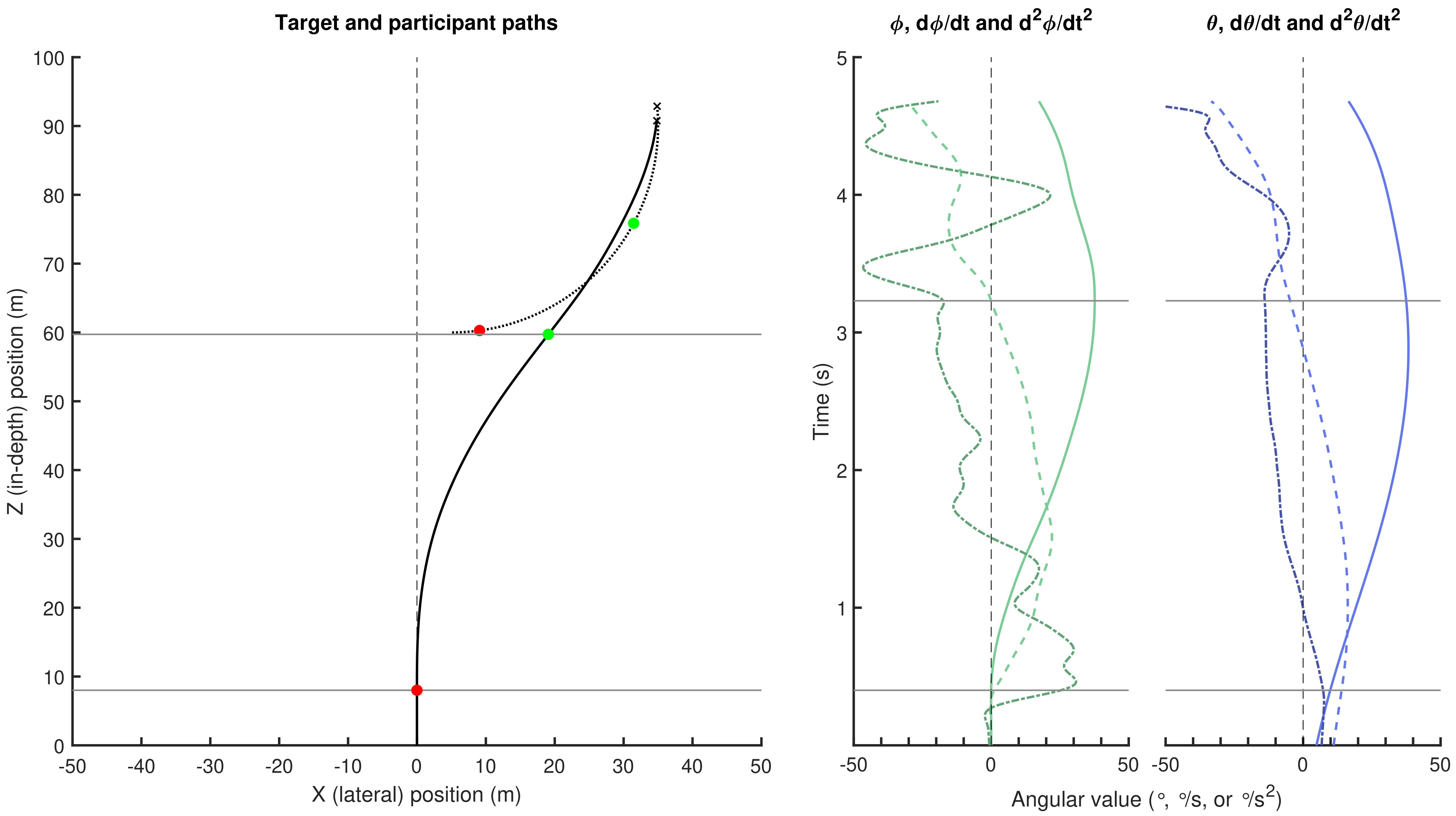

P8/B2
S5/R30-IN

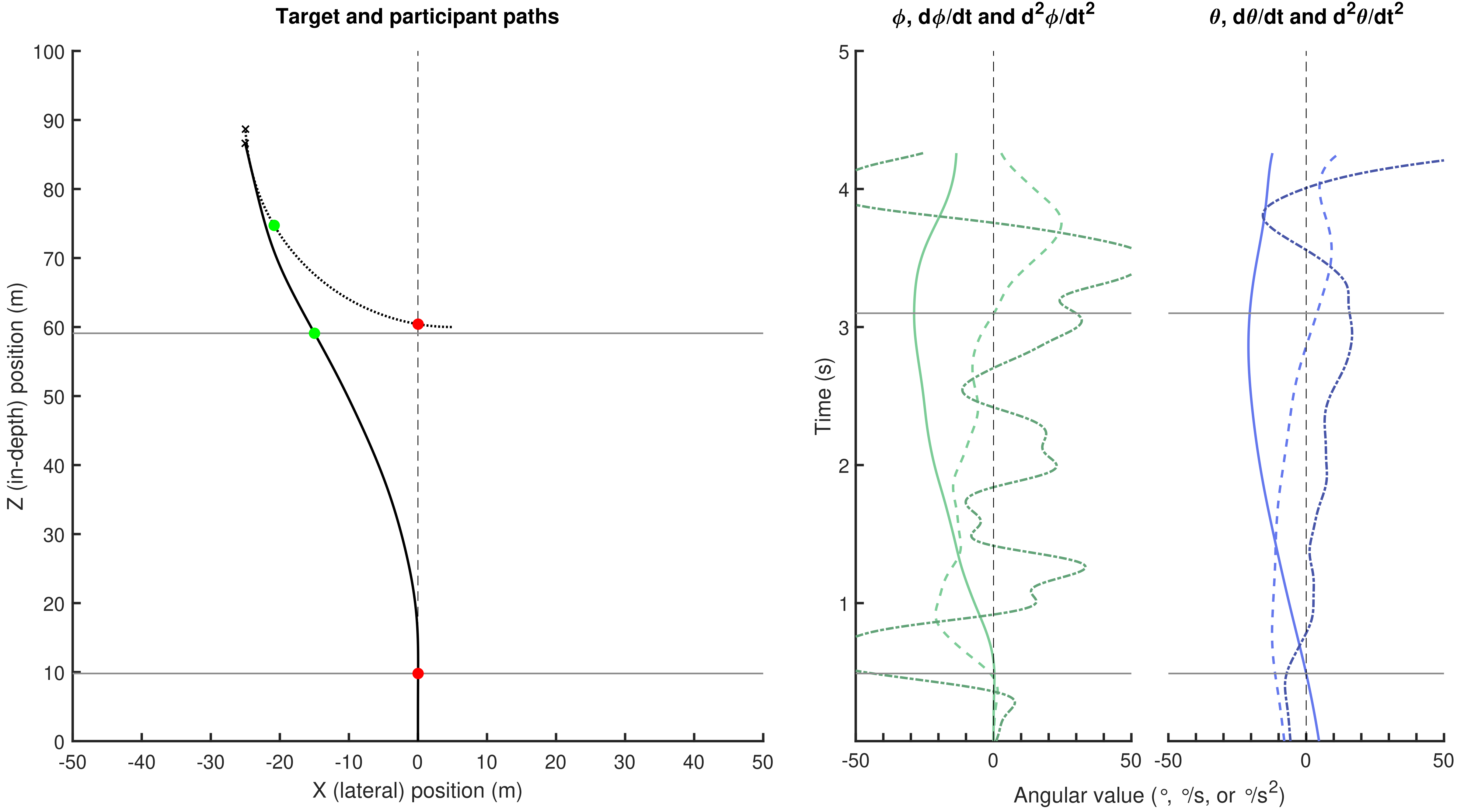

P8/B2
S15/R10-IN

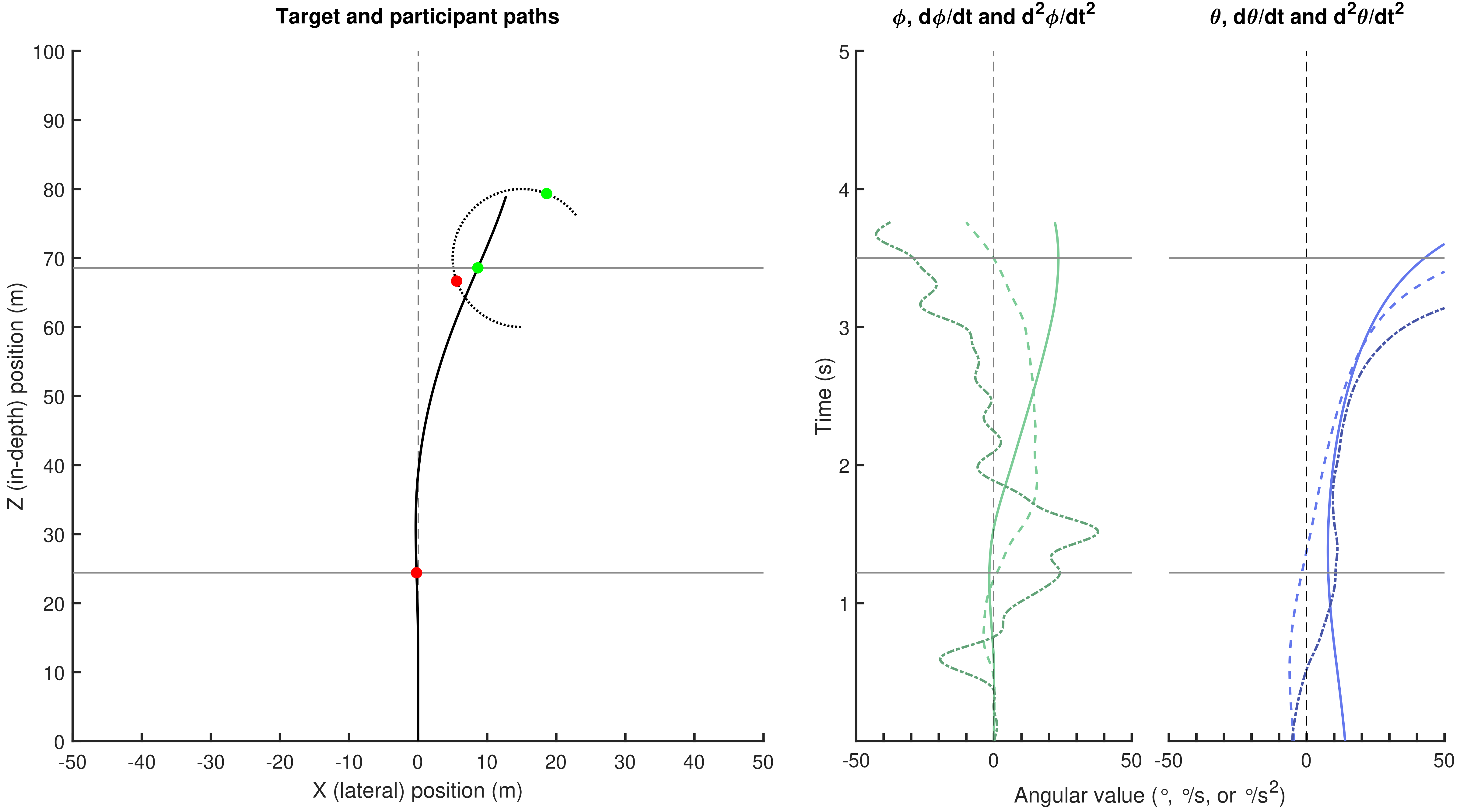

P8/B2
S15/R20-IN

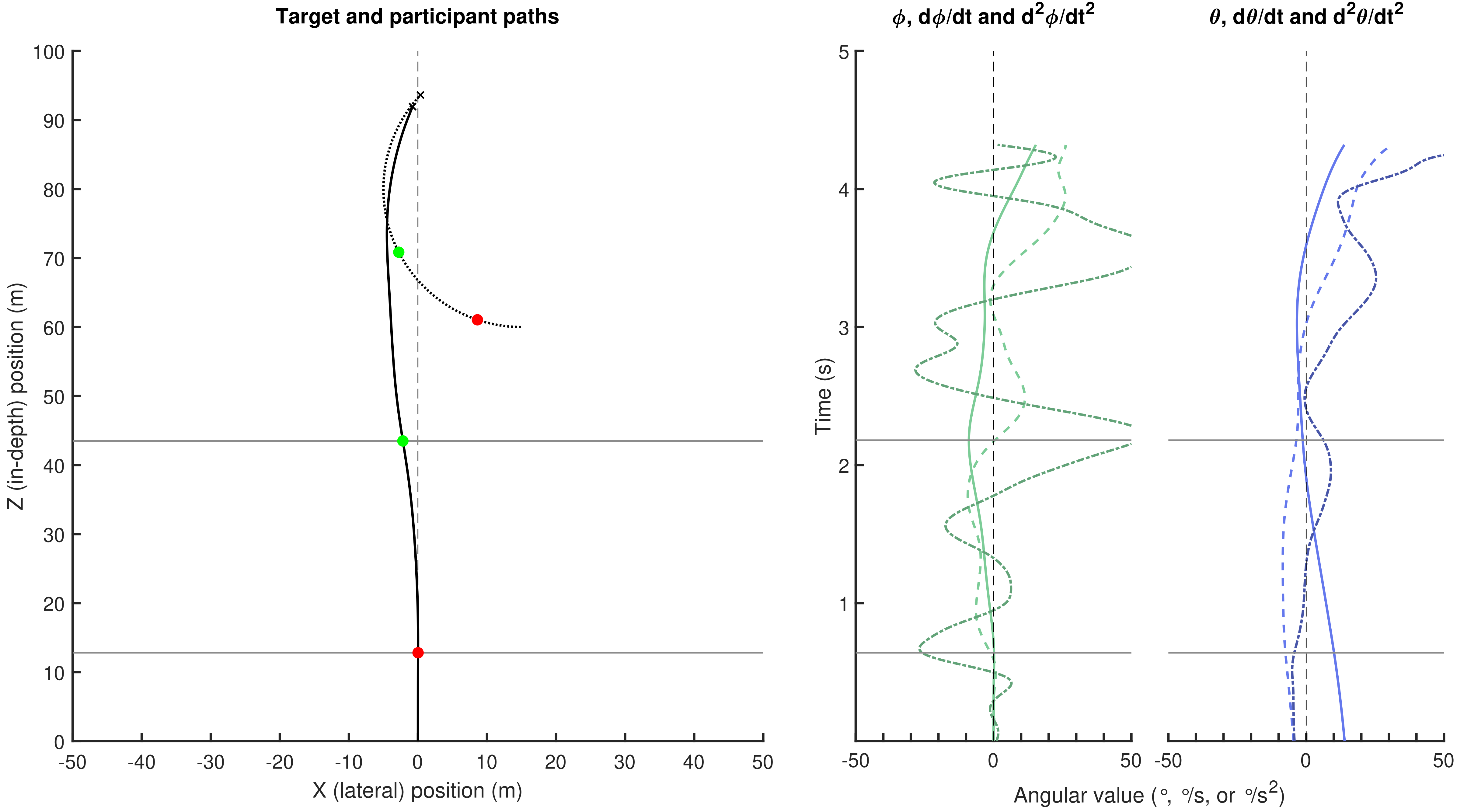

P8/B2
S15/R30-IN

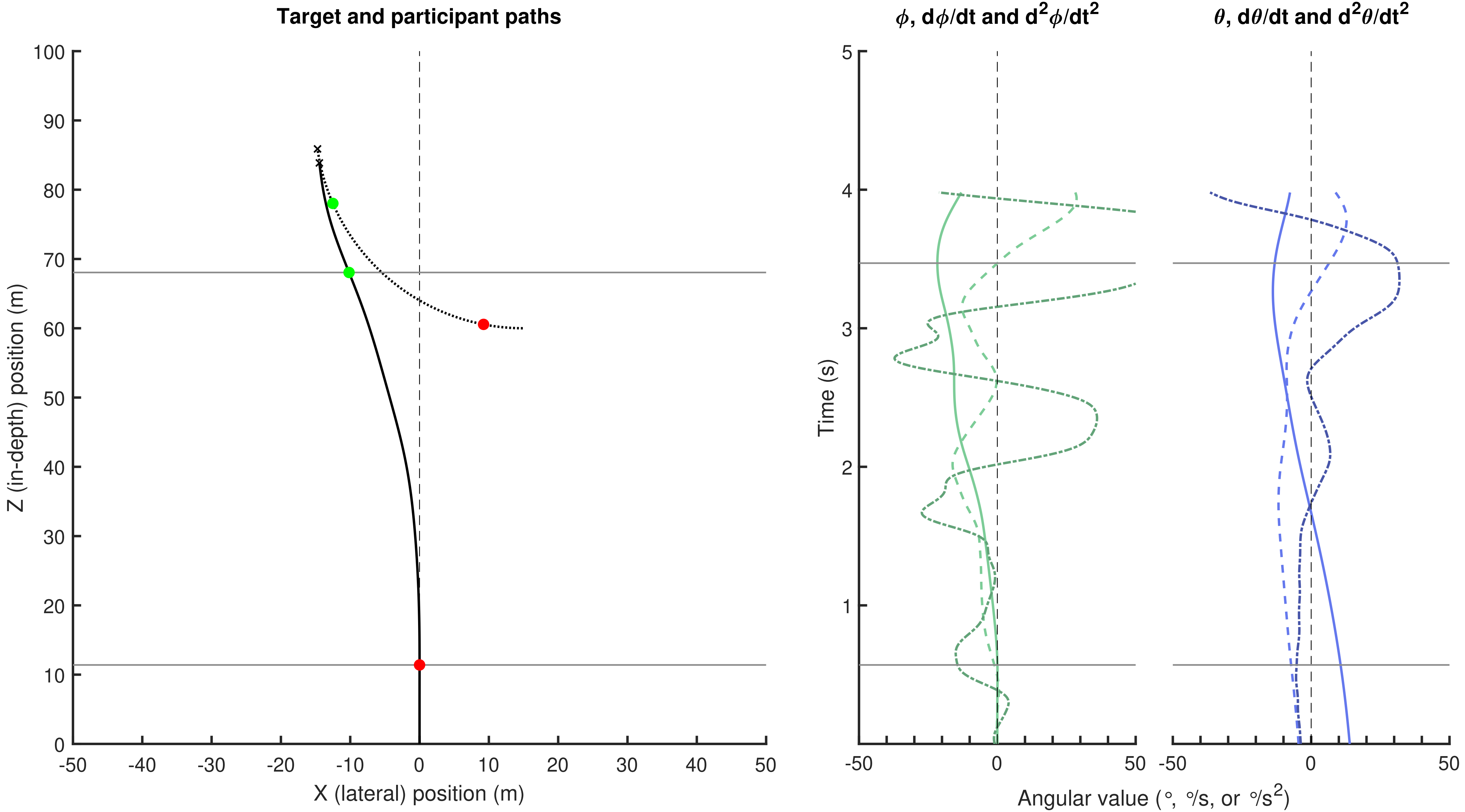

P8/B2
S20/R10-IN

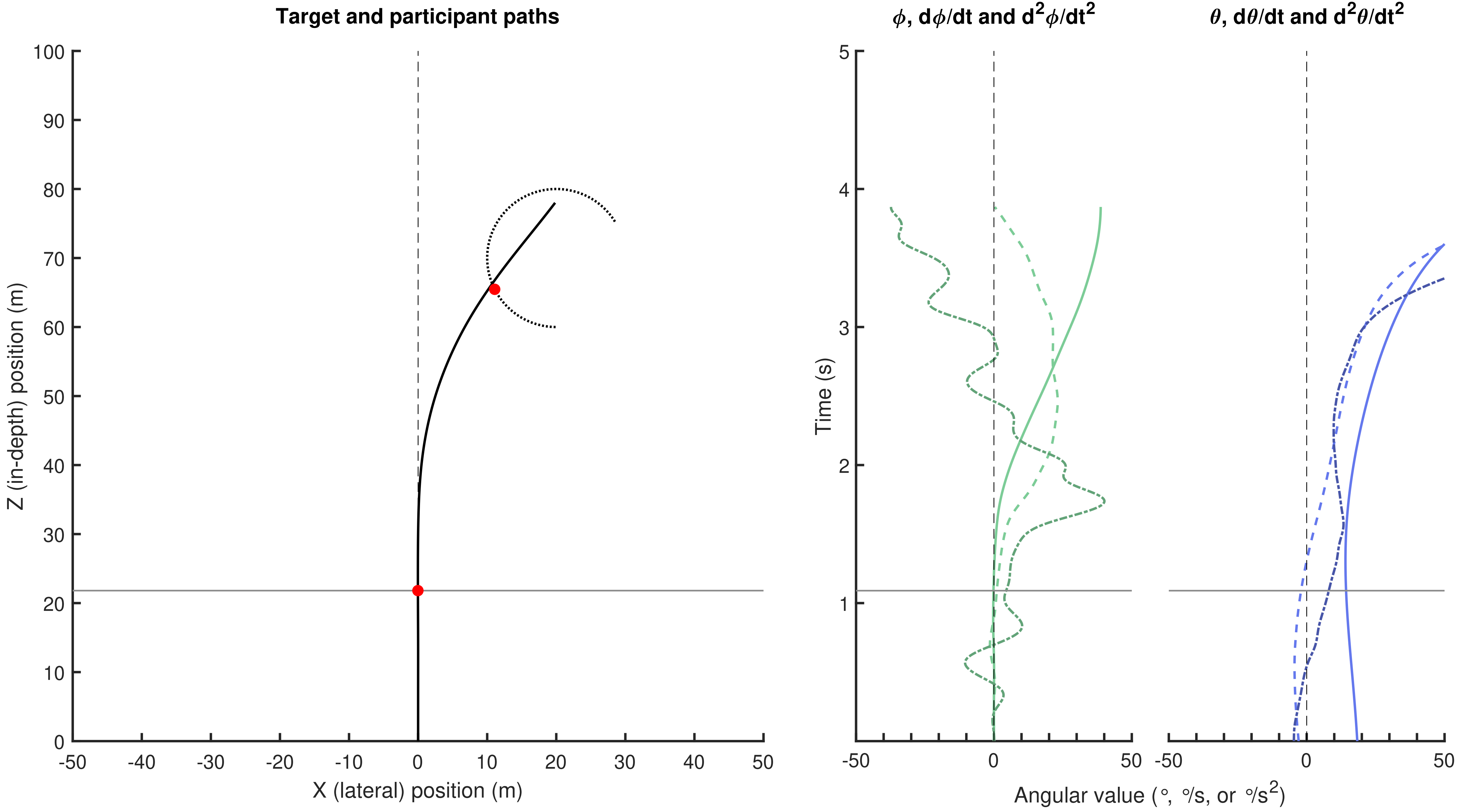

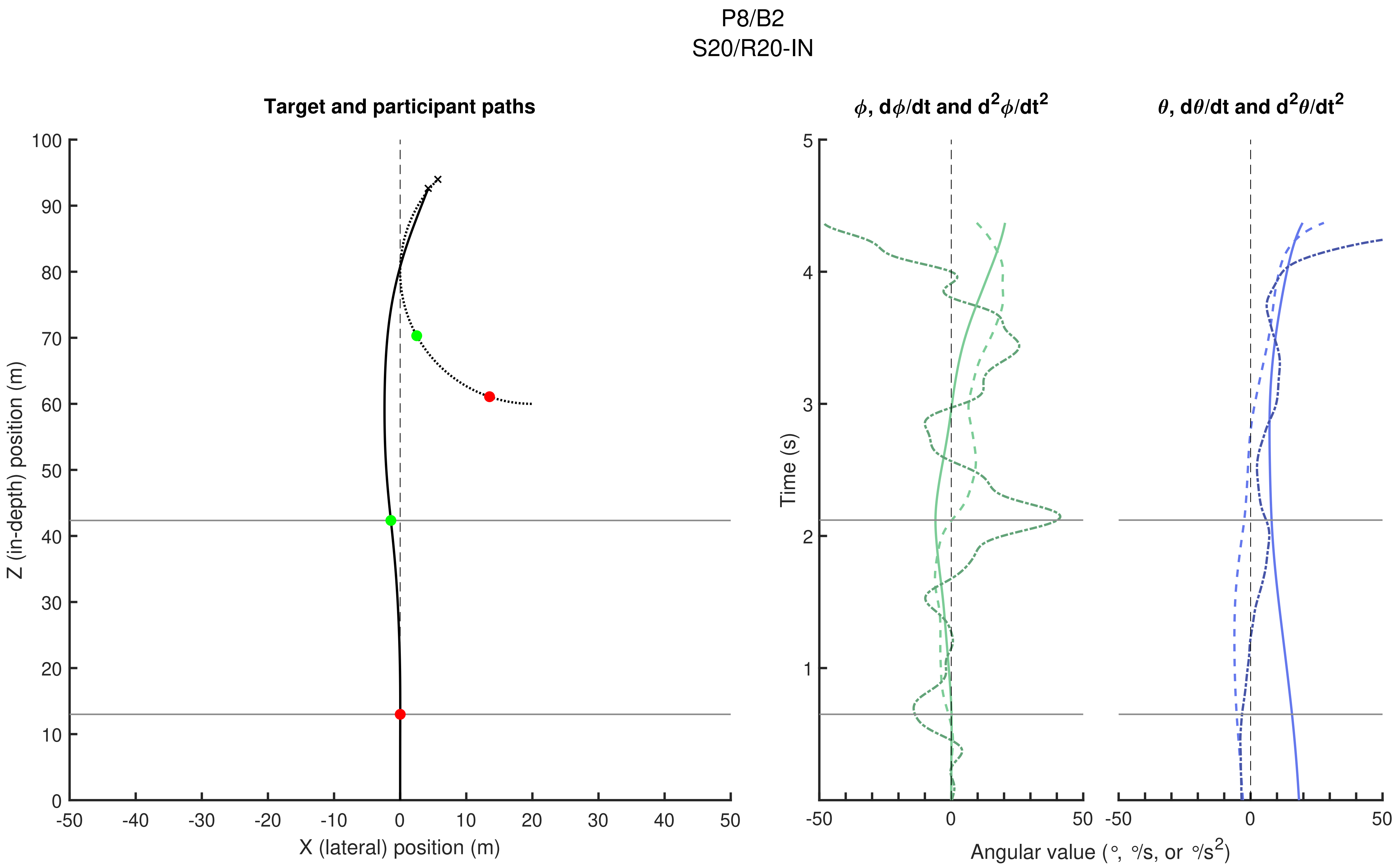

P8/B2
S20/R20-IN
Target and participant paths
ϕ, dϕ/dt and d$^2\phi$/dt^2
θ, dθ/dt and d$^2\theta$/dt^2
Z (in-depth) position (m)
X (lateral) position (m)
Time (s)
Angular value (°, °/s, or °/s^2)

P8/B2
S20/R30-IN

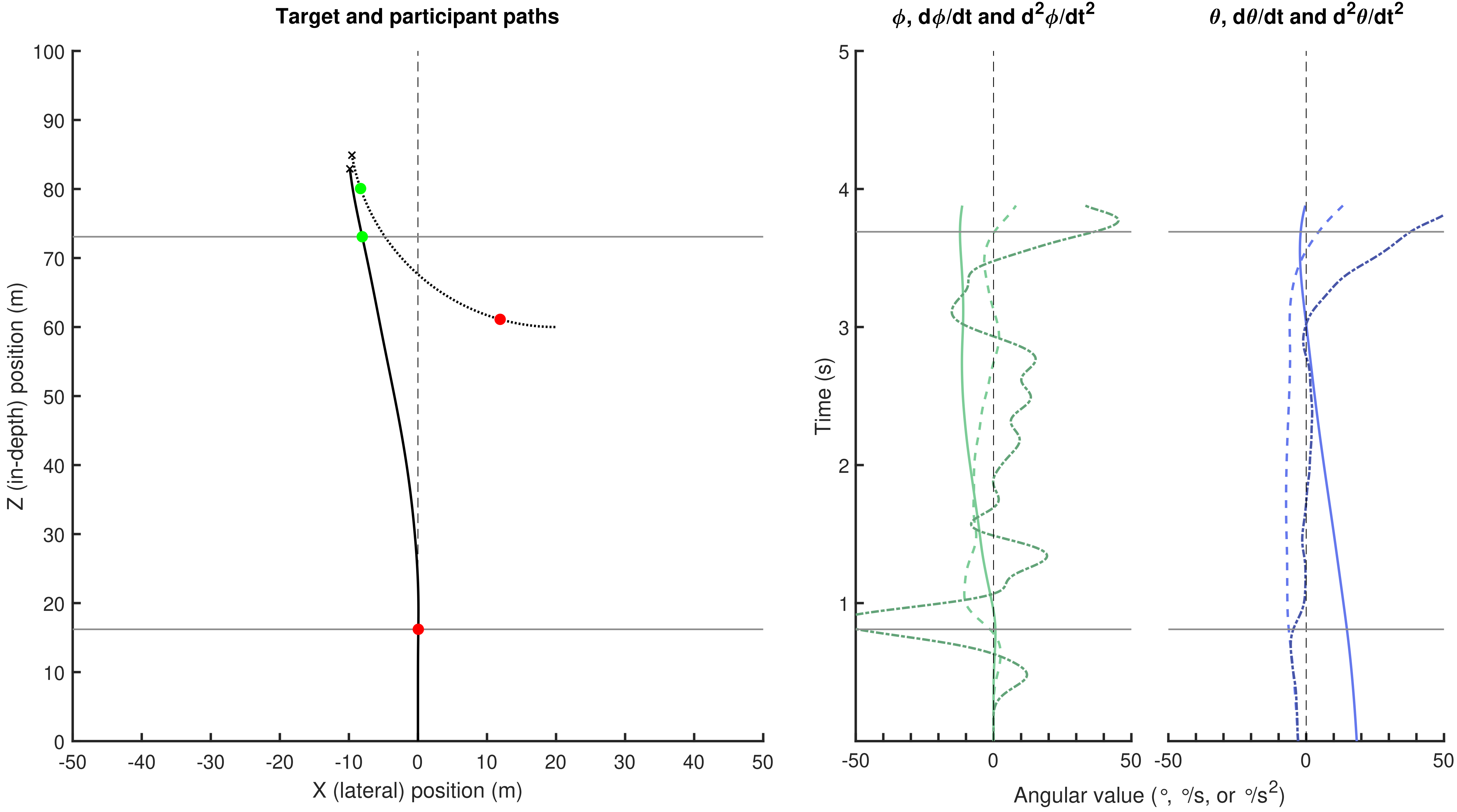

P8/B3
S20/R10-IN

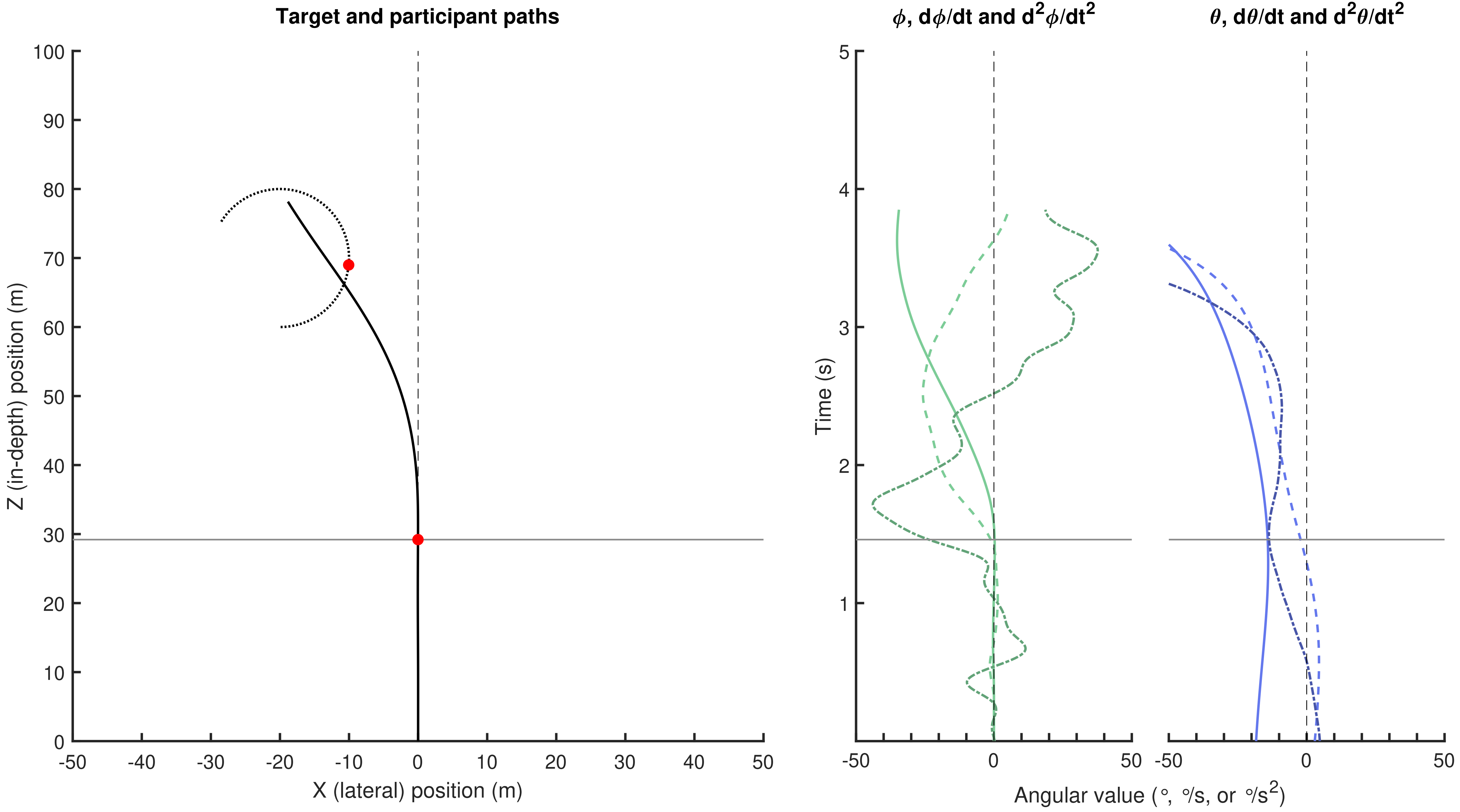

P8/B3
S20/R20-IN

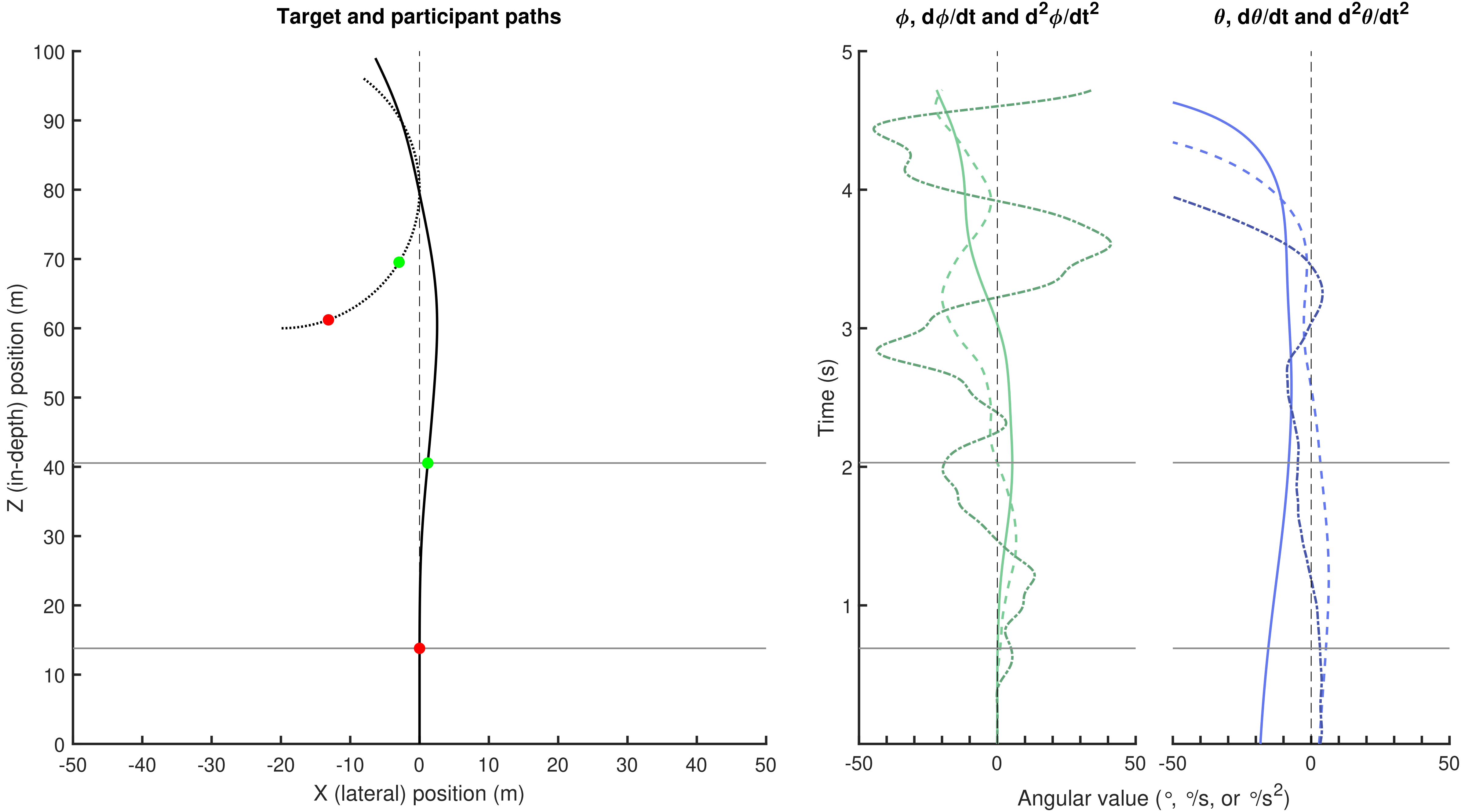

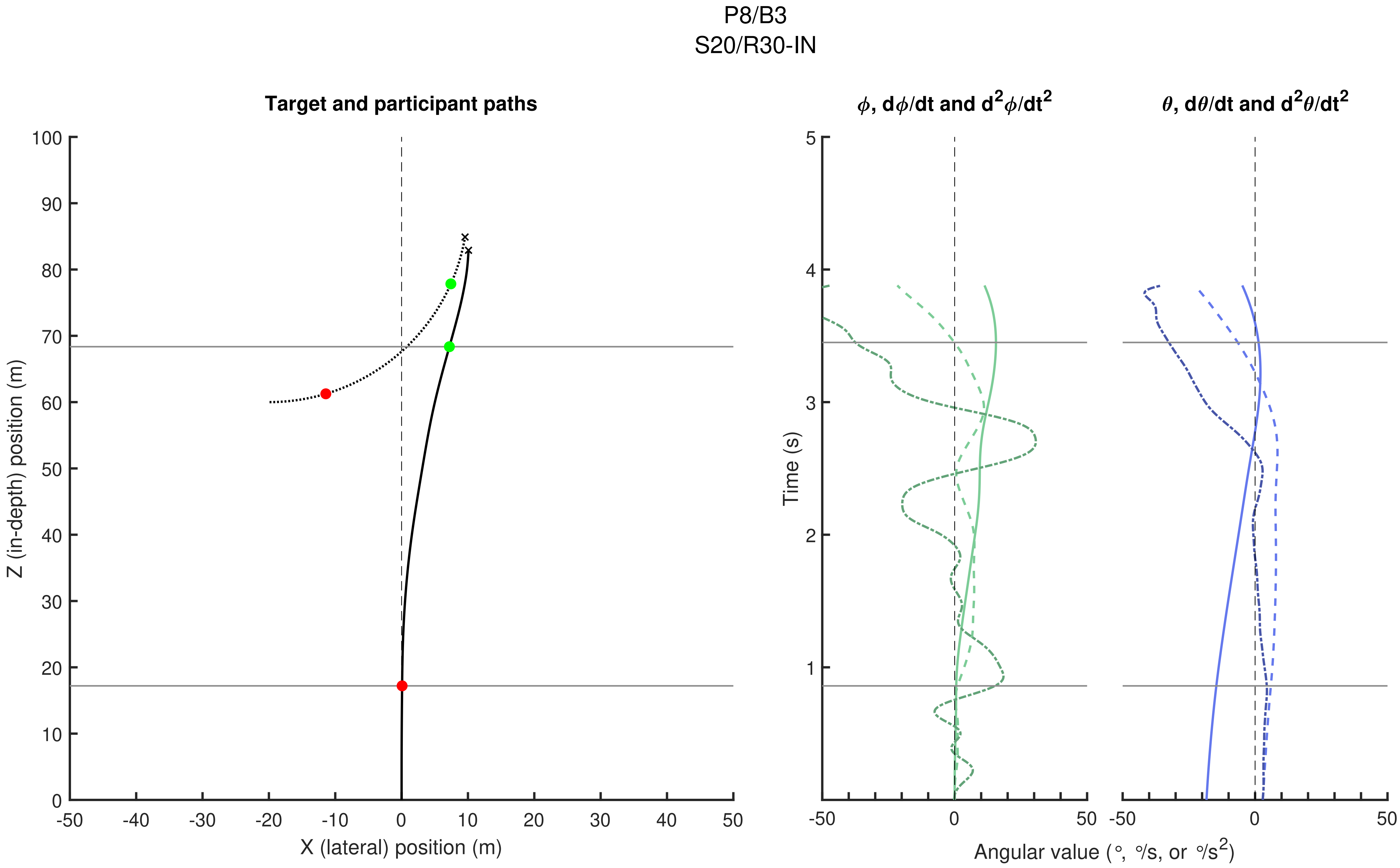

P8/B3
S20/R30-IN
Target and participant paths
ϕ, dϕ/dt and d$^2\phi$/dt^2
θ, dθ/dt and d$^2\theta$/dt^2
Z (in-depth) position (m)
X (lateral) position (m)
Time (s)
Angular value (°, °/s, or °/s^2)
100
90
80
70
60
50
40
30
20
10
0
-50
-40
-30
-20
-10
0
10
20
30
40
50
5
4
3
2
1
-50
0
50
-50
0
50

P8/B3
S15/R10-IN

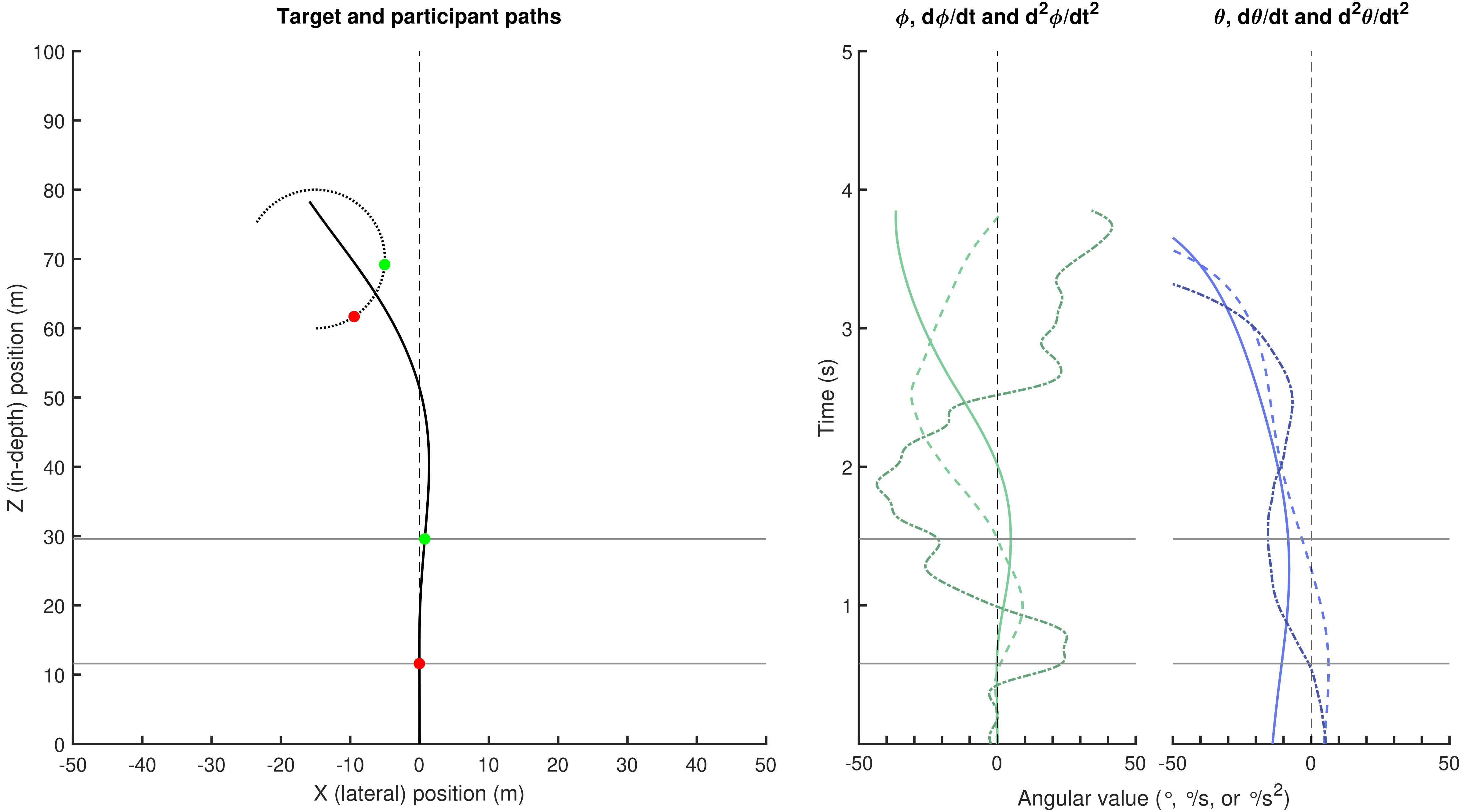

P8/B3
S15/R20-IN

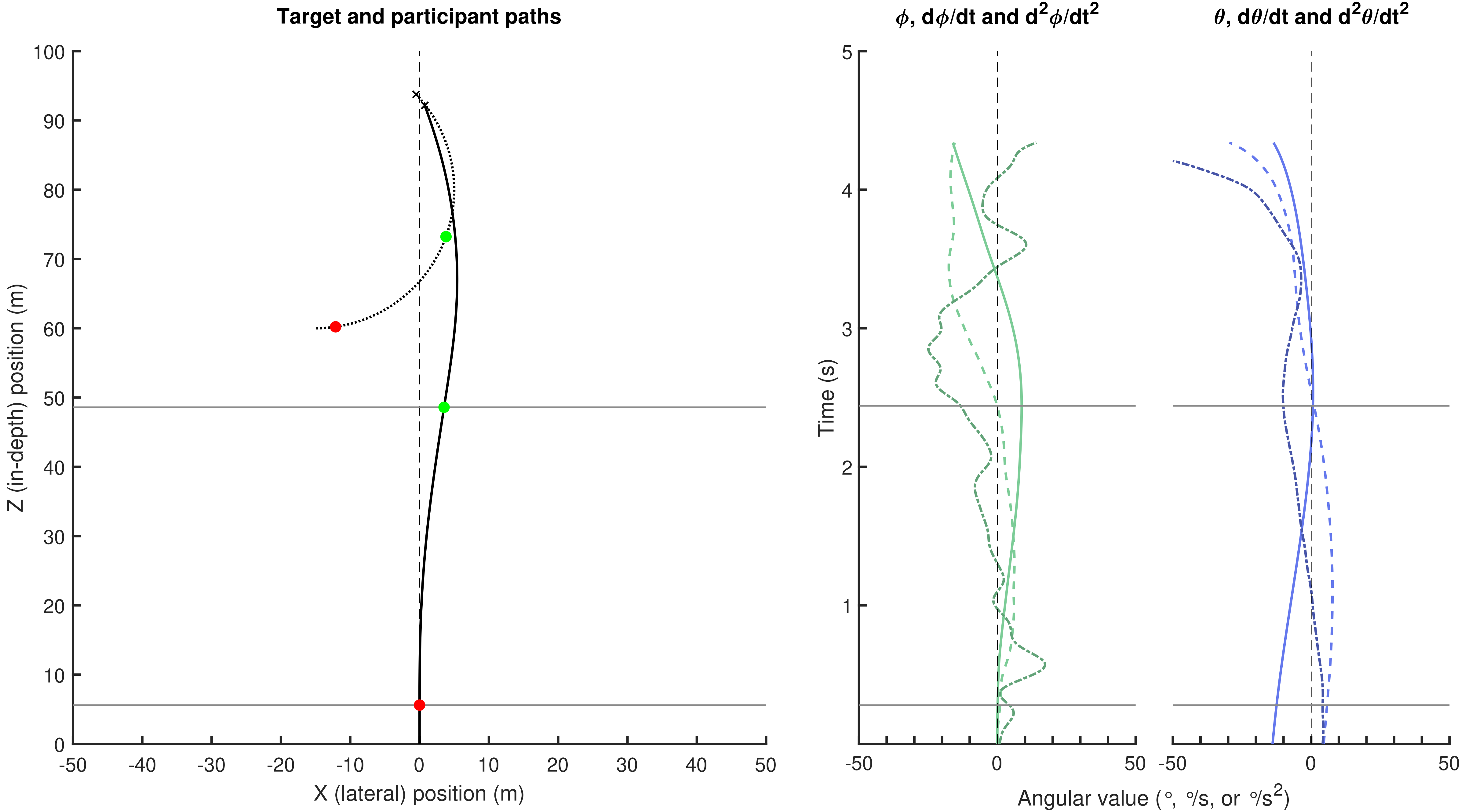

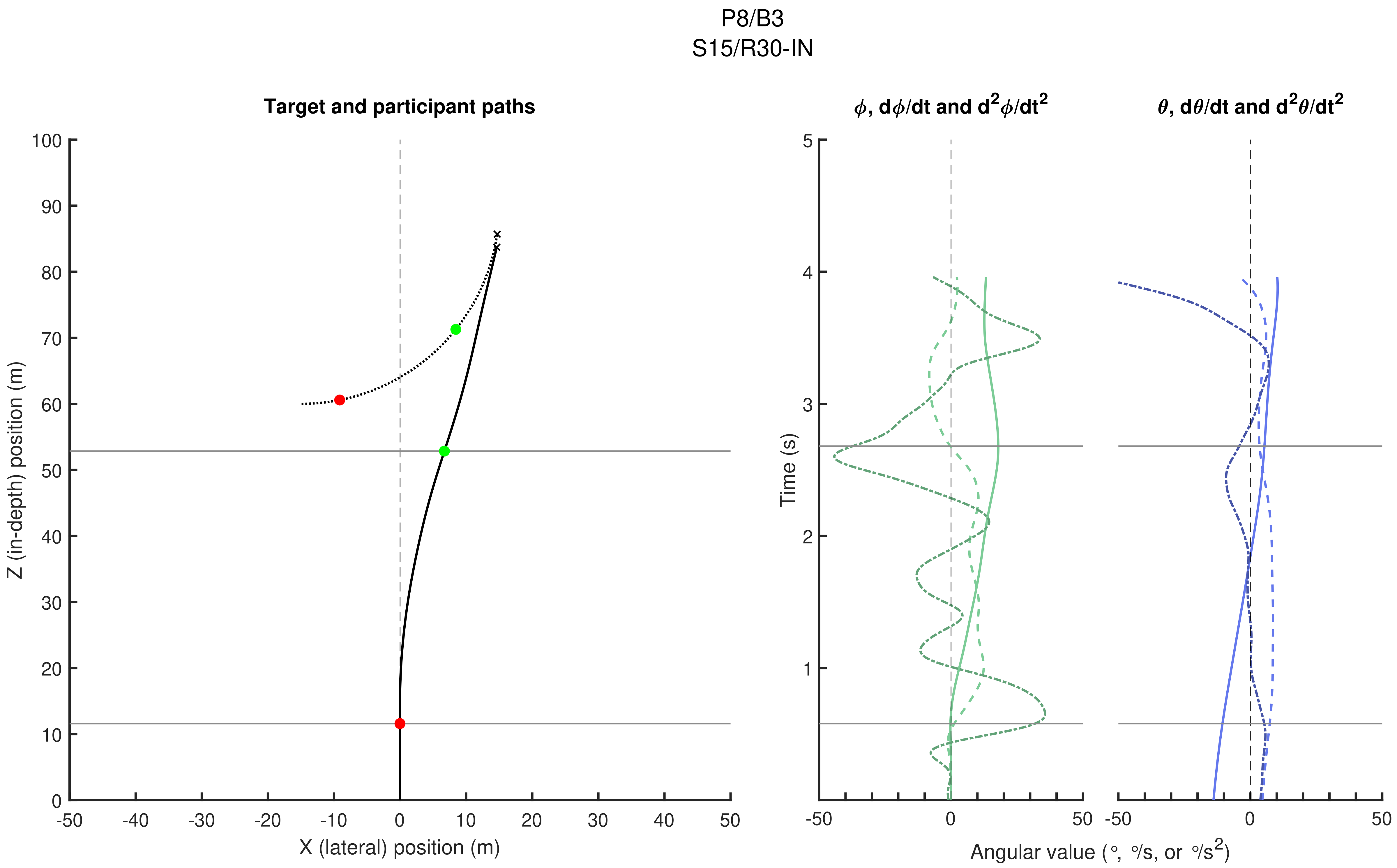

P8/B3
S15/R30-IN
Target and participant paths
Z (in-depth) position (m)
X (lateral) position (m)
100
90
80
70
60
50
40
30
20
10
0
-50
-40
-30
-20
-10
0
10
20
30
40
50
ϕ, dϕ/dt and d²ϕ/dt²
θ, dθ/dt and d²θ/dt²
Time (s)
5
4
3
2
1
-50
0
50
-50
0
50
Angular value (°, °/s, or °/s²)

P8/B3
S5/R10-IN

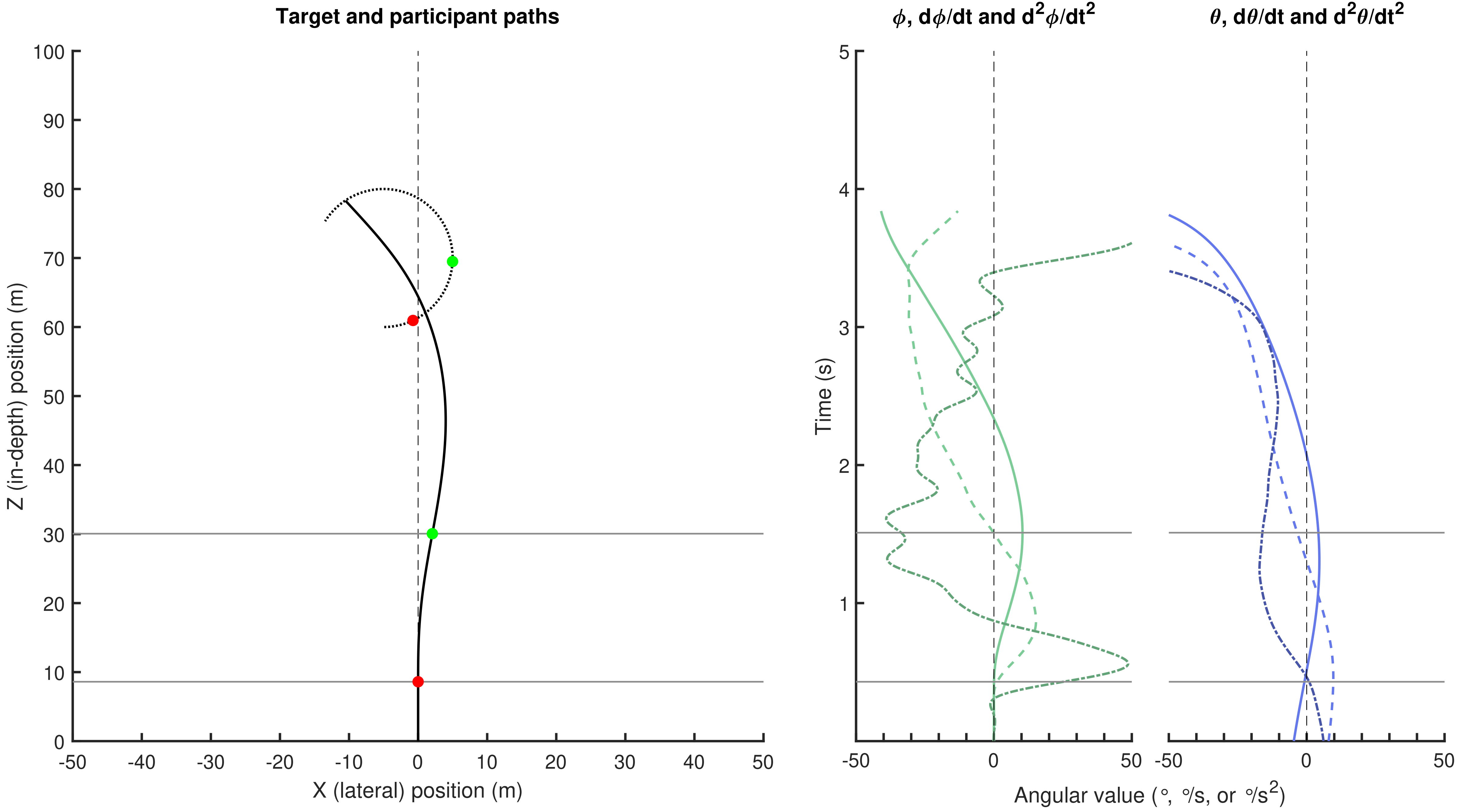

P8/B3
S5/R10-OUT

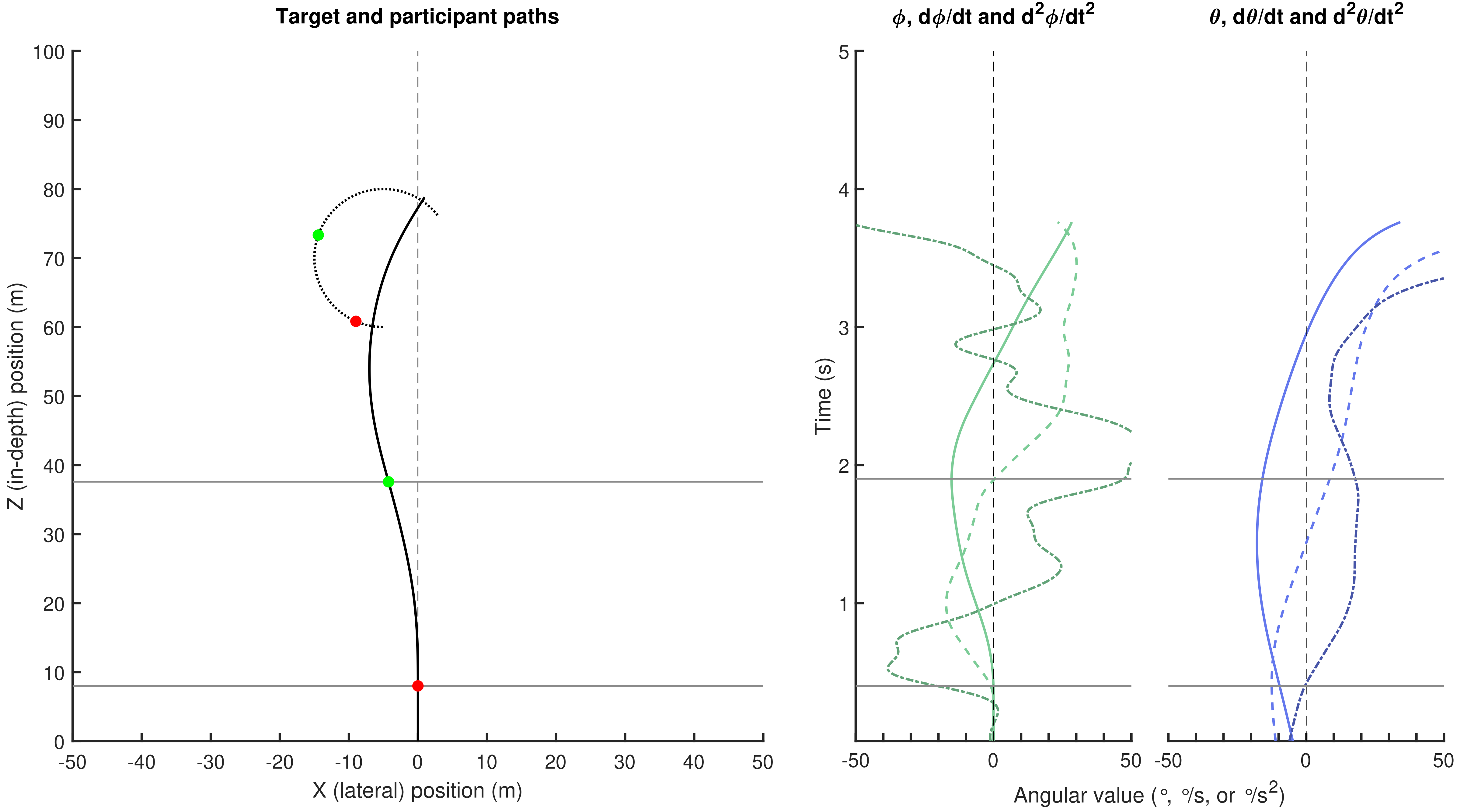

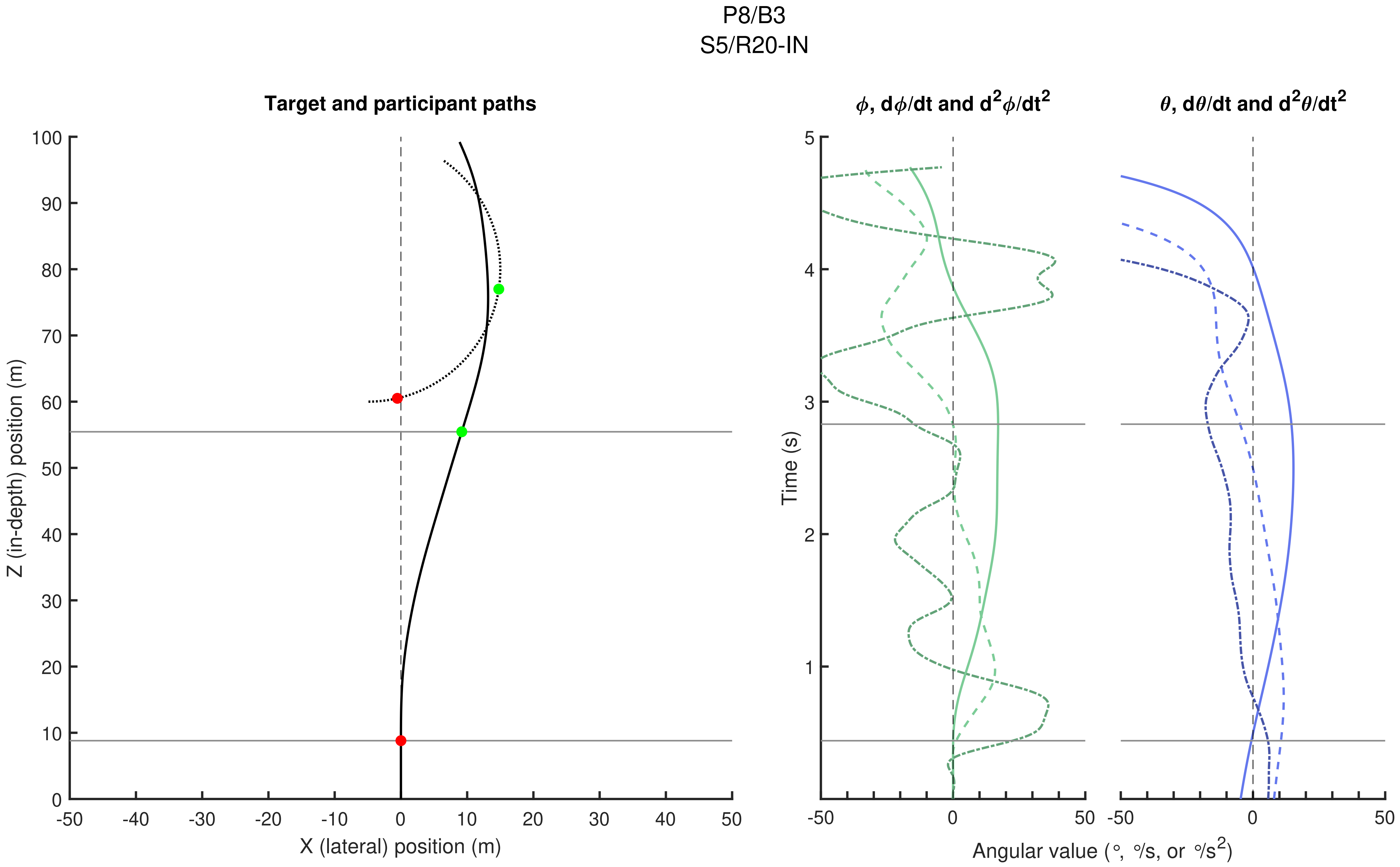
P8/B3
S5/R20-IN
Target and participant paths
Z (in-depth) position (m)
X (lateral) position (m)
ϕ, dϕ/dt and d²ϕ/dt²
θ, dθ/dt and d²θ/dt²
Time (s)
Angular value (°, °/s, or °/s²)

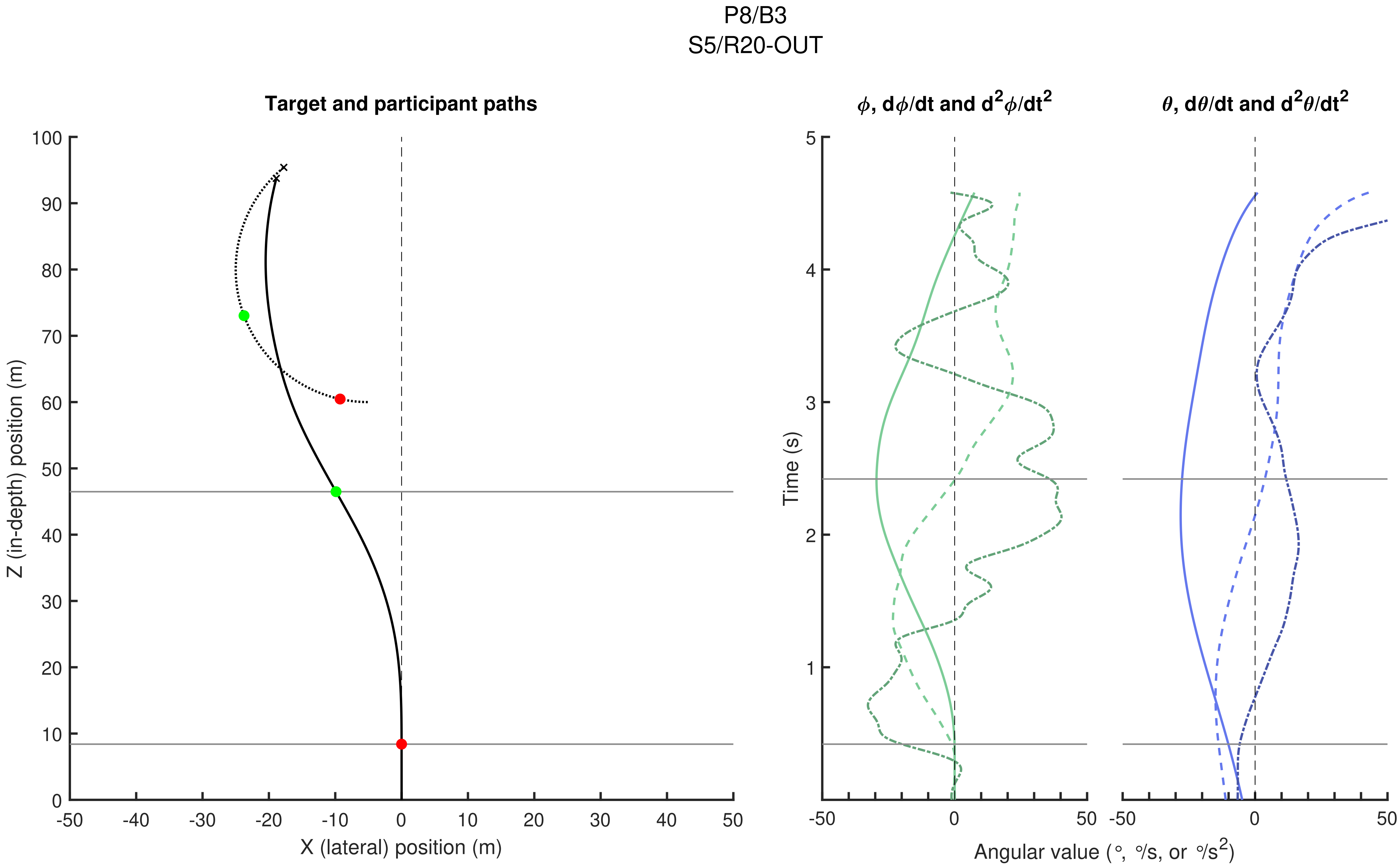
P8/B3
S5/R20-OUT
Target and participant paths
Z (in-depth) position (m)
X (lateral) position (m)
100
90
80
70
60
50
40
30
20
10
0
-50
-40
-30
-20
-10
0
10
20
30
40
50
ϕ, dϕ/dt and d²ϕ/dt²
θ, dθ/dt and d²θ/dt²
Time (s)
5
4
3
2
1
-50
0
50
-50
0
50
Angular value (°, °/s, or °/s²)

P8/B3
S5/R30-IN

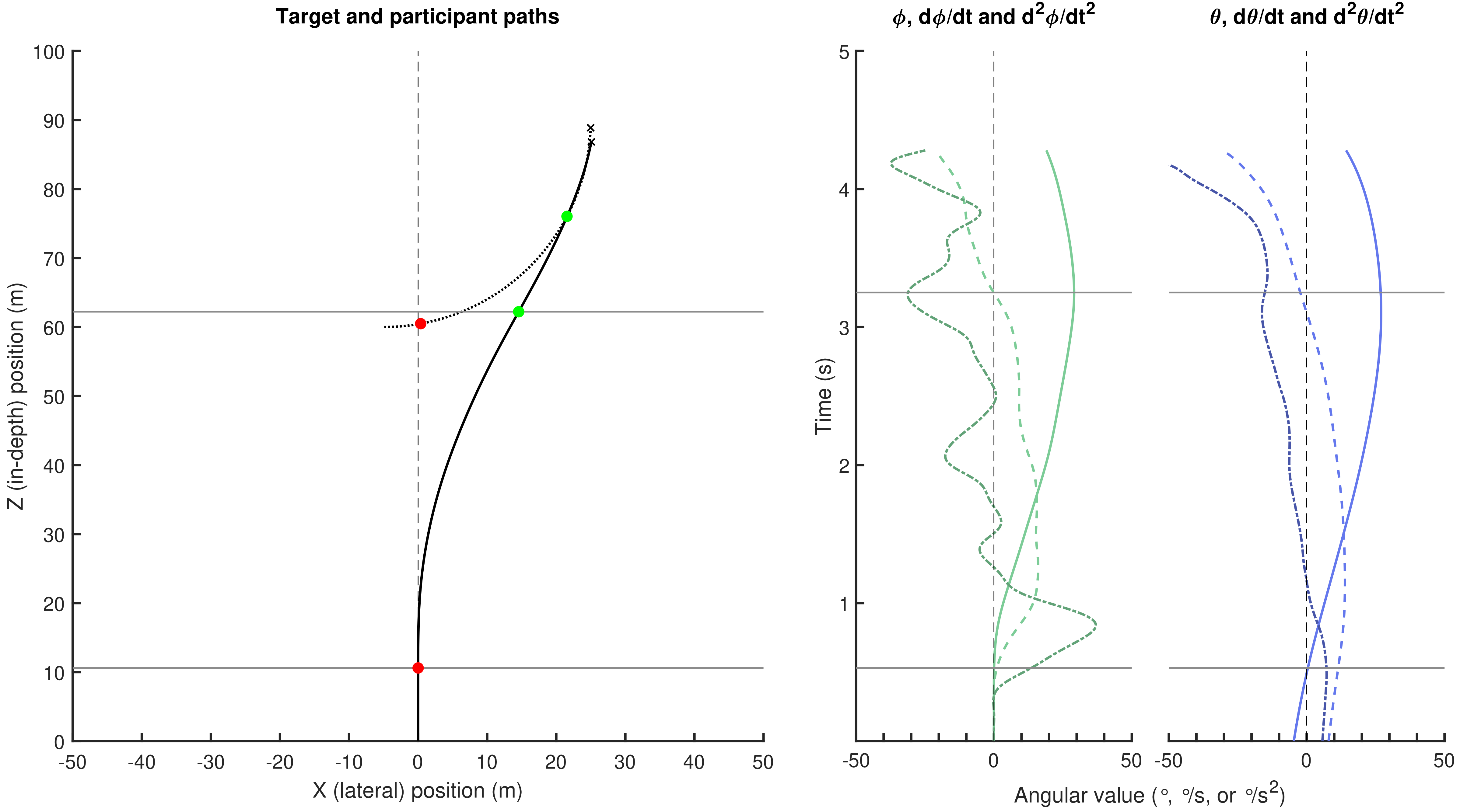

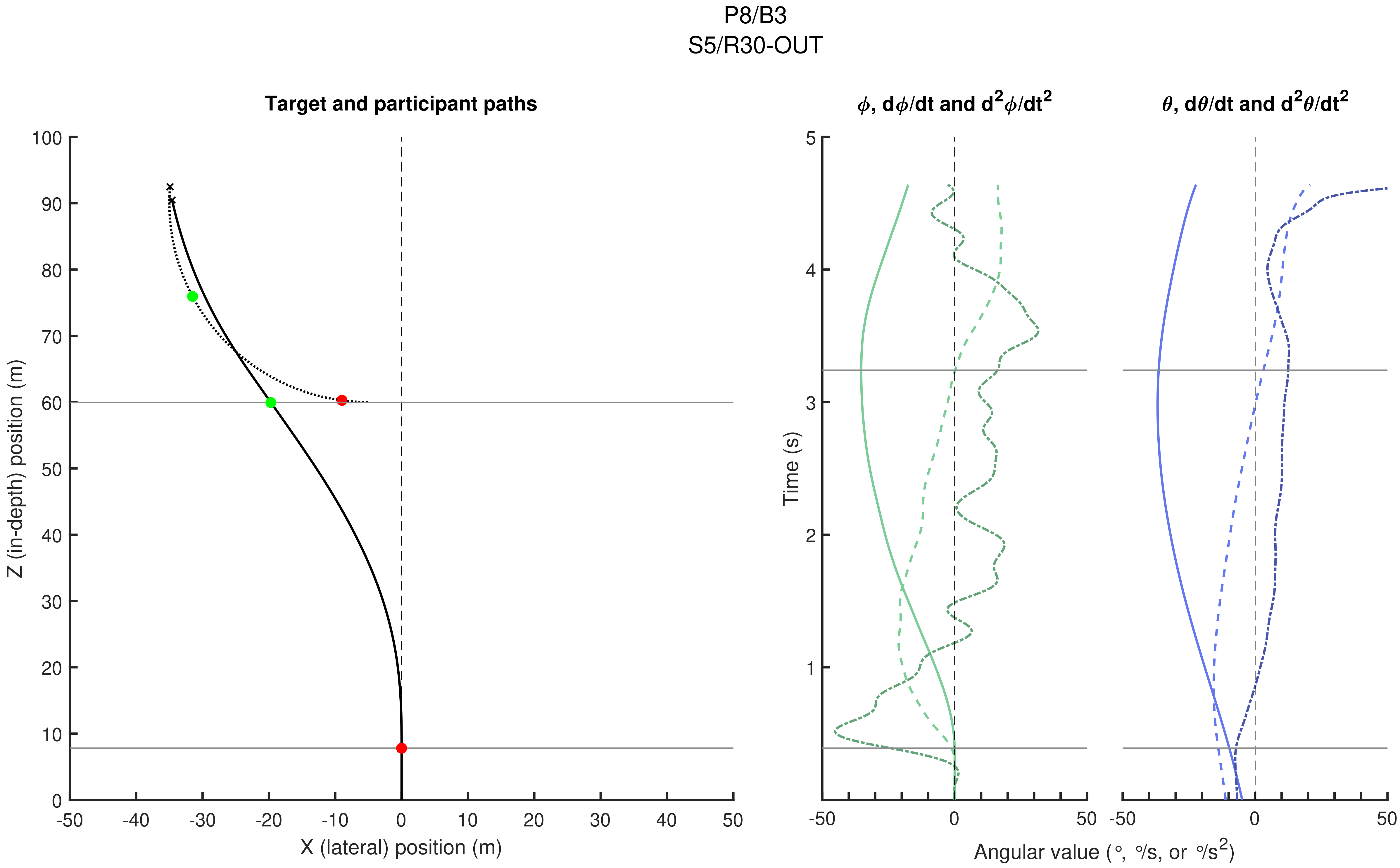
P8/B3
S5/R30-OUT
Target and participant paths
Z (in-depth) position (m)
X (lateral) position (m)
100
90
80
70
60
50
40
30
20
10
0
-50 -40 -30 -20 -10 0 10 20 30 40 50
φ, dφ/dt and d²φ/dt²
θ, dθ/dt and d²θ/dt²
Time (s)
5
4
3
2
1
-50 0 50 -50 0 50
Angular value (°, °/s, or °/s²)

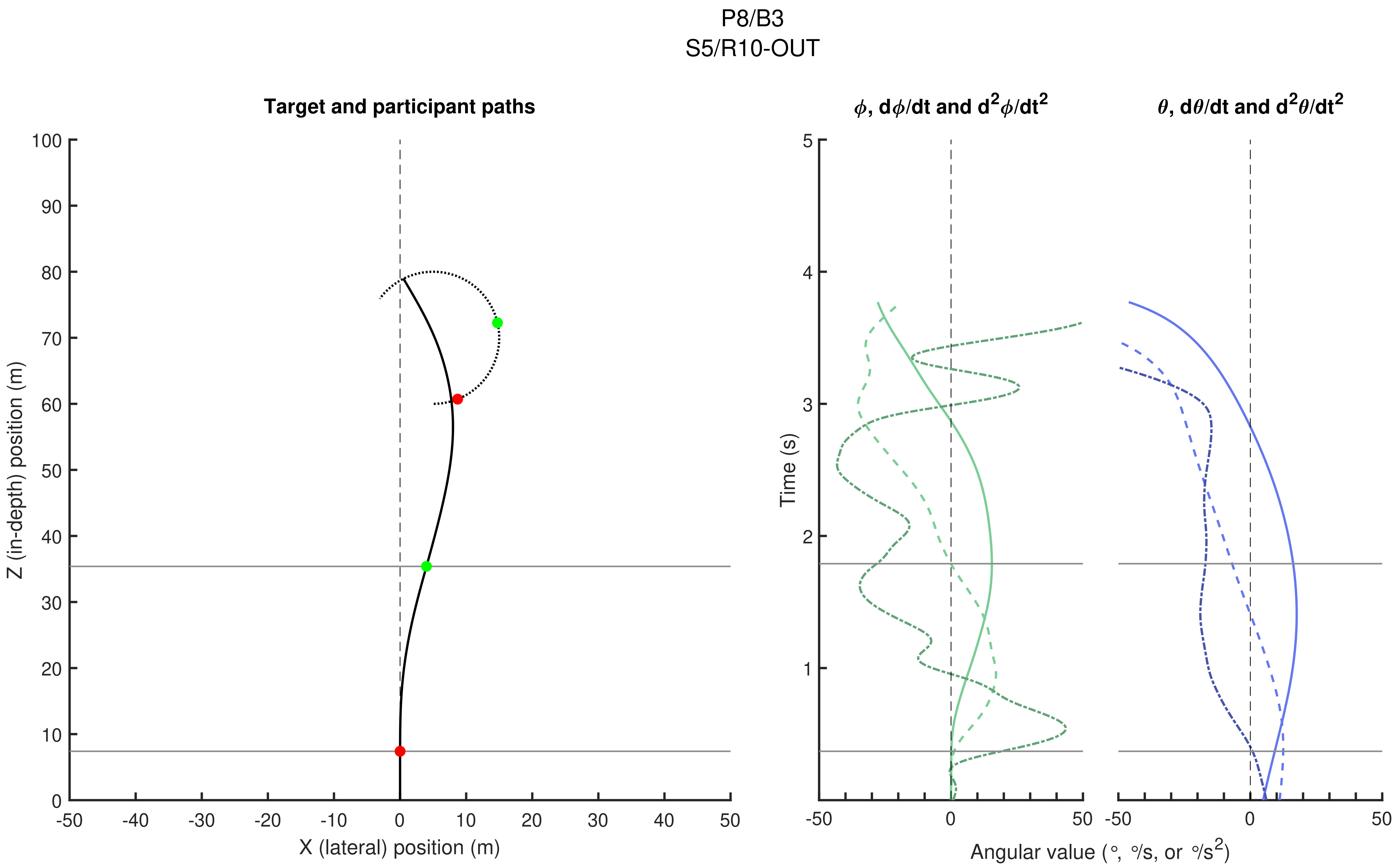
P8/B3
S5/R10-OUT
Target and participant paths
Z (in-depth) position (m)
X (lateral) position (m)
ϕ, dϕ/dt and d$^2\phi$/dt^2
θ, dθ/dt and d$^2\theta$/dt^2
Time (s)
Angular value (°, °/s, or °/s^2)

P8/B3
S5/R10-IN

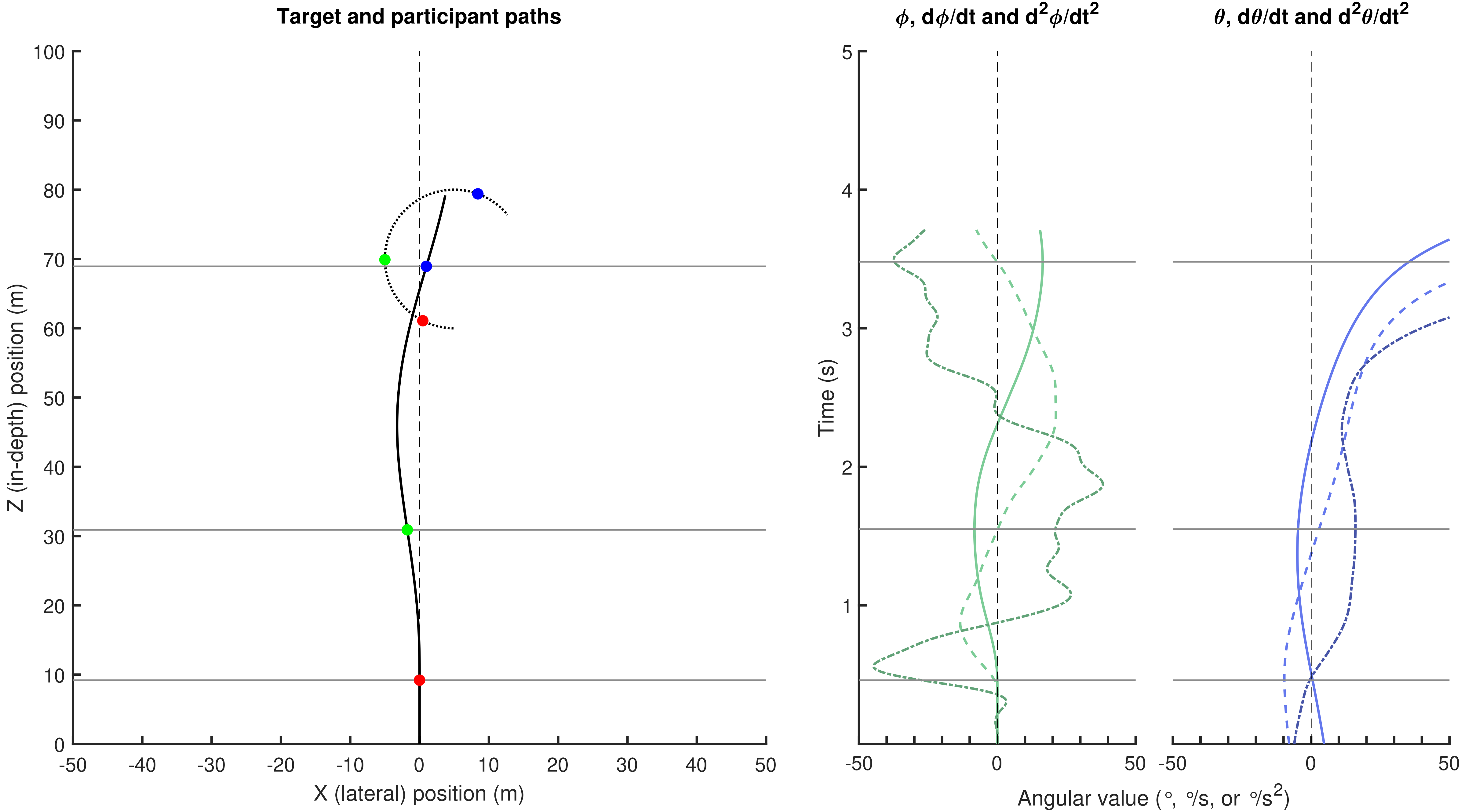

P8/B3
S5/R20-OUT

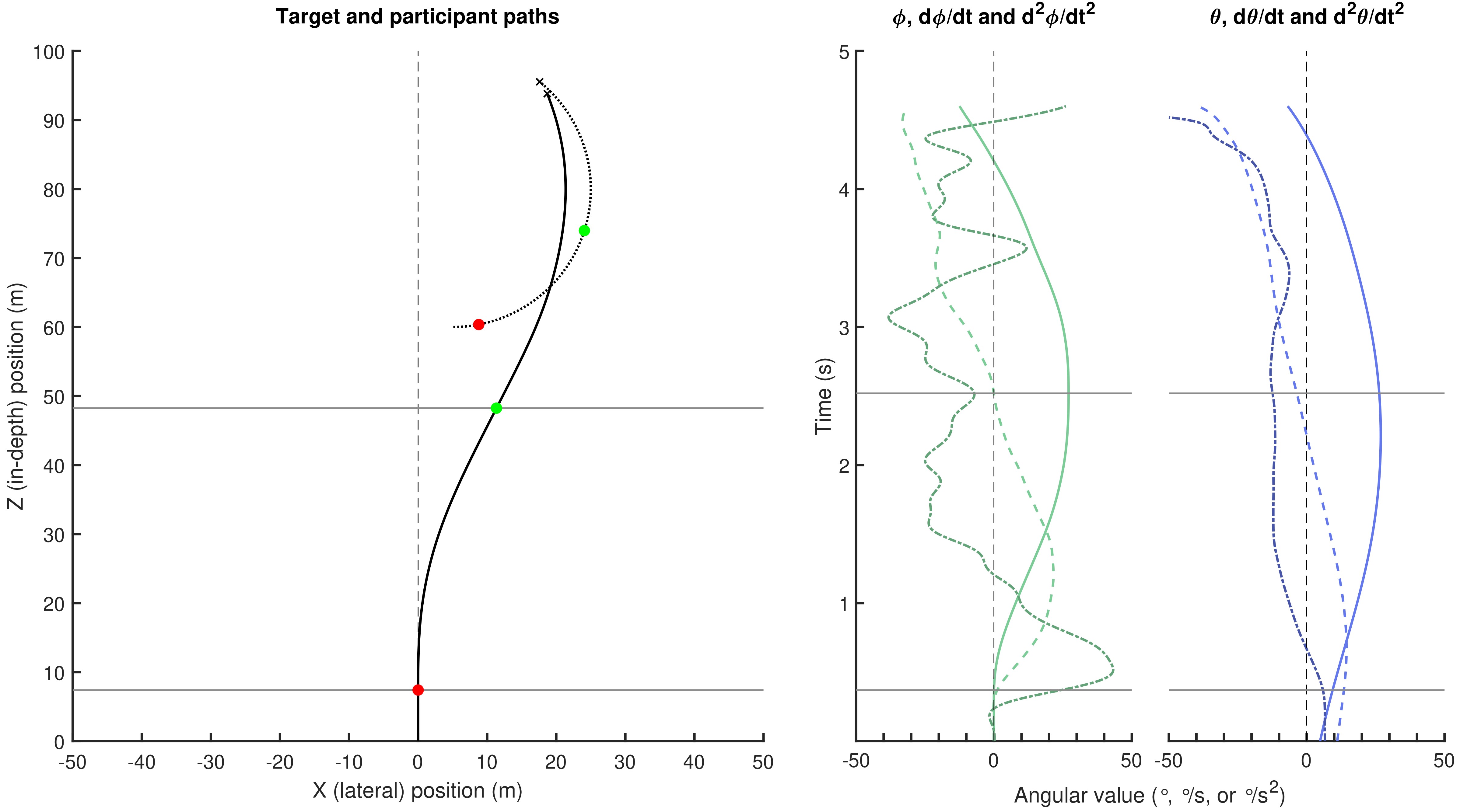

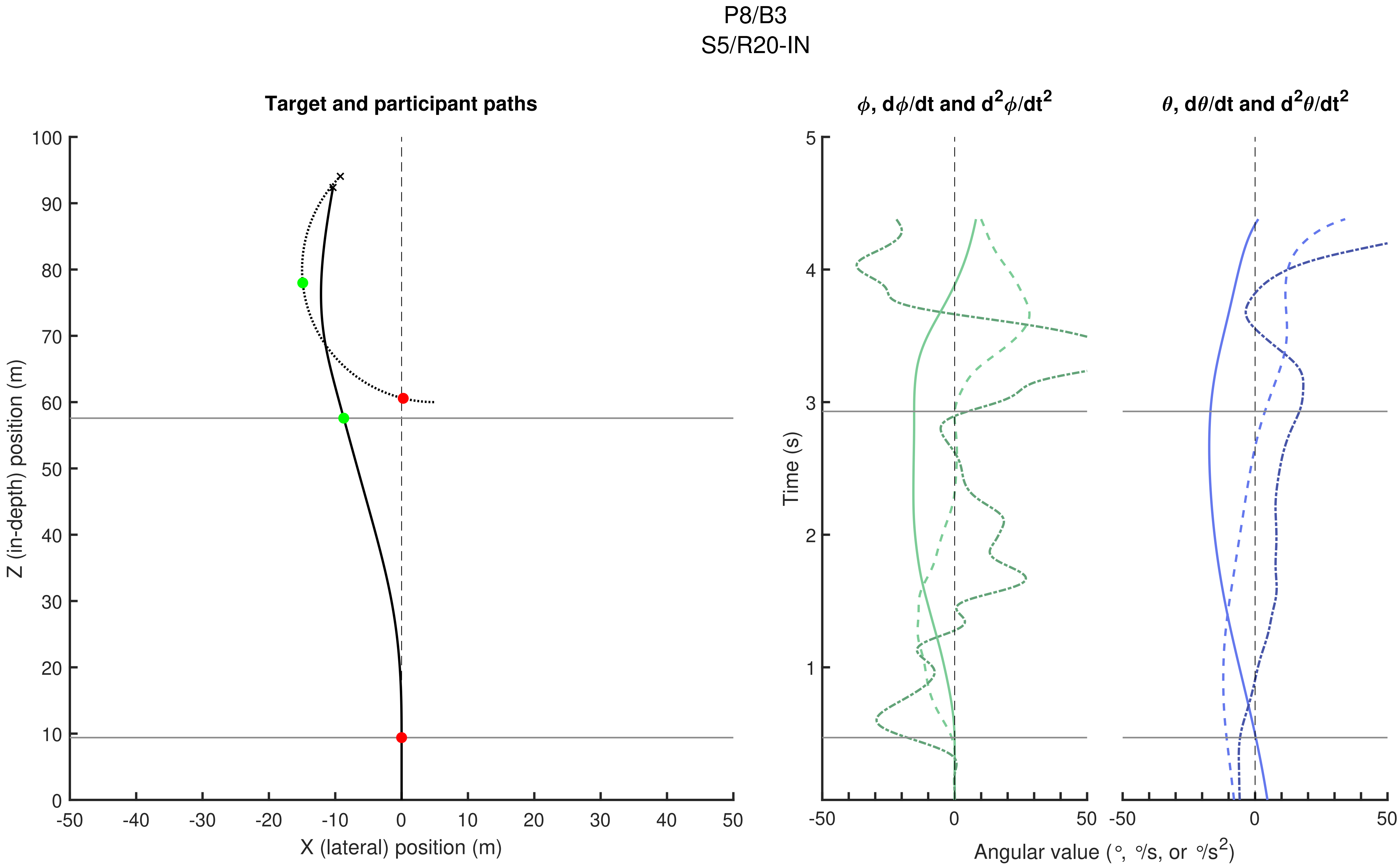
P8/B3
S5/R20-IN
Target and participant paths
Z (in-depth) position (m)
X (lateral) position (m)
100
90
80
70
60
50
40
30
20
10
0
-50
-40
-30
-20
-10
0
10
20
30
40
50
ϕ, dϕ/dt and d$^2\phi$/dt^2
θ, dθ/dt and d$^2\theta$/dt^2
Time (s)
5
4
3
2
1
-50
0
50
-50
0
50
Angular value (°, °/s, or °/s^2)

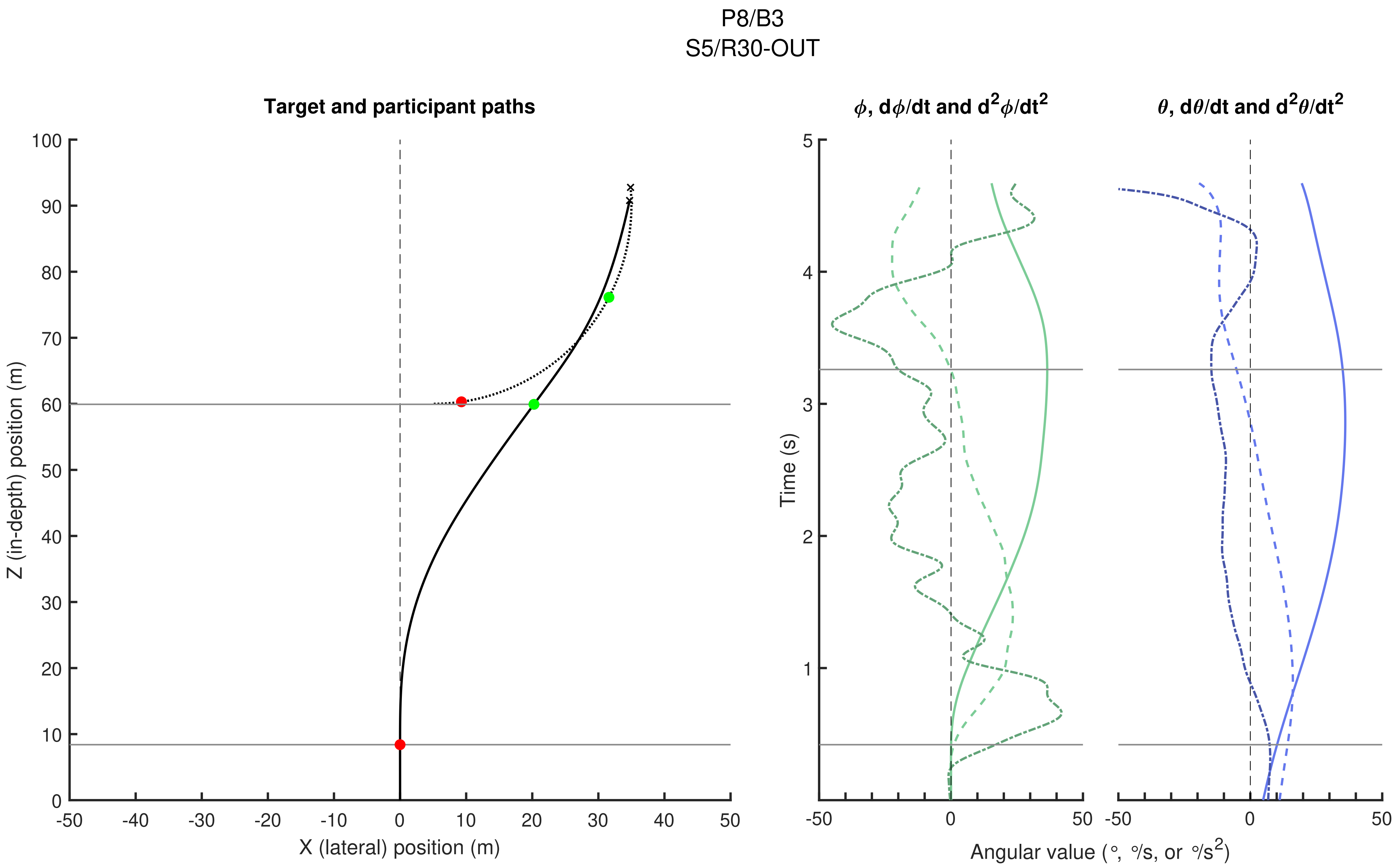
P8/B3
S5/R30-OUT
Target and participant paths
Z (in-depth) position (m)
X (lateral) position (m)
0
10
20
30
40
50
60
70
80
90
100
-50
-40
-30
-20
-10
0
10
20
30
40
50
ϕ, dϕ/dt and d$^2\phi$/dt^2
θ, dθ/dt and d$^2\theta$/dt^2
Time (s)
1
2
3
4
5
-50
0
50
-50
0
50
Angular value (°, °/s, or °/s^2)

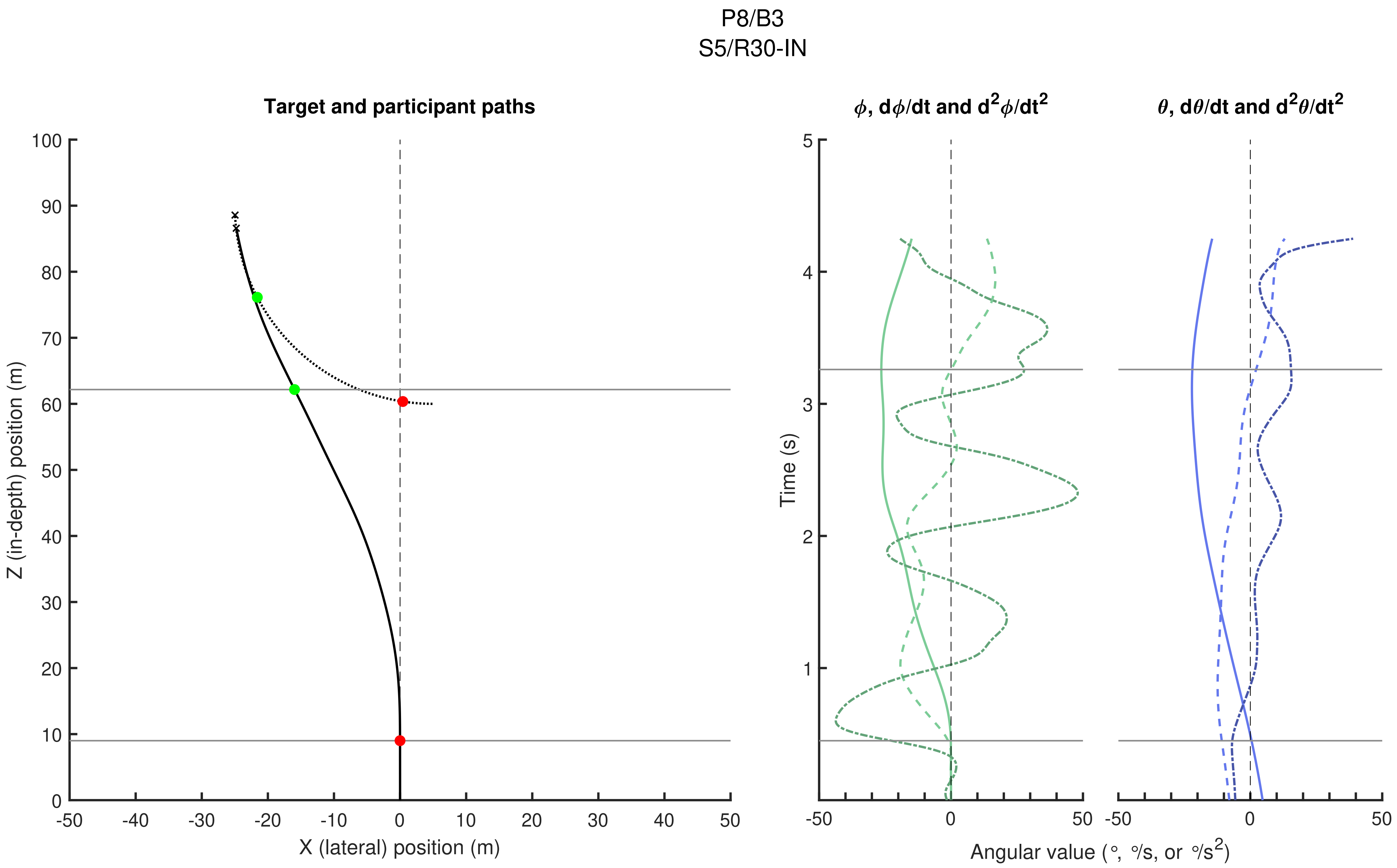

P8/B3
S5/R30-IN
Target and participant paths
Z (in-depth) position (m)
X (lateral) position (m)
ϕ, dϕ/dt and d$^2\phi$/dt^2
θ, dθ/dt and d$^2\theta$/dt^2
Time (s)
Angular value (°, °/s, or °/s^2)

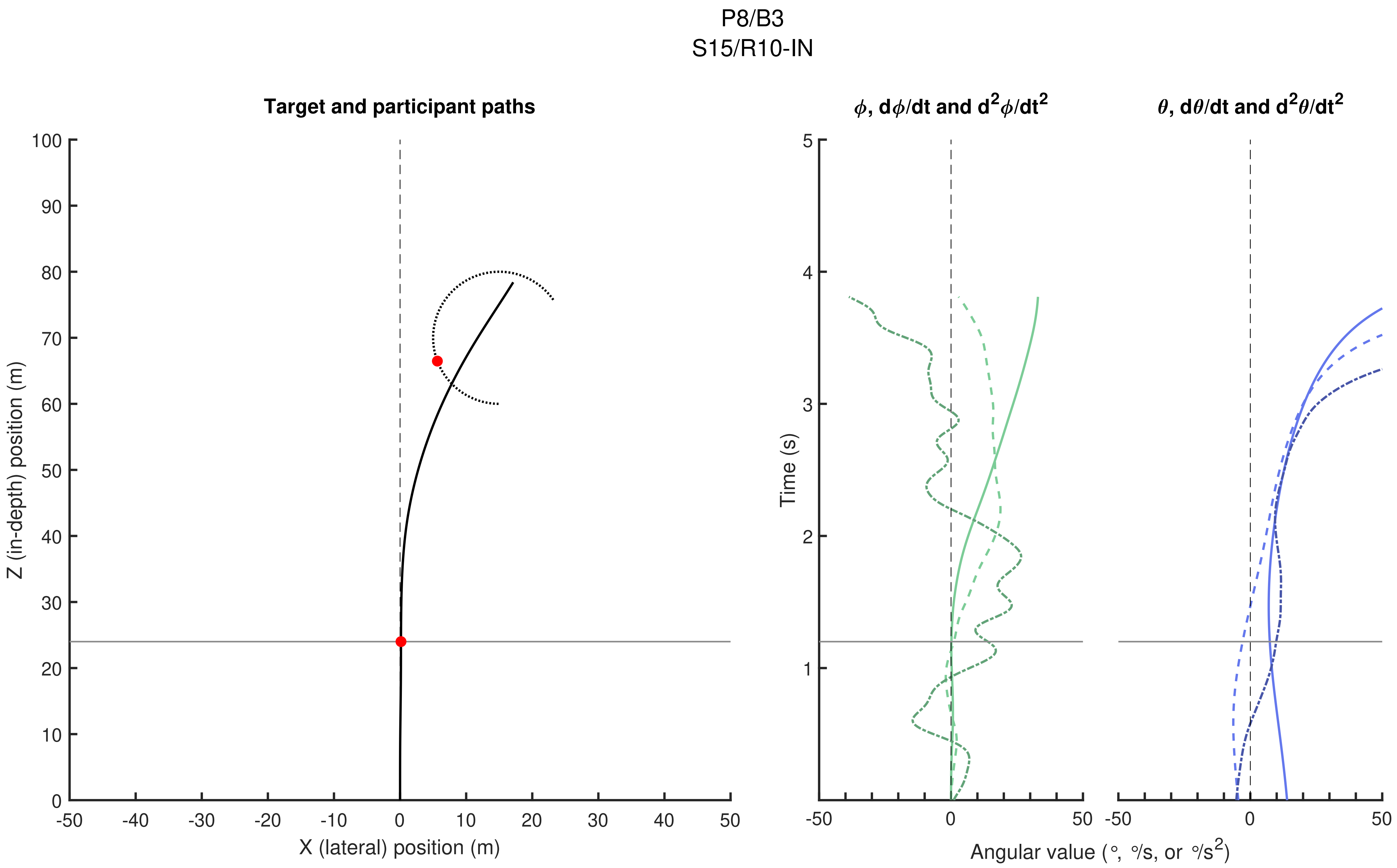
P8/B3
S15/R10-IN
Target and participant paths
Z (in-depth) position (m)
X (lateral) position (m)
ϕ, dϕ/dt and d²ϕ/dt²
θ, dθ/dt and d²θ/dt²
Time (s)
Angular value (°, °/s, or °/s²)

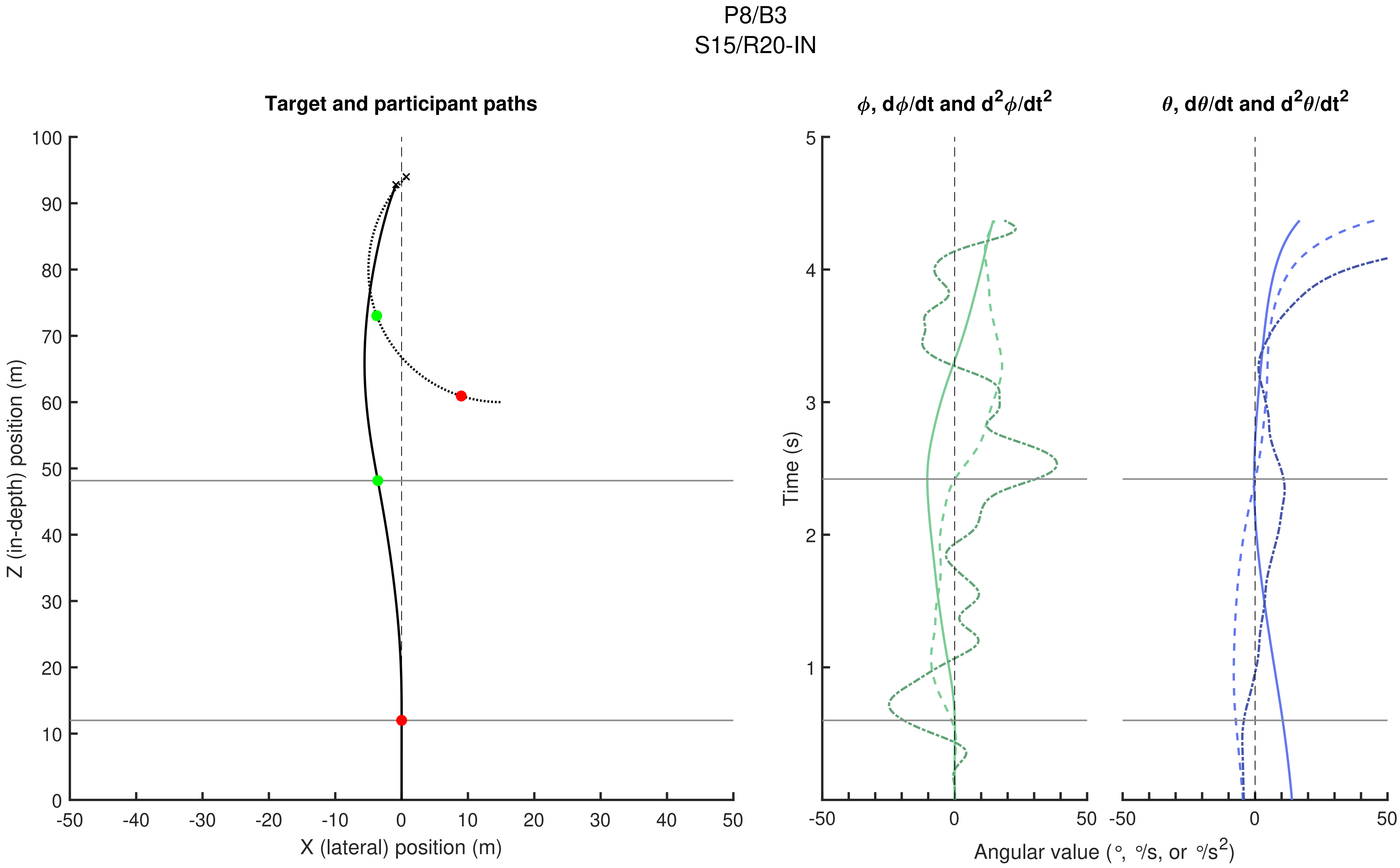
P8/B3
S15/R20-IN
Target and participant paths
Z (in-depth) position (m)
X (lateral) position (m)
100
90
80
70
60
50
40
30
20
10
0
-50
-40
-30
-20
-10
0
10
20
30
40
50
φ, dφ/dt and d²φ/dt²
θ, dθ/dt and d²θ/dt²
Time (s)
5
4
3
2
1
-50
0
50
Angular value (°, °/s, or °/s²)

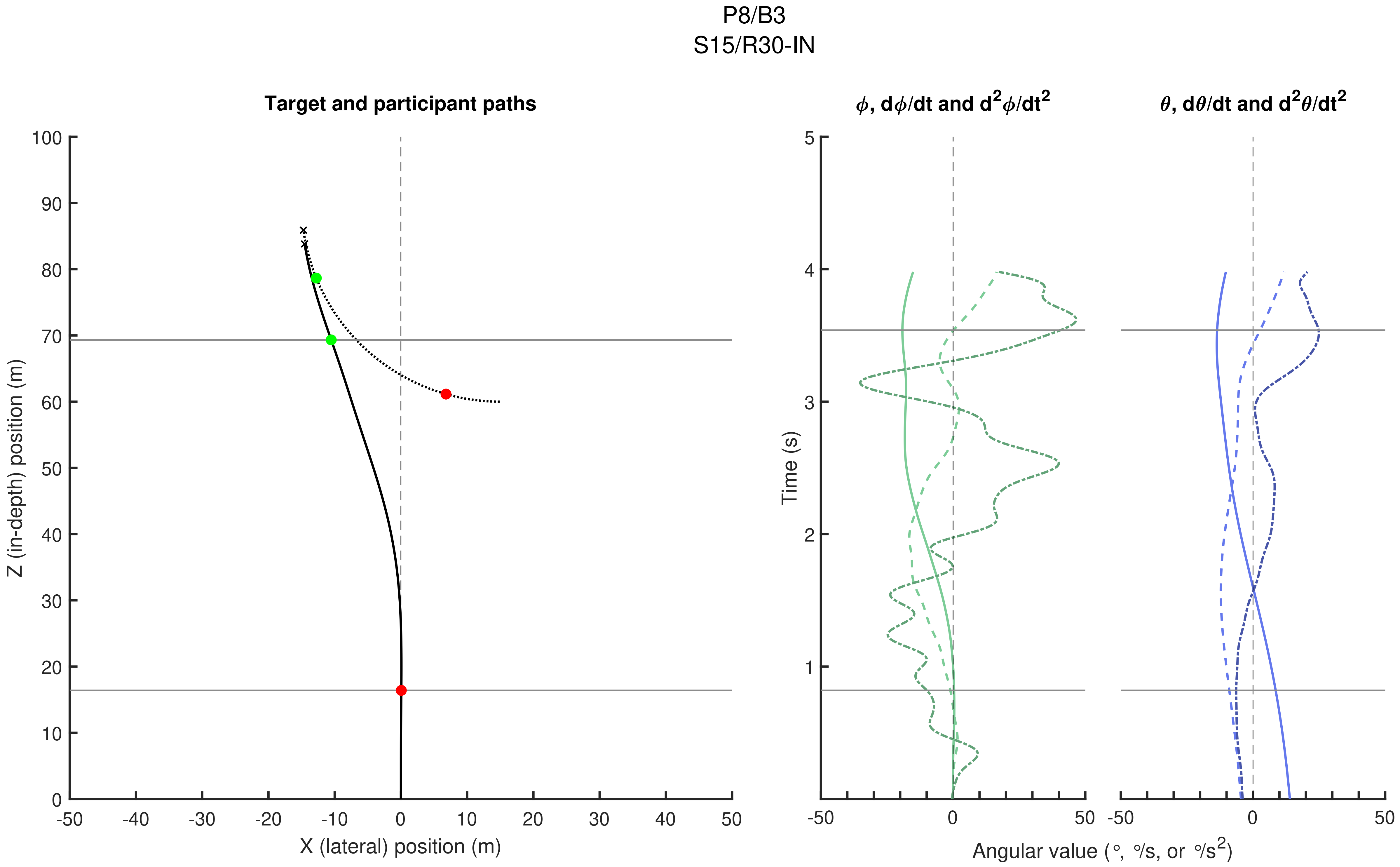
P8/B3
S15/R30-IN
Target and participant paths
Z (in-depth) position (m)
X (lateral) position (m)
ϕ, dϕ/dt and d$^2\phi$/dt^2
θ, dθ/dt and d$^2\theta$/dt^2
Time (s)
Angular value (°, °/s, or °/s^2)

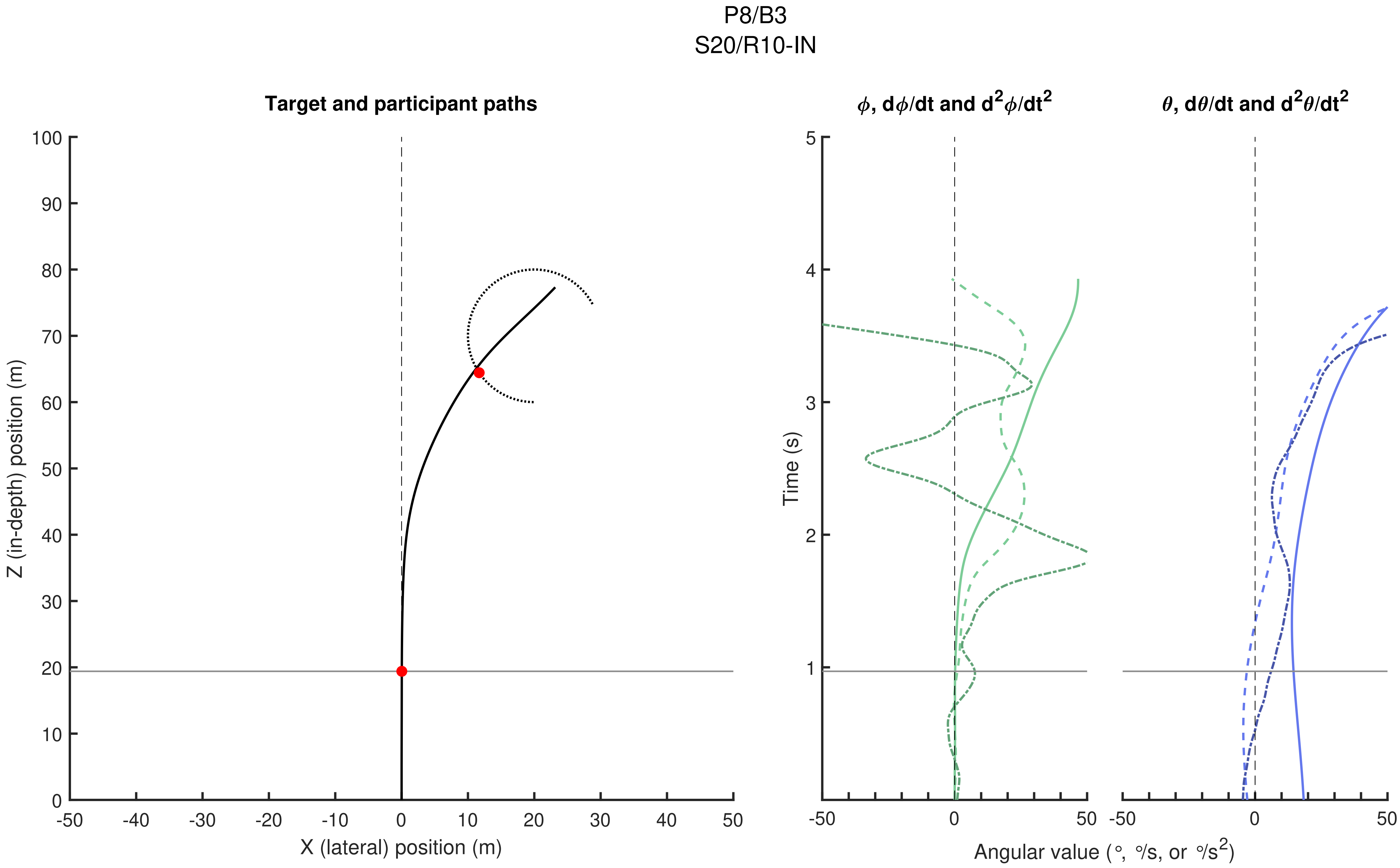
P8/B3
S20/R10-IN
Target and participant paths
Z (in-depth) position (m)
X (lateral) position (m)
100
90
80
70
60
50
40
30
20
10
0
-50
-40
-30
-20
-10
0
10
20
30
40
50
ϕ, dϕ/dt and d$^2\phi$/dt^2
θ, dθ/dt and d$^2\theta$/dt^2
Time (s)
5
4
3
2
1
-50
0
50
-50
0
50
Angular value (°, °/s, or °/s^2)

P8/B3
S20/R20-IN

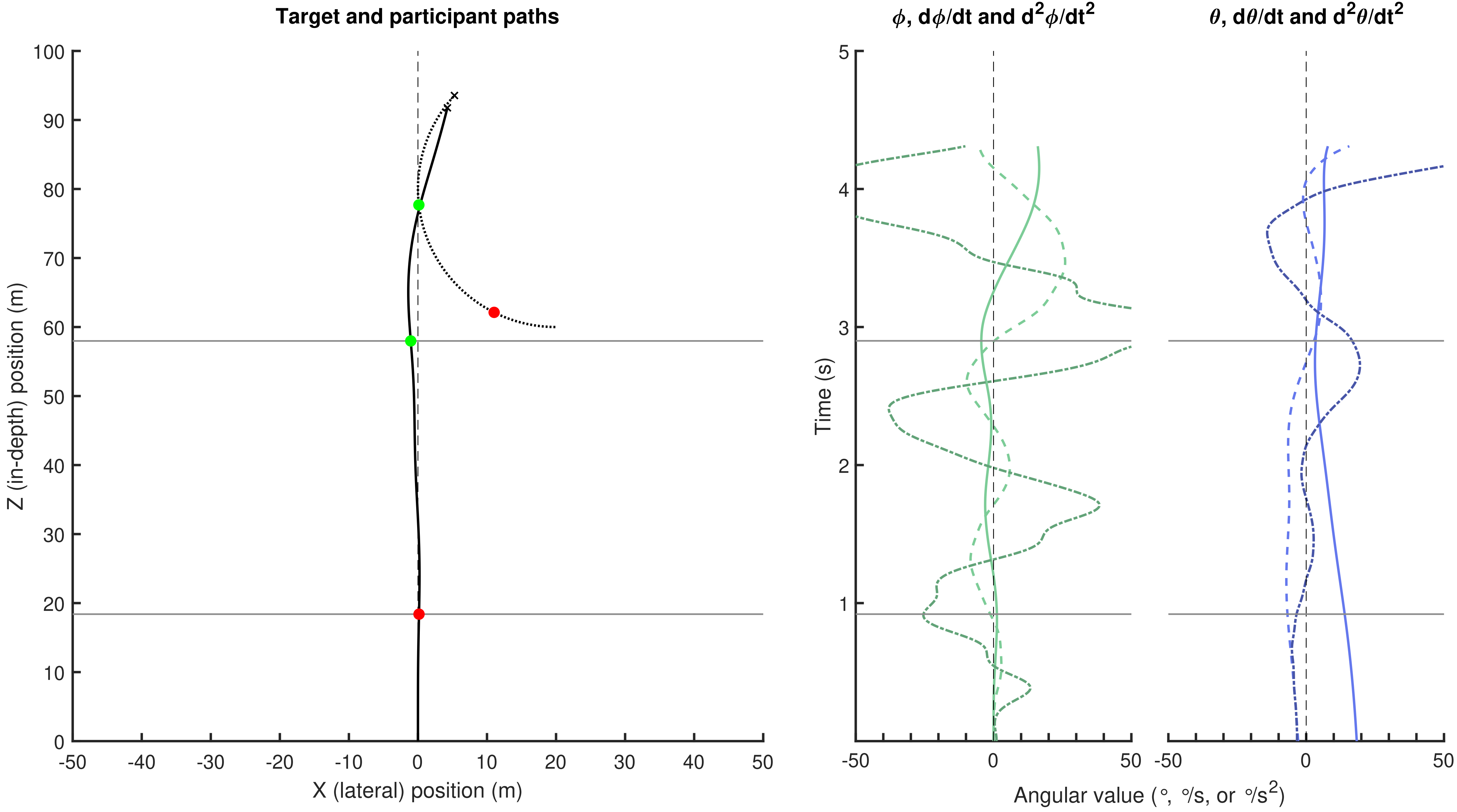

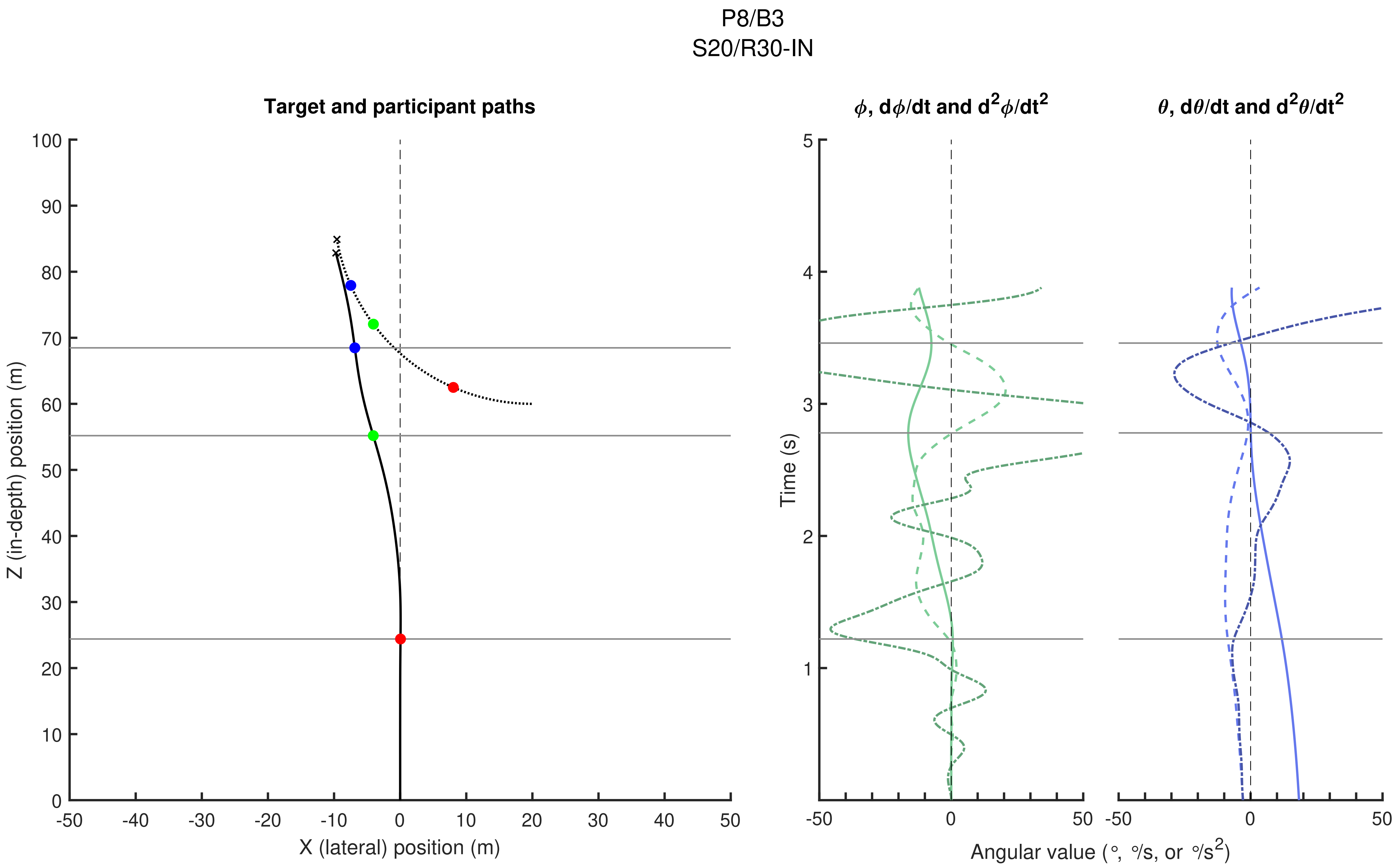
P8/B3
S20/R30-IN
Target and participant paths
Z (in-depth) position (m)
X (lateral) position (m)
ϕ, dϕ/dt and d$^2\phi$/dt^2
θ, dθ/dt and d$^2\theta$/dt^2
Time (s)
Angular value (°, °/s, or °/s^2)

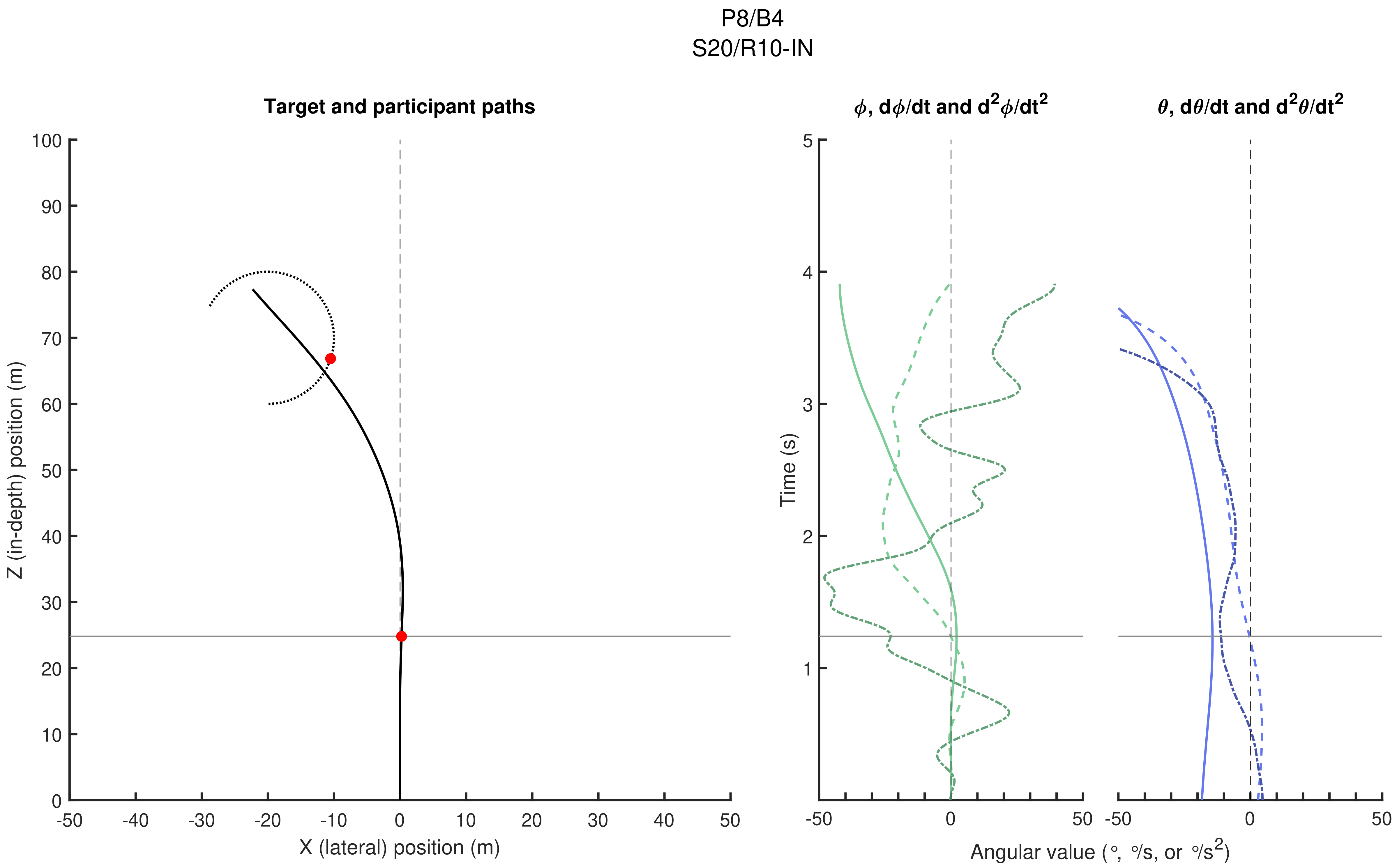

P8/B4
S20/R10-IN
Target and participant paths
ϕ, dϕ/dt and d²ϕ/dt²
θ, dθ/dt and d²θ/dt²
Z (in-depth) position (m)
X (lateral) position (m)
Time (s)
Angular value (°, °/s, or °/s²)

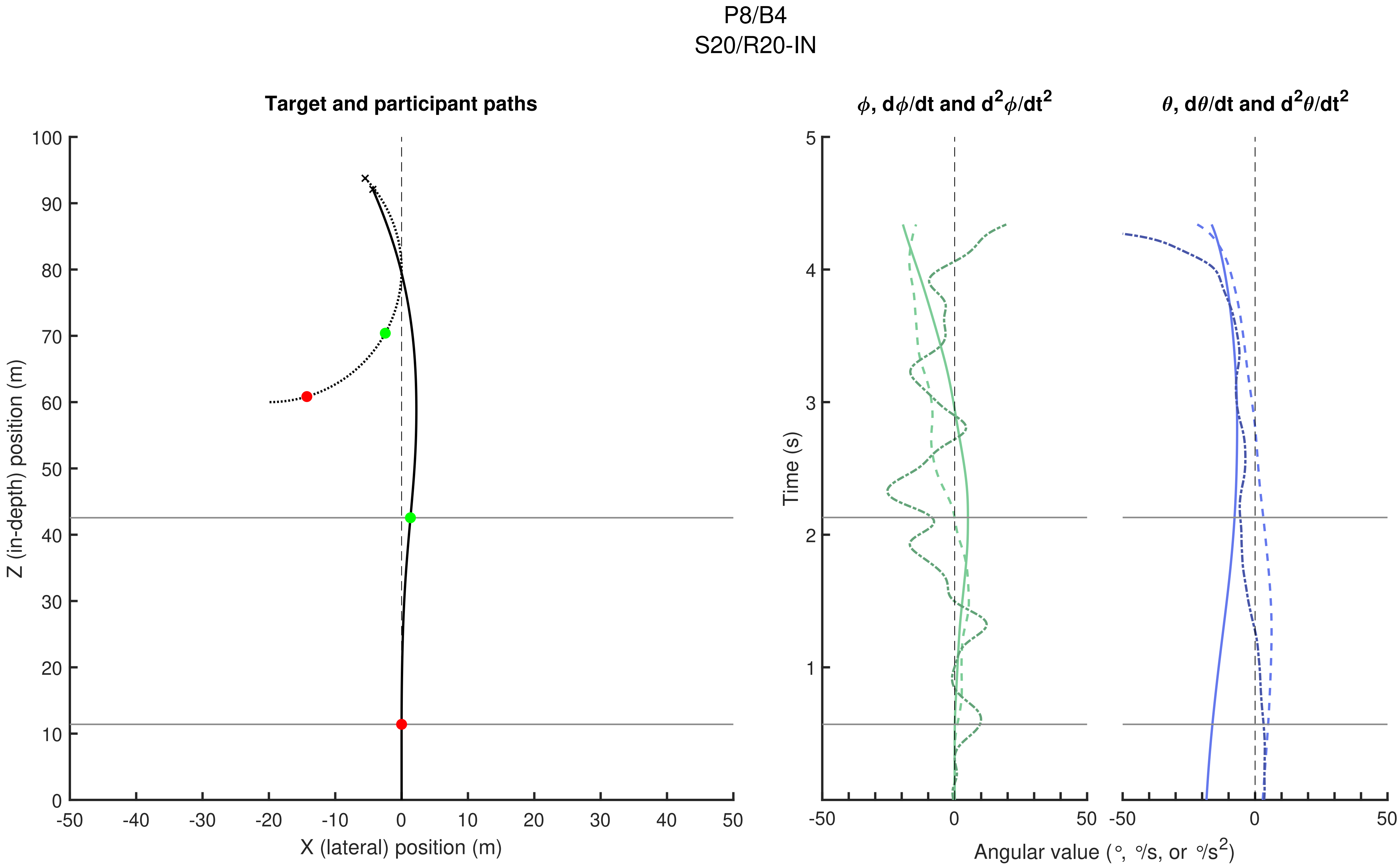
P8/B4
S20/R20-IN
Target and participant paths
Z (in-depth) position (m)
X (lateral) position (m)
ϕ, dϕ/dt and d$^2\phi$/dt^2
θ, dθ/dt and d$^2\theta$/dt^2
Time (s)
Angular value (°, °/s, or °/s^2)

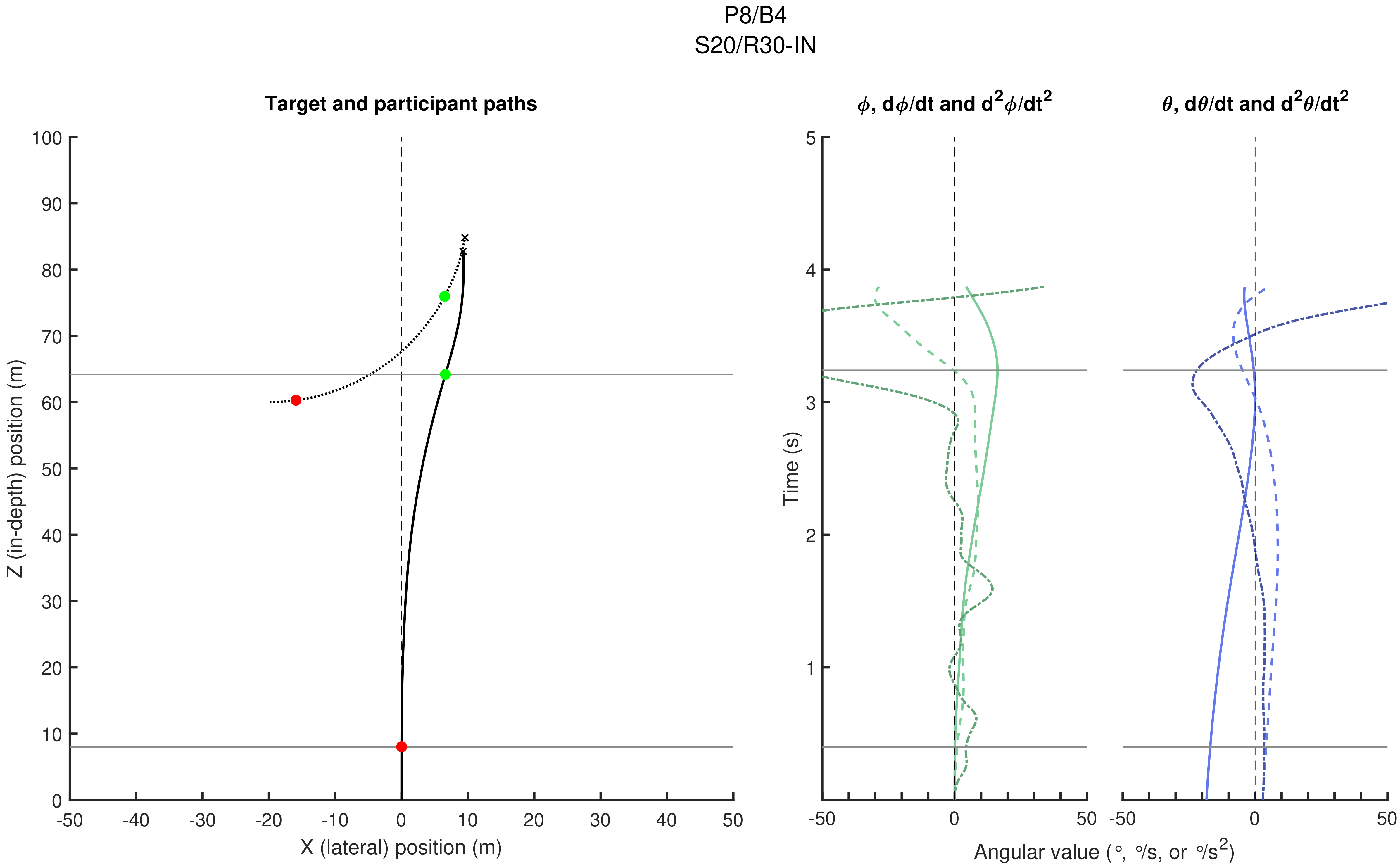

P8/B4
S20/R30-IN
Target and participant paths
Z (in-depth) position (m)
X (lateral) position (m)
φ, dφ/dt and d²φ/dt²
θ, dθ/dt and d²θ/dt²
Time (s)
Angular value (°, °/s, or °/s²)

P8/B4
S15/R10-IN

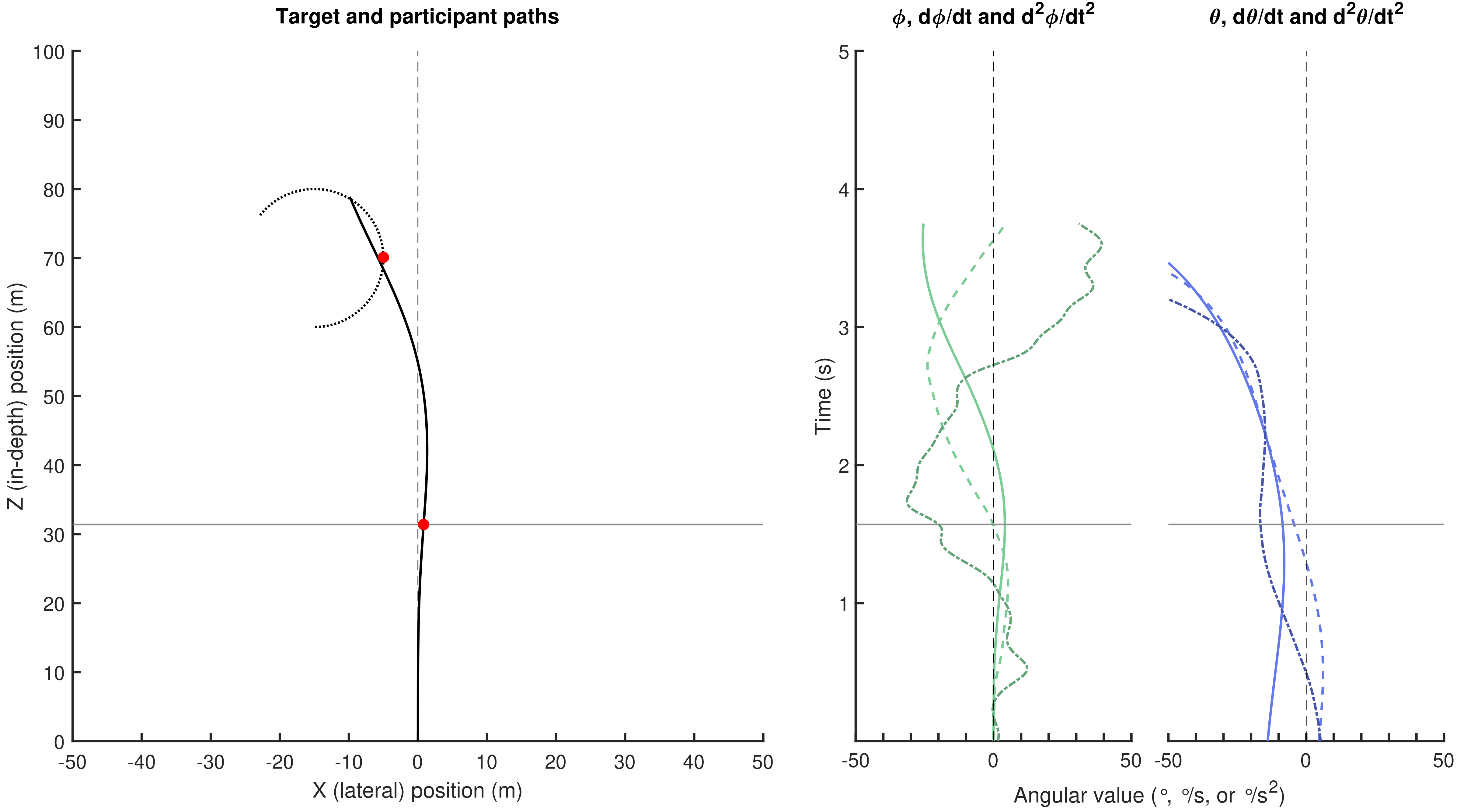

P8/B4
S15/R20-IN

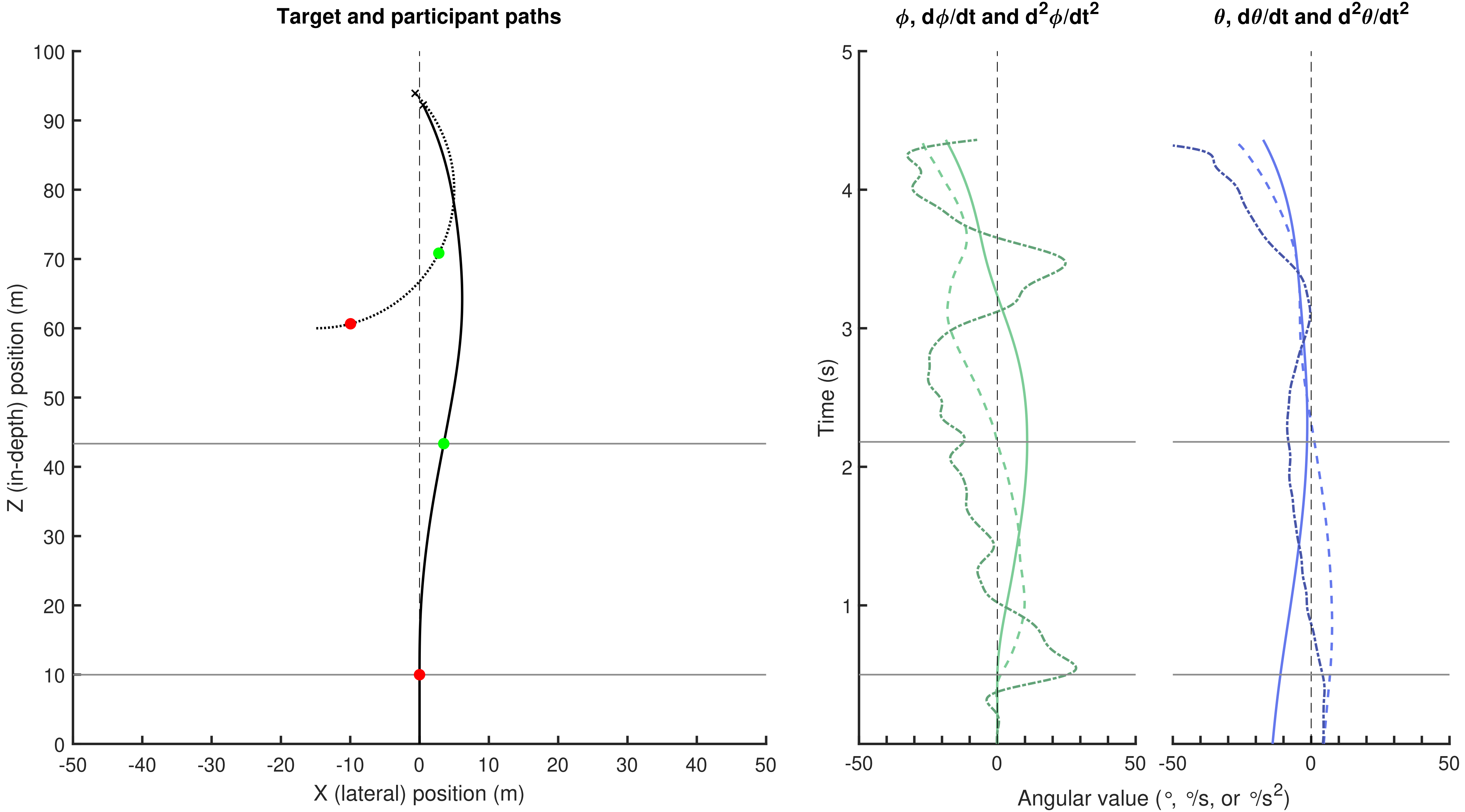

P8/B4
S15/R30-IN

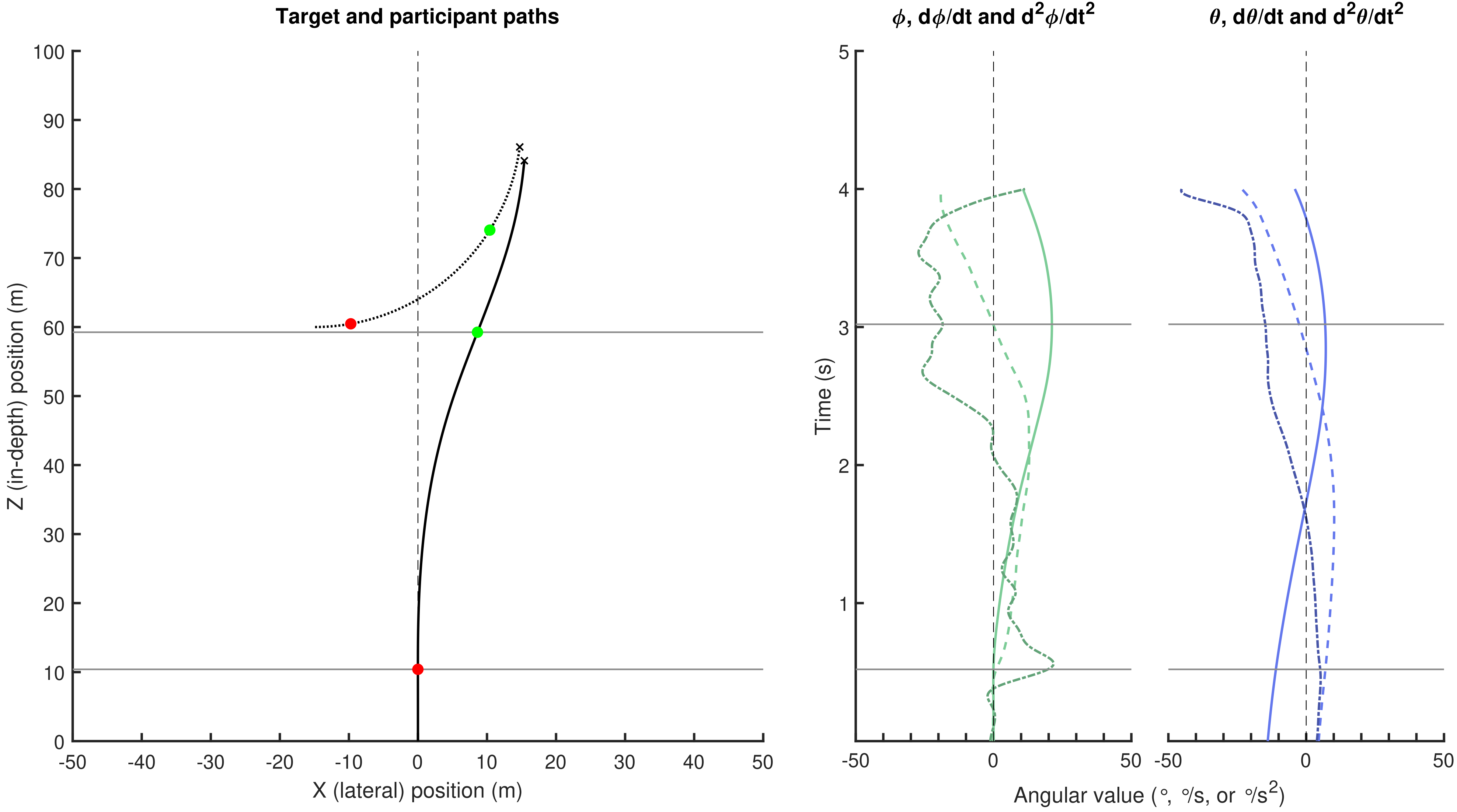

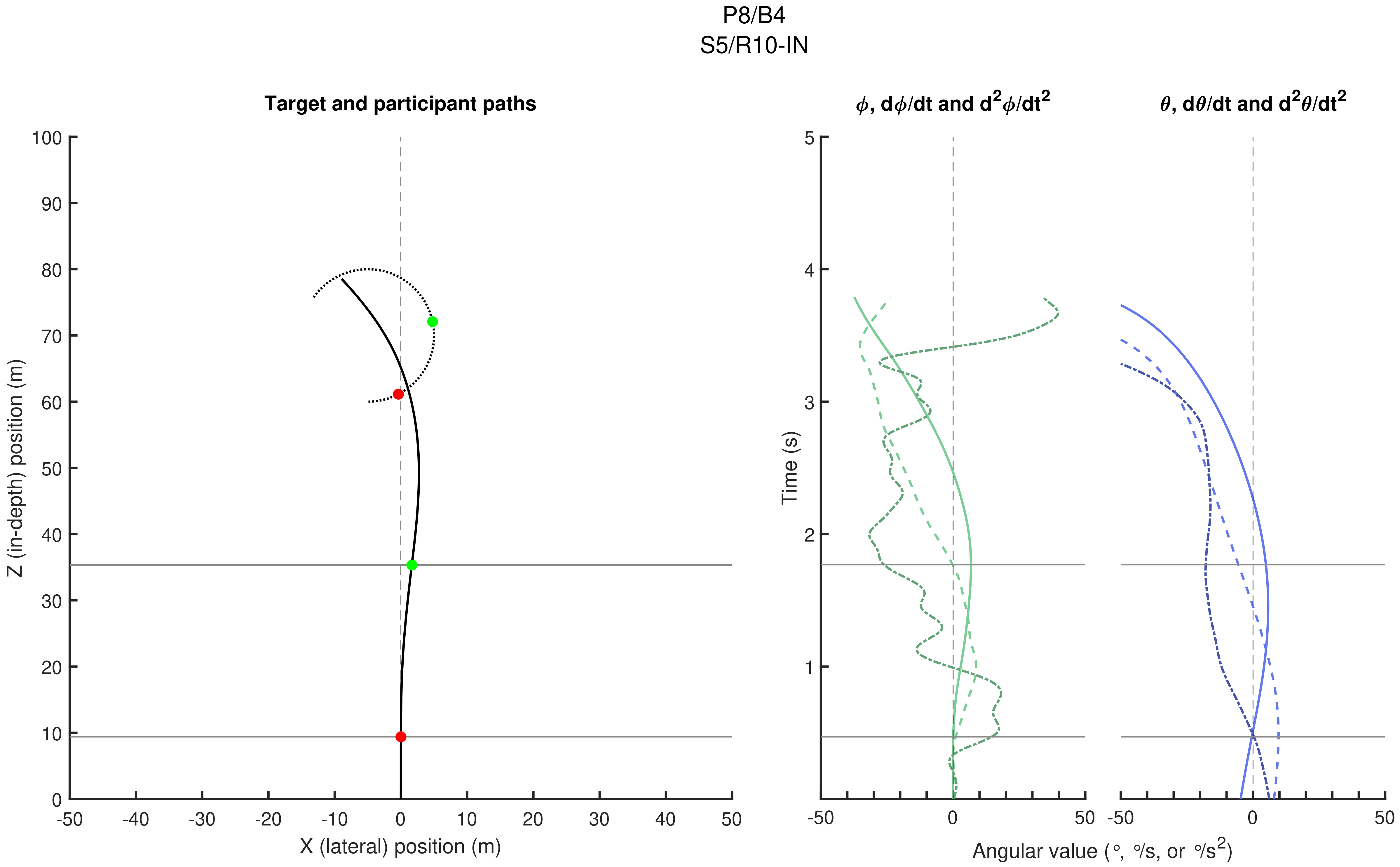
P8/B4
S5/R10-IN
Target and participant paths
Z (in-depth) position (m)
X (lateral) position (m)
ϕ, dϕ/dt and d²ϕ/dt²
θ, dθ/dt and d²θ/dt²
Time (s)
Angular value (°, °/s, or °/s²)

P8/B4
S5/R10-OUT

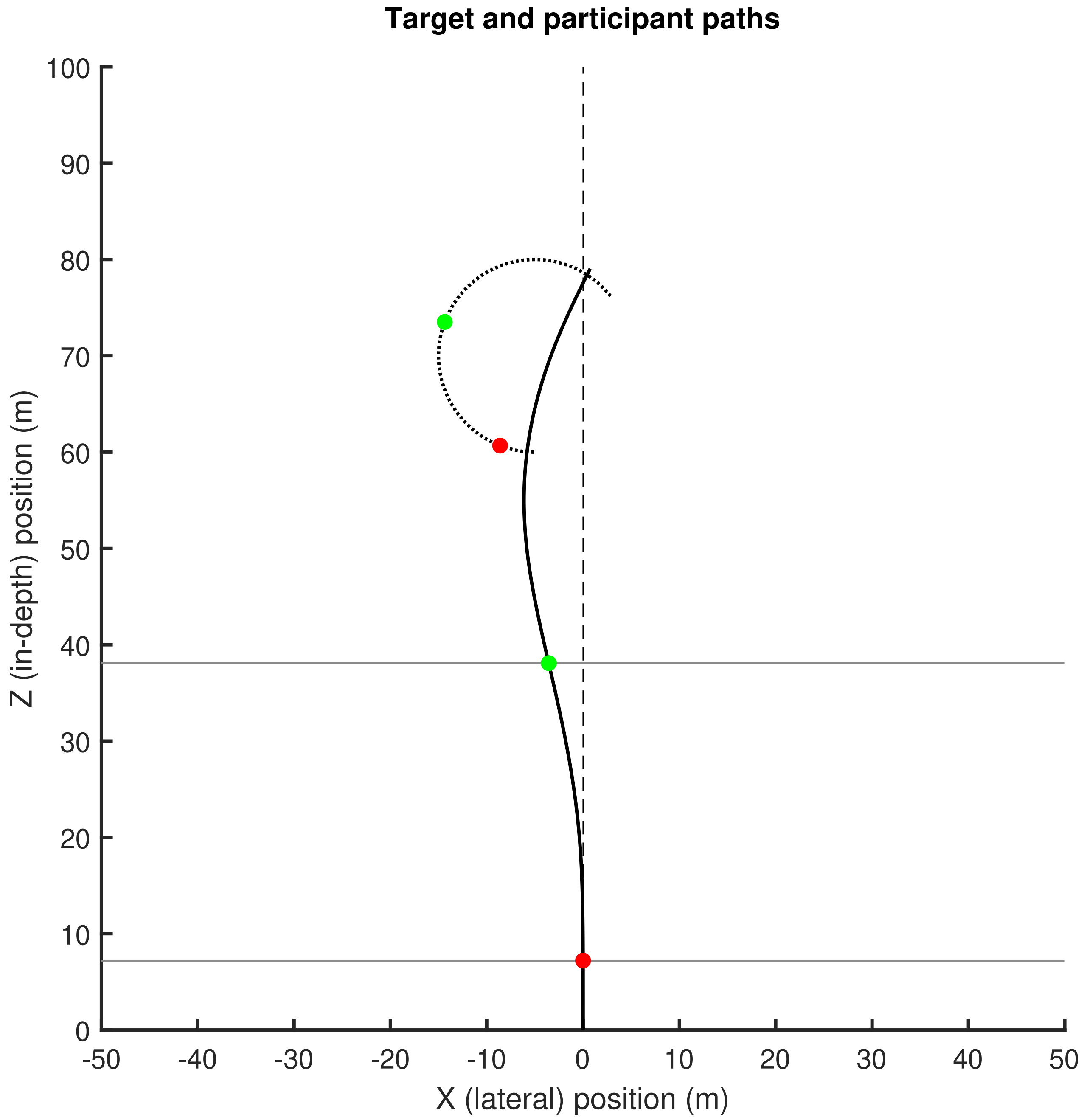

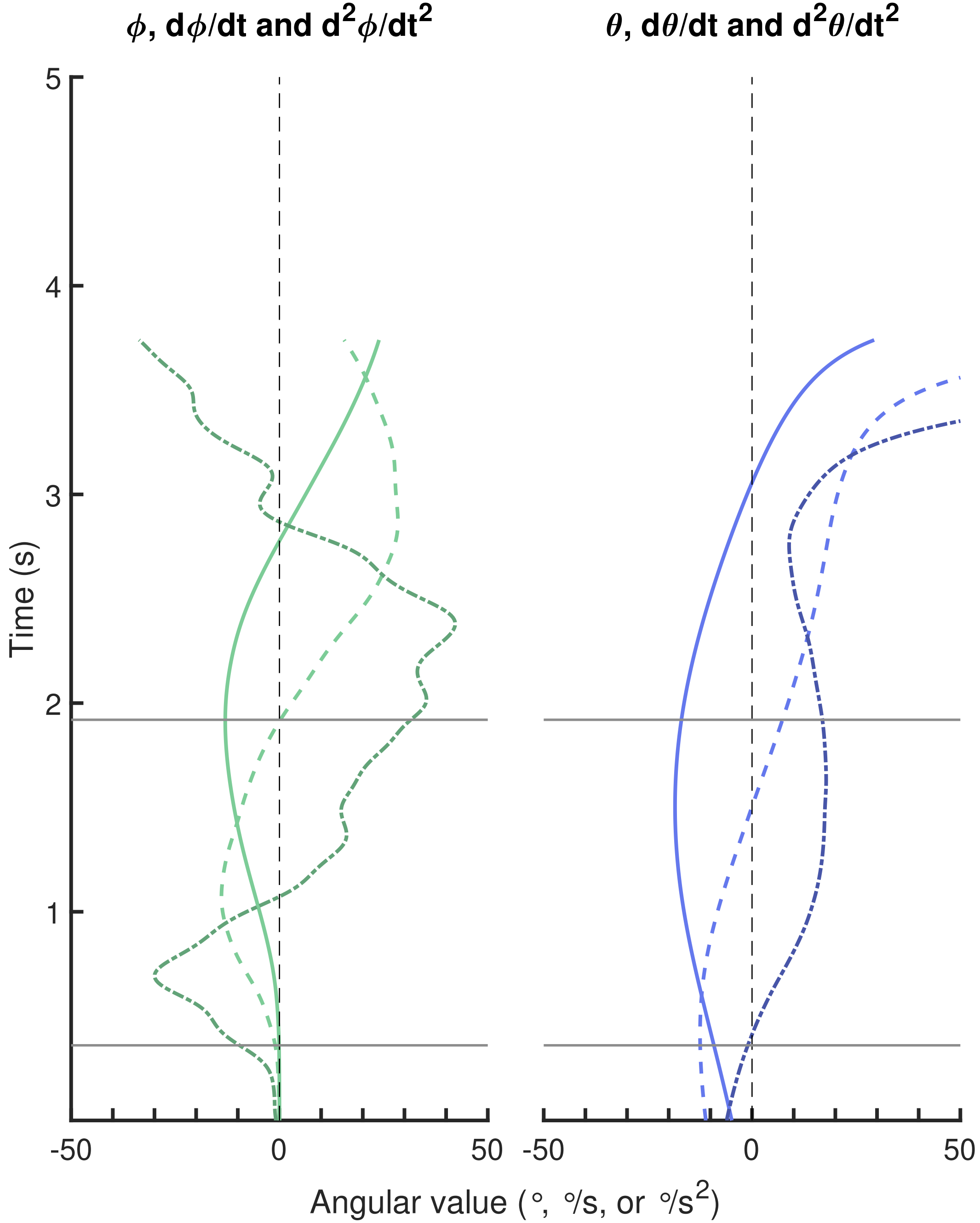

P8/B4
S5/R20-IN

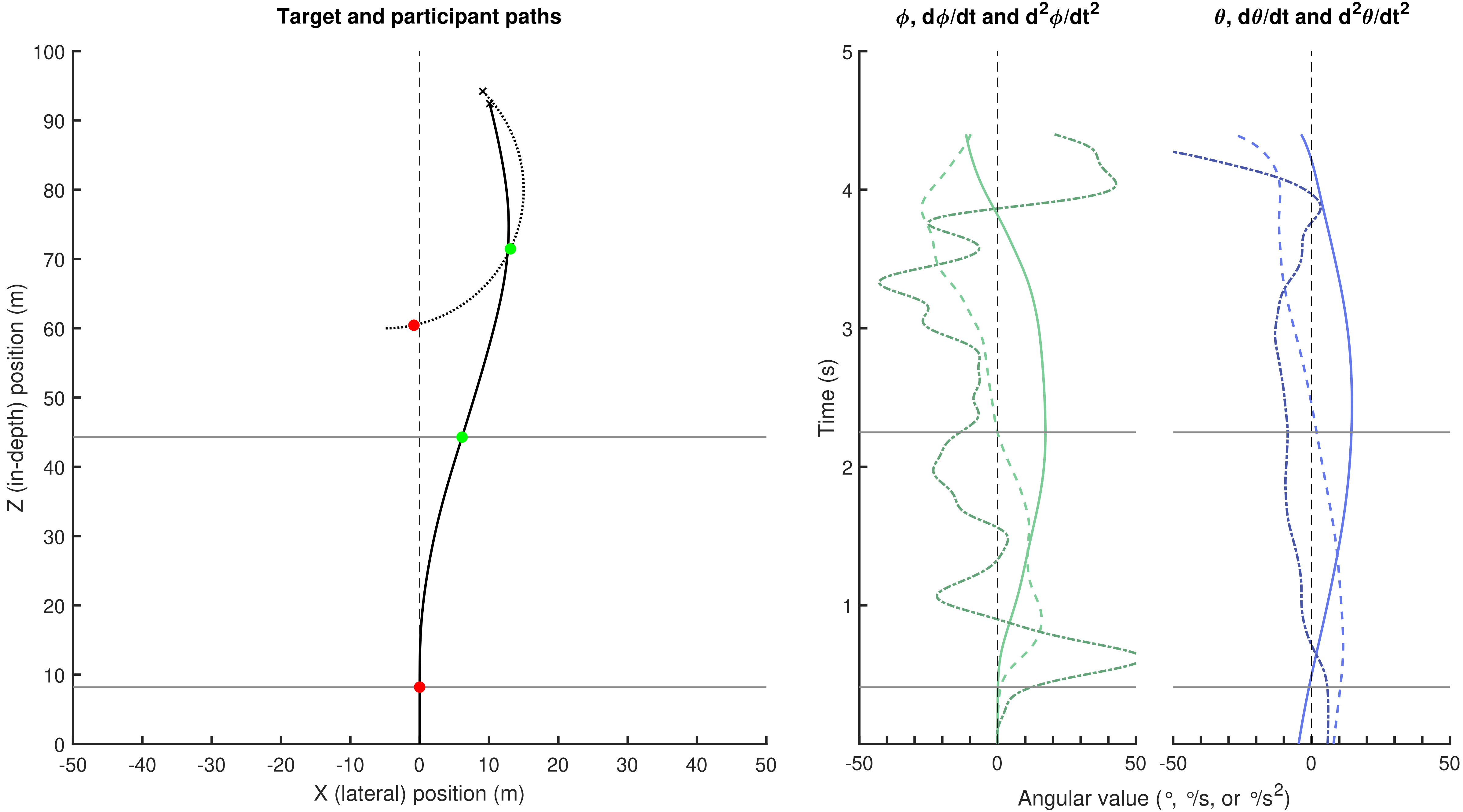

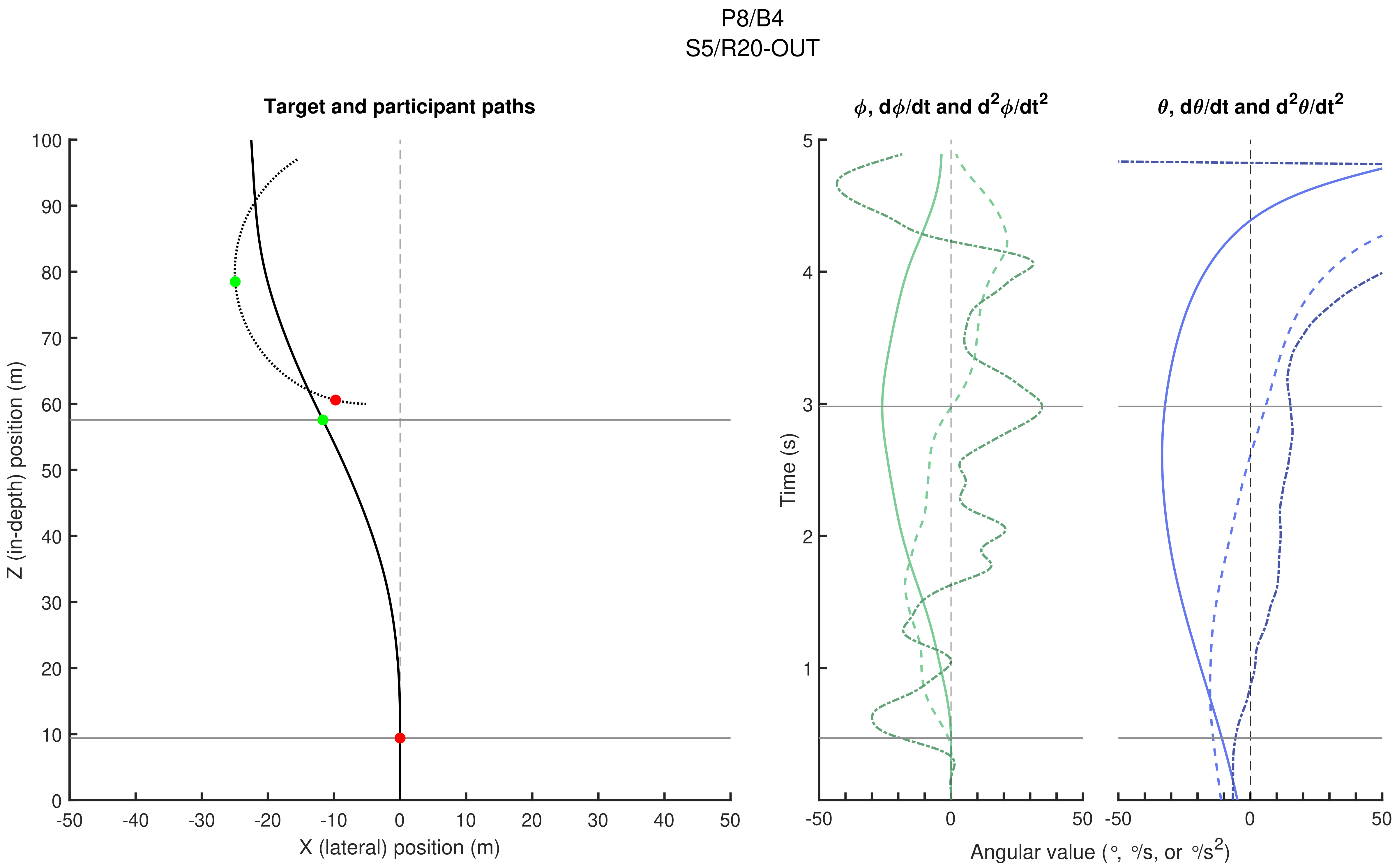
P8/B4
S5/R20-OUT
Target and participant paths
Z (in-depth) position (m)
X (lateral) position (m)
ϕ, dϕ/dt and d$^2\phi$/dt^2
θ, dθ/dt and d$^2\theta$/dt^2
Time (s)
Angular value (°, °/s, or °/s^2)

P8/B4
S5/R30-IN

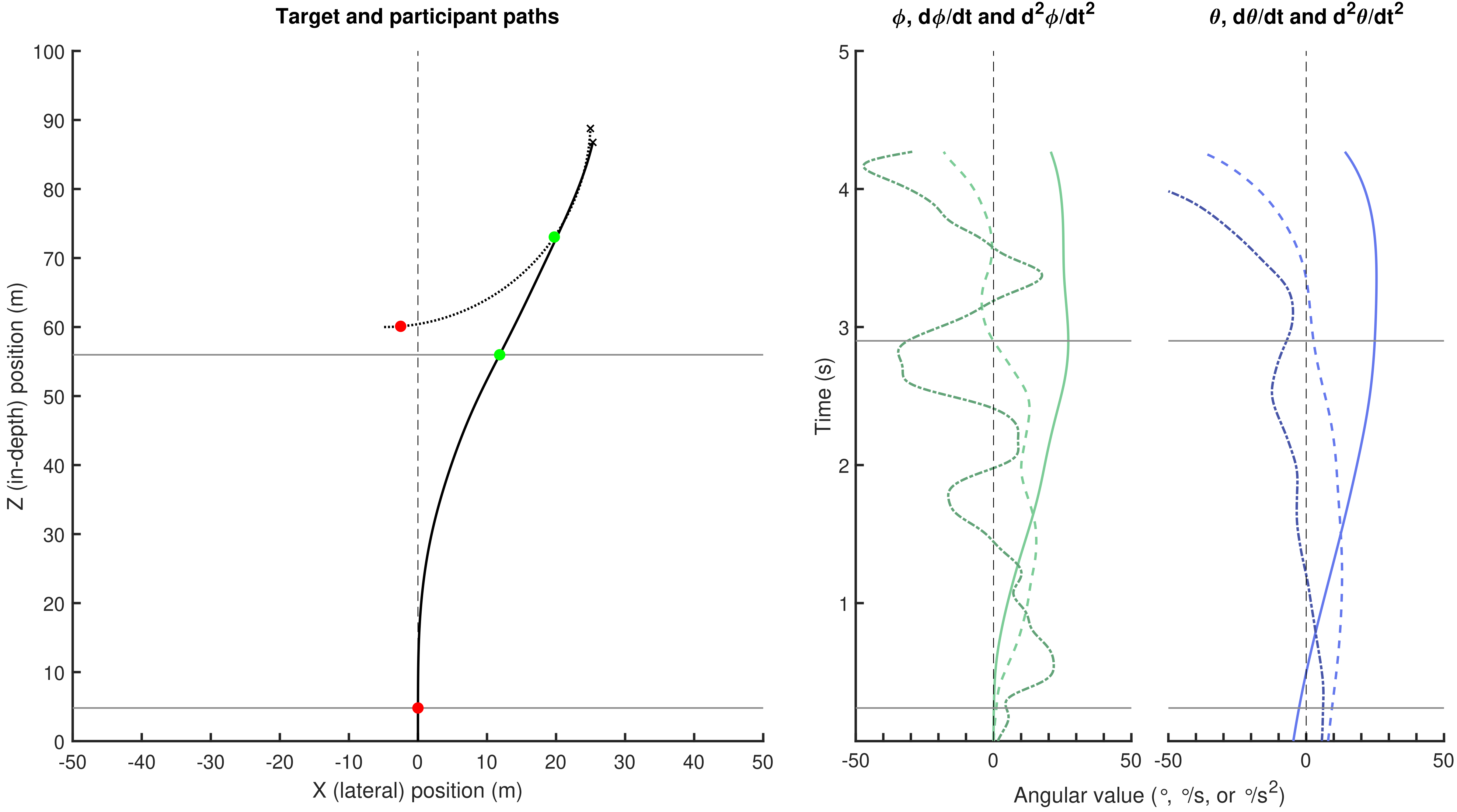

P8/B4
S5/R30-OUT

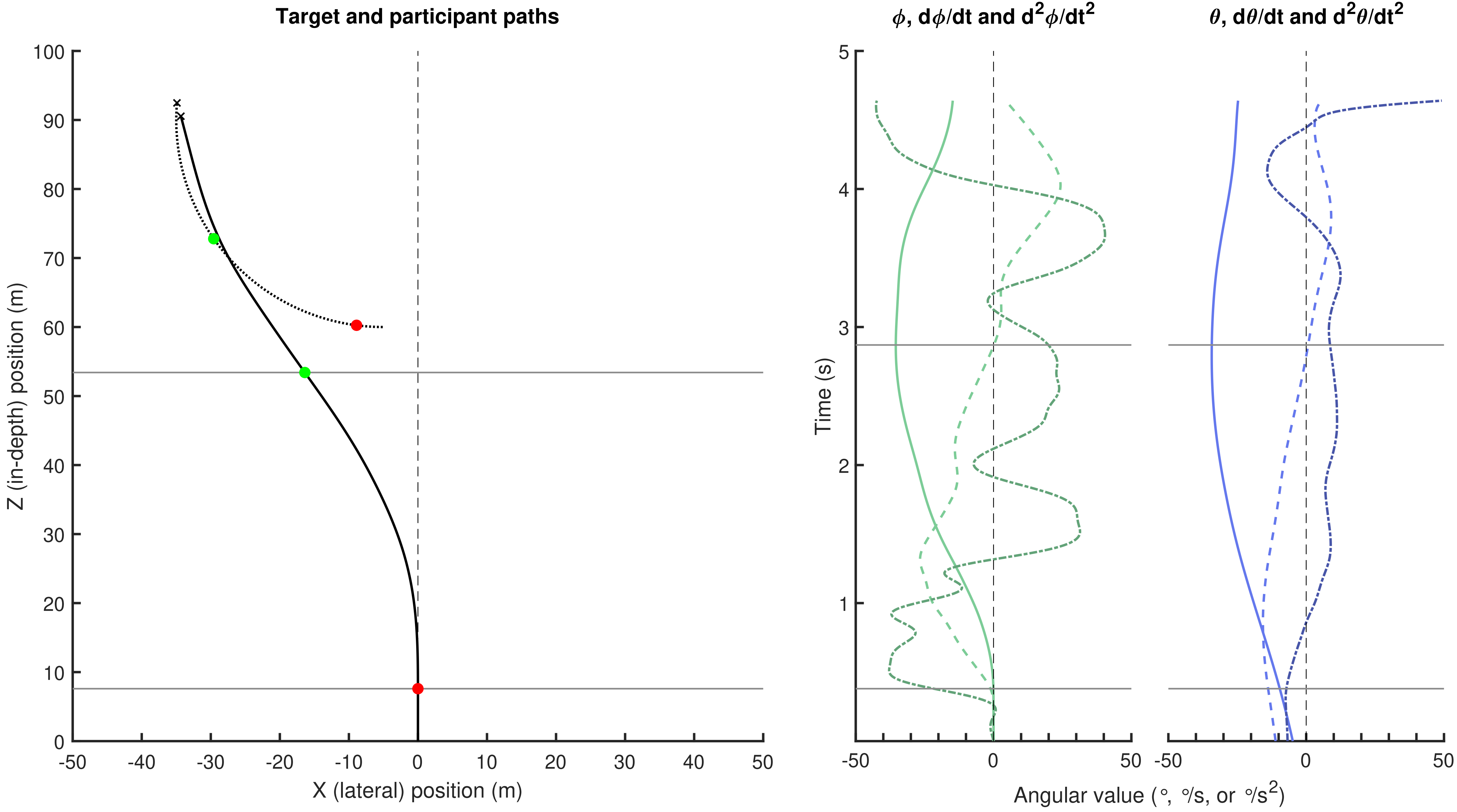

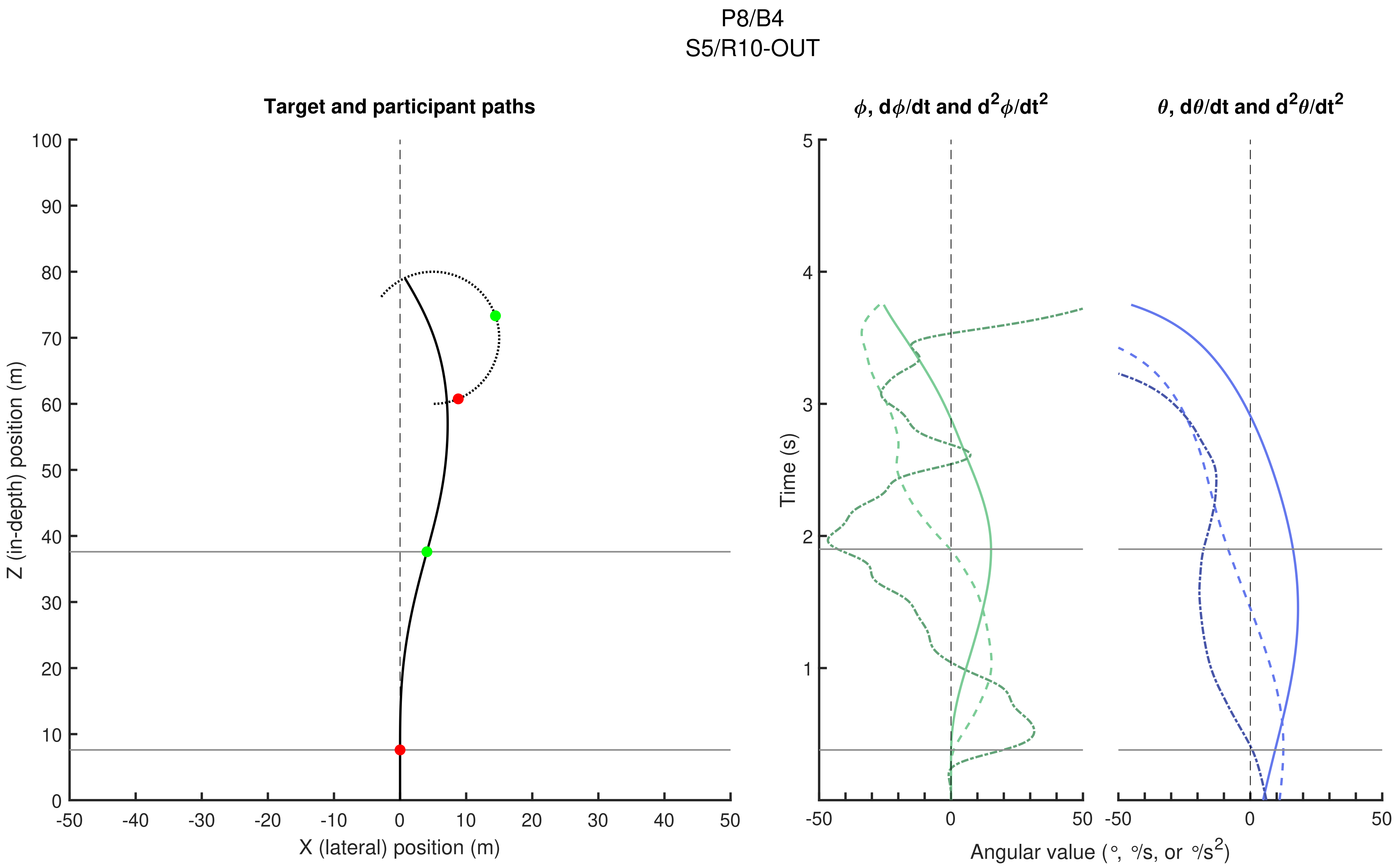

P8/B4
S5/R10-OUT
Target and participant paths
Z (in-depth) position (m)
X (lateral) position (m)
φ, dφ/dt and d²φ/dt²
θ, dθ/dt and d²θ/dt²
Time (s)
Angular value (°, °/s, or °/s²)

P8/B4
S5/R10-IN

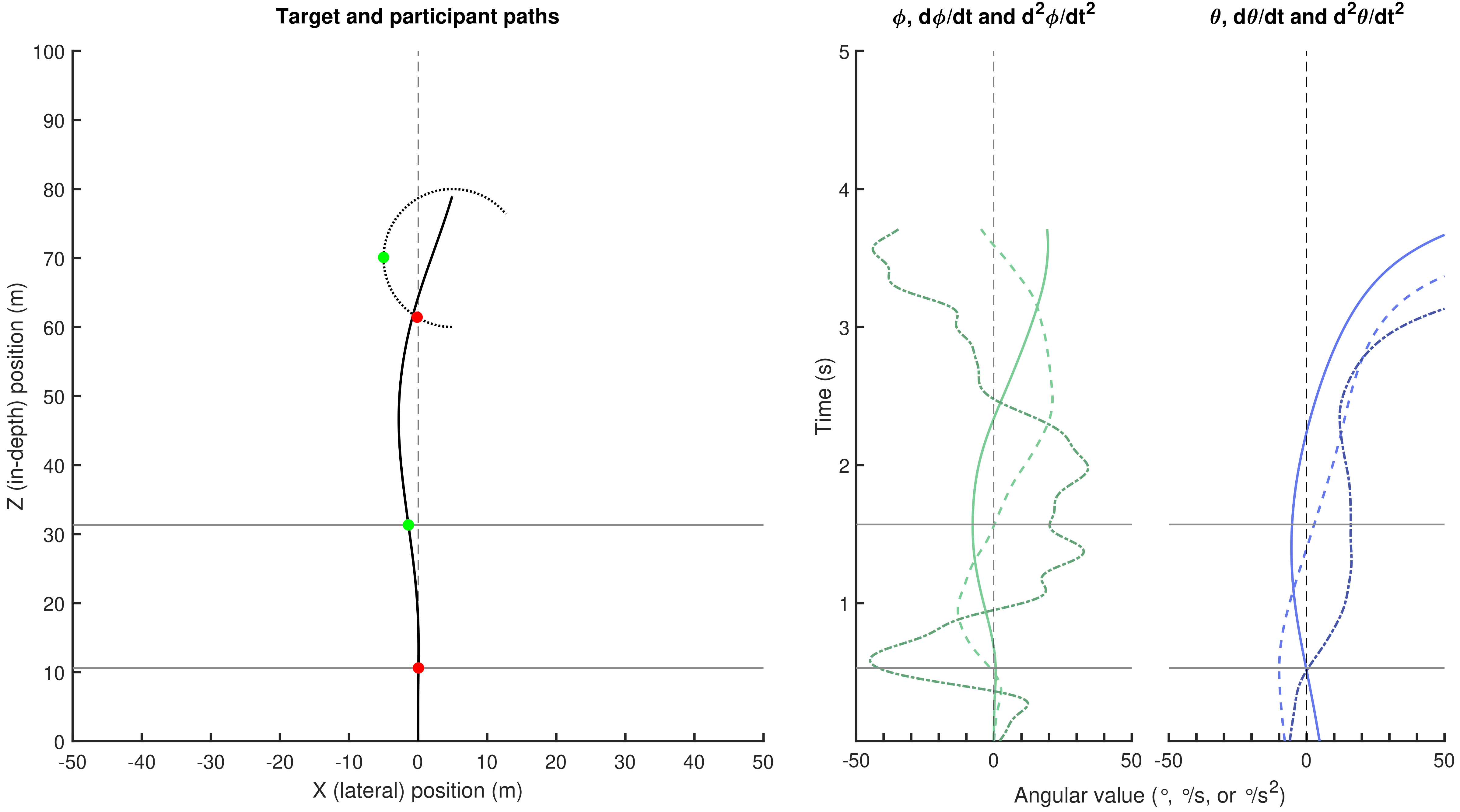

P8/B4
S5/R20-OUT

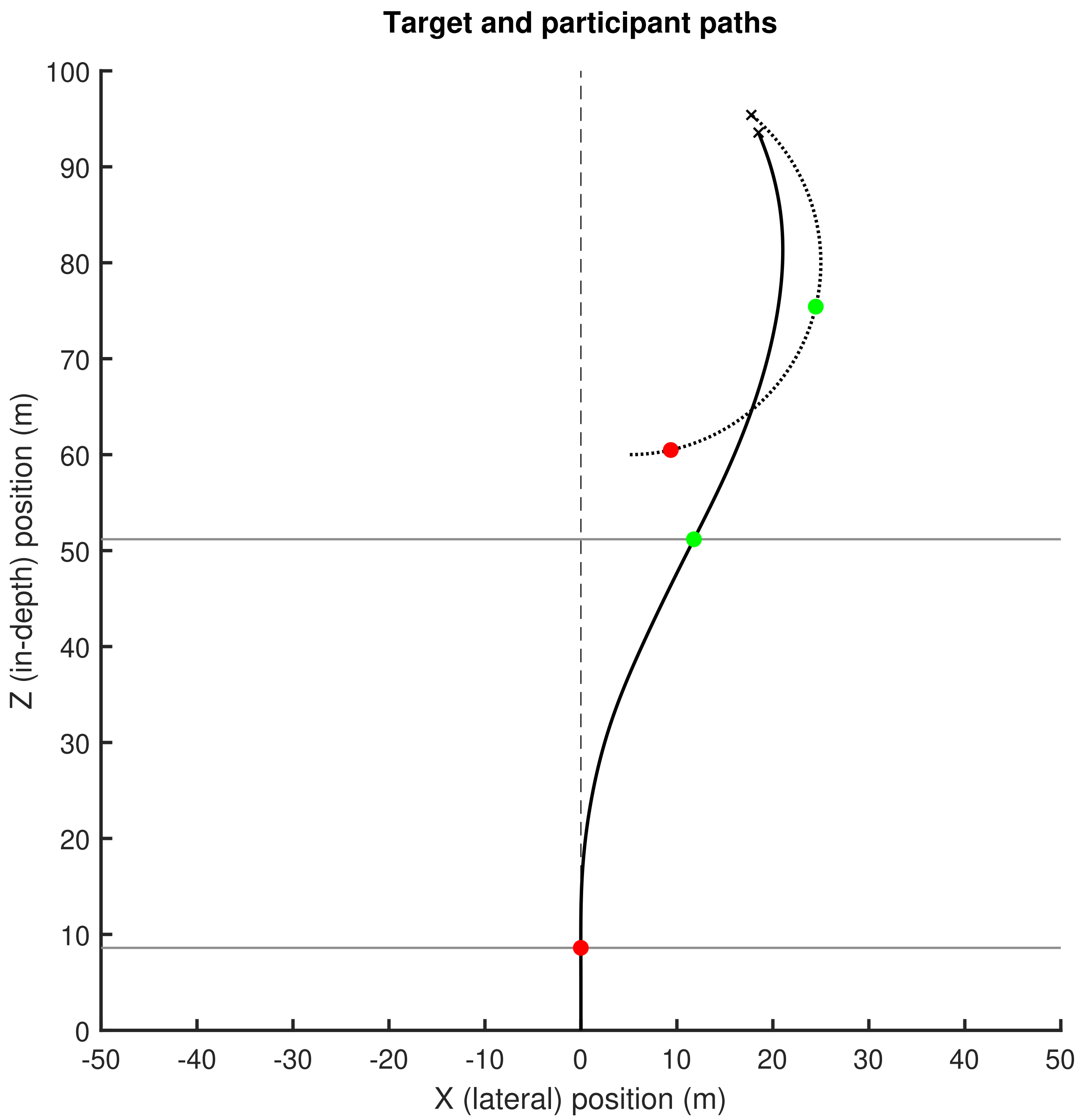

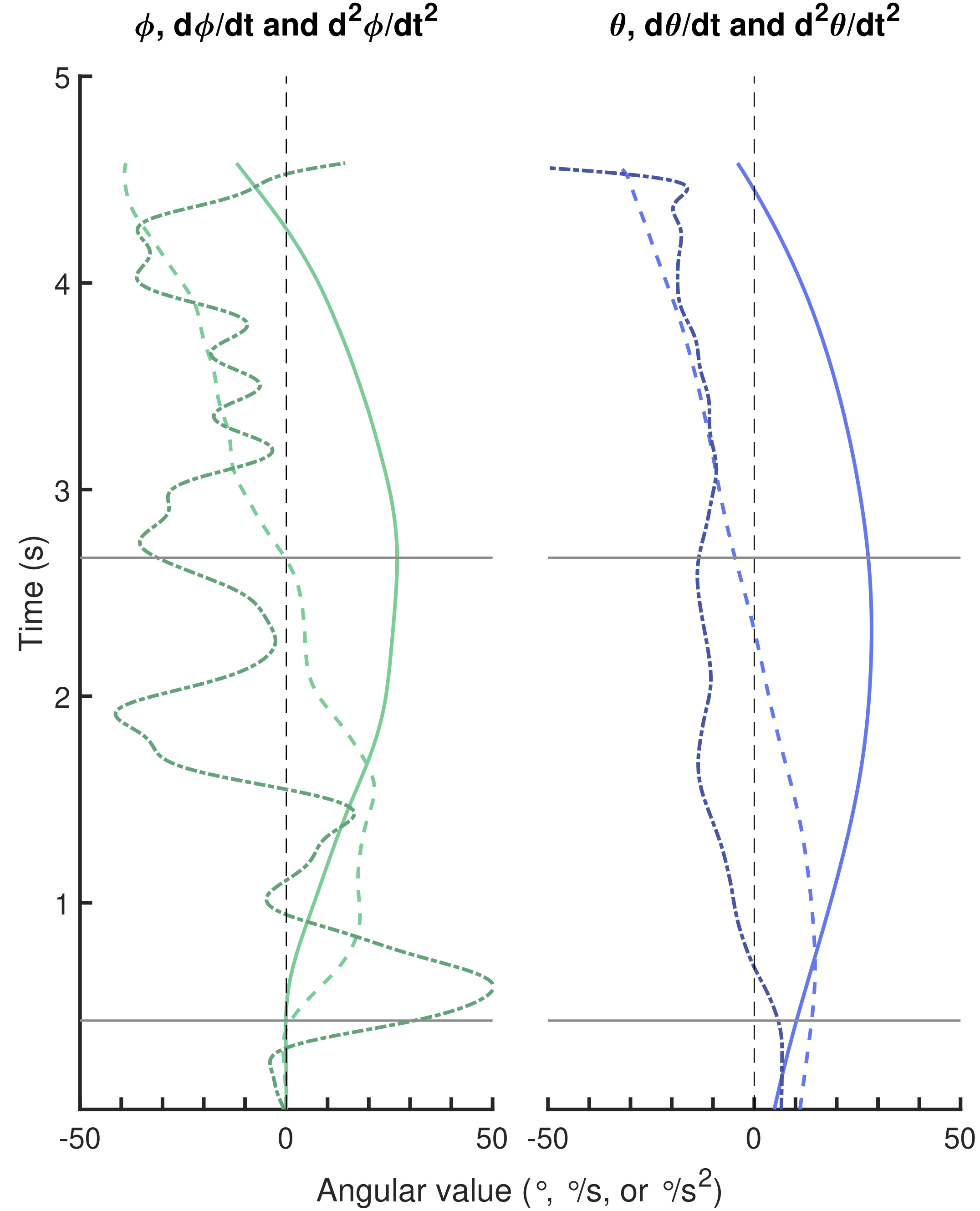

P8/B4
S5/R20-IN

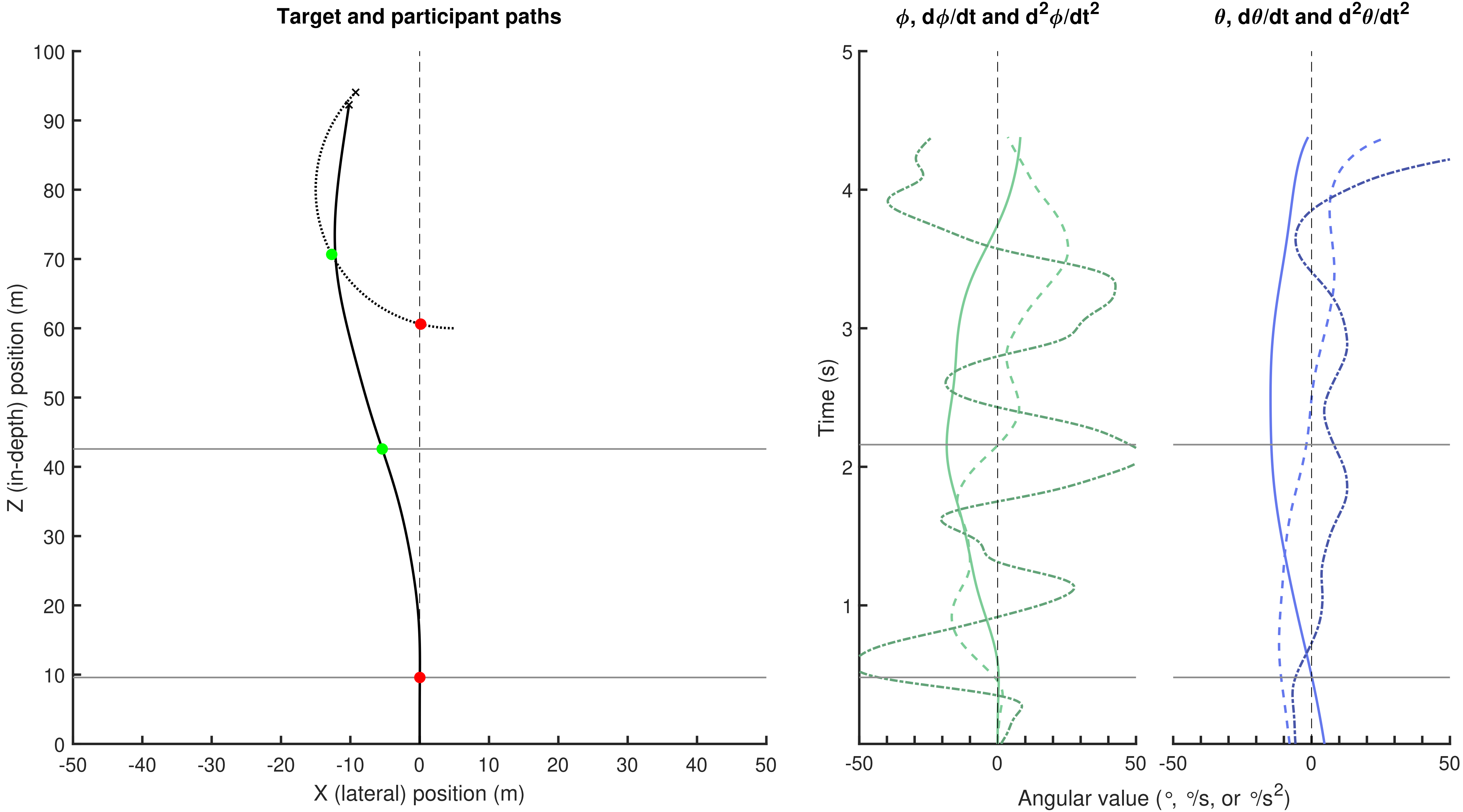

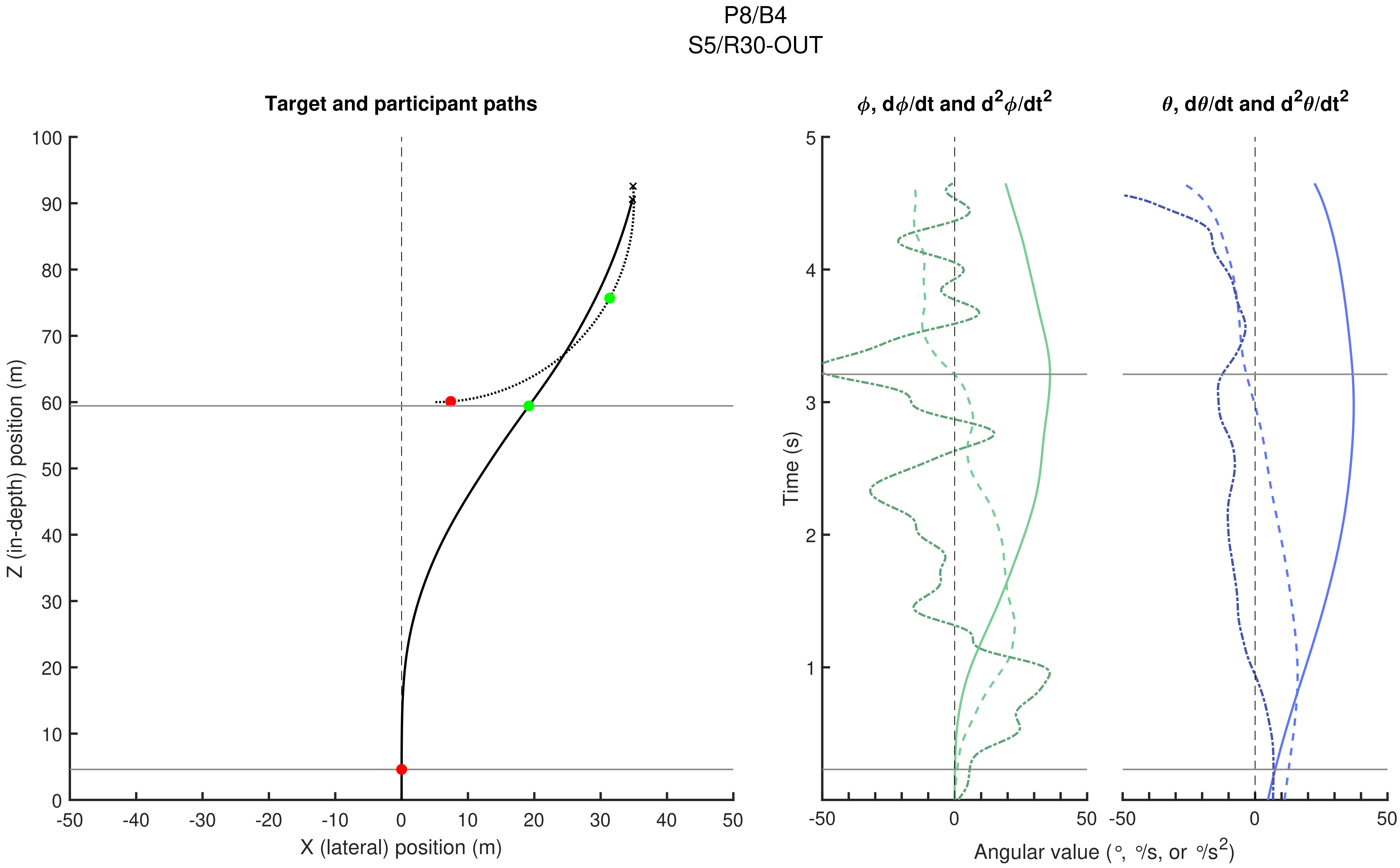
P8/B4
S5/R30-OUT
Target and participant paths
Z (in-depth) position (m)
X (lateral) position (m)
φ, dφ/dt and d²φ/dt²
θ, dθ/dt and d²θ/dt²
Time (s)
Angular value (°, °/s, or °/s²)

P8/B4
S5/R30-IN

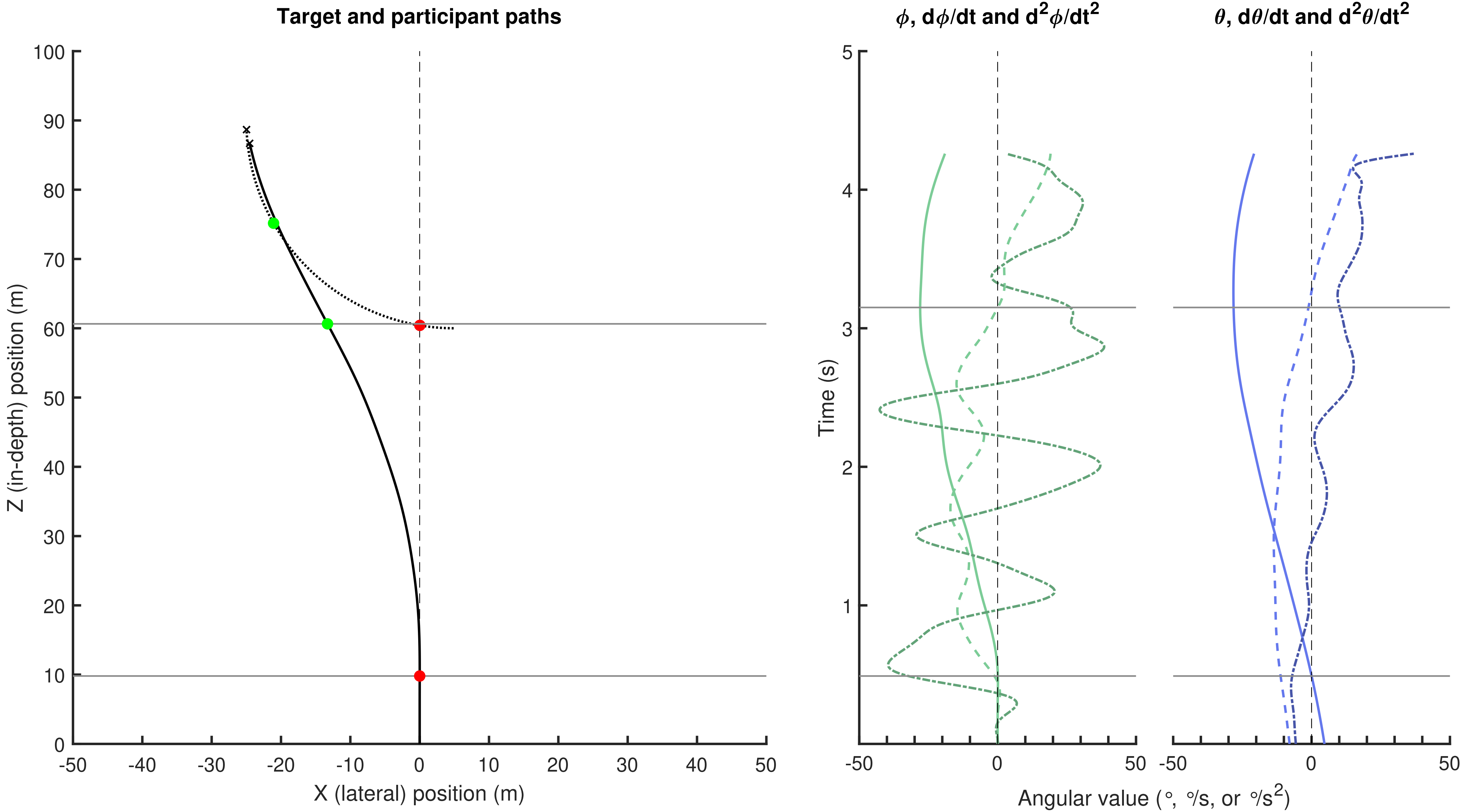

P8/B4
S15/R10-IN

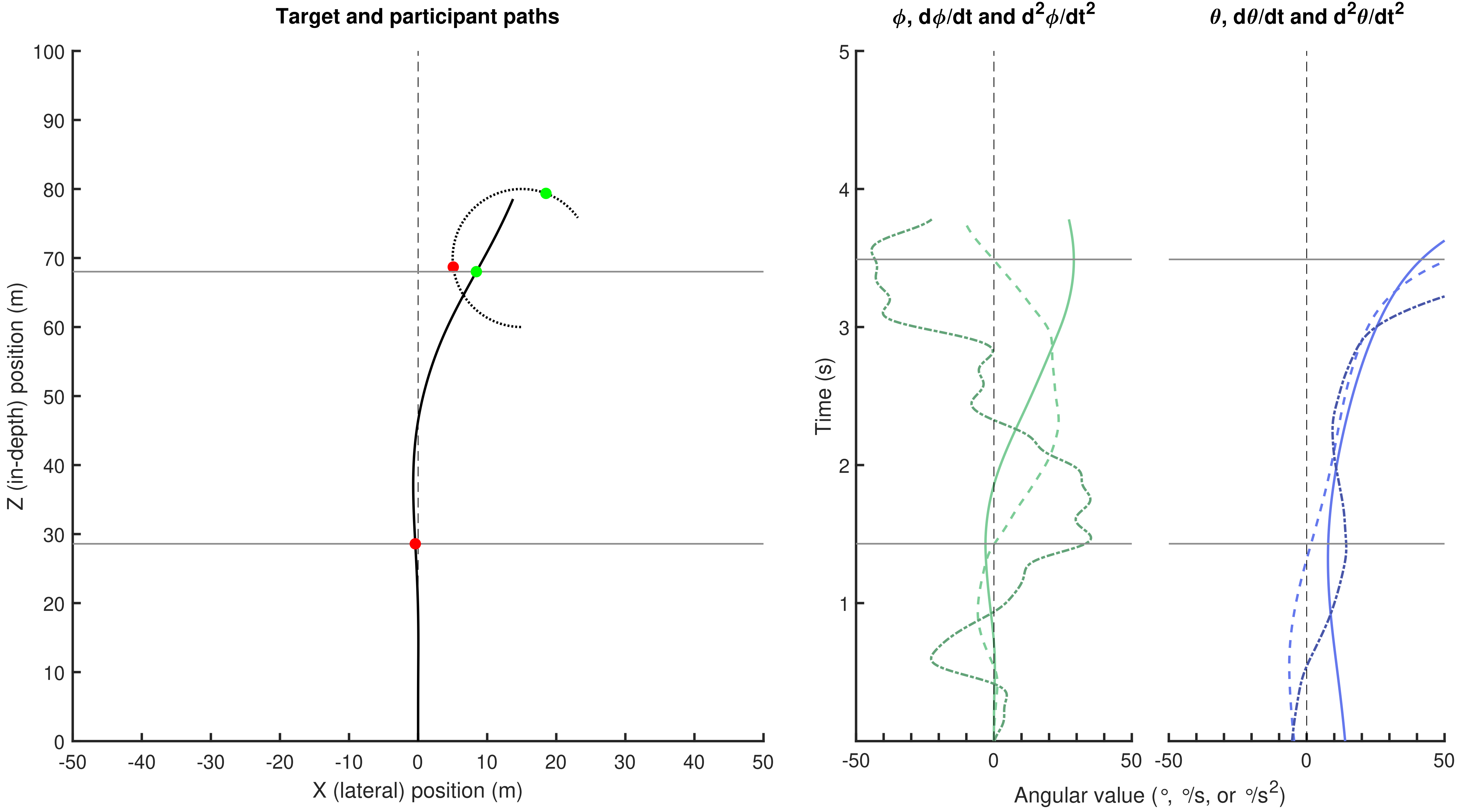

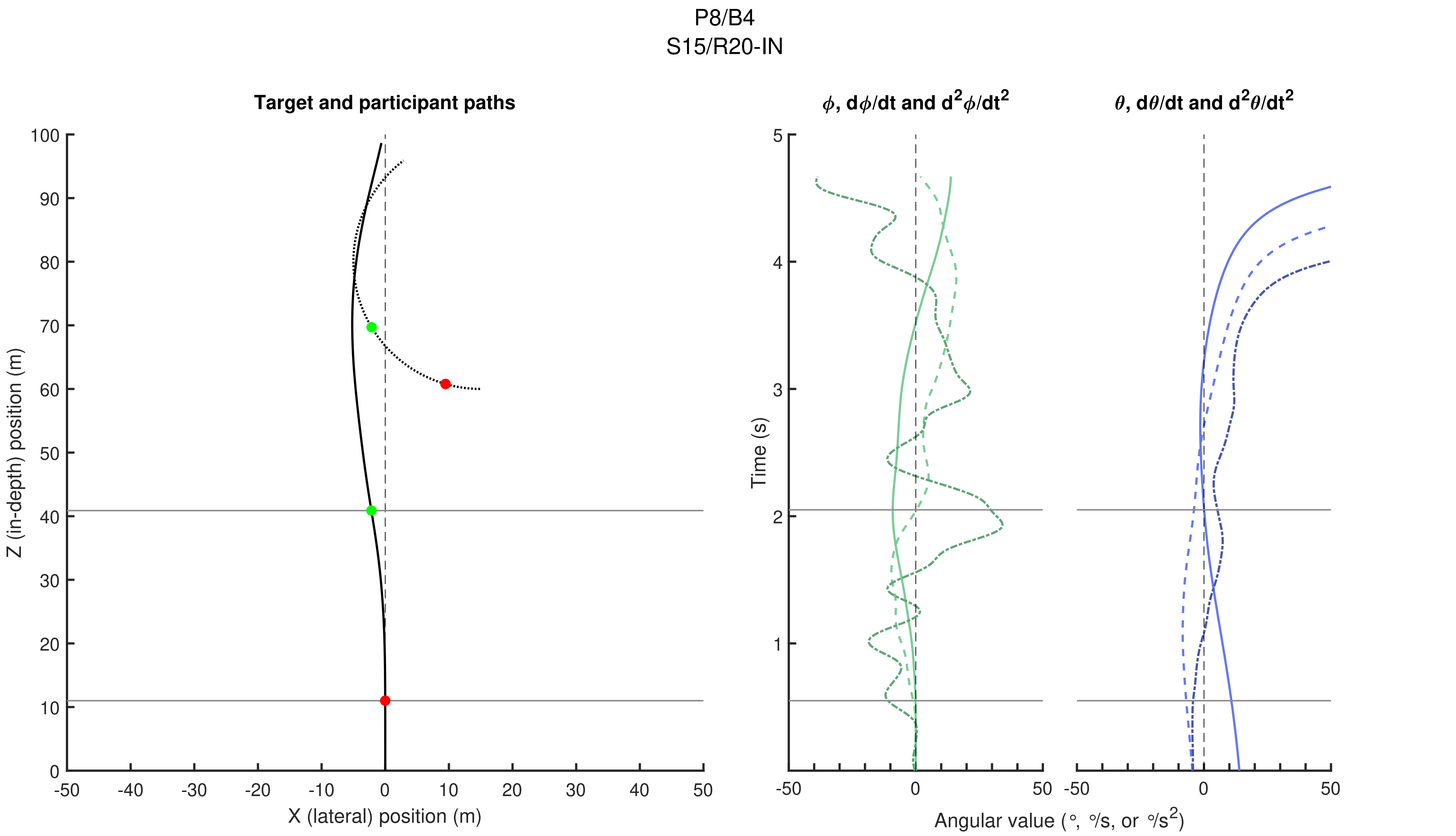
P8/B4
S15/R20-IN
Target and participant paths
Z (in-depth) position (m)
X (lateral) position (m)
ϕ, dϕ/dt and d²ϕ/dt²
θ, dθ/dt and d²θ/dt²
Time (s)
Angular value (°, °/s, or °/s²)

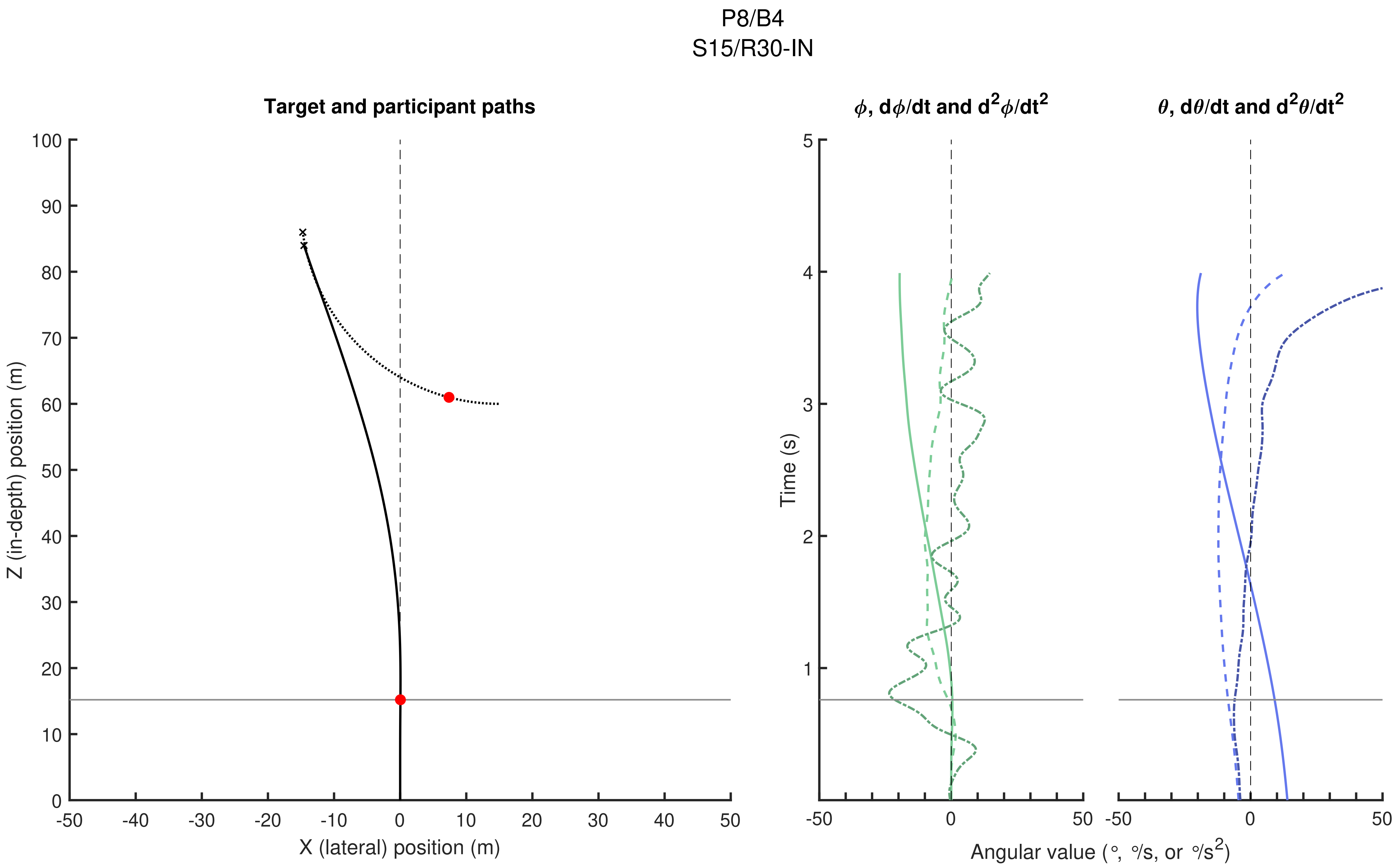
P8/B4
S15/R30-IN
Target and participant paths
Z (in-depth) position (m)
X (lateral) position (m)
100
90
80
70
60
50
40
30
20
10
0
-50
-40
-30
-20
-10
0
10
20
30
40
50
φ, dφ/dt and d²φ/dt²
θ, dθ/dt and d²θ/dt²
Time (s)
5
4
3
2
1
-50
0
50
-50
0
50
Angular value (°, °/s, or °/s²)

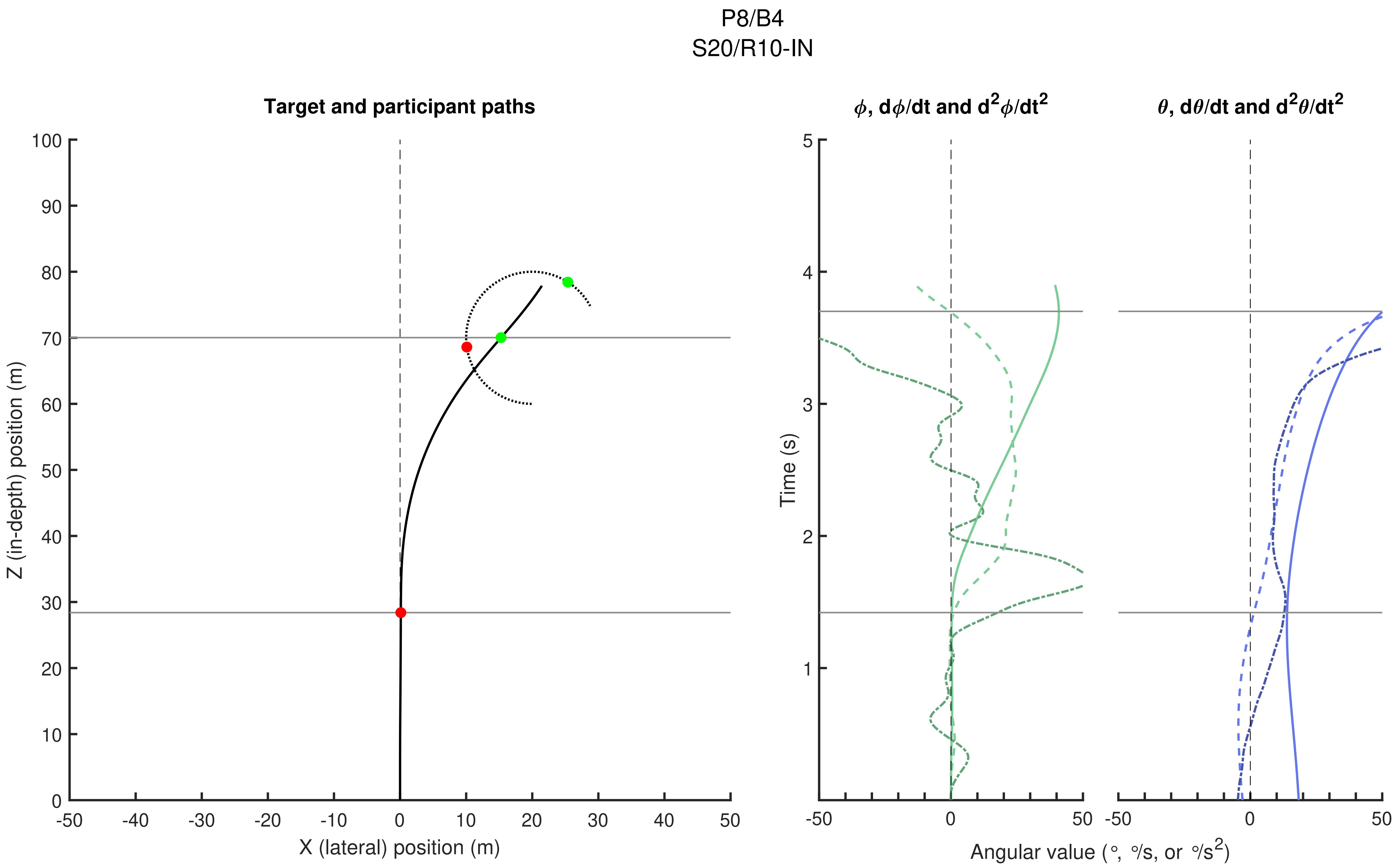
P8/B4
S20/R10-IN
Target and participant paths
Z (in-depth) position (m)
X (lateral) position (m)
ϕ, dϕ/dt and d²ϕ/dt²
θ, dθ/dt and d²θ/dt²
Time (s)
Angular value (°, °/s, or °/s²)

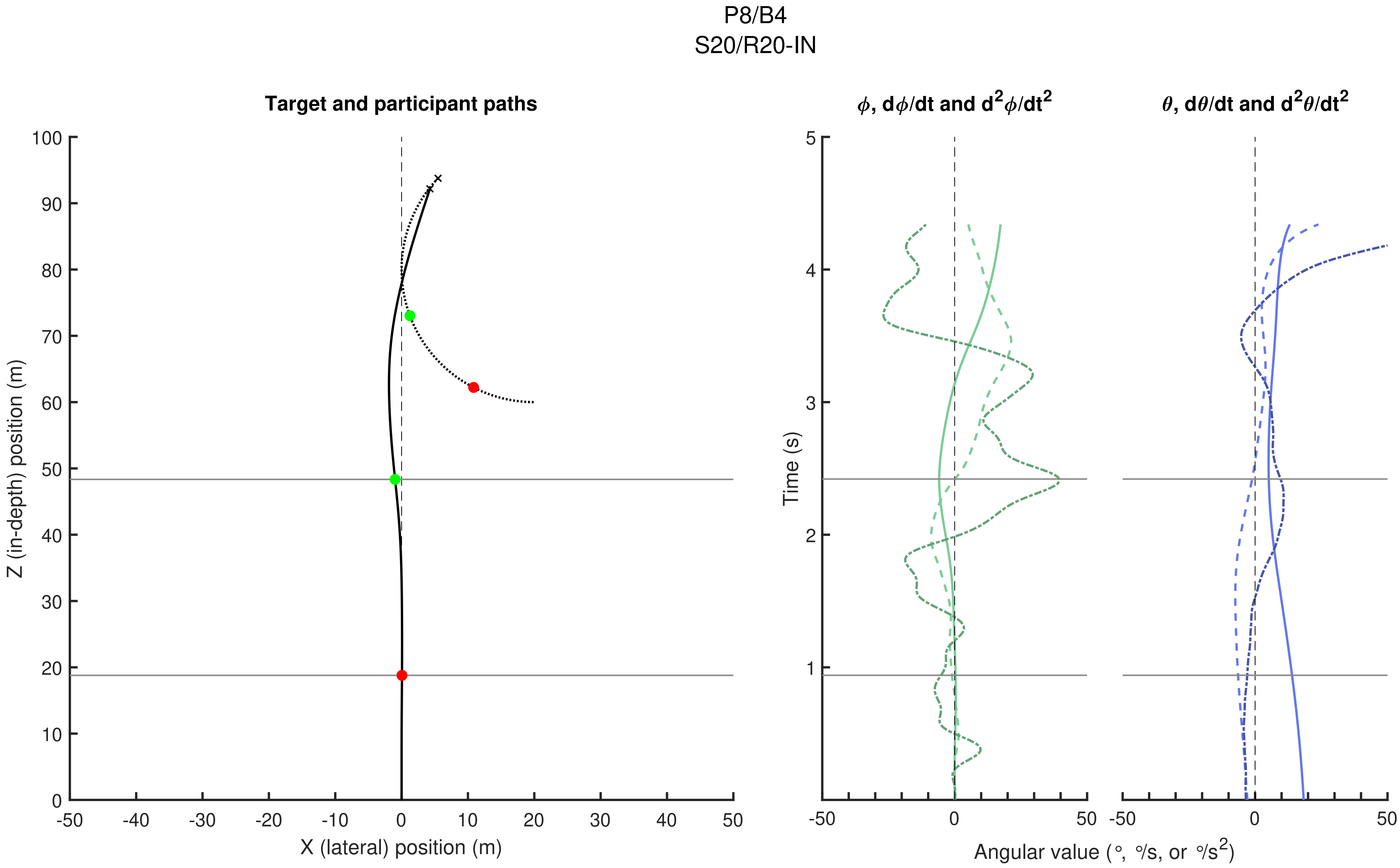

P8/B4
S20/R20-IN
Target and participant paths
Z (in-depth) position (m)
X (lateral) position (m)
ϕ, dϕ/dt and d$^2\phi$/dt^2
θ, dθ/dt and d$^2\theta$/dt^2
Time (s)
Angular value (°, °/s, or °/s^2)

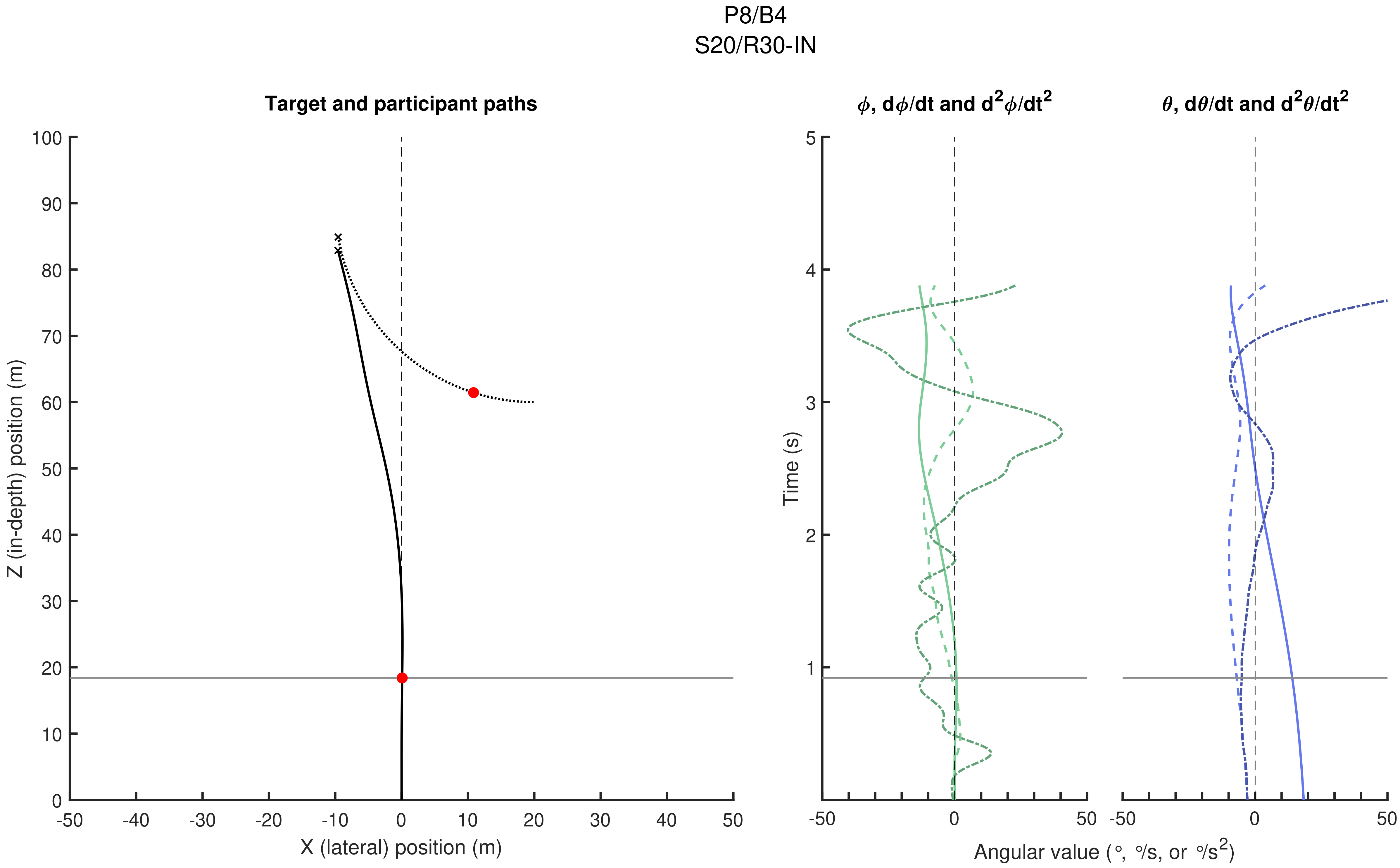
P8/B4
S20/R30-IN
Target and participant paths
ϕ, dϕ/dt and d$^2\phi$/dt^2
θ, dθ/dt and d$^2\theta$/dt^2
Z (in-depth) position (m)
X (lateral) position (m)
Time (s)
Angular value (°, °/s, or °/s^2)
100
90
80
70
60
50
40
30
20
10
0
-50
-40
-30
-20
-10
0
10
20
30
40
50
5
4
3
2
1

P8/B5
S20/R10-IN

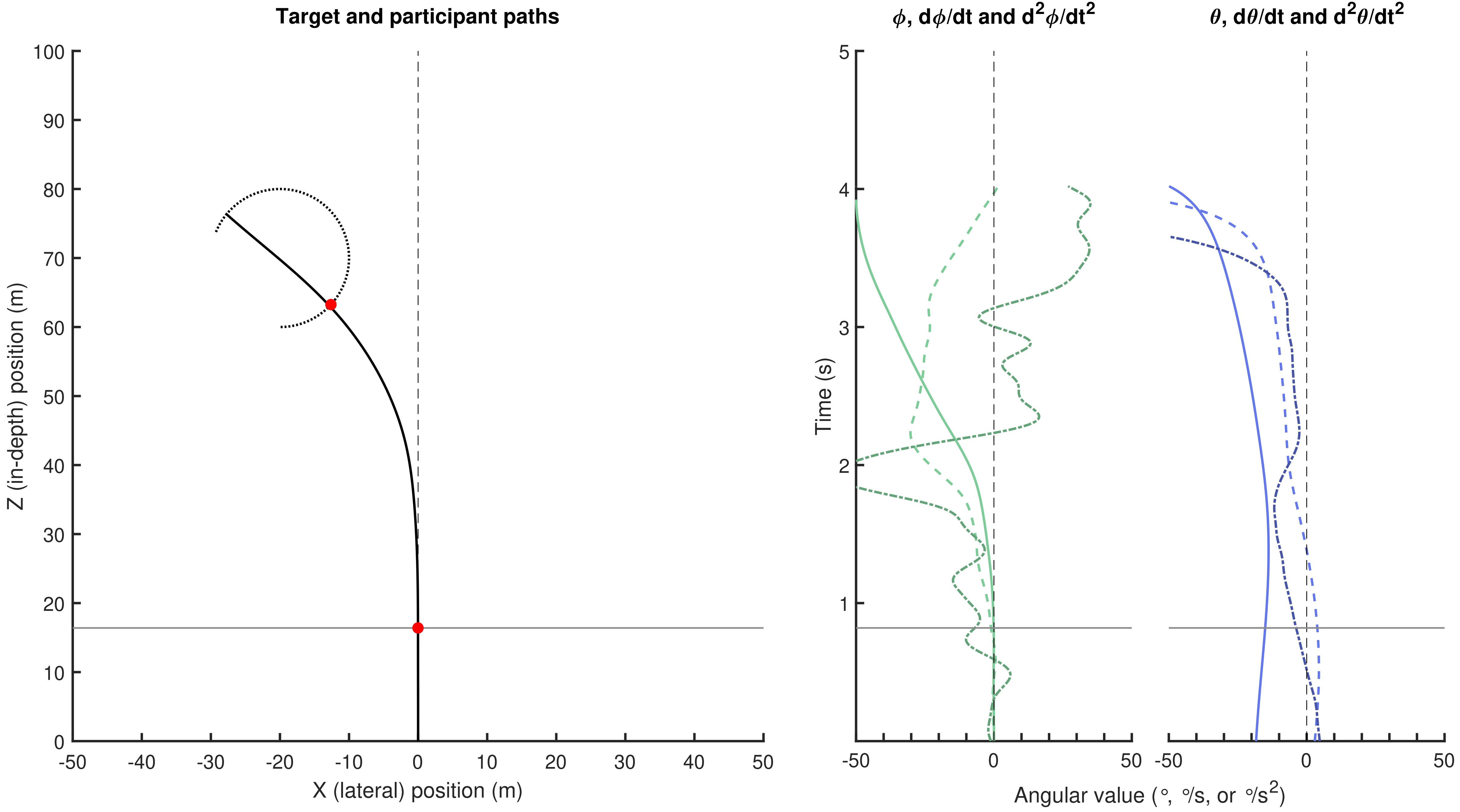

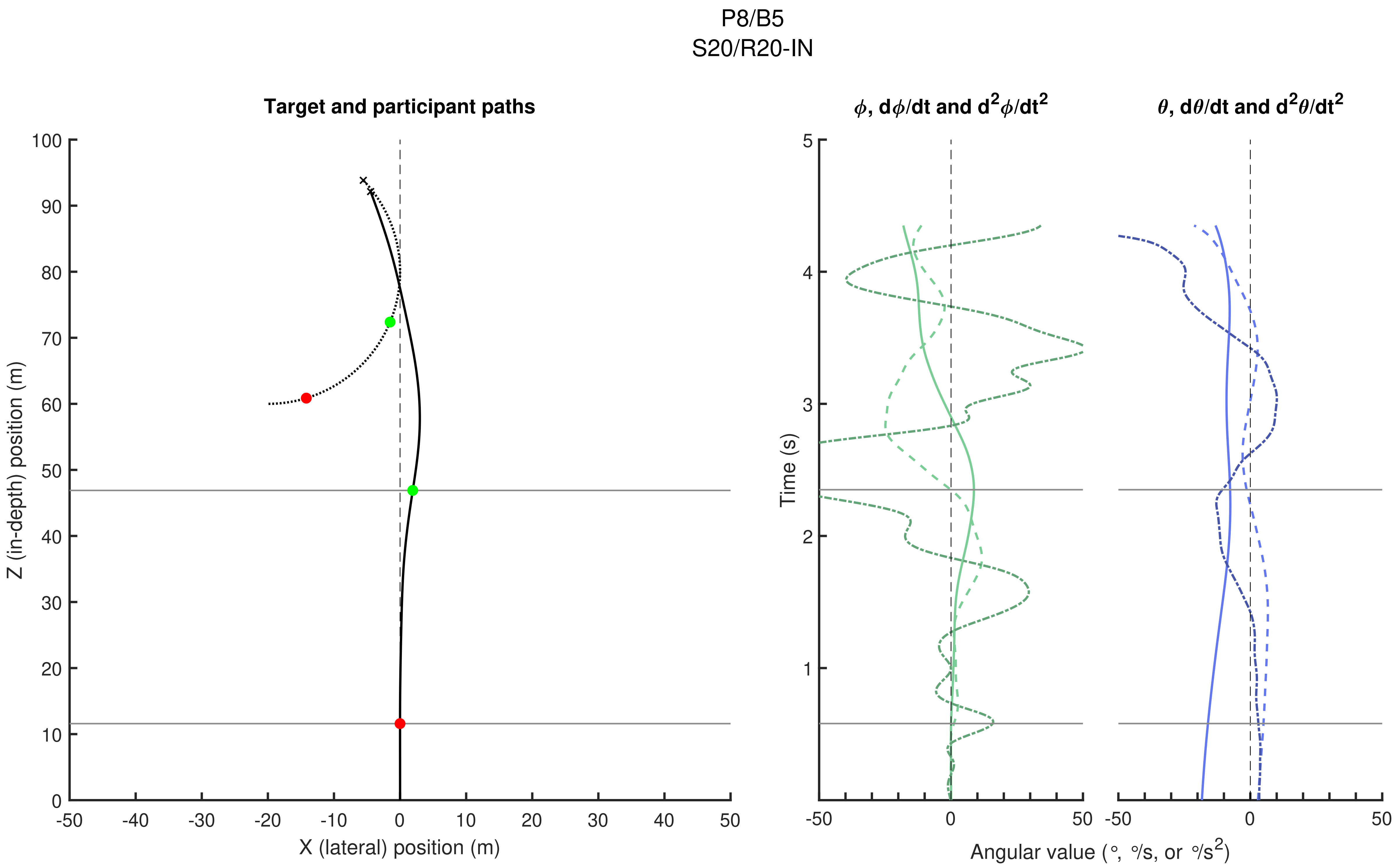

P8/B5
S20/R20-IN
Target and participant paths
Z (in-depth) position (m)
X (lateral) position (m)
ϕ, dϕ/dt and d²ϕ/dt²
θ, dθ/dt and d²θ/dt²
Time (s)
Angular value (°, °/s, or °/s²)

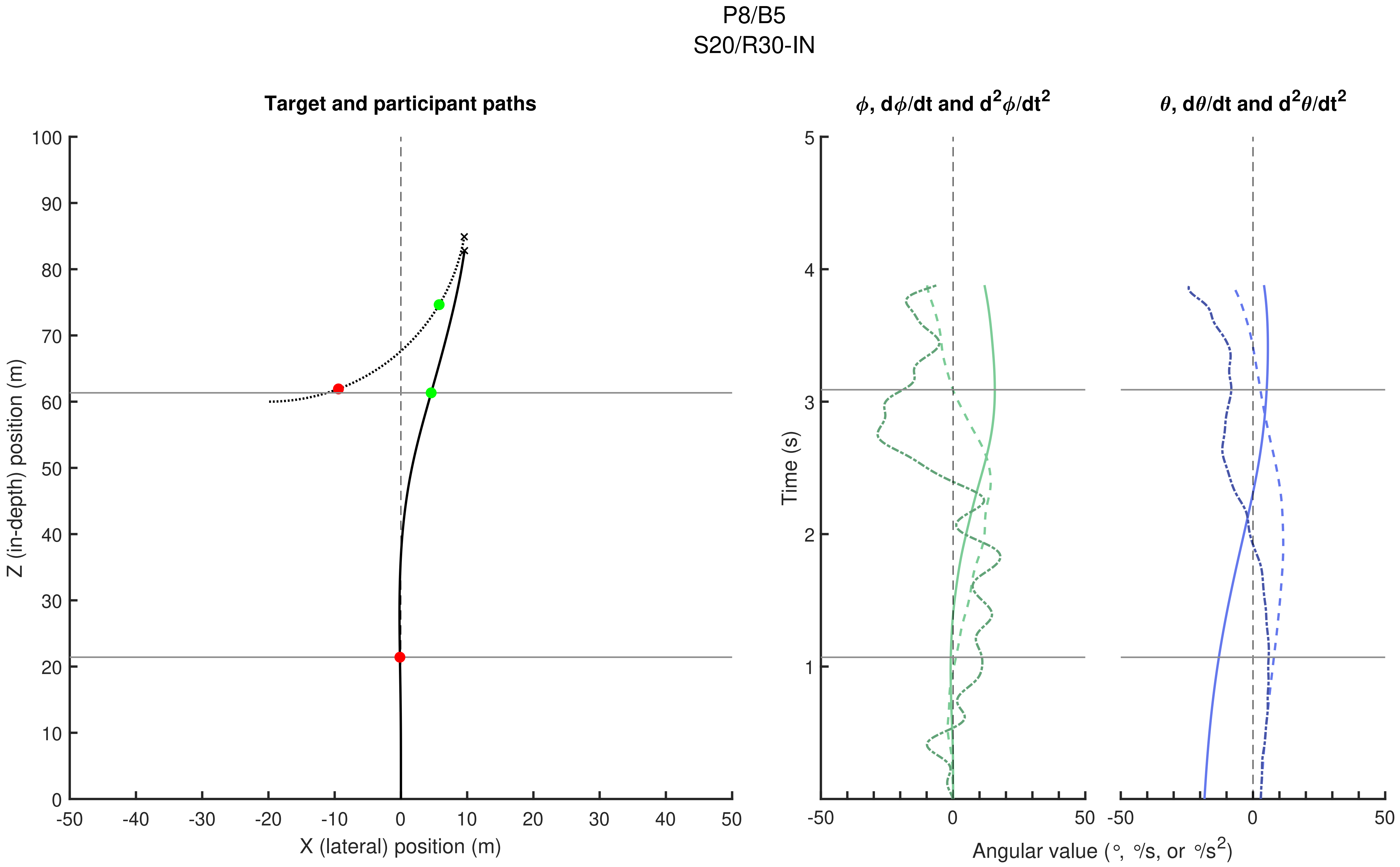
P8/B5
S20/R30-IN
Target and participant paths
Z (in-depth) position (m)
X (lateral) position (m)
0
10
20
30
40
50
60
70
80
90
100
-50
-40
-30
-20
-10
0
10
20
30
40
50
φ, dφ/dt and d²φ/dt²
θ, dθ/dt and d²θ/dt²
Time (s)
1
2
3
4
5
-50
0
50
-50
0
50
Angular value (°, °/s, or °/s²)

P8/B5
S15/R10-IN

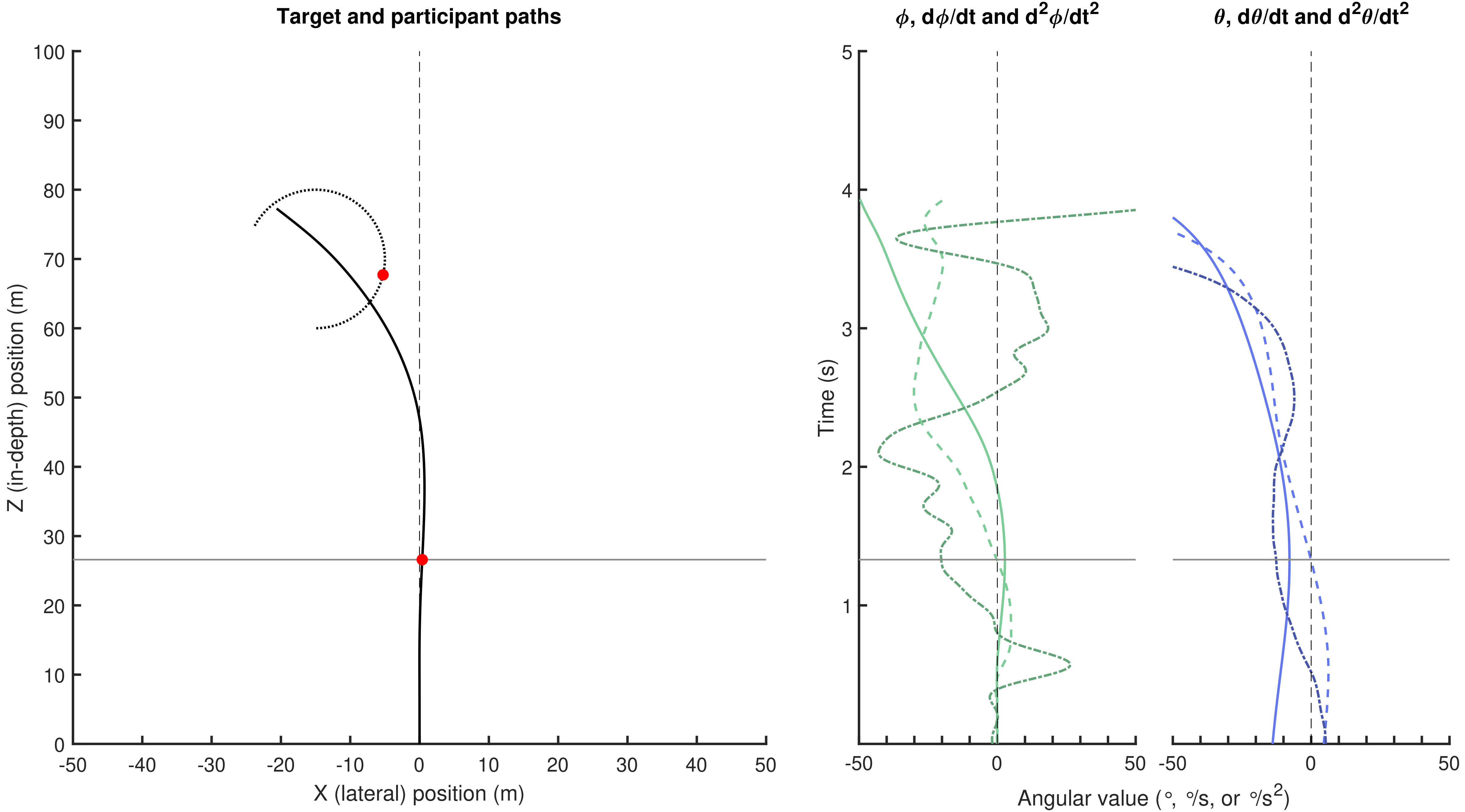

P8/B5
S15/R20-IN

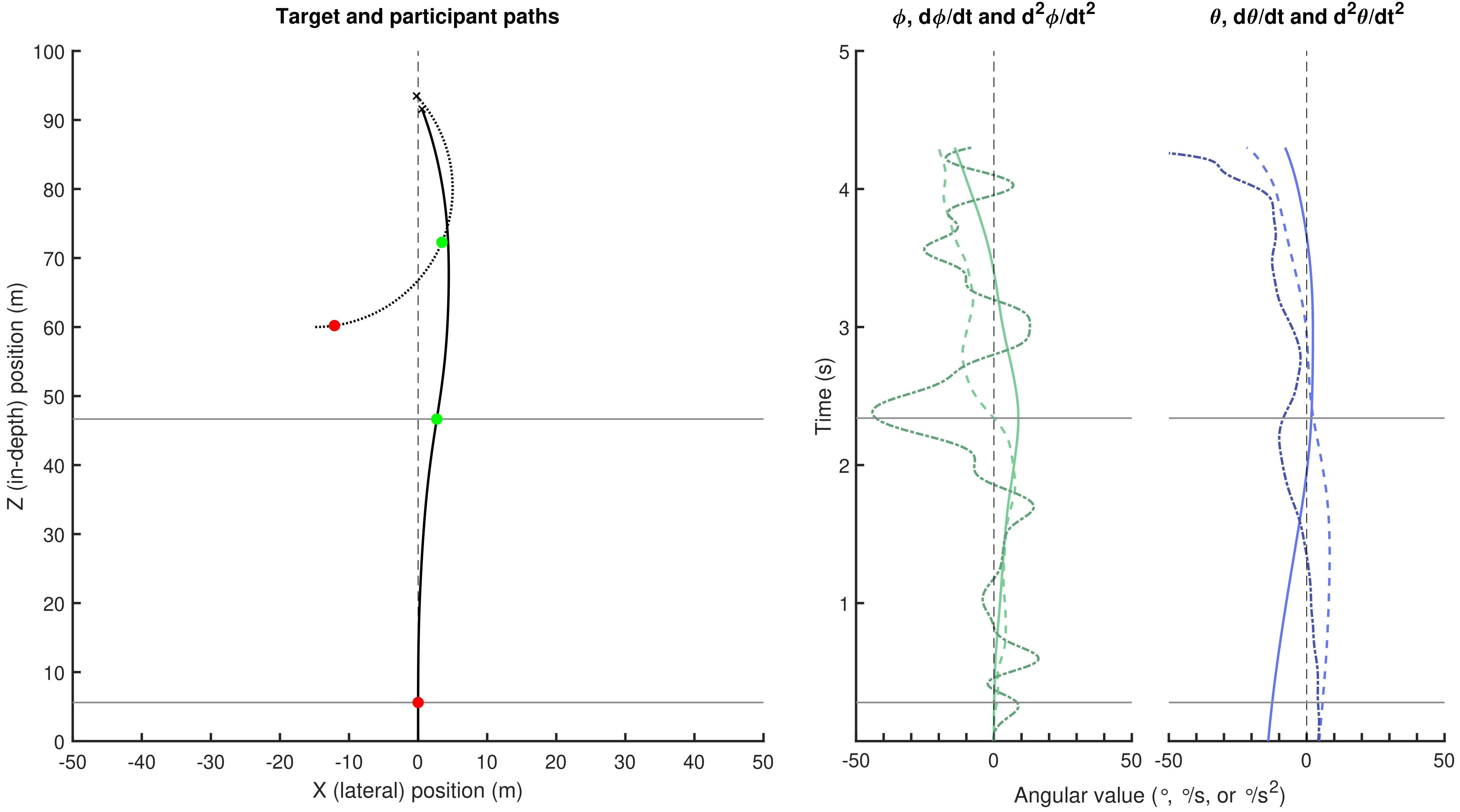

P8/B5
S15/R30-IN

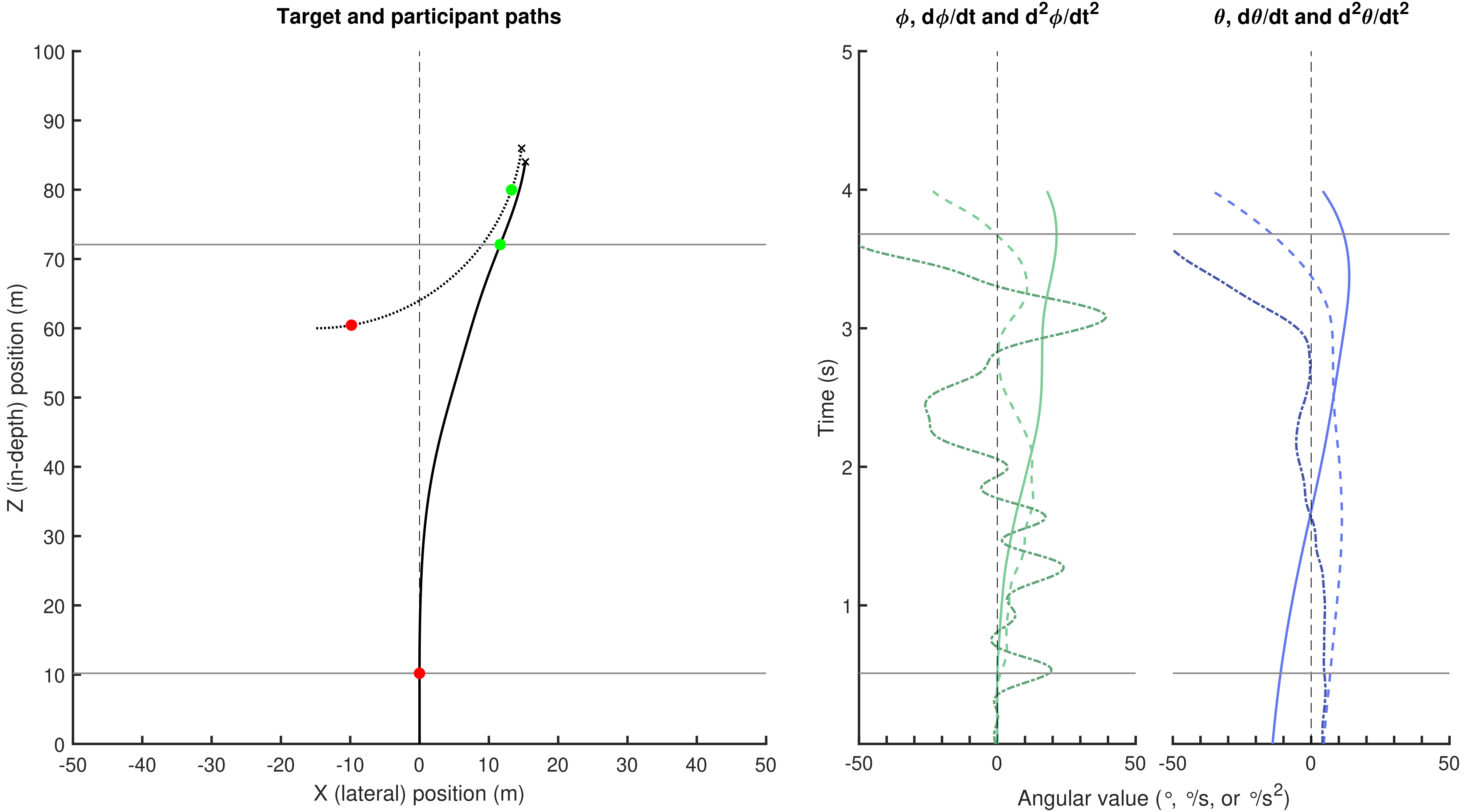

P8/B5
S5/R10-IN

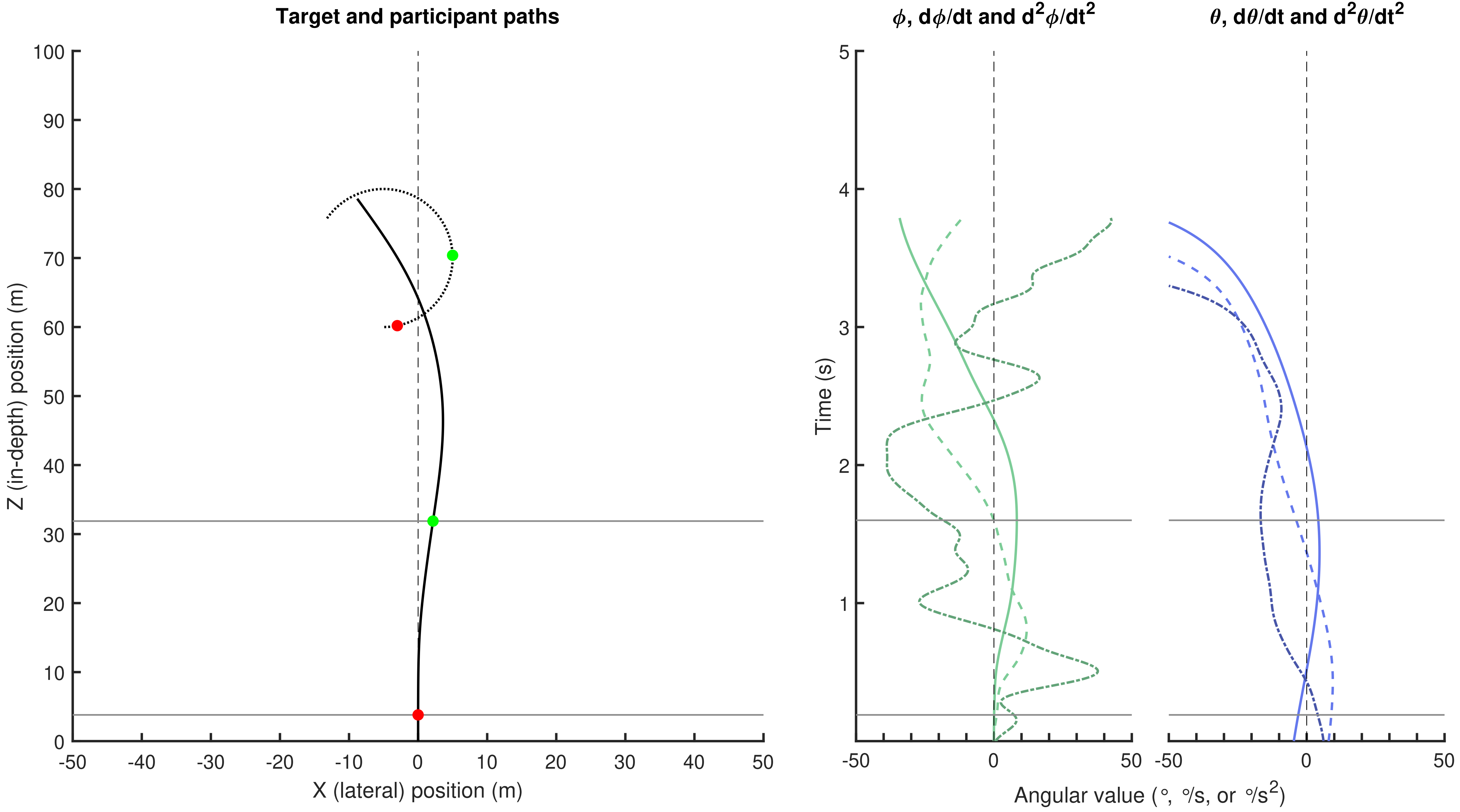

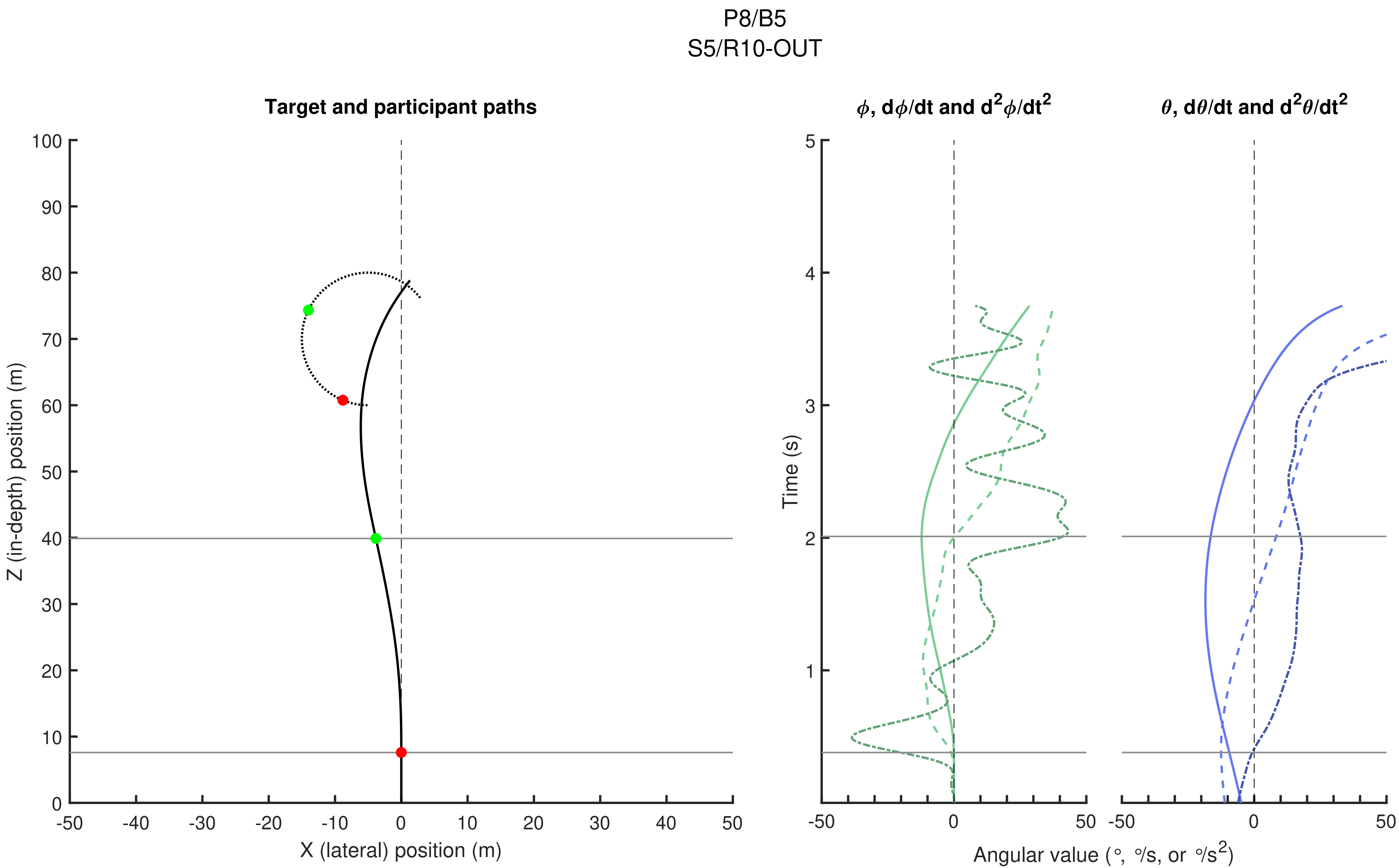

P8/B5
S5/R10-OUT
Target and participant paths
ϕ, dϕ/dt and d$^2\phi$/dt^2
θ, dθ/dt and d$^2\theta$/dt^2
Z (in-depth) position (m)
X (lateral) position (m)
Time (s)
Angular value (°, °/s, or °/s^2)

P8/B5
S5/R20-IN

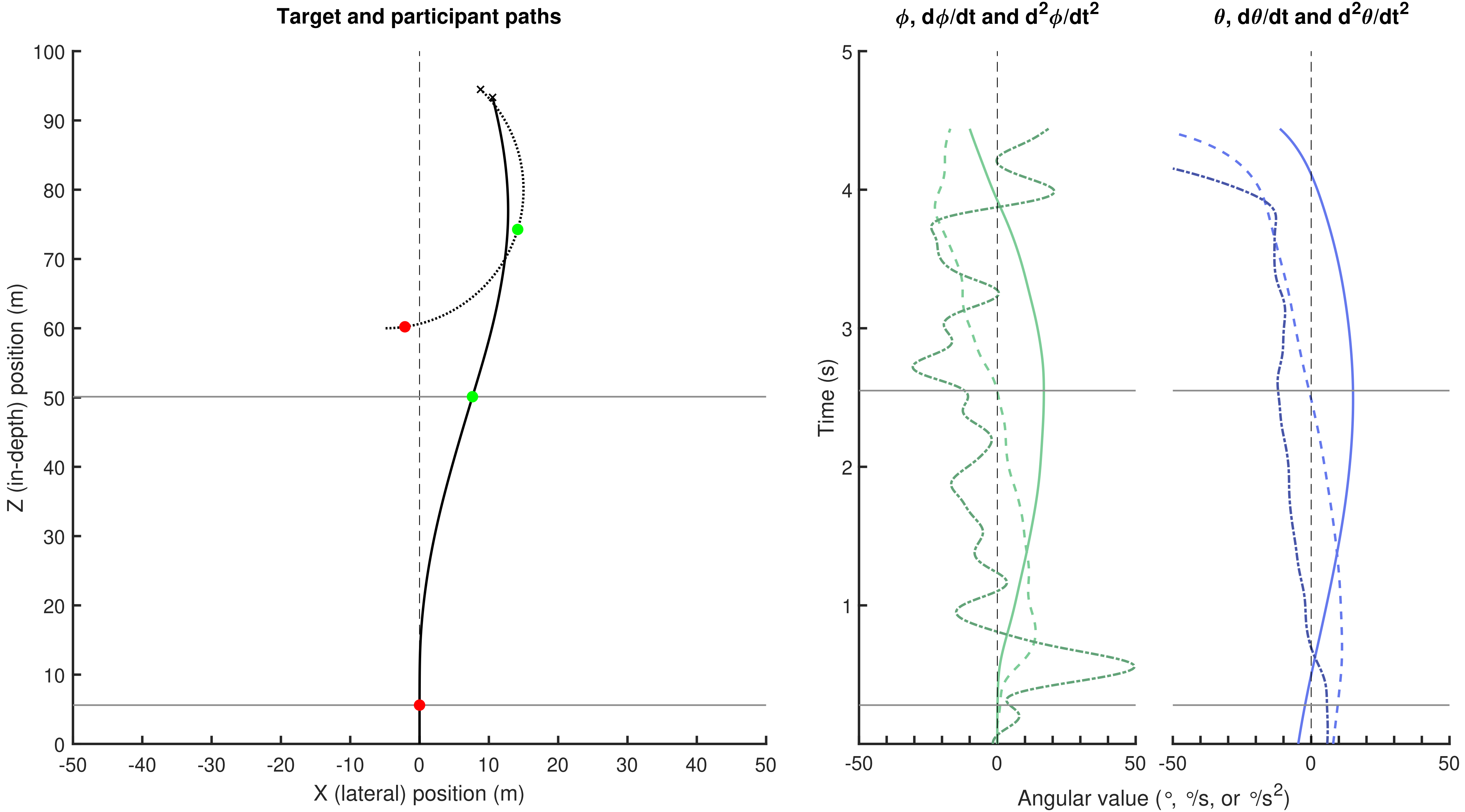

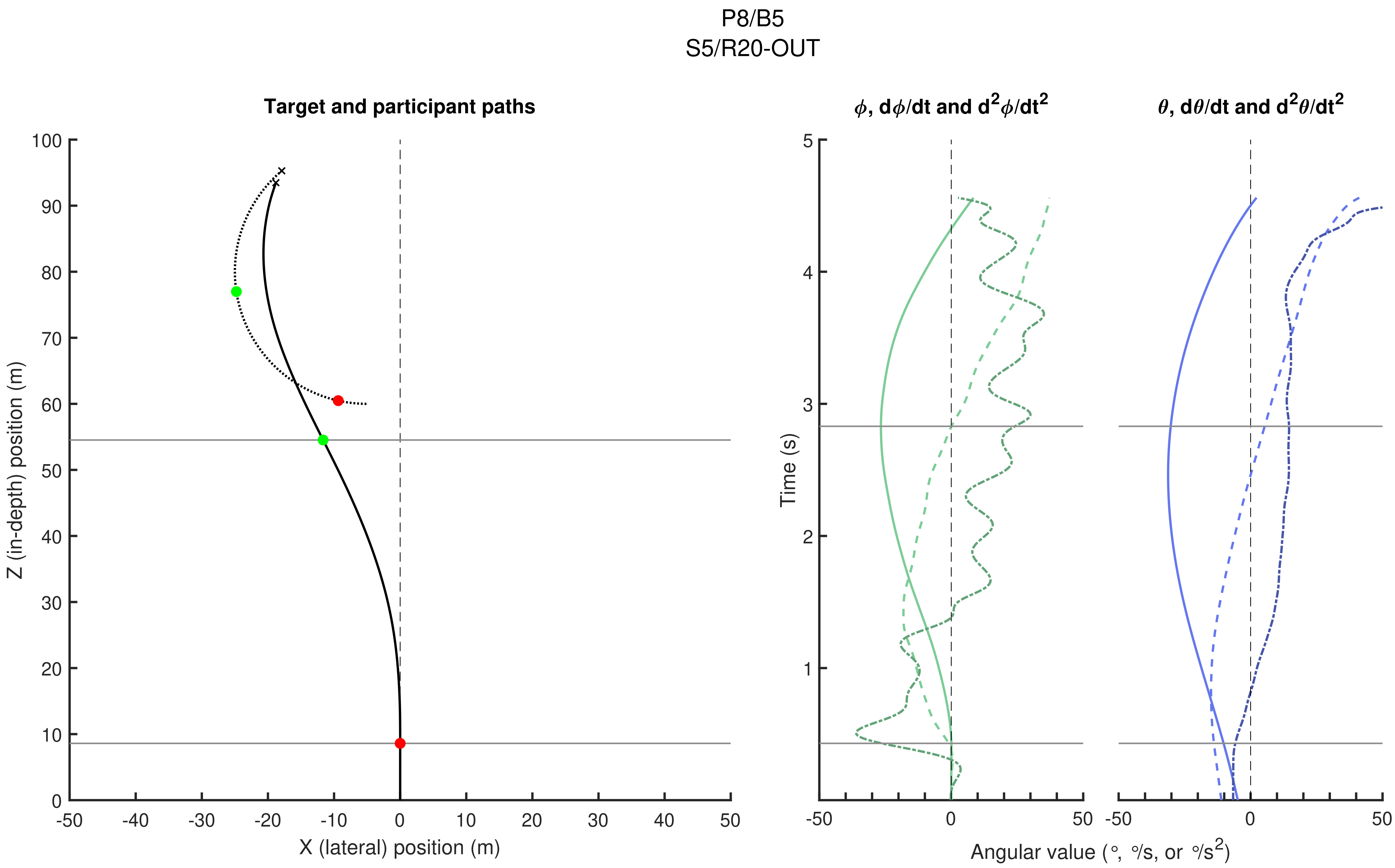

P8/B5
S5/R20-OUT
Target and participant paths
Z (in-depth) position (m)
X (lateral) position (m)
φ, dφ/dt and d²φ/dt²
θ, dθ/dt and d²θ/dt²
Time (s)
Angular value (°, °/s, or °/s²)

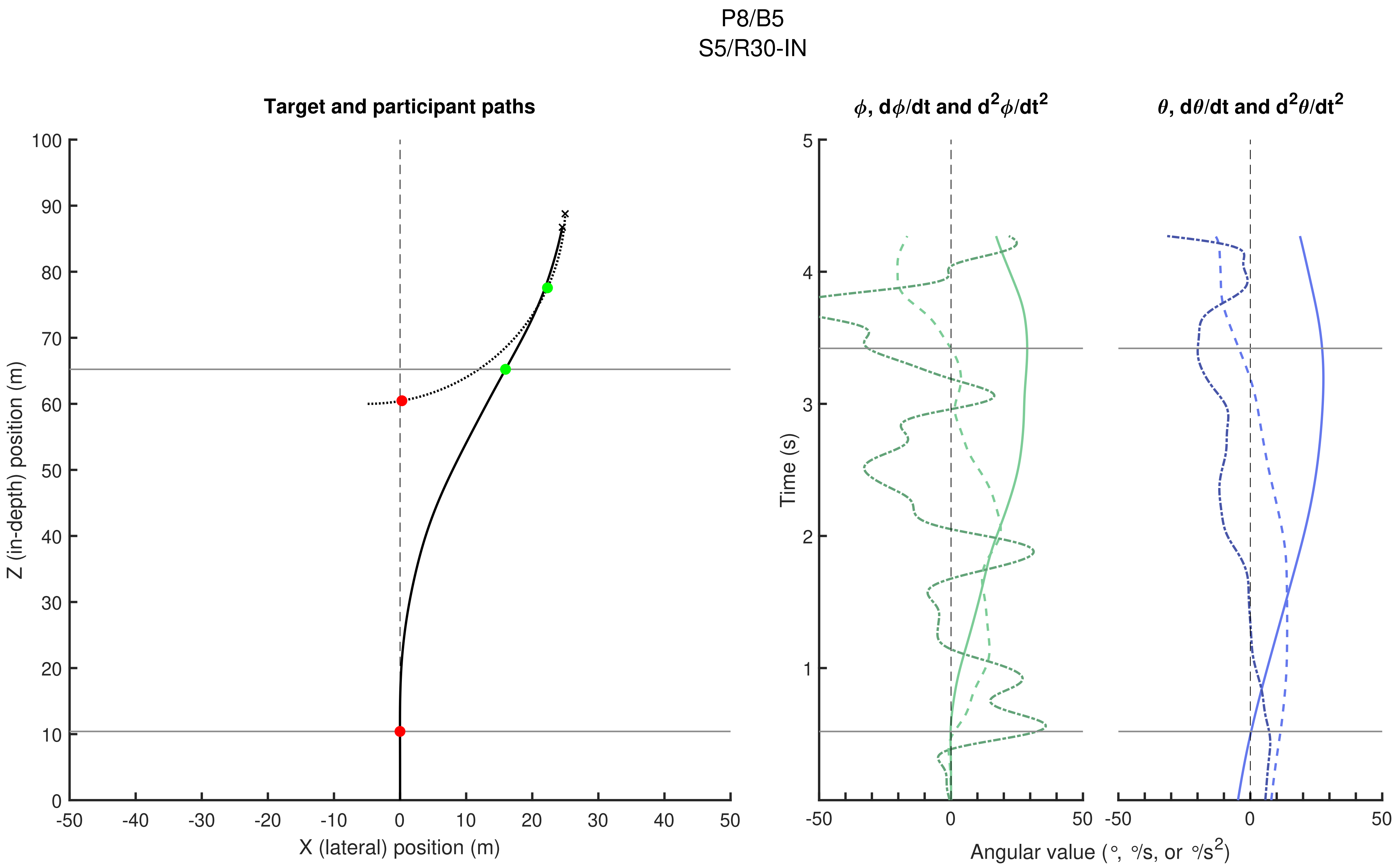
P8/B5
S5/R30-IN
Target and participant paths
Z (in-depth) position (m)
X (lateral) position (m)
100
90
80
70
60
50
40
30
20
10
0
-50
-40
-30
-20
-10
0
10
20
30
40
50
φ, dφ/dt and d²φ/dt²
θ, dθ/dt and d²θ/dt²
Time (s)
5
4
3
2
1
-50
0
50
-50
0
50
Angular value (°, °/s, or °/s²)

P8/B5
S5/R30-OUT

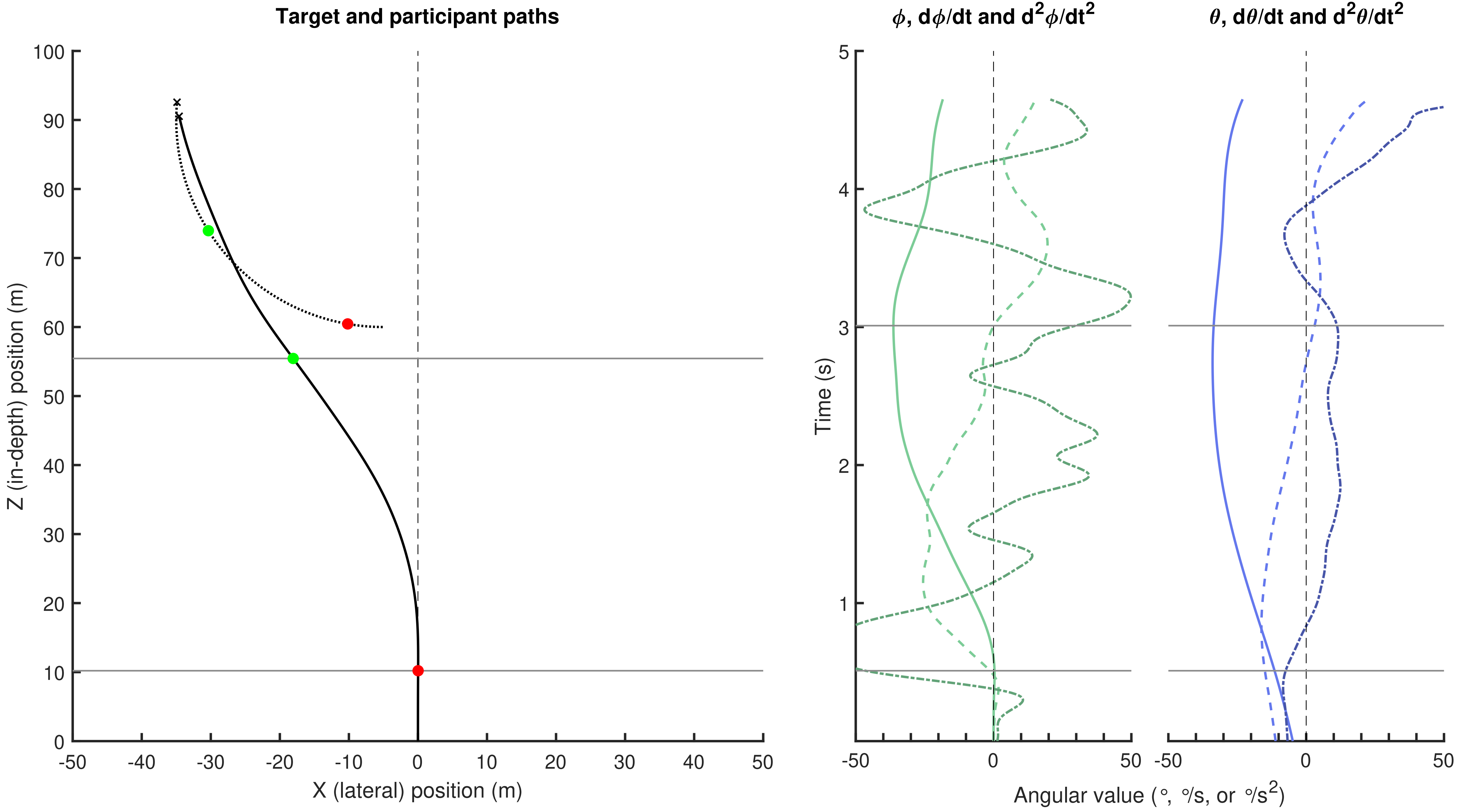

P8/B5
S5/R10-OUT

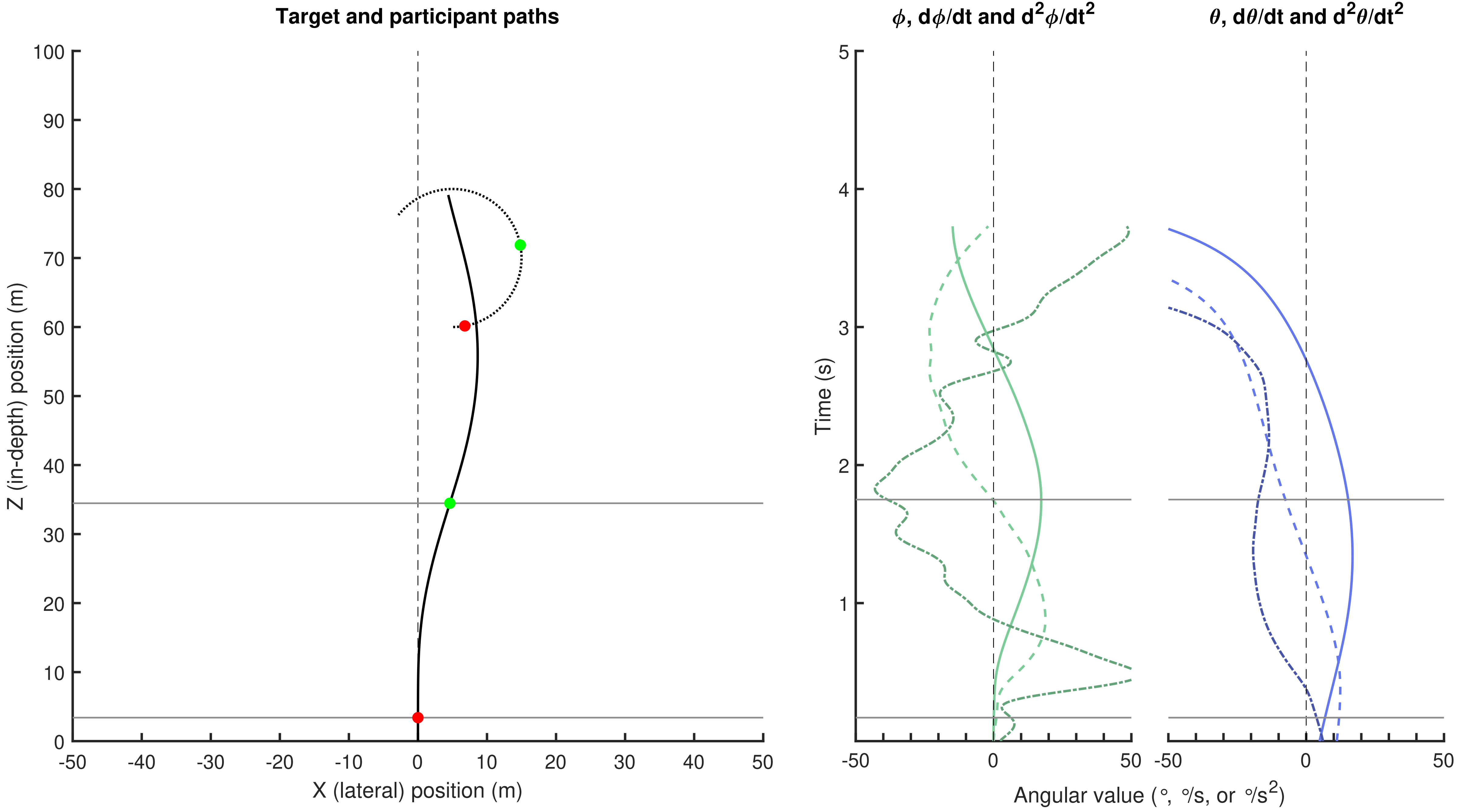

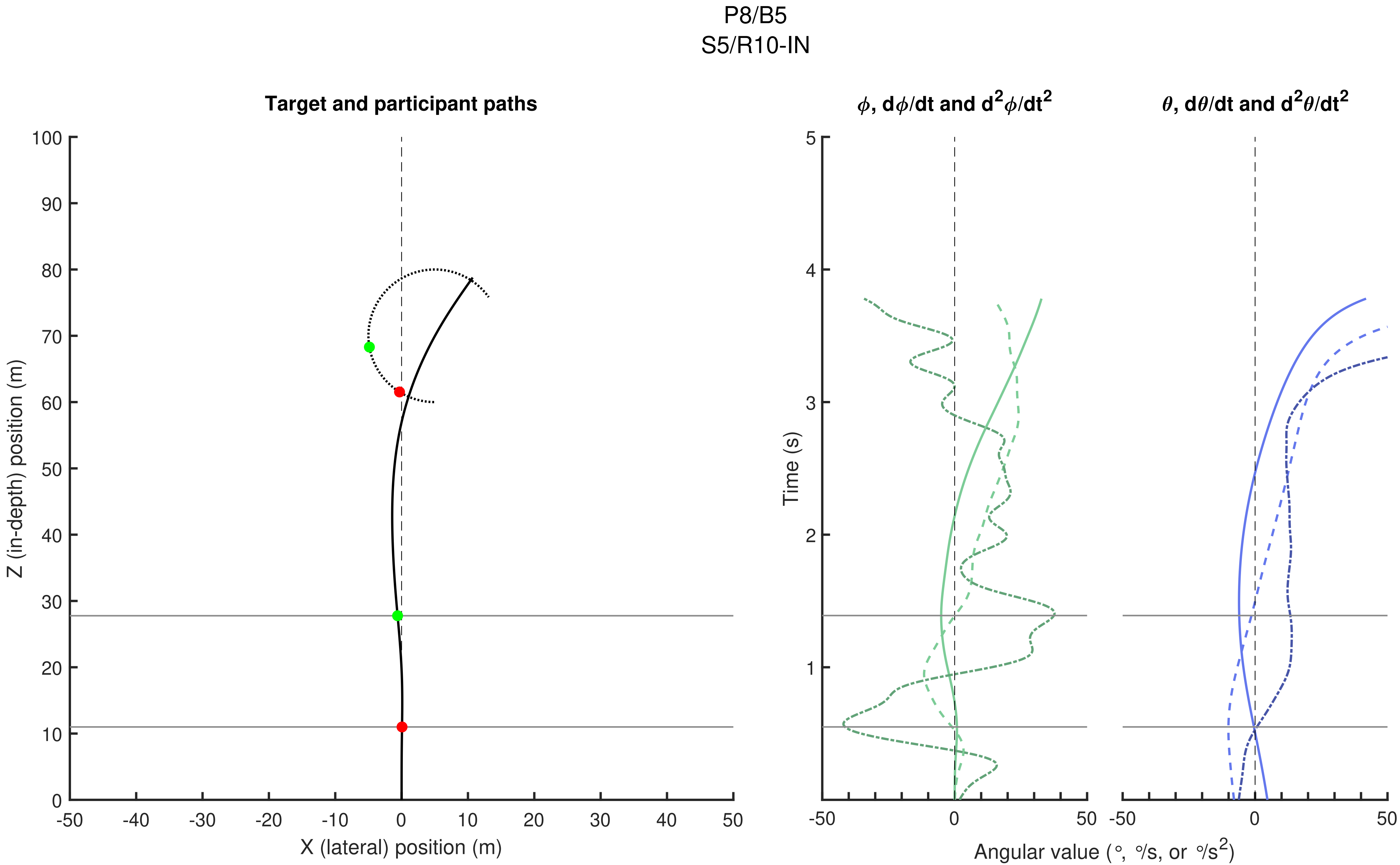
P8/B5
S5/R10-IN
Target and participant paths
Z (in-depth) position (m)
X (lateral) position (m)
0
10
20
30
40
50
60
70
80
90
100
-50
-40
-30
-20
-10
0
10
20
30
40
50
ϕ, dϕ/dt and d²ϕ/dt²
θ, dθ/dt and d²θ/dt²
Time (s)
1
2
3
4
5
-50
0
50
-50
0
50
Angular value (°, °/s, or °/s²)

P8/B5
S5/R20-OUT

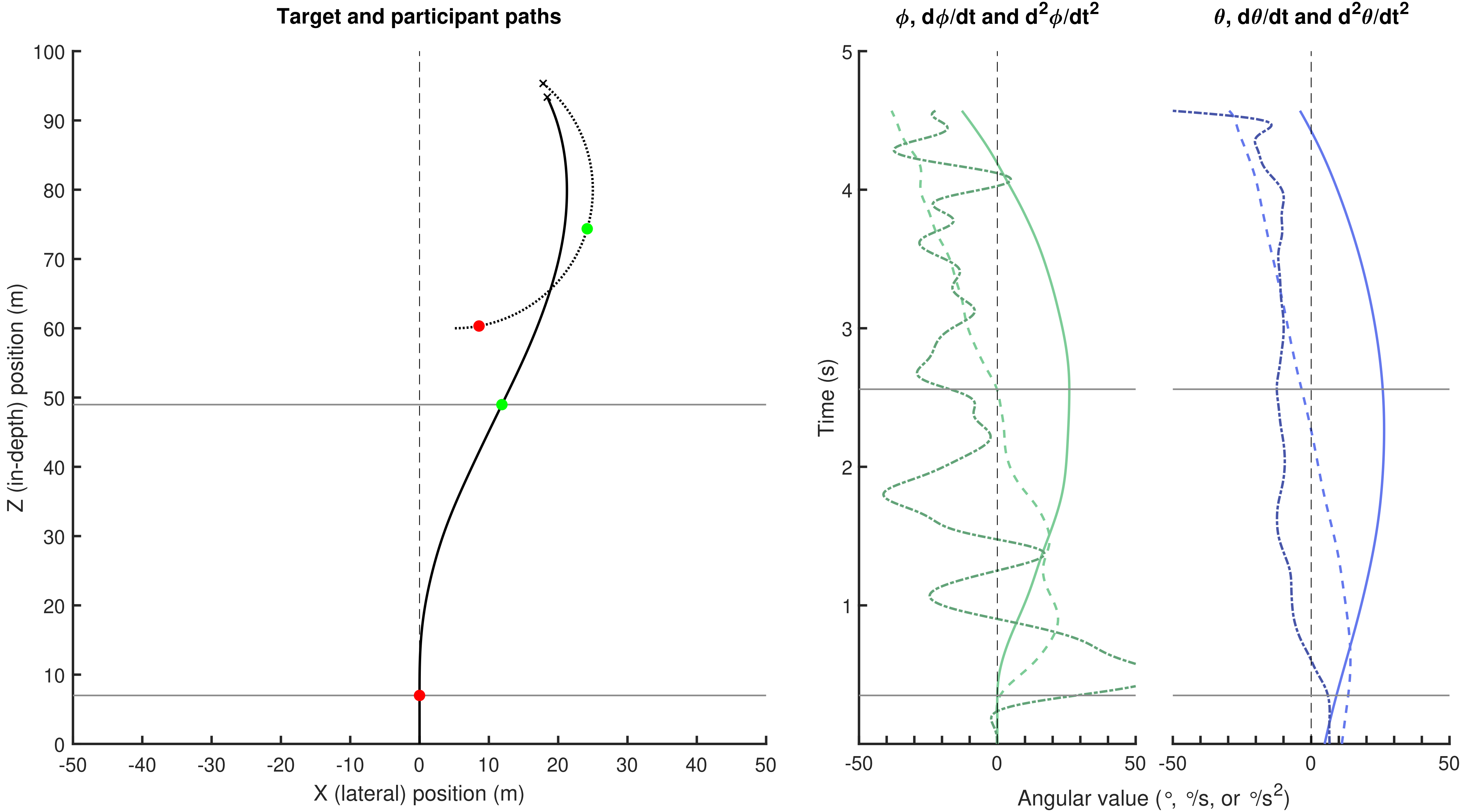

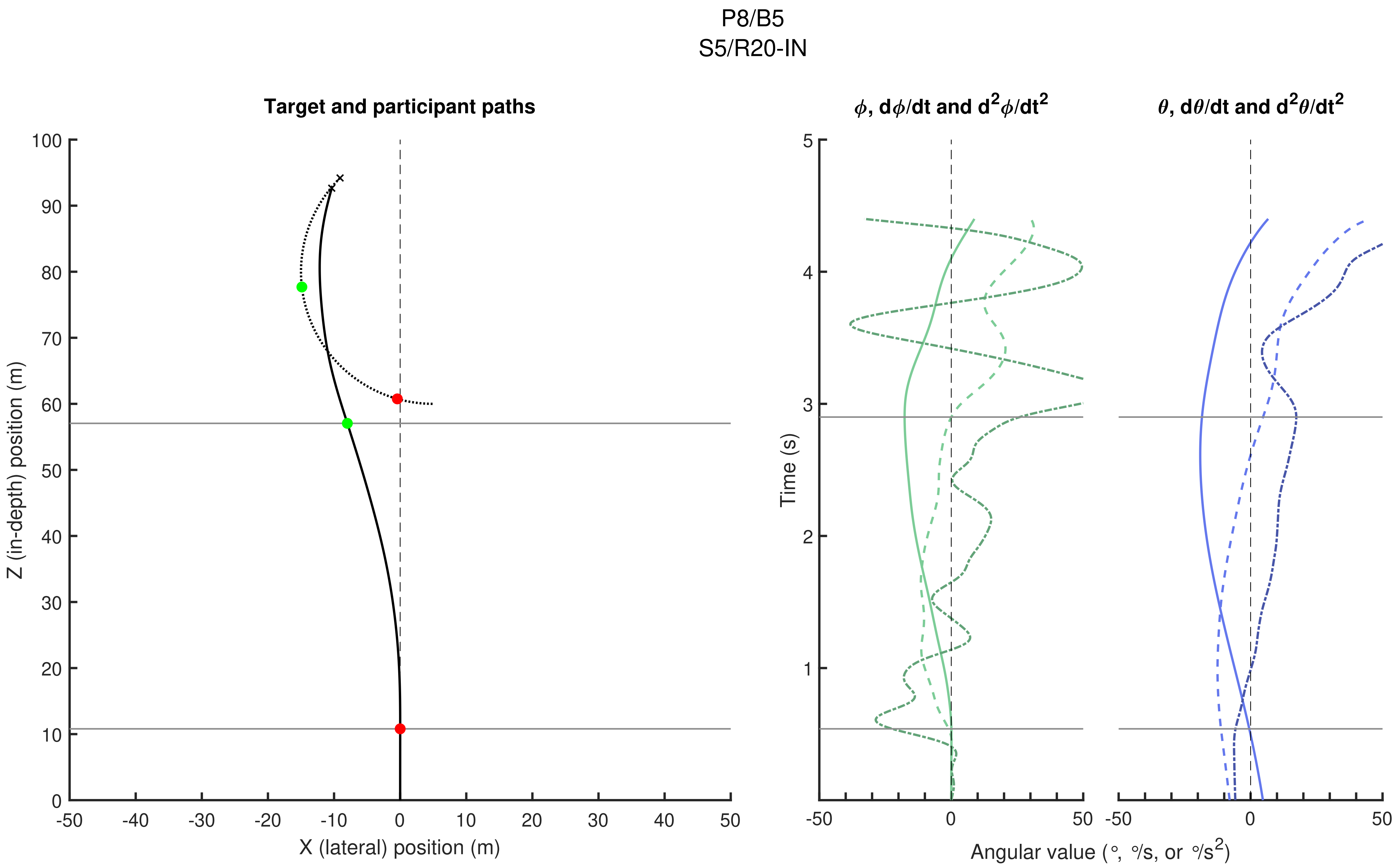

P8/B5
S5/R20-IN
Target and participant paths
ϕ, dϕ/dt and d$^2\phi$/dt^2
θ, dθ/dt and d$^2\theta$/dt^2
Z (in-depth) position (m)
X (lateral) position (m)
Time (s)
Angular value (°, °/s, or °/s^2)

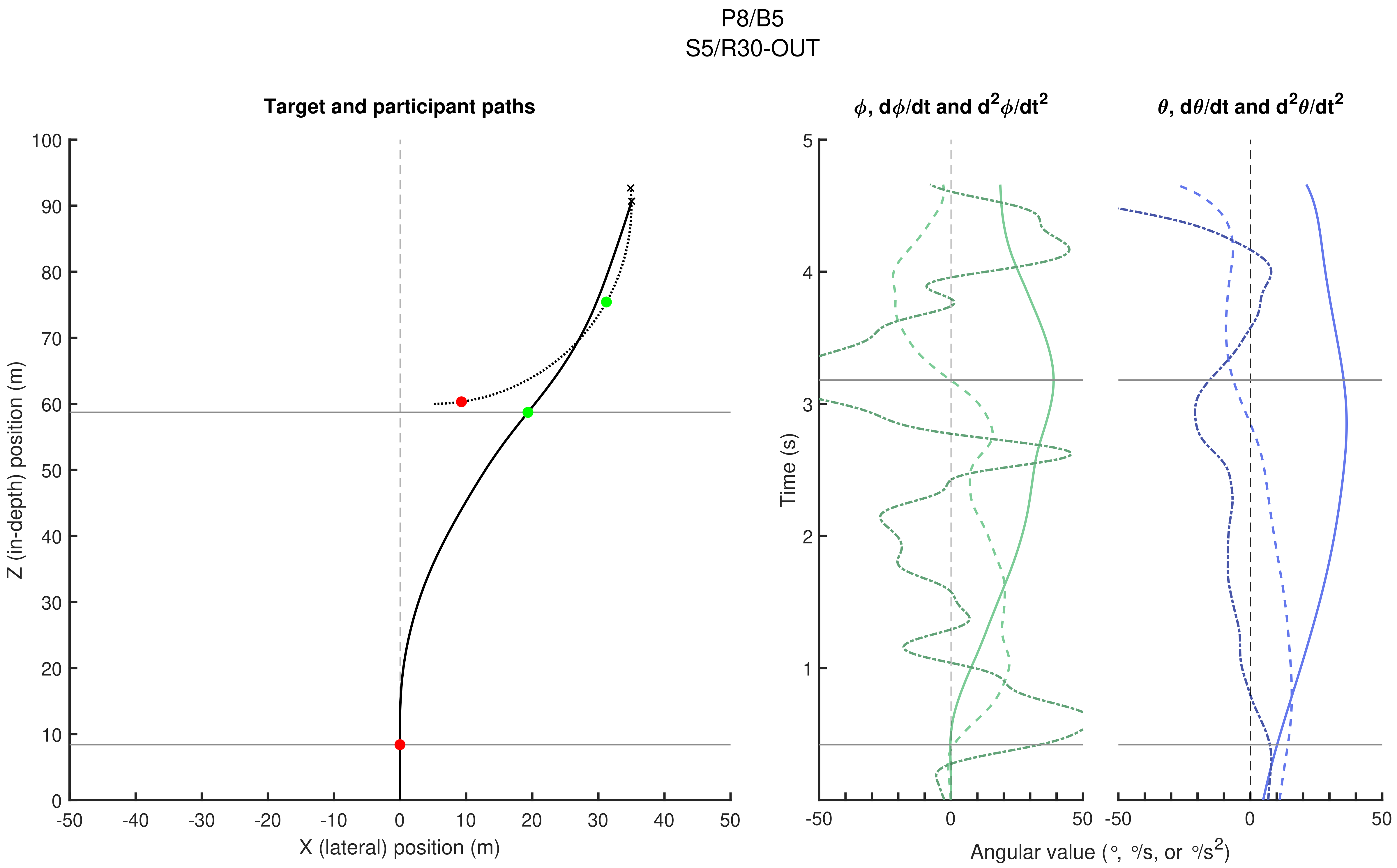

P8/B5
S5/R30-OUT
Target and participant paths
ϕ, dϕ/dt and d$^2\phi$/dt^2
θ, dθ/dt and d$^2\theta$/dt^2
Z (in-depth) position (m)
X (lateral) position (m)
Time (s)
Angular value (°, °/s, or °/s^2)

P8/B5
S5/R30-IN

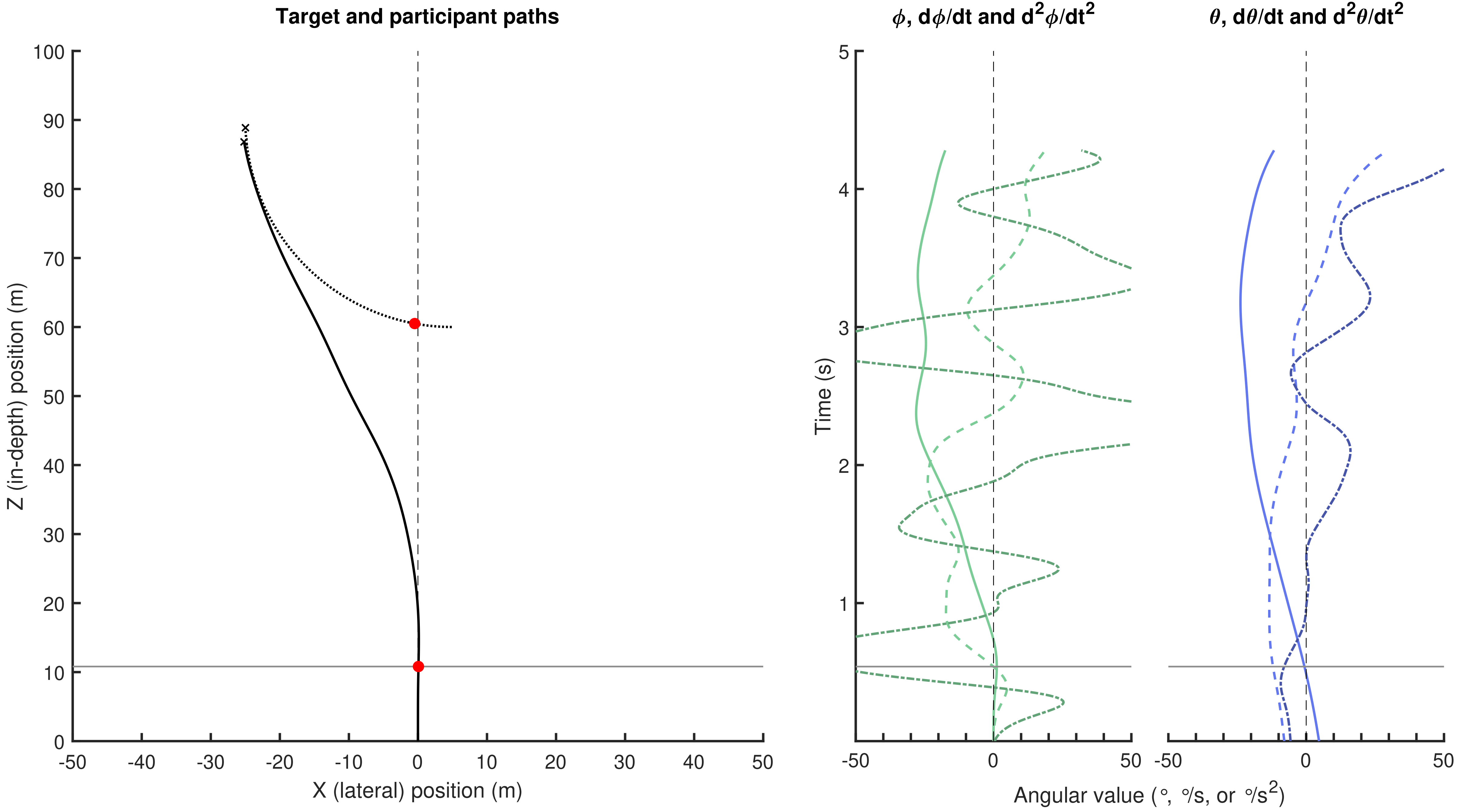

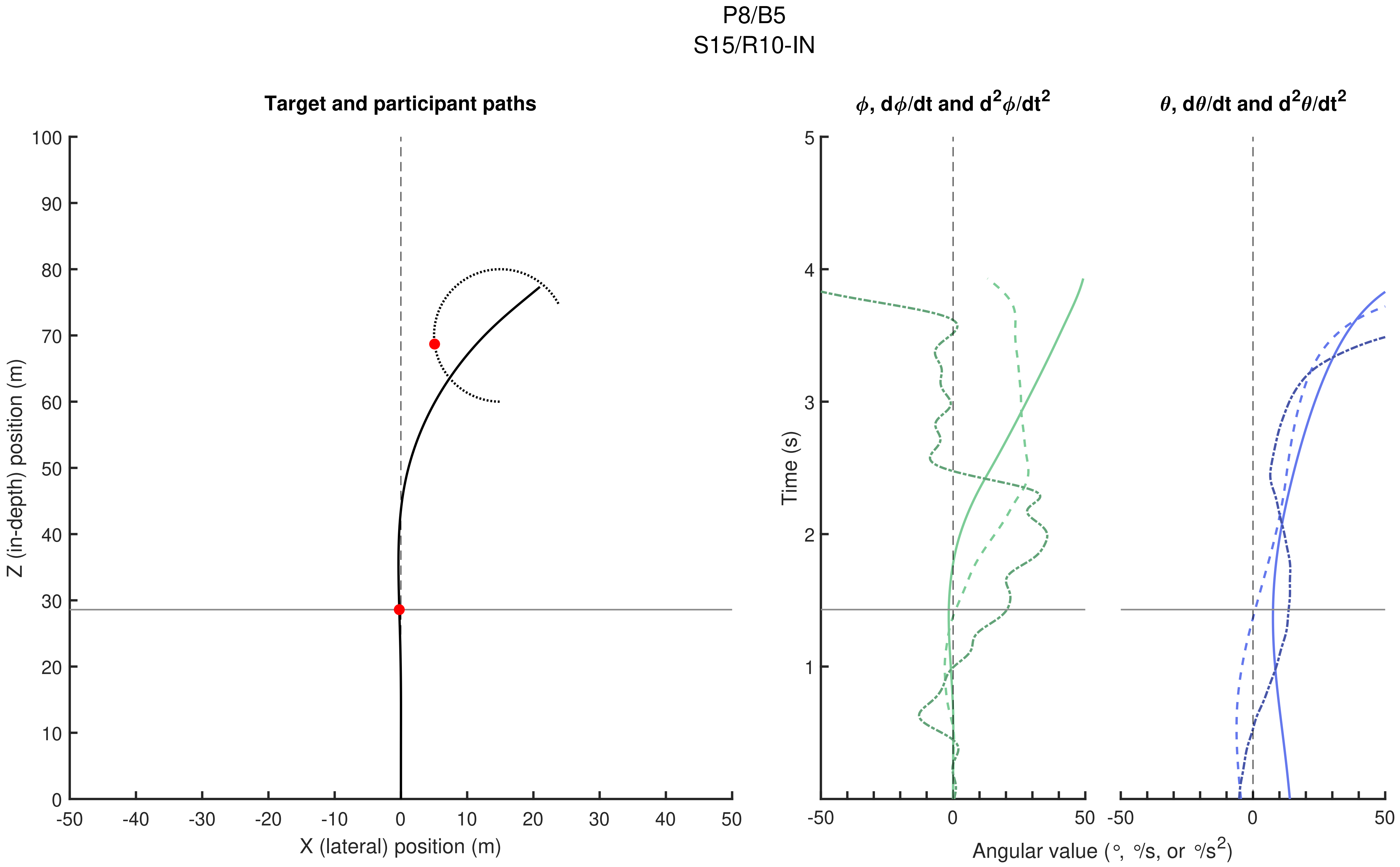
P8/B5
S15/R10-IN
Target and participant paths
Z (in-depth) position (m)
X (lateral) position (m)
ϕ, dϕ/dt and d$^2\phi$/dt^2
θ, dθ/dt and d$^2\theta$/dt^2
Time (s)
Angular value (°, °/s, or °/s^2)

P8/B5
S15/R20-IN

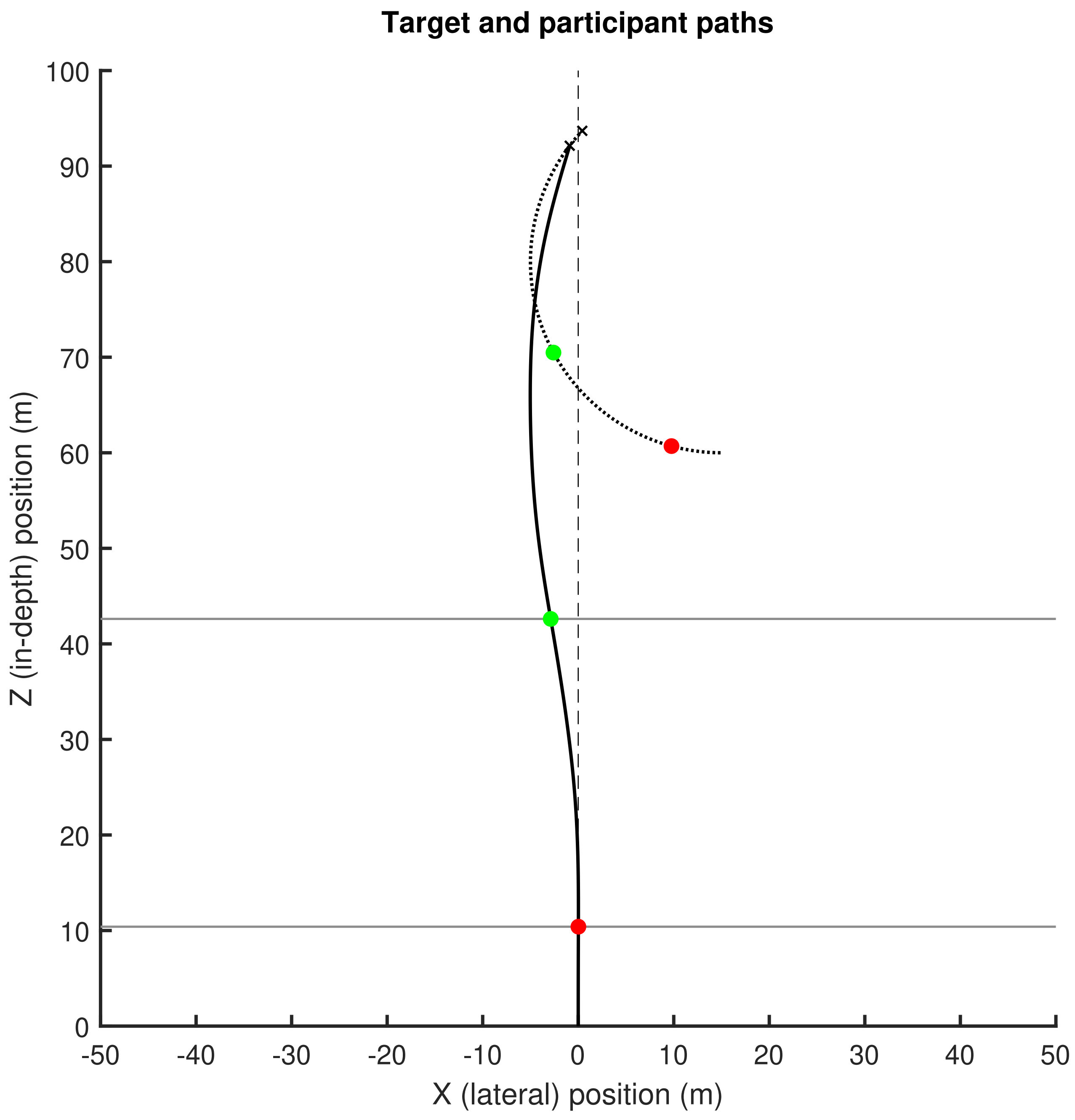

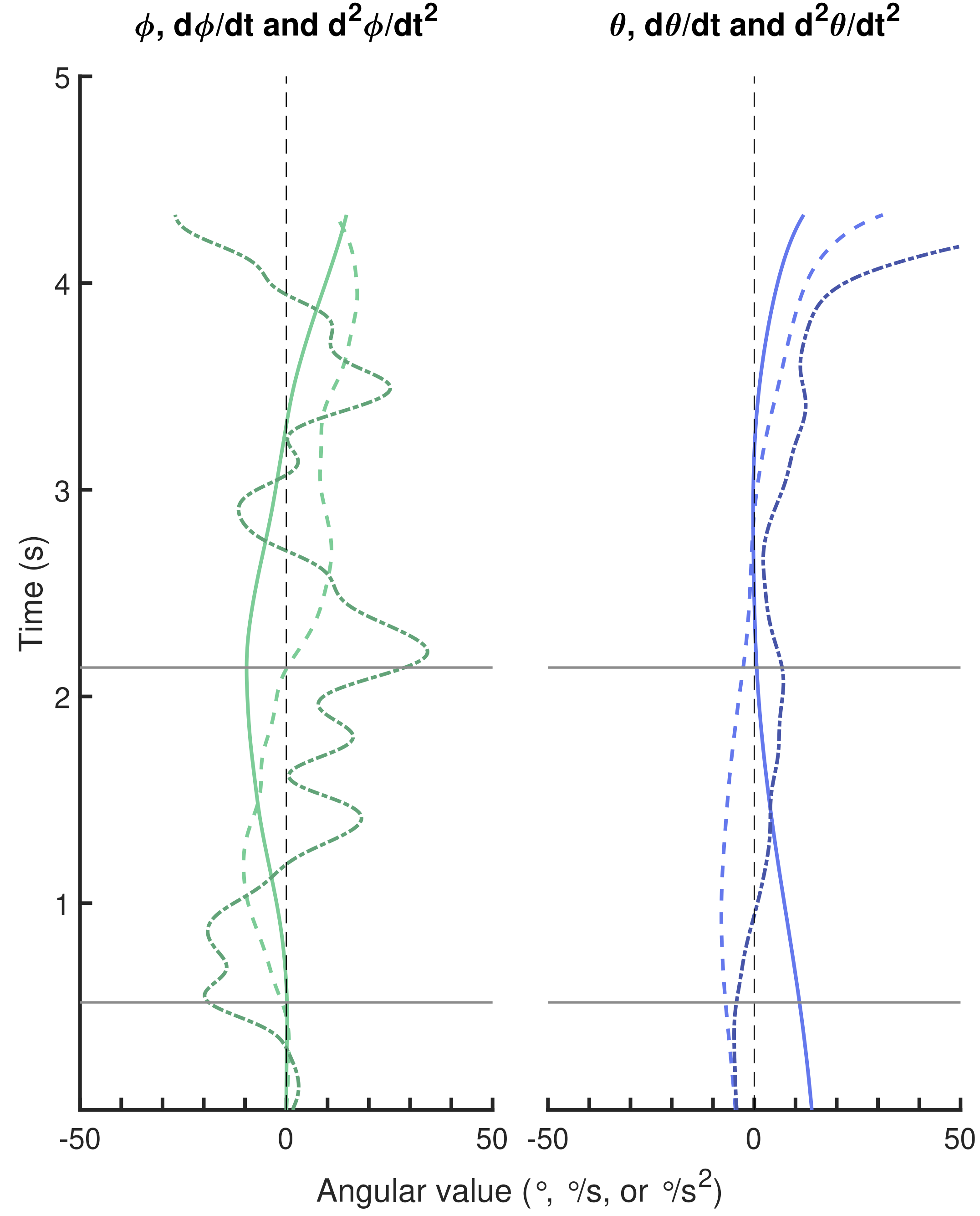

P8/B5
S15/R30-IN

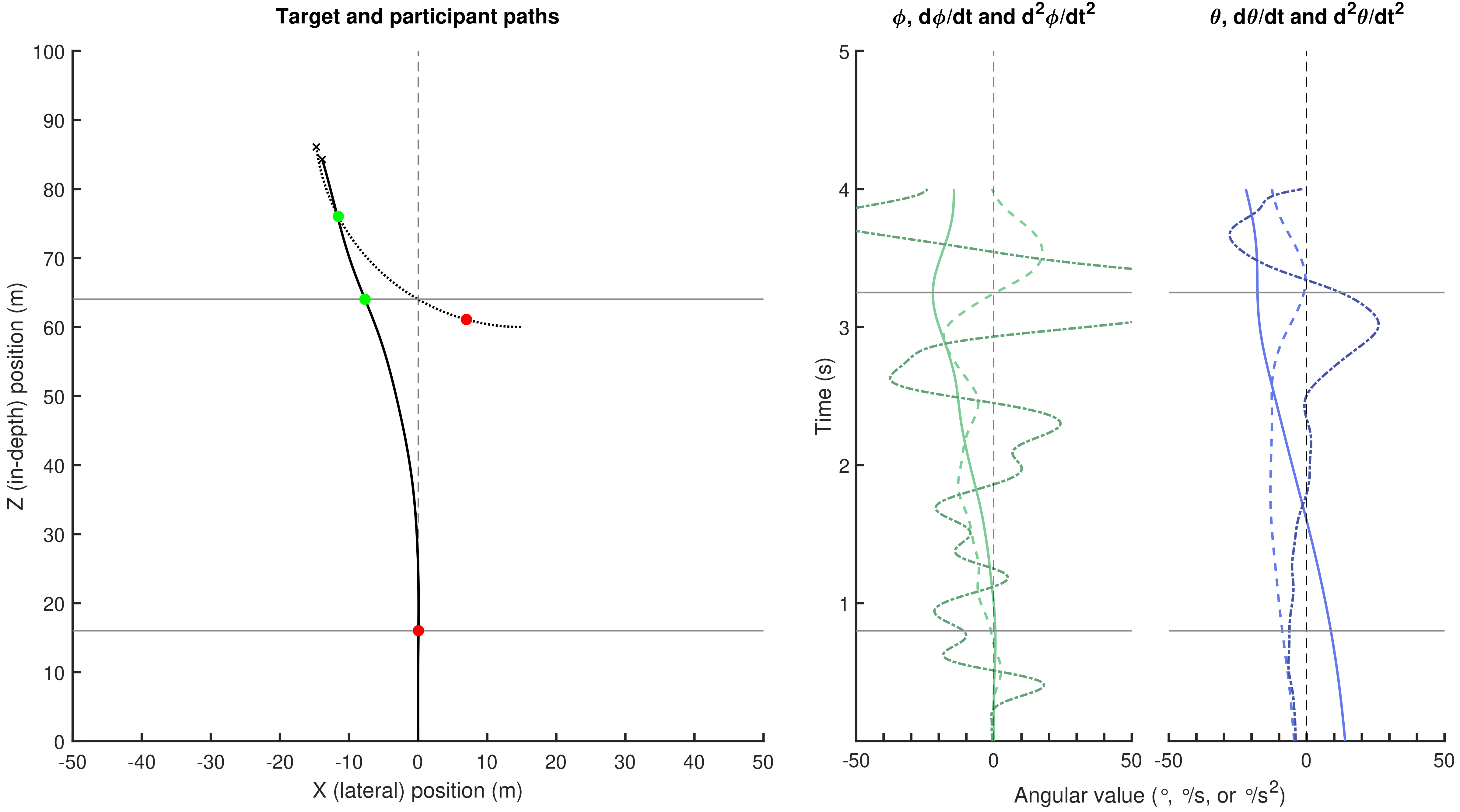

P8/B5
S20/R10-IN

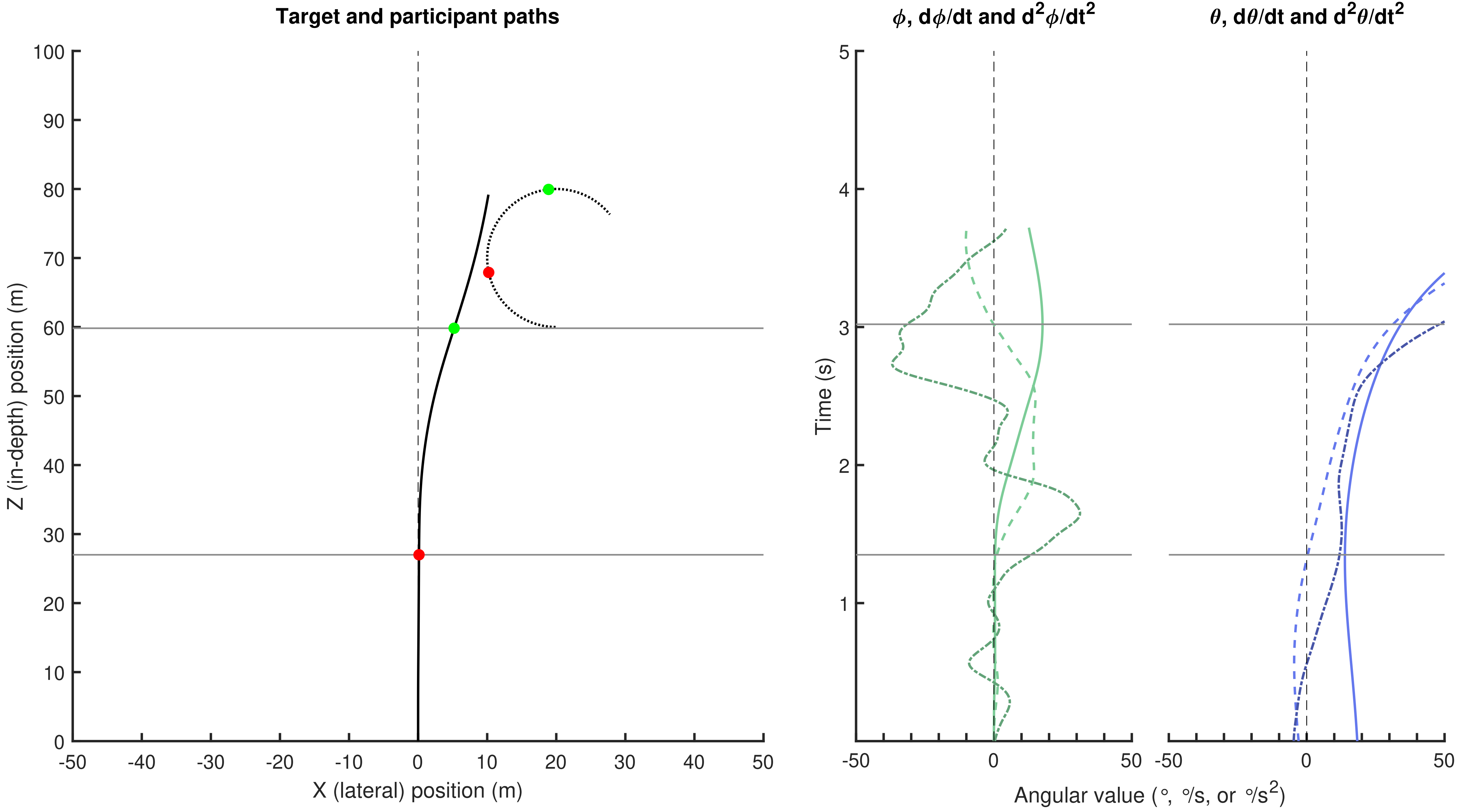

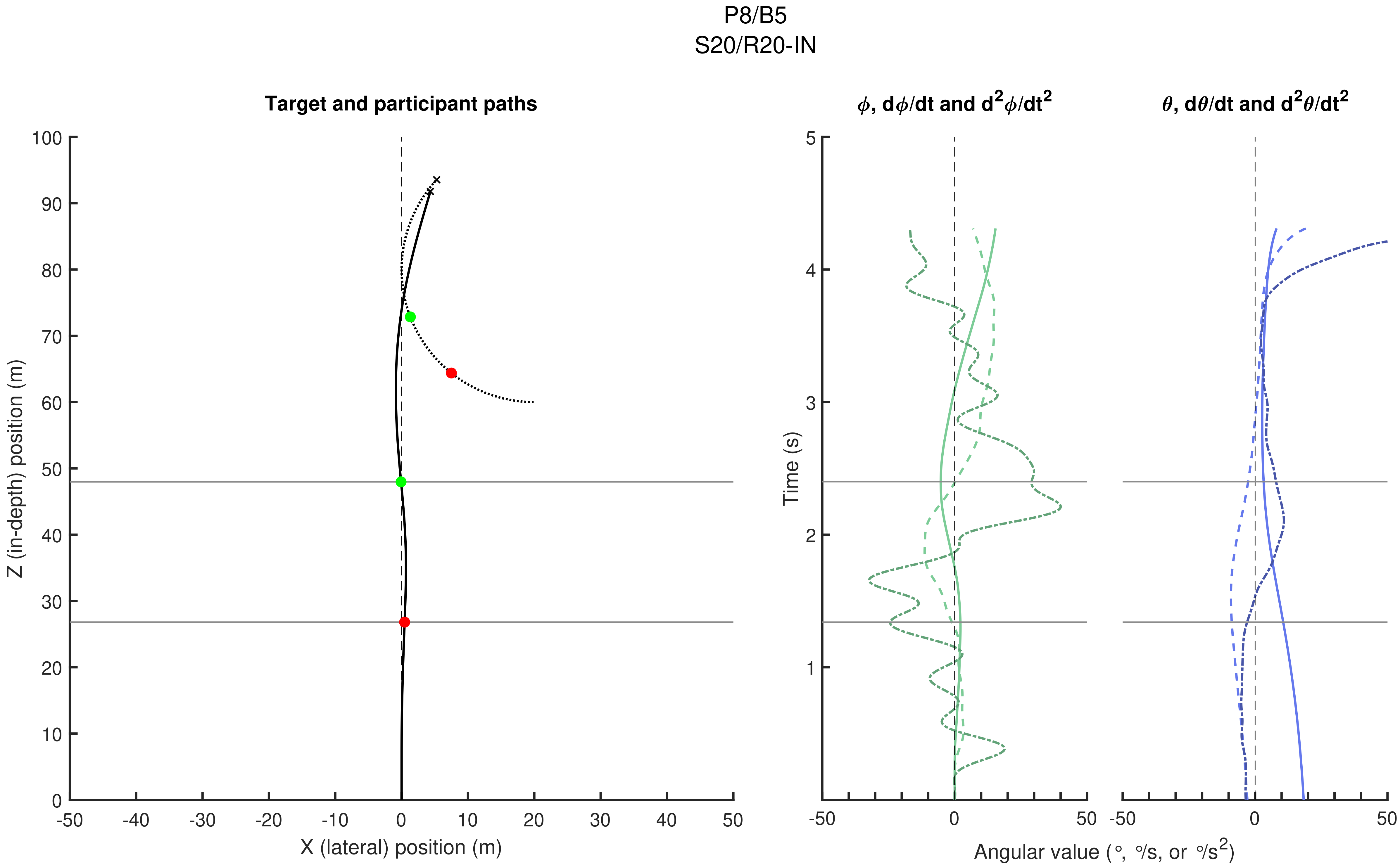
P8/B5
S20/R20-IN
Target and participant paths
ϕ, dϕ/dt and d$^2\phi$/dt^2
θ, dθ/dt and d$^2\theta$/dt^2
Z (in-depth) position (m)
X (lateral) position (m)
Time (s)
Angular value (°, °/s, or °/s^2)
100
90
80
70
60
50
40
30
20
10
0
-50
-40
-30
-20
-10
0
10
20
30
40
50
5
4
3
2
1
-50
0
50
-50
0
50

P8/B5
S20/R30-IN

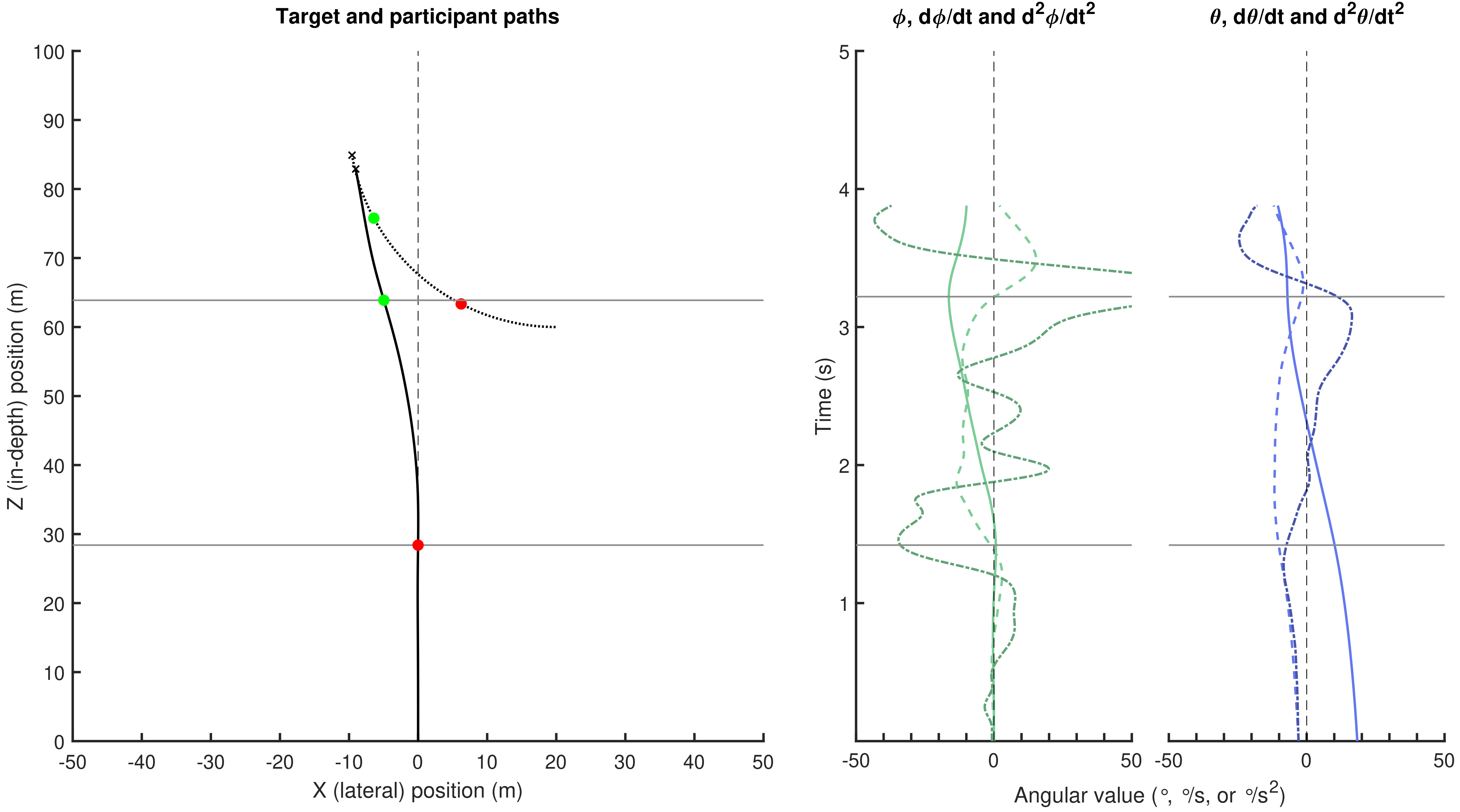

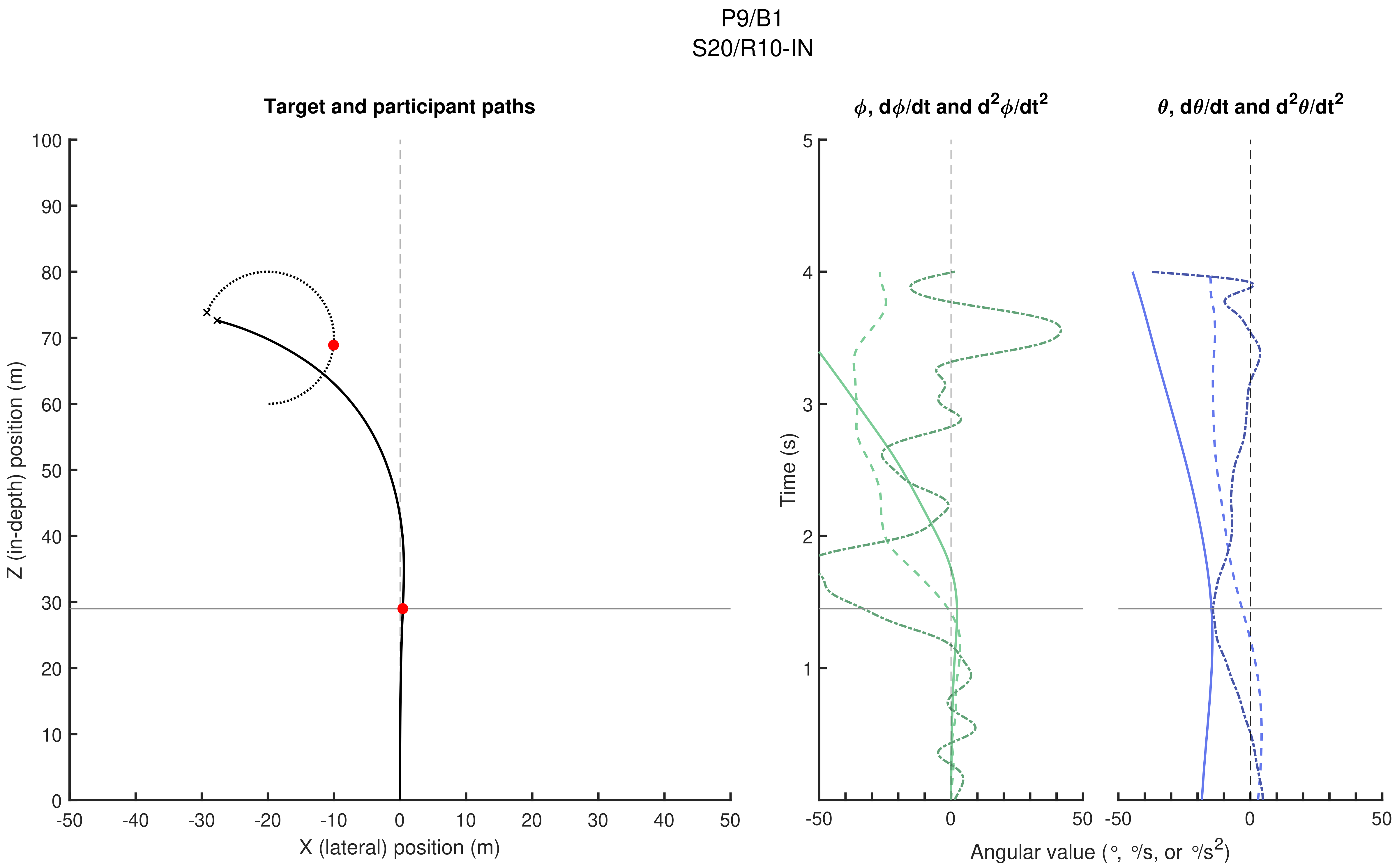
P9/B1
S20/R10-IN
Target and participant paths
Z (in-depth) position (m)
X (lateral) position (m)
ϕ, dϕ/dt and d²ϕ/dt²
θ, dθ/dt and d²θ/dt²
Time (s)
Angular value (°, °/s, or °/s²)

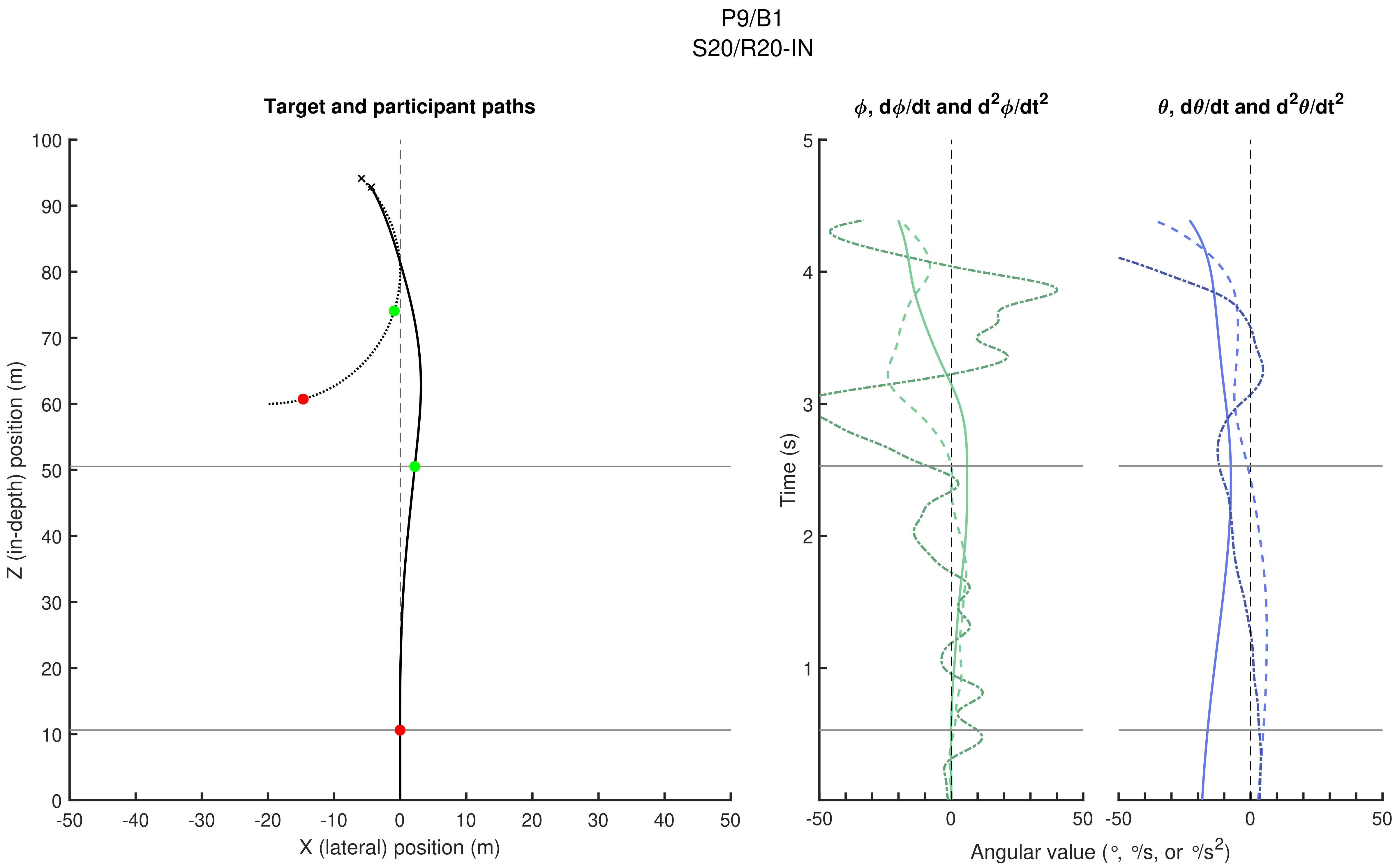

P9/B1
S20/R20-IN
Target and participant paths
Z (in-depth) position (m)
X (lateral) position (m)
100
90
80
70
60
50
40
30
20
10
0
-50
-40
-30
-20
-10
0
10
20
30
40
50
ϕ, dϕ/dt and d²ϕ/dt²
θ, dθ/dt and d²θ/dt²
Time (s)
5
4
3
2
1
-50
0
50
-50
0
50
Angular value (°, °/s, or °/s²)

P9/B1
S20/R30-IN

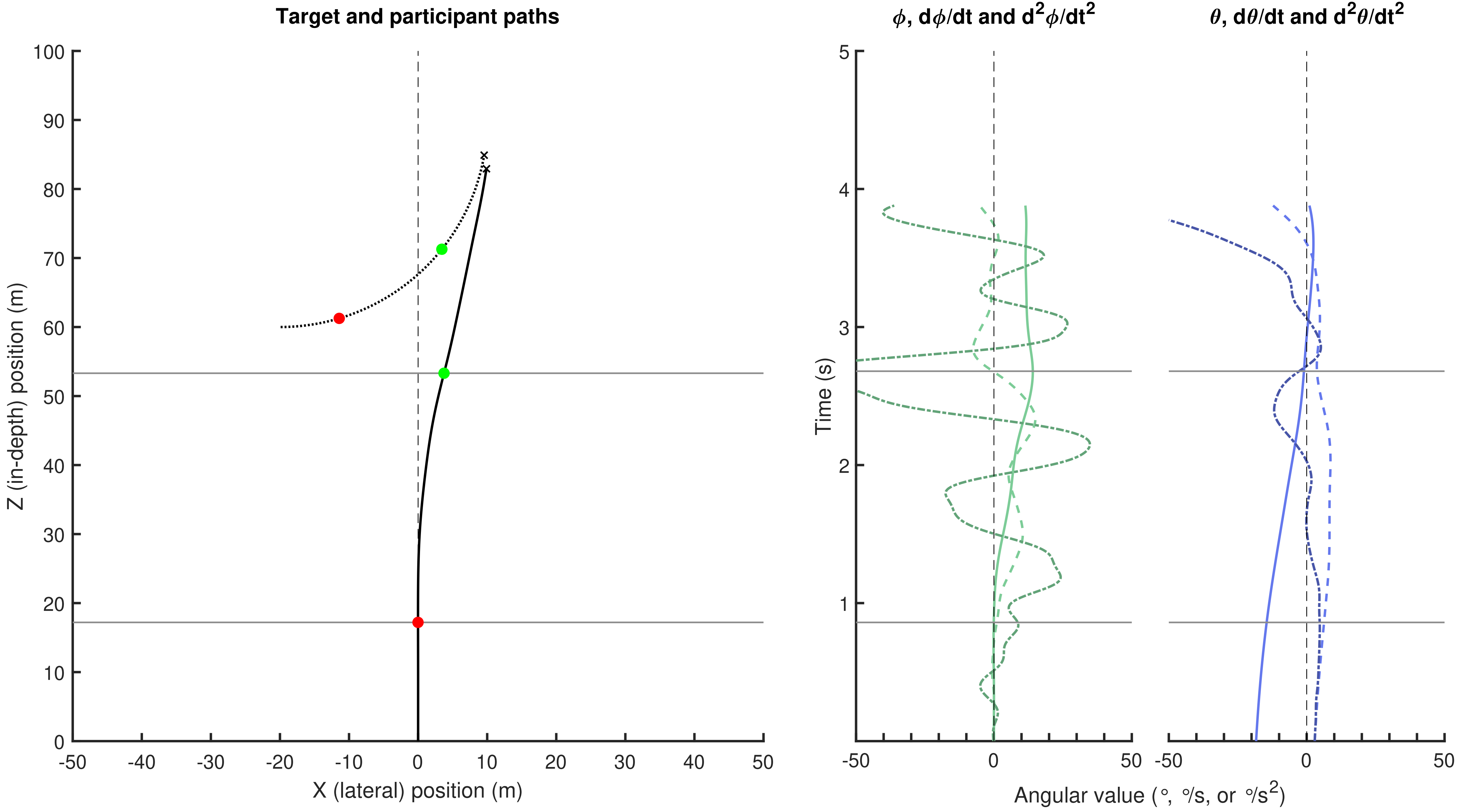

P9/B1
S15/R10-IN

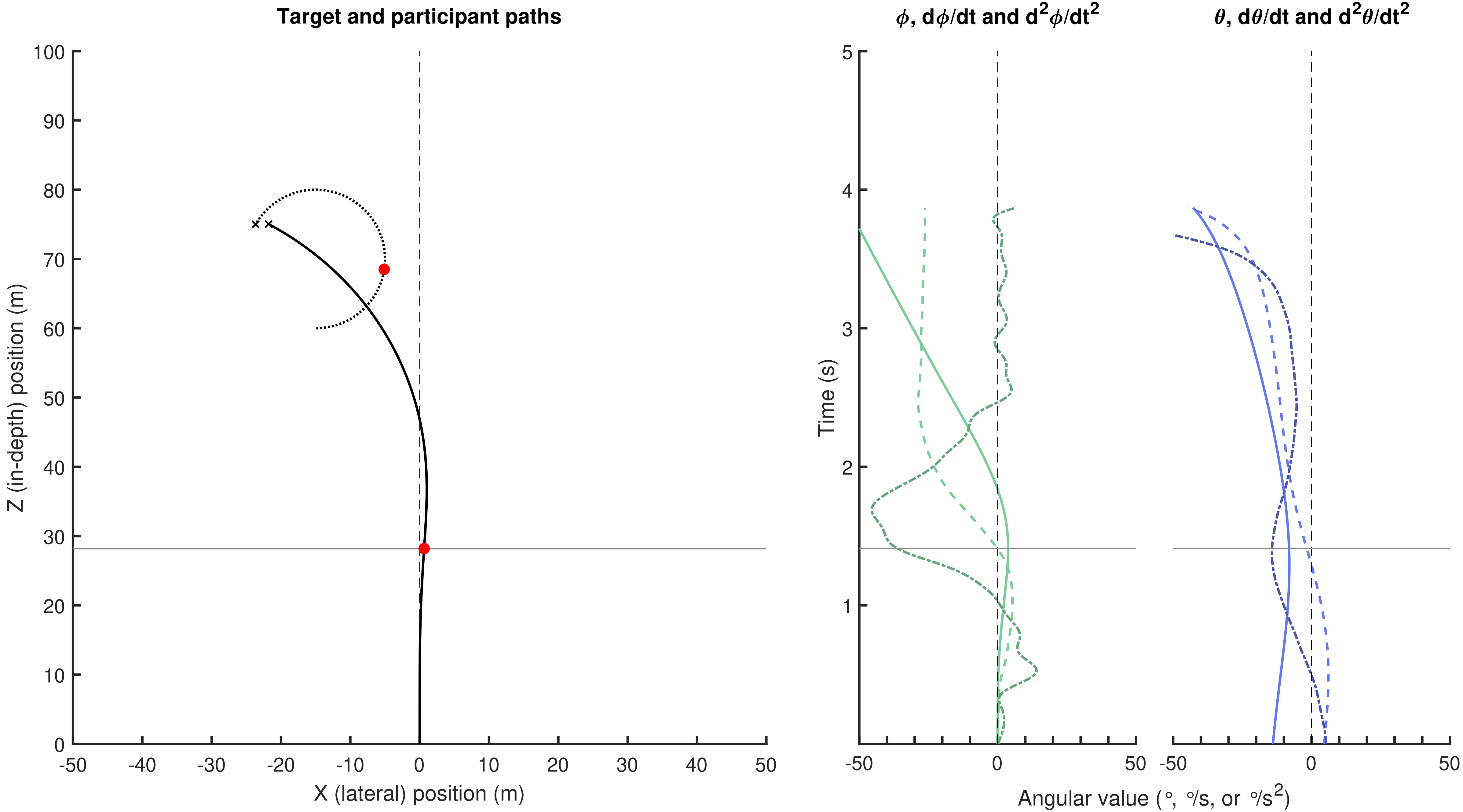

P9/B1
S15/R20-IN

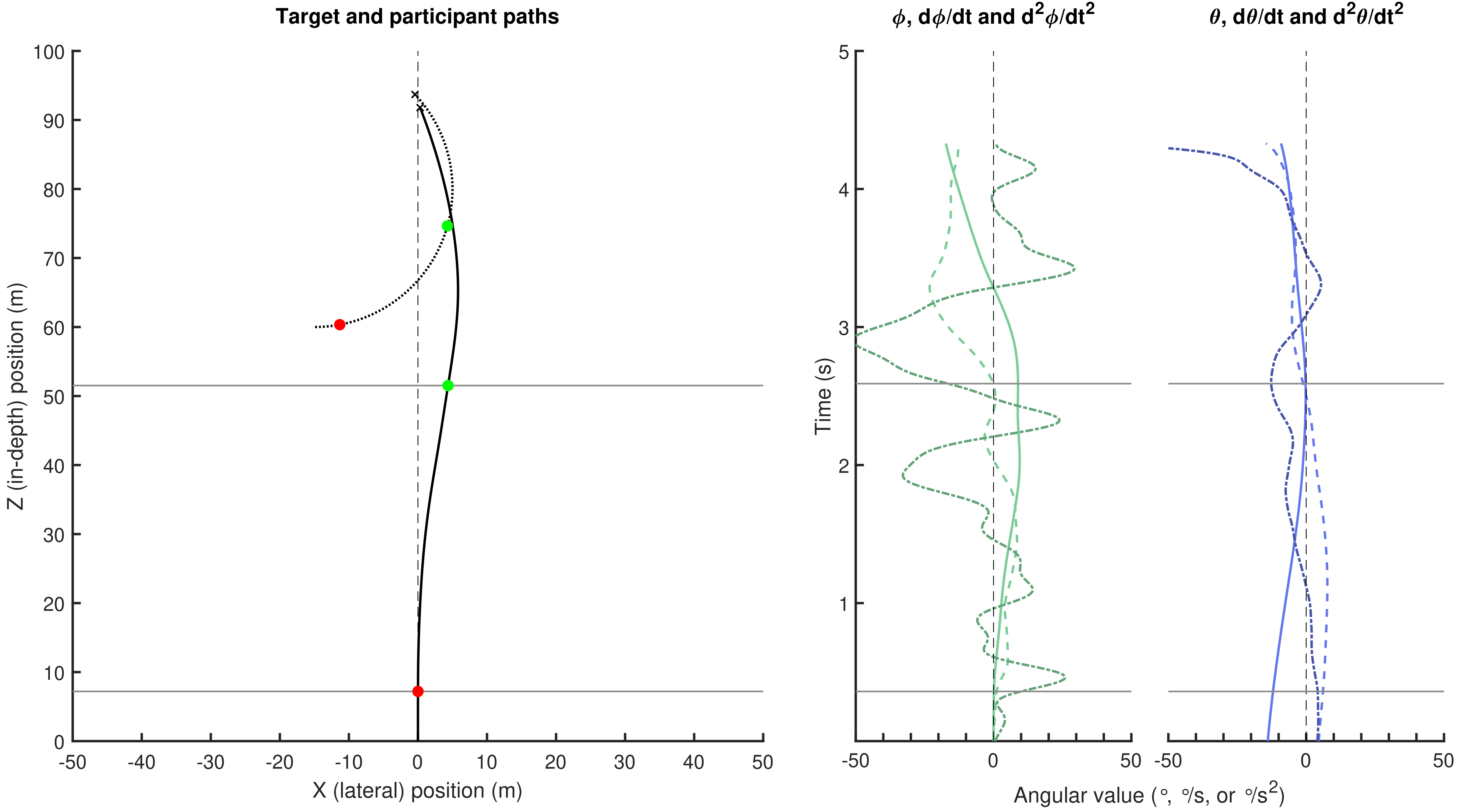

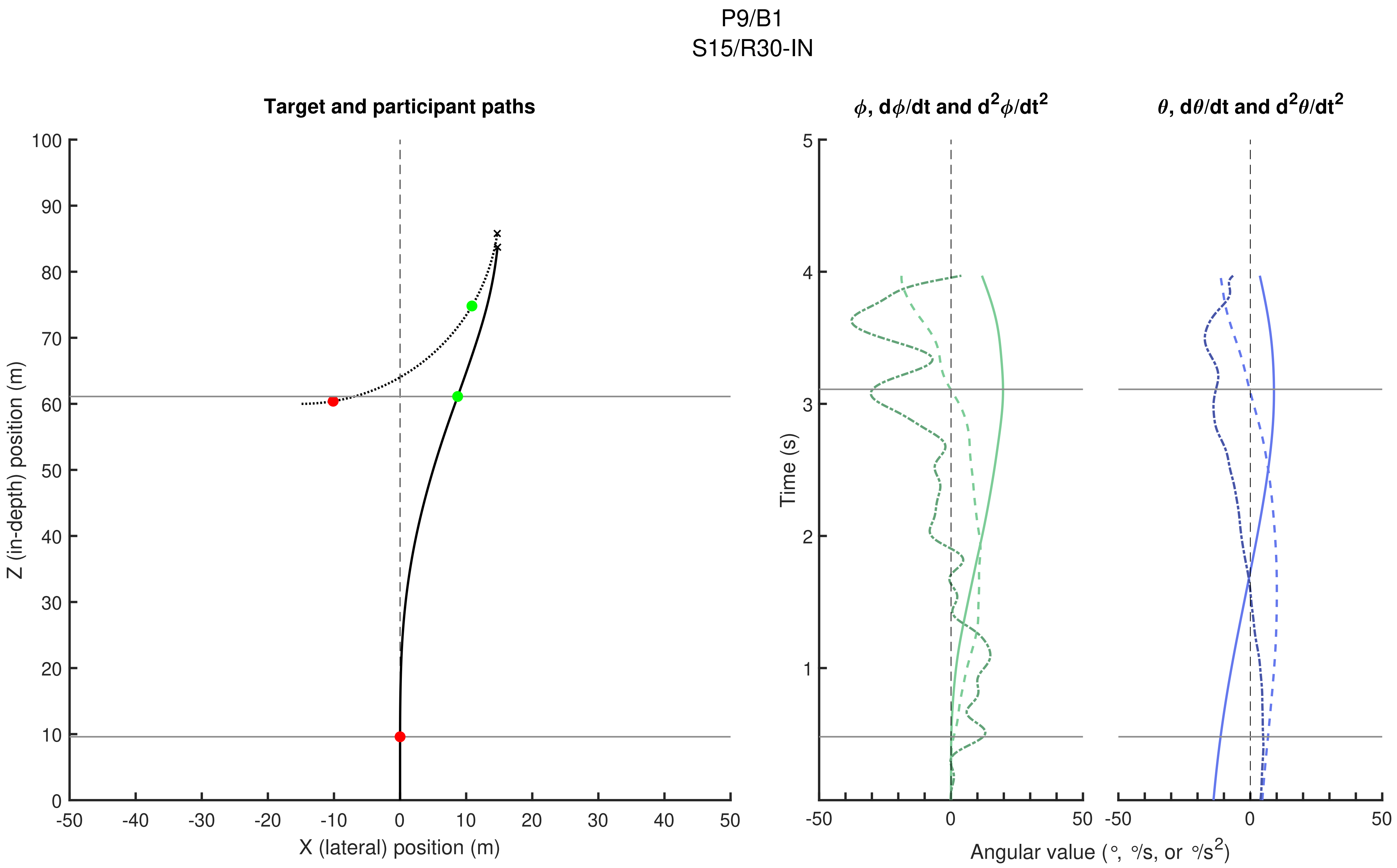

P9/B1
S15/R30-IN
Target and participant paths
Z (in-depth) position (m)
X (lateral) position (m)
ϕ, dϕ/dt and d$^2\phi$/dt^2
θ, dθ/dt and d$^2\theta$/dt^2
Time (s)
Angular value (°, °/s, or °/s^2)

P9/B1
S5/R10-IN

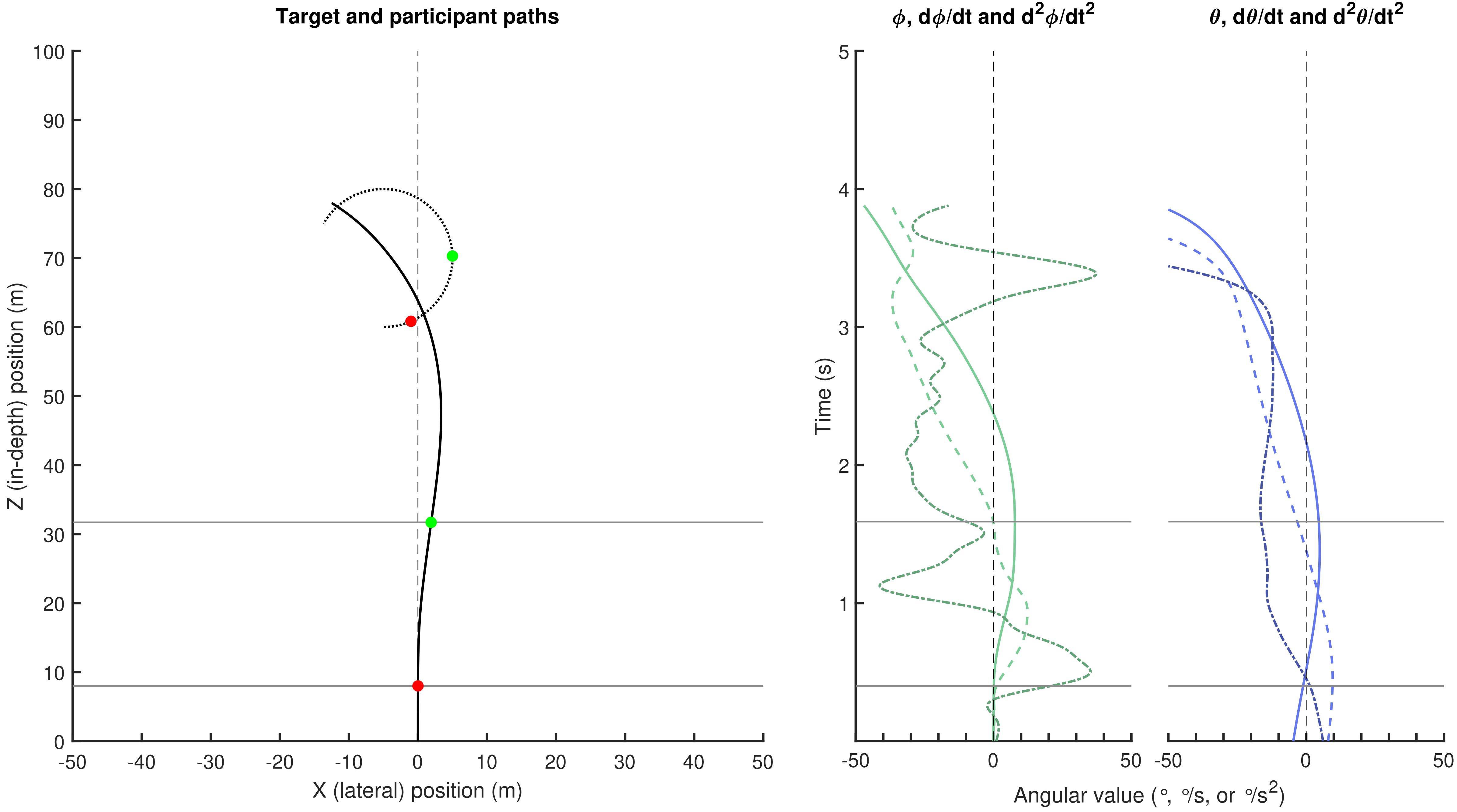

P9/B1
S5/R10-OUT

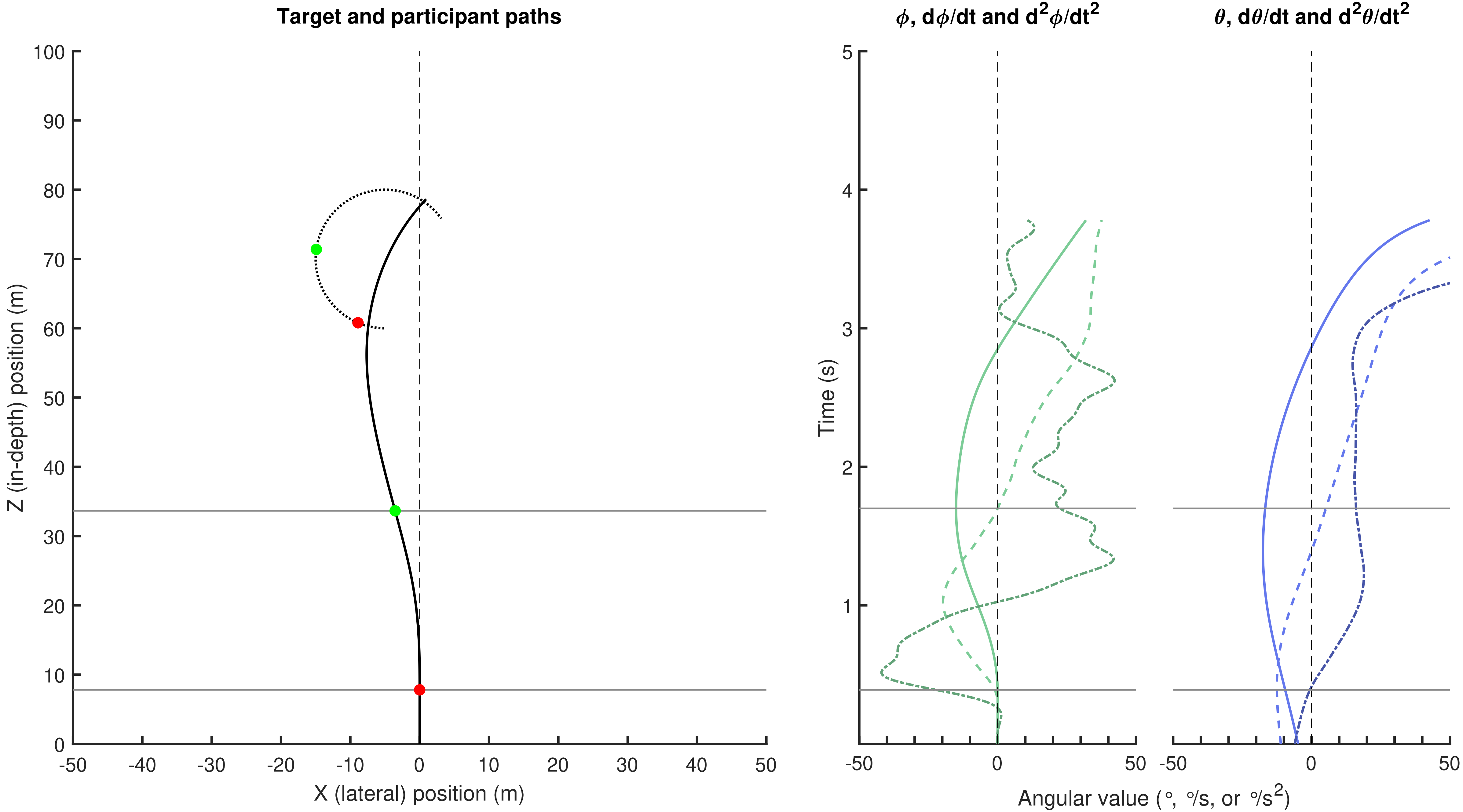

P9/B1
S5/R20-IN

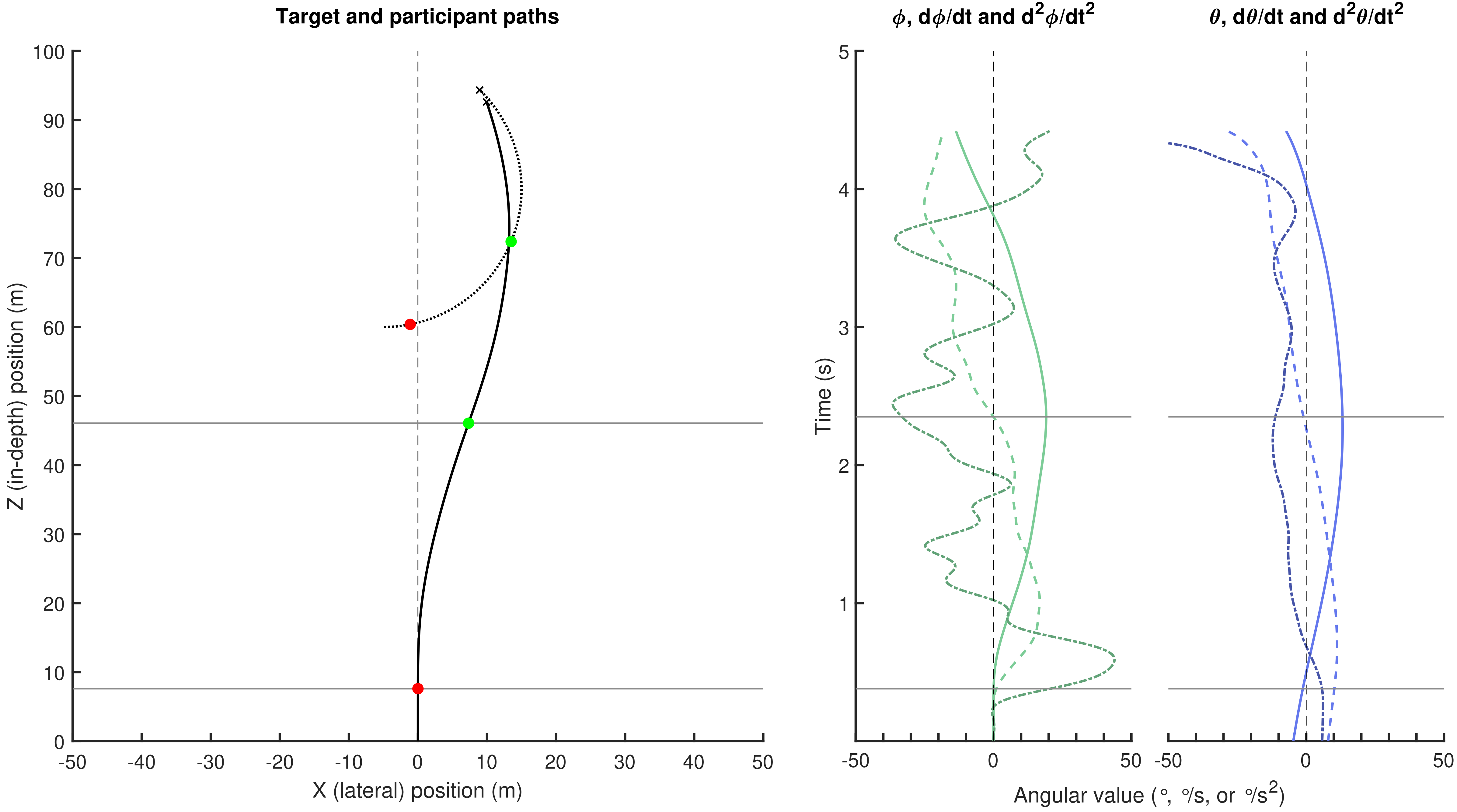

P9/B1
S5/R20-OUT

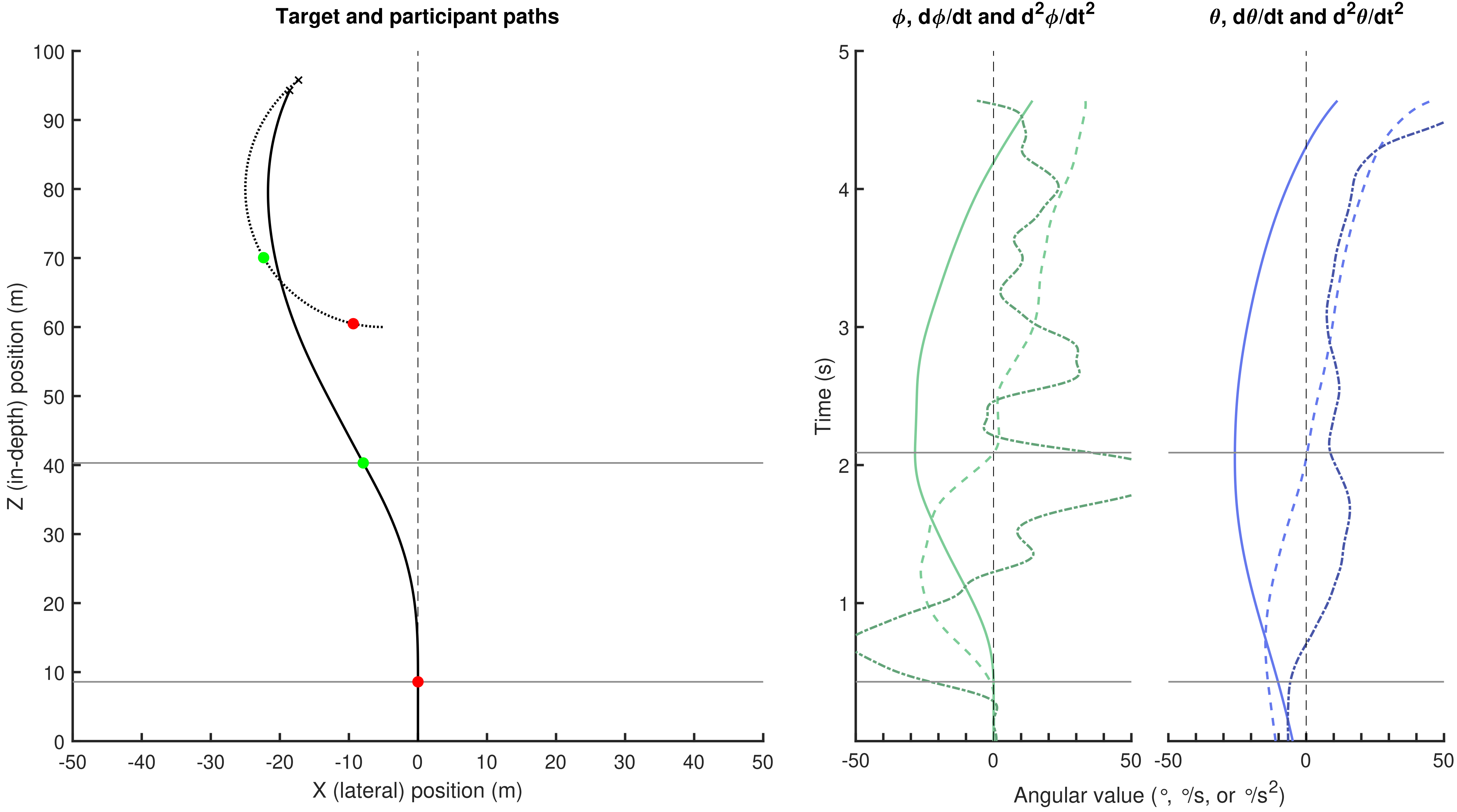

P9/B1
S5/R30-IN

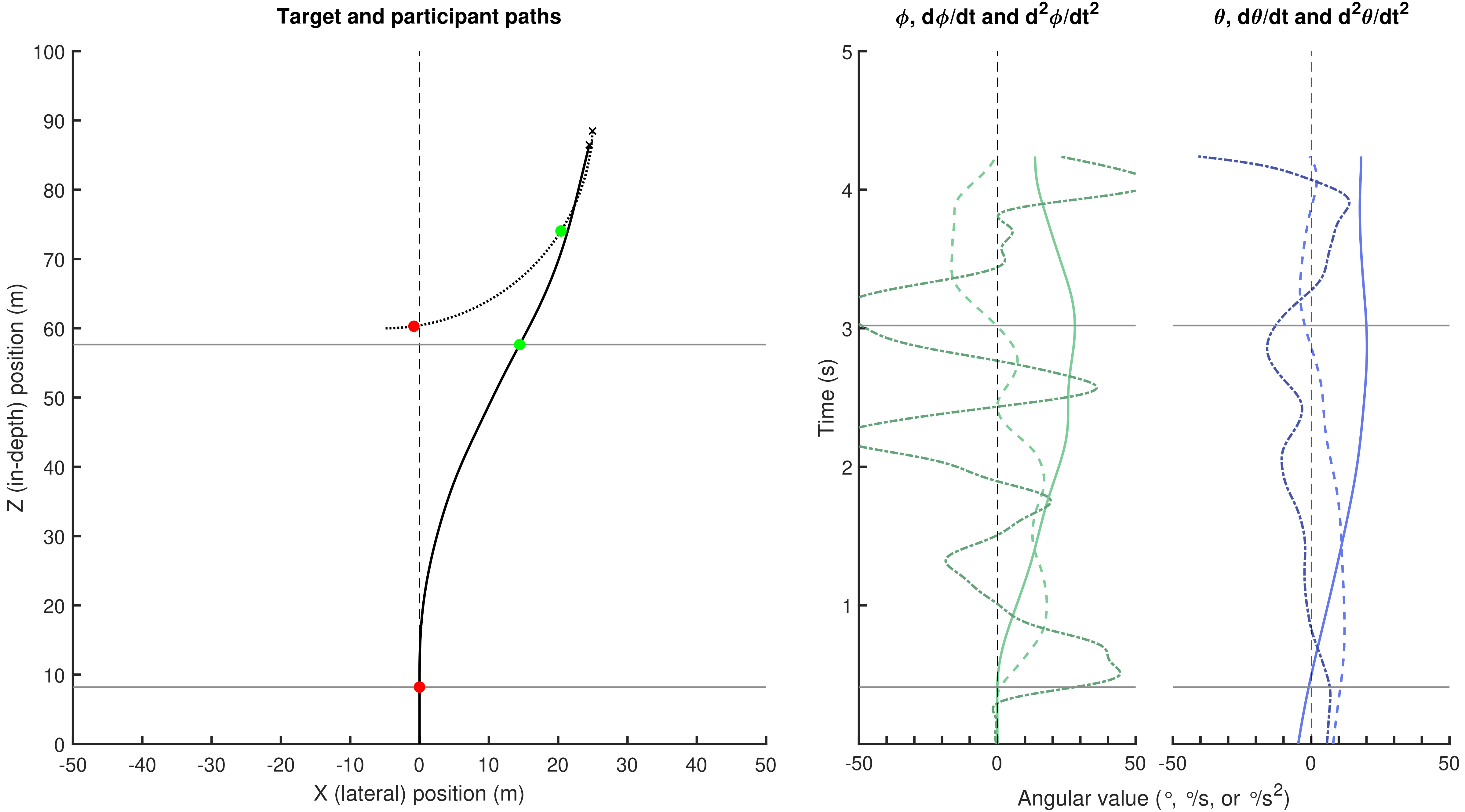

P9/B1
S5/R30-OUT

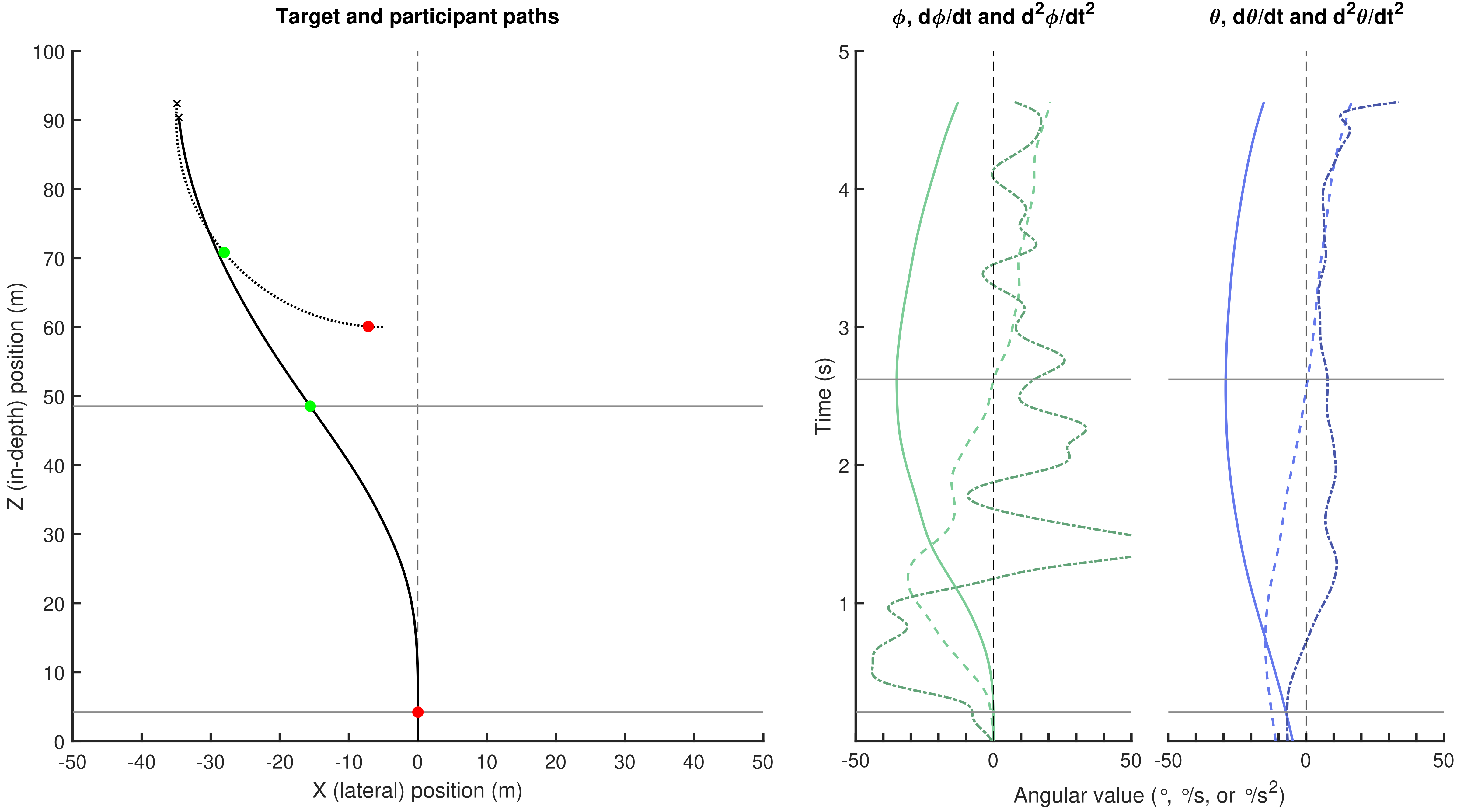

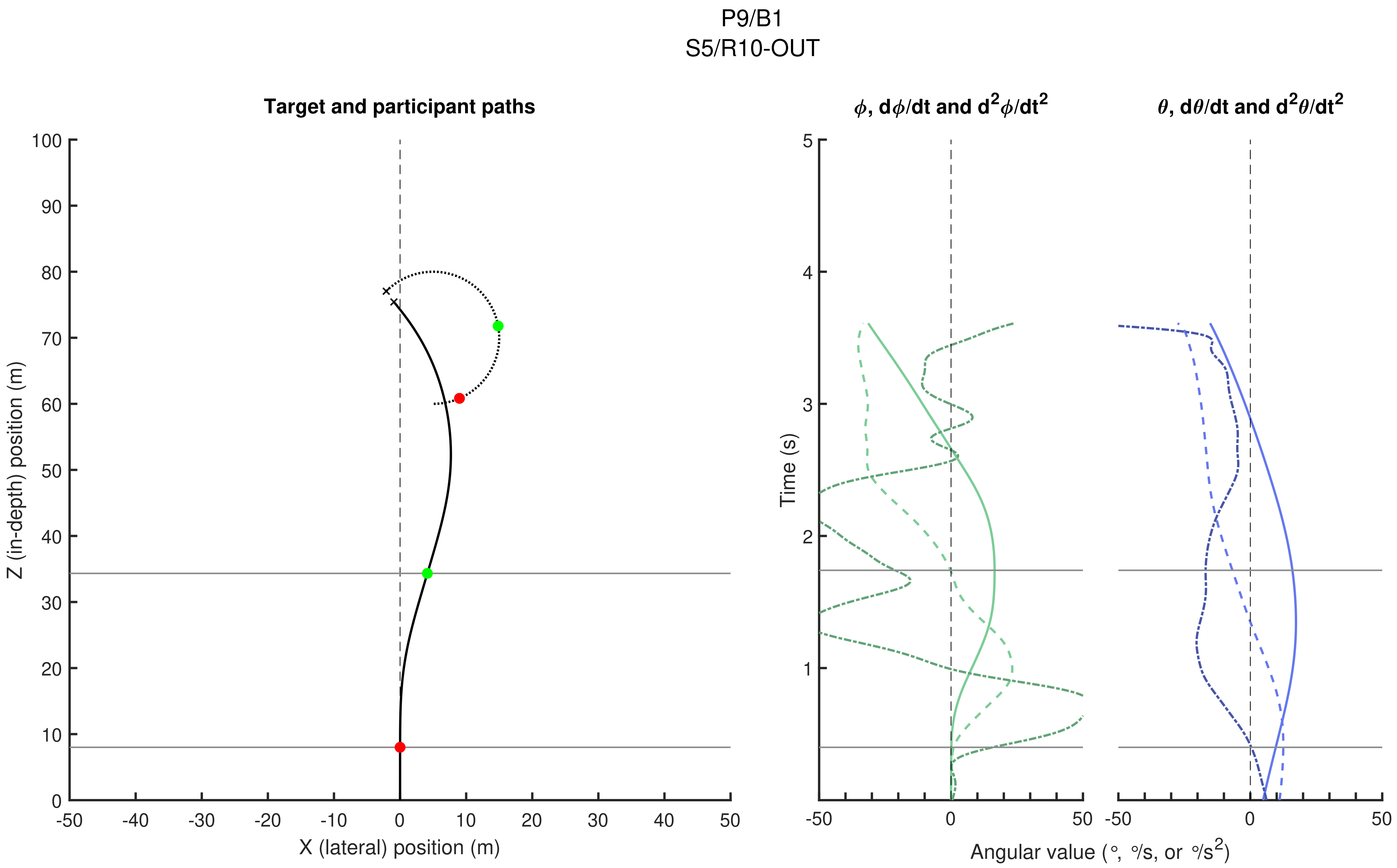
P9/B1
S5/R10-OUT
Target and participant paths
Z (in-depth) position (m)
X (lateral) position (m)
ϕ, dϕ/dt and d$^2\phi$/dt^2
θ, dθ/dt and d$^2\theta$/dt^2
Time (s)
Angular value (°, °/s, or °/s^2)

P9/B1
S5/R10-IN

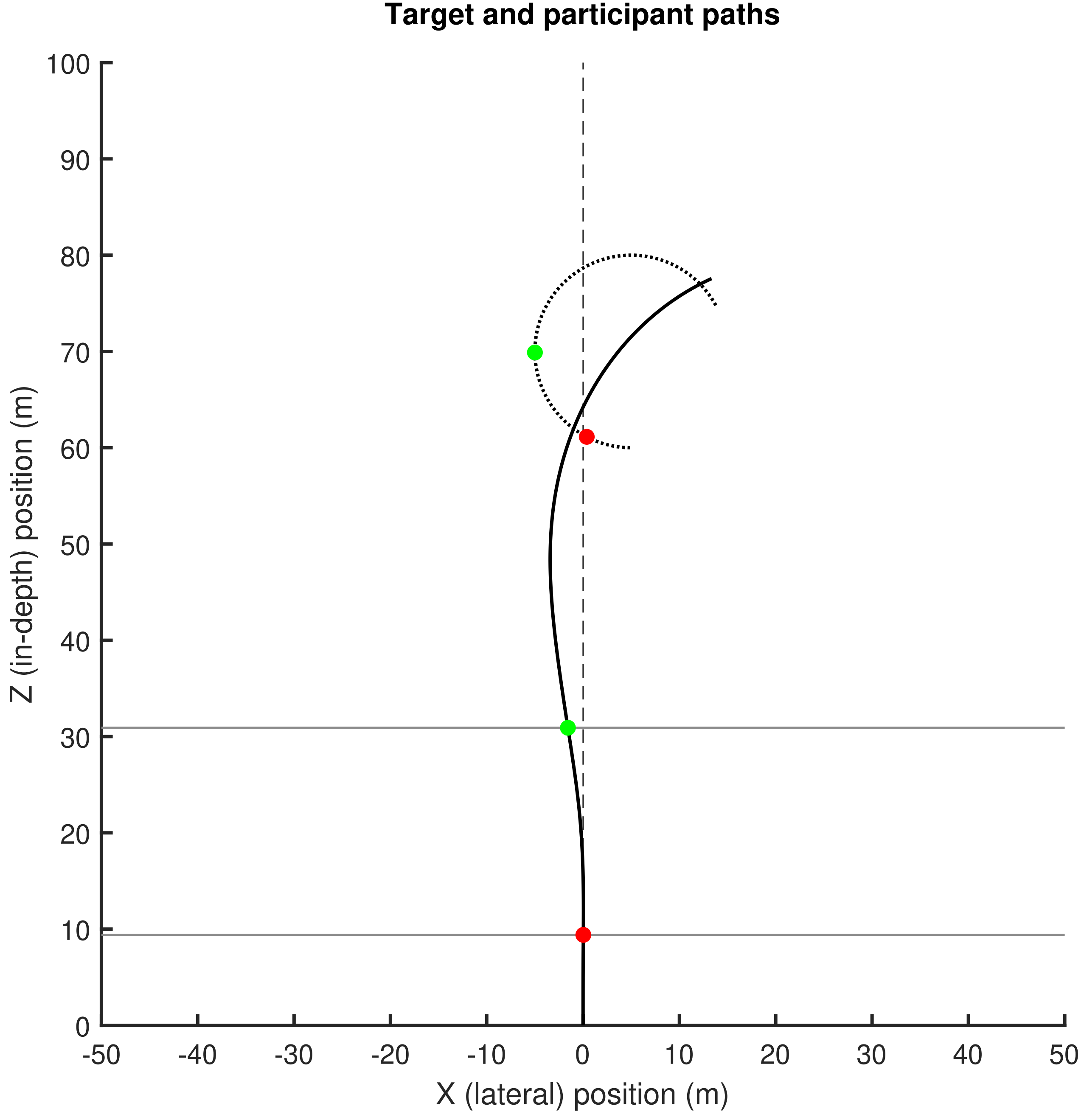

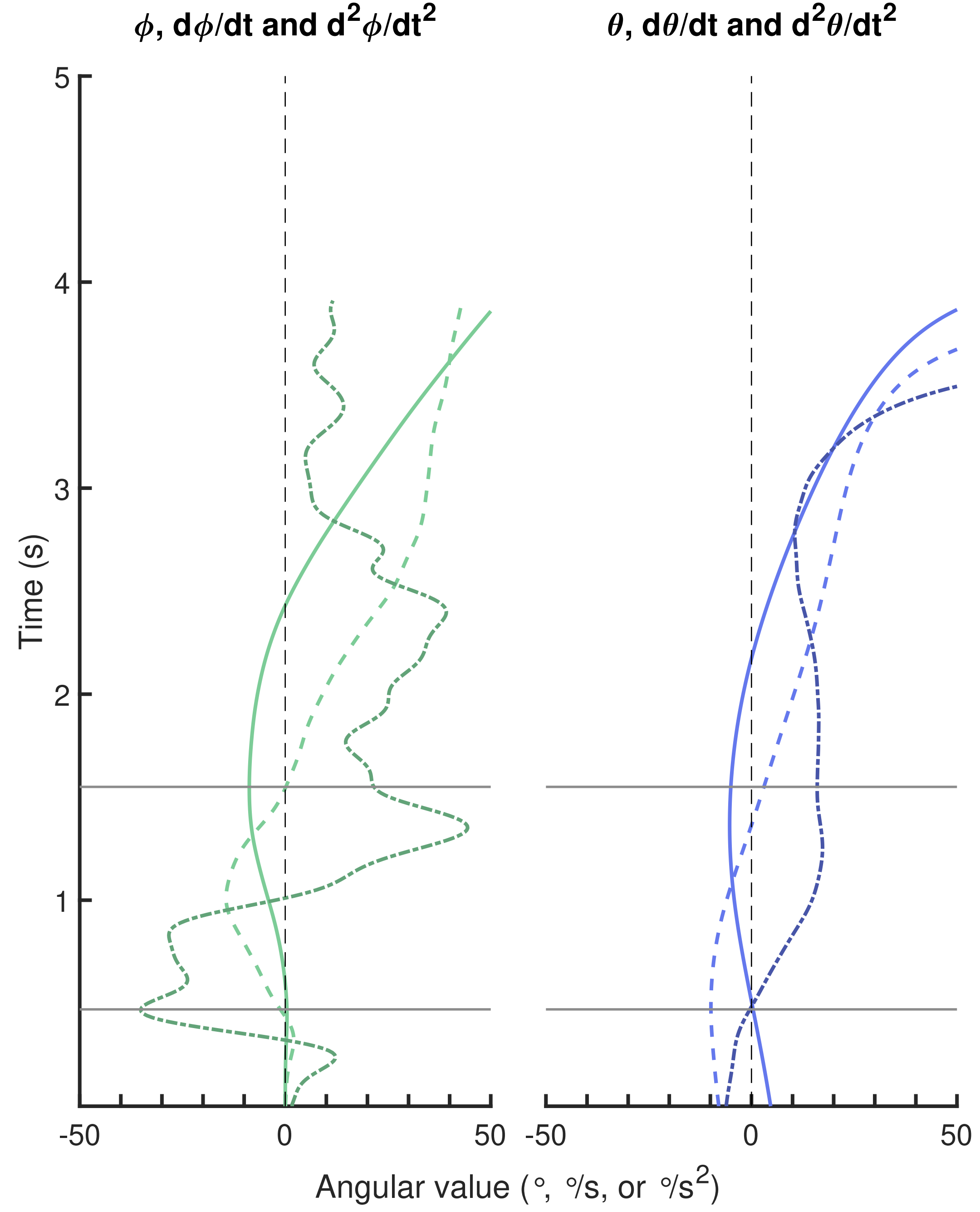

P9/B1
S5/R20-OUT

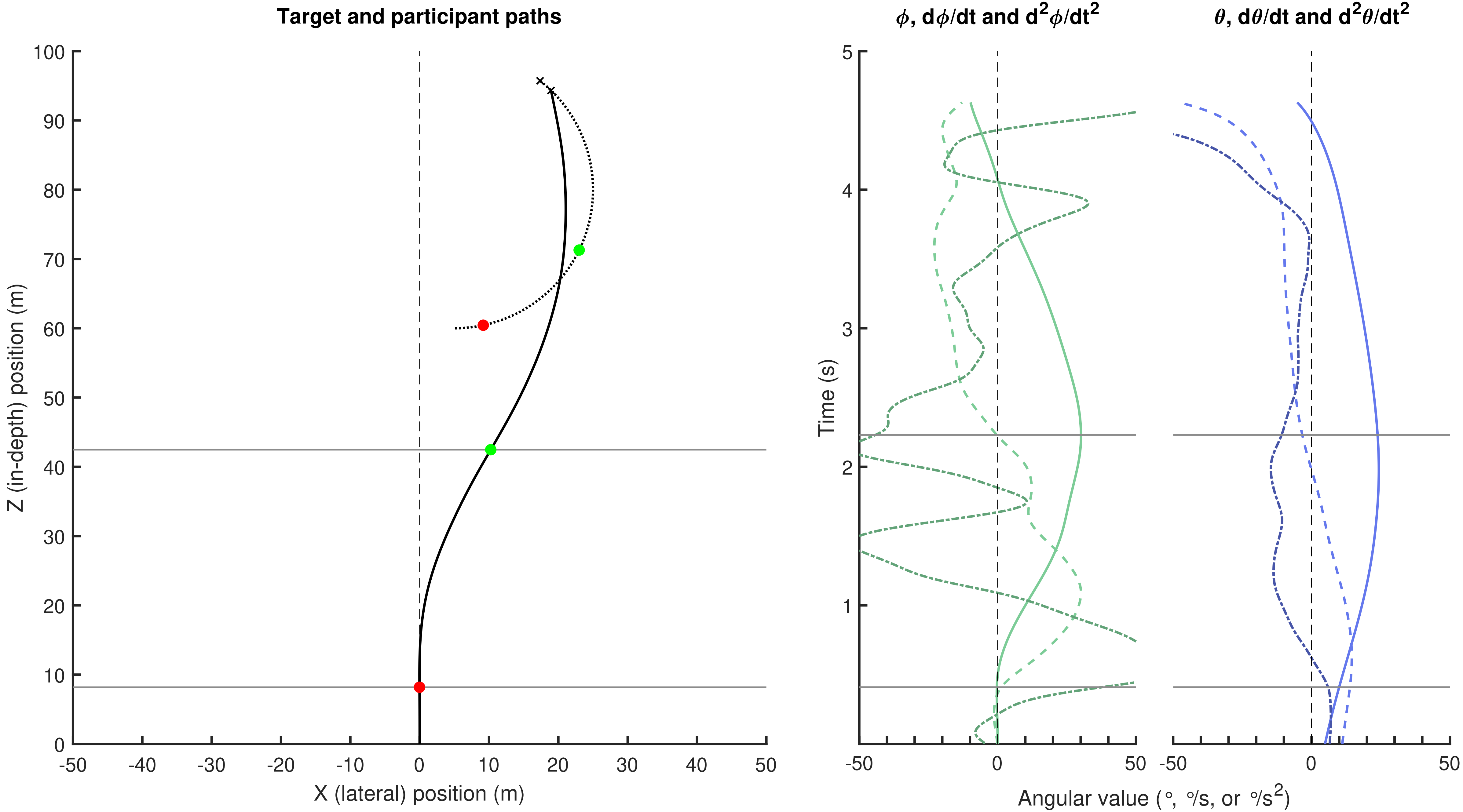

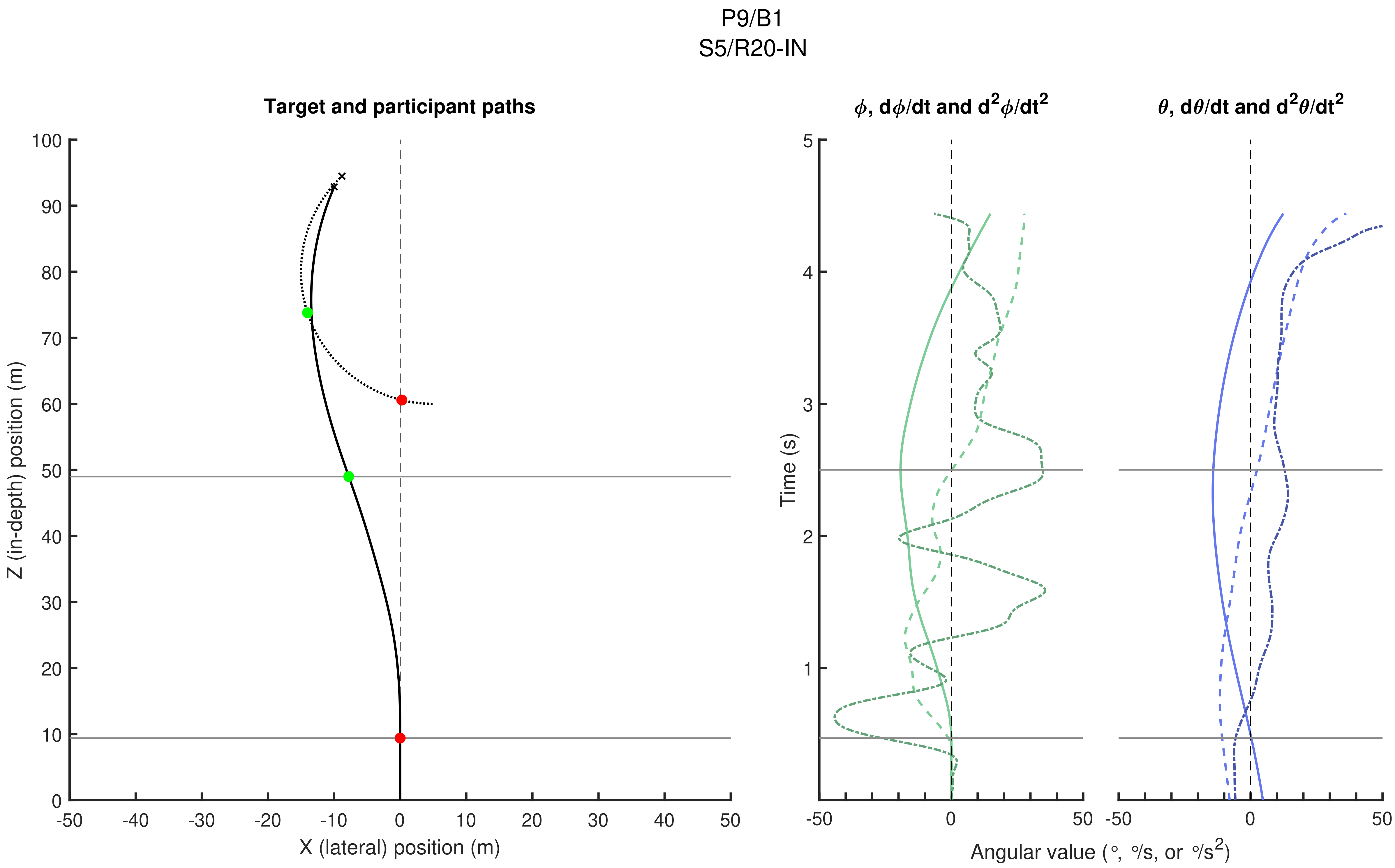
P9/B1
S5/R20-IN
Target and participant paths
Z (in-depth) position (m)
X (lateral) position (m)
100
90
80
70
60
50
40
30
20
10
0
-50
-40
-30
-20
-10
0
10
20
30
40
50
ϕ, dϕ/dt and d$^2\phi$/dt^2
θ, dθ/dt and d$^2\theta$/dt^2
Time (s)
5
4
3
2
1
-50
0
50
-50
0
50
Angular value (°, °/s, or °/s^2)

P9/B1
S5/R30-OUT

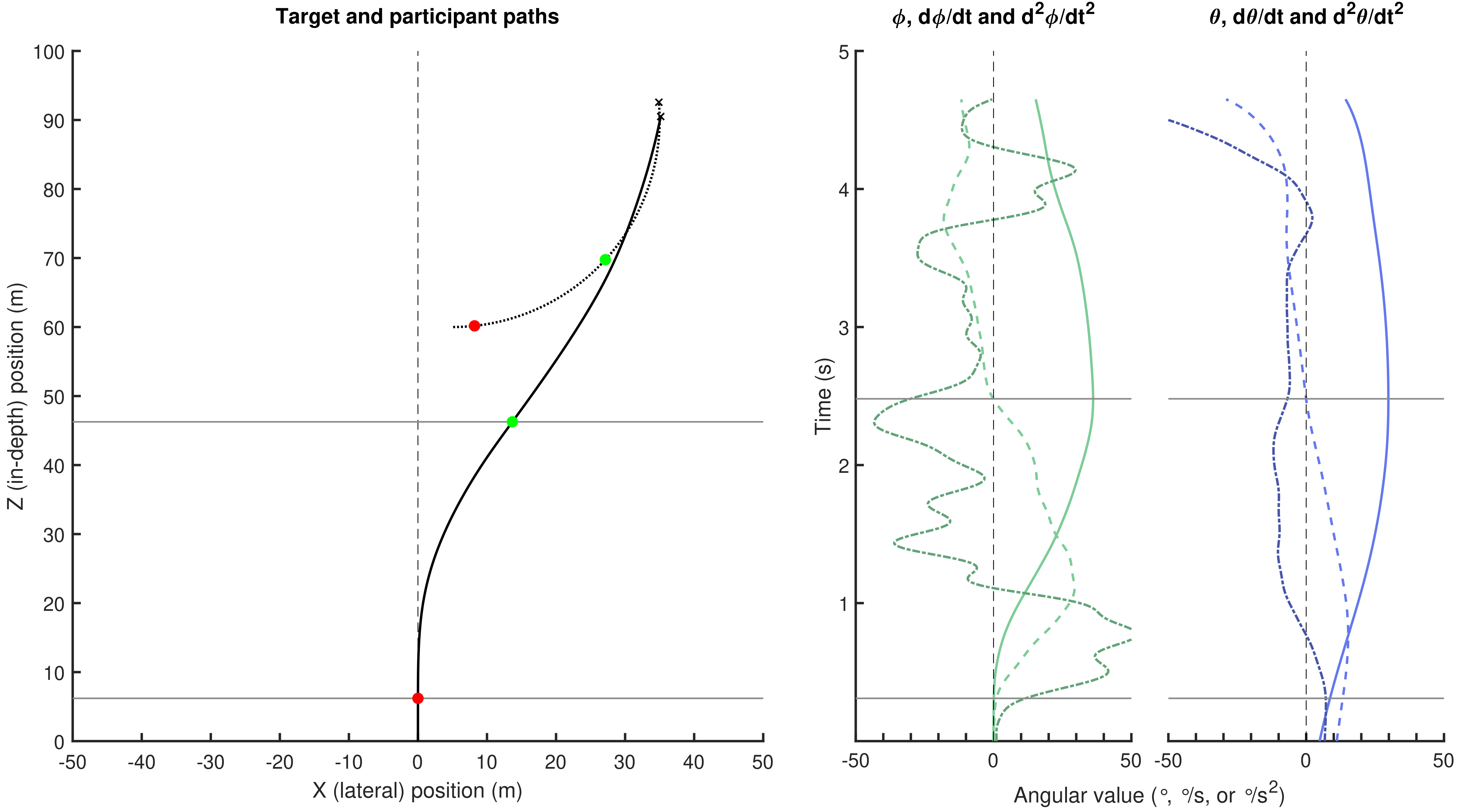

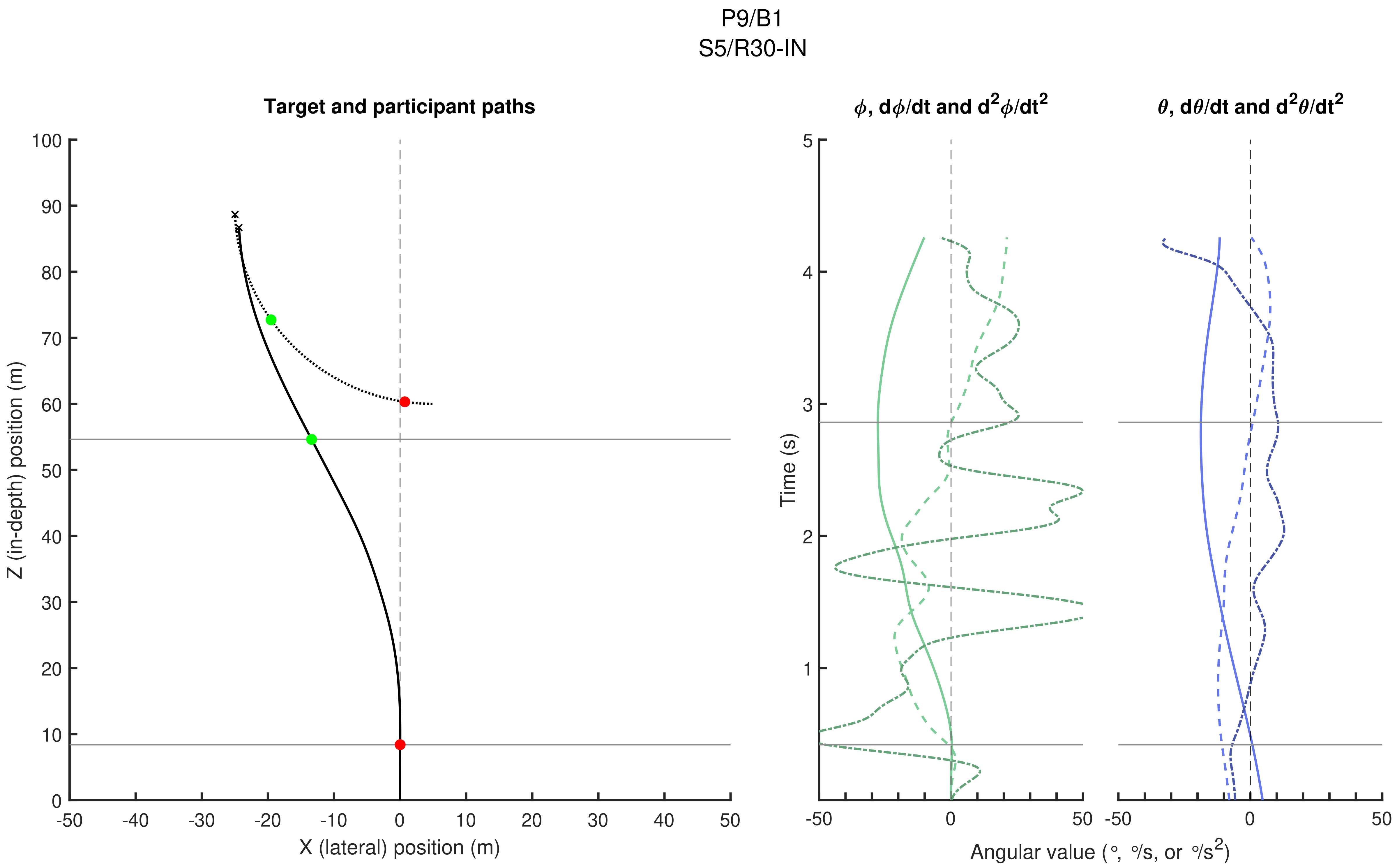
P9/B1
S5/R30-IN
Target and participant paths
Z (in-depth) position (m)
X (lateral) position (m)
ϕ, dϕ/dt and d$^2\phi$/dt^2
θ, dθ/dt and d$^2\theta$/dt^2
Time (s)
Angular value (°, °/s, or °/s^2)

P9/B1
S15/R10-IN

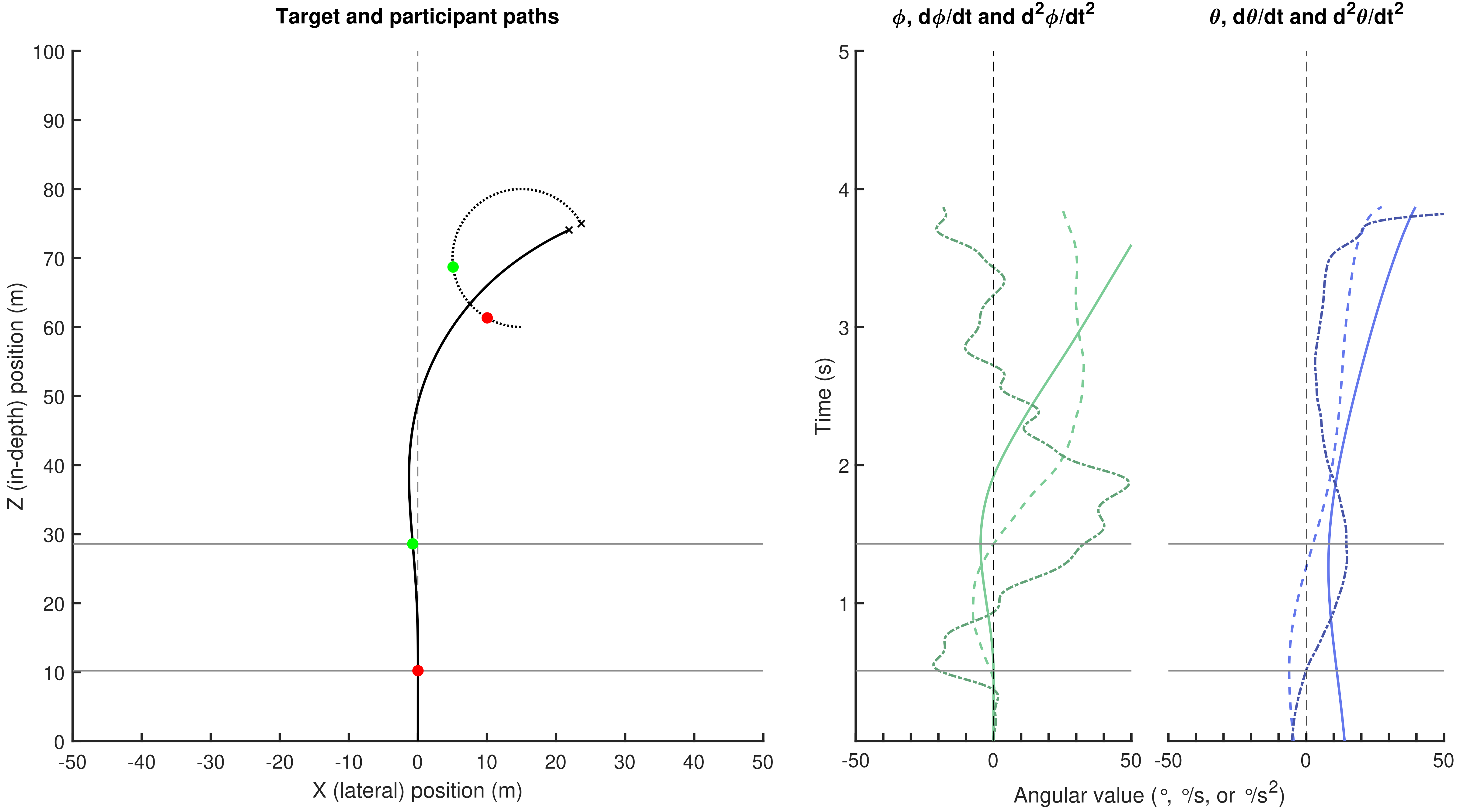

P9/B1
S15/R20-IN

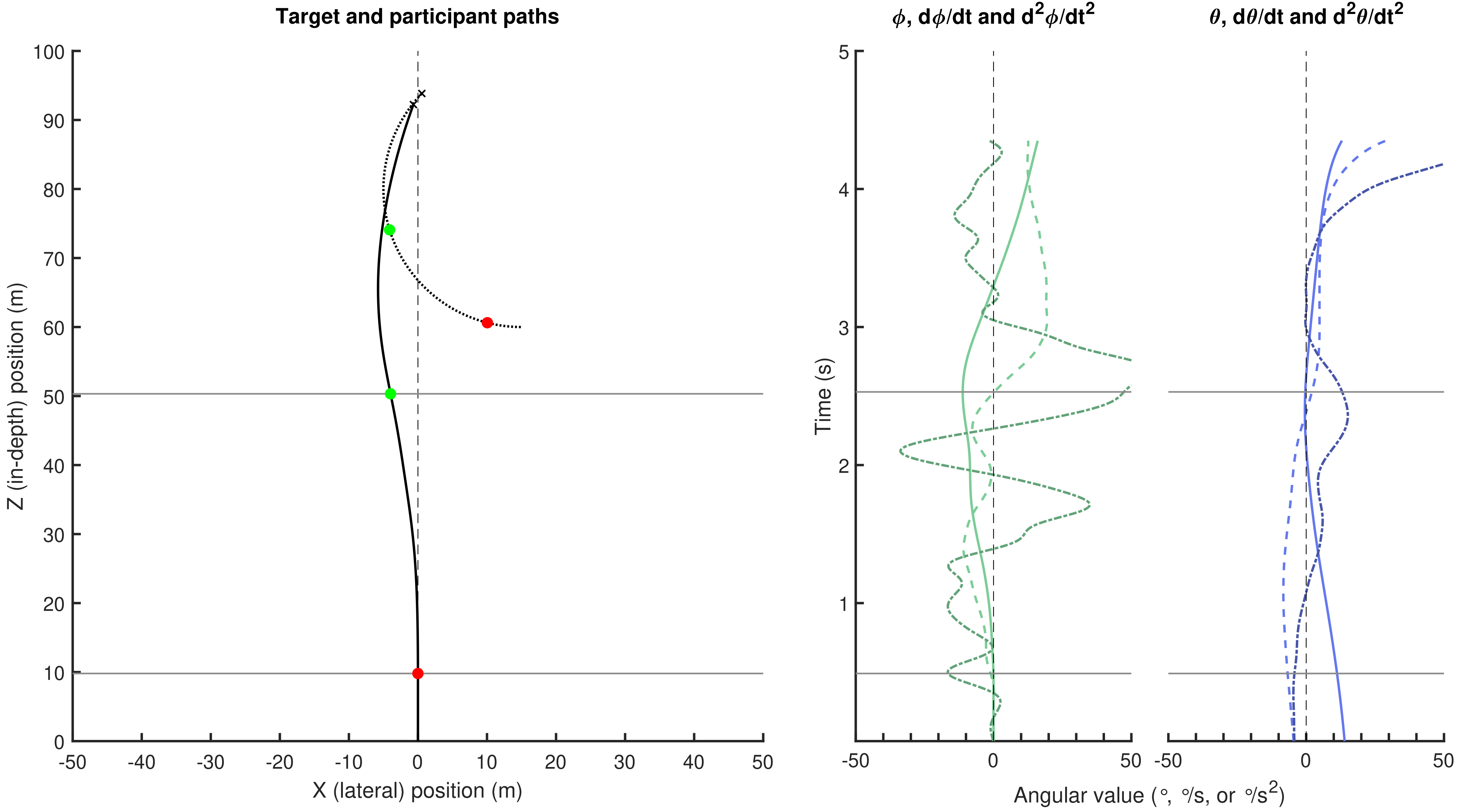

P9/B1
S15/R30-IN

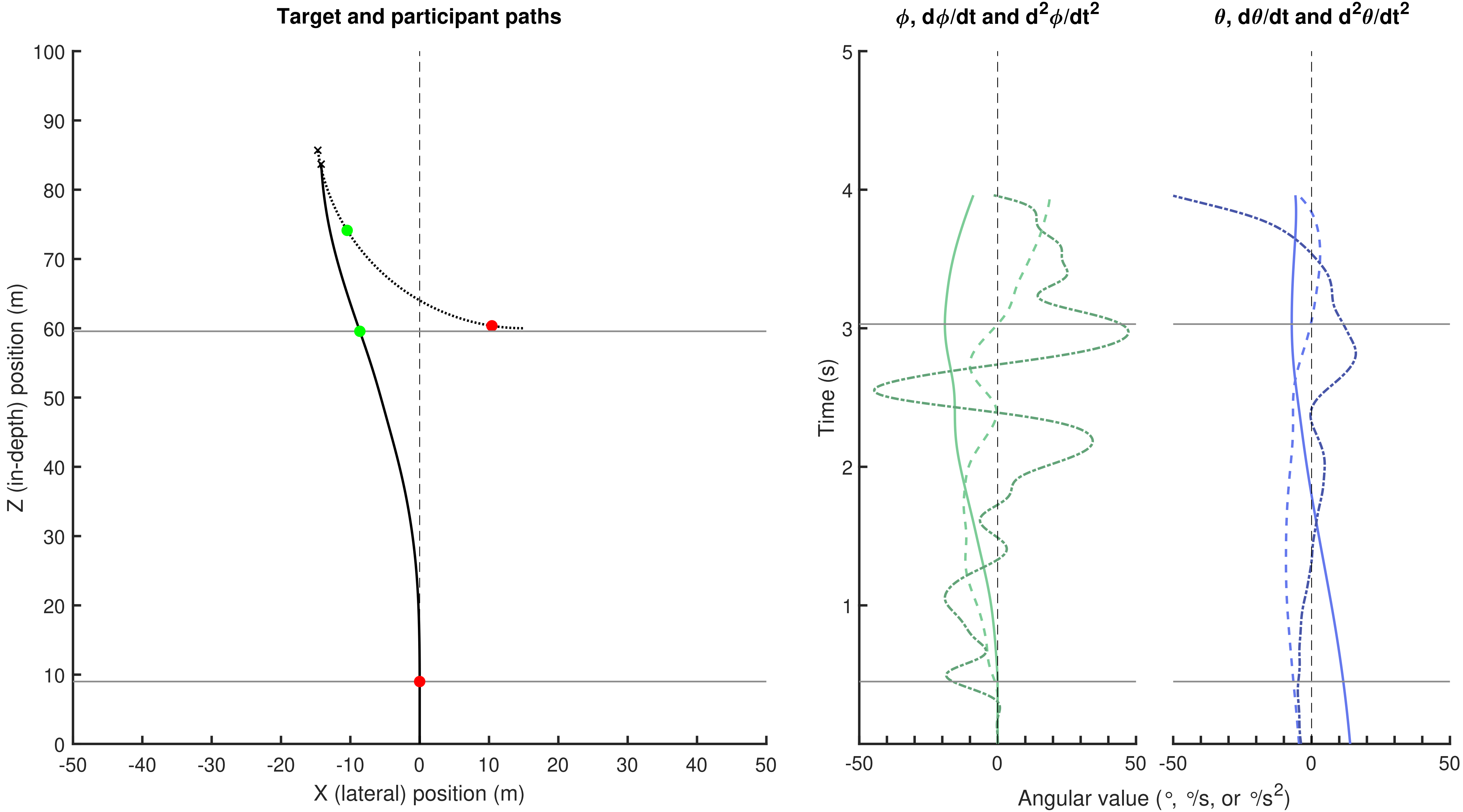

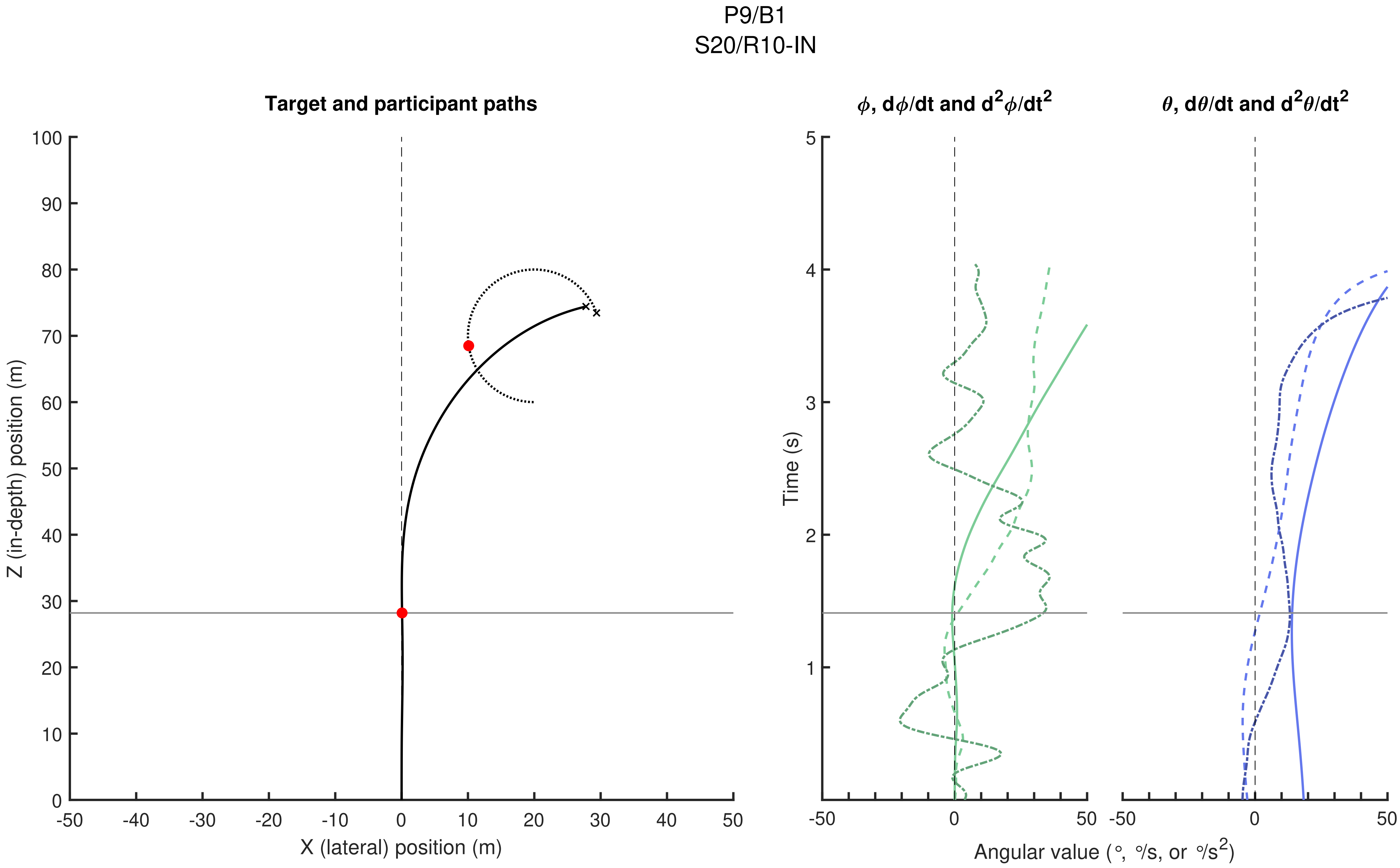

P9/B1
S20/R10-IN
Target and participant paths
Z (in-depth) position (m)
X (lateral) position (m)
ϕ, dϕ/dt and d$^2\phi$/dt^2
θ, dθ/dt and d$^2\theta$/dt^2
Time (s)
Angular value (°, °/s, or °/s^2)

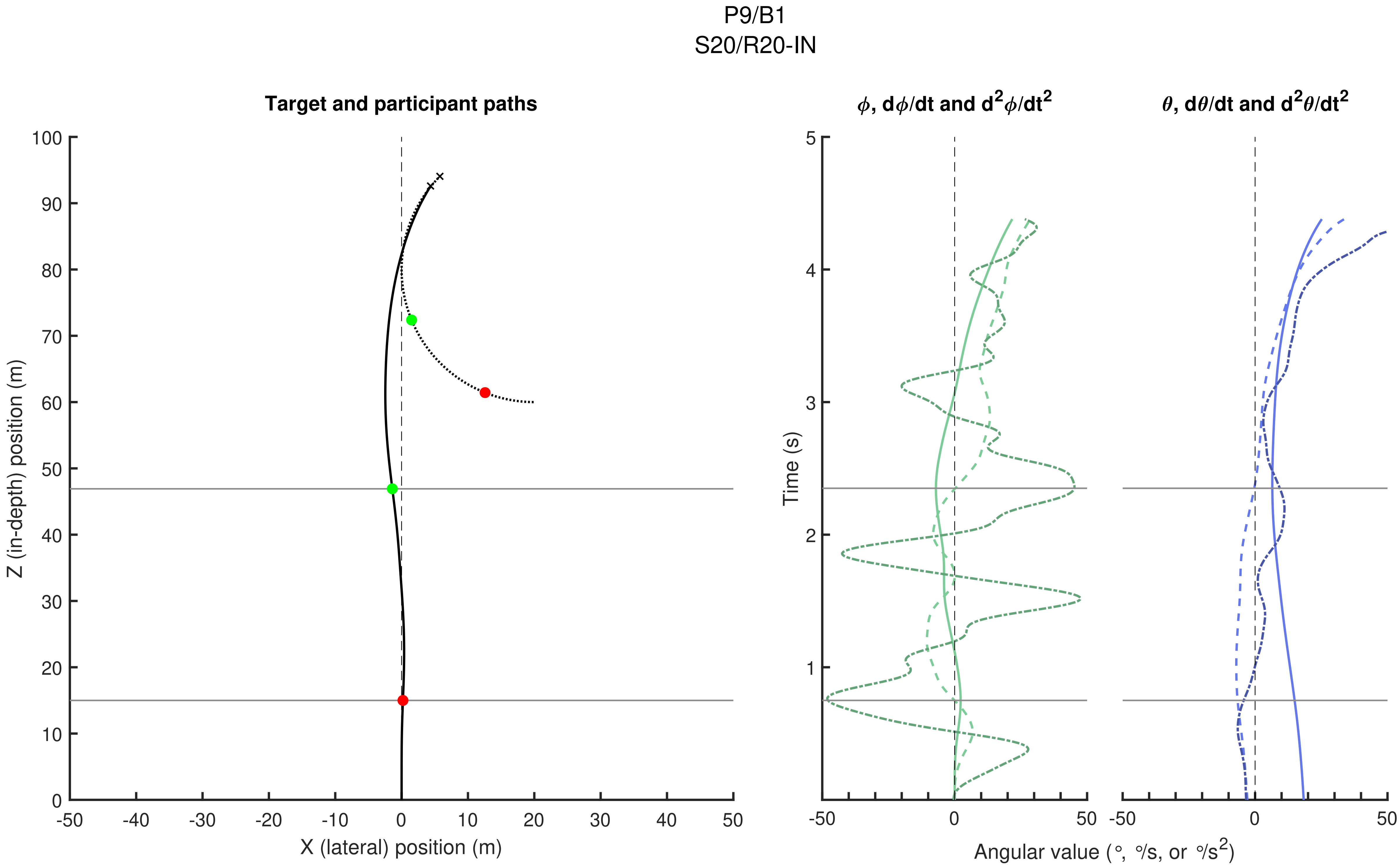
P9/B1
S20/R20-IN
Target and participant paths
Z (in-depth) position (m)
X (lateral) position (m)
ϕ, dϕ/dt and d$^2\phi$/dt^2
θ, dθ/dt and d$^2\theta$/dt^2
Time (s)
Angular value (°, °/s, or °/s^2)

P9/B1
S20/R30-IN

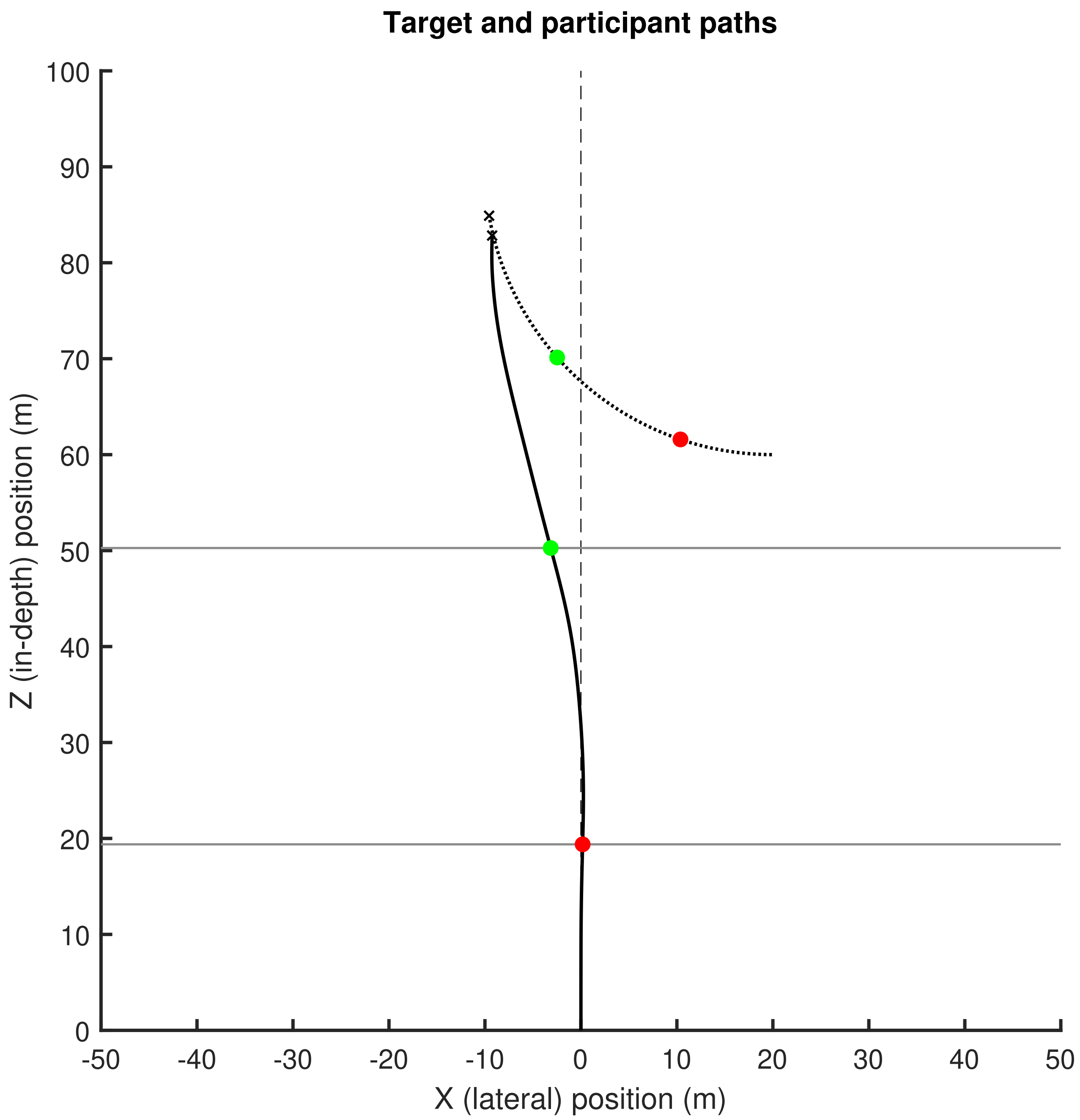

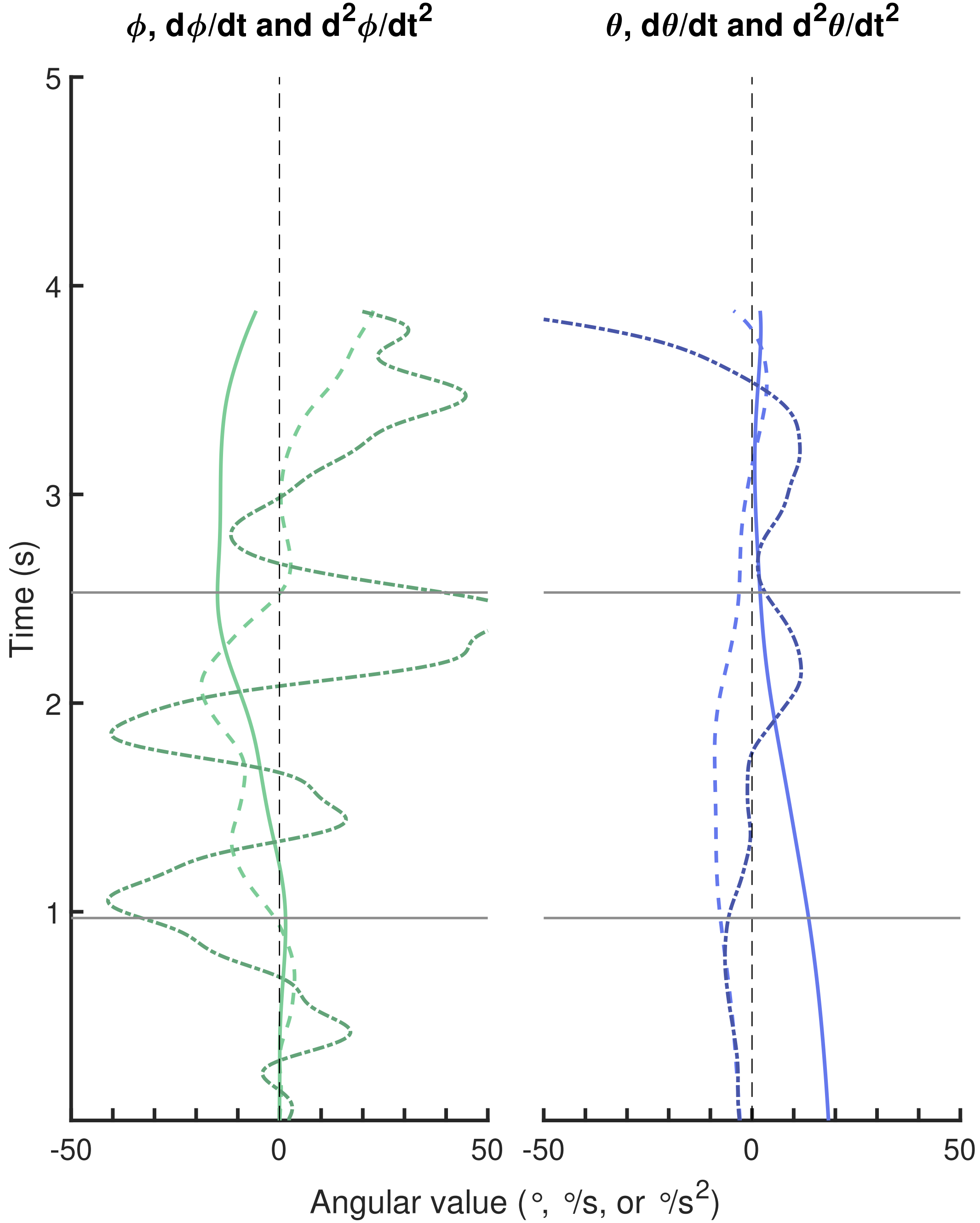

P9/B2
S20/R10-IN

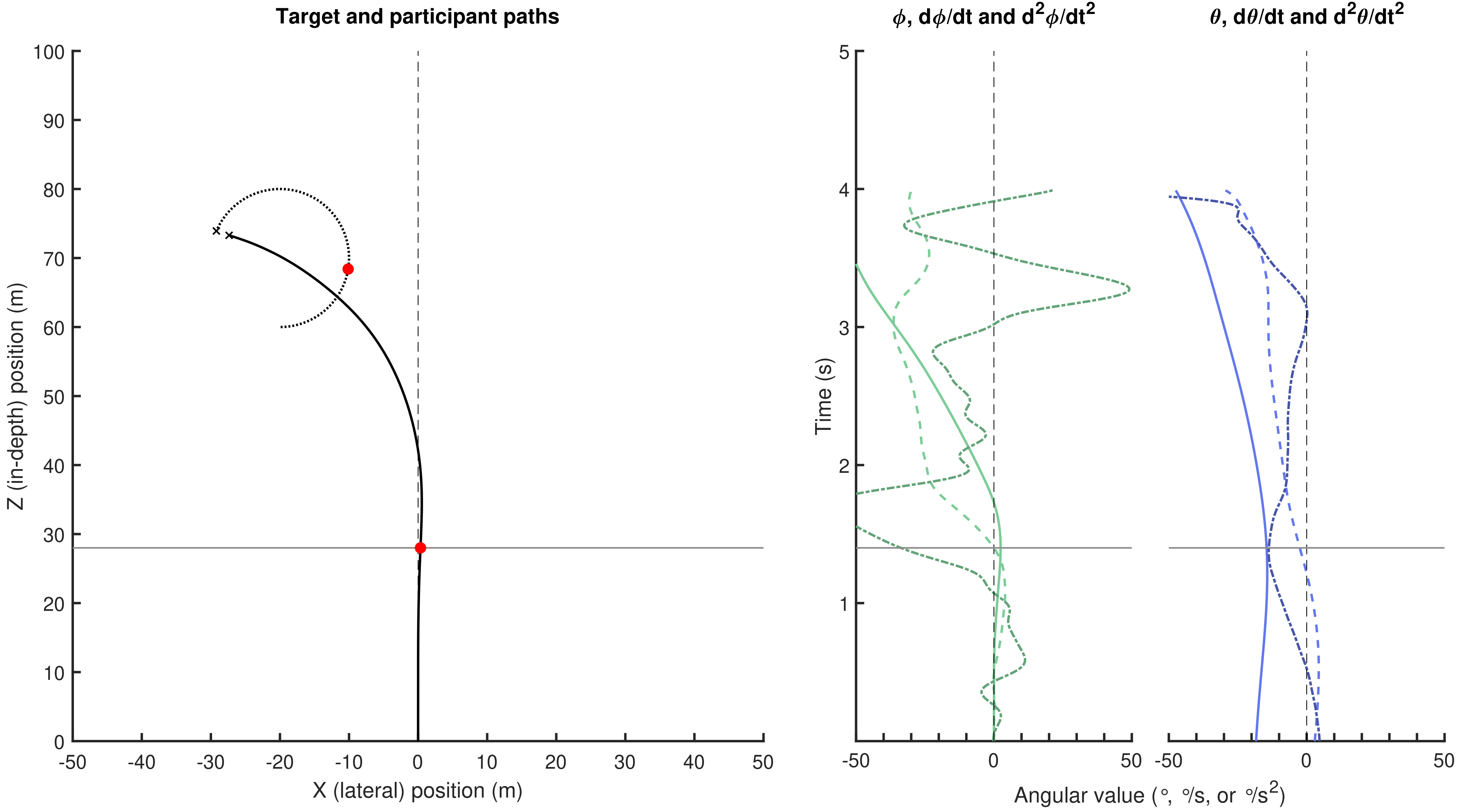

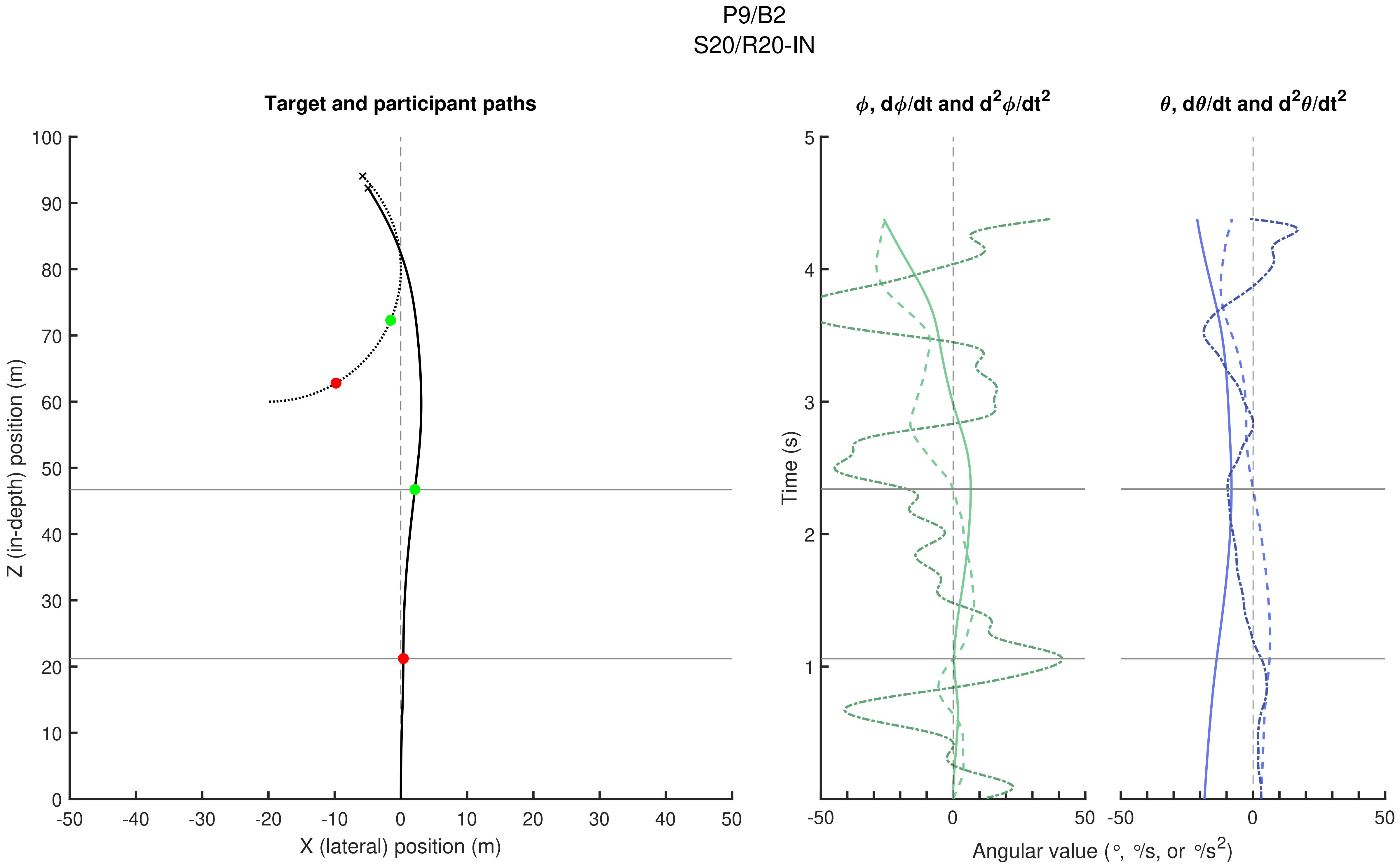

P9/B2
S20/R20-IN
Target and participant paths
Z (in-depth) position (m)
X (lateral) position (m)
ϕ, dϕ/dt and d²ϕ/dt²
θ, dθ/dt and d²θ/dt²
Time (s)
Angular value (°, °/s, or °/s²)

P9/B2
S20/R30-IN

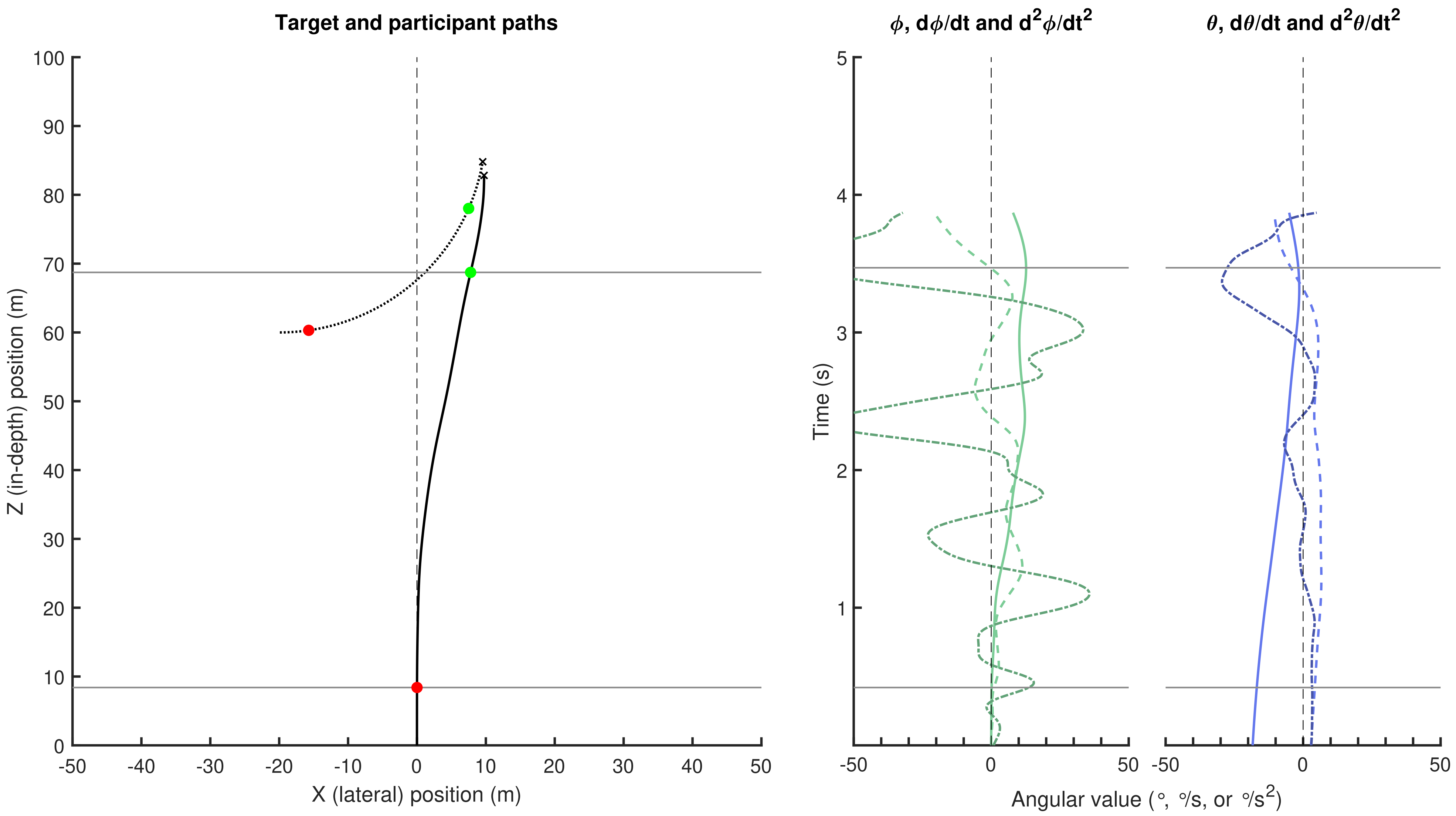

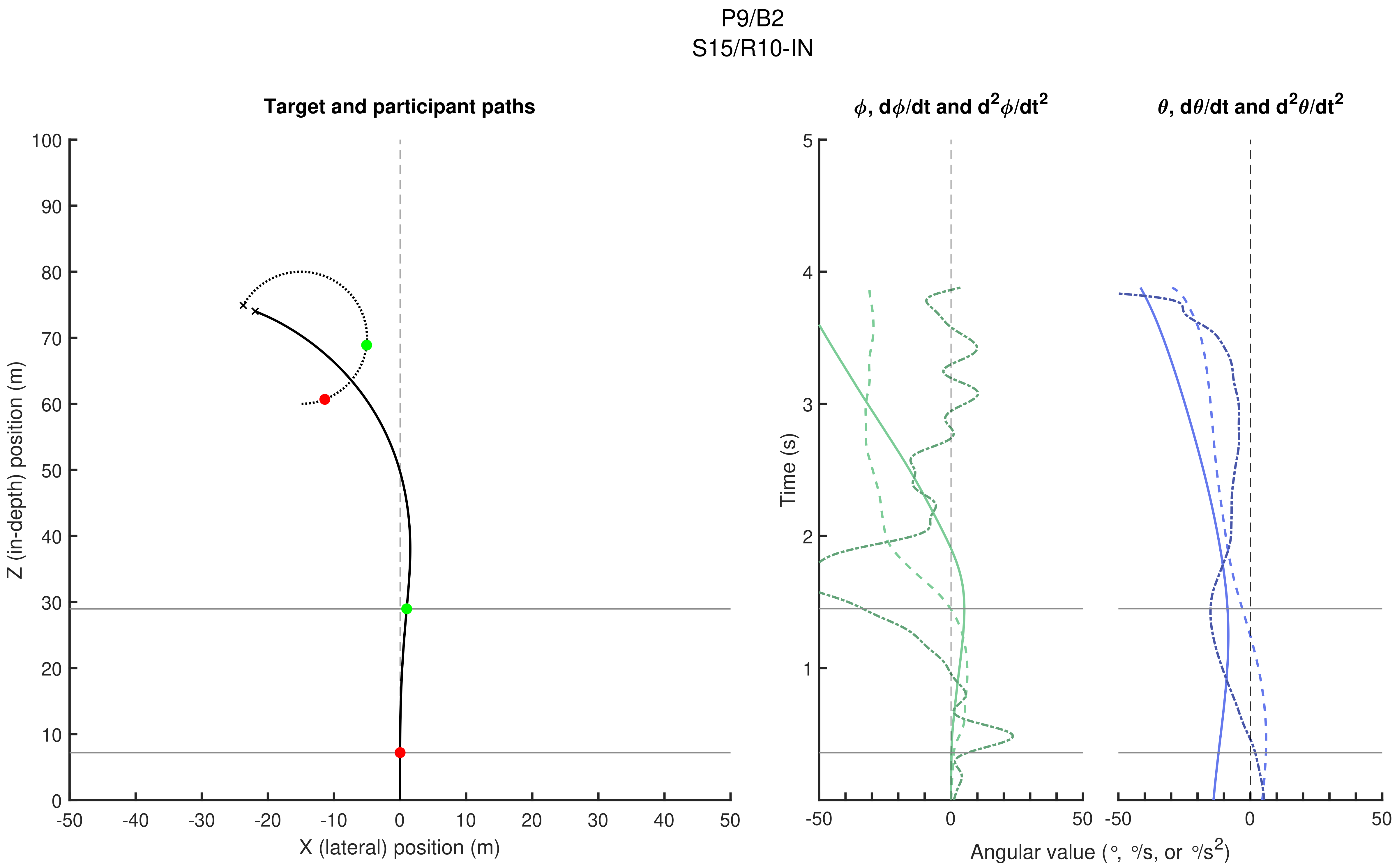

P9/B2
S15/R10-IN
Target and participant paths
Z (in-depth) position (m)
X (lateral) position (m)
100
90
80
70
60
50
40
30
20
10
0
-50
-40
-30
-20
-10
0
10
20
30
40
50
ϕ, dϕ/dt and d$^2\phi$/dt^2
θ, dθ/dt and d$^2\theta$/dt^2
Time (s)
5
4
3
2
1
-50
0
50
-50
0
50
Angular value (°, °/s, or °/s^2)

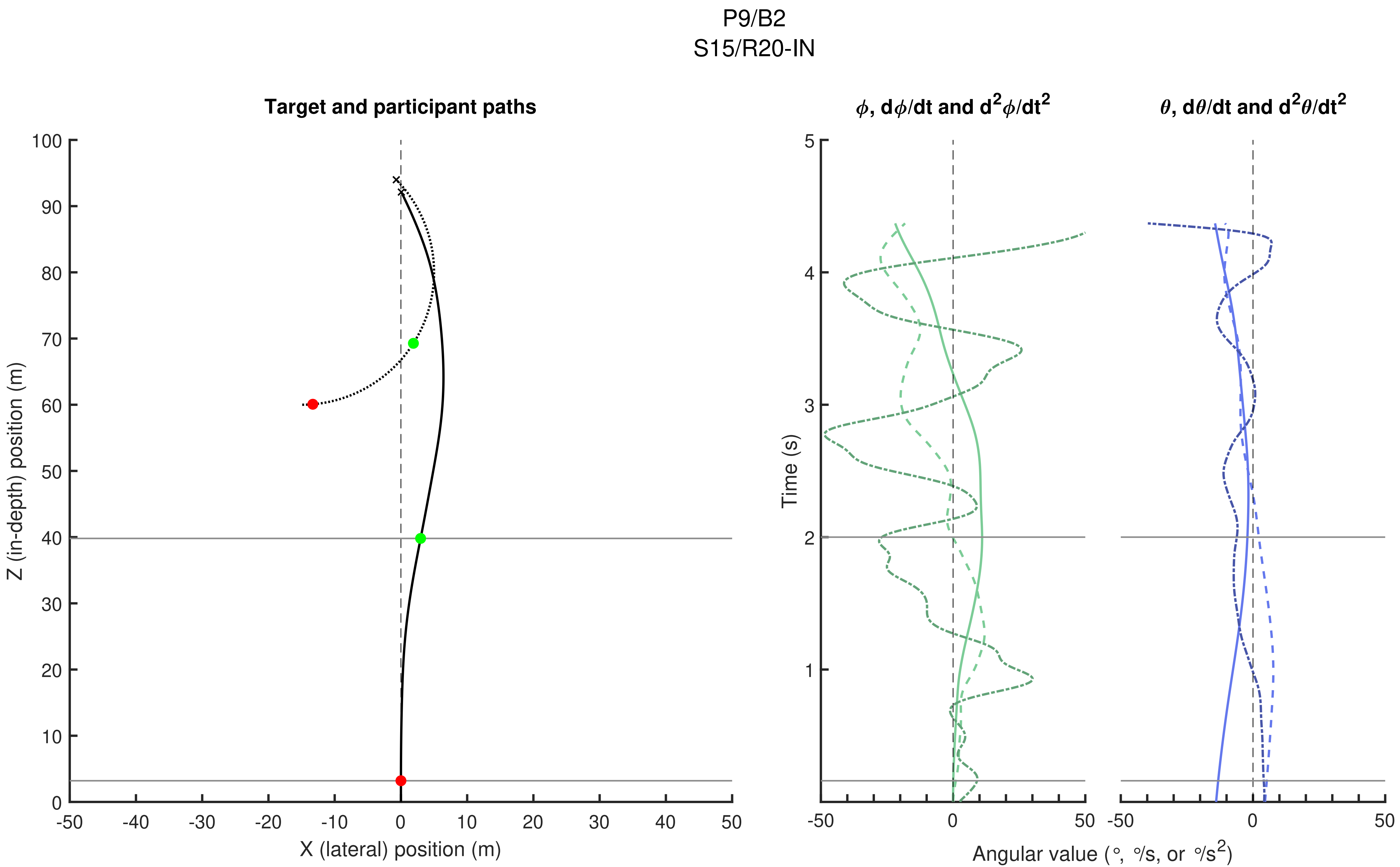
P9/B2
S15/R20-IN
Target and participant paths
Z (in-depth) position (m)
X (lateral) position (m)
ϕ, dϕ/dt and d$^2\phi$/dt^2
θ, dθ/dt and d$^2\theta$/dt^2
Time (s)
Angular value (°, °/s, or °/s^2)
100
90
80
70
60
50
40
30
20
10
0
-50
-40
-30
-20
-10
0
10
20
30
40
50
5
4
3
2
1
-50
0
50
-50
0
50

P9/B2
S15/R30-IN

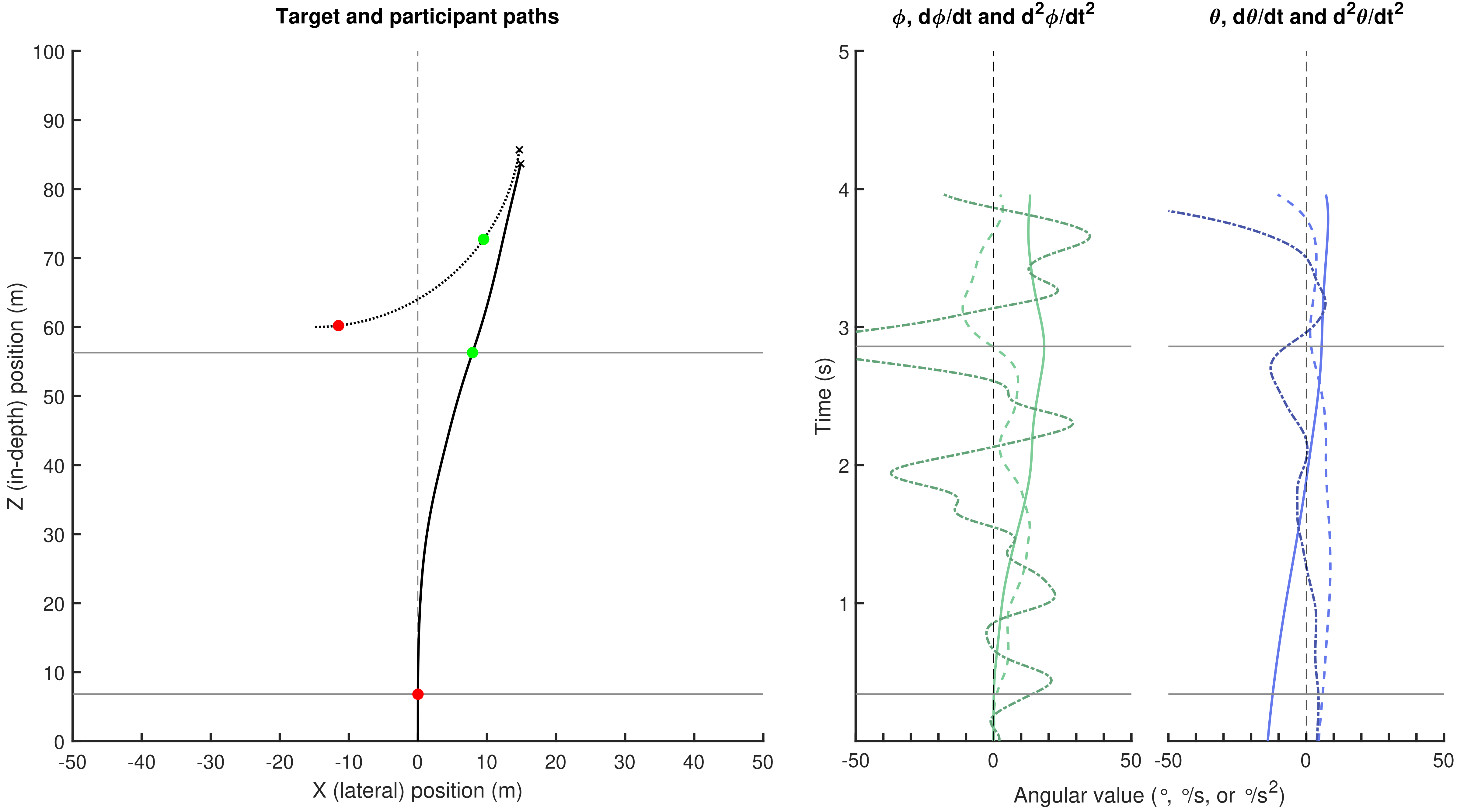

P9/B2
S5/R10-IN

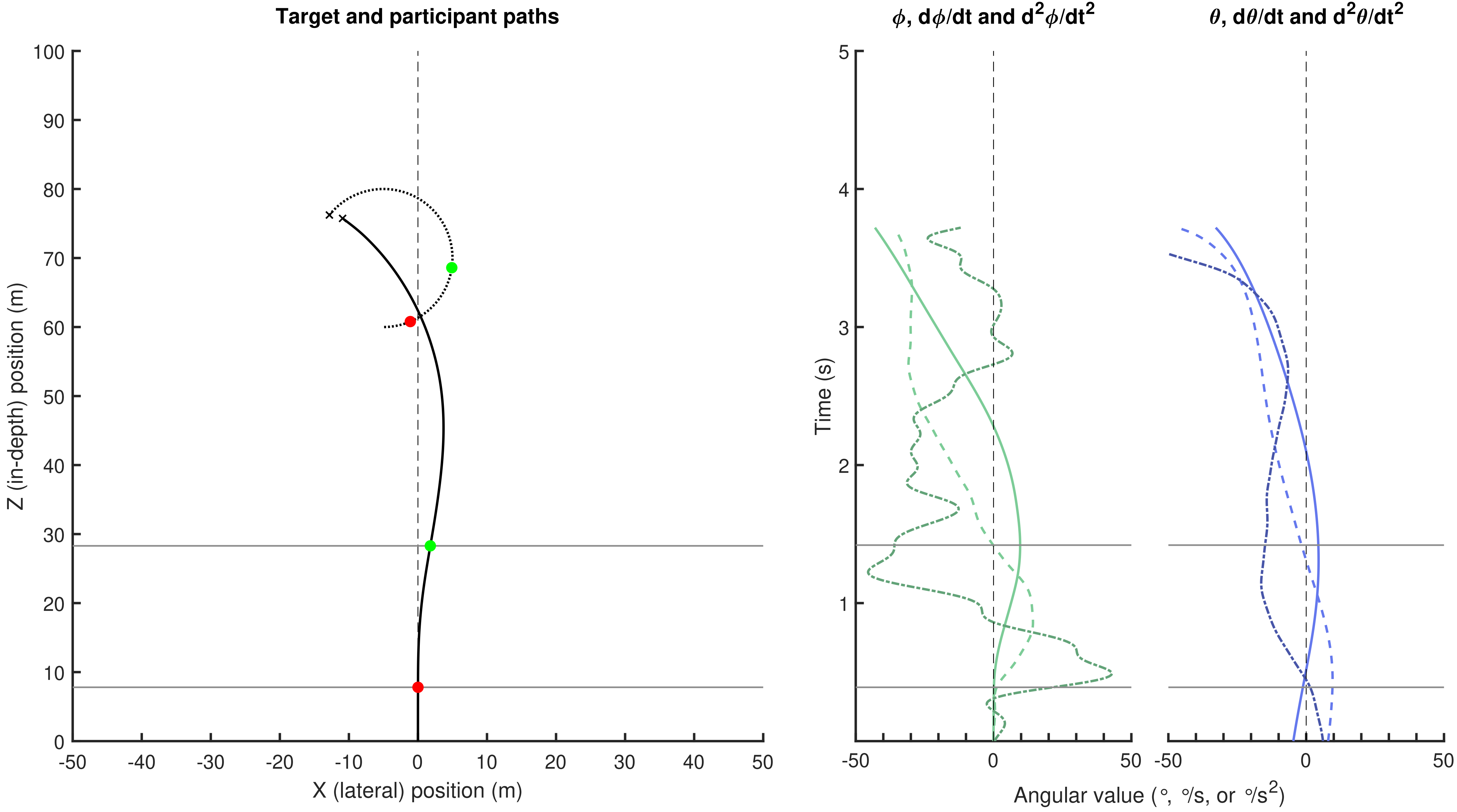

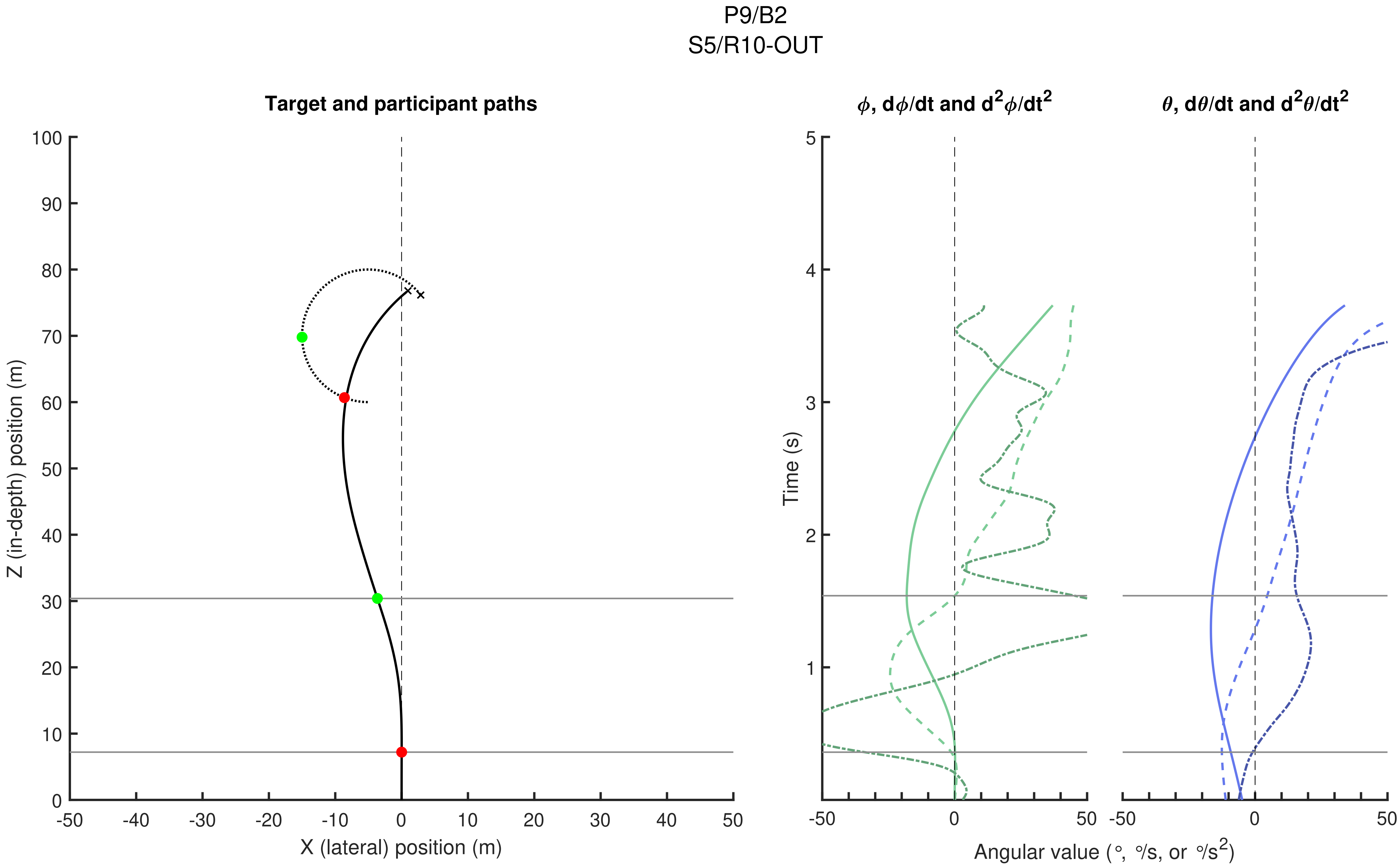
P9/B2
S5/R10-OUT
Target and participant paths
Z (in-depth) position (m)
X (lateral) position (m)
100
90
80
70
60
50
40
30
20
10
0
-50
-40
-30
-20
-10
0
10
20
30
40
50
ϕ, dϕ/dt and d$^2\phi$/dt^2
θ, dθ/dt and d$^2\theta$/dt^2
Time (s)
5
4
3
2
1
-50
0
50
-50
0
50
Angular value (°, °/s, or °/s^2)

P9/B2
S5/R20-IN

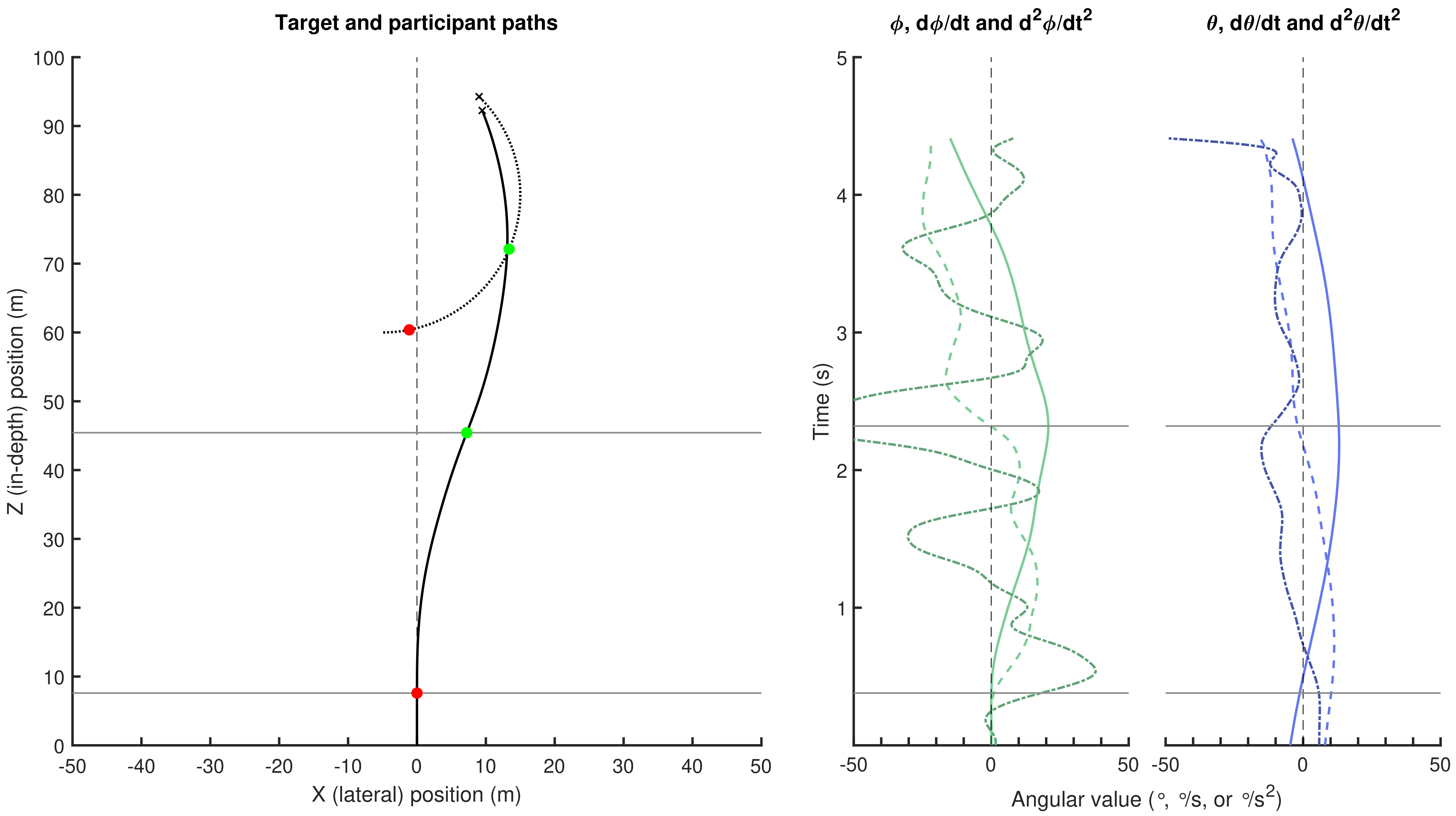

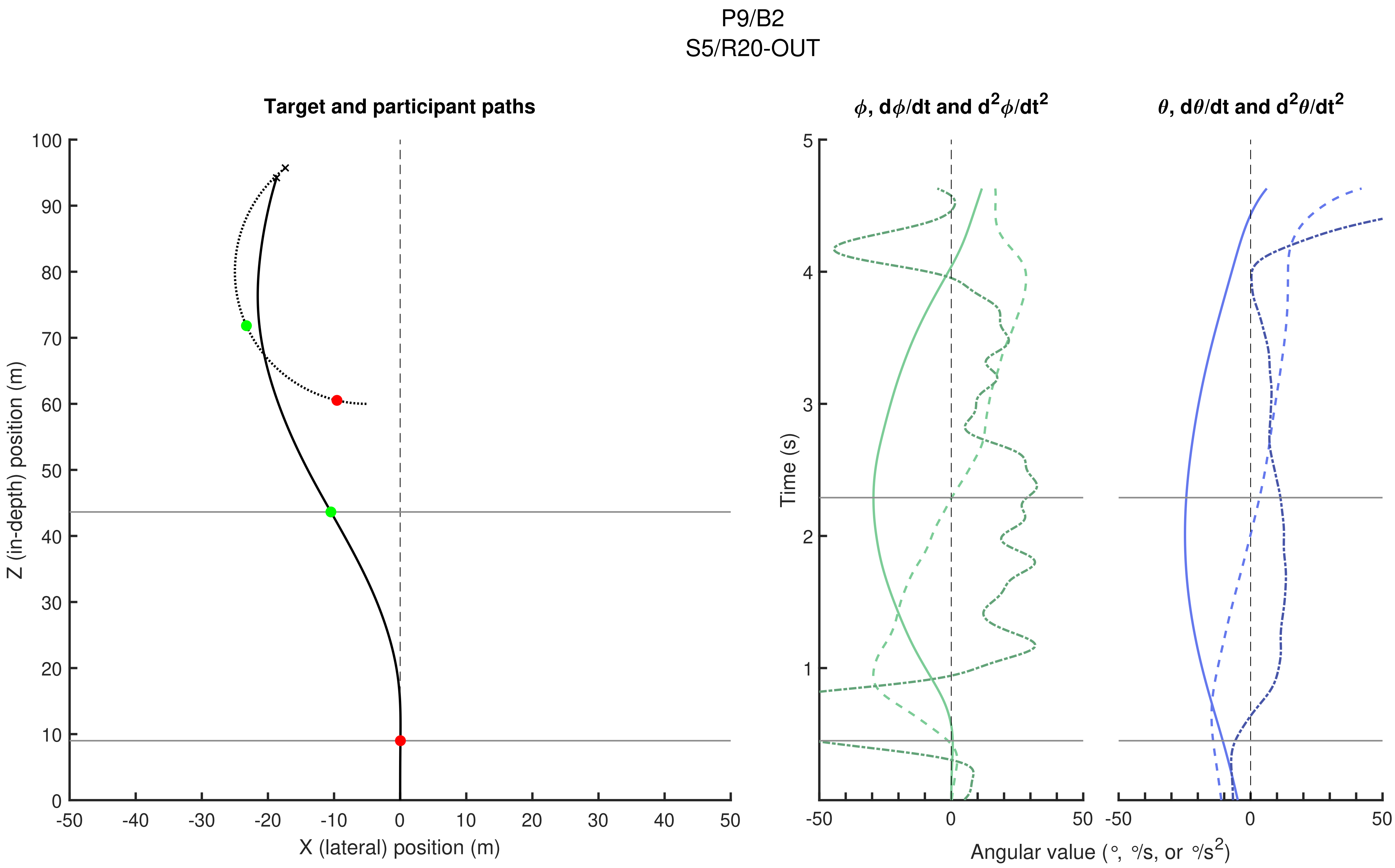

P9/B2
S5/R20-OUT
Target and participant paths
Z (in-depth) position (m)
X (lateral) position (m)
ϕ, dϕ/dt and d²ϕ/dt²
θ, dθ/dt and d²θ/dt²
Time (s)
Angular value (°, °/s, or °/s²)

P9/B2
S5/R30-IN

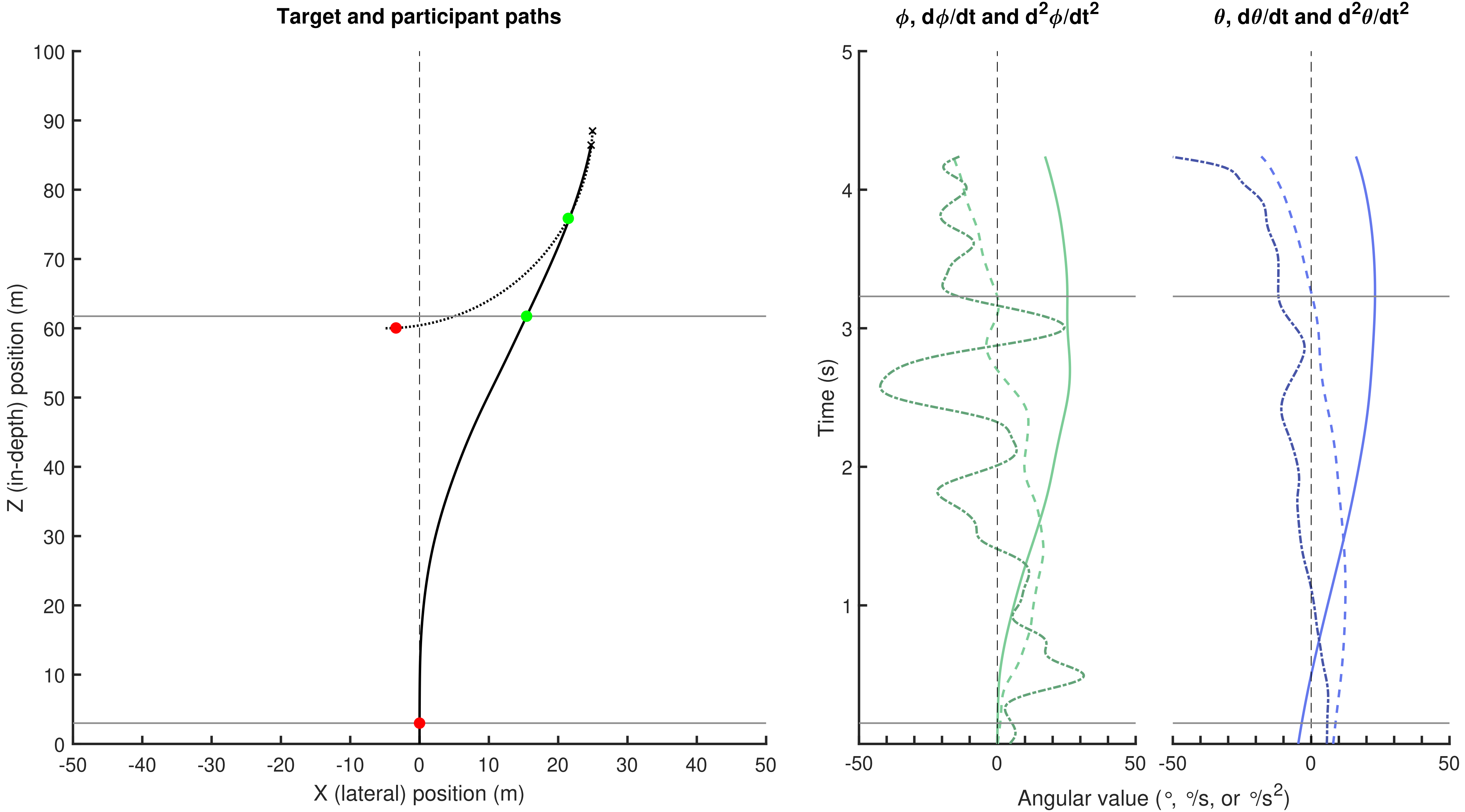

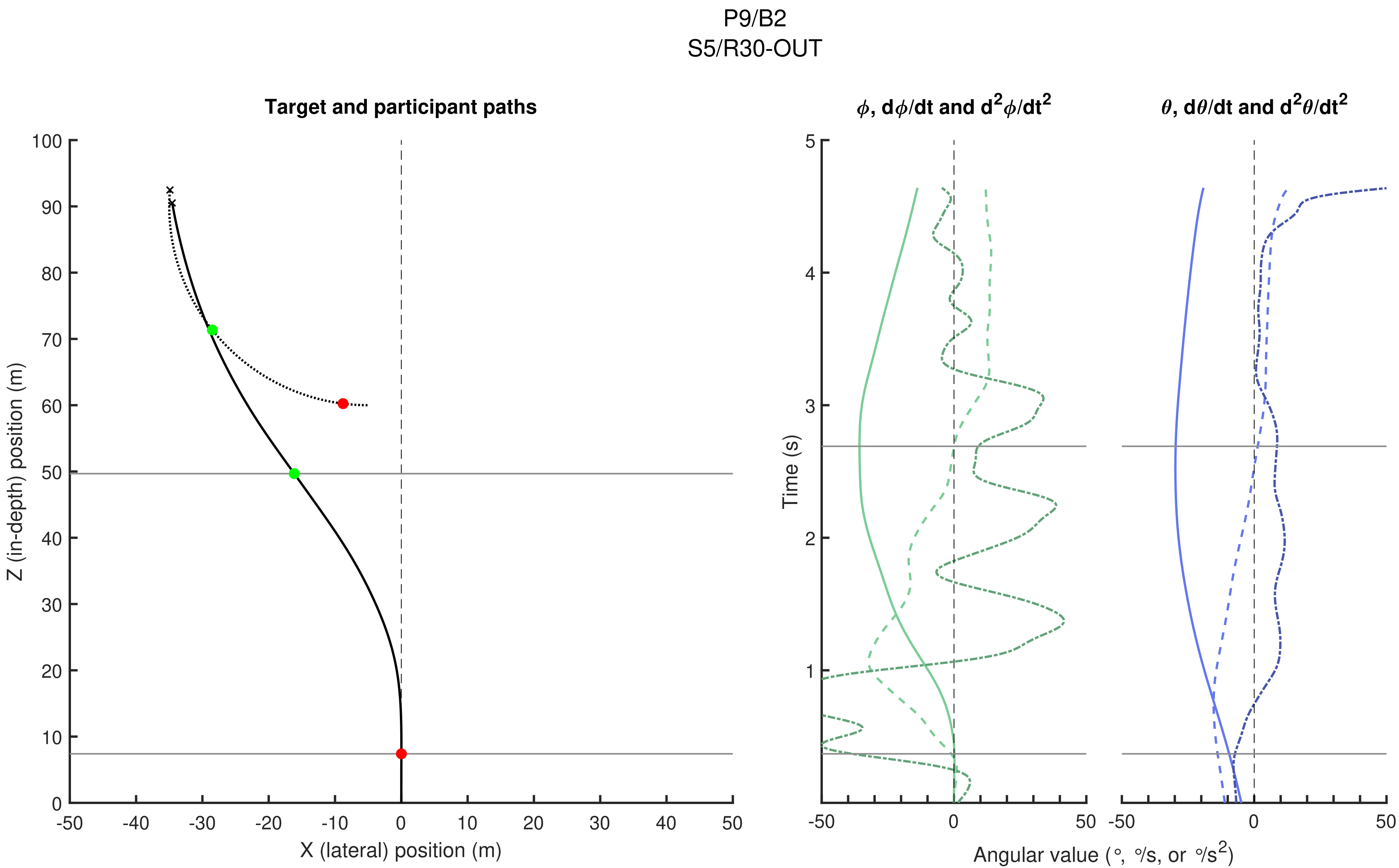

P9/B2
S5/R30-OUT
Target and participant paths
Z (in-depth) position (m)
X (lateral) position (m)
ϕ, dϕ/dt and d$^2\phi$/dt^2
θ, dθ/dt and d$^2\theta$/dt^2
Time (s)
Angular value (°, °/s, or °/s^2)

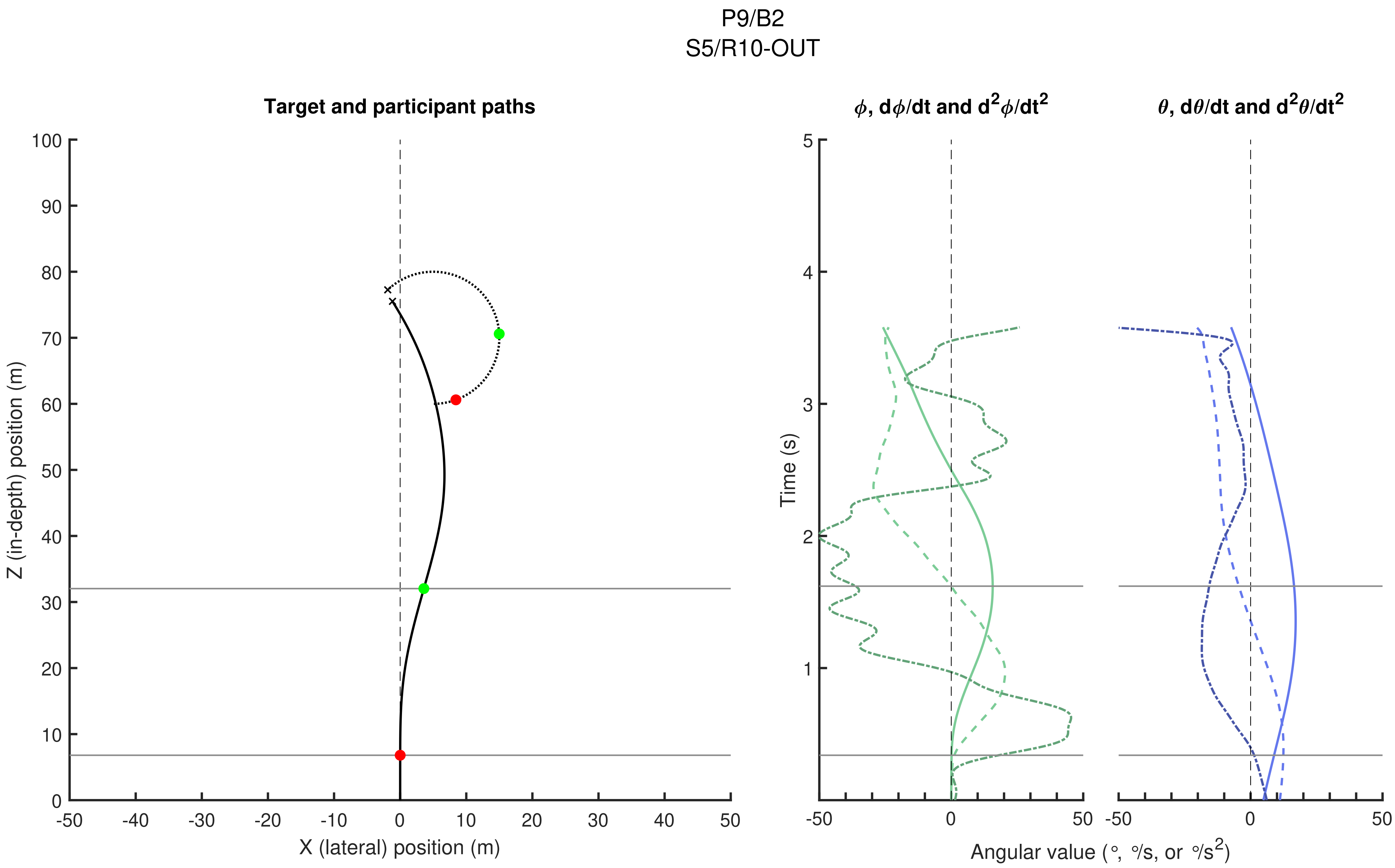

P9/B2
S5/R10-OUT
Target and participant paths
ϕ, dϕ/dt and d$^2\phi$/dt^2
θ, dθ/dt and d$^2\theta$/dt^2
Z (in-depth) position (m)
X (lateral) position (m)
Time (s)
Angular value (°, °/s, or °/s^2)

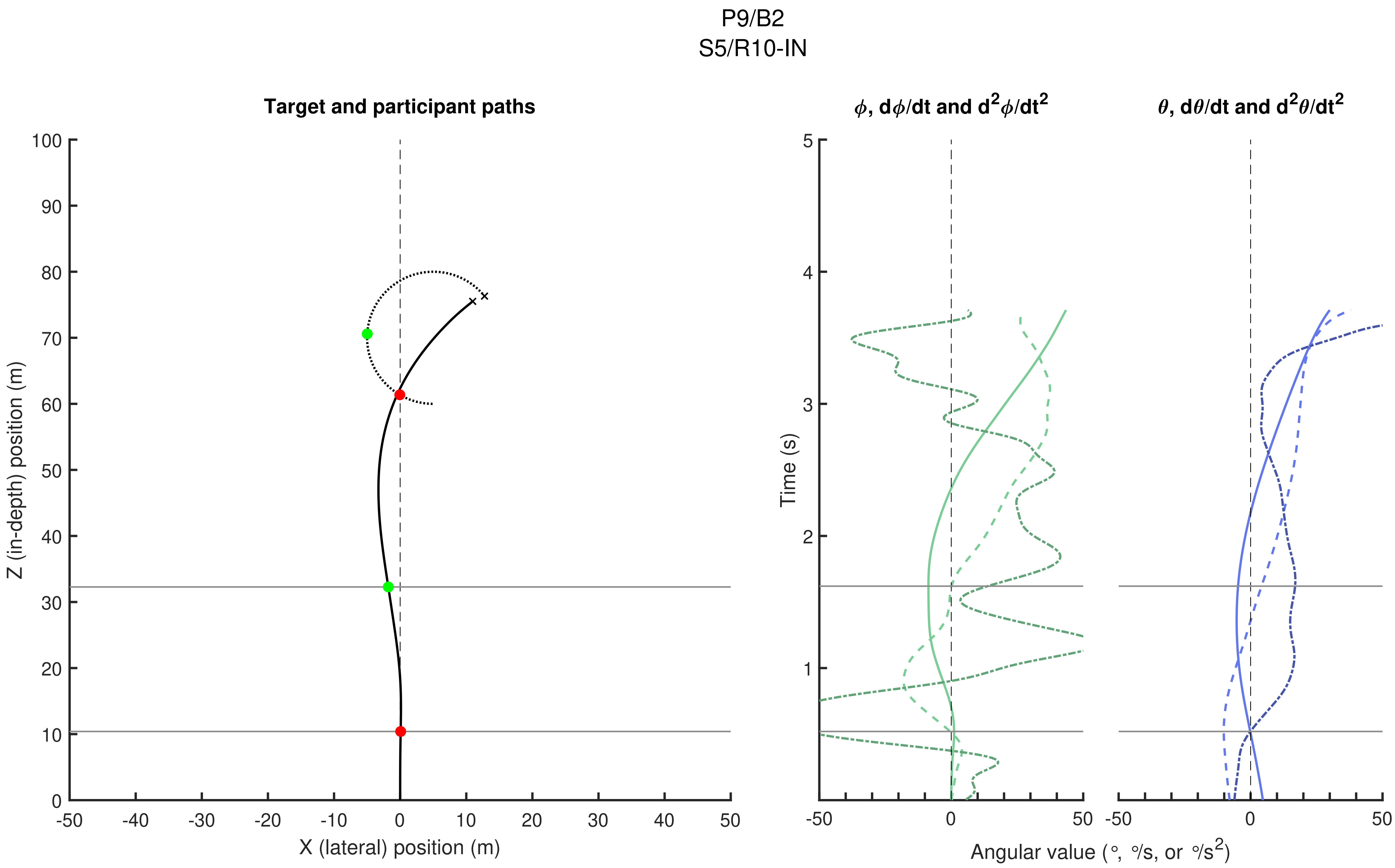
P9/B2
S5/R10-IN
Target and participant paths
Z (in-depth) position (m)
X (lateral) position (m)
100
90
80
70
60
50
40
30
20
10
0
-50
-40
-30
-20
-10
0
10
20
30
40
50
ϕ, dϕ/dt and d²ϕ/dt²
θ, dθ/dt and d²θ/dt²
Time (s)
5
4
3
2
1
-50
0
50
-50
0
50
Angular value (°, °/s, or °/s²)

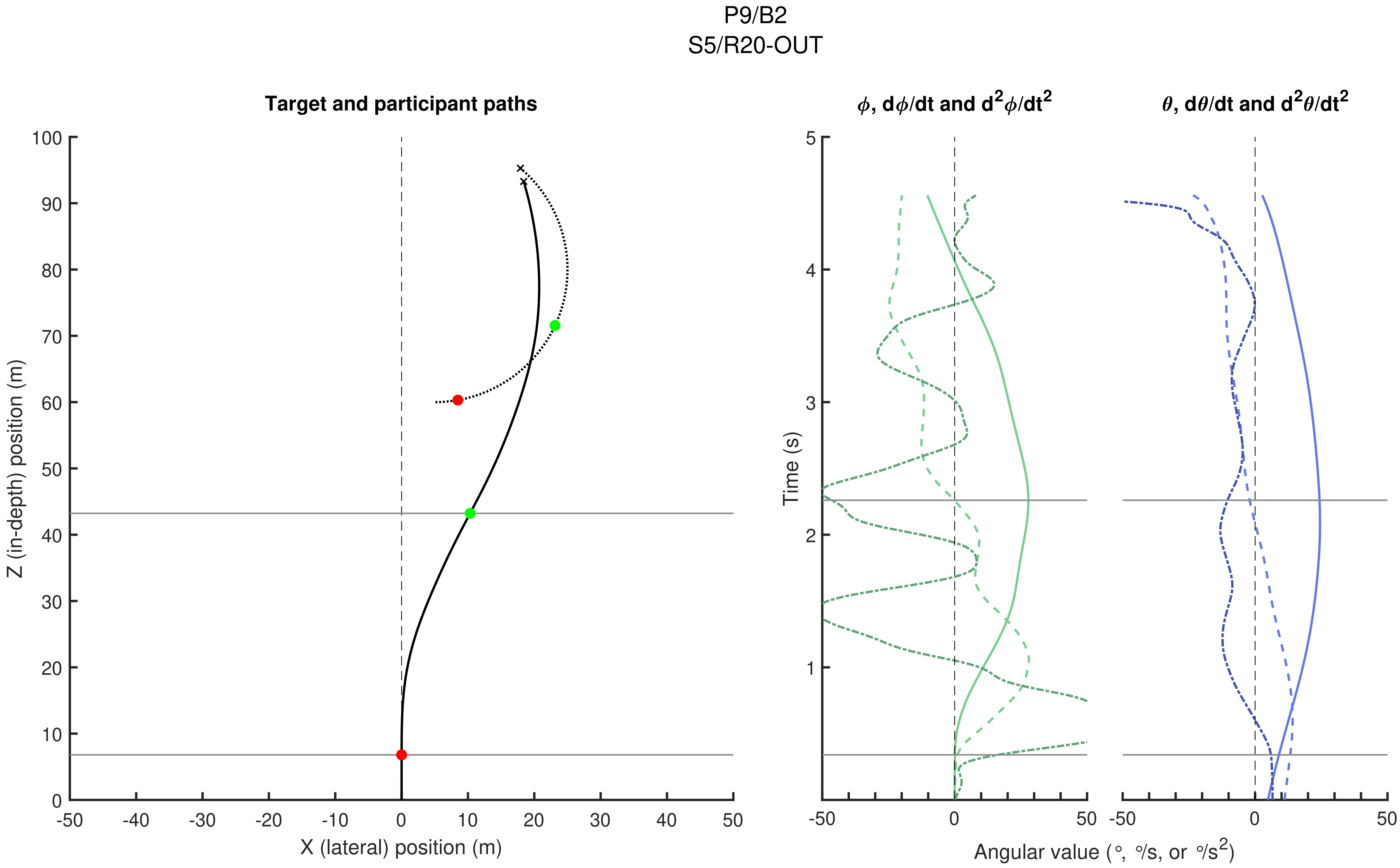
P9/B2
S5/R20-OUT
Target and participant paths
Z (in-depth) position (m)
X (lateral) position (m)
ϕ, dϕ/dt and d$^2\phi$/dt^2
θ, dθ/dt and d$^2\theta$/dt^2
Time (s)
Angular value (°, °/s, or °/s^2)

P9/B2
S5/R20-IN

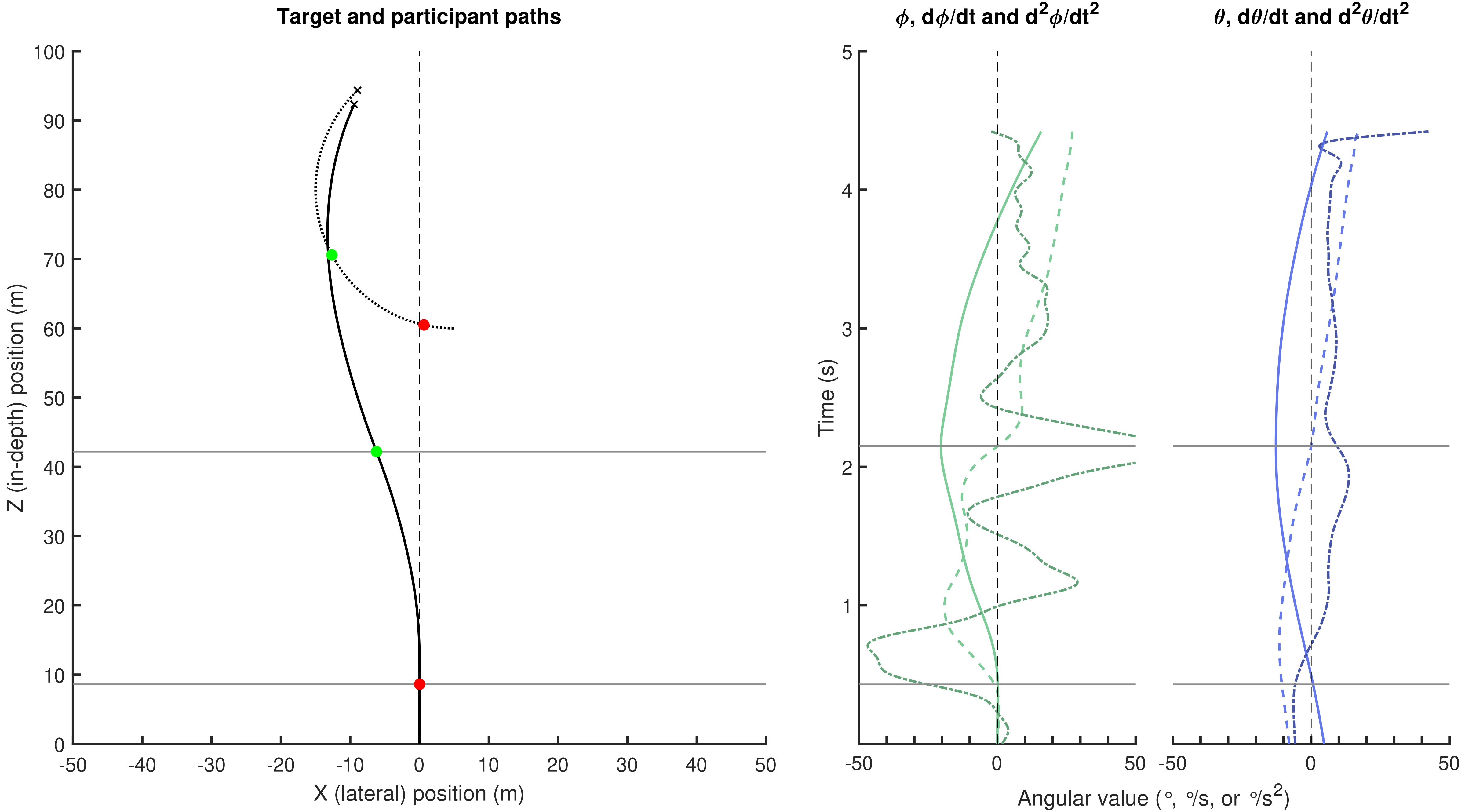

P9/B2
S5/R30-OUT

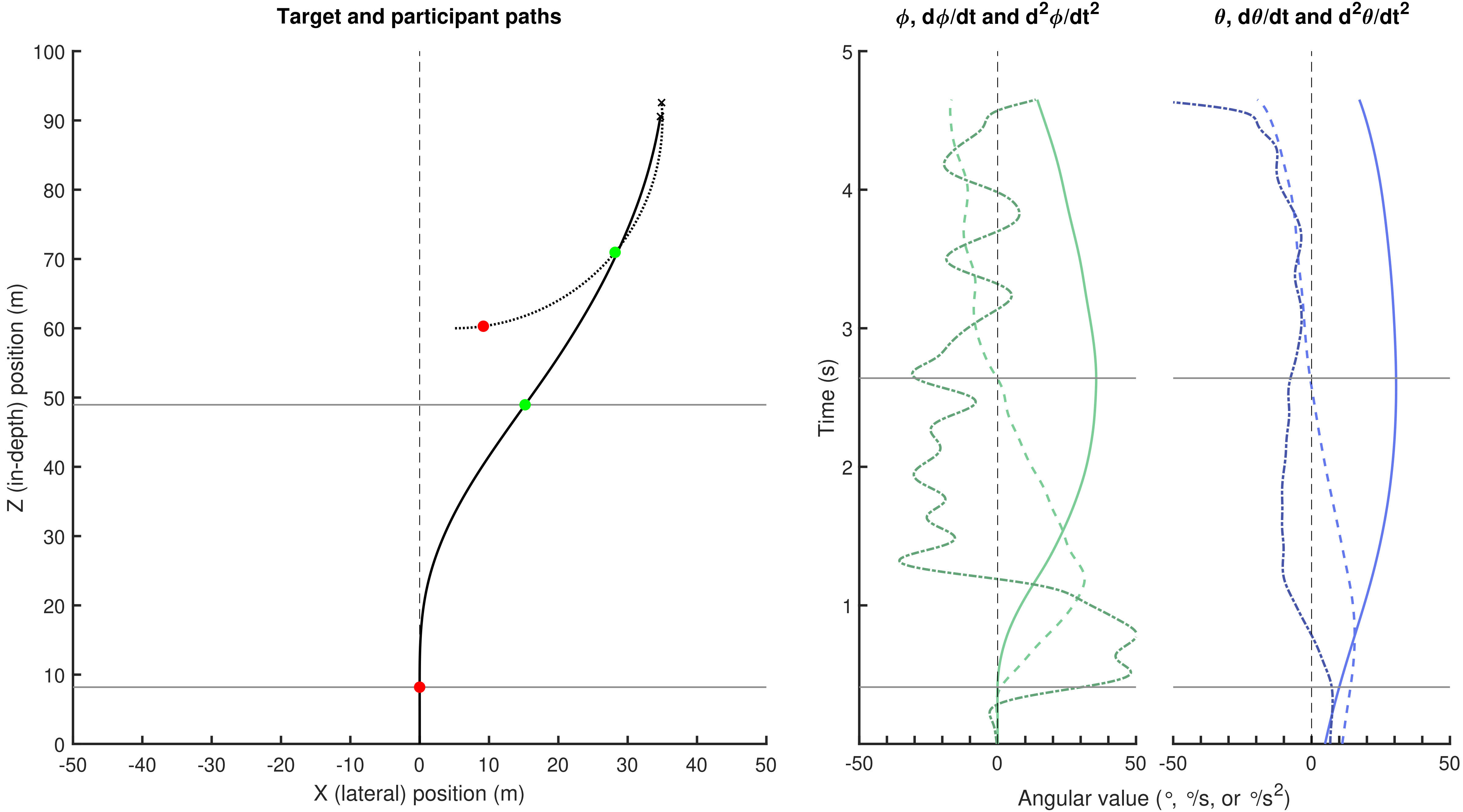

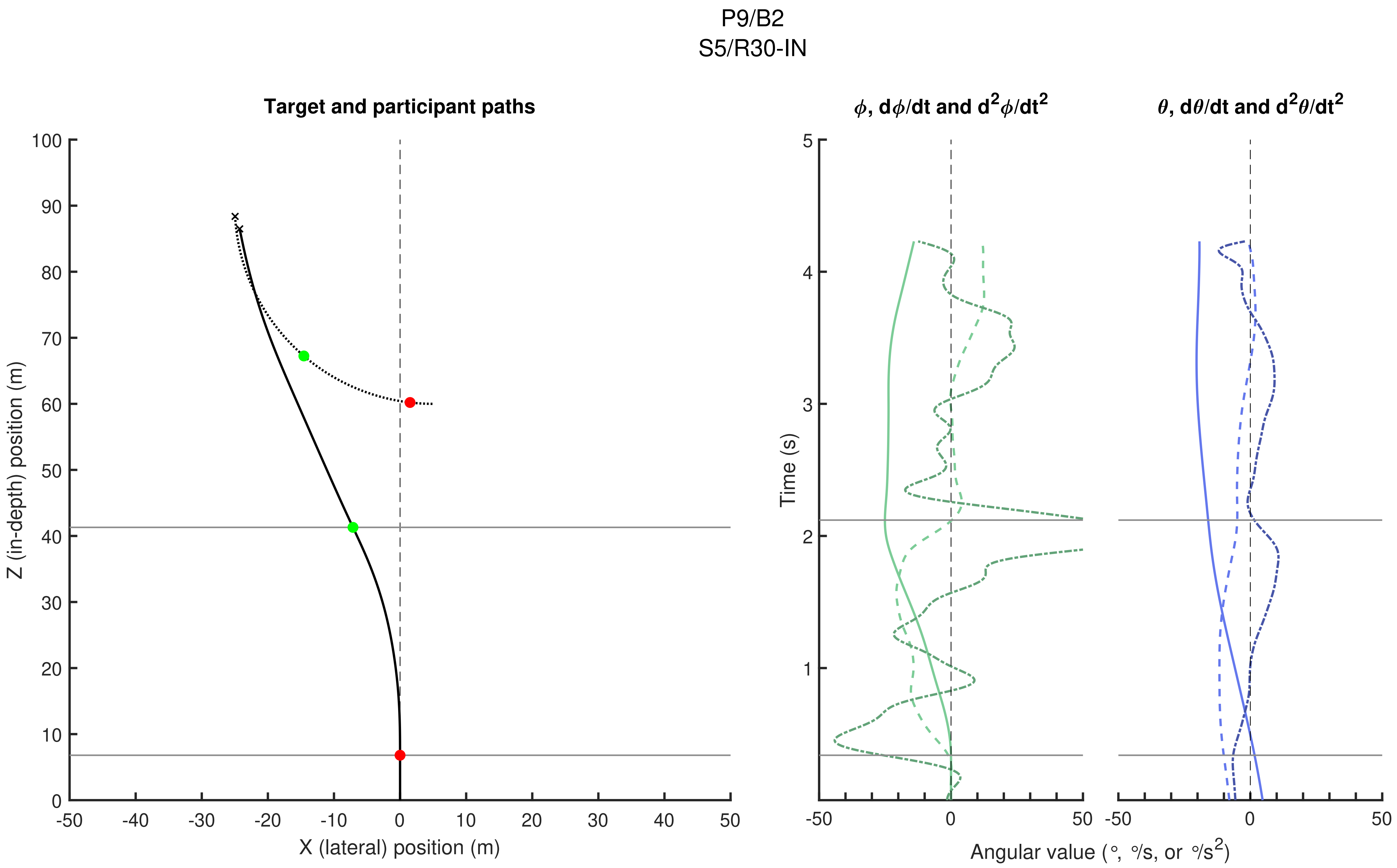
P9/B2
S5/R30-IN
Target and participant paths
Z (in-depth) position (m)
X (lateral) position (m)
100
90
80
70
60
50
40
30
20
10
0
-50
-40
-30
-20
-10
0
10
20
30
40
50
φ, dφ/dt and d²φ/dt²
θ, dθ/dt and d²θ/dt²
Time (s)
5
4
3
2
1
-50
0
50
-50
0
50
Angular value (°, °/s, or °/s²)

P9/B2
S15/R10-IN

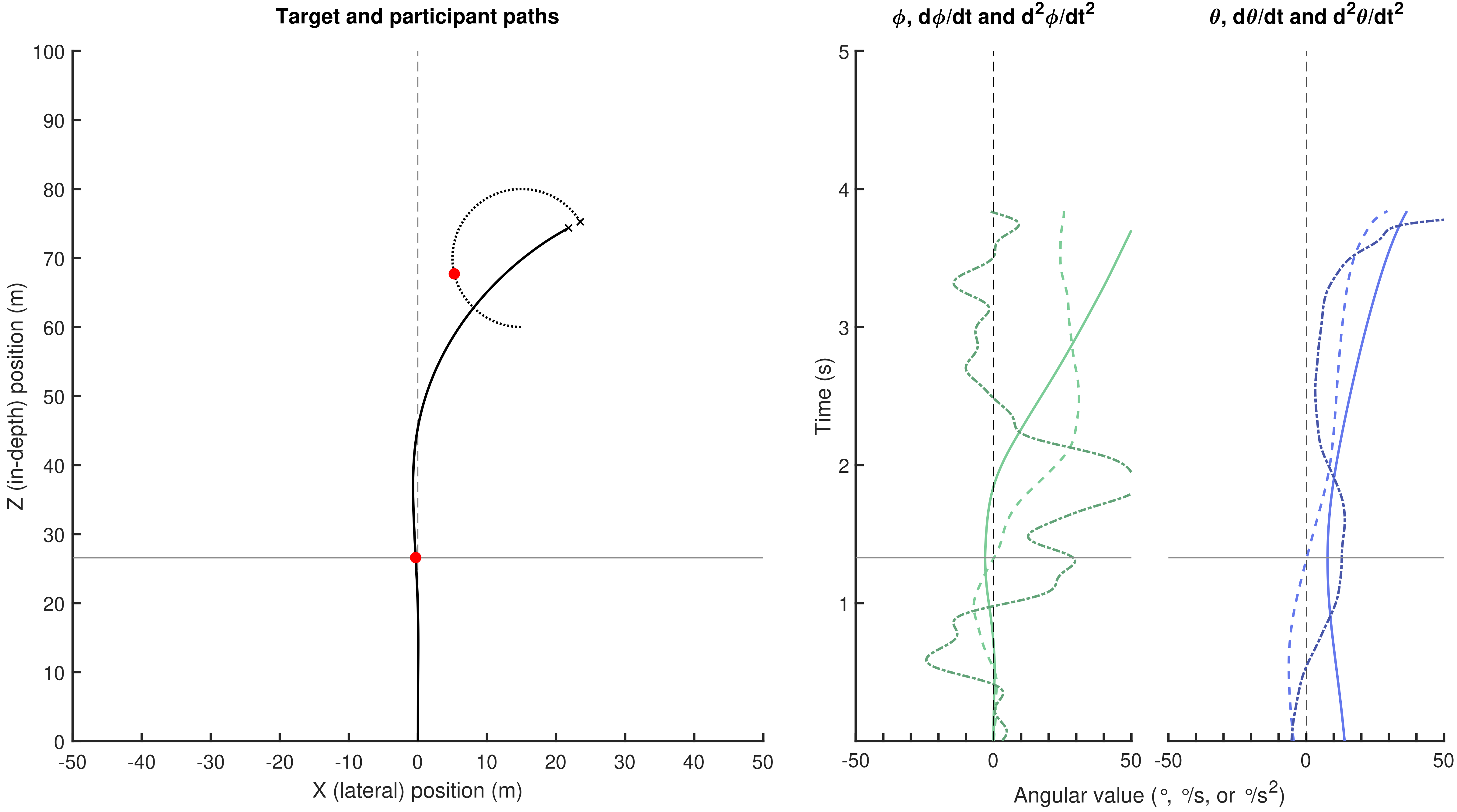

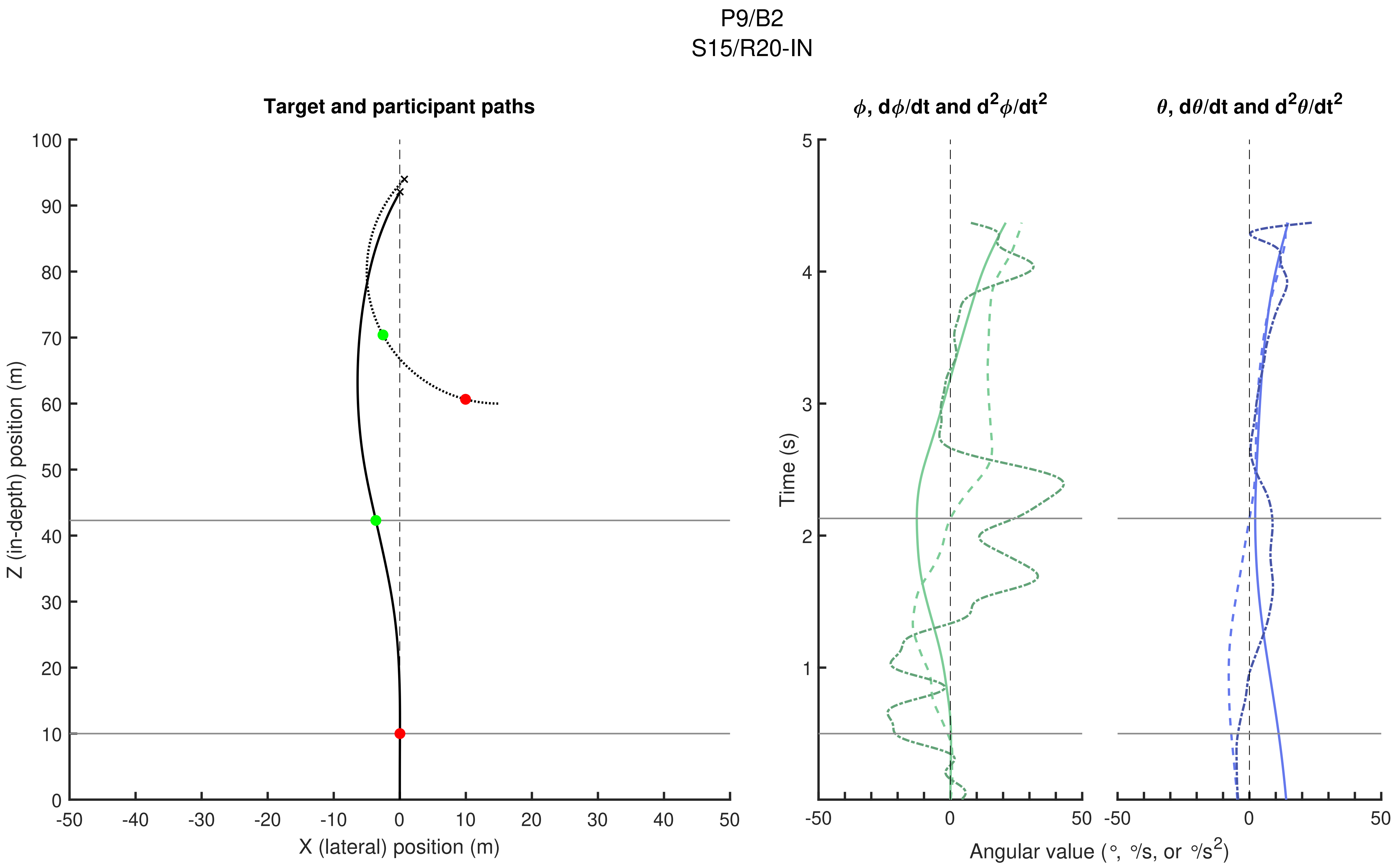

P9/B2
S15/R20-IN
Target and participant paths
Z (in-depth) position (m)
X (lateral) position (m)
ϕ, dϕ/dt and d$^2\phi$/dt^2
θ, dθ/dt and d$^2\theta$/dt^2
Time (s)
Angular value (°, °/s, or °/s^2)

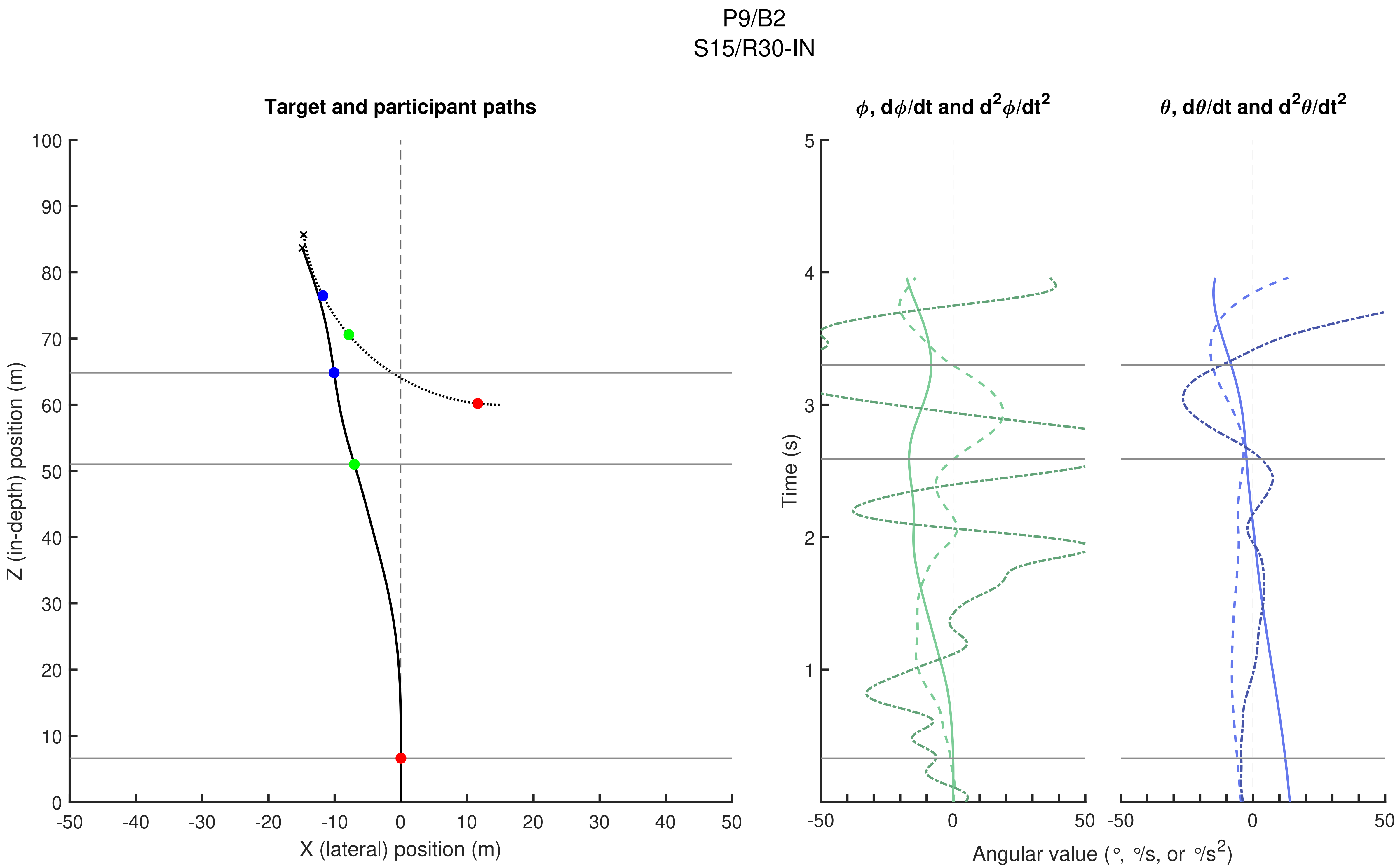

P9/B2
S15/R30-IN
Target and participant paths
Z (in-depth) position (m)
X (lateral) position (m)
0
10
20
30
40
50
60
70
80
90
100
-50
-40
-30
-20
-10
0
10
20
30
40
50
ϕ, dϕ/dt and d$^2\phi$/dt^2
θ, dθ/dt and d$^2\theta$/dt^2
Time (s)
1
2
3
4
5
-50
0
50
-50
0
50
Angular value (°, °/s, or °/s^2)

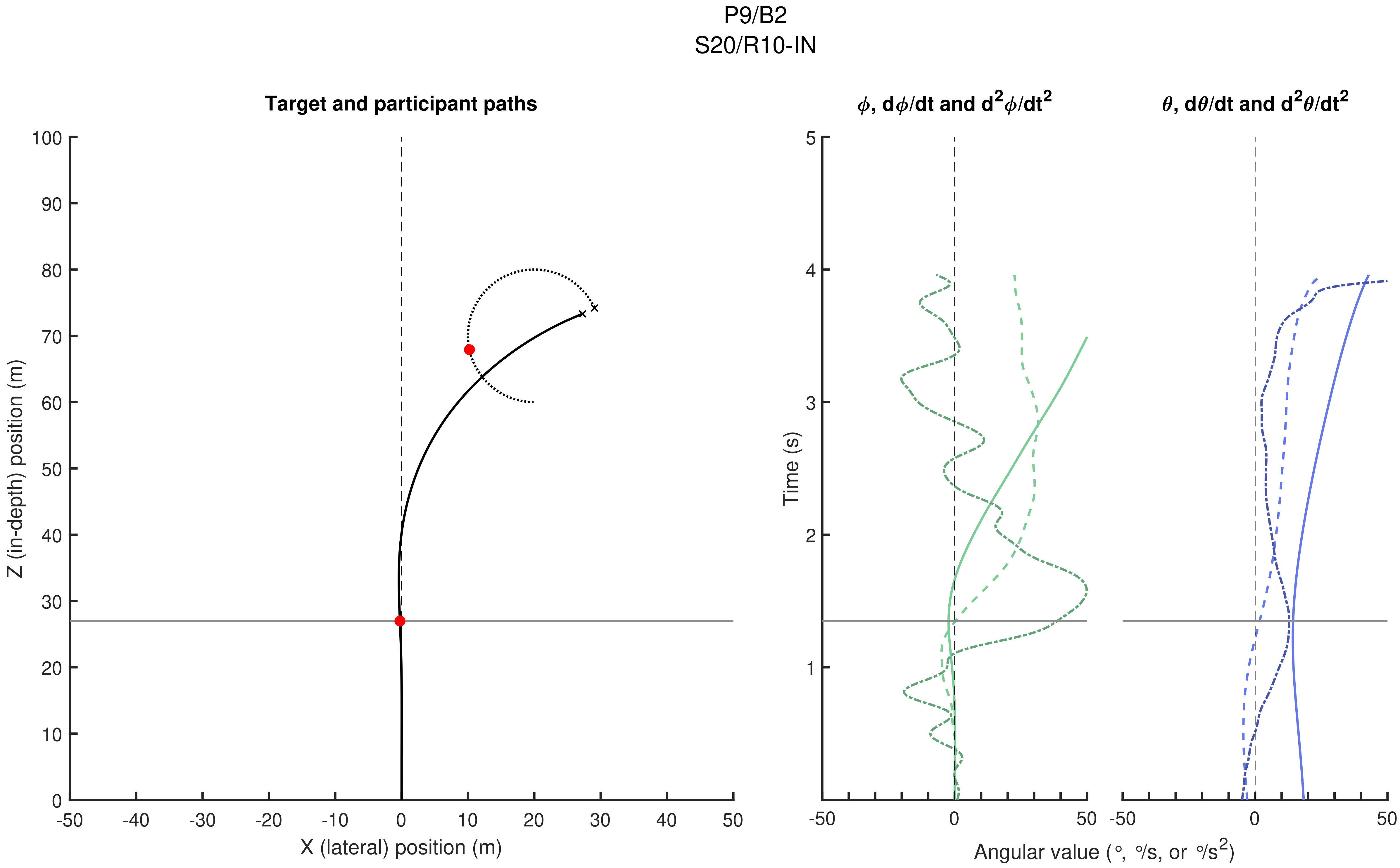
P9/B2
S20/R10-IN
Target and participant paths
Z (in-depth) position (m)
X (lateral) position (m)
100
90
80
70
60
50
40
30
20
10
0
-50
-40
-30
-20
-10
0
10
20
30
40
50
φ, dφ/dt and d²φ/dt²
θ, dθ/dt and d²θ/dt²
Time (s)
5
4
3
2
1
-50
0
50
-50
0
50
Angular value (°, °/s, or °/s²)

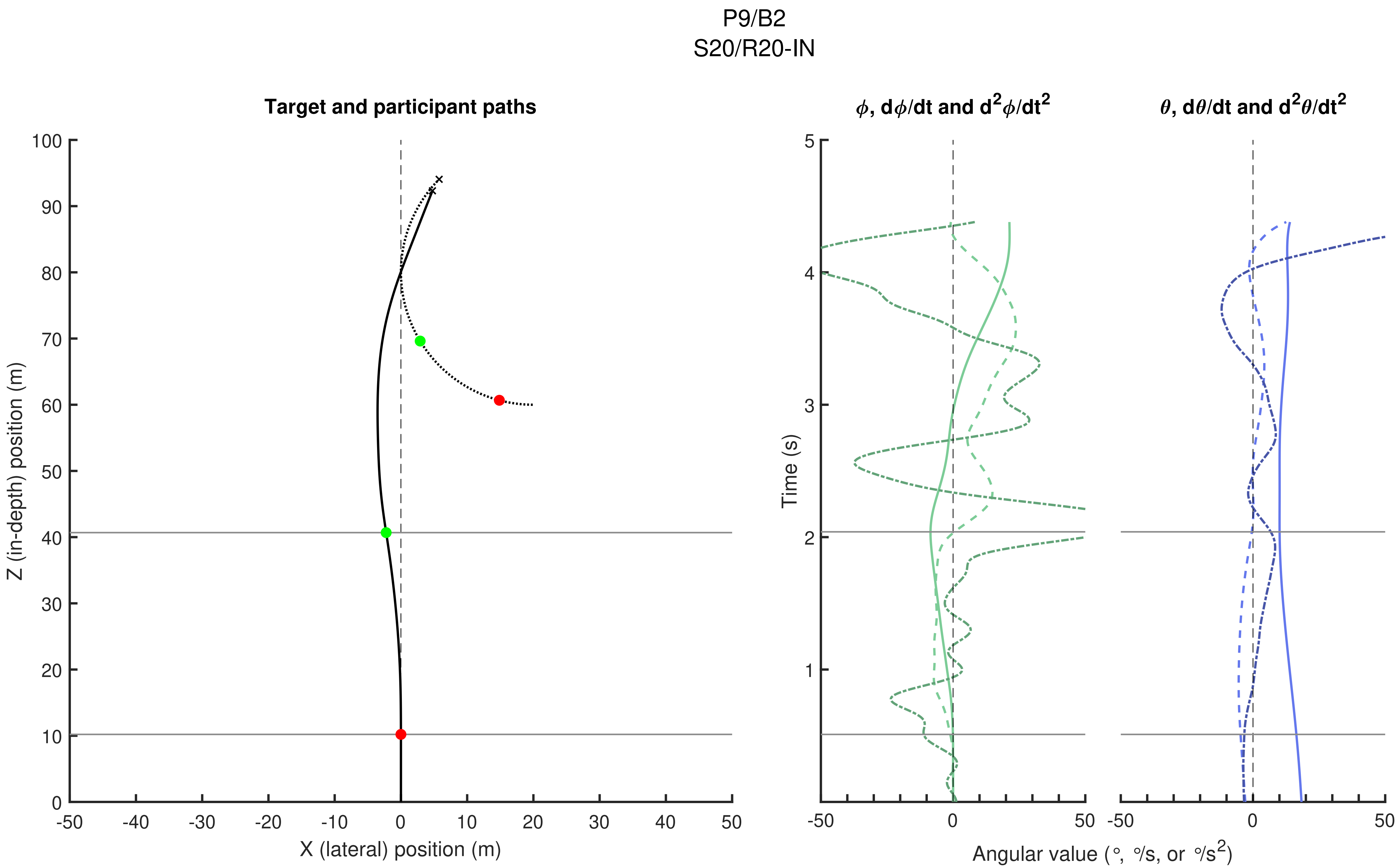
P9/B2
S20/R20-IN
Target and participant paths
ϕ, dϕ/dt and d$^2\phi$/dt^2
θ, dθ/dt and d$^2\theta$/dt^2
Z (in-depth) position (m)
X (lateral) position (m)
Time (s)
Angular value (°, °/s, or °/s^2)
100
90
80
70
60
50
40
30
20
10
0
-50
-40
-30
-20
-10
0
10
20
30
40
50
5
4
3
2
1
-50
0
50
-50
0
50

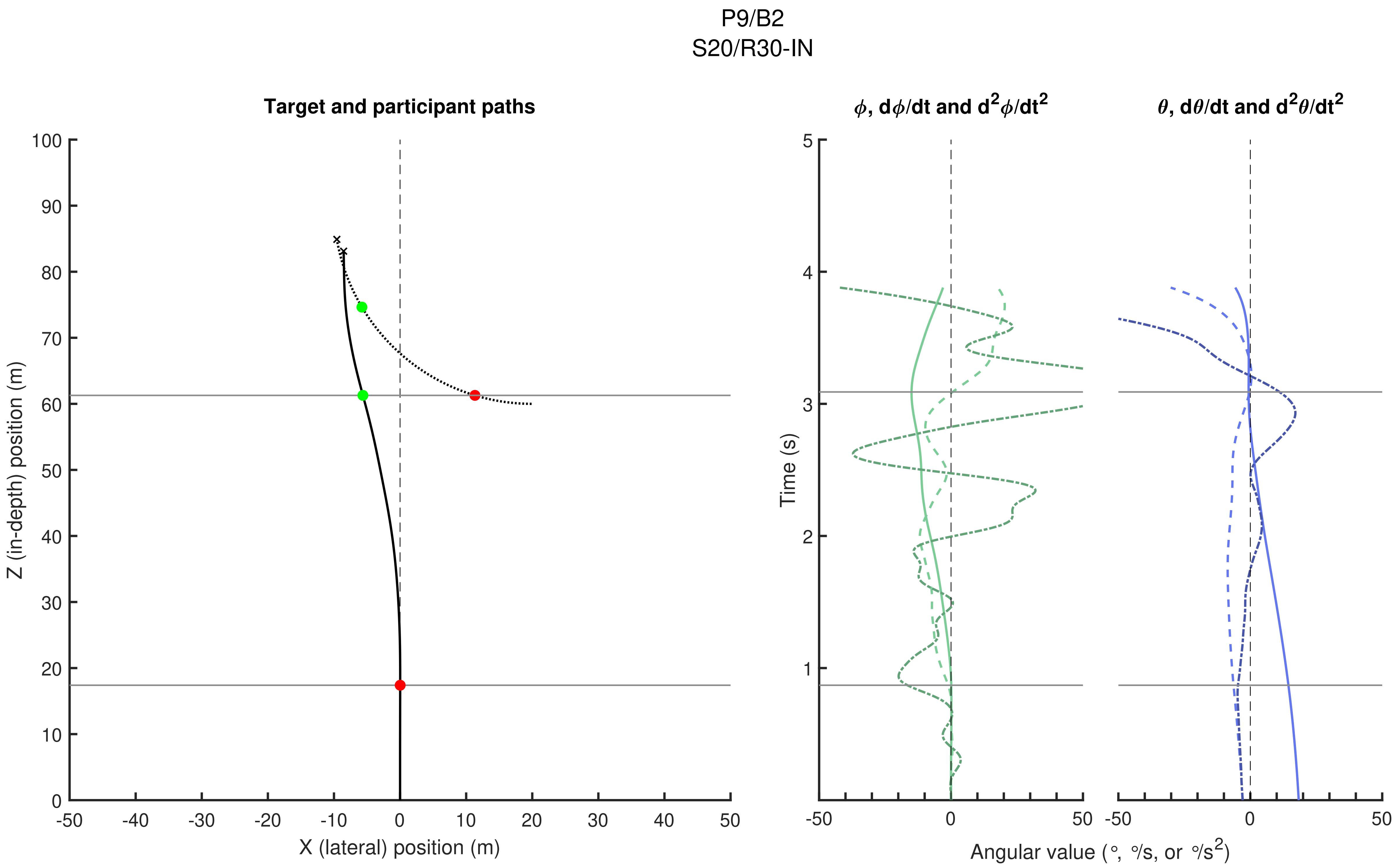
P9/B2
S20/R30-IN
Target and participant paths
Z (in-depth) position (m)
X (lateral) position (m)
ϕ, dϕ/dt and d²ϕ/dt²
θ, dθ/dt and d²θ/dt²
Time (s)
Angular value (°, °/s, or °/s²)

P9/B3
S20/R10-IN

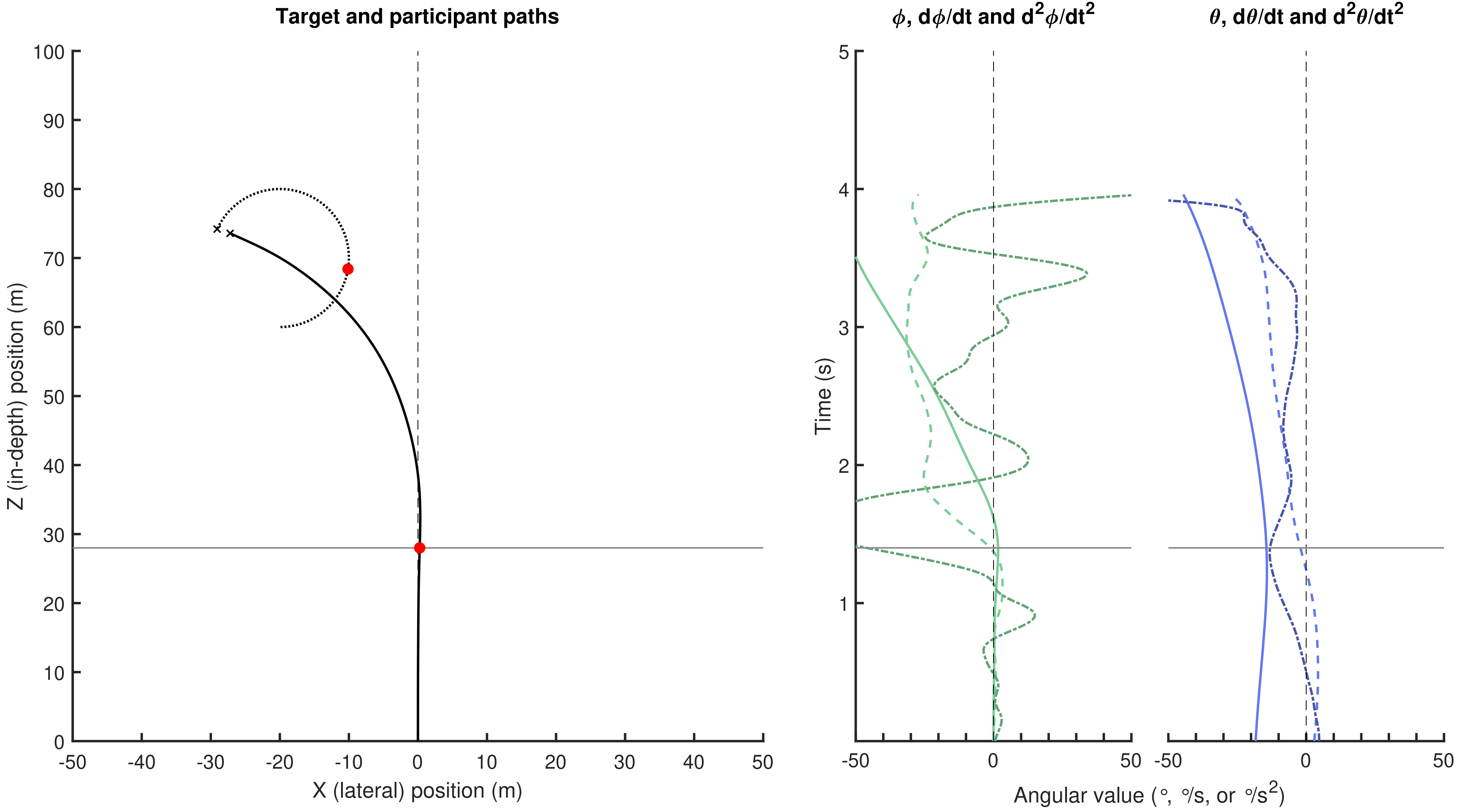

P9/B3
S20/R20-IN

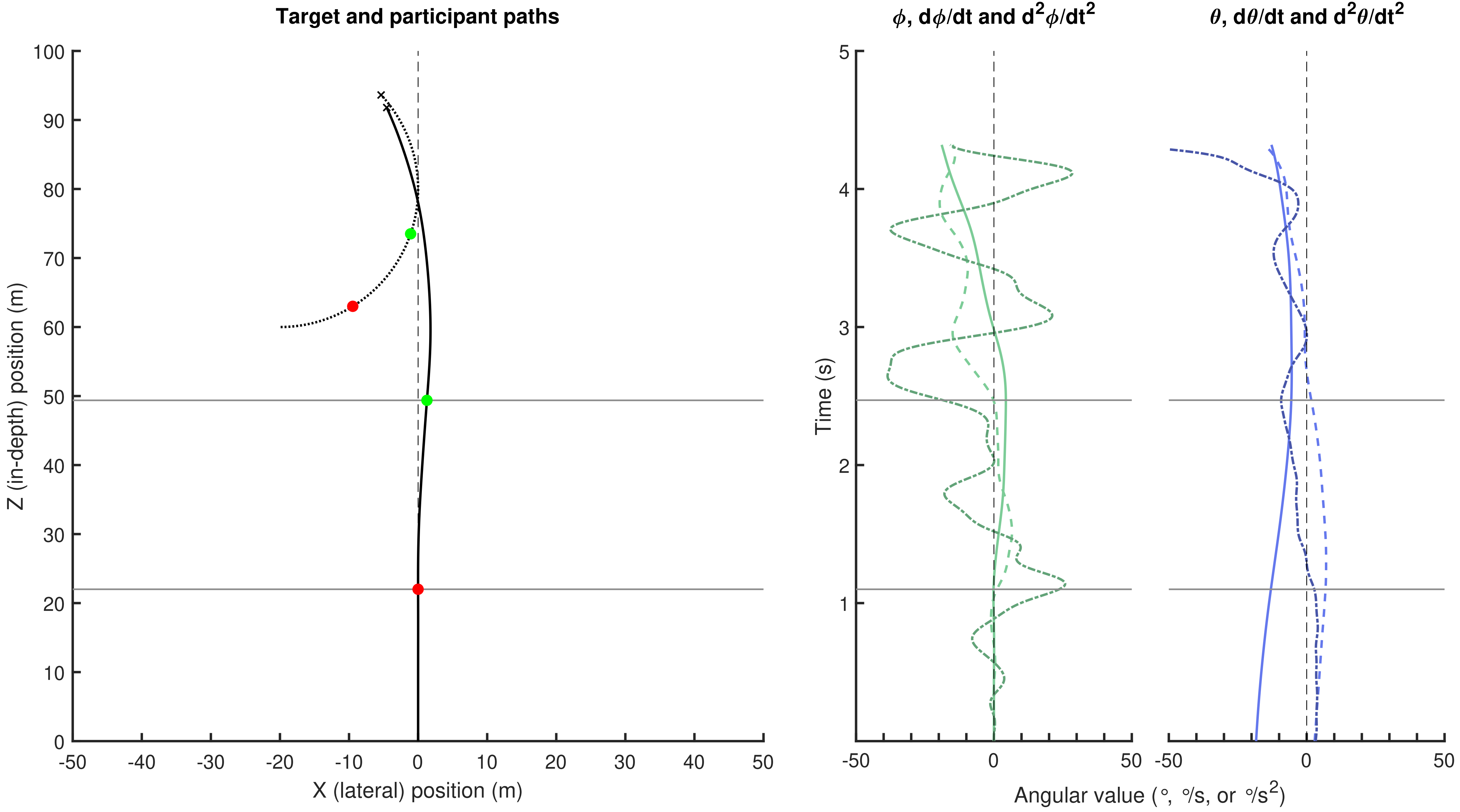

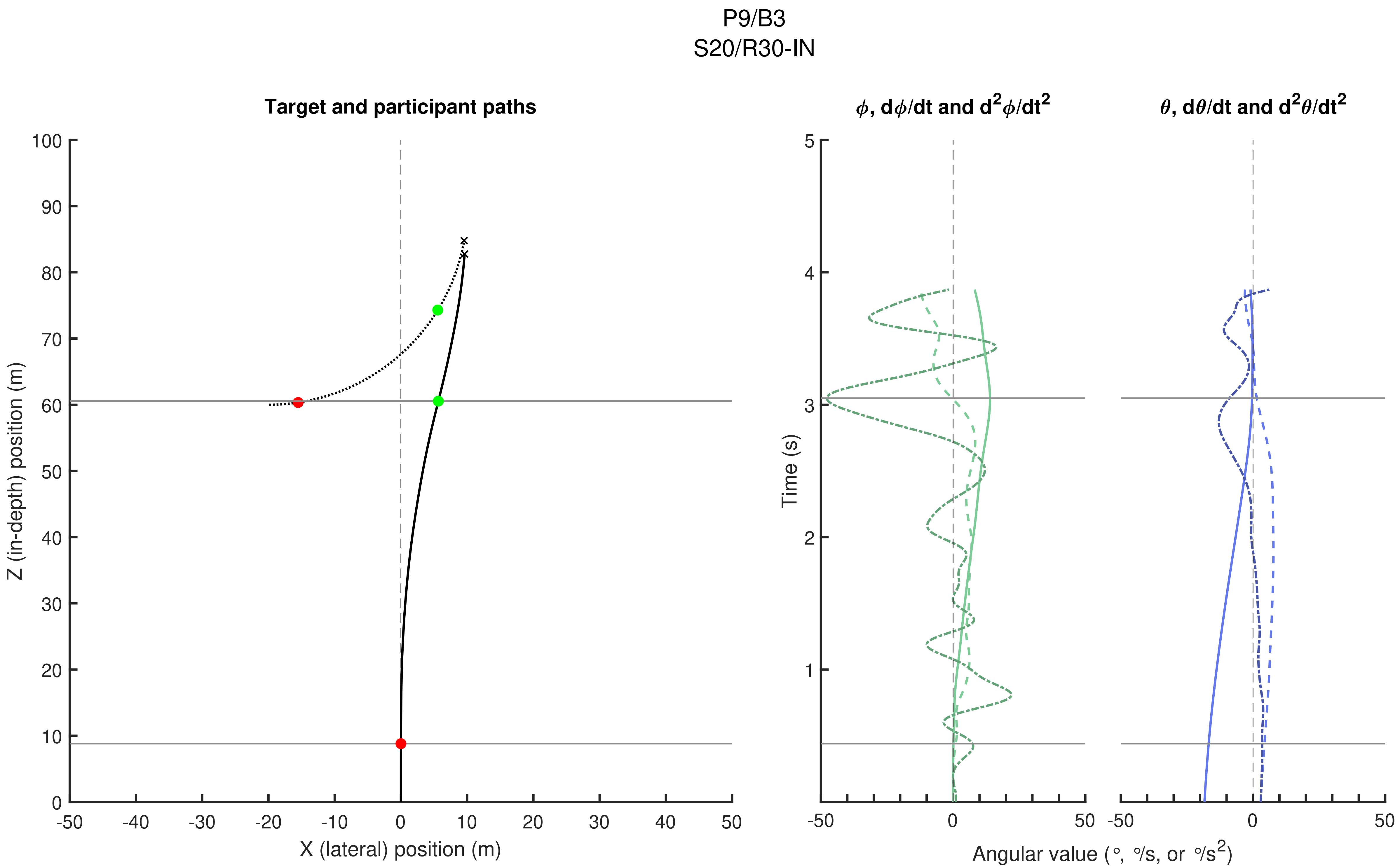

P9/B3
S20/R30-IN
Target and participant paths
Z (in-depth) position (m)
X (lateral) position (m)
ϕ, dϕ/dt and d$^2\phi$/dt^2
θ, dθ/dt and d$^2\theta$/dt^2
Time (s)
Angular value (°, °/s, or °/s^2)

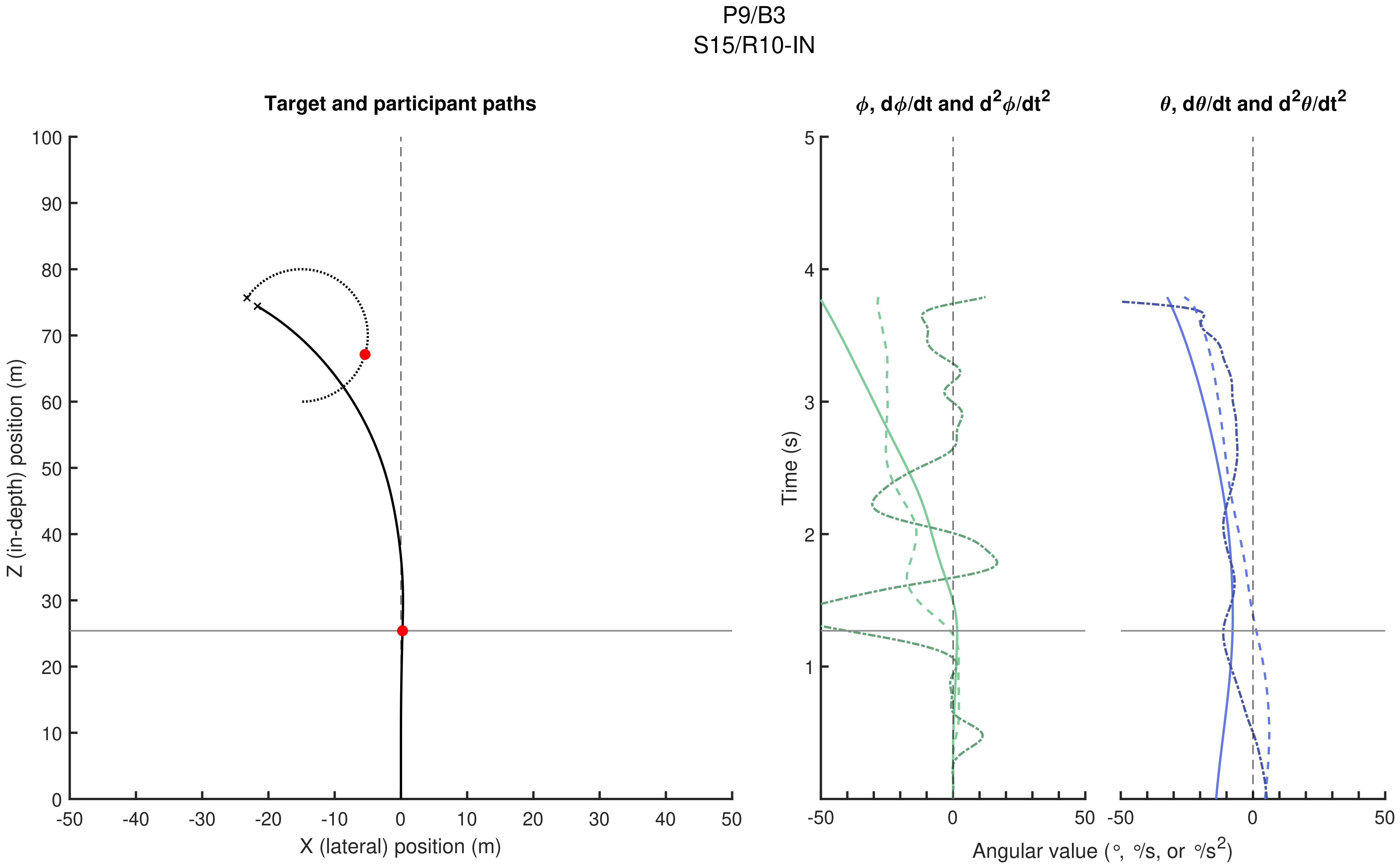
P9/B3
S15/R10-IN
Target and participant paths
Z (in-depth) position (m)
X (lateral) position (m)
100
90
80
70
60
50
40
30
20
10
0
-50
-40
-30
-20
-10
0
10
20
30
40
50
ϕ, dϕ/dt and d$^2\phi$/dt^2
θ, dθ/dt and d$^2\theta$/dt^2
Time (s)
5
4
3
2
1
-50
0
50
-50
0
50
Angular value (°, °/s, or °/s^2)

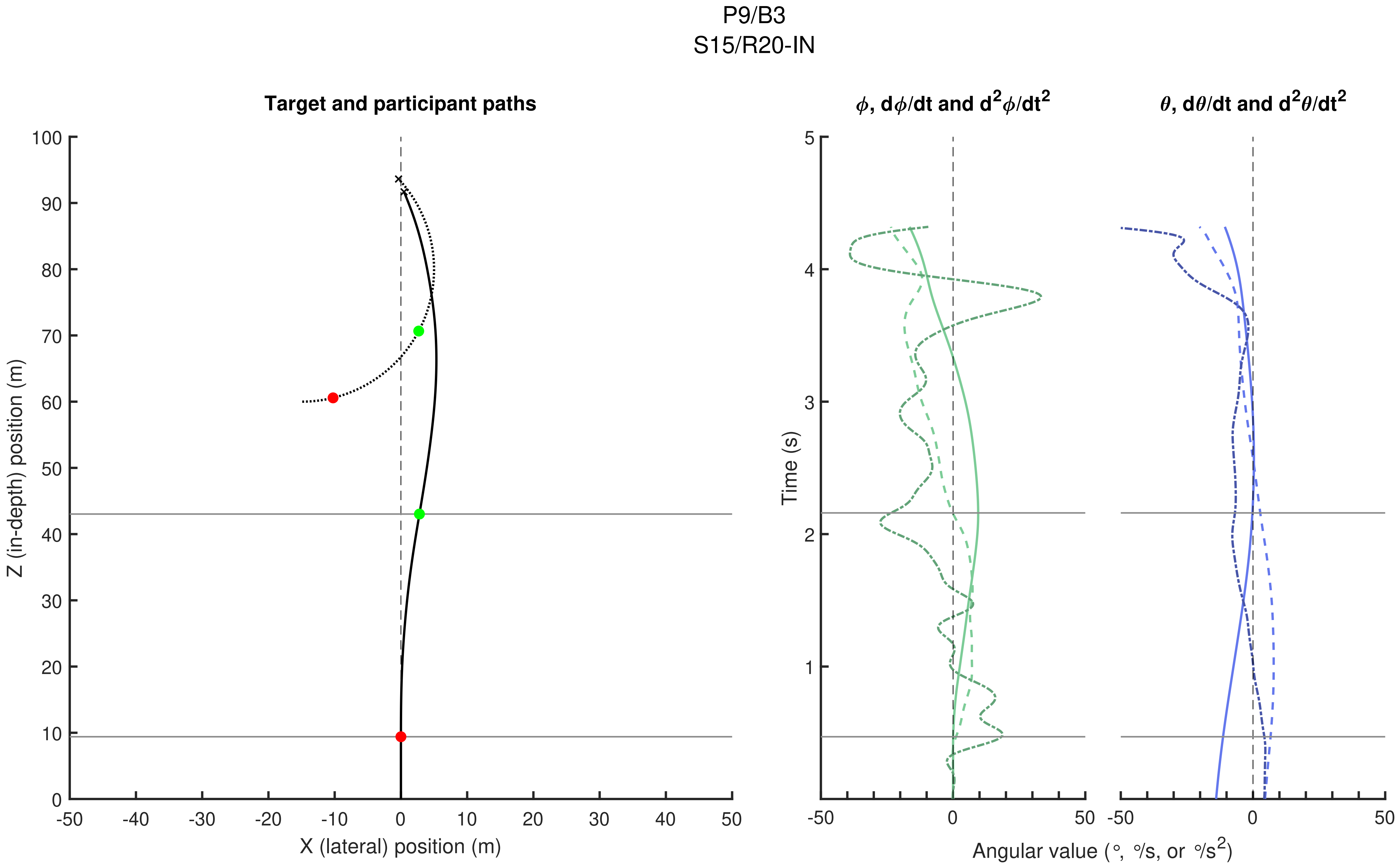
P9/B3
S15/R20-IN
Target and participant paths
Z (in-depth) position (m)
X (lateral) position (m)
φ, dφ/dt and d²φ/dt²
θ, dθ/dt and d²θ/dt²
Time (s)
Angular value (°, °/s, or °/s²)

P9/B3
S15/R30-IN

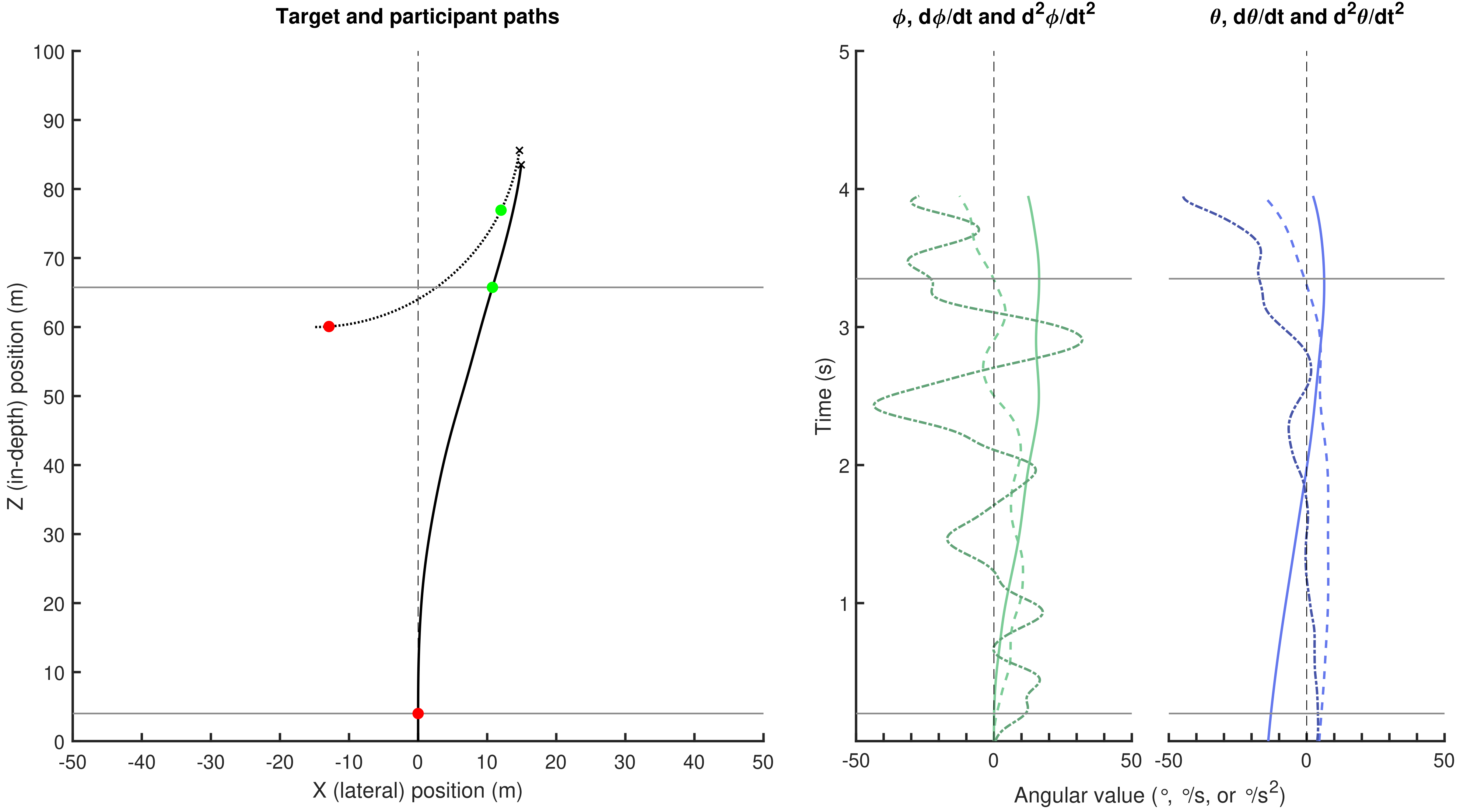

P9/B3
S5/R10-IN

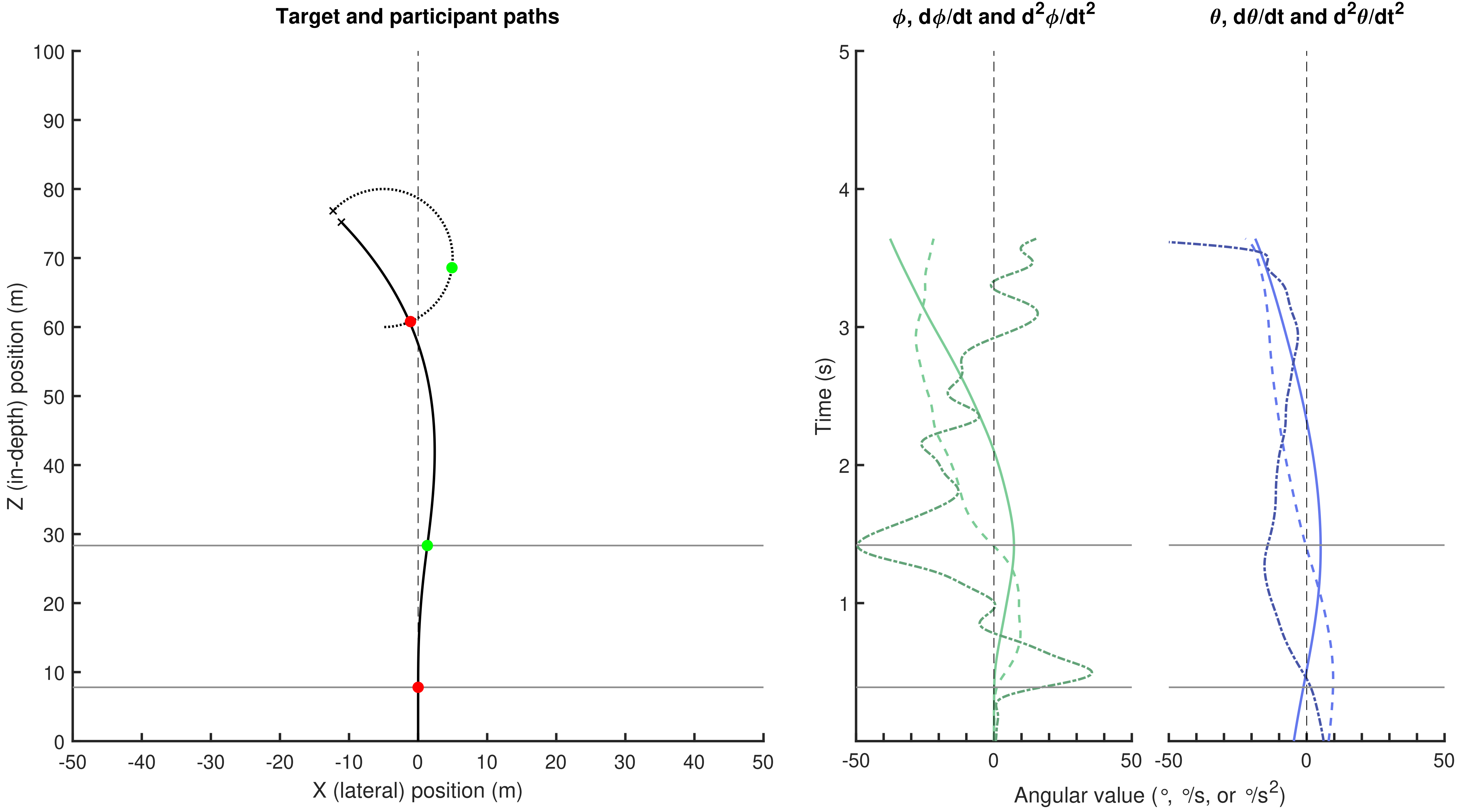

P9/B3
S5/R10-OUT

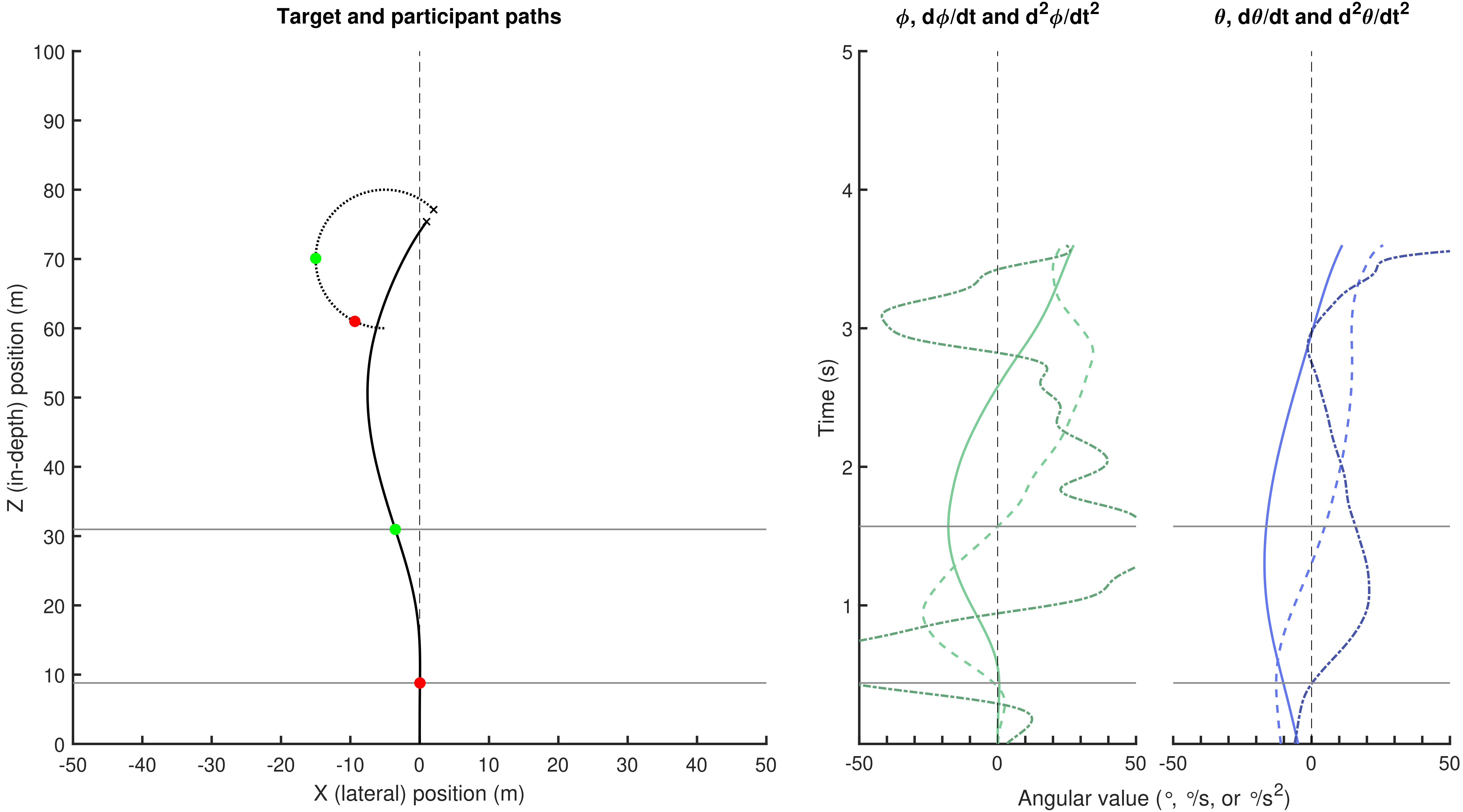

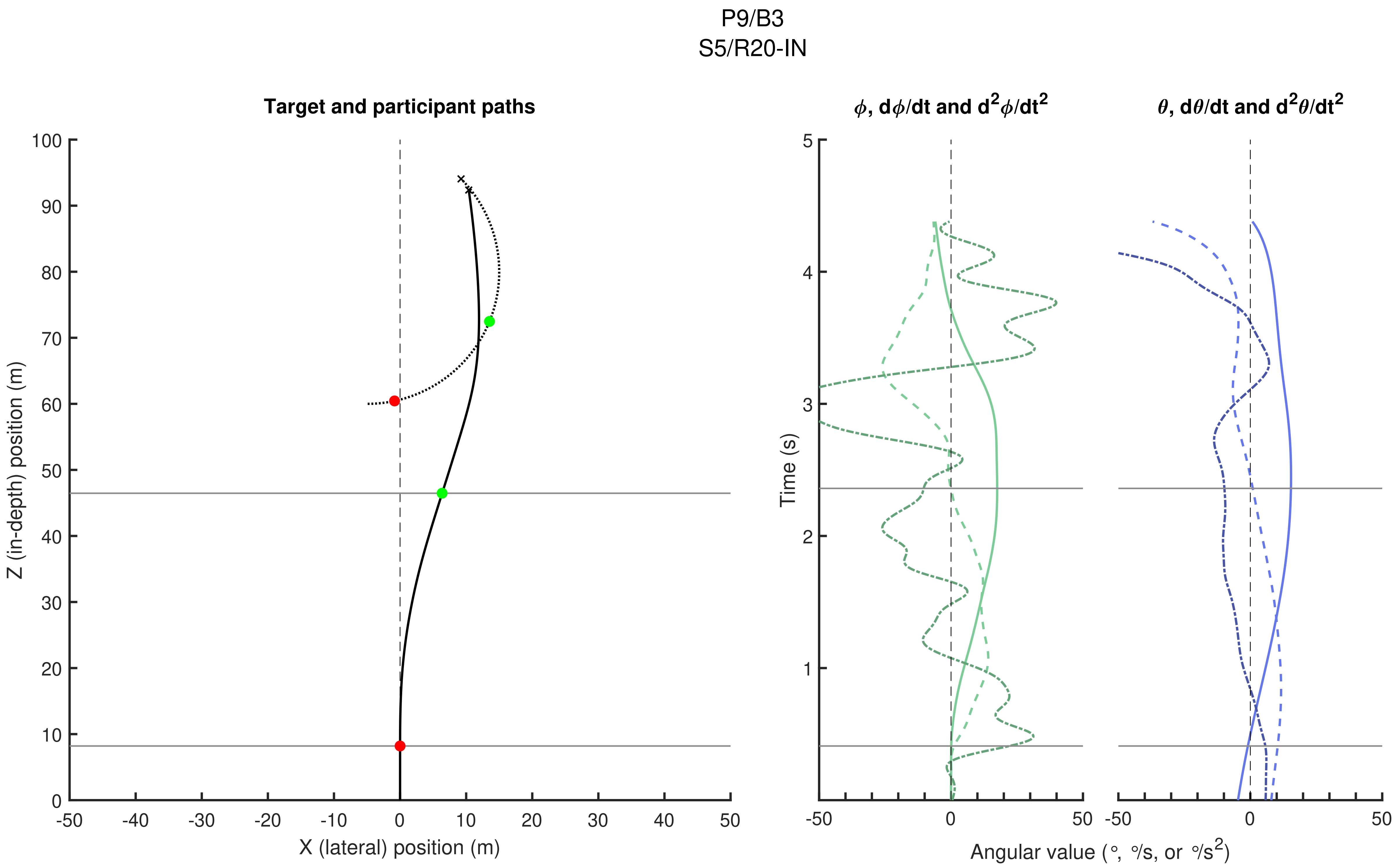
P9/B3
S5/R20-IN
Target and participant paths
ϕ, dϕ/dt and d²ϕ/dt²
θ, dθ/dt and d²θ/dt²
Z (in-depth) position (m)
X (lateral) position (m)
Time (s)
Angular value (°, °/s, or °/s²)

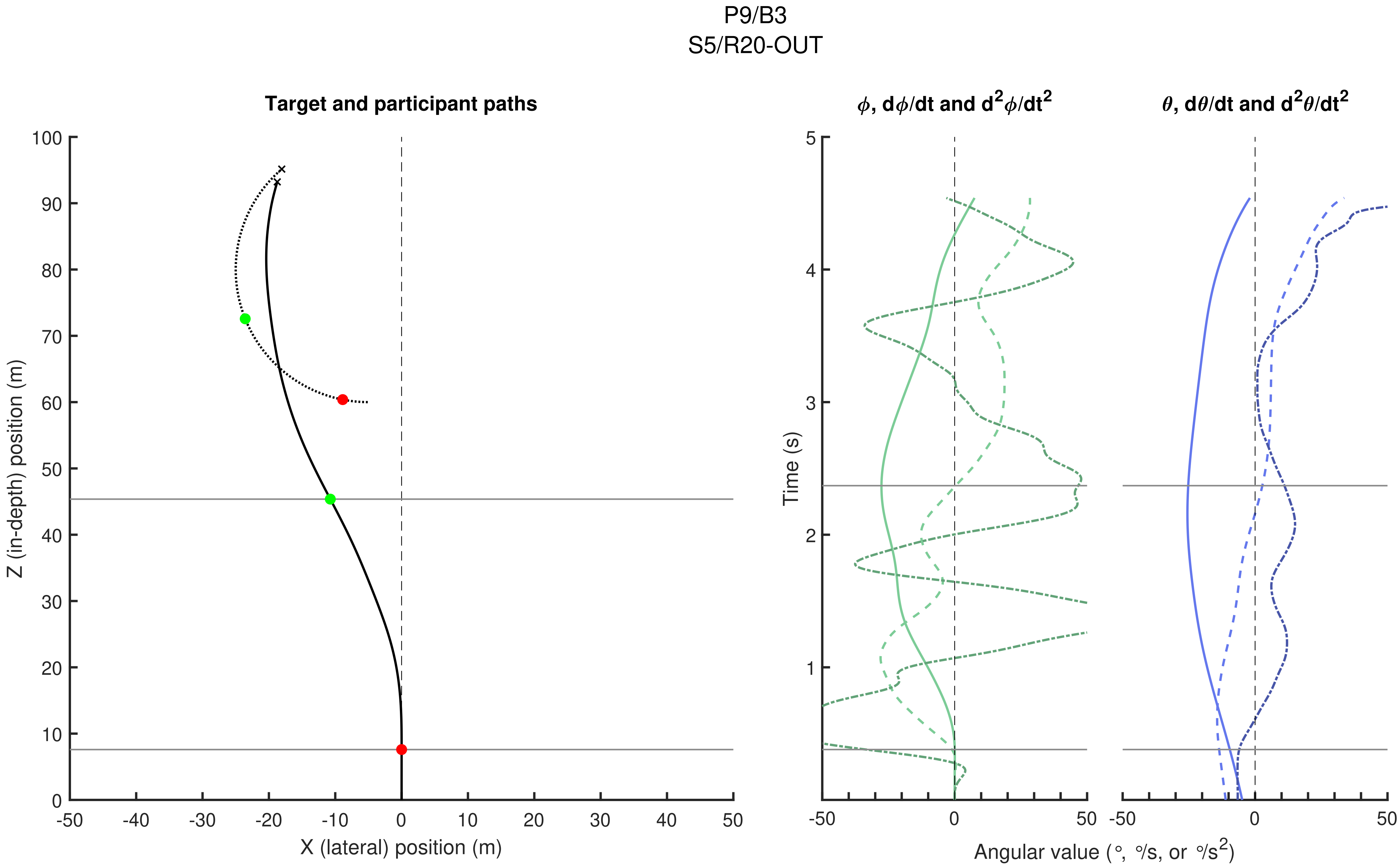
P9/B3
S5/R20-OUT
Target and participant paths
Z (in-depth) position (m)
X (lateral) position (m)
ϕ, dϕ/dt and d$^2\phi$/dt^2
θ, dθ/dt and d$^2\theta$/dt^2
Time (s)
Angular value (°, °/s, or °/s^2)
100
90
80
70
60
50
40
30
20
10
0
-50
-40
-30
-20
-10
0
10
20
30
40
50
5
4
3
2
1
-50
0
50
-50
0
50

P9/B3
S5/R30-IN

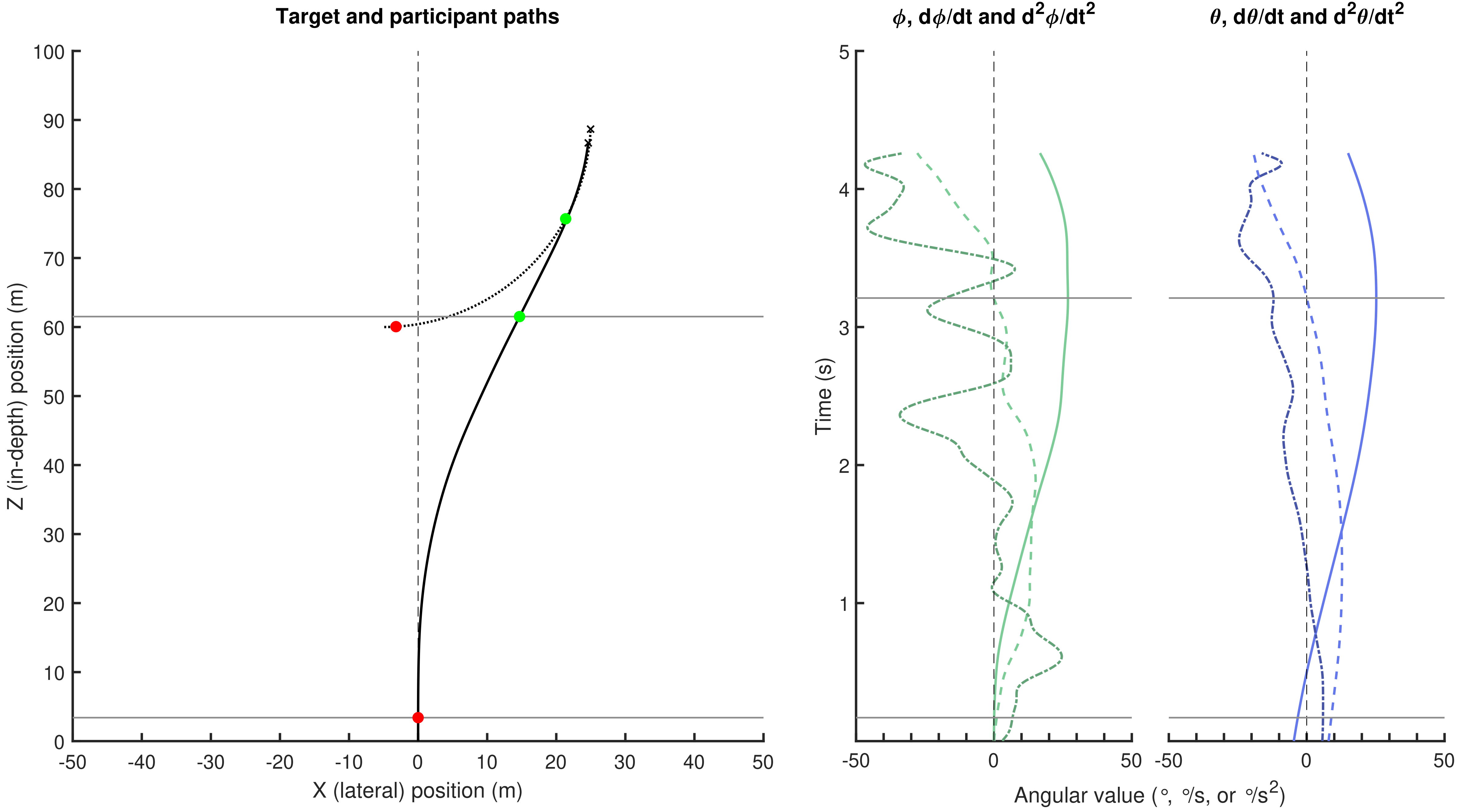

P9/B3
S5/R30-OUT

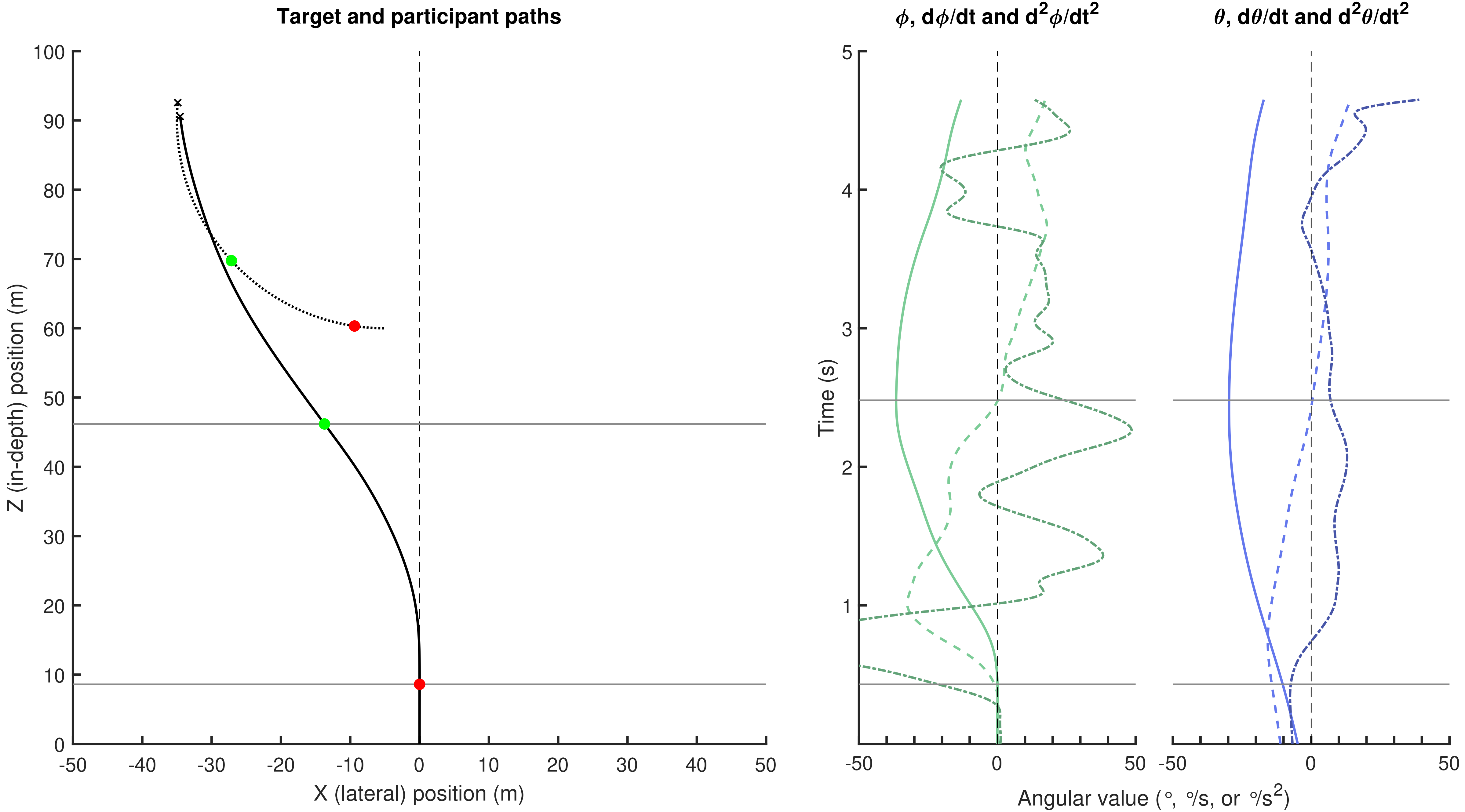

P9/B3
S5/R10-OUT

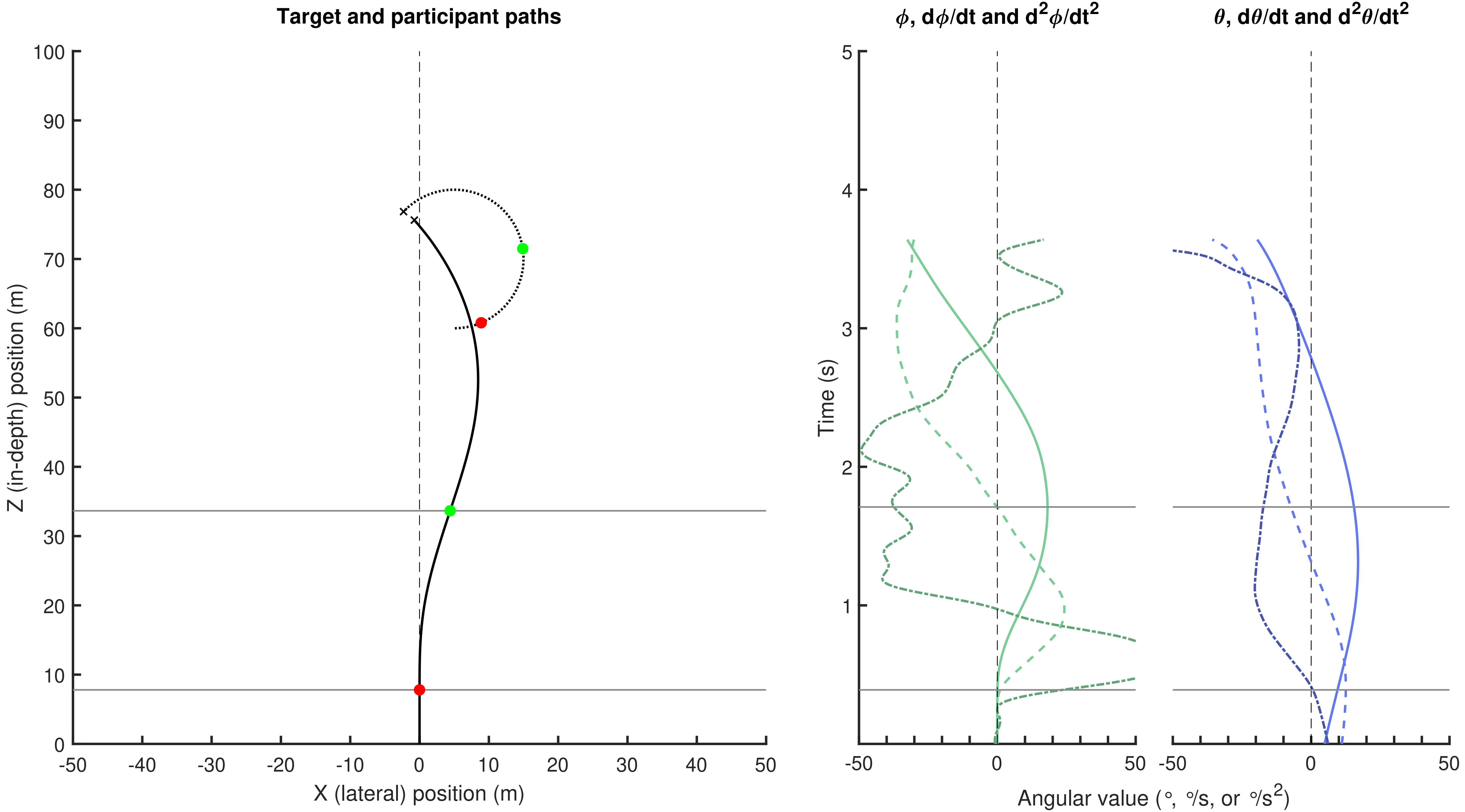

P9/B3
S5/R10-IN

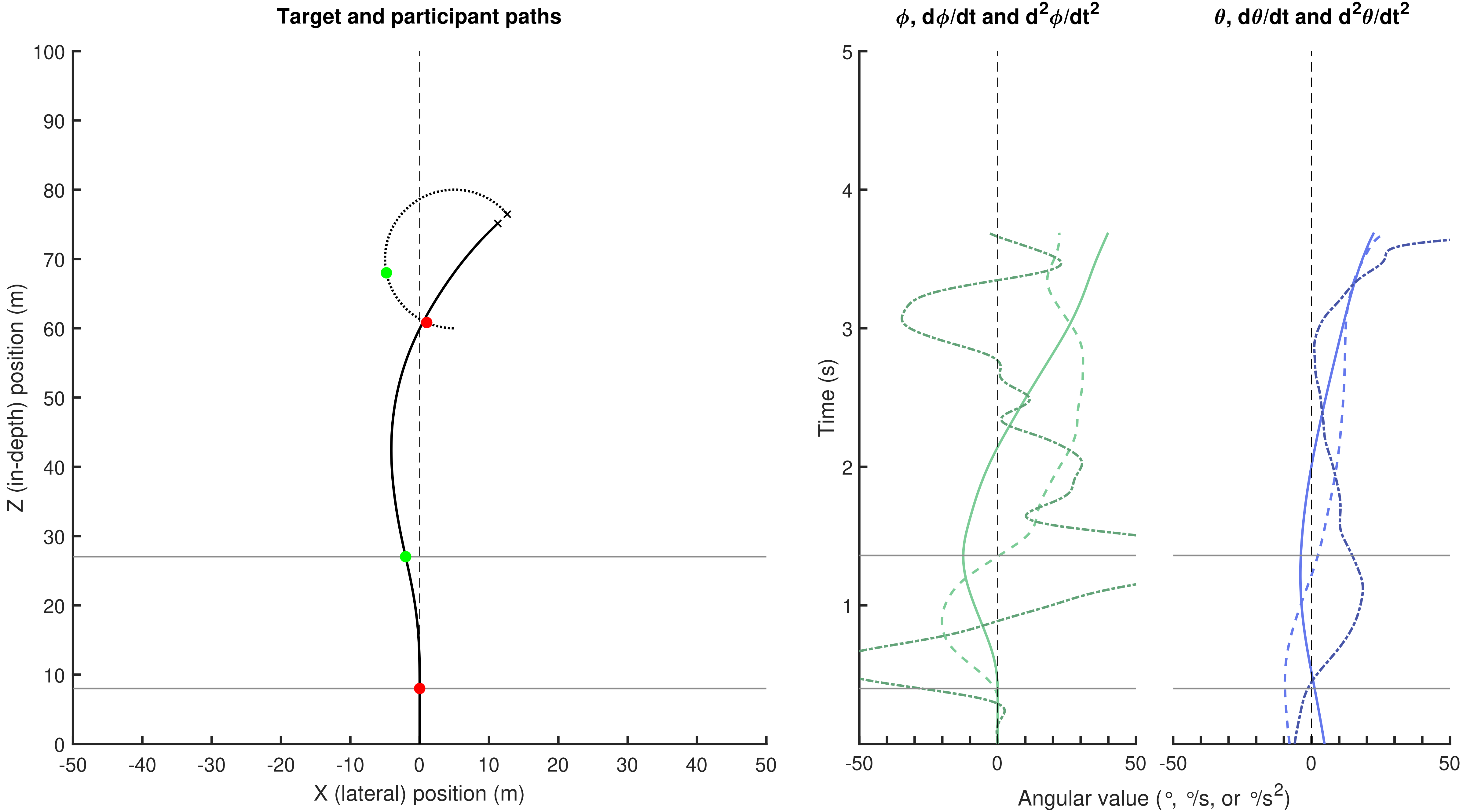

P9/B3
S5/R20-OUT

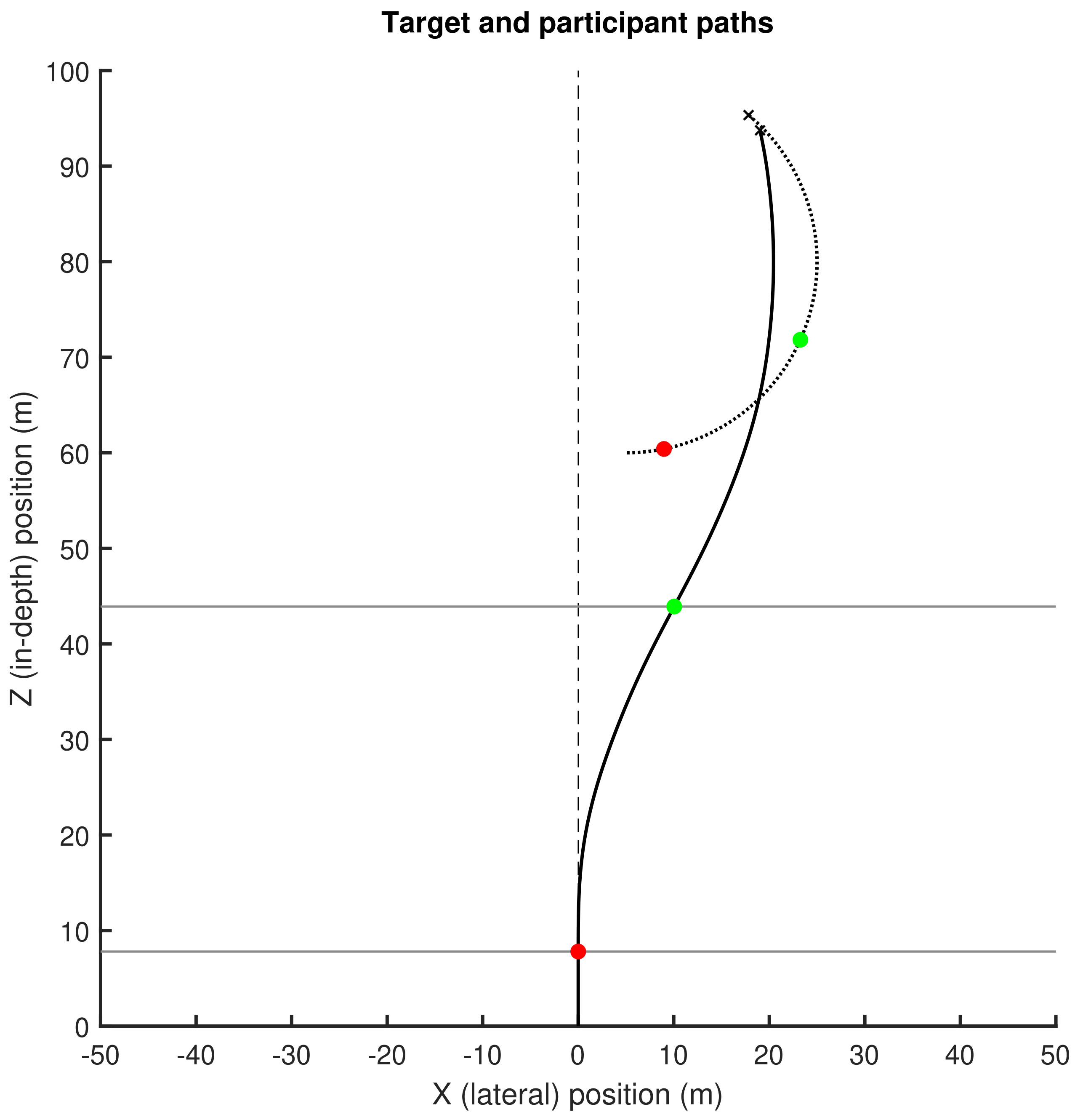

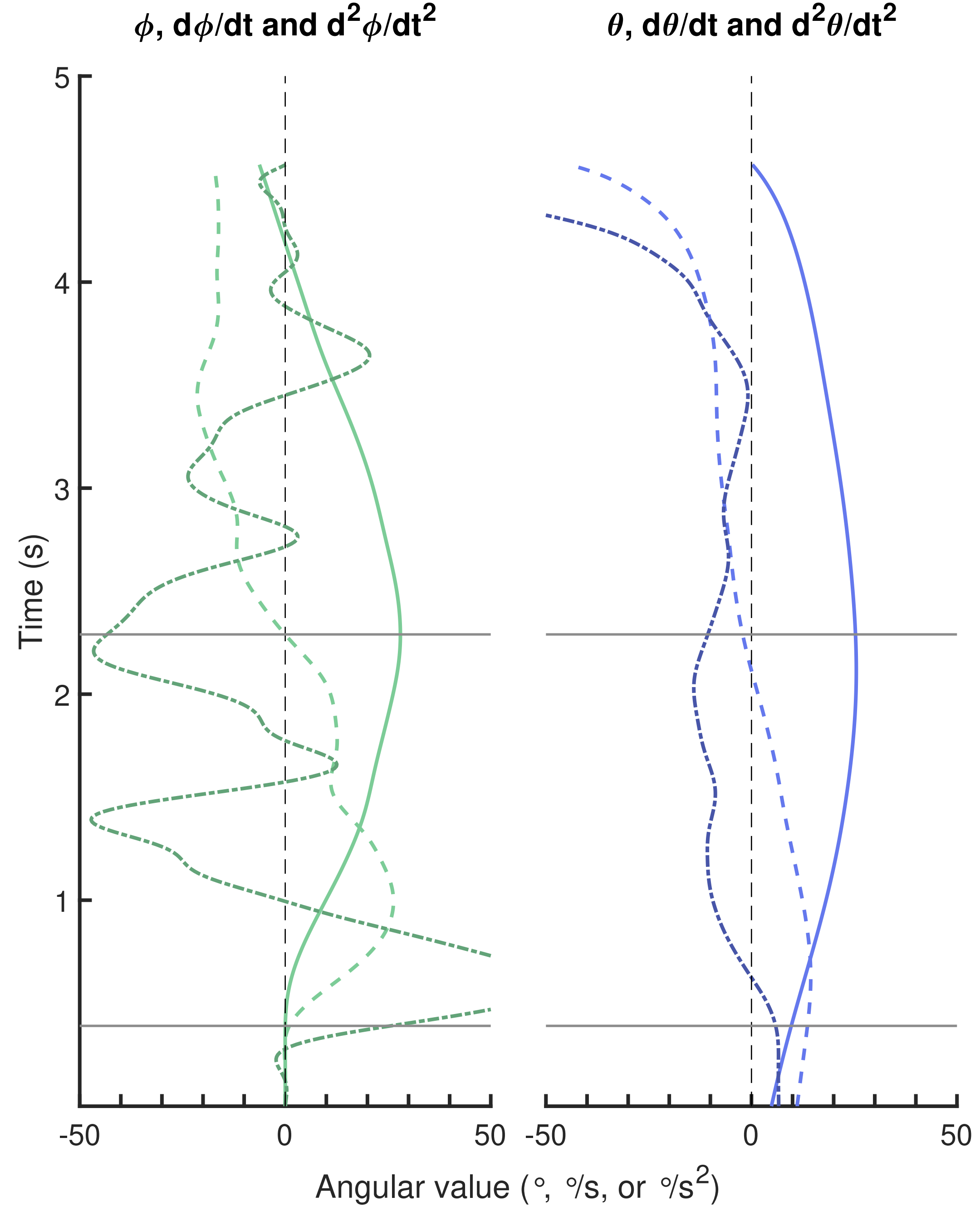

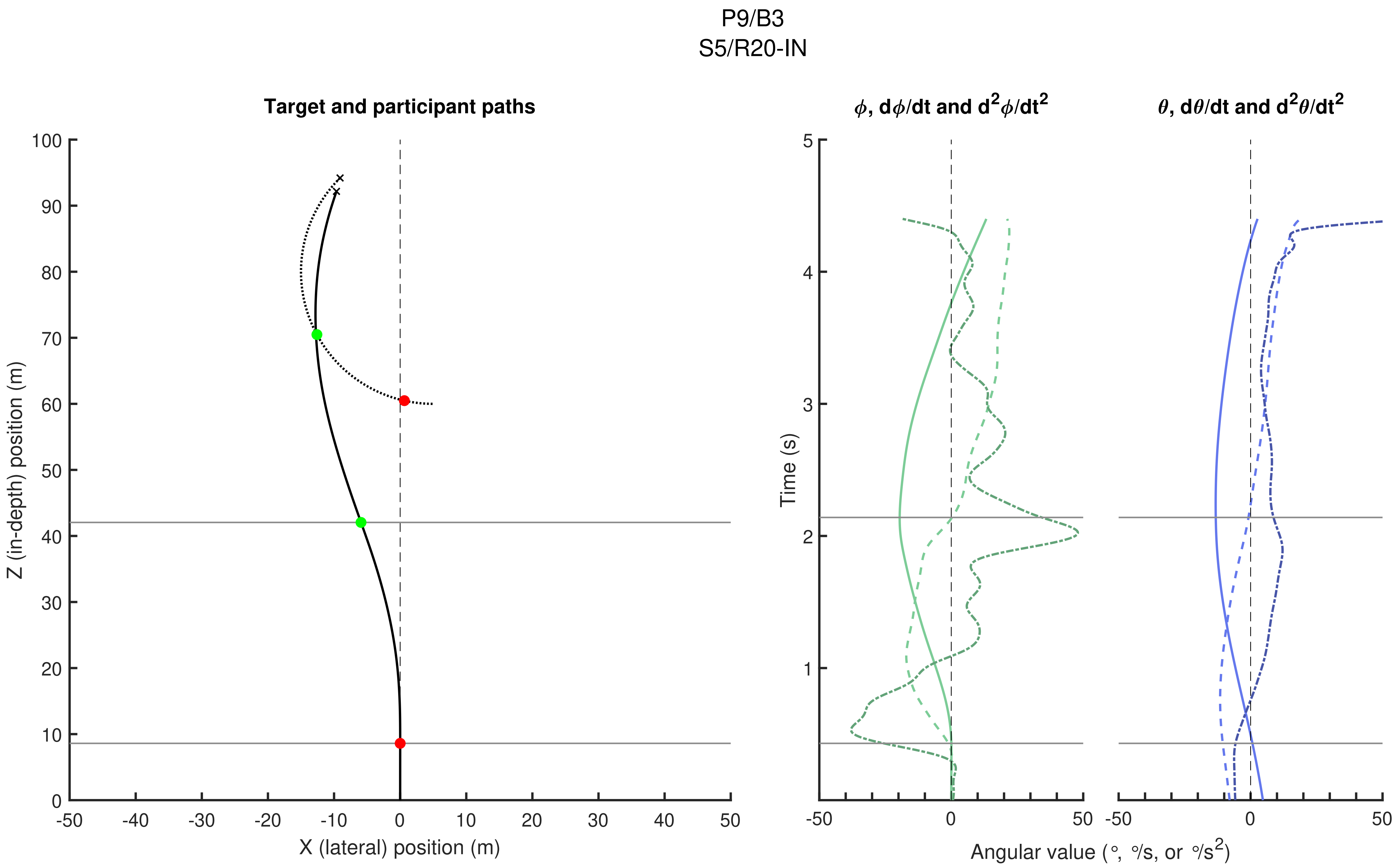

P9/B3
S5/R20-IN
Target and participant paths
ϕ, dϕ/dt and d$^2\phi$/dt^2
θ, dθ/dt and d$^2\theta$/dt^2
Z (in-depth) position (m)
X (lateral) position (m)
Time (s)
Angular value (°, °/s, or °/s^2)

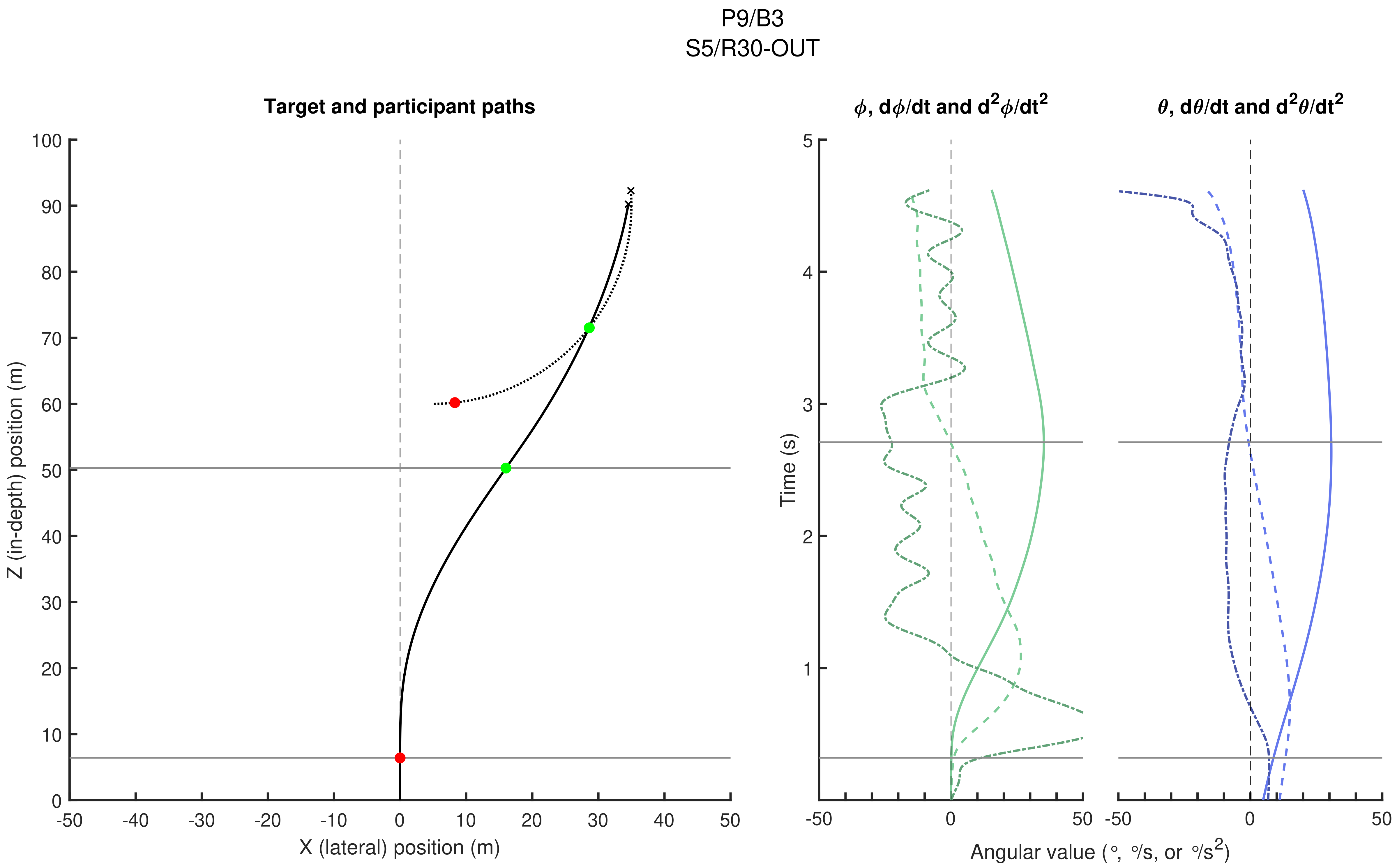
P9/B3
S5/R30-OUT
Target and participant paths
Z (in-depth) position (m)
X (lateral) position (m)
ϕ, dϕ/dt and d$^2\phi$/dt^2
θ, dθ/dt and d$^2\theta$/dt^2
Time (s)
Angular value (°, °/s, or °/s^2)

P9/B3
S5/R30-IN

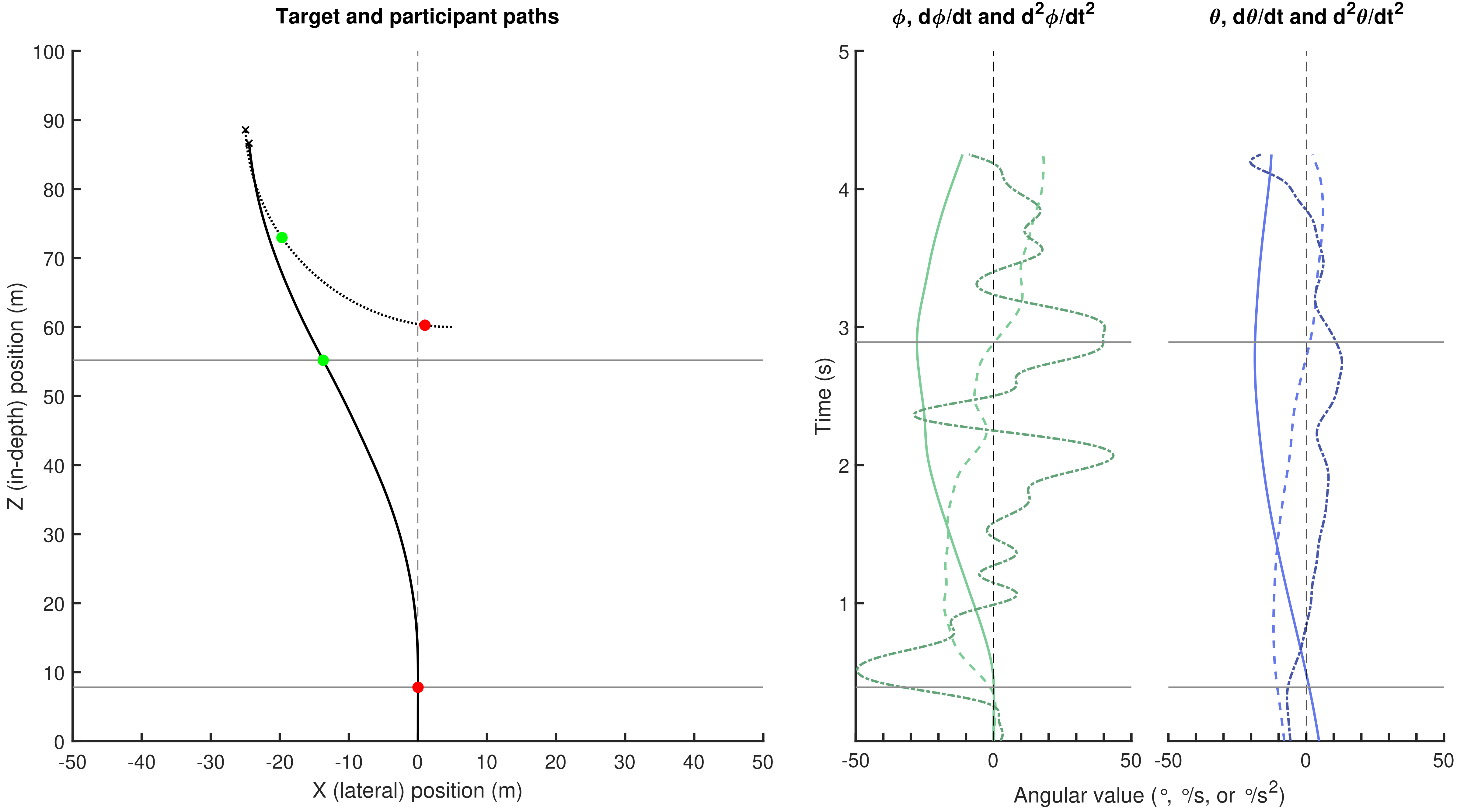

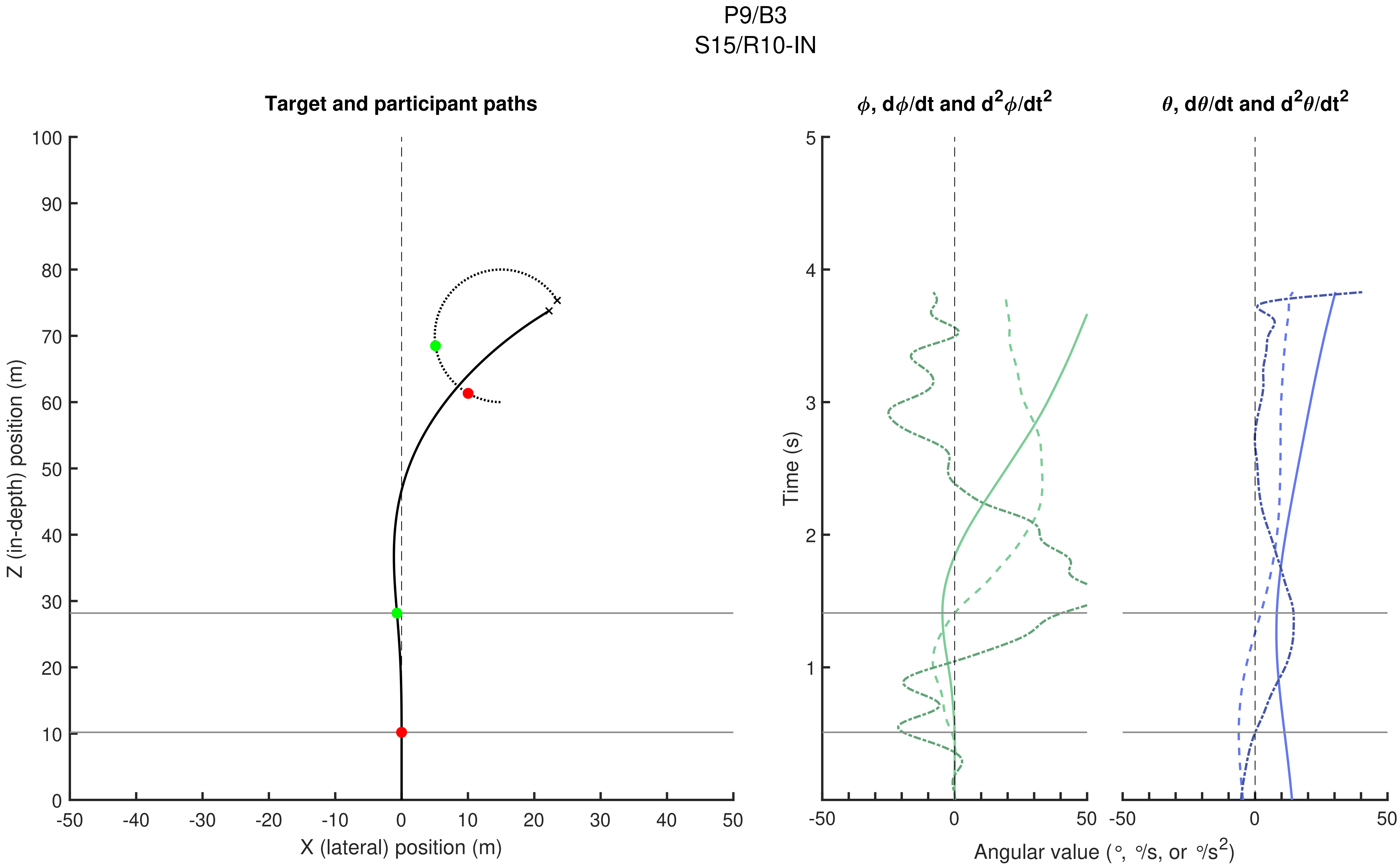
P9/B3
S15/R10-IN
Target and participant paths
Z (in-depth) position (m)
X (lateral) position (m)
φ, dφ/dt and d²φ/dt²
θ, dθ/dt and d²θ/dt²
Time (s)
Angular value (°, °/s, or °/s²)

P9/B3
S15/R20-IN

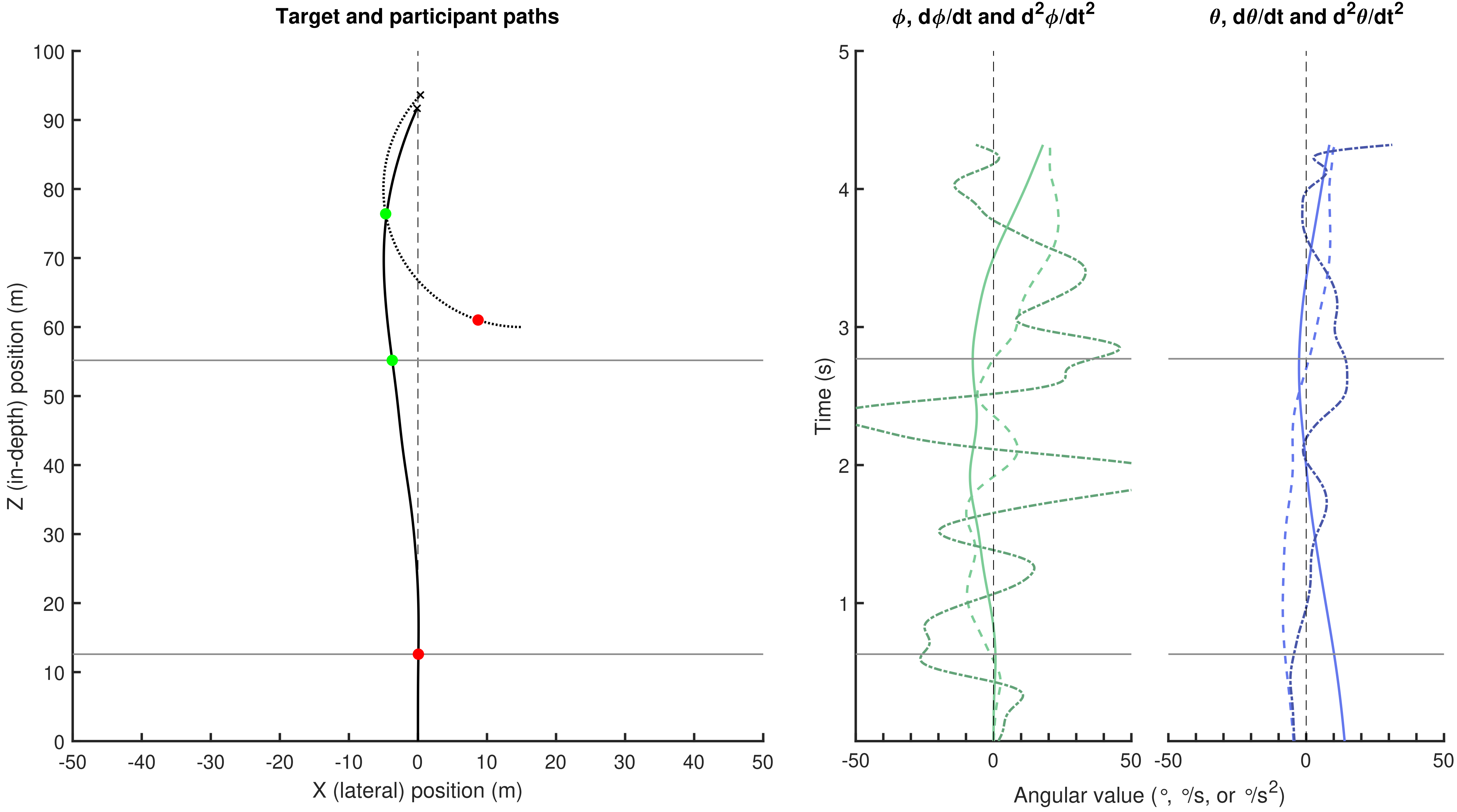

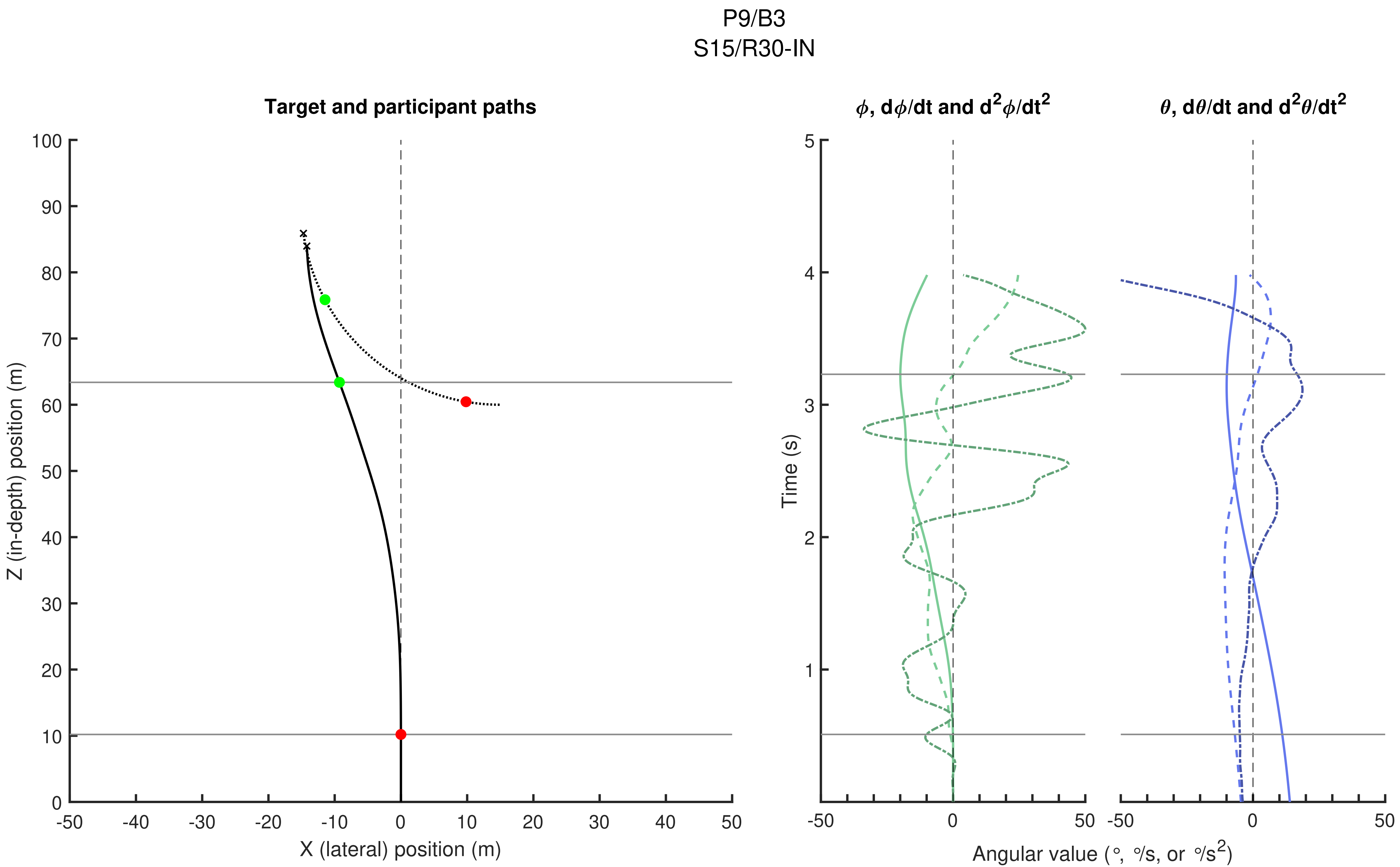

P9/B3
S15/R30-IN
Target and participant paths
Z (in-depth) position (m)
X (lateral) position (m)
ϕ, dϕ/dt and d$^2\phi$/dt^2
θ, dθ/dt and d$^2\theta$/dt^2
Time (s)
Angular value (°, °/s, or °/s^2)

P9/B3
S20/R10-IN

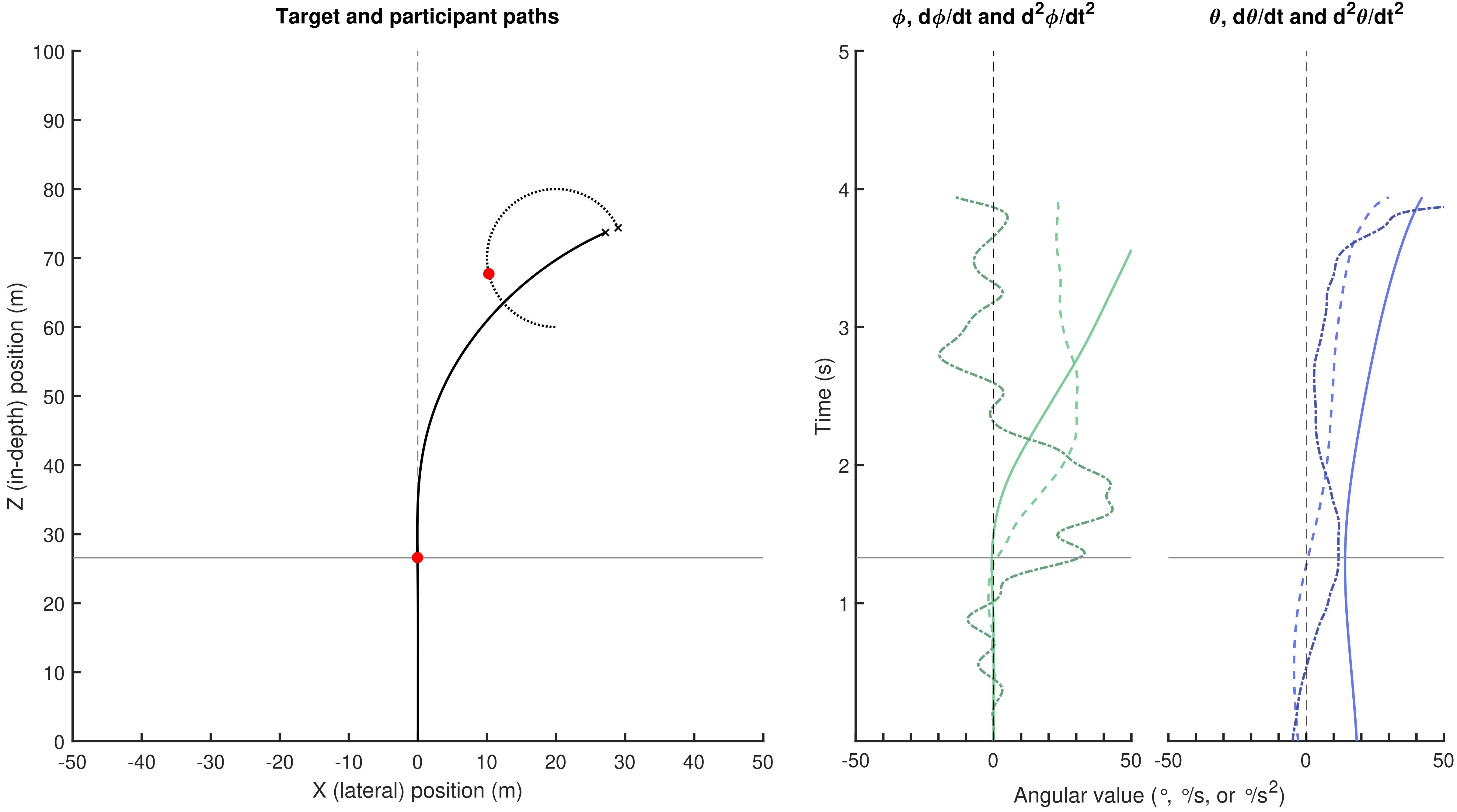

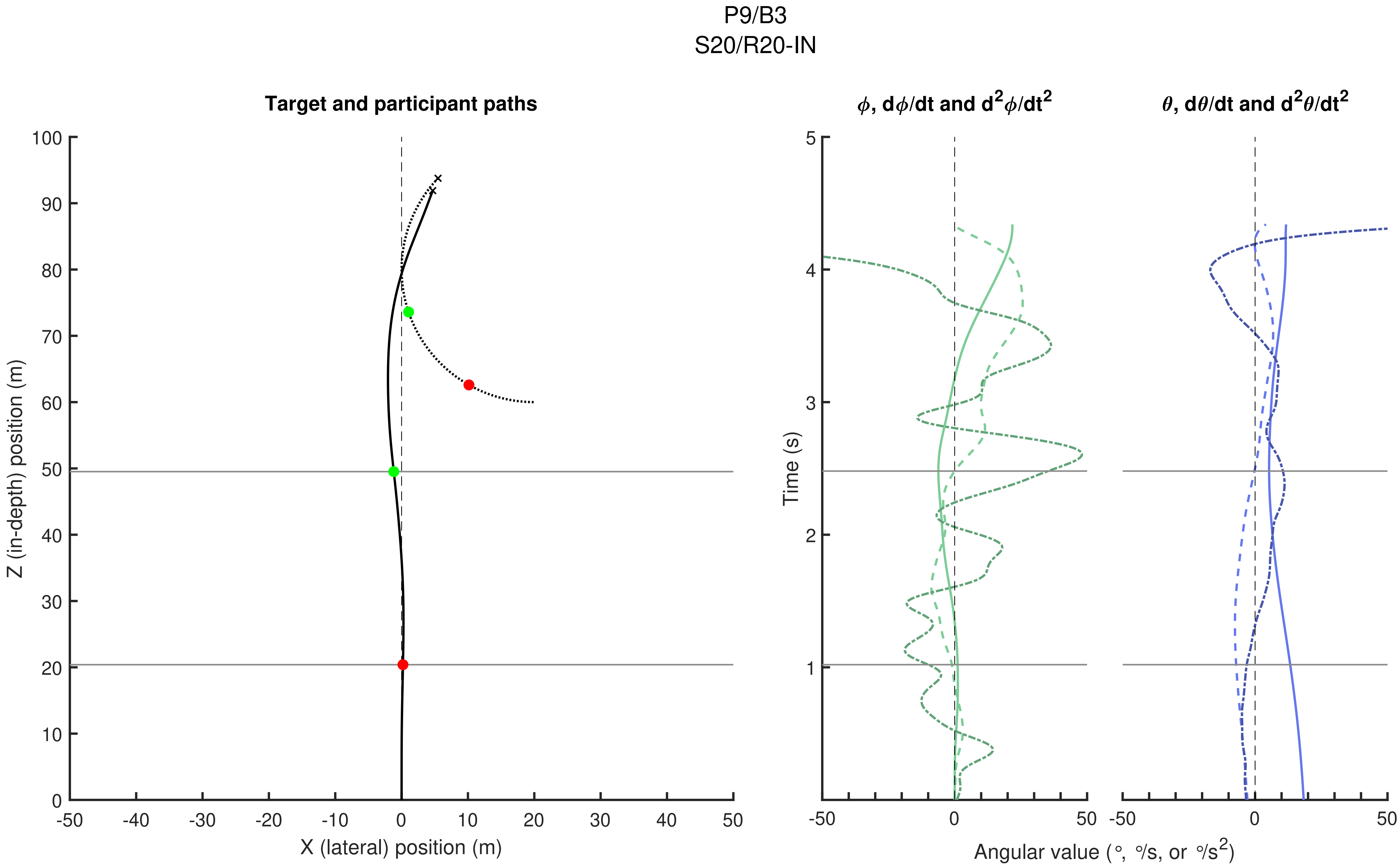

P9/B3
S20/R20-IN
Target and participant paths
Z (in-depth) position (m)
X (lateral) position (m)
φ, dφ/dt and d²φ/dt²
θ, dθ/dt and d²θ/dt²
Time (s)
Angular value (°, °/s, or °/s²)

P9/B3
S20/R30-IN

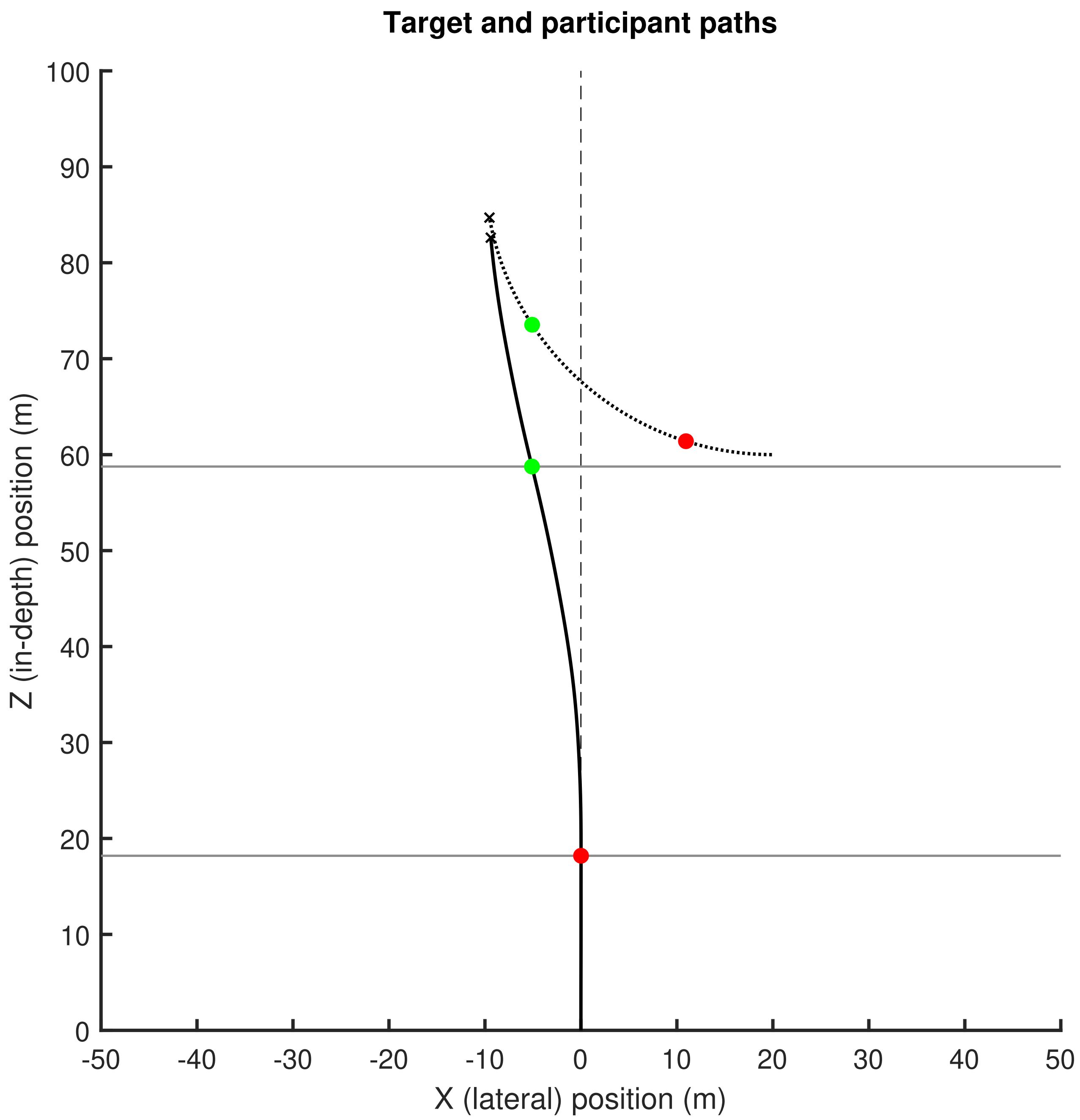

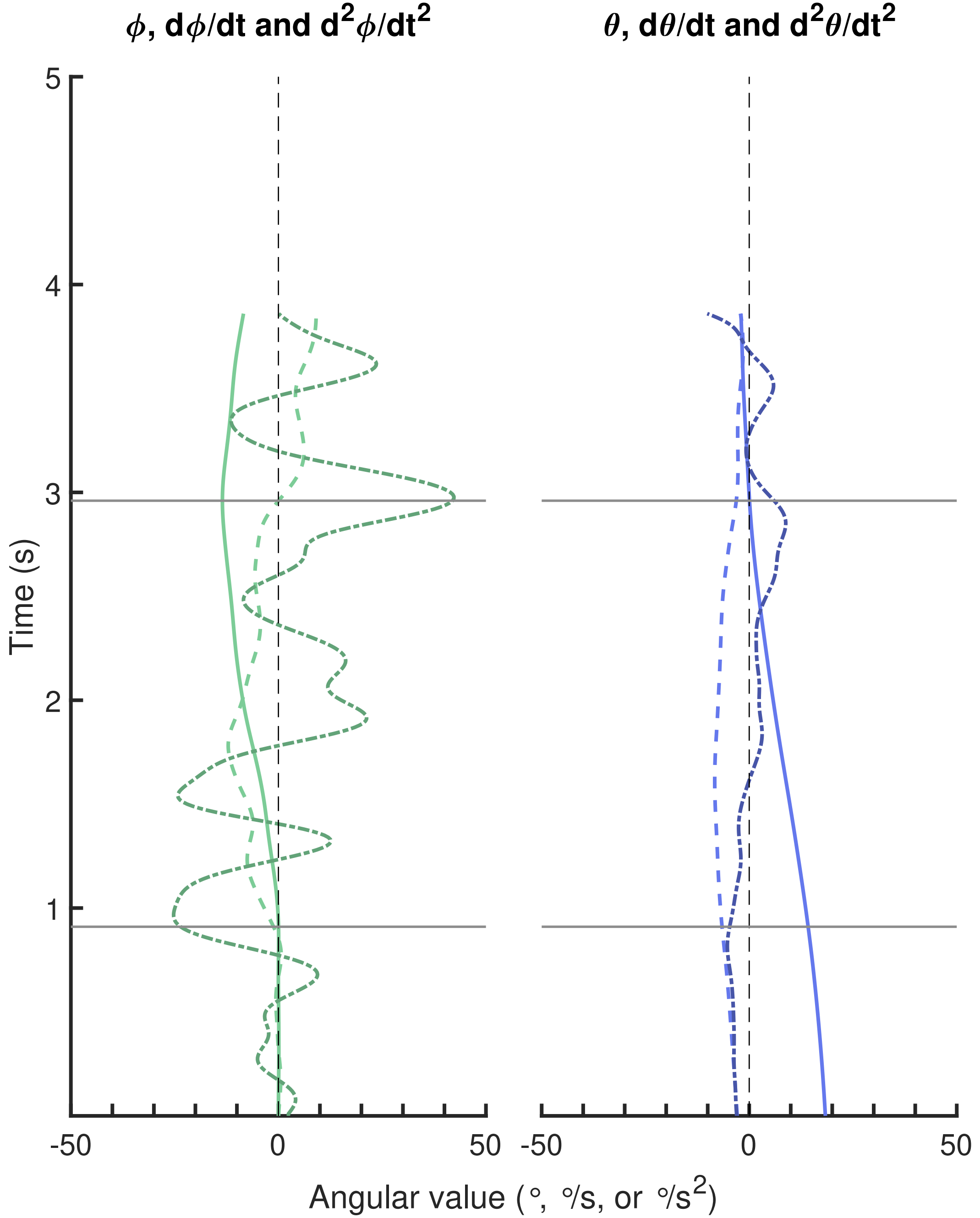

P9/B4
S20/R10-IN

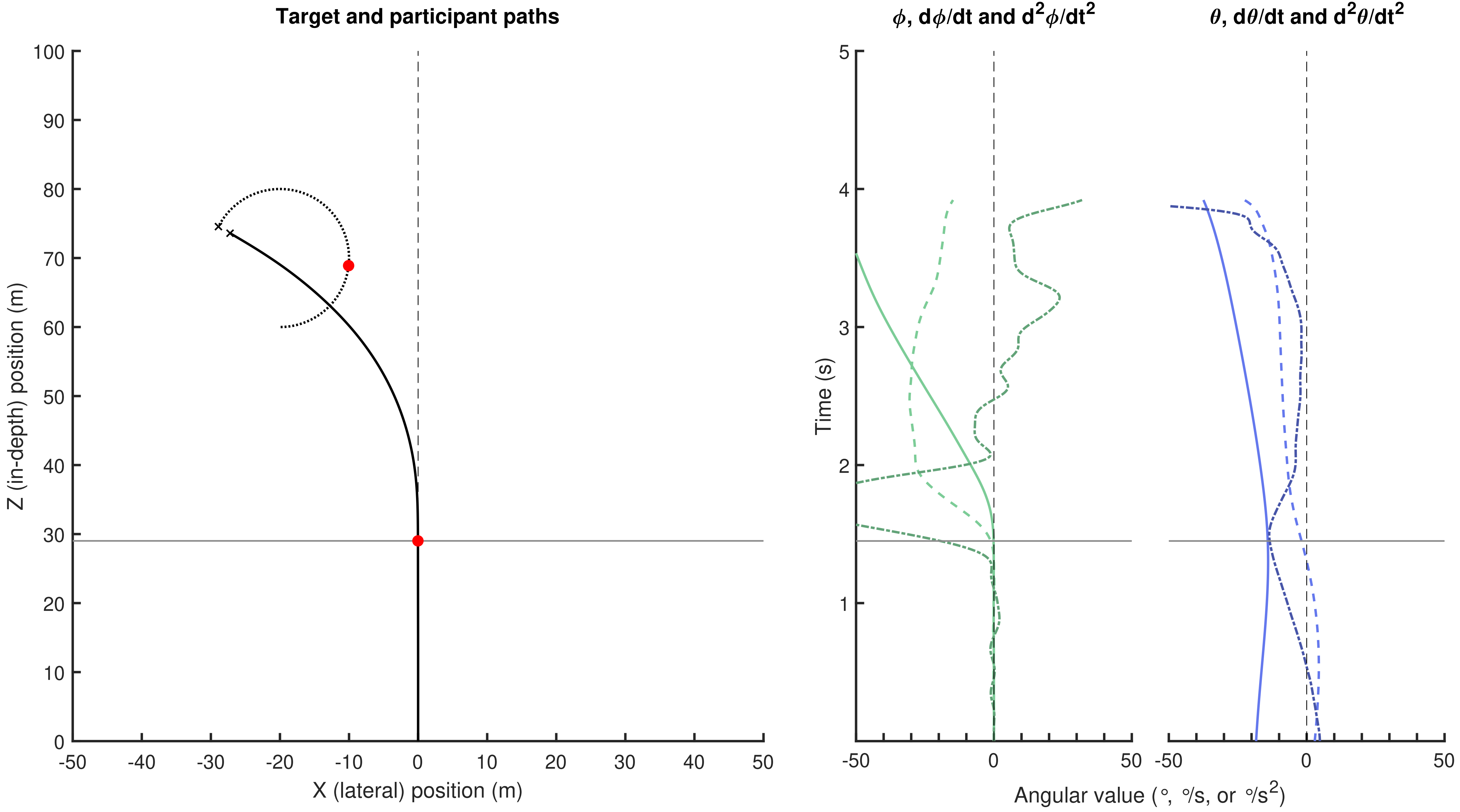

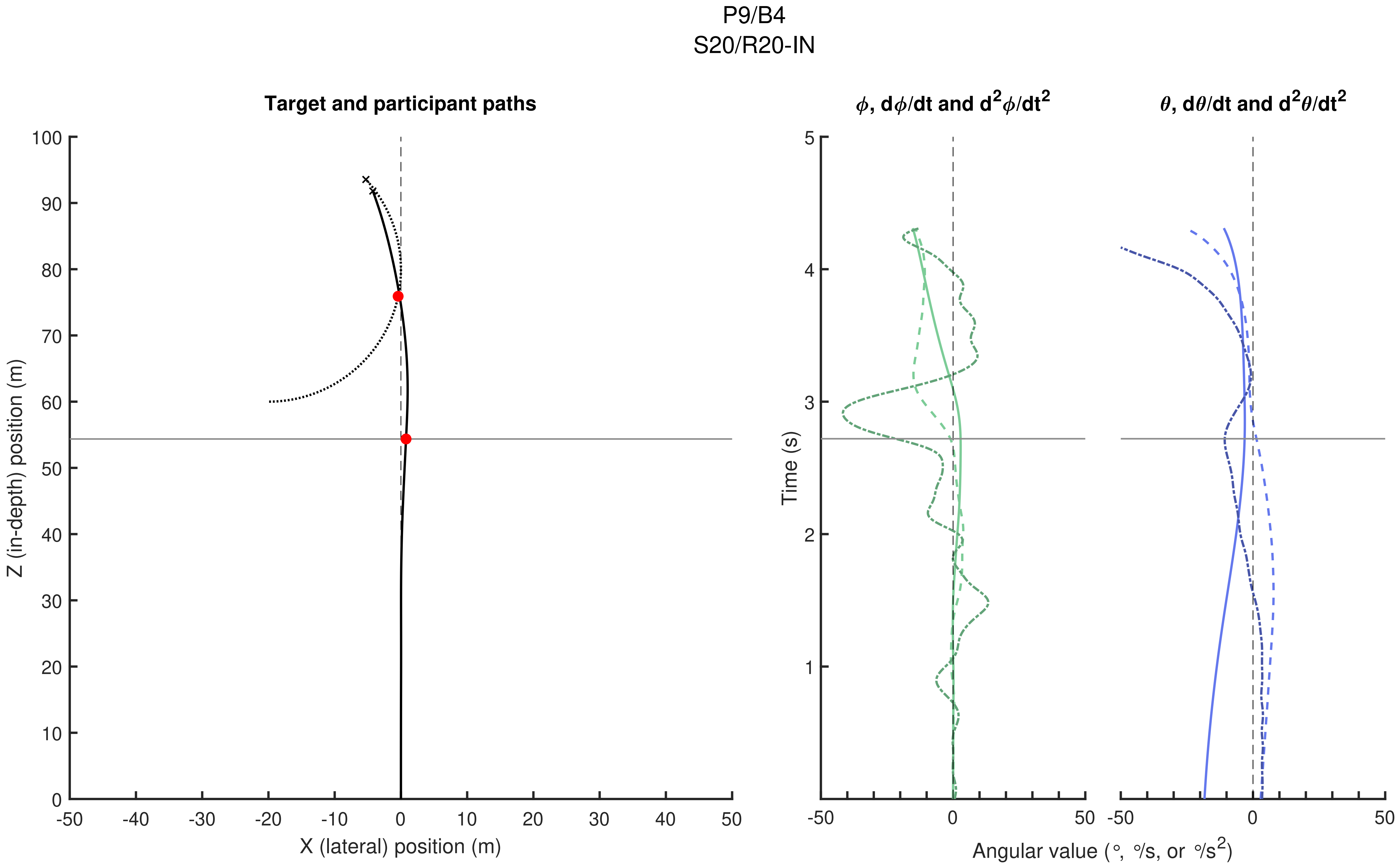

P9/B4
S20/R20-IN
Target and participant paths
ϕ, dϕ/dt and d$^2\phi$/dt^2
θ, dθ/dt and d$^2\theta$/dt^2
Z (in-depth) position (m)
X (lateral) position (m)
Time (s)
Angular value (°, °/s, or °/s^2)
100
90
80
70
60
50
40
30
20
10
0
-50
-40
-30
-20
-10
0
10
20
30
40
50
5
4
3
2
1
-50
0
50
-50
0
50

P9/B4
S20/R30-IN

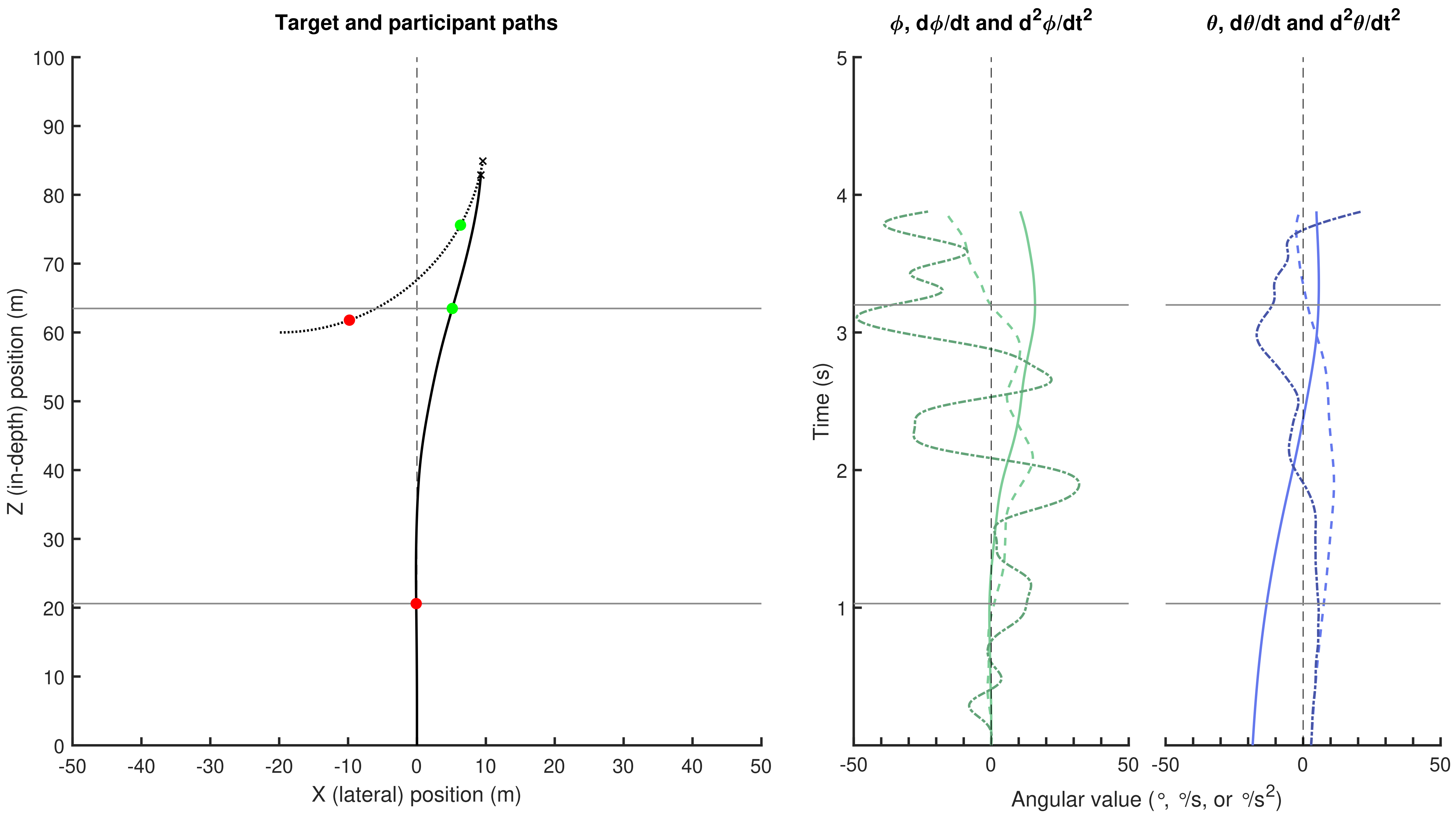

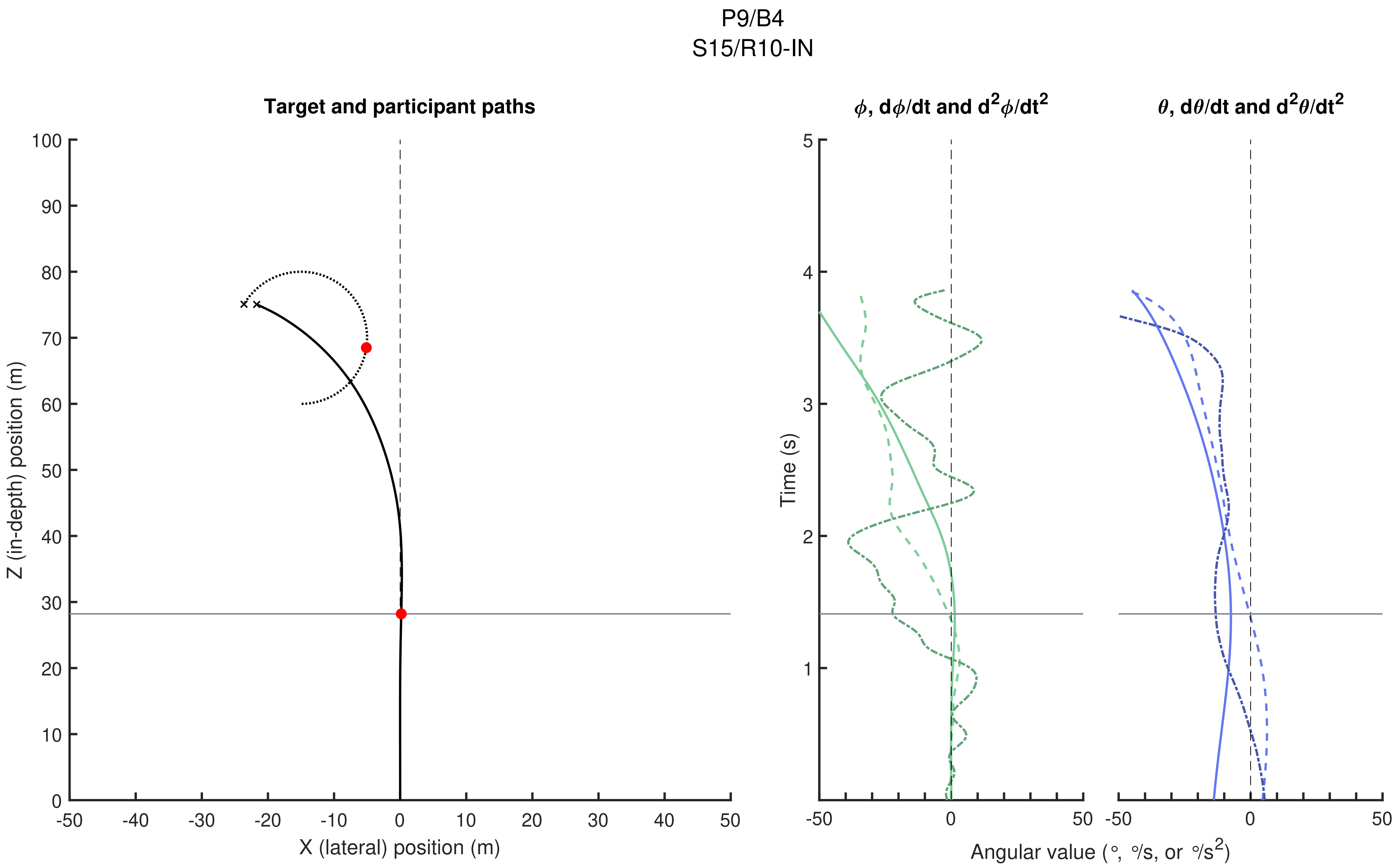

P9/B4
S15/R10-IN
Target and participant paths
Z (in-depth) position (m)
X (lateral) position (m)
100
90
80
70
60
50
40
30
20
10
0
-50
-40
-30
-20
-10
0
10
20
30
40
50
ϕ, dϕ/dt and d$^2\phi$/dt^2
θ, dθ/dt and d$^2\theta$/dt^2
Time (s)
5
4
3
2
1
-50
0
50
-50
0
50
Angular value (°, °/s, or °/s^2)

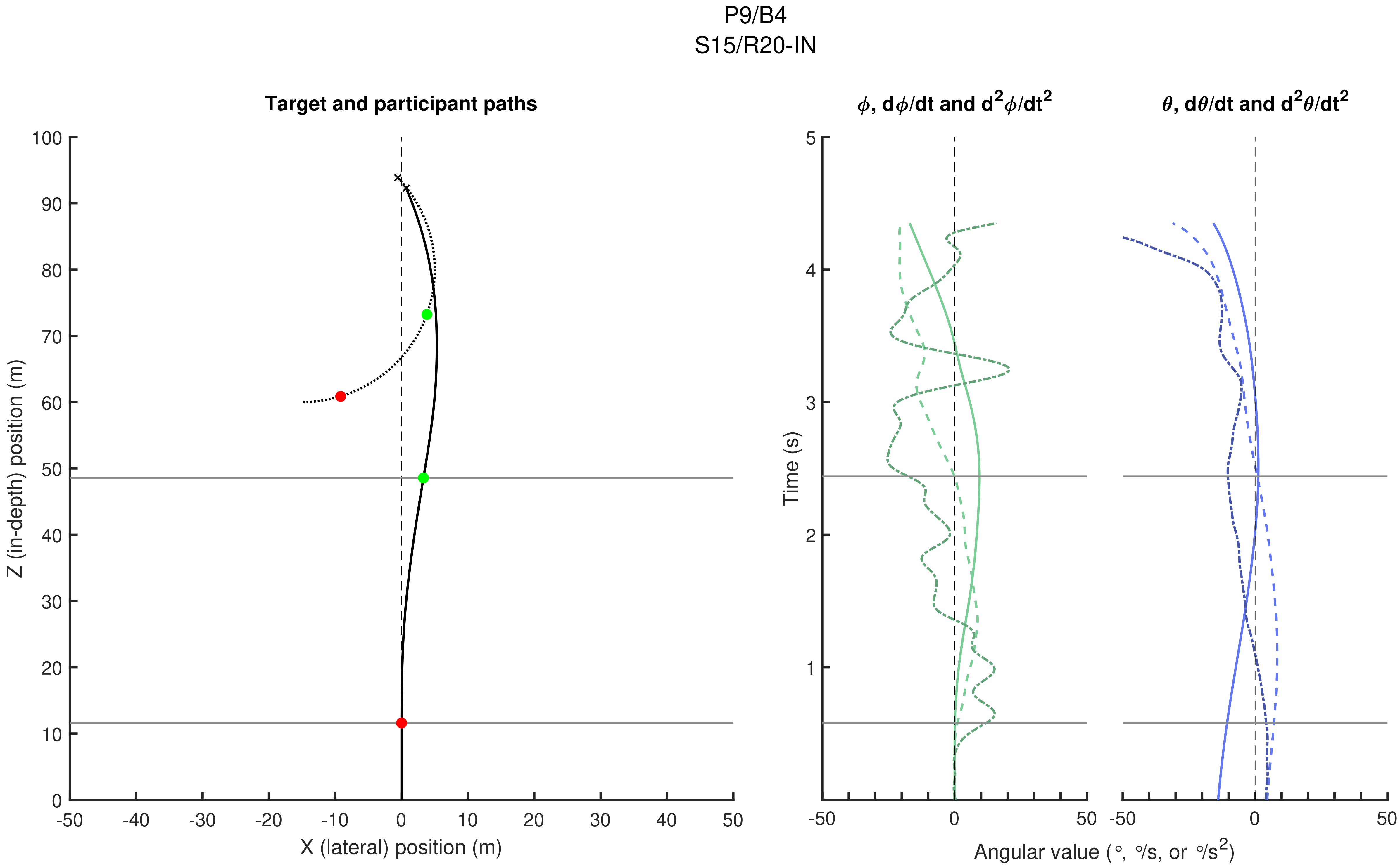
P9/B4
S15/R20-IN
Target and participant paths
ϕ, dϕ/dt and d²ϕ/dt²
θ, dθ/dt and d²θ/dt²
Z (in-depth) position (m)
X (lateral) position (m)
Time (s)
Angular value (°, °/s, or °/s²)

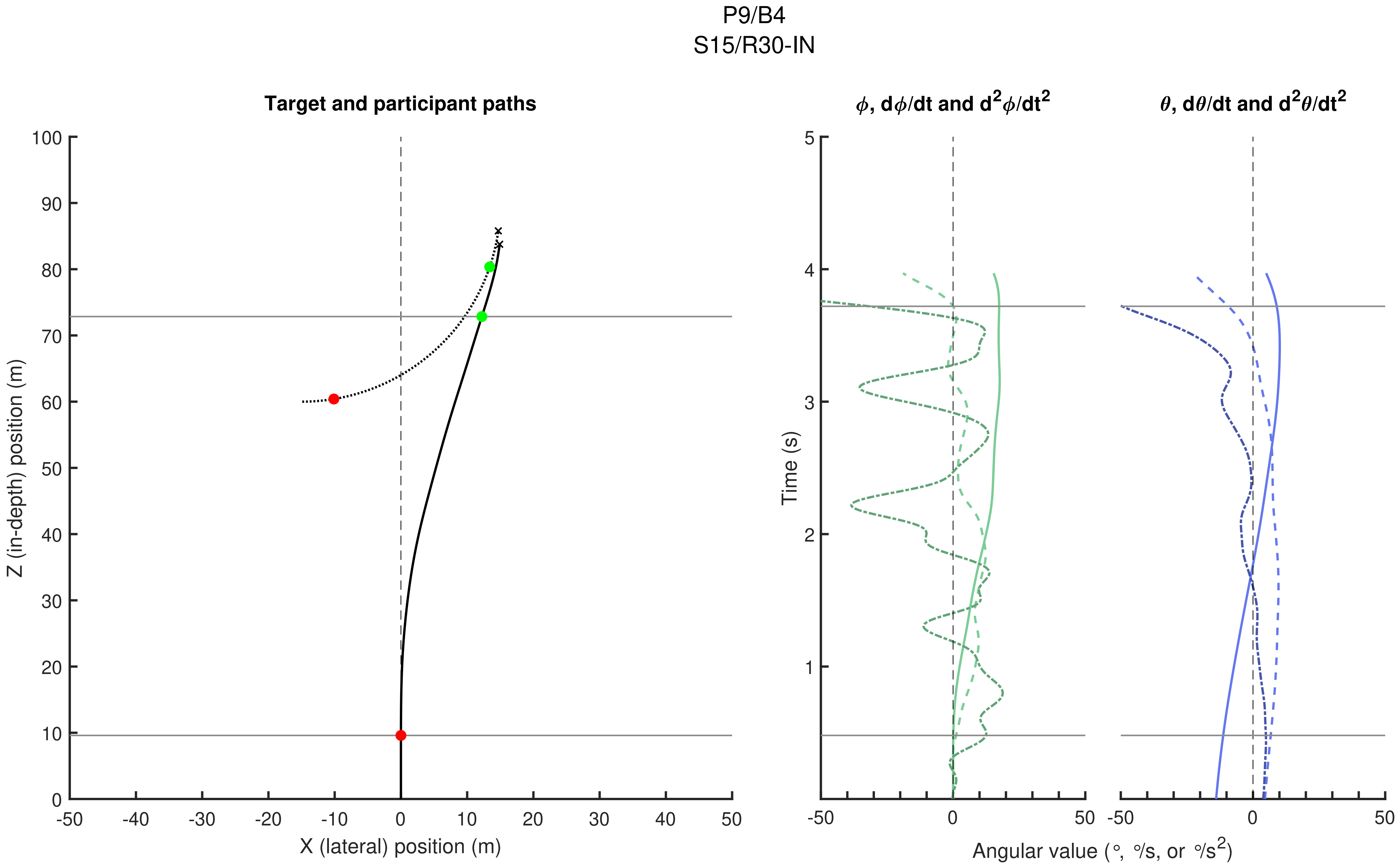
P9/B4
S15/R30-IN
Target and participant paths
Z (in-depth) position (m)
X (lateral) position (m)
φ, dφ/dt and d²φ/dt²
θ, dθ/dt and d²θ/dt²
Time (s)
Angular value (°, °/s, or °/s²)

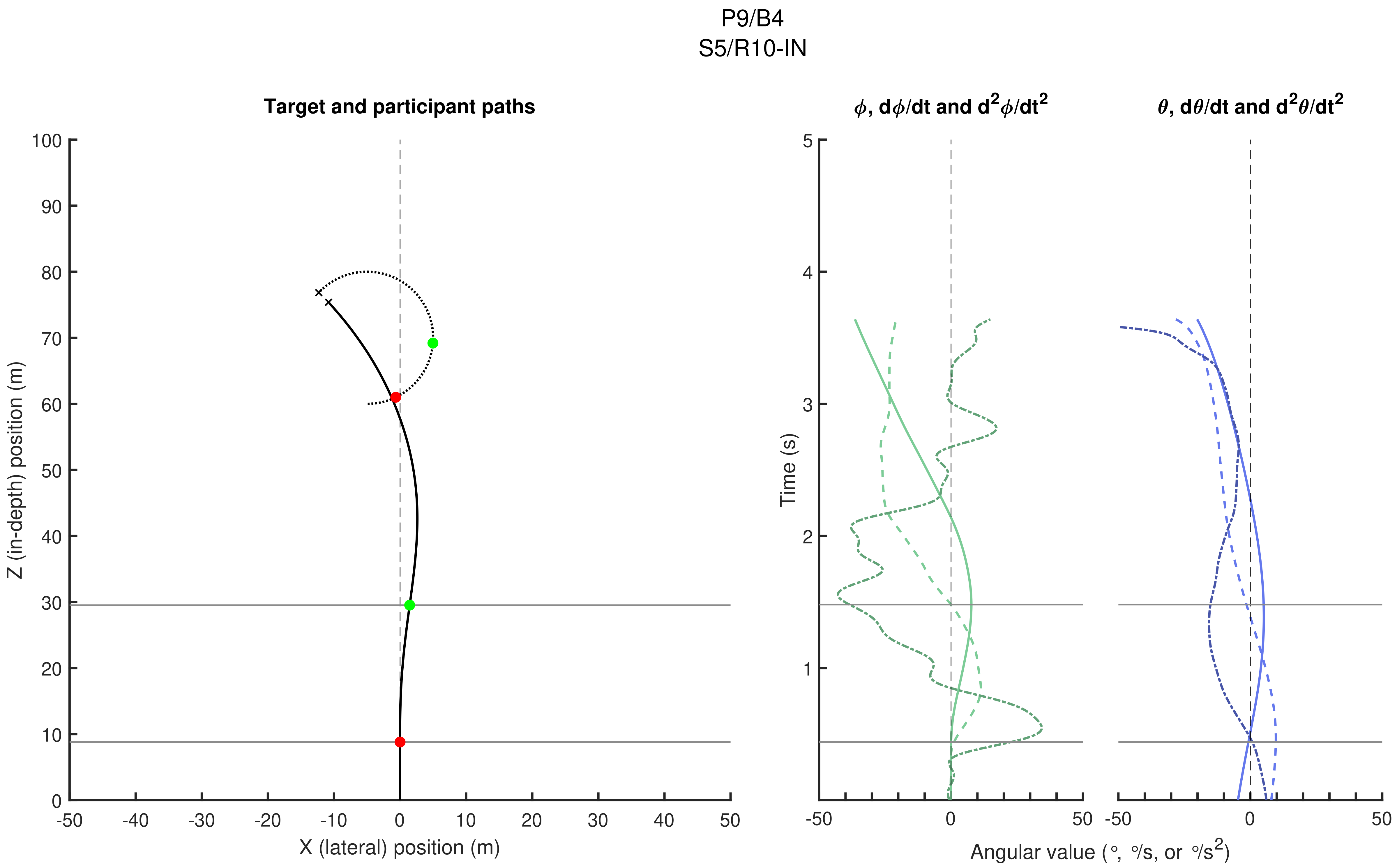
P9/B4
S5/R10-IN
Target and participant paths
Z (in-depth) position (m)
X (lateral) position (m)
ϕ, dϕ/dt and d$^2\phi$/dt^2
θ, dθ/dt and d$^2\theta$/dt^2
Time (s)
Angular value (°, °/s, or °/s^2)

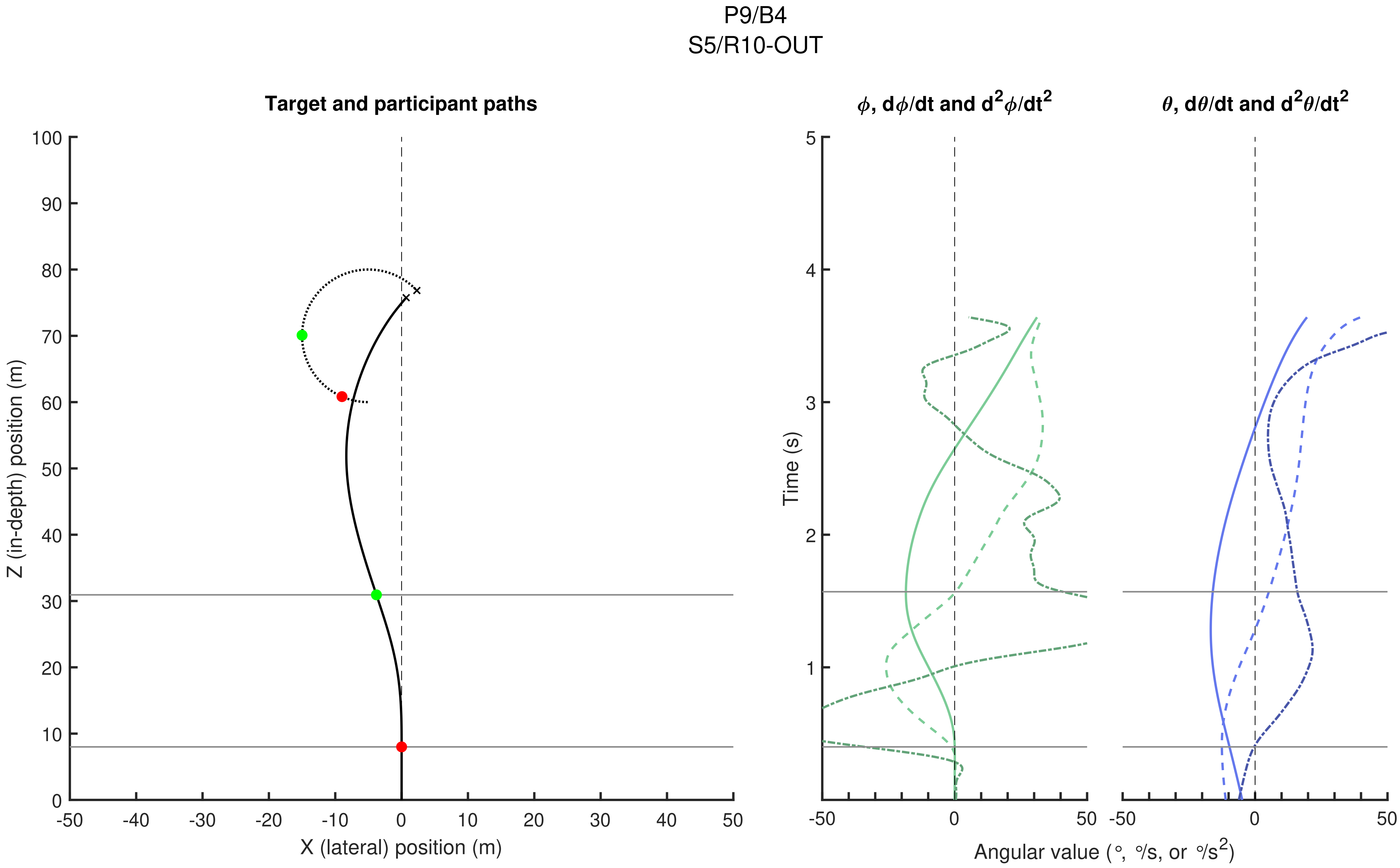
P9/B4
S5/R10-OUT
Target and participant paths
Z (in-depth) position (m)
X (lateral) position (m)
ϕ, dϕ/dt and d$^2\phi$/dt^2
θ, dθ/dt and d$^2\theta$/dt^2
Time (s)
Angular value (°, °/s, or °/s^2)

P9/B4
S5/R20-IN

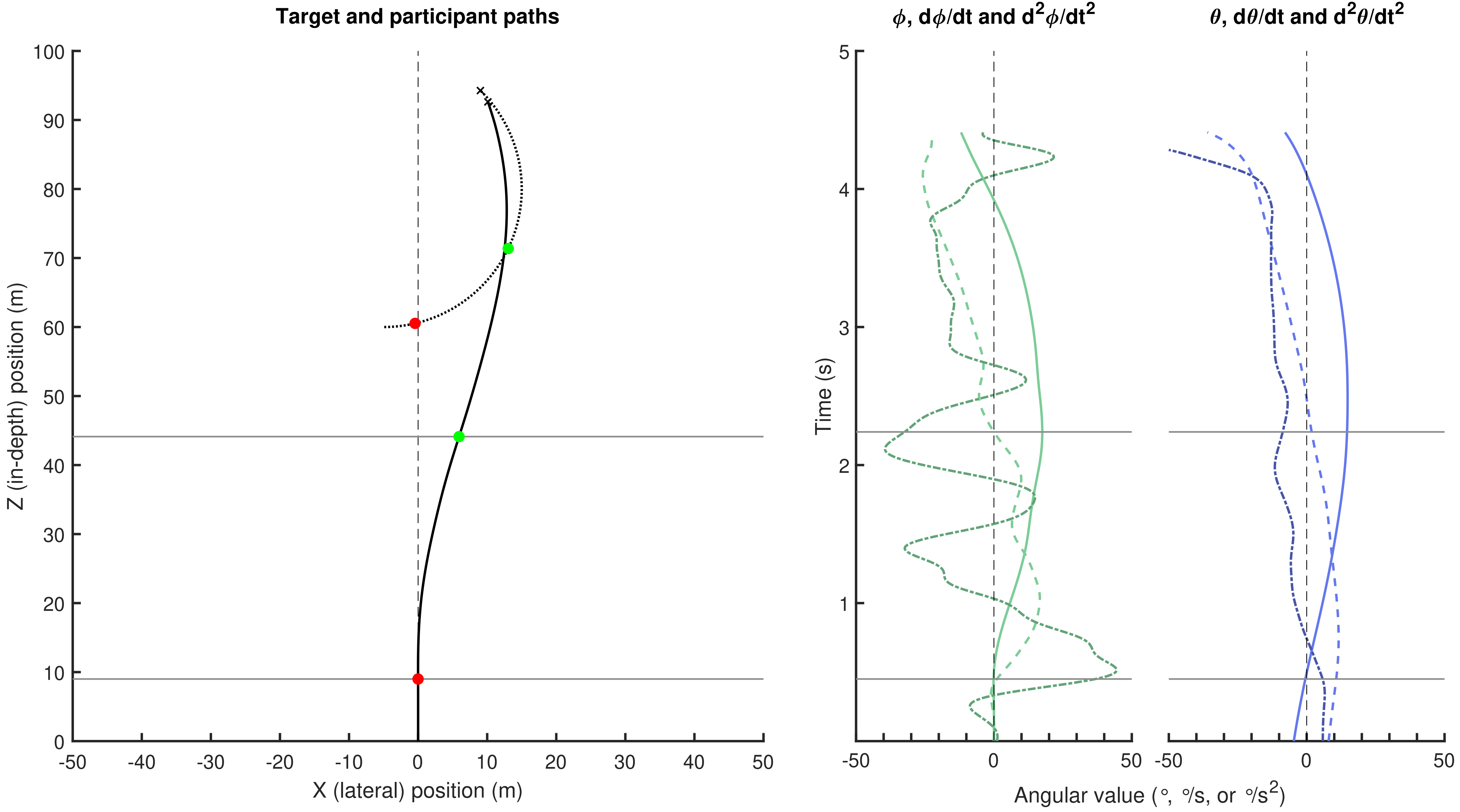

P9/B4
S5/R20-OUT

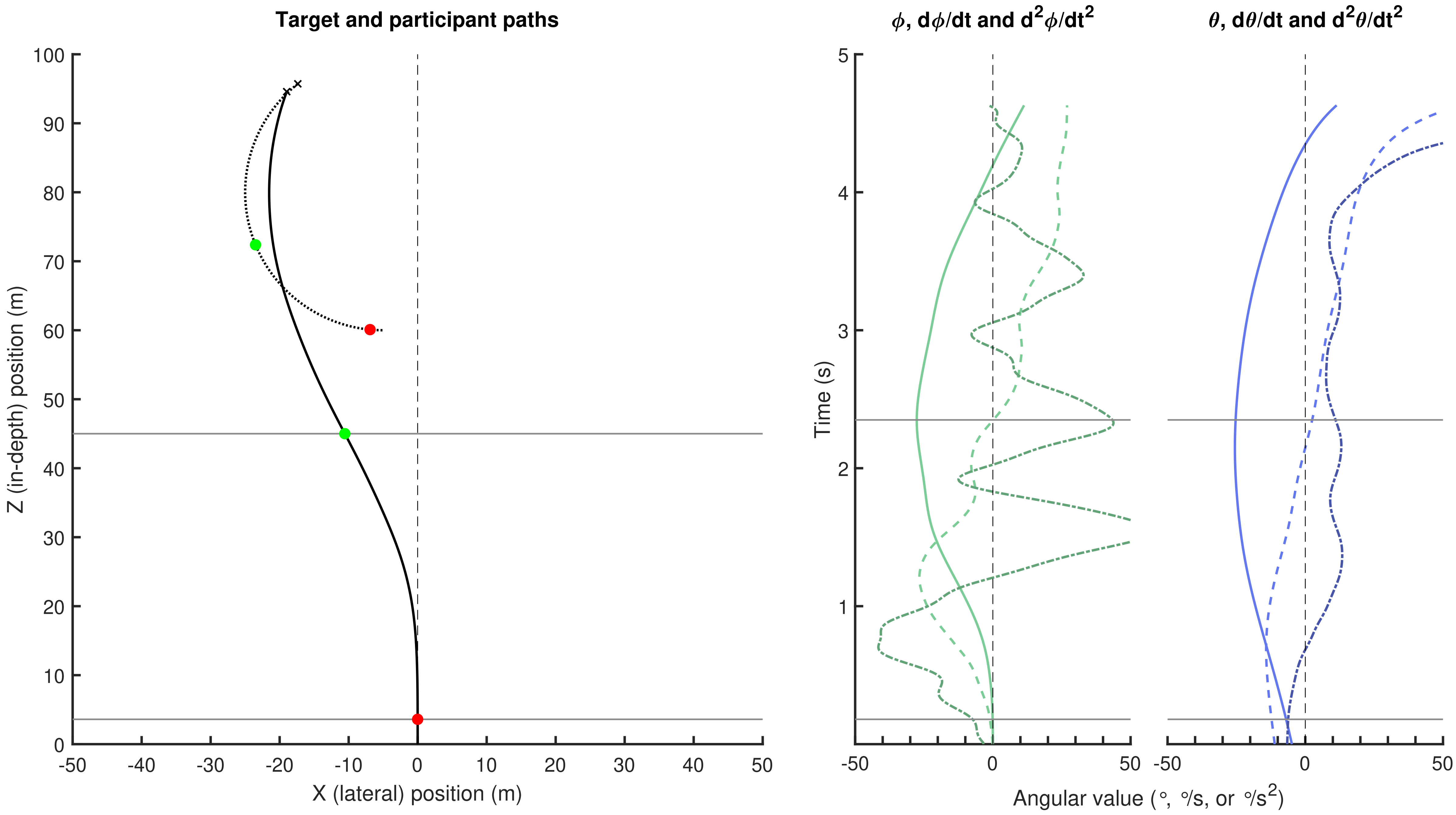

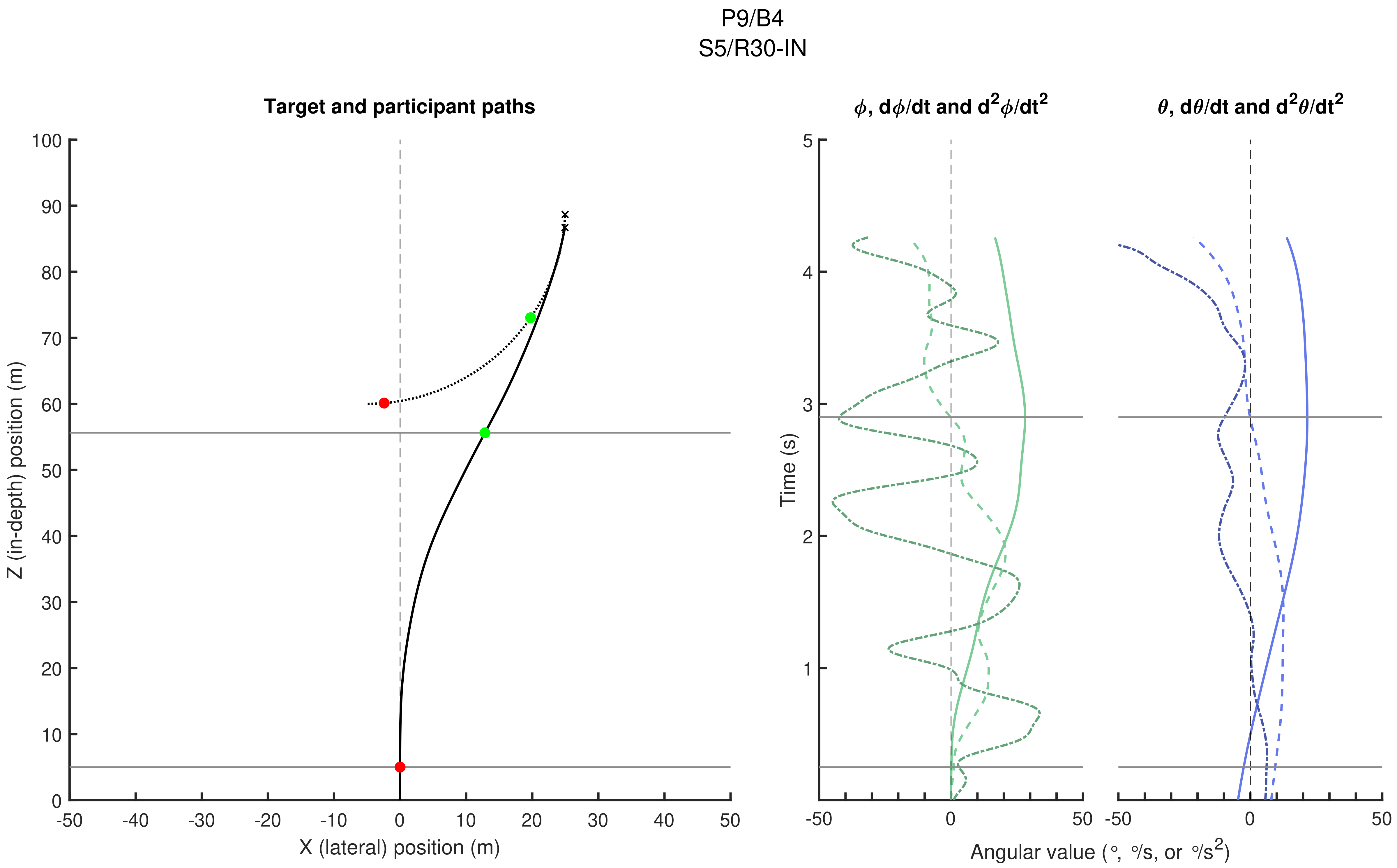
P9/B4
S5/R30-IN
Target and participant paths
Z (in-depth) position (m)
X (lateral) position (m)
ϕ, dϕ/dt and d²ϕ/dt²
θ, dθ/dt and d²θ/dt²
Time (s)
Angular value (°, °/s, or °/s²)

P9/B4
S5/R30-OUT

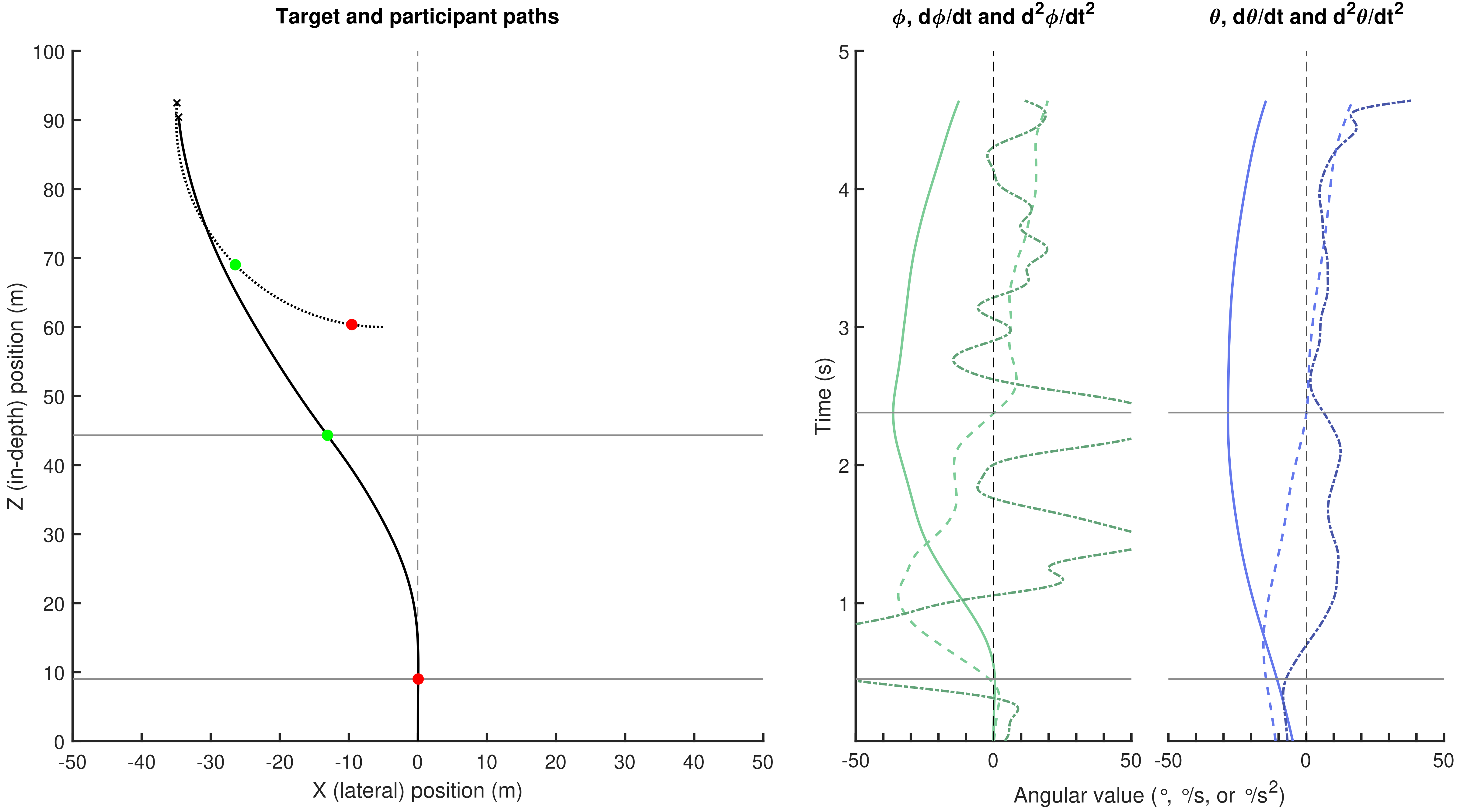

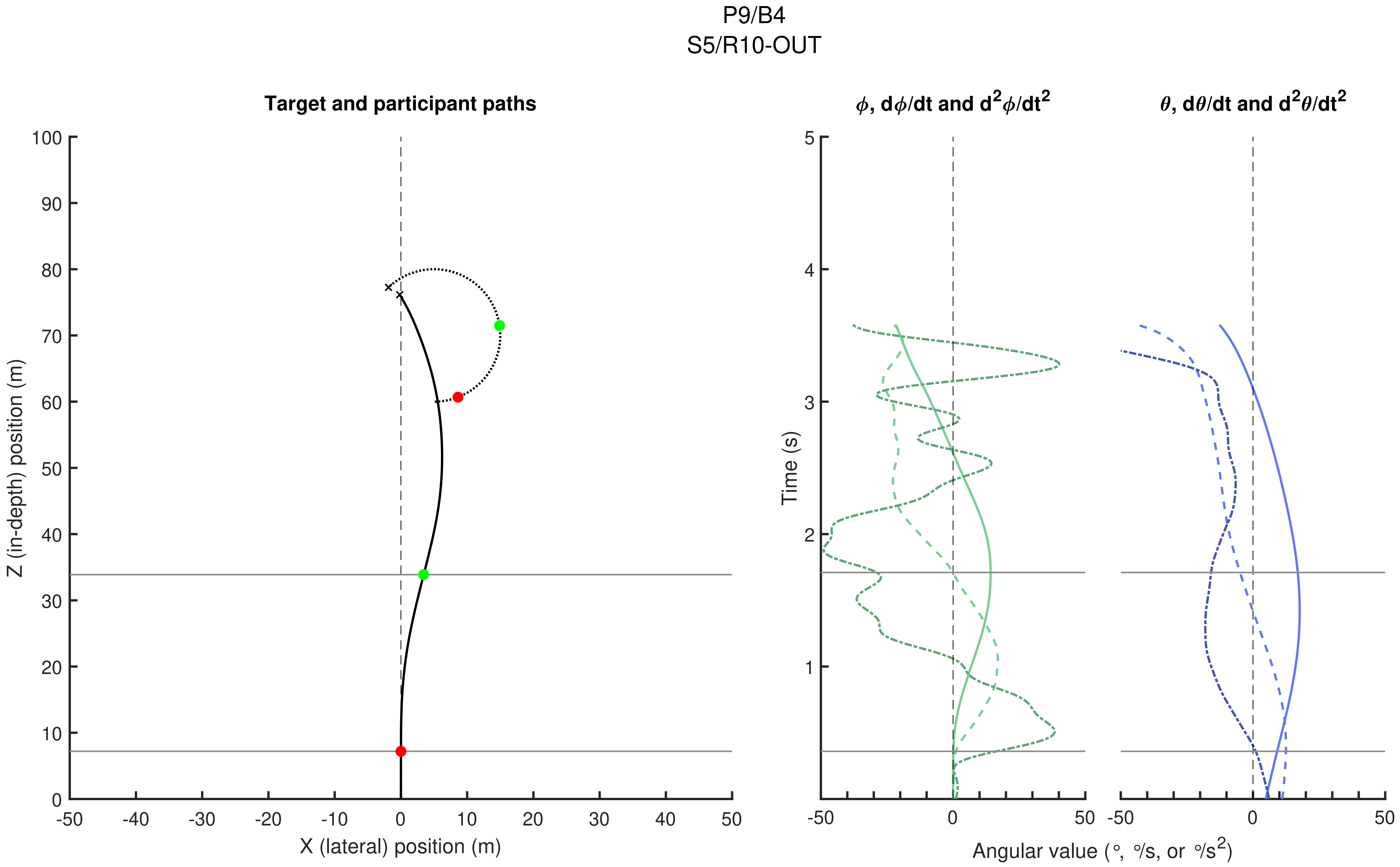
P9/B4
S5/R10-OUT
Target and participant paths
Z (in-depth) position (m)
X (lateral) position (m)
ϕ, dϕ/dt and d$^2\phi$/dt^2
θ, dθ/dt and d$^2\theta$/dt^2
Time (s)
Angular value (°, °/s, or °/s^2)

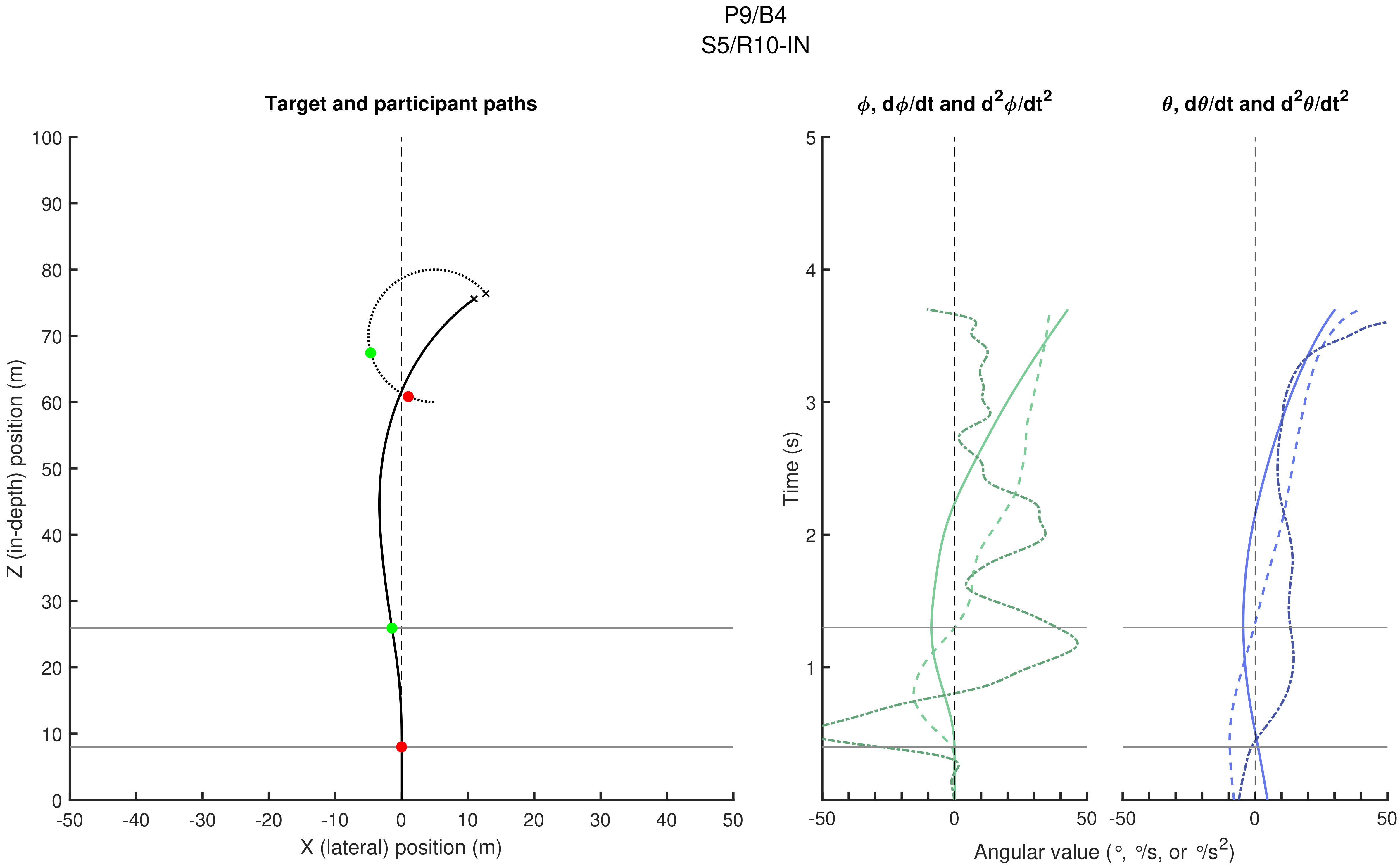

P9/B4
S5/R10-IN
Target and participant paths
Z (in-depth) position (m)
X (lateral) position (m)
φ, dφ/dt and d²φ/dt²
θ, dθ/dt and d²θ/dt²
Time (s)
Angular value (°, °/s, or °/s²)

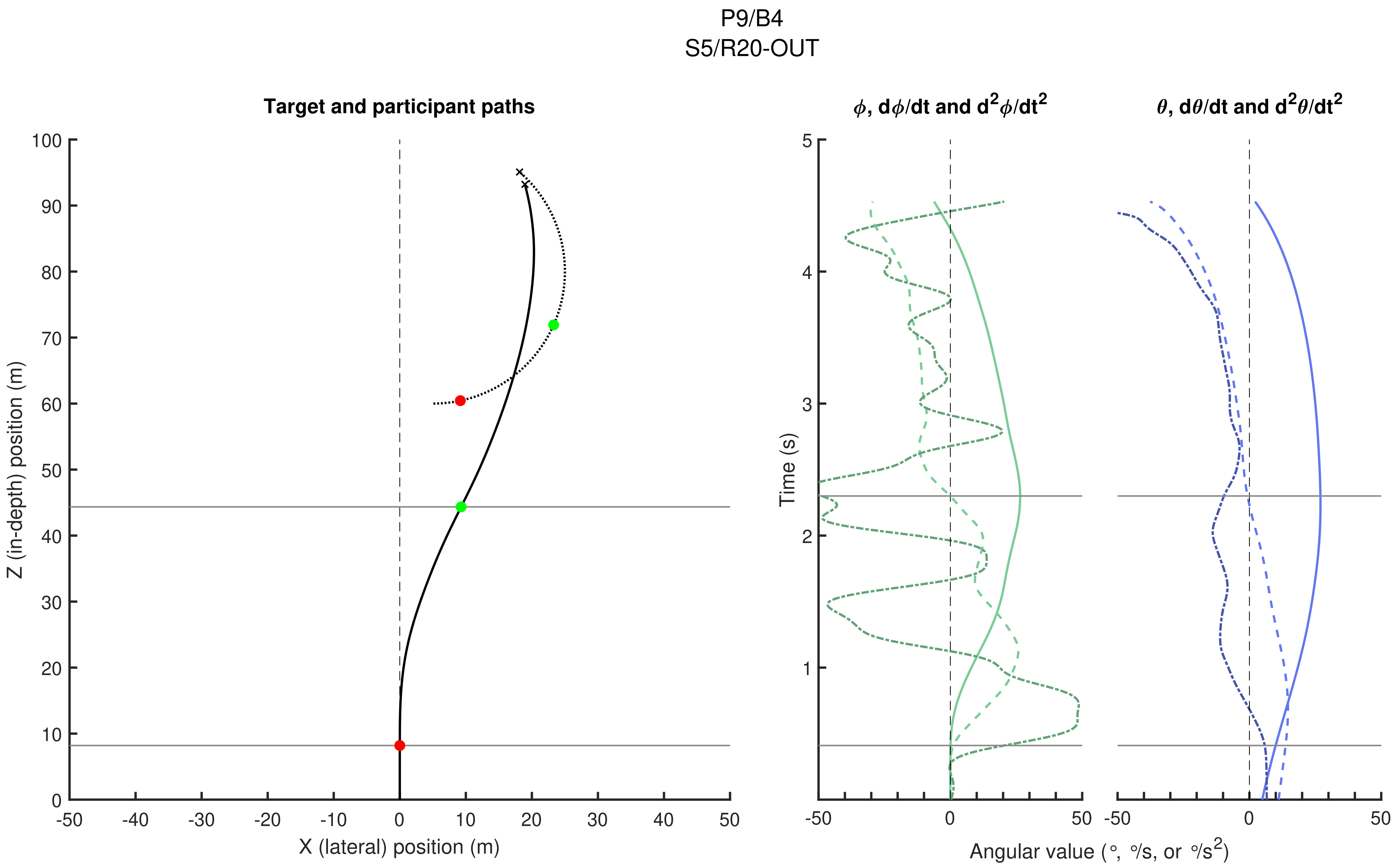
P9/B4
S5/R20-OUT
Target and participant paths
Z (in-depth) position (m)
X (lateral) position (m)
ϕ, dϕ/dt and d$^2\phi$/dt^2
θ, dθ/dt and d$^2\theta$/dt^2
Time (s)
Angular value (°, °/s, or °/s^2)

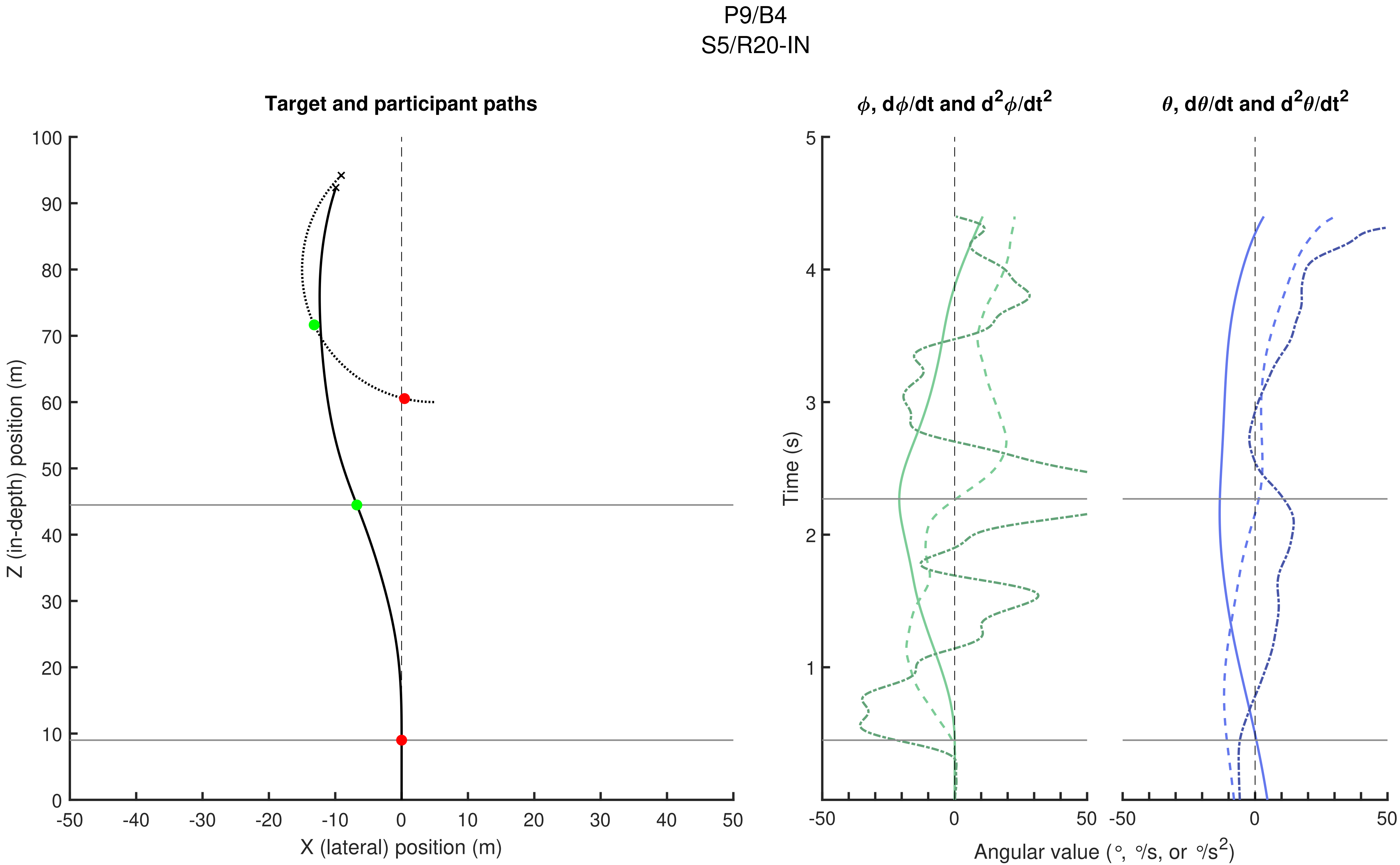
P9/B4
S5/R20-IN
Target and participant paths
Z (in-depth) position (m)
X (lateral) position (m)
φ, dφ/dt and d²φ/dt²
θ, dθ/dt and d²θ/dt²
Time (s)
Angular value (°, °/s, or °/s²)

P9/B4
S5/R30-OUT

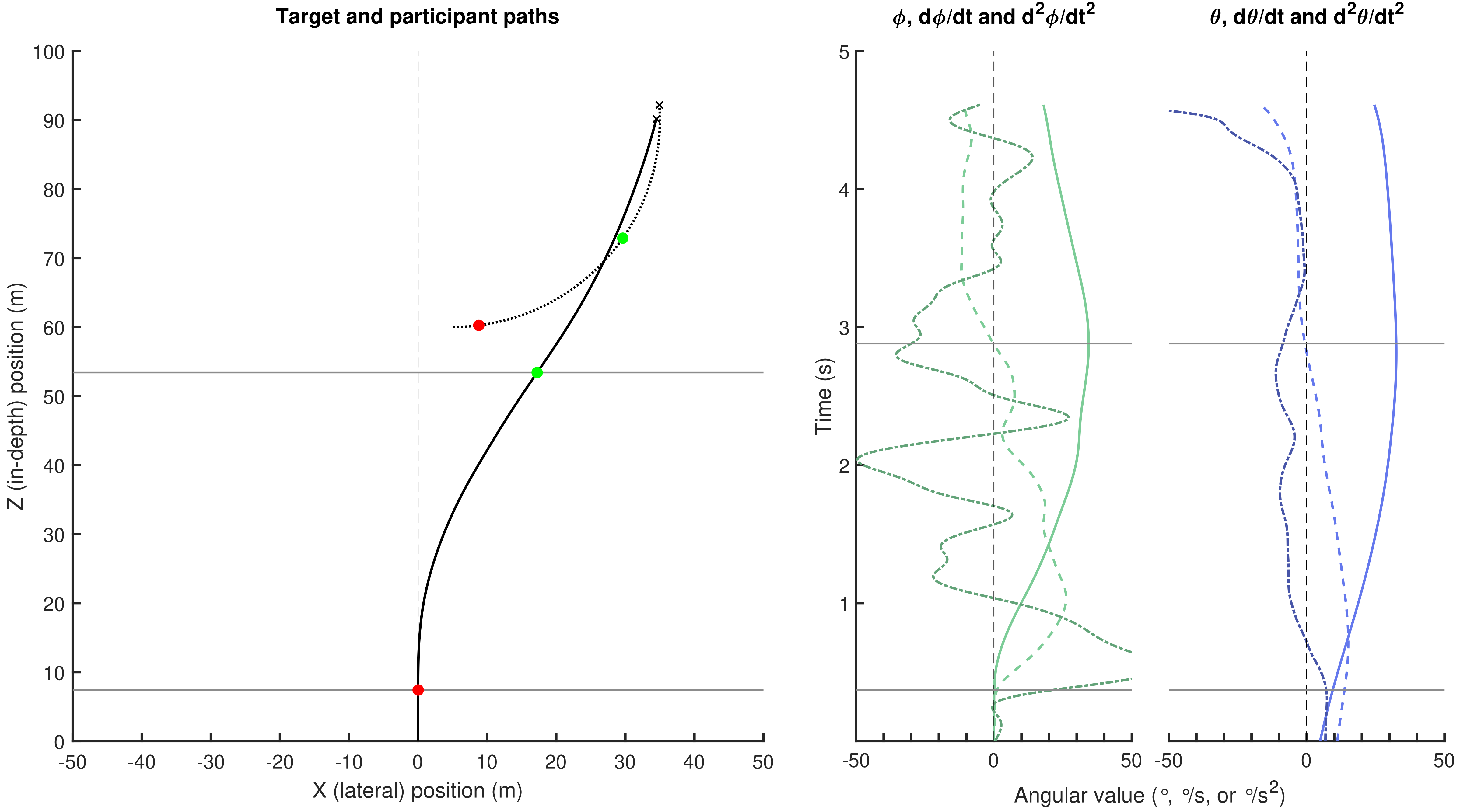

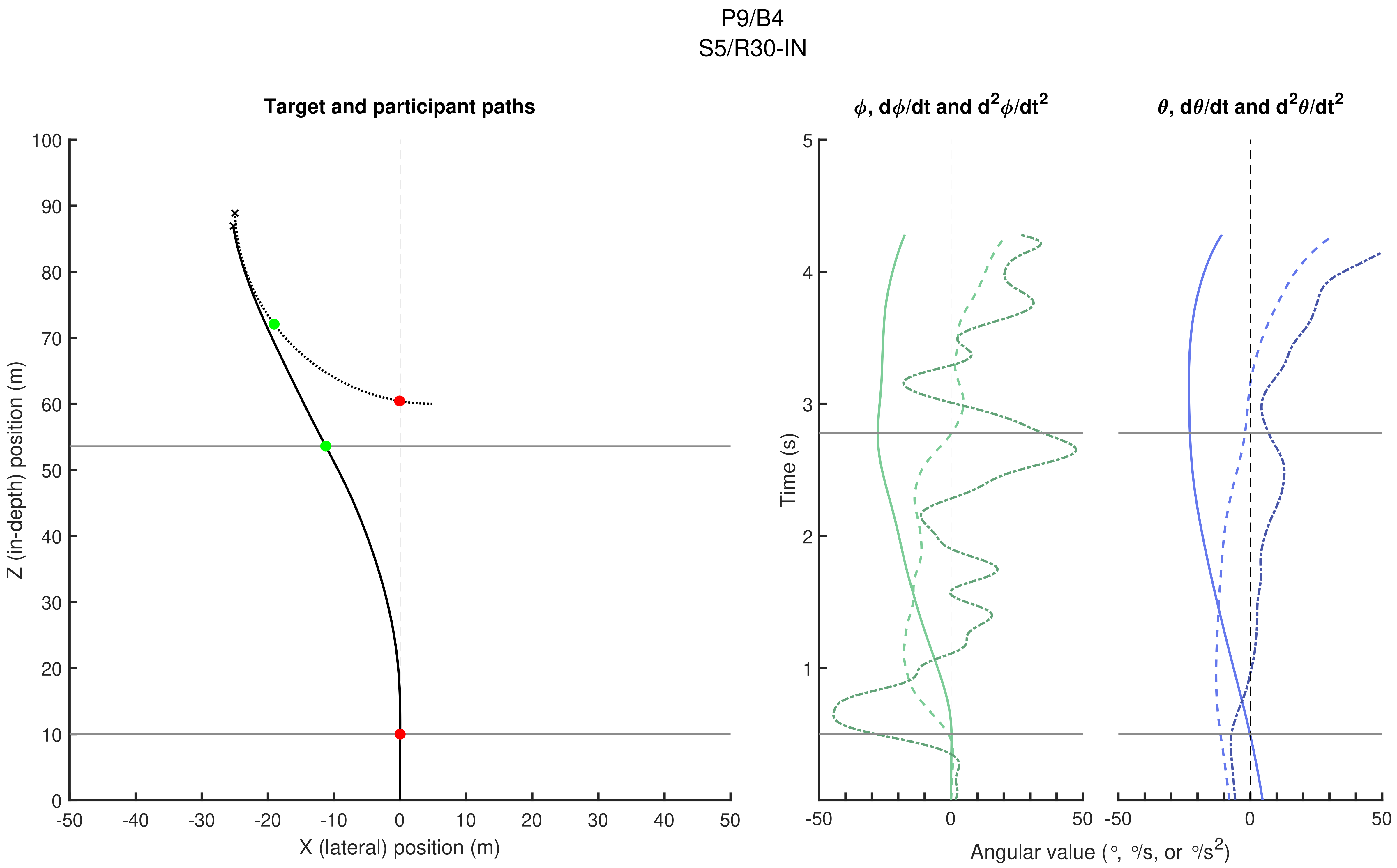
P9/B4
S5/R30-IN
Target and participant paths
ϕ, dϕ/dt and d$^2\phi$/dt^2
θ, dθ/dt and d$^2\theta$/dt^2
Z (in-depth) position (m)
X (lateral) position (m)
Time (s)
Angular value (°, °/s, or °/s^2)
100
90
80
70
60
50
40
30
20
10
0
-50
-40
-30
-20
-10
0
10
20
30
40
50
5
4
3
2
1
-50
0
50
-50
0
50

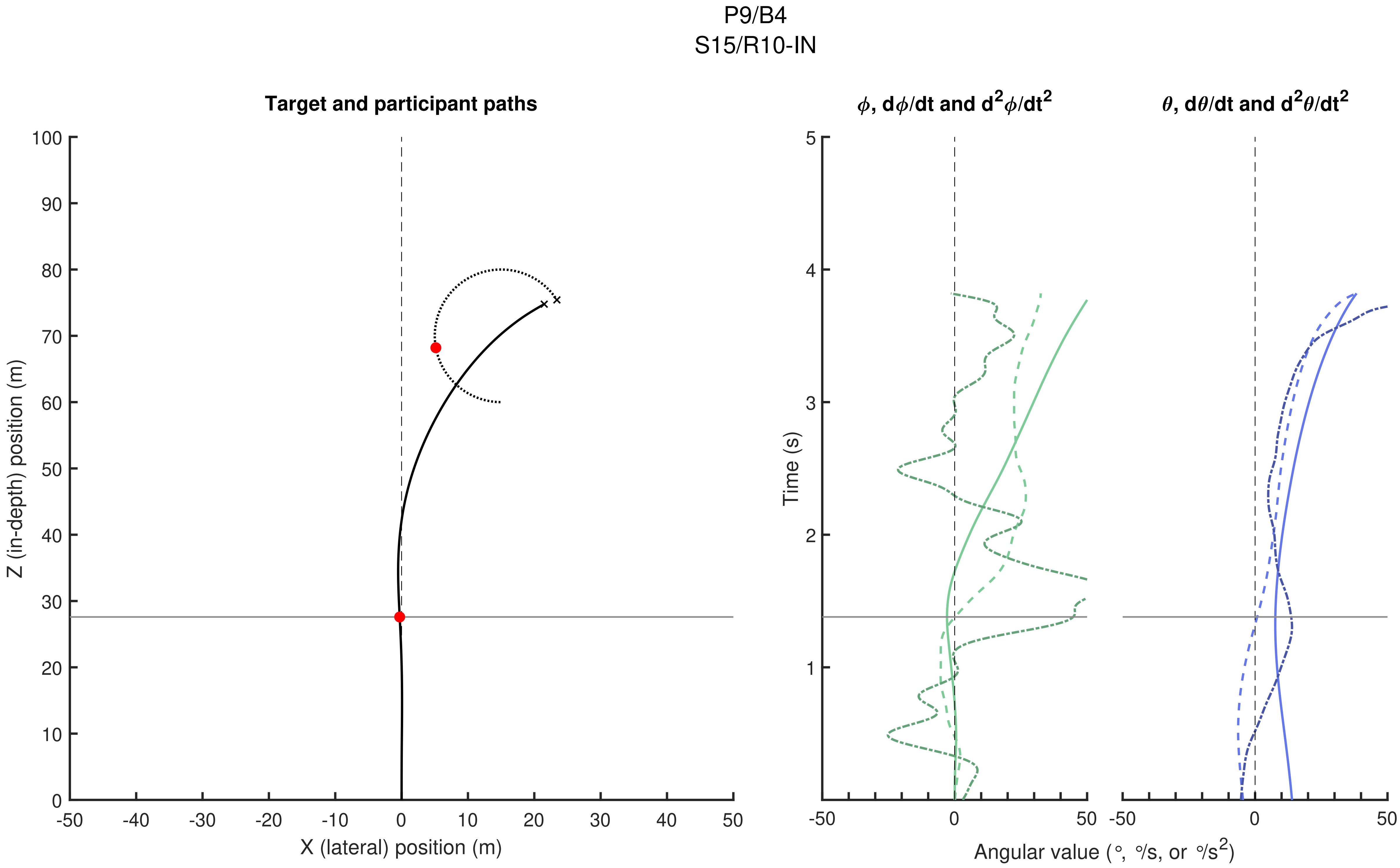
P9/B4
S15/R10-IN
Target and participant paths
Z (in-depth) position (m)
X (lateral) position (m)
φ, dφ/dt and d²φ/dt²
θ, dθ/dt and d²θ/dt²
Time (s)
Angular value (°, °/s, or °/s²)

P9/B4
S15/R20-IN

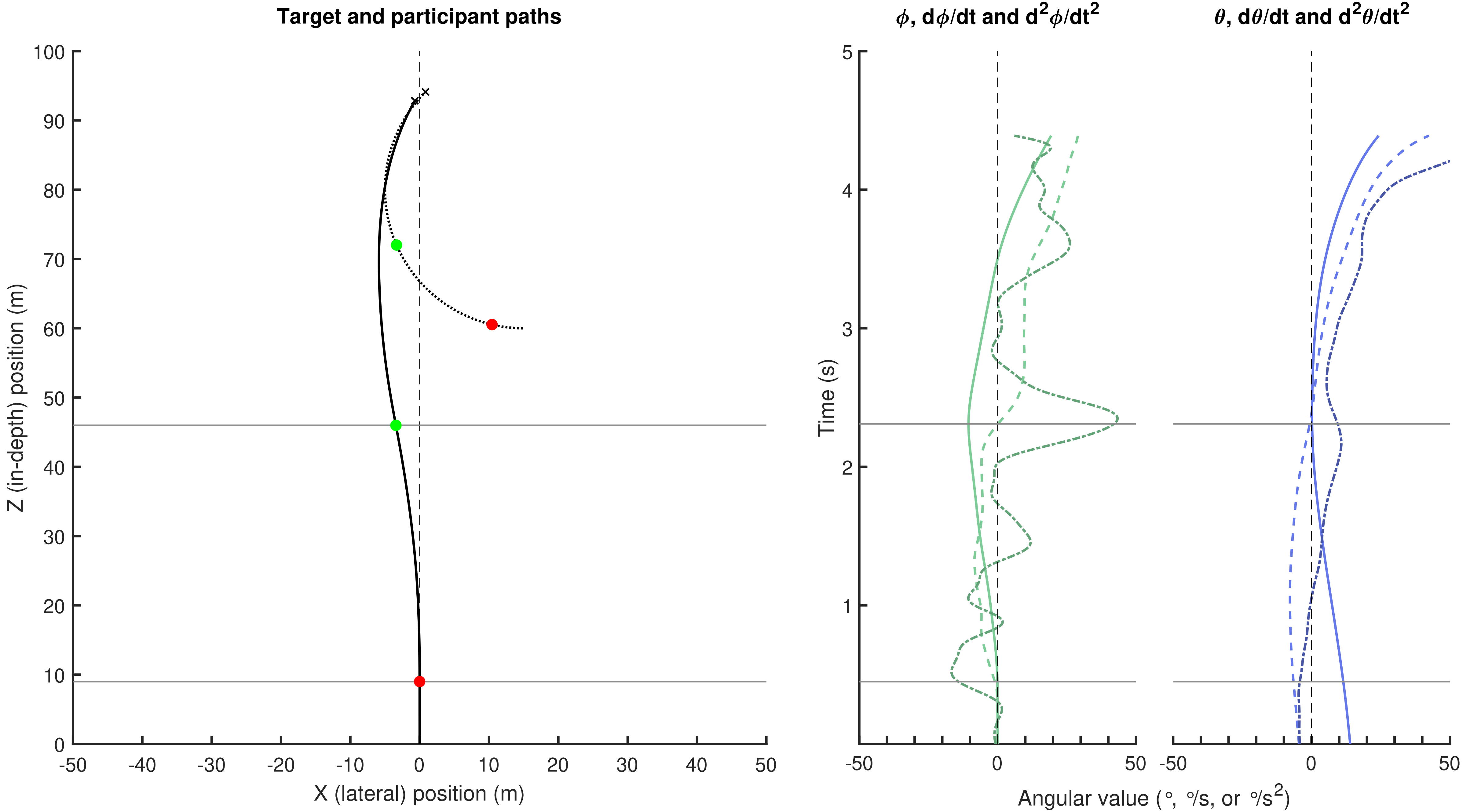

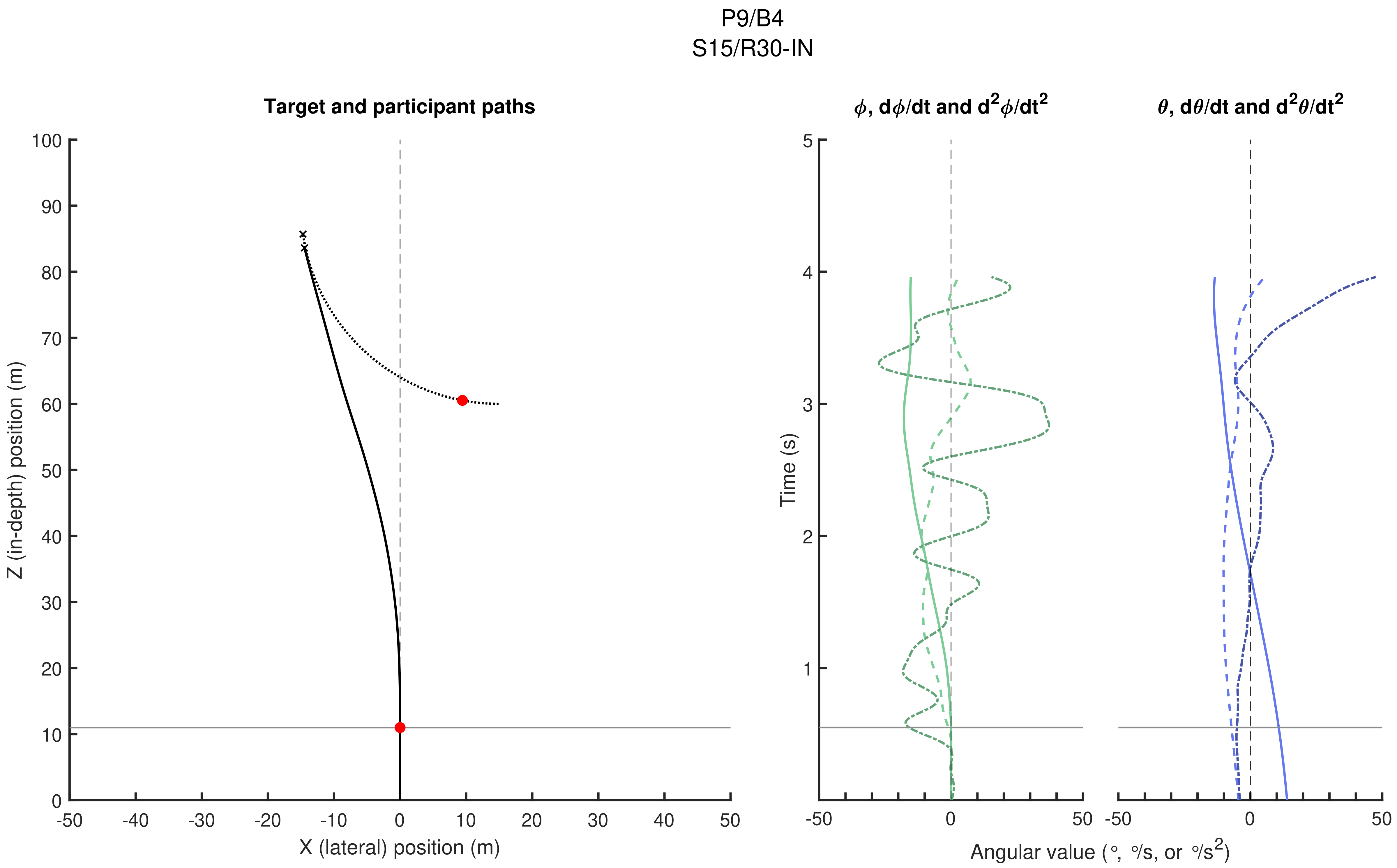

P9/B4
S15/R30-IN
Target and participant paths
Z (in-depth) position (m)
X (lateral) position (m)
ϕ, dϕ/dt and d$^2\phi$/dt^2
θ, dθ/dt and d$^2\theta$/dt^2
Time (s)
Angular value (°, °/s, or °/s^2)

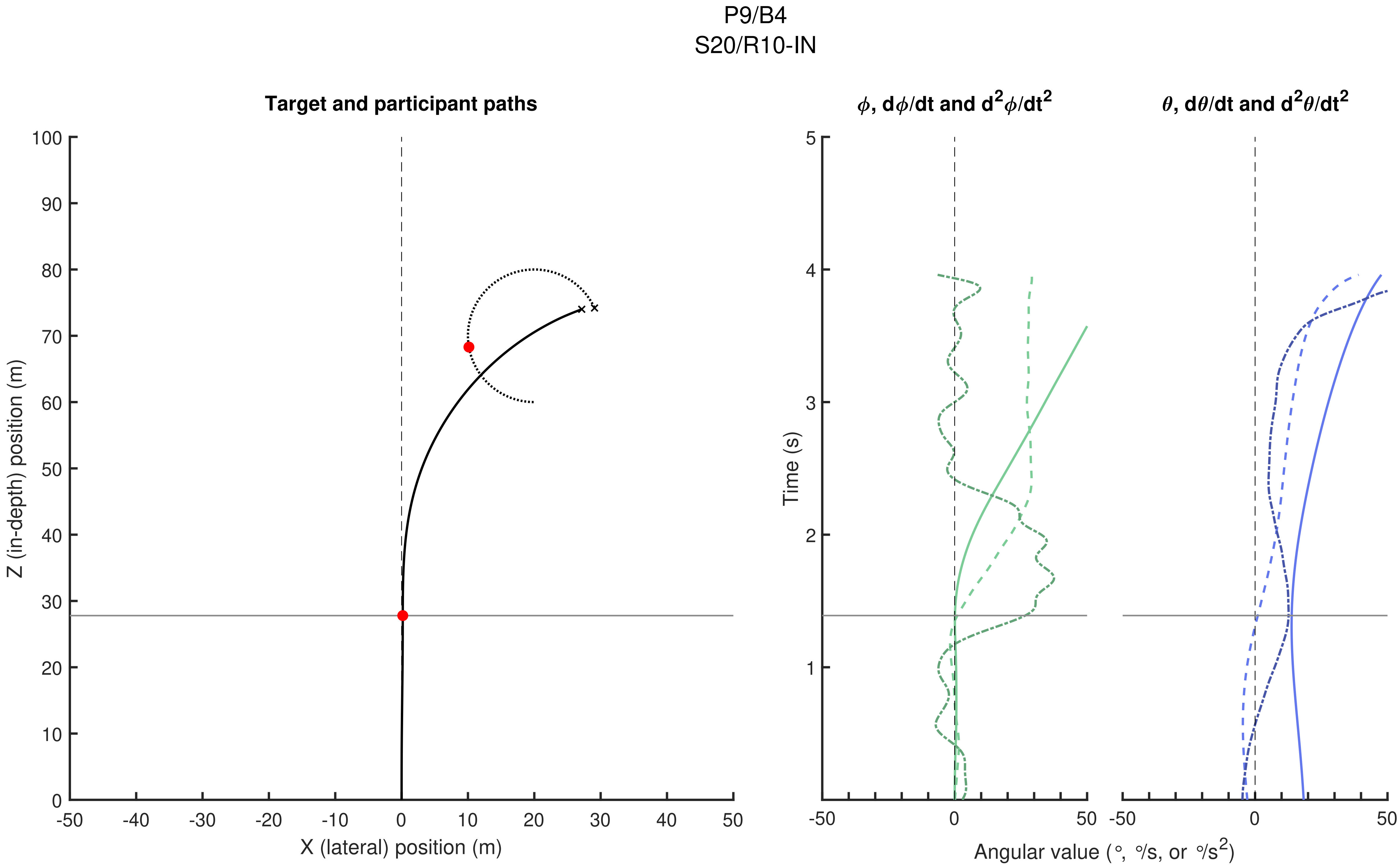
P9/B4
S20/R10-IN
Target and participant paths
Z (in-depth) position (m)
X (lateral) position (m)
100
90
80
70
60
50
40
30
20
10
0
-50
-40
-30
-20
-10
0
10
20
30
40
50
ϕ, dϕ/dt and d$^2\phi$/dt^2
θ, dθ/dt and d$^2\theta$/dt^2
Time (s)
5
4
3
2
1
-50
0
50
-50
0
50
Angular value (°, °/s, or °/s^2)

P9/B4
S20/R20-IN

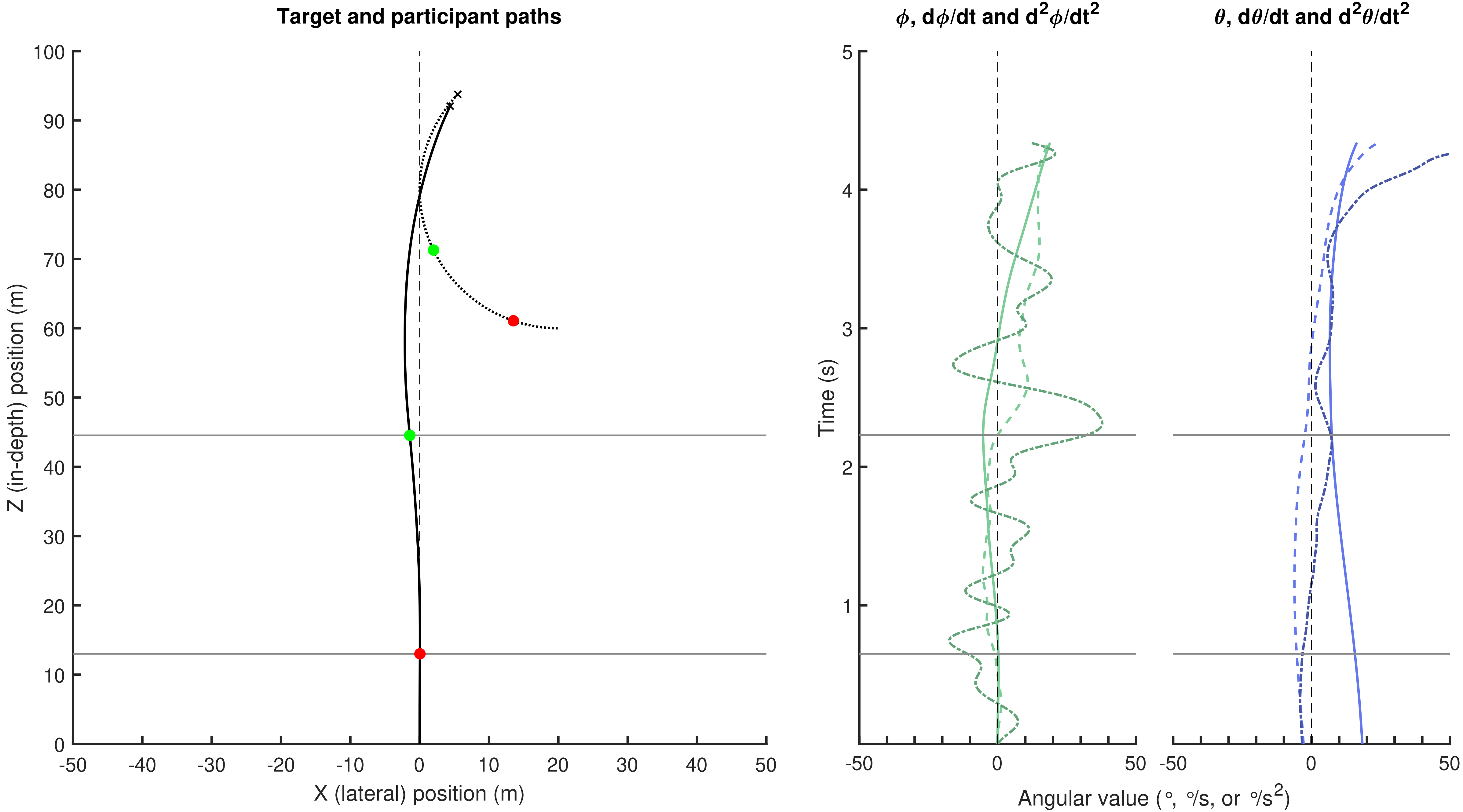

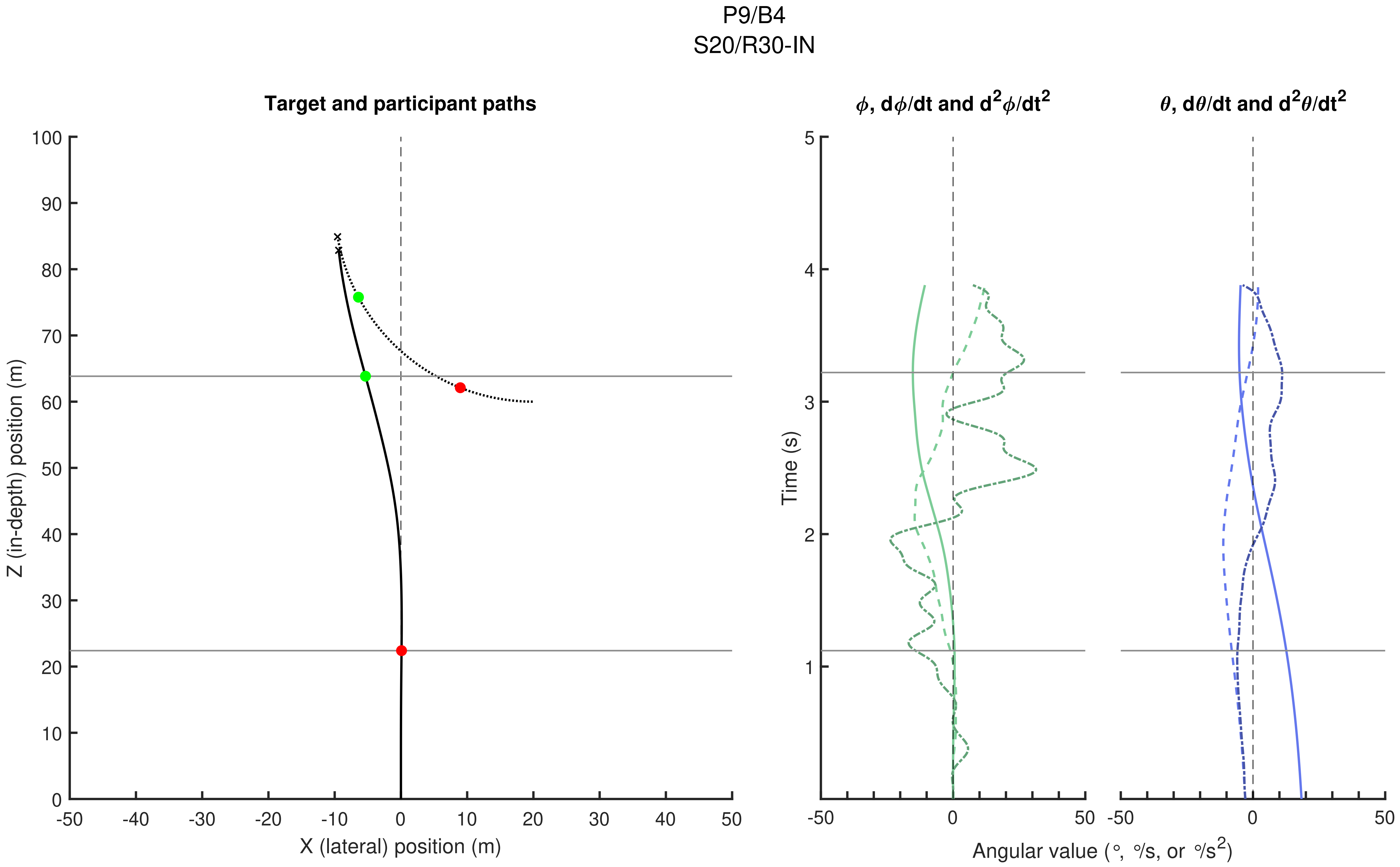
P9/B4
S20/R30-IN
Target and participant paths
Z (in-depth) position (m)
X (lateral) position (m)
φ, dφ/dt and d²φ/dt²
θ, dθ/dt and d²θ/dt²
Time (s)
Angular value (°, °/s, or °/s²)

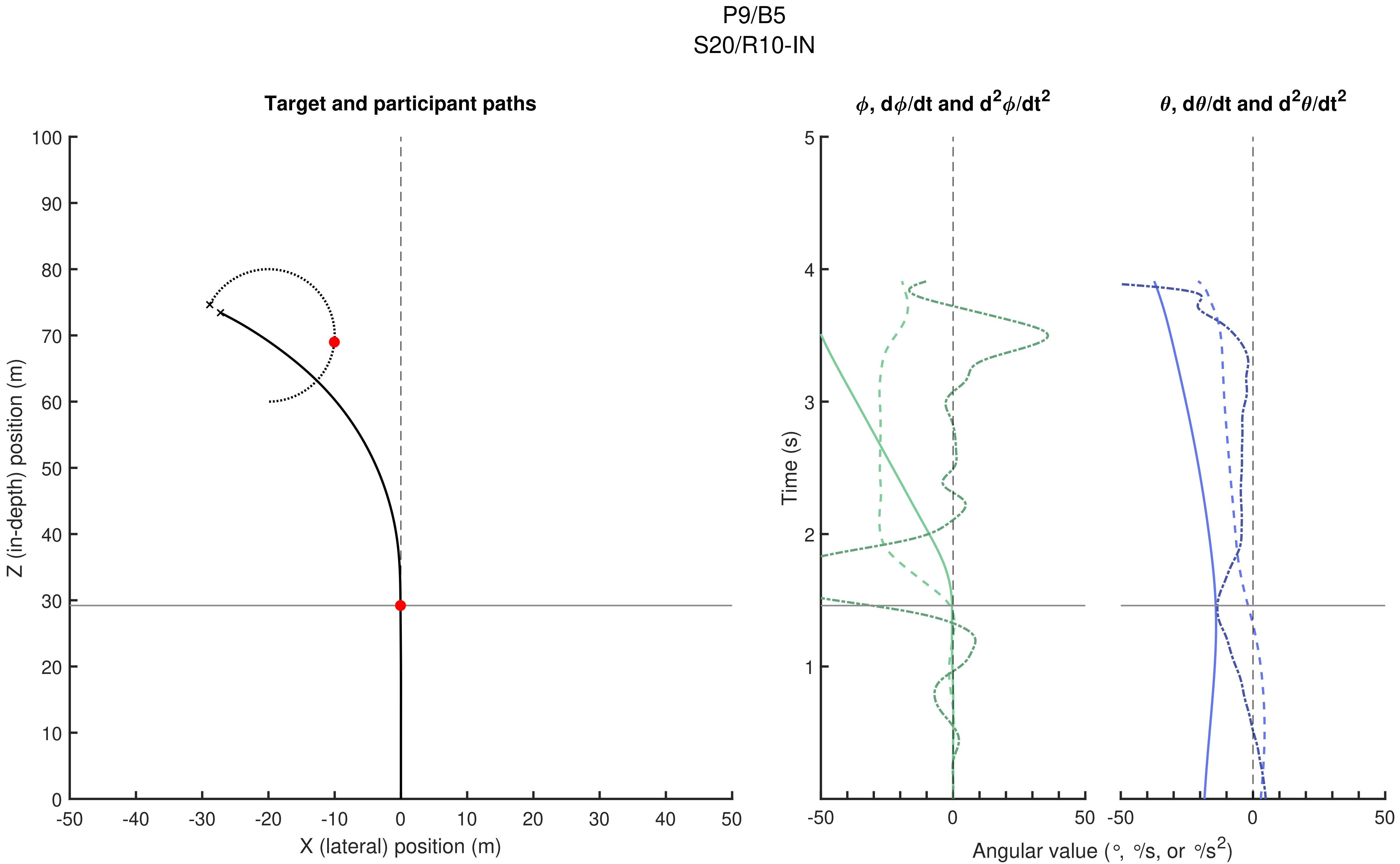
P9/B5
S20/R10-IN
Target and participant paths
Z (in-depth) position (m)
X (lateral) position (m)
100
90
80
70
60
50
40
30
20
10
0
-50
-40
-30
-20
-10
0
10
20
30
40
50
ϕ, dϕ/dt and d²ϕ/dt²
θ, dθ/dt and d²θ/dt²
Time (s)
5
4
3
2
1
-50
0
50
-50
0
50
Angular value (°, °/s, or °/s²)

P9/B5
S20/R20-IN

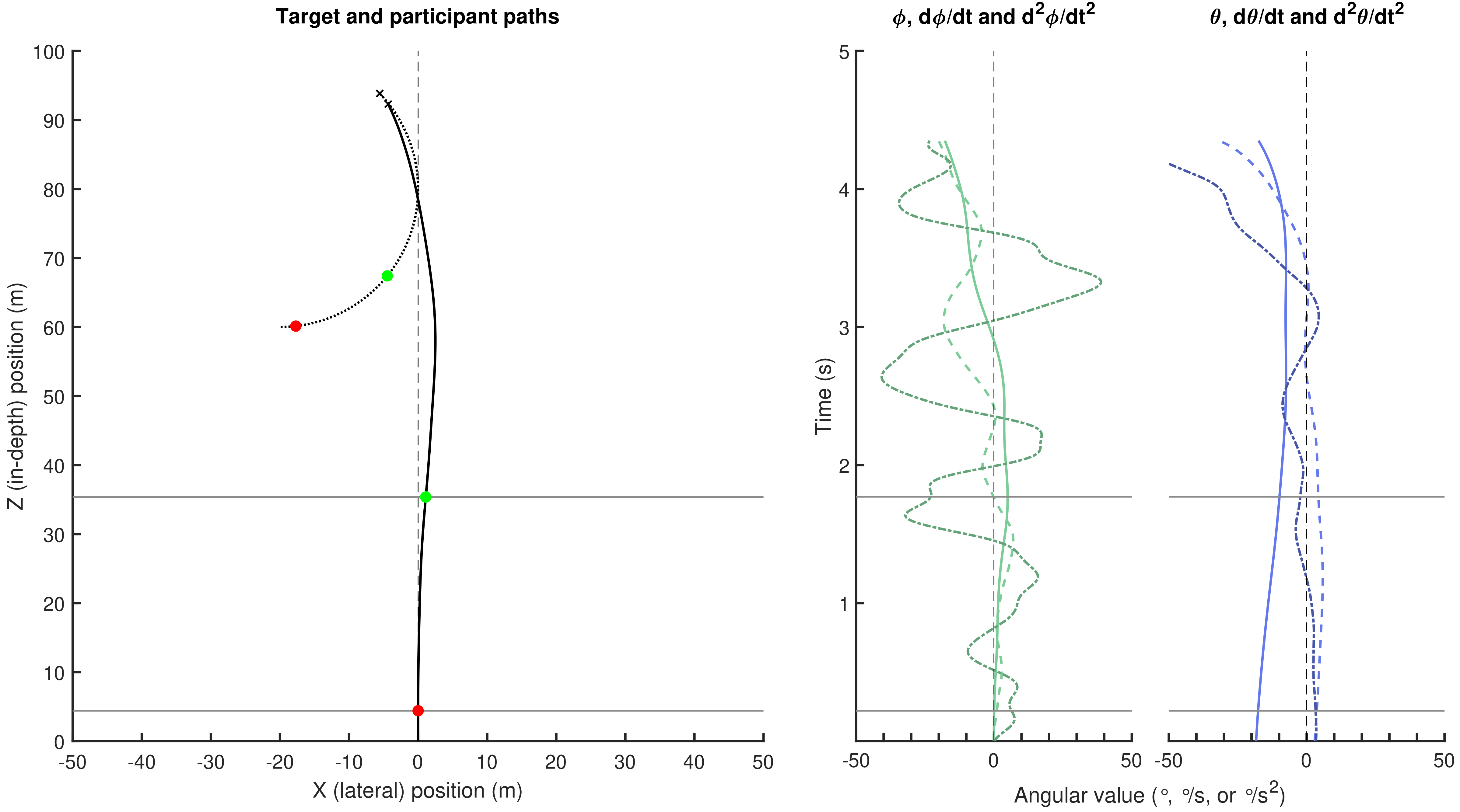

P9/B5
S20/R30-IN

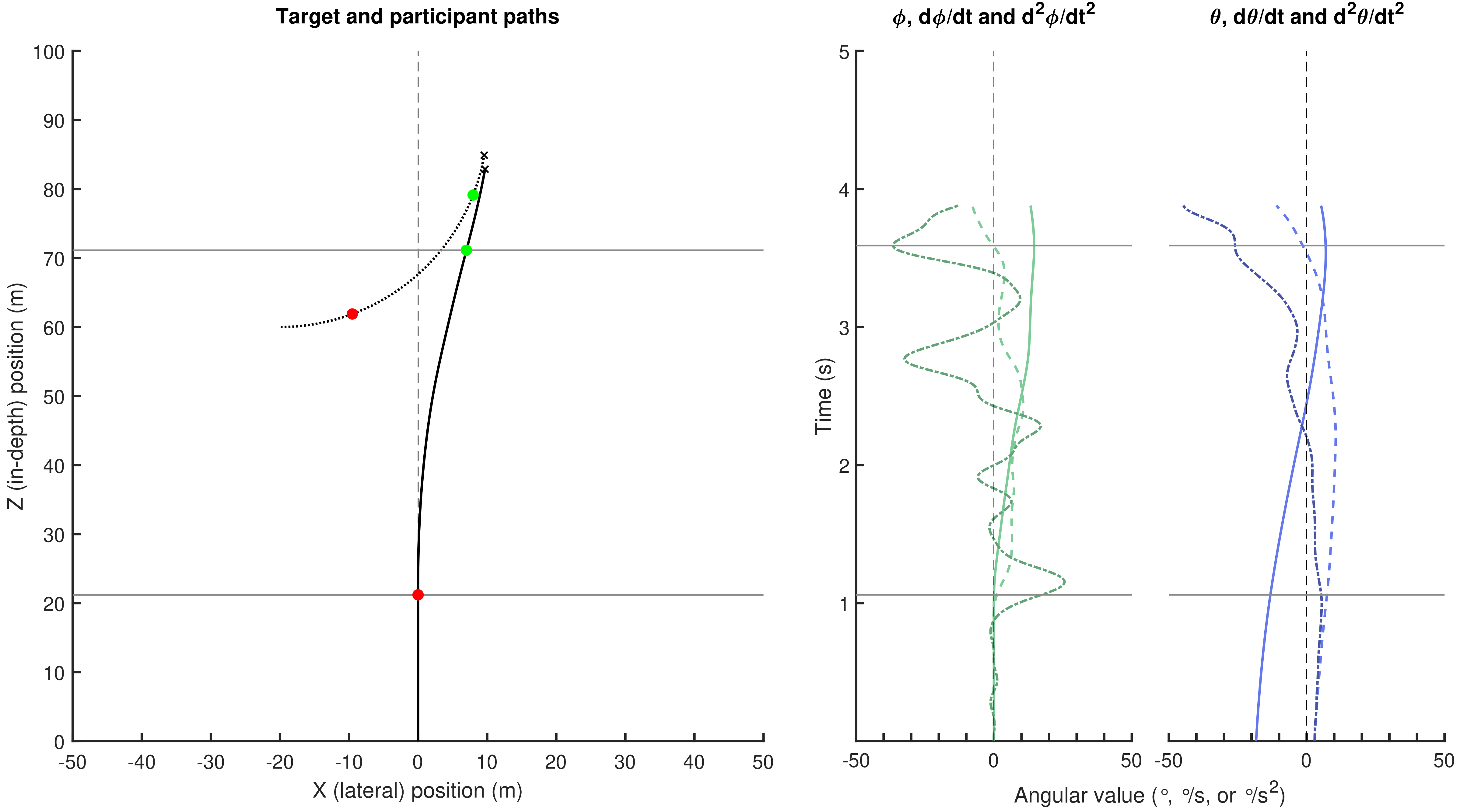

P9/B5
S15/R10-IN

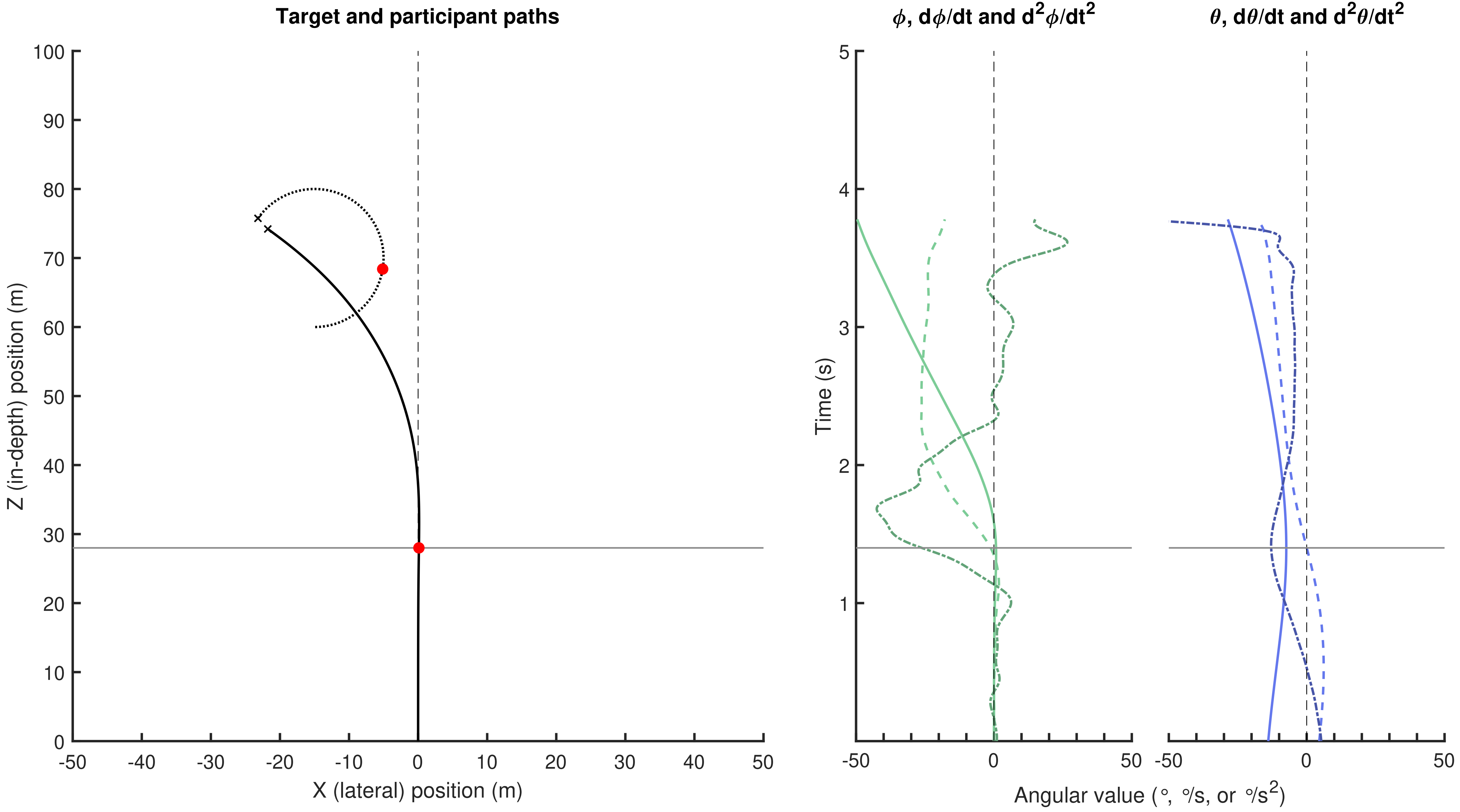

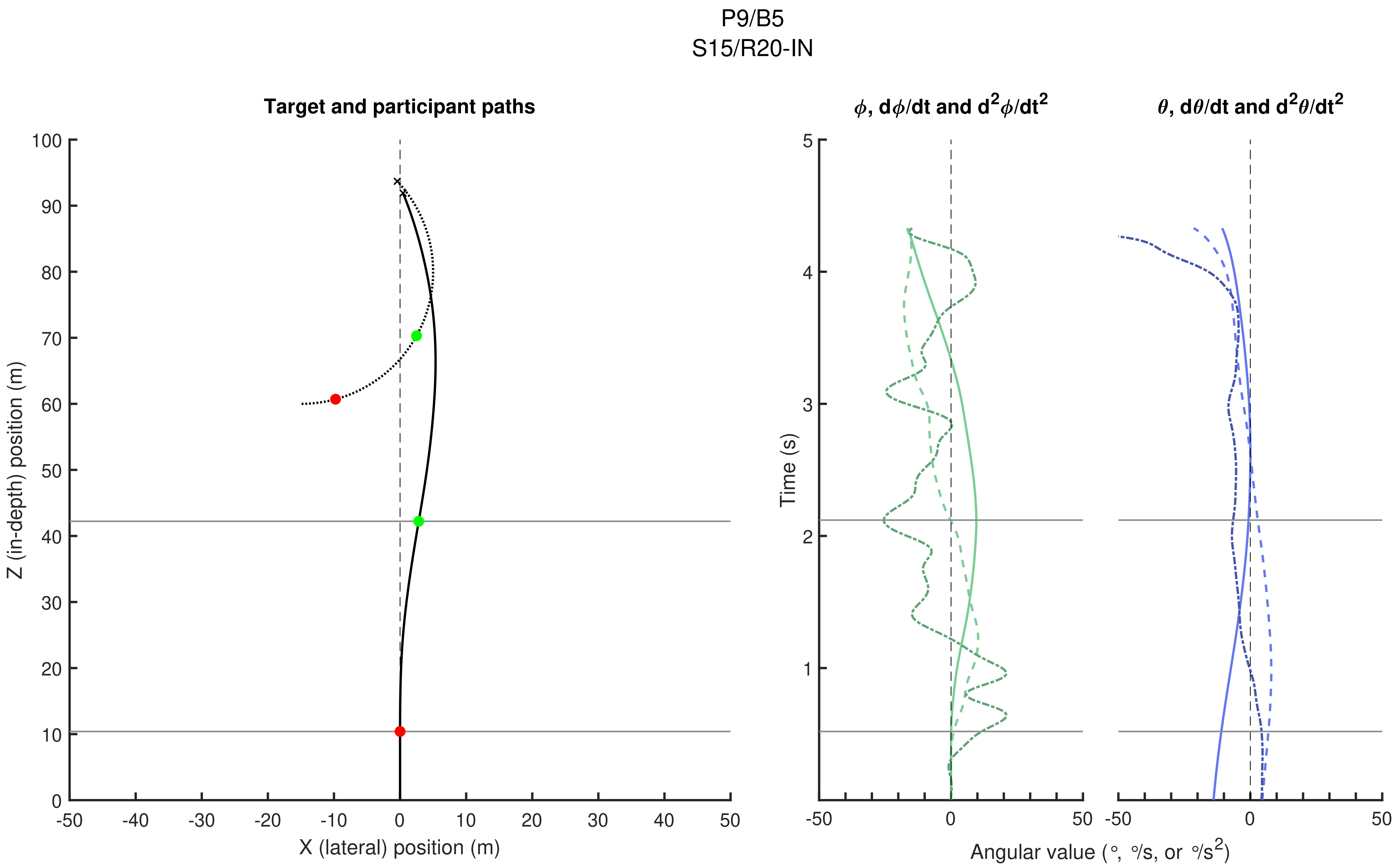
P9/B5
S15/R20-IN
Target and participant paths
Z (in-depth) position (m)
X (lateral) position (m)
0
10
20
30
40
50
60
70
80
90
100
-50
-40
-30
-20
-10
0
10
20
30
40
50
ϕ, dϕ/dt and d$^2\phi$/dt^2
θ, dθ/dt and d$^2\theta$/dt^2
Time (s)
1
2
3
4
5
-50
0
50
-50
0
50
Angular value (°, °/s, or °/s^2)

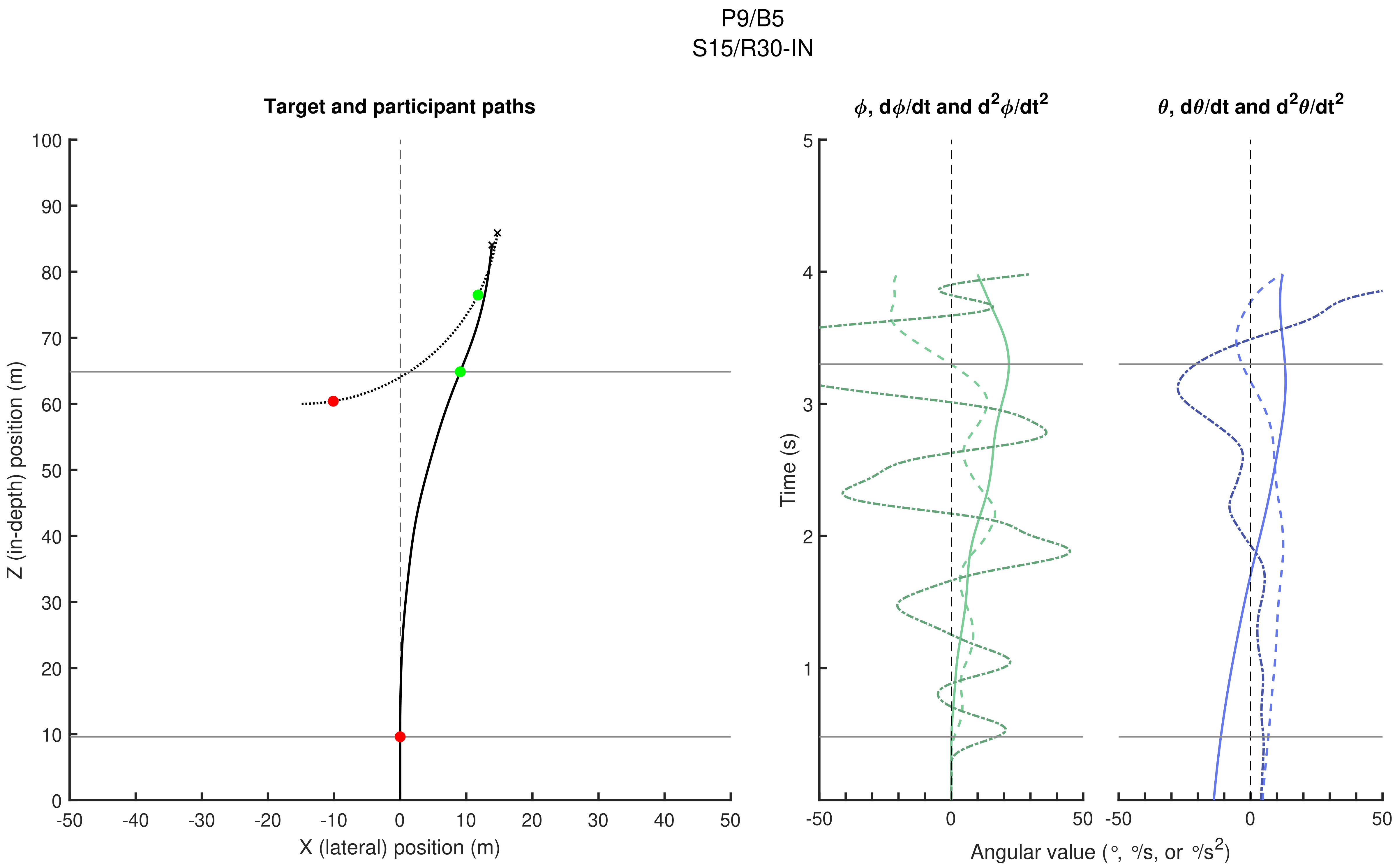
P9/B5
S15/R30-IN
Target and participant paths
Z (in-depth) position (m)
X (lateral) position (m)
φ, dφ/dt and d²φ/dt²
θ, dθ/dt and d²θ/dt²
Time (s)
Angular value (°, °/s, or °/s²)

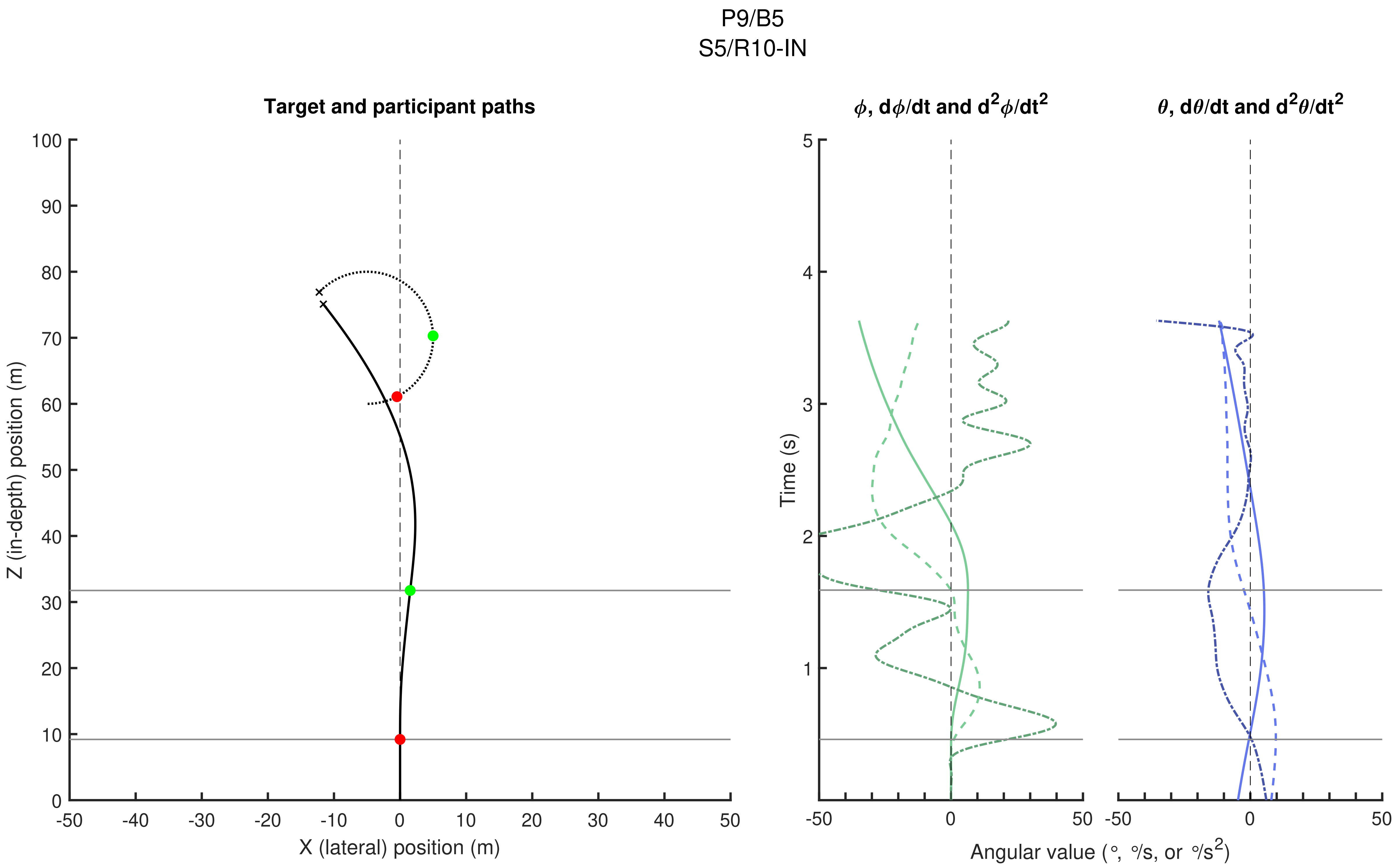
P9/B5
S5/R10-IN
Target and participant paths
Z (in-depth) position (m)
X (lateral) position (m)
φ, dφ/dt and d²φ/dt²
θ, dθ/dt and d²θ/dt²
Time (s)
Angular value (°, °/s, or °/s²)

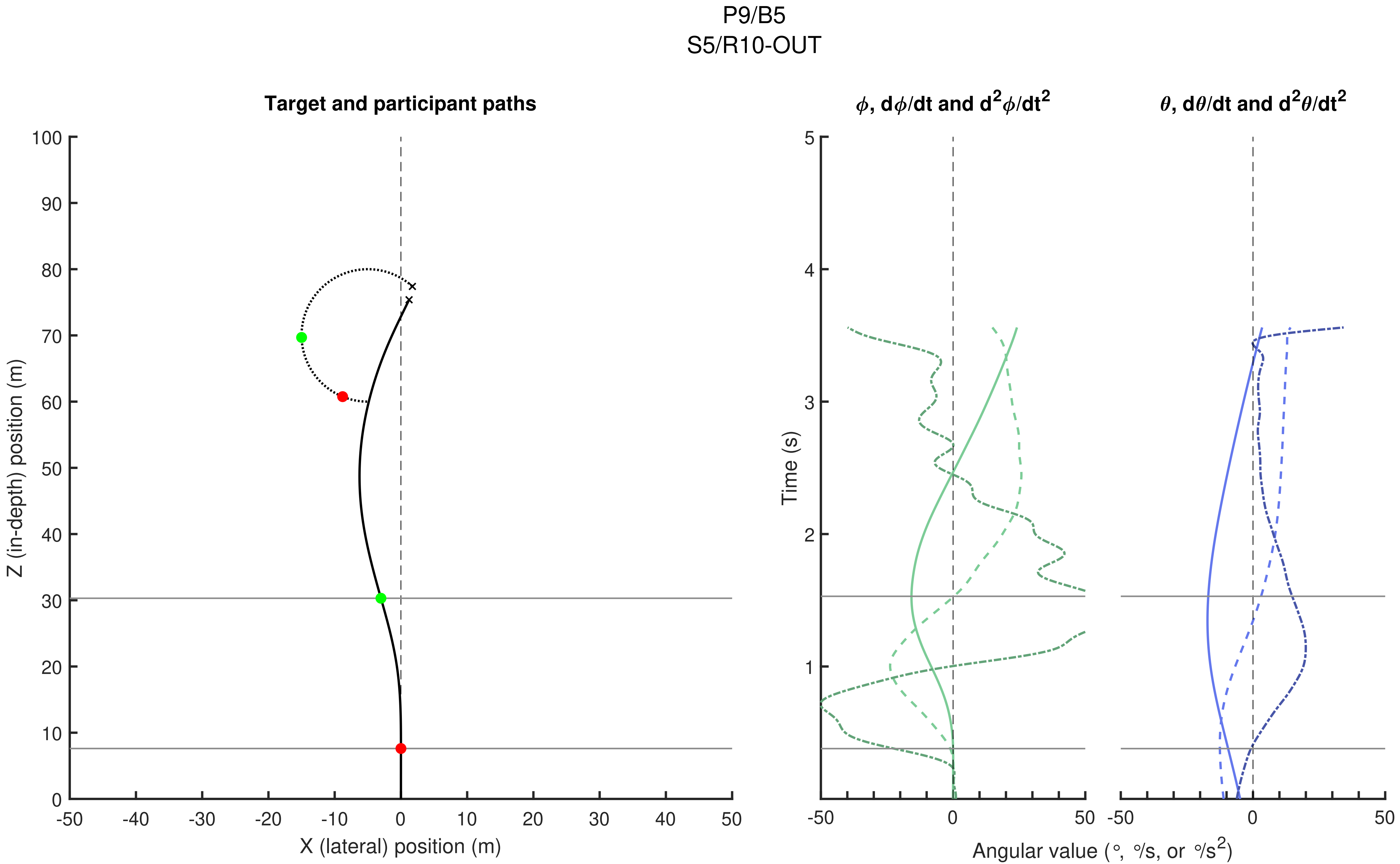
P9/B5
S5/R10-OUT
Target and participant paths
Z (in-depth) position (m)
X (lateral) position (m)
φ, dφ/dt and d²φ/dt²
θ, dθ/dt and d²θ/dt²
Time (s)
Angular value (°, °/s, or °/s²)

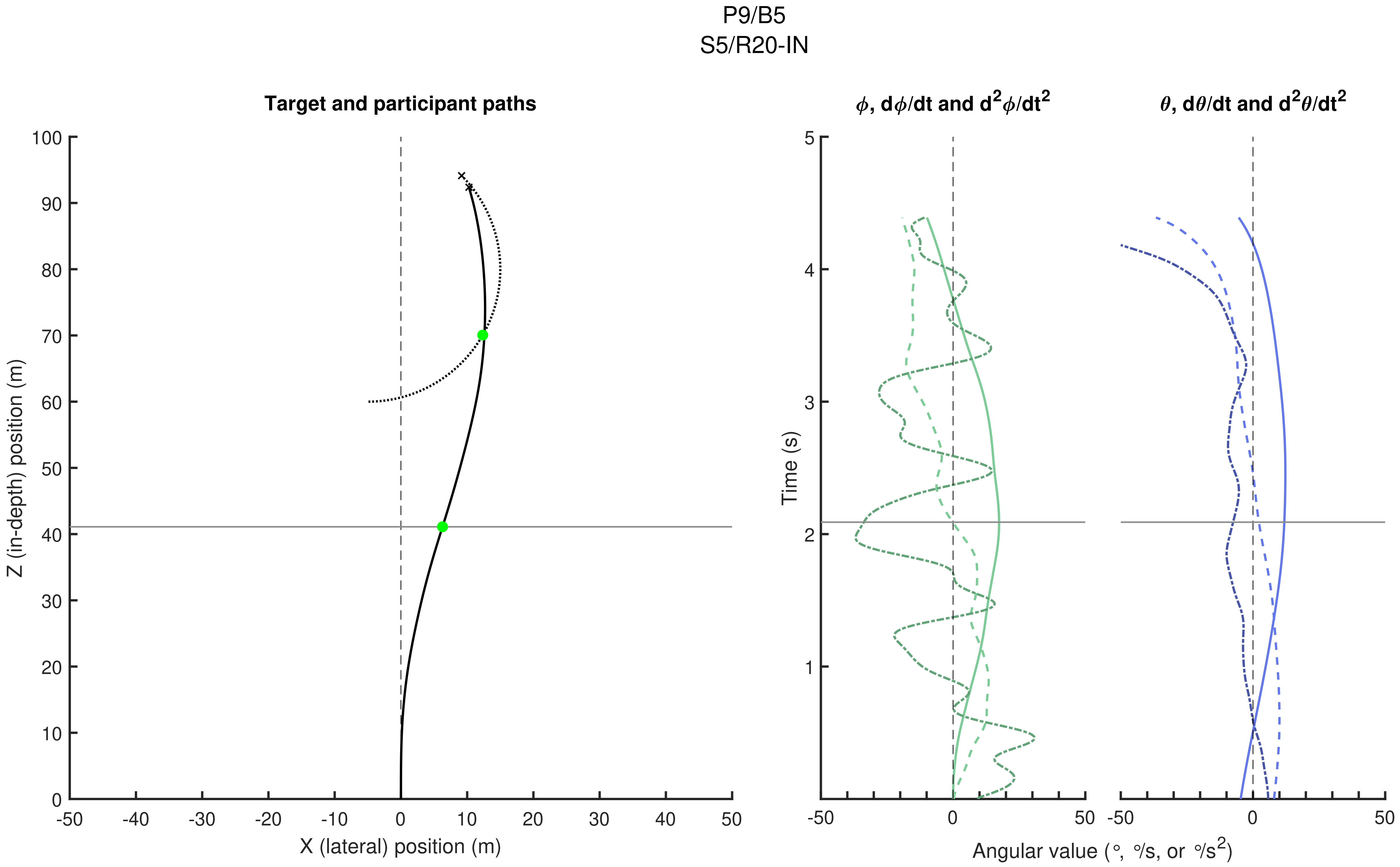
P9/B5
S5/R20-IN
Target and participant paths
Z (in-depth) position (m)
X (lateral) position (m)
ϕ, dϕ/dt and d²ϕ/dt²
θ, dθ/dt and d²θ/dt²
Time (s)
Angular value (°, °/s, or °/s²)

P9/B5
S5/R20-OUT

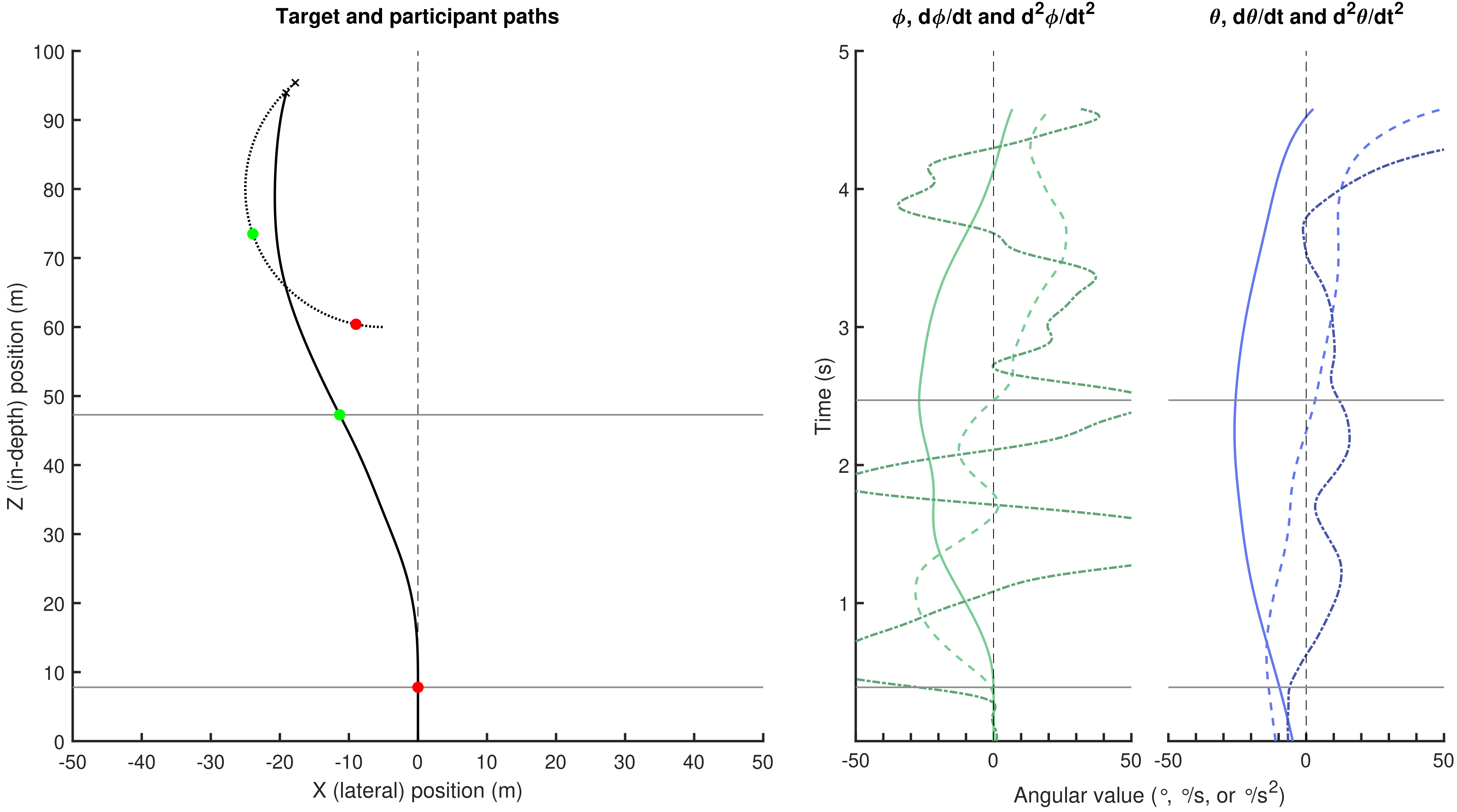

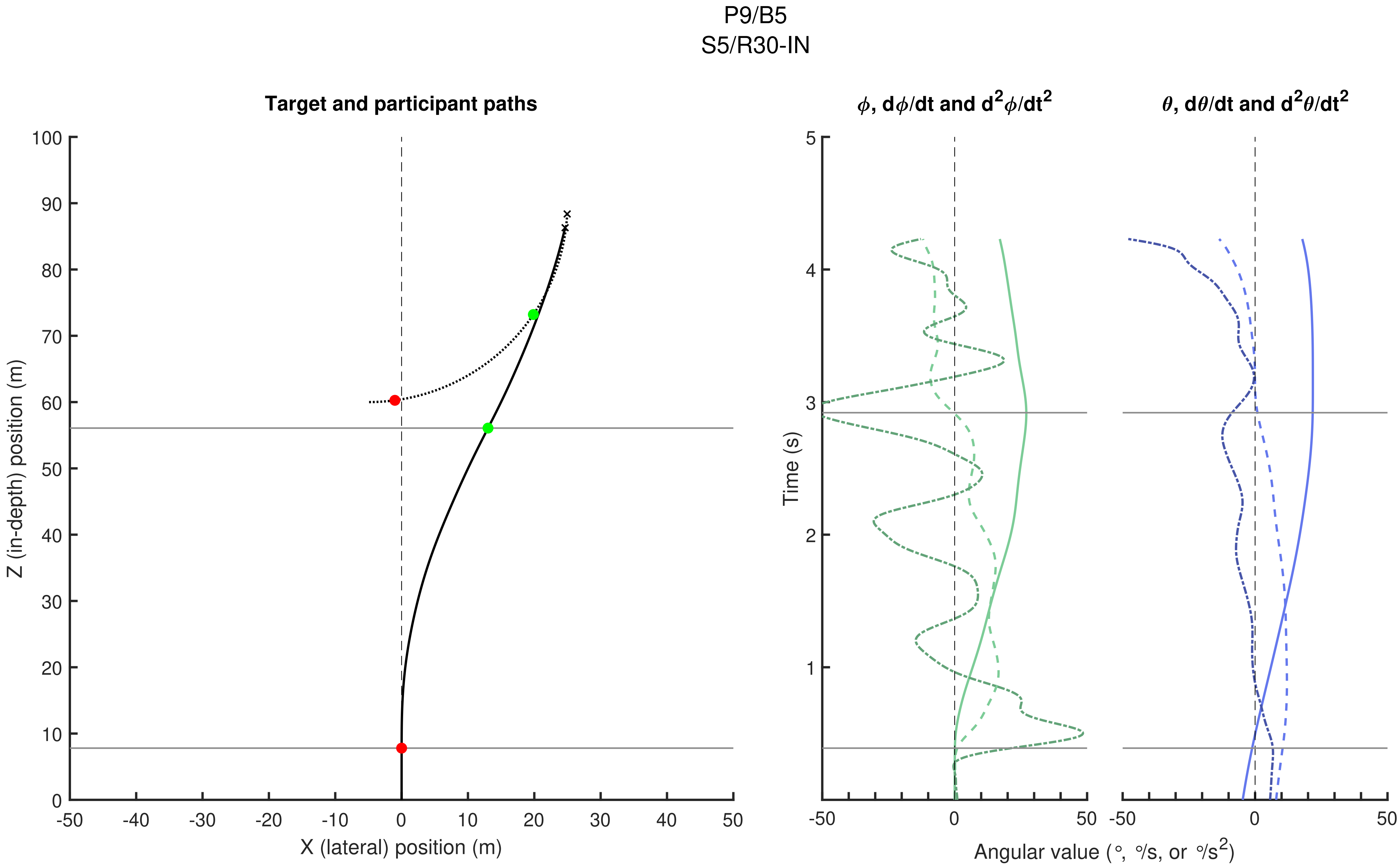
P9/B5
S5/R30-IN
Target and participant paths
Z (in-depth) position (m)
X (lateral) position (m)
φ, dφ/dt and d²φ/dt²
θ, dθ/dt and d²θ/dt²
Time (s)
Angular value (°, °/s, or °/s²)

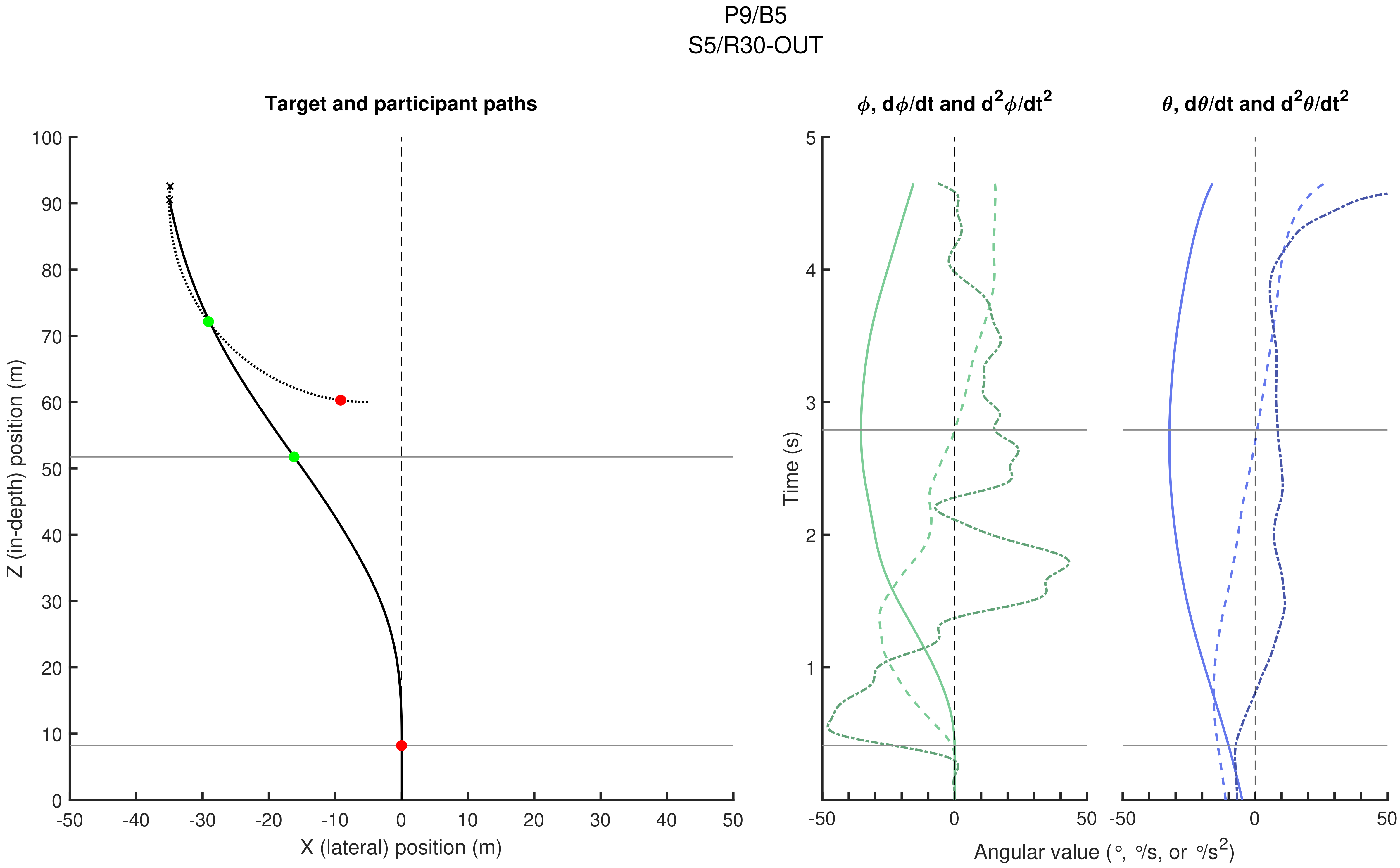

P9/B5
S5/R30-OUT
Target and participant paths
Z (in-depth) position (m)
X (lateral) position (m)
-50
-40
-30
-20
-10
0
10
20
30
40
50
60
70
80
90
100
ϕ, dϕ/dt and d²ϕ/dt²
θ, dθ/dt and d²θ/dt²
Time (s)
1
2
3
4
5
Angular value (°, °/s, or °/s²)

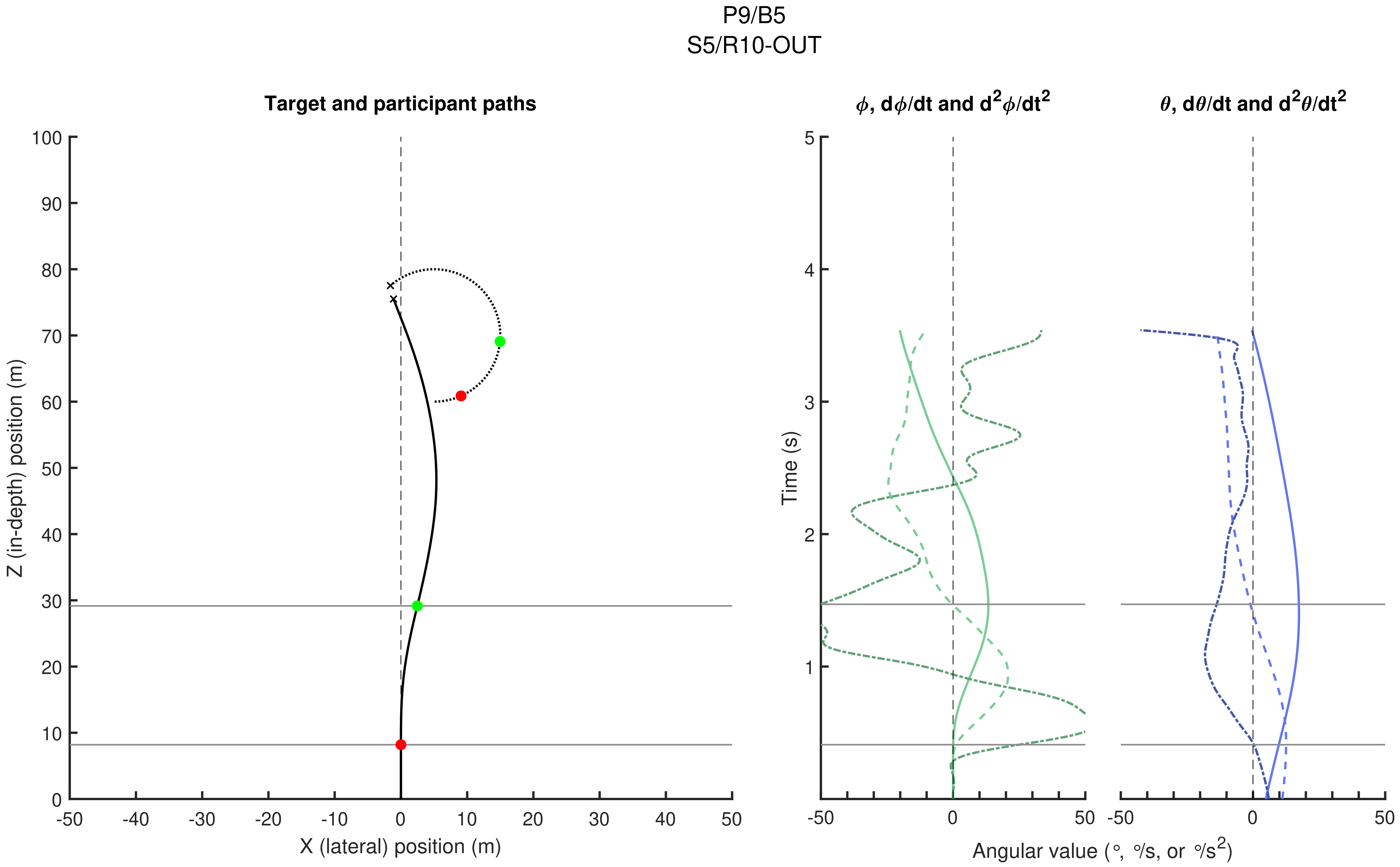
P9/B5
S5/R10-OUT
Target and participant paths
Z (in-depth) position (m)
X (lateral) position (m)
φ, dφ/dt and d²φ/dt²
θ, dθ/dt and d²θ/dt²
Time (s)
Angular value (°, °/s, or °/s²)

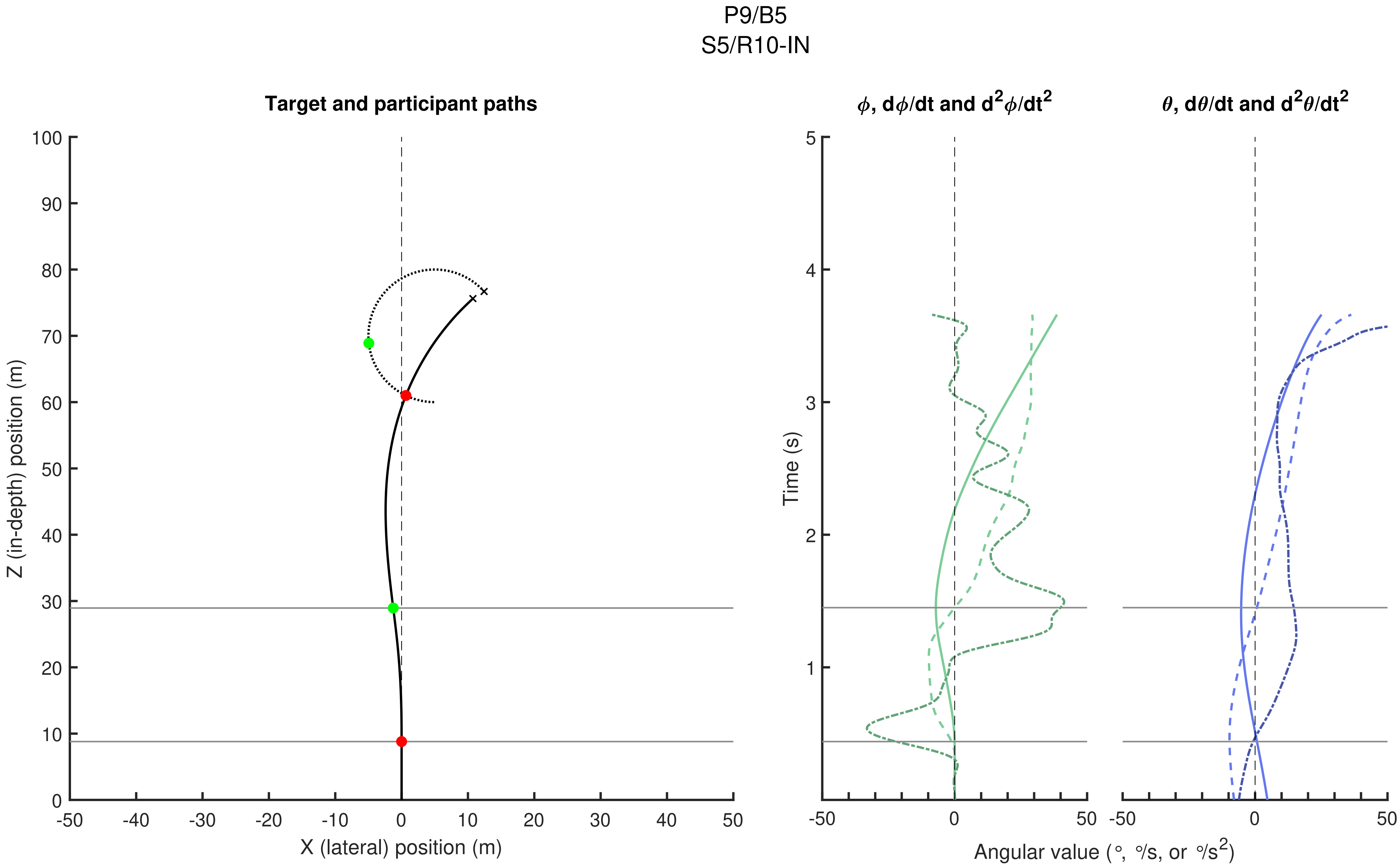

P9/B5
S5/R10-IN
Target and participant paths
Z (in-depth) position (m)
X (lateral) position (m)
100
90
80
70
60
50
40
30
20
10
0
-50
-40
-30
-20
-10
0
10
20
30
40
50
ϕ, dϕ/dt and d$^2\phi$/dt^2
θ, dθ/dt and d$^2\theta$/dt^2
Time (s)
5
4
3
2
1
-50
0
50
-50
0
50
Angular value (°, °/s, or °/s^2)

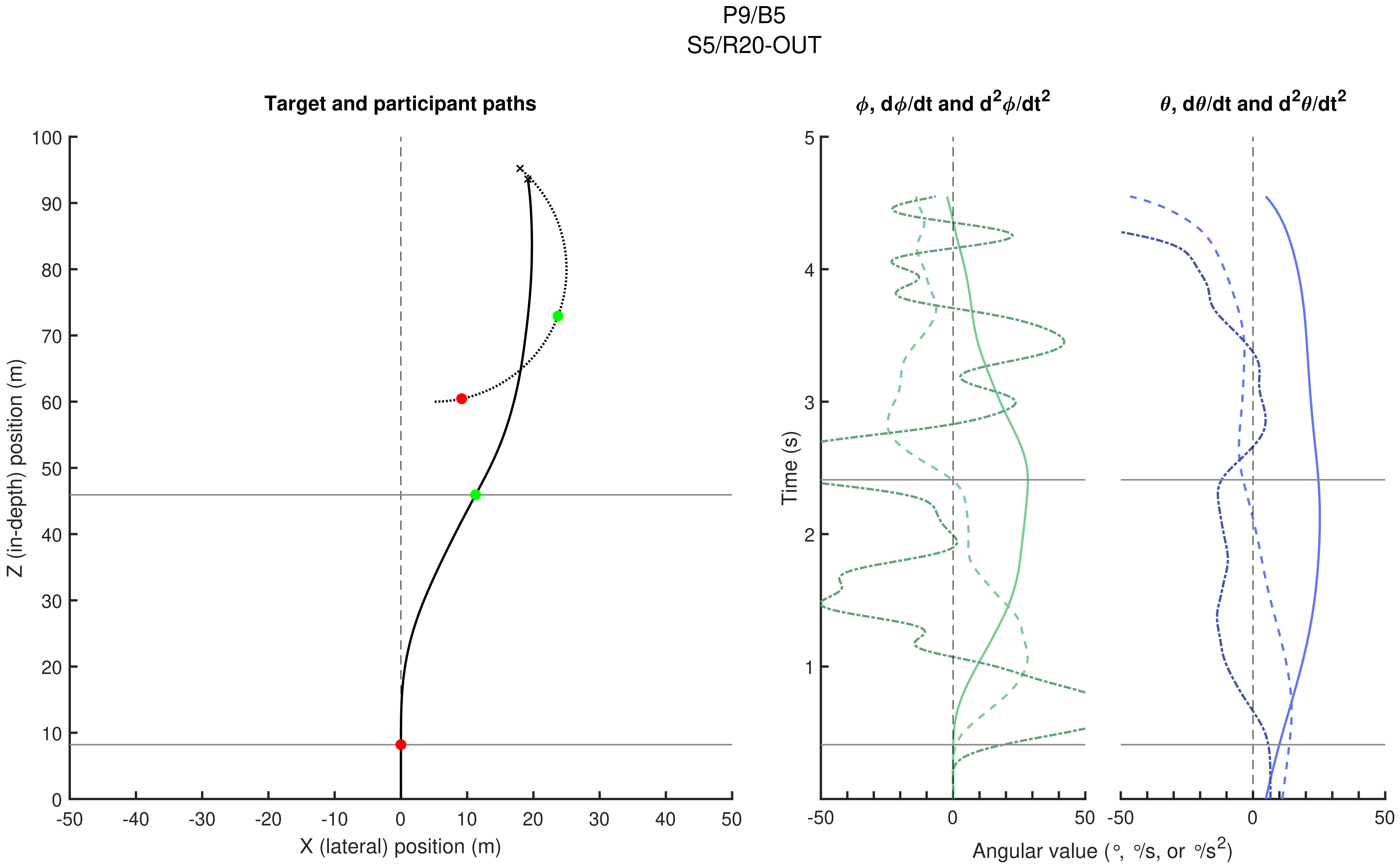

P9/B5
S5/R20-OUT
Target and participant paths
Z (in-depth) position (m)
X (lateral) position (m)
φ, dφ/dt and d²φ/dt²
θ, dθ/dt and d²θ/dt²
Time (s)
Angular value (°, °/s, or °/s²)

P9/B5
S5/R20-IN

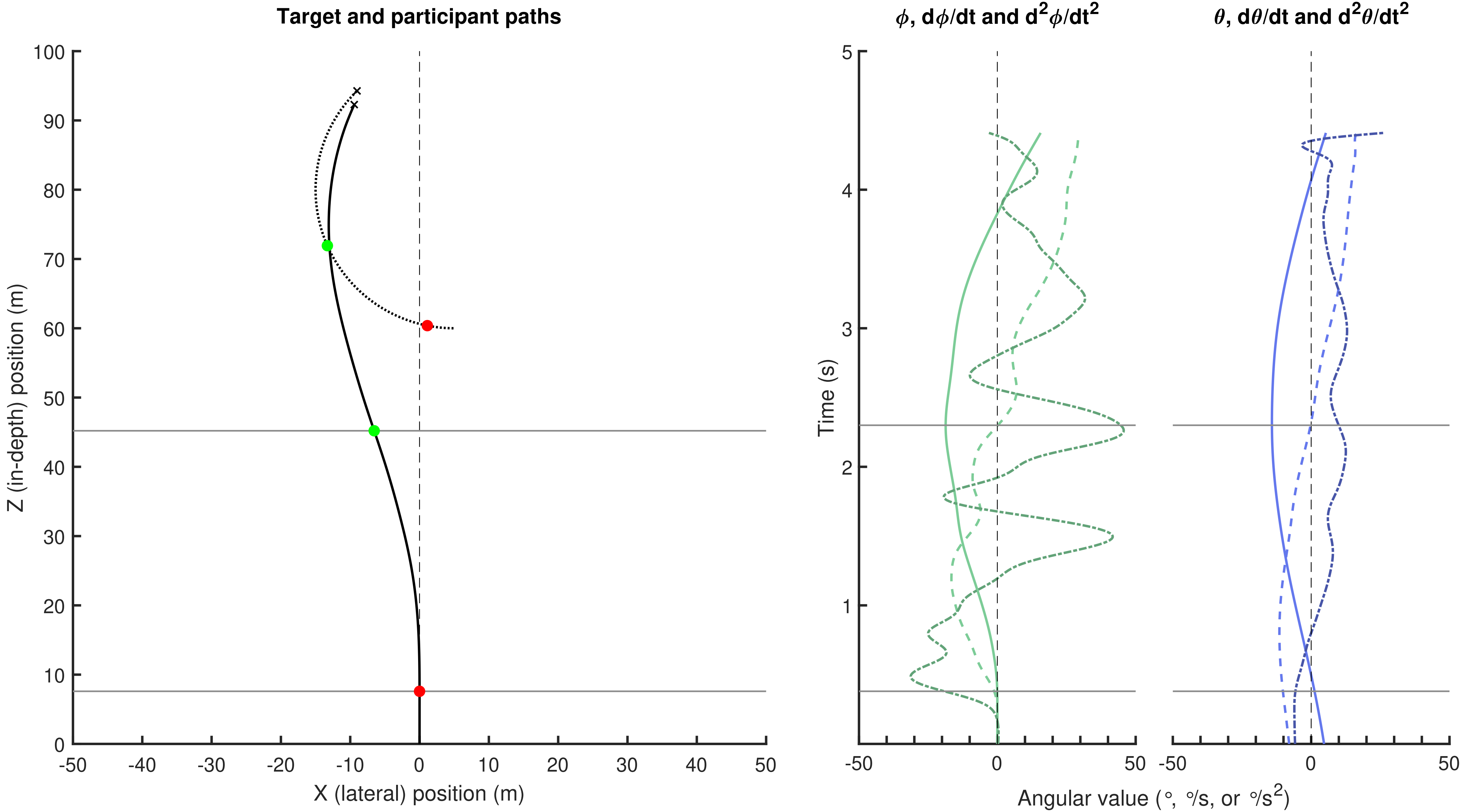

P9/B5
S5/R30-OUT

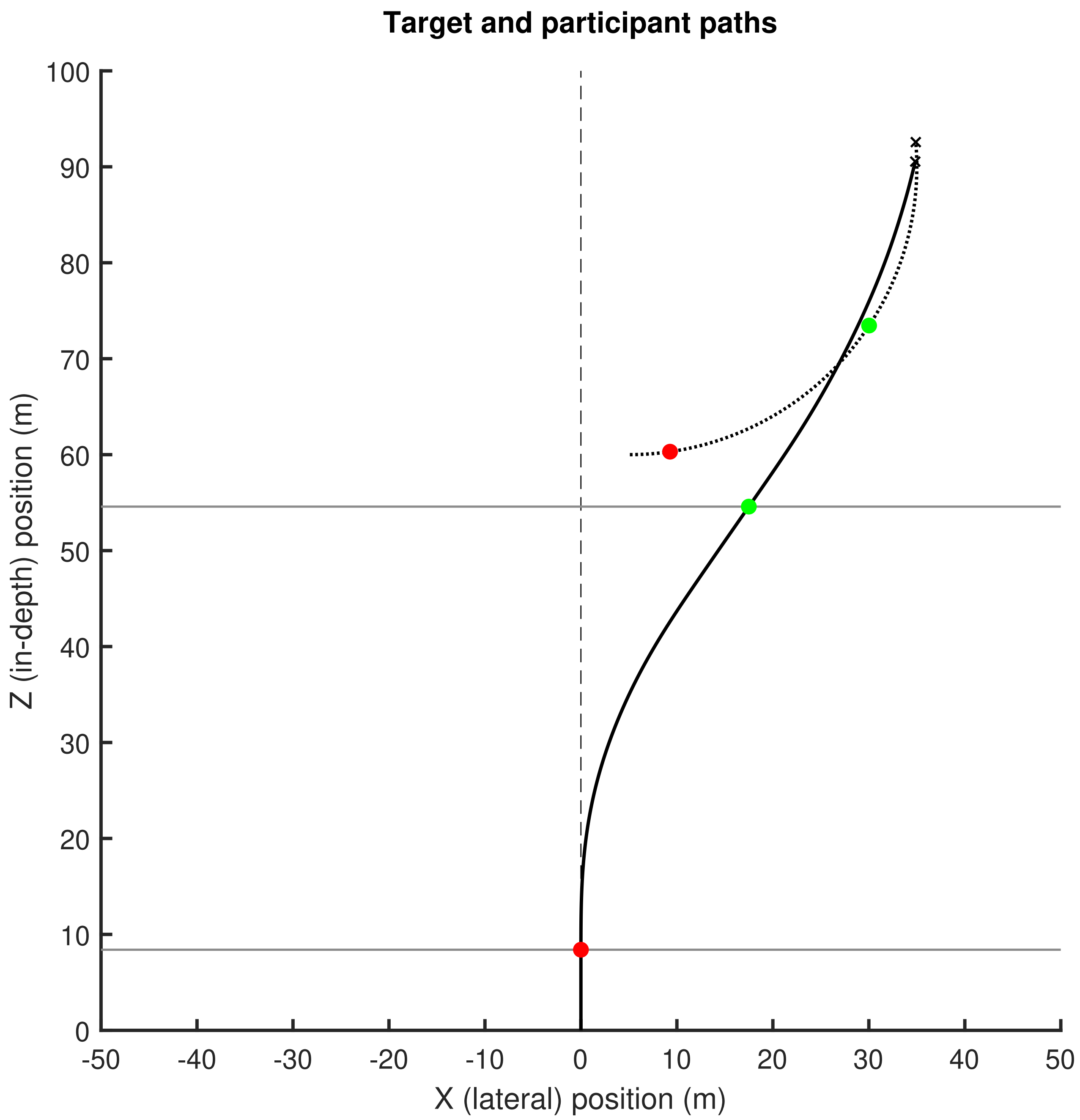

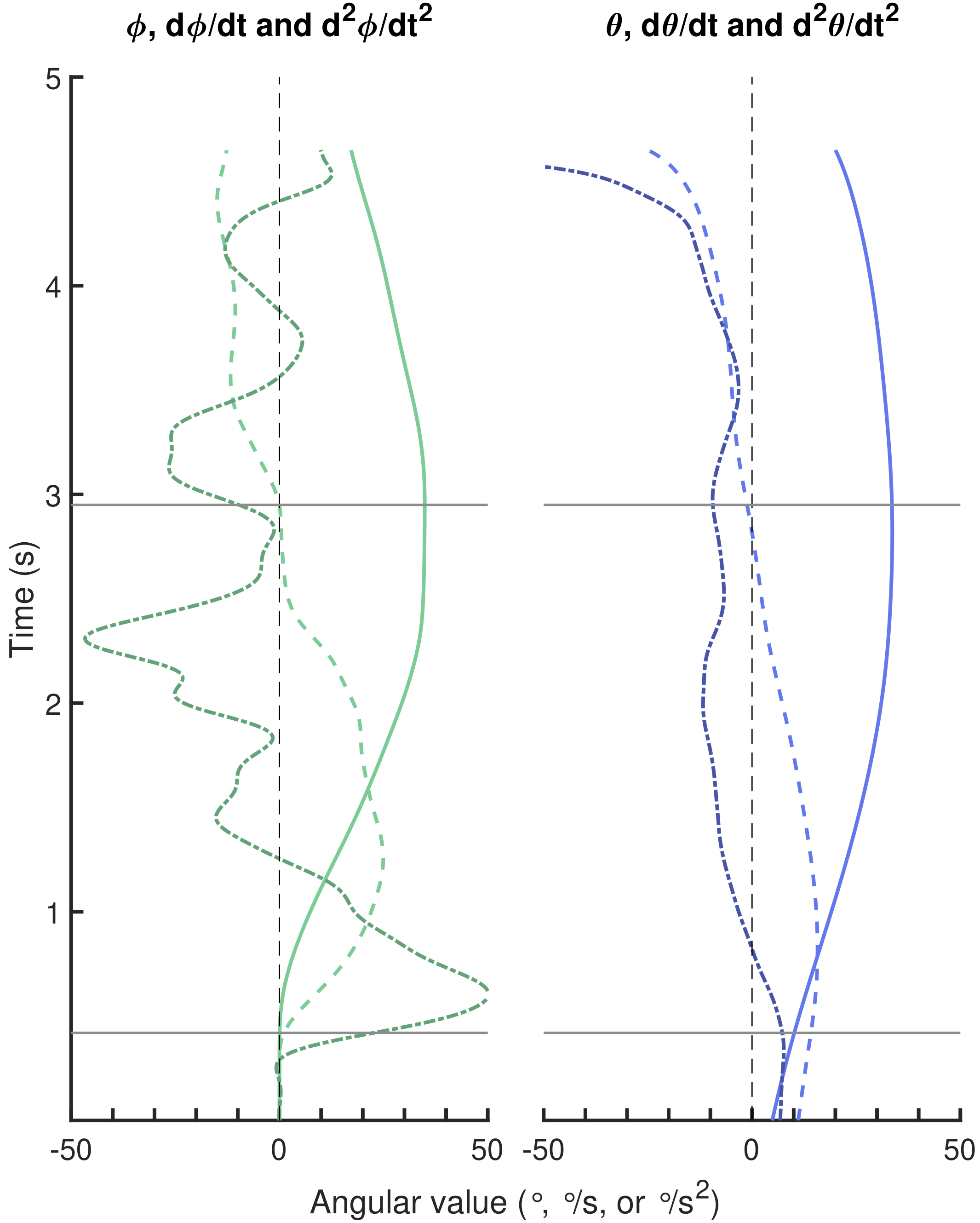

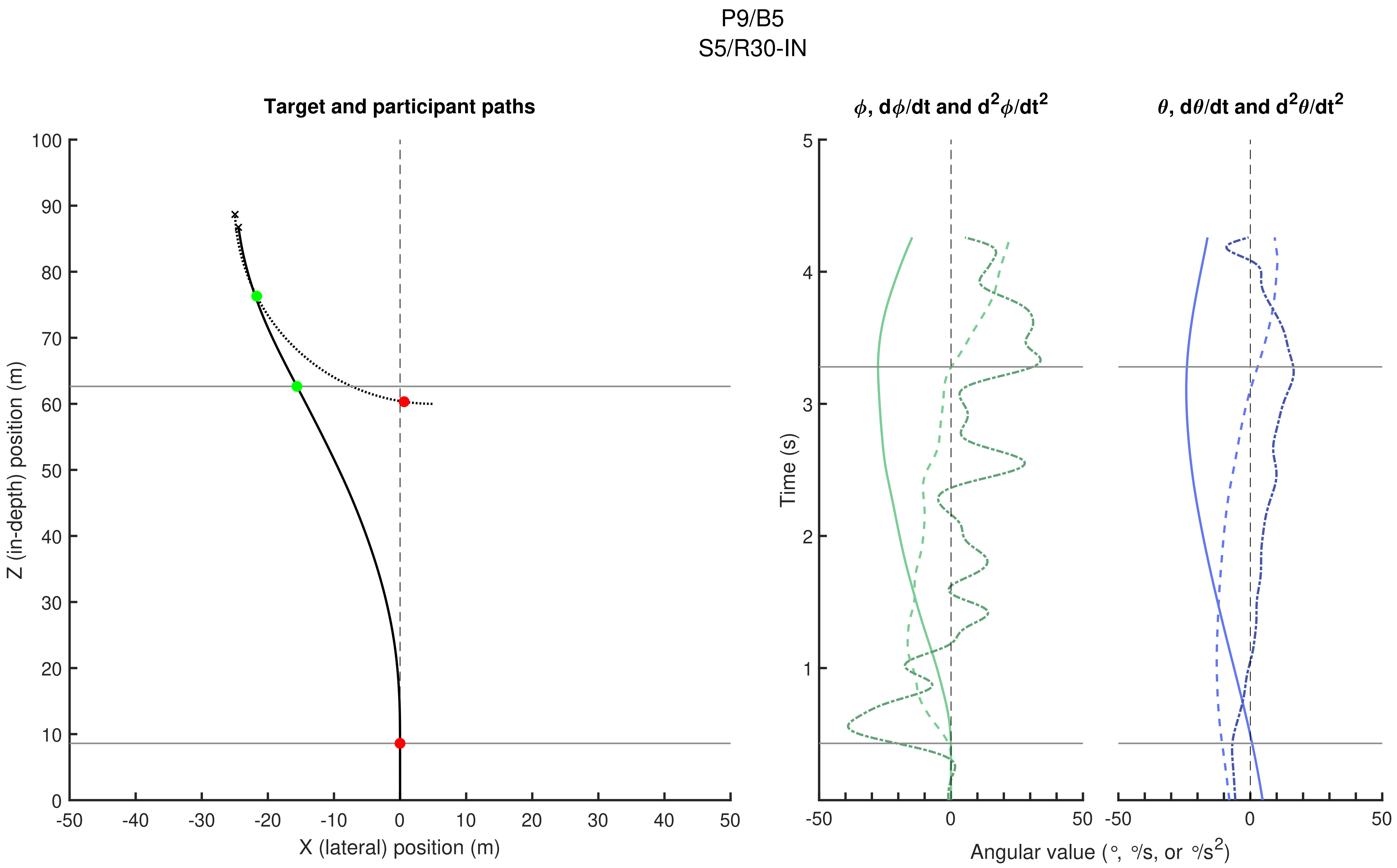
P9/B5
S5/R30-IN
Target and participant paths
Z (in-depth) position (m)
X (lateral) position (m)
100
90
80
70
60
50
40
30
20
10
0
-50
-40
-30
-20
-10
0
10
20
30
40
50
ϕ, dϕ/dt and d²ϕ/dt²
θ, dθ/dt and d²θ/dt²
Time (s)
5
4
3
2
1
-50
0
50
-50
0
50
Angular value (°, °/s, or °/s²)

P9/B5
S15/R10-IN

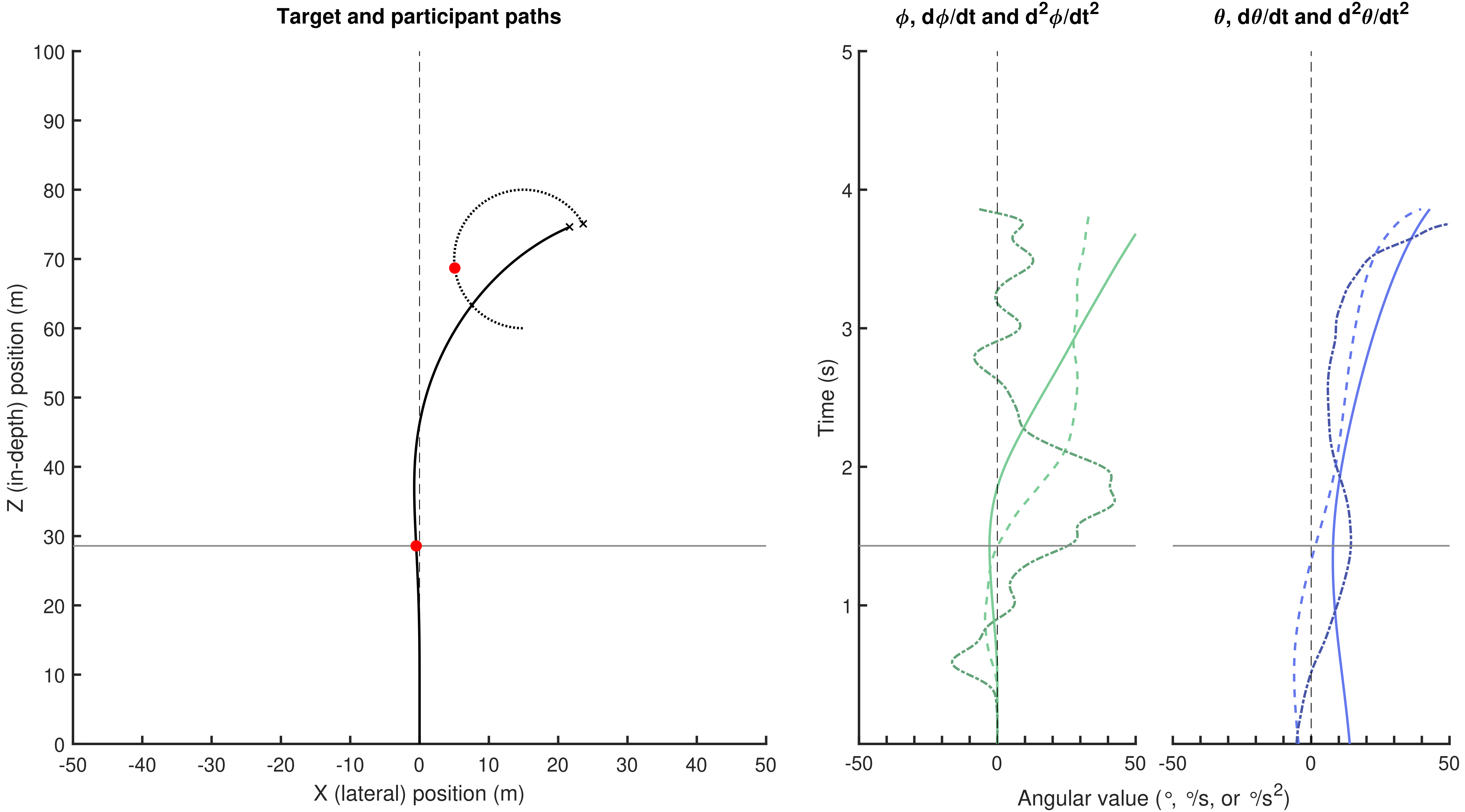

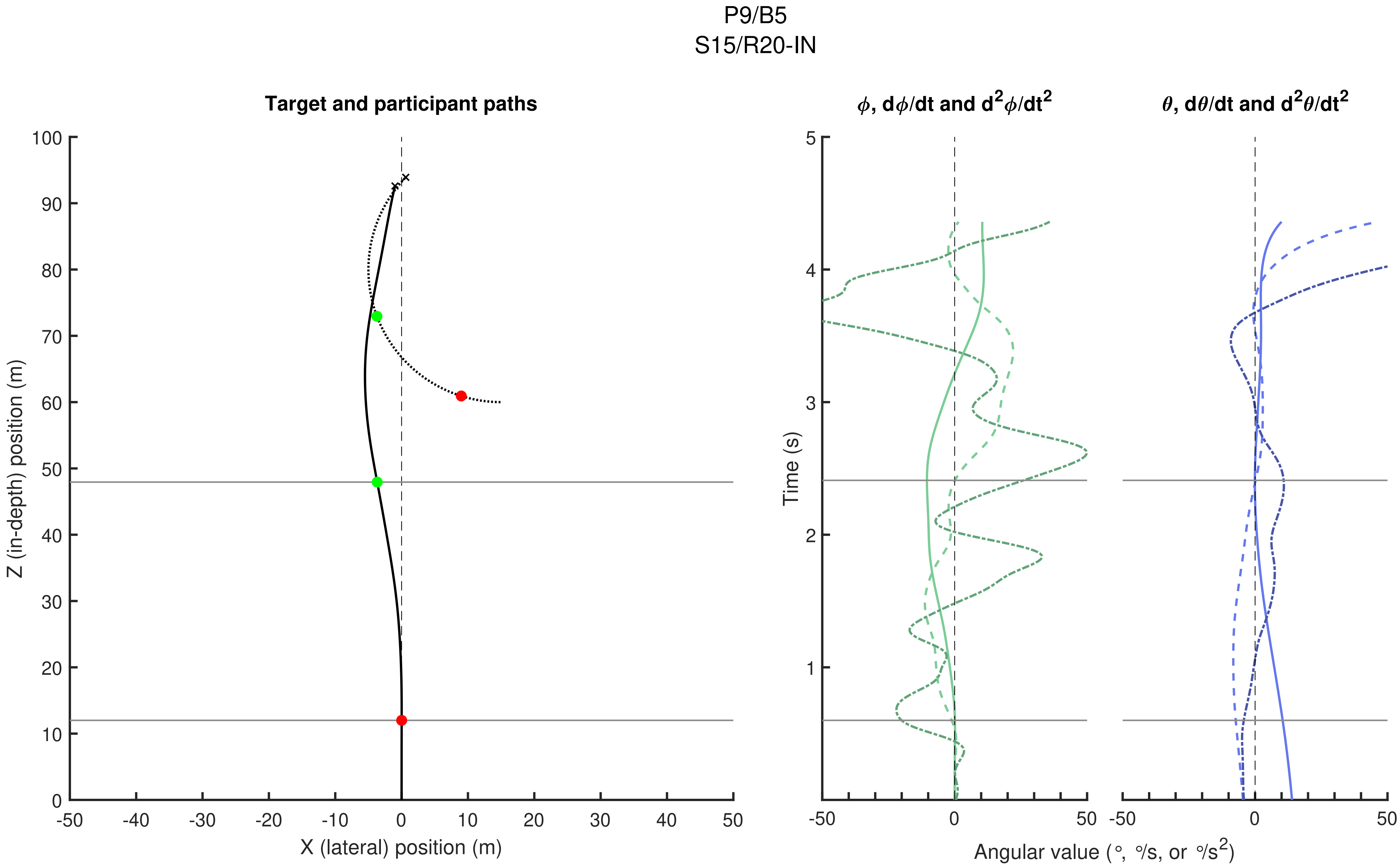

P9/B5
S15/R20-IN
Target and participant paths
Z (in-depth) position (m)
X (lateral) position (m)
100
90
80
70
60
50
40
30
20
10
0
-50
-40
-30
-20
-10
0
10
20
30
40
50
ϕ, dϕ/dt and d$^2\phi$/dt^2
θ, dθ/dt and d$^2\theta$/dt^2
Time (s)
5
4
3
2
1
-50
0
50
-50
0
50
Angular value (°, °/s, or °/s^2)

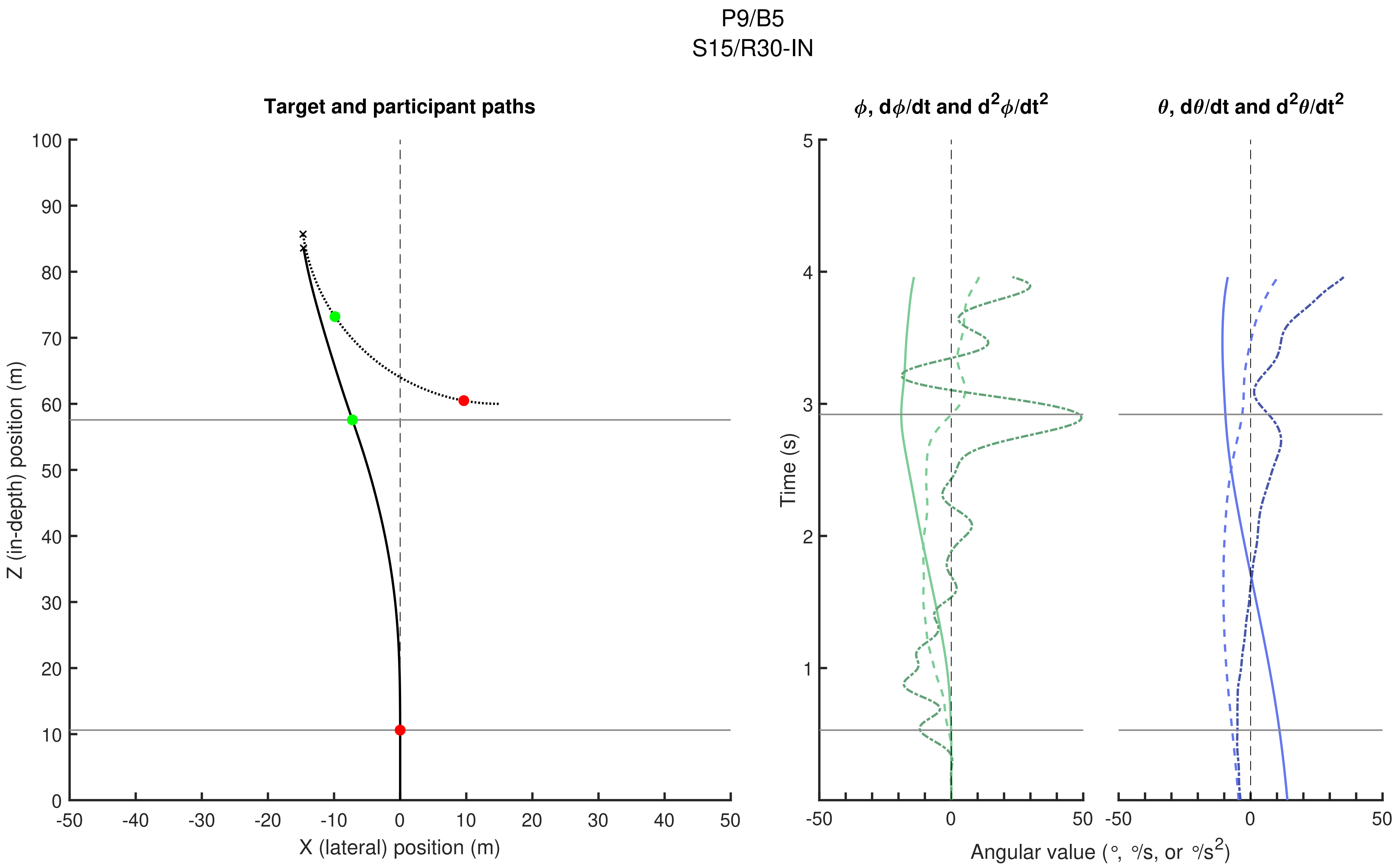

P9/B5
S15/R30-IN
Target and participant paths
Z (in-depth) position (m)
X (lateral) position (m)
ϕ, dϕ/dt and d$^2\phi$/dt^2
θ, dθ/dt and d$^2\theta$/dt^2
Time (s)
Angular value (°, °/s, or °/s^2)

P9/B5
S20/R10-IN

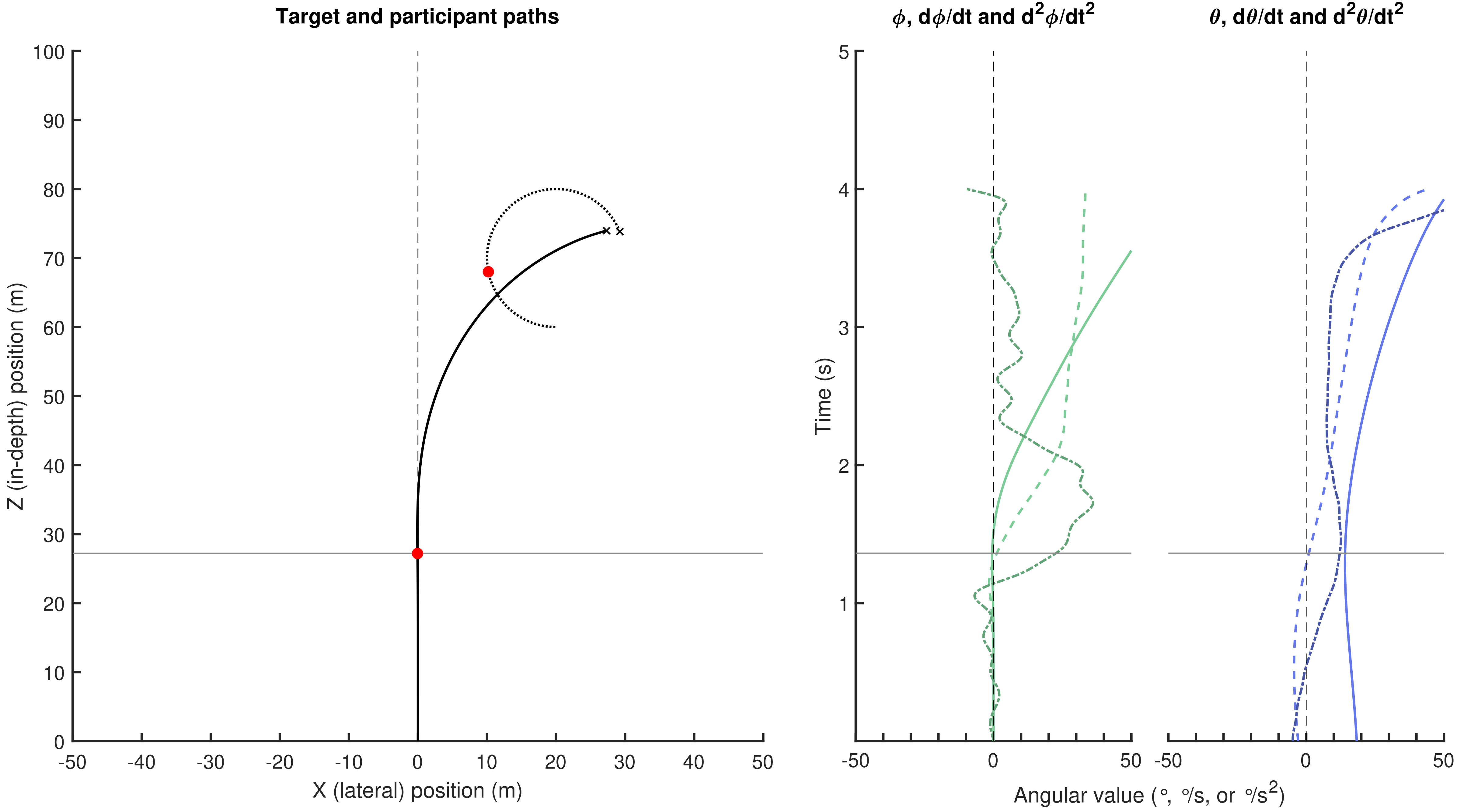

P9/B5
S20/R20-IN

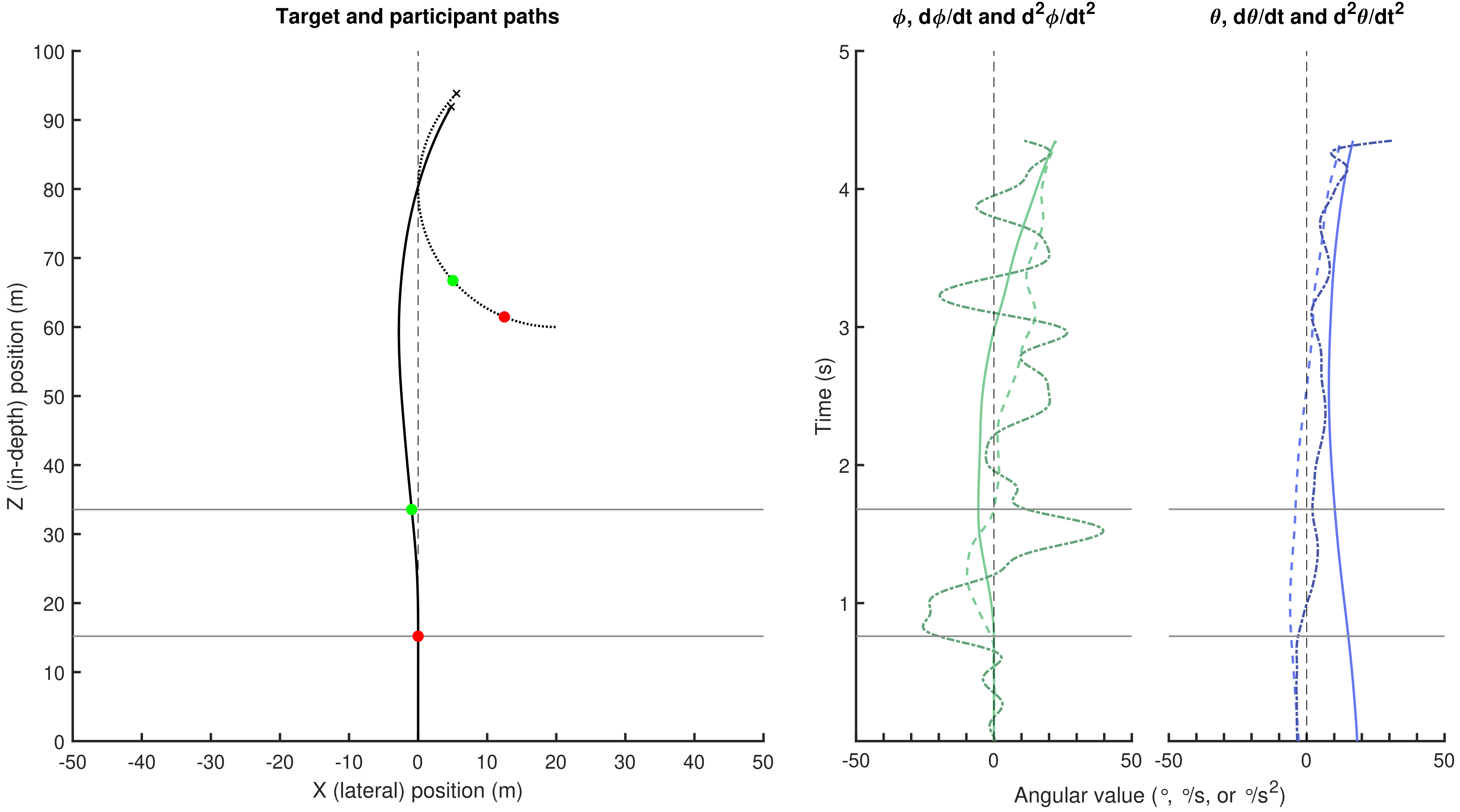

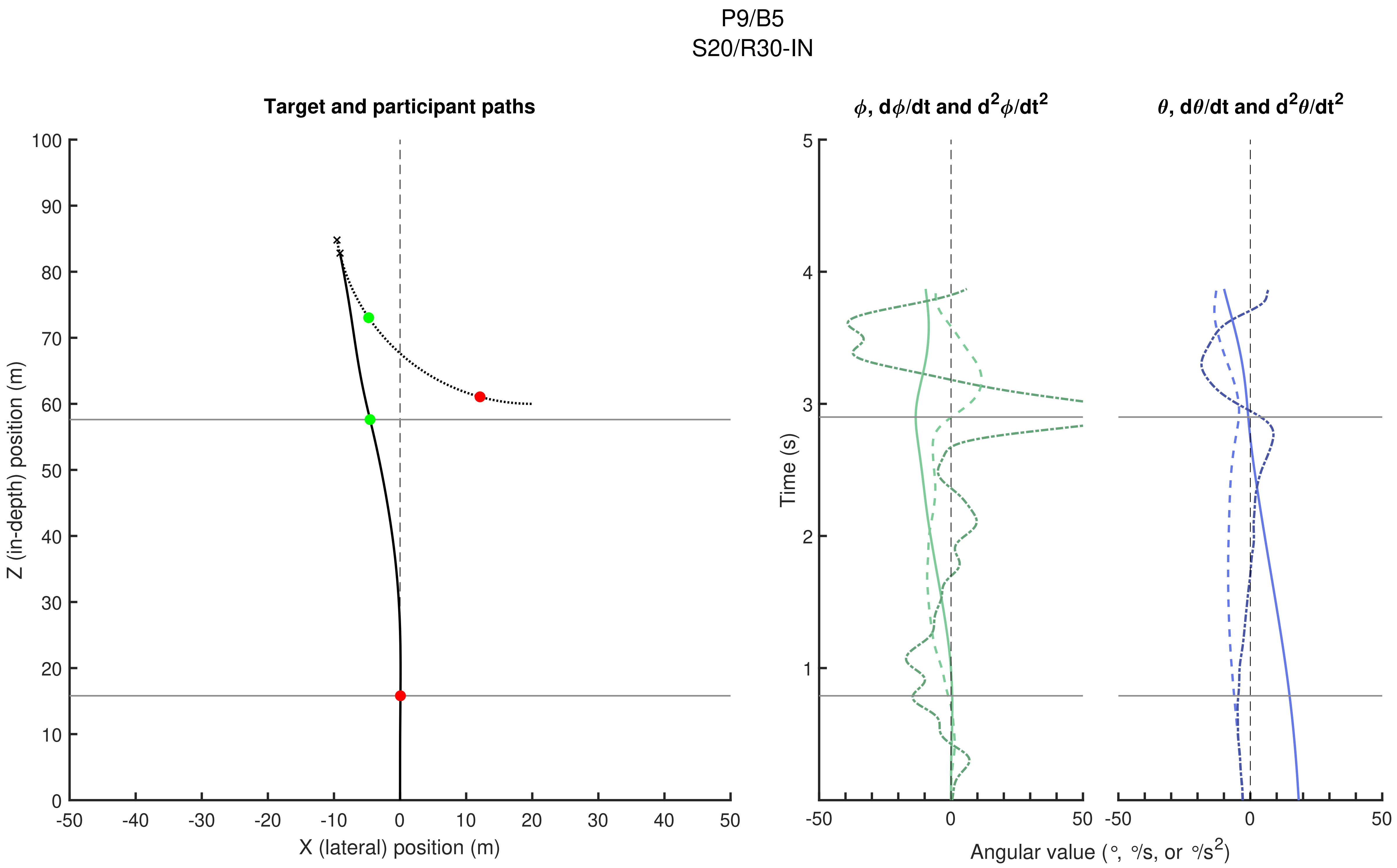

P9/B5
S20/R30-IN
Target and participant paths
Z (in-depth) position (m)
X (lateral) position (m)
100
90
80
70
60
50
40
30
20
10
0
-50
-40
-30
-20
-10
0
10
20
30
40
50
ϕ, dϕ/dt and d$^2\phi$/dt^2
θ, dθ/dt and d$^2\theta$/dt^2
Time (s)
5
4
3
2
1
-50
0
50
-50
0
50
Angular value (°, °/s, or °/s^2)

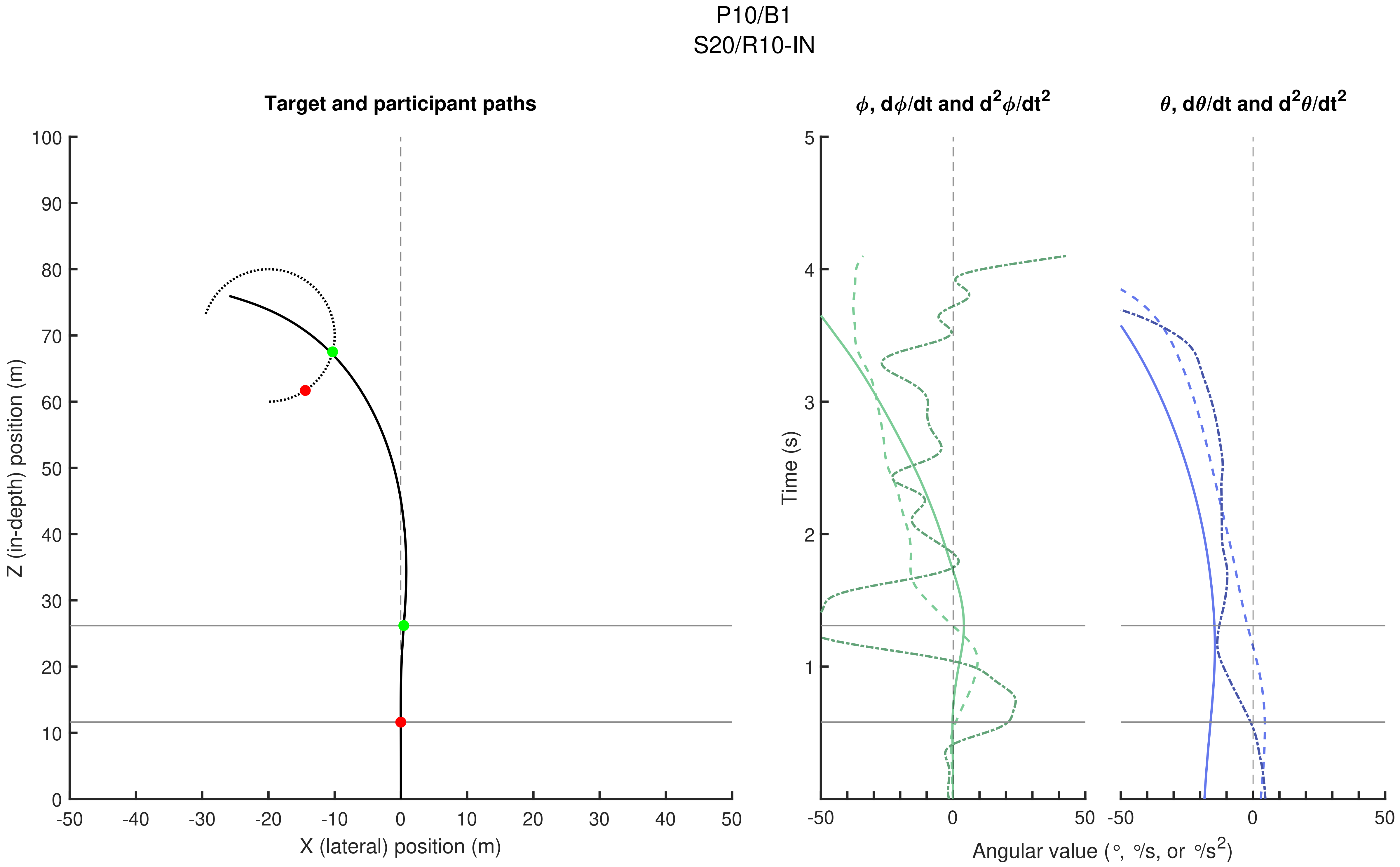

P10/B1
S20/R10-IN
Target and participant paths
ϕ, dϕ/dt and d$^2\phi$/dt^2
θ, dθ/dt and d$^2\theta$/dt^2
Z (in-depth) position (m)
X (lateral) position (m)
Time (s)
Angular value (°, °/s, or °/s^2)

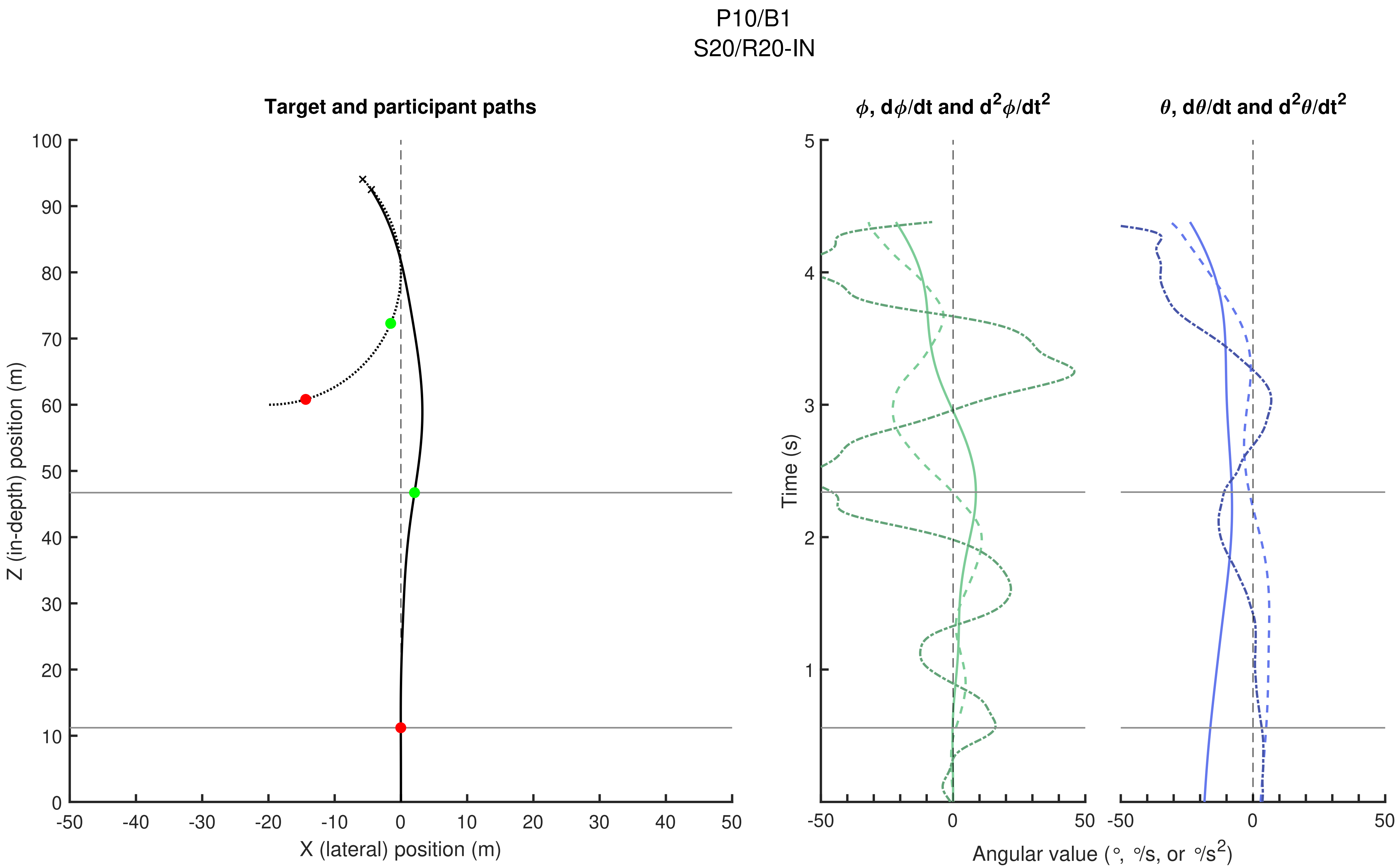

P10/B1
S20/R20-IN
Target and participant paths
Z (in-depth) position (m)
X (lateral) position (m)
100
90
80
70
60
50
40
30
20
10
0
-50
-40
-30
-20
-10
0
10
20
30
40
50
ϕ, dϕ/dt and d²ϕ/dt²
θ, dθ/dt and d²θ/dt²
Time (s)
5
4
3
2
1
-50
0
50
-50
0
50
Angular value (°, °/s, or °/s²)

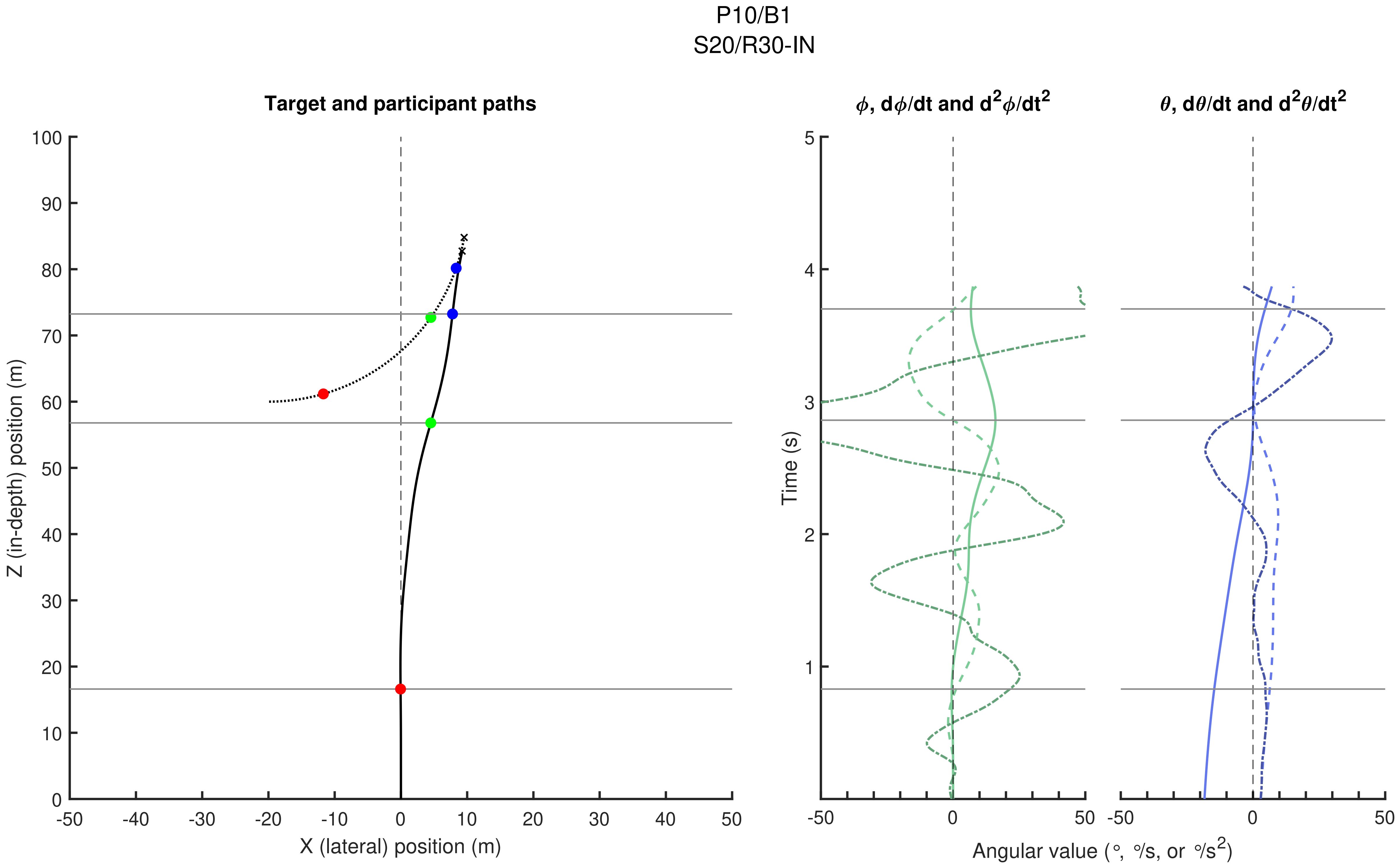
P10/B1
S20/R30-IN
Target and participant paths
ϕ, dϕ/dt and d²ϕ/dt²
θ, dθ/dt and d²θ/dt²
Z (in-depth) position (m)
X (lateral) position (m)
Time (s)
Angular value (°, °/s, or °/s²)
100
90
80
70
60
50
40
30
20
10
0
-50
-40
-30
-20
-10
0
10
20
30
40
50
5
4
3
2
1
-50
0
50
-50
0
50

P10/B1
S15/R10-IN

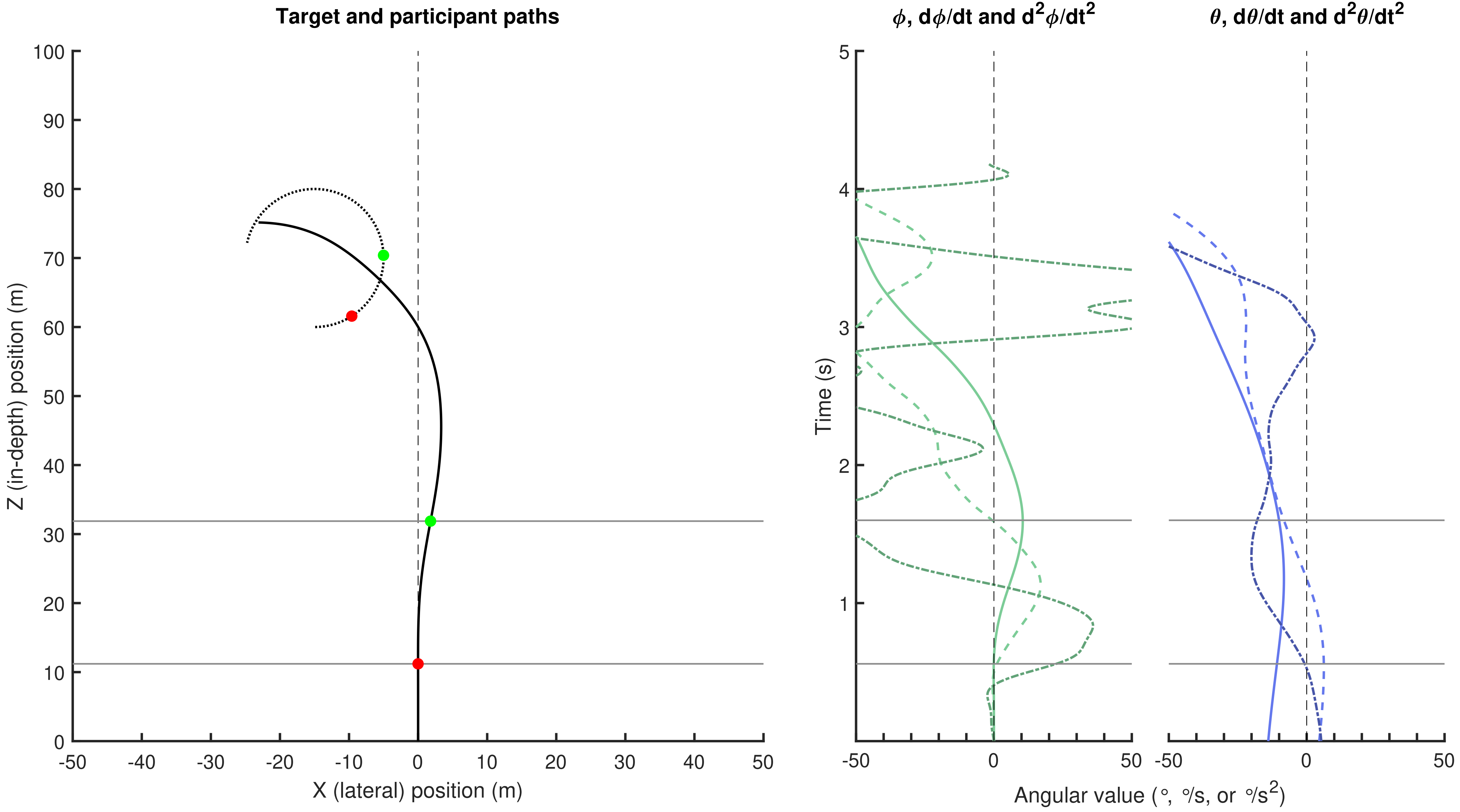

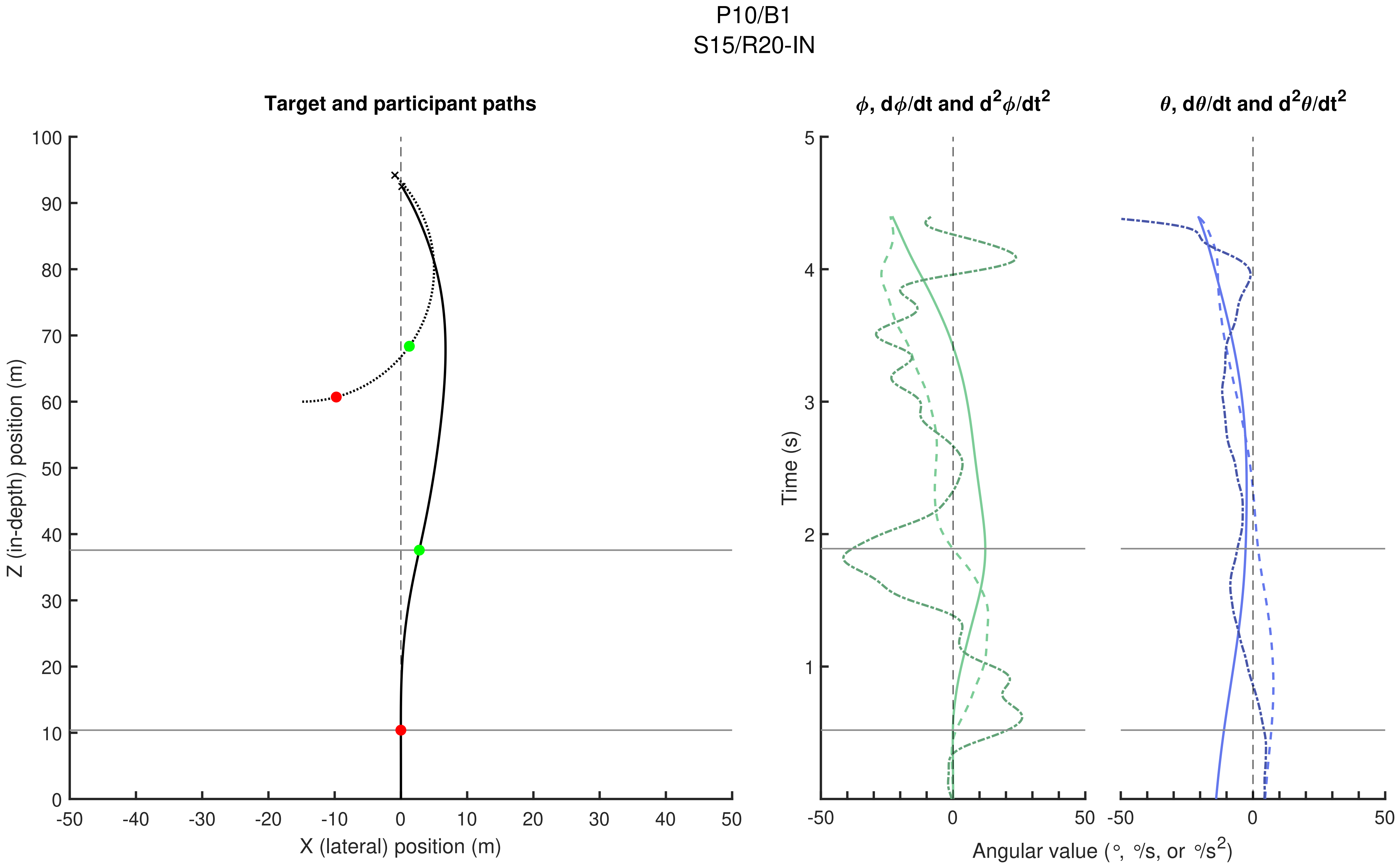

P10/B1
S15/R20-IN
Target and participant paths
Z (in-depth) position (m)
X (lateral) position (m)
φ, dφ/dt and d²φ/dt²
θ, dθ/dt and d²θ/dt²
Time (s)
Angular value (°, °/s, or °/s²)

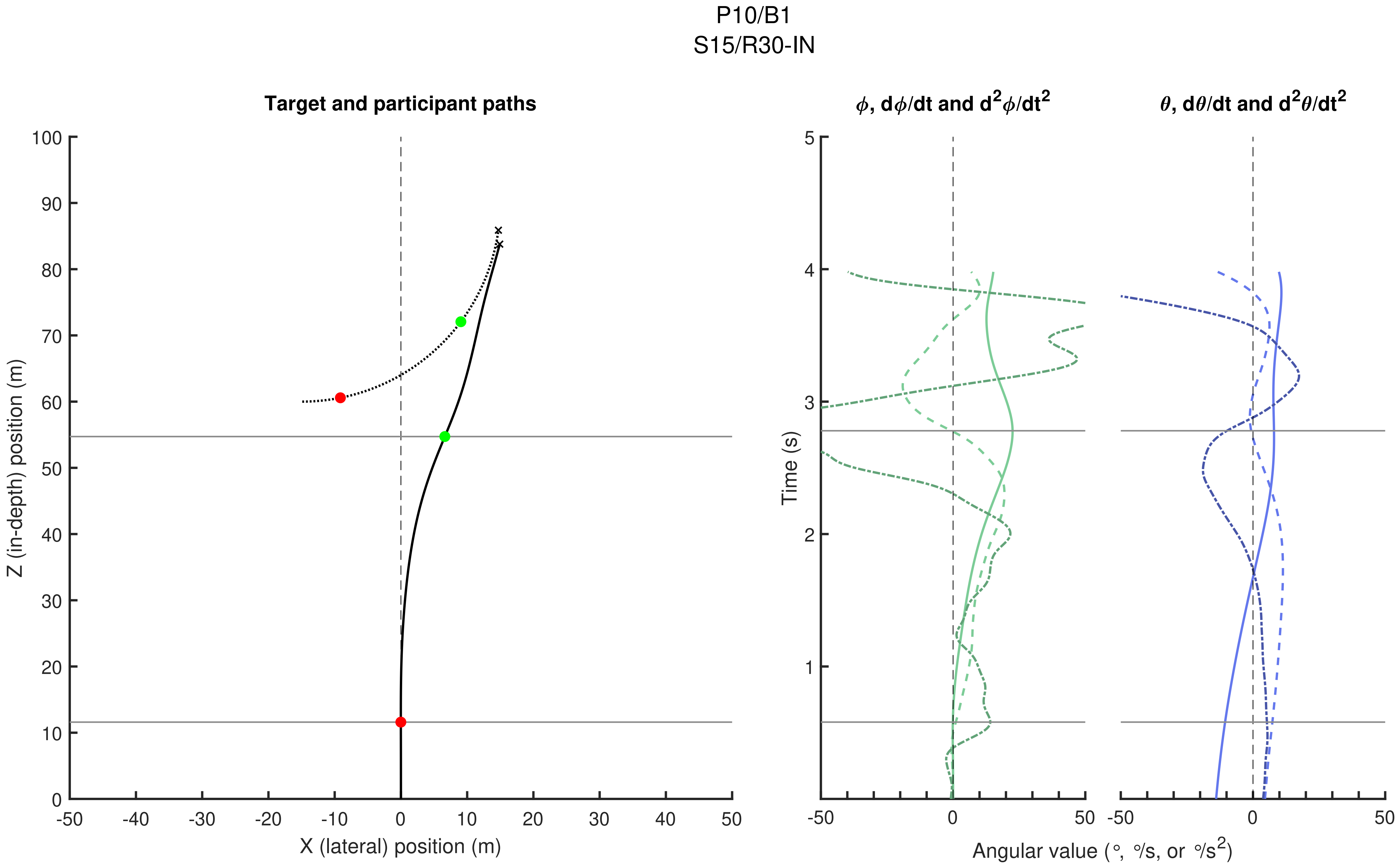
P10/B1
S15/R30-IN
Target and participant paths
Z (in-depth) position (m)
X (lateral) position (m)
ϕ, dϕ/dt and d$^2\phi$/dt^2
θ, dθ/dt and d$^2\theta$/dt^2
Time (s)
Angular value (°, °/s, or °/s^2)

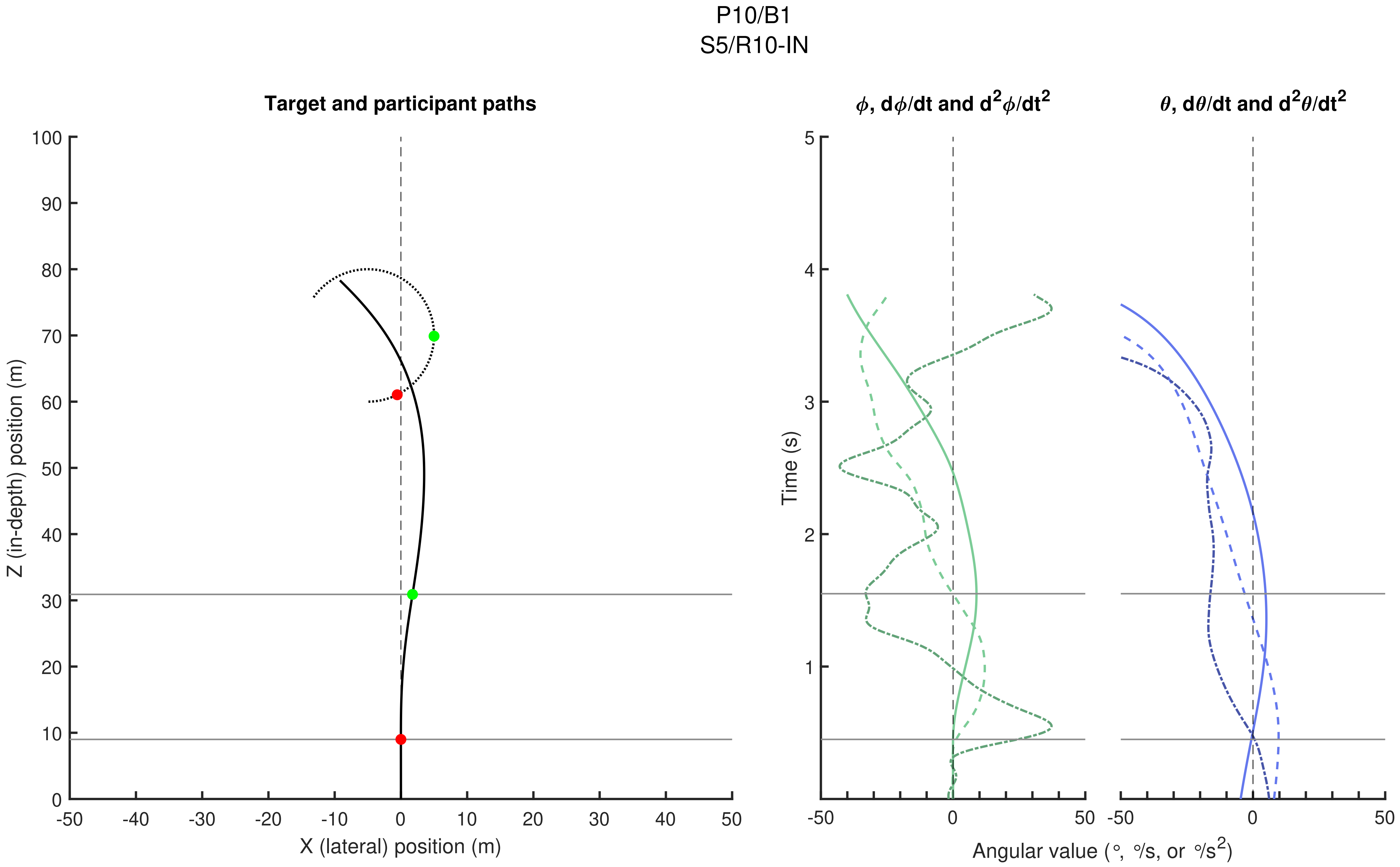
P10/B1
S5/R10-IN
Target and participant paths
Z (in-depth) position (m)
X (lateral) position (m)
100
90
80
70
60
50
40
30
20
10
0
-50
-40
-30
-20
-10
0
10
20
30
40
50
φ, dφ/dt and d²φ/dt²
θ, dθ/dt and d²θ/dt²
Time (s)
5
4
3
2
1
-50
0
50
-50
0
50
Angular value (°, °/s, or °/s²)

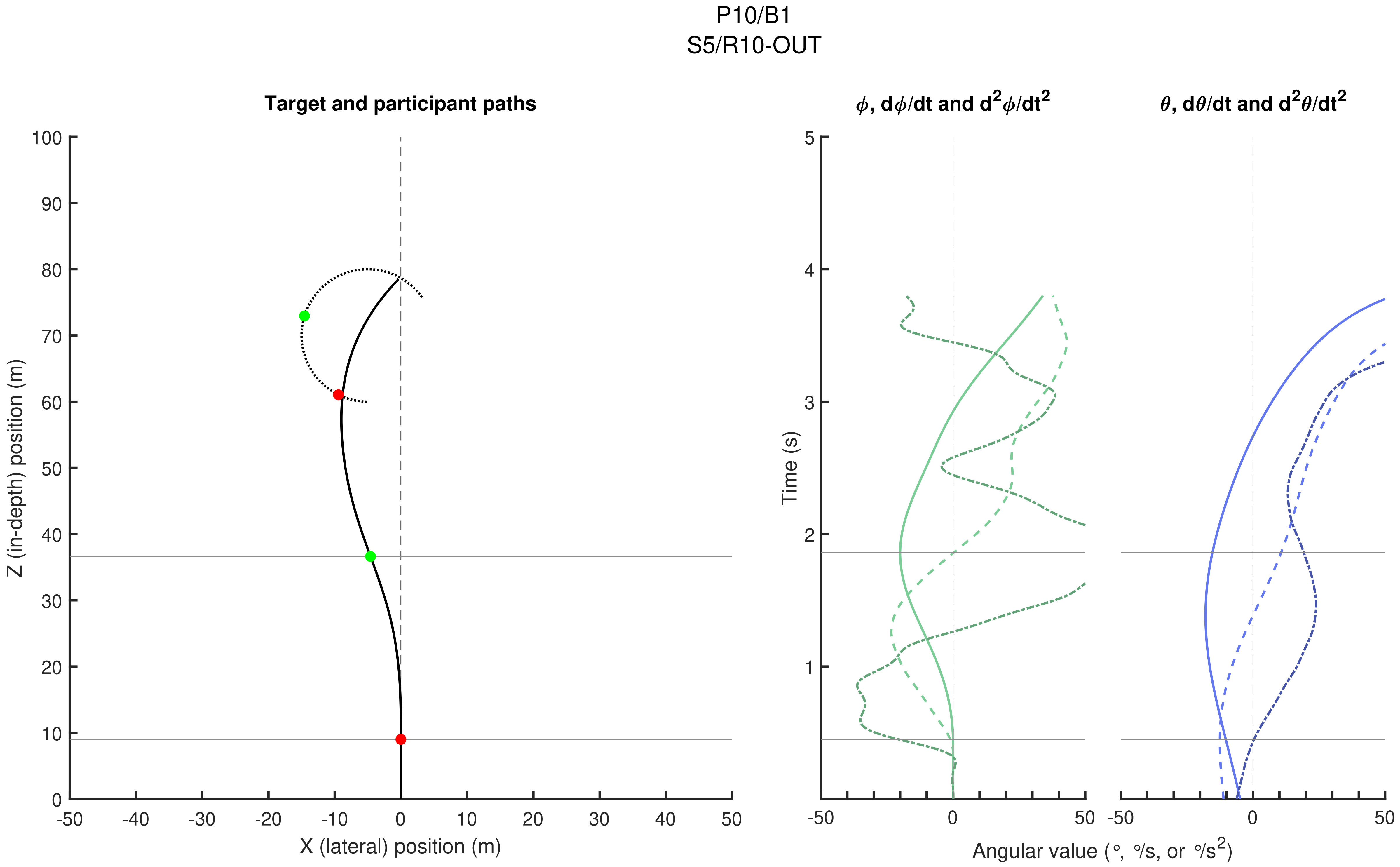

P10/B1
S5/R10-OUT
Target and participant paths
ϕ, dϕ/dt and d$^2\phi$/dt^2
θ, dθ/dt and d$^2\theta$/dt^2
Z (in-depth) position (m)
X (lateral) position (m)
Time (s)
Angular value (°, °/s, or °/s^2)

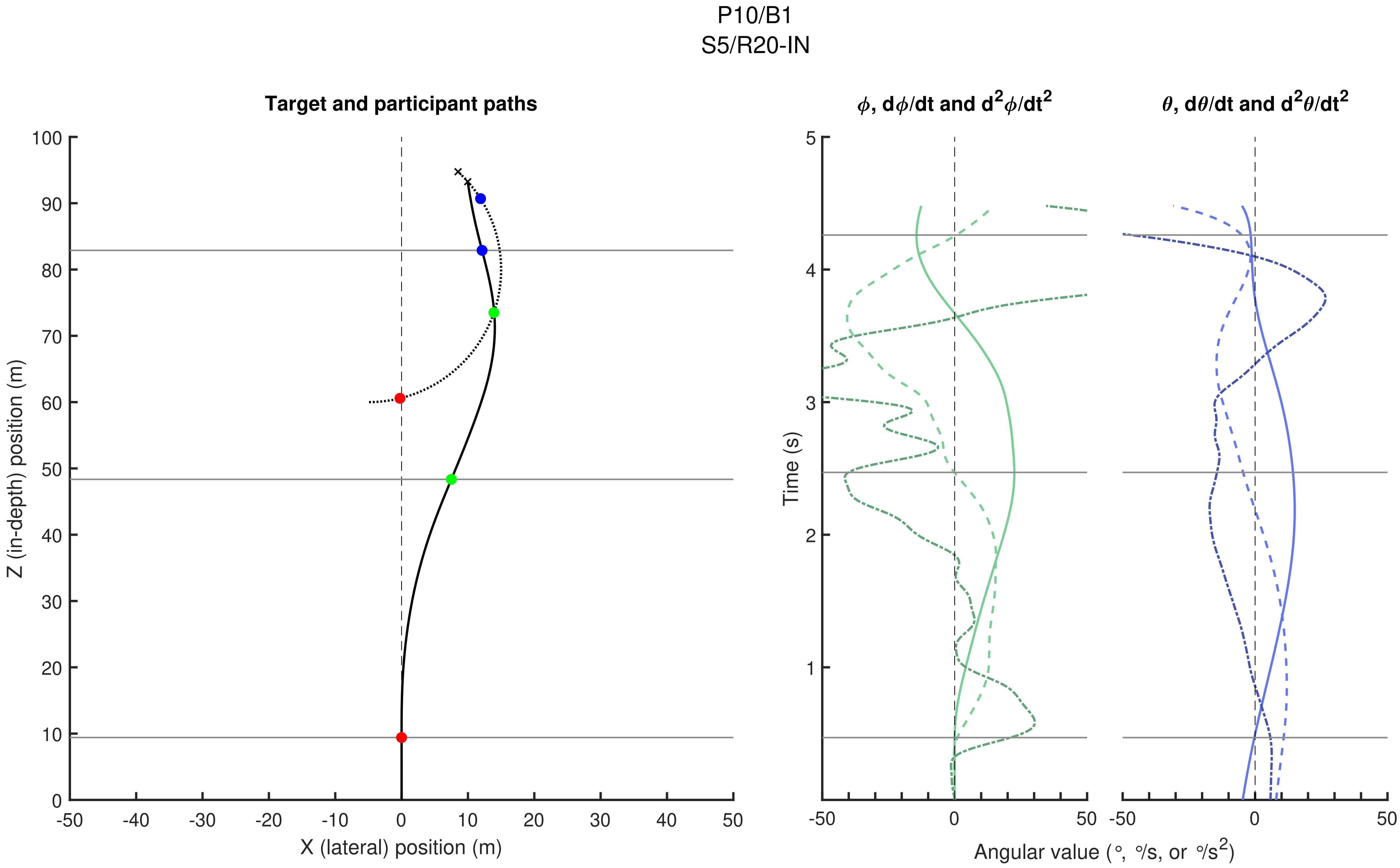

P10/B1
S5/R20-IN
Target and participant paths
Z (in-depth) position (m)
X (lateral) position (m)
ϕ, dϕ/dt and d²ϕ/dt²
θ, dθ/dt and d²θ/dt²
Time (s)
Angular value (°, °/s, or °/s²)

P10/B1
S5/R20-OUT

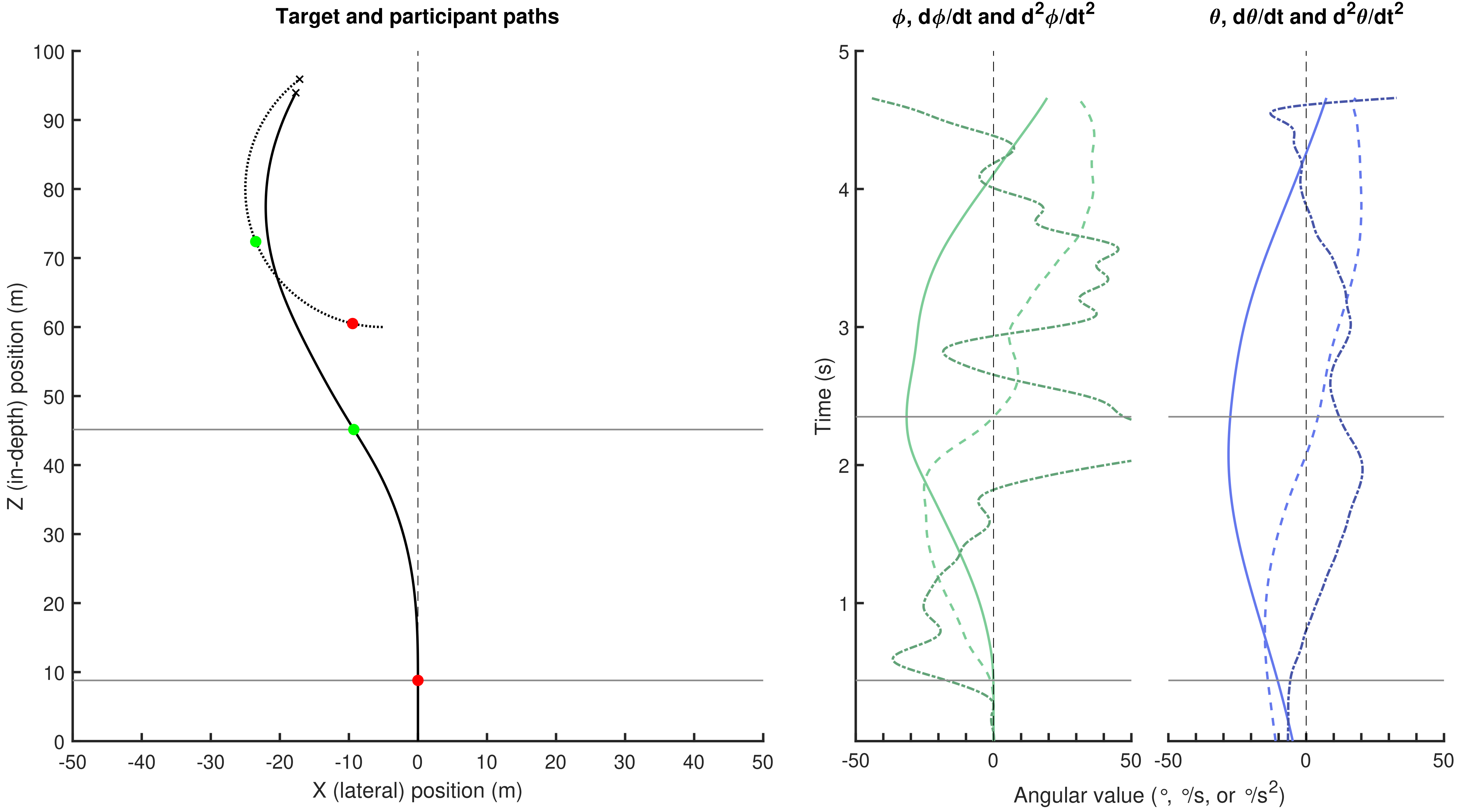

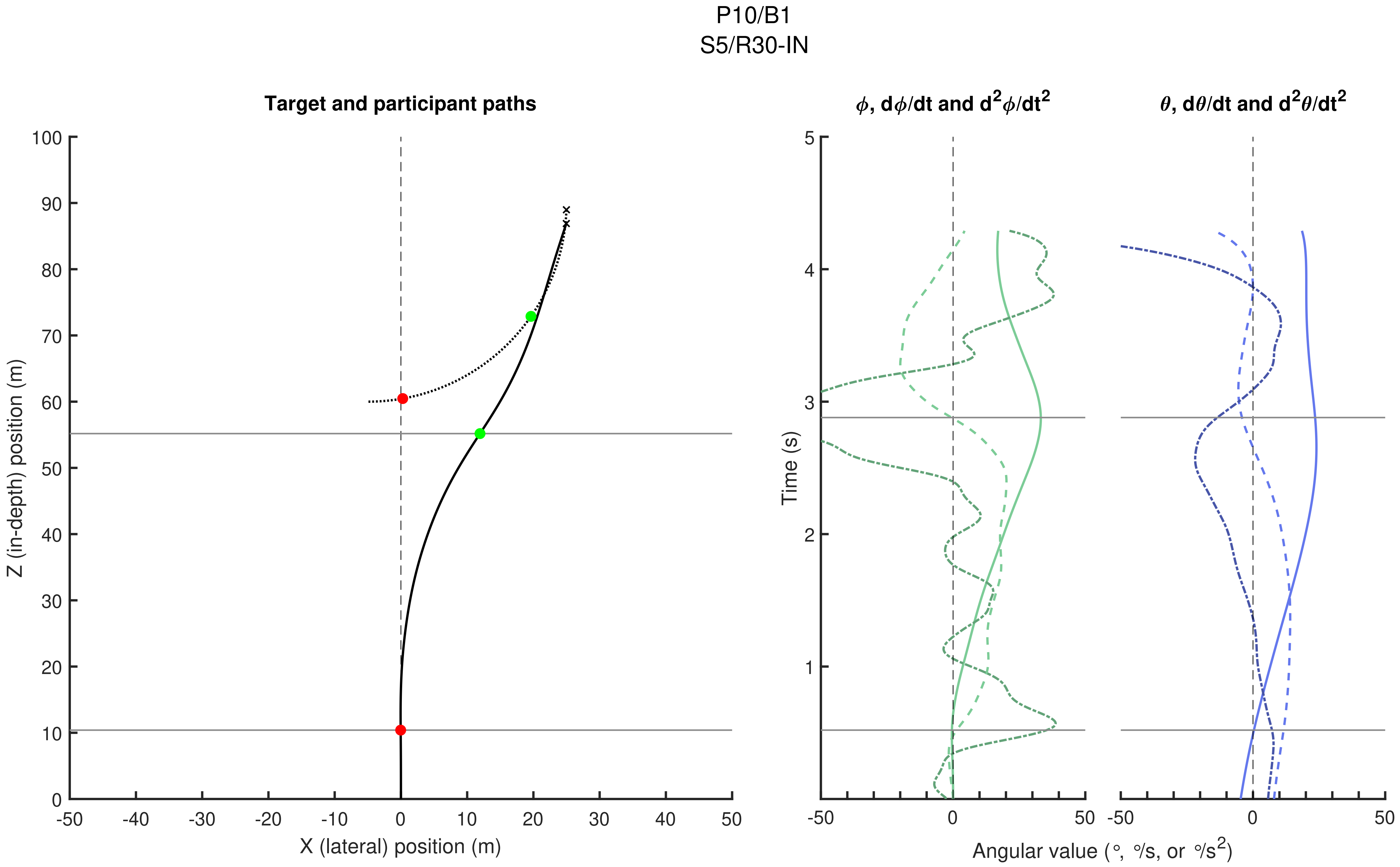
P10/B1
S5/R30-IN
Target and participant paths
Z (in-depth) position (m)
X (lateral) position (m)
ϕ, dϕ/dt and d$^2\phi$/dt^2
θ, dθ/dt and d$^2\theta$/dt^2
Time (s)
Angular value (°, °/s, or °/s^2)

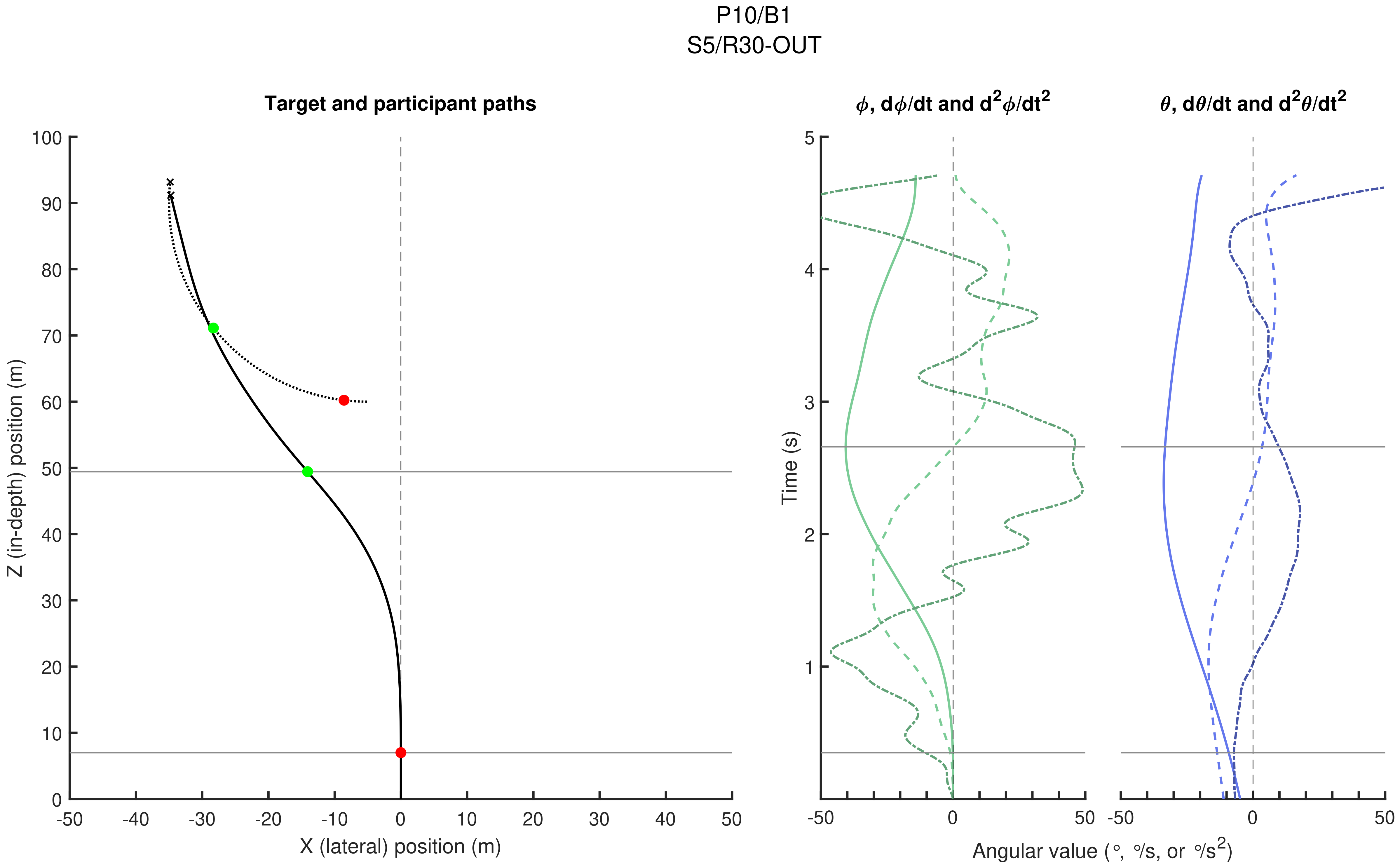

P10/B1
S5/R30-OUT
Target and participant paths
Z (in-depth) position (m)
X (lateral) position (m)
ϕ, dϕ/dt and d²ϕ/dt²
θ, dθ/dt and d²θ/dt²
Time (s)
Angular value (°, °/s, or °/s²)

P10/B1
S5/R10-OUT

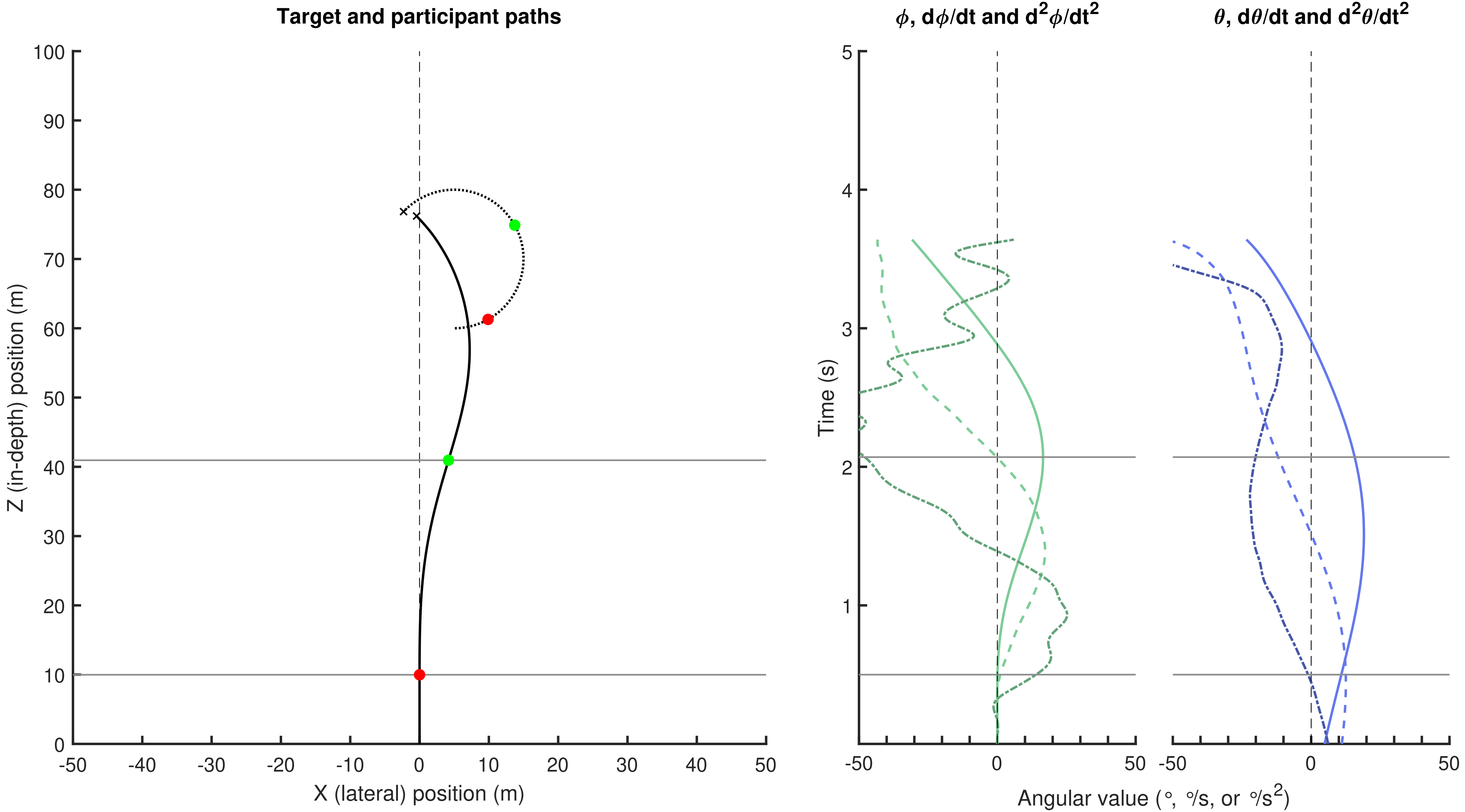

P10/B1
S5/R10-IN

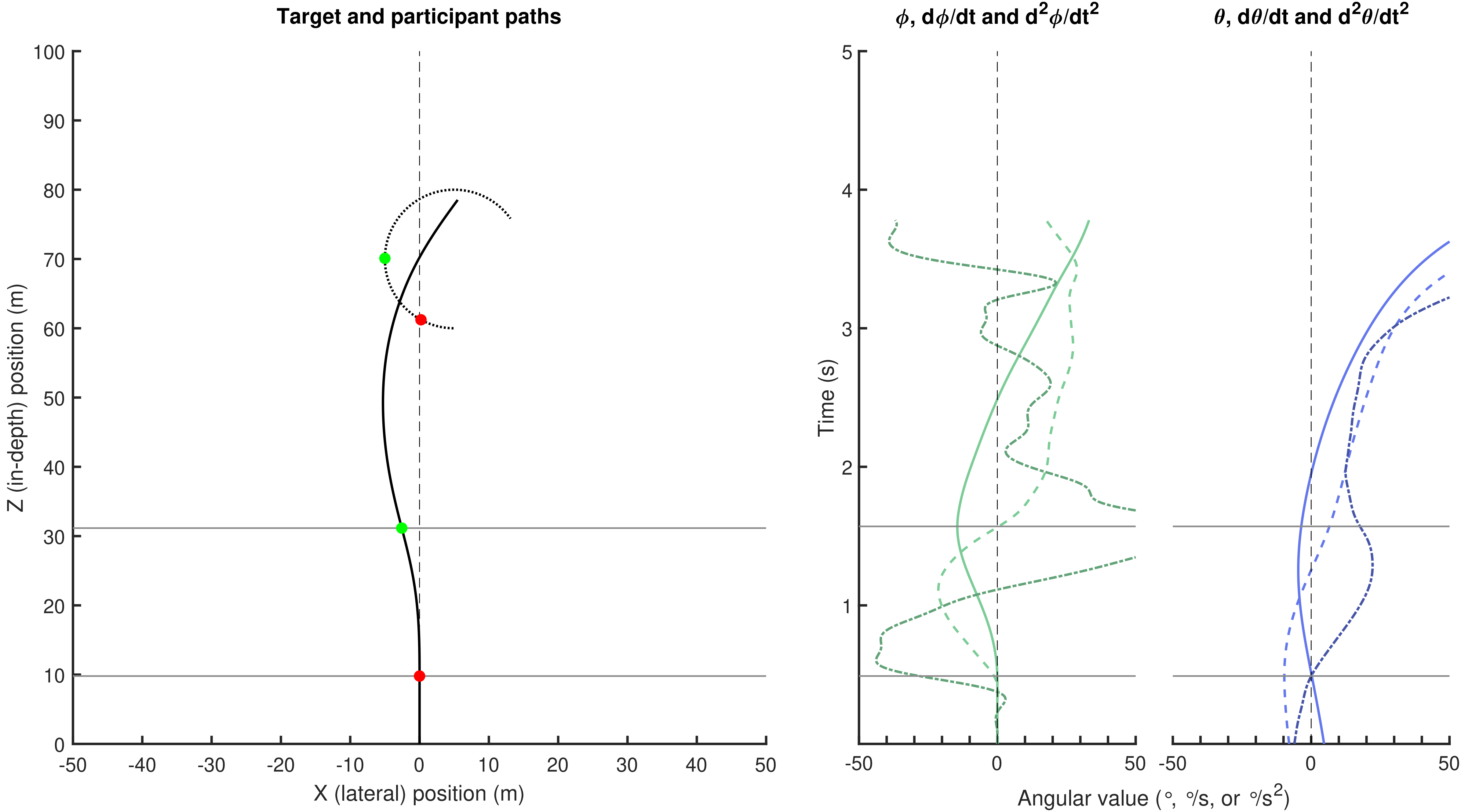

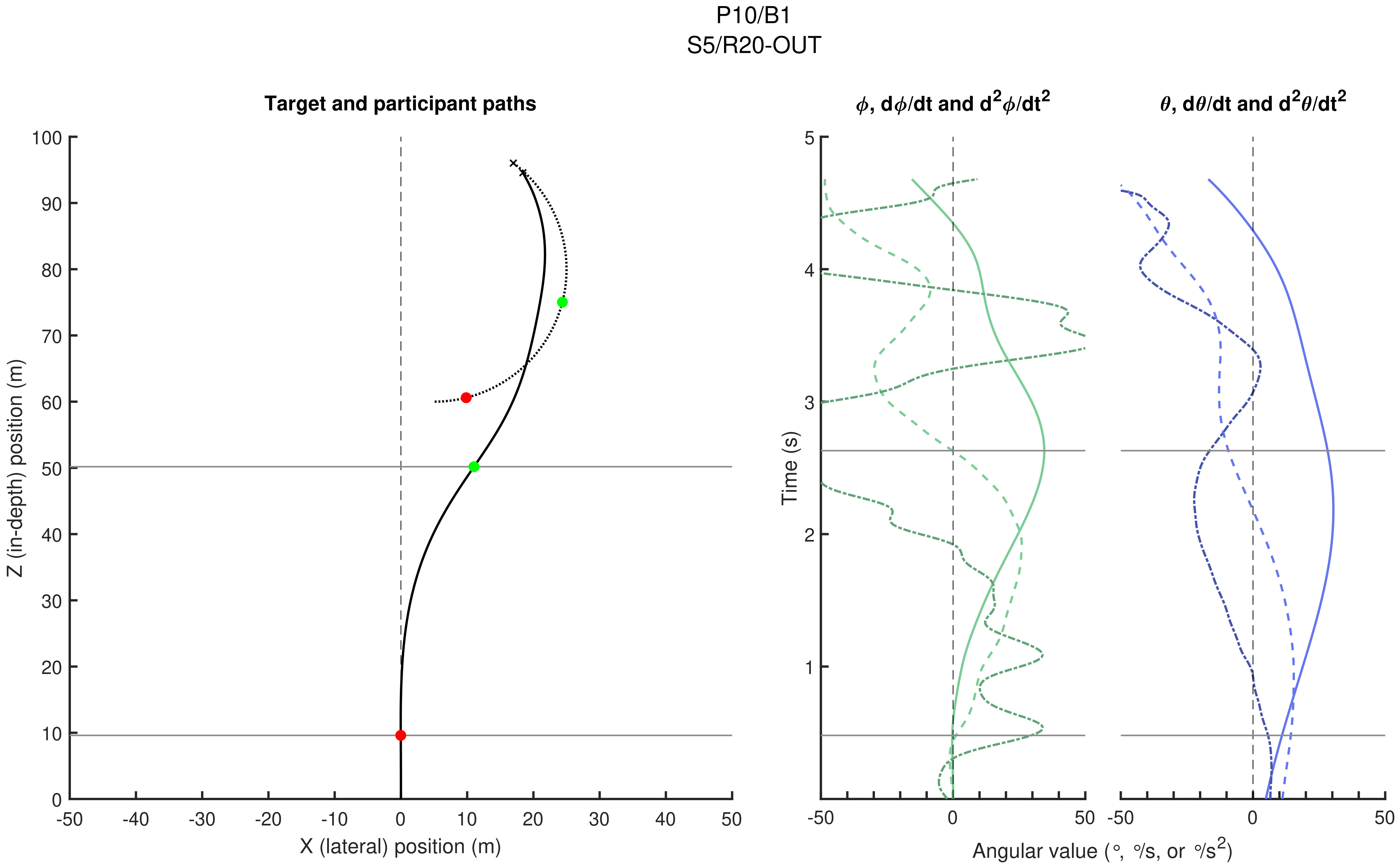
P10/B1
S5/R20-OUT
Target and participant paths
Z (in-depth) position (m)
X (lateral) position (m)
φ, dφ/dt and d²φ/dt²
θ, dθ/dt and d²θ/dt²
Time (s)
Angular value (°, °/s, or °/s²)

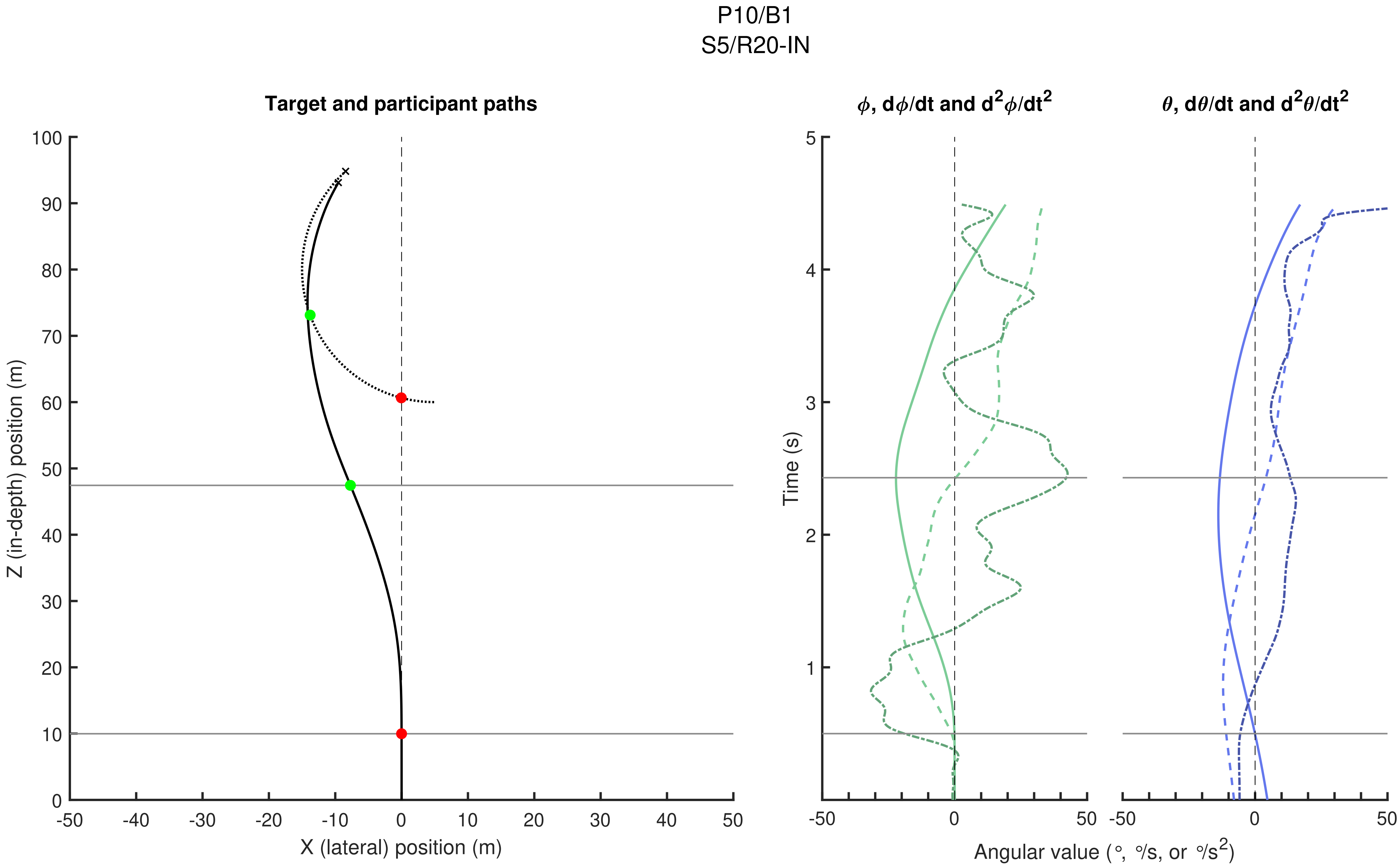
P10/B1
S5/R20-IN
Target and participant paths
Z (in-depth) position (m)
X (lateral) position (m)
ϕ, dϕ/dt and d$^2\phi$/dt^2
θ, dθ/dt and d$^2\theta$/dt^2
Time (s)
Angular value (°, °/s, or °/s^2)

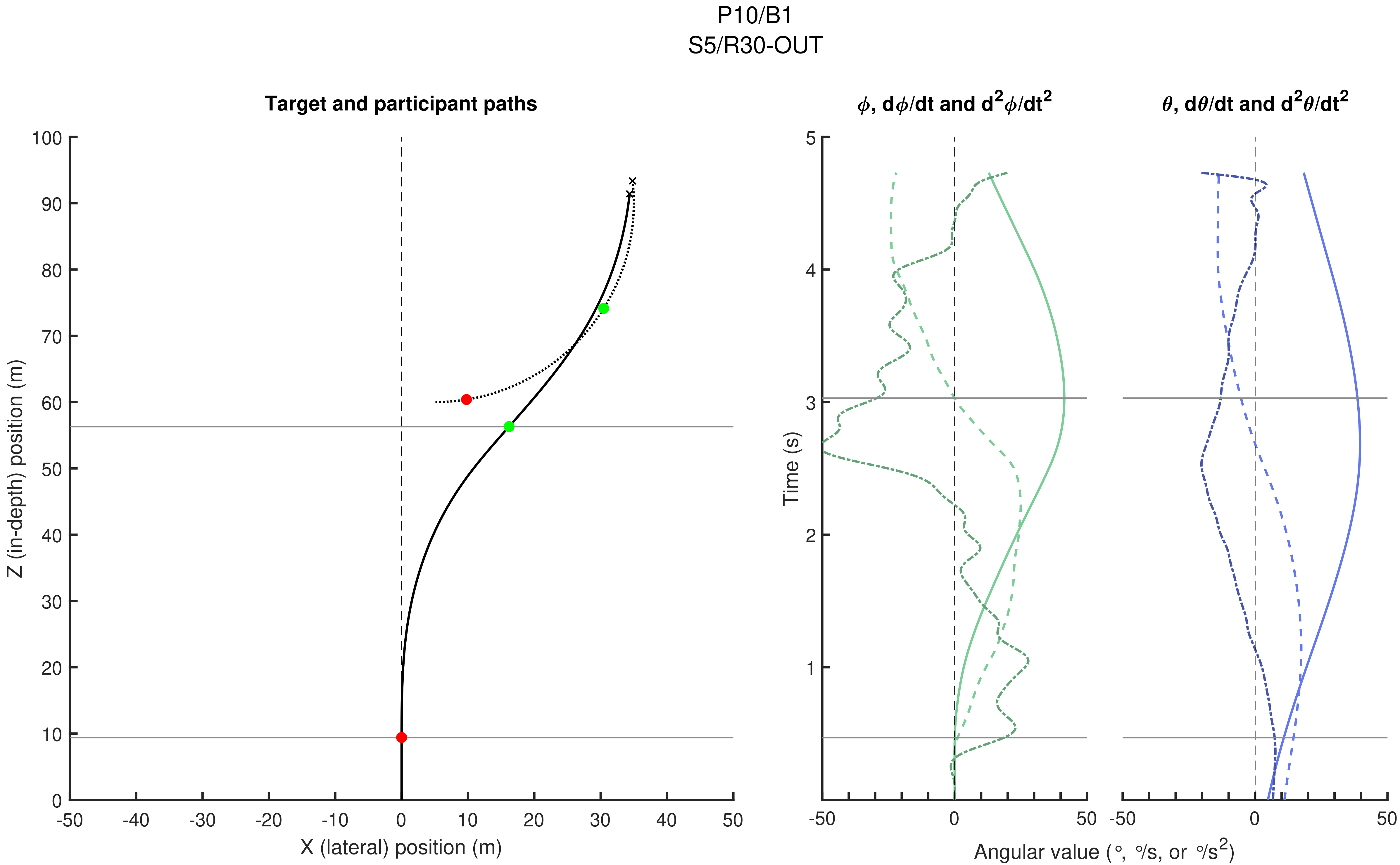
P10/B1
S5/R30-OUT
Target and participant paths
Z (in-depth) position (m)
X (lateral) position (m)
φ, dφ/dt and d²φ/dt²
θ, dθ/dt and d²θ/dt²
Time (s)
Angular value (°, °/s, or °/s²)
100
90
80
70
60
50
40
30
20
10
0
-50
-40
-30
-20
-10
0
10
20
30
40
50
5
4
3
2
1
-50
0
50
-50
0
50

P10/B1
S5/R30-IN

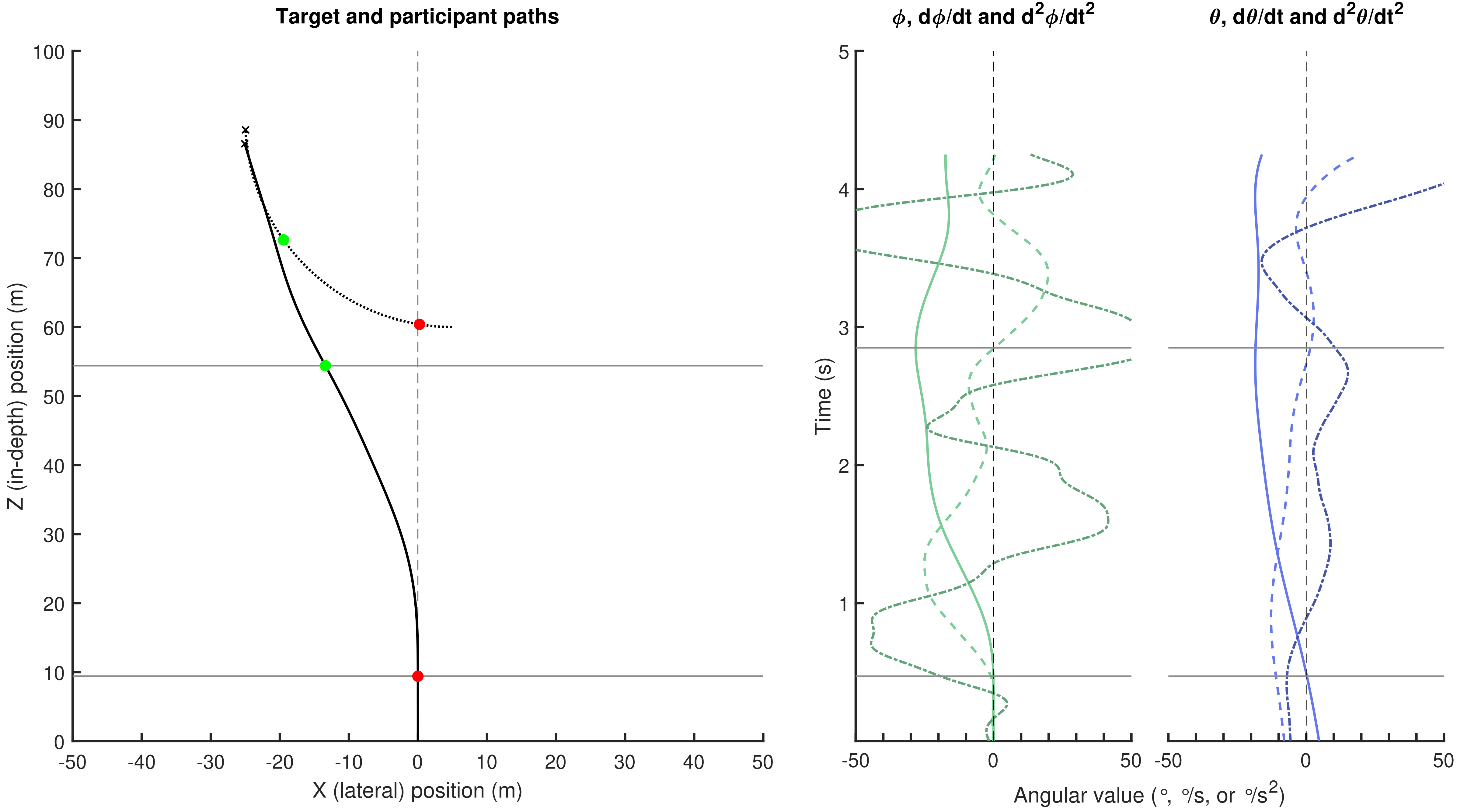

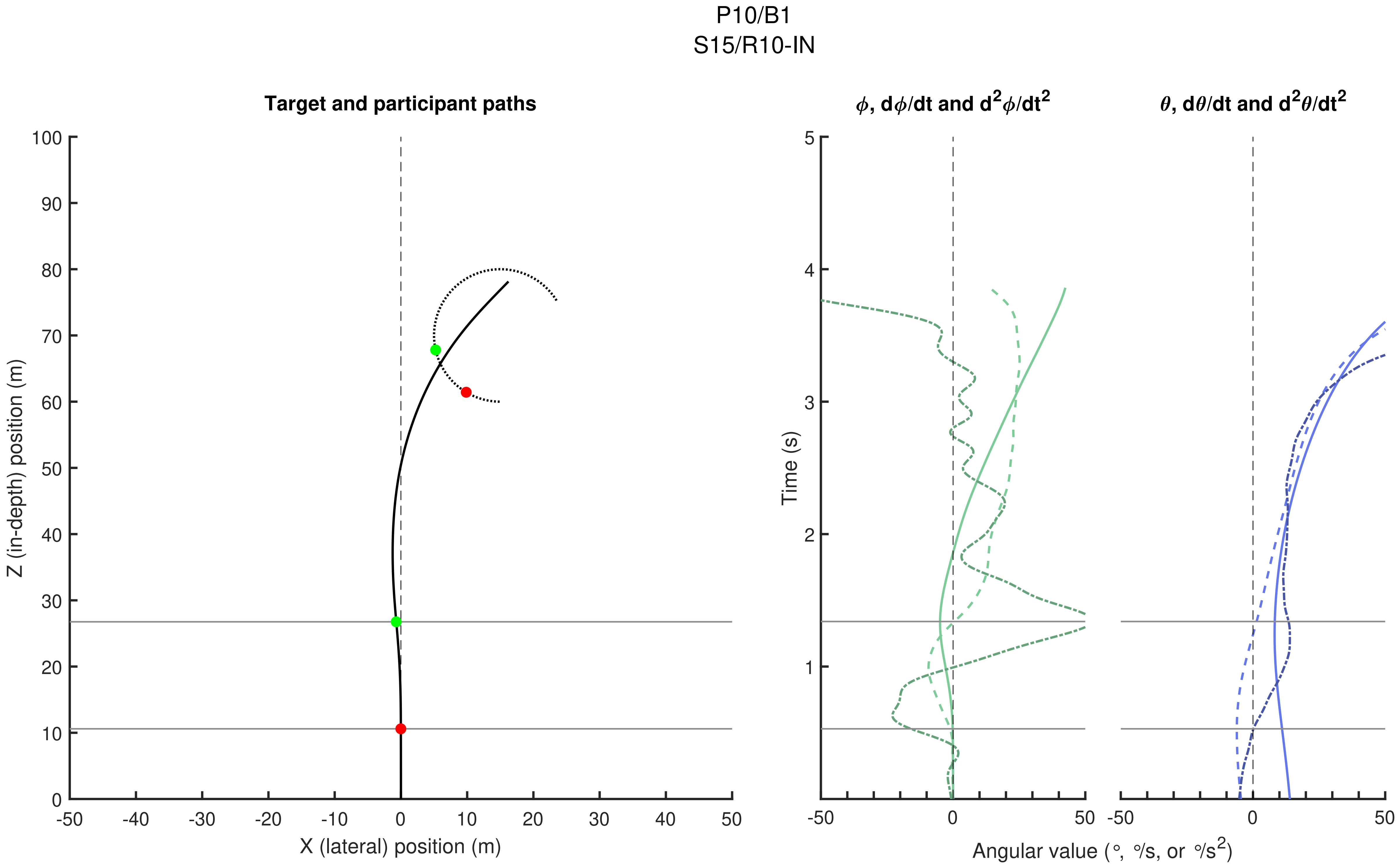

P10/B1
S15/R10-IN
Target and participant paths
Z (in-depth) position (m)
X (lateral) position (m)
φ, dφ/dt and d²φ/dt²
θ, dθ/dt and d²θ/dt²
Time (s)
Angular value (°, °/s, or °/s²)

P10/B1
S15/R20-IN

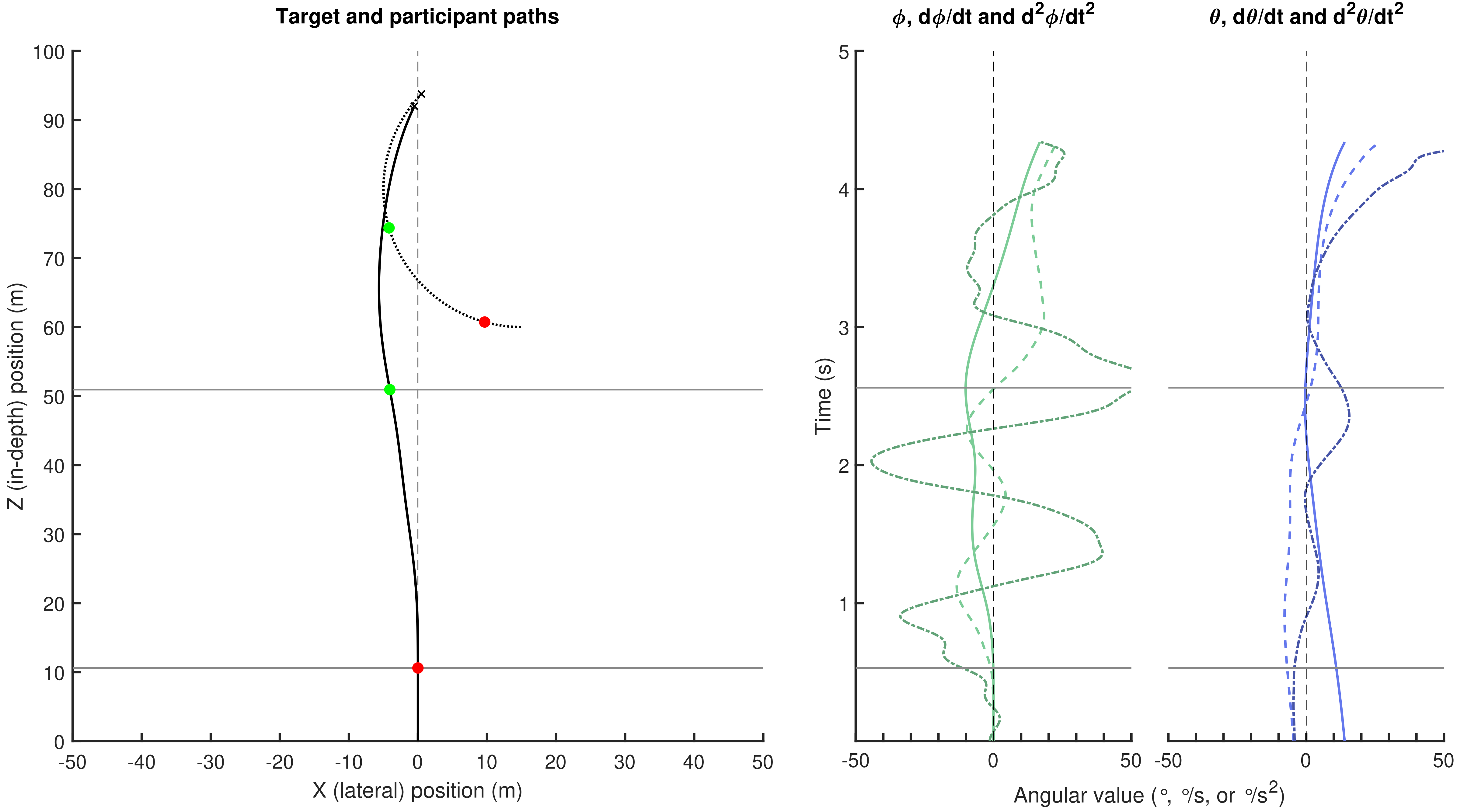

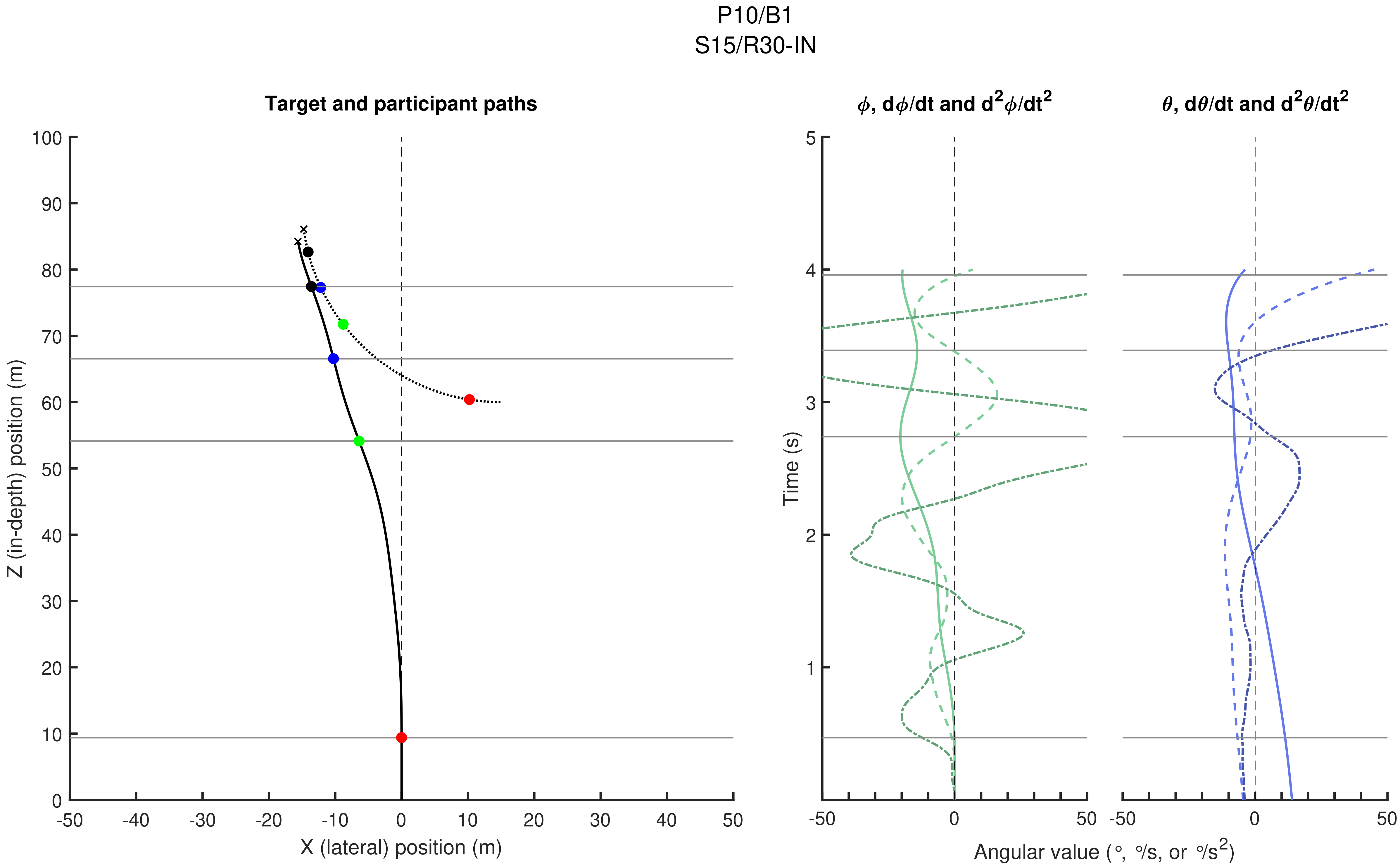

P10/B1
S15/R30-IN
Target and participant paths
Z (in-depth) position (m)
X (lateral) position (m)
ϕ, dϕ/dt and d$^2\phi$/dt^2
θ, dθ/dt and d$^2\theta$/dt^2
Time (s)
Angular value (°, °/s, or °/s^2)

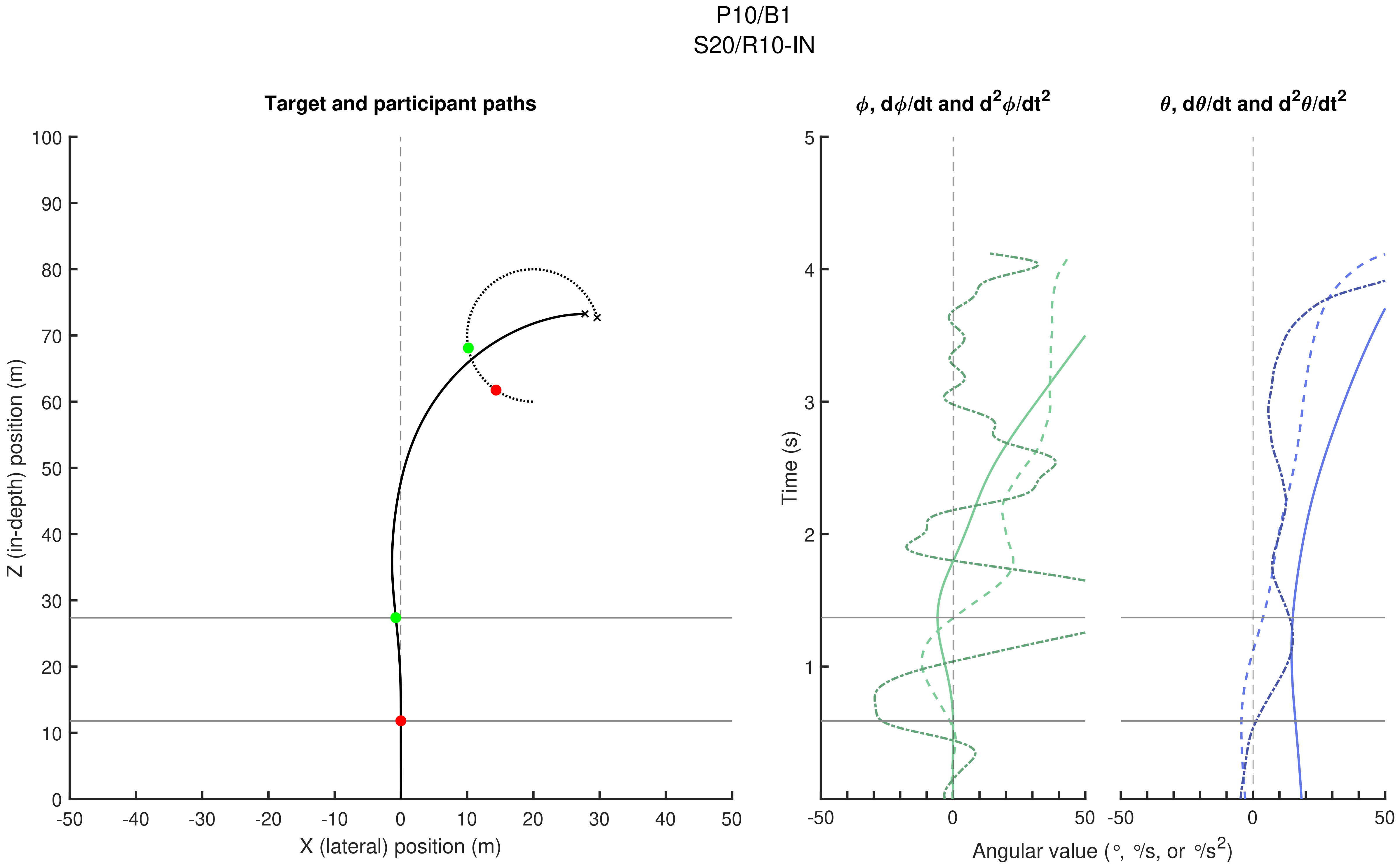

P10/B1
S20/R10-IN
Target and participant paths
Z (in-depth) position (m)
X (lateral) position (m)
φ, dφ/dt and d²φ/dt²
θ, dθ/dt and d²θ/dt²
Time (s)
Angular value (°, °/s, or °/s²)

P10/B1
S20/R20-IN

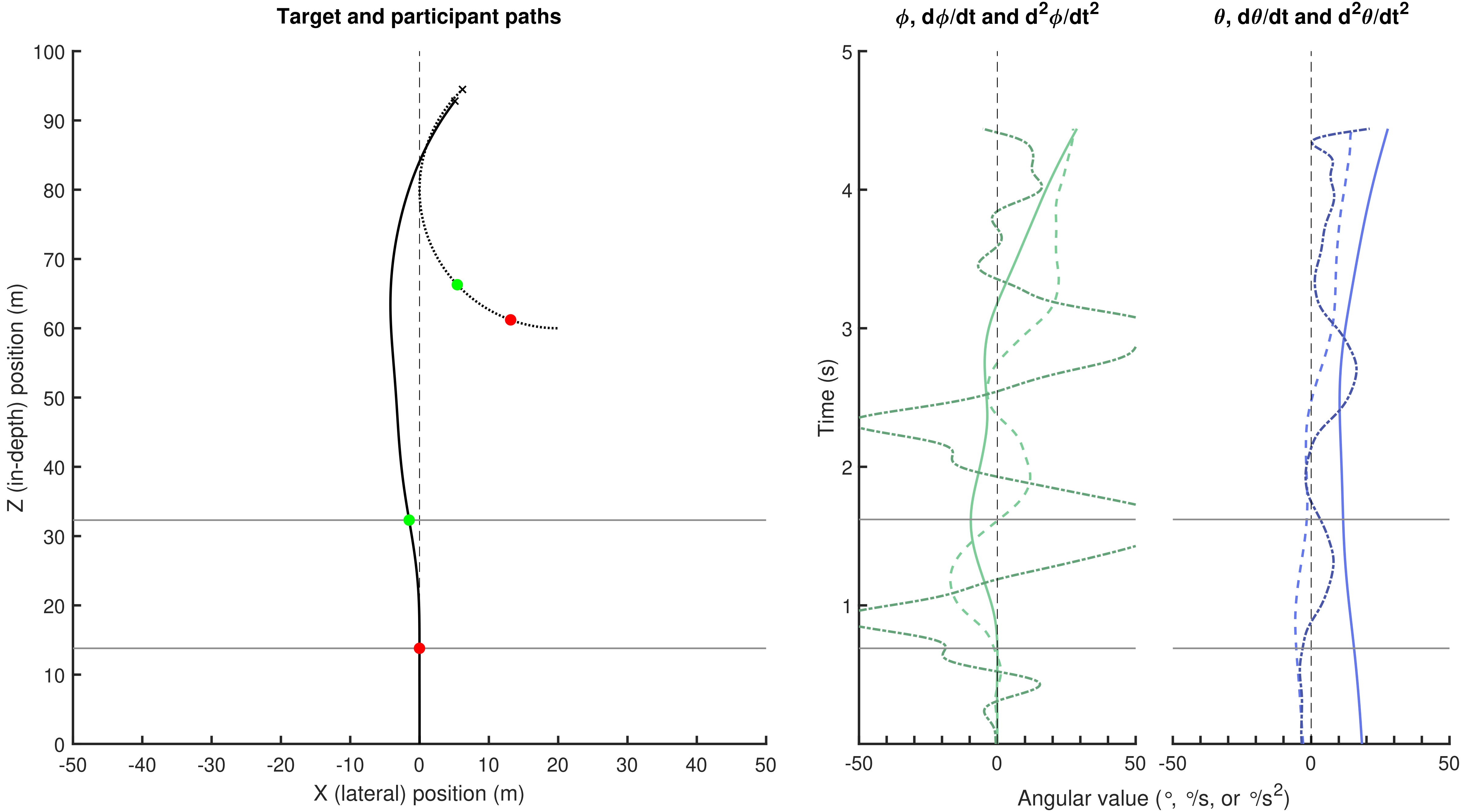

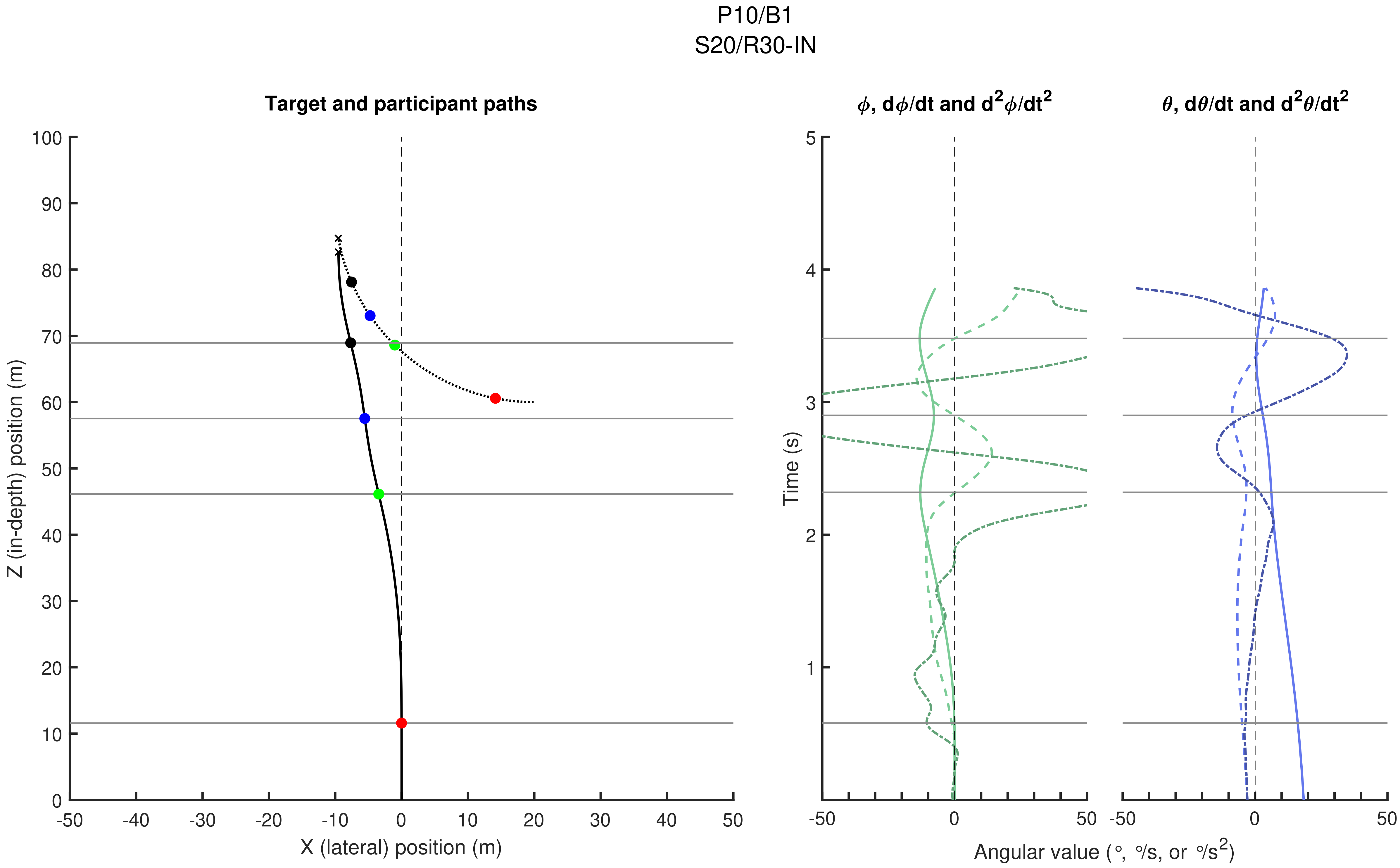

P10/B1
S20/R30-IN
Target and participant paths
Z (in-depth) position (m)
X (lateral) position (m)
ϕ, dϕ/dt and d$^2\phi$/dt^2
θ, dθ/dt and d$^2\theta$/dt^2
Time (s)
Angular value (°, °/s, or °/s^2)

P10/B2
S20/R10-IN

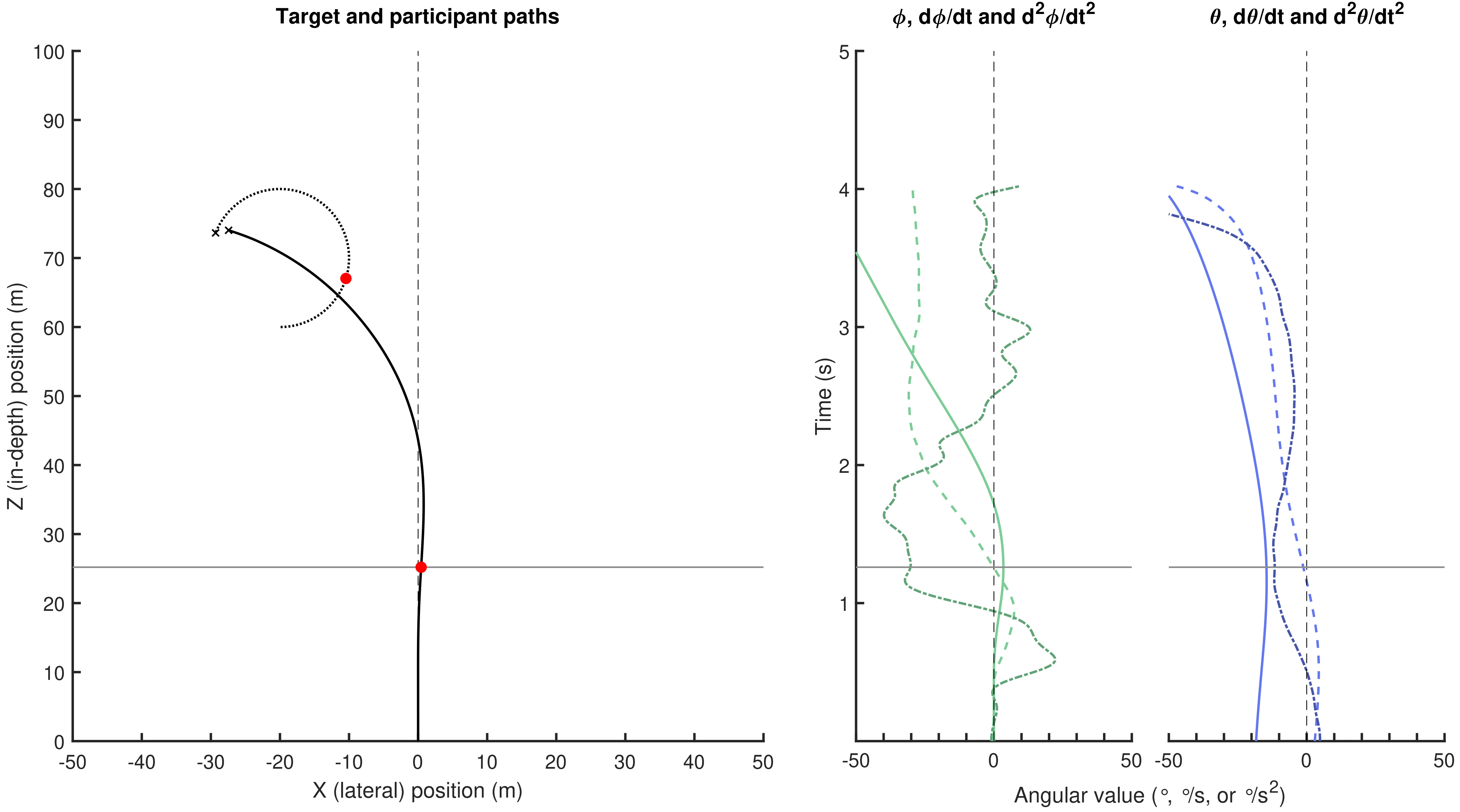

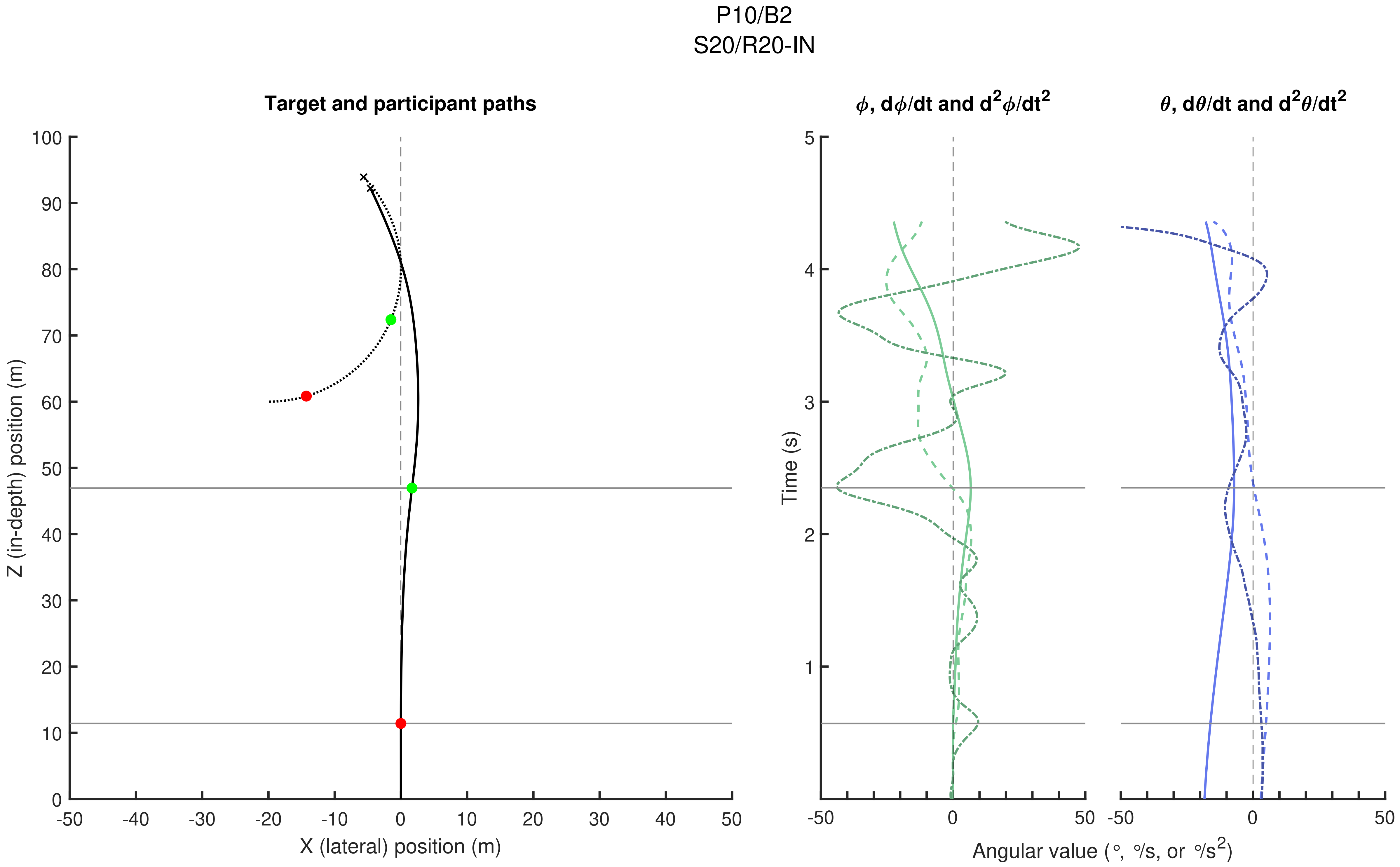
P10/B2
S20/R20-IN
Target and participant paths
Z (in-depth) position (m)
X (lateral) position (m)
ϕ, dϕ/dt and d$^2\phi$/dt^2
θ, dθ/dt and d$^2\theta$/dt^2
Time (s)
Angular value (°, °/s, or °/s^2)

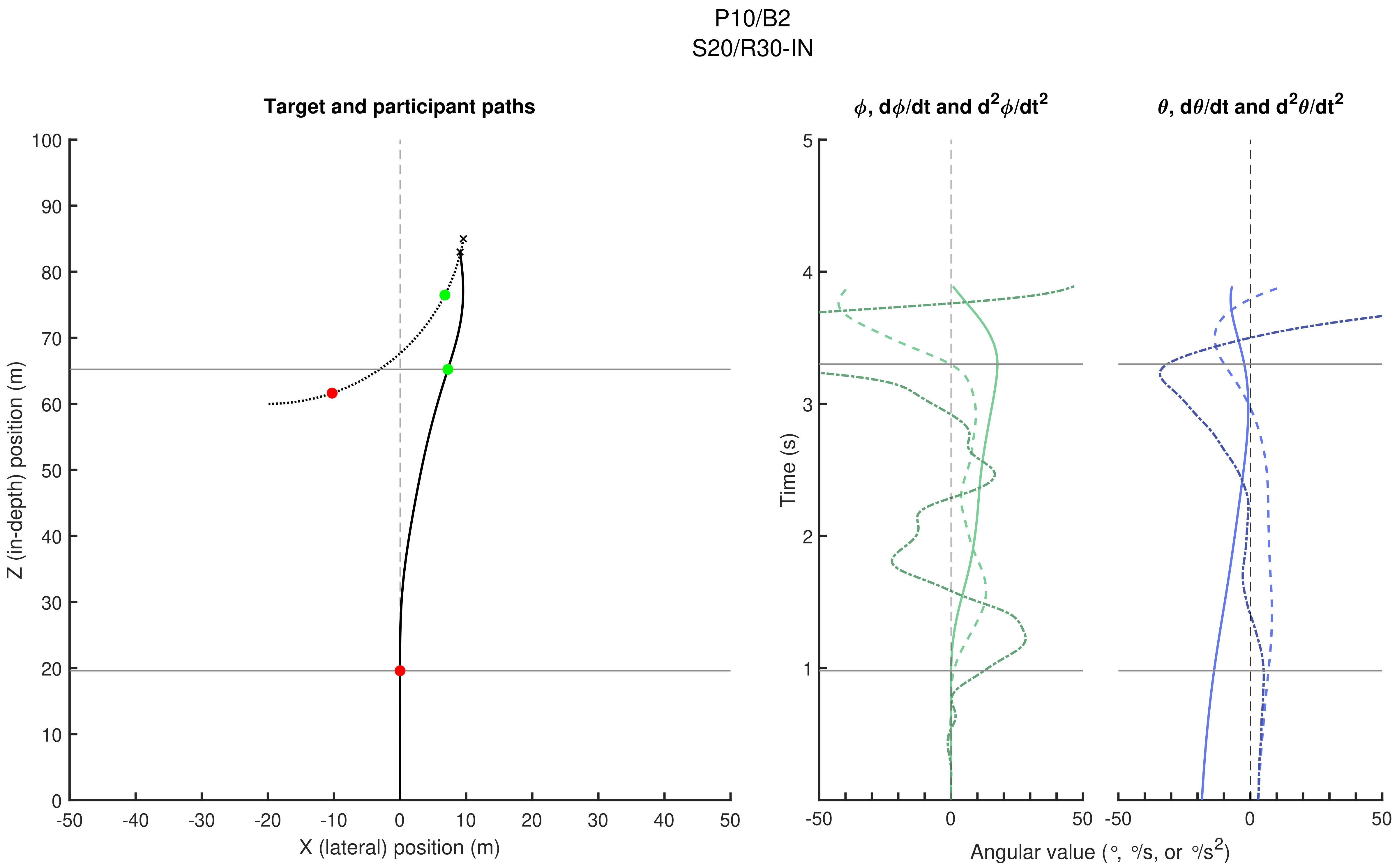

P10/B2
S20/R30-IN
Target and participant paths
Z (in-depth) position (m)
X (lateral) position (m)
ϕ, dϕ/dt and d$^2\phi$/dt^2
θ, dθ/dt and d$^2\theta$/dt^2
Time (s)
Angular value (°, °/s, or °/s^2)

P10/B2
S15/R10-IN

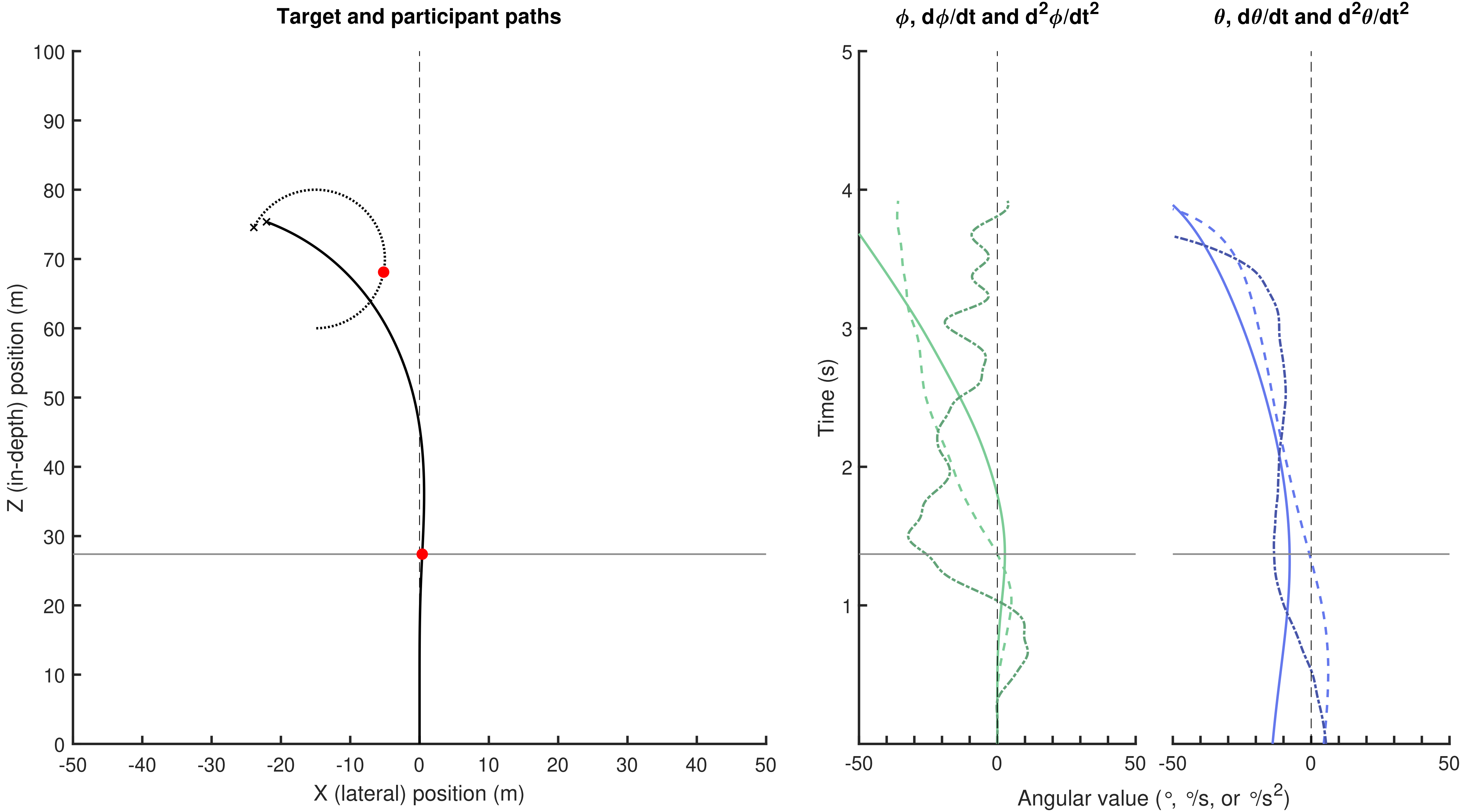

P10/B2
S15/R20-IN

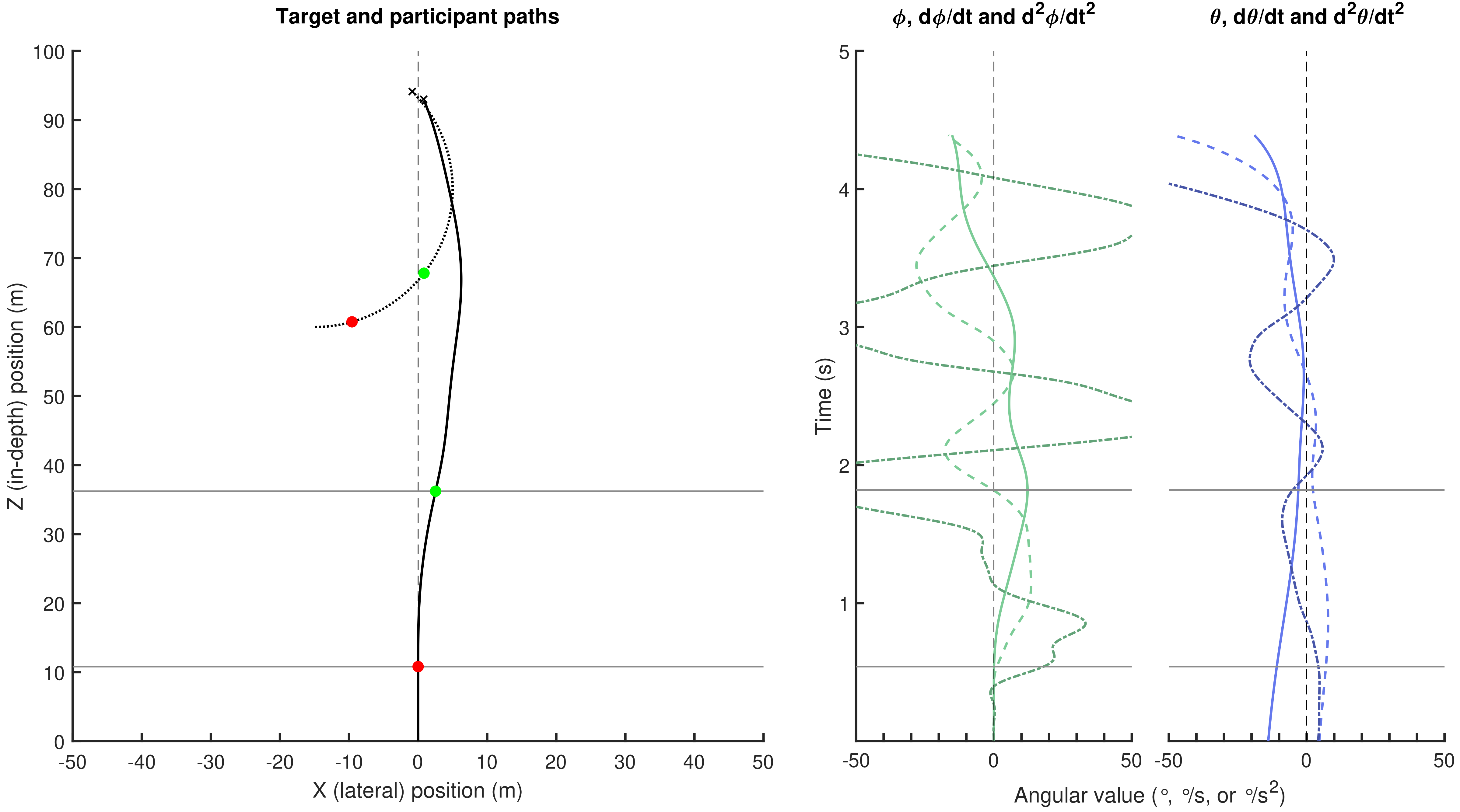

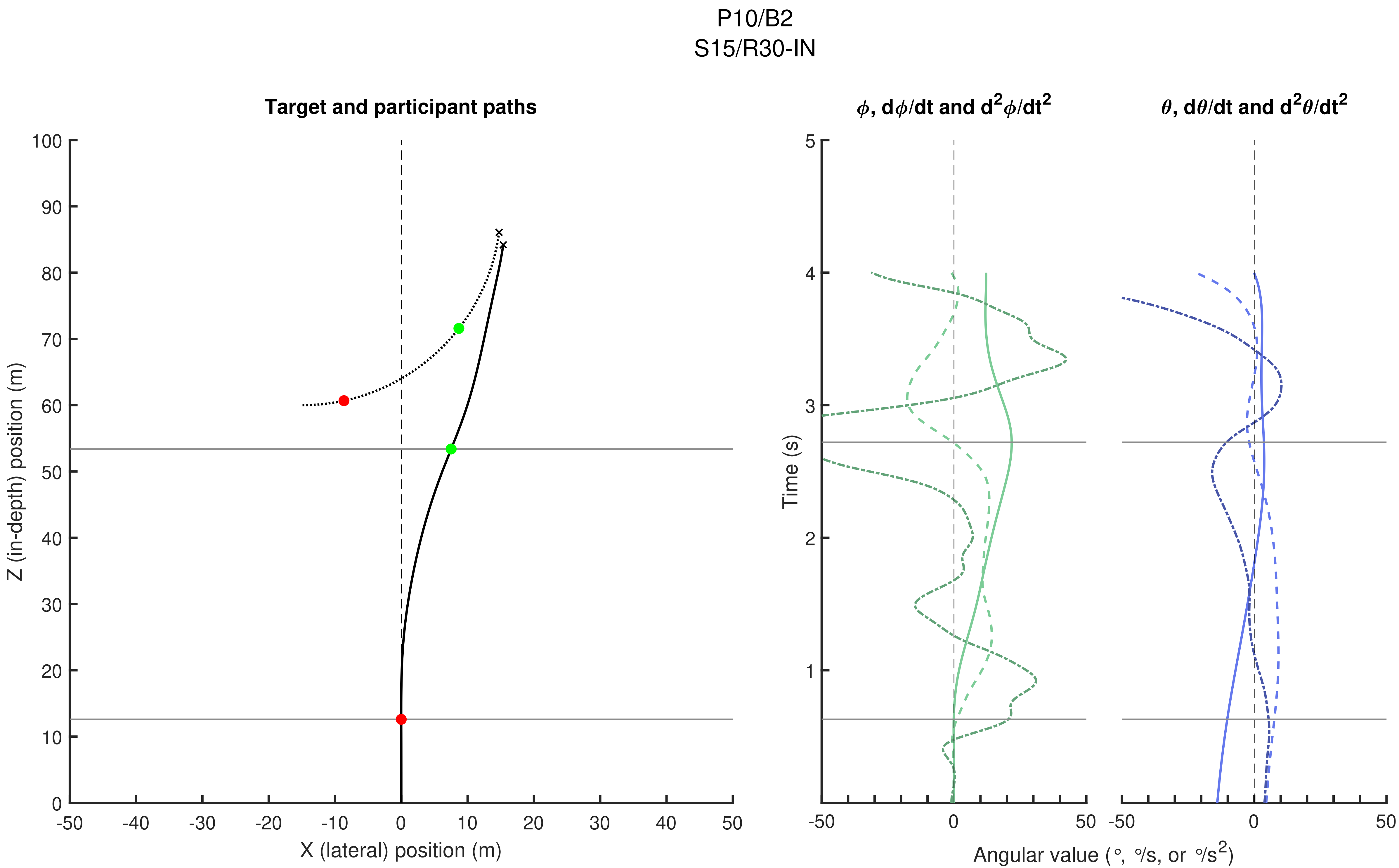
P10/B2
S15/R30-IN
Target and participant paths
Z (in-depth) position (m)
X (lateral) position (m)
ϕ, dϕ/dt and d$^2\phi$/dt^2
θ, dθ/dt and d$^2\theta$/dt^2
Time (s)
Angular value (°, °/s, or °/s^2)

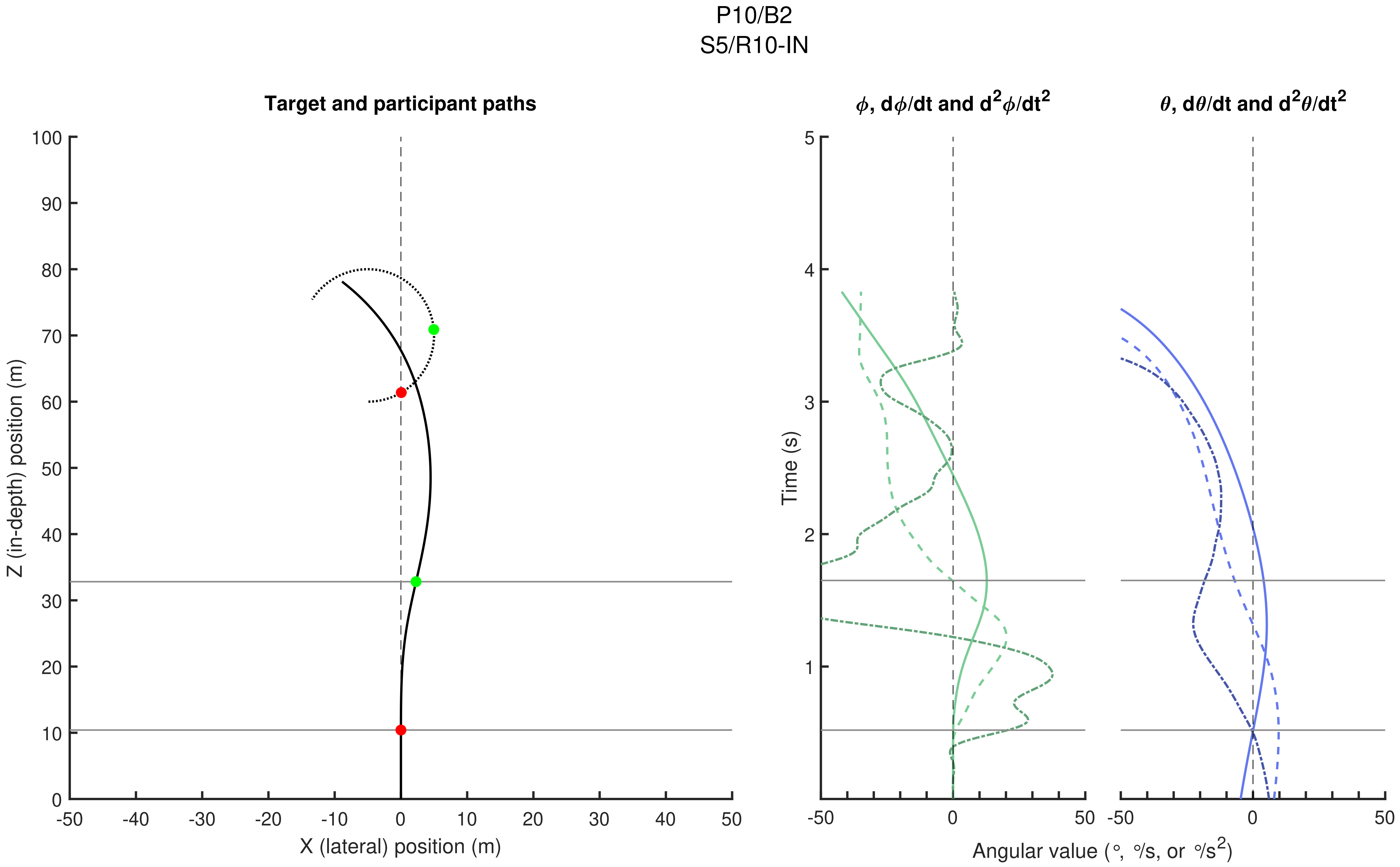

P10/B2
S5/R10-IN
Target and participant paths
Z (in-depth) position (m)
X (lateral) position (m)
100
90
80
70
60
50
40
30
20
10
0
-50
-40
-30
-20
-10
0
10
20
30
40
50
ϕ, dϕ/dt and d$^2\phi$/dt^2
θ, dθ/dt and d$^2\theta$/dt^2
Time (s)
5
4
3
2
1
-50
0
50
Angular value (°, °/s, or °/s^2)

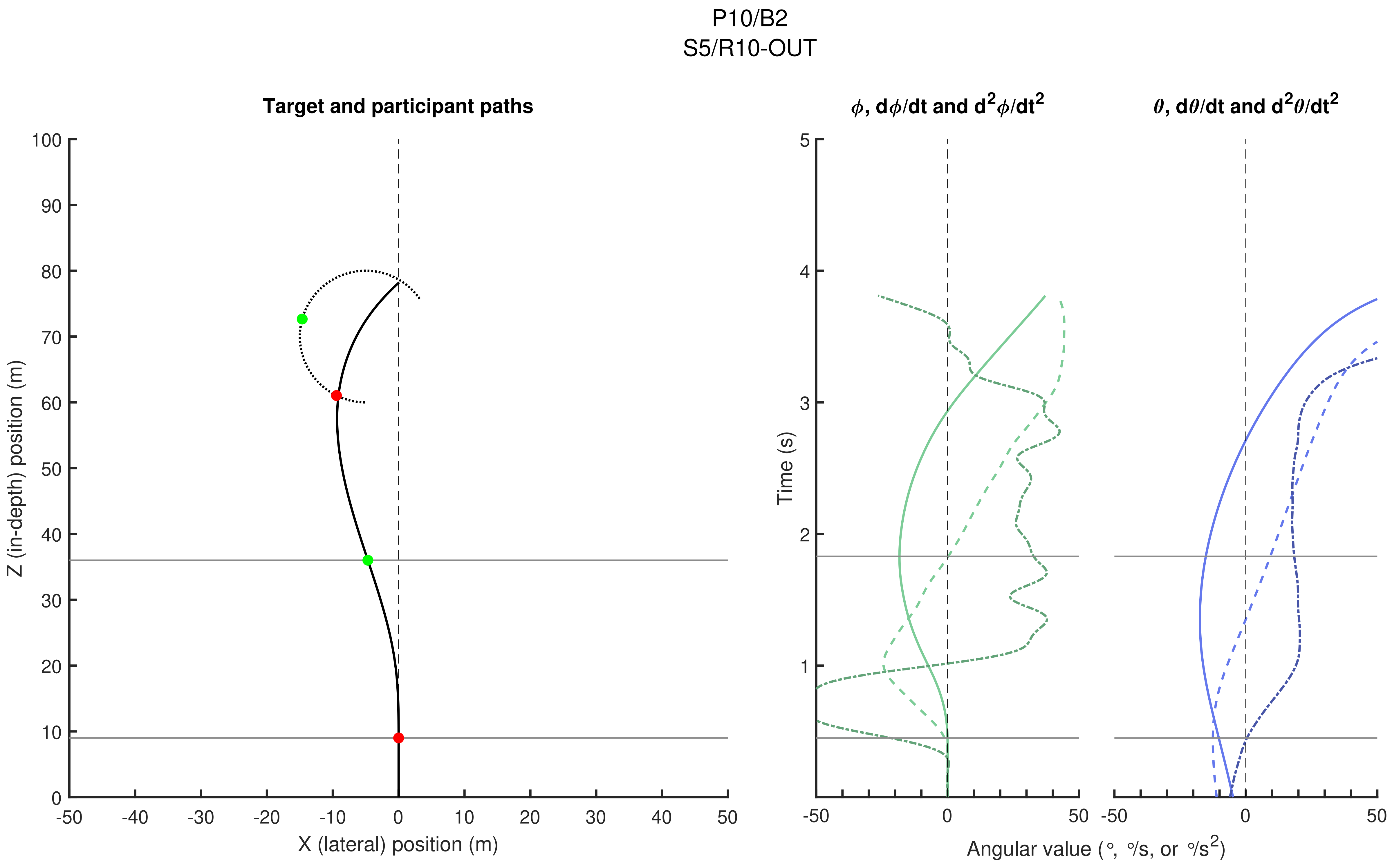
P10/B2
S5/R10-OUT
Target and participant paths
Z (in-depth) position (m)
X (lateral) position (m)
ϕ, dϕ/dt and d²ϕ/dt²
θ, dθ/dt and d²θ/dt²
Time (s)
Angular value (°, °/s, or °/s²)

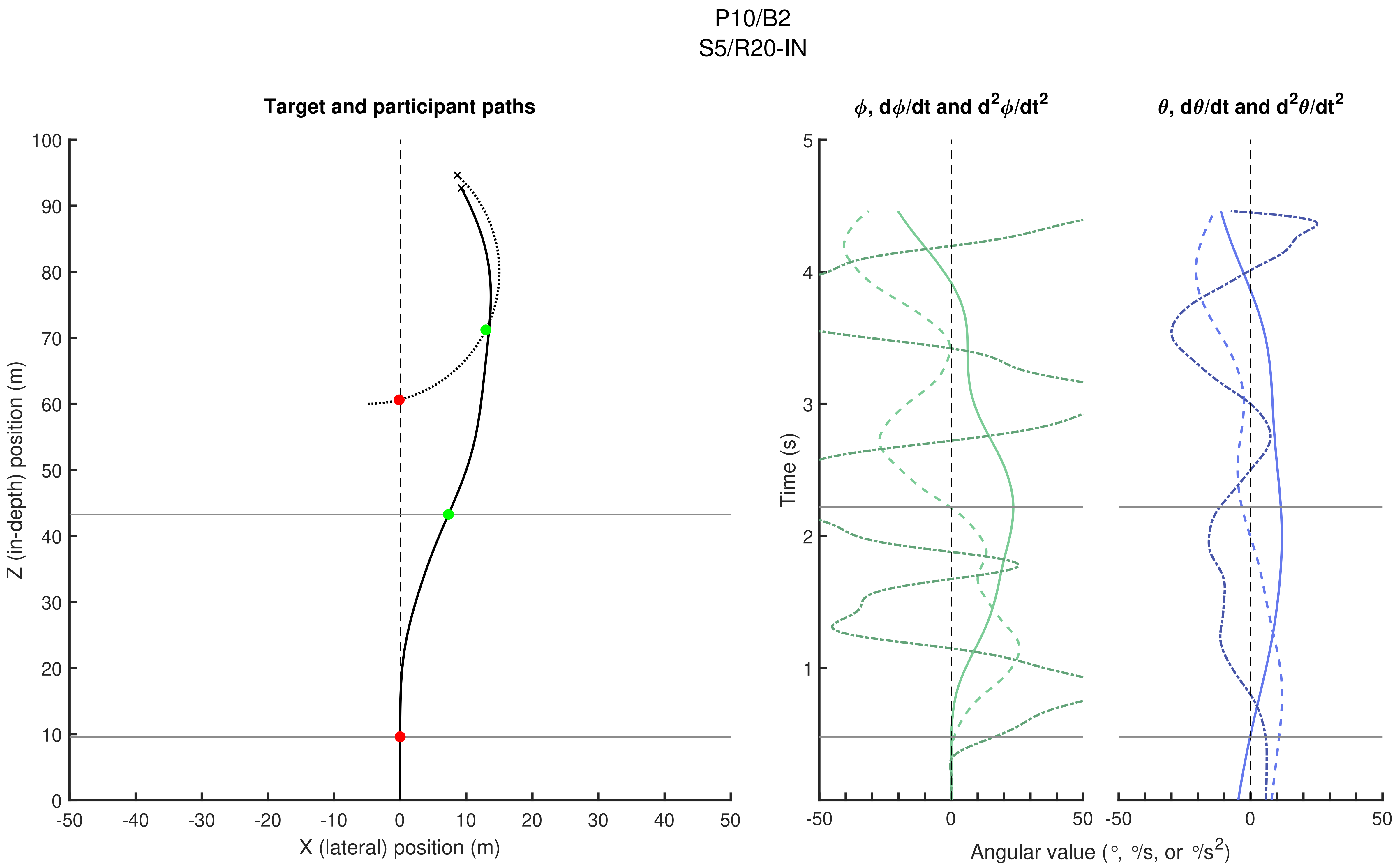

P10/B2
S5/R20-IN
Target and participant paths
Z (in-depth) position (m)
X (lateral) position (m)
ϕ, dϕ/dt and d$^2\phi$/dt^2
θ, dθ/dt and d$^2\theta$/dt^2
Time (s)
Angular value (°, °/s, or °/s^2)

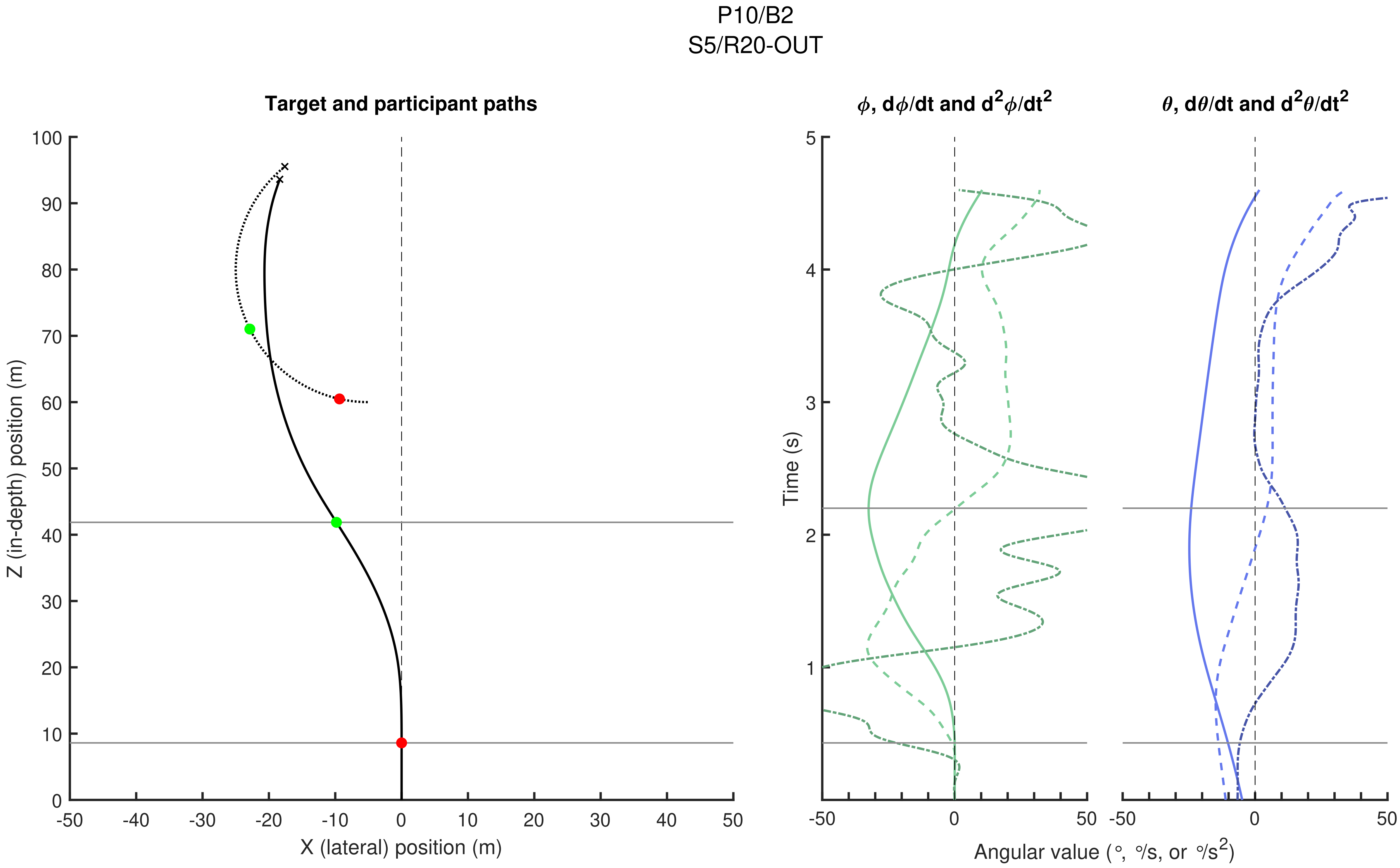

P10/B2
S5/R20-OUT
Target and participant paths
Z (in-depth) position (m)
X (lateral) position (m)
100
90
80
70
60
50
40
30
20
10
0
-50
-40
-30
-20
-10
0
10
20
30
40
50
ϕ, dϕ/dt and d2ϕ/dt2
θ, dθ/dt and d2θ/dt2
Time (s)
5
4
3
2
1
-50
0
50
Angular value (°, °/s, or °/s2)

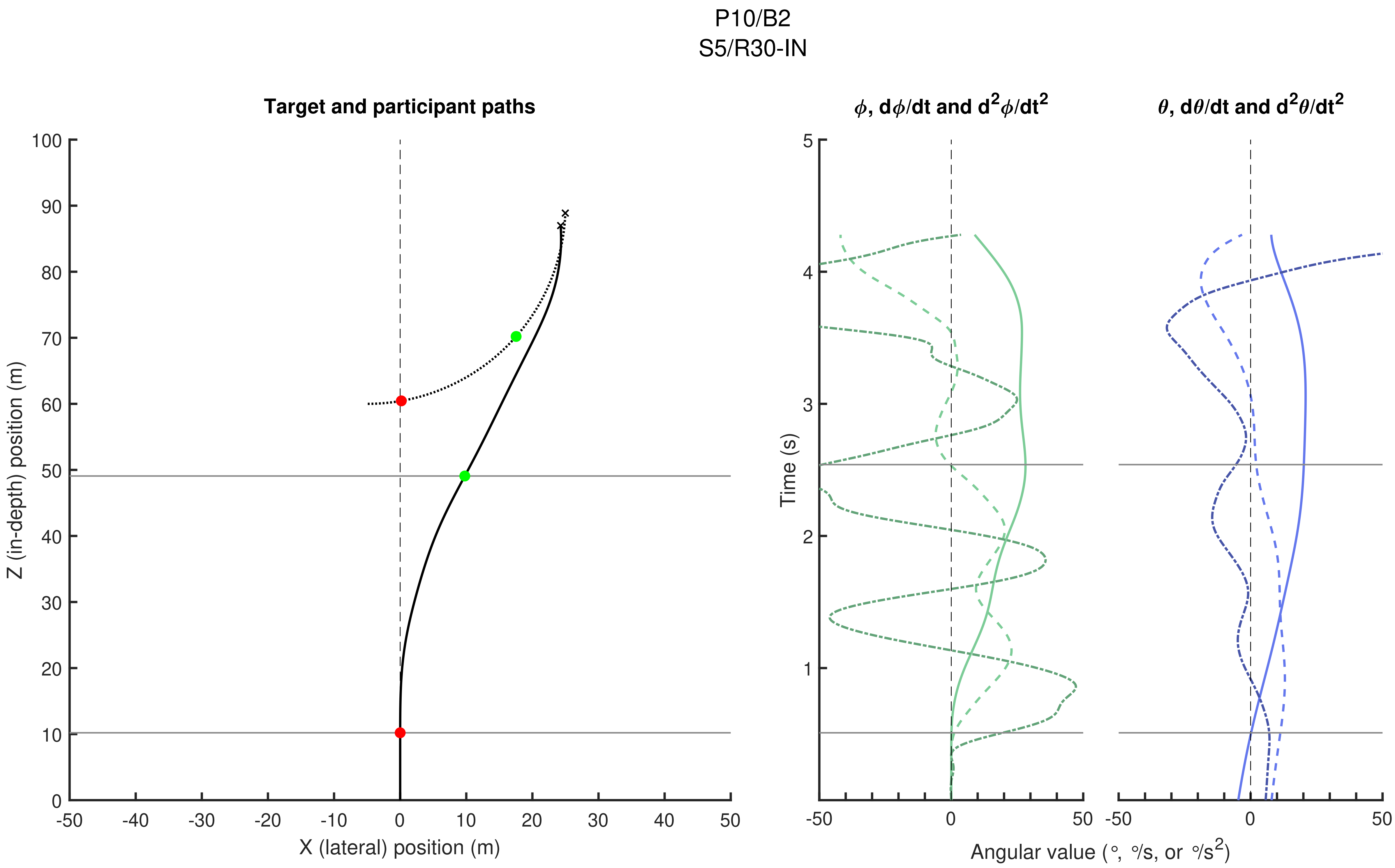
P10/B2
S5/R30-IN
Target and participant paths
Z (in-depth) position (m)
X (lateral) position (m)
100
90
80
70
60
50
40
30
20
10
0
-50
-40
-30
-20
-10
0
10
20
30
40
50
ϕ, dϕ/dt and d²ϕ/dt²
θ, dθ/dt and d²θ/dt²
Time (s)
5
4
3
2
1
-50
0
50
-50
0
50
Angular value (°, °/s, or °/s²)

P10/B2
S5/R30-OUT

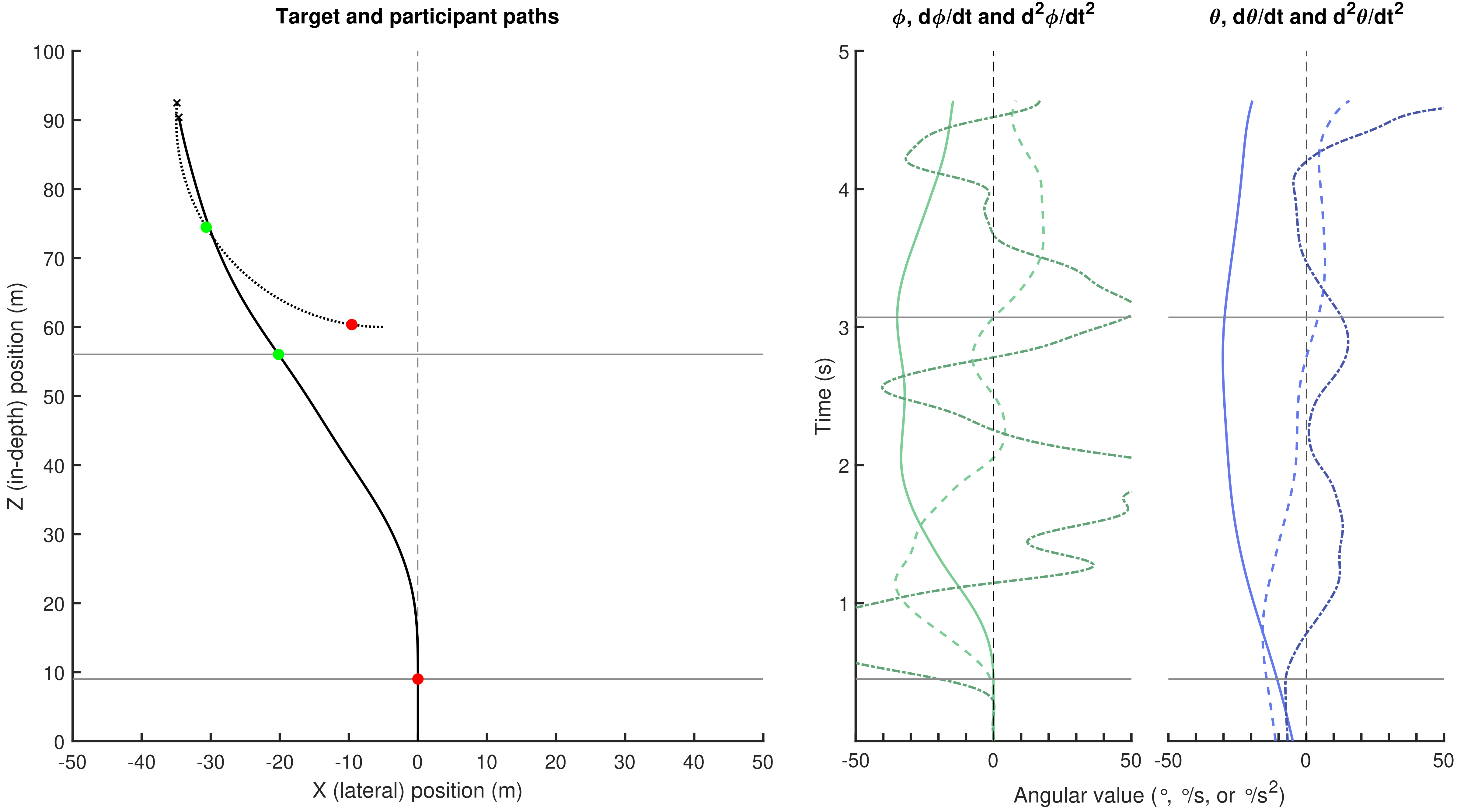

P10/B2
S5/R10-OUT

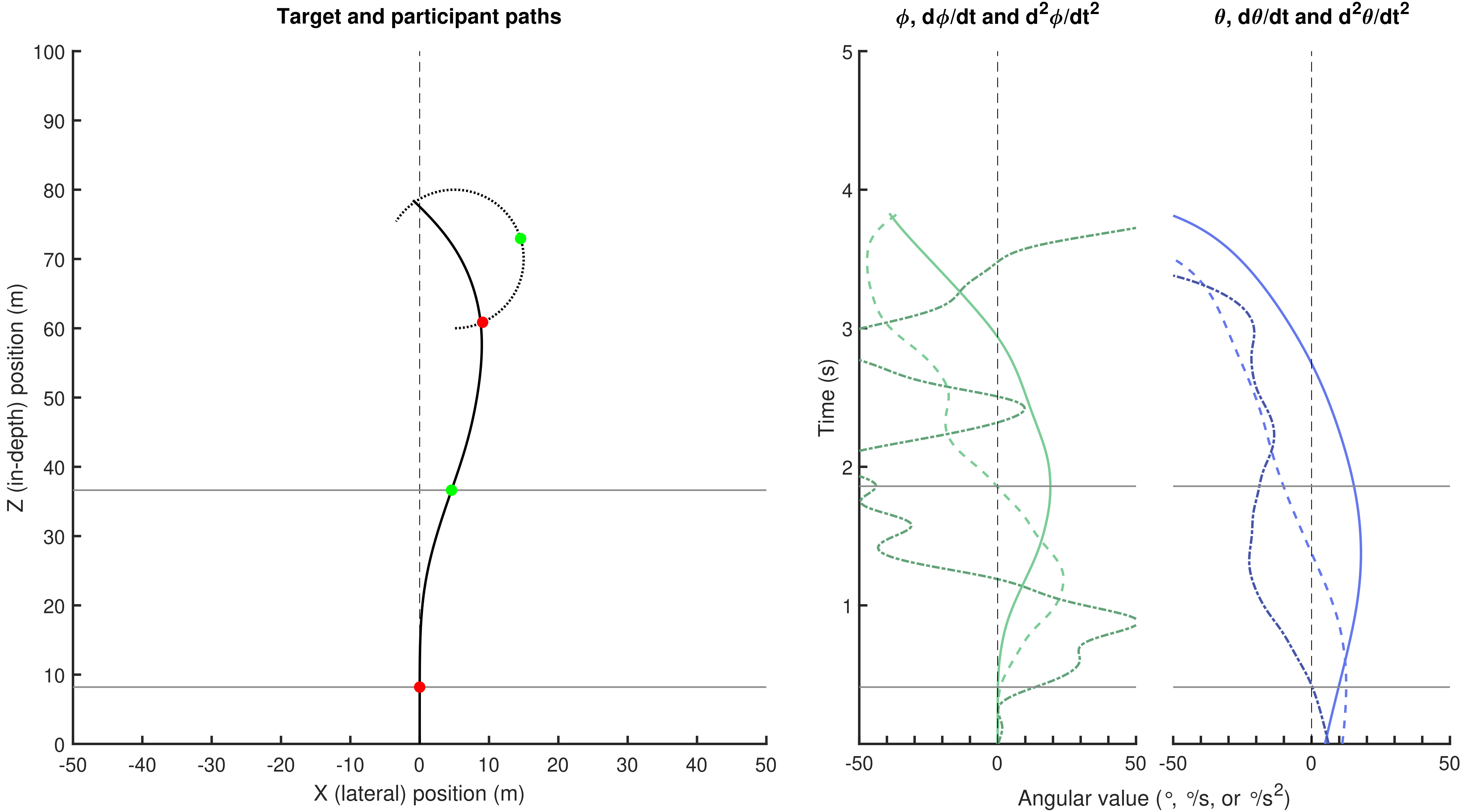

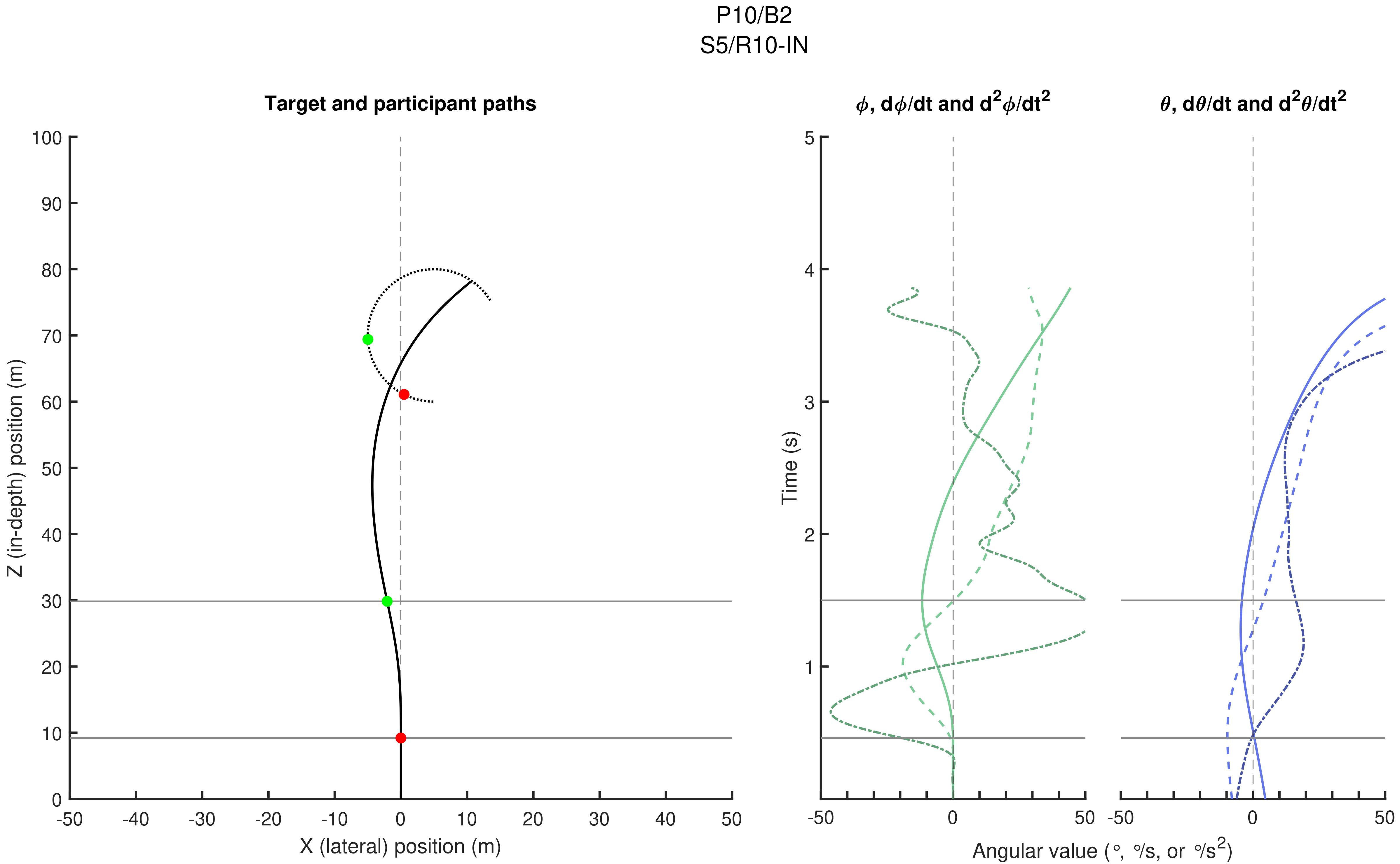

P10/B2
S5/R10-IN
Target and participant paths
ϕ, dϕ/dt and d²ϕ/dt²
θ, dθ/dt and d²θ/dt²
Z (in-depth) position (m)
X (lateral) position (m)
Time (s)
Angular value (°, °/s, or °/s²)

P10/B2
S5/R20-OUT

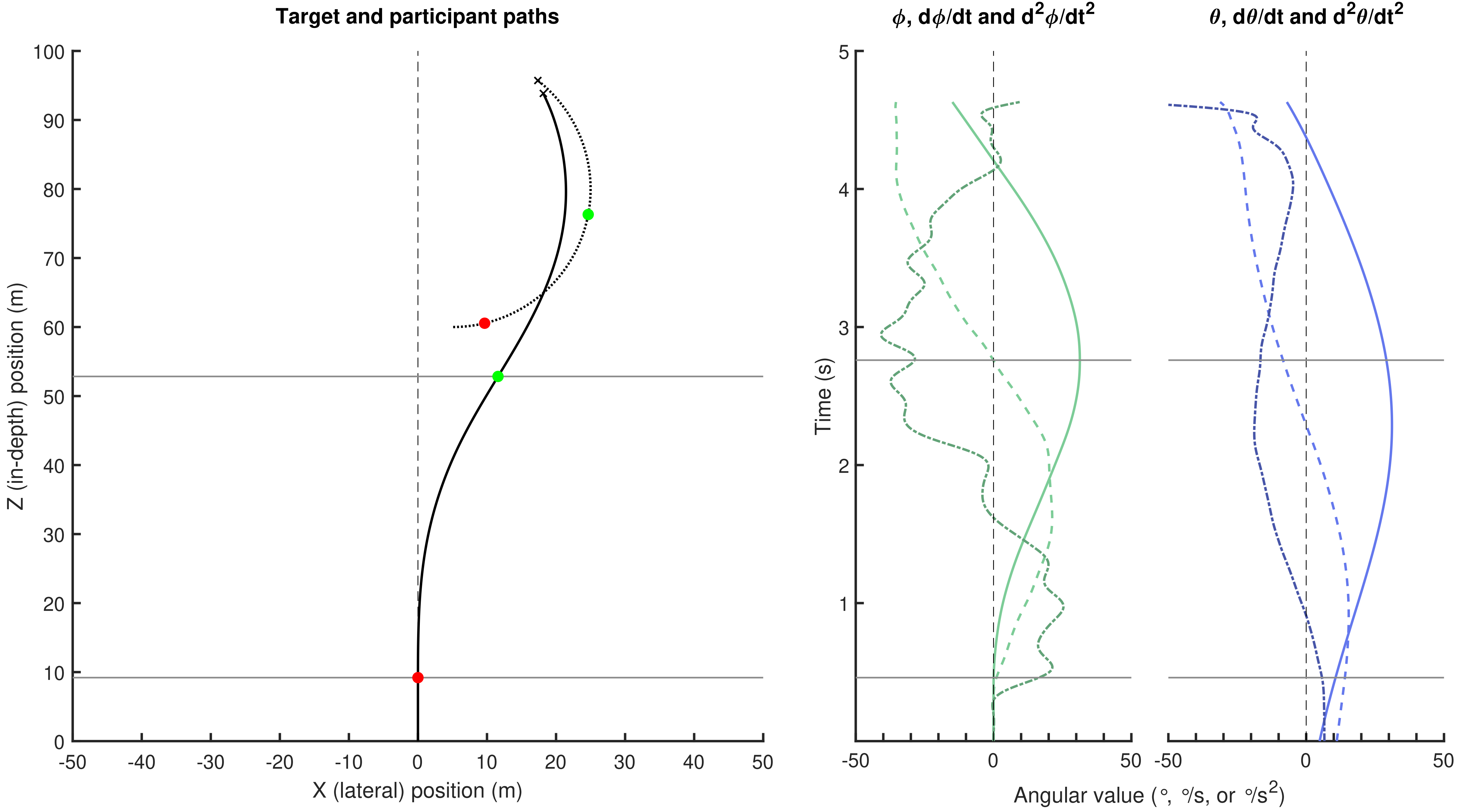

P10/B2
S5/R20-IN

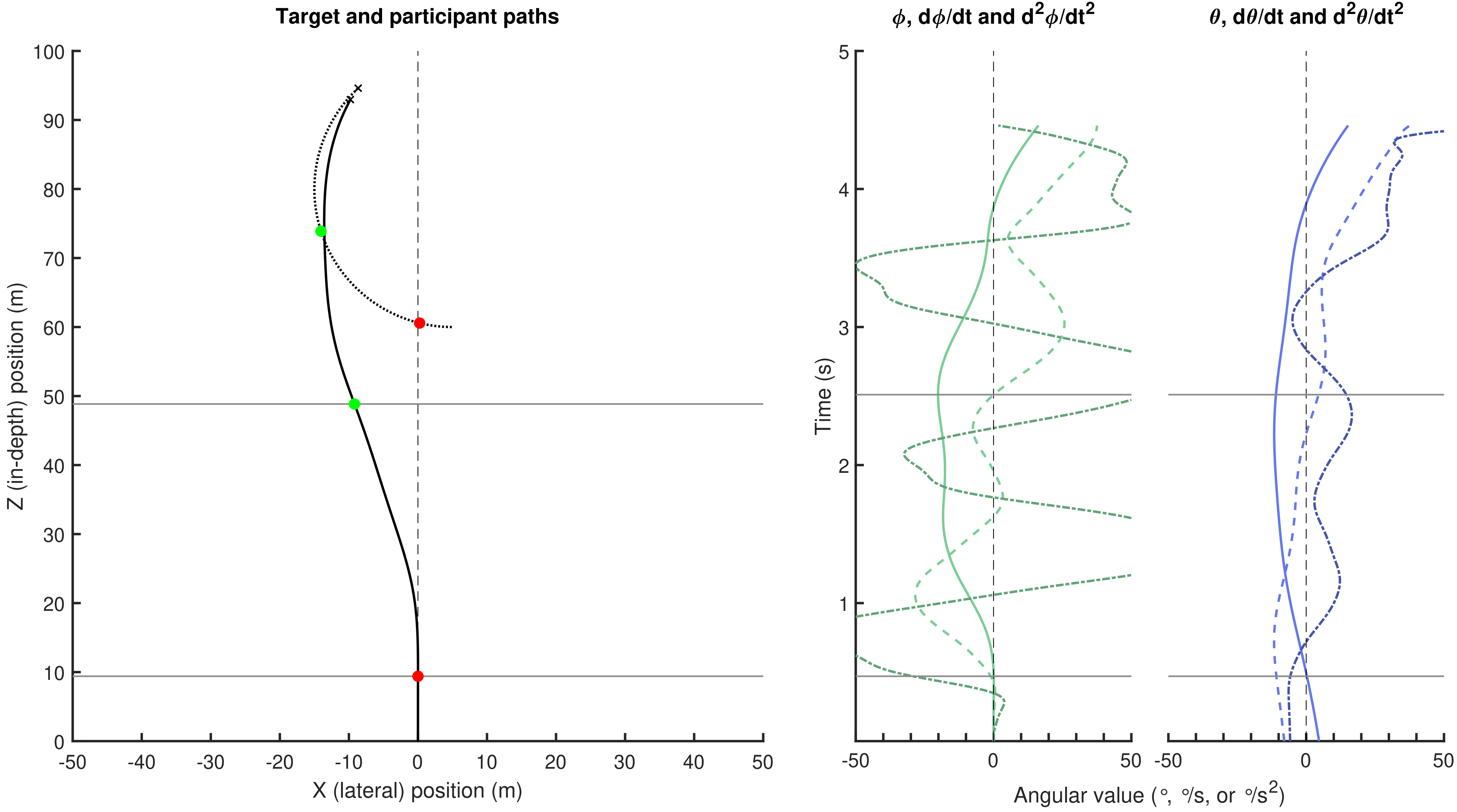

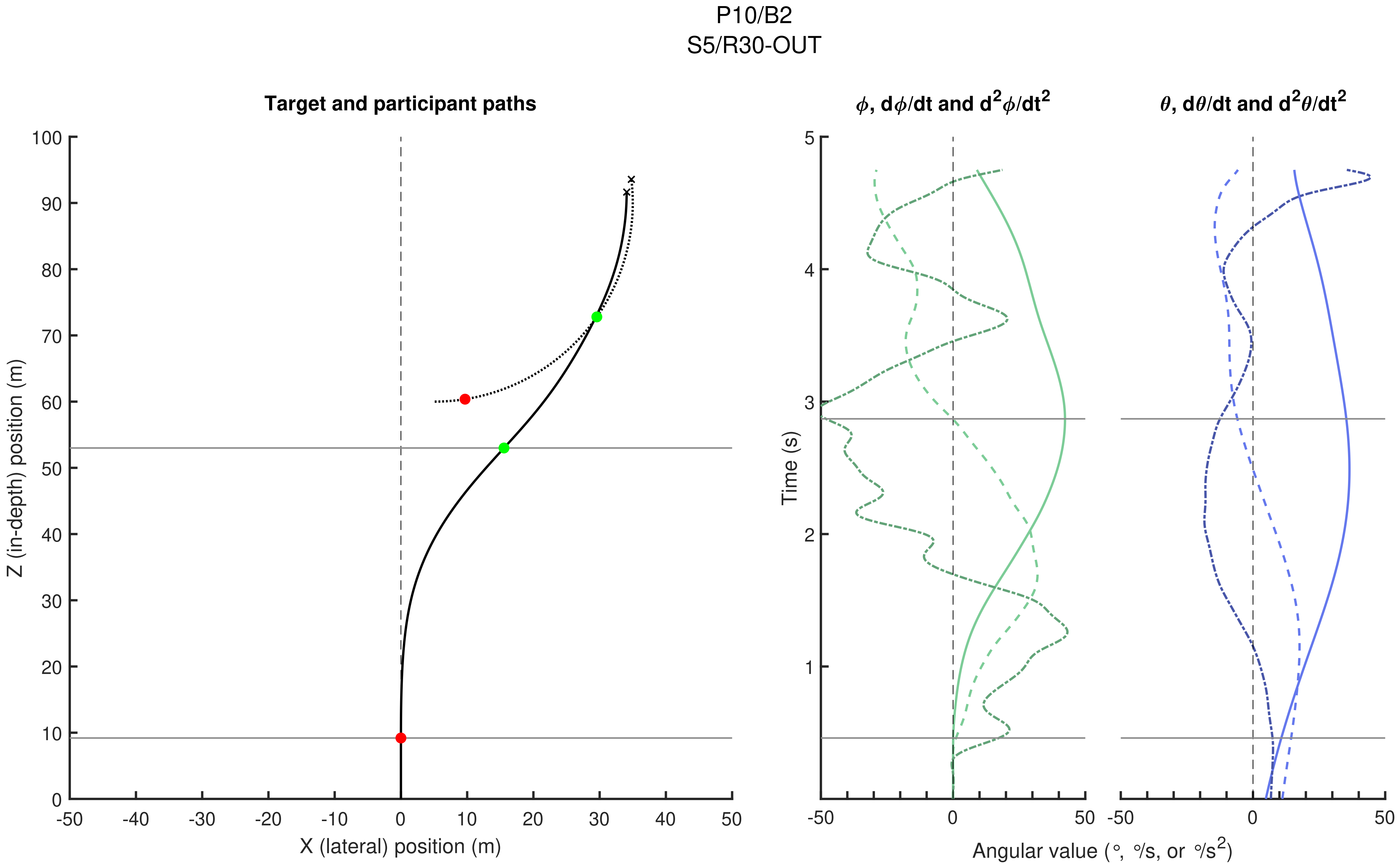
P10/B2
S5/R30-OUT
Target and participant paths
Z (in-depth) position (m)
X (lateral) position (m)
ϕ, dϕ/dt and d$^2\phi$/dt^2
θ, dθ/dt and d$^2\theta$/dt^2
Time (s)
Angular value (°, °/s, or °/s^2)

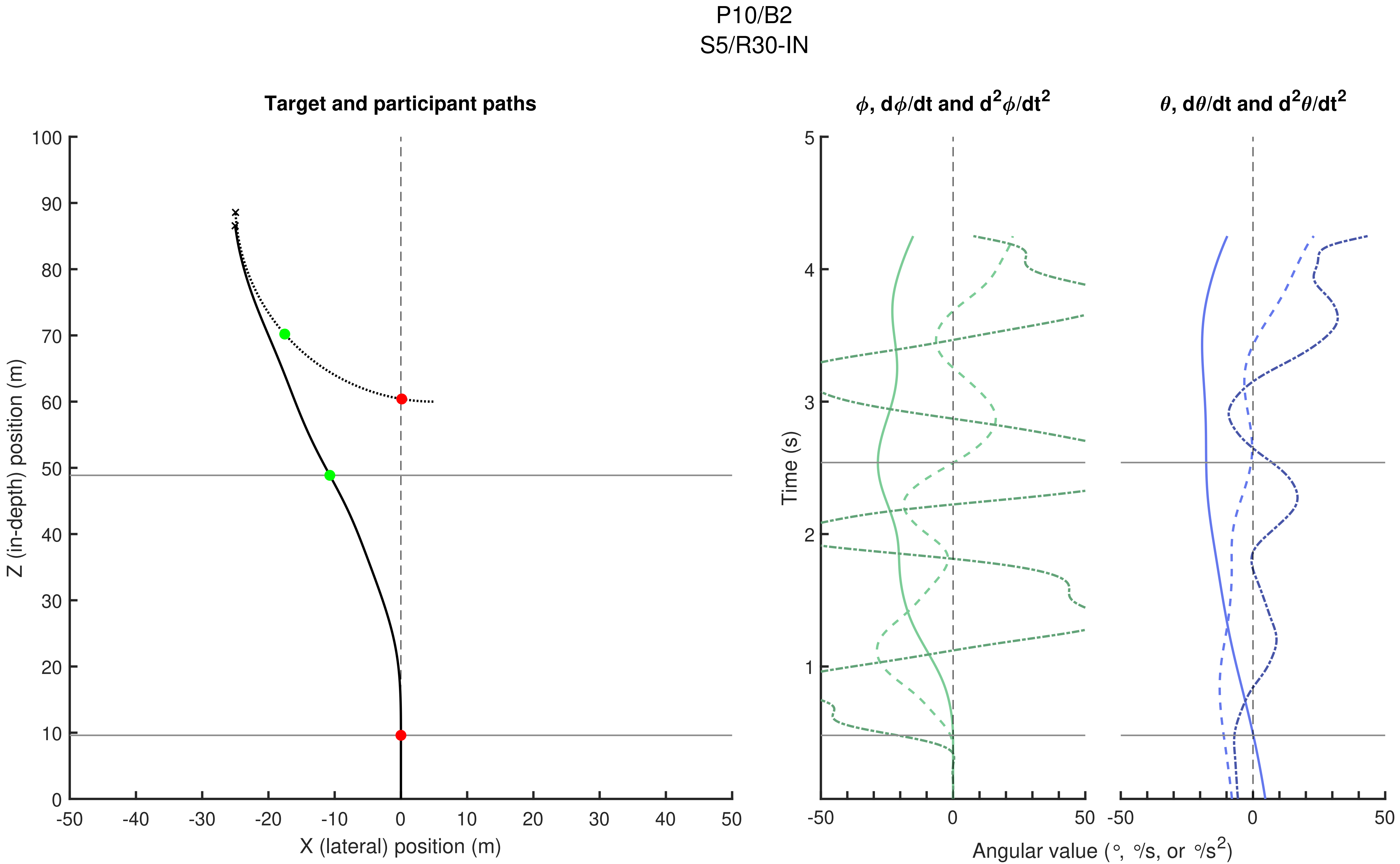

P10/B2
S5/R30-IN
Target and participant paths
Z (in-depth) position (m)
X (lateral) position (m)
φ, dφ/dt and d²φ/dt²
θ, dθ/dt and d²θ/dt²
Time (s)
Angular value (°, °/s, or °/s²)

P10/B2
S15/R10-IN

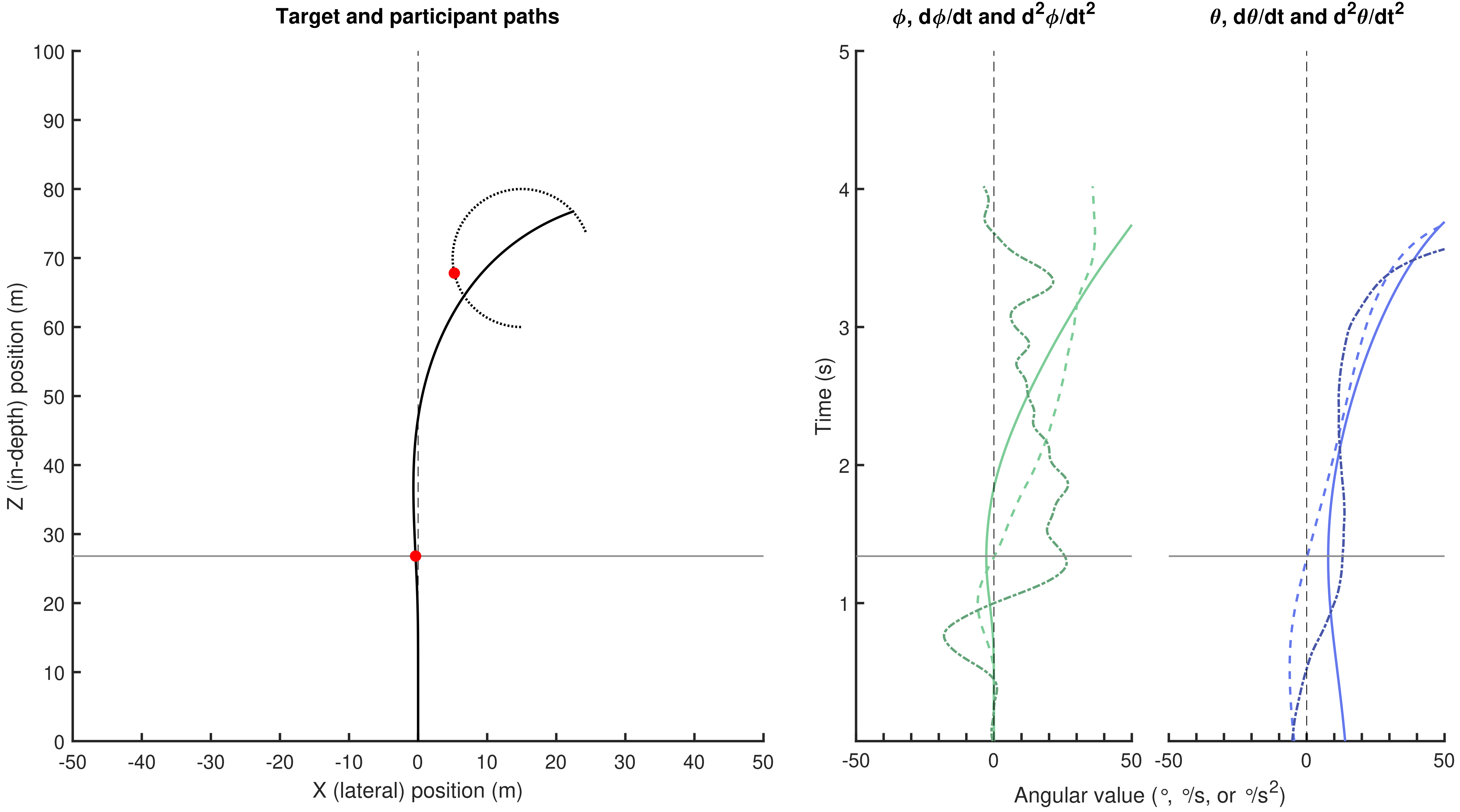

P10/B2
S15/R20-IN

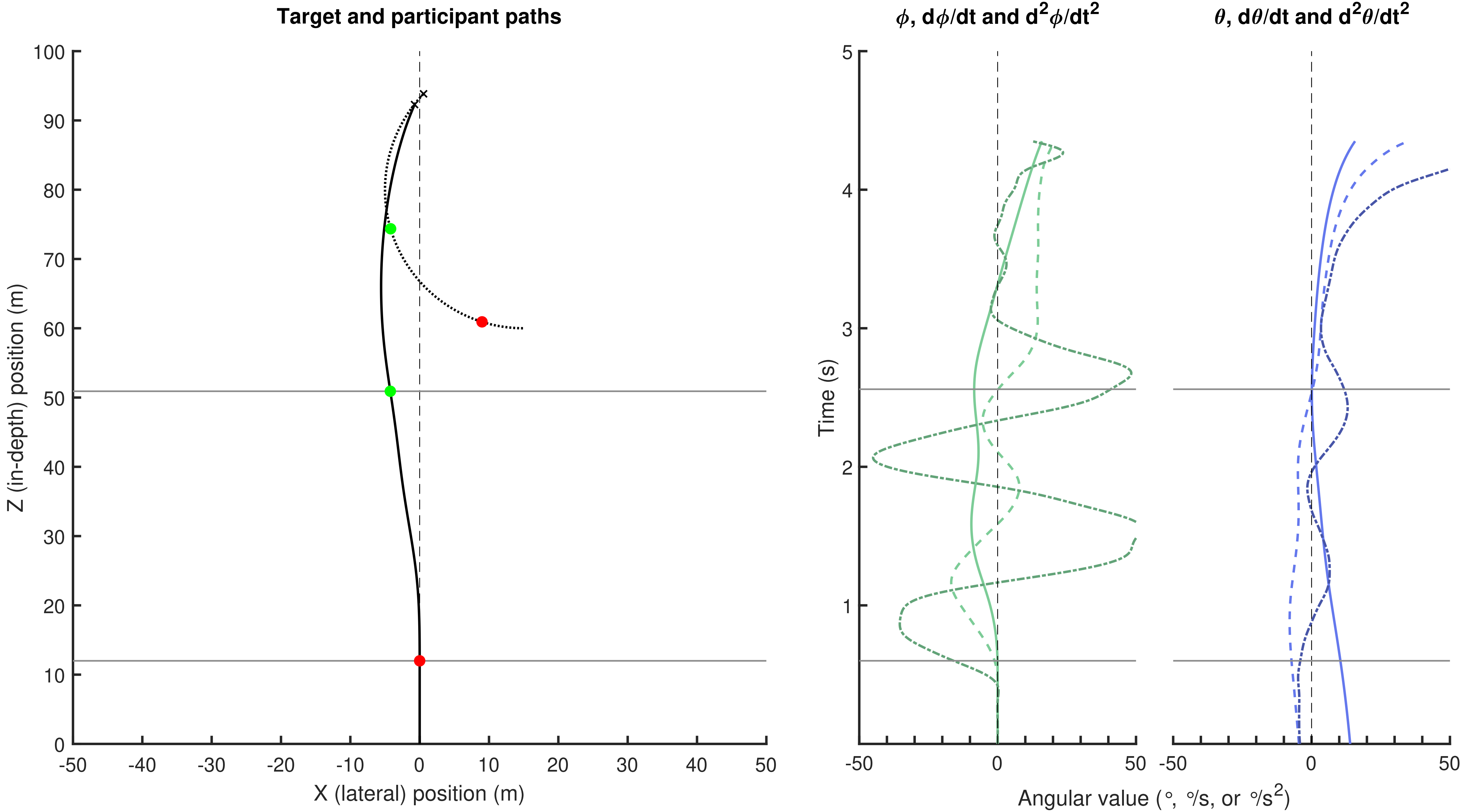

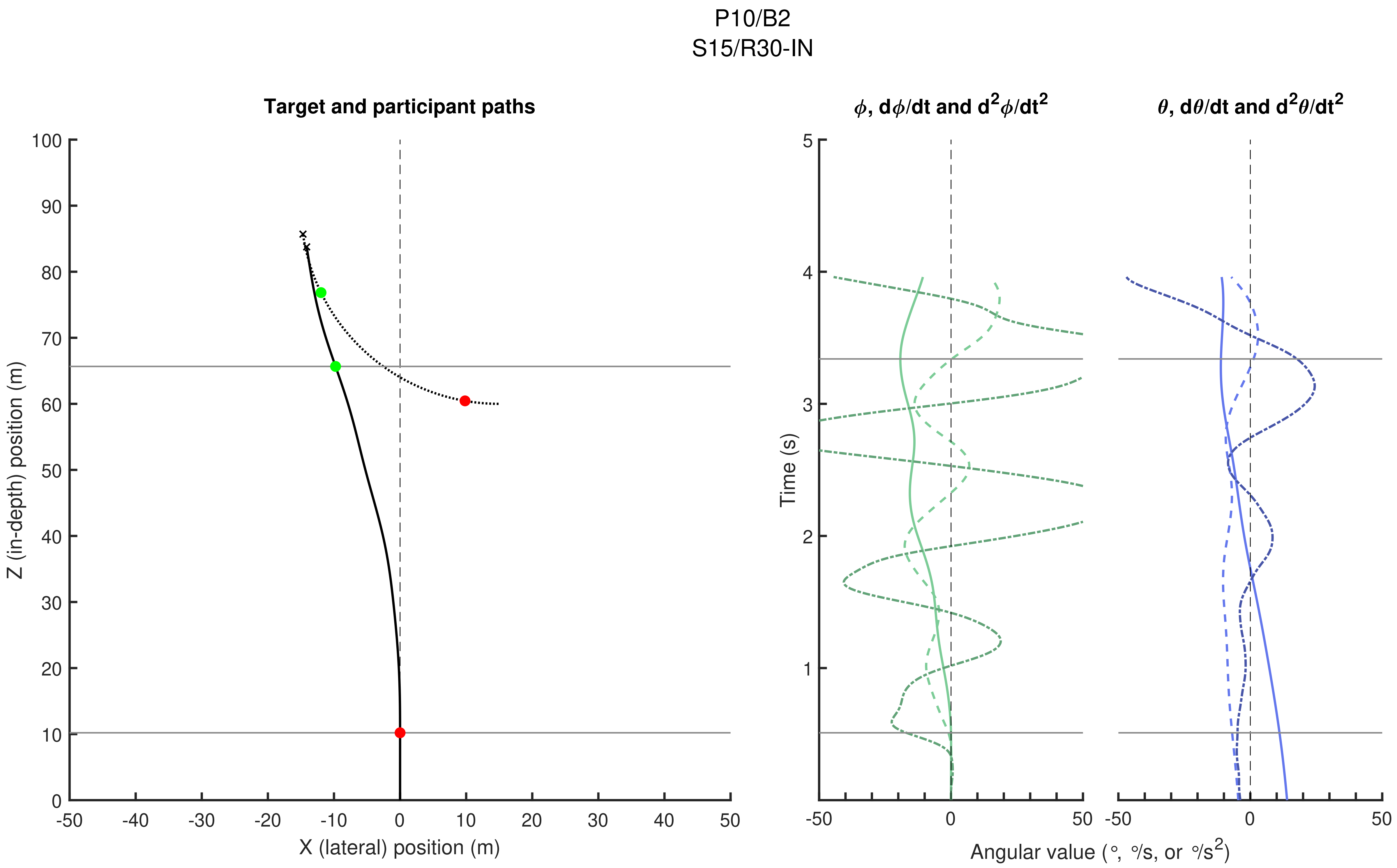
P10/B2
S15/R30-IN
Target and participant paths
Z (in-depth) position (m)
X (lateral) position (m)
0
10
20
30
40
50
60
70
80
90
100
-50
-40
-30
-20
-10
0
10
20
30
40
50
ϕ, dϕ/dt and d$^2\phi$/dt^2
θ, dθ/dt and d$^2\theta$/dt^2
Time (s)
1
2
3
4
5
-50
0
50
-50
0
50
Angular value (°, °/s, or °/s^2)

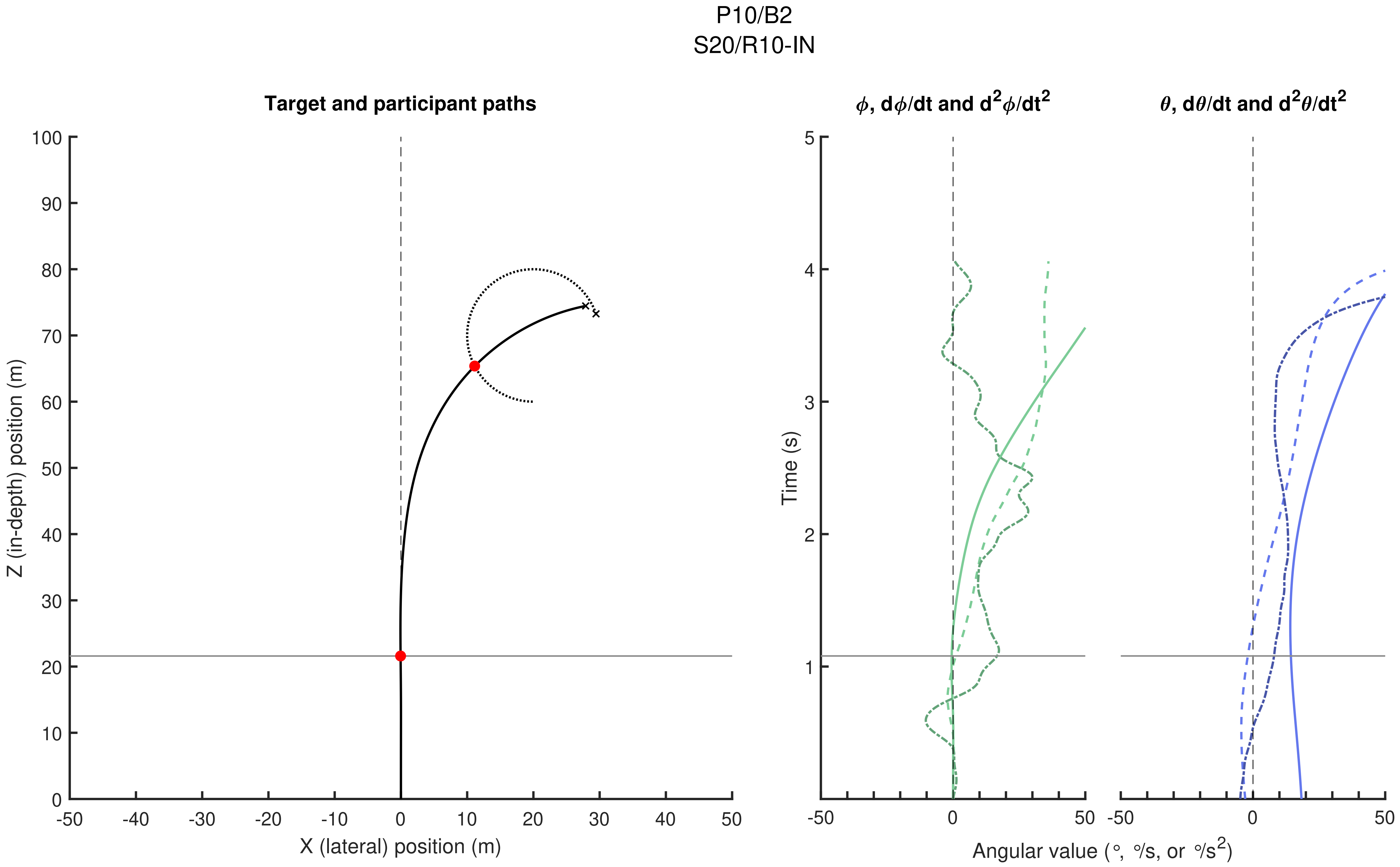

P10/B2
S20/R10-IN
Target and participant paths
ϕ, dϕ/dt and d$^2\phi$/dt^2
θ, dθ/dt and d$^2\theta$/dt^2
Z (in-depth) position (m)
X (lateral) position (m)
Time (s)
Angular value (°, °/s, or °/s^2)

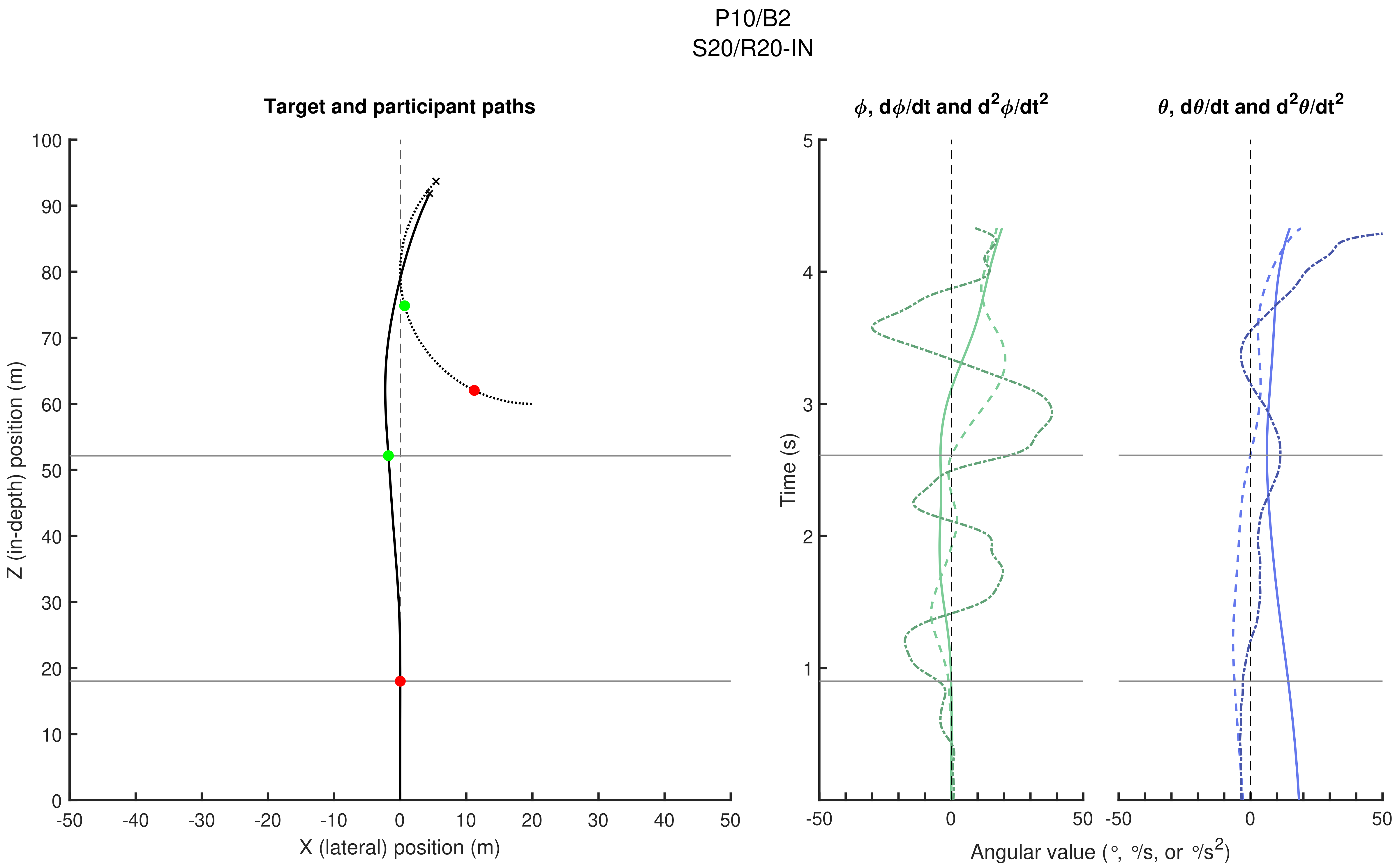
P10/B2
S20/R20-IN
Target and participant paths
Z (in-depth) position (m)
X (lateral) position (m)
ϕ, dϕ/dt and d$^2\phi$/dt^2
θ, dθ/dt and d$^2\theta$/dt^2
Time (s)
Angular value (°, °/s, or °/s^2)

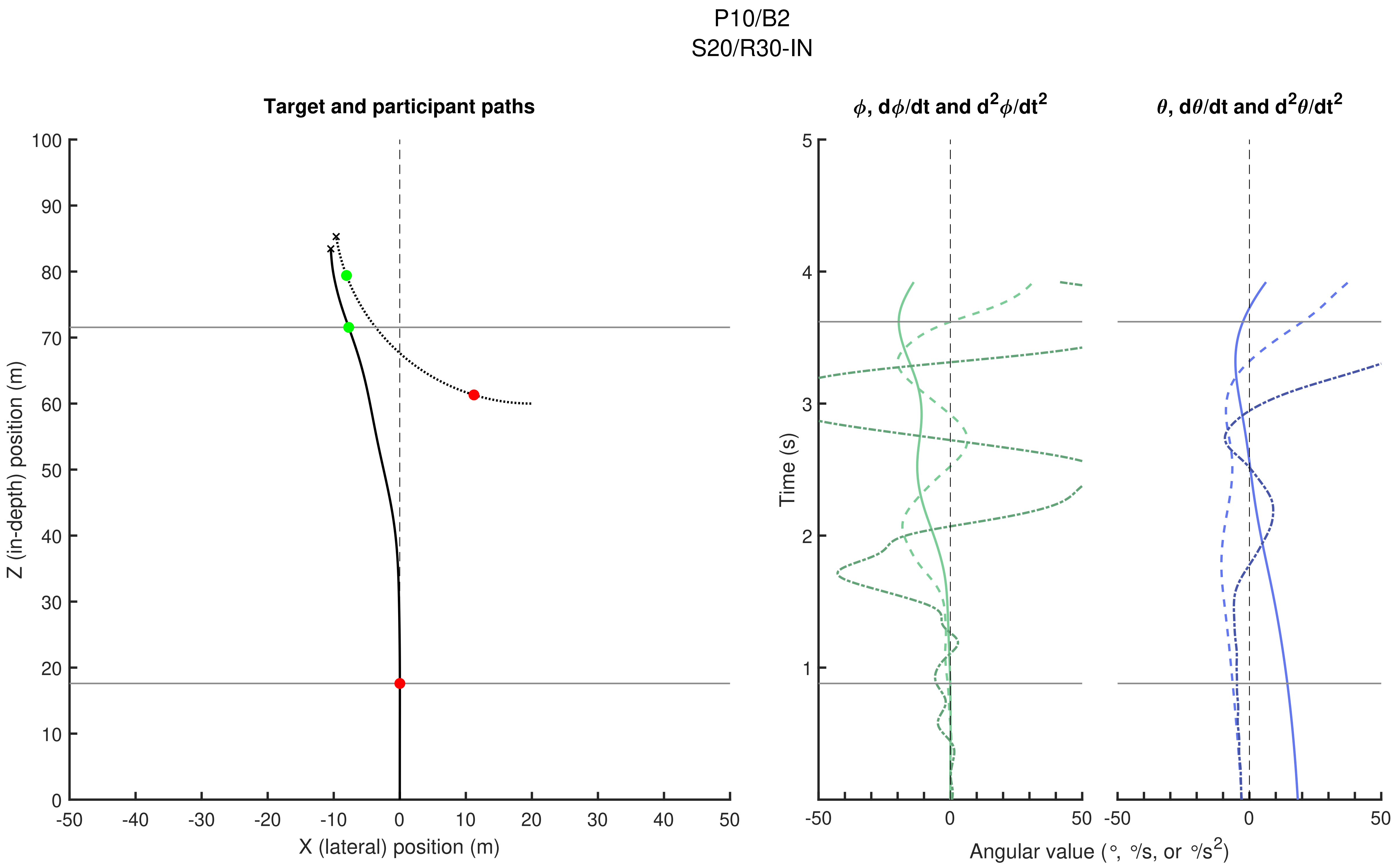

P10/B2
S20/R30-IN
Target and participant paths
ϕ, dϕ/dt and d$^2\phi$/dt^2
θ, dθ/dt and d$^2\theta$/dt^2
Z (in-depth) position (m)
X (lateral) position (m)
Time (s)
Angular value (°, °/s, or °/s^2)

P10/B3
S20/R10-IN

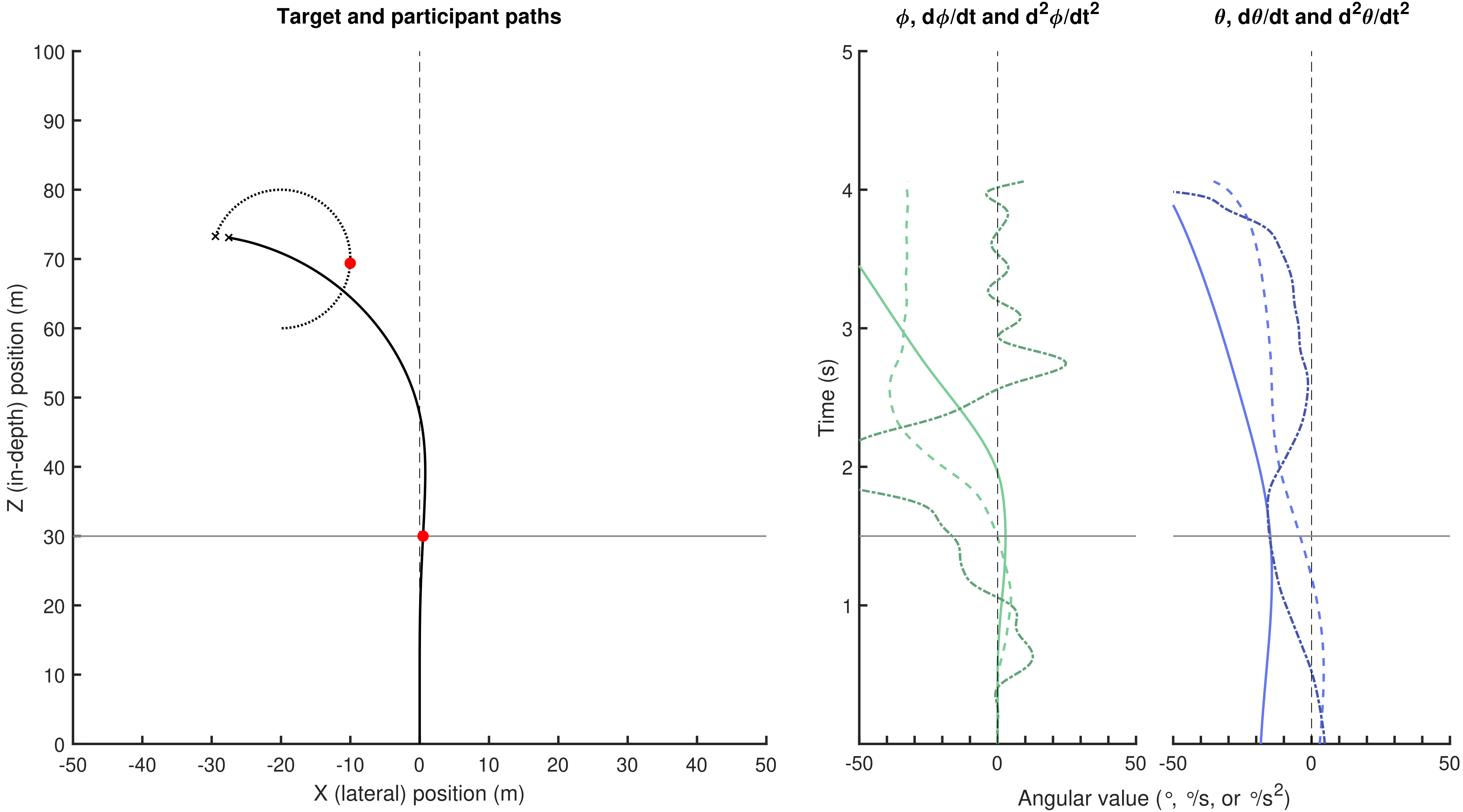

P10/B3
S20/R20-IN

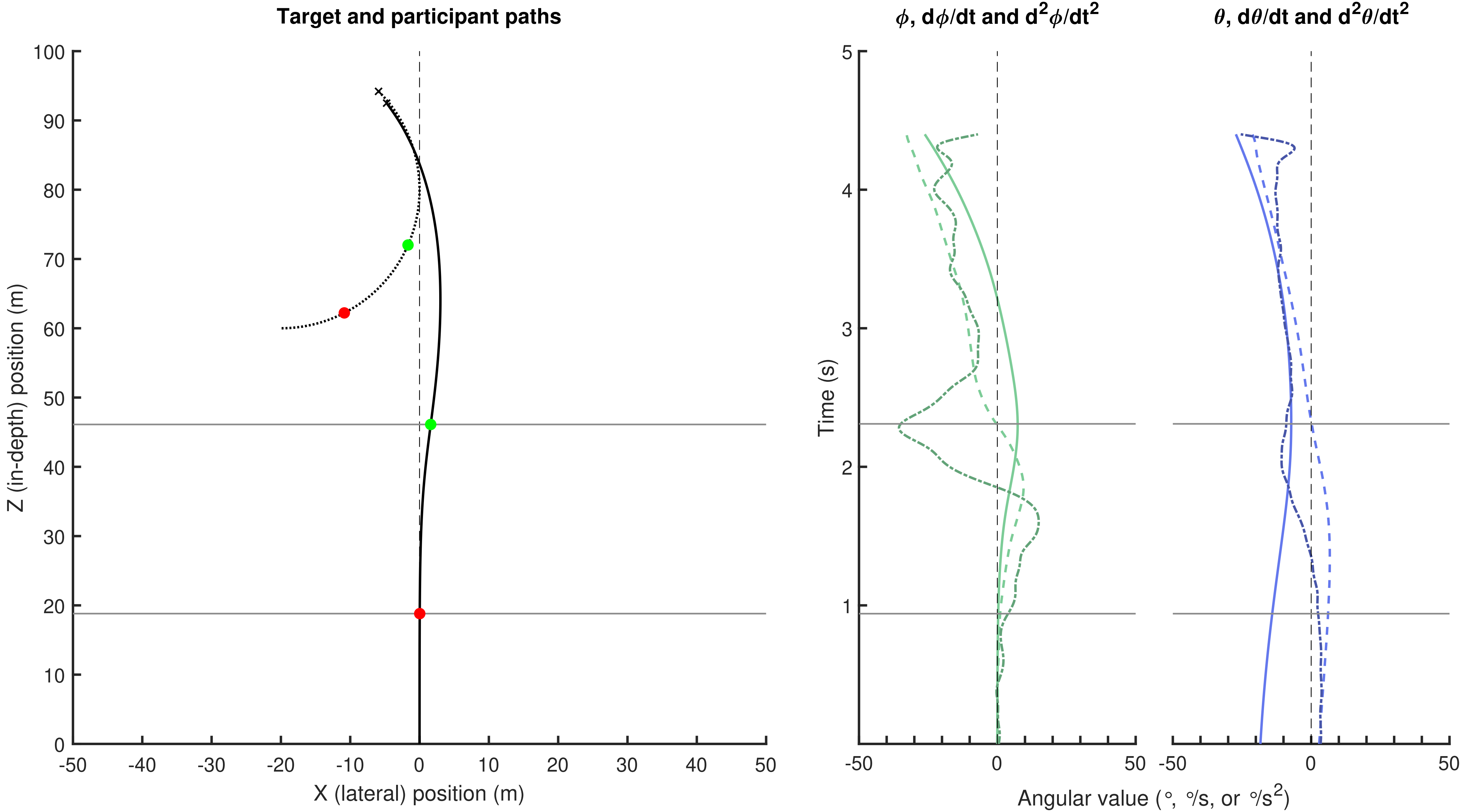

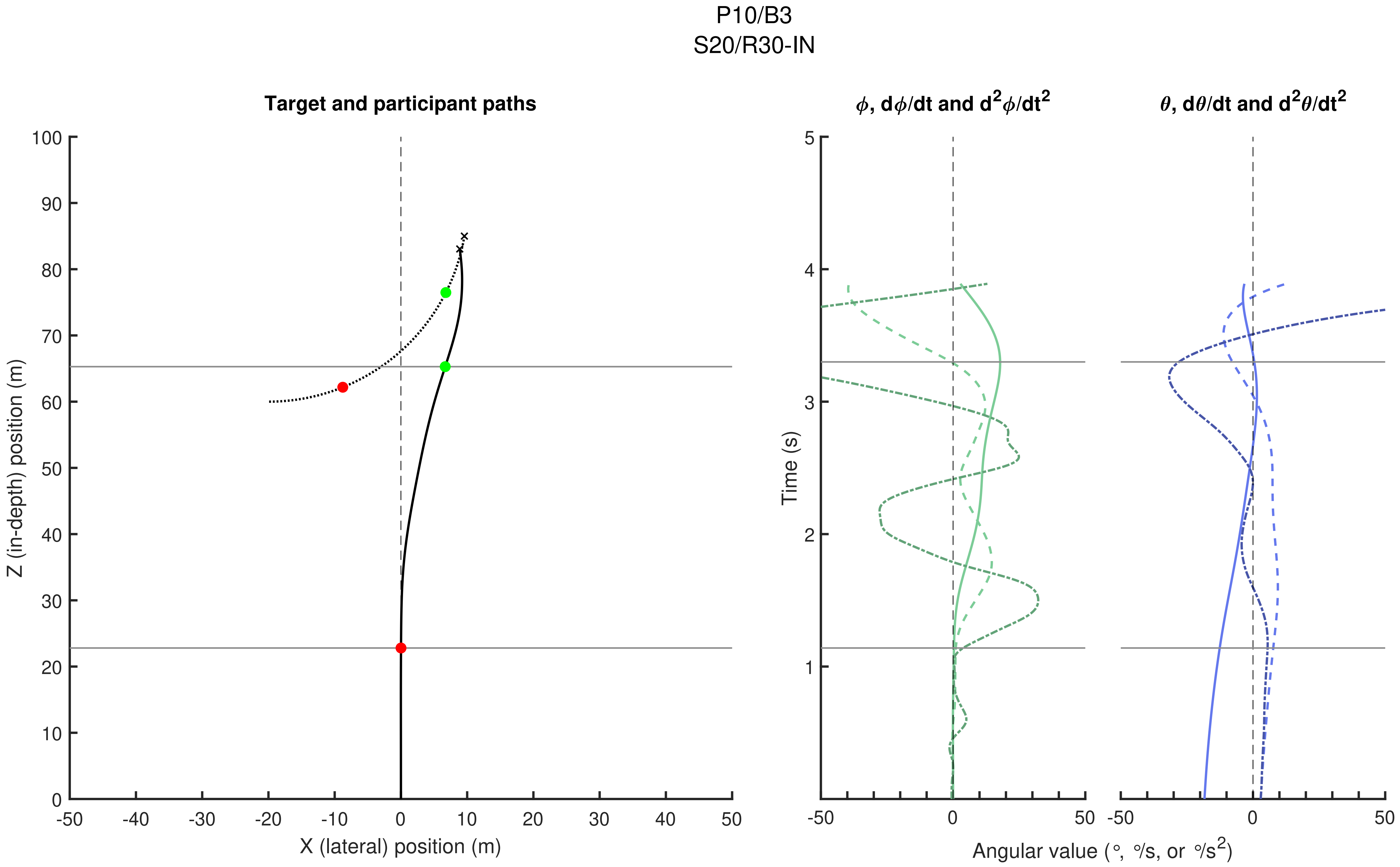
P10/B3
S20/R30-IN
Target and participant paths
Z (in-depth) position (m)
X (lateral) position (m)
φ, dφ/dt and d²φ/dt²
θ, dθ/dt and d²θ/dt²
Time (s)
Angular value (°, °/s, or °/s²)

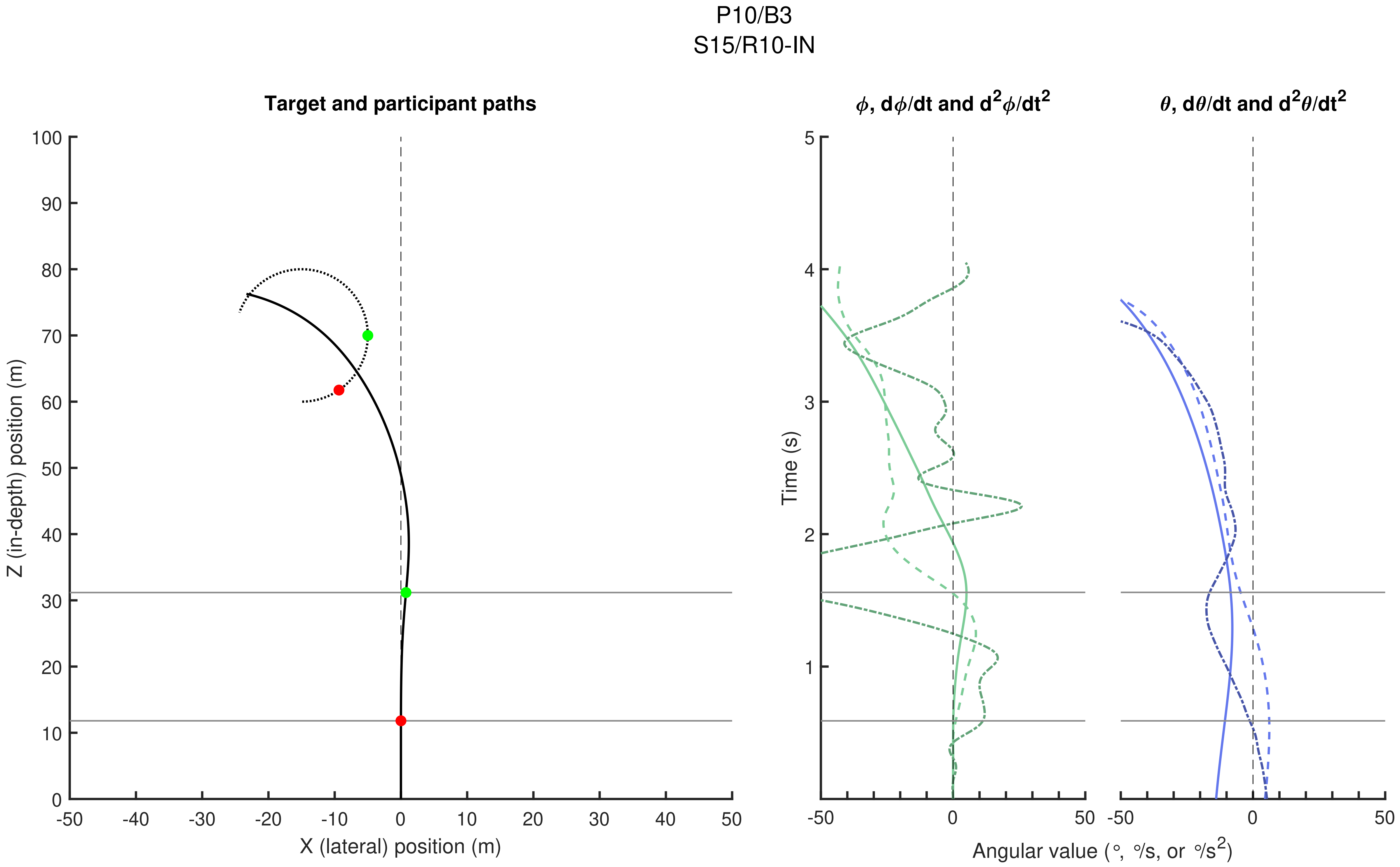
P10/B3
S15/R10-IN
Target and participant paths
Z (in-depth) position (m)
X (lateral) position (m)
100
90
80
70
60
50
40
30
20
10
0
-50
-40
-30
-20
-10
0
10
20
30
40
50
ϕ, dϕ/dt and d$^2\phi$/dt^2
θ, dθ/dt and d$^2\theta$/dt^2
Time (s)
5
4
3
2
1
-50
0
50
-50
0
50
Angular value (°, °/s, or °/s^2)

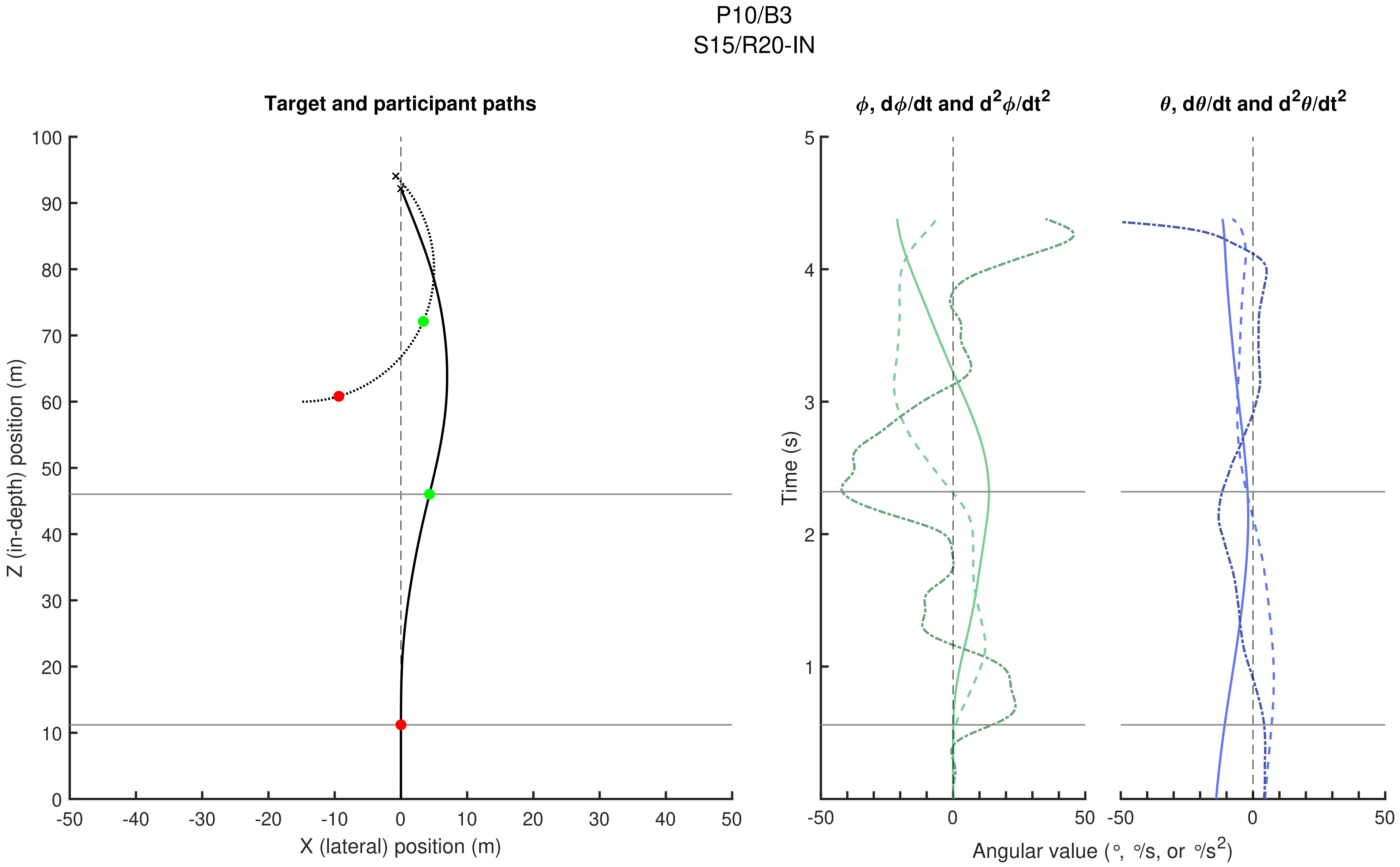
P10/B3
S15/R20-IN
Target and participant paths
Z (in-depth) position (m)
X (lateral) position (m)
100
90
80
70
60
50
40
30
20
10
0
-50
-40
-30
-20
-10
0
10
20
30
40
50
ϕ, dϕ/dt and d$^2\phi$/dt^2
θ, dθ/dt and d$^2\theta$/dt^2
Time (s)
5
4
3
2
1
-50
0
50
-50
0
50
Angular value (°, °/s, or °/s^2)

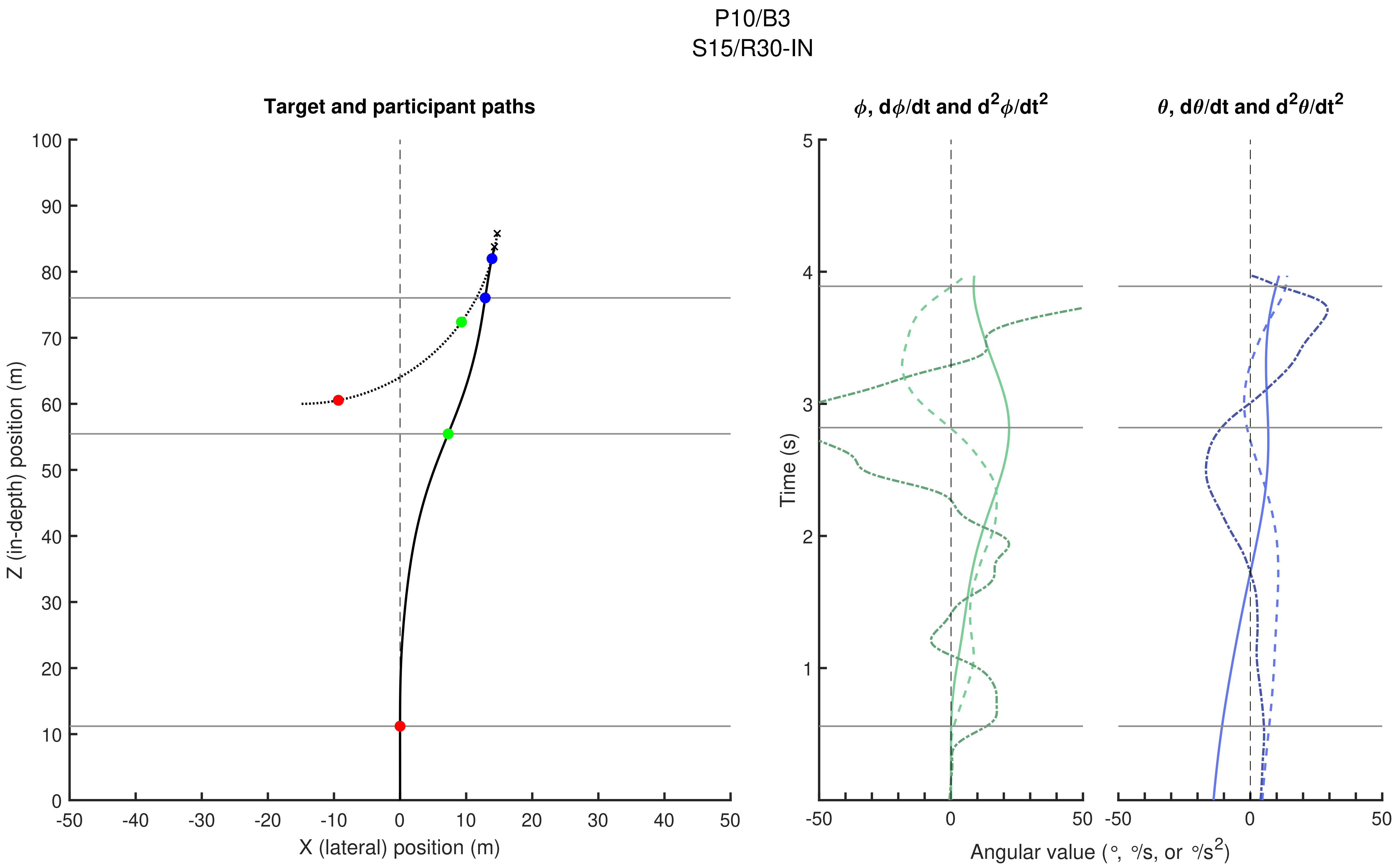
P10/B3
S15/R30-IN
Target and participant paths
Z (in-depth) position (m)
X (lateral) position (m)
100
90
80
70
60
50
40
30
20
10
0
-50
-40
-30
-20
-10
0
10
20
30
40
50
ϕ, dϕ/dt and d$^2\phi$/dt^2
θ, dθ/dt and d$^2\theta$/dt^2
Time (s)
5
4
3
2
1
Angular value (°, °/s, or °/s^2)

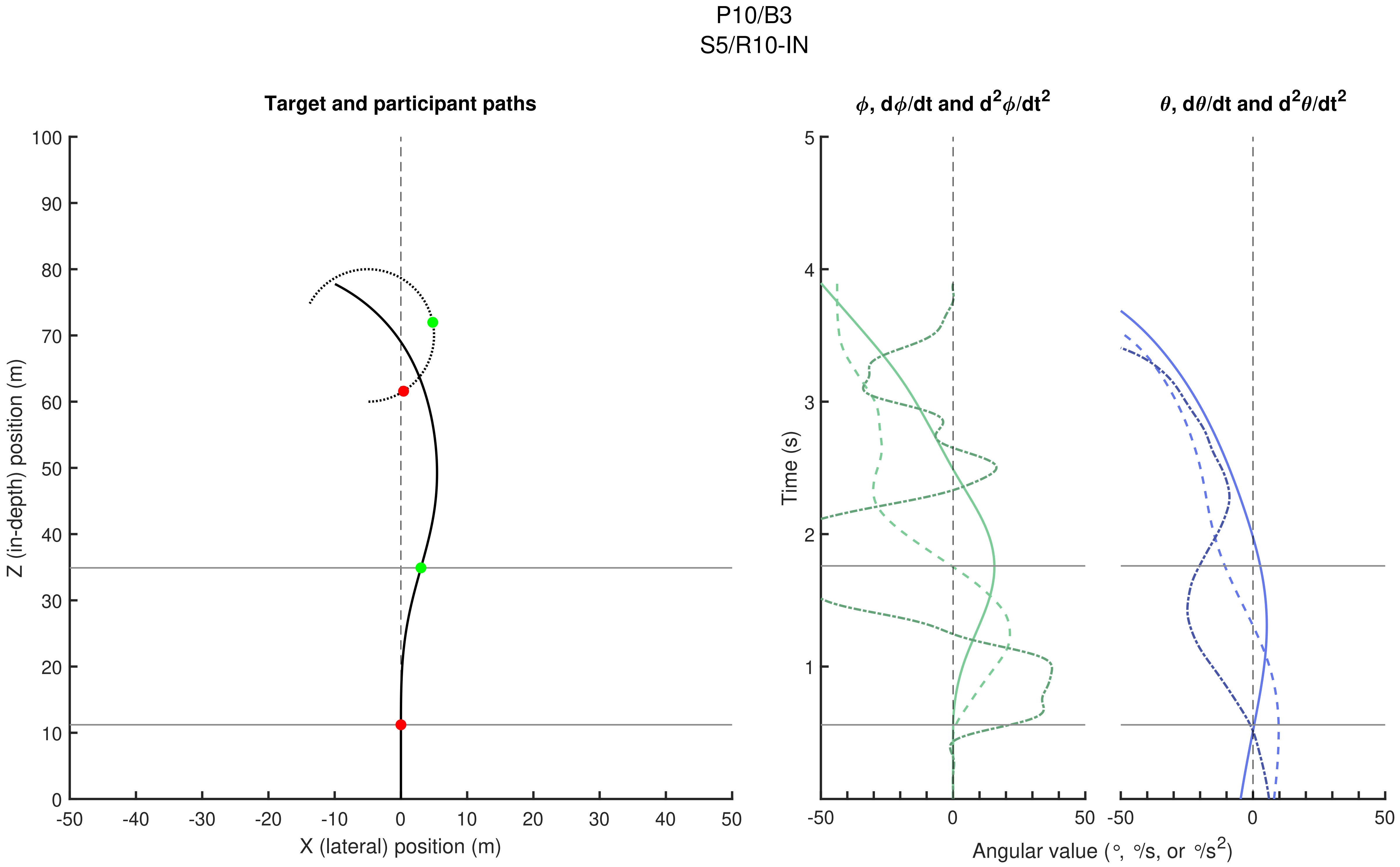

P10/B3
S5/R10-IN
Target and participant paths
ϕ, dϕ/dt and d$^2\phi$/dt^2
θ, dθ/dt and d$^2\theta$/dt^2
Z (in-depth) position (m)
X (lateral) position (m)
Time (s)
Angular value (°, °/s, or °/s^2)

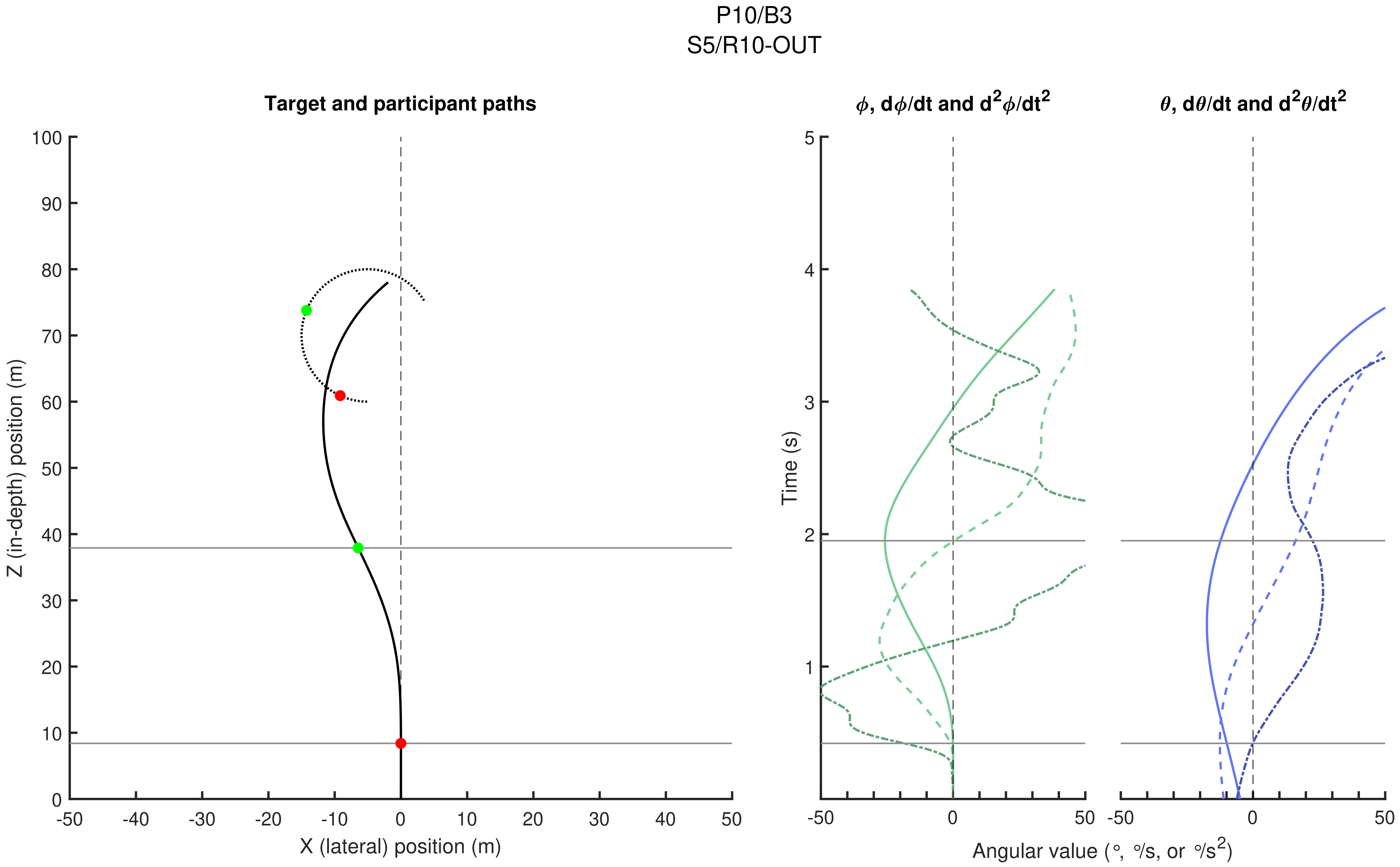
P10/B3
S5/R10-OUT
Target and participant paths
Z (in-depth) position (m)
X (lateral) position (m)
100
90
80
70
60
50
40
30
20
10
0
-50
-40
-30
-20
-10
0
10
20
30
40
50
ϕ, dϕ/dt and d²ϕ/dt²
θ, dθ/dt and d²θ/dt²
Time (s)
5
4
3
2
1
-50
0
50
-50
0
50
Angular value (°, °/s, or °/s²)

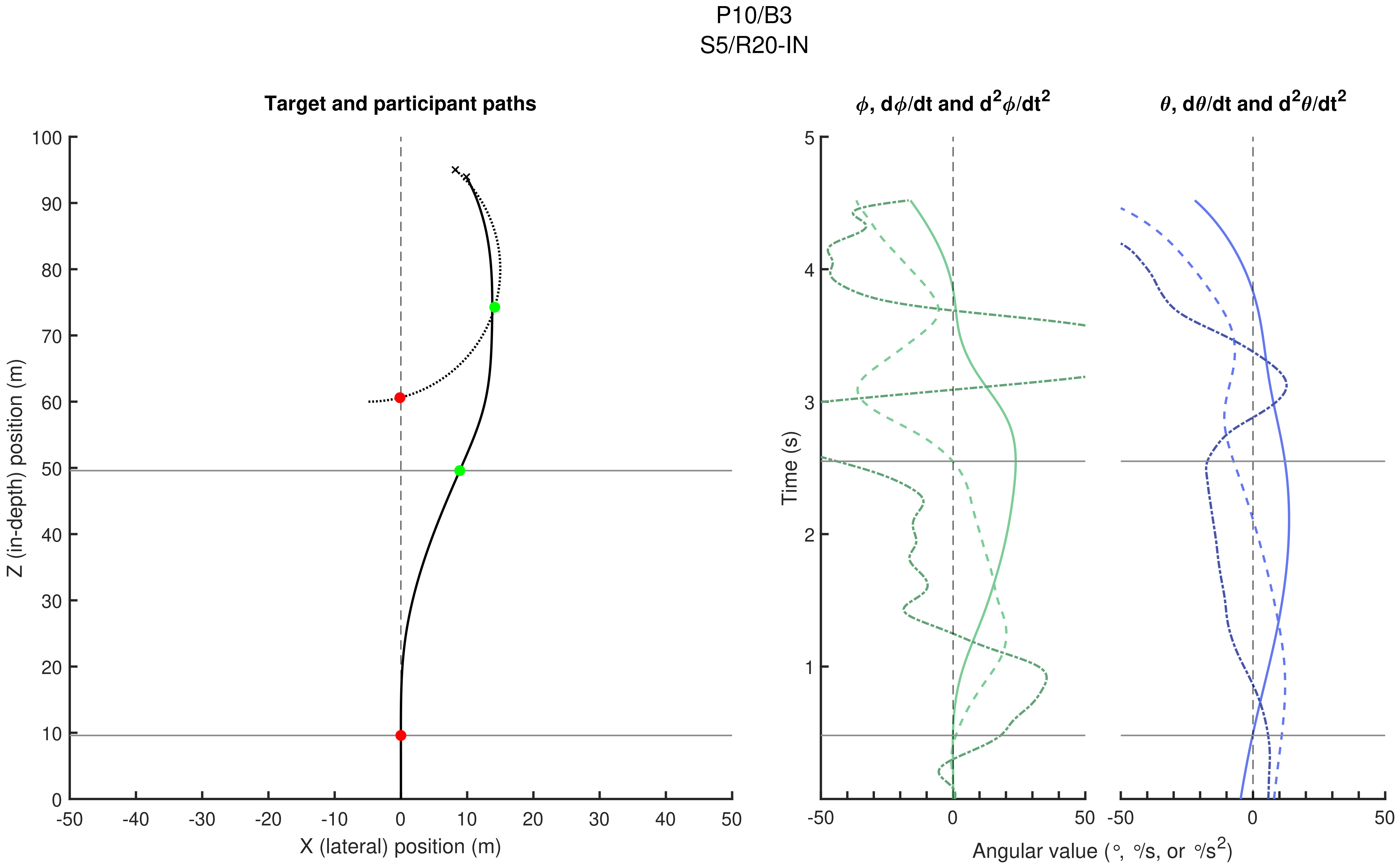

P10/B3
S5/R20-IN
Target and participant paths
Z (in-depth) position (m)
X (lateral) position (m)
0
10
20
30
40
50
60
70
80
90
100
-50
-40
-30
-20
-10
0
10
20
30
40
50
ϕ, dϕ/dt and d²ϕ/dt²
θ, dθ/dt and d²θ/dt²
Time (s)
1
2
3
4
5
-50
0
50
-50
0
50
Angular value (°, °/s, or °/s²)

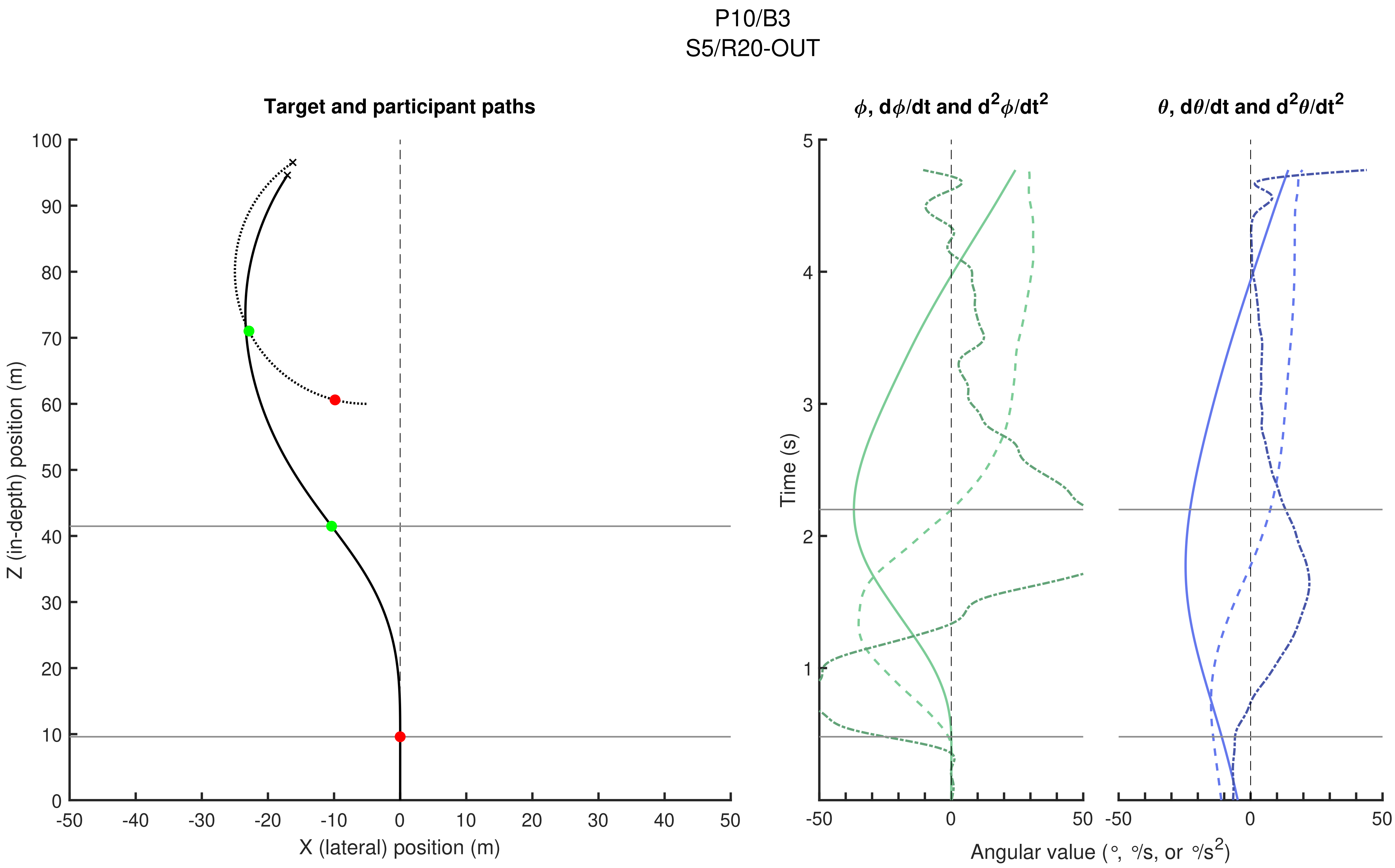

P10/B3
S5/R20-OUT
Target and participant paths
Z (in-depth) position (m)
X (lateral) position (m)
φ, dφ/dt and d²φ/dt²
θ, dθ/dt and d²θ/dt²
Time (s)
Angular value (°, °/s, or °/s²)

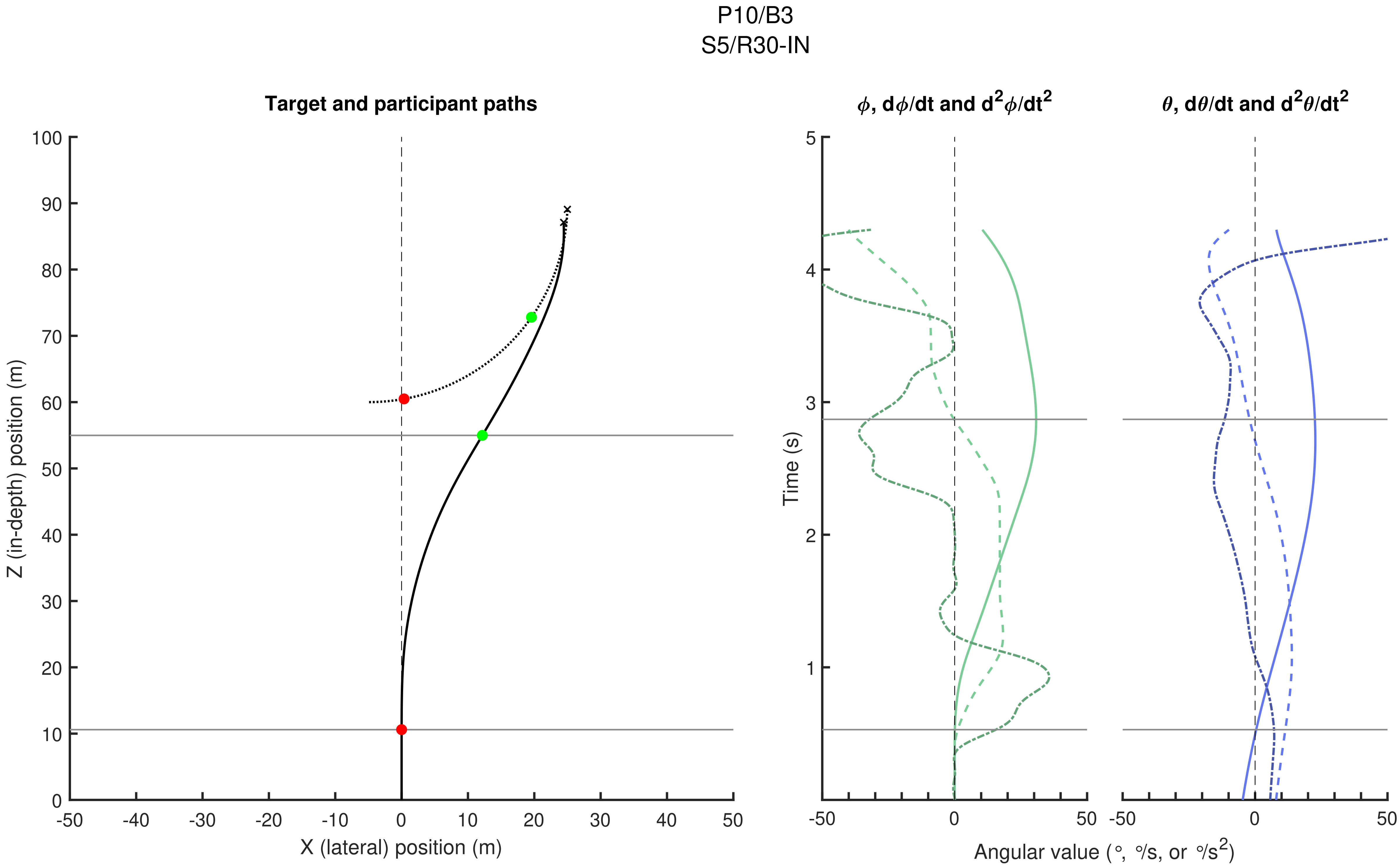
P10/B3
S5/R30-IN
Target and participant paths
Z (in-depth) position (m)
X (lateral) position (m)
ϕ, dϕ/dt and d²ϕ/dt²
θ, dθ/dt and d²θ/dt²
Time (s)
Angular value (°, °/s, or °/s²)

P10/B3
S5/R30-OUT

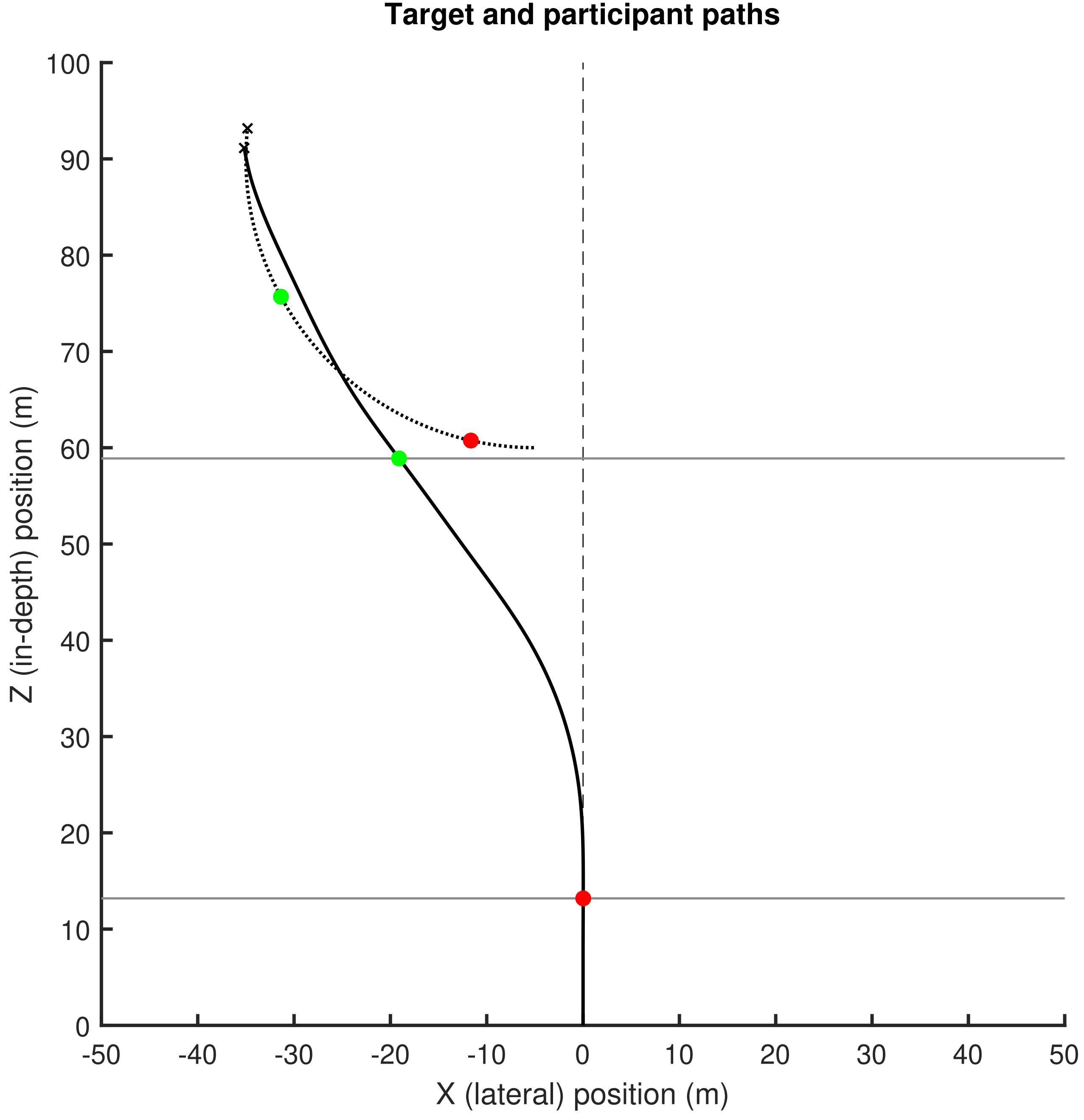

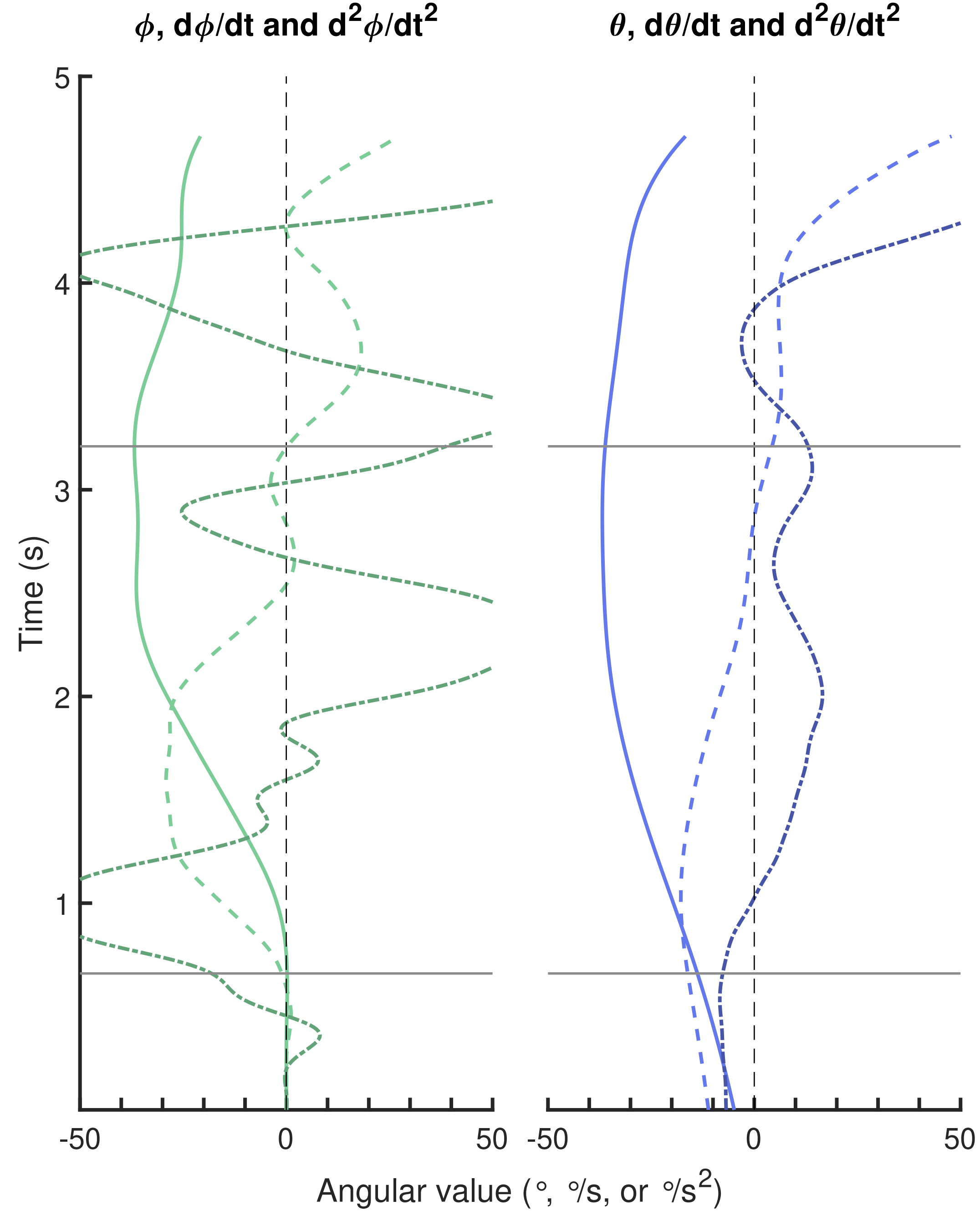

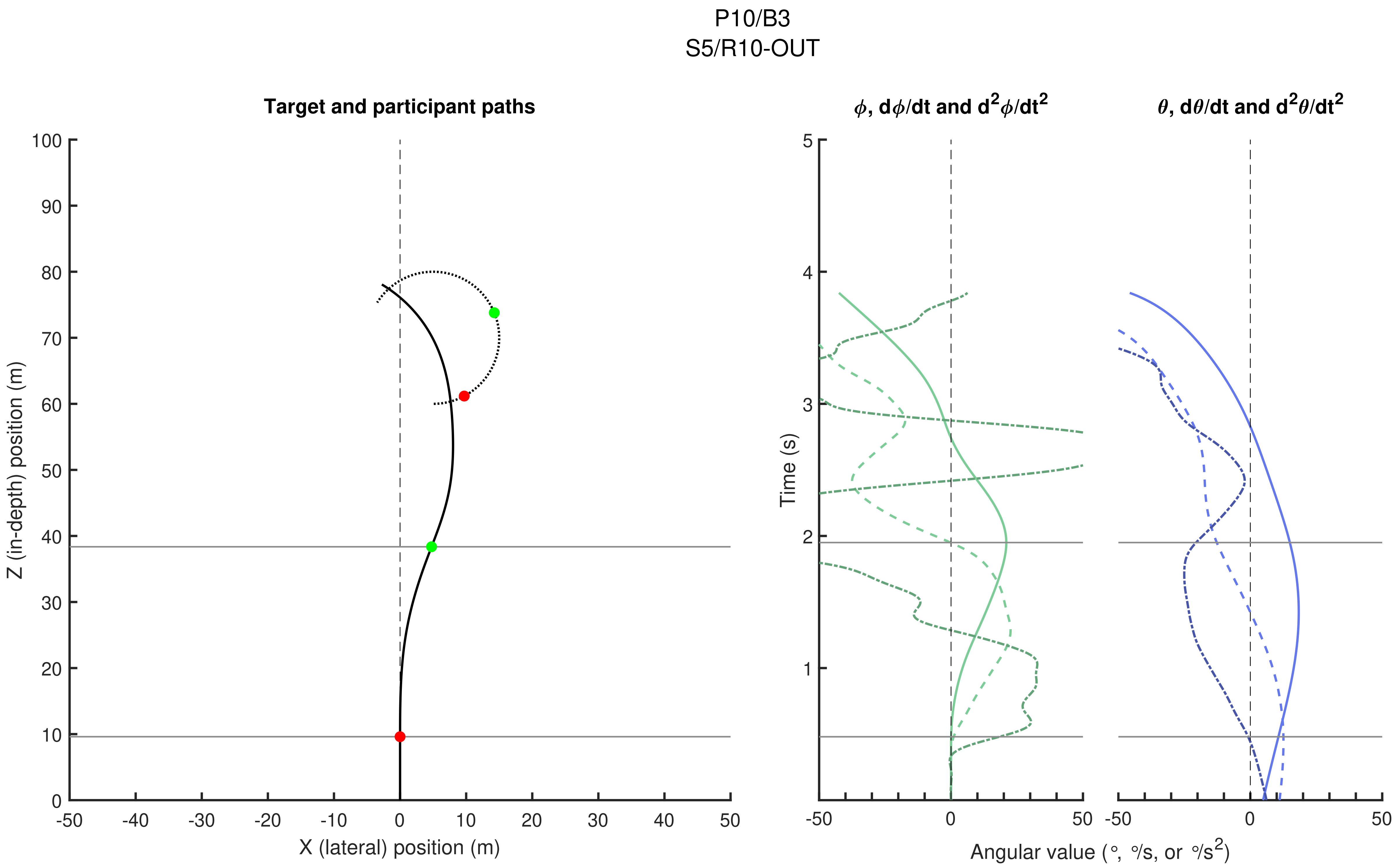
P10/B3
S5/R10-OUT
Target and participant paths
Z (in-depth) position (m)
X (lateral) position (m)
100
90
80
70
60
50
40
30
20
10
0
-50
-40
-30
-20
-10
0
10
20
30
40
50
ϕ, dϕ/dt and d²ϕ/dt²
θ, dθ/dt and d²θ/dt²
Time (s)
5
4
3
2
1
-50
0
50
-50
0
50
Angular value (°, °/s, or °/s²)

P10/B3
S5/R10-IN

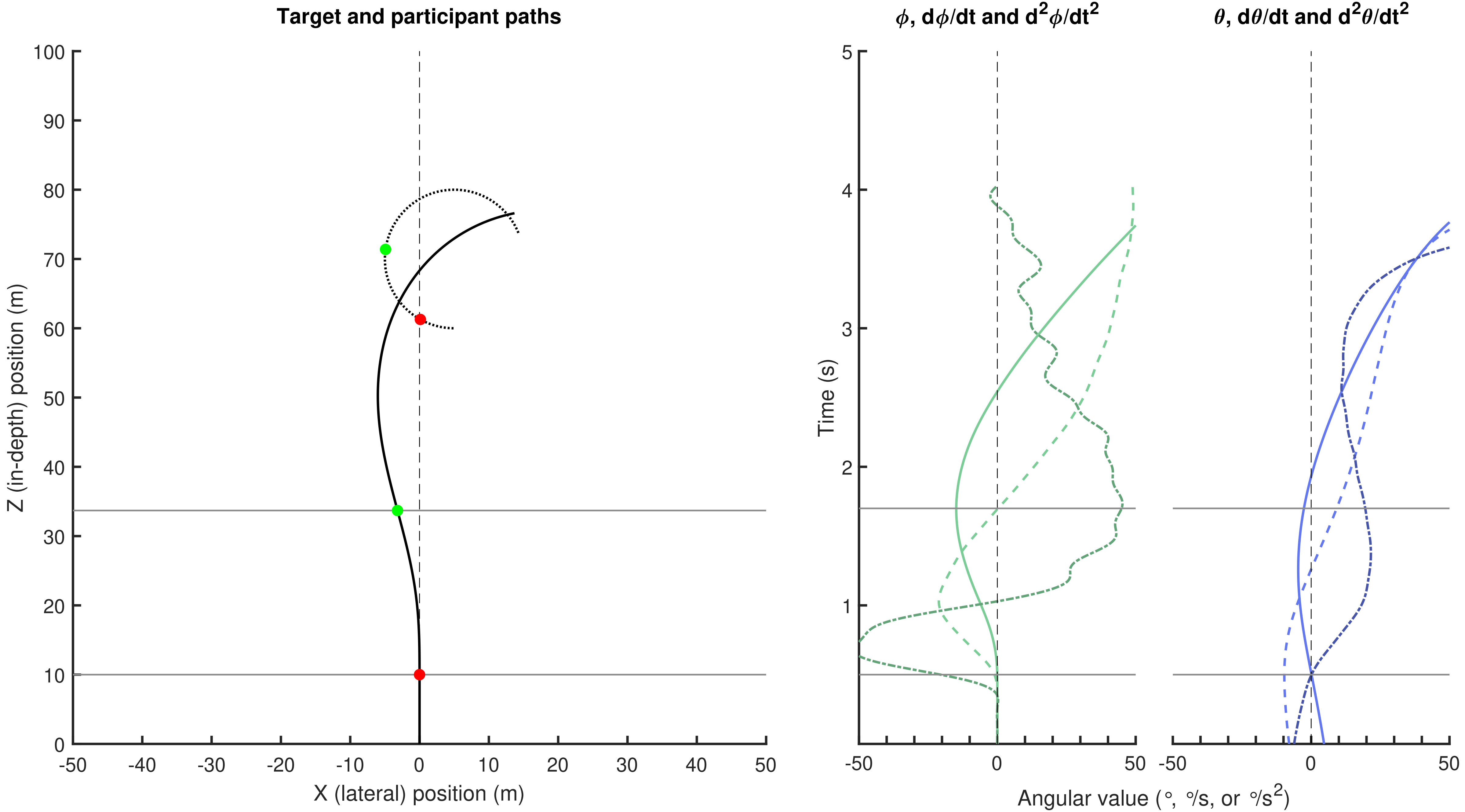

P10/B3
S5/R20-OUT

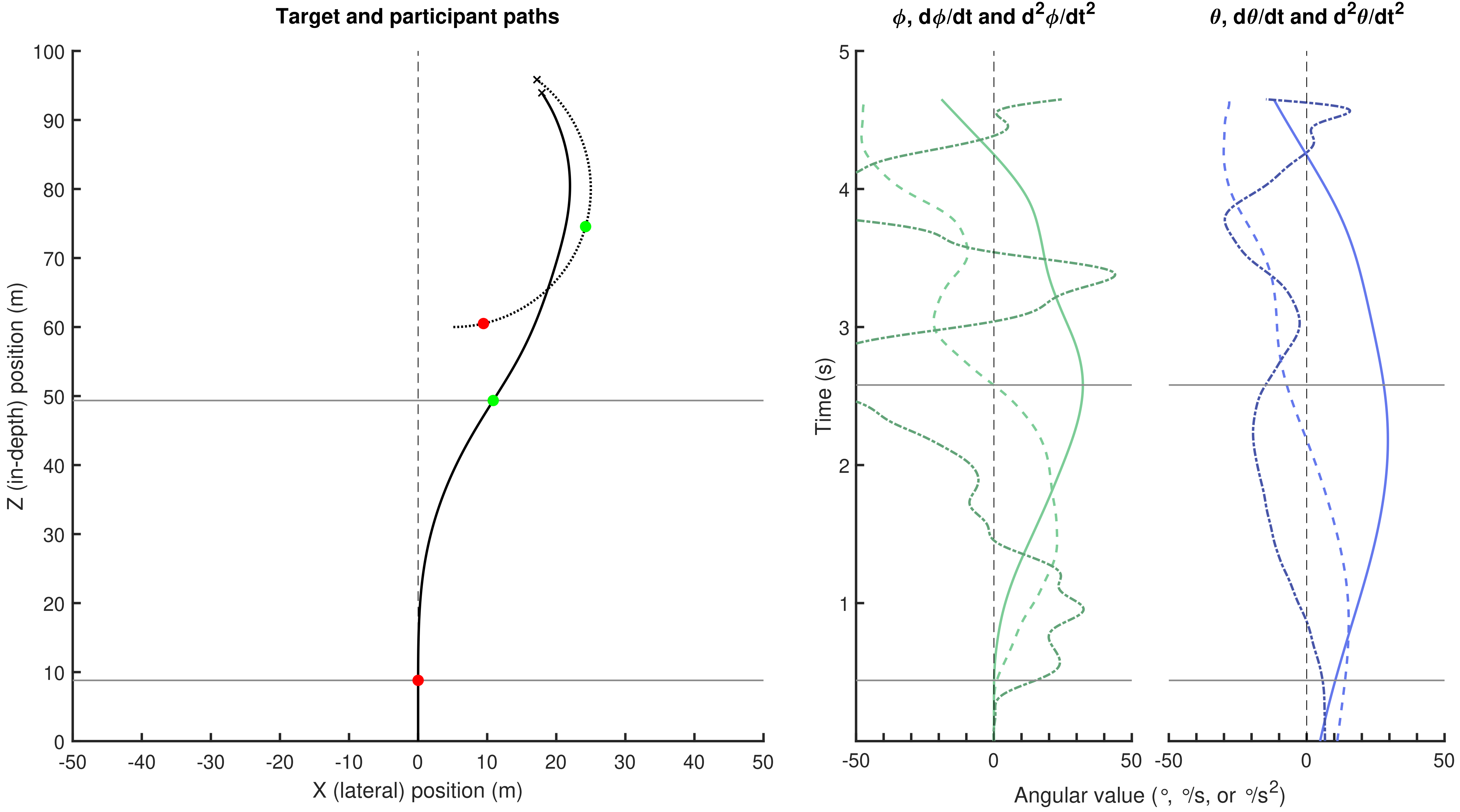

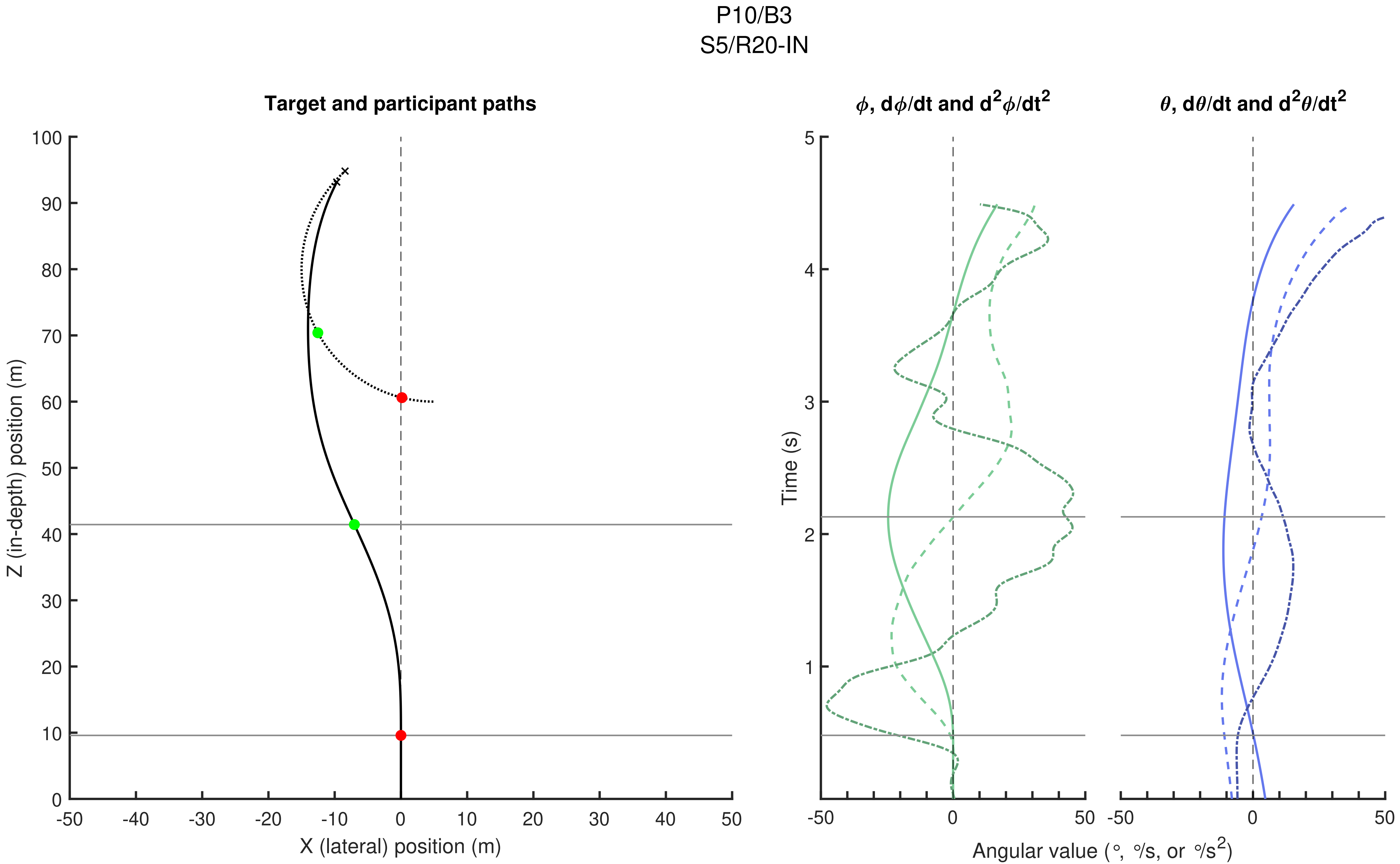
P10/B3
S5/R20-IN
Target and participant paths
Z (in-depth) position (m)
X (lateral) position (m)
ϕ, dϕ/dt and d$^2\phi$/dt^2
θ, dθ/dt and d$^2\theta$/dt^2
Time (s)
Angular value (°, °/s, or °/s^2)

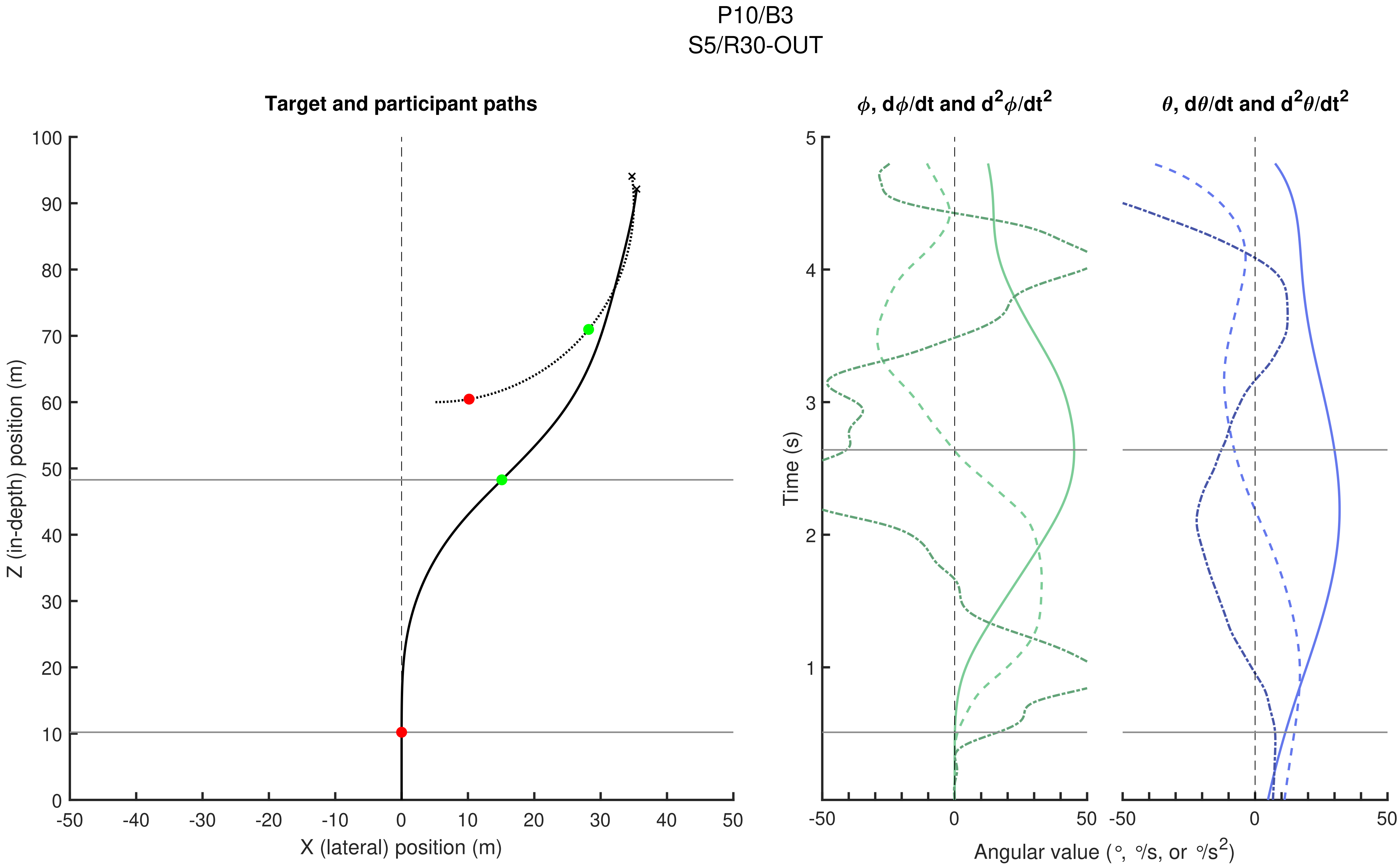

P10/B3
S5/R30-OUT
Target and participant paths
Z (in-depth) position (m)
X (lateral) position (m)
ϕ, dϕ/dt and d$^2\phi$/dt^2
θ, dθ/dt and d$^2\theta$/dt^2
Time (s)
Angular value (°, °/s, or °/s^2)

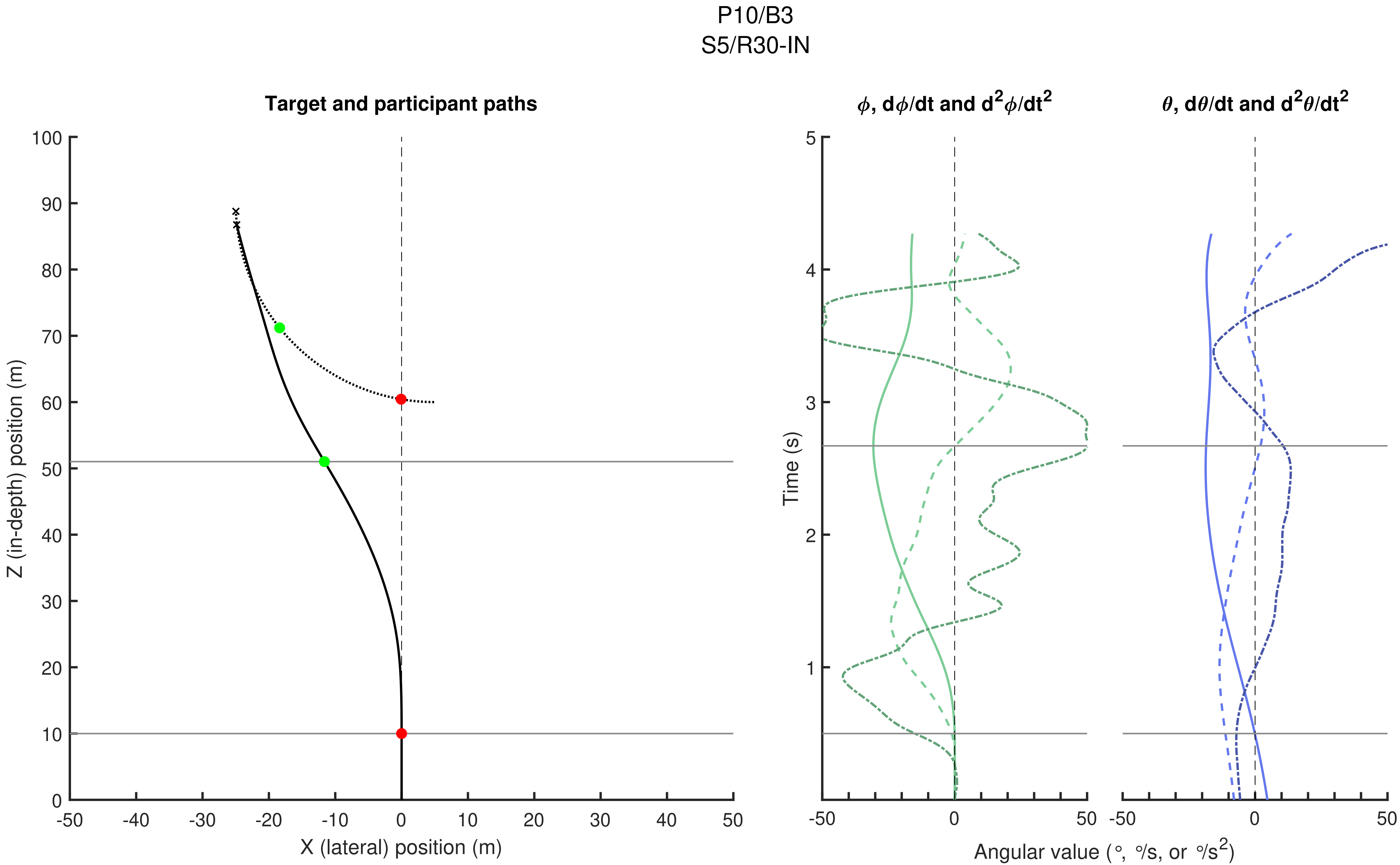

P10/B3
S5/R30-IN
Target and participant paths
Z (in-depth) position (m)
X (lateral) position (m)
φ, dφ/dt and d²φ/dt²
θ, dθ/dt and d²θ/dt²
Time (s)
Angular value (°, °/s, or °/s²)

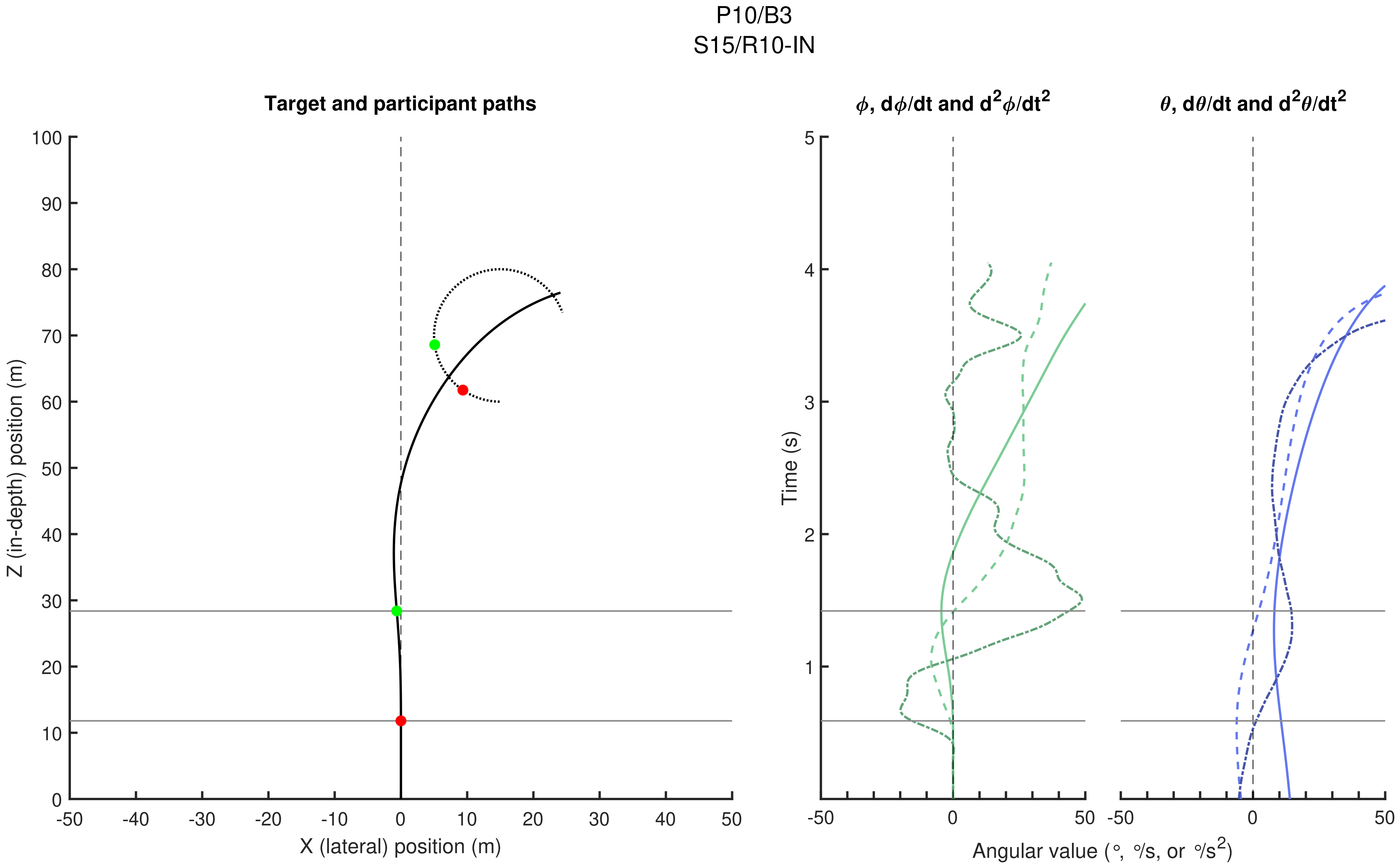
P10/B3
S15/R10-IN
Target and participant paths
ϕ, dϕ/dt and d$^2\phi$/dt^2
θ, dθ/dt and d$^2\theta$/dt^2
Z (in-depth) position (m)
X (lateral) position (m)
Time (s)
Angular value (°, °/s, or °/s^2)
100
90
80
70
60
50
40
30
20
10
0
-50
-40
-30
-20
-10
0
10
20
30
40
50
5
4
3
2
1
-50
0
50
-50
0
50

P10/B3
S15/R20-IN

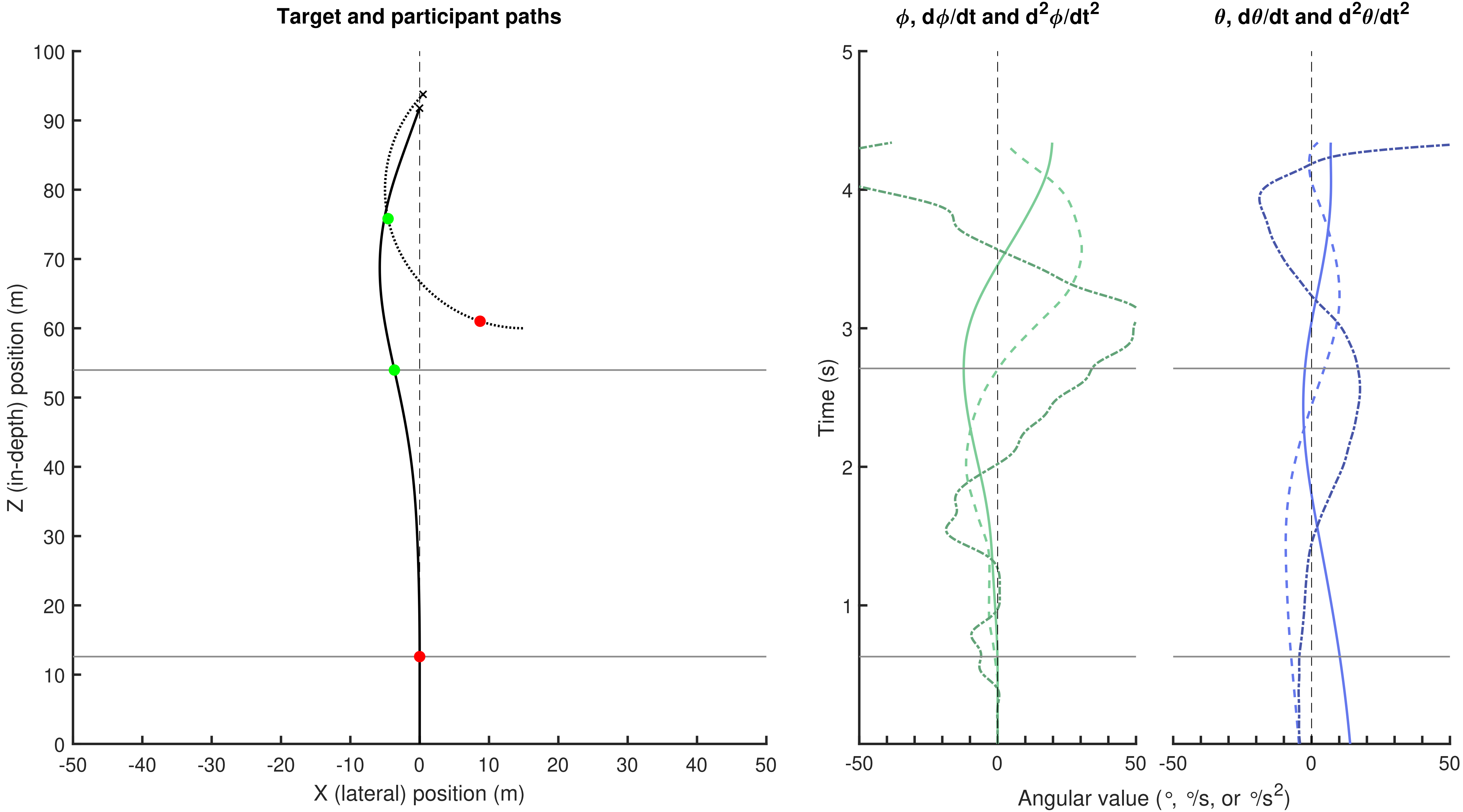

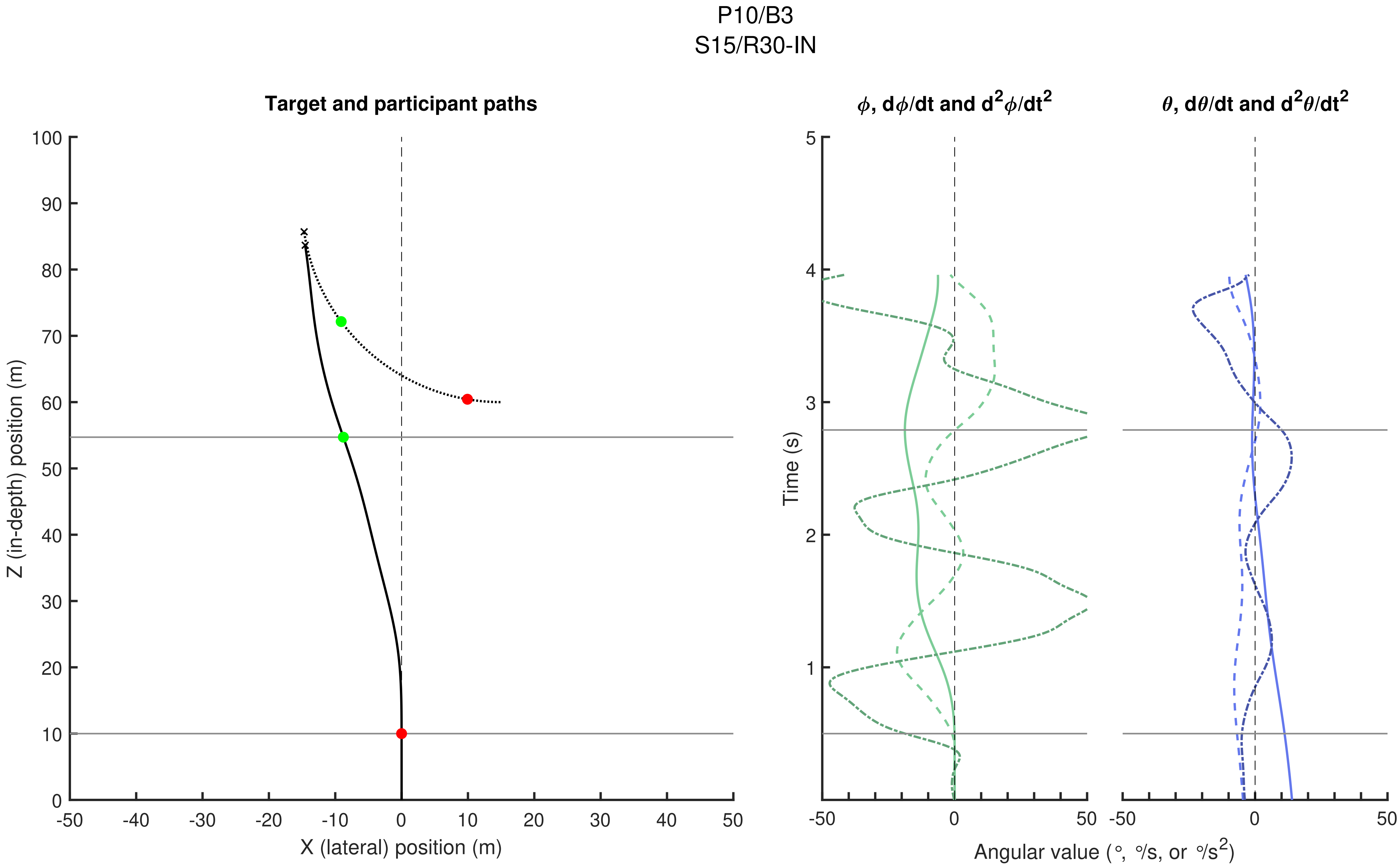

P10/B3
S15/R30-IN
Target and participant paths
Z (in-depth) position (m)
X (lateral) position (m)
100
90
80
70
60
50
40
30
20
10
0
-50
-40
-30
-20
-10
0
10
20
30
40
50
ϕ, dϕ/dt and d²ϕ/dt²
θ, dθ/dt and d²θ/dt²
Time (s)
5
4
3
2
1
-50
0
50
Angular value (°, °/s, or °/s²)

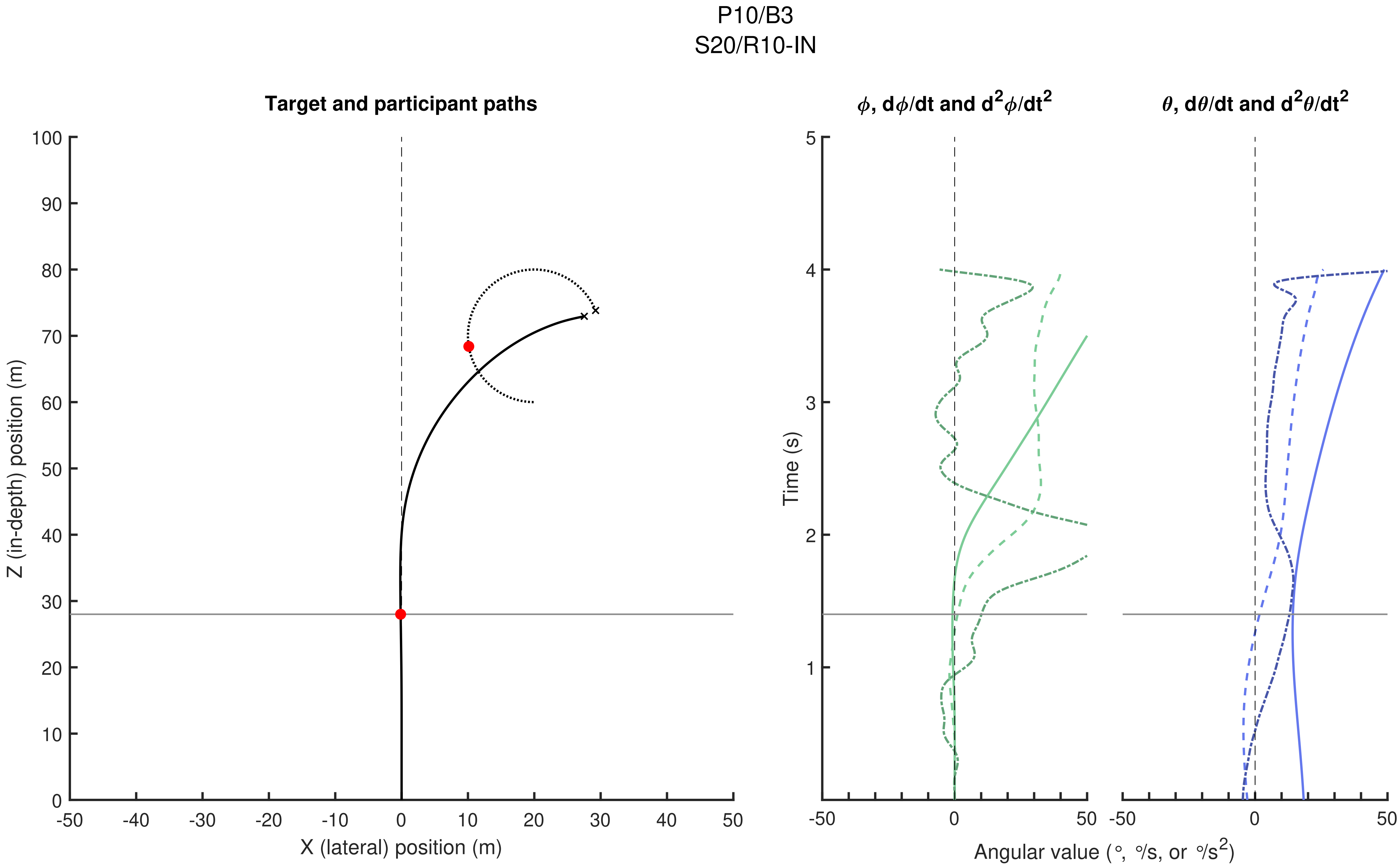
P10/B3
S20/R10-IN
Target and participant paths
Z (in-depth) position (m)
X (lateral) position (m)
φ, dφ/dt and d²φ/dt²
θ, dθ/dt and d²θ/dt²
Time (s)
Angular value (°, °/s, or °/s²)

P10/B3
S20/R20-IN

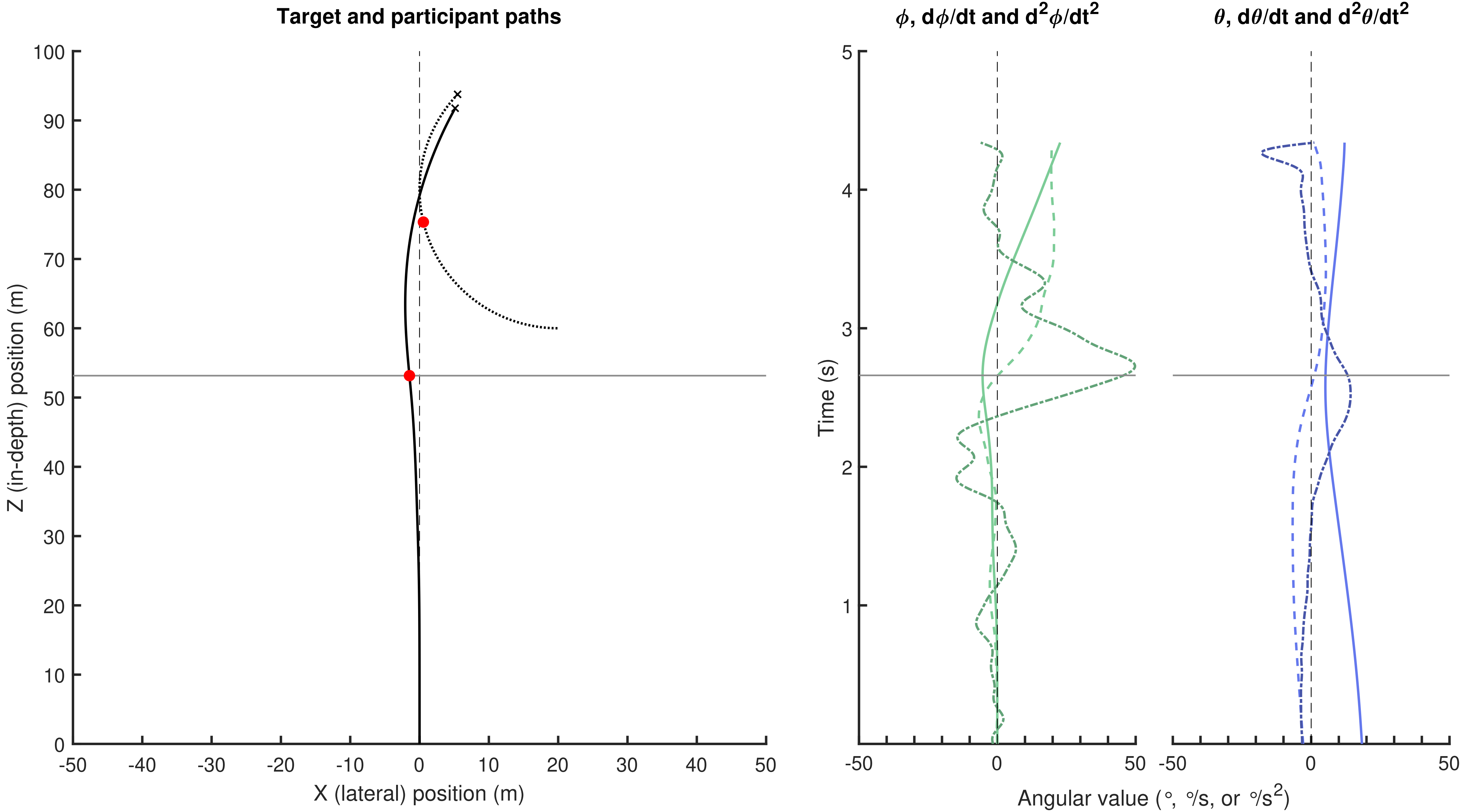

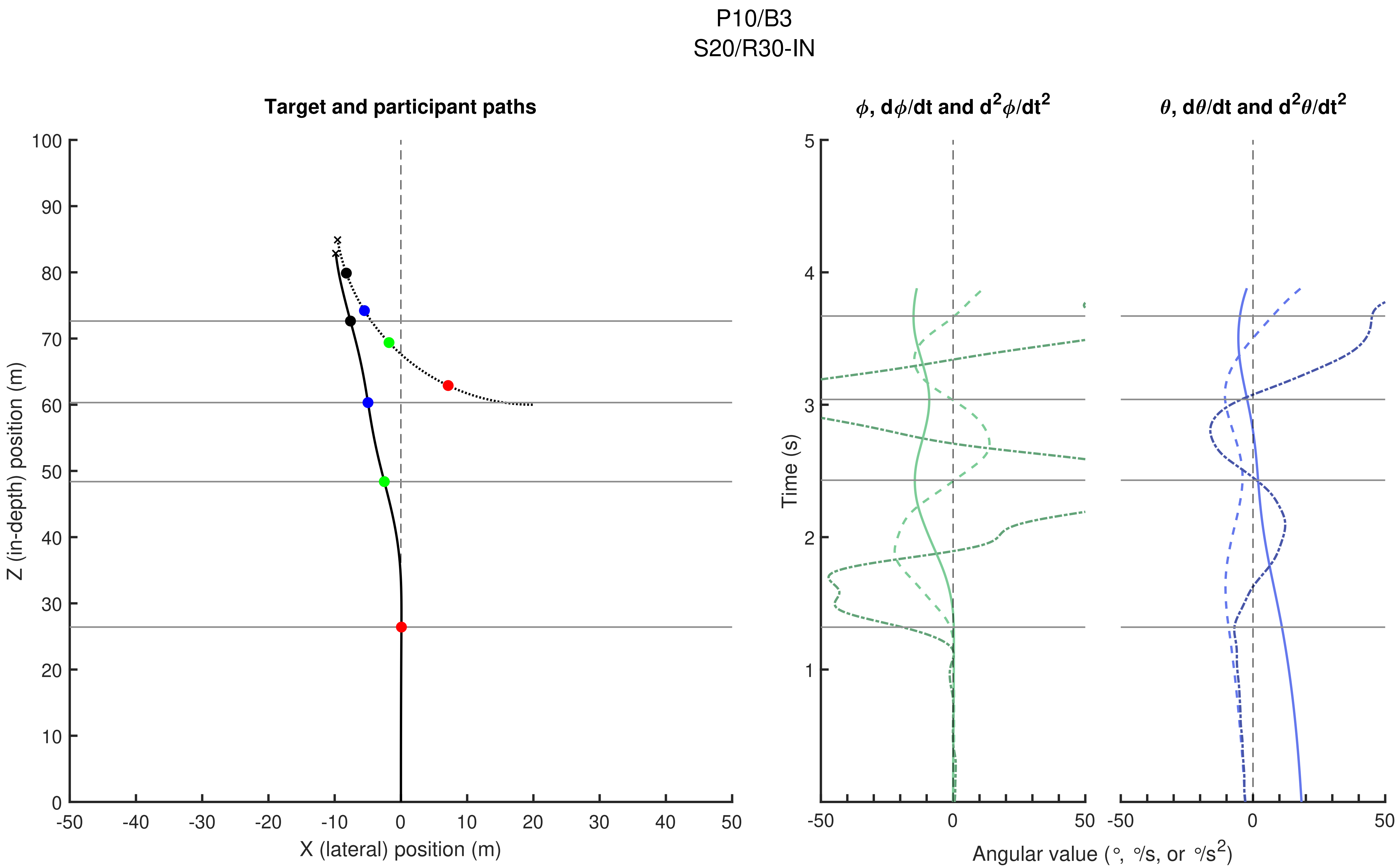
P10/B3
S20/R30-IN
Target and participant paths
Z (in-depth) position (m)
X (lateral) position (m)
φ, dφ/dt and d²φ/dt²
θ, dθ/dt and d²θ/dt²
Time (s)
Angular value (°, °/s, or °/s²)

P10/B4
S20/R10-IN

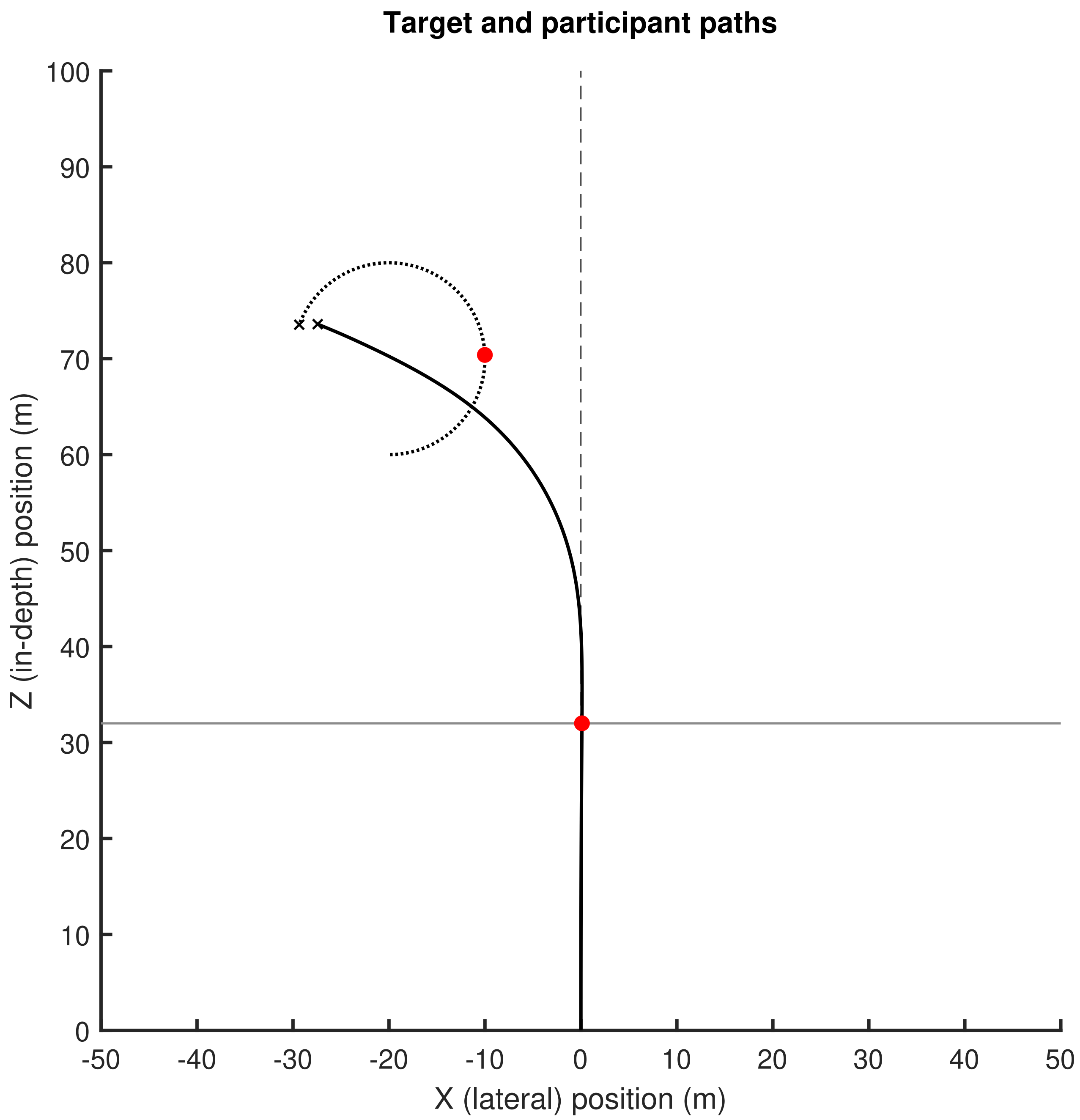

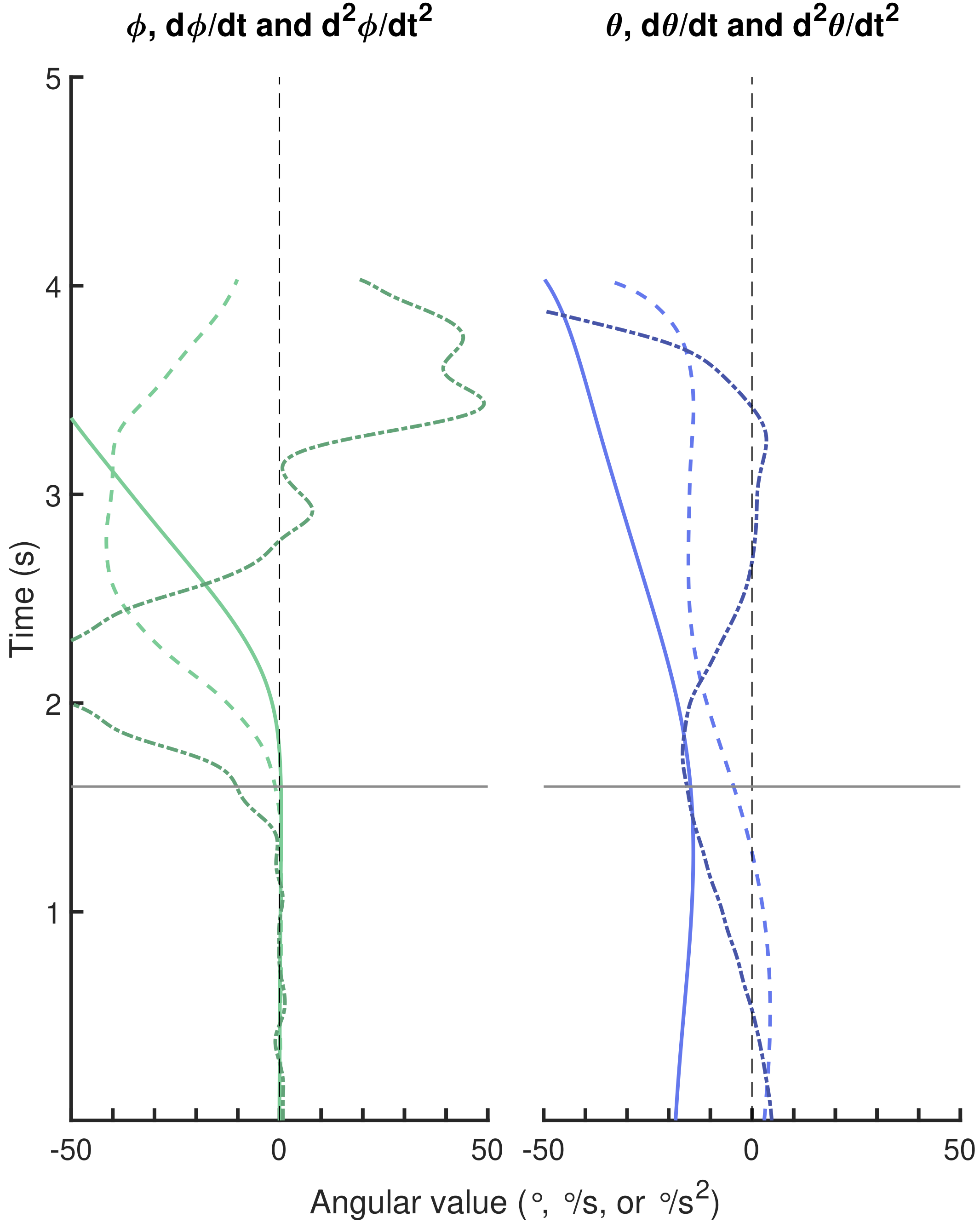

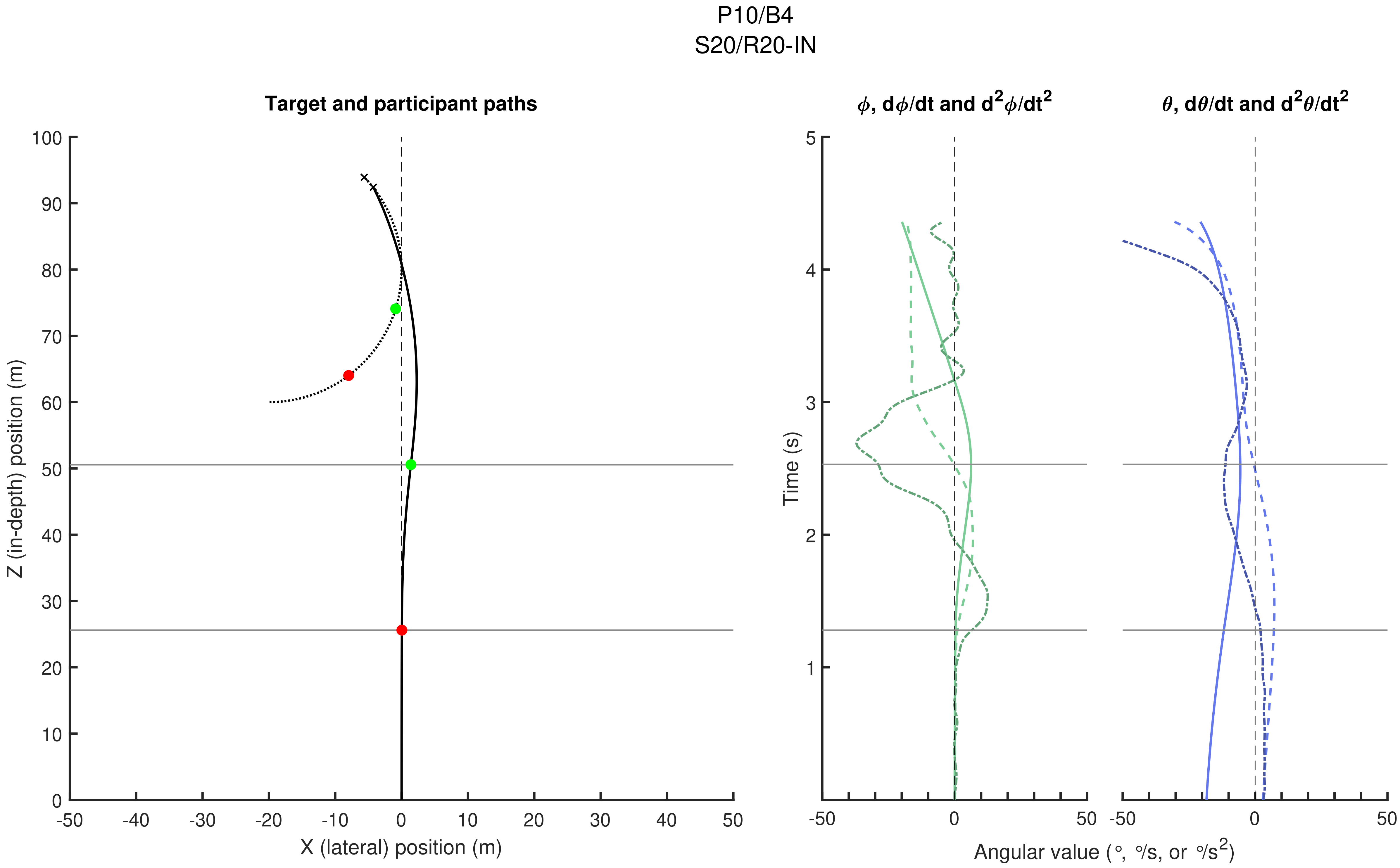

P10/B4
S20/R20-IN
Target and participant paths
Z (in-depth) position (m)
X (lateral) position (m)
100
90
80
70
60
50
40
30
20
10
0
-50
-40
-30
-20
-10
0
10
20
30
40
50
φ, dφ/dt and d²φ/dt²
θ, dθ/dt and d²θ/dt²
Time (s)
5
4
3
2
1
-50
0
50
Angular value (°, °/s, or °/s²)

P10/B4
S20/R30-IN

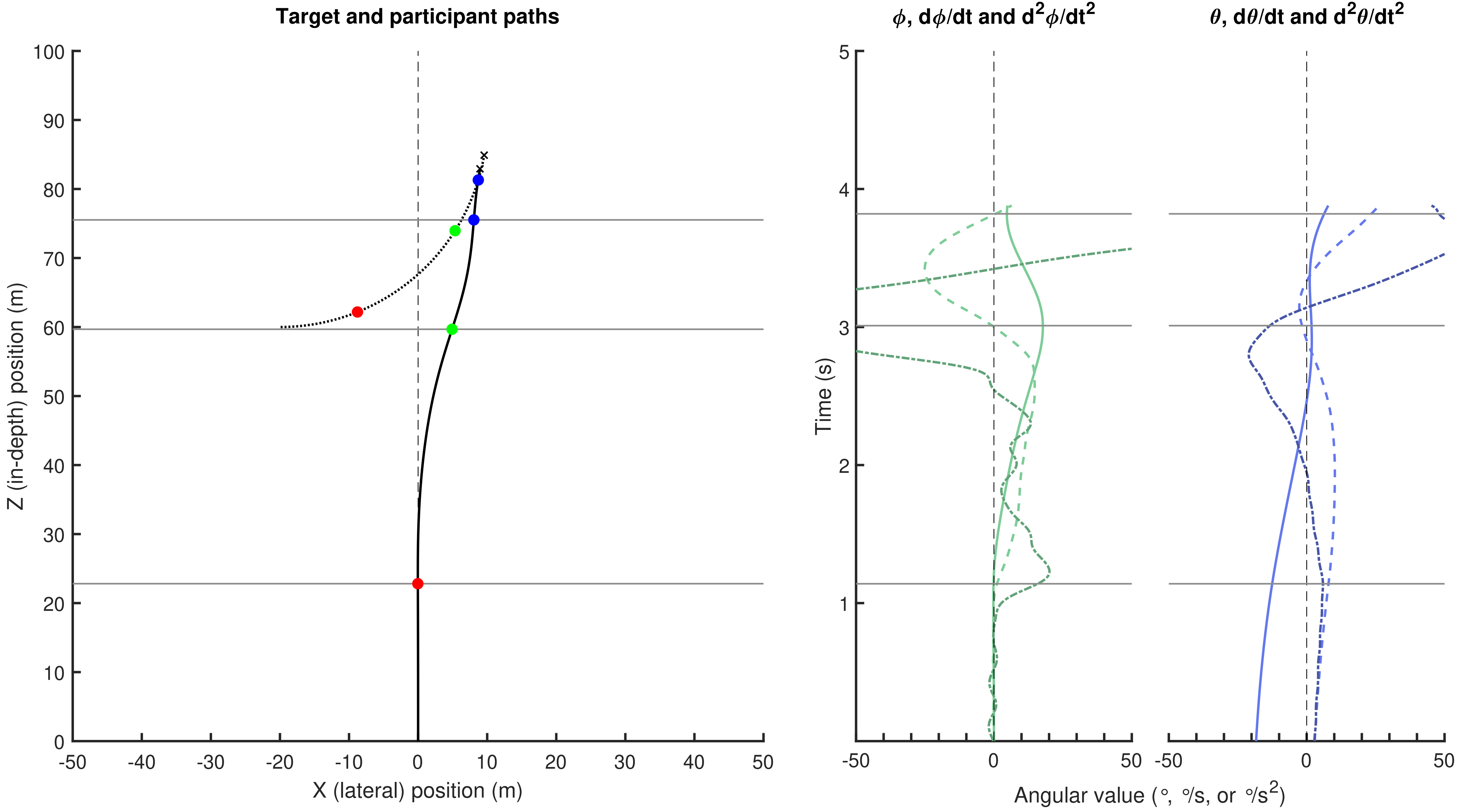

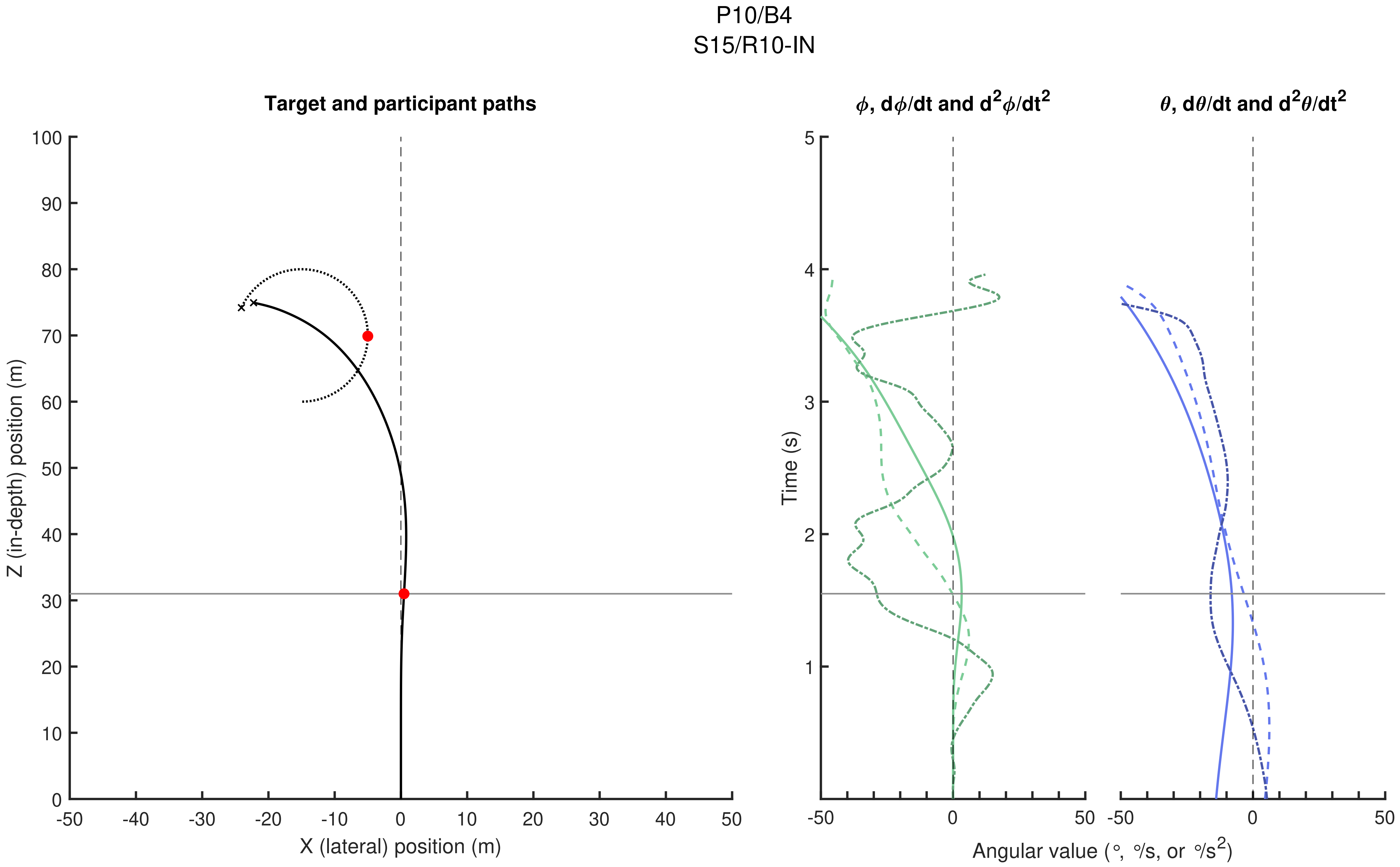
P10/B4
S15/R10-IN
Target and participant paths
Z (in-depth) position (m)
X (lateral) position (m)
φ, dφ/dt and d²φ/dt²
θ, dθ/dt and d²θ/dt²
Time (s)
Angular value (°, °/s, or °/s²)

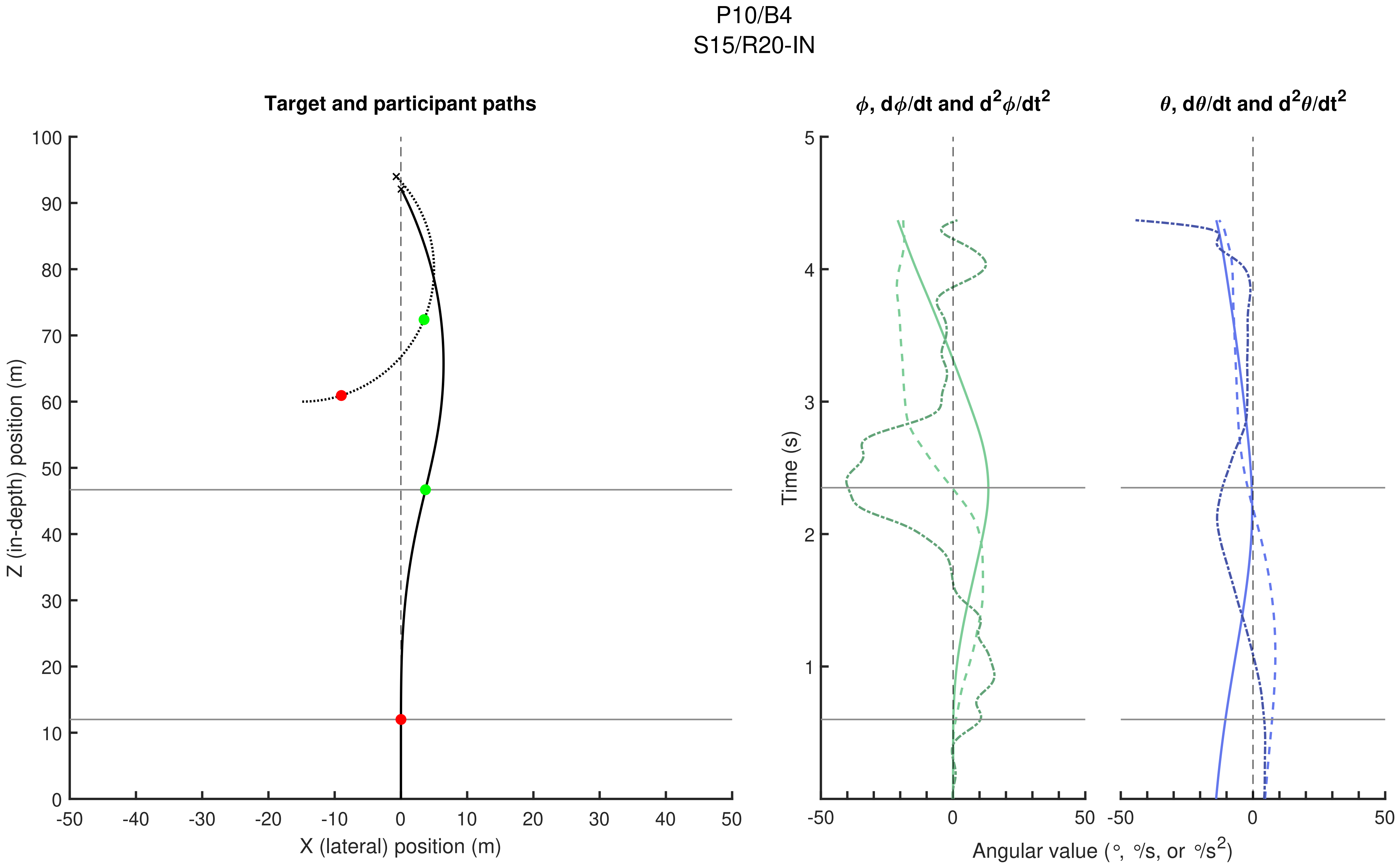
P10/B4
S15/R20-IN
Target and participant paths
ϕ, dϕ/dt and d$^2\phi$/dt^2
θ, dθ/dt and d$^2\theta$/dt^2
Z (in-depth) position (m)
X (lateral) position (m)
Time (s)
Angular value (°, °/s, or °/s^2)
100
90
80
70
60
50
40
30
20
10
0
-50
-40
-30
-20
-10
0
10
20
30
40
50
5
4
3
2
1

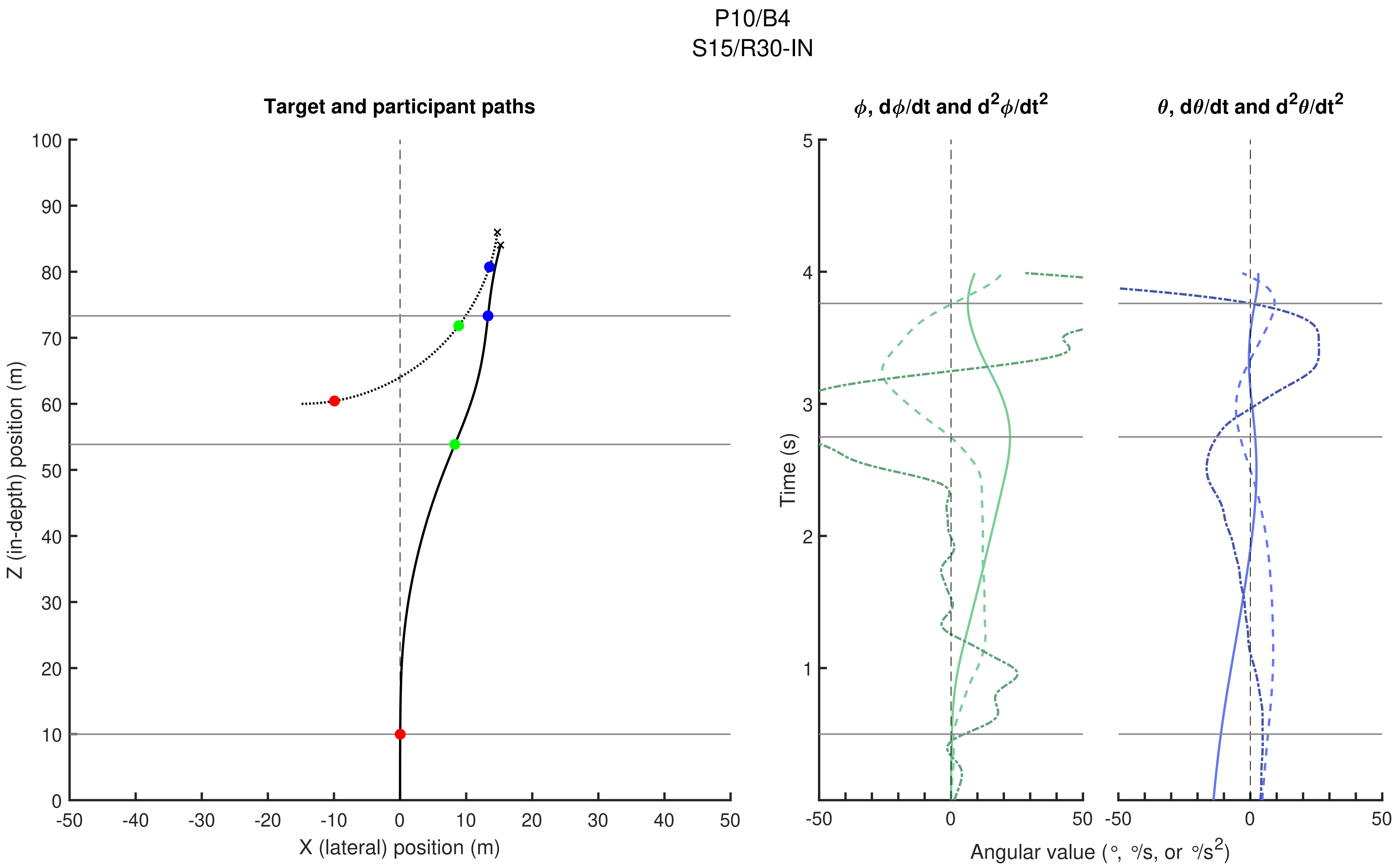
P10/B4
S15/R30-IN
Target and participant paths
Z (in-depth) position (m)
X (lateral) position (m)
φ, dφ/dt and d²φ/dt²
θ, dθ/dt and d²θ/dt²
Time (s)
Angular value (°, °/s, or °/s²)

P10/B4
S5/R10-IN

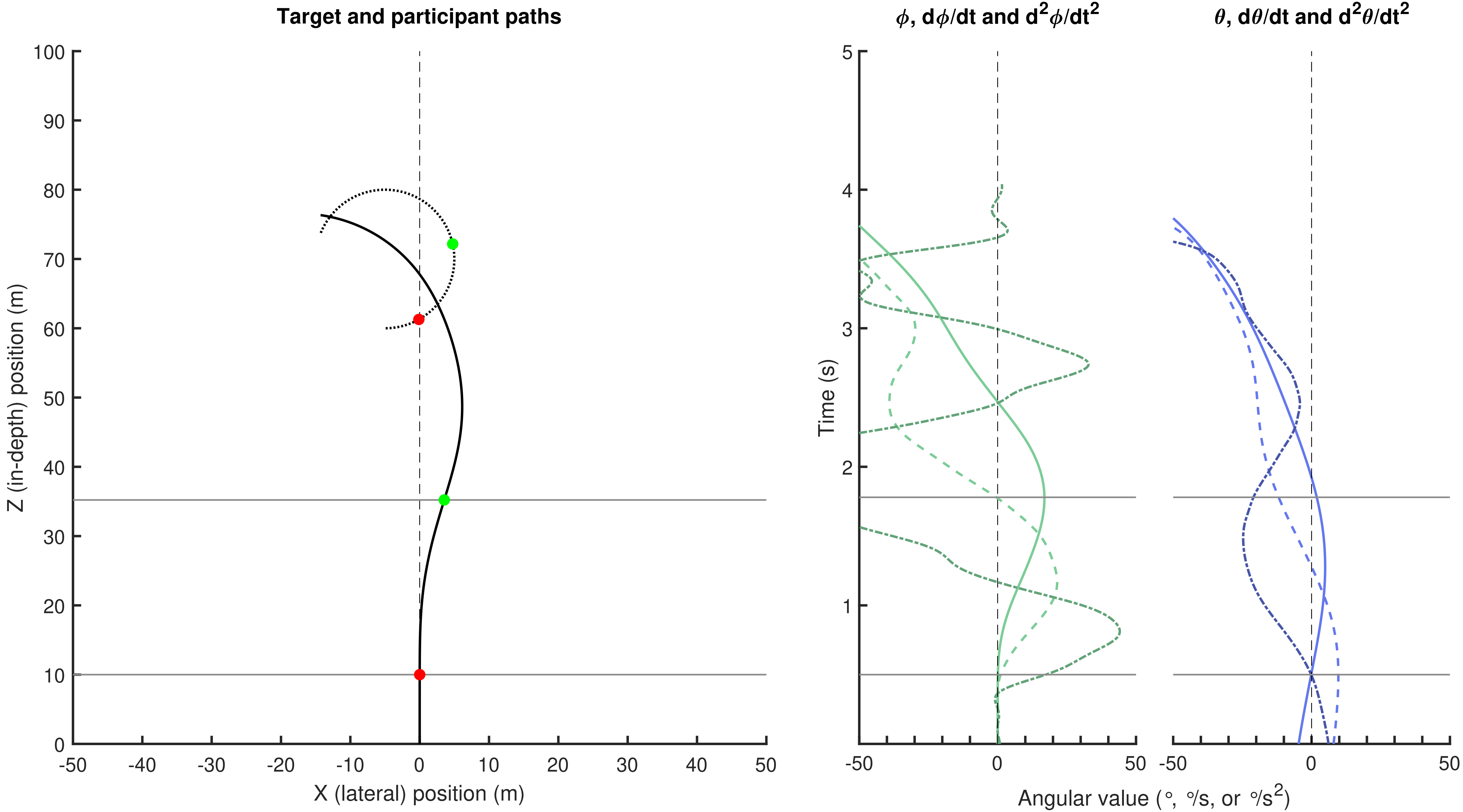

P10/B4
S5/R10-OUT

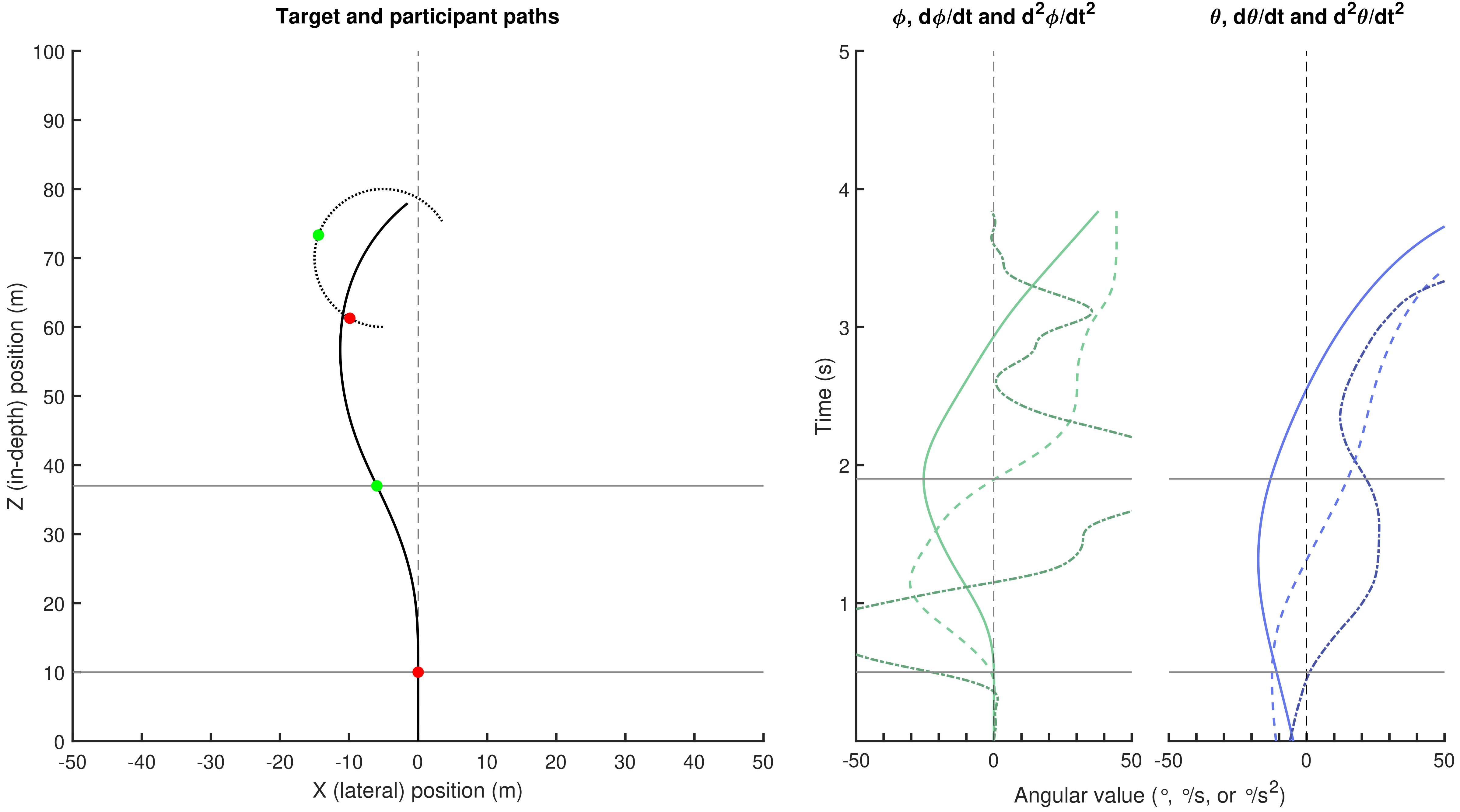

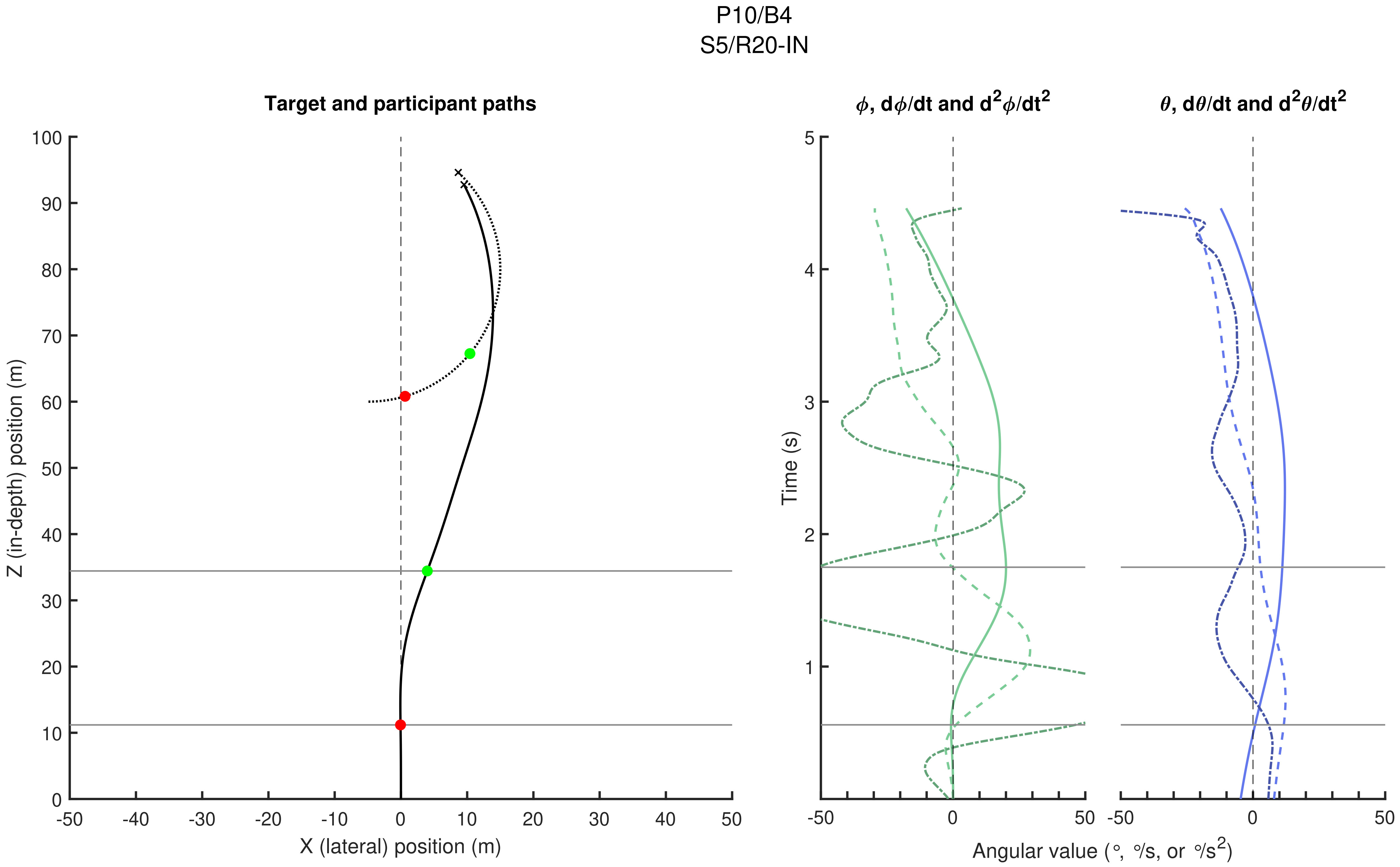
P10/B4
S5/R20-IN
Target and participant paths
Z (in-depth) position (m)
X (lateral) position (m)
100
90
80
70
60
50
40
30
20
10
0
-50
-40
-30
-20
-10
0
10
20
30
40
50
ϕ, dϕ/dt and d²ϕ/dt²
θ, dθ/dt and d²θ/dt²
Time (s)
5
4
3
2
1
-50
0
50
-50
0
50
Angular value (°, °/s, or °/s²)

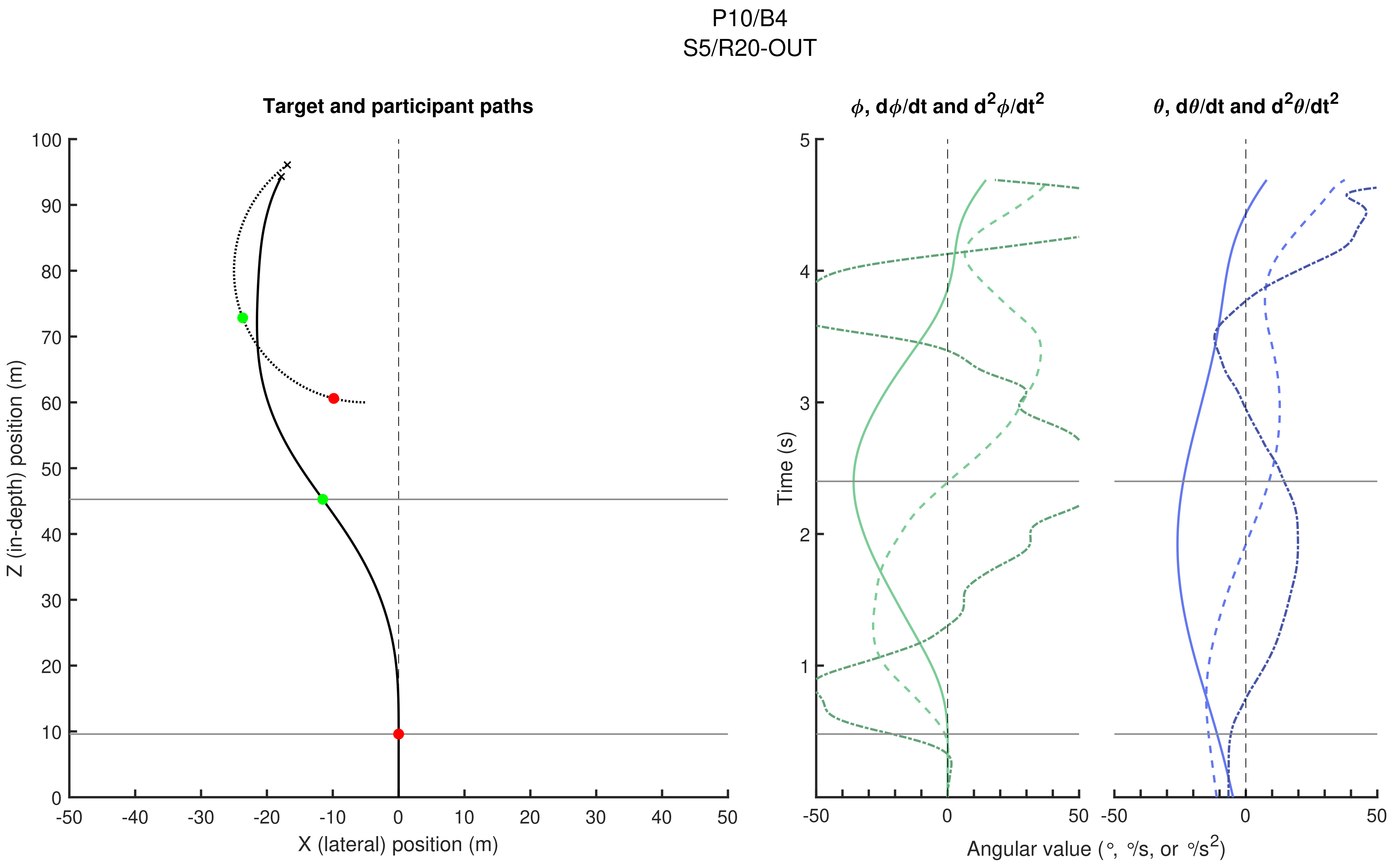

P10/B4
S5/R20-OUT
Target and participant paths
ϕ, dϕ/dt and d$^2\phi$/dt^2
θ, dθ/dt and d$^2\theta$/dt^2
Z (in-depth) position (m)
X (lateral) position (m)
Time (s)
Angular value (°, °/s, or °/s^2)

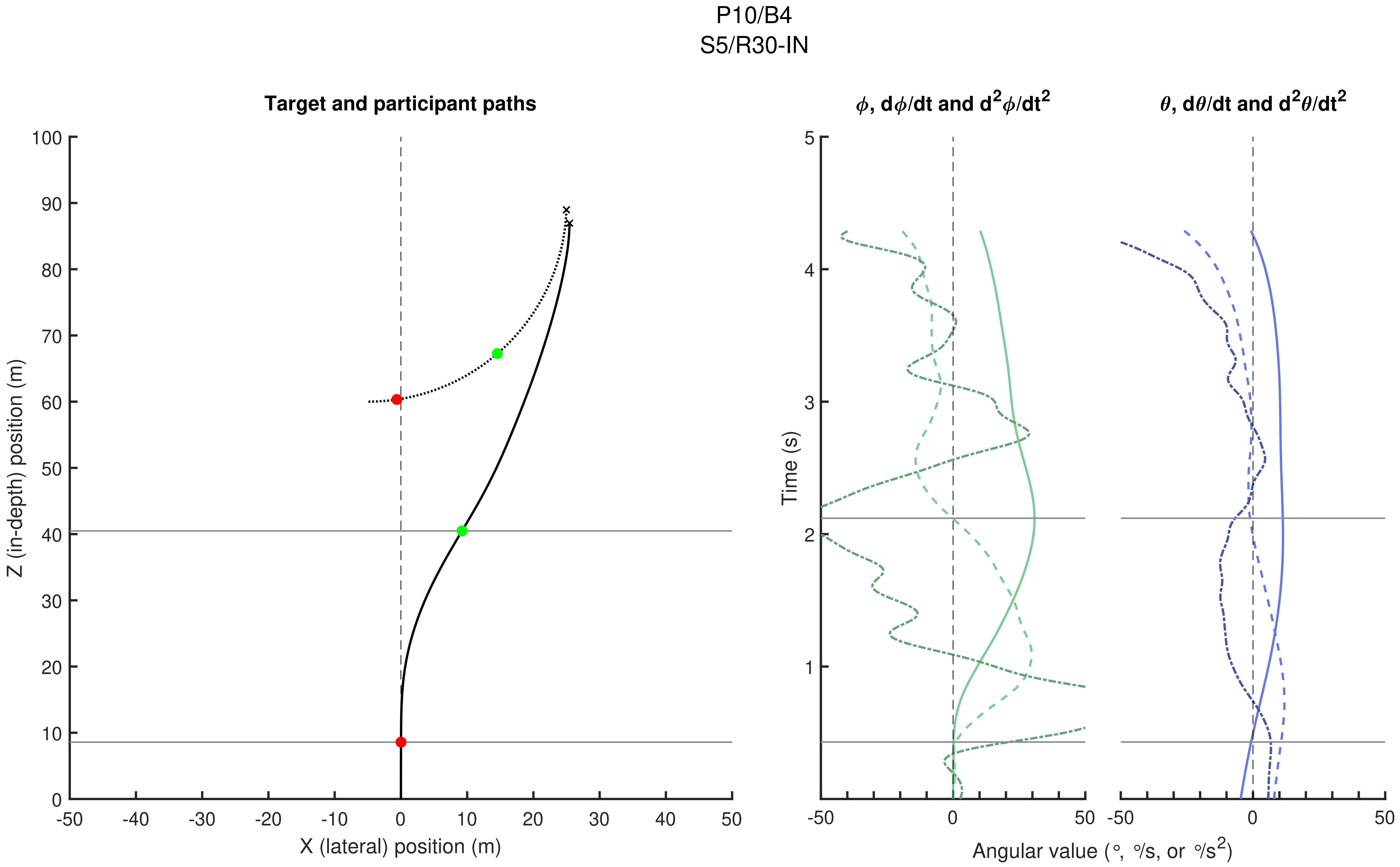

P10/B4
S5/R30-IN
Target and participant paths
Z (in-depth) position (m)
X (lateral) position (m)
φ, dφ/dt and d²φ/dt²
θ, dθ/dt and d²θ/dt²
Time (s)
Angular value (°, °/s, or °/s²)

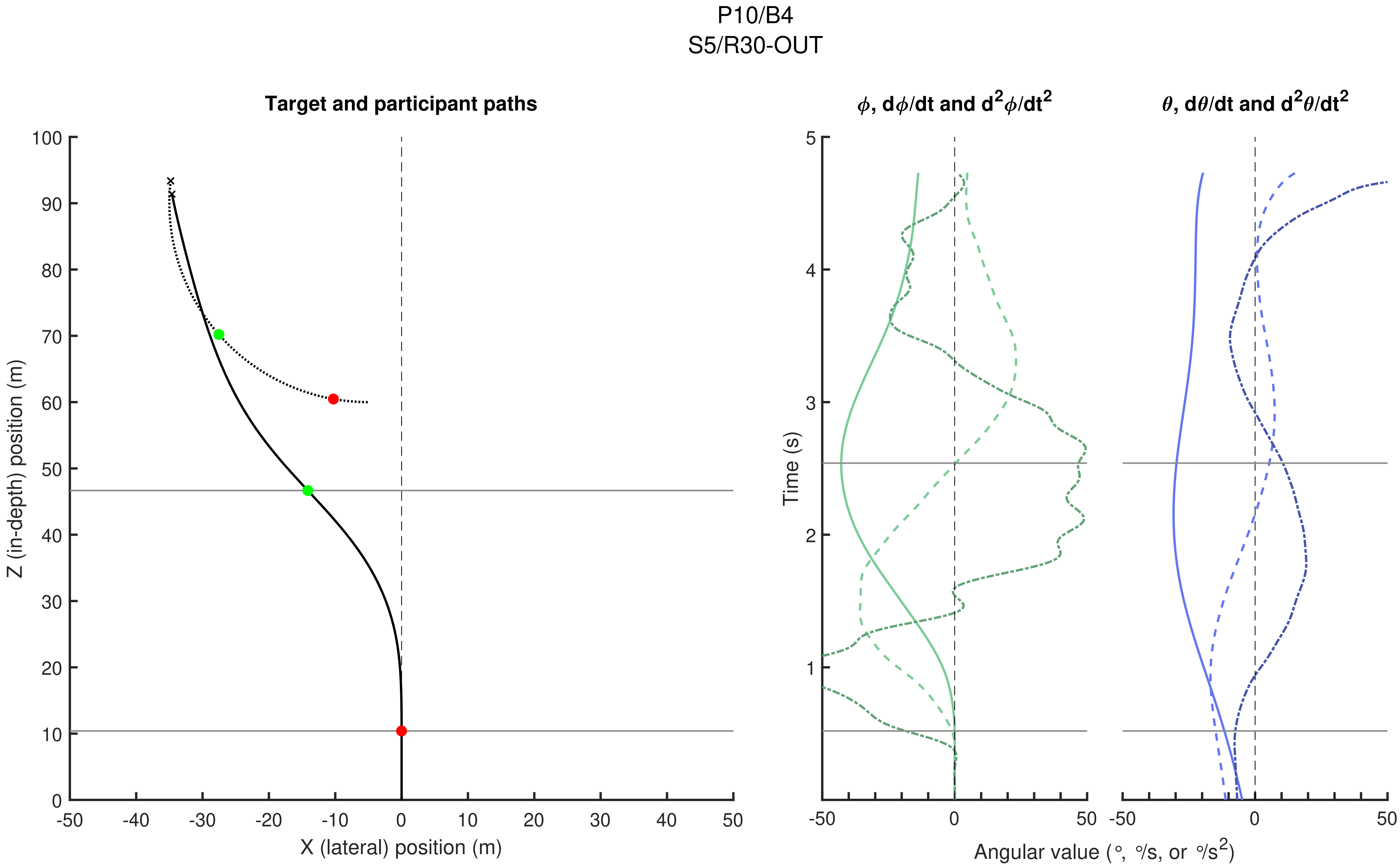
P10/B4
S5/R30-OUT
Target and participant paths
Z (in-depth) position (m)
X (lateral) position (m)
100
90
80
70
60
50
40
30
20
10
0
-50
-40
-30
-20
-10
0
10
20
30
40
50
φ, dφ/dt and d²φ/dt²
θ, dθ/dt and d²θ/dt²
Time (s)
5
4
3
2
1
-50
0
50
-50
0
50
Angular value (°, °/s, or °/s²)

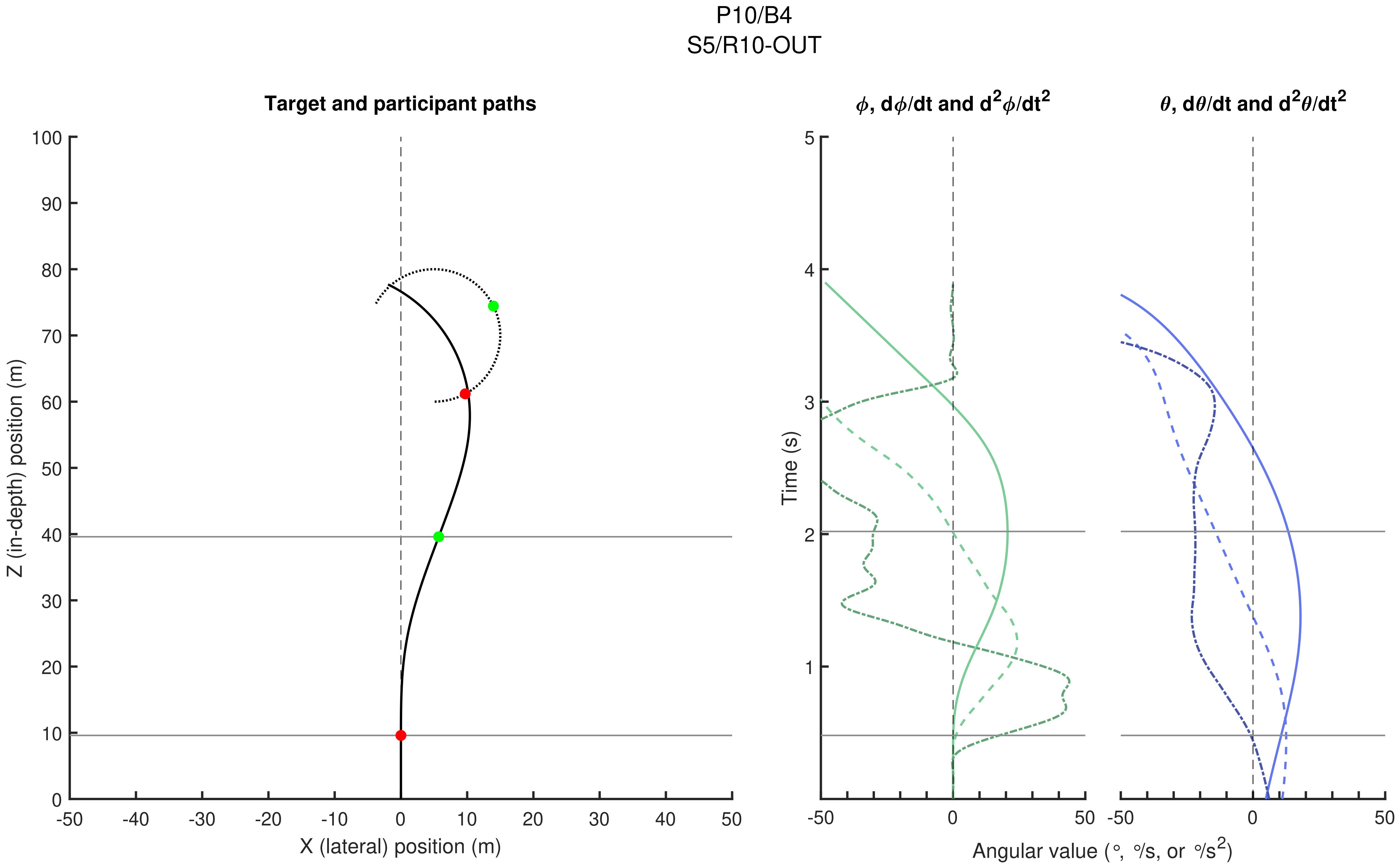

P10/B4
S5/R10-OUT
Target and participant paths
Z (in-depth) position (m)
X (lateral) position (m)
100
90
80
70
60
50
40
30
20
10
0
-50
-40
-30
-20
-10
0
10
20
30
40
50
ϕ, dϕ/dt and d$^2\phi$/dt^2
θ, dθ/dt and d$^2\theta$/dt^2
Time (s)
5
4
3
2
1
-50
0
50
-50
0
50
Angular value (°, °/s, or °/s^2)

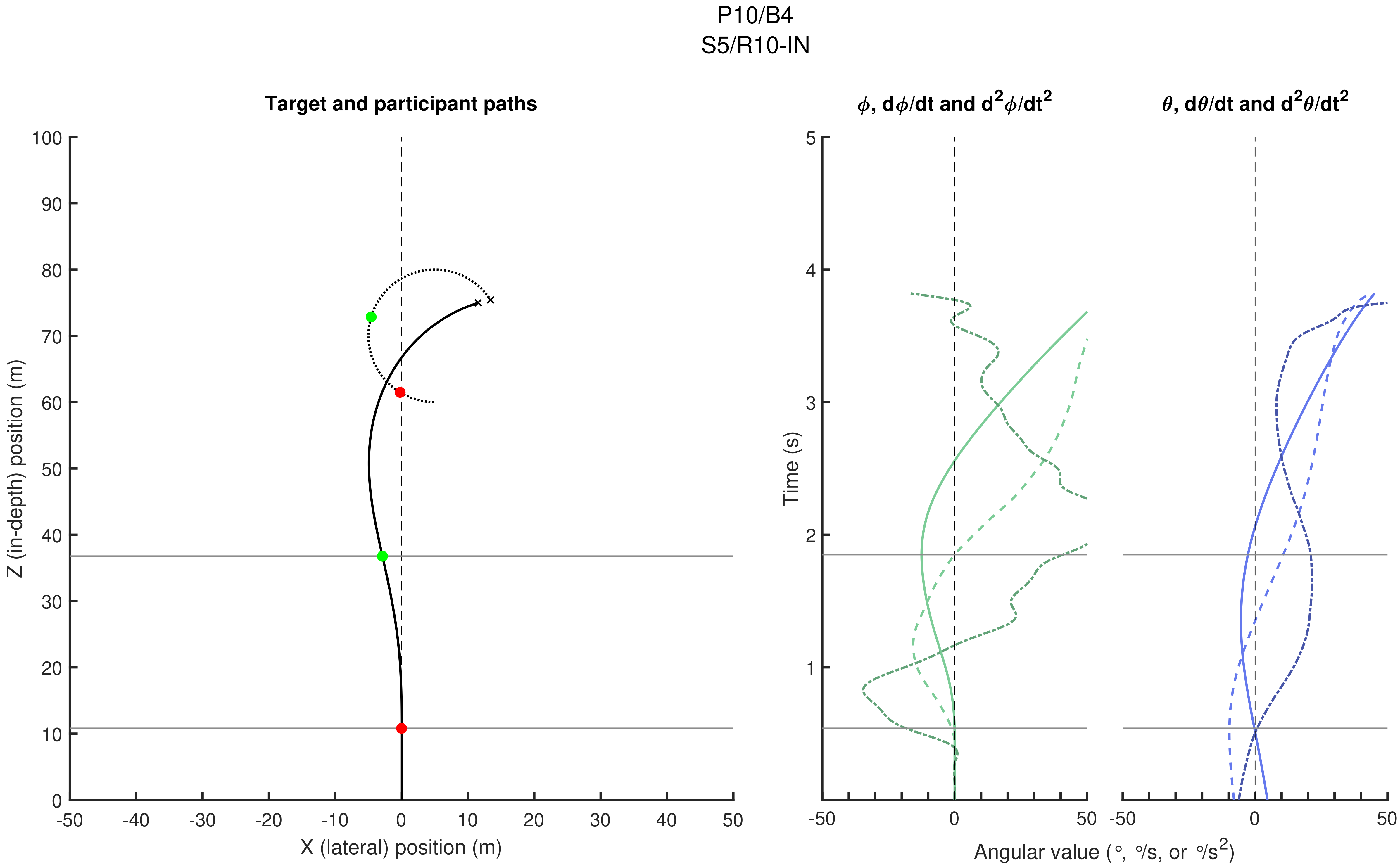

P10/B4
S5/R10-IN
Target and participant paths
Z (in-depth) position (m)
X (lateral) position (m)
-50
-40
-30
-20
-10
0
10
20
30
40
50
100
90
80
70
60
50
40
30
20
10
0
φ, dφ/dt and d²φ/dt²
θ, dθ/dt and d²θ/dt²
Time (s)
5
4
3
2
1
-50
0
50
-50
0
50
Angular value (°, °/s, or °/s²)

P10/B4
S5/R20-OUT

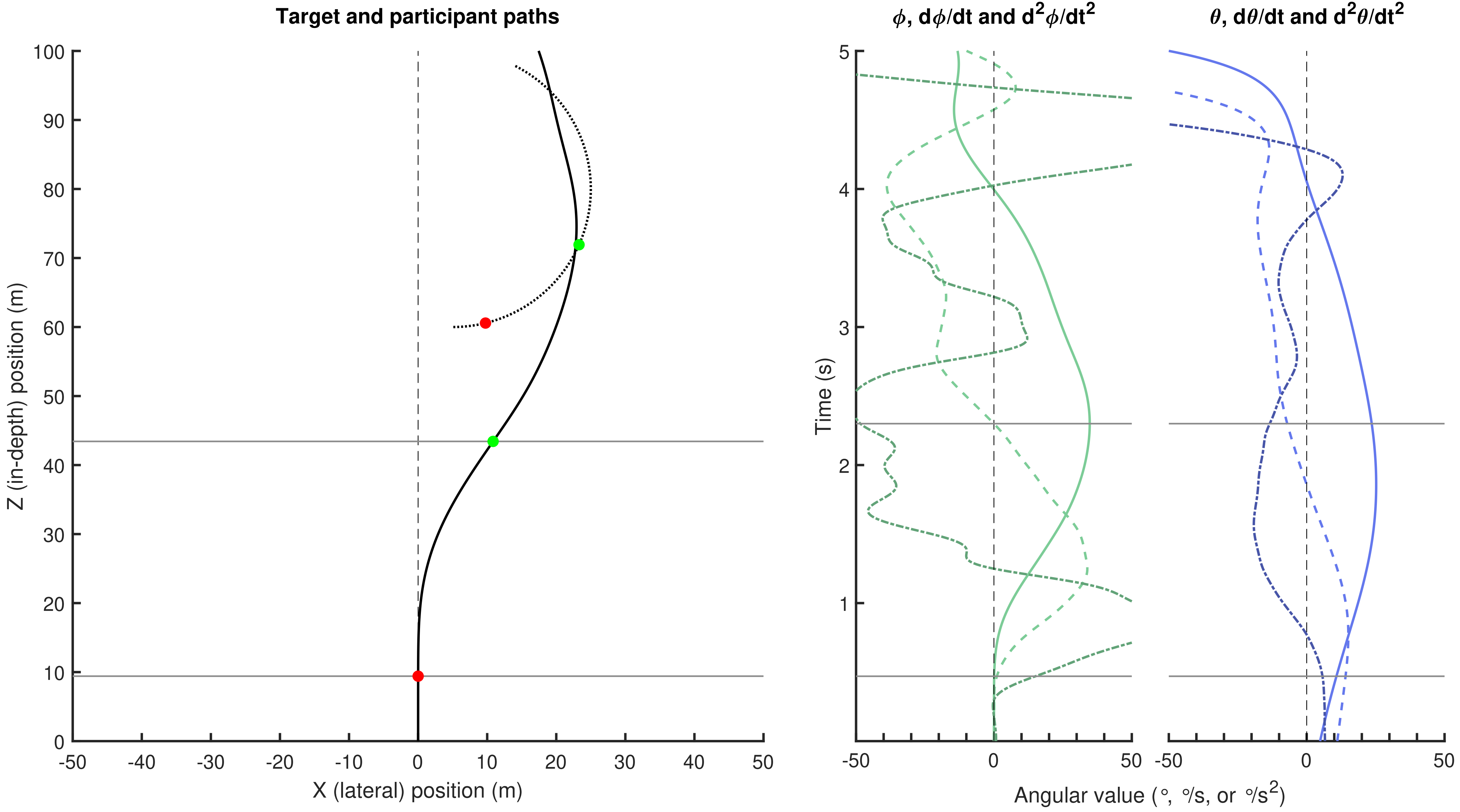

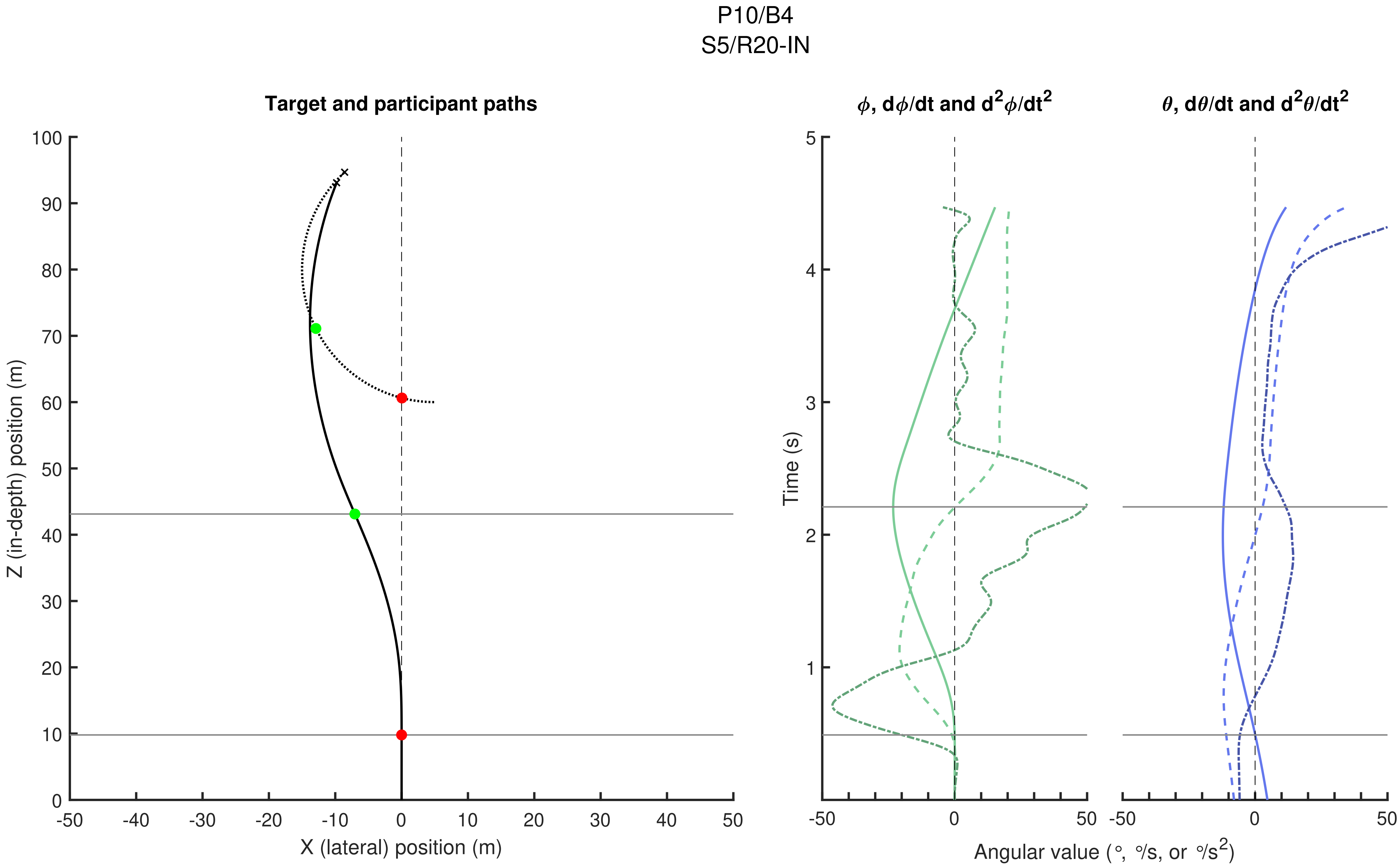

P10/B4
S5/R20-IN
Target and participant paths
Z (in-depth) position (m)
X (lateral) position (m)
φ, dφ/dt and d²φ/dt²
θ, dθ/dt and d²θ/dt²
Time (s)
Angular value (°, °/s, or °/s²)

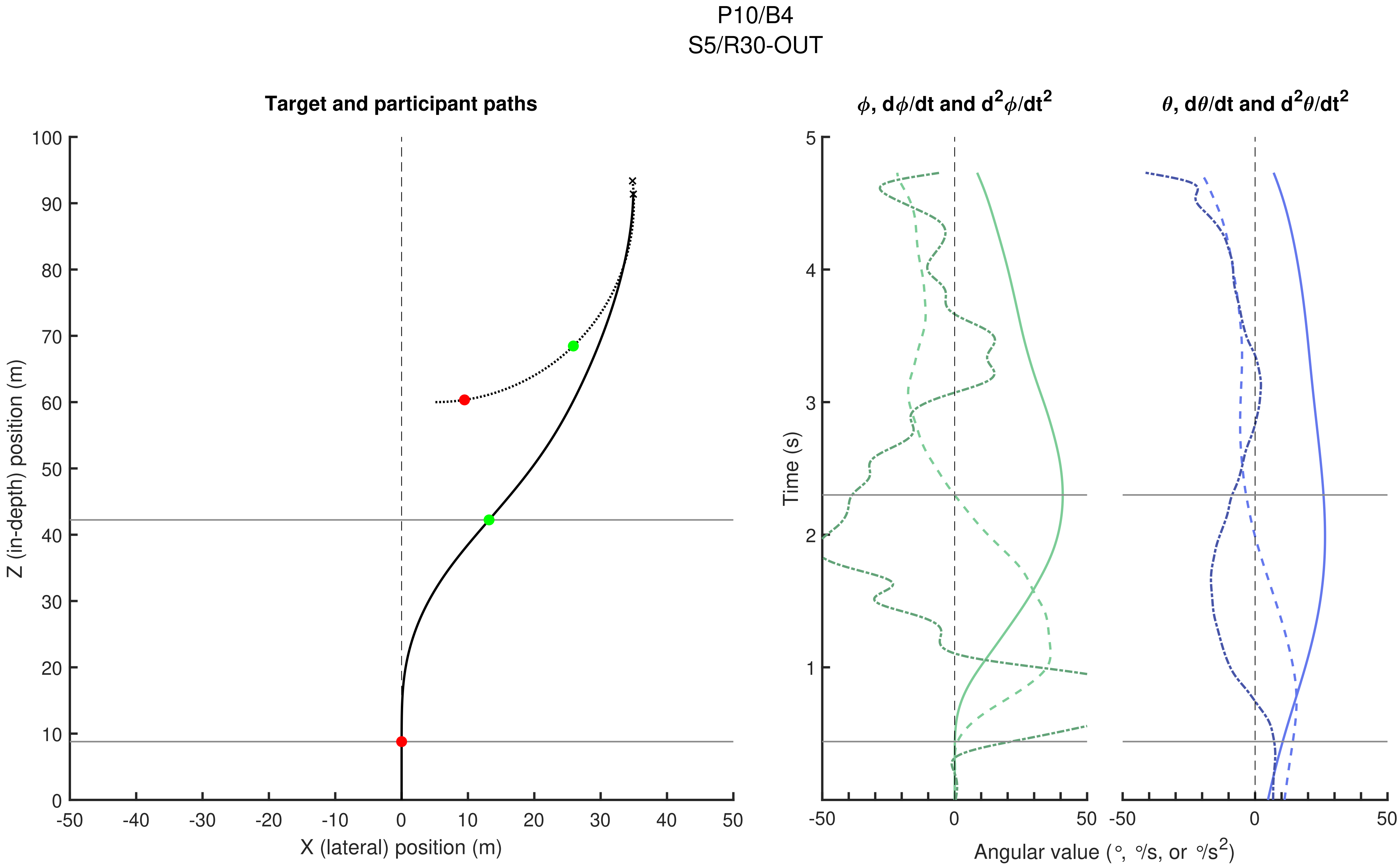

P10/B4
S5/R30-OUT
Target and participant paths
ϕ, dϕ/dt and d$^2\phi$/dt^2
θ, dθ/dt and d$^2\theta$/dt^2
Z (in-depth) position (m)
X (lateral) position (m)
Time (s)
Angular value (°, °/s, or °/s^2)
0
10
20
30
40
50
60
70
80
90
100
-50
-40
-30
-20
-10
0
10
20
30
40
50
1
2
3
4
5
-50
0
50
-50
0
50

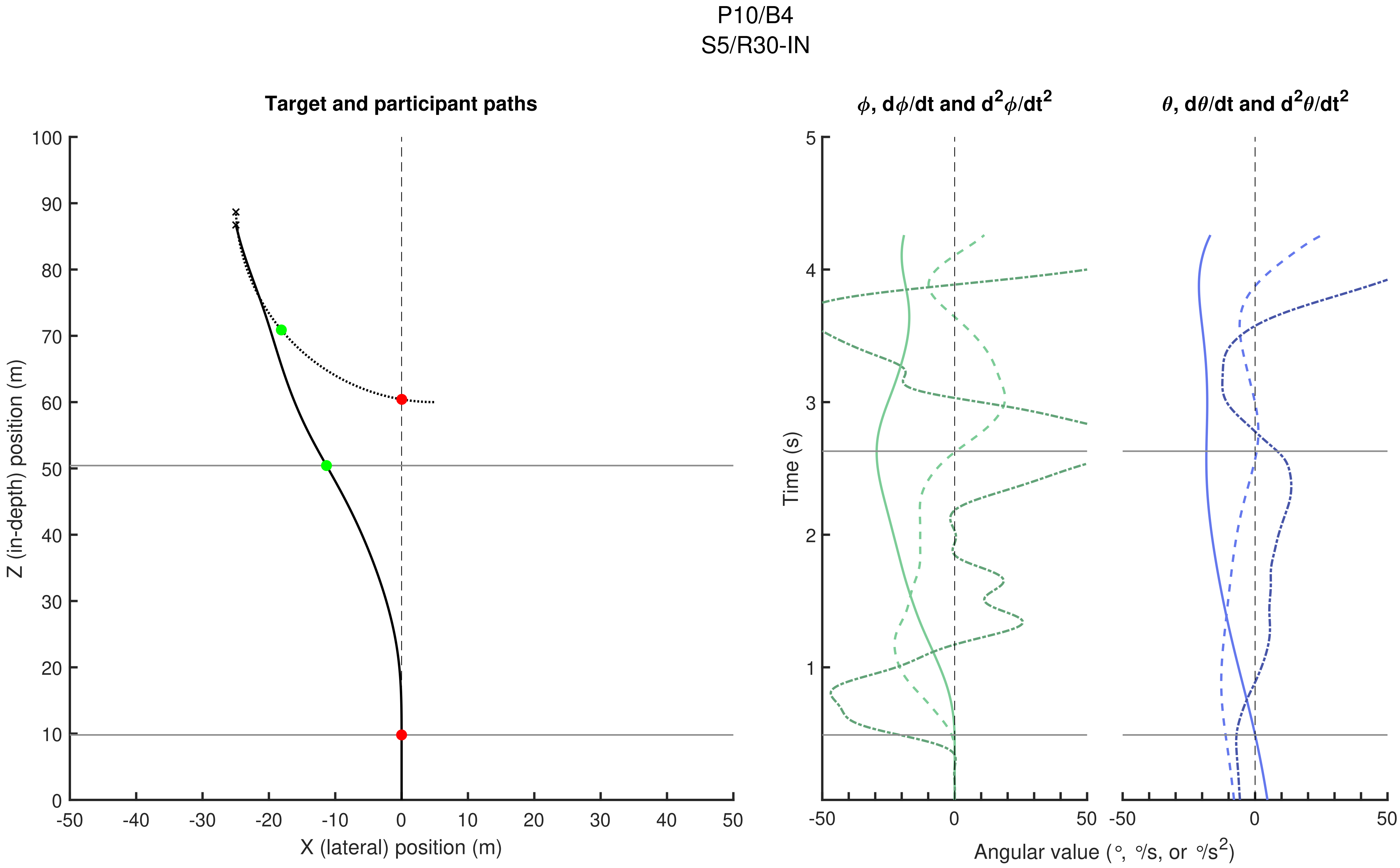

P10/B4
S5/R30-IN
Target and participant paths
Z (in-depth) position (m)
X (lateral) position (m)
100
90
80
70
60
50
40
30
20
10
0
-50
-40
-30
-20
-10
0
10
20
30
40
50
φ, dφ/dt and d²φ/dt²
θ, dθ/dt and d²θ/dt²
Time (s)
5
4
3
2
1
-50
0
50
-50
0
50
Angular value (°, °/s, or °/s²)

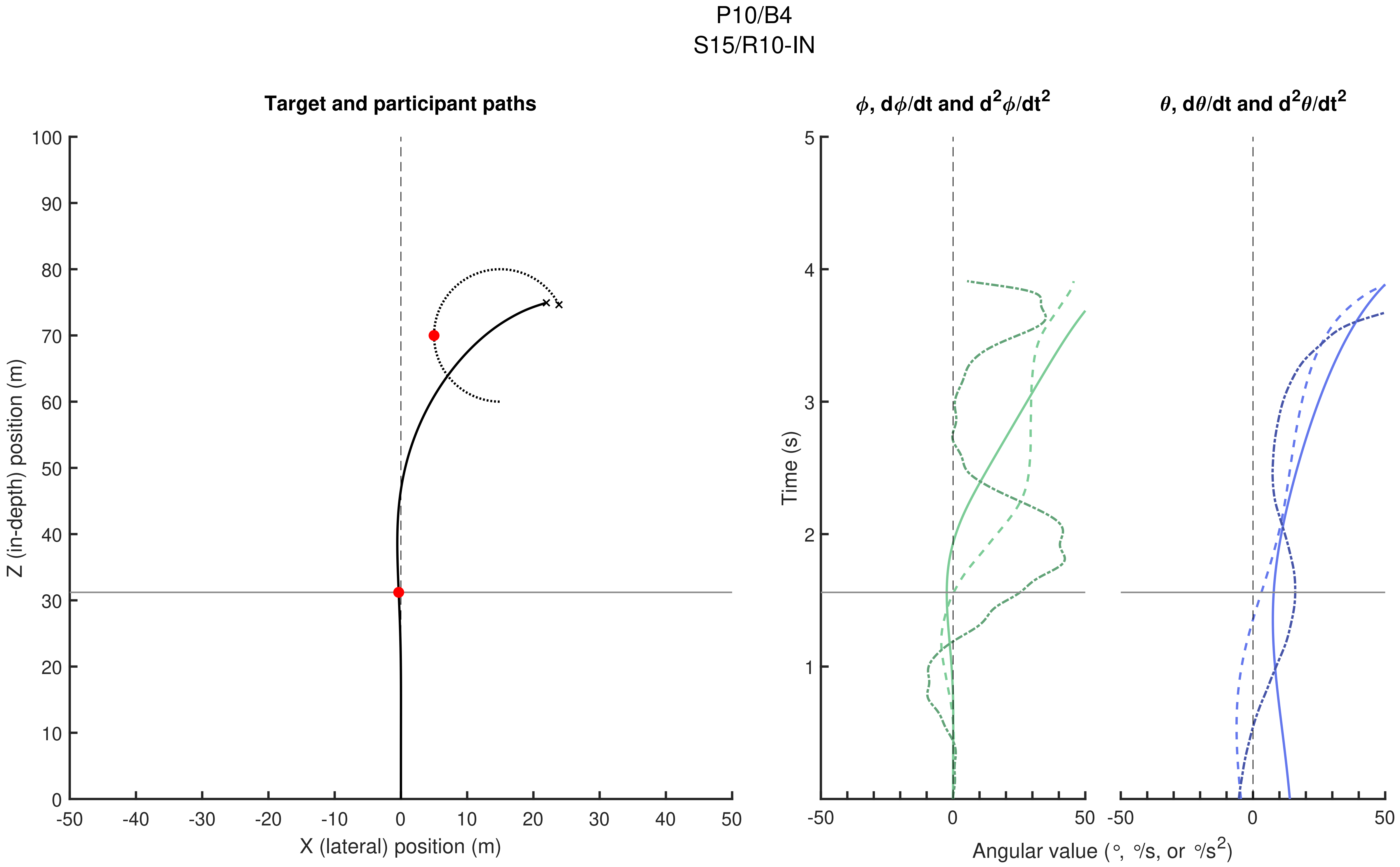
P10/B4
S15/R10-IN
Target and participant paths
ϕ, dϕ/dt and d$^2\phi$/dt^2
θ, dθ/dt and d$^2\theta$/dt^2
Z (in-depth) position (m)
X (lateral) position (m)
Time (s)
Angular value (°, °/s, or °/s^2)
100
90
80
70
60
50
40
30
20
10
0
-50
-40
-30
-20
-10
0
10
20
30
40
50
5
4
3
2
1
-50
0
50
-50
0
50

P10/B4
S15/R20-IN

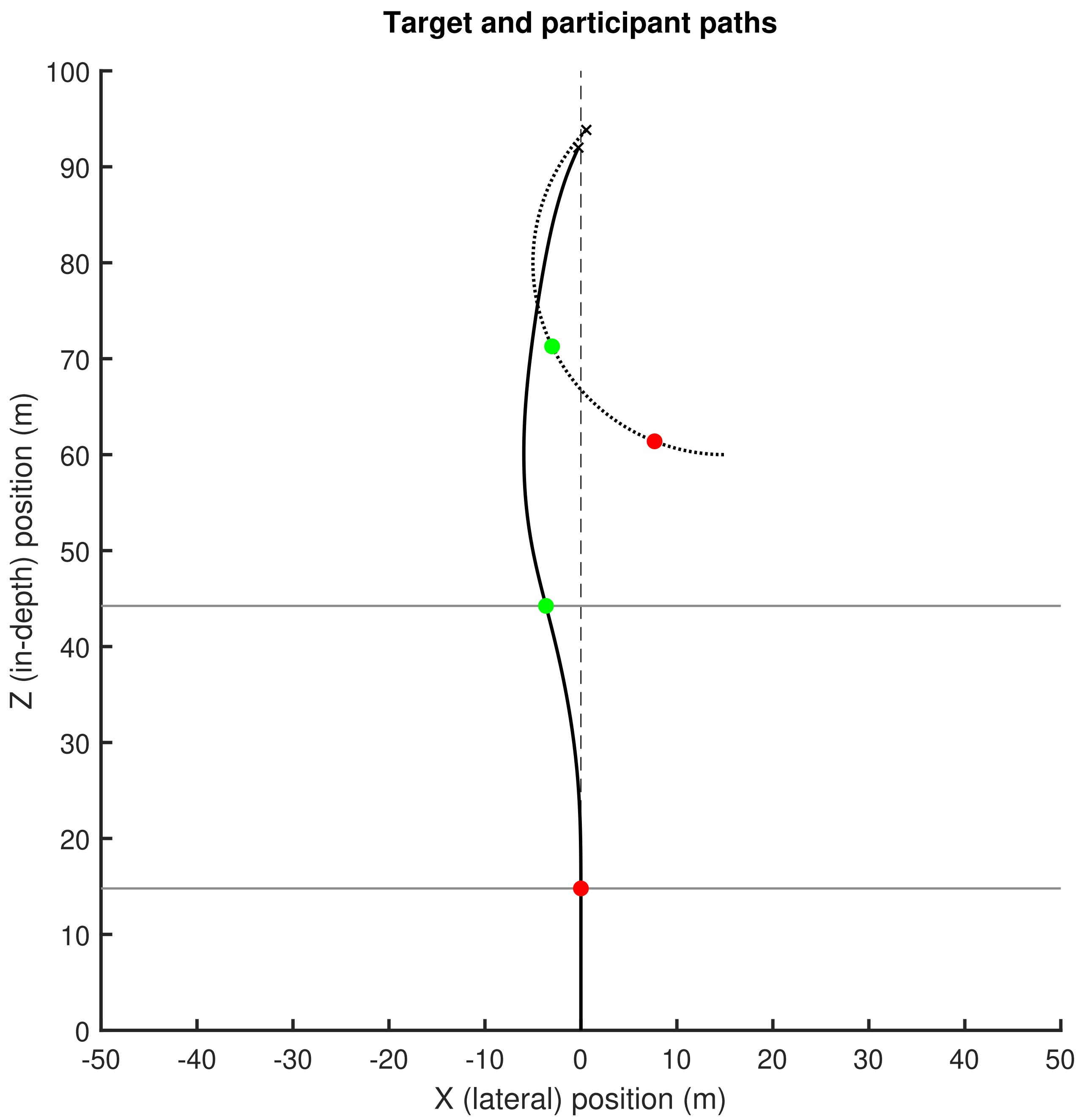

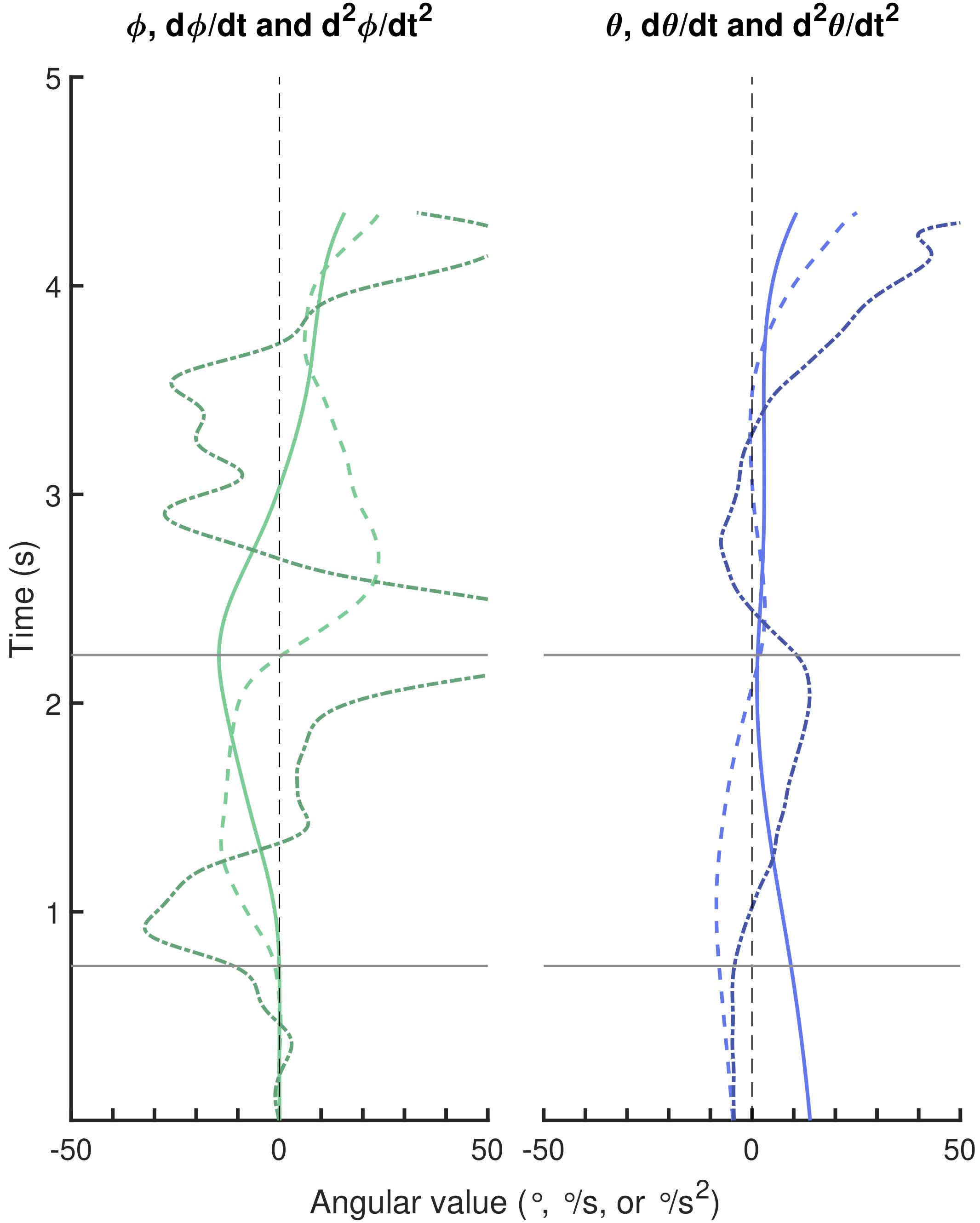

P10/B4
S15/R30-IN

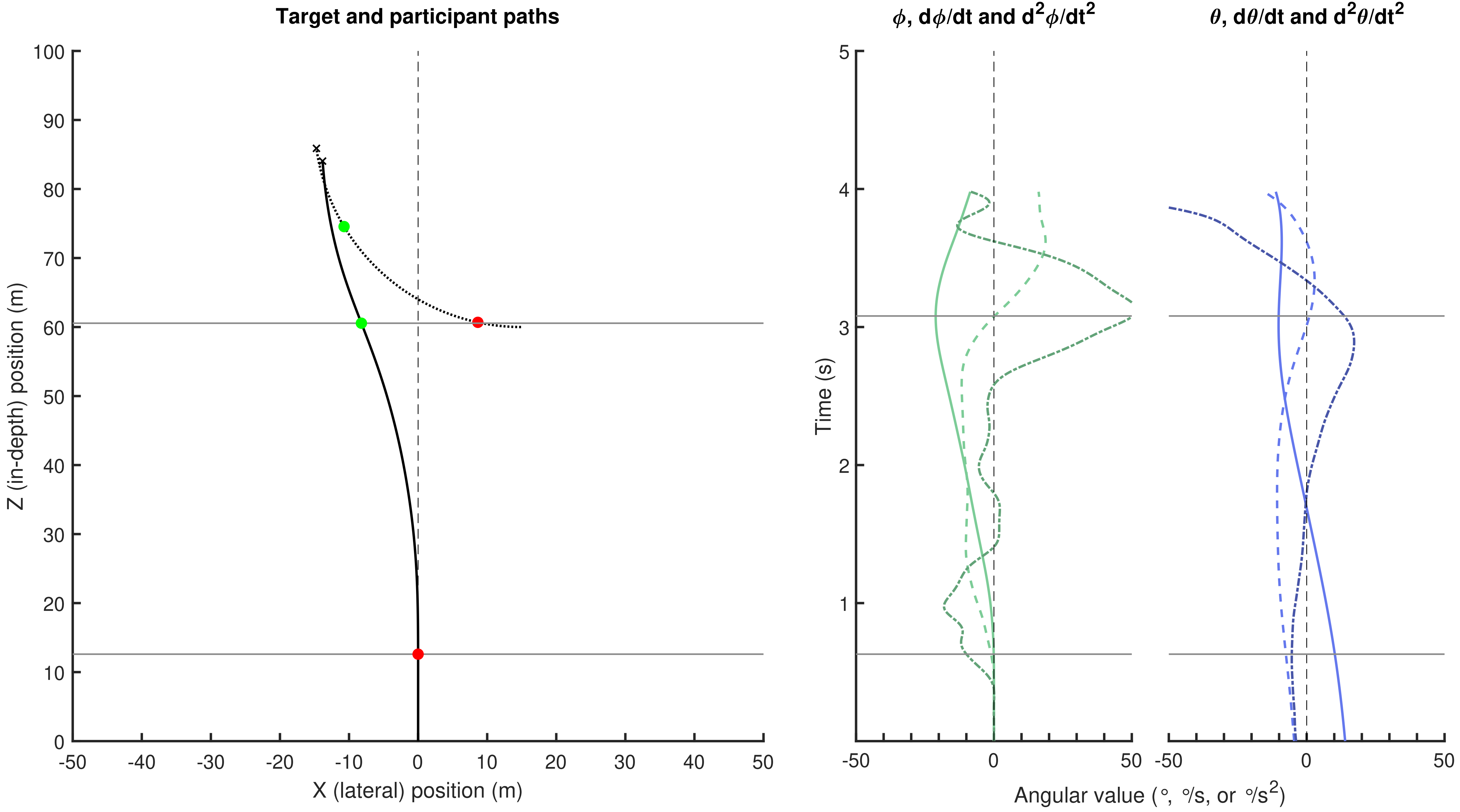

P10/B4
S20/R10-IN

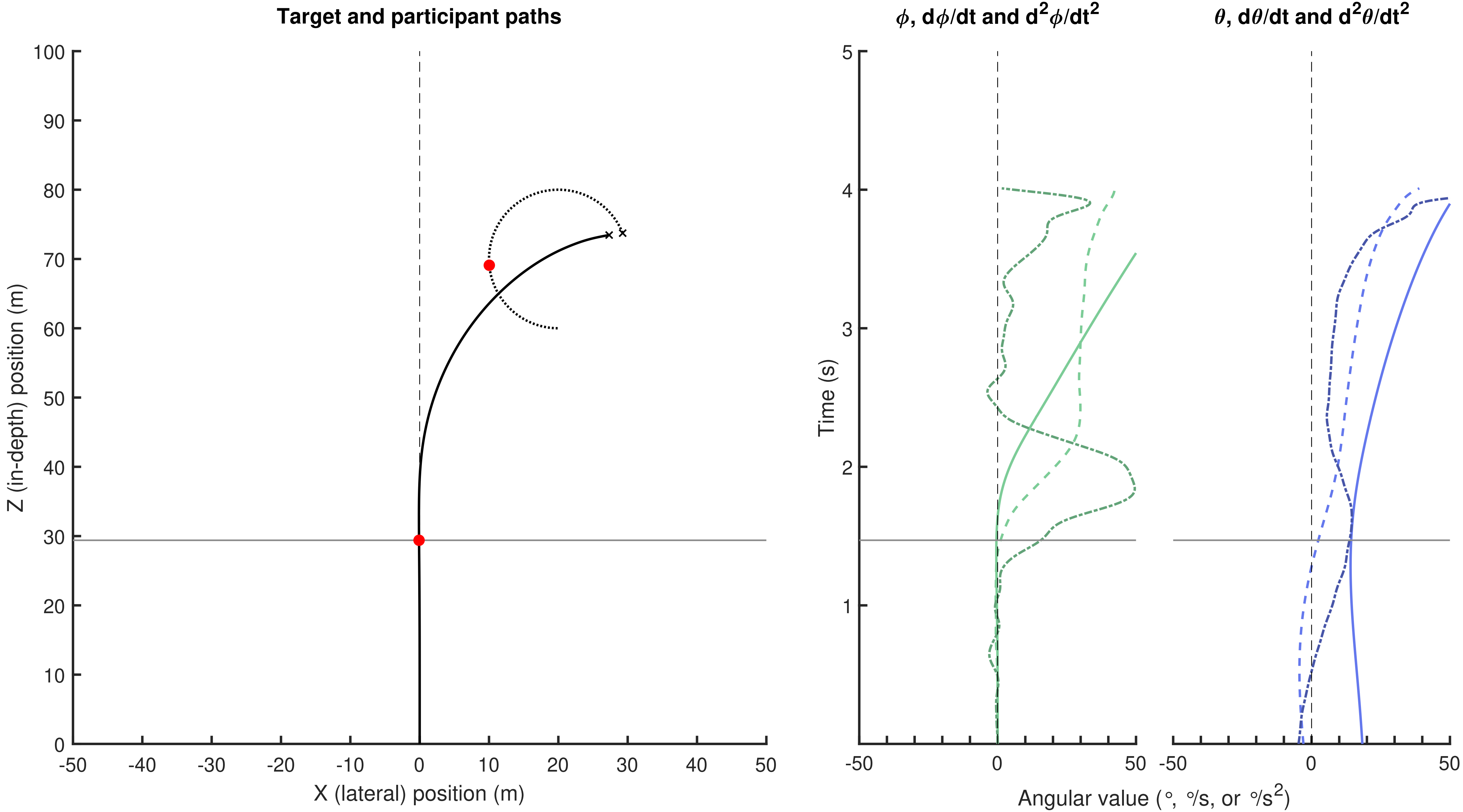

P10/B4
S20/R20-IN

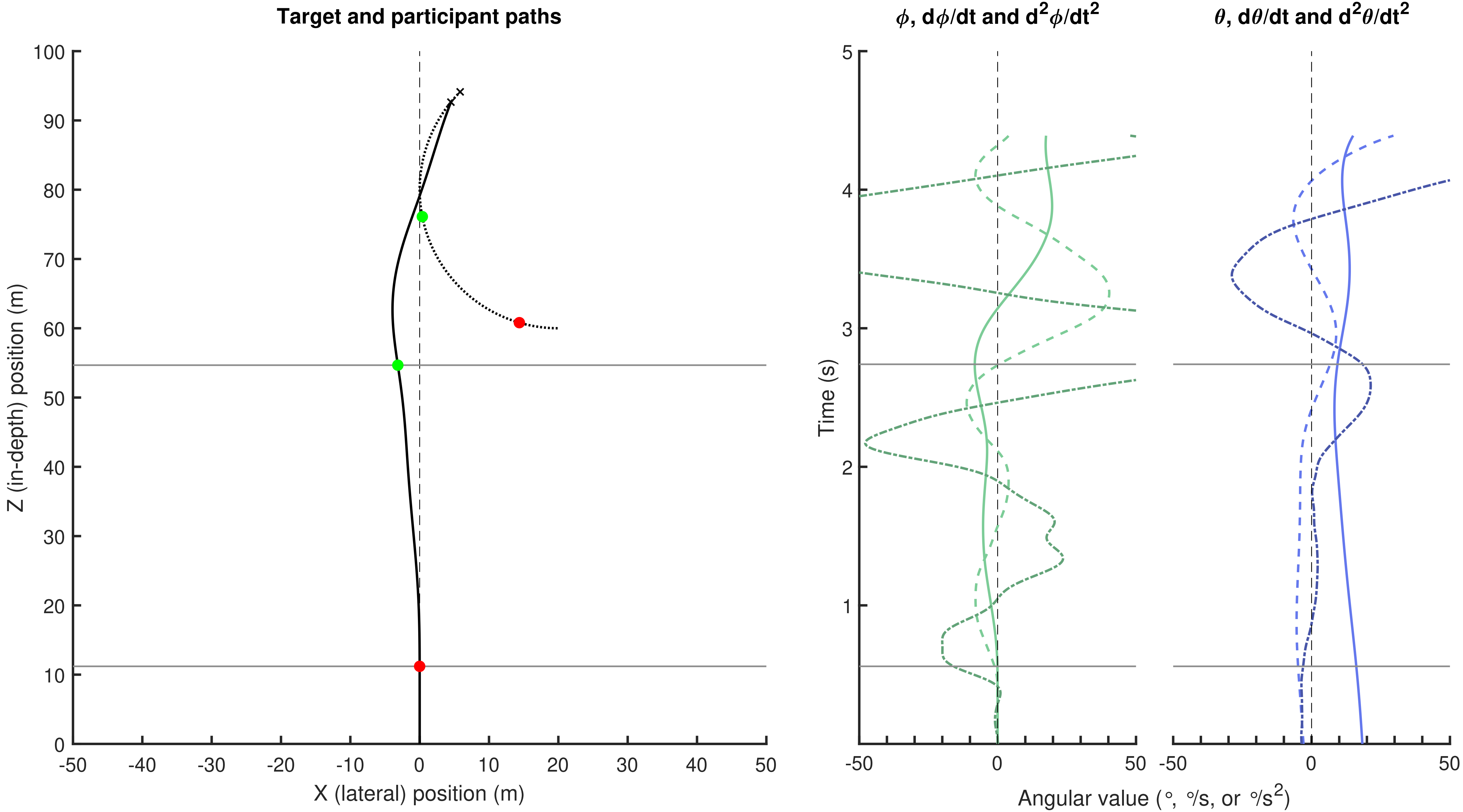

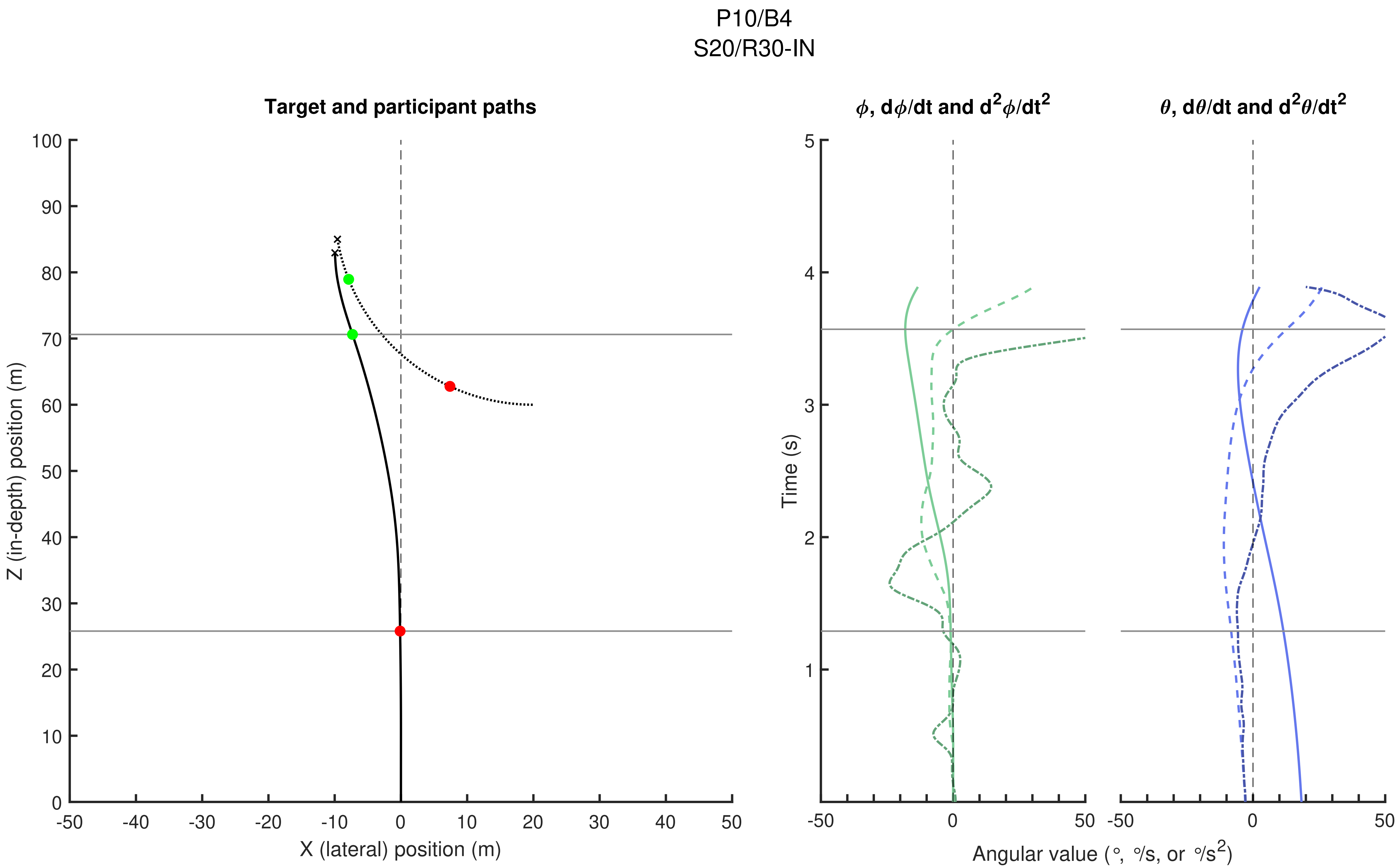

P10/B4
S20/R30-IN
Target and participant paths
Z (in-depth) position (m)
X (lateral) position (m)
100
90
80
70
60
50
40
30
20
10
0
-50
-40
-30
-20
-10
0
10
20
30
40
50
ϕ, dϕ/dt and d²ϕ/dt²
θ, dθ/dt and d²θ/dt²
Time (s)
5
4
3
2
1
-50
0
50
-50
0
50
Angular value (°, °/s, or °/s²)

P10/B5
S20/R10-IN

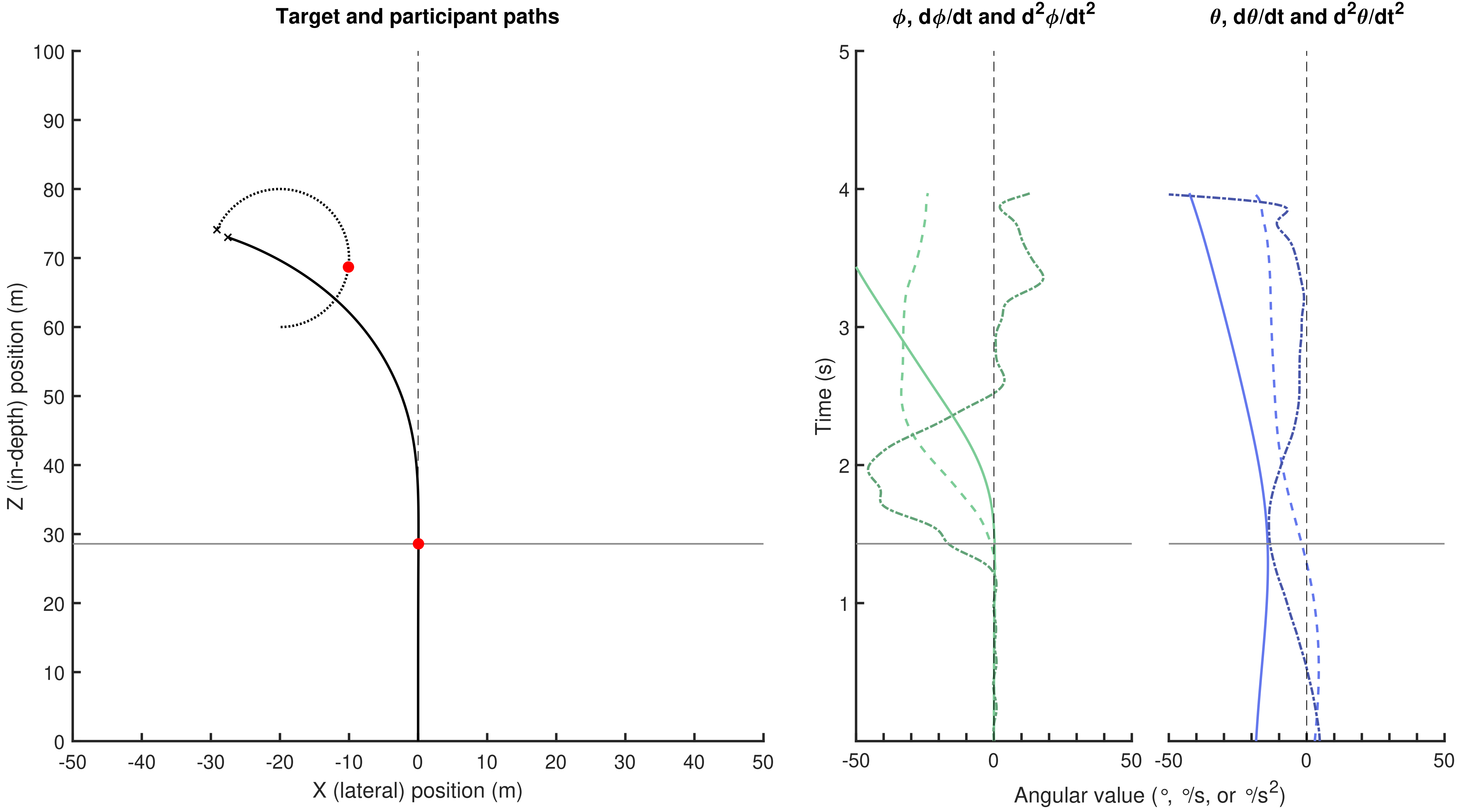

P10/B5
S20/R20-IN

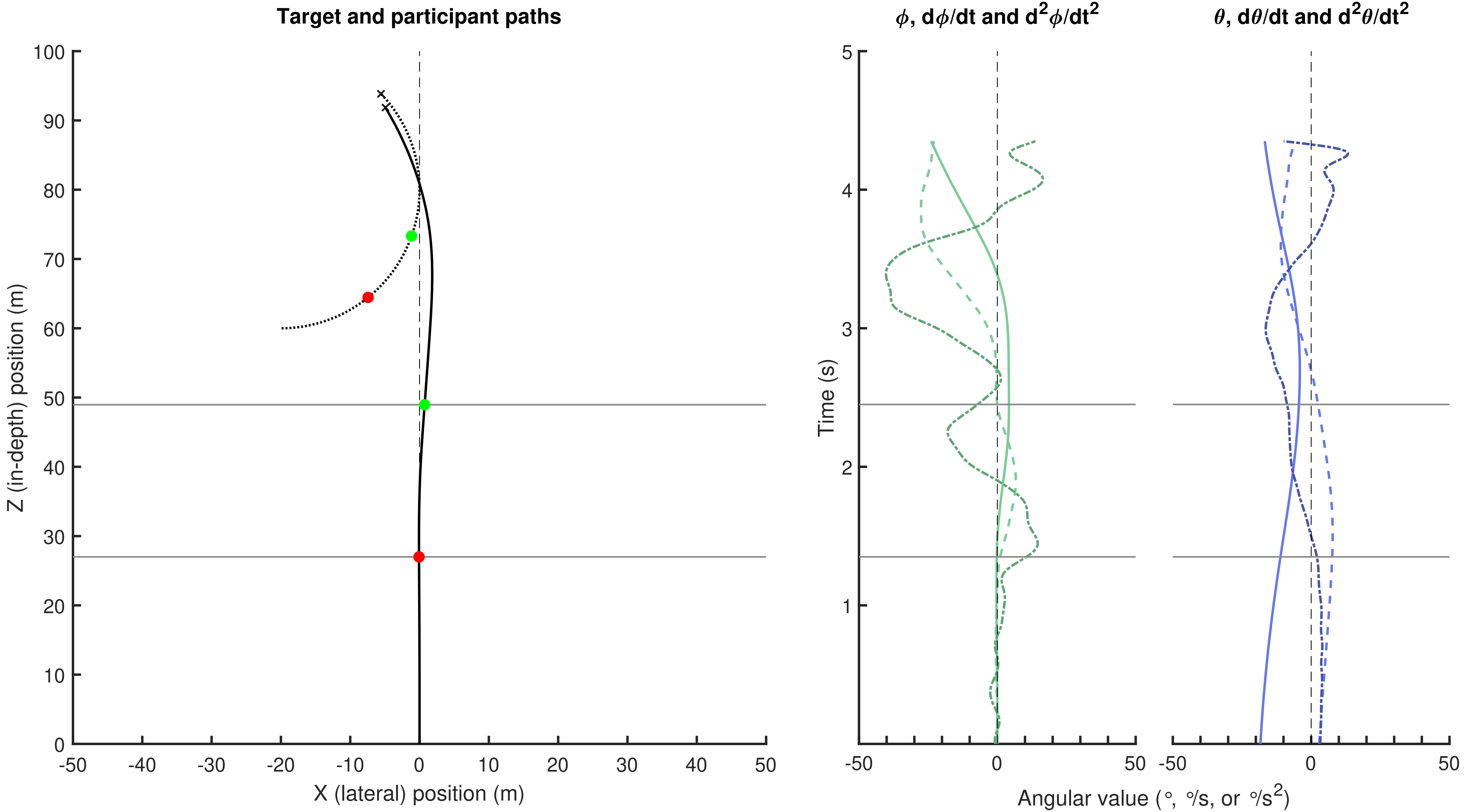

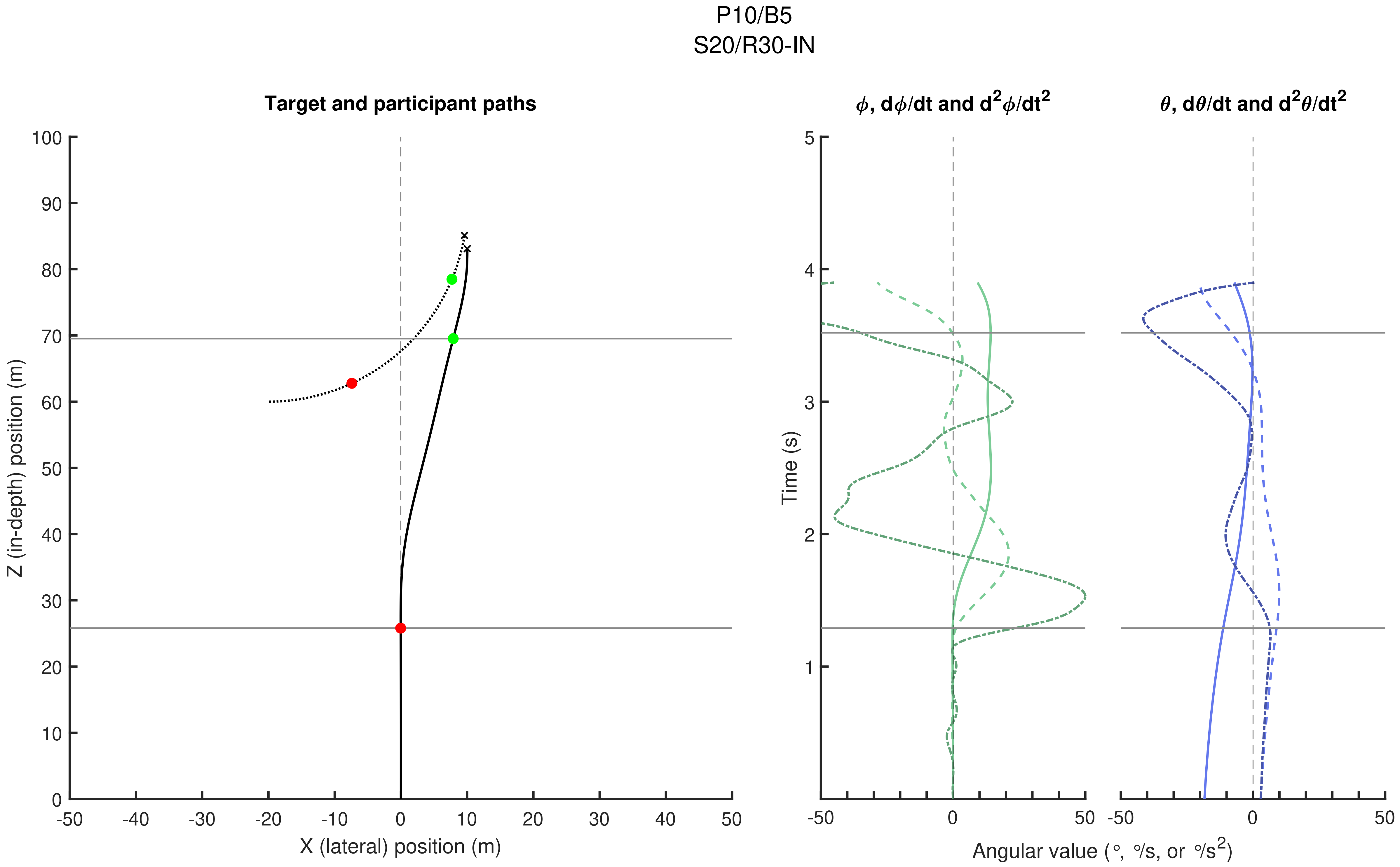
P10/B5
S20/R30-IN
Target and participant paths
Z (in-depth) position (m)
X (lateral) position (m)
ϕ, dϕ/dt and d$^2\phi$/dt^2
θ, dθ/dt and d$^2\theta$/dt^2
Time (s)
Angular value (°, °/s, or °/s^2)

P10/B5
S15/R10-IN

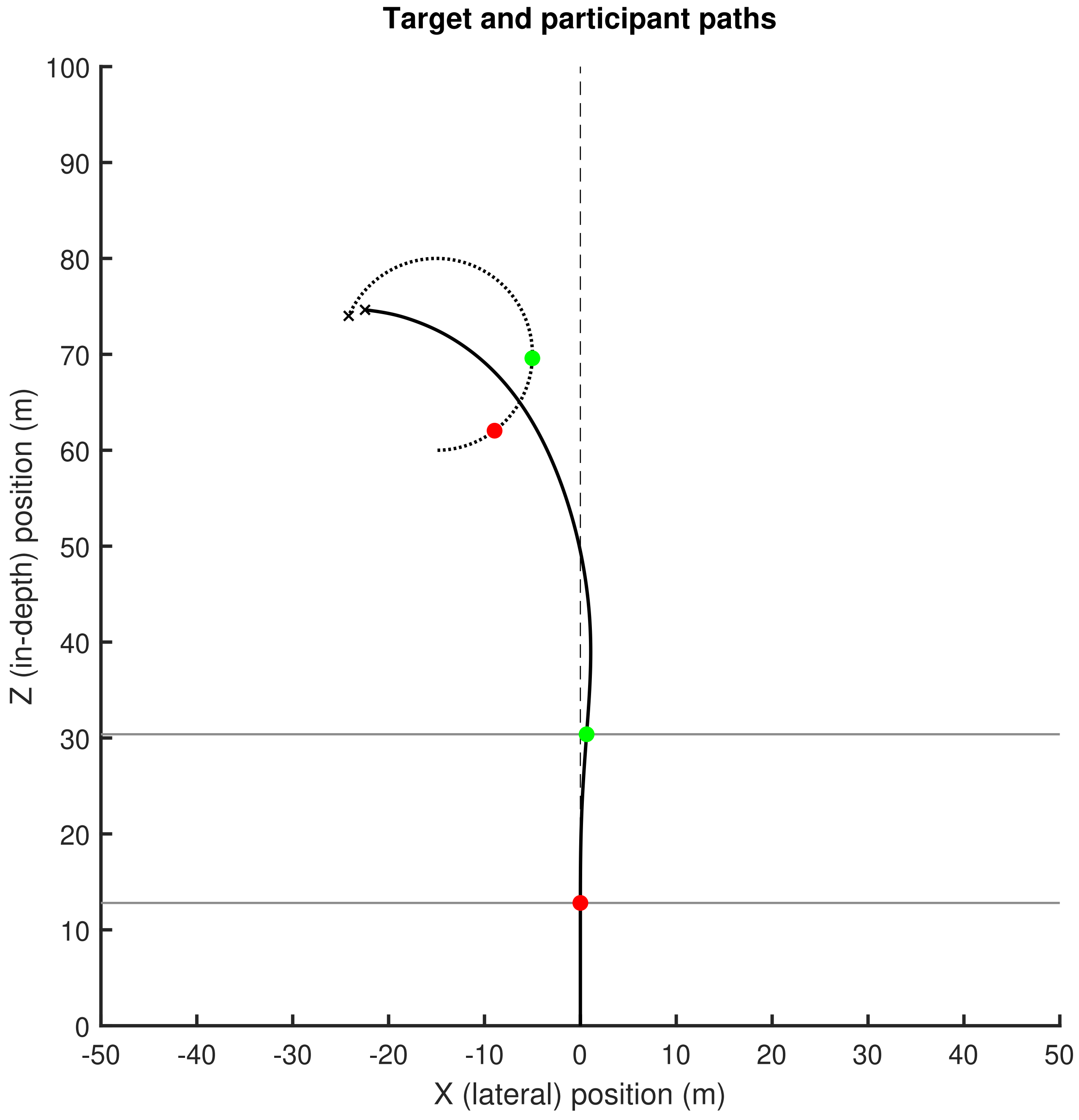

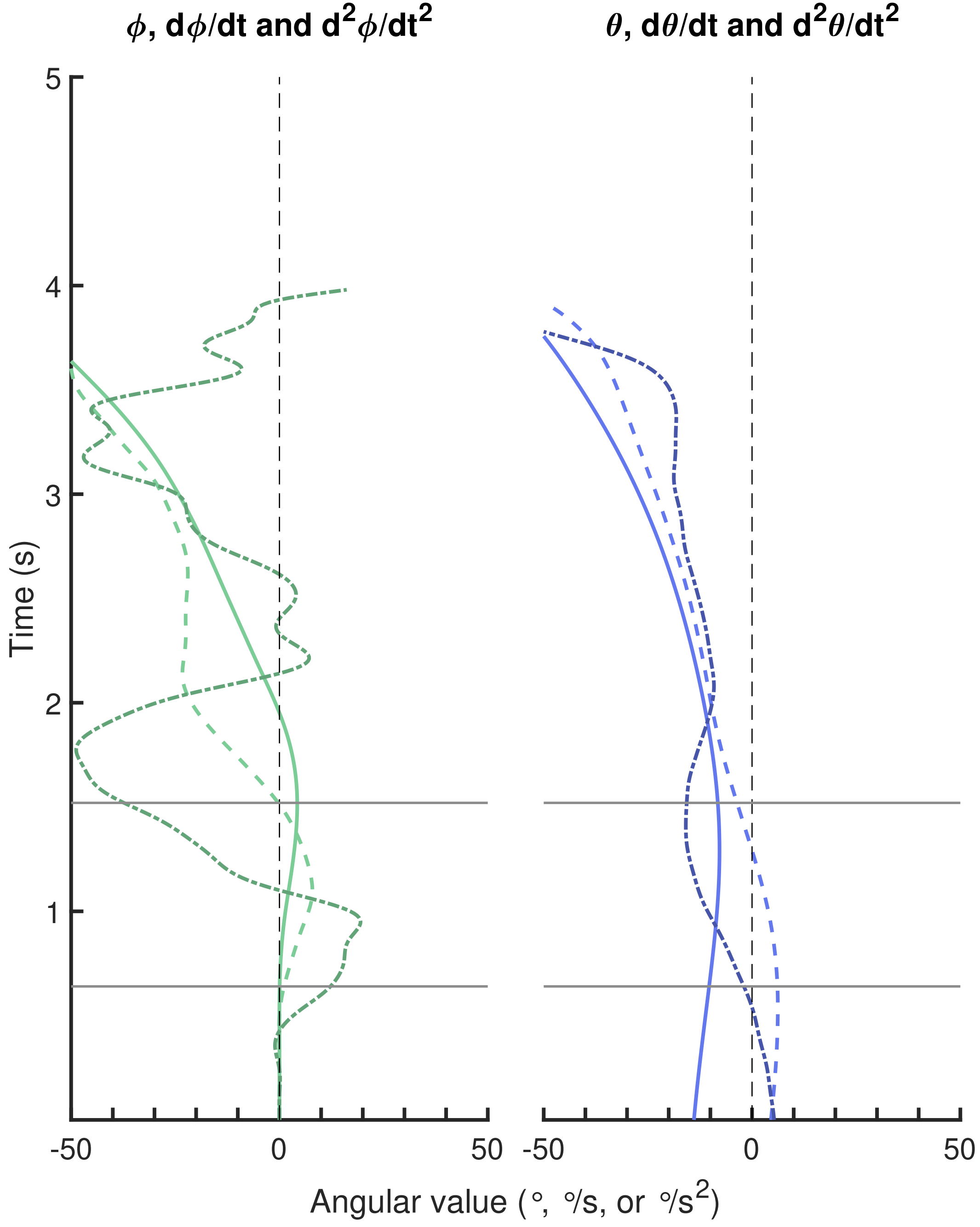

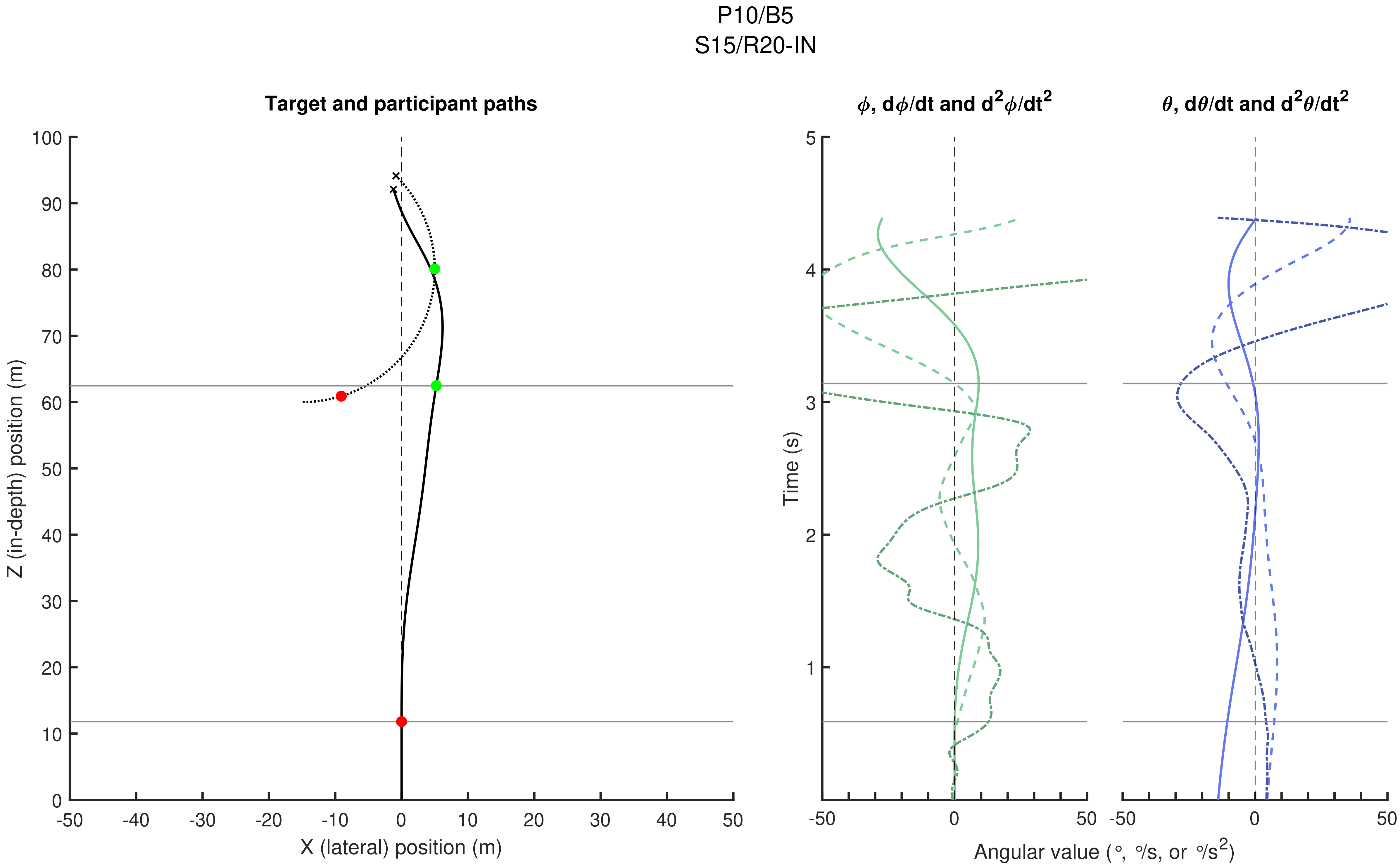
P10/B5
S15/R20-IN
Target and participant paths
ϕ, dϕ/dt and d$^2\phi$/dt^2
θ, dθ/dt and d$^2\theta$/dt^2
Z (in-depth) position (m)
X (lateral) position (m)
Time (s)
Angular value (°, °/s, or °/s^2)

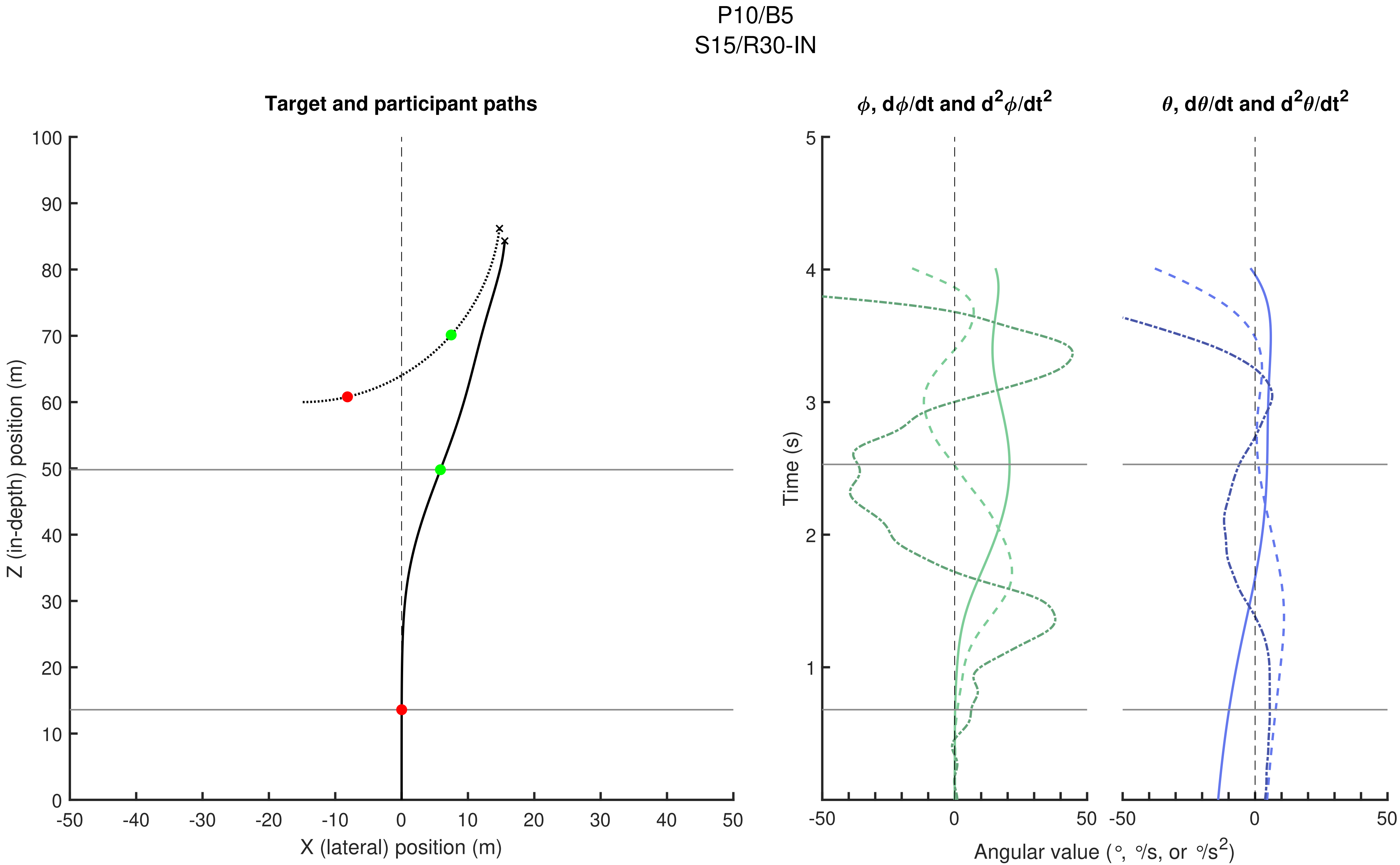
P10/B5
S15/R30-IN
Target and participant paths
Z (in-depth) position (m)
X (lateral) position (m)
100
90
80
70
60
50
40
30
20
10
0
-50
-40
-30
-20
-10
0
10
20
30
40
50
ϕ, dϕ/dt and d$^2\phi$/dt^2
θ, dθ/dt and d$^2\theta$/dt^2
Time (s)
5
4
3
2
1
-50
0
50
-50
0
50
Angular value (°, °/s, or °/s^2)

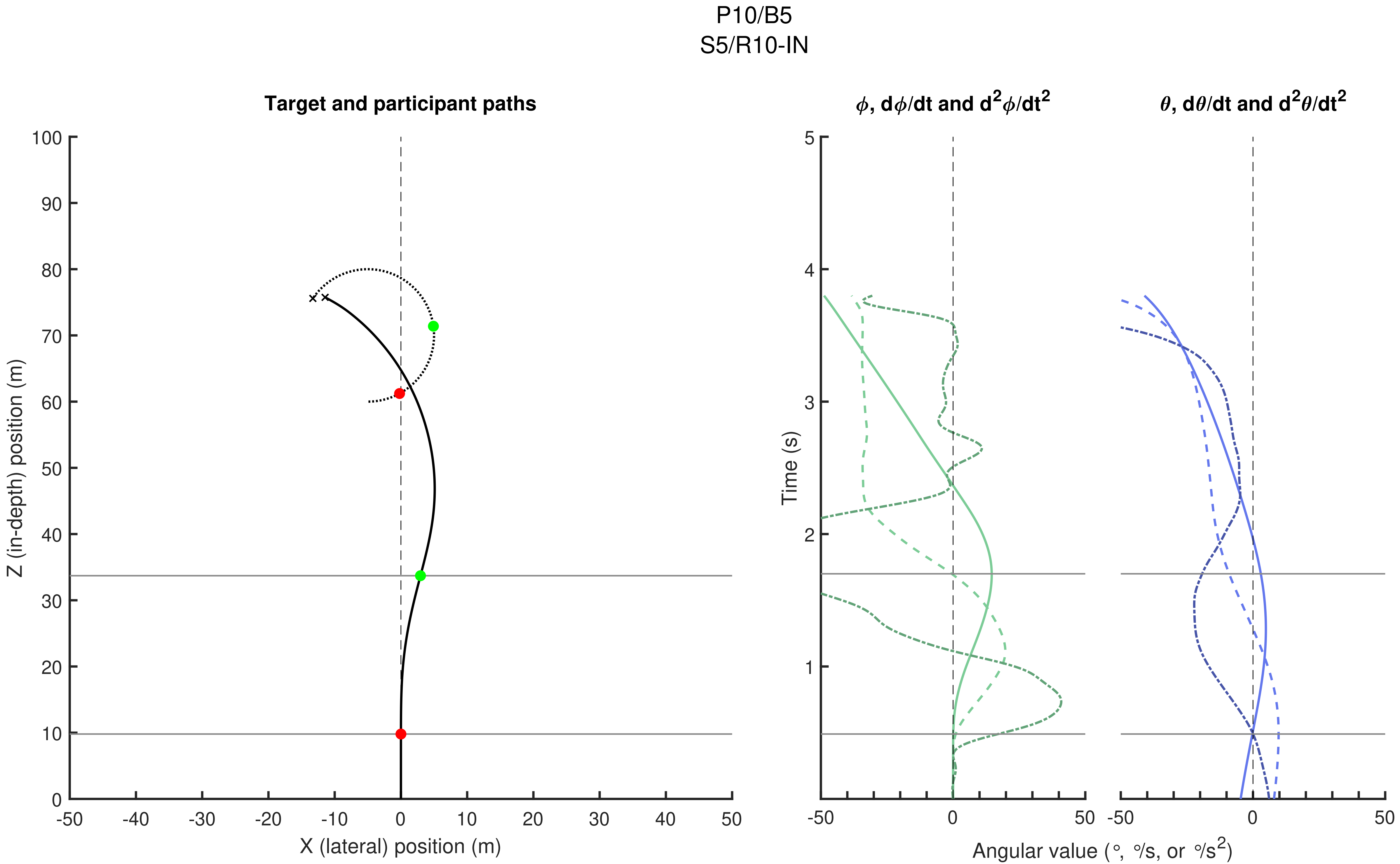
P10/B5
S5/R10-IN
Target and participant paths
Z (in-depth) position (m)
X (lateral) position (m)
ϕ, dϕ/dt and d²ϕ/dt²
θ, dθ/dt and d²θ/dt²
Time (s)
Angular value (°, °/s, or °/s²)

P10/B5
S5/R10-OUT

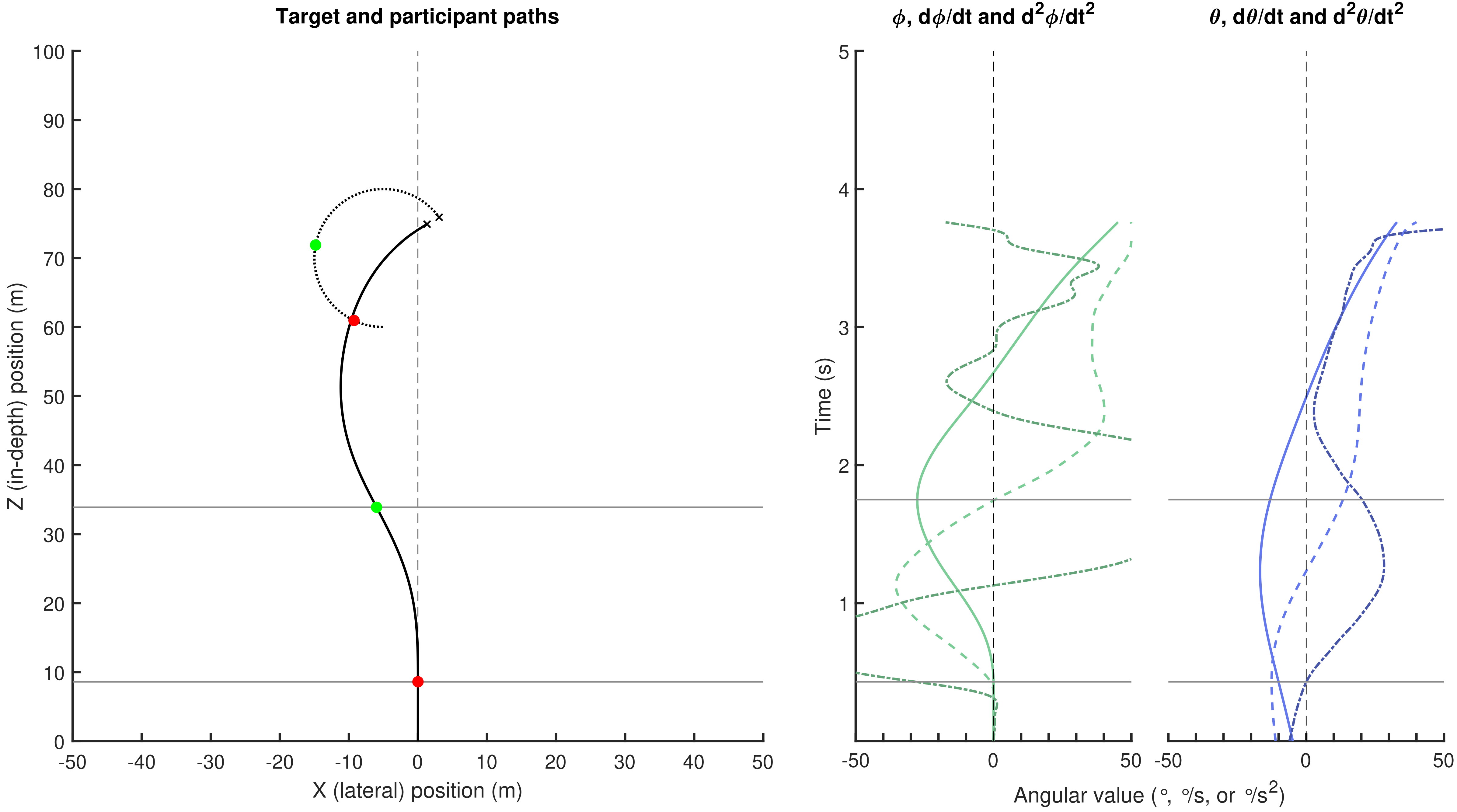

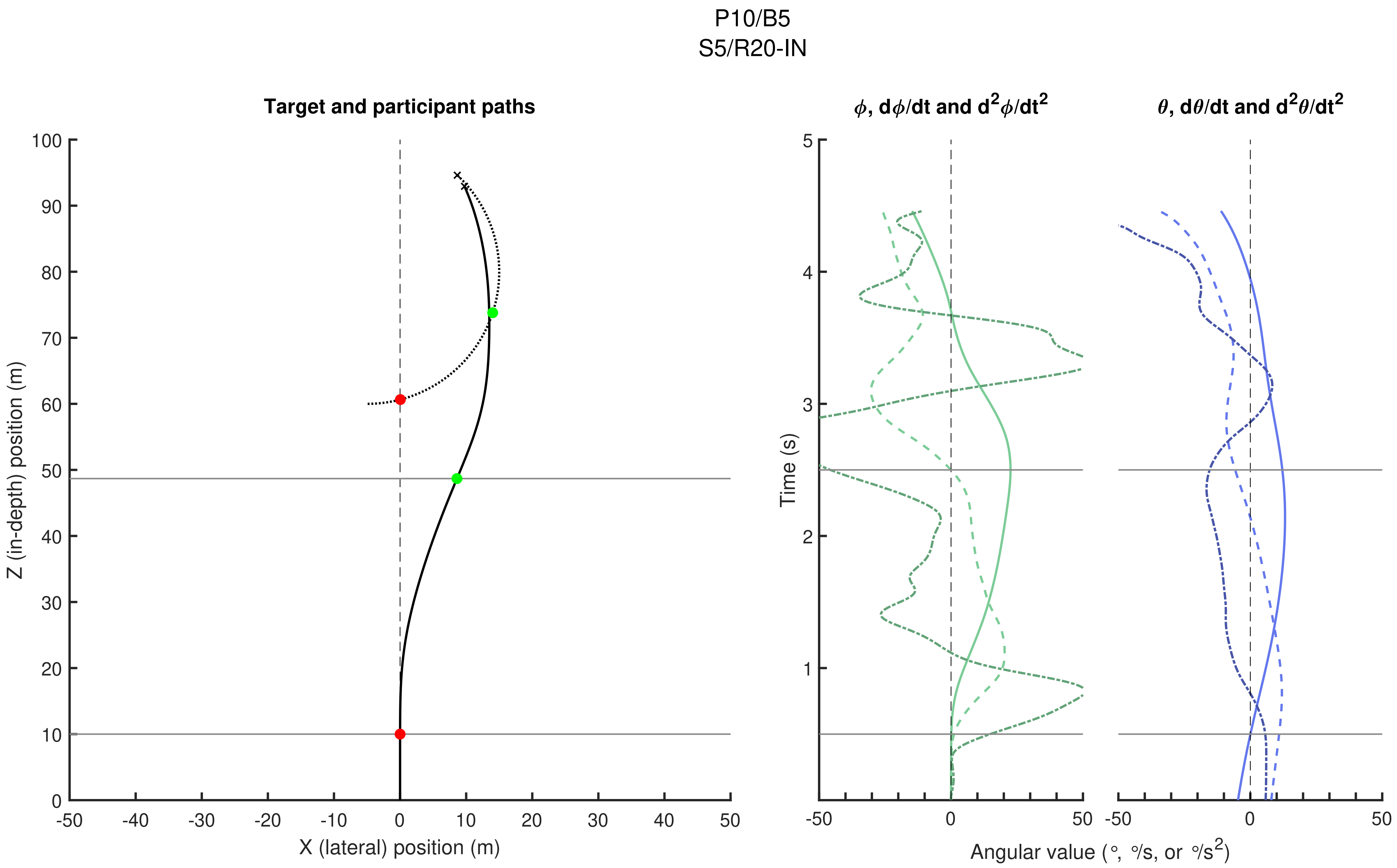
P10/B5
S5/R20-IN
Target and participant paths
Z (in-depth) position (m)
X (lateral) position (m)
φ, dφ/dt and d²φ/dt²
θ, dθ/dt and d²θ/dt²
Time (s)
Angular value (°, °/s, or °/s²)

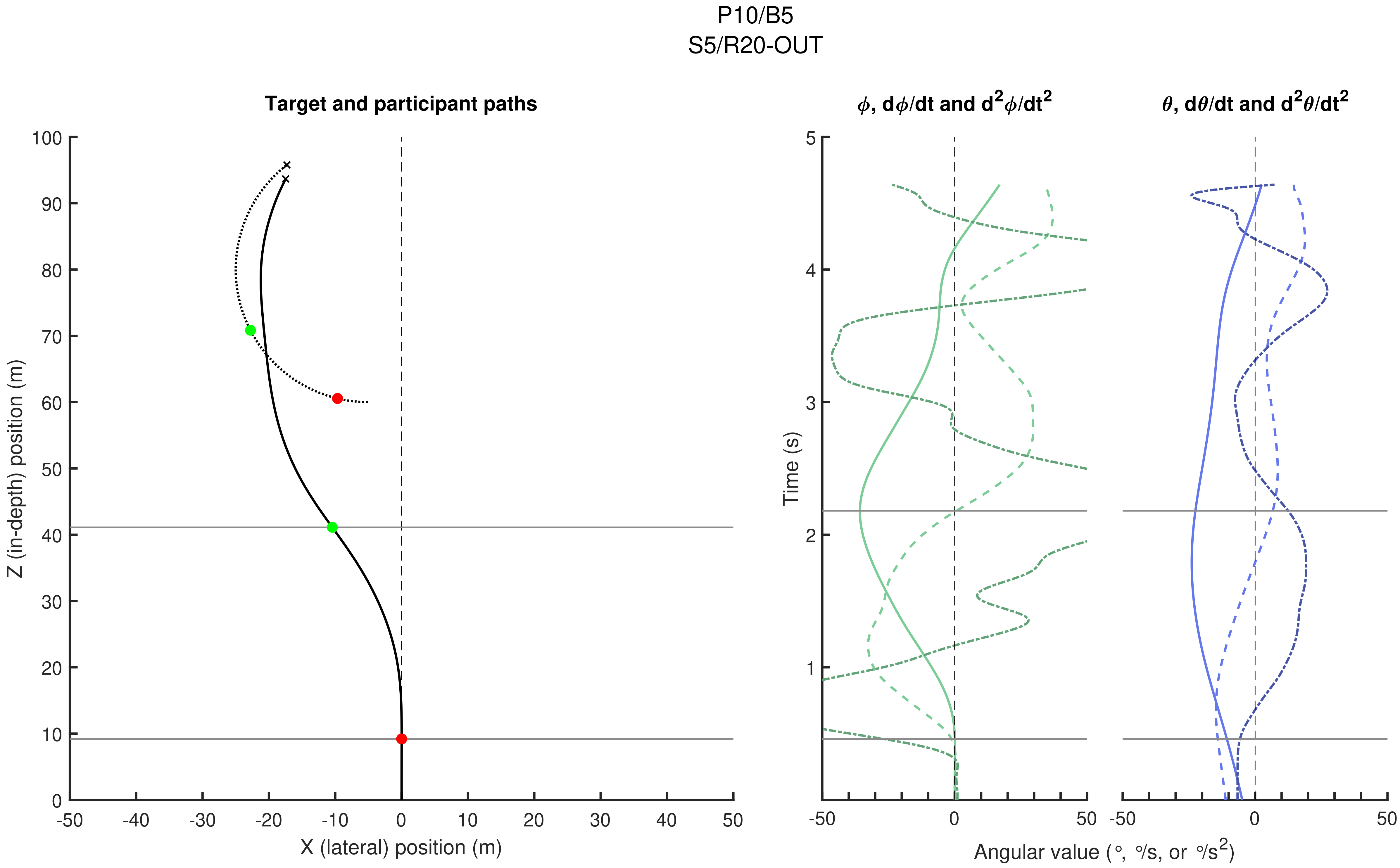
P10/B5
S5/R20-OUT
Target and participant paths
Z (in-depth) position (m)
X (lateral) position (m)
100
90
80
70
60
50
40
30
20
10
0
-50
-40
-30
-20
-10
0
10
20
30
40
50
φ, dφ/dt and d²φ/dt²
θ, dθ/dt and d²θ/dt²
Time (s)
5
4
3
2
1
-50
0
50
Angular value (°, °/s, or °/s²)

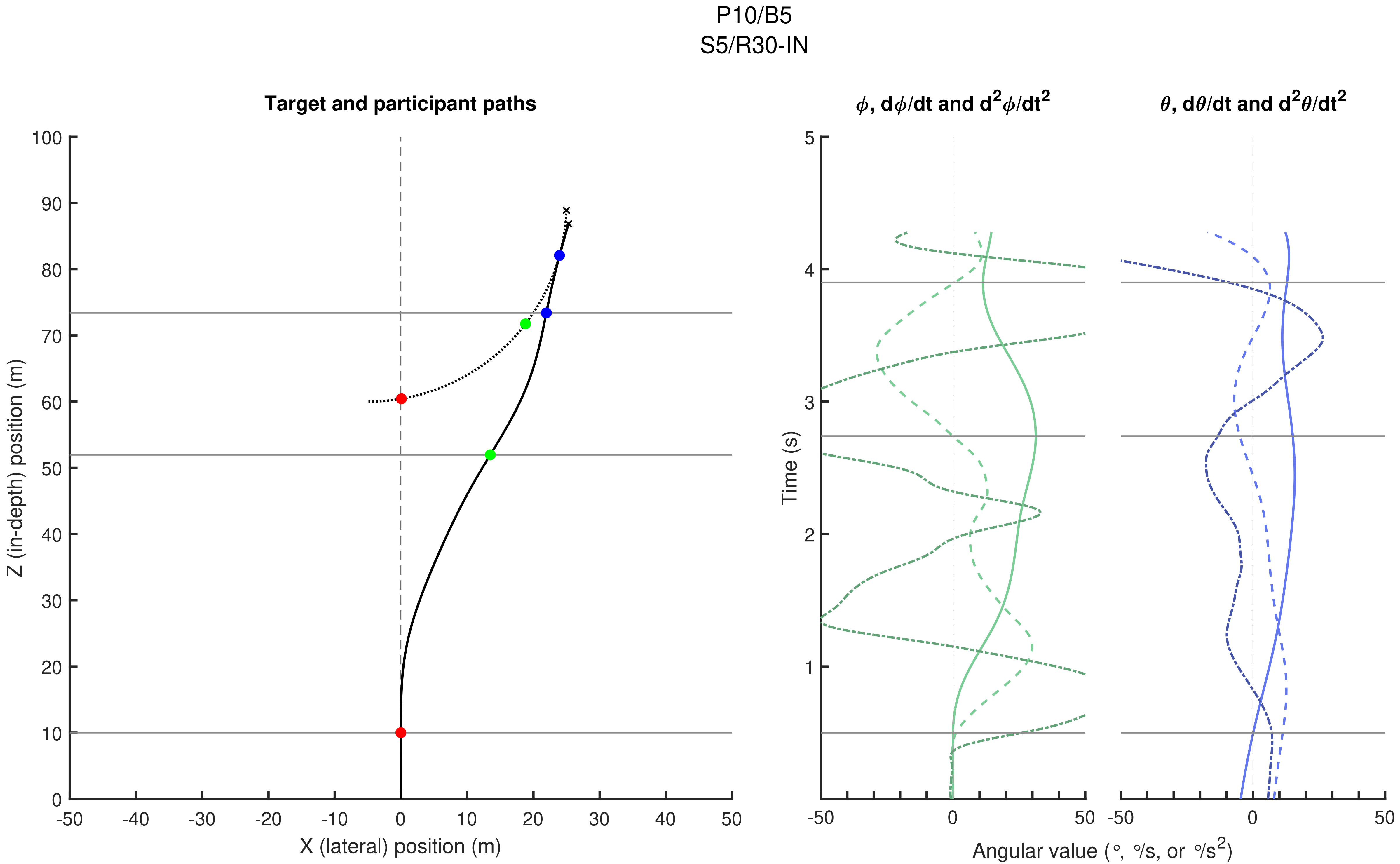

P10/B5
S5/R30-IN
Target and participant paths
Z (in-depth) position (m)
X (lateral) position (m)
100
90
80
70
60
50
40
30
20
10
0
-50
-40
-30
-20
-10
0
10
20
30
40
50
ϕ, dϕ/dt and d²ϕ/dt²
θ, dθ/dt and d²θ/dt²
Time (s)
5
4
3
2
1
-50
0
50
-50
0
50
Angular value (°, °/s, or °/s²)

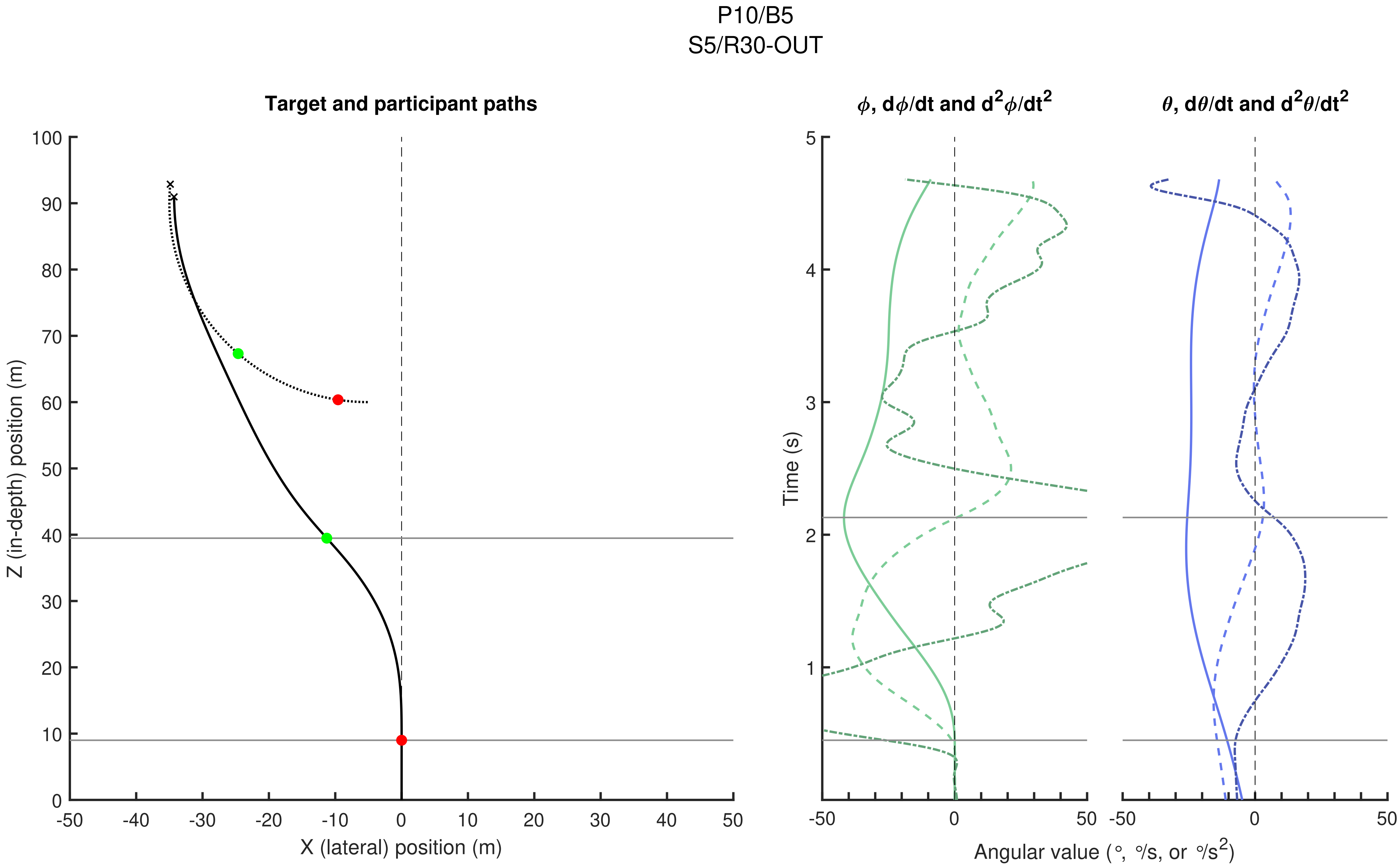
P10/B5
S5/R30-OUT
Target and participant paths
Z (in-depth) position (m)
X (lateral) position (m)
ϕ, dϕ/dt and d$^2\phi$/dt^2
θ, dθ/dt and d$^2\theta$/dt^2
Time (s)
Angular value (°, °/s, or °/s^2)

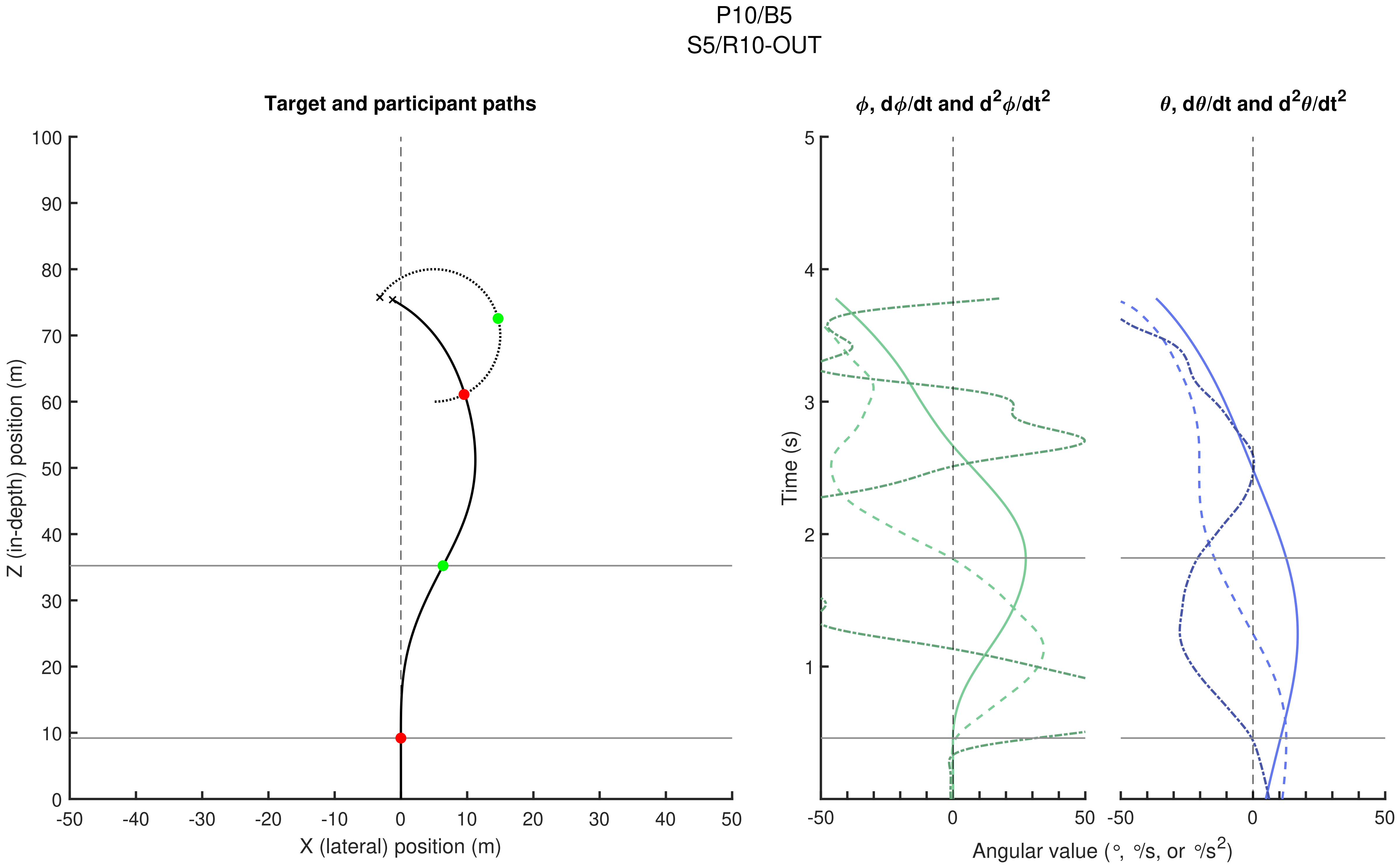
P10/B5
S5/R10-OUT
Target and participant paths
Z (in-depth) position (m)
X (lateral) position (m)
φ, dφ/dt and d²φ/dt²
θ, dθ/dt and d²θ/dt²
Time (s)
Angular value (°, °/s, or °/s²)

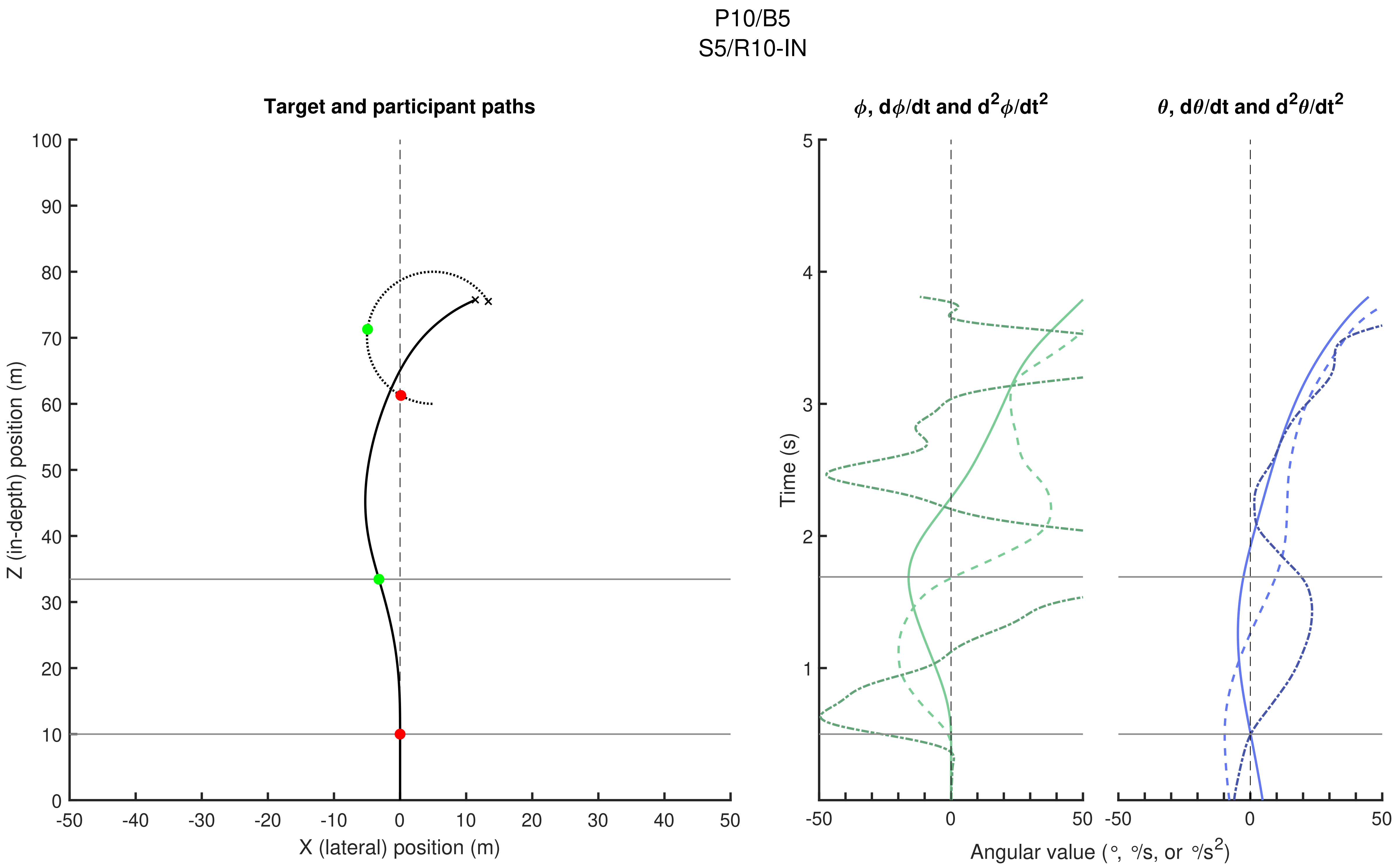
P10/B5
S5/R10-IN
Target and participant paths
Z (in-depth) position (m)
X (lateral) position (m)
ϕ, dϕ/dt and d$^2\phi$/dt^2
θ, dθ/dt and d$^2\theta$/dt^2
Time (s)
Angular value (°, °/s, or °/s^2)

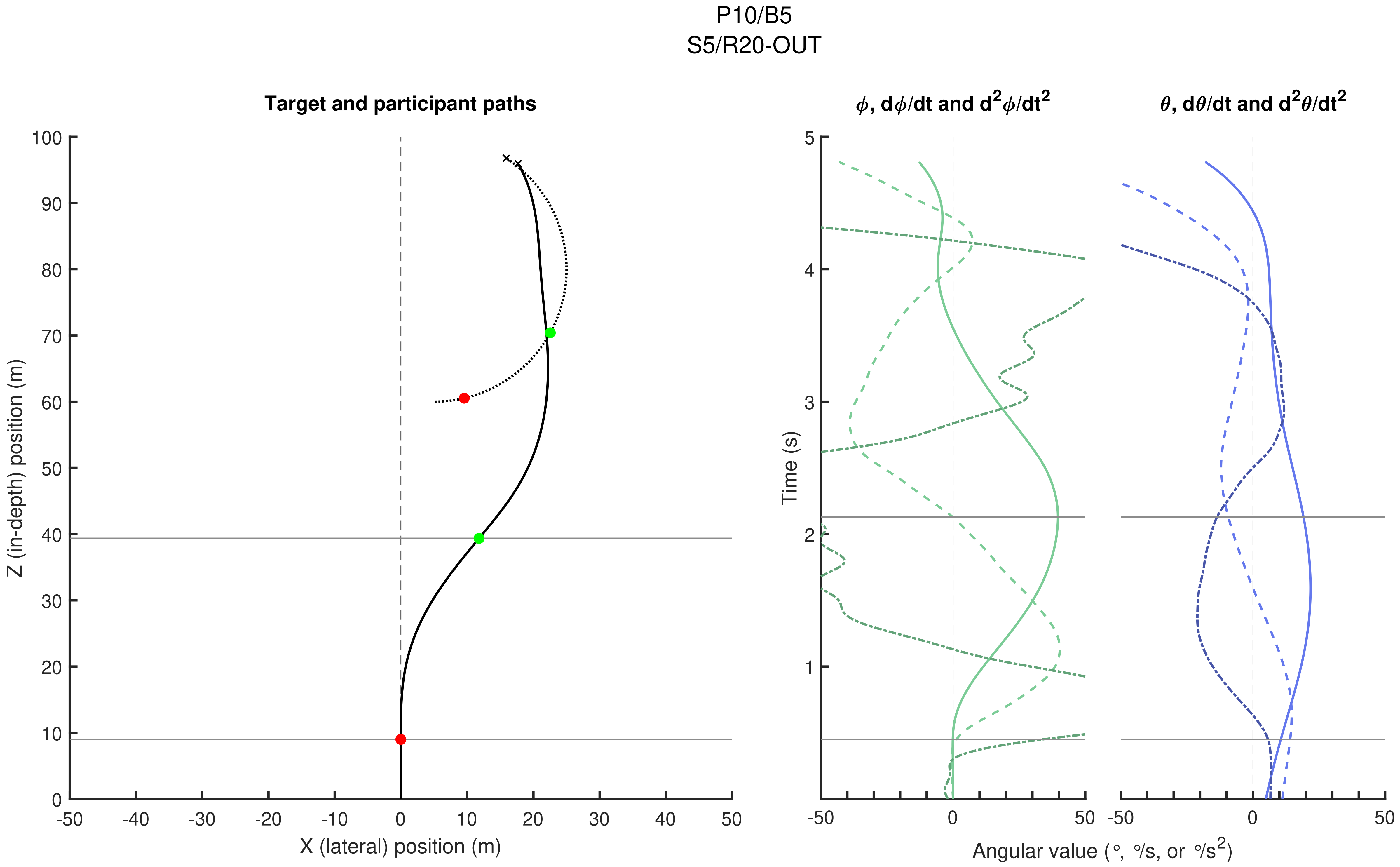
P10/B5
S5/R20-OUT
Target and participant paths
ϕ, dϕ/dt and d$^2\phi$/dt^2
θ, dθ/dt and d$^2\theta$/dt^2
Z (in-depth) position (m)
X (lateral) position (m)
Time (s)
Angular value (°, °/s, or °/s^2)

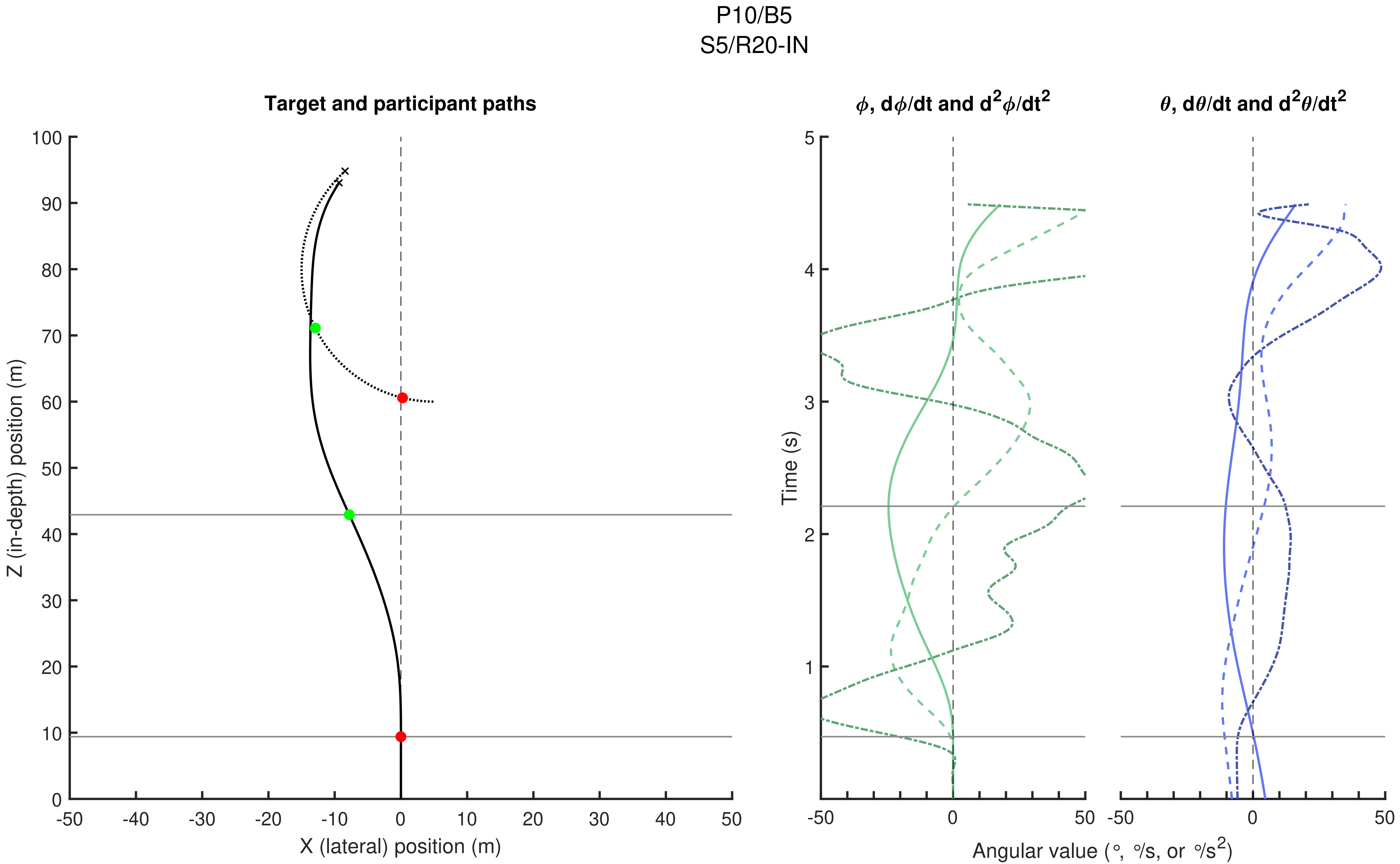
P10/B5
S5/R20-IN
Target and participant paths
Z (in-depth) position (m)
X (lateral) position (m)
ϕ, dϕ/dt and d$^2\phi$/dt^2
θ, dθ/dt and d$^2\theta$/dt^2
Time (s)
Angular value (°, °/s, or °/s^2)

P10/B5
S5/R30-OUT

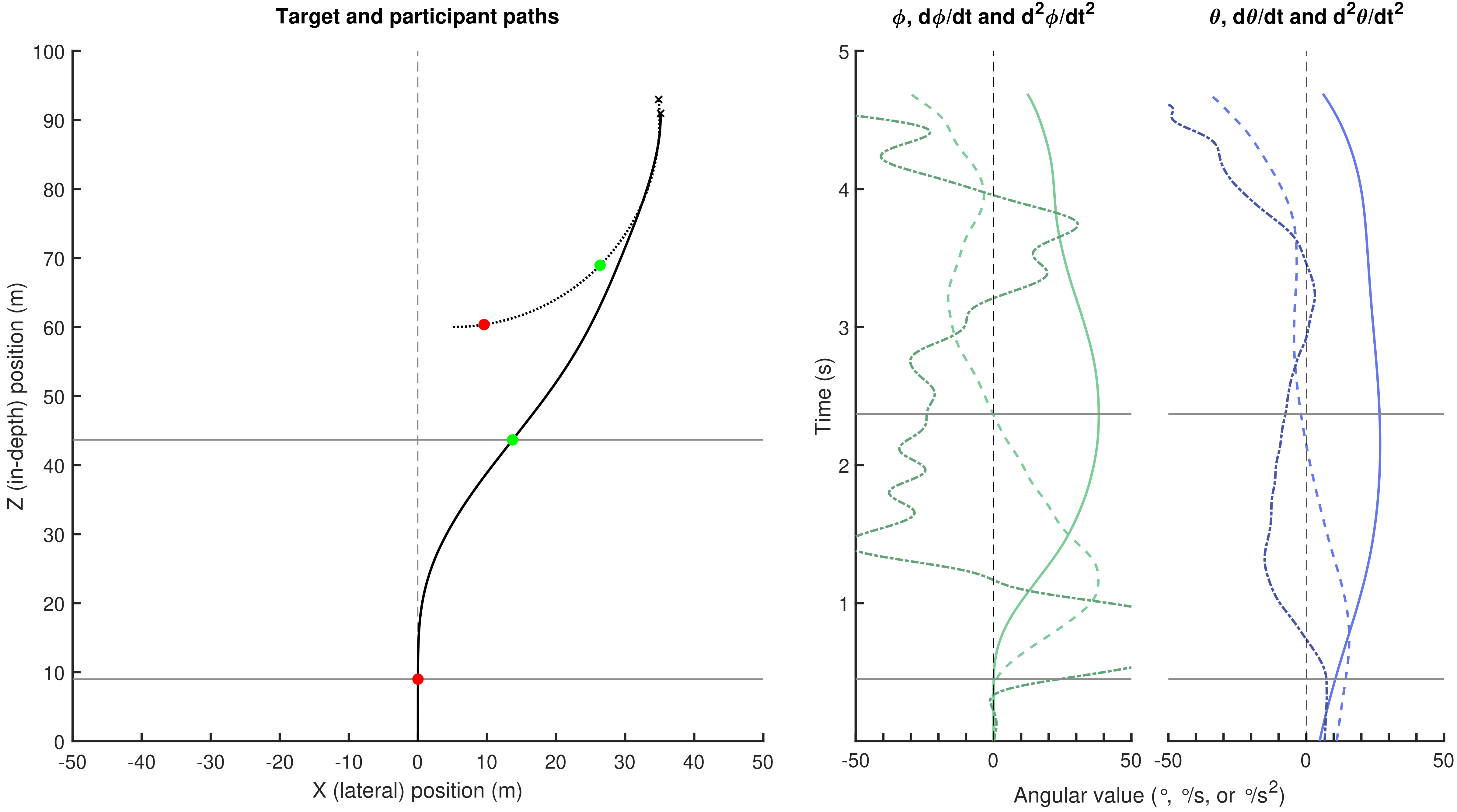

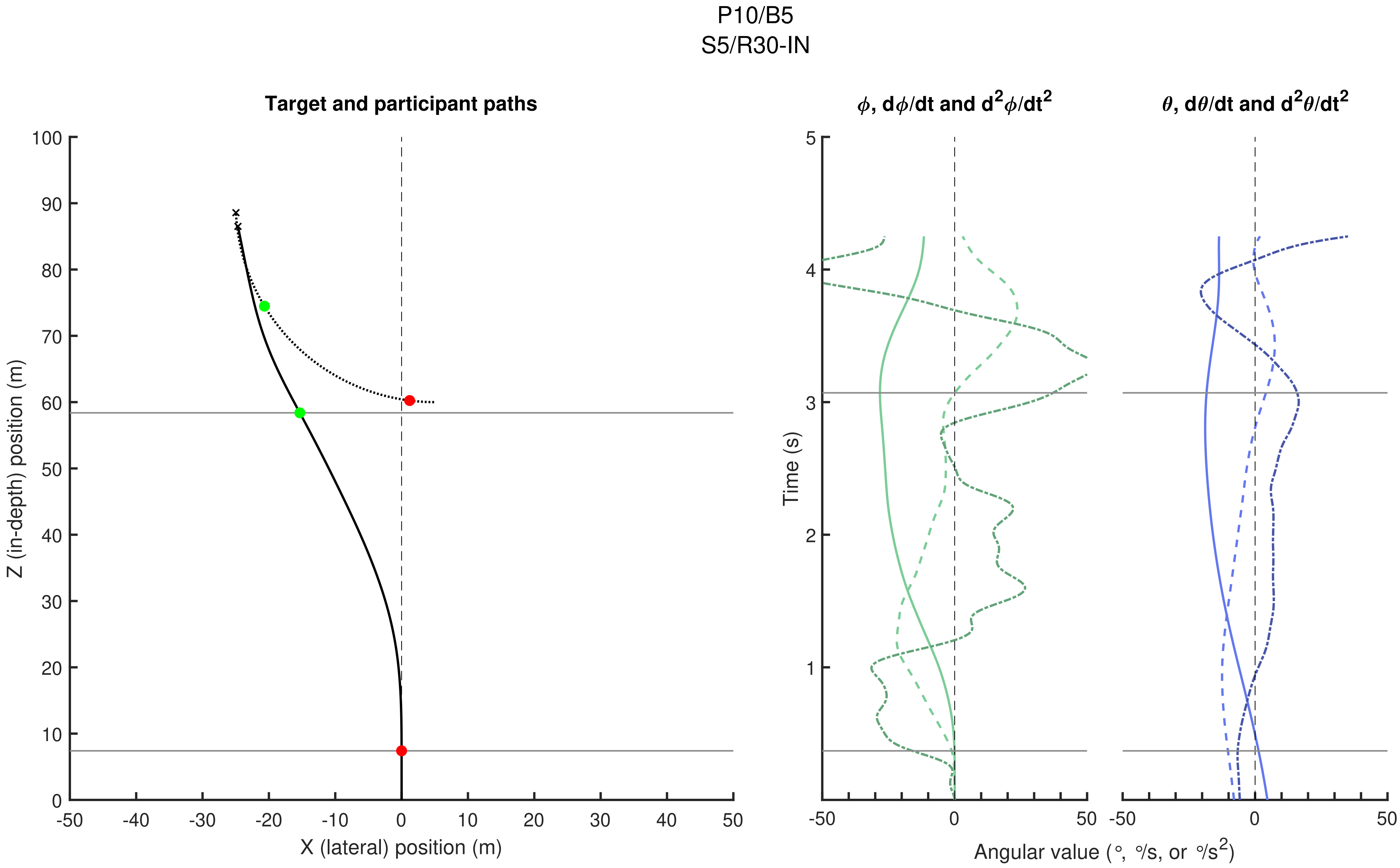
P10/B5
S5/R30-IN
Target and participant paths
Z (in-depth) position (m)
X (lateral) position (m)
100
90
80
70
60
50
40
30
20
10
0
-50
-40
-30
-20
-10
0
10
20
30
40
50
ϕ, dϕ/dt and d²ϕ/dt²
θ, dθ/dt and d²θ/dt²
Time (s)
5
4
3
2
1
-50
0
50
-50
0
50
Angular value (°, °/s, or °/s²)

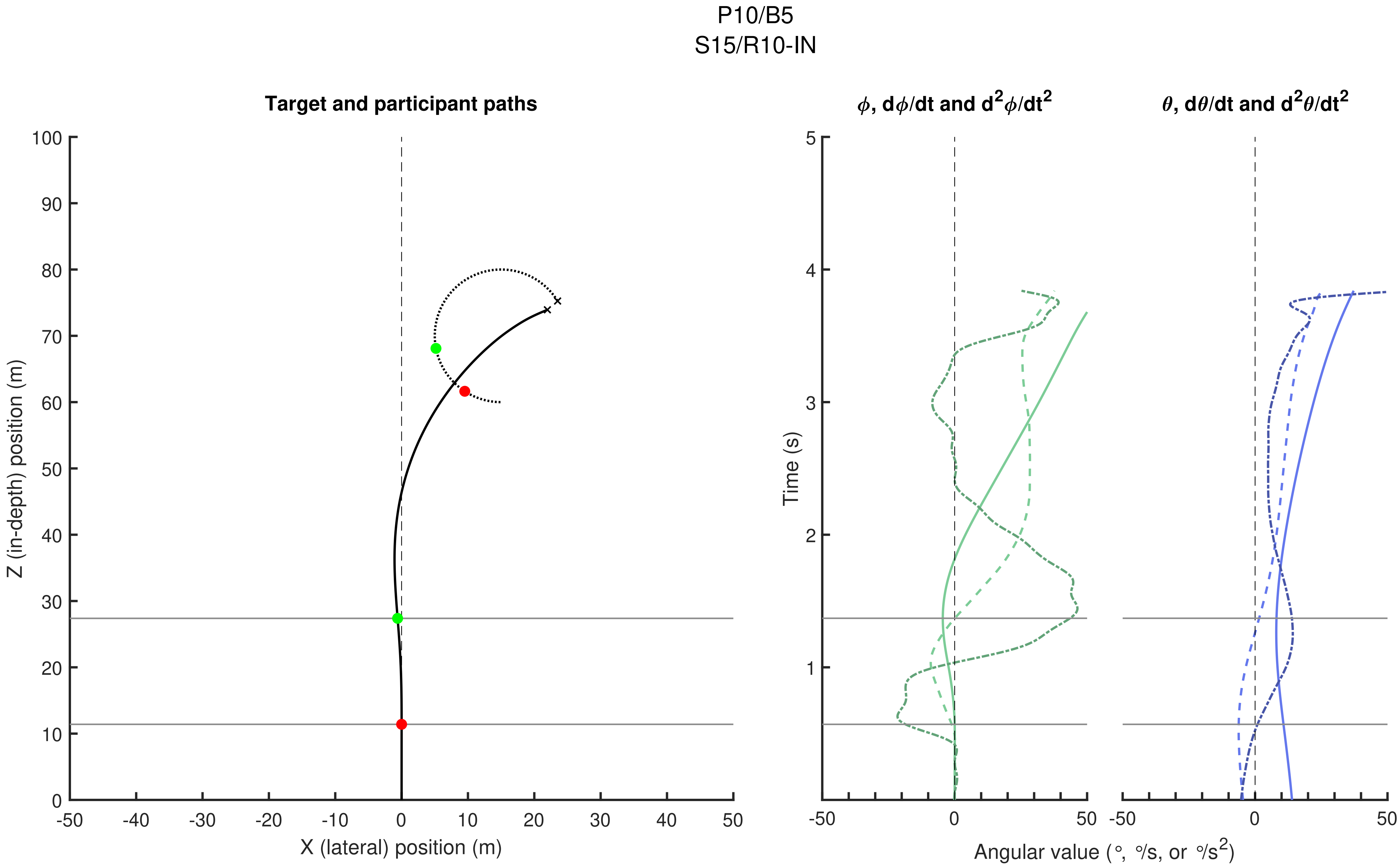

P10/B5
S15/R10-IN
Target and participant paths
Z (in-depth) position (m)
X (lateral) position (m)
100
90
80
70
60
50
40
30
20
10
0
-50
-40
-30
-20
-10
0
10
20
30
40
50
ϕ, dϕ/dt and d$^2\phi$/dt^2
θ, dθ/dt and d$^2\theta$/dt^2
Time (s)
5
4
3
2
1
-50
0
50
-50
0
50
Angular value (°, °/s, or °/s^2)

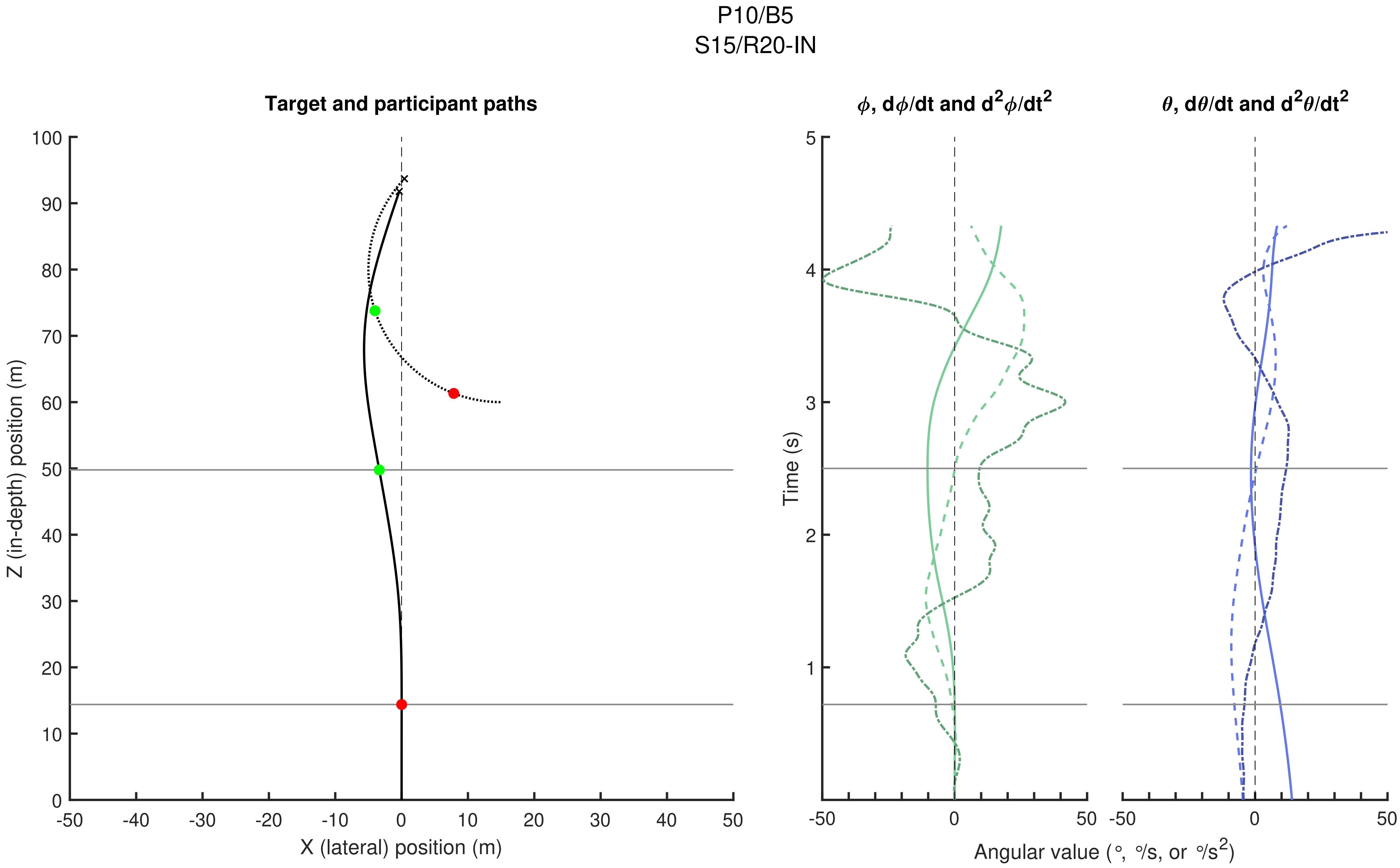

P10/B5
S15/R20-IN
Target and participant paths
Z (in-depth) position (m)
X (lateral) position (m)
ϕ, dϕ/dt and d²ϕ/dt²
θ, dθ/dt and d²θ/dt²
Time (s)
Angular value (°, °/s, or °/s²)

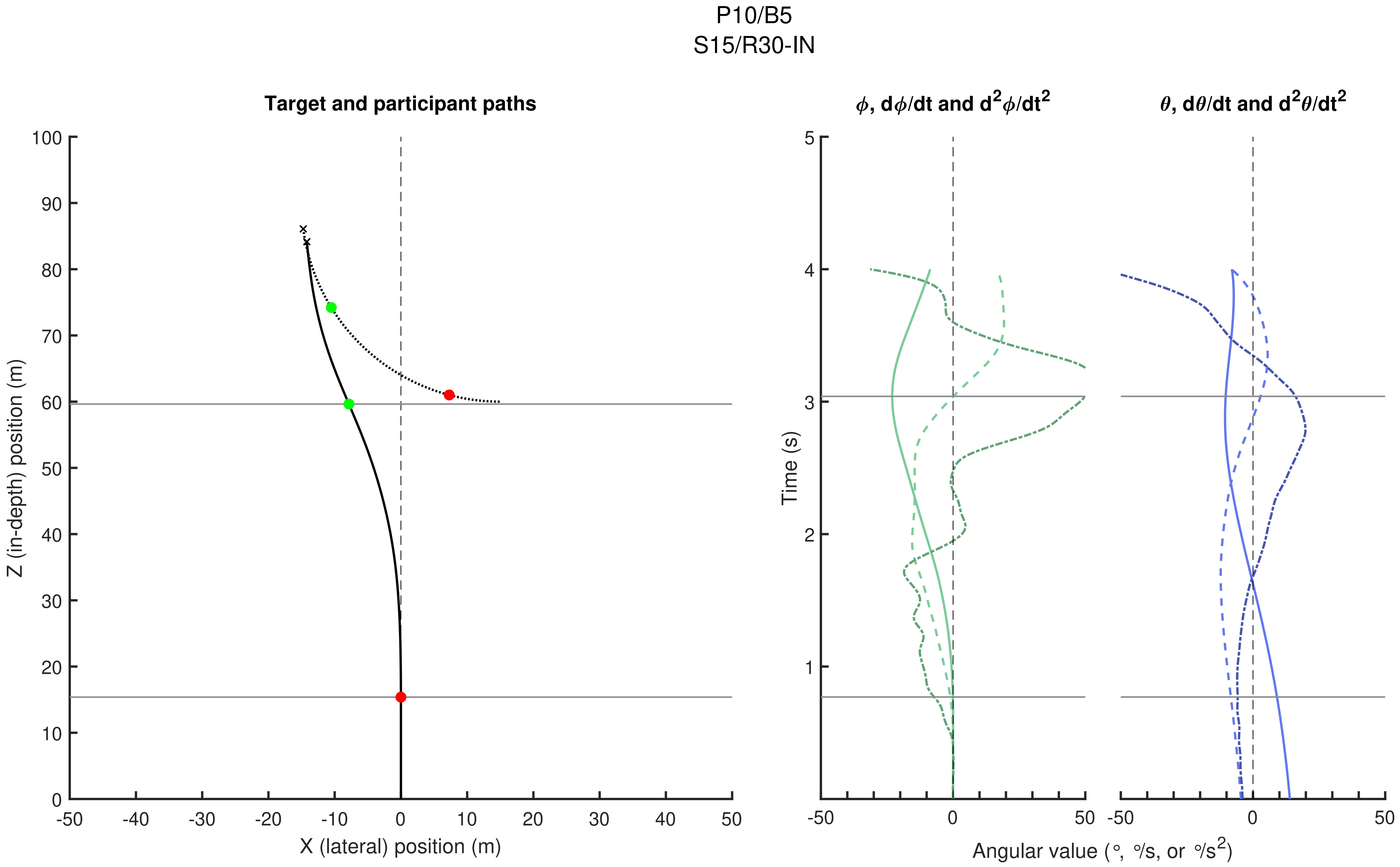
P10/B5
S15/R30-IN
Target and participant paths
Z (in-depth) position (m)
X (lateral) position (m)
ϕ, dϕ/dt and d$^2\phi$/dt^2
θ, dθ/dt and d$^2\theta$/dt^2
Time (s)
Angular value (°, °/s, or °/s^2)

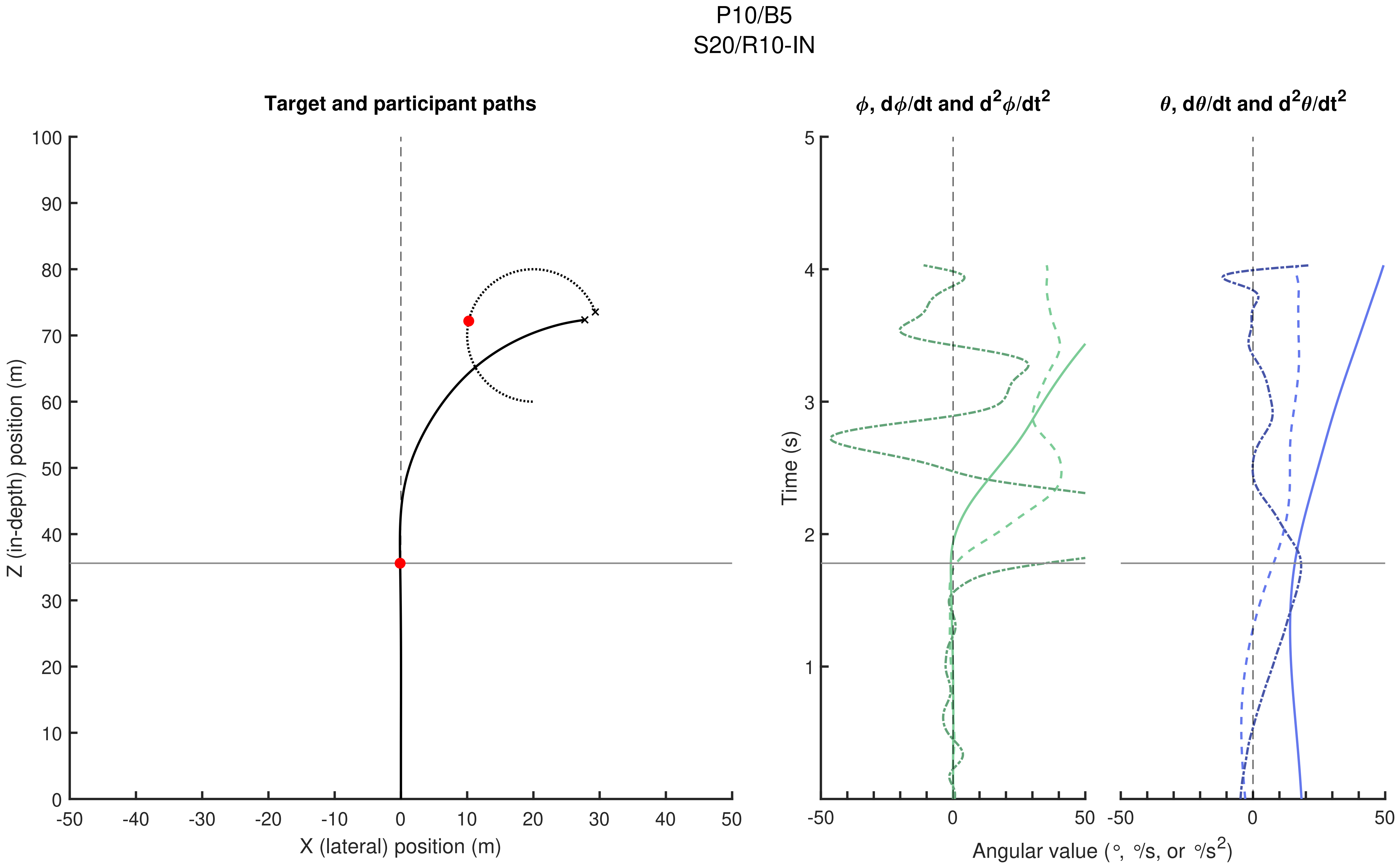
P10/B5
S20/R10-IN
Target and participant paths
Z (in-depth) position (m)
X (lateral) position (m)
100
90
80
70
60
50
40
30
20
10
0
-50
-40
-30
-20
-10
0
10
20
30
40
50
ϕ, dϕ/dt and d$^2\phi$/dt^2
θ, dθ/dt and d$^2\theta$/dt^2
Time (s)
5
4
3
2
1
-50
0
50
-50
0
50
Angular value (°, °/s, or °/s^2)

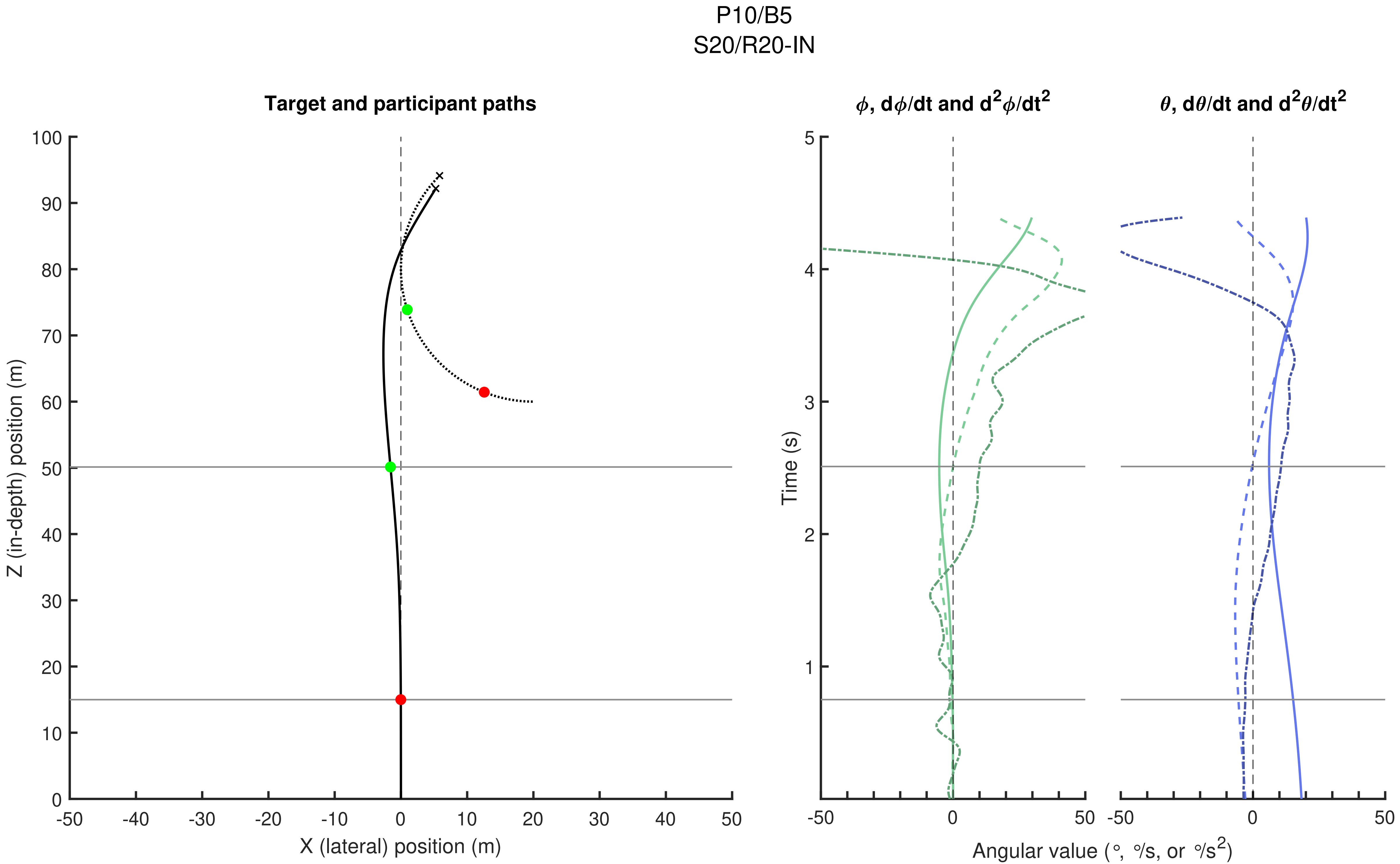
P10/B5
S20/R20-IN
Target and participant paths
Z (in-depth) position (m)
X (lateral) position (m)
φ, dφ/dt and d²φ/dt²
θ, dθ/dt and d²θ/dt²
Time (s)
Angular value (°, °/s, or °/s²)

P10/B5
S20/R30-IN

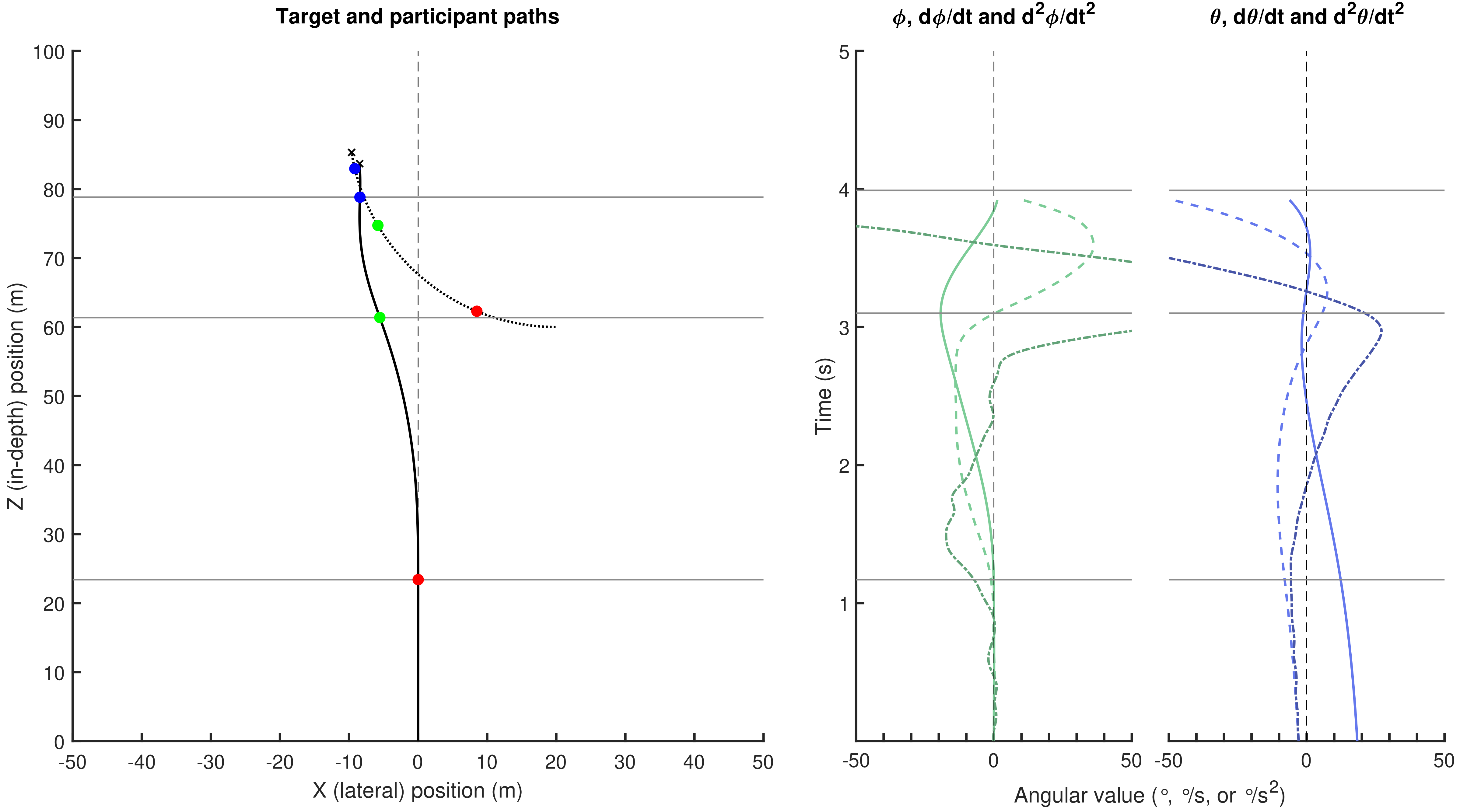

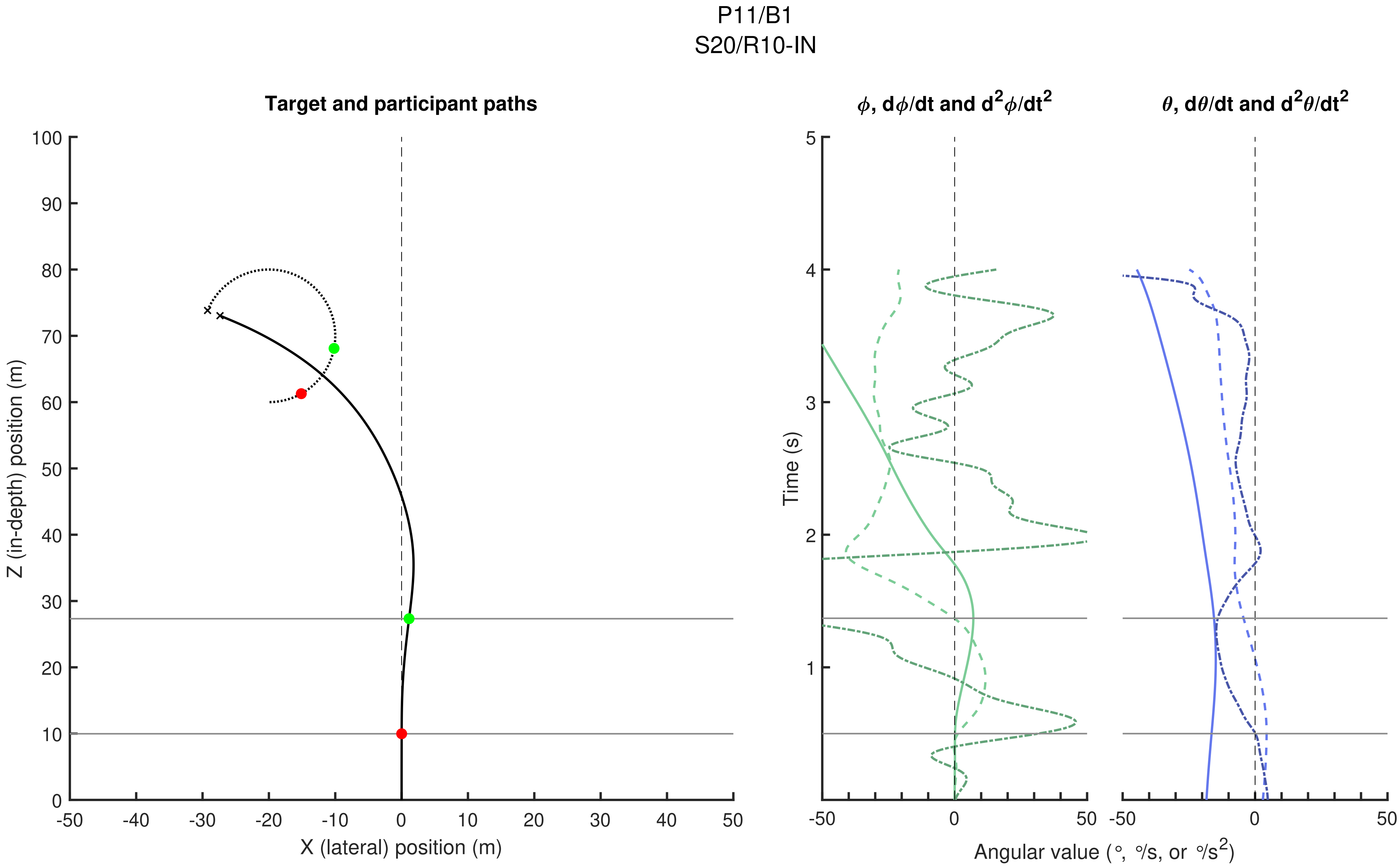
P11/B1
S20/R10-IN
Target and participant paths
ϕ, dϕ/dt and d$^2\phi$/dt^2
θ, dθ/dt and d$^2\theta$/dt^2
Z (in-depth) position (m)
X (lateral) position (m)
Time (s)
Angular value (°, °/s, or °/s^2)
100
90
80
70
60
50
40
30
20
10
0
-50
-40
-30
-20
-10
0
10
20
30
40
50
5
4
3
2
1
-50
0
50
-50
0
50

P11/B1
S20/R20-IN

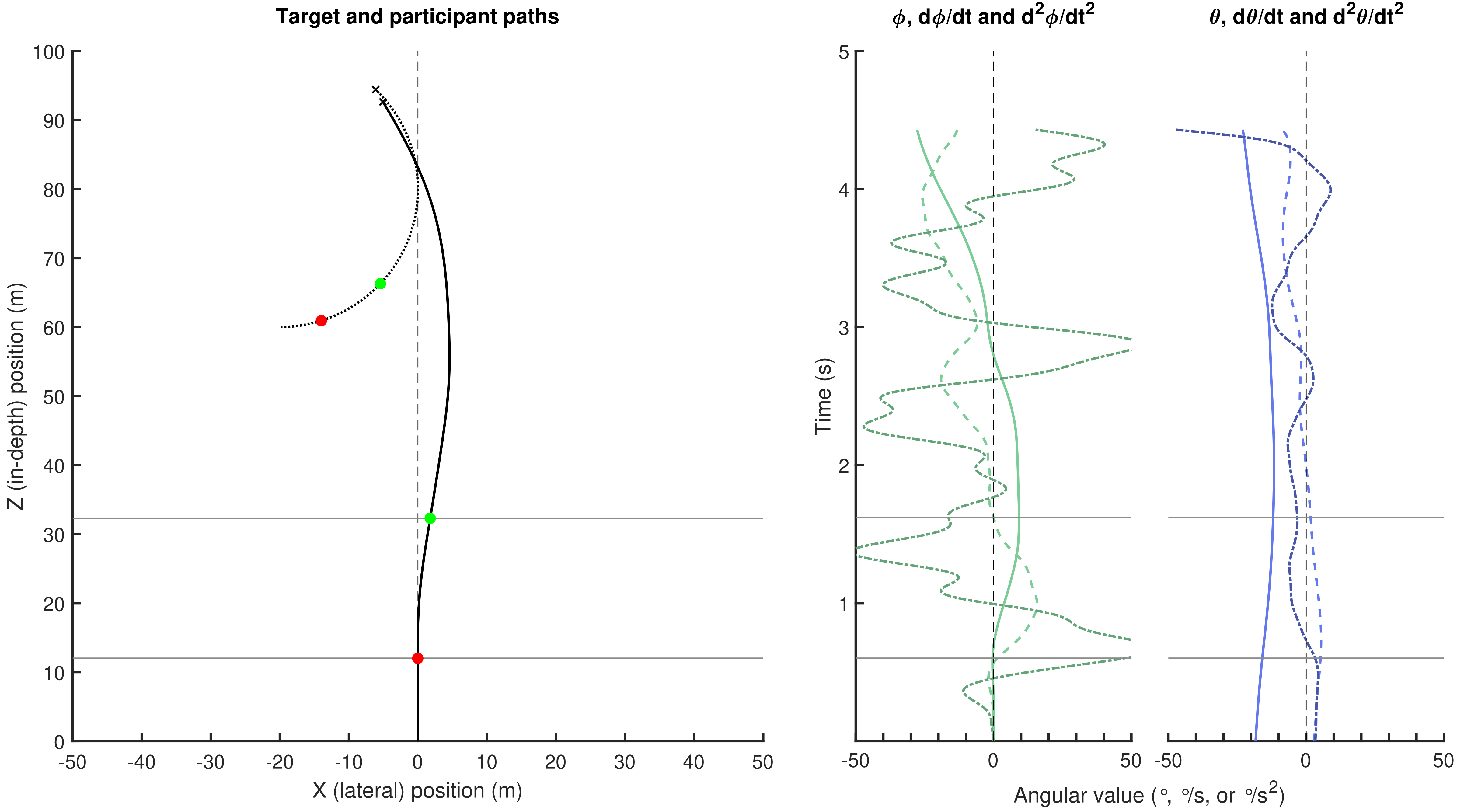

P11/B1
S20/R30-IN

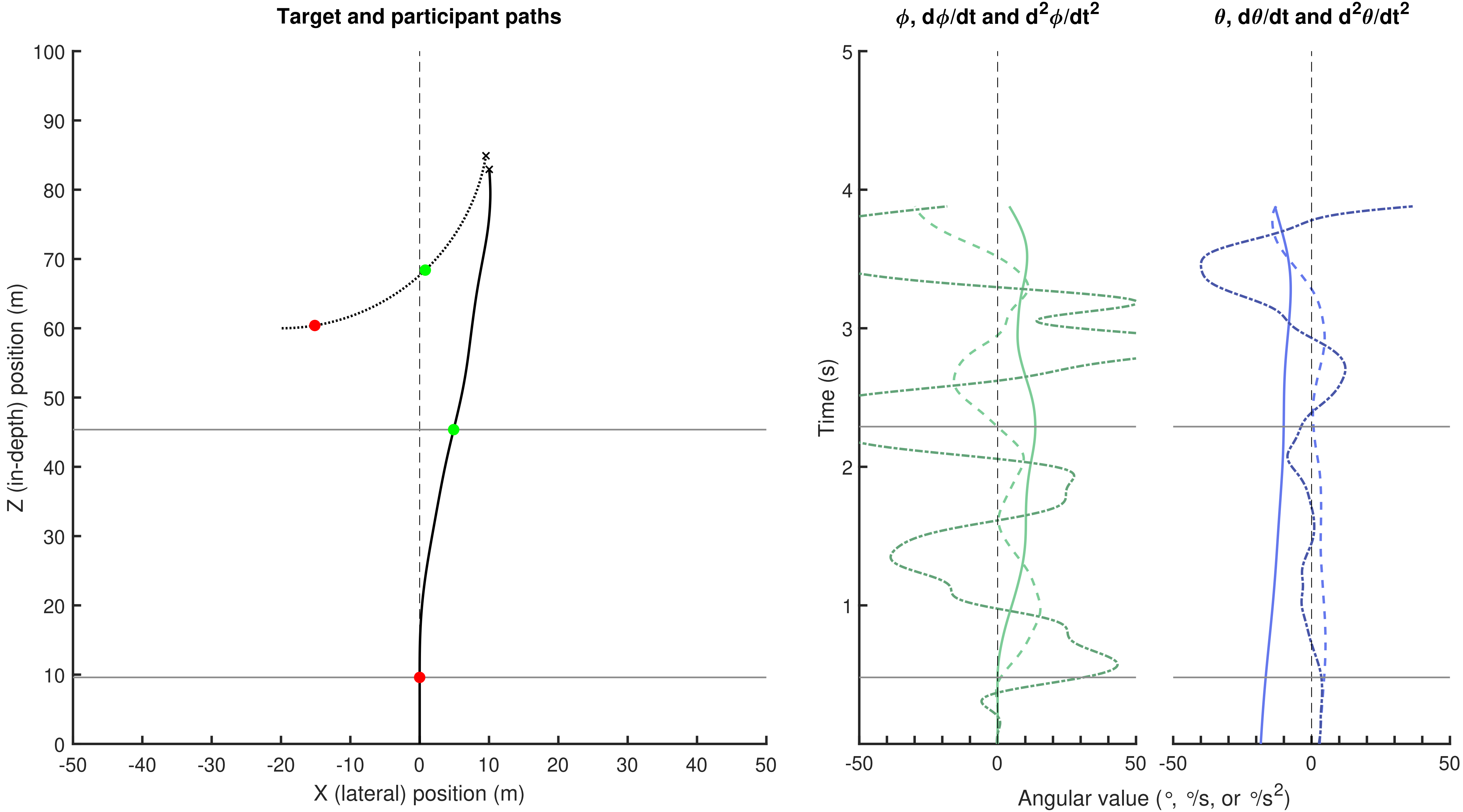

P11/B1
S15/R10-IN

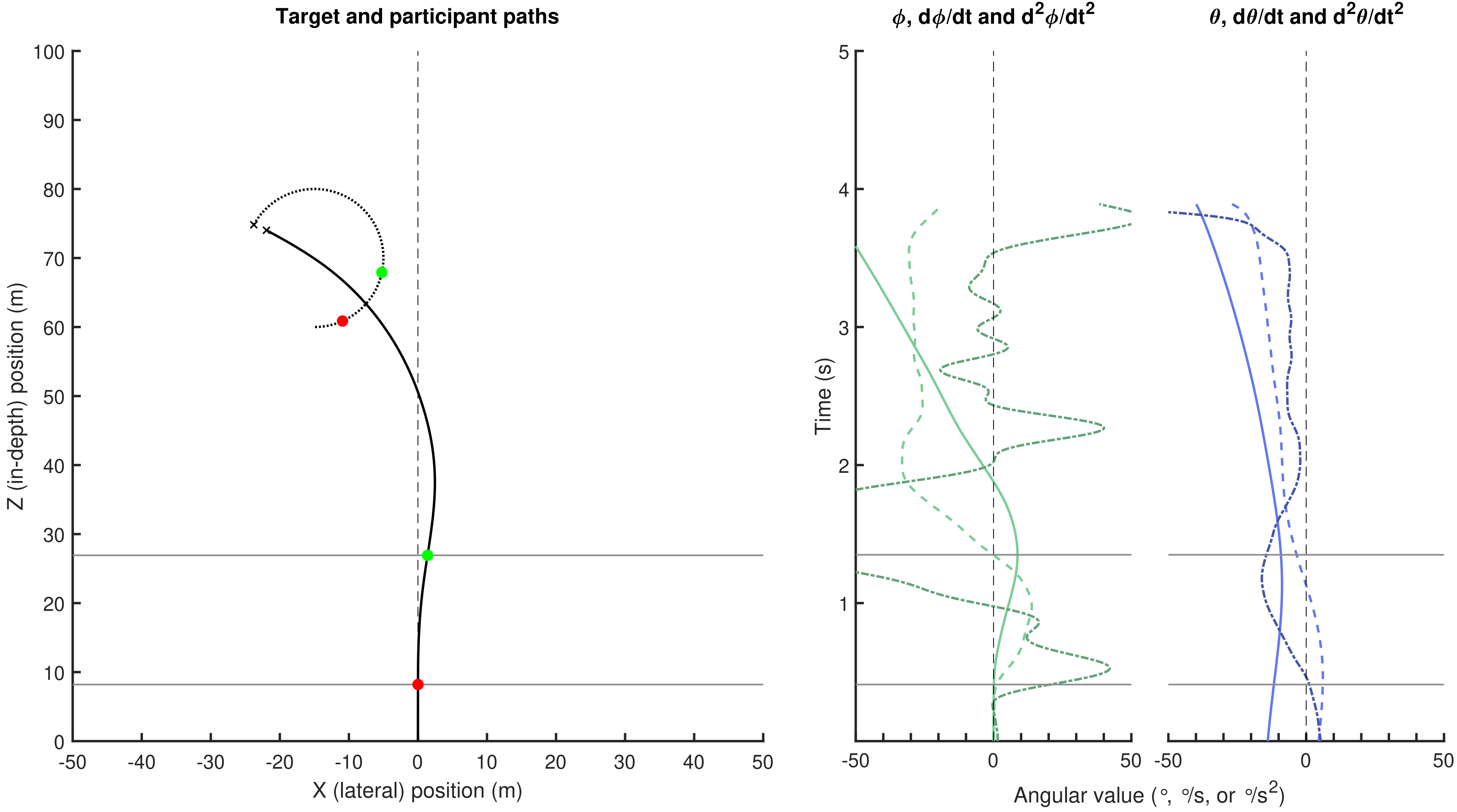

P11/B1
S15/R20-IN

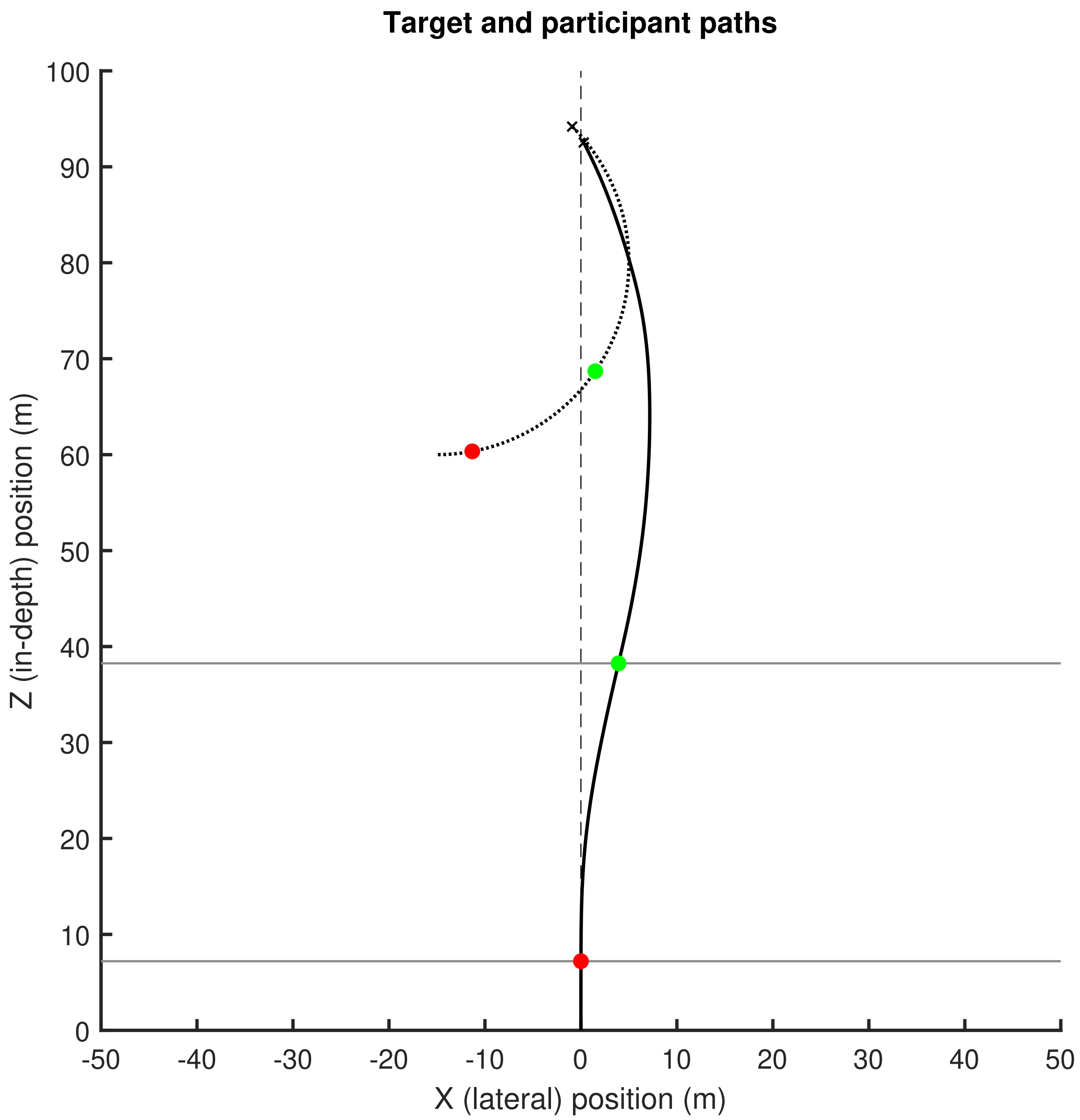

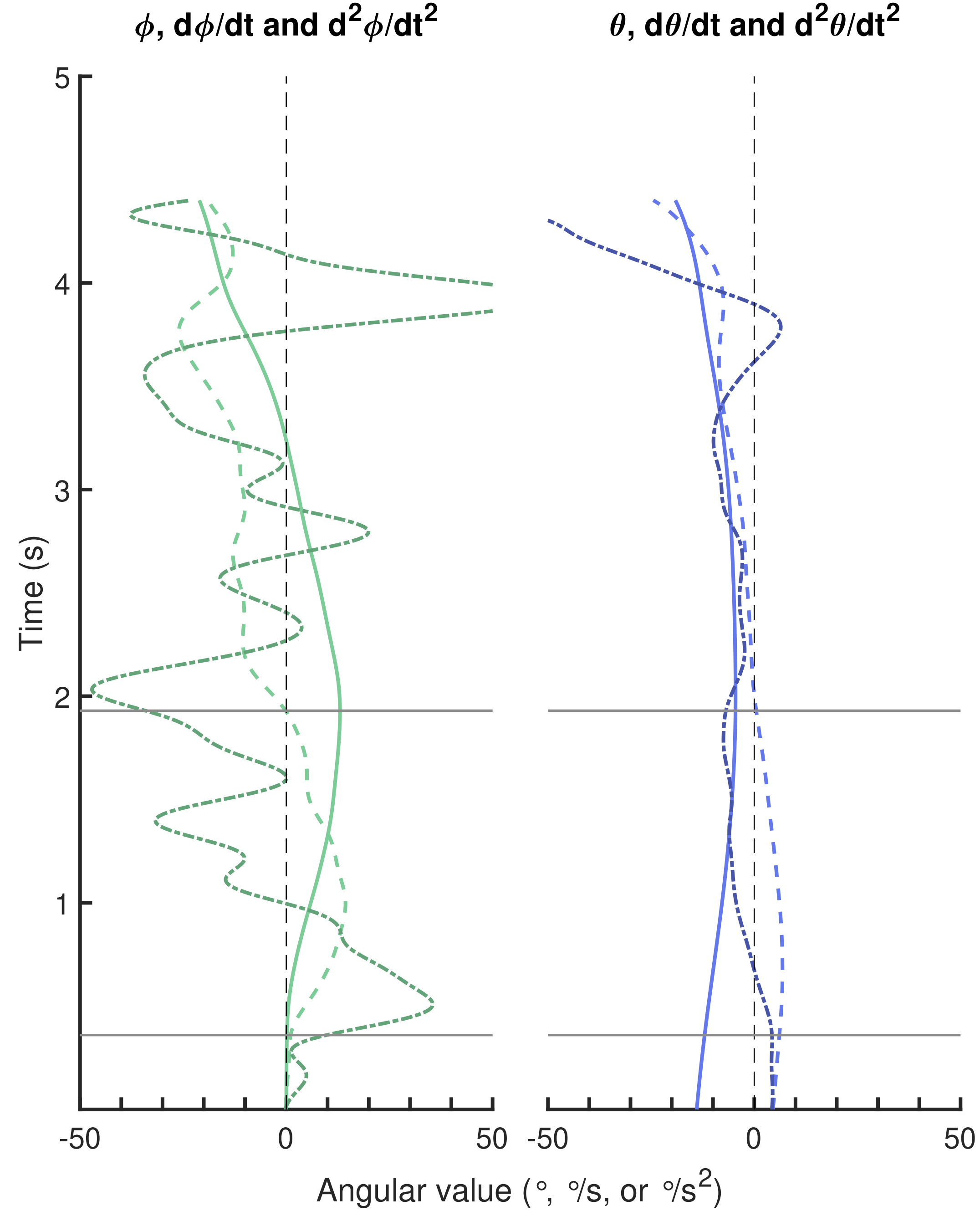

P11/B1
S15/R30-IN

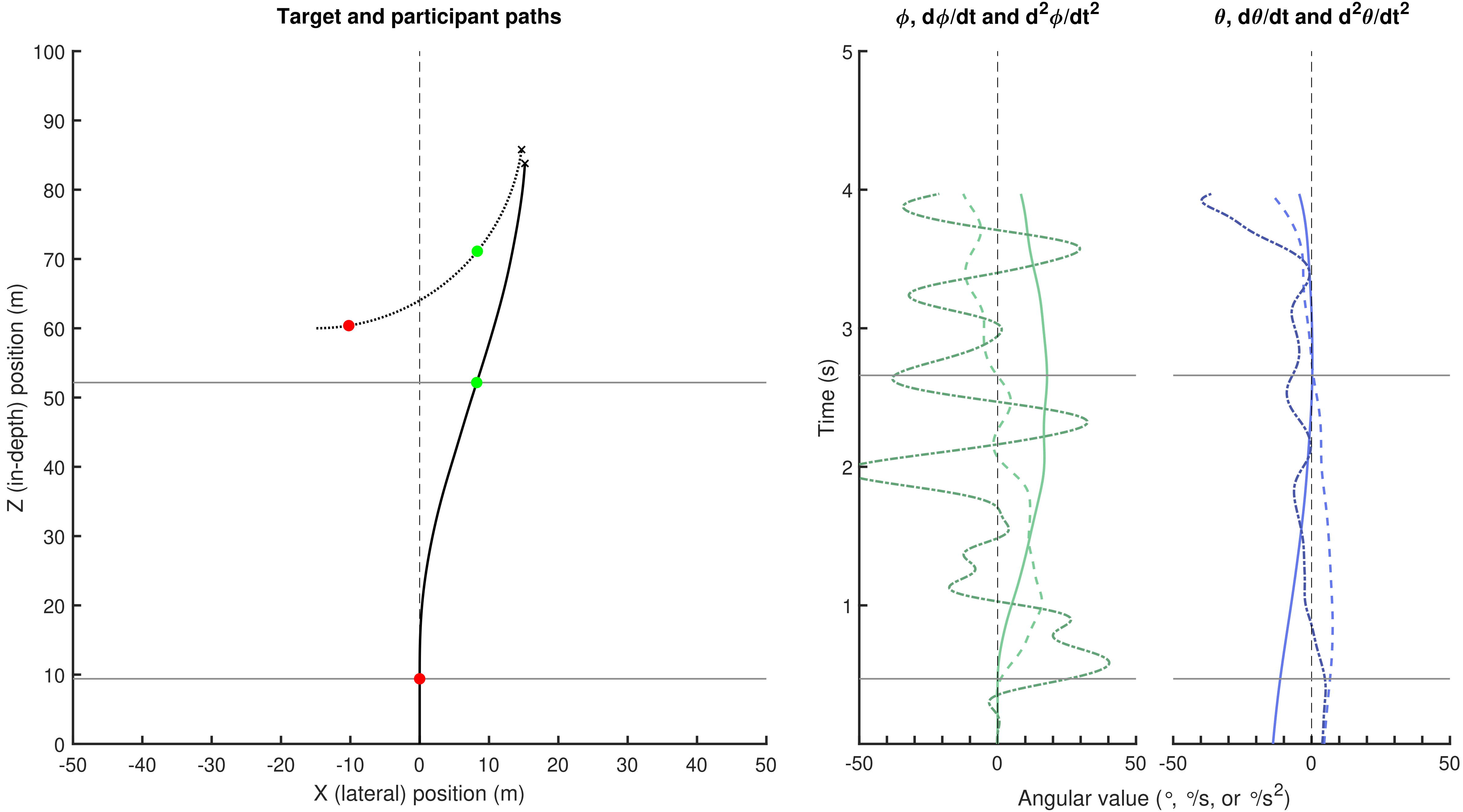

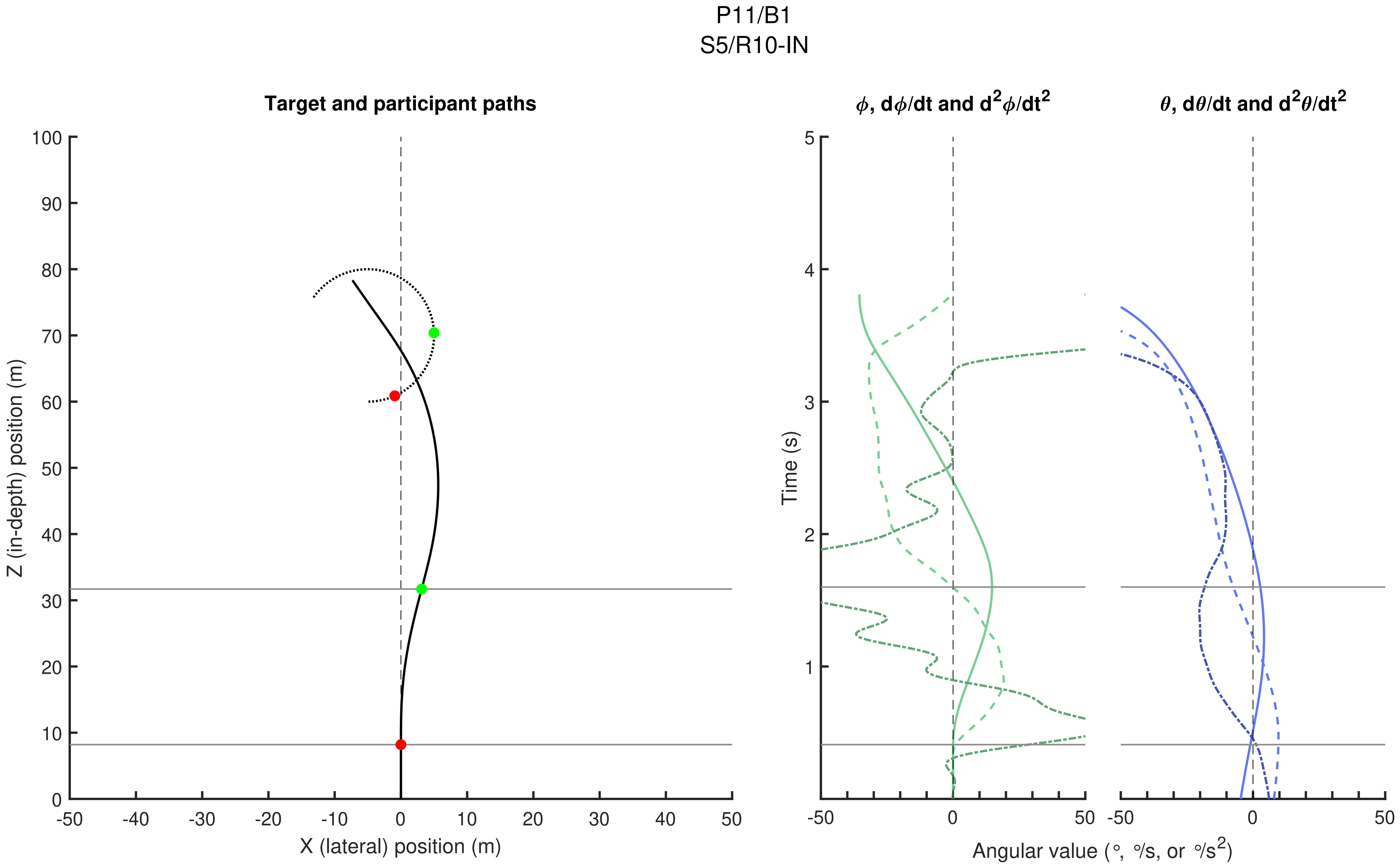
P11/B1
S5/R10-IN
Target and participant paths
Z (in-depth) position (m)
X (lateral) position (m)
-50
-40
-30
-20
-10
0
10
20
30
40
50
100
90
80
70
60
50
40
30
20
10
0
ϕ, dϕ/dt and d$^2\phi$/dt^2
θ, dθ/dt and d$^2\theta$/dt^2
Time (s)
5
4
3
2
1
-50
0
50
-50
0
50
Angular value (°, °/s, or °/s^2)

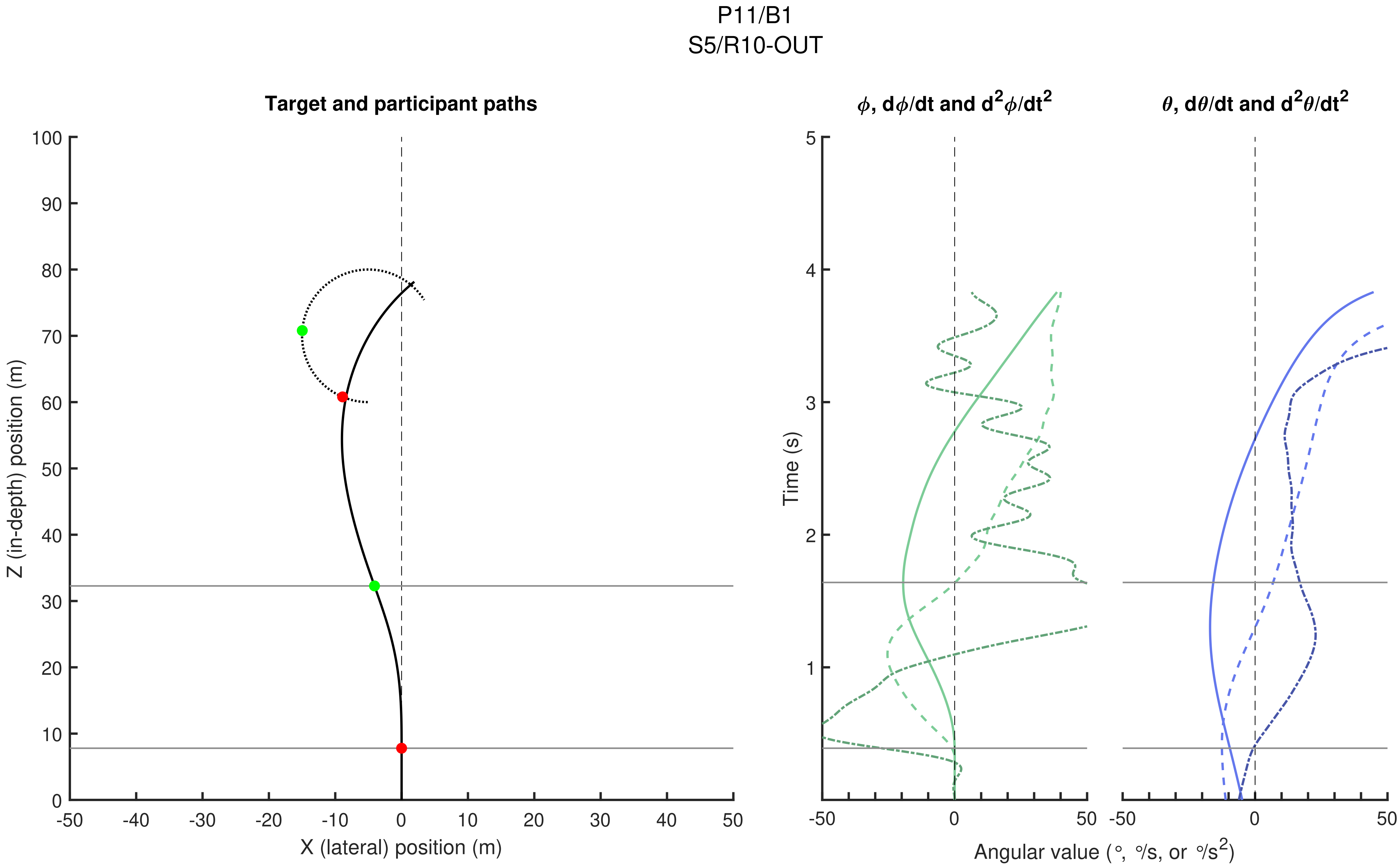

P11/B1
S5/R10-OUT
Target and participant paths
Z (in-depth) position (m)
X (lateral) position (m)
ϕ, dϕ/dt and d$^2\phi$/dt^2
θ, dθ/dt and d$^2\theta$/dt^2
Time (s)
Angular value (°, °/s, or °/s^2)

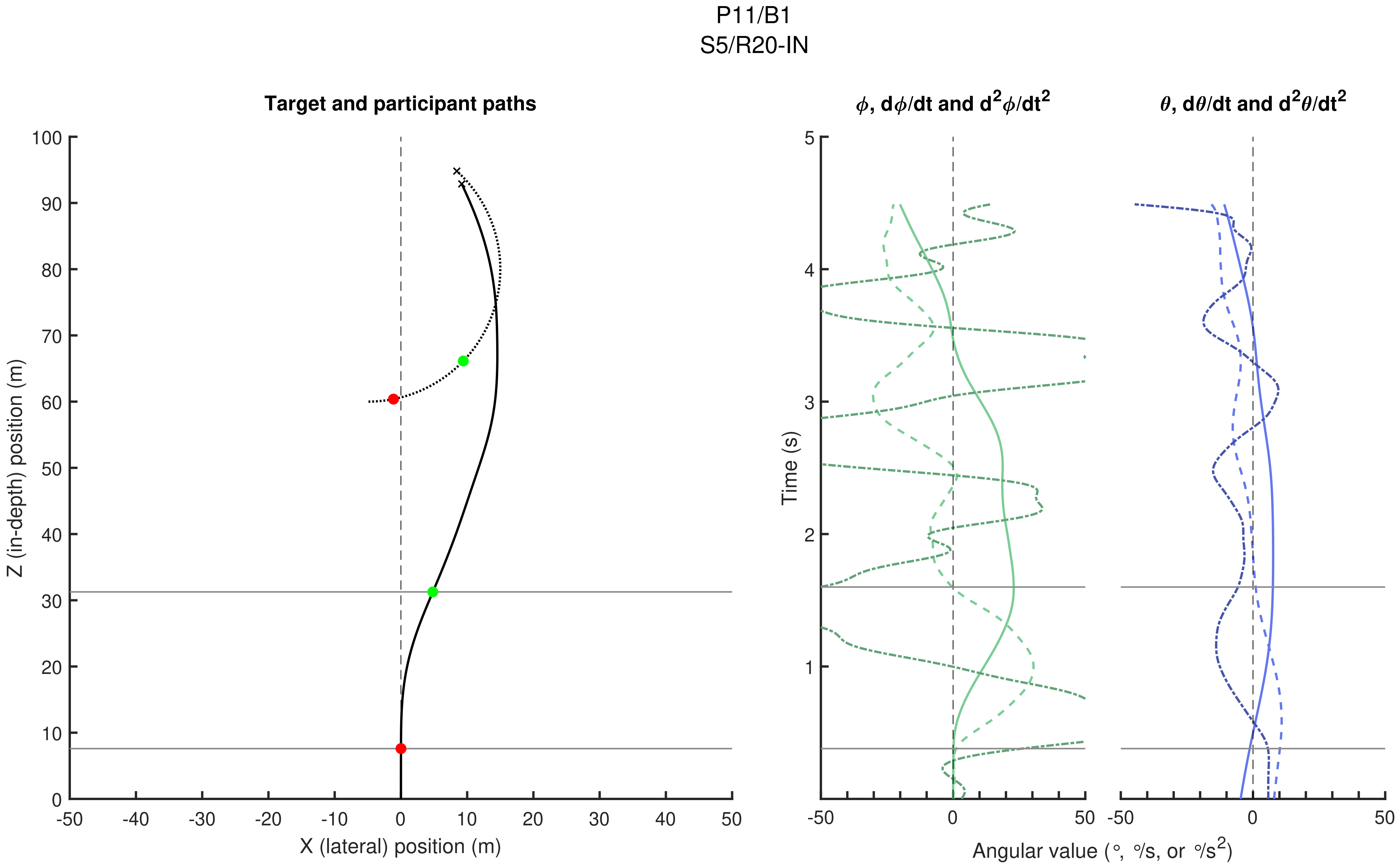
P11/B1
S5/R20-IN
Target and participant paths
Z (in-depth) position (m)
X (lateral) position (m)
φ, dφ/dt and d²φ/dt²
θ, dθ/dt and d²θ/dt²
Time (s)
Angular value (°, °/s, or °/s²)
100
90
80
70
60
50
40
30
20
10
0
-50
-40
-30
-20
-10
0
10
20
30
40
50
5
4
3
2
1
-50
0
50
-50
0
50

P11/B1
S5/R20-OUT

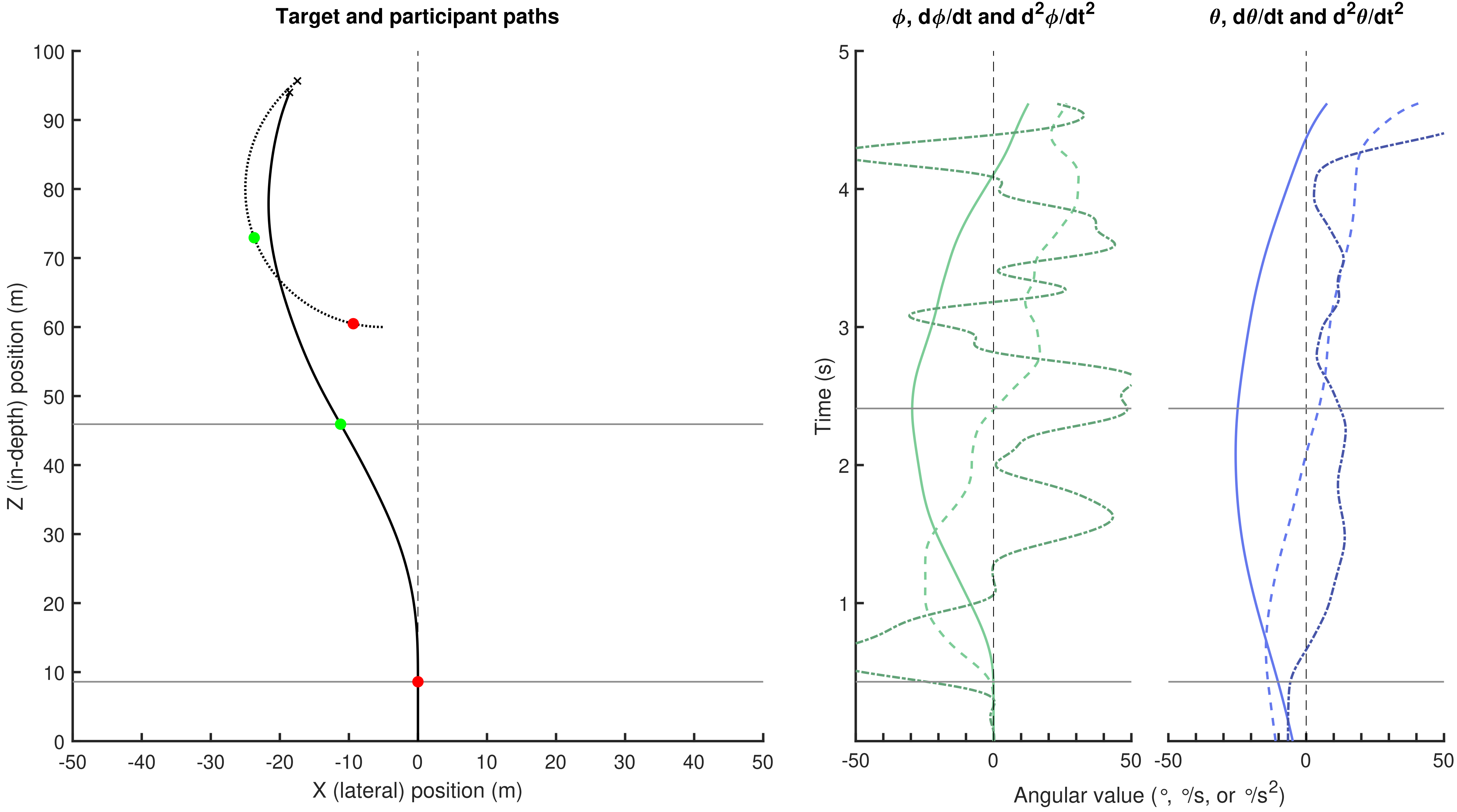

P11/B1
S5/R30-IN

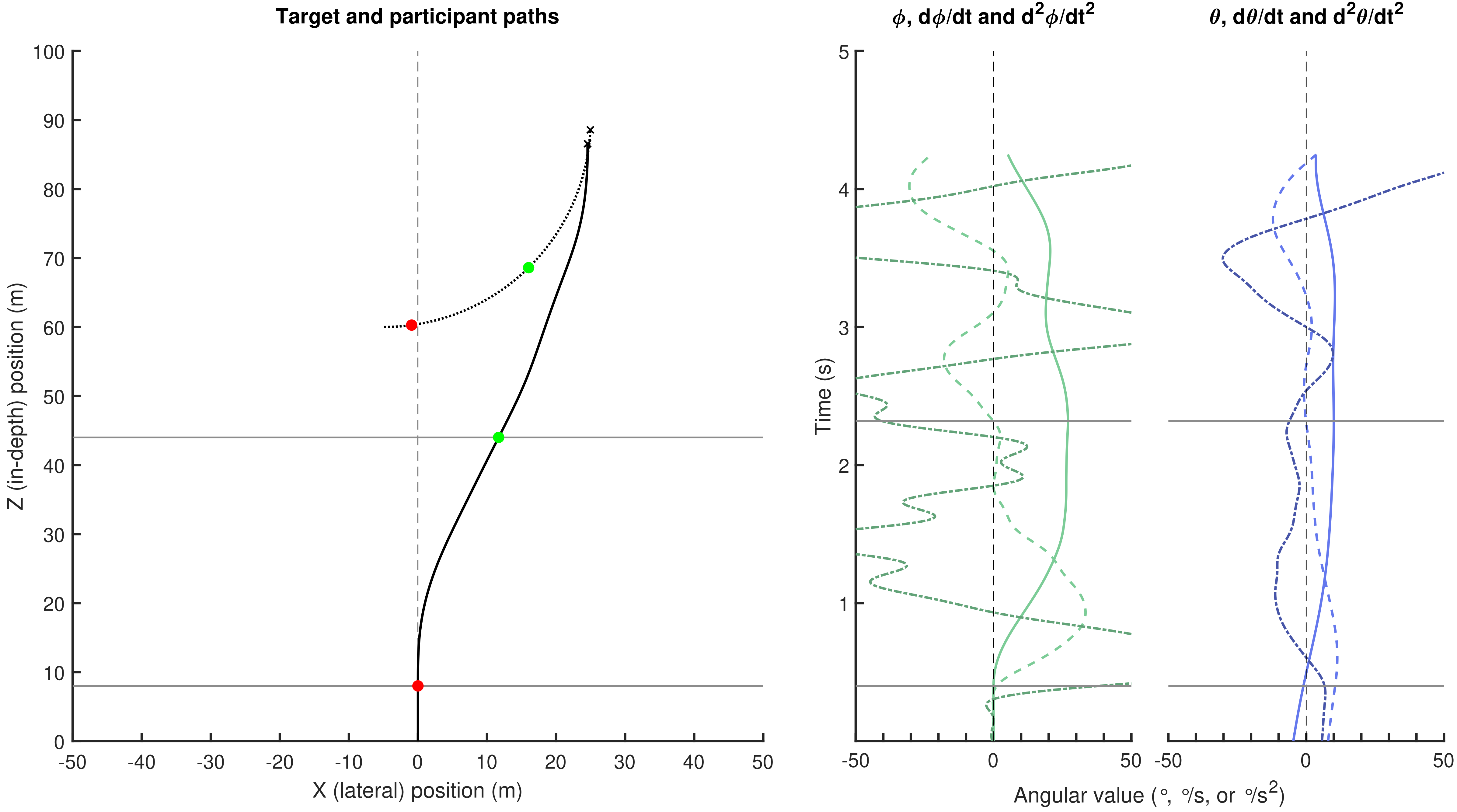

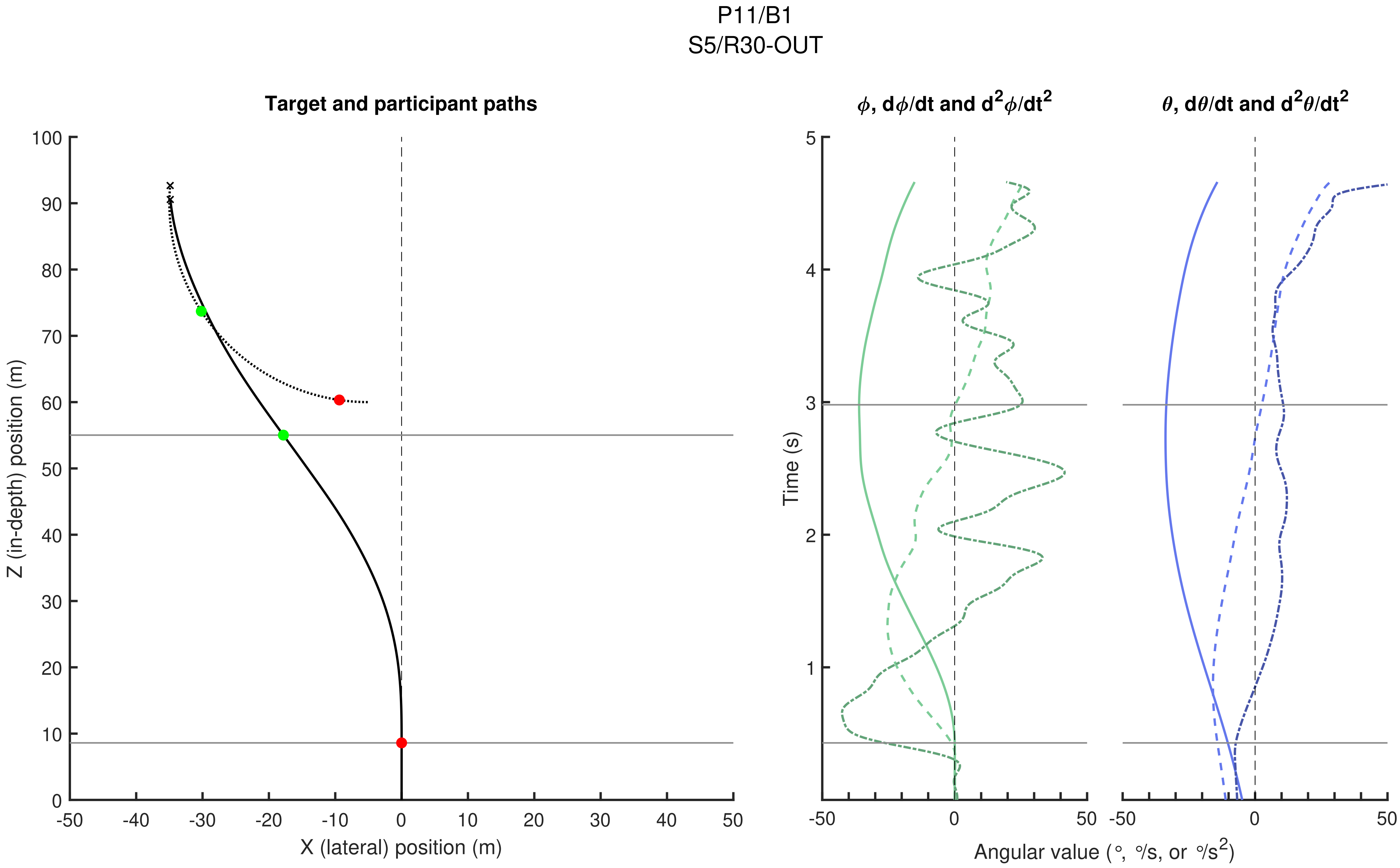

P11/B1
S5/R30-OUT
Target and participant paths
Z (in-depth) position (m)
X (lateral) position (m)
ϕ, dϕ/dt and d$^2\phi$/dt^2
θ, dθ/dt and d$^2\theta$/dt^2
Time (s)
Angular value (°, °/s, or °/s^2)

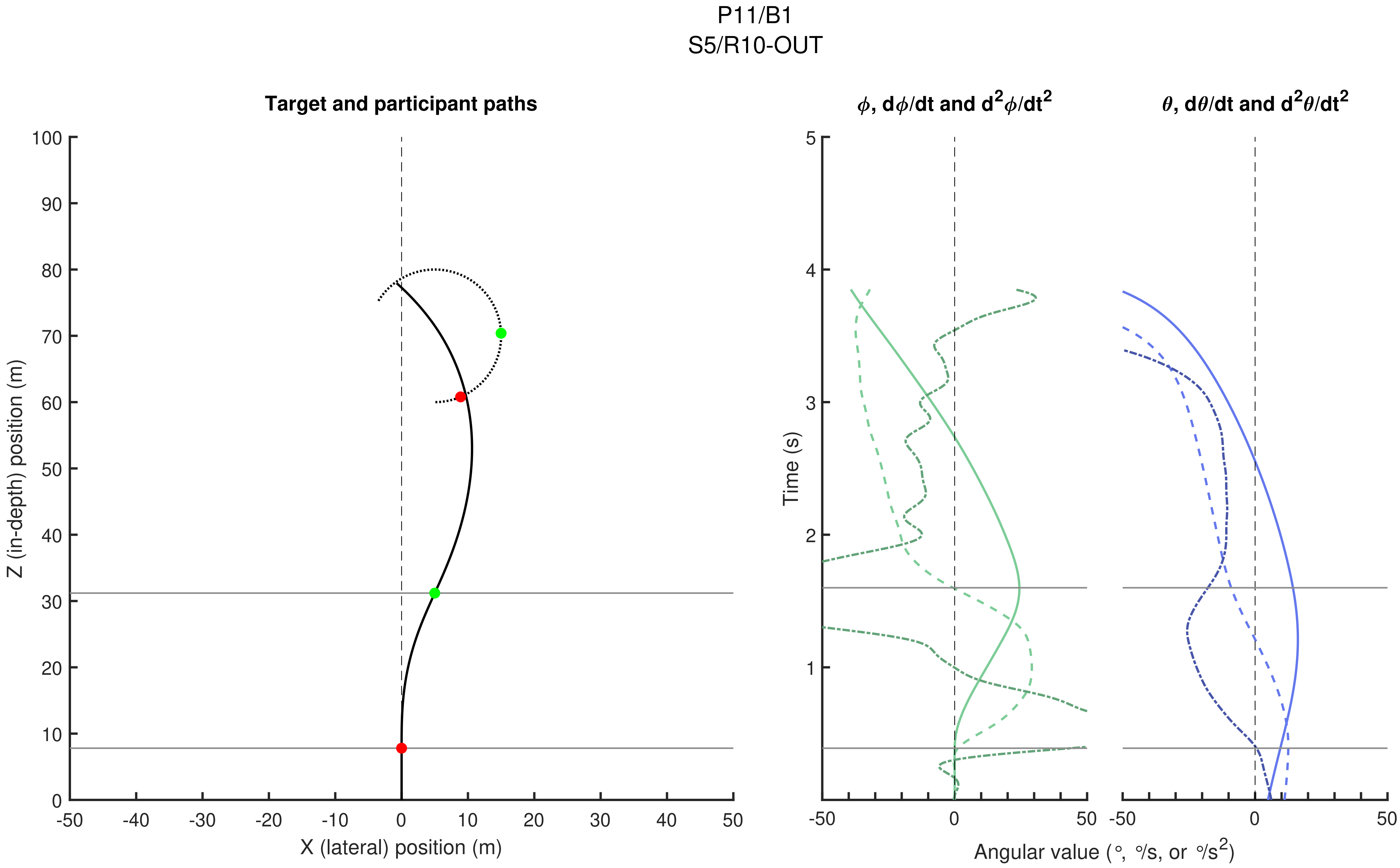
P11/B1
S5/R10-OUT
Target and participant paths
ϕ, dϕ/dt and d$^2\phi$/dt^2
θ, dθ/dt and d$^2\theta$/dt^2
Z (in-depth) position (m)
X (lateral) position (m)
Time (s)
Angular value (°, °/s, or °/s^2)
100
90
80
70
60
50
40
30
20
10
0
-50
-40
-30
-20
-10
0
10
20
30
40
50
5
4
3
2
1
-50
0
50
-50
0
50

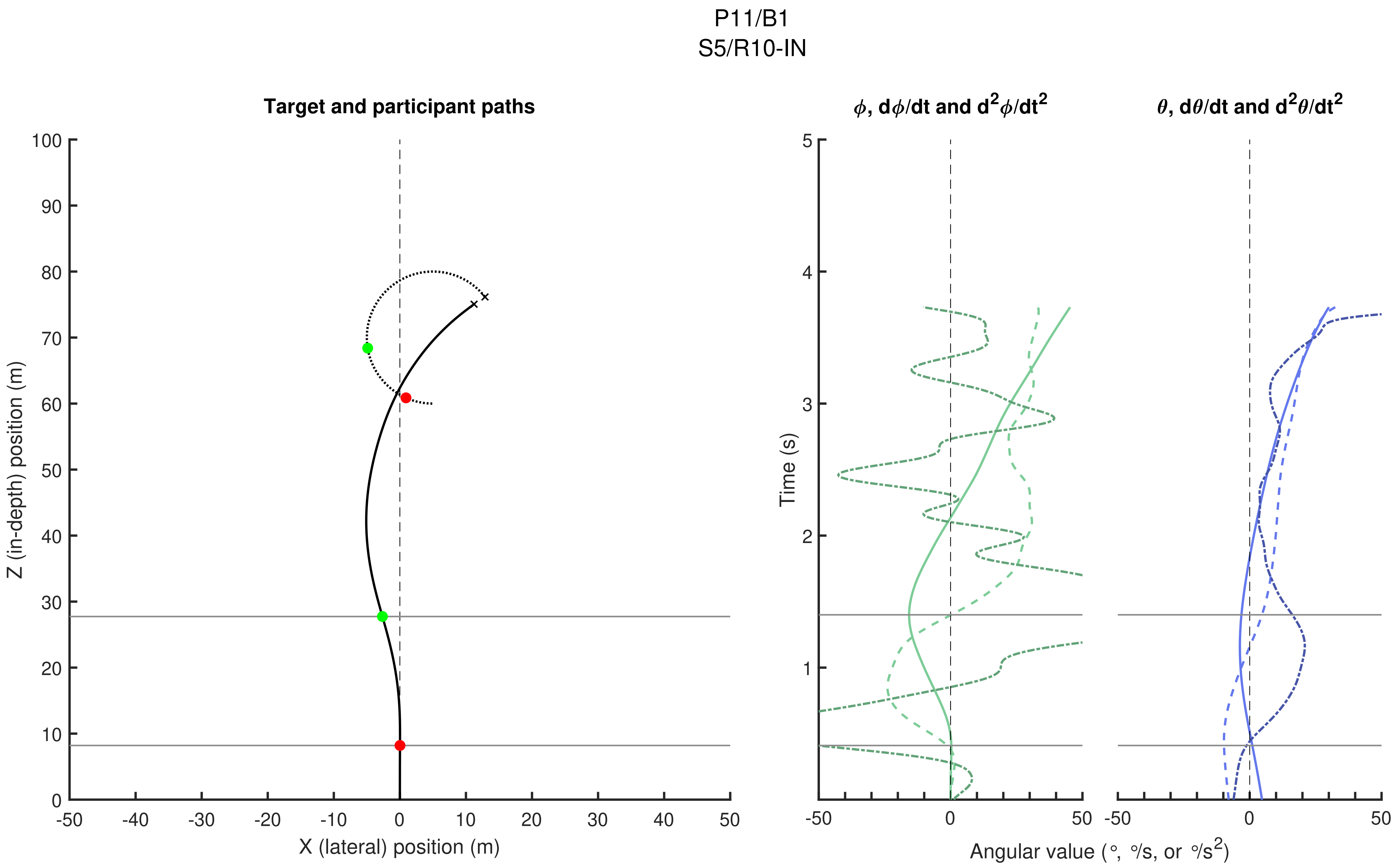
P11/B1
S5/R10-IN
Target and participant paths
Z (in-depth) position (m)
X (lateral) position (m)
ϕ, dϕ/dt and d$^2\phi$/dt^2
θ, dθ/dt and d$^2\theta$/dt^2
Time (s)
Angular value (°, °/s, or °/s^2)

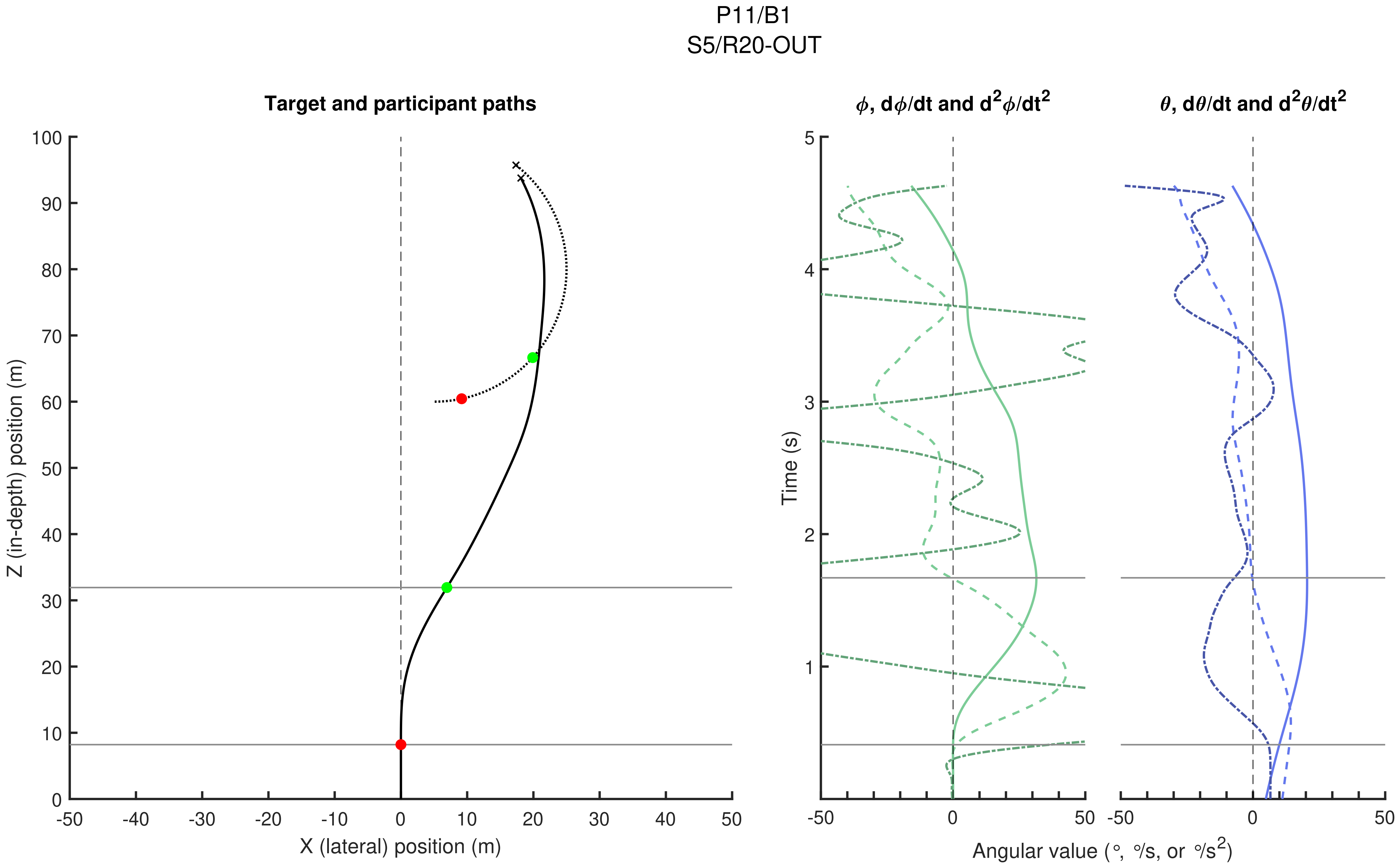
P11/B1
S5/R20-OUT
Target and participant paths
Z (in-depth) position (m)
X (lateral) position (m)
ϕ, dϕ/dt and d²ϕ/dt²
θ, dθ/dt and d²θ/dt²
Time (s)
Angular value (°, °/s, or °/s²)

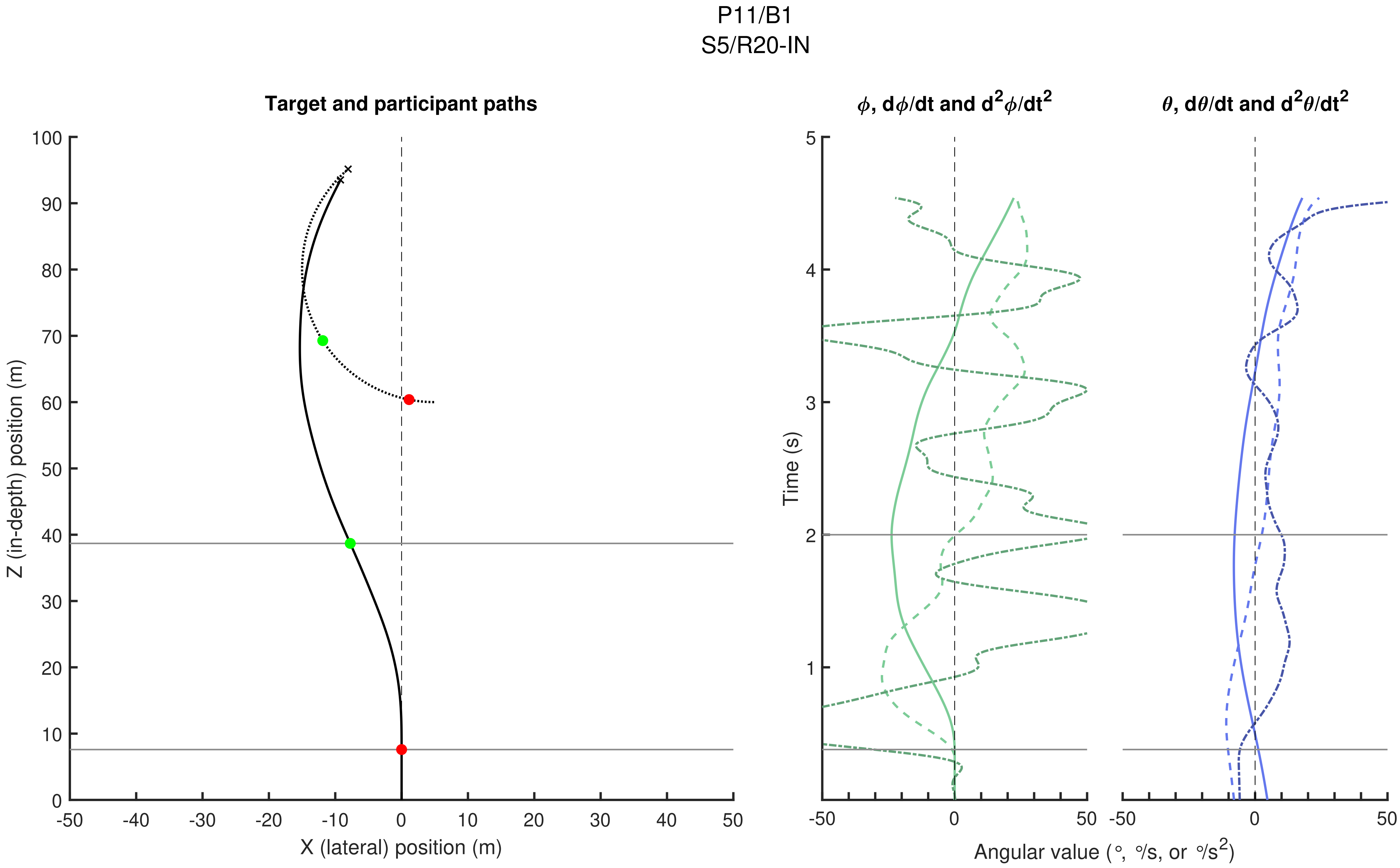
P11/B1
S5/R20-IN
Target and participant paths
Z (in-depth) position (m)
X (lateral) position (m)
100
90
80
70
60
50
40
30
20
10
0
-50
-40
-30
-20
-10
0
10
20
30
40
50
ϕ, dϕ/dt and d$^2\phi$/dt^2
θ, dθ/dt and d$^2\theta$/dt^2
Time (s)
5
4
3
2
1
-50
0
50
-50
0
50
Angular value (°, °/s, or °/s^2)

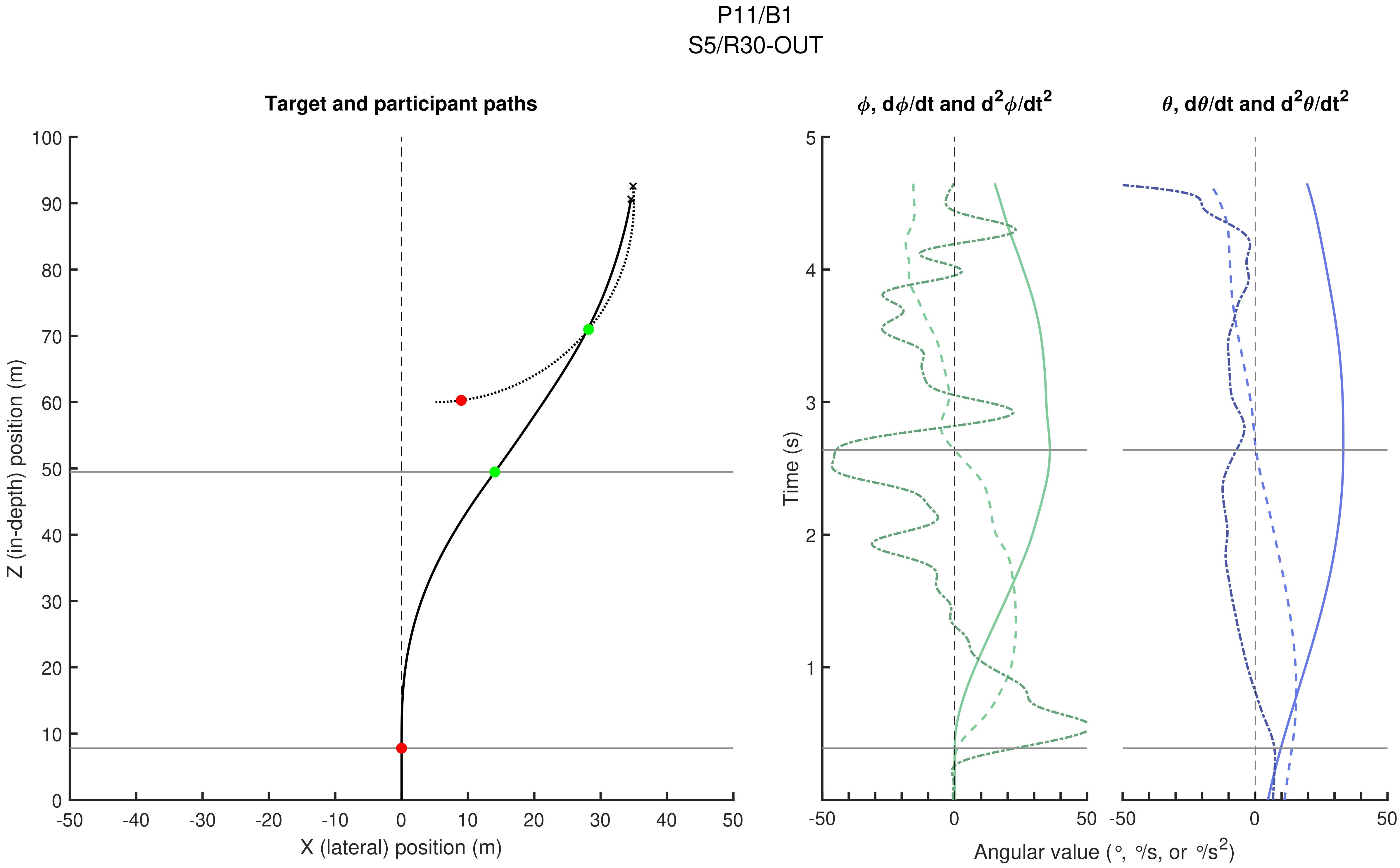
P11/B1
S5/R30-OUT
Target and participant paths
Z (in-depth) position (m)
X (lateral) position (m)
100
90
80
70
60
50
40
30
20
10
0
-50
-40
-30
-20
-10
0
10
20
30
40
50
ϕ, dϕ/dt and d²ϕ/dt²
θ, dθ/dt and d²θ/dt²
Time (s)
5
4
3
2
1
-50
0
50
-50
0
50
Angular value (°, °/s, or °/s²)

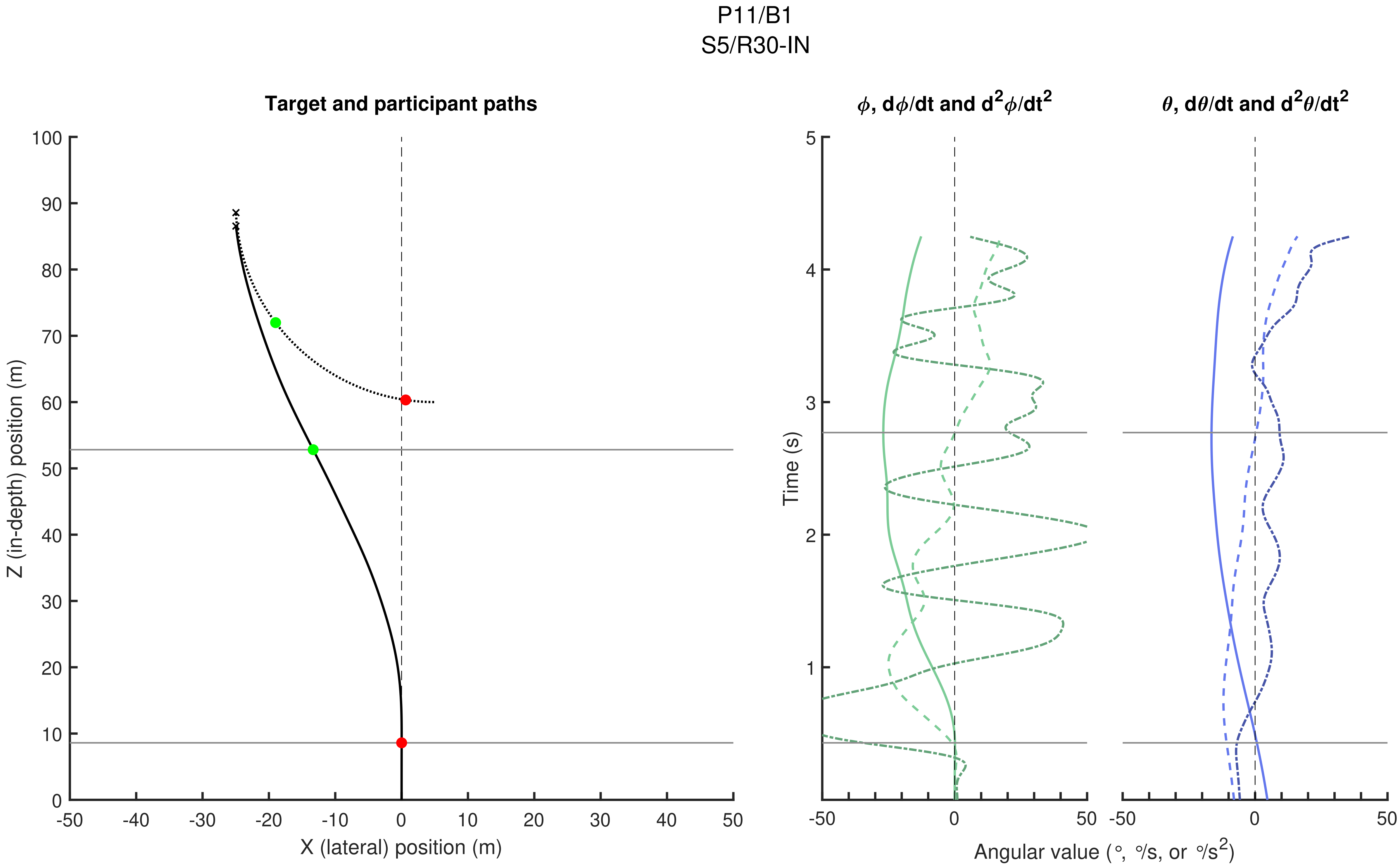
P11/B1
S5/R30-IN
Target and participant paths
Z (in-depth) position (m)
X (lateral) position (m)
ϕ, dϕ/dt and d²ϕ/dt²
θ, dθ/dt and d²θ/dt²
Time (s)
Angular value (°, °/s, or °/s²)

P11/B1
S15/R10-IN

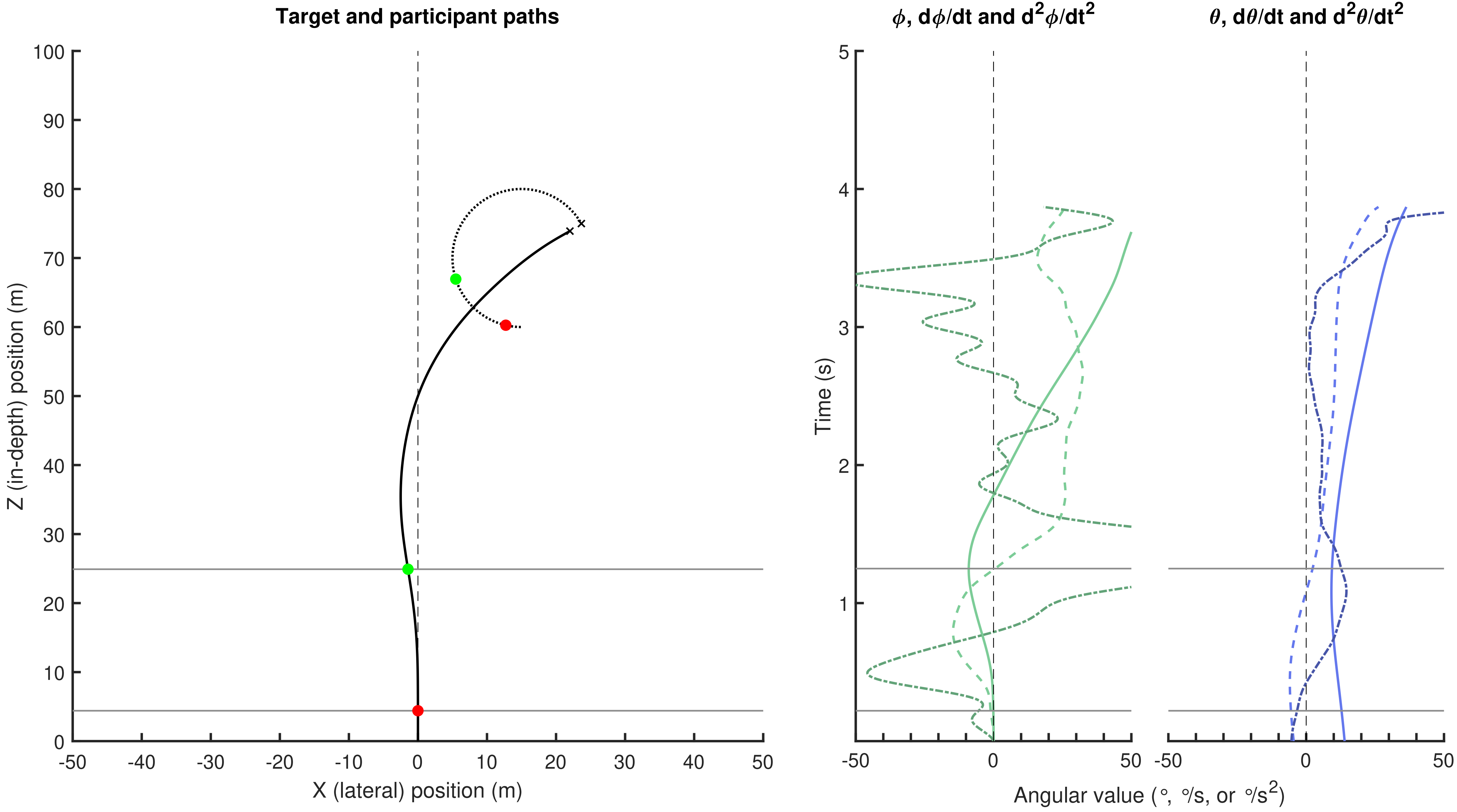

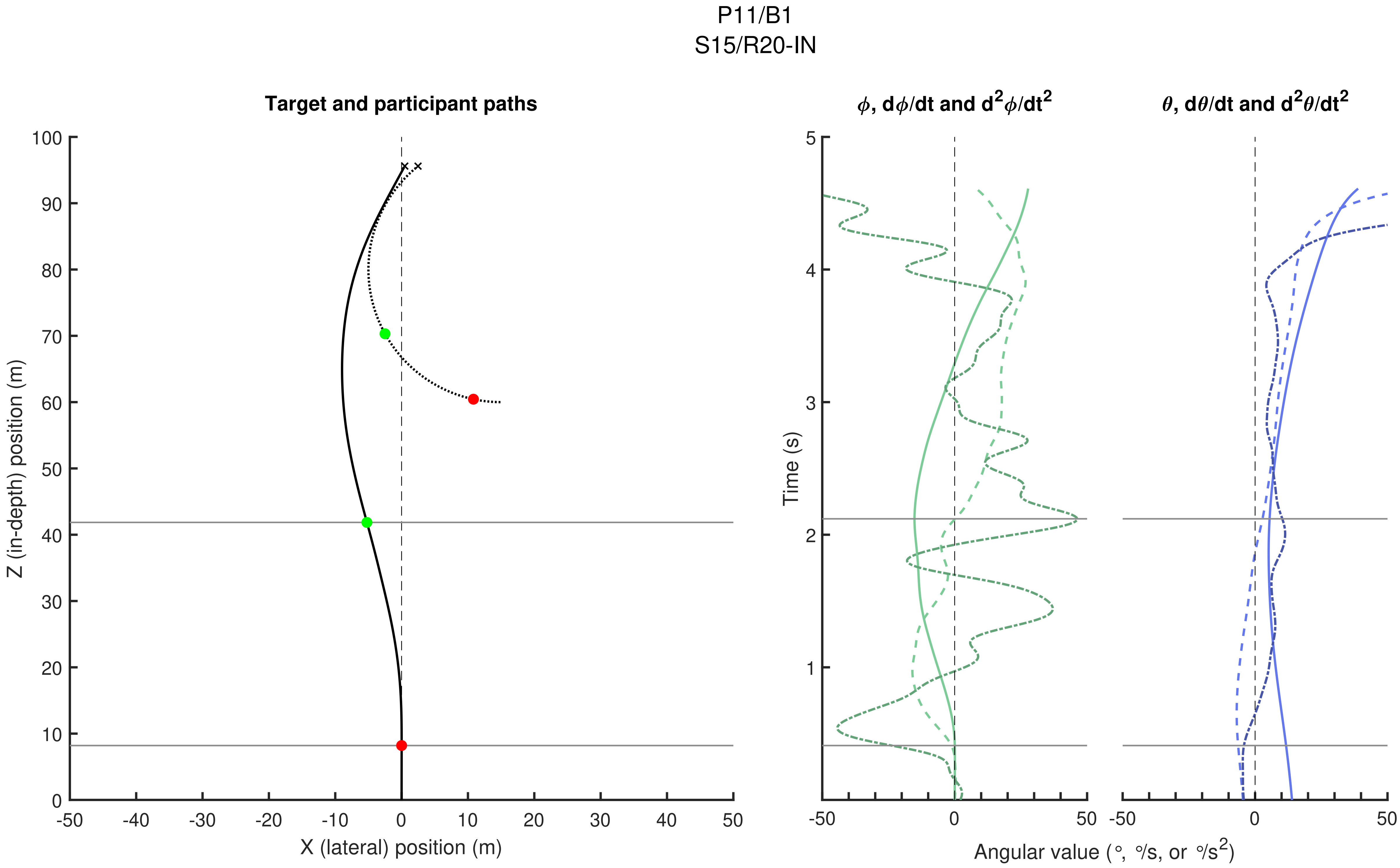
P11/B1
S15/R20-IN
Target and participant paths
Z (in-depth) position (m)
X (lateral) position (m)
ϕ, dϕ/dt and d$^2\phi$/dt^2
θ, dθ/dt and d$^2\theta$/dt^2
Time (s)
Angular value (°, °/s, or °/s^2)

P11/B1
S15/R30-IN

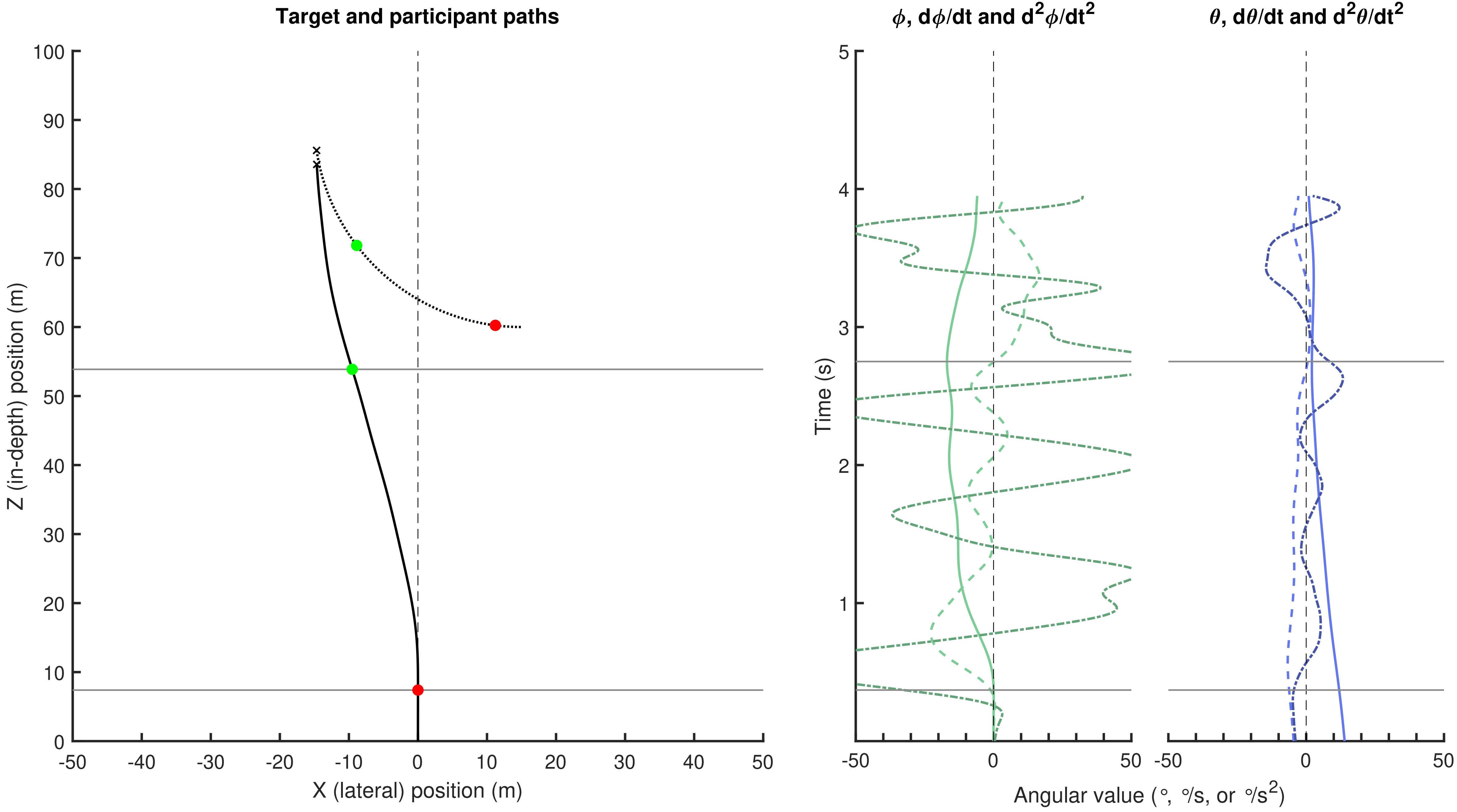

P11/B1
S20/R10-IN

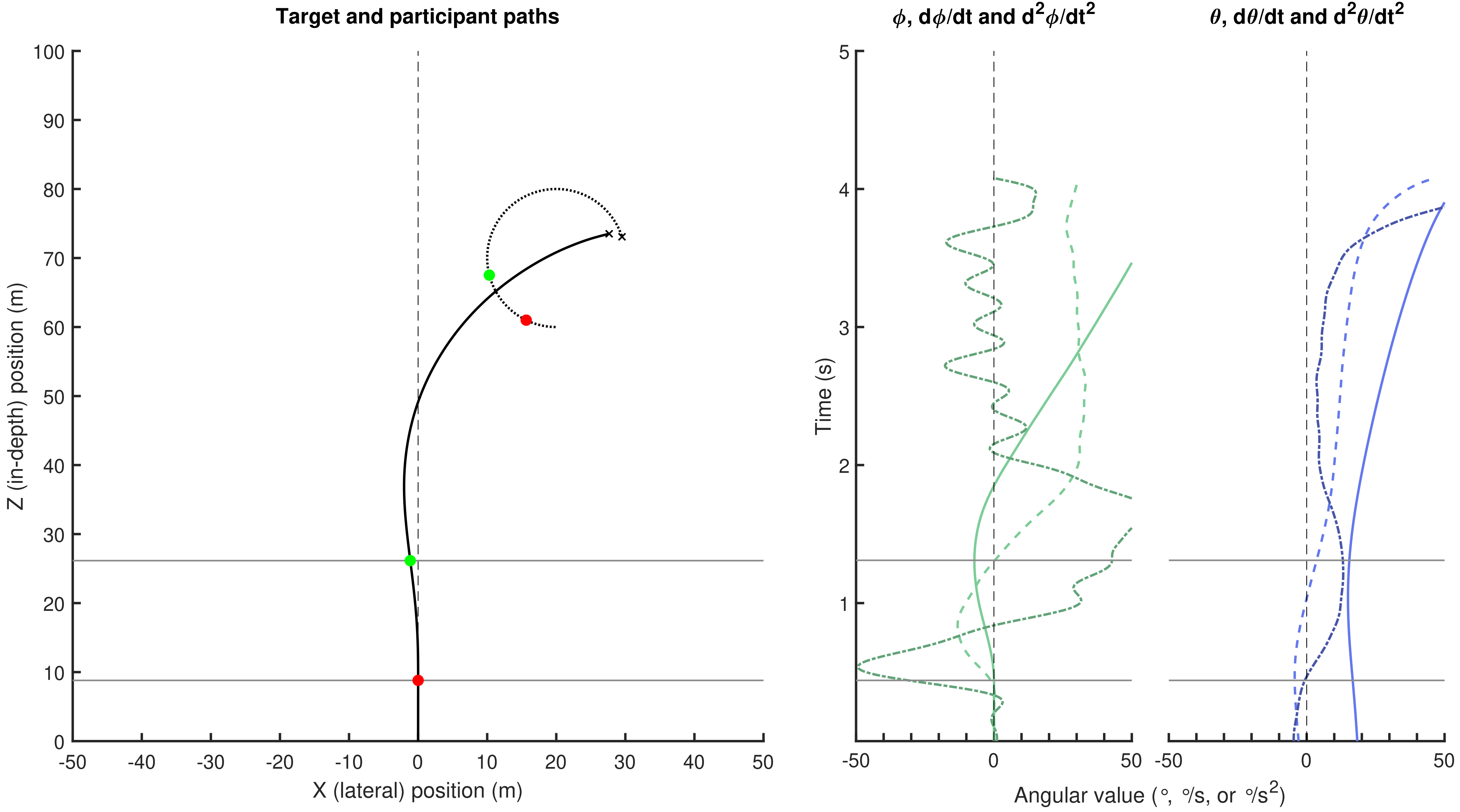

P11/B1
S20/R20-IN

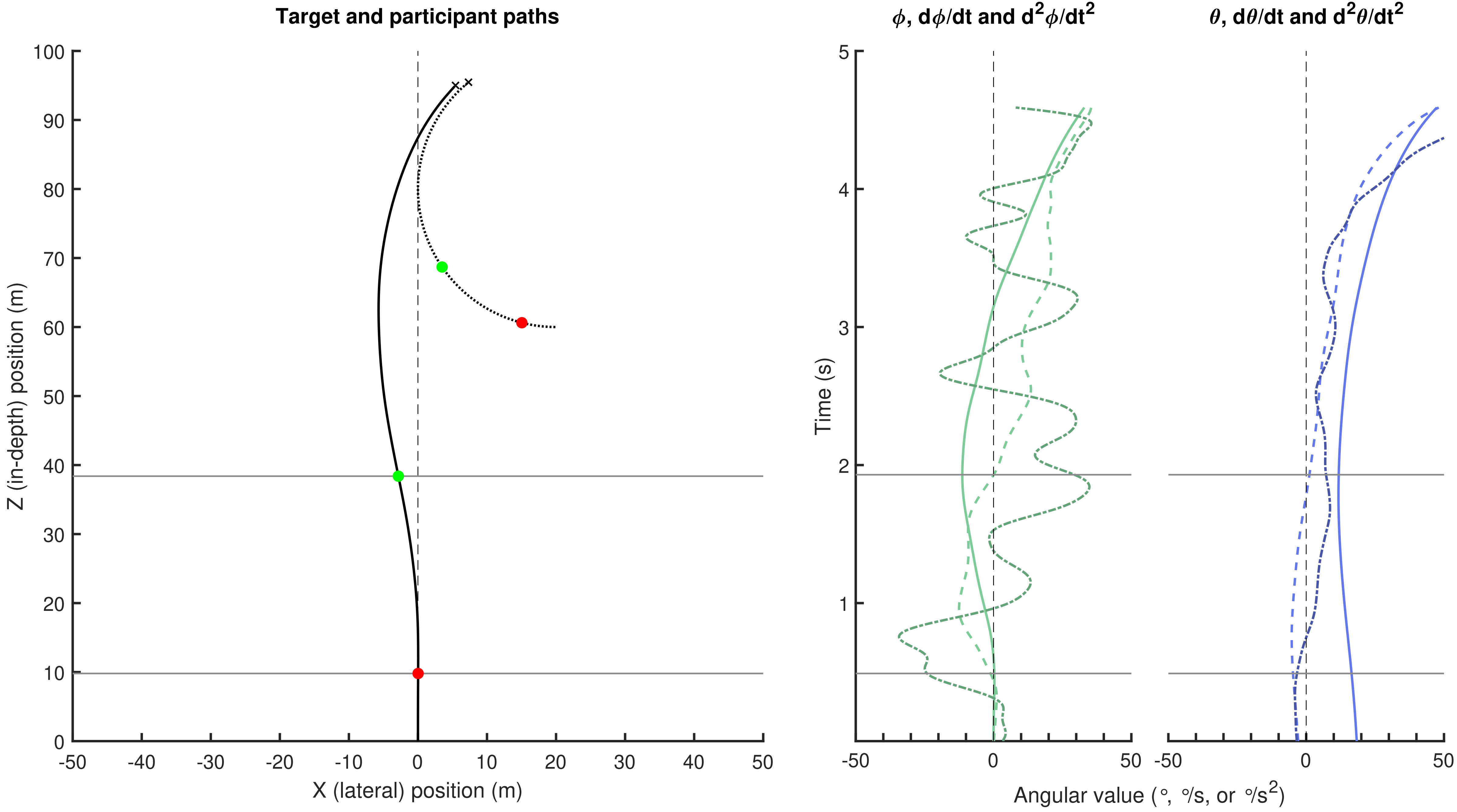

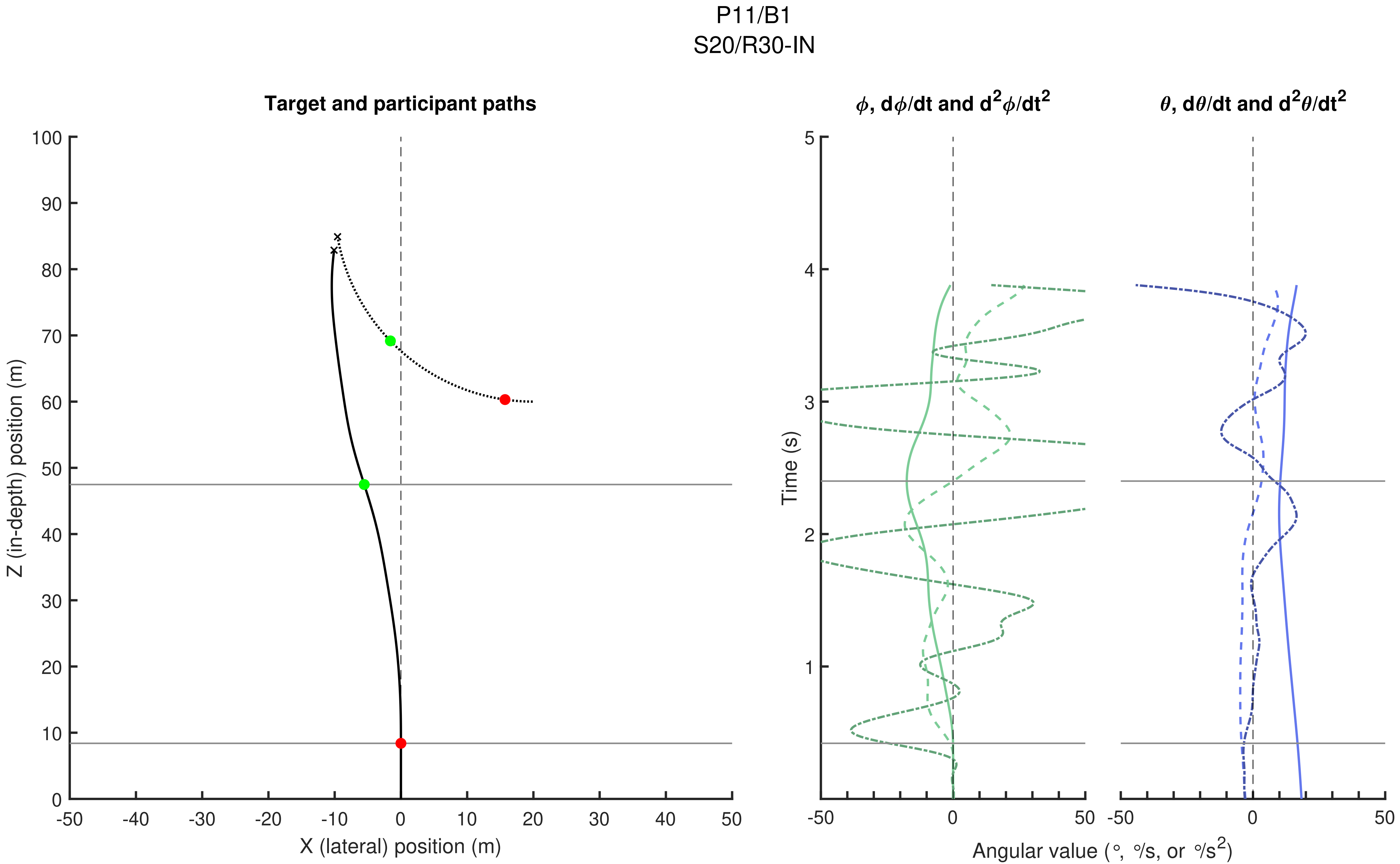

P11/B1
S20/R30-IN
Target and participant paths
ϕ, dϕ/dt and d$^2\phi$/dt^2
θ, dθ/dt and d$^2\theta$/dt^2
Z (in-depth) position (m)
X (lateral) position (m)
Time (s)
Angular value (°, °/s, or °/s^2)

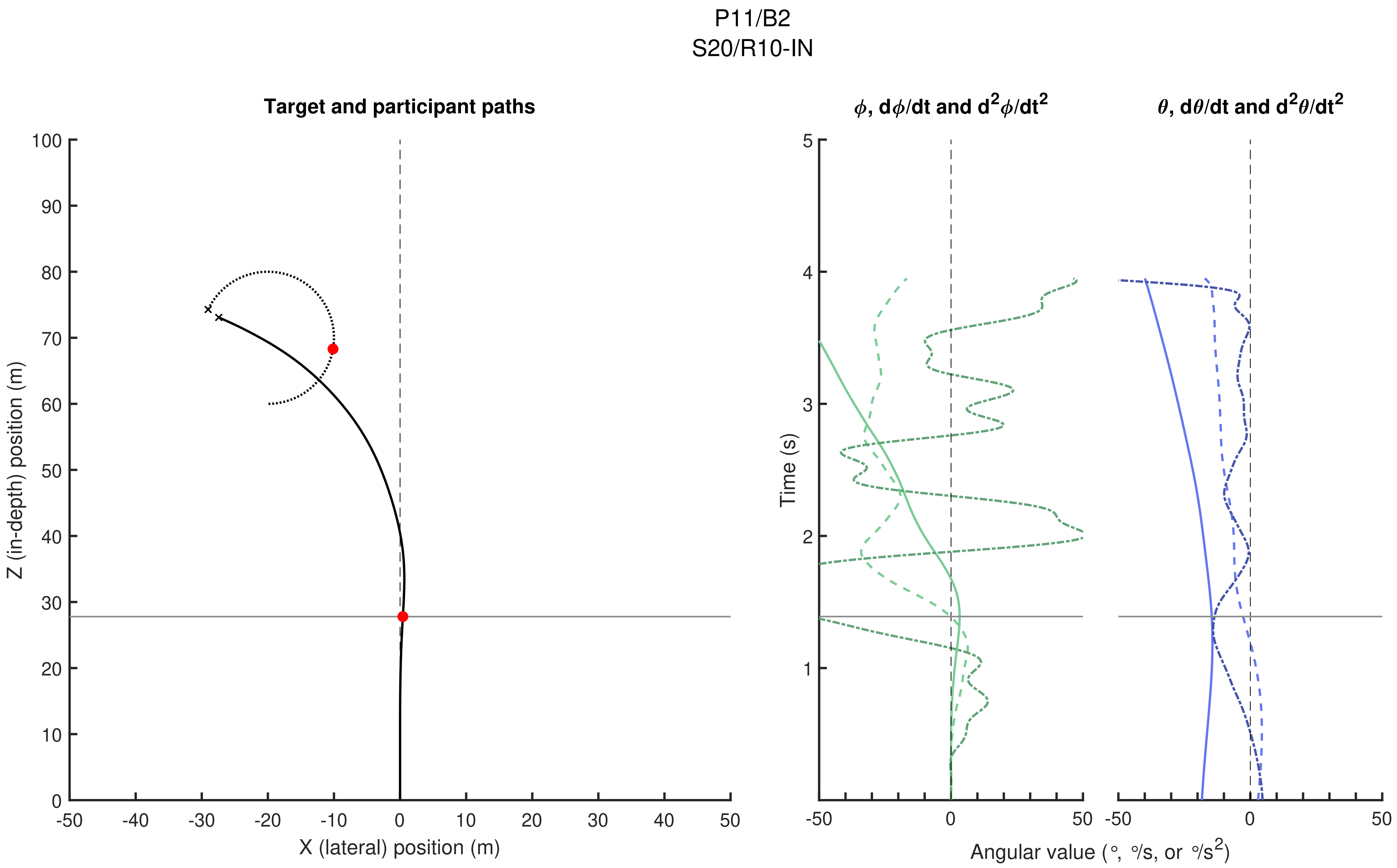
P11/B2
S20/R10-IN
Target and participant paths
ϕ, dϕ/dt and d$^2\phi$/dt^2
θ, dθ/dt and d$^2\theta$/dt^2
Z (in-depth) position (m)
X (lateral) position (m)
Time (s)
Angular value (°, °/s, or °/s^2)
100
90
80
70
60
50
40
30
20
10
0
-50
-40
-30
-20
-10
0
10
20
30
40
50
5
4
3
2
1

P11/B2
S20/R20-IN

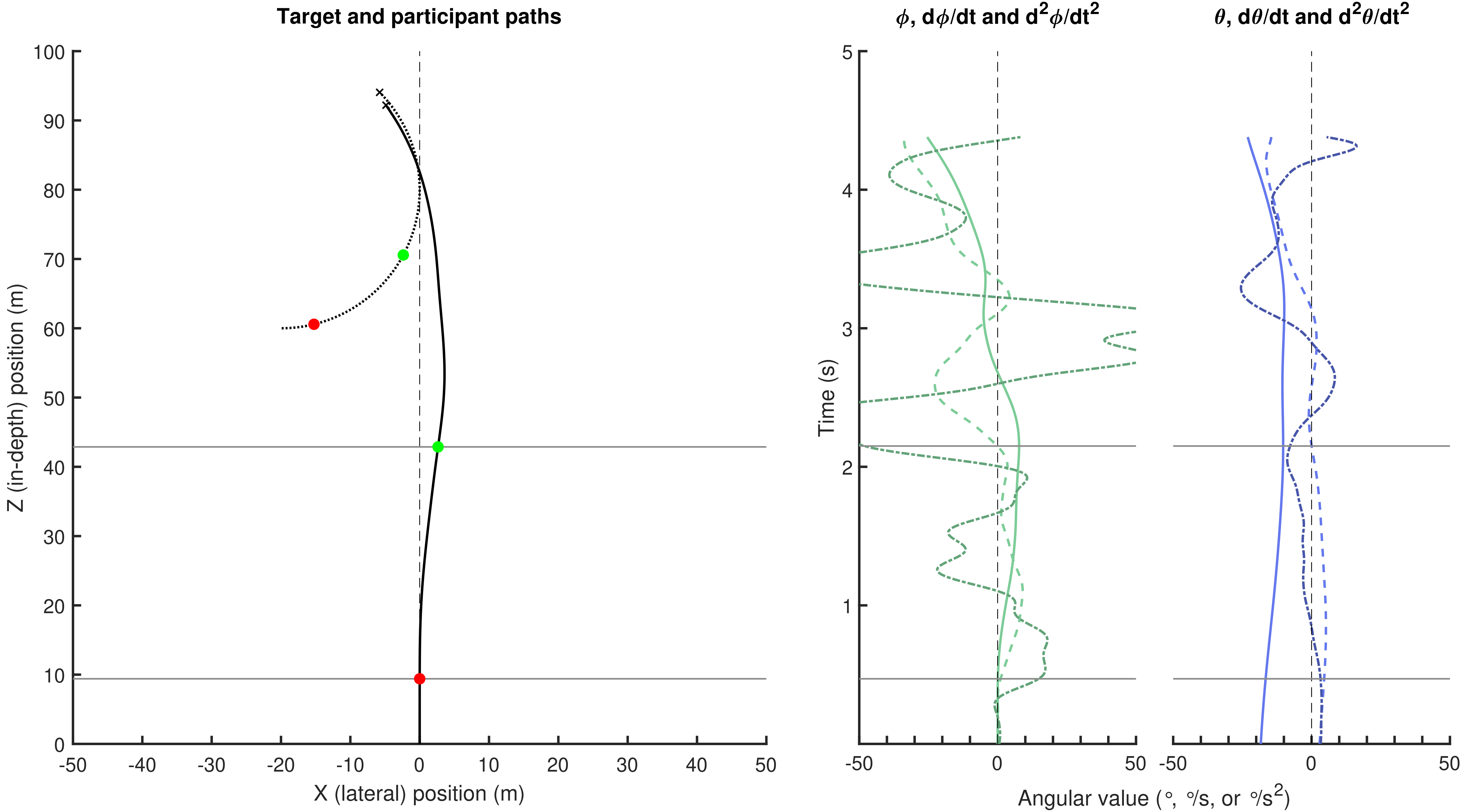

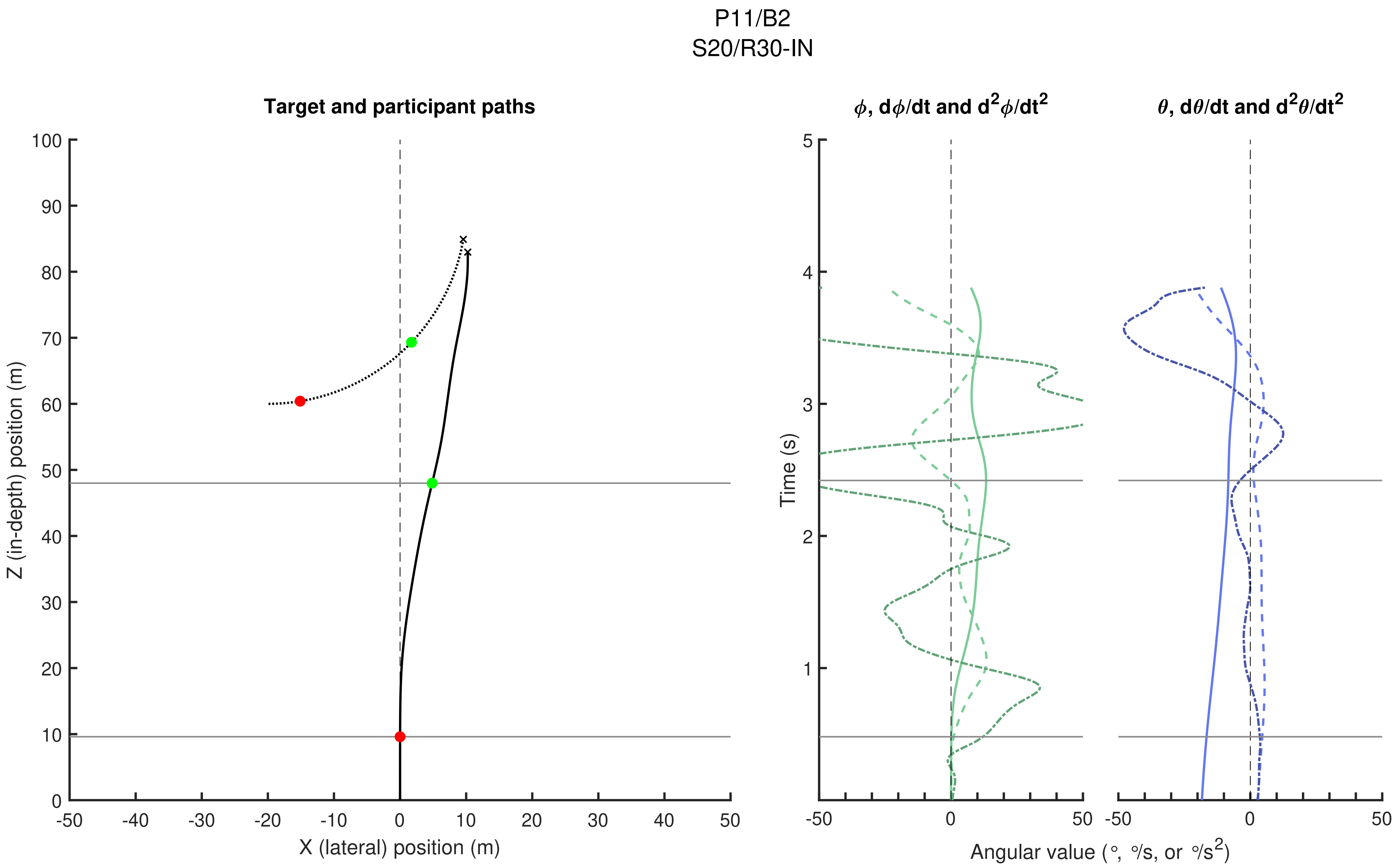

P11/B2
S20/R30-IN
Target and participant paths
Z (in-depth) position (m)
X (lateral) position (m)
100
90
80
70
60
50
40
30
20
10
0
-50
-40
-30
-20
-10
0
10
20
30
40
50
ϕ, dϕ/dt and d$^2\phi$/dt^2
θ, dθ/dt and d$^2\theta$/dt^2
Time (s)
5
4
3
2
1
-50
0
50
-50
0
50
Angular value (°, °/s, or °/s^2)

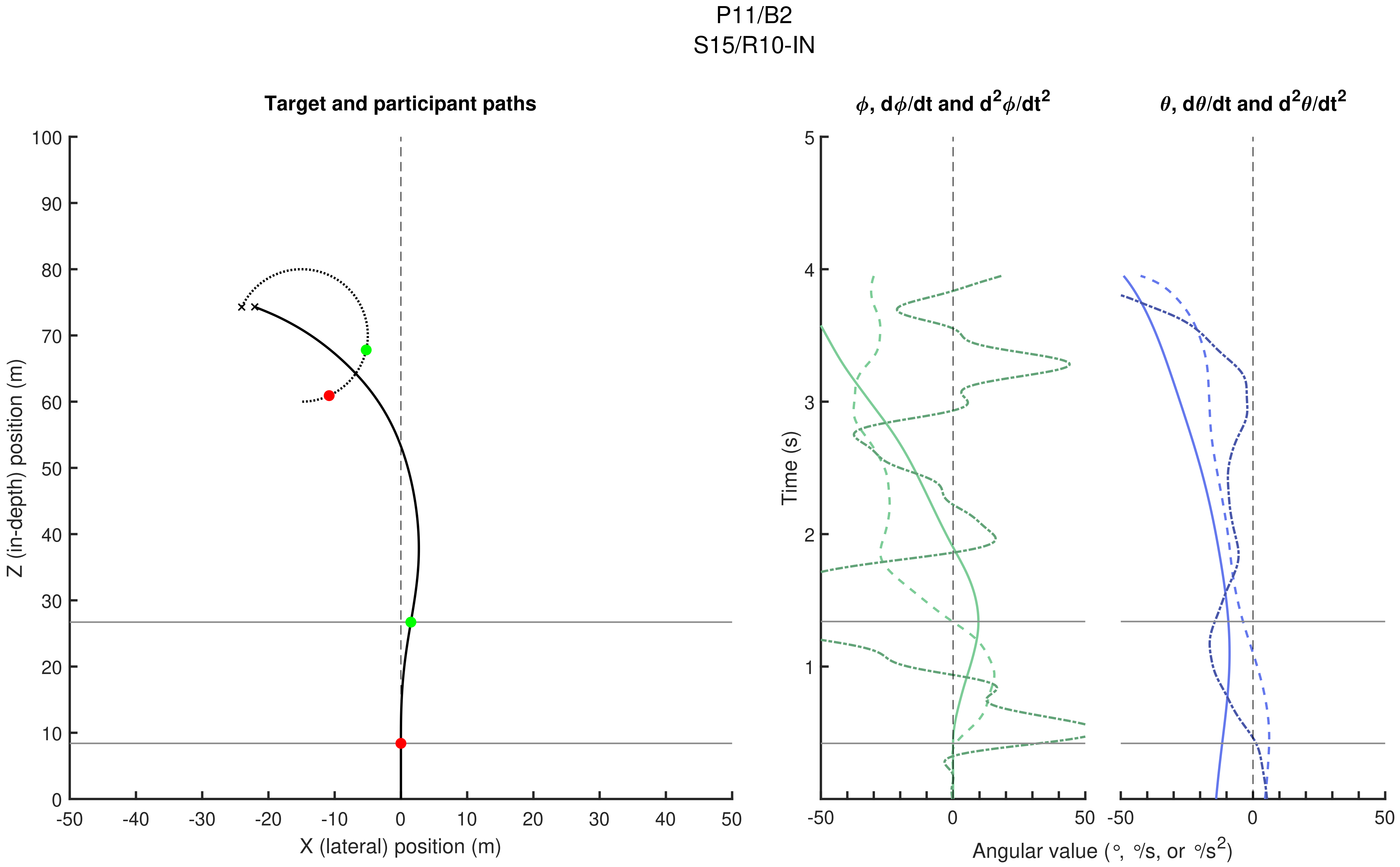
P11/B2
S15/R10-IN
Target and participant paths
Z (in-depth) position (m)
X (lateral) position (m)
100
90
80
70
60
50
40
30
20
10
0
-50
-40
-30
-20
-10
0
10
20
30
40
50
ϕ, dϕ/dt and d$^2\phi$/dt^2
θ, dθ/dt and d$^2\theta$/dt^2
Time (s)
5
4
3
2
1
-50
0
50
-50
0
50
Angular value (°, °/s, or °/s^2)

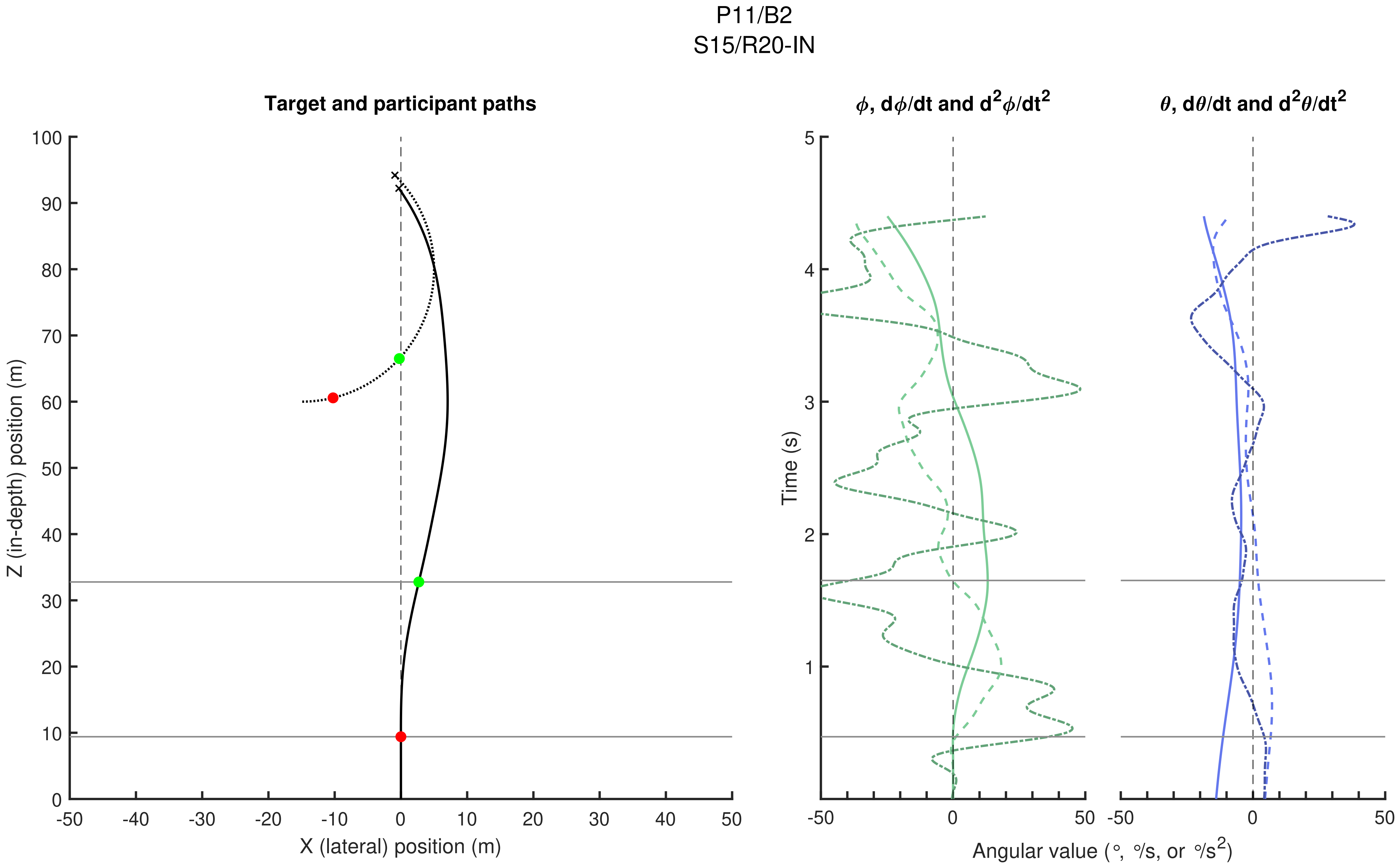
P11/B2
S15/R20-IN
Target and participant paths
Z (in-depth) position (m)
X (lateral) position (m)
φ, dφ/dt and d²φ/dt²
θ, dθ/dt and d²θ/dt²
Time (s)
Angular value (°, °/s, or °/s²)

P11/B2
S15/R30-IN

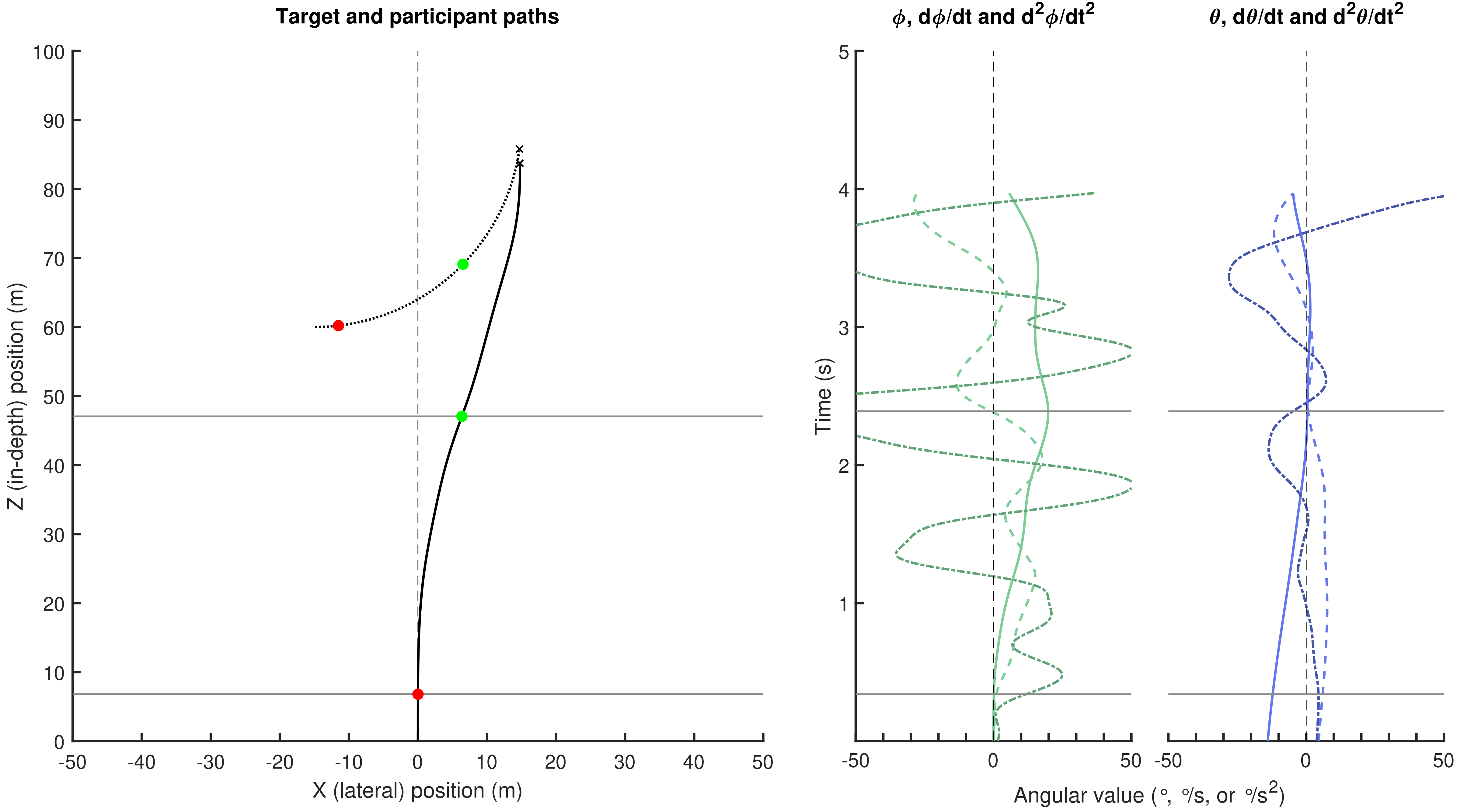

P11/B2
S5/R10-IN

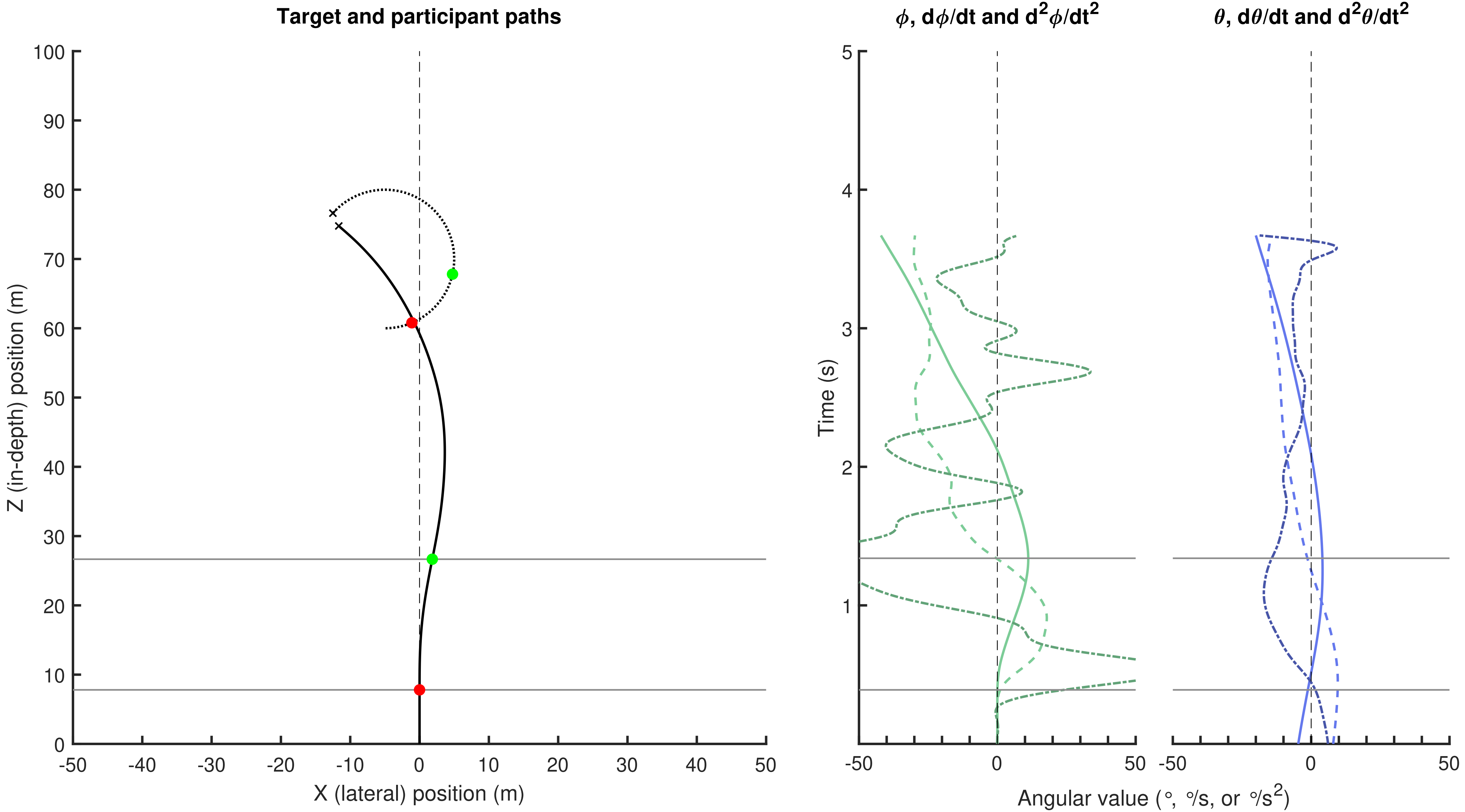

P11/B2
S5/R10-OUT

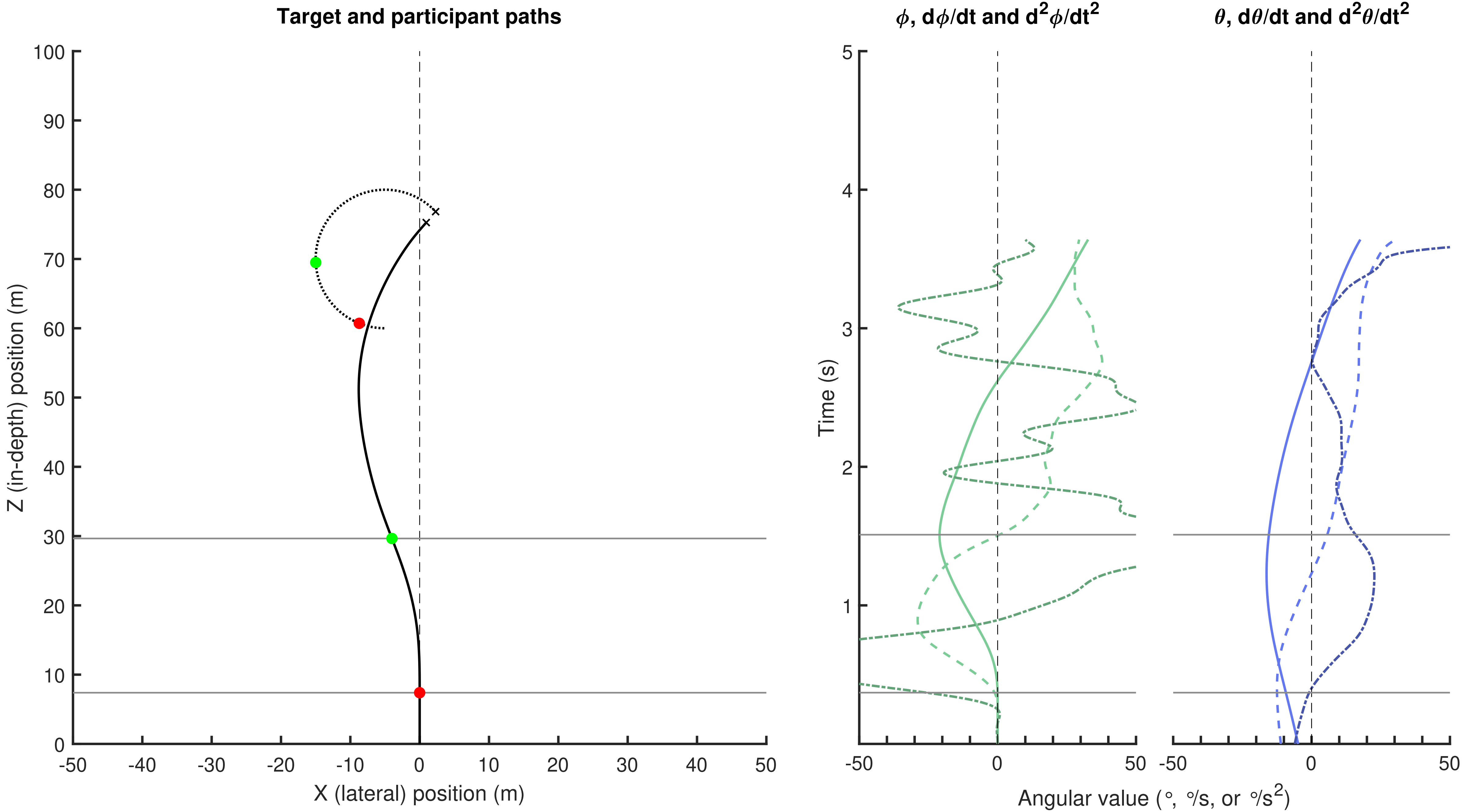

P11/B2
S5/R20-IN

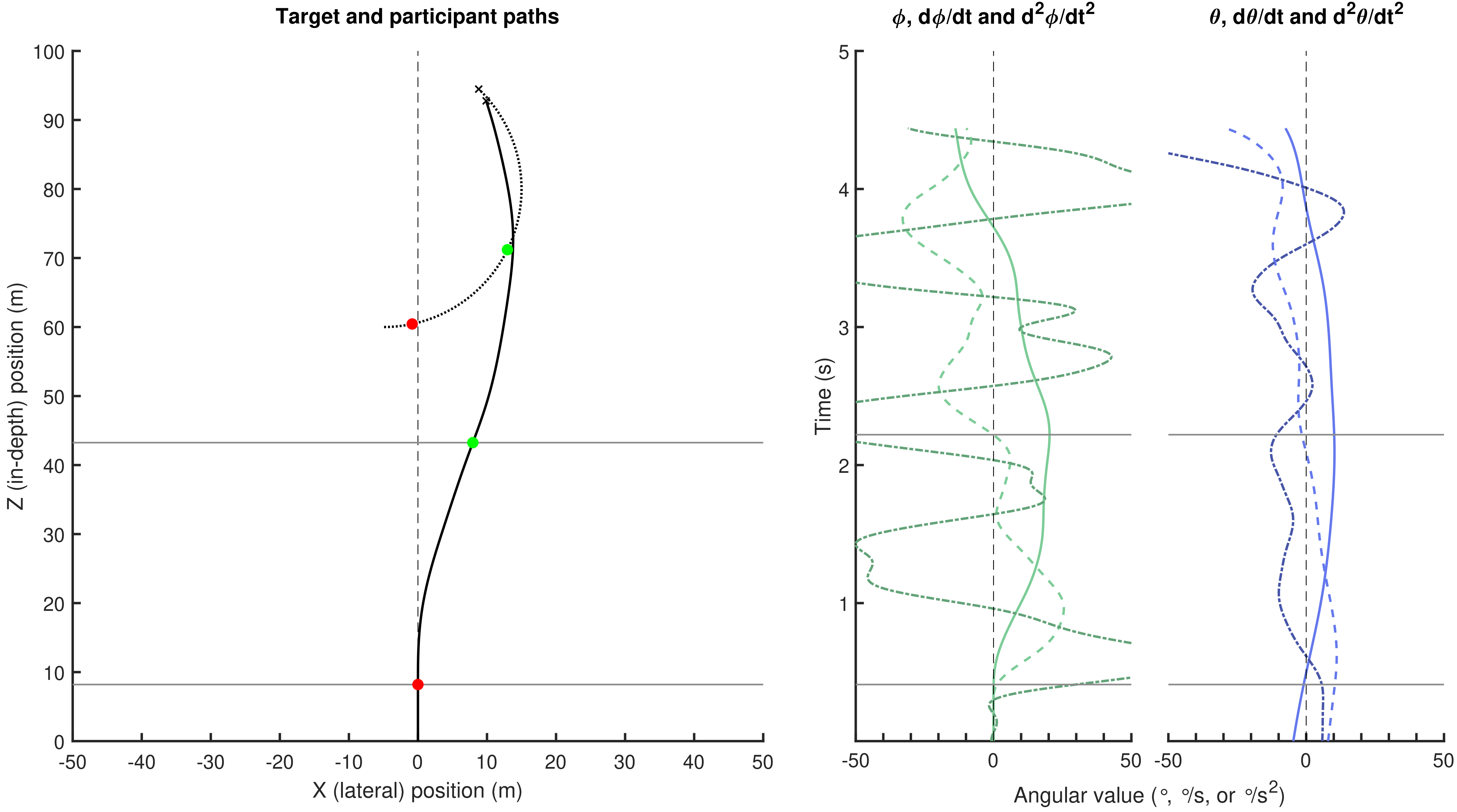

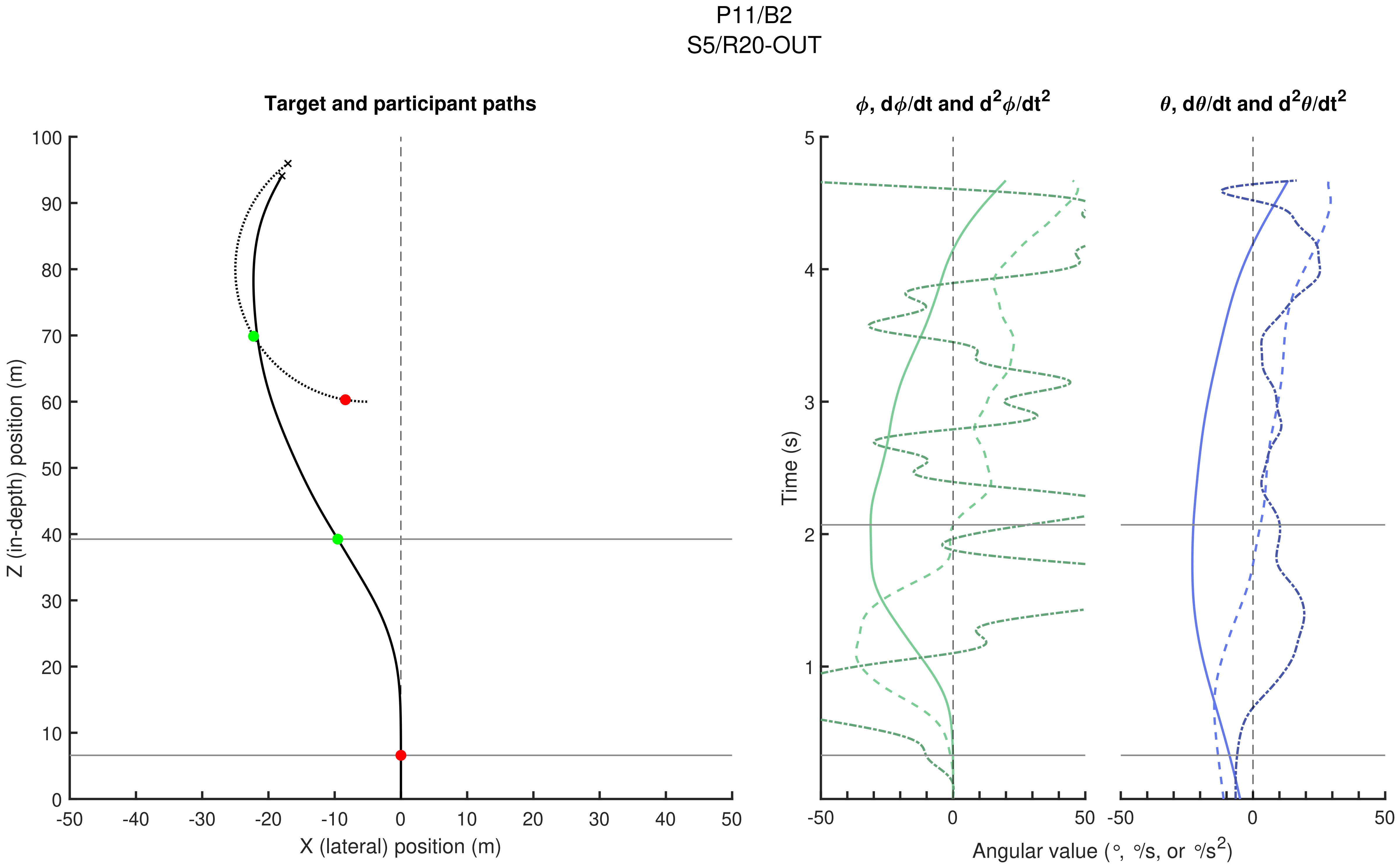
P11/B2
S5/R20-OUT
Target and participant paths
Z (in-depth) position (m)
X (lateral) position (m)
ϕ, dϕ/dt and d²ϕ/dt²
θ, dθ/dt and d²θ/dt²
Time (s)
Angular value (°, °/s, or °/s²)

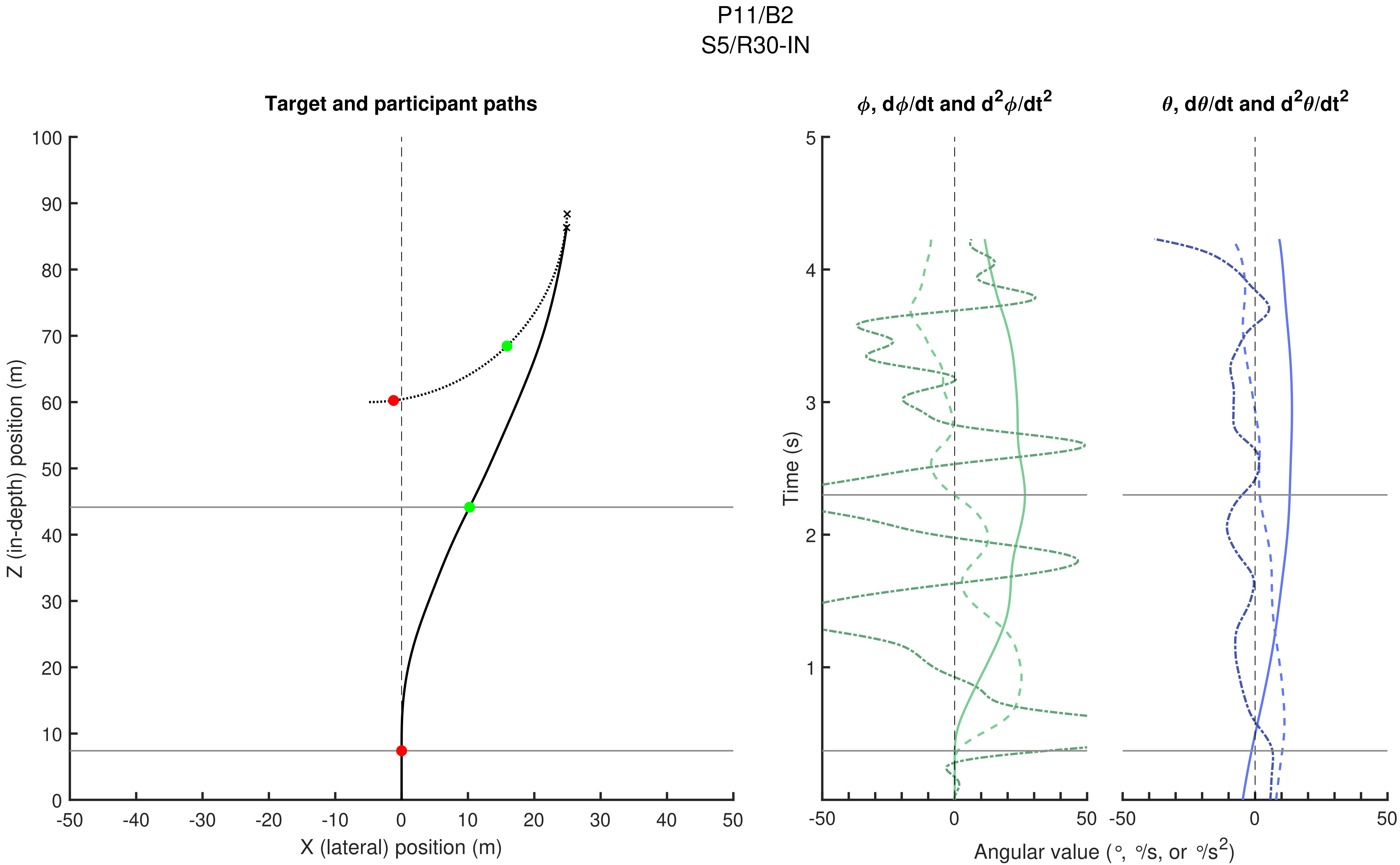
P11/B2
S5/R30-IN
Target and participant paths
Z (in-depth) position (m)
X (lateral) position (m)
100
90
80
70
60
50
40
30
20
10
0
-50
-40
-30
-20
-10
0
10
20
30
40
50
ϕ, dϕ/dt and d$^2\phi$/dt^2
θ, dθ/dt and d$^2\theta$/dt^2
Time (s)
5
4
3
2
1
-50
0
50
-50
0
50
Angular value (°, °/s, or °/s^2)

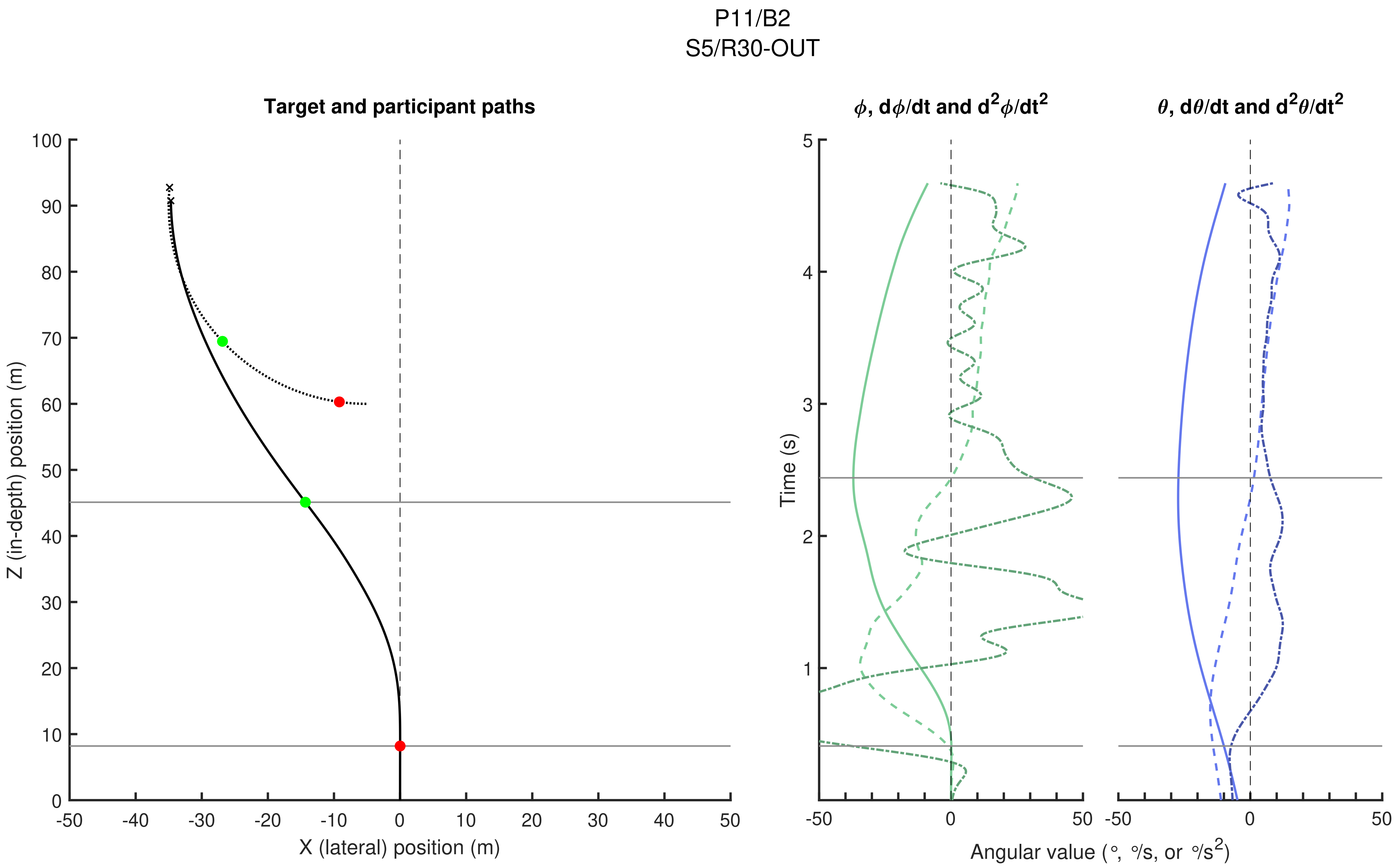

P11/B2
S5/R30-OUT
Target and participant paths
ϕ, dϕ/dt and d$^2\phi$/dt^2
θ, dθ/dt and d$^2\theta$/dt^2
Z (in-depth) position (m)
X (lateral) position (m)
Time (s)
Angular value (°, °/s, or °/s^2)

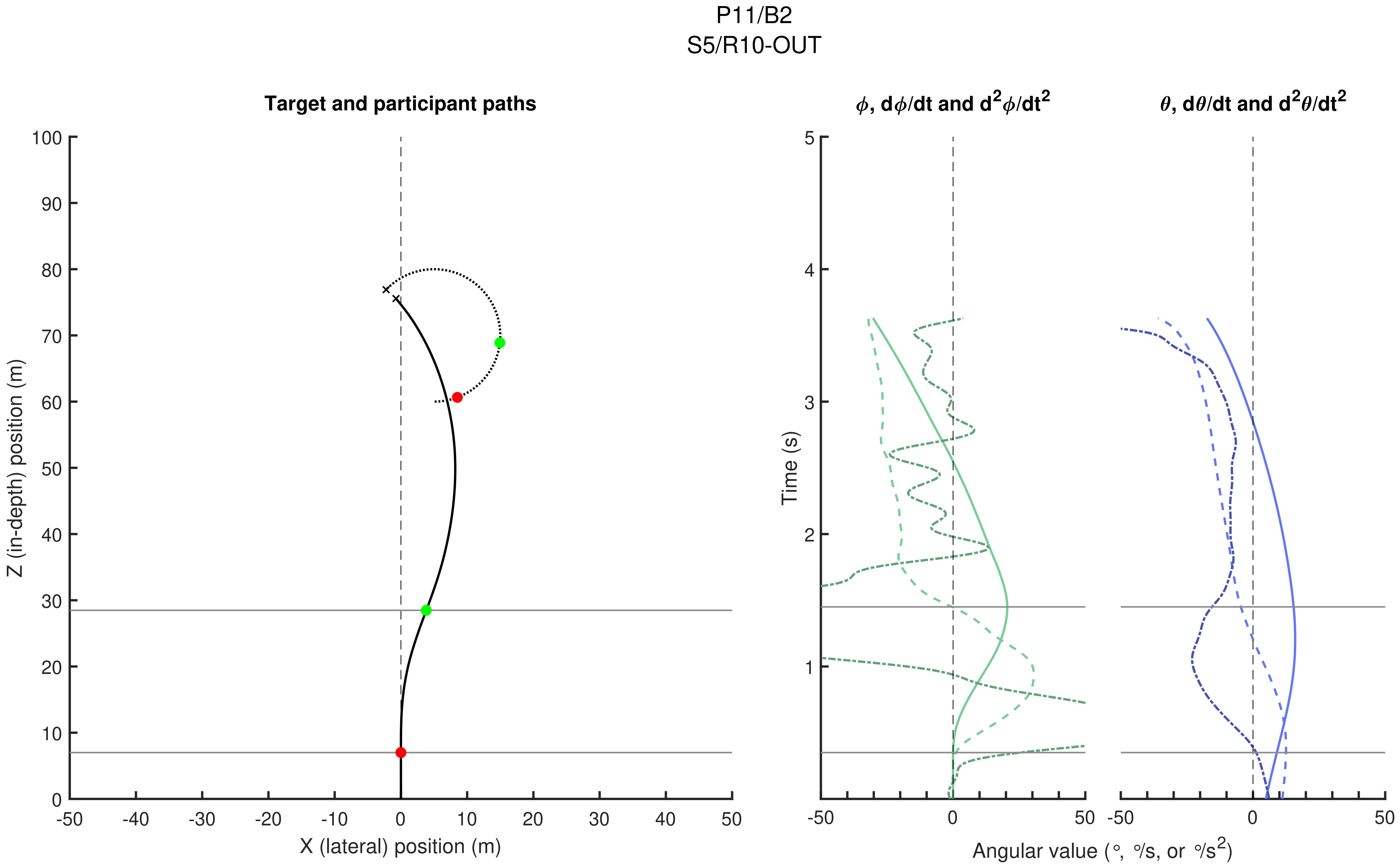
P11/B2
S5/R10-OUT
Target and participant paths
Z (in-depth) position (m)
X (lateral) position (m)
φ, dφ/dt and d²φ/dt²
θ, dθ/dt and d²θ/dt²
Time (s)
Angular value (°, °/s, or °/s²)

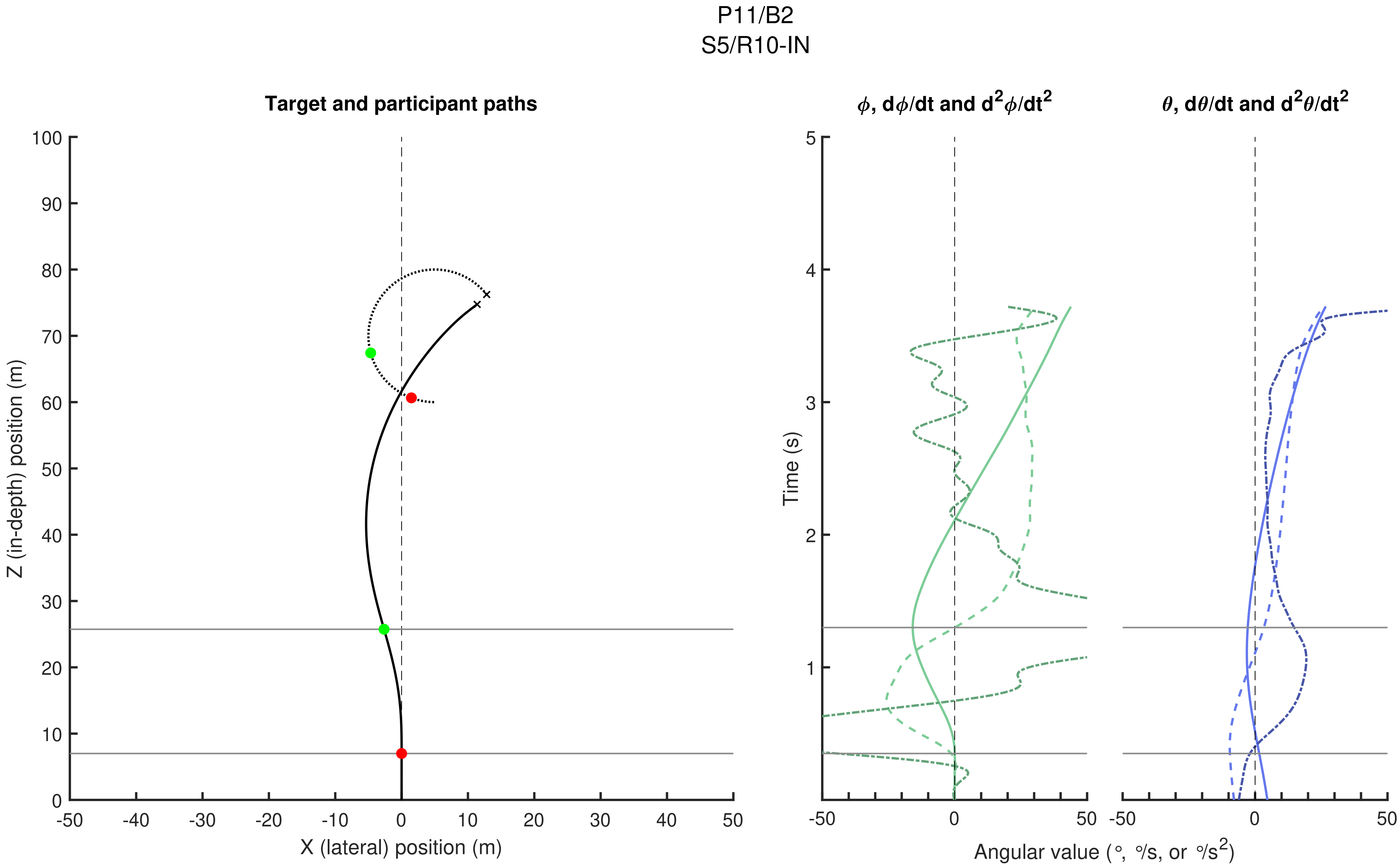
P11/B2
S5/R10-IN
Target and participant paths
ϕ, dϕ/dt and d$^2\phi$/dt^2
θ, dθ/dt and d$^2\theta$/dt^2
Z (in-depth) position (m)
X (lateral) position (m)
Time (s)
Angular value (°, °/s, or °/s^2)
100
90
80
70
60
50
40
30
20
10
0
-50
-40
-30
-20
-10
0
10
20
30
40
50
5
4
3
2
1
-50
0
50
-50
0
50

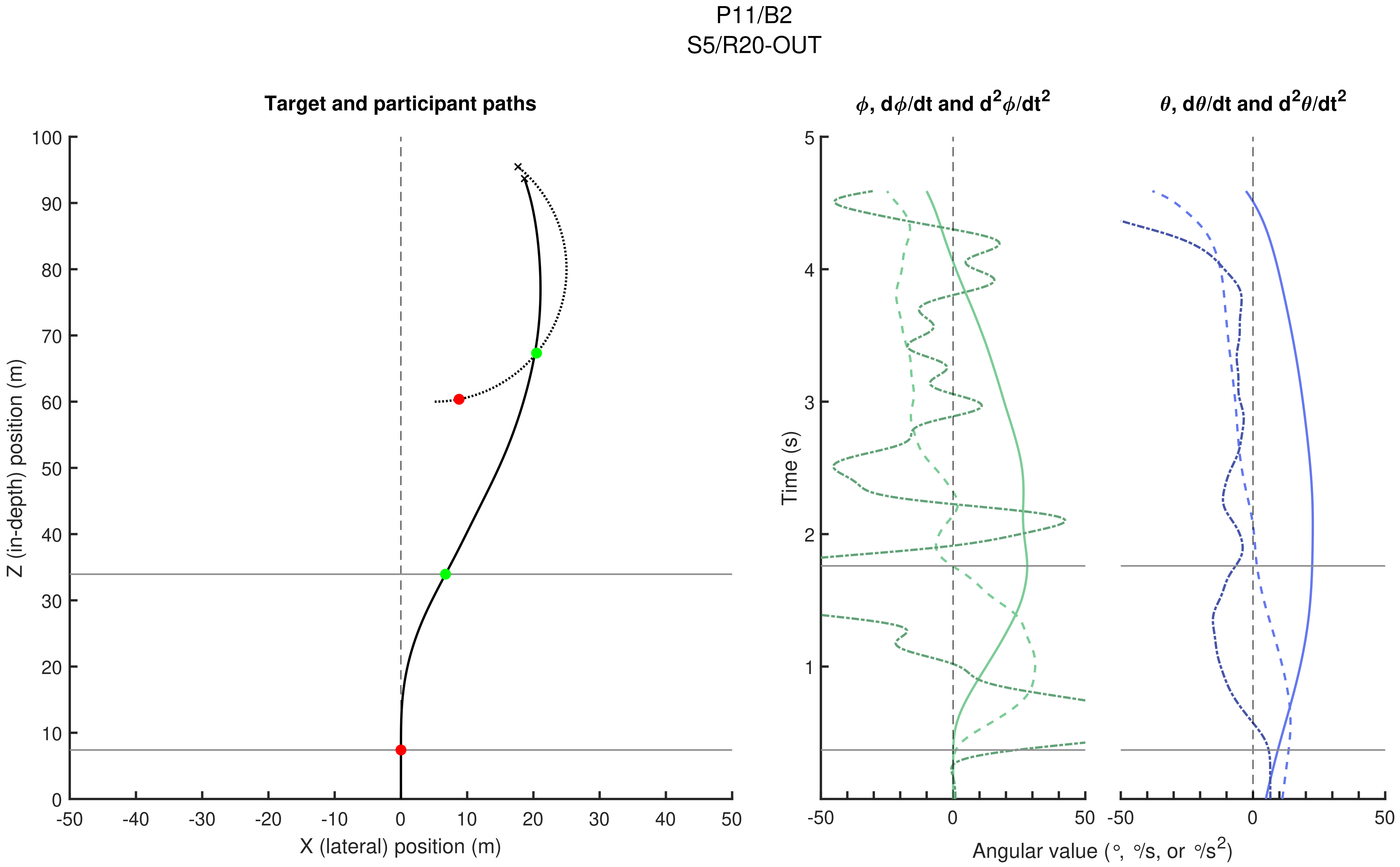
P11/B2
S5/R20-OUT
Target and participant paths
Z (in-depth) position (m)
X (lateral) position (m)
100
90
80
70
60
50
40
30
20
10
0
-50
-40
-30
-20
-10
0
10
20
30
40
50
ϕ, dϕ/dt and d²ϕ/dt²
θ, dθ/dt and d²θ/dt²
Time (s)
5
4
3
2
1
-50
0
50
-50
0
50
Angular value (°, °/s, or °/s²)

P11/B2
S5/R20-IN

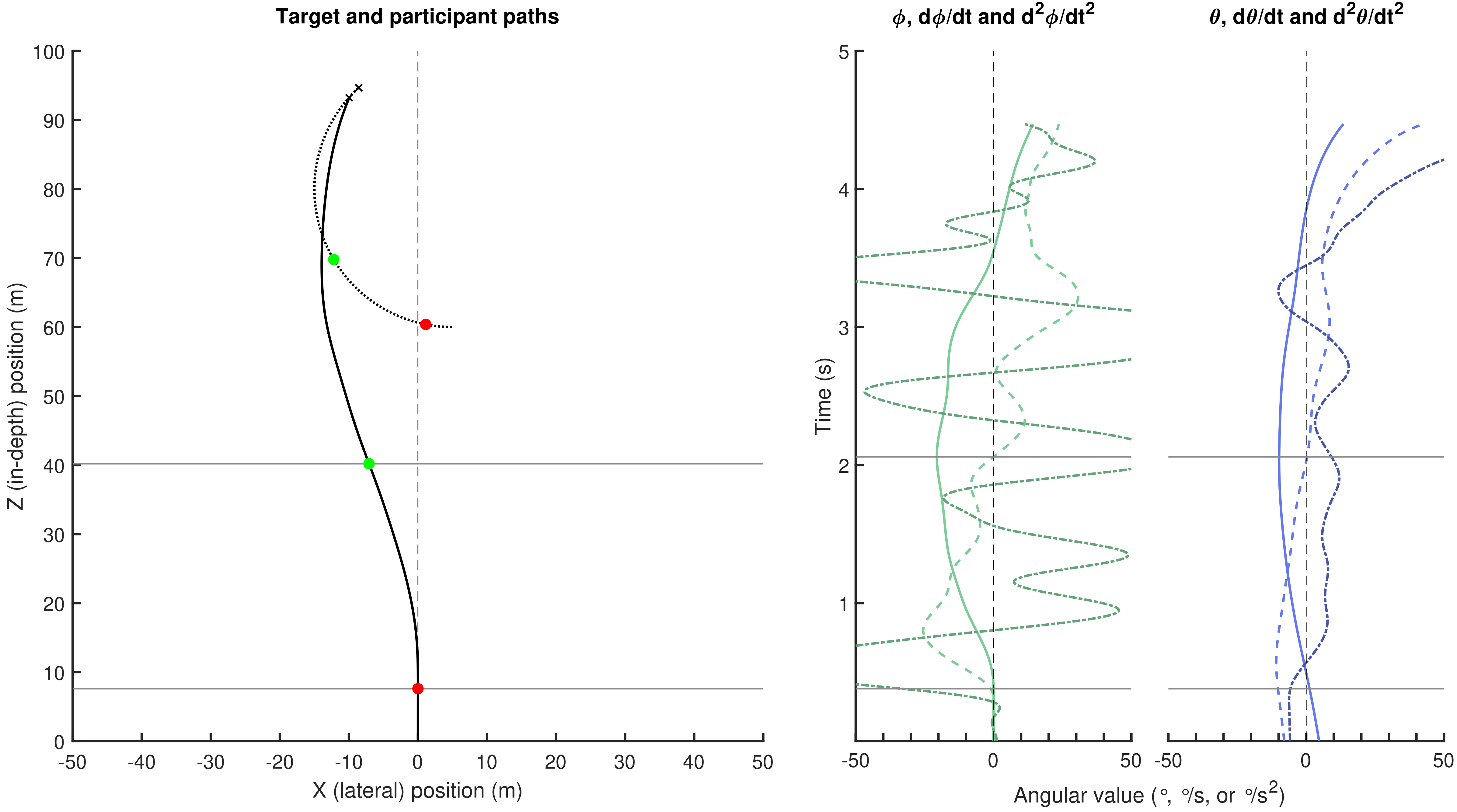

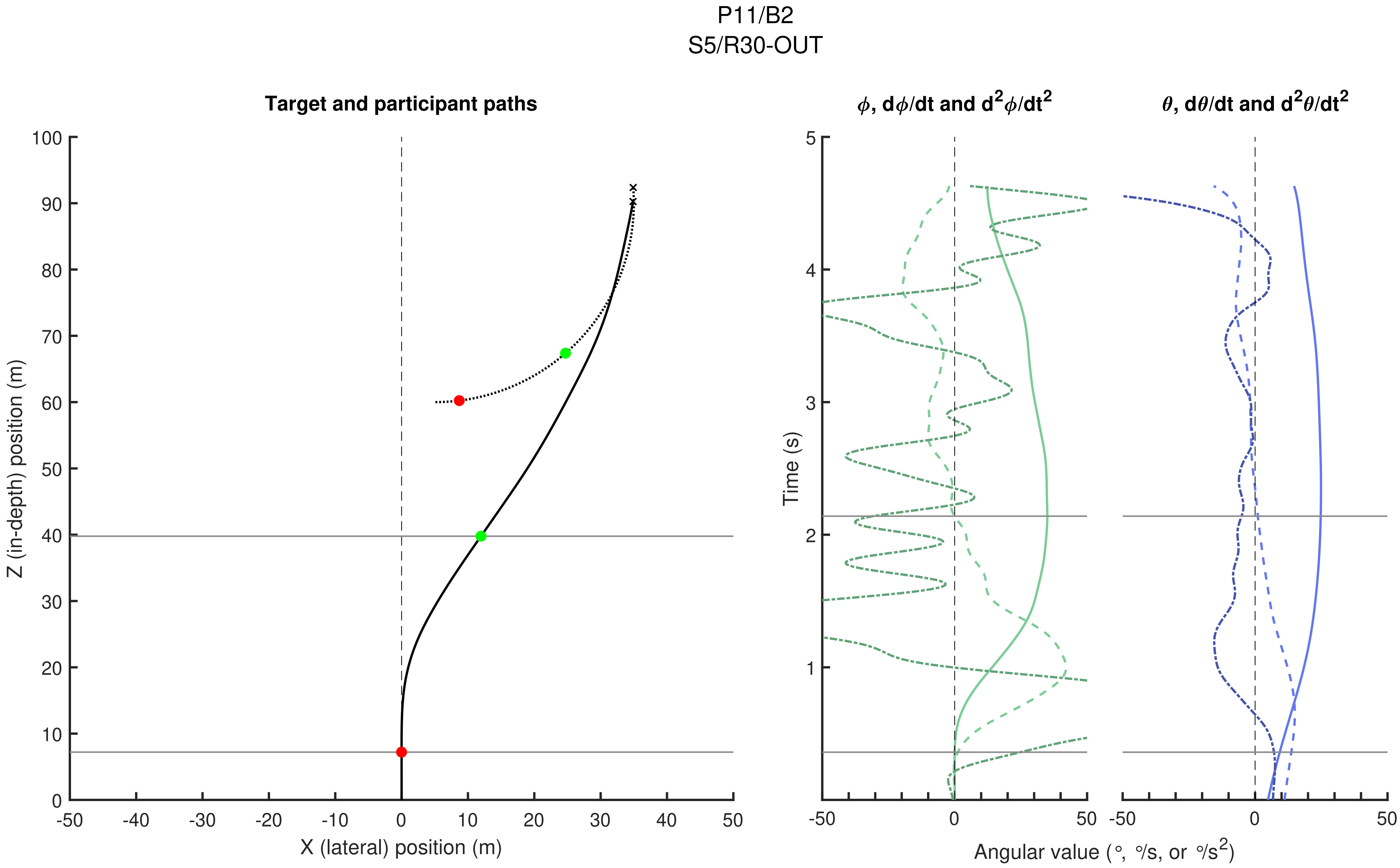
P11/B2
S5/R30-OUT
Target and participant paths
Z (in-depth) position (m)
X (lateral) position (m)
0
10
20
30
40
50
60
70
80
90
100
-50
-40
-30
-20
-10
0
10
20
30
40
50
ϕ, dϕ/dt and d$^2\phi$/dt^2
θ, dθ/dt and d$^2\theta$/dt^2
Time (s)
1
2
3
4
5
-50
0
50
-50
0
50
Angular value (°, °/s, or °/s^2)

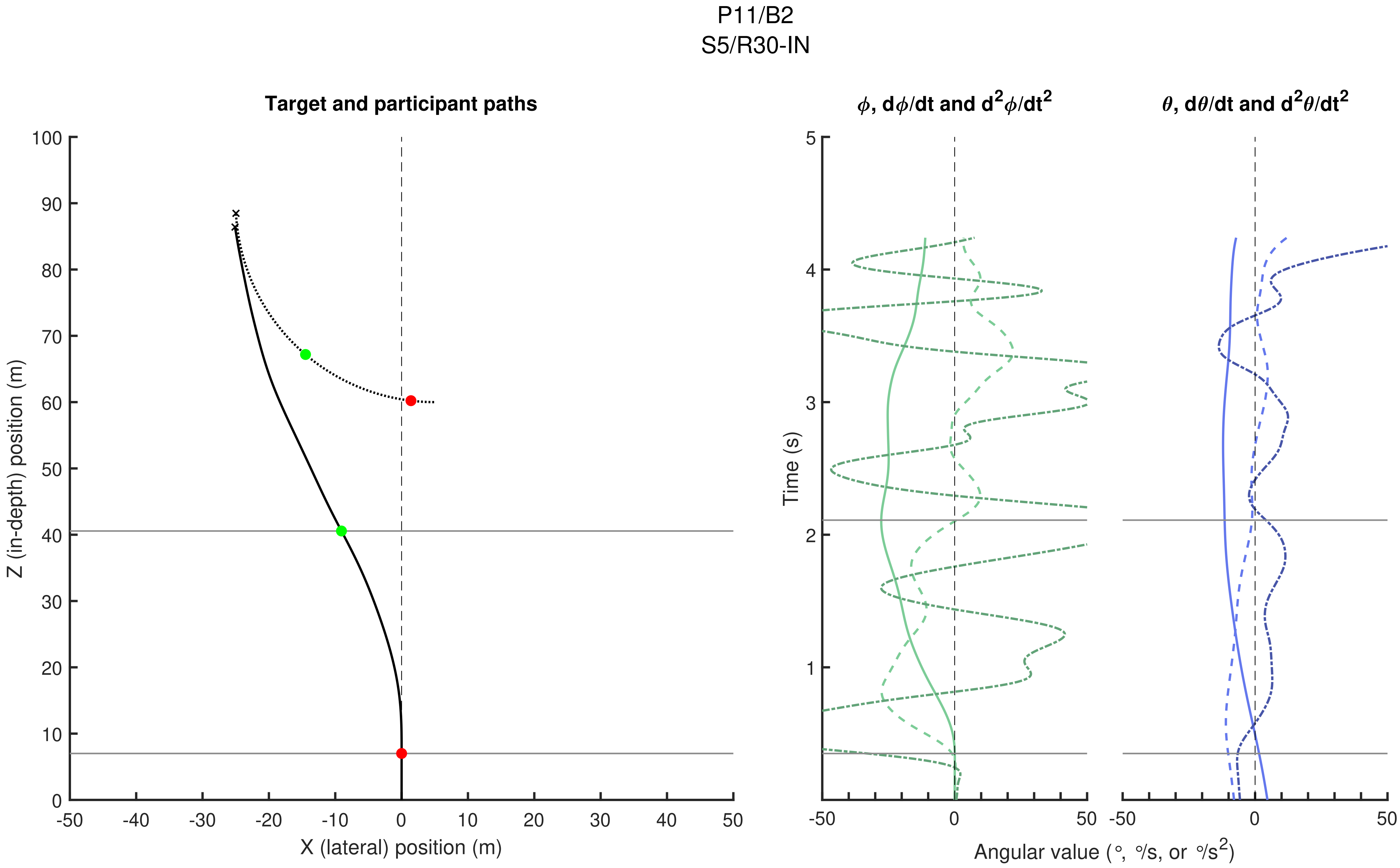
P11/B2
S5/R30-IN
Target and participant paths
ϕ, dϕ/dt and d$^2\phi$/dt^2
θ, dθ/dt and d$^2\theta$/dt^2
Z (in-depth) position (m)
X (lateral) position (m)
Time (s)
Angular value (°, °/s, or °/s^2)
100
90
80
70
60
50
40
30
20
10
0
-50
-40
-30
-20
-10
0
10
20
30
40
50
5
4
3
2
1
-50
0
50
-50
0
50

P11/B2
S15/R10-IN

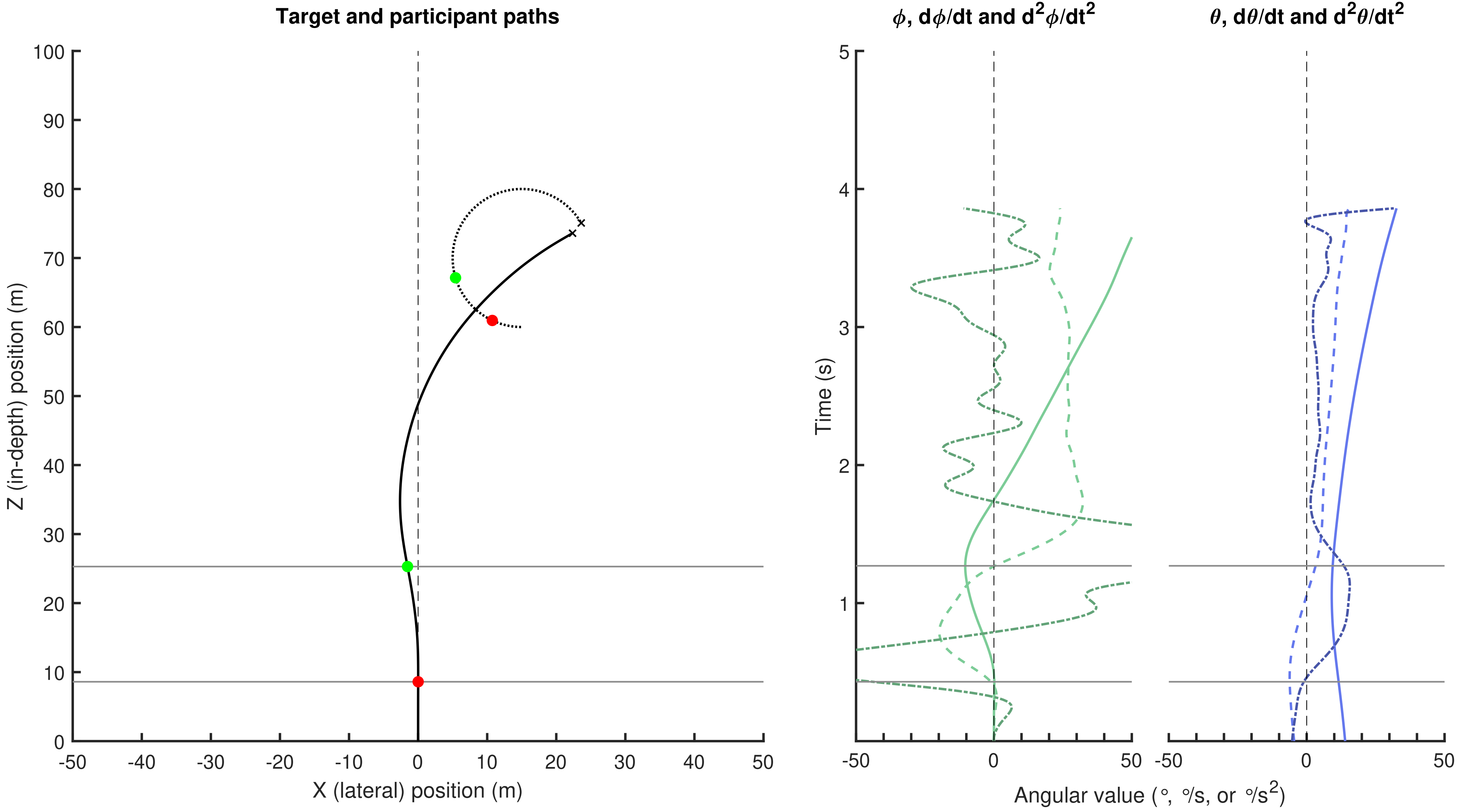

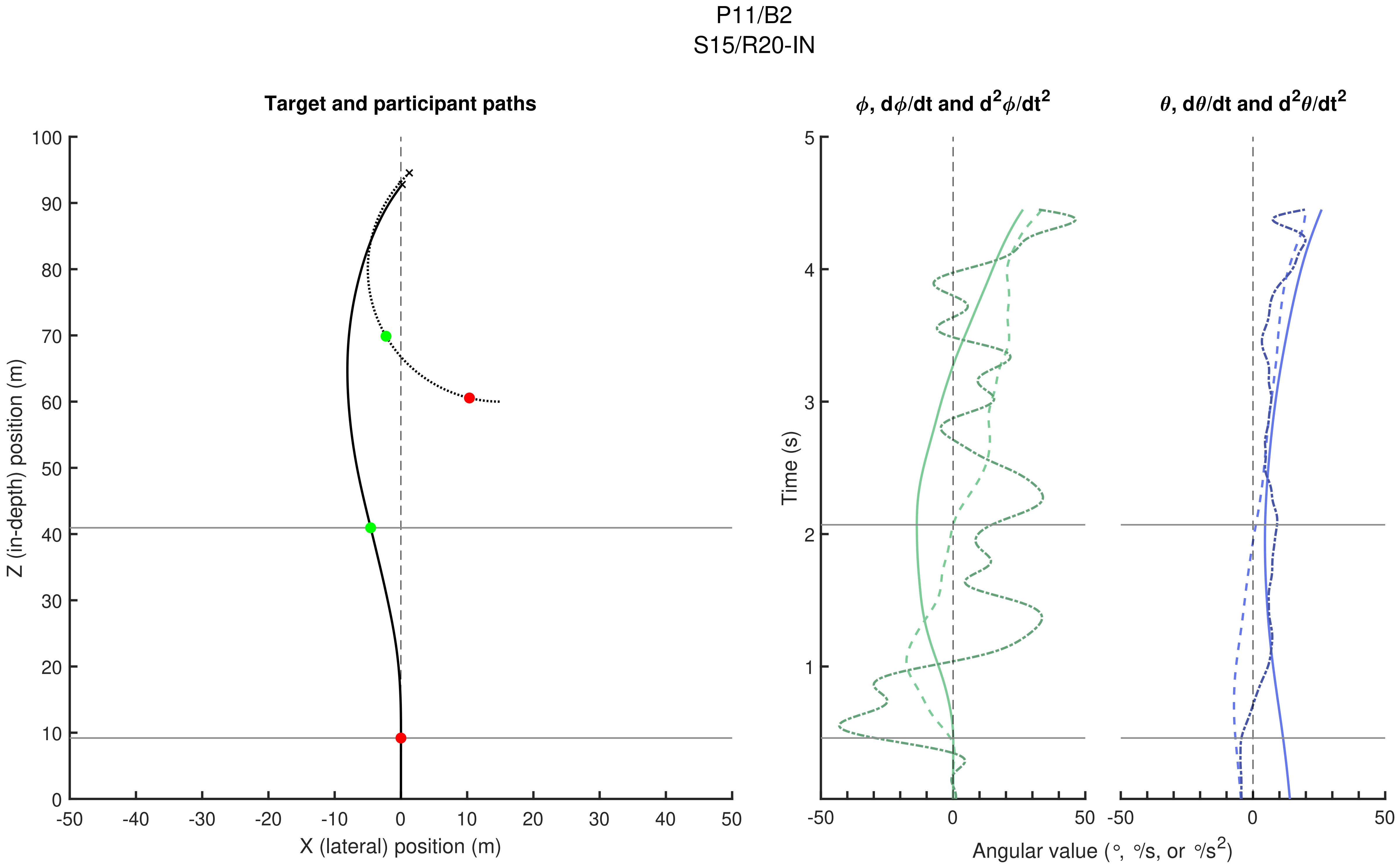
P11/B2
S15/R20-IN
Target and participant paths
Z (in-depth) position (m)
X (lateral) position (m)
ϕ, dϕ/dt and d²ϕ/dt²
θ, dθ/dt and d²θ/dt²
Time (s)
Angular value (°, °/s, or °/s²)

P11/B2
S15/R30-IN

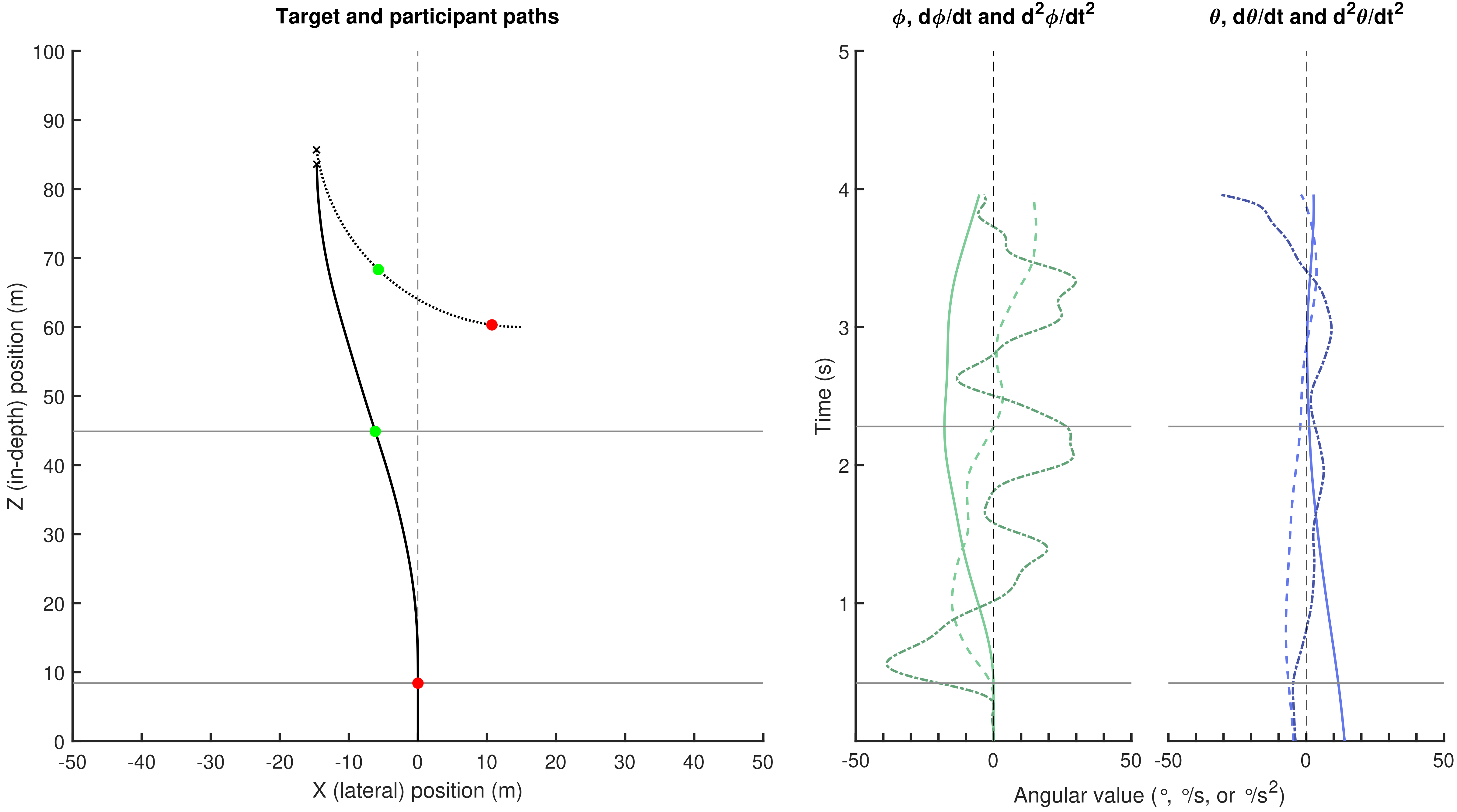

P11/B2
S20/R10-IN

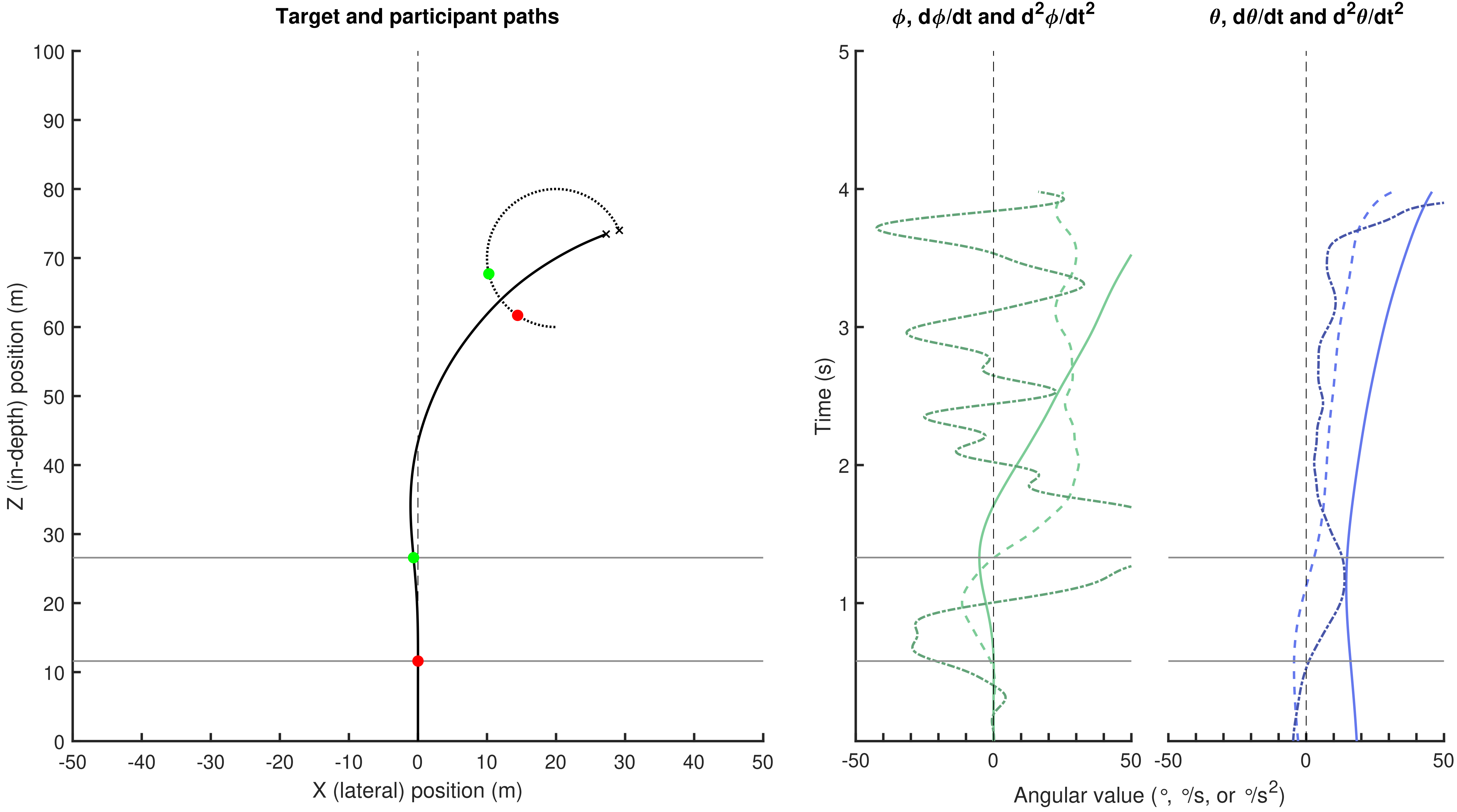

P11/B2
S20/R20-IN

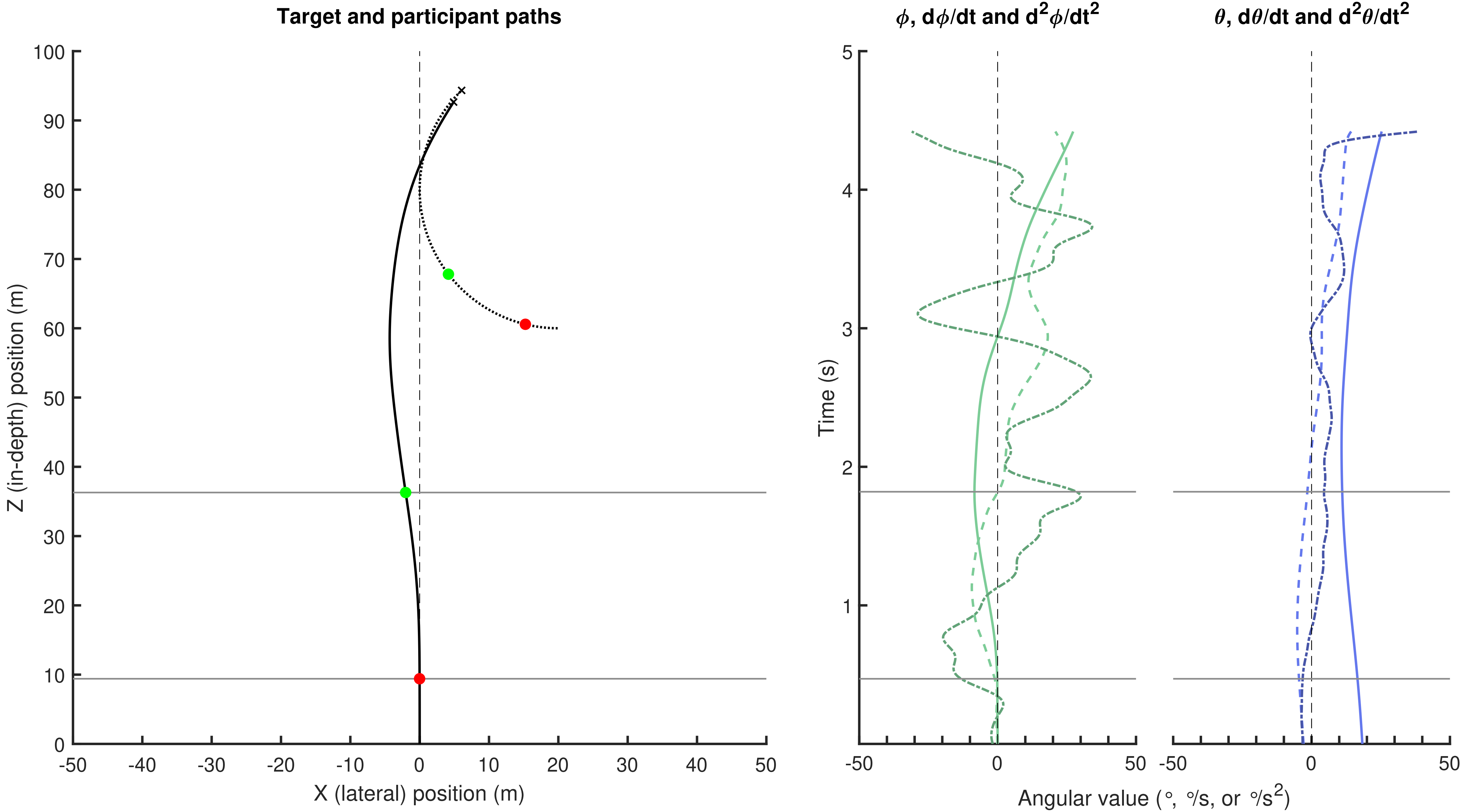

P11/B2
S20/R30-IN

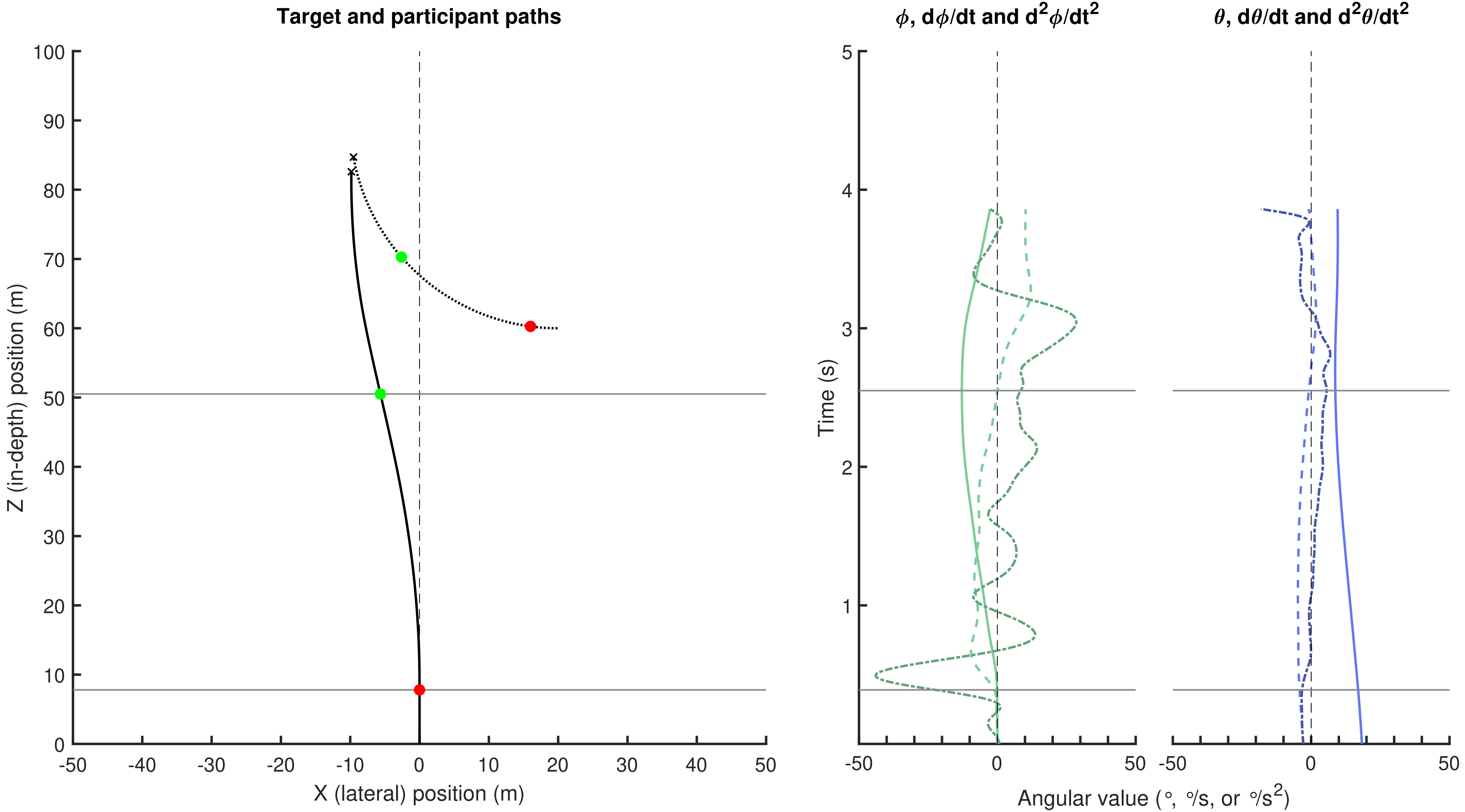

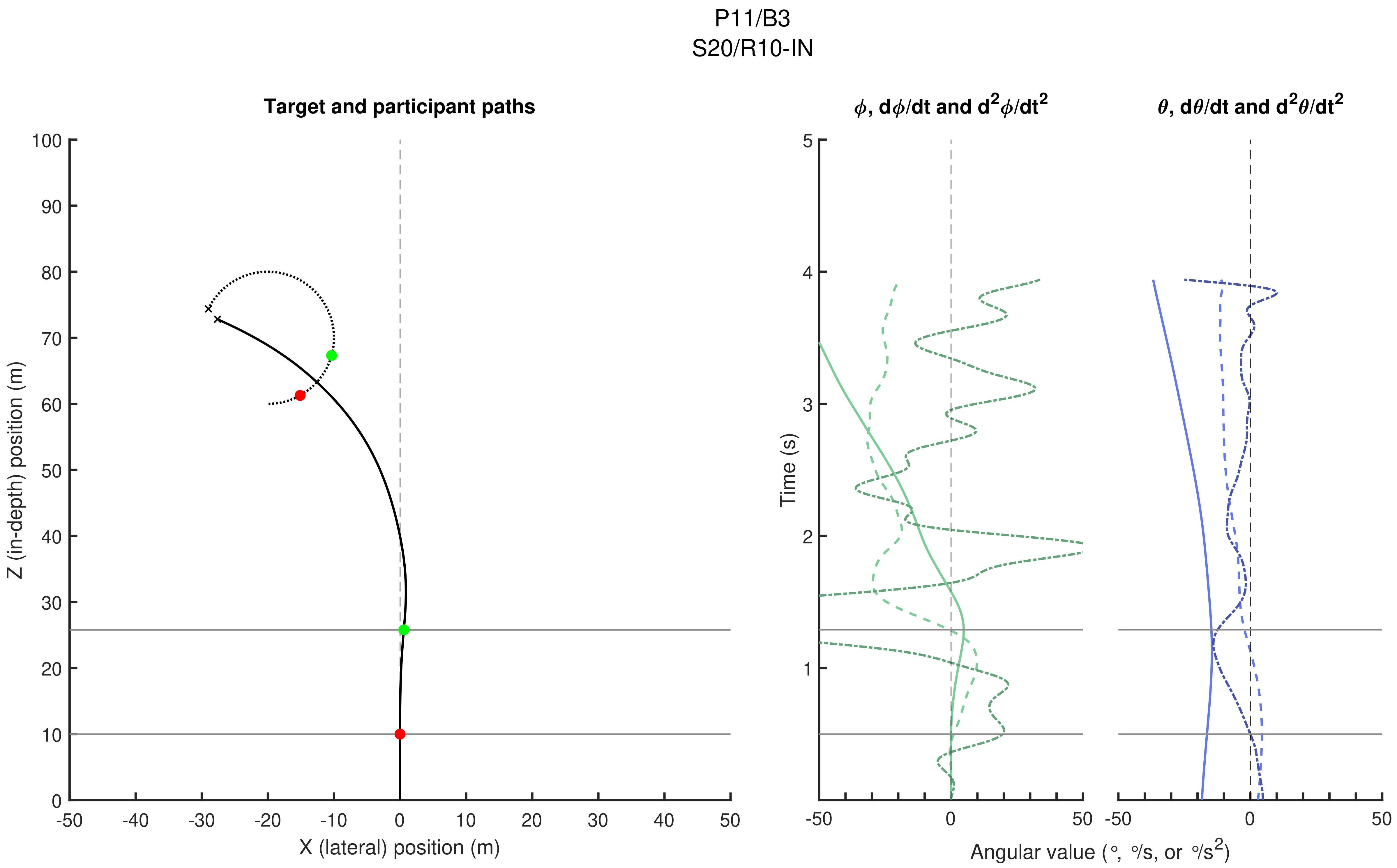
P11/B3
S20/R10-IN
Target and participant paths
Z (in-depth) position (m)
X (lateral) position (m)
φ, dφ/dt and d²φ/dt²
θ, dθ/dt and d²θ/dt²
Time (s)
Angular value (°, °/s, or °/s²)

P11/B3
S20/R20-IN

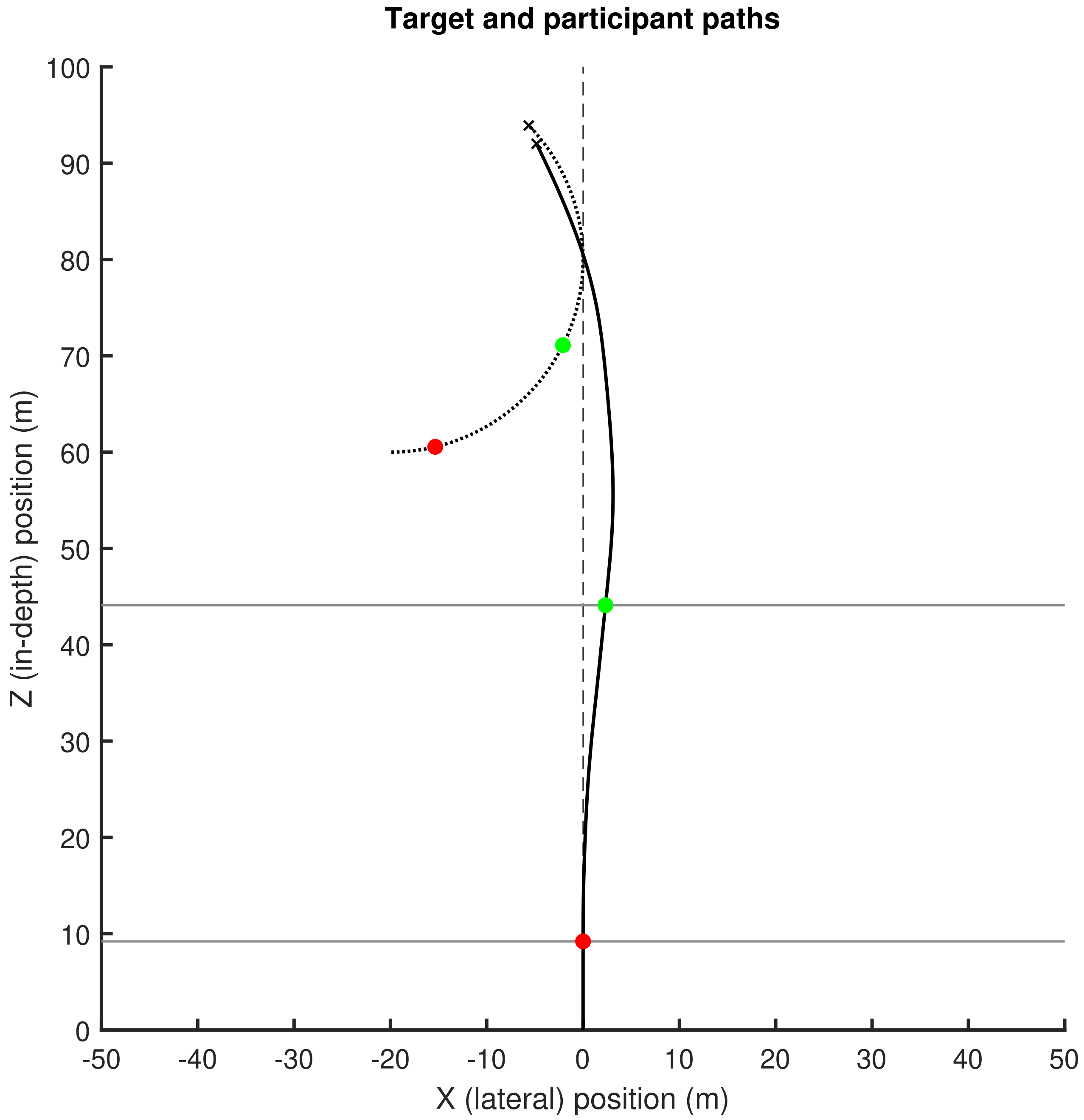

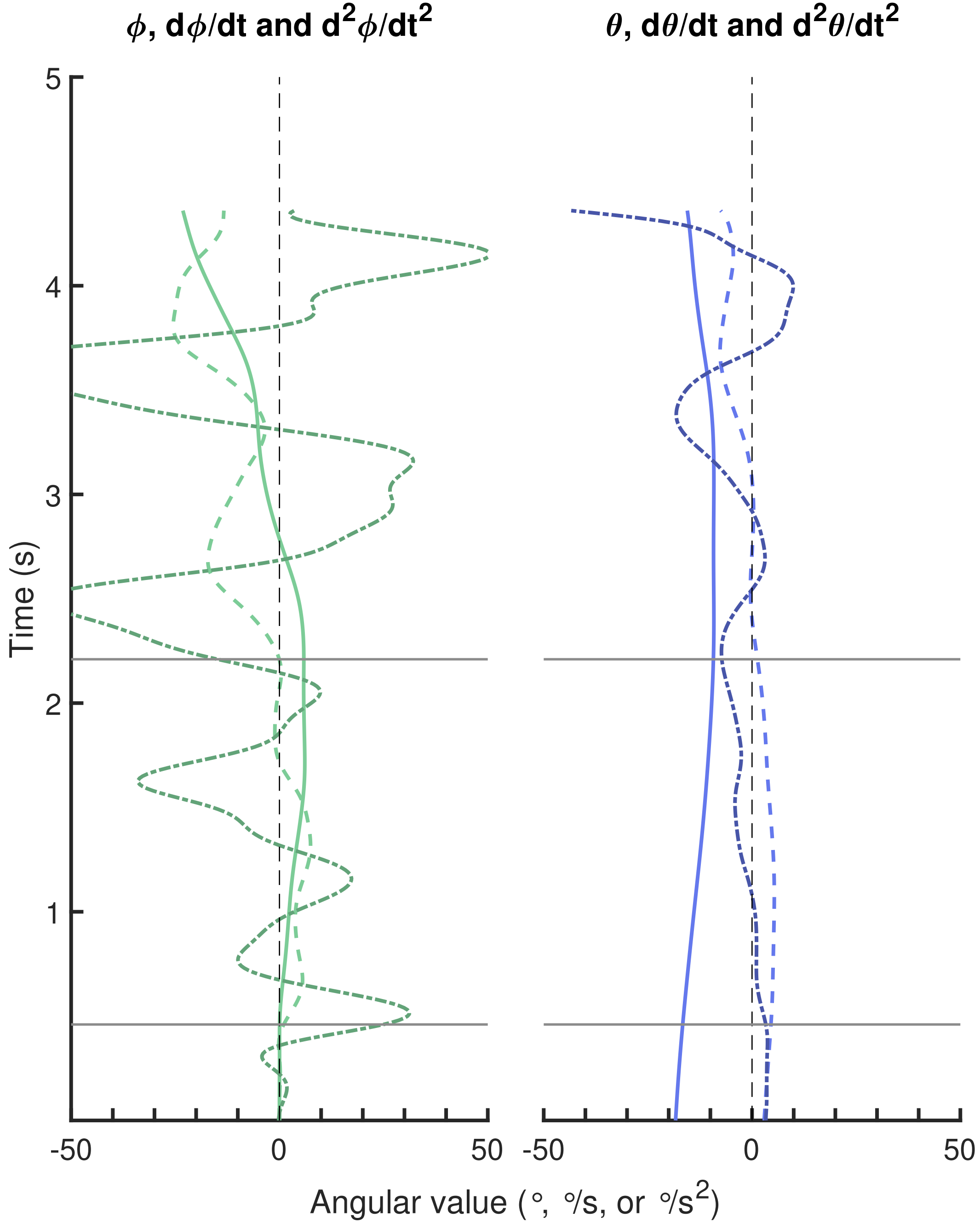

P11/B3
S20/R30-IN

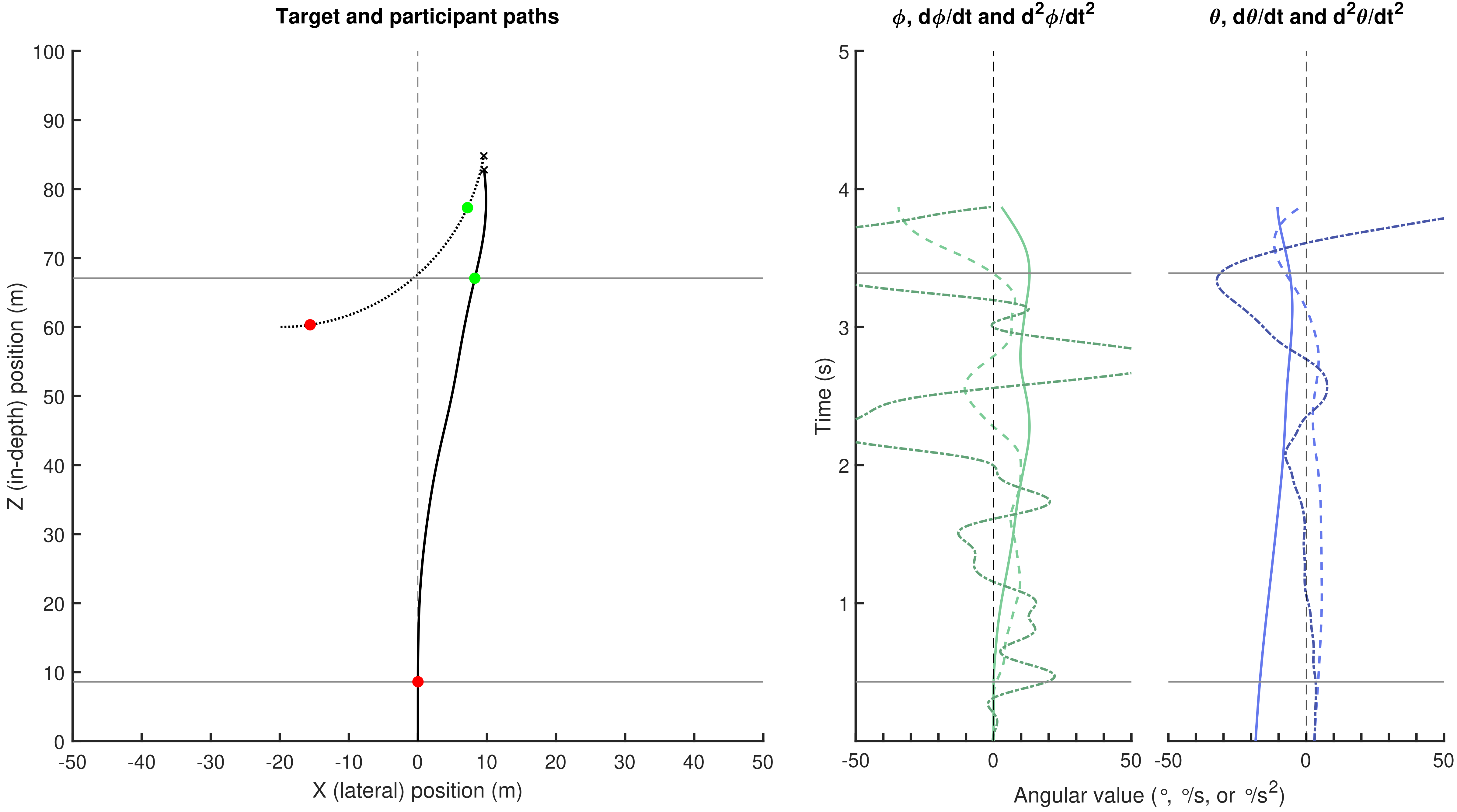

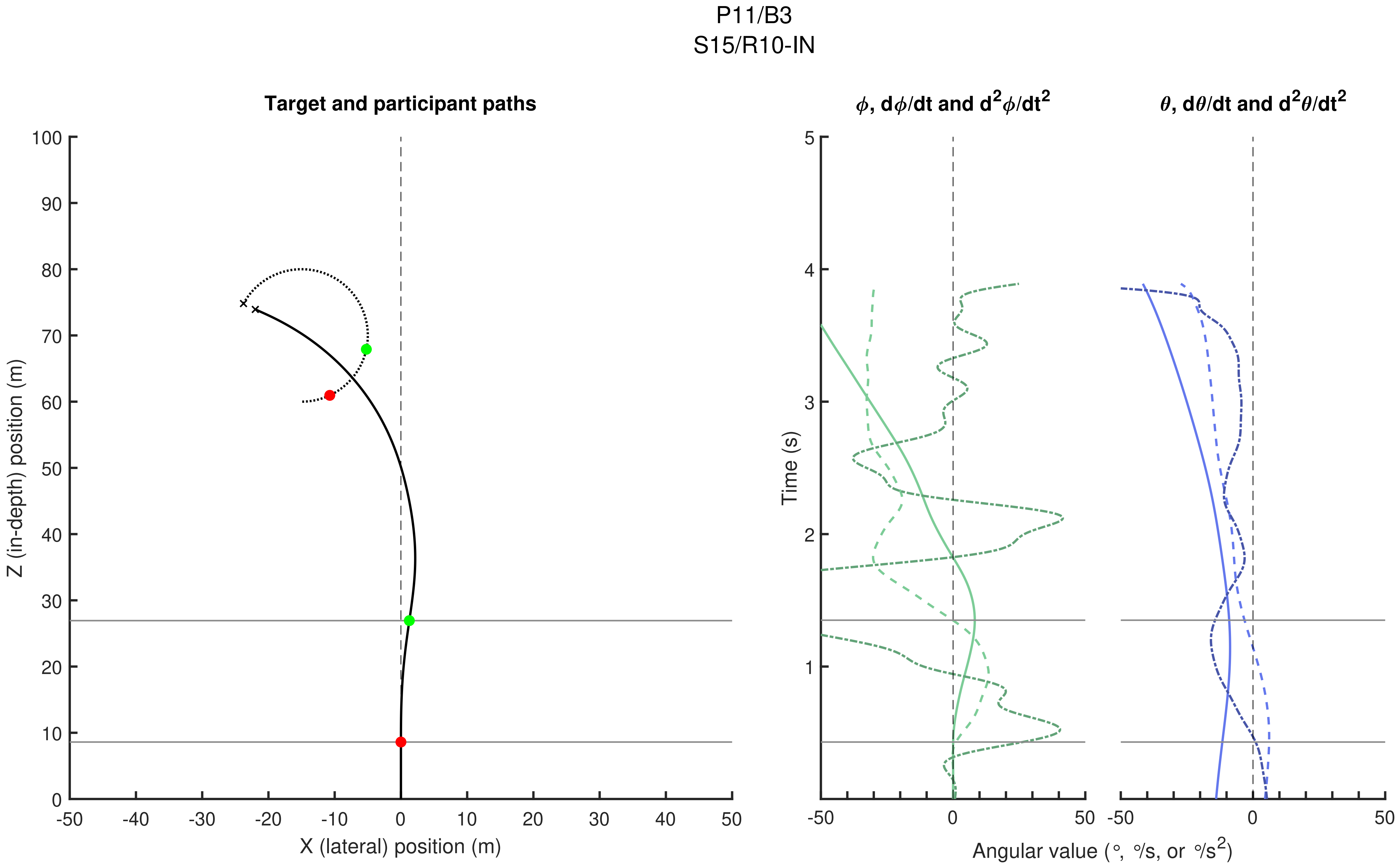
P11/B3
S15/R10-IN
Target and participant paths
Z (in-depth) position (m)
X (lateral) position (m)
ϕ, dϕ/dt and d²ϕ/dt²
θ, dθ/dt and d²θ/dt²
Time (s)
Angular value (°, °/s, or °/s²)

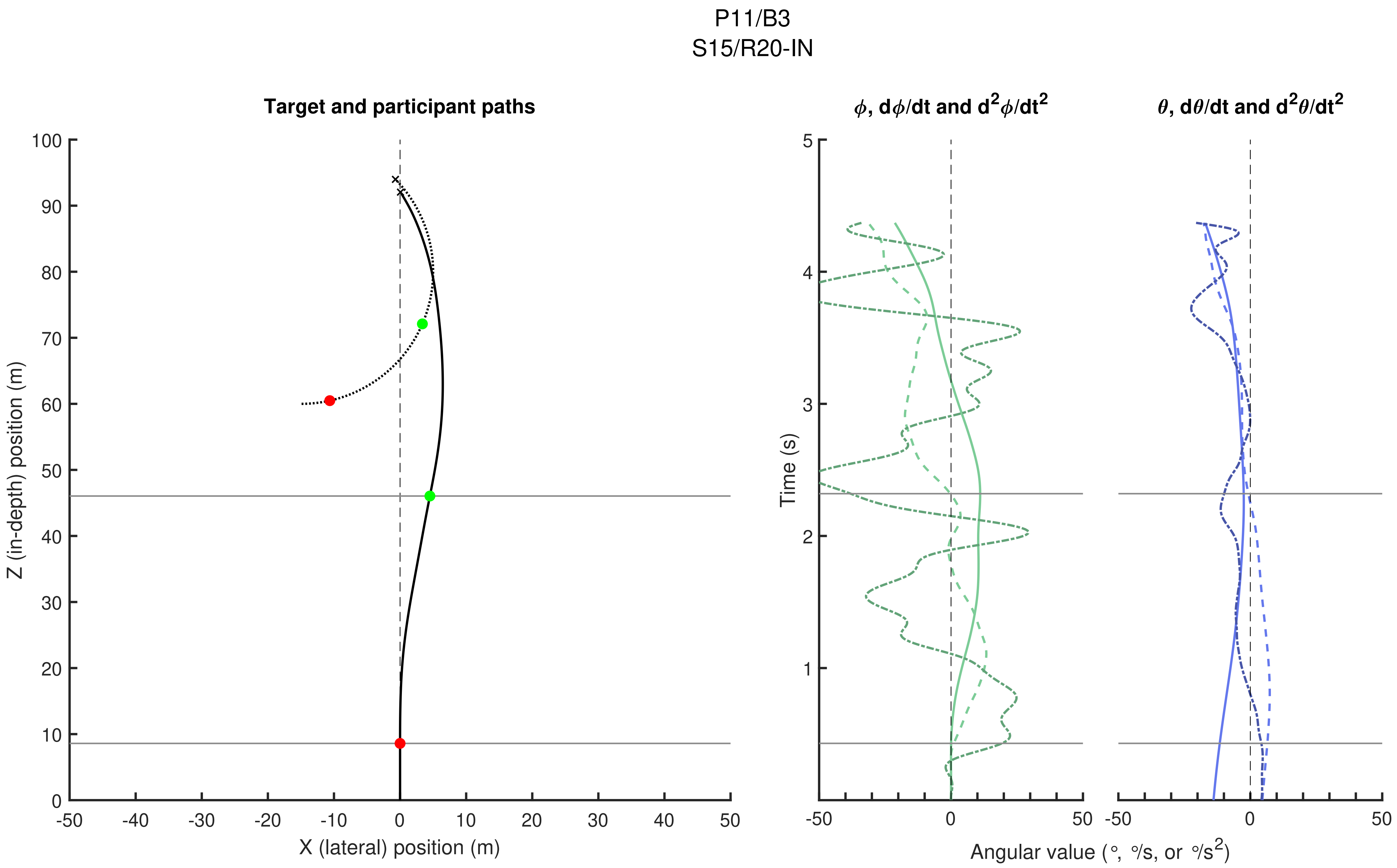

P11/B3
S15/R20-IN
Target and participant paths
Z (in-depth) position (m)
X (lateral) position (m)
ϕ, dϕ/dt and d$^2\phi$/dt^2
θ, dθ/dt and d$^2\theta$/dt^2
Time (s)
Angular value (°, °/s, or °/s^2)

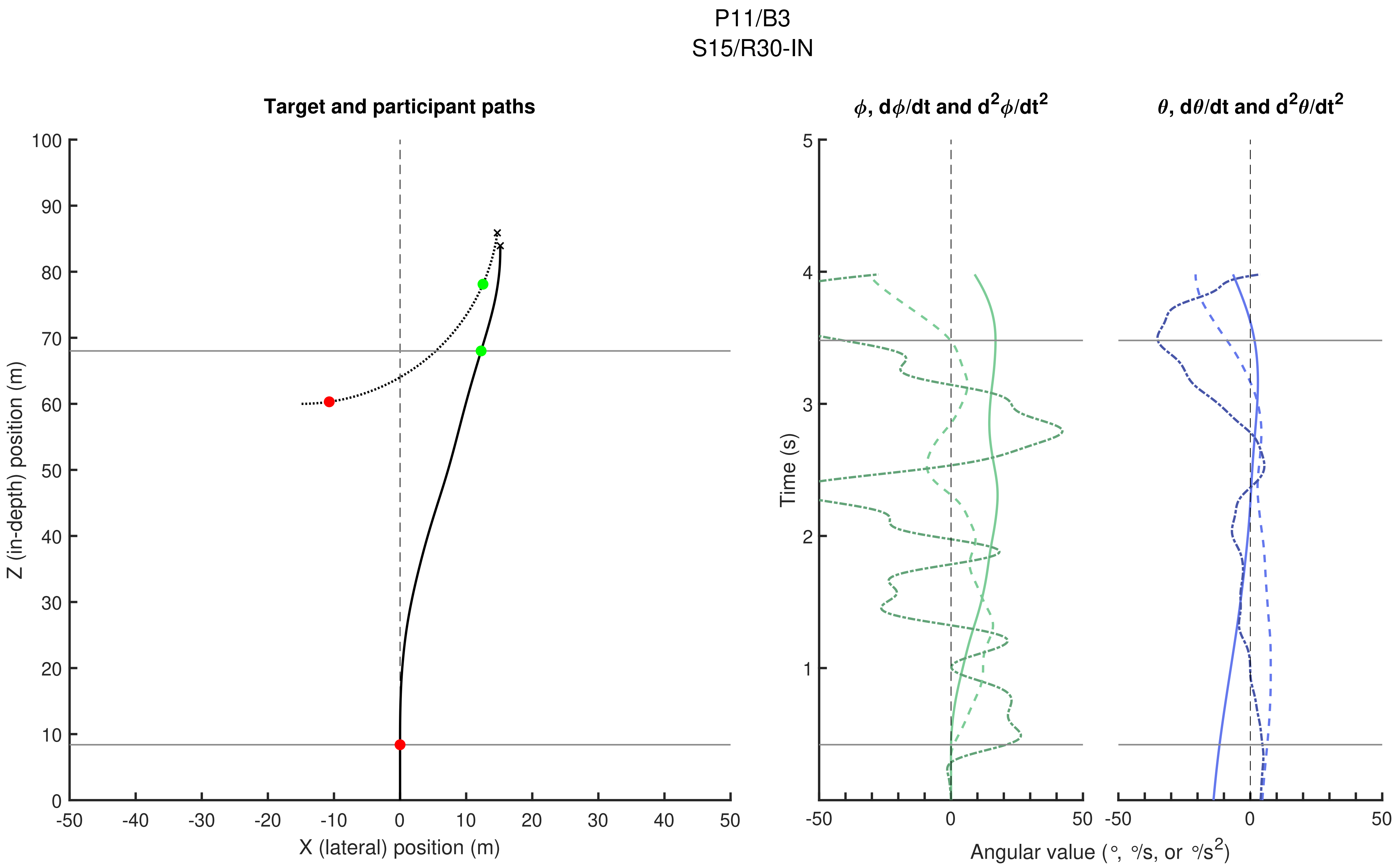
P11/B3
S15/R30-IN
Target and participant paths
Z (in-depth) position (m)
X (lateral) position (m)
100
90
80
70
60
50
40
30
20
10
0
-50
-40
-30
-20
-10
0
10
20
30
40
50
ϕ, dϕ/dt and d²ϕ/dt²
θ, dθ/dt and d²θ/dt²
Time (s)
5
4
3
2
1
-50
0
50
-50
0
50
Angular value (°, °/s, or °/s²)

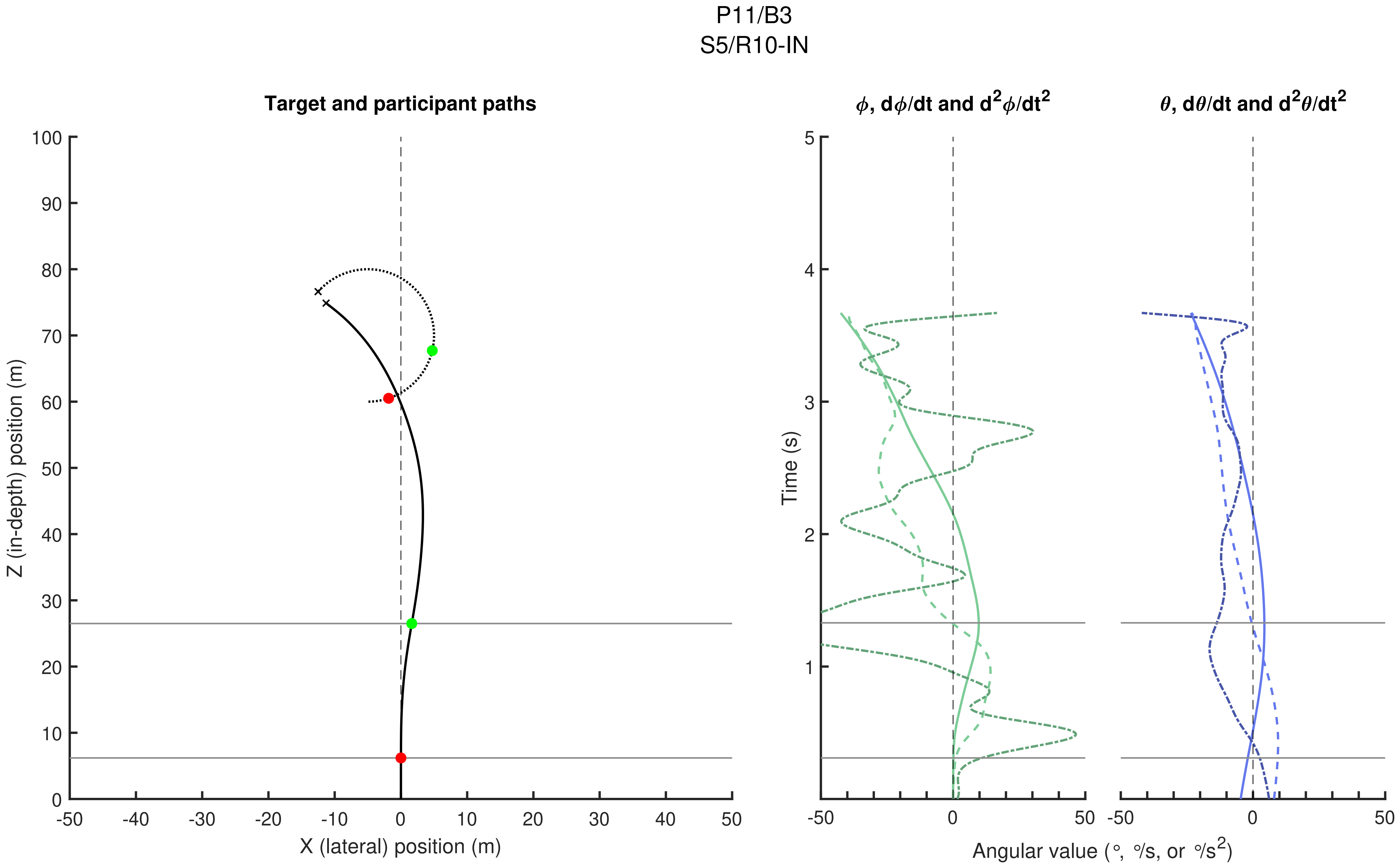

P11/B3
S5/R10-IN
Target and participant paths
Z (in-depth) position (m)
X (lateral) position (m)
ϕ, dϕ/dt and d$^2\phi$/dt^2
θ, dθ/dt and d$^2\theta$/dt^2
Time (s)
Angular value (°, °/s, or °/s^2)

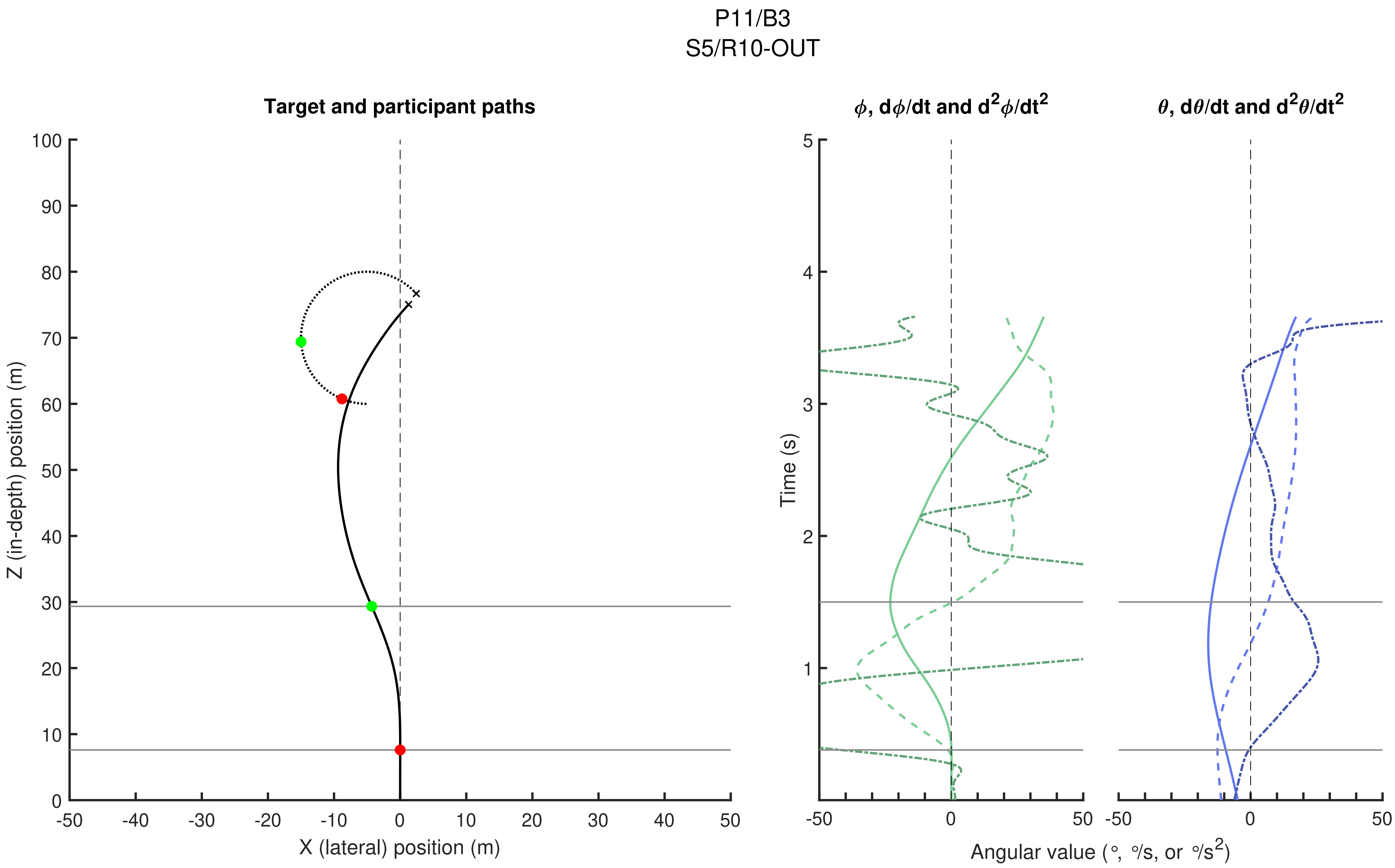

P11/B3
S5/R10-OUT
Target and participant paths
Z (in-depth) position (m)
X (lateral) position (m)
φ, dφ/dt and d²φ/dt²
θ, dθ/dt and d²θ/dt²
Time (s)
Angular value (°, °/s, or °/s²)

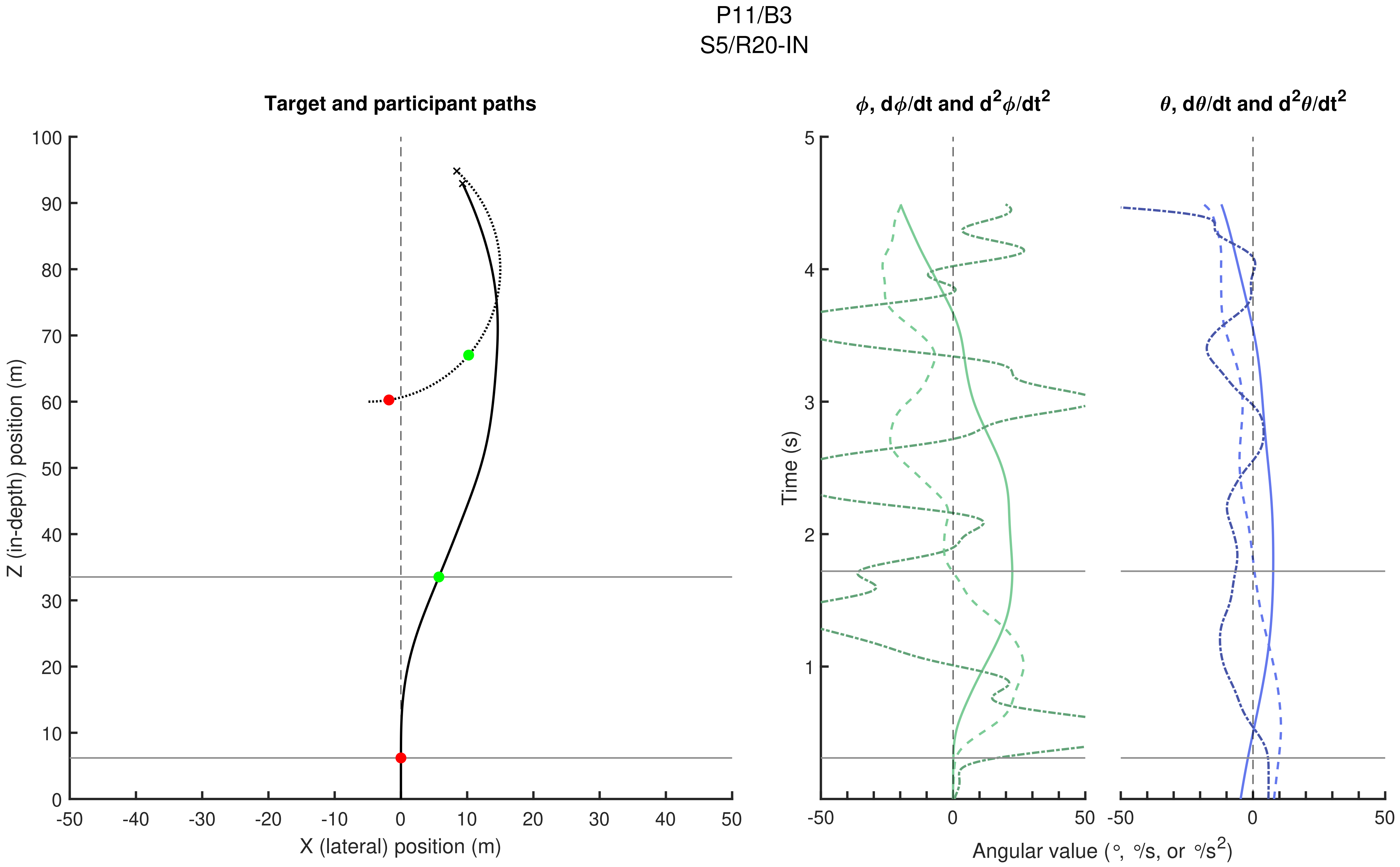
P11/B3
S5/R20-IN
Target and participant paths
Z (in-depth) position (m)
X (lateral) position (m)
φ, dφ/dt and d²φ/dt²
θ, dθ/dt and d²θ/dt²
Time (s)
Angular value (°, °/s, or °/s²)

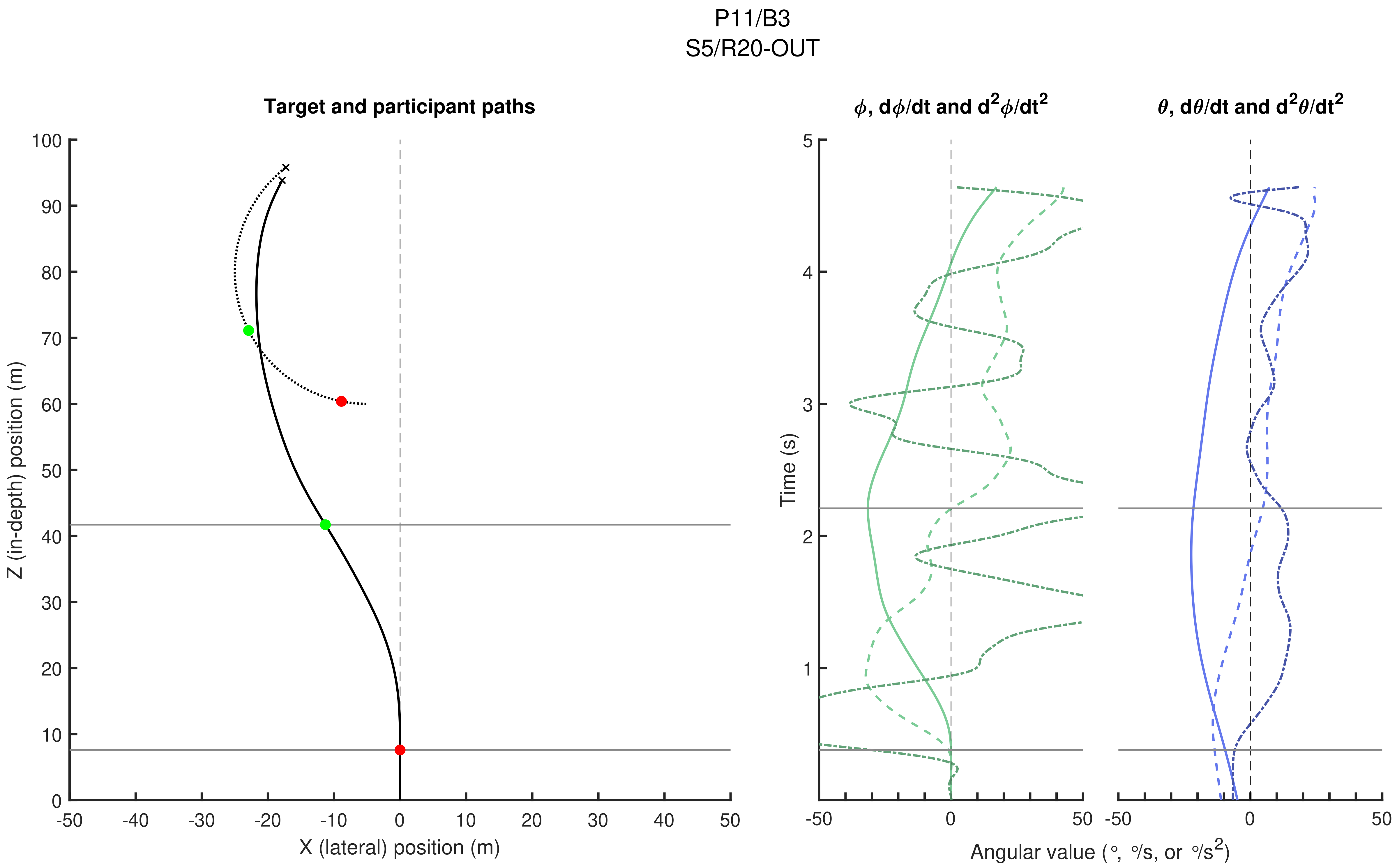
P11/B3
S5/R20-OUT
Target and participant paths
Z (in-depth) position (m)
X (lateral) position (m)
φ, dφ/dt and d²φ/dt²
θ, dθ/dt and d²θ/dt²
Time (s)
Angular value (°, °/s, or °/s²)

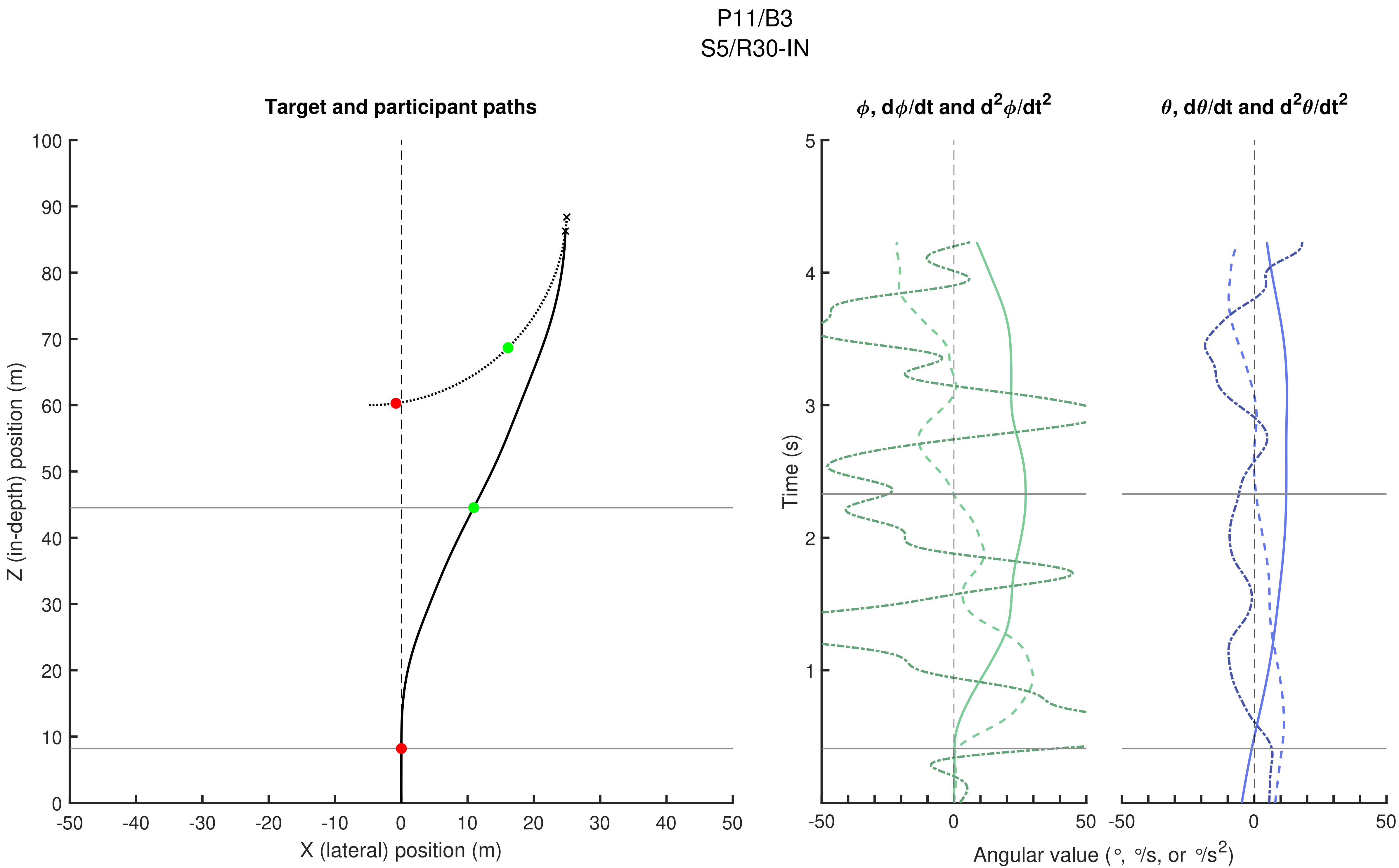

P11/B3
S5/R30-IN
Target and participant paths
Z (in-depth) position (m)
X (lateral) position (m)
ϕ, dϕ/dt and d$^2\phi$/dt^2
θ, dθ/dt and d$^2\theta$/dt^2
Time (s)
Angular value (°, °/s, or °/s^2)

P11/B3
S5/R30-OUT

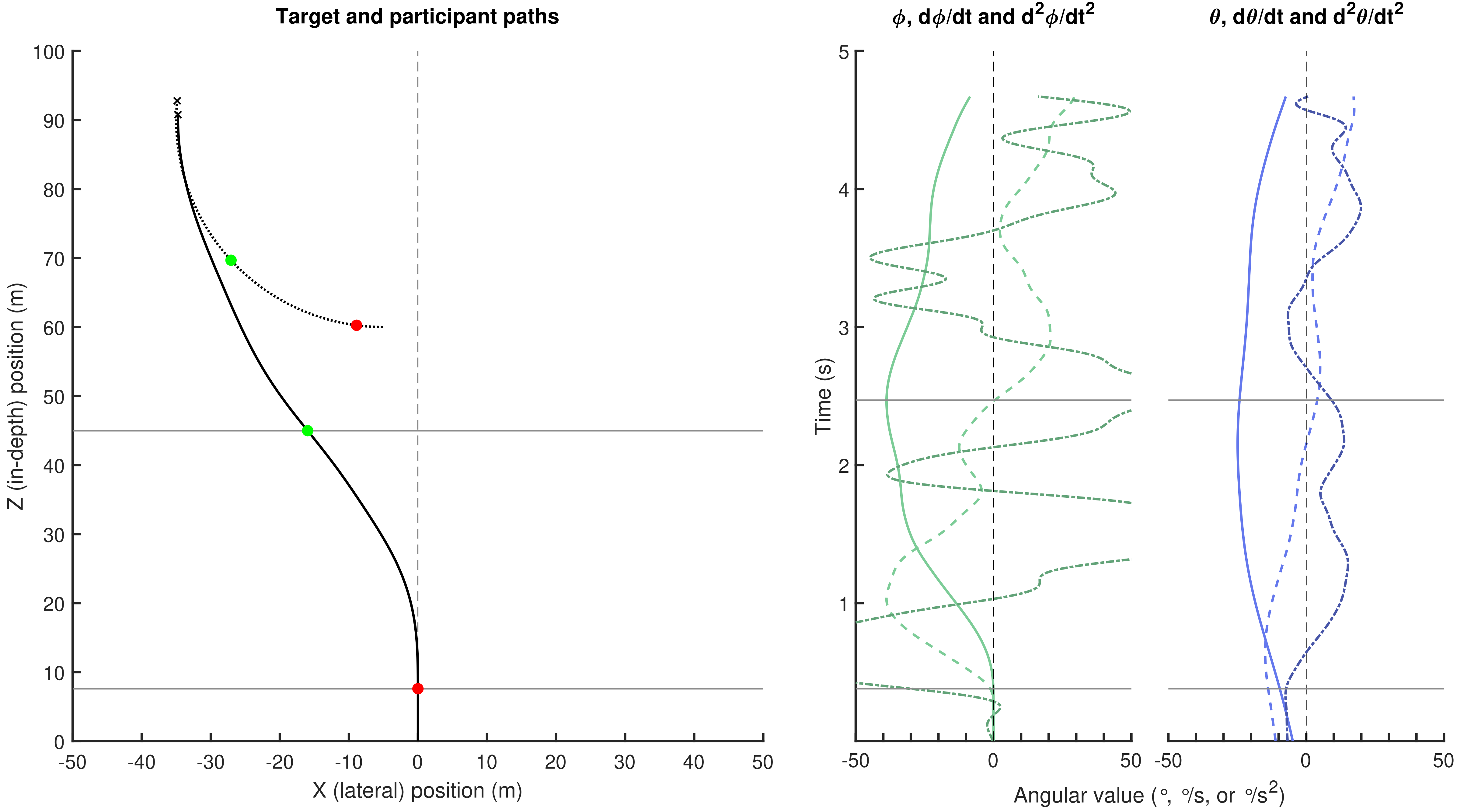

P11/B3
S5/R10-OUT

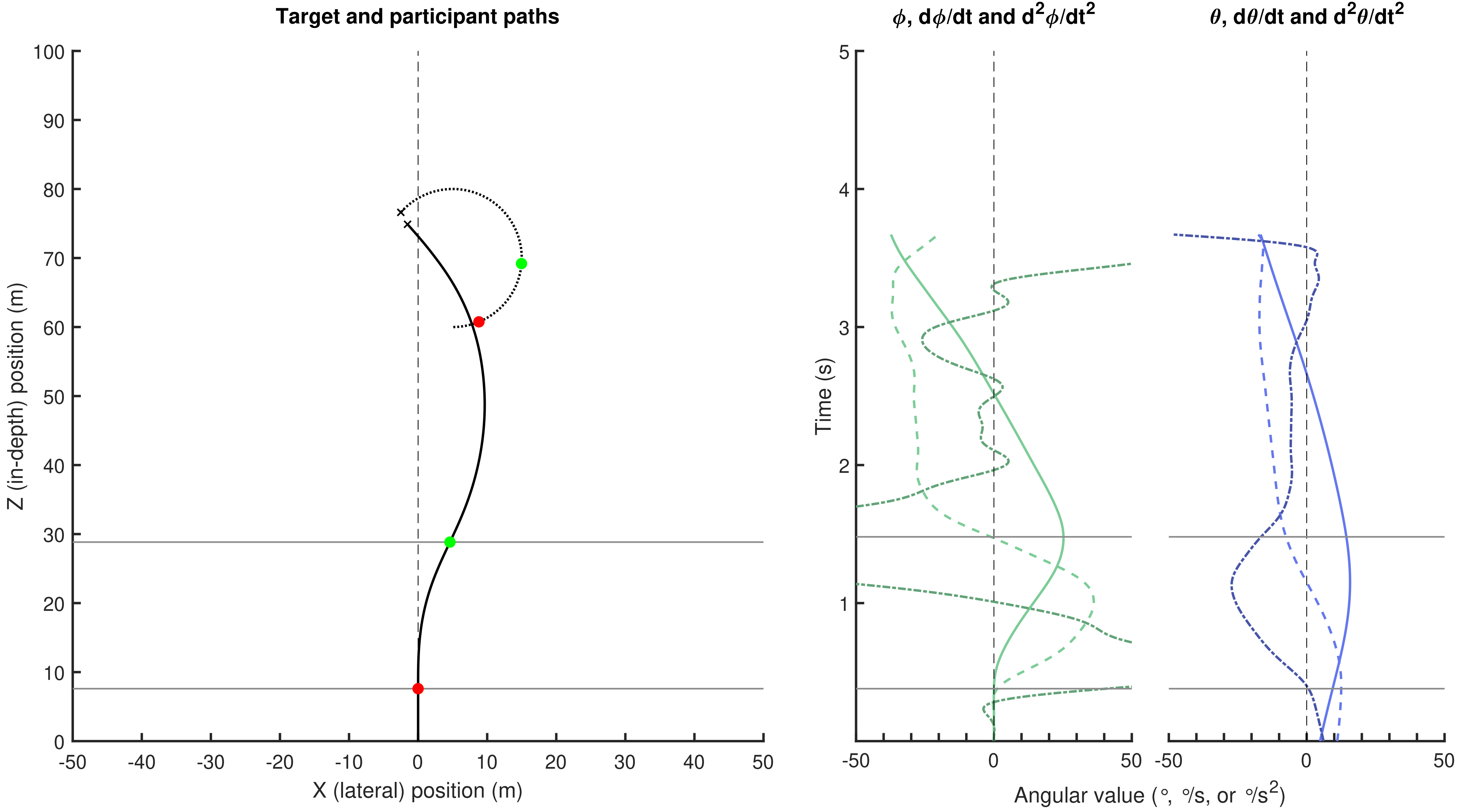

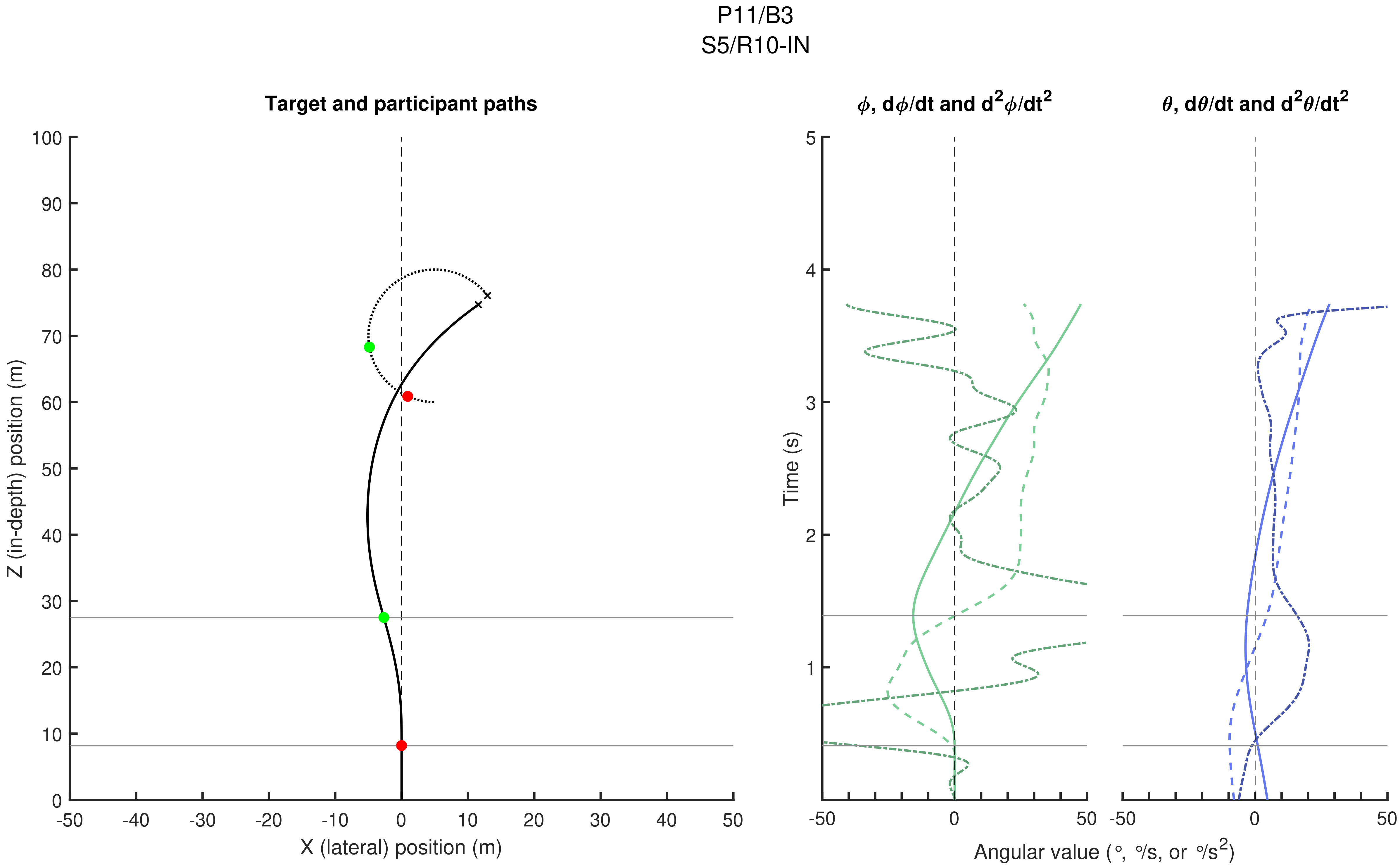
P11/B3
S5/R10-IN
Target and participant paths
Z (in-depth) position (m)
X (lateral) position (m)
100
90
80
70
60
50
40
30
20
10
0
-50
-40
-30
-20
-10
0
10
20
30
40
50
ϕ, dϕ/dt and d²ϕ/dt²
θ, dθ/dt and d²θ/dt²
Time (s)
5
4
3
2
1
-50
0
50
-50
0
50
Angular value (°, °/s, or °/s²)

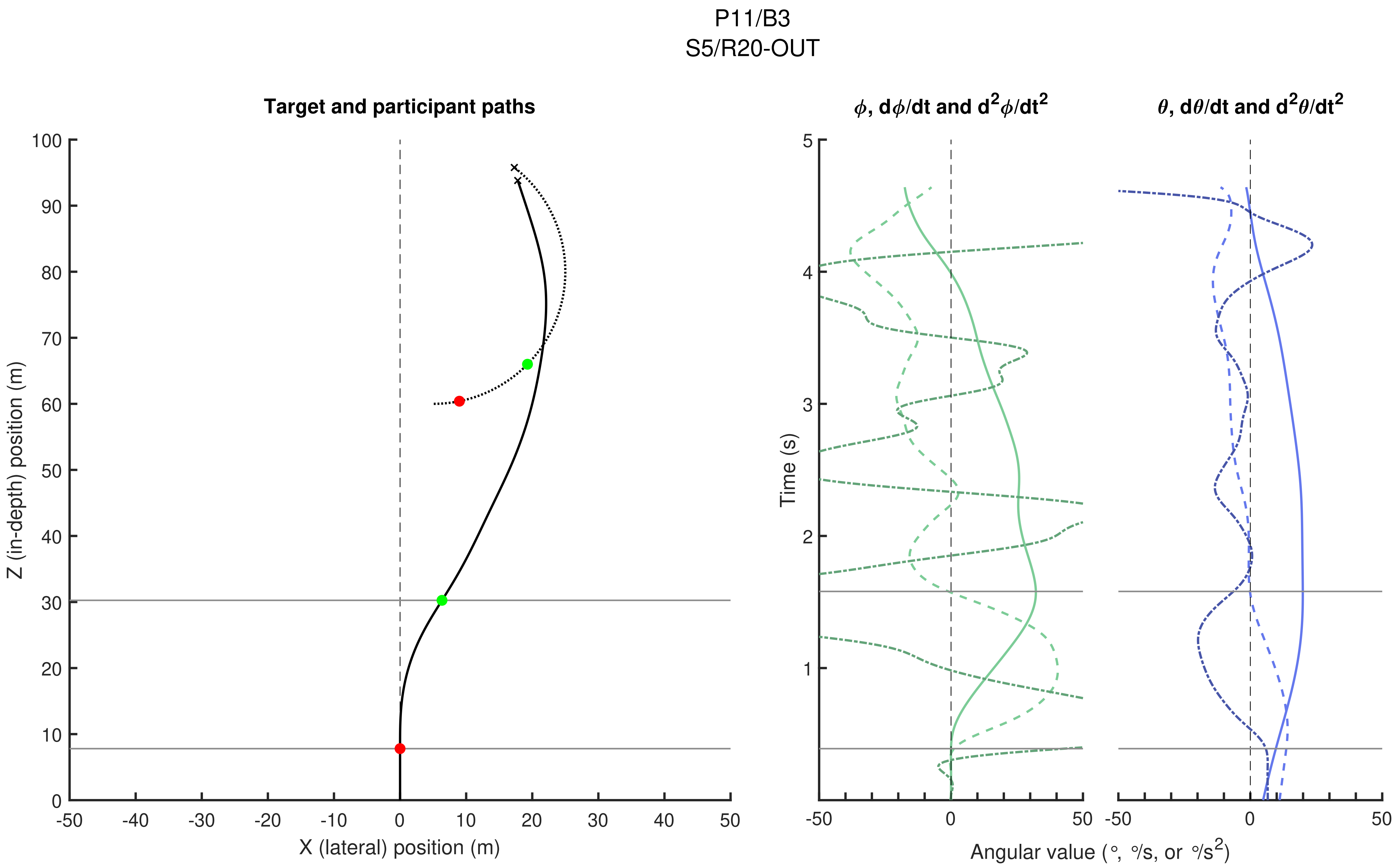

P11/B3
S5/R20-OUT
Target and participant paths
Z (in-depth) position (m)
X (lateral) position (m)
100
90
80
70
60
50
40
30
20
10
0
-50
-40
-30
-20
-10
0
10
20
30
40
50
ϕ, dϕ/dt and d$^2\phi$/dt^2
θ, dθ/dt and d$^2\theta$/dt^2
Time (s)
5
4
3
2
1
-50
0
50
-50
0
50
Angular value (°, °/s, or °/s^2)

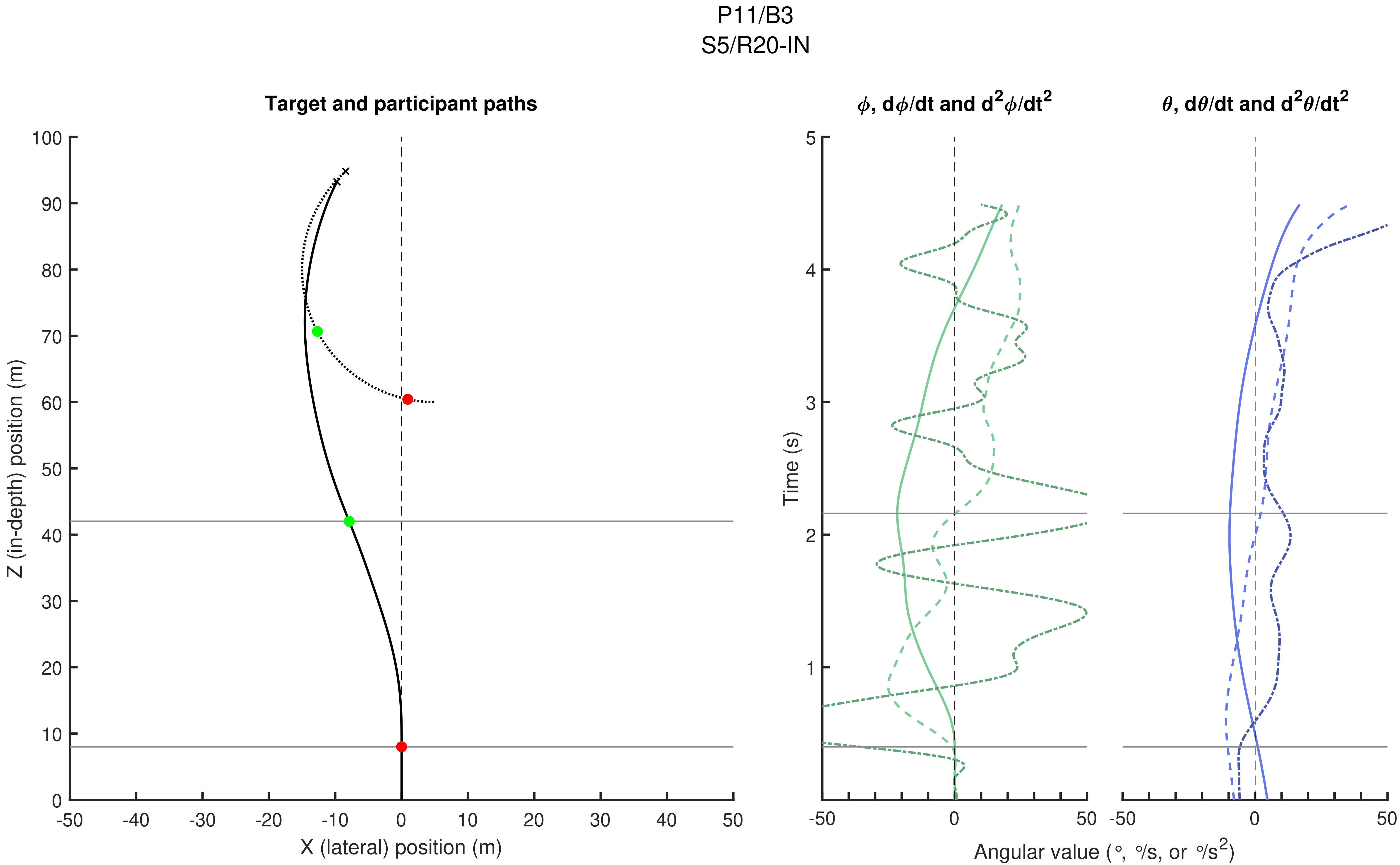
P11/B3
S5/R20-IN
Target and participant paths
Z (in-depth) position (m)
X (lateral) position (m)
ϕ, dϕ/dt and d$^2\phi$/dt^2
θ, dθ/dt and d$^2\theta$/dt^2
Time (s)
Angular value (°, °/s, or °/s^2)

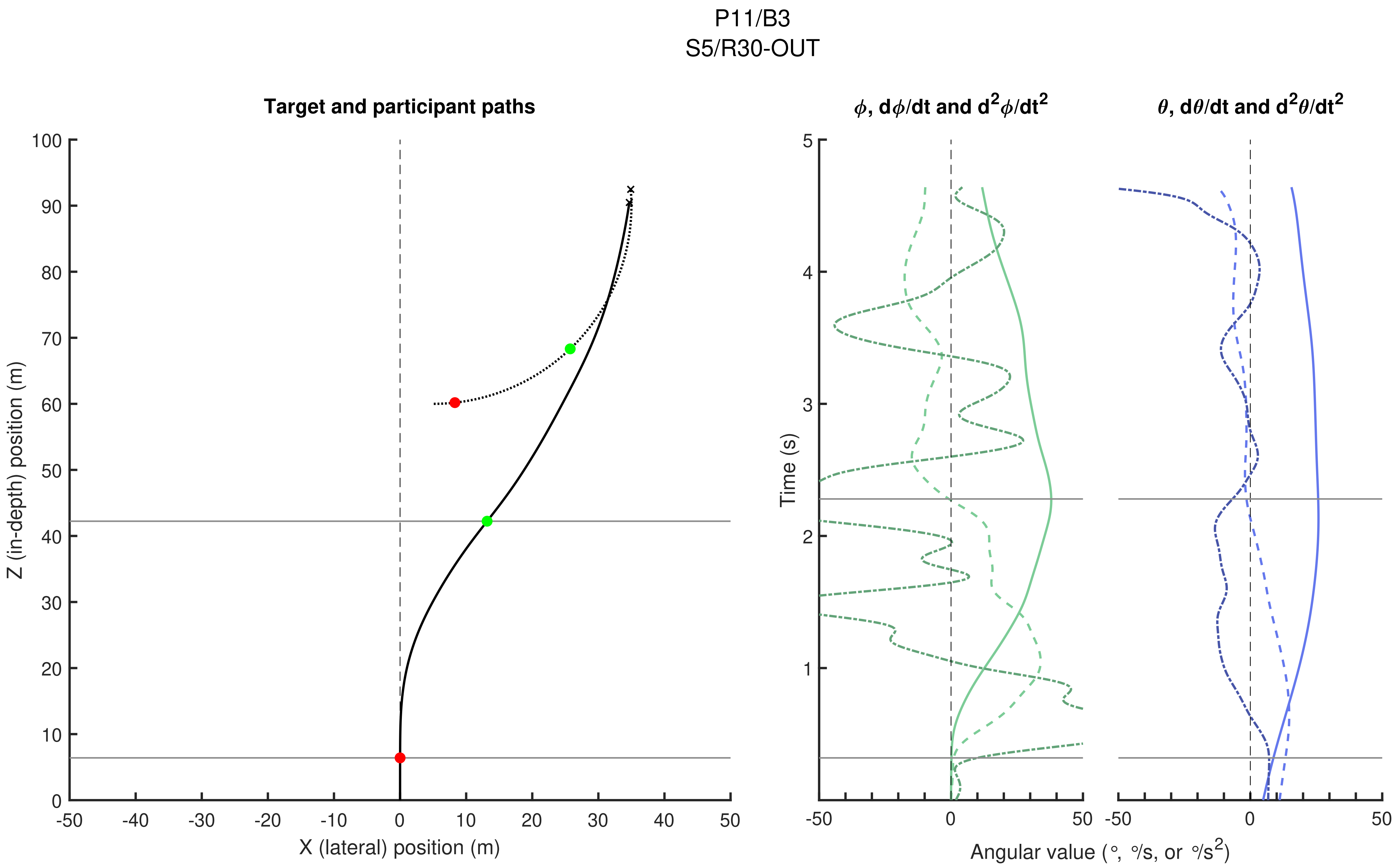
P11/B3
S5/R30-OUT
Target and participant paths
Z (in-depth) position (m)
X (lateral) position (m)
100
90
80
70
60
50
40
30
20
10
0
-50
-40
-30
-20
-10
0
10
20
30
40
50
φ, dφ/dt and d²φ/dt²
θ, dθ/dt and d²θ/dt²
Time (s)
5
4
3
2
1
-50
0
50
Angular value (°, °/s, or °/s²)

P11/B3
S5/R30-IN

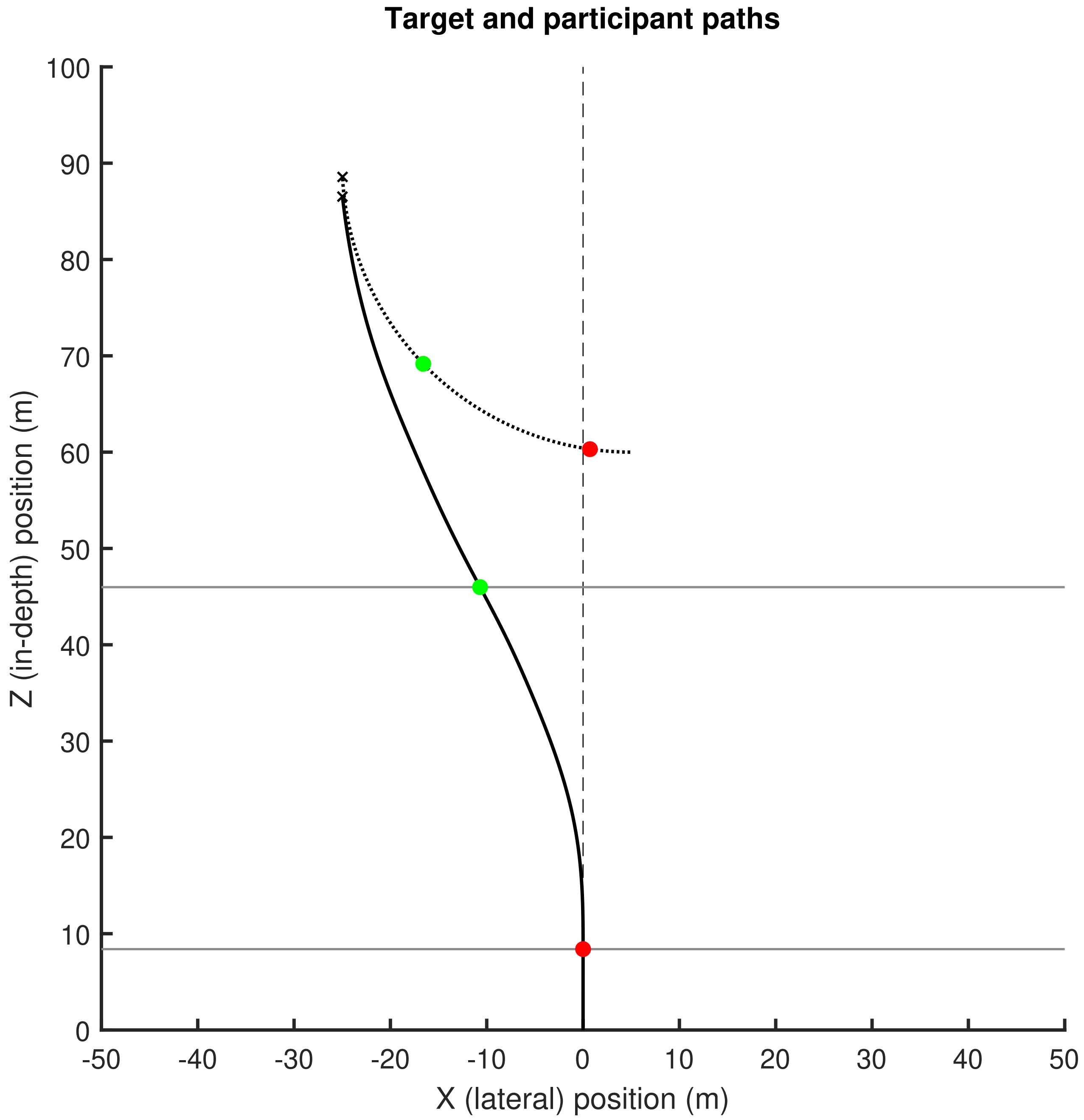

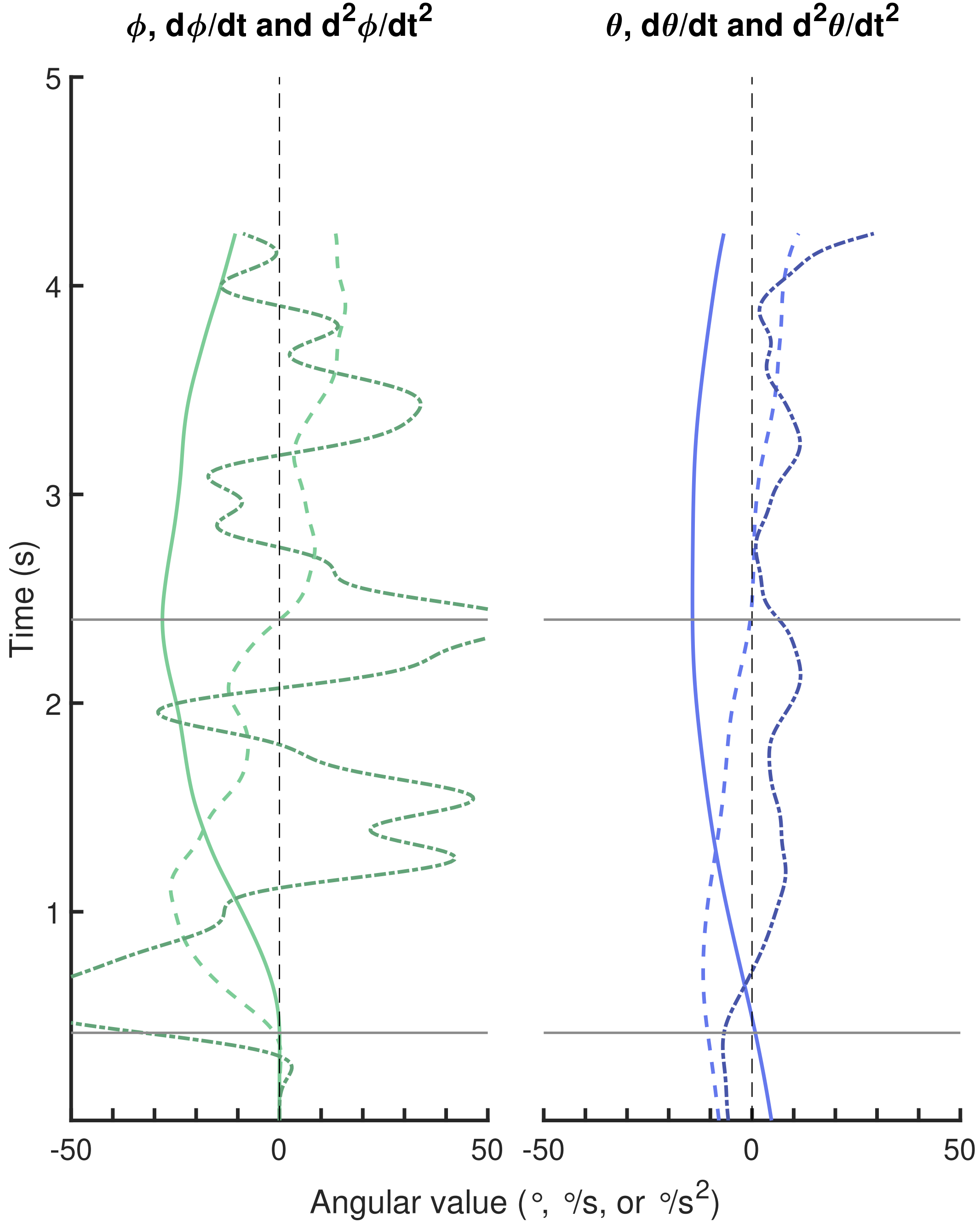

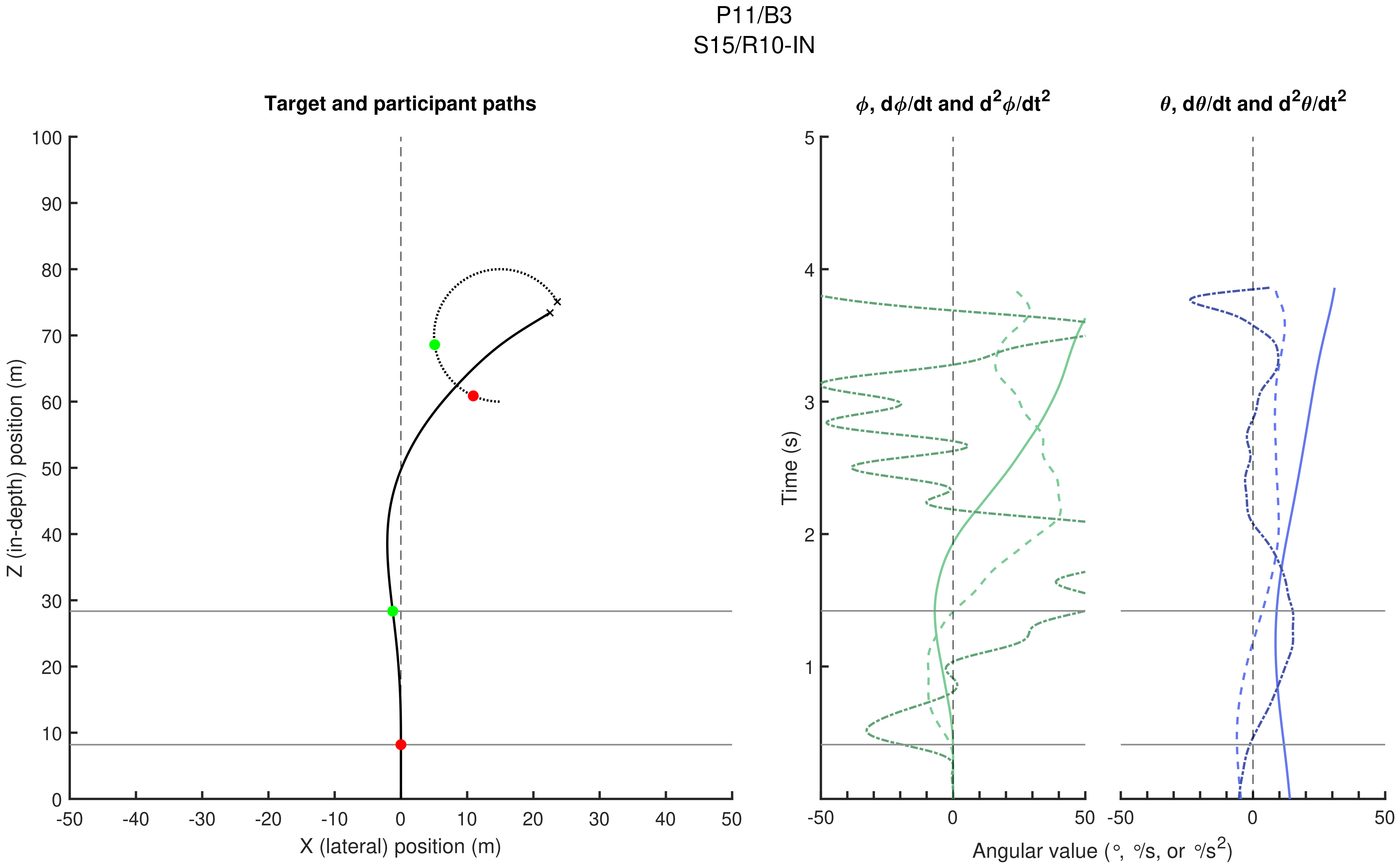
P11/B3
S15/R10-IN
Target and participant paths
φ, dφ/dt and d²φ/dt²
θ, dθ/dt and d²θ/dt²
Z (in-depth) position (m)
X (lateral) position (m)
Time (s)
Angular value (°, °/s, or °/s²)
100
90
80
70
60
50
40
30
20
10
0
-50
-40
-30
-20
-10
0
10
20
30
40
50
5
4
3
2
1

P11/B3
S15/R20-IN

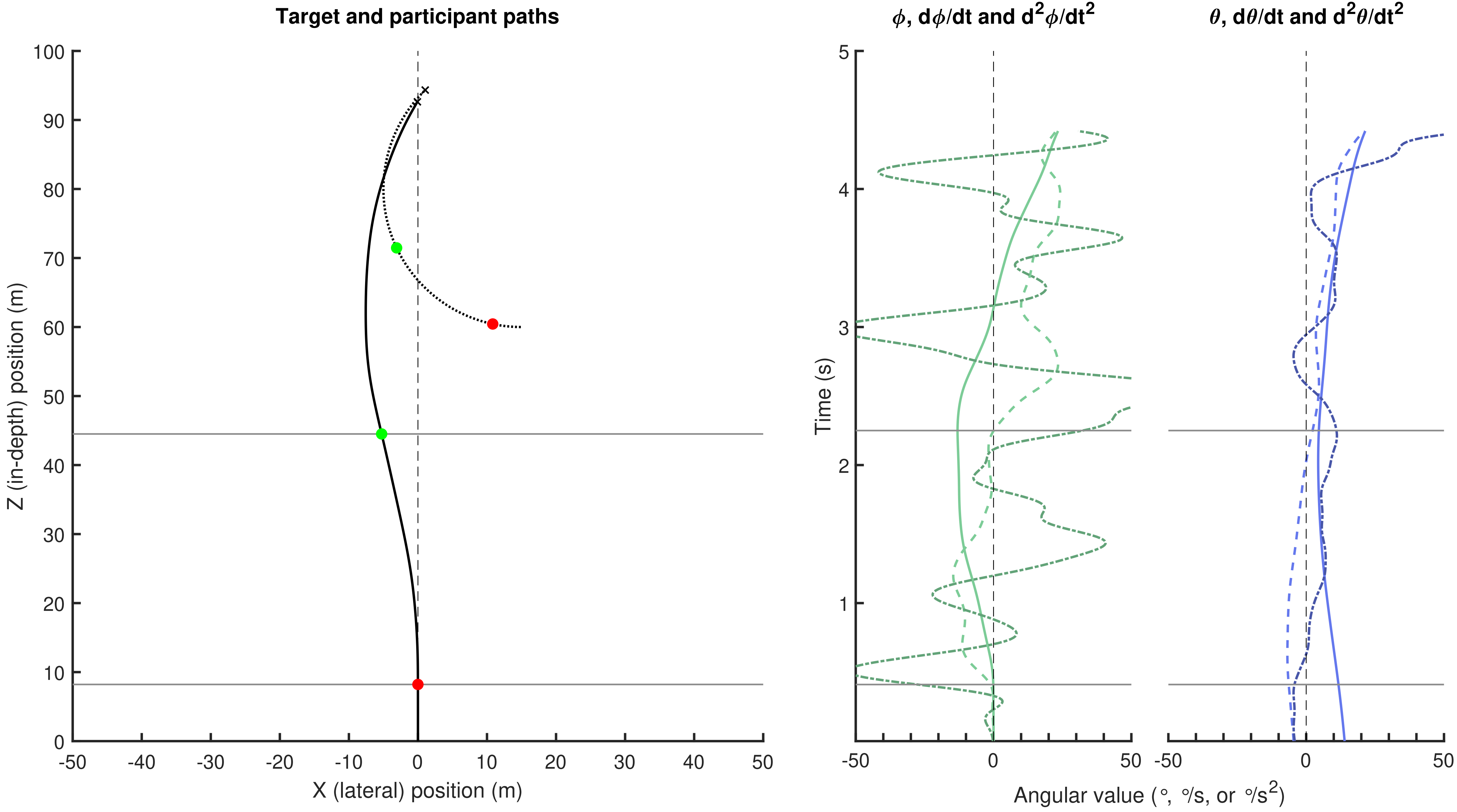

P11/B3
S15/R30-IN

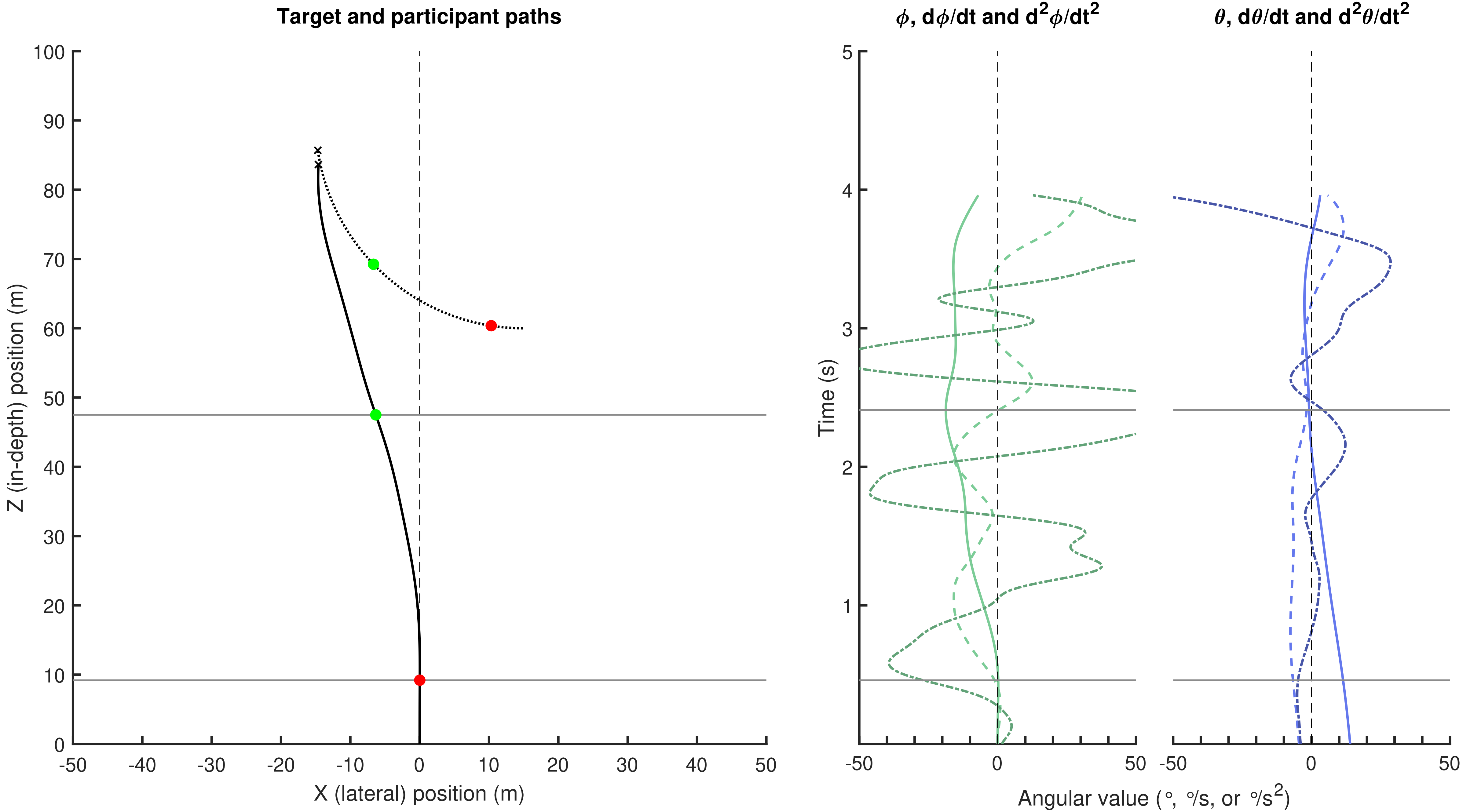

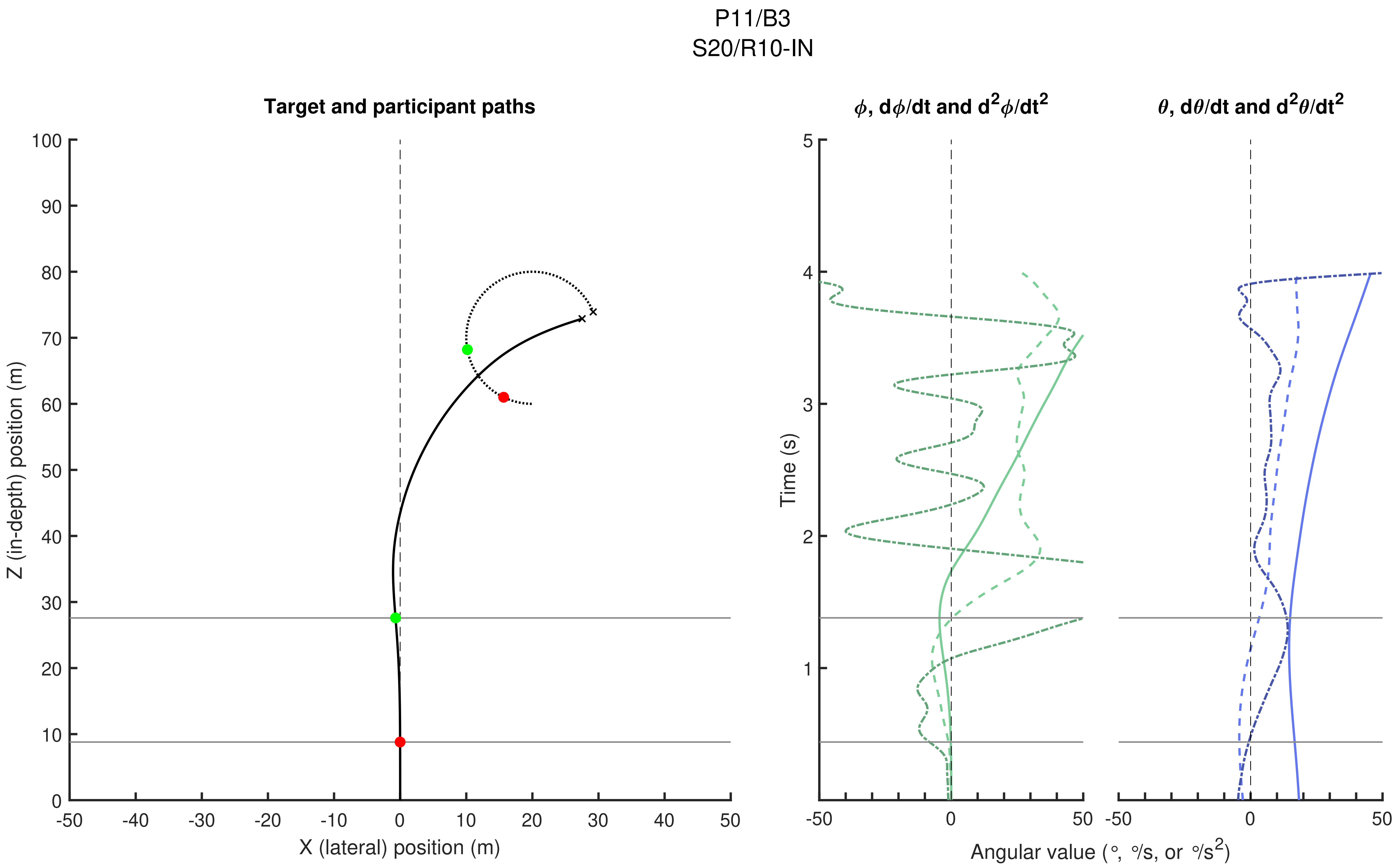
P11/B3
S20/R10-IN
Target and participant paths
ϕ, dϕ/dt and d$^2\phi$/dt^2
θ, dθ/dt and d$^2\theta$/dt^2
Z (in-depth) position (m)
X (lateral) position (m)
Time (s)
Angular value (°, °/s, or °/s^2)
100
90
80
70
60
50
40
30
20
10
0
-50
-40
-30
-20
-10
0
10
20
30
40
50
5
4
3
2
1
-50
0
50
-50
0
50

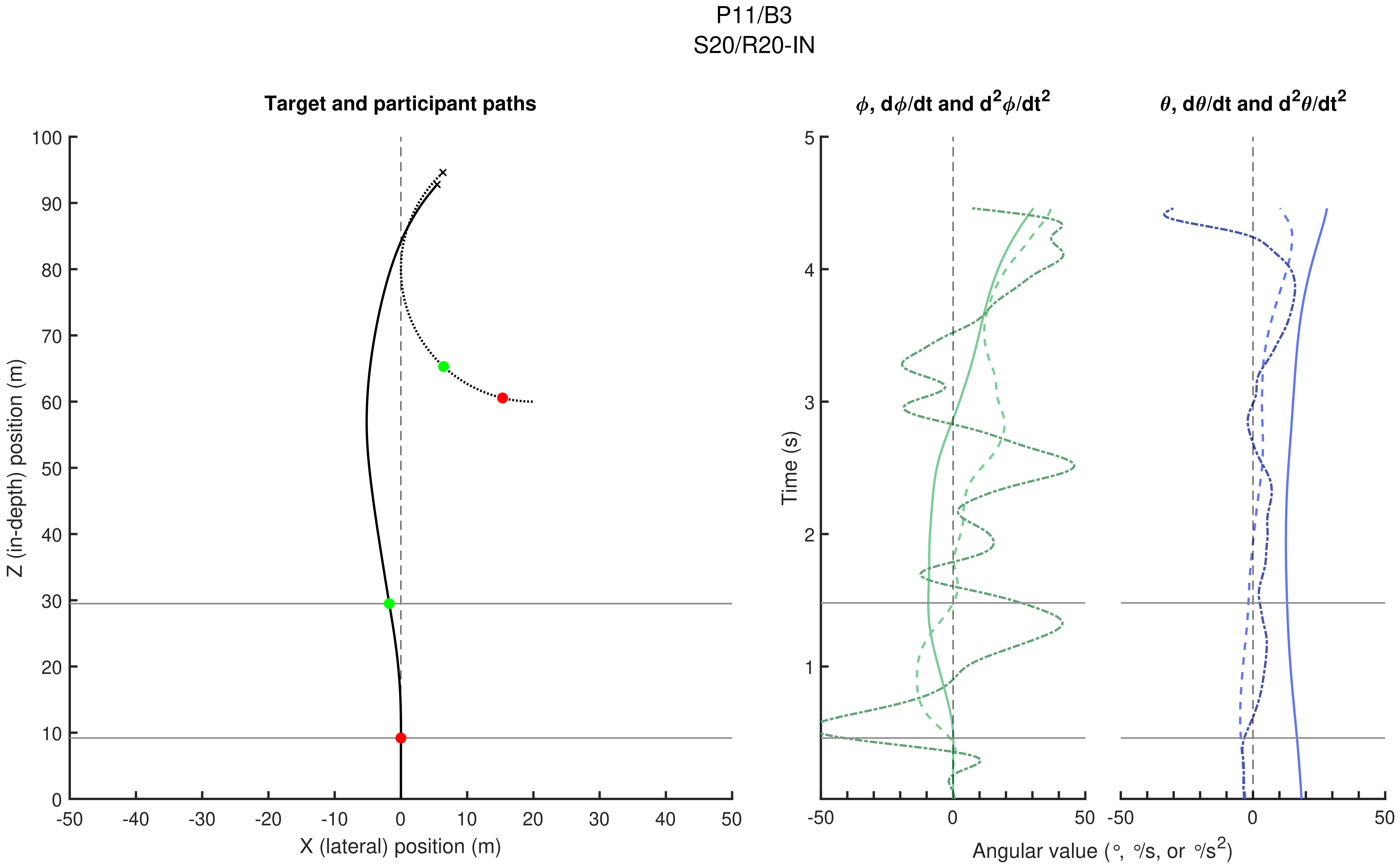
P11/B3
S20/R20-IN
Target and participant paths
Z (in-depth) position (m)
X (lateral) position (m)
ϕ, dϕ/dt and d$^2\phi$/dt^2
θ, dθ/dt and d$^2\theta$/dt^2
Time (s)
Angular value (°, °/s, or °/s^2)

P11/B3
S20/R30-IN

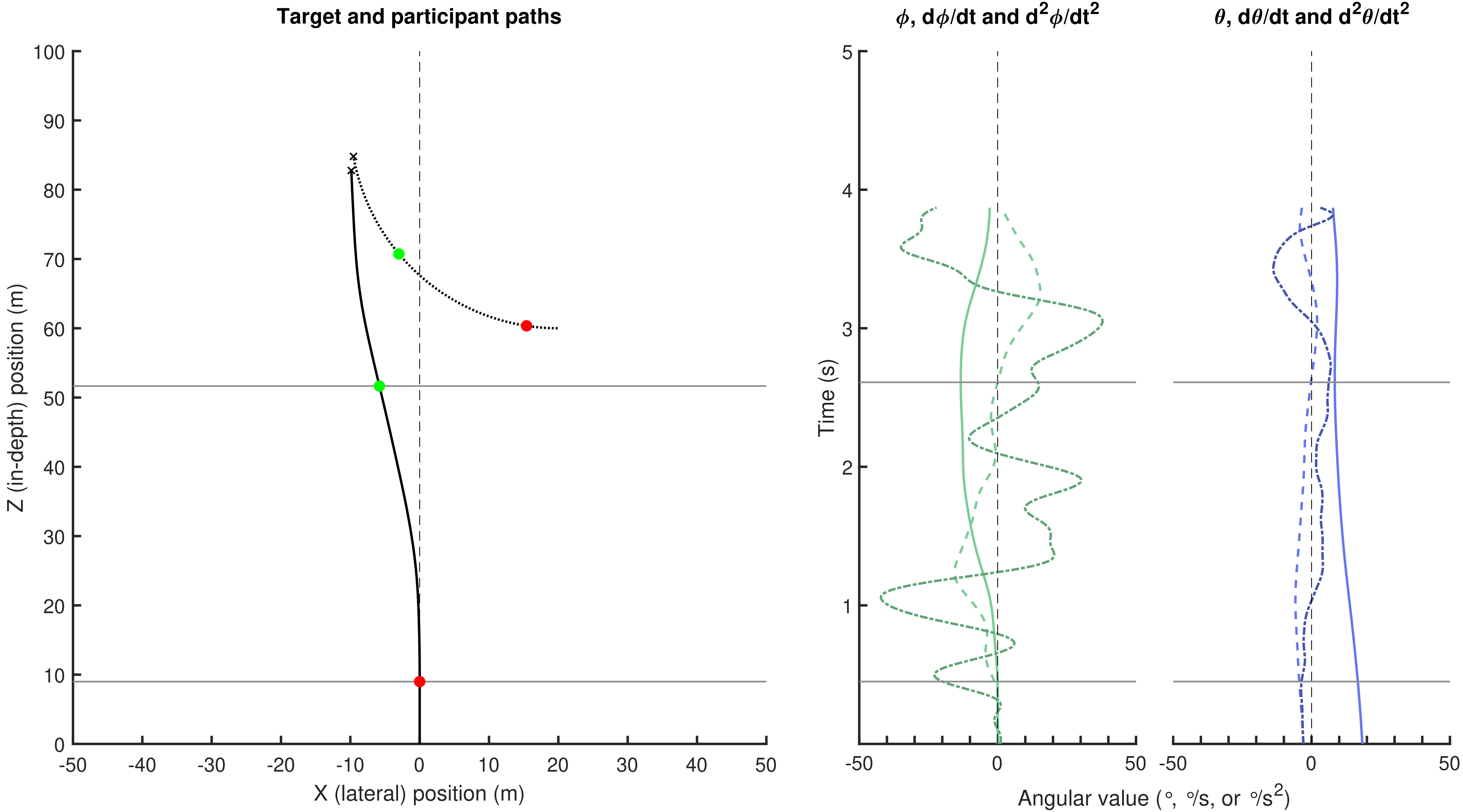

P11/B4
S20/R10-IN

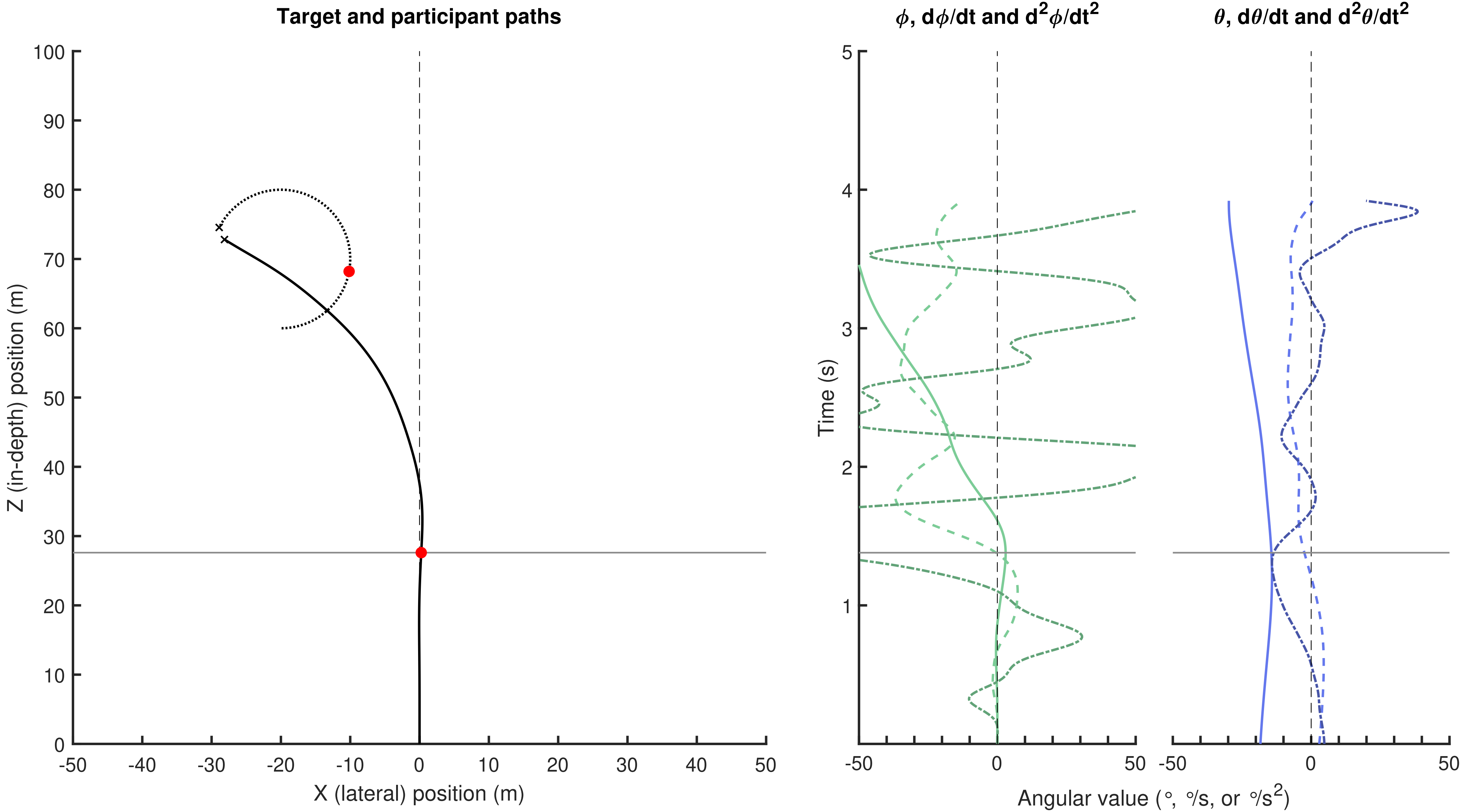

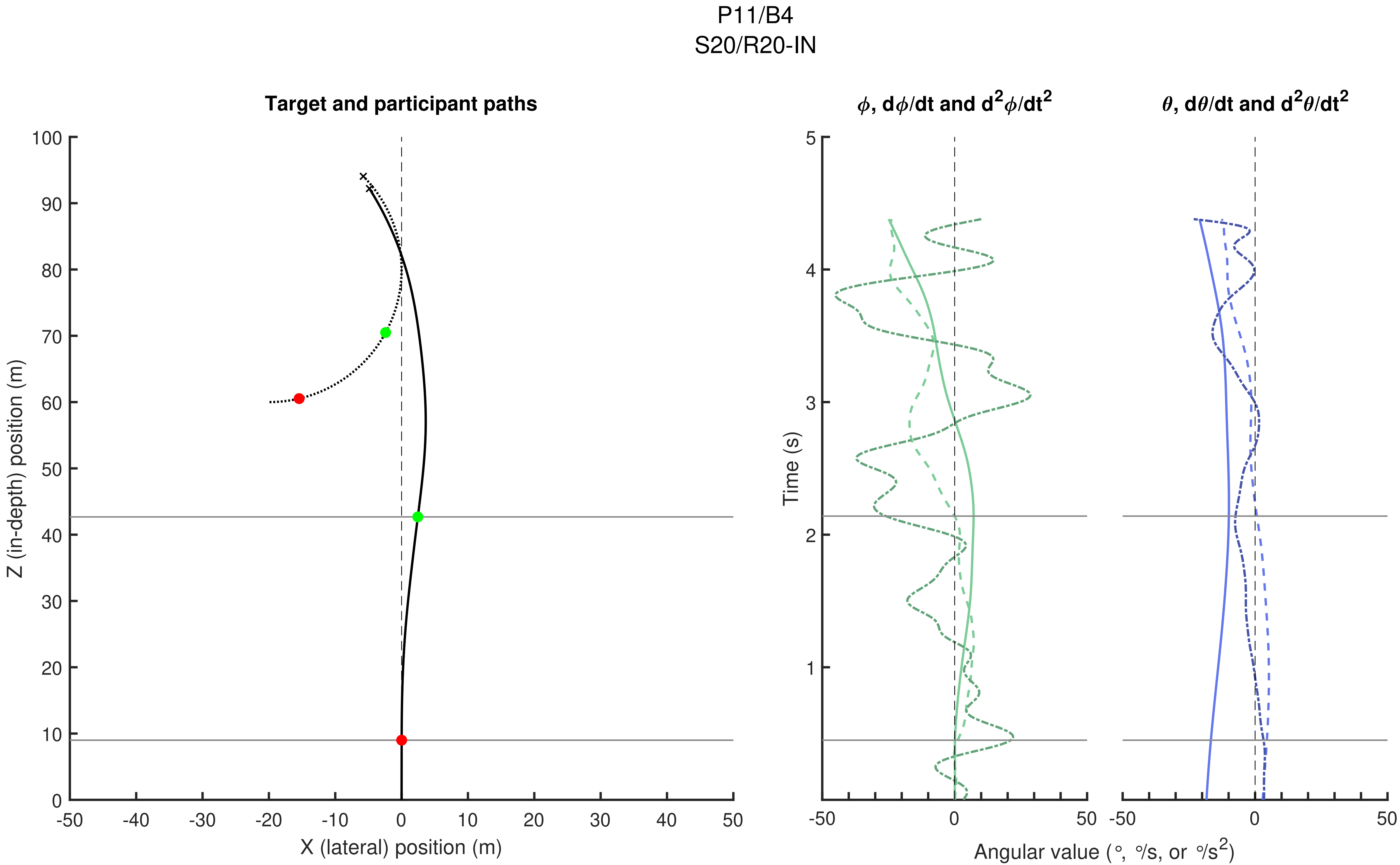
P11/B4
S20/R20-IN
Target and participant paths
Z (in-depth) position (m)
X (lateral) position (m)
ϕ, dϕ/dt and d²ϕ/dt²
θ, dθ/dt and d²θ/dt²
Time (s)
Angular value (°, °/s, or °/s²)

P11/B4
S20/R30-IN

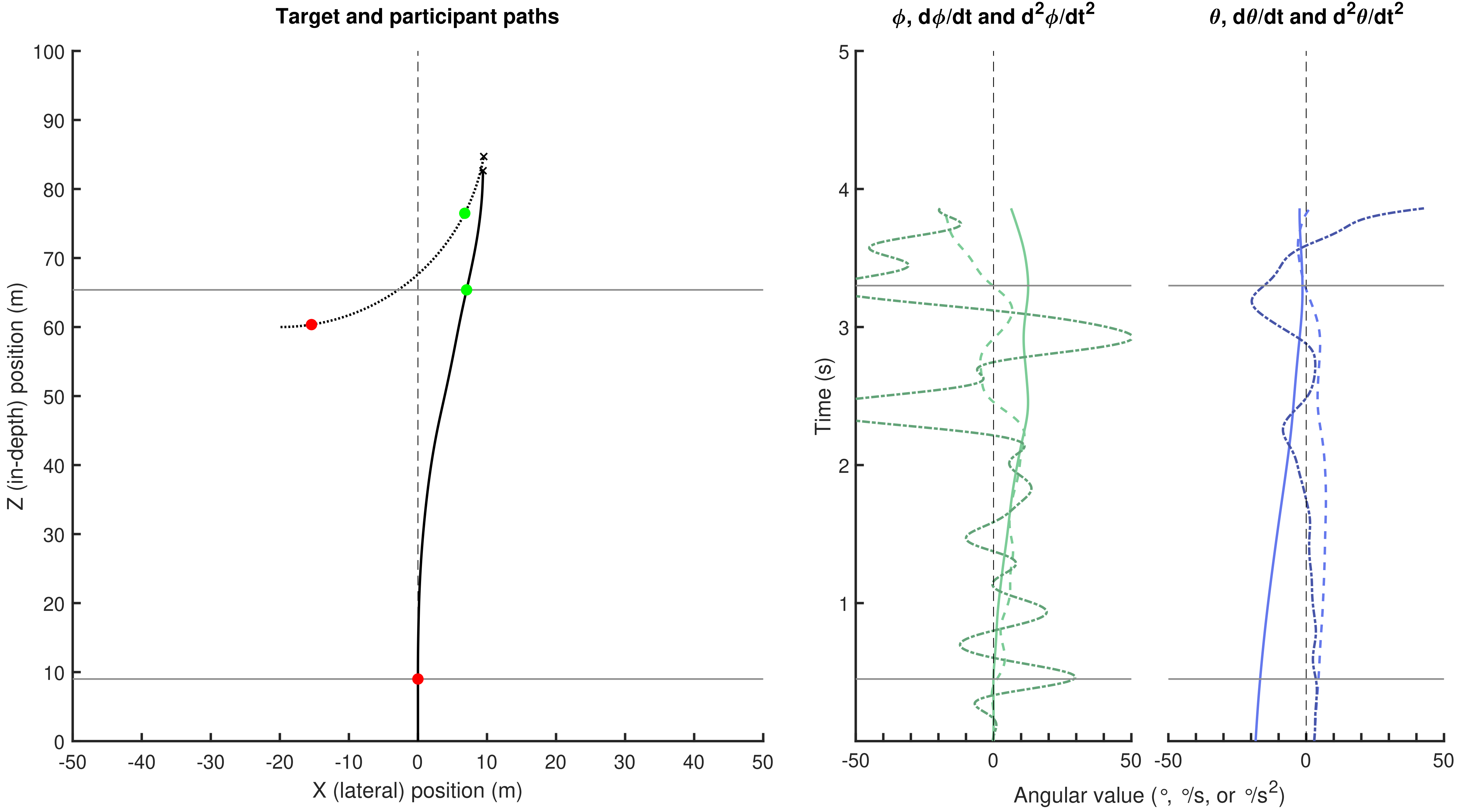

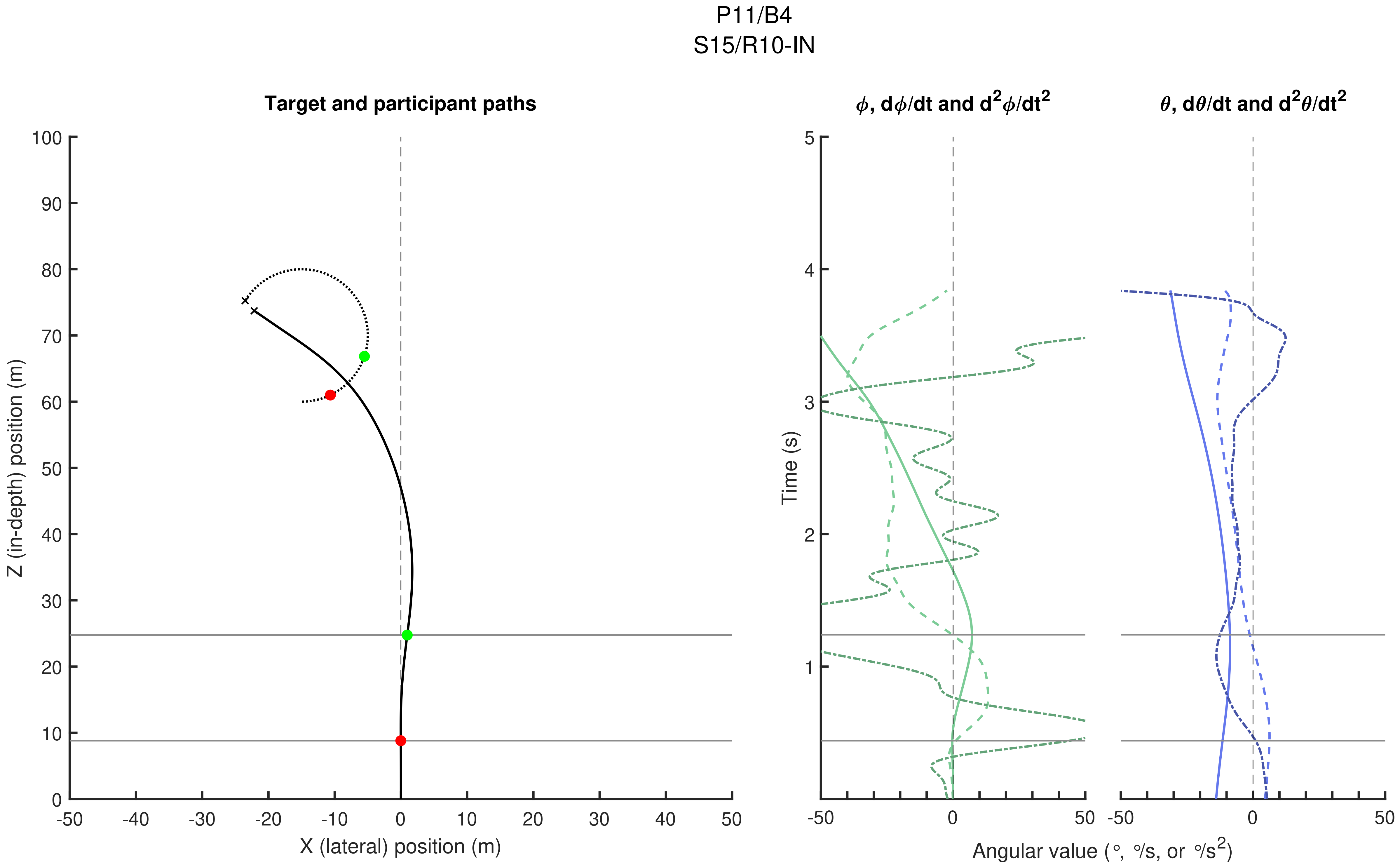
P11/B4
S15/R10-IN
Target and participant paths
Z (in-depth) position (m)
X (lateral) position (m)
ϕ, dϕ/dt and d$^2\phi$/dt^2
θ, dθ/dt and d$^2\theta$/dt^2
Time (s)
Angular value (°, °/s, or °/s^2)

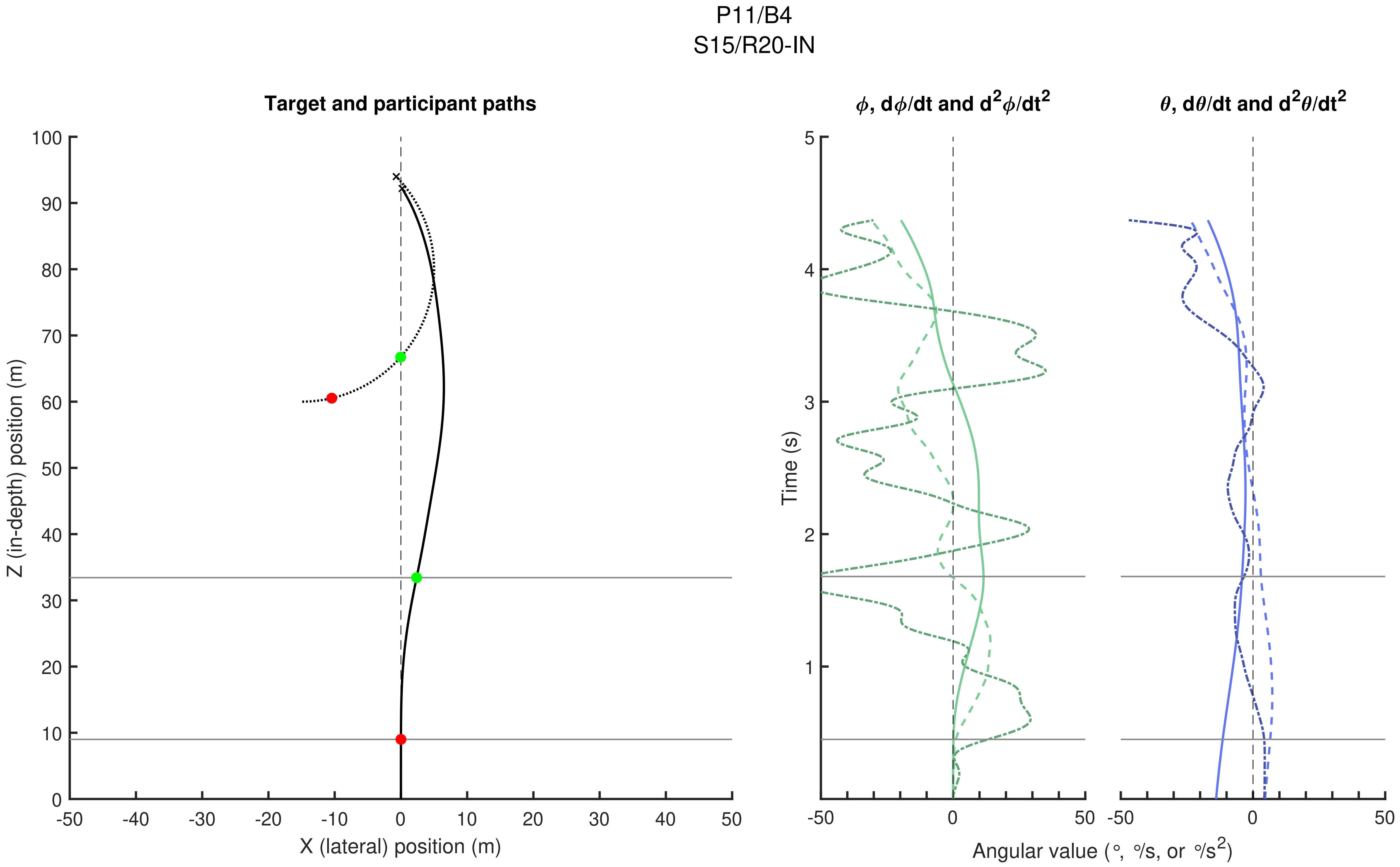
P11/B4
S15/R20-IN
Target and participant paths
Z (in-depth) position (m)
X (lateral) position (m)
ϕ, dϕ/dt and d²ϕ/dt²
θ, dθ/dt and d²θ/dt²
Time (s)
Angular value (°, °/s, or °/s²)

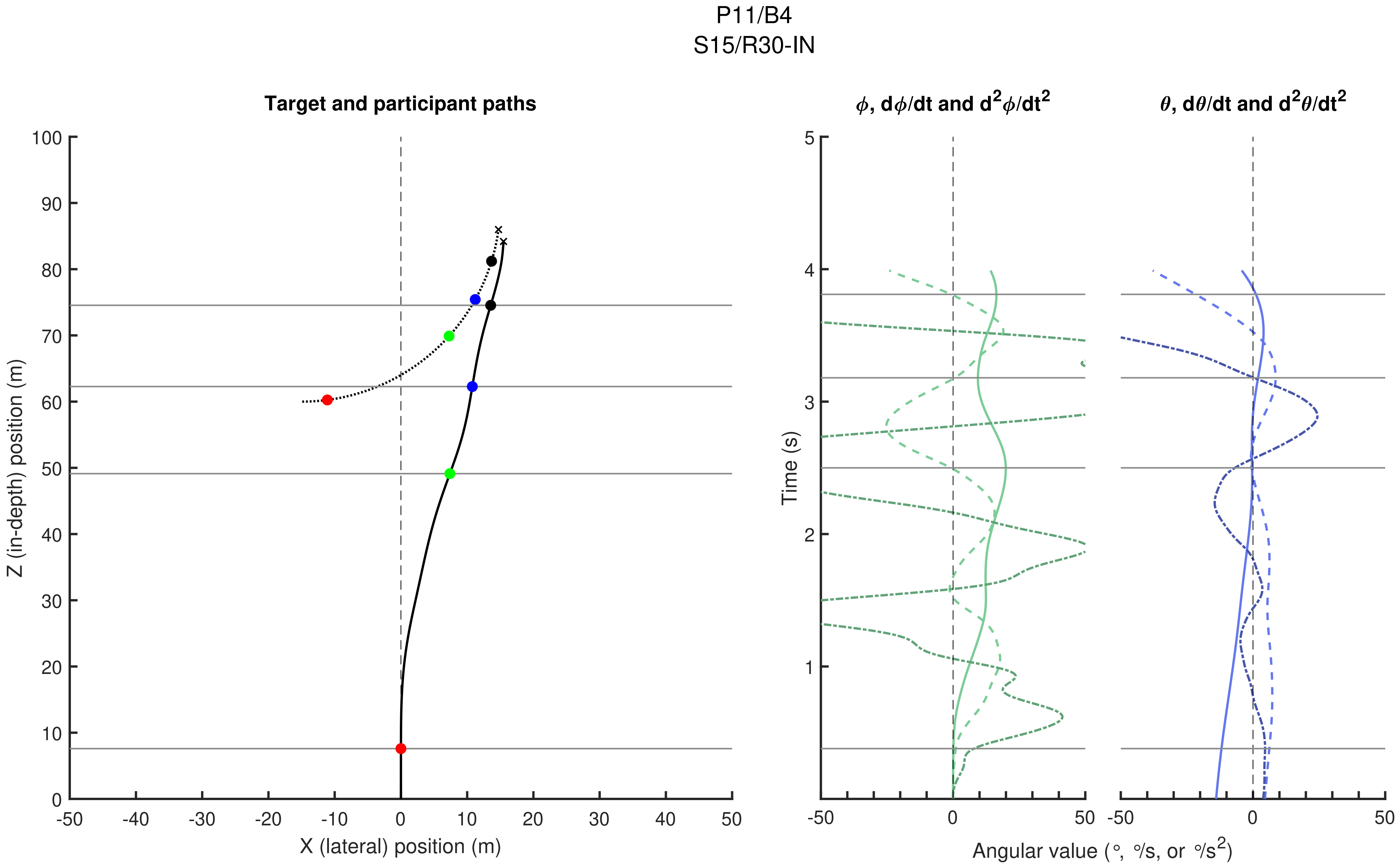

P11/B4
S15/R30-IN
Target and participant paths
Z (in-depth) position (m)
X (lateral) position (m)
100
90
80
70
60
50
40
30
20
10
0
-50
-40
-30
-20
-10
0
10
20
30
40
50
φ, dφ/dt and d²φ/dt²
θ, dθ/dt and d²θ/dt²
Time (s)
5
4
3
2
1
-50
0
50
-50
0
50
Angular value (°, °/s, or °/s²)

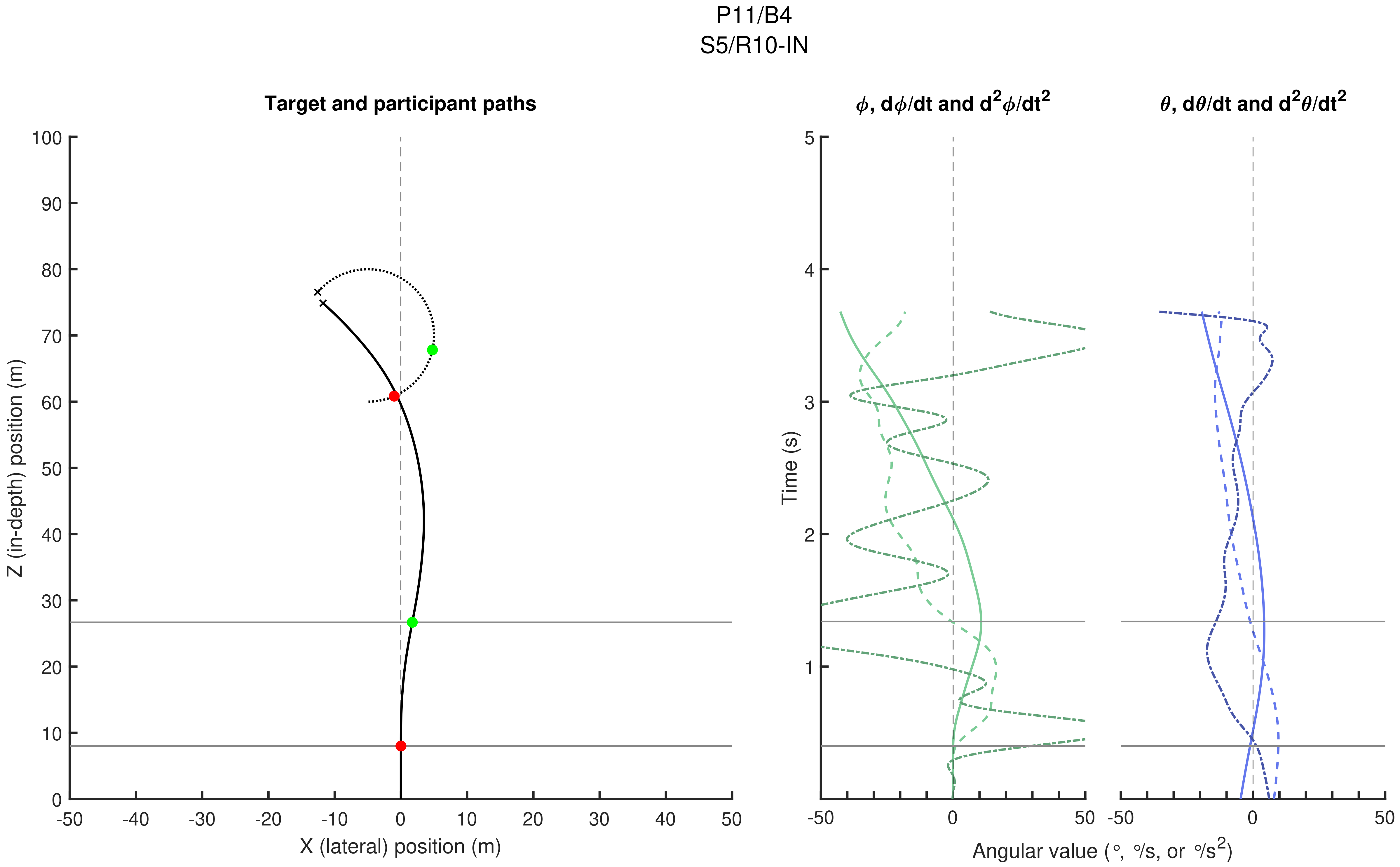

P11/B4
S5/R10-IN
Target and participant paths
Z (in-depth) position (m)
X (lateral) position (m)
φ, dφ/dt and d²φ/dt²
θ, dθ/dt and d²θ/dt²
Time (s)
Angular value (°, °/s, or °/s²)

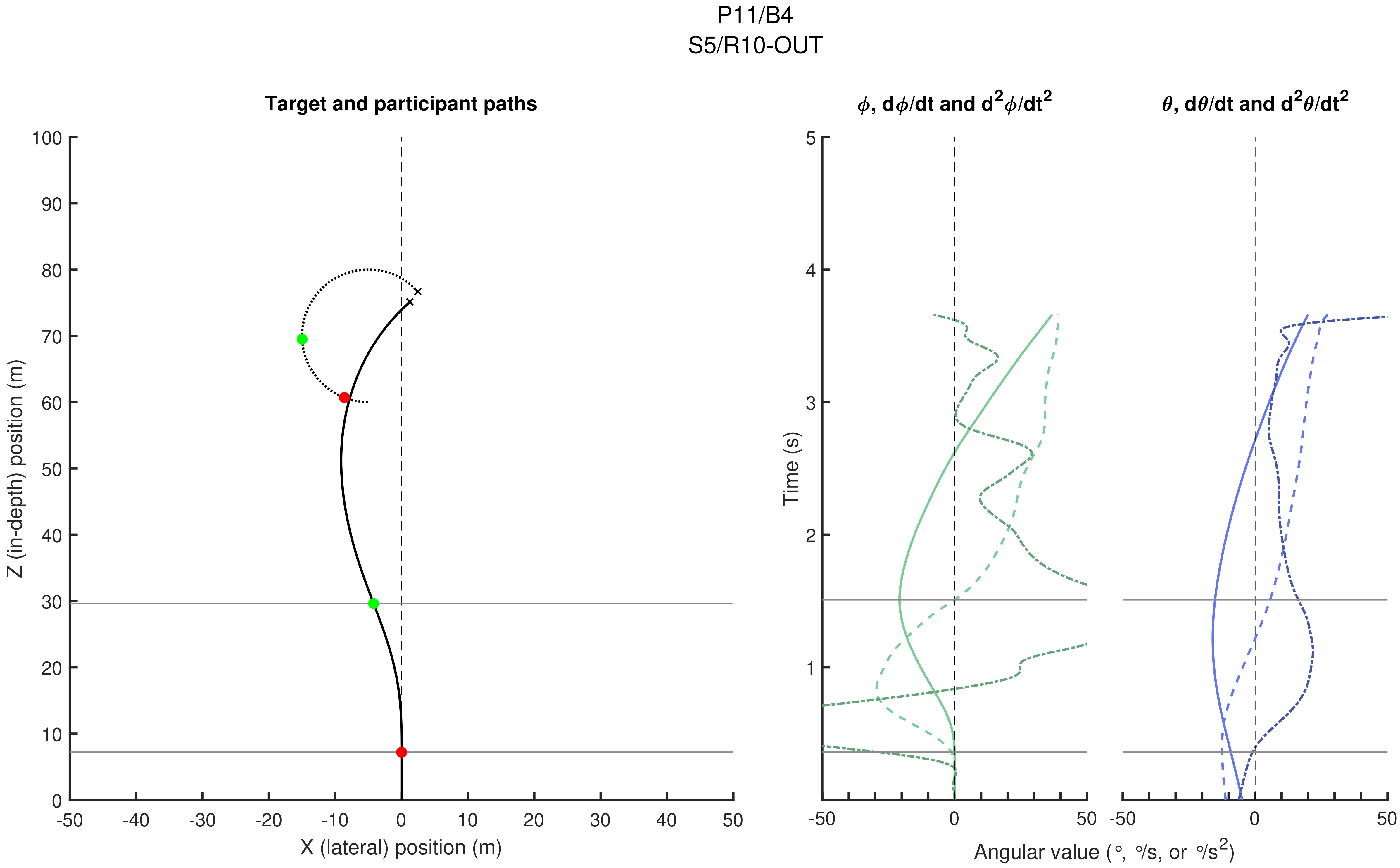

P11/B4
S5/R10-OUT
Target and participant paths
Z (in-depth) position (m)
X (lateral) position (m)
100
90
80
70
60
50
40
30
20
10
0
-50
-40
-30
-20
-10
0
10
20
30
40
50
ϕ, dϕ/dt and d$^2\phi$/dt^2
θ, dθ/dt and d$^2\theta$/dt^2
Time (s)
5
4
3
2
1
-50
0
50
-50
0
50
Angular value (°, °/s, or °/s^2)

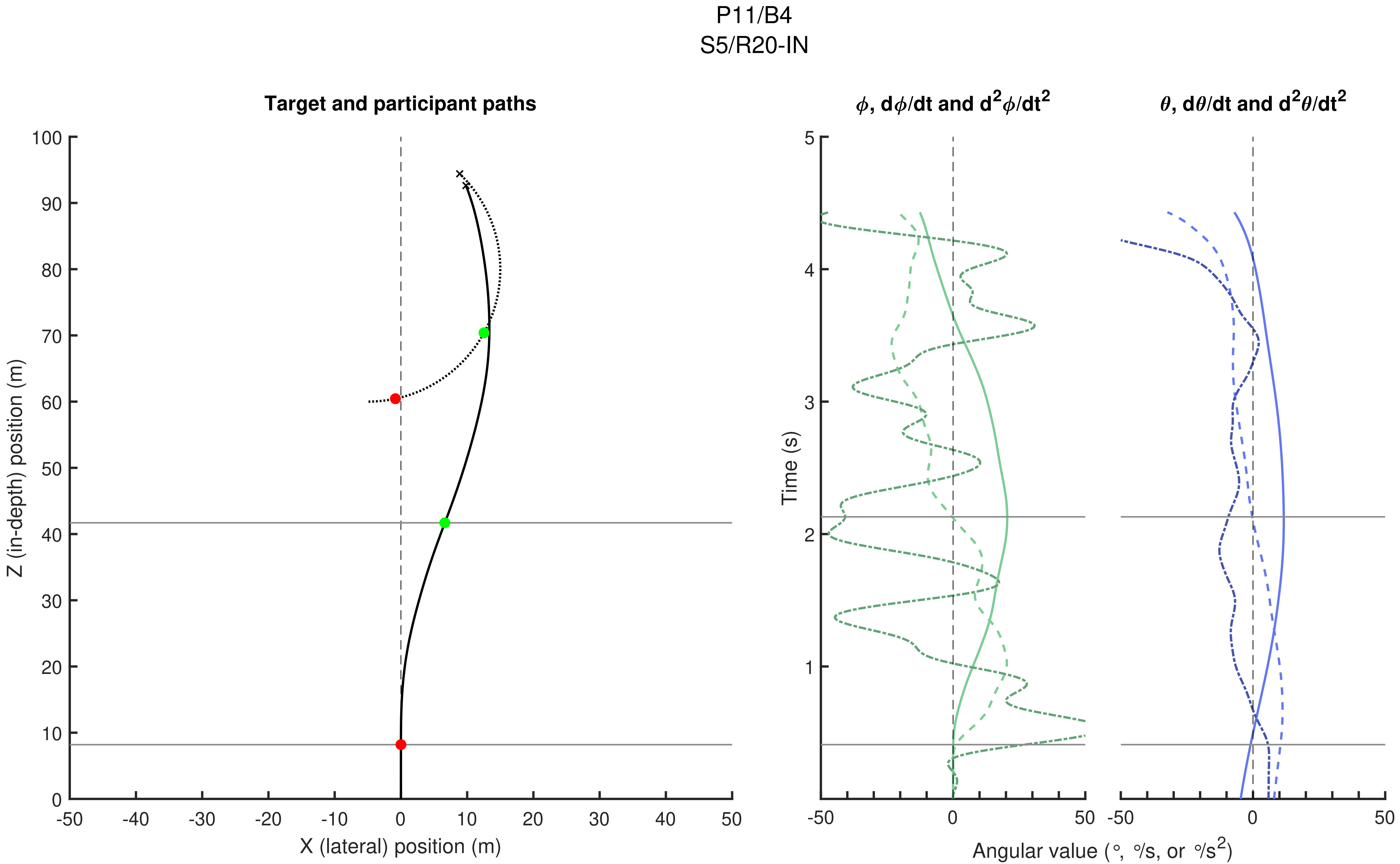

P11/B4
S5/R20-IN
Target and participant paths
Z (in-depth) position (m)
X (lateral) position (m)
ϕ, dϕ/dt and d$^2\phi$/dt^2
θ, dθ/dt and d$^2\theta$/dt^2
Time (s)
Angular value (°, °/s, or °/s^2)

P11/B4
S5/R20-OUT

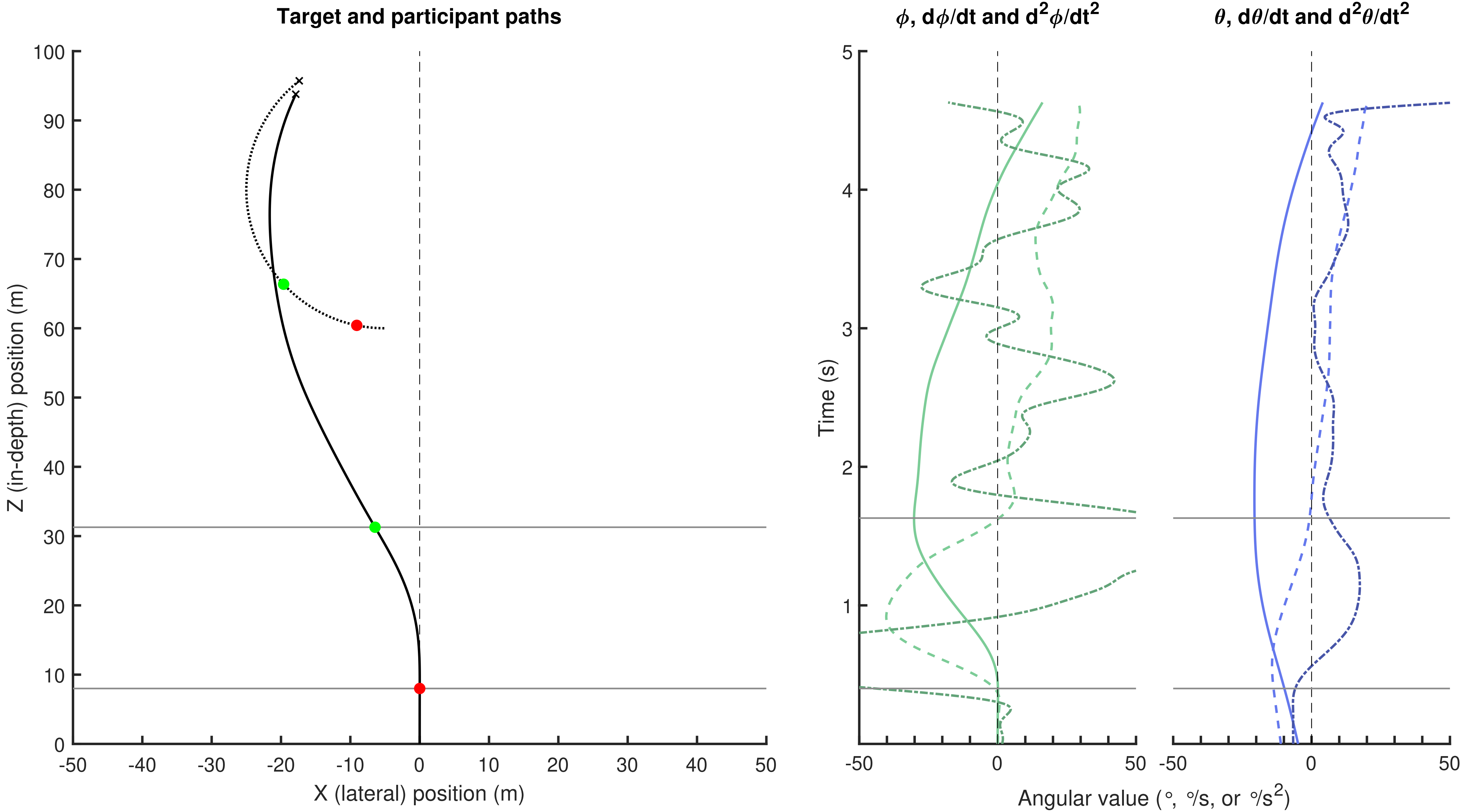

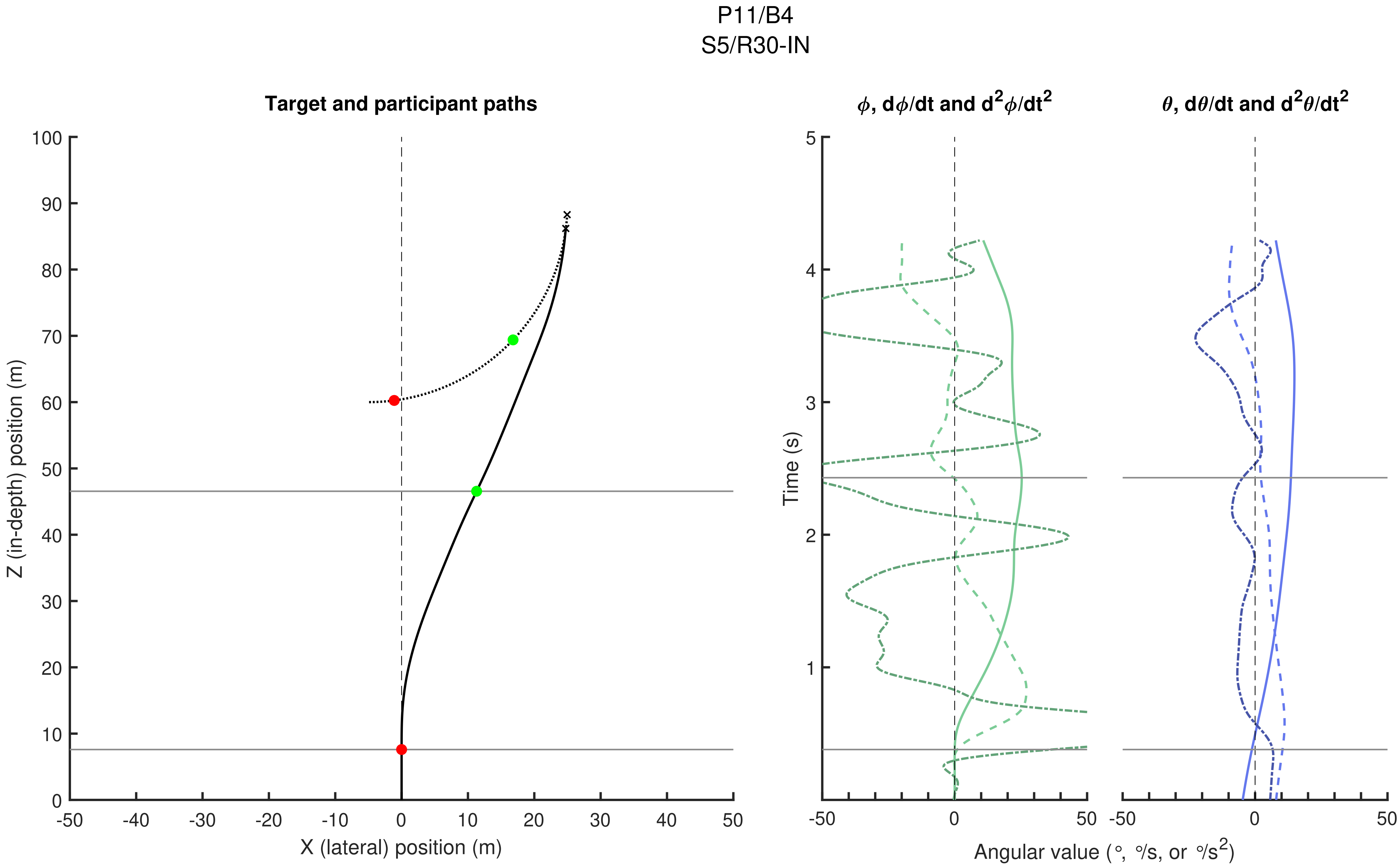
P11/B4
S5/R30-IN
Target and participant paths
Z (in-depth) position (m)
X (lateral) position (m)
φ, dφ/dt and d²φ/dt²
θ, dθ/dt and d²θ/dt²
Time (s)
Angular value (°, °/s, or °/s²)

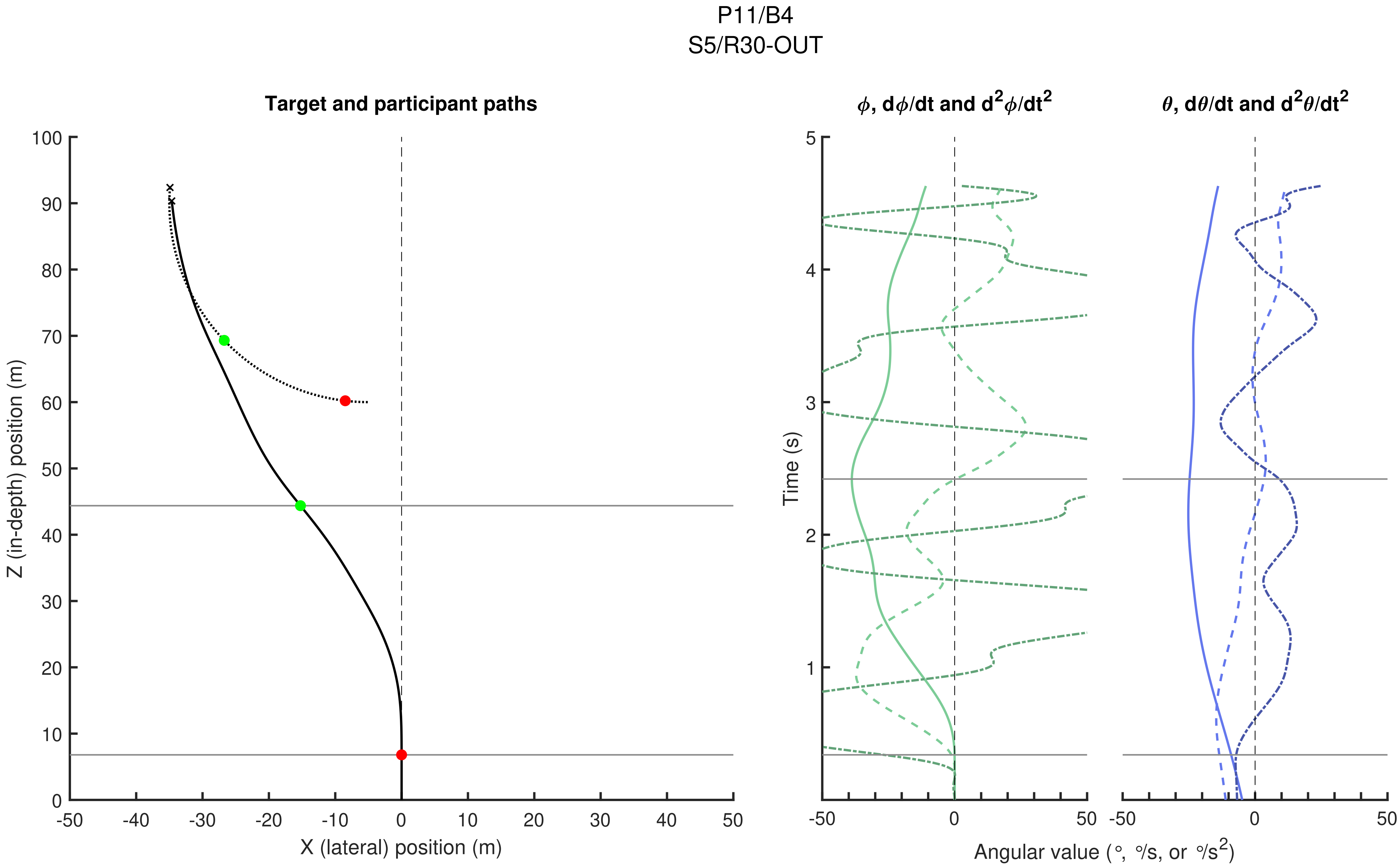
P11/B4
S5/R30-OUT
Target and participant paths
Z (in-depth) position (m)
X (lateral) position (m)
φ, dφ/dt and d²φ/dt²
θ, dθ/dt and d²θ/dt²
Time (s)
Angular value (°, °/s, or °/s²)

P11/B4
S5/R10-OUT

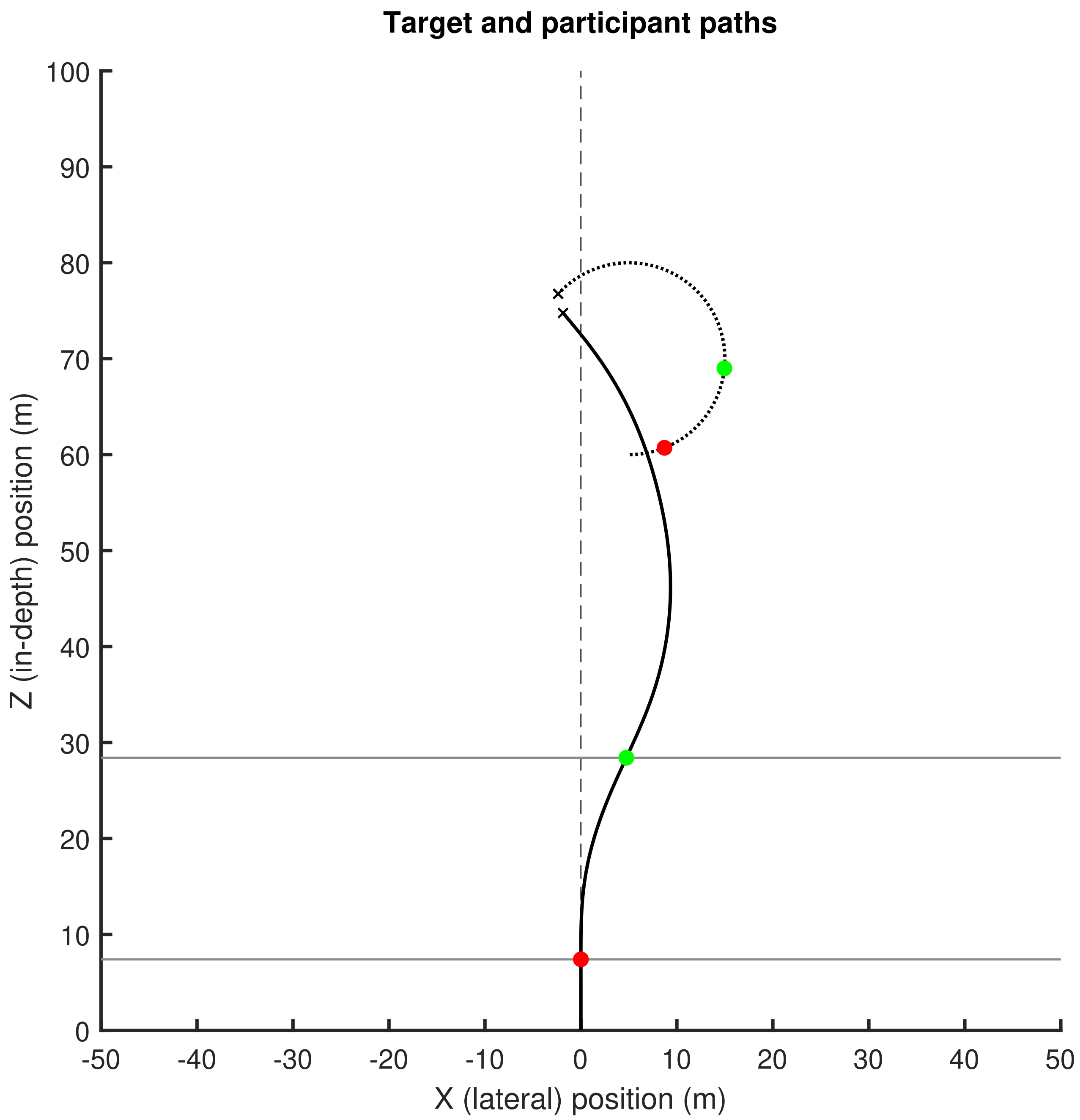

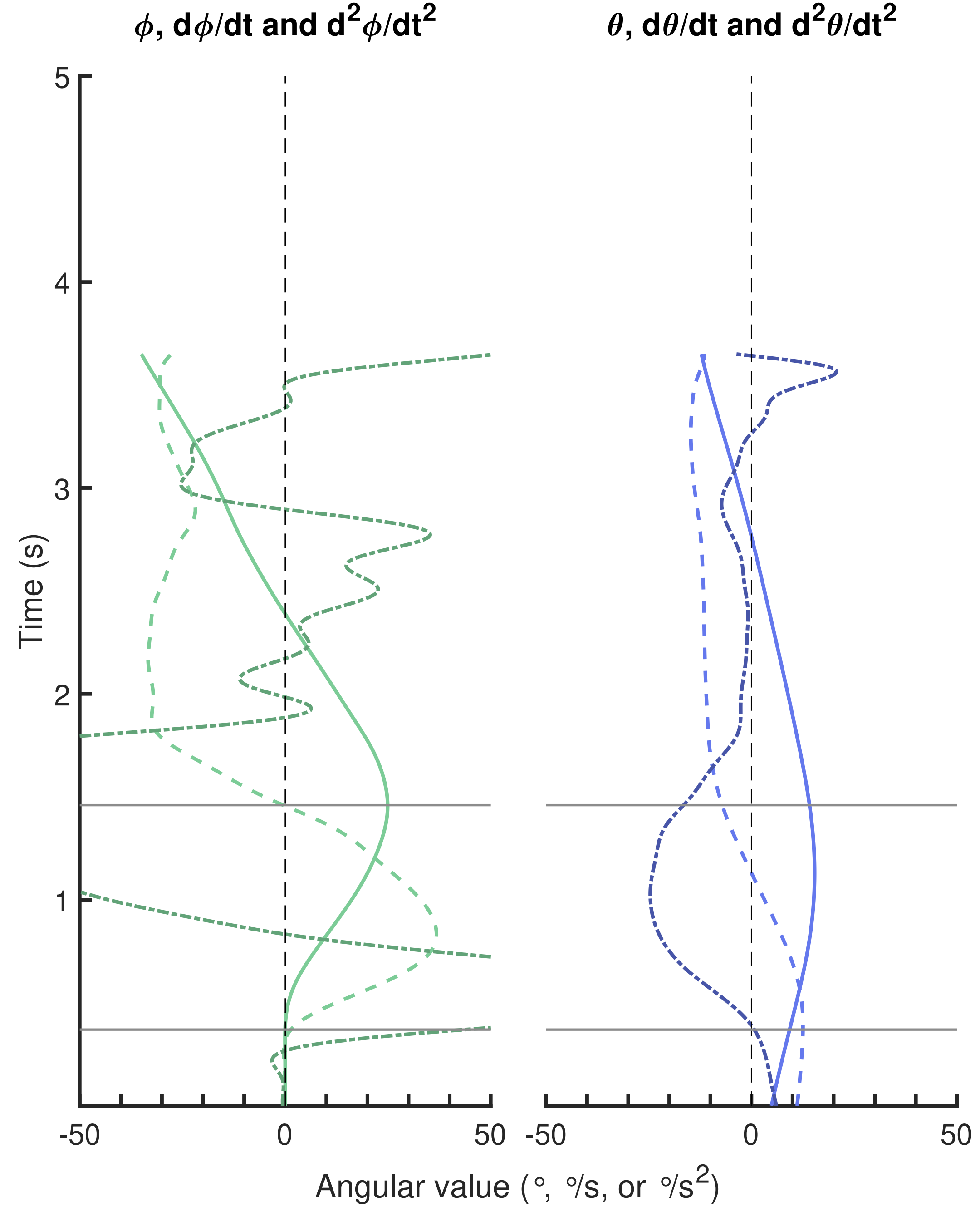

P11/B4
S5/R10-IN

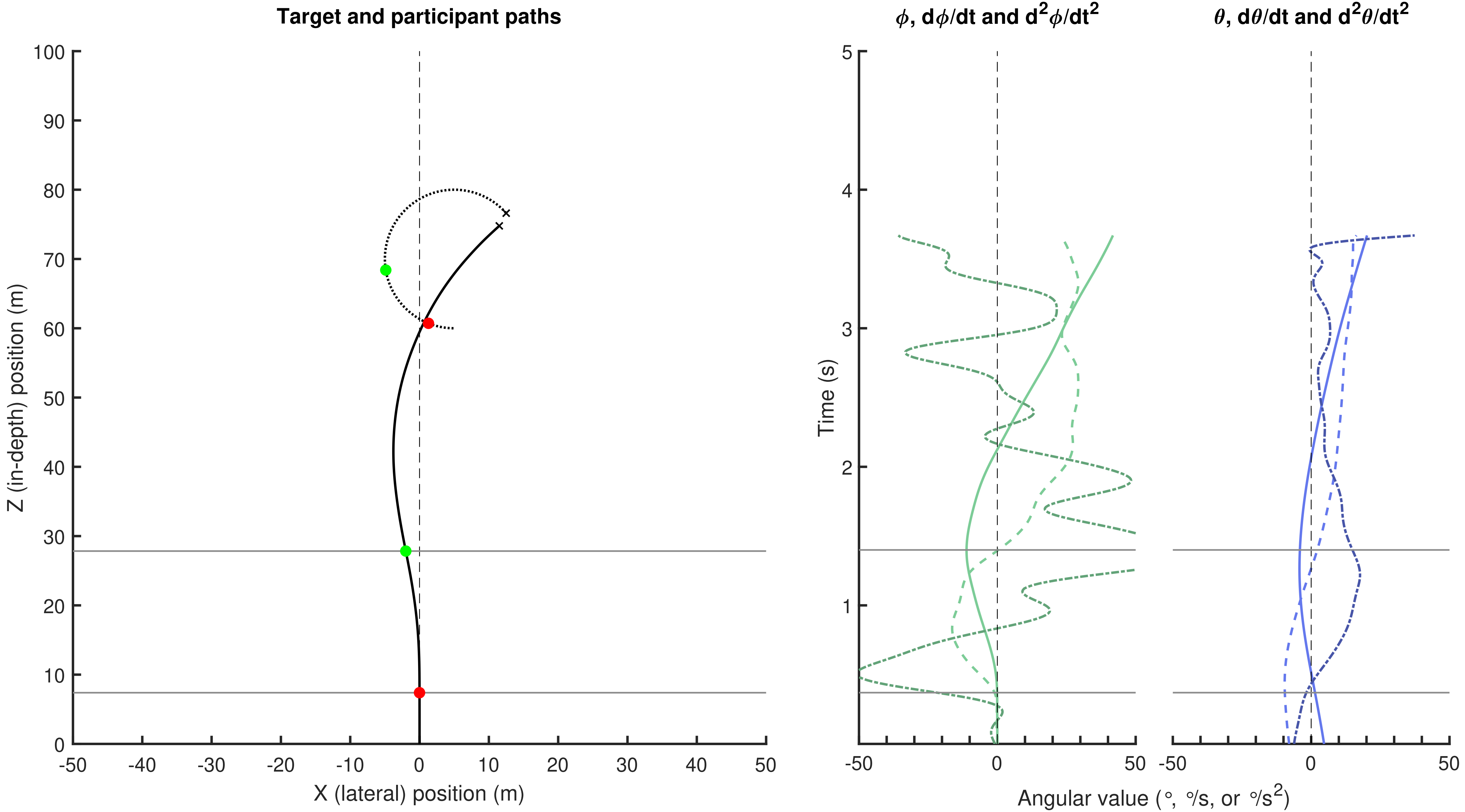

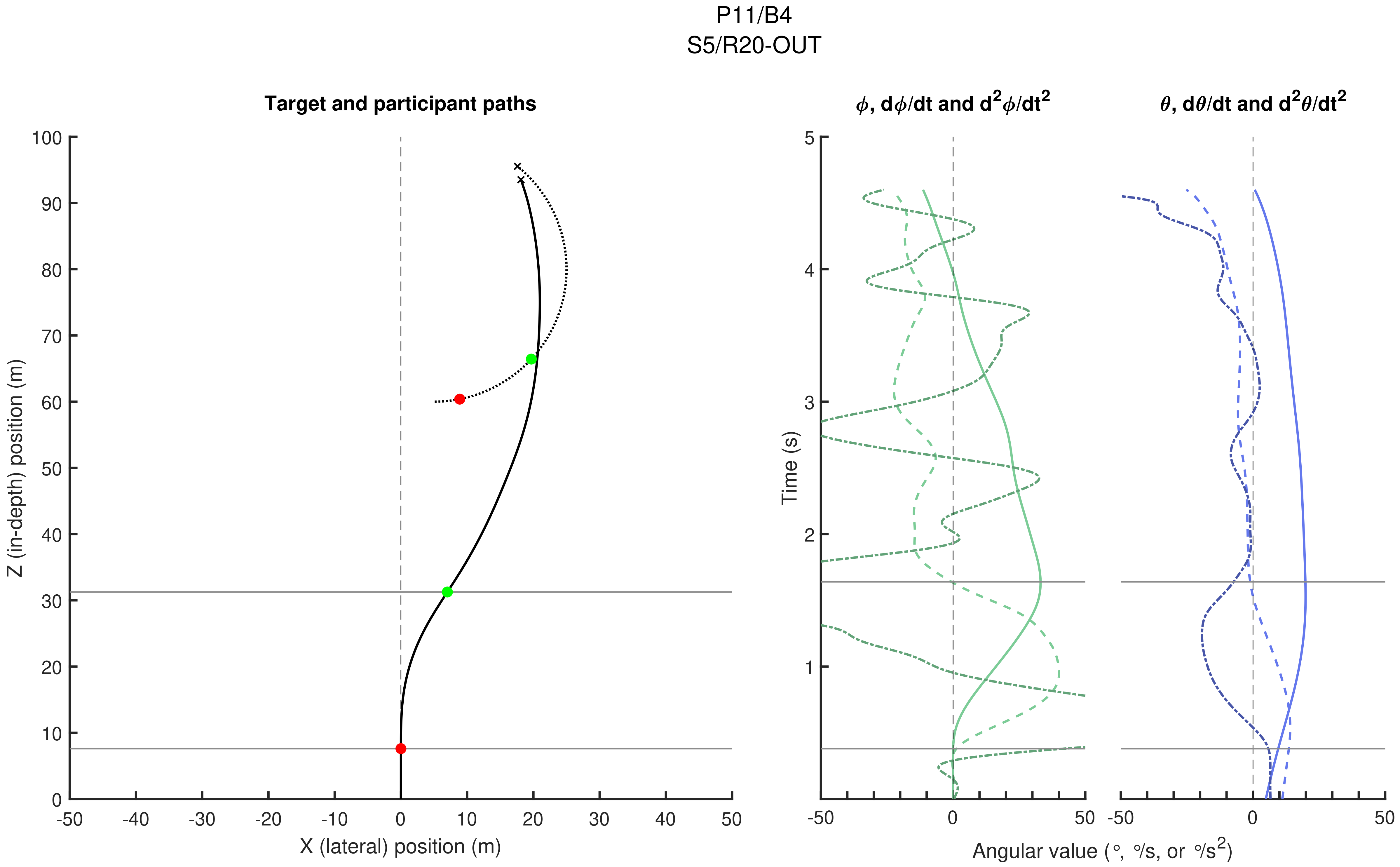

P11/B4
S5/R20-OUT
Target and participant paths
Z (in-depth) position (m)
X (lateral) position (m)
ϕ, dϕ/dt and d²ϕ/dt²
θ, dθ/dt and d²θ/dt²
Time (s)
Angular value (°, °/s, or °/s²)

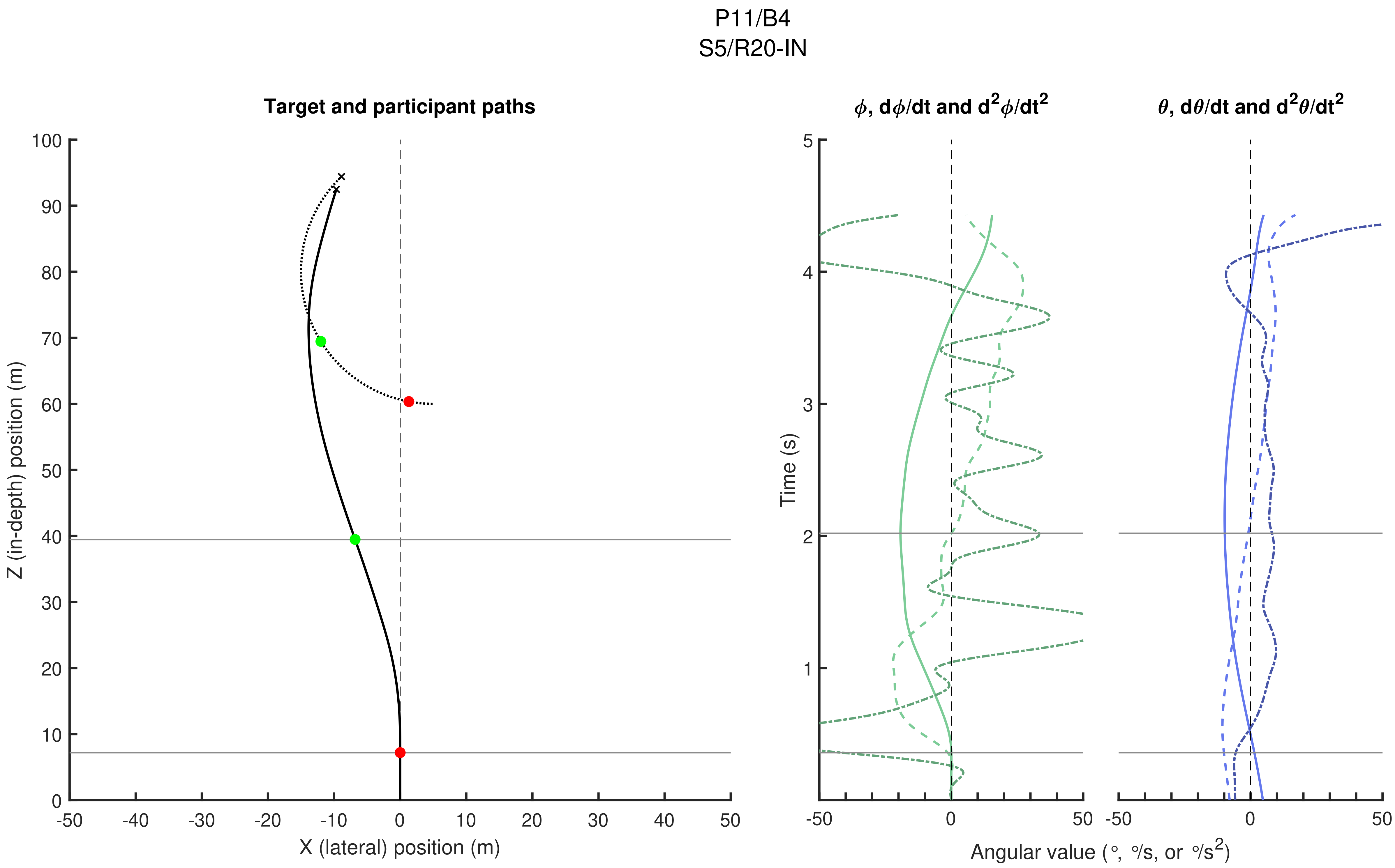

P11/B4
S5/R20-IN
Target and participant paths
Z (in-depth) position (m)
X (lateral) position (m)
ϕ, dϕ/dt and d$^2\phi$/dt^2
θ, dθ/dt and d$^2\theta$/dt^2
Time (s)
Angular value (°, °/s, or °/s^2)

P11/B4
S5/R30-OUT

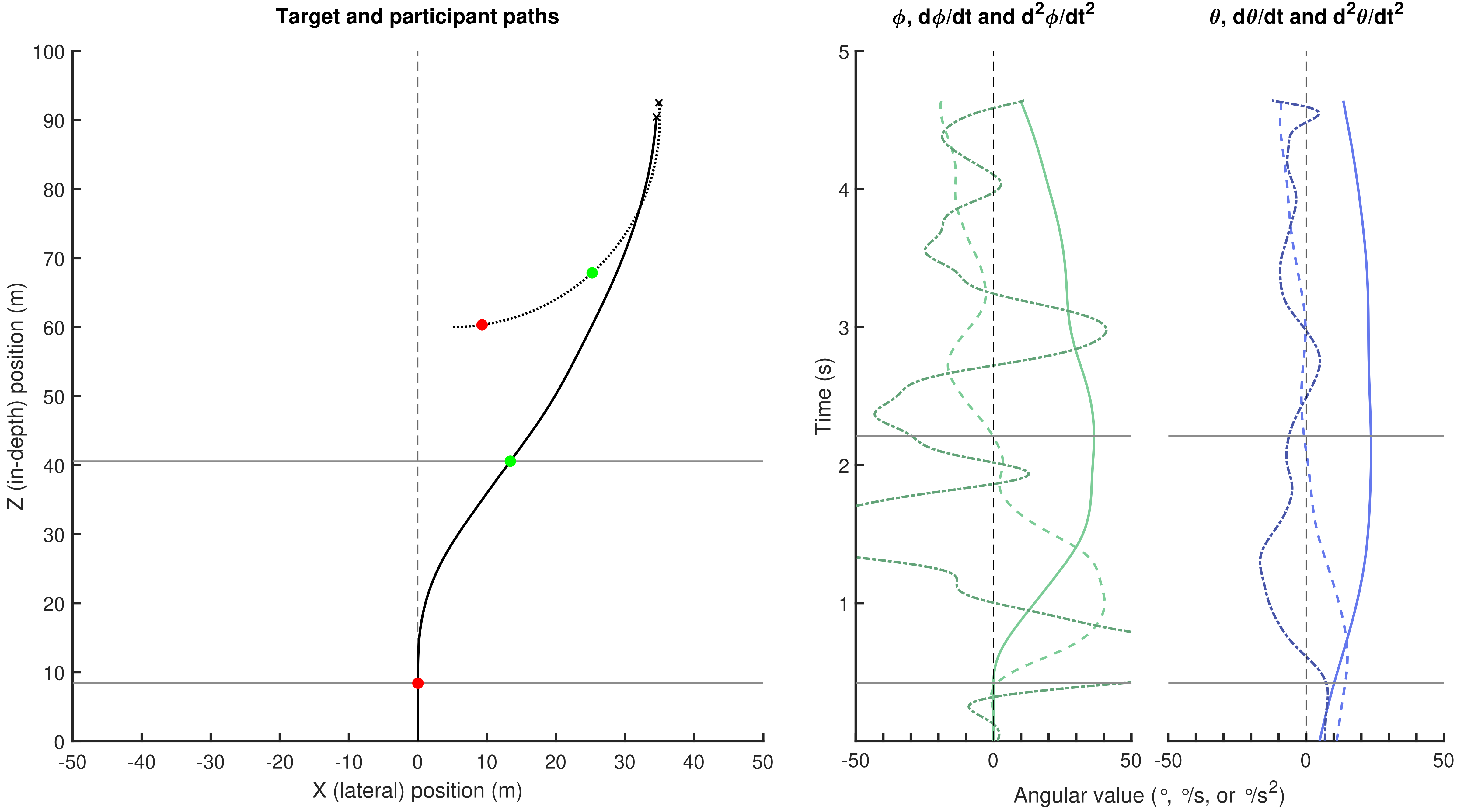

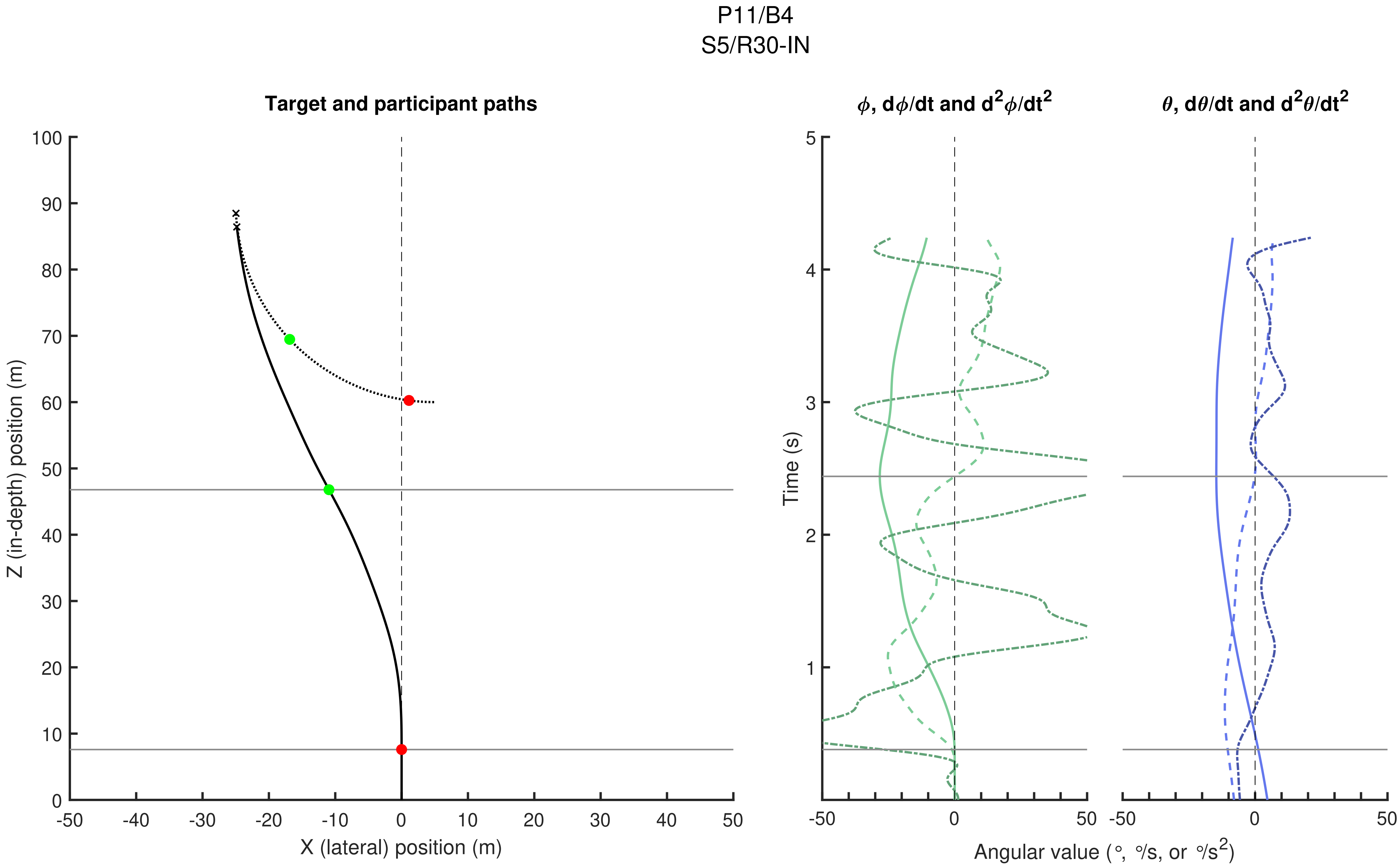

P11/B4
S5/R30-IN
Target and participant paths
ϕ, dϕ/dt and d$^2\phi$/dt^2
θ, dθ/dt and d$^2\theta$/dt^2
Z (in-depth) position (m)
X (lateral) position (m)
Time (s)
Angular value (°, °/s, or °/s^2)
100
90
80
70
60
50
40
30
20
10
0
-50
-40
-30
-20
-10
0
10
20
30
40
50
5
4
3
2
1
-50
0
50
-50
0
50

P11/B4
S15/R10-IN

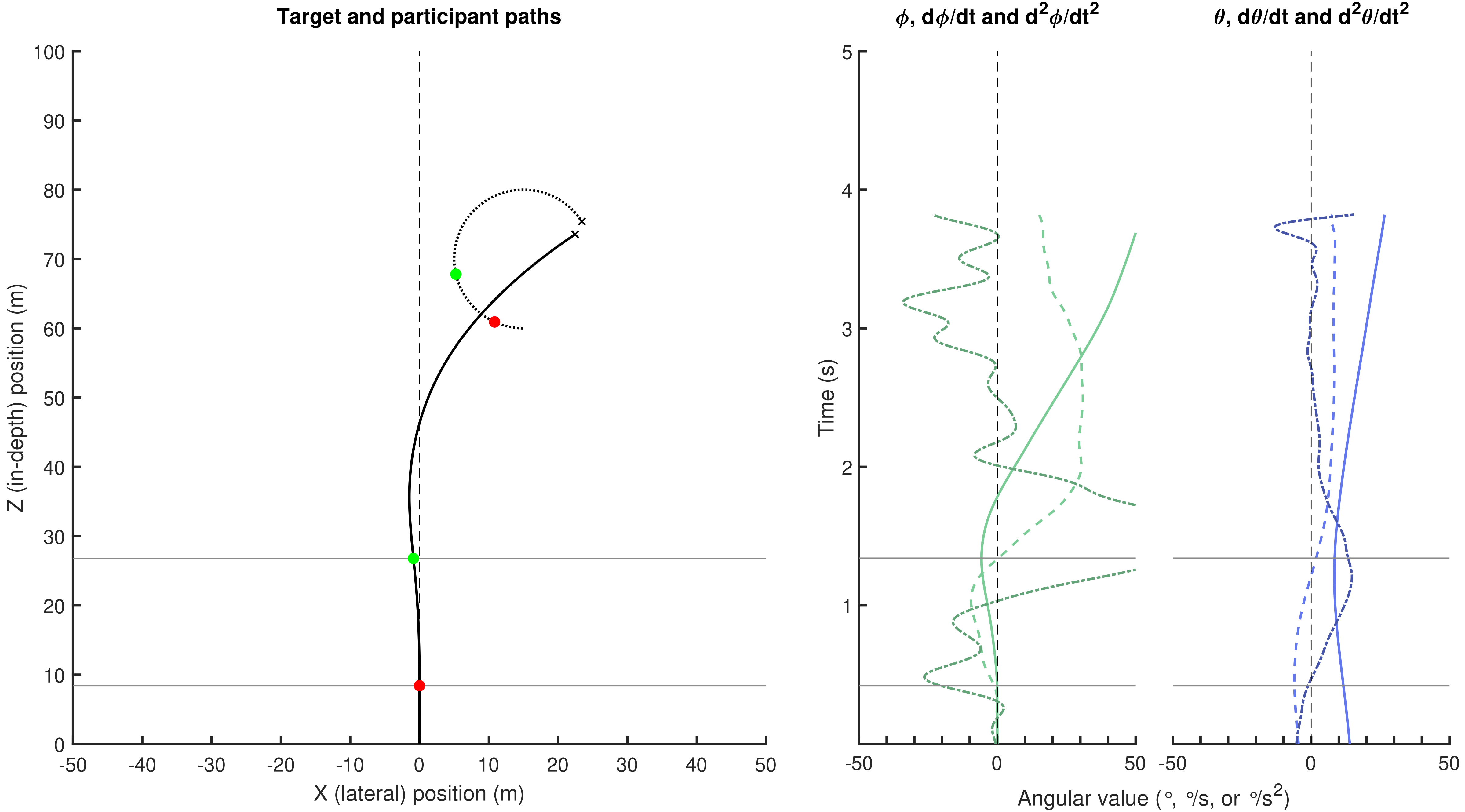

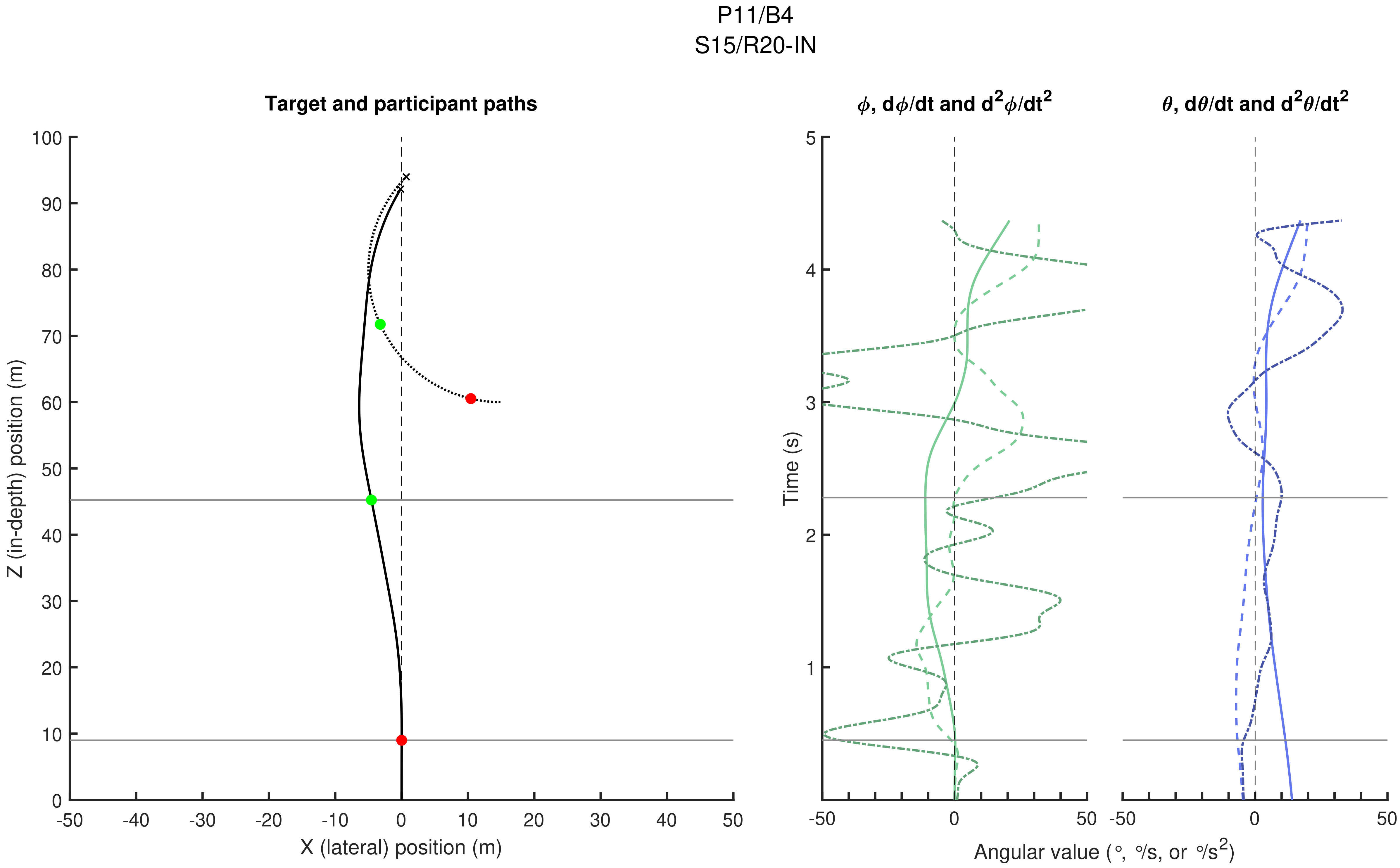

P11/B4
S15/R20-IN
Target and participant paths
Z (in-depth) position (m)
X (lateral) position (m)
ϕ, dϕ/dt and d²ϕ/dt²
θ, dθ/dt and d²θ/dt²
Time (s)
Angular value (°, °/s, or °/s²)

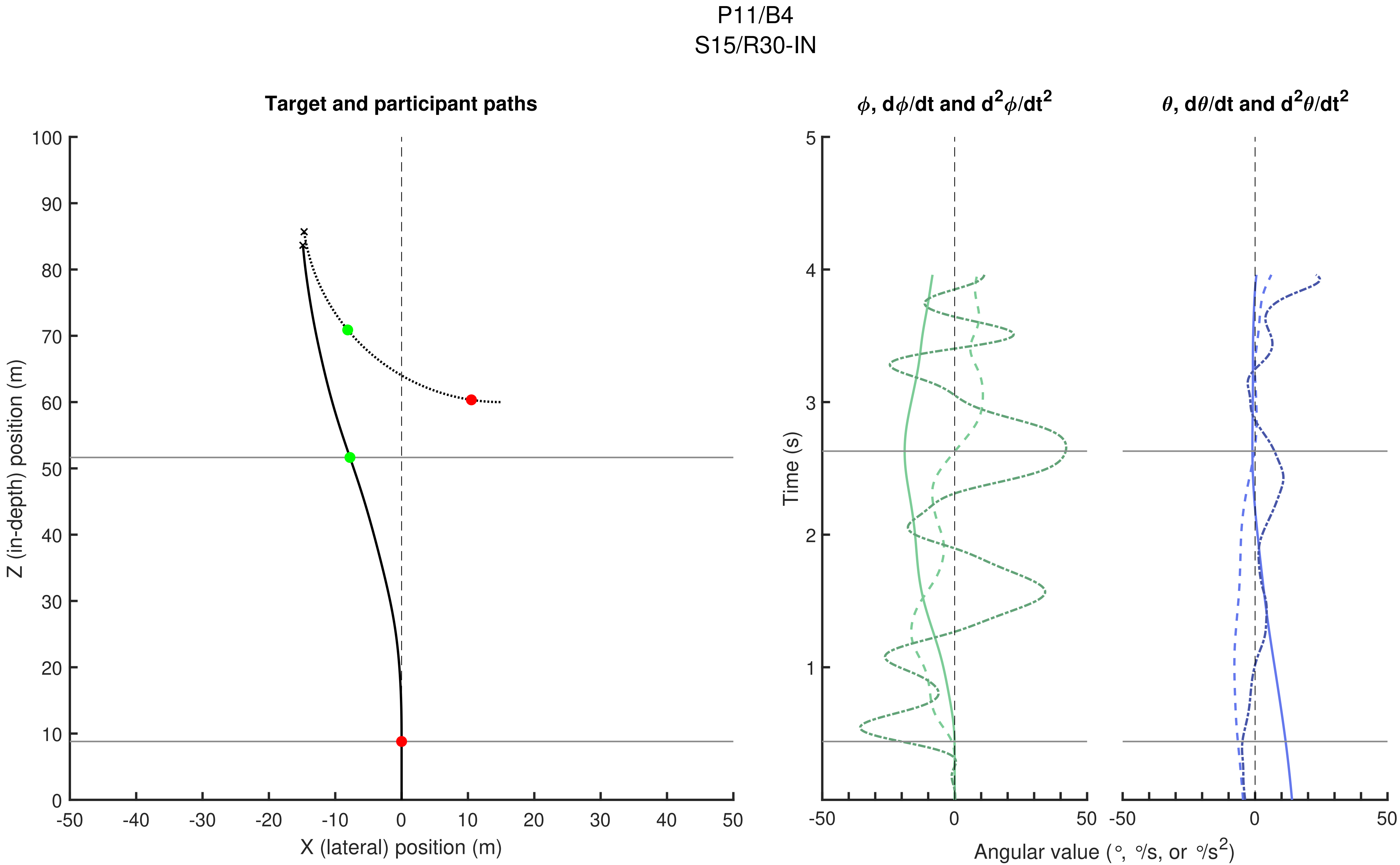
P11/B4
S15/R30-IN
Target and participant paths
Z (in-depth) position (m)
X (lateral) position (m)
ϕ, dϕ/dt and d$^2\phi$/dt^2
θ, dθ/dt and d$^2\theta$/dt^2
Time (s)
Angular value (°, °/s, or °/s^2)

P11/B4
S20/R10-IN

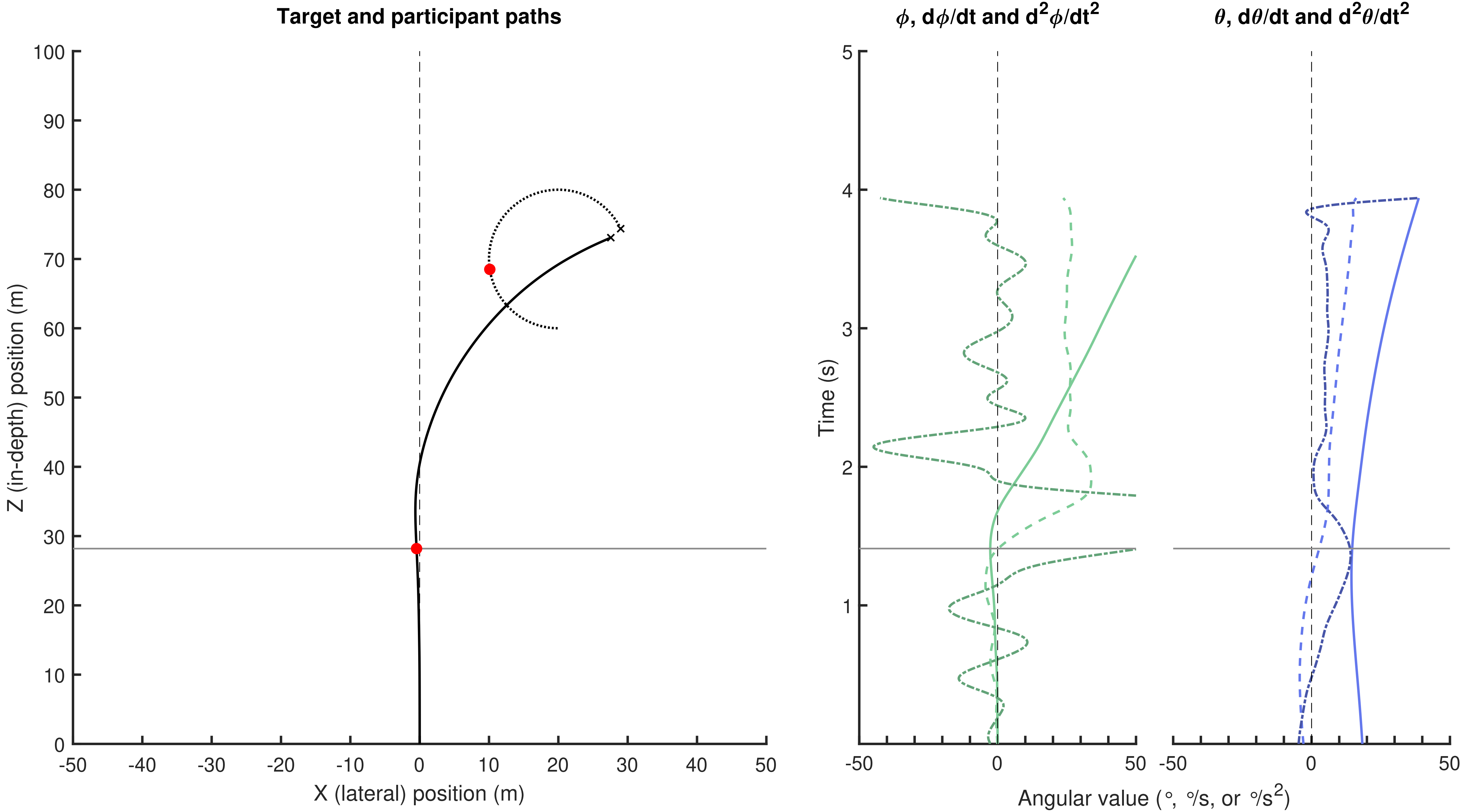

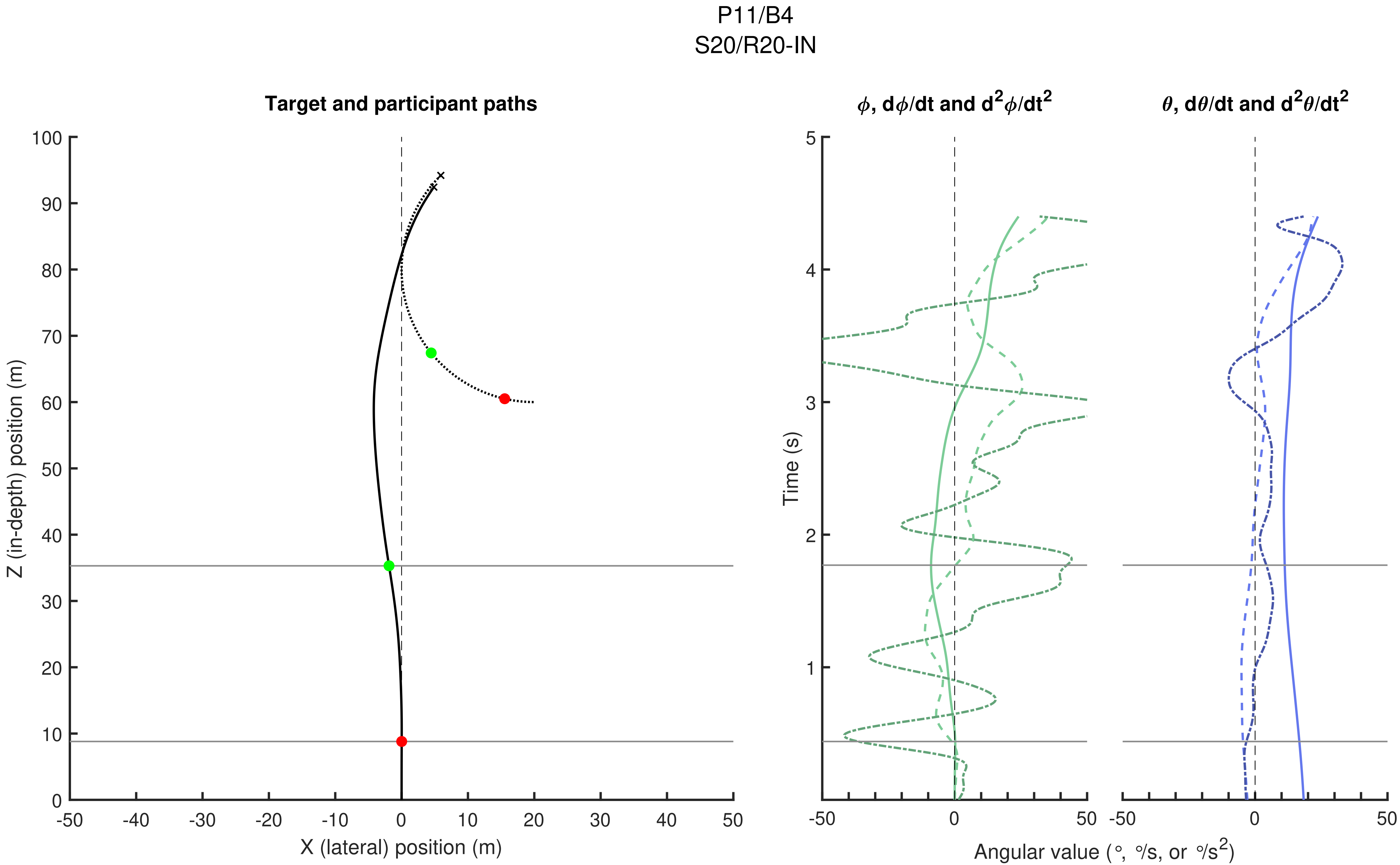
P11/B4
S20/R20-IN
Target and participant paths
Z (in-depth) position (m)
X (lateral) position (m)
100
90
80
70
60
50
40
30
20
10
0
-50
-40
-30
-20
-10
0
10
20
30
40
50
ϕ, dϕ/dt and d$^2\phi$/dt^2
θ, dθ/dt and d$^2\theta$/dt^2
Time (s)
5
4
3
2
1
-50
0
50
-50
0
50
Angular value (°, °/s, or °/s^2)

P11/B4
S20/R30-IN

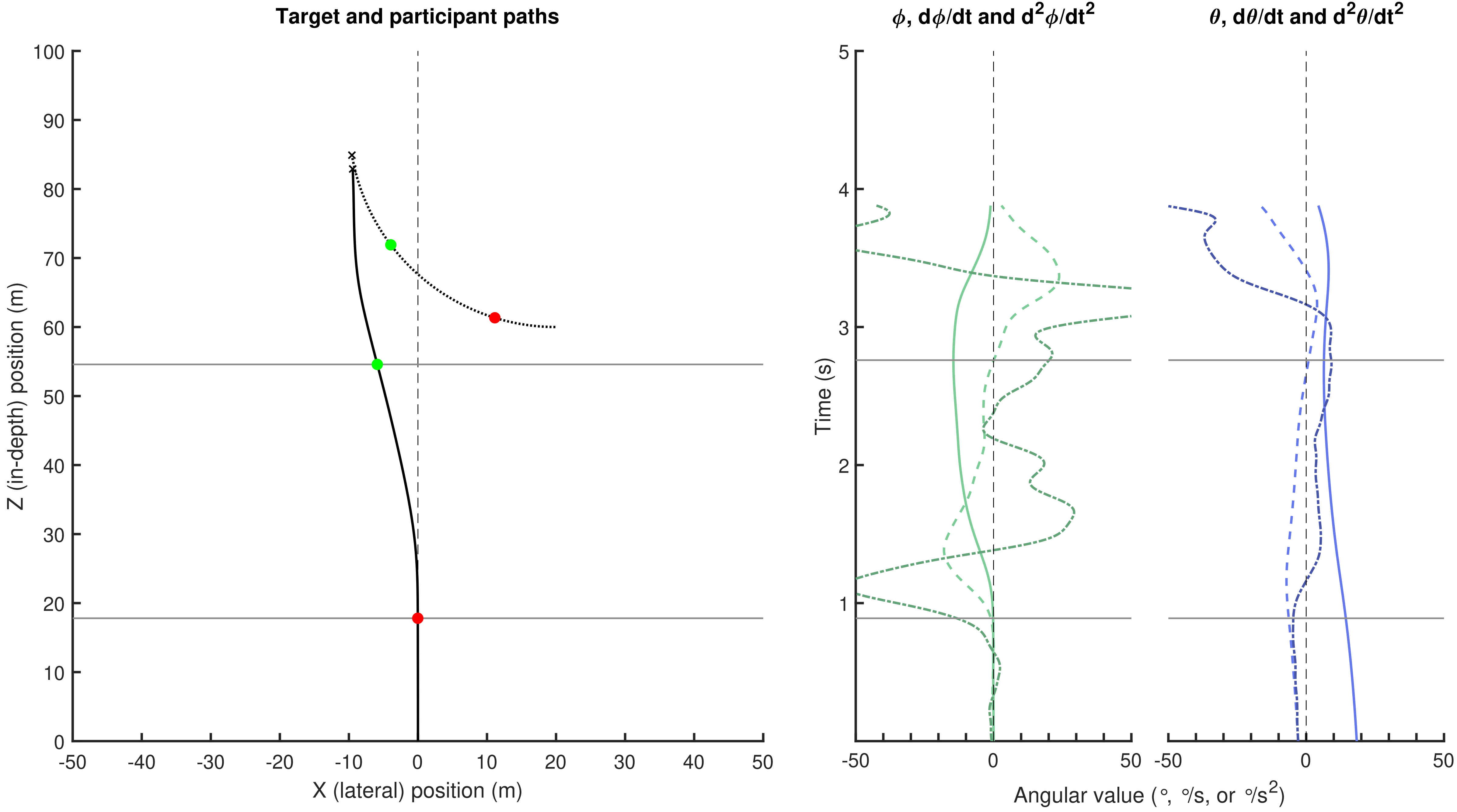

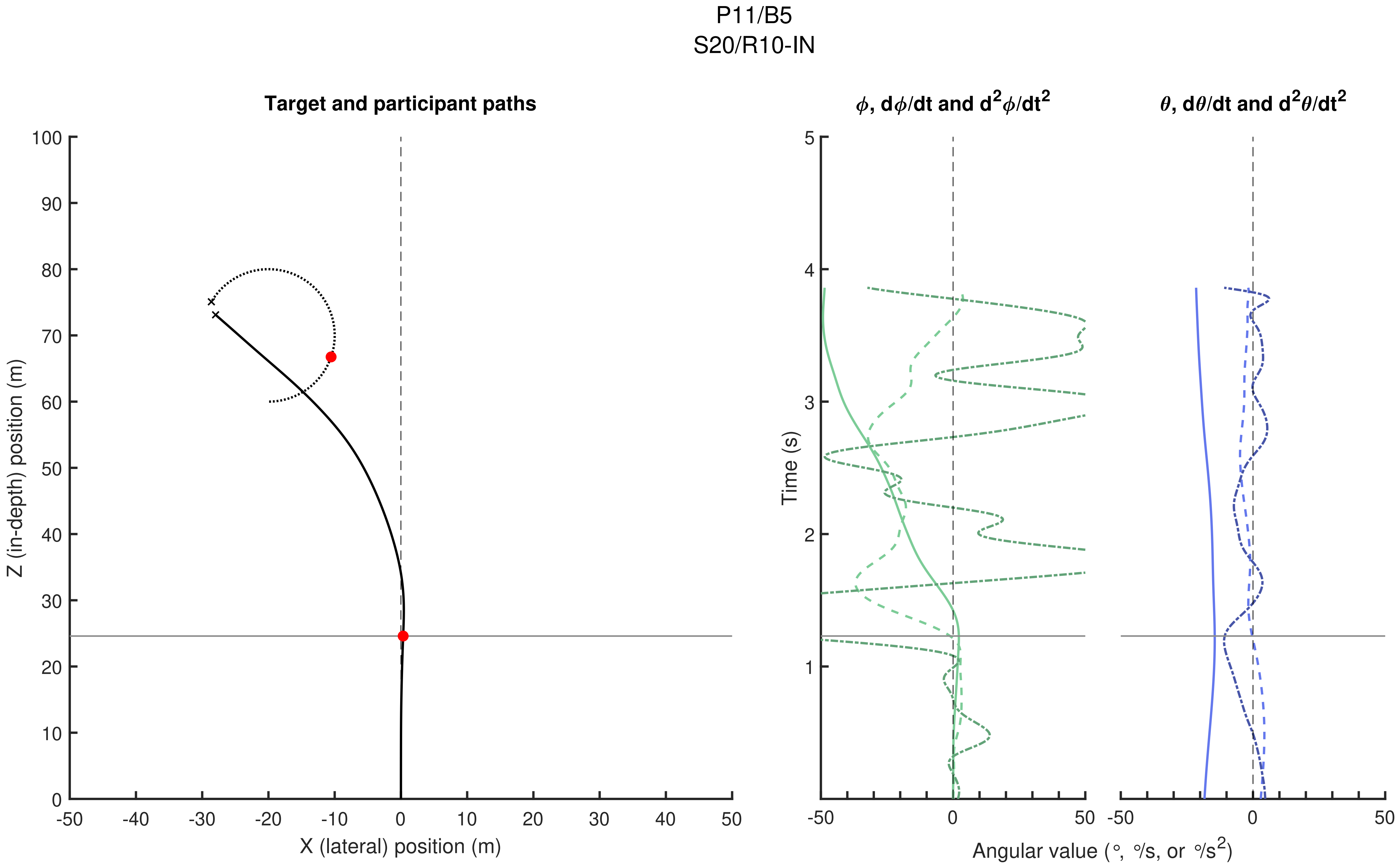
P11/B5
S20/R10-IN
Target and participant paths
Z (in-depth) position (m)
X (lateral) position (m)
100
90
80
70
60
50
40
30
20
10
0
-50
-40
-30
-20
-10
0
10
20
30
40
50
φ, dφ/dt and d²φ/dt²
θ, dθ/dt and d²θ/dt²
Time (s)
5
4
3
2
1
-50
0
50
-50
0
50
Angular value (°, °/s, or °/s²)

P11/B5
S20/R20-IN

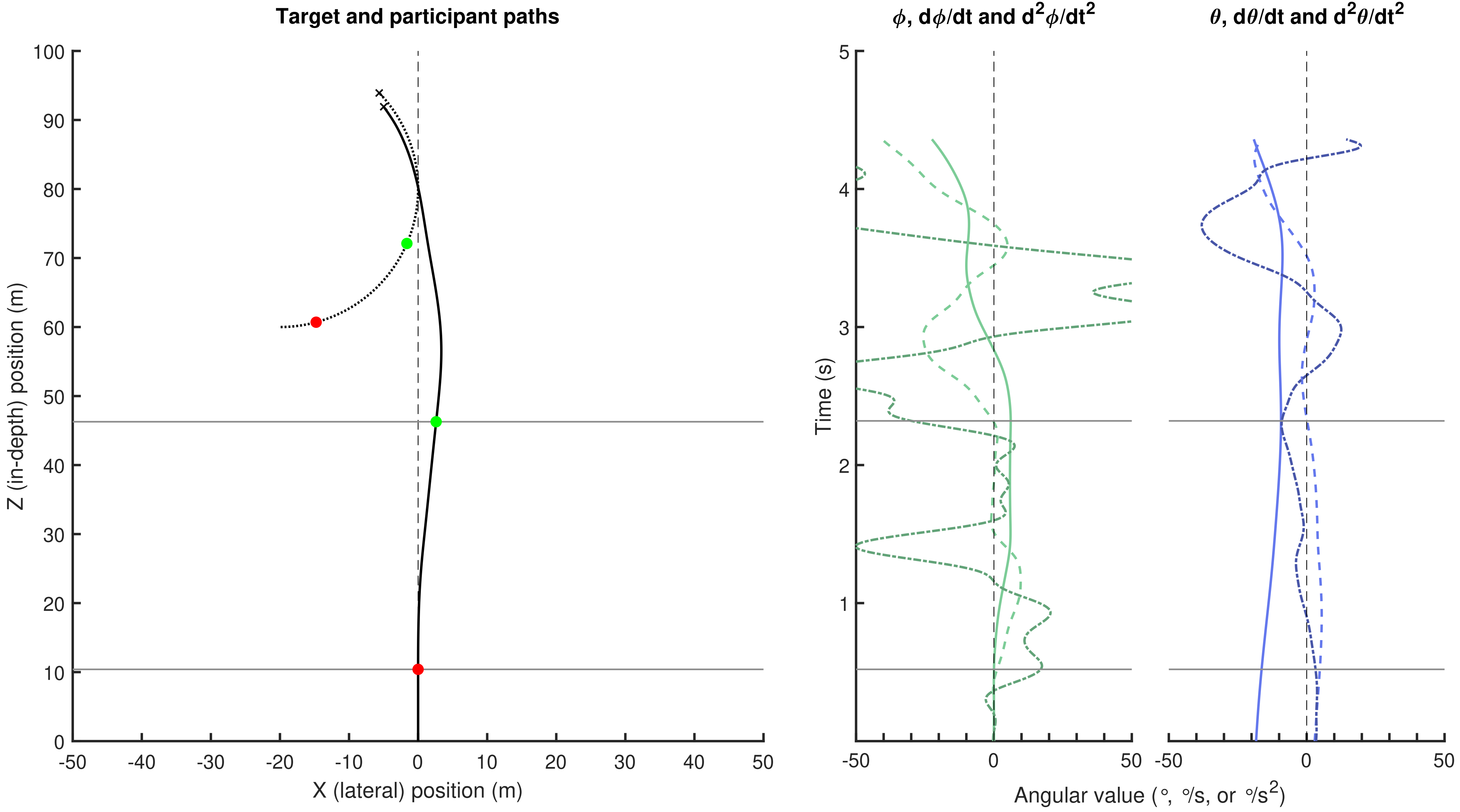

P11/B5
S20/R30-IN

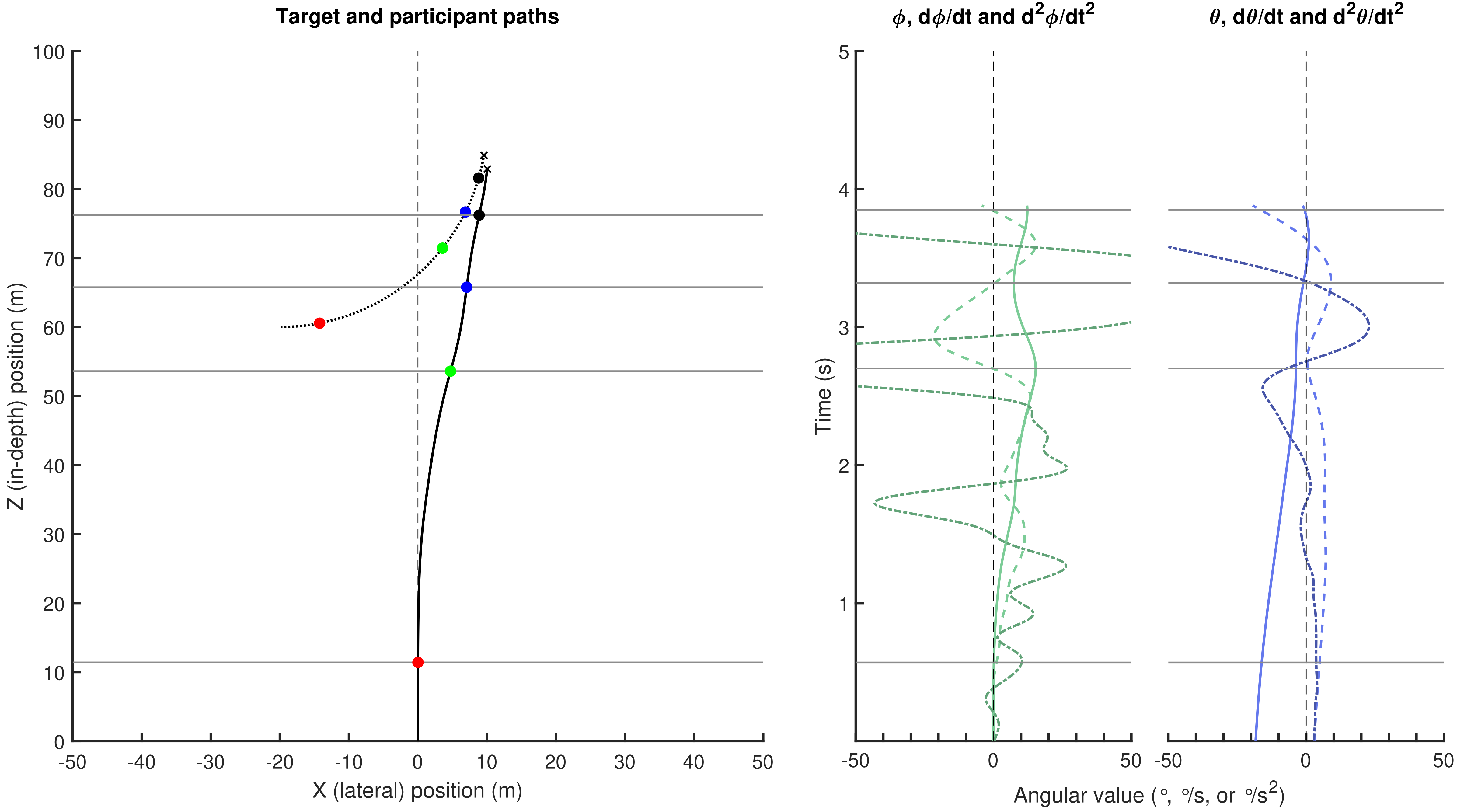

P11/B5
S15/R10-IN

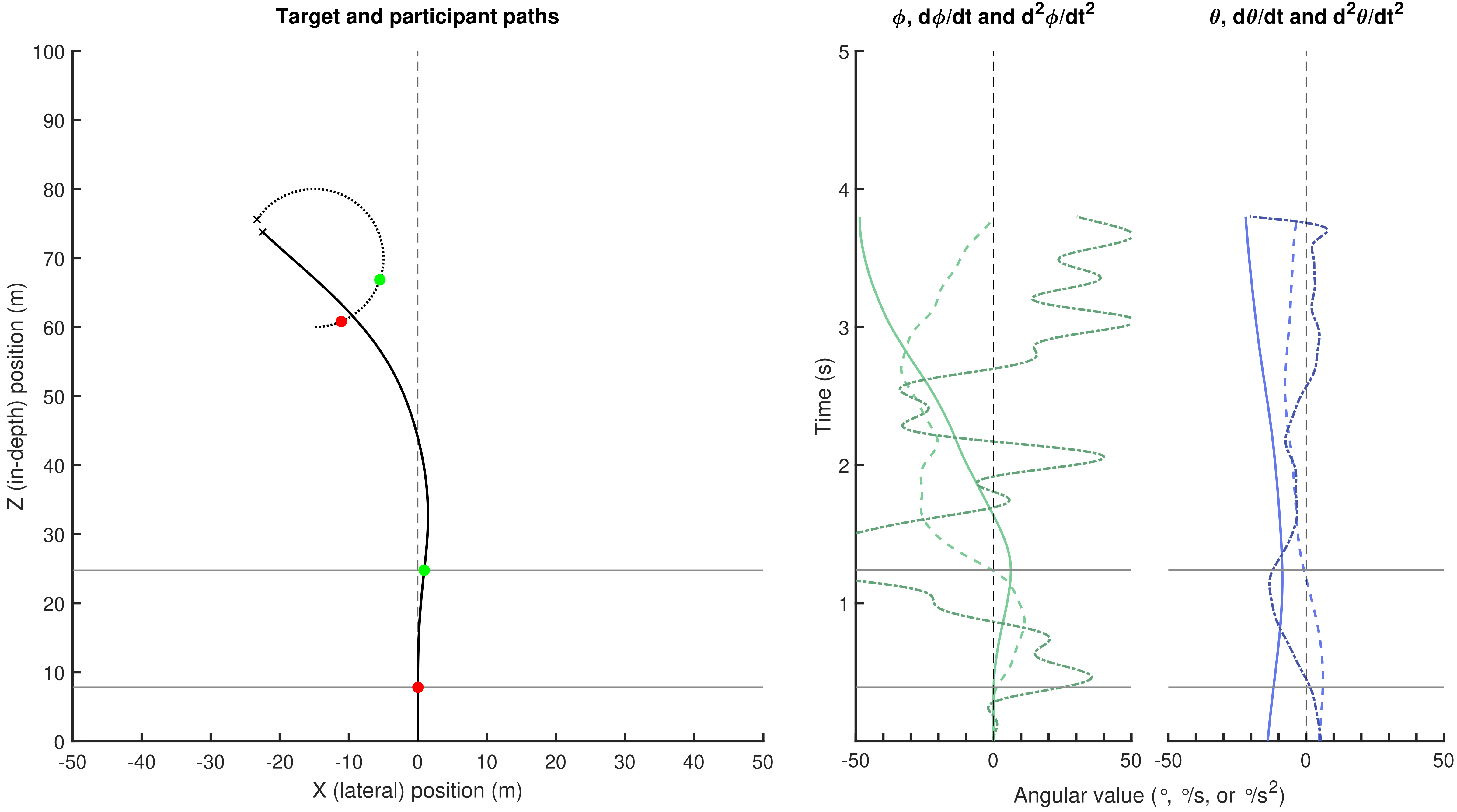

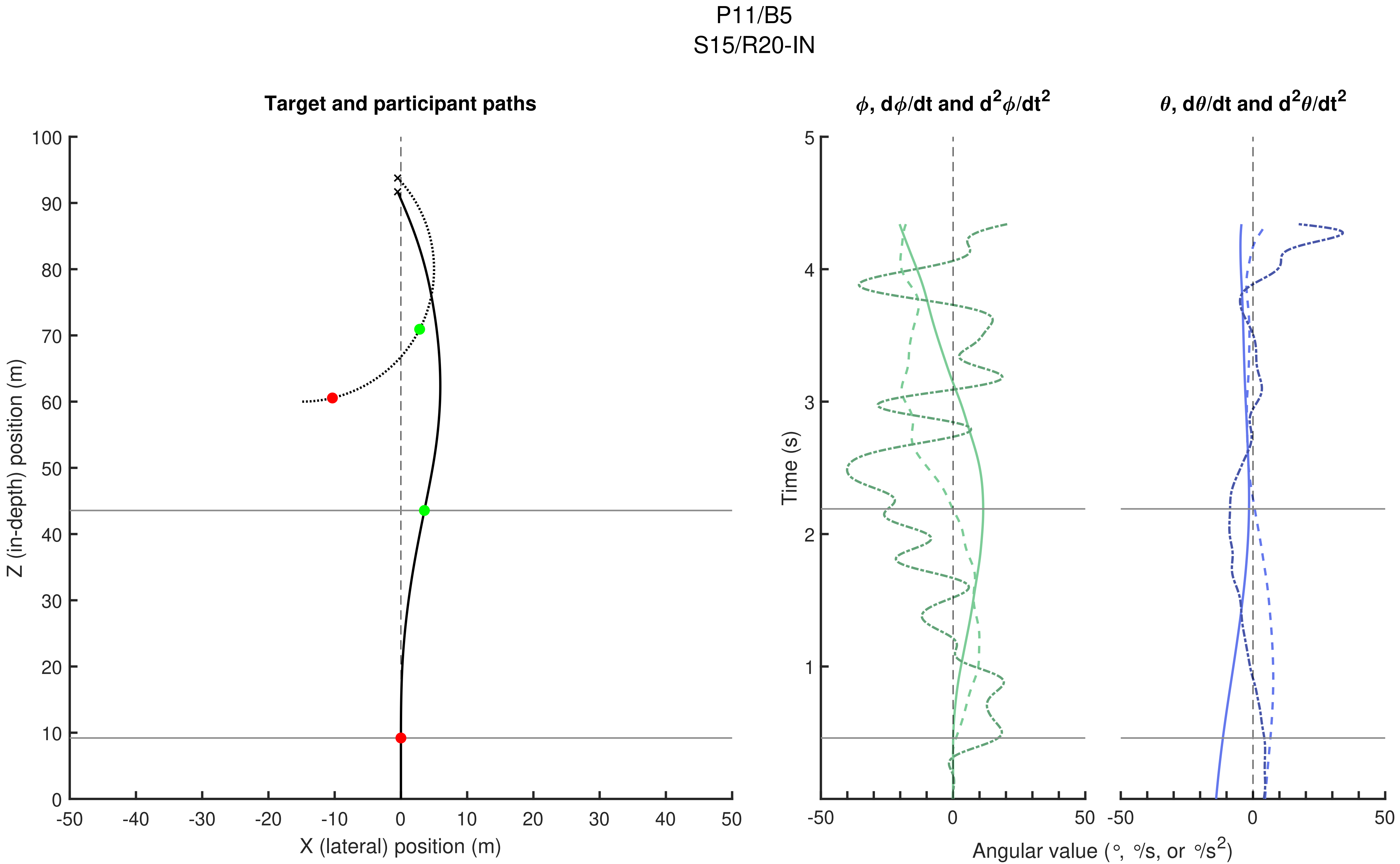
P11/B5
S15/R20-IN
Target and participant paths
Z (in-depth) position (m)
X (lateral) position (m)
ϕ, dϕ/dt and d$^2\phi$/dt^2
θ, dθ/dt and d$^2\theta$/dt^2
Time (s)
Angular value (°, °/s, or °/s^2)

P11/B5
S15/R30-IN

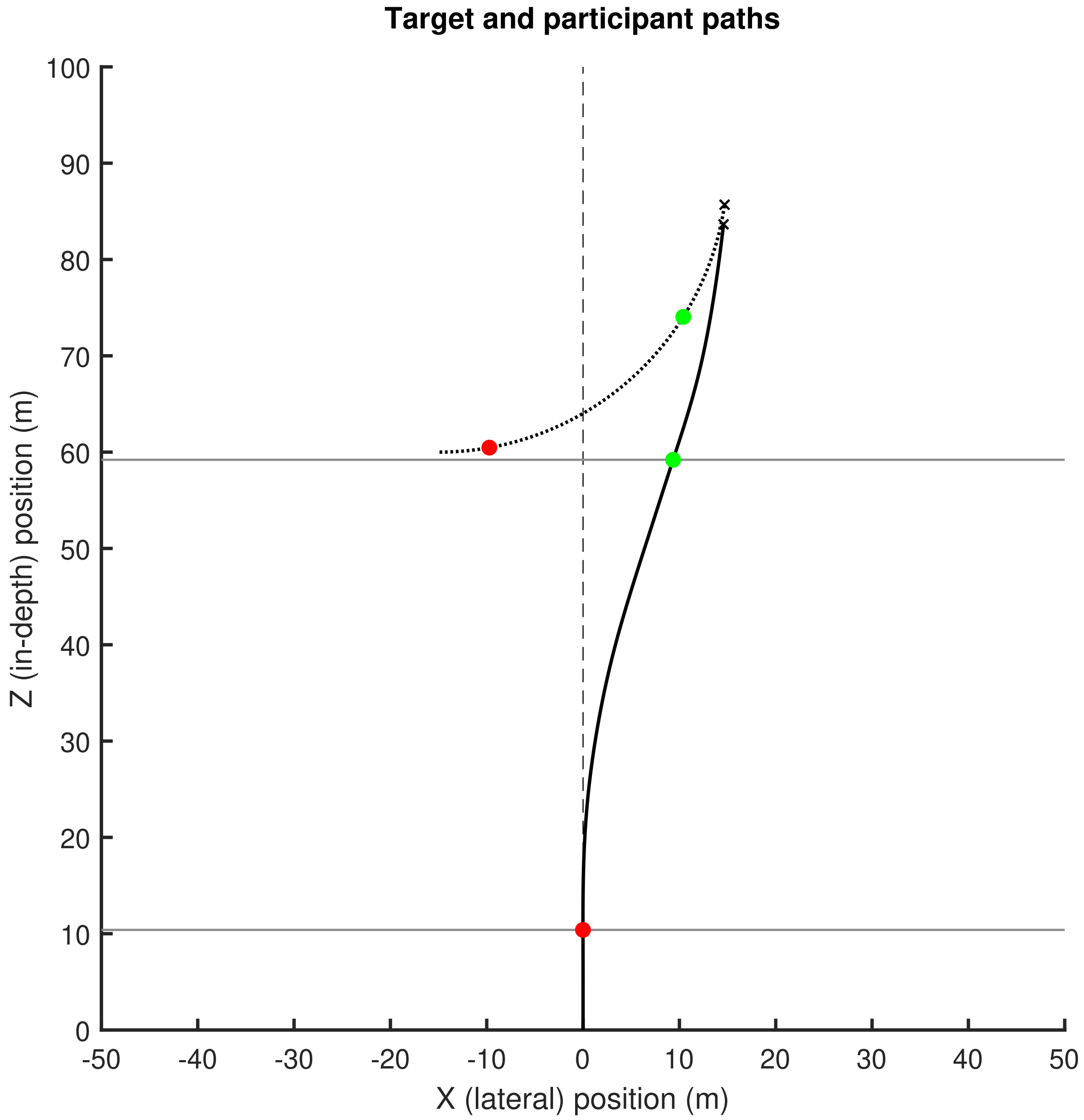

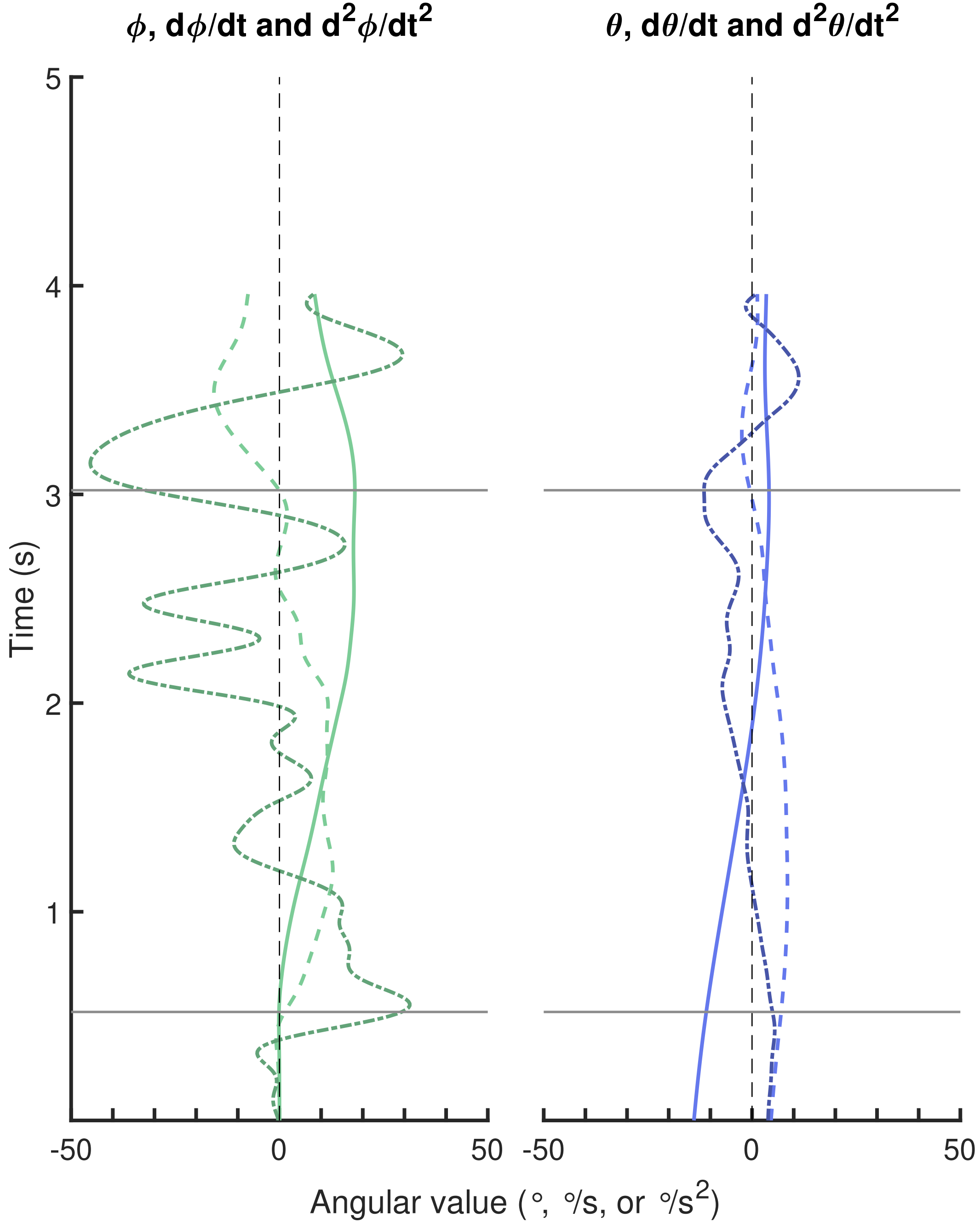

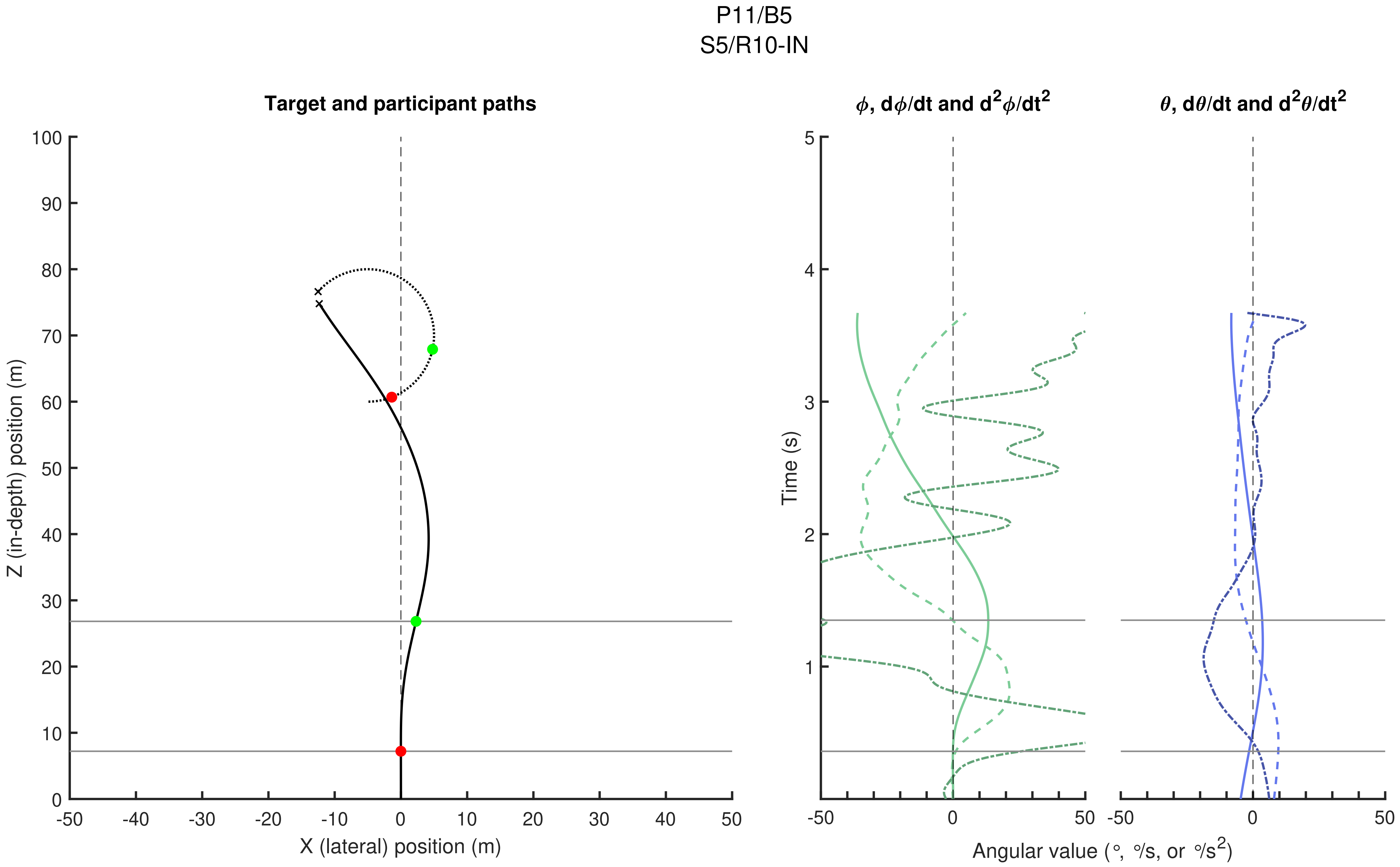
P11/B5
S5/R10-IN
Target and participant paths
Z (in-depth) position (m)
X (lateral) position (m)
φ, dφ/dt and d²φ/dt²
θ, dθ/dt and d²θ/dt²
Time (s)
Angular value (°, °/s, or °/s²)

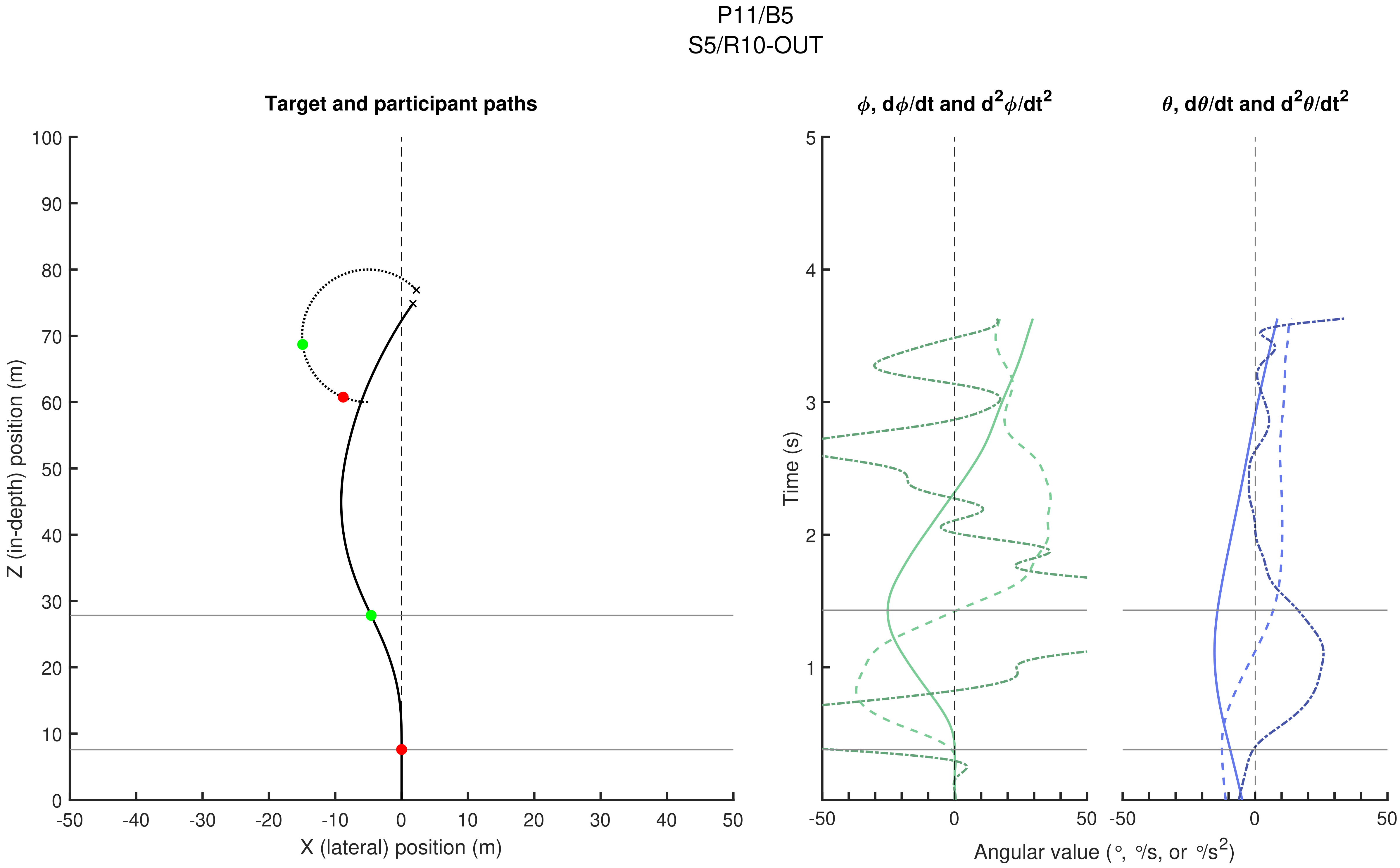

P11/B5
S5/R10-OUT
Target and participant paths
ϕ, dϕ/dt and d$^2\phi$/dt^2
θ, dθ/dt and d$^2\theta$/dt^2
Z (in-depth) position (m)
X (lateral) position (m)
Time (s)
Angular value (°, °/s, or °/s^2)
100
90
80
70
60
50
40
30
20
10
0
-50
-40
-30
-20
-10
0
10
20
30
40
50
5
4
3
2
1
-50
0
50
-50
0
50

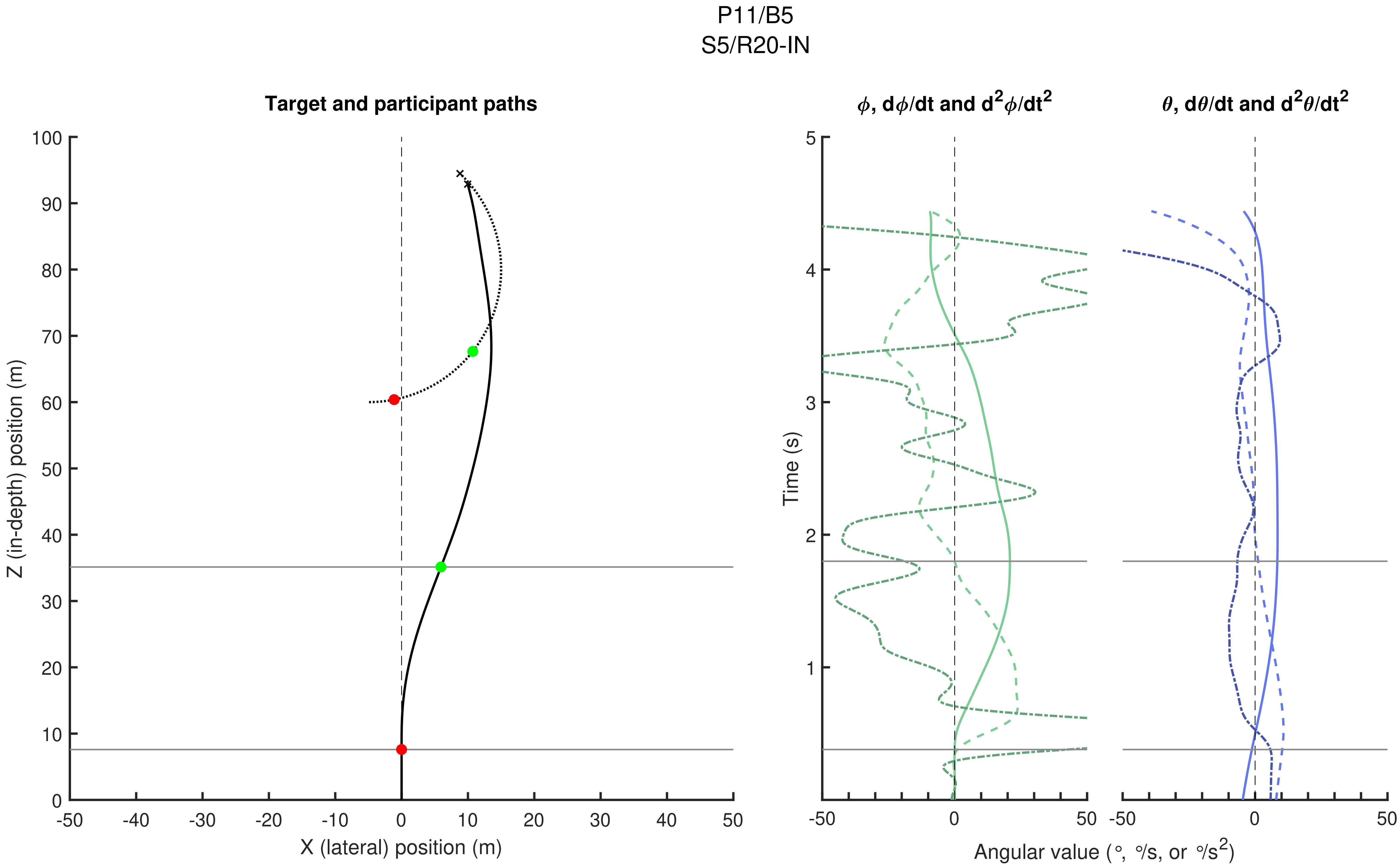
P11/B5
S5/R20-IN
Target and participant paths
ϕ, dϕ/dt and d$^2\phi$/dt^2
θ, dθ/dt and d$^2\theta$/dt^2
Z (in-depth) position (m)
X (lateral) position (m)
Time (s)
Angular value (°, °/s, or °/s^2)

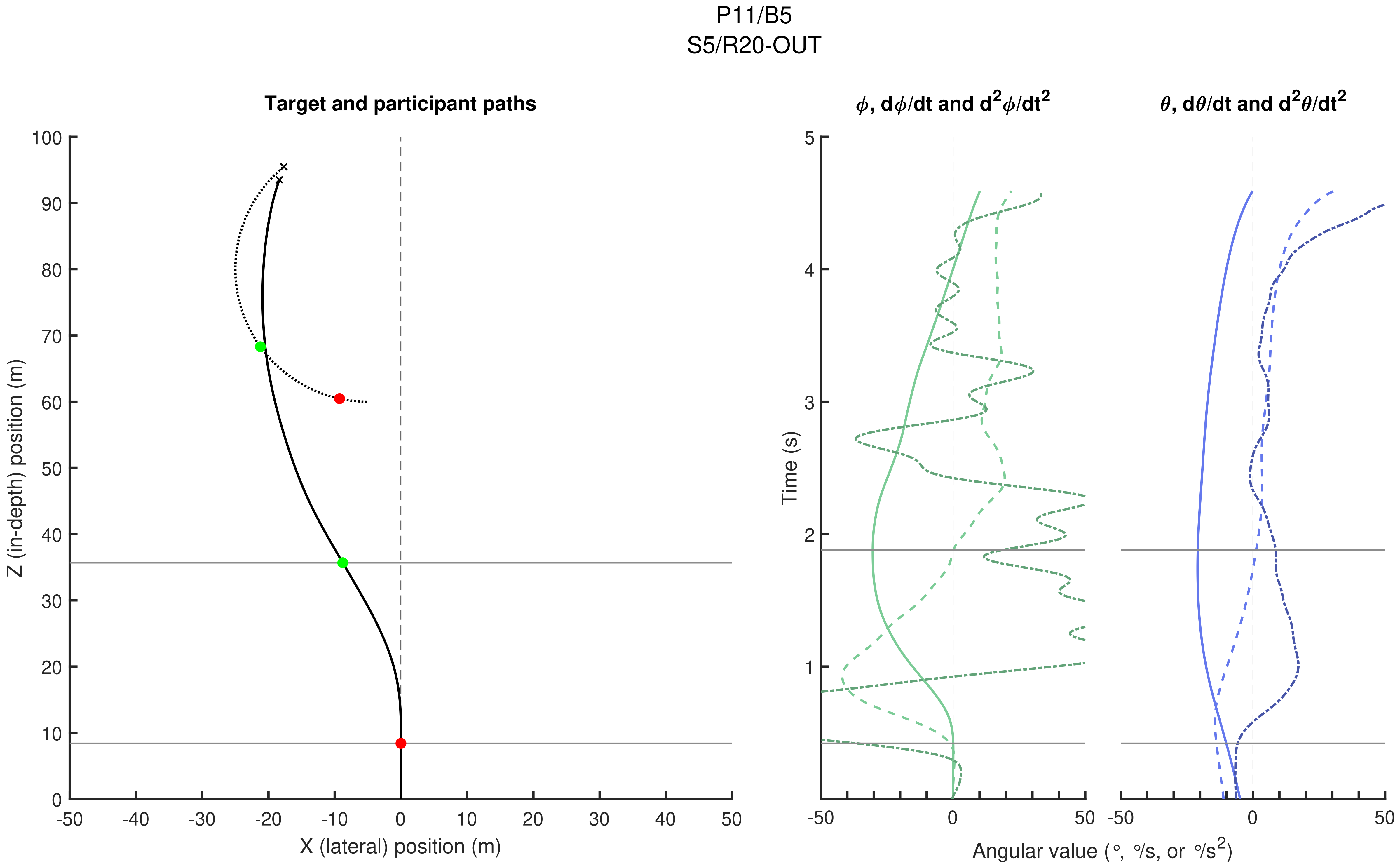
P11/B5
S5/R20-OUT
Target and participant paths
Z (in-depth) position (m)
X (lateral) position (m)
ϕ, dϕ/dt and d$^2\phi$/dt^2
θ, dθ/dt and d$^2\theta$/dt^2
Time (s)
Angular value (°, °/s, or °/s^2)

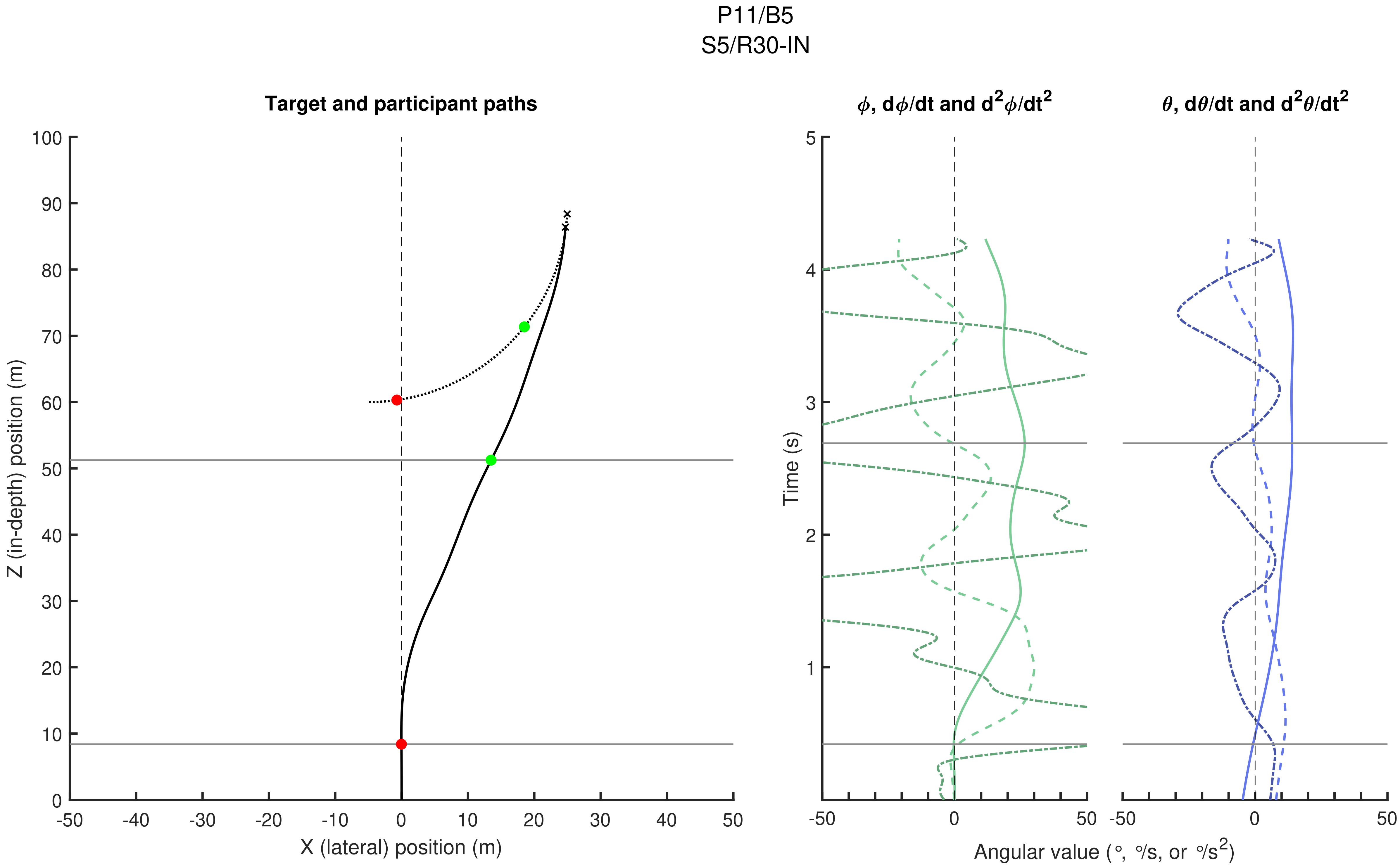
P11/B5
S5/R30-IN
Target and participant paths
Z (in-depth) position (m)
X (lateral) position (m)
ϕ, dϕ/dt and d$^2\phi$/dt^2
θ, dθ/dt and d$^2\theta$/dt^2
Time (s)
Angular value (°, °/s, or °/s^2)

P11/B5
S5/R30-OUT

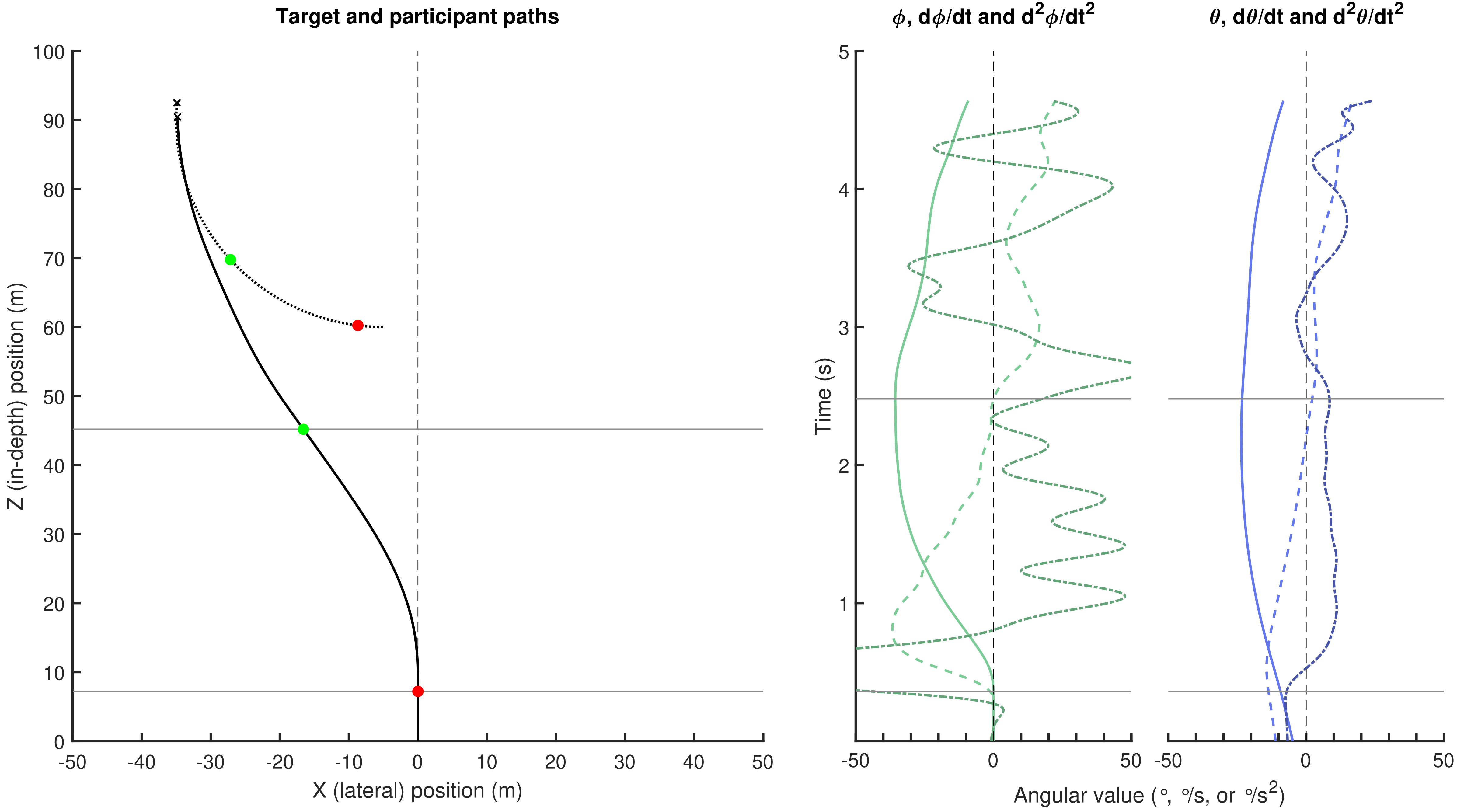

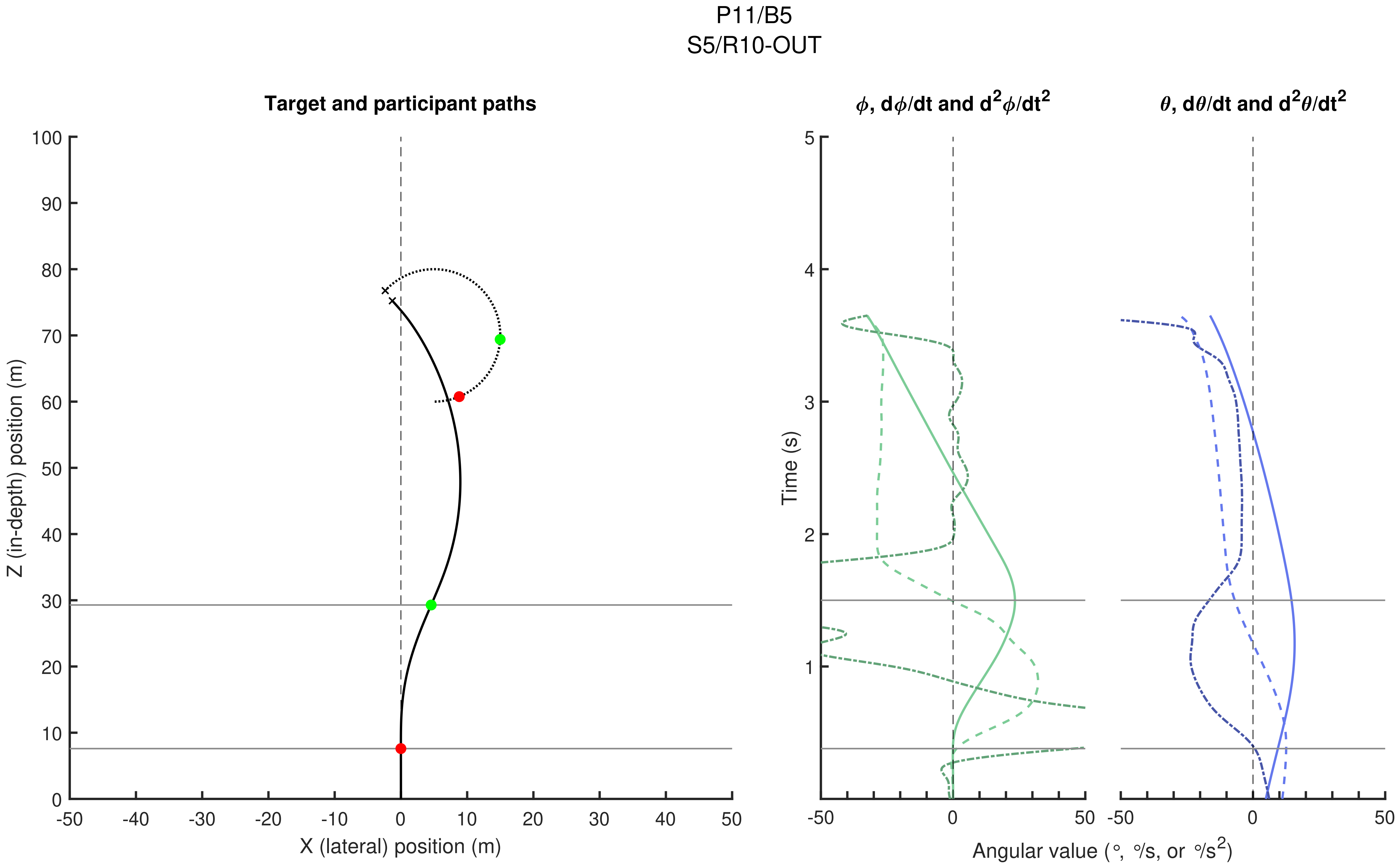
P11/B5
S5/R10-OUT
Target and participant paths
Z (in-depth) position (m)
X (lateral) position (m)
100
90
80
70
60
50
40
30
20
10
0
-50
-40
-30
-20
-10
0
10
20
30
40
50
φ, dφ/dt and d²φ/dt²
θ, dθ/dt and d²θ/dt²
Time (s)
5
4
3
2
1
-50
0
50
-50
0
50
Angular value (°, °/s, or °/s²)

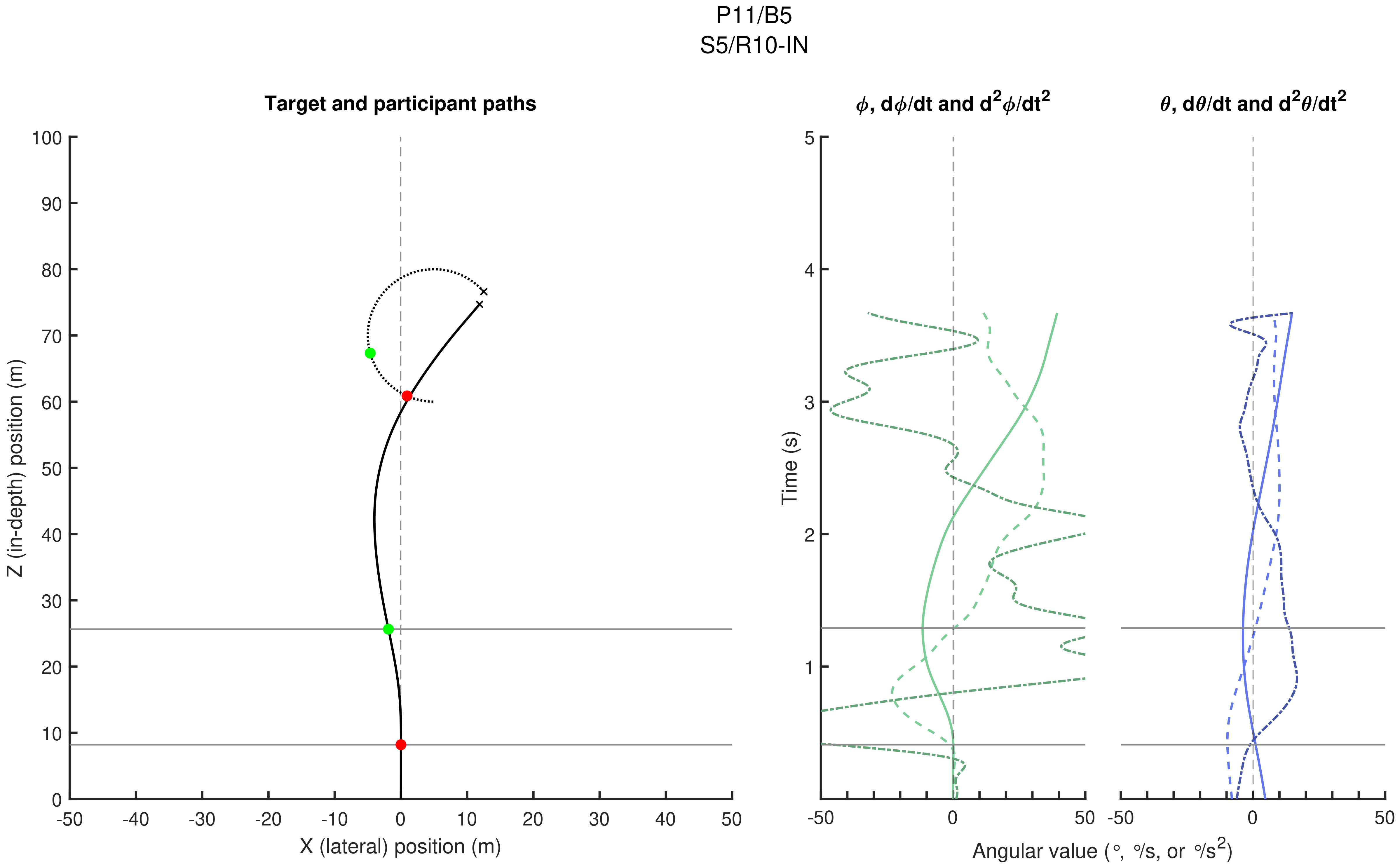

P11/B5
S5/R10-IN
Target and participant paths
Z (in-depth) position (m)
X (lateral) position (m)
100
90
80
70
60
50
40
30
20
10
0
-50
-40
-30
-20
-10
0
10
20
30
40
50
ϕ, dϕ/dt and d²ϕ/dt²
θ, dθ/dt and d²θ/dt²
Time (s)
5
4
3
2
1
-50
0
50
-50
0
50
Angular value (°, °/s, or °/s²)

P11/B5
S5/R20-OUT

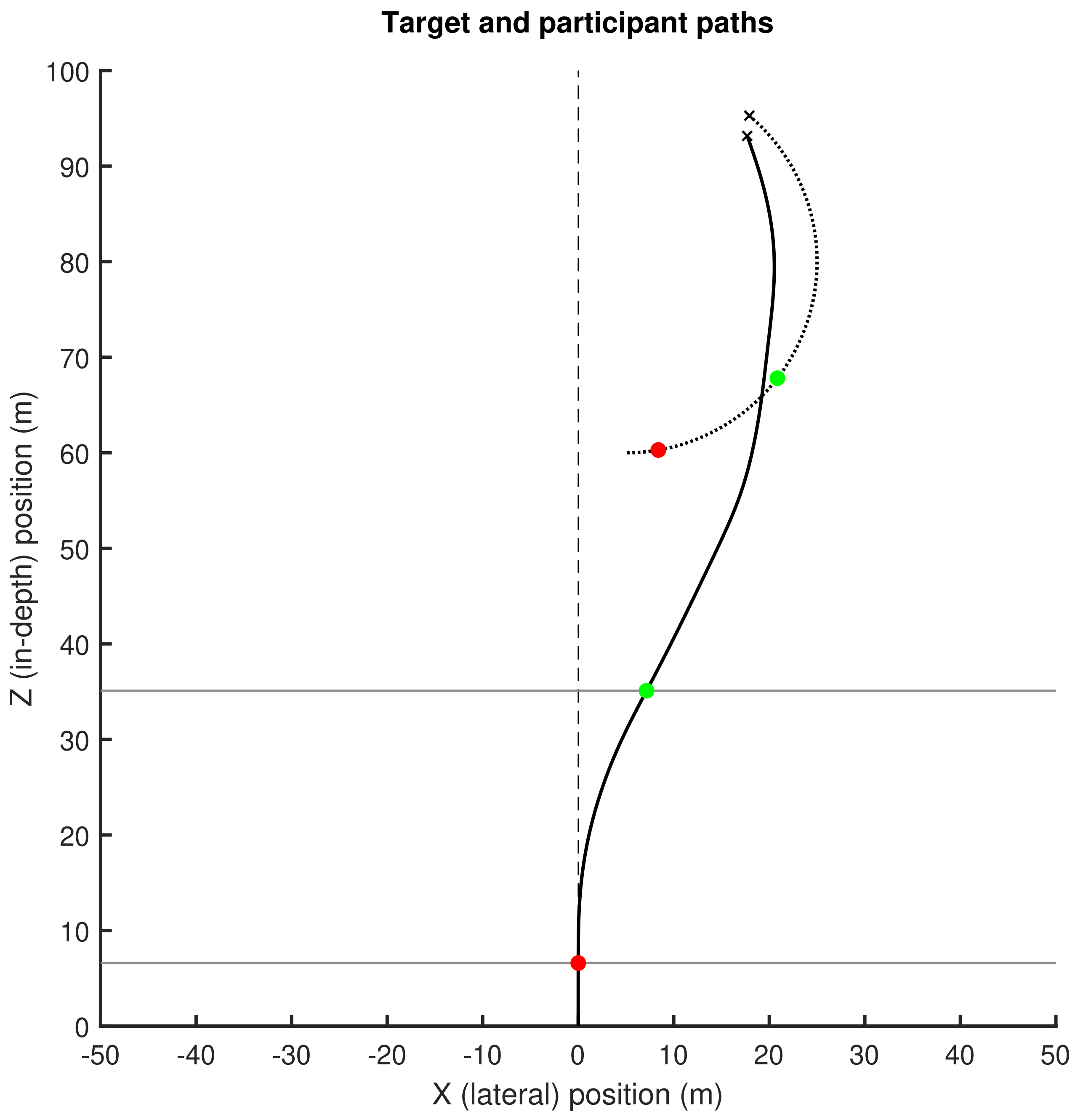

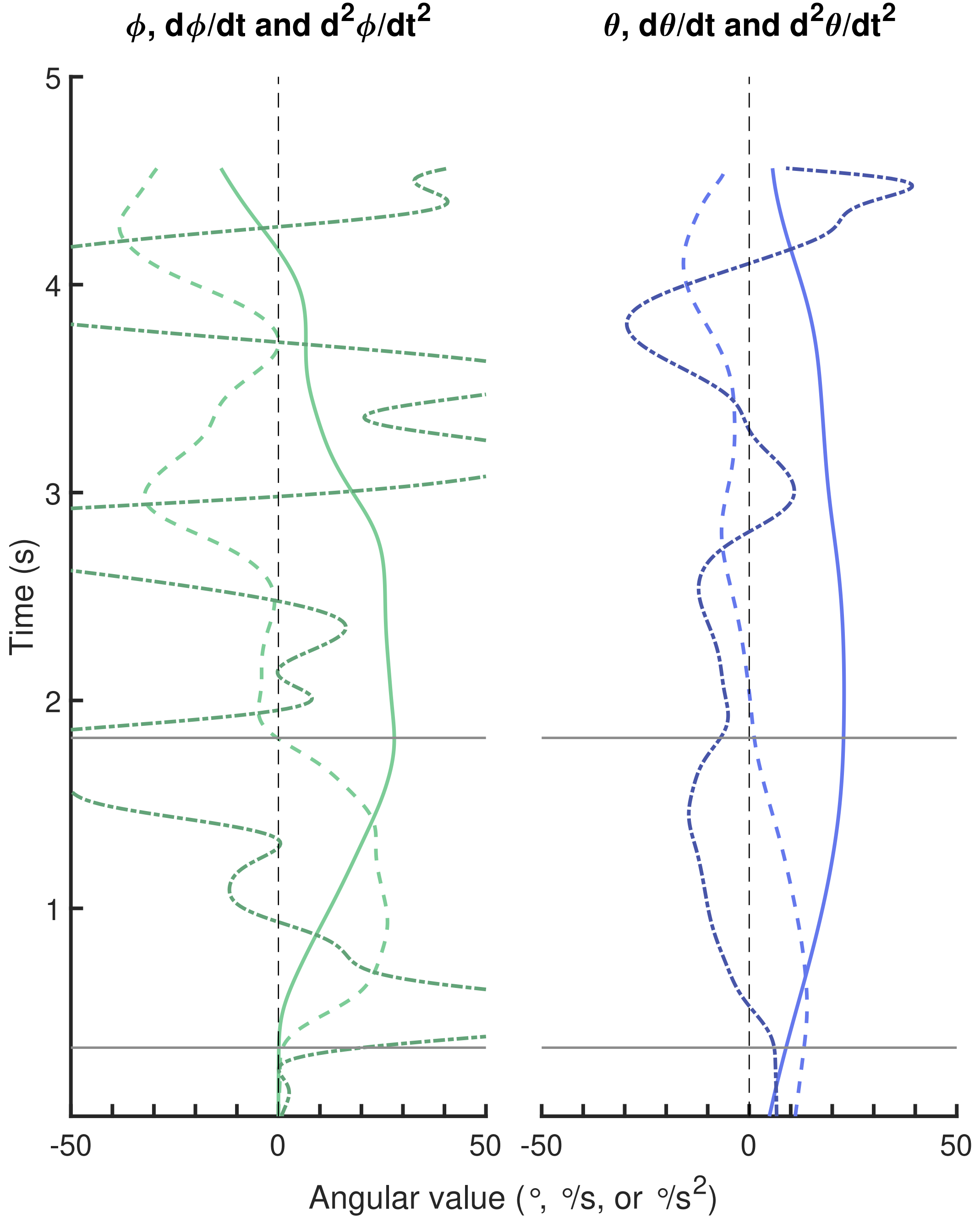

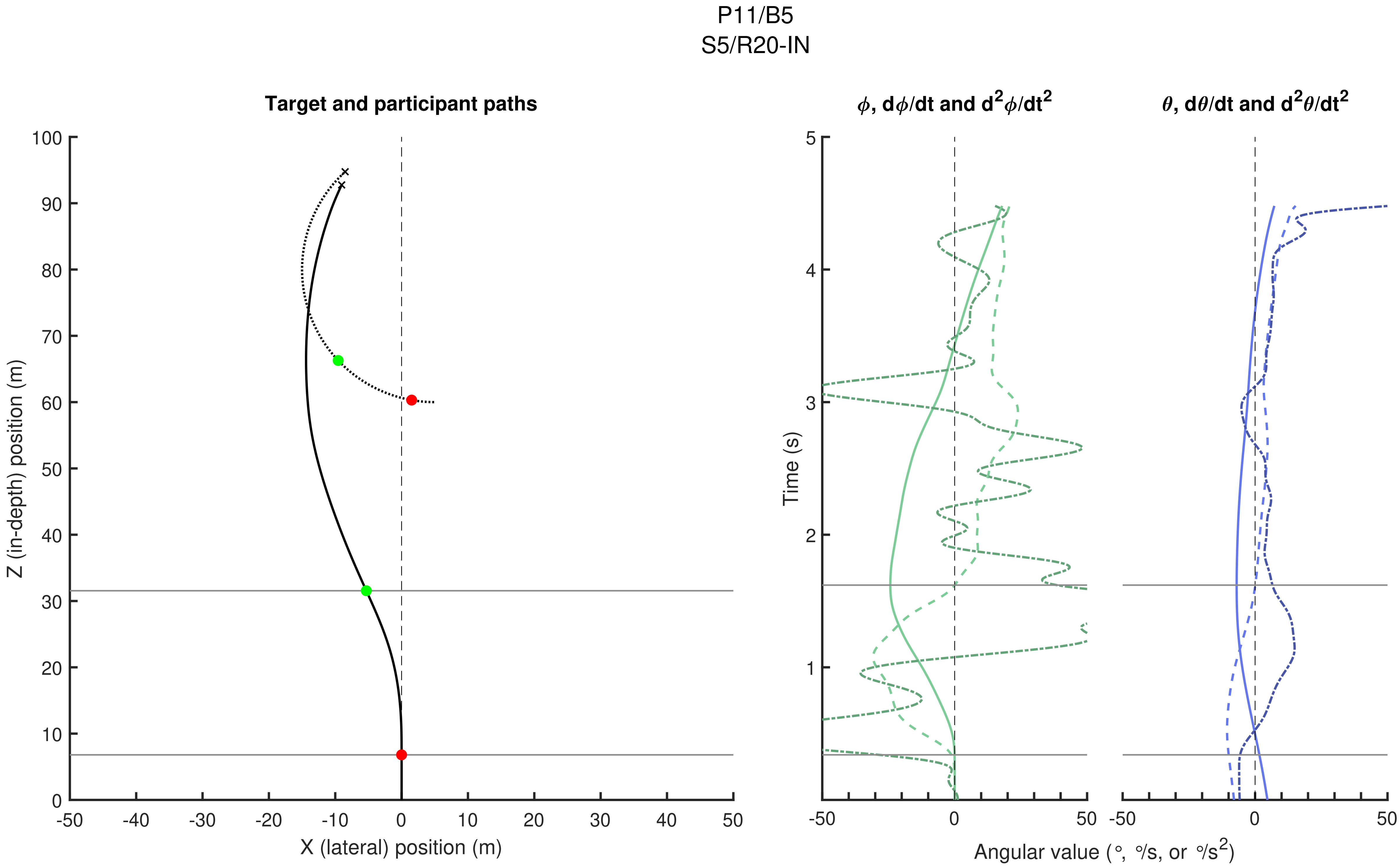
P11/B5
S5/R20-IN
Target and participant paths
Z (in-depth) position (m)
X (lateral) position (m)
ϕ, dϕ/dt and d²ϕ/dt²
θ, dθ/dt and d²θ/dt²
Time (s)
Angular value (°, °/s, or °/s²)

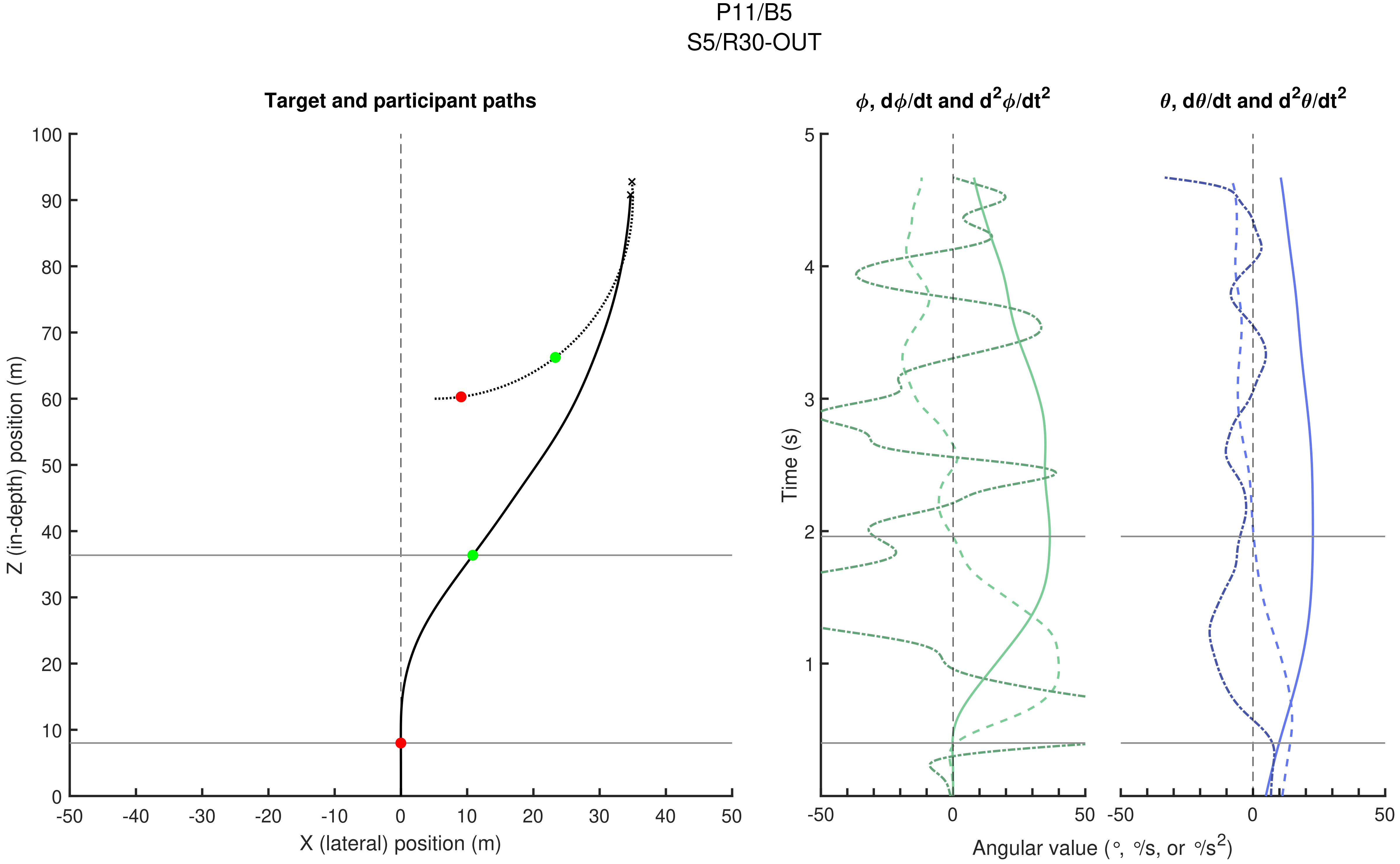
P11/B5
S5/R30-OUT
Target and participant paths
Z (in-depth) position (m)
X (lateral) position (m)
ϕ, dϕ/dt and d²ϕ/dt²
θ, dθ/dt and d²θ/dt²
Time (s)
Angular value (°, °/s, or °/s²)

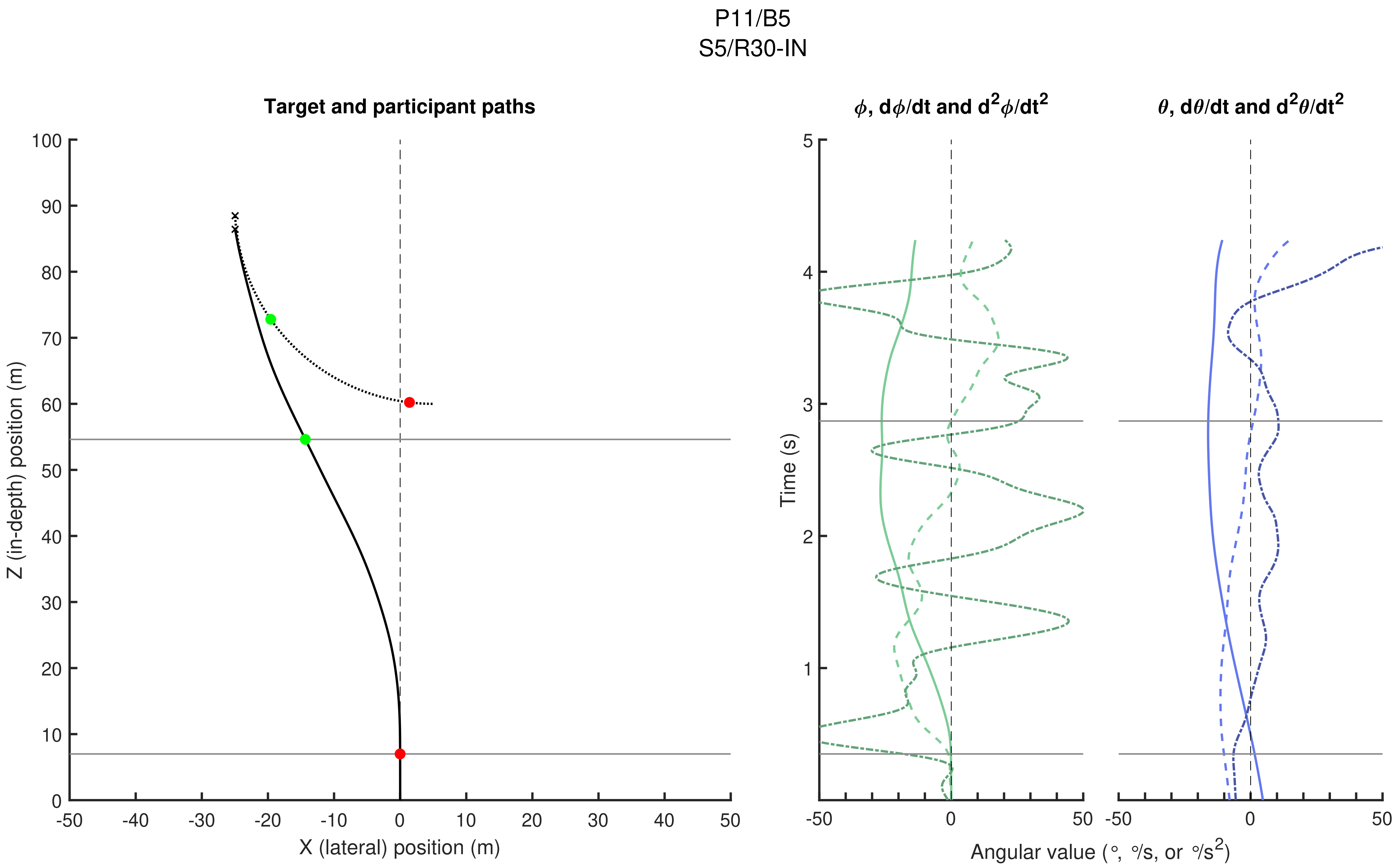
P11/B5
S5/R30-IN
Target and participant paths
Z (in-depth) position (m)
X (lateral) position (m)
φ, dφ/dt and d²φ/dt²
θ, dθ/dt and d²θ/dt²
Time (s)
Angular value (°, °/s, or °/s²)

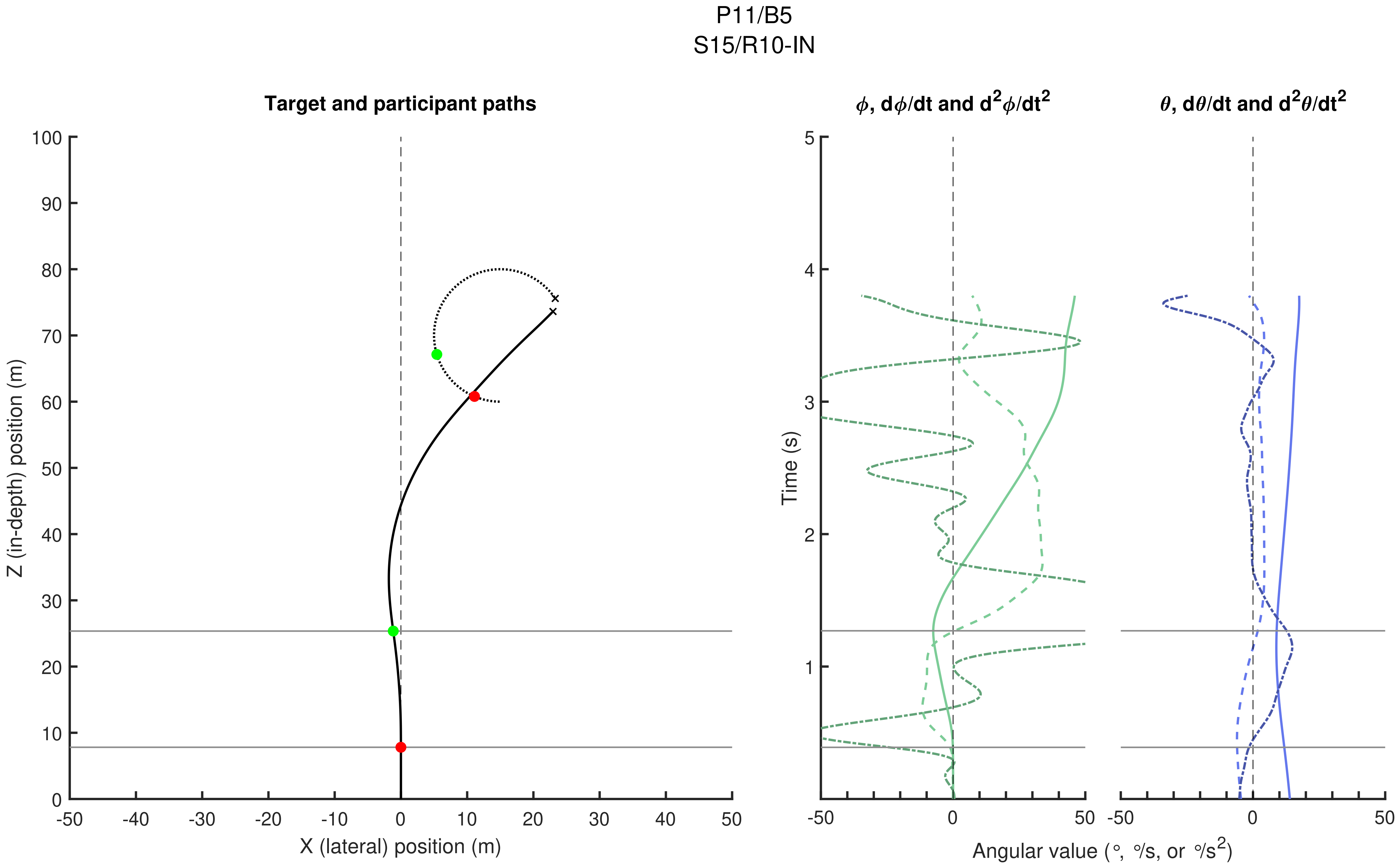
P11/B5
S15/R10-IN
Target and participant paths
Z (in-depth) position (m)
X (lateral) position (m)
φ, dφ/dt and d²φ/dt²
θ, dθ/dt and d²θ/dt²
Time (s)
Angular value (°, °/s, or °/s²)

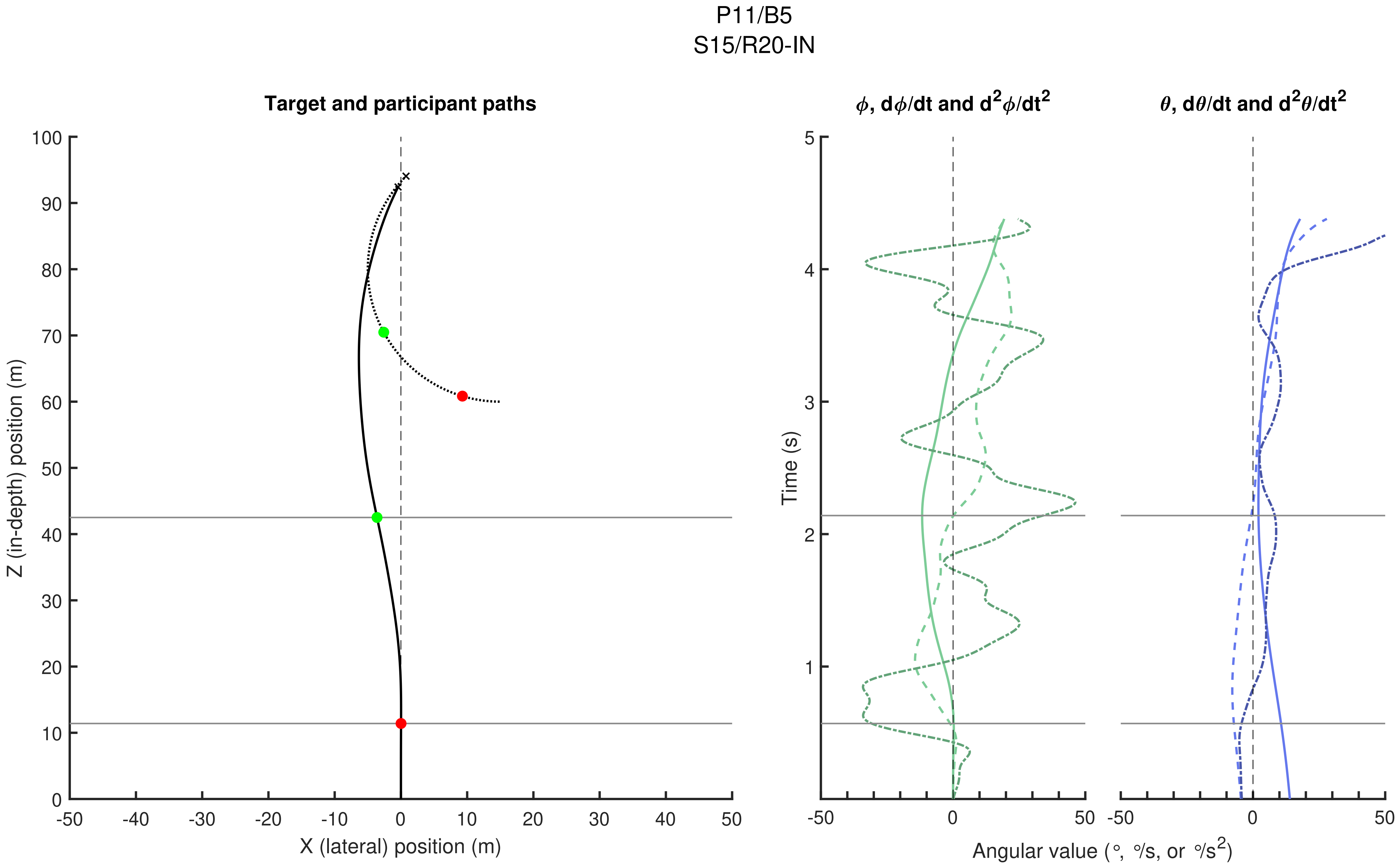

P11/B5
S15/R20-IN
Target and participant paths
ϕ, dϕ/dt and d$^2\phi$/dt^2
θ, dθ/dt and d$^2\theta$/dt^2
Z (in-depth) position (m)
X (lateral) position (m)
Time (s)
Angular value (°, °/s, or °/s^2)

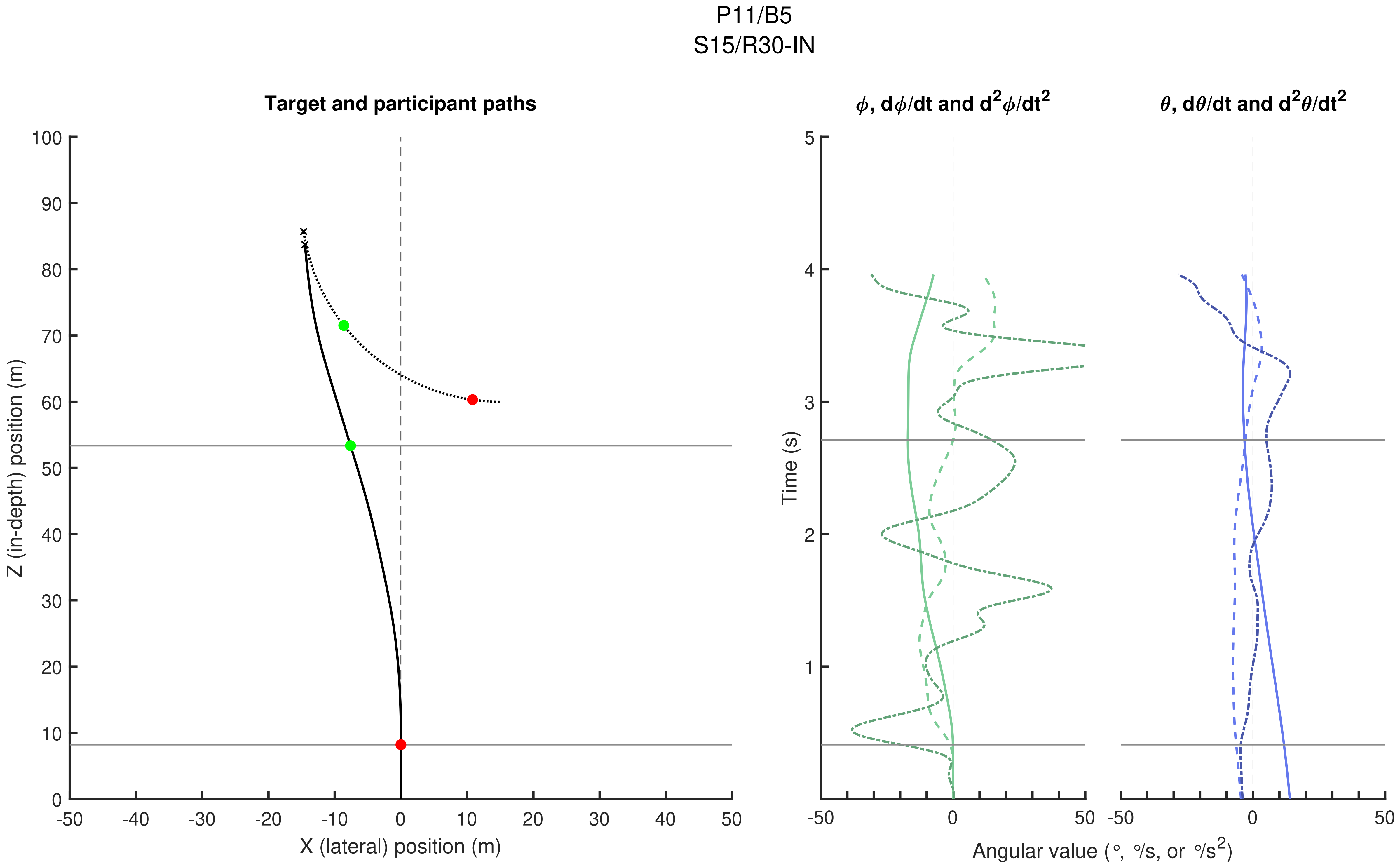

P11/B5
S15/R30-IN
Target and participant paths
Z (in-depth) position (m)
X (lateral) position (m)
ϕ, dϕ/dt and d²ϕ/dt²
θ, dθ/dt and d²θ/dt²
Time (s)
Angular value (°, °/s, or °/s²)

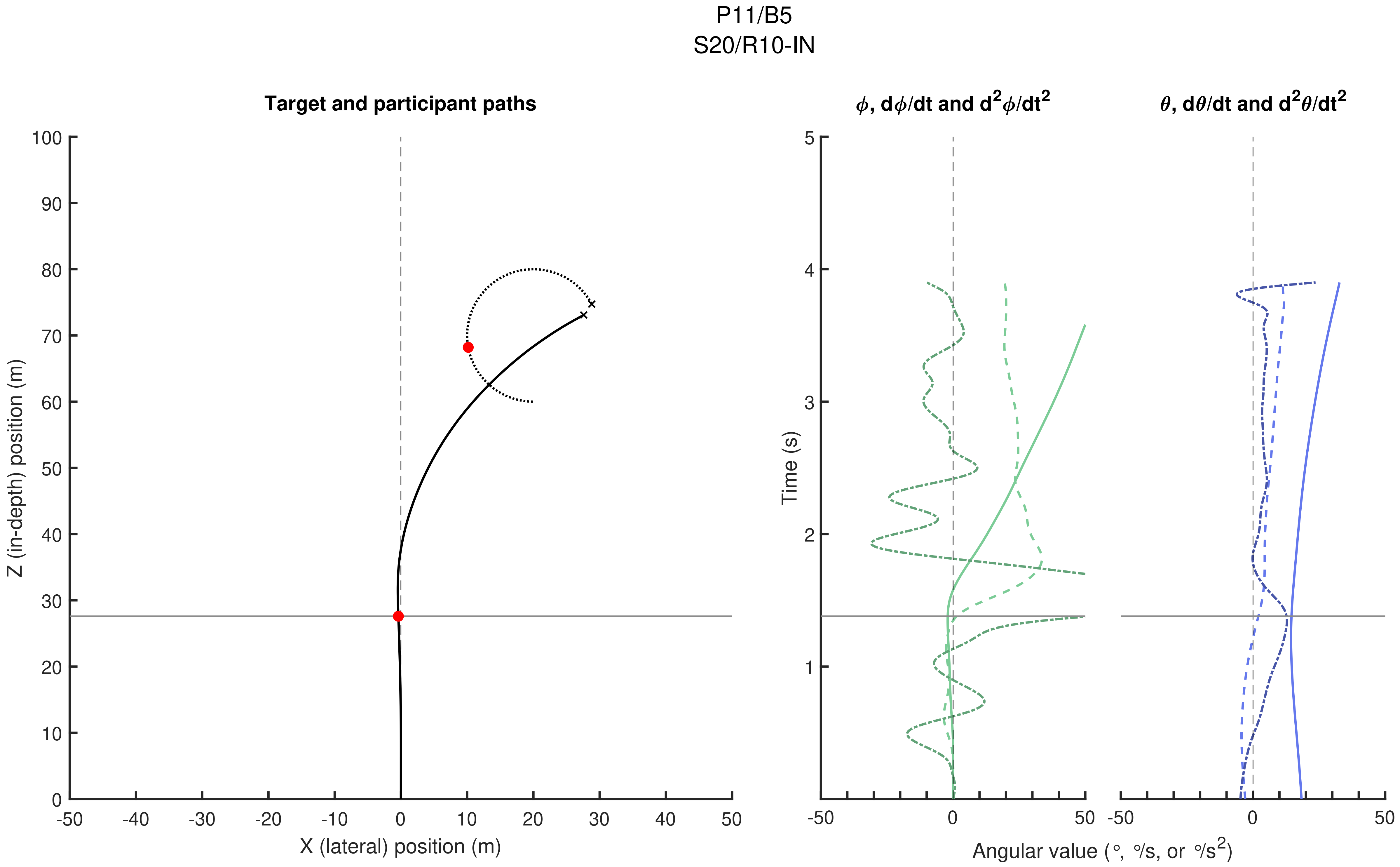
P11/B5
S20/R10-IN
Target and participant paths
Z (in-depth) position (m)
X (lateral) position (m)
ϕ, dϕ/dt and d$^2\phi$/dt^2
θ, dθ/dt and d$^2\theta$/dt^2
Time (s)
Angular value (°, °/s, or °/s^2)

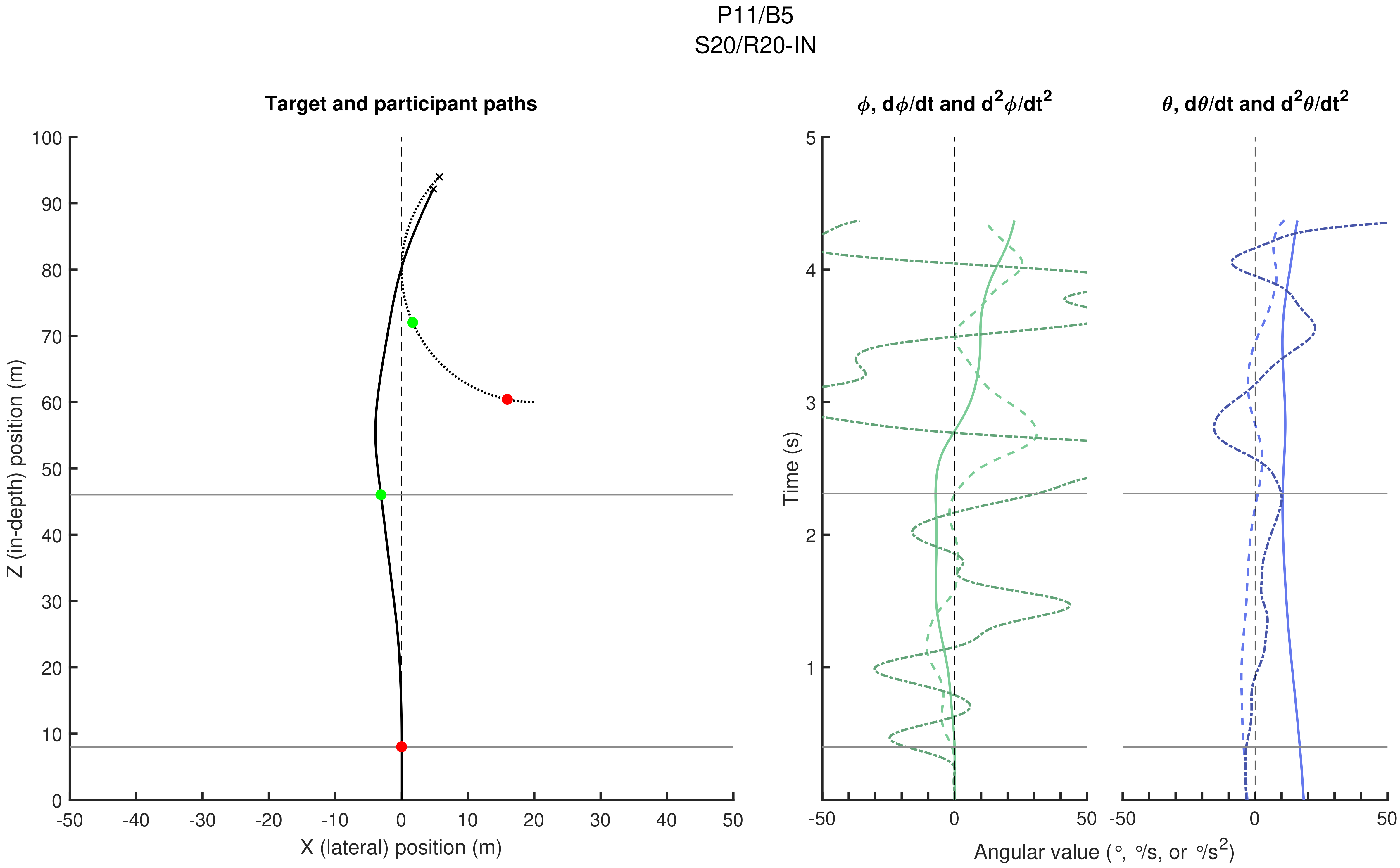
P11/B5
S20/R20-IN
Target and participant paths
Z (in-depth) position (m)
X (lateral) position (m)
100
90
80
70
60
50
40
30
20
10
0
-50
-40
-30
-20
-10
0
10
20
30
40
50
ϕ, dϕ/dt and d²ϕ/dt²
θ, dθ/dt and d²θ/dt²
Time (s)
5
4
3
2
1
-50
0
50
-50
0
50
Angular value (°, °/s, or °/s²)

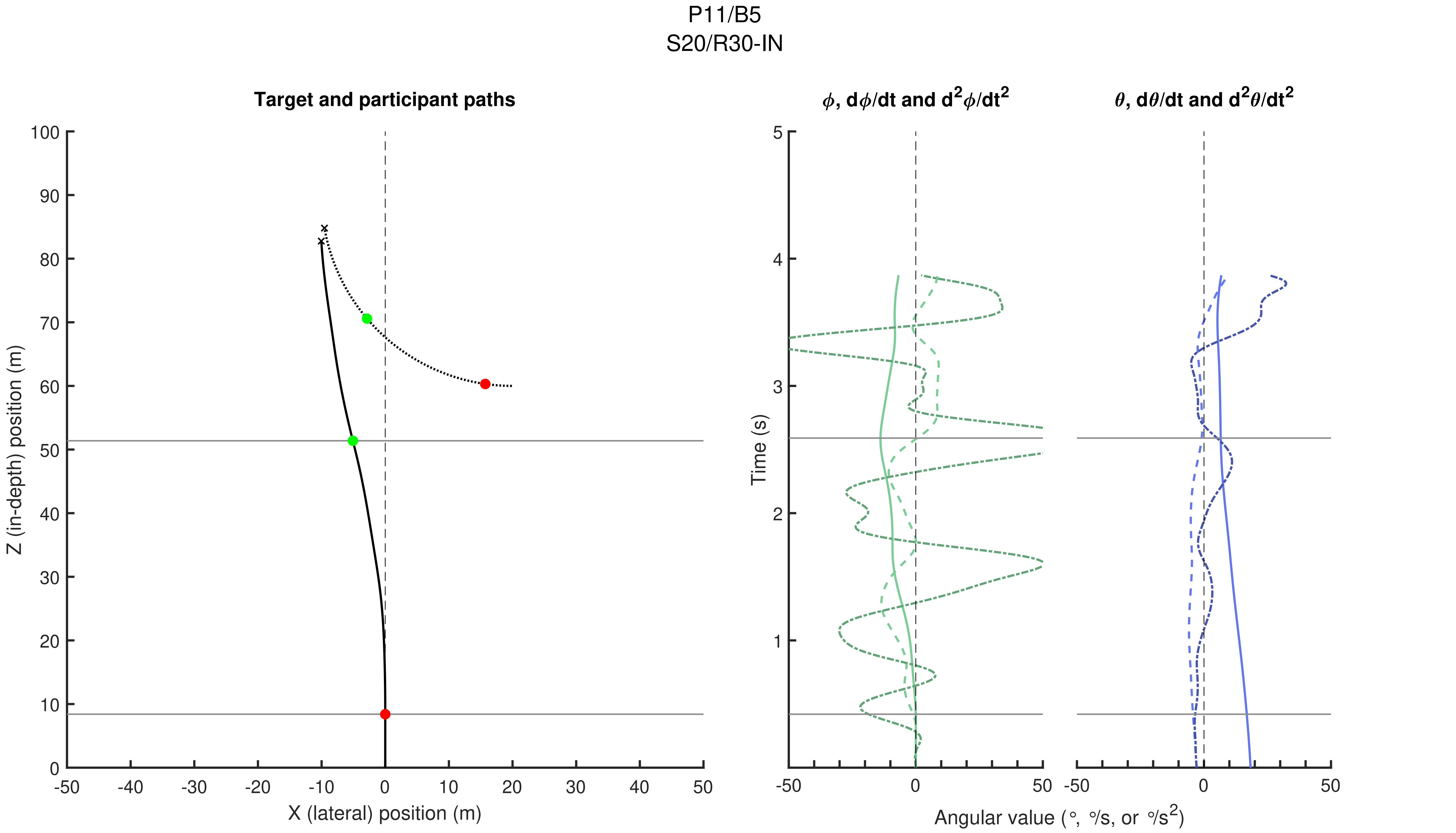
P11/B5
S20/R30-IN
Target and participant paths
Z (in-depth) position (m)
X (lateral) position (m)
ϕ, dϕ/dt and d$^2\phi$/dt^2
θ, dθ/dt and d$^2\theta$/dt^2
Time (s)
Angular value (°, °/s, or °/s^2)

P12/B1
S20/R10-IN

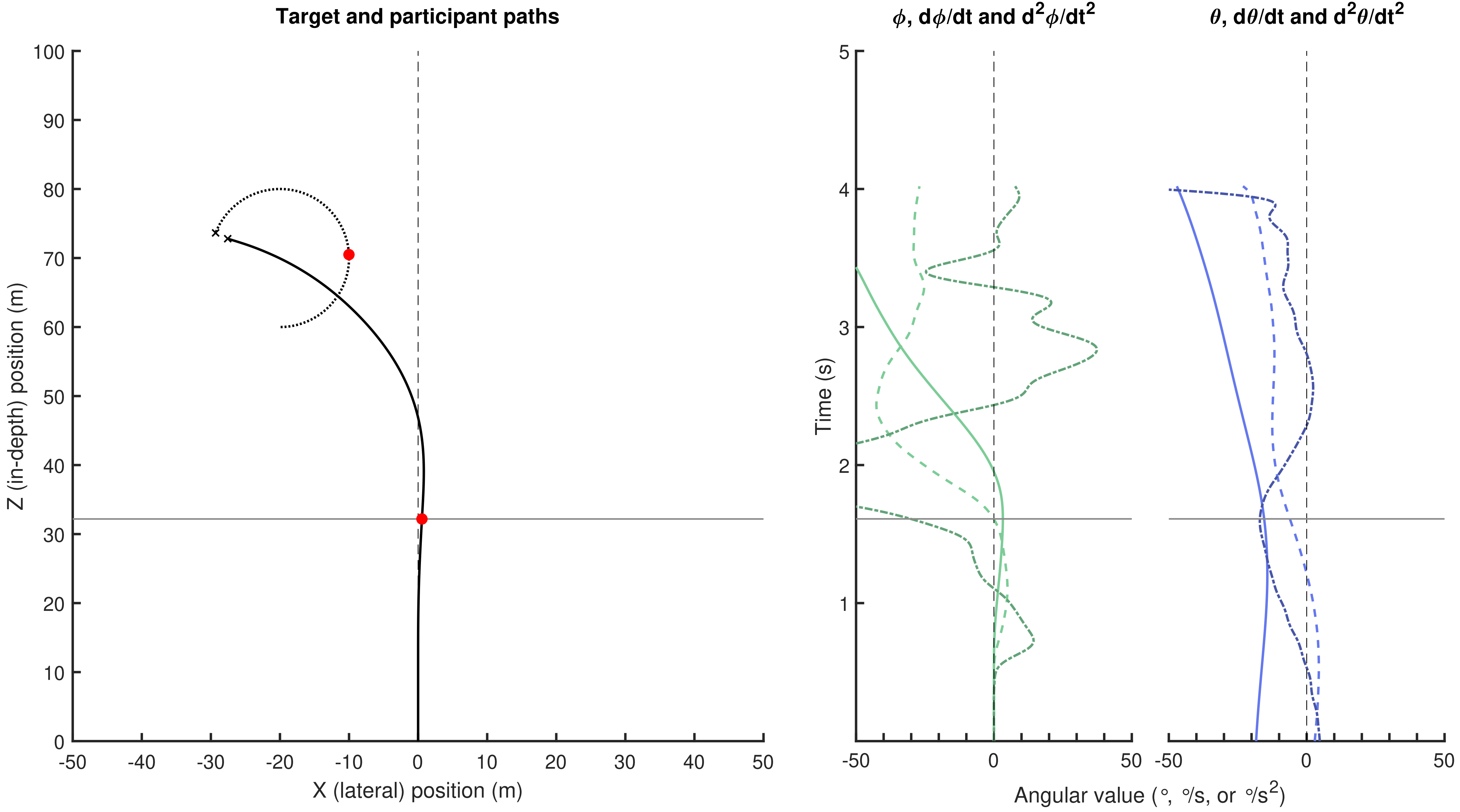

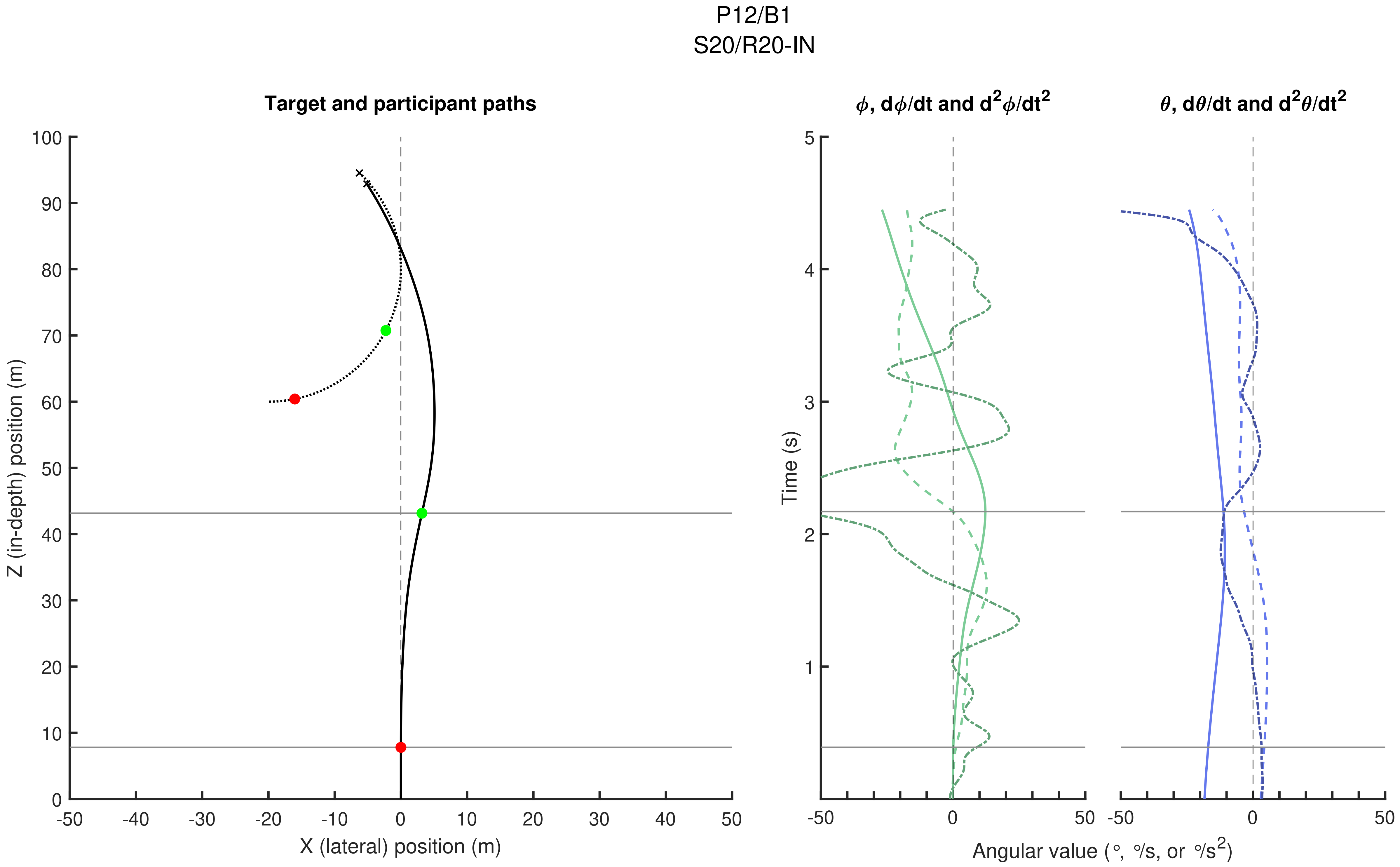
P12/B1
S20/R20-IN
Target and participant paths
ϕ, dϕ/dt and d$^2\phi$/dt^2
θ, dθ/dt and d$^2\theta$/dt^2
Z (in-depth) position (m)
X (lateral) position (m)
Time (s)
Angular value (°, °/s, or °/s^2)

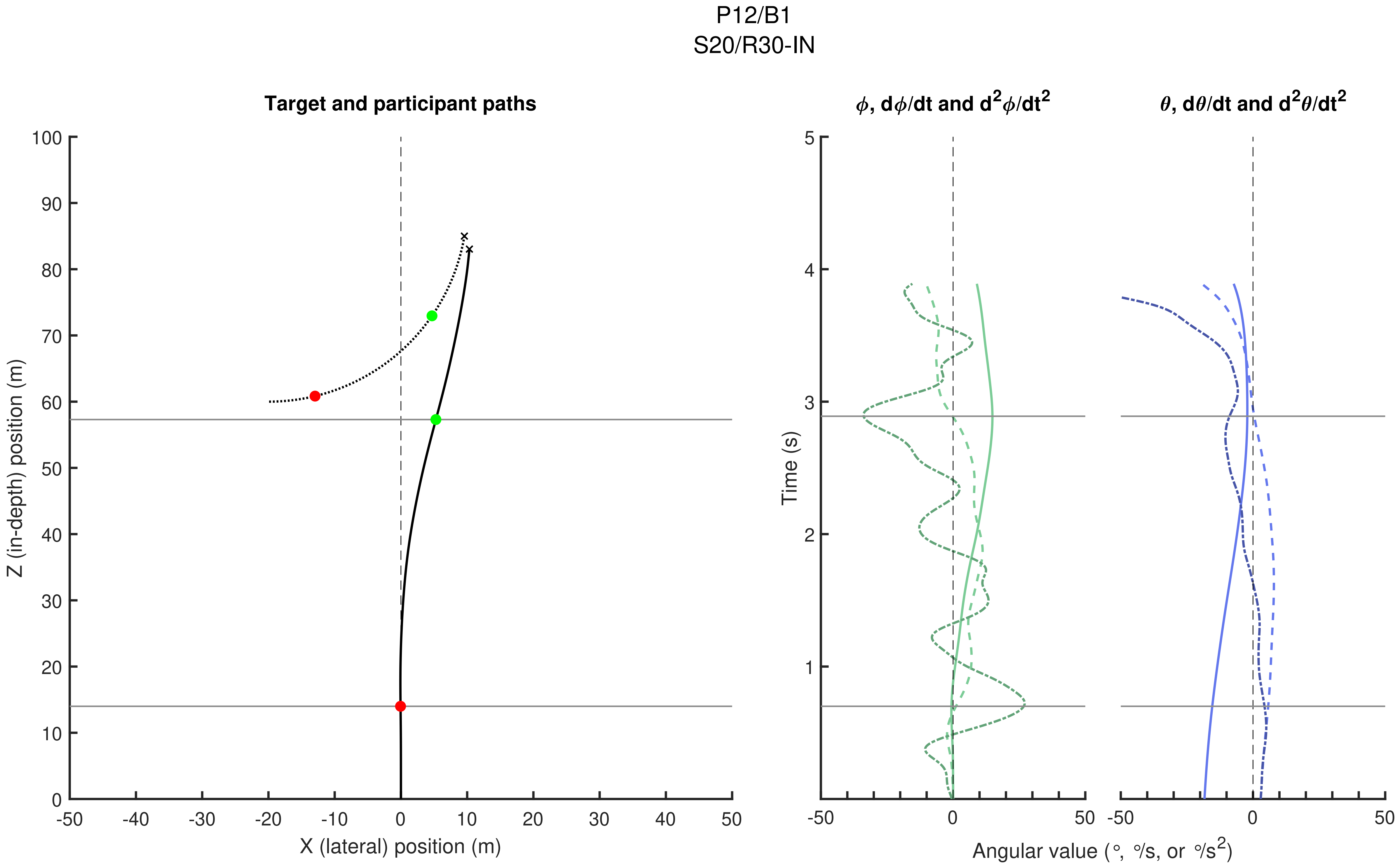
P12/B1
S20/R30-IN
Target and participant paths
Z (in-depth) position (m)
X (lateral) position (m)
ϕ, dϕ/dt and d$^2\phi$/dt^2
θ, dθ/dt and d$^2\theta$/dt^2
Time (s)
Angular value (°, °/s, or °/s^2)

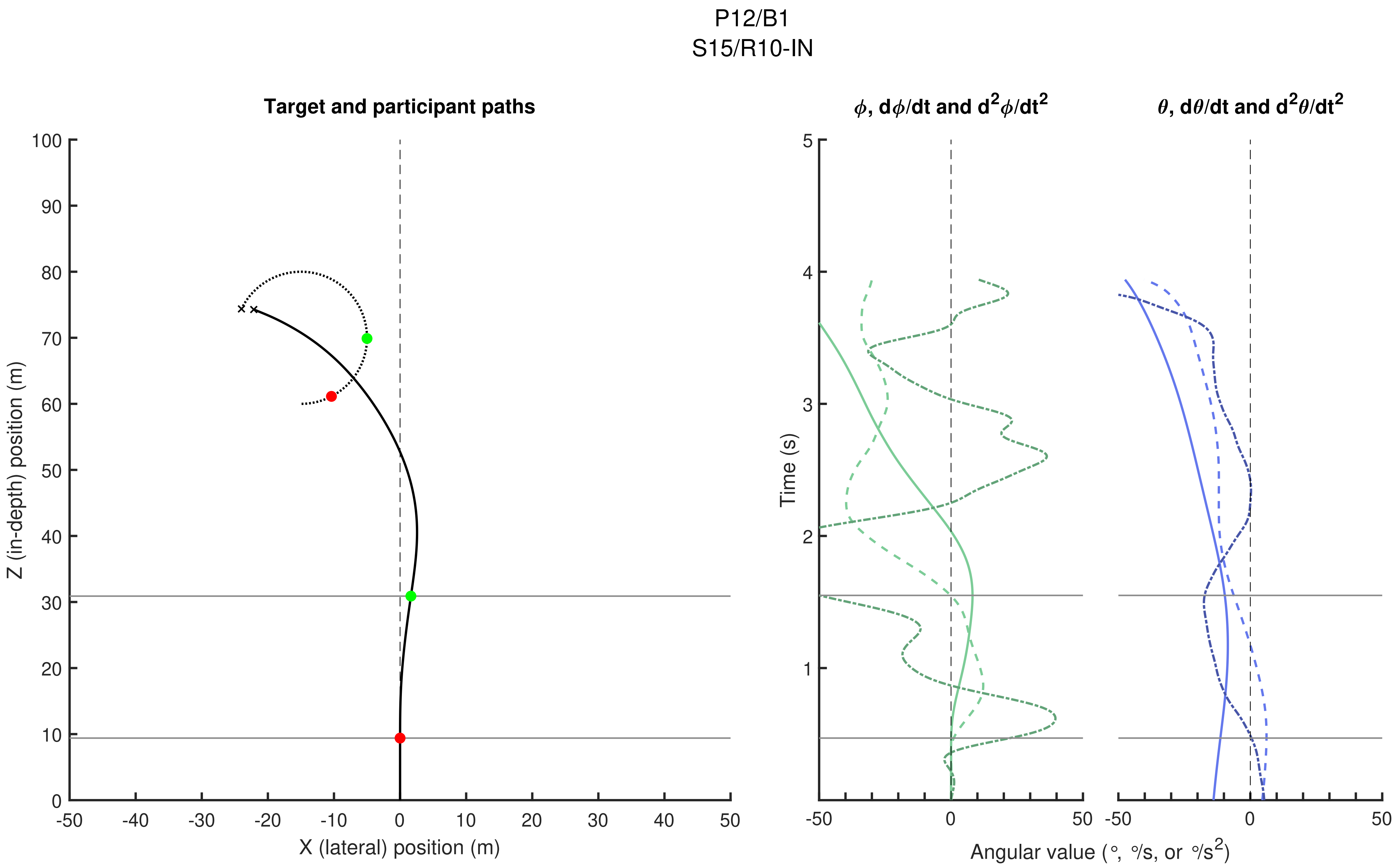
P12/B1
S15/R10-IN
Target and participant paths
ϕ, dϕ/dt and d$^2\phi$/dt^2
θ, dθ/dt and d$^2\theta$/dt^2
Z (in-depth) position (m)
X (lateral) position (m)
Time (s)
Angular value (°, °/s, or °/s^2)

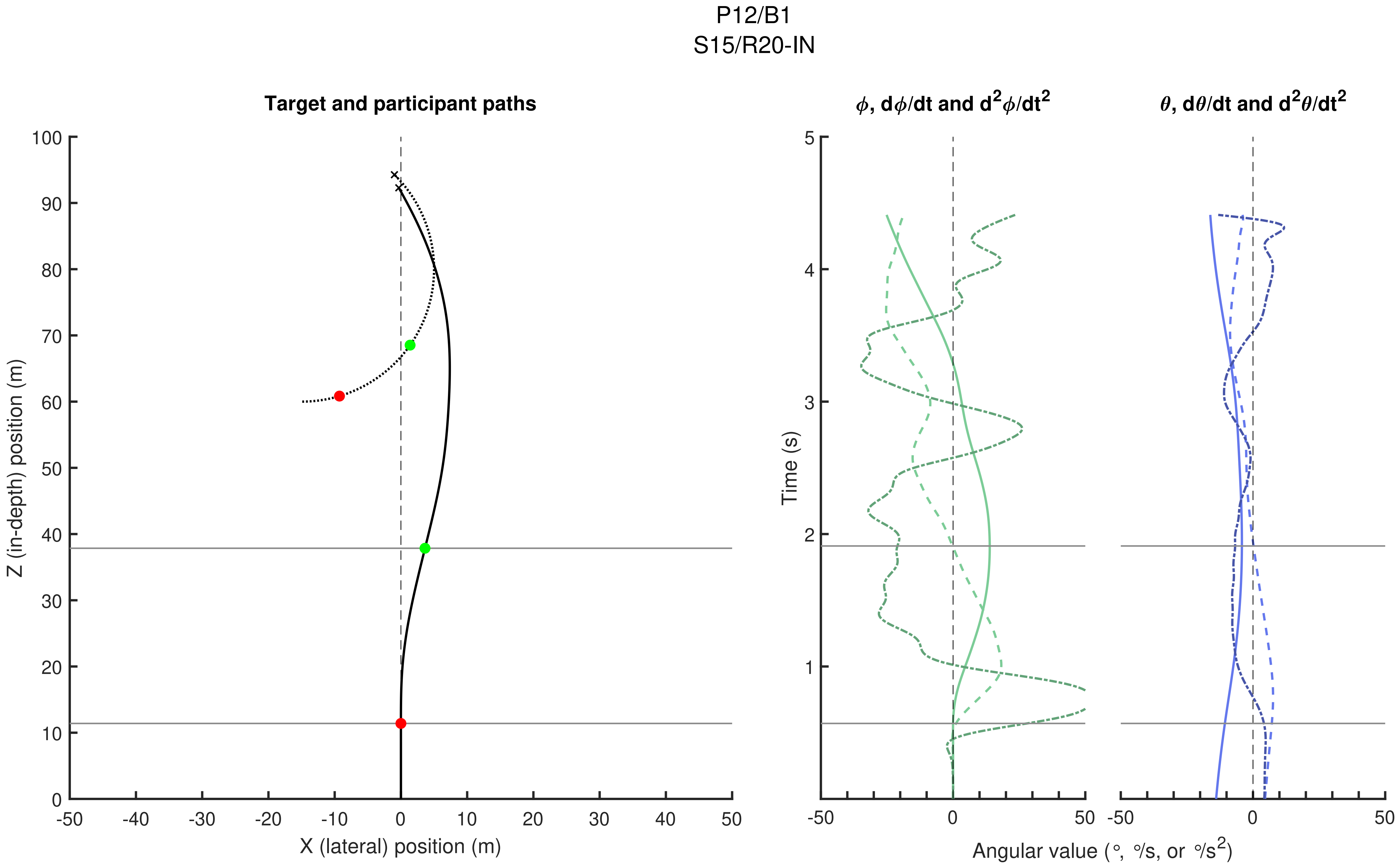

P12/B1
S15/R20-IN
Target and participant paths
Z (in-depth) position (m)
X (lateral) position (m)
0
10
20
30
40
50
60
70
80
90
100
-50
-40
-30
-20
-10
0
10
20
30
40
50
ϕ, dϕ/dt and d²ϕ/dt²
θ, dθ/dt and d²θ/dt²
Time (s)
1
2
3
4
5
-50
0
50
-50
0
50
Angular value (°, °/s, or °/s²)

P12/B1
S15/R30-IN

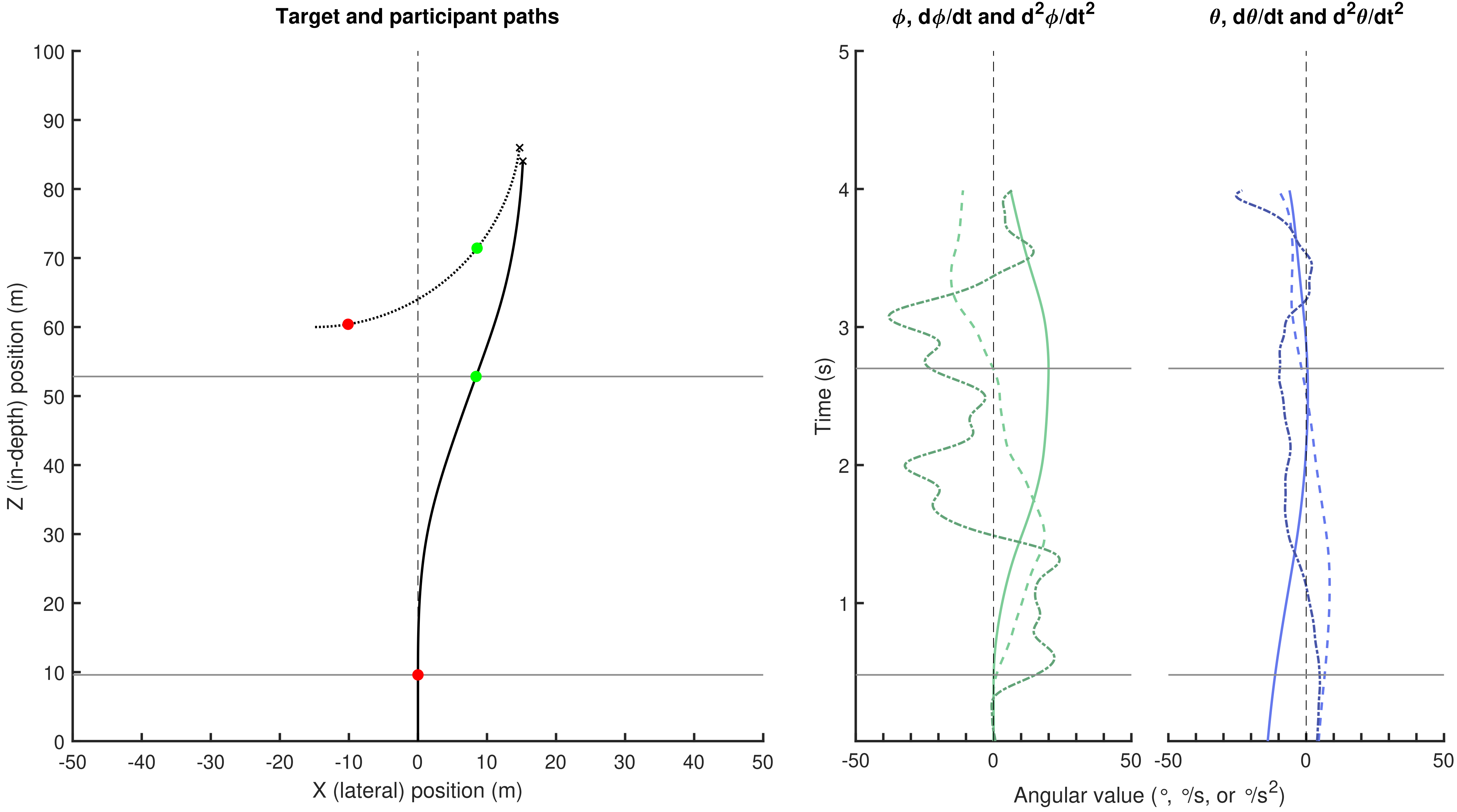

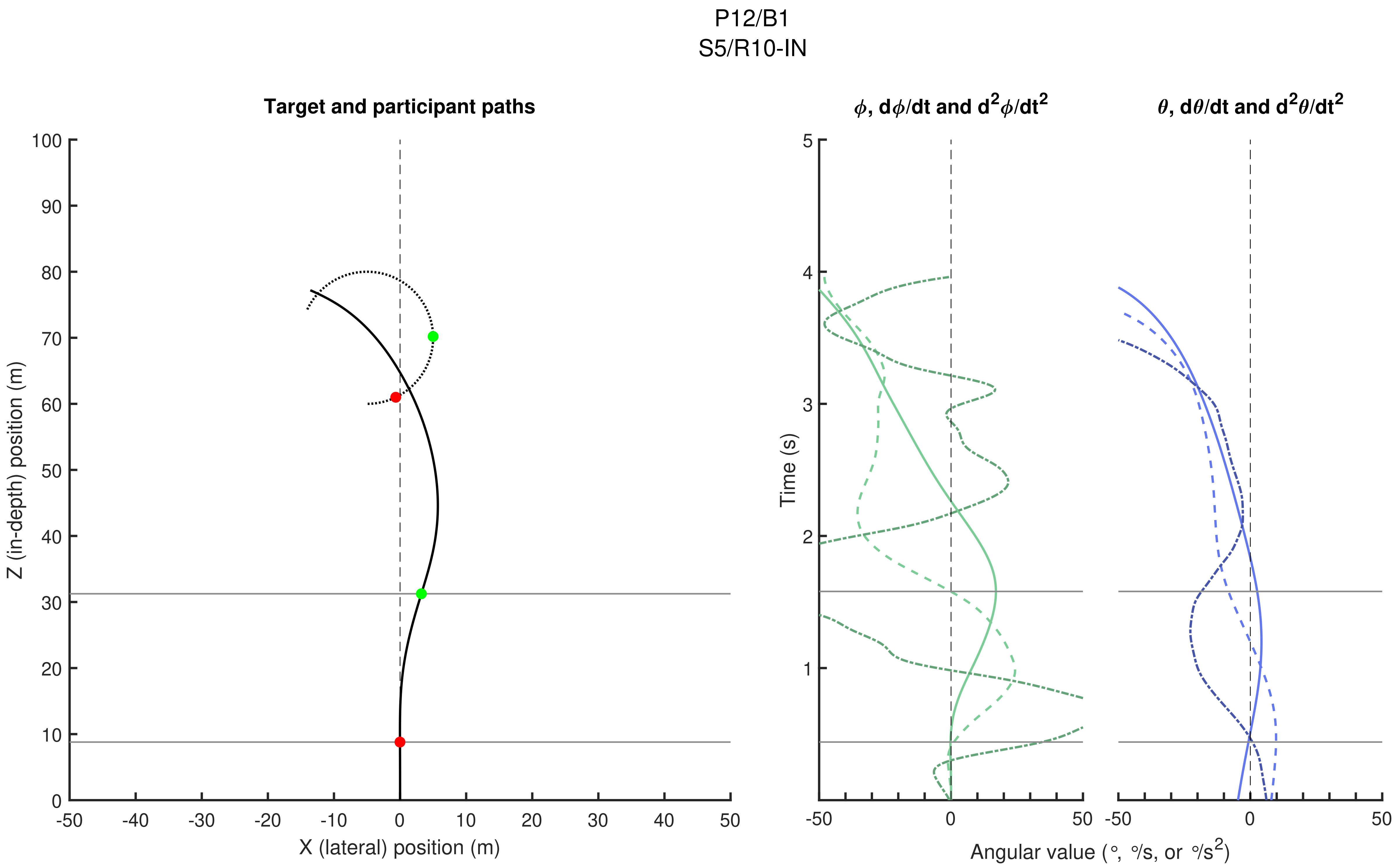
P12/B1
S5/R10-IN
Target and participant paths
ϕ, dϕ/dt and d$^2\phi$/dt^2
θ, dθ/dt and d$^2\theta$/dt^2
Z (in-depth) position (m)
X (lateral) position (m)
Time (s)
Angular value (°, °/s, or °/s^2)

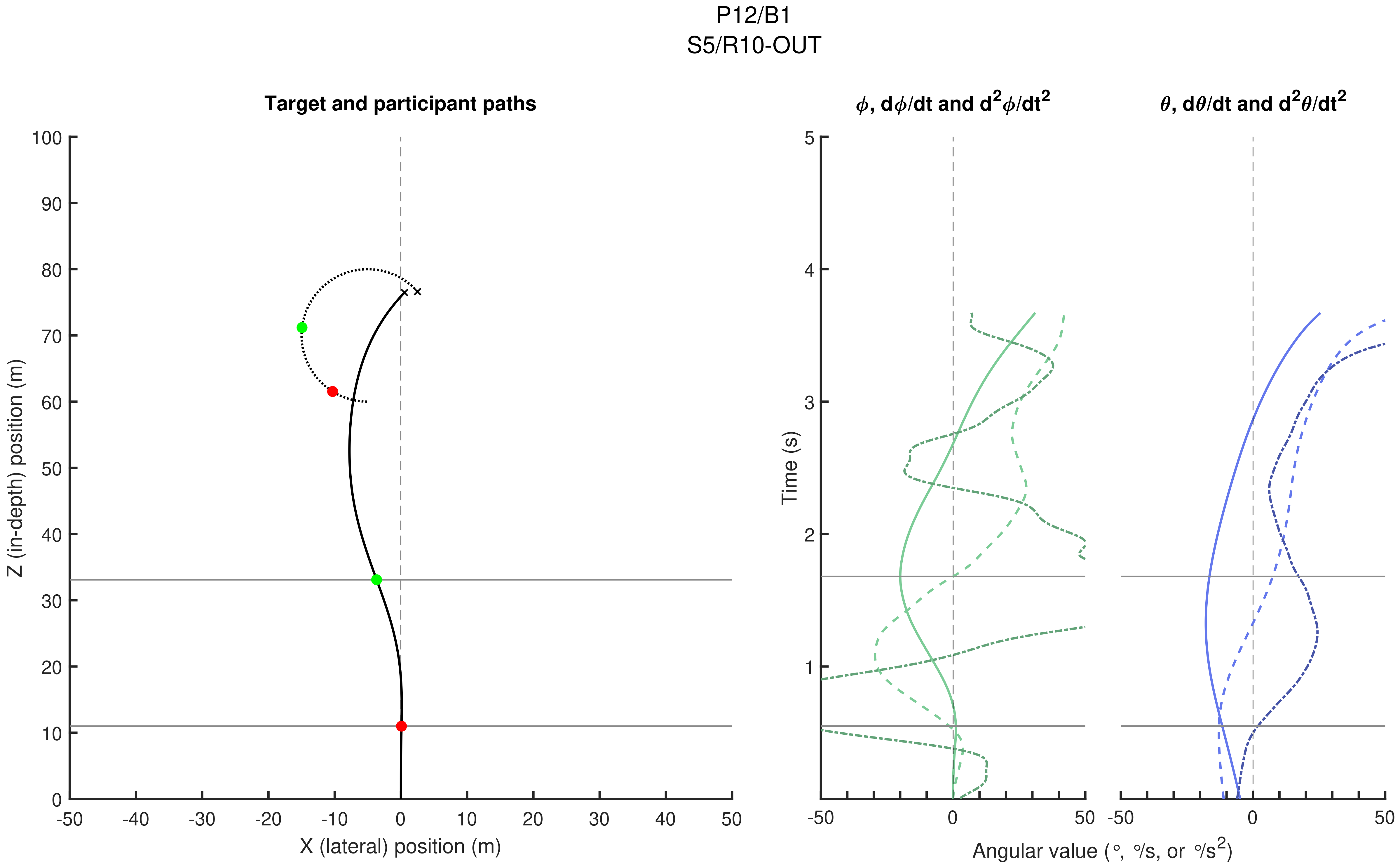

P12/B1
S5/R10-OUT
Target and participant paths
Z (in-depth) position (m)
X (lateral) position (m)
100
90
80
70
60
50
40
30
20
10
0
-50
-40
-30
-20
-10
0
10
20
30
40
50
φ, dφ/dt and d²φ/dt²
θ, dθ/dt and d²θ/dt²
Time (s)
5
4
3
2
1
-50
0
50
-50
0
50
Angular value (°, °/s, or °/s²)

P12/B1
S5/R20-IN

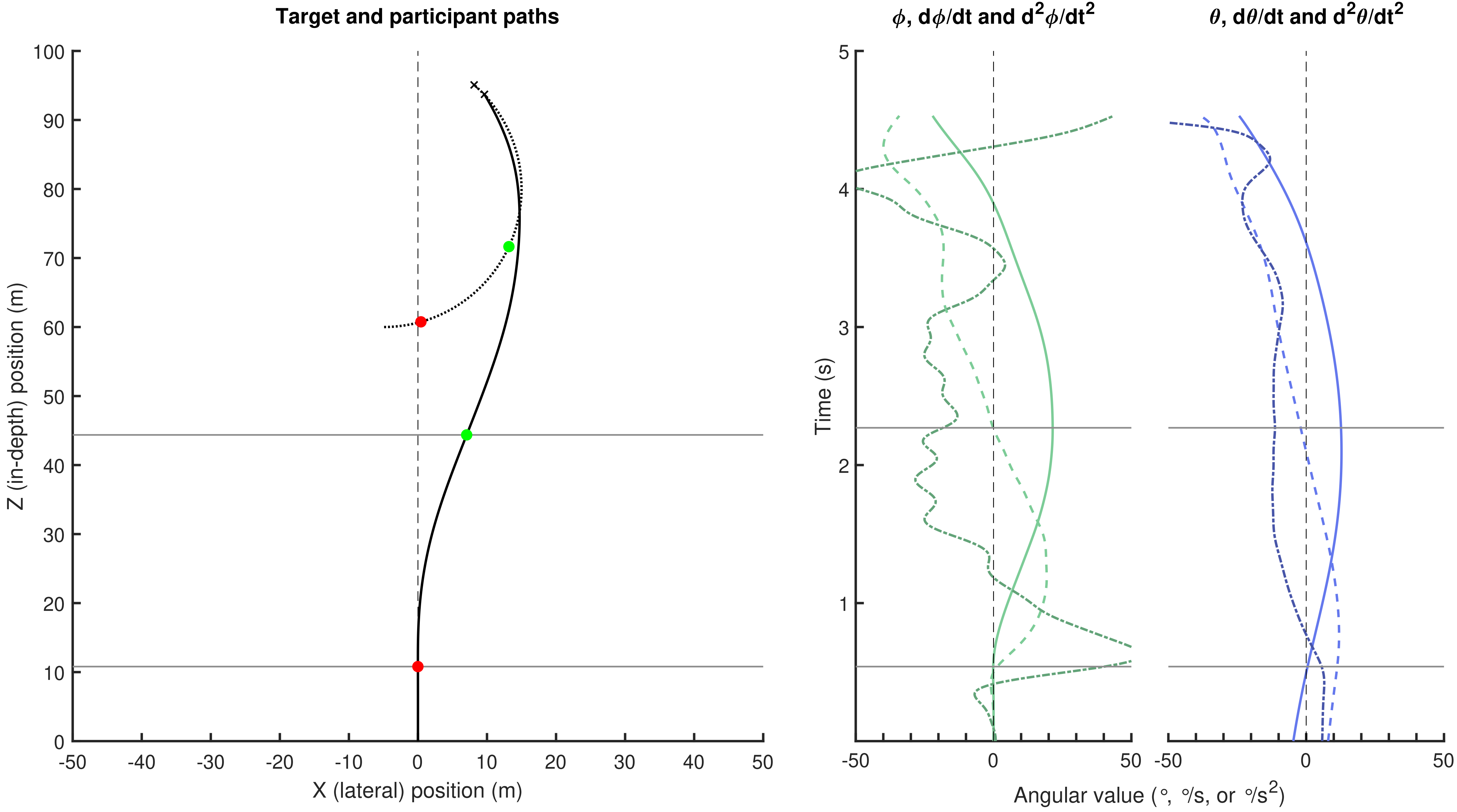

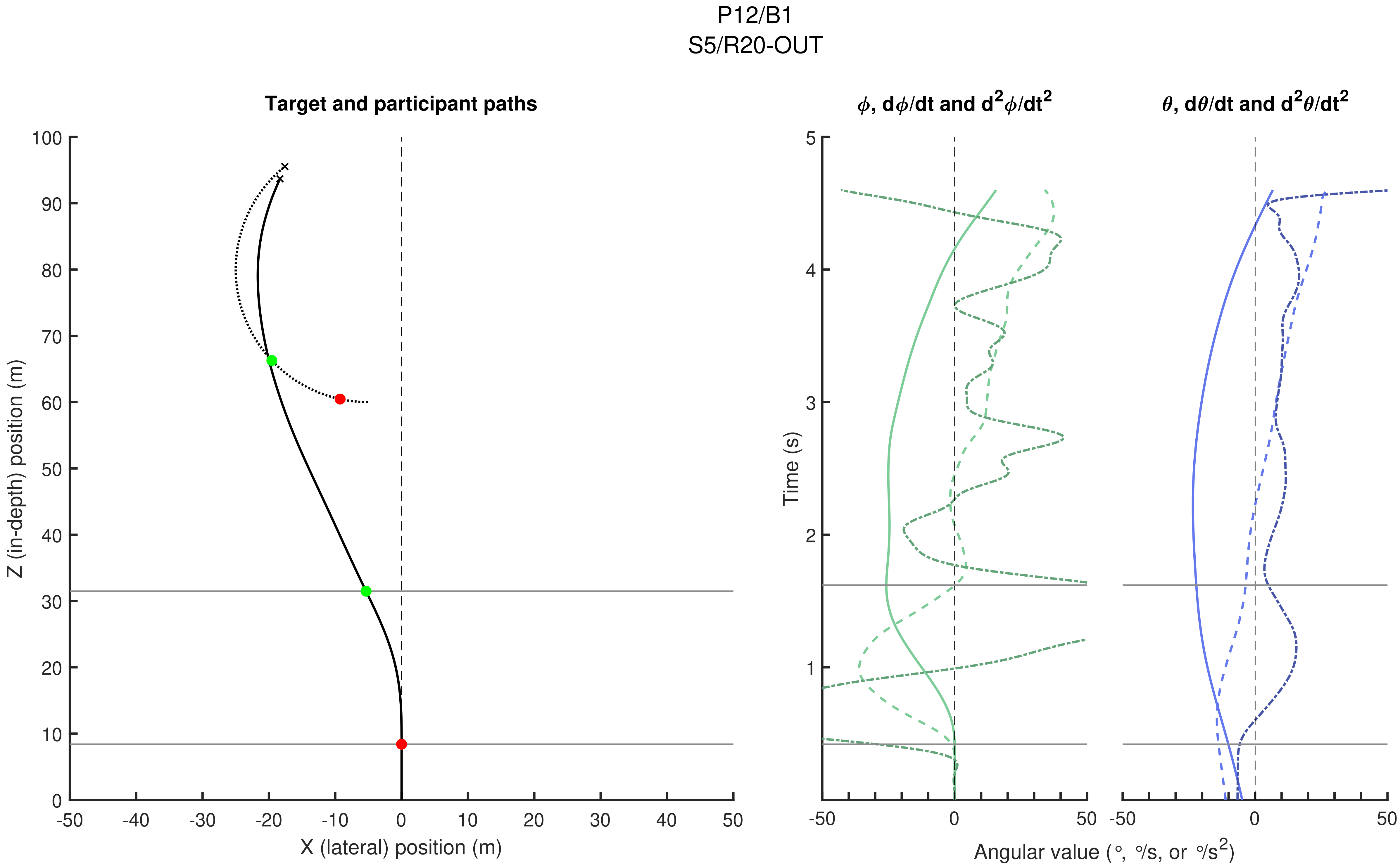

P12/B1
S5/R20-OUT
Target and participant paths
Z (in-depth) position (m)
X (lateral) position (m)
ϕ, dϕ/dt and d$^2\phi$/dt^2
θ, dθ/dt and d$^2\theta$/dt^2
Time (s)
Angular value (°, °/s, or °/s^2)

P12/B1
S5/R30-IN

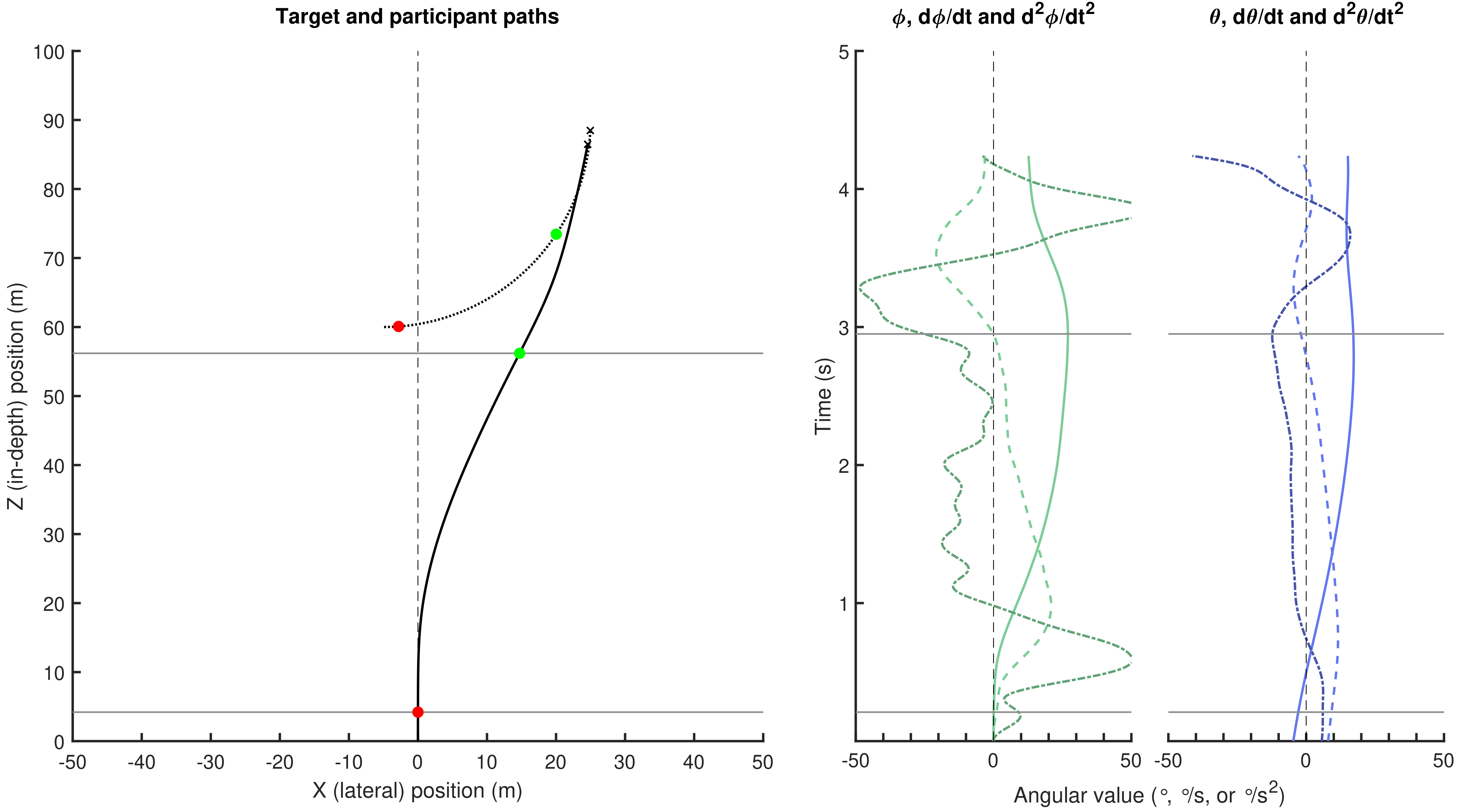

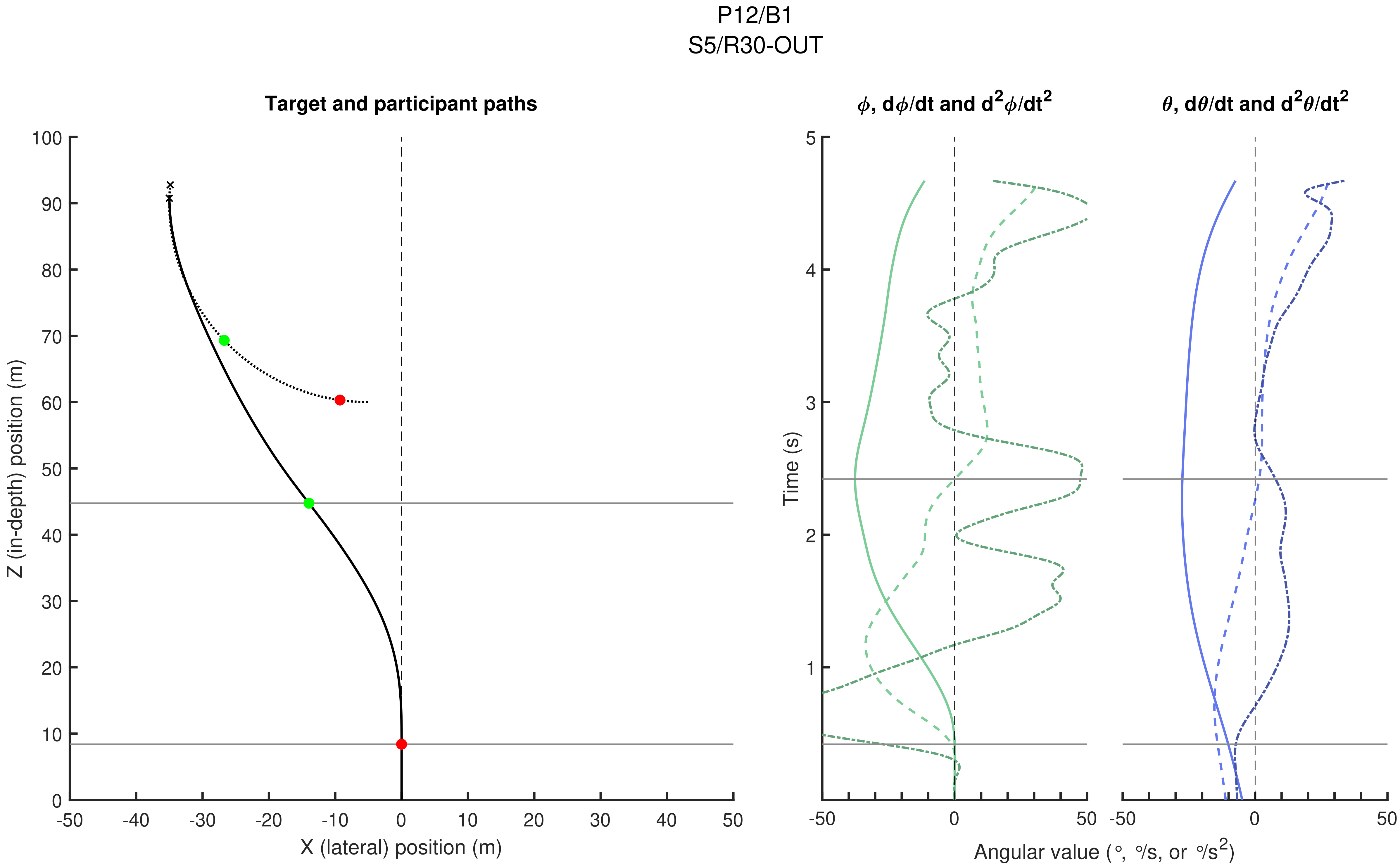

P12/B1
S5/R30-OUT
Target and participant paths
Z (in-depth) position (m)
X (lateral) position (m)
ϕ, dϕ/dt and d$^2\phi$/dt^2
θ, dθ/dt and d$^2\theta$/dt^2
Time (s)
Angular value (°, °/s, or °/s^2)

P12/B1
S5/R10-OUT

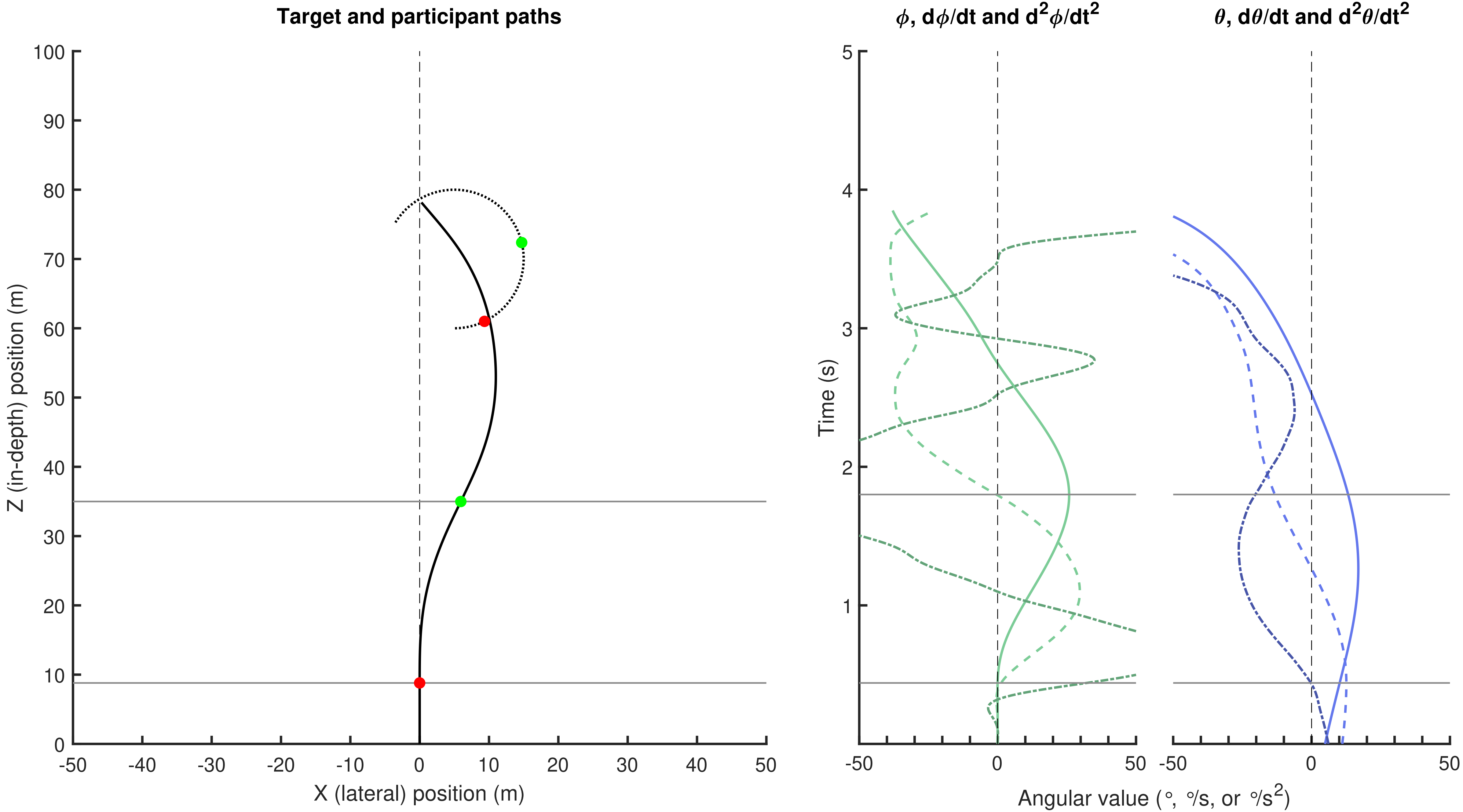

P12/B1
S5/R10-IN

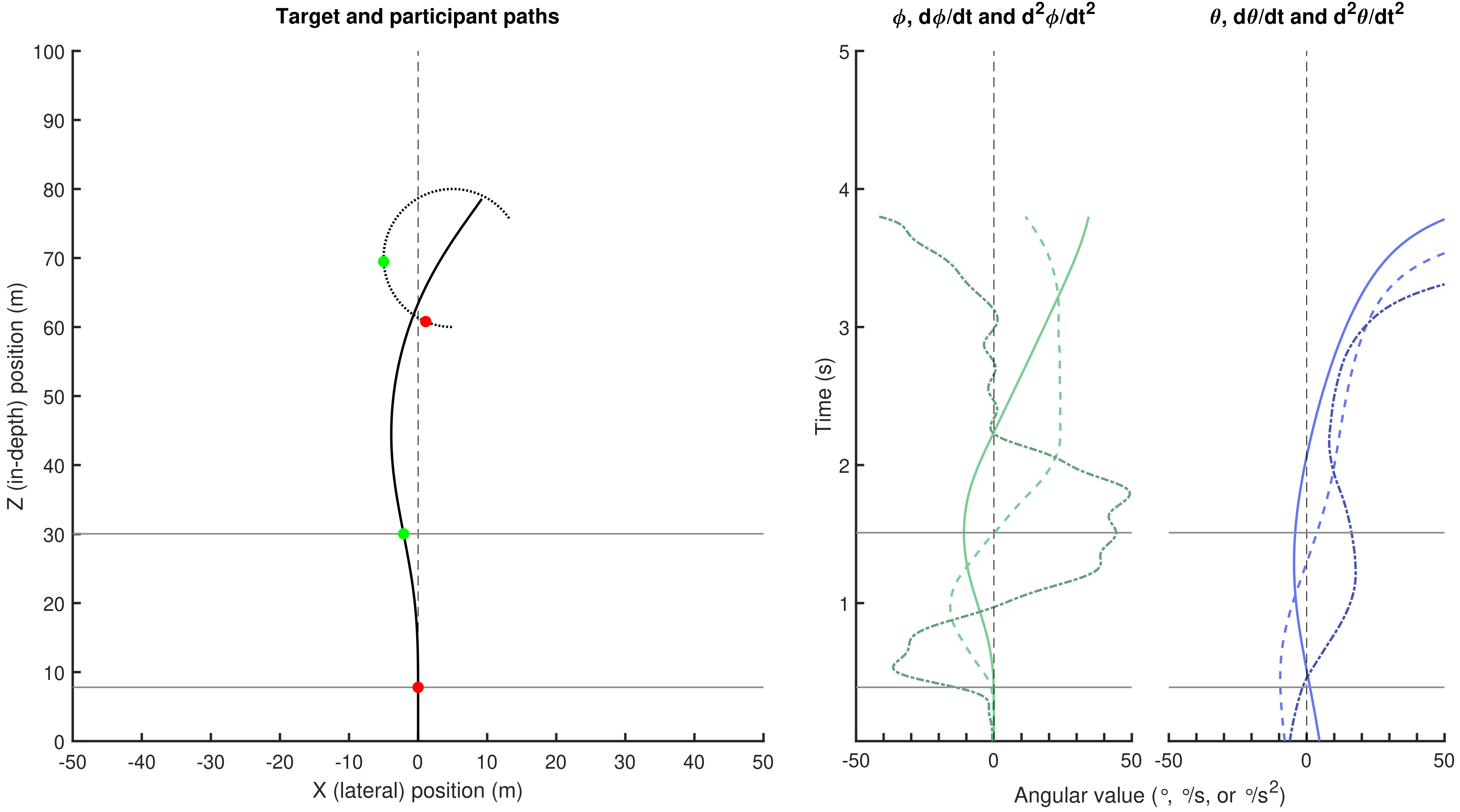

P12/B1
S5/R20-OUT

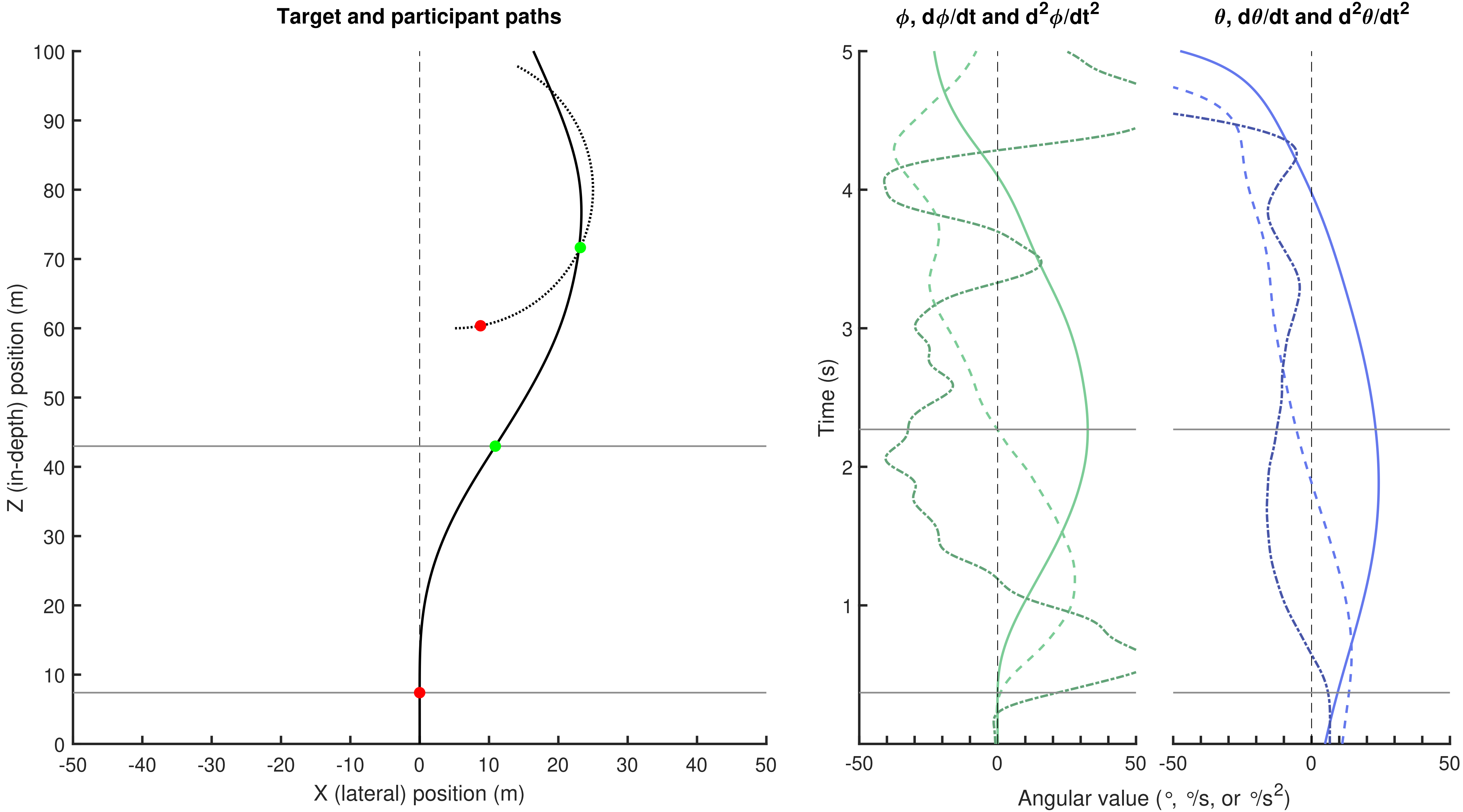

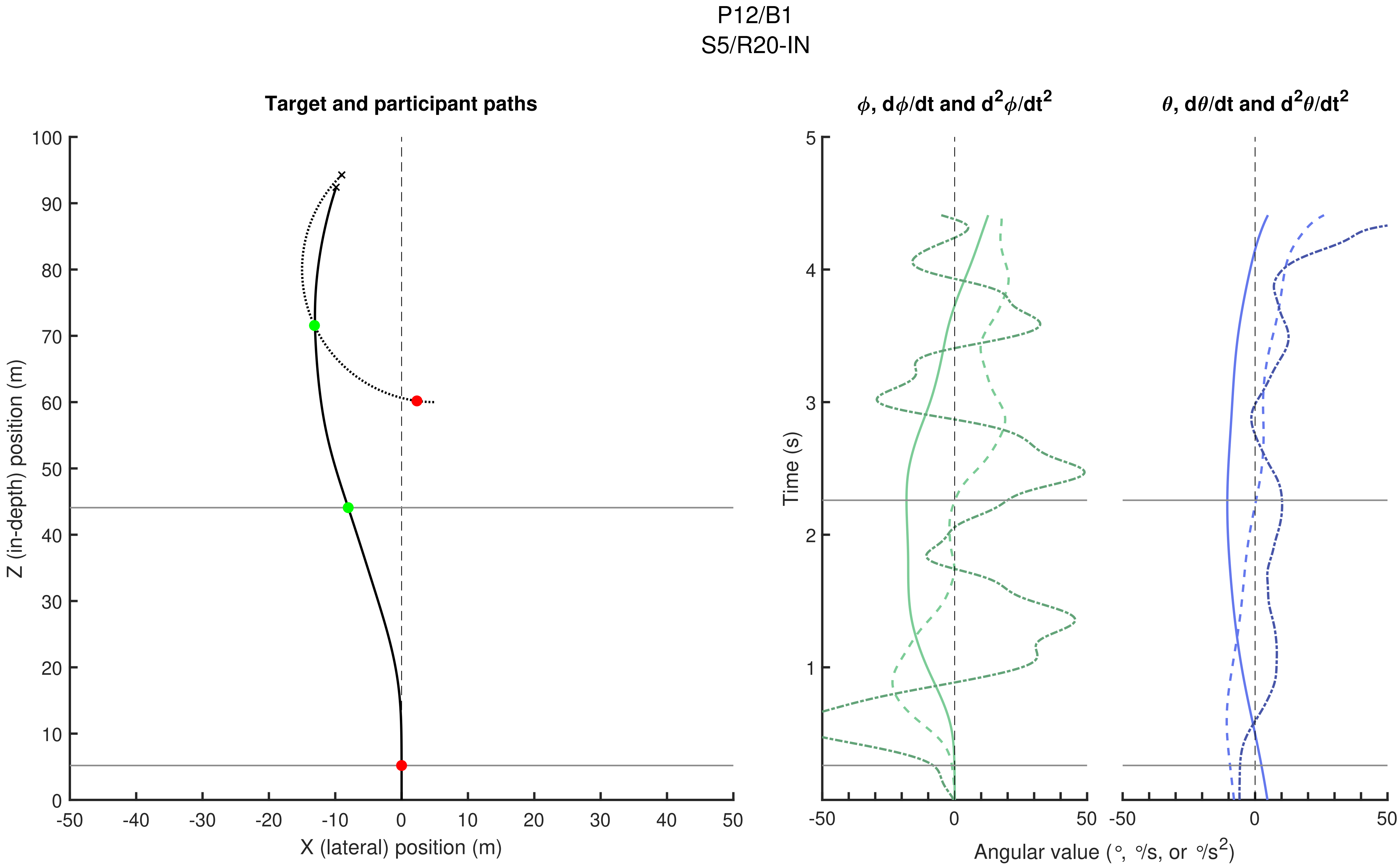
P12/B1
S5/R20-IN
Target and participant paths
Z (in-depth) position (m)
X (lateral) position (m)
ϕ, dϕ/dt and d$^2\phi$/dt^2
θ, dθ/dt and d$^2\theta$/dt^2
Time (s)
Angular value (°, °/s, or °/s^2)

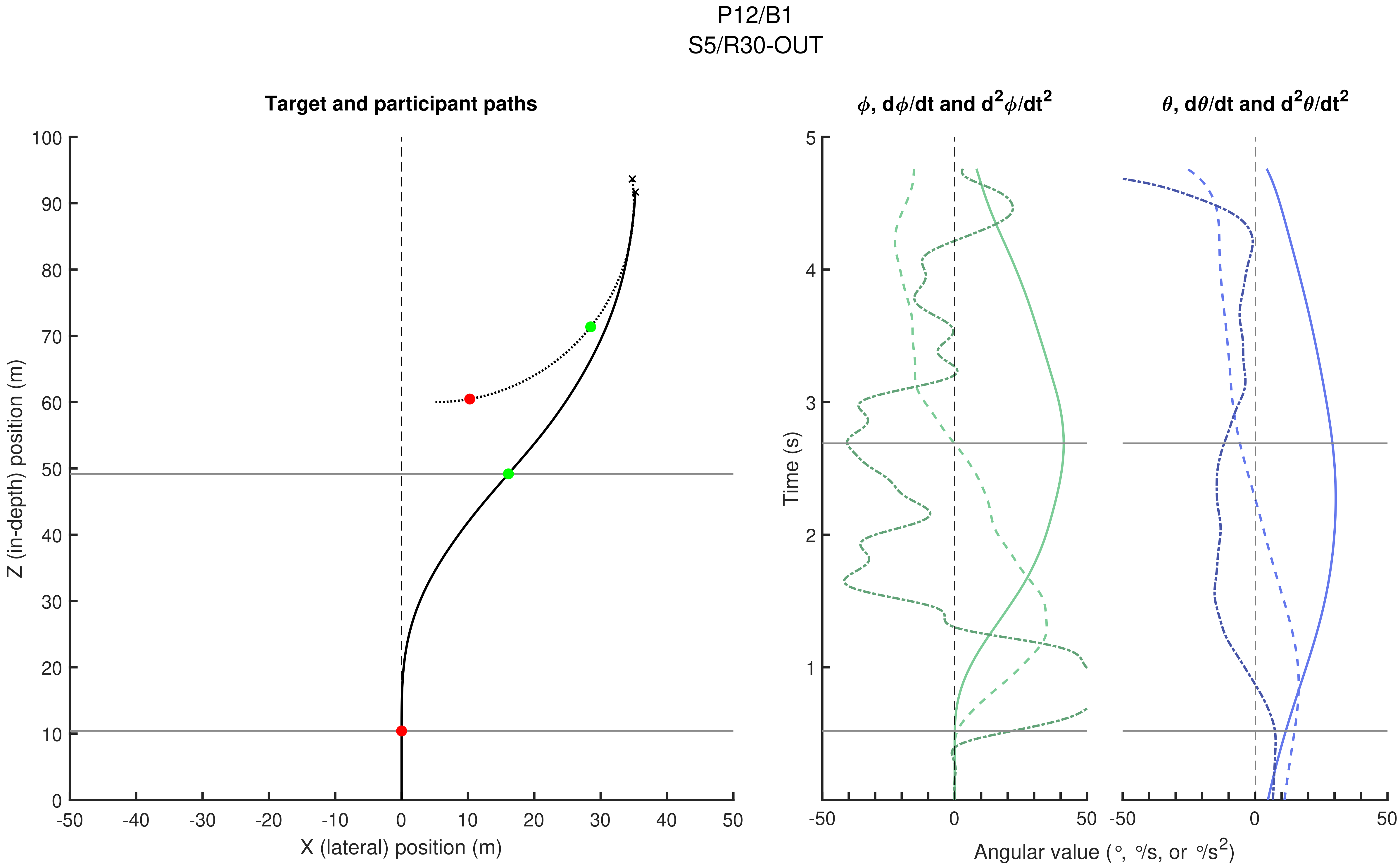
P12/B1
S5/R30-OUT
Target and participant paths
ϕ, dϕ/dt and d$^2\phi$/dt^2
θ, dθ/dt and d$^2\theta$/dt^2
Z (in-depth) position (m)
X (lateral) position (m)
Time (s)
Angular value (°, °/s, or °/s^2)
100
90
80
70
60
50
40
30
20
10
0
-50
-40
-30
-20
-10
0
10
20
30
40
50
5
4
3
2
1
-50
0
50
-50
0
50

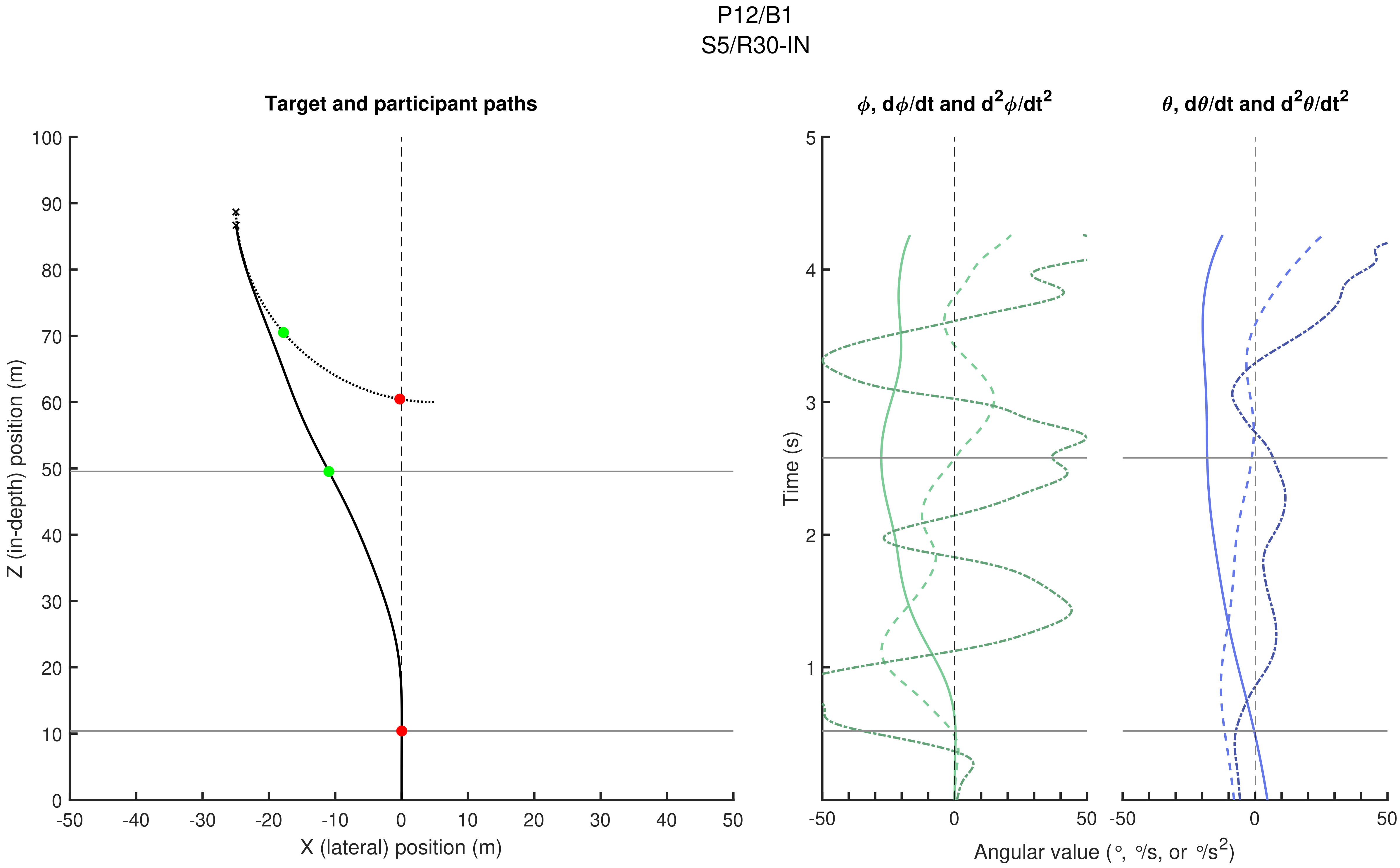
P12/B1
S5/R30-IN
Target and participant paths
Z (in-depth) position (m)
X (lateral) position (m)
0
10
20
30
40
50
60
70
80
90
100
-50
-40
-30
-20
-10
0
10
20
30
40
50
φ, dφ/dt and d²φ/dt²
θ, dθ/dt and d²θ/dt²
Time (s)
1
2
3
4
5
-50
0
50
-50
0
50
Angular value (°, °/s, or °/s²)

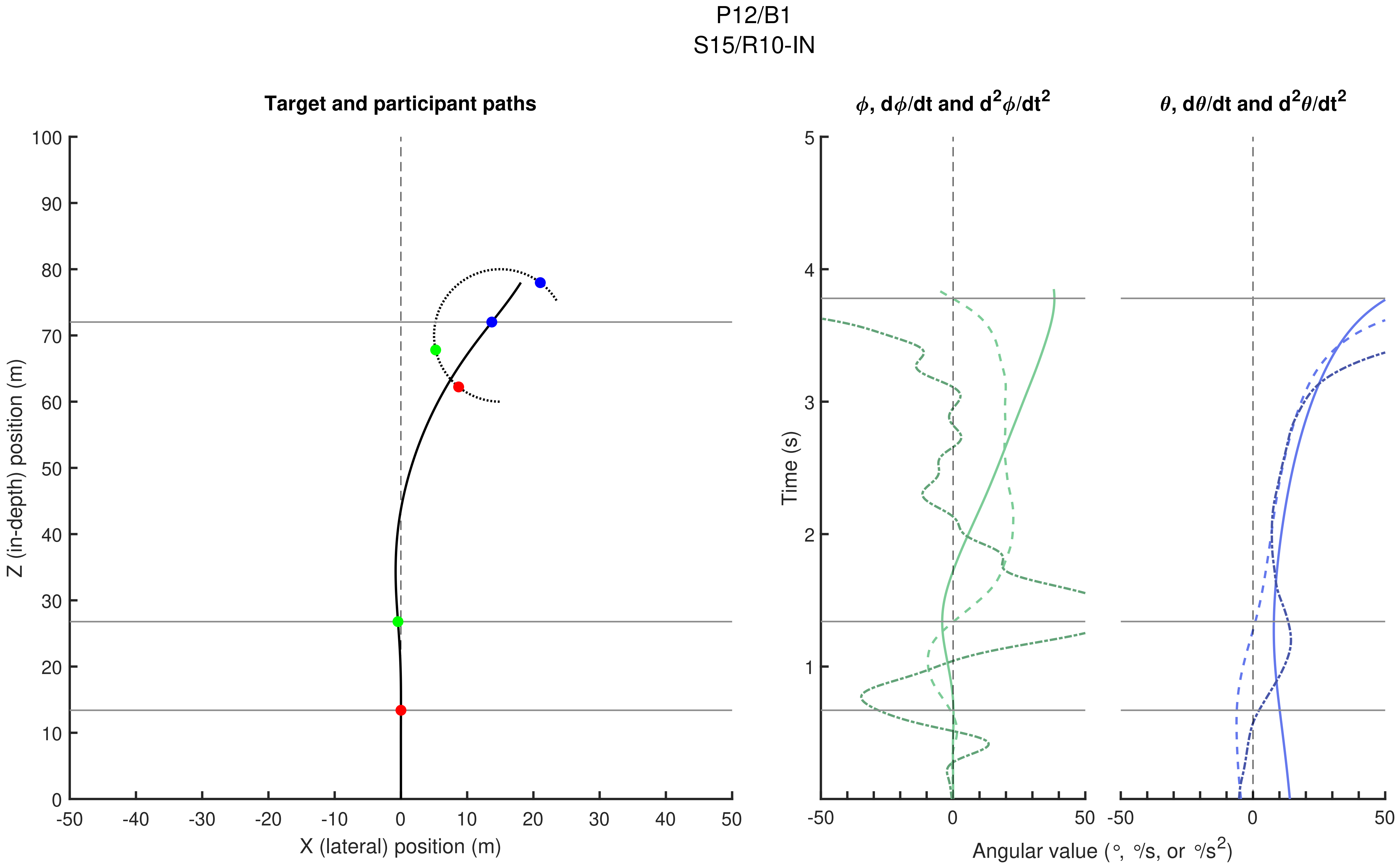

P12/B1
S15/R10-IN
Target and participant paths
ϕ, dϕ/dt and d$^2\phi$/dt^2
θ, dθ/dt and d$^2\theta$/dt^2
Z (in-depth) position (m)
X (lateral) position (m)
Time (s)
Angular value (°, °/s, or °/s^2)

P12/B1
S15/R20-IN

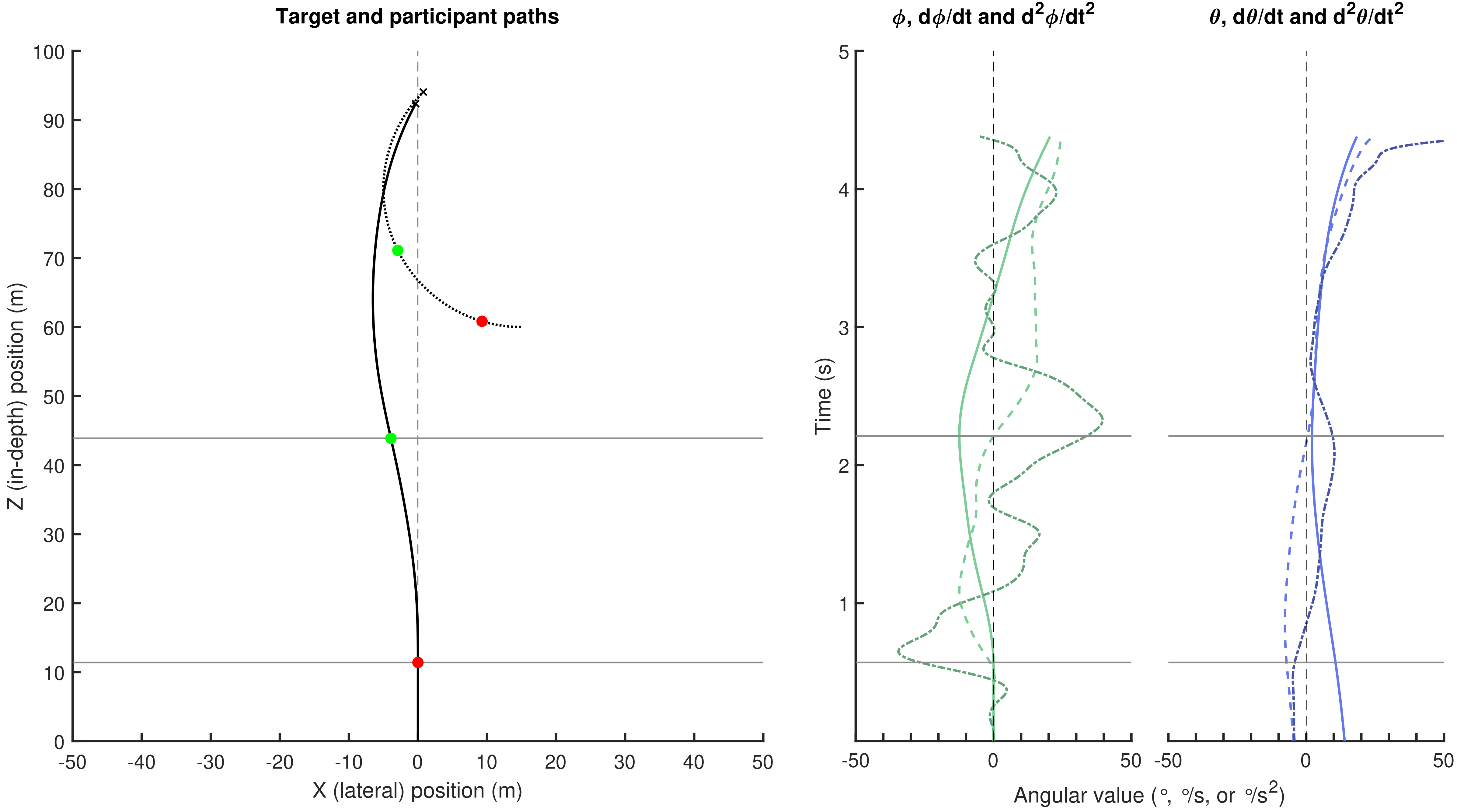

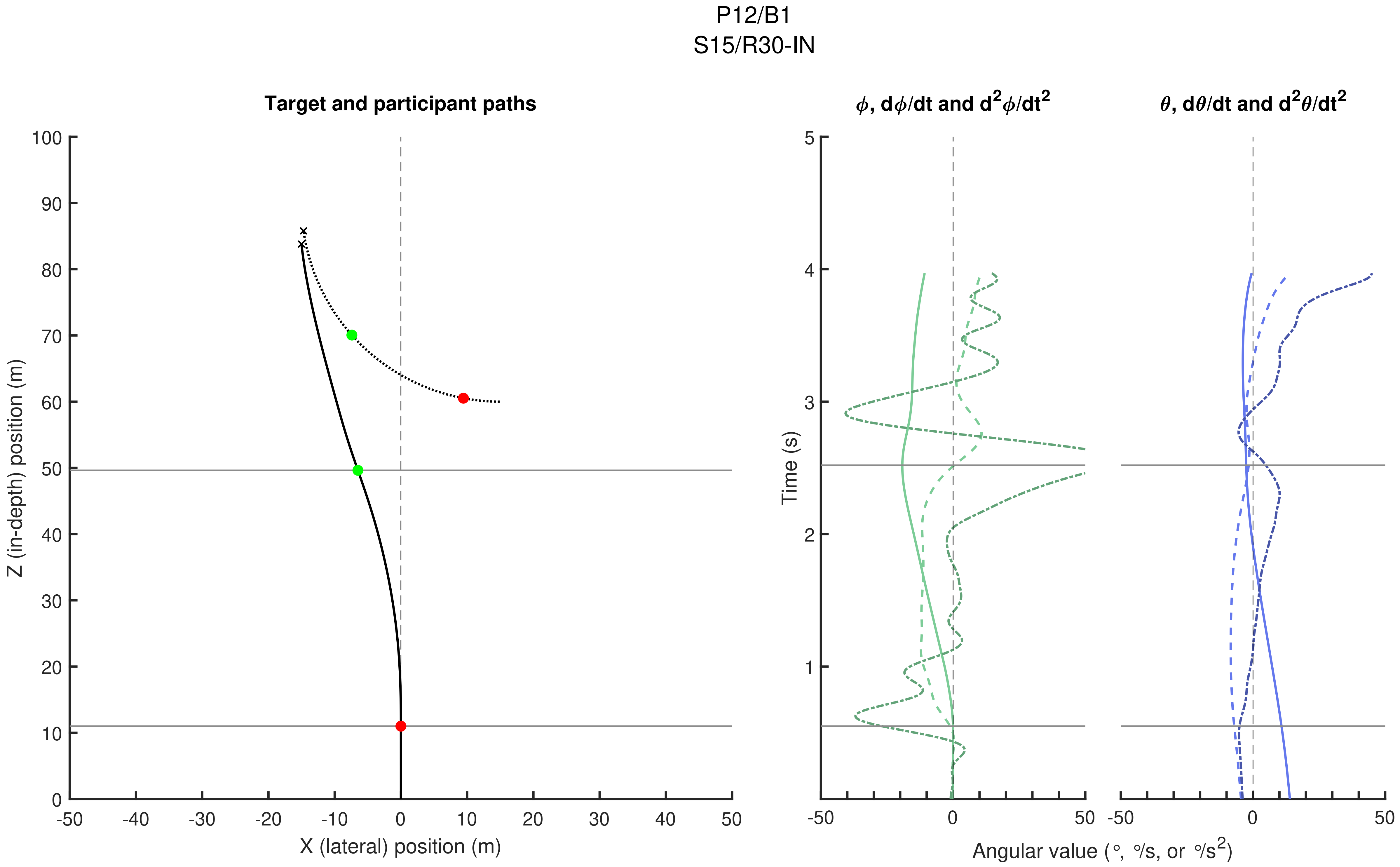

P12/B1
S15/R30-IN
Target and participant paths
Z (in-depth) position (m)
X (lateral) position (m)
ϕ, dϕ/dt and d$^2\phi$/dt^2
θ, dθ/dt and d$^2\theta$/dt^2
Time (s)
Angular value (°, °/s, or °/s^2)

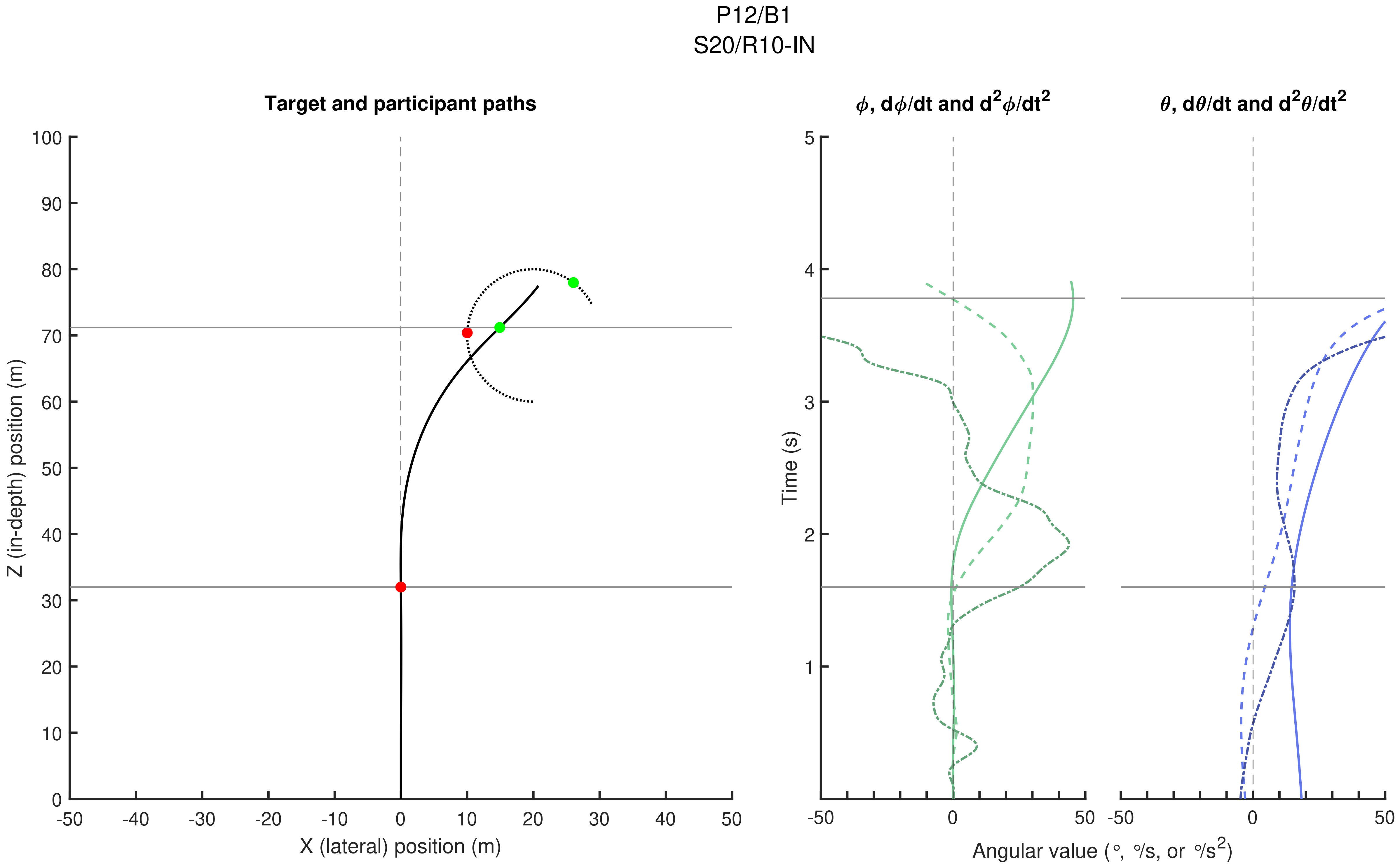
P12/B1
S20/R10-IN
Target and participant paths
Z (in-depth) position (m)
X (lateral) position (m)
ϕ, dϕ/dt and d$^2\phi$/dt^2
θ, dθ/dt and d$^2\theta$/dt^2
Time (s)
Angular value (°, °/s, or °/s^2)

P12/B1
S20/R20-IN

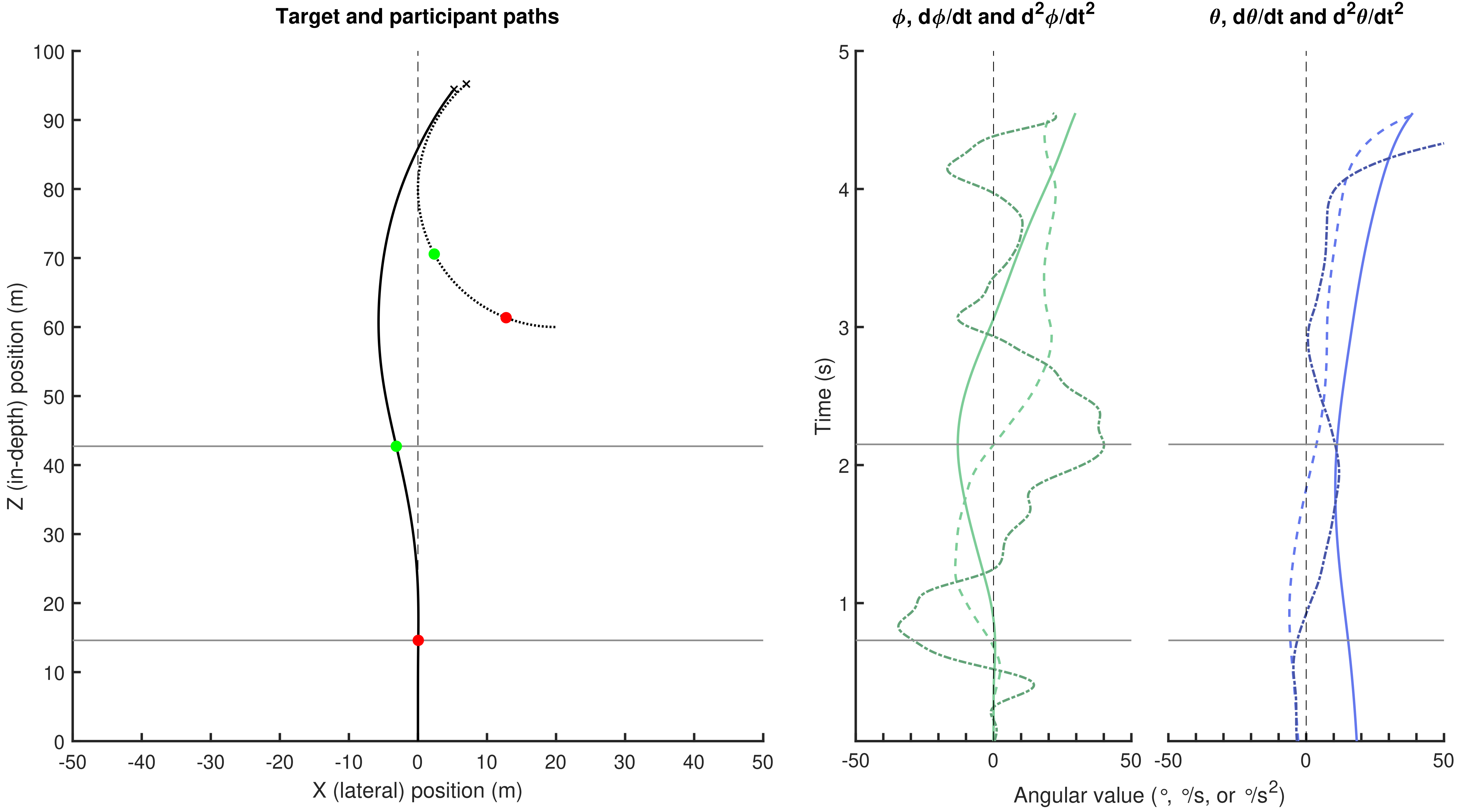

P12/B1
S20/R30-IN

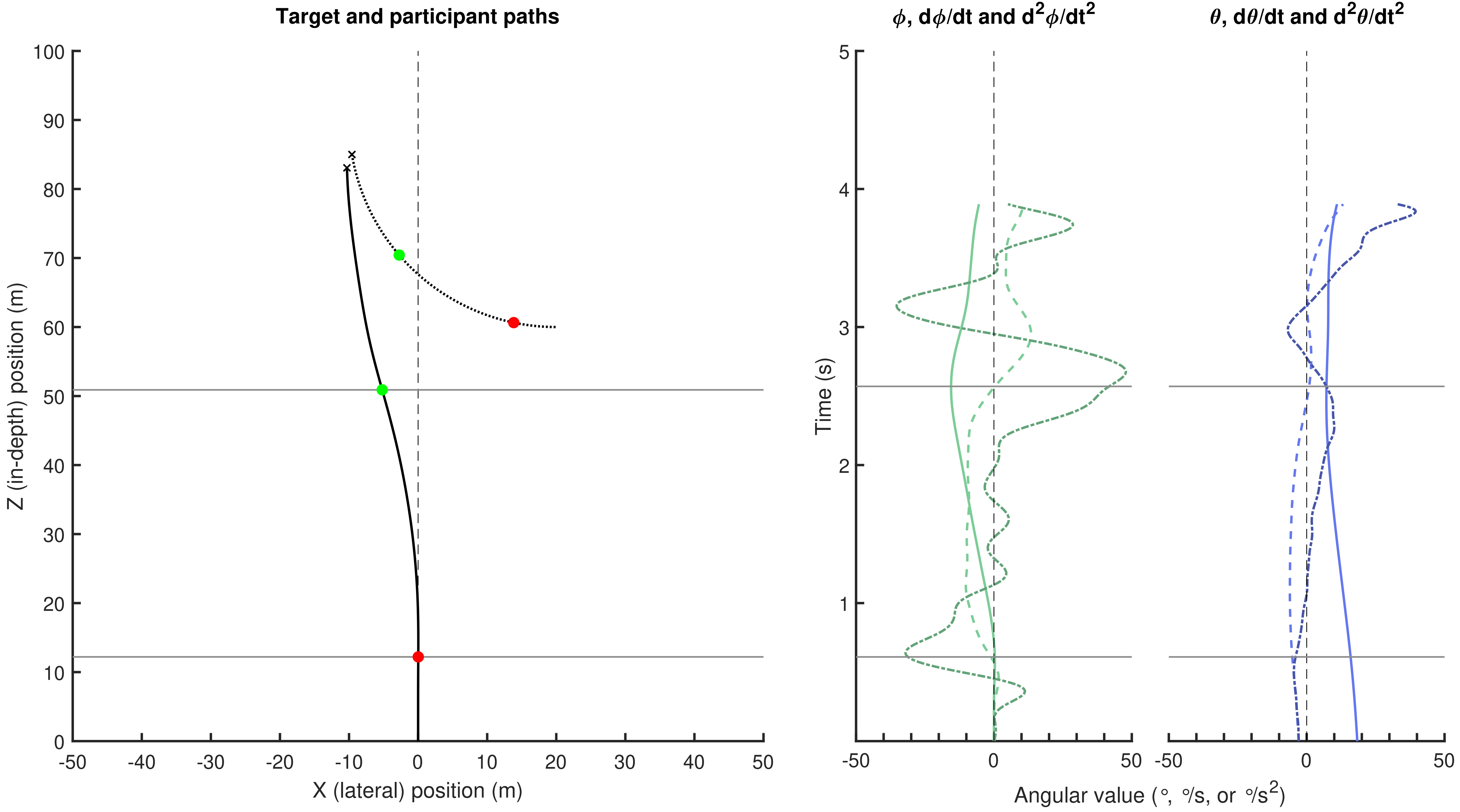

P12/B2
S20/R10-IN

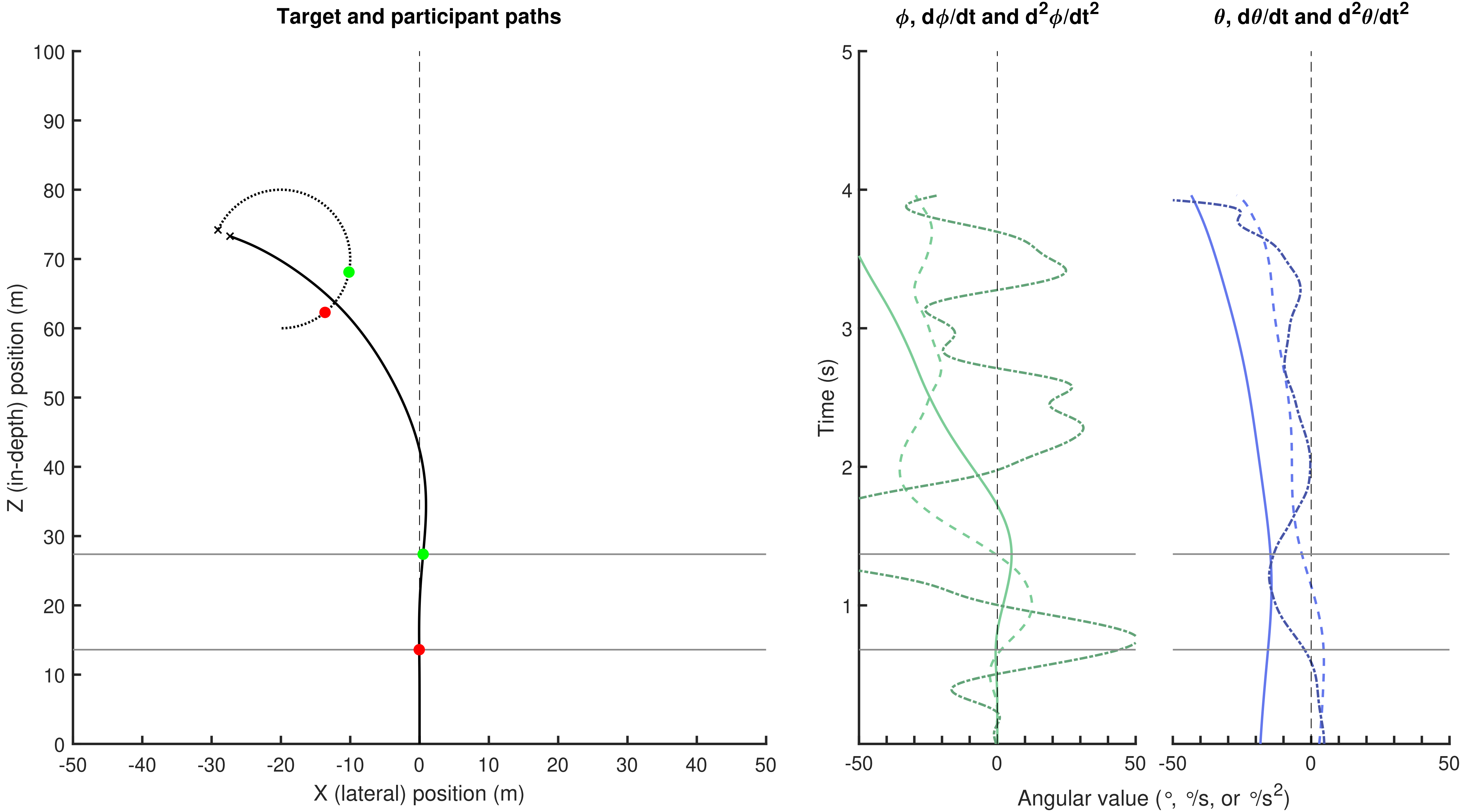

P12/B2
S20/R20-IN

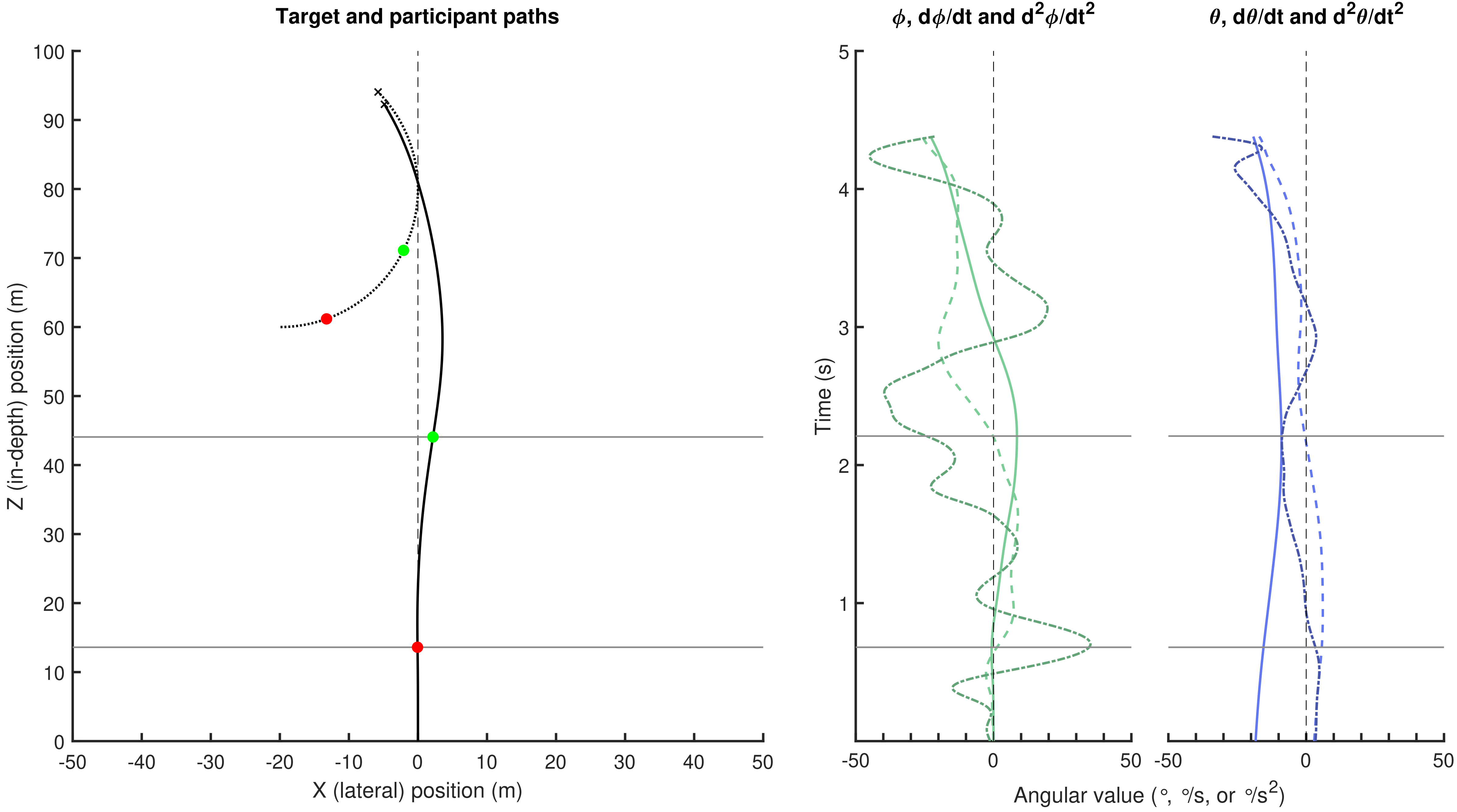

P12/B2
S20/R30-IN

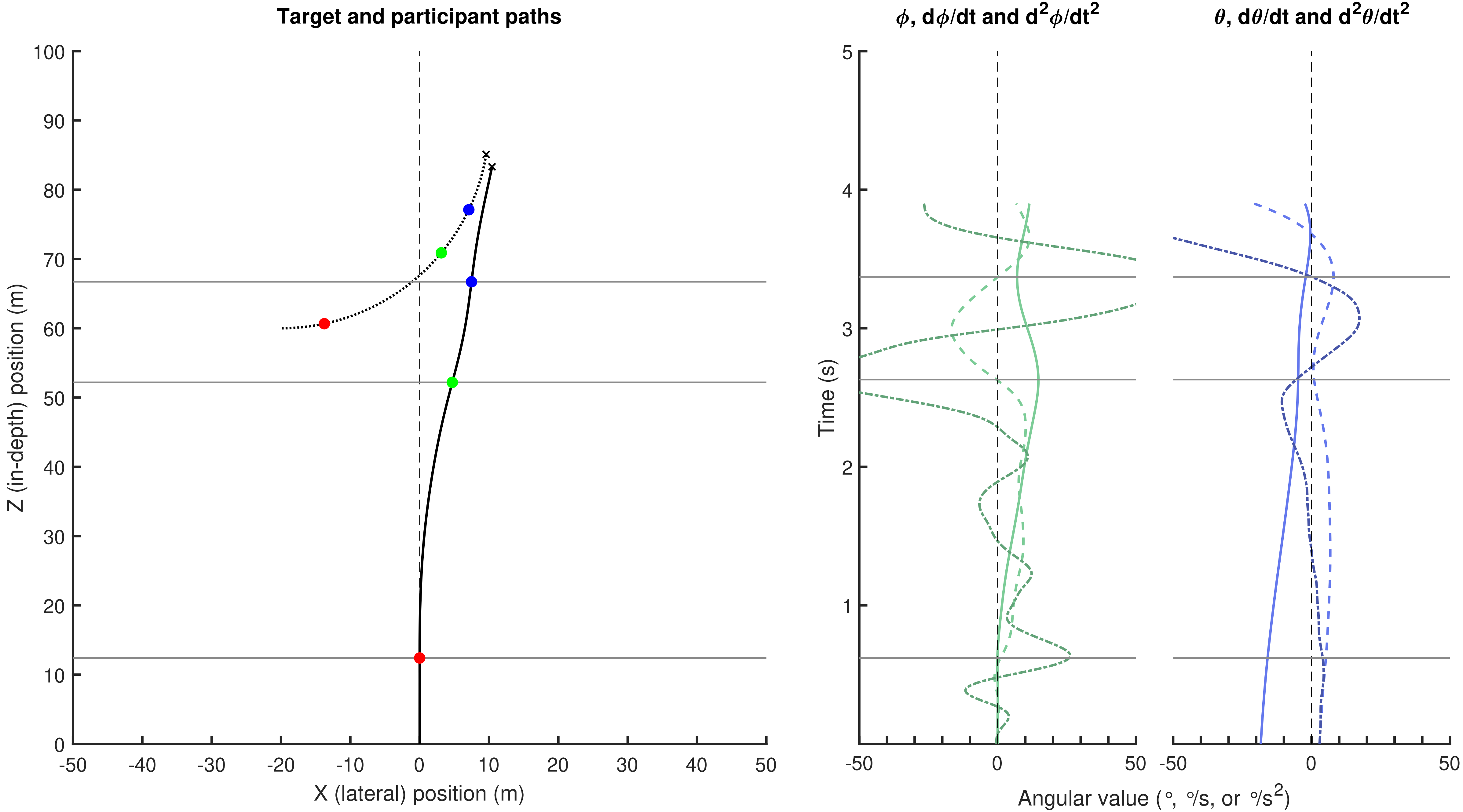

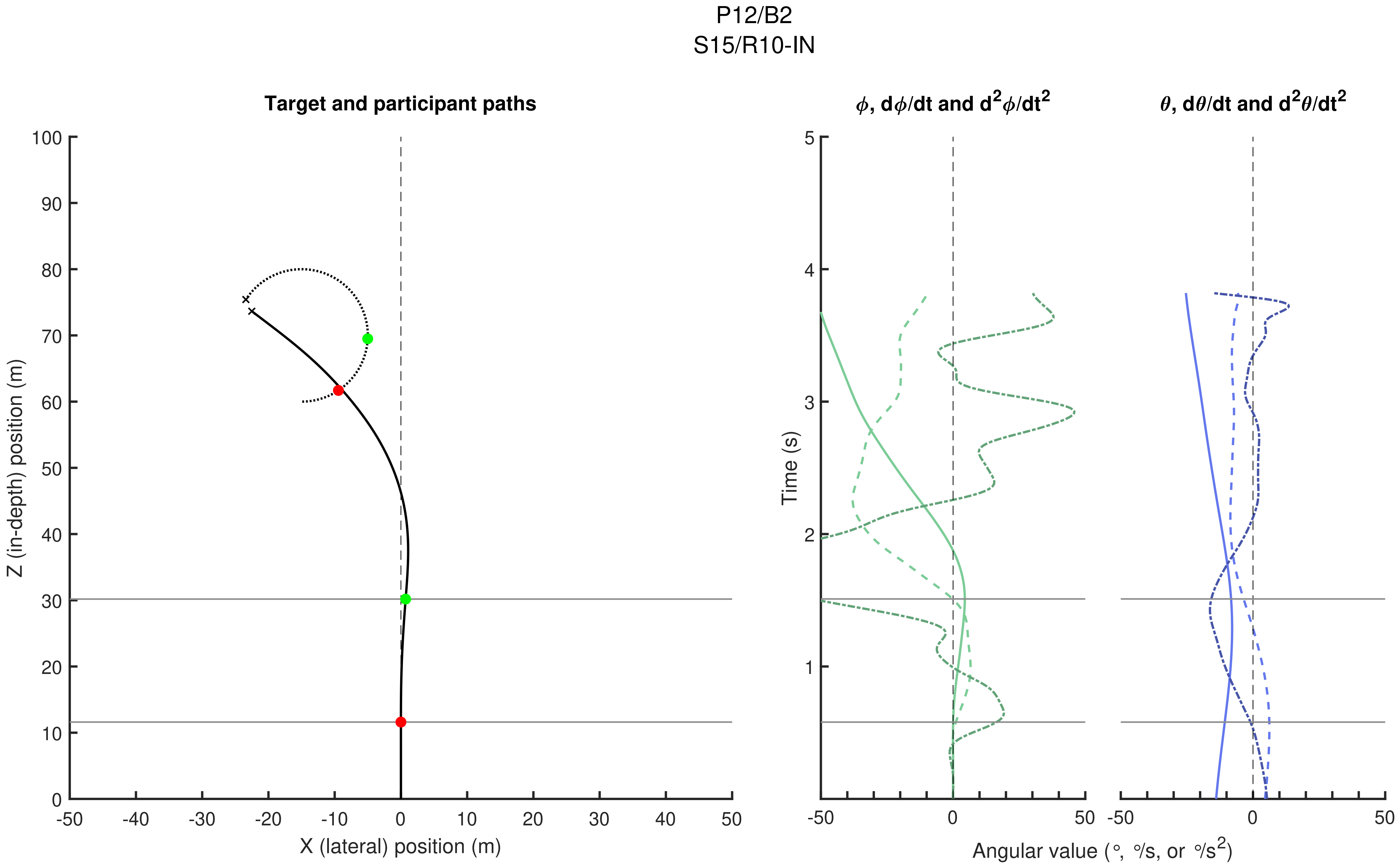
P12/B2
S15/R10-IN
Target and participant paths
Z (in-depth) position (m)
X (lateral) position (m)
100
90
80
70
60
50
40
30
20
10
0
-50
-40
-30
-20
-10
0
10
20
30
40
50
ϕ, dϕ/dt and d²ϕ/dt²
θ, dθ/dt and d²θ/dt²
Time (s)
5
4
3
2
1
-50
0
50
-50
0
50
Angular value (°, °/s, or °/s²)

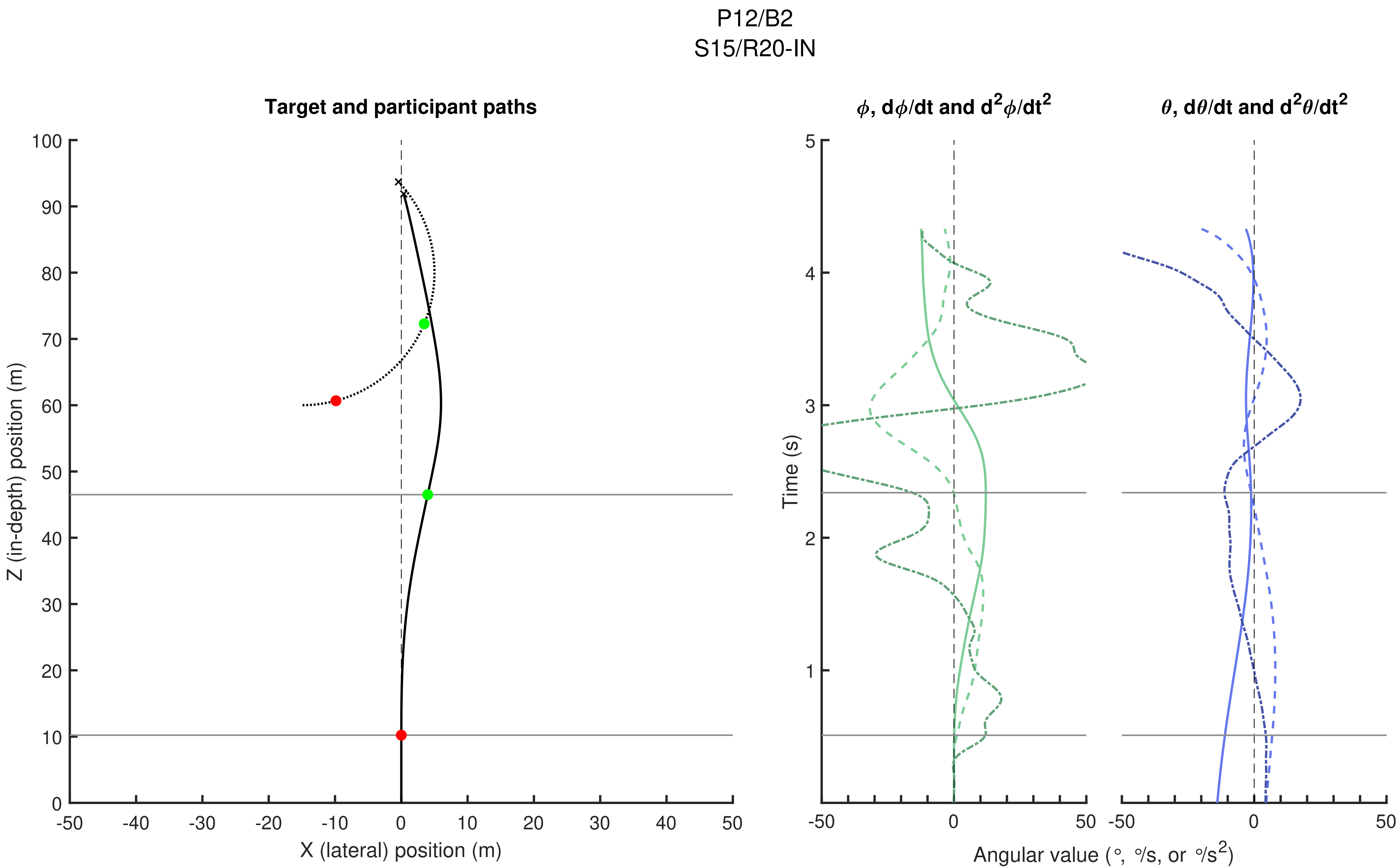

P12/B2
S15/R20-IN
Target and participant paths
Z (in-depth) position (m)
X (lateral) position (m)
φ, dφ/dt and d²φ/dt²
θ, dθ/dt and d²θ/dt²
Time (s)
Angular value (°, °/s, or °/s²)

P12/B2
S15/R30-IN

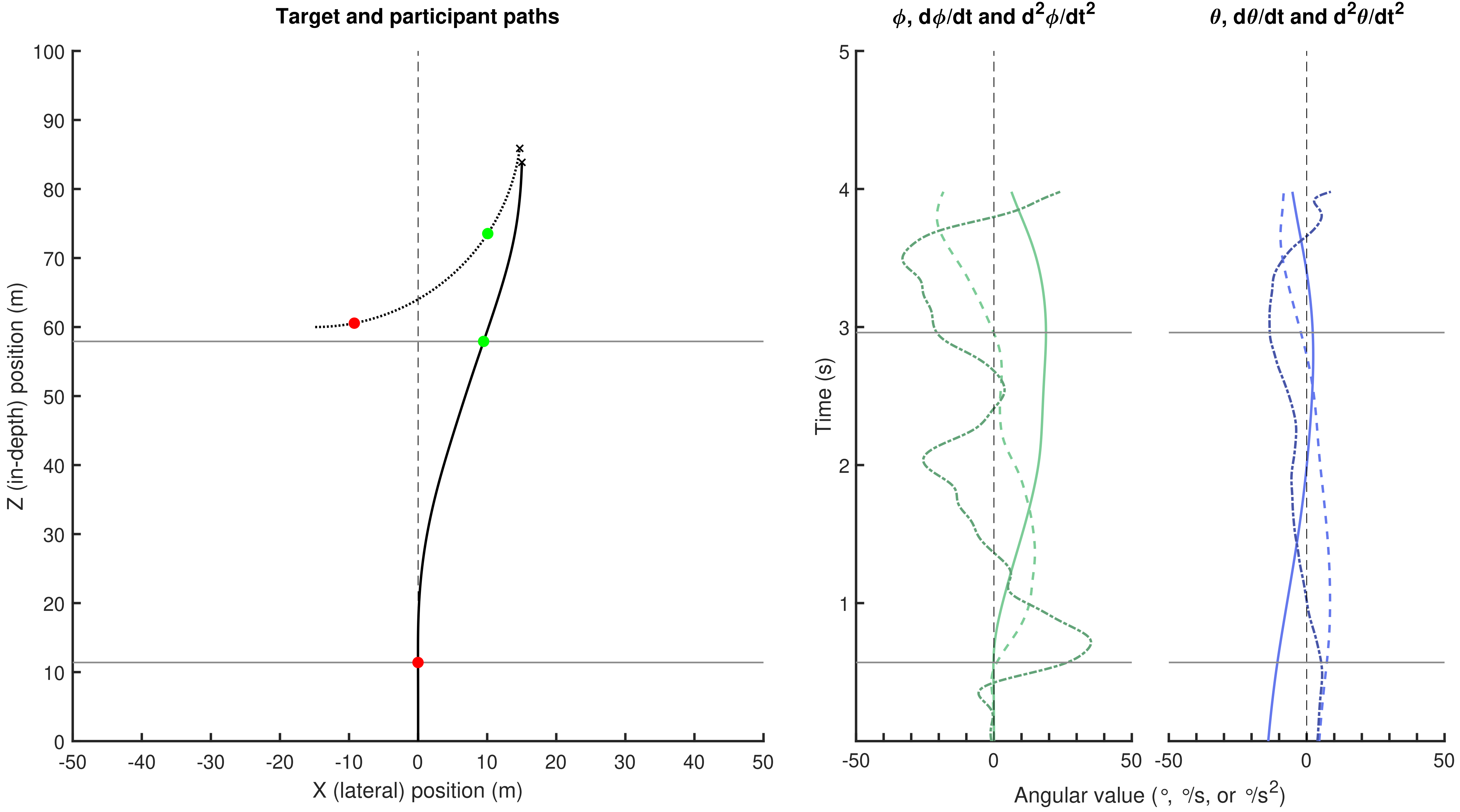

P12/B2
S5/R10-IN

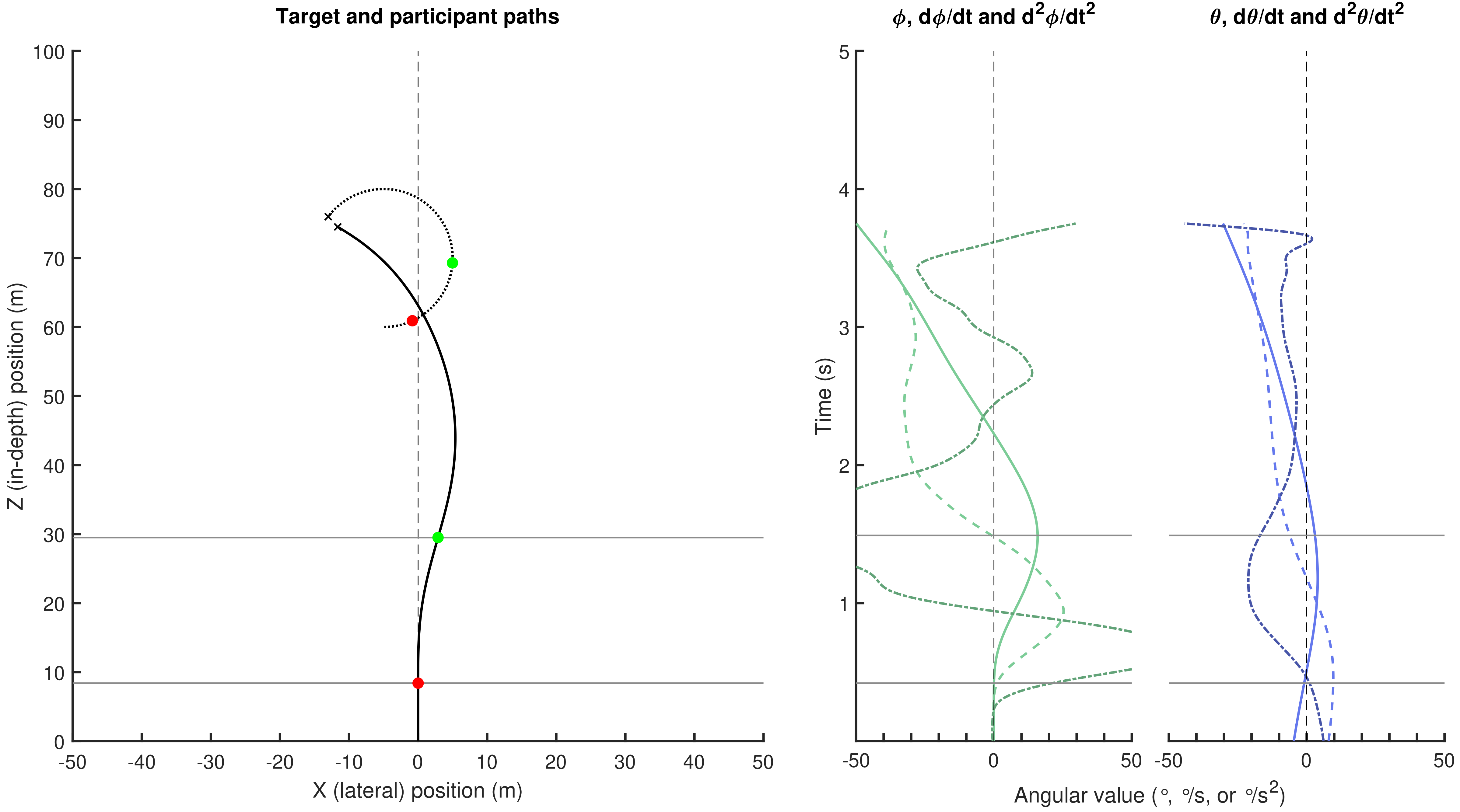

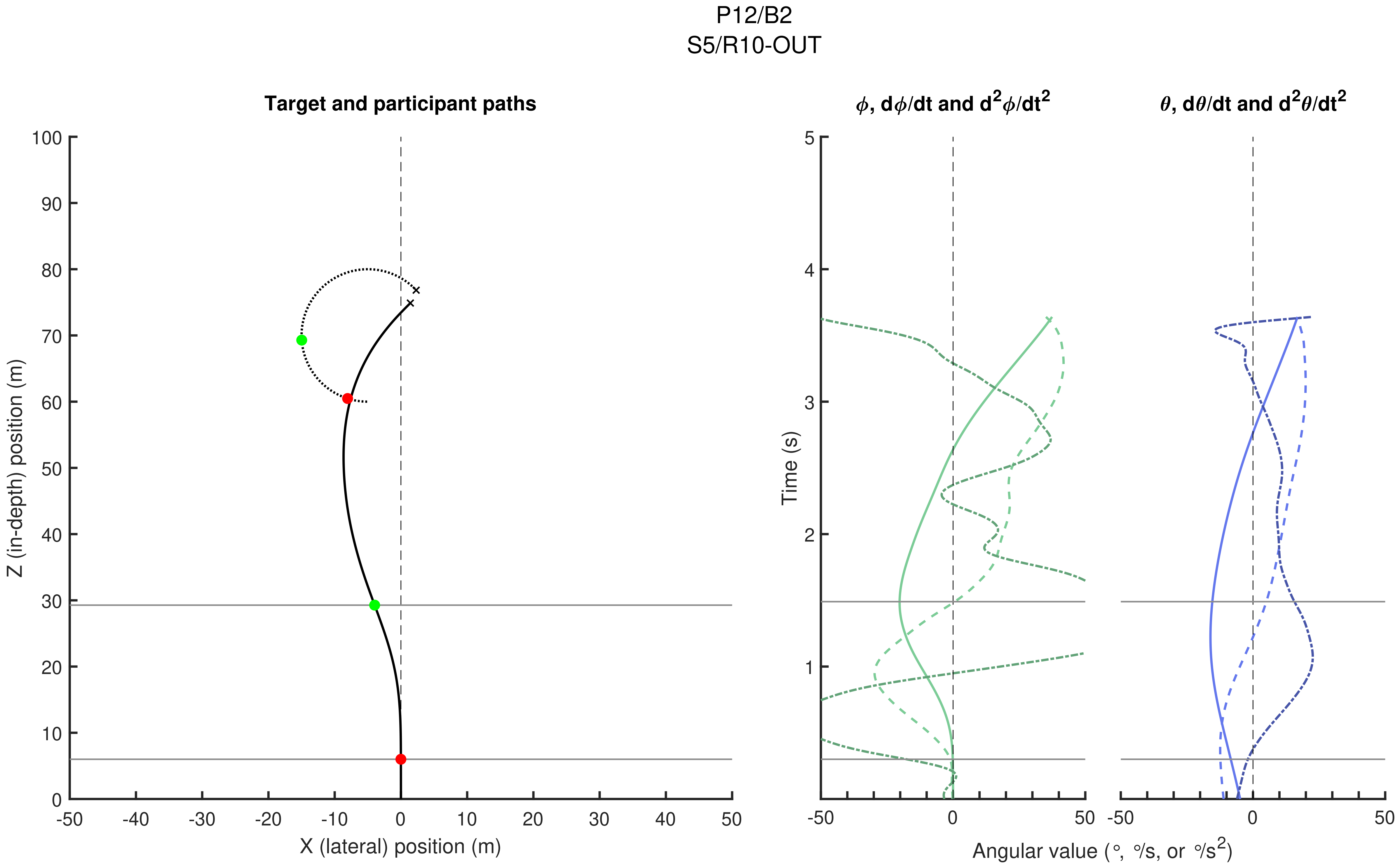

P12/B2
S5/R10-OUT
Target and participant paths
Z (in-depth) position (m)
X (lateral) position (m)
100
90
80
70
60
50
40
30
20
10
0
-50
-40
-30
-20
-10
0
10
20
30
40
50
ϕ, dϕ/dt and d$^2\phi$/dt^2
θ, dθ/dt and d$^2\theta$/dt^2
Time (s)
5
4
3
2
1
-50
0
50
-50
0
50
Angular value (°, °/s, or °/s^2)

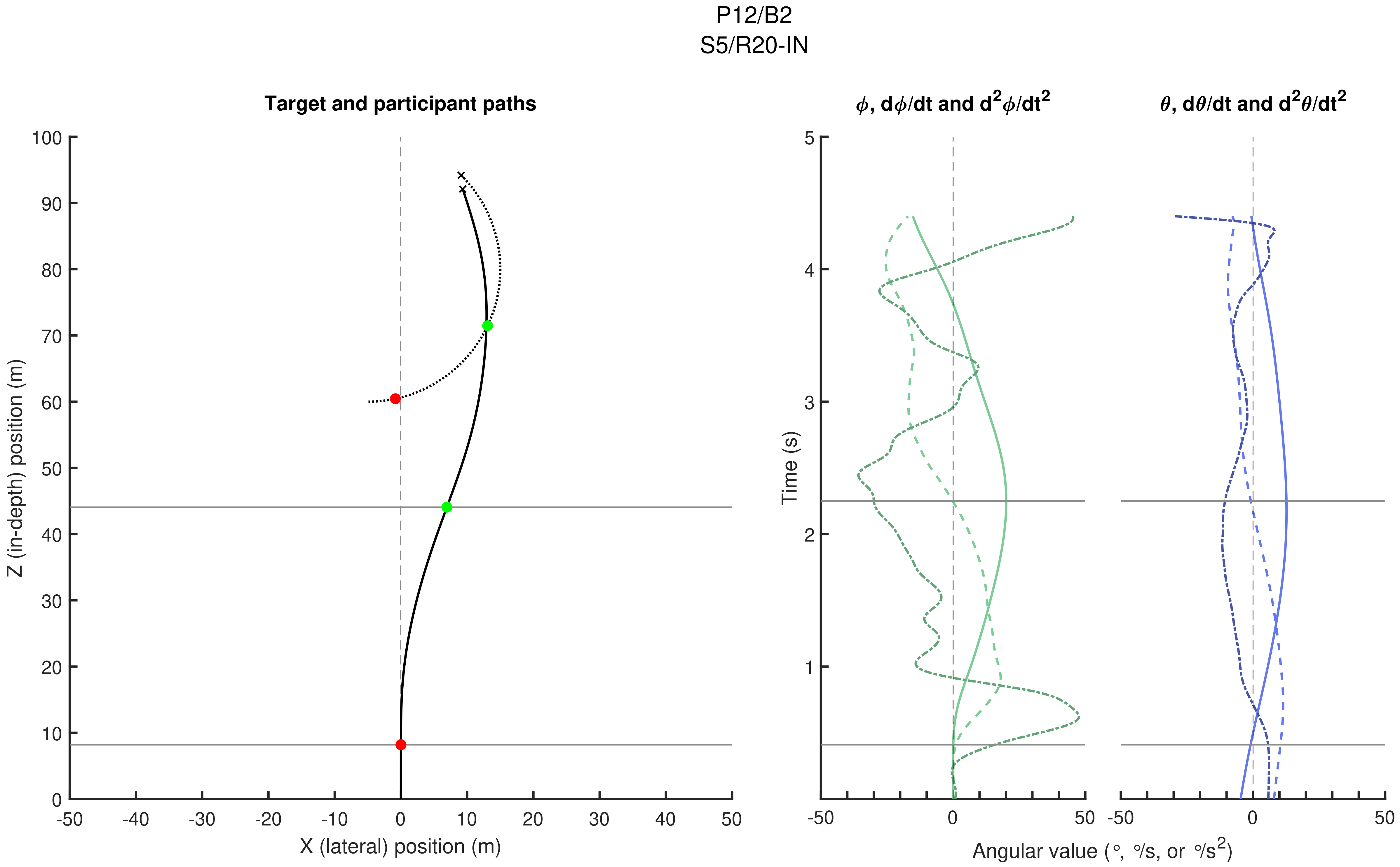
P12/B2
S5/R20-IN
Target and participant paths
Z (in-depth) position (m)
X (lateral) position (m)
ϕ, dϕ/dt and d$^2\phi$/dt^2
θ, dθ/dt and d$^2\theta$/dt^2
Time (s)
Angular value (°, °/s, or °/s^2)

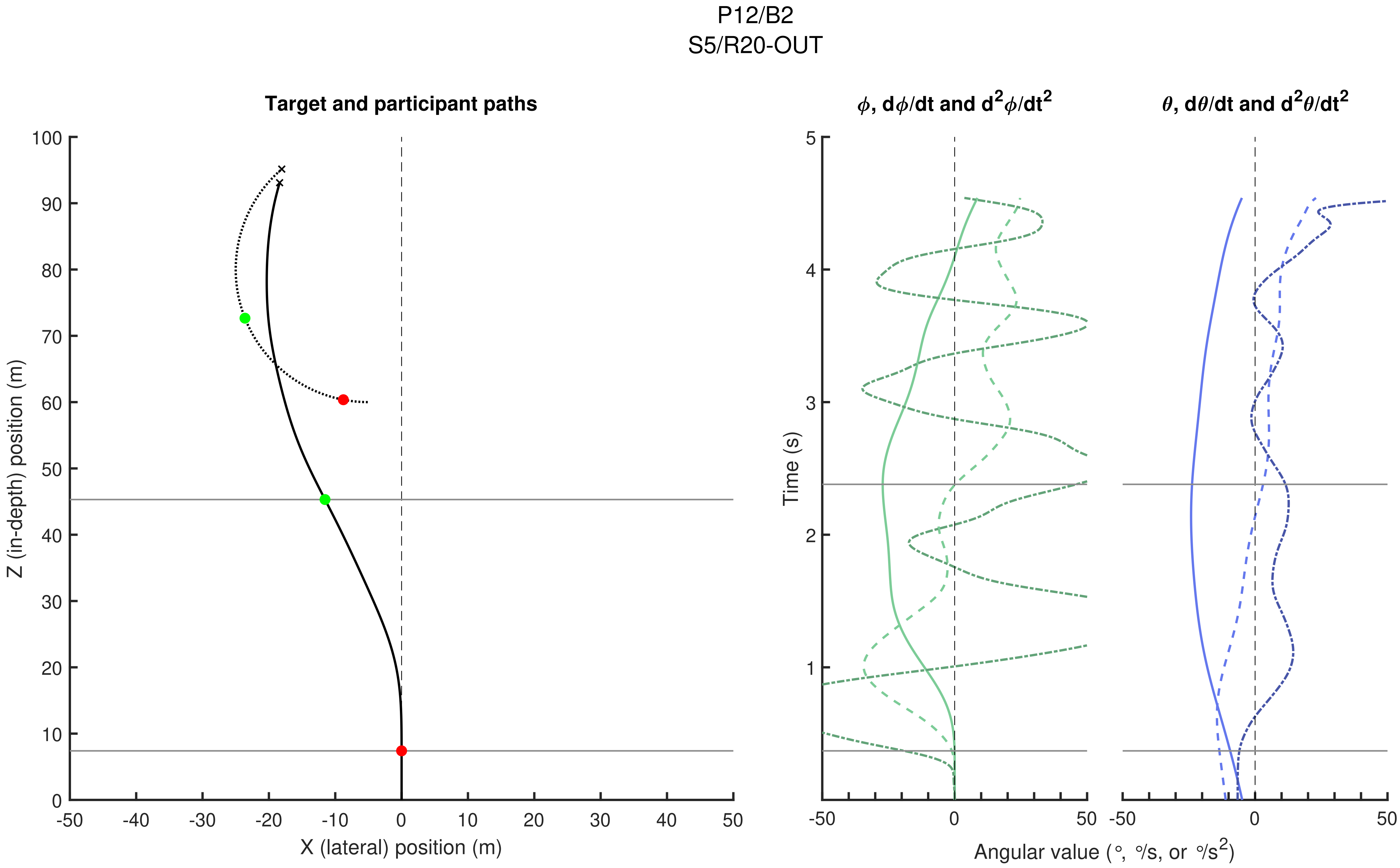
P12/B2
S5/R20-OUT
Target and participant paths
ϕ, dϕ/dt and d$^2\phi$/dt^2
θ, dθ/dt and d$^2\theta$/dt^2
Z (in-depth) position (m)
X (lateral) position (m)
Time (s)
Angular value (°, °/s, or °/s^2)
100
90
80
70
60
50
40
30
20
10
0
-50
-40
-30
-20
-10
0
10
20
30
40
50
5
4
3
2
1
-50
0
50
-50
0
50

P12/B2
S5/R30-IN

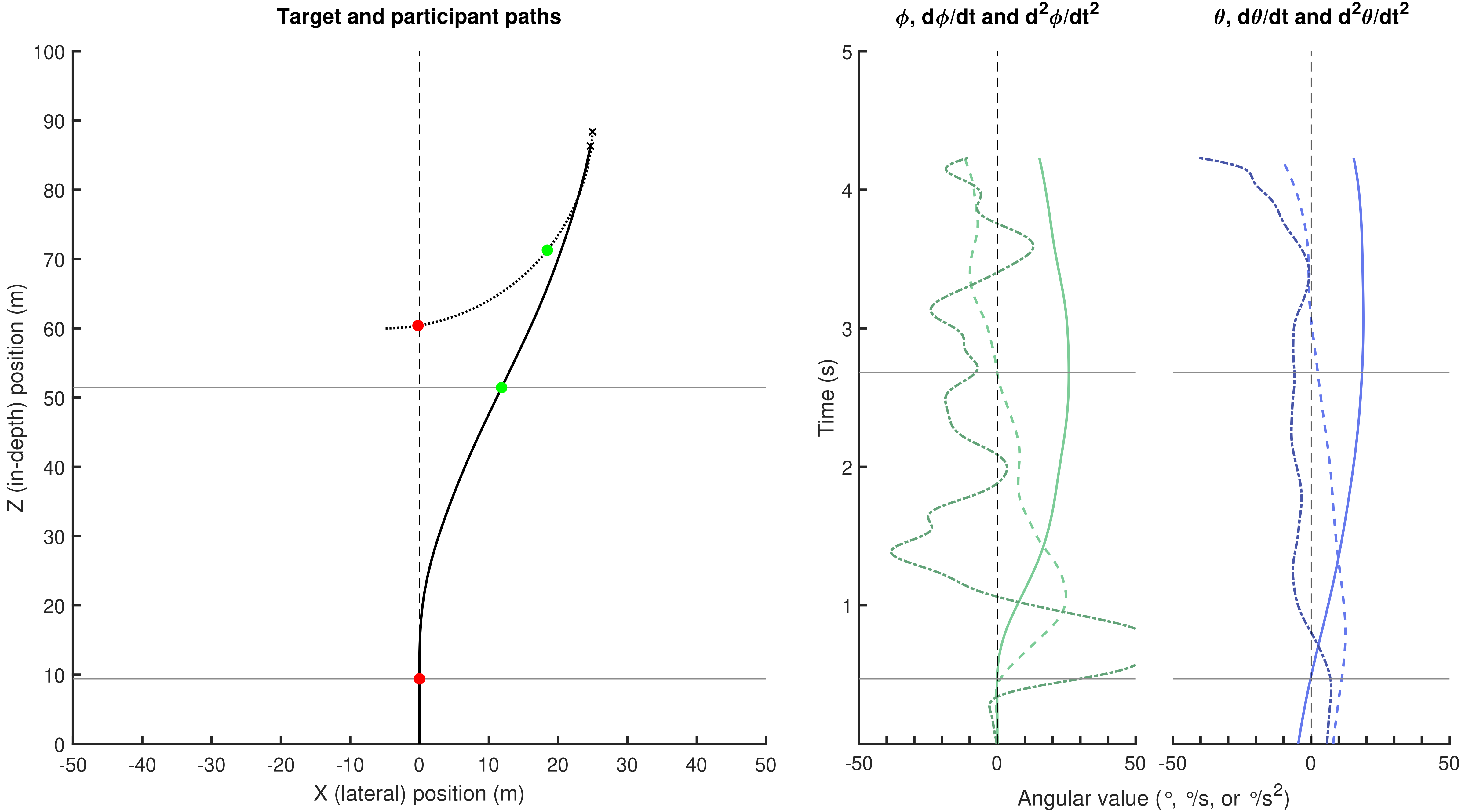

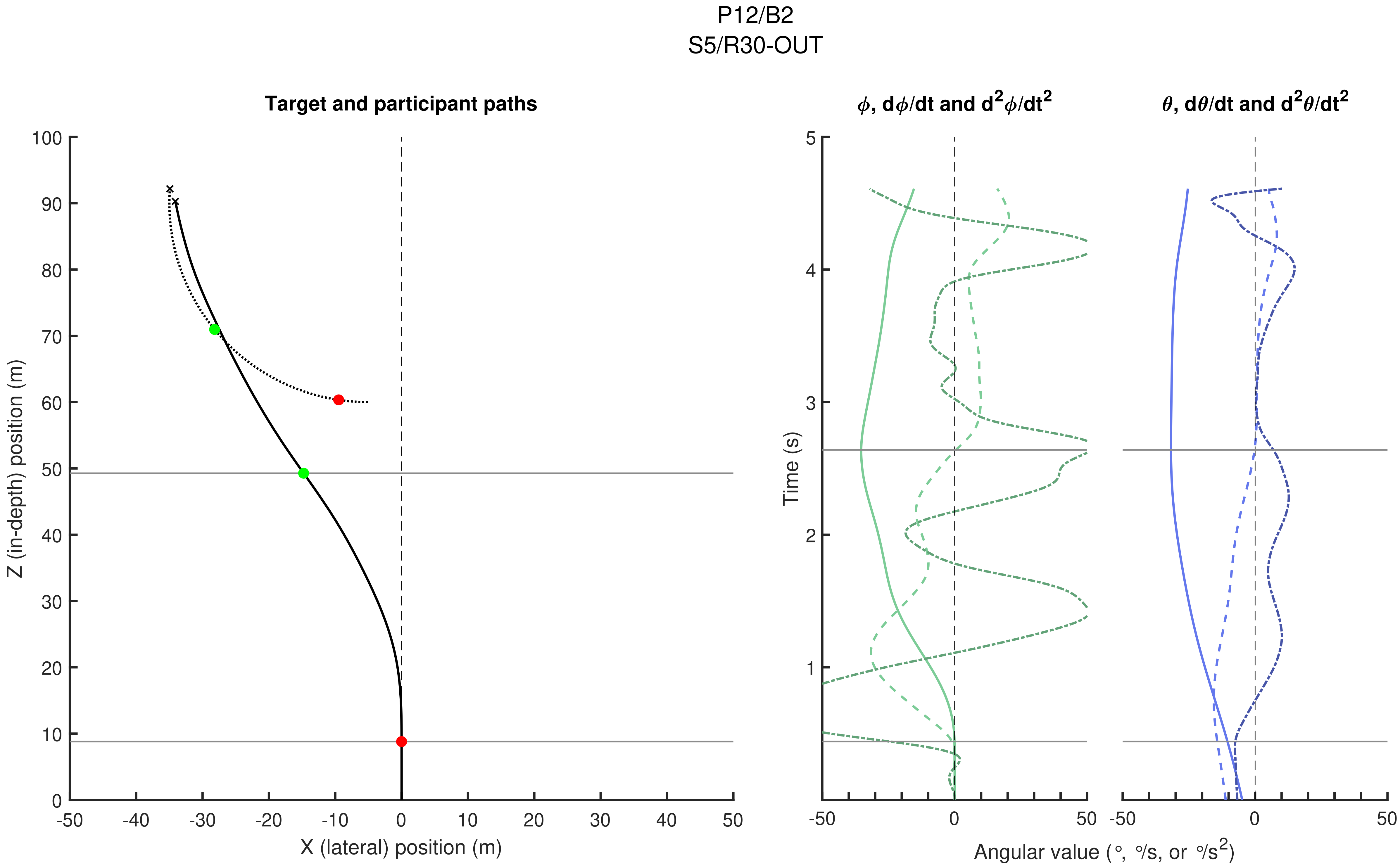

P12/B2
S5/R30-OUT
Target and participant paths
Z (in-depth) position (m)
X (lateral) position (m)
ϕ, dϕ/dt and d$^2\phi$/dt^2
θ, dθ/dt and d$^2\theta$/dt^2
Time (s)
Angular value (°, °/s, or °/s^2)

P12/B2
S5/R10-OUT

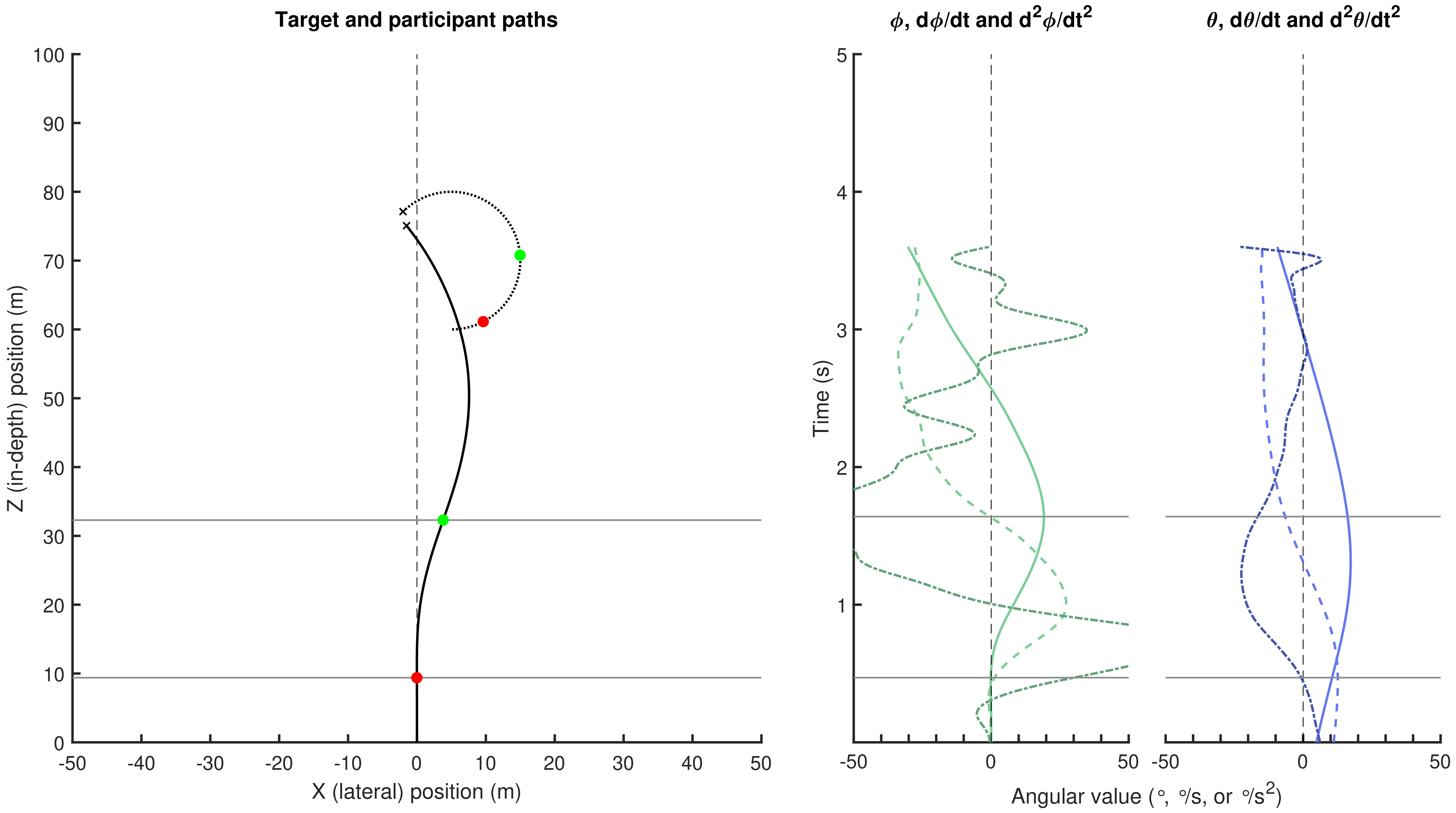

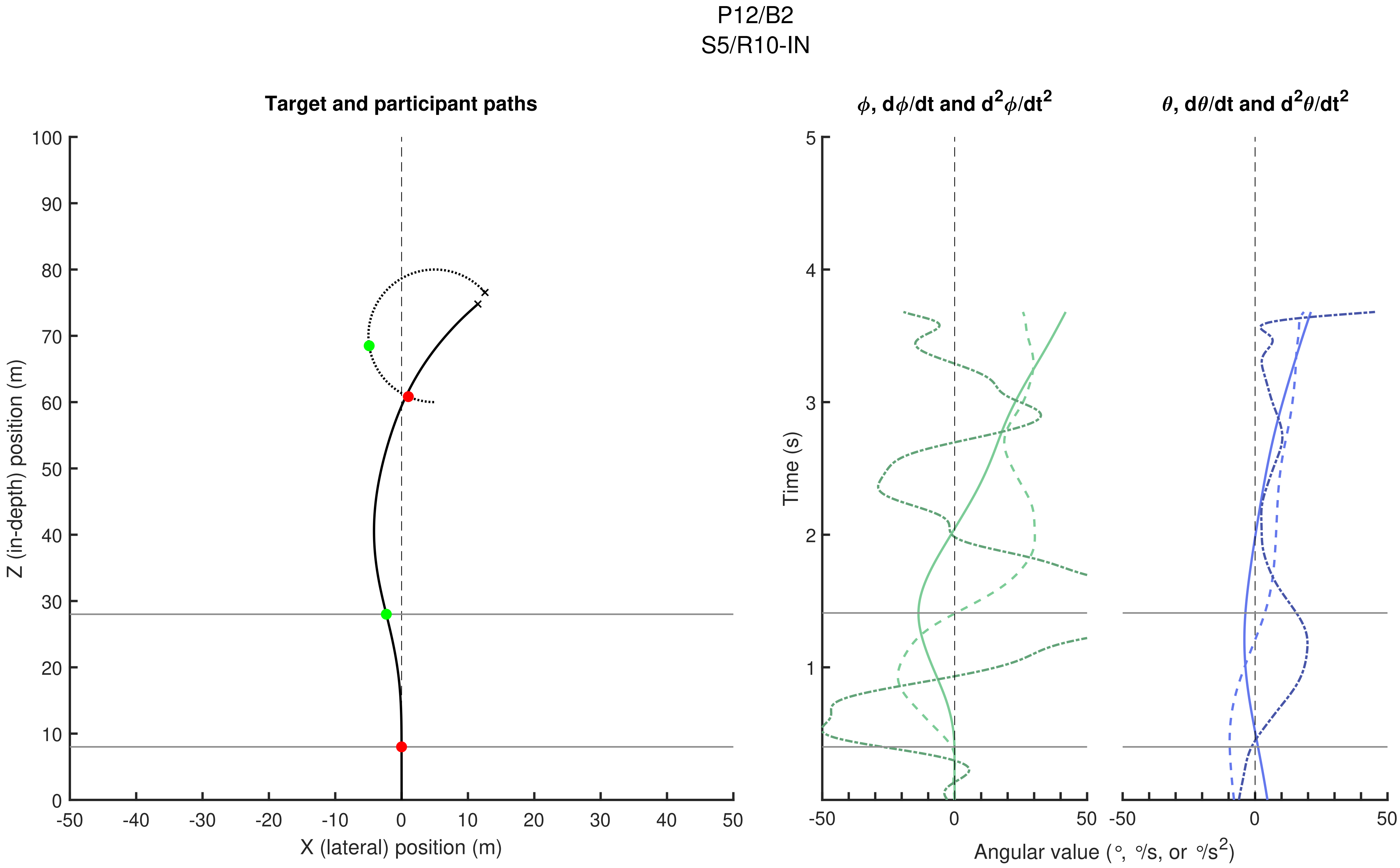
P12/B2
S5/R10-IN
Target and participant paths
ϕ, dϕ/dt and d$^2\phi$/dt^2
θ, dθ/dt and d$^2\theta$/dt^2
Z (in-depth) position (m)
X (lateral) position (m)
Time (s)
Angular value (°, °/s, or °/s^2)
100
90
80
70
60
50
40
30
20
10
0
-50
-40
-30
-20
-10
0
10
20
30
40
50
5
4
3
2
1
-50
0
50
-50
0
50

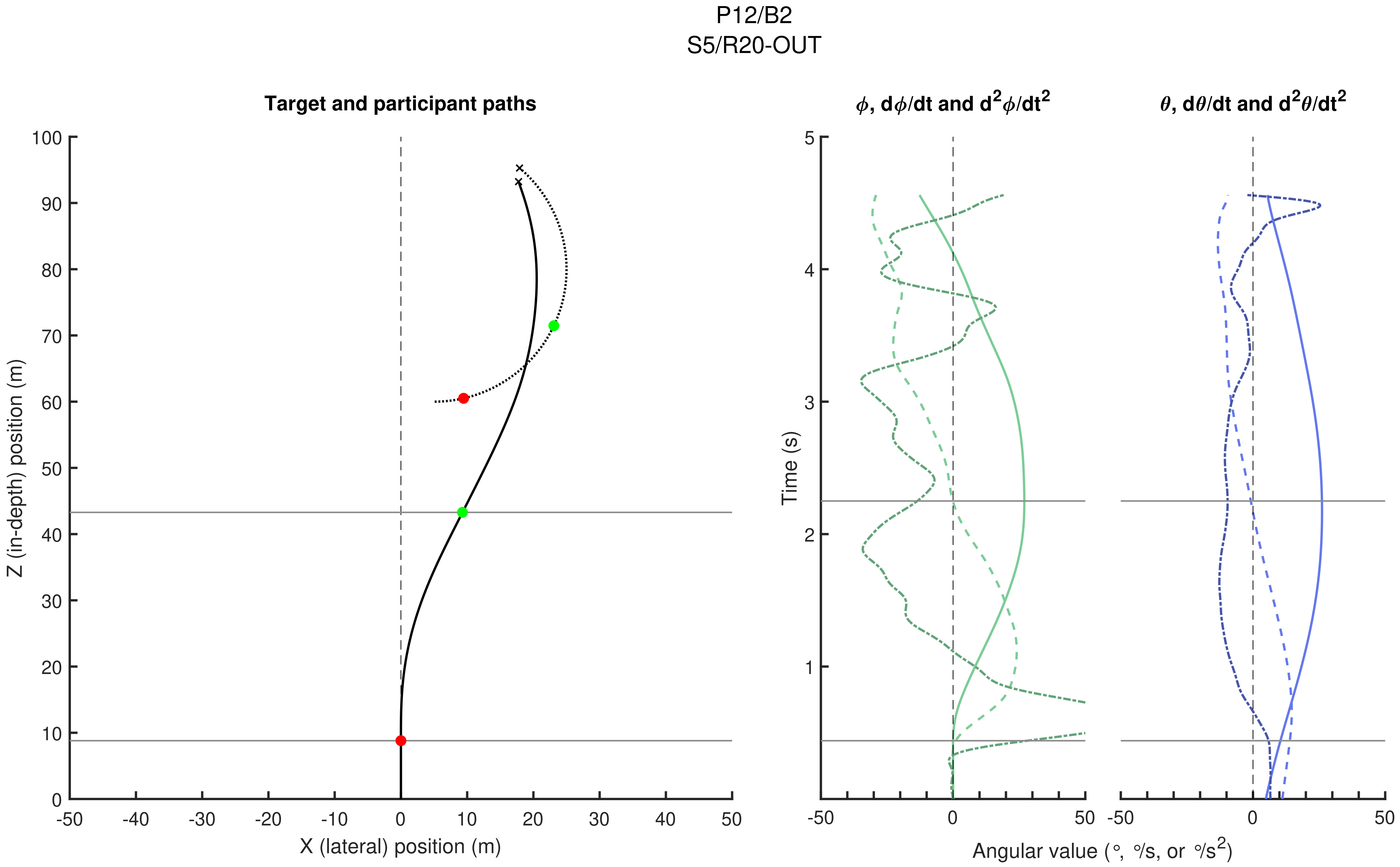
P12/B2
S5/R20-OUT
Target and participant paths
Z (in-depth) position (m)
X (lateral) position (m)
ϕ, dϕ/dt and d²ϕ/dt²
θ, dθ/dt and d²θ/dt²
Time (s)
Angular value (°, °/s, or °/s²)

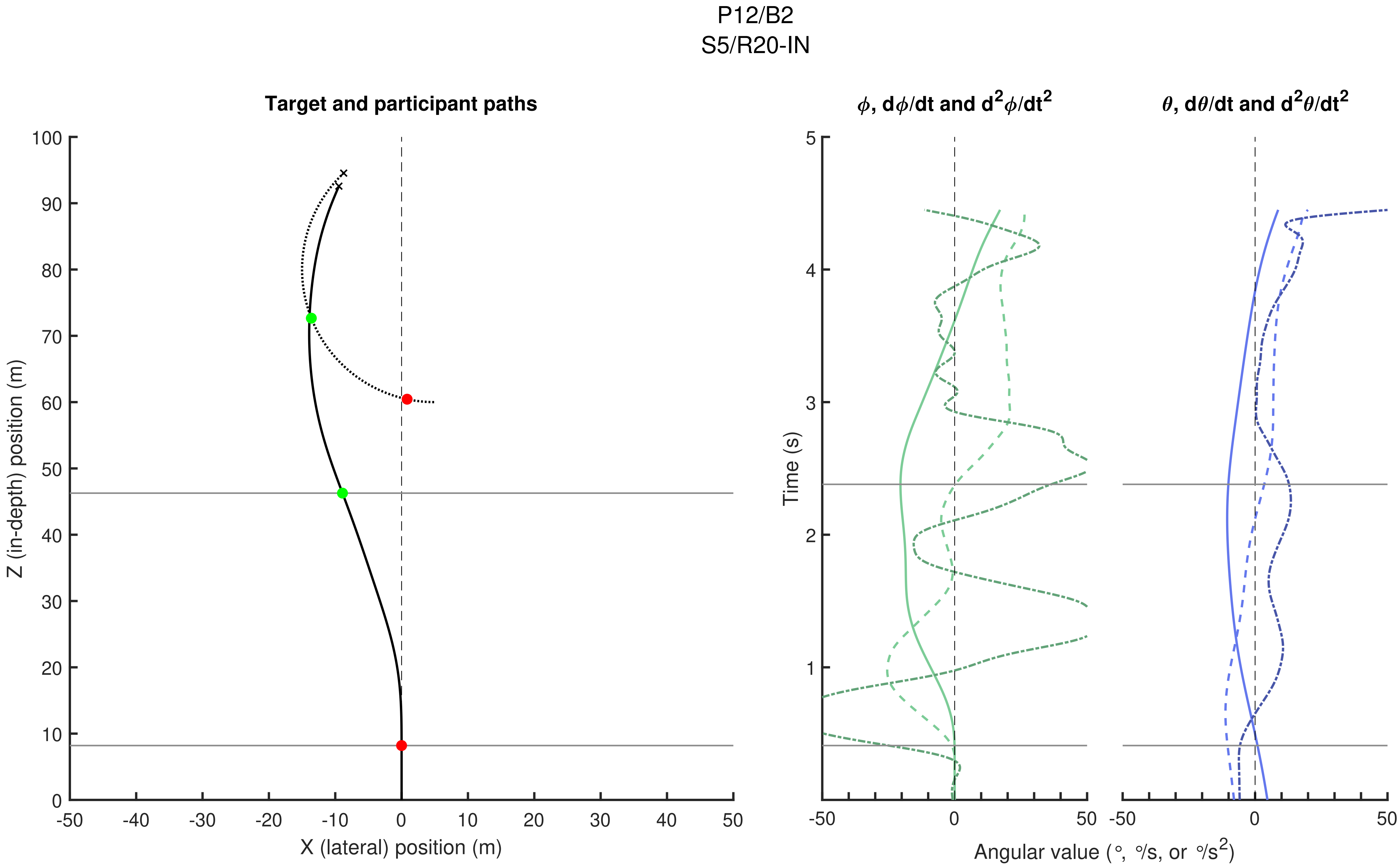

P12/B2
S5/R20-IN
Target and participant paths
Z (in-depth) position (m)
X (lateral) position (m)
ϕ, dϕ/dt and d$^2\phi$/dt^2
θ, dθ/dt and d$^2\theta$/dt^2
Time (s)
Angular value (°, °/s, or °/s^2)

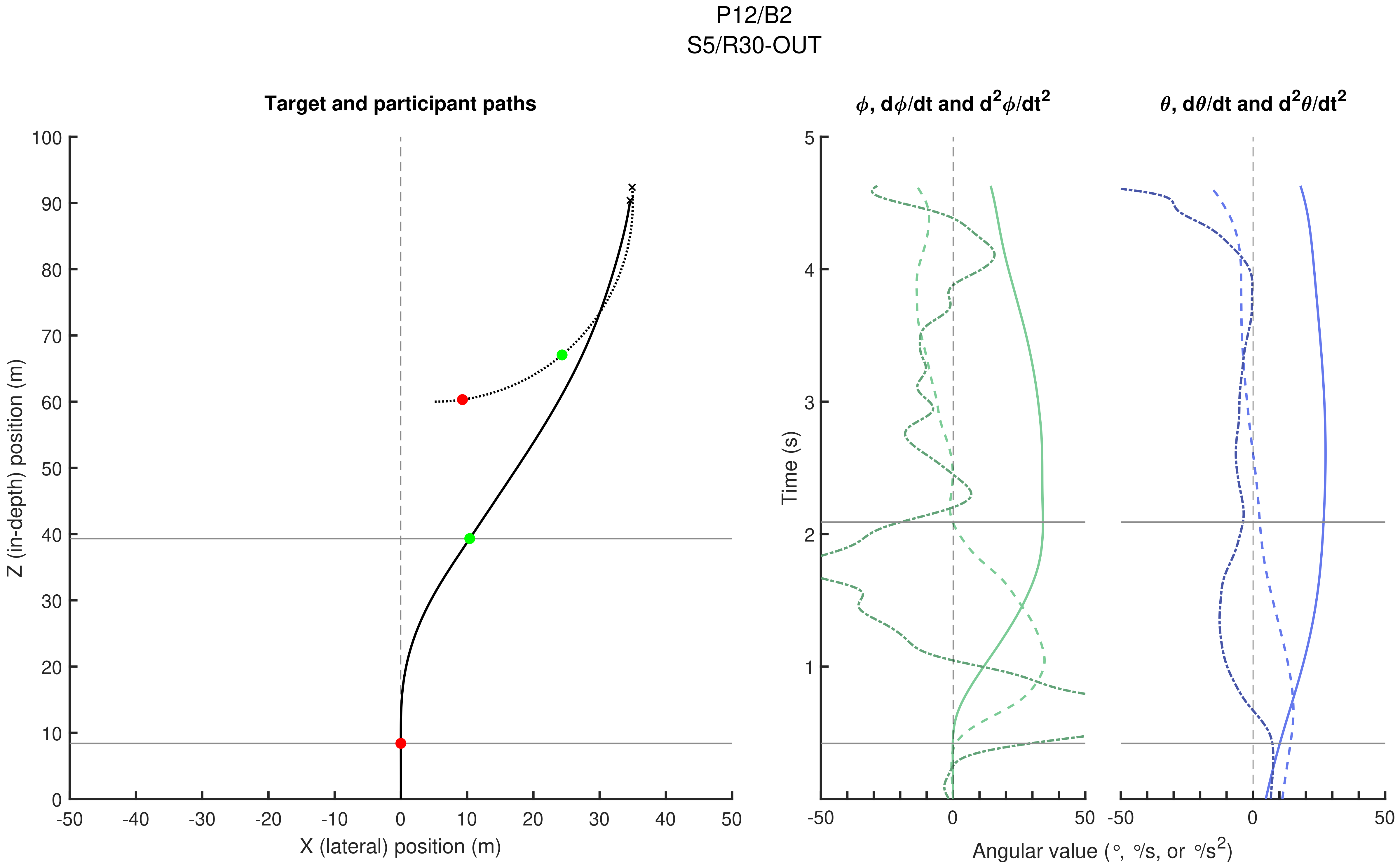

P12/B2
S5/R30-OUT
Target and participant paths
Z (in-depth) position (m)
X (lateral) position (m)
ϕ, dϕ/dt and d²ϕ/dt²
θ, dθ/dt and d²θ/dt²
Time (s)
Angular value (°, °/s, or °/s²)

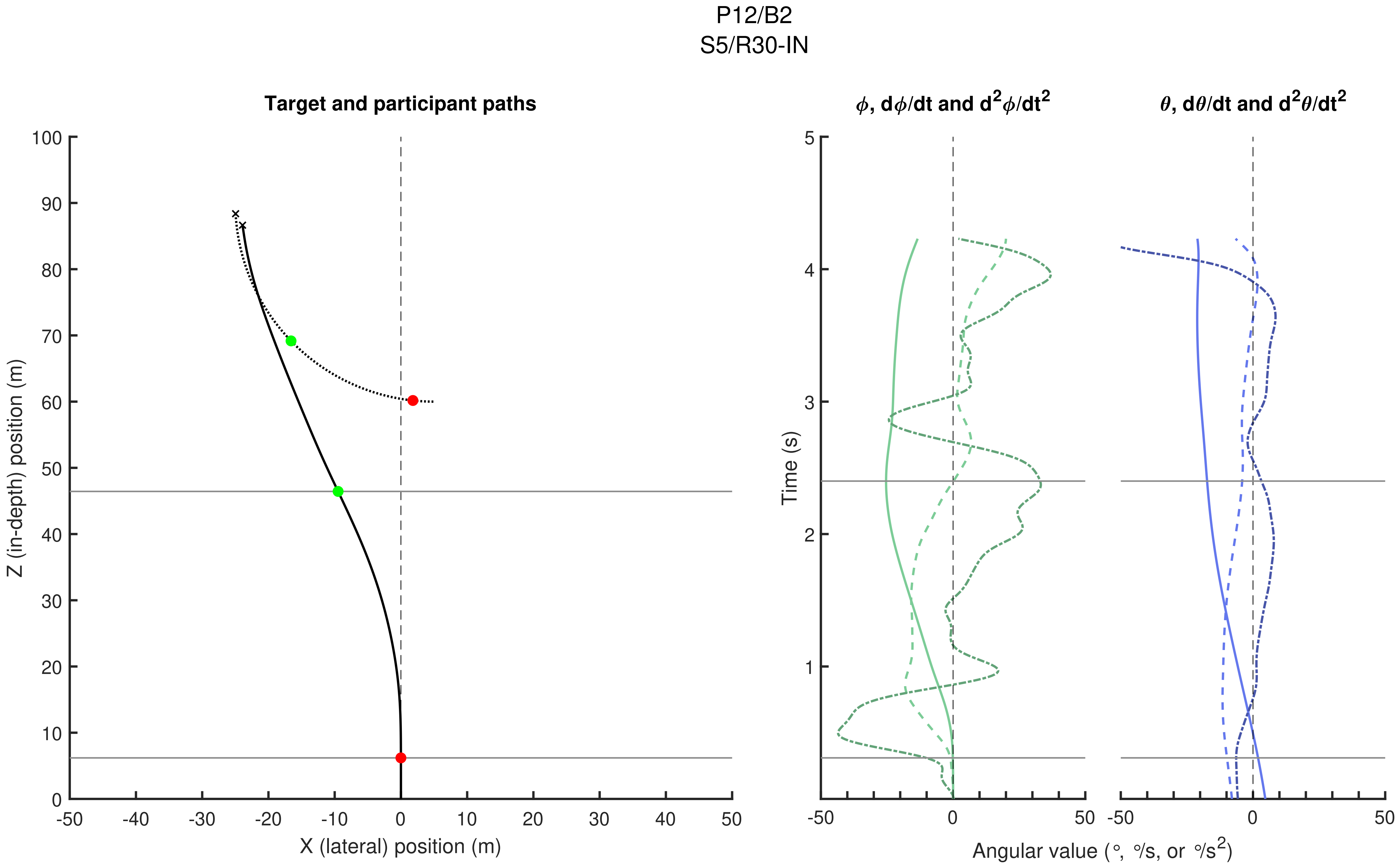
P12/B2
S5/R30-IN
Target and participant paths
Z (in-depth) position (m)
X (lateral) position (m)
100
90
80
70
60
50
40
30
20
10
0
-50
-40
-30
-20
-10
0
10
20
30
40
50
ϕ, dϕ/dt and d²ϕ/dt²
θ, dθ/dt and d²θ/dt²
Time (s)
5
4
3
2
1
-50
0
50
-50
0
50
Angular value (°, °/s, or °/s²)

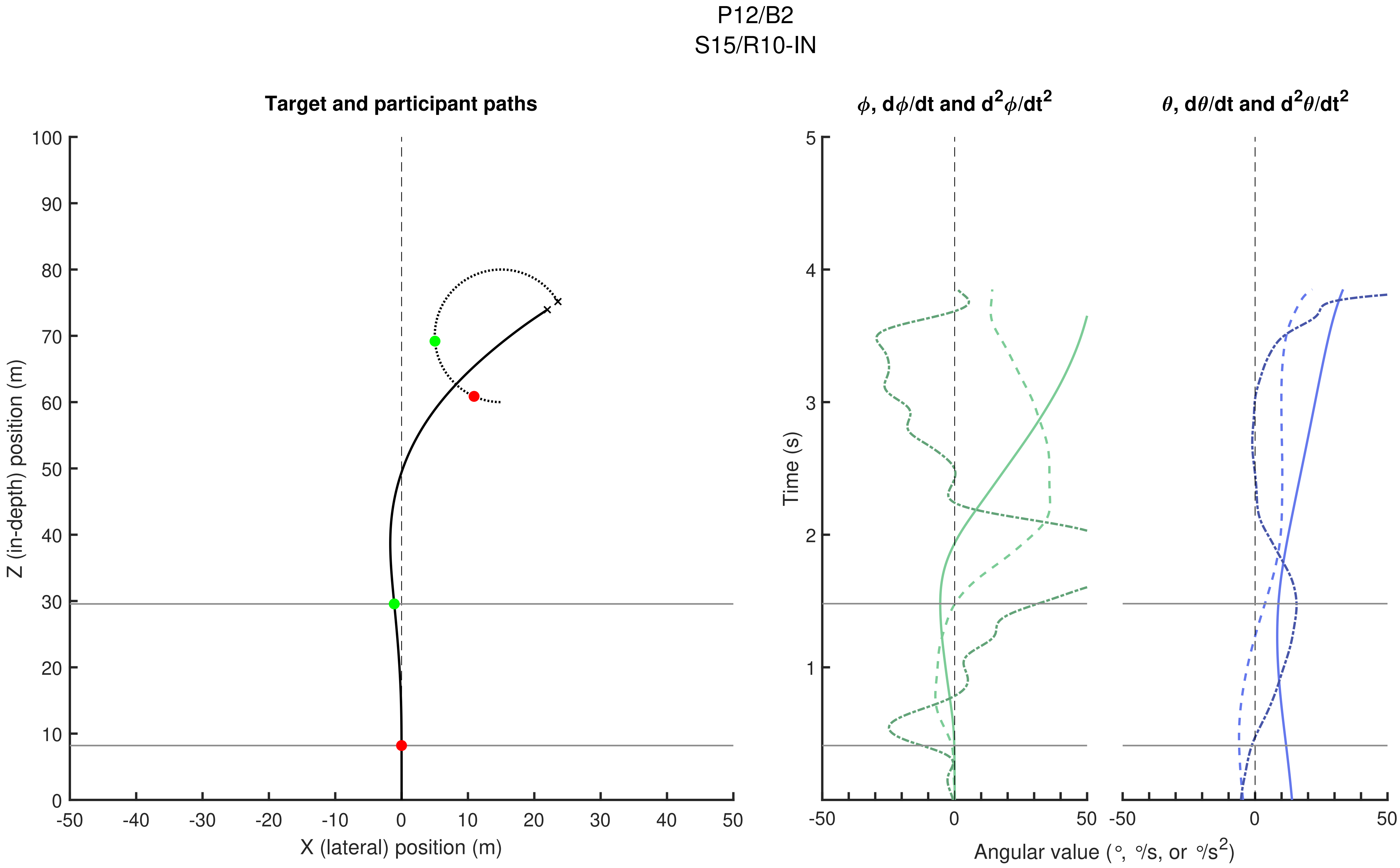
P12/B2
S15/R10-IN
Target and participant paths
ϕ, dϕ/dt and d²ϕ/dt²
θ, dθ/dt and d²θ/dt²
Z (in-depth) position (m)
X (lateral) position (m)
Time (s)
Angular value (°, °/s, or °/s²)
100
90
80
70
60
50
40
30
20
10
0
-50
-40
-30
-20
-10
0
10
20
30
40
50
5
4
3
2
1
-50
0
50
-50
0
50

P12/B2
S15/R20-IN

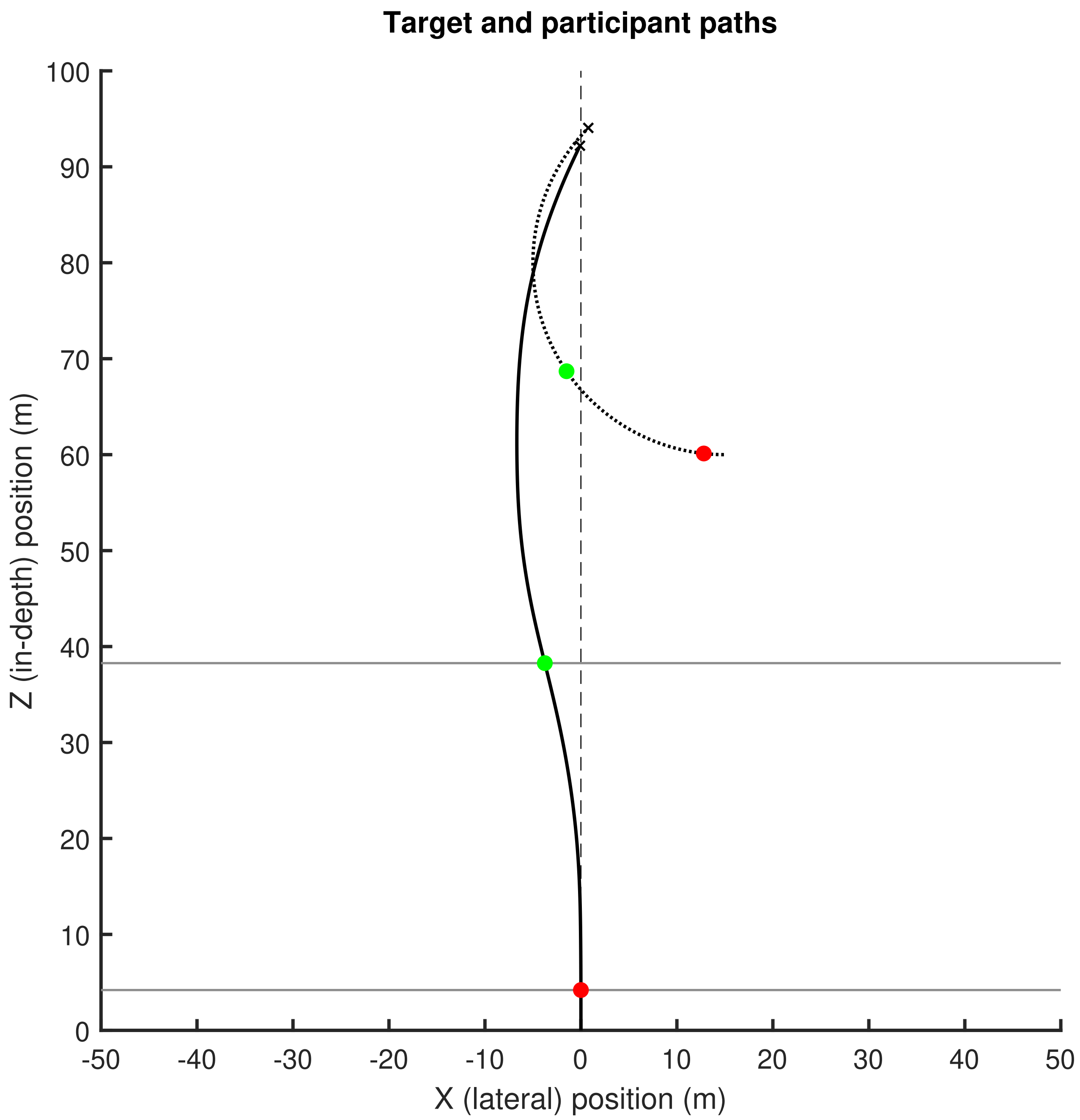

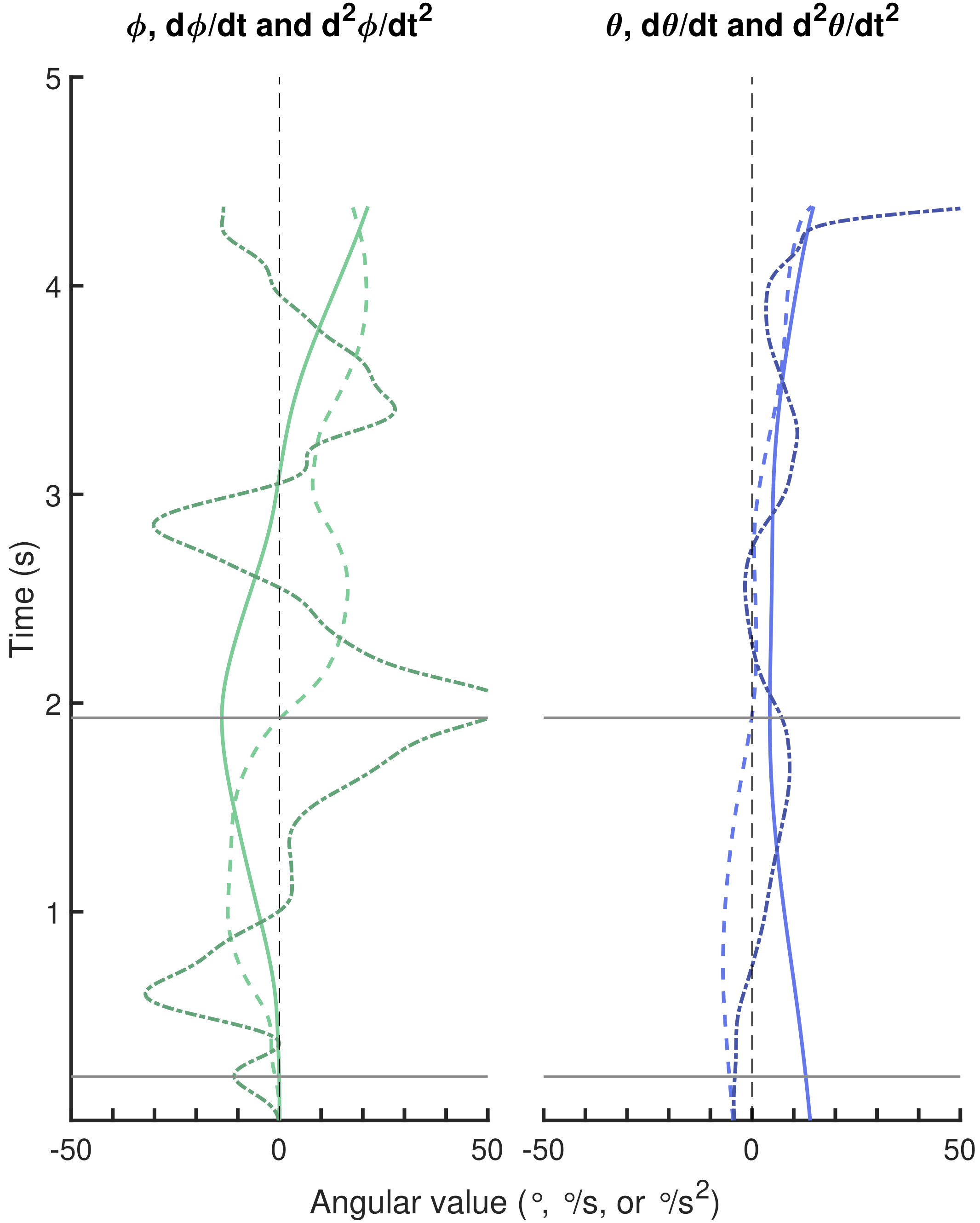

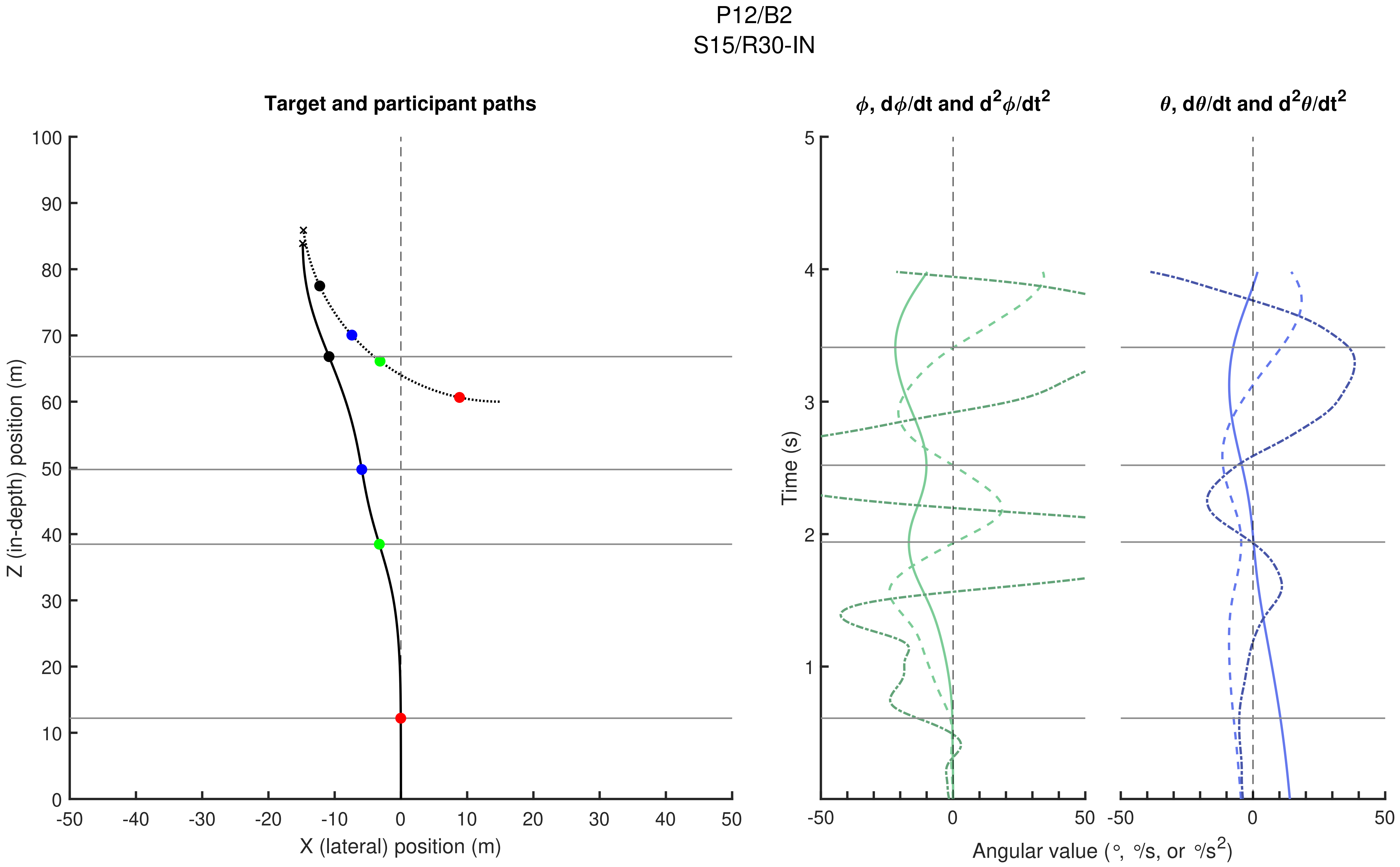
P12/B2
S15/R30-IN
Target and participant paths
Z (in-depth) position (m)
X (lateral) position (m)
100
90
80
70
60
50
40
30
20
10
0
-50
-40
-30
-20
-10
0
10
20
30
40
50
φ, dφ/dt and d²φ/dt²
θ, dθ/dt and d²θ/dt²
Time (s)
5
4
3
2
1
Angular value (°, °/s, or °/s²)

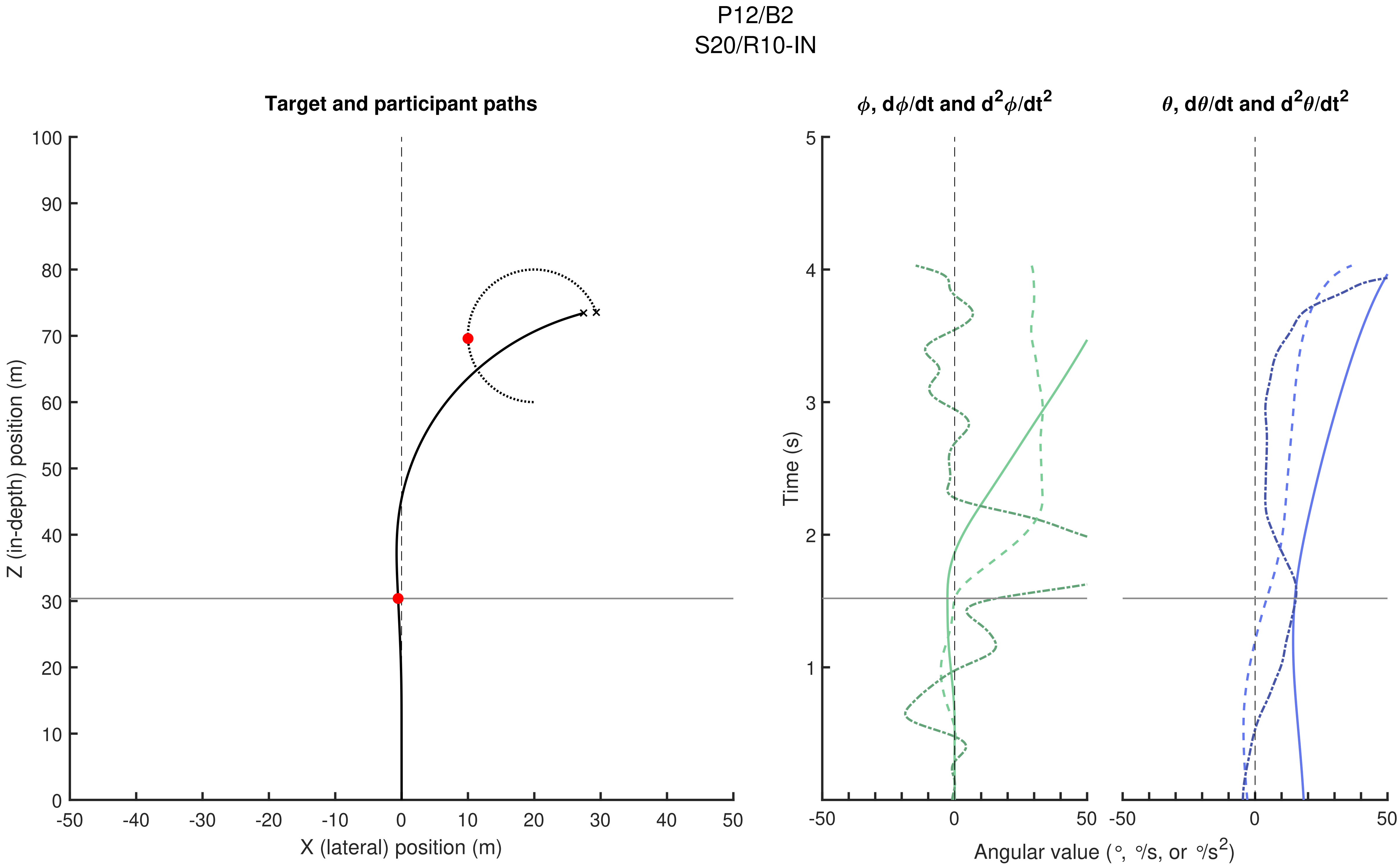
P12/B2
S20/R10-IN
Target and participant paths
Z (in-depth) position (m)
X (lateral) position (m)
ϕ, dϕ/dt and d²ϕ/dt²
θ, dθ/dt and d²θ/dt²
Time (s)
Angular value (°, °/s, or °/s²)
100
90
80
70
60
50
40
30
20
10
0
-50
-40
-30
-20
-10
0
10
20
30
40
50
5
4
3
2
1
-50
0
50
-50
0
50

P12/B2
S20/R20-IN

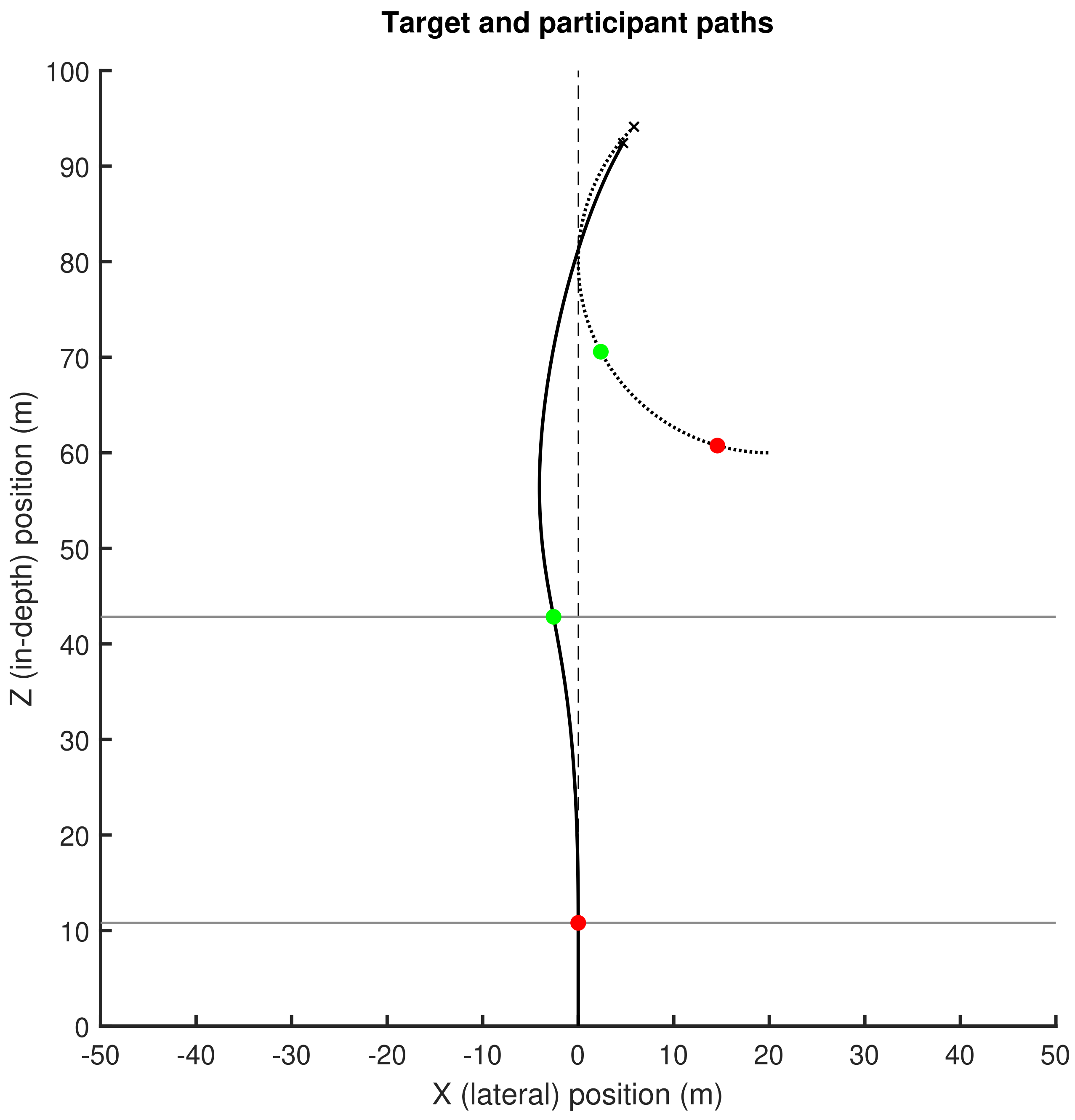

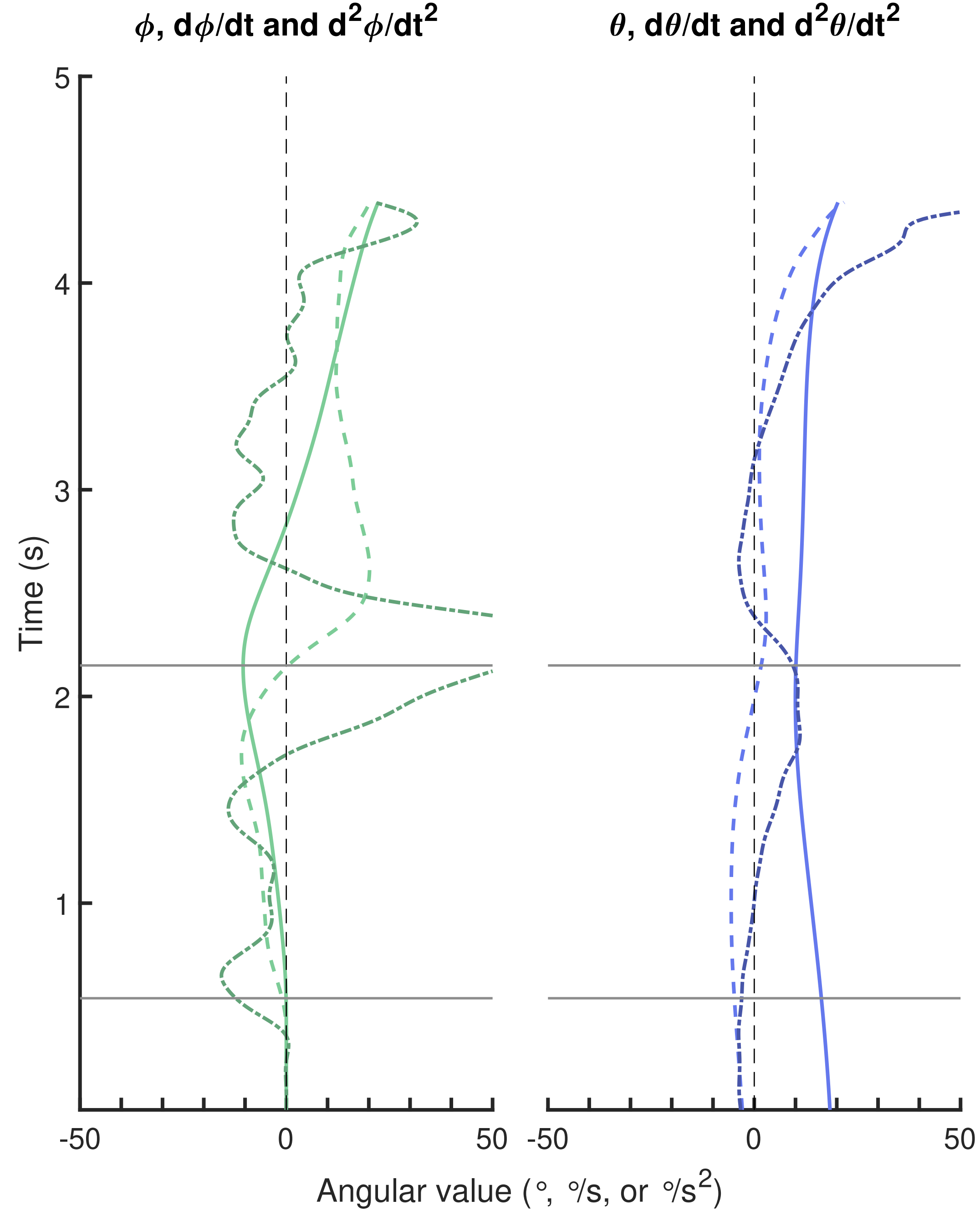

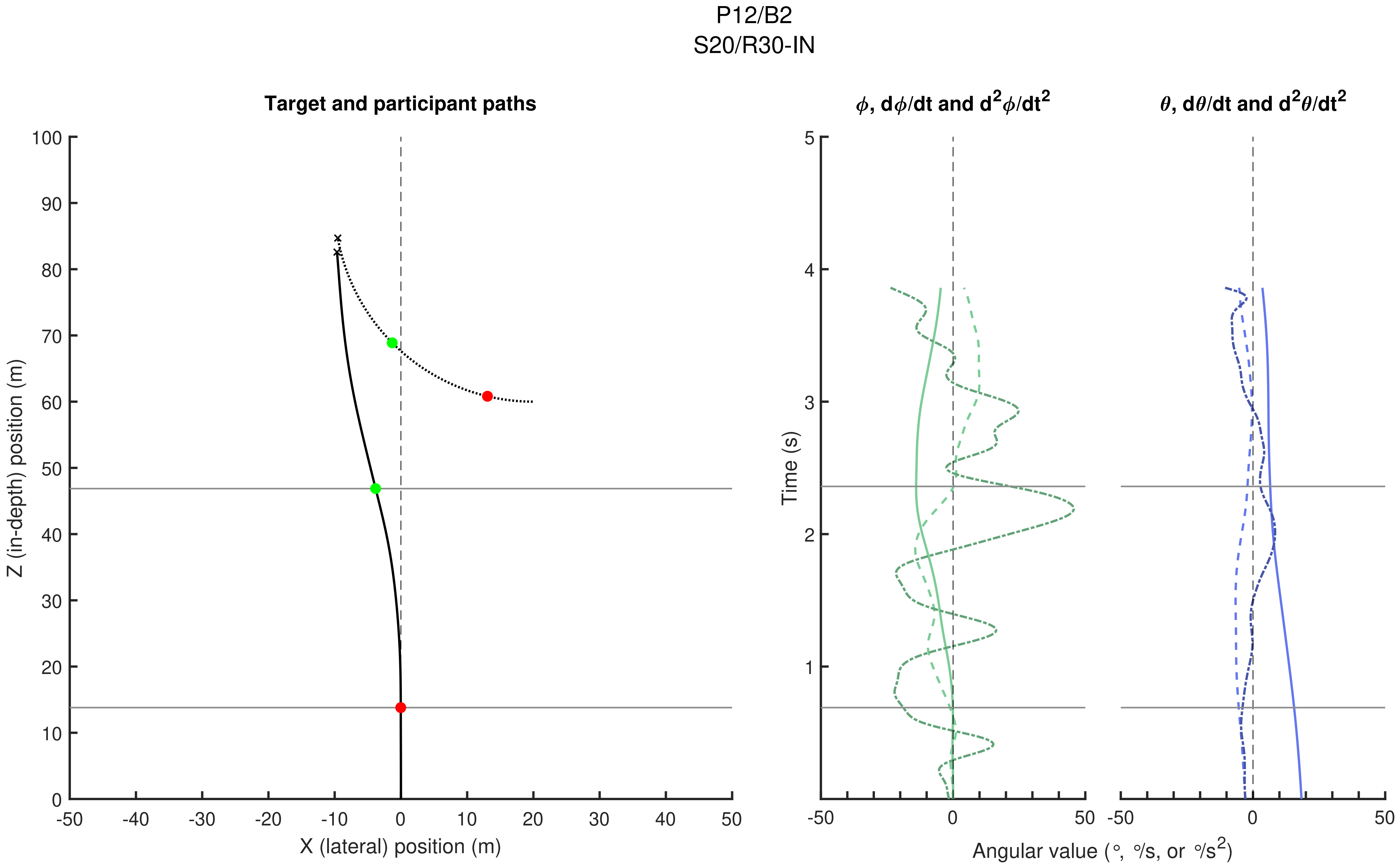
P12/B2
S20/R30-IN
Target and participant paths
Z (in-depth) position (m)
X (lateral) position (m)
ϕ, dϕ/dt and d$^2\phi$/dt^2
θ, dθ/dt and d$^2\theta$/dt^2
Time (s)
Angular value (°, °/s, or °/s^2)

P12/B3
S20/R10-IN

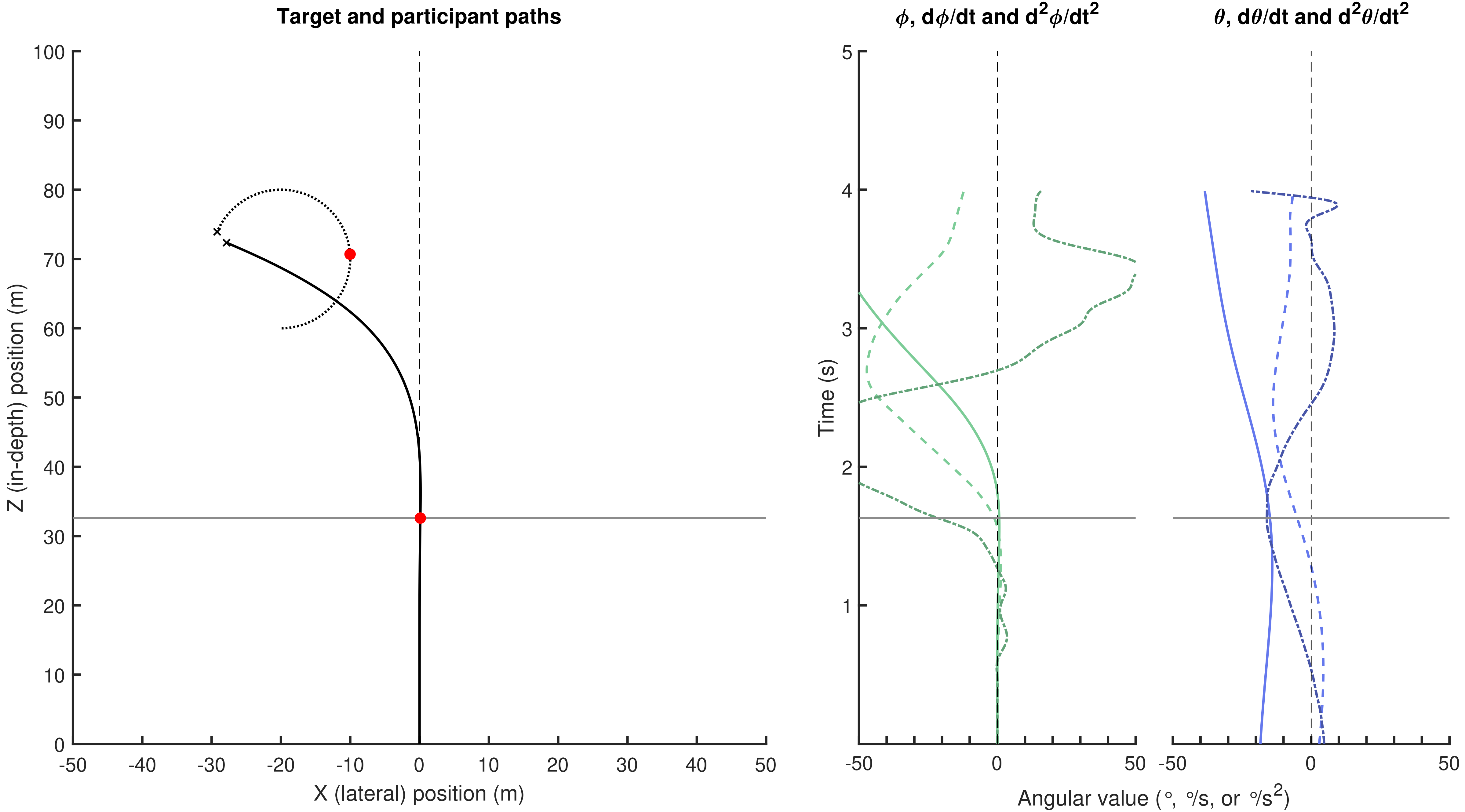

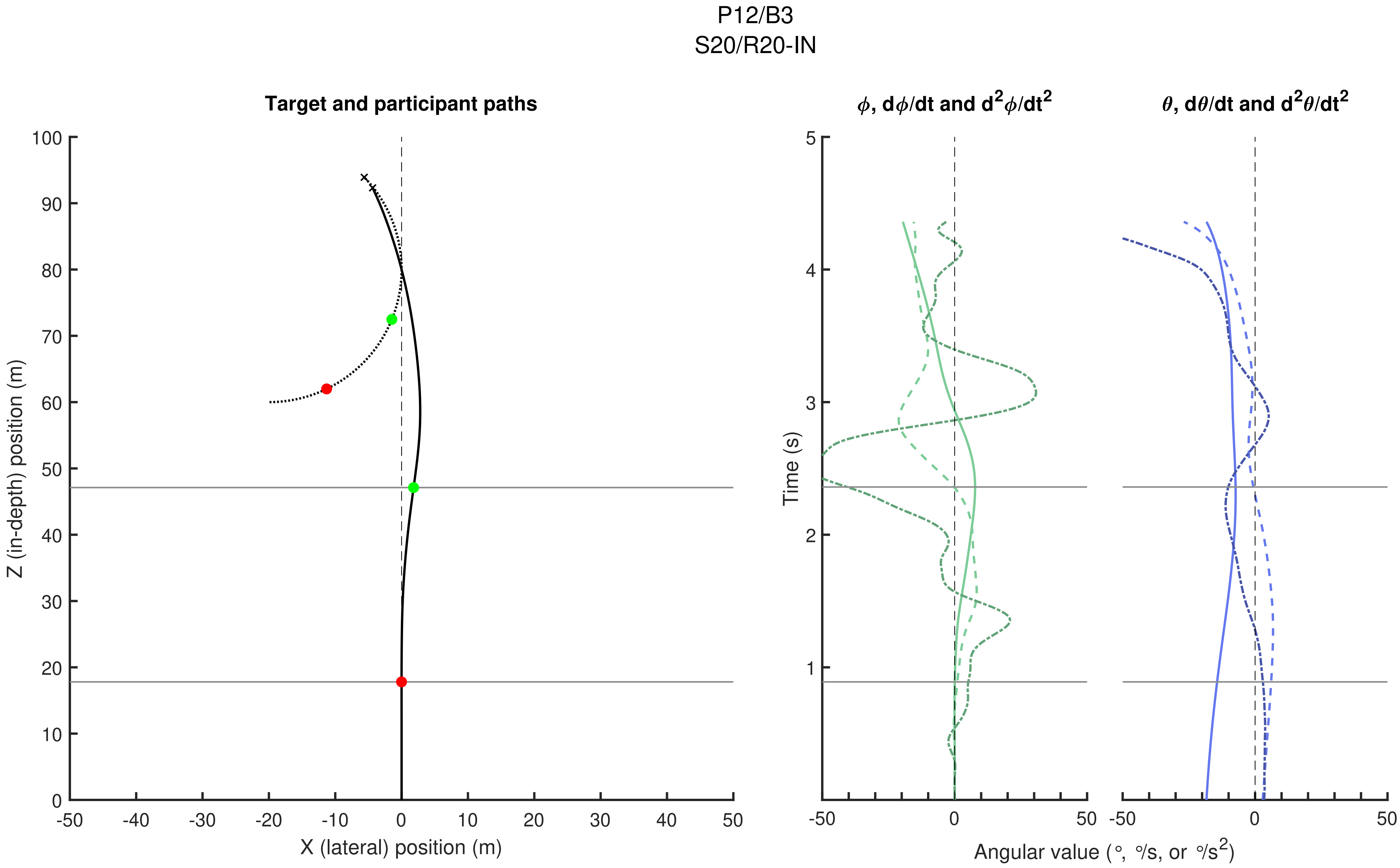
P12/B3
S20/R20-IN
Target and participant paths
Z (in-depth) position (m)
X (lateral) position (m)
ϕ, dϕ/dt and d$^2\phi$/dt^2
θ, dθ/dt and d$^2\theta$/dt^2
Time (s)
Angular value (°, °/s, or °/s^2)

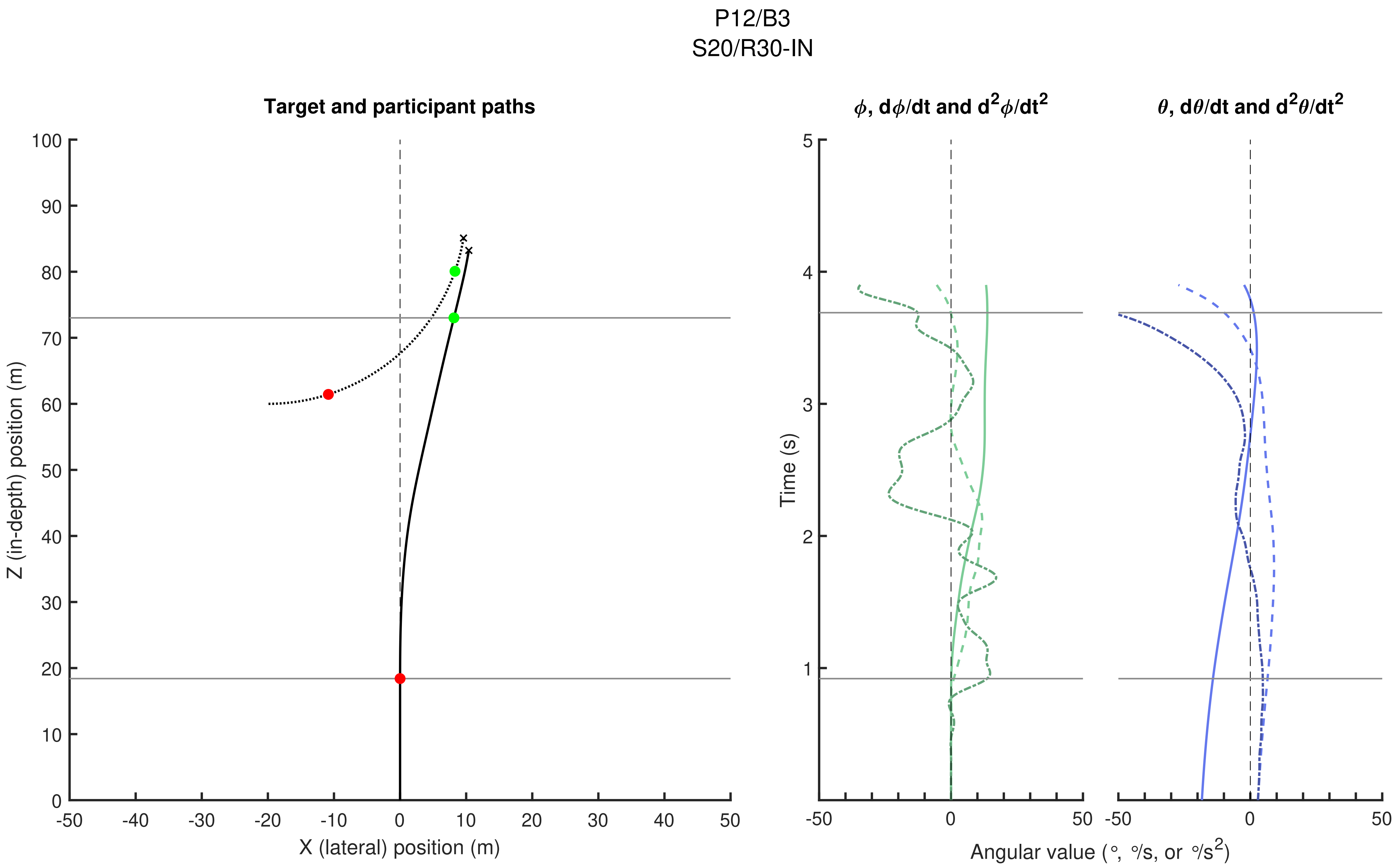

P12/B3
S20/R30-IN
Target and participant paths
Z (in-depth) position (m)
X (lateral) position (m)
ϕ, dϕ/dt and d$^2\phi$/dt^2
θ, dθ/dt and d$^2\theta$/dt^2
Time (s)
Angular value (°, °/s, or °/s^2)

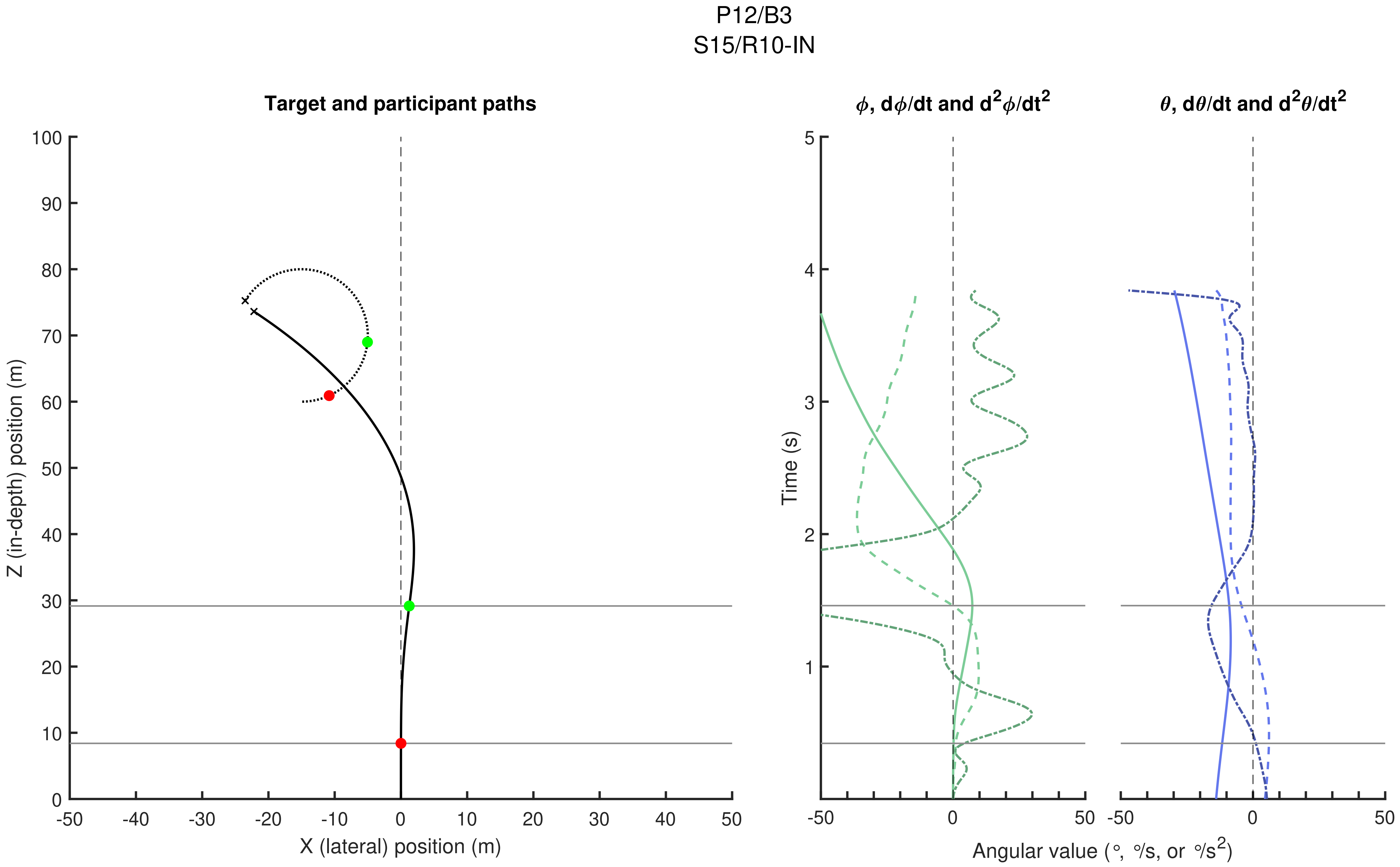
P12/B3
S15/R10-IN
Target and participant paths
Z (in-depth) position (m)
X (lateral) position (m)
ϕ, dϕ/dt and d²ϕ/dt²
θ, dθ/dt and d²θ/dt²
Time (s)
Angular value (°, °/s, or °/s²)

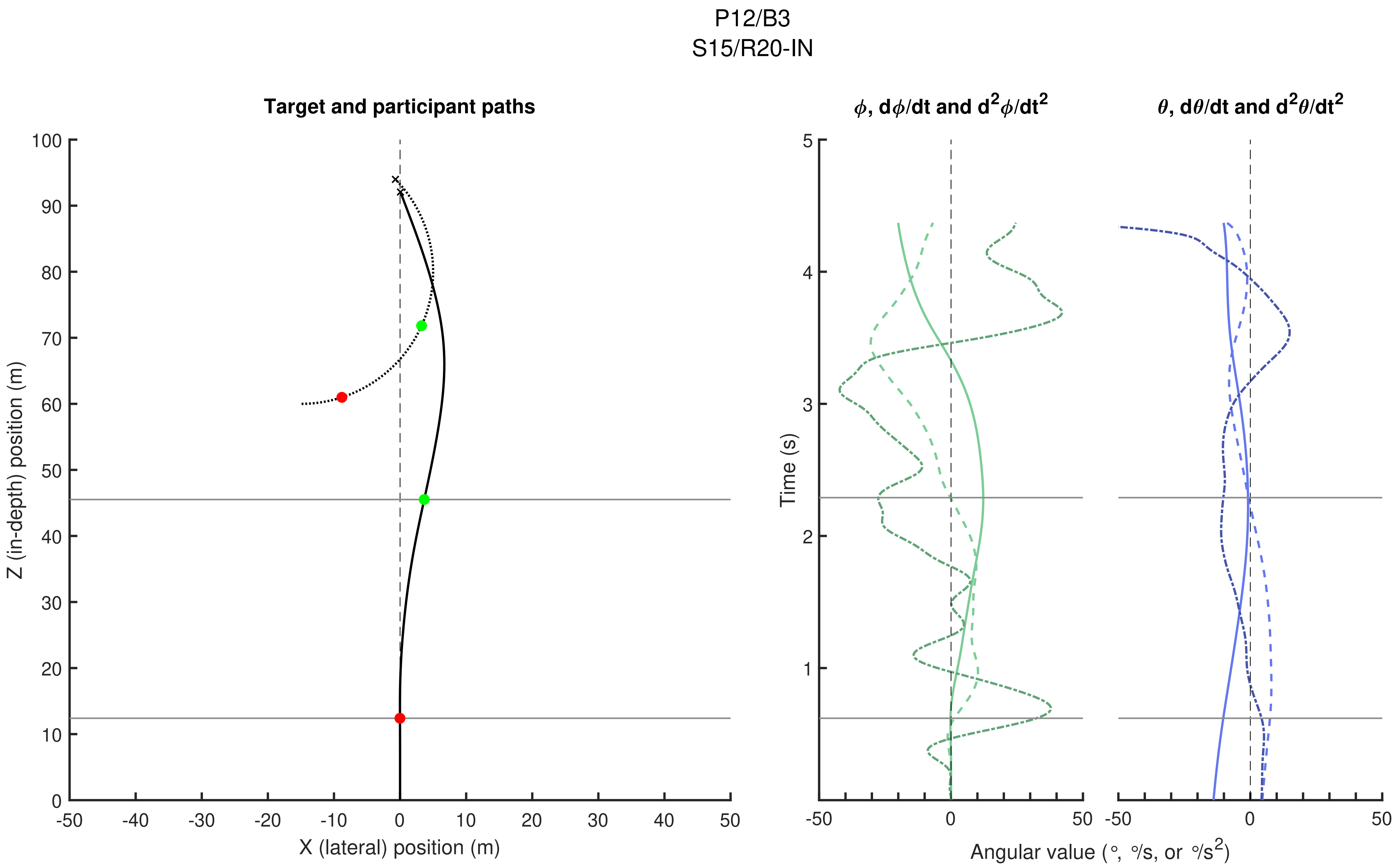
P12/B3
S15/R20-IN
Target and participant paths
Z (in-depth) position (m)
X (lateral) position (m)
100
90
80
70
60
50
40
30
20
10
0
-50
-40
-30
-20
-10
0
10
20
30
40
50
φ, dφ/dt and d²φ/dt²
θ, dθ/dt and d²θ/dt²
Time (s)
5
4
3
2
1
-50
0
50
Angular value (°, °/s, or °/s²)

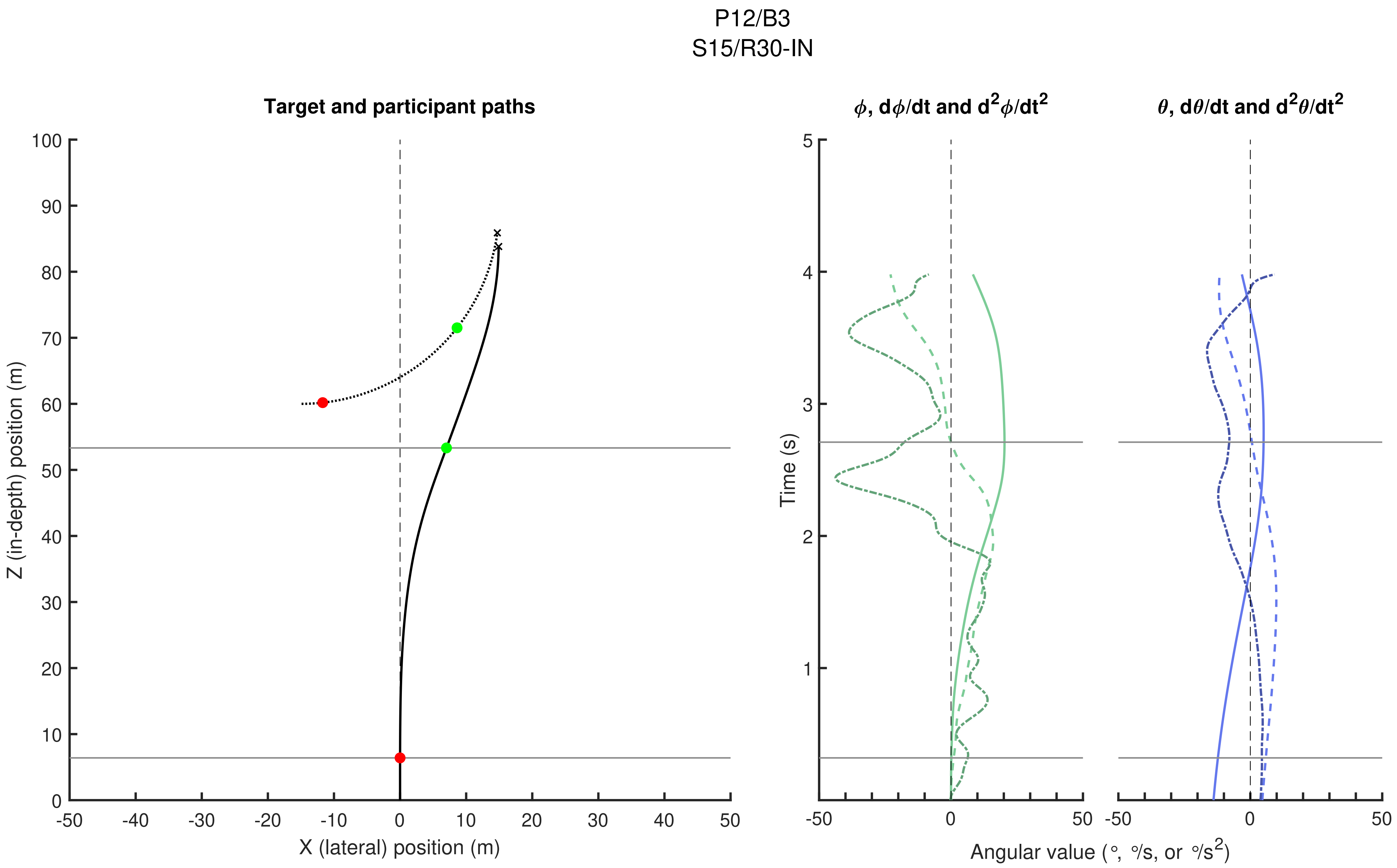

P12/B3
S15/R30-IN
Target and participant paths
ϕ, dϕ/dt and d$^2\phi$/dt^2
θ, dθ/dt and d$^2\theta$/dt^2
Z (in-depth) position (m)
X (lateral) position (m)
Time (s)
Angular value (°, °/s, or °/s^2)

P12/B3
S5/R10-IN

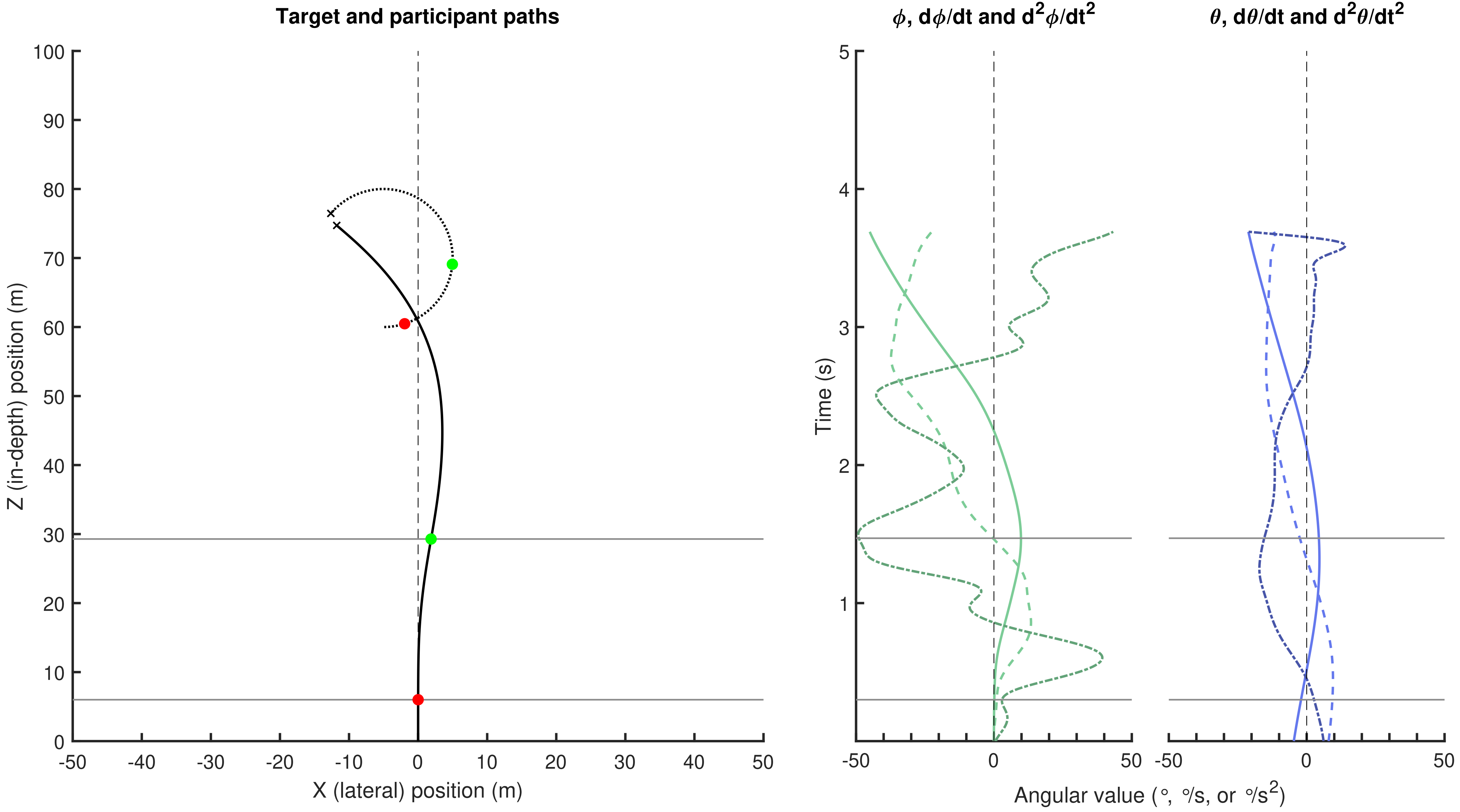

P12/B3
S5/R10-OUT

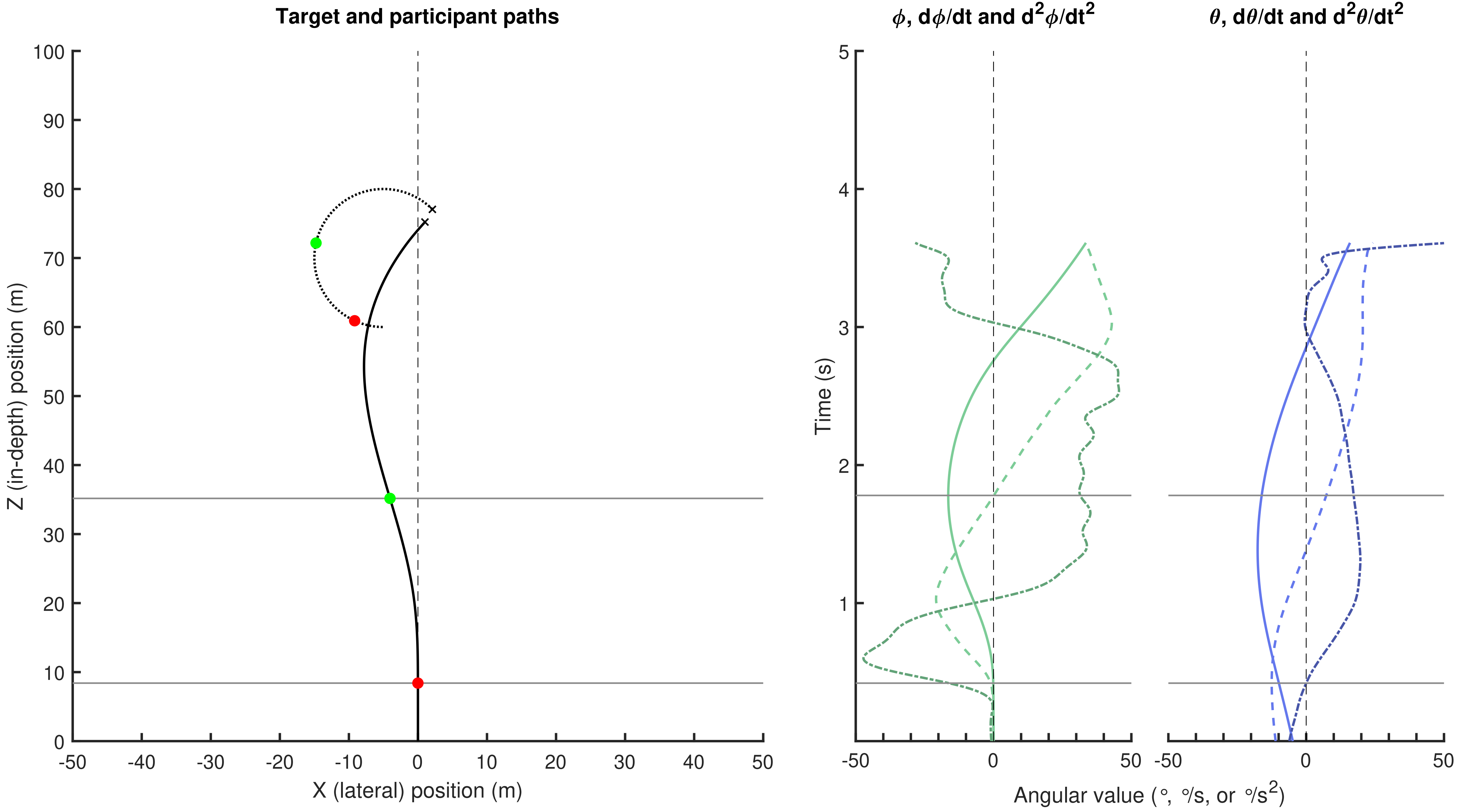

P12/B3
S5/R20-IN

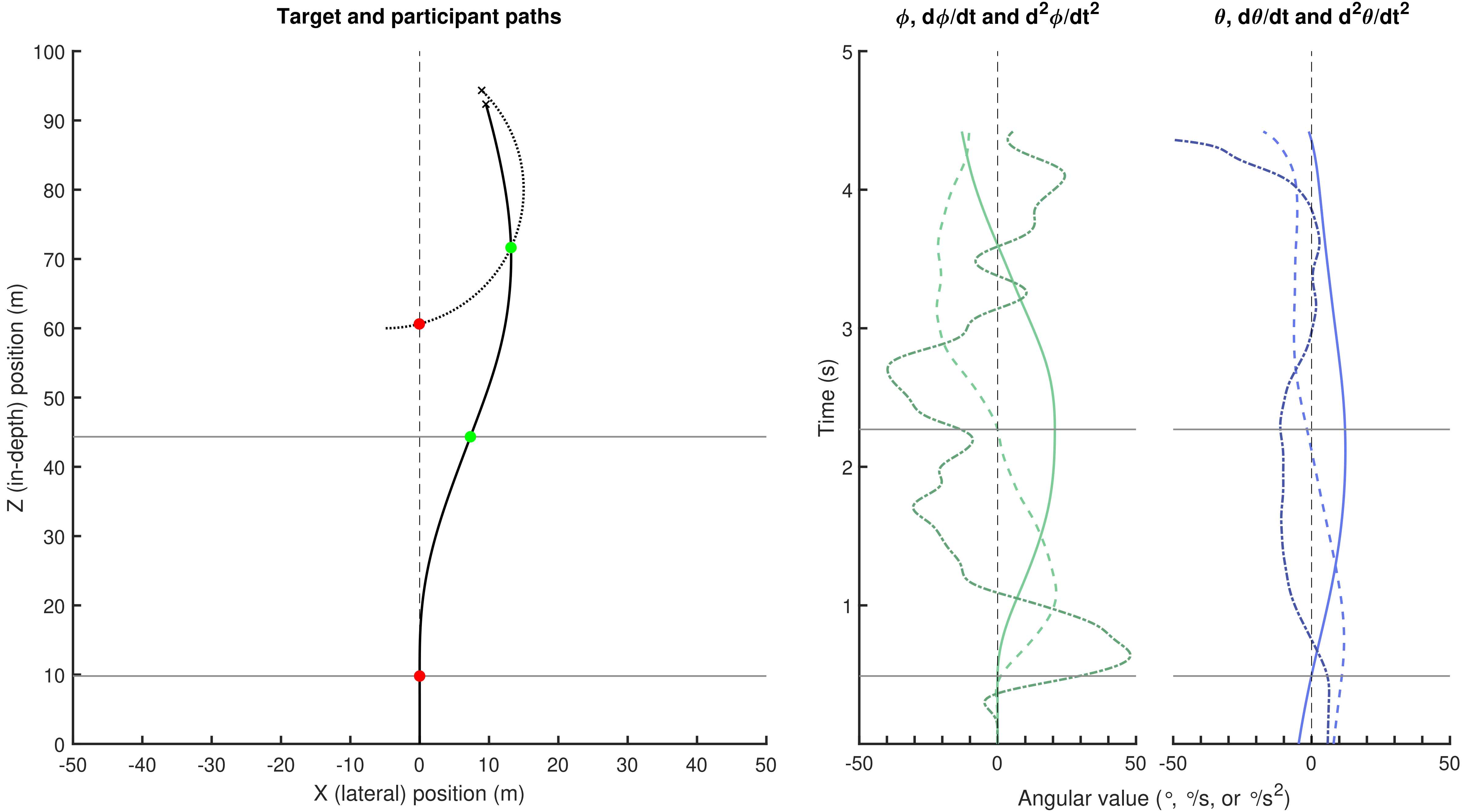

P12/B3
S5/R20-OUT

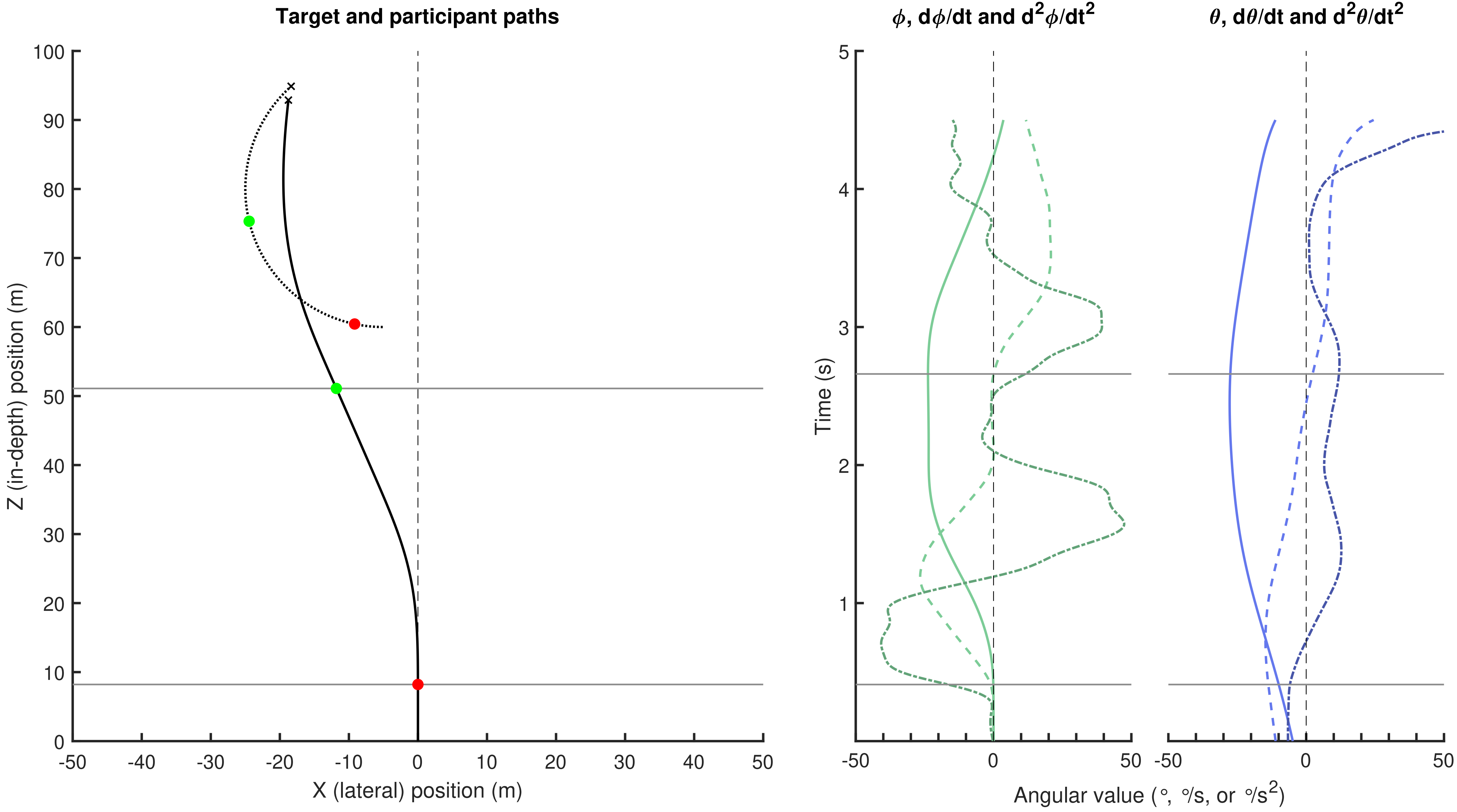

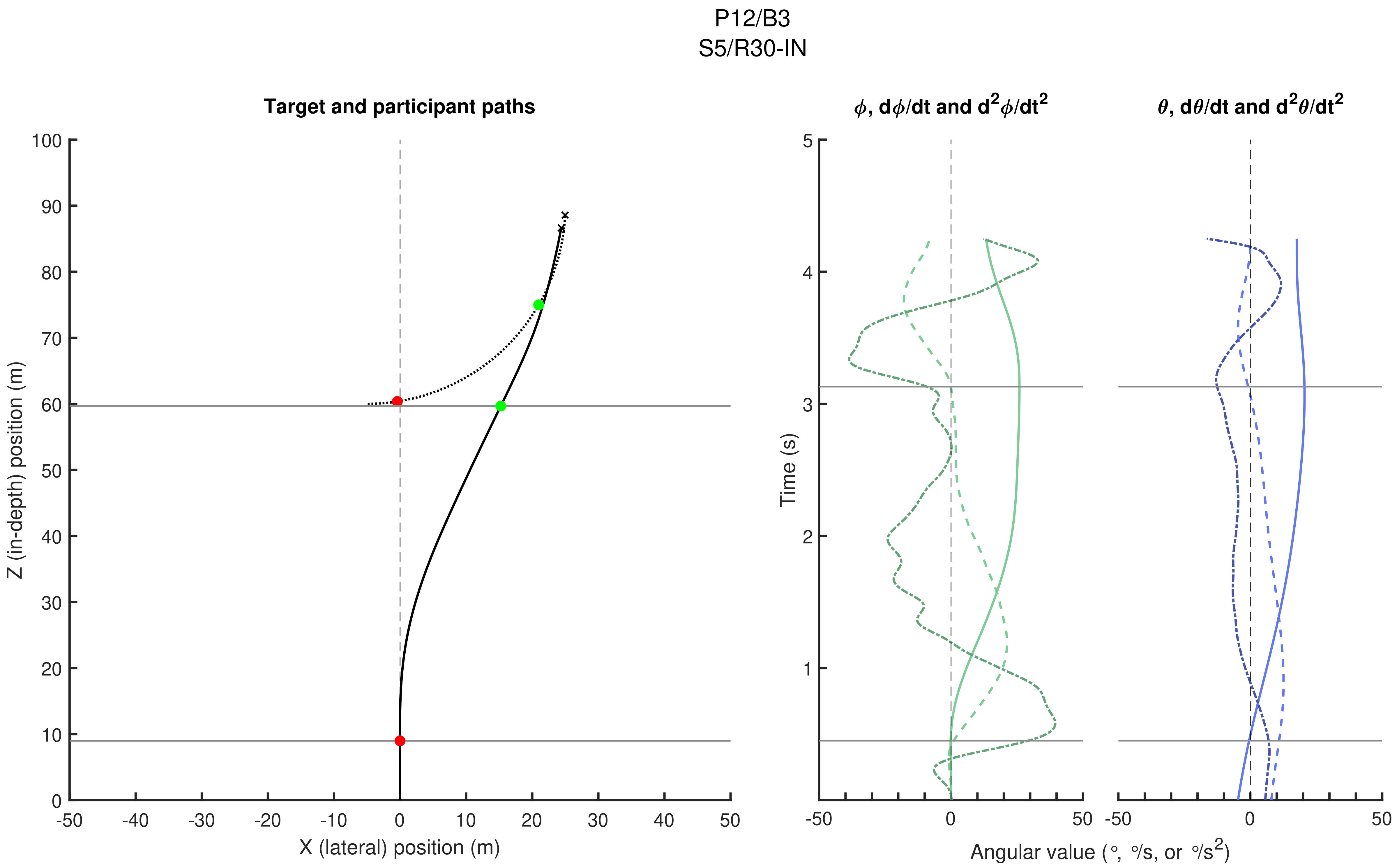
P12/B3
S5/R30-IN
Target and participant paths
Z (in-depth) position (m)
X (lateral) position (m)
φ, dφ/dt and d²φ/dt²
θ, dθ/dt and d²θ/dt²
Time (s)
Angular value (°, °/s, or °/s²)

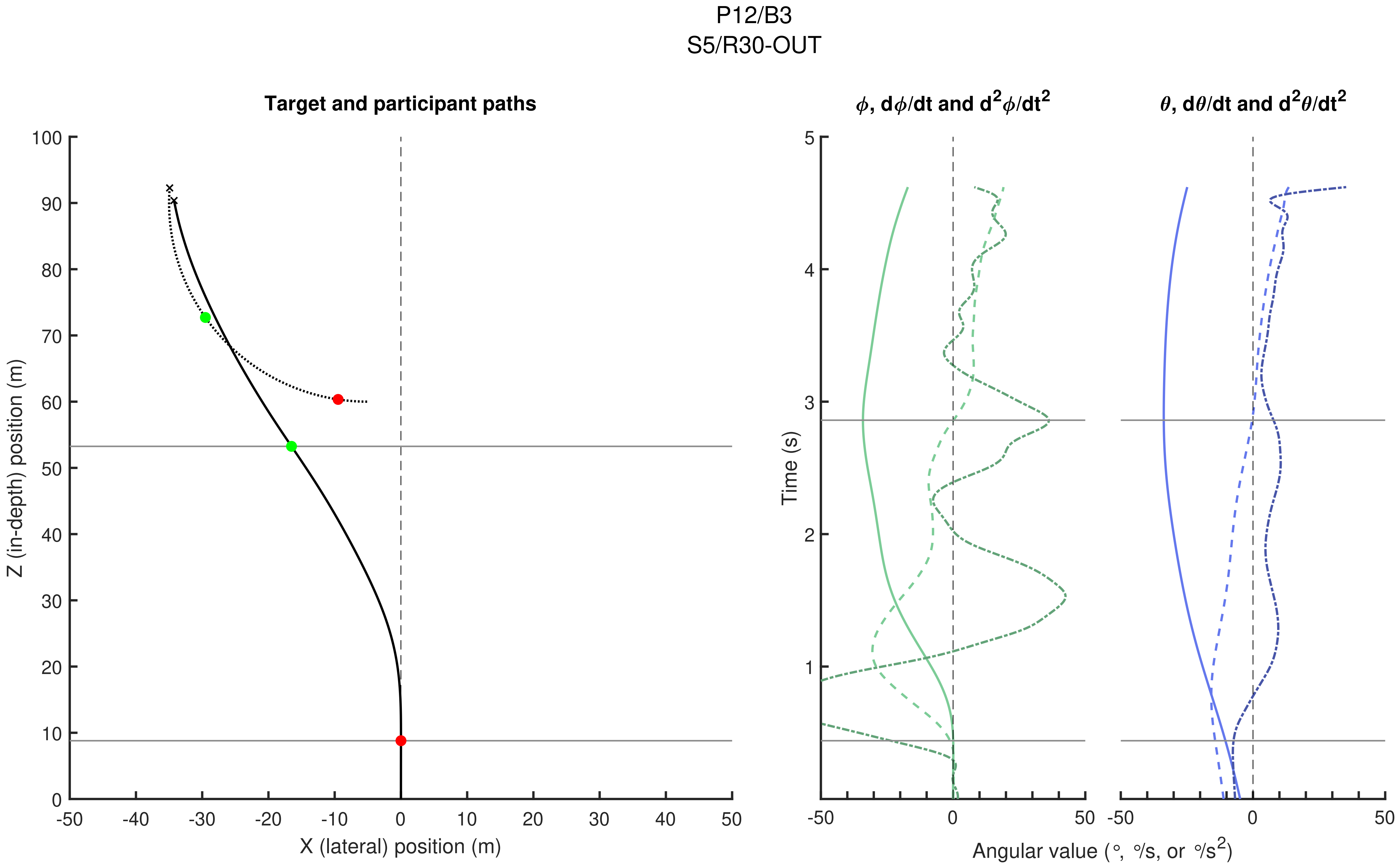
P12/B3
S5/R30-OUT
Target and participant paths
Z (in-depth) position (m)
X (lateral) position (m)
ϕ, dϕ/dt and d$^2\phi$/dt^2
θ, dθ/dt and d$^2\theta$/dt^2
Time (s)
Angular value (°, °/s, or °/s^2)

P12/B3
S5/R10-OUT

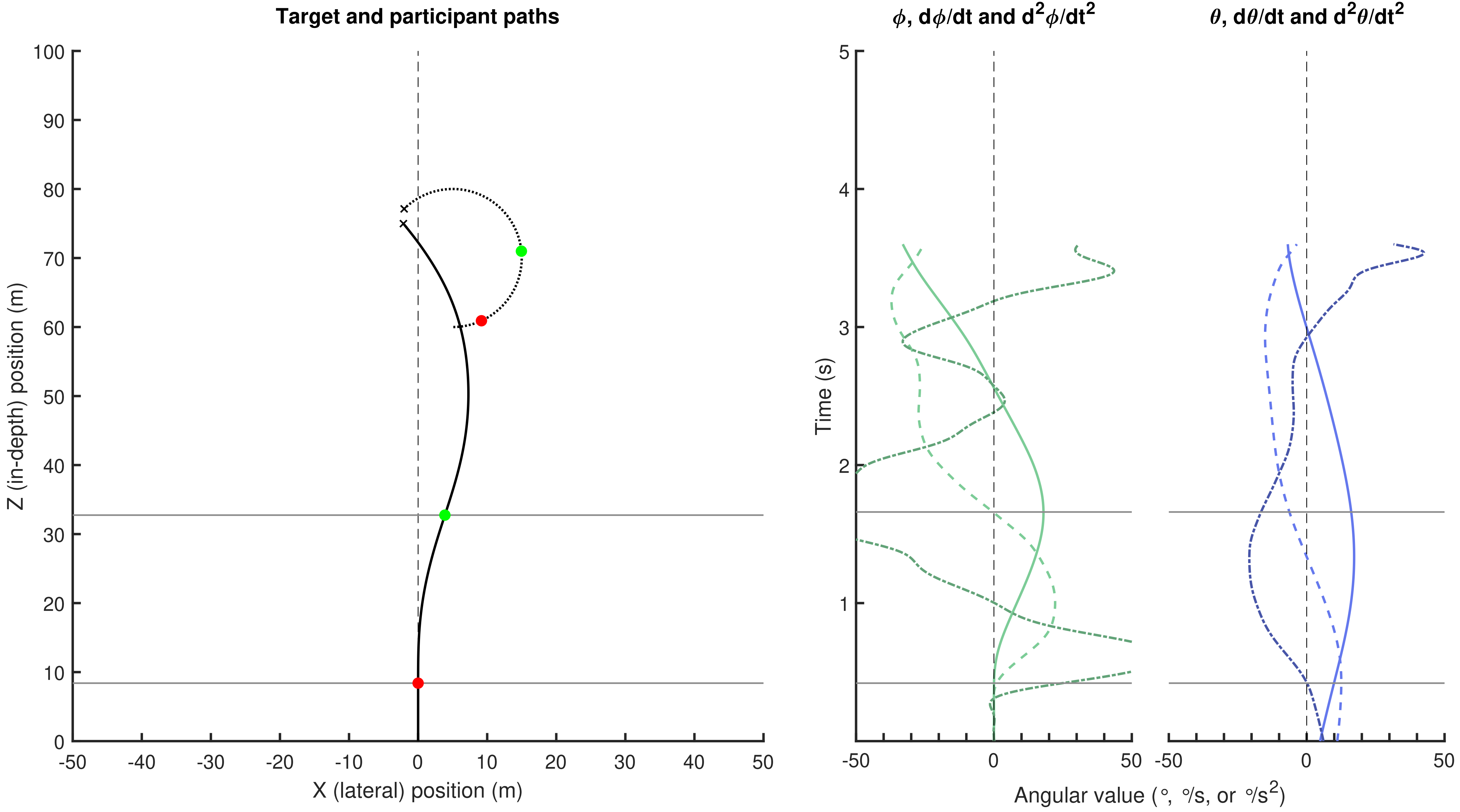

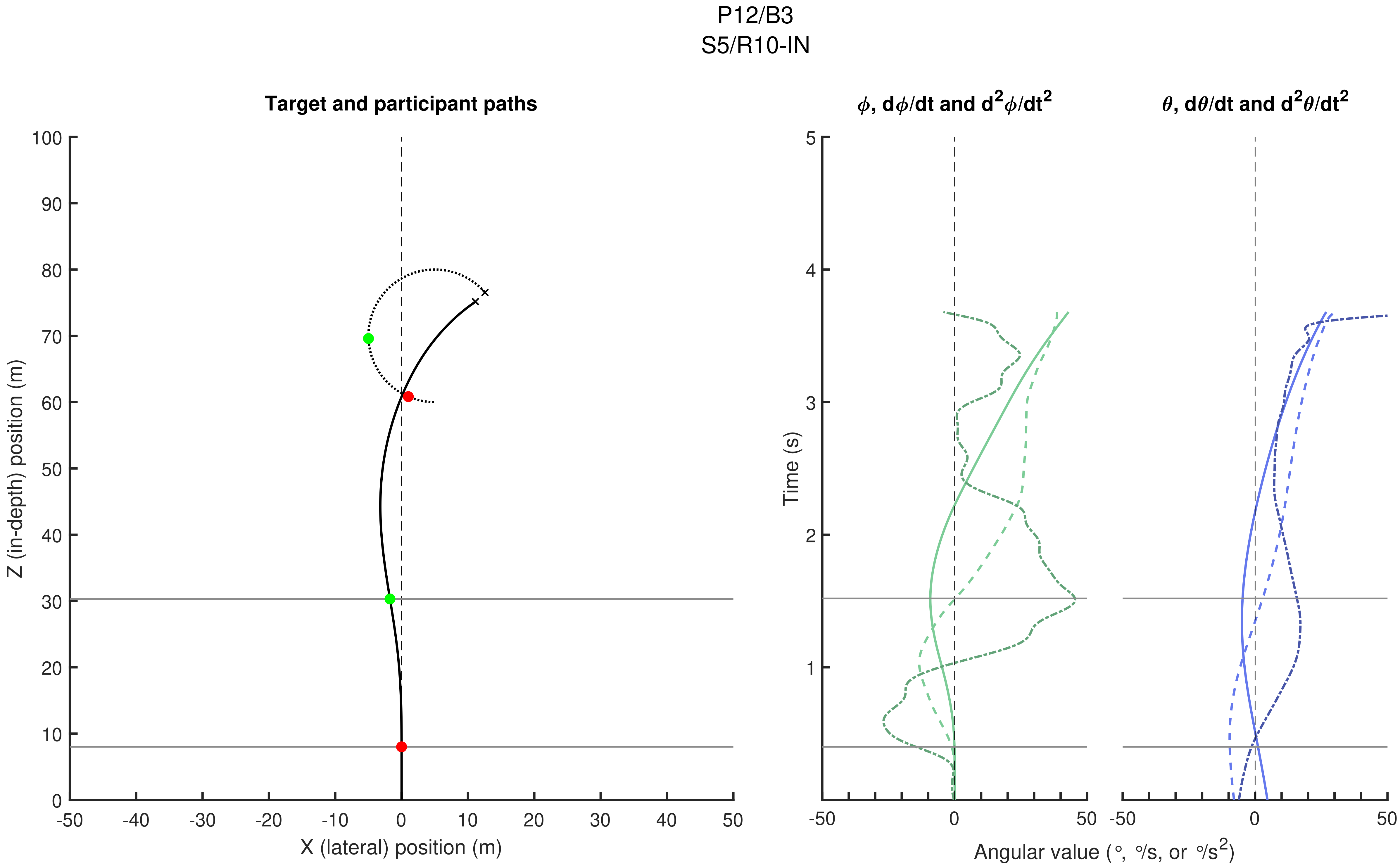

P12/B3
S5/R10-IN
Target and participant paths
Z (in-depth) position (m)
X (lateral) position (m)
ϕ, dϕ/dt and d$^2\phi$/dt^2
θ, dθ/dt and d$^2\theta$/dt^2
Time (s)
Angular value (°, °/s, or °/s^2)

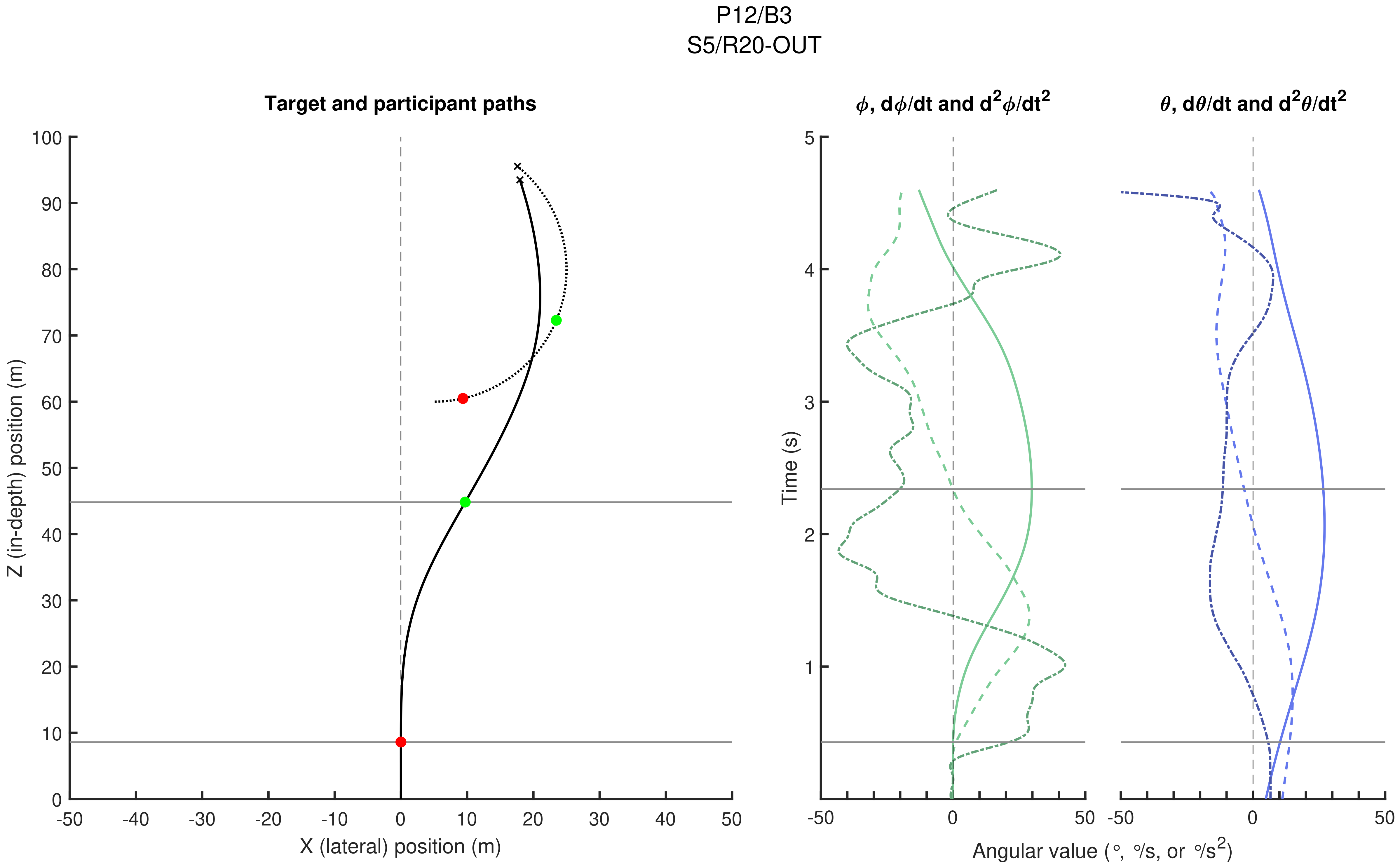

P12/B3
S5/R20-OUT
Target and participant paths
Z (in-depth) position (m)
X (lateral) position (m)
-50
-40
-30
-20
-10
0
10
20
30
40
50
60
70
80
90
100
ϕ, dϕ/dt and d²ϕ/dt²
θ, dθ/dt and d²θ/dt²
Time (s)
1
2
3
4
5
Angular value (°, °/s, or °/s²)

P12/B3
S5/R20-IN

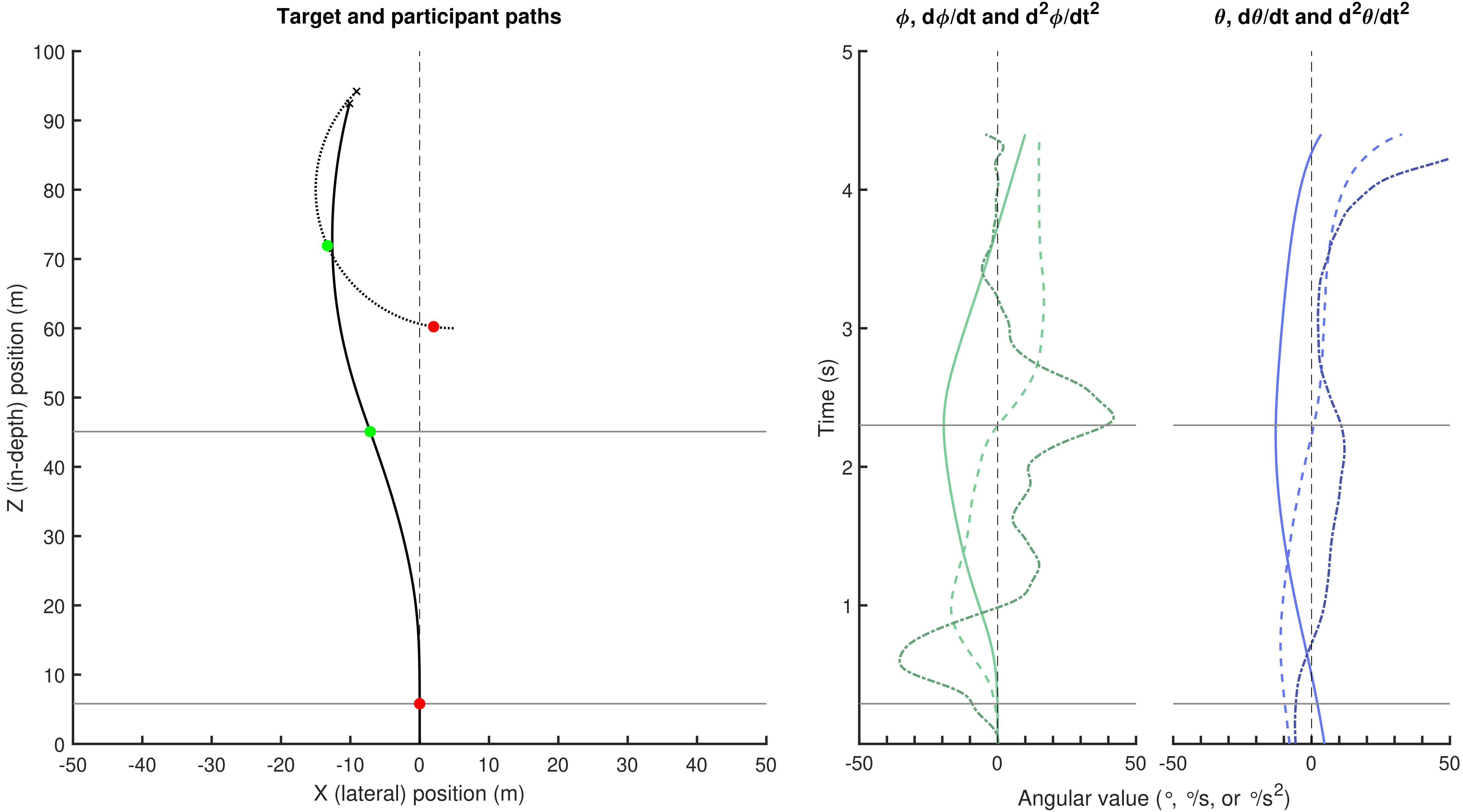

P12/B3
S5/R30-OUT

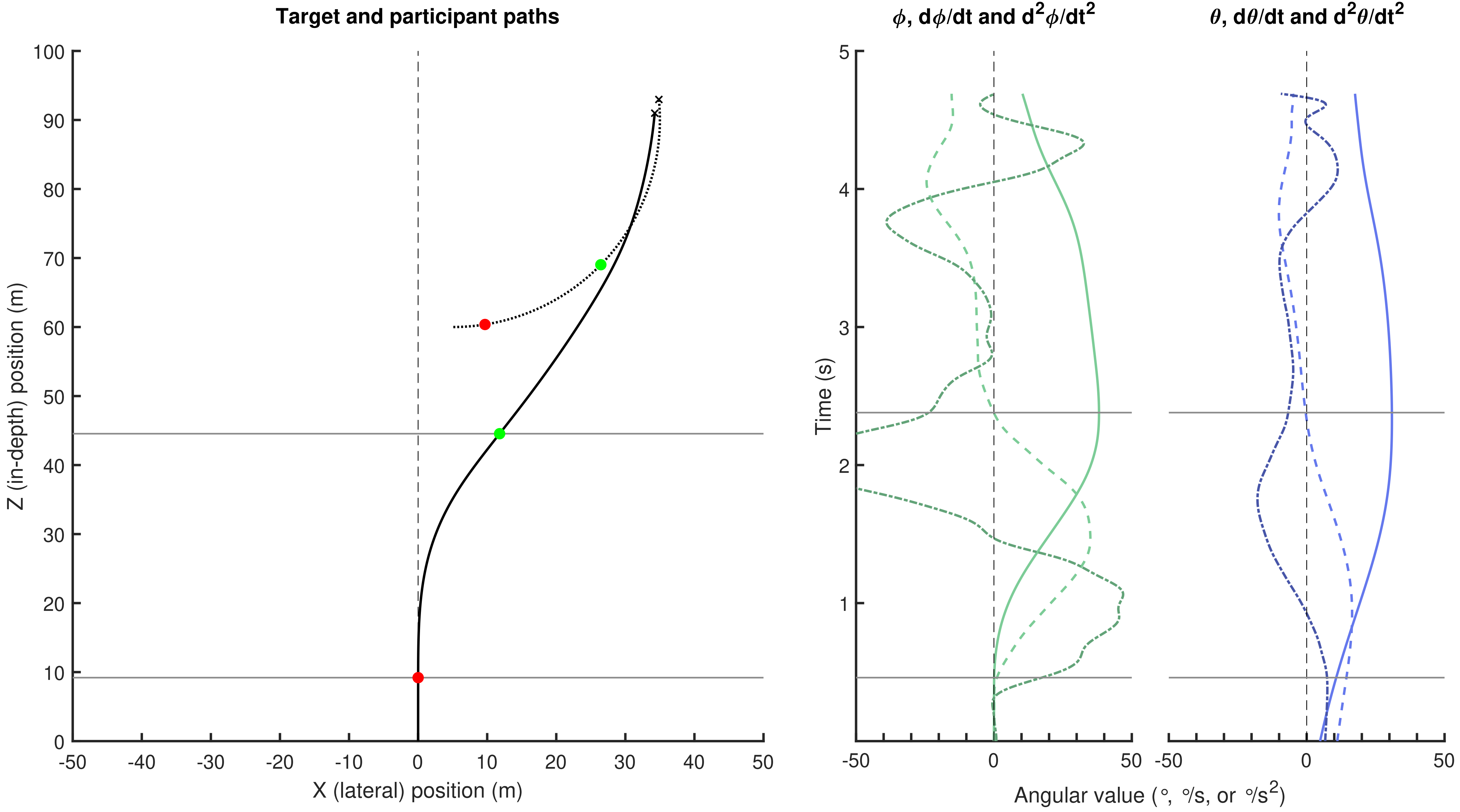

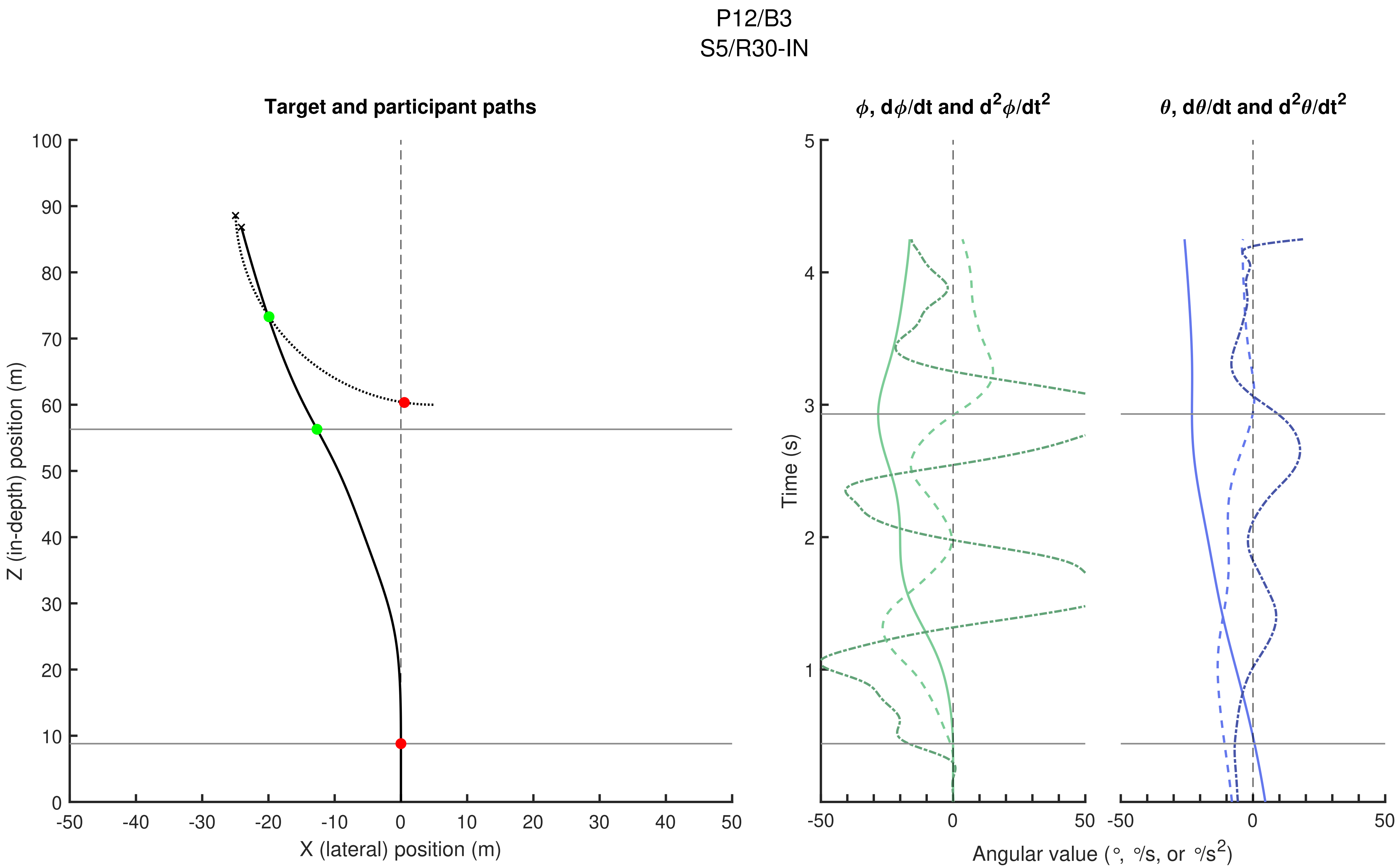
P12/B3
S5/R30-IN
Target and participant paths
Z (in-depth) position (m)
X (lateral) position (m)
ϕ, dϕ/dt and d$^2\phi$/dt^2
θ, dθ/dt and d$^2\theta$/dt^2
Time (s)
Angular value (°, °/s, or °/s^2)

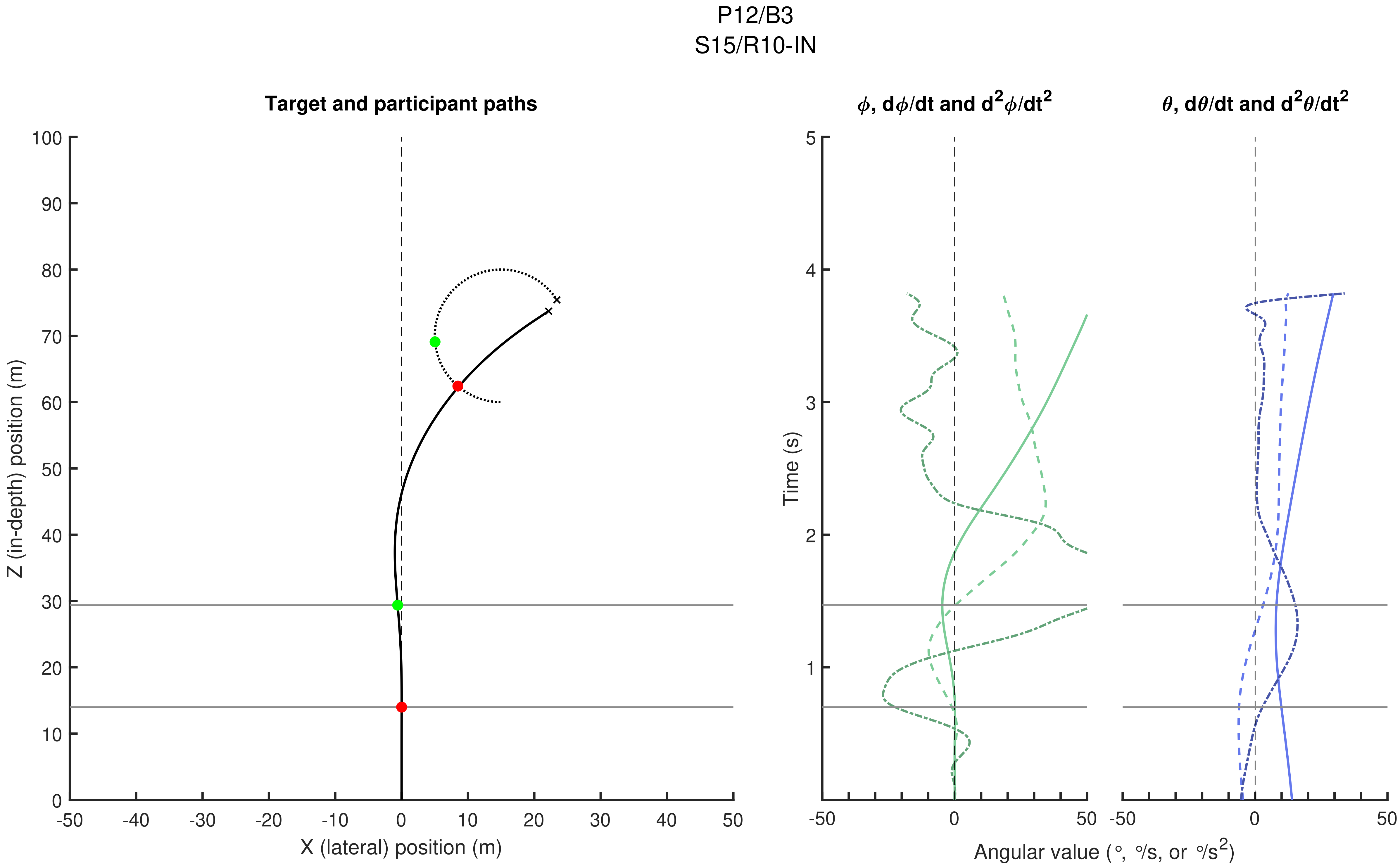
P12/B3
S15/R10-IN
Target and participant paths
Z (in-depth) position (m)
X (lateral) position (m)
100
90
80
70
60
50
40
30
20
10
0
-50
-40
-30
-20
-10
0
10
20
30
40
50
ϕ, dϕ/dt and d$^2\phi$/dt^2
θ, dθ/dt and d$^2\theta$/dt^2
Time (s)
5
4
3
2
1
-50
0
50
-50
0
50
Angular value (°, °/s, or °/s^2)

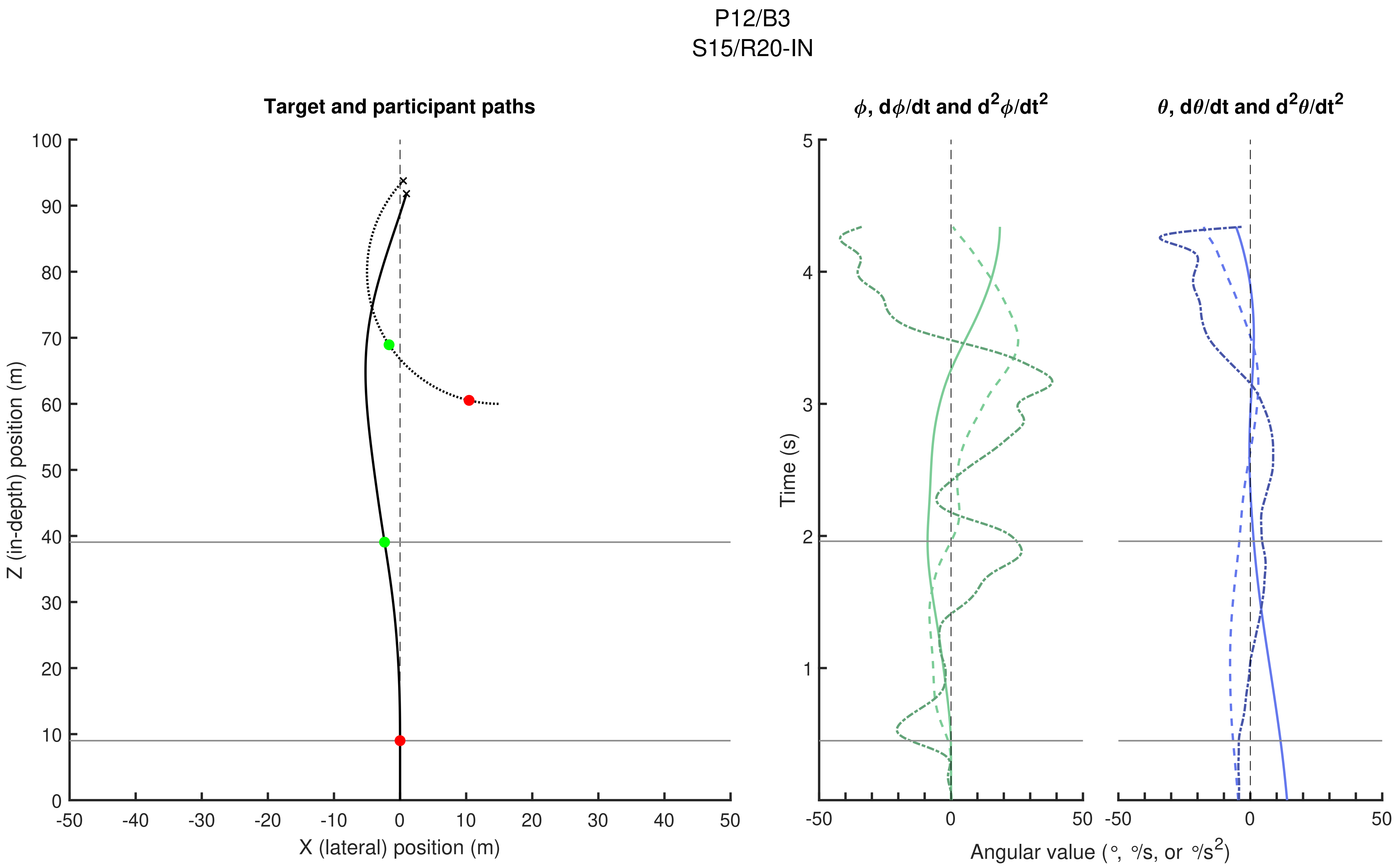
P12/B3
S15/R20-IN
Target and participant paths
ϕ, dϕ/dt and d$^2\phi$/dt^2
θ, dθ/dt and d$^2\theta$/dt^2
Z (in-depth) position (m)
X (lateral) position (m)
Time (s)
Angular value (°, °/s, or °/s^2)

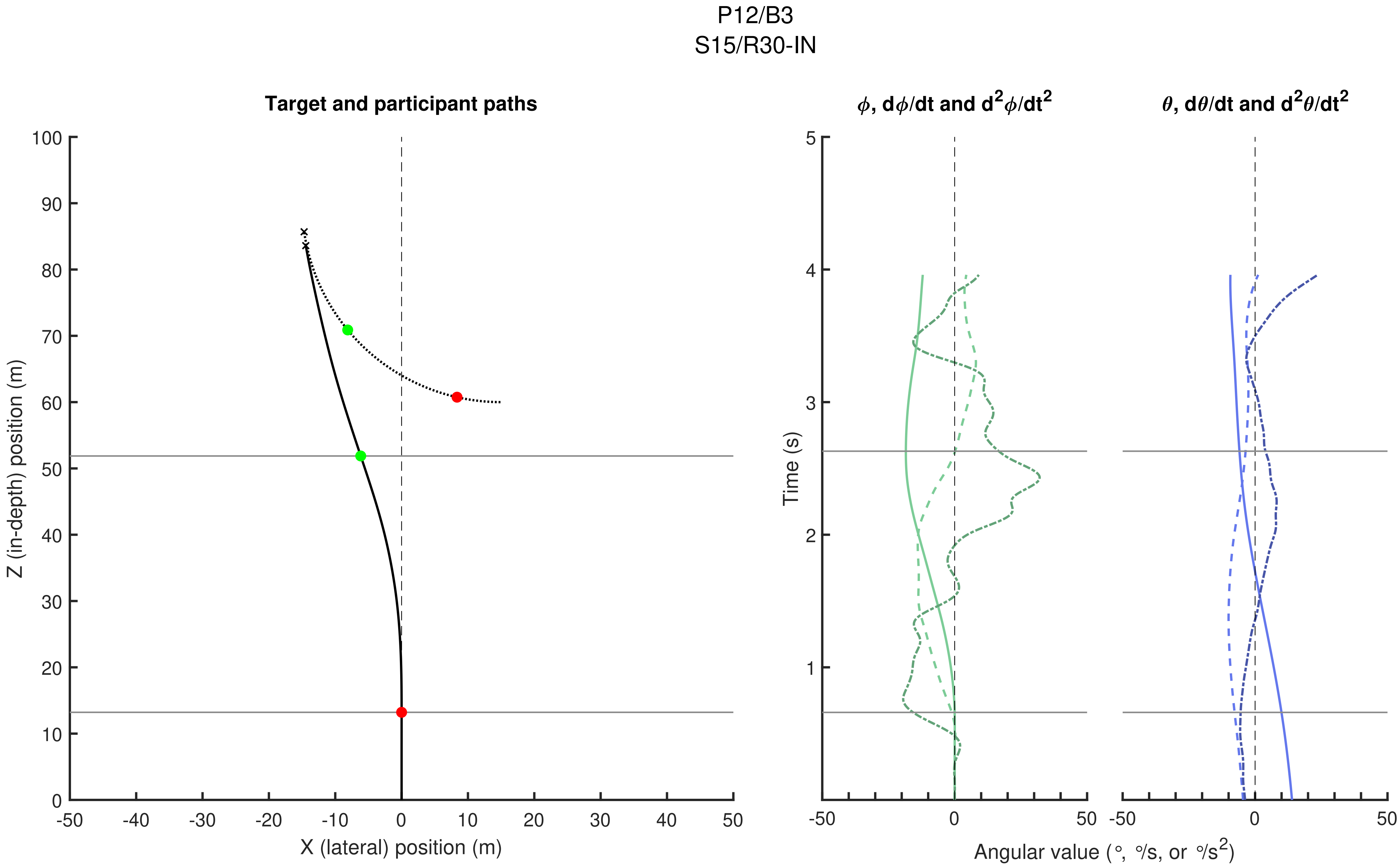
P12/B3
S15/R30-IN
Target and participant paths
ϕ, dϕ/dt and d$^2\phi$/dt^2
θ, dθ/dt and d$^2\theta$/dt^2
Z (in-depth) position (m)
X (lateral) position (m)
Time (s)
Angular value (°, °/s, or °/s^2)
100
90
80
70
60
50
40
30
20
10
0
-50
-40
-30
-20
-10
0
10
20
30
40
50
5
4
3
2
1
-50
0
50
-50
0
50

P12/B3
S20/R10-IN

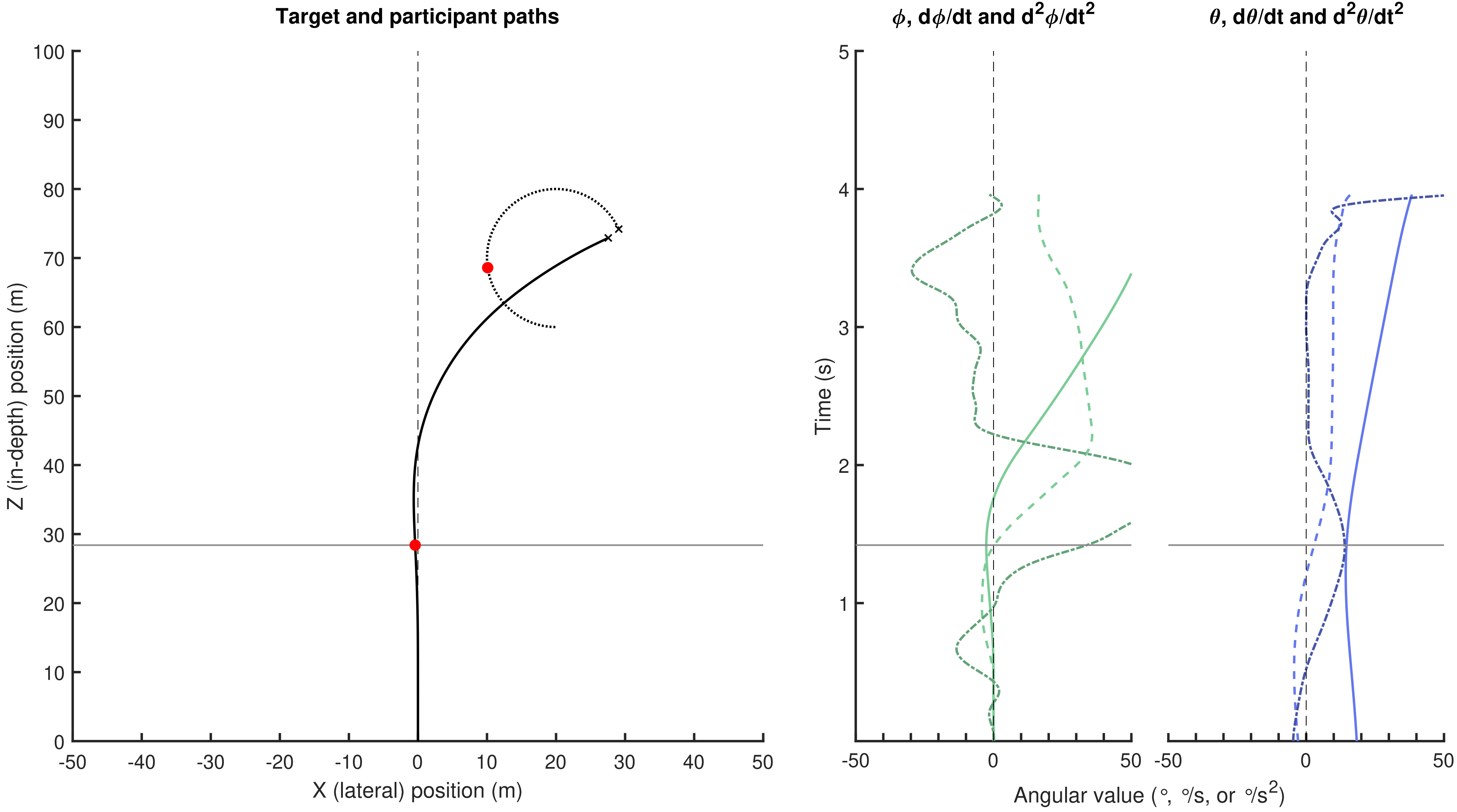

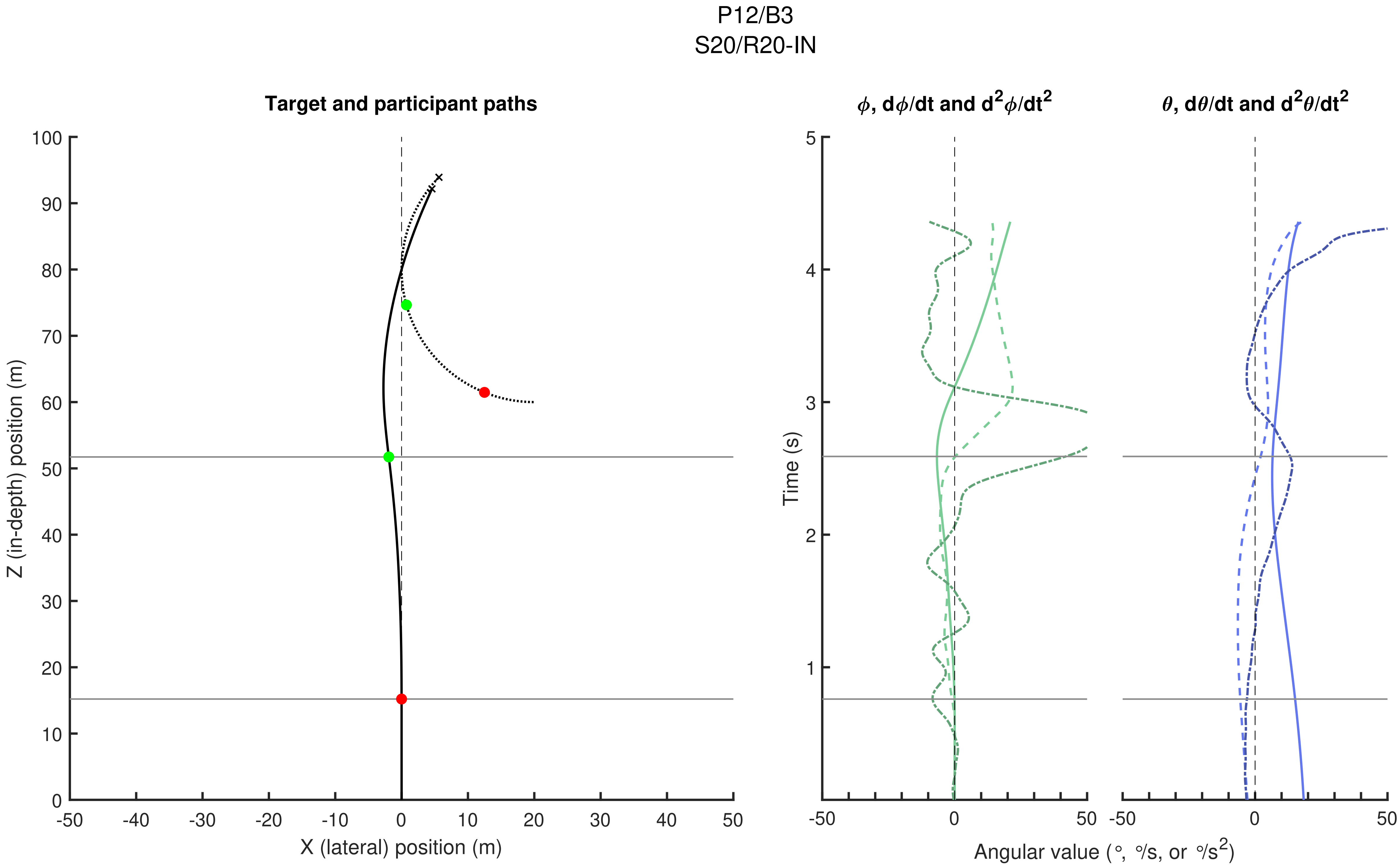

P12/B3
S20/R20-IN
Target and participant paths
Z (in-depth) position (m)
X (lateral) position (m)
ϕ, dϕ/dt and d²ϕ/dt²
θ, dθ/dt and d²θ/dt²
Time (s)
Angular value (°, °/s, or °/s²)

P12/B3
S20/R30-IN

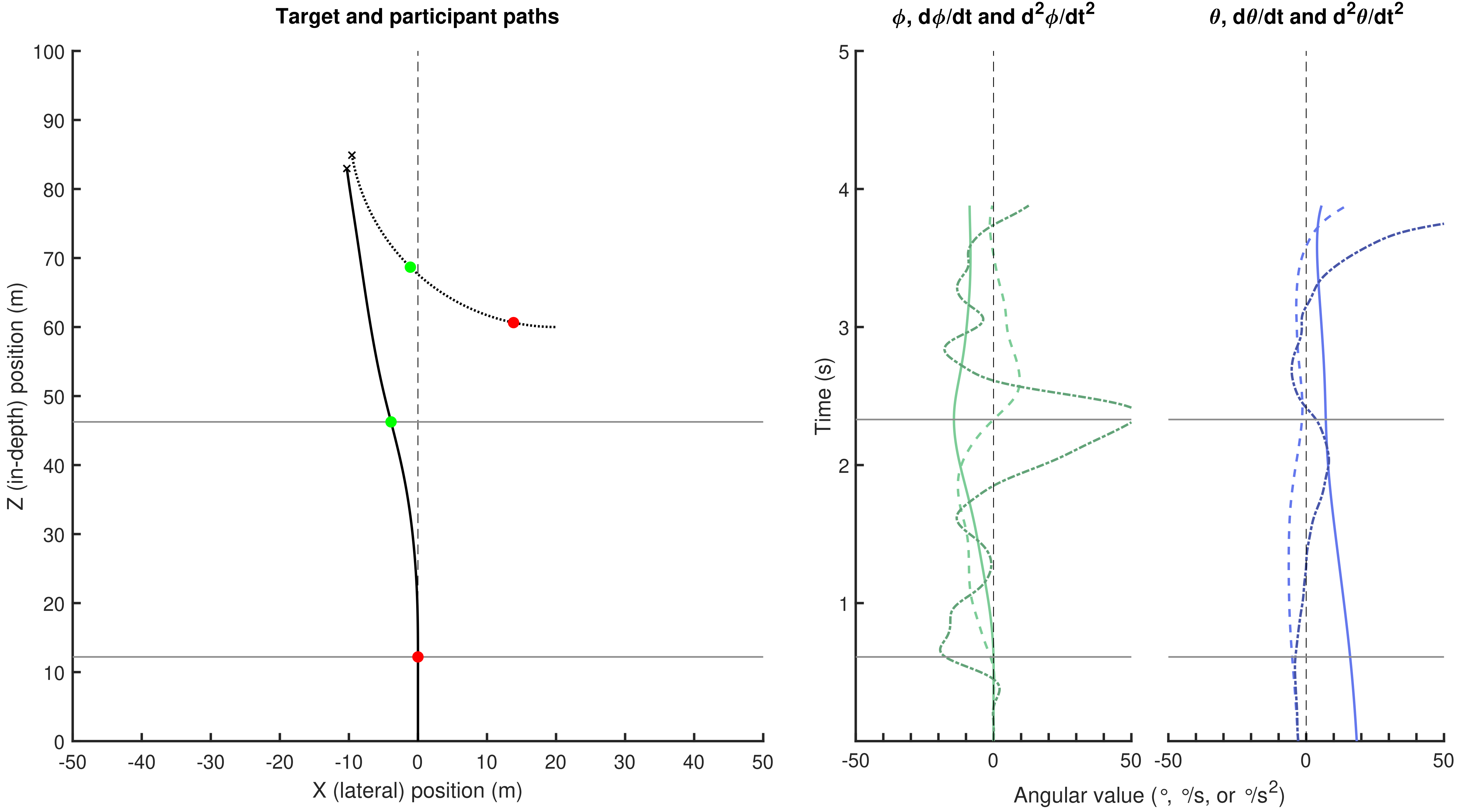

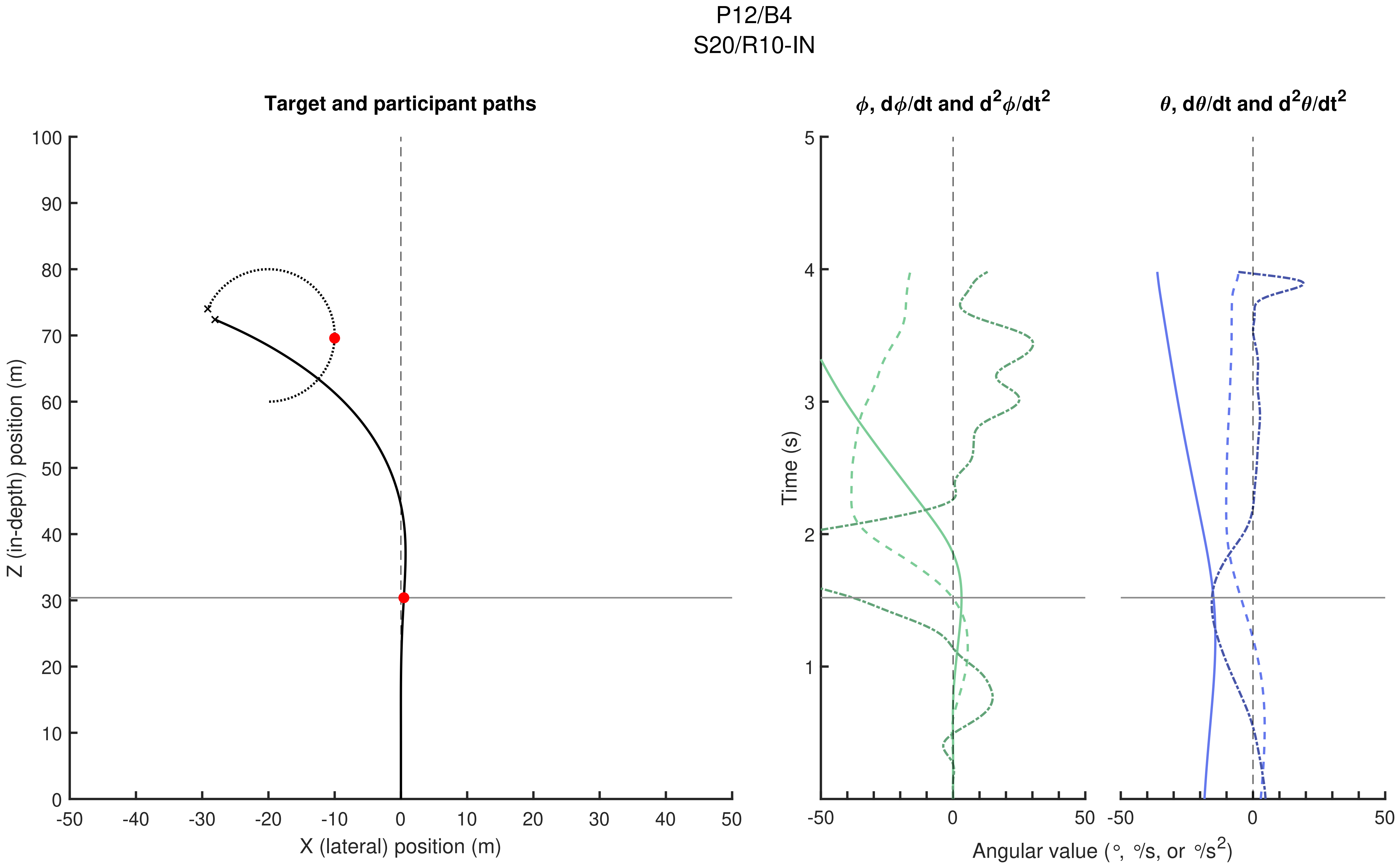

P12/B4
S20/R10-IN
Target and participant paths
Z (in-depth) position (m)
X (lateral) position (m)
100
90
80
70
60
50
40
30
20
10
0
-50
-40
-30
-20
-10
0
10
20
30
40
50
ϕ, dϕ/dt and d²ϕ/dt²
θ, dθ/dt and d²θ/dt²
Time (s)
5
4
3
2
1
-50
0
50
-50
0
50
Angular value (°, °/s, or °/s²)

P12/B4
S20/R20-IN

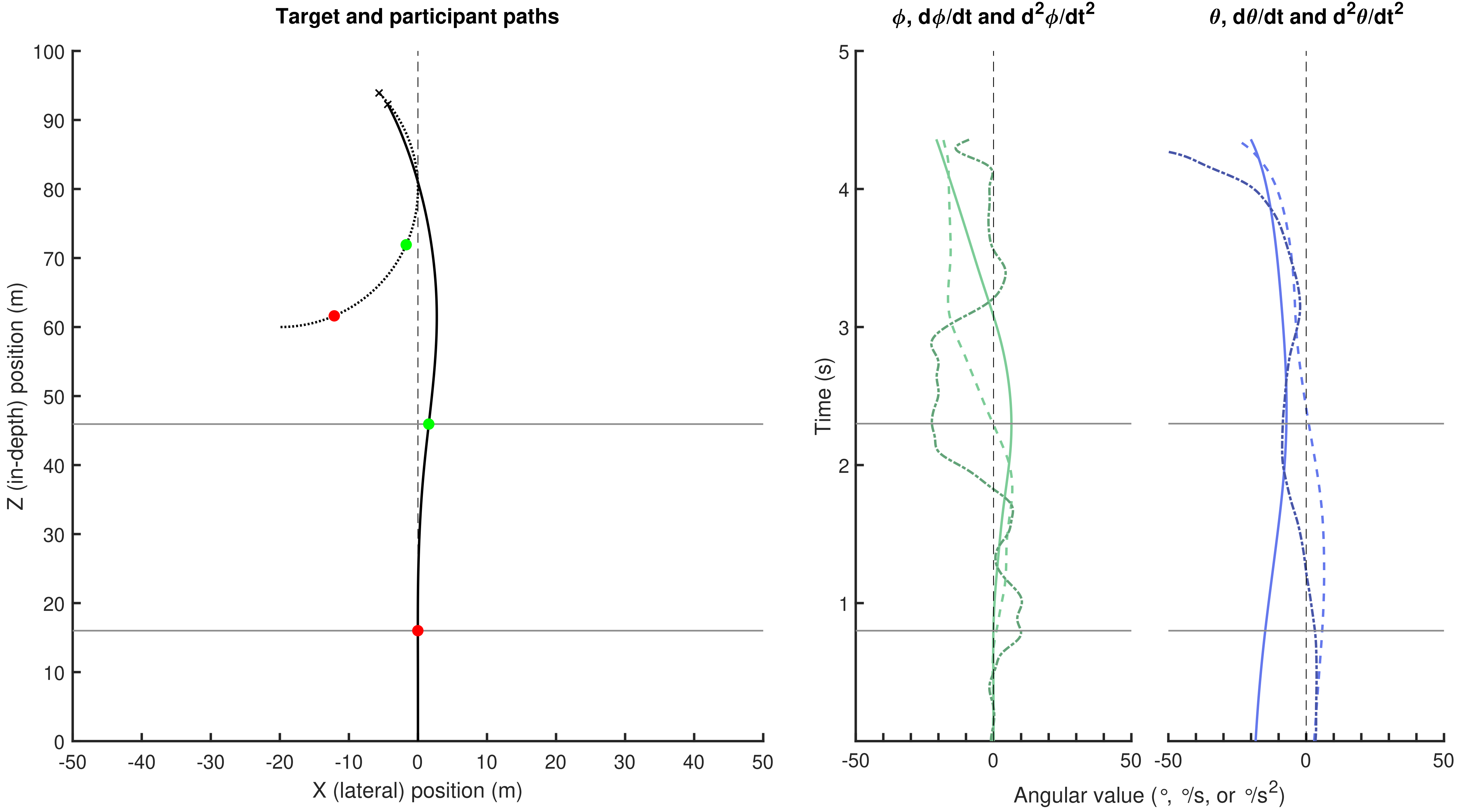

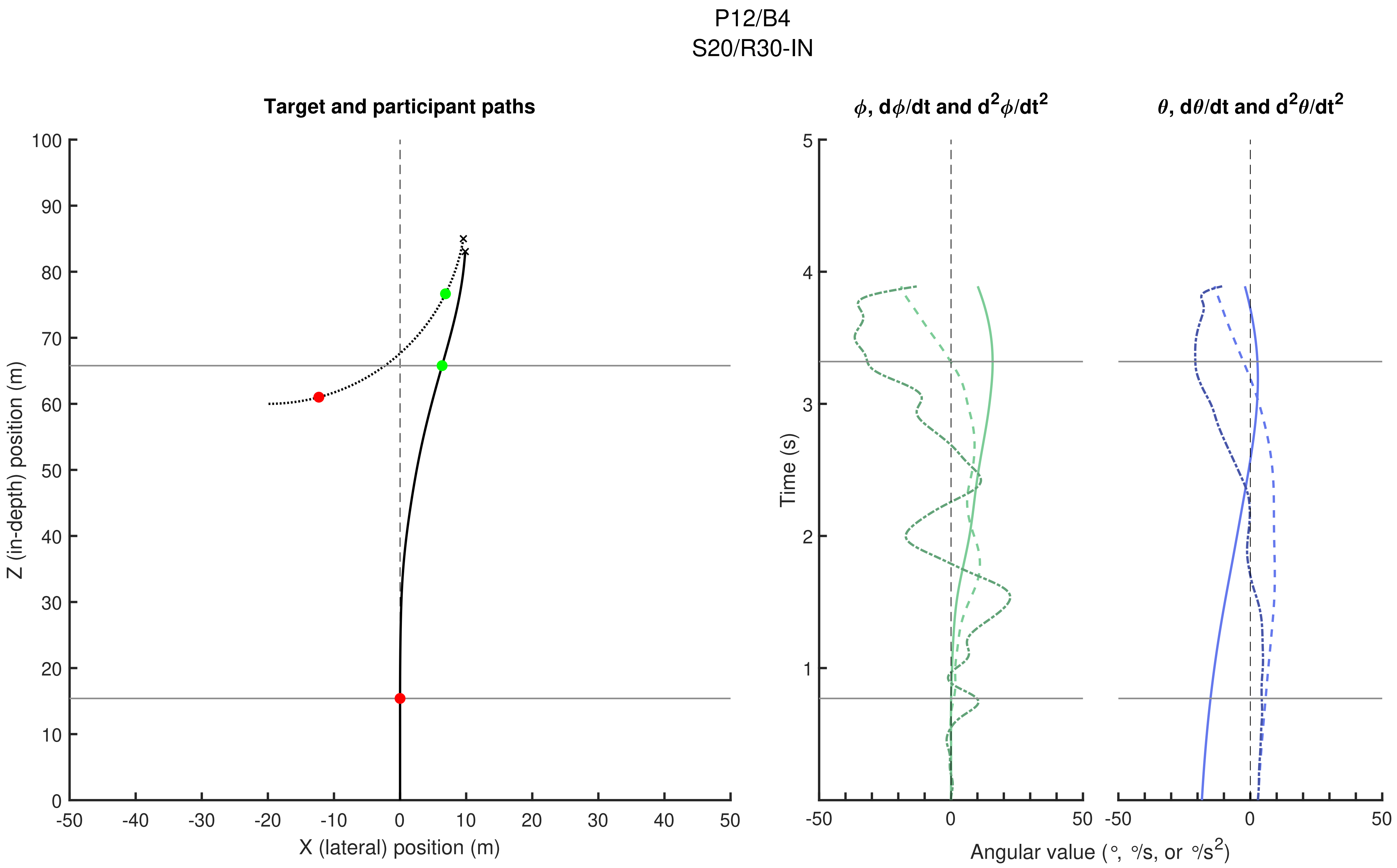
P12/B4
S20/R30-IN
Target and participant paths
Z (in-depth) position (m)
X (lateral) position (m)
φ, dφ/dt and d²φ/dt²
θ, dθ/dt and d²θ/dt²
Time (s)
Angular value (°, °/s, or °/s²)

P12/B4
S15/R10-IN

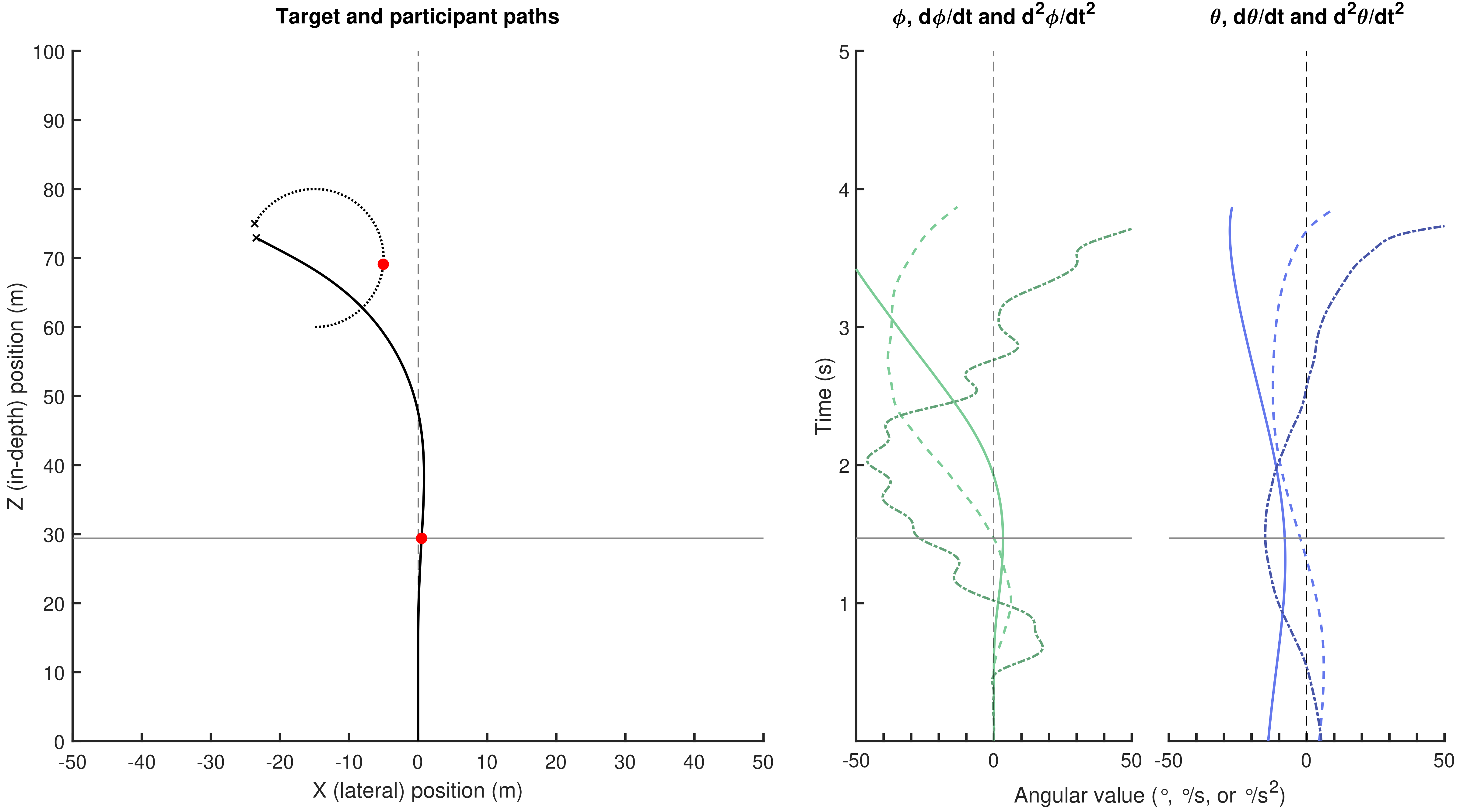

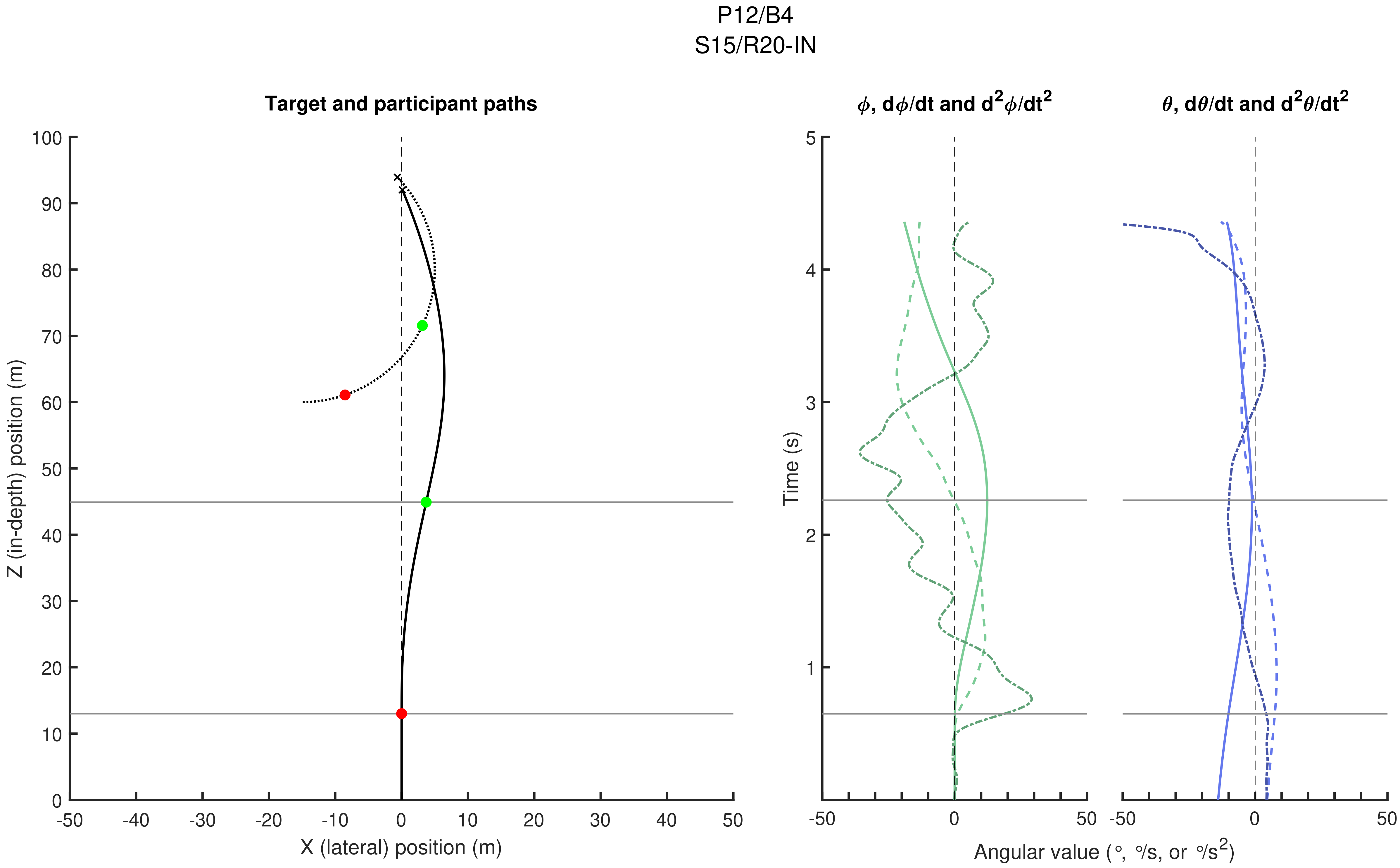
P12/B4
S15/R20-IN
Target and participant paths
Z (in-depth) position (m)
X (lateral) position (m)
ϕ, dϕ/dt and d²ϕ/dt²
θ, dθ/dt and d²θ/dt²
Time (s)
Angular value (°, °/s, or °/s²)
100
90
80
70
60
50
40
30
20
10
0
-50
-40
-30
-20
-10
0
10
20
30
40
50
5
4
3
2
1

P12/B4
S15/R30-IN

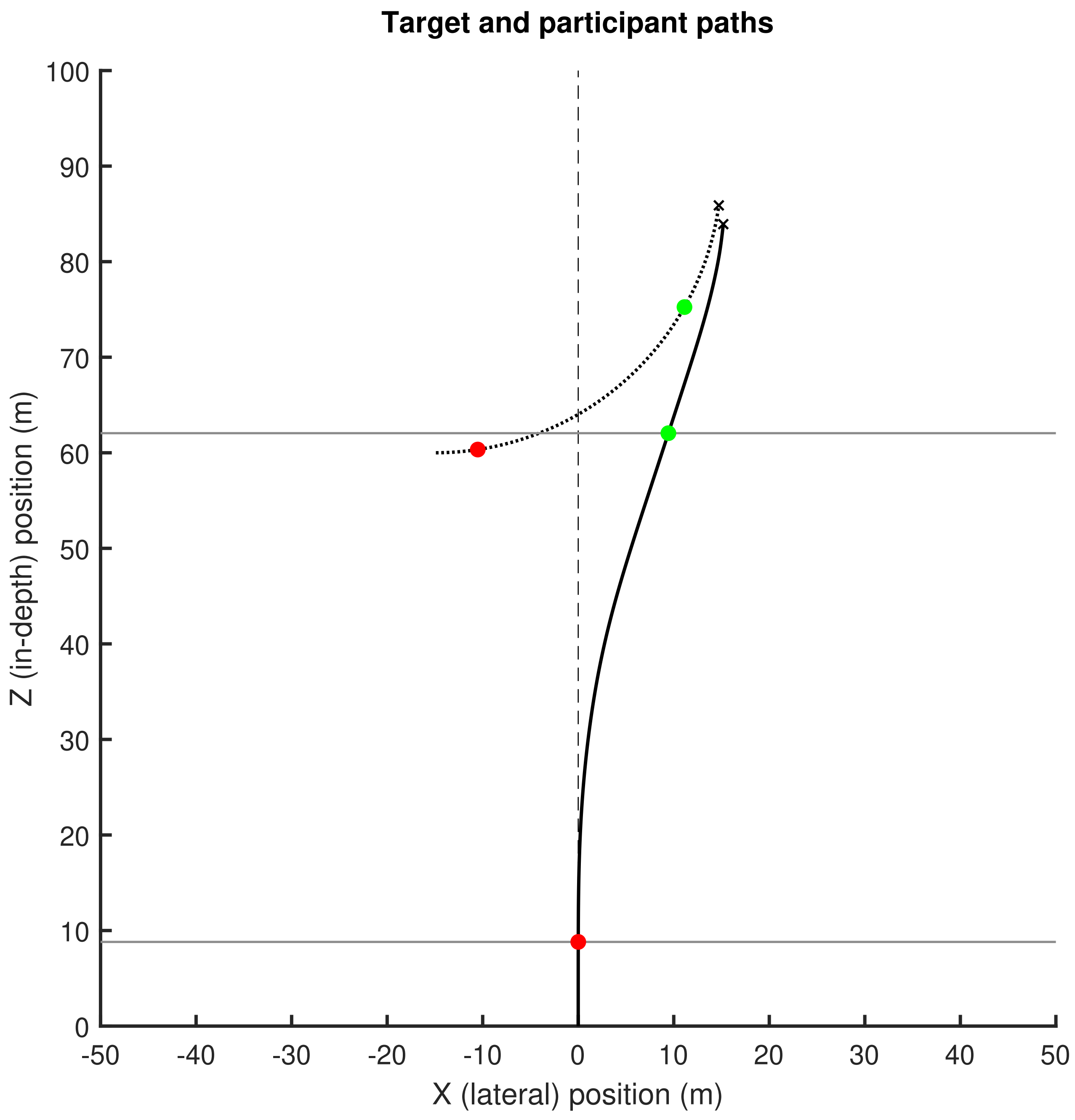

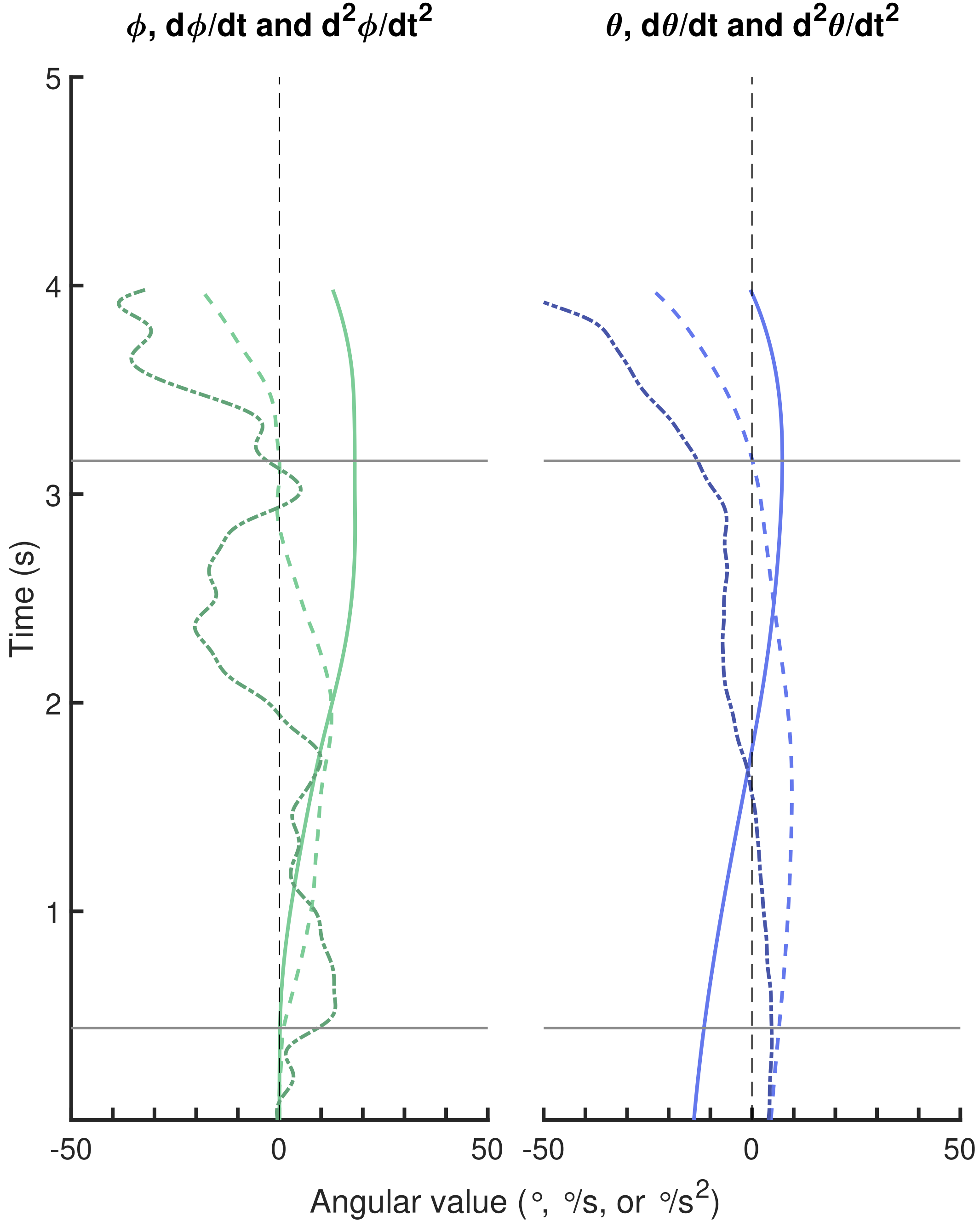

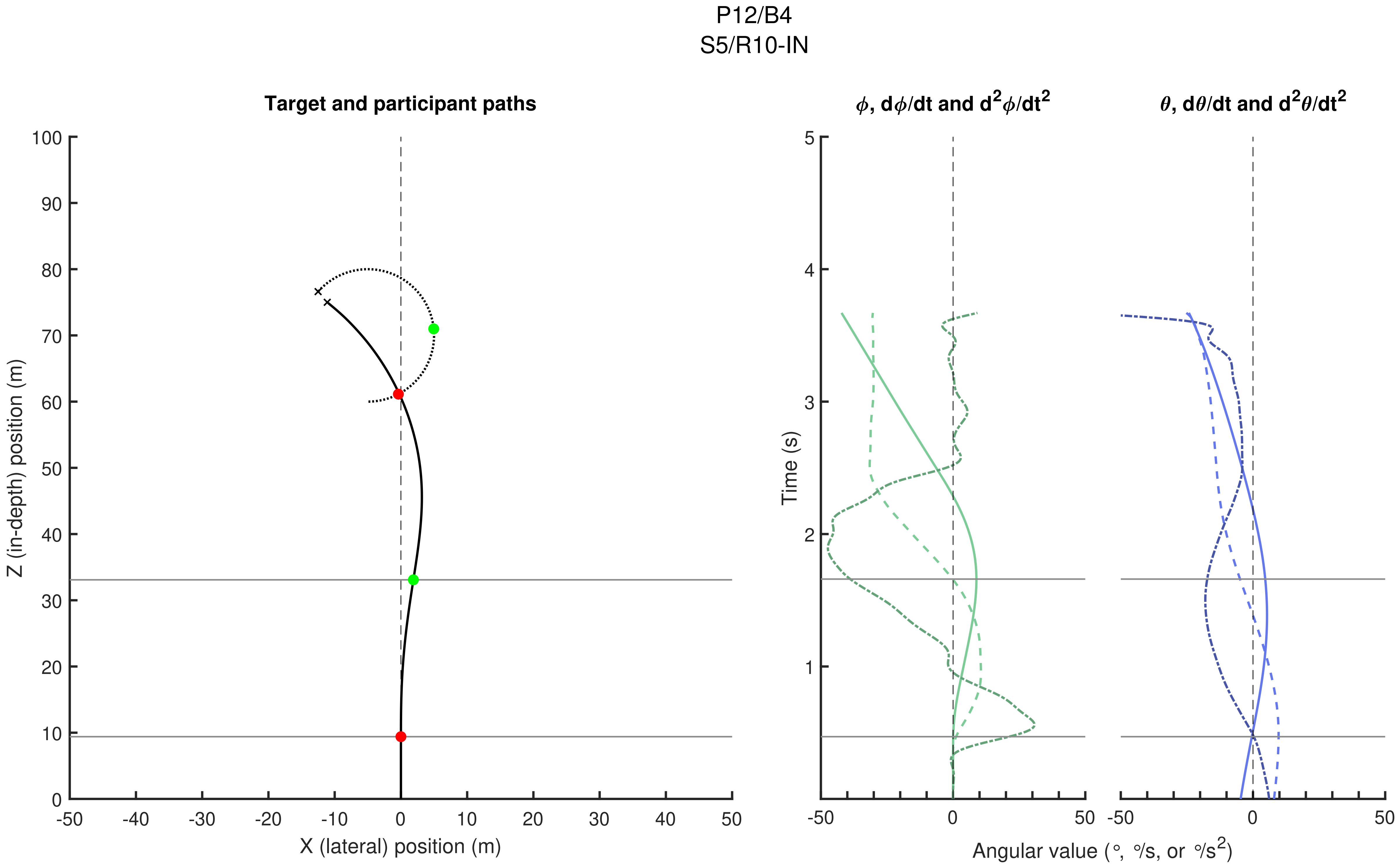

P12/B4
S5/R10-IN
Target and participant paths
ϕ, dϕ/dt and d$^2\phi$/dt^2
θ, dθ/dt and d$^2\theta$/dt^2
Z (in-depth) position (m)
X (lateral) position (m)
Time (s)
Angular value (°, °/s, or °/s^2)

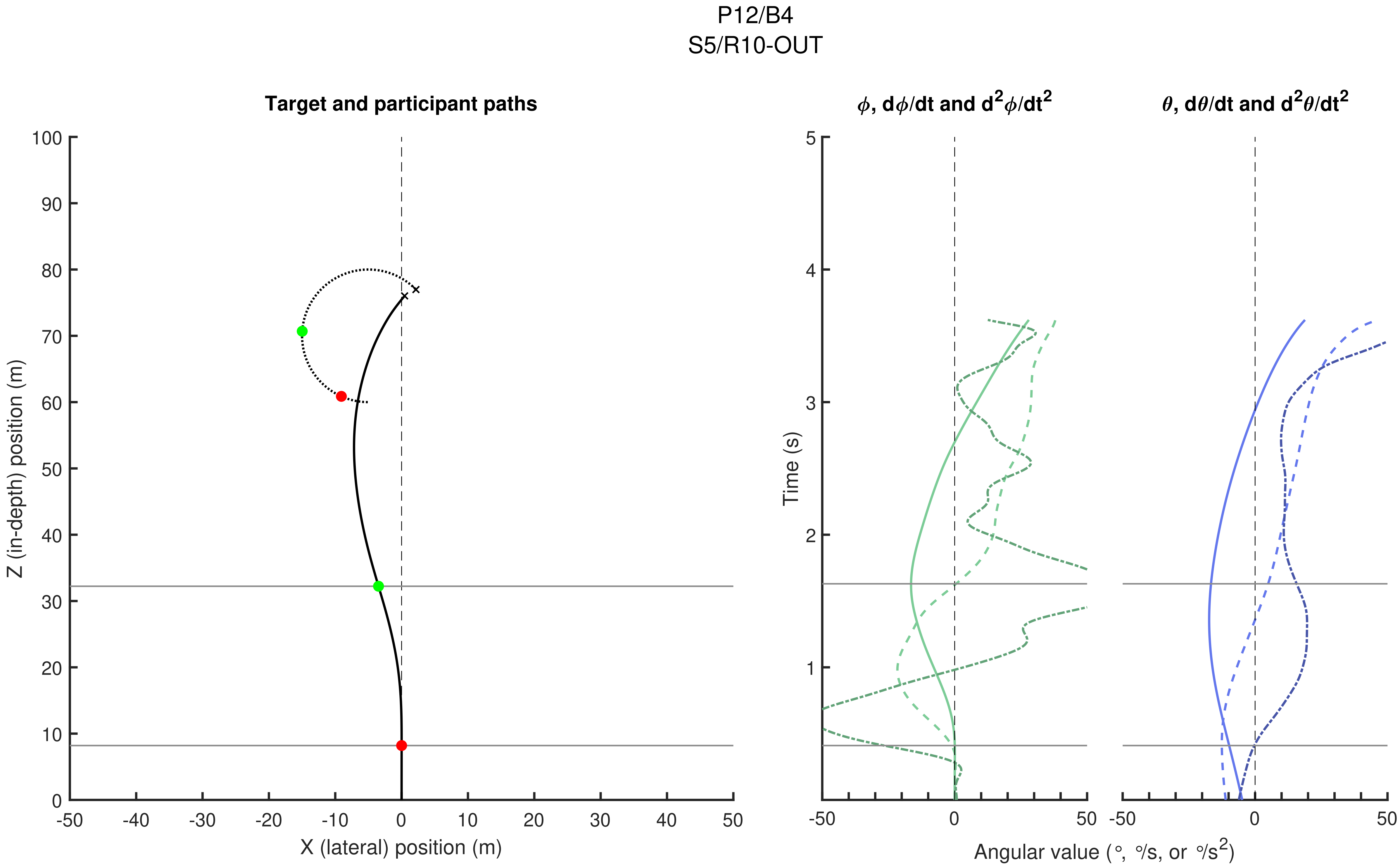
P12/B4
S5/R10-OUT
Target and participant paths
Z (in-depth) position (m)
X (lateral) position (m)
100
90
80
70
60
50
40
30
20
10
0
-50
-40
-30
-20
-10
0
10
20
30
40
50
ϕ, dϕ/dt and d²ϕ/dt²
θ, dθ/dt and d²θ/dt²
Time (s)
5
4
3
2
1
-50
0
50
-50
0
50
Angular value (°, °/s, or °/s²)

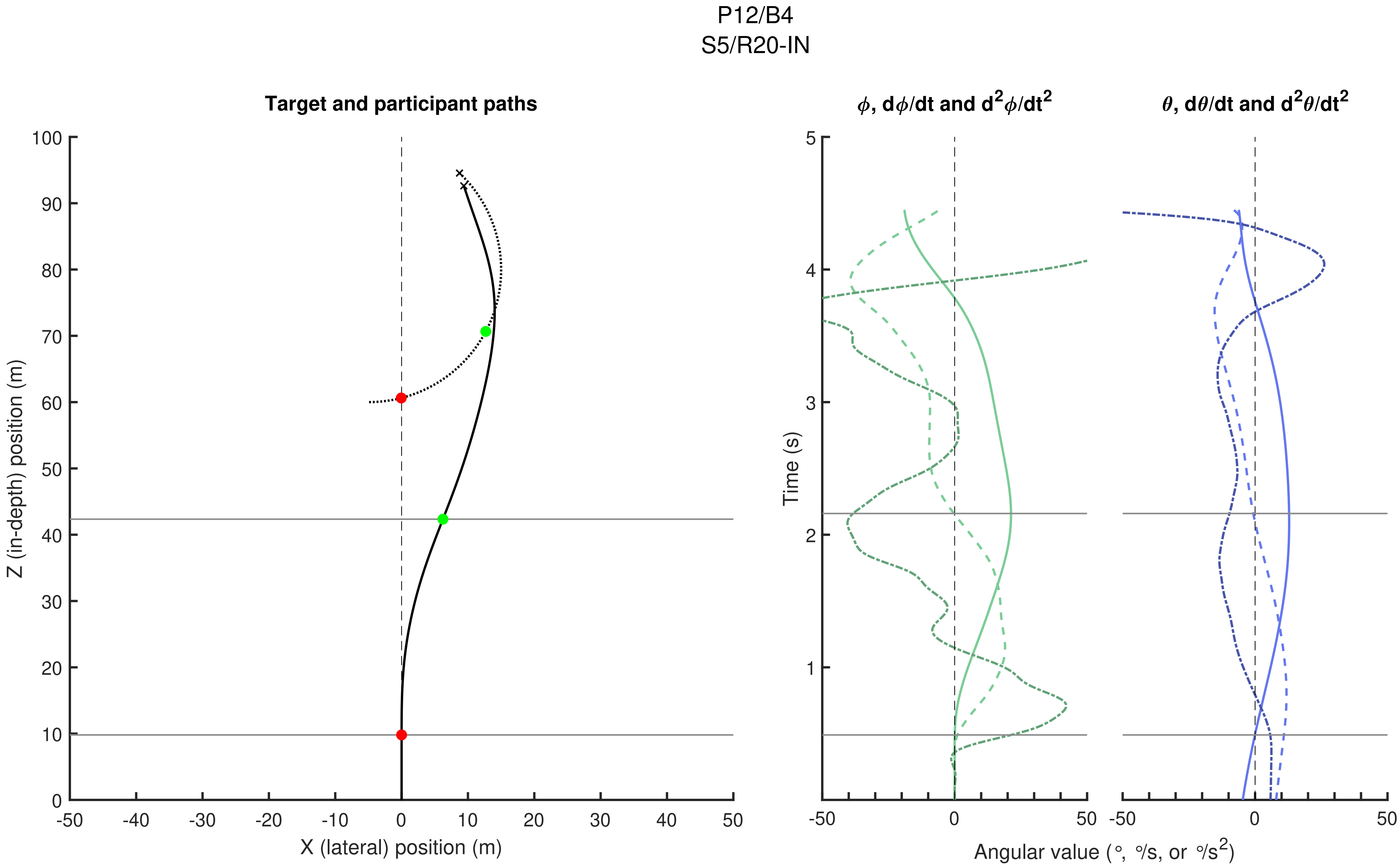

P12/B4
S5/R20-IN
Target and participant paths
Z (in-depth) position (m)
X (lateral) position (m)
100
90
80
70
60
50
40
30
20
10
0
-50
-40
-30
-20
-10
0
10
20
30
40
50
ϕ, dϕ/dt and d$^2\phi$/dt^2
θ, dθ/dt and d$^2\theta$/dt^2
Time (s)
5
4
3
2
1
-50
0
50
-50
0
50
Angular value (°, °/s, or °/s^2)

P12/B4
S5/R20-OUT

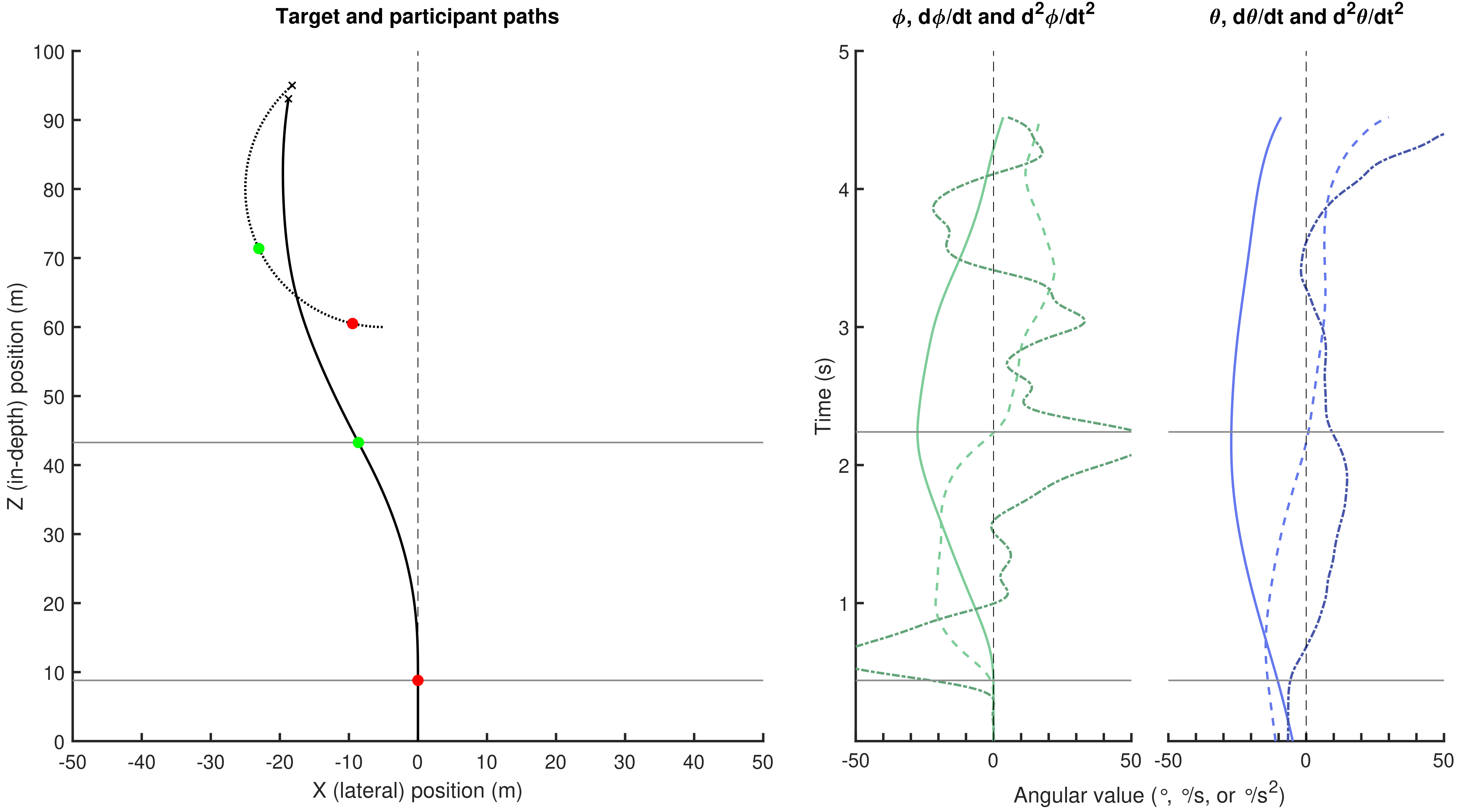

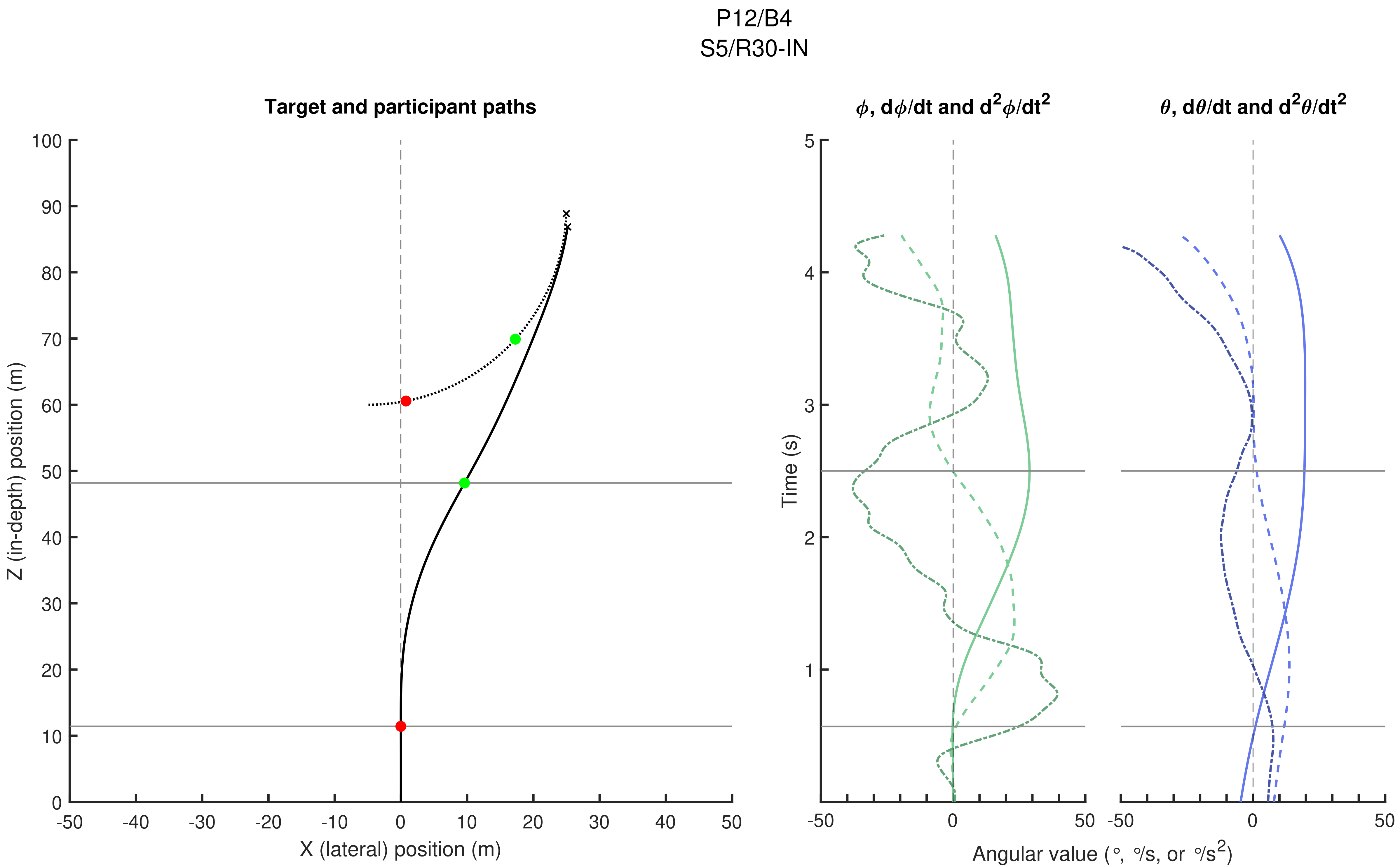

P12/B4
S5/R30-IN
Target and participant paths
Z (in-depth) position (m)
X (lateral) position (m)
100
90
80
70
60
50
40
30
20
10
0
-50
-40
-30
-20
-10
0
10
20
30
40
50
ϕ, dϕ/dt and d²ϕ/dt²
θ, dθ/dt and d²θ/dt²
Time (s)
5
4
3
2
1
-50
0
50
-50
0
50
Angular value (°, °/s, or °/s²)

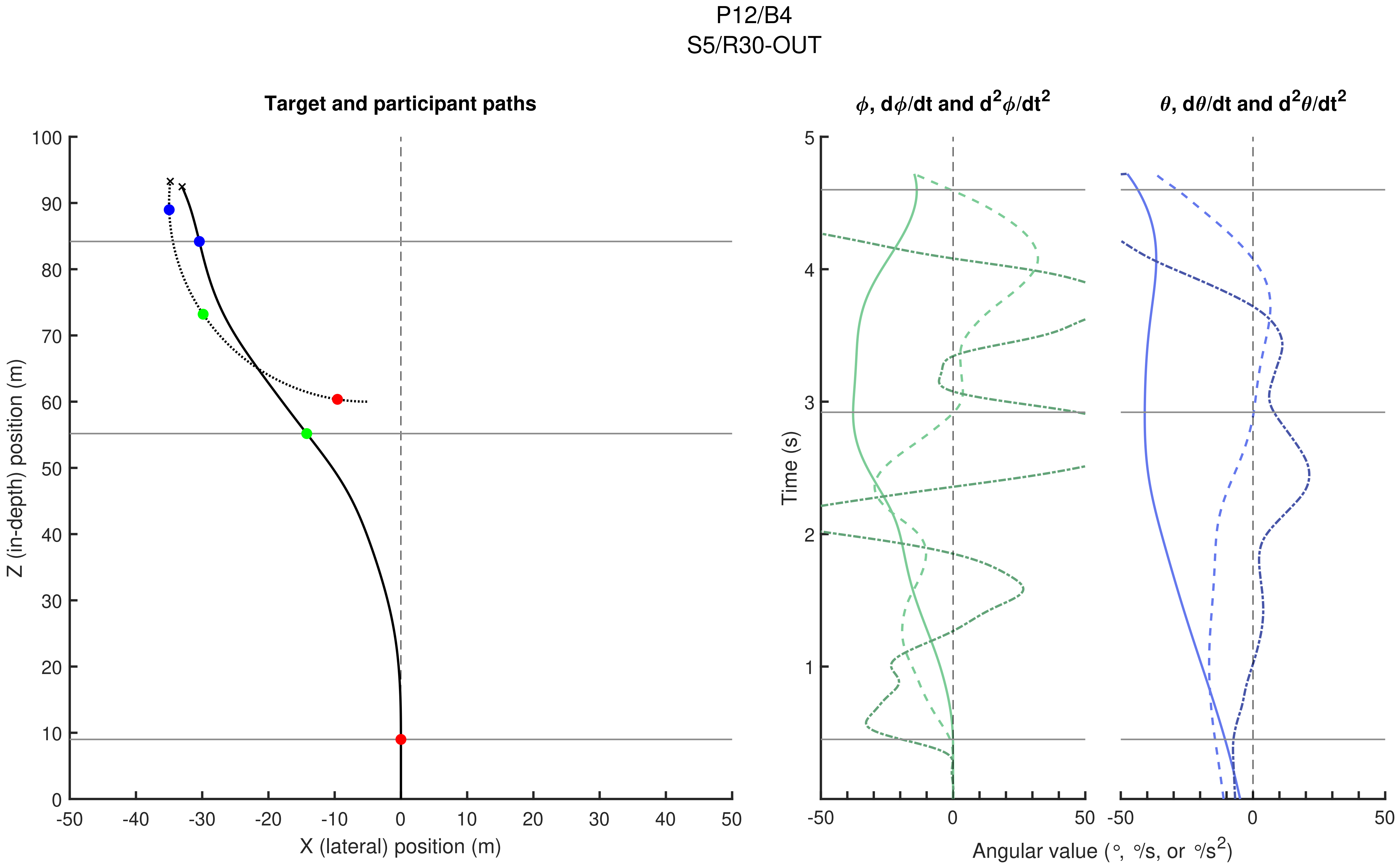
P12/B4
S5/R30-OUT
Target and participant paths
Z (in-depth) position (m)
X (lateral) position (m)
ϕ, dϕ/dt and d$^2\phi$/dt^2
θ, dθ/dt and d$^2\theta$/dt^2
Time (s)
Angular value (°, °/s, or °/s^2)

P12/B4
S5/R10-OUT

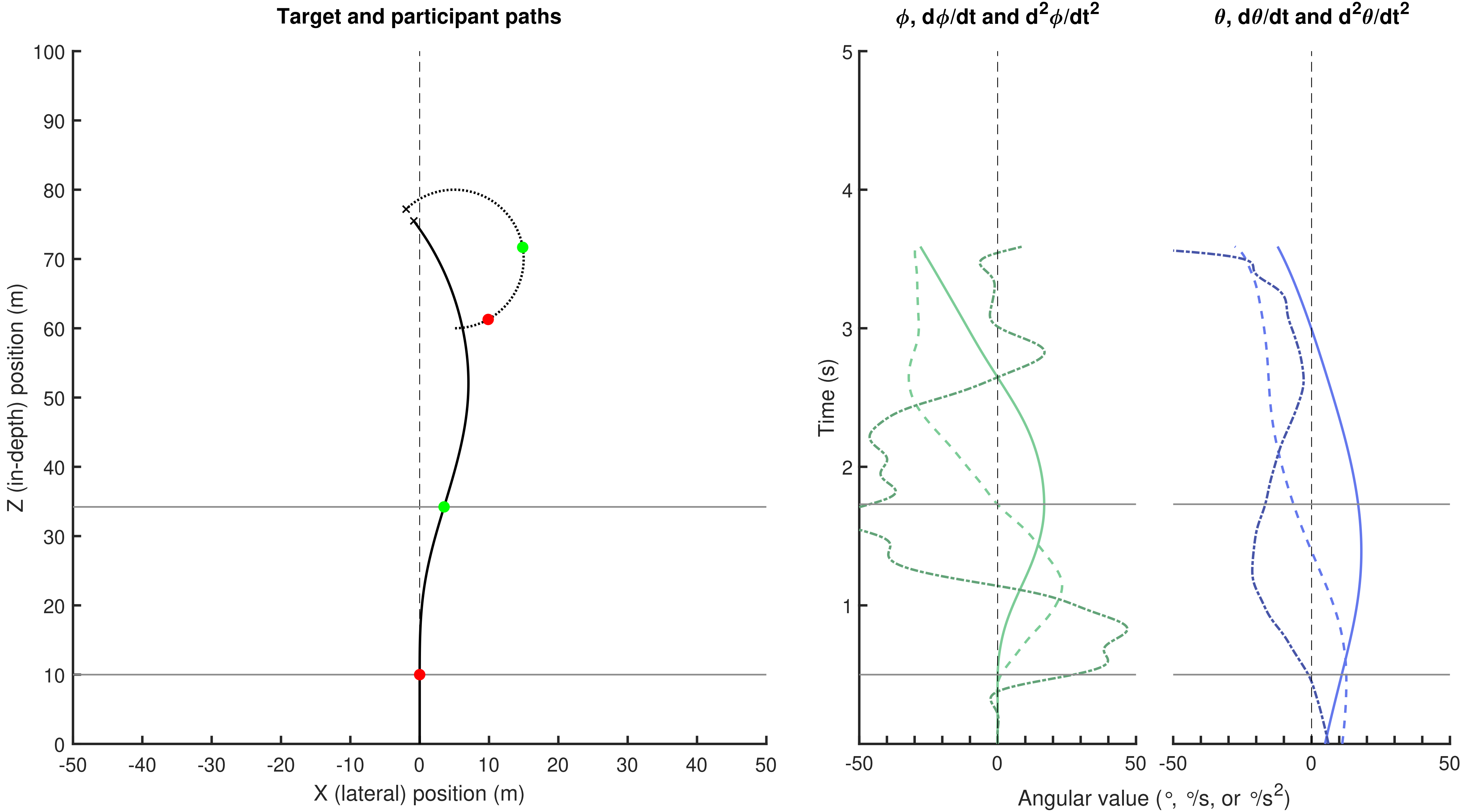

P12/B4
S5/R10-IN

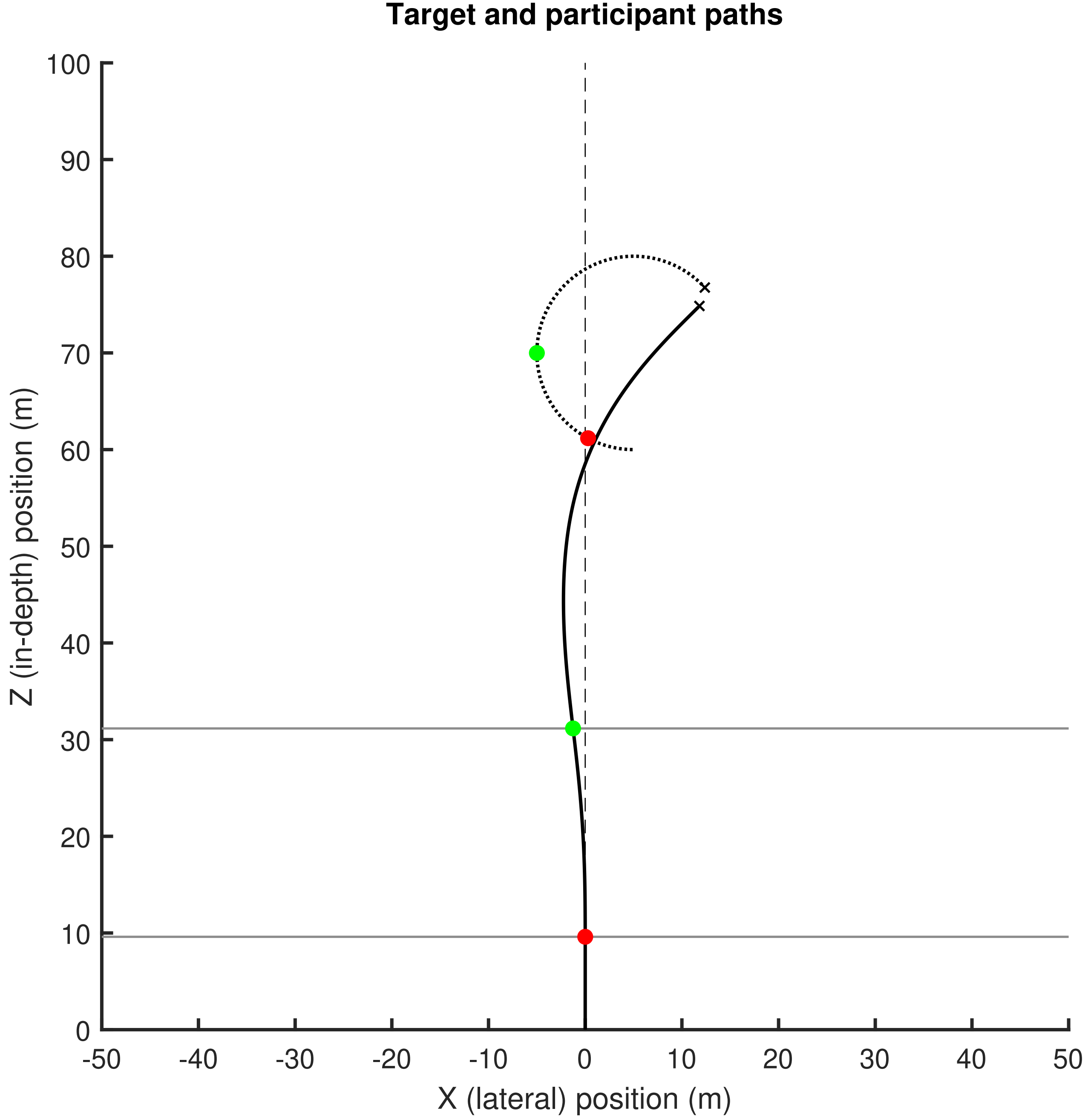

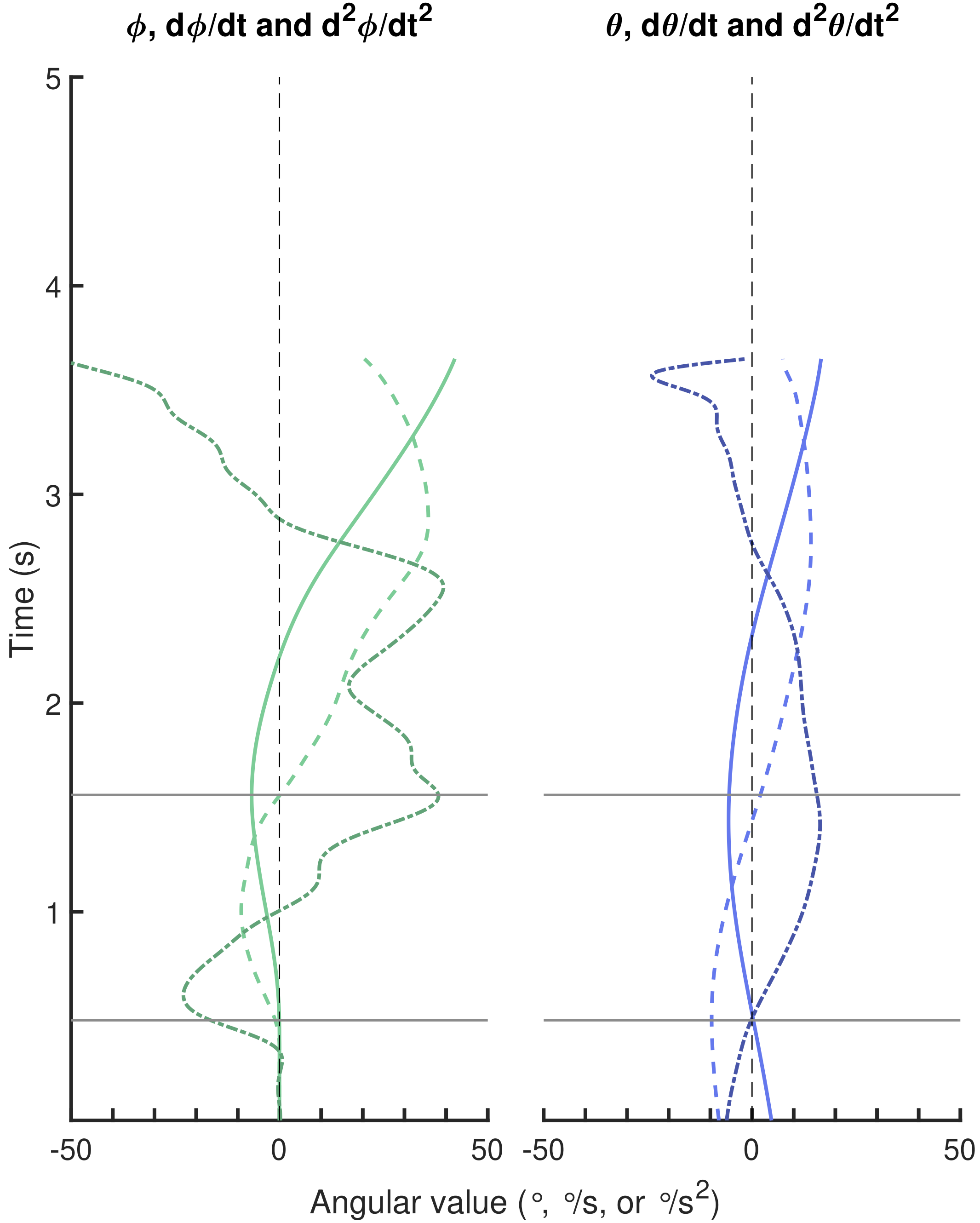

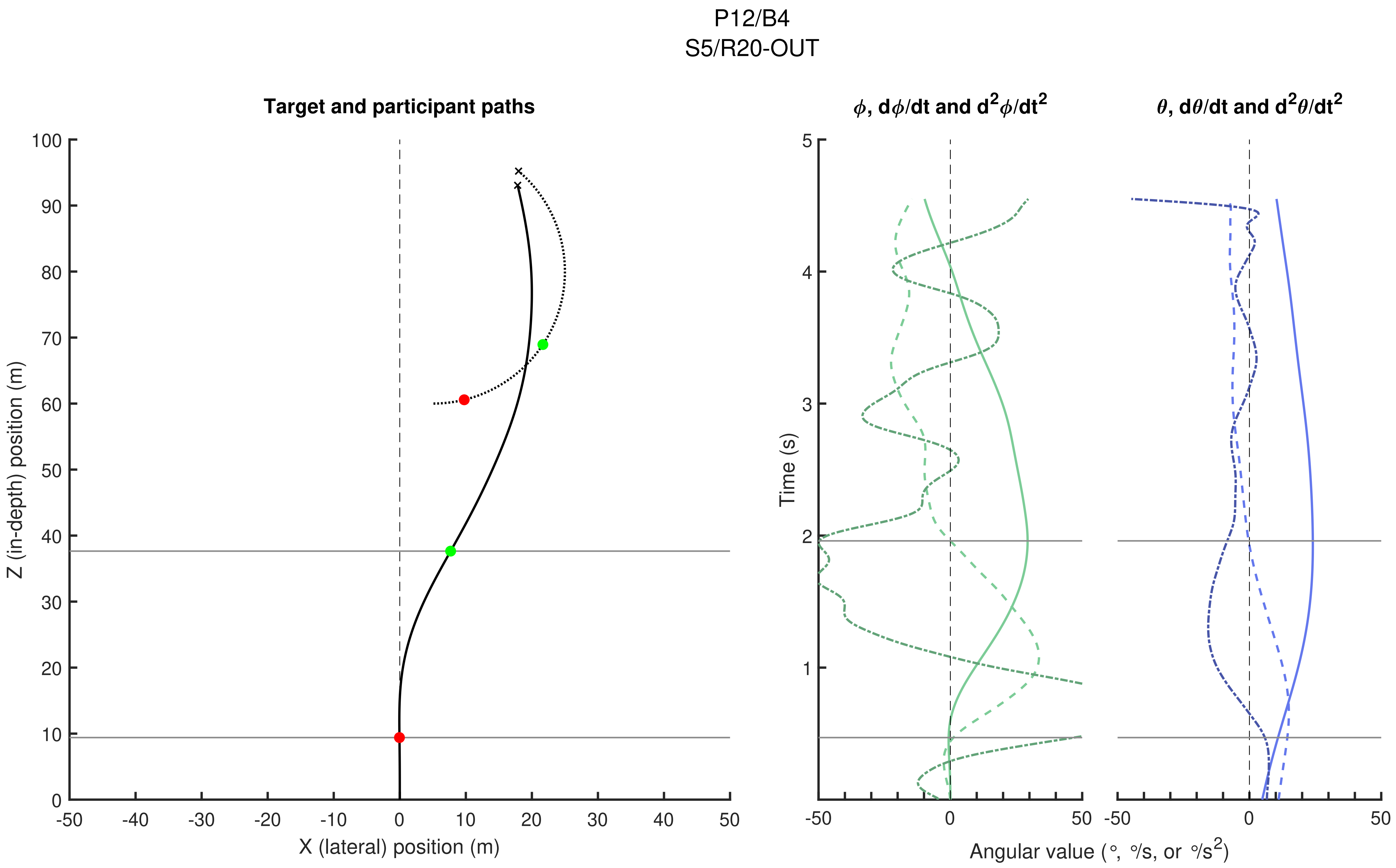
P12/B4
S5/R20-OUT
Target and participant paths
Z (in-depth) position (m)
X (lateral) position (m)
0
10
20
30
40
50
60
70
80
90
100
-50
-40
-30
-20
-10
0
10
20
30
40
50
ϕ, dϕ/dt and d$^2\phi$/dt^2
θ, dθ/dt and d$^2\theta$/dt^2
Time (s)
1
2
3
4
5
-50
0
50
-50
0
50
Angular value (°, °/s, or °/s^2)

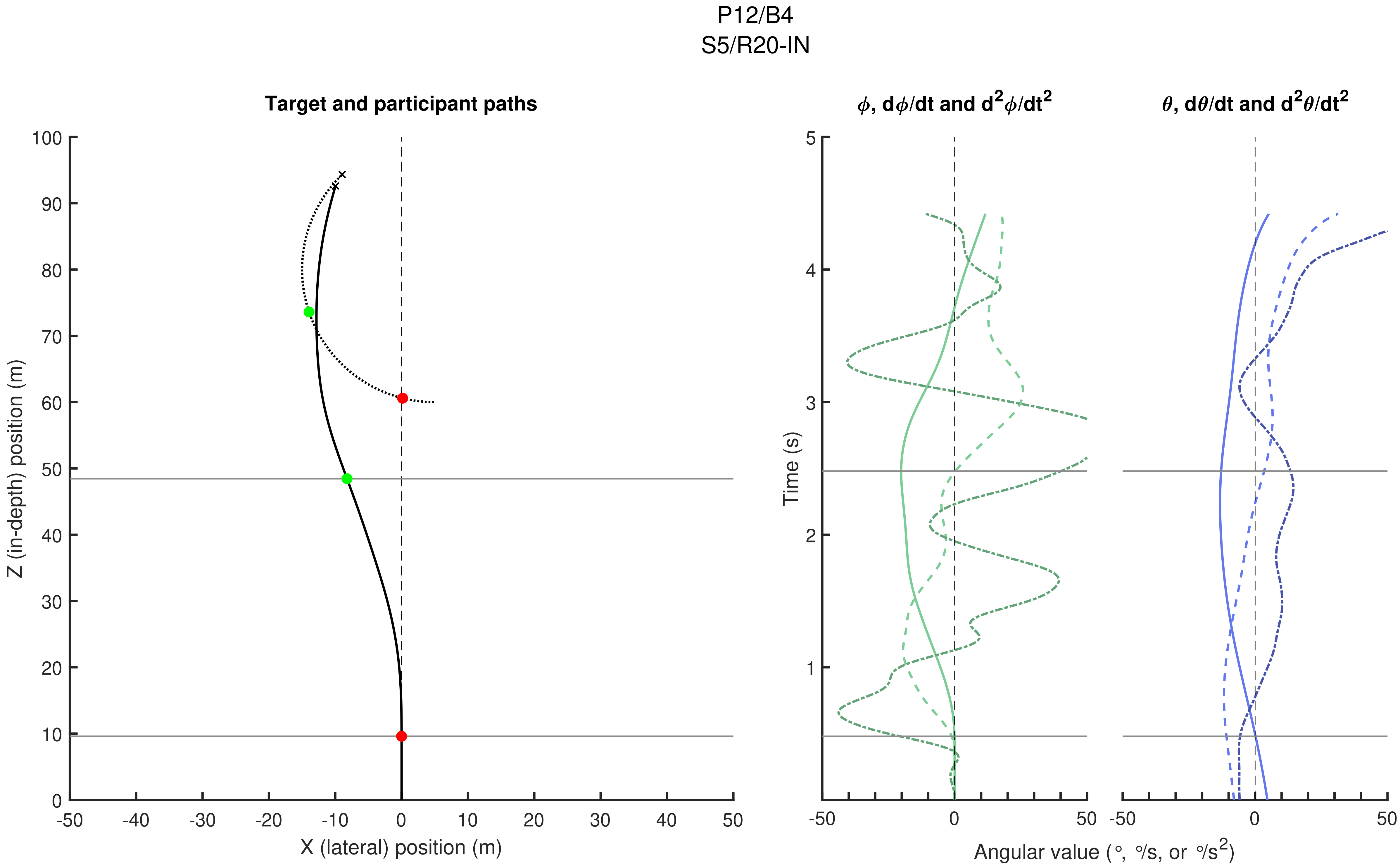

P12/B4
S5/R20-IN
Target and participant paths
ϕ, dϕ/dt and d$^2\phi$/dt^2
θ, dθ/dt and d$^2\theta$/dt^2
Z (in-depth) position (m)
X (lateral) position (m)
Time (s)
Angular value (°, °/s, or °/s^2)
100
90
80
70
60
50
40
30
20
10
0
-50
-40
-30
-20
-10
0
10
20
30
40
50
5
4
3
2
1
-50
0
50
-50
0
50

P12/B4
S5/R30-OUT

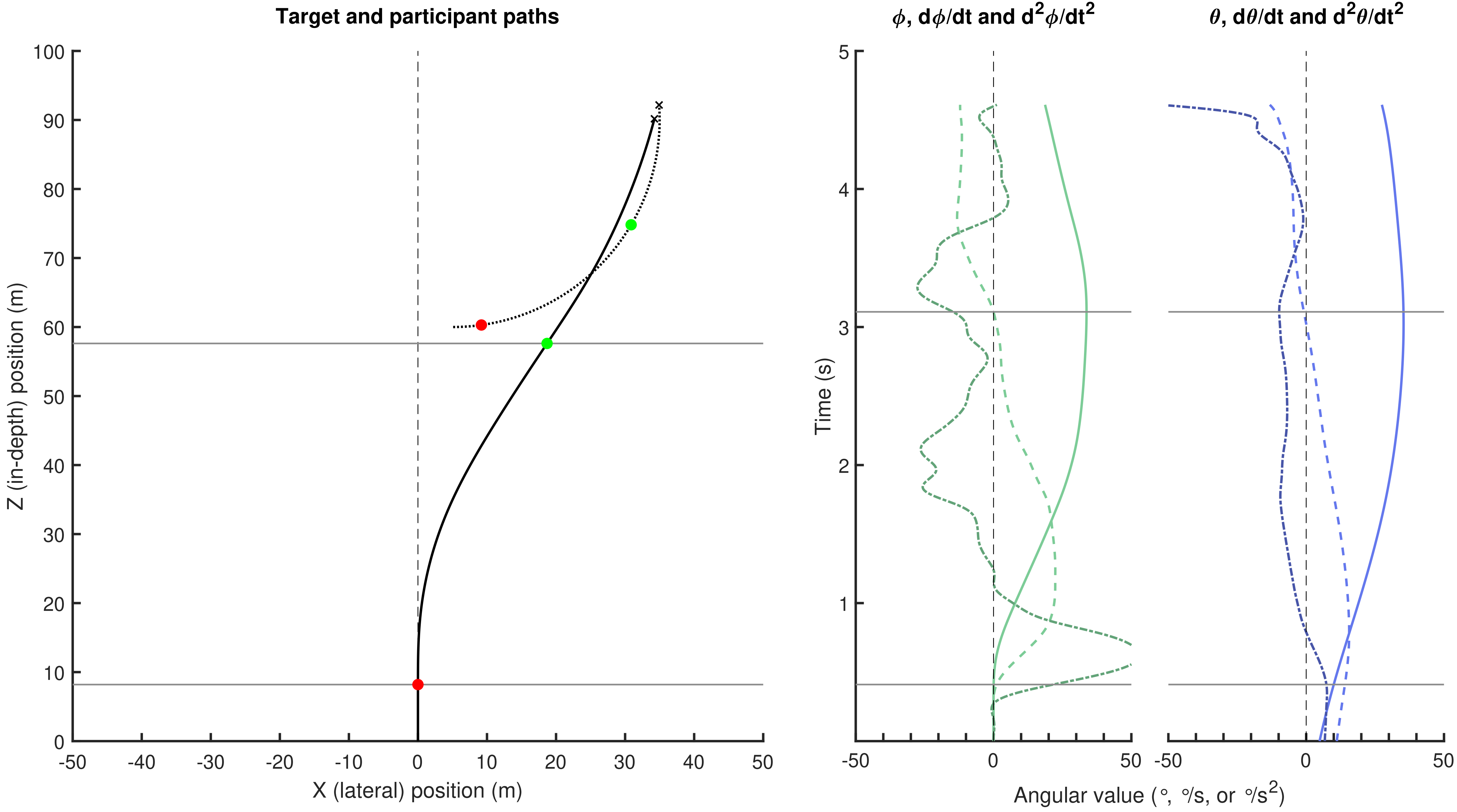

P12/B4
S5/R30-IN

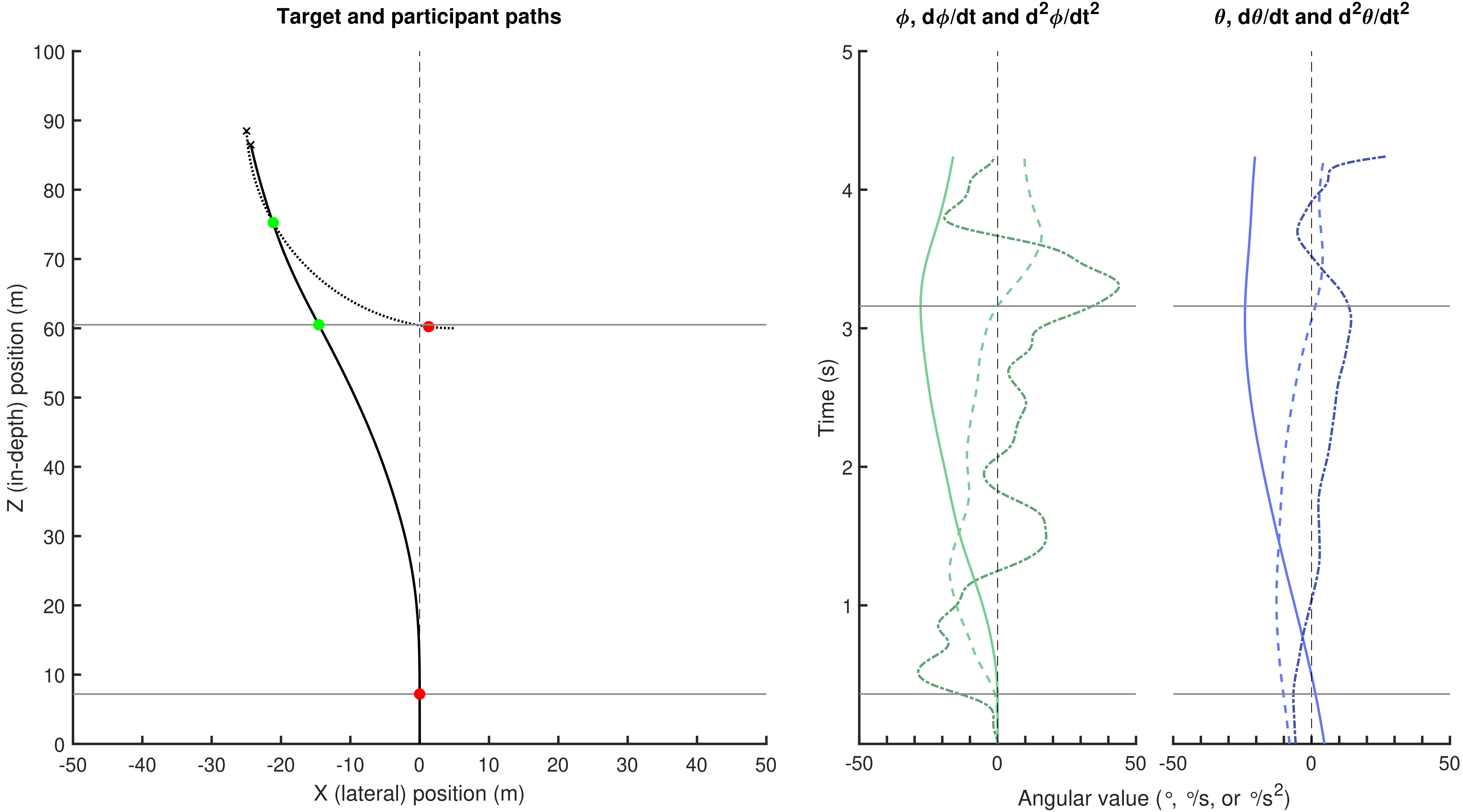

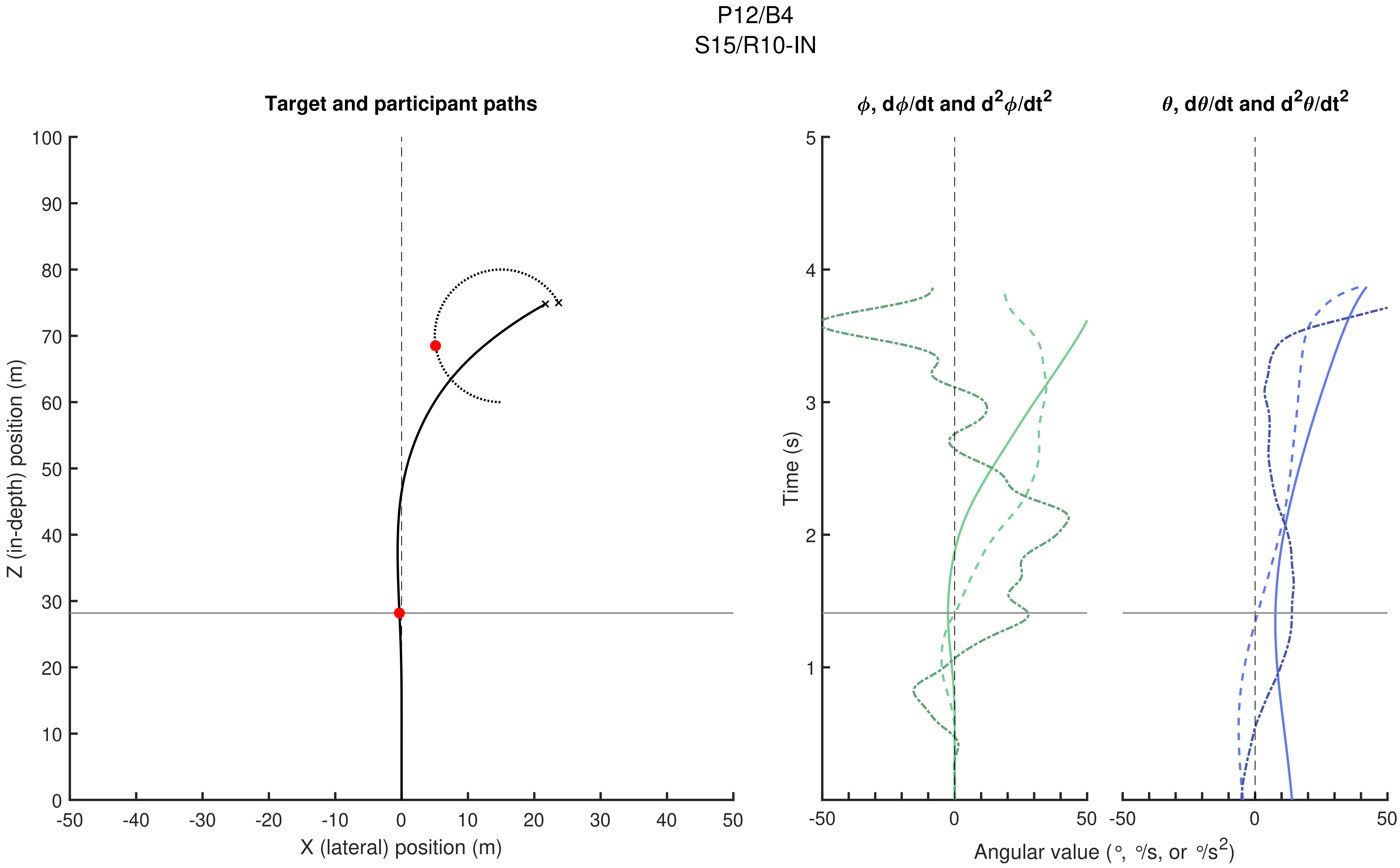
P12/B4
S15/R10-IN
Target and participant paths
Z (in-depth) position (m)
X (lateral) position (m)
ϕ, dϕ/dt and d$^2\phi$/dt^2
θ, dθ/dt and d$^2\theta$/dt^2
Time (s)
Angular value (°, °/s, or °/s^2)

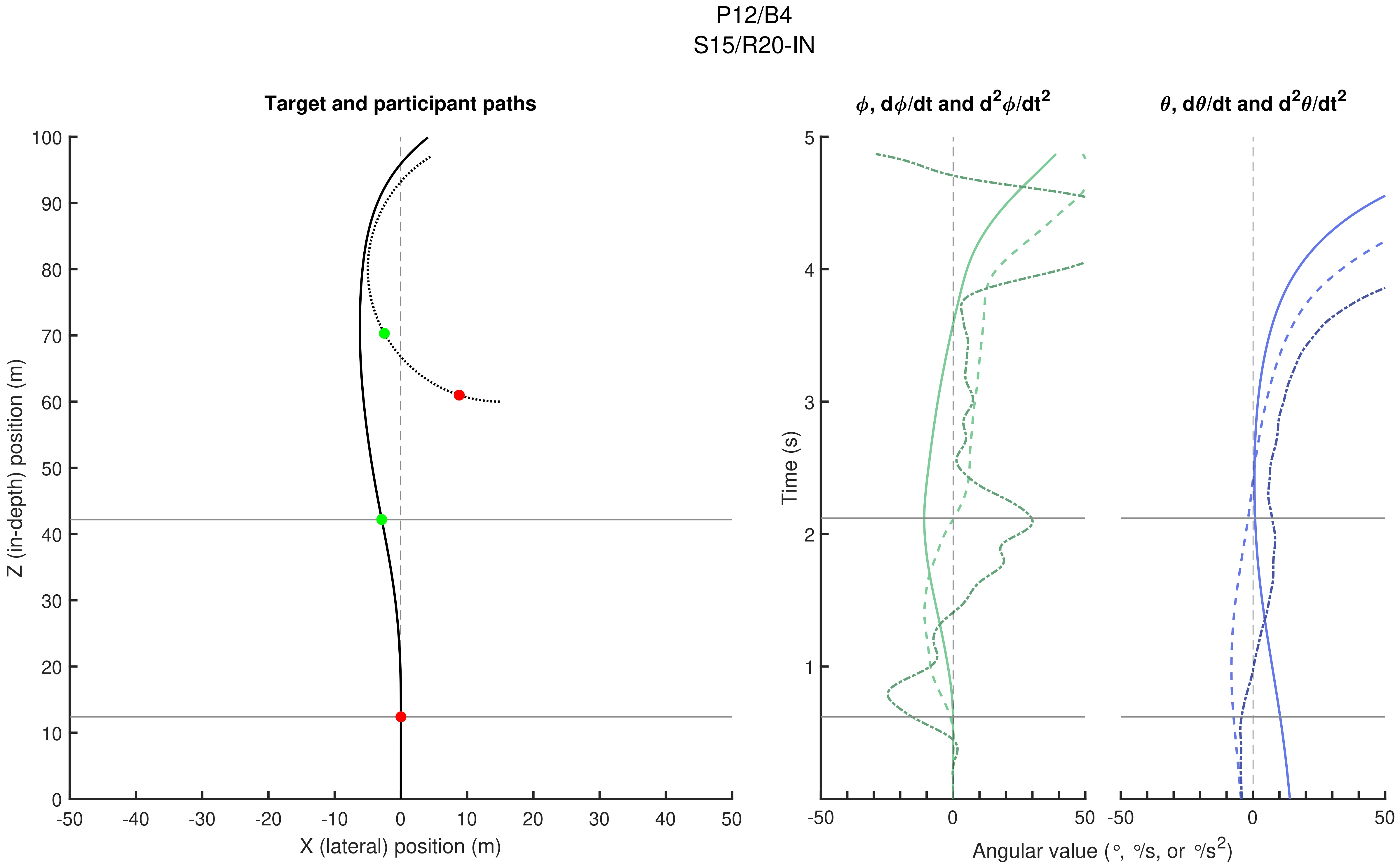
P12/B4
S15/R20-IN
Target and participant paths
Z (in-depth) position (m)
X (lateral) position (m)
ϕ, dϕ/dt and d$^2\phi$/dt^2
θ, dθ/dt and d$^2\theta$/dt^2
Time (s)
Angular value (°, °/s, or °/s^2)

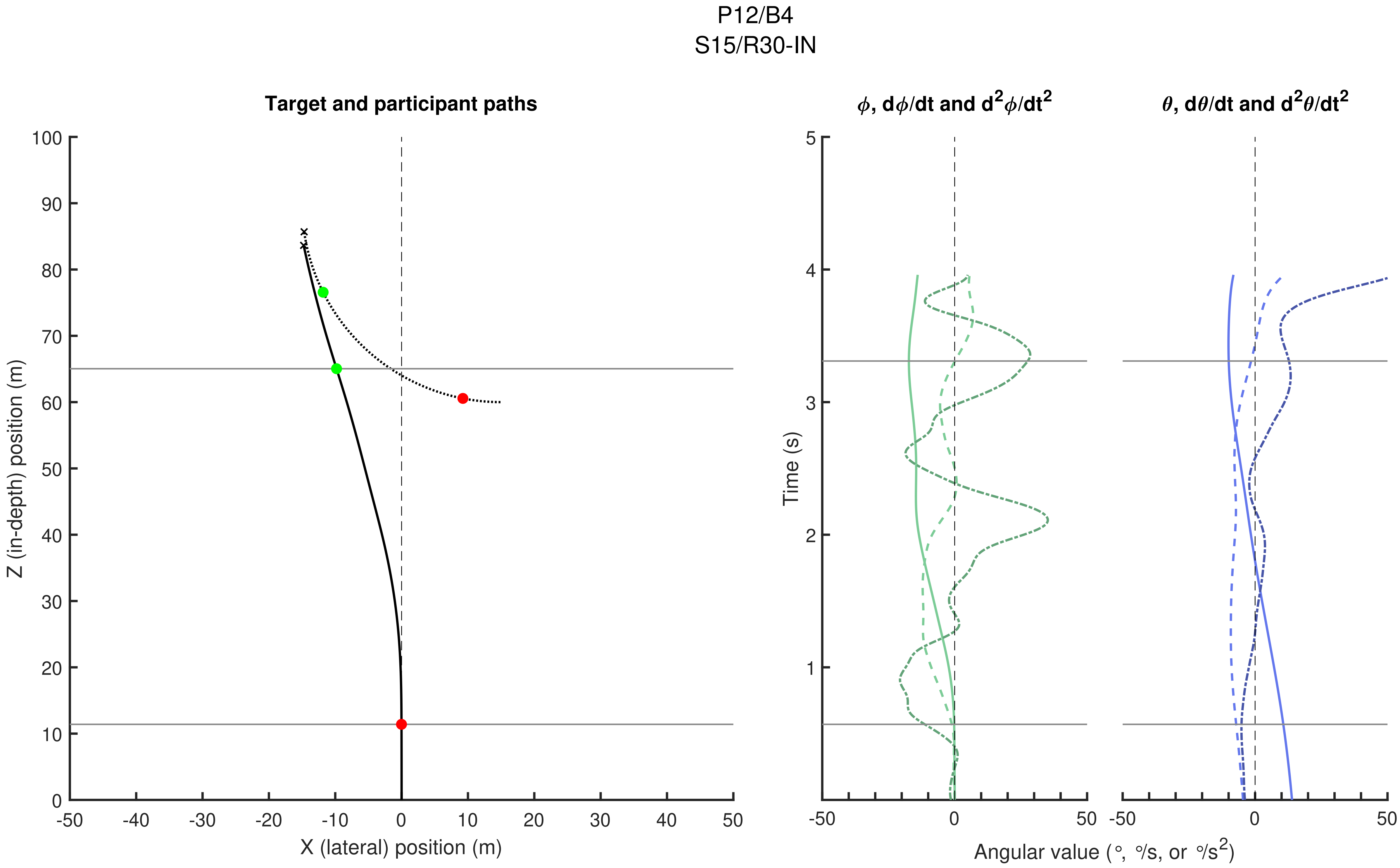

P12/B4
S15/R30-IN
Target and participant paths
Z (in-depth) position (m)
X (lateral) position (m)
ϕ, dϕ/dt and d$^2\phi$/dt^2
θ, dθ/dt and d$^2\theta$/dt^2
Time (s)
Angular value (°, °/s, or °/s^2)

P12/B4
S20/R10-IN

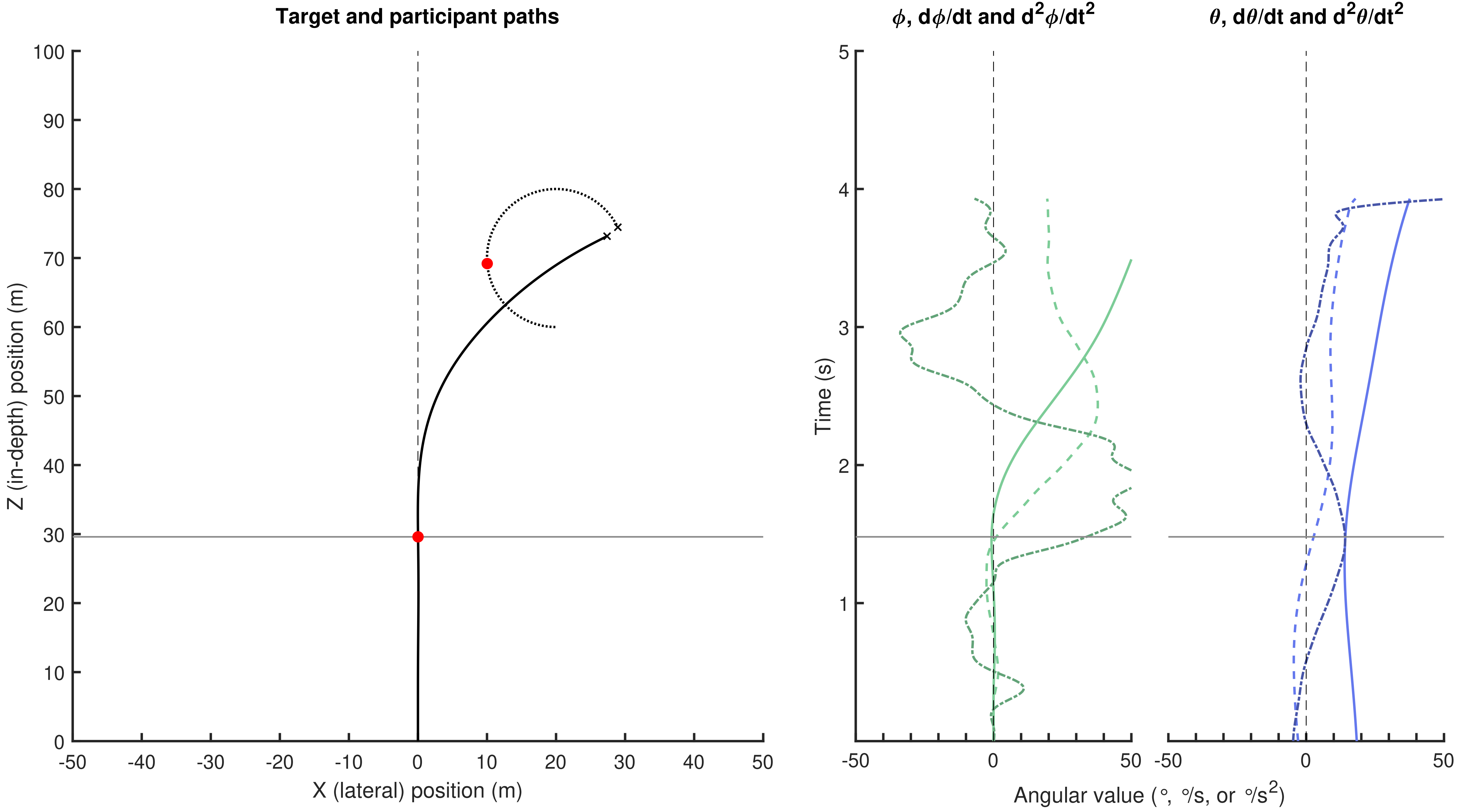

P12/B4
S20/R20-IN

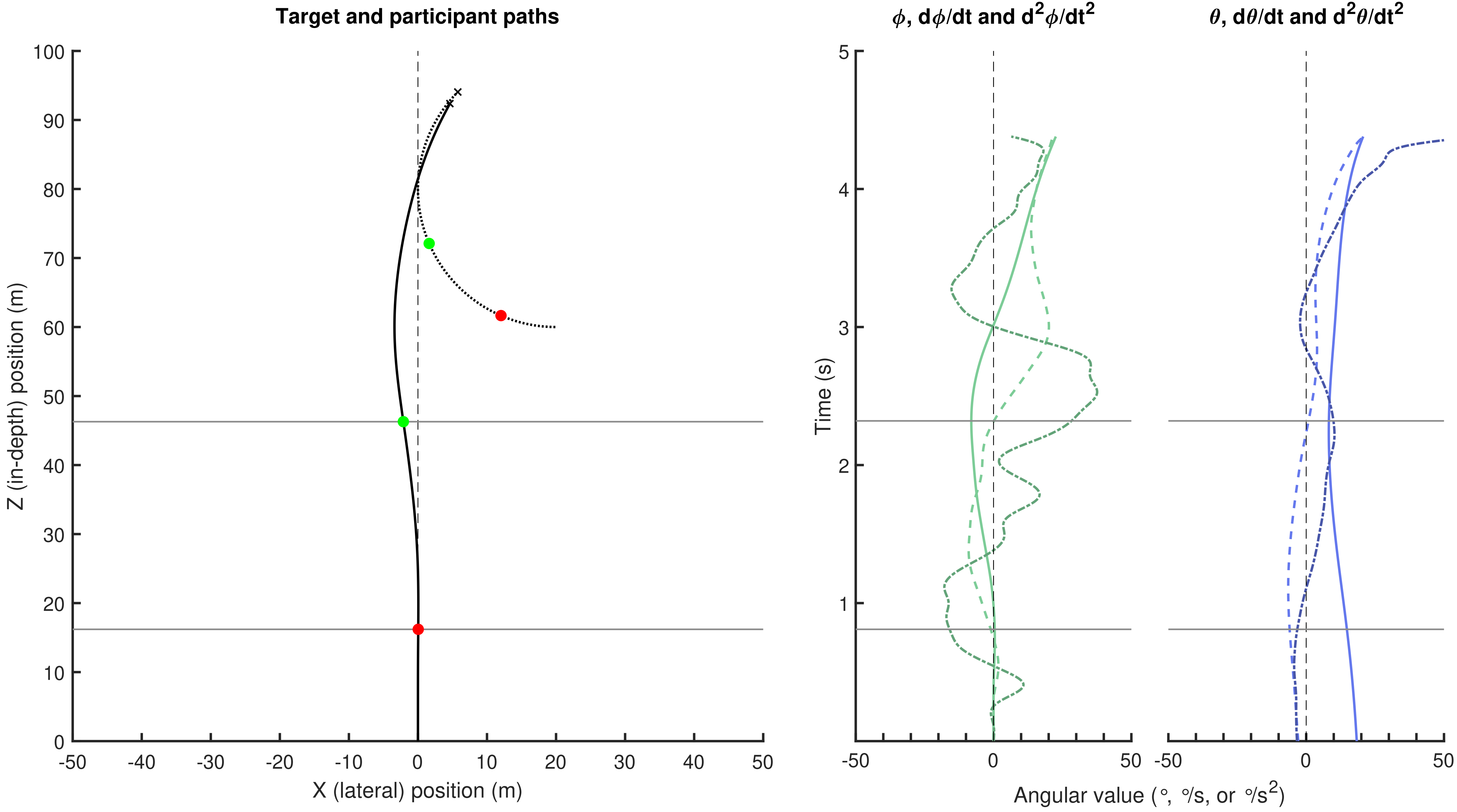

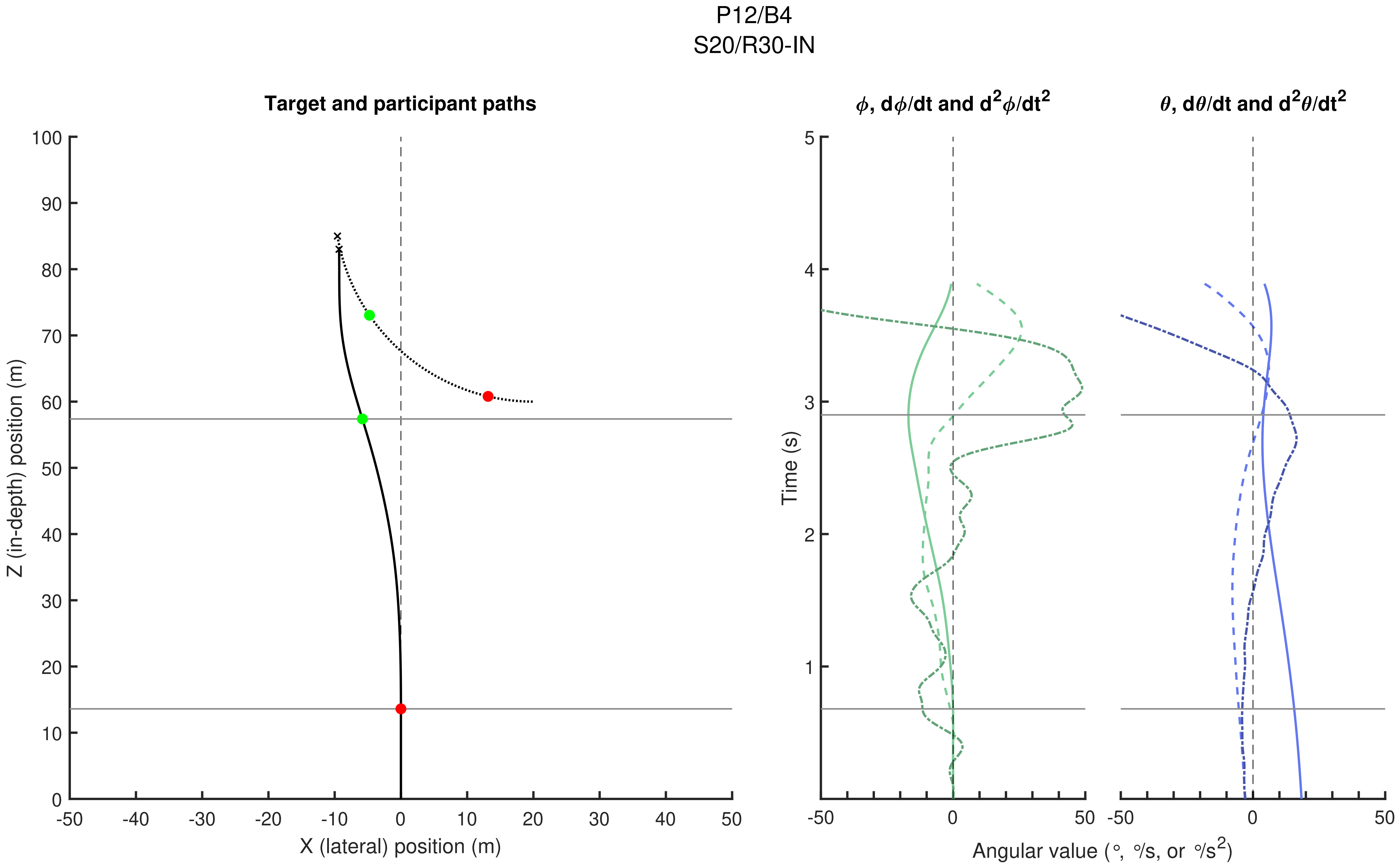

P12/B4
S20/R30-IN
Target and participant paths
Z (in-depth) position (m)
X (lateral) position (m)
ϕ, dϕ/dt and d$^2\phi$/dt^2
θ, dθ/dt and d$^2\theta$/dt^2
Time (s)
Angular value (°, °/s, or °/s^2)

P12/B5
S20/R10-IN

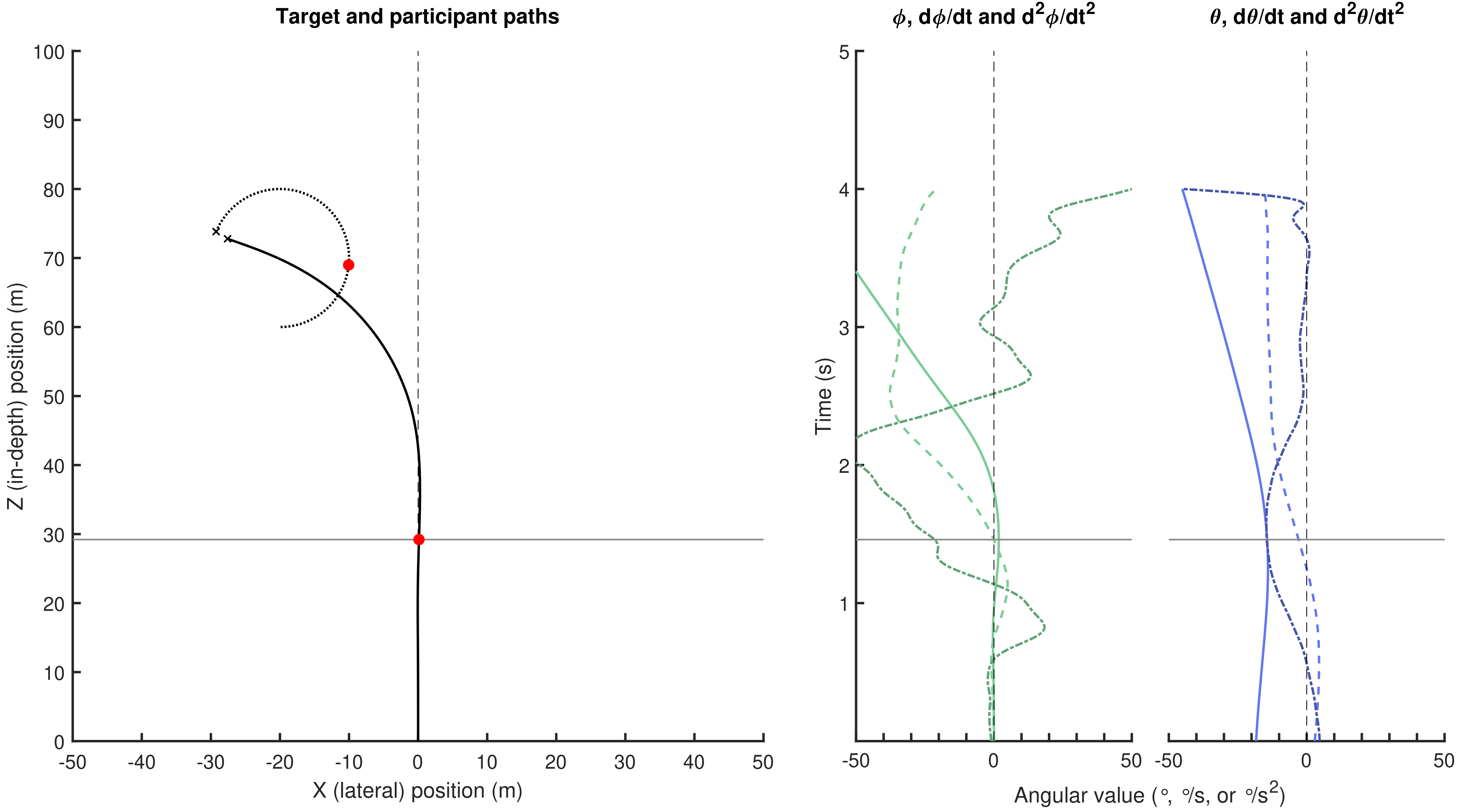

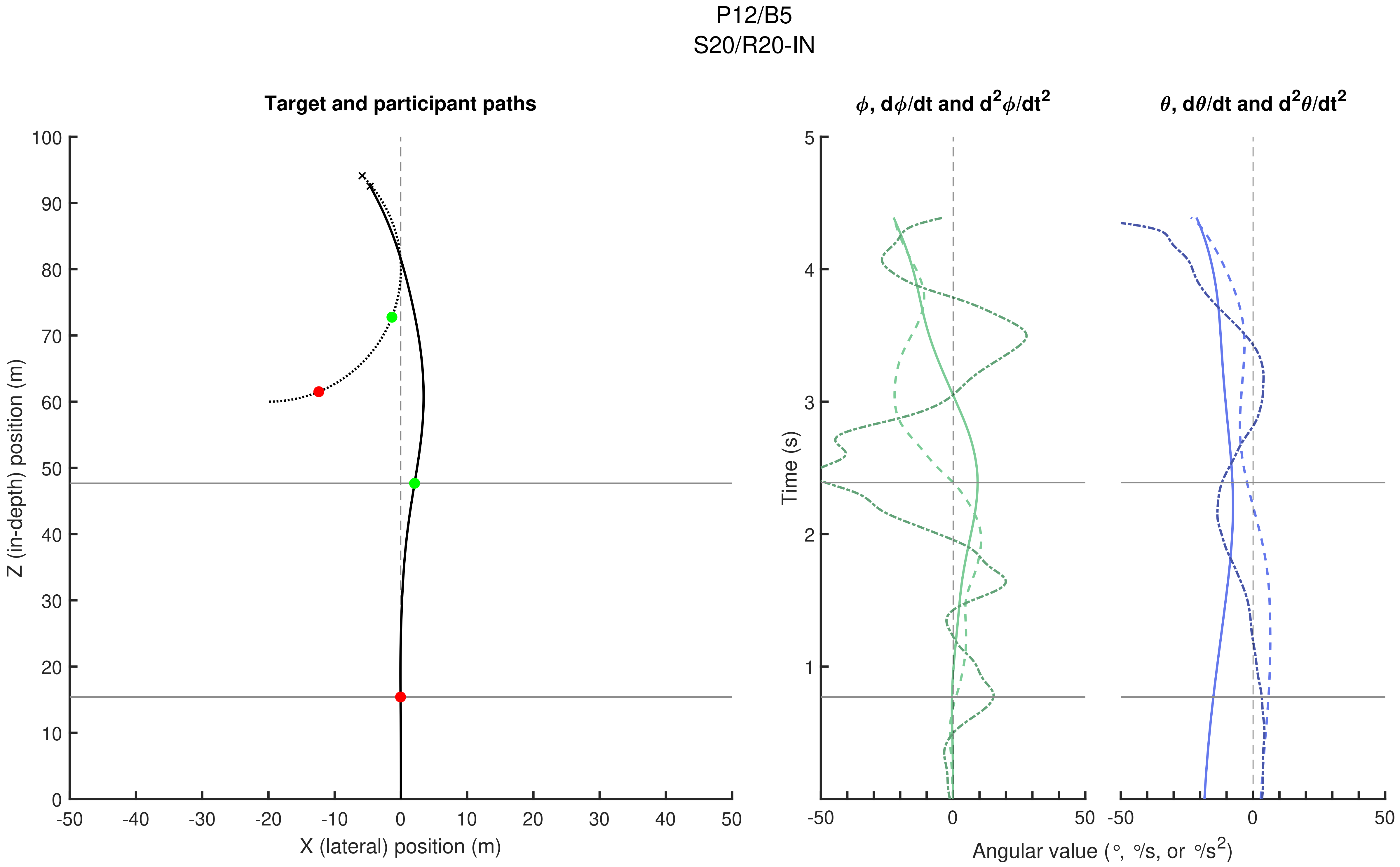
P12/B5
S20/R20-IN
Target and participant paths
Z (in-depth) position (m)
X (lateral) position (m)
ϕ, dϕ/dt and d$^2\phi$/dt^2
θ, dθ/dt and d$^2\theta$/dt^2
Time (s)
Angular value (°, °/s, or °/s^2)

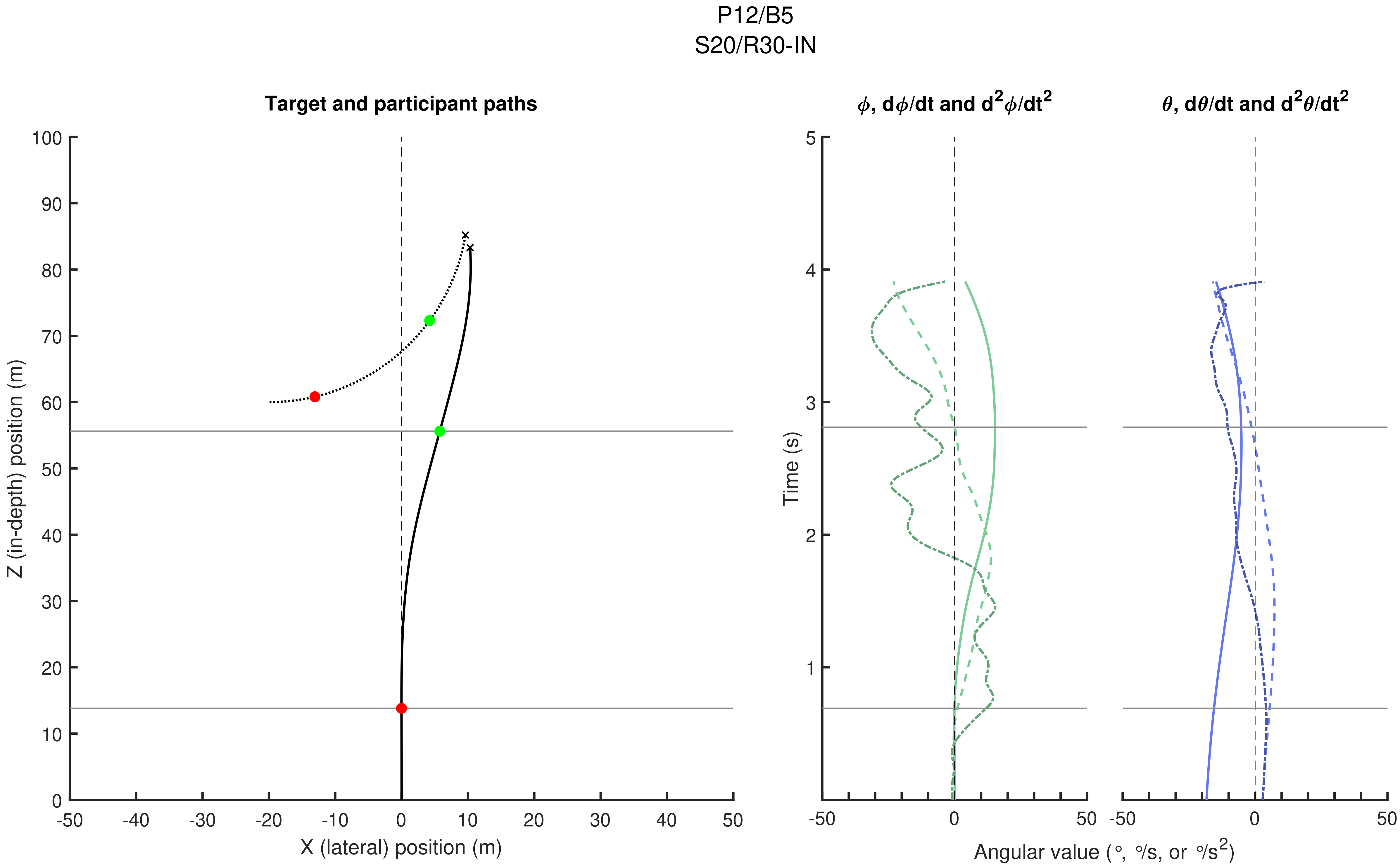
P12/B5
S20/R30-IN
Target and participant paths
Z (in-depth) position (m)
X (lateral) position (m)
100
90
80
70
60
50
40
30
20
10
0
-50
-40
-30
-20
-10
0
10
20
30
40
50
φ, dφ/dt and d²φ/dt²
θ, dθ/dt and d²θ/dt²
Time (s)
5
4
3
2
1
-50
0
50
-50
0
50
Angular value (°, °/s, or °/s²)

P12/B5
S15/R10-IN

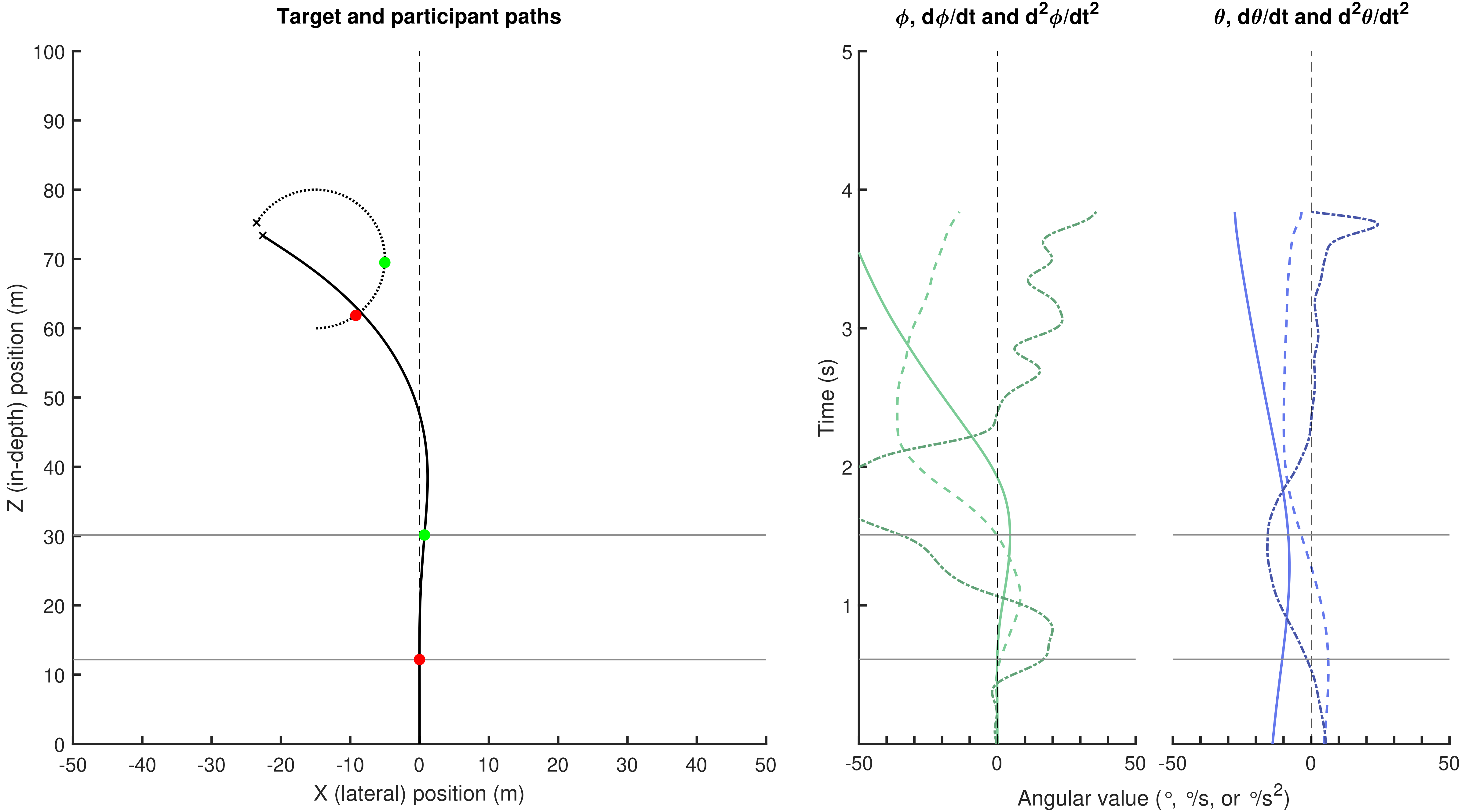

P12/B5
S15/R20-IN

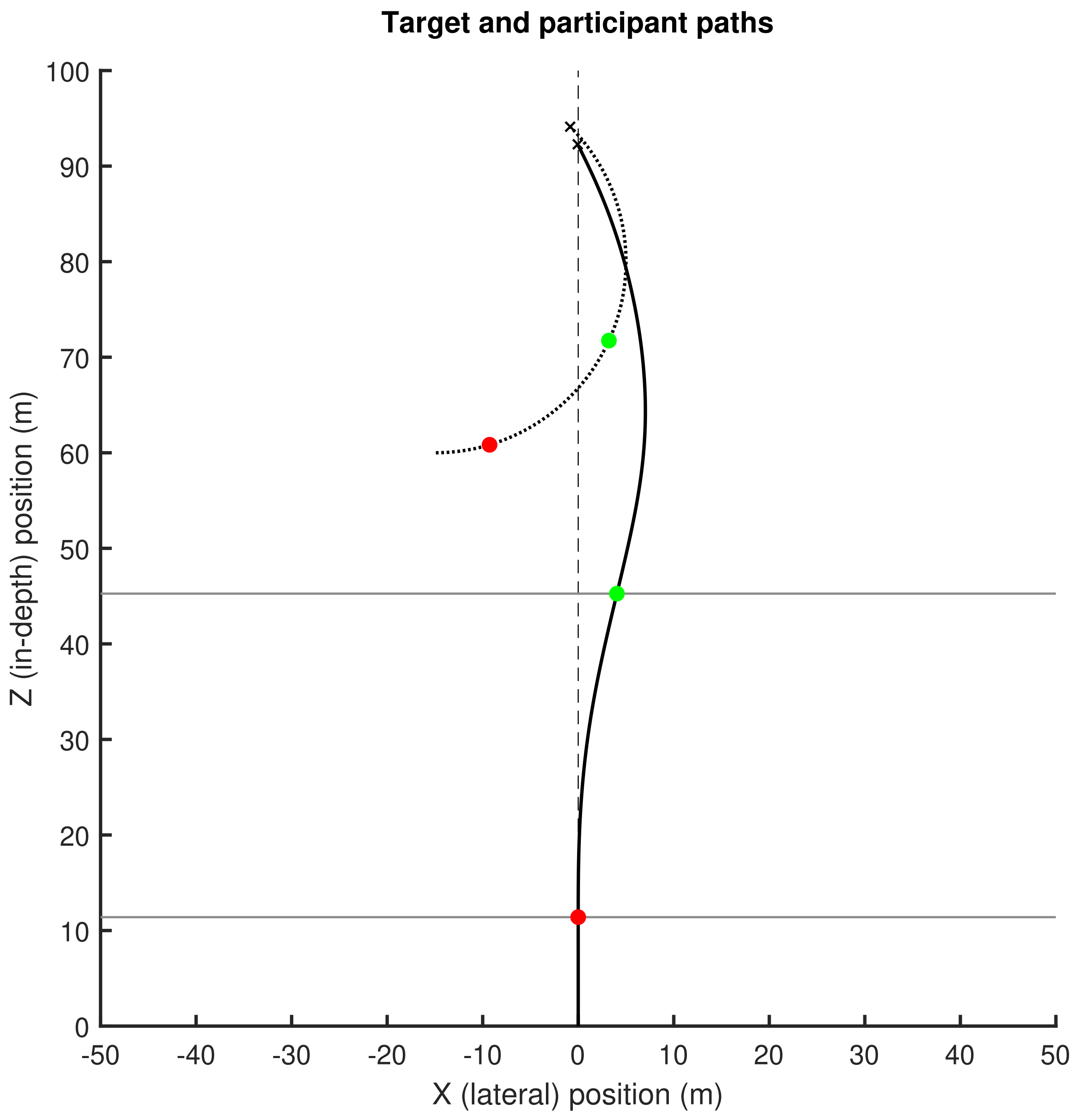

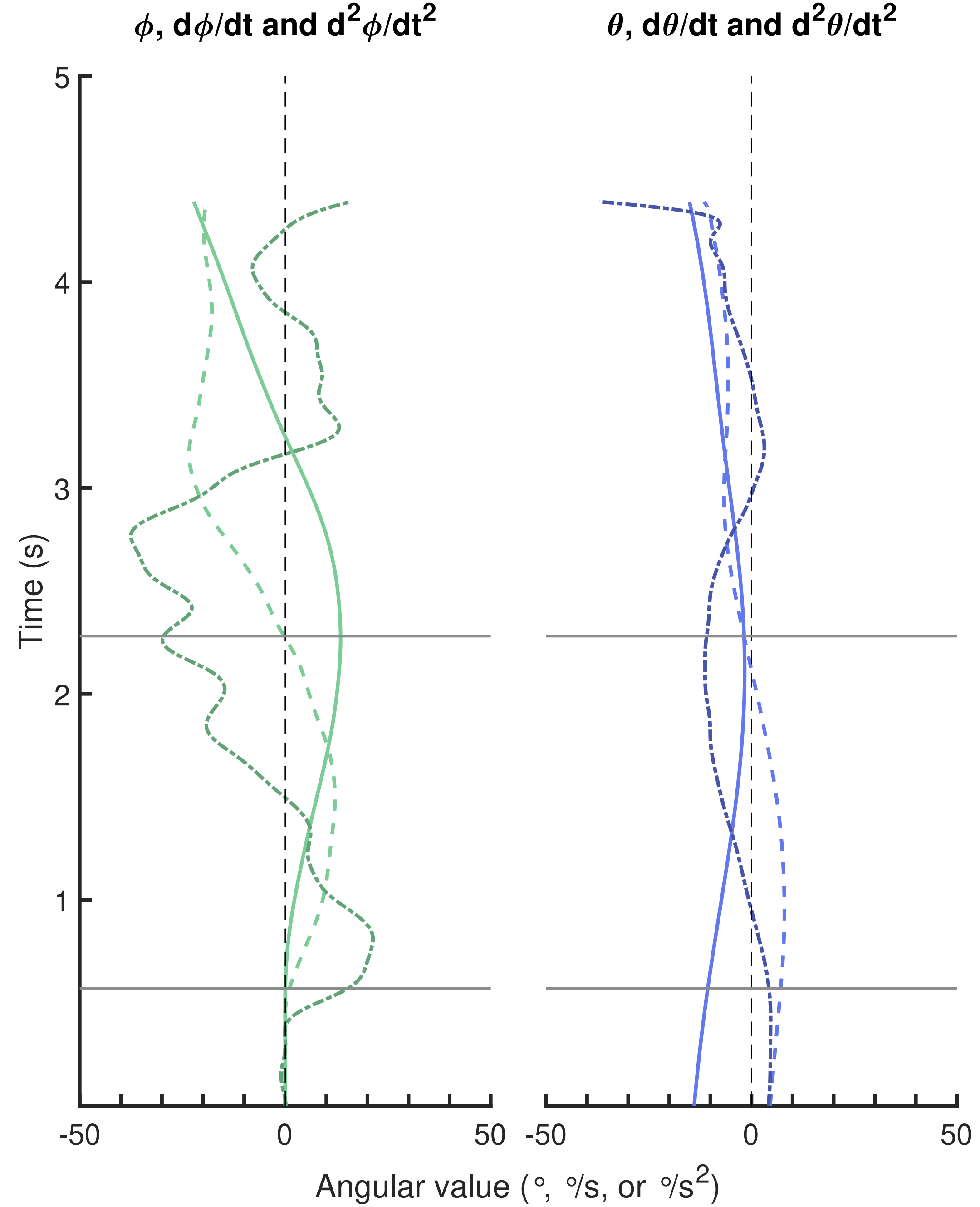

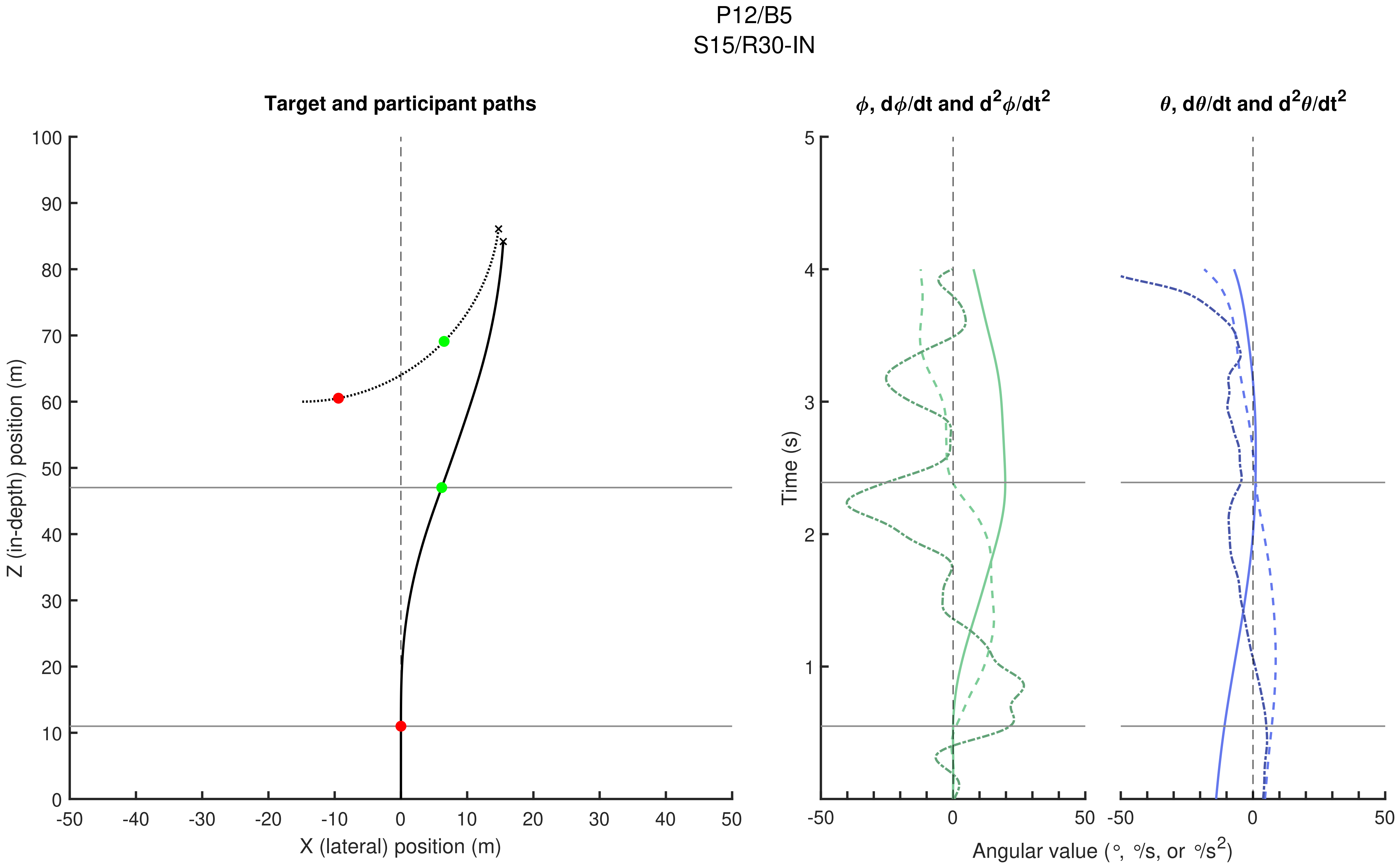

P12/B5
S15/R30-IN
Target and participant paths
Z (in-depth) position (m)
X (lateral) position (m)
φ, dφ/dt and d²φ/dt²
θ, dθ/dt and d²θ/dt²
Time (s)
Angular value (°, °/s, or °/s²)

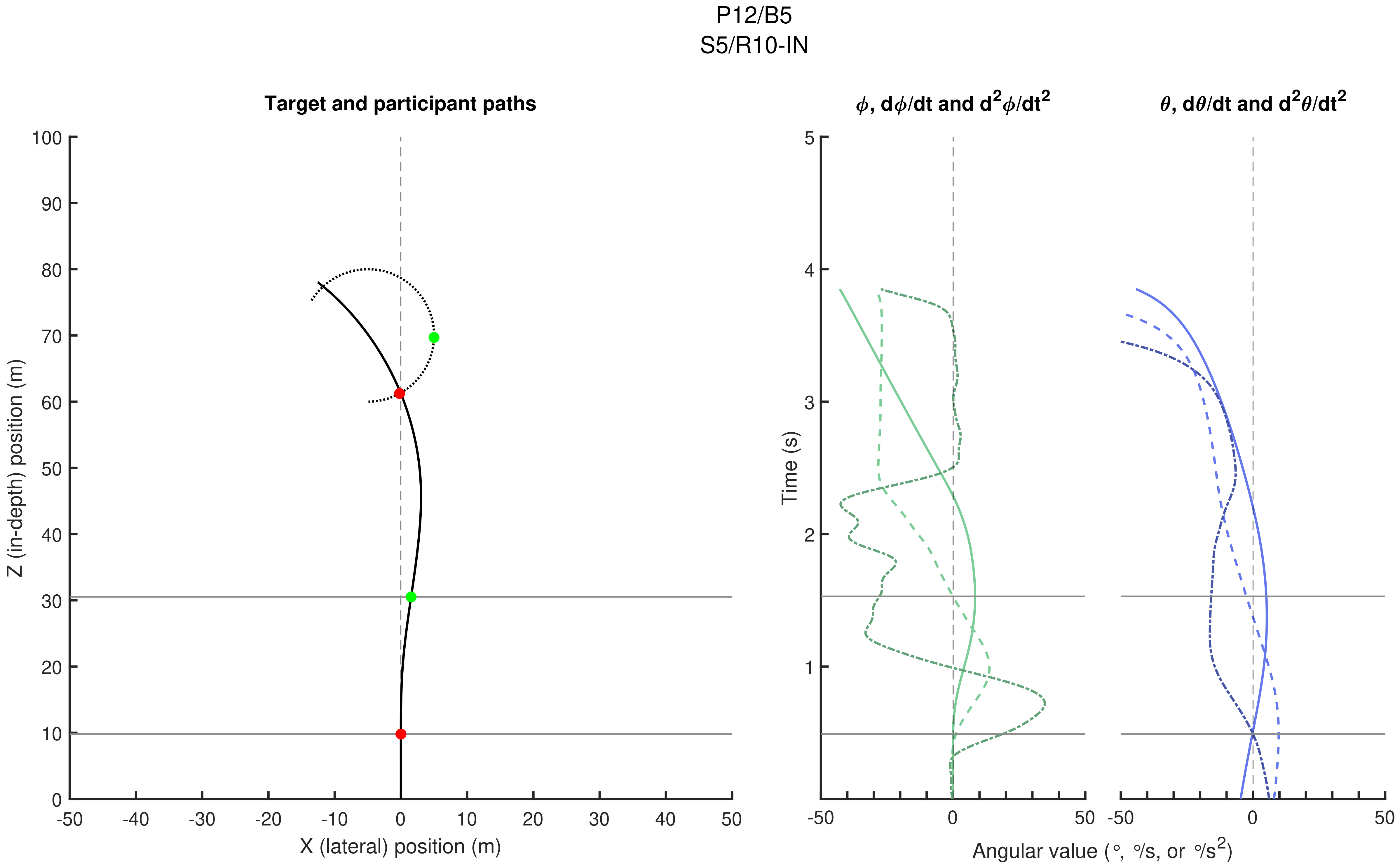

P12/B5
S5/R10-IN
Target and participant paths
Z (in-depth) position (m)
X (lateral) position (m)
0
10
20
30
40
50
60
70
80
90
100
-50
-40
-30
-20
-10
0
10
20
30
40
50
ϕ, dϕ/dt and d$^2\phi$/dt^2
θ, dθ/dt and d$^2\theta$/dt^2
Time (s)
1
2
3
4
5
-50
0
50
-50
0
50
Angular value (°, °/s, or °/s^2)

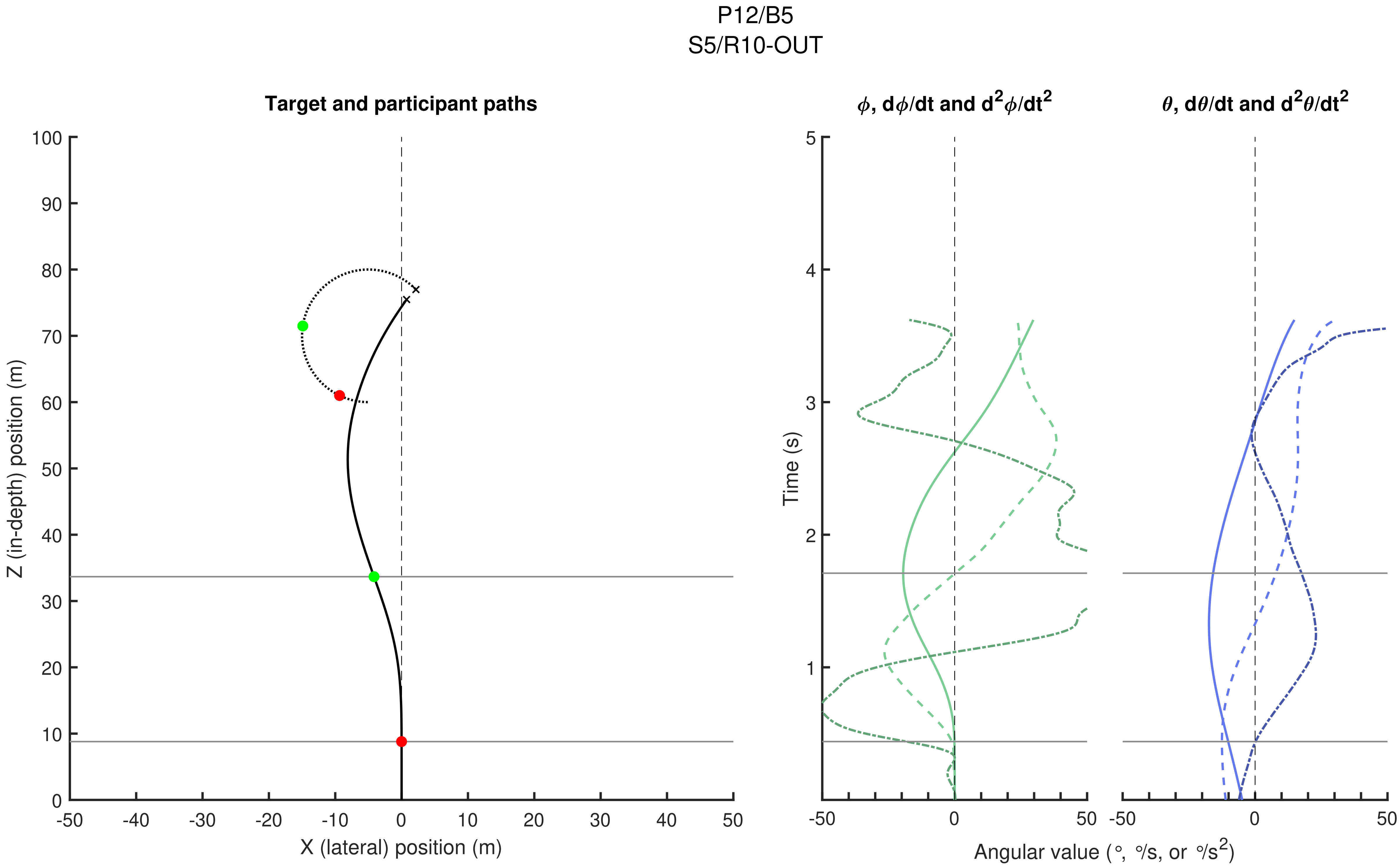
P12/B5
S5/R10-OUT
Target and participant paths
Z (in-depth) position (m)
X (lateral) position (m)
ϕ, dϕ/dt and d$^2\phi$/dt^2
θ, dθ/dt and d$^2\theta$/dt^2
Time (s)
Angular value (°, °/s, or °/s^2)

P12/B5
S5/R20-IN

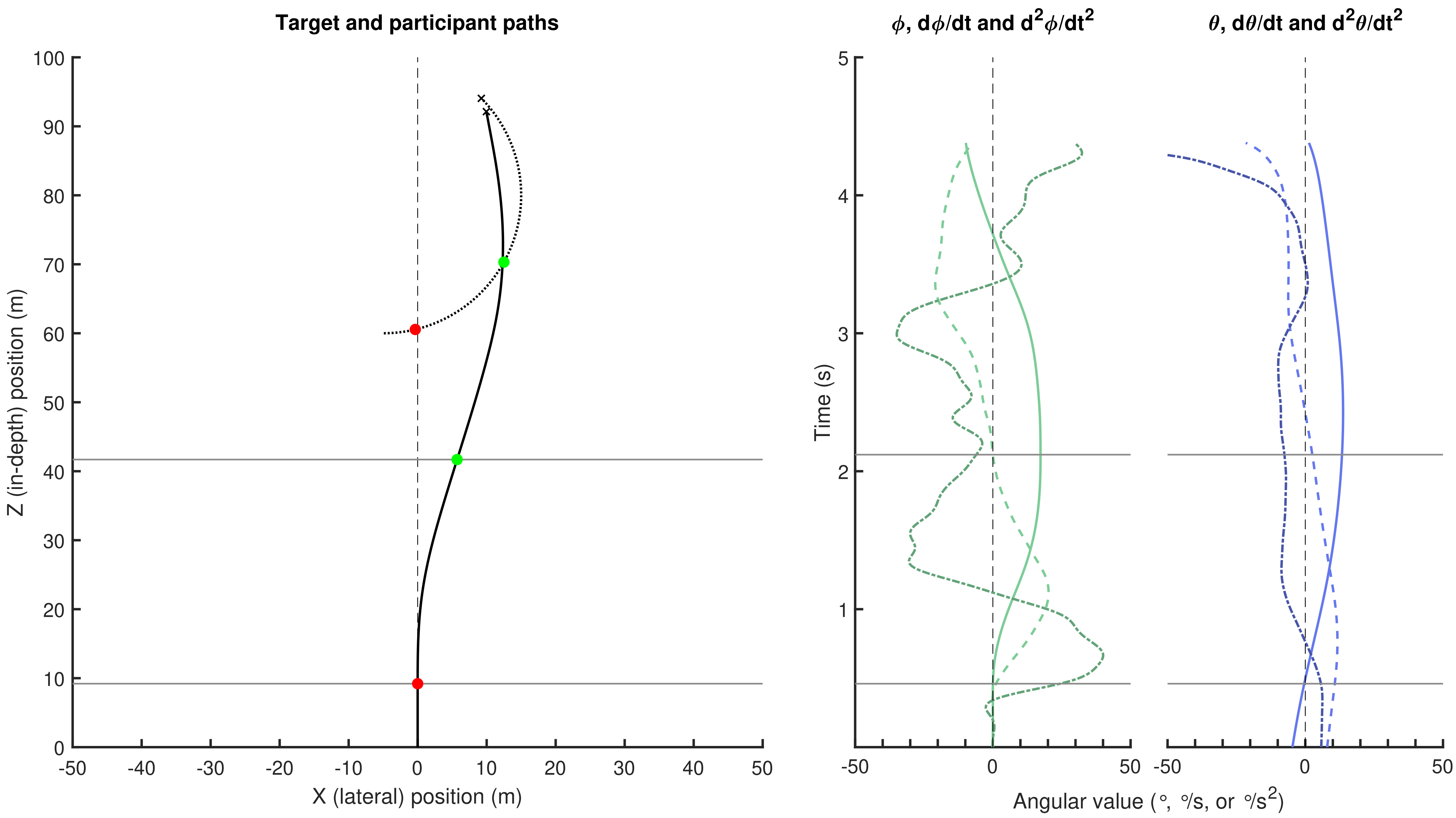

P12/B5
S5/R20-OUT

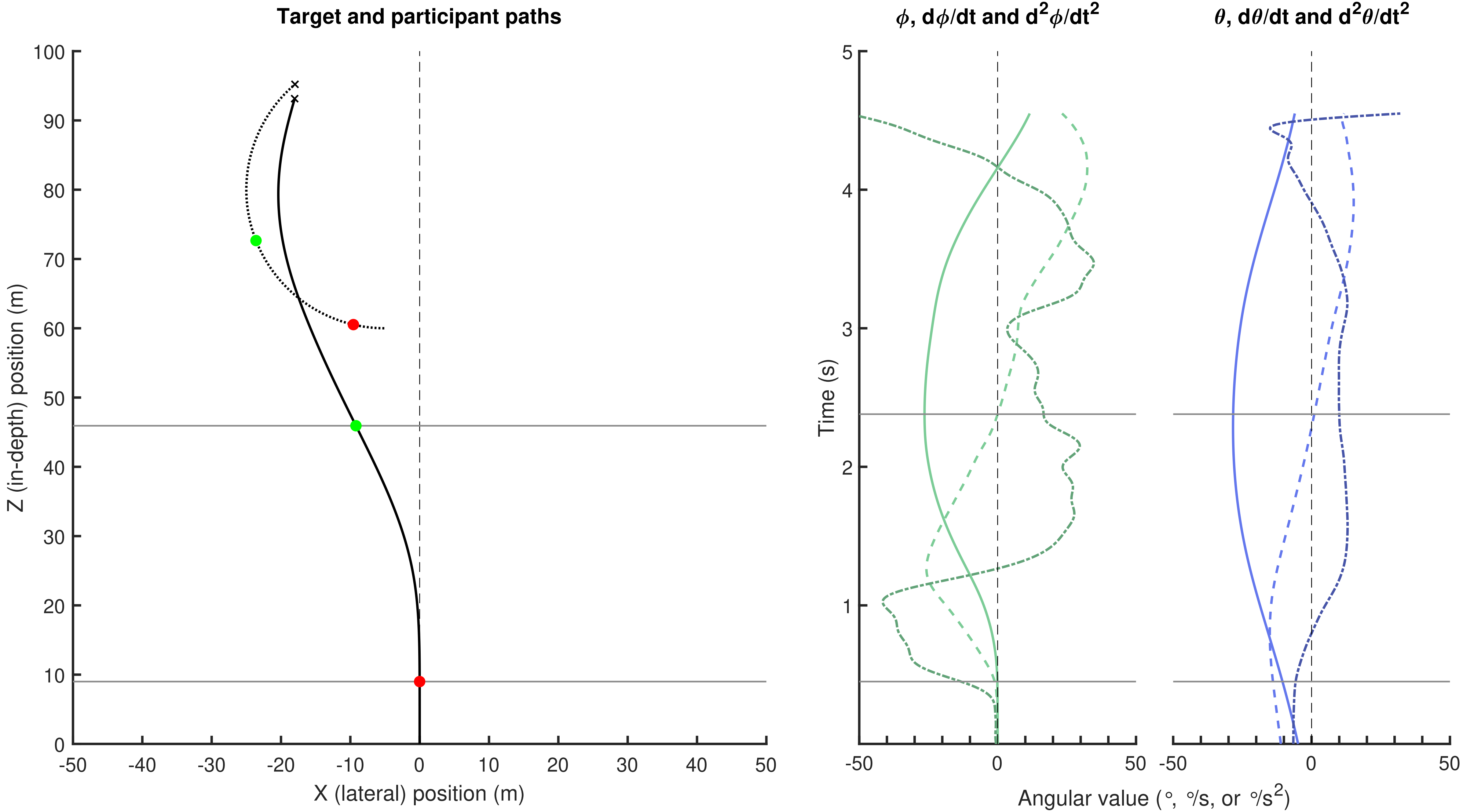

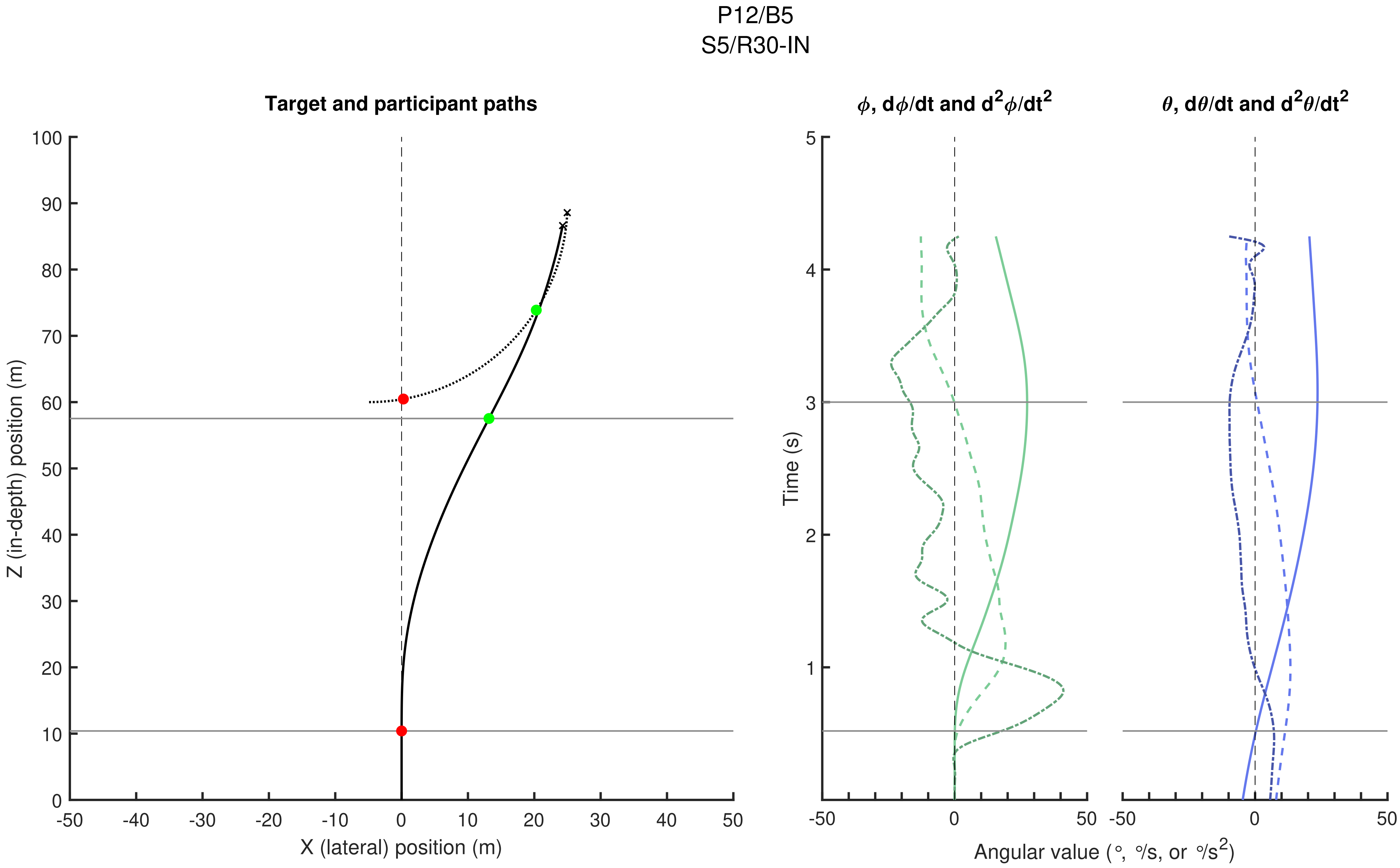

P12/B5
S5/R30-IN
Target and participant paths
Z (in-depth) position (m)
X (lateral) position (m)
ϕ, dϕ/dt and d$^2\phi$/dt^2
θ, dθ/dt and d$^2\theta$/dt^2
Time (s)
Angular value (°, °/s, or °/s^2)

P12/B5
S5/R30-OUT

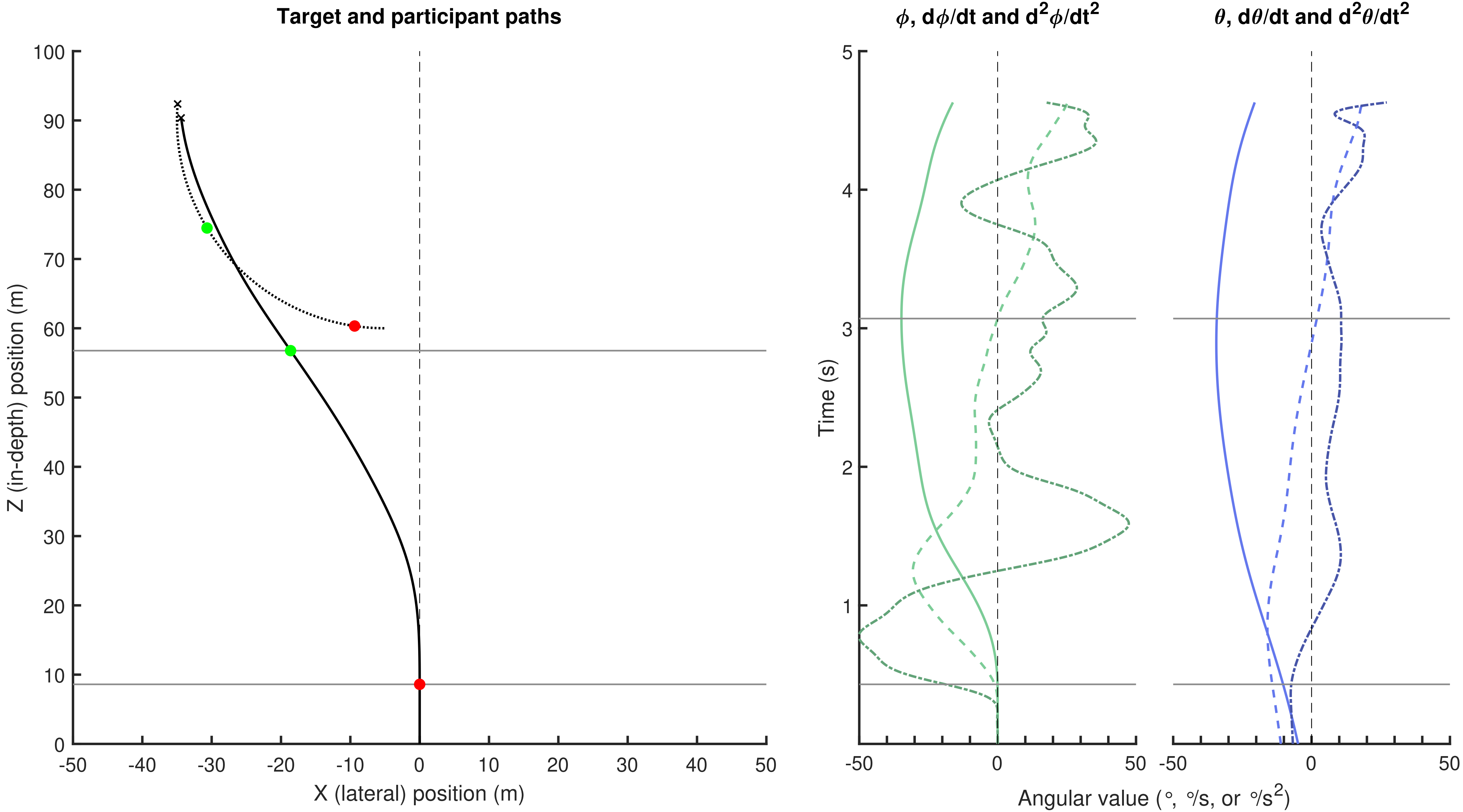

P12/B5
S5/R10-OUT

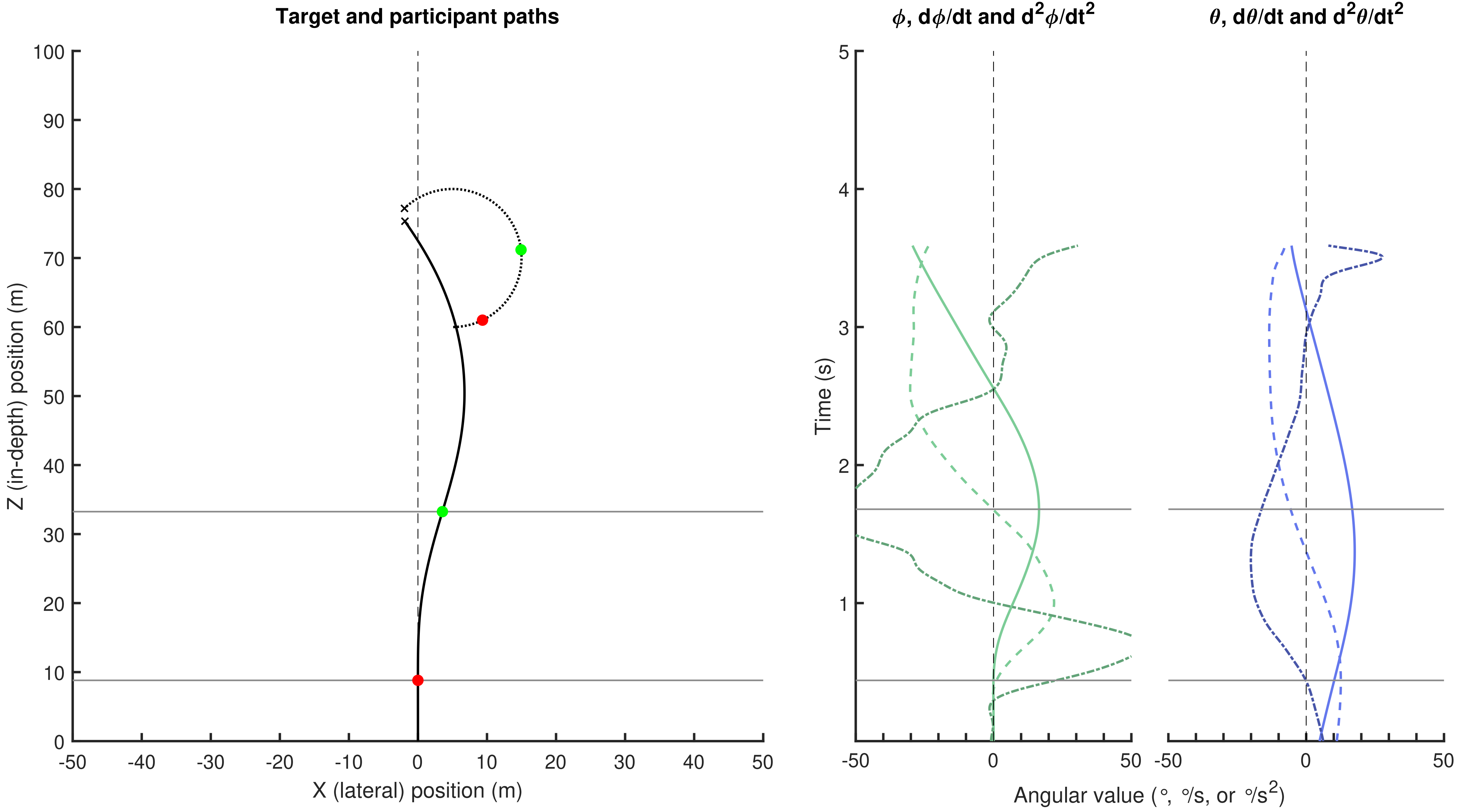

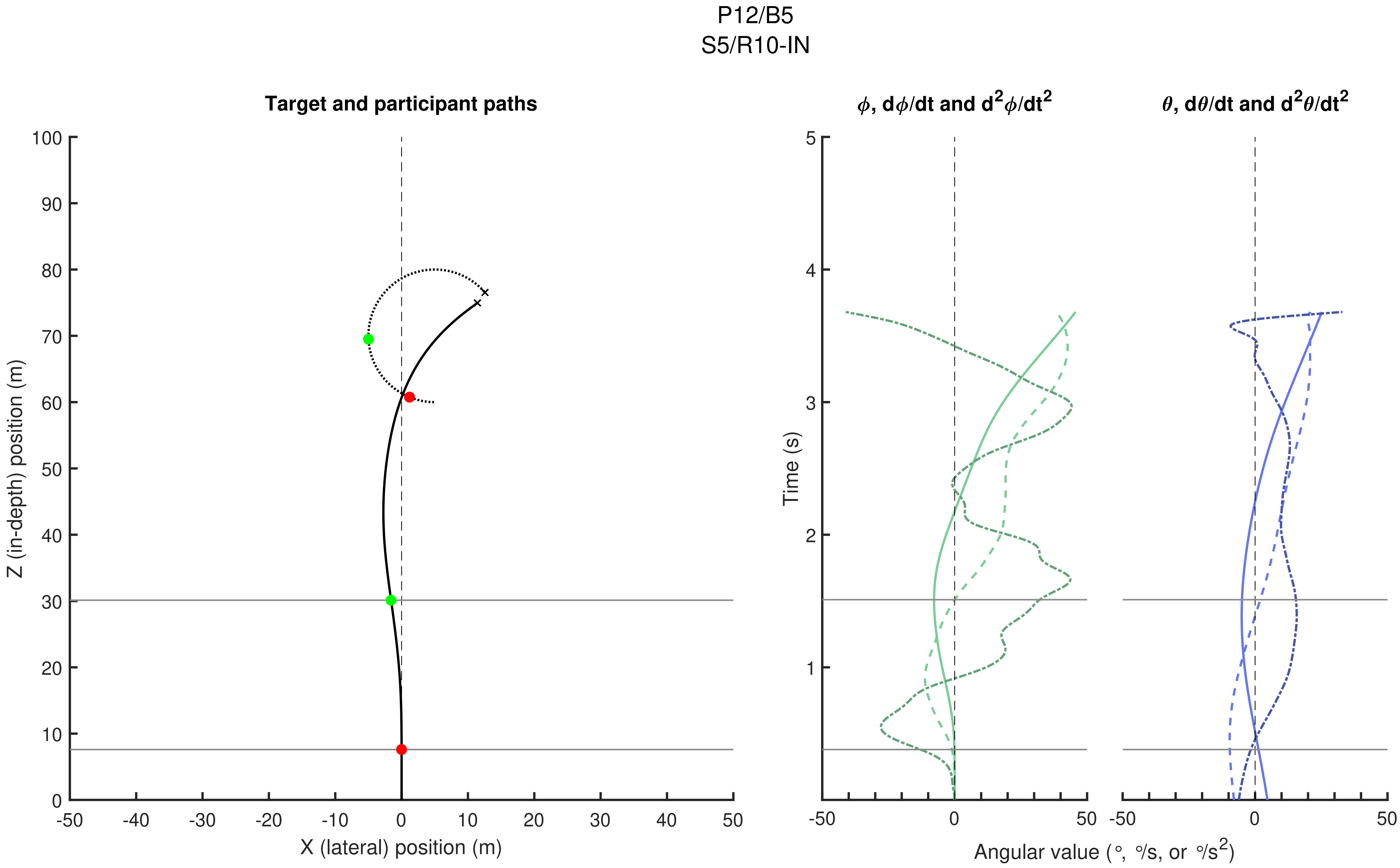
P12/B5
S5/R10-IN
Target and participant paths
Z (in-depth) position (m)
X (lateral) position (m)
100
90
80
70
60
50
40
30
20
10
0
-50
-40
-30
-20
-10
0
10
20
30
40
50
ϕ, dϕ/dt and d²ϕ/dt²
θ, dθ/dt and d²θ/dt²
Time (s)
5
4
3
2
1
-50
0
50
-50
0
50
Angular value (°, °/s, or °/s²)

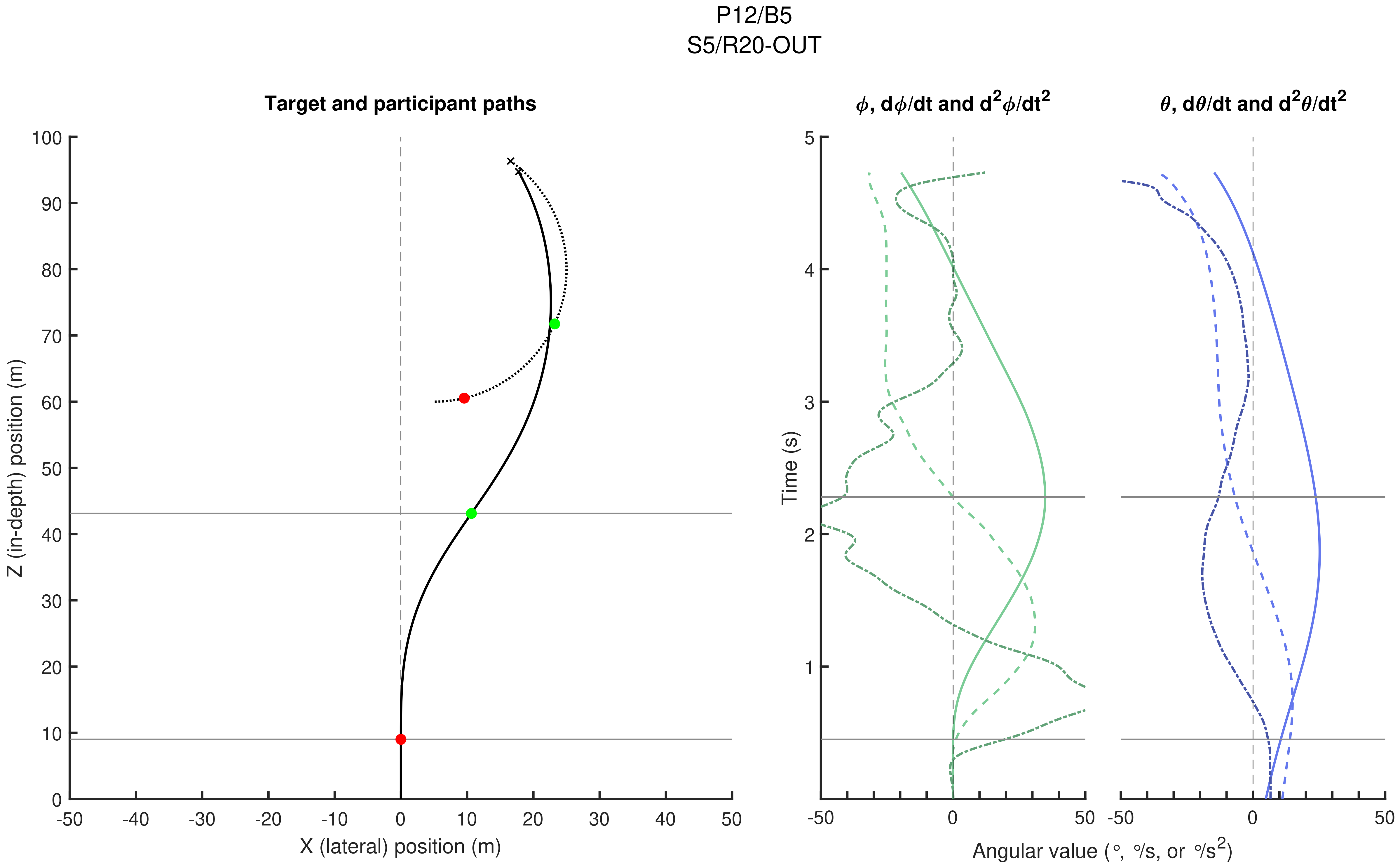

P12/B5
S5/R20-OUT
Target and participant paths
Z (in-depth) position (m)
X (lateral) position (m)
100
90
80
70
60
50
40
30
20
10
0
-50
-40
-30
-20
-10
0
10
20
30
40
50
ϕ, dϕ/dt and d²ϕ/dt²
θ, dθ/dt and d²θ/dt²
Time (s)
5
4
3
2
1
-50
0
50
-50
0
50
Angular value (°, °/s, or °/s²)

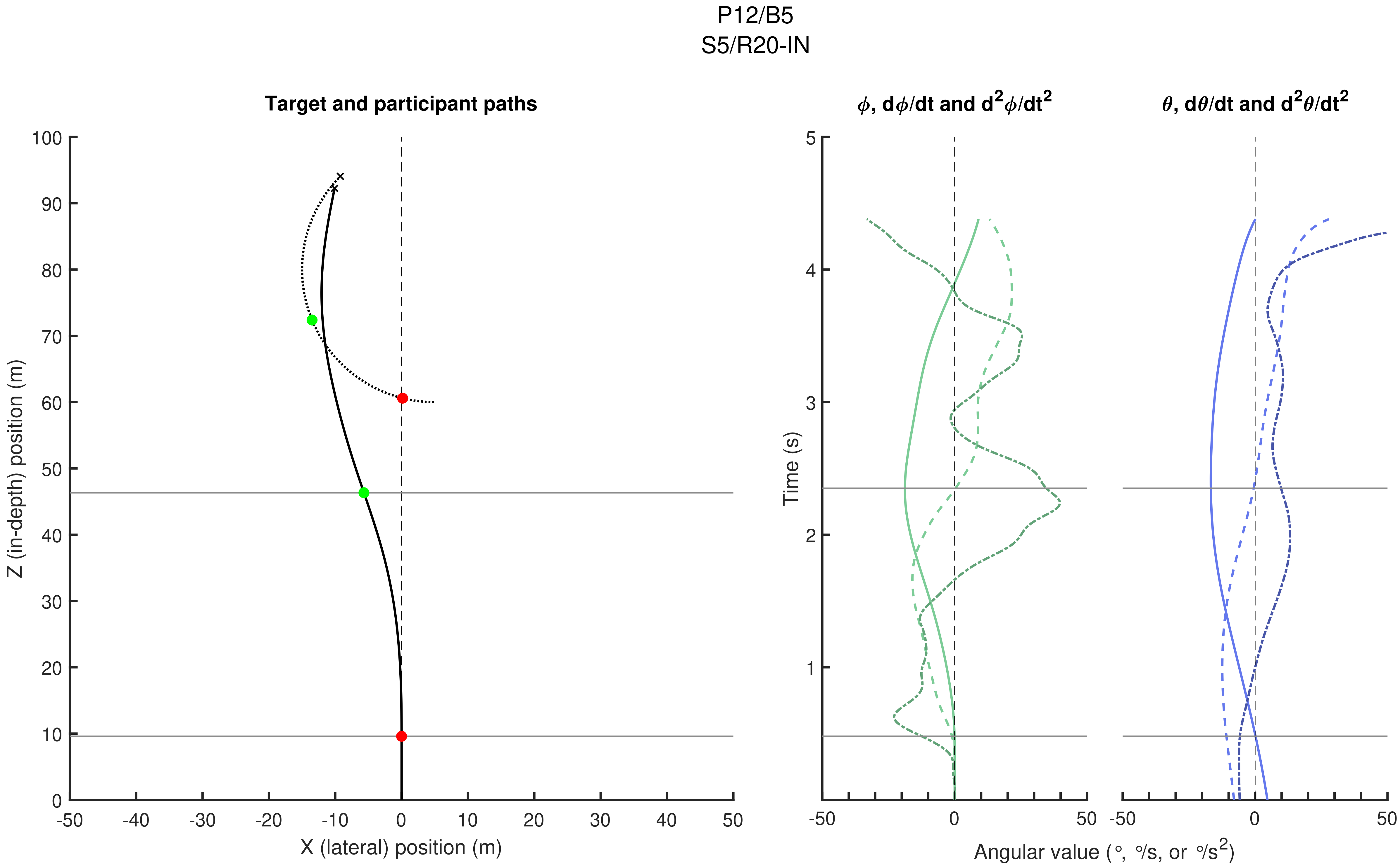
P12/B5
S5/R20-IN
Target and participant paths
Z (in-depth) position (m)
X (lateral) position (m)
ϕ, dϕ/dt and d²ϕ/dt²
θ, dθ/dt and d²θ/dt²
Time (s)
Angular value (°, °/s, or °/s²)

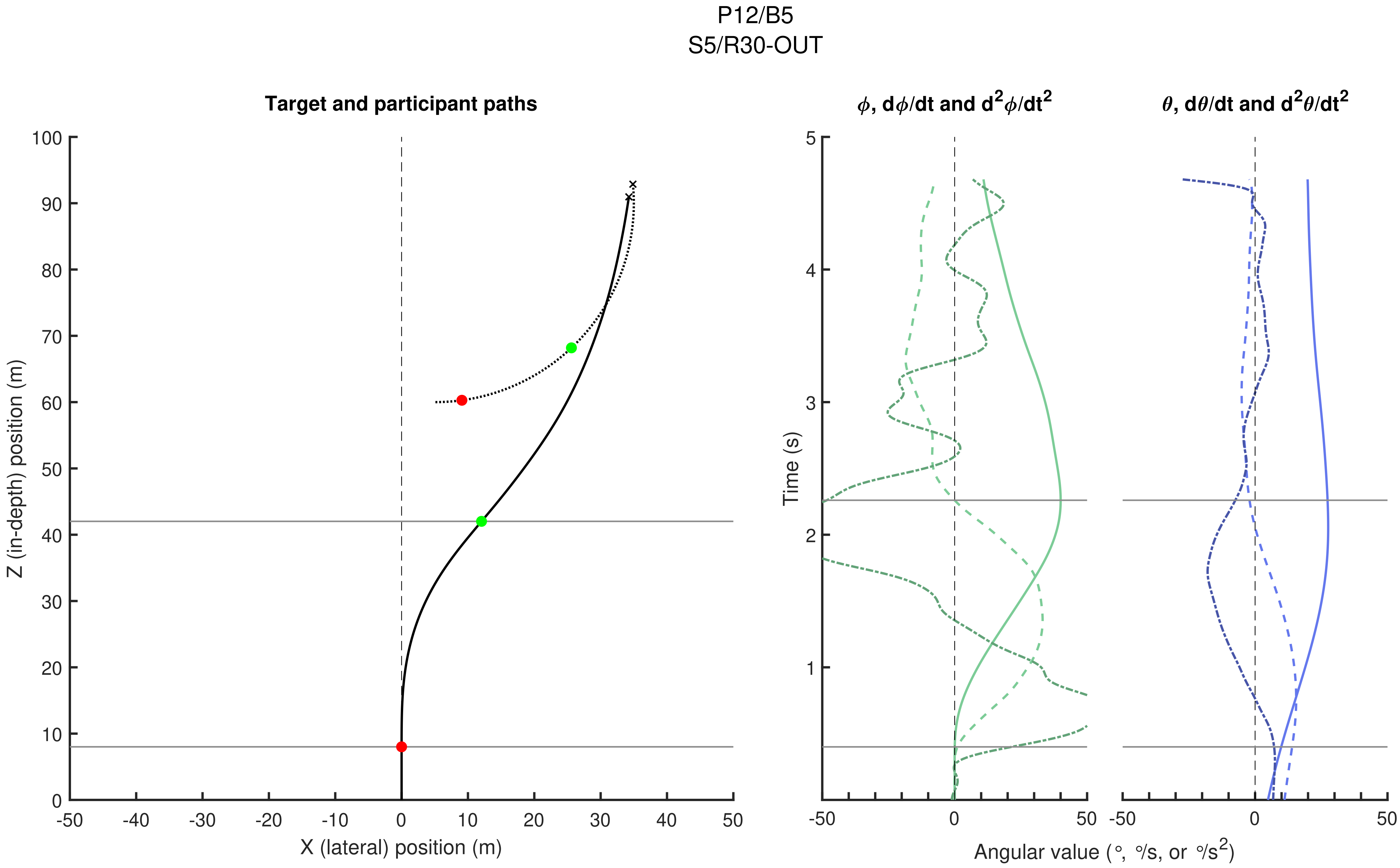
P12/B5
S5/R30-OUT
Target and participant paths
Z (in-depth) position (m)
X (lateral) position (m)
100
90
80
70
60
50
40
30
20
10
0
-50
-40
-30
-20
-10
0
10
20
30
40
50
ϕ, dϕ/dt and d²ϕ/dt²
θ, dθ/dt and d²θ/dt²
Time (s)
5
4
3
2
1
-50
0
50
-50
0
50
Angular value (°, °/s, or °/s²)

P12/B5
S5/R30-IN

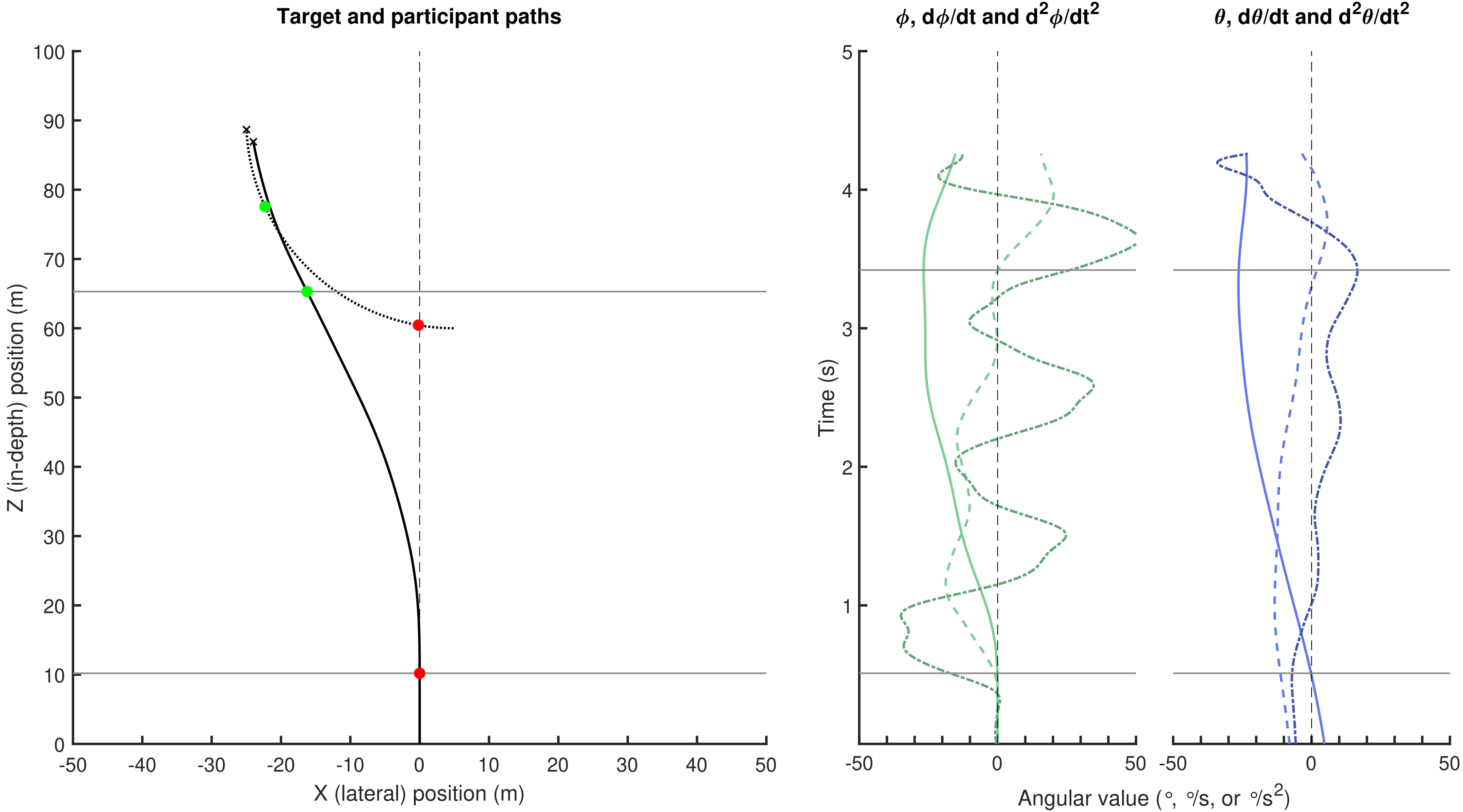

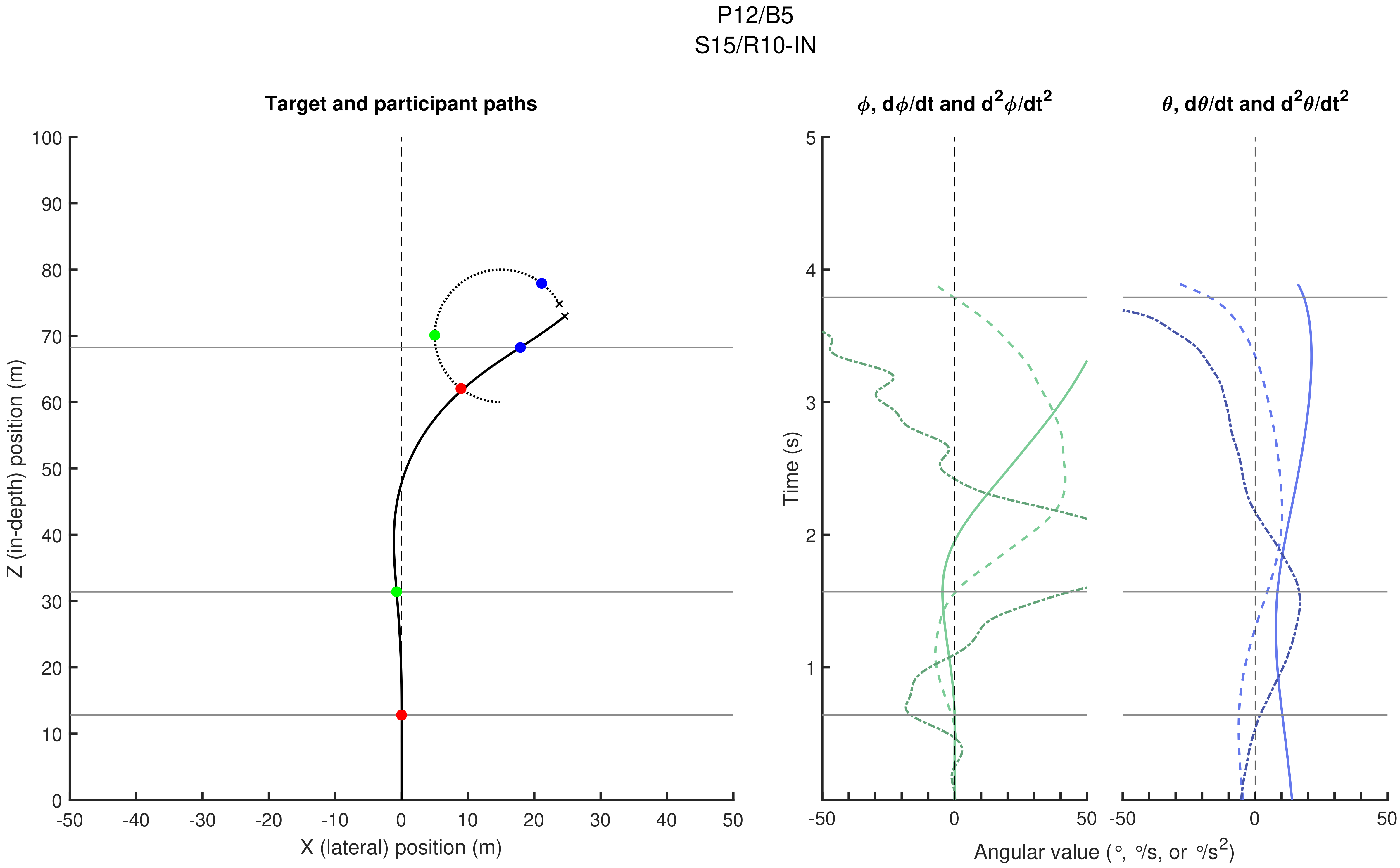

P12/B5
S15/R10-IN
Target and participant paths
ϕ, dϕ/dt and d$^2\phi$/dt^2
θ, dθ/dt and d$^2\theta$/dt^2
Z (in-depth) position (m)
X (lateral) position (m)
Time (s)
Angular value (°, °/s, or °/s^2)

P12/B5
S15/R20-IN

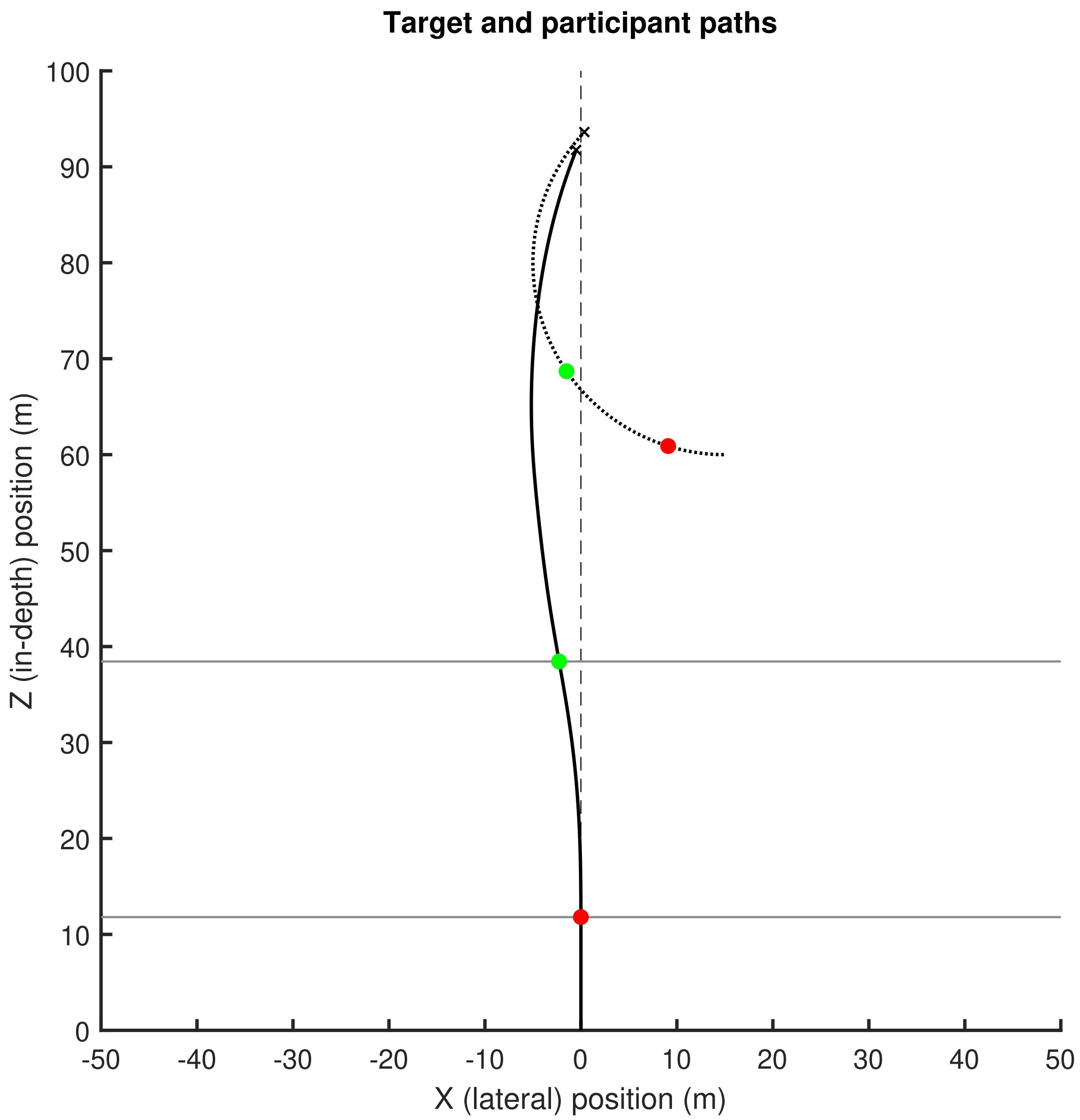

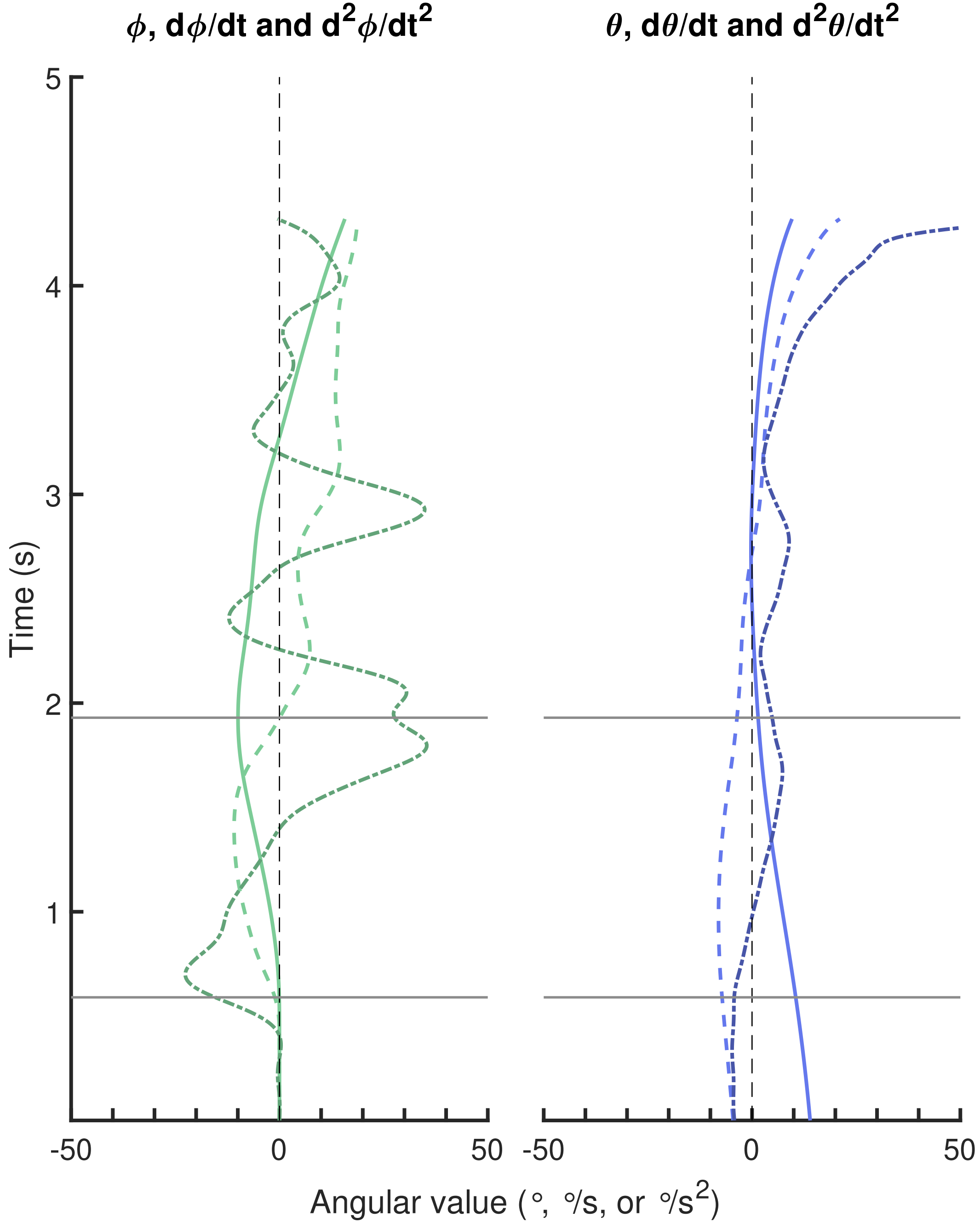

P12/B5
S15/R30-IN

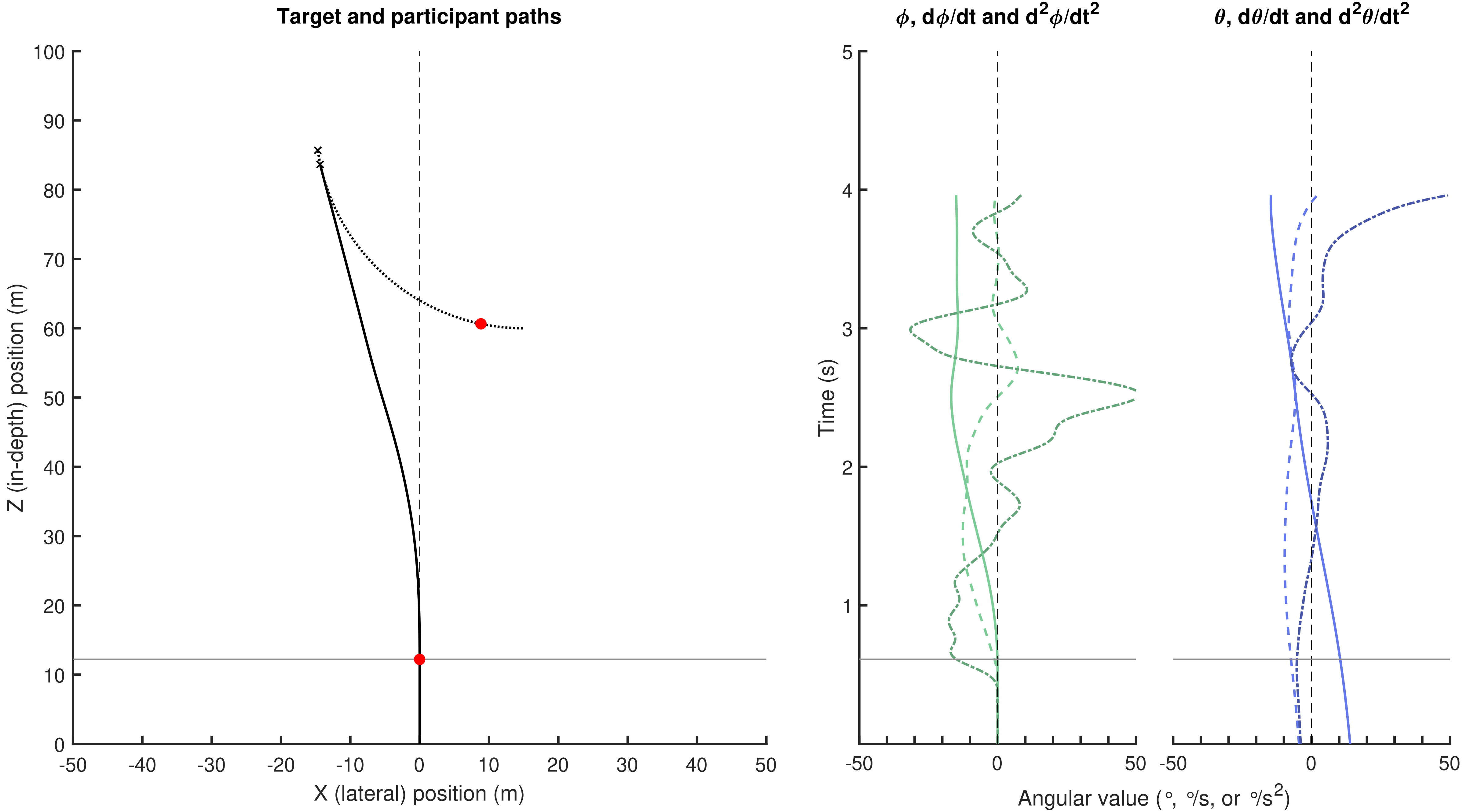

P12/B5
S20/R10-IN

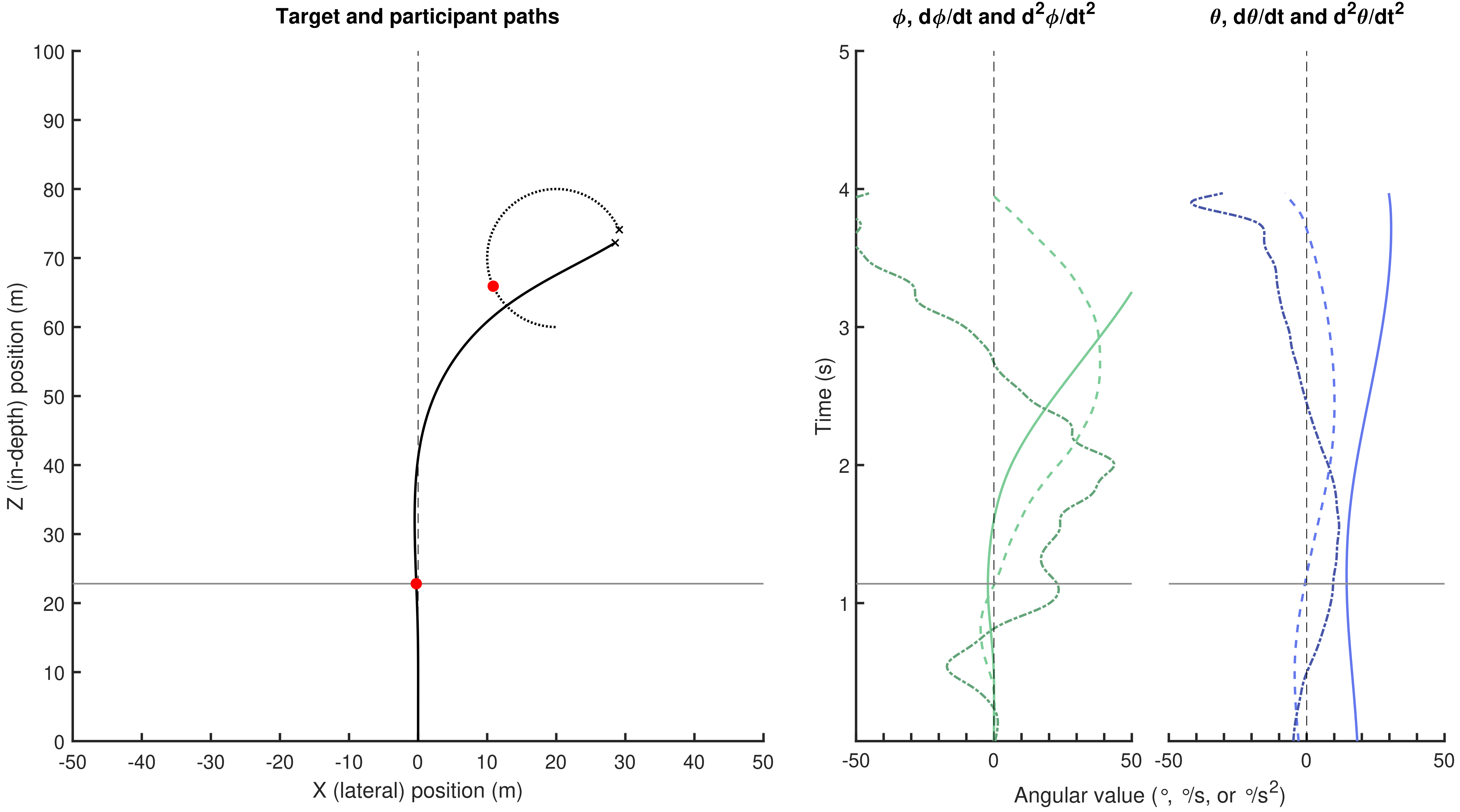

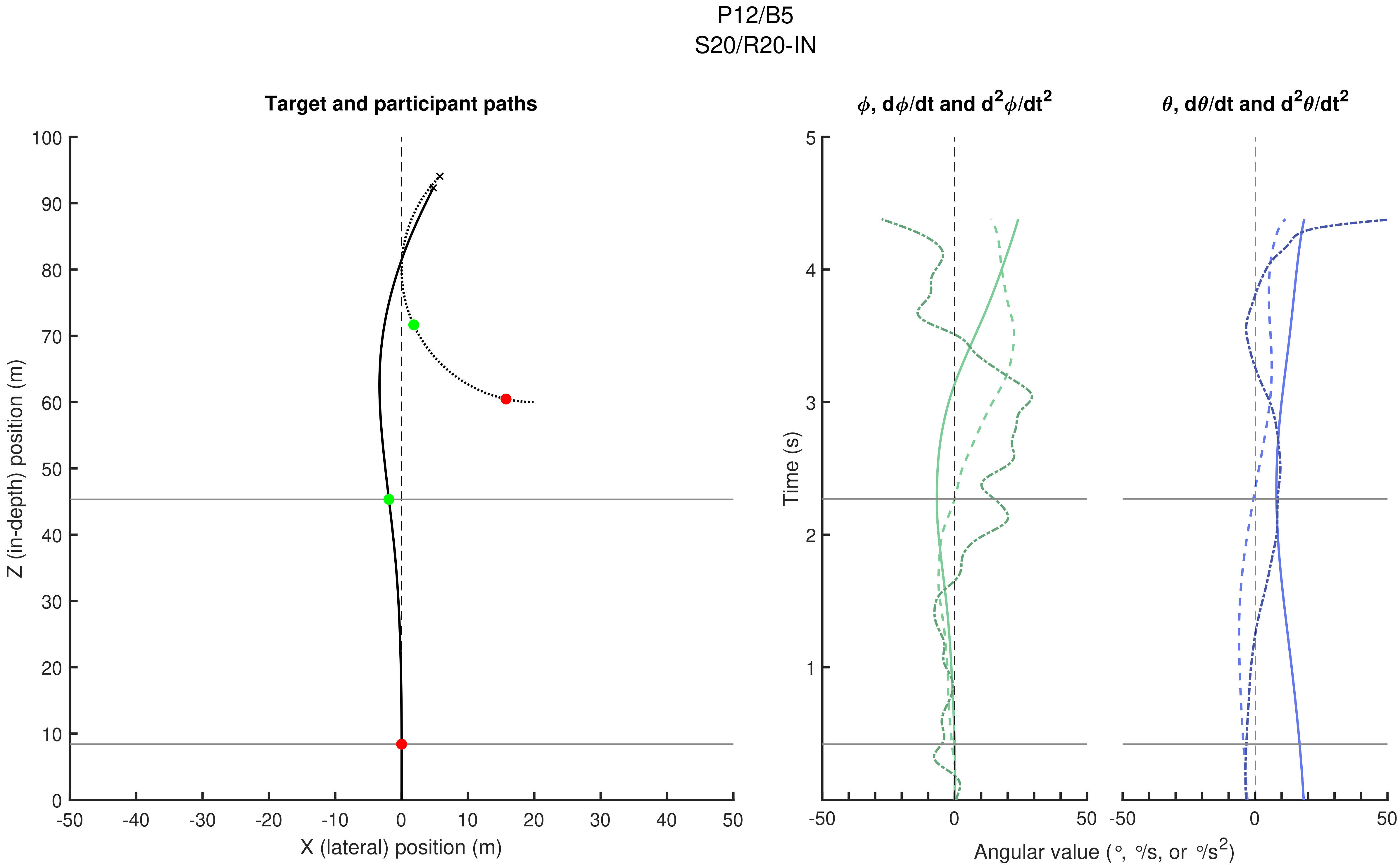
P12/B5
S20/R20-IN
Target and participant paths
ϕ, dϕ/dt and d$^2\phi$/dt^2
θ, dθ/dt and d$^2\theta$/dt^2
Z (in-depth) position (m)
X (lateral) position (m)
Time (s)
Angular value (°, °/s, or °/s^2)
100
90
80
70
60
50
40
30
20
10
0
-50
-40
-30
-20
-10
0
10
20
30
40
50
5
4
3
2
1
-50
0
50
-50
0
50

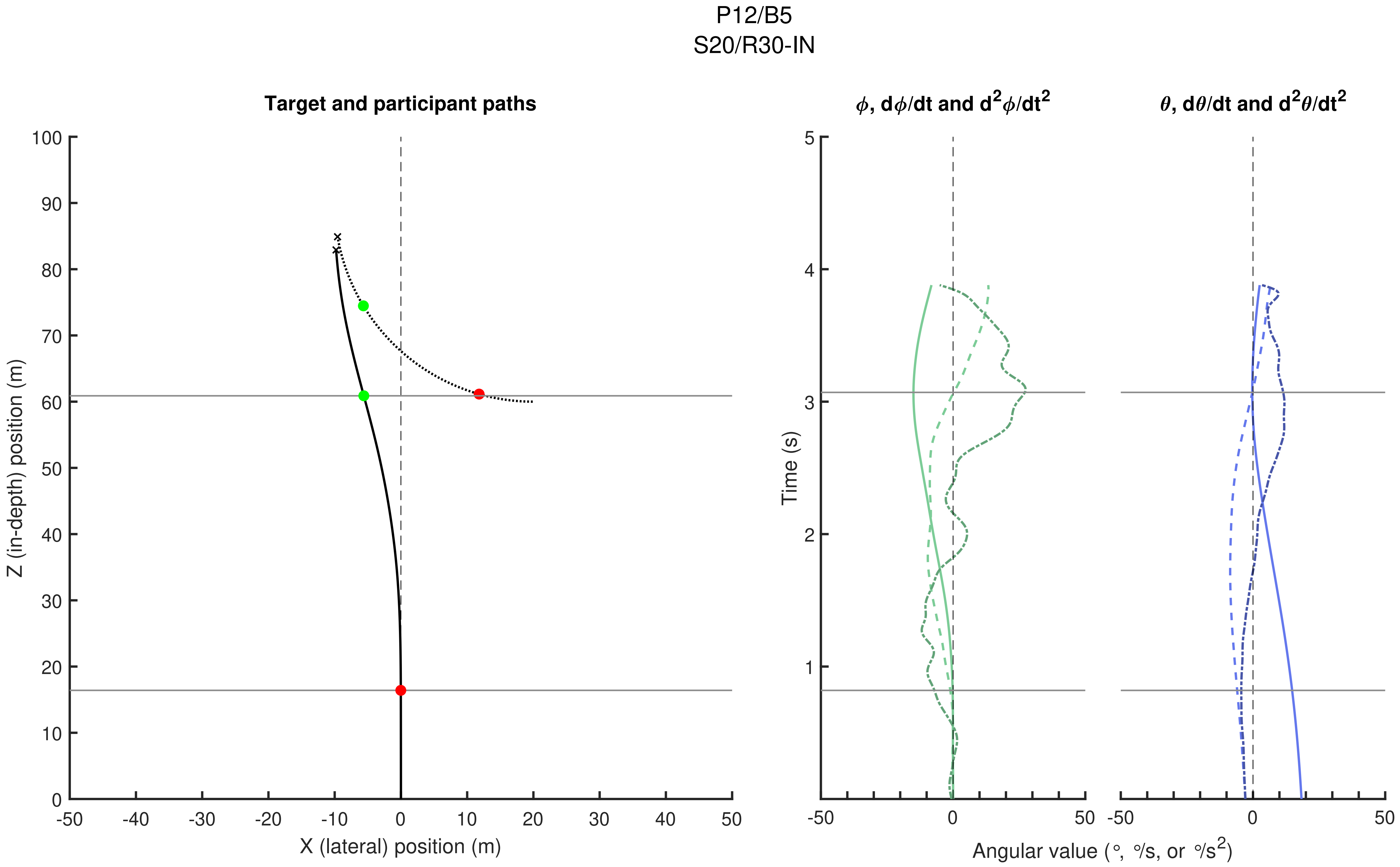

P12/B5
S20/R30-IN
Target and participant paths
Z (in-depth) position (m)
X (lateral) position (m)
100
90
80
70
60
50
40
30
20
10
0
-50
-40
-30
-20
-10
0
10
20
30
40
50
ϕ, dϕ/dt and d$^2\phi$/dt^2
θ, dθ/dt and d$^2\theta$/dt^2
Time (s)
5
4
3
2
1
-50
0
50
-50
0
50
Angular value (°, °/s, or °/s^2)

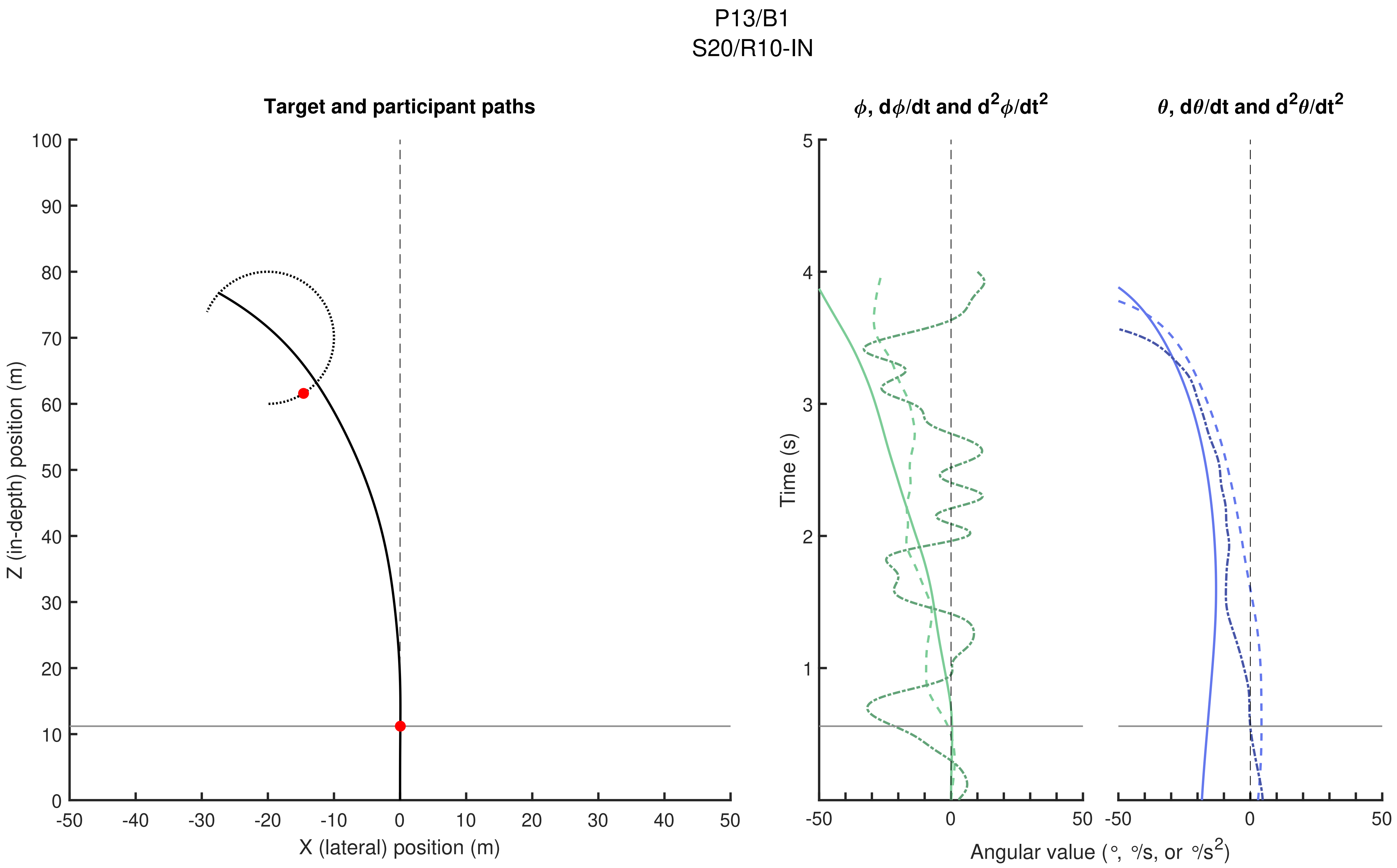

P13/B1
S20/R10-IN
Target and participant paths
Z (in-depth) position (m)
X (lateral) position (m)
100
90
80
70
60
50
40
30
20
10
0
-50
-40
-30
-20
-10
0
10
20
30
40
50
ϕ, dϕ/dt and d$^2\phi$/dt^2
θ, dθ/dt and d$^2\theta$/dt^2
Time (s)
5
4
3
2
1
-50
0
50
Angular value (°, °/s, or °/s^2)

P13/B1
S20/R20-IN

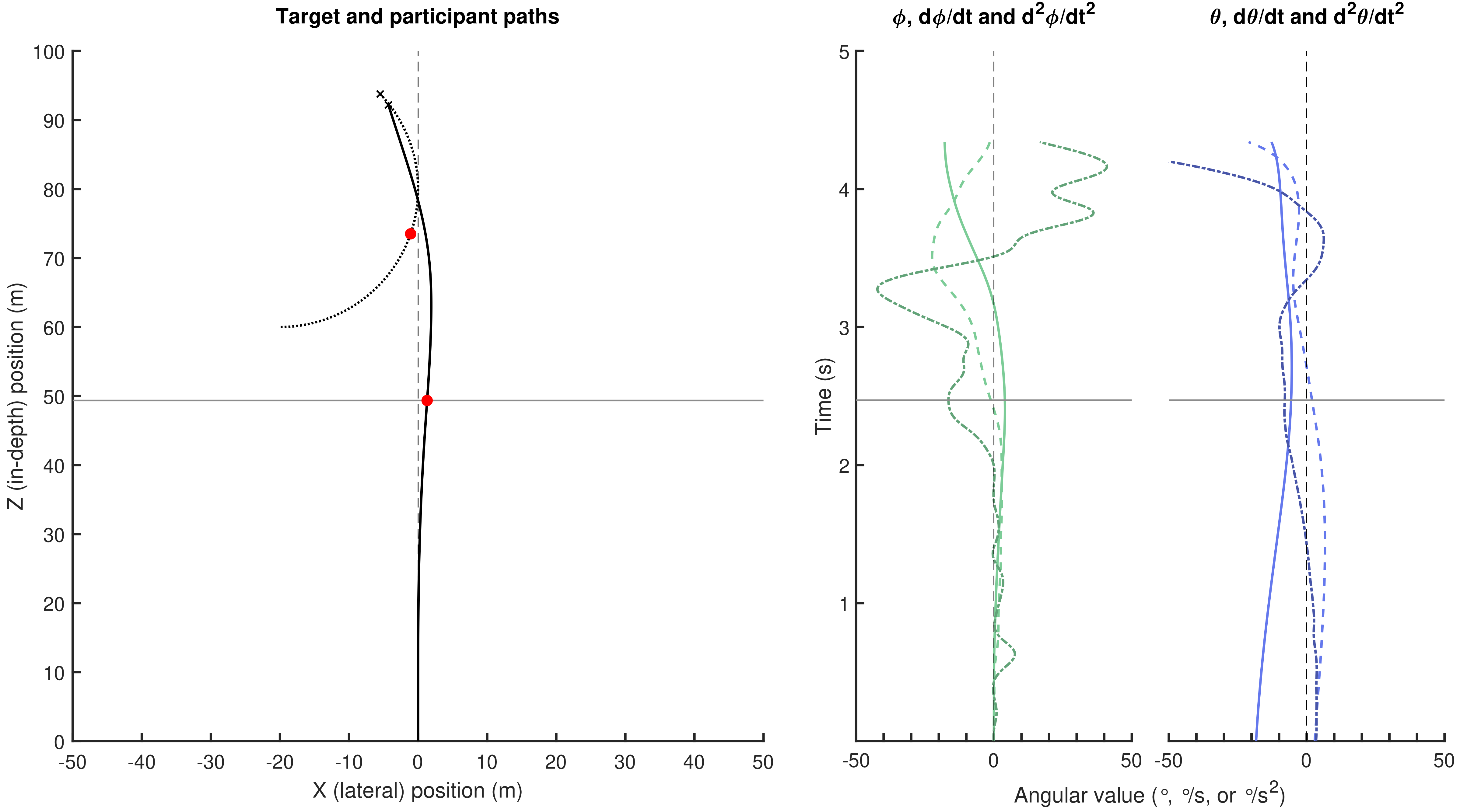

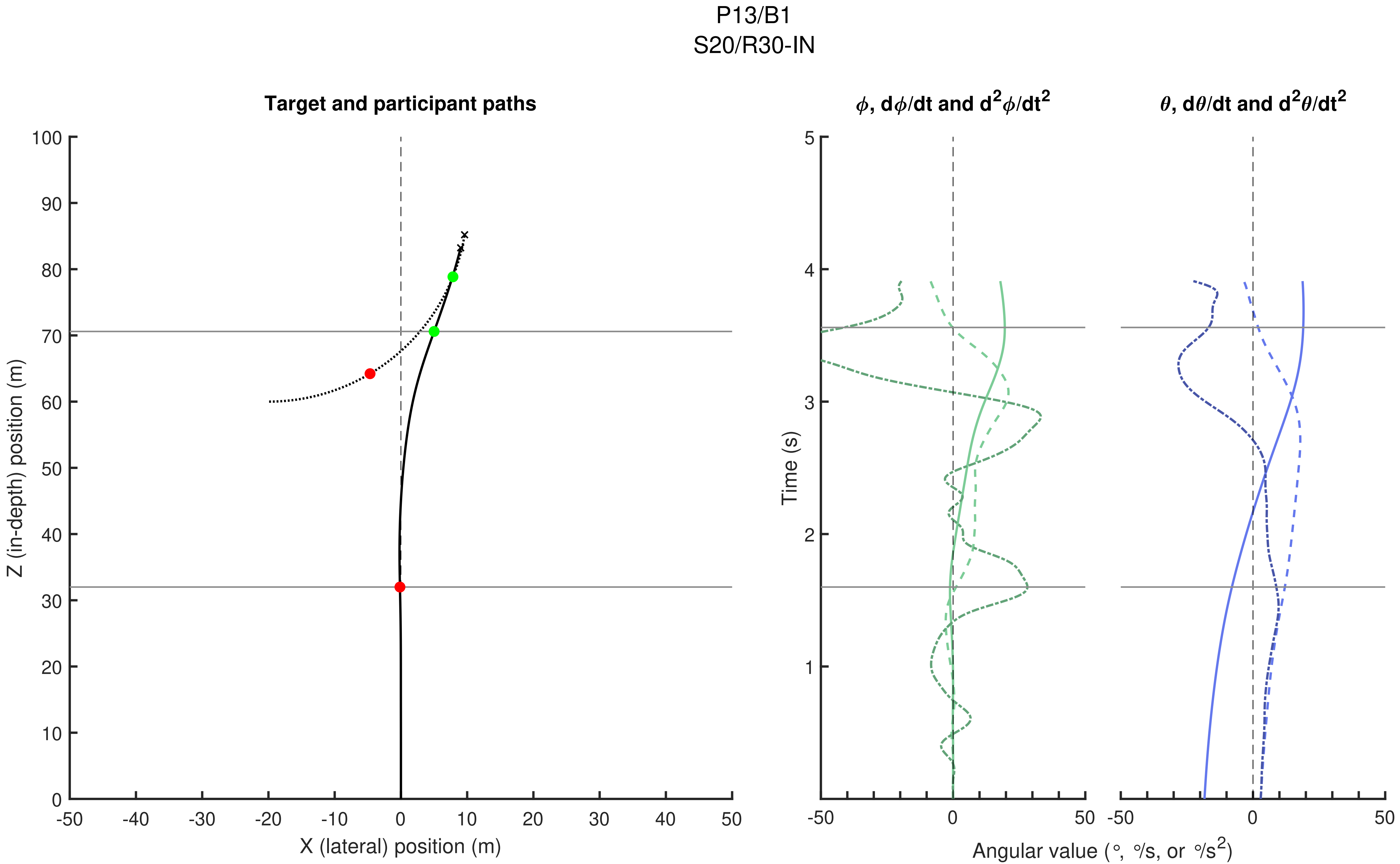

P13/B1
S20/R30-IN
Target and participant paths
Z (in-depth) position (m)
X (lateral) position (m)
ϕ, dϕ/dt and d$^2\phi$/dt^2
θ, dθ/dt and d$^2\theta$/dt^2
Time (s)
Angular value (°, °/s, or °/s^2)

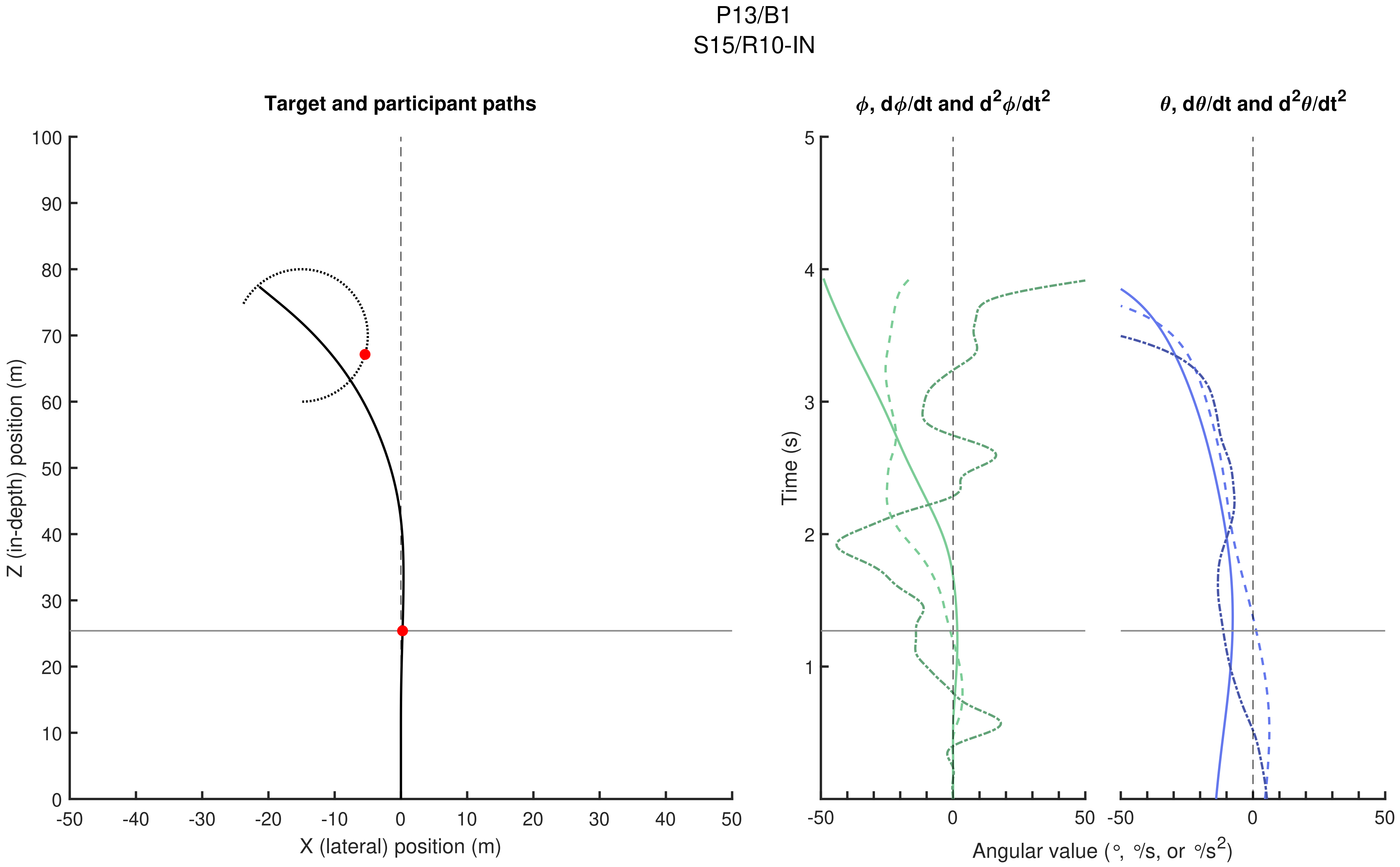
P13/B1
S15/R10-IN
Target and participant paths
ϕ, dϕ/dt and d$^2\phi$/dt^2
θ, dθ/dt and d$^2\theta$/dt^2
Z (in-depth) position (m)
X (lateral) position (m)
Time (s)
Angular value (°, °/s, or °/s^2)
100
90
80
70
60
50
40
30
20
10
0
-50
-40
-30
-20
-10
0
10
20
30
40
50
5
4
3
2
1
-50
0
50
-50
0
50

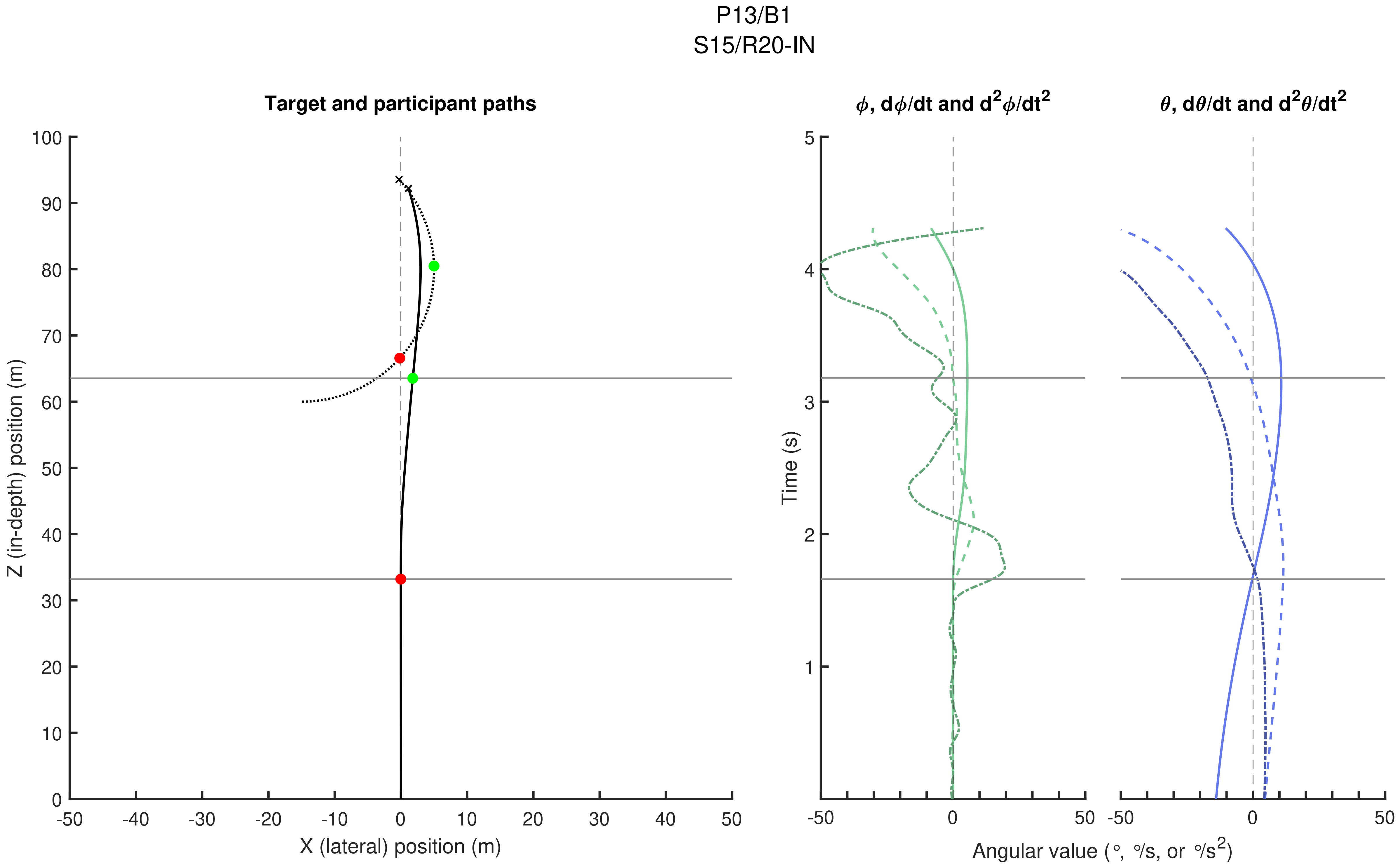
P13/B1
S15/R20-IN
Target and participant paths
Z (in-depth) position (m)
X (lateral) position (m)
ϕ, dϕ/dt and d²ϕ/dt²
θ, dθ/dt and d²θ/dt²
Time (s)
Angular value (°, °/s, or °/s²)

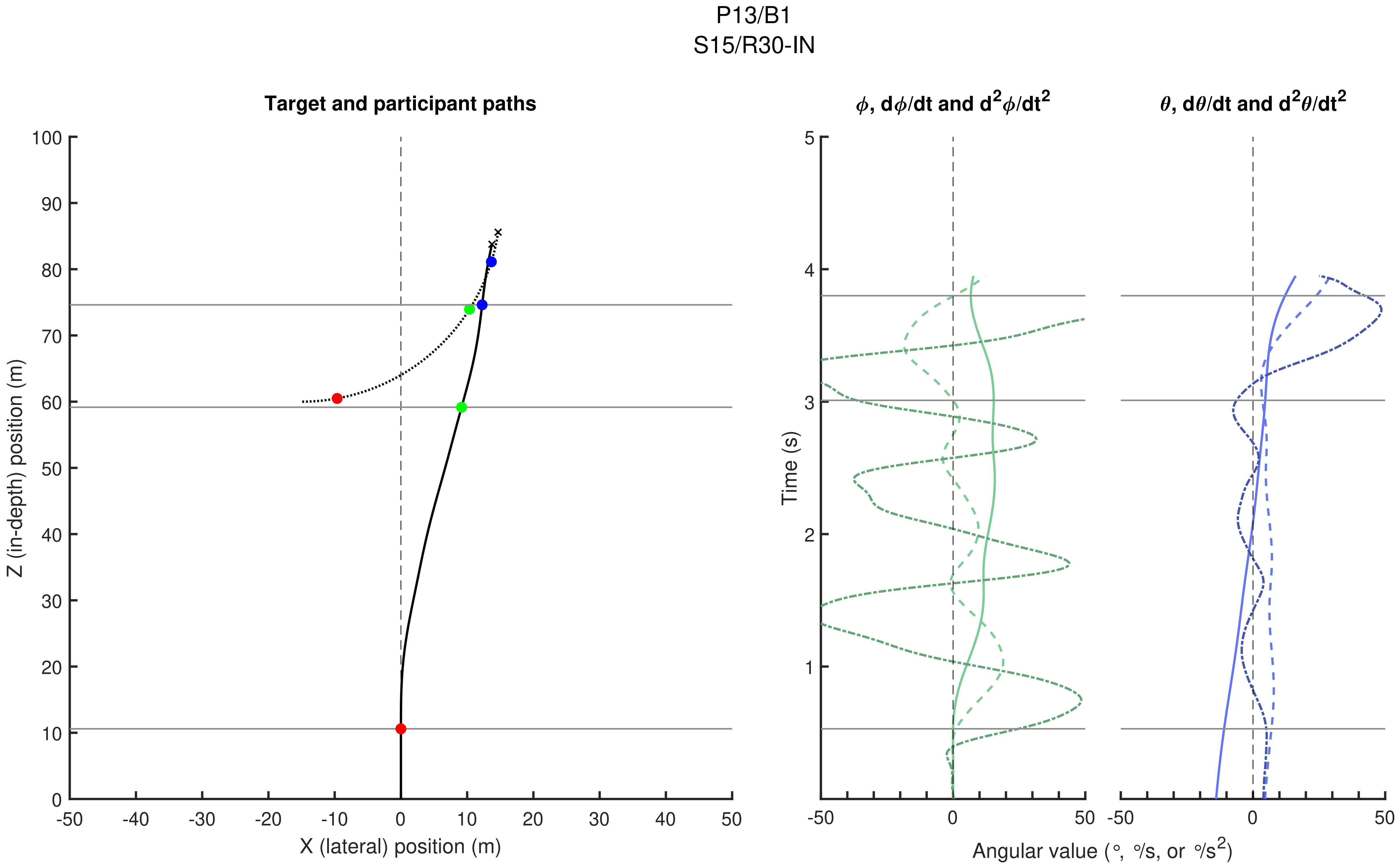
P13/B1
S15/R30-IN
Target and participant paths
Z (in-depth) position (m)
X (lateral) position (m)
ϕ, dϕ/dt and d²ϕ/dt²
θ, dθ/dt and d²θ/dt²
Time (s)
Angular value (°, °/s, or °/s²)

P13/B1
S5/R10-IN

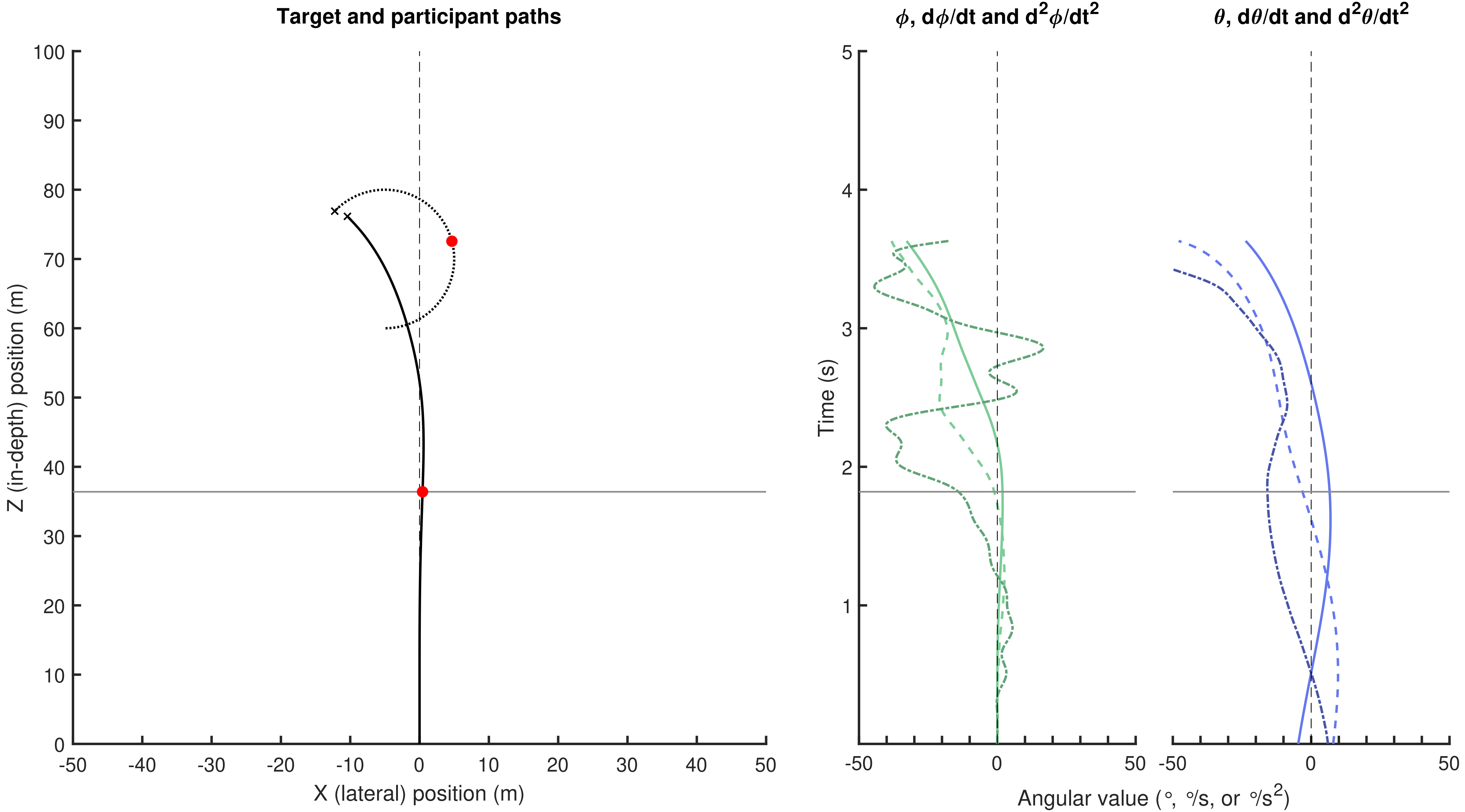

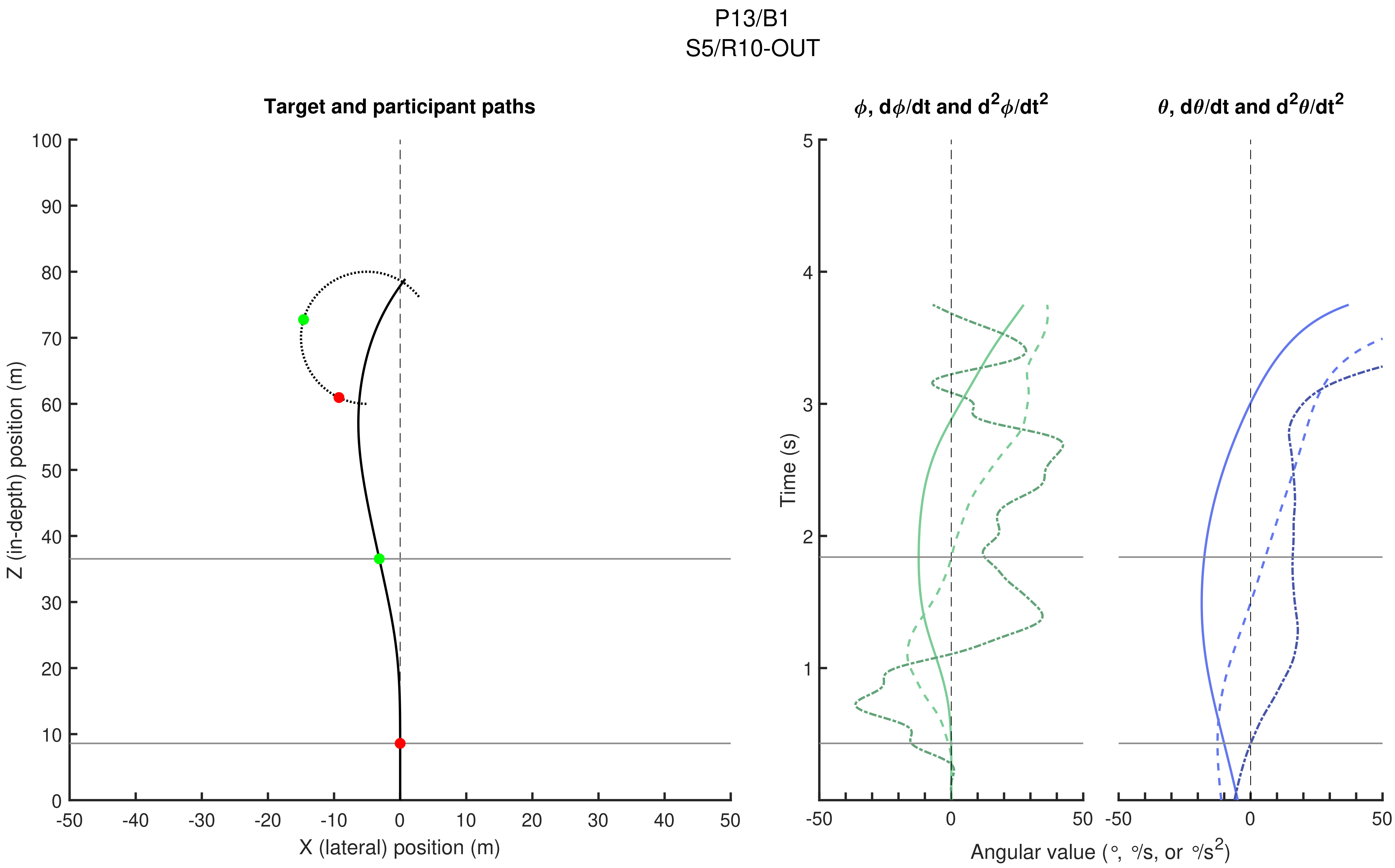

P13/B1
S5/R10-OUT
Target and participant paths
ϕ, dϕ/dt and d$^2\phi$/dt^2
θ, dθ/dt and d$^2\theta$/dt^2
Z (in-depth) position (m)
X (lateral) position (m)
Time (s)
Angular value (°, °/s, or °/s^2)
100
90
80
70
60
50
40
30
20
10
0
-50
-40
-30
-20
-10
0
10
20
30
40
50
5
4
3
2
1
-50
0
50
-50
0
50

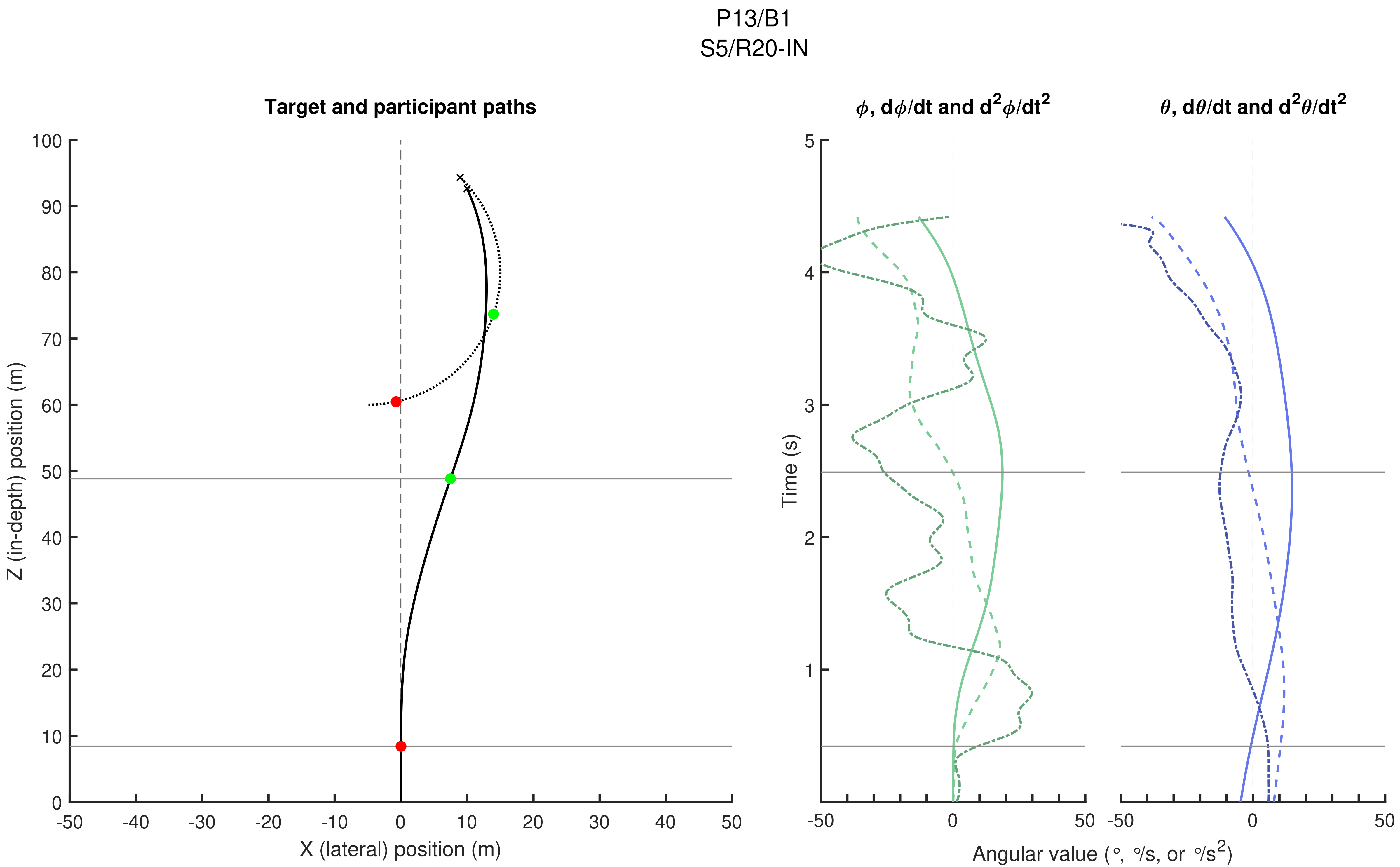
P13/B1
S5/R20-IN
Target and participant paths
Z (in-depth) position (m)
X (lateral) position (m)
ϕ, dϕ/dt and d$^2\phi$/dt^2
θ, dθ/dt and d$^2\theta$/dt^2
Time (s)
Angular value (°, °/s, or °/s^2)

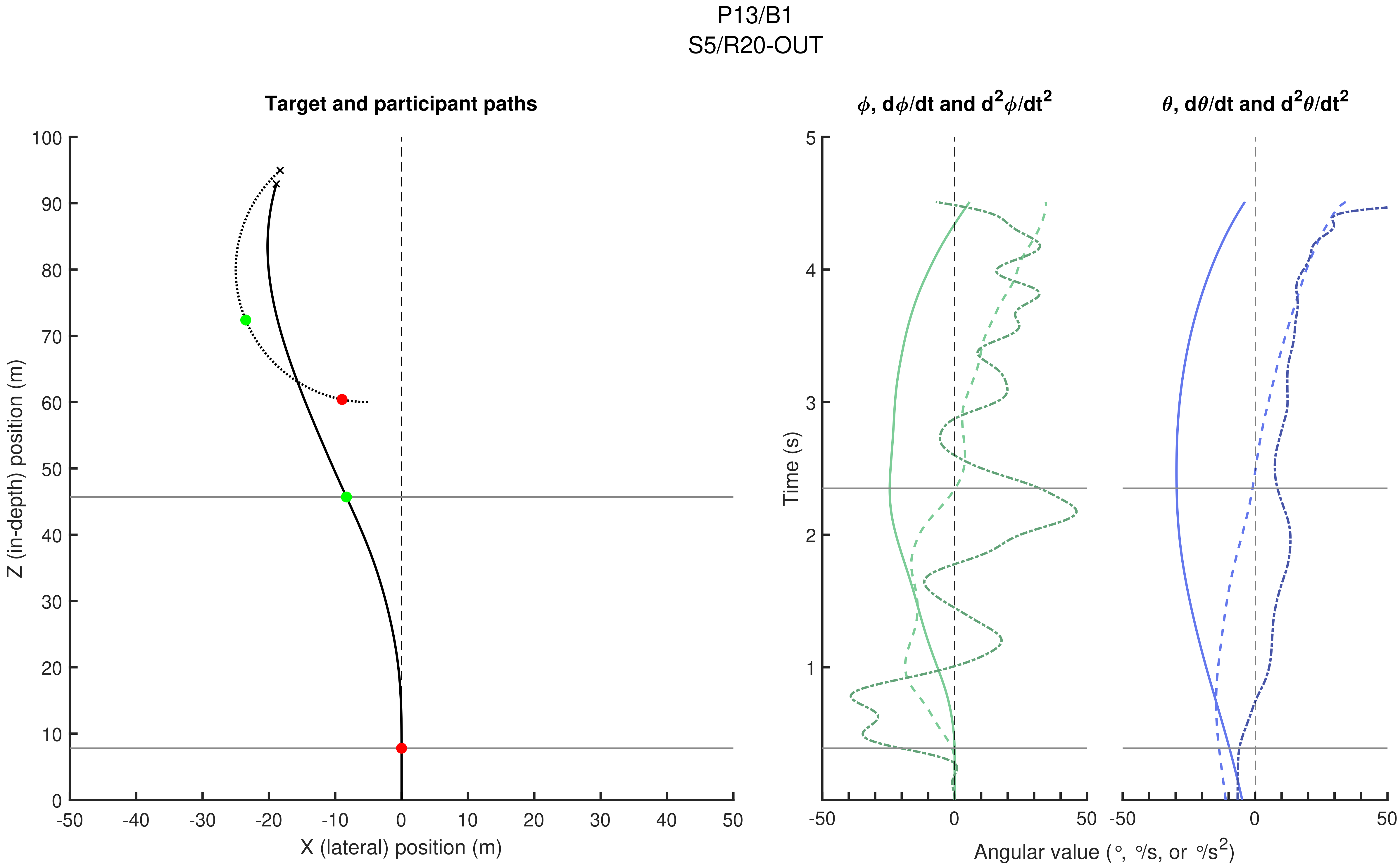

P13/B1
S5/R20-OUT
Target and participant paths
Z (in-depth) position (m)
X (lateral) position (m)
ϕ, dϕ/dt and d²ϕ/dt²
θ, dθ/dt and d²θ/dt²
Time (s)
Angular value (°, °/s, or °/s²)

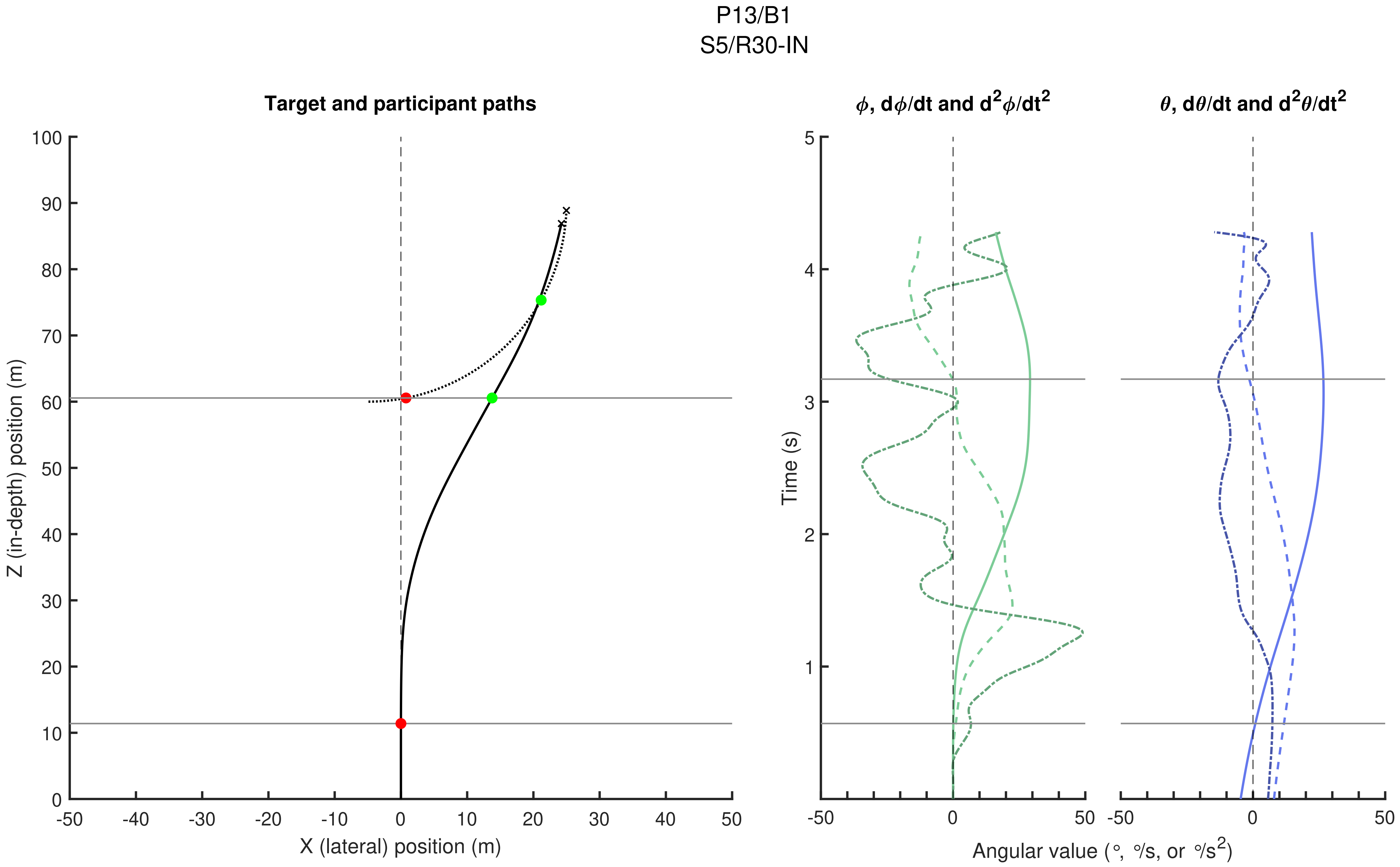

P13/B1
S5/R30-IN
Target and participant paths
Z (in-depth) position (m)
X (lateral) position (m)
ϕ, dϕ/dt and d$^2\phi$/dt^2
θ, dθ/dt and d$^2\theta$/dt^2
Time (s)
Angular value (°, °/s, or °/s^2)

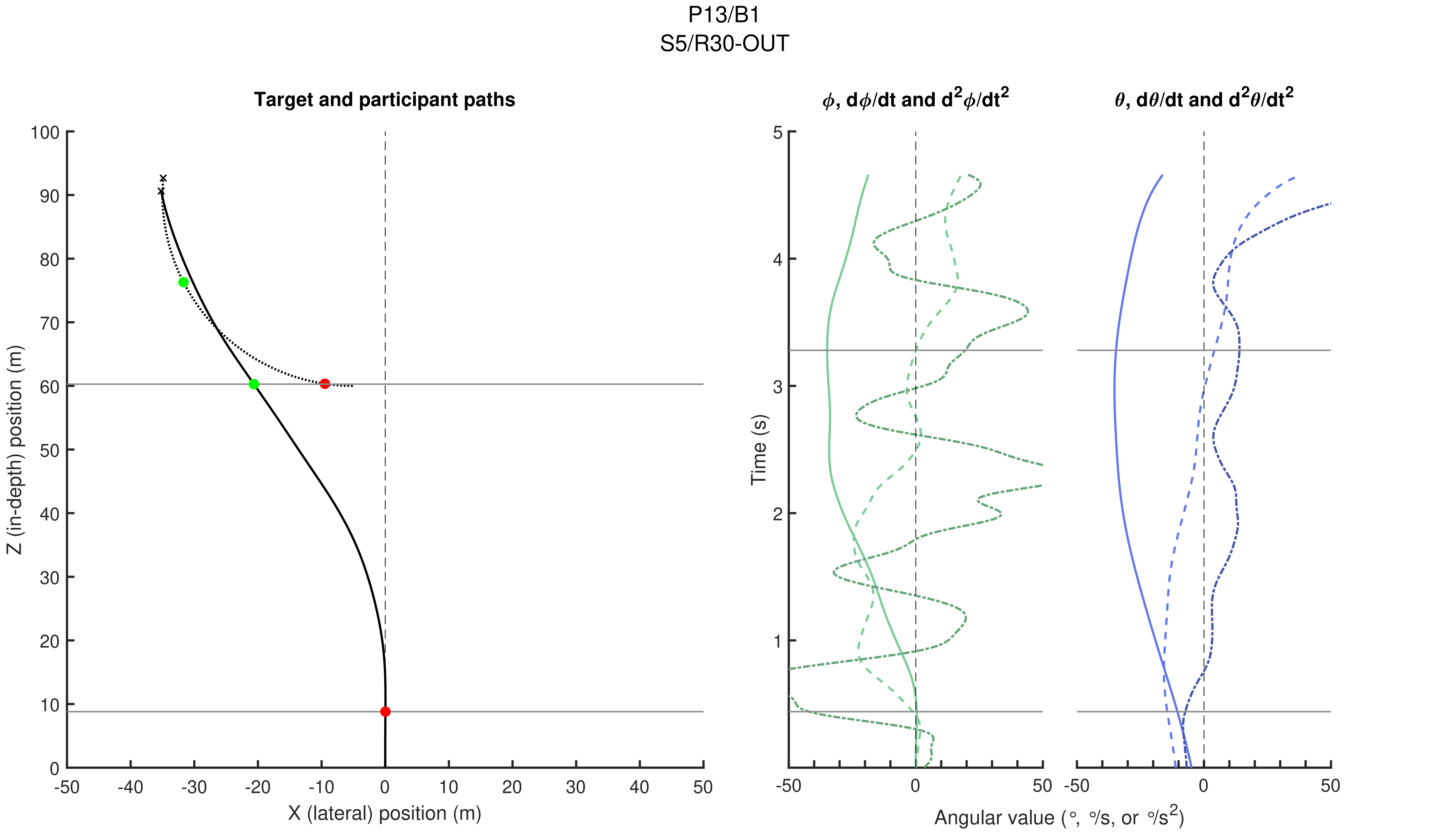

P13/B1
S5/R30-OUT
Target and participant paths
Z (in-depth) position (m)
X (lateral) position (m)
0
10
20
30
40
50
60
70
80
90
100
-50
-40
-30
-20
-10
0
10
20
30
40
50
ϕ, dϕ/dt and d²ϕ/dt²
θ, dθ/dt and d²θ/dt²
Time (s)
1
2
3
4
5
-50
0
50
-50
0
50
Angular value (°, °/s, or °/s²)

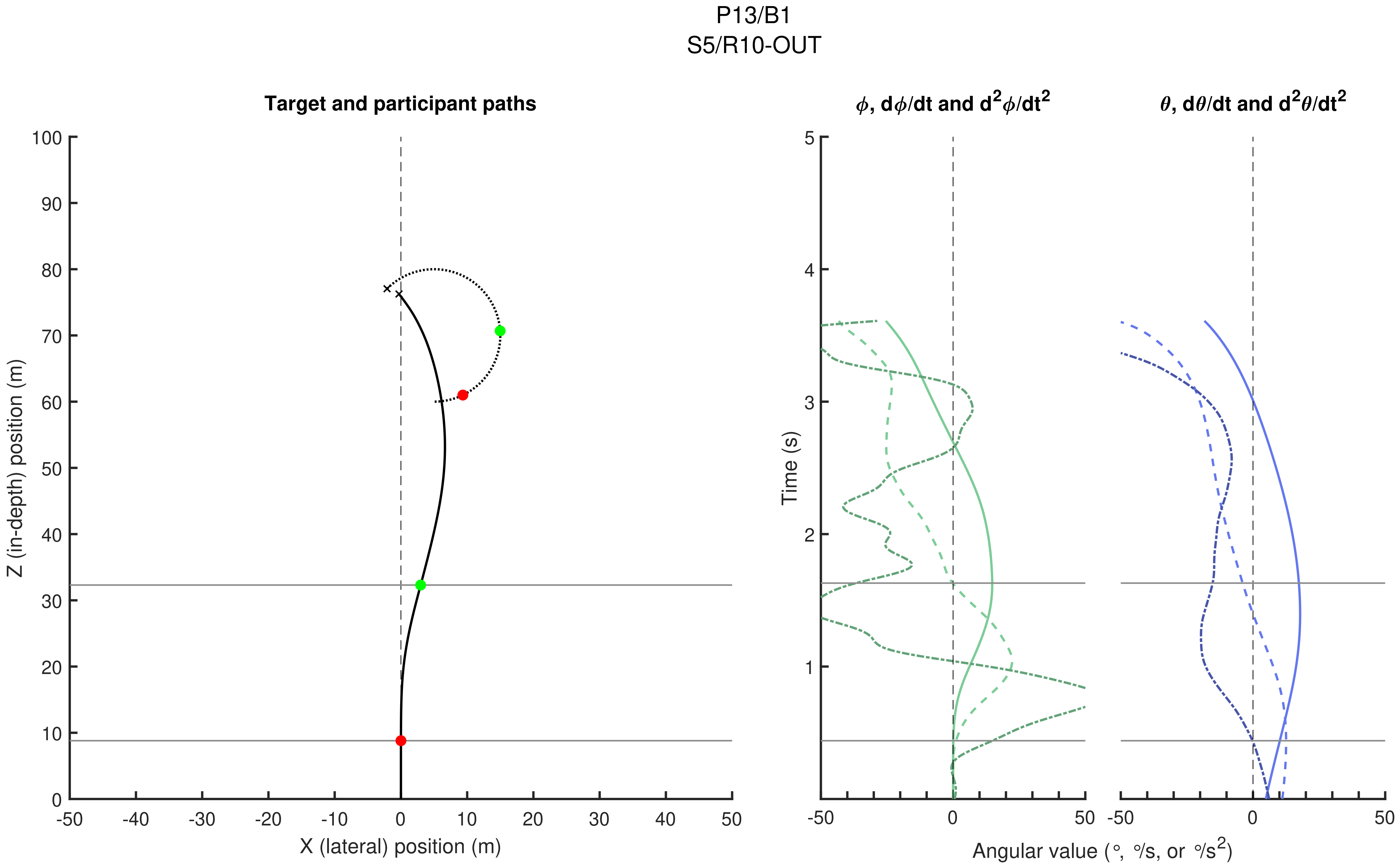
P13/B1
S5/R10-OUT
Target and participant paths
Z (in-depth) position (m)
X (lateral) position (m)
ϕ, dϕ/dt and d$^2\phi$/dt^2
θ, dθ/dt and d$^2\theta$/dt^2
Time (s)
Angular value (°, °/s, or °/s^2)

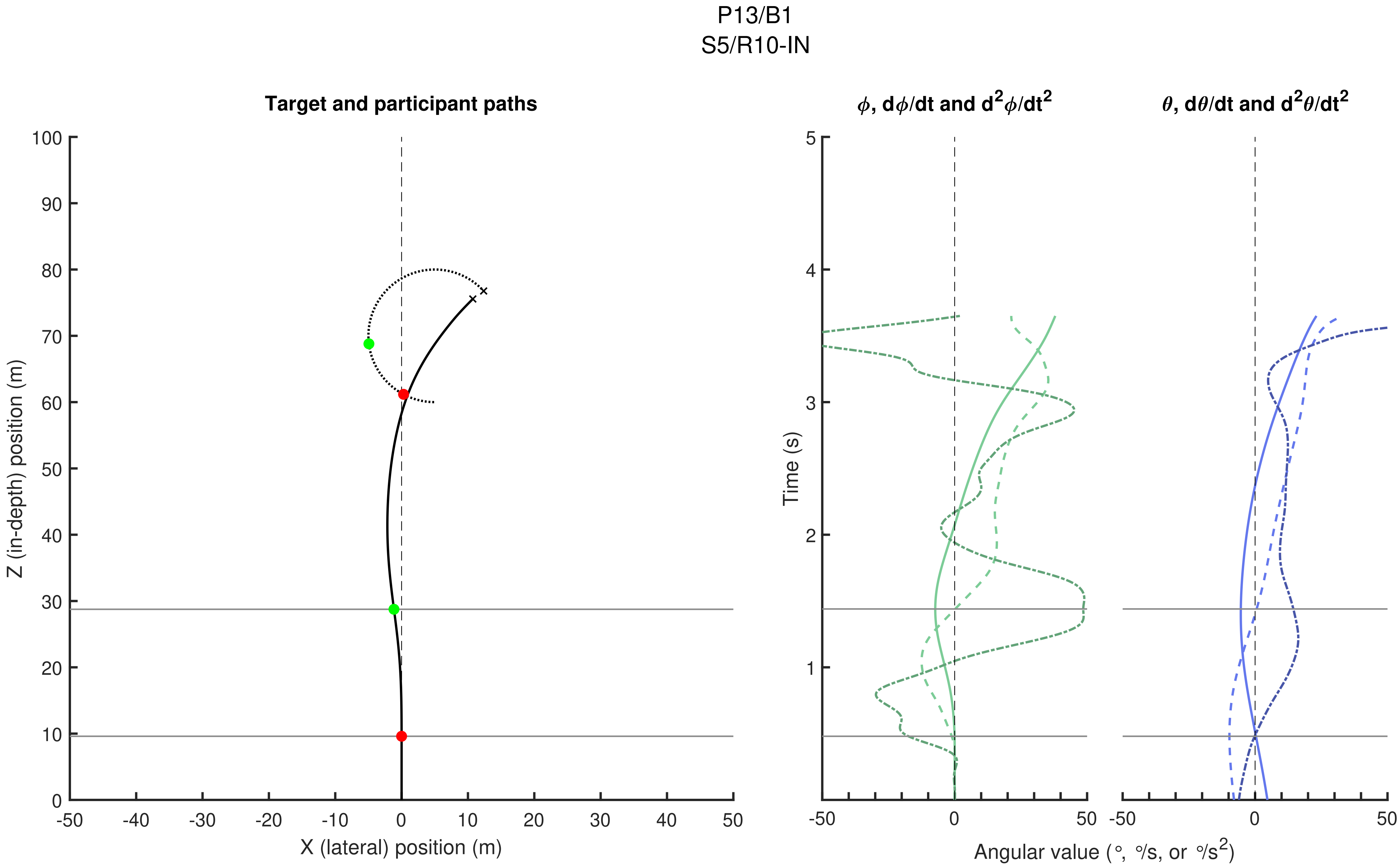
P13/B1
S5/R10-IN
Target and participant paths
φ, dφ/dt and d²φ/dt²
θ, dθ/dt and d²θ/dt²
Z (in-depth) position (m)
X (lateral) position (m)
Time (s)
Angular value (°, °/s, or °/s²)
100
90
80
70
60
50
40
30
20
10
0
-50
-40
-30
-20
-10
0
10
20
30
40
50
5
4
3
2
1
-50
0
50
-50
0
50

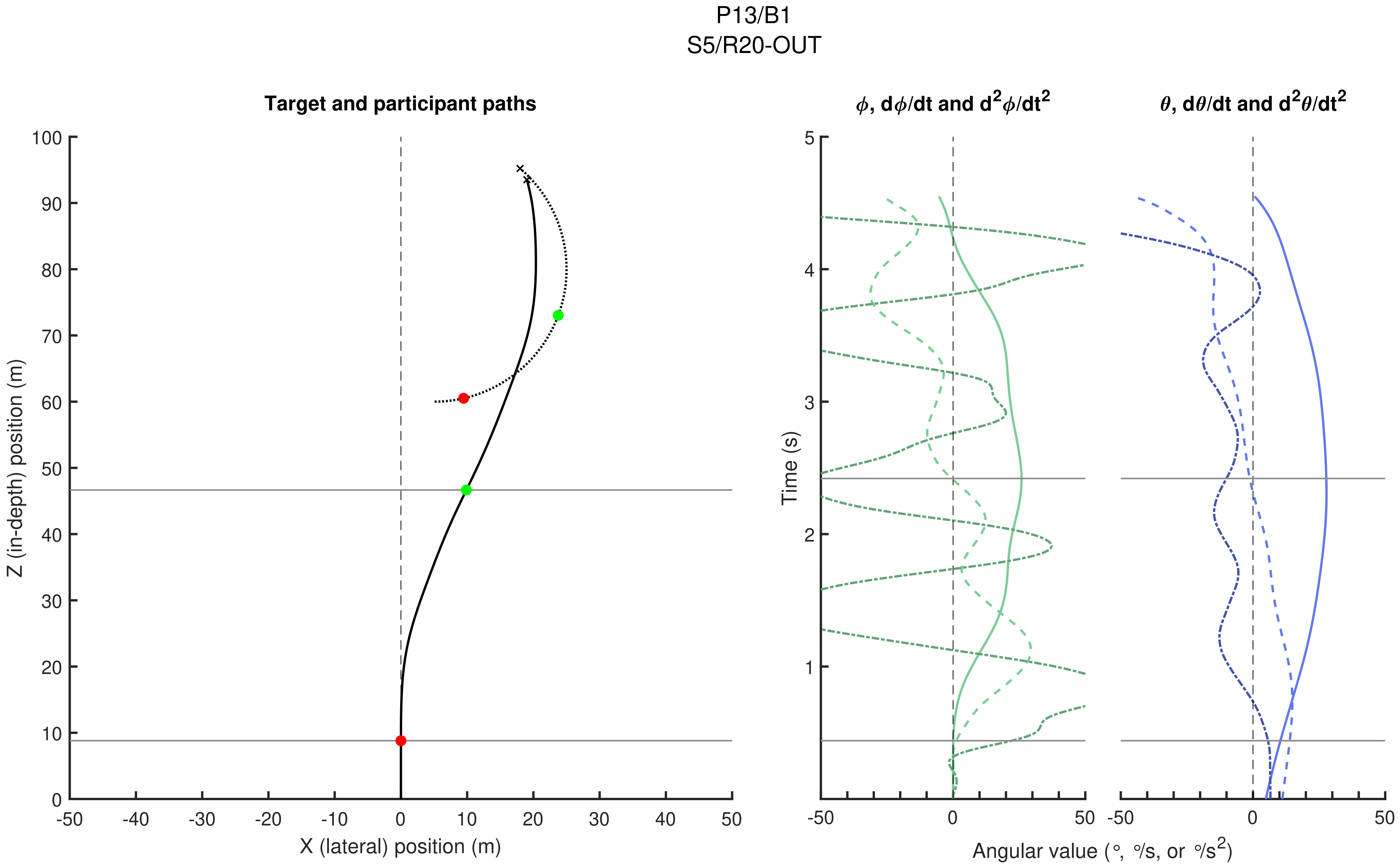

P13/B1
S5/R20-OUT
Target and participant paths
ϕ, dϕ/dt and d$^2\phi$/dt^2
θ, dθ/dt and d$^2\theta$/dt^2
Z (in-depth) position (m)
X (lateral) position (m)
Time (s)
Angular value (°, °/s, or °/s^2)

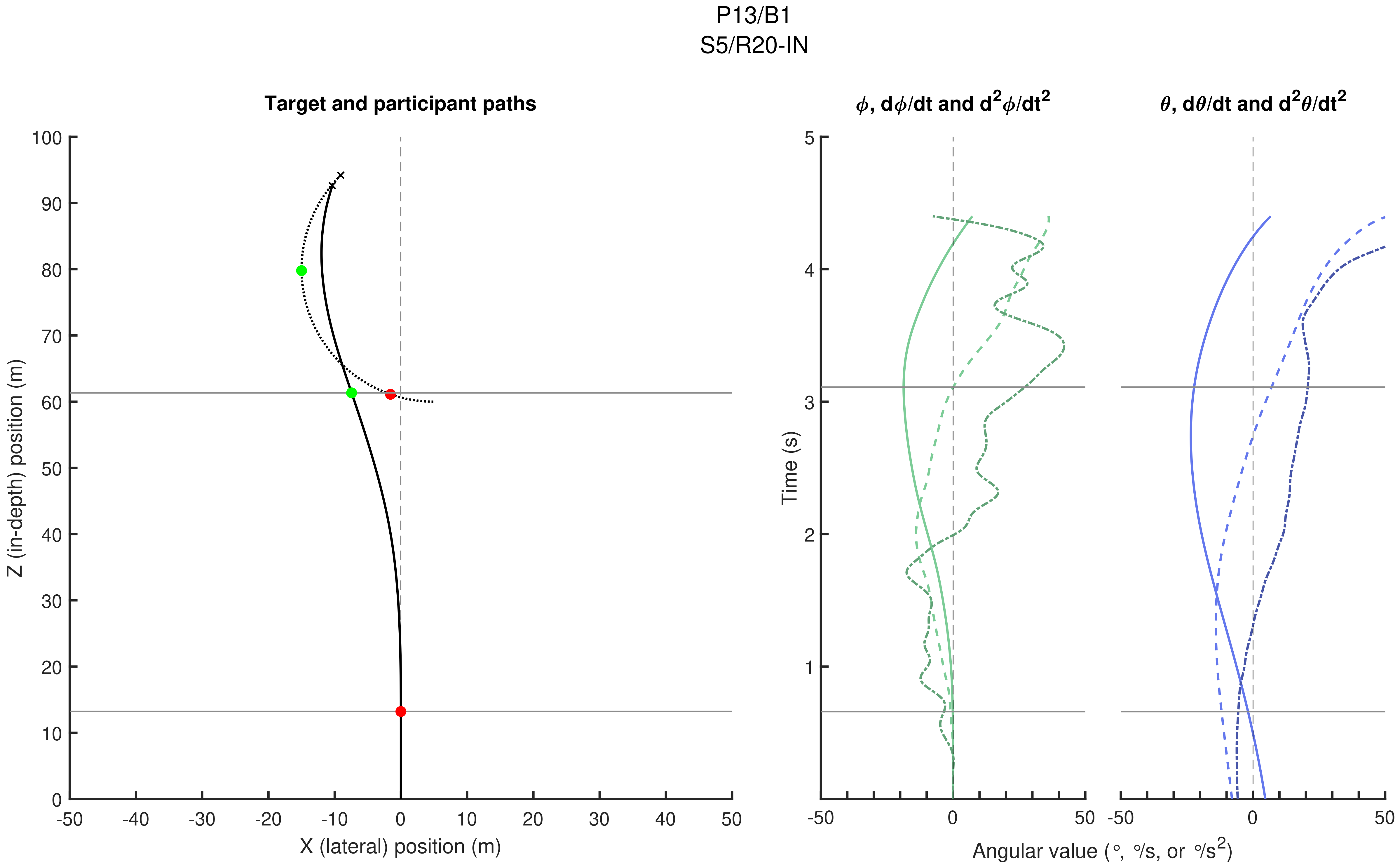
P13/B1
S5/R20-IN
Target and participant paths
ϕ, dϕ/dt and d$^2\phi$/dt^2
θ, dθ/dt and d$^2\theta$/dt^2
Z (in-depth) position (m)
X (lateral) position (m)
Time (s)
Angular value (°, °/s, or °/s^2)
100
90
80
70
60
50
40
30
20
10
0
-50
-40
-30
-20
-10
0
10
20
30
40
50
5
4
3
2
1
-50
0
50
-50
0
50

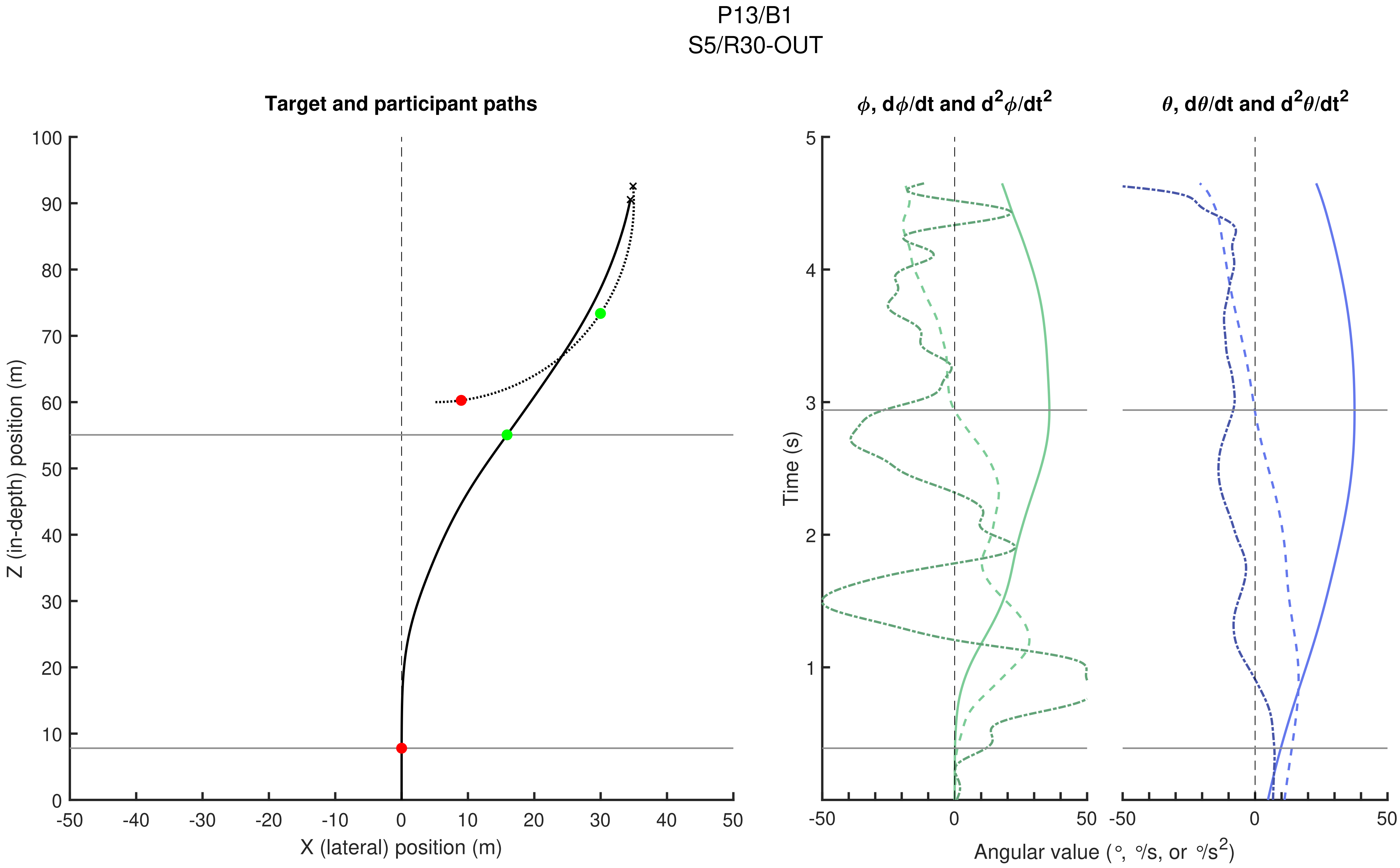
P13/B1
S5/R30-OUT
Target and participant paths
Z (in-depth) position (m)
X (lateral) position (m)
φ, dφ/dt and d²φ/dt²
θ, dθ/dt and d²θ/dt²
Time (s)
Angular value (°, °/s, or °/s²)
100
90
80
70
60
50
40
30
20
10
0
-50
-40
-30
-20
-10
0
10
20
30
40
50
5
4
3
2
1
-50
0
50
-50
0
50

P13/B1
S5/R30-IN

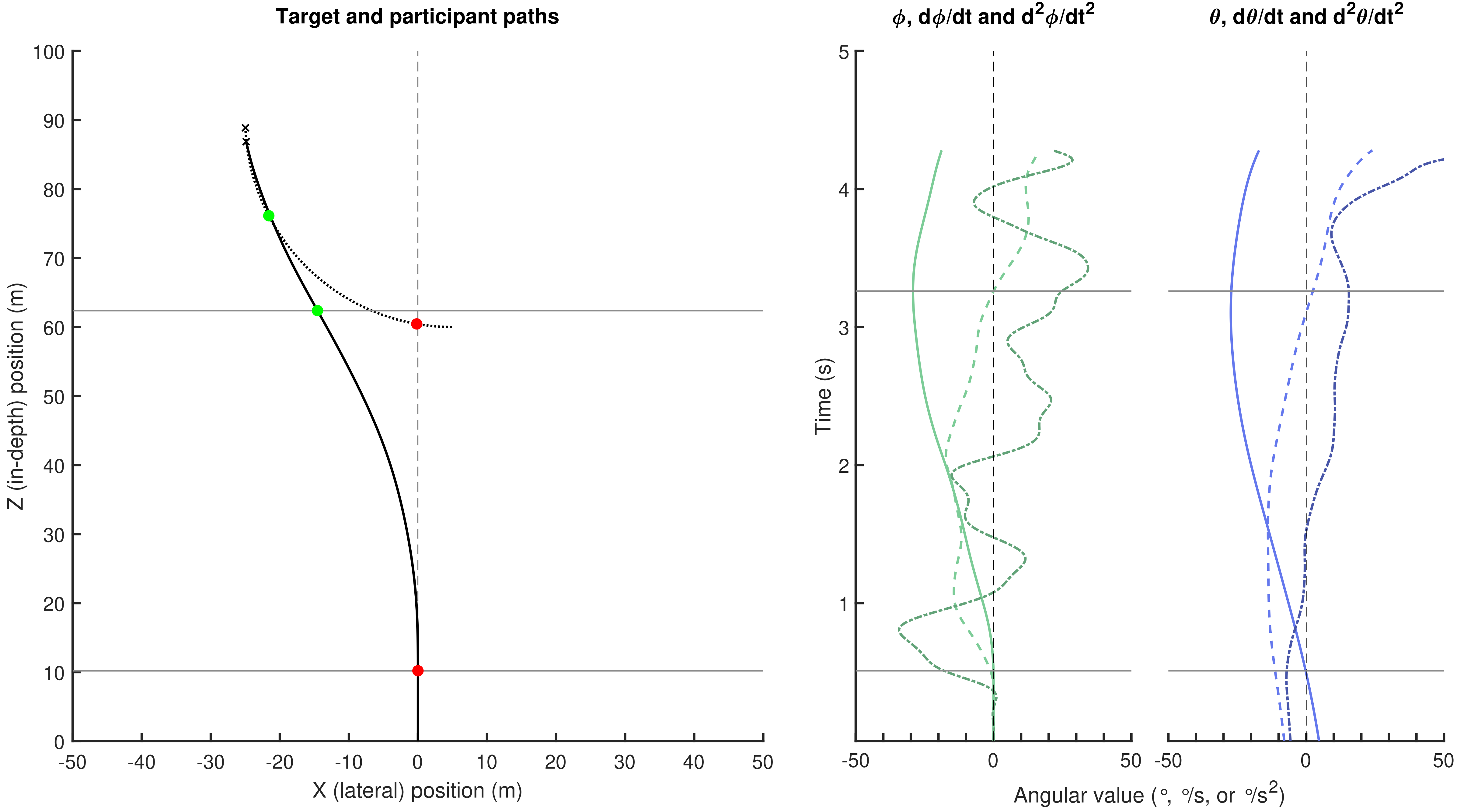

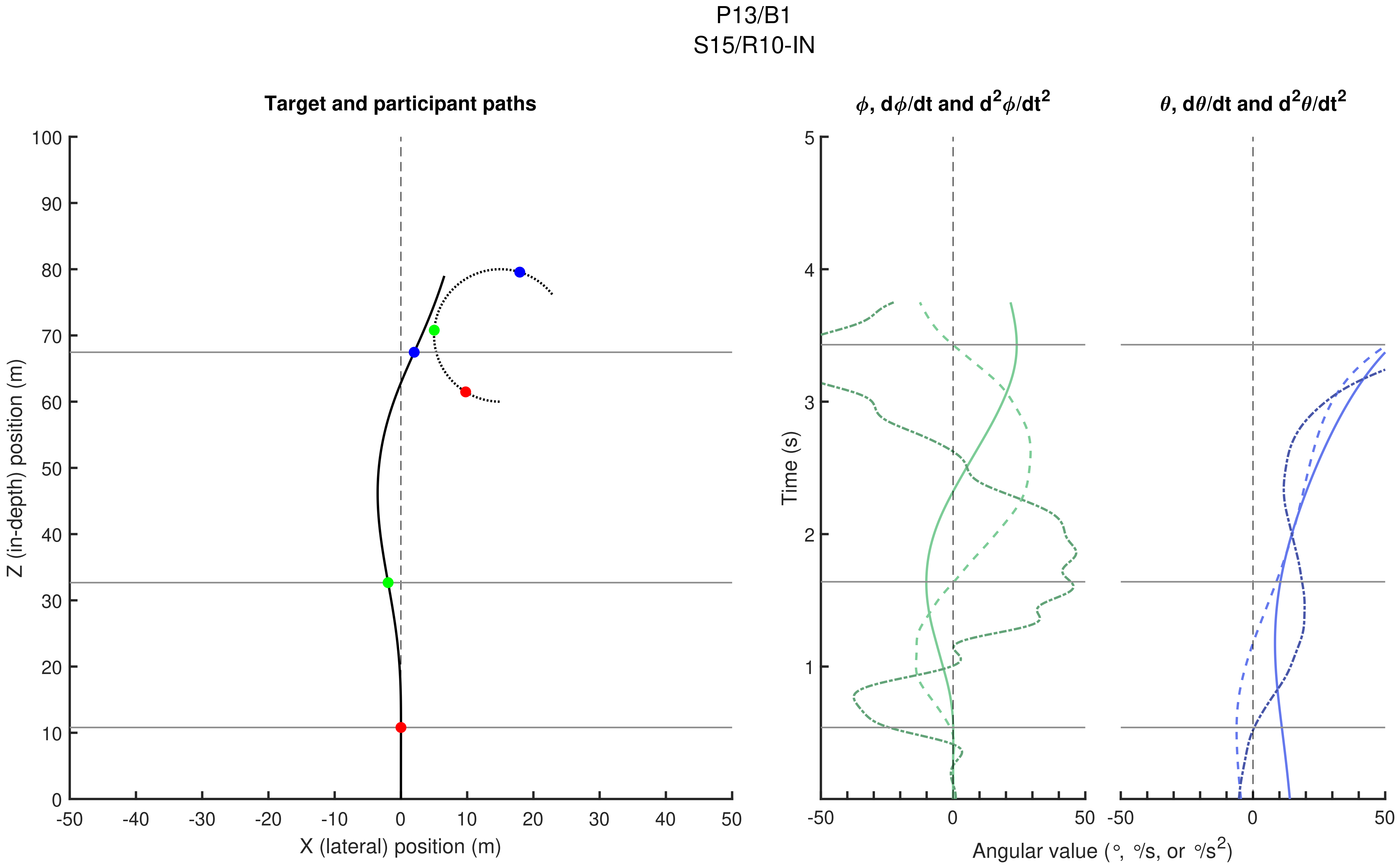

P13/B1
S15/R10-IN
Target and participant paths
ϕ, dϕ/dt and d$^2\phi$/dt^2
θ, dθ/dt and d$^2\theta$/dt^2
Z (in-depth) position (m)
X (lateral) position (m)
Time (s)
Angular value (°, °/s, or °/s^2)
100
90
80
70
60
50
40
30
20
10
0
-50
-40
-30
-20
-10
0
10
20
30
40
50
5
4
3
2
1
-50
0
50
-50
0
50

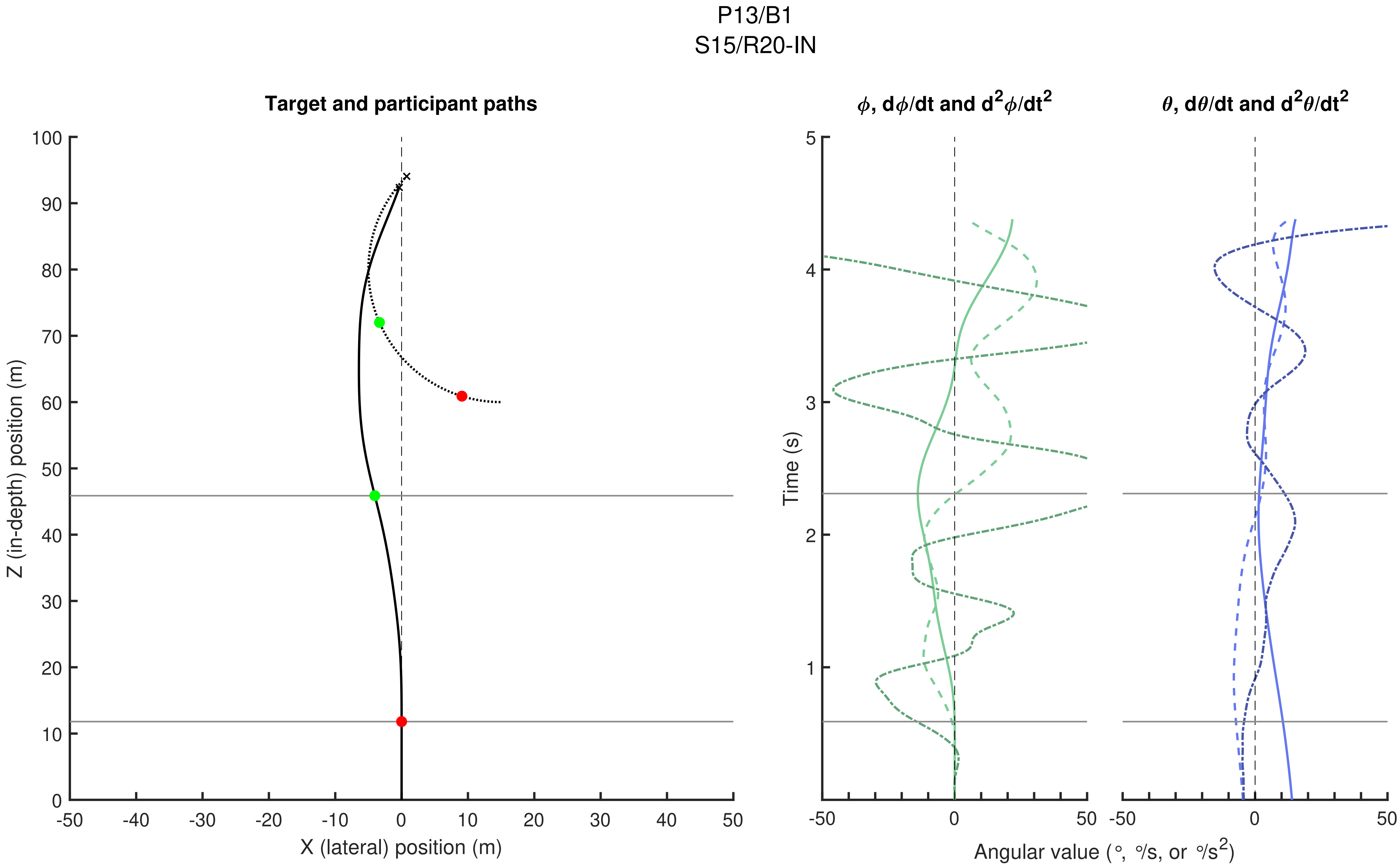
P13/B1
S15/R20-IN
Target and participant paths
Z (in-depth) position (m)
X (lateral) position (m)
ϕ, dϕ/dt and d$^2\phi$/dt^2
θ, dθ/dt and d$^2\theta$/dt^2
Time (s)
Angular value (°, °/s, or °/s^2)

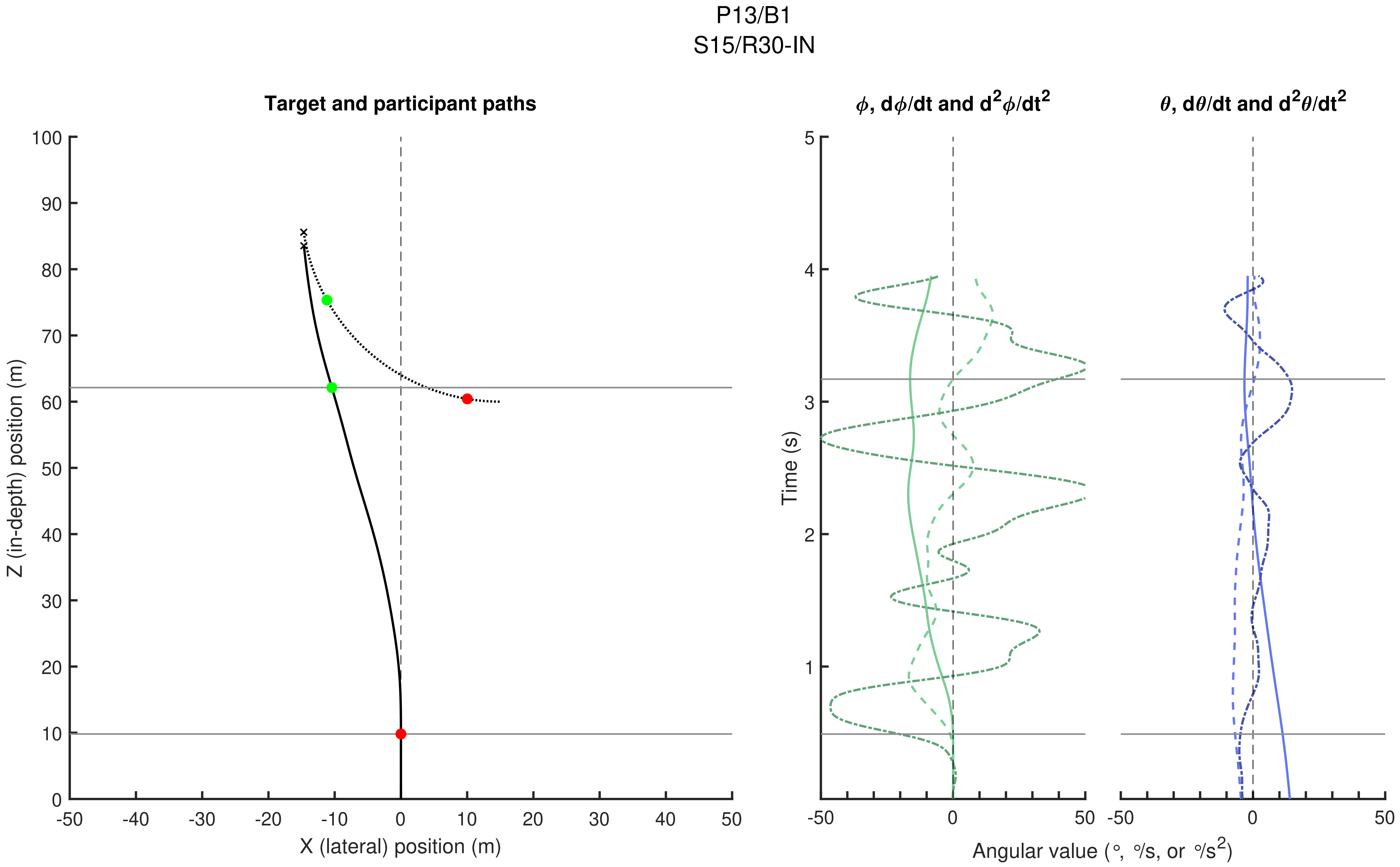
P13/B1
S15/R30-IN
Target and participant paths
Z (in-depth) position (m)
X (lateral) position (m)
φ, dφ/dt and d²φ/dt²
θ, dθ/dt and d²θ/dt²
Time (s)
Angular value (°, °/s, or °/s²)

P13/B1
S20/R10-IN

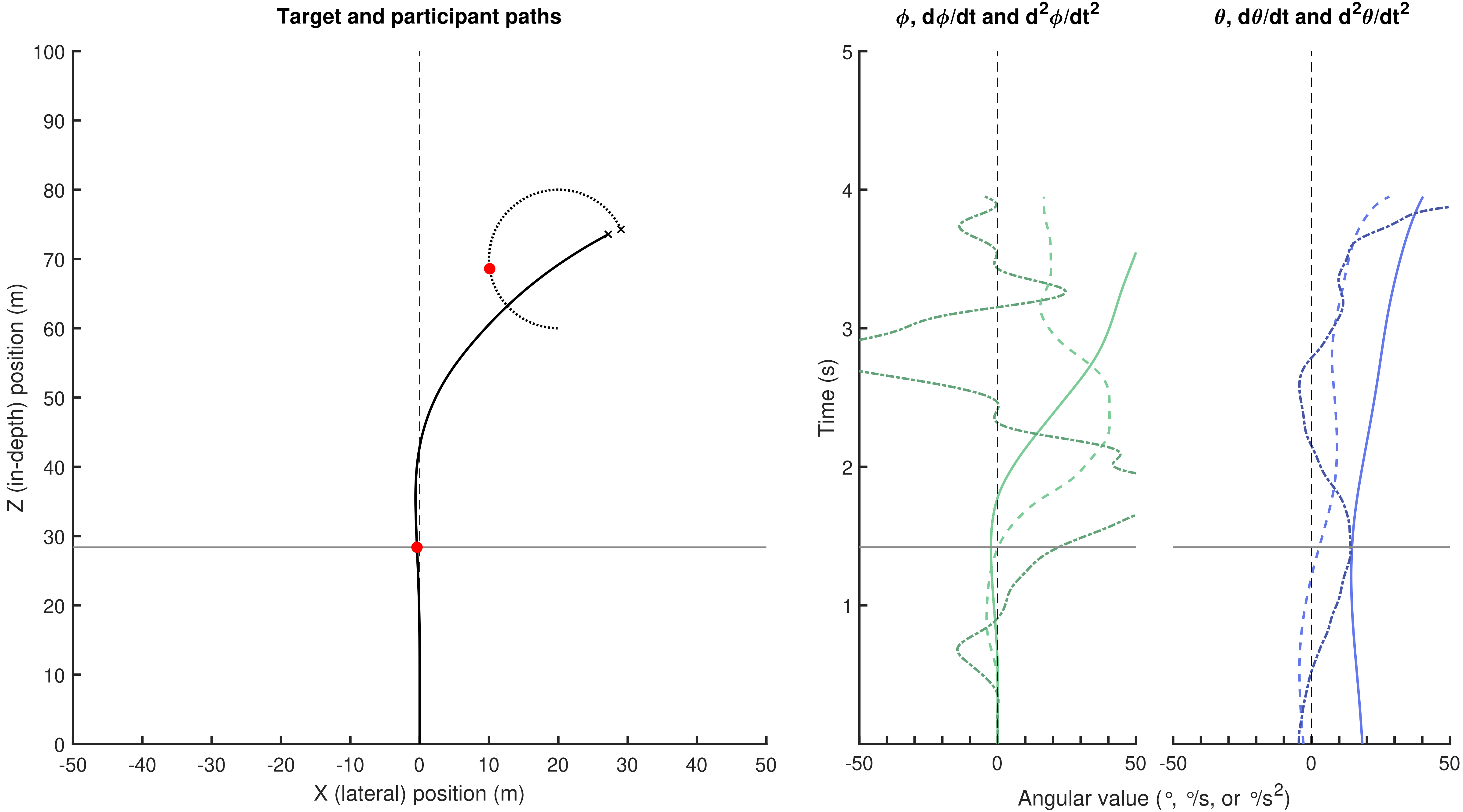

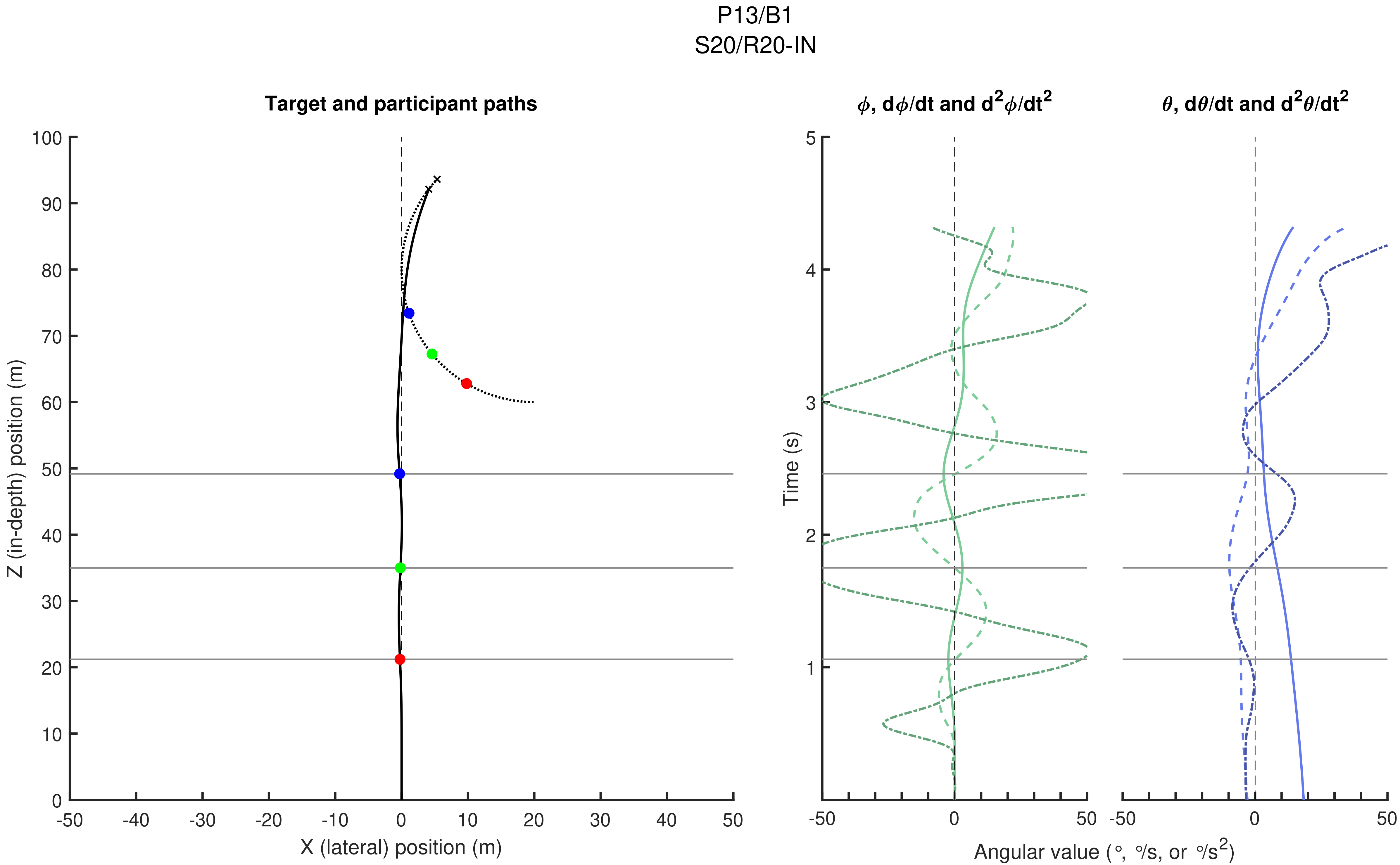

P13/B1
S20/R20-IN
Target and participant paths
ϕ, dϕ/dt and d$^2\phi$/dt^2
θ, dθ/dt and d$^2\theta$/dt^2
Z (in-depth) position (m)
X (lateral) position (m)
Time (s)
Angular value (°, °/s, or °/s^2)
0
10
20
30
40
50
60
70
80
90
100
-50
-40
-30
-20
-10
0
10
20
30
40
50
1
2
3
4
5
-50
0
50
-50
0
50

P13/B1
S20/R30-IN

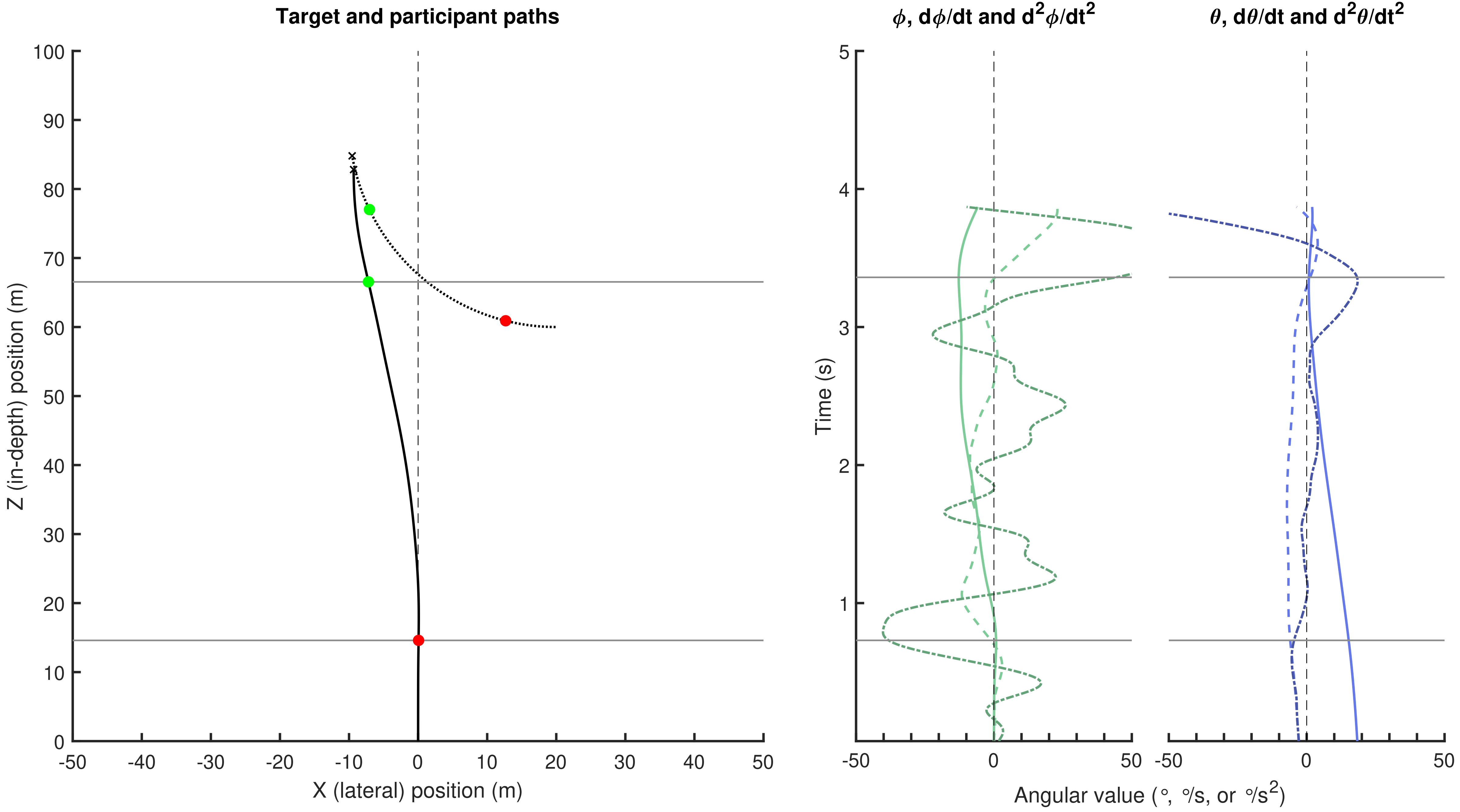

P13/B2
S20/R10-IN

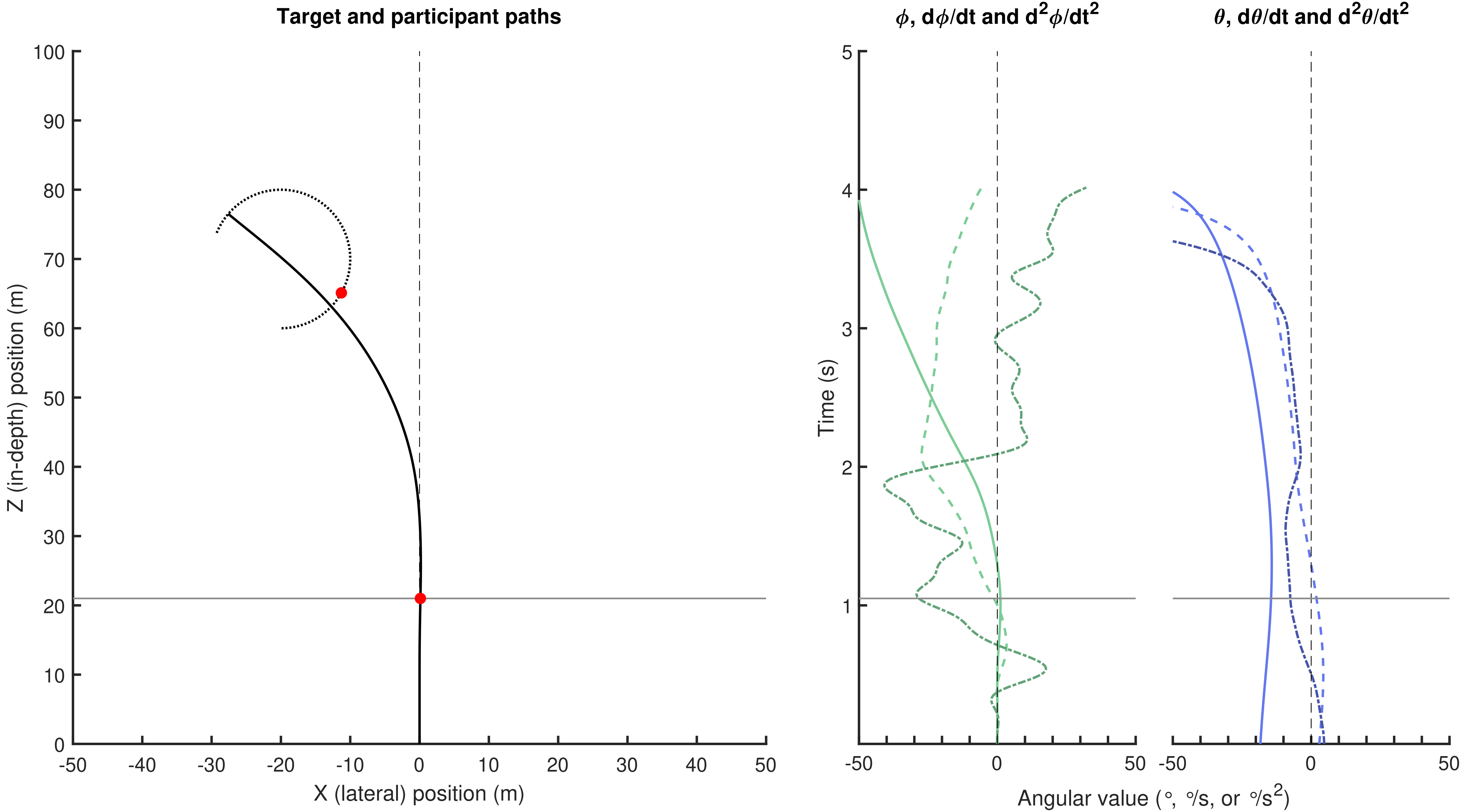

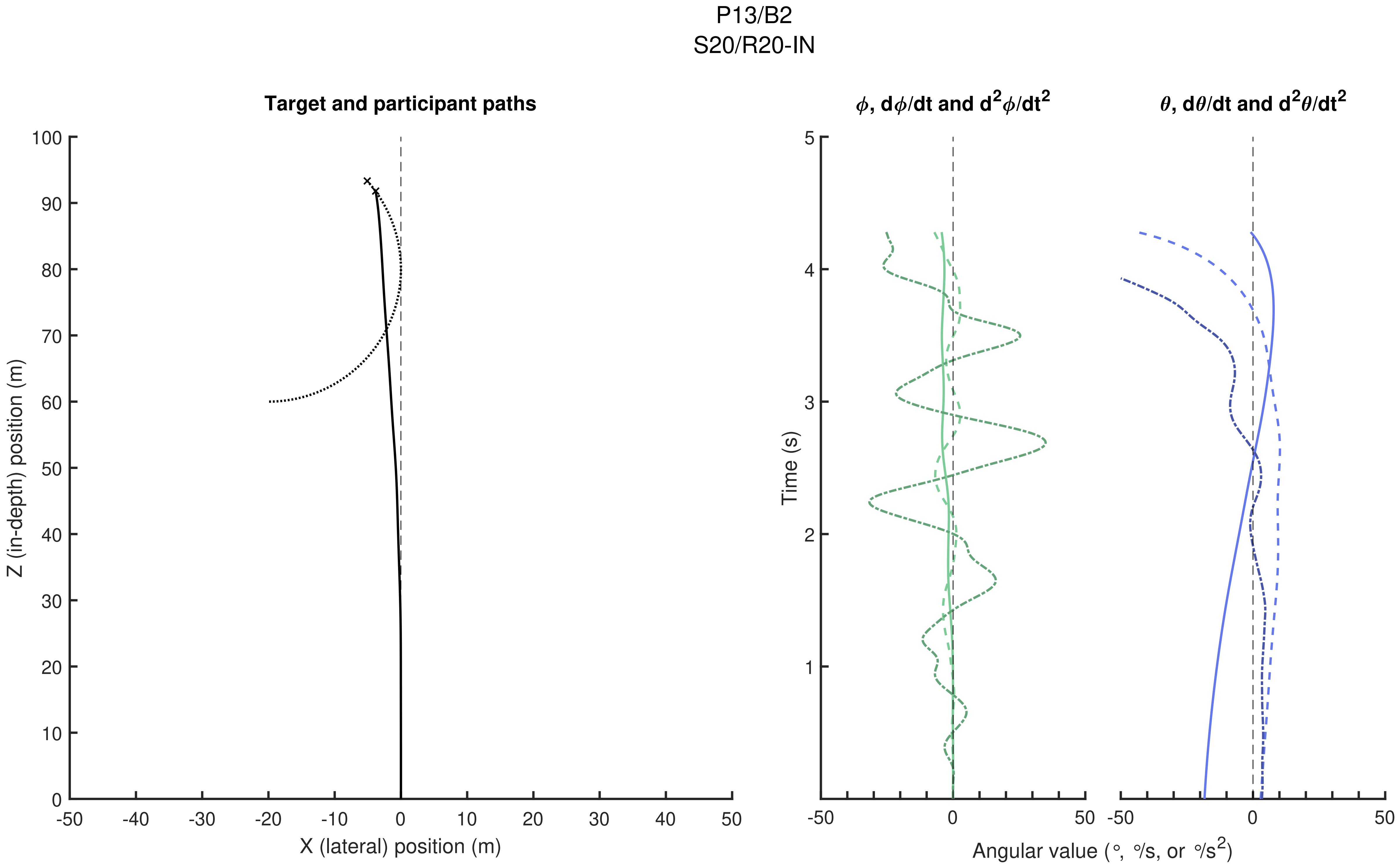
P13/B2
S20/R20-IN
Target and participant paths
Z (in-depth) position (m)
X (lateral) position (m)
100
90
80
70
60
50
40
30
20
10
0
-50
-40
-30
-20
-10
0
10
20
30
40
50
ϕ, dϕ/dt and d$^2\phi$/dt^2
θ, dθ/dt and d$^2\theta$/dt^2
Time (s)
5
4
3
2
1
-50
0
50
-50
0
50
Angular value (°, °/s, or °/s^2)

P13/B2
S20/R30-IN

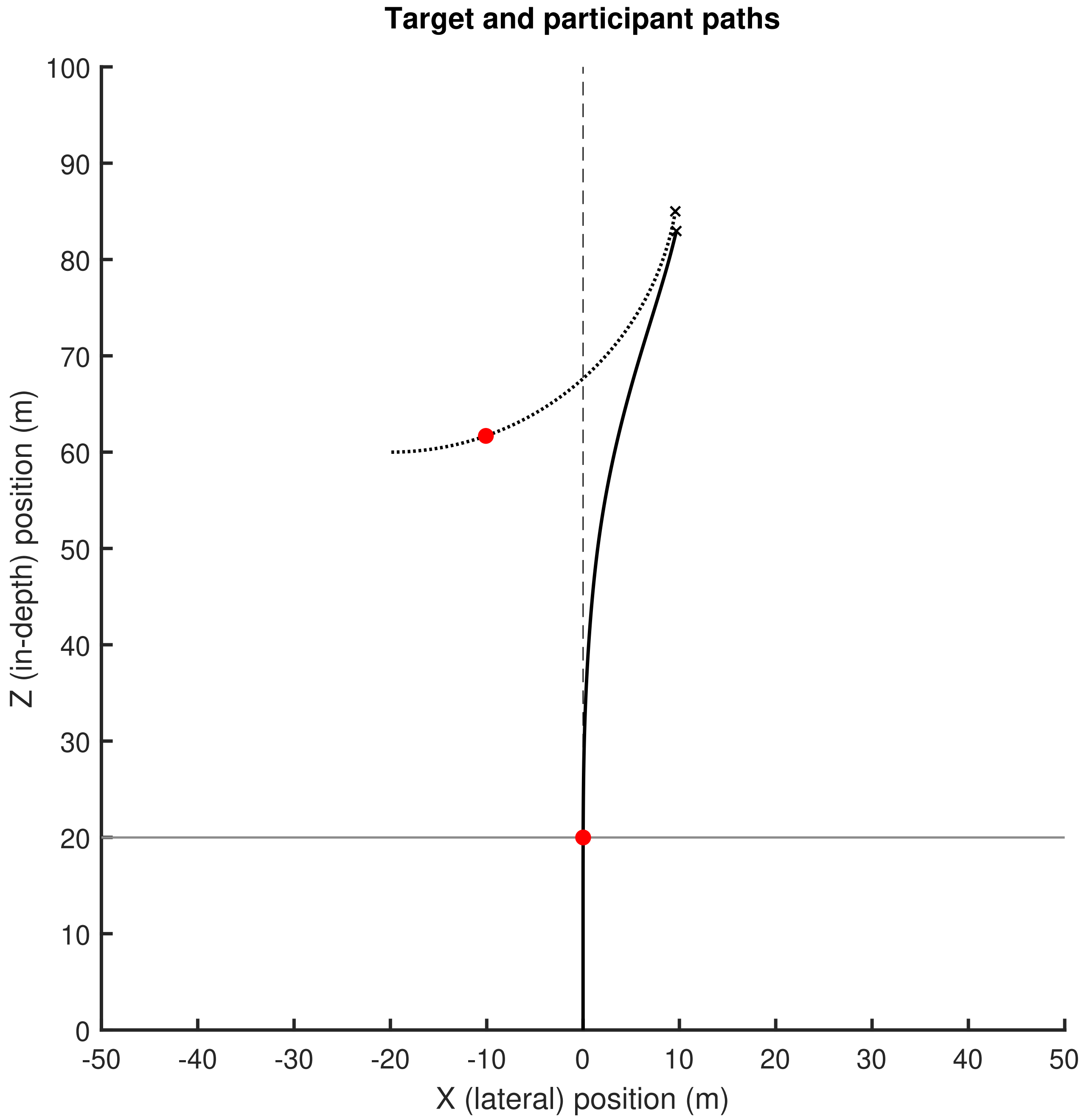

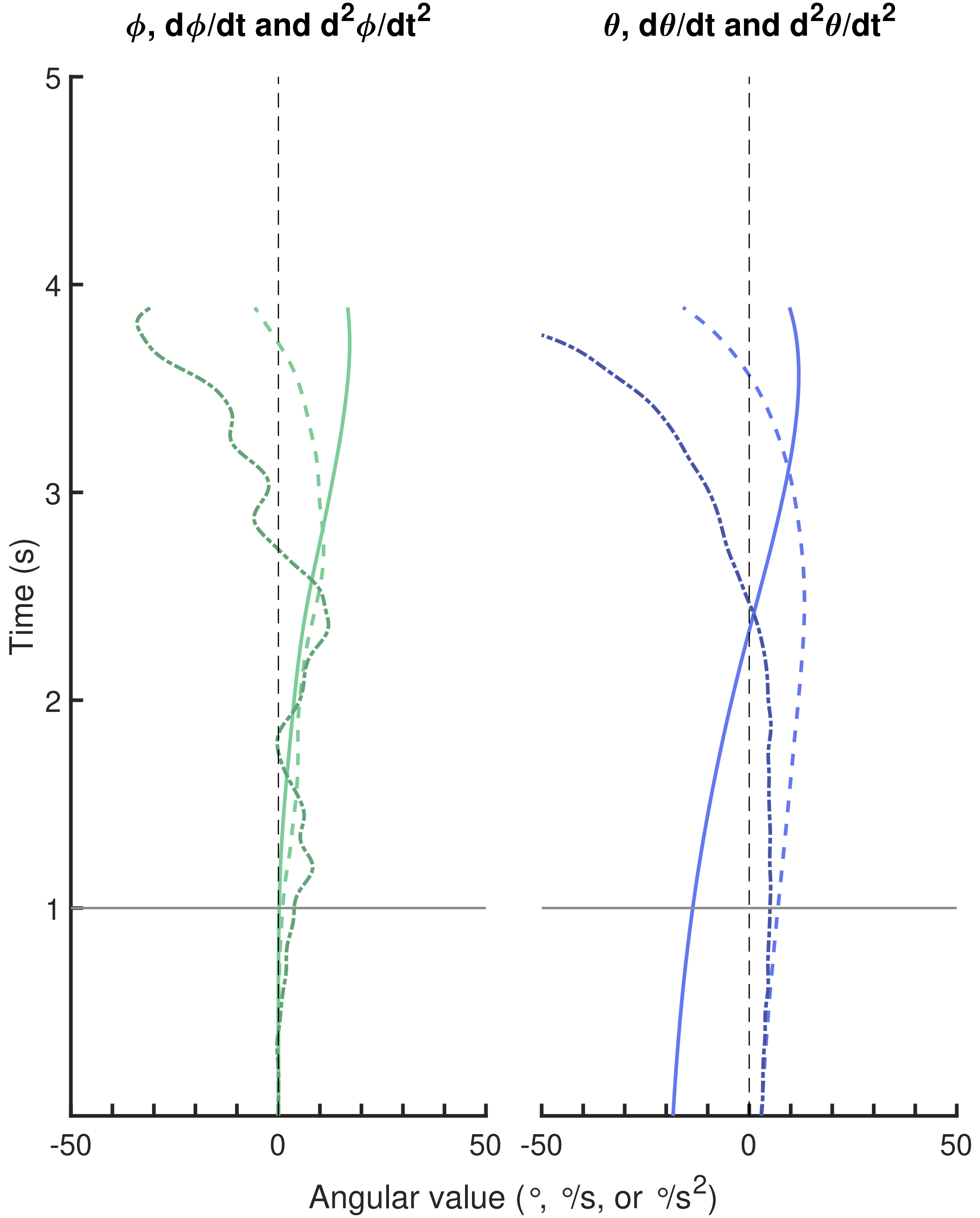

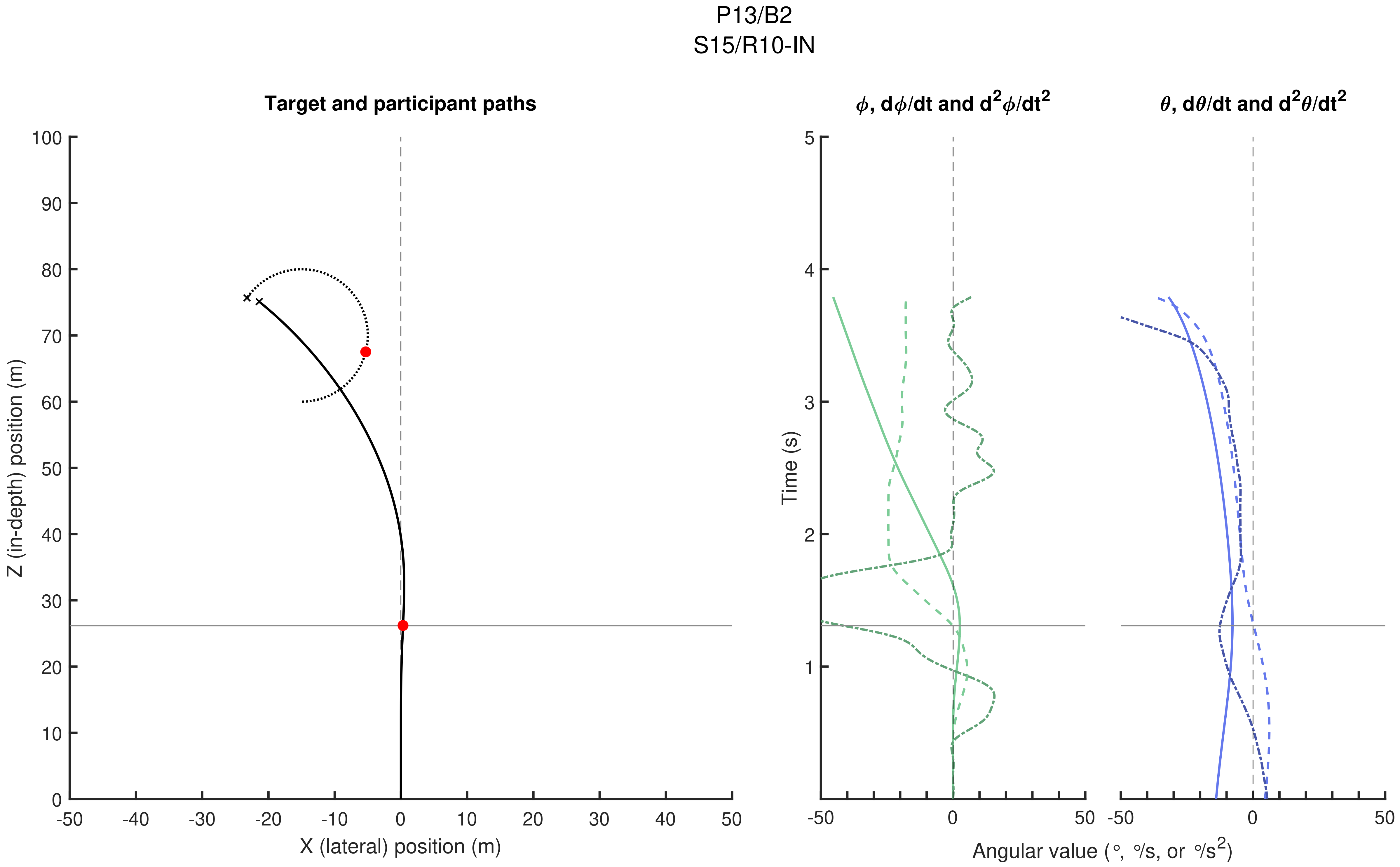
P13/B2
S15/R10-IN
Target and participant paths
Z (in-depth) position (m)
X (lateral) position (m)
100
90
80
70
60
50
40
30
20
10
0
-50
-40
-30
-20
-10
0
10
20
30
40
50
φ, dφ/dt and d²φ/dt²
θ, dθ/dt and d²θ/dt²
Time (s)
5
4
3
2
1
-50
0
50
-50
0
50
Angular value (°, °/s, or °/s²)

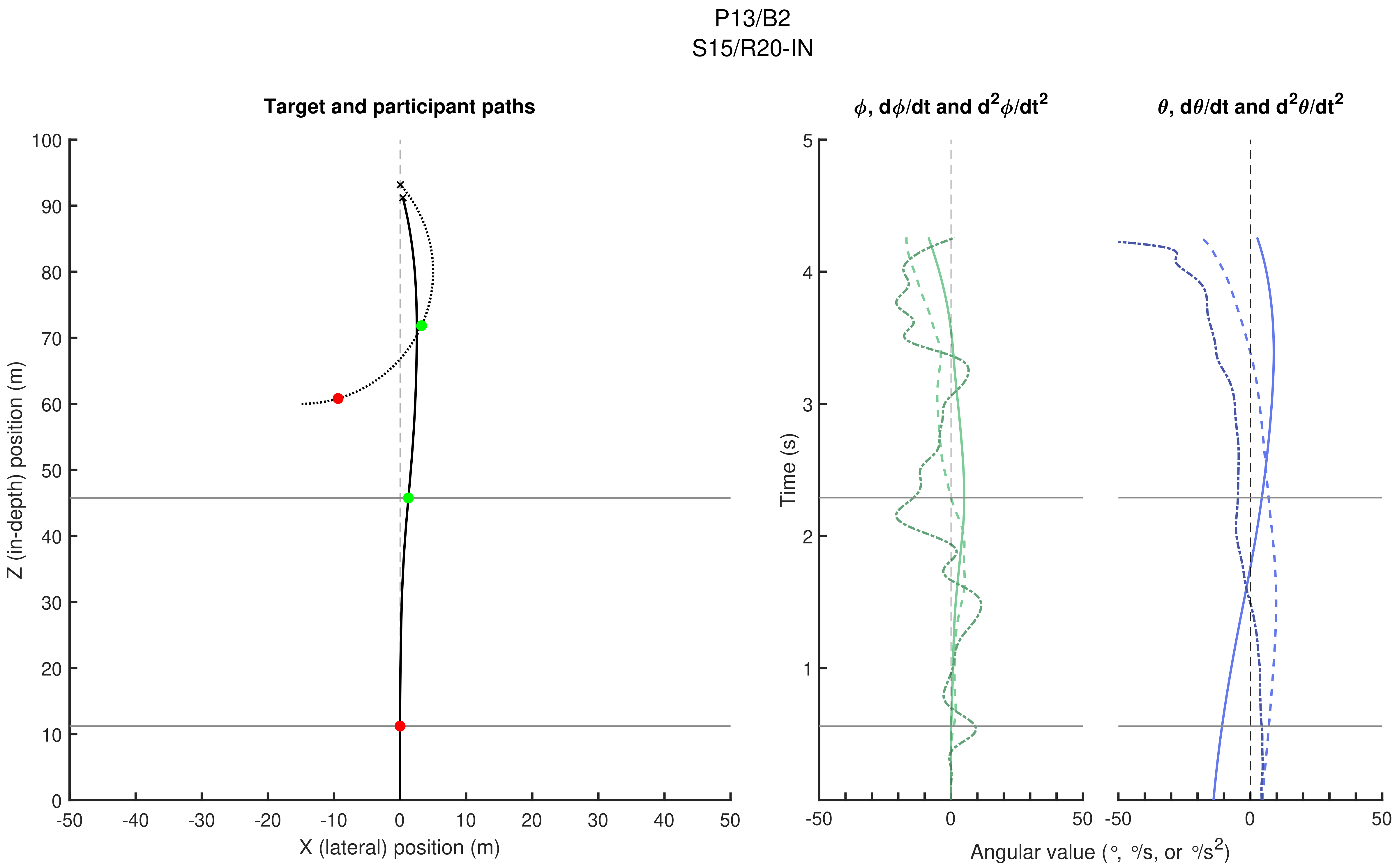
P13/B2
S15/R20-IN
Target and participant paths
Z (in-depth) position (m)
X (lateral) position (m)
100
90
80
70
60
50
40
30
20
10
0
-50
-40
-30
-20
-10
0
10
20
30
40
50
ϕ, dϕ/dt and d$^2\phi$/dt^2
θ, dθ/dt and d$^2\theta$/dt^2
Time (s)
5
4
3
2
1
-50
0
50
-50
0
50
Angular value (°, °/s, or °/s^2)

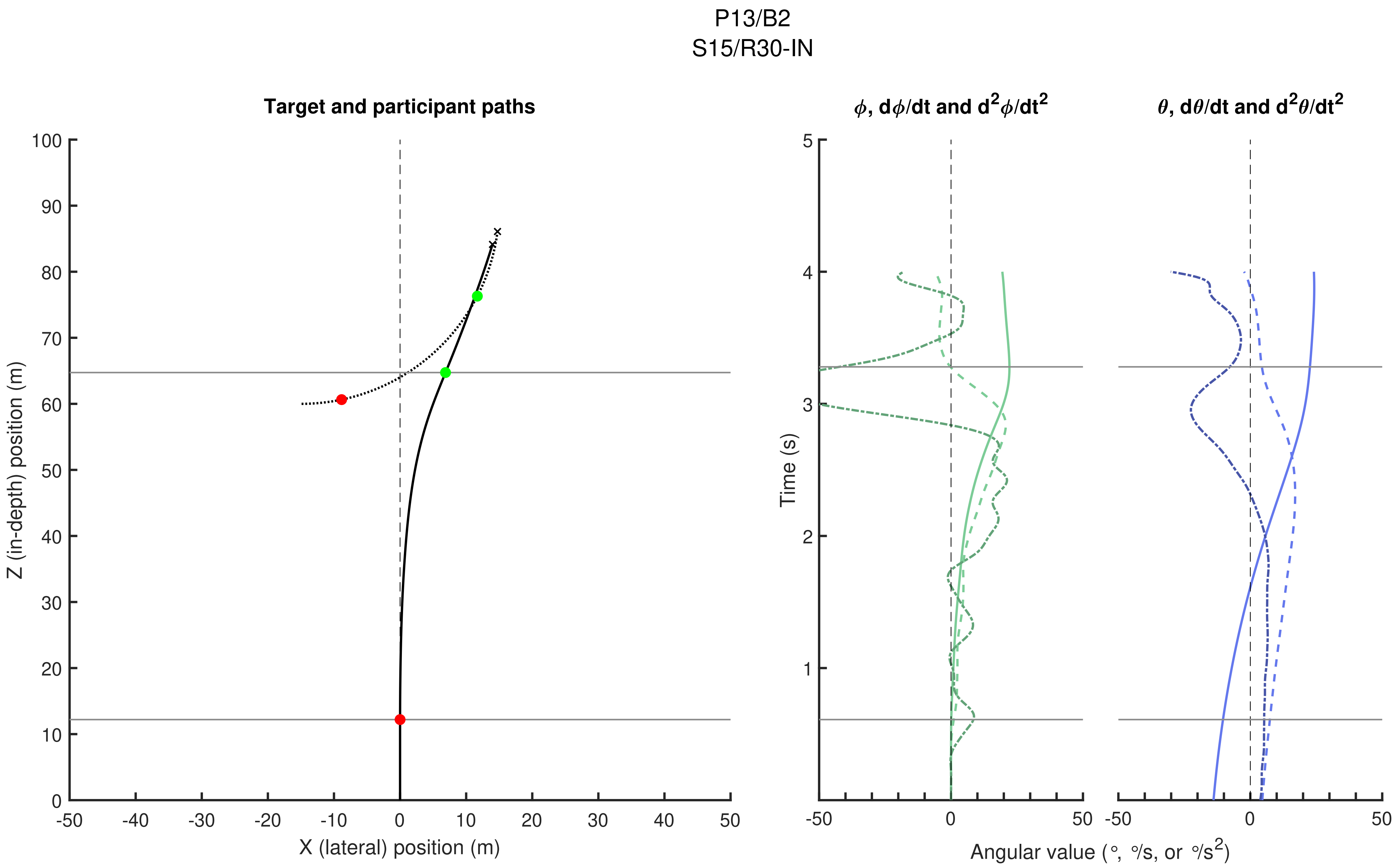

P13/B2
S15/R30-IN
Target and participant paths
Z (in-depth) position (m)
X (lateral) position (m)
φ, dφ/dt and d²φ/dt²
θ, dθ/dt and d²θ/dt²
Time (s)
Angular value (°, °/s, or °/s²)

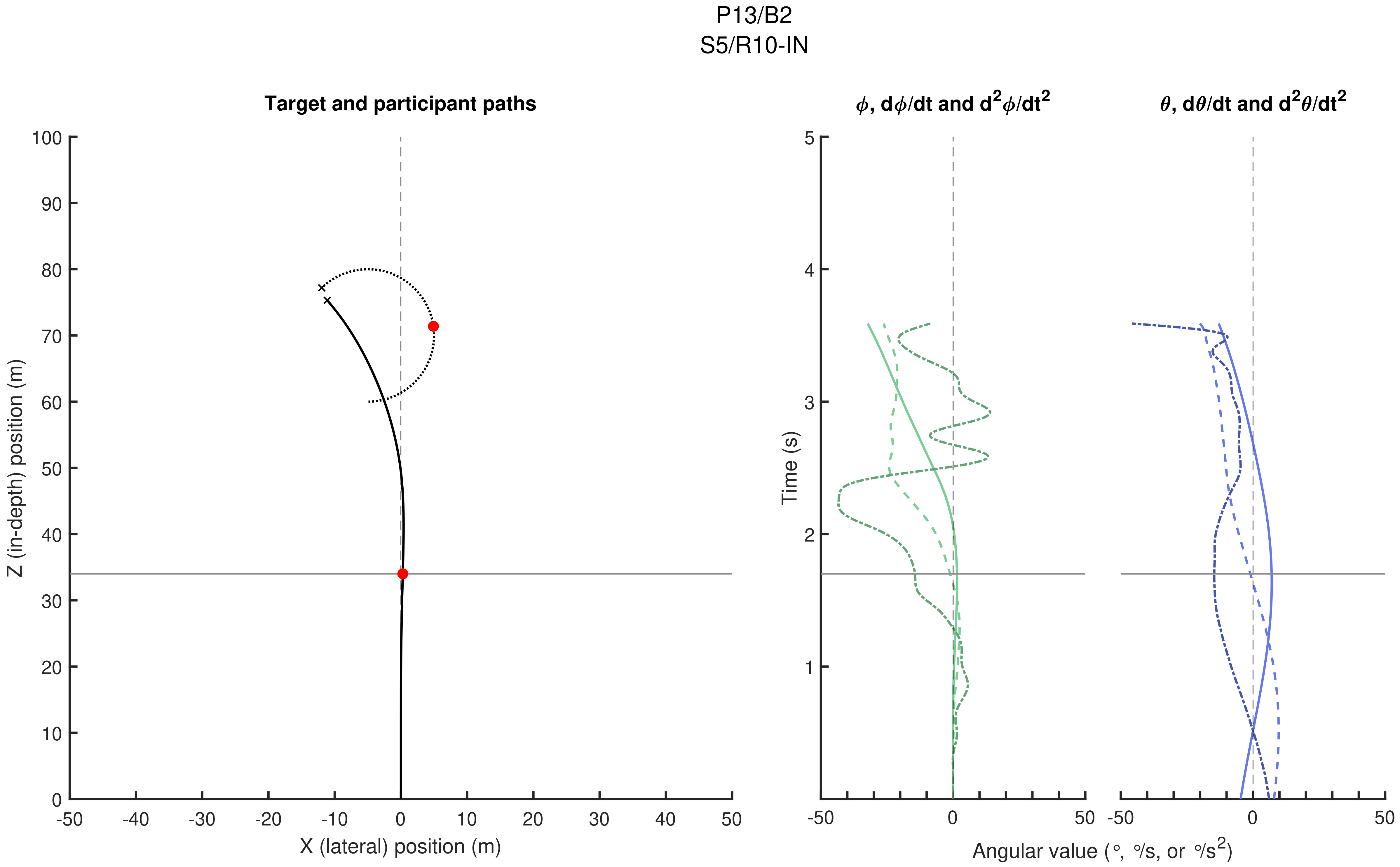
P13/B2
S5/R10-IN
Target and participant paths
Z (in-depth) position (m)
X (lateral) position (m)
φ, dφ/dt and d²φ/dt²
θ, dθ/dt and d²θ/dt²
Time (s)
Angular value (°, °/s, or °/s²)

P13/B2
S5/R10-OUT

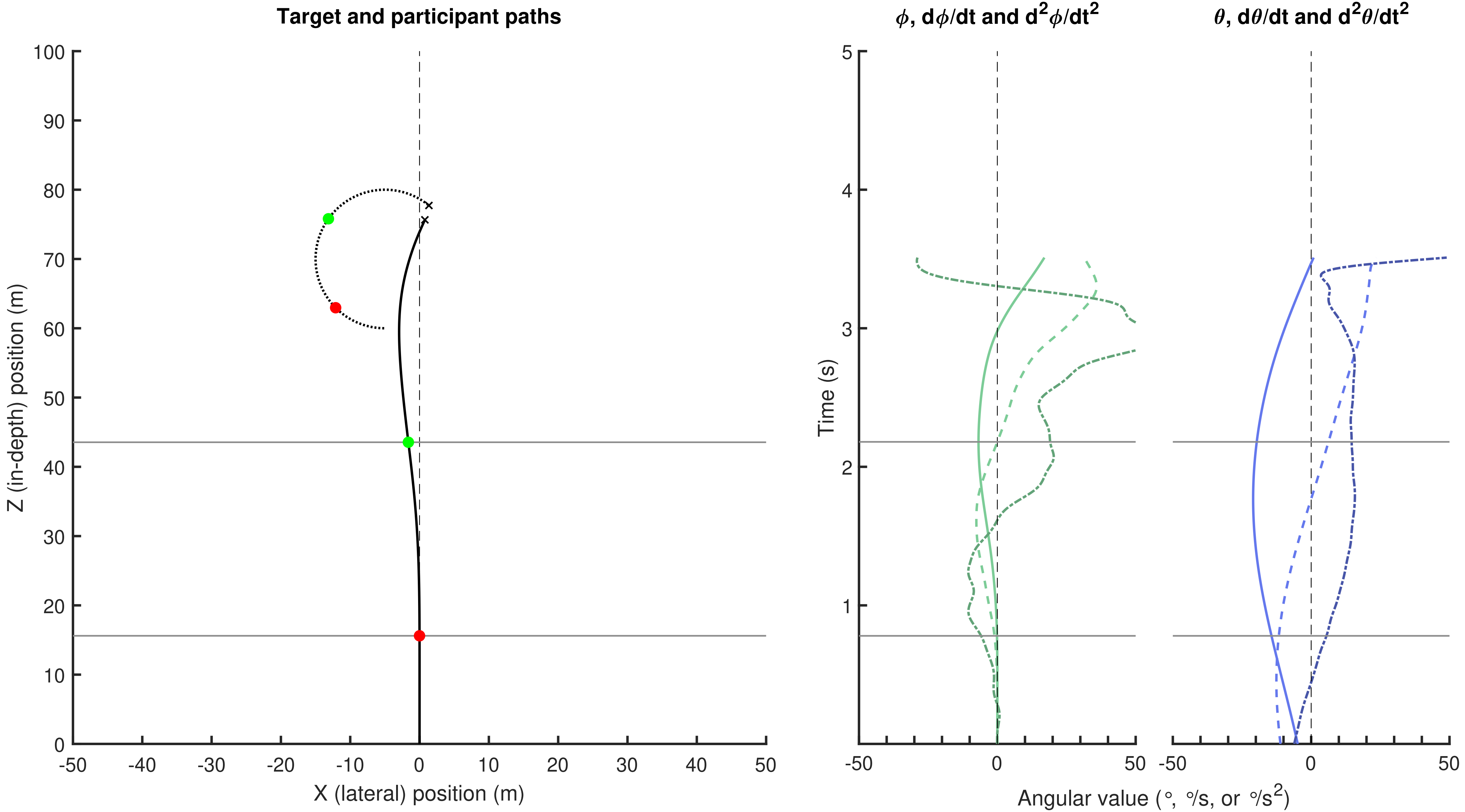

P13/B2
S5/R20-IN

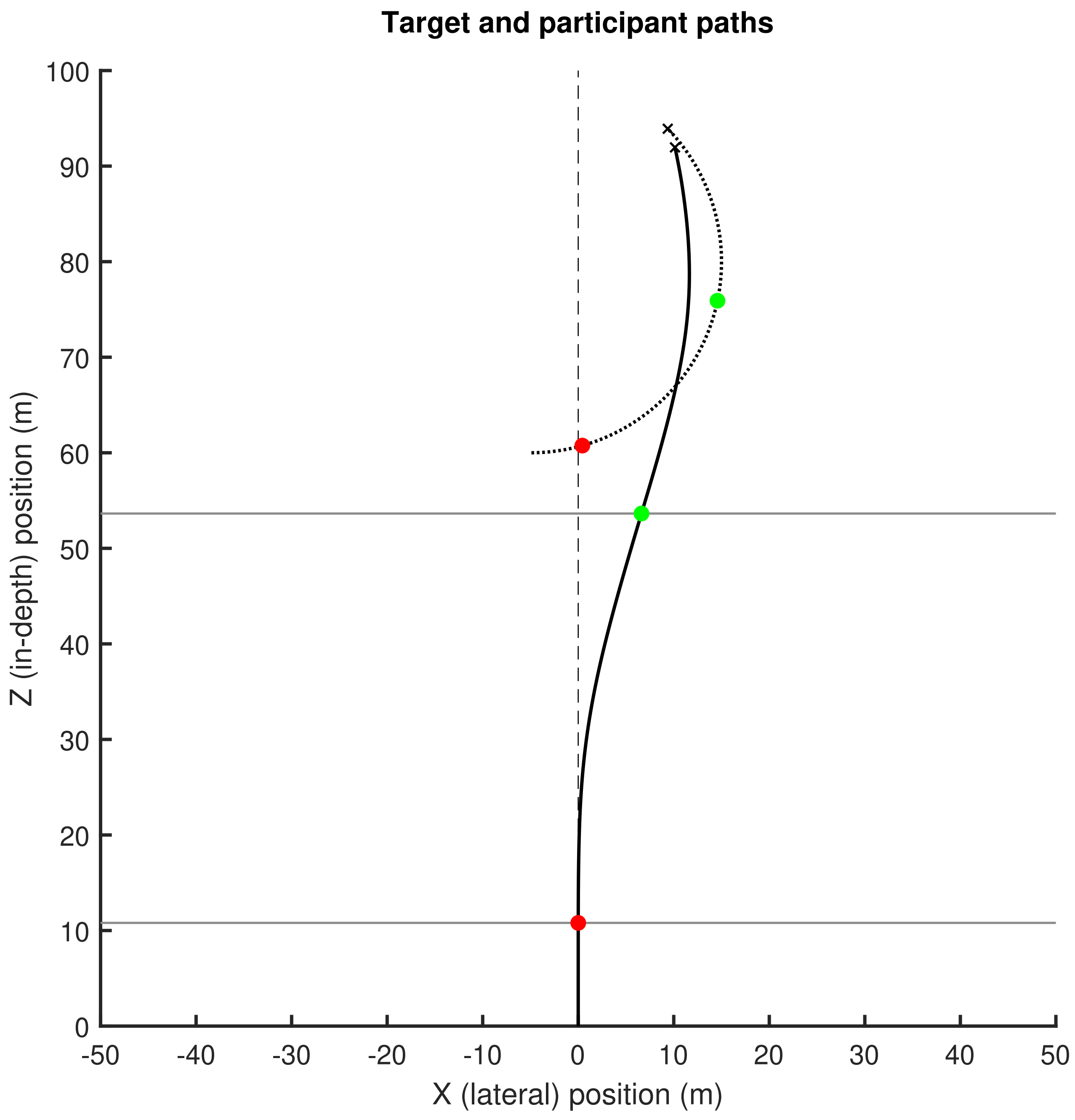

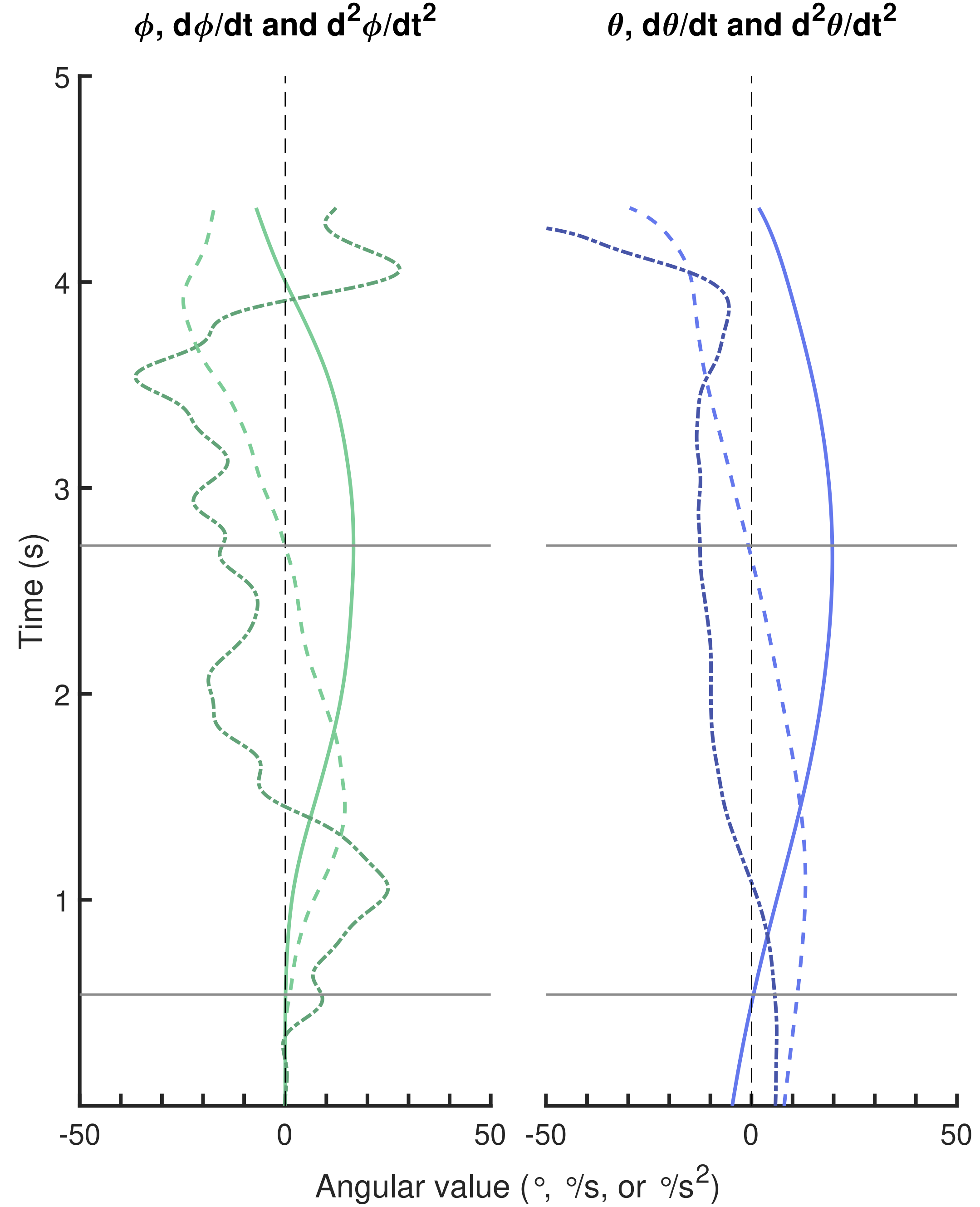

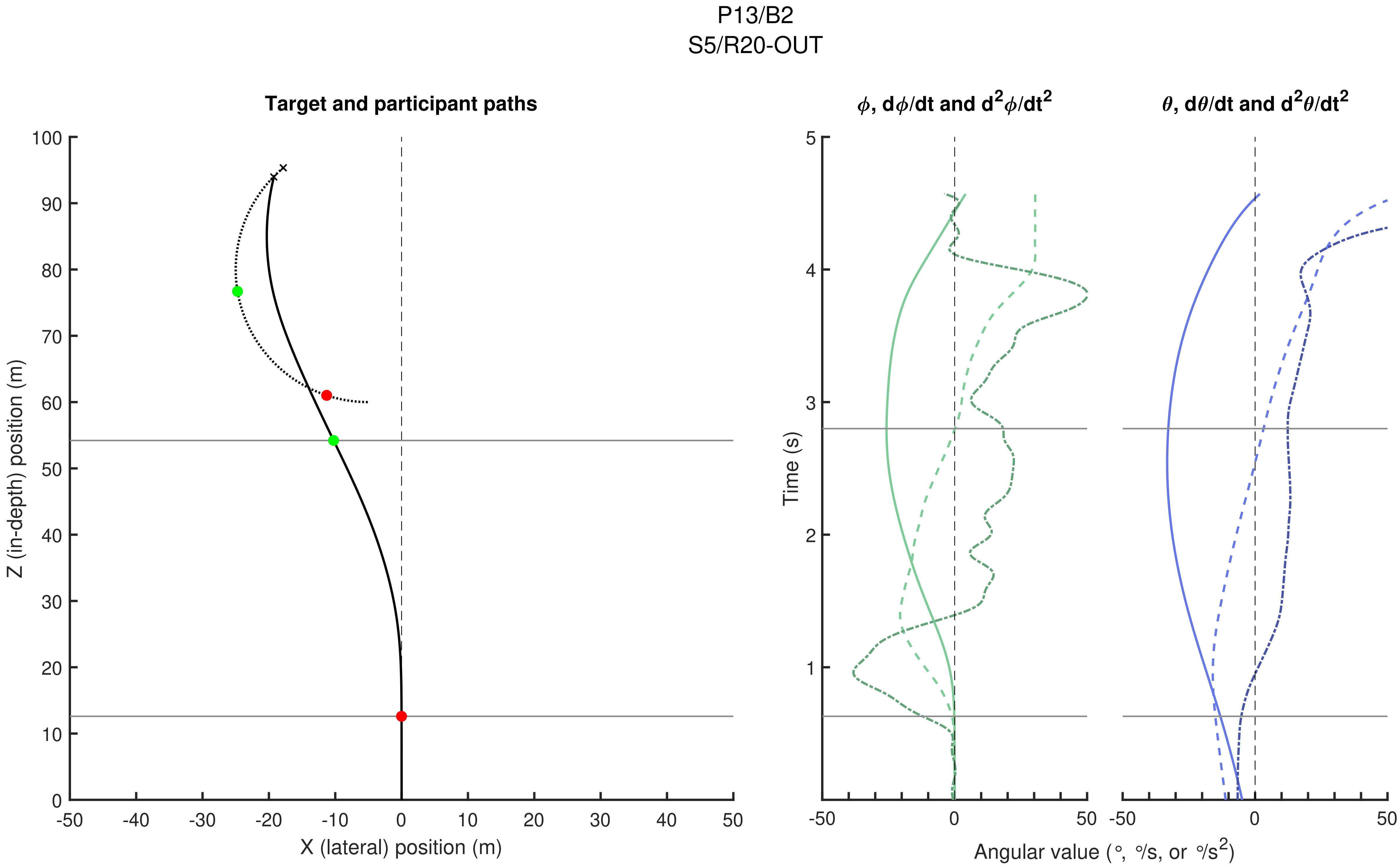
P13/B2
S5/R20-OUT
Target and participant paths
Z (in-depth) position (m)
X (lateral) position (m)
100
90
80
70
60
50
40
30
20
10
0
-50
-40
-30
-20
-10
0
10
20
30
40
50
ϕ, dϕ/dt and d²ϕ/dt²
θ, dθ/dt and d²θ/dt²
Time (s)
5
4
3
2
1
-50
0
50
Angular value (°, °/s, or °/s²)

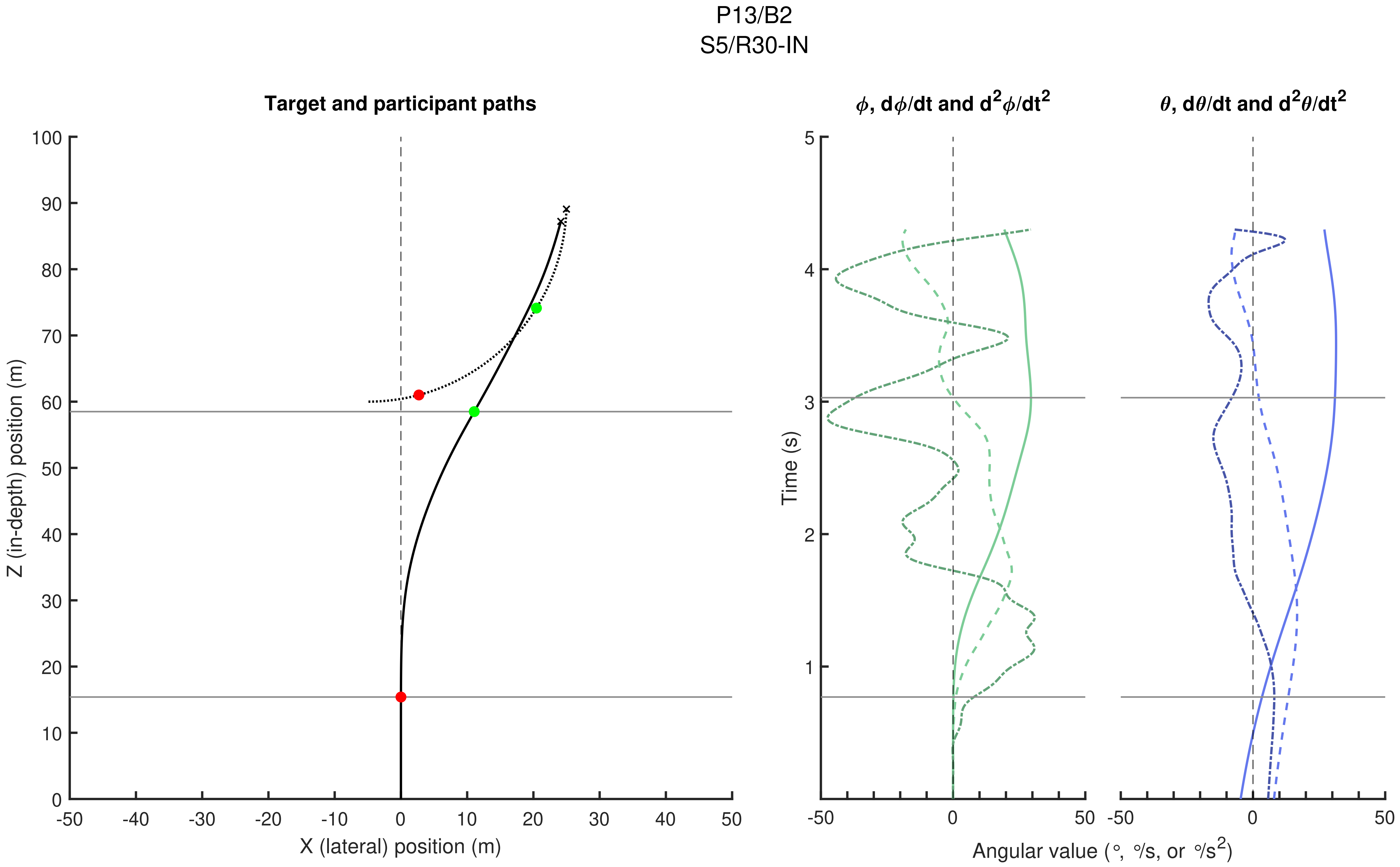

P13/B2
S5/R30-IN
Target and participant paths
Z (in-depth) position (m)
X (lateral) position (m)
φ, dφ/dt and d²φ/dt²
θ, dθ/dt and d²θ/dt²
Time (s)
Angular value (°, °/s, or °/s²)

P13/B2
S5/R30-OUT

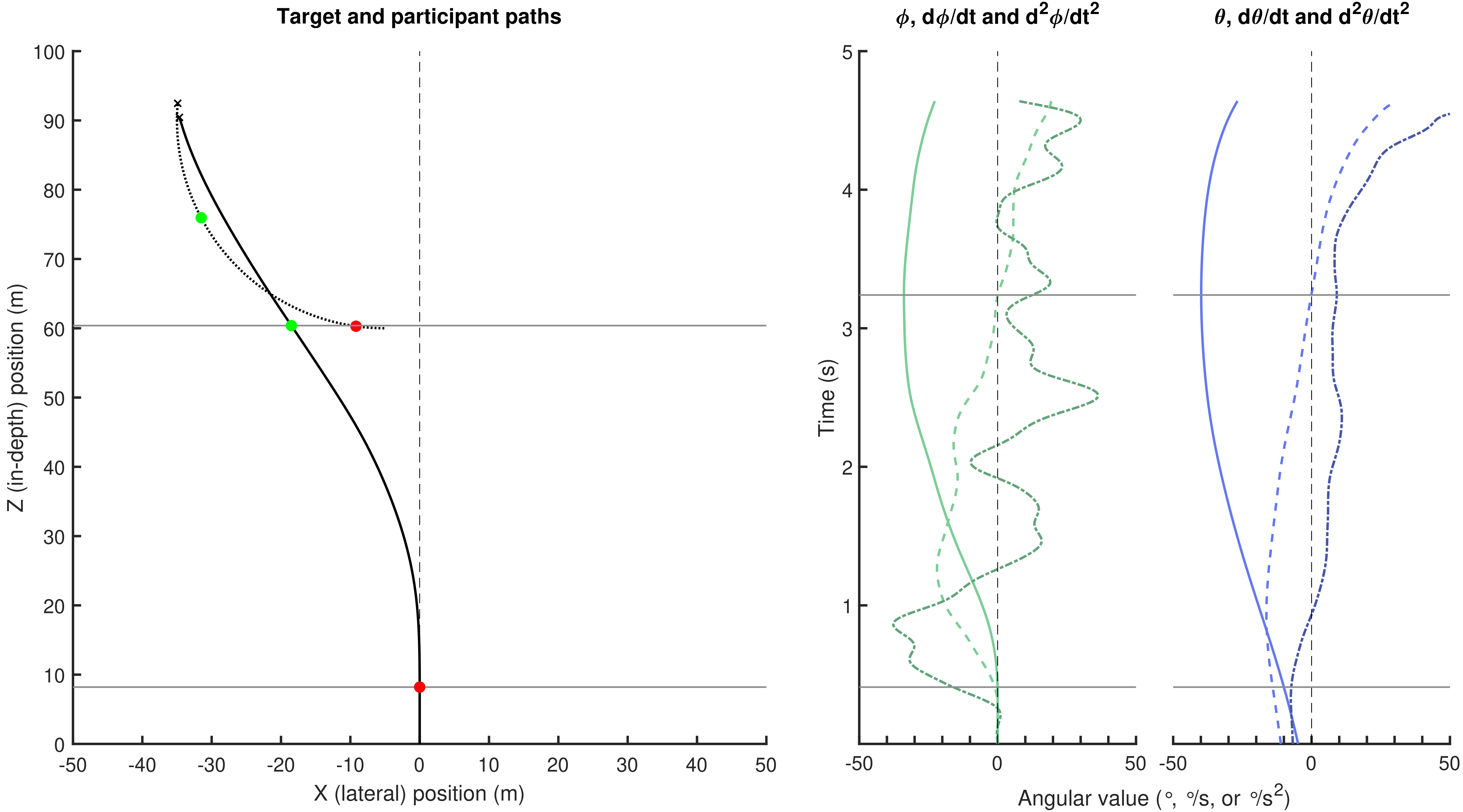

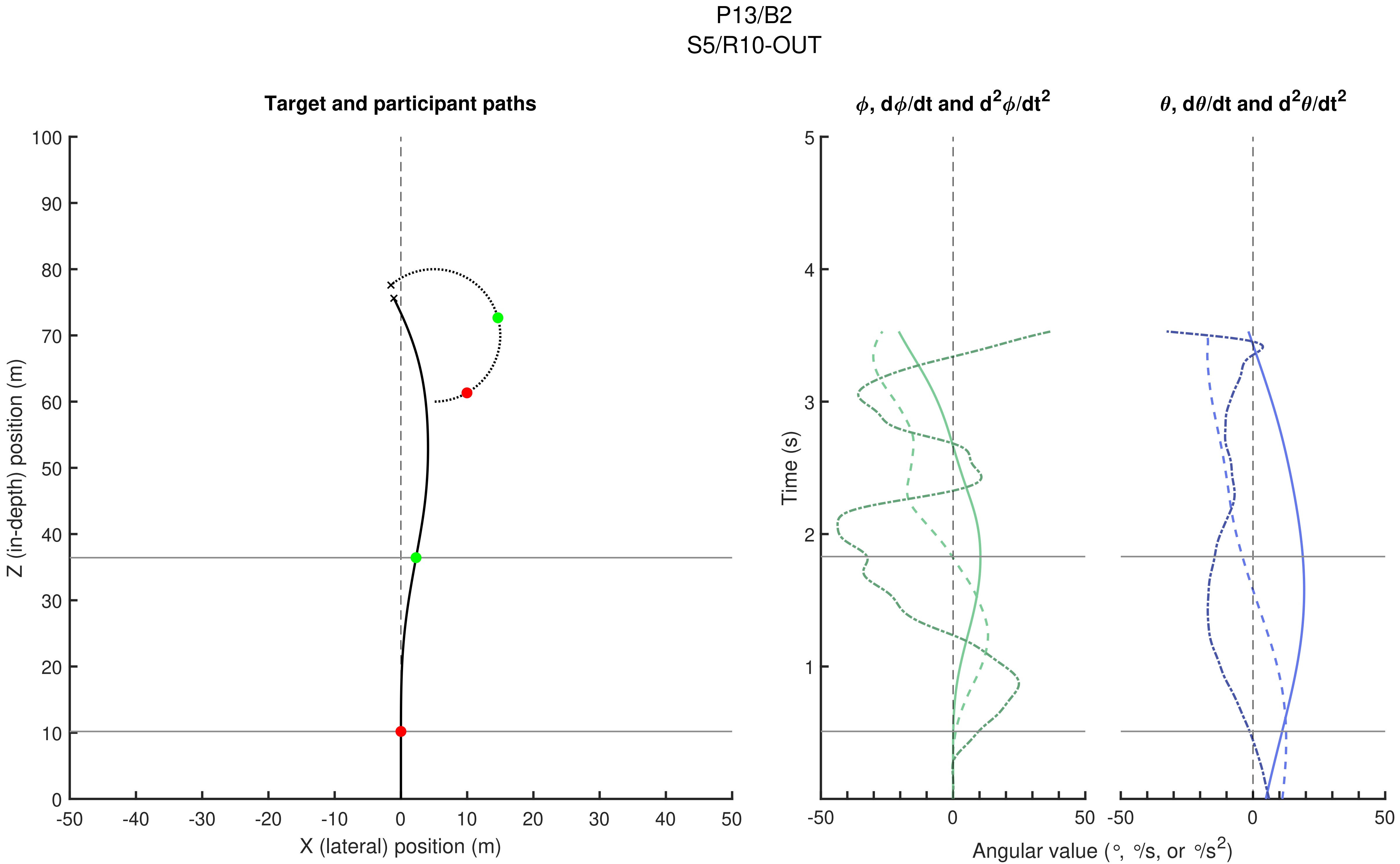
P13/B2
S5/R10-OUT
Target and participant paths
Z (in-depth) position (m)
X (lateral) position (m)
ϕ, dϕ/dt and d²ϕ/dt²
θ, dθ/dt and d²θ/dt²
Time (s)
Angular value (°, °/s, or °/s²)

P13/B2
S5/R10-IN

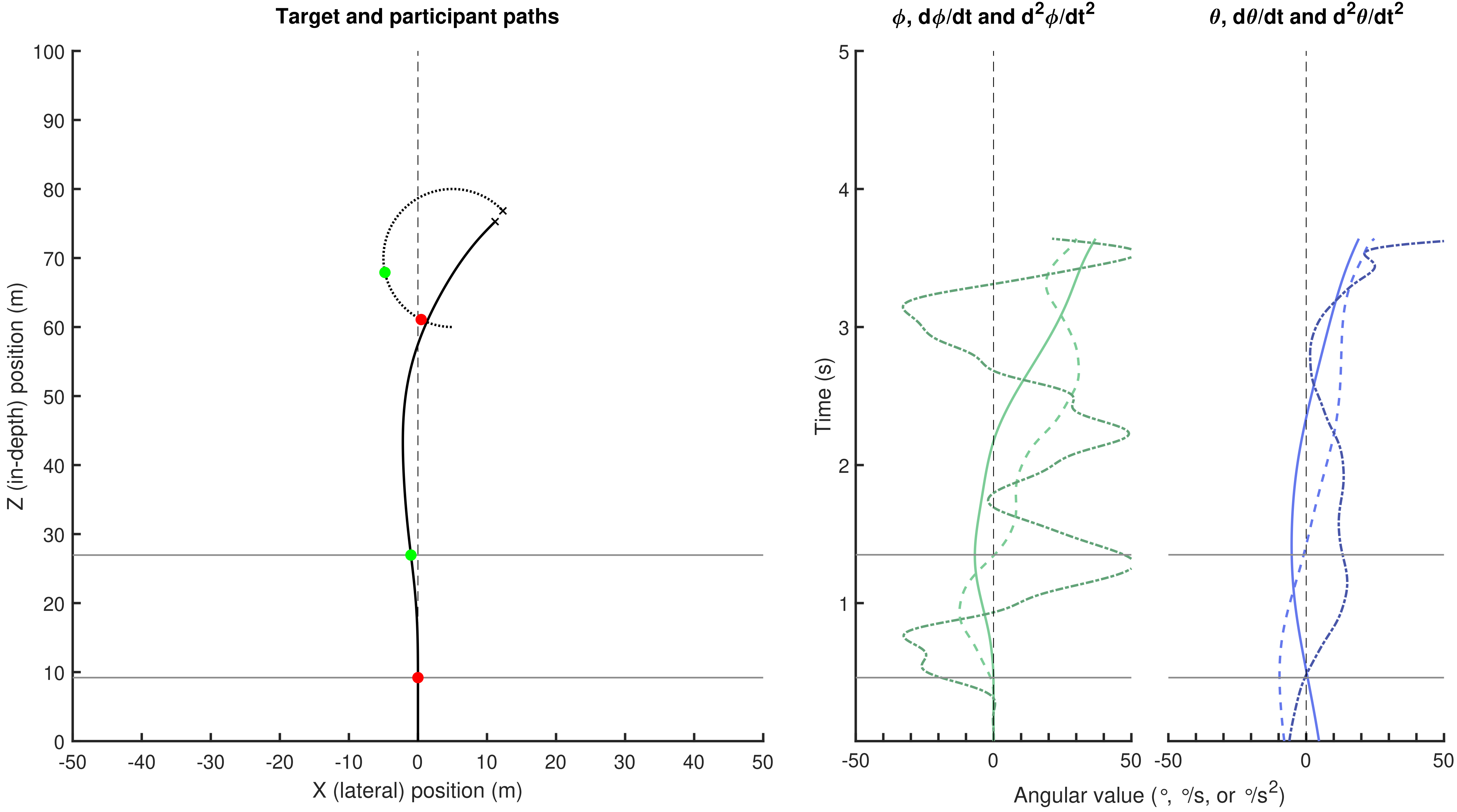

P13/B2
S5/R20-OUT

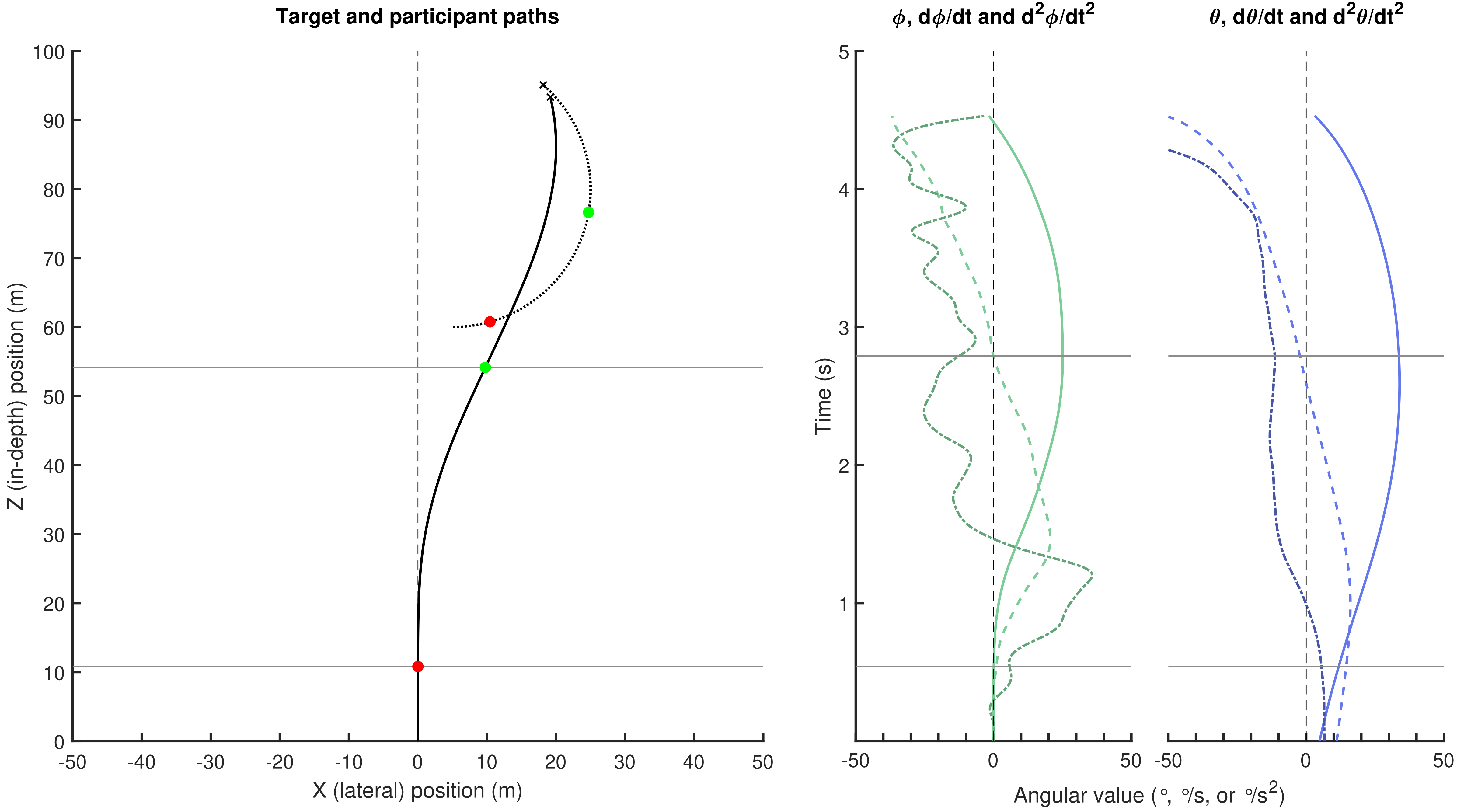

P13/B2
S5/R20-IN

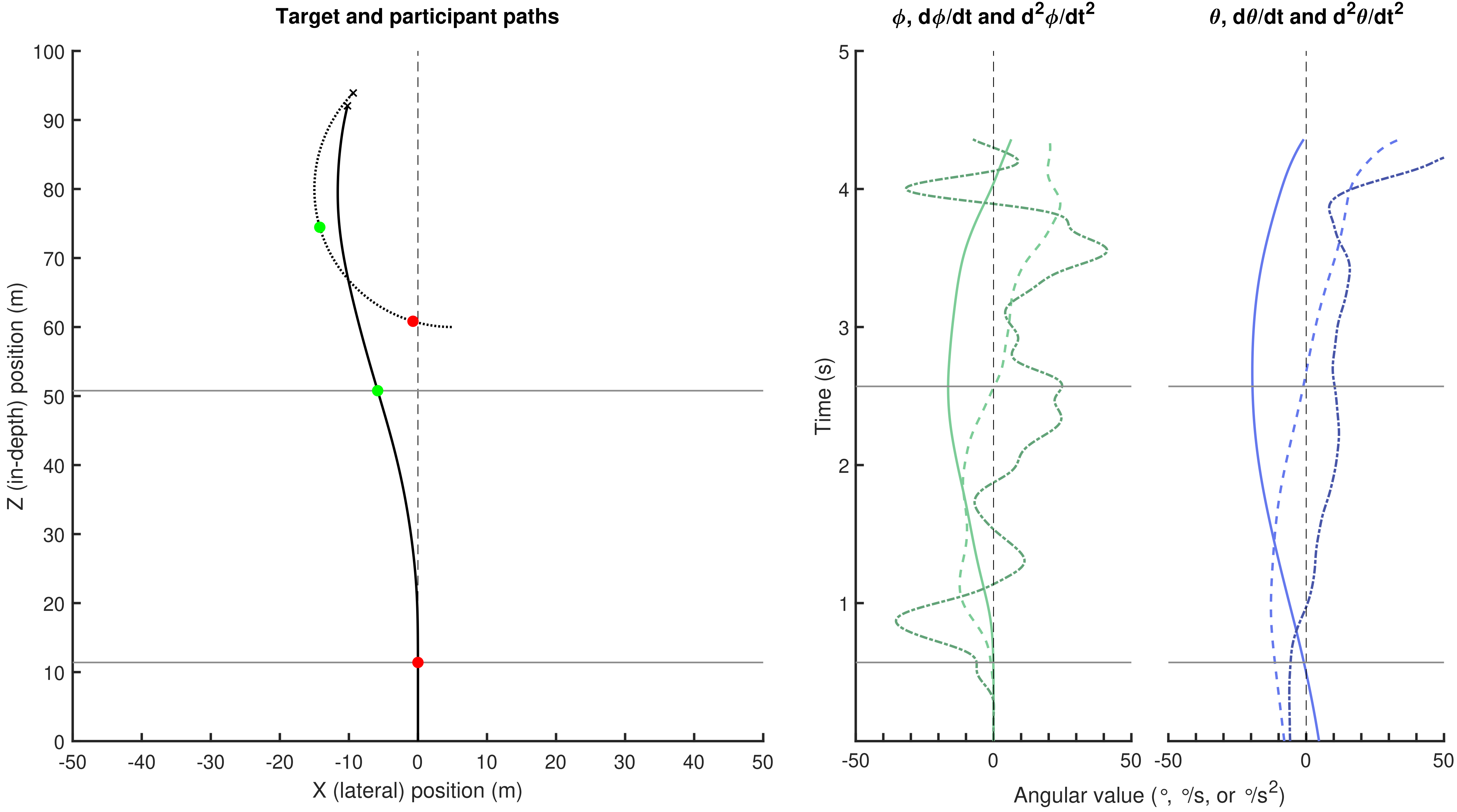

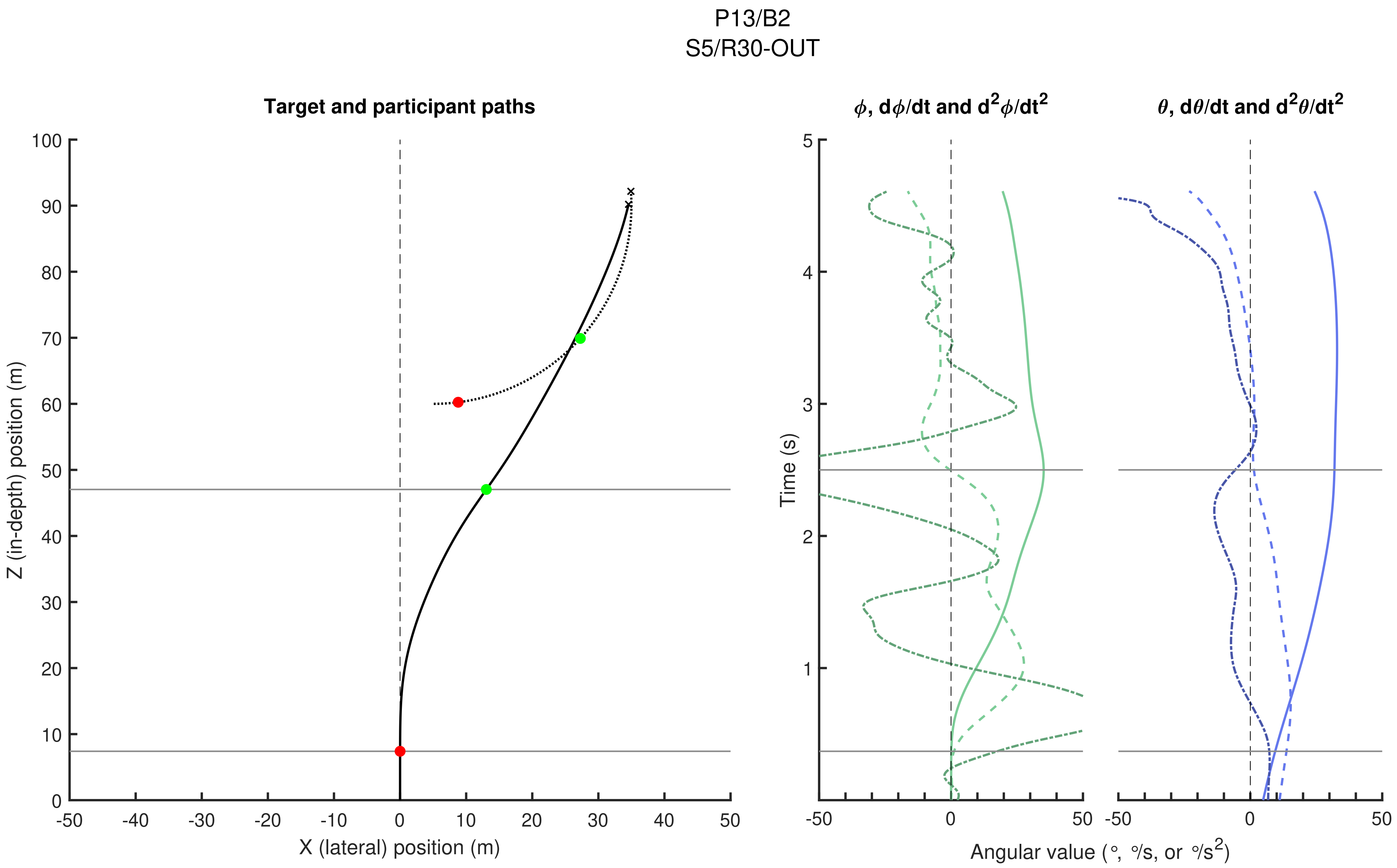
P13/B2
S5/R30-OUT
Target and participant paths
Z (in-depth) position (m)
X (lateral) position (m)
φ, dφ/dt and d²φ/dt²
θ, dθ/dt and d²θ/dt²
Time (s)
Angular value (°, °/s, or °/s²)

P13/B2
S5/R30-IN

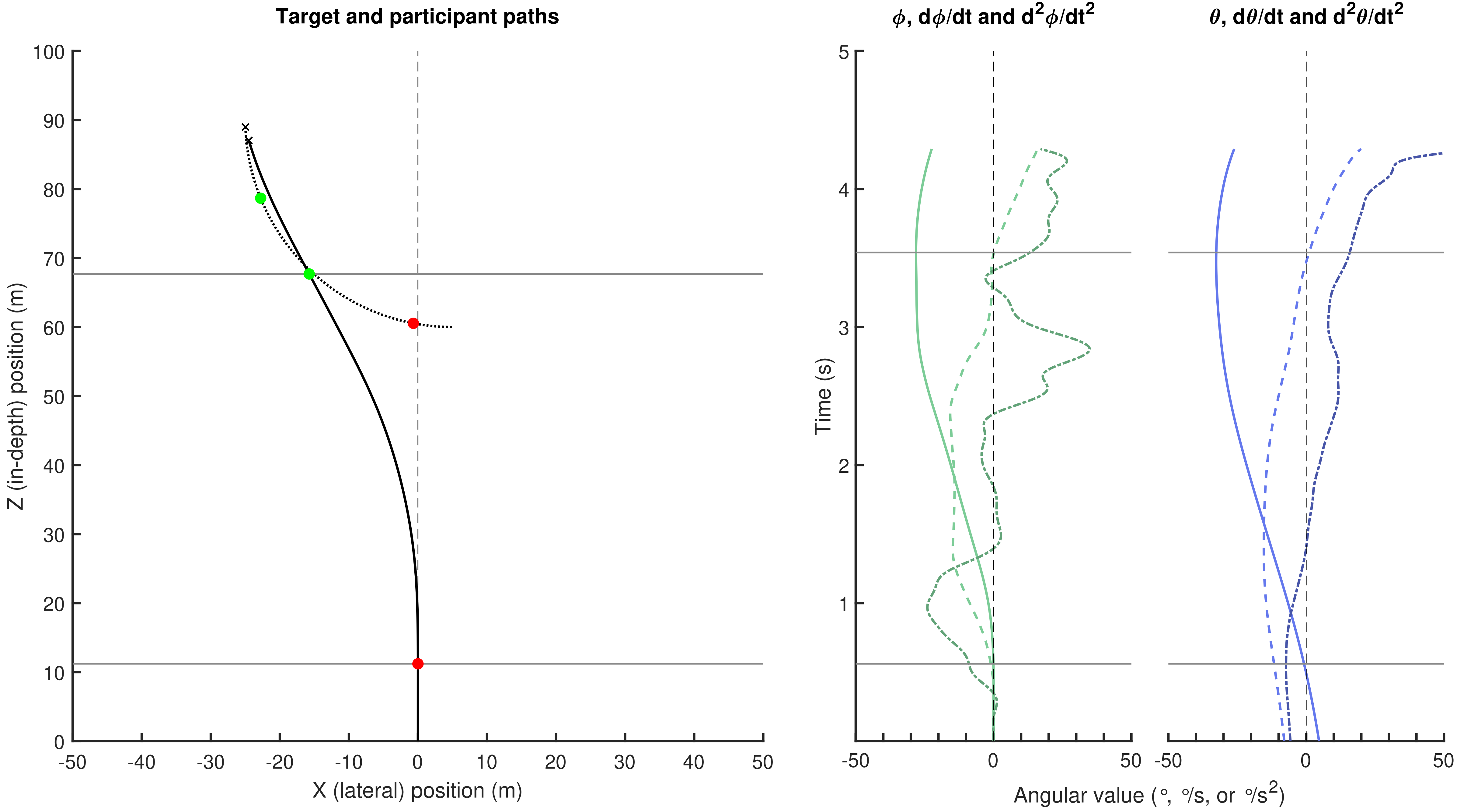

P13/B2
S15/R10-IN

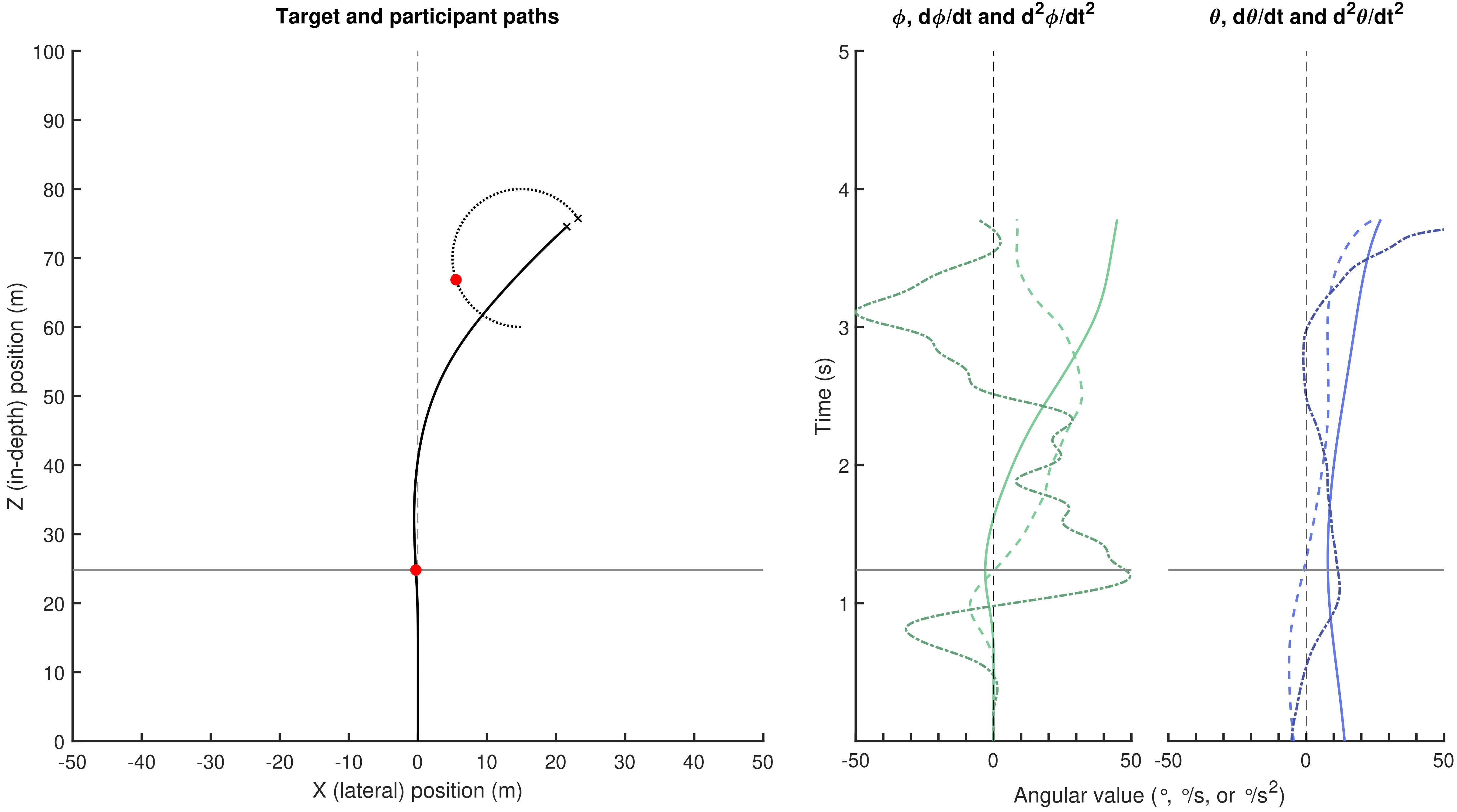

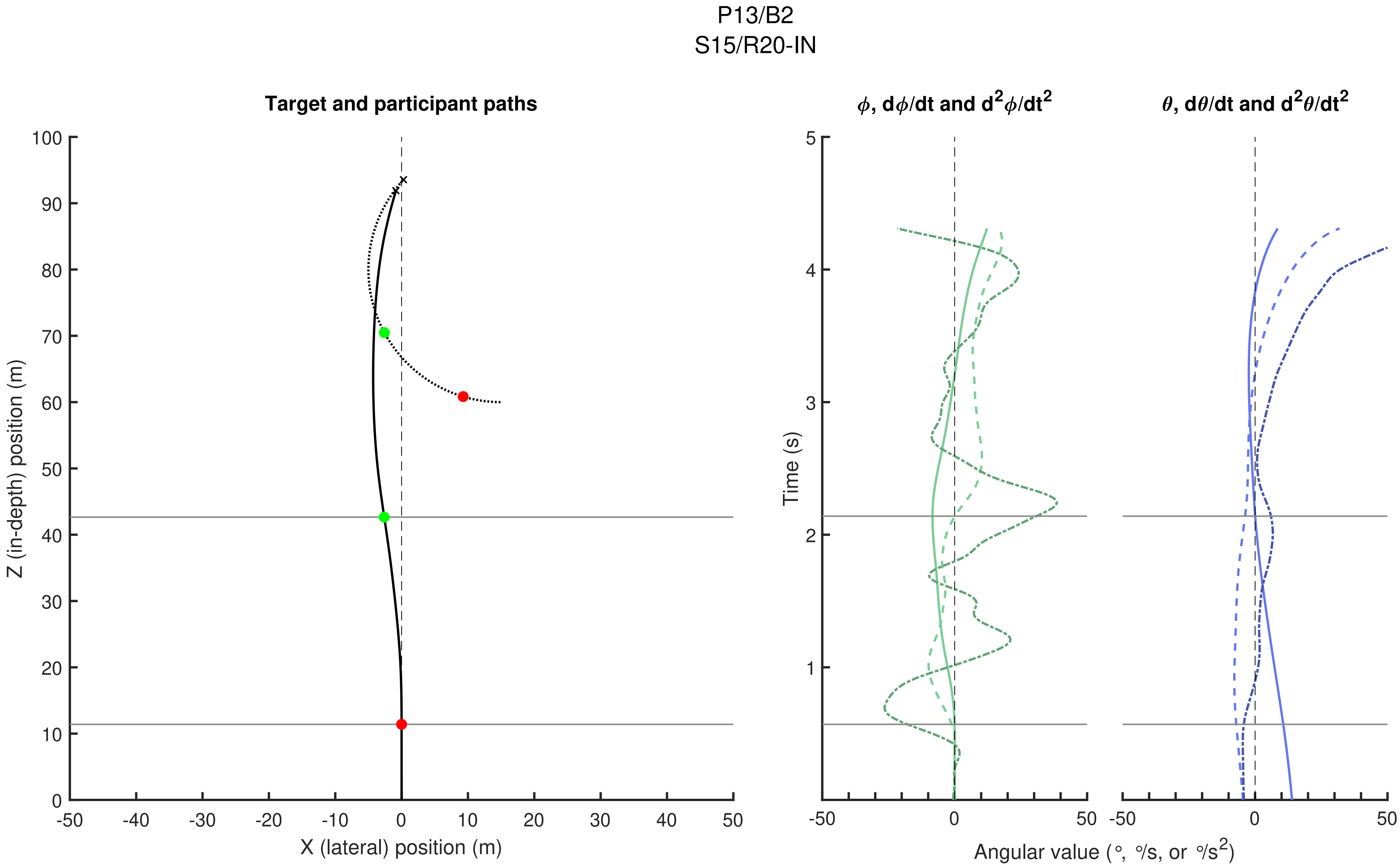
P13/B2
S15/R20-IN
Target and participant paths
ϕ, dϕ/dt and d$^2\phi$/dt^2
θ, dθ/dt and d$^2\theta$/dt^2
Z (in-depth) position (m)
X (lateral) position (m)
Time (s)
Angular value (°, °/s, or °/s^2)

P13/B2
S15/R30-IN

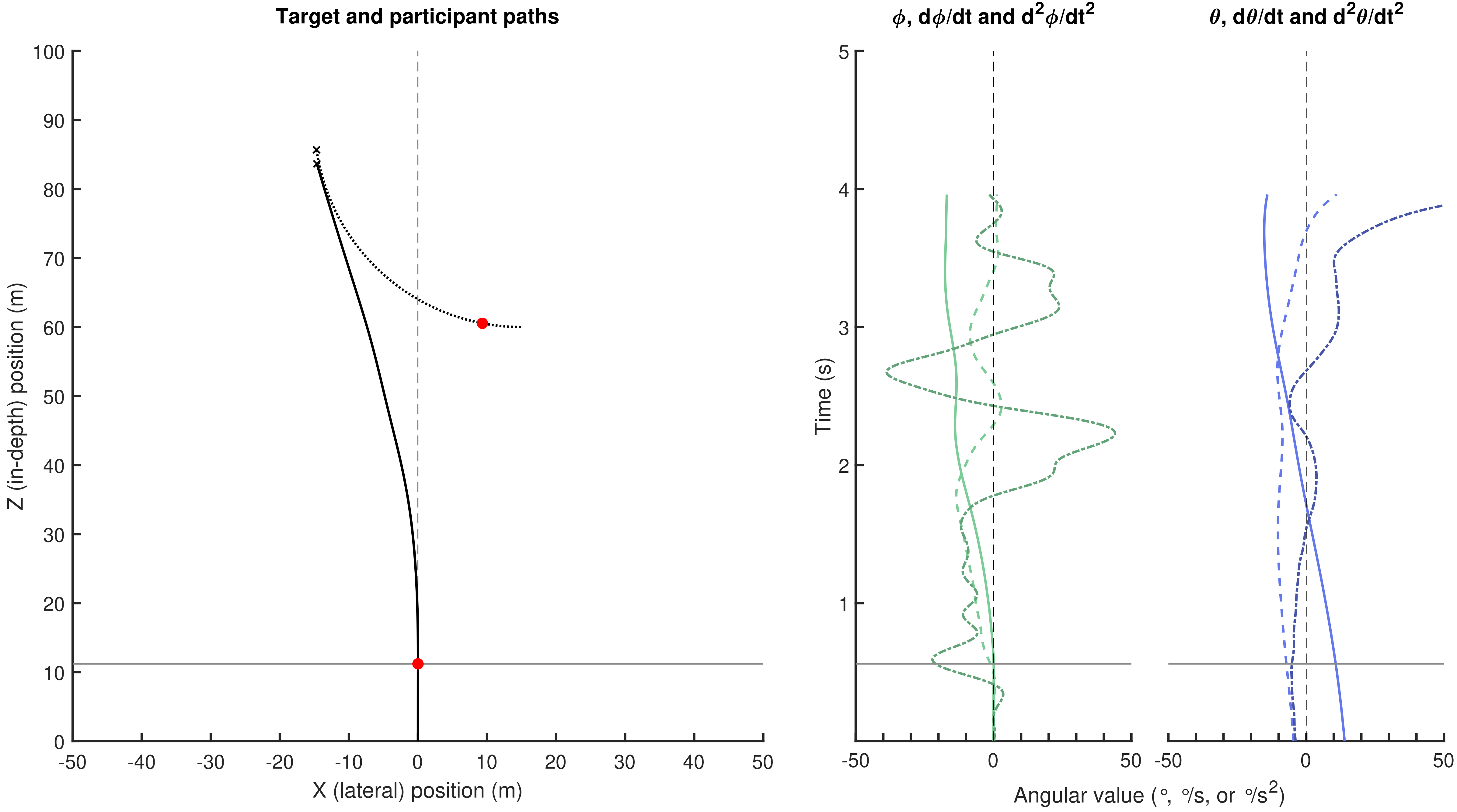

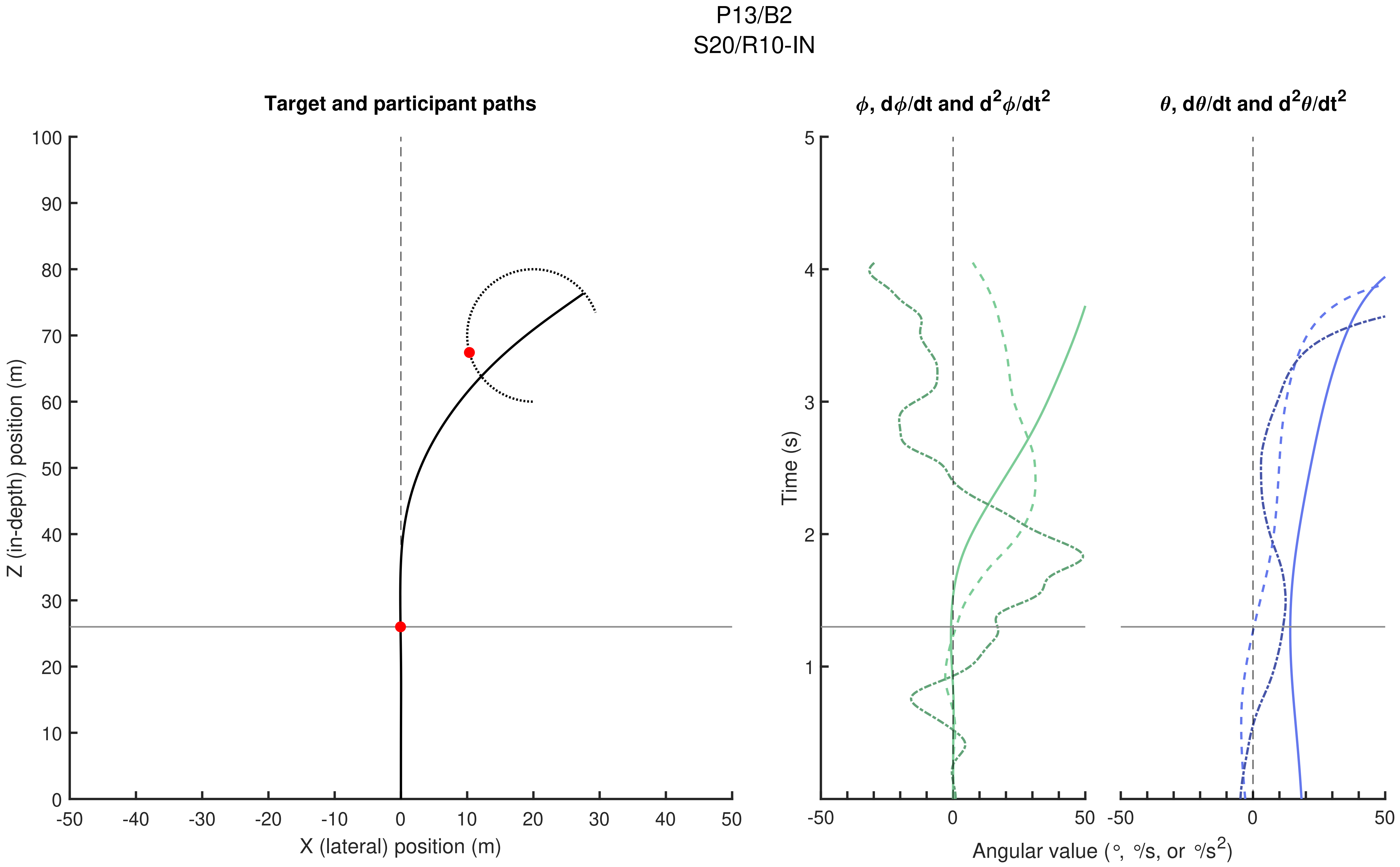
P13/B2
S20/R10-IN
Target and participant paths
Z (in-depth) position (m)
X (lateral) position (m)
ϕ, dϕ/dt and d$^2\phi$/dt^2
θ, dθ/dt and d$^2\theta$/dt^2
Time (s)
Angular value (°, °/s, or °/s^2)

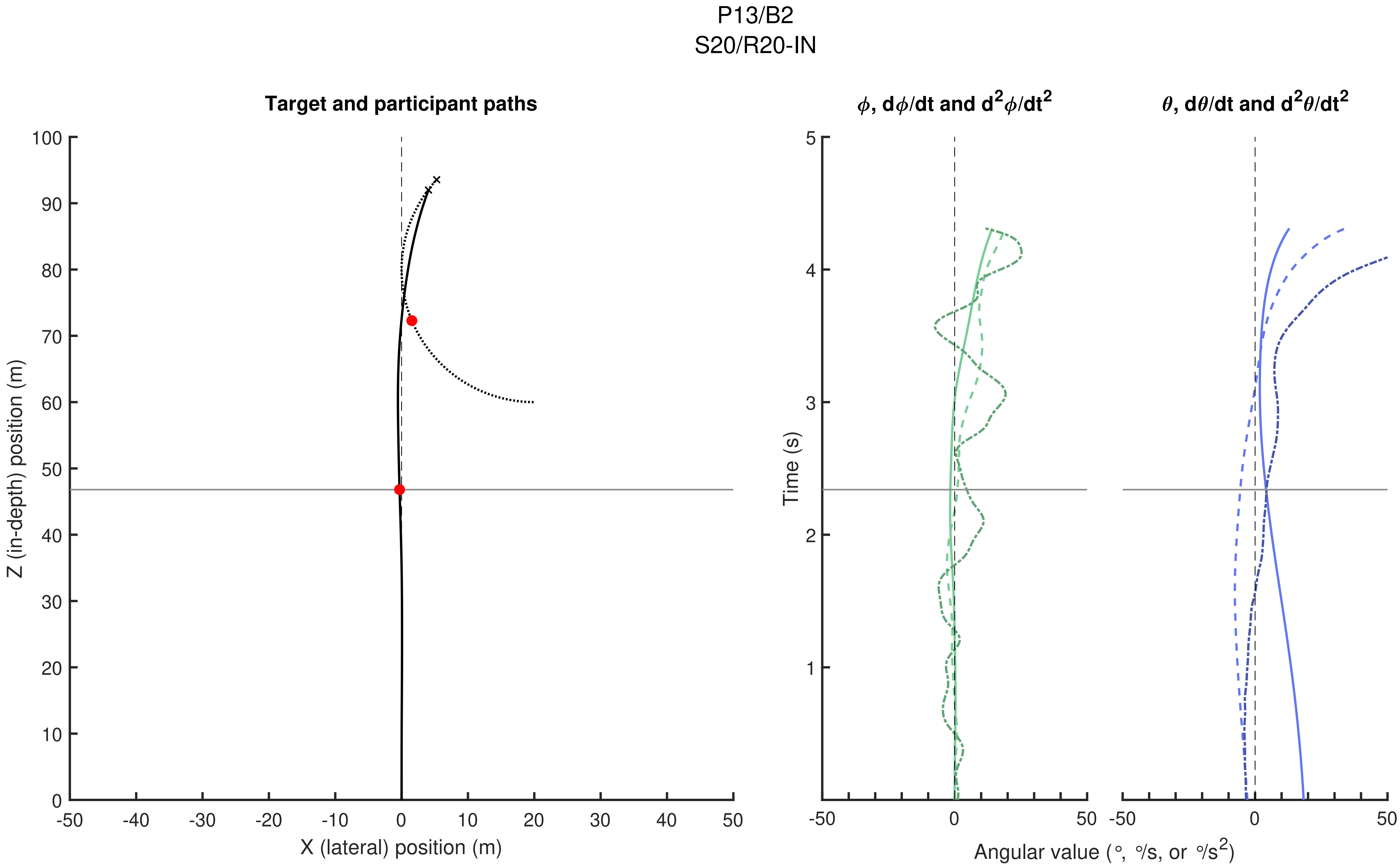

P13/B2
S20/R20-IN
Target and participant paths
φ, dφ/dt and d²φ/dt²
θ, dθ/dt and d²θ/dt²
Z (in-depth) position (m)
X (lateral) position (m)
Time (s)
Angular value (°, °/s, or °/s²)

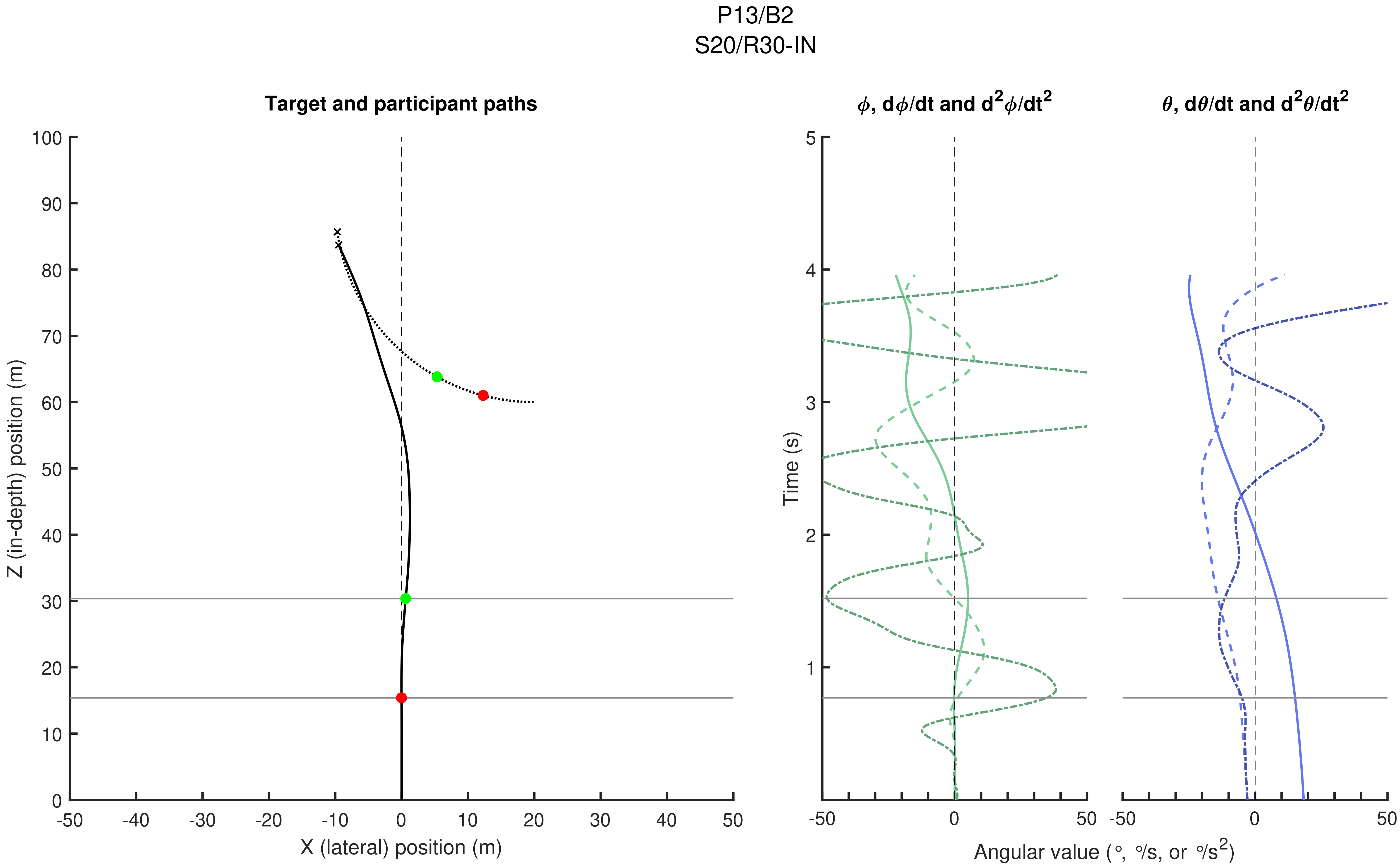
P13/B2
S20/R30-IN
Target and participant paths
ϕ, dϕ/dt and d$^2\phi$/dt^2
θ, dθ/dt and d$^2\theta$/dt^2
Z (in-depth) position (m)
X (lateral) position (m)
Time (s)
Angular value (°, °/s, or °/s^2)

P13/B3
S20/R10-IN

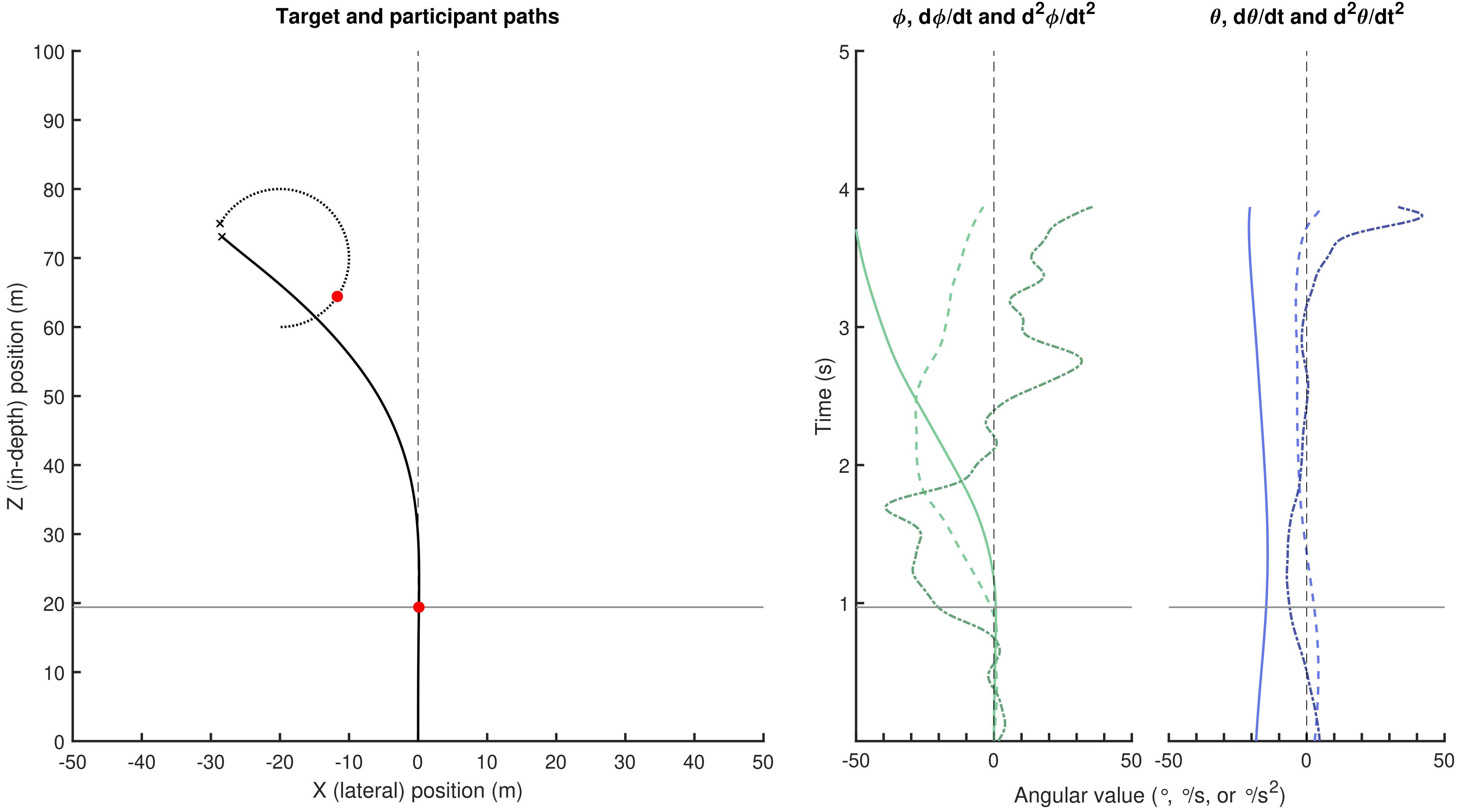

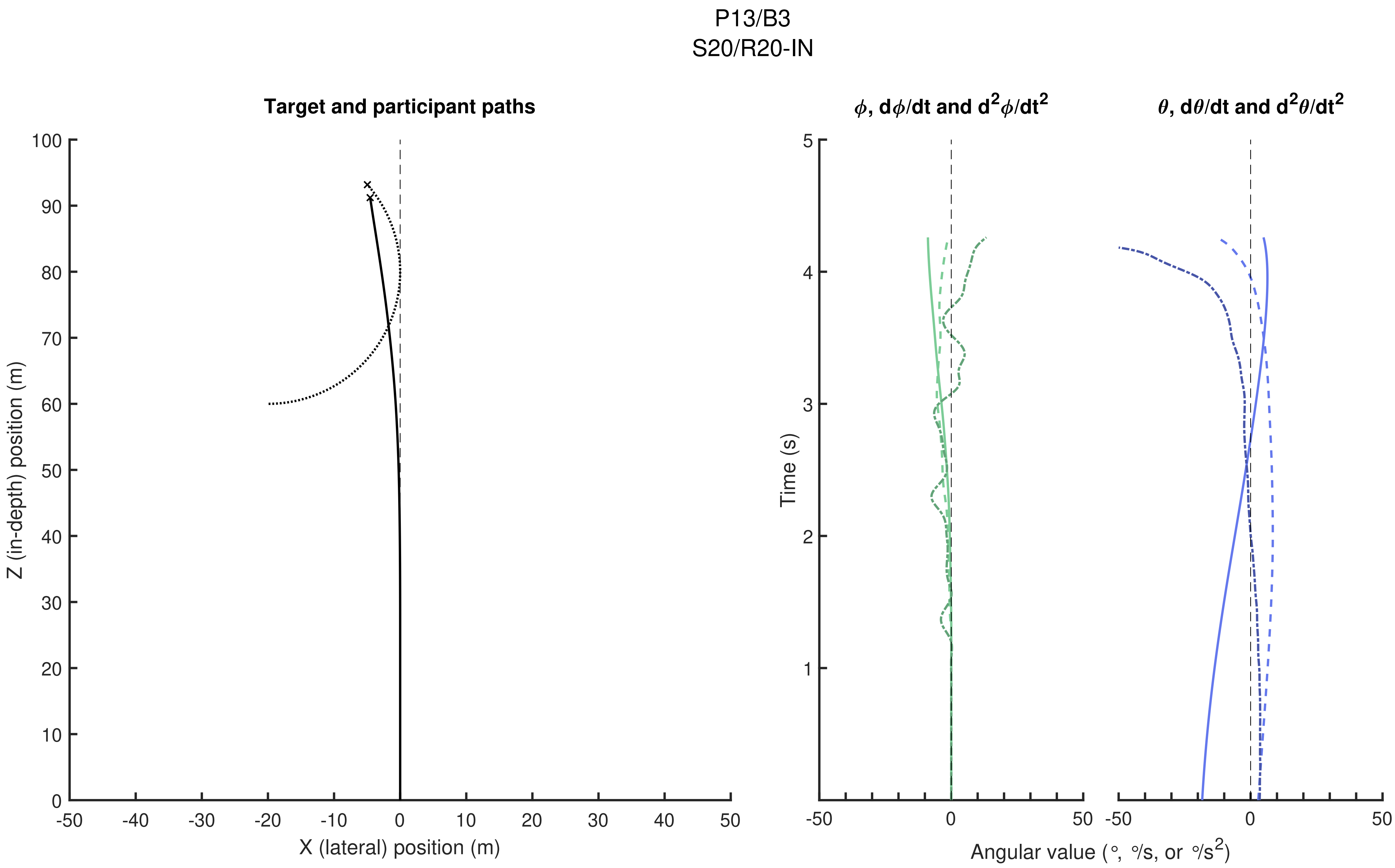
P13/B3
S20/R20-IN
Target and participant paths
ϕ, dϕ/dt and d²ϕ/dt²
θ, dθ/dt and d²θ/dt²
Z (in-depth) position (m)
X (lateral) position (m)
Time (s)
Angular value (°, °/s, or °/s²)
100
90
80
70
60
50
40
30
20
10
0
-50
-40
-30
-20
-10
0
10
20
30
40
50
5
4
3
2
1
-50
0
50
-50
0
50

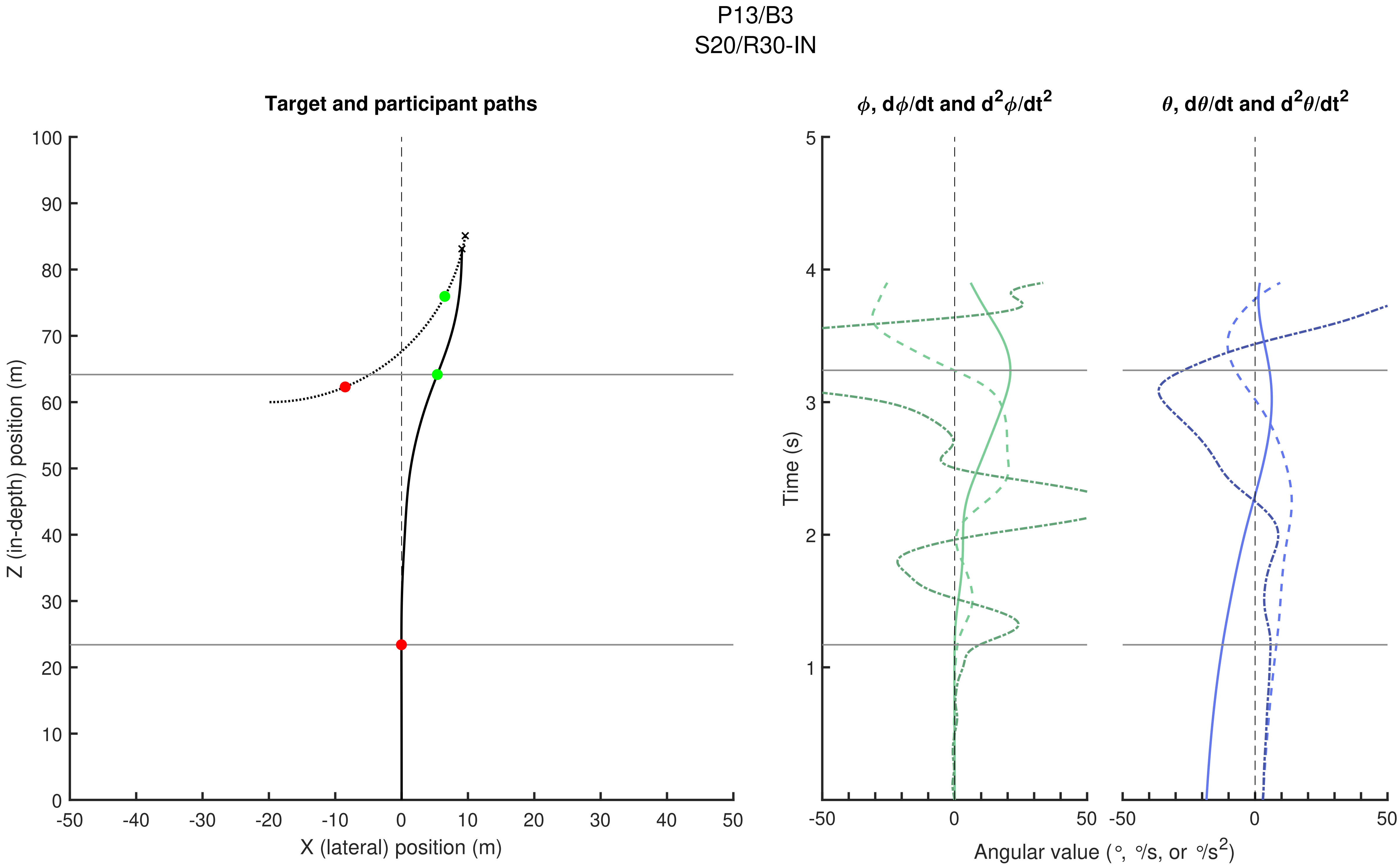
P13/B3
S20/R30-IN
Target and participant paths
ϕ, dϕ/dt and d$^2\phi$/dt^2
θ, dθ/dt and d$^2\theta$/dt^2
Z (in-depth) position (m)
X (lateral) position (m)
Time (s)
Angular value (°, °/s, or °/s^2)
100
90
80
70
60
50
40
30
20
10
0
-50
-40
-30
-20
-10
0
10
20
30
40
50
5
4
3
2
1

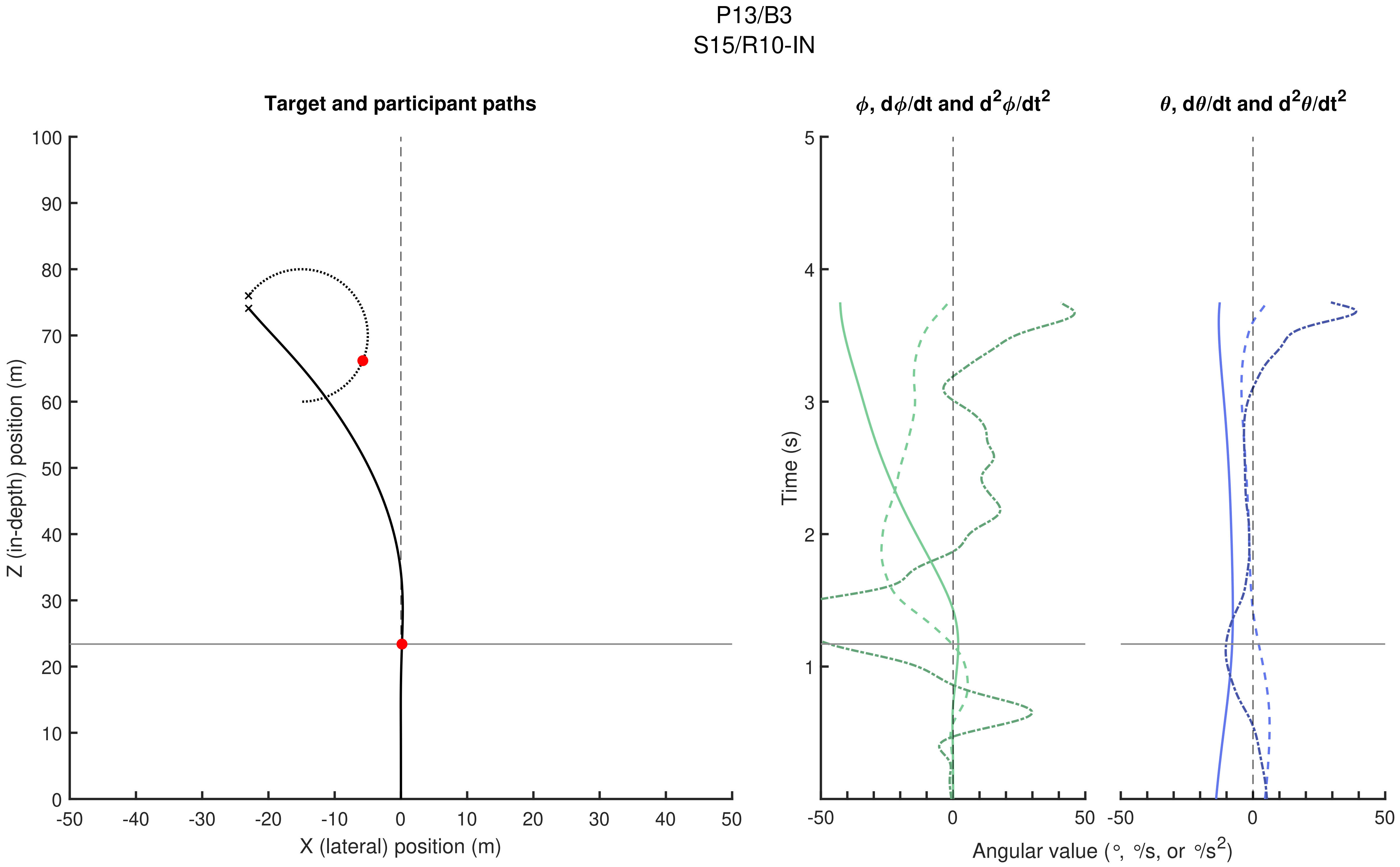

P13/B3
S15/R10-IN
Target and participant paths
Z (in-depth) position (m)
X (lateral) position (m)
ϕ, dϕ/dt and d²ϕ/dt²
θ, dθ/dt and d²θ/dt²
Time (s)
Angular value (°, °/s, or °/s²)

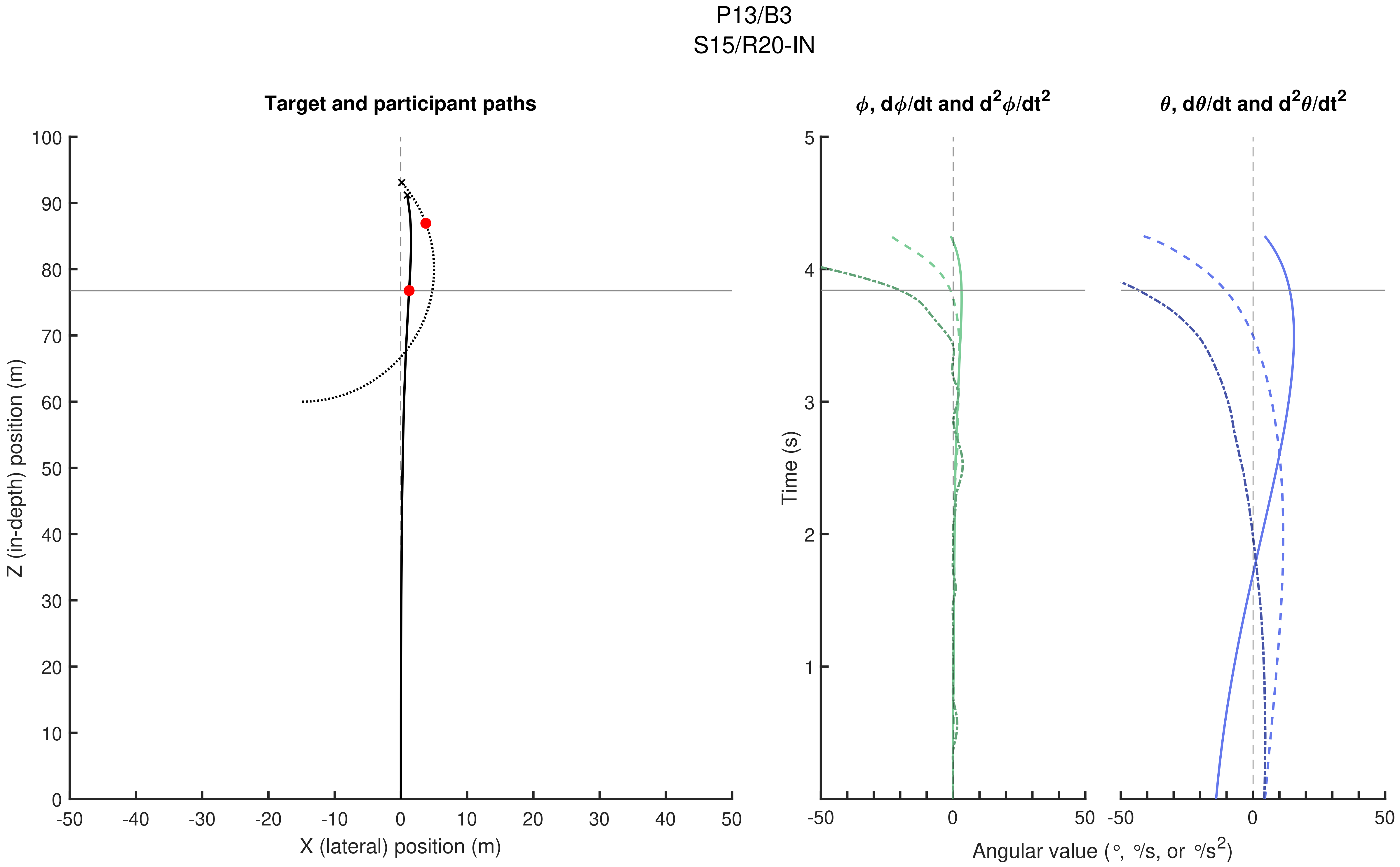

P13/B3
S15/R20-IN
Target and participant paths
ϕ, dϕ/dt and d$^2\phi$/dt^2
θ, dθ/dt and d$^2\theta$/dt^2
Z (in-depth) position (m)
X (lateral) position (m)
Time (s)
Angular value (°, °/s, or °/s^2)

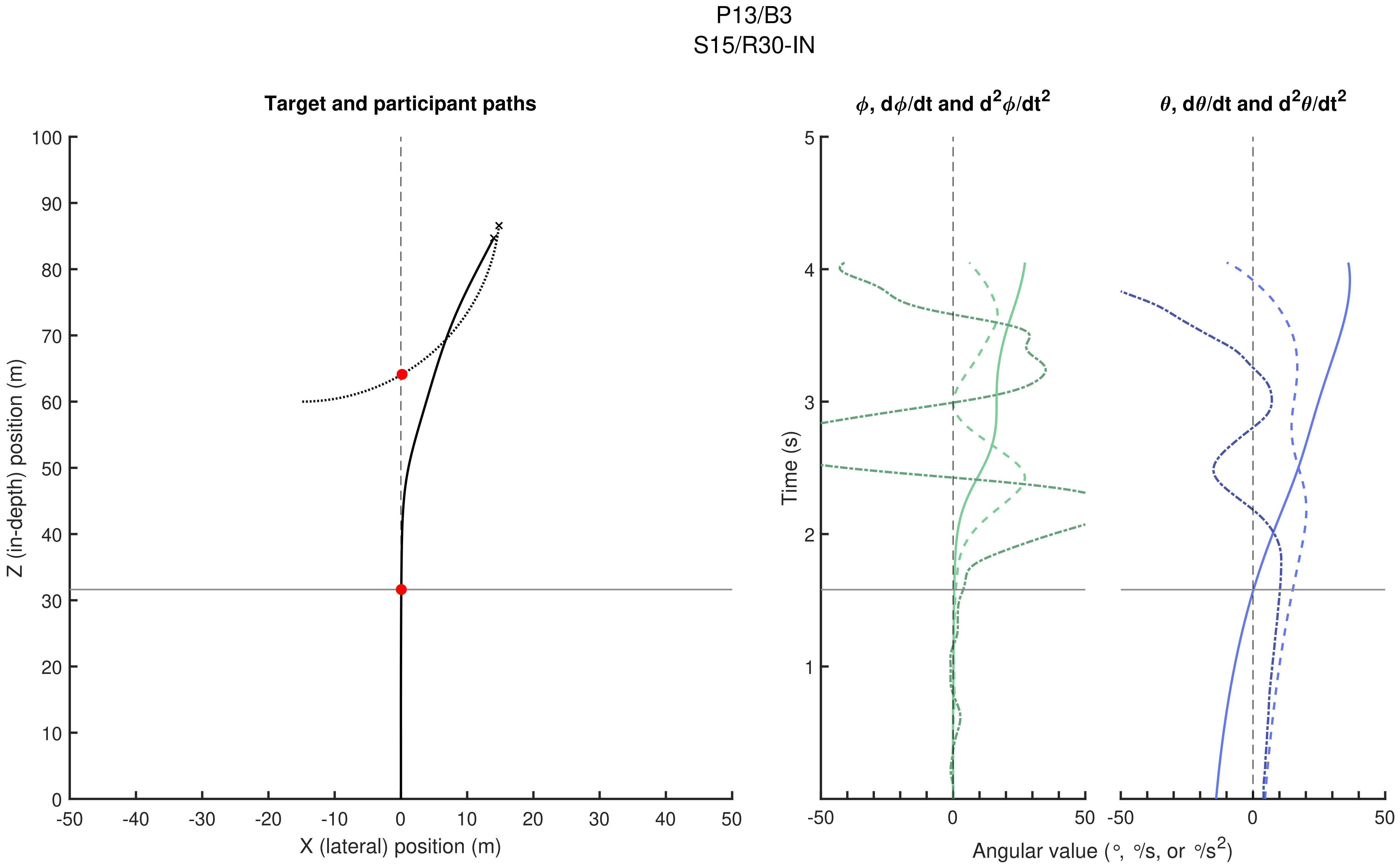

P13/B3
S15/R30-IN
Target and participant paths
Z (in-depth) position (m)
X (lateral) position (m)
ϕ, dϕ/dt and d$^2\phi$/dt^2
θ, dθ/dt and d$^2\theta$/dt^2
Time (s)
Angular value (°, °/s, or °/s^2)

P13/B3
S5/R10-IN

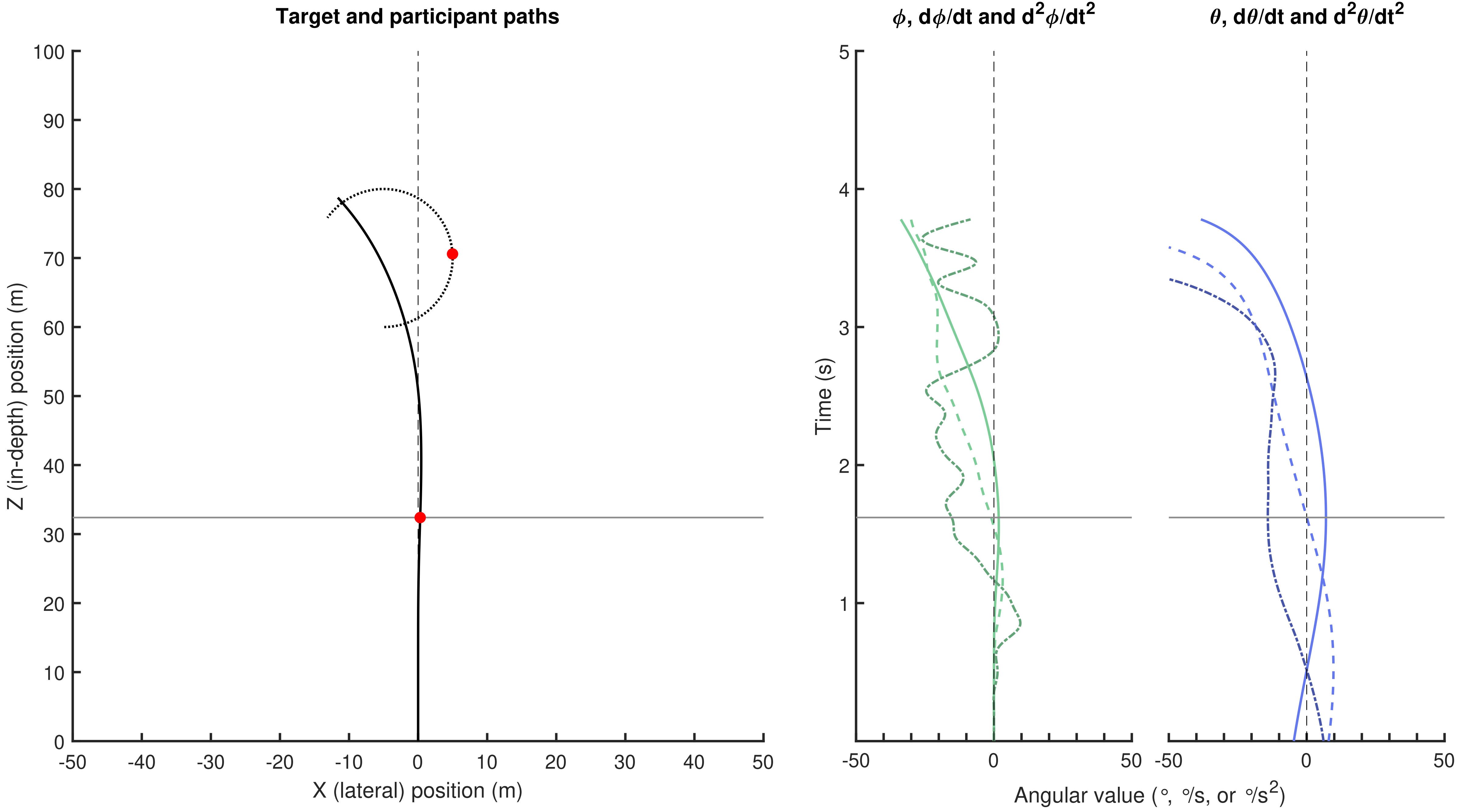

P13/B3
S5/R10-OUT

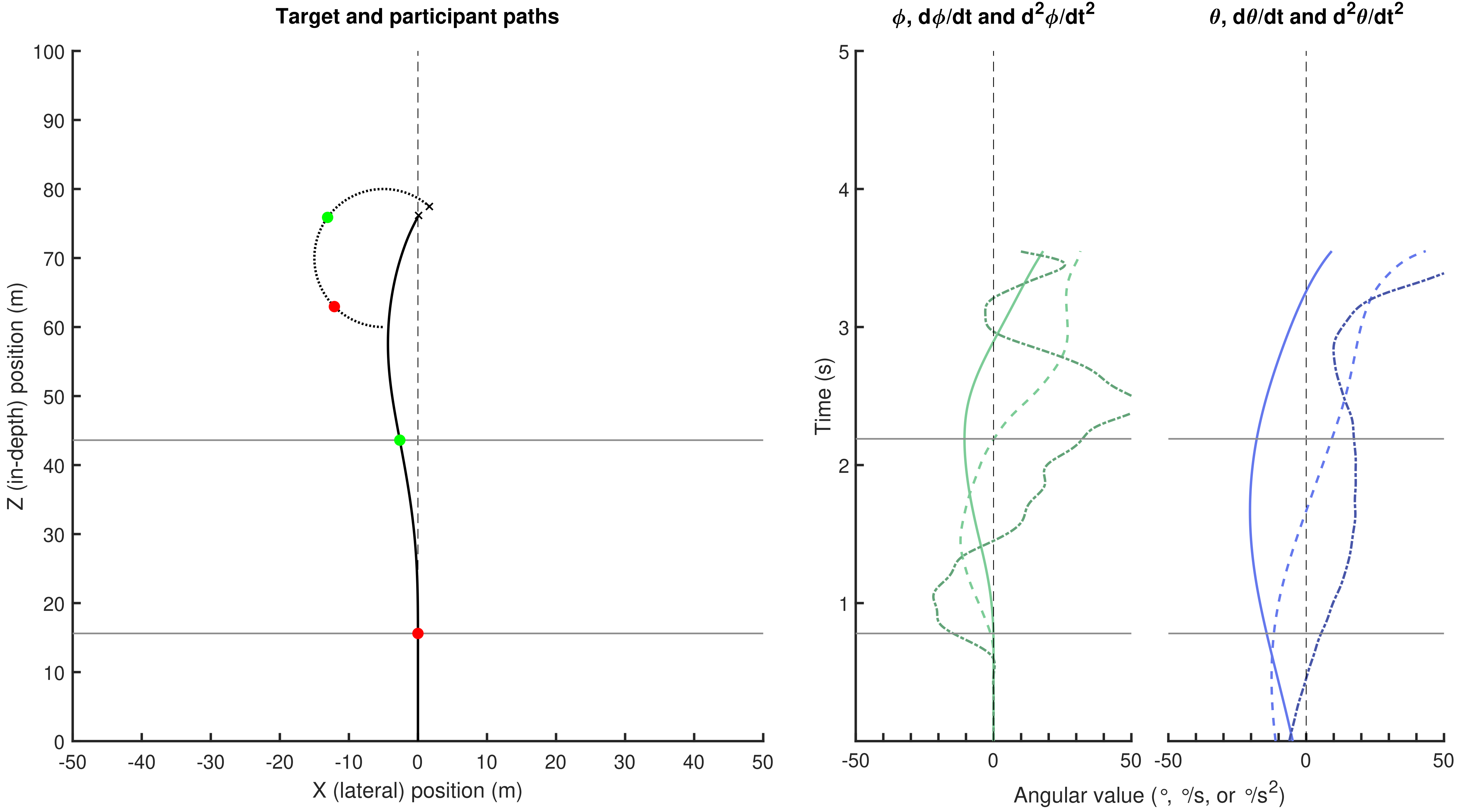

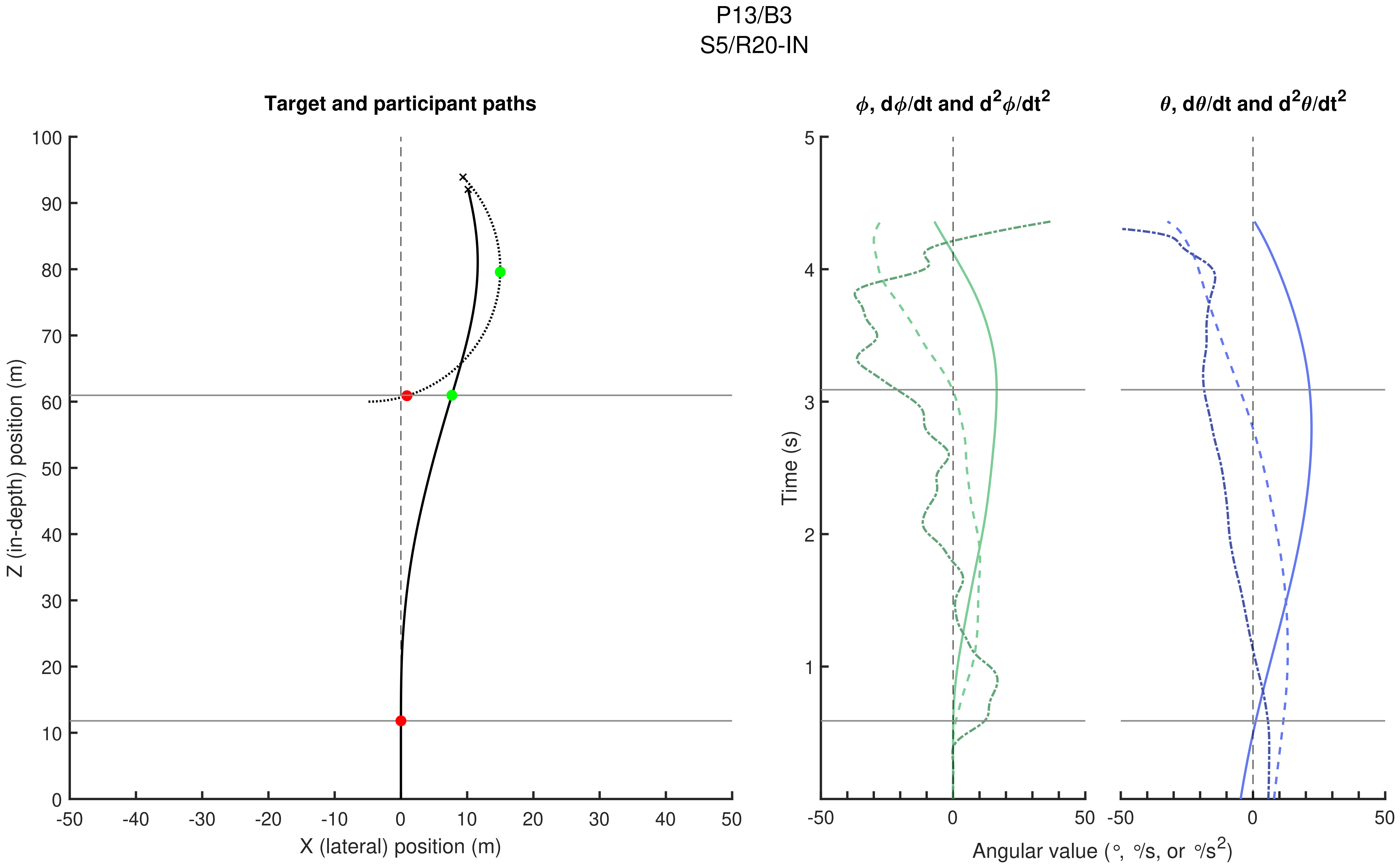
P13/B3
S5/R20-IN
Target and participant paths
Z (in-depth) position (m)
X (lateral) position (m)
-50
-40
-30
-20
-10
0
10
20
30
40
50
100
90
80
70
60
50
40
30
20
10
0
ϕ, dϕ/dt and d$^2\phi$/dt^2
θ, dθ/dt and d$^2\theta$/dt^2
Time (s)
5
4
3
2
1
-50
0
50
-50
0
50
Angular value (°, °/s, or °/s^2)

P13/B3
S5/R20-OUT

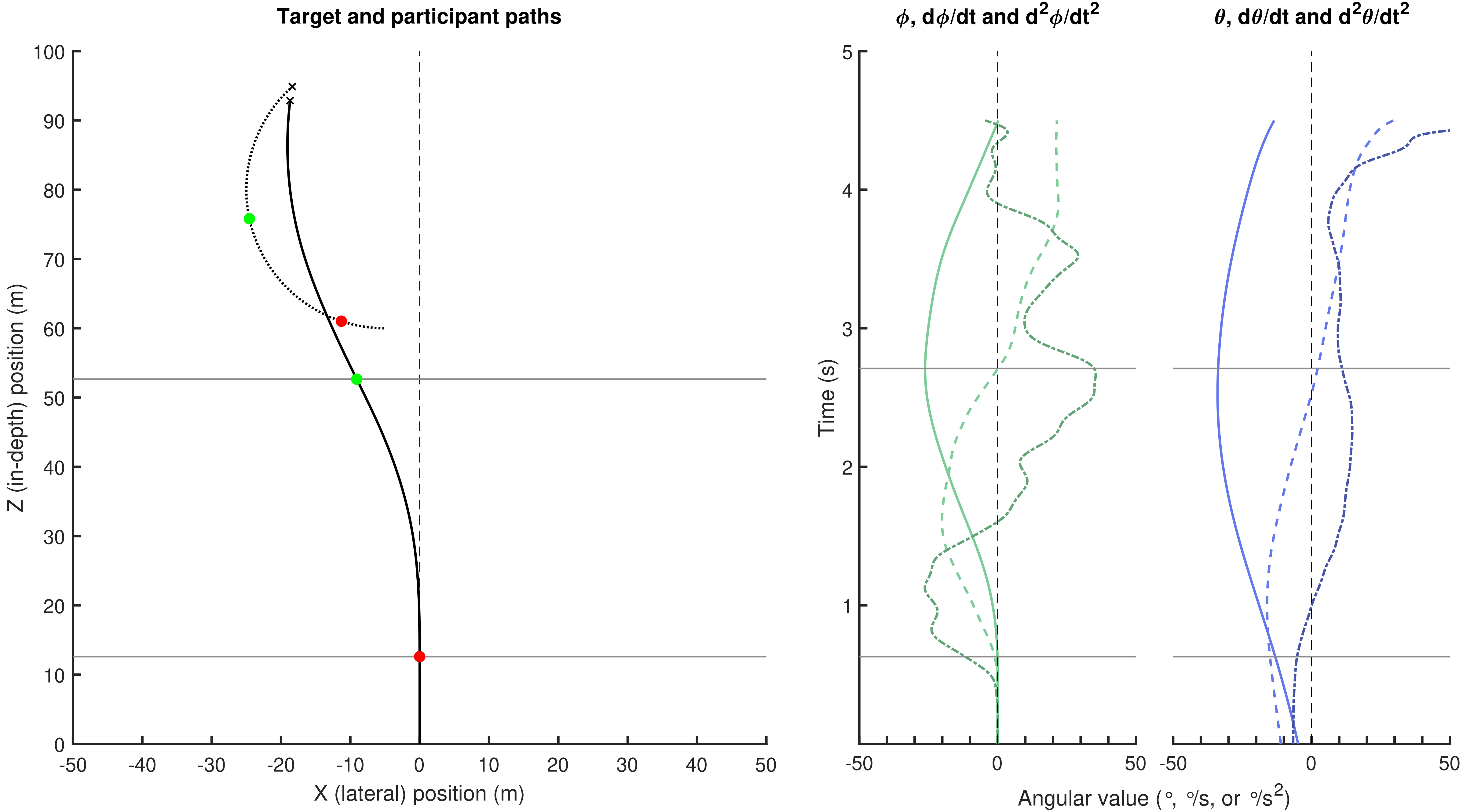

P13/B3
S5/R30-IN

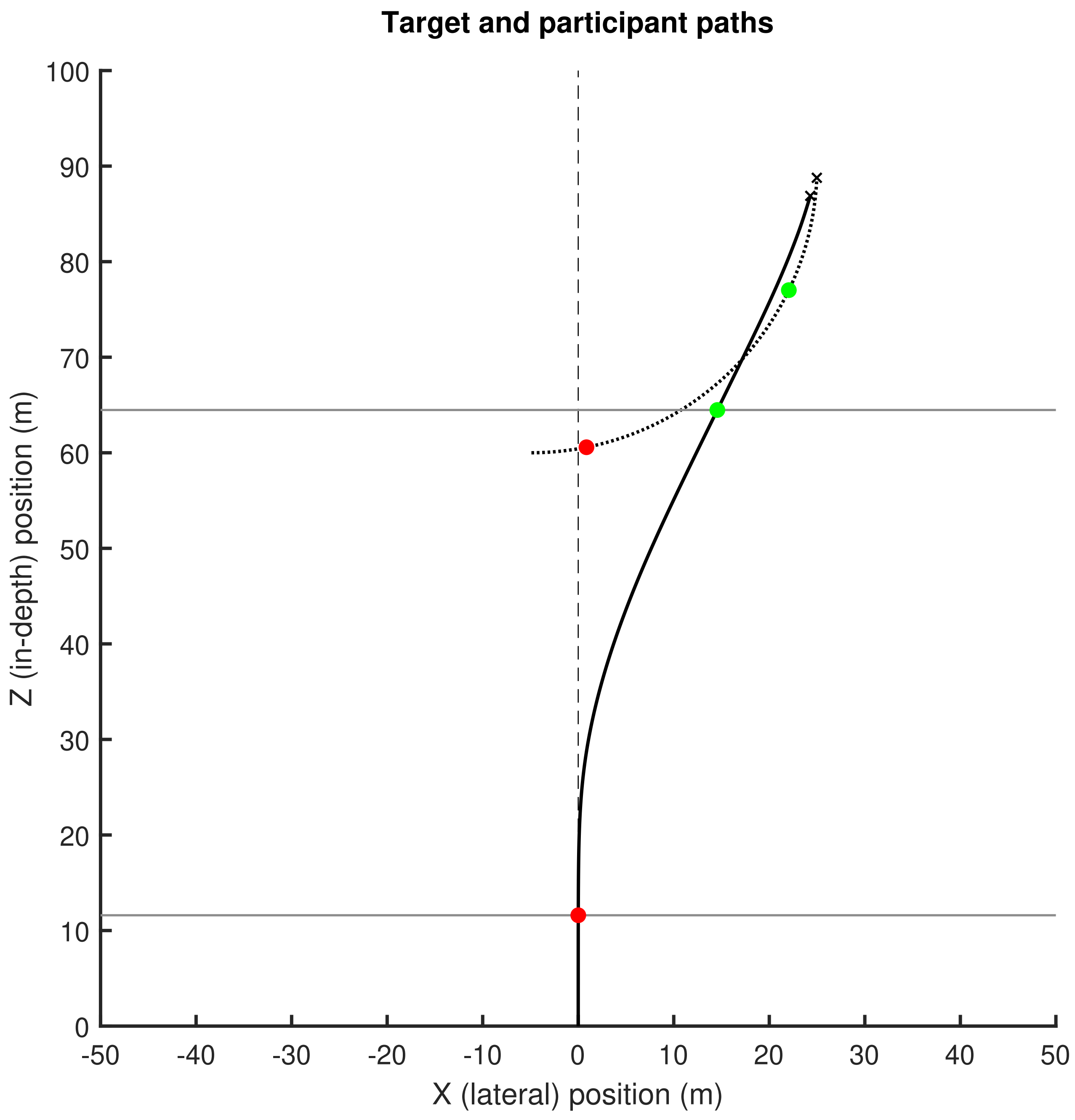

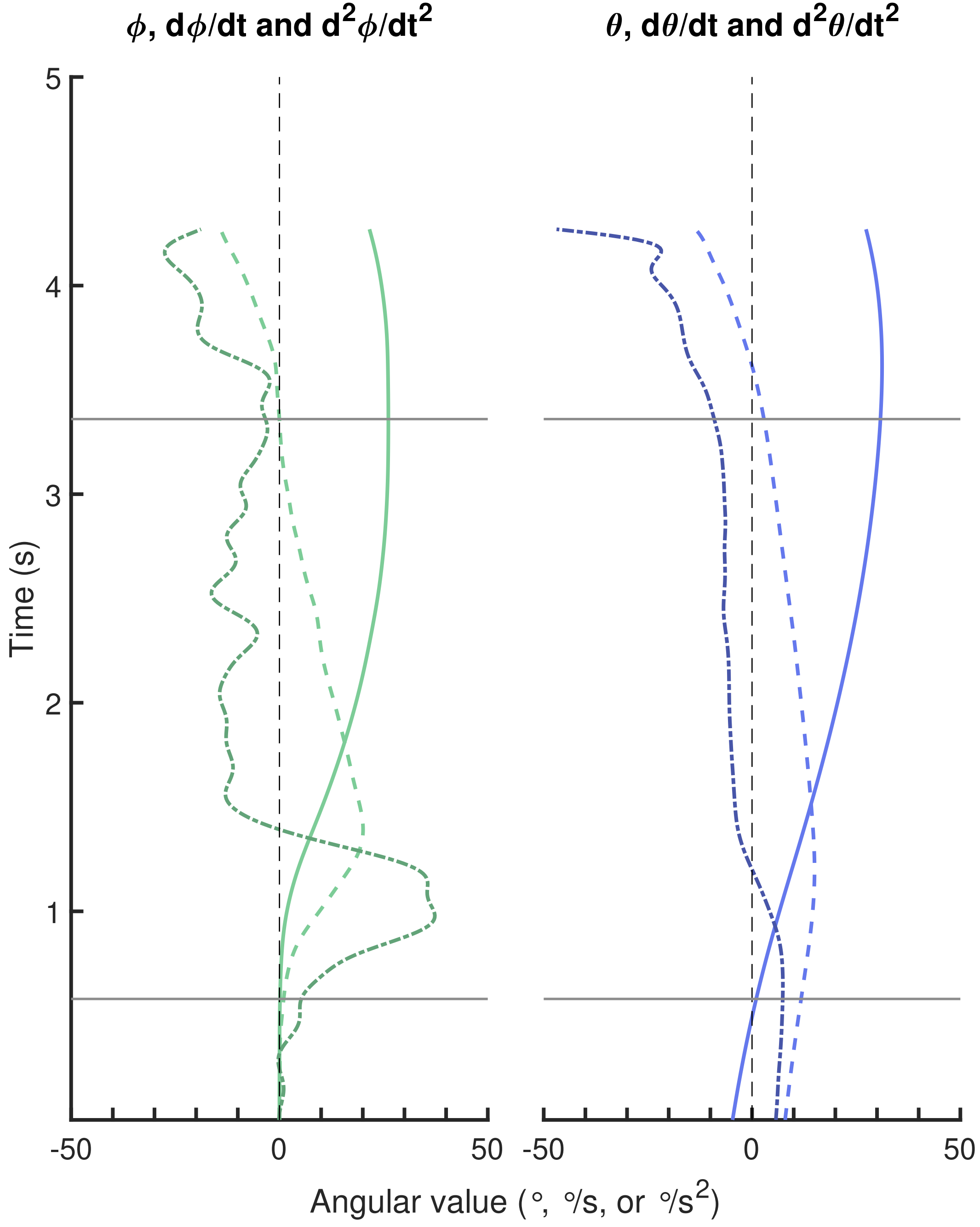

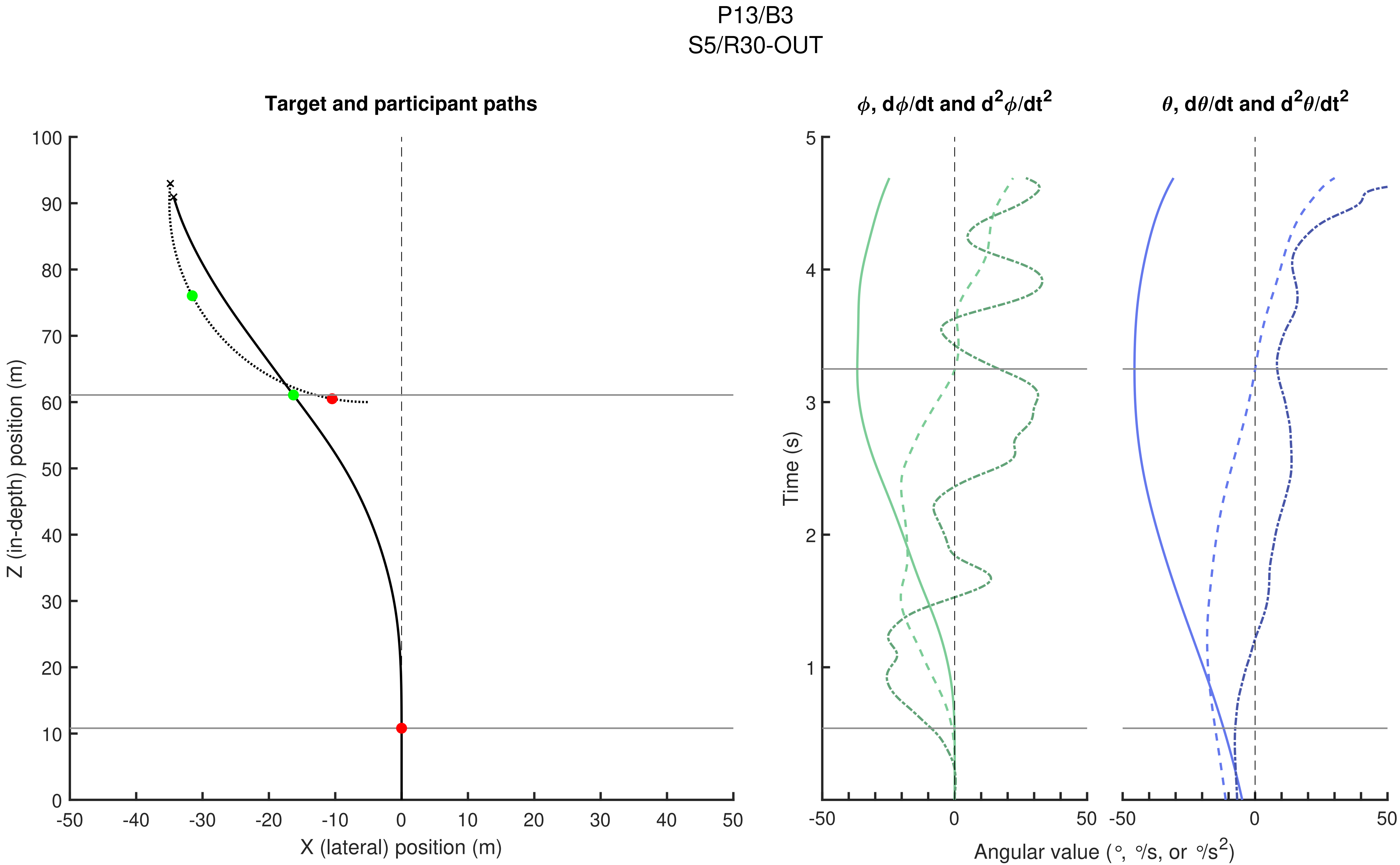
P13/B3
S5/R30-OUT
Target and participant paths
Z (in-depth) position (m)
X (lateral) position (m)
0
10
20
30
40
50
60
70
80
90
100
-50
-40
-30
-20
-10
0
10
20
30
40
50
ϕ, dϕ/dt and d²ϕ/dt²
θ, dθ/dt and d²θ/dt²
Time (s)
1
2
3
4
5
-50
0
50
-50
0
50
Angular value (°, °/s, or °/s²)

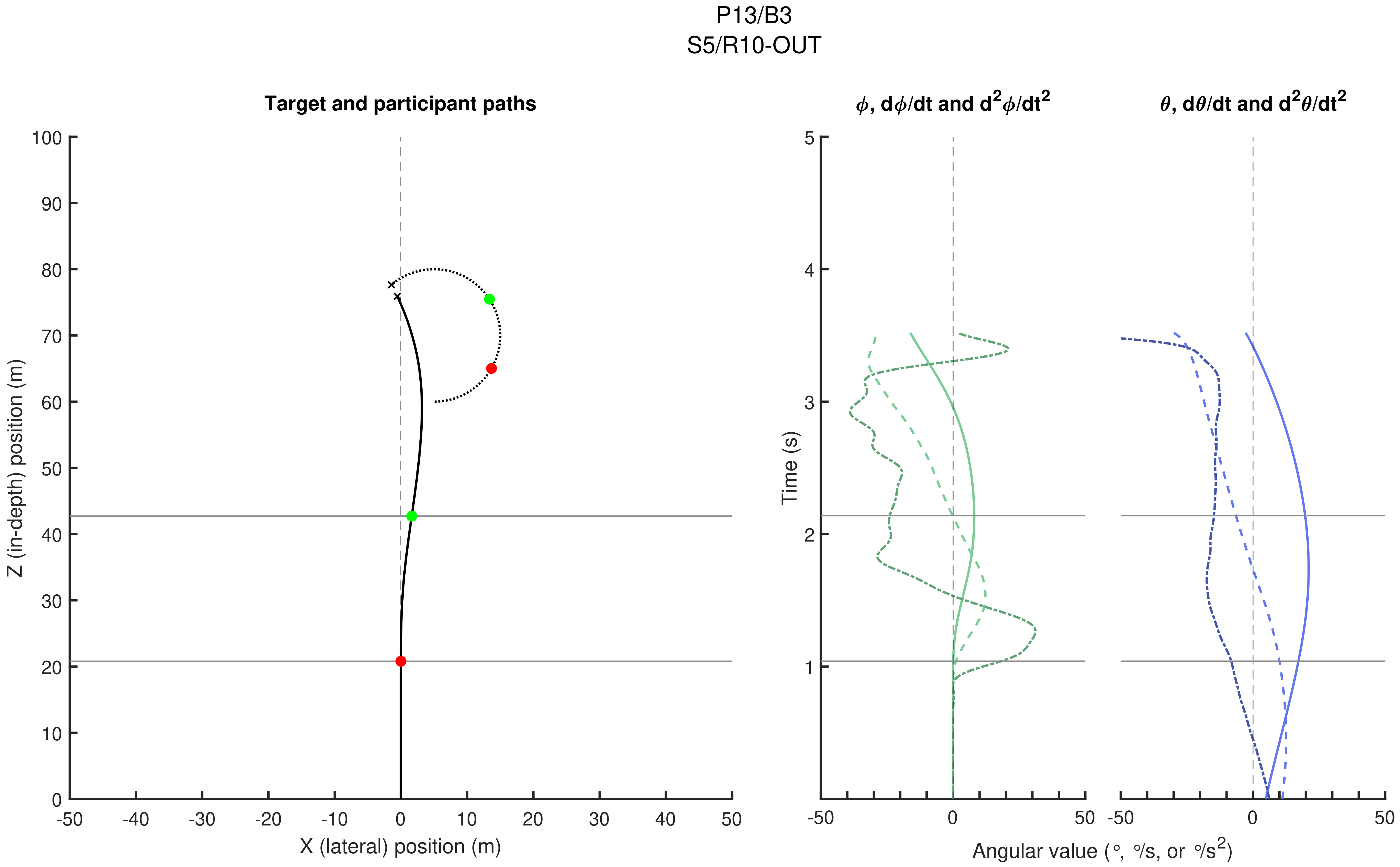

P13/B3
S5/R10-OUT
Target and participant paths
Z (in-depth) position (m)
X (lateral) position (m)
100
90
80
70
60
50
40
30
20
10
0
-50
-40
-30
-20
-10
0
10
20
30
40
50
ϕ, dϕ/dt and d$^2\phi$/dt^2
θ, dθ/dt and d$^2\theta$/dt^2
Time (s)
5
4
3
2
1
-50
0
50
-50
0
50
Angular value (°, °/s, or °/s^2)

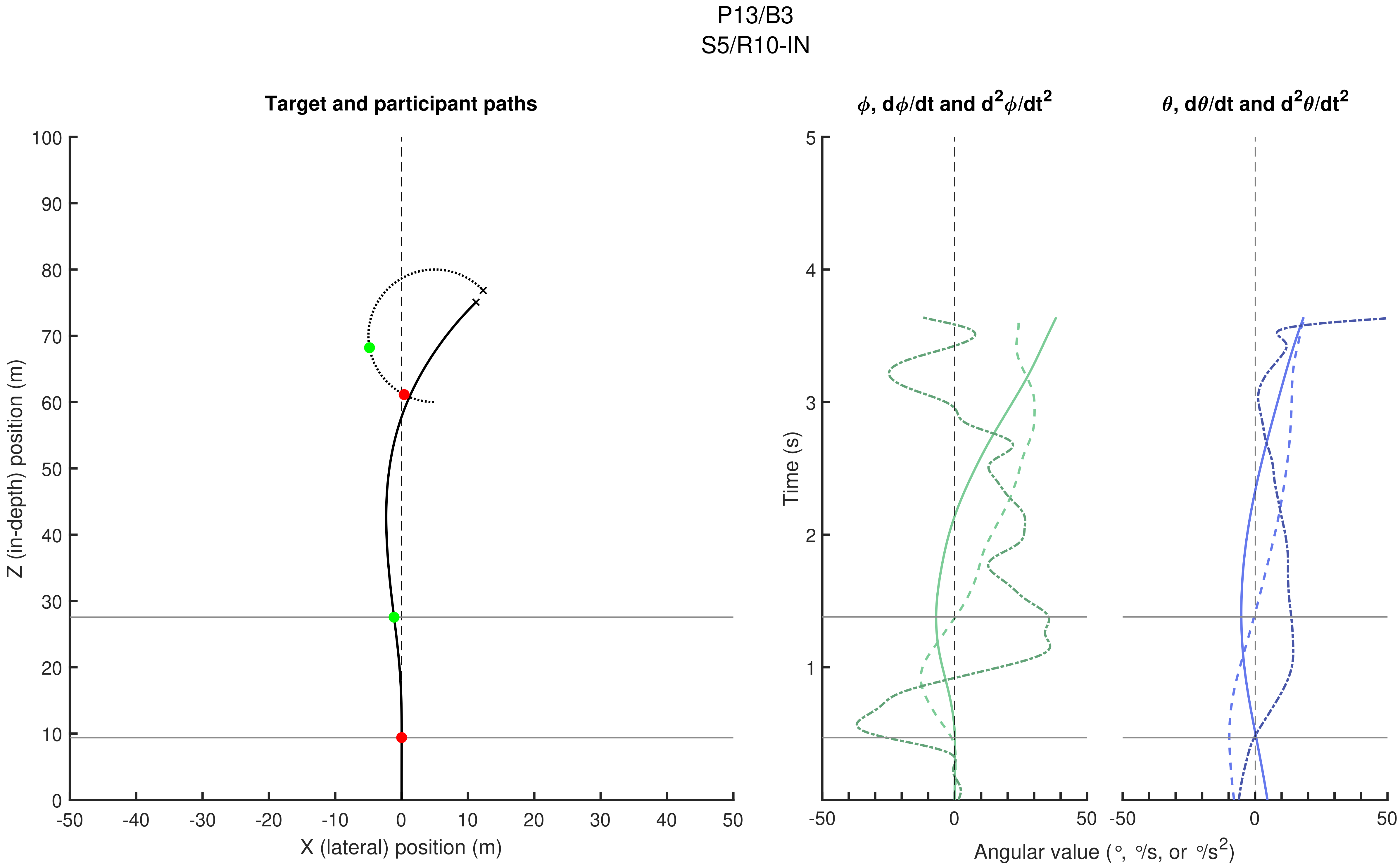
P13/B3
S5/R10-IN
Target and participant paths
ϕ, dϕ/dt and d²ϕ/dt²
θ, dθ/dt and d²θ/dt²
Z (in-depth) position (m)
X (lateral) position (m)
Time (s)
Angular value (°, °/s, or °/s²)
100
90
80
70
60
50
40
30
20
10
0
-50
-40
-30
-20
-10
0
10
20
30
40
50
5
4
3
2
1
-50
0
50
-50
0
50

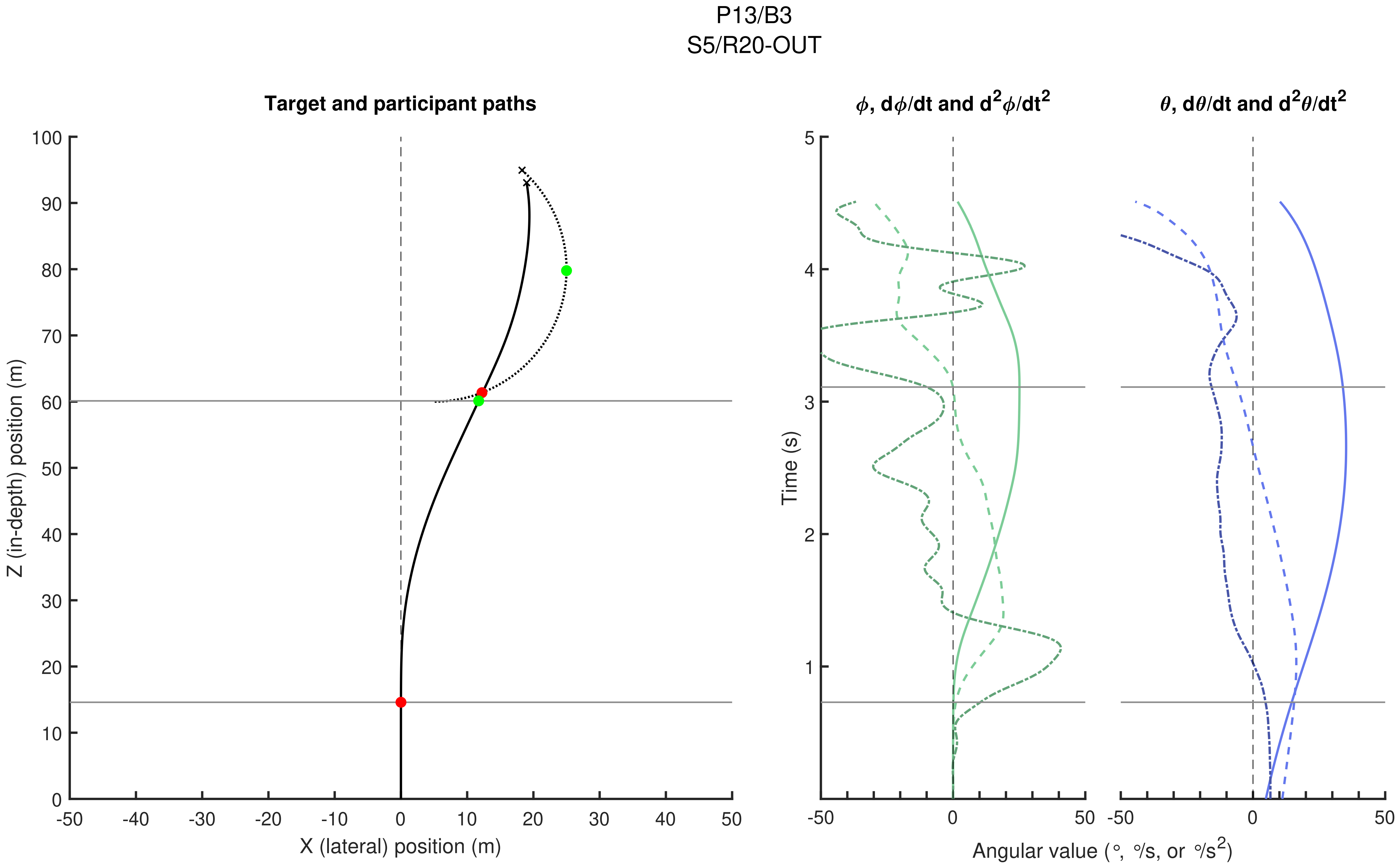

P13/B3
S5/R20-OUT
Target and participant paths
Z (in-depth) position (m)
X (lateral) position (m)
100
90
80
70
60
50
40
30
20
10
0
-50
-40
-30
-20
-10
0
10
20
30
40
50
ϕ, dϕ/dt and d$^2\phi$/dt^2
θ, dθ/dt and d$^2\theta$/dt^2
Time (s)
5
4
3
2
1
-50
0
50
-50
0
50
Angular value (°, °/s, or °/s^2)

P13/B3
S5/R20-IN

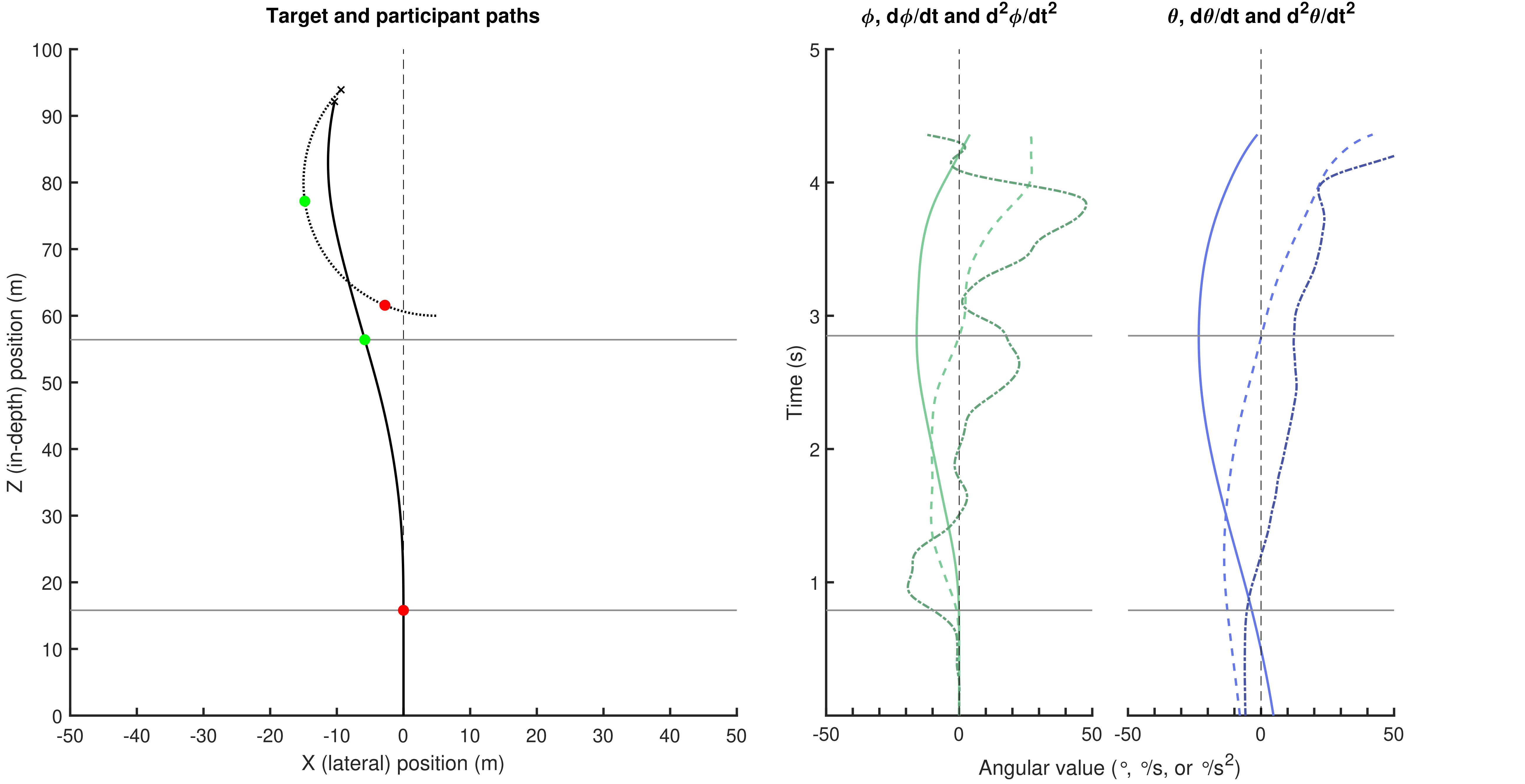

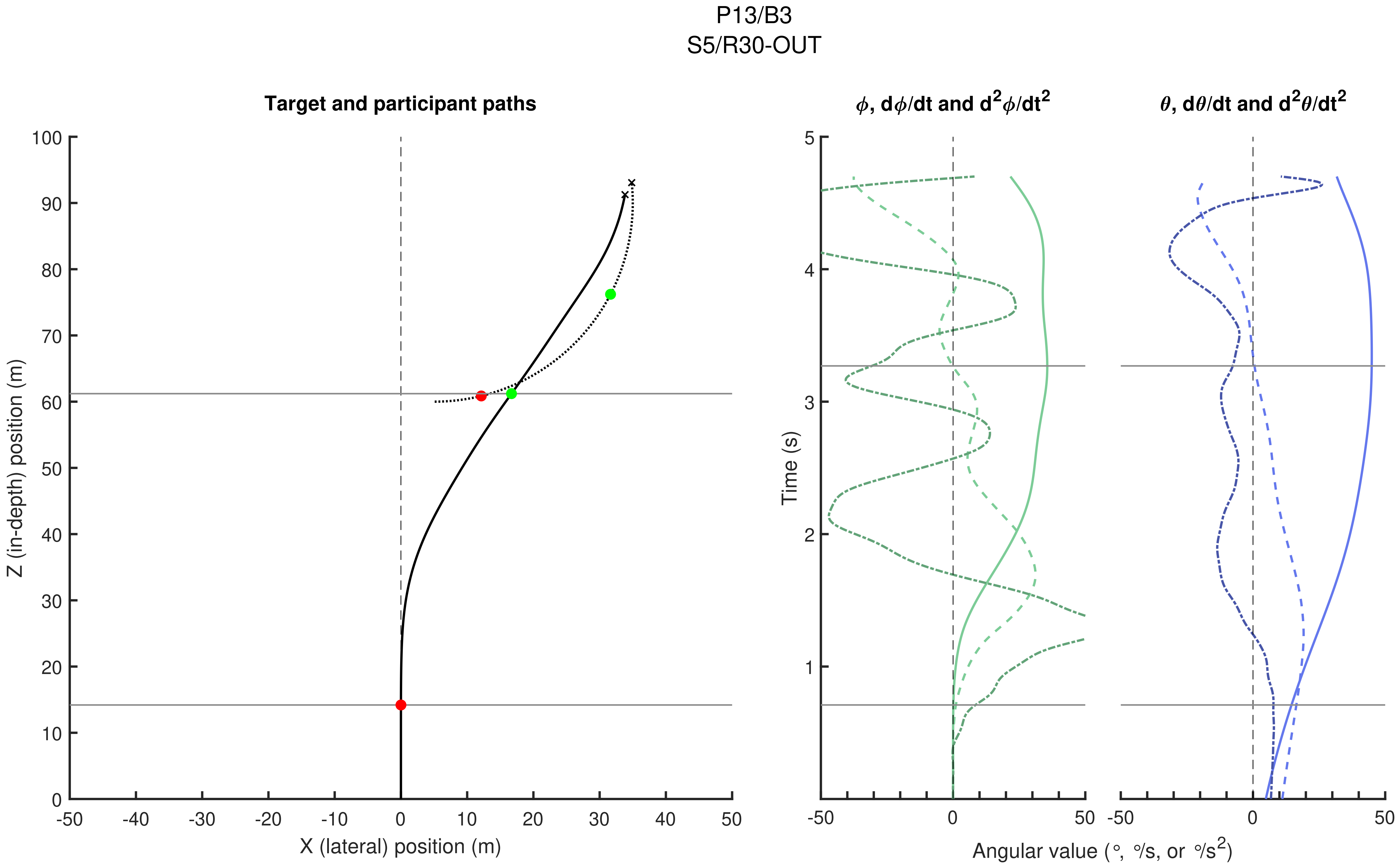
P13/B3
S5/R30-OUT
Target and participant paths
Z (in-depth) position (m)
X (lateral) position (m)
ϕ, dϕ/dt and d$^2\phi$/dt^2
θ, dθ/dt and d$^2\theta$/dt^2
Time (s)
Angular value (°, °/s, or °/s^2)

P13/B3
S5/R30-IN

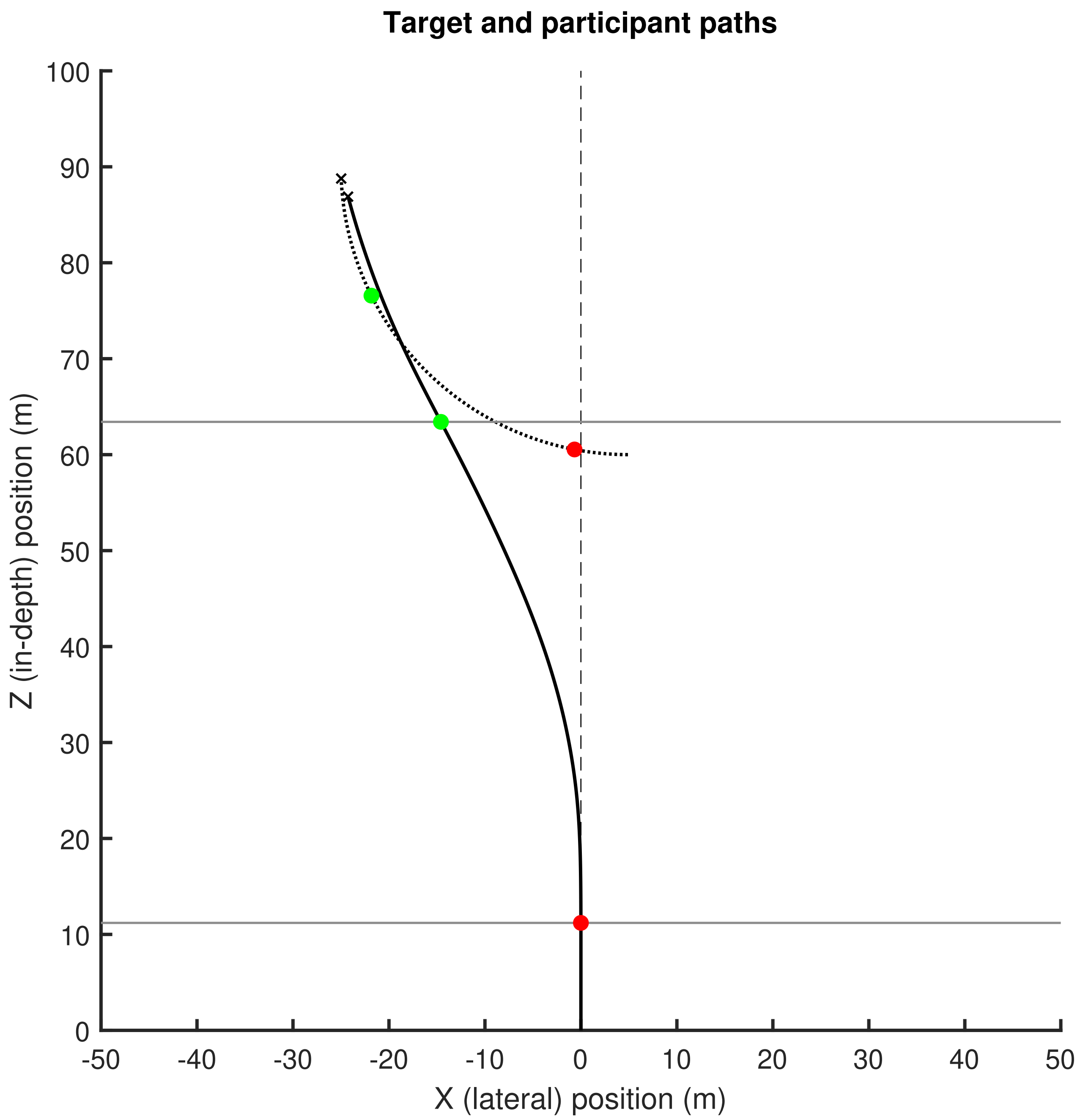

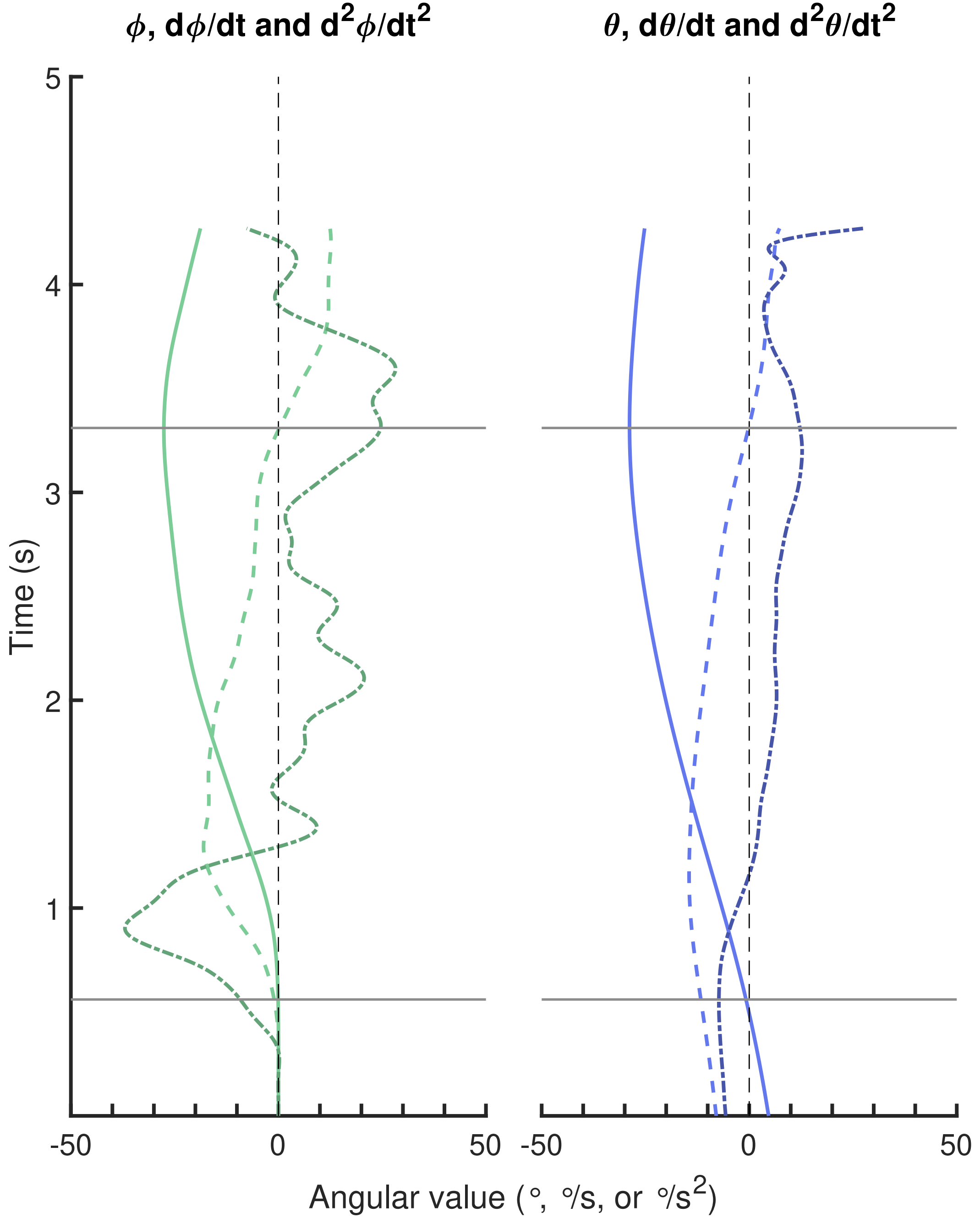

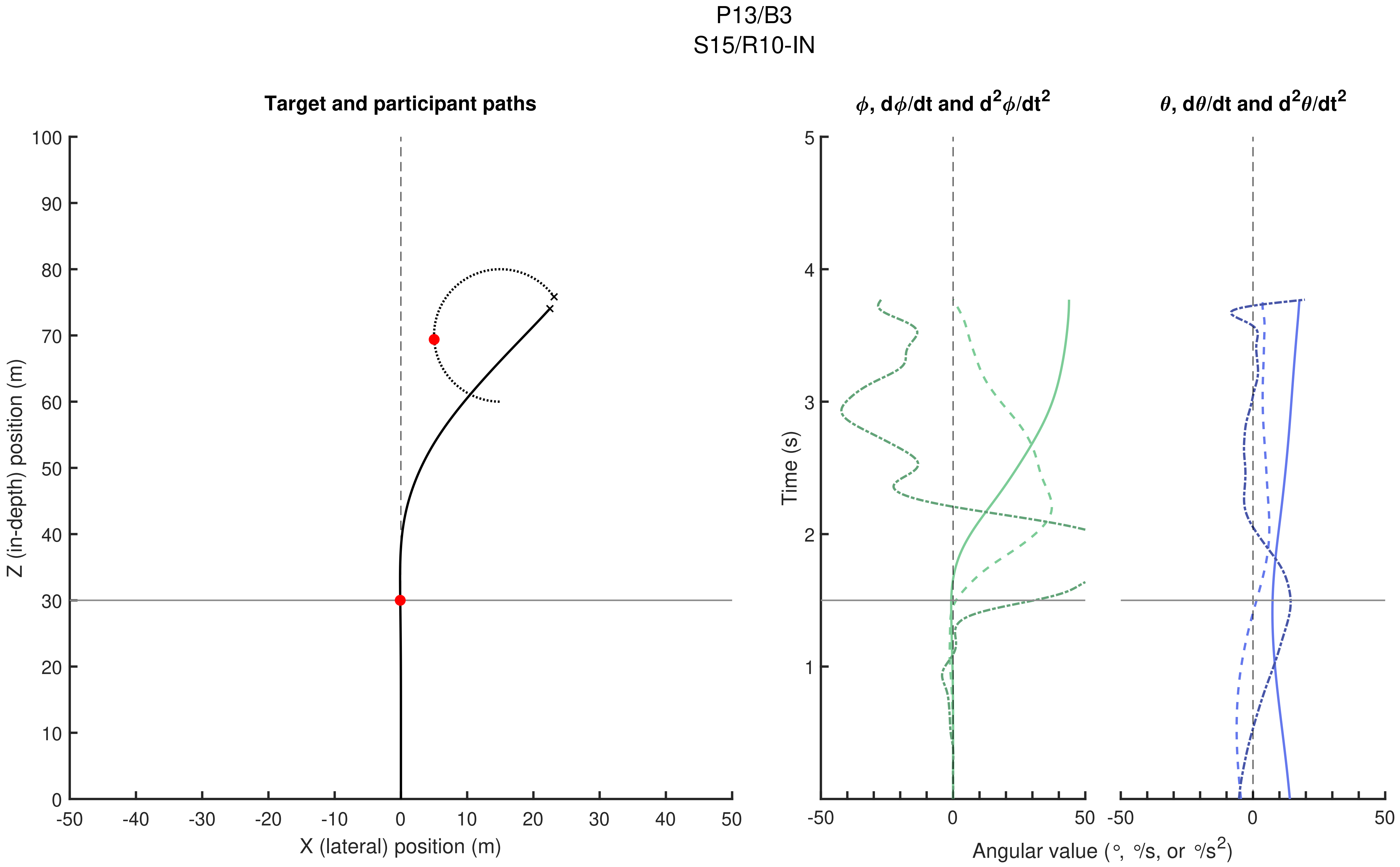

P13/B3
S15/R10-IN
Target and participant paths
Z (in-depth) position (m)
X (lateral) position (m)
φ, dφ/dt and d²φ/dt²
θ, dθ/dt and d²θ/dt²
Time (s)
Angular value (°, °/s, or °/s²)

P13/B3
S15/R20-IN

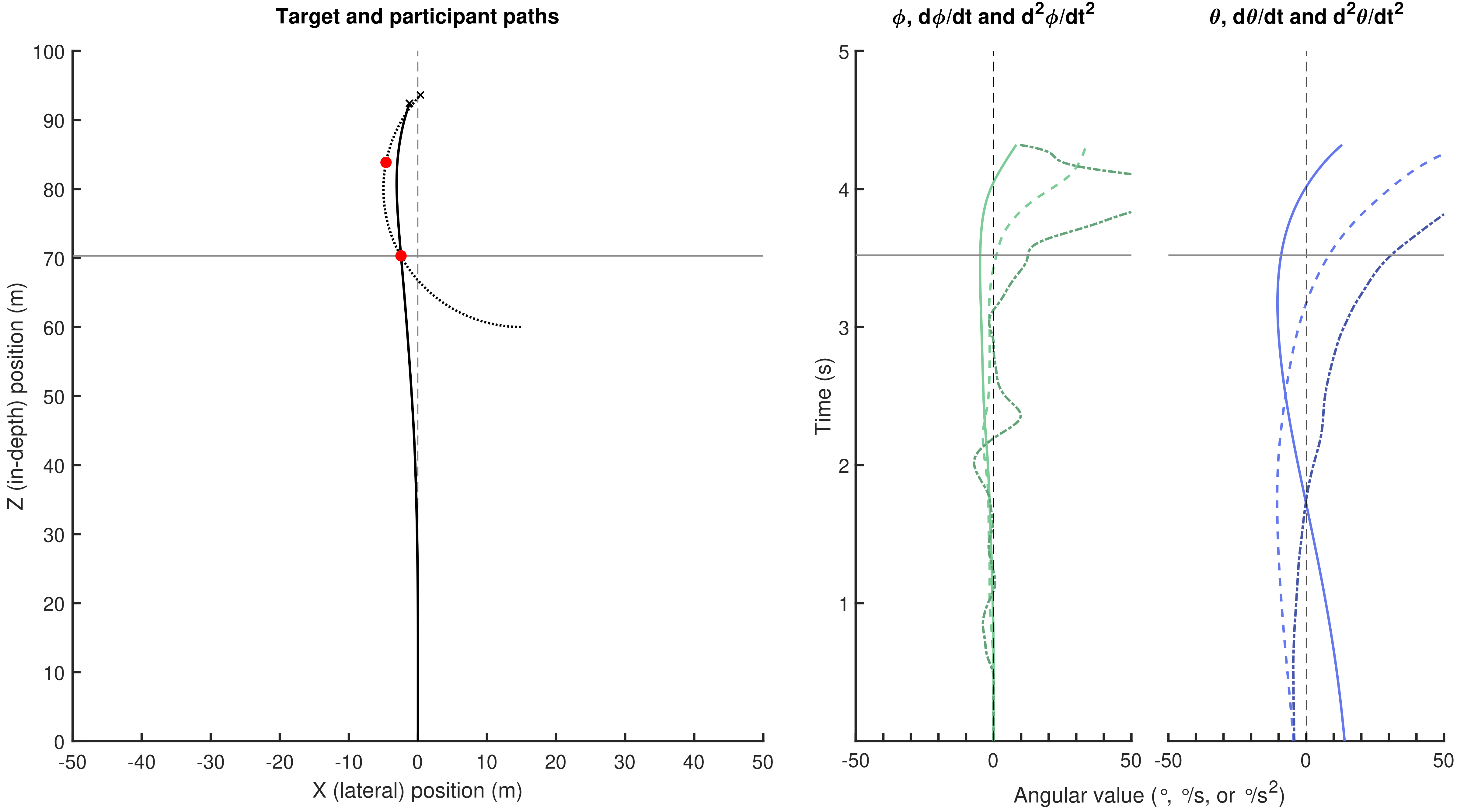

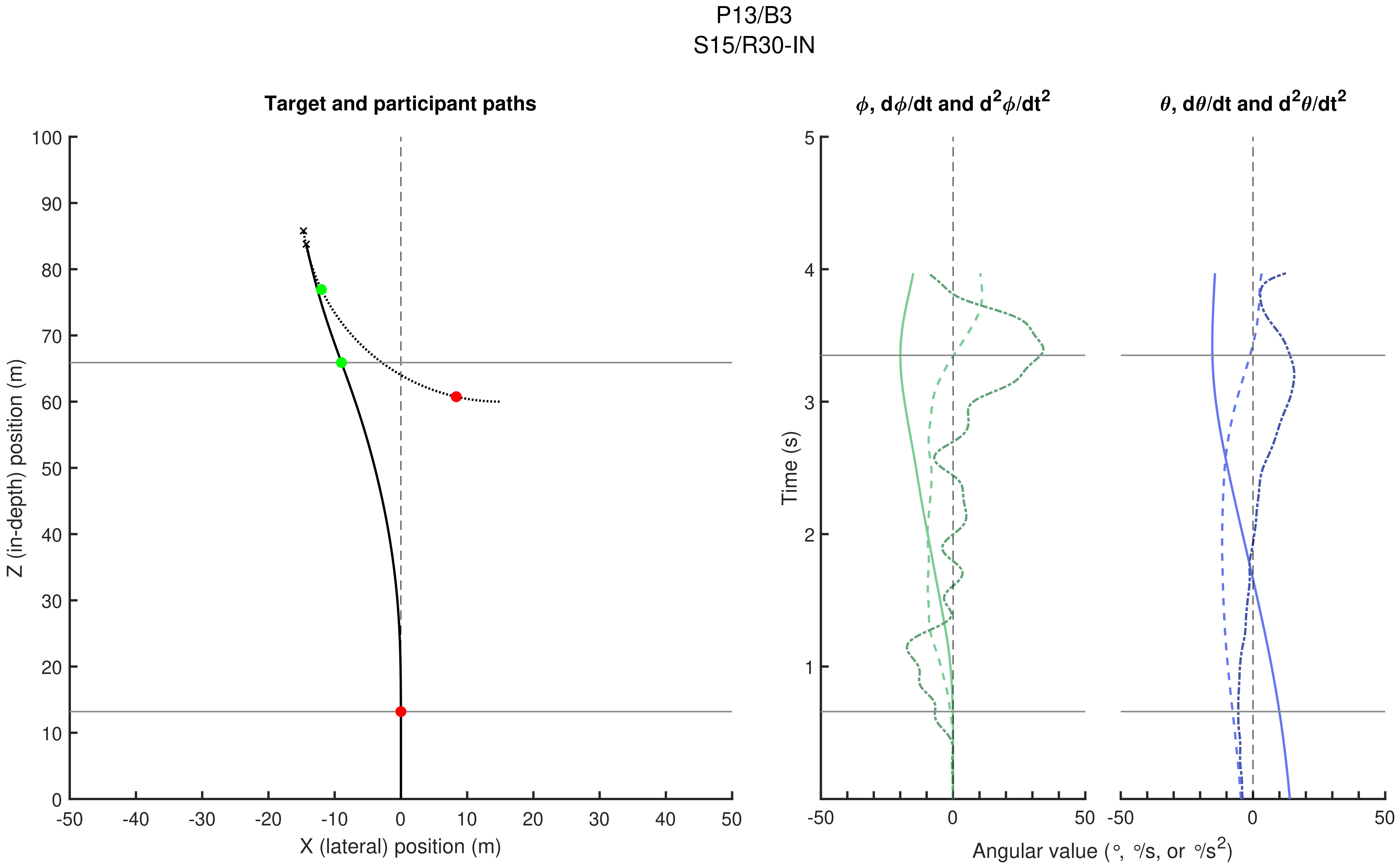
P13/B3
S15/R30-IN
Target and participant paths
Z (in-depth) position (m)
X (lateral) position (m)
ϕ, dϕ/dt and d$^2\phi$/dt^2
θ, dθ/dt and d$^2\theta$/dt^2
Time (s)
Angular value (°, °/s, or °/s^2)

P13/B3
S20/R10-IN

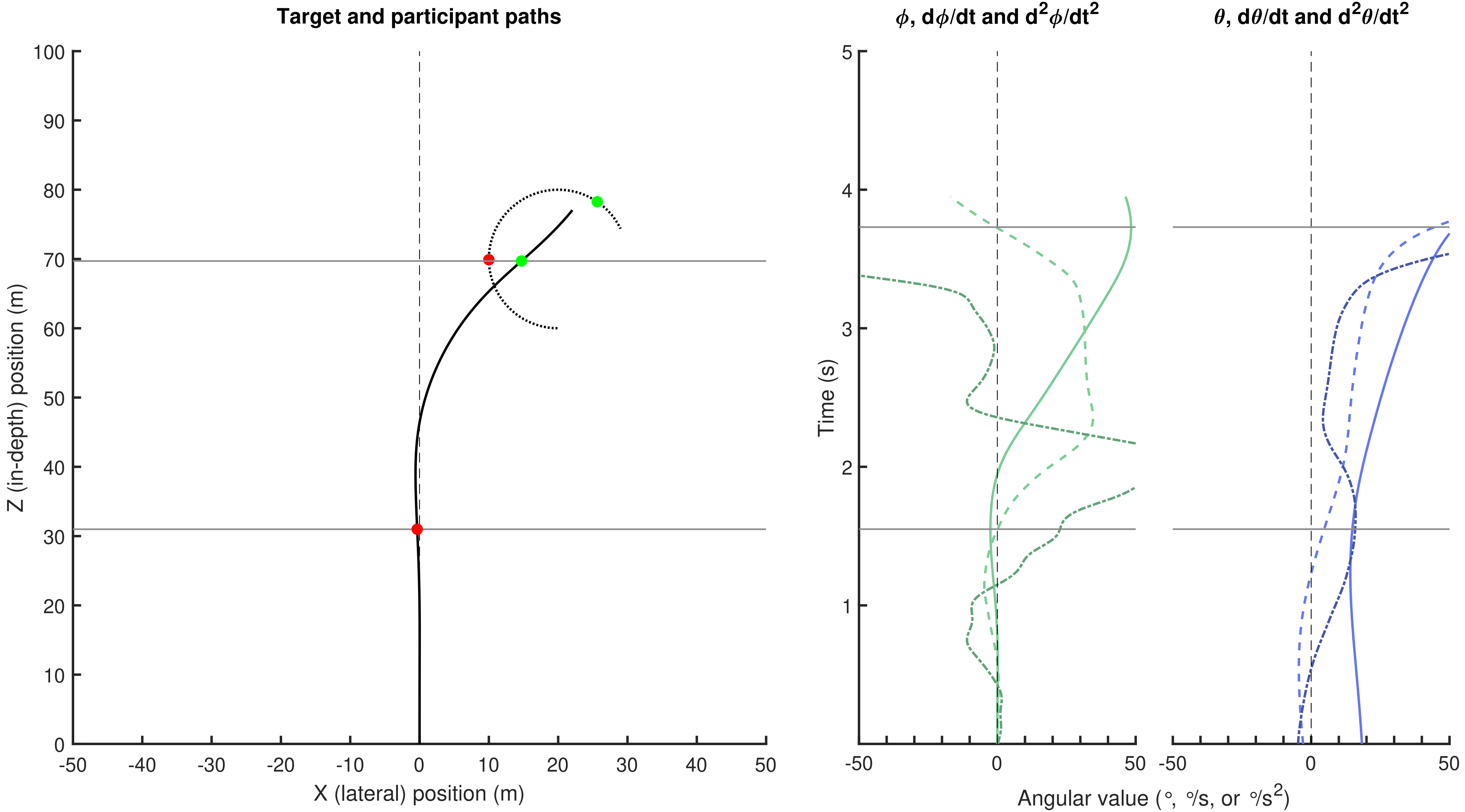

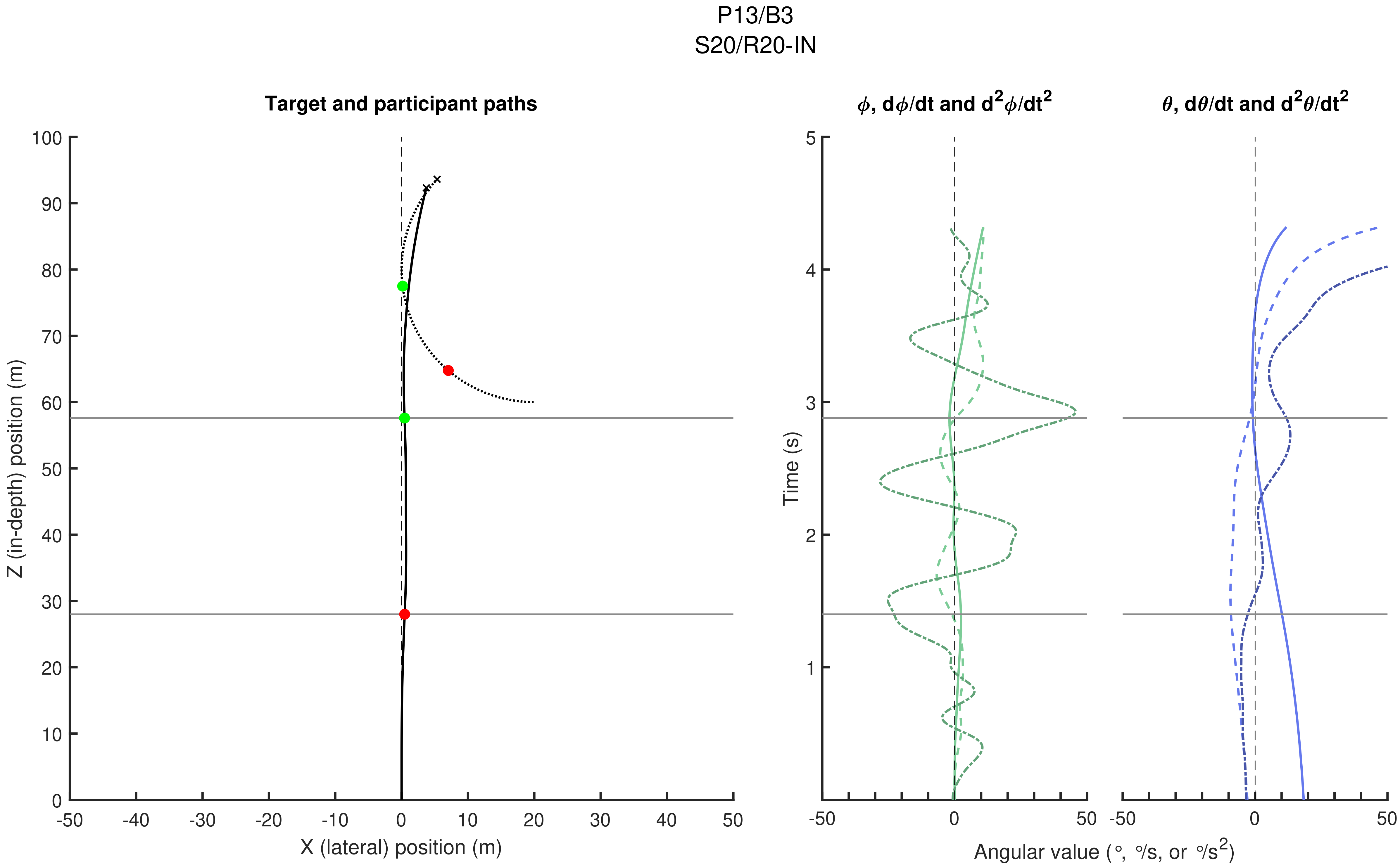
P13/B3
S20/R20-IN
Target and participant paths
Z (in-depth) position (m)
X (lateral) position (m)
ϕ, dϕ/dt and d$^2\phi$/dt^2
θ, dθ/dt and d$^2\theta$/dt^2
Time (s)
Angular value (°, °/s, or °/s^2)

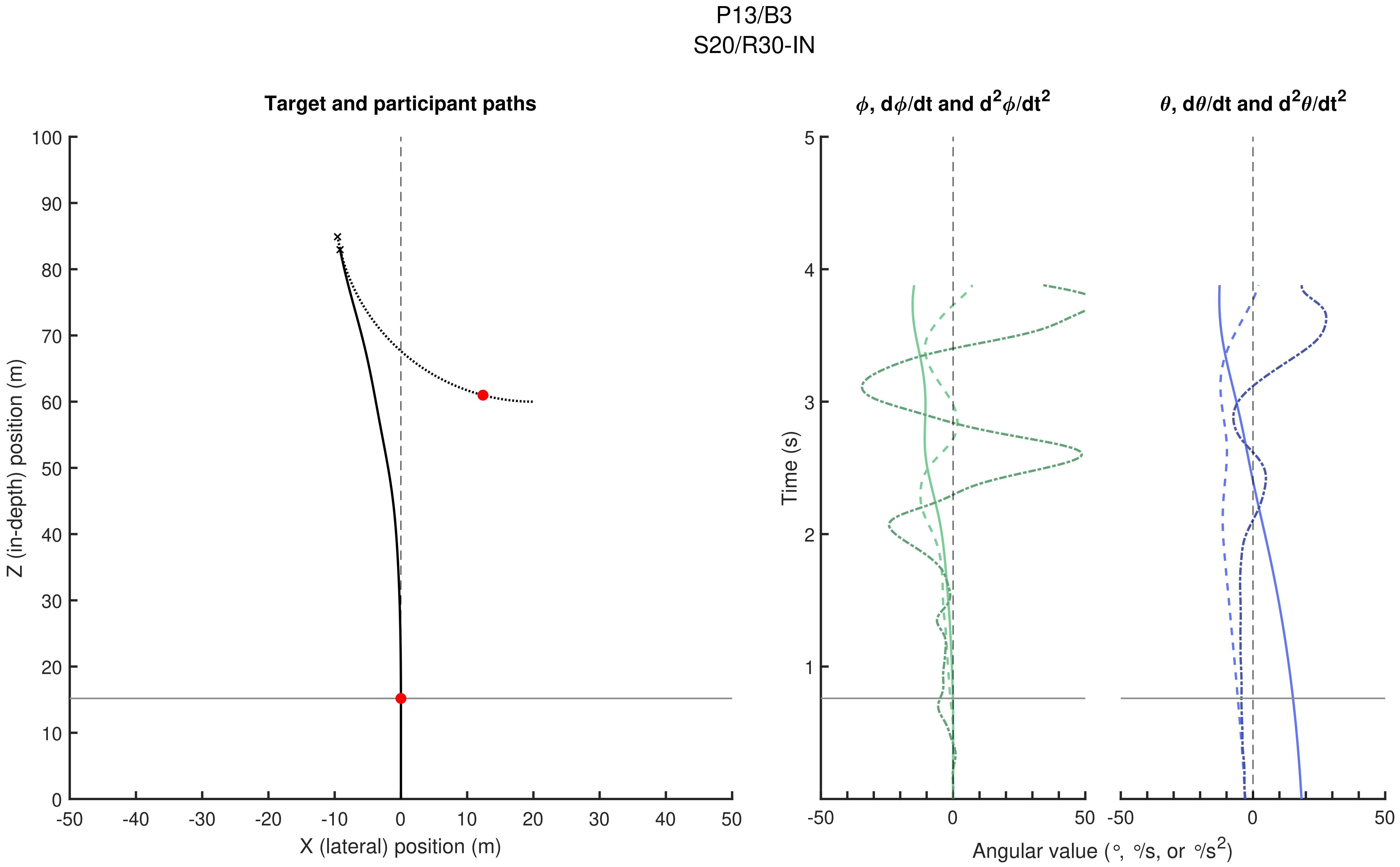
P13/B3
S20/R30-IN
Target and participant paths
Z (in-depth) position (m)
X (lateral) position (m)
ϕ, dϕ/dt and d$^2\phi$/dt^2
θ, dθ/dt and d$^2\theta$/dt^2
Time (s)
Angular value (°, °/s, or °/s^2)

P13/B4
S20/R10-IN

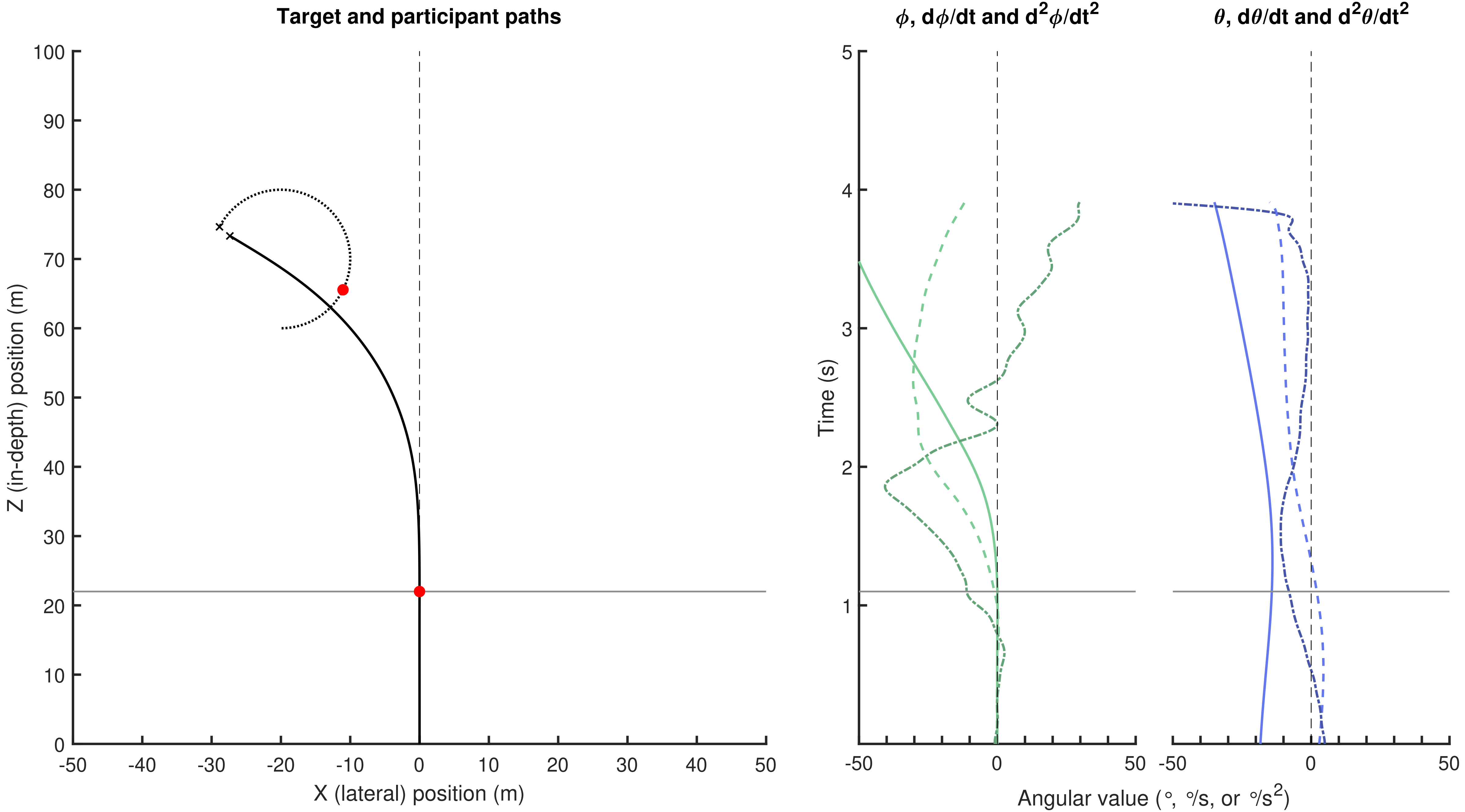

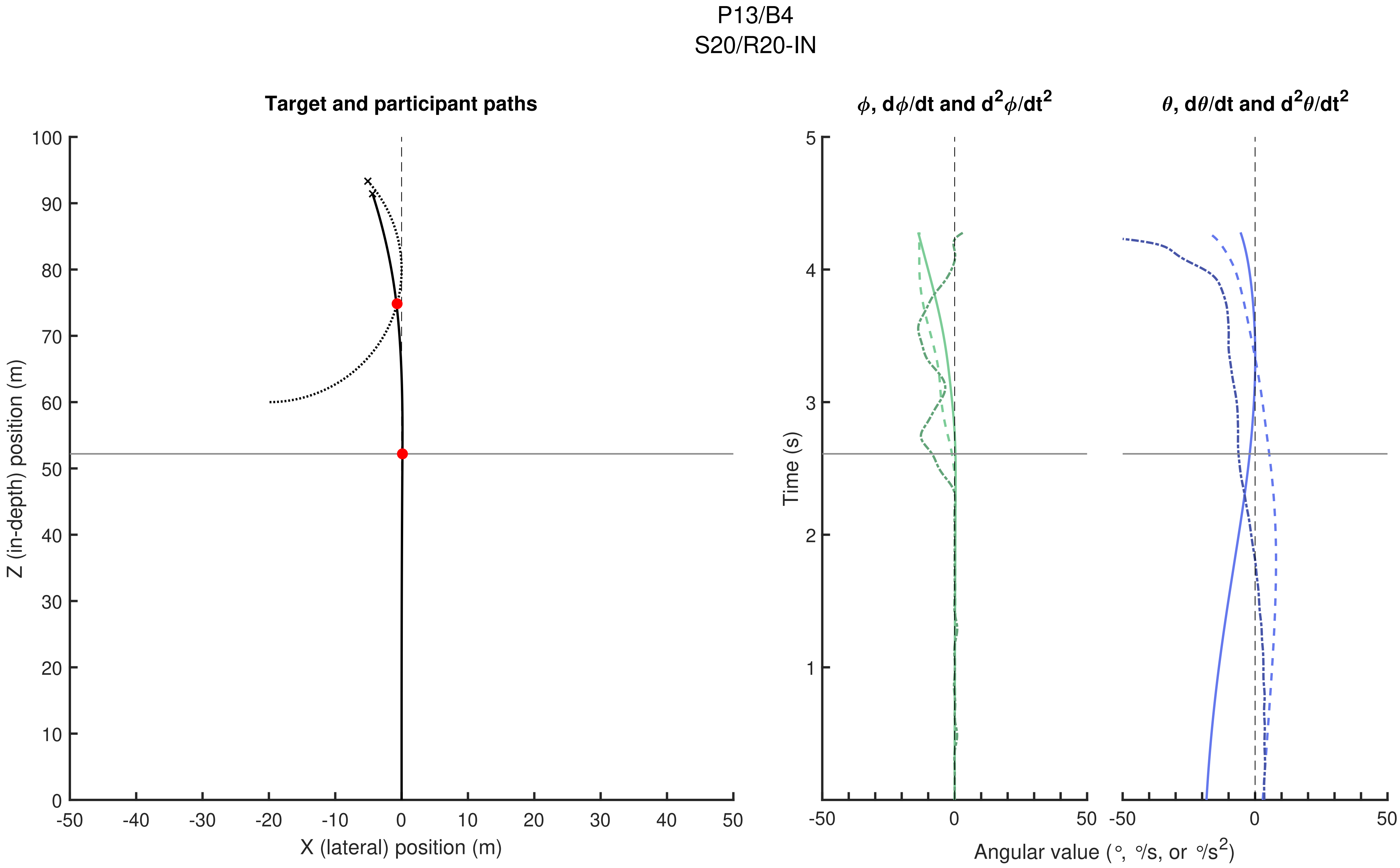
P13/B4
S20/R20-IN
Target and participant paths
Z (in-depth) position (m)
X (lateral) position (m)
ϕ, dϕ/dt and d$^2\phi$/dt^2
θ, dθ/dt and d$^2\theta$/dt^2
Time (s)
Angular value (°, °/s, or °/s^2)

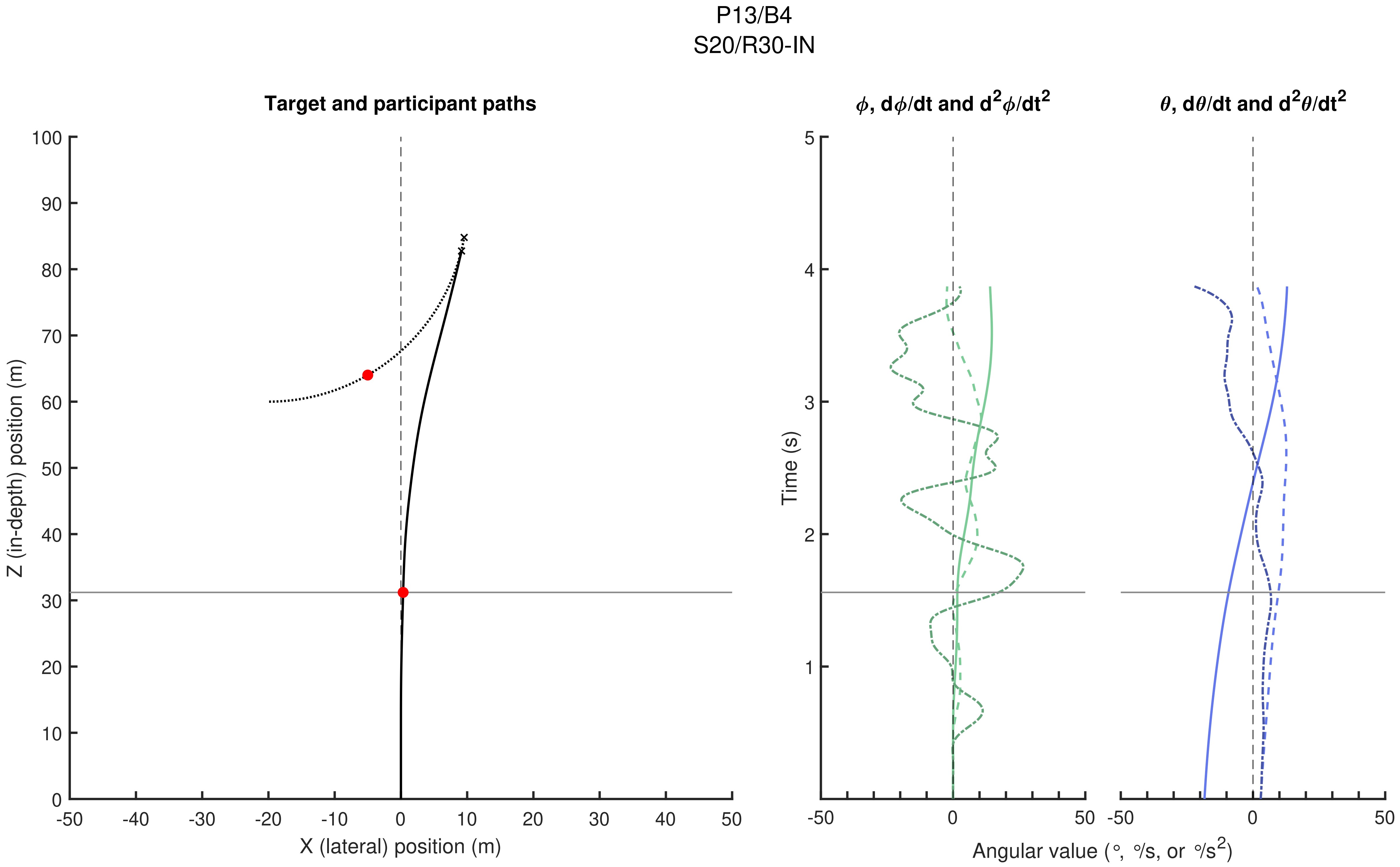

P13/B4
S20/R30-IN
Target and participant paths
Z (in-depth) position (m)
X (lateral) position (m)
100
90
80
70
60
50
40
30
20
10
0
-50
-40
-30
-20
-10
0
10
20
30
40
50
ϕ, dϕ/dt and d$^2\phi$/dt^2
θ, dθ/dt and d$^2\theta$/dt^2
Time (s)
5
4
3
2
1
-50
0
50
-50
0
50
Angular value (°, °/s, or °/s^2)

P13/B4
S15/R10-IN

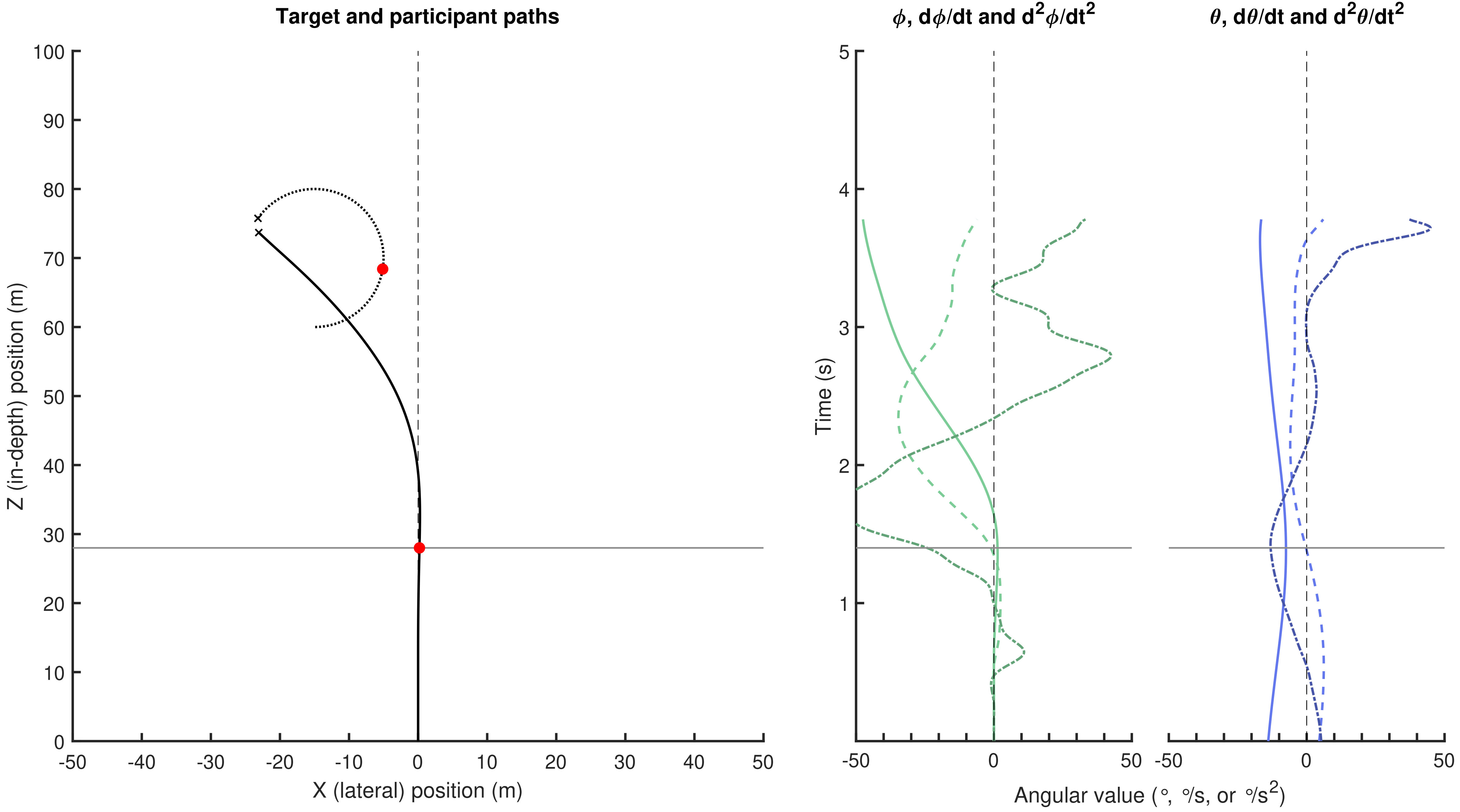

P13/B4
S15/R20-IN

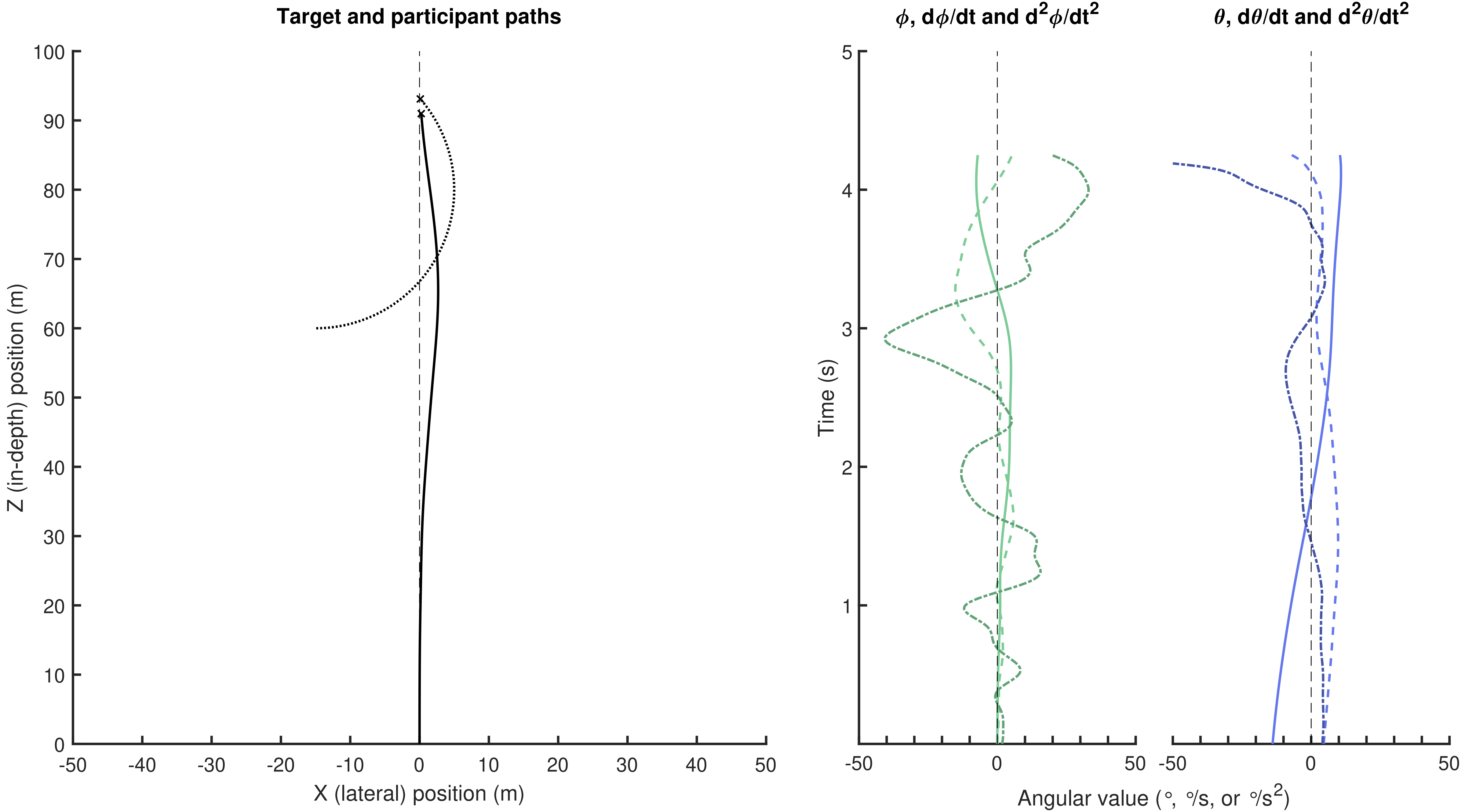

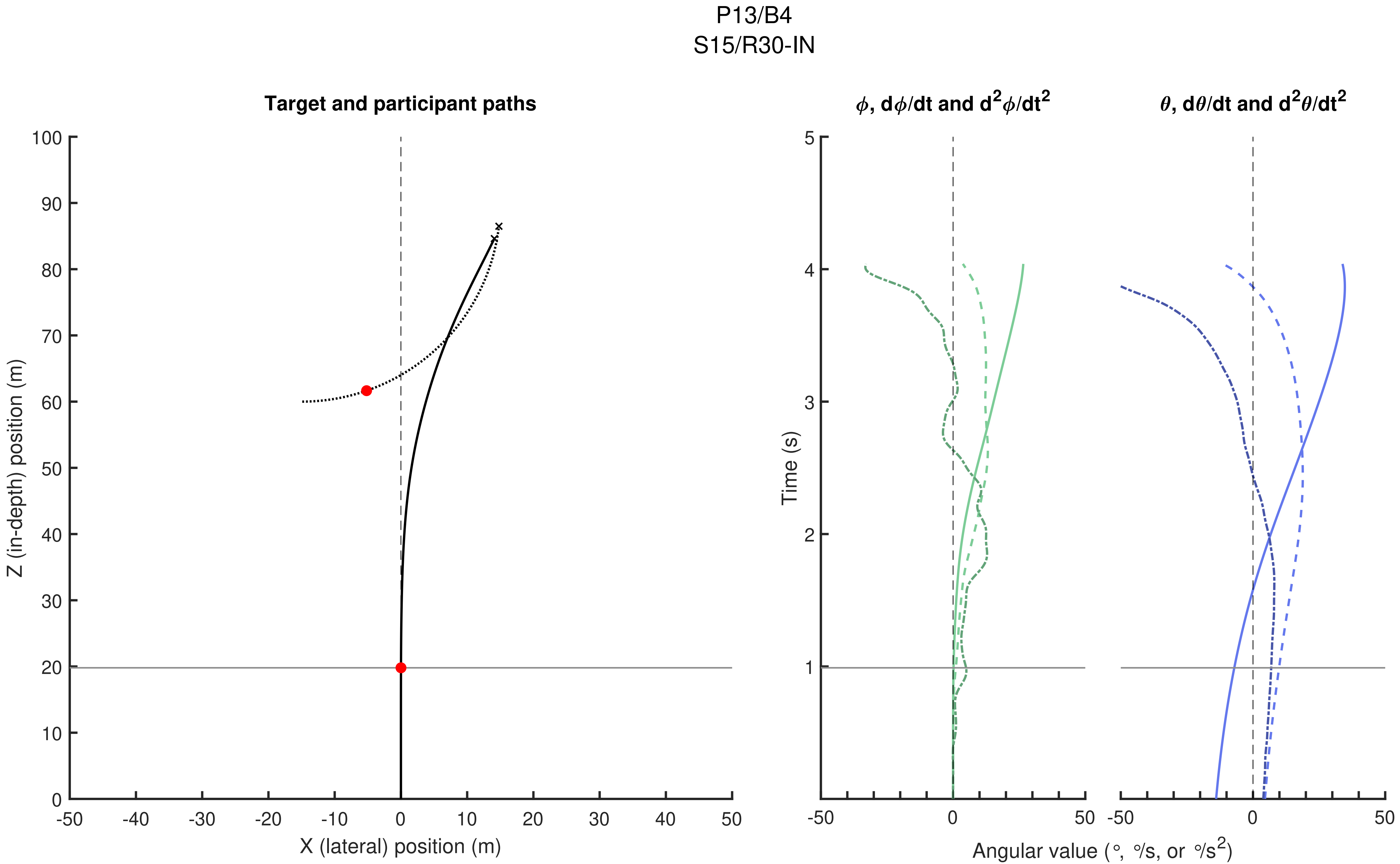

P13/B4
S15/R30-IN
Target and participant paths
Z (in-depth) position (m)
X (lateral) position (m)
100
90
80
70
60
50
40
30
20
10
0
-50
-40
-30
-20
-10
0
10
20
30
40
50
ϕ, dϕ/dt and d$^2\phi$/dt^2
θ, dθ/dt and d$^2\theta$/dt^2
Time (s)
5
4
3
2
1
-50
0
50
-50
0
50
Angular value (°, °/s, or °/s^2)

P13/B4
S5/R10-IN

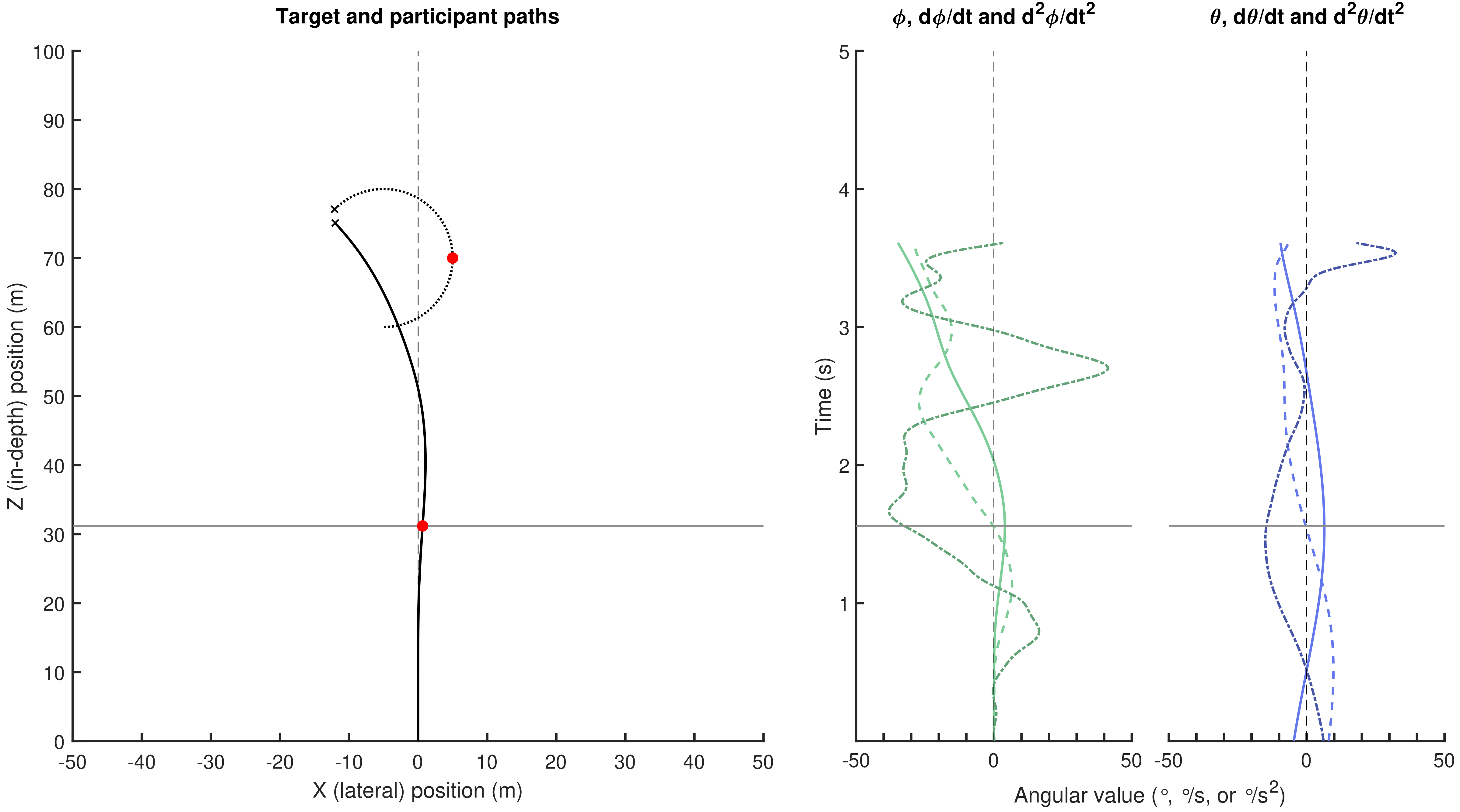

P13/B4
S5/R10-OUT

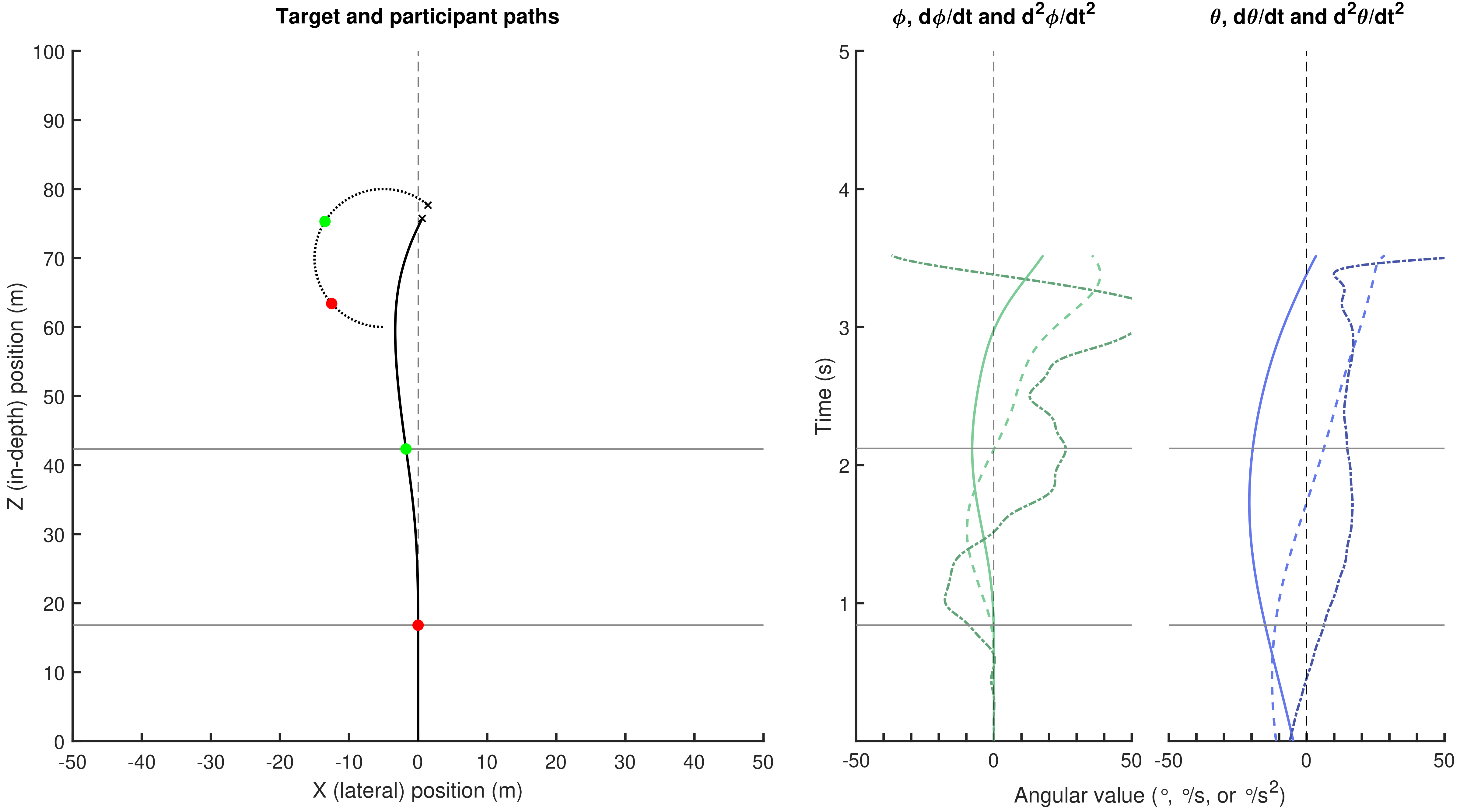

P13/B4
S5/R20-IN

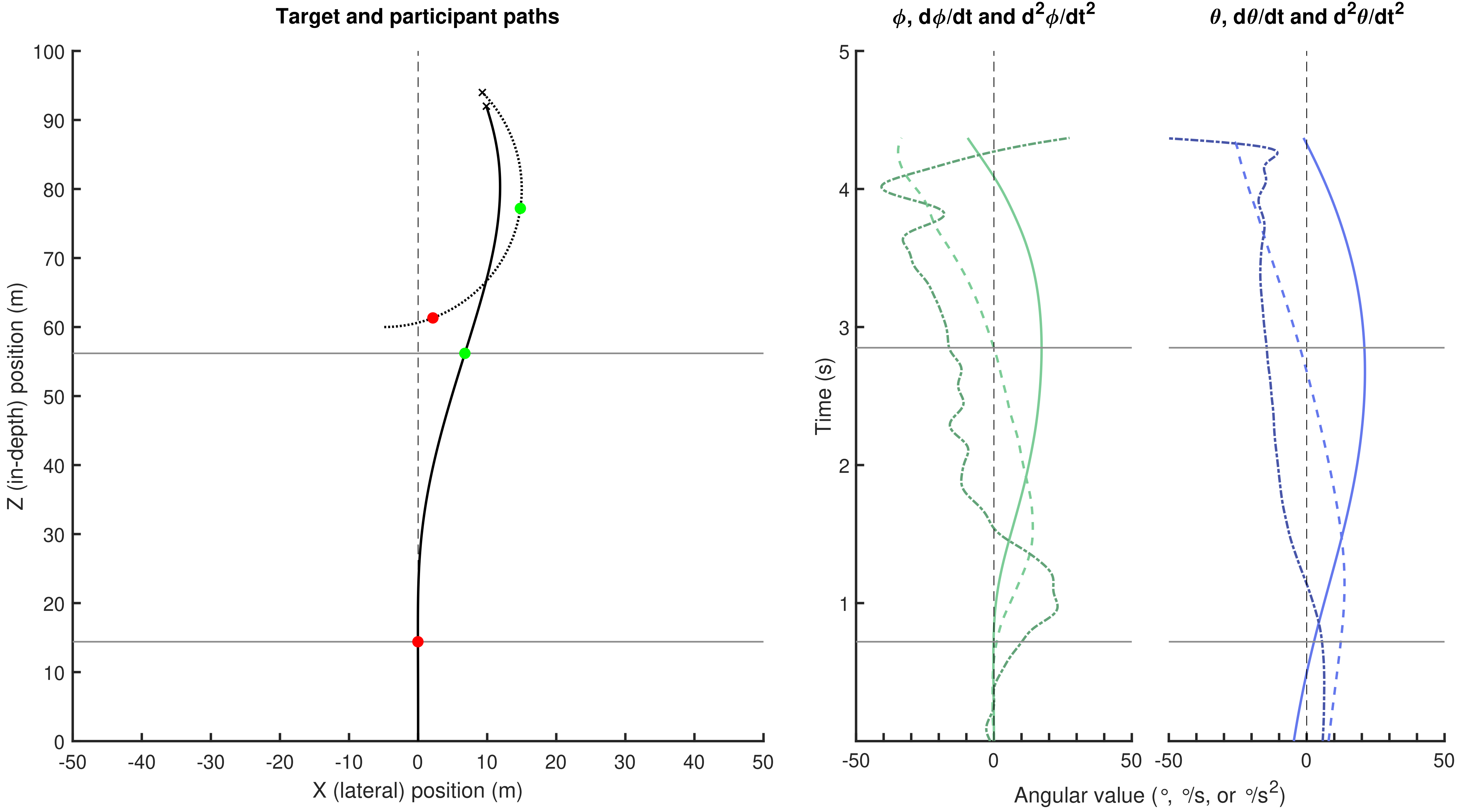

P13/B4
S5/R20-OUT

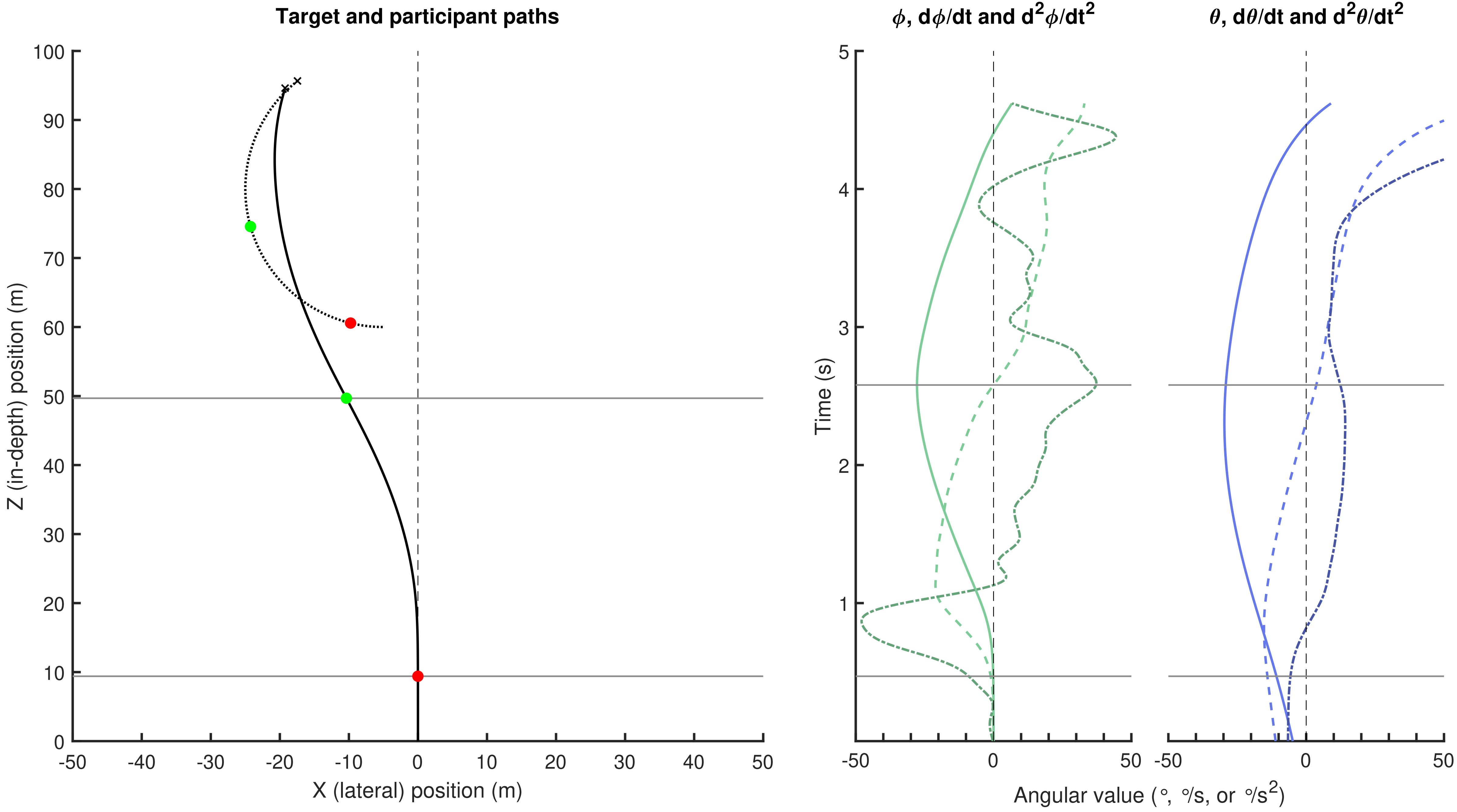

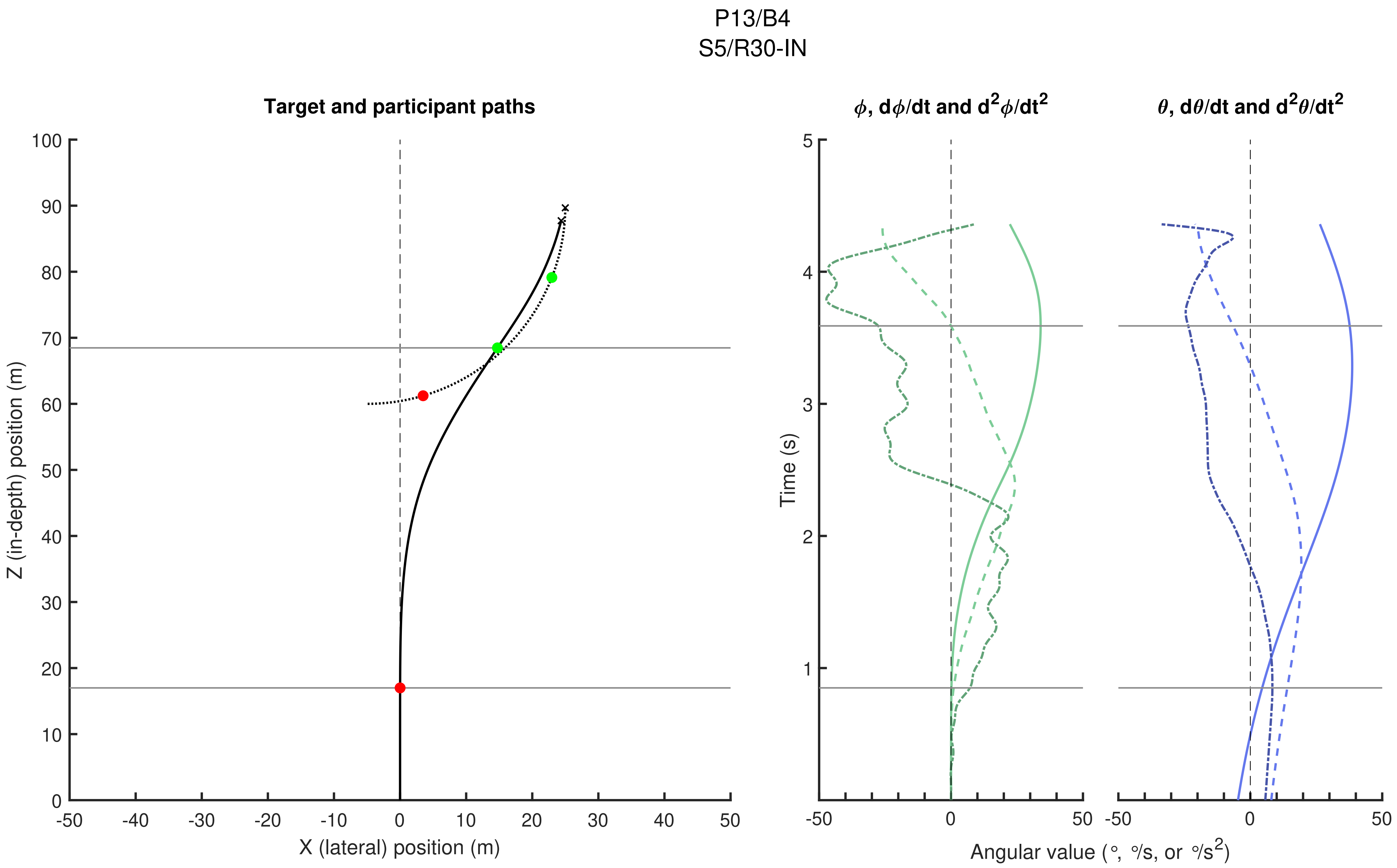

P13/B4
S5/R30-IN
Target and participant paths
Z (in-depth) position (m)
X (lateral) position (m)
ϕ, dϕ/dt and d$^2\phi$/dt^2
θ, dθ/dt and d$^2\theta$/dt^2
Time (s)
Angular value (°, °/s, or °/s^2)

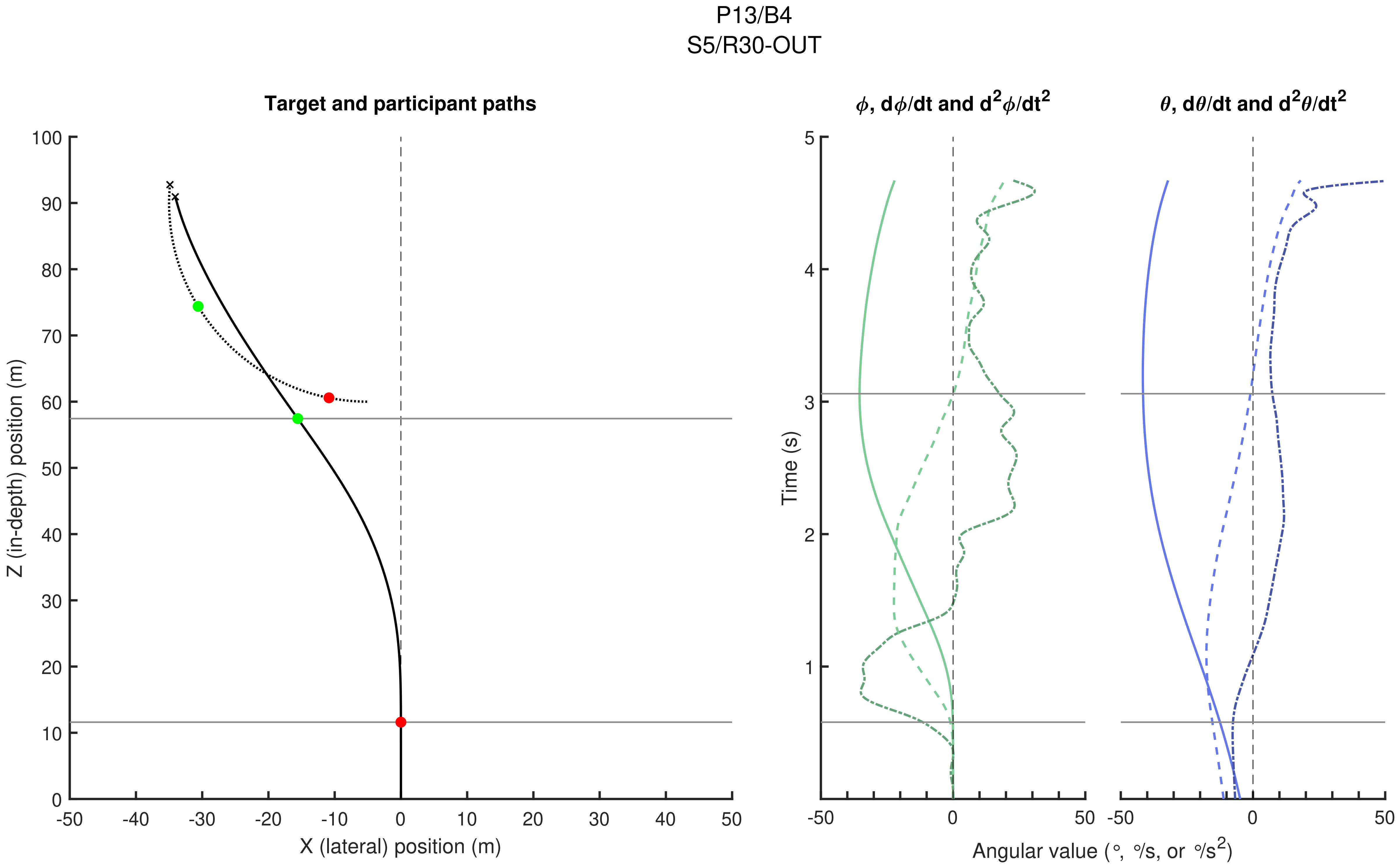
P13/B4
S5/R30-OUT
Target and participant paths
Z (in-depth) position (m)
X (lateral) position (m)
100
90
80
70
60
50
40
30
20
10
0
-50
-40
-30
-20
-10
0
10
20
30
40
50
ϕ, dϕ/dt and d²ϕ/dt²
θ, dθ/dt and d²θ/dt²
Time (s)
5
4
3
2
1
-50
0
50
-50
0
50
Angular value (°, °/s, or °/s²)

P13/B4
S5/R10-OUT

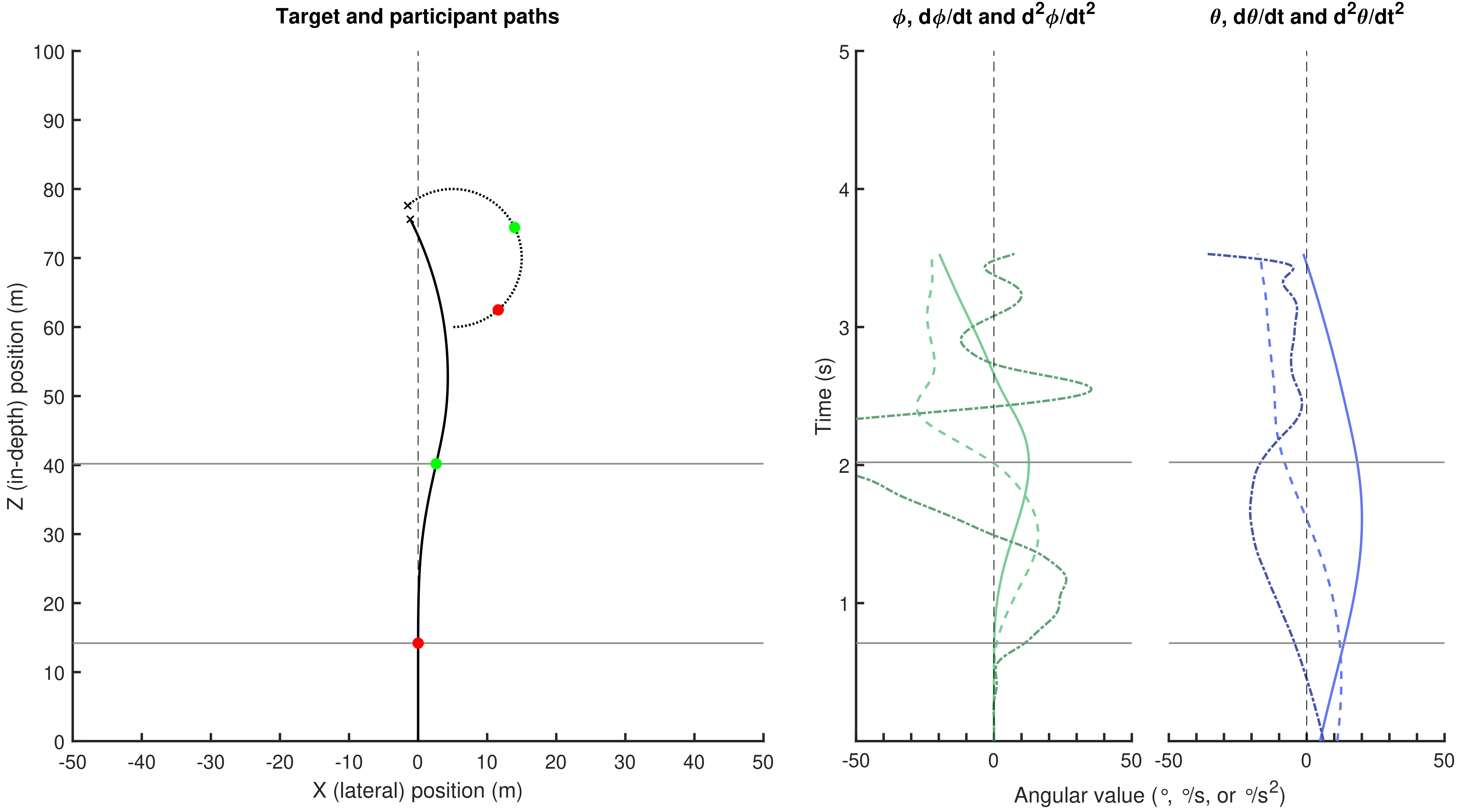

P13/B4
S5/R10-IN

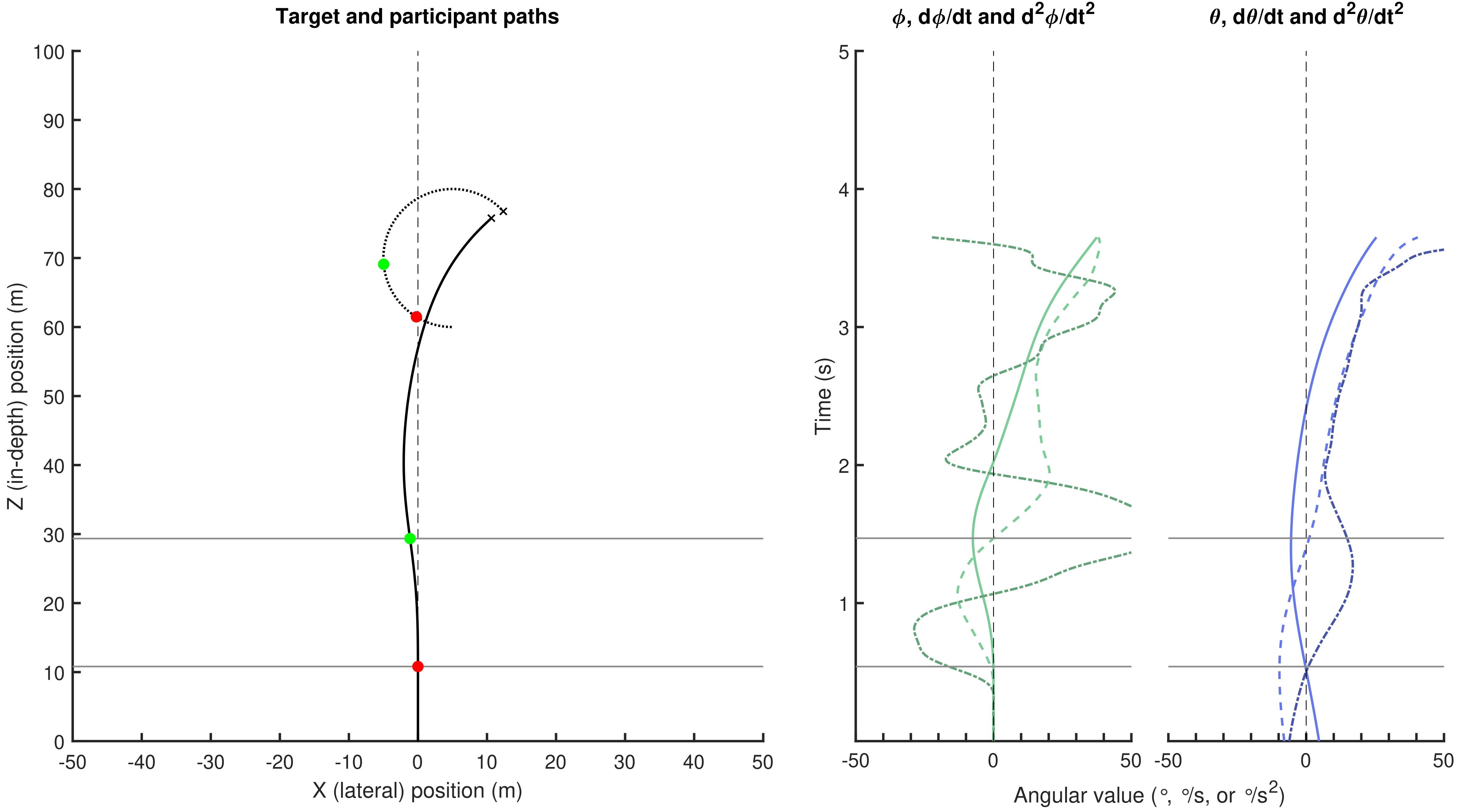

P13/B4
S5/R20-OUT

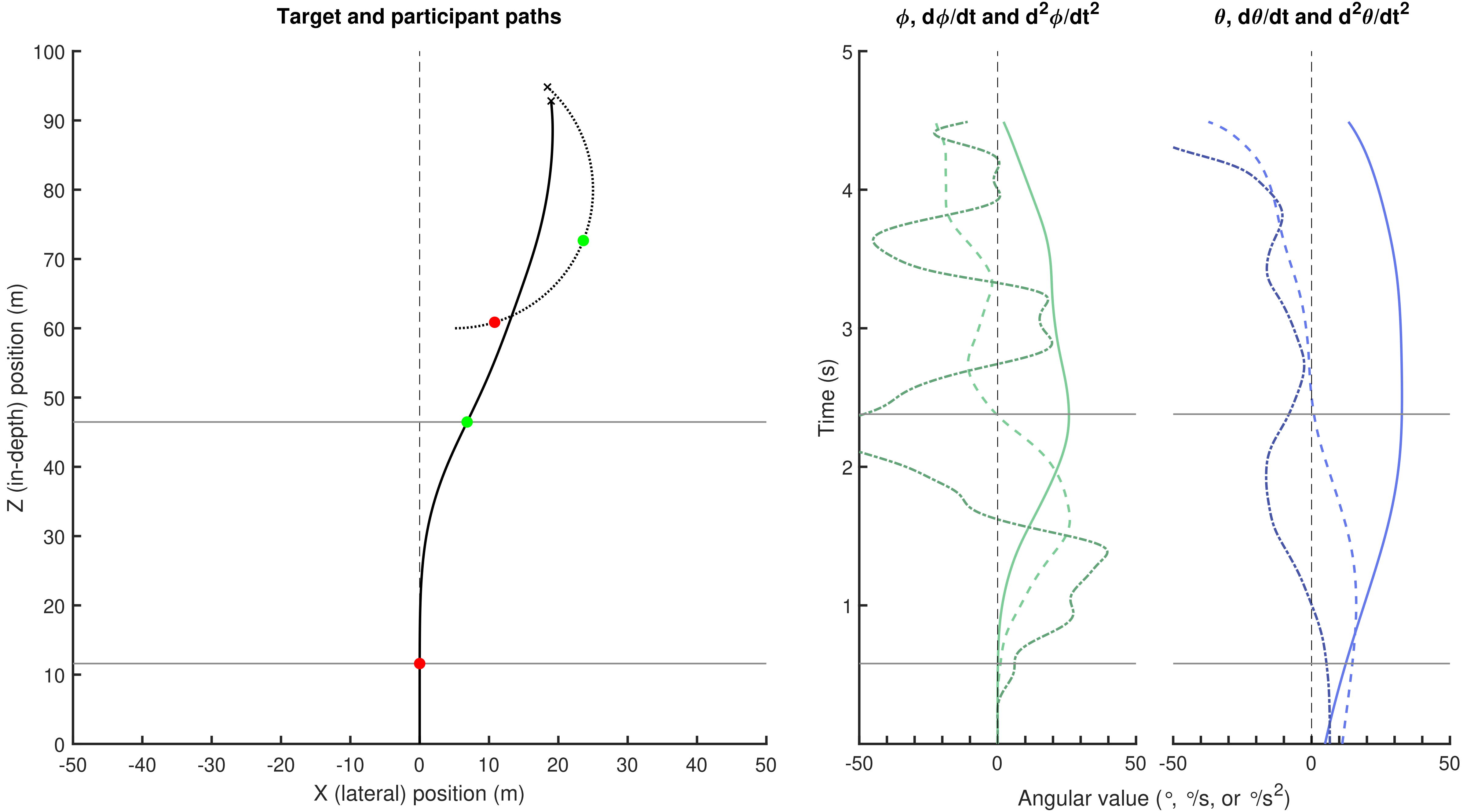

P13/B4
S5/R20-IN

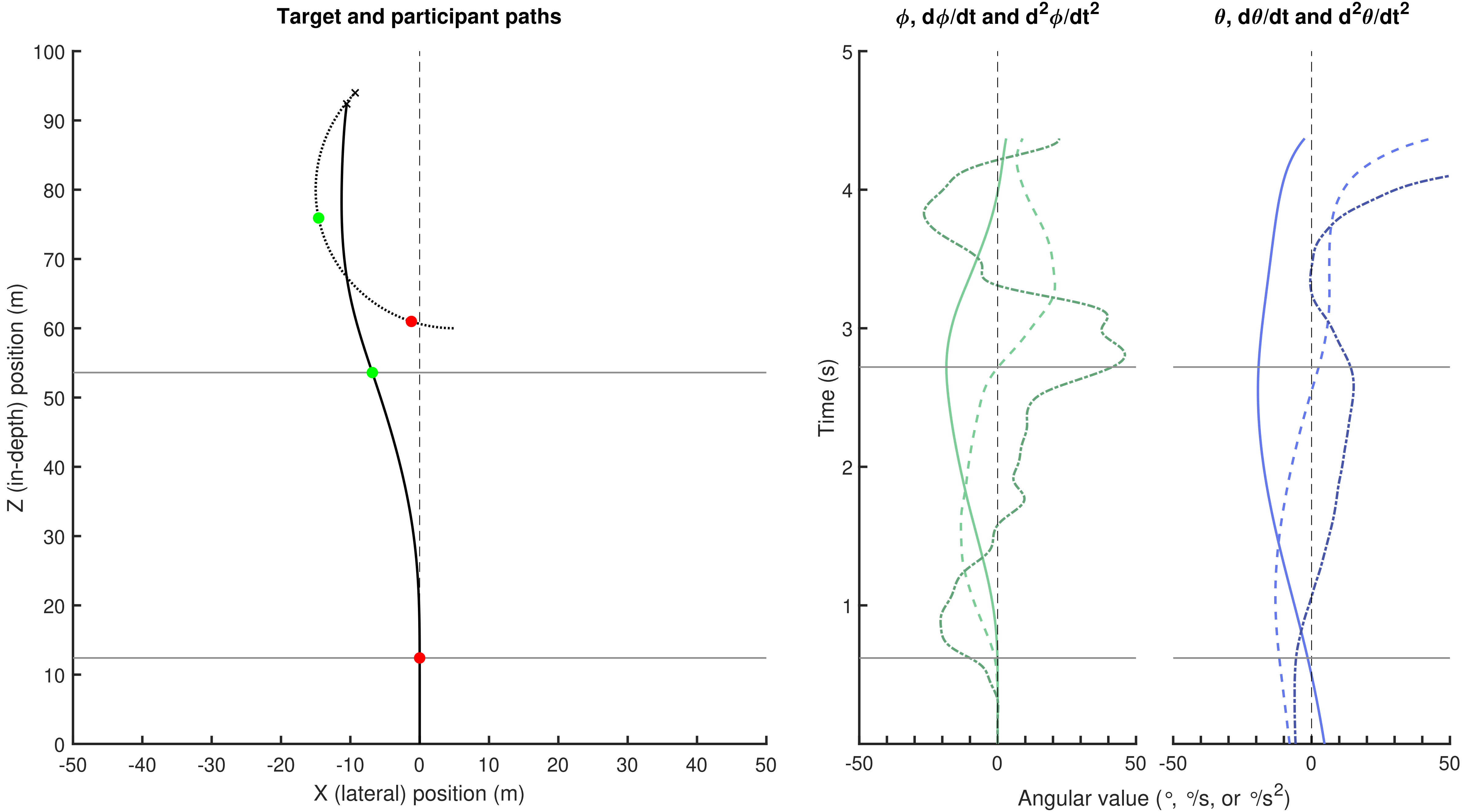

P13/B4
S5/R30-OUT

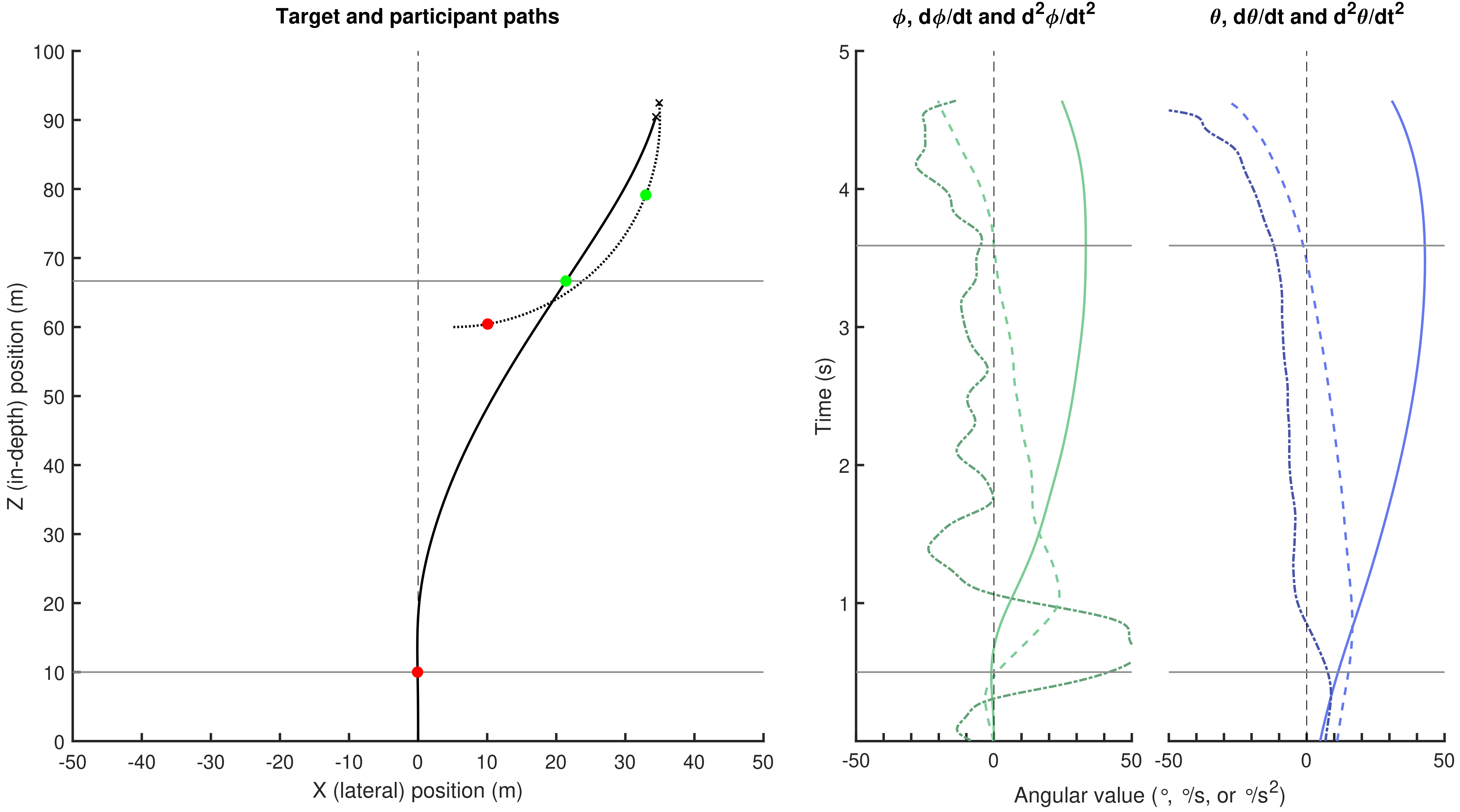

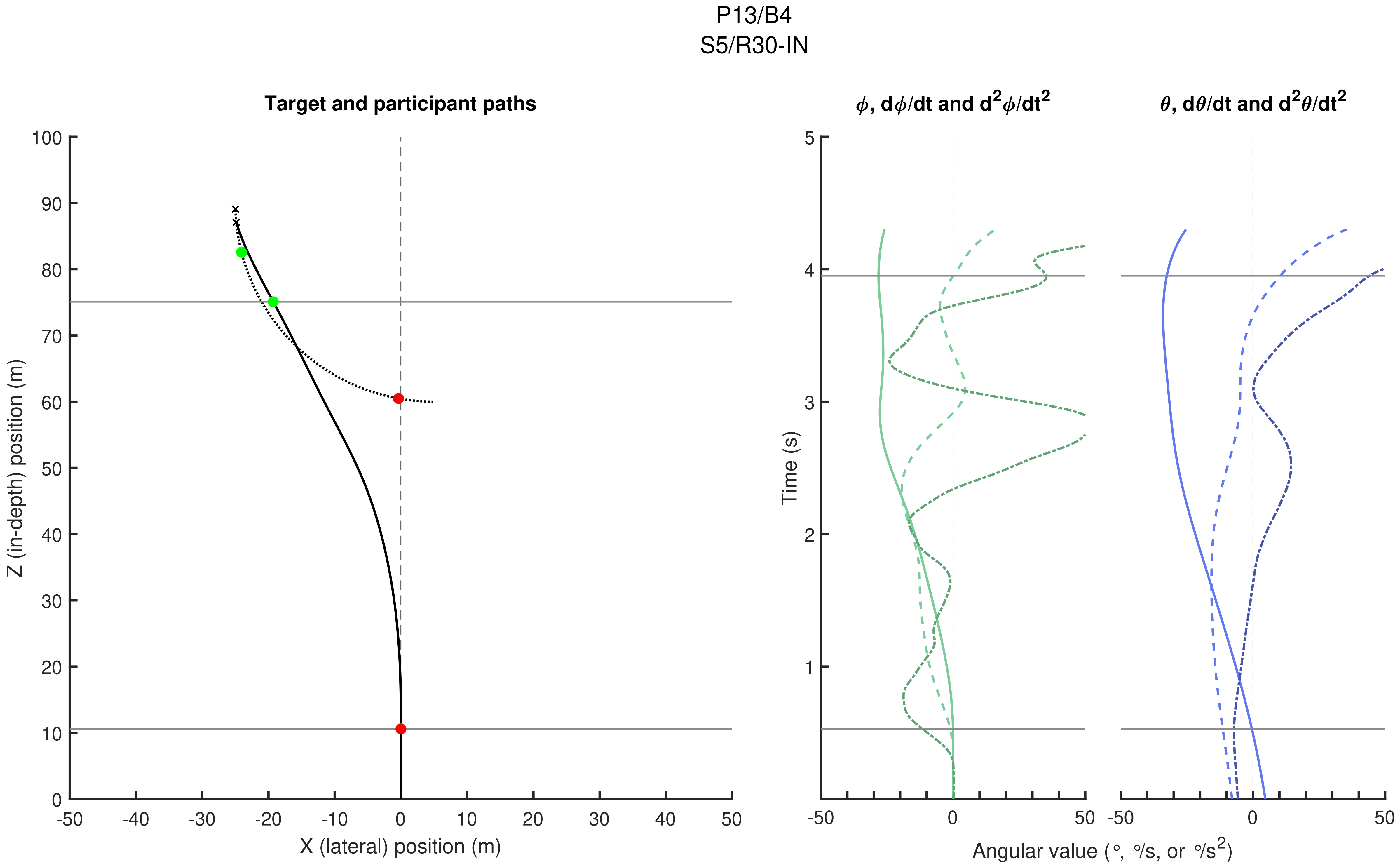
P13/B4
S5/R30-IN
Target and participant paths
Z (in-depth) position (m)
X (lateral) position (m)
-50
-40
-30
-20
-10
0
10
20
30
40
50
0
10
20
30
40
50
60
70
80
90
100
ϕ, dϕ/dt and d$^2\phi$/dt^2
θ, dθ/dt and d$^2\theta$/dt^2
Time (s)
1
2
3
4
5
Angular value (°, °/s, or °/s^2)

P13/B4
S15/R10-IN

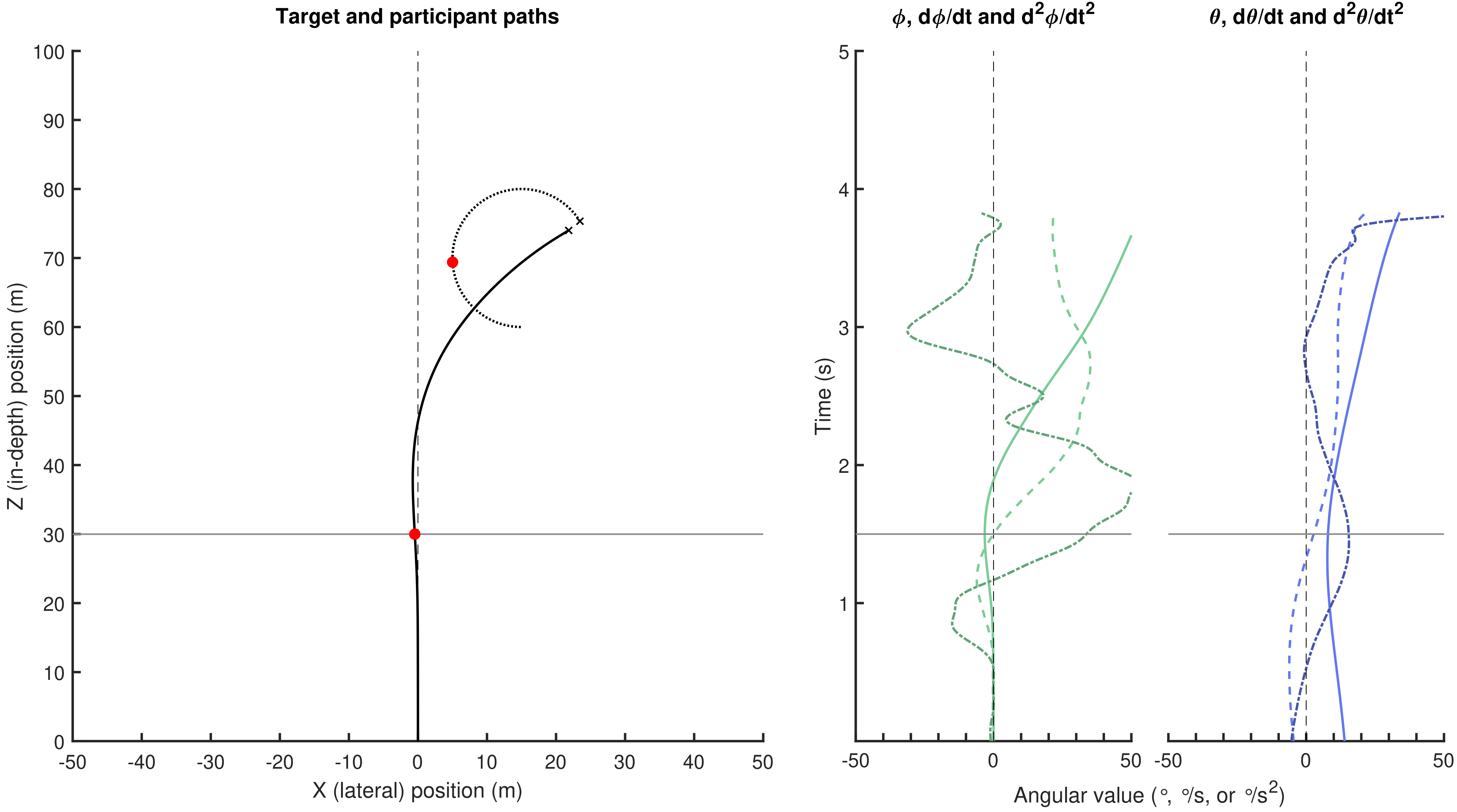

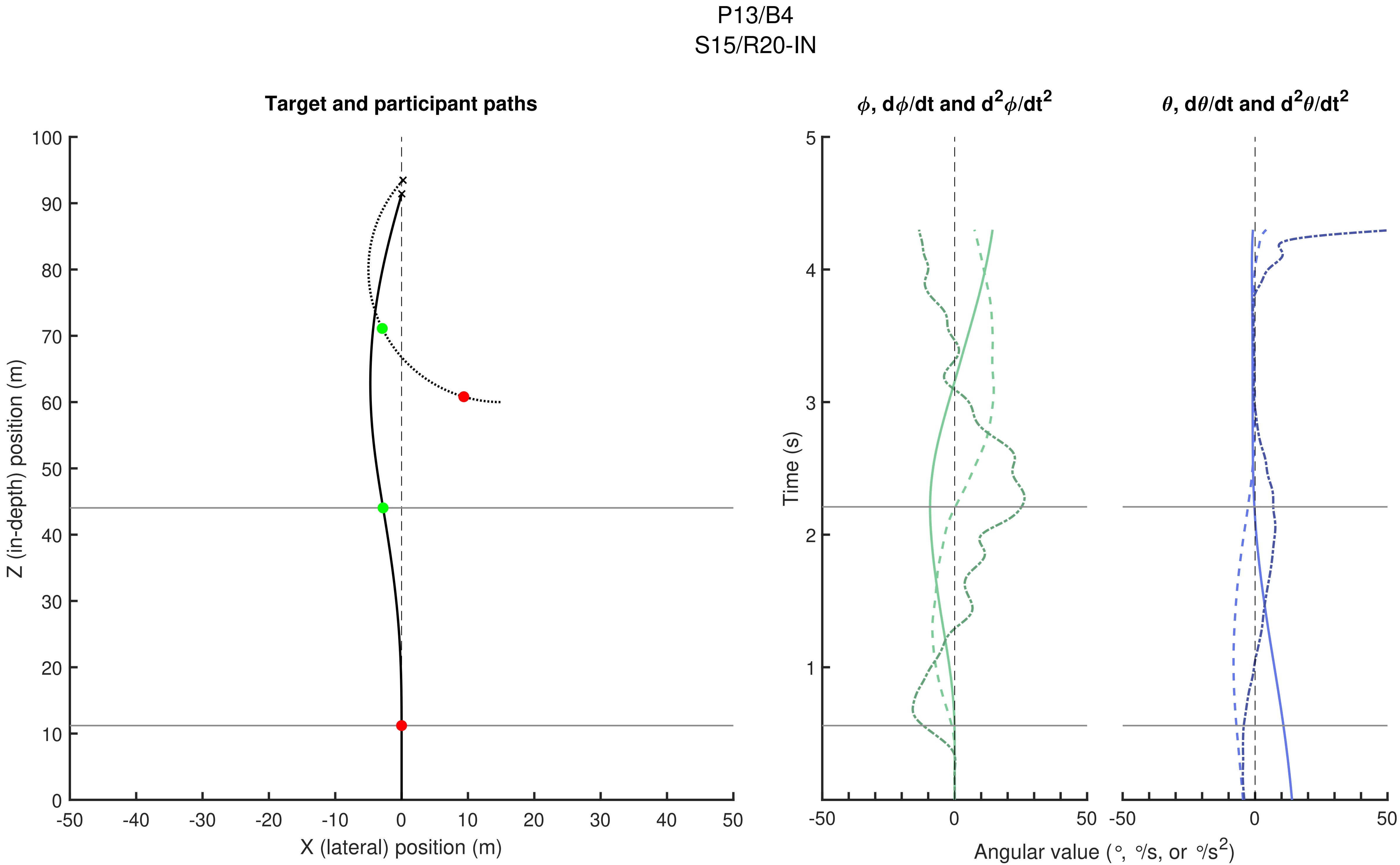

P13/B4
S15/R20-IN
Target and participant paths
Z (in-depth) position (m)
X (lateral) position (m)
100
90
80
70
60
50
40
30
20
10
0
-50
-40
-30
-20
-10
0
10
20
30
40
50
φ, dφ/dt and d²φ/dt²
θ, dθ/dt and d²θ/dt²
Time (s)
5
4
3
2
1
-50
0
50
-50
0
50
Angular value (°, °/s, or °/s²)

P13/B4
S15/R30-IN

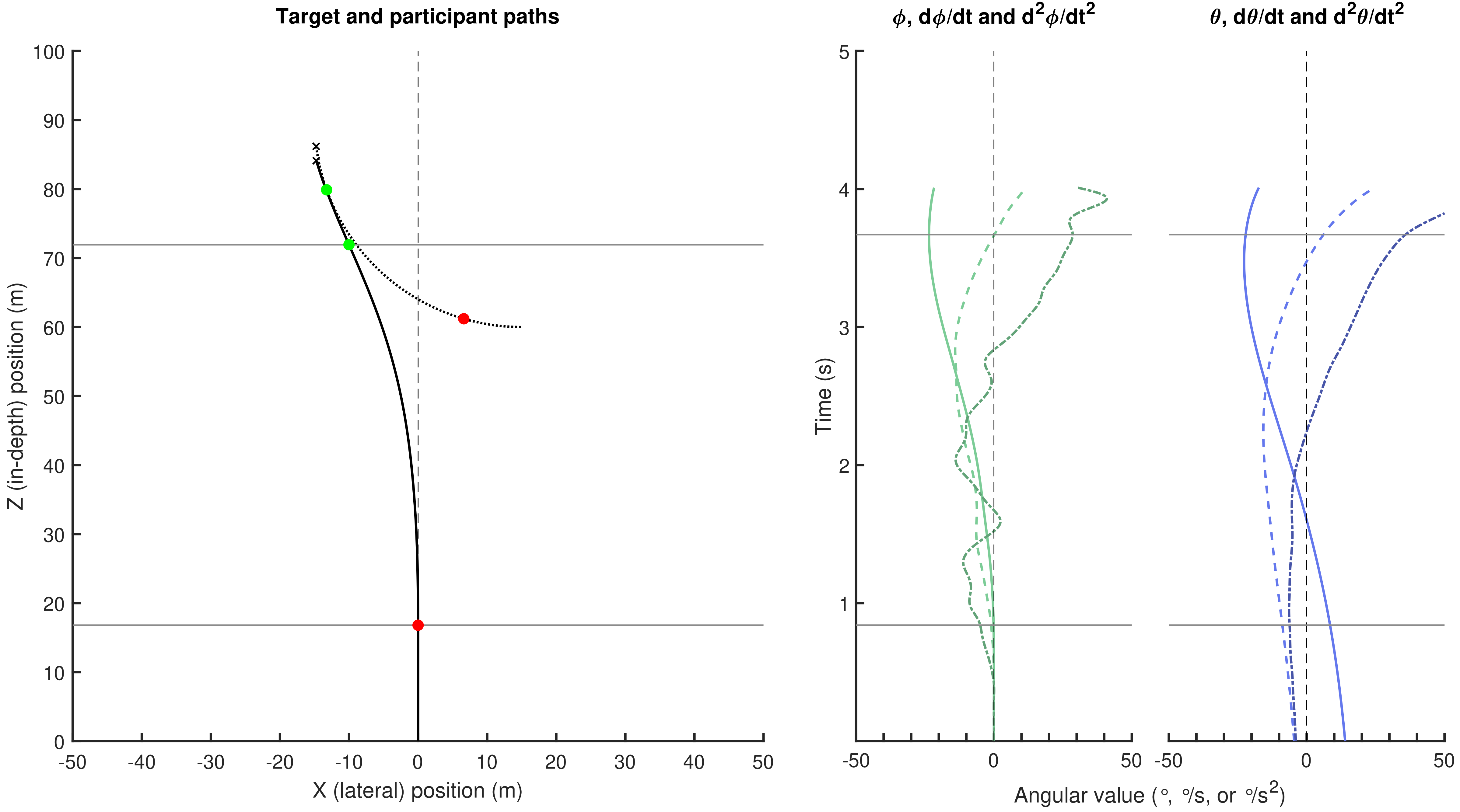

P13/B4
S20/R10-IN

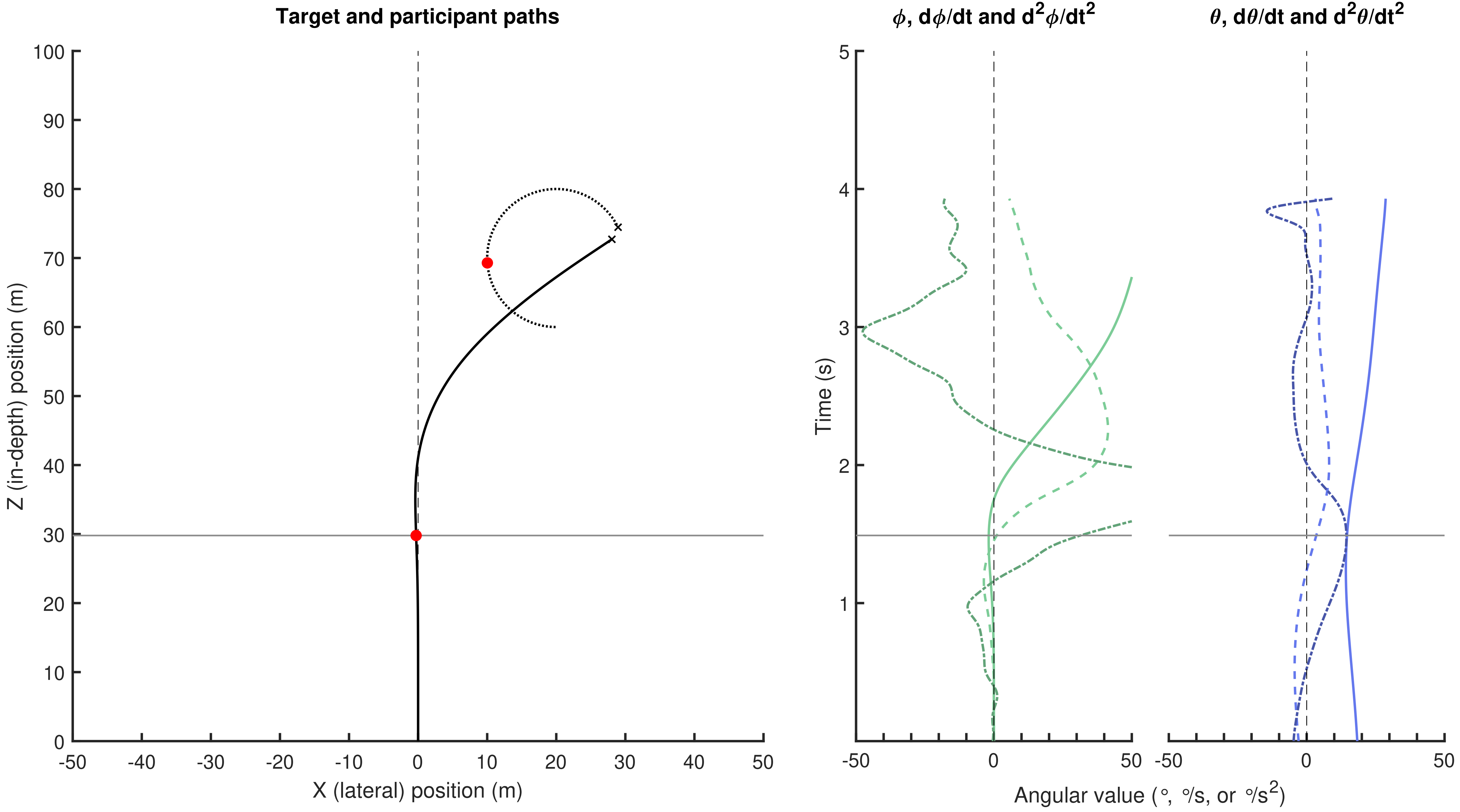

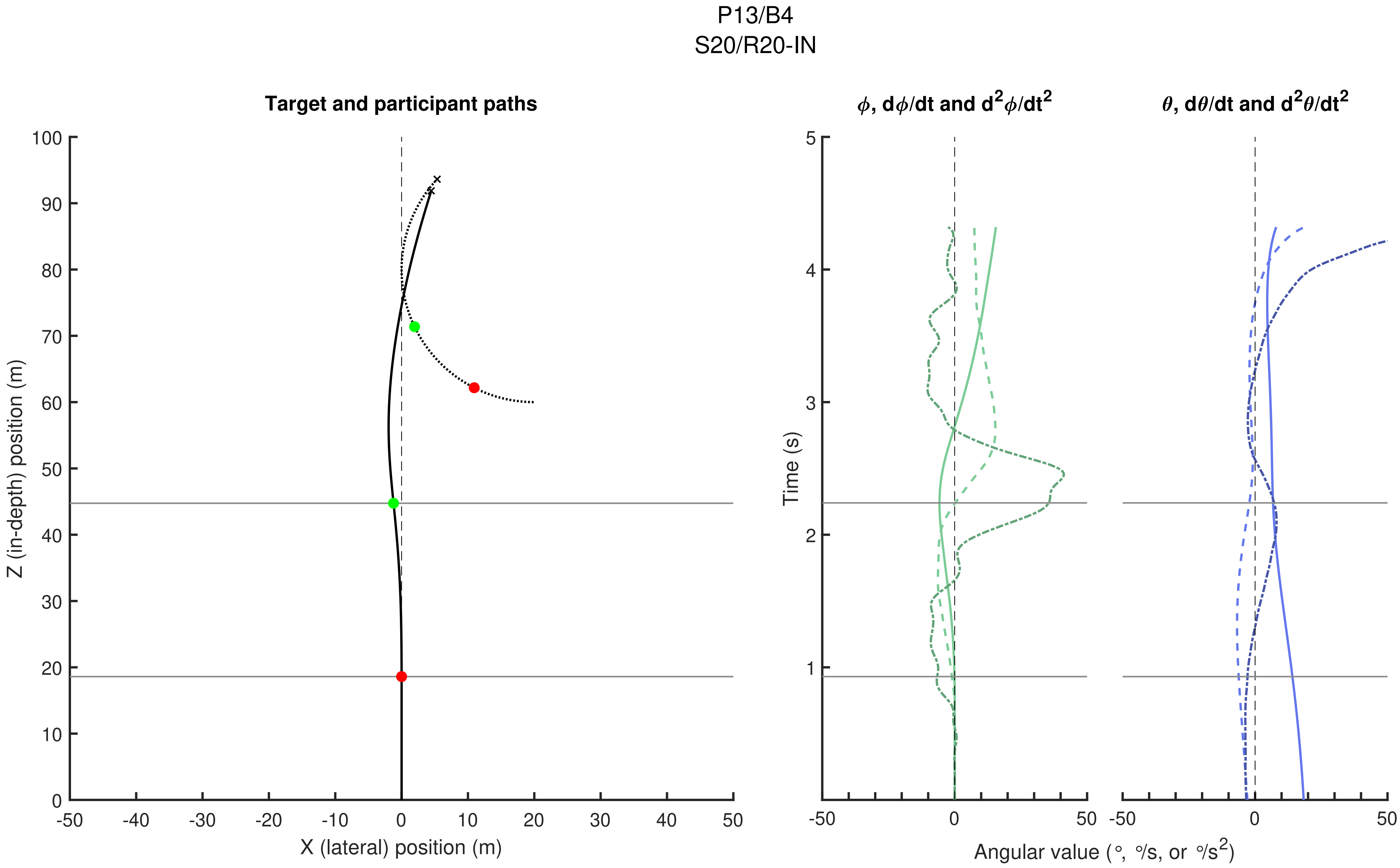
P13/B4
S20/R20-IN
Target and participant paths
Z (in-depth) position (m)
X (lateral) position (m)
ϕ, dϕ/dt and d$^2\phi$/dt^2
θ, dθ/dt and d$^2\theta$/dt^2
Time (s)
Angular value (°, °/s, or °/s^2)

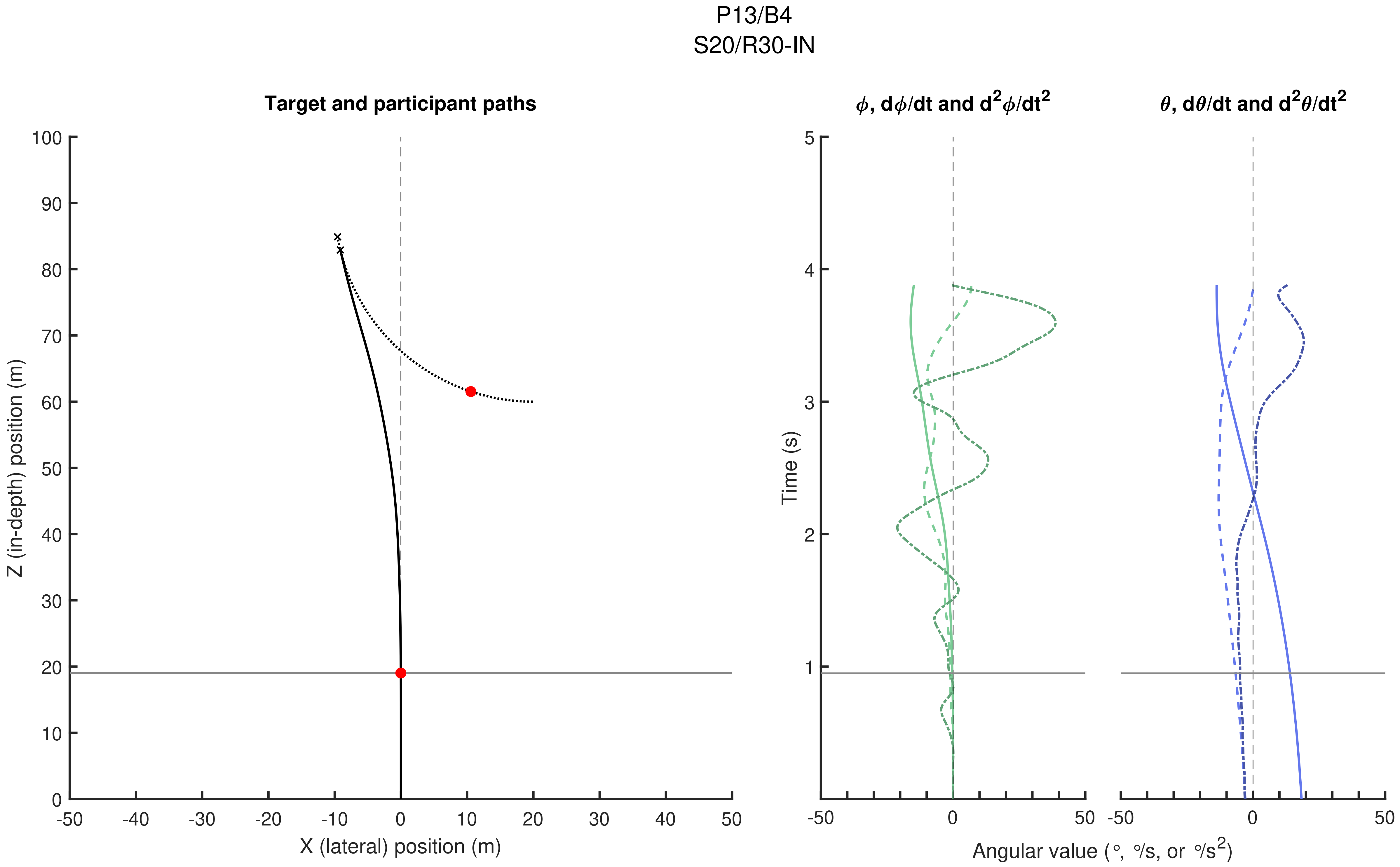

P13/B4
S20/R30-IN
Target and participant paths
ϕ, dϕ/dt and d$^2\phi$/dt^2
θ, dθ/dt and d$^2\theta$/dt^2
Z (in-depth) position (m)
X (lateral) position (m)
Time (s)
Angular value (°, °/s, or °/s^2)

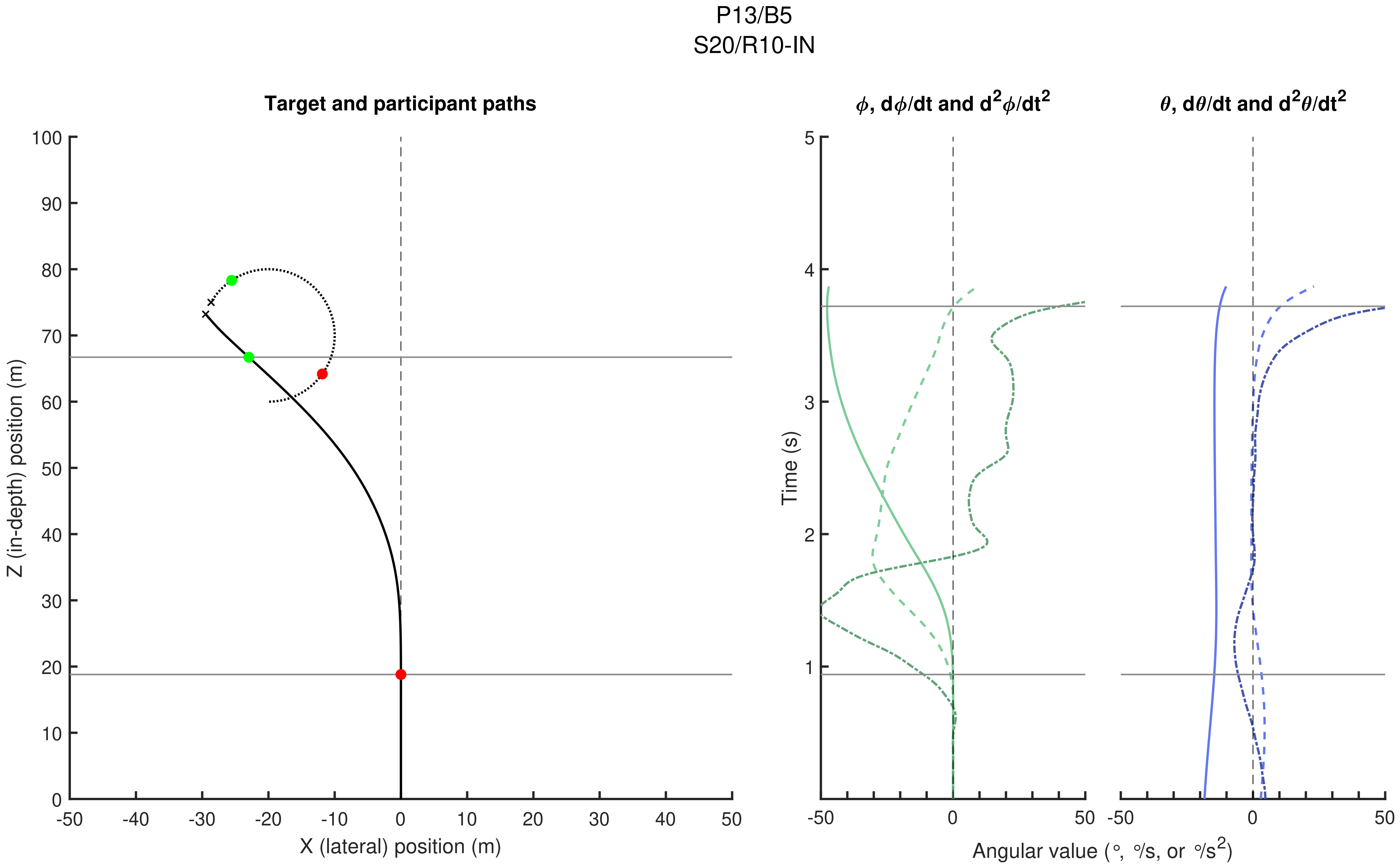

P13/B5
S20/R10-IN
Target and participant paths
Z (in-depth) position (m)
X (lateral) position (m)
100
90
80
70
60
50
40
30
20
10
0
-50
-40
-30
-20
-10
0
10
20
30
40
50
ϕ, dϕ/dt and d$^2\phi$/dt^2
θ, dθ/dt and d$^2\theta$/dt^2
Time (s)
5
4
3
2
1
-50
0
50
-50
0
50
Angular value (°, °/s, or °/s^2)

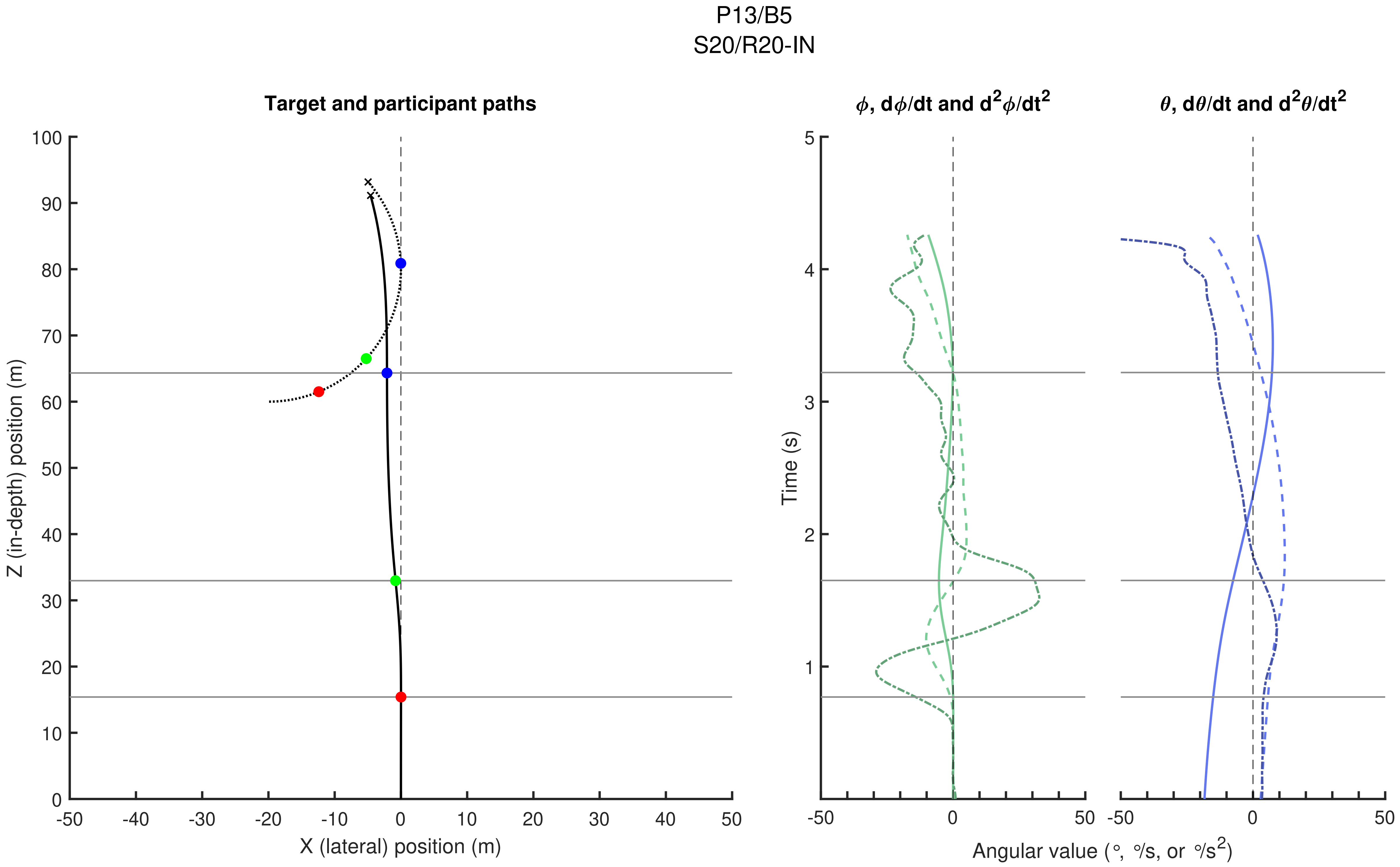

P13/B5
S20/R20-IN
Target and participant paths
Z (in-depth) position (m)
X (lateral) position (m)
ϕ, dϕ/dt and d$^2\phi$/dt^2
θ, dθ/dt and d$^2\theta$/dt^2
Time (s)
Angular value (°, °/s, or °/s^2)

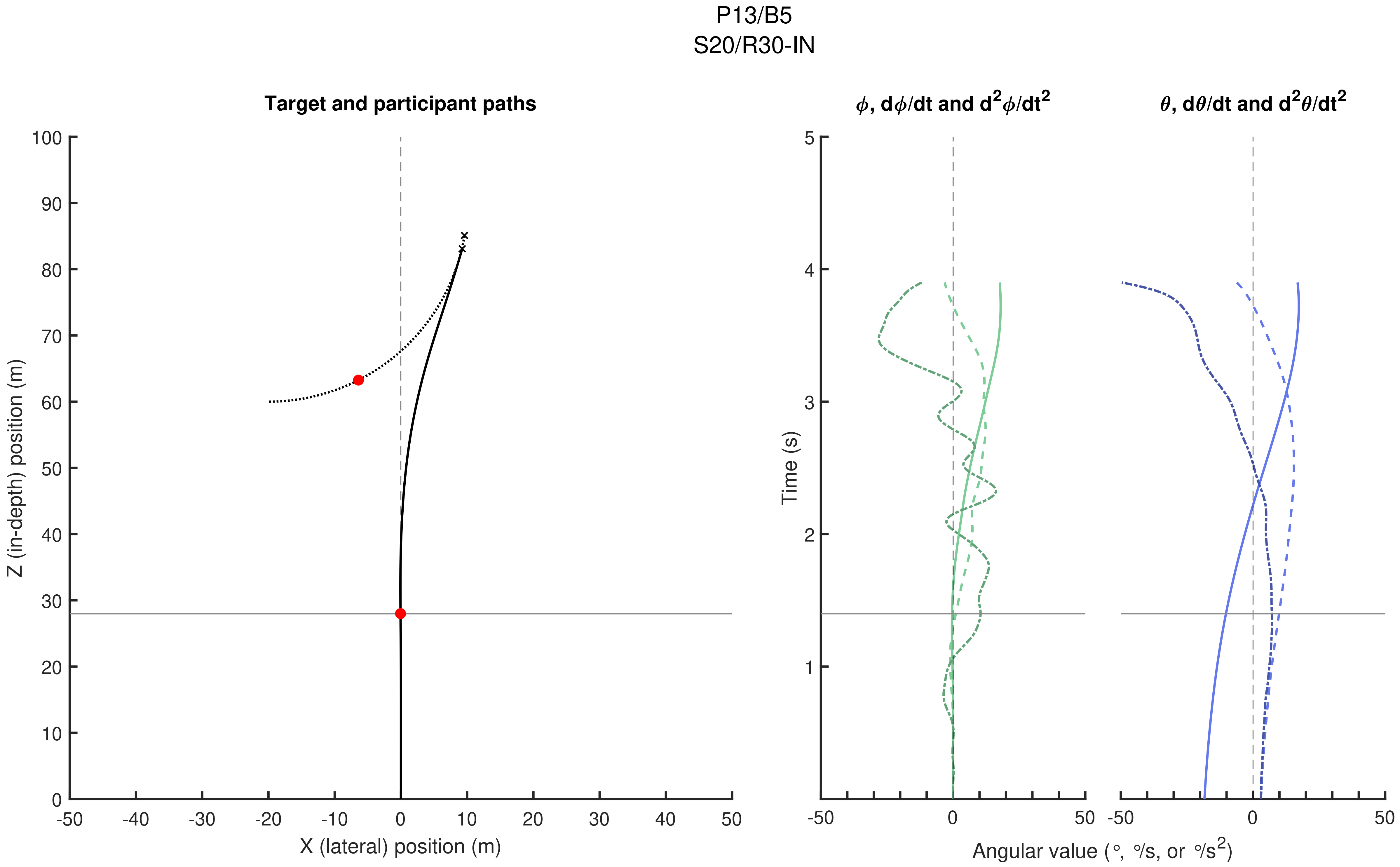

P13/B5
S20/R30-IN
Target and participant paths
ϕ, dϕ/dt and d$^2\phi$/dt^2
θ, dθ/dt and d$^2\theta$/dt^2
Z (in-depth) position (m)
X (lateral) position (m)
Time (s)
Angular value (°, °/s, or °/s^2)

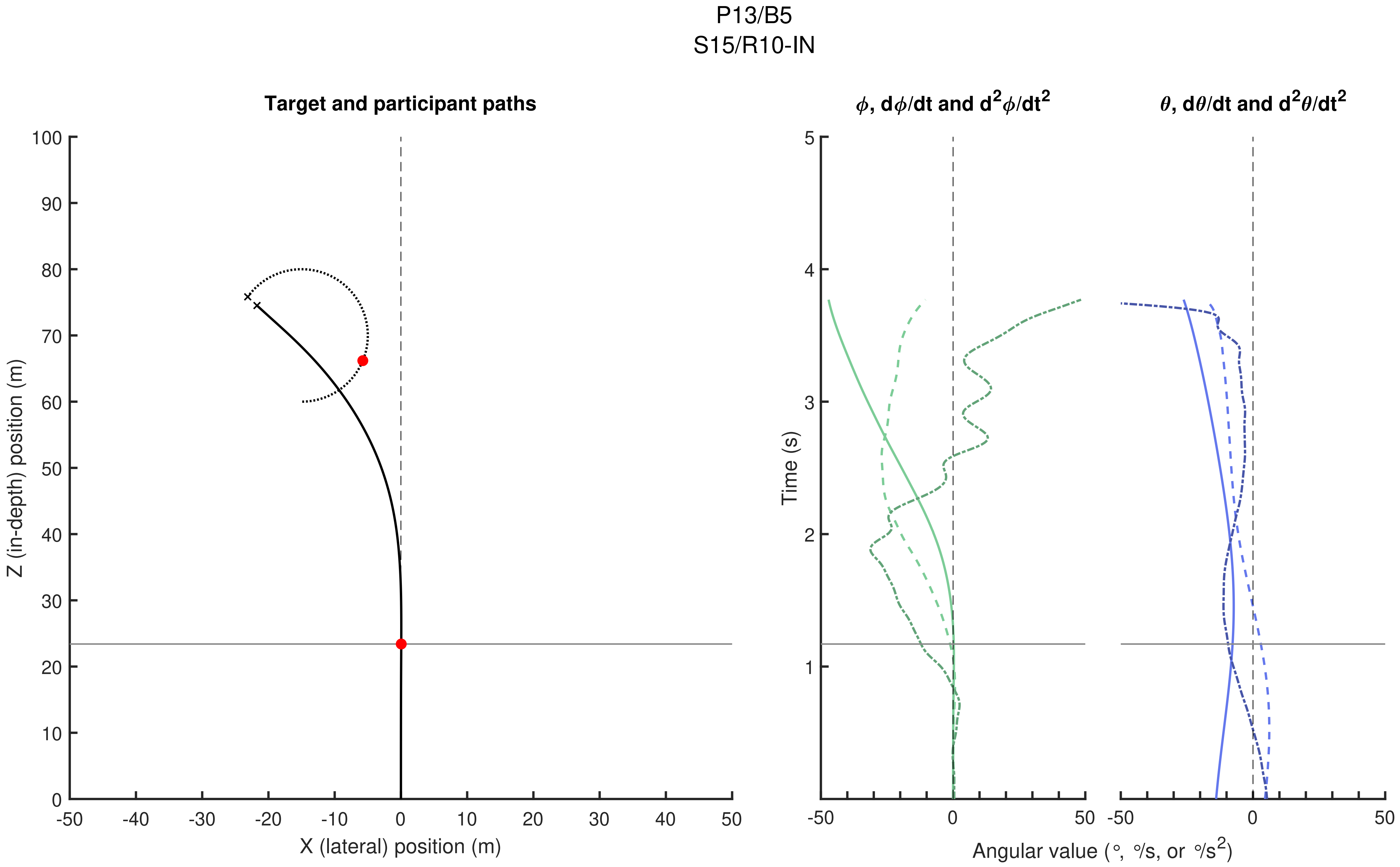

P13/B5
S15/R10-IN
Target and participant paths
Z (in-depth) position (m)
X (lateral) position (m)
100
90
80
70
60
50
40
30
20
10
0
-50
-40
-30
-20
-10
0
10
20
30
40
50
ϕ, dϕ/dt and d²ϕ/dt²
θ, dθ/dt and d²θ/dt²
Time (s)
5
4
3
2
1
-50
0
50
-50
0
50
Angular value (°, °/s, or °/s²)

P13/B5
S15/R20-IN

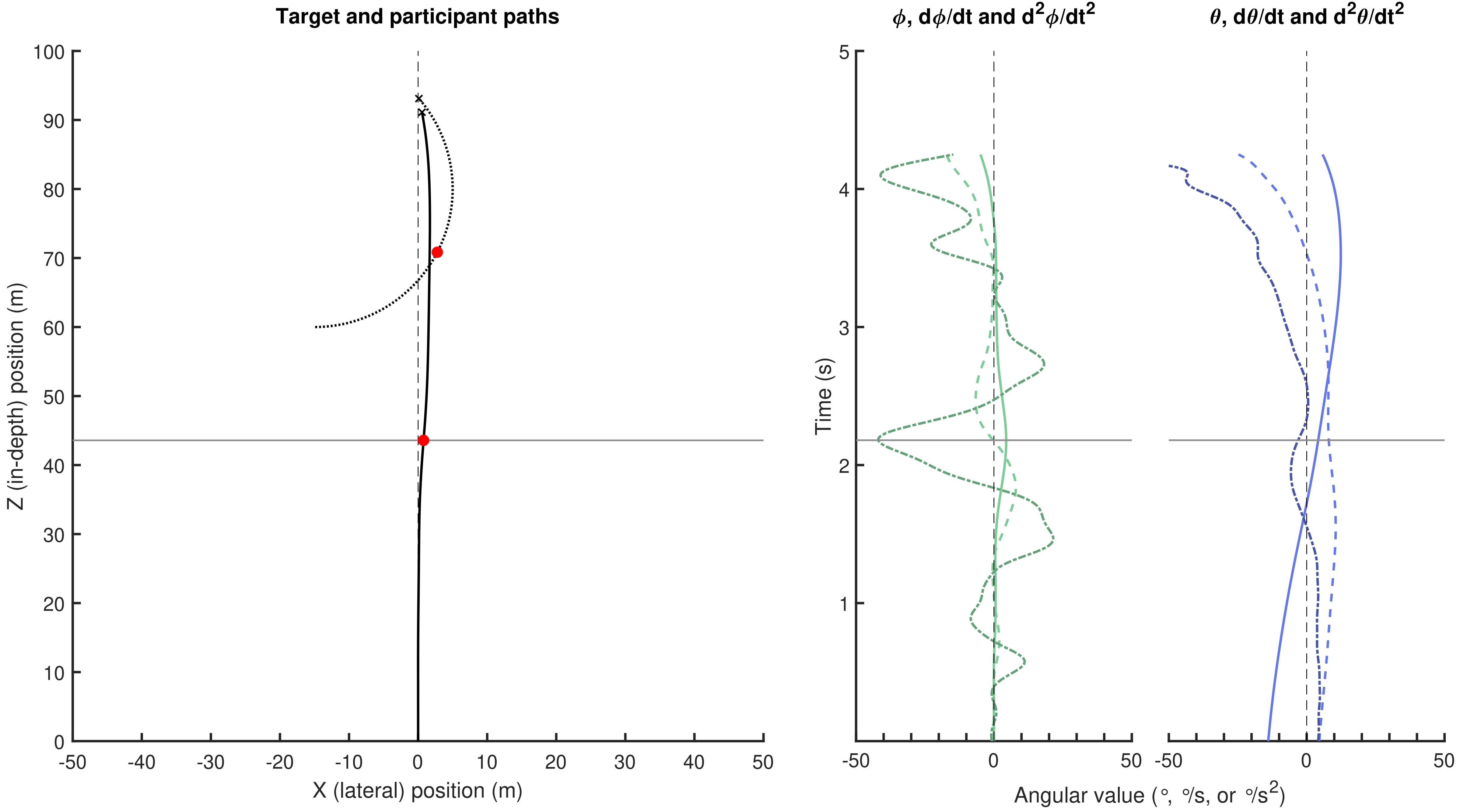

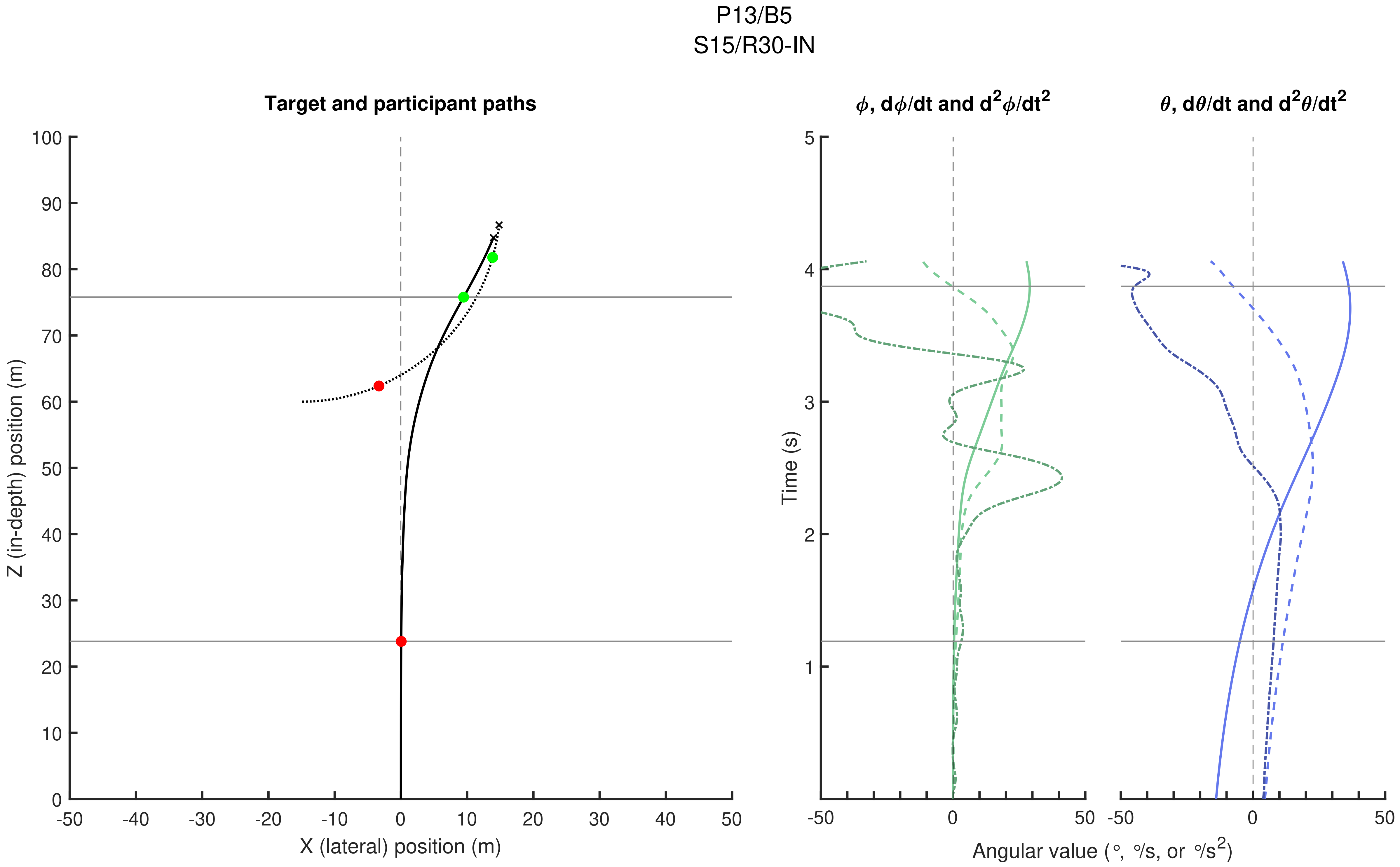

P13/B5
S15/R30-IN
Target and participant paths
Z (in-depth) position (m)
X (lateral) position (m)
ϕ, dϕ/dt and d$^2\phi$/dt^2
θ, dθ/dt and d$^2\theta$/dt^2
Time (s)
Angular value (°, °/s, or °/s^2)

P13/B5
S5/R10-IN

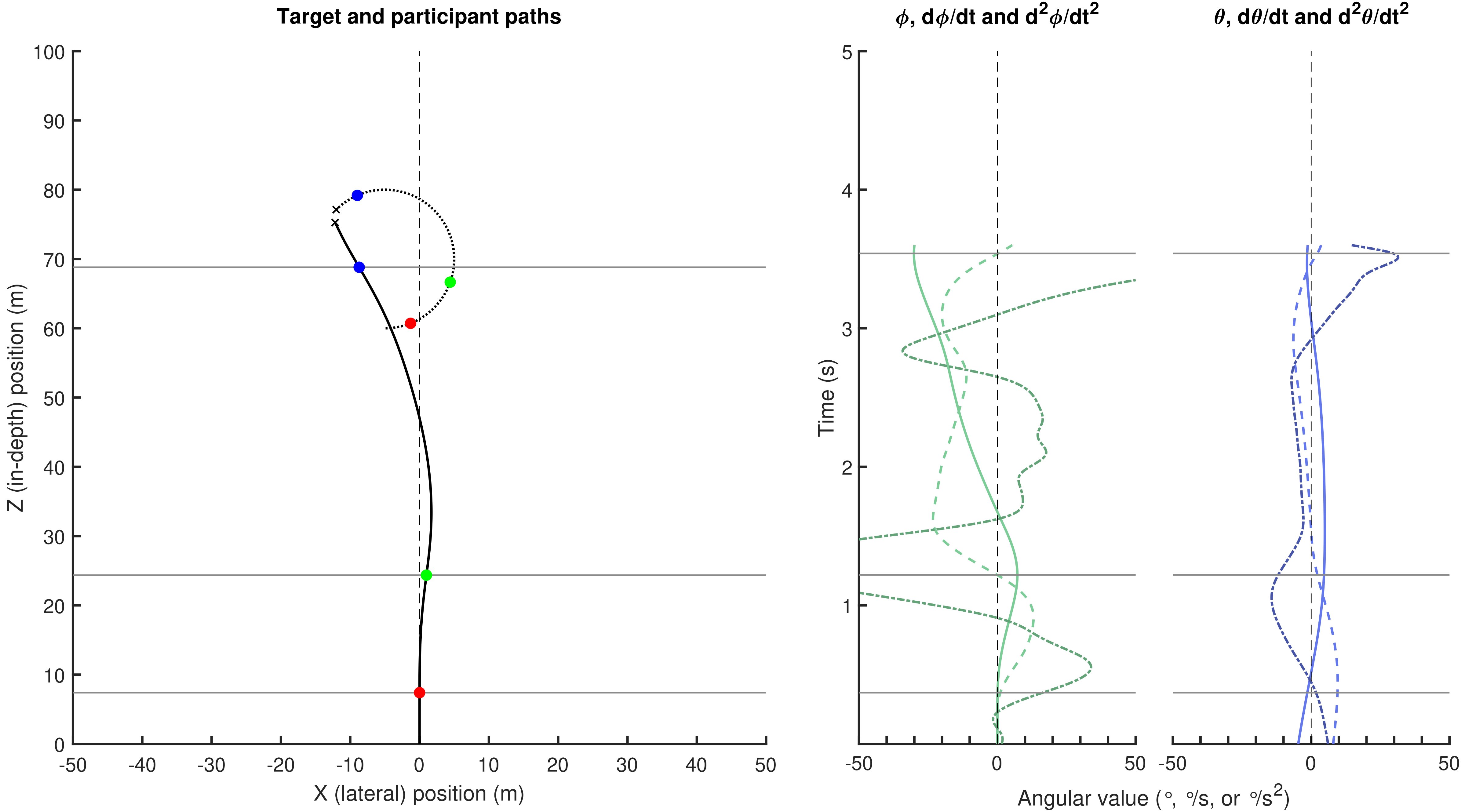

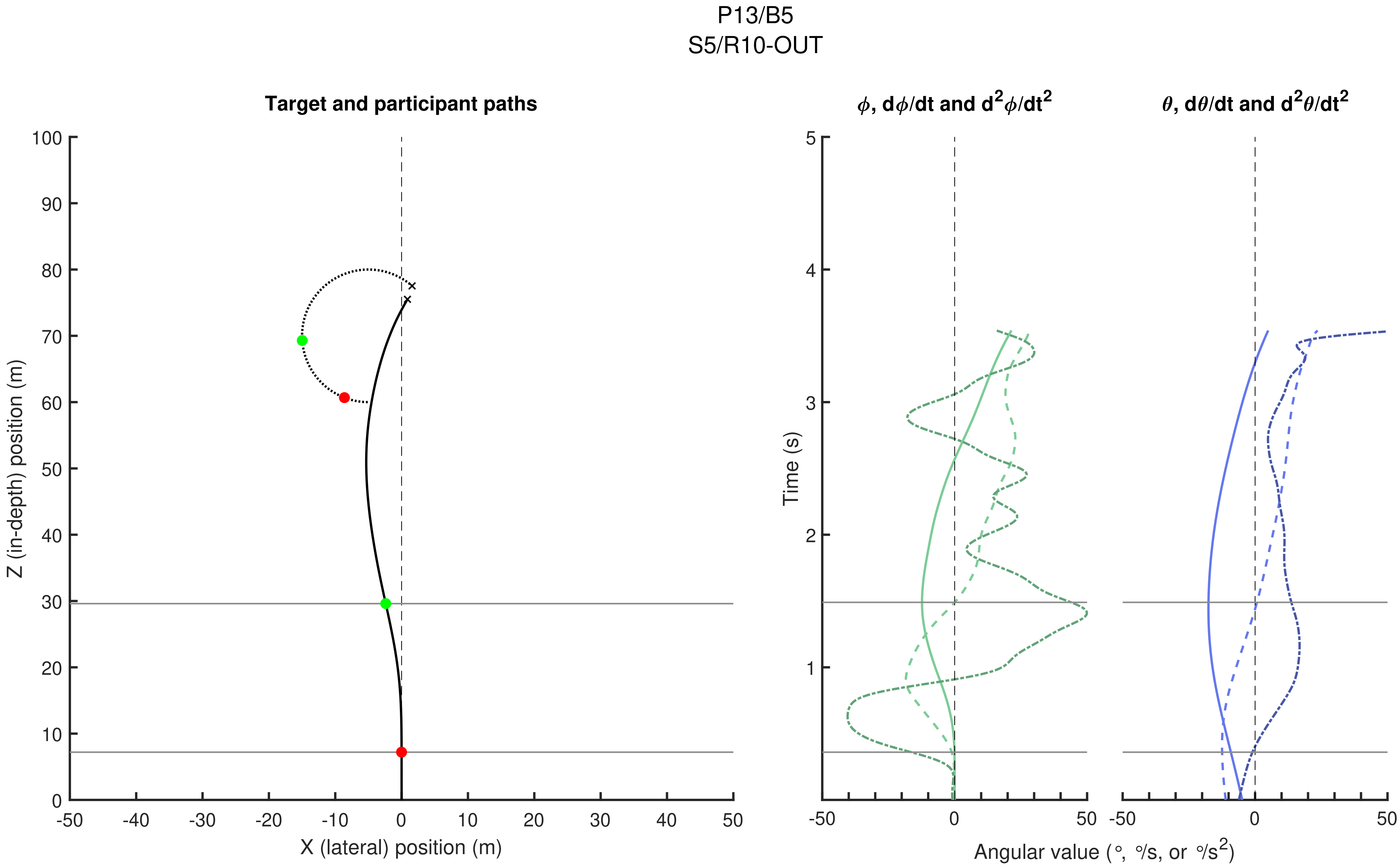
P13/B5
S5/R10-OUT
Target and participant paths
Z (in-depth) position (m)
X (lateral) position (m)
ϕ, dϕ/dt and d$^2\phi$/dt^2
θ, dθ/dt and d$^2\theta$/dt^2
Time (s)
Angular value (°, °/s, or °/s^2)

P13/B5
S5/R20-IN

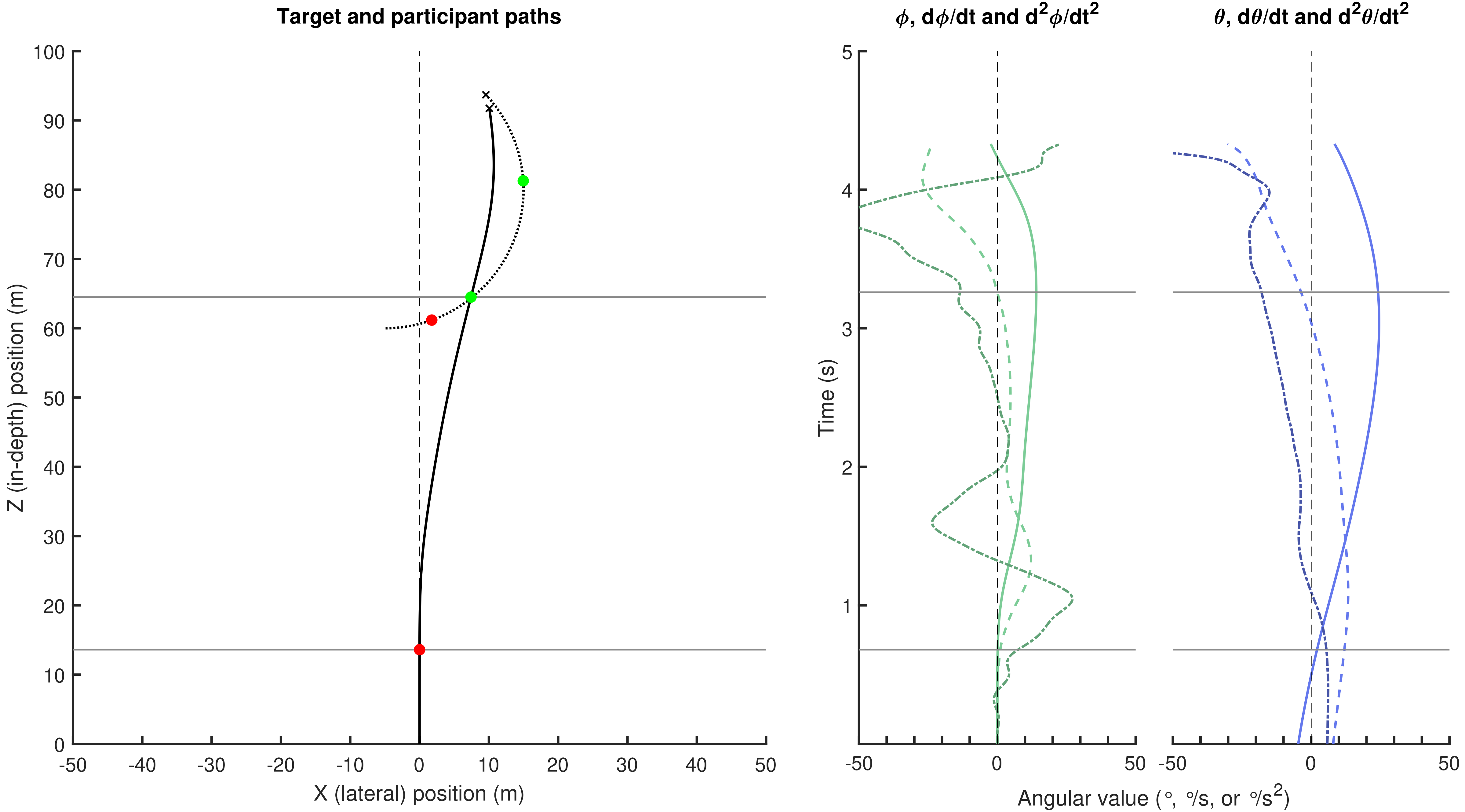

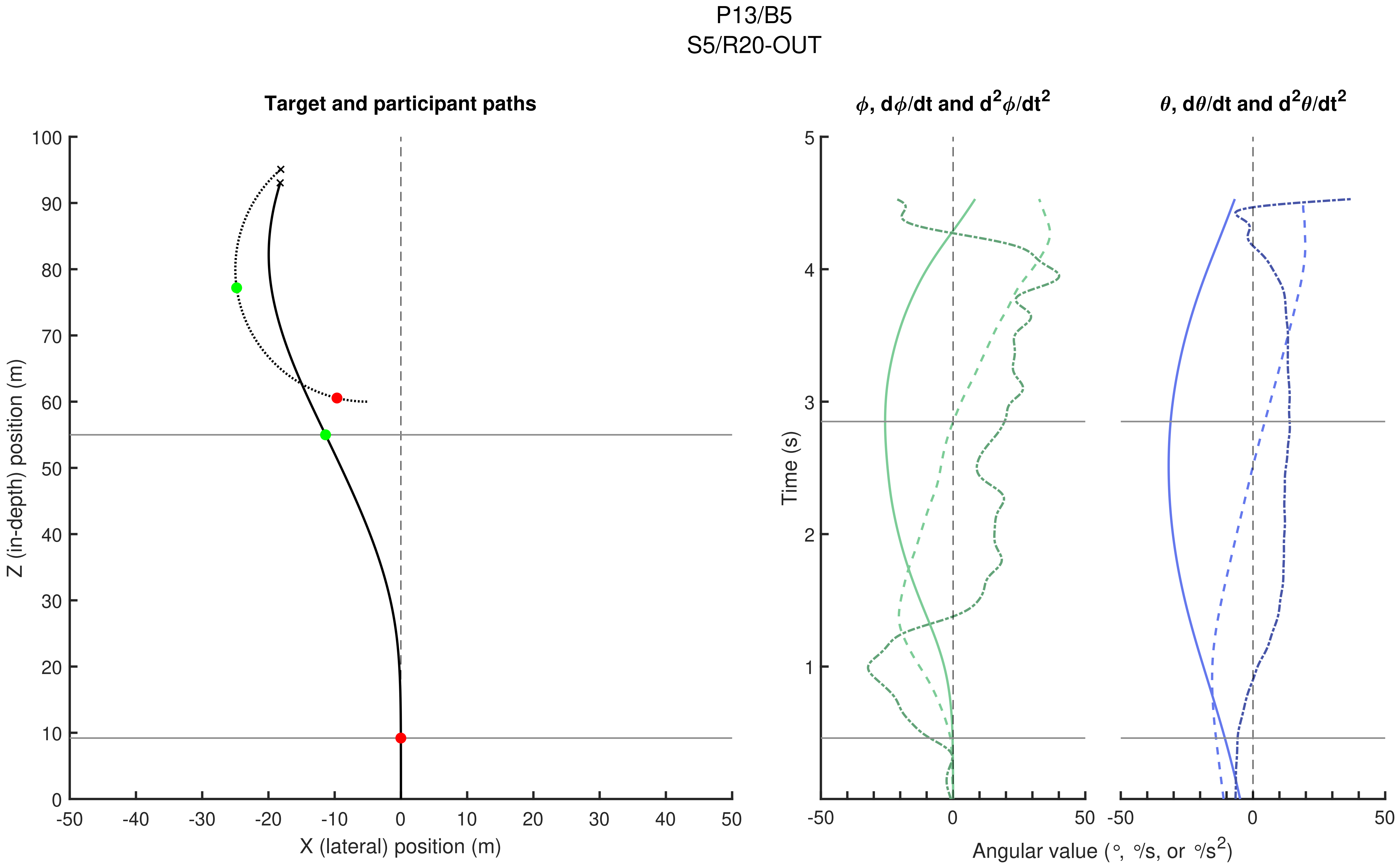

P13/B5
S5/R20-OUT
Target and participant paths
Z (in-depth) position (m)
X (lateral) position (m)
ϕ, dϕ/dt and d$^2\phi$/dt^2
θ, dθ/dt and d$^2\theta$/dt^2
Time (s)
Angular value (°, °/s, or °/s^2)

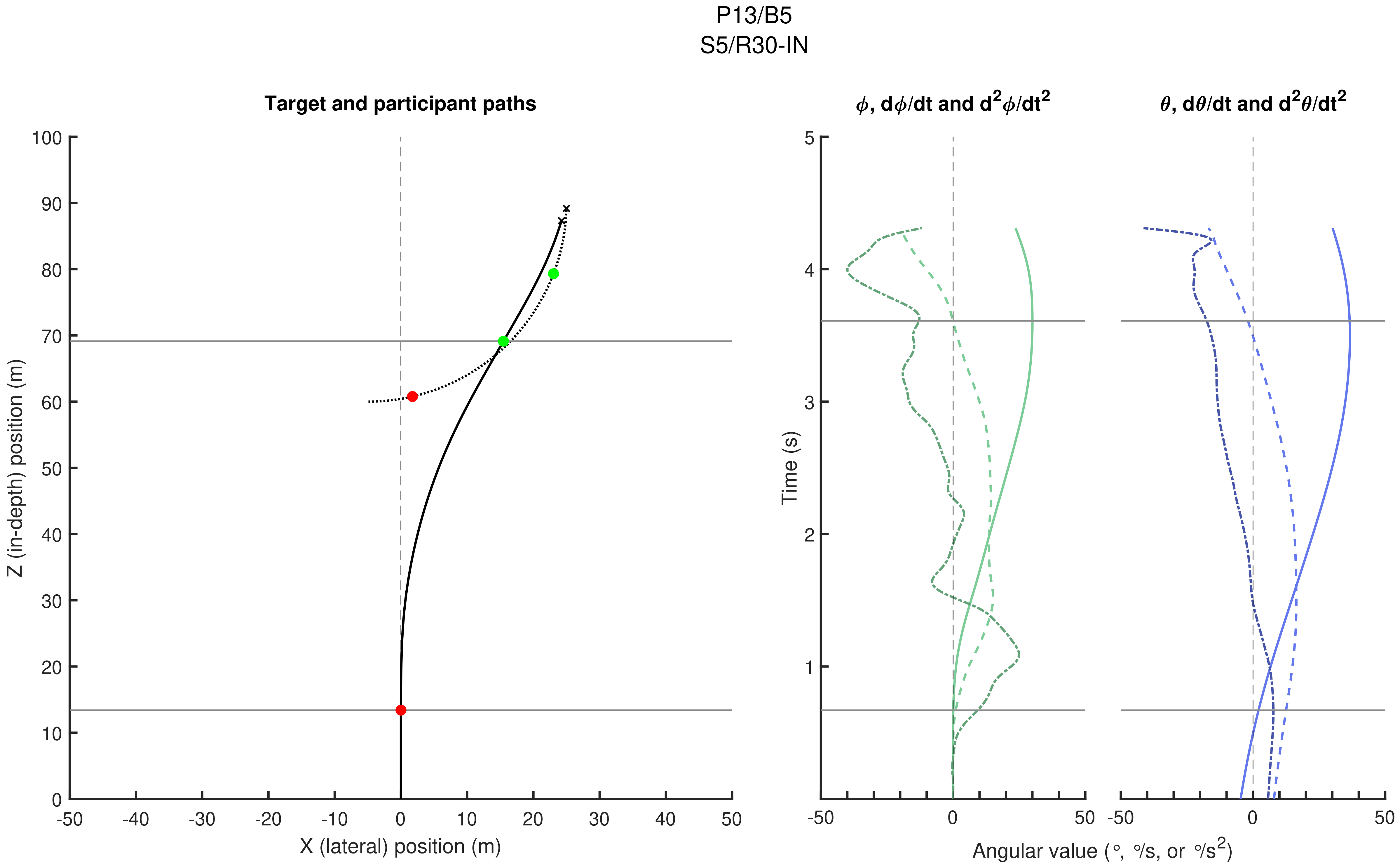

P13/B5
S5/R30-IN
Target and participant paths
Z (in-depth) position (m)
X (lateral) position (m)
ϕ, dϕ/dt and d$^2\phi$/dt^2
θ, dθ/dt and d$^2\theta$/dt^2
Time (s)
Angular value (°, °/s, or °/s^2)

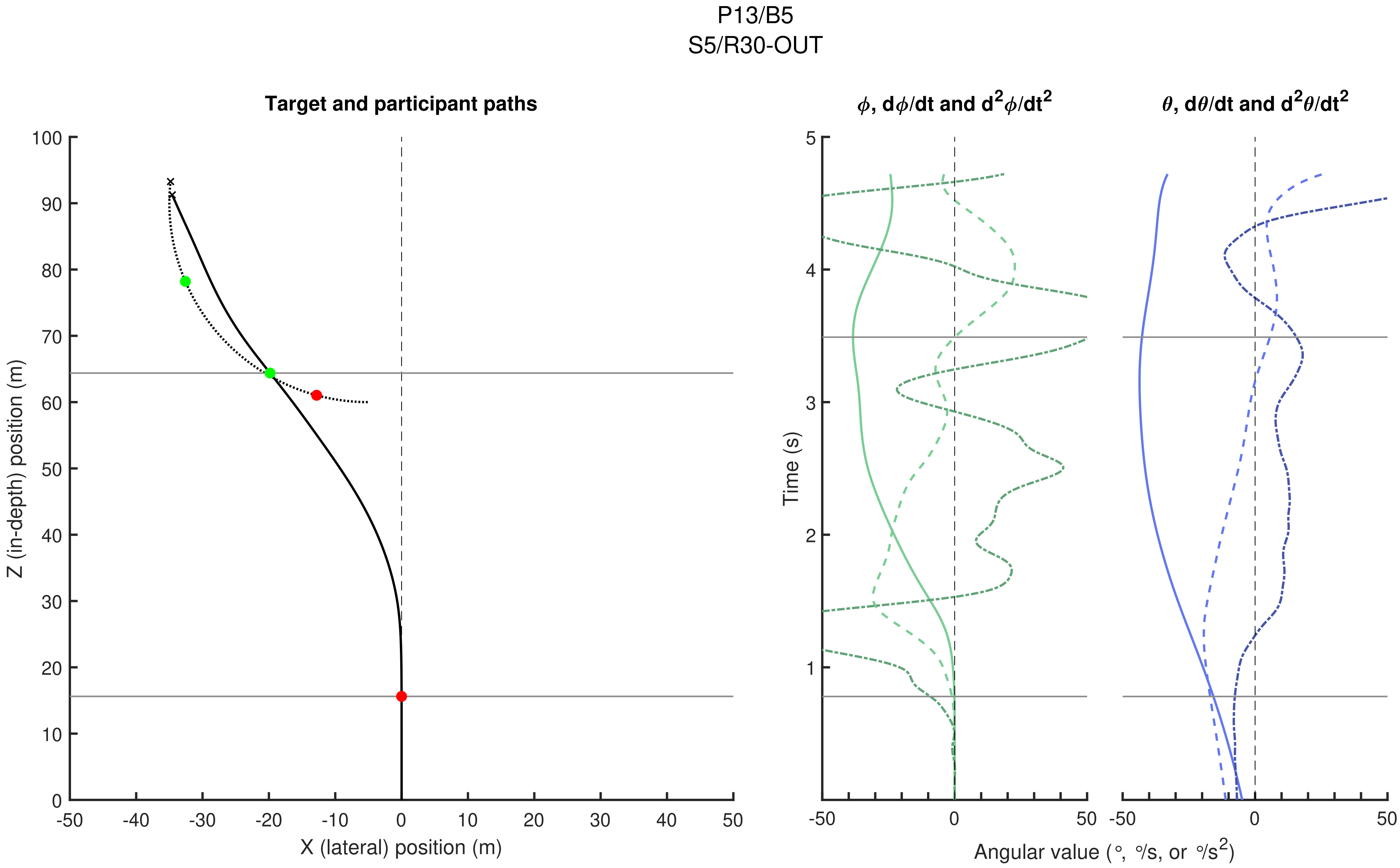
P13/B5
S5/R30-OUT
Target and participant paths
Z (in-depth) position (m)
X (lateral) position (m)
100
90
80
70
60
50
40
30
20
10
0
-50
-40
-30
-20
-10
0
10
20
30
40
50
ϕ, dϕ/dt and d$^2\phi$/dt^2
θ, dθ/dt and d$^2\theta$/dt^2
Time (s)
5
4
3
2
1
-50
0
50
-50
0
50
Angular value (°, °/s, or °/s^2)

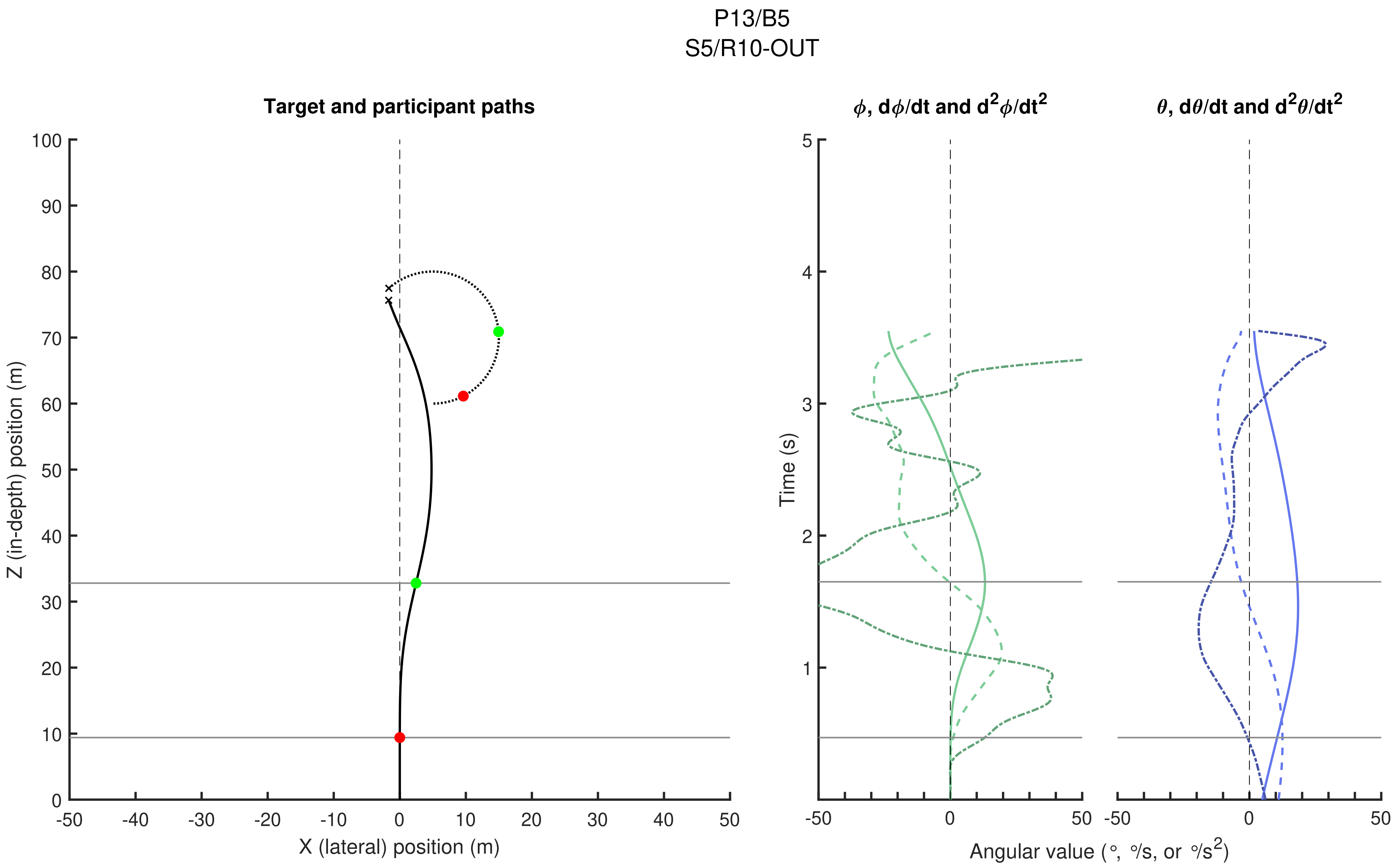
P13/B5
S5/R10-OUT
Target and participant paths
ϕ, dϕ/dt and d$^2\phi$/dt^2
θ, dθ/dt and d$^2\theta$/dt^2
Z (in-depth) position (m)
X (lateral) position (m)
Time (s)
Angular value (°, °/s, or °/s^2)
100
90
80
70
60
50
40
30
20
10
0
-50
-40
-30
-20
-10
0
10
20
30
40
50
5
4
3
2
1
-50
0
50
-50
0
50

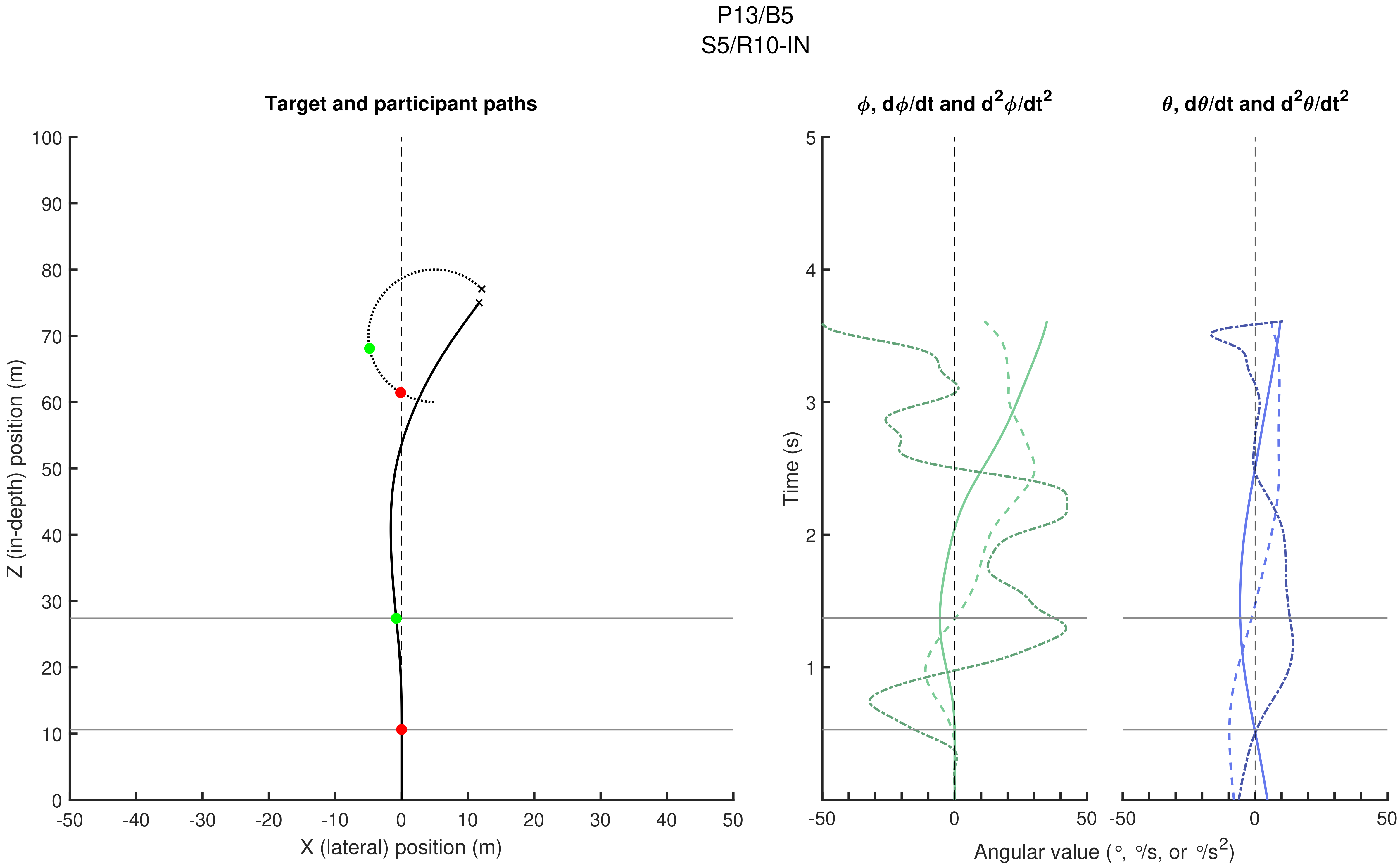
P13/B5
S5/R10-IN
Target and participant paths
ϕ, dϕ/dt and d²ϕ/dt²
θ, dθ/dt and d²θ/dt²
Z (in-depth) position (m)
X (lateral) position (m)
Time (s)
Angular value (°, °/s, or °/s²)
100
90
80
70
60
50
40
30
20
10
0
-50
-40
-30
-20
-10
0
10
20
30
40
50
5
4
3
2
1
-50
0
50
-50
0
50

P13/B5
S5/R20-OUT

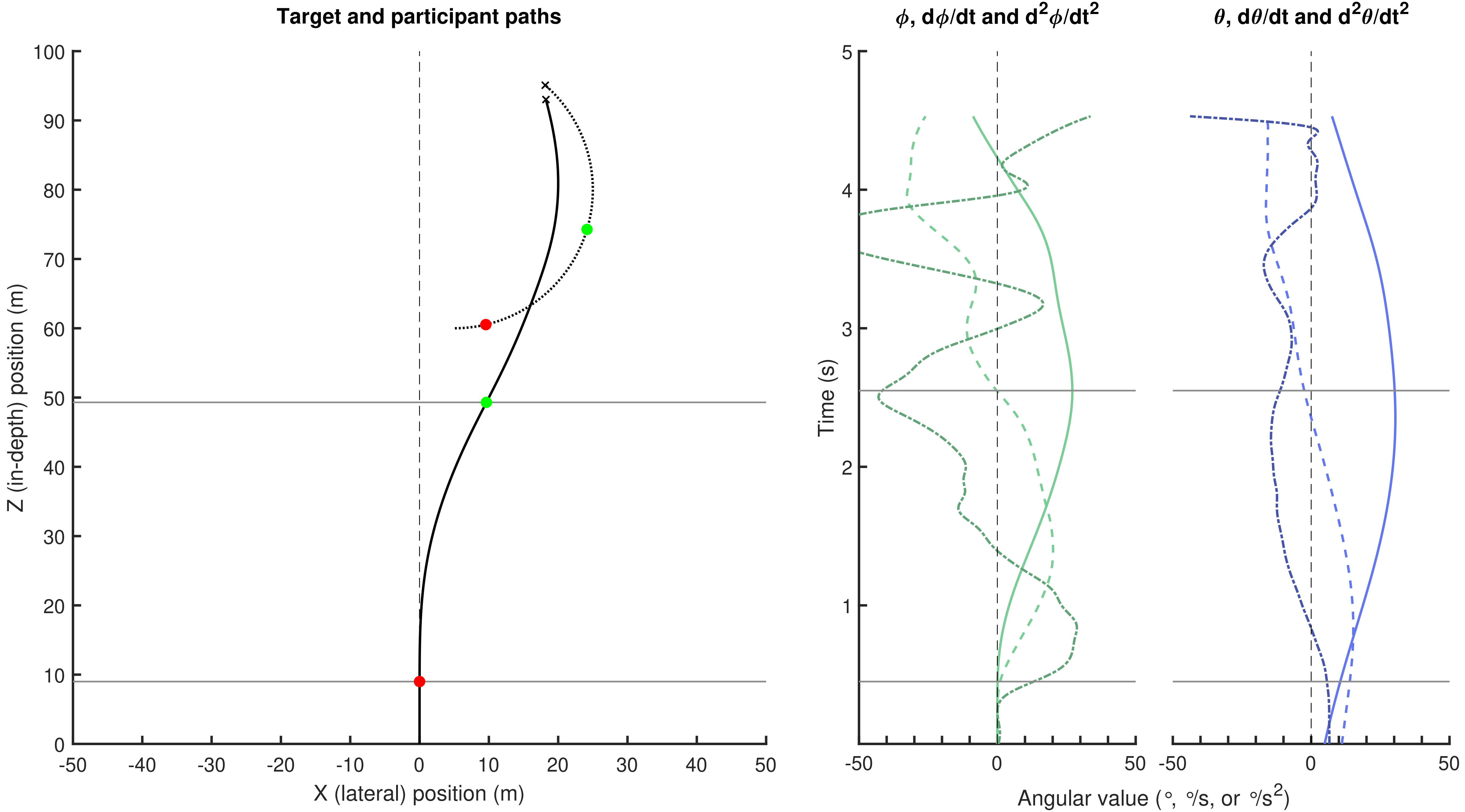

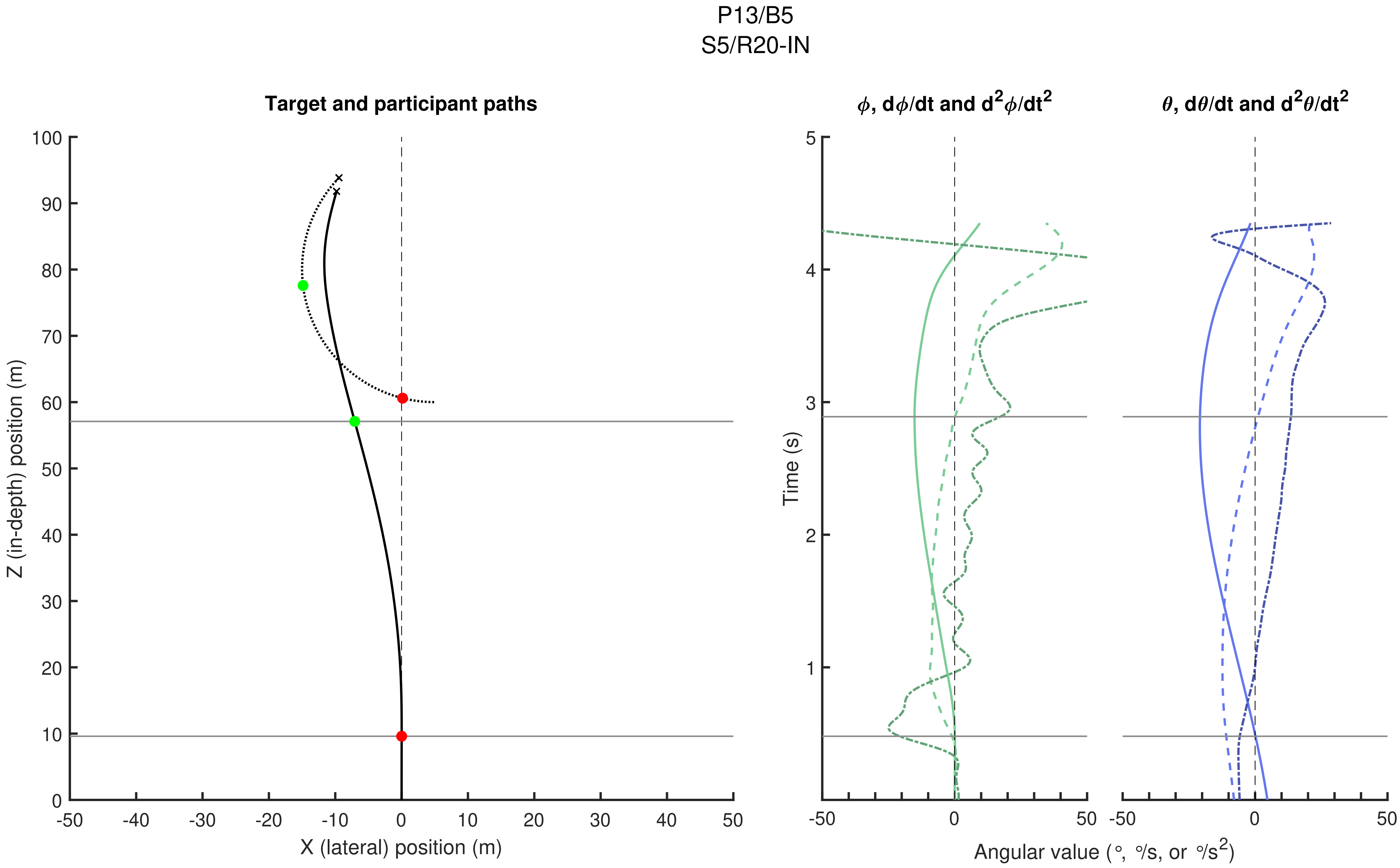

P13/B5
S5/R20-IN
Target and participant paths
Z (in-depth) position (m)
X (lateral) position (m)
ϕ, dϕ/dt and d$^2\phi$/dt^2
θ, dθ/dt and d$^2\theta$/dt^2
Time (s)
Angular value (°, °/s, or °/s^2)

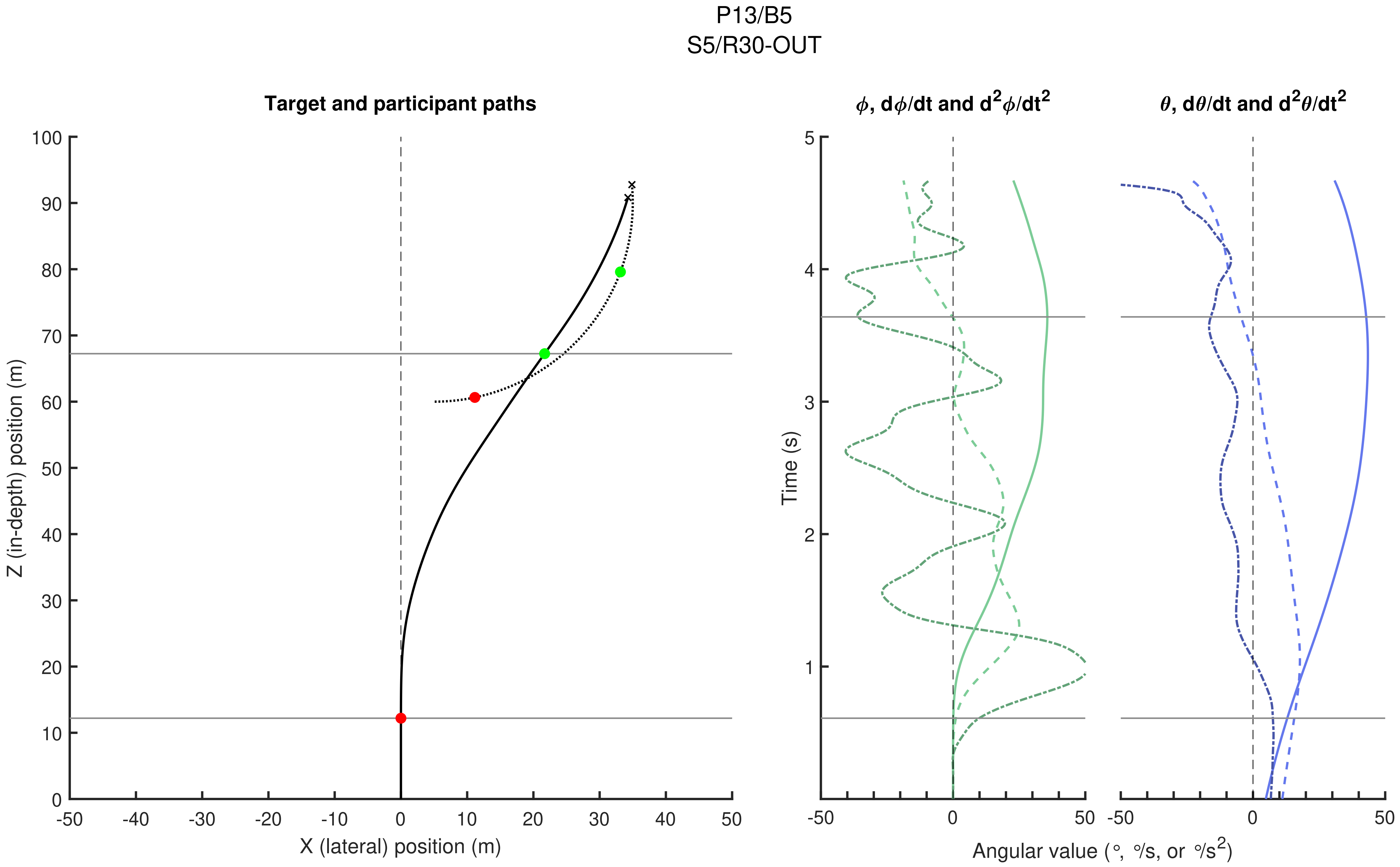

P13/B5
S5/R30-OUT
Target and participant paths
Z (in-depth) position (m)
X (lateral) position (m)
100
90
80
70
60
50
40
30
20
10
0
-50
-40
-30
-20
-10
0
10
20
30
40
50
ϕ, dϕ/dt and d$^2\phi$/dt^2
θ, dθ/dt and d$^2\theta$/dt^2
Time (s)
5
4
3
2
1
-50
0
50
-50
0
50
Angular value (°, °/s, or °/s^2)

P13/B5
S5/R30-IN

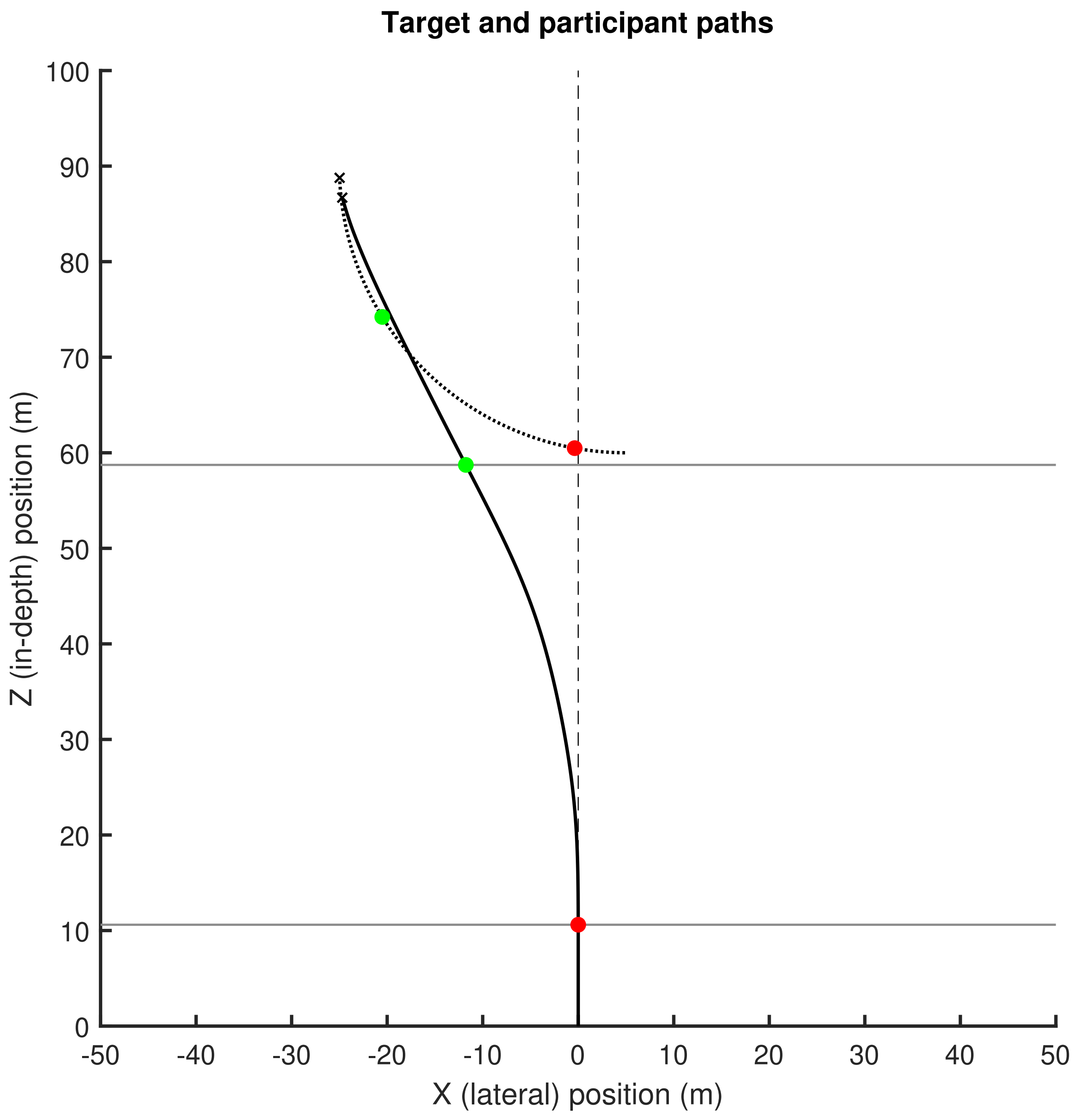

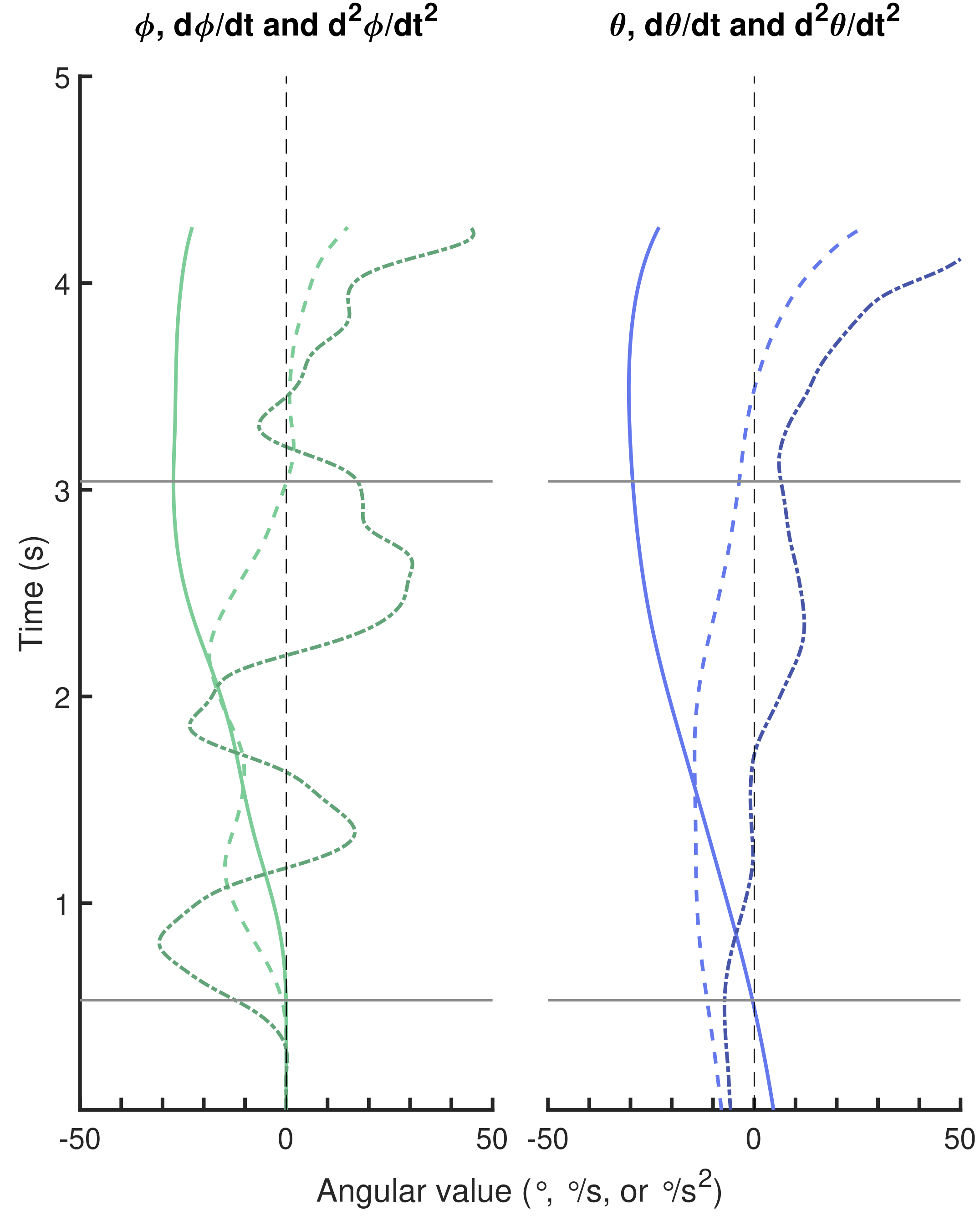

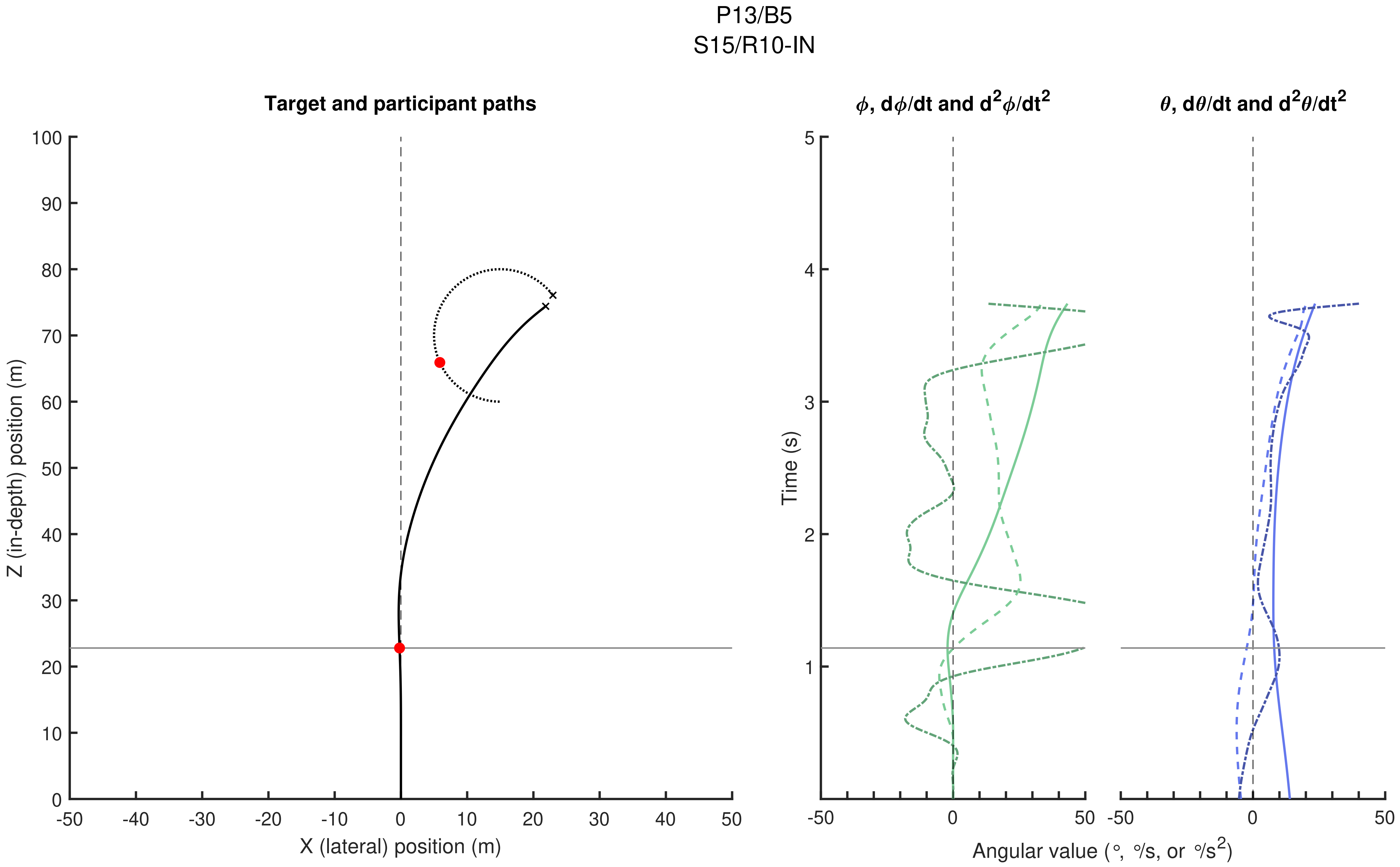
P13/B5
S15/R10-IN
Target and participant paths
Z (in-depth) position (m)
X (lateral) position (m)
ϕ, dϕ/dt and d²ϕ/dt²
θ, dθ/dt and d²θ/dt²
Time (s)
Angular value (°, °/s, or °/s²)

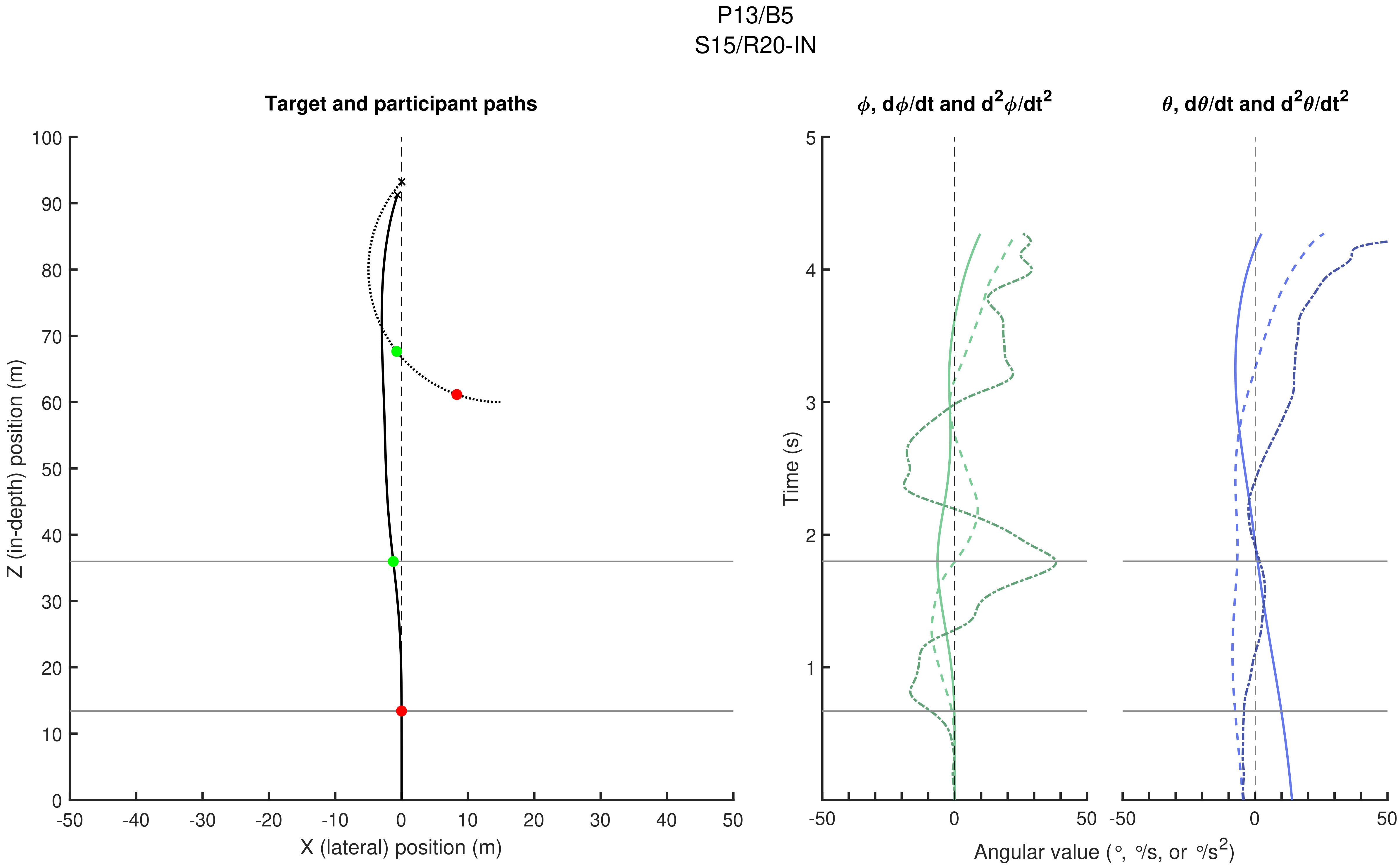
P13/B5
S15/R20-IN
Target and participant paths
Z (in-depth) position (m)
X (lateral) position (m)
ϕ, dϕ/dt and d$^2\phi$/dt^2
θ, dθ/dt and d$^2\theta$/dt^2
Time (s)
Angular value (°, °/s, or °/s^2)

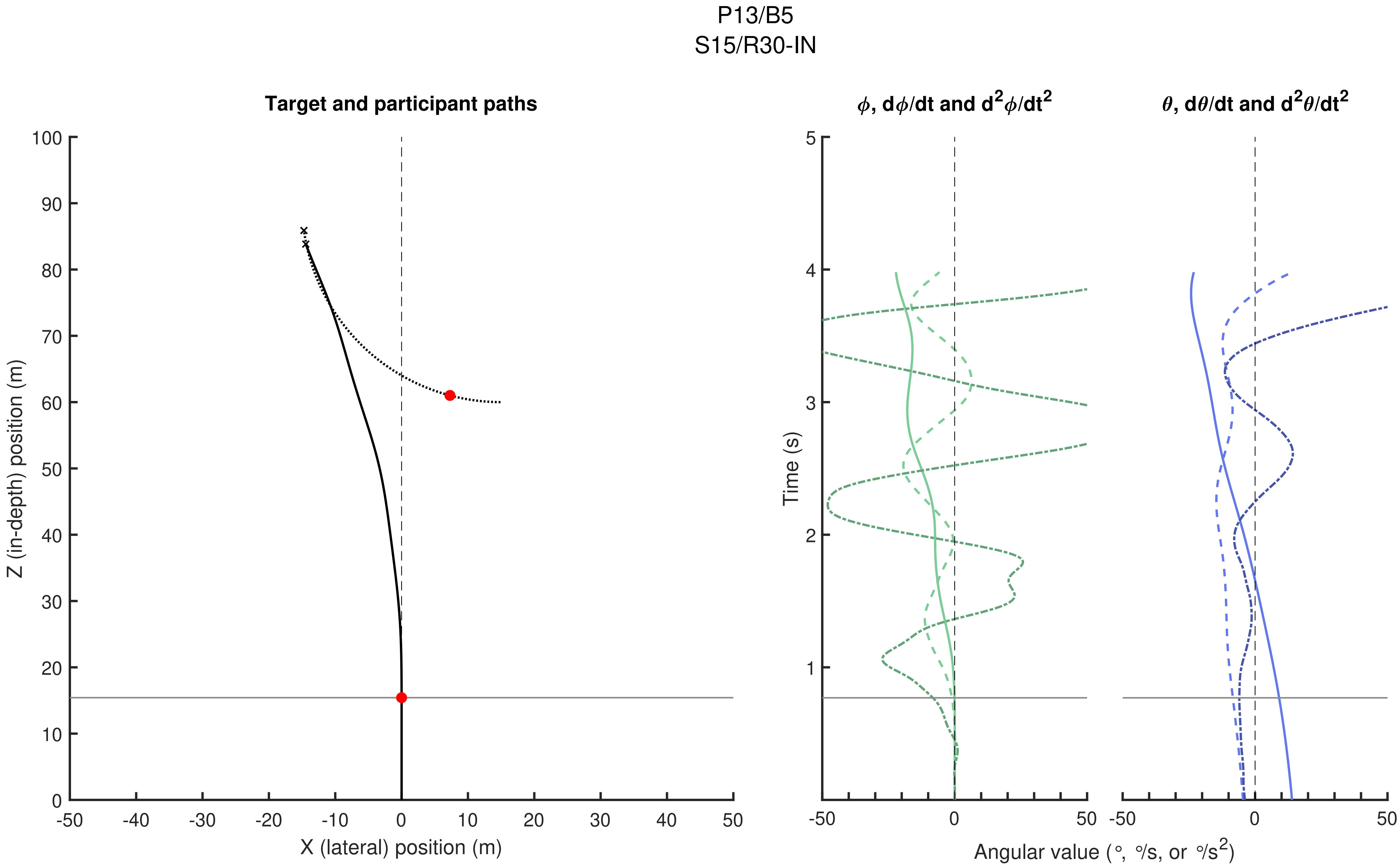
P13/B5
S15/R30-IN
Target and participant paths
Z (in-depth) position (m)
X (lateral) position (m)
φ, dφ/dt and d²φ/dt²
θ, dθ/dt and d²θ/dt²
Time (s)
Angular value (°, °/s, or °/s²)

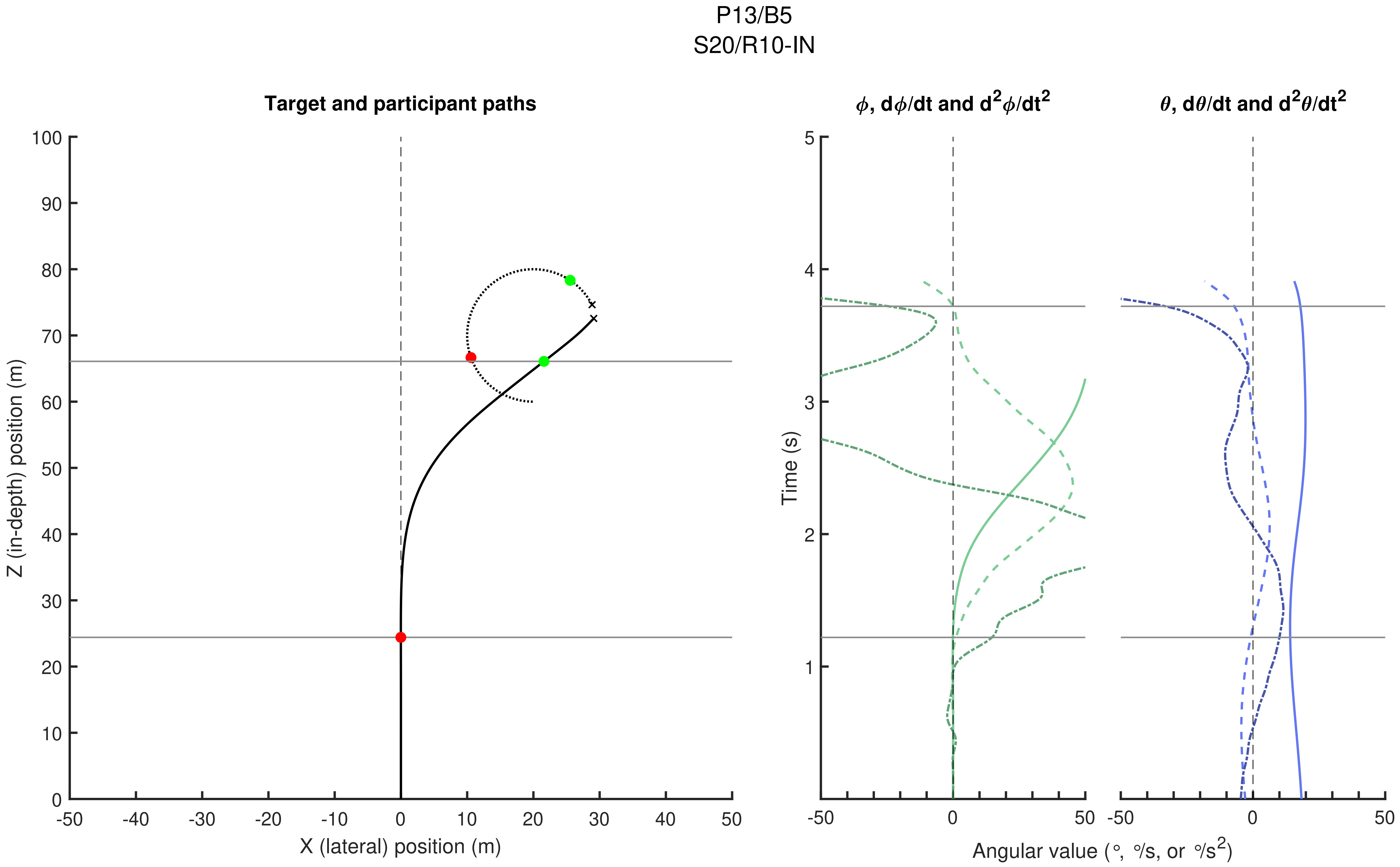
P13/B5
S20/R10-IN
Target and participant paths
Z (in-depth) position (m)
X (lateral) position (m)
100
90
80
70
60
50
40
30
20
10
0
-50
-40
-30
-20
-10
0
10
20
30
40
50
ϕ, dϕ/dt and d$^2\phi$/dt^2
θ, dθ/dt and d$^2\theta$/dt^2
Time (s)
5
4
3
2
1
-50
0
50
-50
0
50
Angular value (°, °/s, or °/s^2)

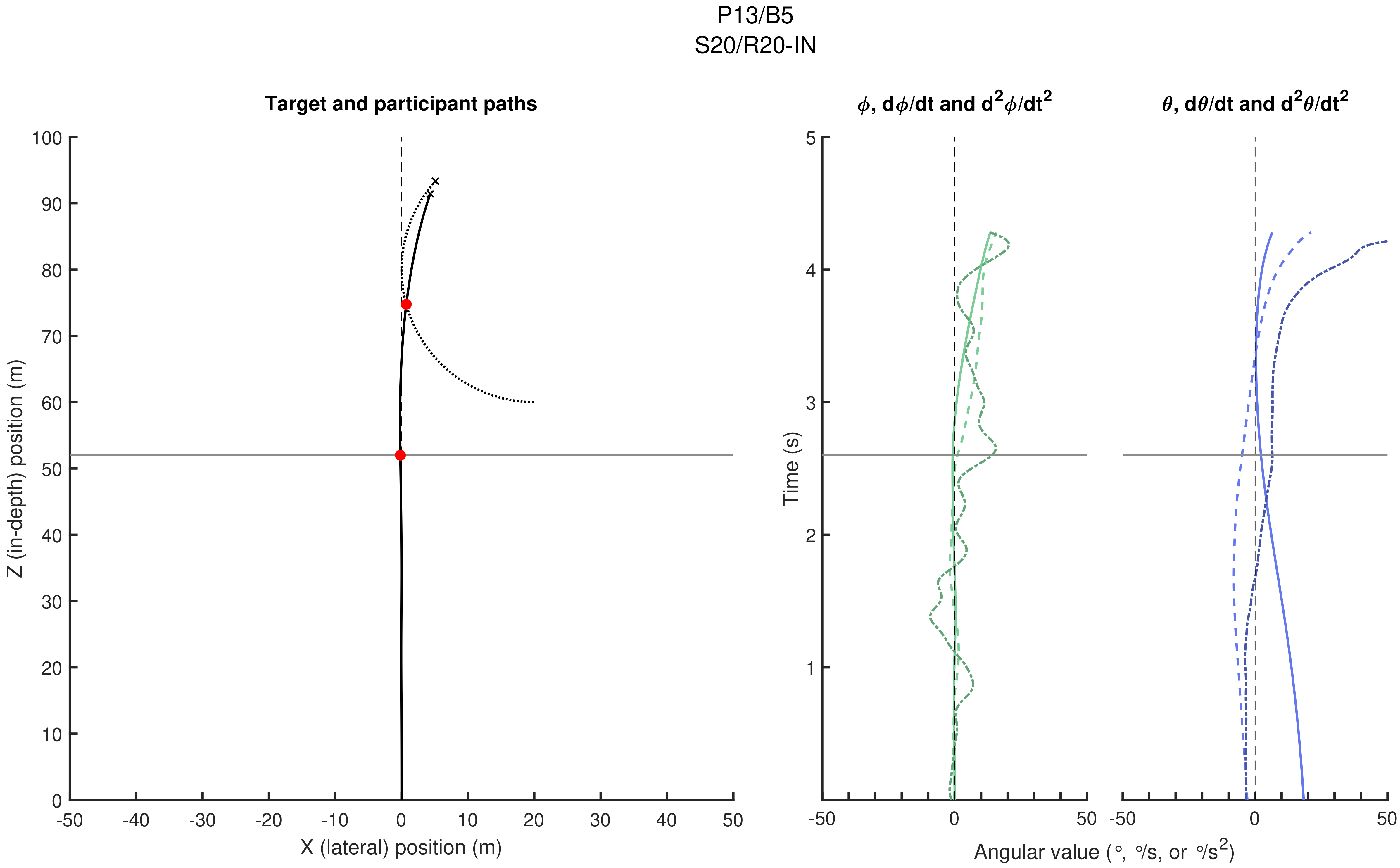
P13/B5
S20/R20-IN
Target and participant paths
ϕ, dϕ/dt and d$^2\phi$/dt^2
θ, dθ/dt and d$^2\theta$/dt^2
Z (in-depth) position (m)
X (lateral) position (m)
Time (s)
Angular value (°, °/s, or °/s^2)
100
90
80
70
60
50
40
30
20
10
0
-50
-40
-30
-20
-10
0
10
20
30
40
50
5
4
3
2
1
-50
0
50
-50
0
50

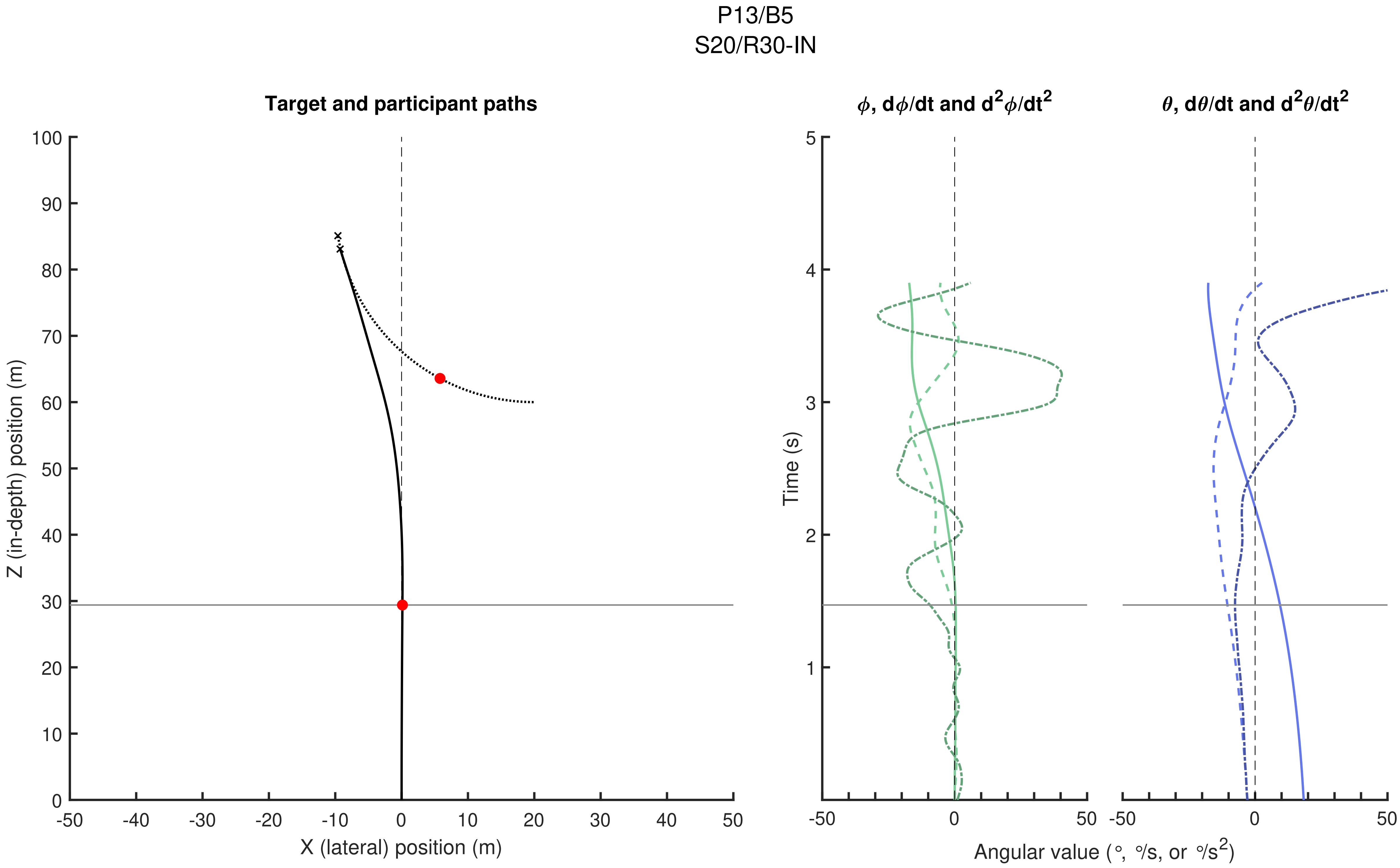
P13/B5
S20/R30-IN
Target and participant paths
Z (in-depth) position (m)
X (lateral) position (m)
ϕ, dϕ/dt and d²ϕ/dt²
θ, dθ/dt and d²θ/dt²
Time (s)
Angular value (°, °/s, or °/s²)

P14/B1
S20/R10-IN

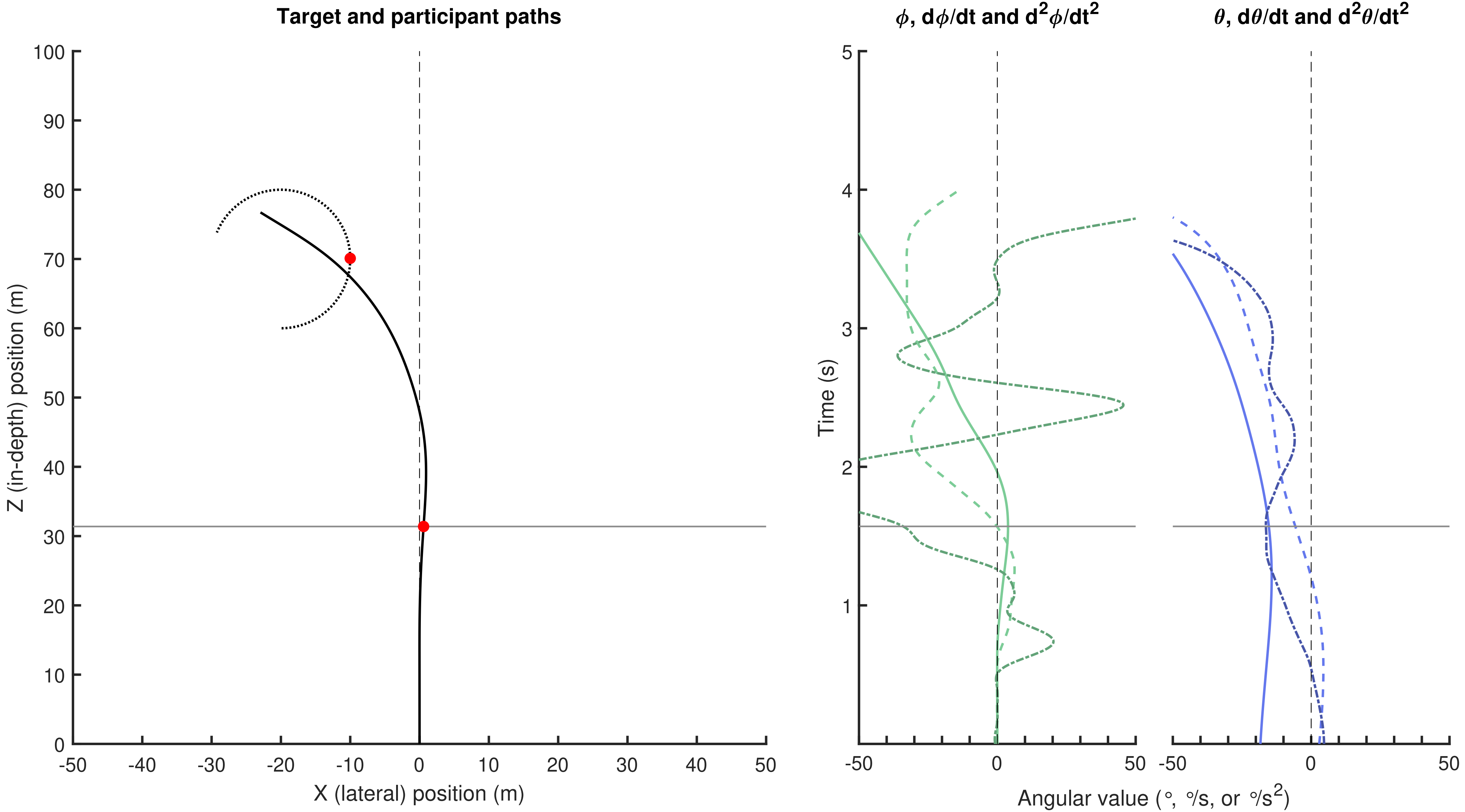

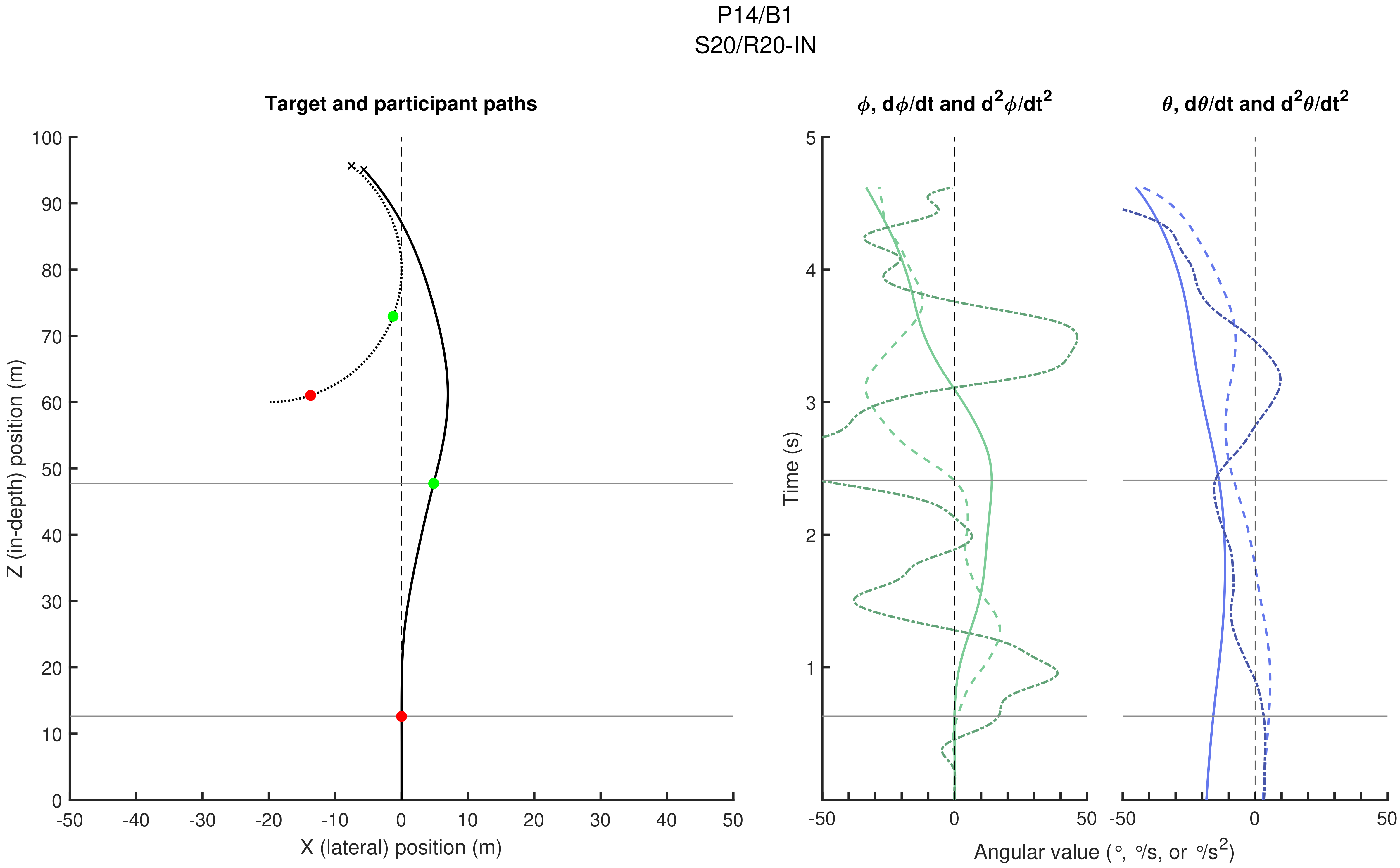

P14/B1
S20/R20-IN
Target and participant paths
Z (in-depth) position (m)
X (lateral) position (m)
ϕ, dϕ/dt and d$^2\phi$/dt^2
θ, dθ/dt and d$^2\theta$/dt^2
Time (s)
Angular value (°, °/s, or °/s^2)

P14/B1
S20/R30-IN

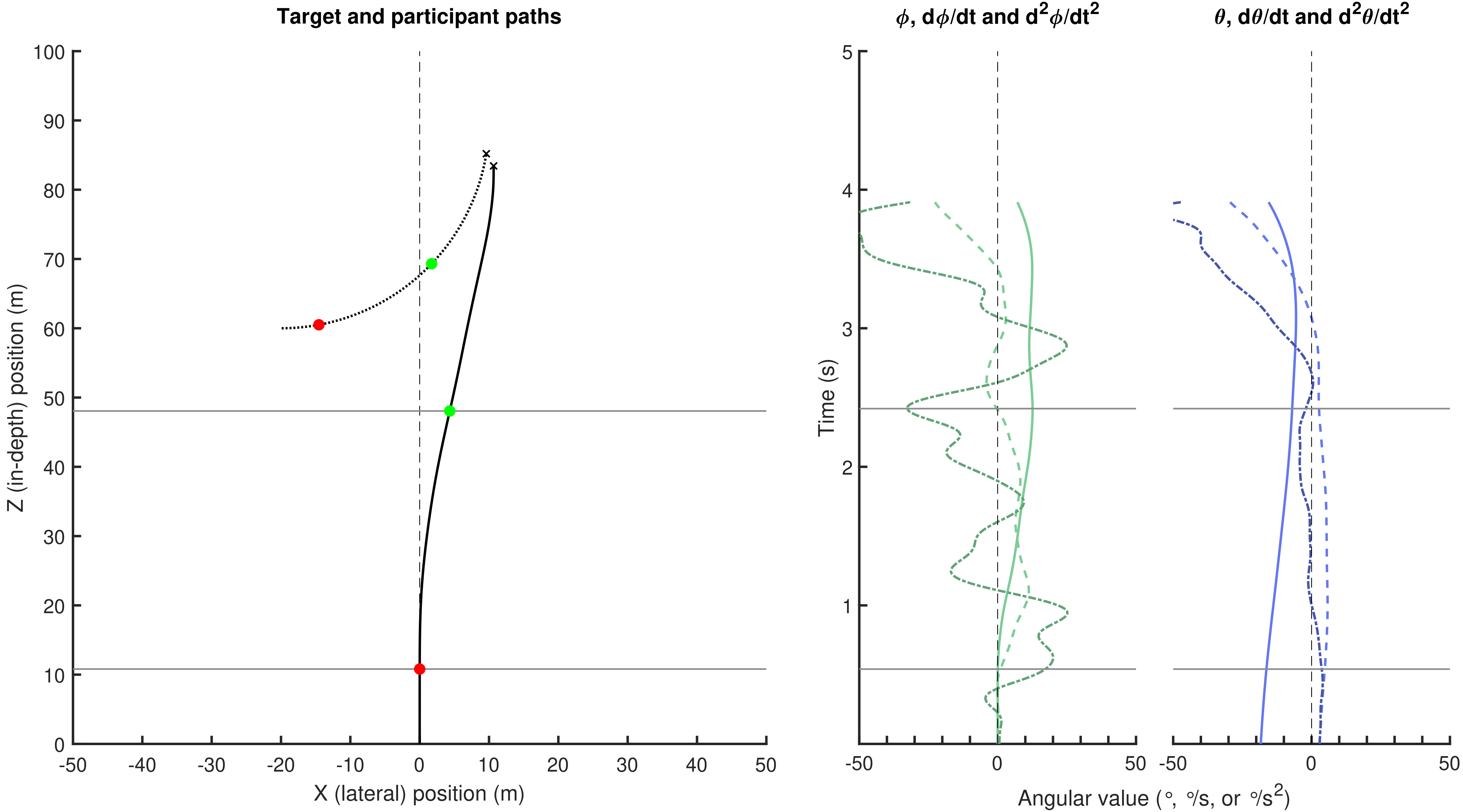

P14/B1
S15/R10-IN

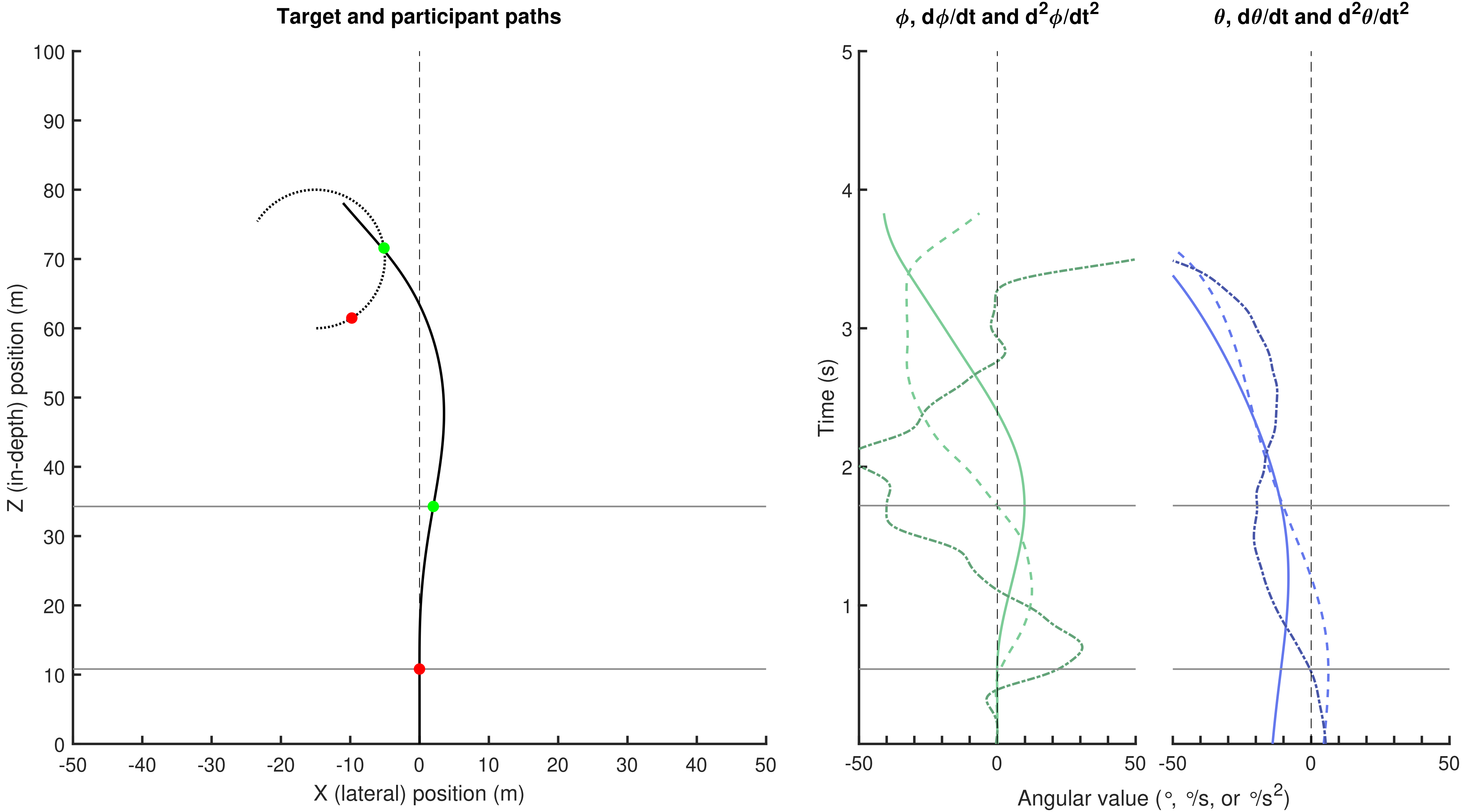

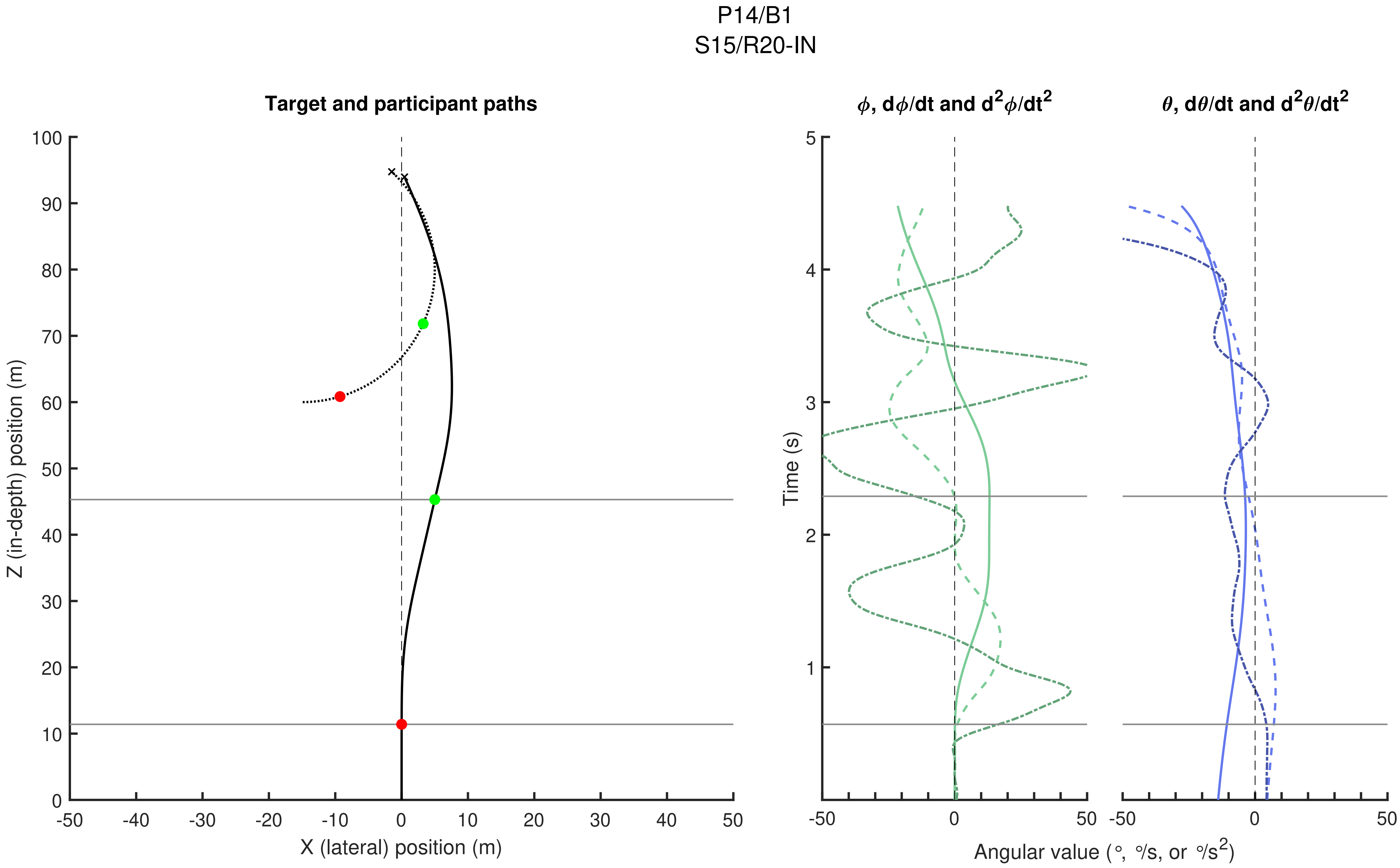

P14/B1
S15/R20-IN
Target and participant paths
Z (in-depth) position (m)
X (lateral) position (m)
φ, dφ/dt and d²φ/dt²
θ, dθ/dt and d²θ/dt²
Time (s)
Angular value (°, °/s, or °/s²)

P14/B1
S15/R30-IN

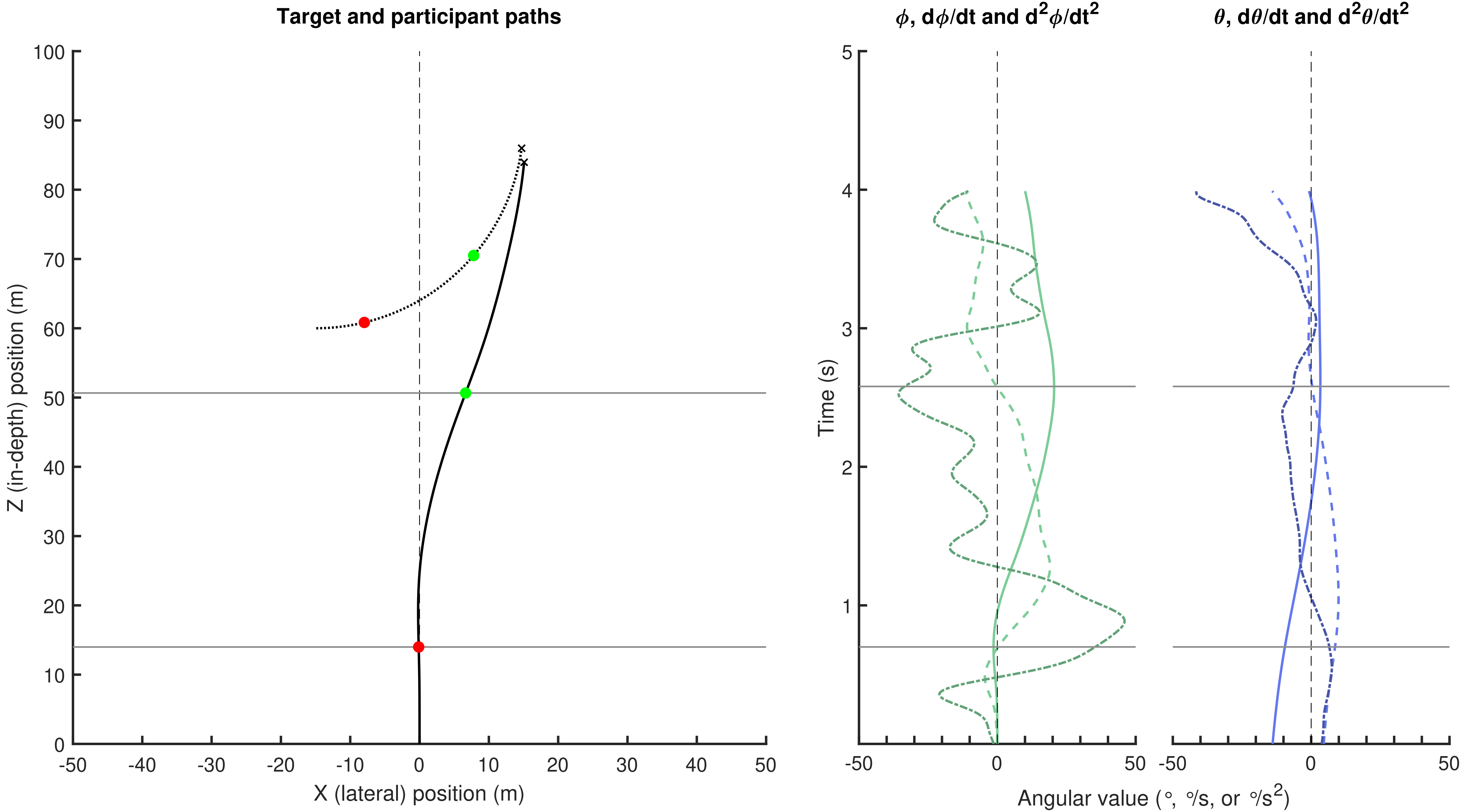

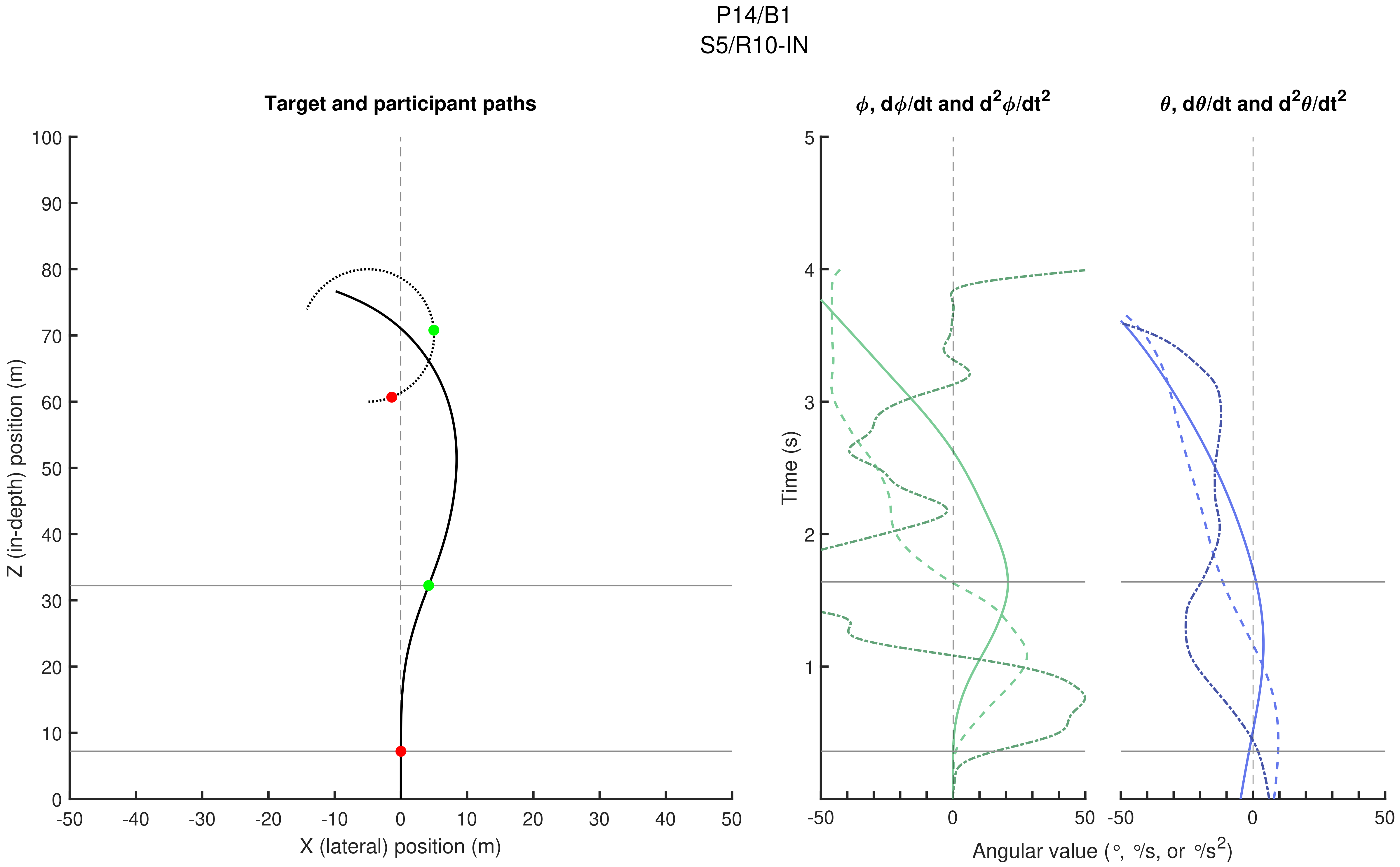
P14/B1
S5/R10-IN
Target and participant paths
Z (in-depth) position (m)
X (lateral) position (m)
ϕ, dϕ/dt and d$^2\phi$/dt^2
θ, dθ/dt and d$^2\theta$/dt^2
Time (s)
Angular value (°, °/s, or °/s^2)

P14/B1
S5/R10-OUT

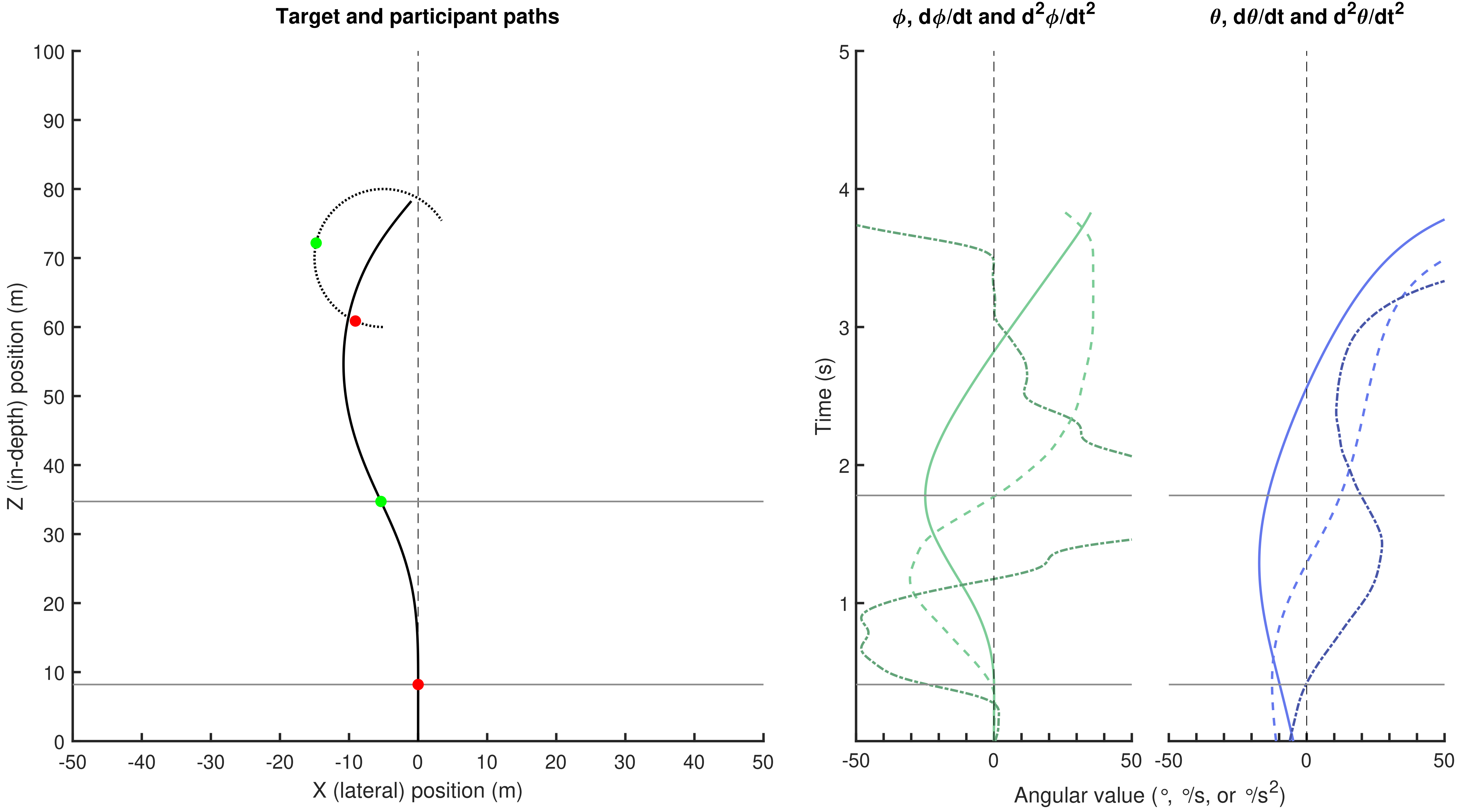

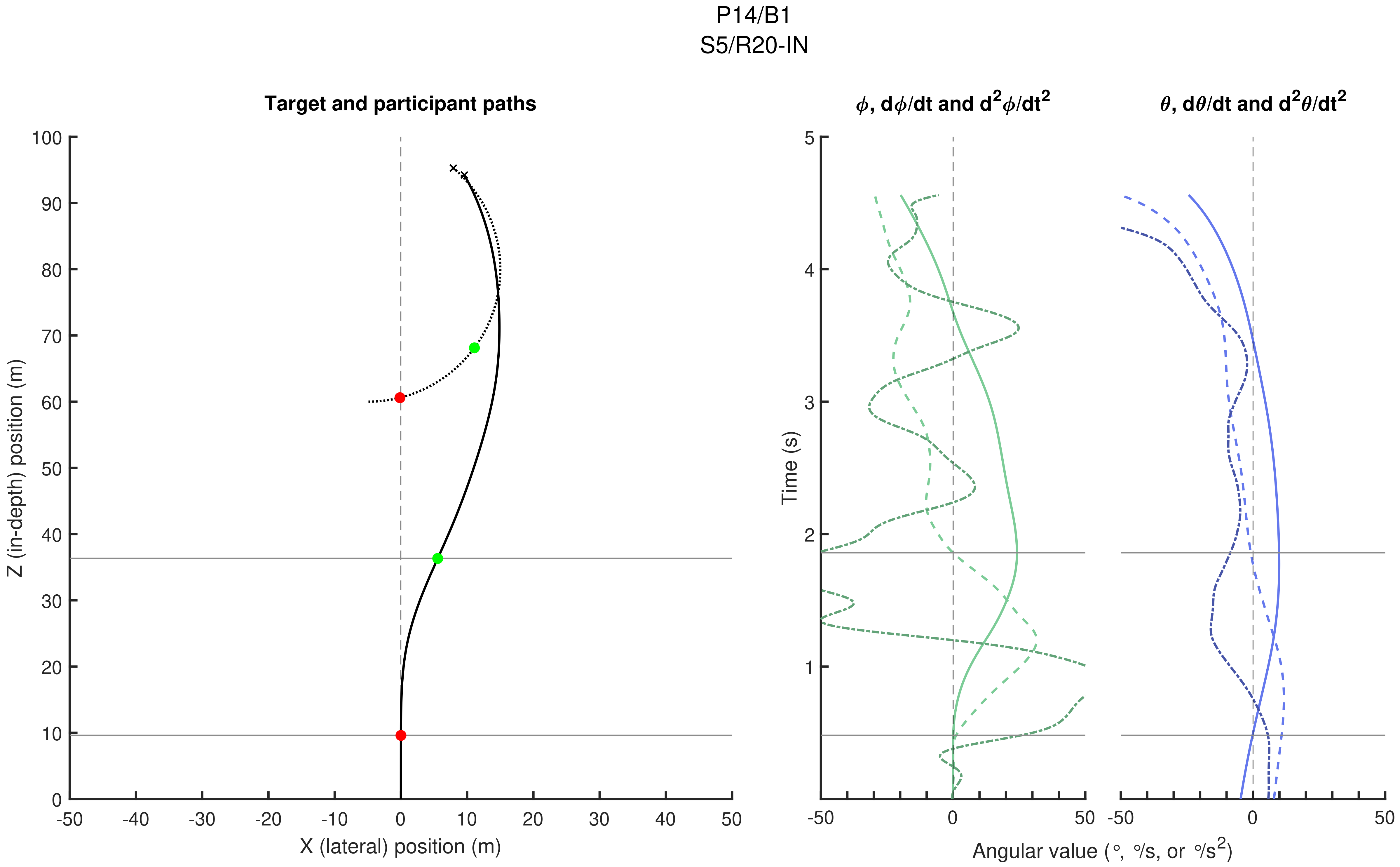
P14/B1
S5/R20-IN
Target and participant paths
Z (in-depth) position (m)
X (lateral) position (m)
ϕ, dϕ/dt and d$^2\phi$/dt^2
θ, dθ/dt and d$^2\theta$/dt^2
Time (s)
Angular value (°, °/s, or °/s^2)

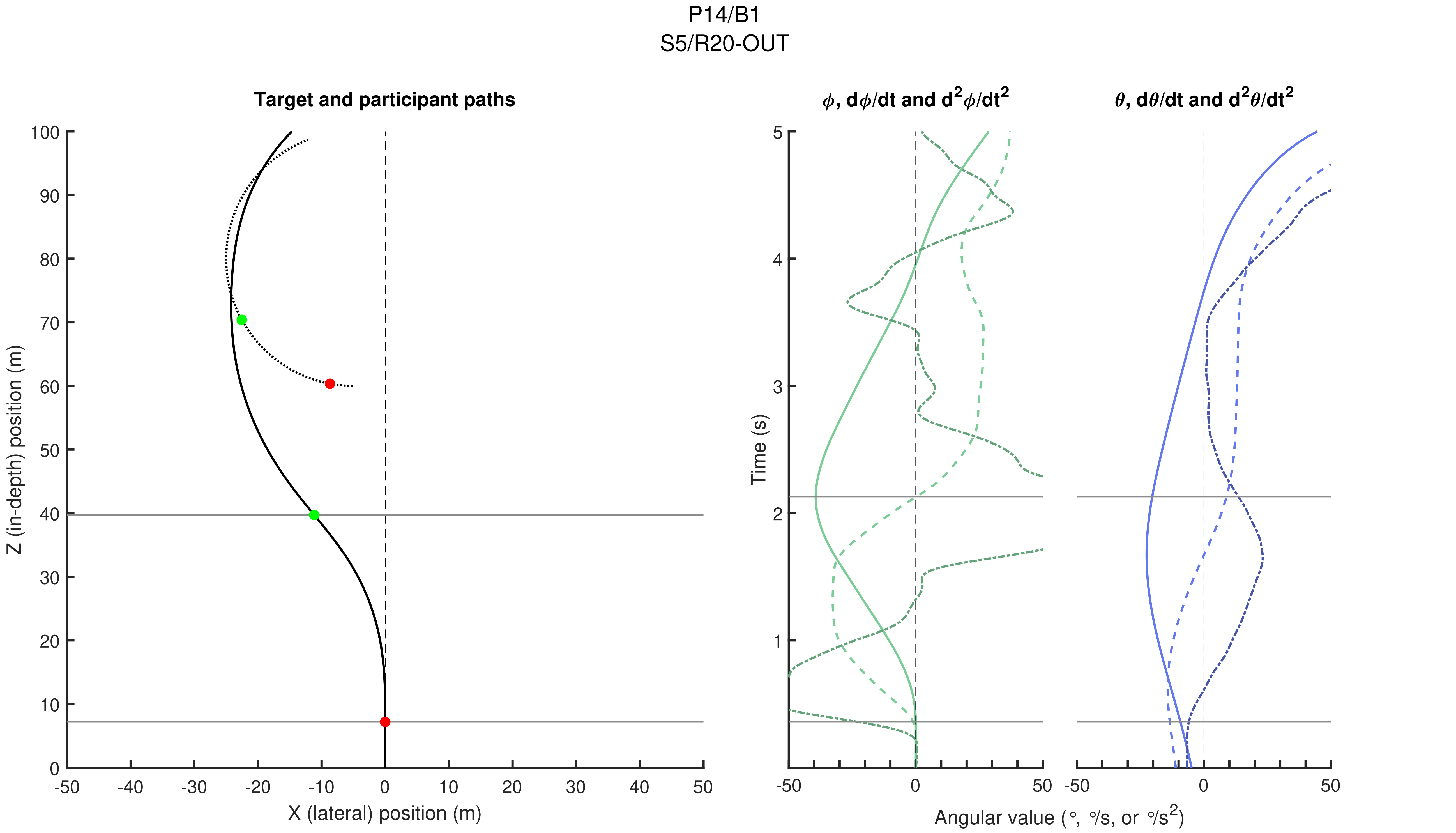
P14/B1
S5/R20-OUT
Target and participant paths
Z (in-depth) position (m)
X (lateral) position (m)
φ, dφ/dt and d²φ/dt²
θ, dθ/dt and d²θ/dt²
Time (s)
Angular value (°, °/s, or °/s²)

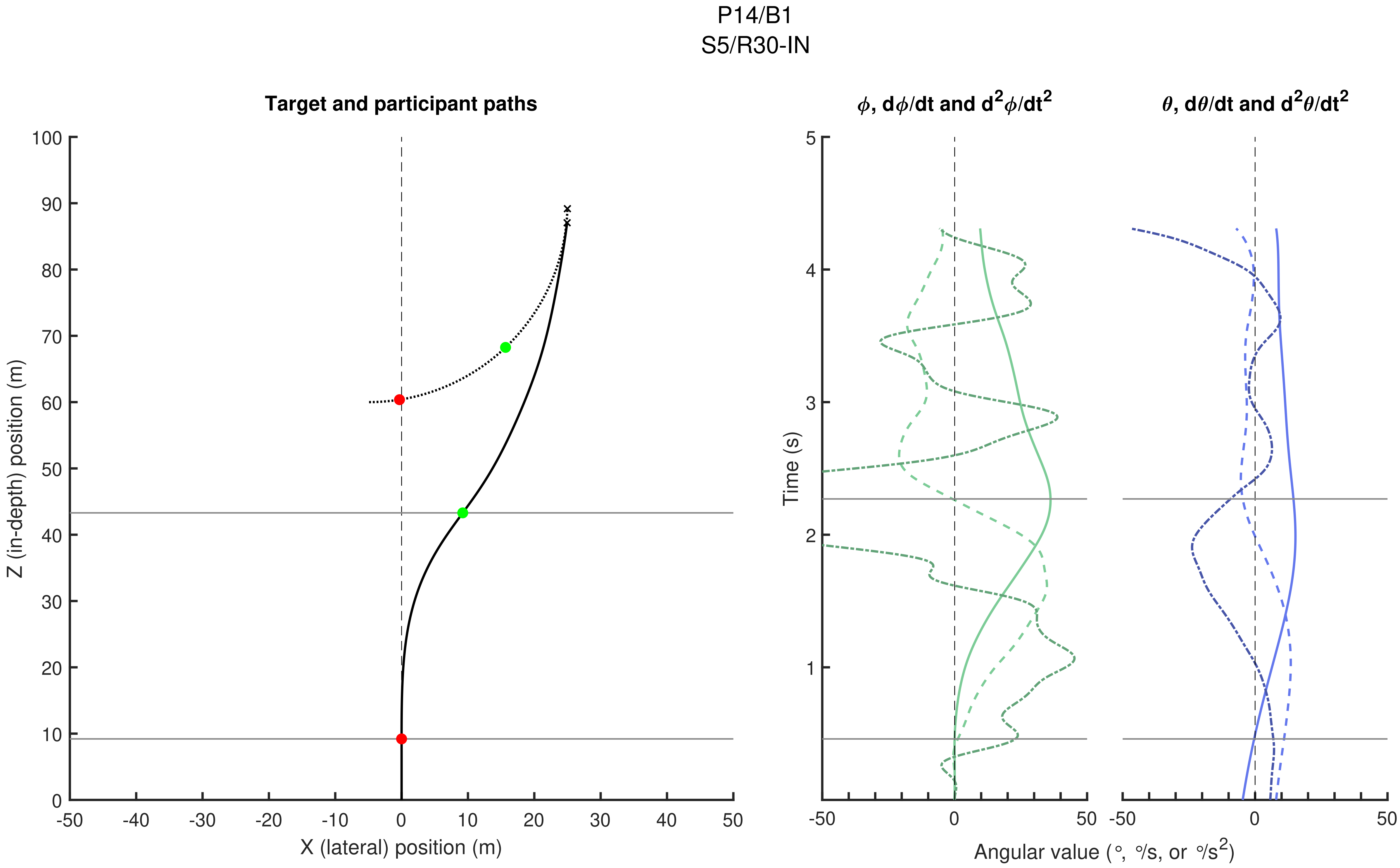
P14/B1
S5/R30-IN
Target and participant paths
Z (in-depth) position (m)
X (lateral) position (m)
ϕ, dϕ/dt and d$^2\phi$/dt^2
θ, dθ/dt and d$^2\theta$/dt^2
Time (s)
Angular value (°, °/s, or °/s^2)

P14/B1
S5/R30-OUT

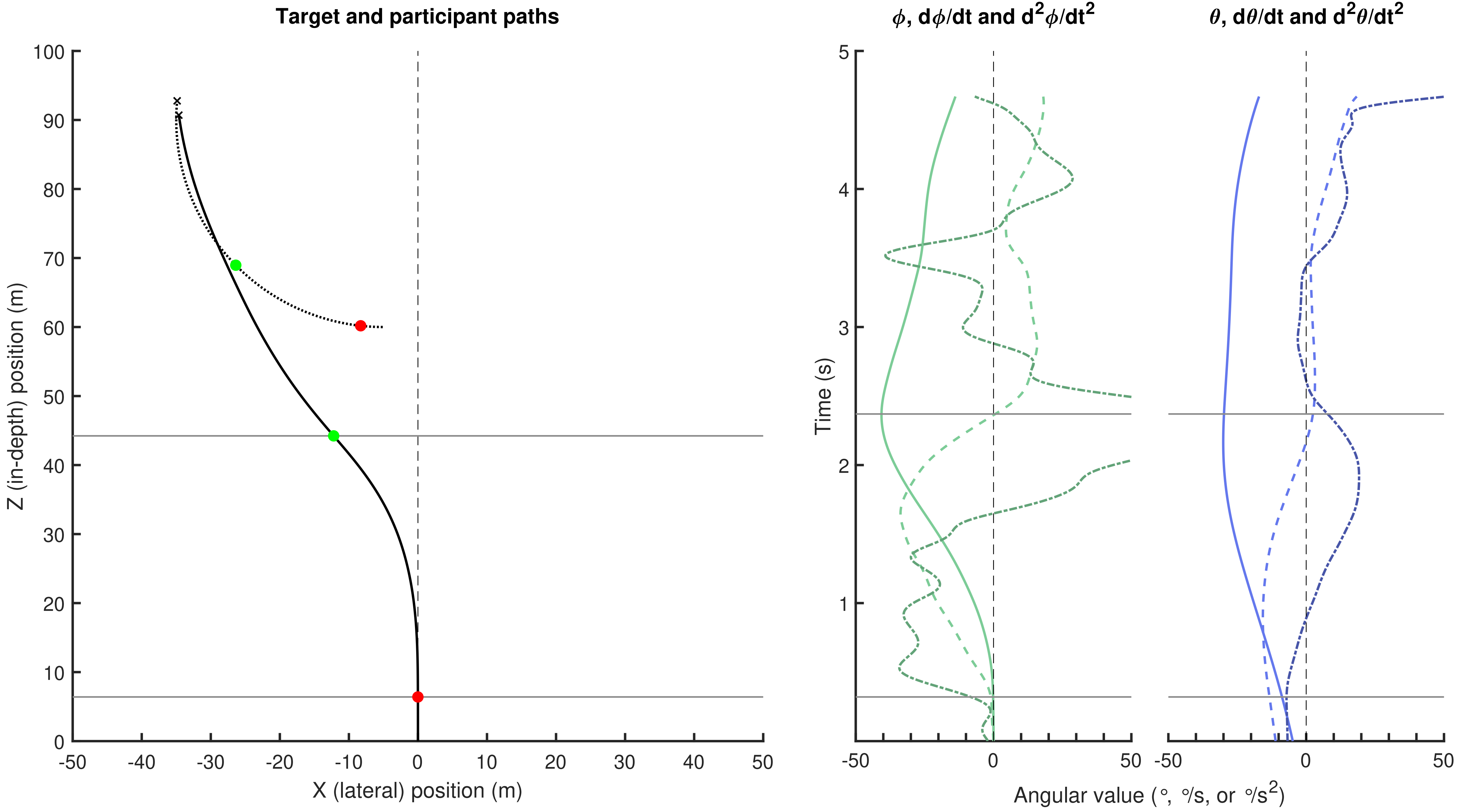

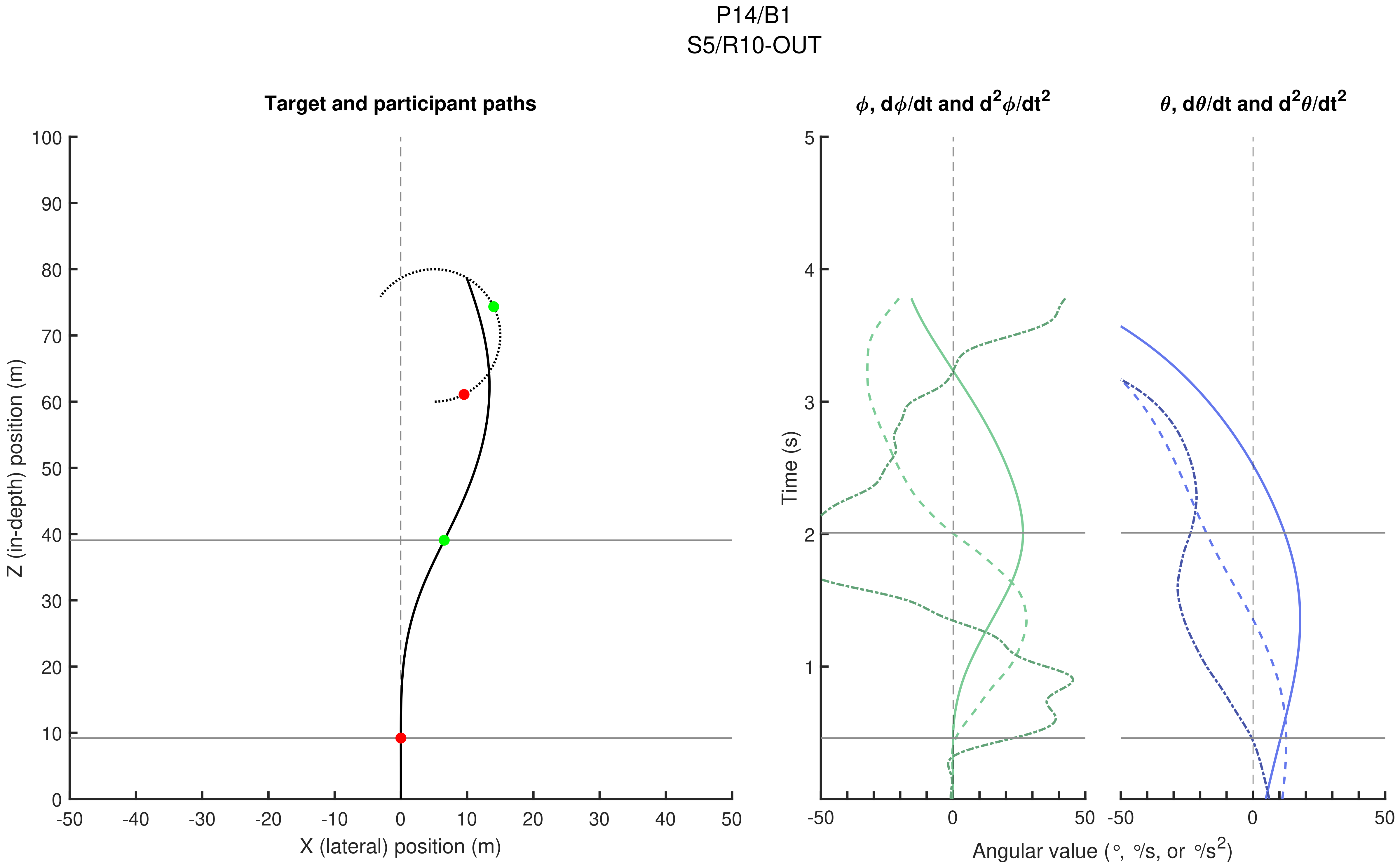
P14/B1
S5/R10-OUT
Target and participant paths
Z (in-depth) position (m)
X (lateral) position (m)
φ, dφ/dt and d²φ/dt²
θ, dθ/dt and d²θ/dt²
Time (s)
Angular value (°, °/s, or °/s²)

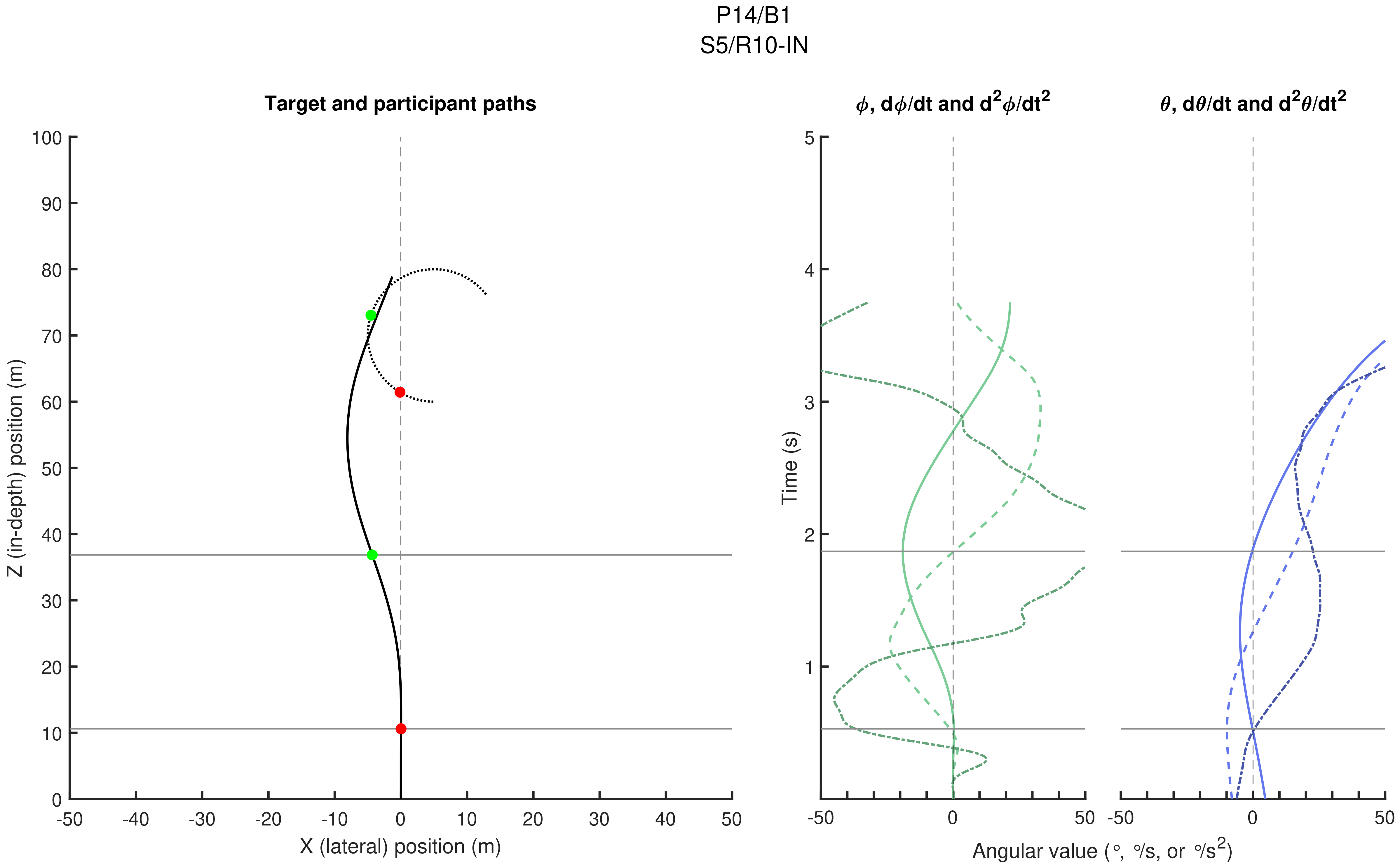
P14/B1
S5/R10-IN
Target and participant paths
Z (in-depth) position (m)
X (lateral) position (m)
ϕ, dϕ/dt and d²ϕ/dt²
θ, dθ/dt and d²θ/dt²
Time (s)
Angular value (°, °/s, or °/s²)

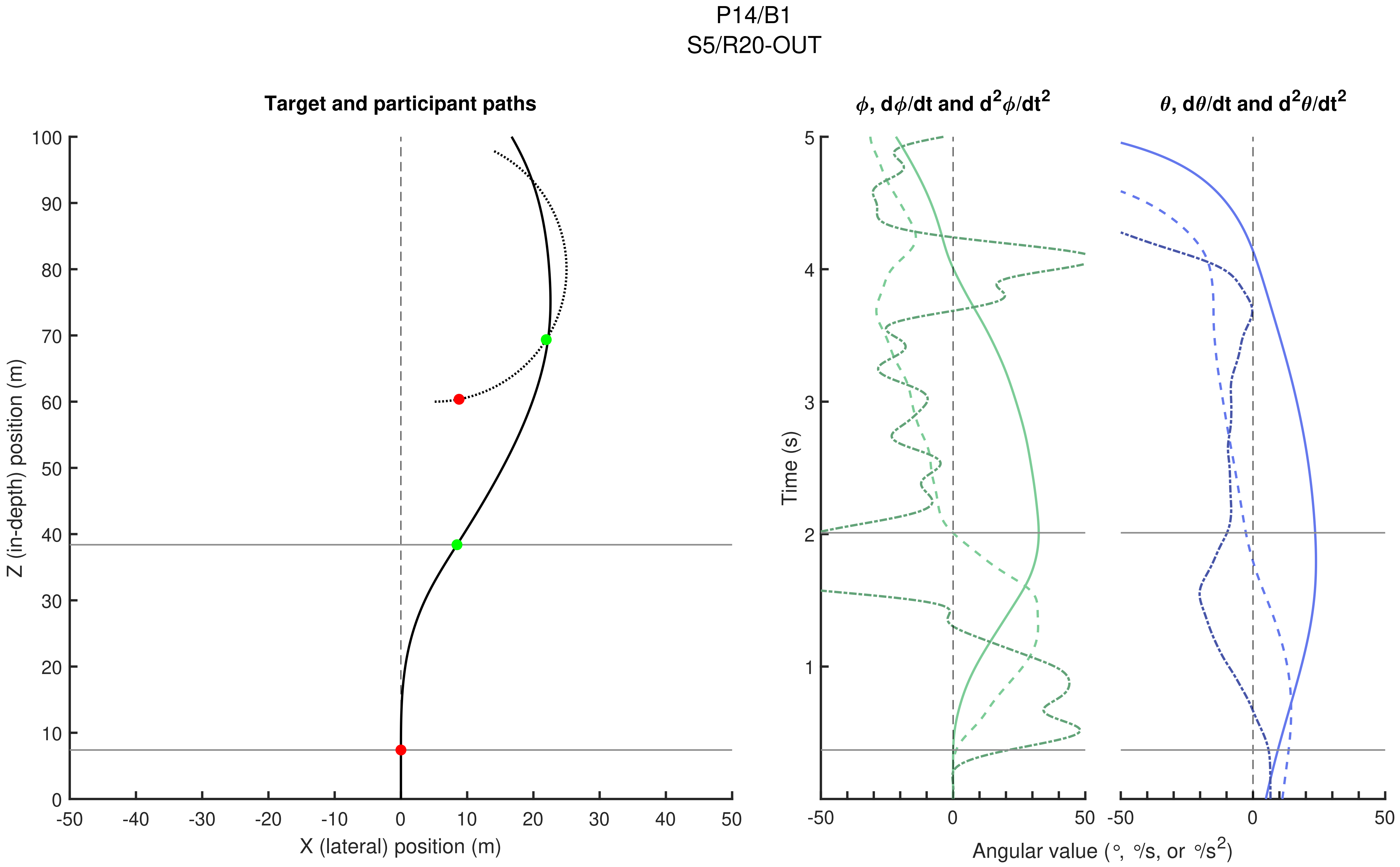

P14/B1
S5/R20-OUT
Target and participant paths
Z (in-depth) position (m)
X (lateral) position (m)
ϕ, dϕ/dt and d$^2\phi$/dt^2
θ, dθ/dt and d$^2\theta$/dt^2
Time (s)
Angular value (°, °/s, or °/s^2)

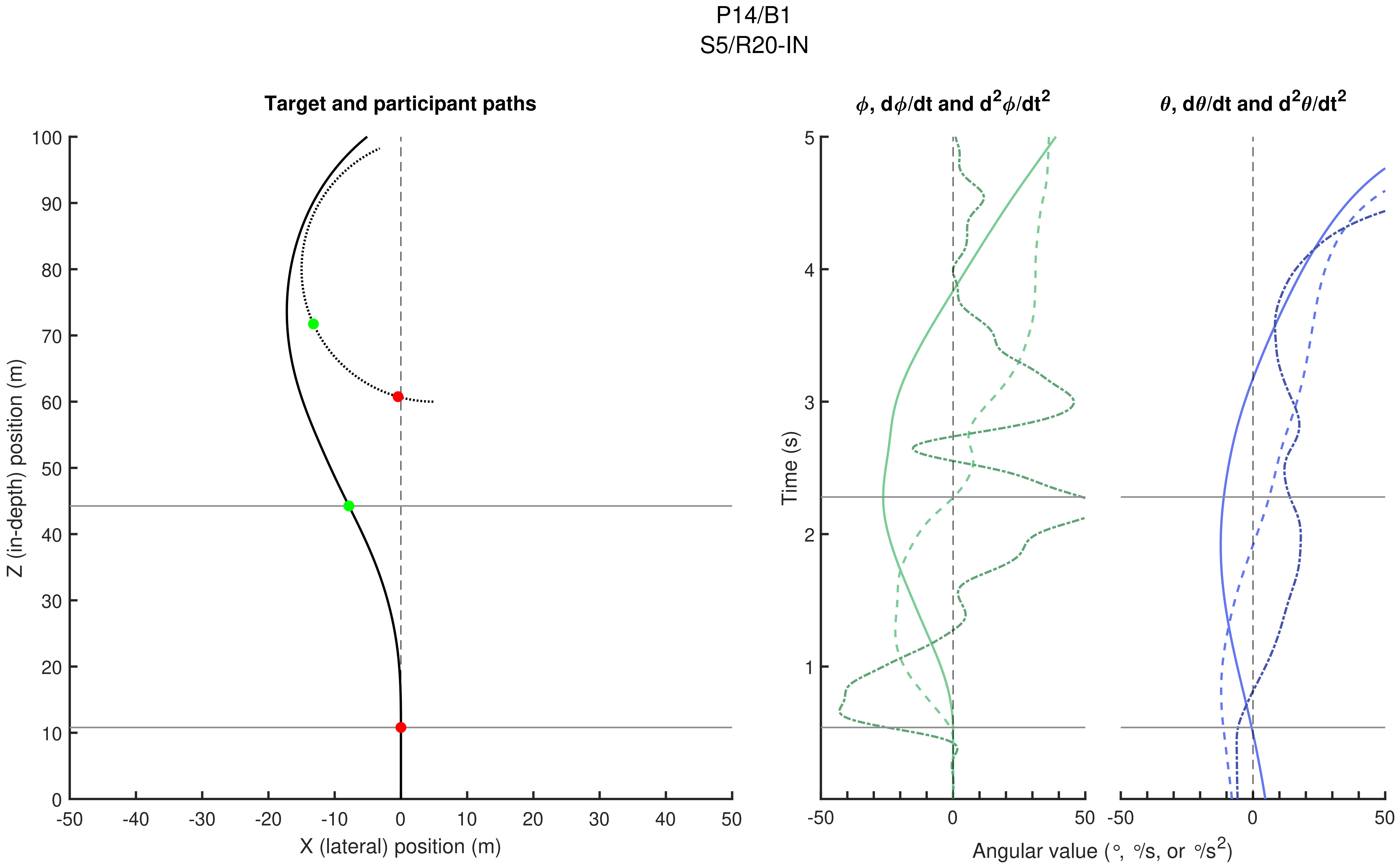

P14/B1
S5/R20-IN
Target and participant paths
φ, dφ/dt and d²φ/dt²
θ, dθ/dt and d²θ/dt²
Z (in-depth) position (m)
X (lateral) position (m)
Time (s)
Angular value (°, °/s, or °/s²)
100
90
80
70
60
50
40
30
20
10
0
-50
-40
-30
-20
-10
0
10
20
30
40
50
5
4
3
2
1

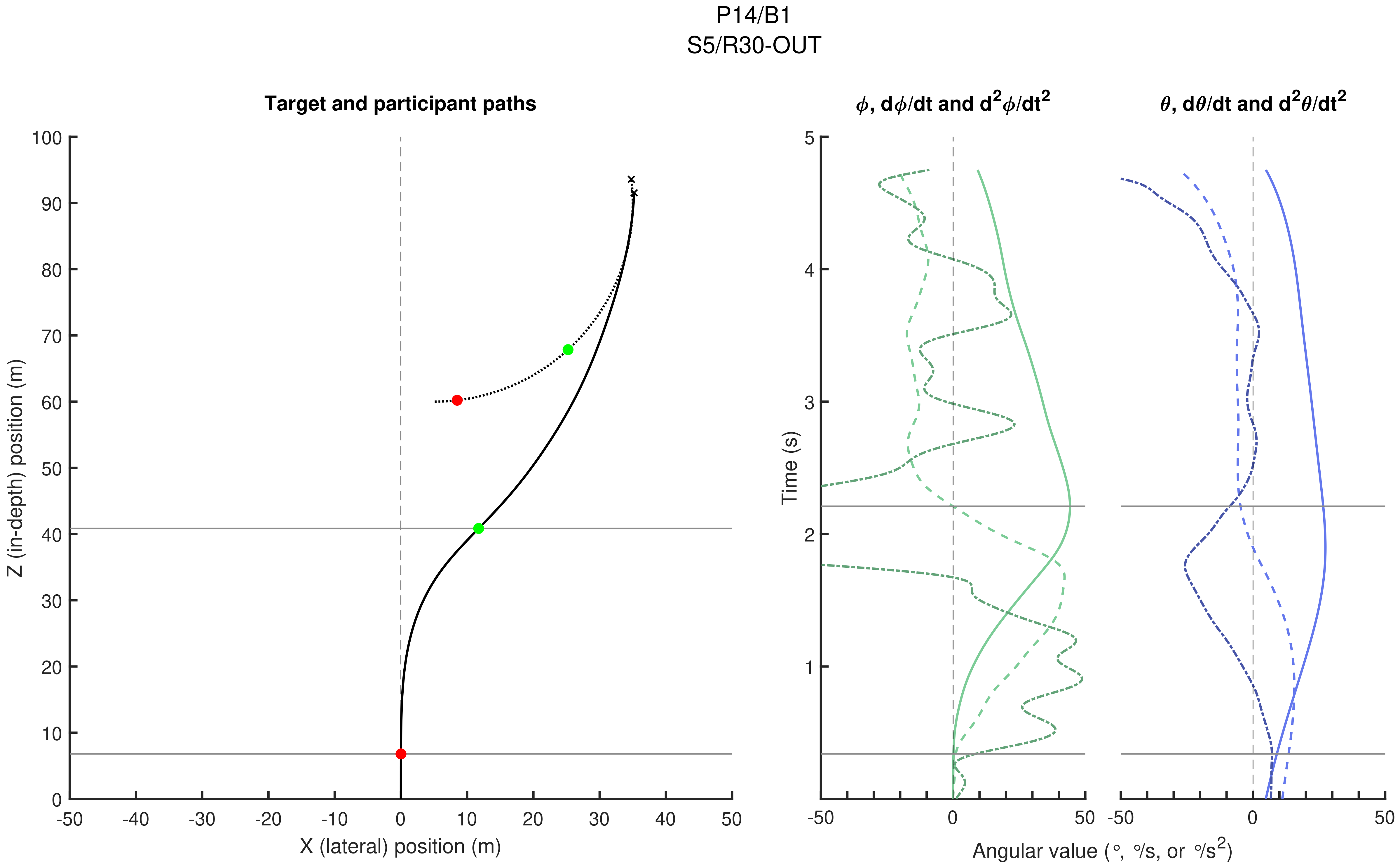
P14/B1
S5/R30-OUT
Target and participant paths
Z (in-depth) position (m)
X (lateral) position (m)
ϕ, dϕ/dt and d²ϕ/dt²
θ, dθ/dt and d²θ/dt²
Time (s)
Angular value (°, °/s, or °/s²)

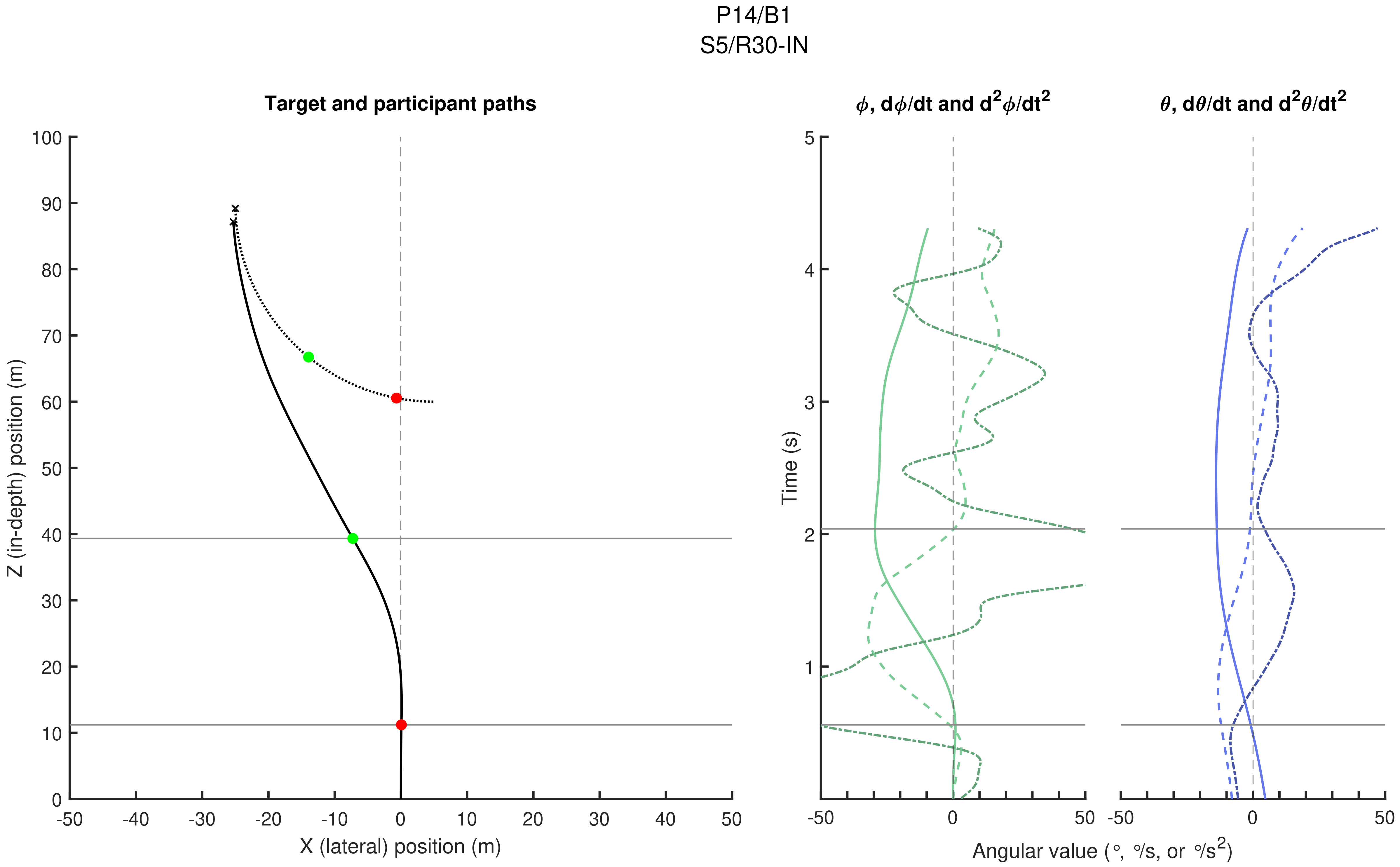
P14/B1
S5/R30-IN
Target and participant paths
Z (in-depth) position (m)
X (lateral) position (m)
ϕ, dϕ/dt and d²ϕ/dt²
θ, dθ/dt and d²θ/dt²
Time (s)
Angular value (°, °/s, or °/s²)

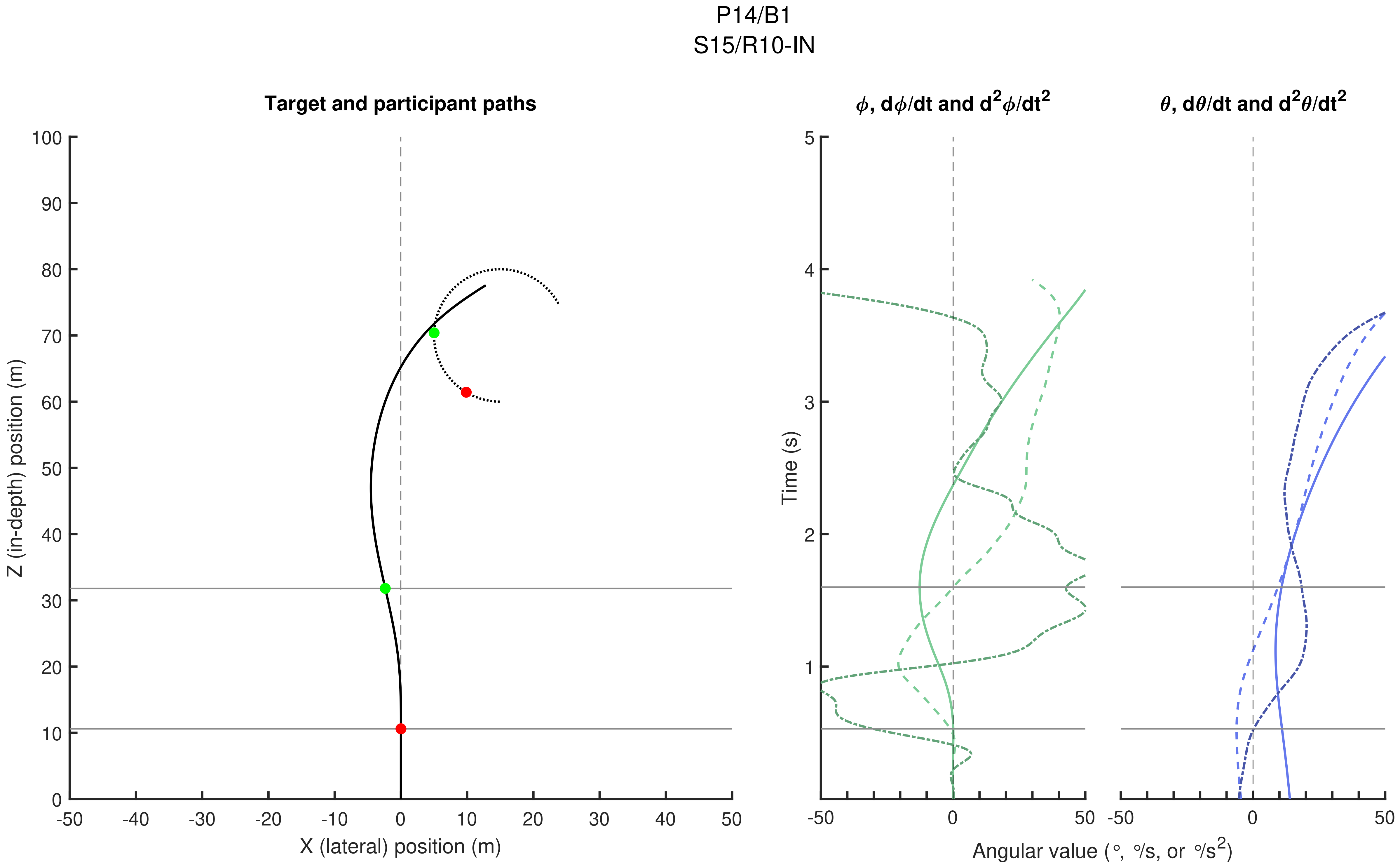
P14/B1
S15/R10-IN
Target and participant paths
Z (in-depth) position (m)
X (lateral) position (m)
100
90
80
70
60
50
40
30
20
10
0
-50
-40
-30
-20
-10
0
10
20
30
40
50
ϕ, dϕ/dt and d²ϕ/dt²
θ, dθ/dt and d²θ/dt²
Time (s)
5
4
3
2
1
-50
0
50
-50
0
50
Angular value (°, °/s, or °/s²)

P14/B1
S15/R20-IN

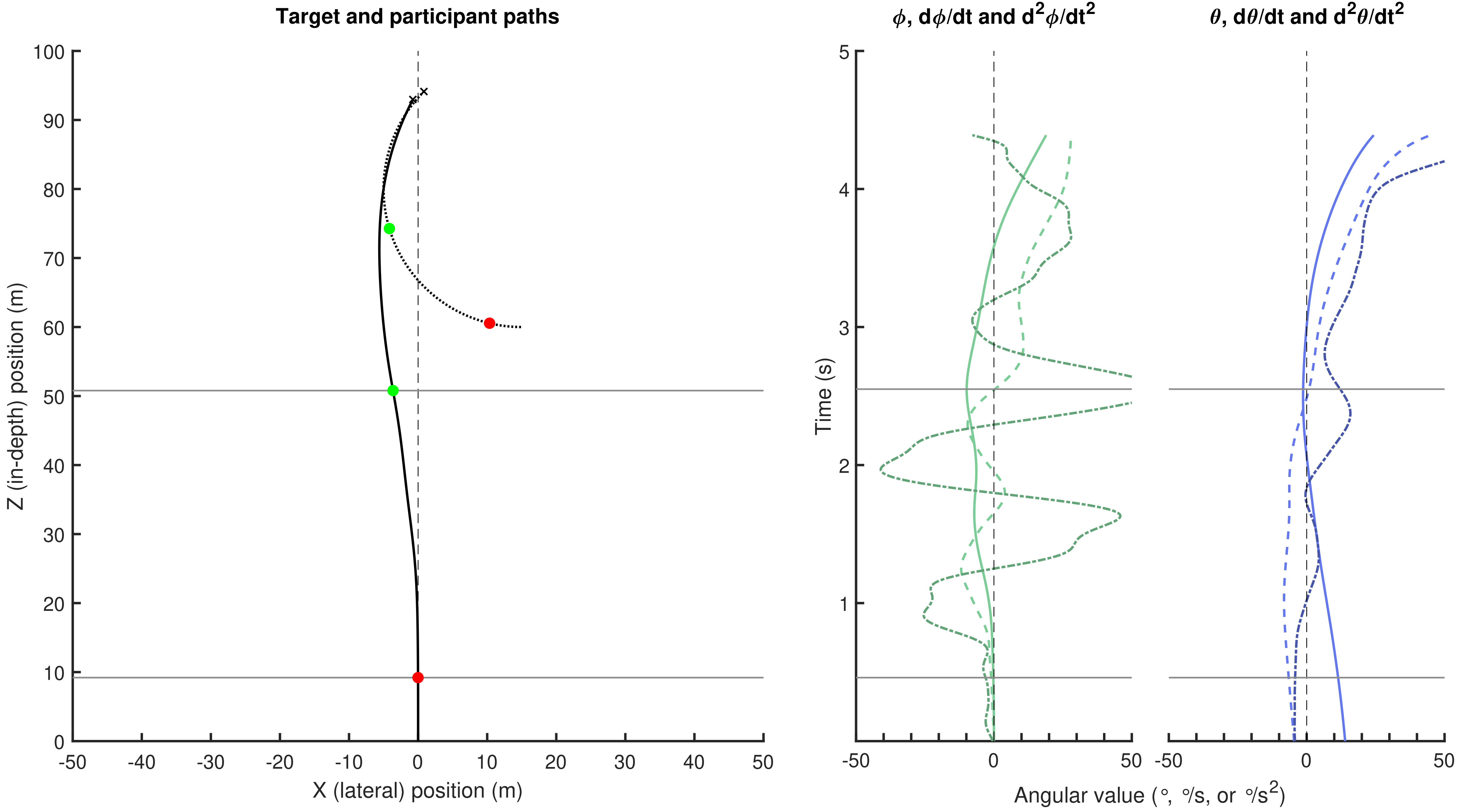

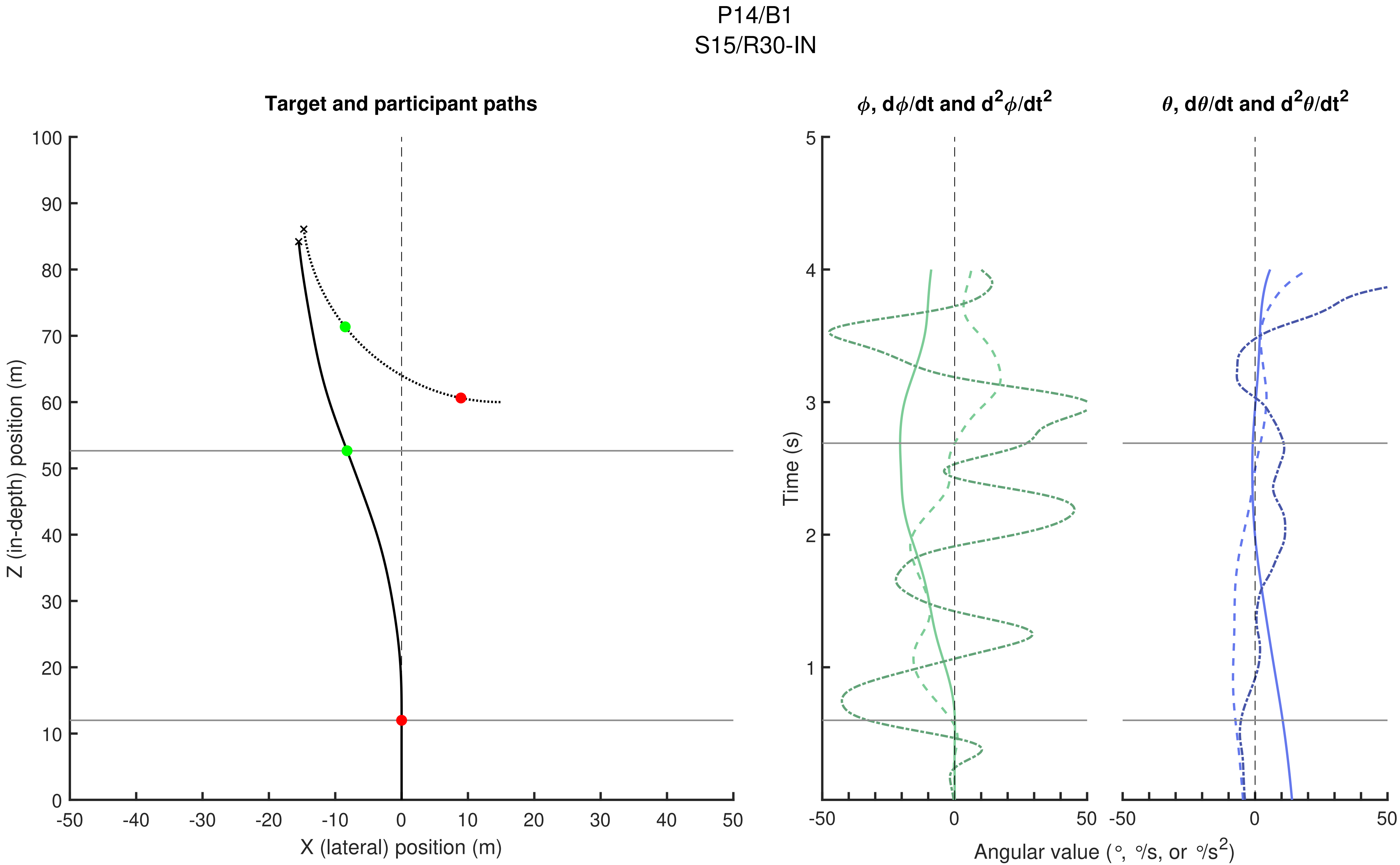
P14/B1
S15/R30-IN
Target and participant paths
ϕ, dϕ/dt and d$^2\phi$/dt^2
θ, dθ/dt and d$^2\theta$/dt^2
Z (in-depth) position (m)
X (lateral) position (m)
Time (s)
Angular value (°, °/s, or °/s^2)

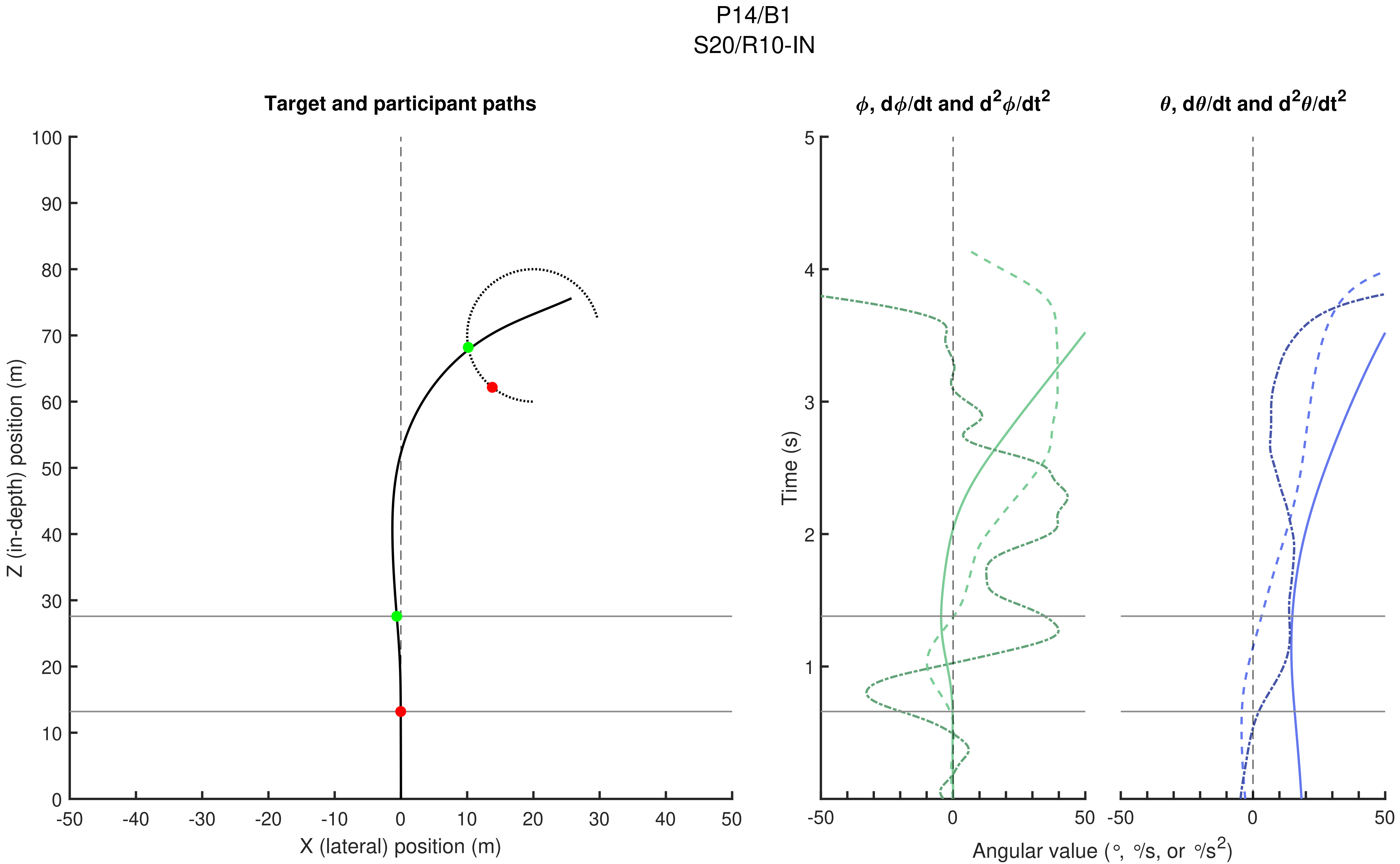
P14/B1
S20/R10-IN
Target and participant paths
Z (in-depth) position (m)
X (lateral) position (m)
ϕ, dϕ/dt and d$^2\phi$/dt^2
θ, dθ/dt and d$^2\theta$/dt^2
Time (s)
Angular value (°, °/s, or °/s^2)

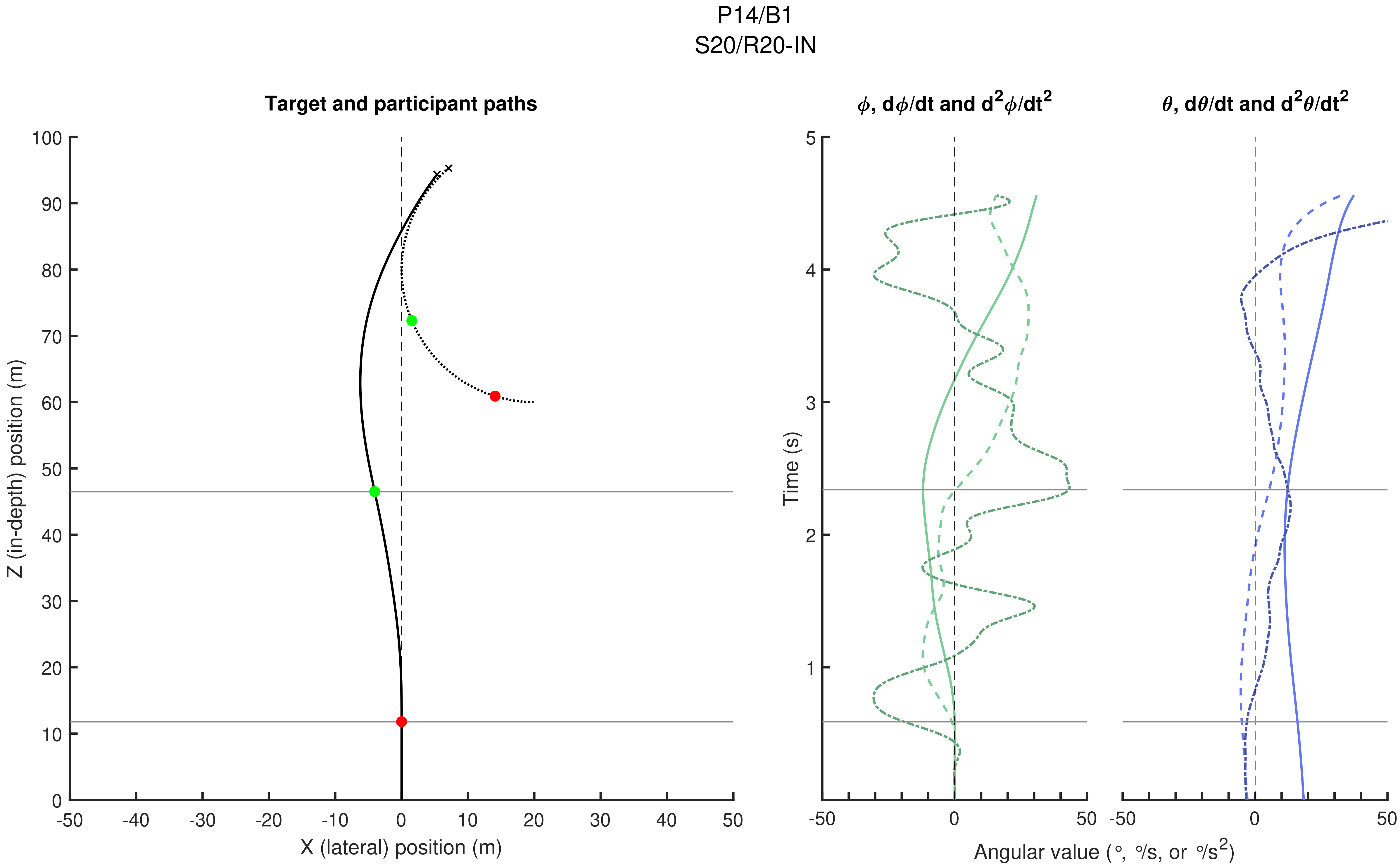
P14/B1
S20/R20-IN
Target and participant paths
Z (in-depth) position (m)
X (lateral) position (m)
0
10
20
30
40
50
60
70
80
90
100
-50
-40
-30
-20
-10
0
10
20
30
40
50
ϕ, dϕ/dt and d$^2\phi$/dt^2
θ, dθ/dt and d$^2\theta$/dt^2
Time (s)
1
2
3
4
5
-50
0
50
-50
0
50
Angular value (°, °/s, or °/s^2)

P14/B1
S20/R30-IN

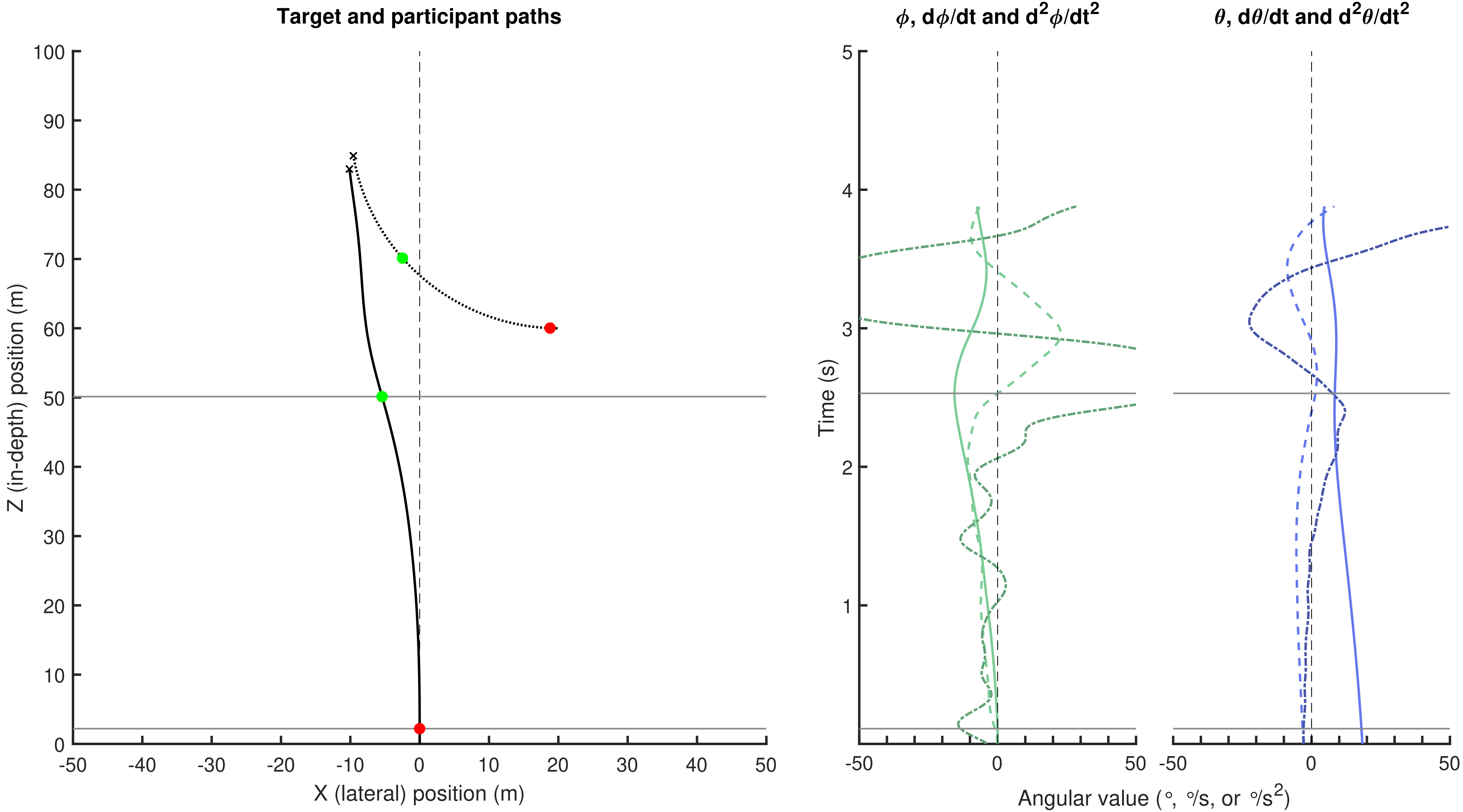

P14/B2
S20/R10-IN

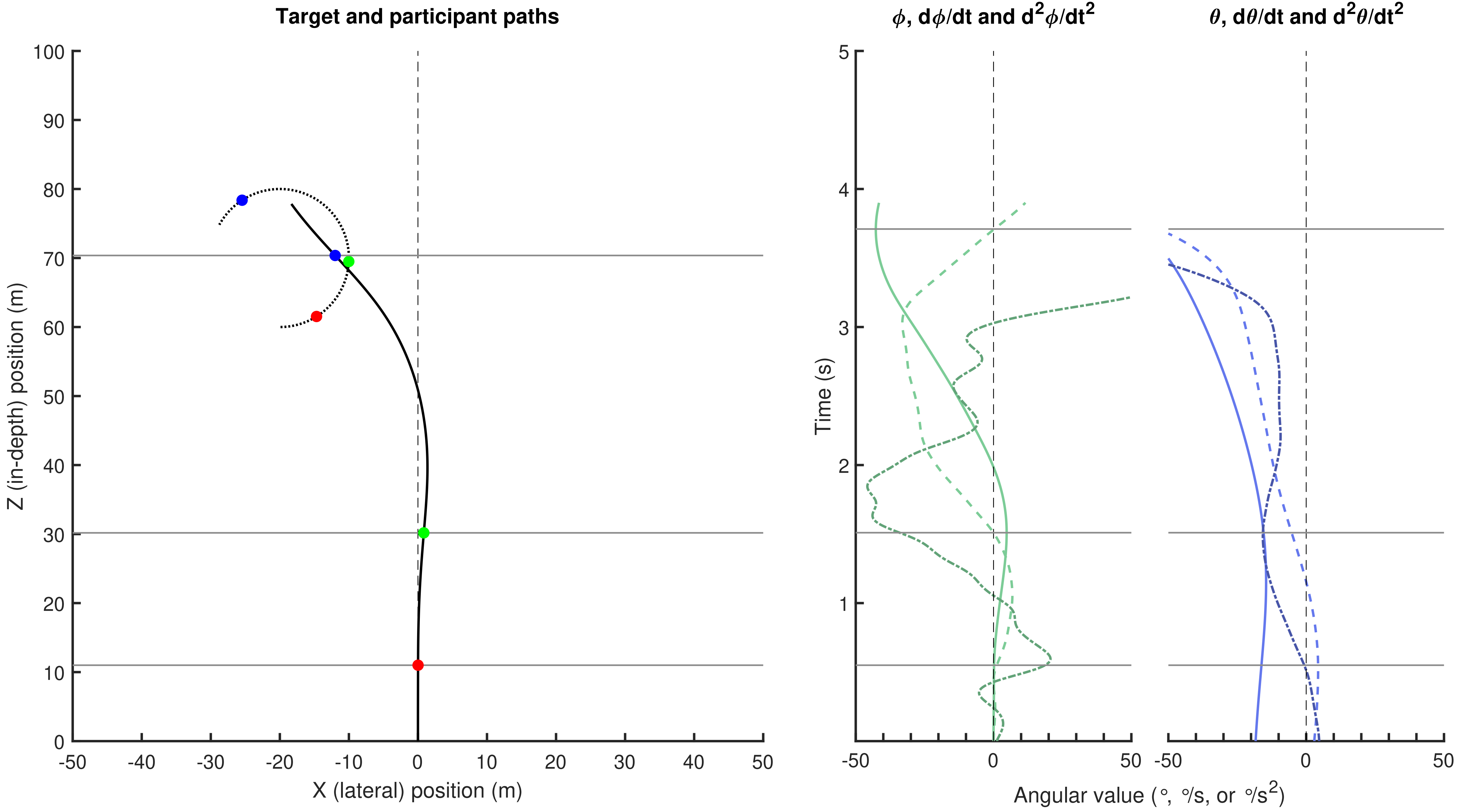

P14/B2
S20/R20-IN

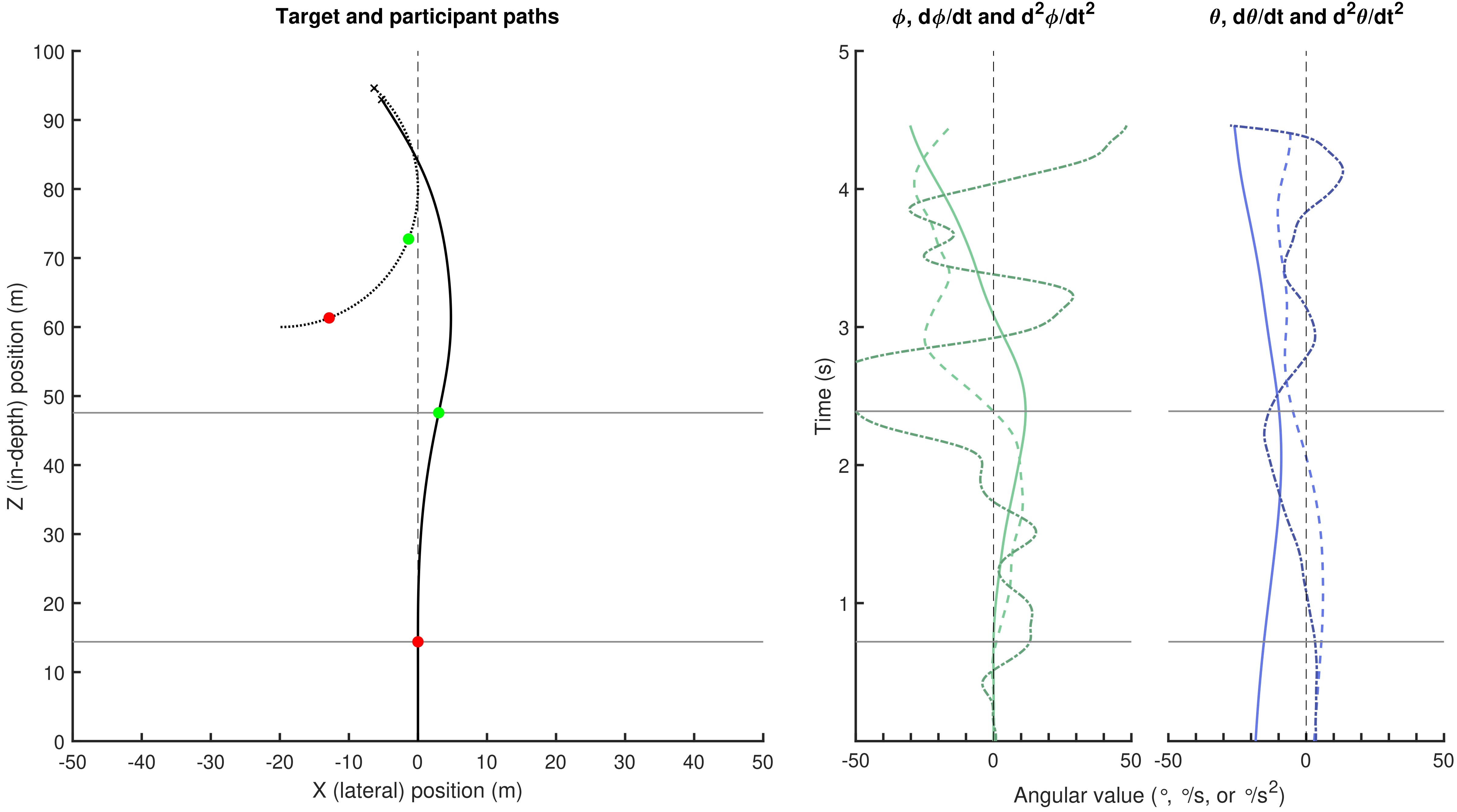

P14/B2
S20/R30-IN

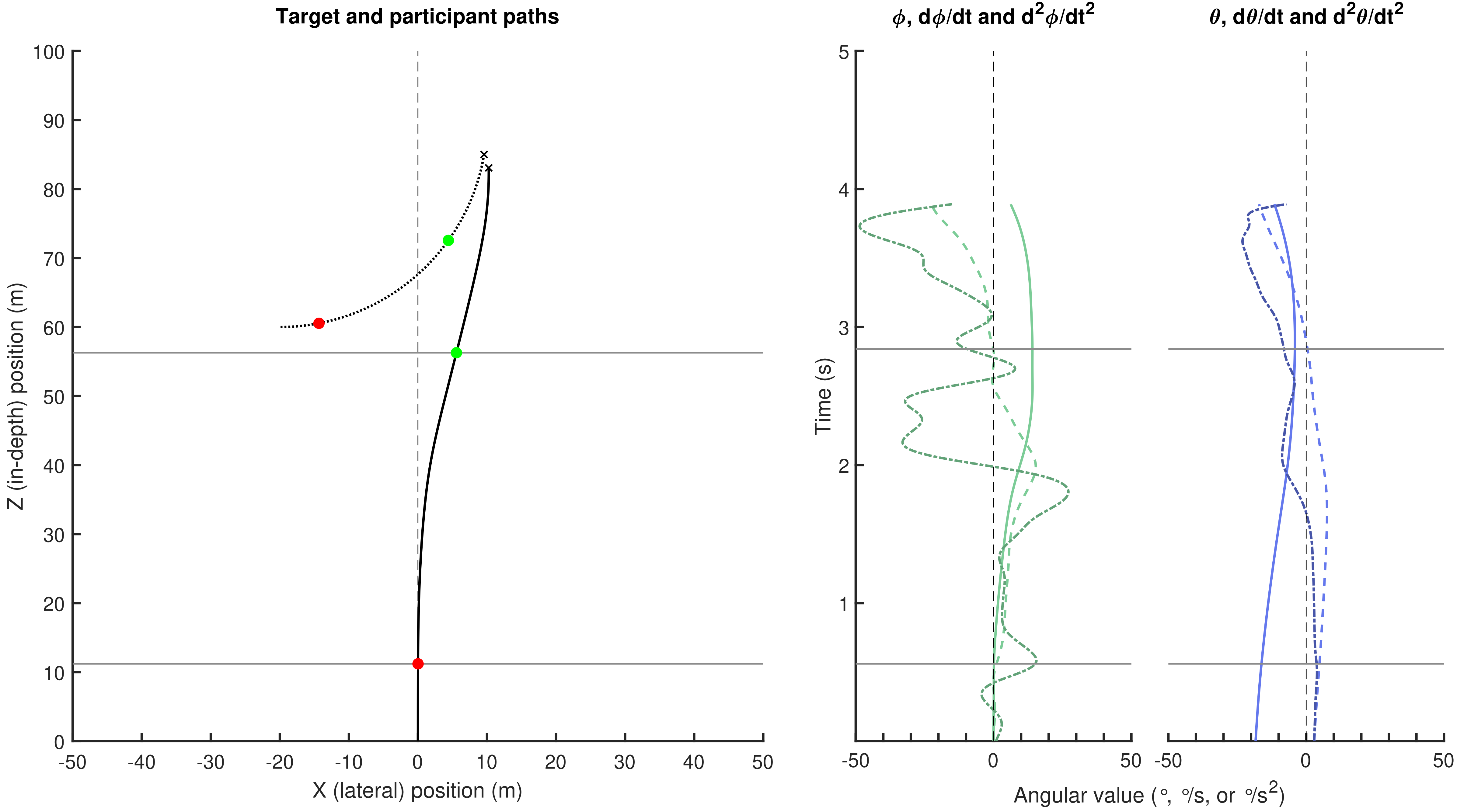

P14/B2
S15/R10-IN

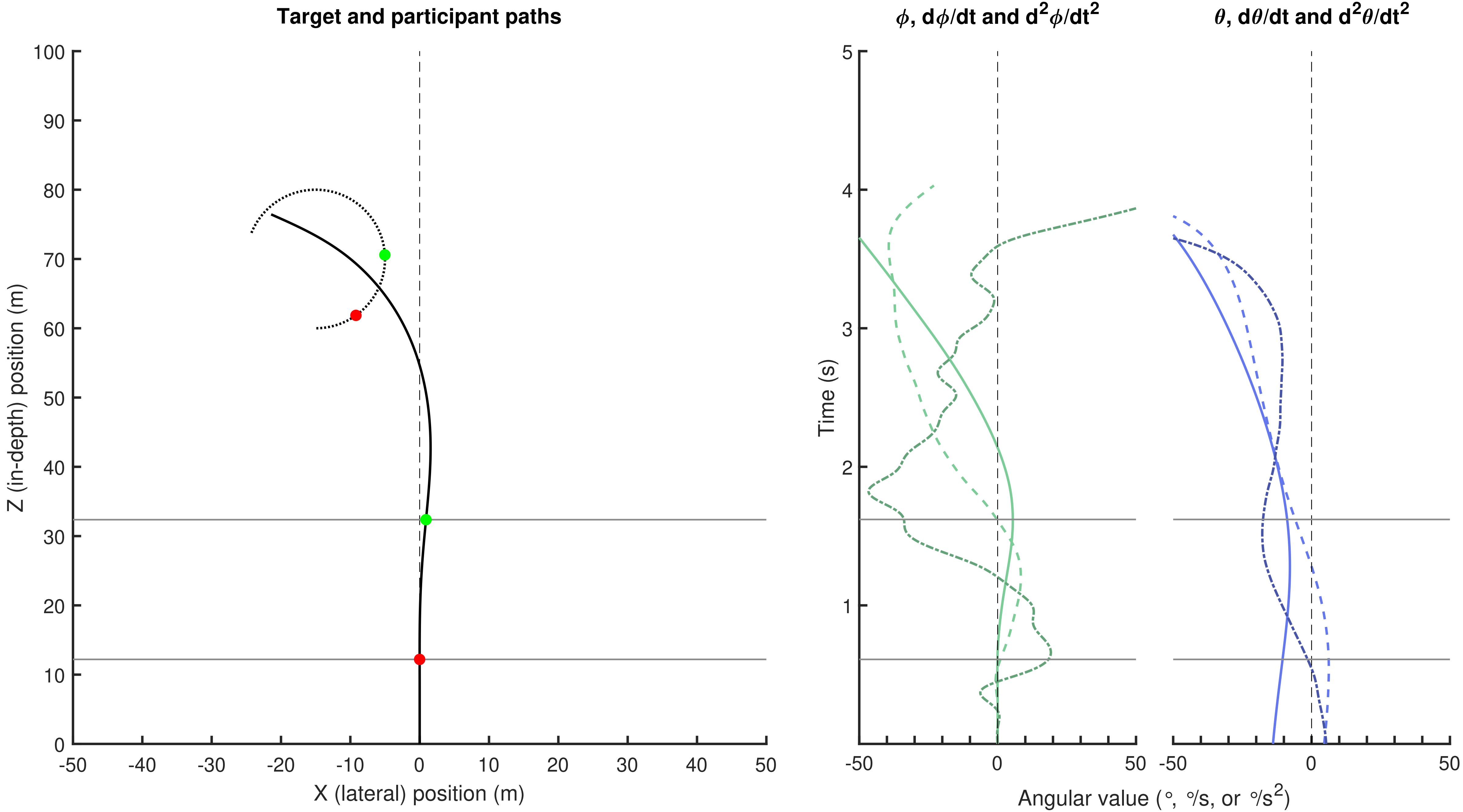

P14/B2
S15/R20-IN

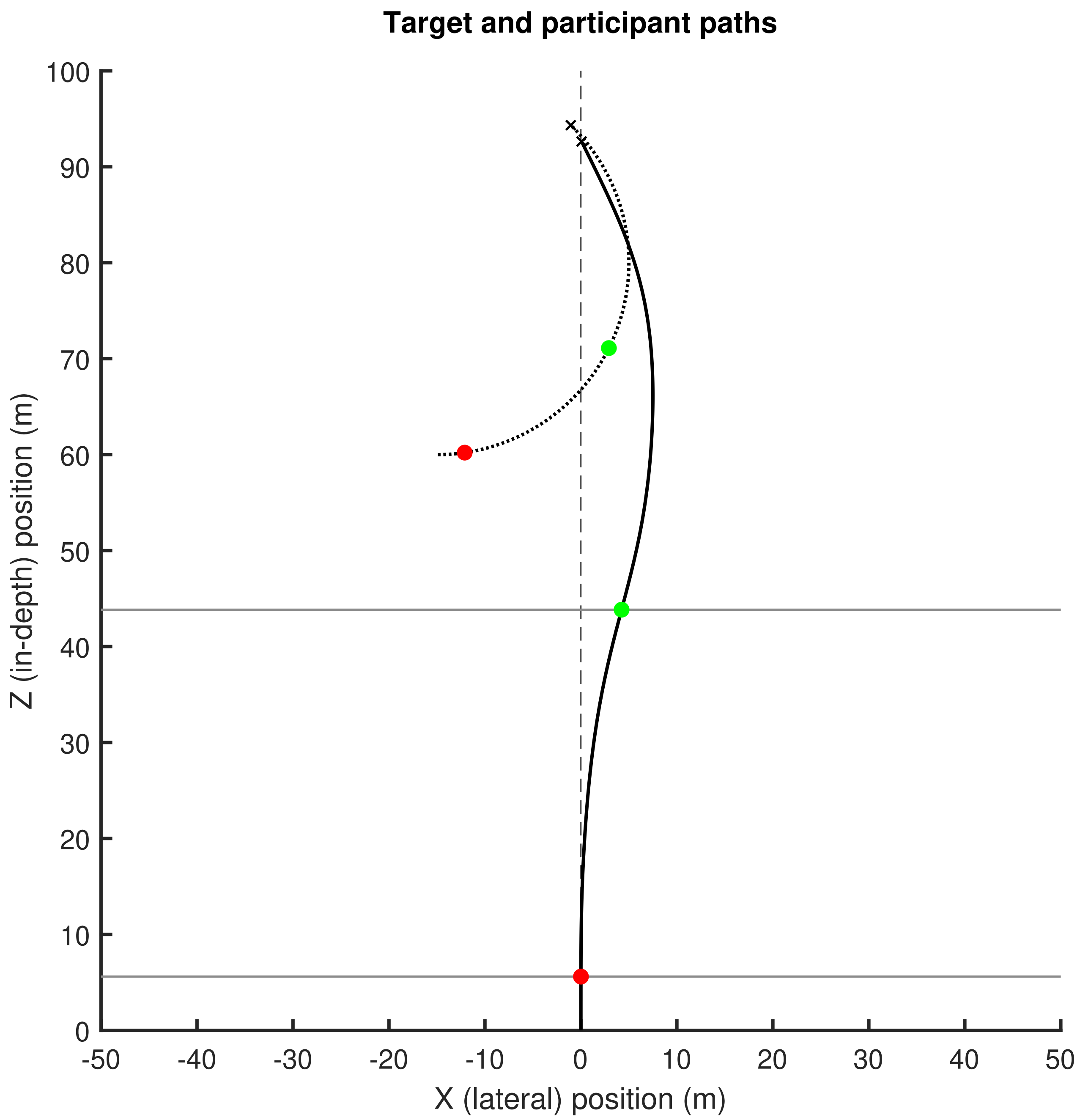

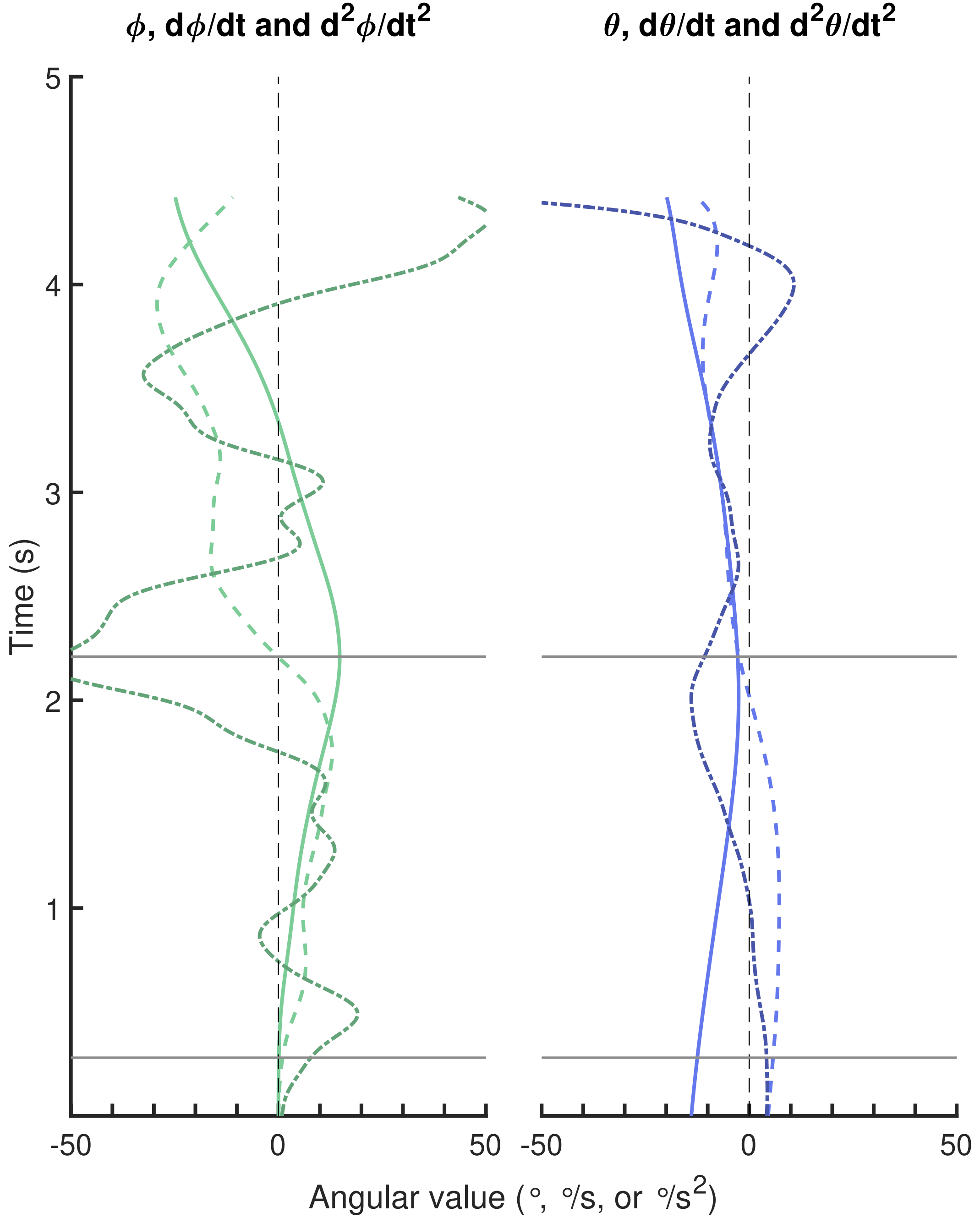

P14/B2
S15/R30-IN

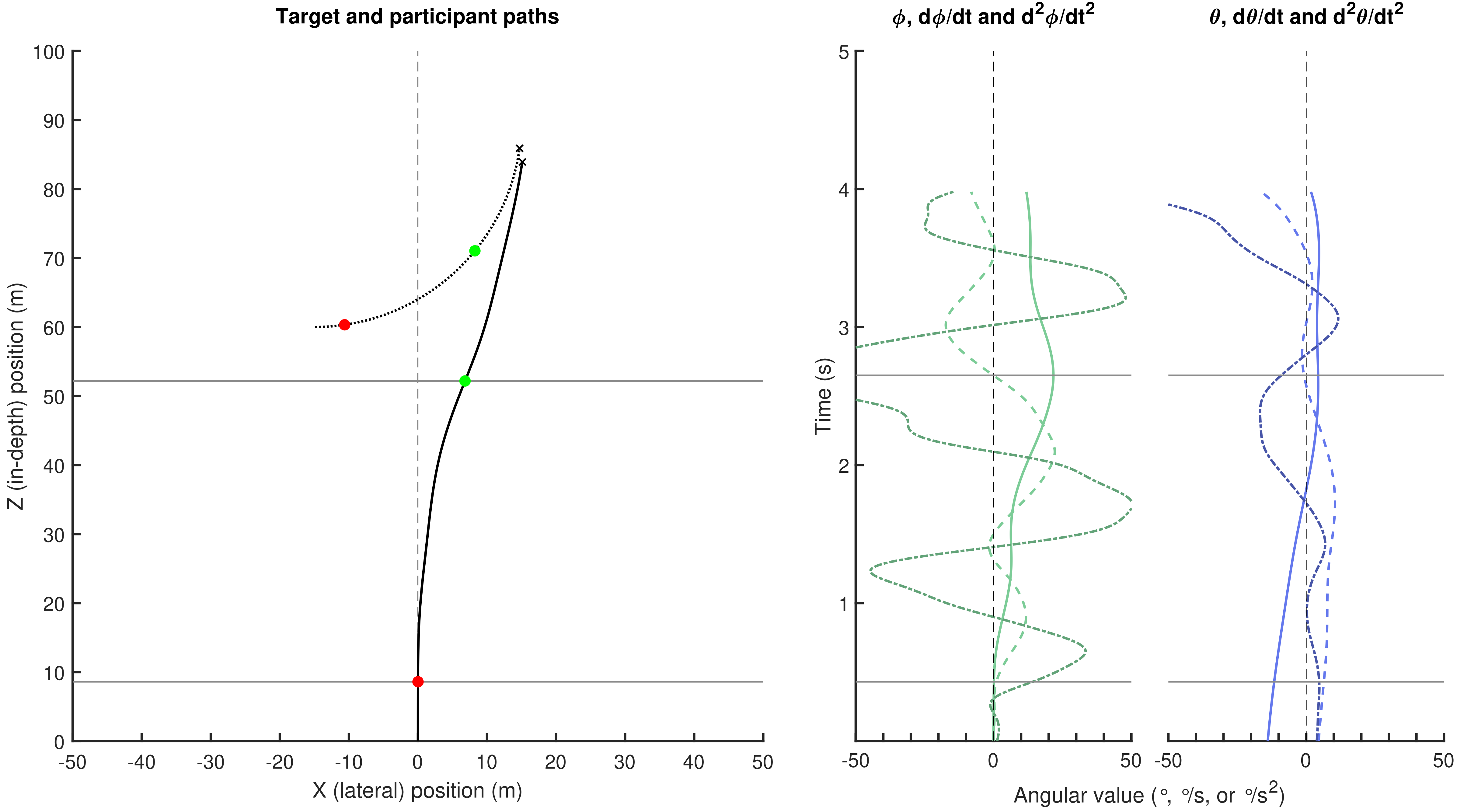

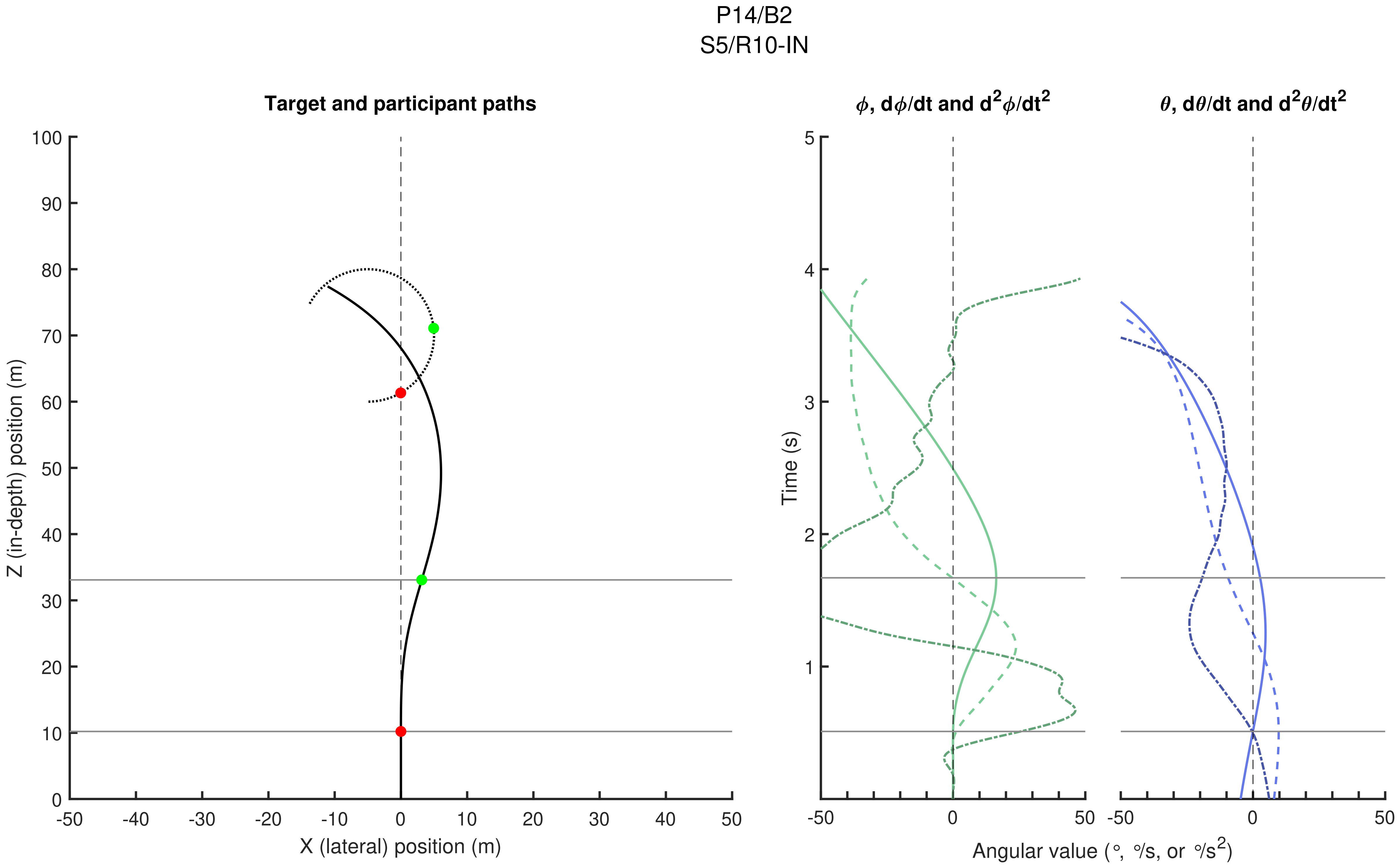
P14/B2
S5/R10-IN
Target and participant paths
Z (in-depth) position (m)
X (lateral) position (m)
φ, dφ/dt and d²φ/dt²
θ, dθ/dt and d²θ/dt²
Time (s)
Angular value (°, °/s, or °/s²)

P14/B2
S5/R10-OUT

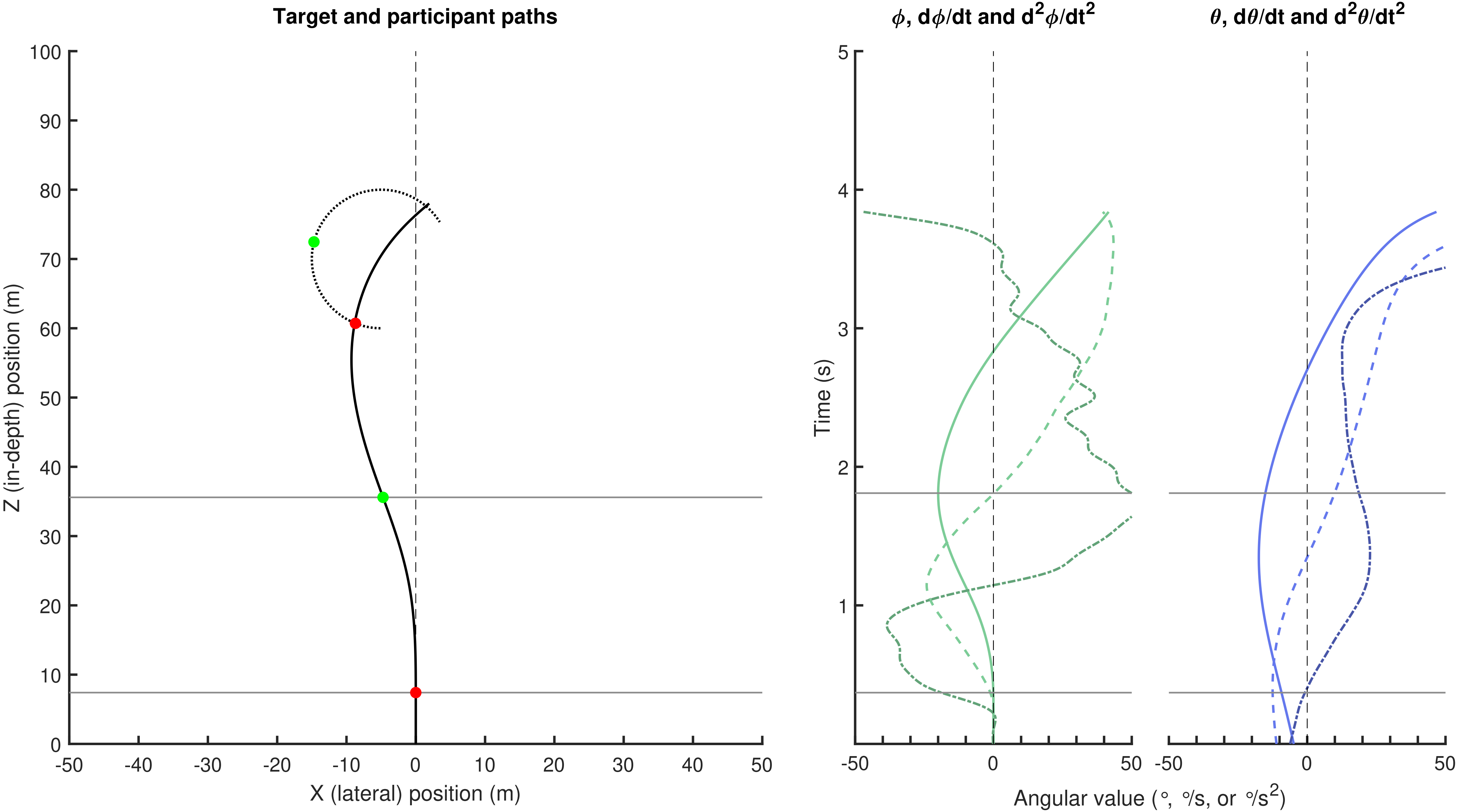

P14/B2
S5/R20-IN

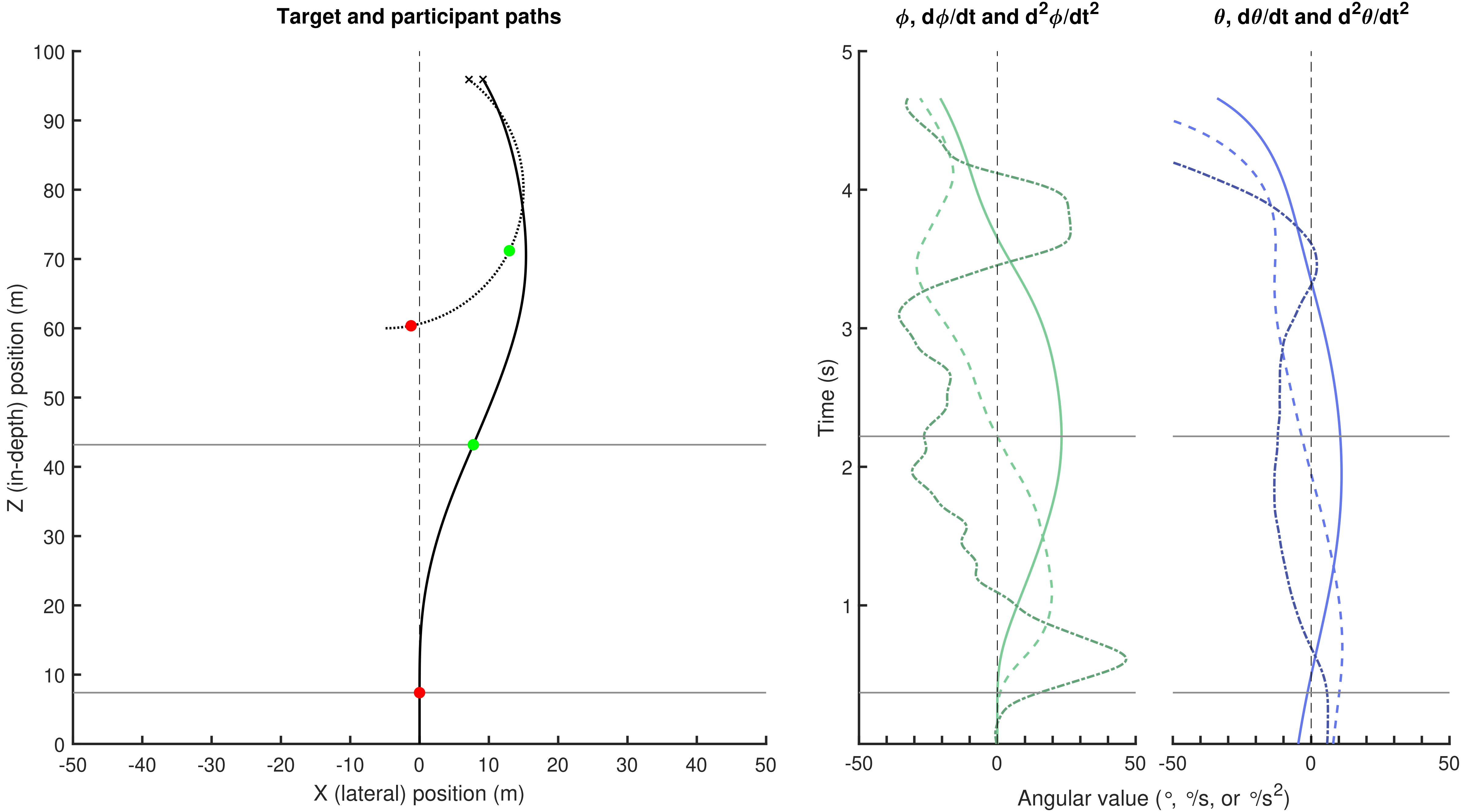

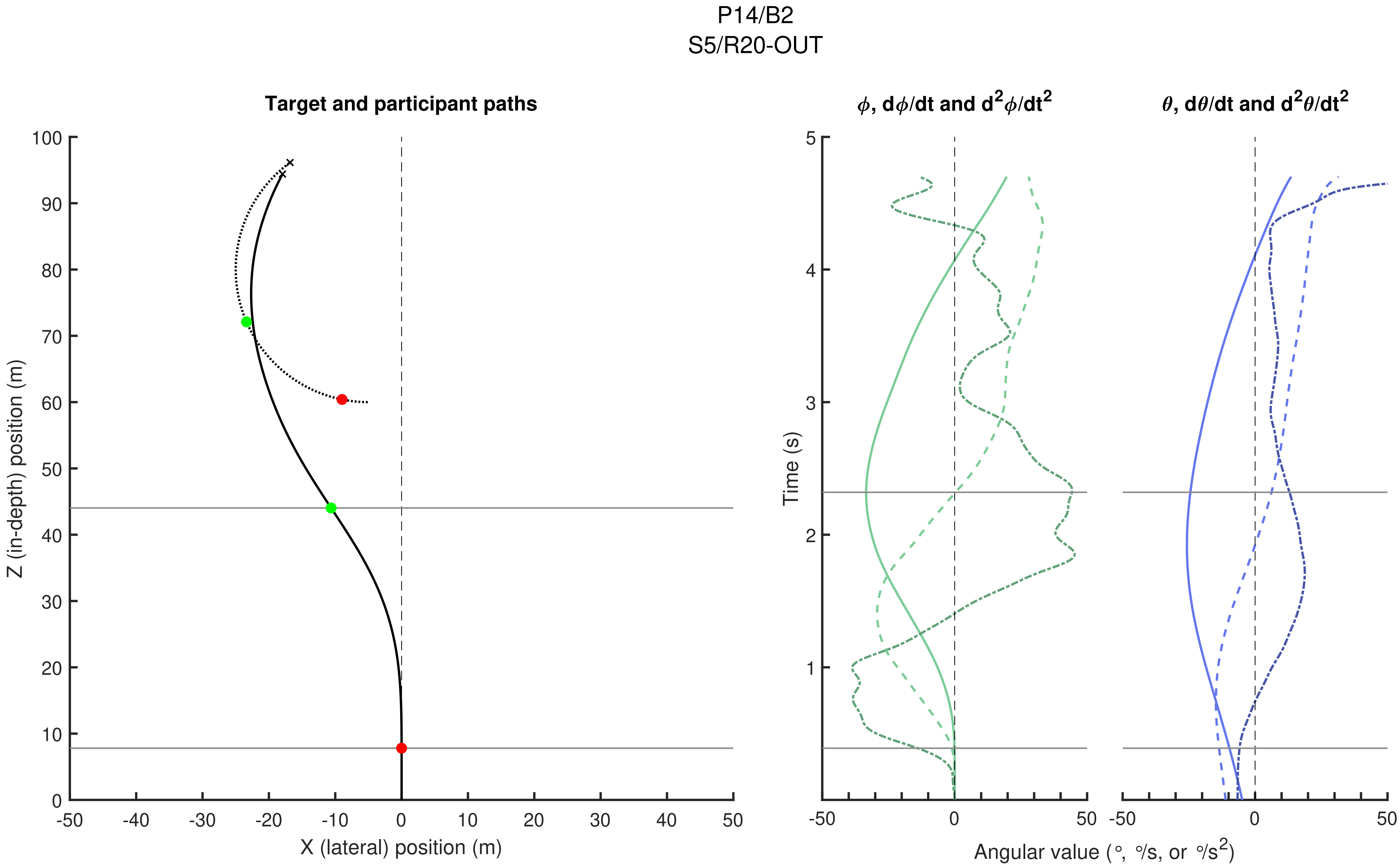

P14/B2
S5/R20-OUT
Target and participant paths
Z (in-depth) position (m)
X (lateral) position (m)
100
90
80
70
60
50
40
30
20
10
0
-50
-40
-30
-20
-10
0
10
20
30
40
50
ϕ, dϕ/dt and d²ϕ/dt²
θ, dθ/dt and d²θ/dt²
Time (s)
5
4
3
2
1
-50
0
50
-50
0
50
Angular value (°, °/s, or °/s²)

P14/B2
S5/R30-IN

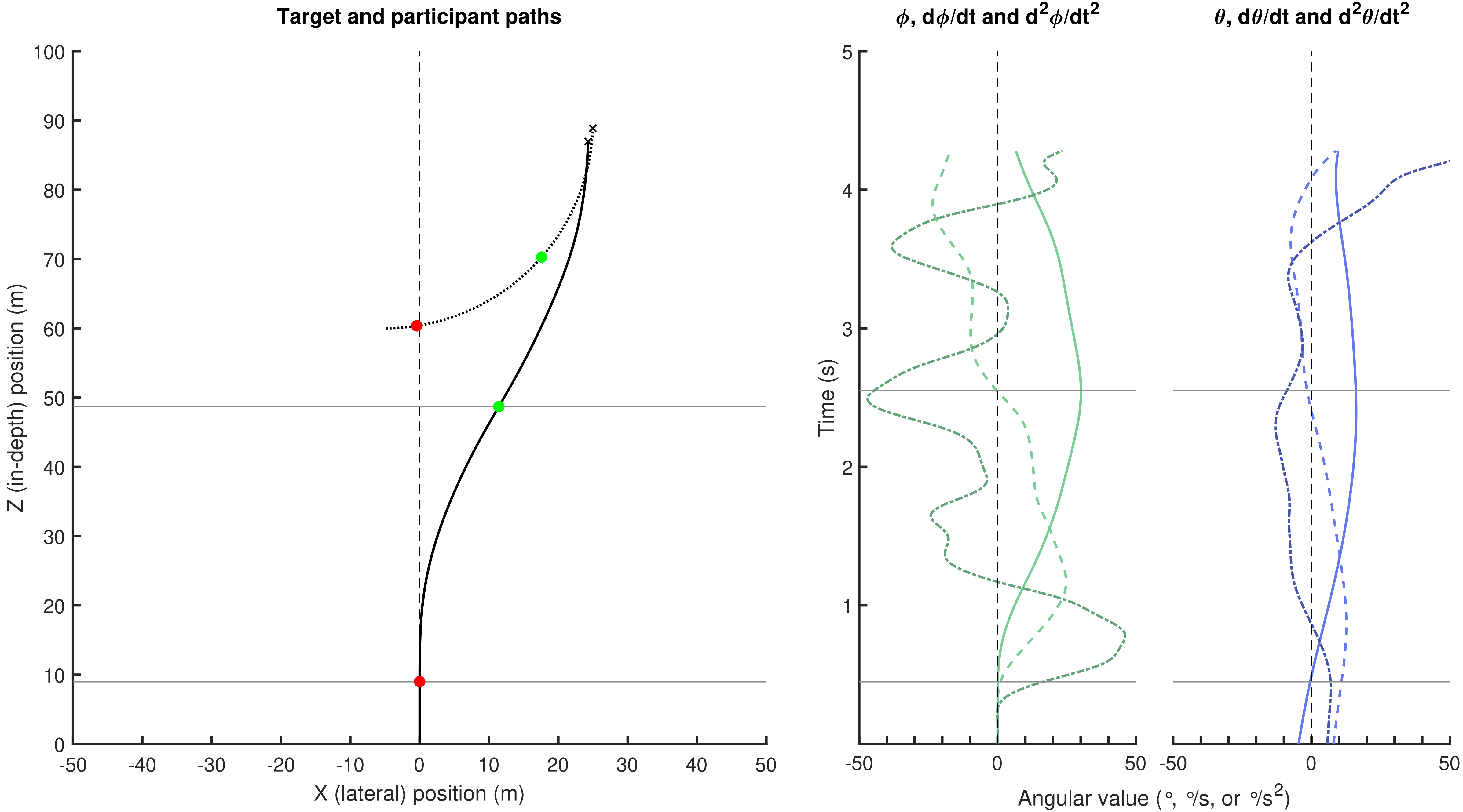

P14/B2
S5/R30-OUT

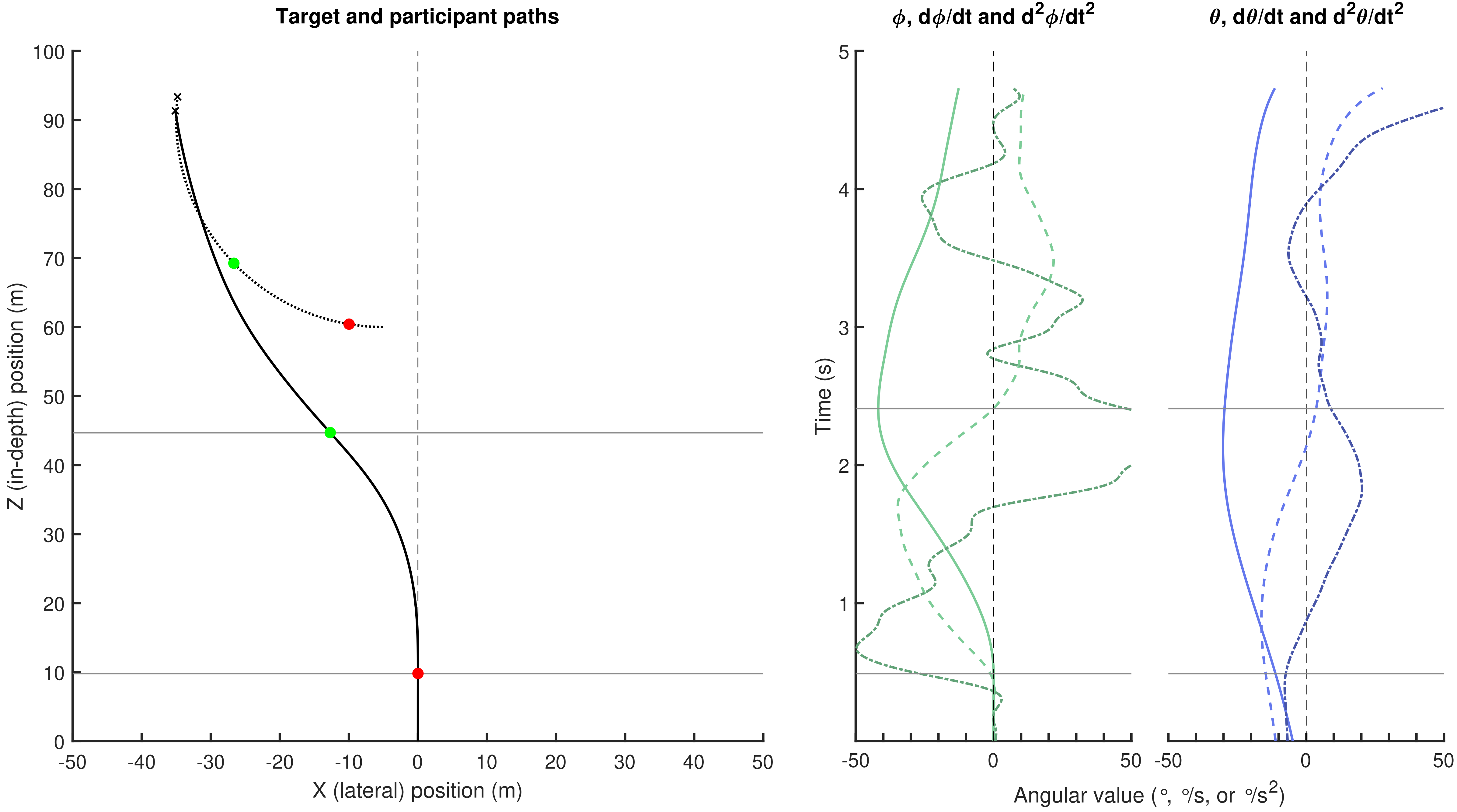

P14/B2
S5/R10-OUT

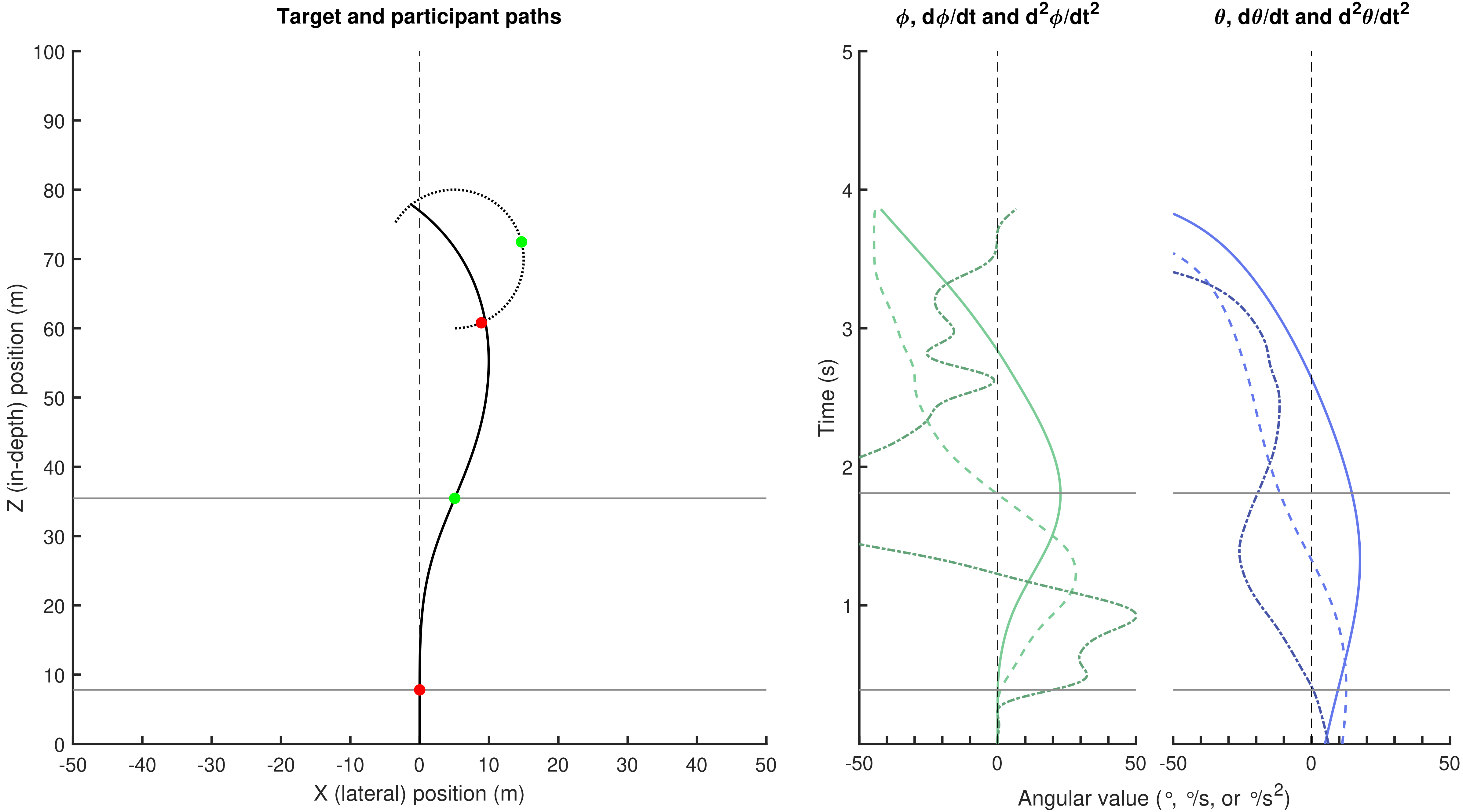

P14/B2
S5/R10-IN

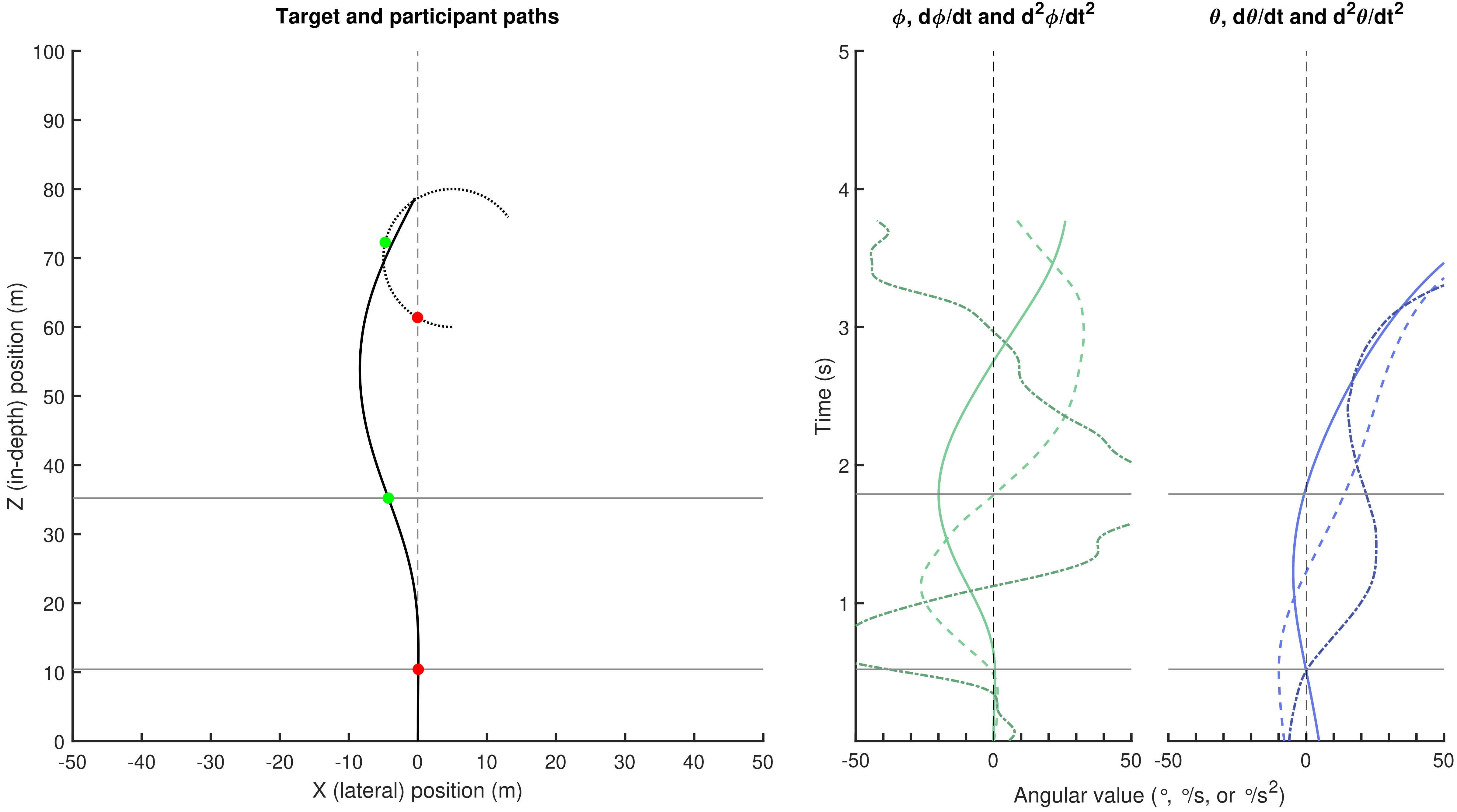

P14/B2
S5/R20-OUT

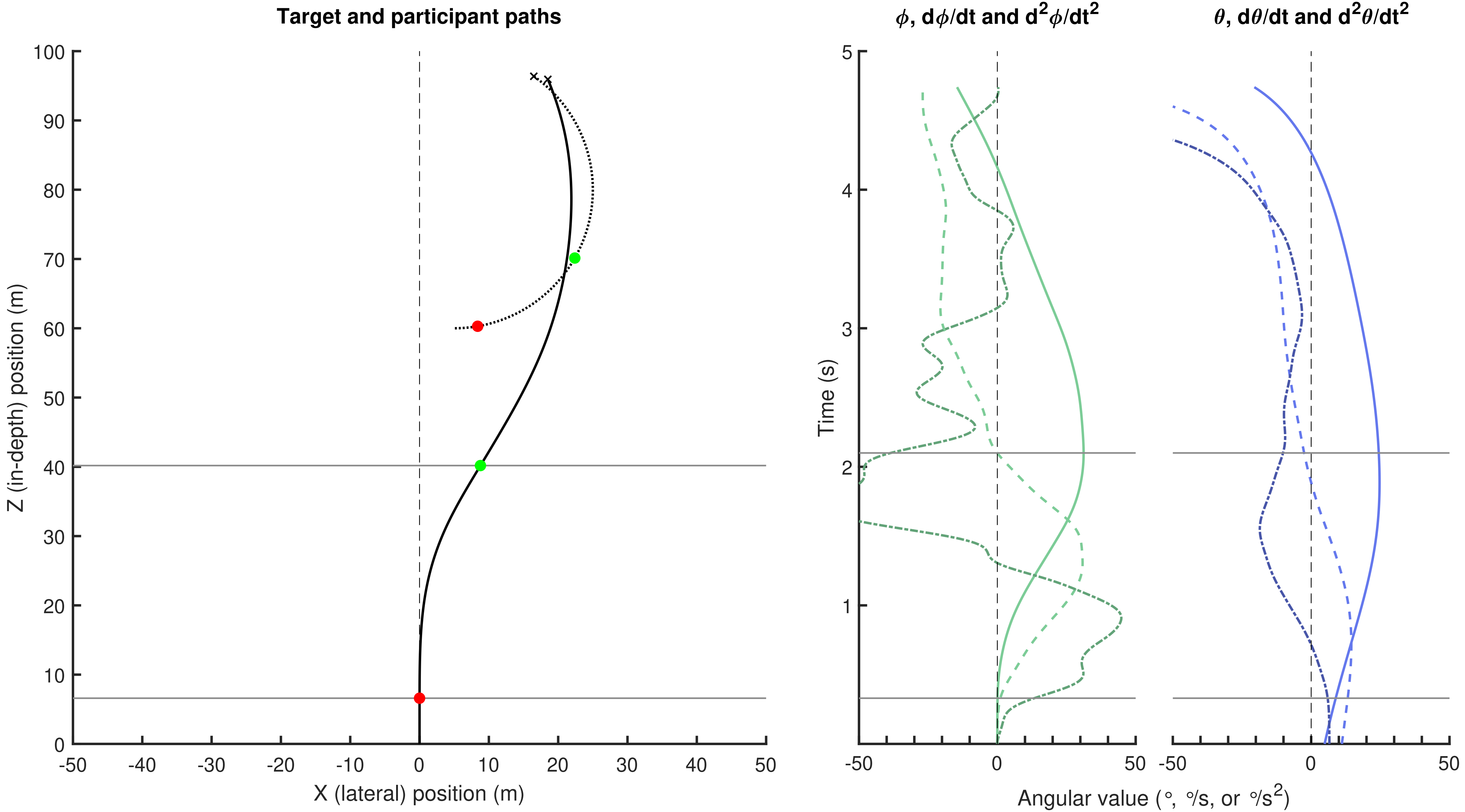

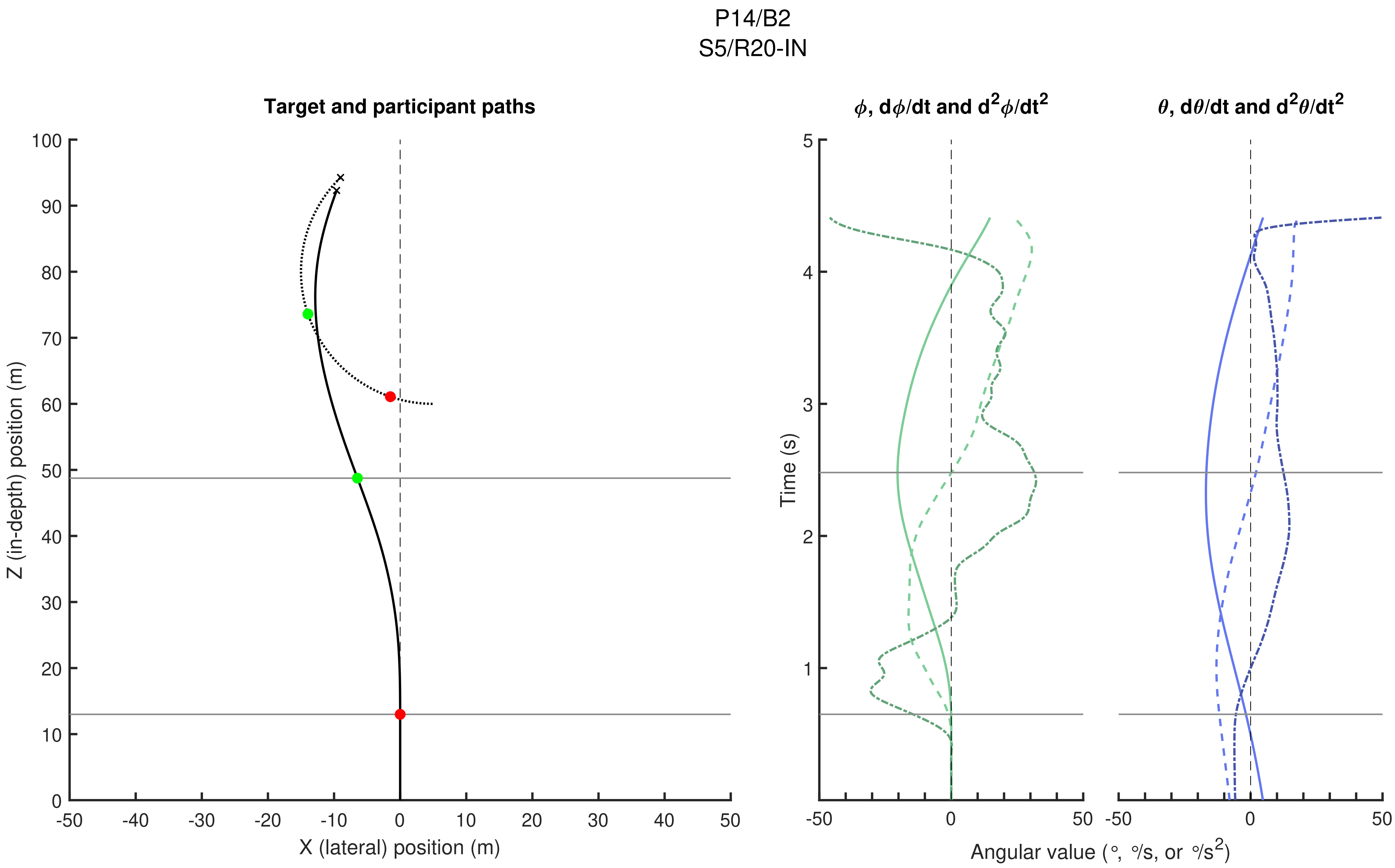
P14/B2
S5/R20-IN
Target and participant paths
Z (in-depth) position (m)
X (lateral) position (m)
100
90
80
70
60
50
40
30
20
10
0
-50
-40
-30
-20
-10
0
10
20
30
40
50
ϕ, dϕ/dt and d²ϕ/dt²
θ, dθ/dt and d²θ/dt²
Time (s)
5
4
3
2
1
-50
0
50
-50
0
50
Angular value (°, °/s, or °/s²)

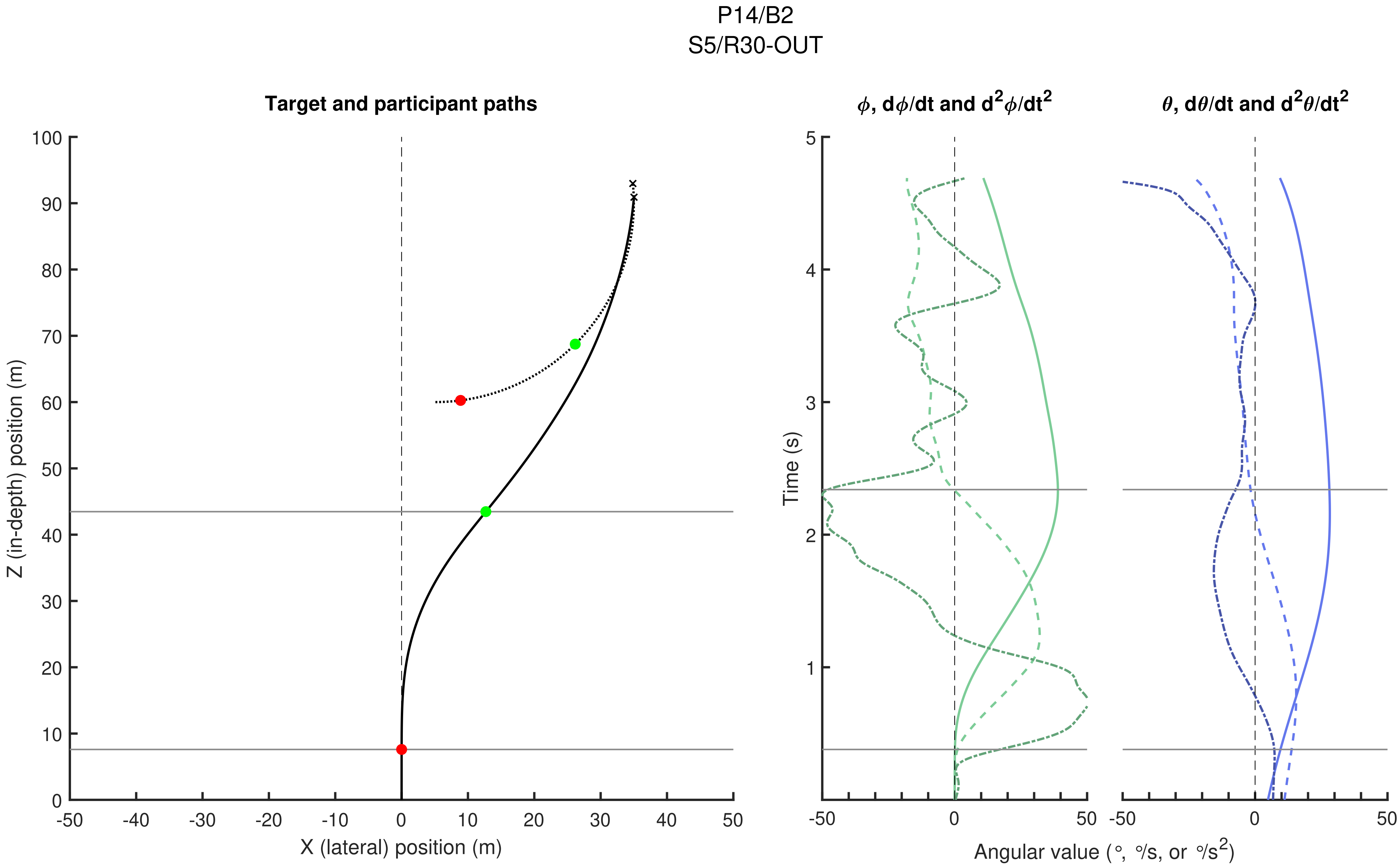
P14/B2
S5/R30-OUT
Target and participant paths
Z (in-depth) position (m)
X (lateral) position (m)
100
90
80
70
60
50
40
30
20
10
0
-50
-40
-30
-20
-10
0
10
20
30
40
50
ϕ, dϕ/dt and d$^2\phi$/dt^2
θ, dθ/dt and d$^2\theta$/dt^2
Time (s)
5
4
3
2
1
-50
0
50
-50
0
50
Angular value (°, °/s, or °/s^2)

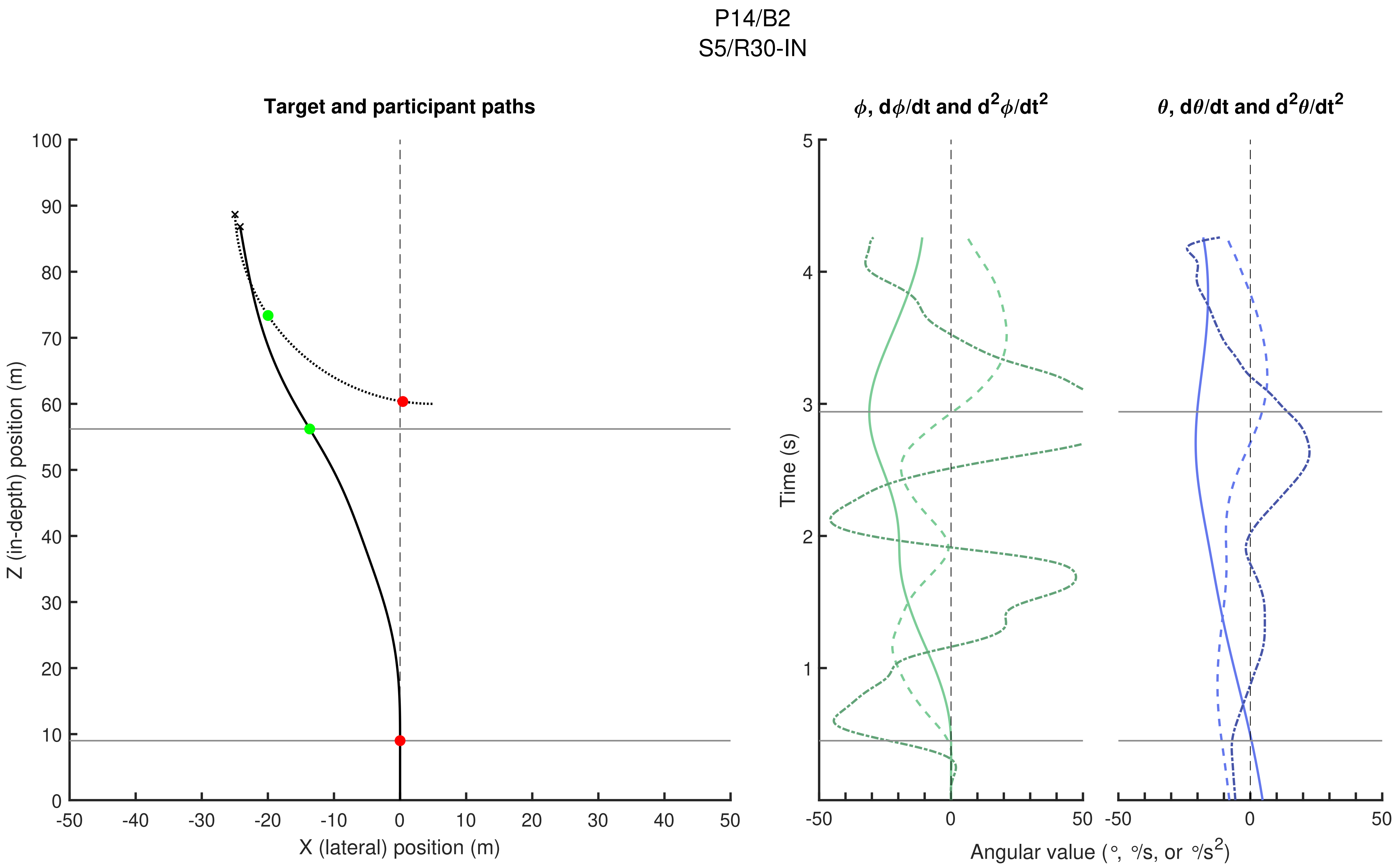

P14/B2
S5/R30-IN
Target and participant paths
Z (in-depth) position (m)
X (lateral) position (m)
ϕ, dϕ/dt and d$^2\phi$/dt^2
θ, dθ/dt and d$^2\theta$/dt^2
Time (s)
Angular value (°, °/s, or °/s^2)

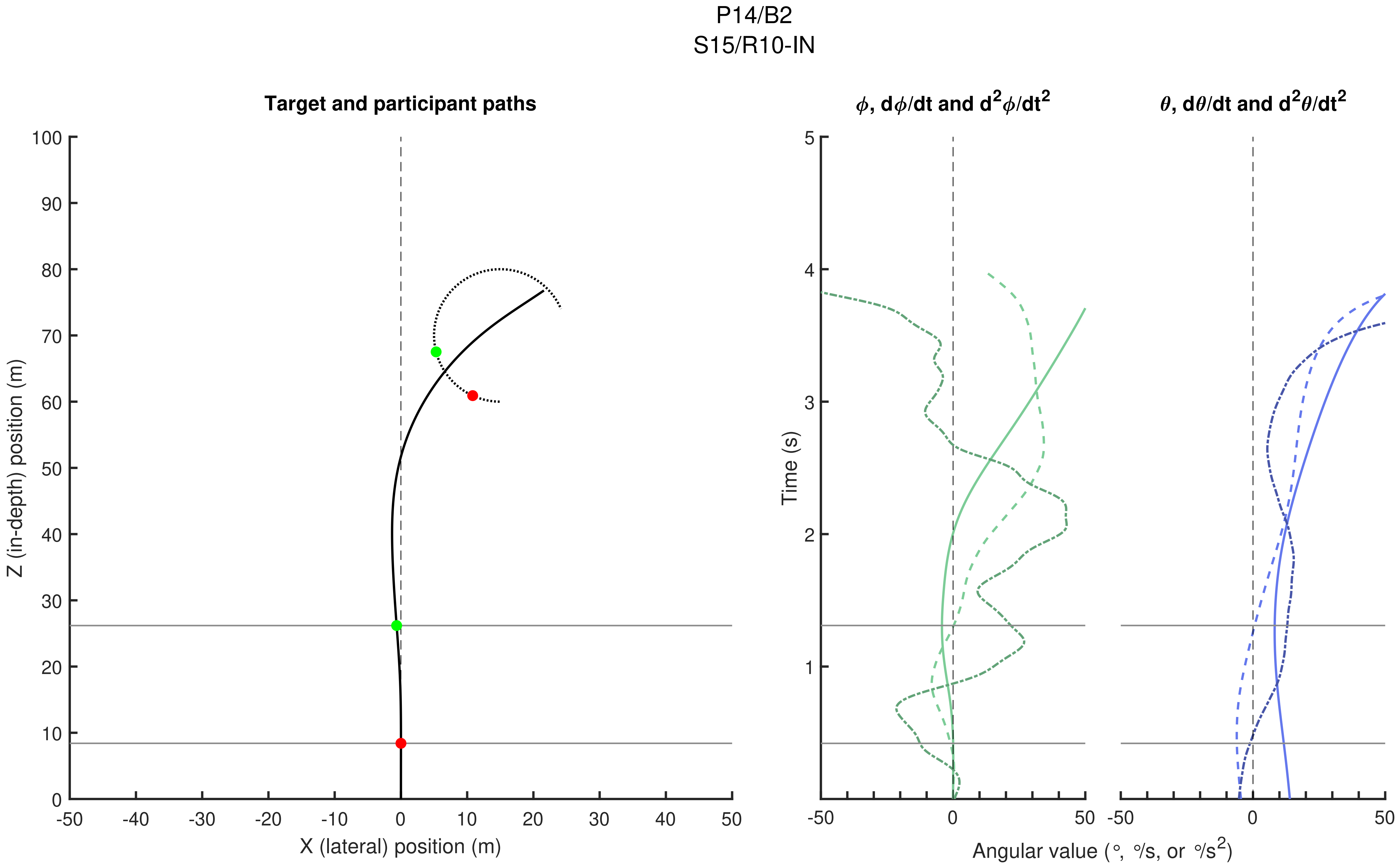
P14/B2
S15/R10-IN
Target and participant paths
Z (in-depth) position (m)
X (lateral) position (m)
ϕ, dϕ/dt and d$^2\phi$/dt^2
θ, dθ/dt and d$^2\theta$/dt^2
Time (s)
Angular value (°, °/s, or °/s^2)

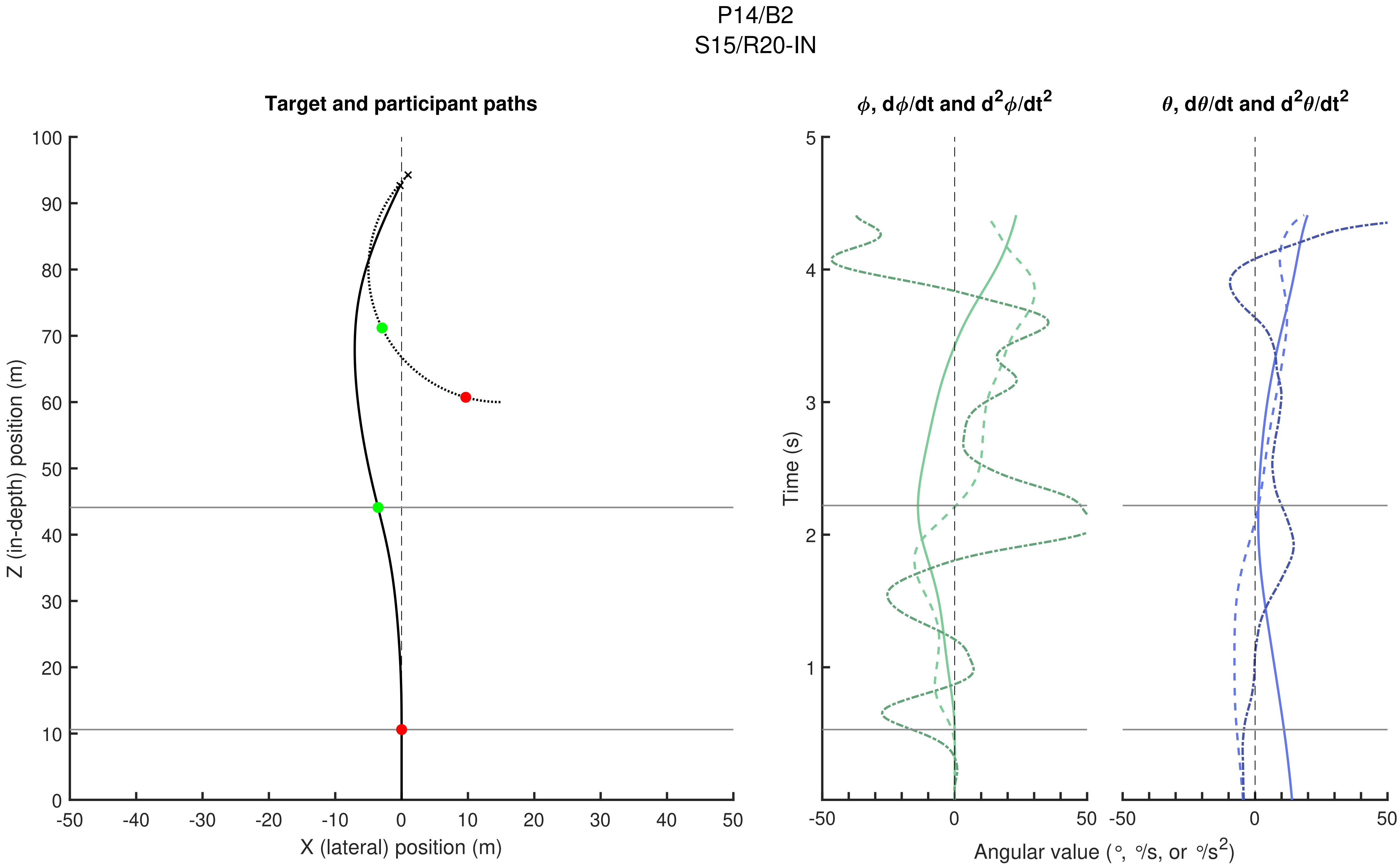

P14/B2
S15/R20-IN
Target and participant paths
Z (in-depth) position (m)
X (lateral) position (m)
ϕ, dϕ/dt and d²ϕ/dt²
θ, dθ/dt and d²θ/dt²
Time (s)
Angular value (°, °/s, or °/s²)

P14/B2
S15/R30-IN

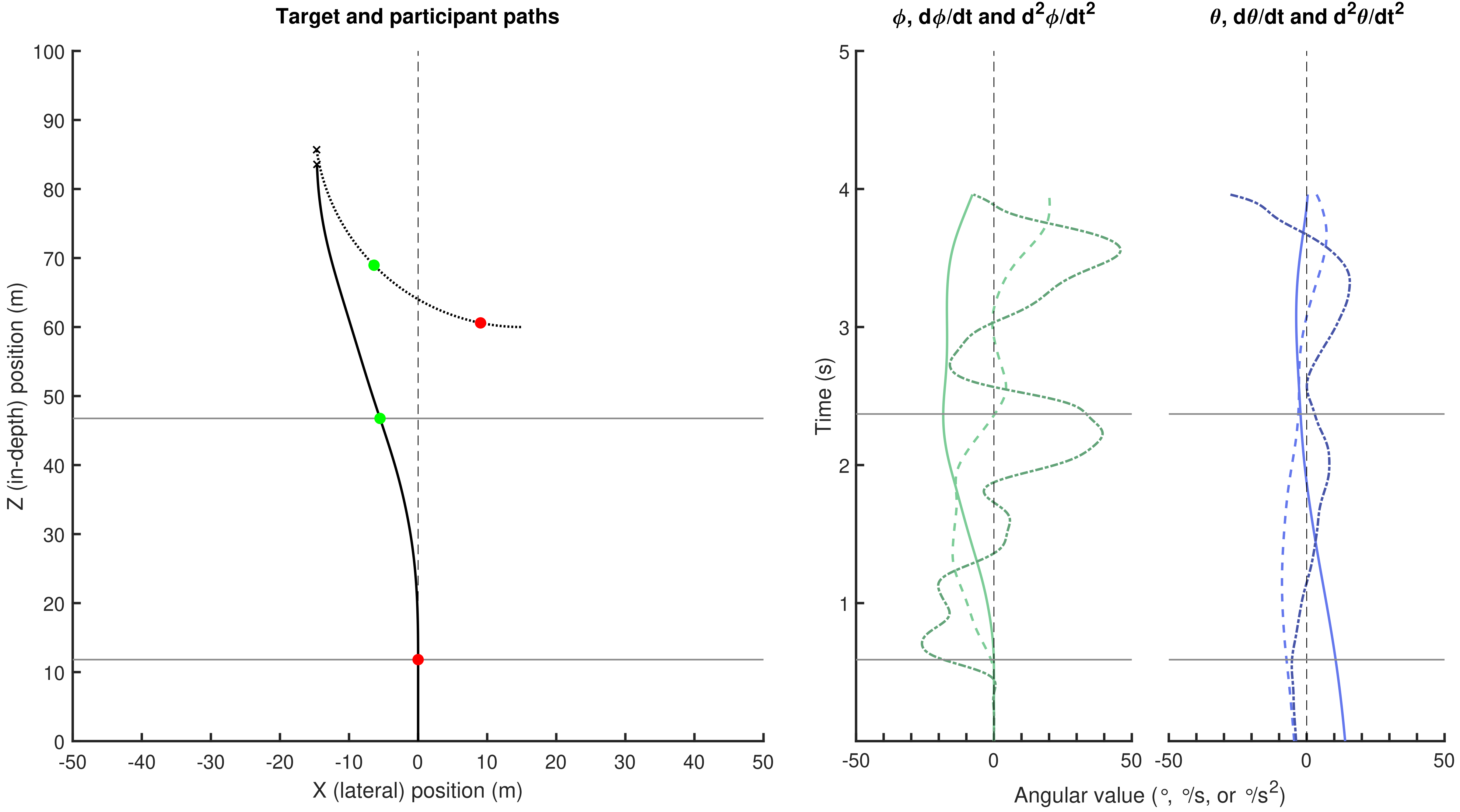

P14/B2
S20/R10-IN

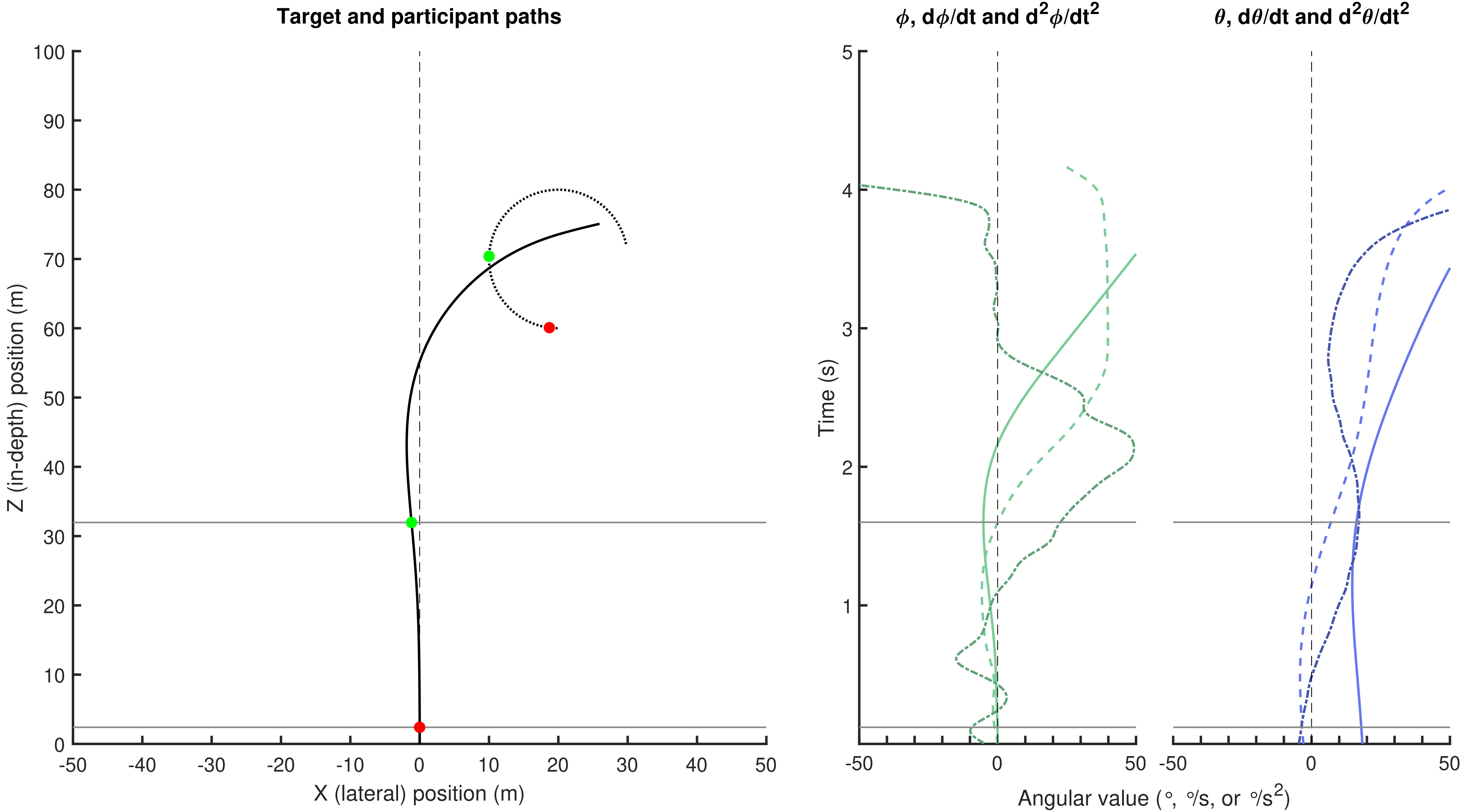

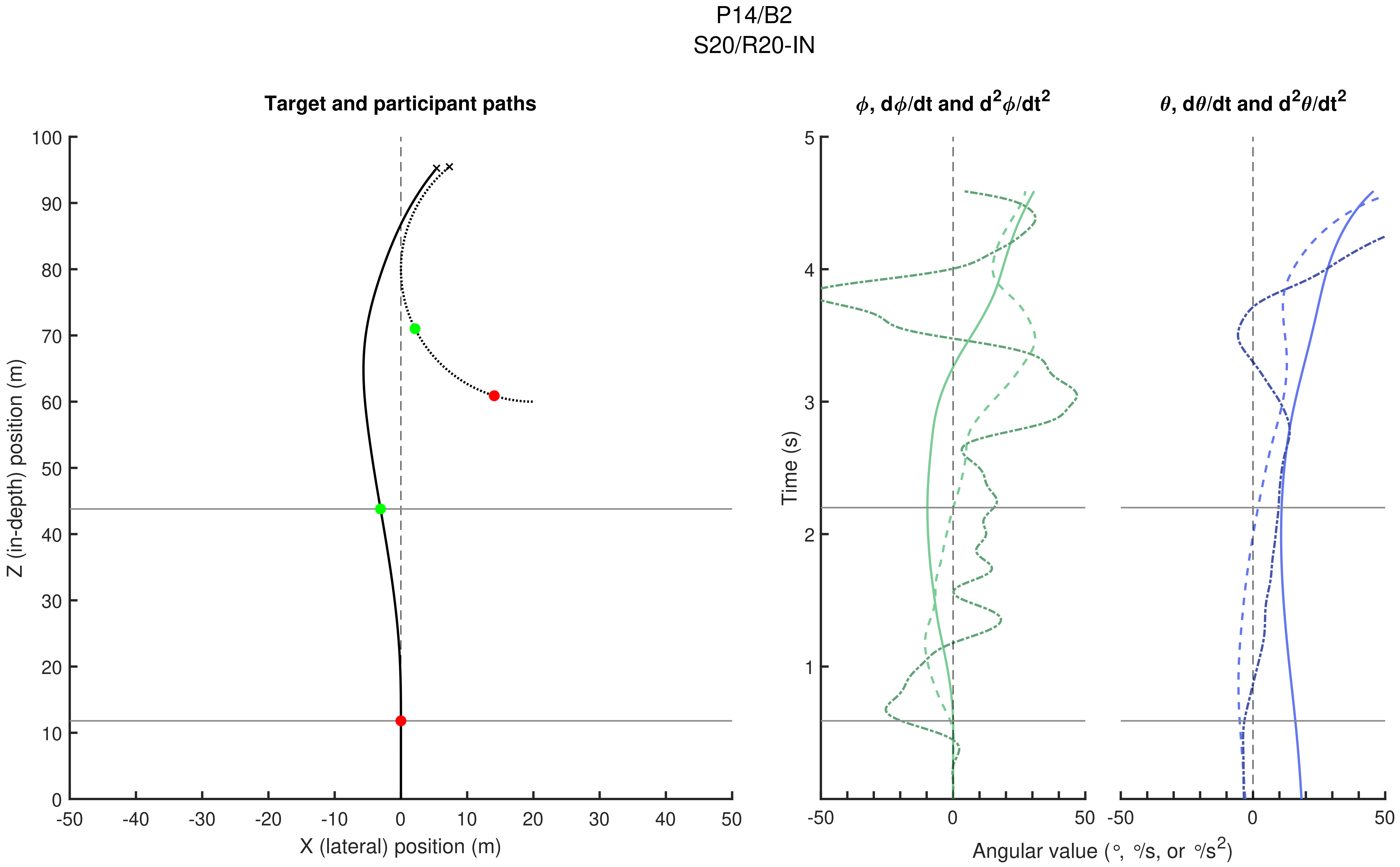

P14/B2
S20/R20-IN
Target and participant paths
Z (in-depth) position (m)
X (lateral) position (m)
100
90
80
70
60
50
40
30
20
10
0
-50
-40
-30
-20
-10
0
10
20
30
40
50
φ, dφ/dt and d²φ/dt²
θ, dθ/dt and d²θ/dt²
Time (s)
5
4
3
2
1
-50
0
50
-50
0
50
Angular value (°, °/s, or °/s²)

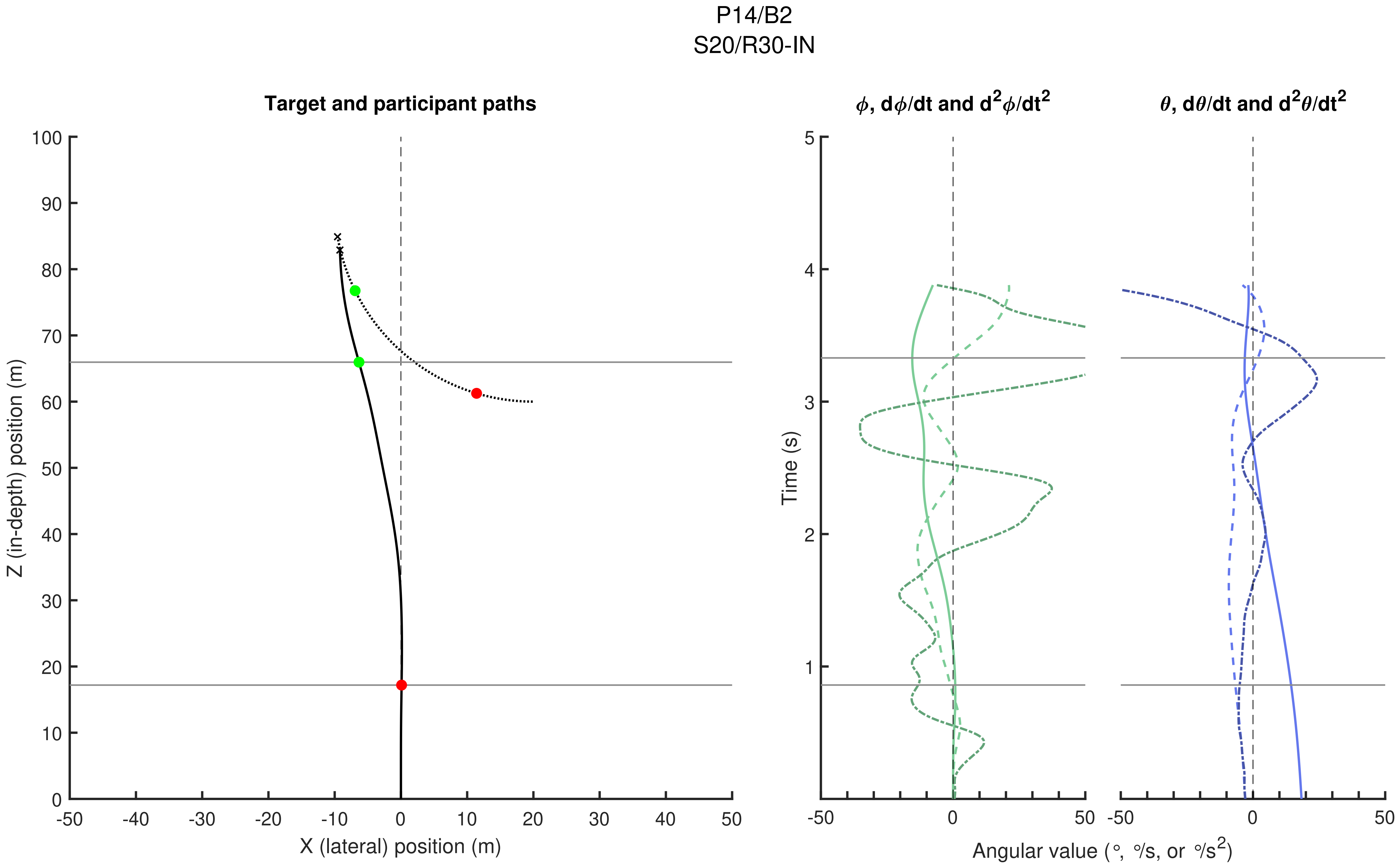
P14/B2
S20/R30-IN
Target and participant paths
Z (in-depth) position (m)
X (lateral) position (m)
ϕ, dϕ/dt and d$^2\phi$/dt^2
θ, dθ/dt and d$^2\theta$/dt^2
Time (s)
Angular value (°, °/s, or °/s^2)

P14/B3
S20/R10-IN

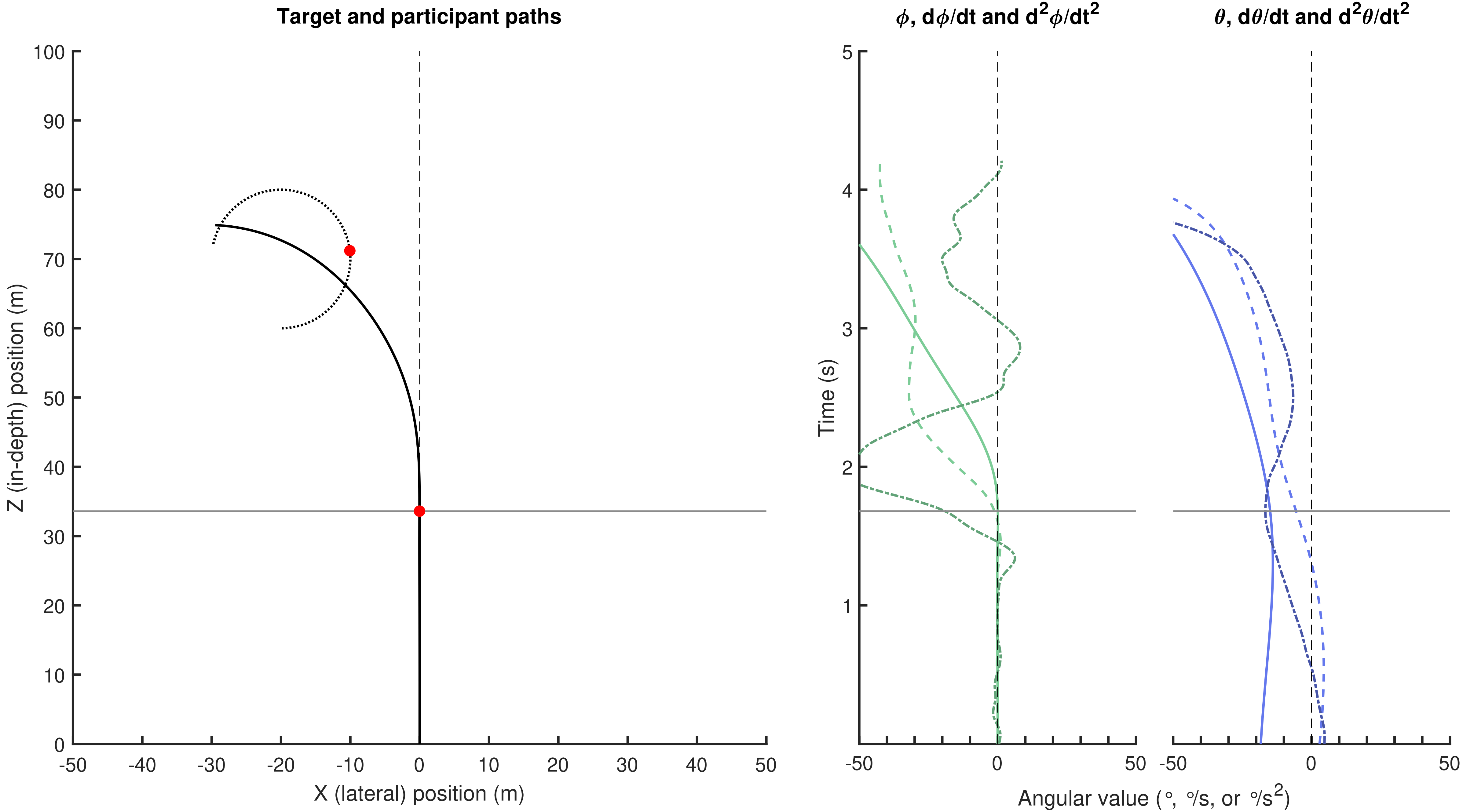

P14/B3
S20/R20-IN

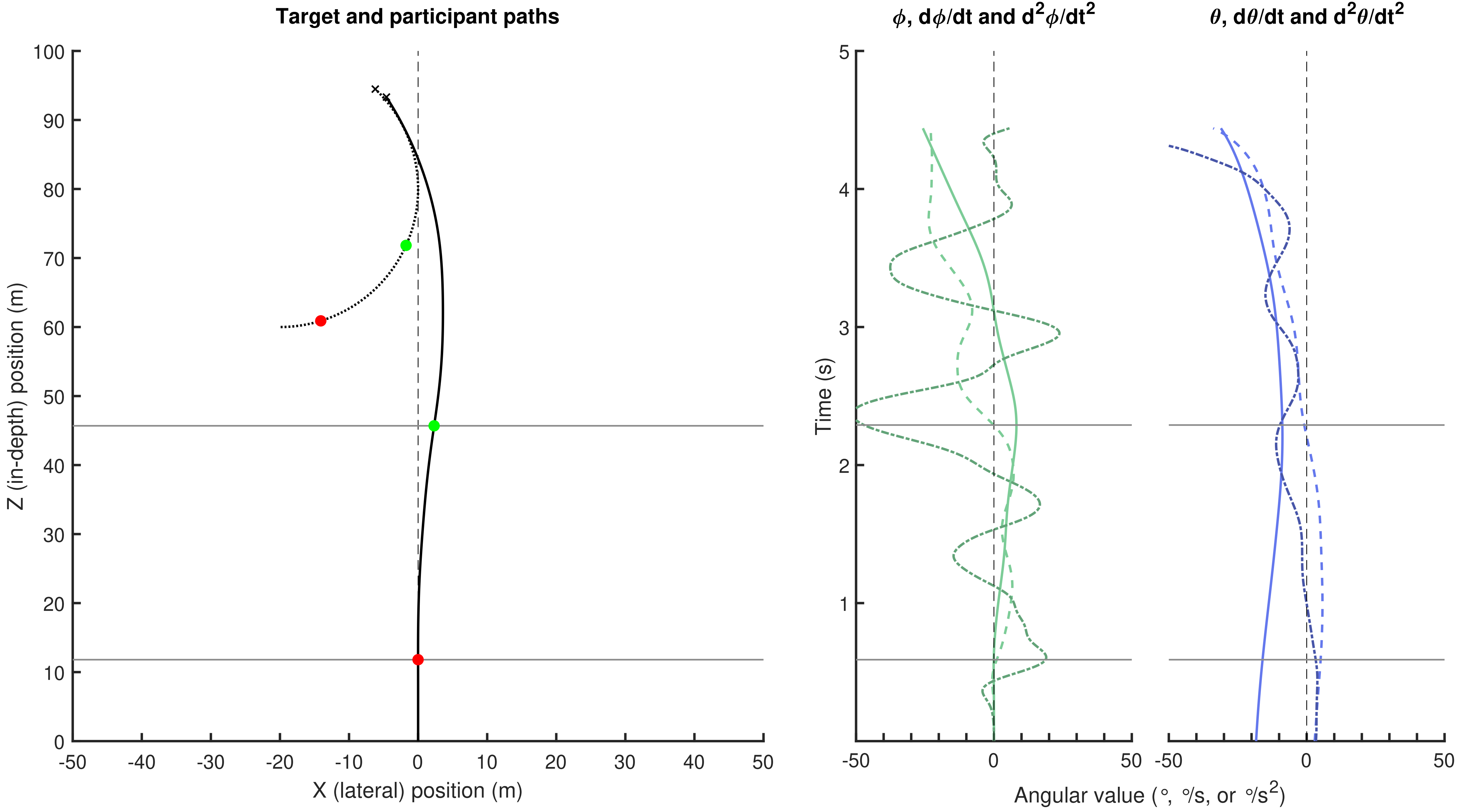

P14/B3
S20/R30-IN

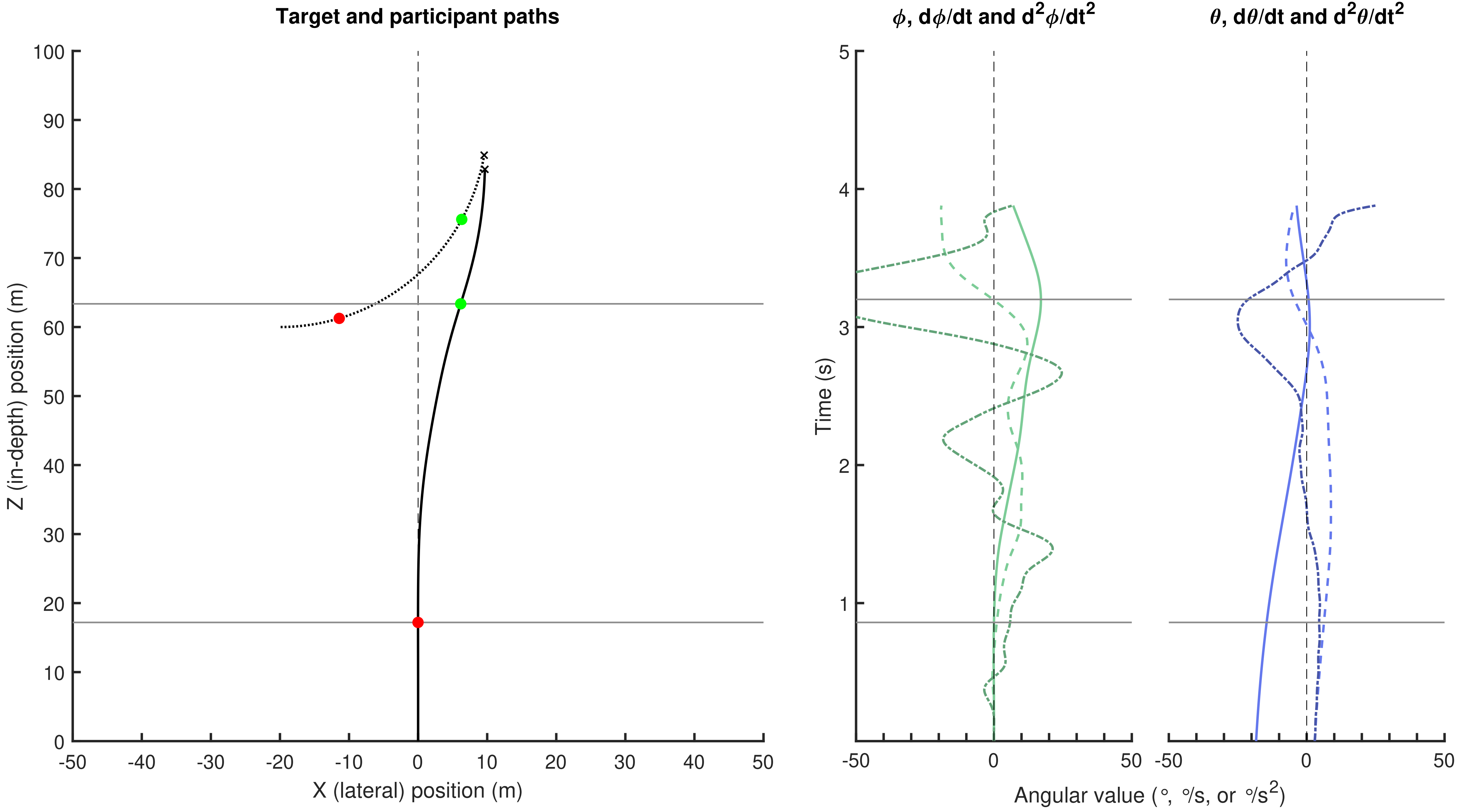

P14/B3
S15/R10-IN

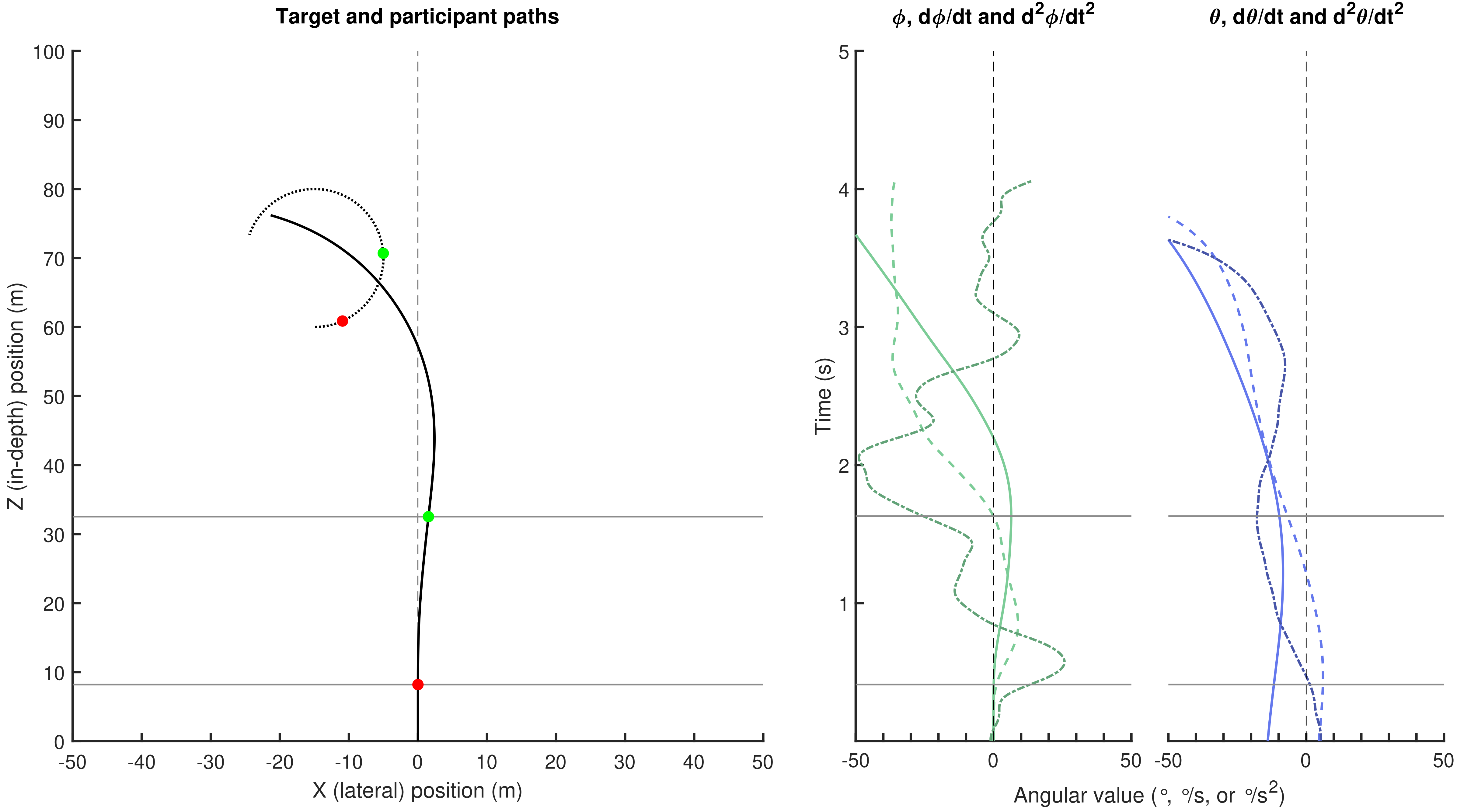

P14/B3
S15/R20-IN

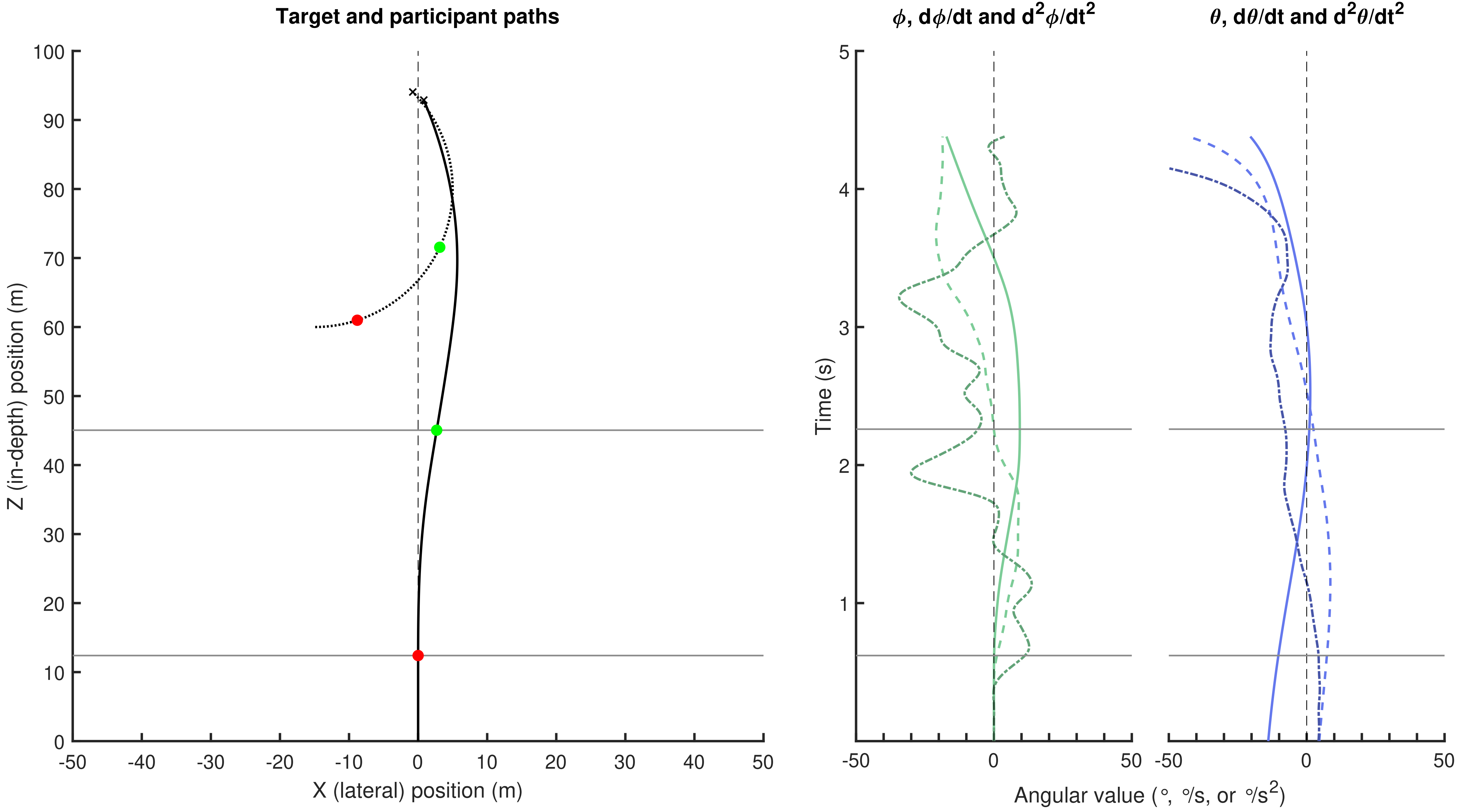

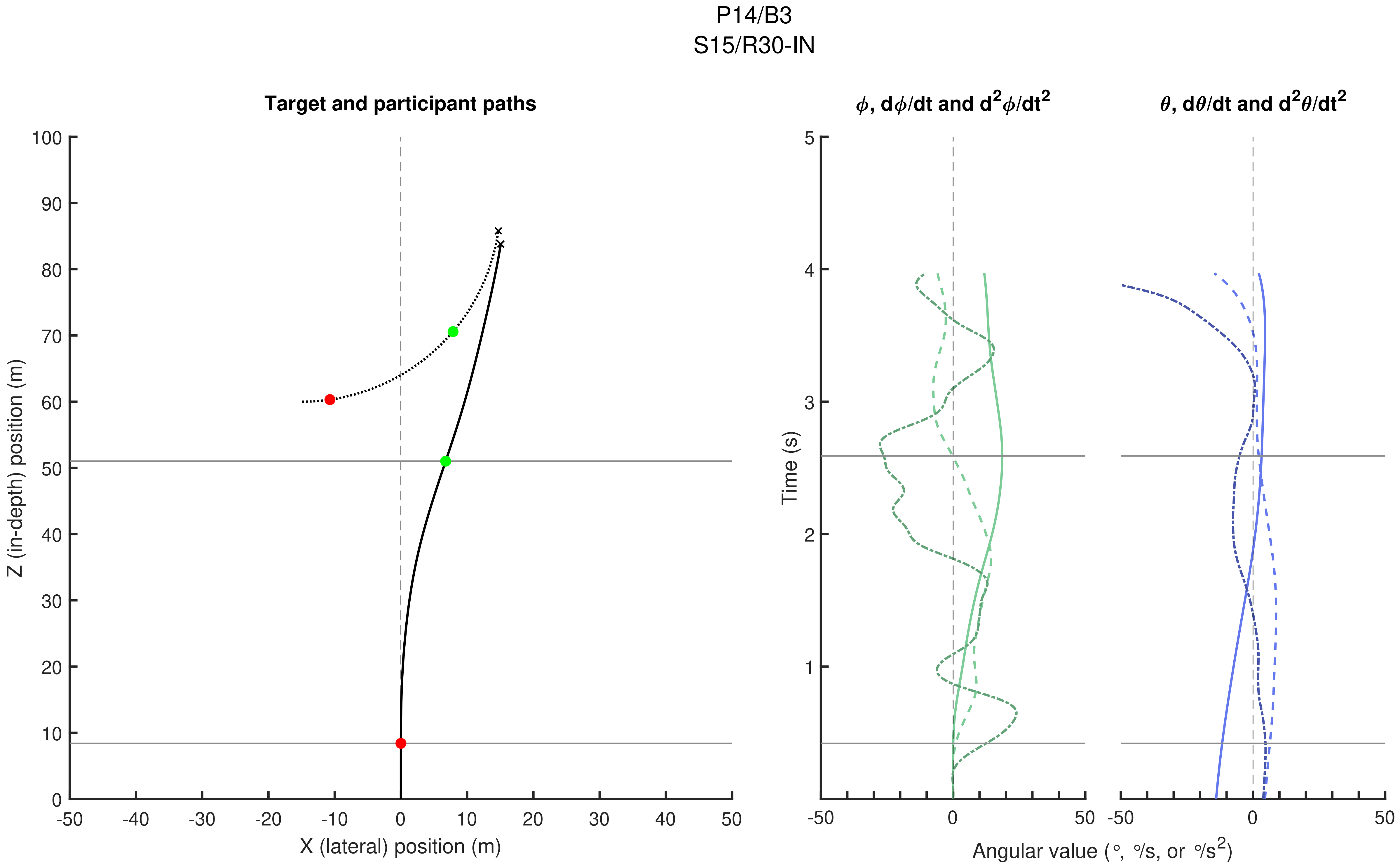
P14/B3
S15/R30-IN
Target and participant paths
ϕ, dϕ/dt and d$^2\phi$/dt^2
θ, dθ/dt and d$^2\theta$/dt^2
Z (in-depth) position (m)
X (lateral) position (m)
Time (s)
Angular value (°, °/s, or °/s^2)
100
90
80
70
60
50
40
30
20
10
0
-50
-40
-30
-20
-10
0
10
20
30
40
50
5
4
3
2
1
-50
0
50
-50
0
50

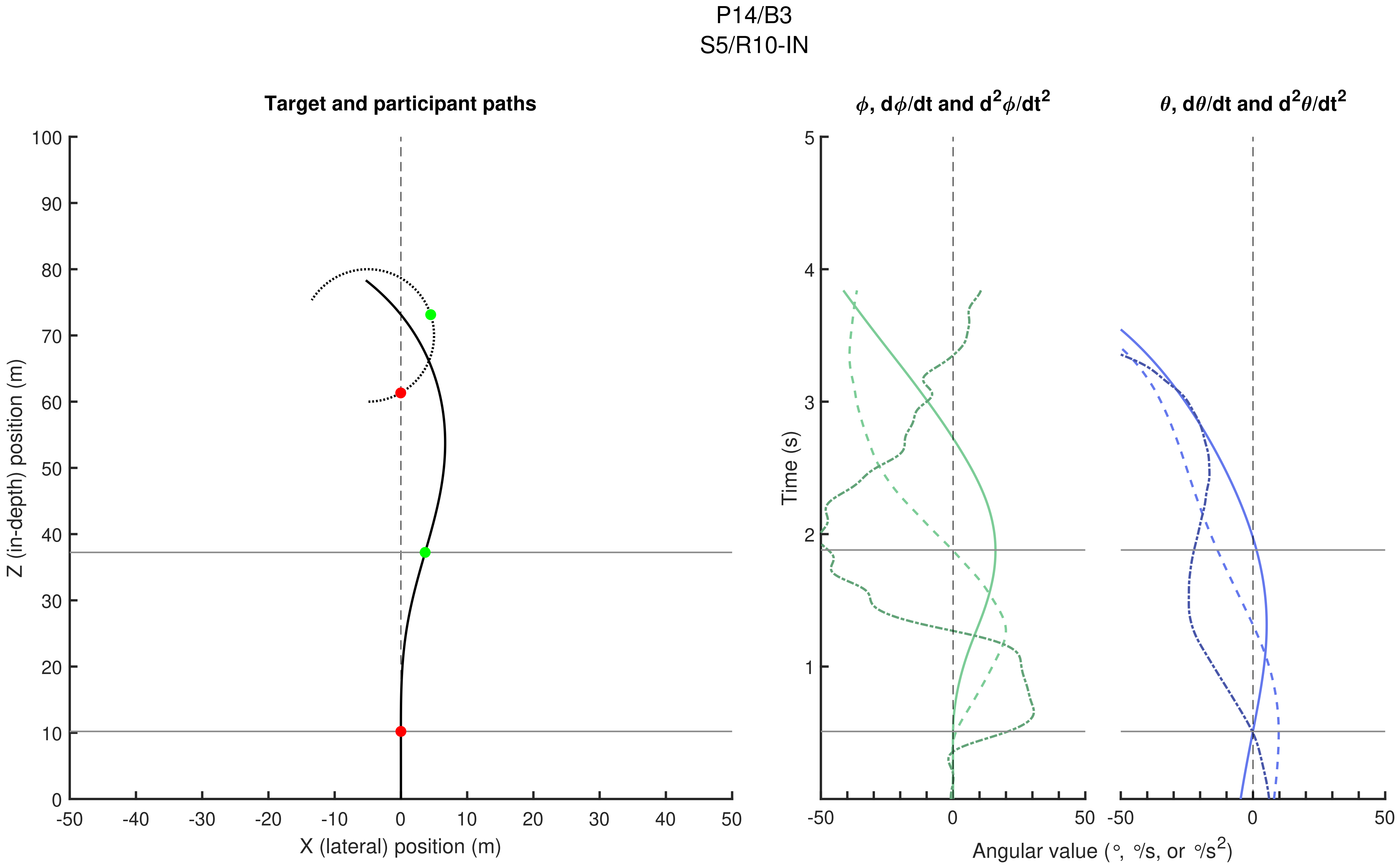

P14/B3
S5/R10-IN
Target and participant paths
Z (in-depth) position (m)
X (lateral) position (m)
100
90
80
70
60
50
40
30
20
10
0
-50
-40
-30
-20
-10
0
10
20
30
40
50
ϕ, dϕ/dt and d$^2\phi$/dt^2
θ, dθ/dt and d$^2\theta$/dt^2
Time (s)
5
4
3
2
1
-50
0
50
-50
0
50
Angular value (°, °/s, or °/s^2)

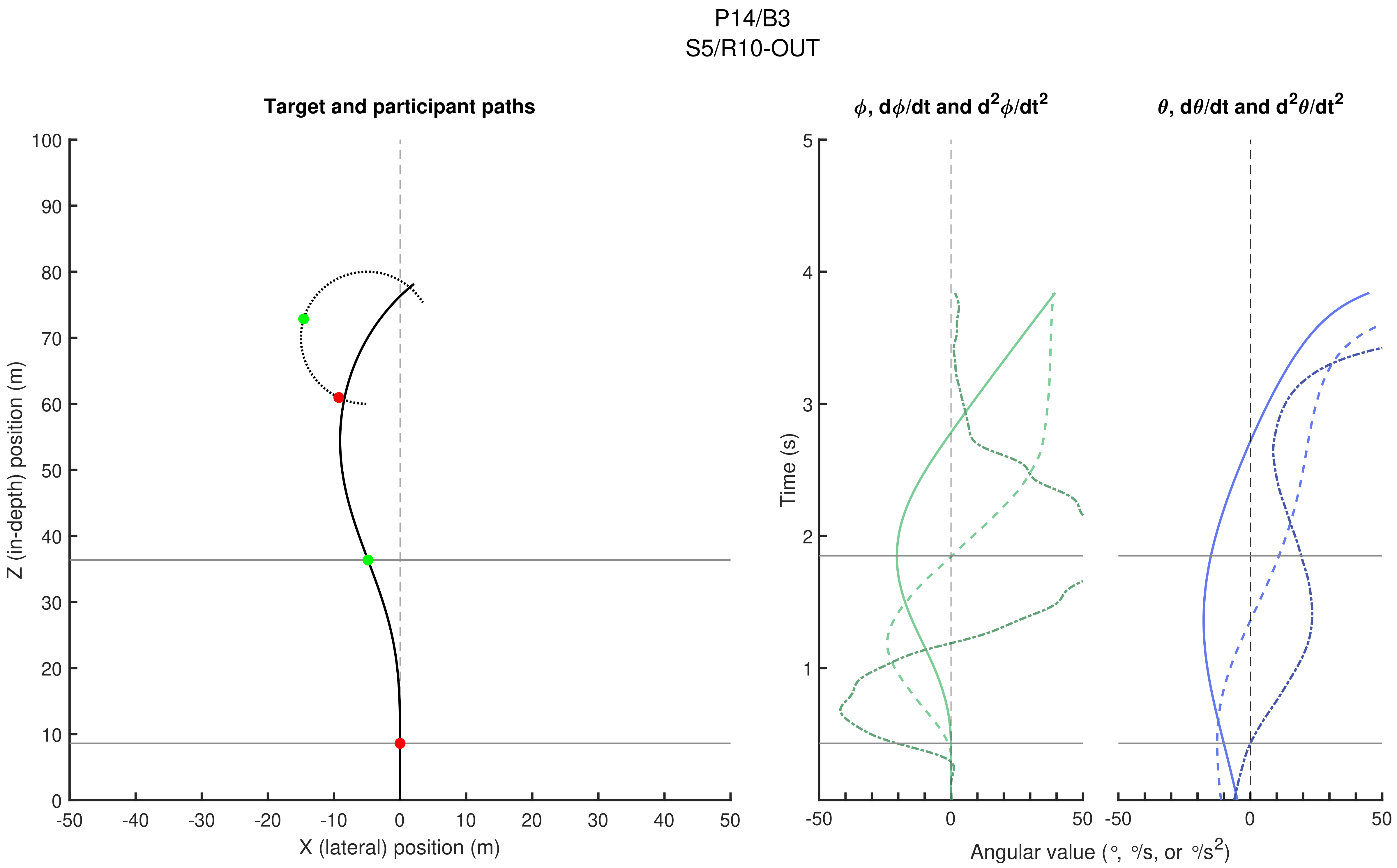

P14/B3
S5/R10-OUT
Target and participant paths
Z (in-depth) position (m)
X (lateral) position (m)
100
90
80
70
60
50
40
30
20
10
0
-50
-40
-30
-20
-10
0
10
20
30
40
50
φ, dφ/dt and d²φ/dt²
θ, dθ/dt and d²θ/dt²
Time (s)
5
4
3
2
1
-50
0
50
-50
0
50
Angular value (°, °/s, or °/s²)

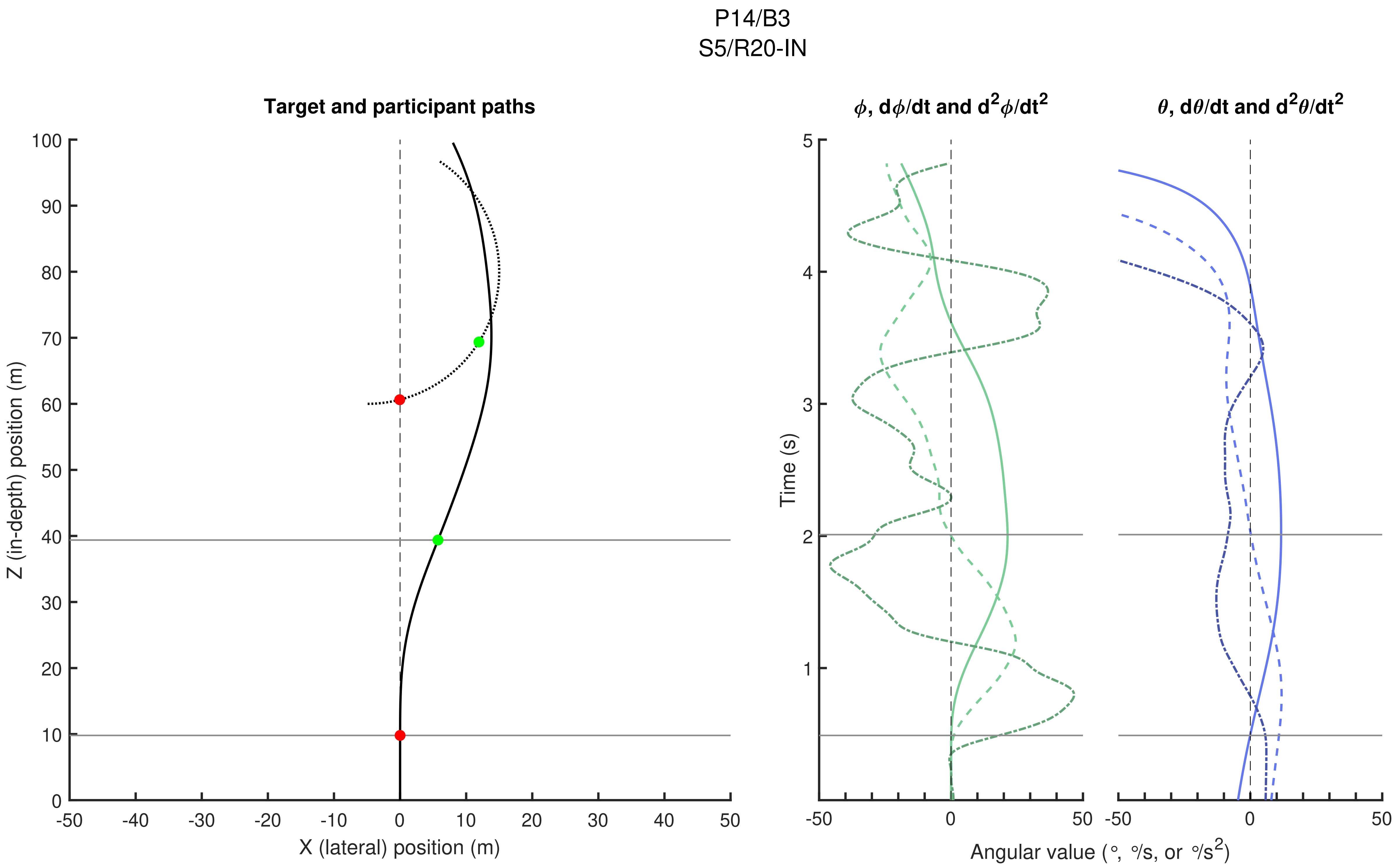
P14/B3
S5/R20-IN
Target and participant paths
Z (in-depth) position (m)
X (lateral) position (m)
ϕ, dϕ/dt and d$^2\phi$/dt^2
θ, dθ/dt and d$^2\theta$/dt^2
Time (s)
Angular value (°, °/s, or °/s^2)

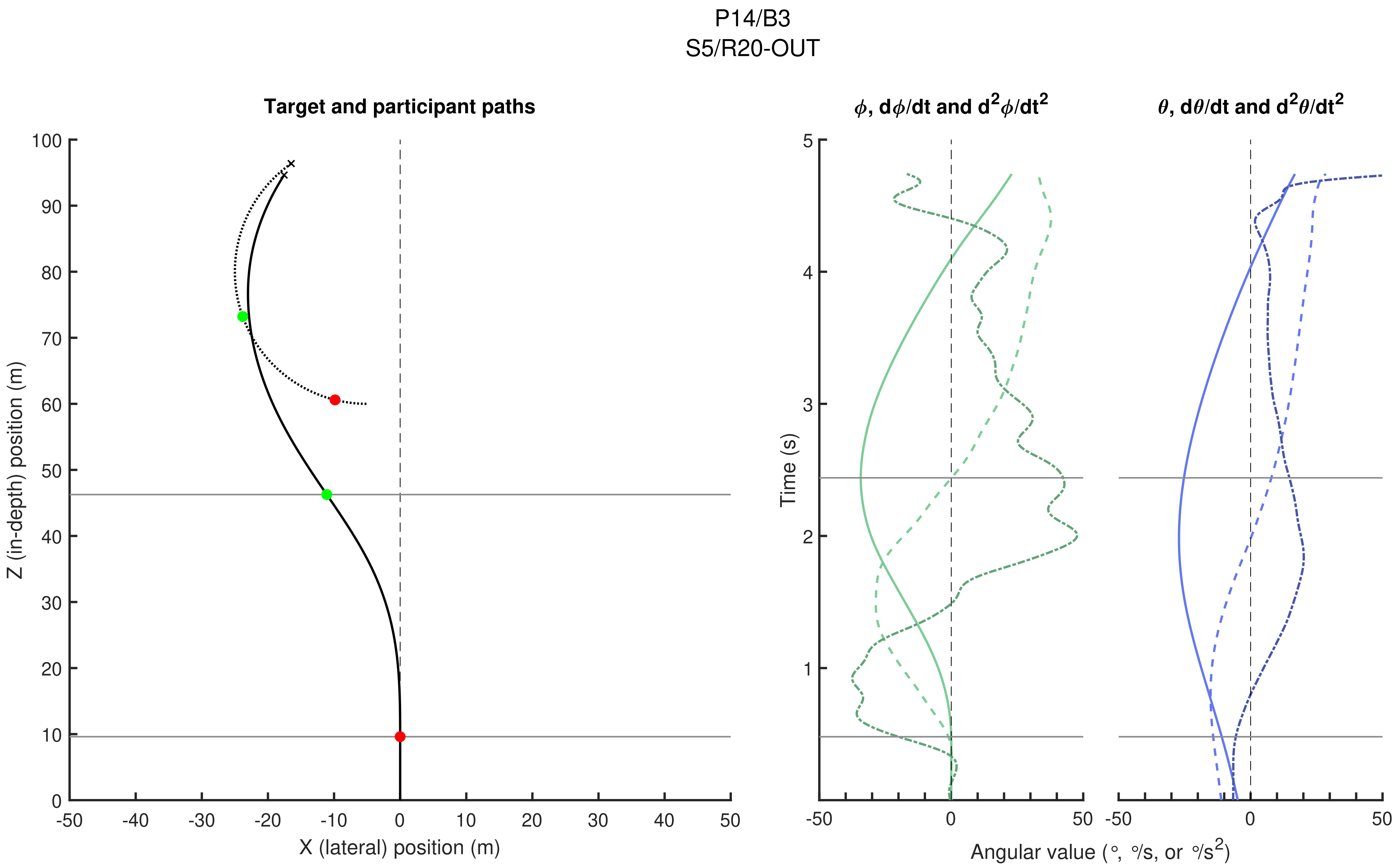

P14/B3
S5/R20-OUT
Target and participant paths
Z (in-depth) position (m)
X (lateral) position (m)
φ, dφ/dt and d²φ/dt²
θ, dθ/dt and d²θ/dt²
Time (s)
Angular value (°, °/s, or °/s²)

P14/B3
S5/R30-IN

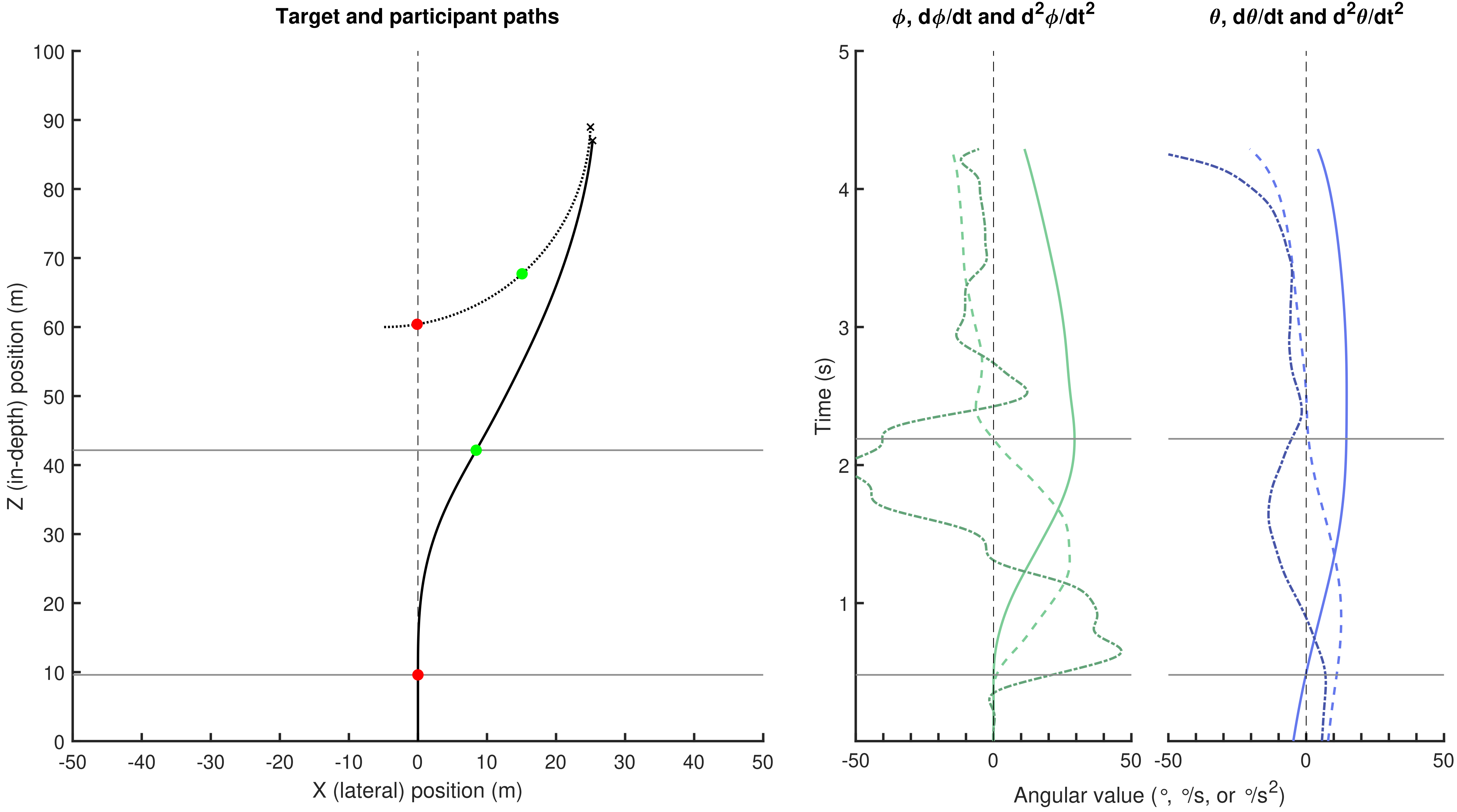

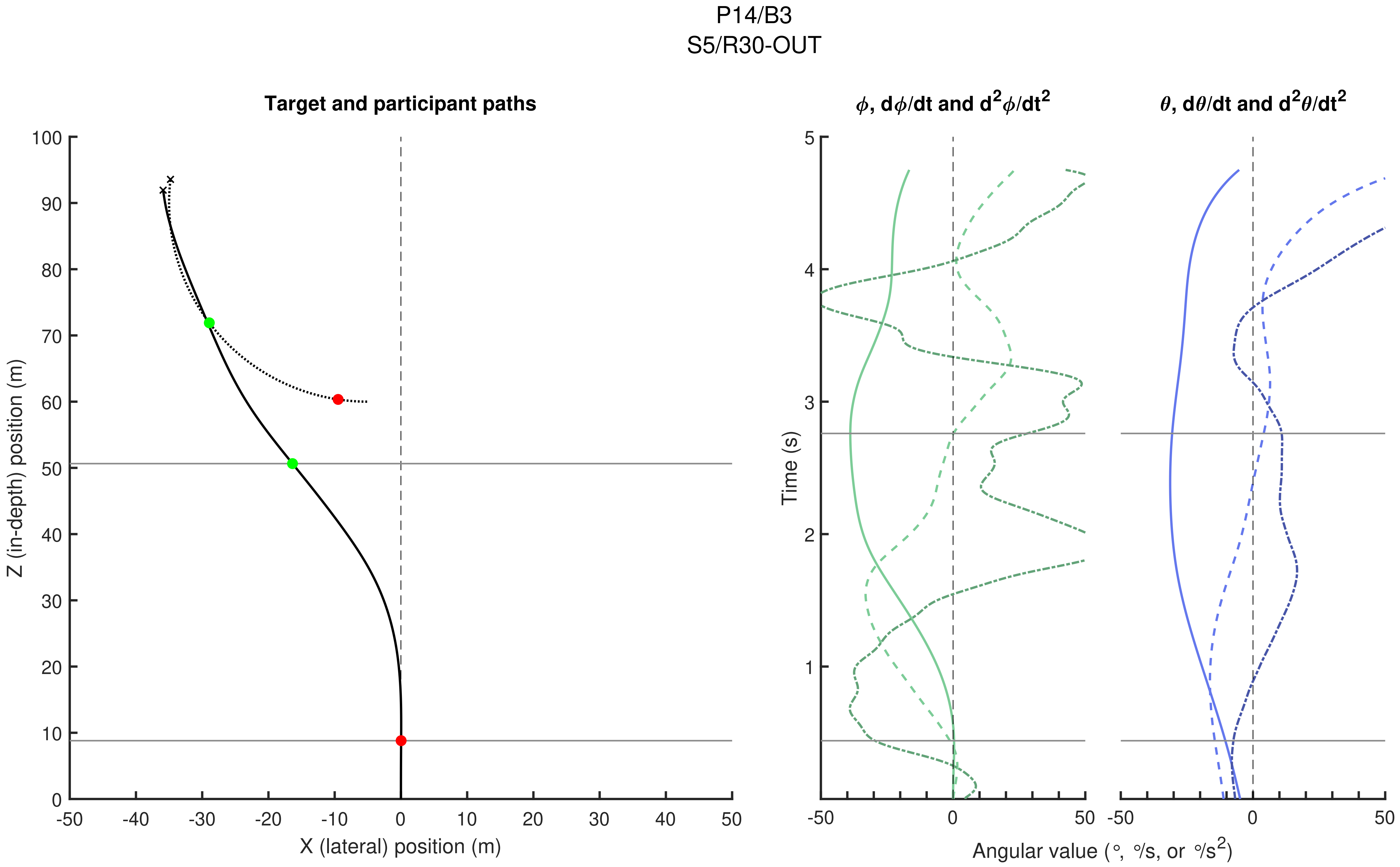

P14/B3
S5/R30-OUT
Target and participant paths
Z (in-depth) position (m)
X (lateral) position (m)
φ, dφ/dt and d²φ/dt²
θ, dθ/dt and d²θ/dt²
Time (s)
Angular value (°, °/s, or °/s²)

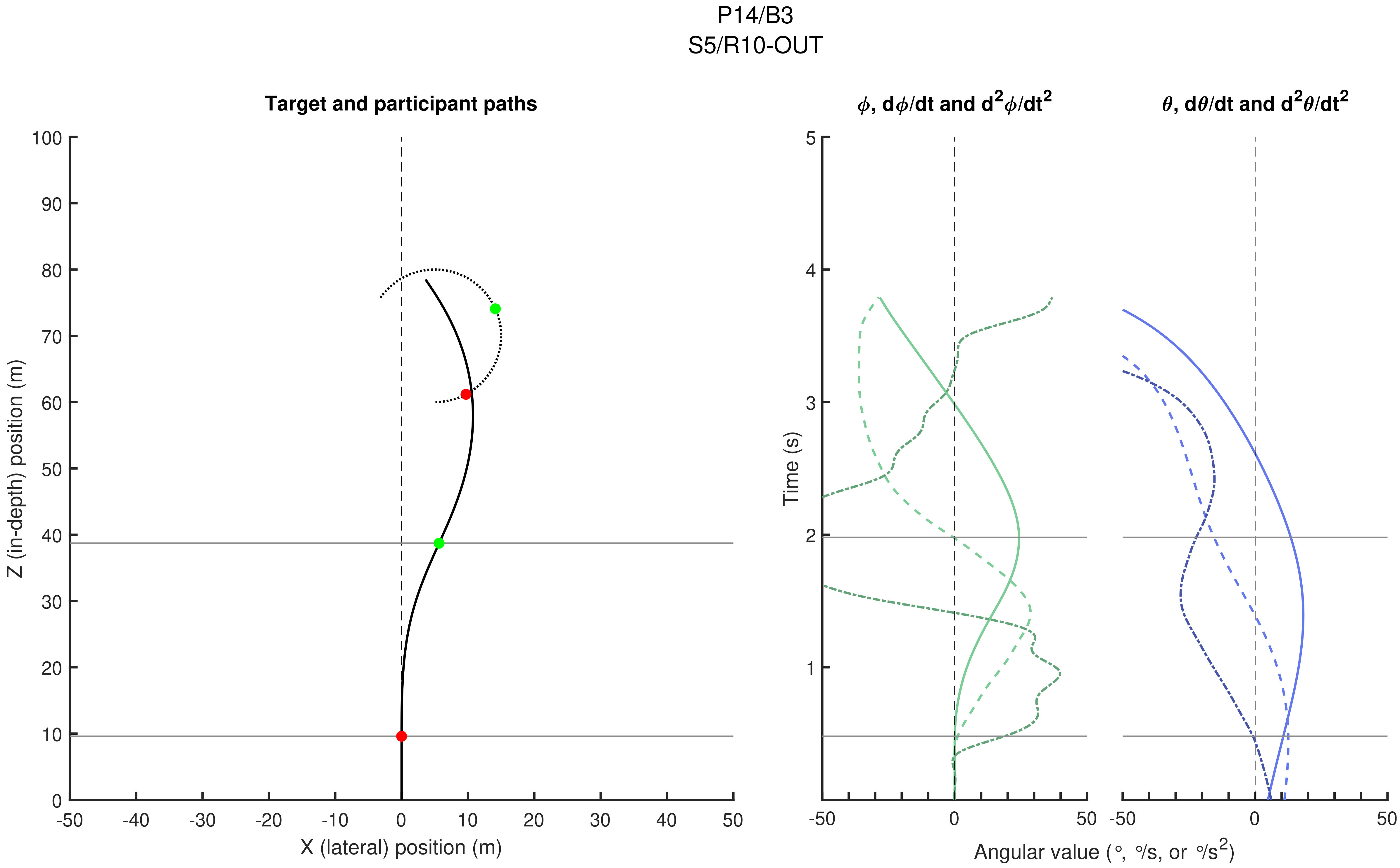

P14/B3
S5/R10-OUT
Target and participant paths
Z (in-depth) position (m)
X (lateral) position (m)
100
90
80
70
60
50
40
30
20
10
0
-50
-40
-30
-20
-10
0
10
20
30
40
50
φ, dφ/dt and d²φ/dt²
θ, dθ/dt and d²θ/dt²
Time (s)
5
4
3
2
1
-50
0
50
-50
0
50
Angular value (°, °/s, or °/s²)

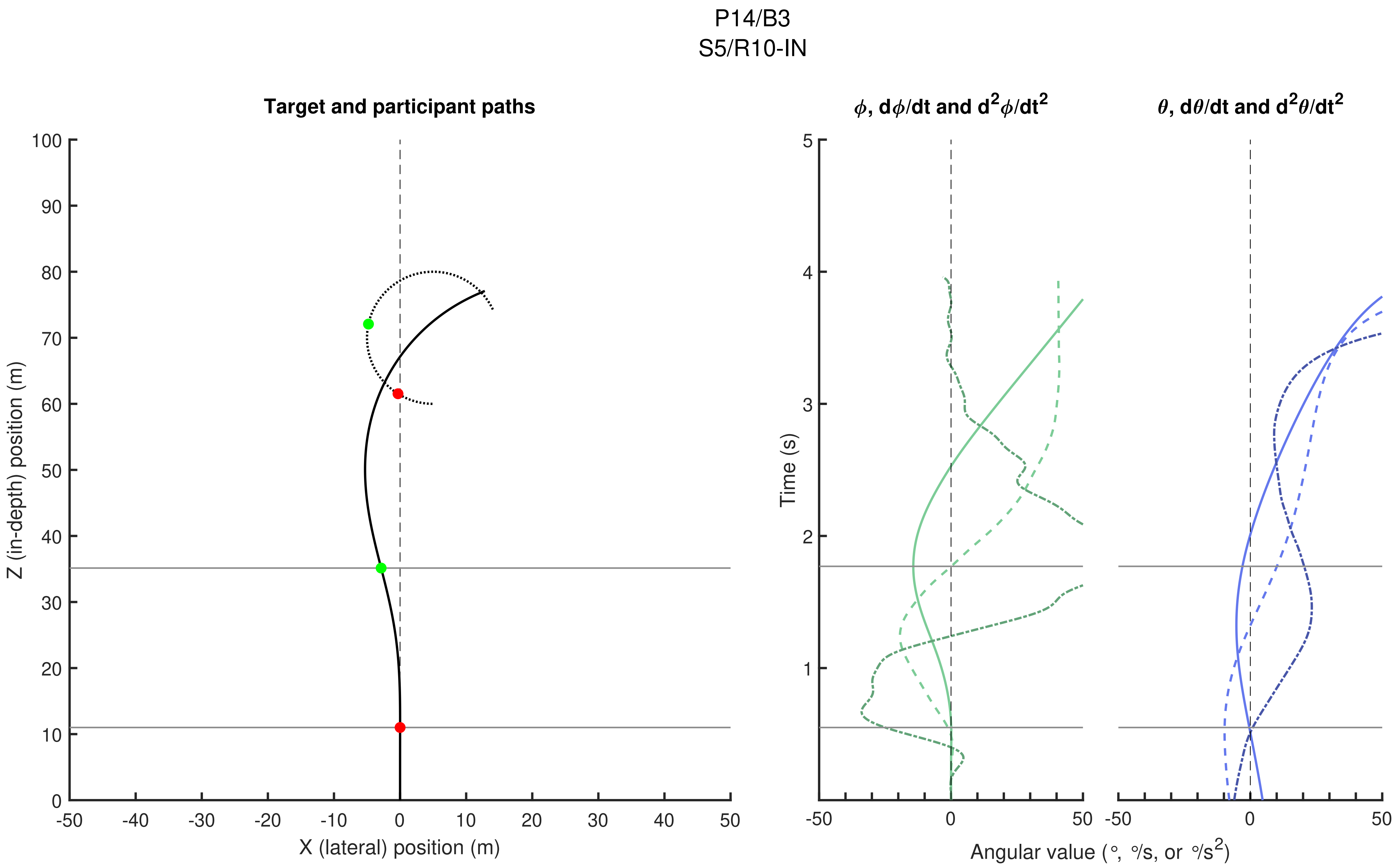
P14/B3
S5/R10-IN
Target and participant paths
Z (in-depth) position (m)
X (lateral) position (m)
φ, dφ/dt and d²φ/dt²
θ, dθ/dt and d²θ/dt²
Time (s)
Angular value (°, °/s, or °/s²)

P14/B3
S5/R20-OUT

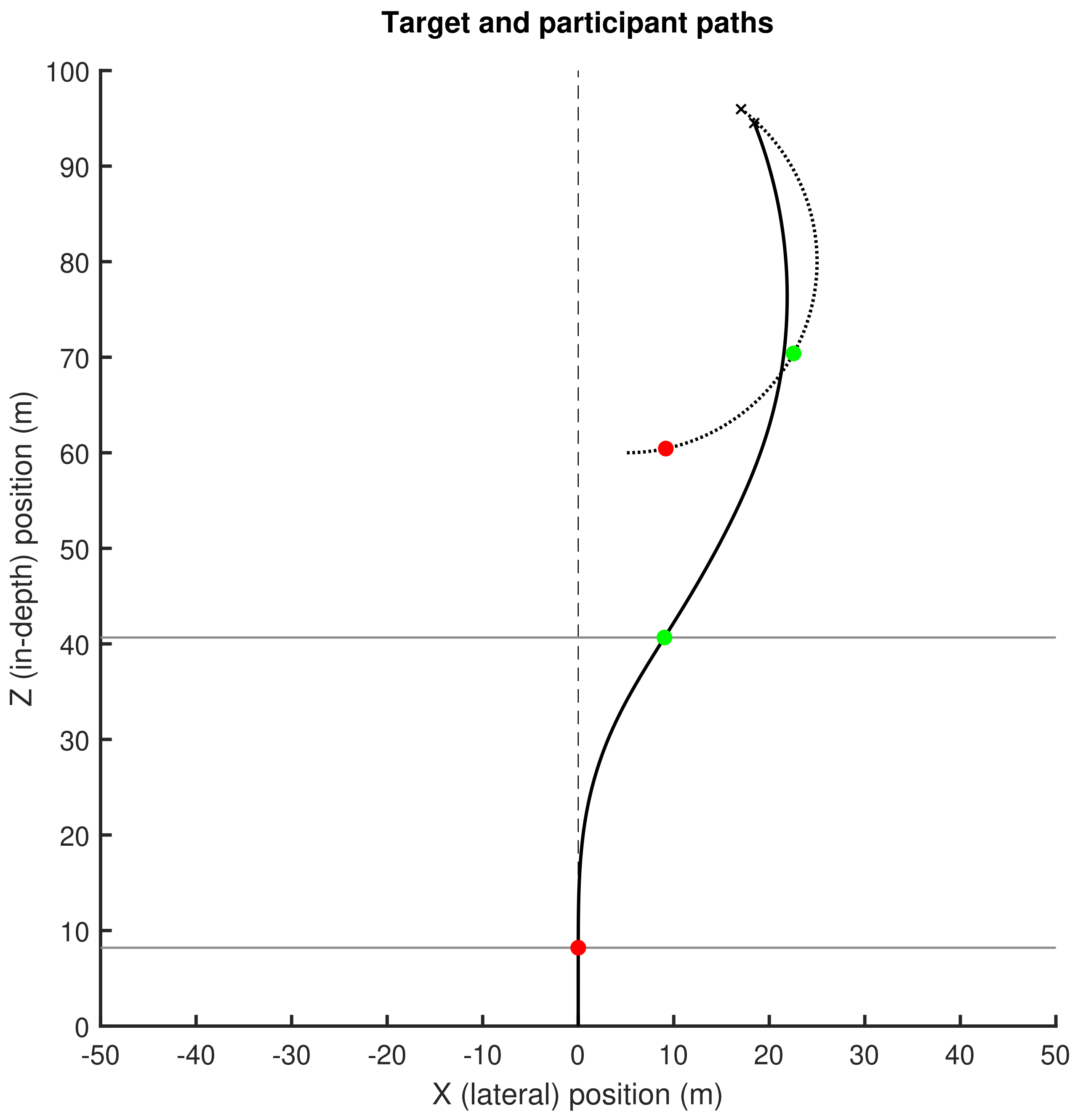

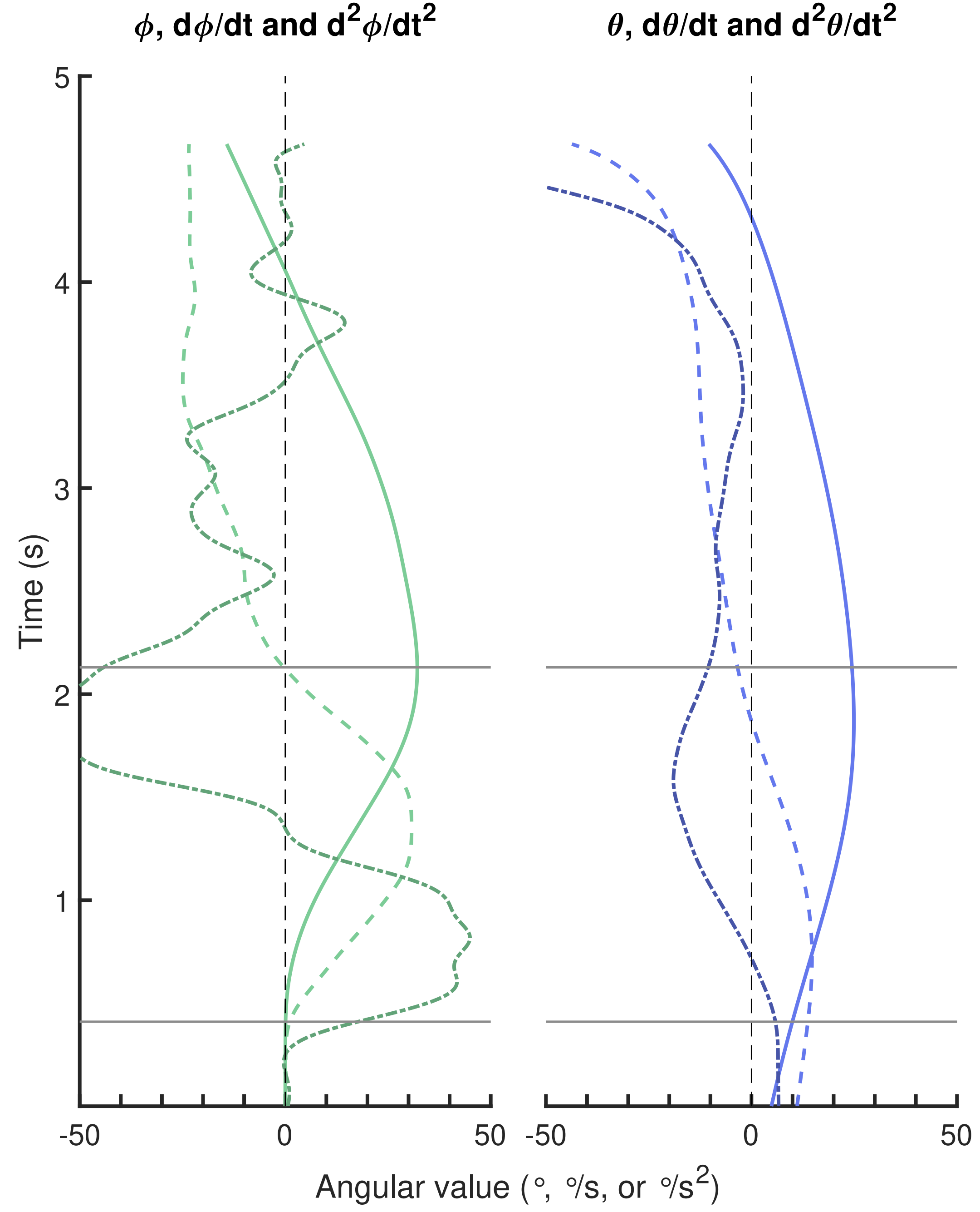

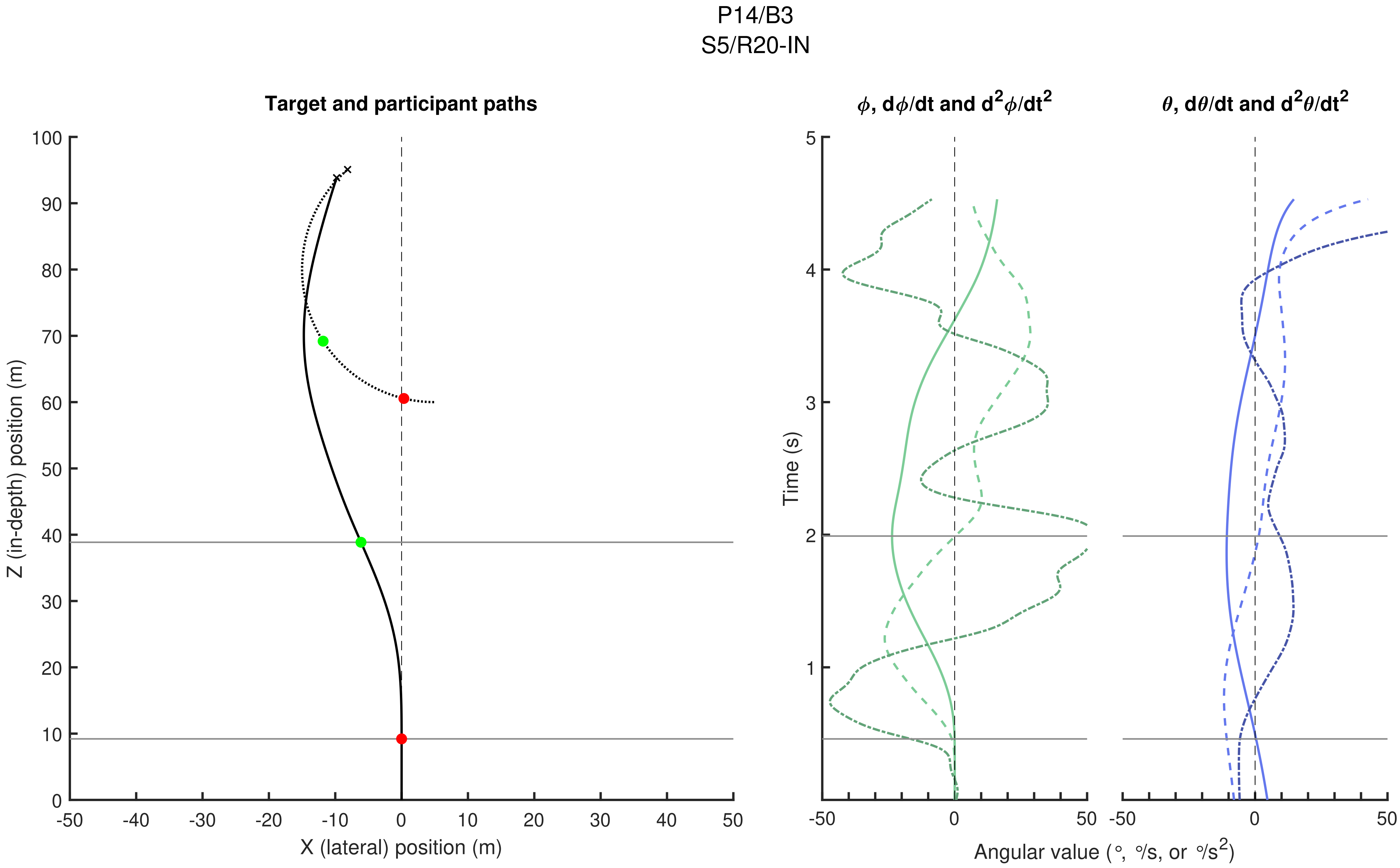

P14/B3
S5/R20-IN
Target and participant paths
Z (in-depth) position (m)
X (lateral) position (m)
ϕ, dϕ/dt and d$^2\phi$/dt^2
θ, dθ/dt and d$^2\theta$/dt^2
Time (s)
Angular value (°, °/s, or °/s^2)

P14/B3
S5/R30-OUT

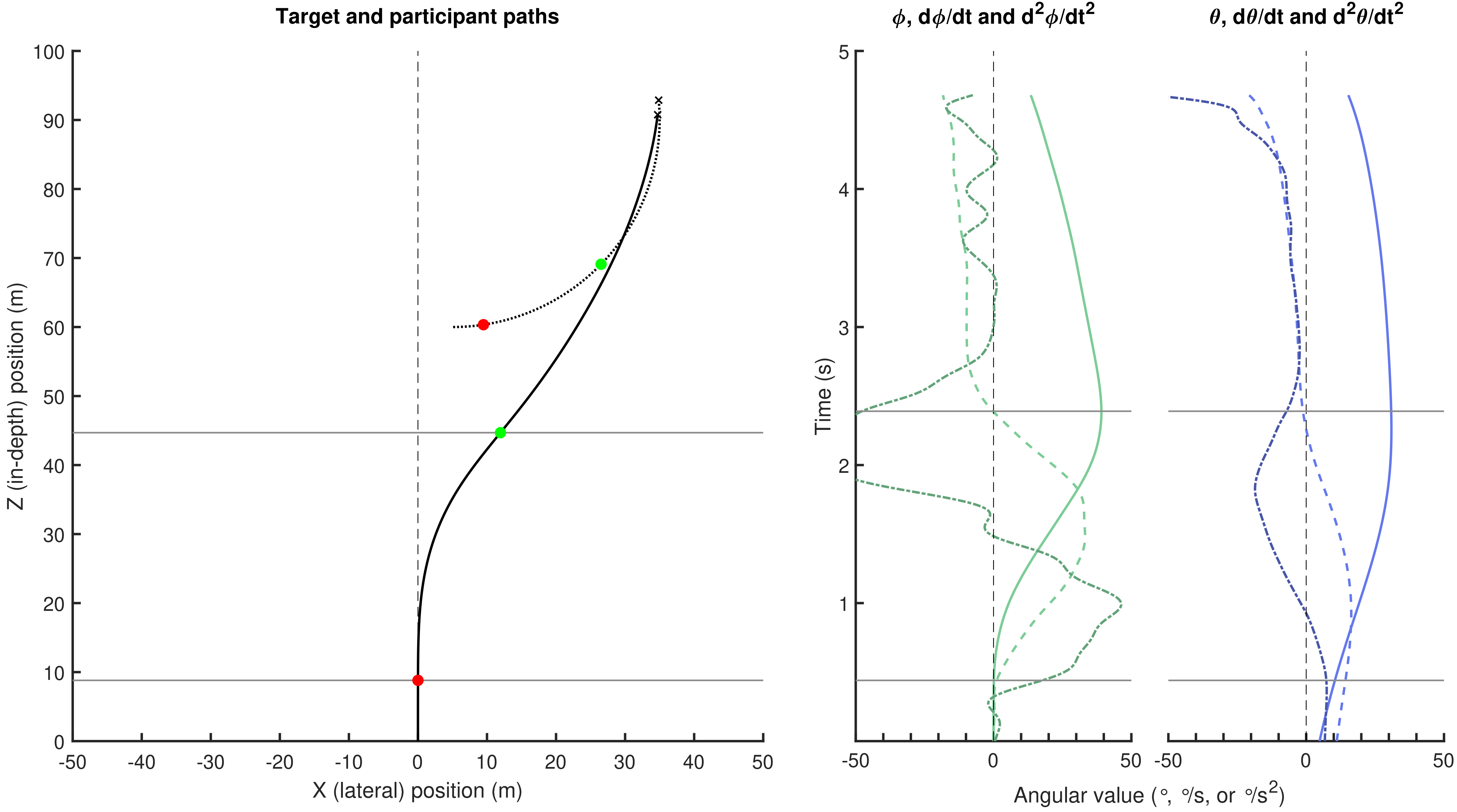

P14/B3
S5/R30-IN

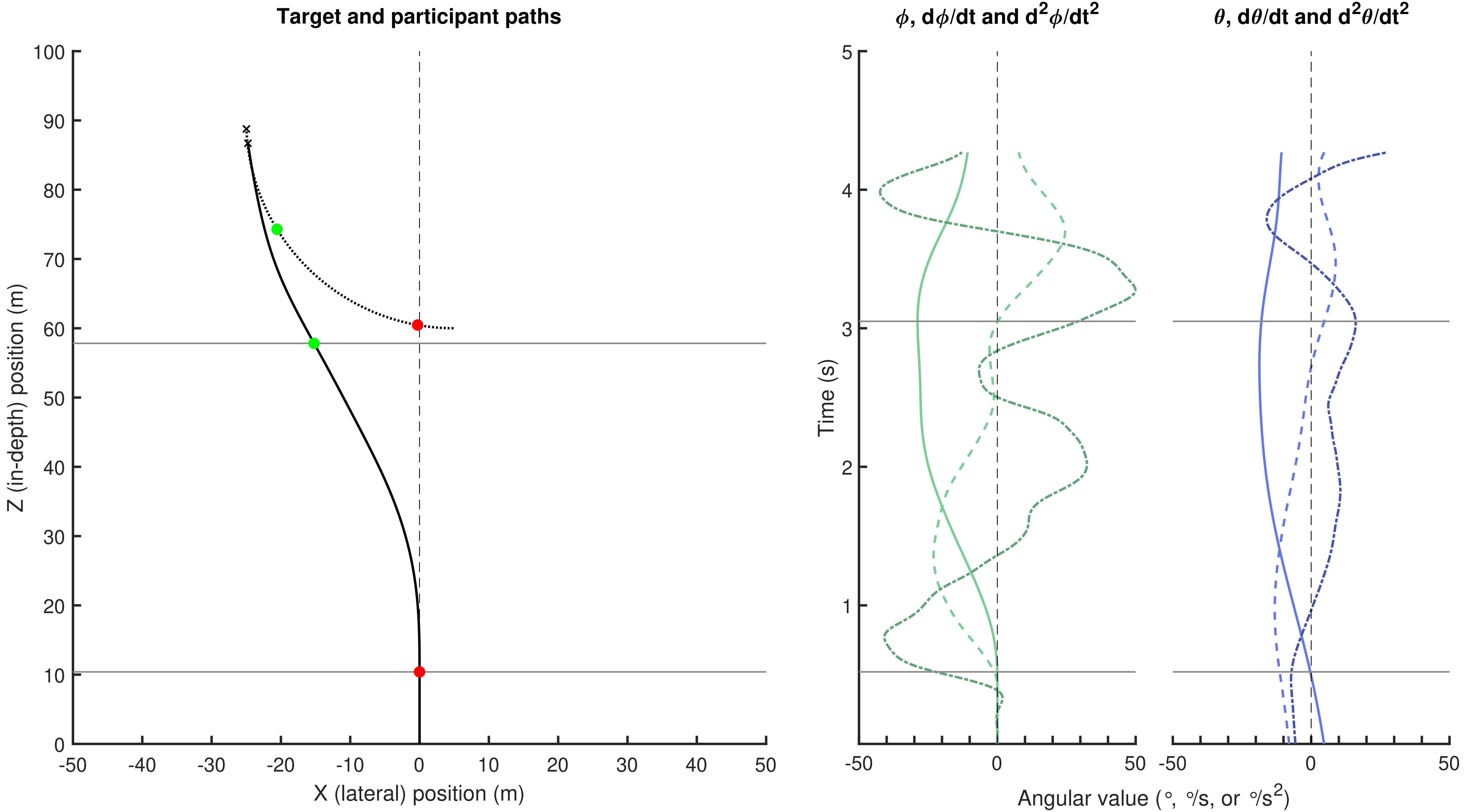

P14/B3
S15/R10-IN

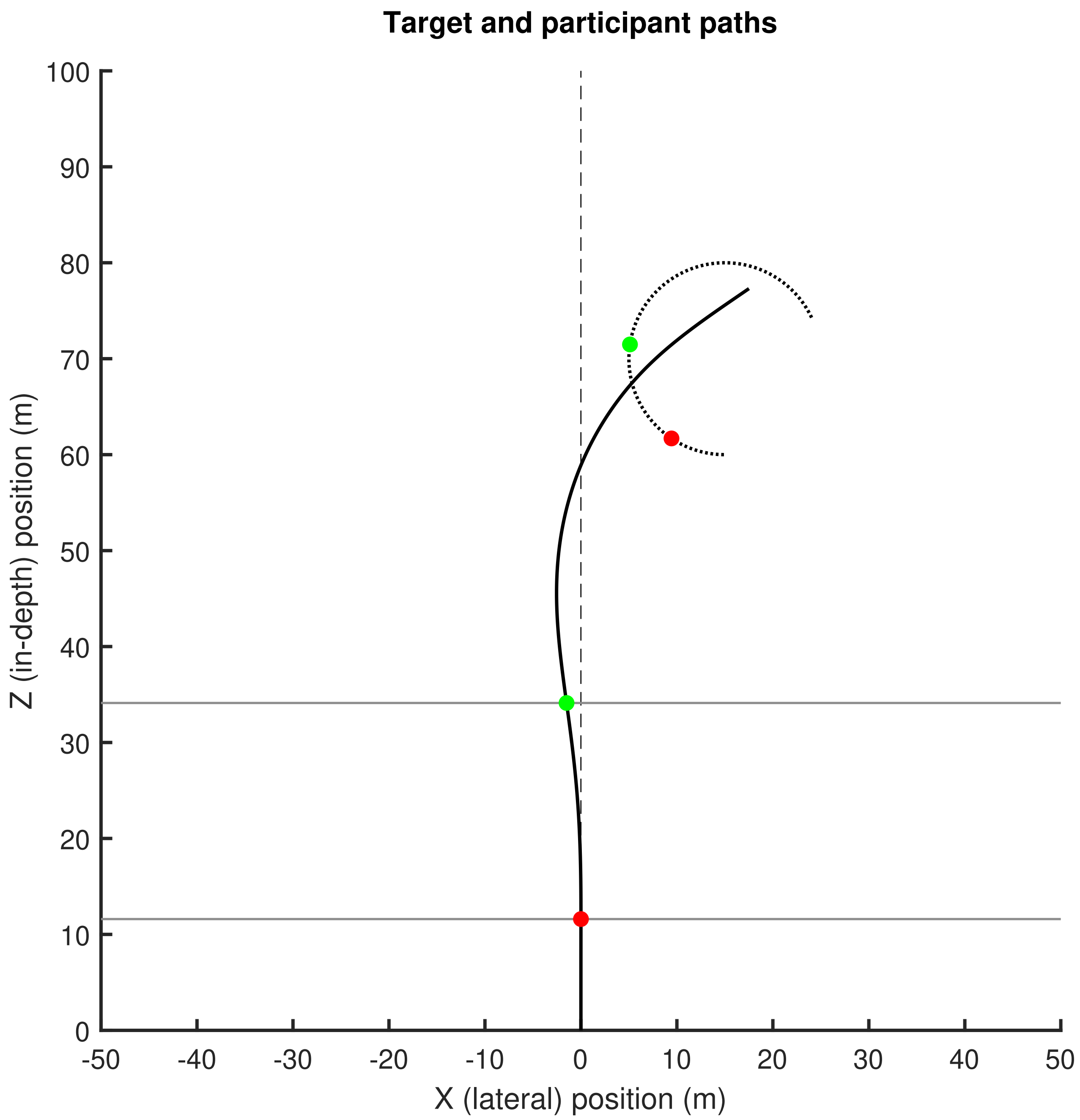

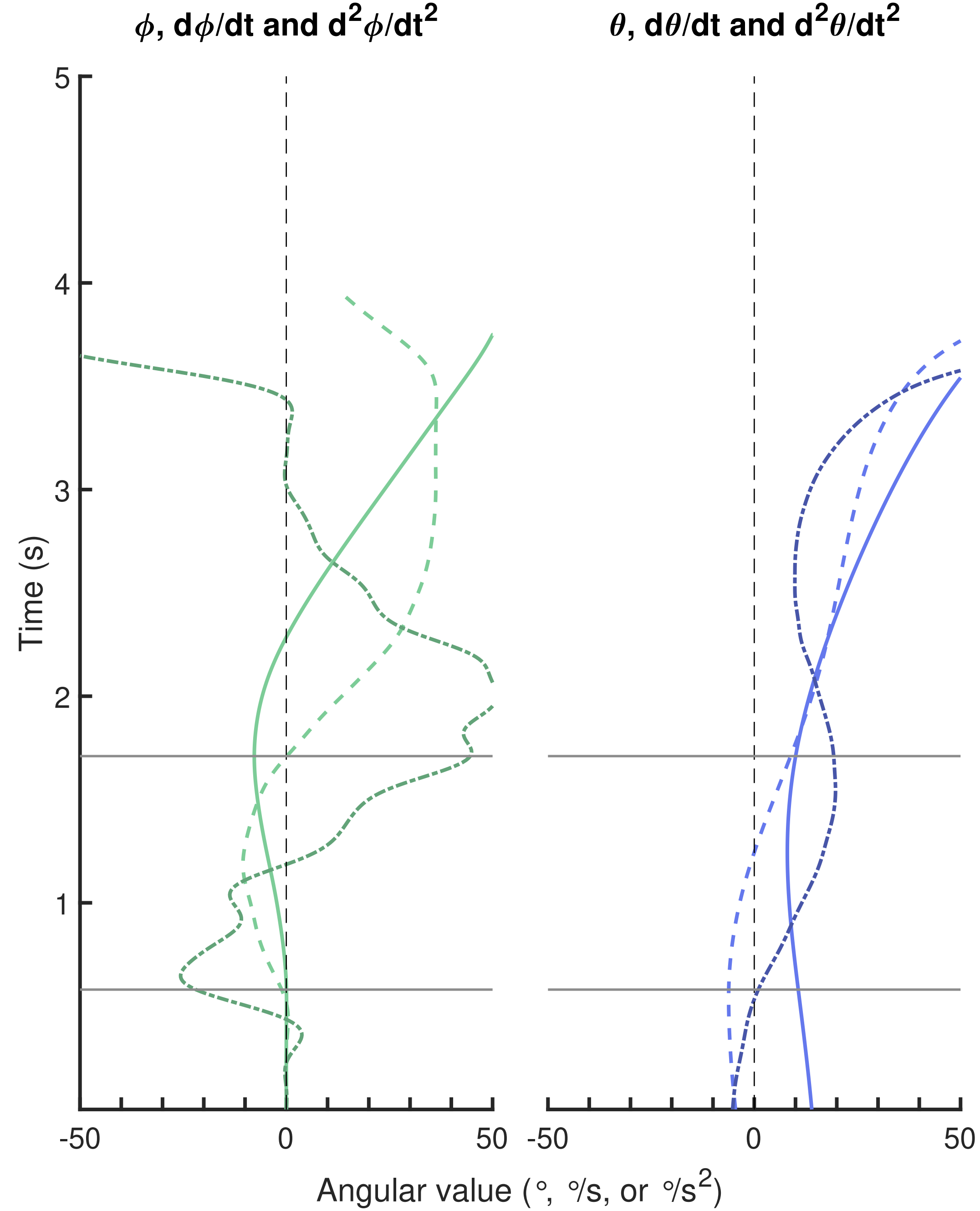

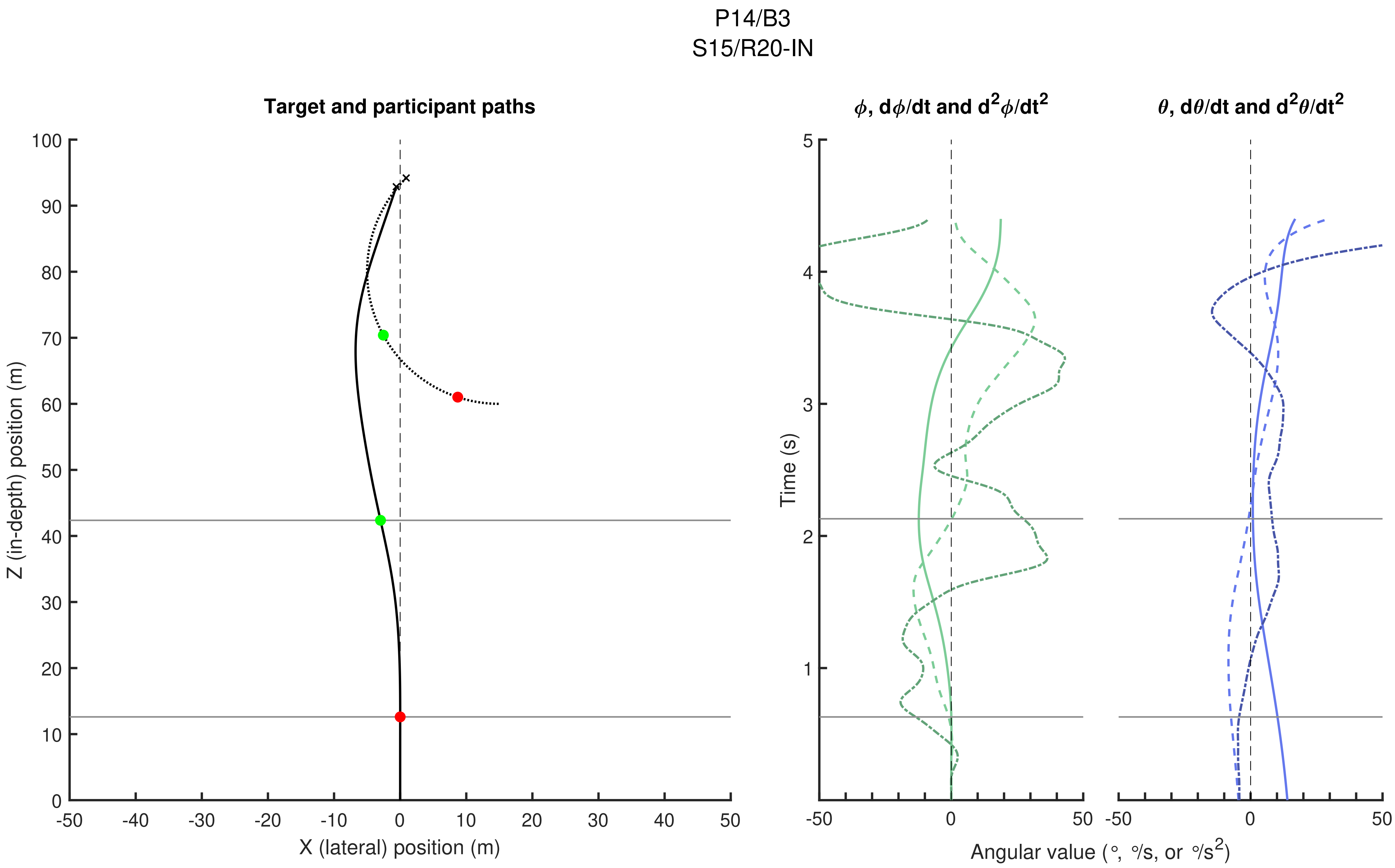
P14/B3
S15/R20-IN
Target and participant paths
Z (in-depth) position (m)
X (lateral) position (m)
ϕ, dϕ/dt and d$^2\phi$/dt^2
θ, dθ/dt and d$^2\theta$/dt^2
Time (s)
Angular value (°, °/s, or °/s^2)

P14/B3
S15/R30-IN

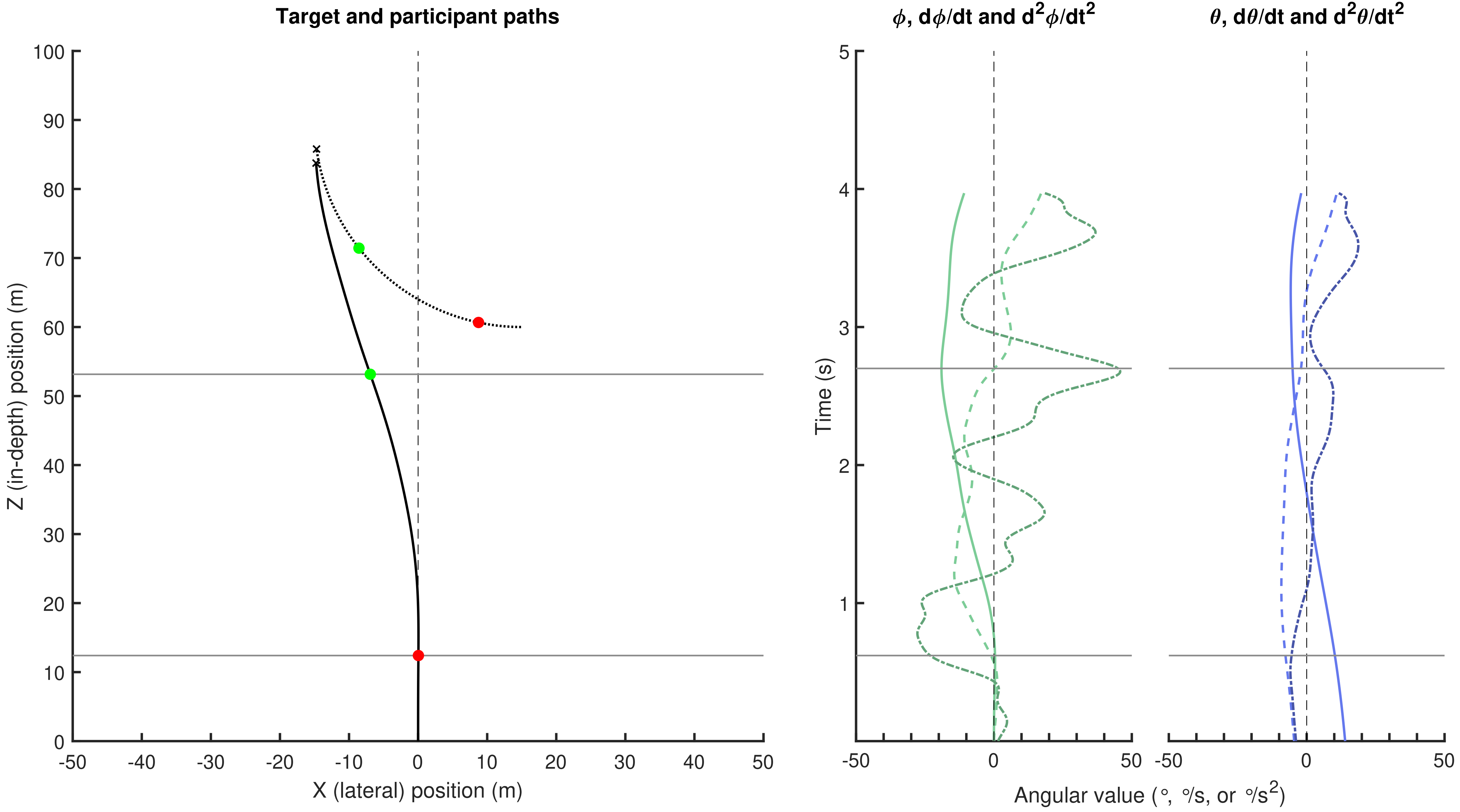

P14/B3
S20/R10-IN

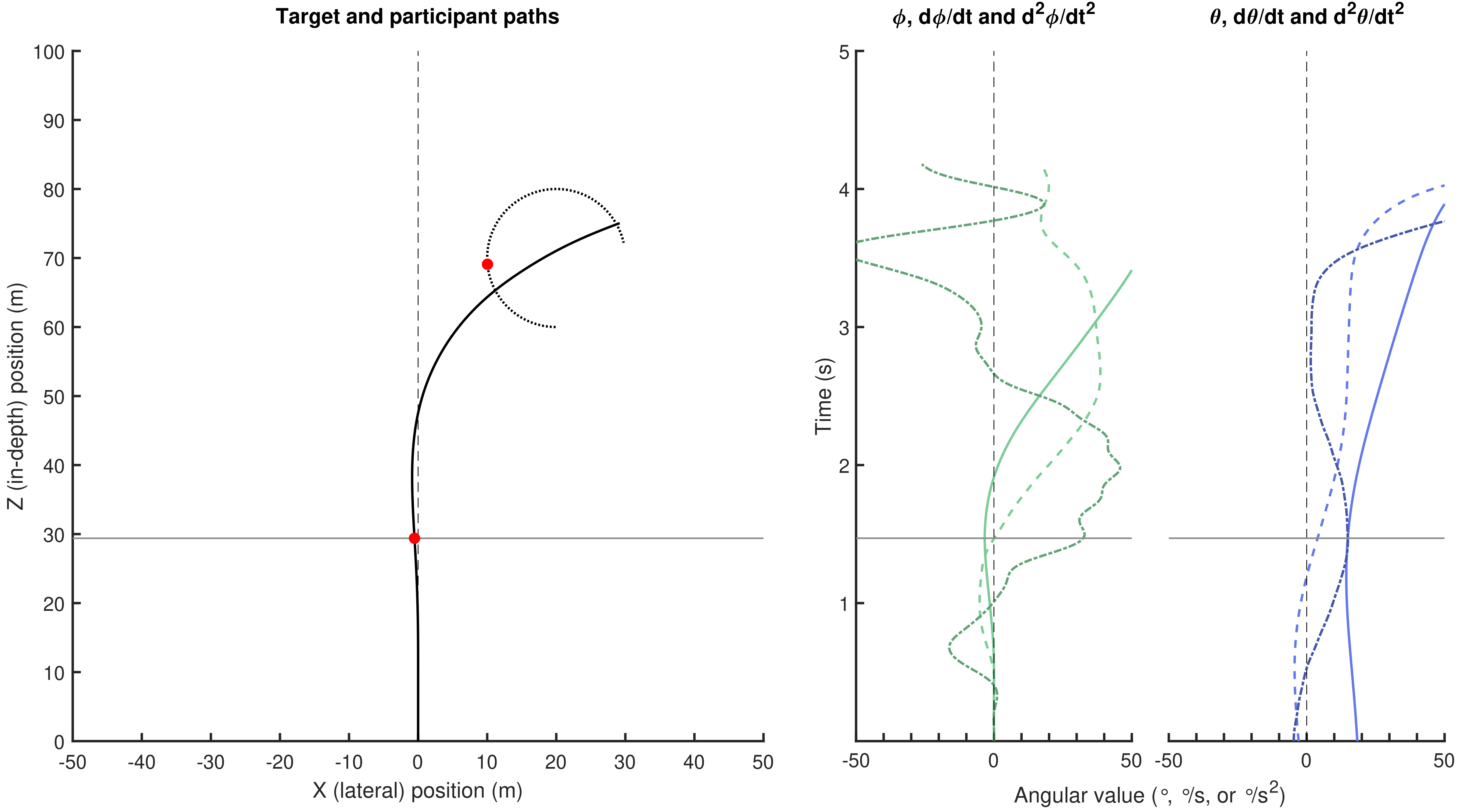

P14/B3
S20/R20-IN

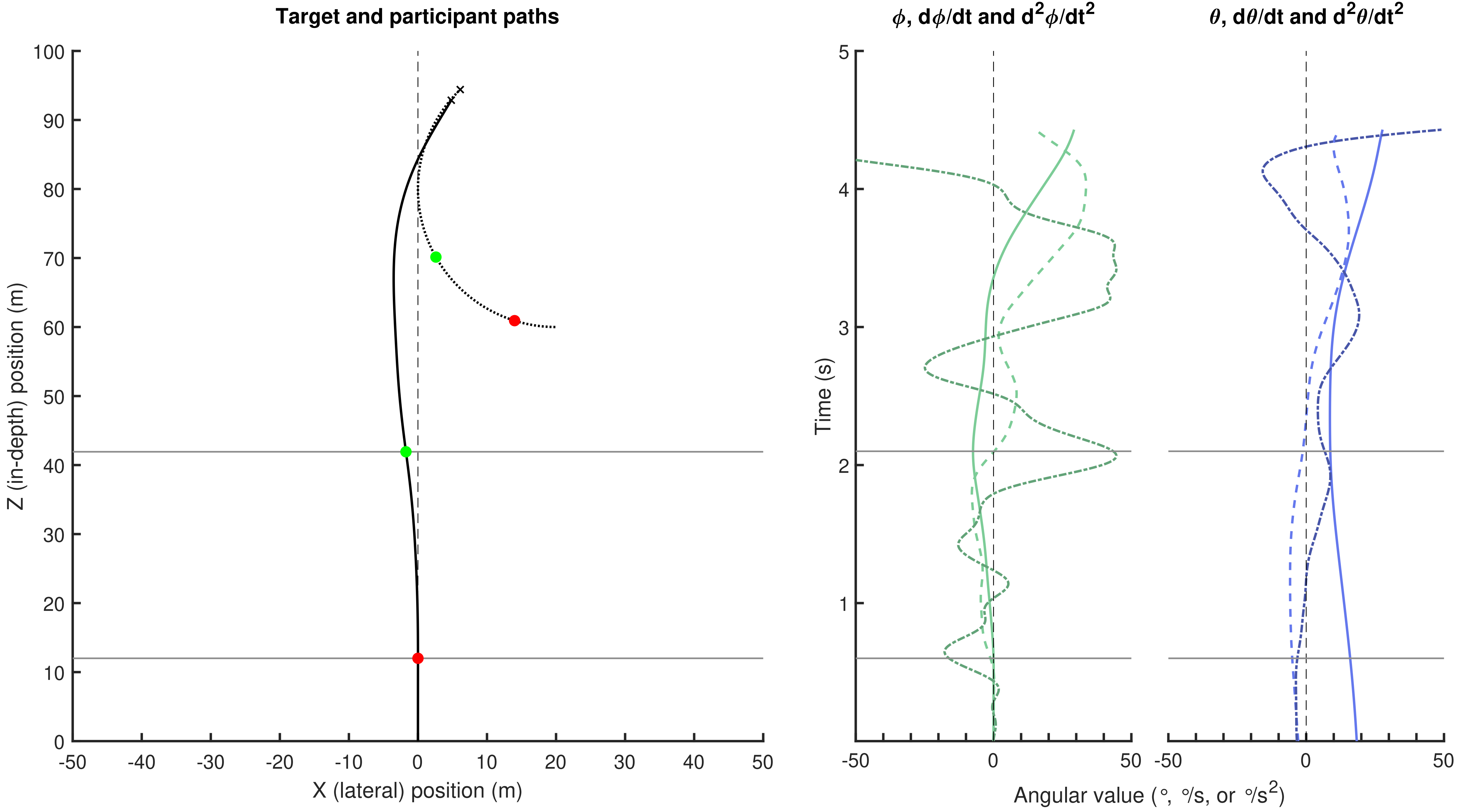

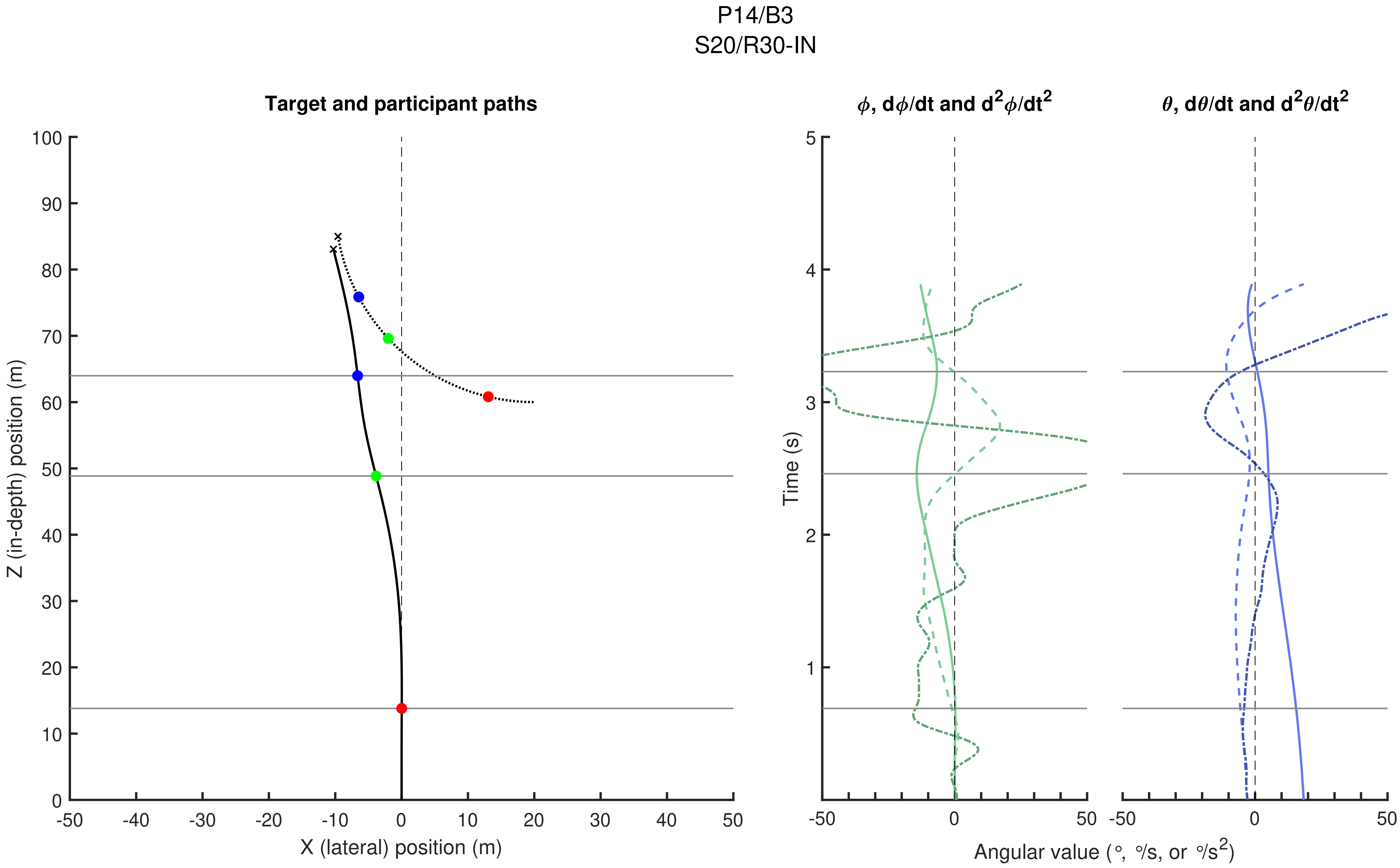
P14/B3
S20/R30-IN
Target and participant paths
Z (in-depth) position (m)
X (lateral) position (m)
100
90
80
70
60
50
40
30
20
10
0
-50
-40
-30
-20
-10
0
10
20
30
40
50
ϕ, dϕ/dt and d$^2\phi$/dt^2
θ, dθ/dt and d$^2\theta$/dt^2
Time (s)
5
4
3
2
1
-50
0
50
-50
0
50
Angular value (°, °/s, or °/s^2)

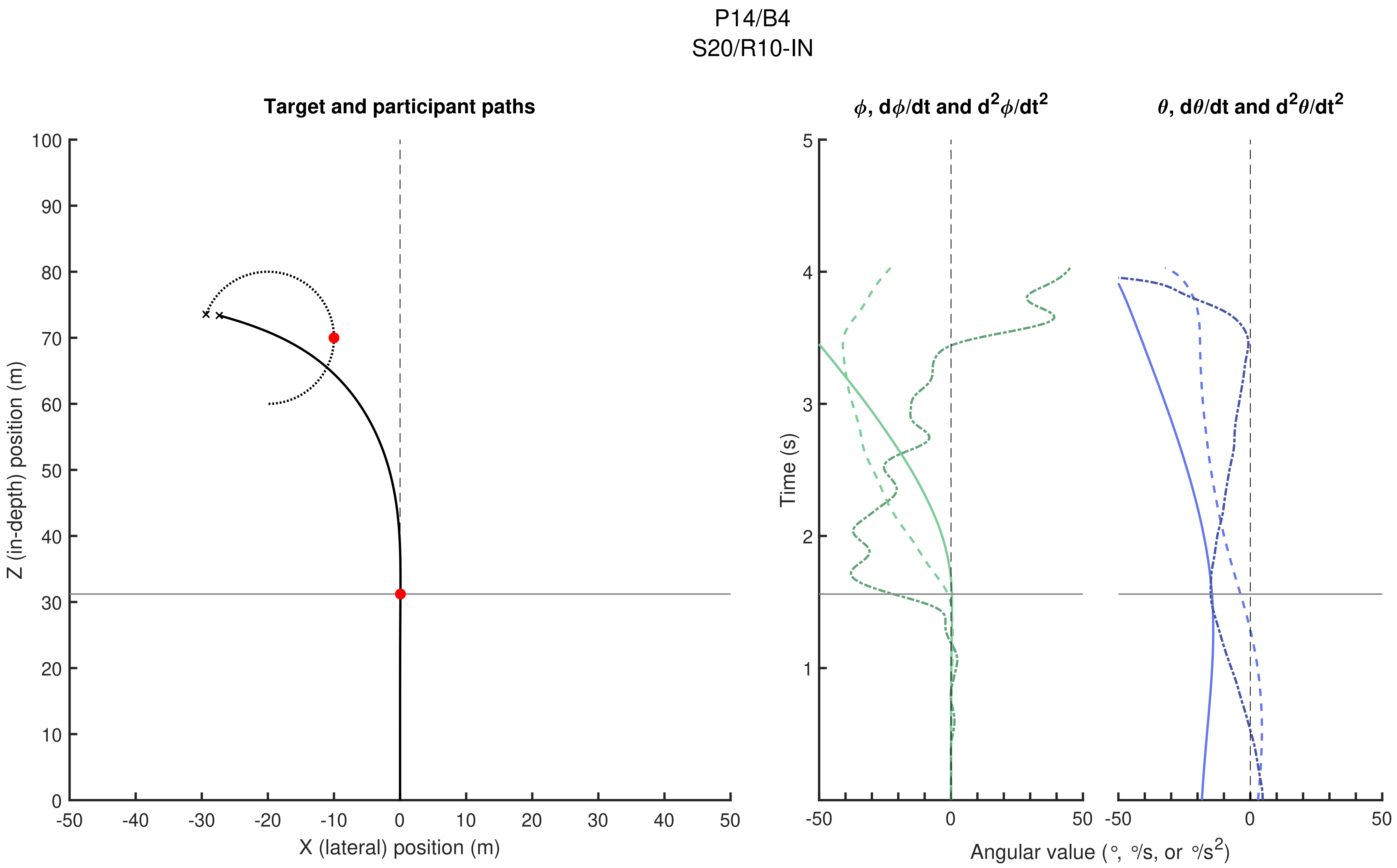

P14/B4
S20/R10-IN
Target and participant paths
Z (in-depth) position (m)
X (lateral) position (m)
ϕ, dϕ/dt and d²ϕ/dt²
θ, dθ/dt and d²θ/dt²
Time (s)
Angular value (°, °/s, or °/s²)

P14/B4
S20/R20-IN

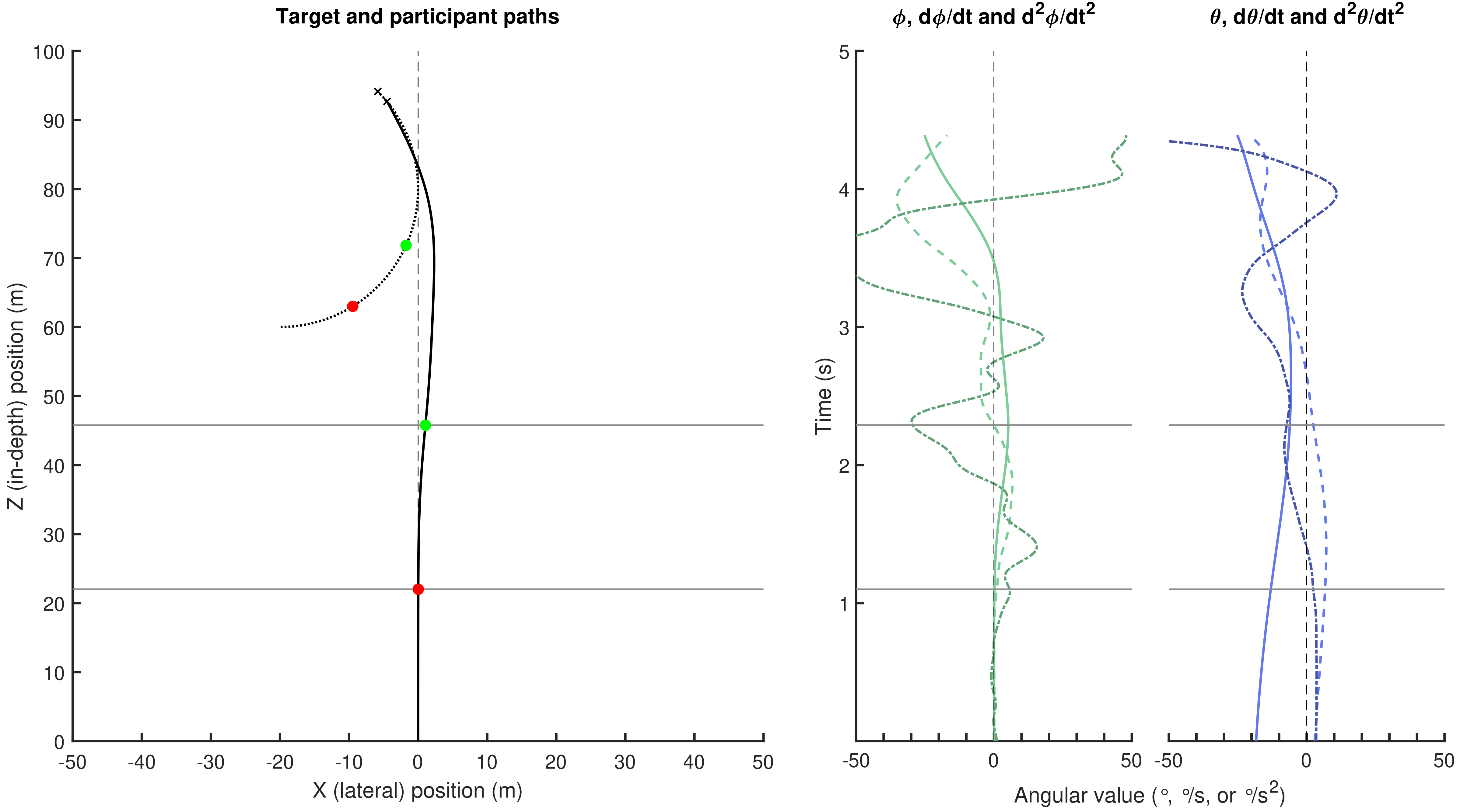

P14/B4
S20/R30-IN

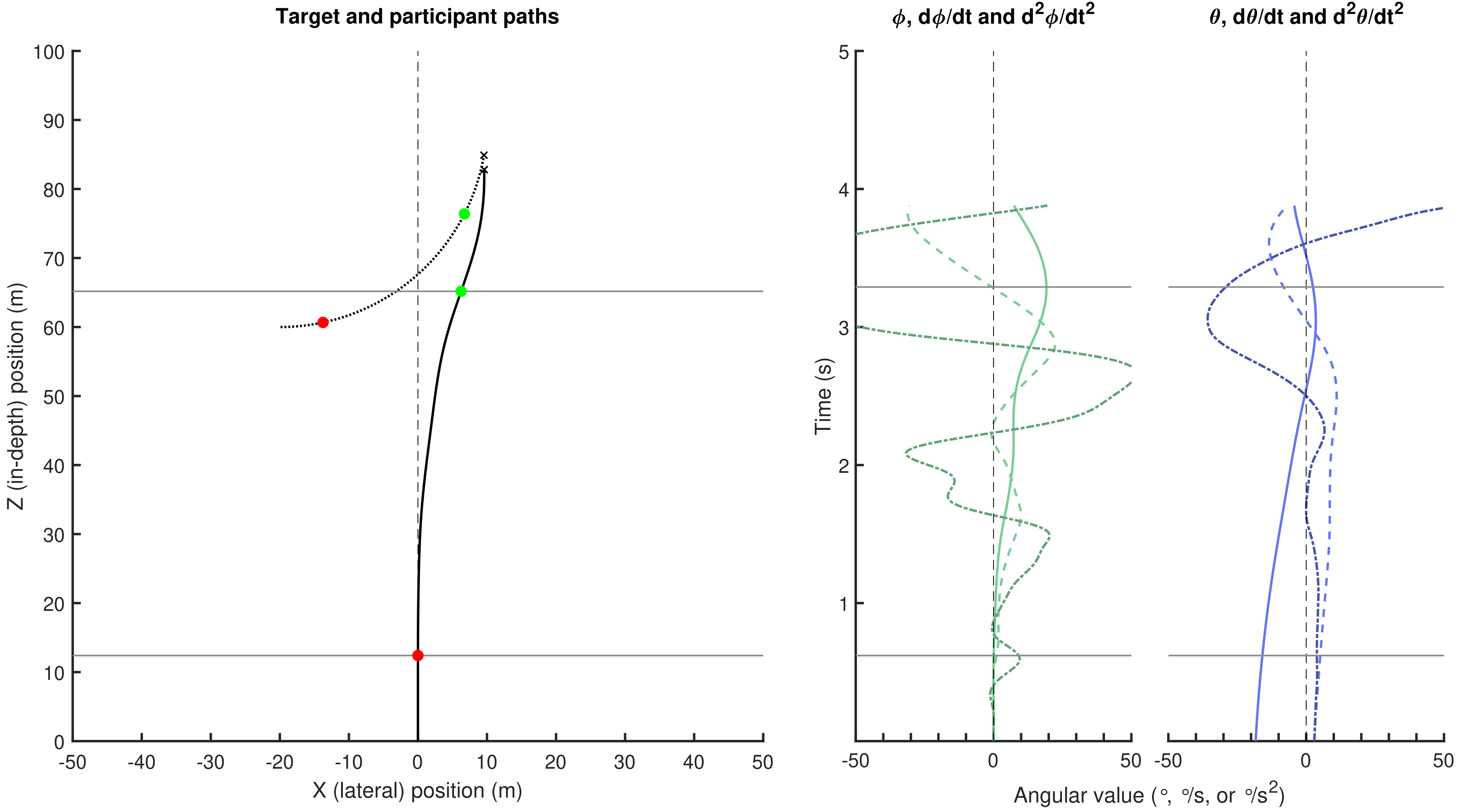

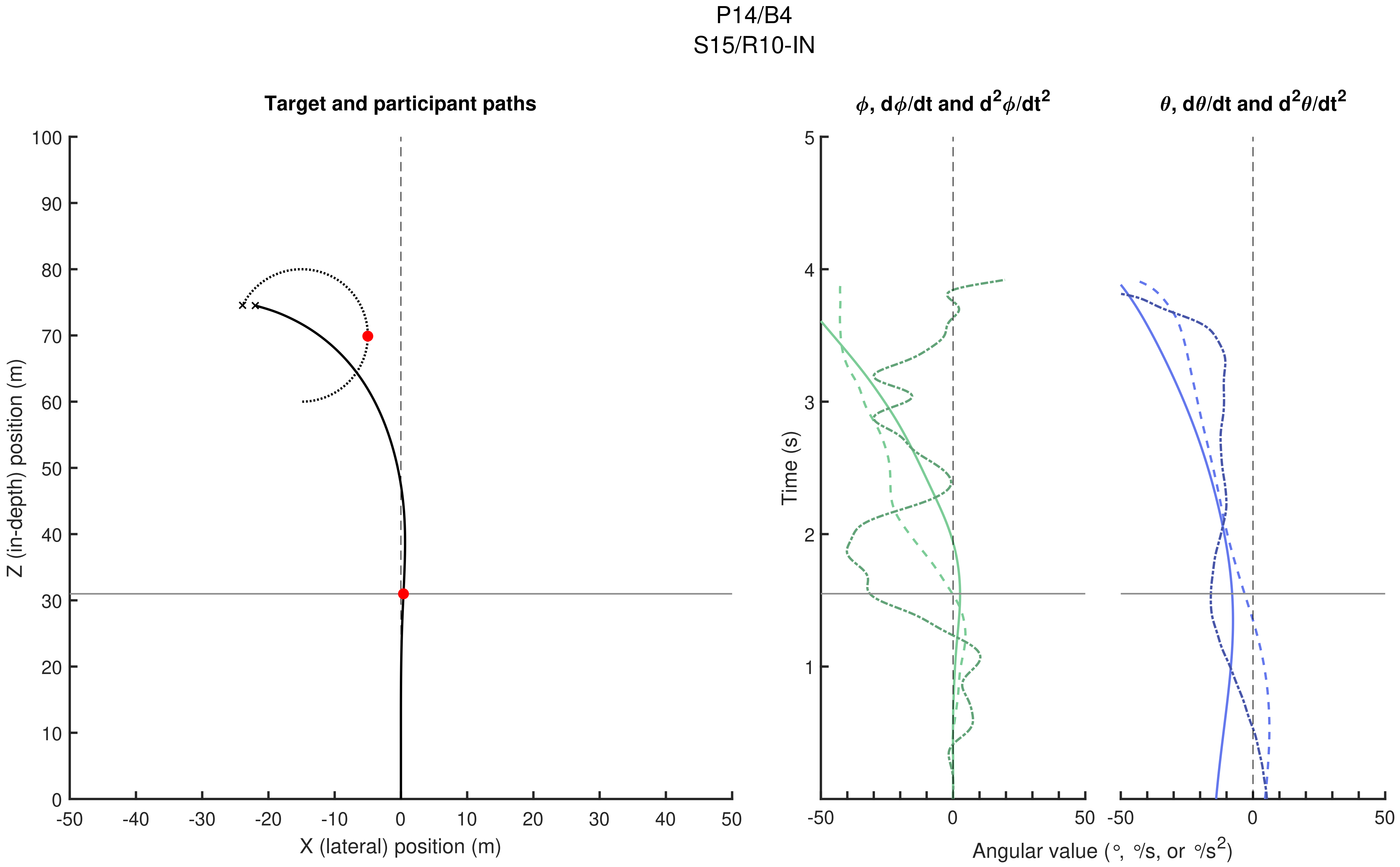
P14/B4
S15/R10-IN
Target and participant paths
Z (in-depth) position (m)
X (lateral) position (m)
ϕ, dϕ/dt and d$^2\phi$/dt^2
θ, dθ/dt and d$^2\theta$/dt^2
Time (s)
Angular value (°, °/s, or °/s^2)

P14/B4
S15/R20-IN

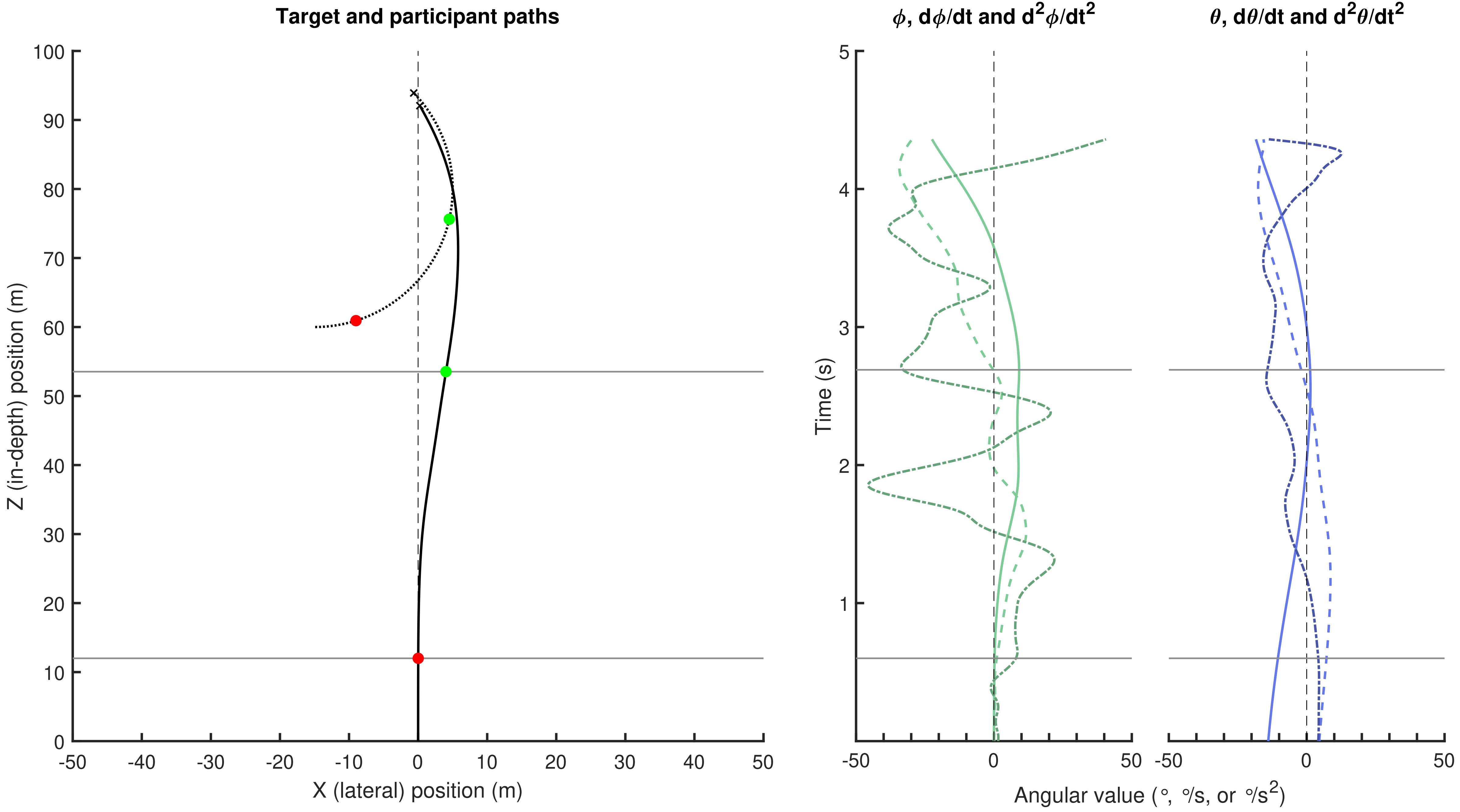

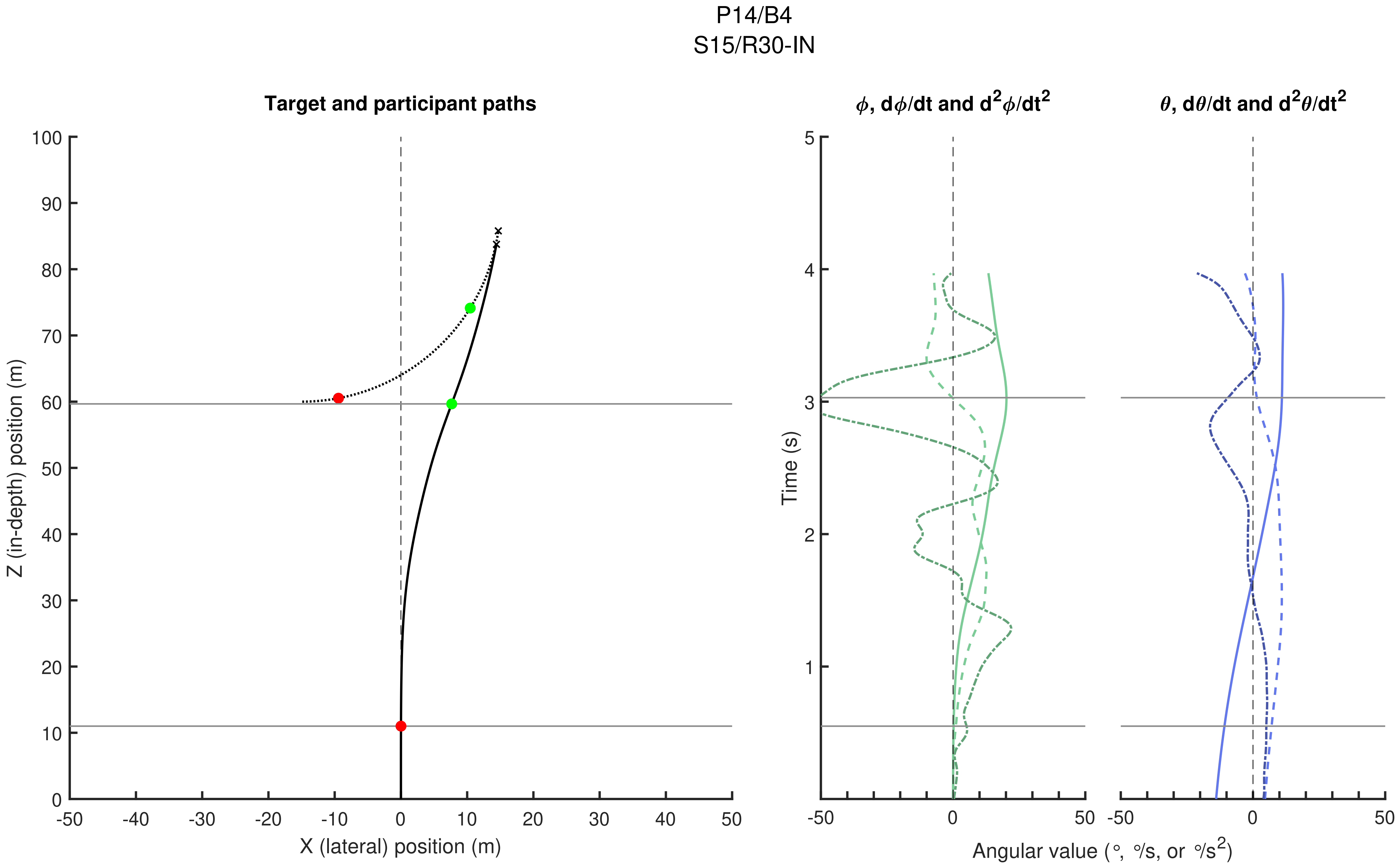
P14/B4
S15/R30-IN
Target and participant paths
ϕ, dϕ/dt and d$^2\phi$/dt^2
θ, dθ/dt and d$^2\theta$/dt^2
Z (in-depth) position (m)
X (lateral) position (m)
Time (s)
Angular value (°, °/s, or °/s^2)
100
90
80
70
60
50
40
30
20
10
0
-50
-40
-30
-20
-10
0
10
20
30
40
50
5
4
3
2
1
-50
0
50
-50
0
50

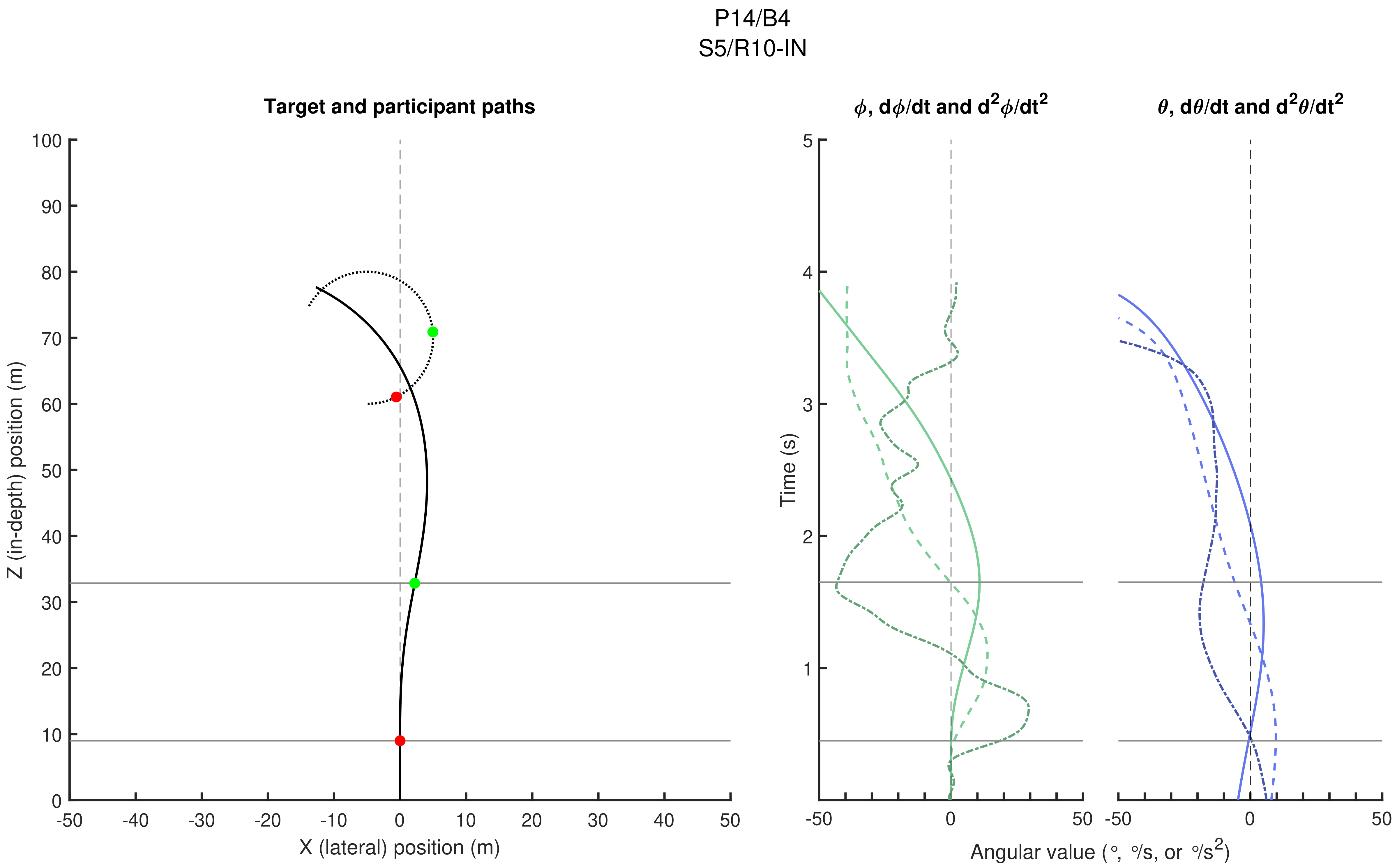
P14/B4
S5/R10-IN
Target and participant paths
ϕ, dϕ/dt and d$^2\phi$/dt^2
θ, dθ/dt and d$^2\theta$/dt^2
Z (in-depth) position (m)
X (lateral) position (m)
Time (s)
Angular value (°, °/s, or °/s^2)

P14/B4
S5/R10-OUT

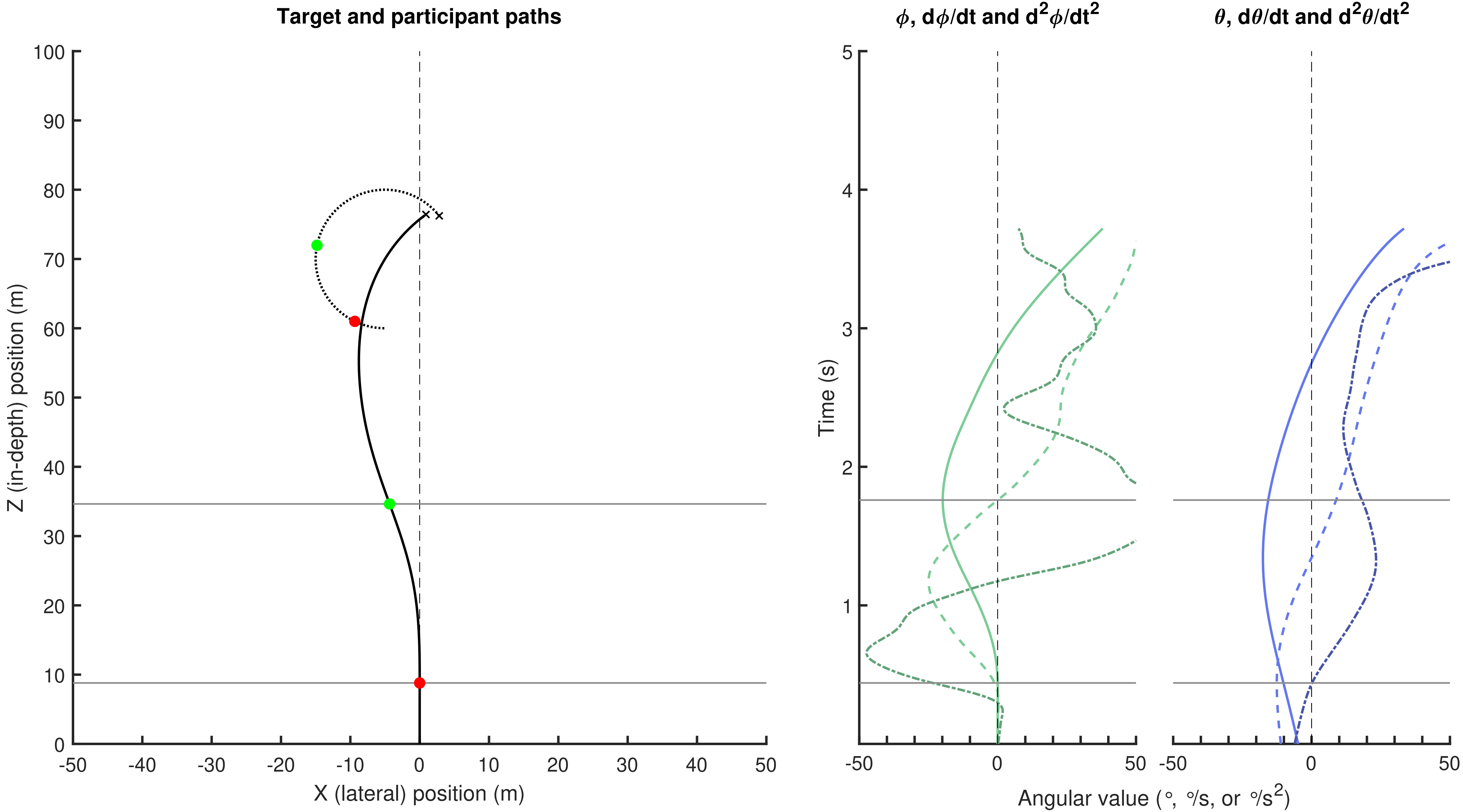

P14/B4
S5/R20-IN

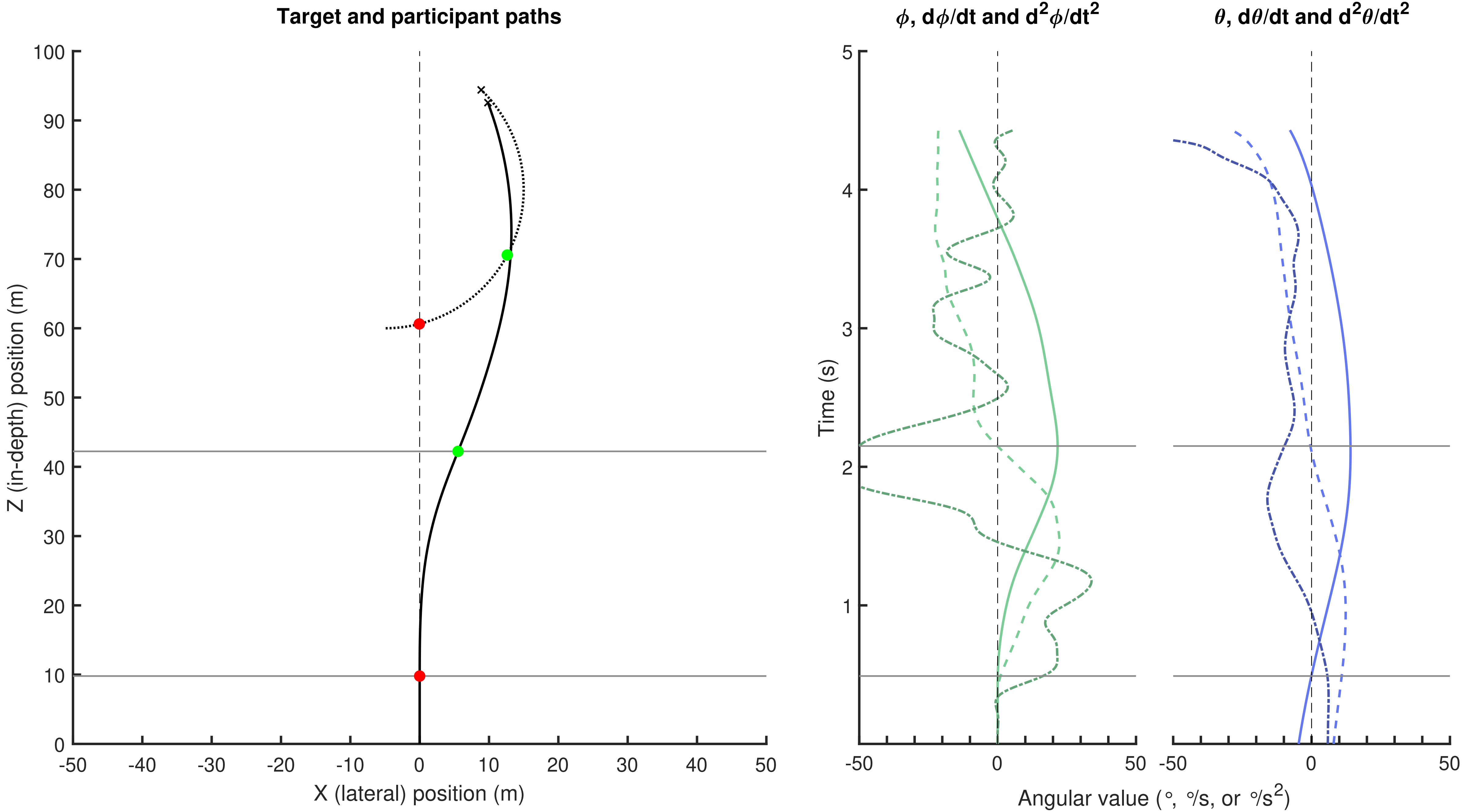

P14/B4
S5/R20-OUT

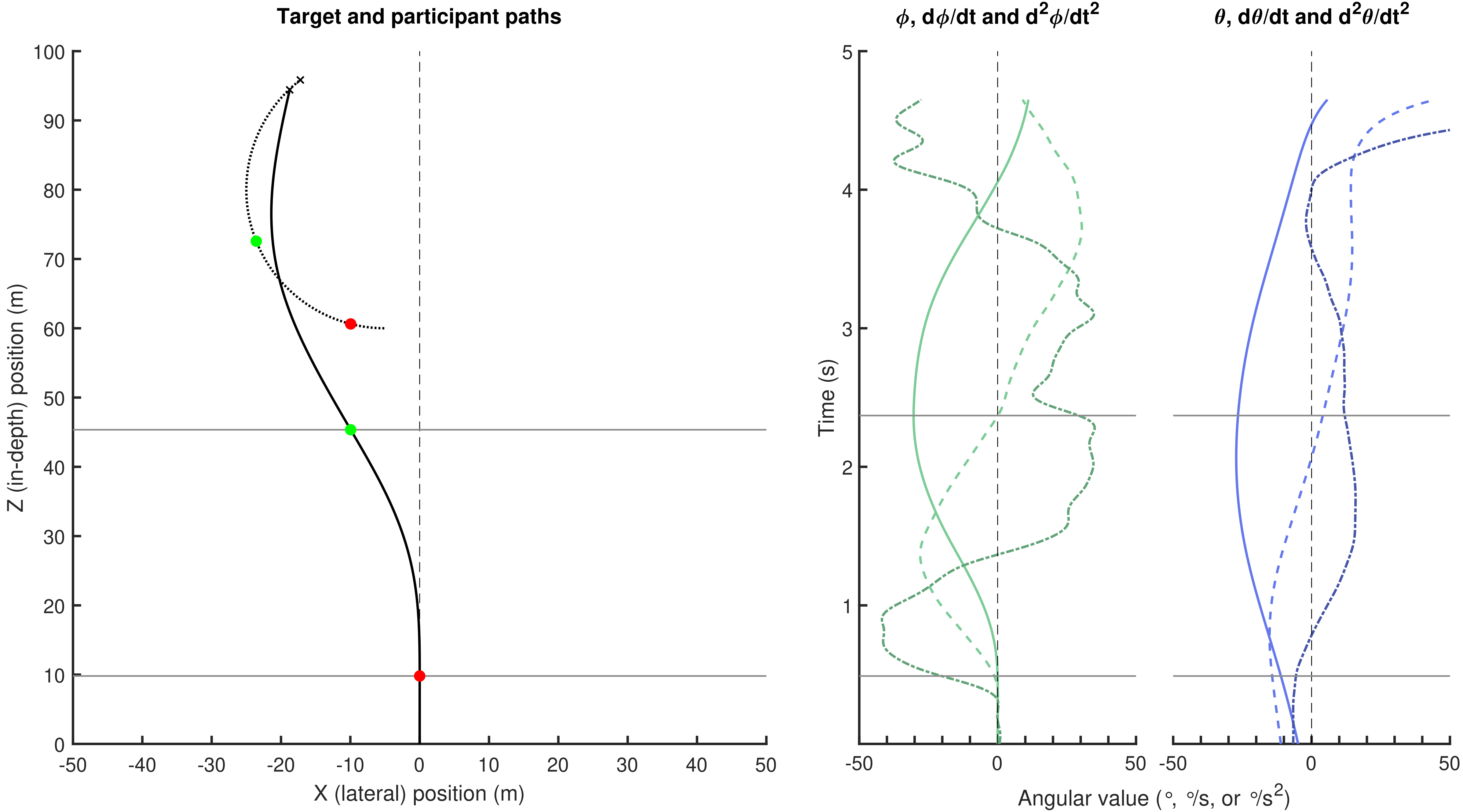

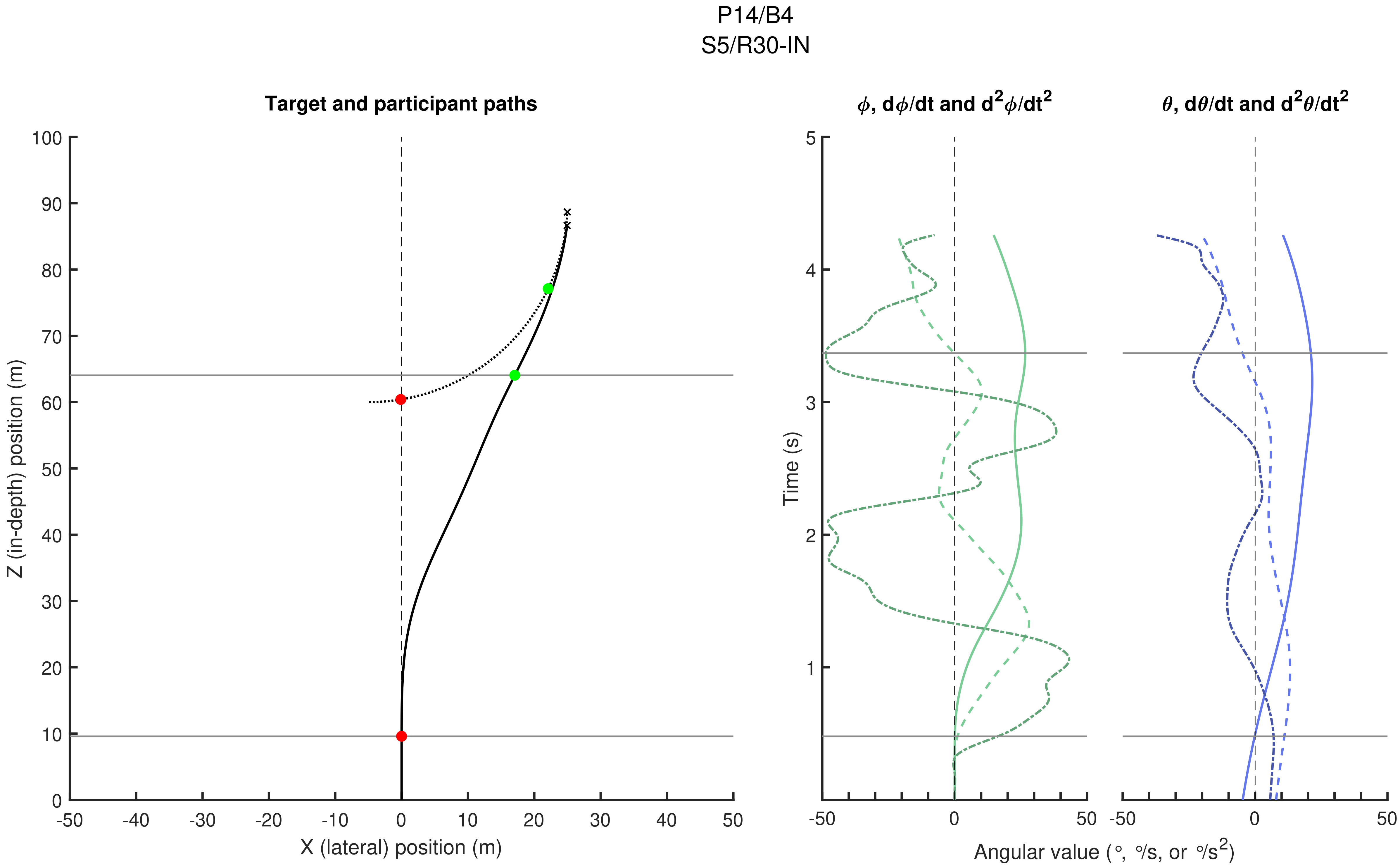

P14/B4
S5/R30-IN
Target and participant paths
ϕ, dϕ/dt and d$^2\phi$/dt^2
θ, dθ/dt and d$^2\theta$/dt^2
Z (in-depth) position (m)
X (lateral) position (m)
Time (s)
Angular value (°, °/s, or °/s^2)

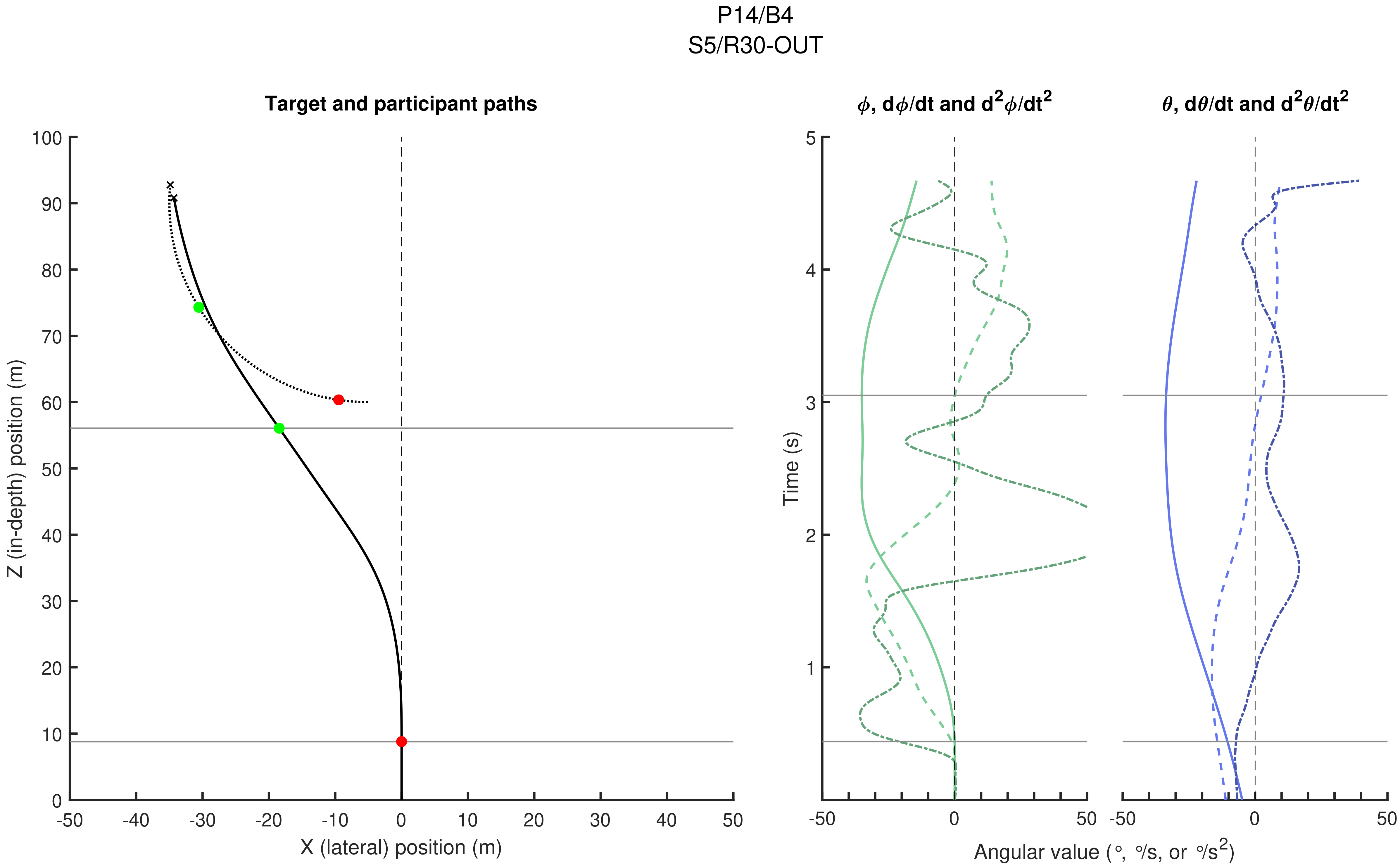
P14/B4
S5/R30-OUT
Target and participant paths
Z (in-depth) position (m)
X (lateral) position (m)
100
90
80
70
60
50
40
30
20
10
0
-50
-40
-30
-20
-10
0
10
20
30
40
50
ϕ, dϕ/dt and d$^2\phi$/dt^2
θ, dθ/dt and d$^2\theta$/dt^2
Time (s)
5
4
3
2
1
-50
0
50
-50
0
50
Angular value (°, °/s, or °/s^2)

P14/B4
S5/R10-OUT

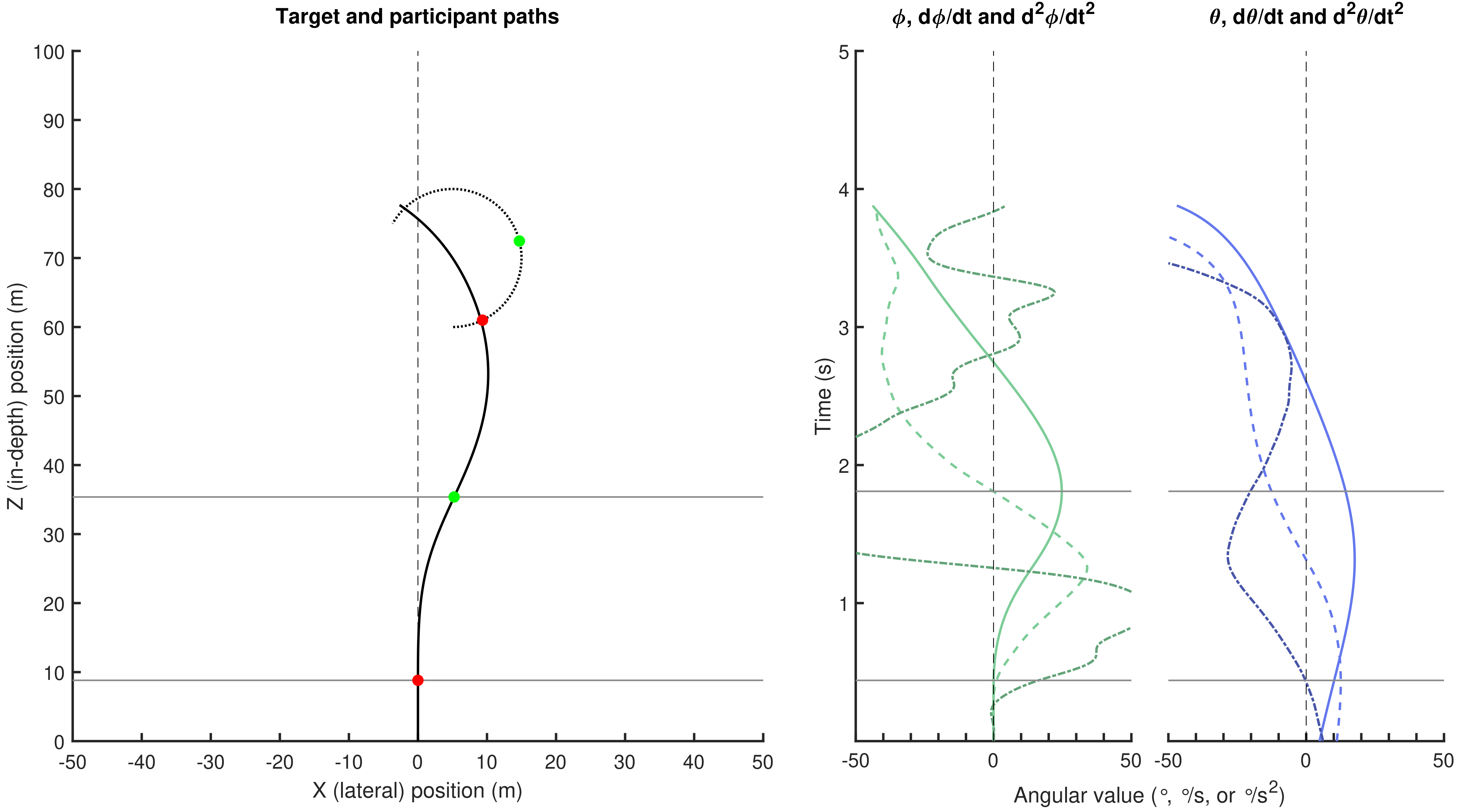

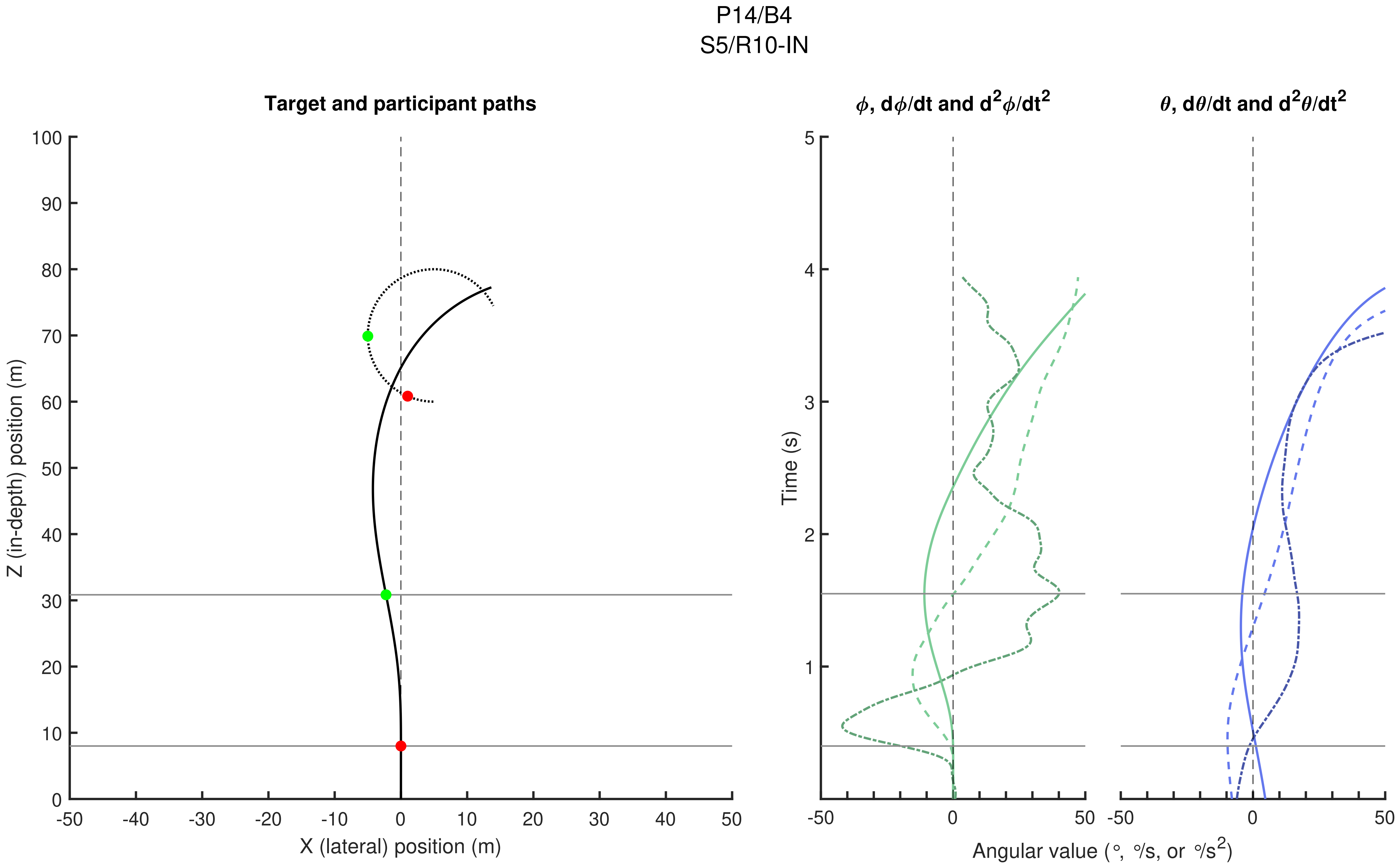
P14/B4
S5/R10-IN
Target and participant paths
Z (in-depth) position (m)
X (lateral) position (m)
100
90
80
70
60
50
40
30
20
10
0
-50
-40
-30
-20
-10
0
10
20
30
40
50
ϕ, dϕ/dt and d$^2\phi$/dt^2
θ, dθ/dt and d$^2\theta$/dt^2
Time (s)
5
4
3
2
1
-50
0
50
-50
0
50
Angular value (°, °/s, or °/s^2)

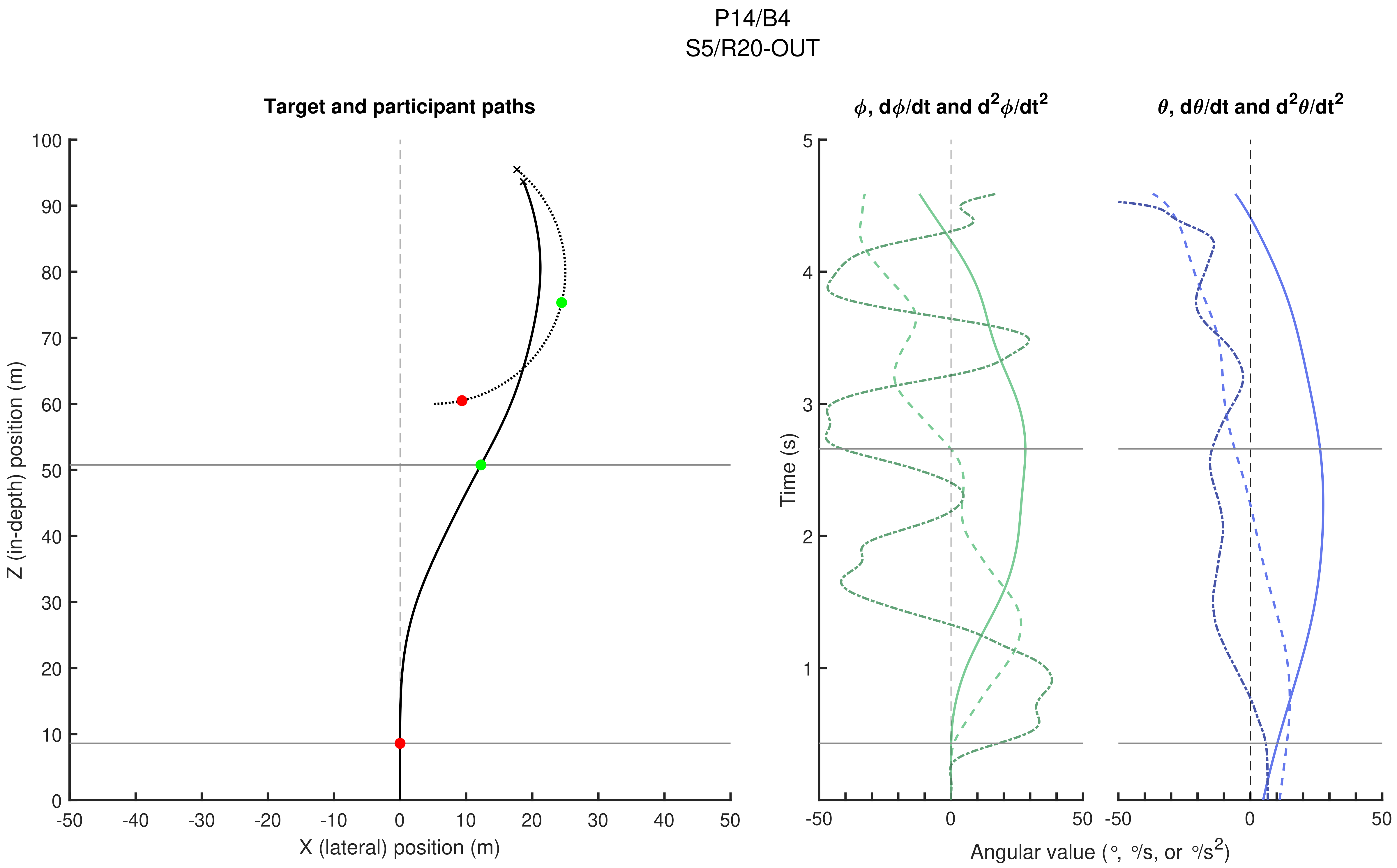
P14/B4
S5/R20-OUT
Target and participant paths
Z (in-depth) position (m)
X (lateral) position (m)
ϕ, dϕ/dt and d²ϕ/dt²
θ, dθ/dt and d²θ/dt²
Time (s)
Angular value (°, °/s, or °/s²)

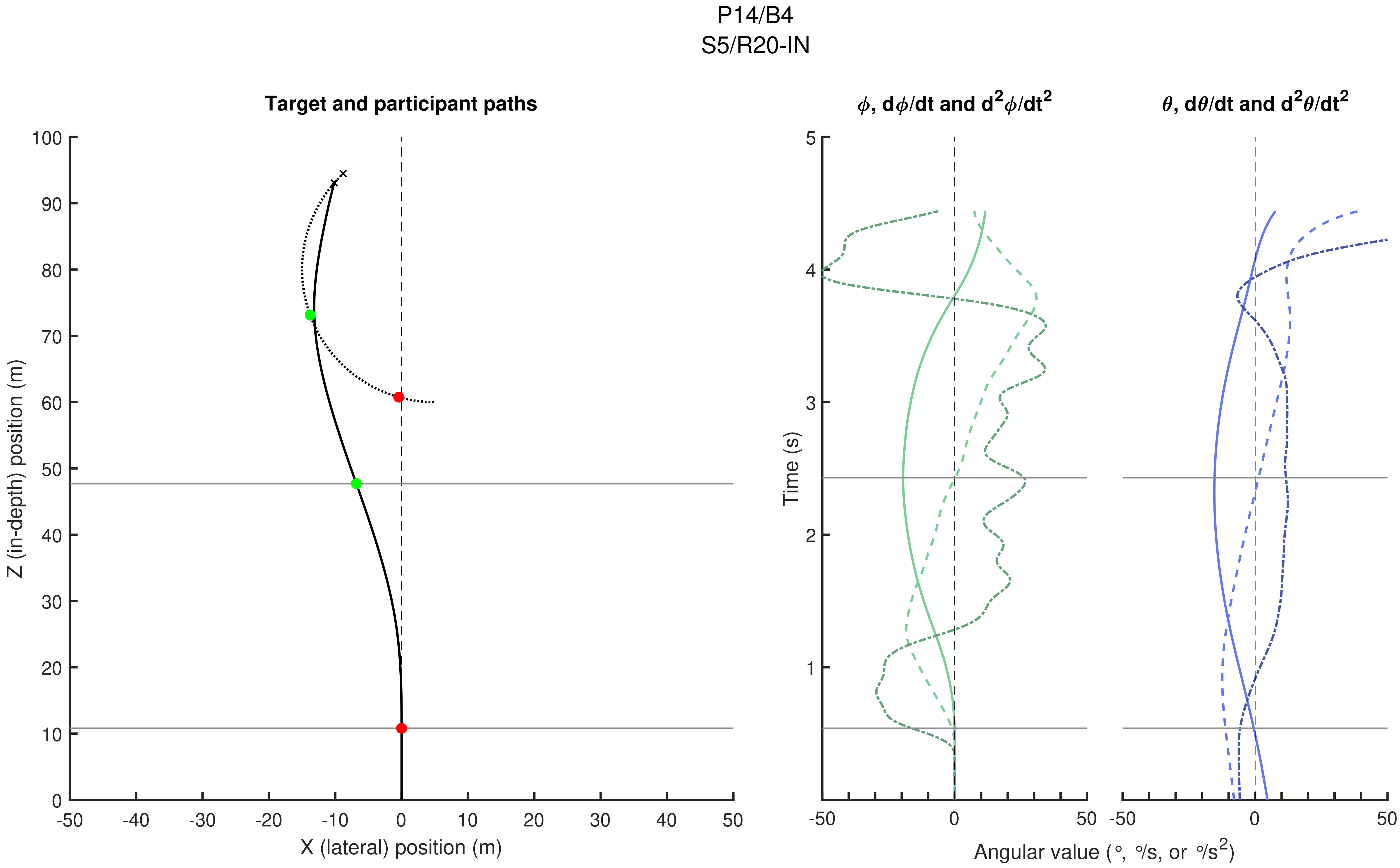

P14/B4
S5/R20-IN
Target and participant paths
Z (in-depth) position (m)
X (lateral) position (m)
ϕ, dϕ/dt and d²ϕ/dt²
θ, dθ/dt and d²θ/dt²
Time (s)
Angular value (°, °/s, or °/s²)

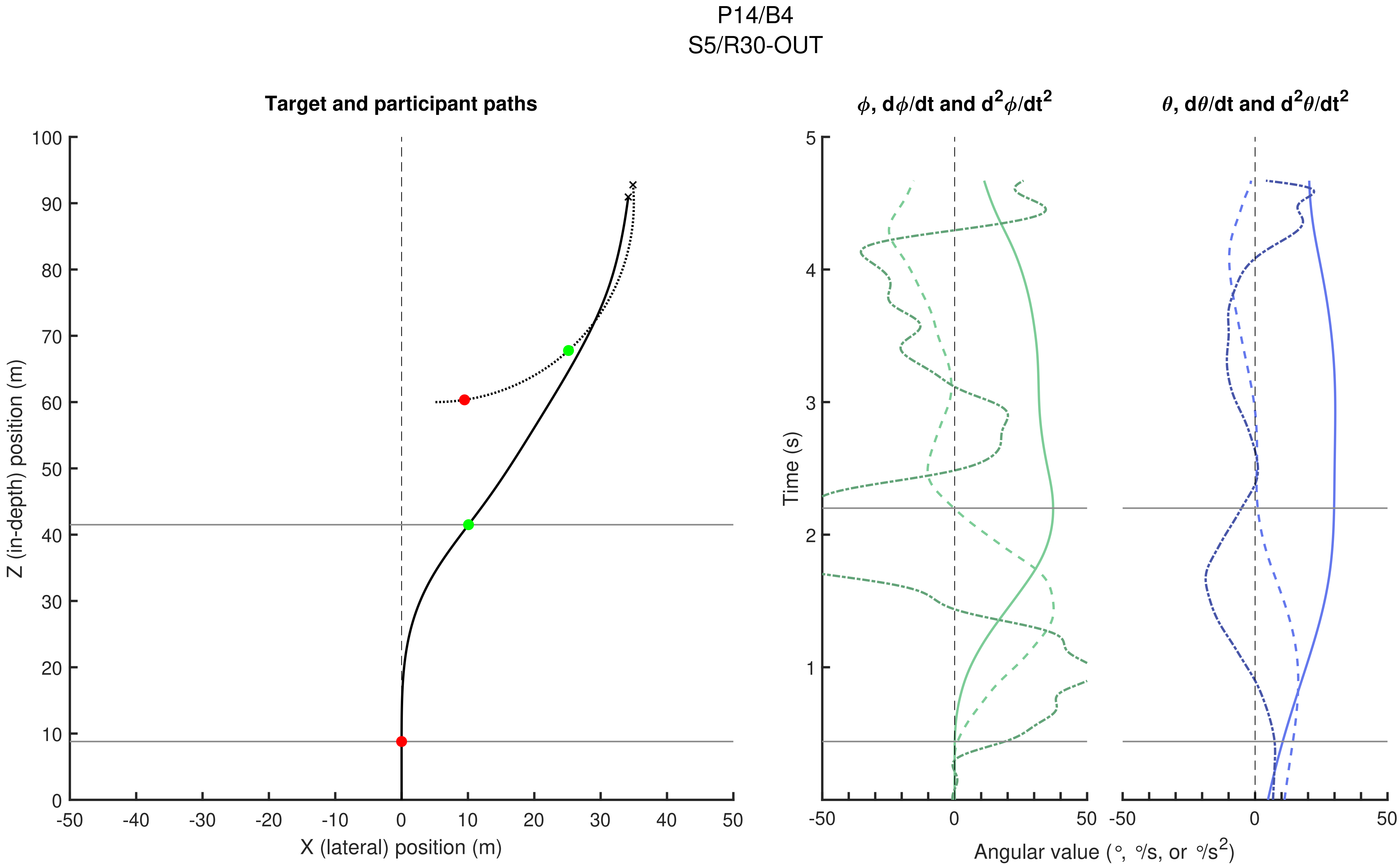
P14/B4
S5/R30-OUT
Target and participant paths
Z (in-depth) position (m)
X (lateral) position (m)
ϕ, dϕ/dt and d$^2\phi$/dt^2
θ, dθ/dt and d$^2\theta$/dt^2
Time (s)
Angular value (°, °/s, or °/s^2)

P14/B4
S5/R30-IN

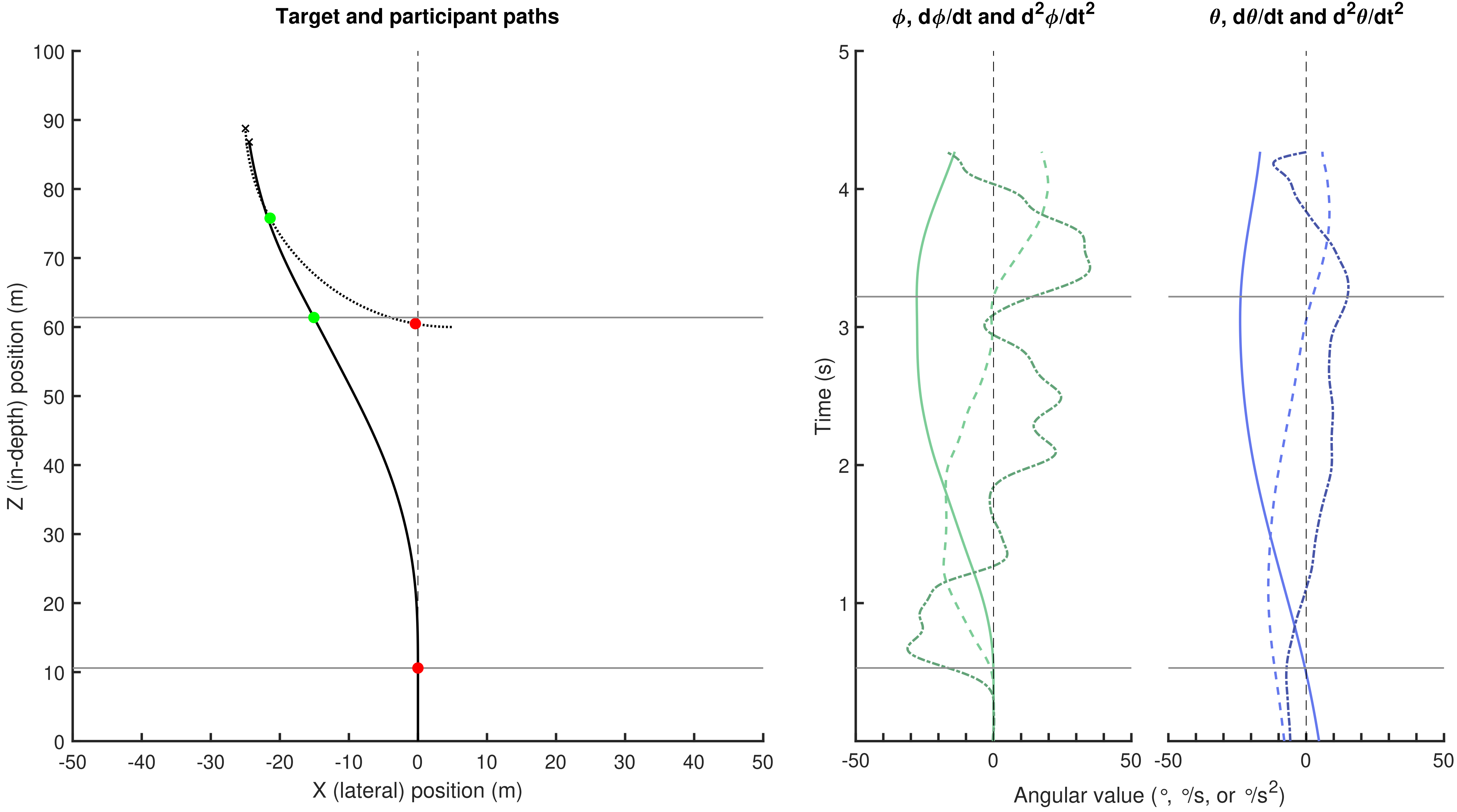

P14/B4
S15/R10-IN

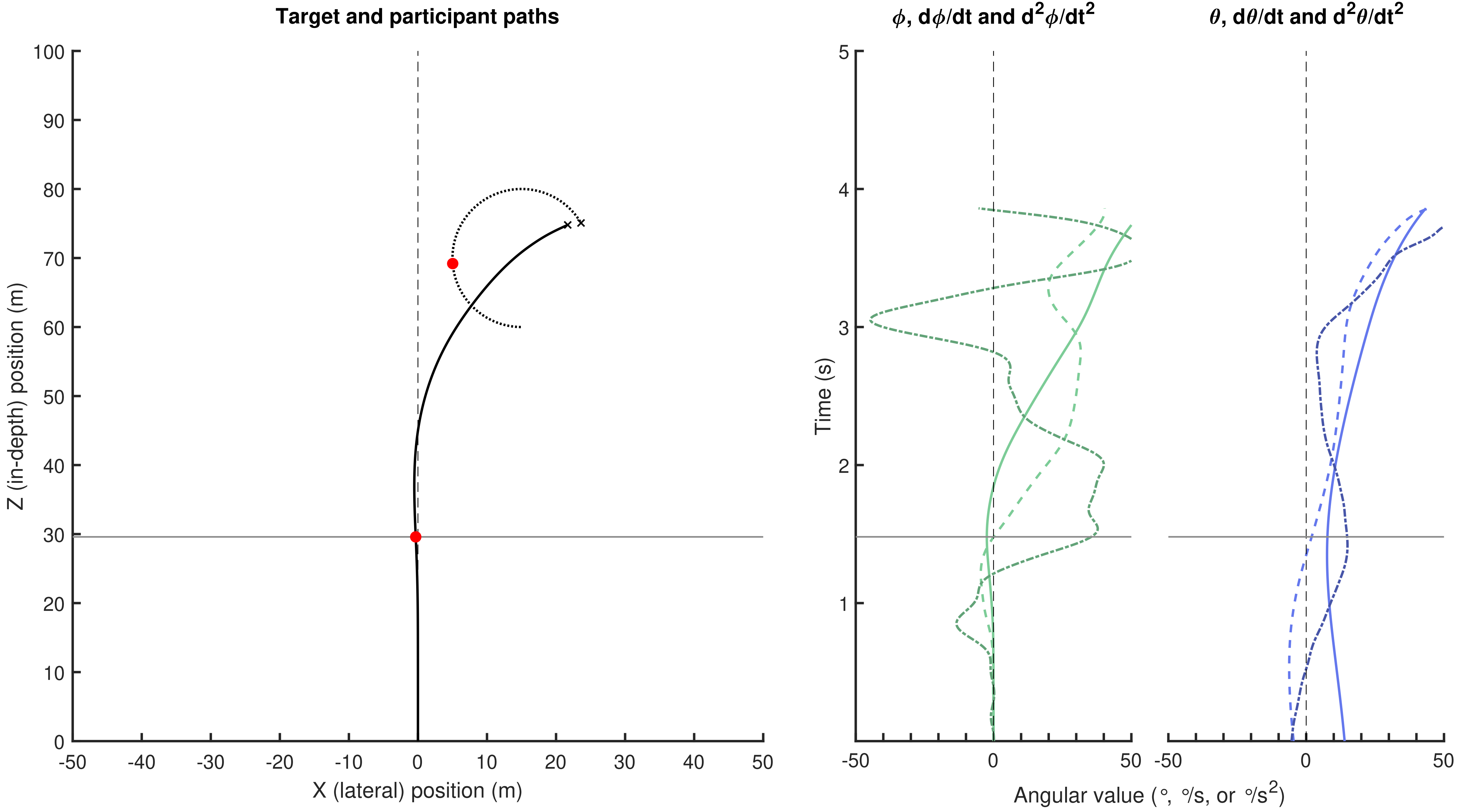

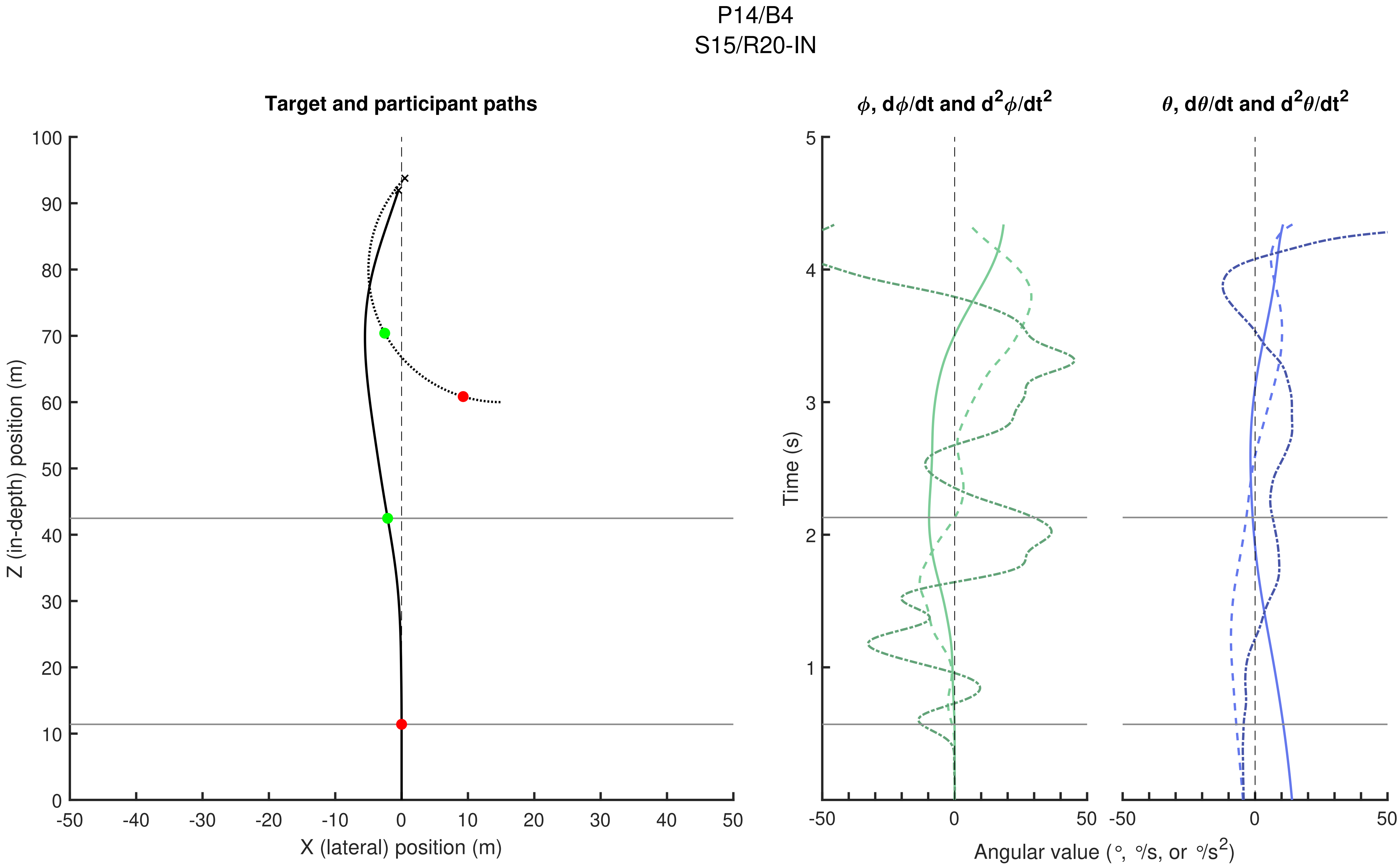

P14/B4
S15/R20-IN
Target and participant paths
Z (in-depth) position (m)
X (lateral) position (m)
ϕ, dϕ/dt and d²ϕ/dt²
θ, dθ/dt and d²θ/dt²
Time (s)
Angular value (°, °/s, or °/s²)

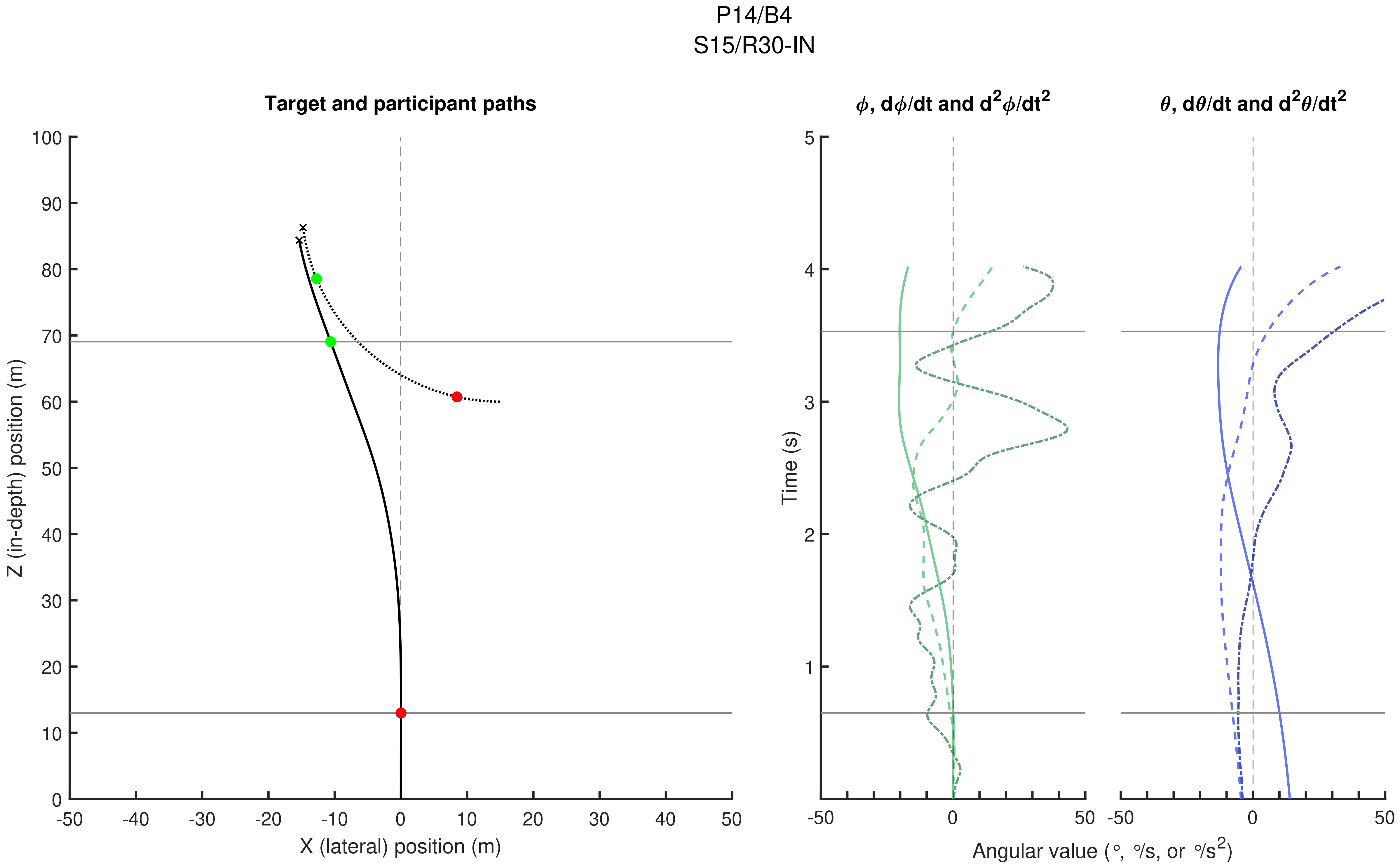

P14/B4
S15/R30-IN
Target and participant paths
Z (in-depth) position (m)
X (lateral) position (m)
100
90
80
70
60
50
40
30
20
10
0
-50
-40
-30
-20
-10
0
10
20
30
40
50
ϕ, dϕ/dt and d$^2\phi$/dt^2
θ, dθ/dt and d$^2\theta$/dt^2
Time (s)
5
4
3
2
1
-50
0
50
-50
0
50
Angular value (°, °/s, or °/s^2)

P14/B4
S20/R10-IN

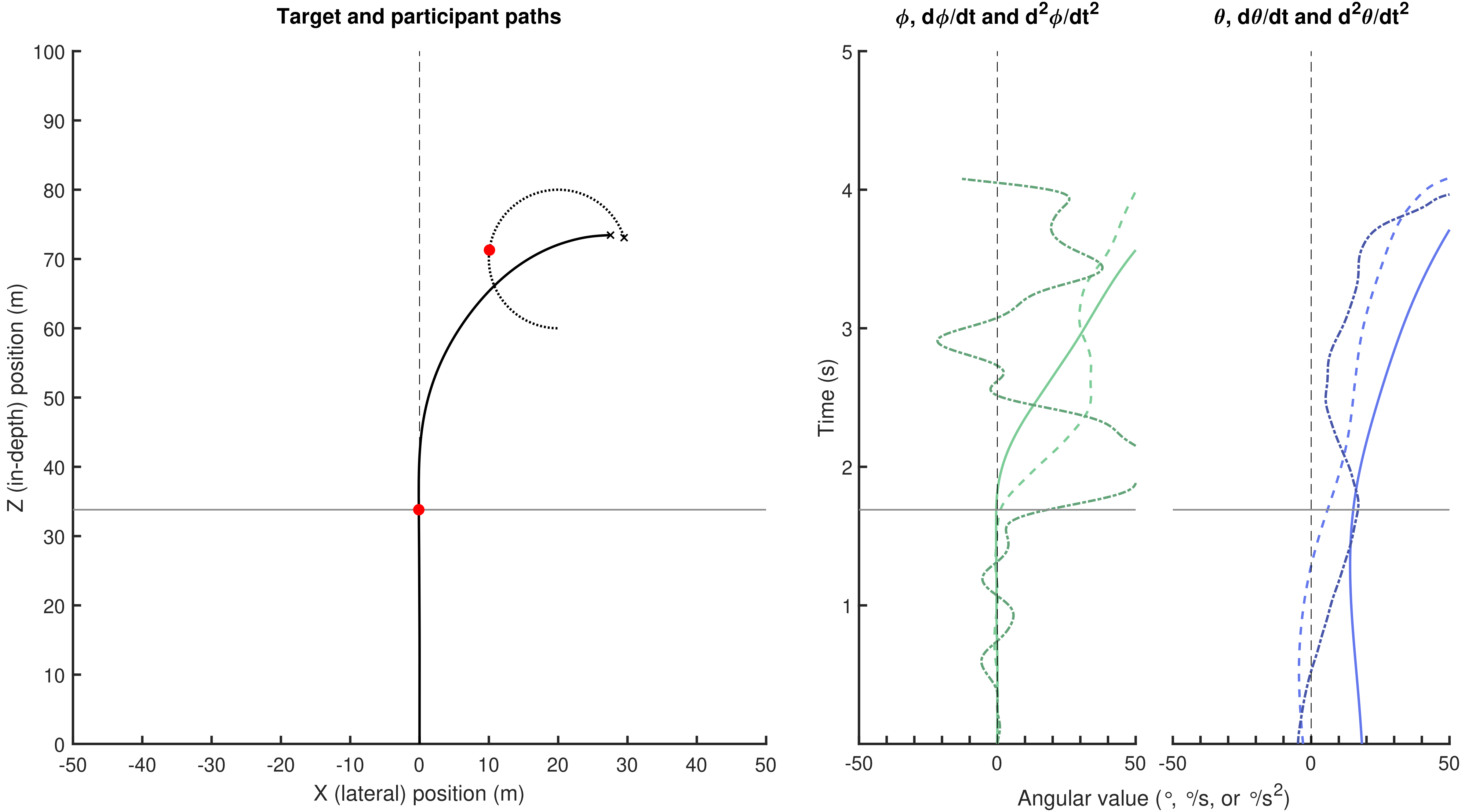

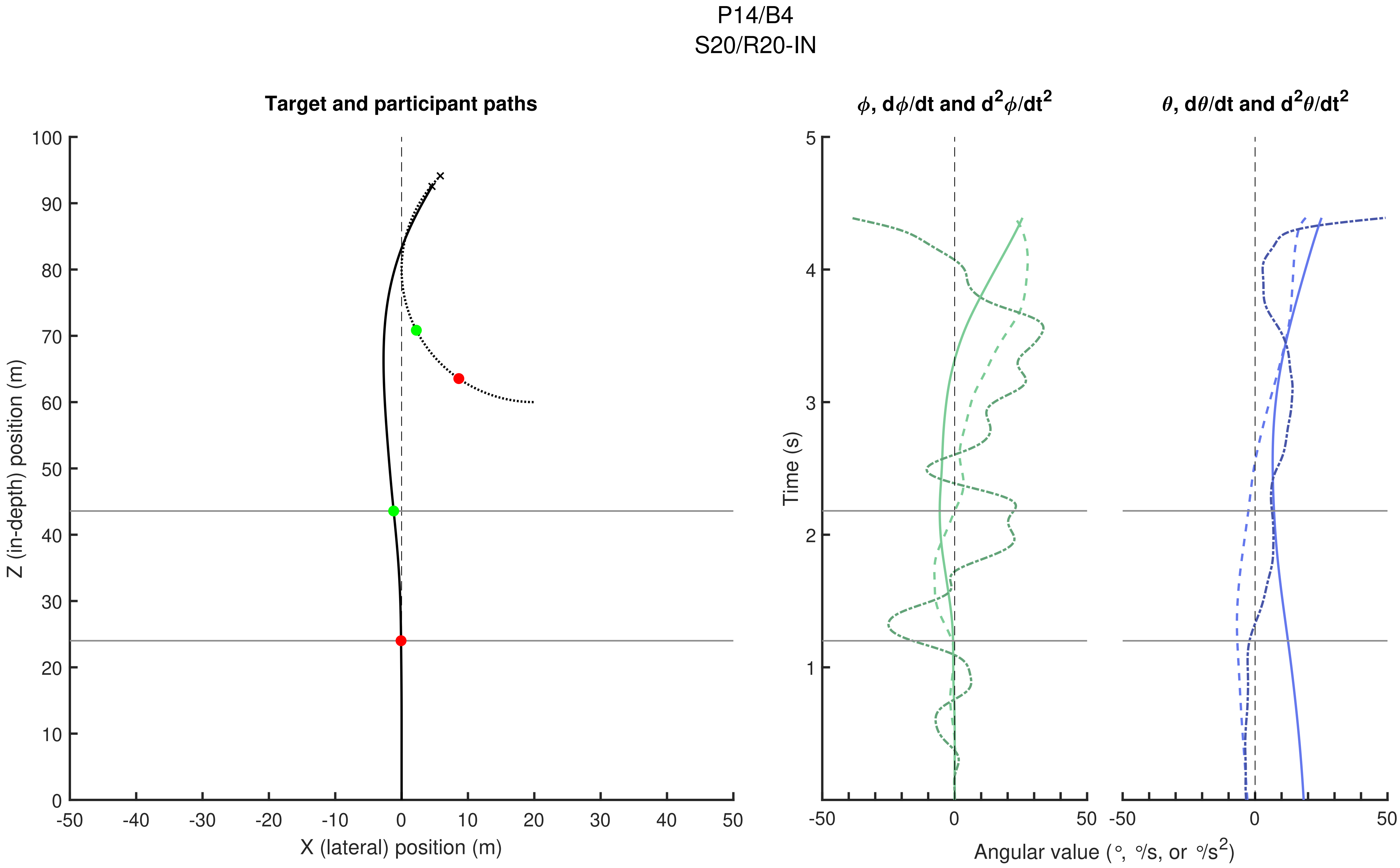

P14/B4
S20/R20-IN
Target and participant paths
Z (in-depth) position (m)
X (lateral) position (m)
ϕ, dϕ/dt and d$^2\phi$/dt^2
θ, dθ/dt and d$^2\theta$/dt^2
Time (s)
Angular value (°, °/s, or °/s^2)

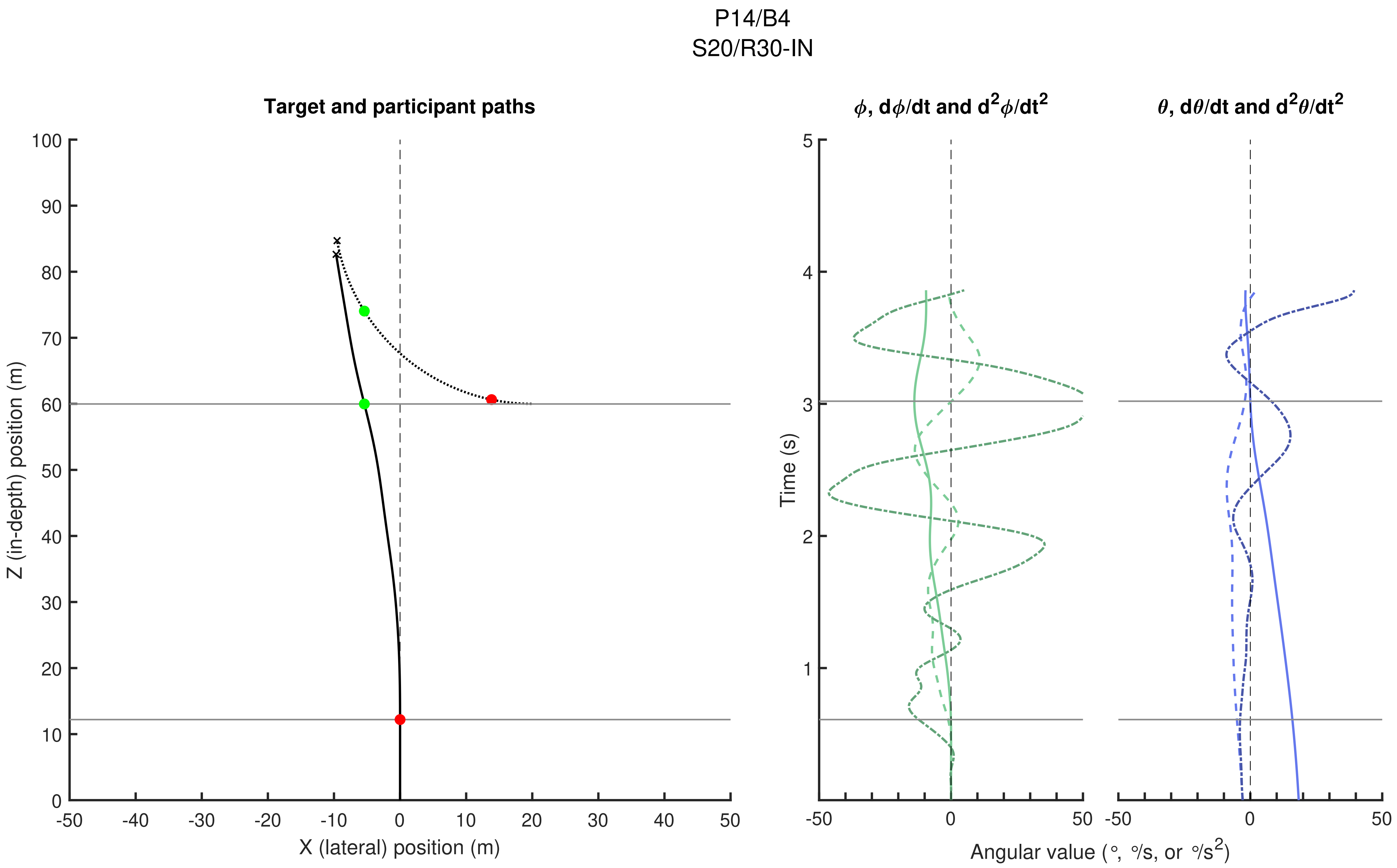
P14/B4
S20/R30-IN
Target and participant paths
Z (in-depth) position (m)
X (lateral) position (m)
ϕ, dϕ/dt and d$^2\phi$/dt^2
θ, dθ/dt and d$^2\theta$/dt^2
Time (s)
Angular value (°, °/s, or °/s^2)

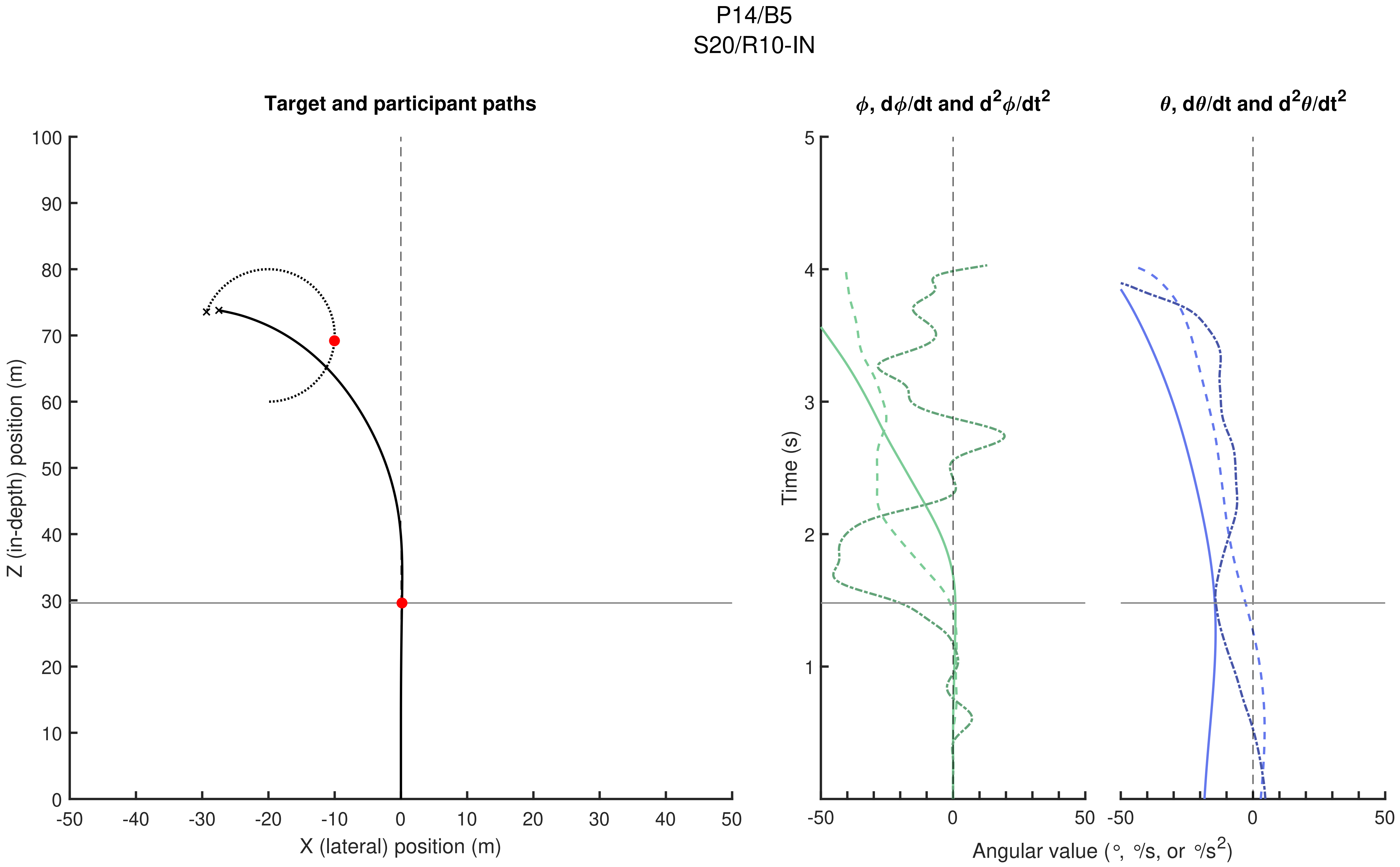
P14/B5
S20/R10-IN
Target and participant paths
Z (in-depth) position (m)
X (lateral) position (m)
φ, dφ/dt and d²φ/dt²
θ, dθ/dt and d²θ/dt²
Time (s)
Angular value (°, °/s, or °/s²)

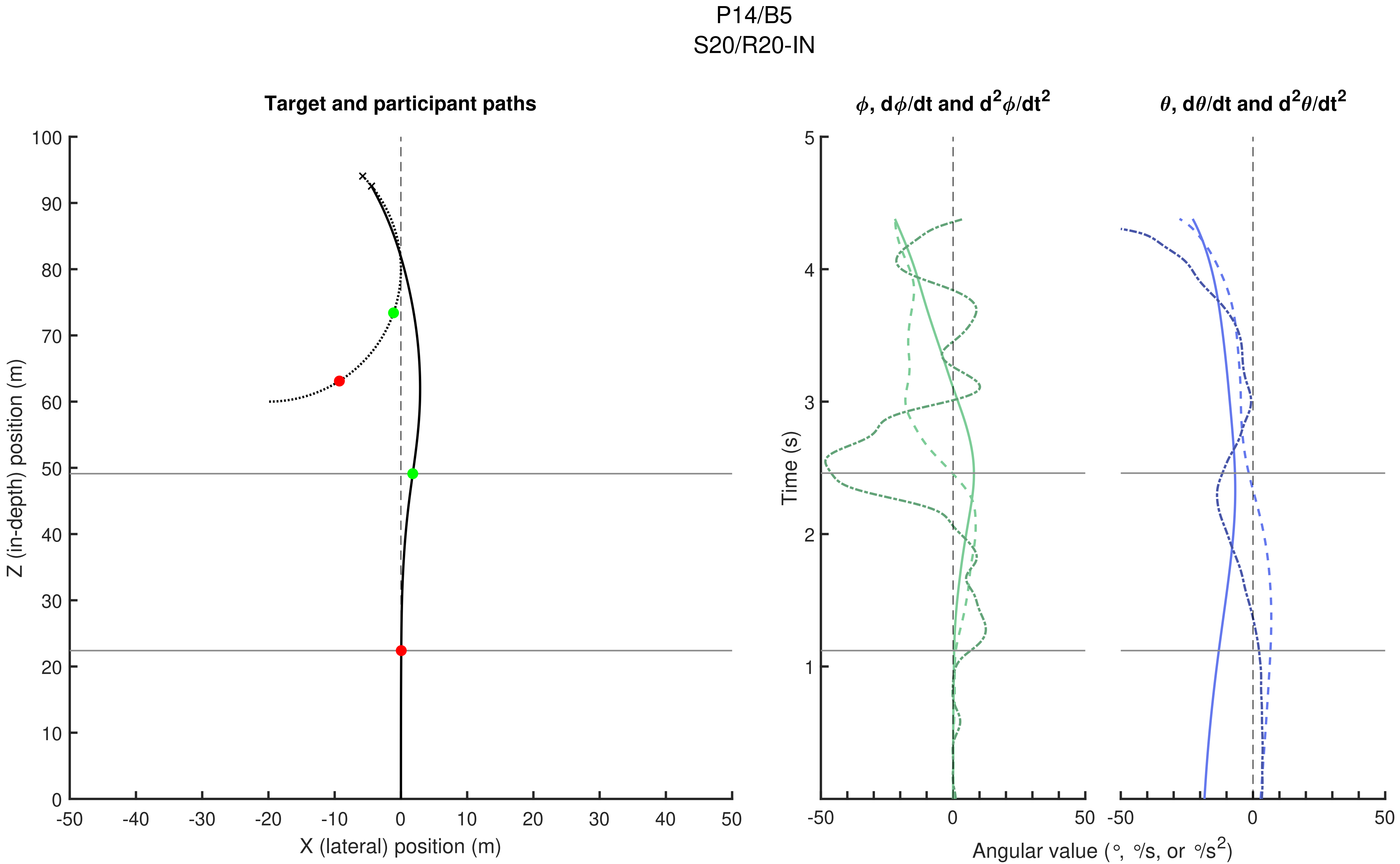

P14/B5
S20/R20-IN
Target and participant paths
Z (in-depth) position (m)
X (lateral) position (m)
100
90
80
70
60
50
40
30
20
10
0
-50
-40
-30
-20
-10
0
10
20
30
40
50
ϕ, dϕ/dt and d²ϕ/dt²
θ, dθ/dt and d²θ/dt²
Time (s)
5
4
3
2
1
-50
0
50
-50
0
50
Angular value (°, °/s, or °/s²)

P14/B5
S20/R30-IN

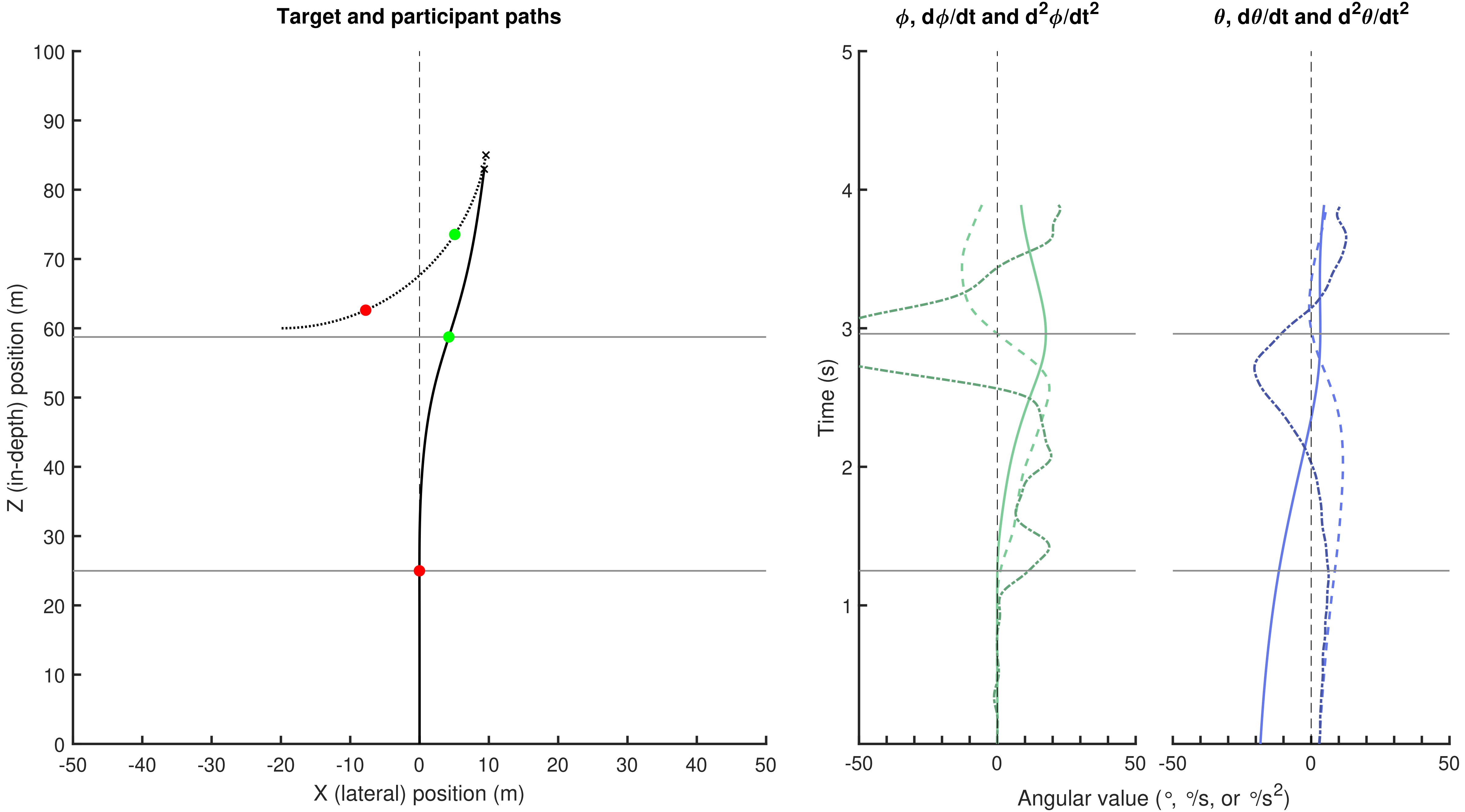

P14/B5
S15/R10-IN

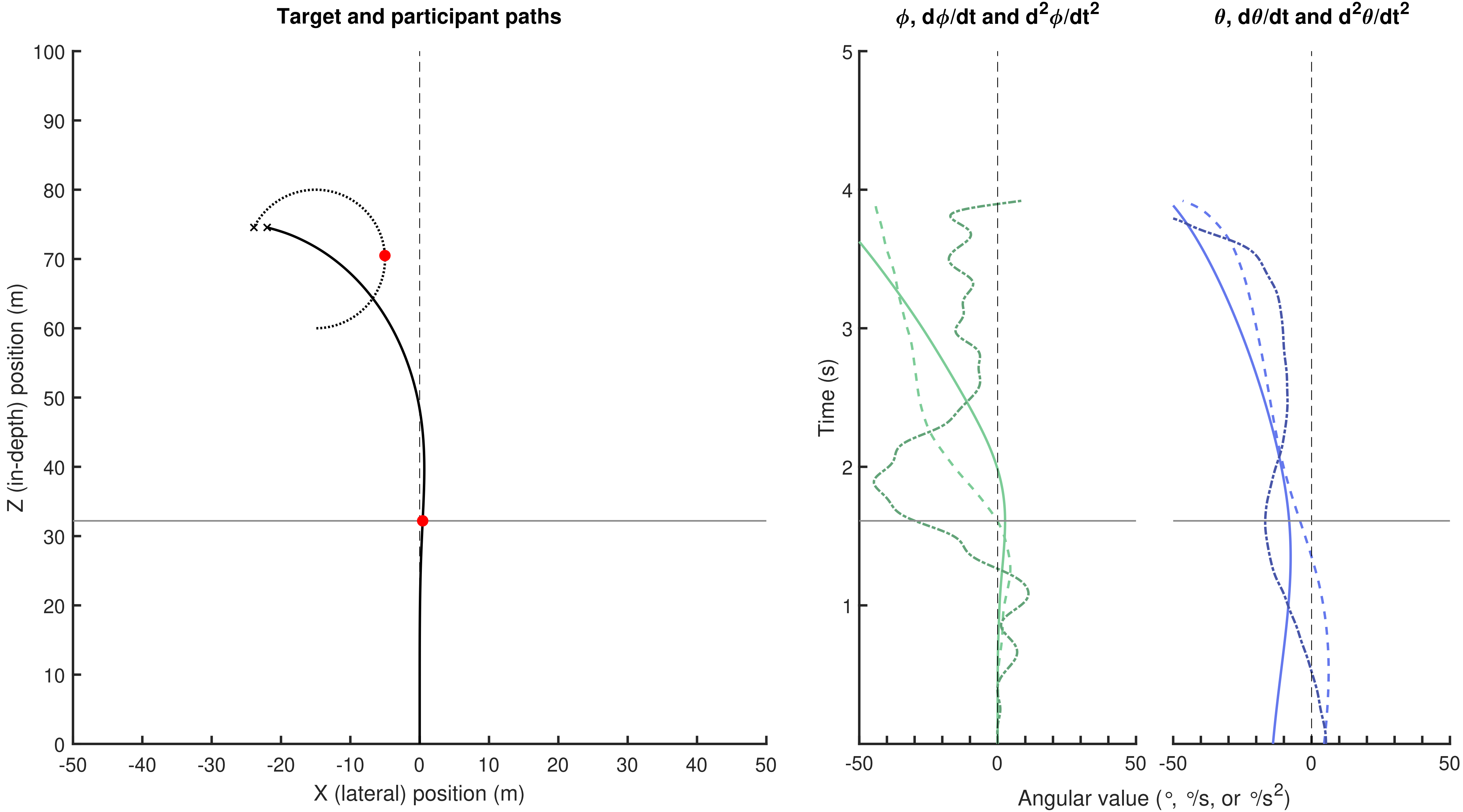

P14/B5
S15/R20-IN

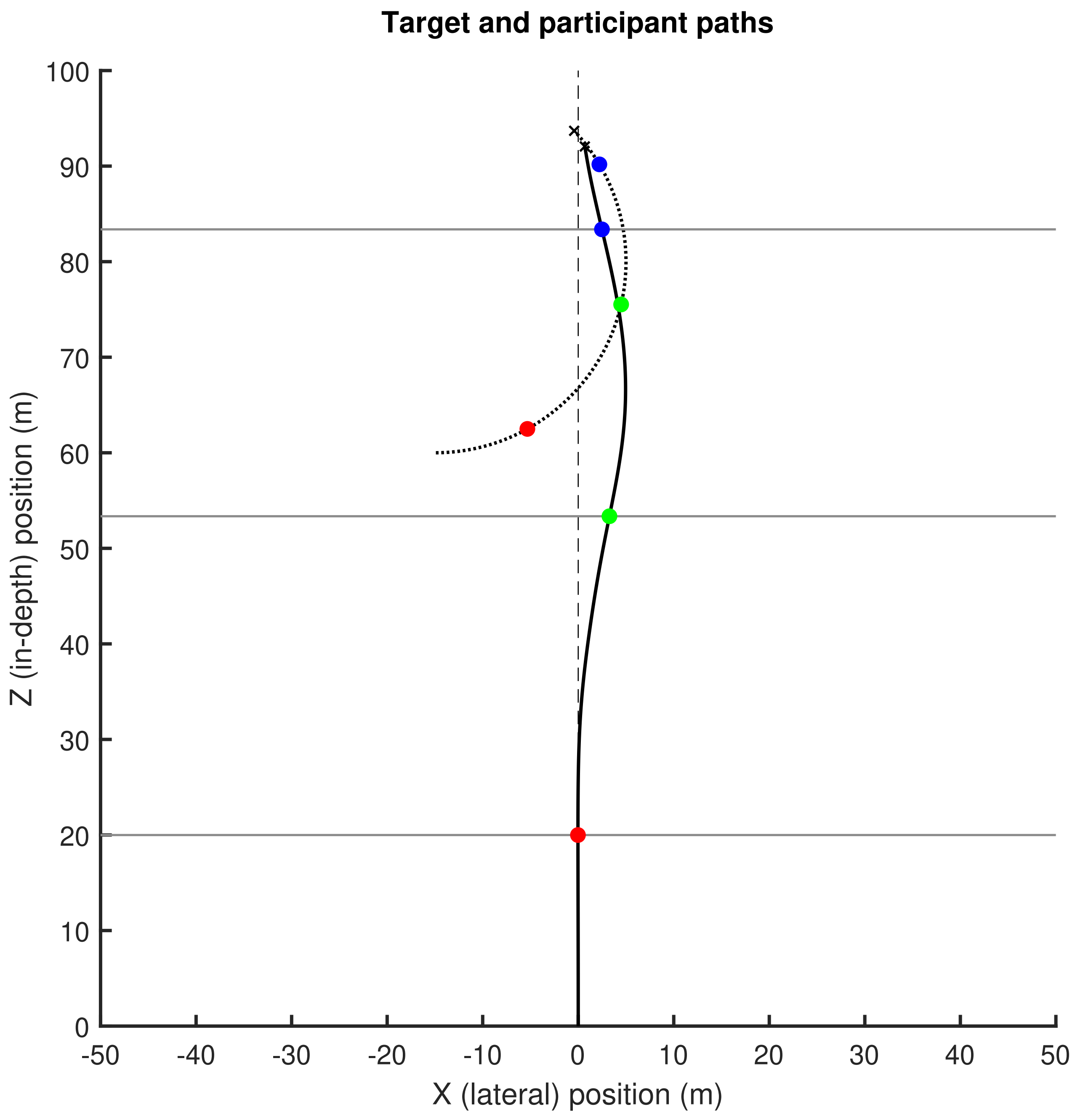

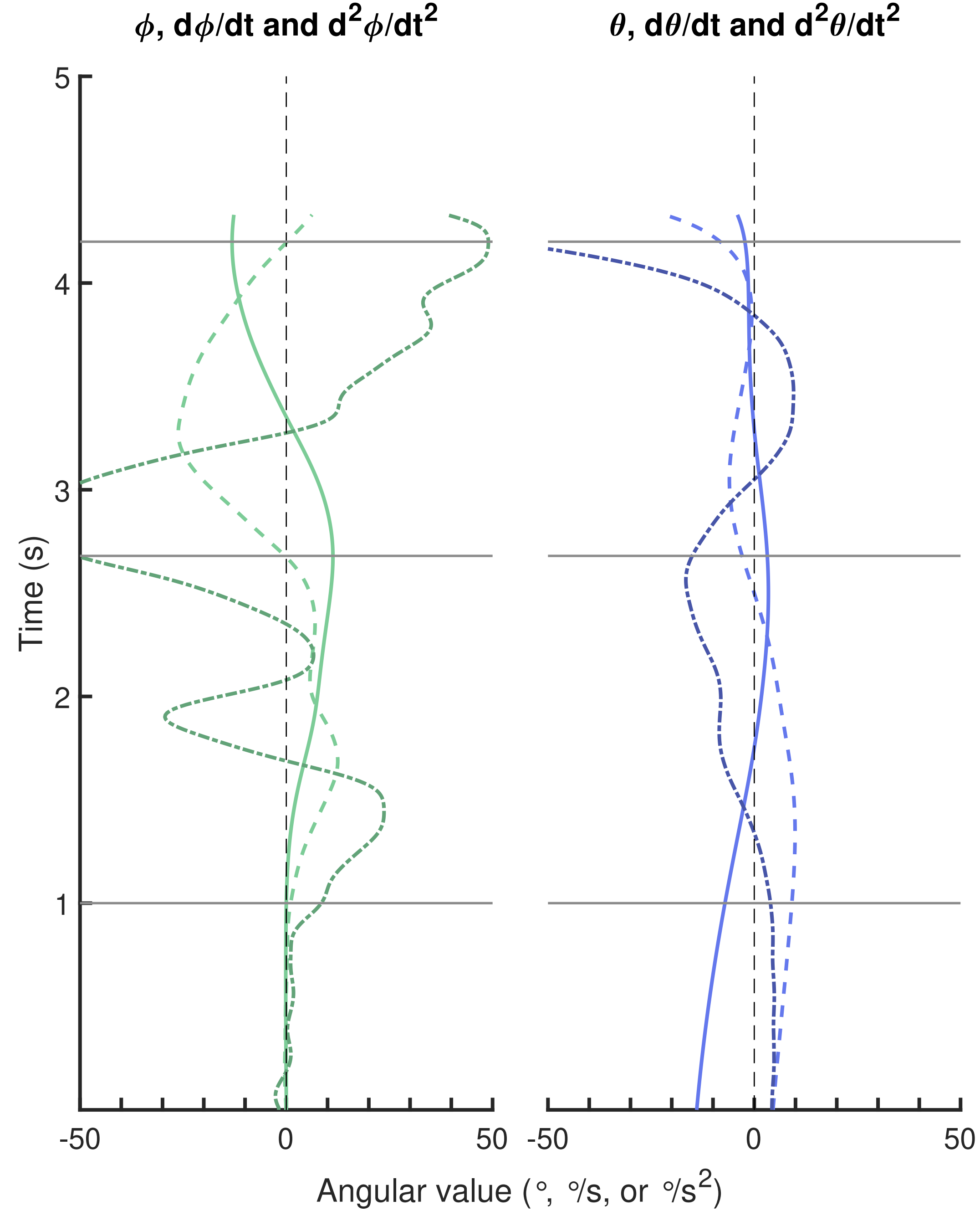

P14/B5
S15/R30-IN

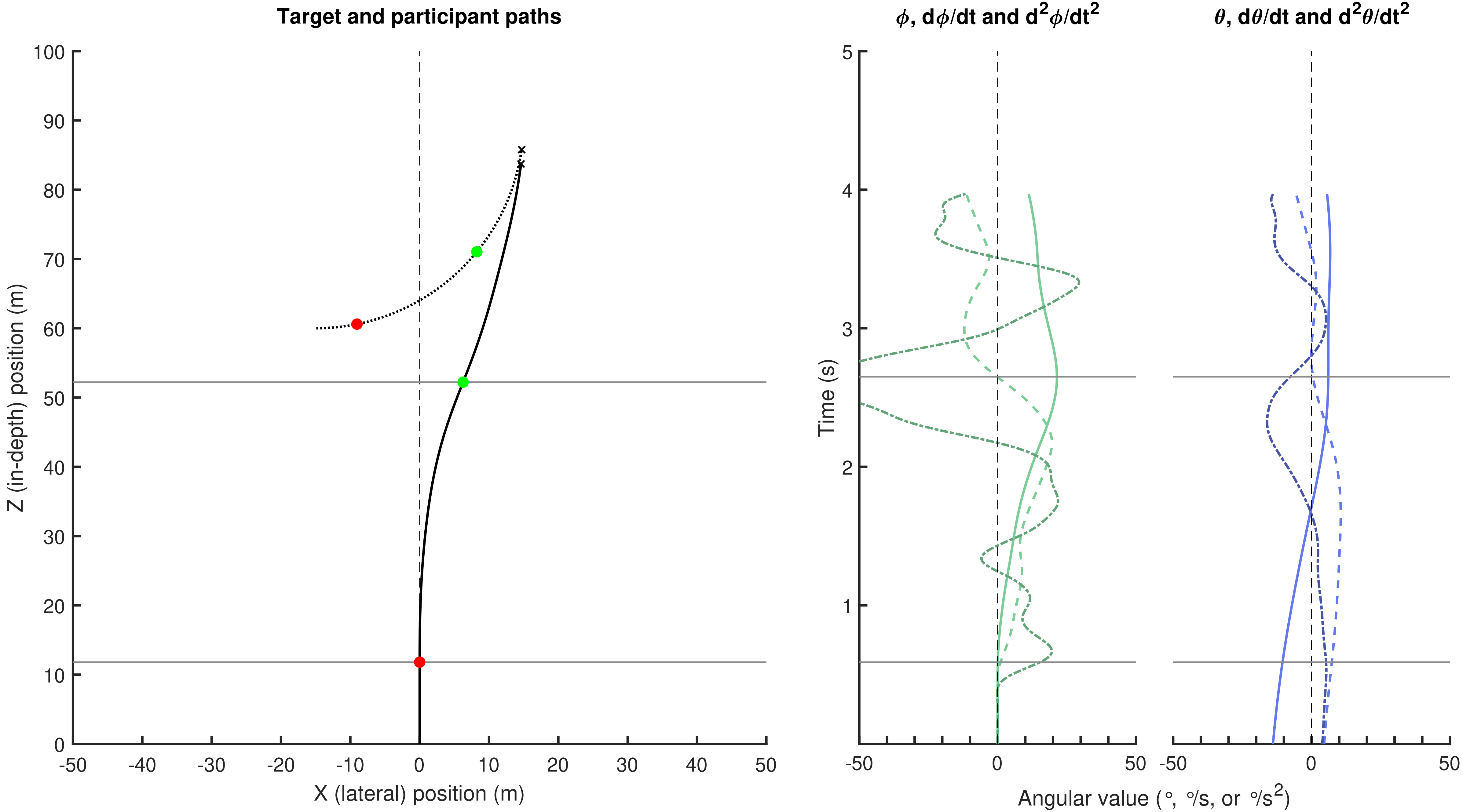

P14/B5
S5/R10-IN

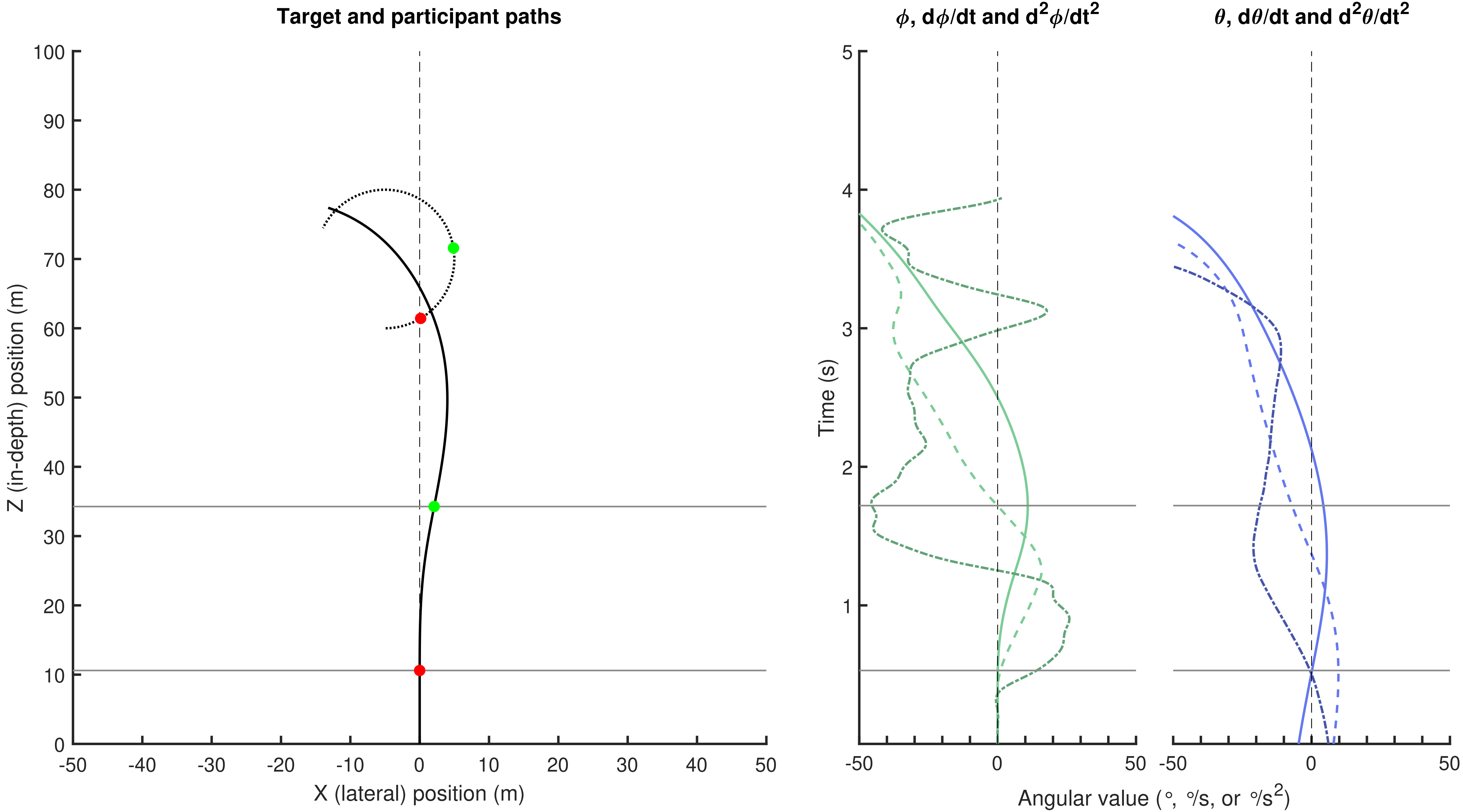

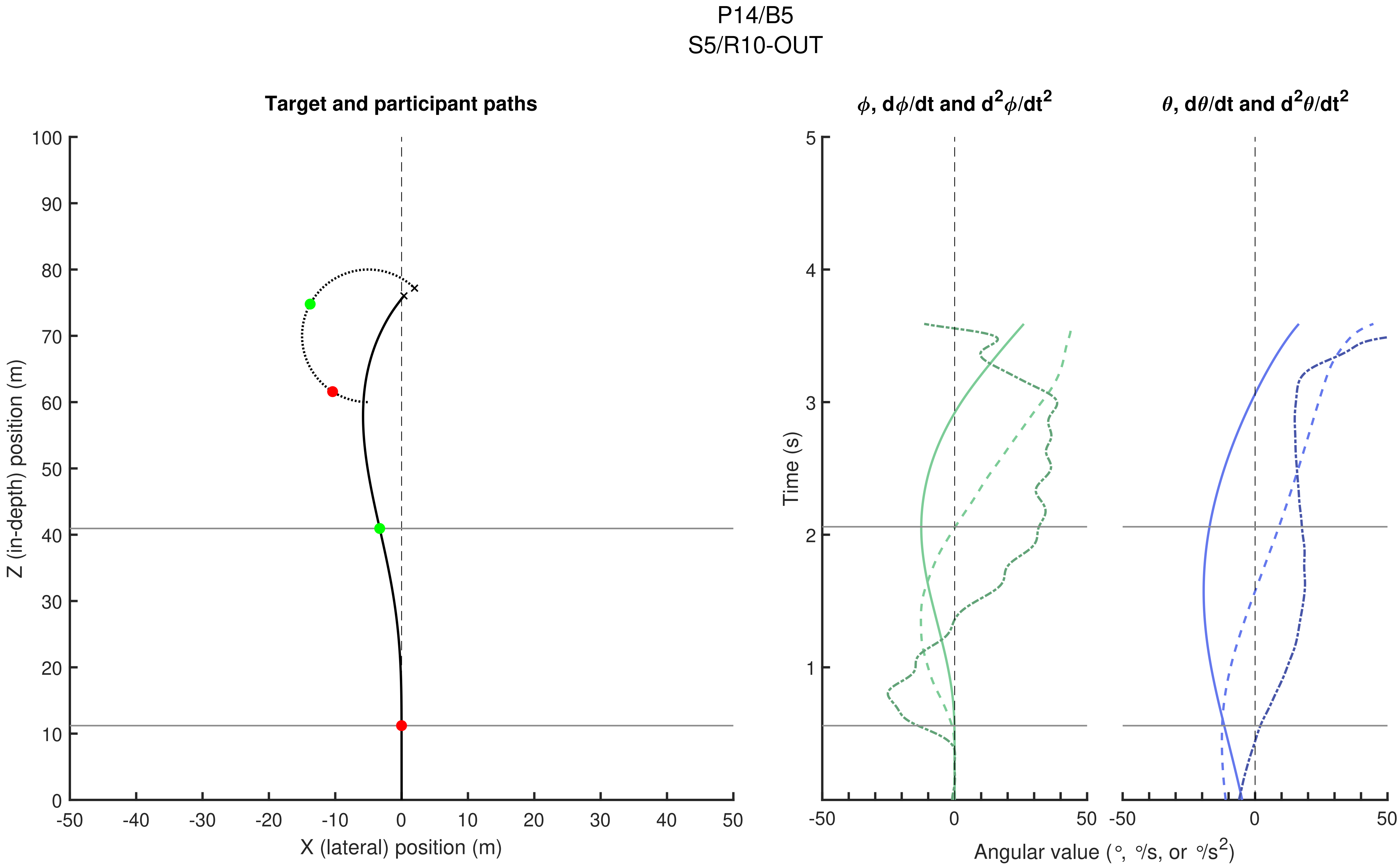
P14/B5
S5/R10-OUT
Target and participant paths
ϕ, dϕ/dt and d$^2\phi$/dt^2
θ, dθ/dt and d$^2\theta$/dt^2
Z (in-depth) position (m)
X (lateral) position (m)
Time (s)
Angular value (°, °/s, or °/s^2)
100
90
80
70
60
50
40
30
20
10
0
-50
-40
-30
-20
-10
0
10
20
30
40
50
5
4
3
2
1
-50
0
50
-50
0
50

P14/B5
S5/R20-IN

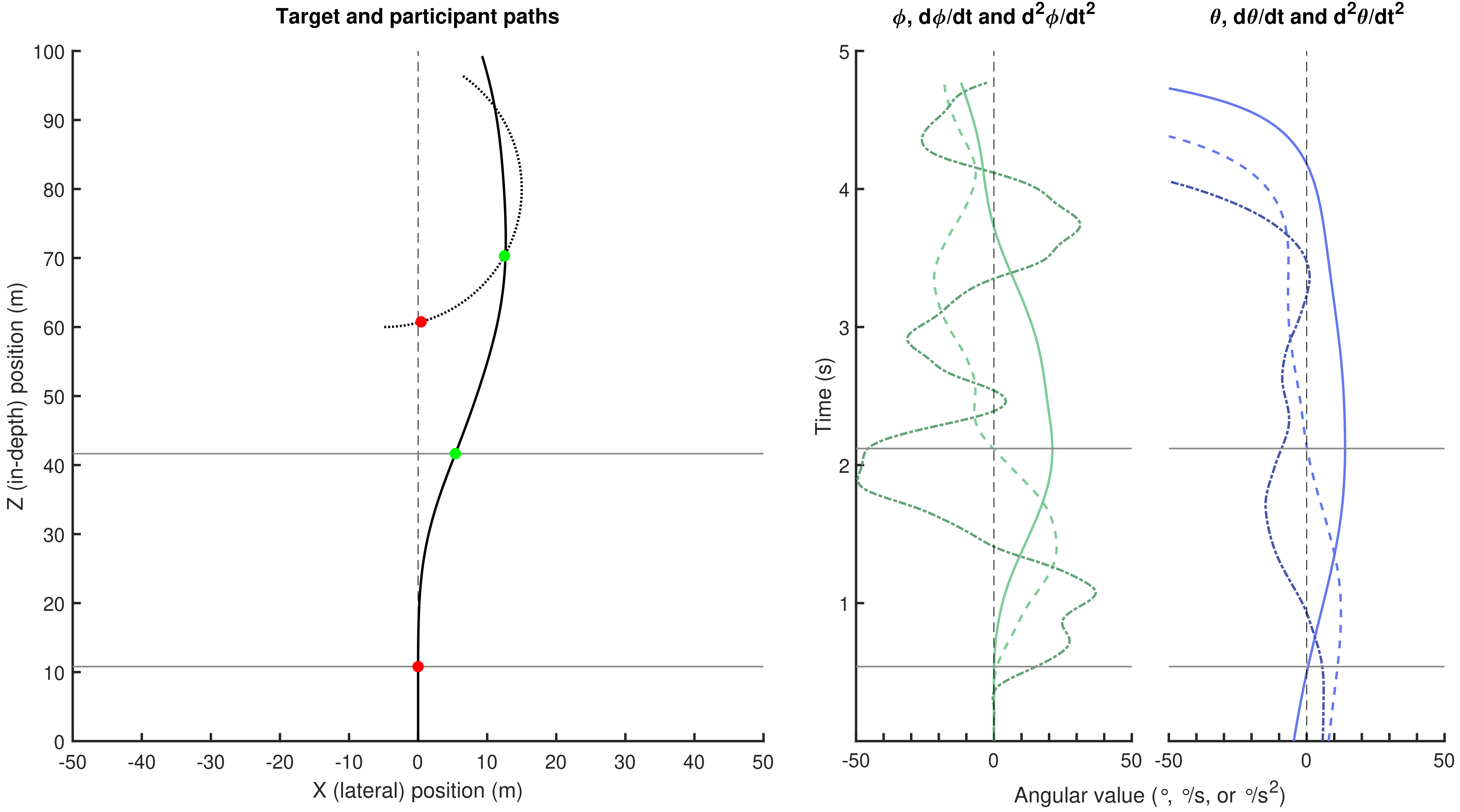

P14/B5
S5/R20-OUT

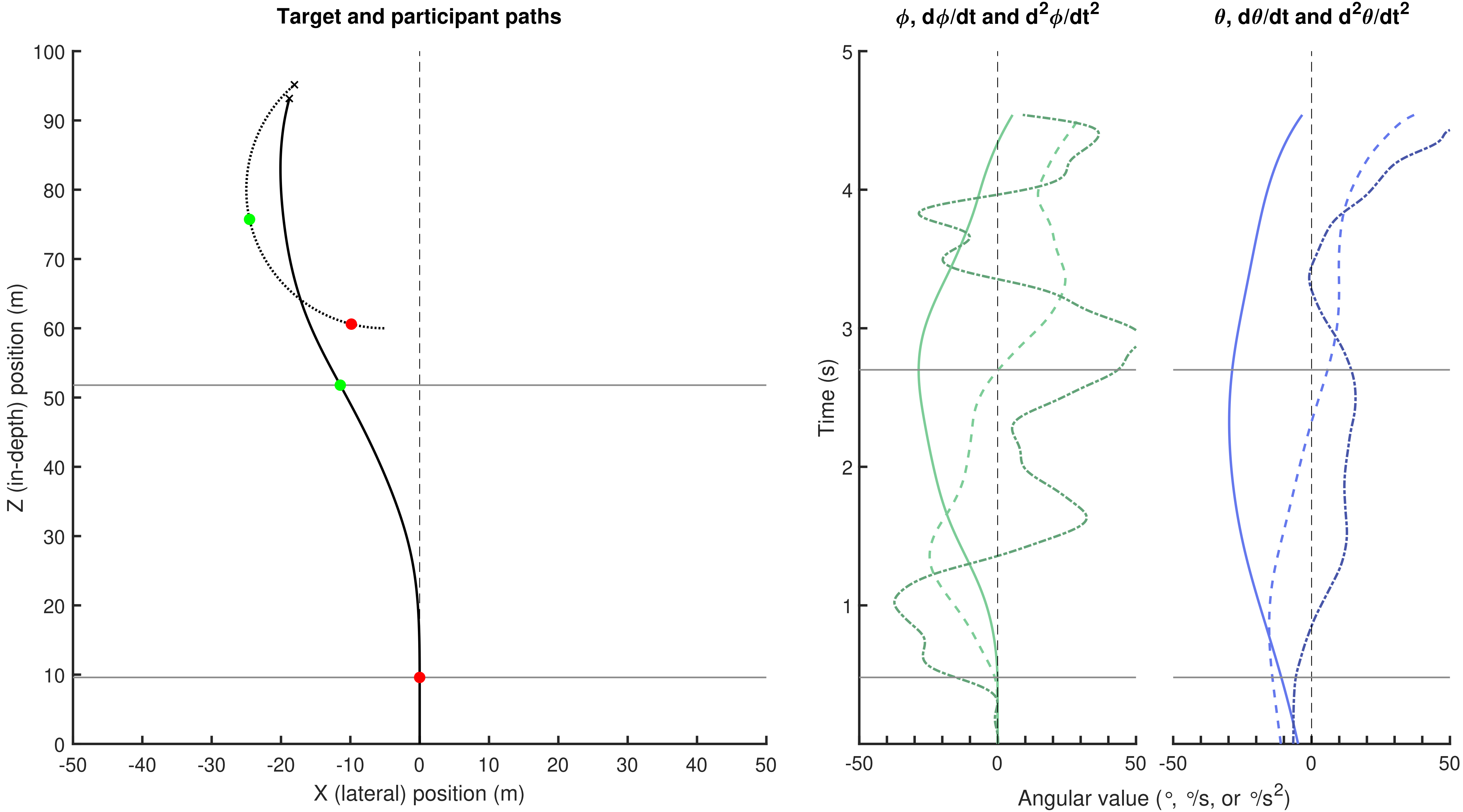

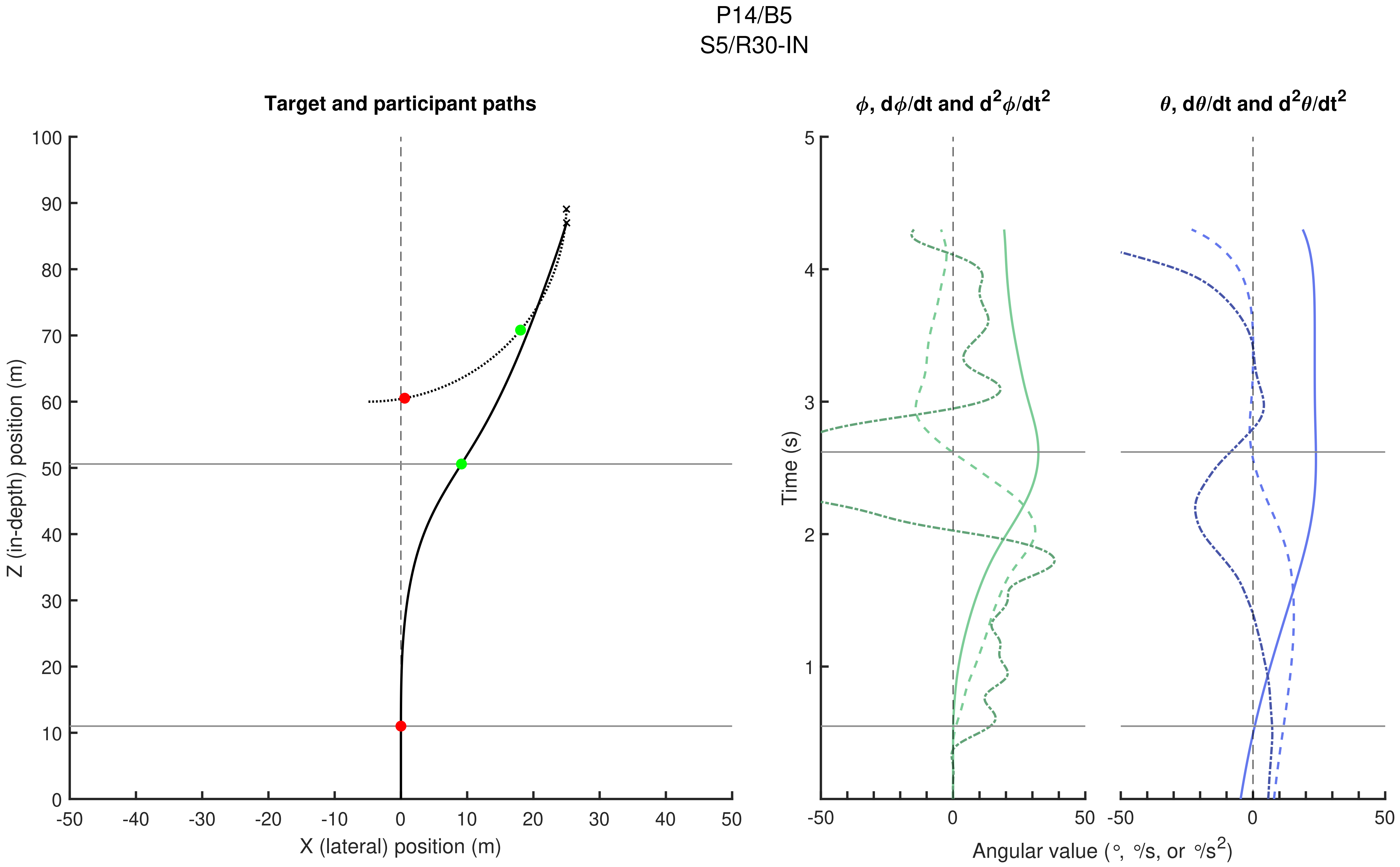
P14/B5
S5/R30-IN
Target and participant paths
Z (in-depth) position (m)
X (lateral) position (m)
100
90
80
70
60
50
40
30
20
10
0
-50
-40
-30
-20
-10
0
10
20
30
40
50
ϕ, dϕ/dt and d$^2\phi$/dt^2
θ, dθ/dt and d$^2\theta$/dt^2
Time (s)
5
4
3
2
1
-50
0
50
-50
0
50
Angular value (°, °/s, or °/s^2)

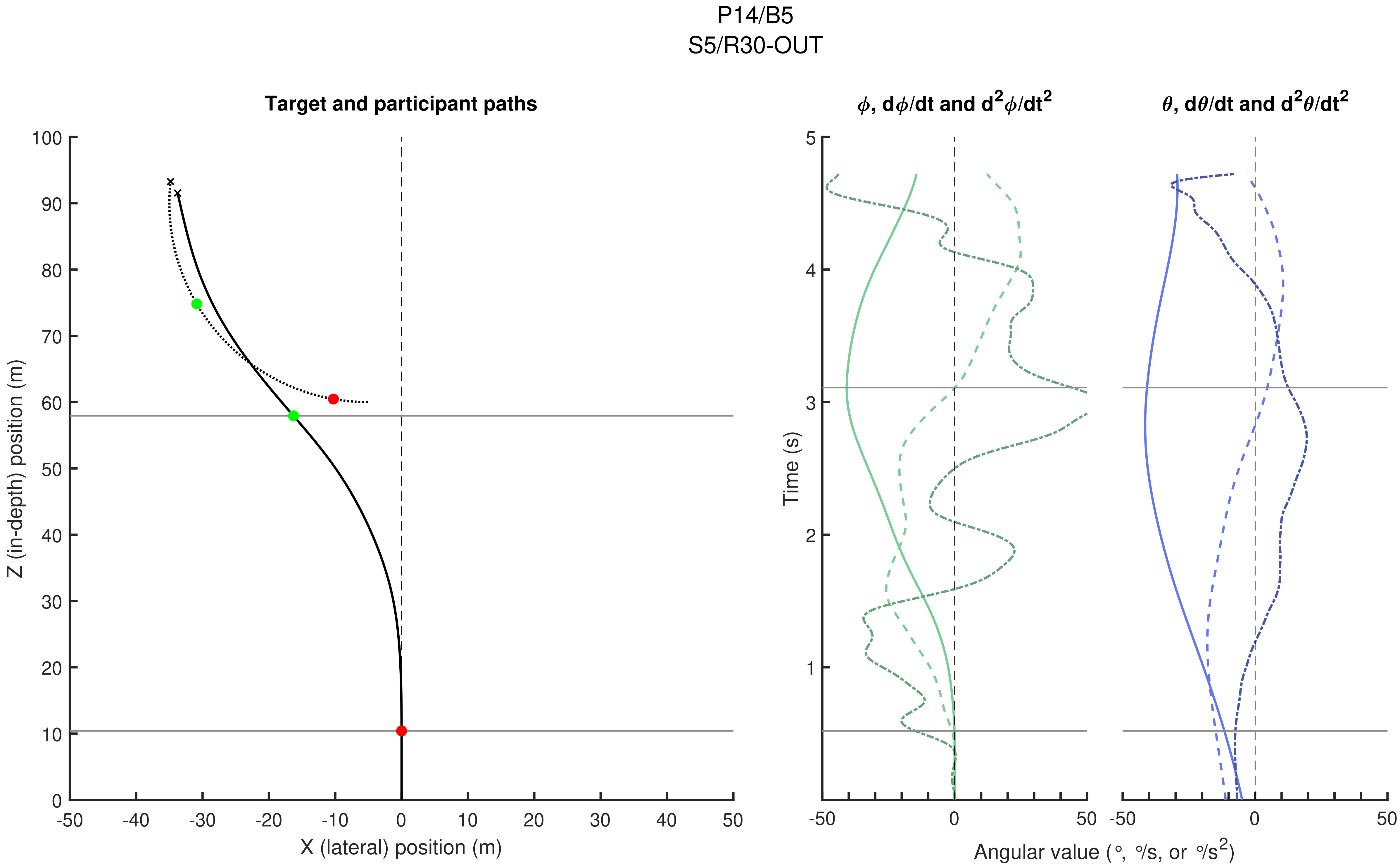
P14/B5
S5/R30-OUT
Target and participant paths
Z (in-depth) position (m)
X (lateral) position (m)
φ, dφ/dt and d²φ/dt²
θ, dθ/dt and d²θ/dt²
Time (s)
Angular value (°, °/s, or °/s²)

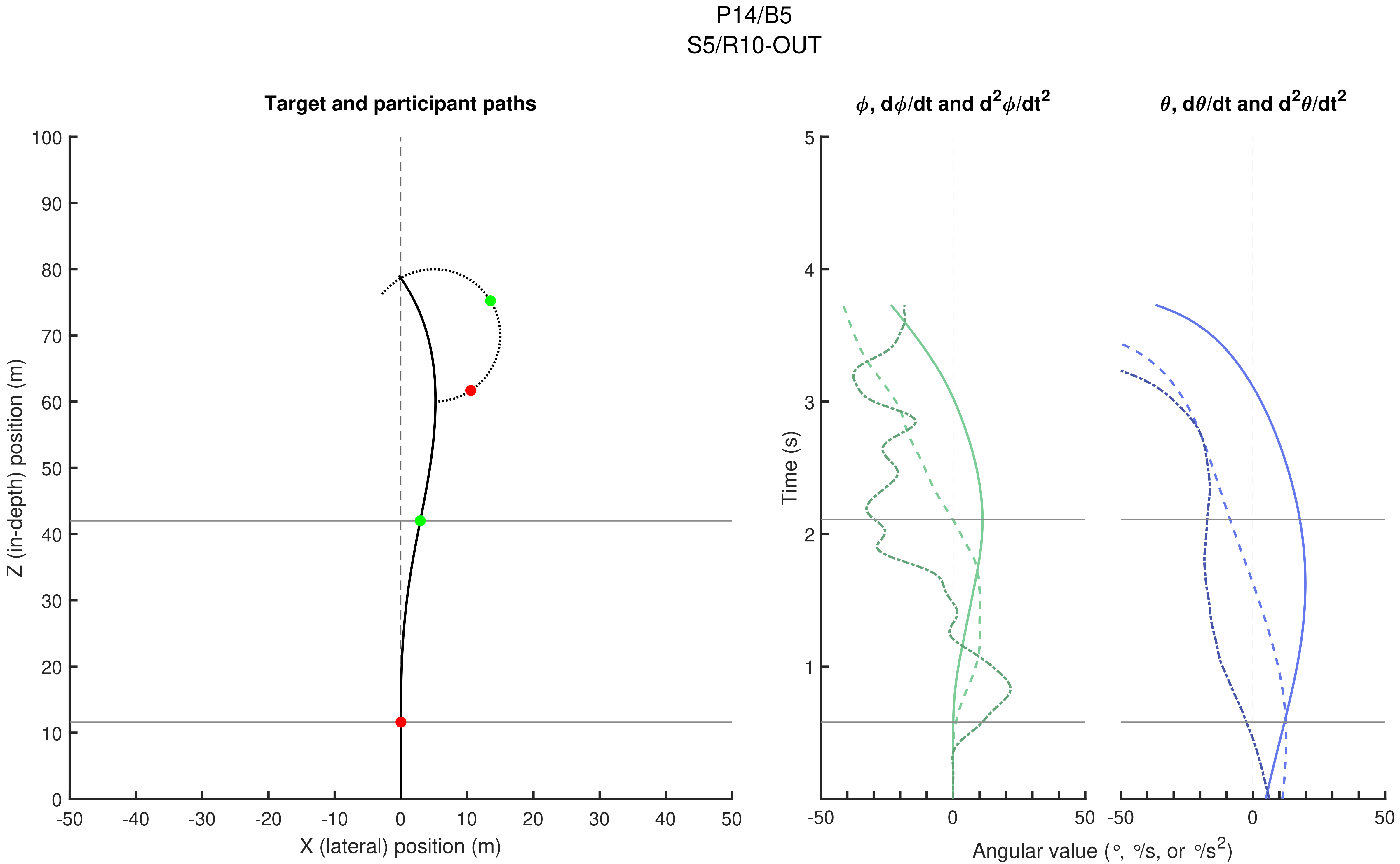

P14/B5
S5/R10-OUT
Target and participant paths
Z (in-depth) position (m)
X (lateral) position (m)
φ, dφ/dt and d²φ/dt²
θ, dθ/dt and d²θ/dt²
Time (s)
Angular value (°, °/s, or °/s²)

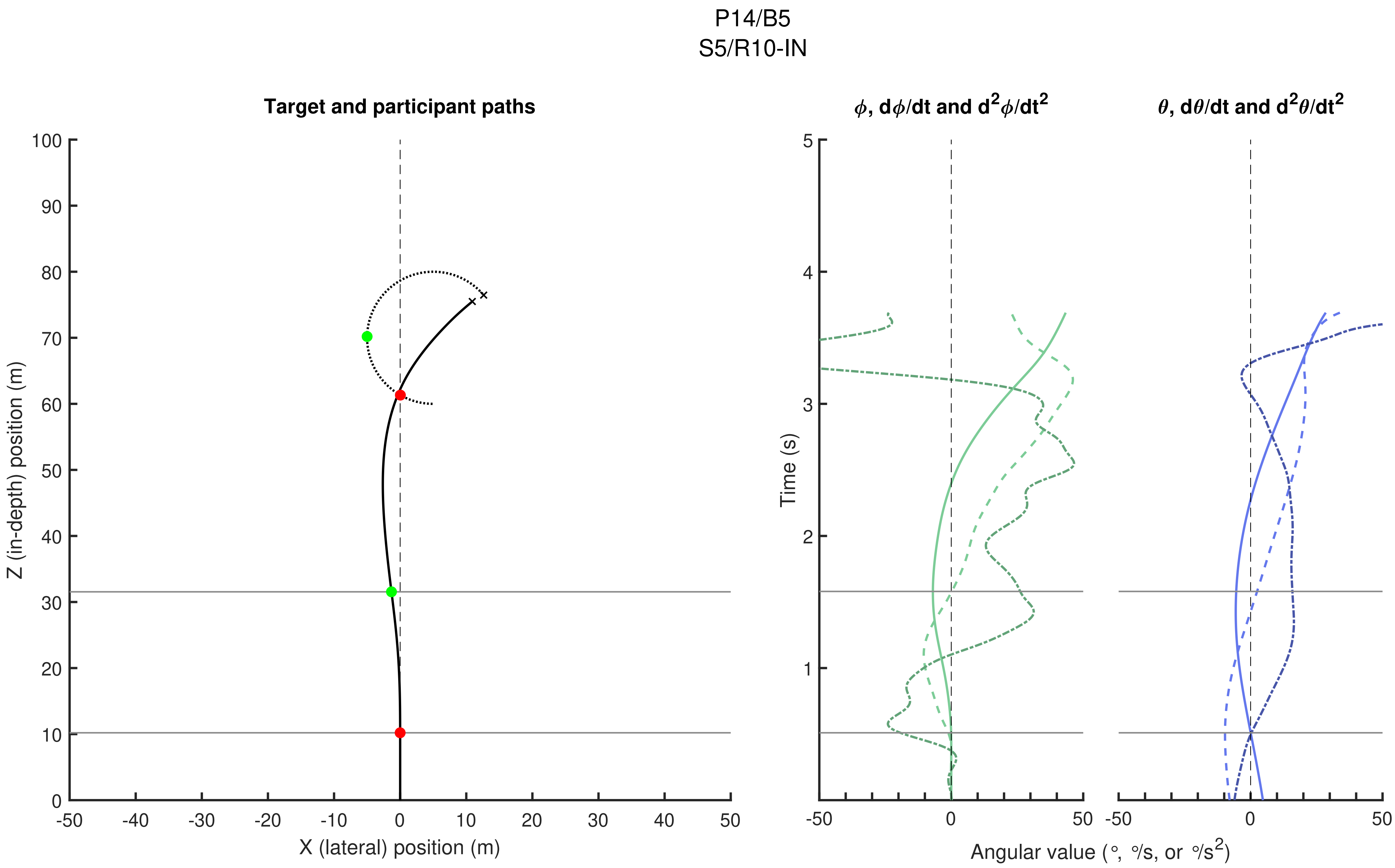

P14/B5
S5/R10-IN
Target and participant paths
ϕ, dϕ/dt and d$^2\phi$/dt^2
θ, dθ/dt and d$^2\theta$/dt^2
Z (in-depth) position (m)
X (lateral) position (m)
Time (s)
Angular value (°, °/s, or °/s^2)

P14/B5
S5/R20-OUT

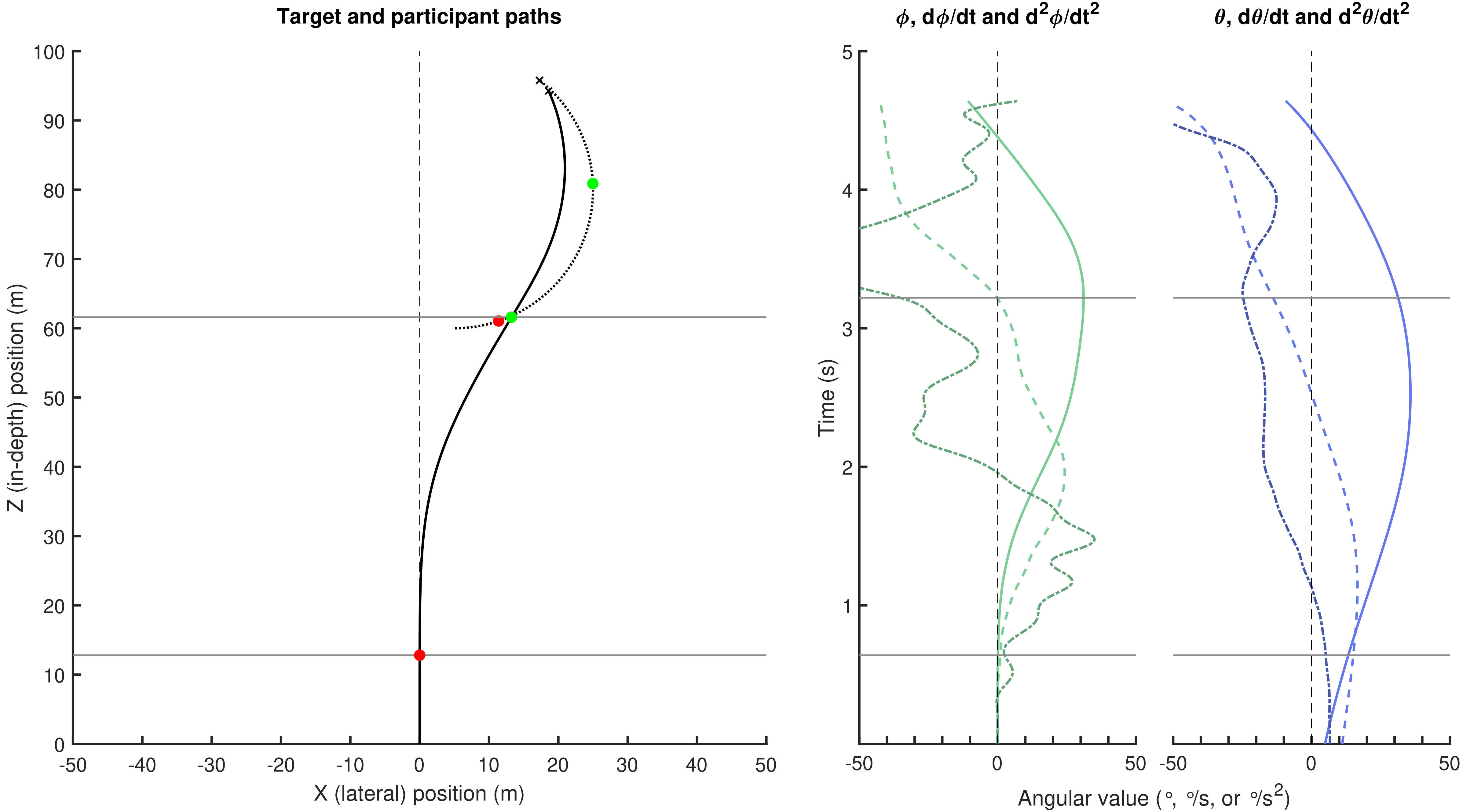

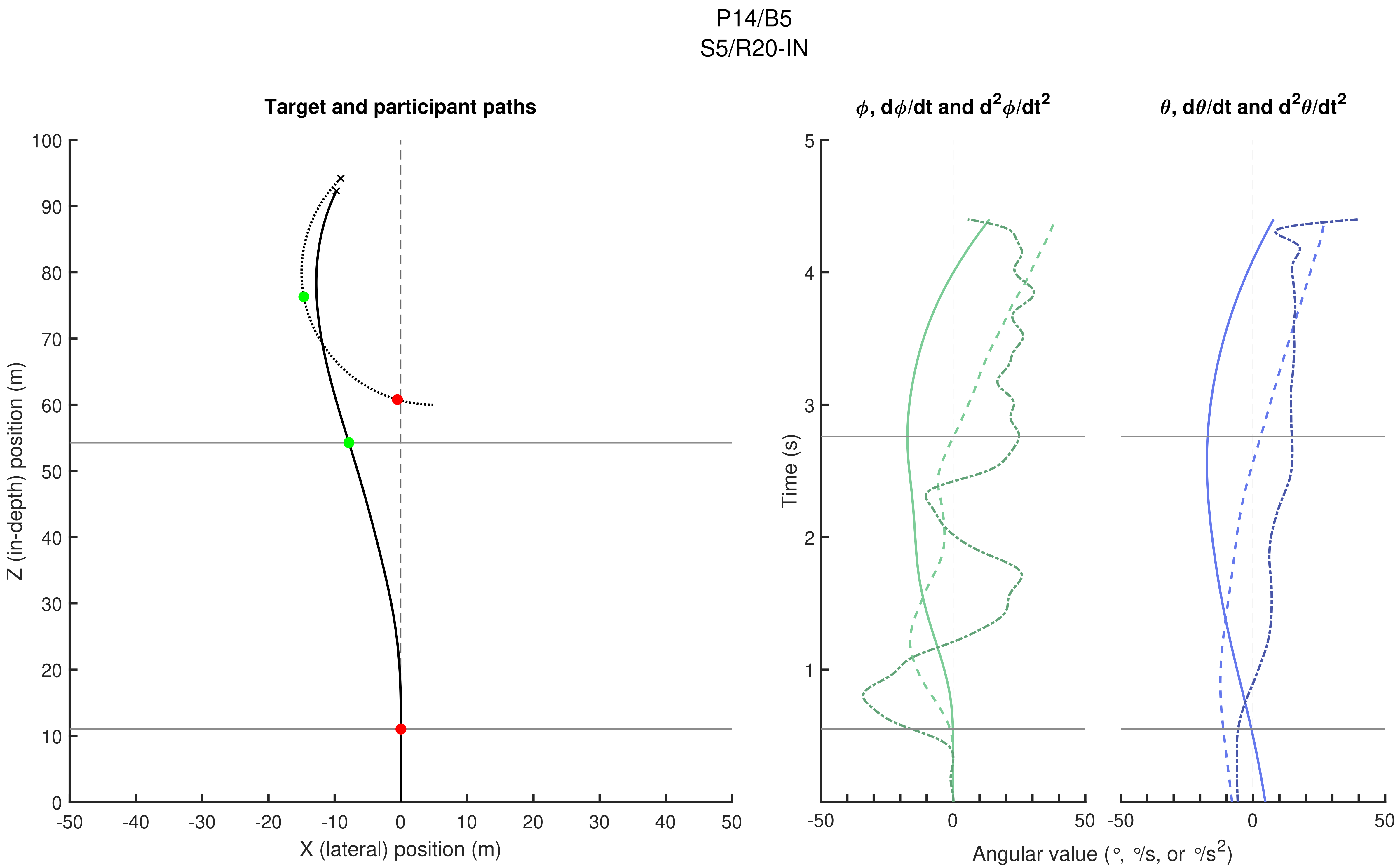

P14/B5
S5/R20-IN
Target and participant paths
Z (in-depth) position (m)
X (lateral) position (m)
100
90
80
70
60
50
40
30
20
10
0
-50
-40
-30
-20
-10
0
10
20
30
40
50
ϕ, dϕ/dt and d$^2\phi$/dt^2
θ, dθ/dt and d$^2\theta$/dt^2
Time (s)
5
4
3
2
1
-50
0
50
-50
0
50
Angular value (°, °/s, or °/s^2)

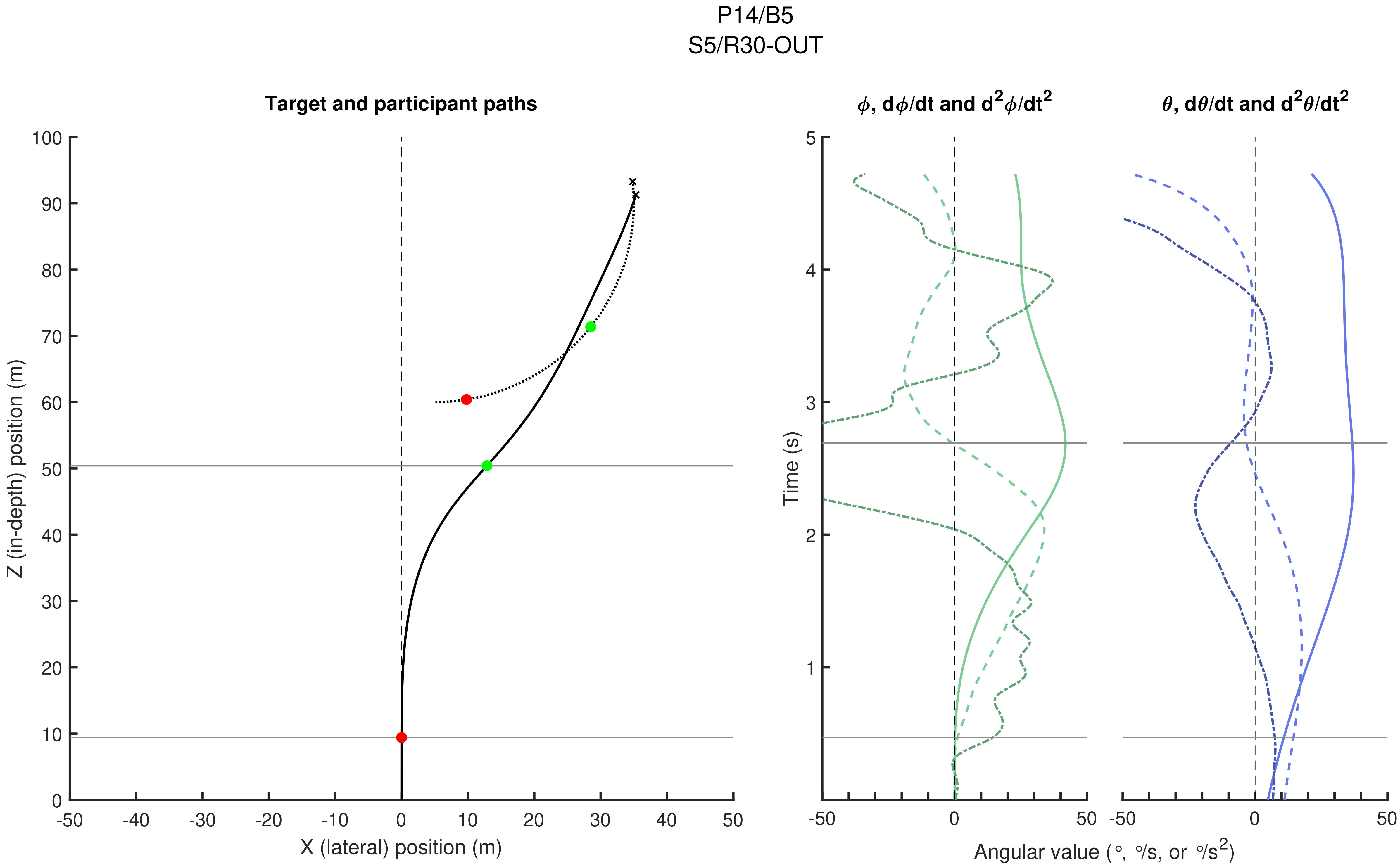

P14/B5
S5/R30-OUT
Target and participant paths
ϕ, dϕ/dt and d$^2\phi$/dt^2
θ, dθ/dt and d$^2\theta$/dt^2
Z (in-depth) position (m)
X (lateral) position (m)
Time (s)
Angular value (°, °/s, or °/s^2)

P14/B5
S5/R30-IN

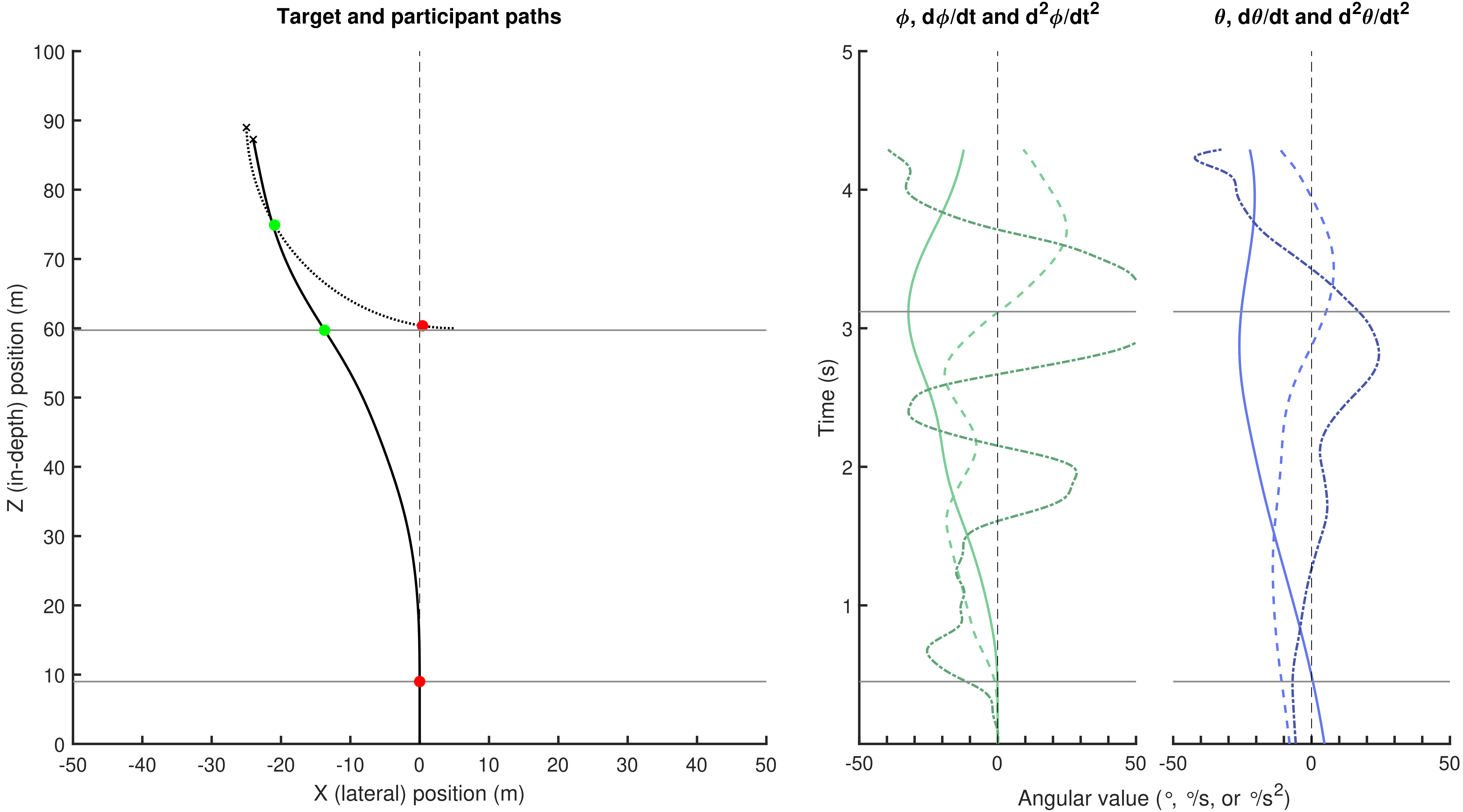

P14/B5
S15/R10-IN

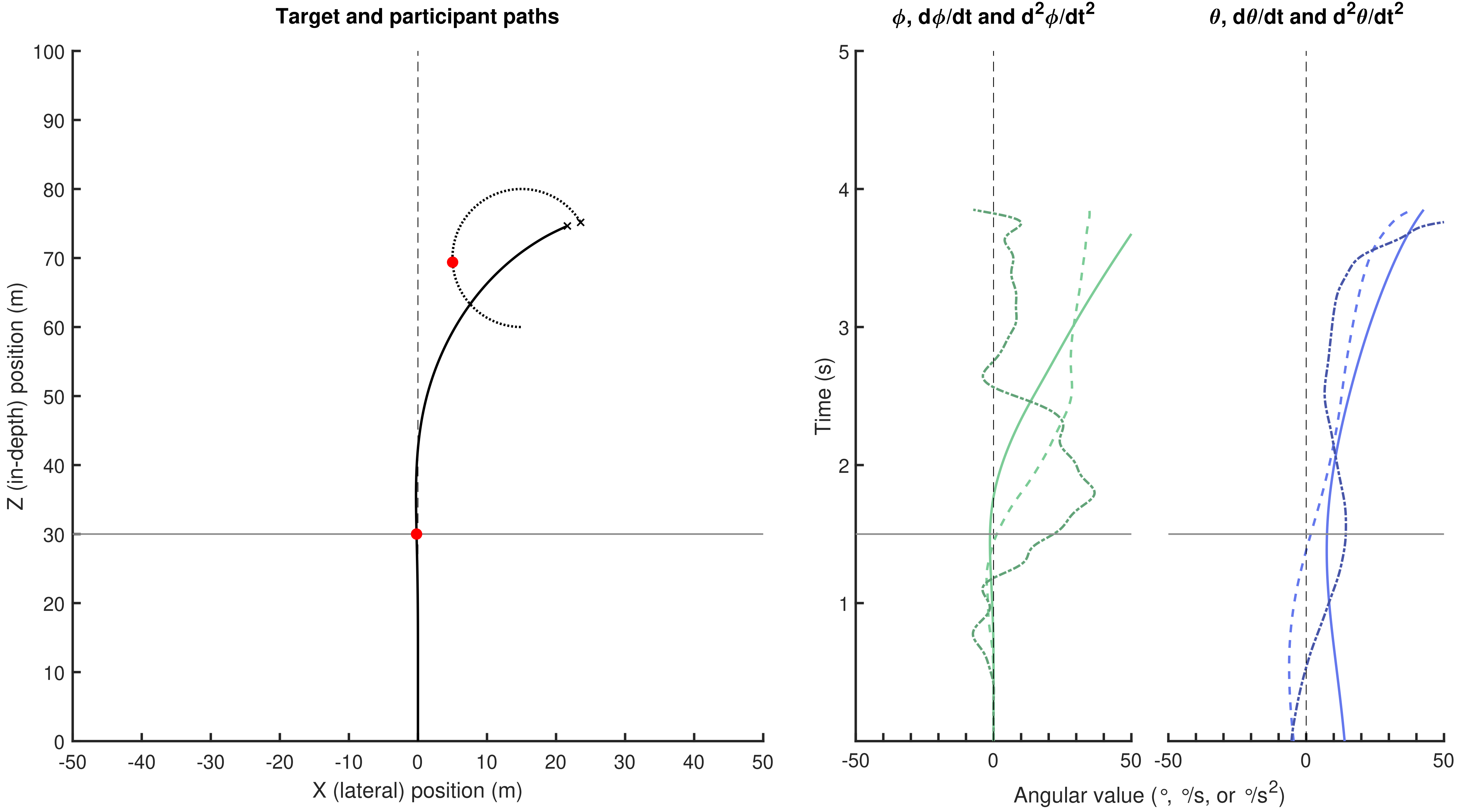

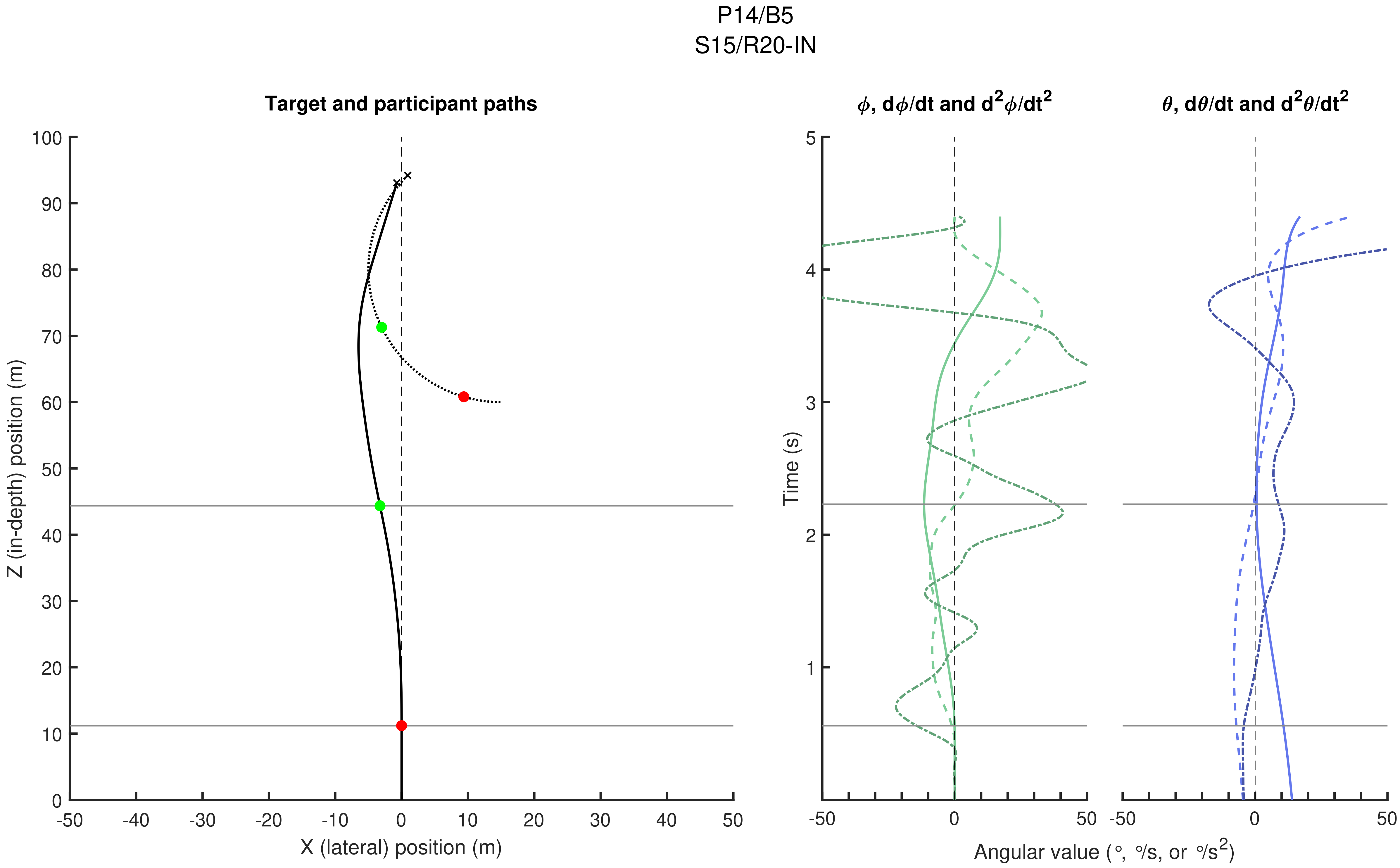
P14/B5
S15/R20-IN
Target and participant paths
Z (in-depth) position (m)
X (lateral) position (m)
φ, dφ/dt and d²φ/dt²
θ, dθ/dt and d²θ/dt²
Time (s)
Angular value (°, °/s, or °/s²)

P14/B5
S15/R30-IN

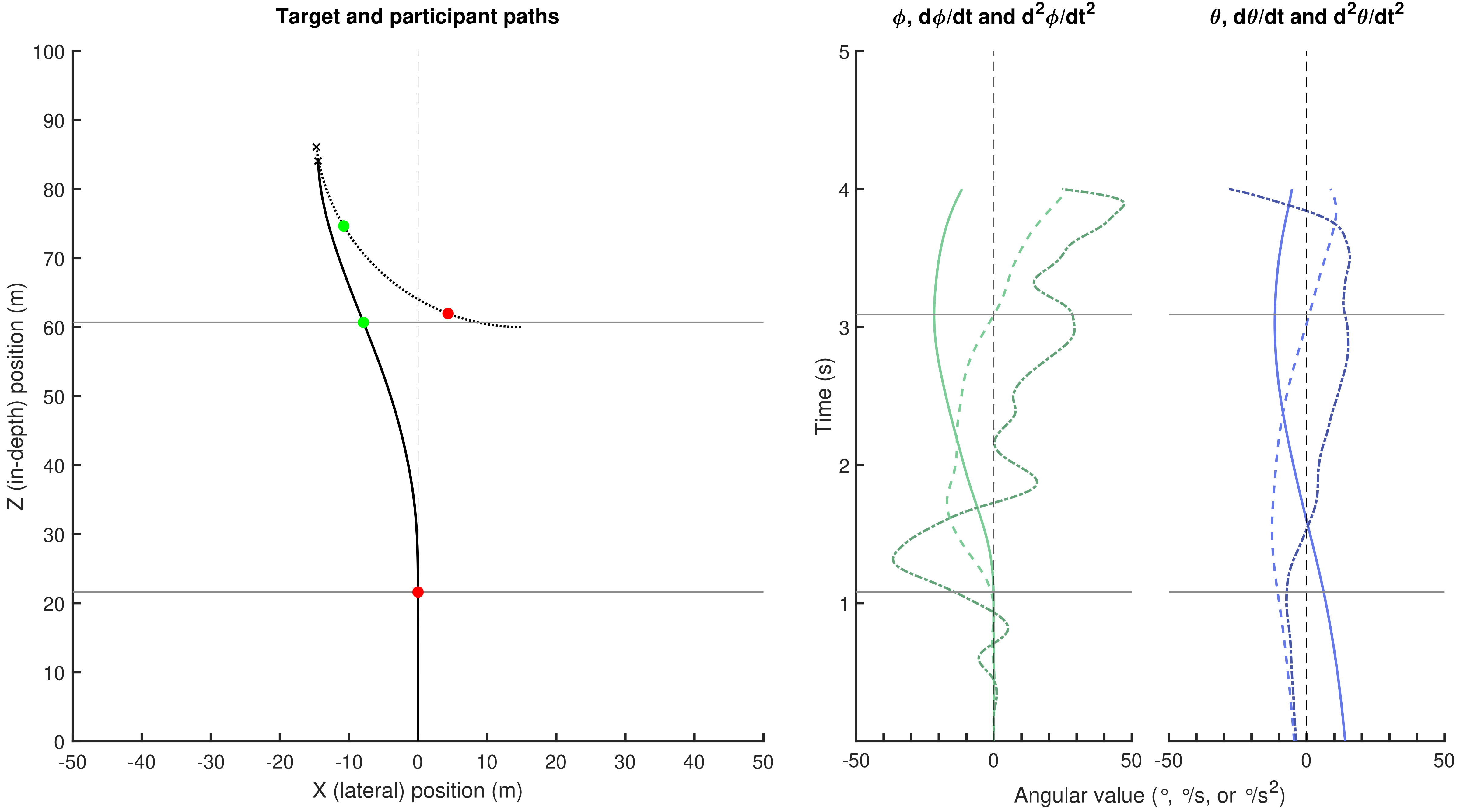

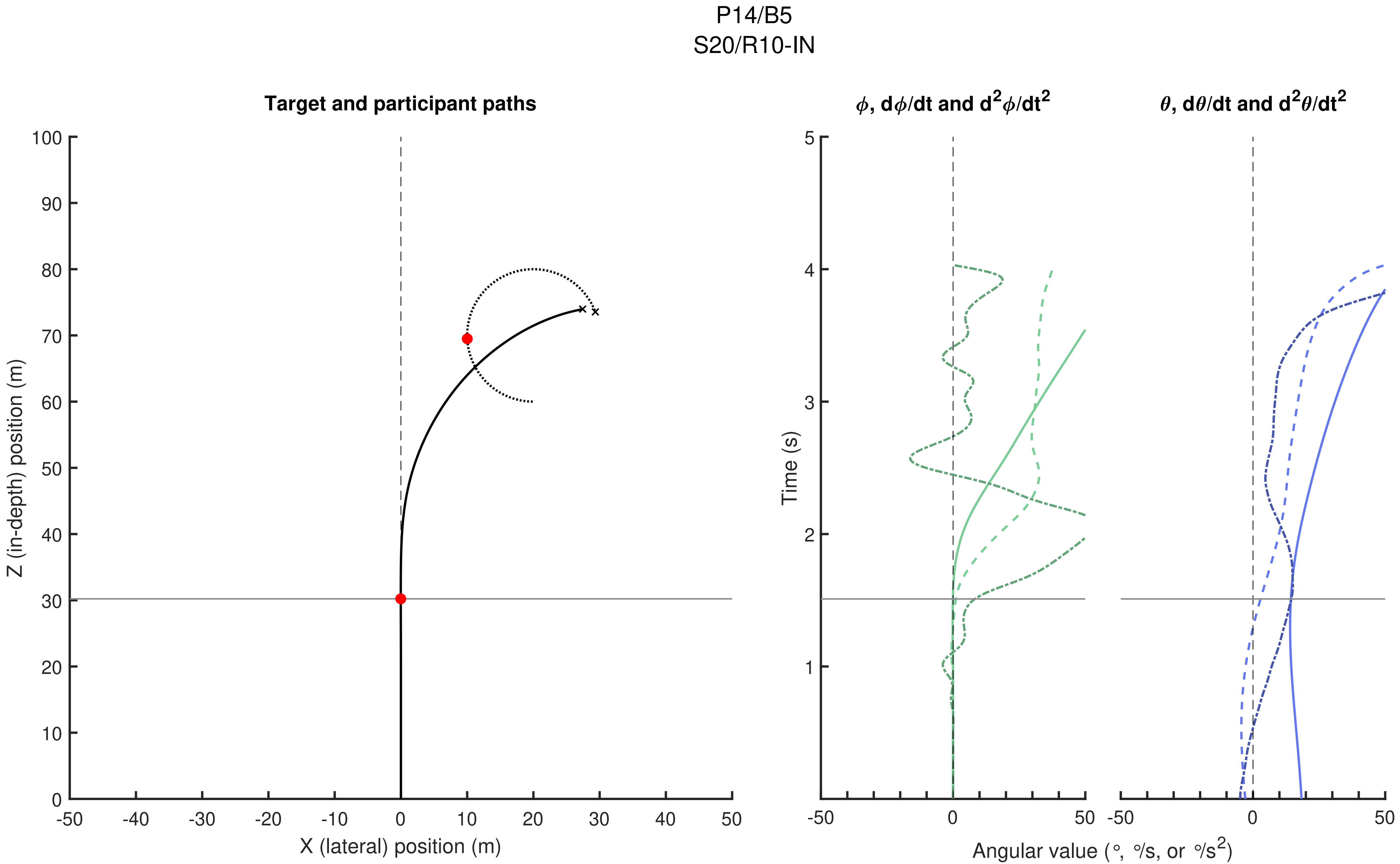
P14/B5
S20/R10-IN
Target and participant paths
Z (in-depth) position (m)
X (lateral) position (m)
ϕ, dϕ/dt and d$^2\phi$/dt^2
θ, dθ/dt and d$^2\theta$/dt^2
Time (s)
Angular value (°, °/s, or °/s^2)

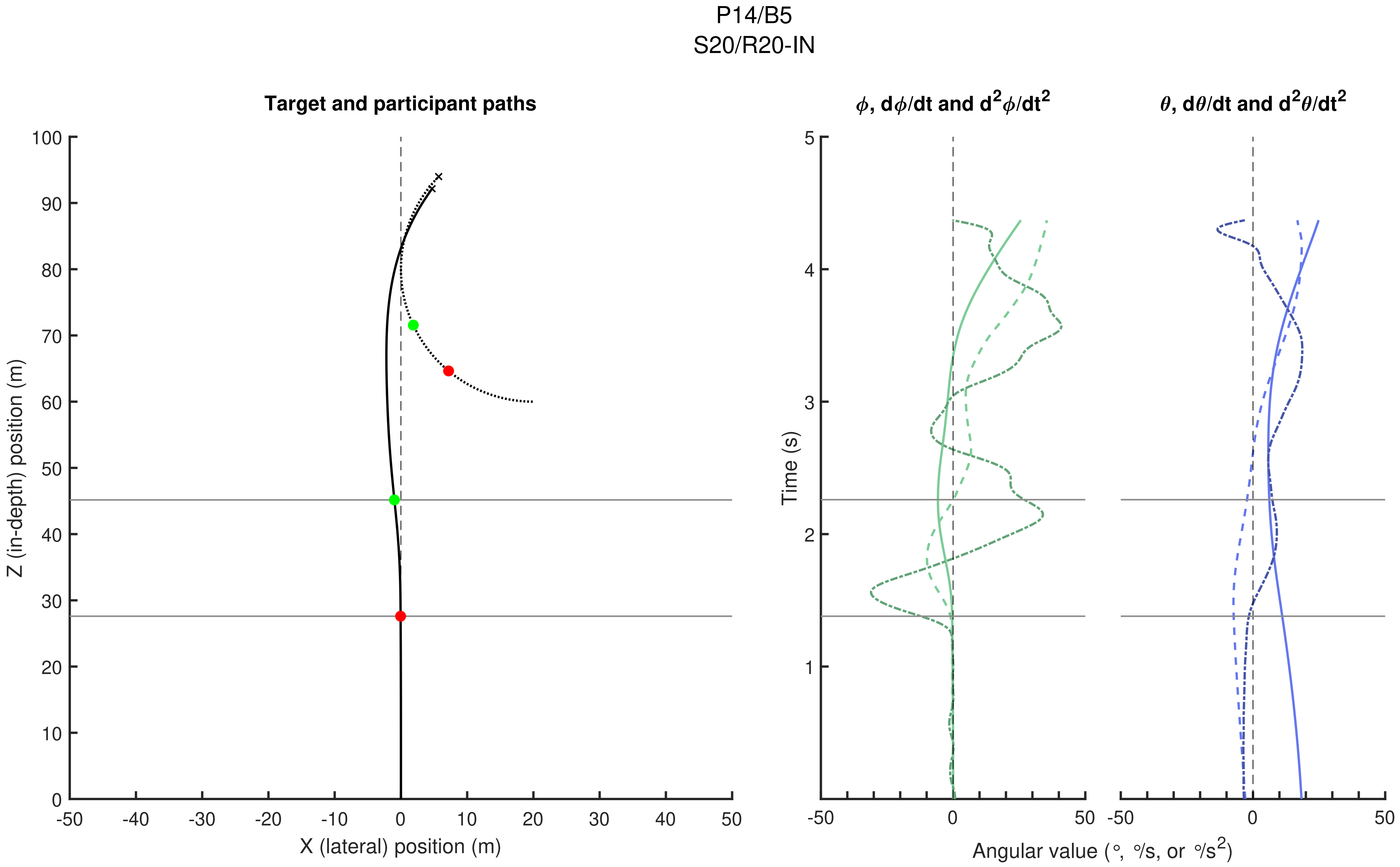

P14/B5
S20/R20-IN
Target and participant paths
Z (in-depth) position (m)
X (lateral) position (m)
100
90
80
70
60
50
40
30
20
10
0
-50
-40
-30
-20
-10
0
10
20
30
40
50
φ, dφ/dt and d²φ/dt²
θ, dθ/dt and d²θ/dt²
Time (s)
5
4
3
2
1
-50
0
50
-50
0
50
Angular value (°, °/s, or °/s²)

P14/B5
S20/R30-IN

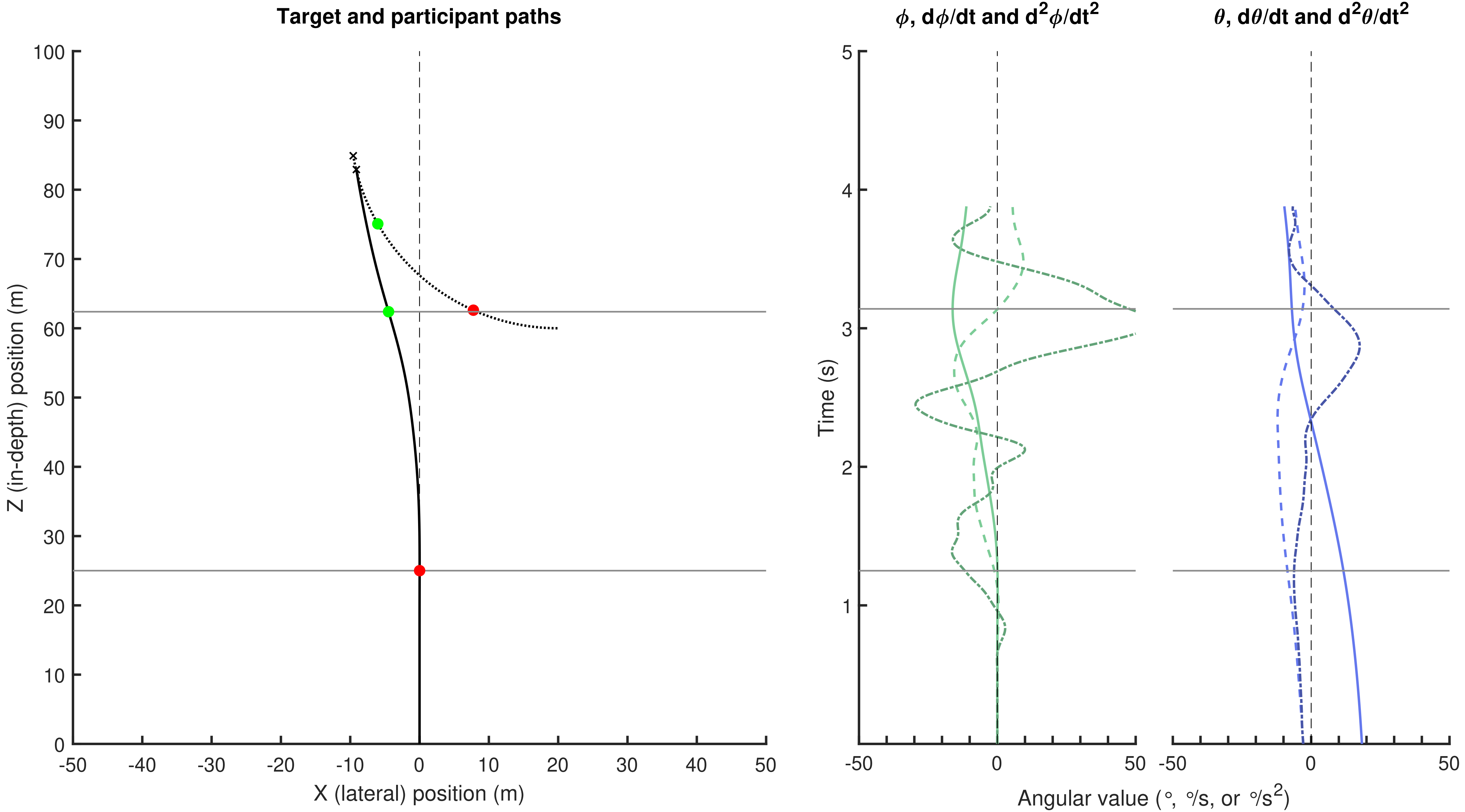

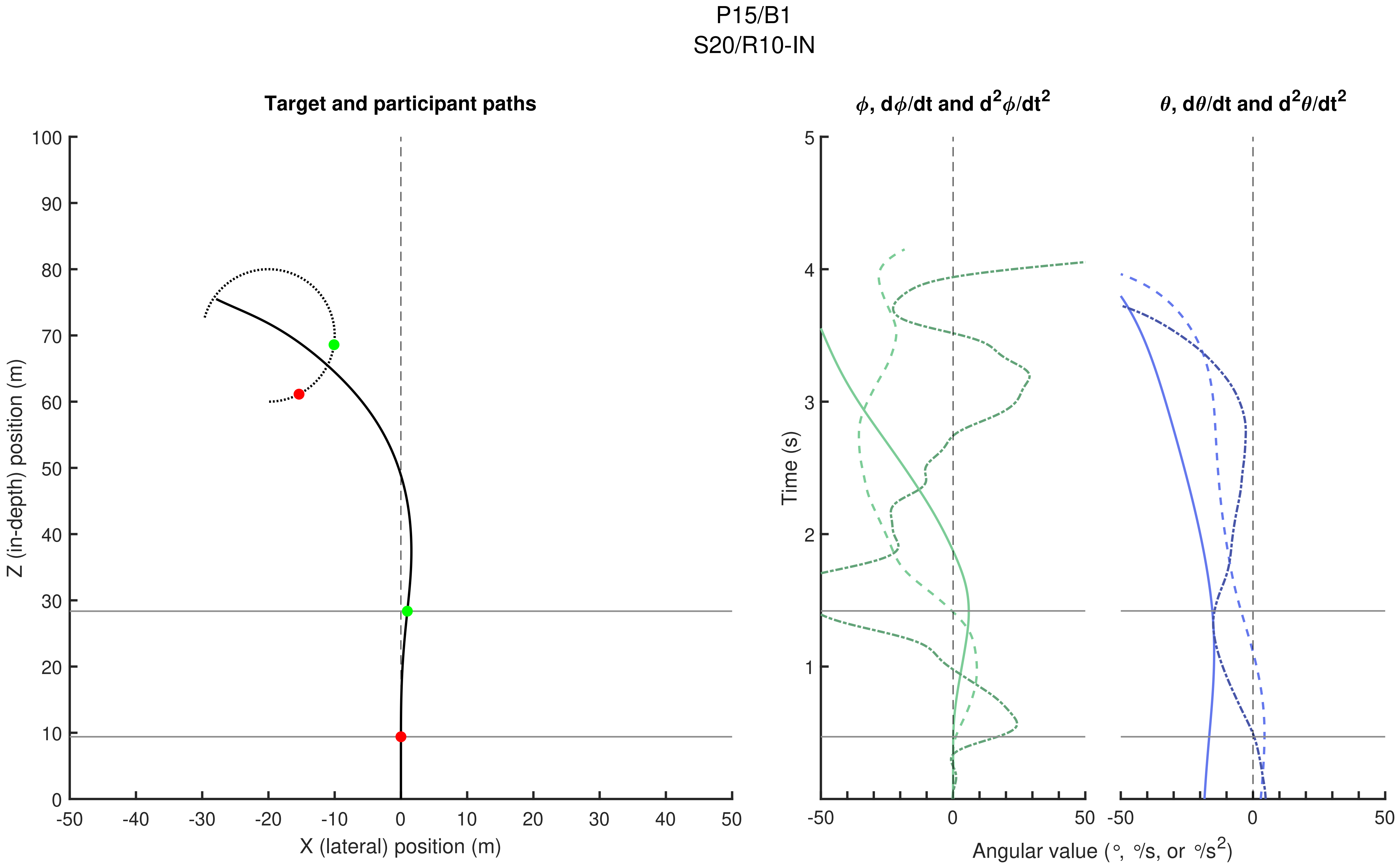

P15/B1
S20/R10-IN
Target and participant paths
ϕ, dϕ/dt and d²ϕ/dt²
θ, dθ/dt and d²θ/dt²
Z (in-depth) position (m)
X (lateral) position (m)
Time (s)
Angular value (°, °/s, or °/s²)
100
90
80
70
60
50
40
30
20
10
0
-50
-40
-30
-20
-10
0
10
20
30
40
50
5
4
3
2
1
-50
0
50
-50
0
50

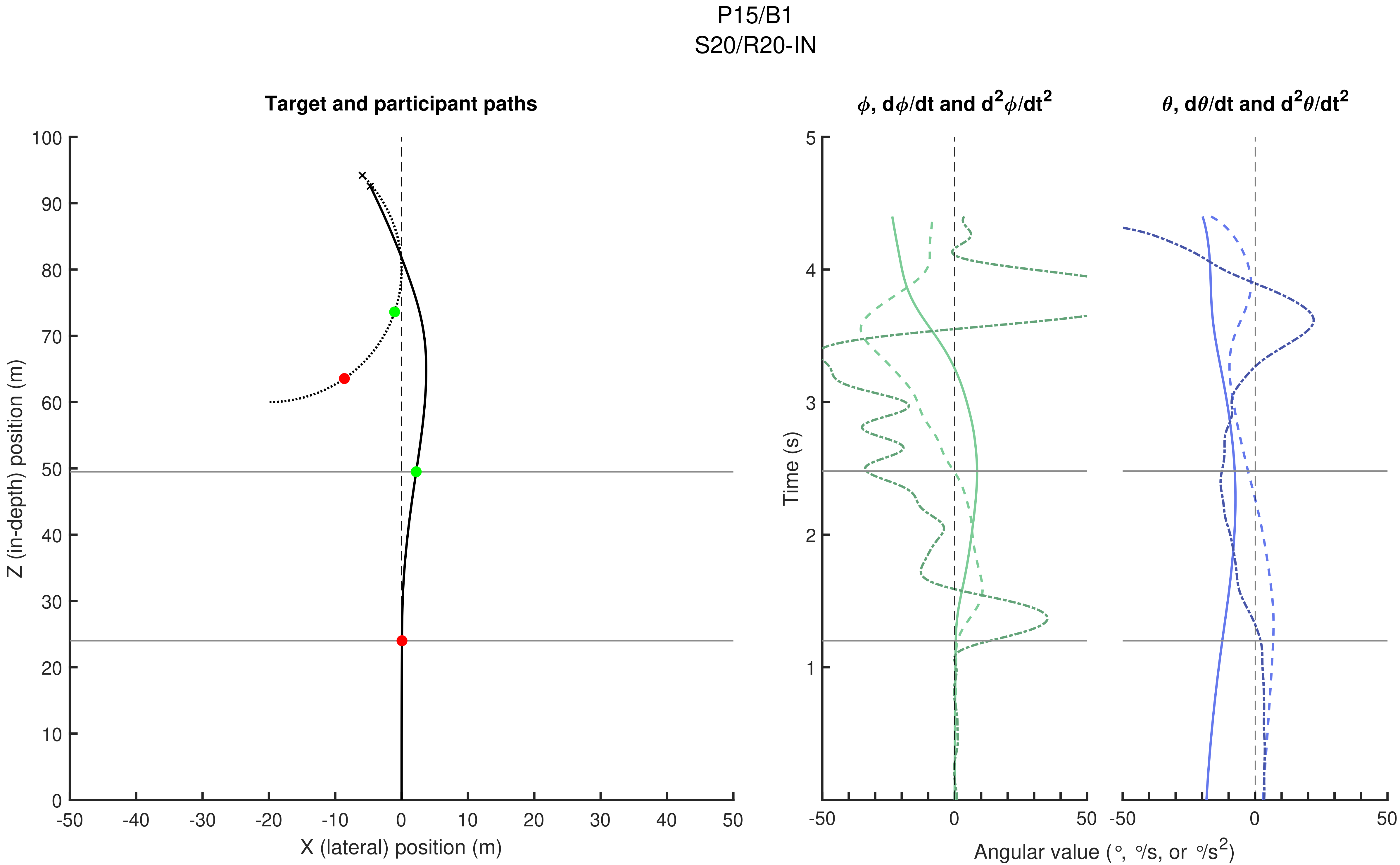
P15/B1
S20/R20-IN
Target and participant paths
Z (in-depth) position (m)
X (lateral) position (m)
100
90
80
70
60
50
40
30
20
10
0
-50
-40
-30
-20
-10
0
10
20
30
40
50
φ, dφ/dt and d²φ/dt²
θ, dθ/dt and d²θ/dt²
Time (s)
5
4
3
2
1
-50
0
50
-50
0
50
Angular value (°, °/s, or °/s²)

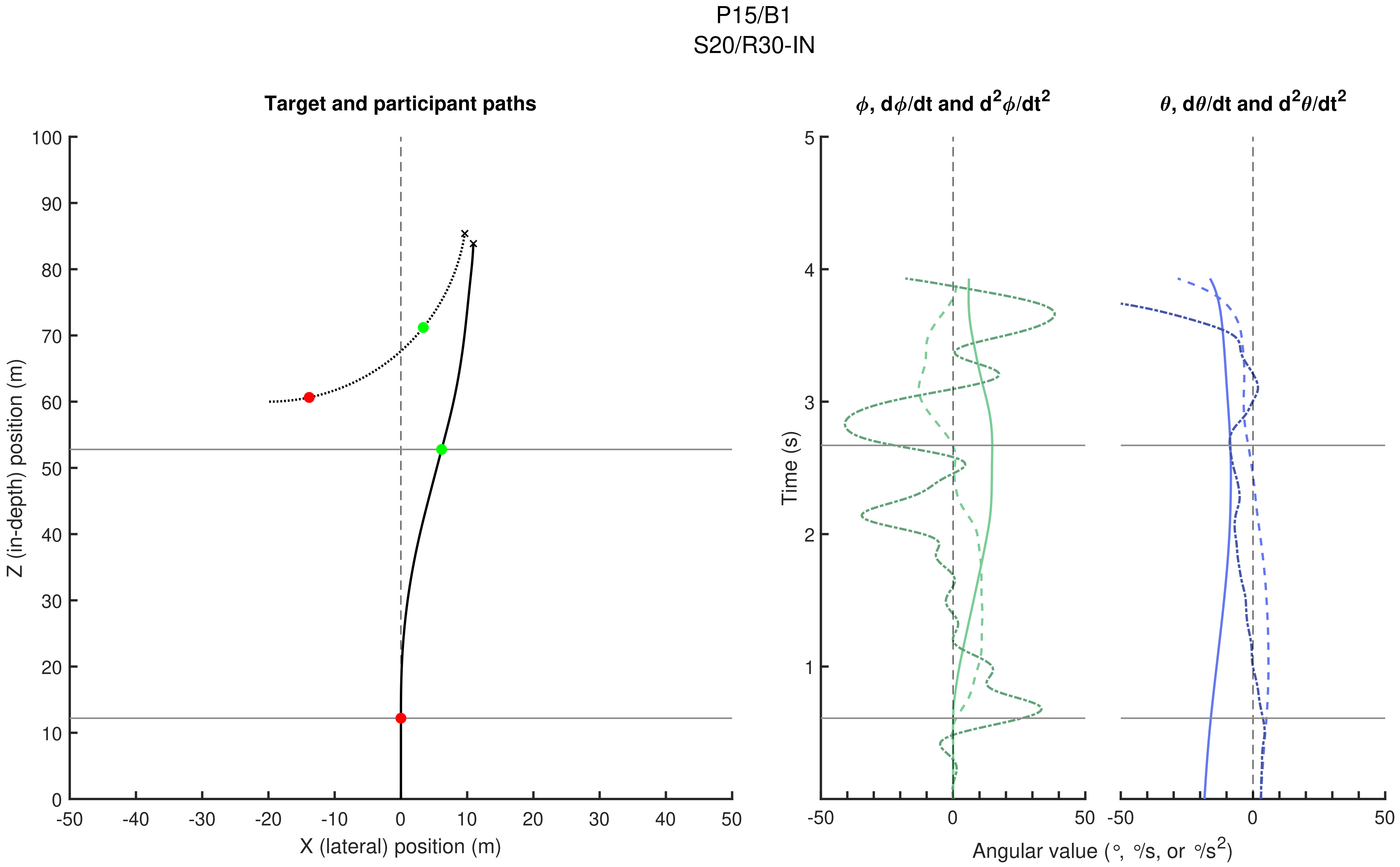
P15/B1
S20/R30-IN
Target and participant paths
Z (in-depth) position (m)
X (lateral) position (m)
ϕ, dϕ/dt and d$^2\phi$/dt^2
θ, dθ/dt and d$^2\theta$/dt^2
Time (s)
Angular value (°, °/s, or °/s^2)

P15/B1
S15/R10-IN

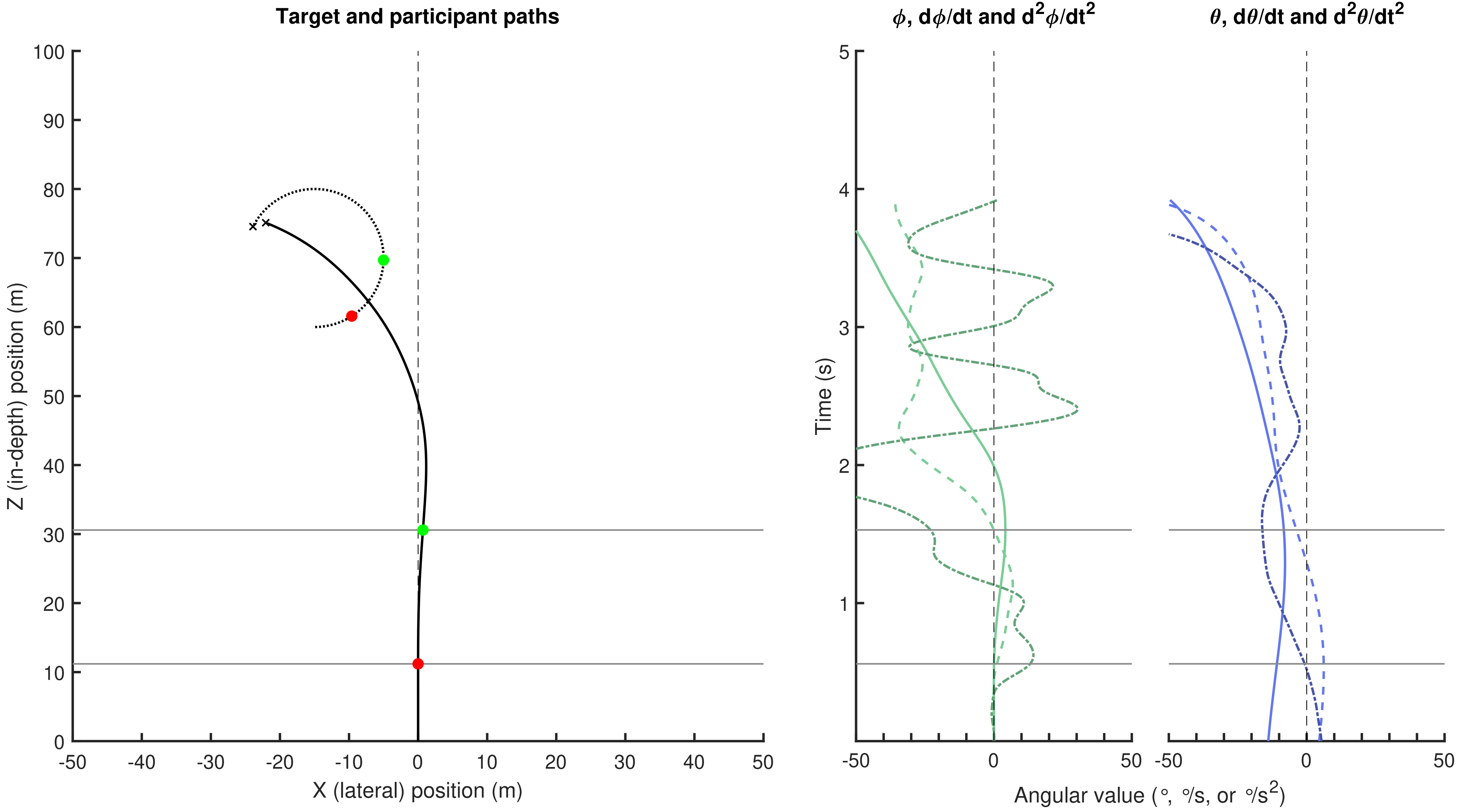

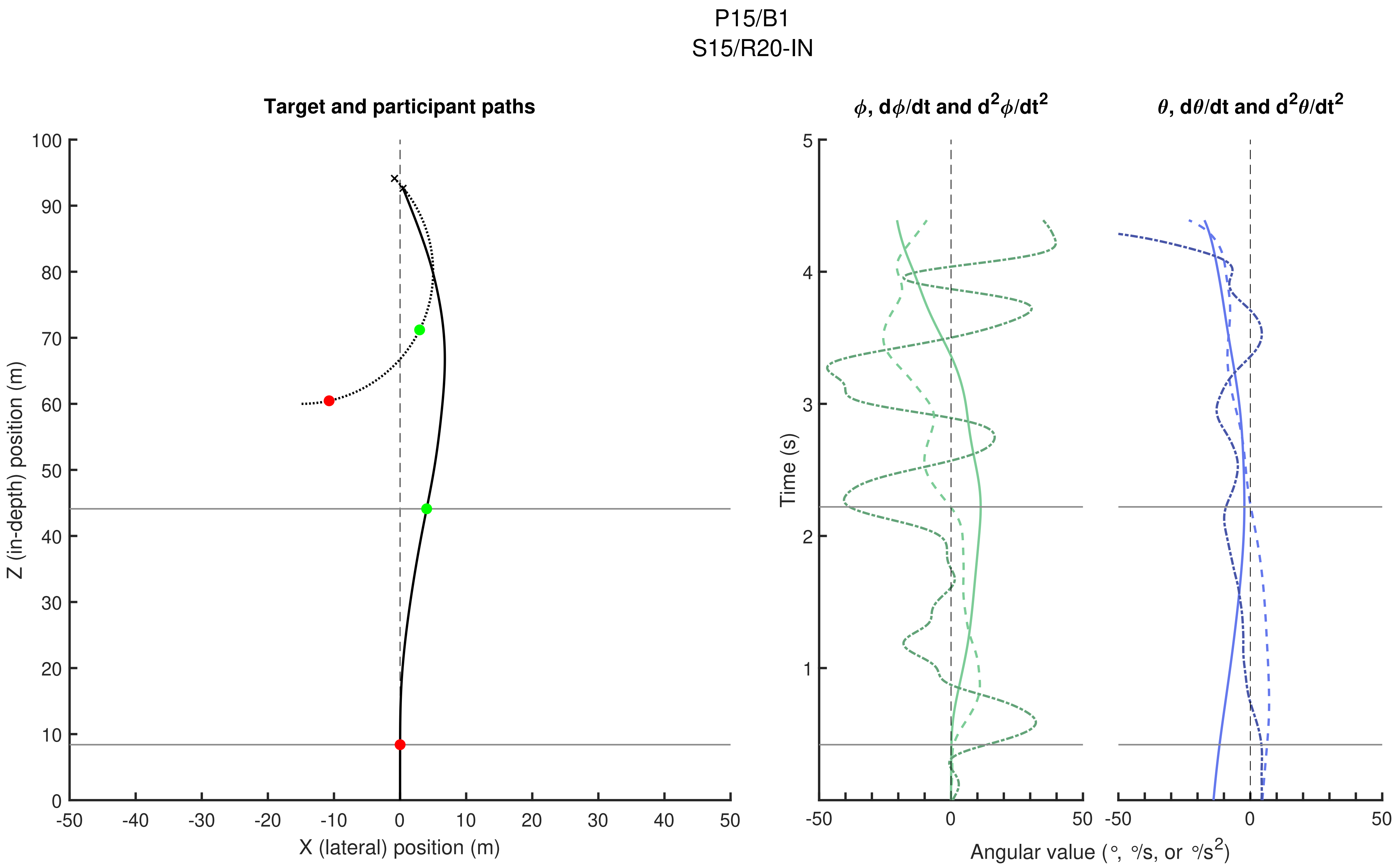

P15/B1
S15/R20-IN
Target and participant paths
Z (in-depth) position (m)
X (lateral) position (m)
ϕ, dϕ/dt and d$^2\phi$/dt^2
θ, dθ/dt and d$^2\theta$/dt^2
Time (s)
Angular value (°, °/s, or °/s^2)

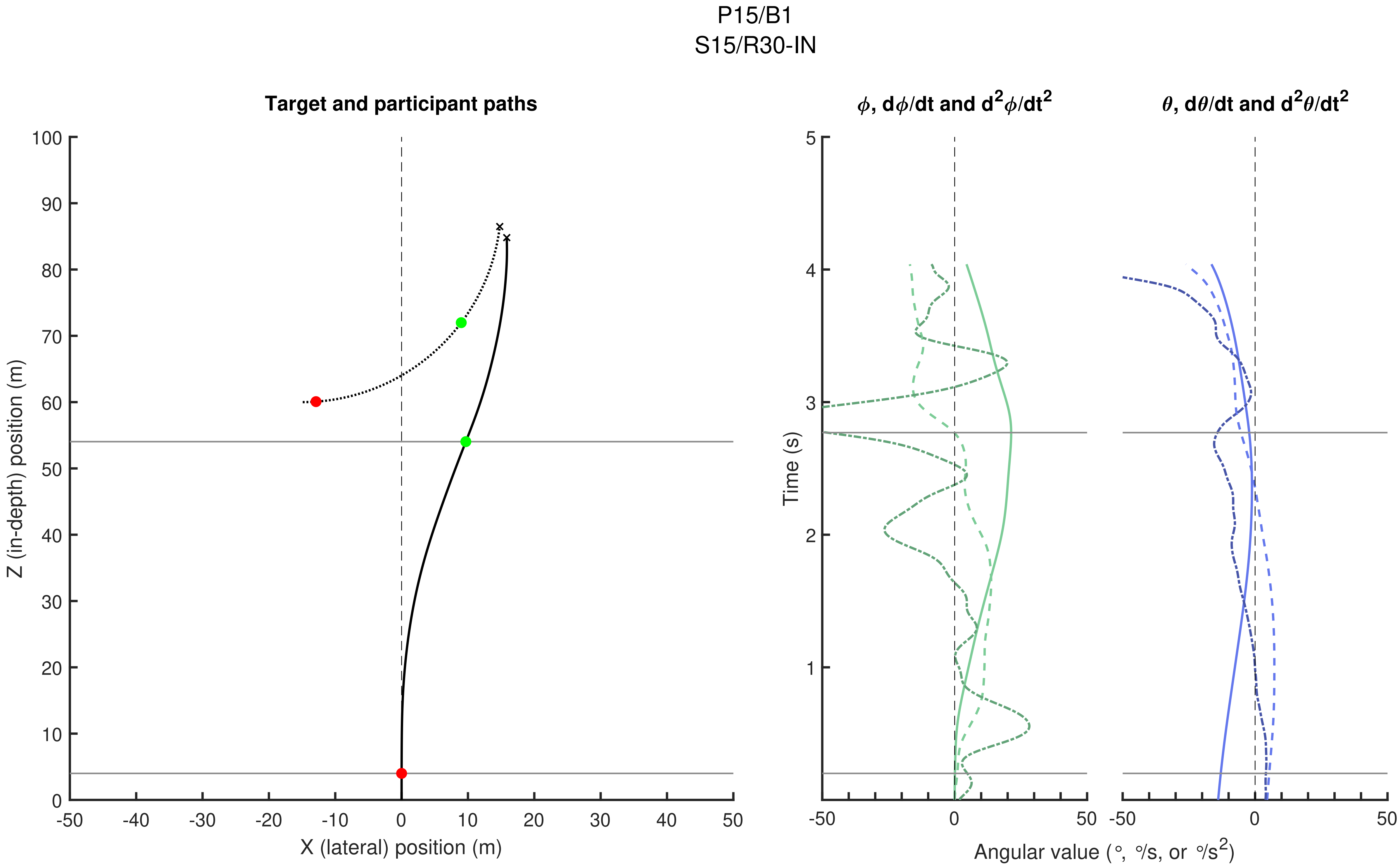

P15/B1
S15/R30-IN
Target and participant paths
Z (in-depth) position (m)
X (lateral) position (m)
100
90
80
70
60
50
40
30
20
10
0
-50
-40
-30
-20
-10
0
10
20
30
40
50
φ, dφ/dt and d²φ/dt²
θ, dθ/dt and d²θ/dt²
Time (s)
5
4
3
2
1
-50
0
50
-50
0
50
Angular value (°, °/s, or °/s²)

P15/B1
S5/R10-IN

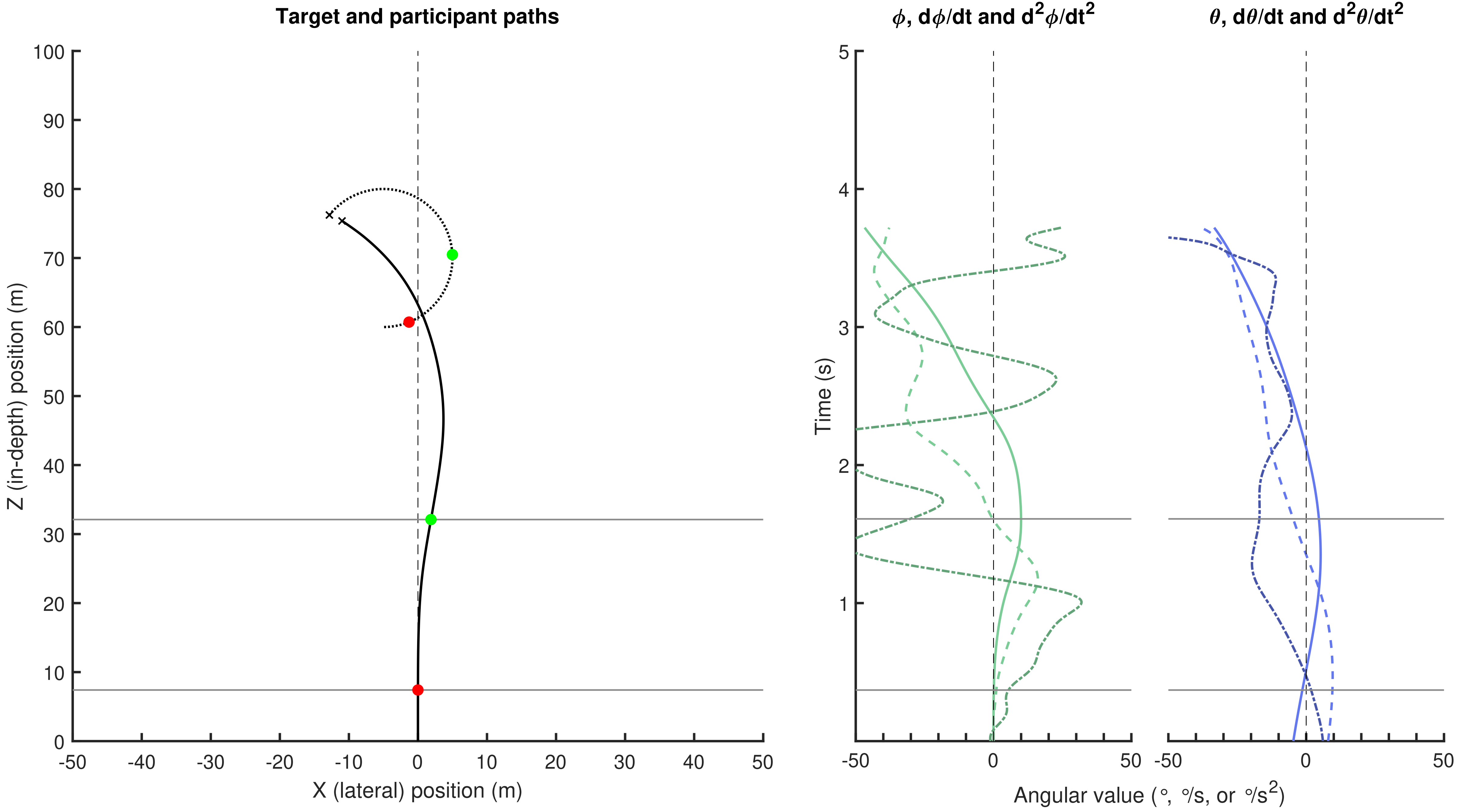

P15/B1
S5/R10-OUT

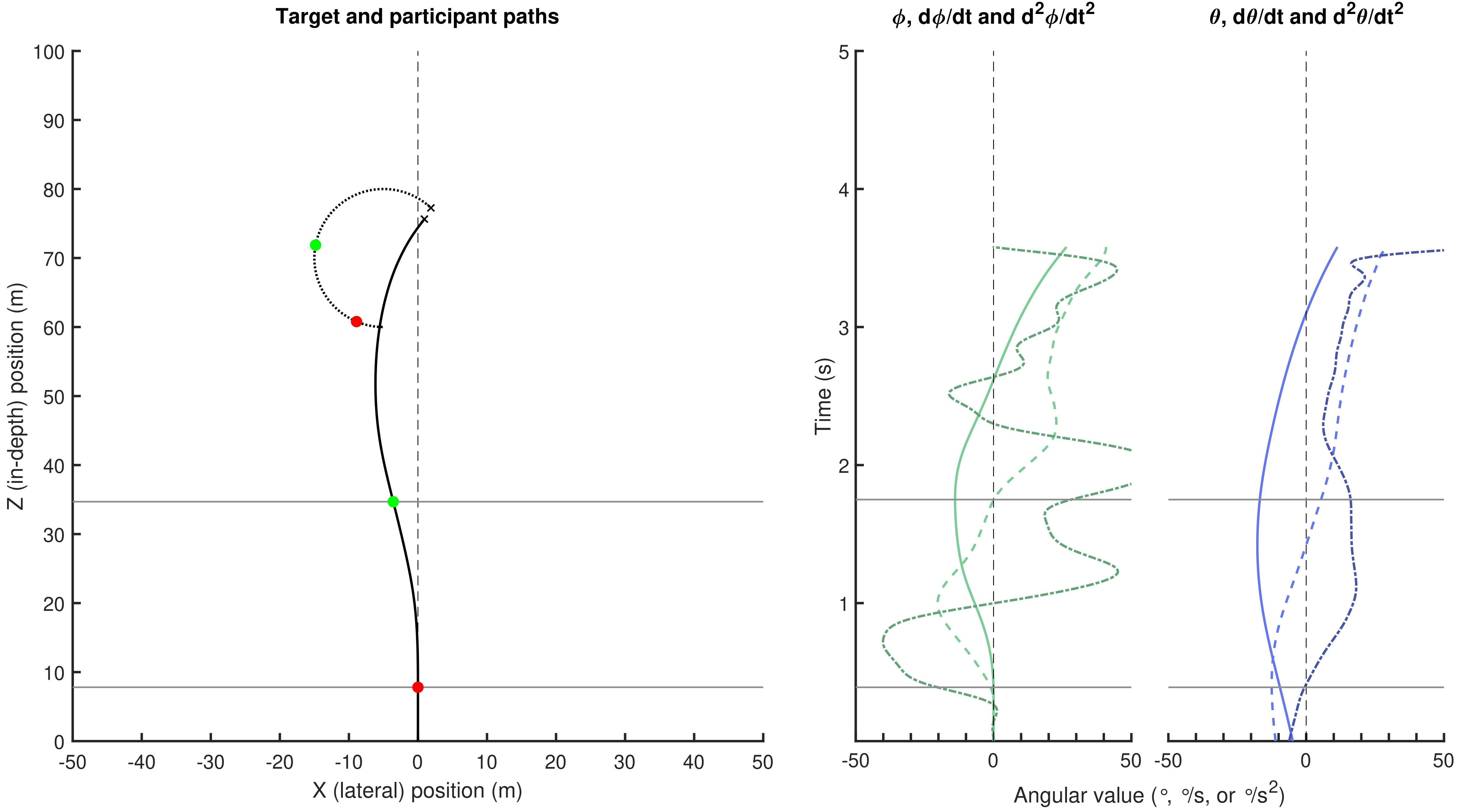

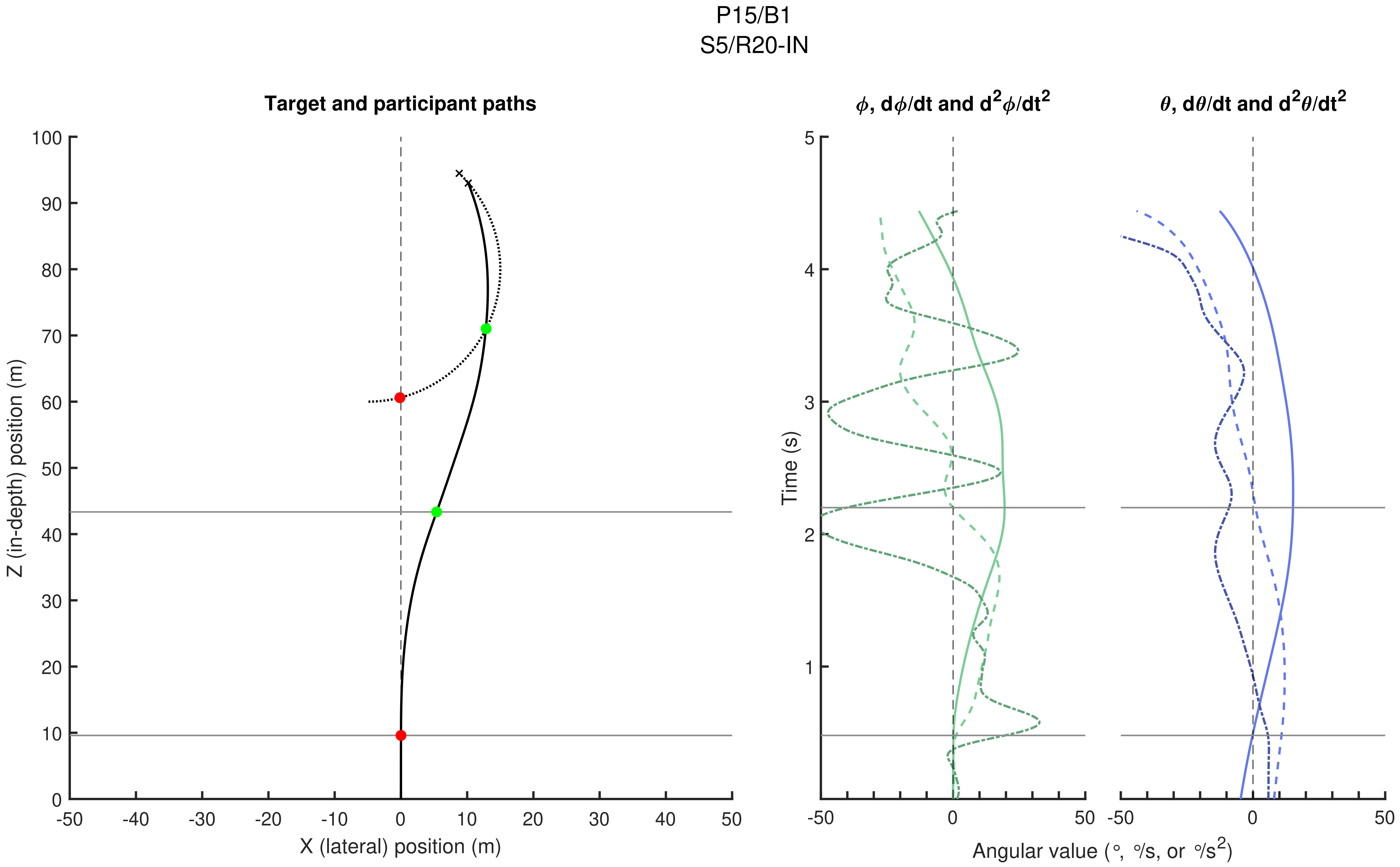

P15/B1
S5/R20-IN
Target and participant paths
Z (in-depth) position (m)
X (lateral) position (m)
100
90
80
70
60
50
40
30
20
10
0
-50
-40
-30
-20
-10
0
10
20
30
40
50
ϕ, dϕ/dt and d²ϕ/dt²
θ, dθ/dt and d²θ/dt²
Time (s)
5
4
3
2
1
-50
0
50
-50
0
50
Angular value (°, °/s, or °/s²)

P15/B1
S5/R20-OUT

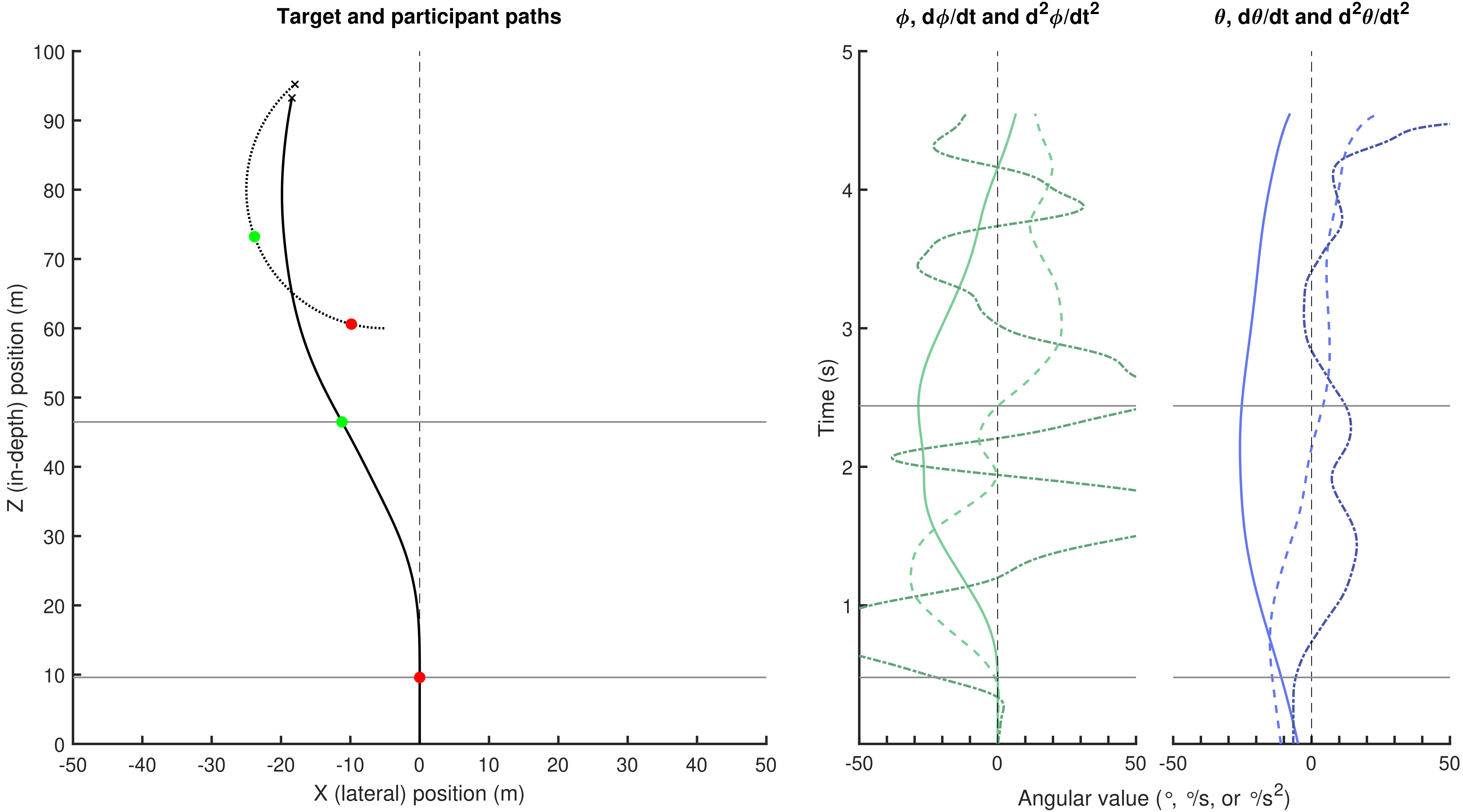

P15/B1
S5/R30-IN

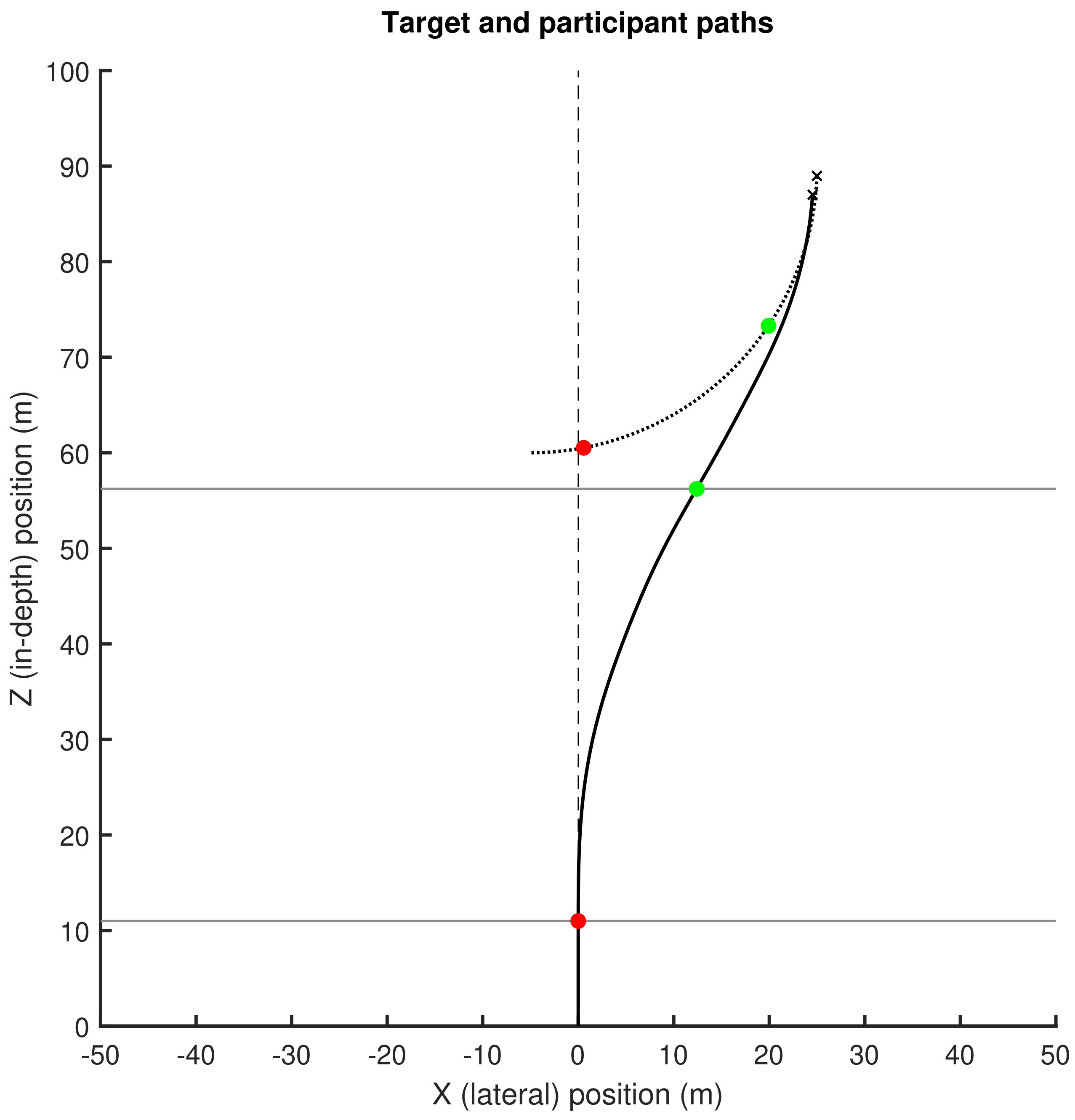

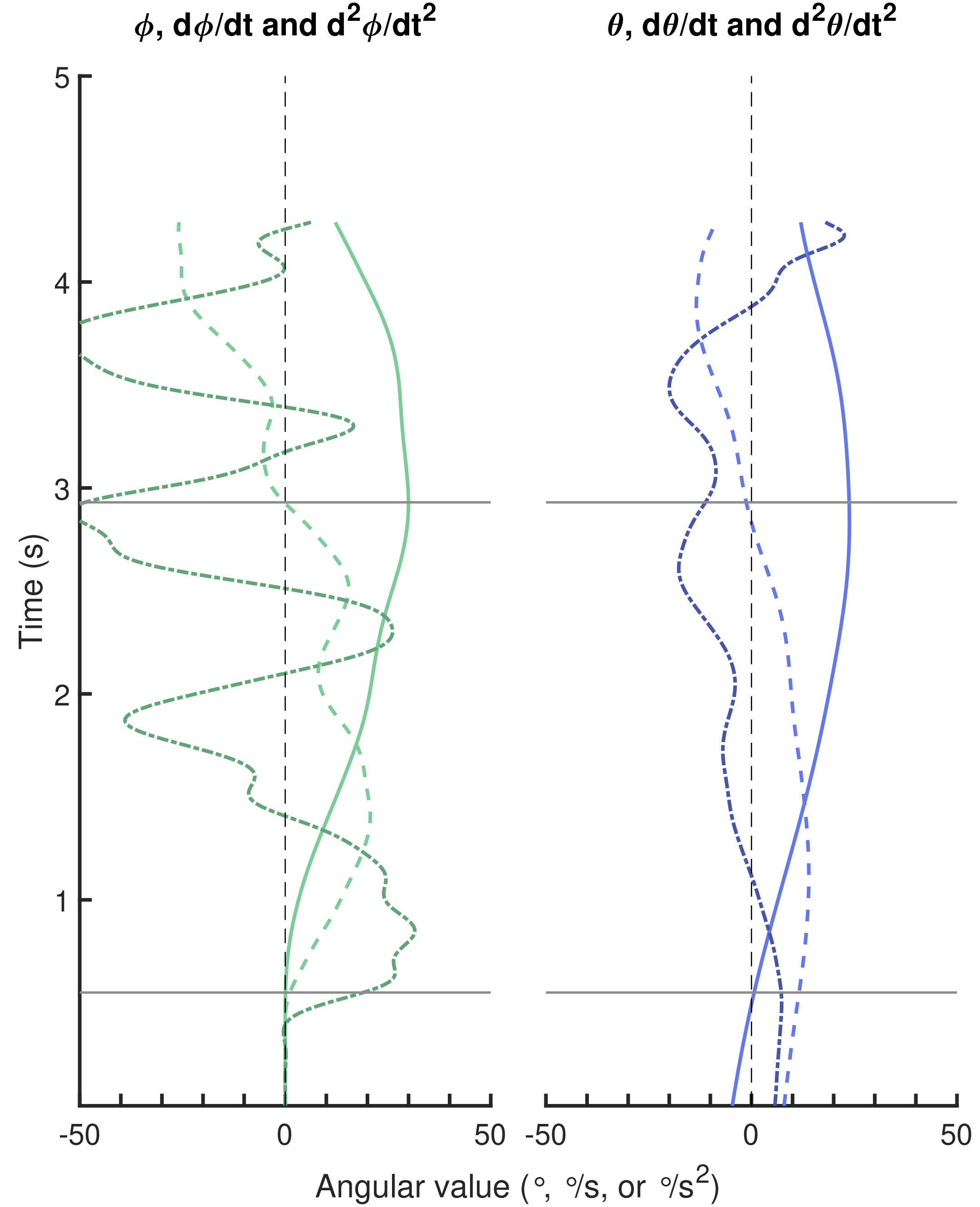

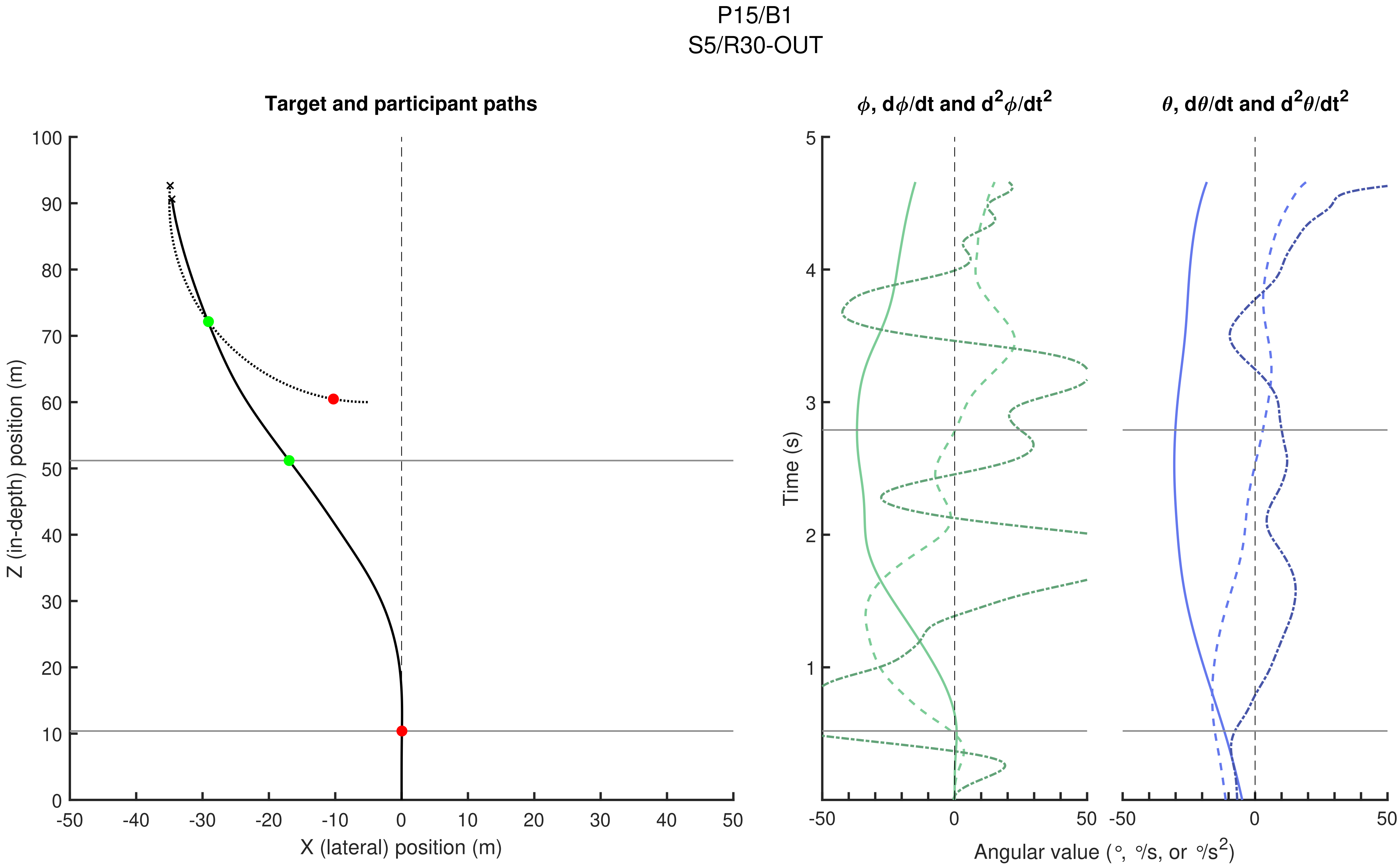

P15/B1
S5/R30-OUT
Target and participant paths
ϕ, dϕ/dt and d$^2\phi$/dt^2
θ, dθ/dt and d$^2\theta$/dt^2
Z (in-depth) position (m)
X (lateral) position (m)
Time (s)
Angular value (°, °/s, or °/s^2)

P15/B1
S5/R10-OUT

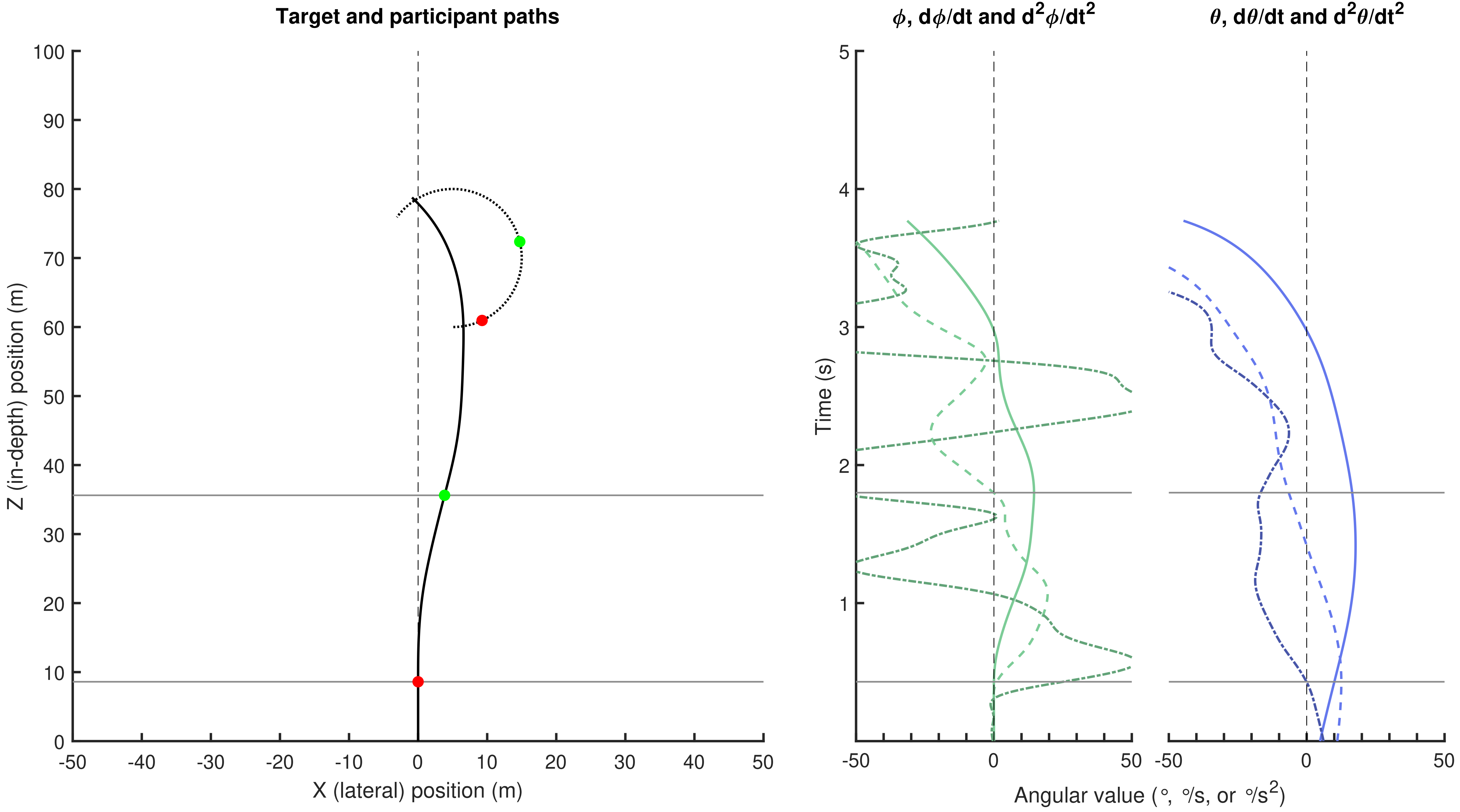

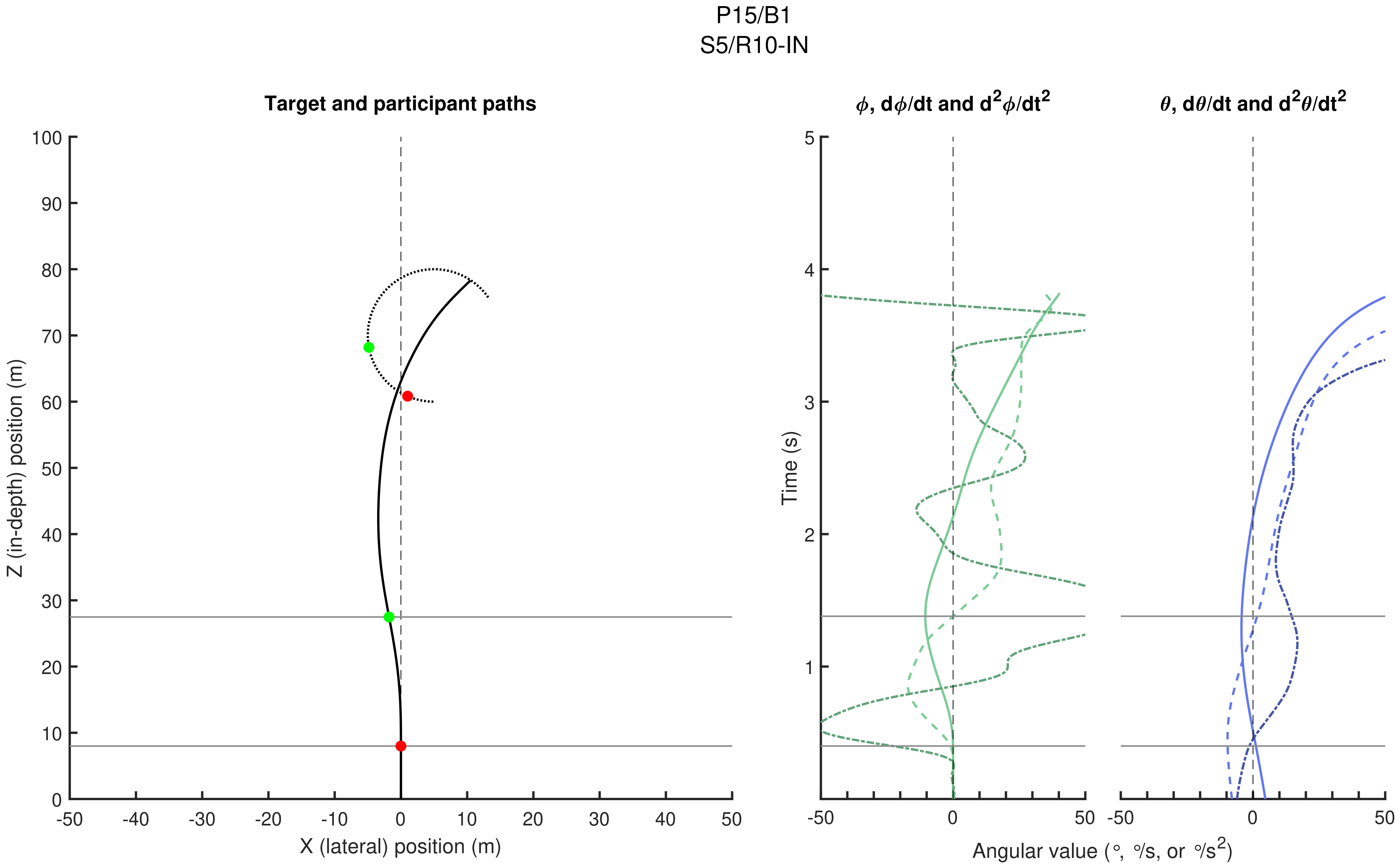

P15/B1
S5/R10-IN
Target and participant paths
Z (in-depth) position (m)
X (lateral) position (m)
φ, dφ/dt and d²φ/dt²
θ, dθ/dt and d²θ/dt²
Time (s)
Angular value (°, °/s, or °/s²)

P15/B1
S5/R20-OUT

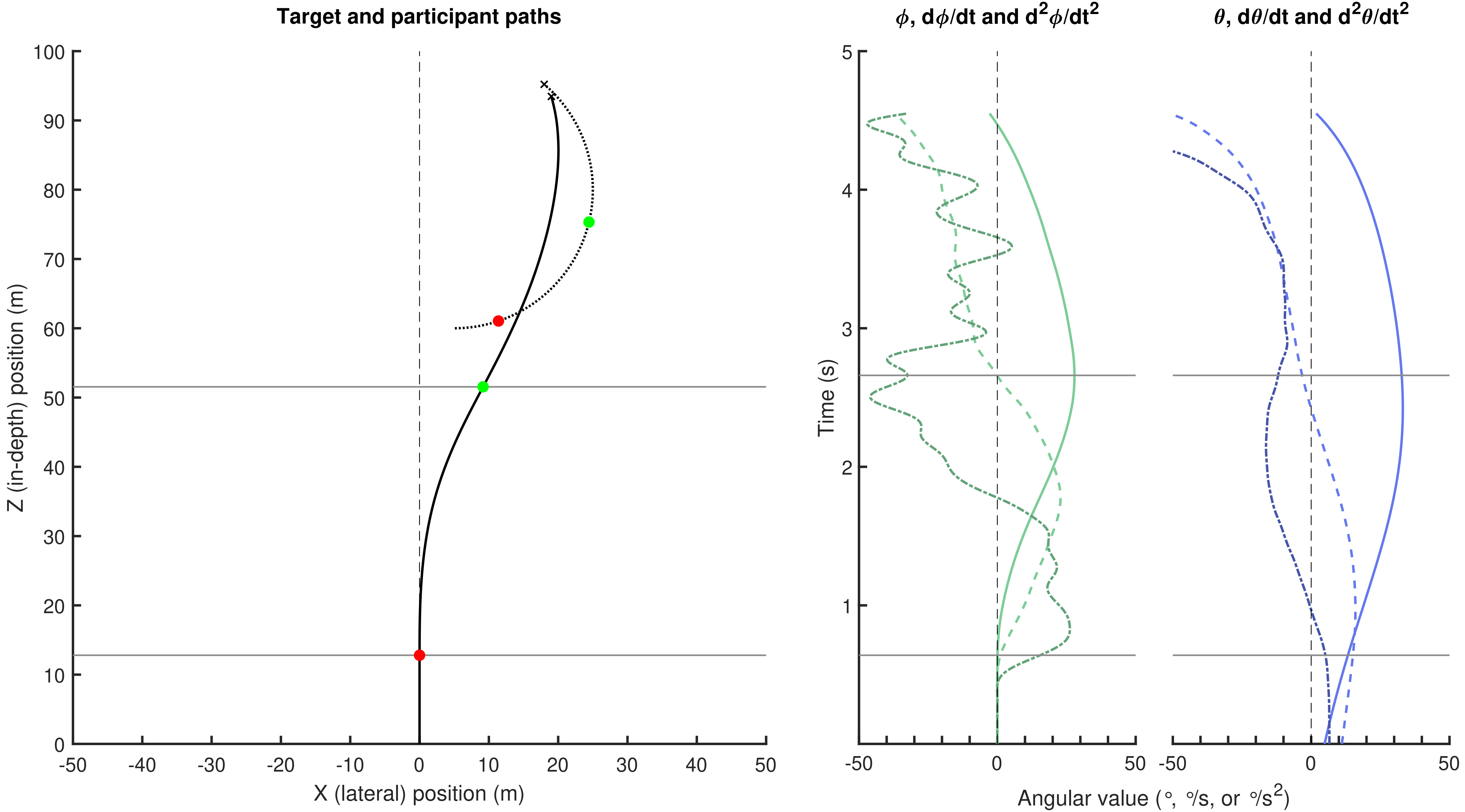

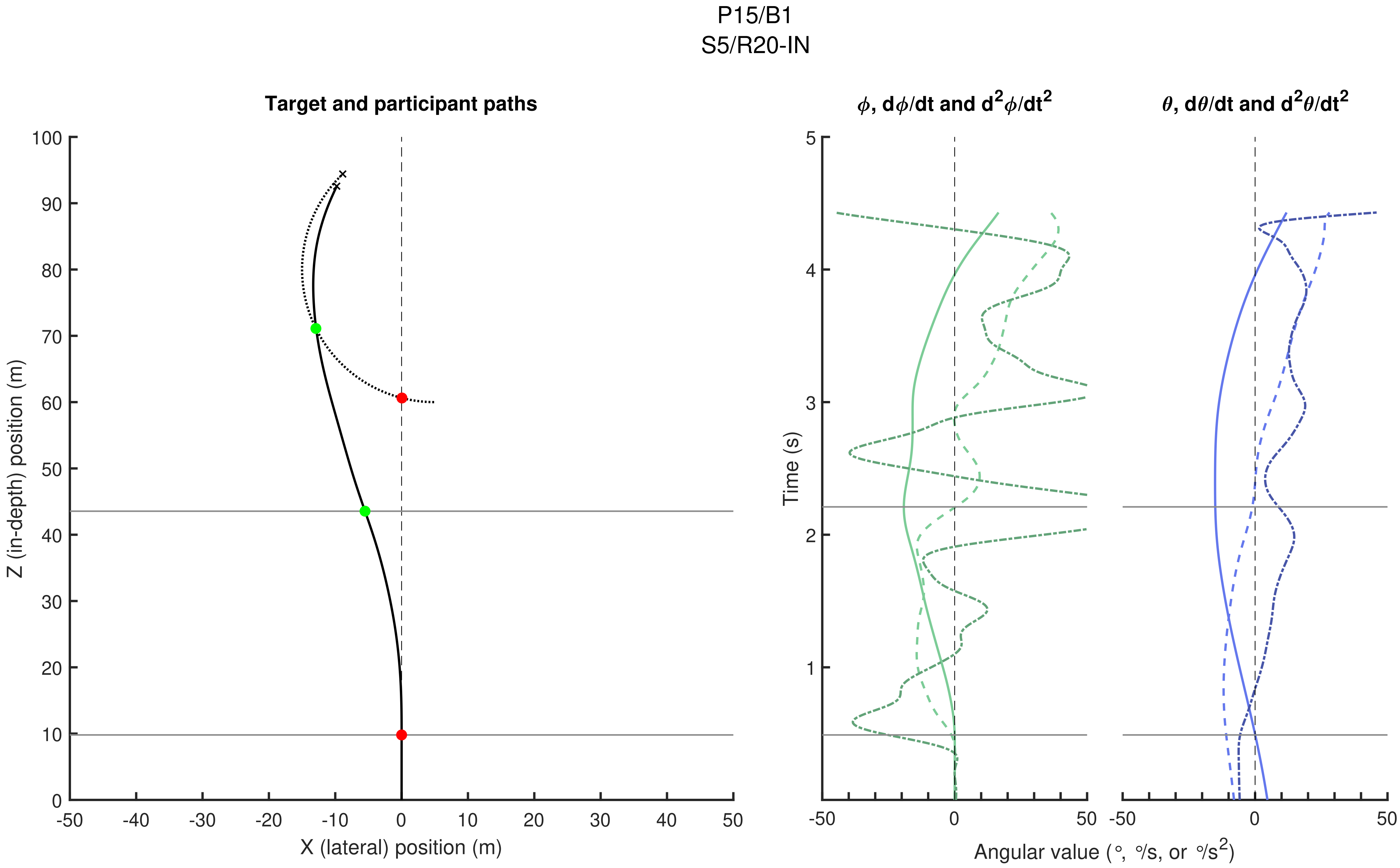

P15/B1
S5/R20-IN
Target and participant paths
Z (in-depth) position (m)
X (lateral) position (m)
φ, dφ/dt and d²φ/dt²
θ, dθ/dt and d²θ/dt²
Time (s)
Angular value (°, °/s, or °/s²)

P15/B1
S5/R30-OUT

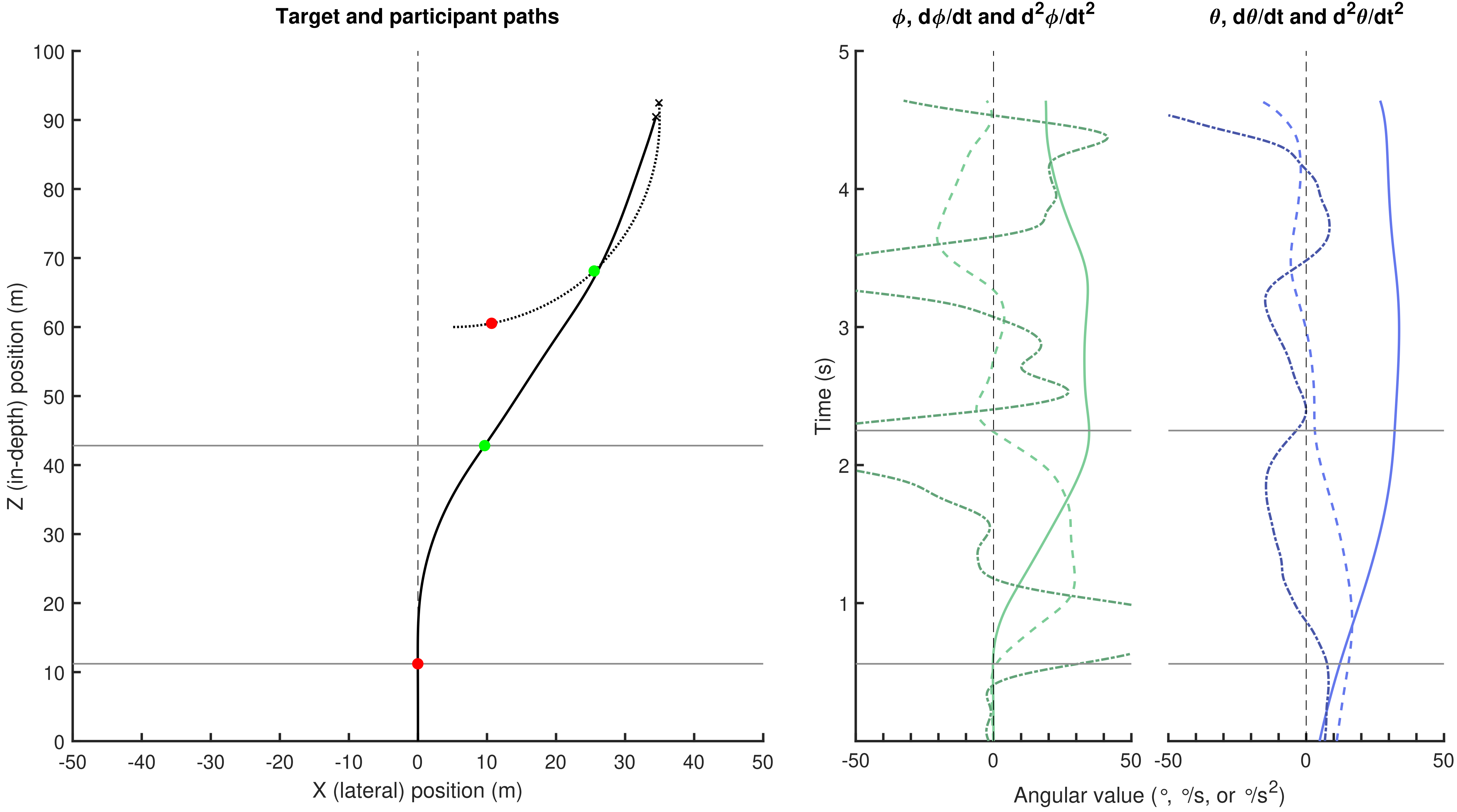

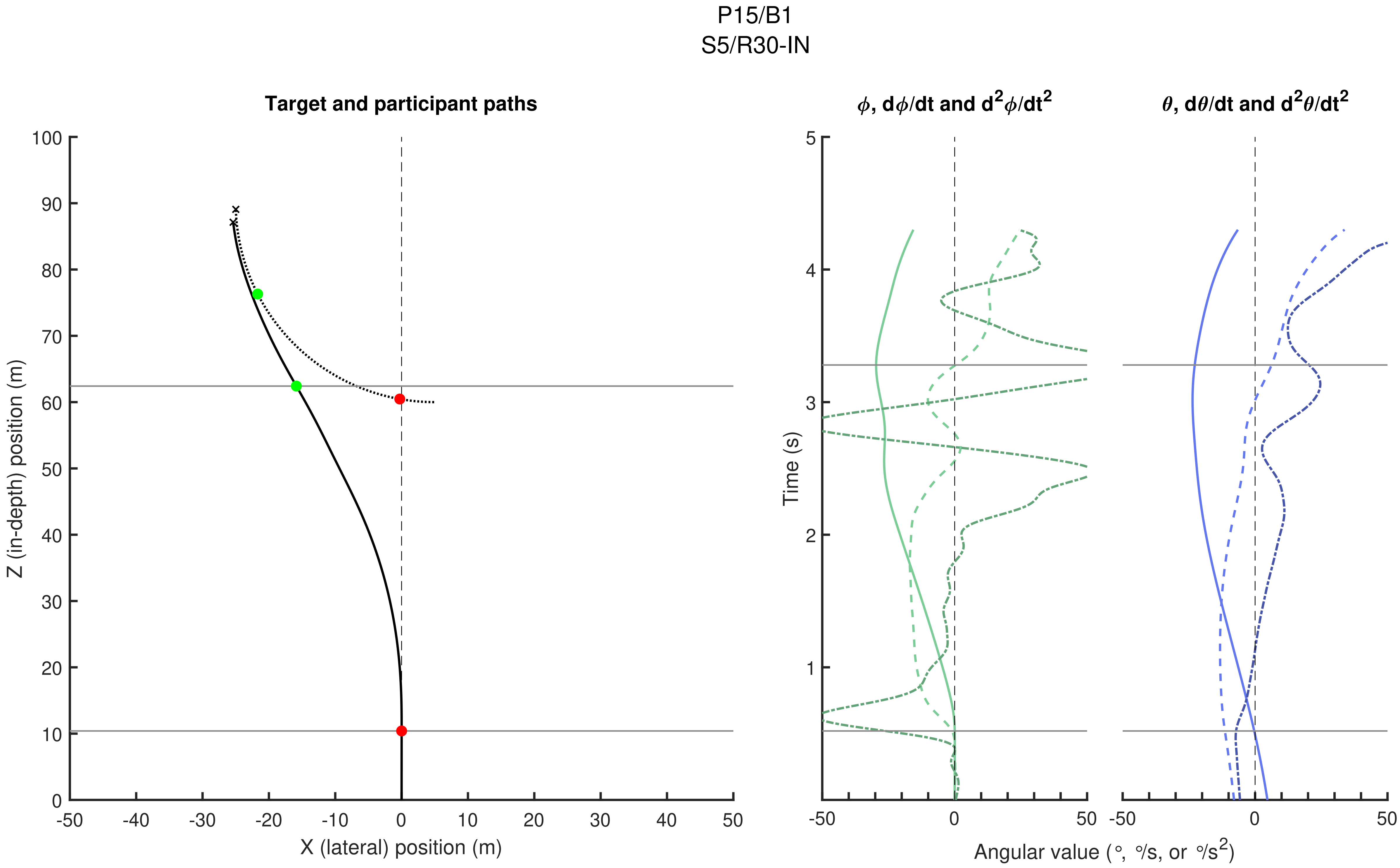

P15/B1
S5/R30-IN
Target and participant paths
Z (in-depth) position (m)
X (lateral) position (m)
ϕ, dϕ/dt and d²ϕ/dt²
θ, dθ/dt and d²θ/dt²
Time (s)
Angular value (°, °/s, or °/s²)

P15/B1
S15/R10-IN

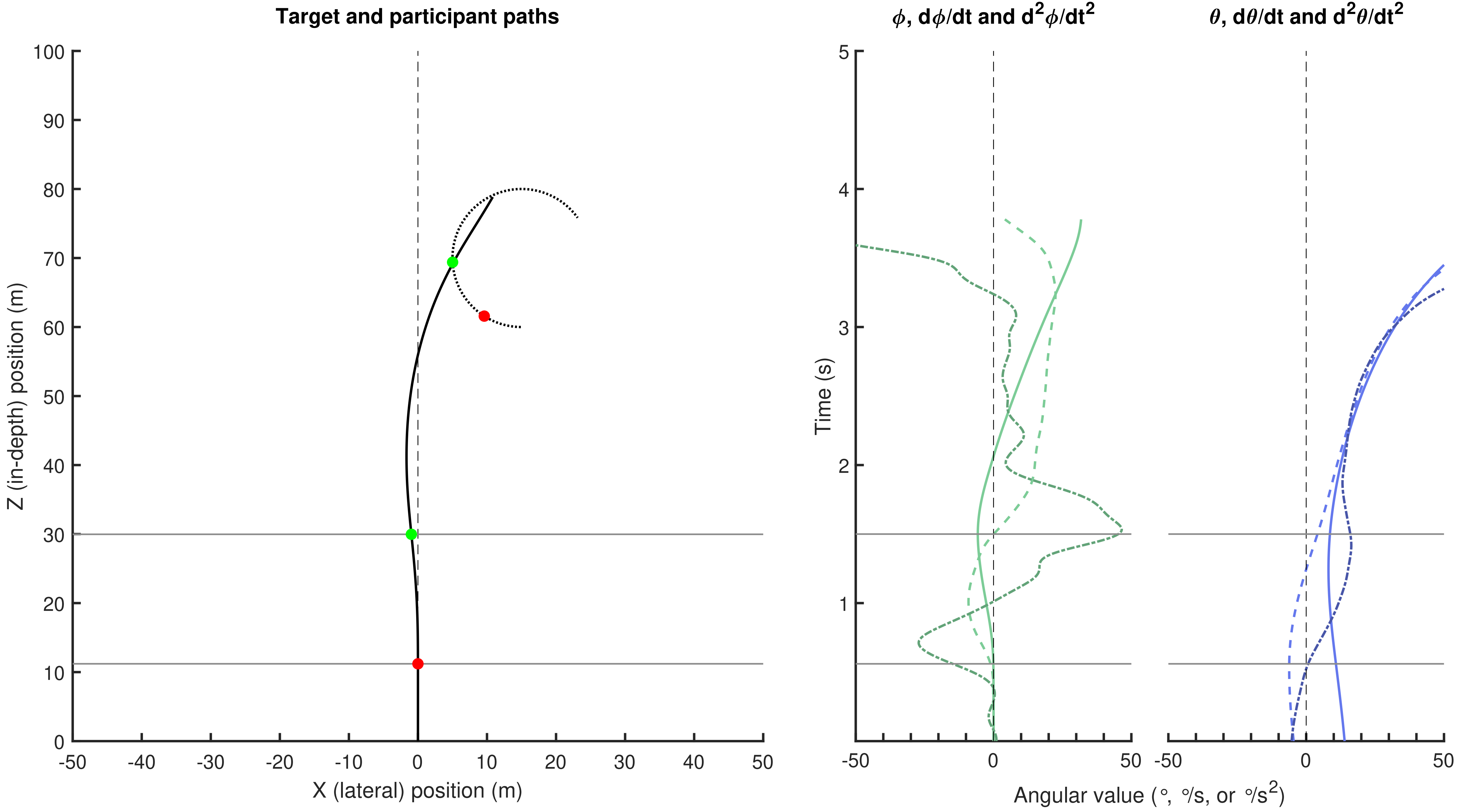

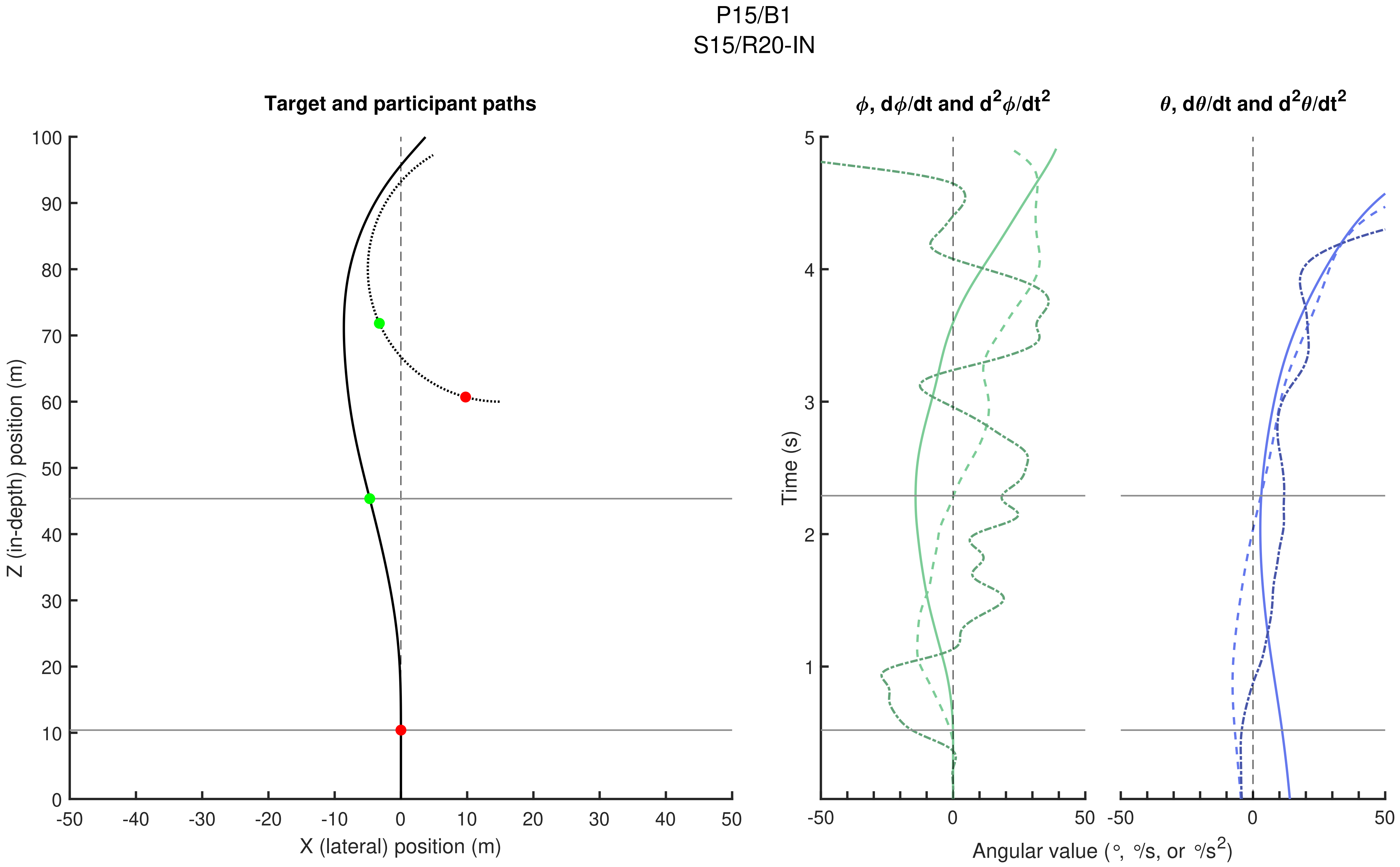

P15/B1
S15/R20-IN
Target and participant paths
Z (in-depth) position (m)
X (lateral) position (m)
ϕ, dϕ/dt and d$^2\phi$/dt^2
θ, dθ/dt and d$^2\theta$/dt^2
Time (s)
Angular value (°, °/s, or °/s^2)

P15/B1
S15/R30-IN

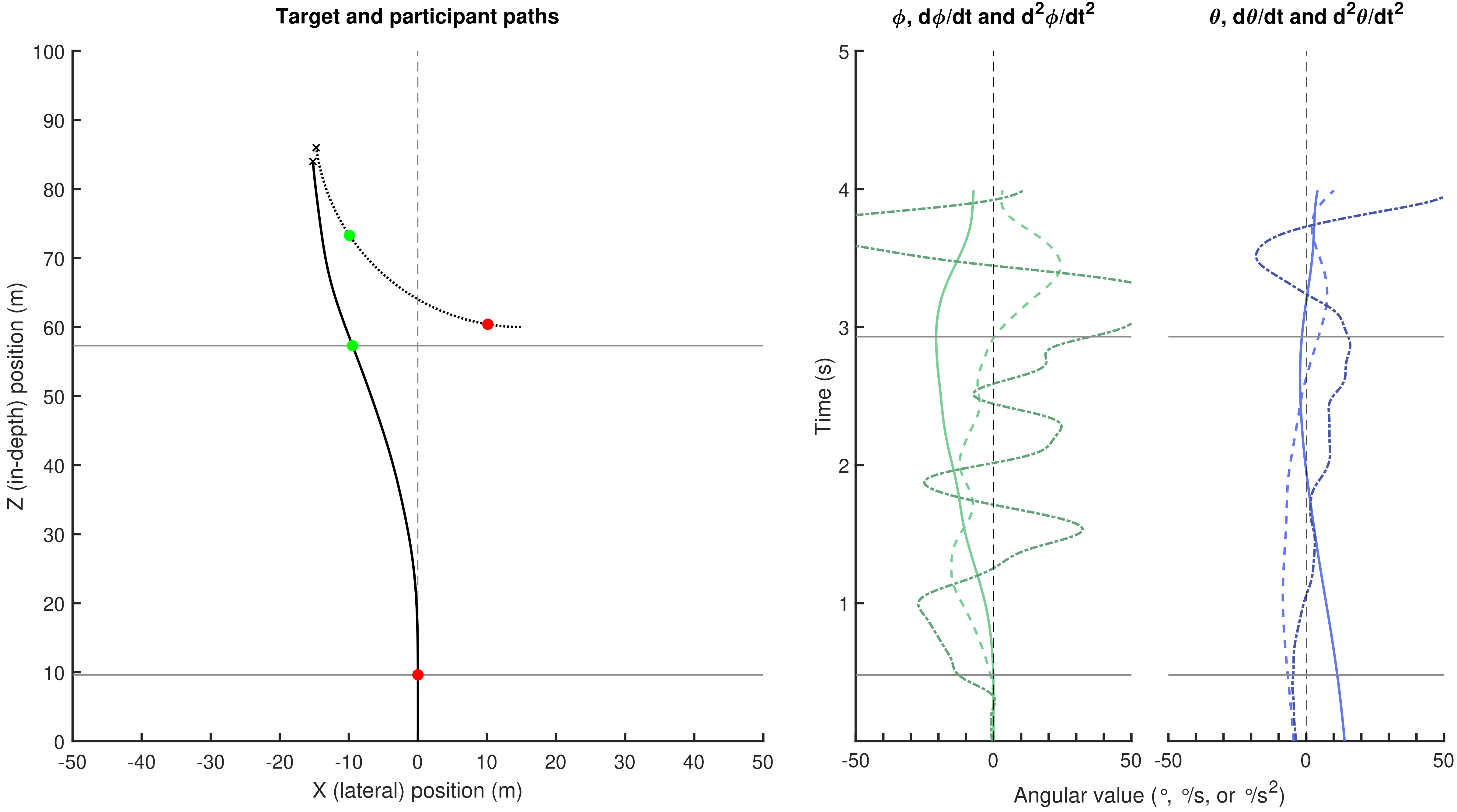

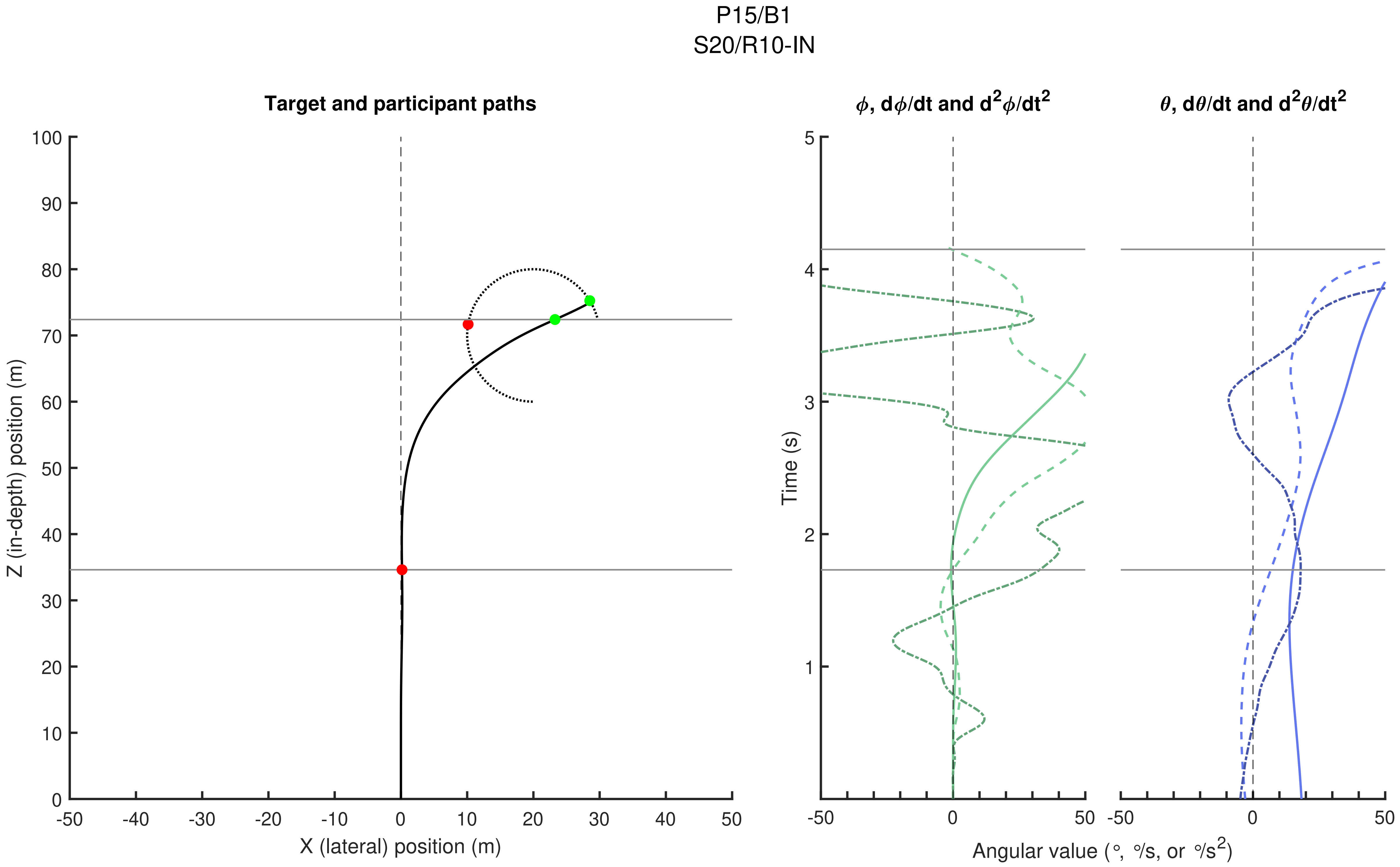
P15/B1
S20/R10-IN
Target and participant paths
Z (in-depth) position (m)
X (lateral) position (m)
100
90
80
70
60
50
40
30
20
10
0
-50
-40
-30
-20
-10
0
10
20
30
40
50
ϕ, dϕ/dt and d$^2\phi$/dt^2
θ, dθ/dt and d$^2\theta$/dt^2
Time (s)
5
4
3
2
1
-50
0
50
-50
0
50
Angular value (°, °/s, or °/s^2)

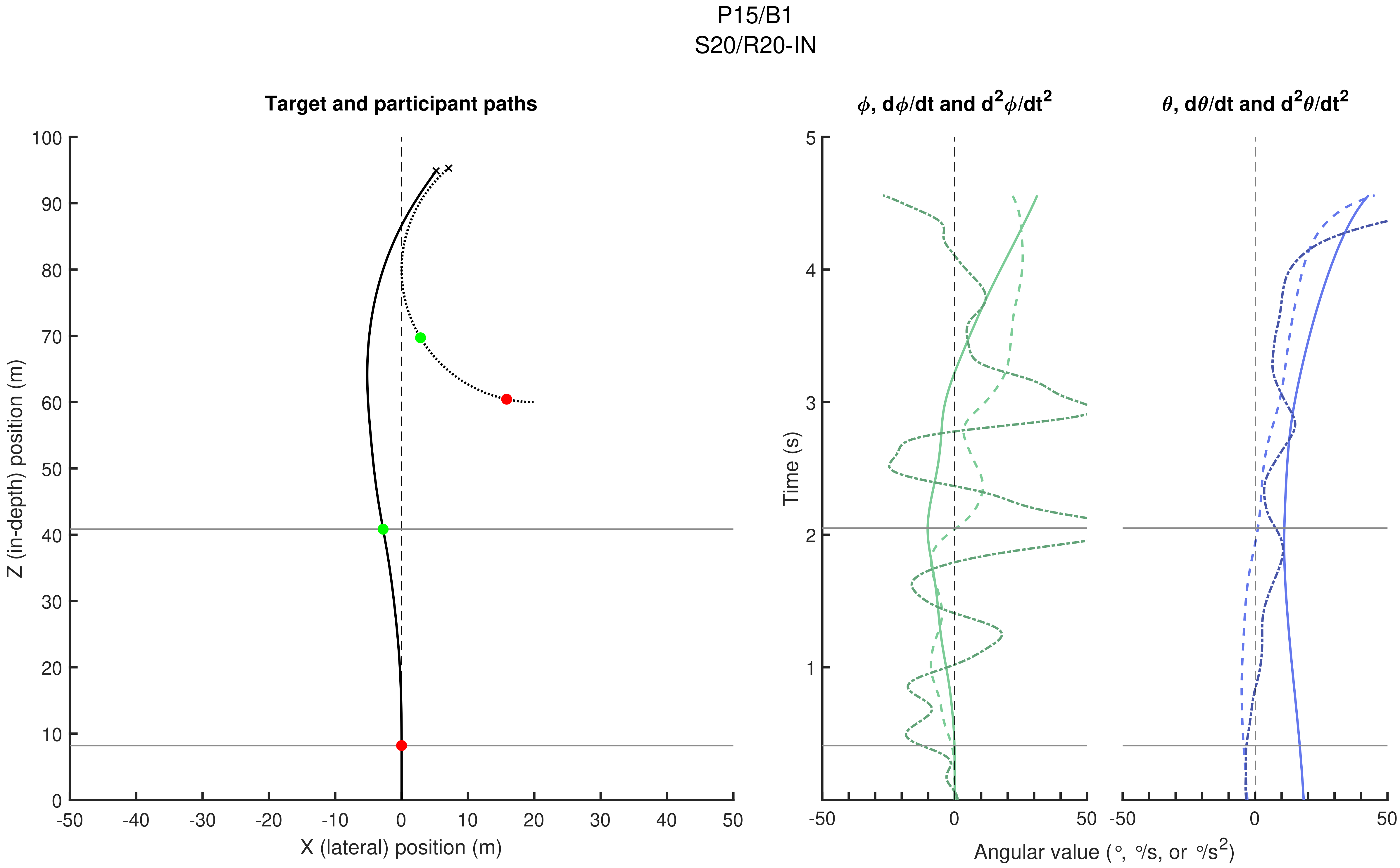
P15/B1
S20/R20-IN
Target and participant paths
ϕ, dϕ/dt and d$^2\phi$/dt^2
θ, dθ/dt and d$^2\theta$/dt^2
Z (in-depth) position (m)
X (lateral) position (m)
Time (s)
Angular value (°, °/s, or °/s^2)
100
90
80
70
60
50
40
30
20
10
0
-50
-40
-30
-20
-10
0
10
20
30
40
50
5
4
3
2
1
-50
0
50
-50
0
50

P15/B1
S20/R30-IN

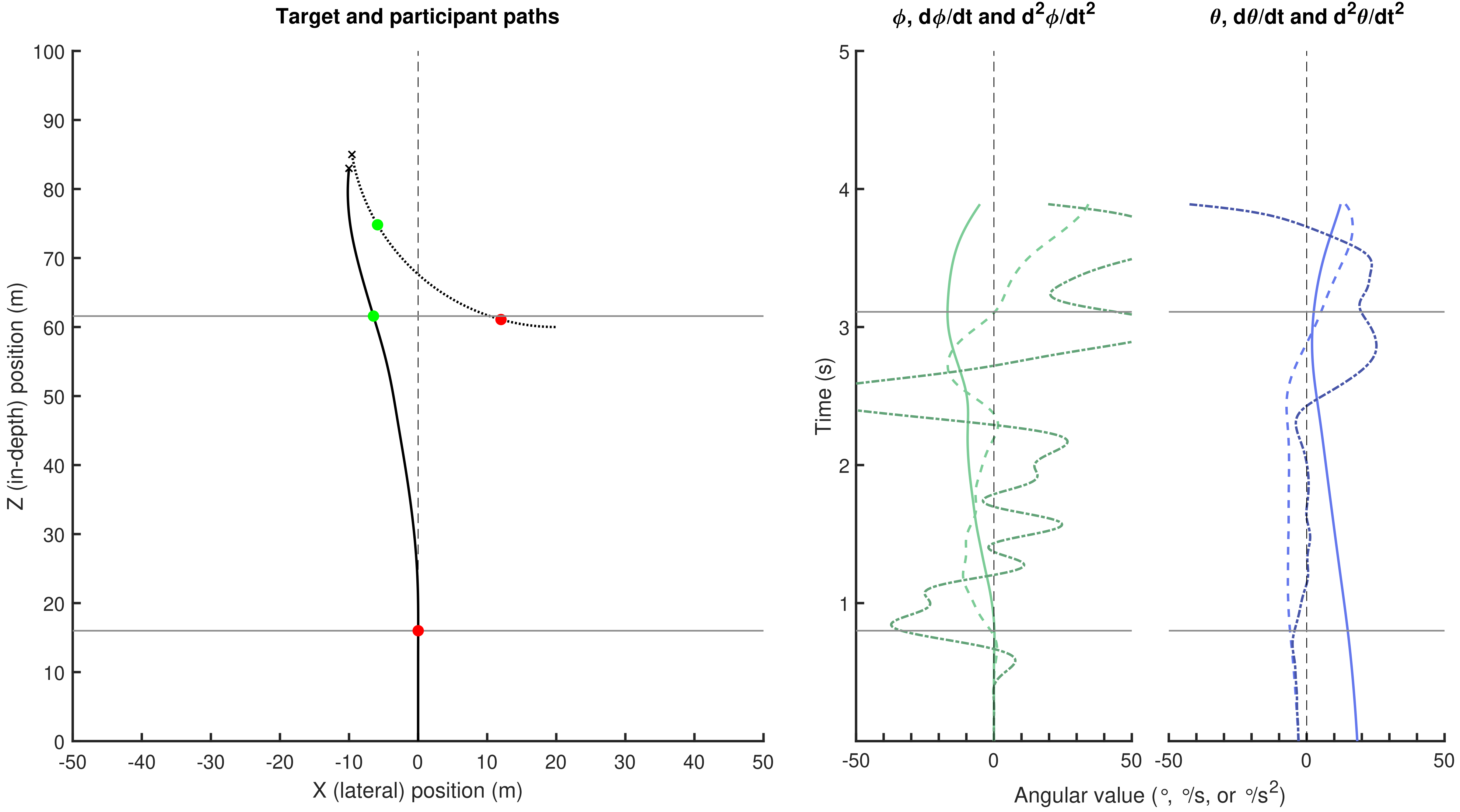

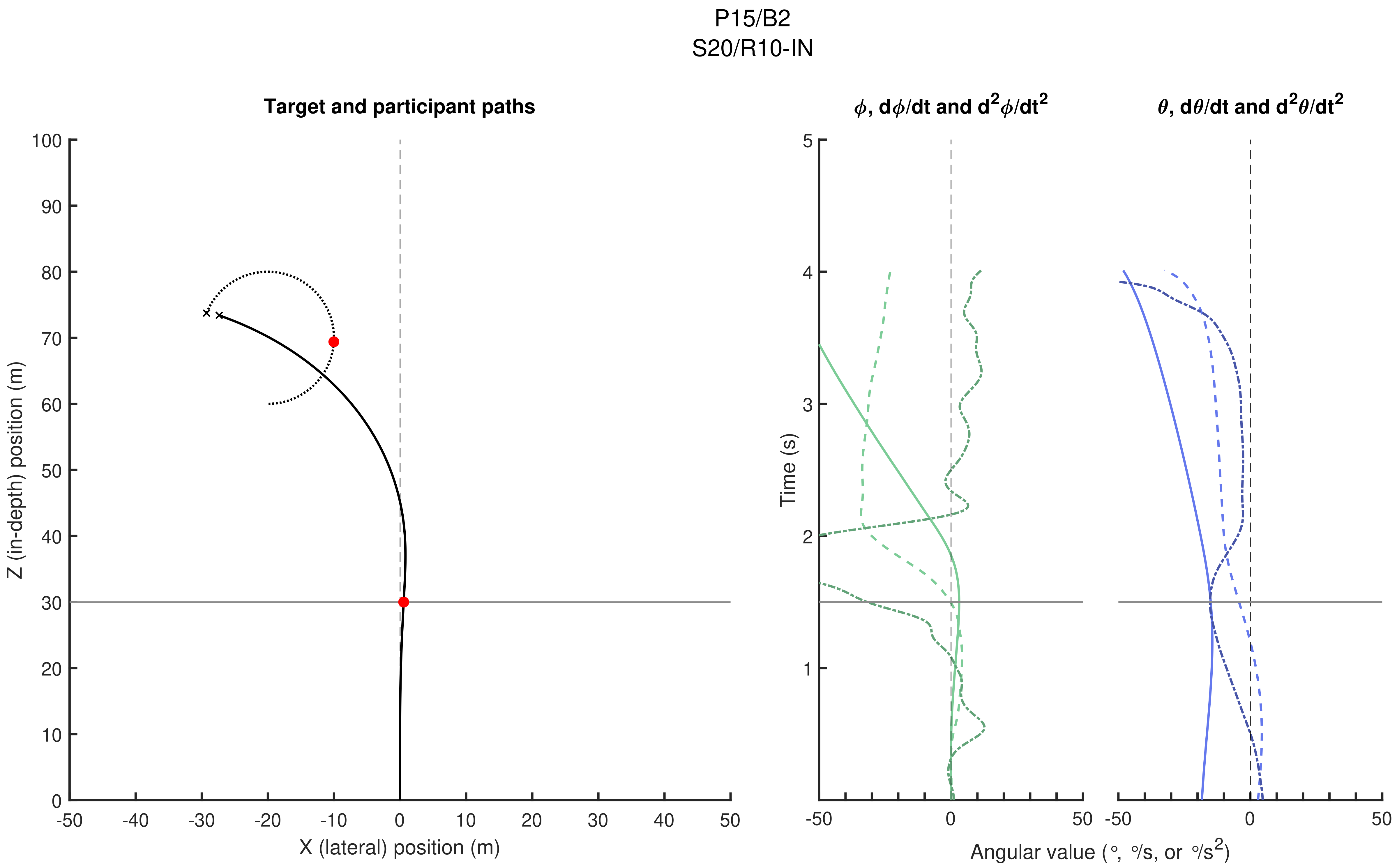
P15/B2
S20/R10-IN
Target and participant paths
ϕ, dϕ/dt and d²ϕ/dt²
θ, dθ/dt and d²θ/dt²
100
90
80
70
60
50
40
30
20
10
0
-50
-40
-30
-20
-10
0
10
20
30
40
50
Z (in-depth) position (m)
X (lateral) position (m)
5
4
3
2
1
Time (s)
-50
0
50
-50
0
50
Angular value (°, °/s, or °/s²)

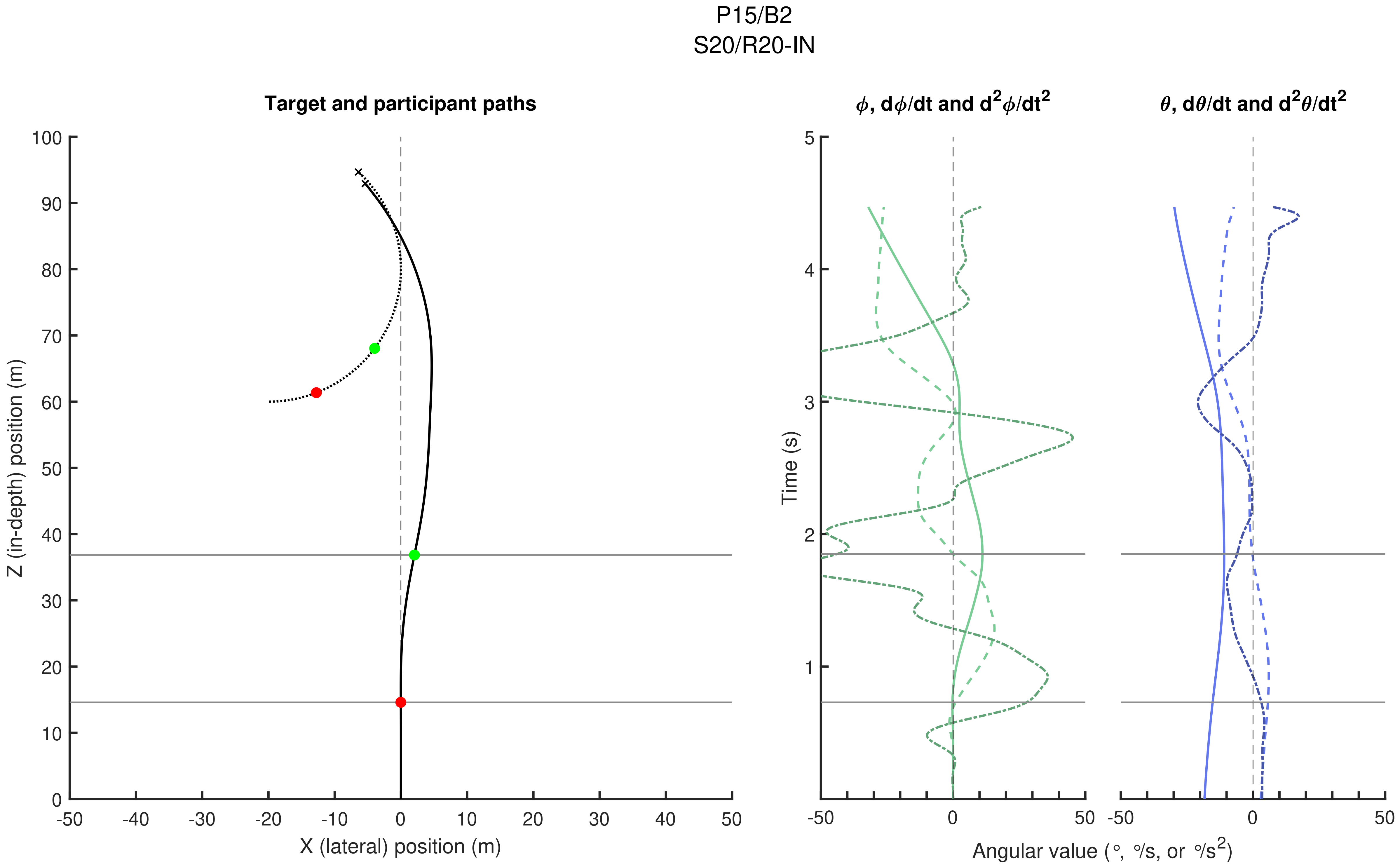
P15/B2
S20/R20-IN
Target and participant paths
Z (in-depth) position (m)
X (lateral) position (m)
0
10
20
30
40
50
60
70
80
90
100
-50
-40
-30
-20
-10
0
10
20
30
40
50
ϕ, dϕ/dt and d²ϕ/dt²
θ, dθ/dt and d²θ/dt²
Time (s)
1
2
3
4
5
-50
0
50
-50
0
50
Angular value (°, °/s, or °/s²)

P15/B2
S20/R30-IN

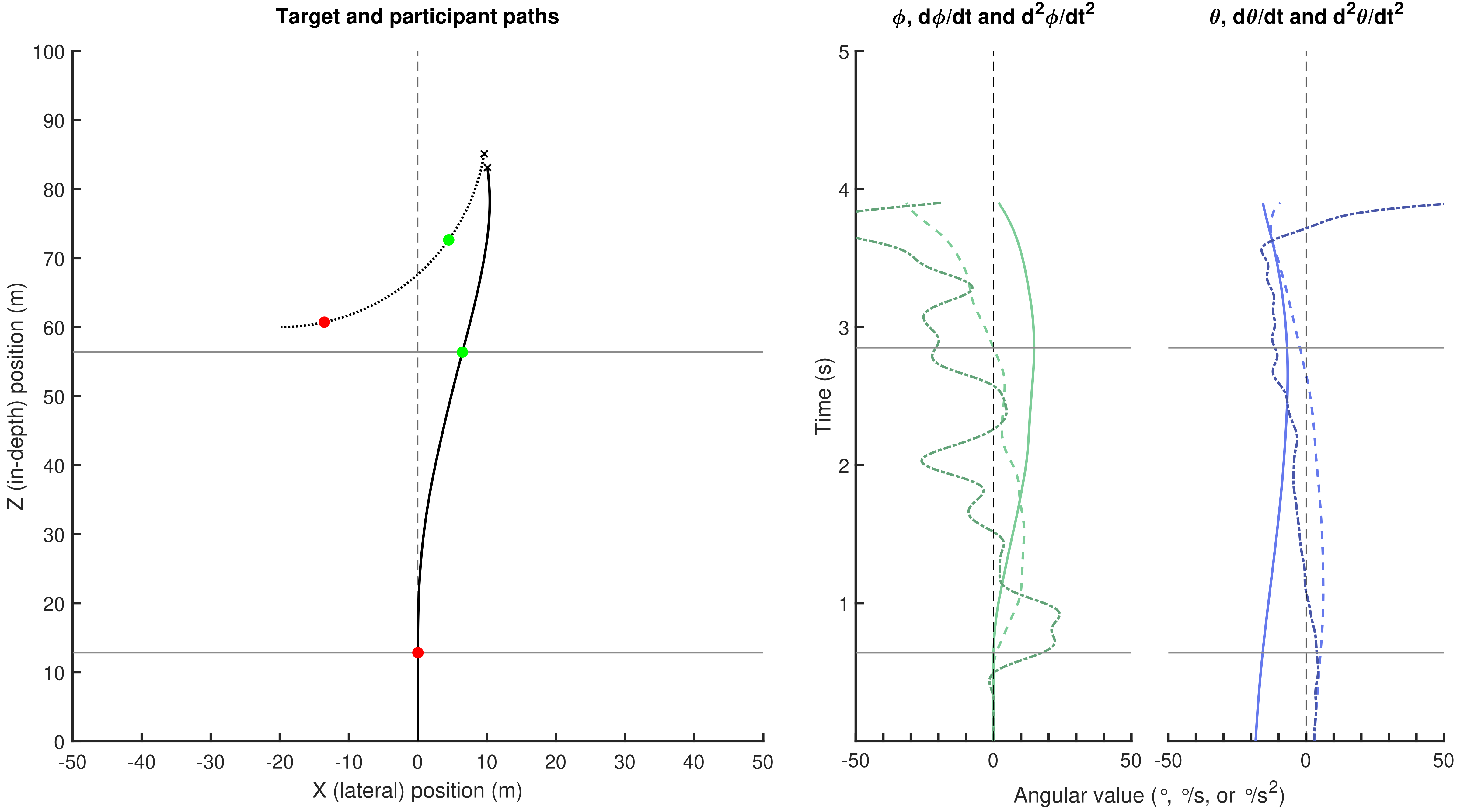

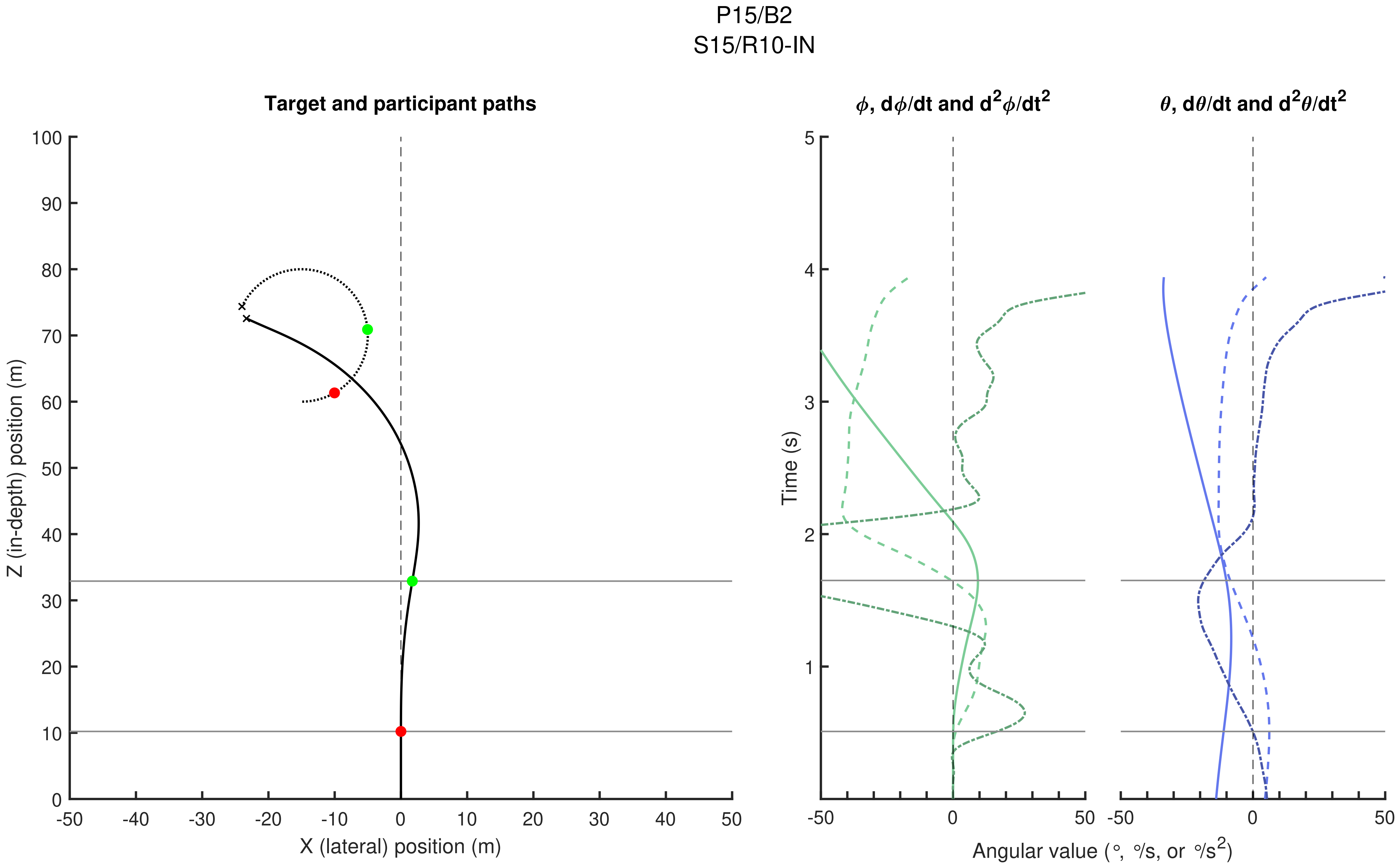

P15/B2
S15/R10-IN
Target and participant paths
Z (in-depth) position (m)
X (lateral) position (m)
ϕ, dϕ/dt and d²ϕ/dt²
θ, dθ/dt and d²θ/dt²
Time (s)
Angular value (°, °/s, or °/s²)

P15/B2
S15/R20-IN

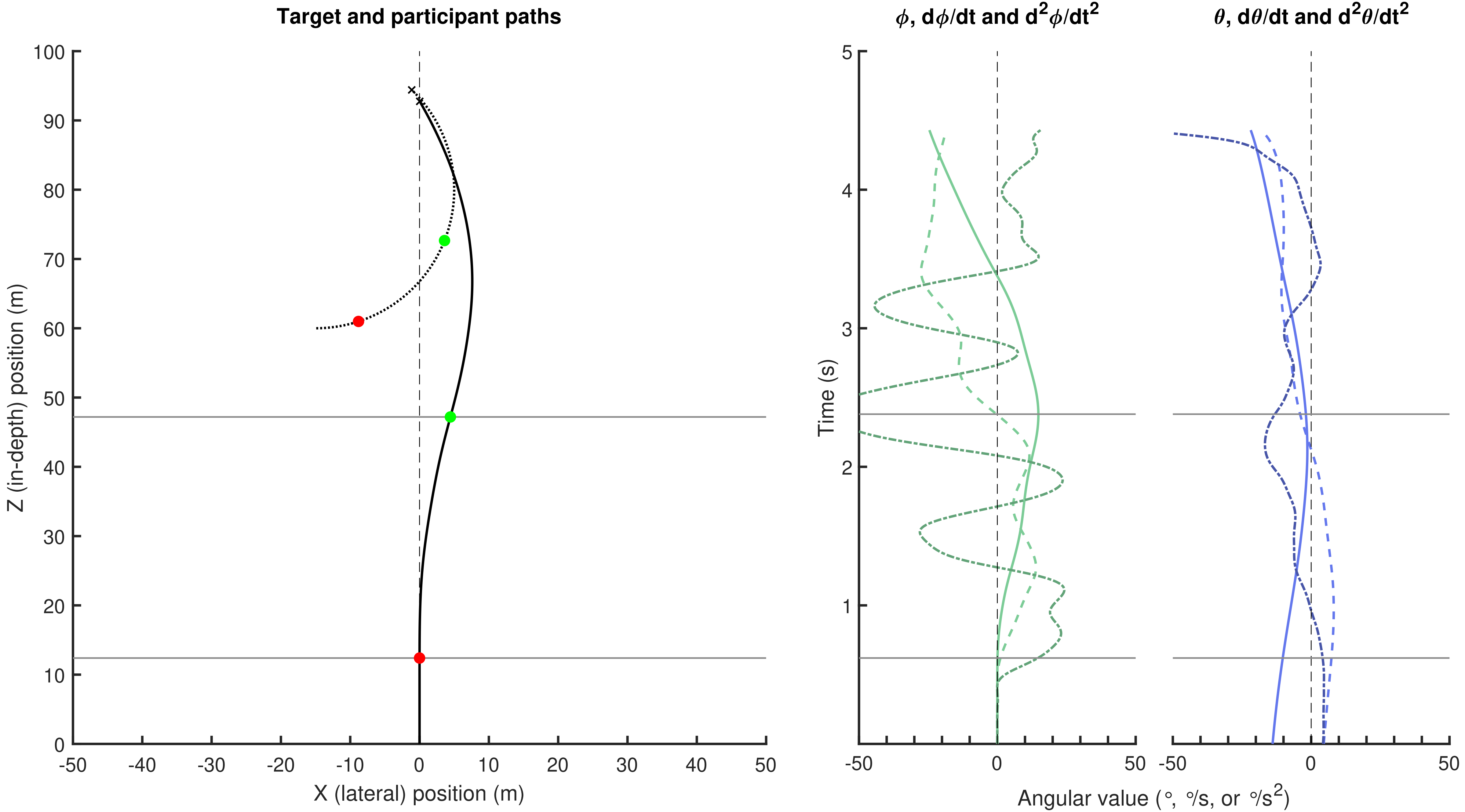

P15/B2
S15/R30-IN

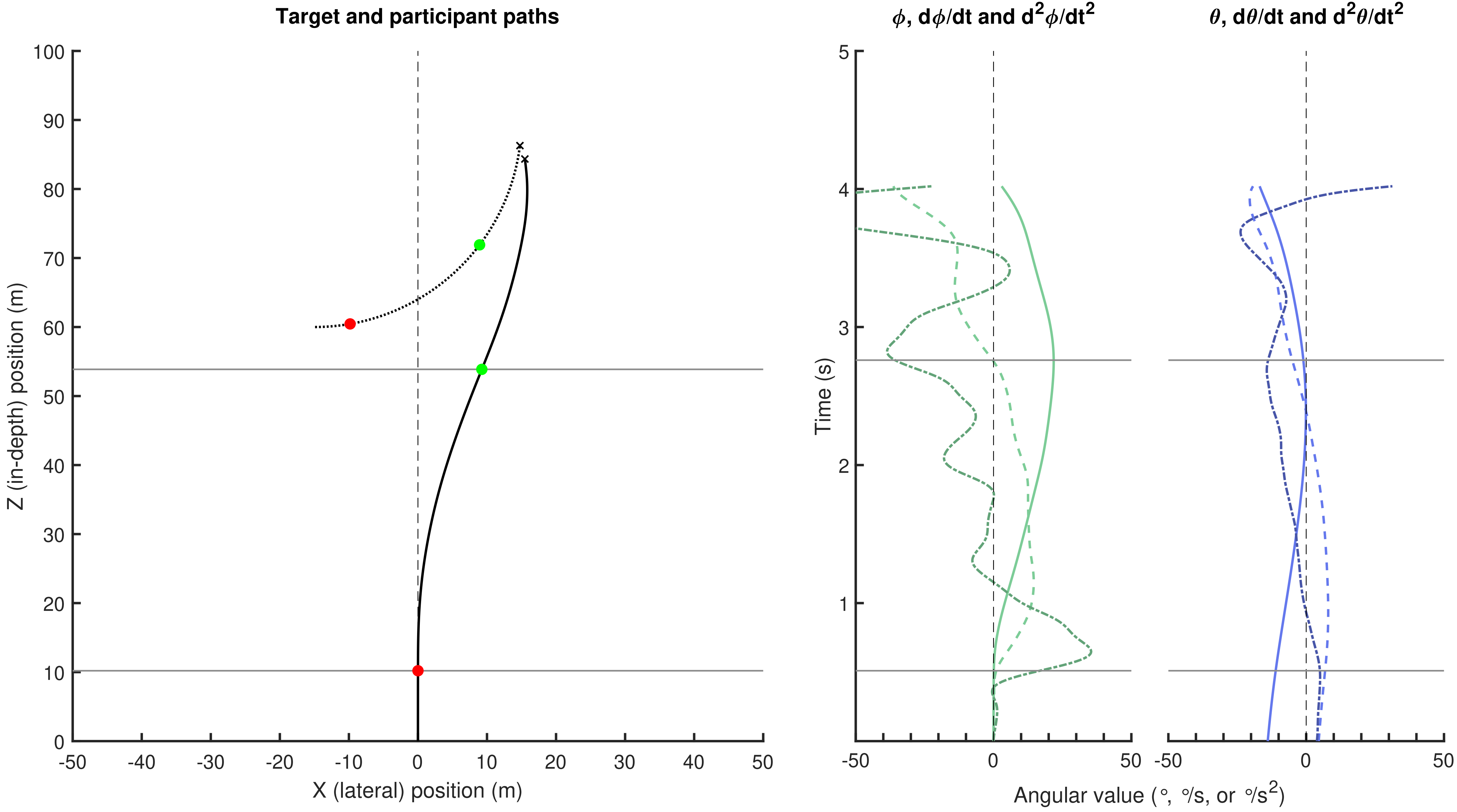

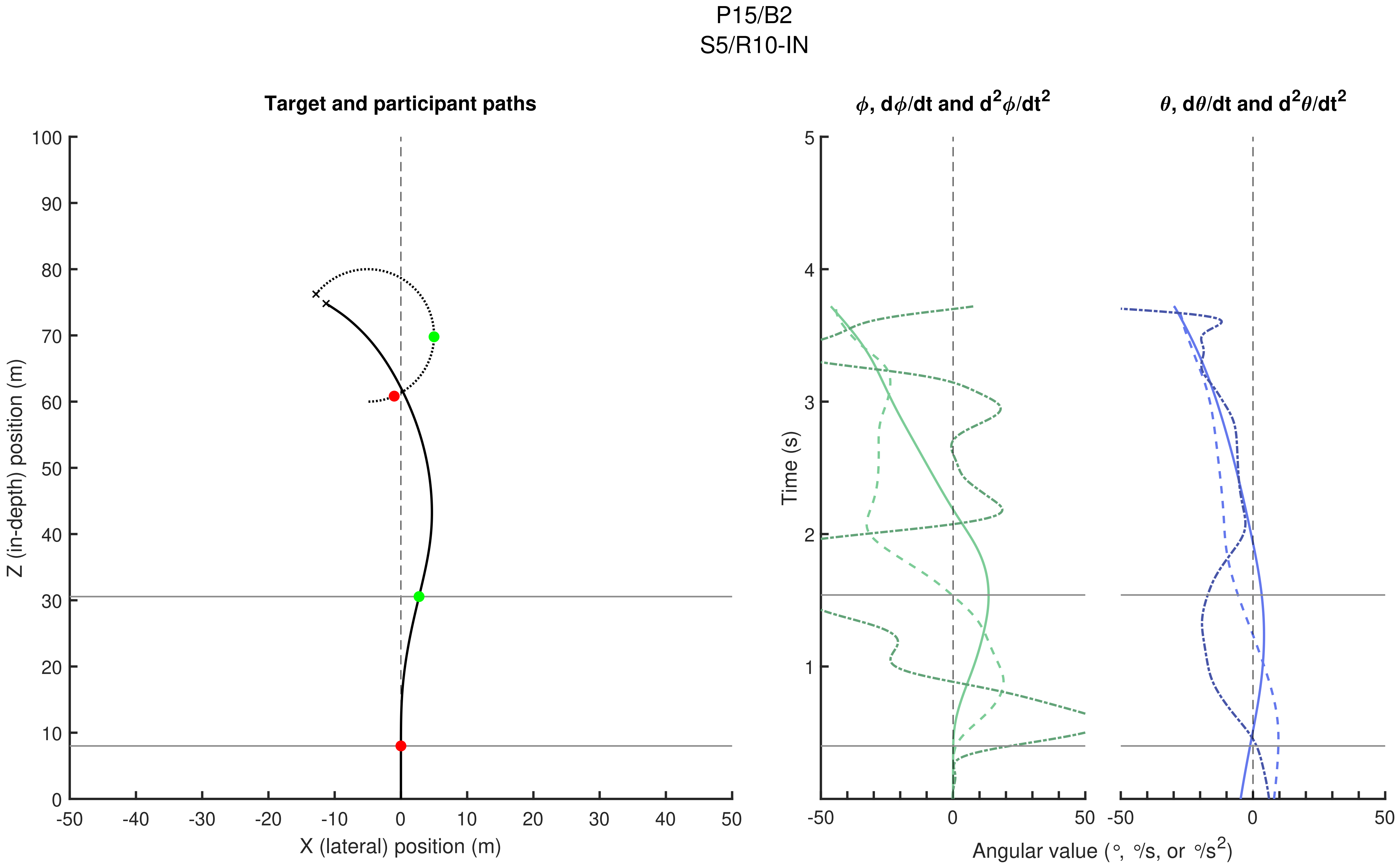
P15/B2
S5/R10-IN
Target and participant paths
Z (in-depth) position (m)
X (lateral) position (m)
ϕ, dϕ/dt and d²ϕ/dt²
θ, dθ/dt and d²θ/dt²
Time (s)
Angular value (°, °/s, or °/s²)

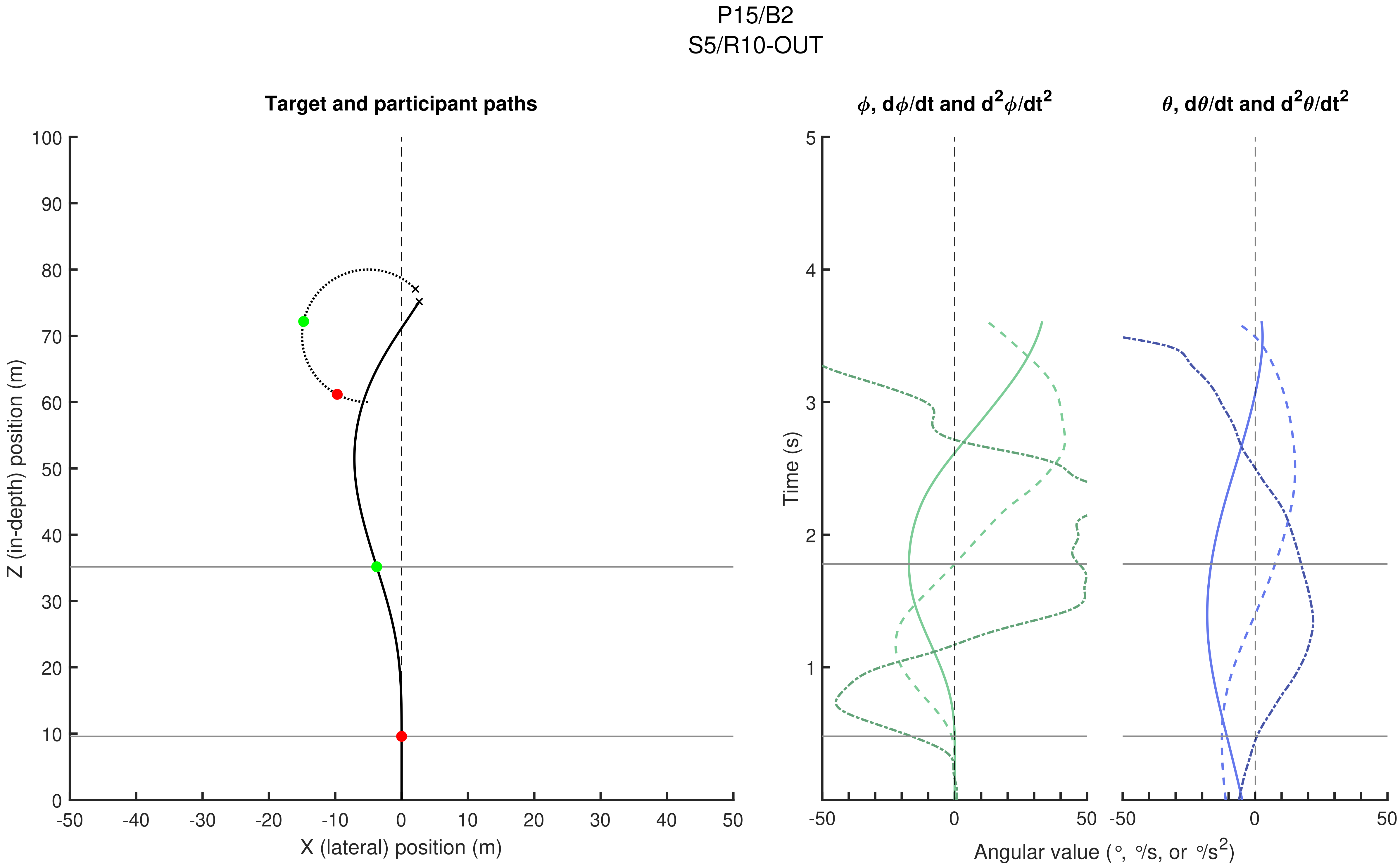
P15/B2
S5/R10-OUT
Target and participant paths
ϕ, dϕ/dt and d$^2\phi$/dt^2
θ, dθ/dt and d$^2\theta$/dt^2
Z (in-depth) position (m)
X (lateral) position (m)
Time (s)
Angular value (°, °/s, or °/s^2)

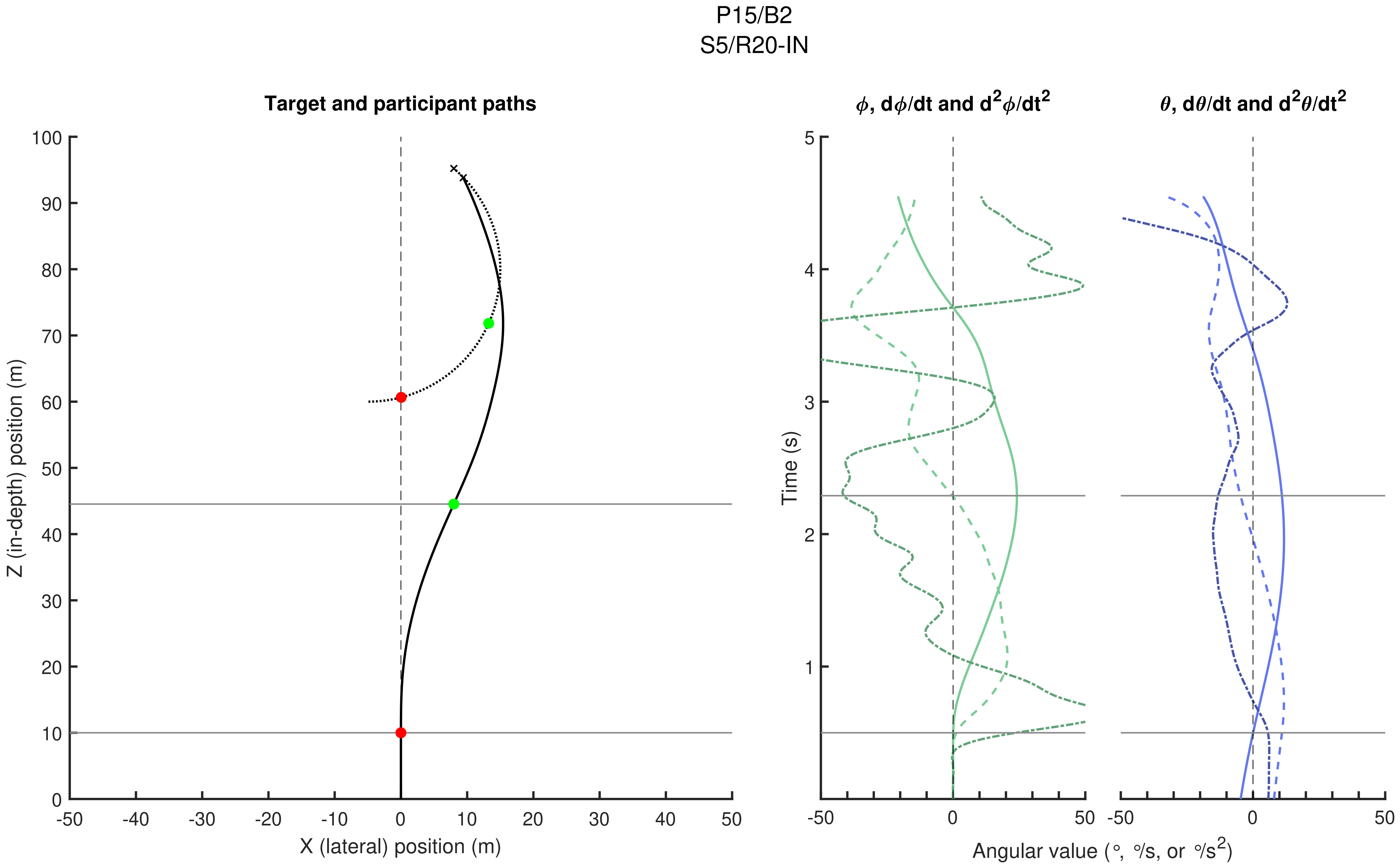
P15/B2
S5/R20-IN
Target and participant paths
Z (in-depth) position (m)
X (lateral) position (m)
0
10
20
30
40
50
60
70
80
90
100
-50
-40
-30
-20
-10
0
10
20
30
40
50
ϕ, dϕ/dt and d²ϕ/dt²
θ, dθ/dt and d²θ/dt²
Time (s)
1
2
3
4
5
-50
0
50
-50
0
50
Angular value (°, °/s, or °/s²)

P15/B2
S5/R20-OUT

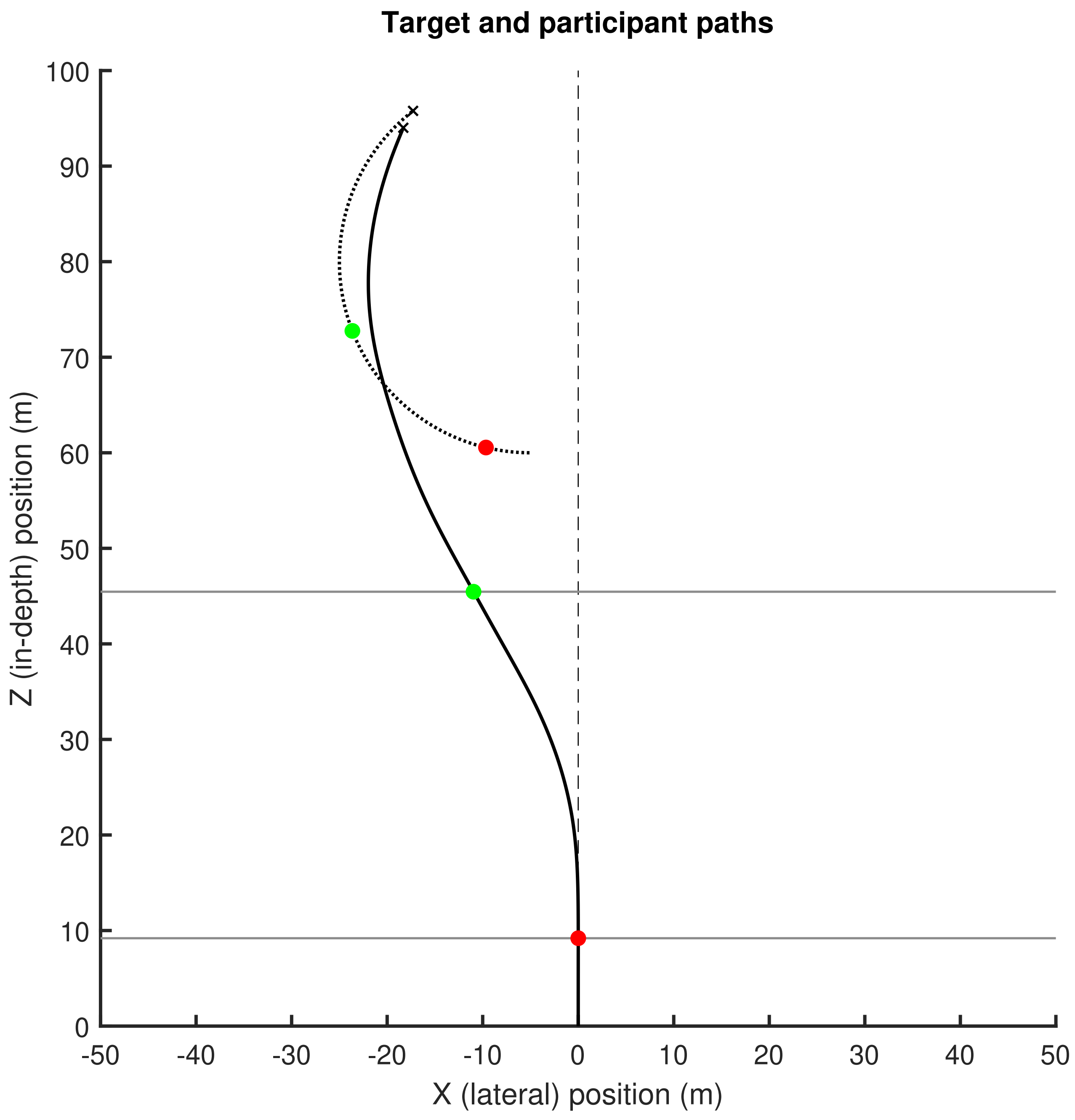

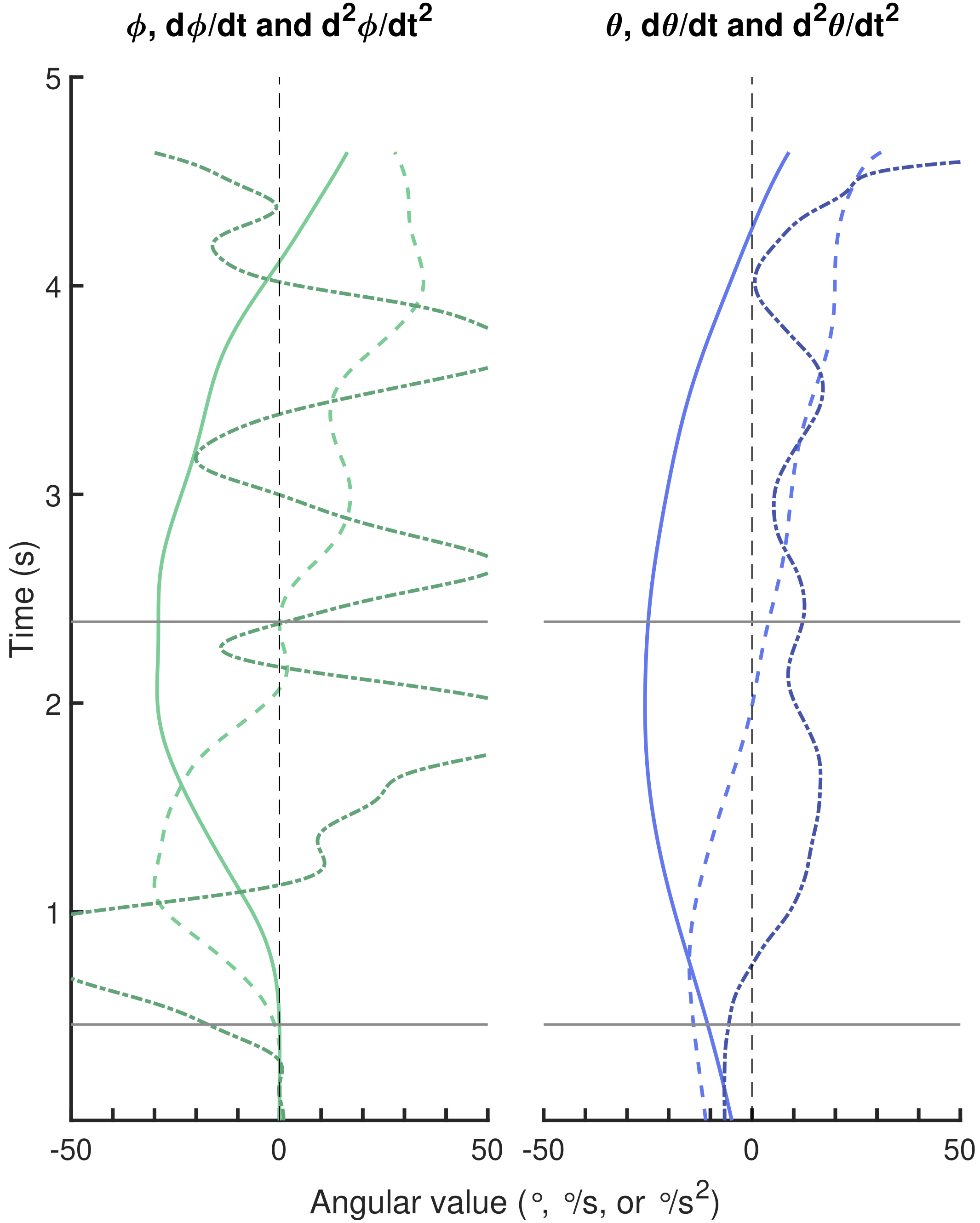

P15/B2
S5/R30-IN

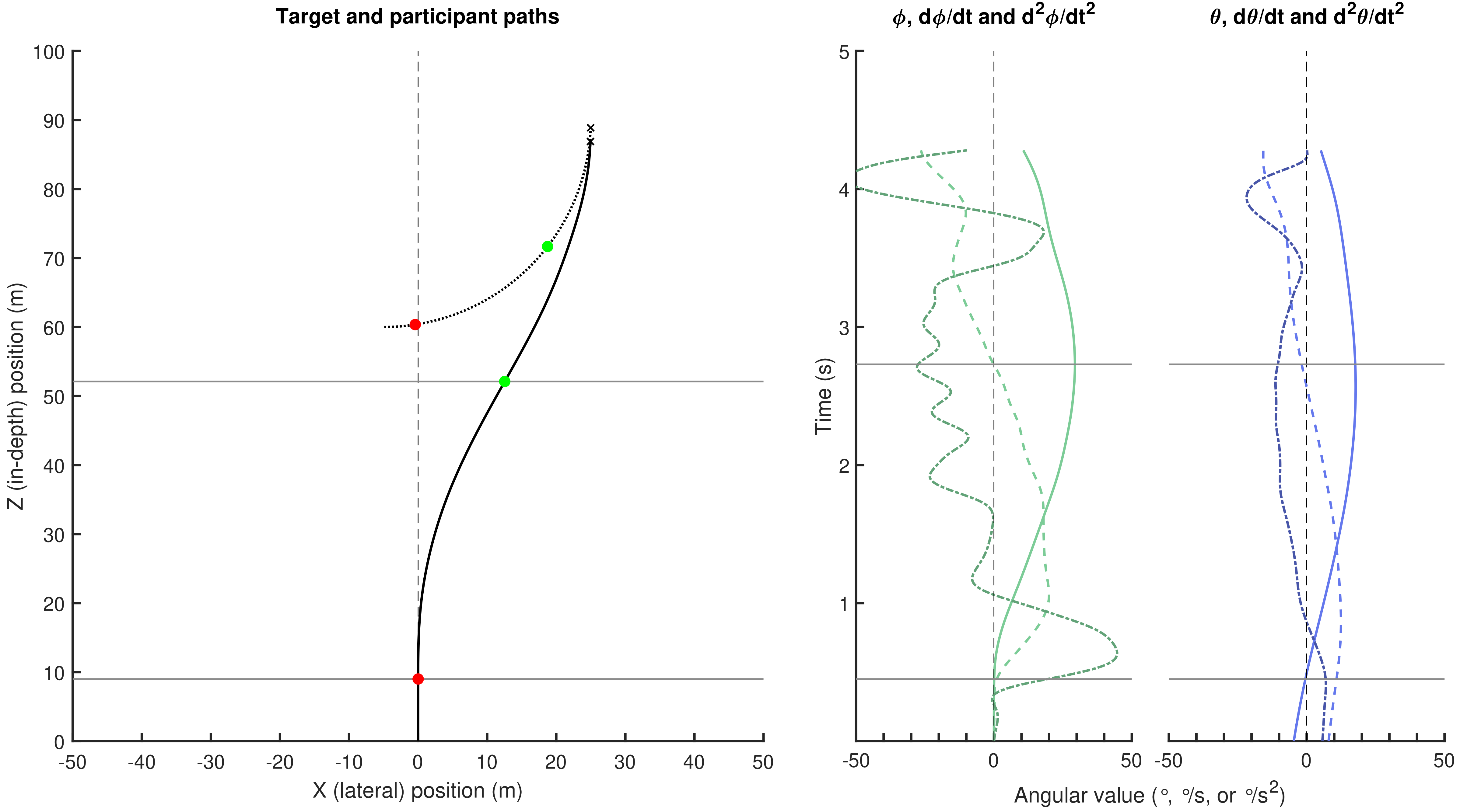

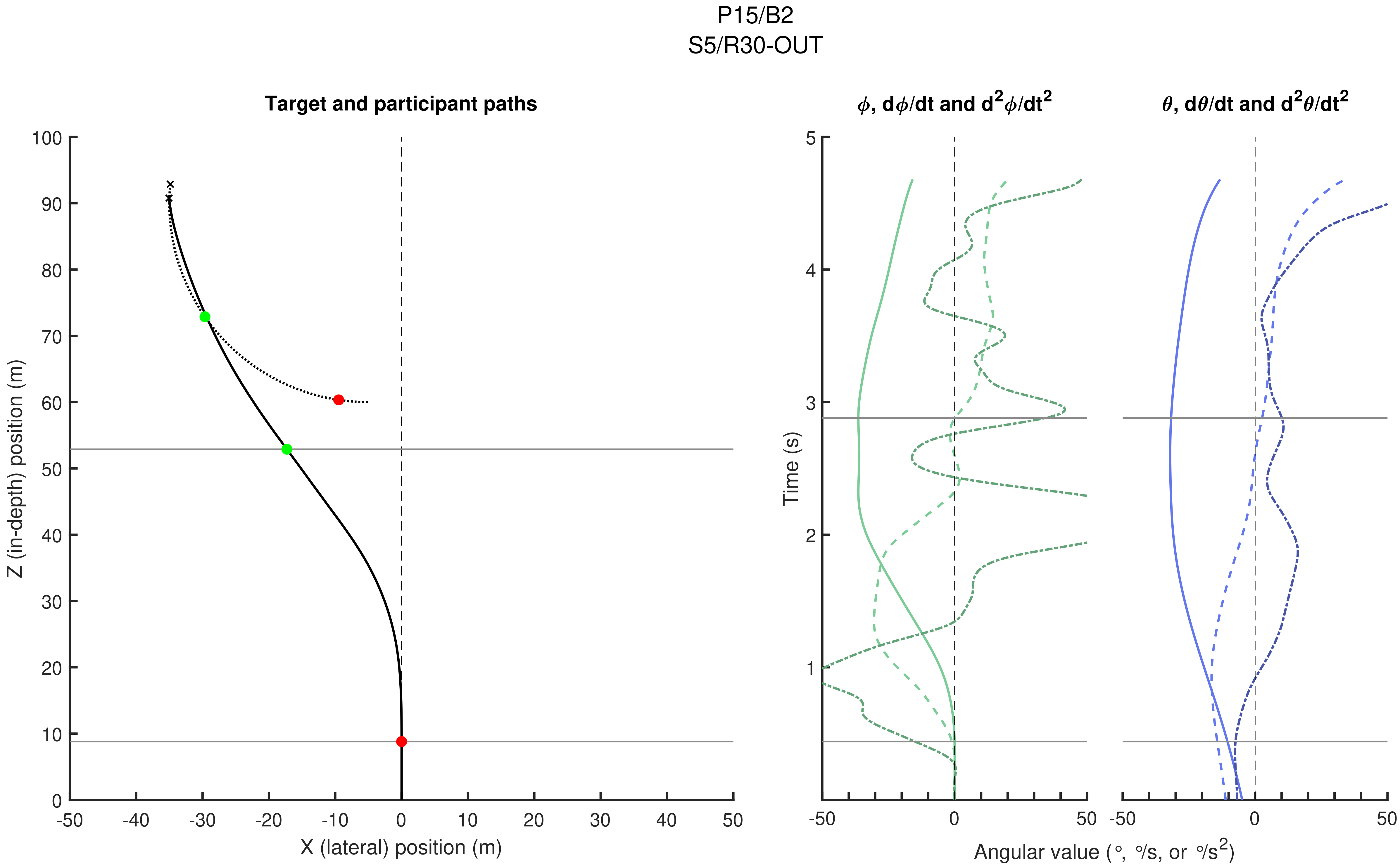
P15/B2
S5/R30-OUT
Target and participant paths
Z (in-depth) position (m)
X (lateral) position (m)
100
90
80
70
60
50
40
30
20
10
0
-50
-40
-30
-20
-10
0
10
20
30
40
50
ϕ, dϕ/dt and d²ϕ/dt²
θ, dθ/dt and d²θ/dt²
Time (s)
5
4
3
2
1
-50
0
50
-50
0
50
Angular value (°, °/s, or °/s²)

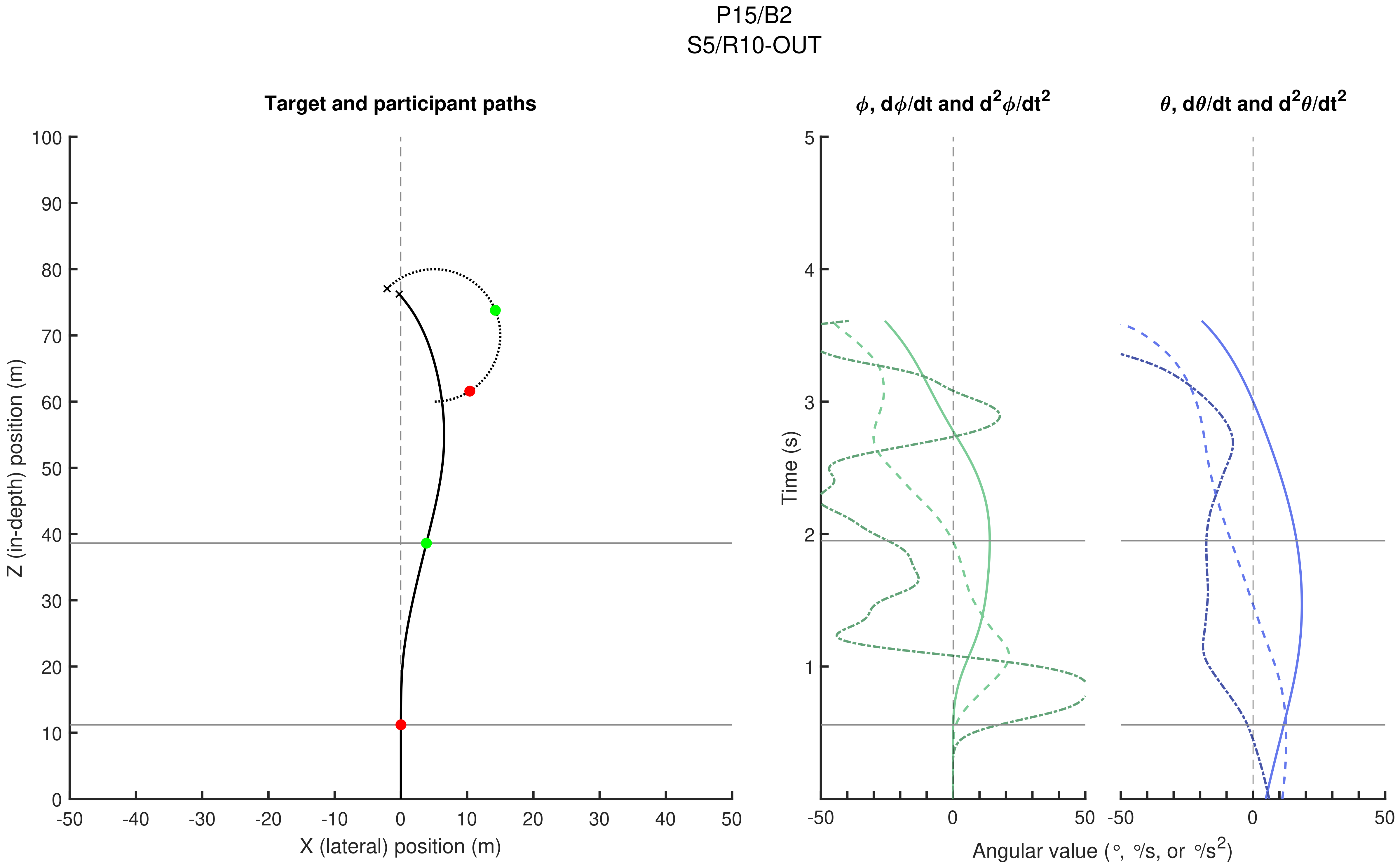
P15/B2
S5/R10-OUT
Target and participant paths
Z (in-depth) position (m)
X (lateral) position (m)
ϕ, dϕ/dt and d$^2\phi$/dt^2
θ, dθ/dt and d$^2\theta$/dt^2
Time (s)
Angular value (°, °/s, or °/s^2)

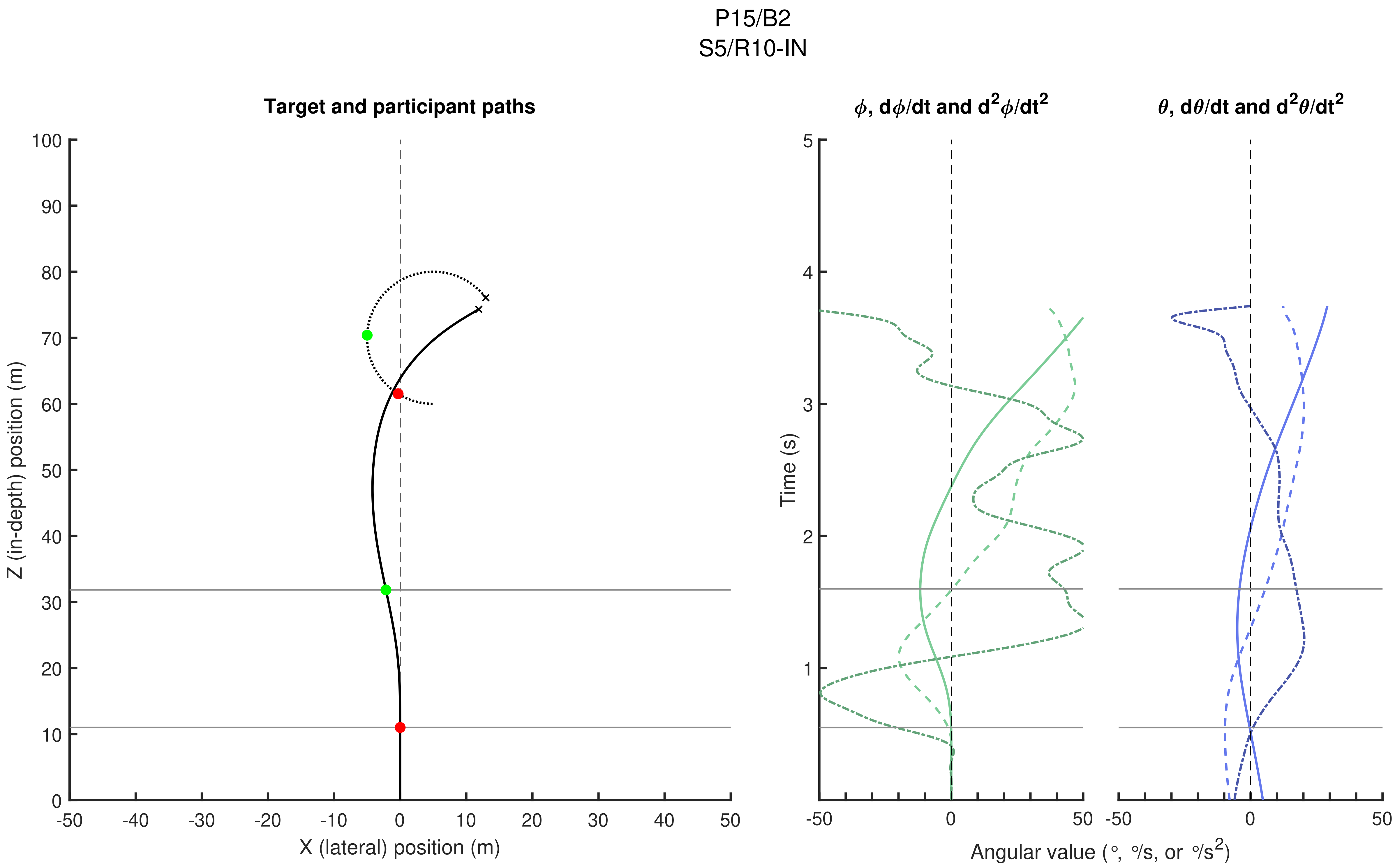

P15/B2
S5/R10-IN
Target and participant paths
Z (in-depth) position (m)
X (lateral) position (m)
ϕ, dϕ/dt and d²ϕ/dt²
θ, dθ/dt and d²θ/dt²
Time (s)
Angular value (°, °/s, or °/s²)

P15/B2
S5/R20-OUT

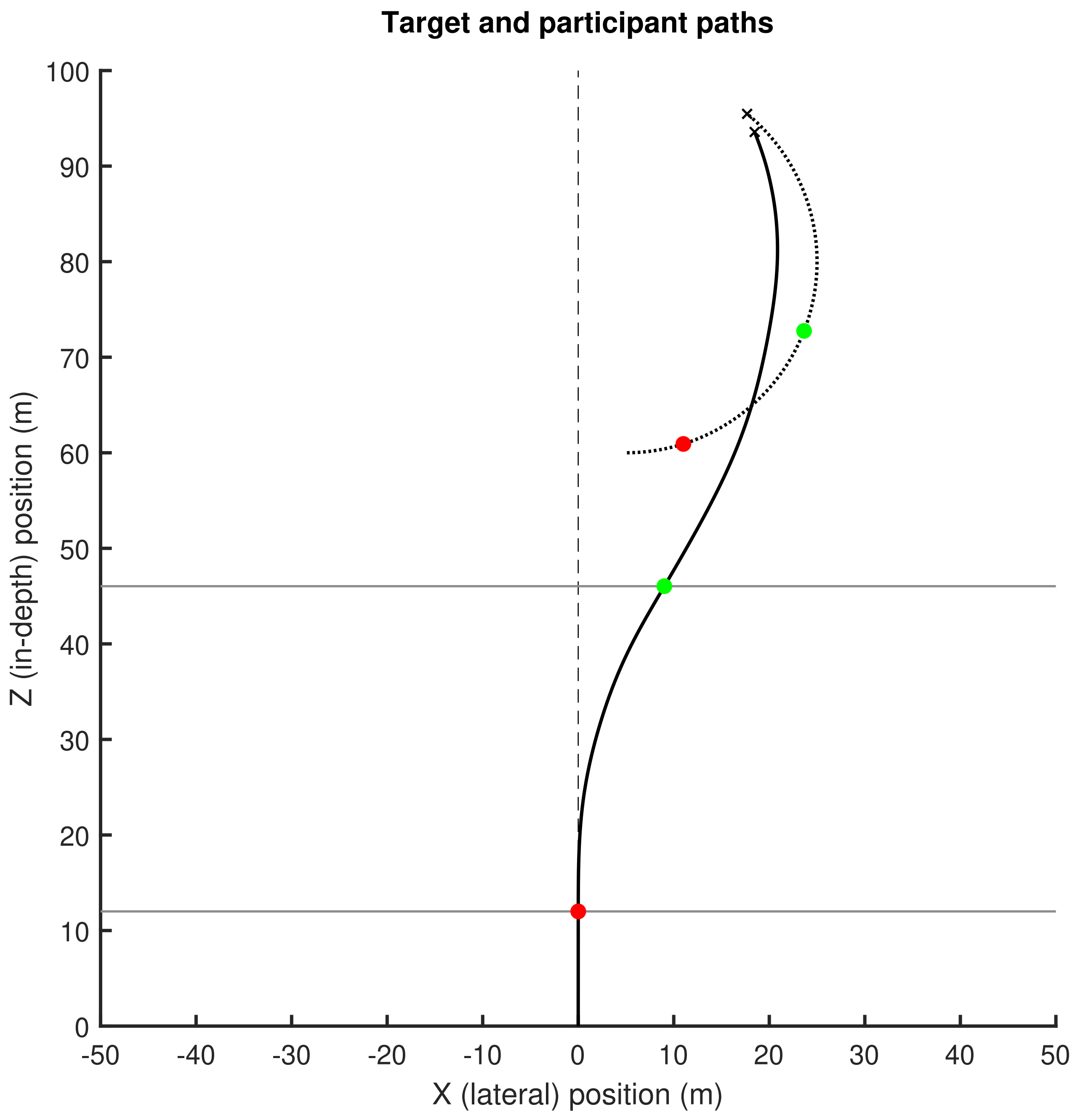

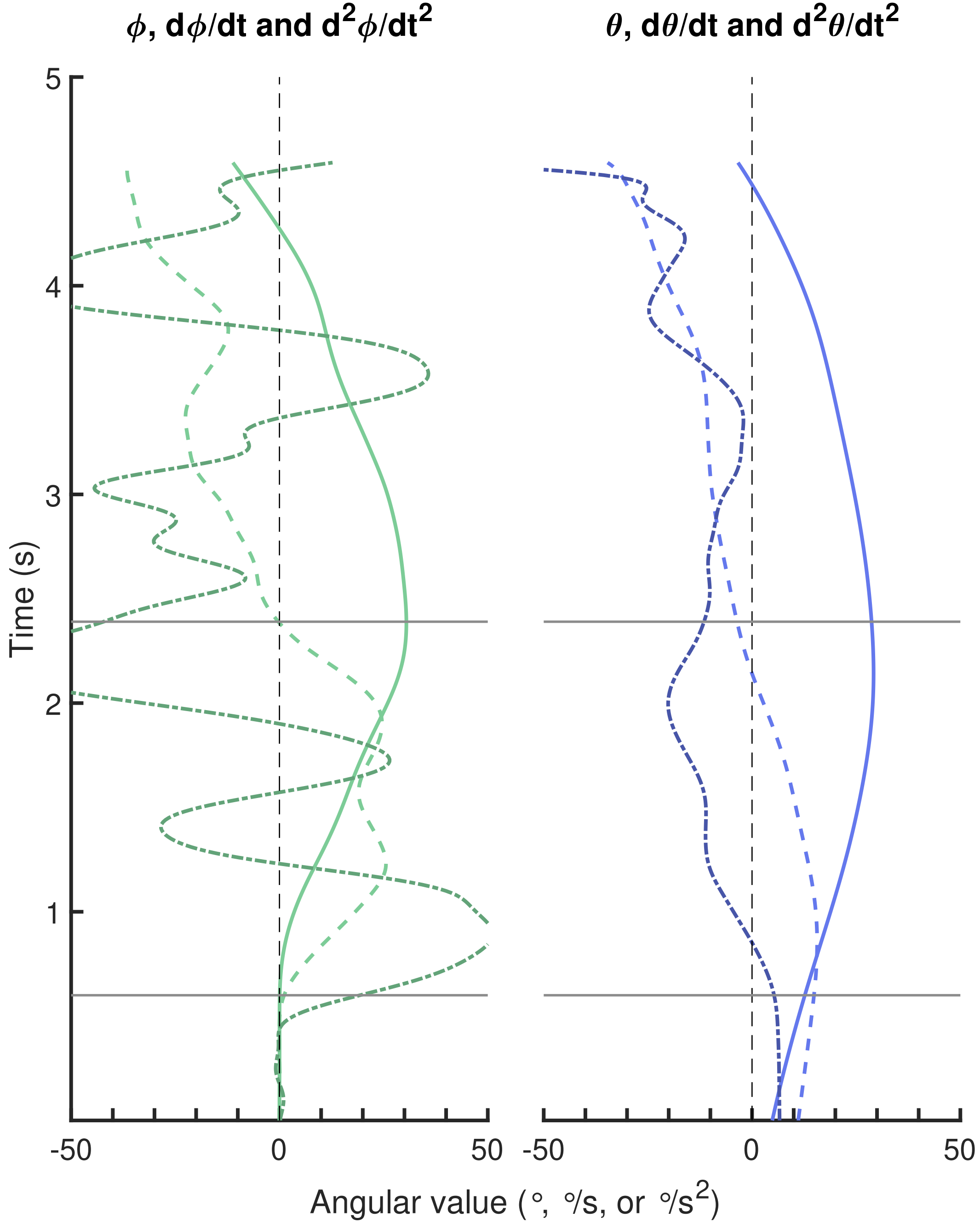

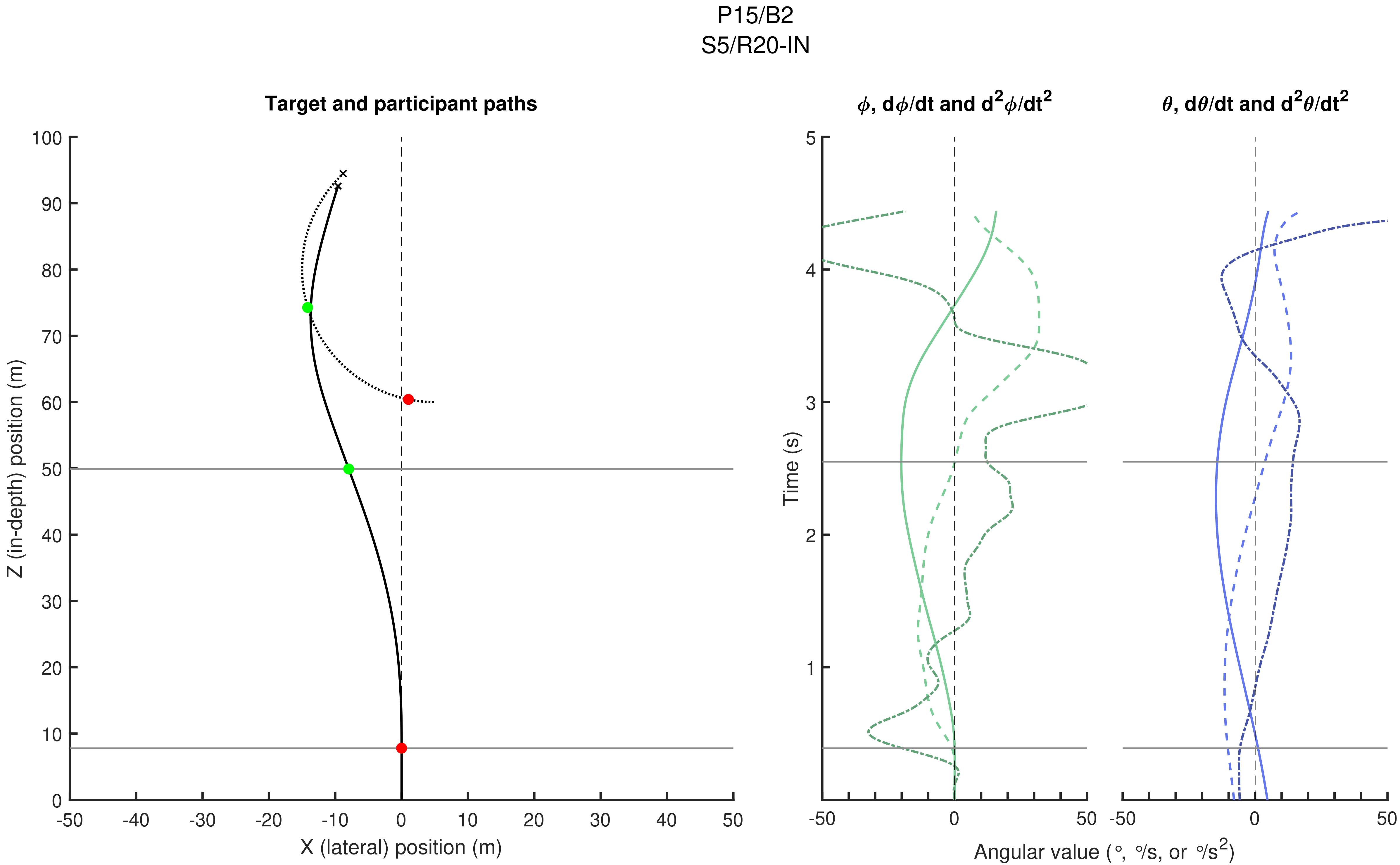
P15/B2
S5/R20-IN
Target and participant paths
Z (in-depth) position (m)
X (lateral) position (m)
ϕ, dϕ/dt and d²ϕ/dt²
θ, dθ/dt and d²θ/dt²
Time (s)
Angular value (°, °/s, or °/s²)

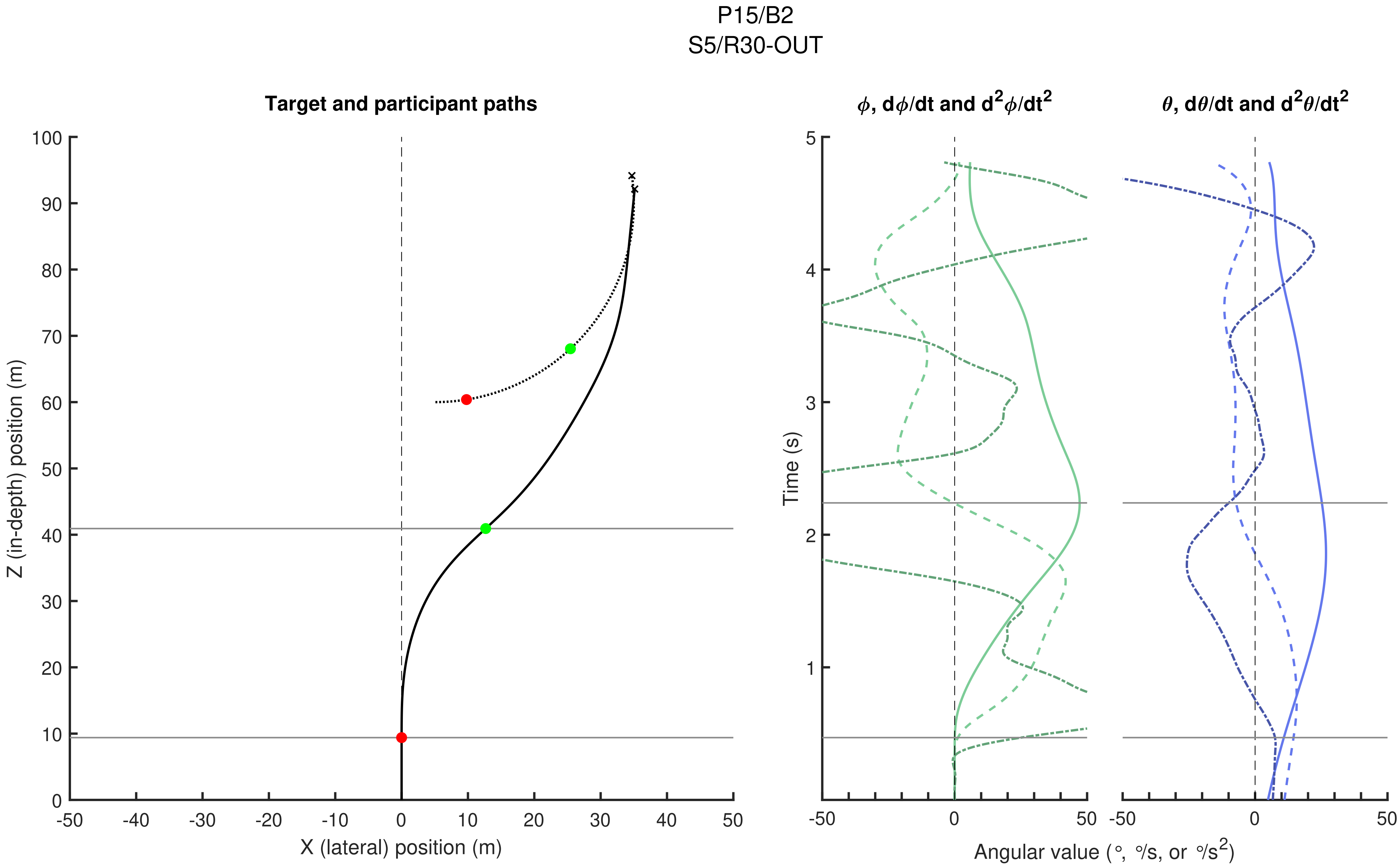

P15/B2
S5/R30-OUT
Target and participant paths
Z (in-depth) position (m)
X (lateral) position (m)
100
90
80
70
60
50
40
30
20
10
0
-50
-40
-30
-20
-10
0
10
20
30
40
50
ϕ, dϕ/dt and d$^2\phi$/dt^2
θ, dθ/dt and d$^2\theta$/dt^2
Time (s)
5
4
3
2
1
-50
0
50
-50
0
50
Angular value (°, °/s, or °/s^2)

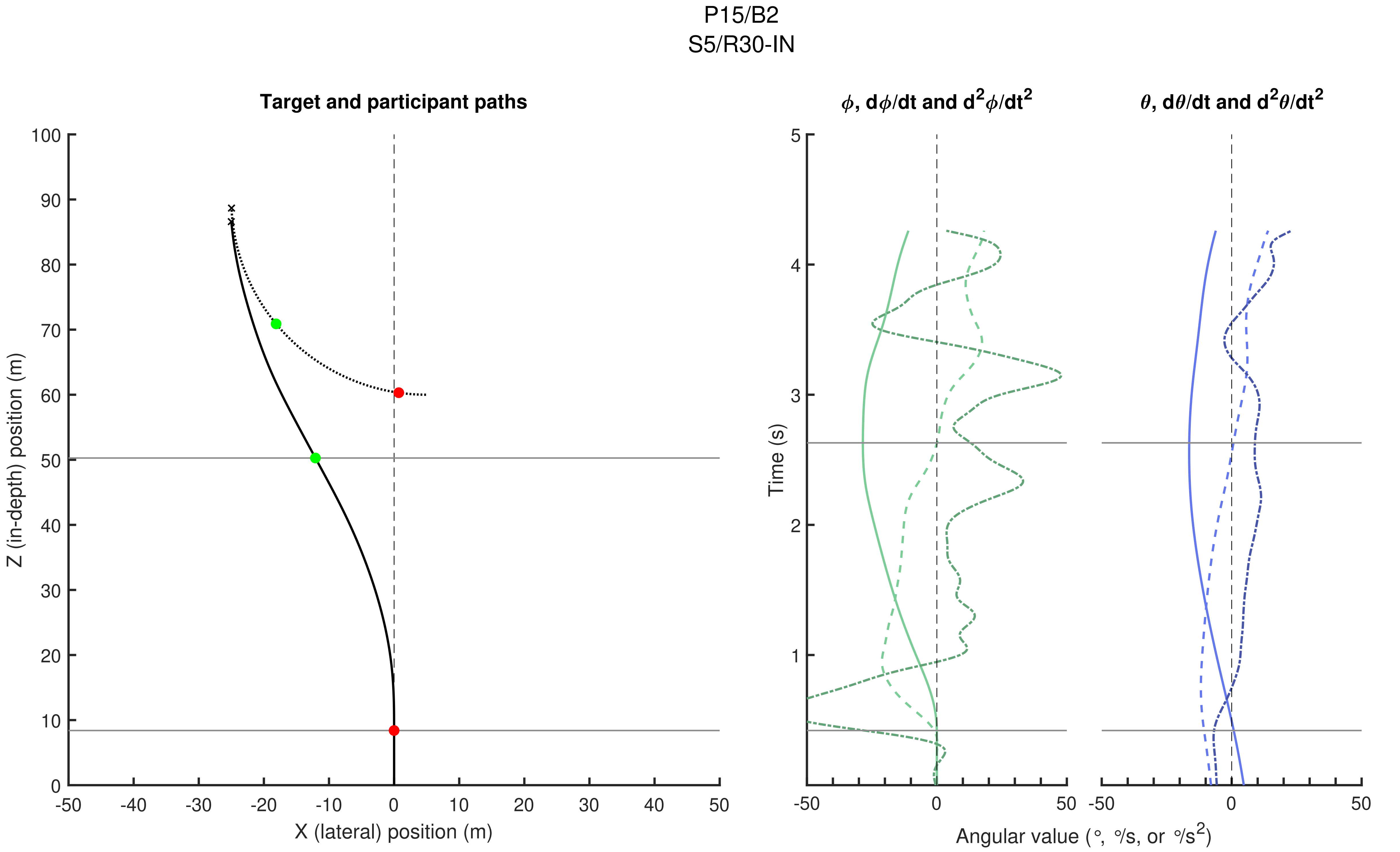
P15/B2
S5/R30-IN
Target and participant paths
Z (in-depth) position (m)
X (lateral) position (m)
ϕ, dϕ/dt and d²ϕ/dt²
θ, dθ/dt and d²θ/dt²
Time (s)
Angular value (°, °/s, or °/s²)

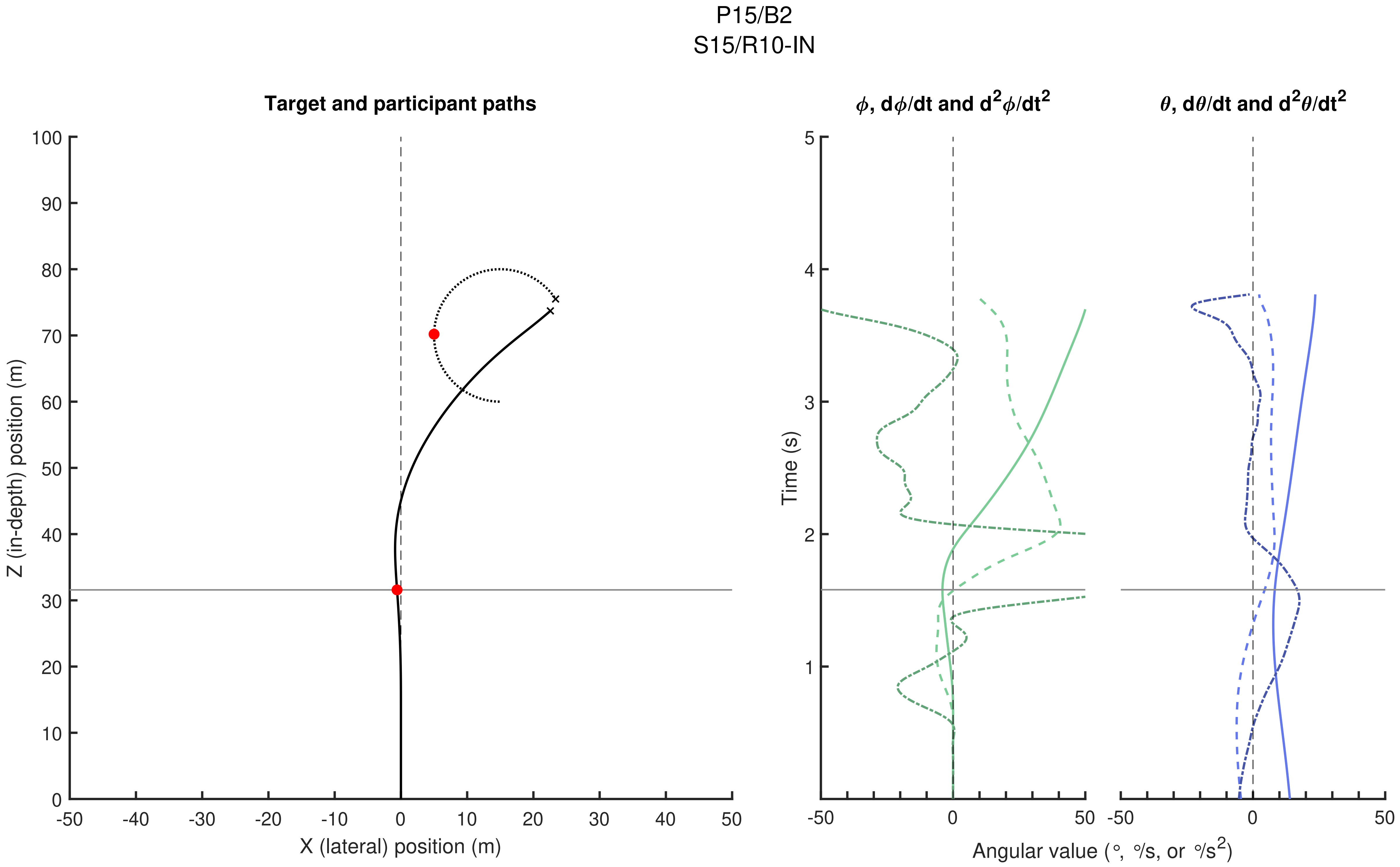

P15/B2
S15/R10-IN
Target and participant paths
Z (in-depth) position (m)
X (lateral) position (m)
ϕ, dϕ/dt and d²ϕ/dt²
θ, dθ/dt and d²θ/dt²
Time (s)
Angular value (°, °/s, or °/s²)

P15/B2
S15/R20-IN

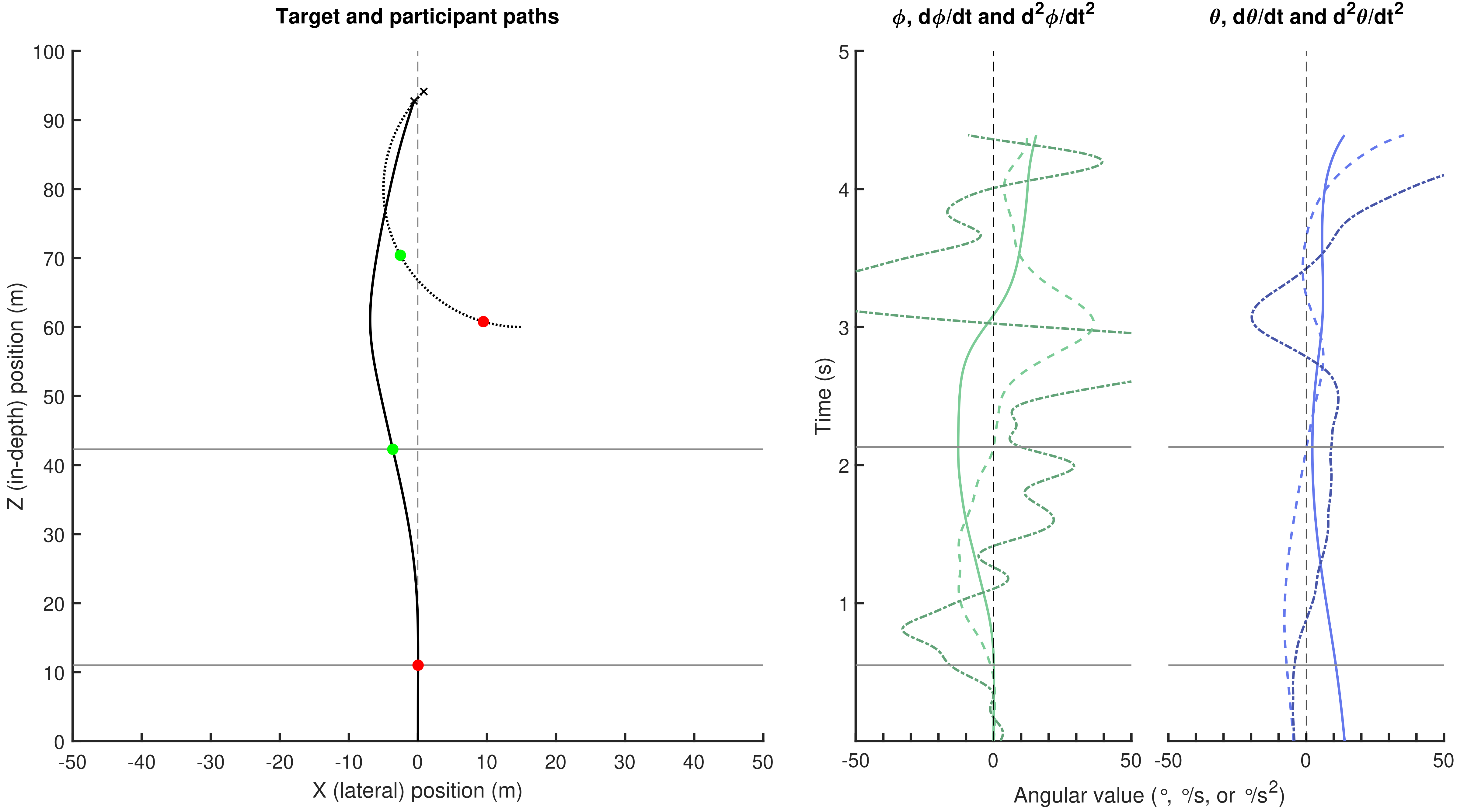

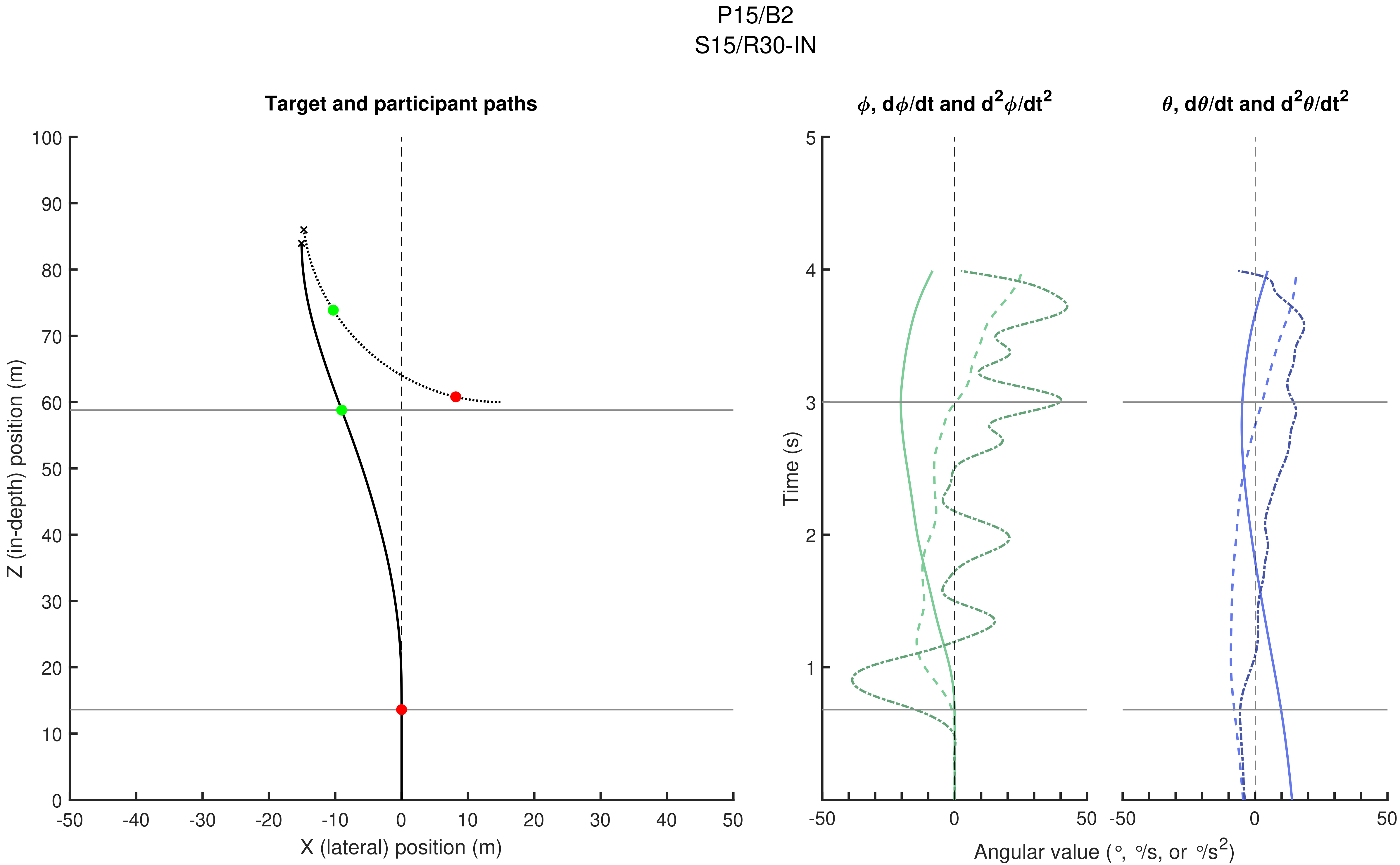

P15/B2
S15/R30-IN
Target and participant paths
Z (in-depth) position (m)
X (lateral) position (m)
ϕ, dϕ/dt and d²ϕ/dt²
θ, dθ/dt and d²θ/dt²
Time (s)
Angular value (°, °/s, or °/s²)

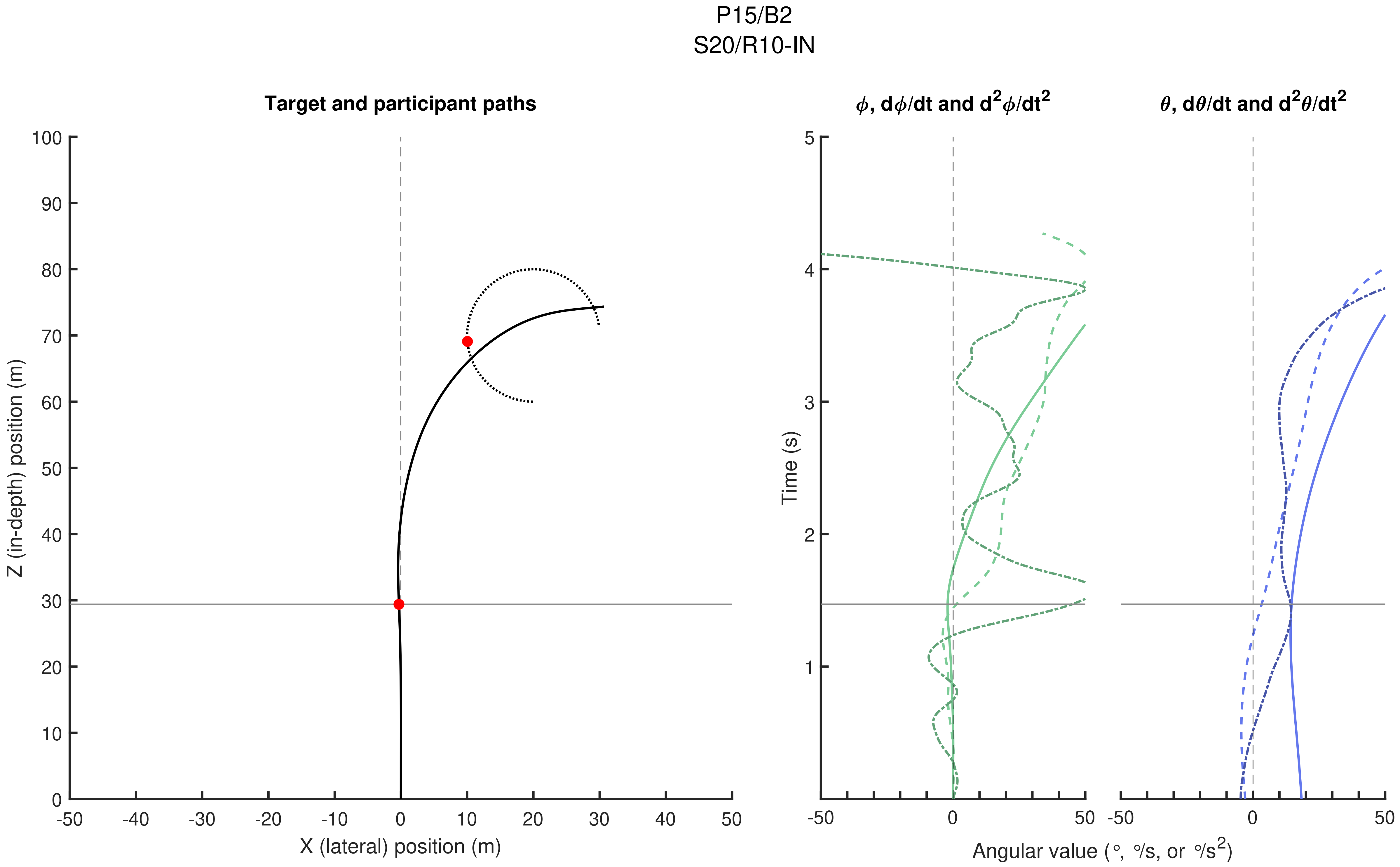
P15/B2
S20/R10-IN
Target and participant paths
ϕ, dϕ/dt and d$^2\phi$/dt^2
θ, dθ/dt and d$^2\theta$/dt^2
Z (in-depth) position (m)
X (lateral) position (m)
Time (s)
Angular value (°, °/s, or °/s^2)
100
90
80
70
60
50
40
30
20
10
0
-50
-40
-30
-20
-10
0
10
20
30
40
50
5
4
3
2
1
-50
0
50
-50
0
50

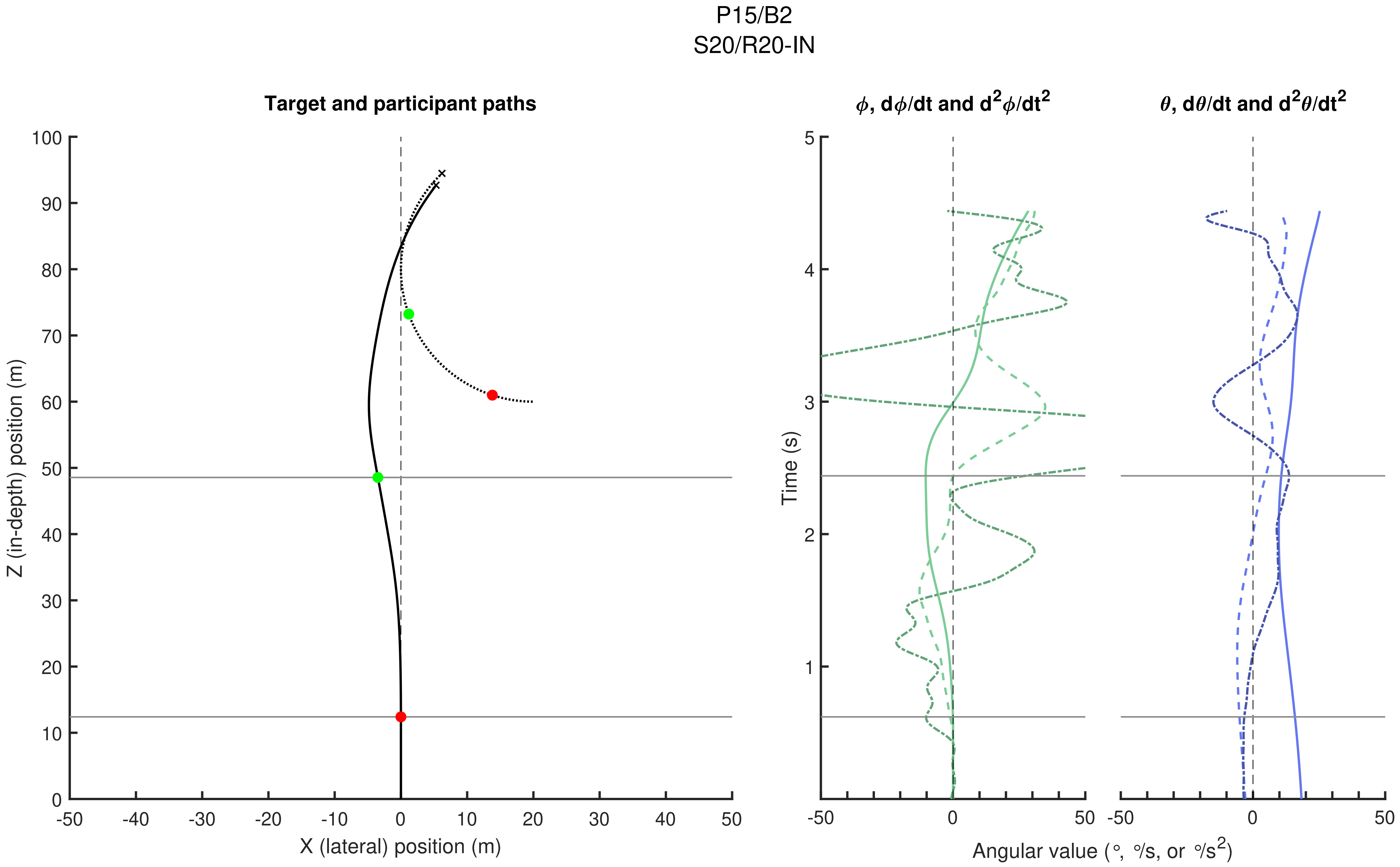
P15/B2
S20/R20-IN
Target and participant paths
Z (in-depth) position (m)
X (lateral) position (m)
ϕ, dϕ/dt and d$^2\phi$/dt^2
θ, dθ/dt and d$^2\theta$/dt^2
Time (s)
Angular value (°, °/s, or °/s^2)

P15/B2
S20/R30-IN

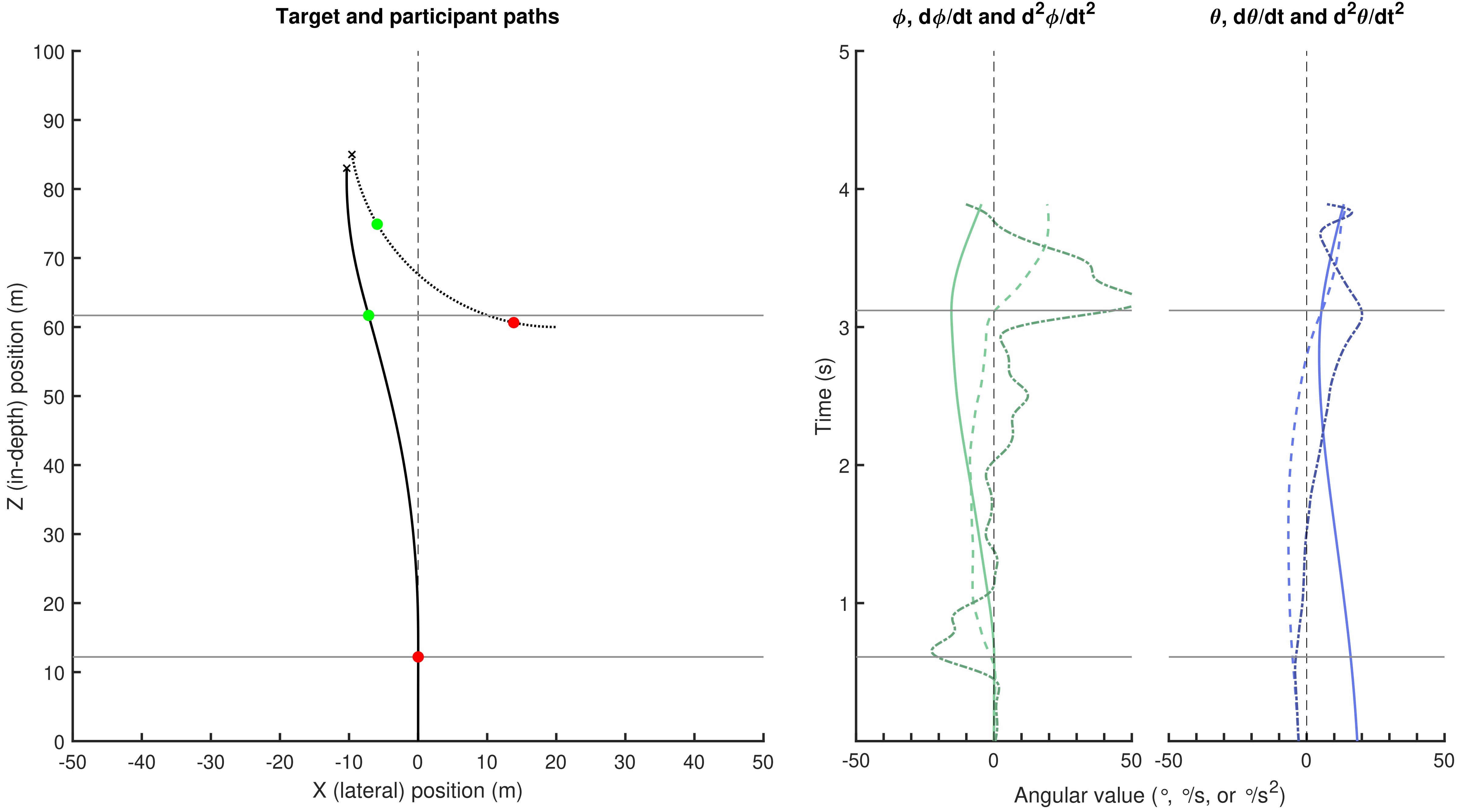

P15/B3
S20/R10-IN

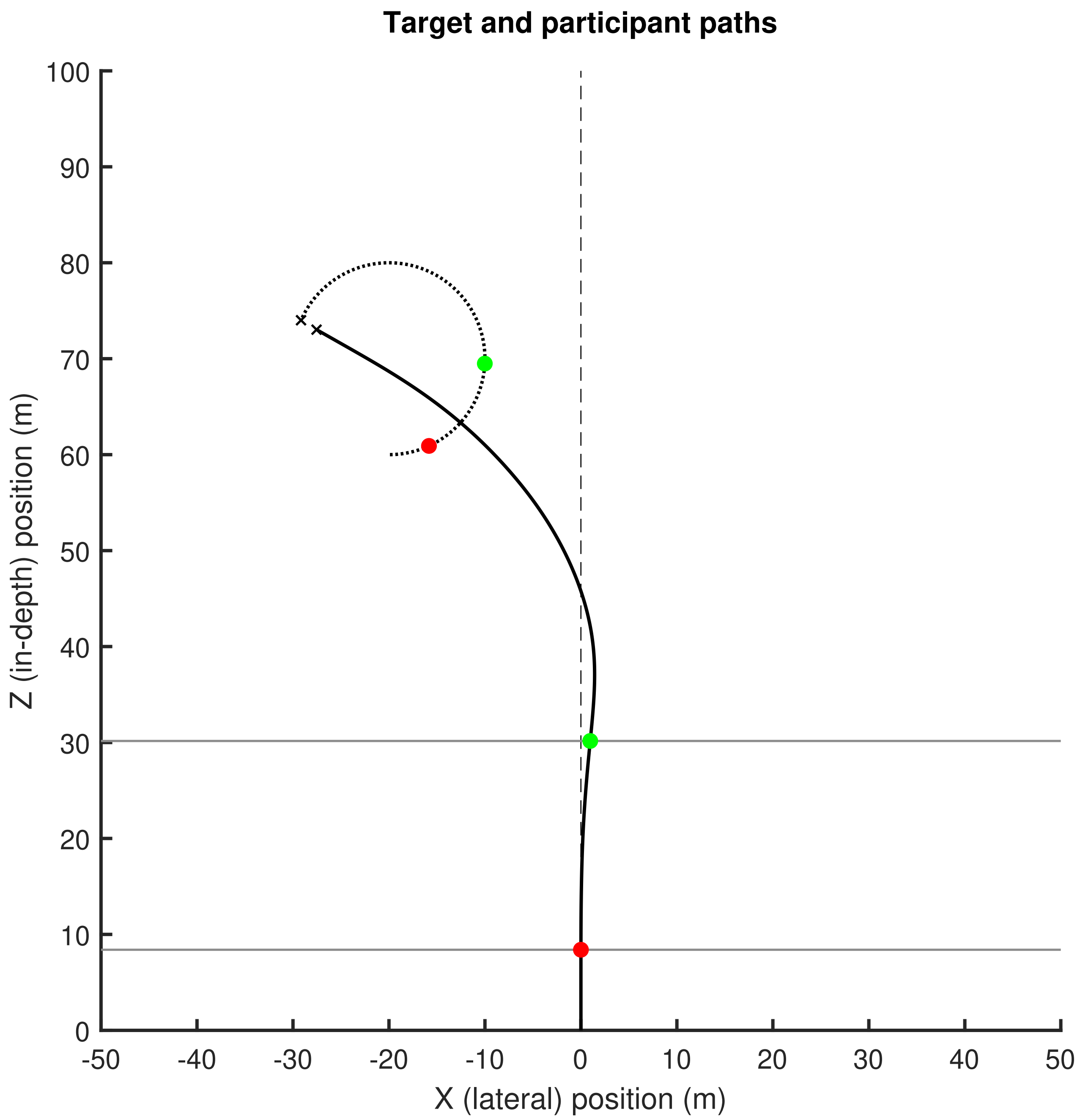

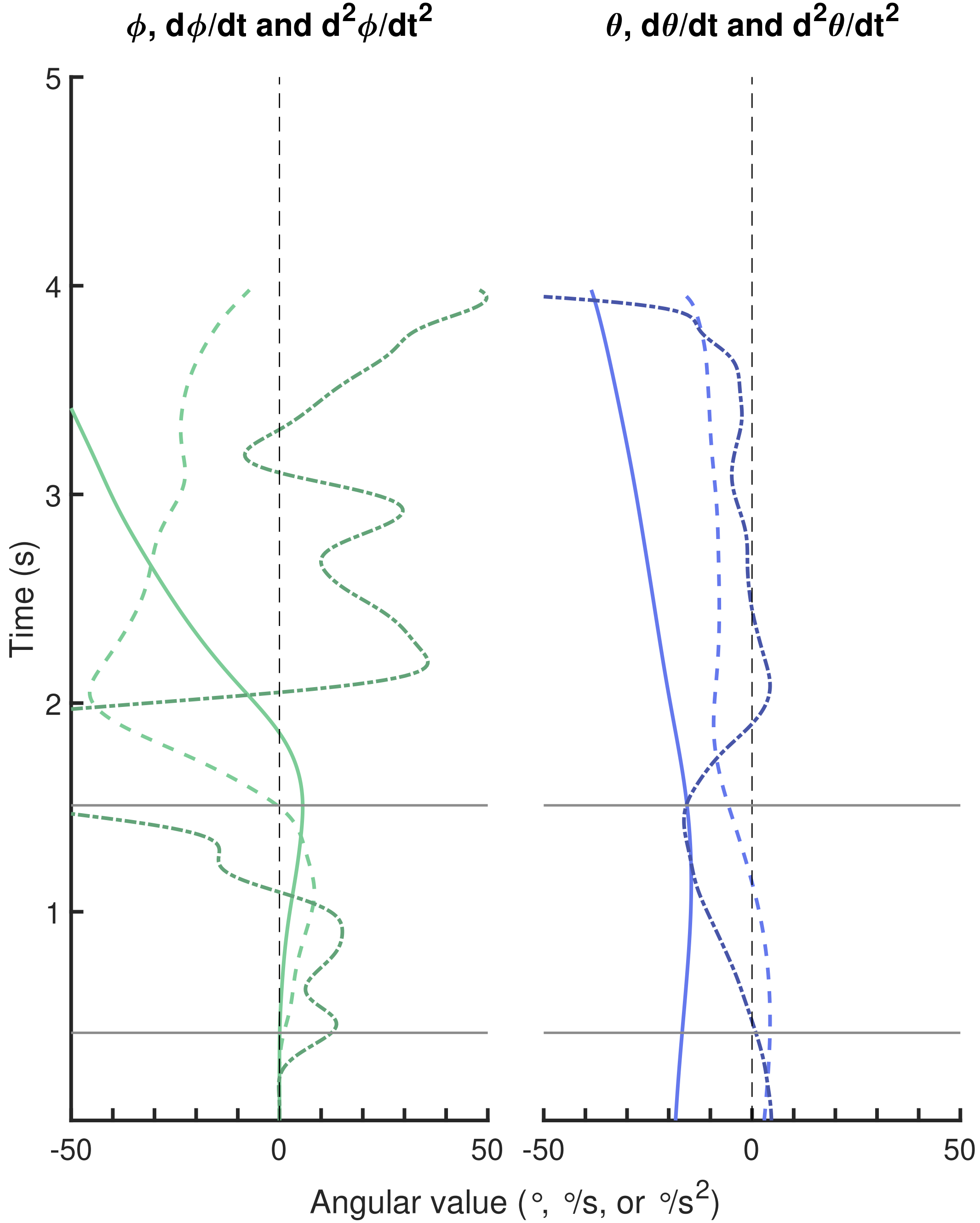

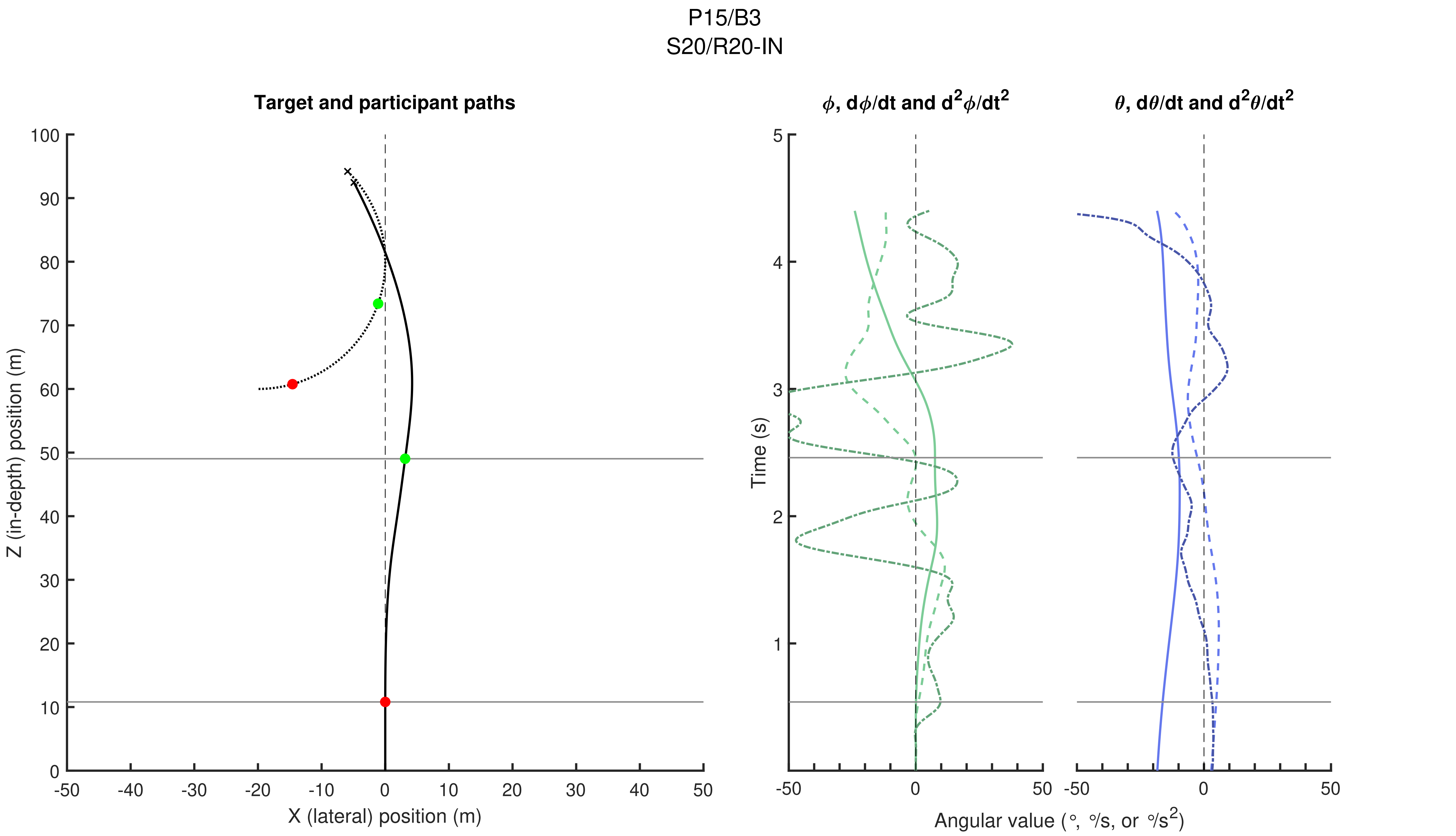
P15/B3
S20/R20-IN
Target and participant paths
Z (in-depth) position (m)
X (lateral) position (m)
100
90
80
70
60
50
40
30
20
10
0
-50
-40
-30
-20
-10
0
10
20
30
40
50
ϕ, dϕ/dt and d$^2\phi$/dt^2
θ, dθ/dt and d$^2\theta$/dt^2
Time (s)
5
4
3
2
1
-50
0
50
-50
0
50
Angular value (°, °/s, or °/s^2)

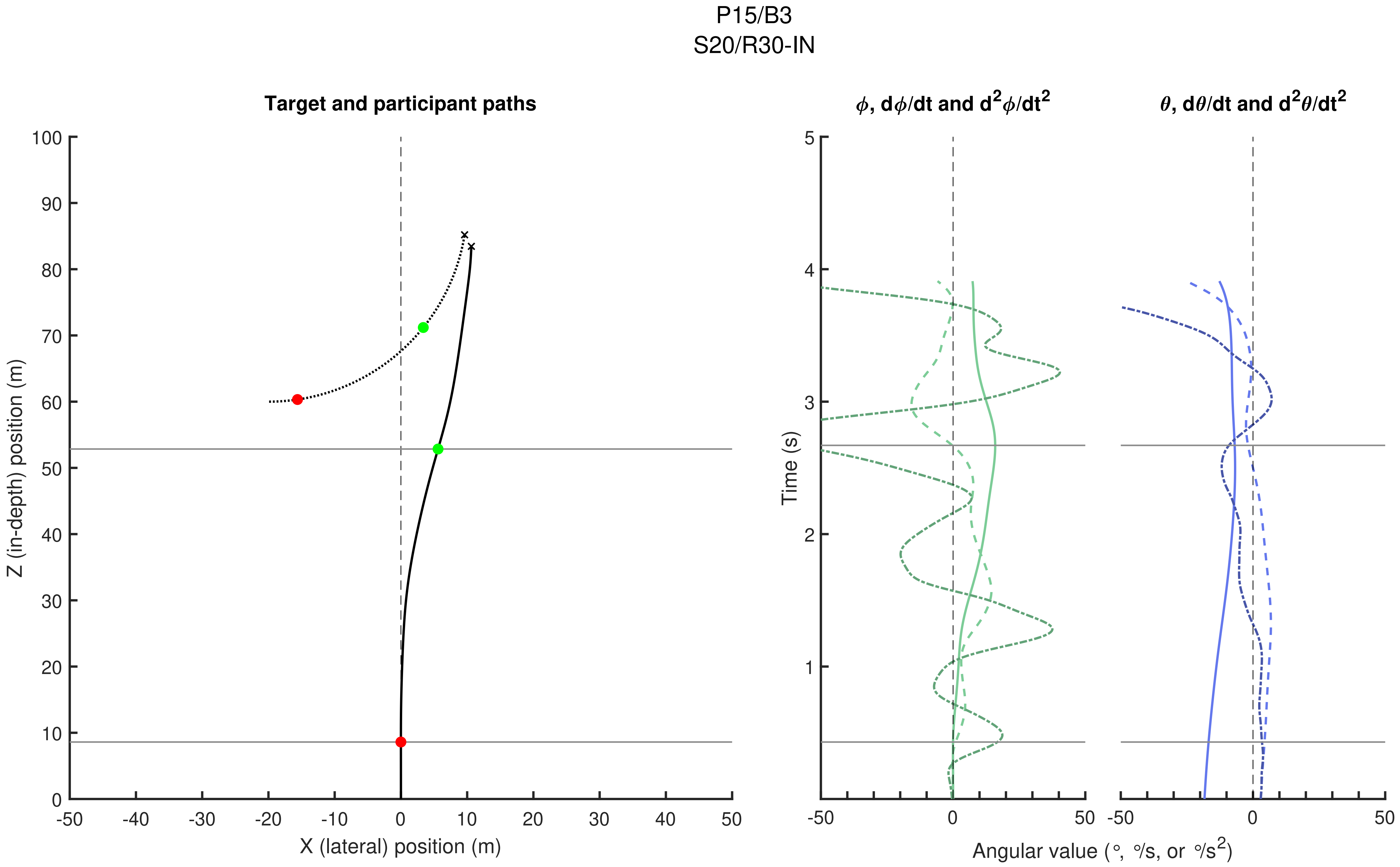
P15/B3
S20/R30-IN
Target and participant paths
Z (in-depth) position (m)
X (lateral) position (m)
100
90
80
70
60
50
40
30
20
10
0
-50
-40
-30
-20
-10
0
10
20
30
40
50
ϕ, dϕ/dt and d²ϕ/dt²
θ, dθ/dt and d²θ/dt²
Time (s)
5
4
3
2
1
-50
0
50
-50
0
50
Angular value (°, °/s, or °/s²)

P15/B3
S15/R10-IN

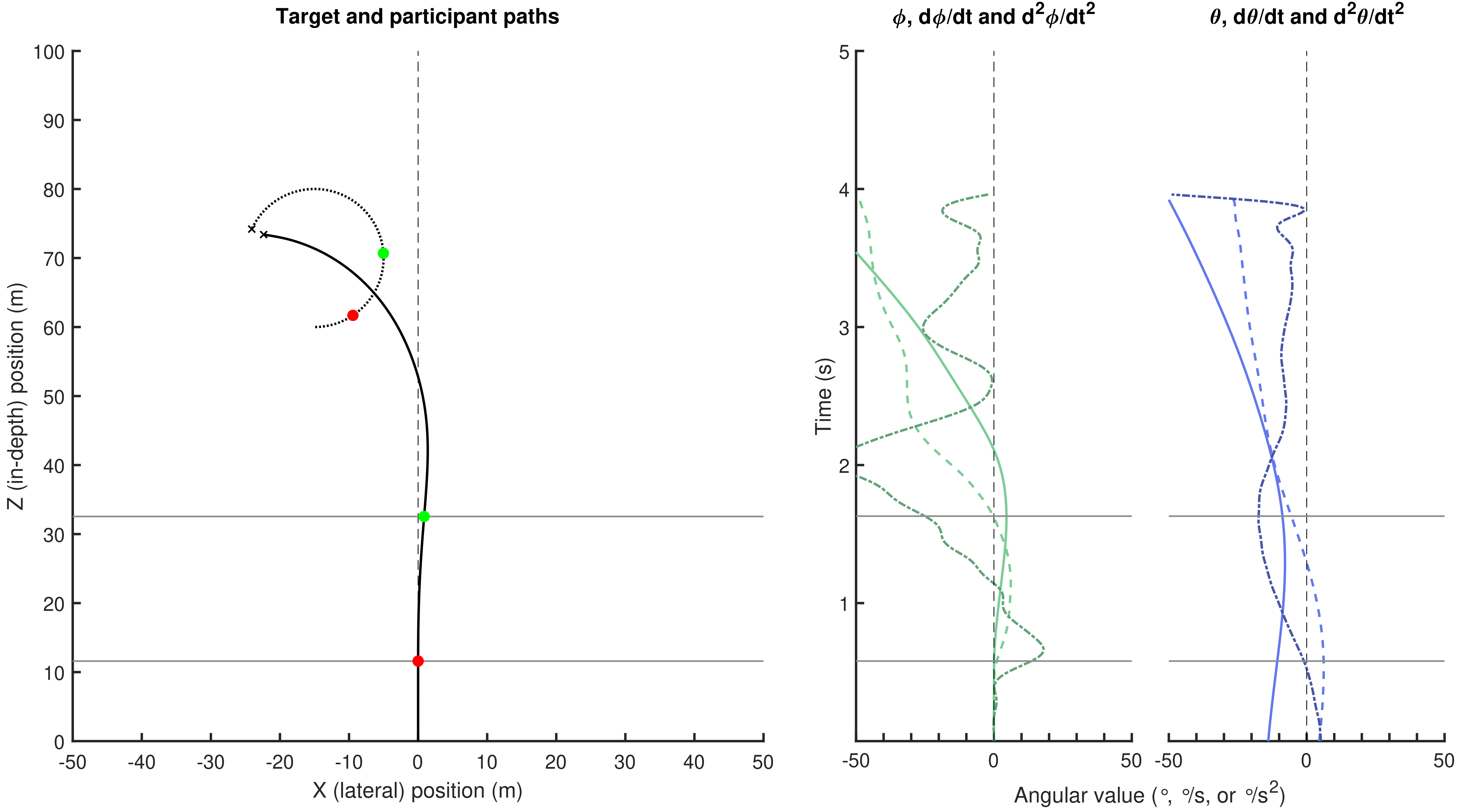

P15/B3
S15/R20-IN

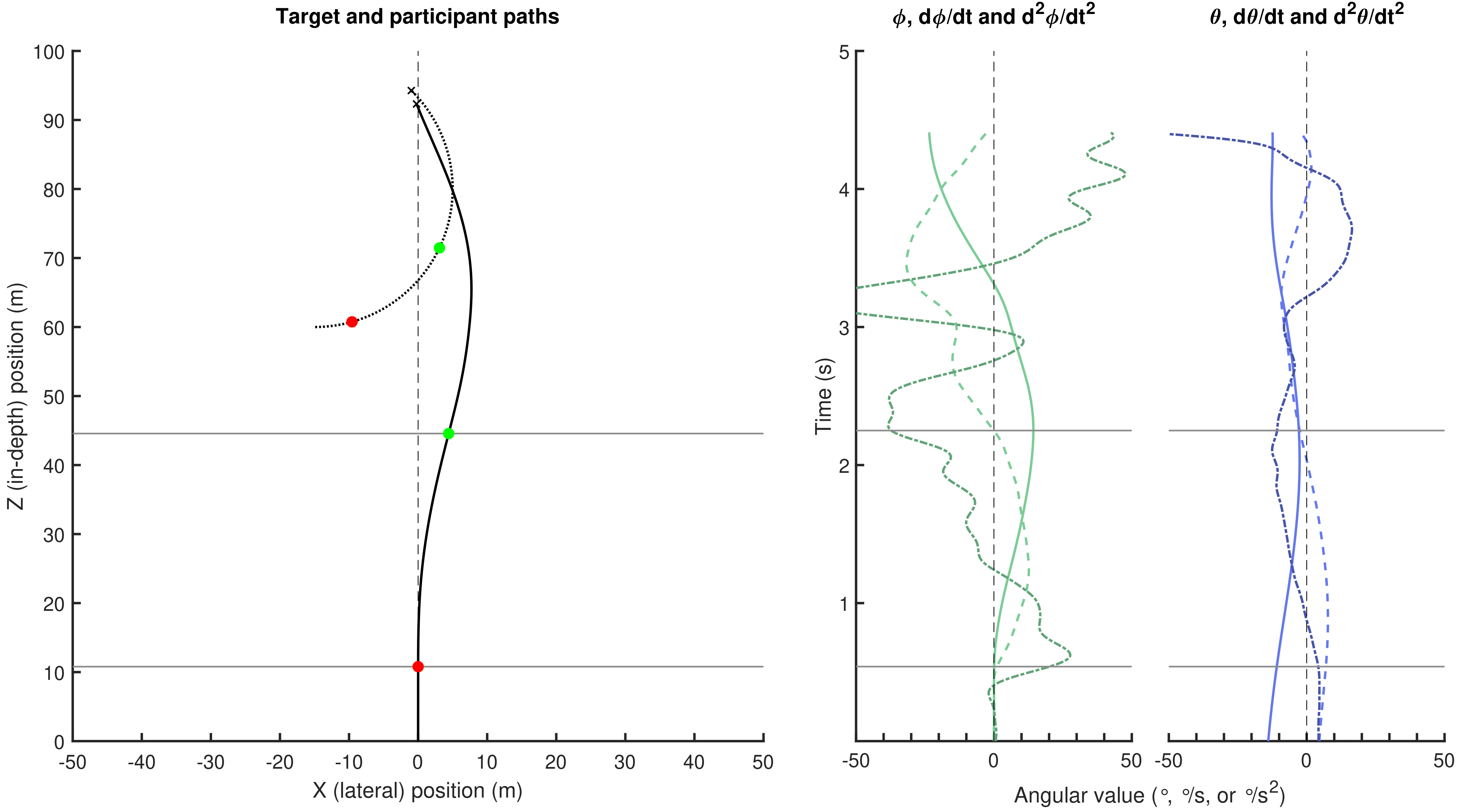

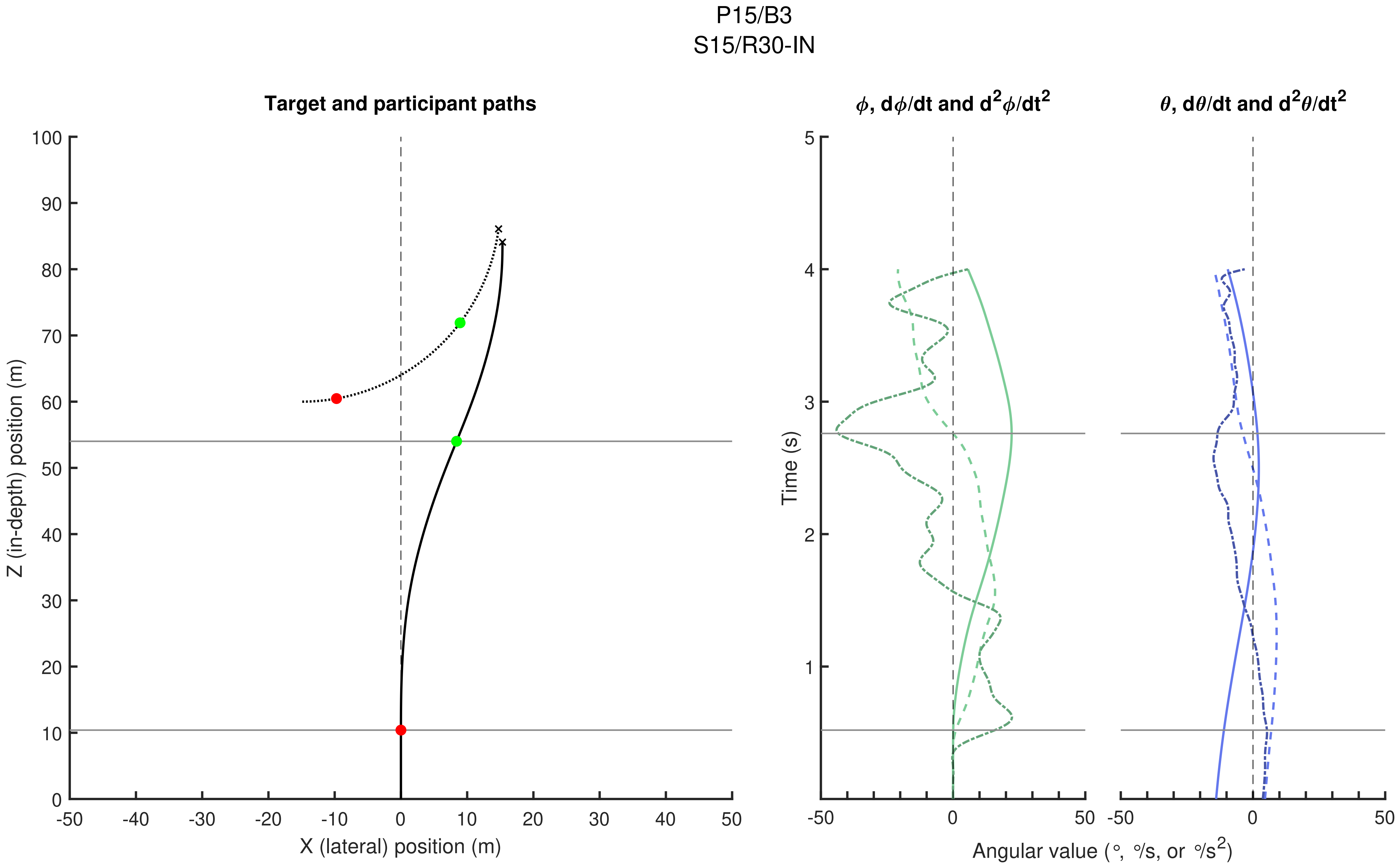
P15/B3
S15/R30-IN
Target and participant paths
Z (in-depth) position (m)
X (lateral) position (m)
φ, dφ/dt and d²φ/dt²
θ, dθ/dt and d²θ/dt²
Time (s)
Angular value (°, °/s, or °/s²)

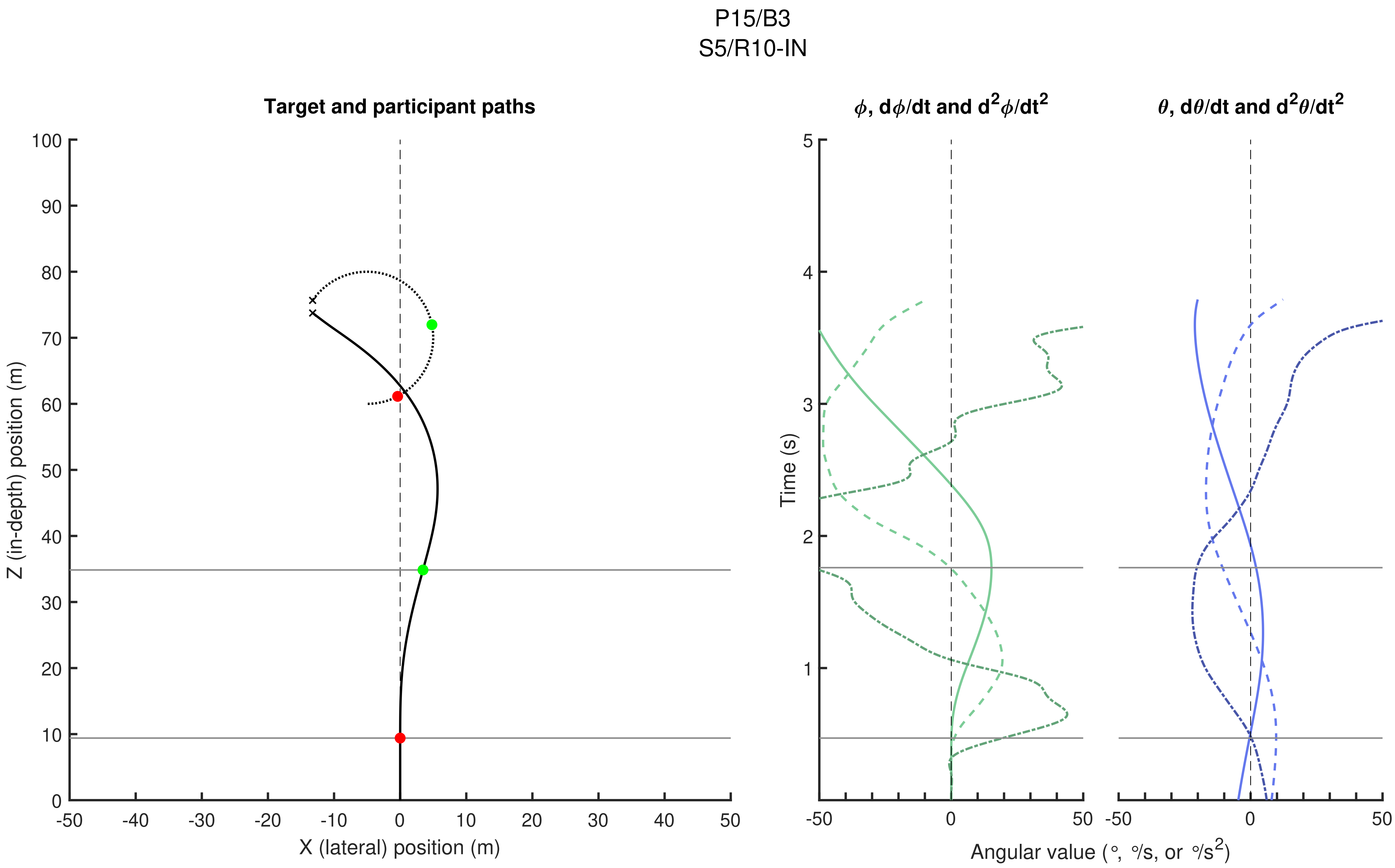
P15/B3
S5/R10-IN
Target and participant paths
ϕ, dϕ/dt and d$^2\phi$/dt^2
θ, dθ/dt and d$^2\theta$/dt^2
Z (in-depth) position (m)
X (lateral) position (m)
Time (s)
Angular value (°, °/s, or °/s^2)

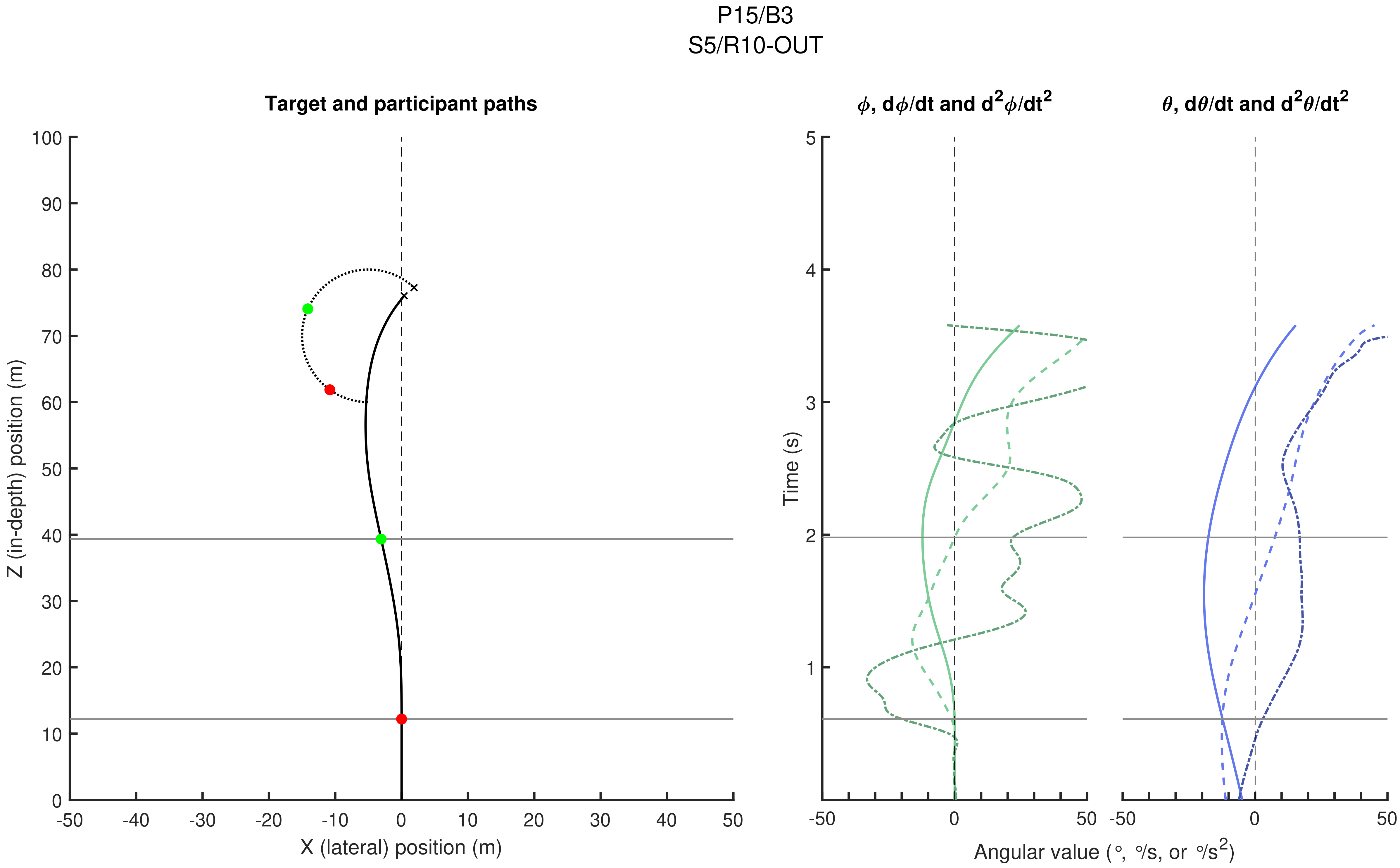
P15/B3
S5/R10-OUT
Target and participant paths
ϕ, dϕ/dt and d$^2\phi$/dt^2
θ, dθ/dt and d$^2\theta$/dt^2
Z (in-depth) position (m)
X (lateral) position (m)
Time (s)
Angular value (°, °/s, or °/s^2)

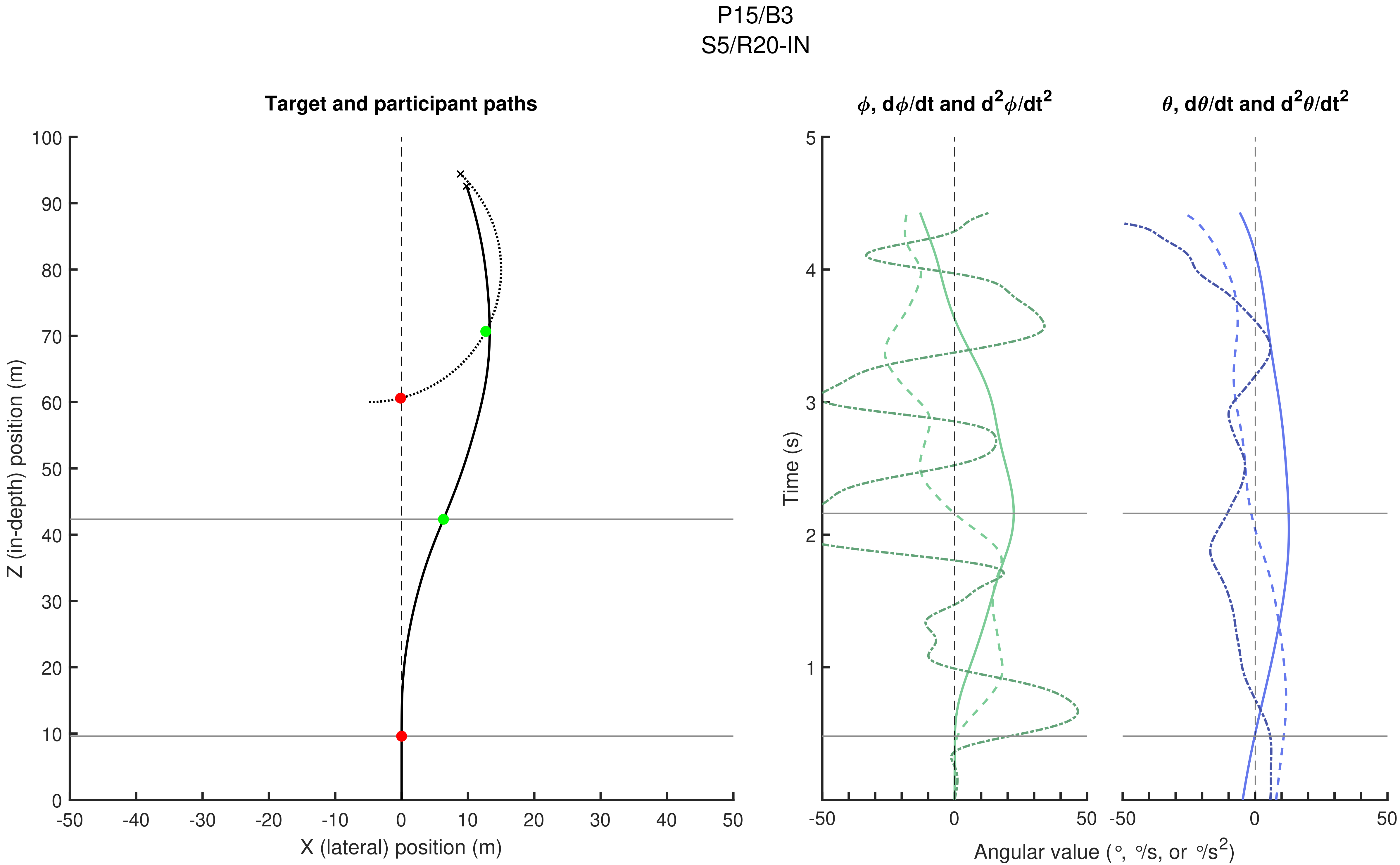
P15/B3
S5/R20-IN
Target and participant paths
Z (in-depth) position (m)
X (lateral) position (m)
0
10
20
30
40
50
60
70
80
90
100
-50
-40
-30
-20
-10
0
10
20
30
40
50
ϕ, dϕ/dt and d²ϕ/dt²
θ, dθ/dt and d²θ/dt²
Time (s)
1
2
3
4
5
-50
0
50
-50
0
50
Angular value (°, °/s, or °/s²)

P15/B3
S5/R20-OUT

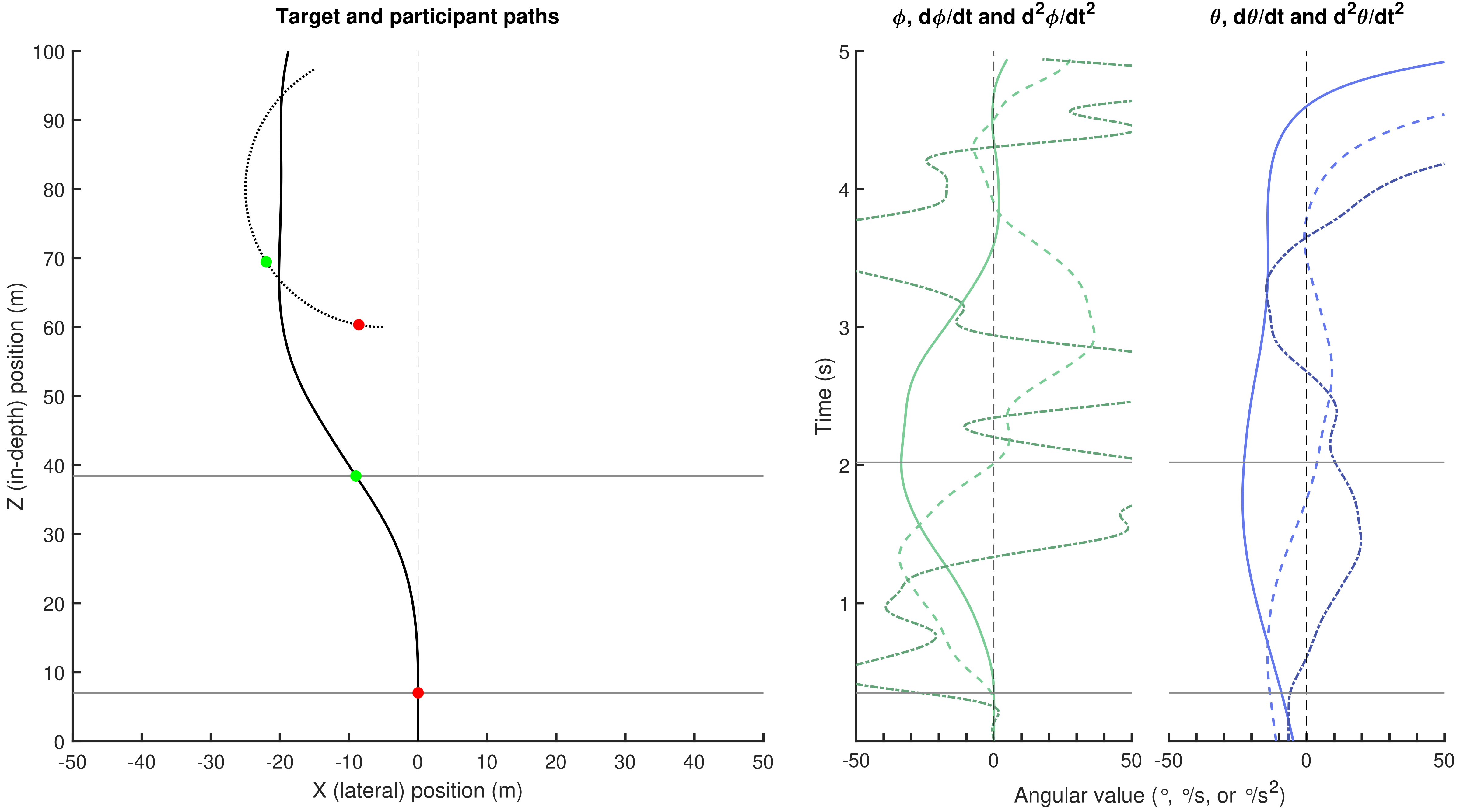

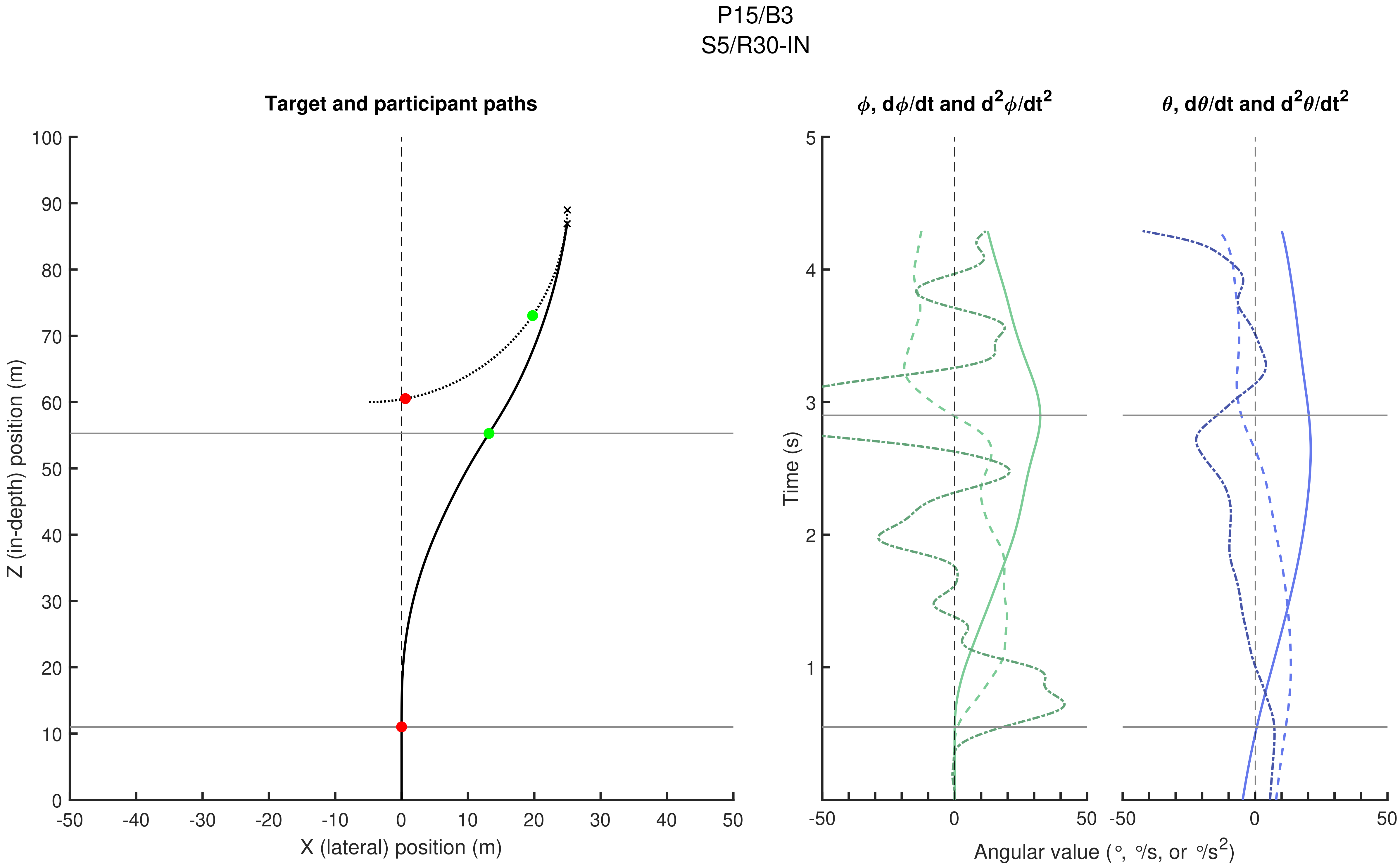
P15/B3
S5/R30-IN
Target and participant paths
Z (in-depth) position (m)
X (lateral) position (m)
ϕ, dϕ/dt and d²ϕ/dt²
θ, dθ/dt and d²θ/dt²
Time (s)
Angular value (°, °/s, or °/s²)
100
90
80
70
60
50
40
30
20
10
0
-50
-40
-30
-20
-10
0
10
20
30
40
50
5
4
3
2
1
-50
0
50
-50
0
50

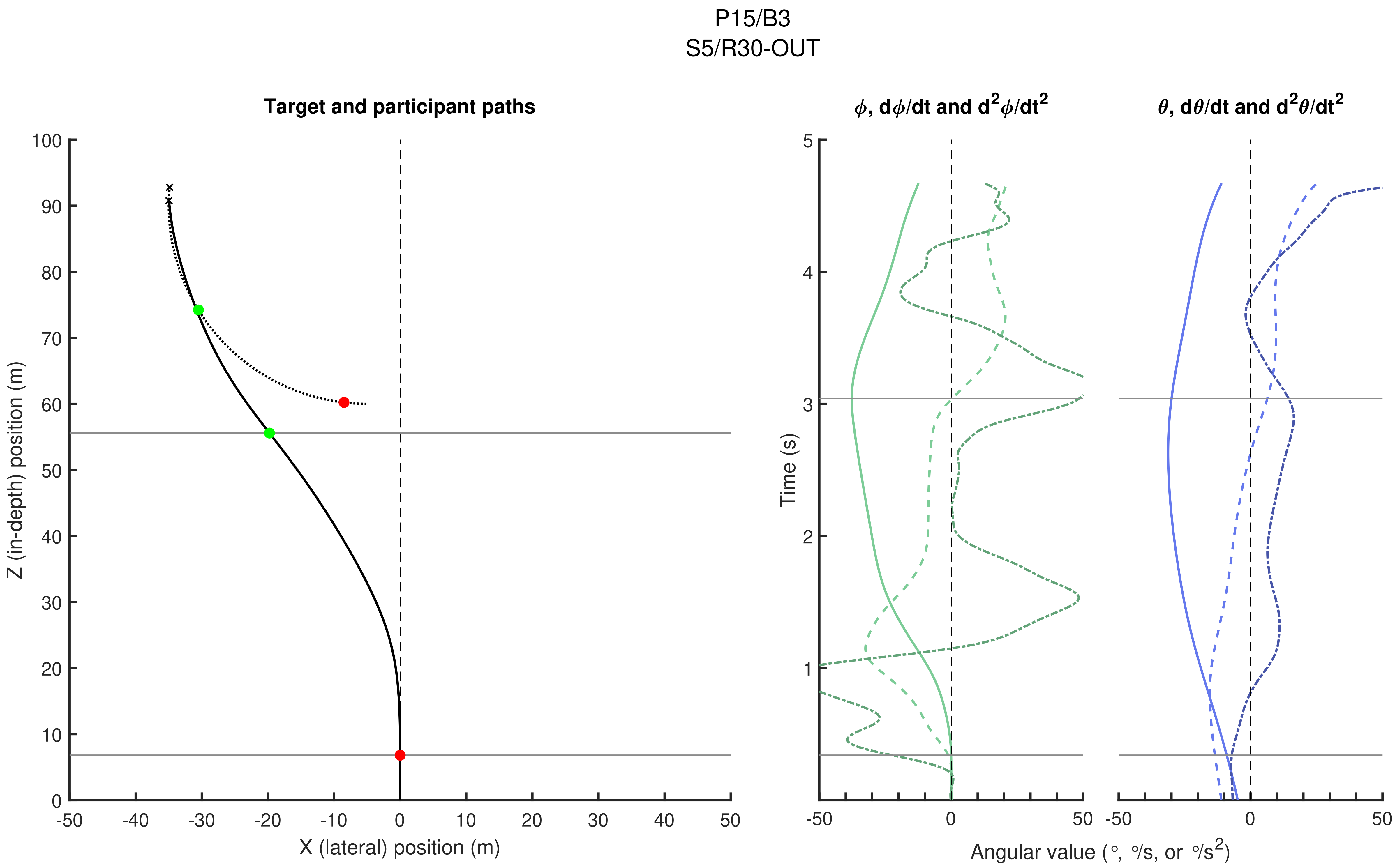

P15/B3
S5/R30-OUT
Target and participant paths
Z (in-depth) position (m)
X (lateral) position (m)
100
90
80
70
60
50
40
30
20
10
0
-50
-40
-30
-20
-10
0
10
20
30
40
50
ϕ, dϕ/dt and d$^2\phi$/dt^2
θ, dθ/dt and d$^2\theta$/dt^2
Time (s)
5
4
3
2
1
-50
0
50
-50
0
50
Angular value (°, °/s, or °/s^2)

P15/B3
S5/R10-OUT

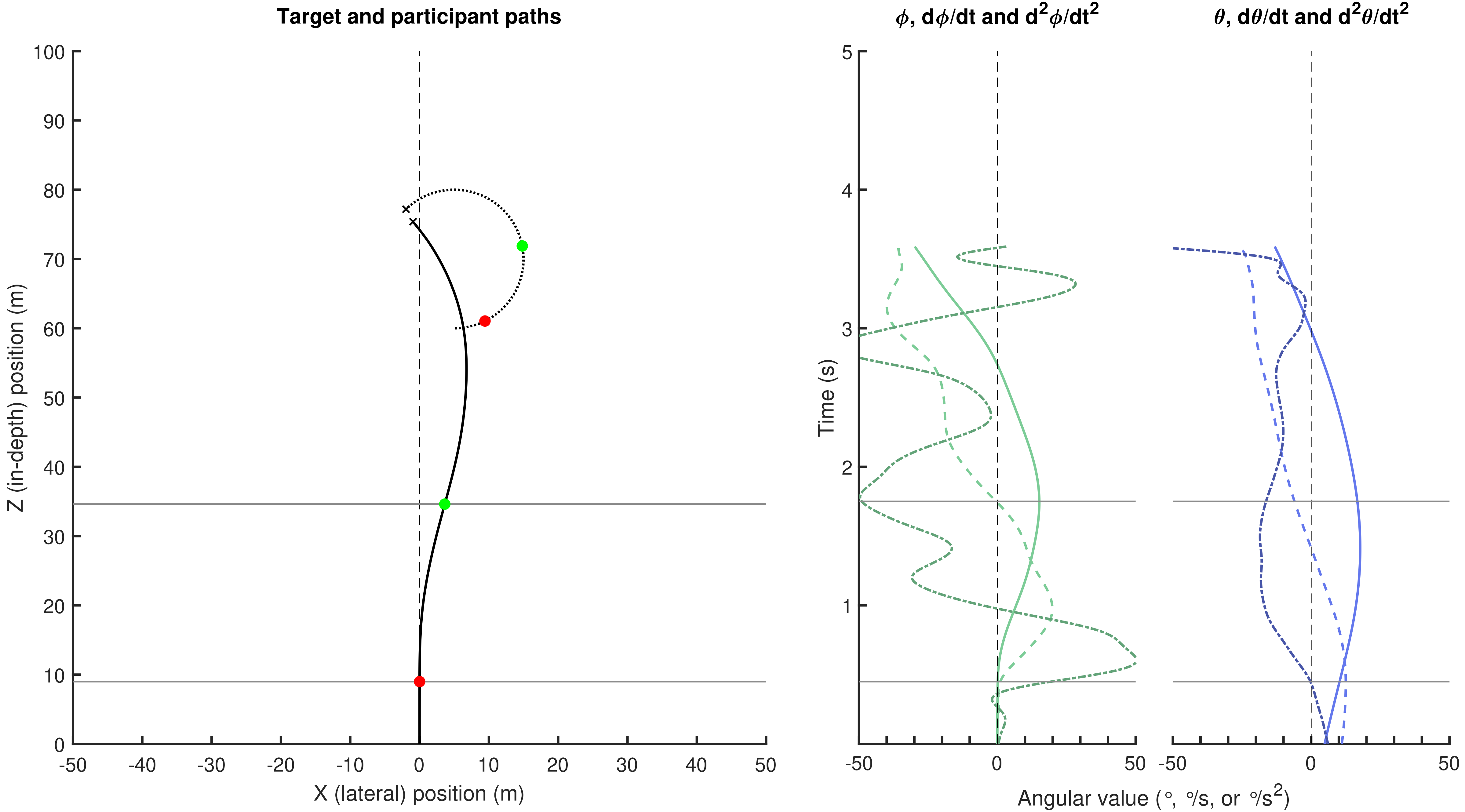

P15/B3
S5/R10-IN

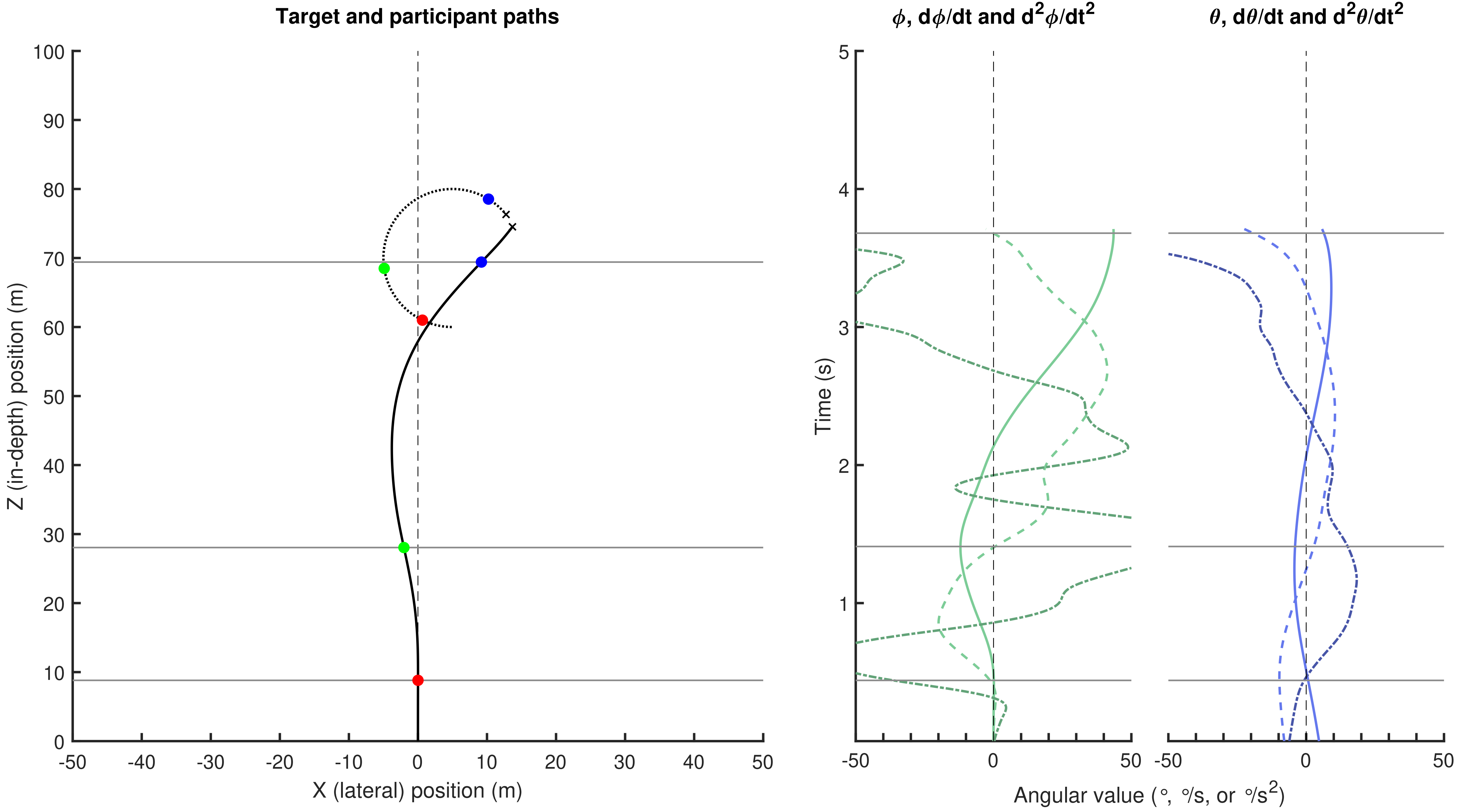

P15/B3
S5/R20-OUT

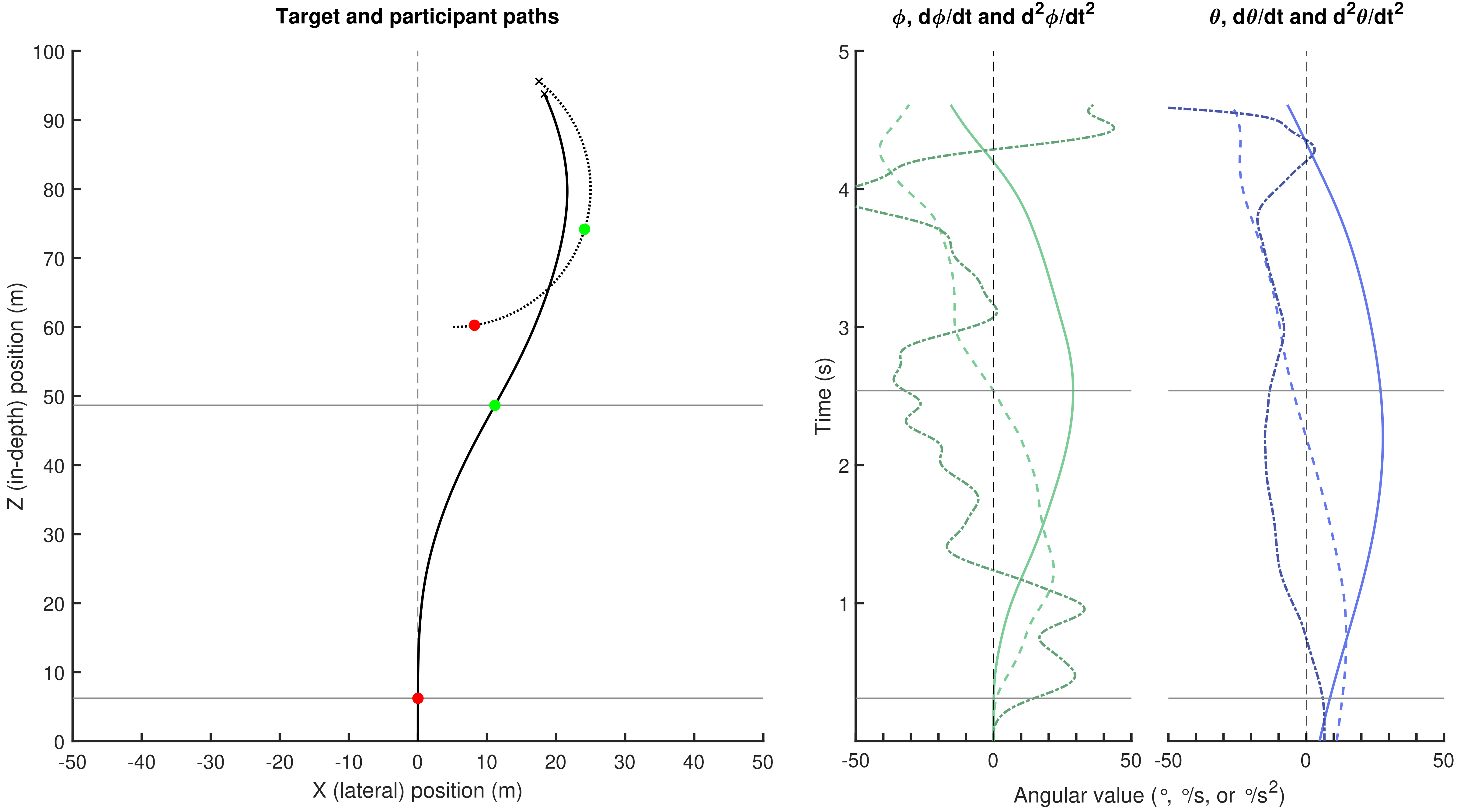

P15/B3
S5/R20-IN

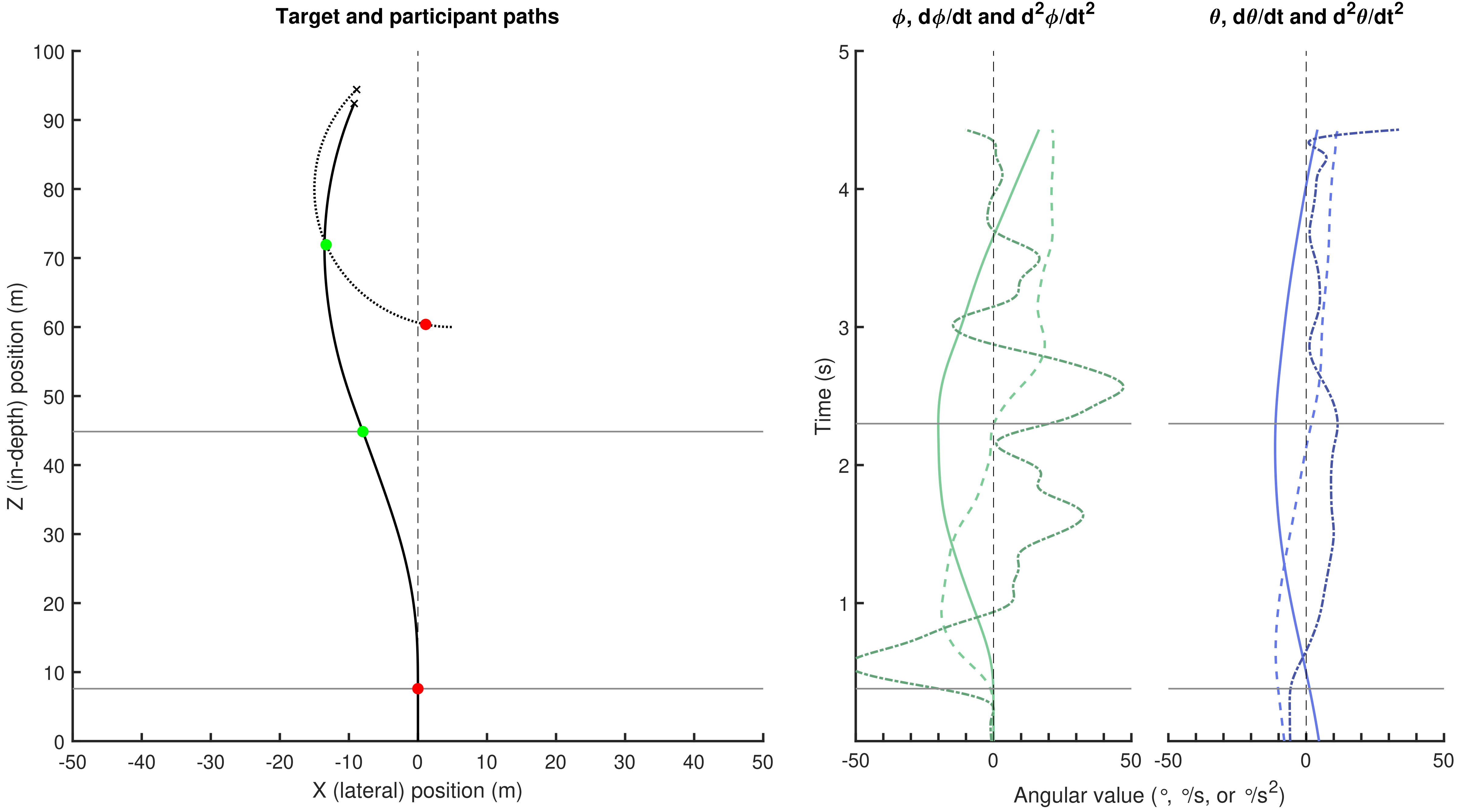

P15/B3
S5/R30-OUT

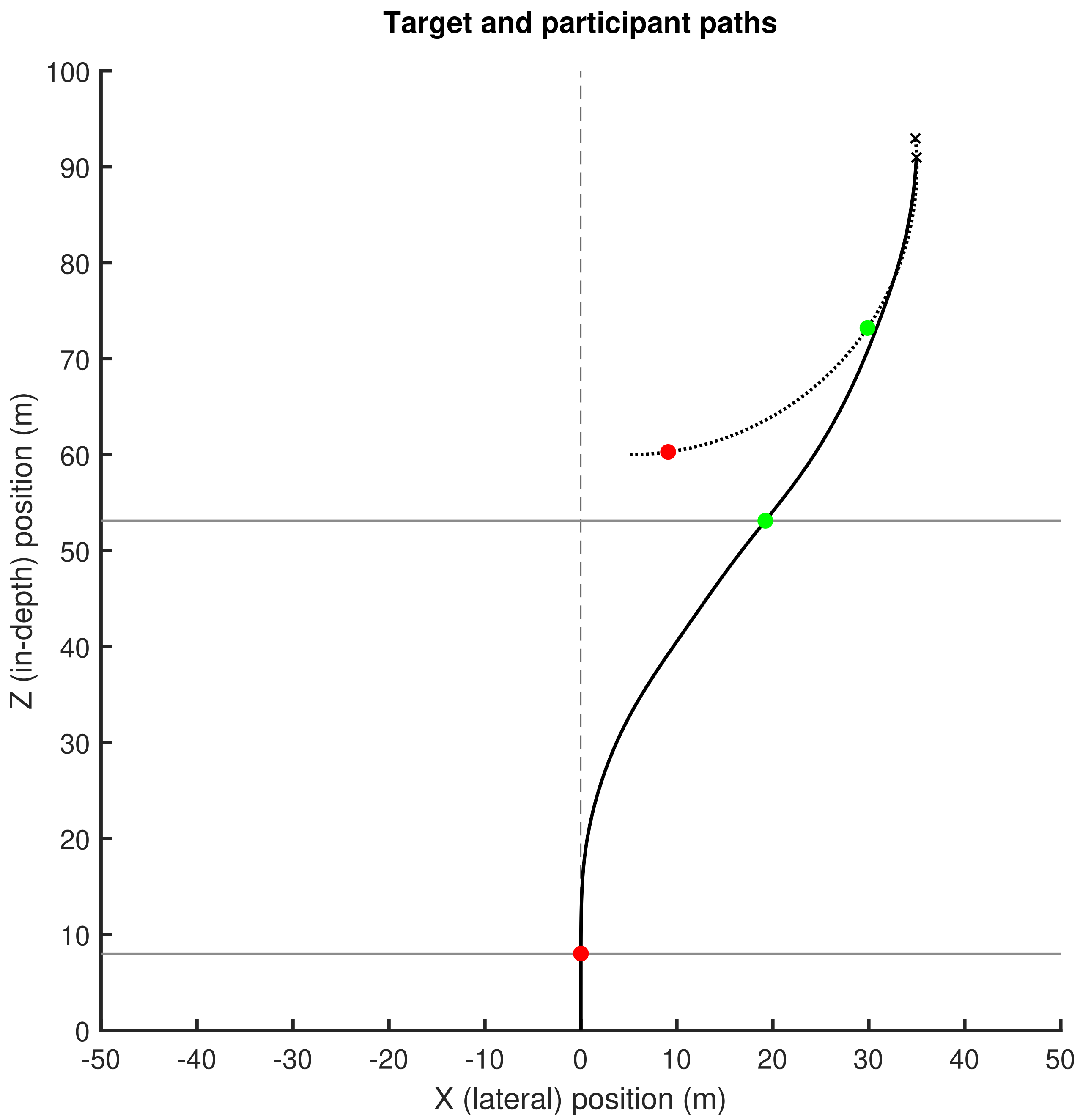

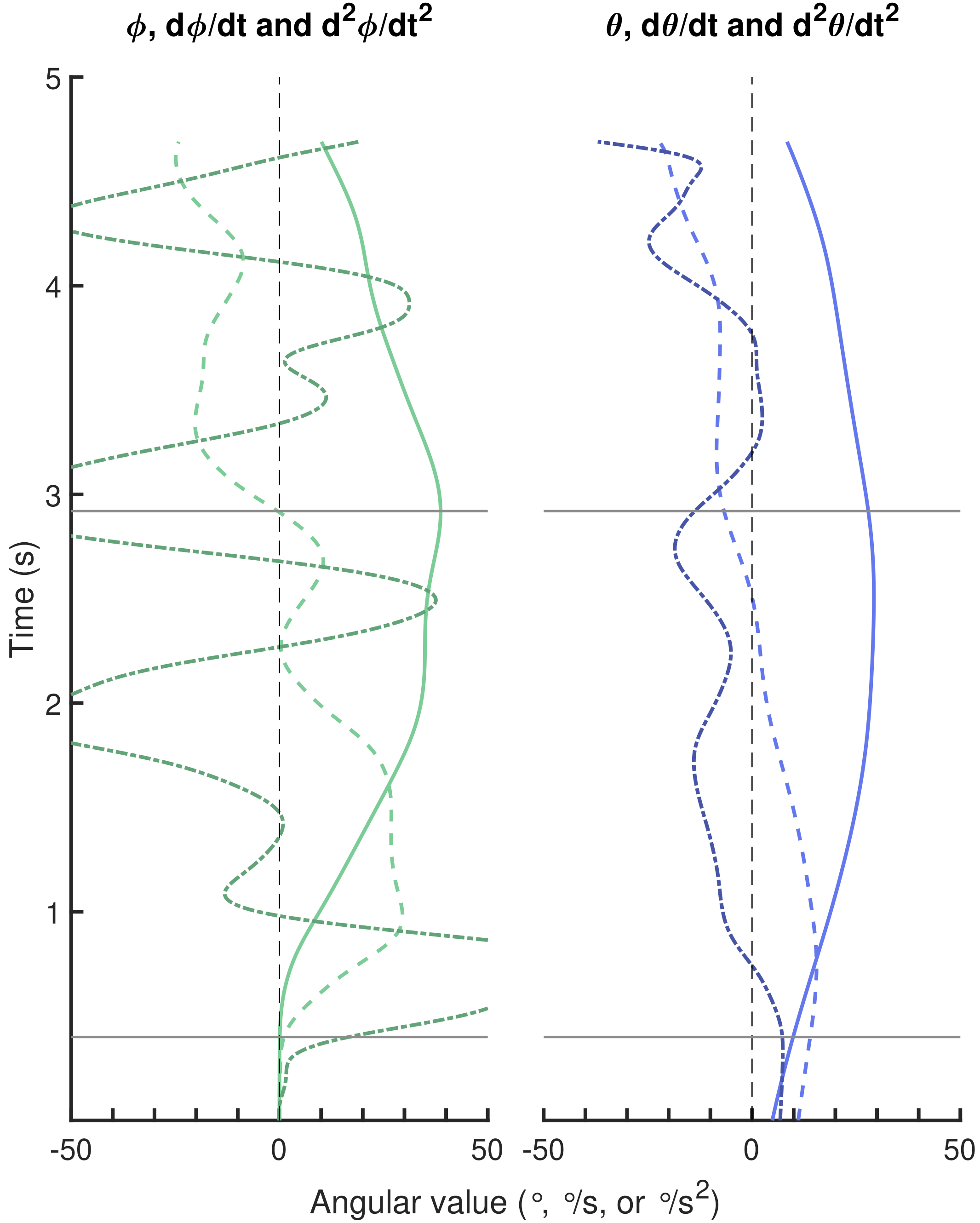

P15/B3
S5/R30-IN

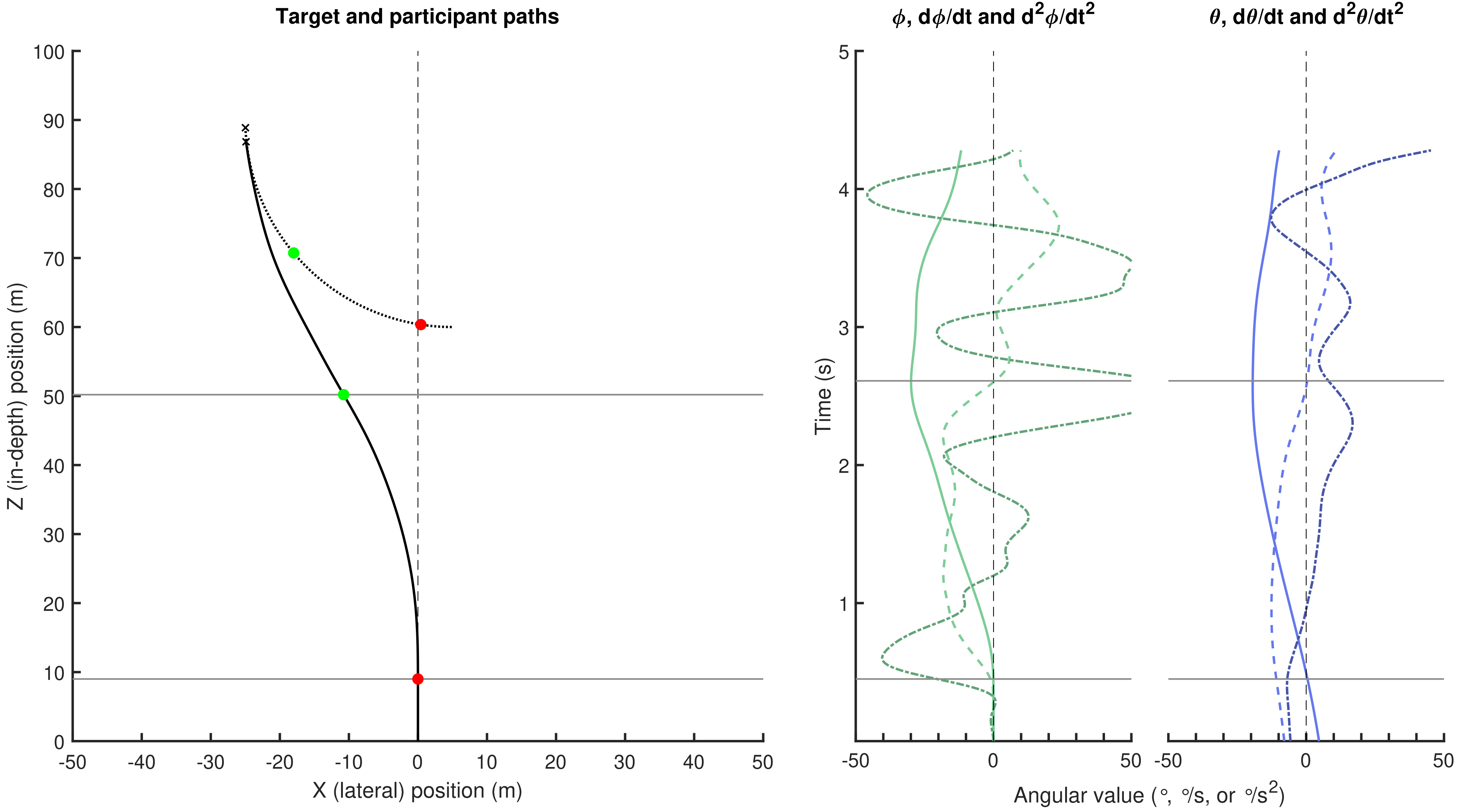

P15/B3
S15/R10-IN

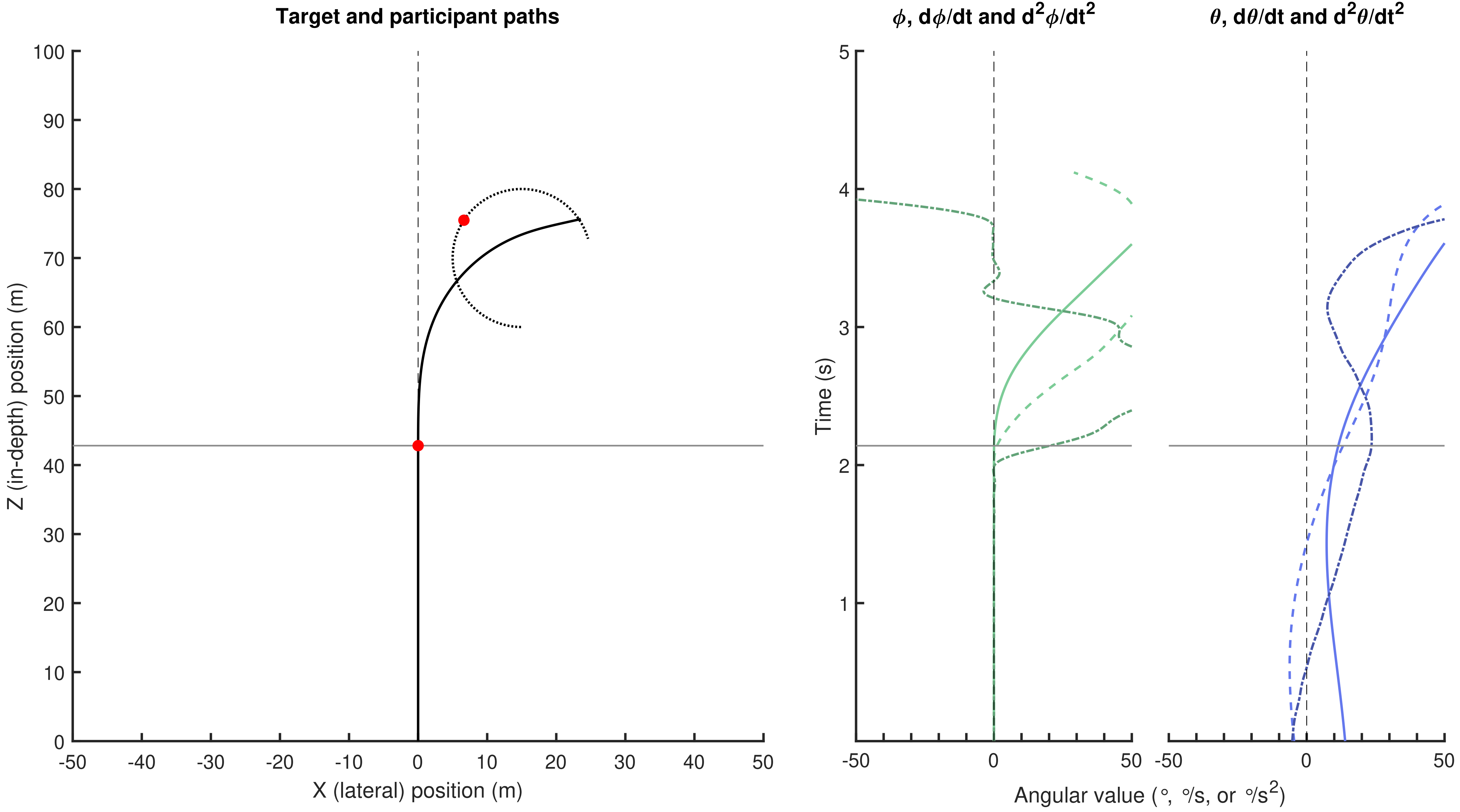

P15/B3
S15/R20-IN

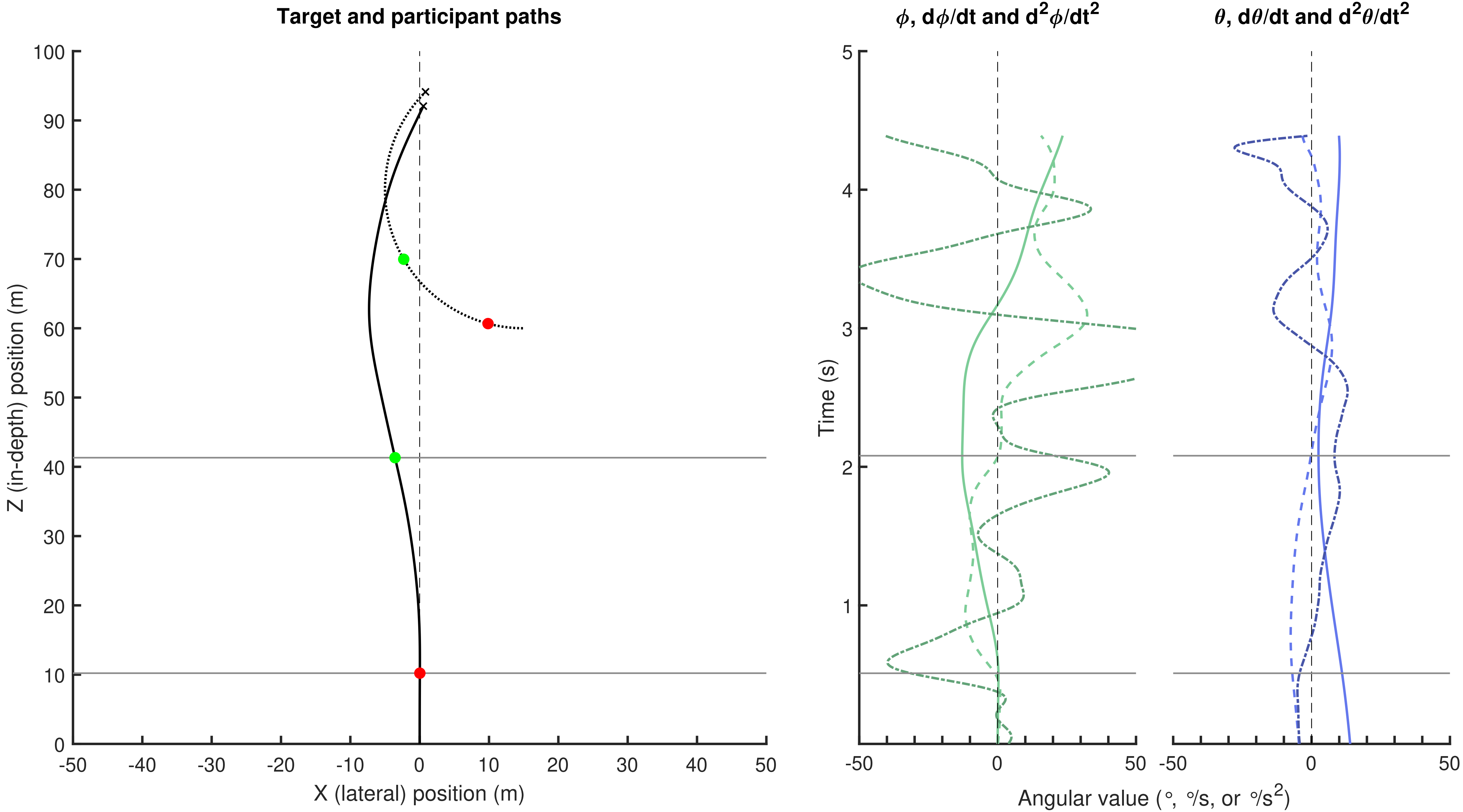

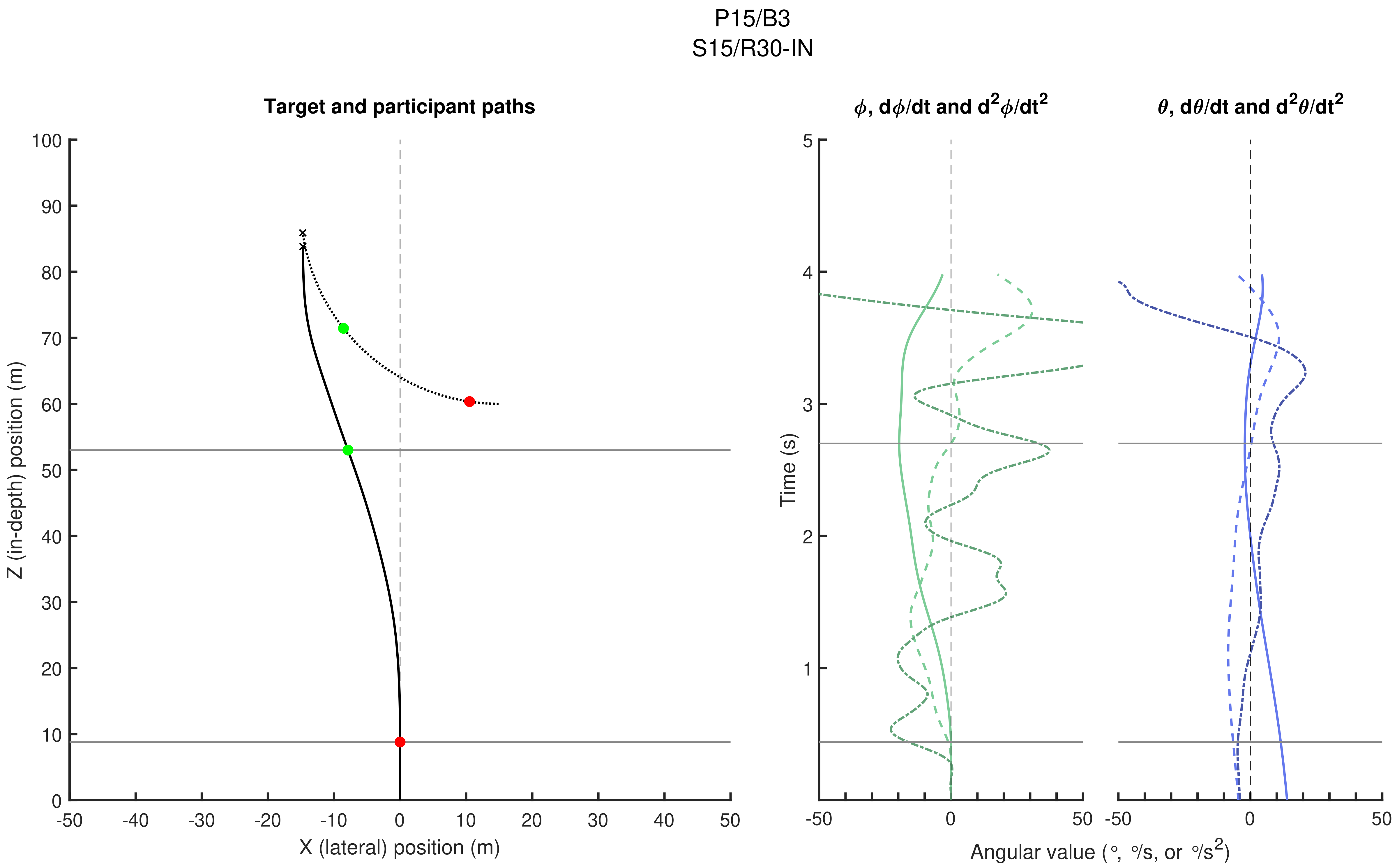

P15/B3
S15/R30-IN
Target and participant paths
Z (in-depth) position (m)
X (lateral) position (m)
ϕ, dϕ/dt and d$^2\phi$/dt^2
θ, dθ/dt and d$^2\theta$/dt^2
Time (s)
Angular value (°, °/s, or °/s^2)

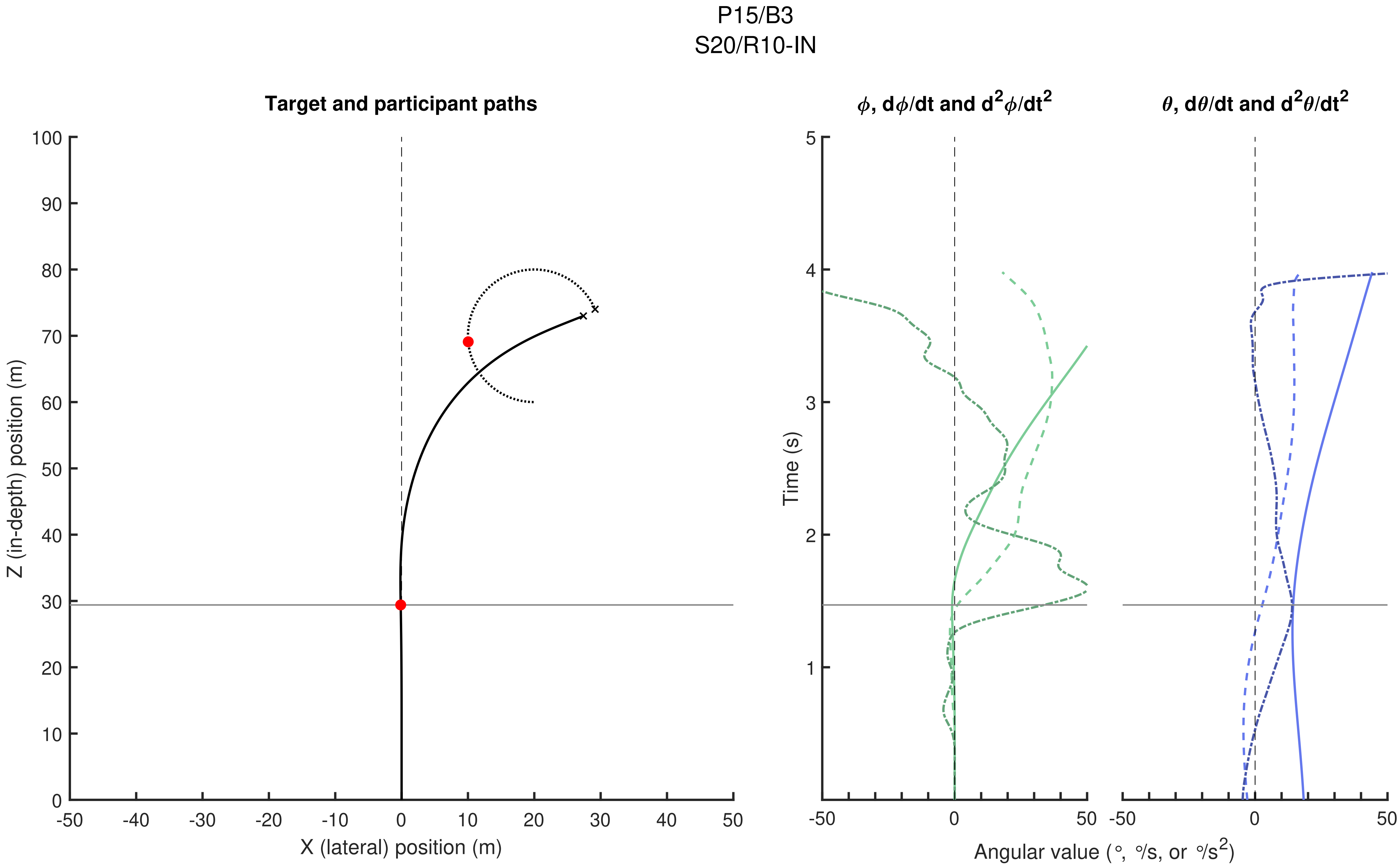

P15/B3
S20/R10-IN
Target and participant paths
Z (in-depth) position (m)
X (lateral) position (m)
ϕ, dϕ/dt and d$^2\phi$/dt^2
θ, dθ/dt and d$^2\theta$/dt^2
Time (s)
Angular value (°, °/s, or °/s^2)

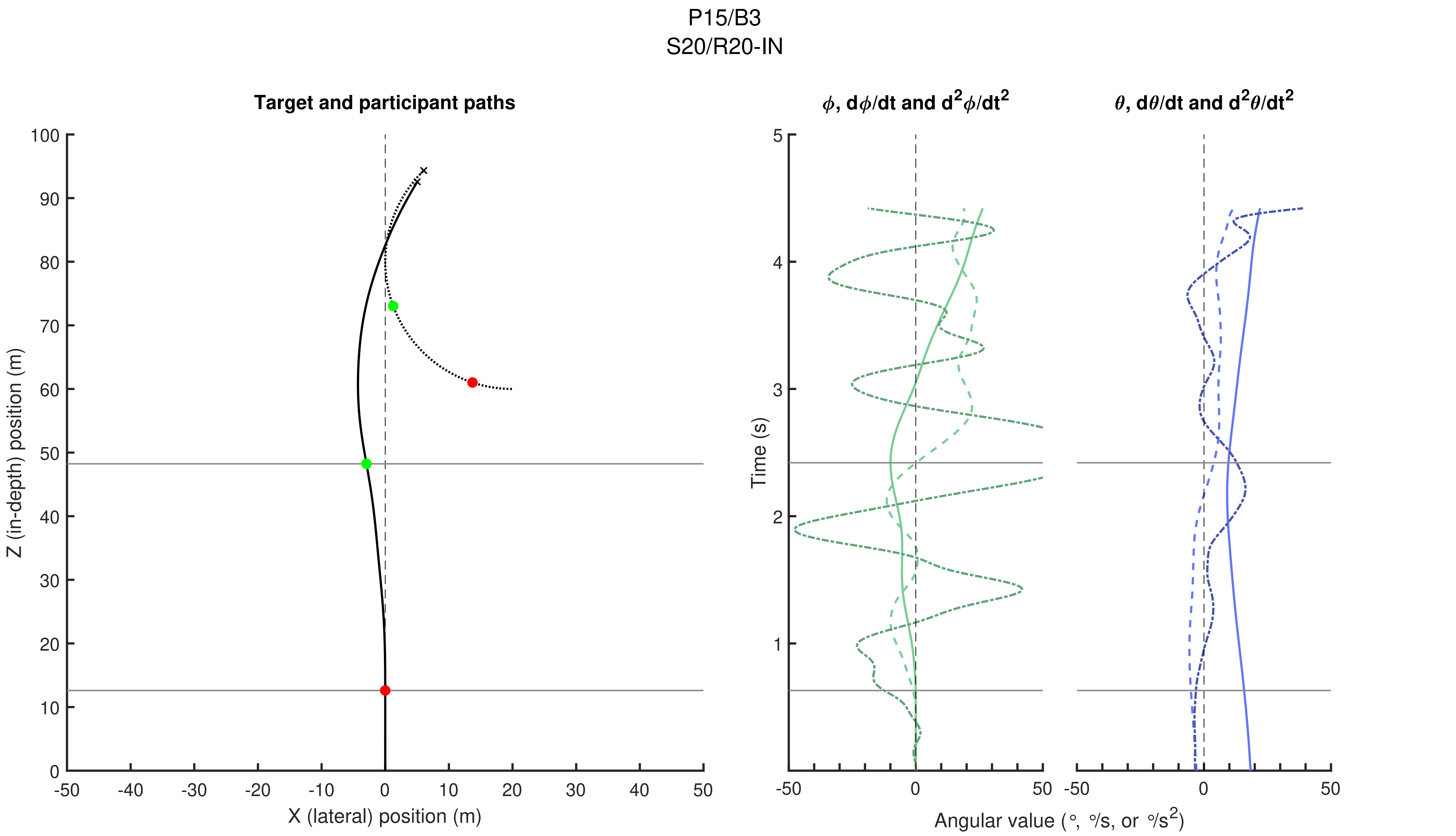
P15/B3
S20/R20-IN
Target and participant paths
Z (in-depth) position (m)
X (lateral) position (m)
ϕ, dϕ/dt and d$^2\phi$/dt^2
θ, dθ/dt and d$^2\theta$/dt^2
Time (s)
Angular value (°, °/s, or °/s^2)

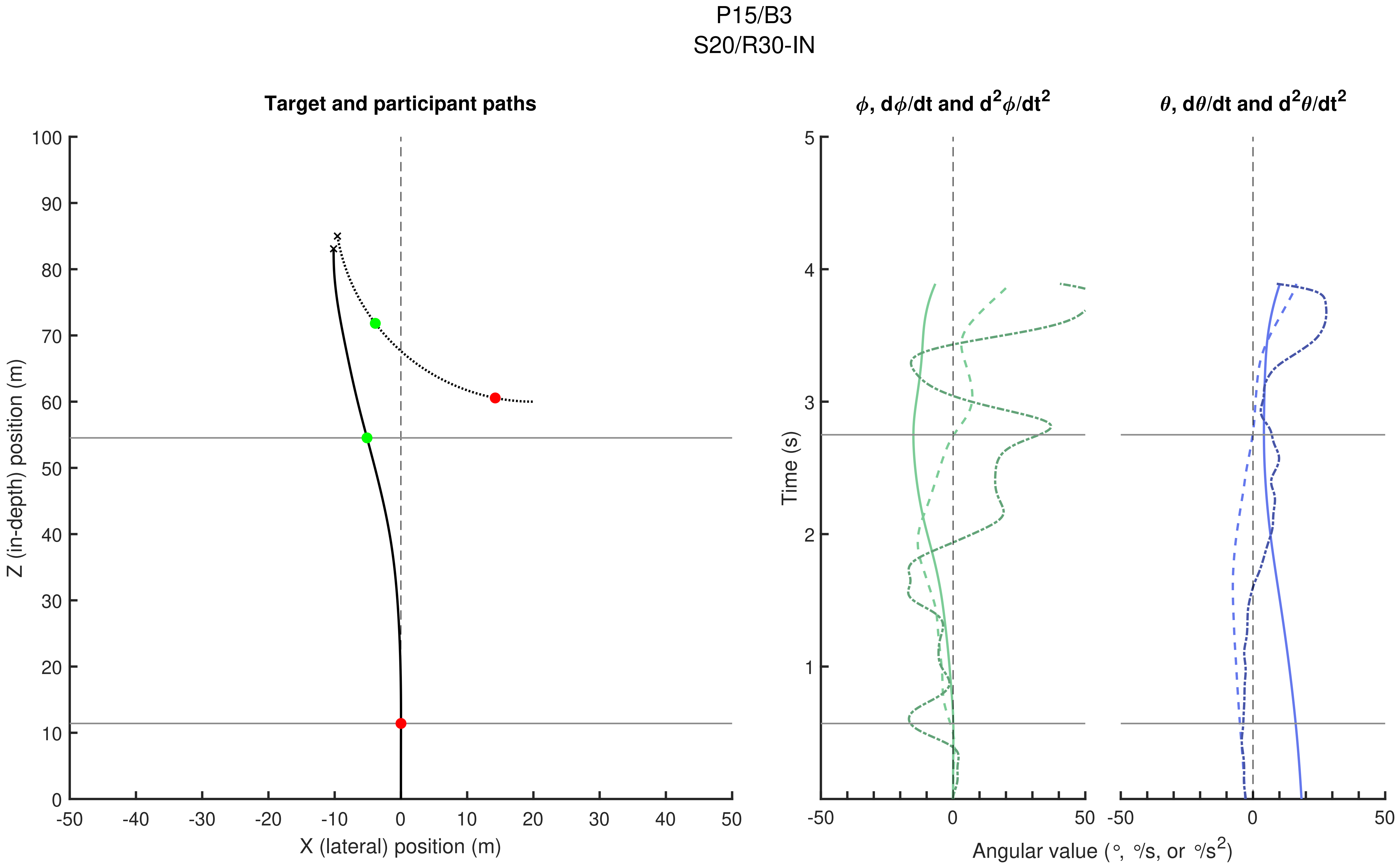
P15/B3
S20/R30-IN
Target and participant paths
Z (in-depth) position (m)
X (lateral) position (m)
φ, dφ/dt and d²φ/dt²
θ, dθ/dt and d²θ/dt²
Time (s)
Angular value (°, °/s, or °/s²)

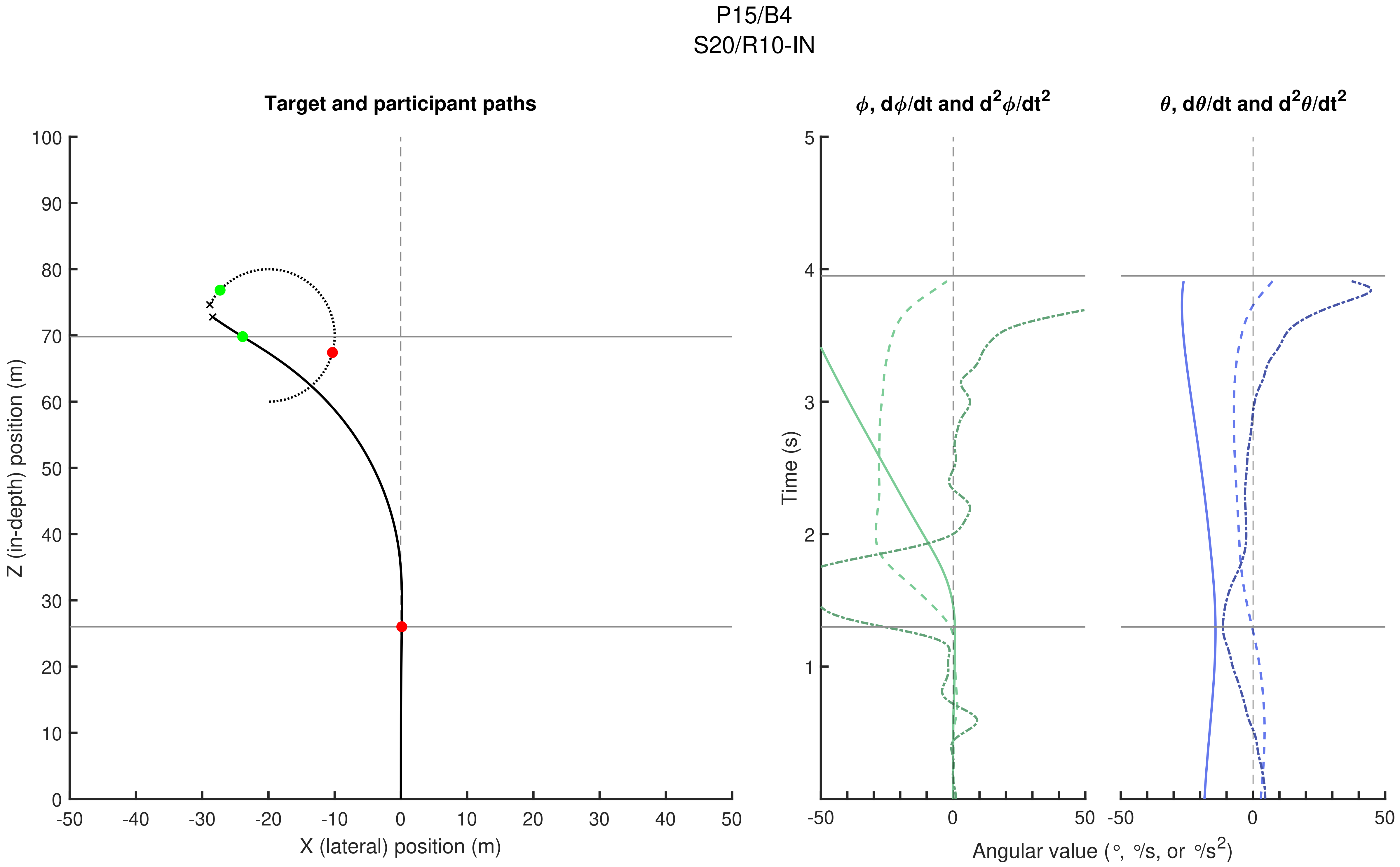
P15/B4
S20/R10-IN
Target and participant paths
Z (in-depth) position (m)
X (lateral) position (m)
100
90
80
70
60
50
40
30
20
10
0
-50
-40
-30
-20
-10
0
10
20
30
40
50
ϕ, dϕ/dt and d$^2\phi$/dt^2
θ, dθ/dt and d$^2\theta$/dt^2
Time (s)
5
4
3
2
1
-50
0
50
-50
0
50
Angular value (°, °/s, or °/s^2)

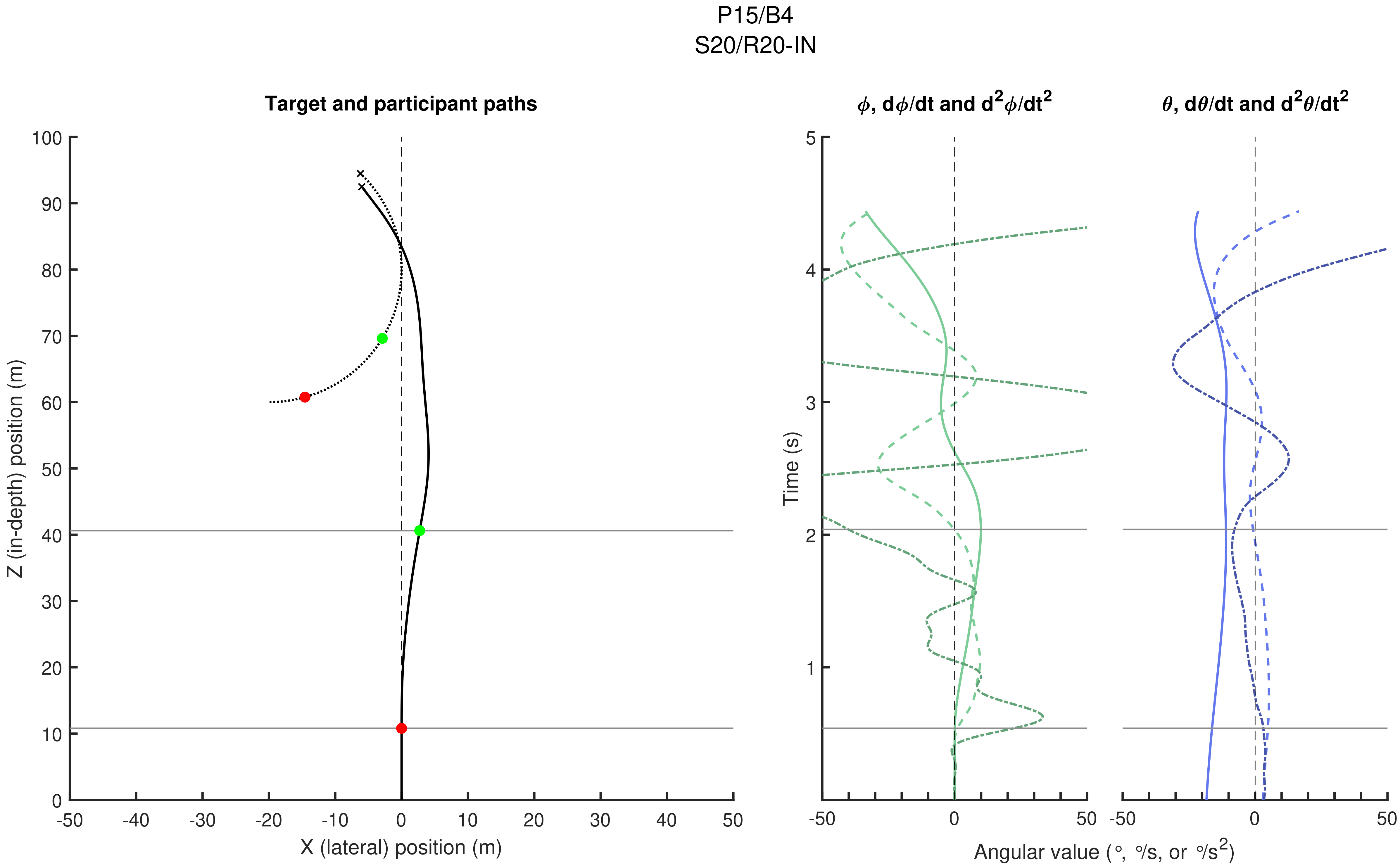
P15/B4
S20/R20-IN
Target and participant paths
ϕ, dϕ/dt and d²ϕ/dt²
θ, dθ/dt and d²θ/dt²
Z (in-depth) position (m)
X (lateral) position (m)
Time (s)
Angular value (°, °/s, or °/s²)
100
90
80
70
60
50
40
30
20
10
0
-50
-40
-30
-20
-10
0
10
20
30
40
50
5
4
3
2
1
-50
0
50
-50
0
50

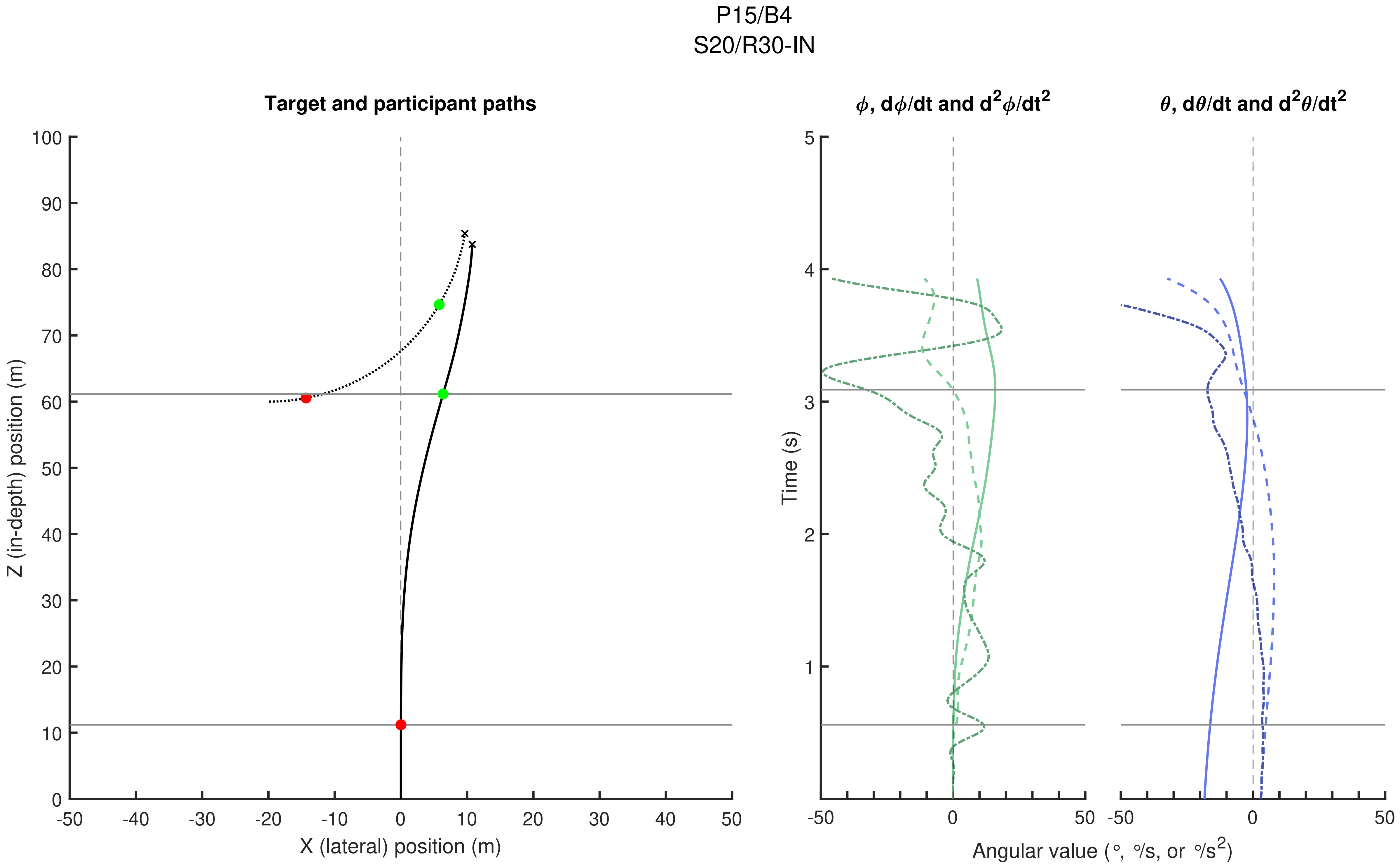

P15/B4
S20/R30-IN
Target and participant paths
φ, dφ/dt and d²φ/dt²
θ, dθ/dt and d²θ/dt²
Z (in-depth) position (m)
X (lateral) position (m)
Time (s)
Angular value (°, °/s, or °/s²)
100
90
80
70
60
50
40
30
20
10
0
-50
-40
-30
-20
-10
0
10
20
30
40
50
5
4
3
2
1
-50
0
50
-50
0
50

P15/B4
S15/R10-IN

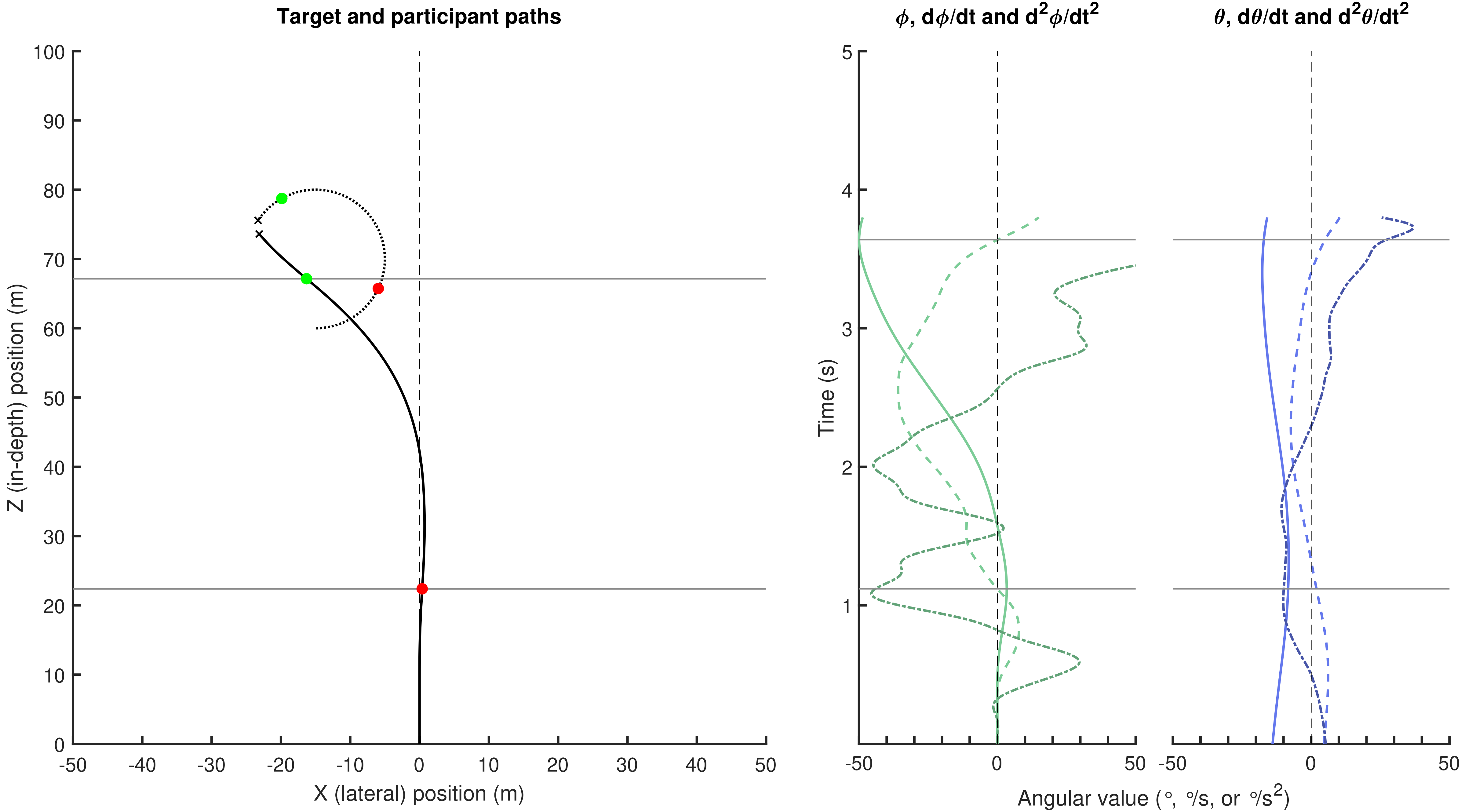

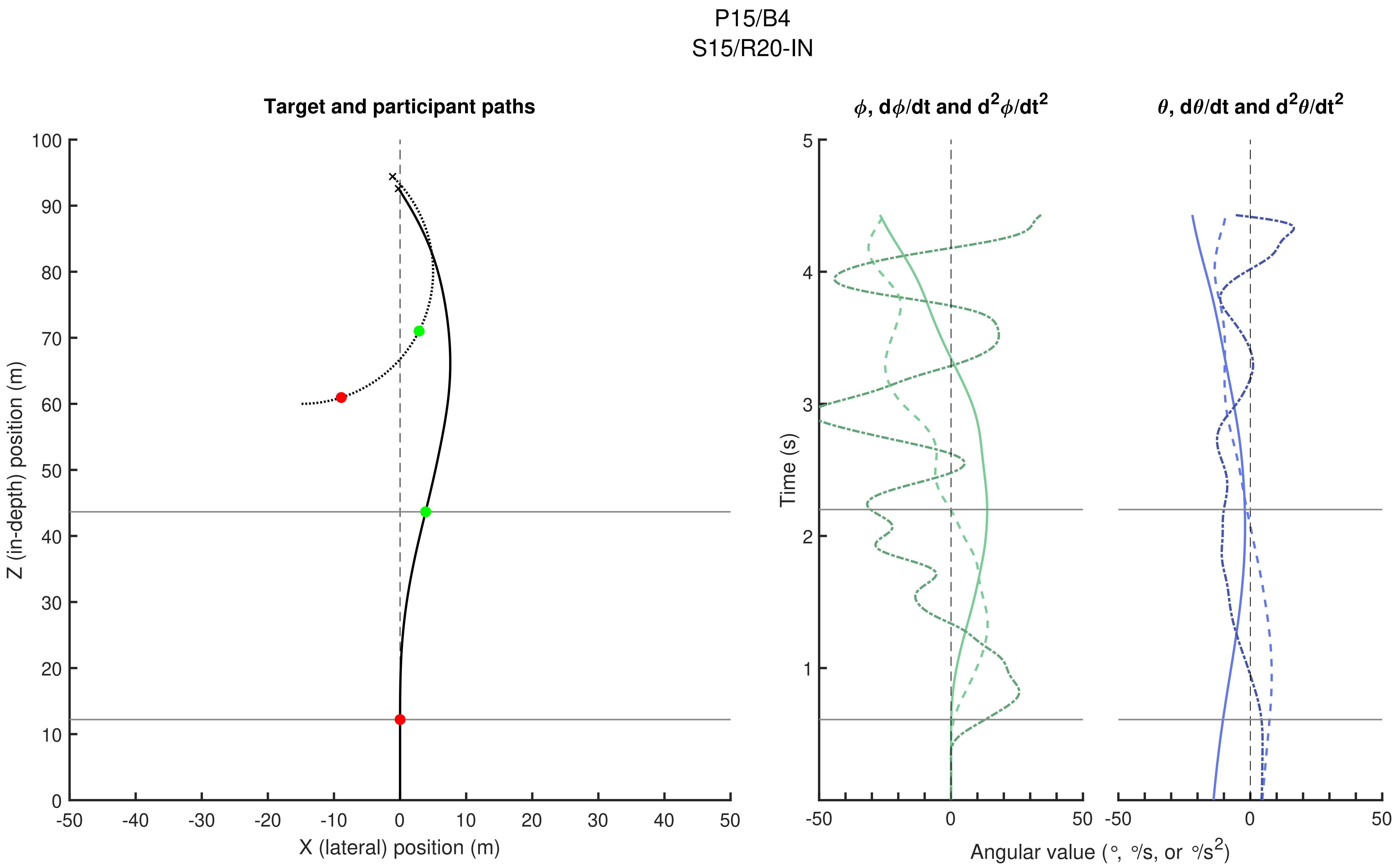

P15/B4
S15/R20-IN
Target and participant paths
ϕ, dϕ/dt and d²ϕ/dt²
θ, dθ/dt and d²θ/dt²
Z (in-depth) position (m)
X (lateral) position (m)
Time (s)
Angular value (°, °/s, or °/s²)
100
90
80
70
60
50
40
30
20
10
0
-50
-40
-30
-20
-10
0
10
20
30
40
50
5
4
3
2
1
-50
0
50
-50
0
50

P15/B4
S15/R30-IN

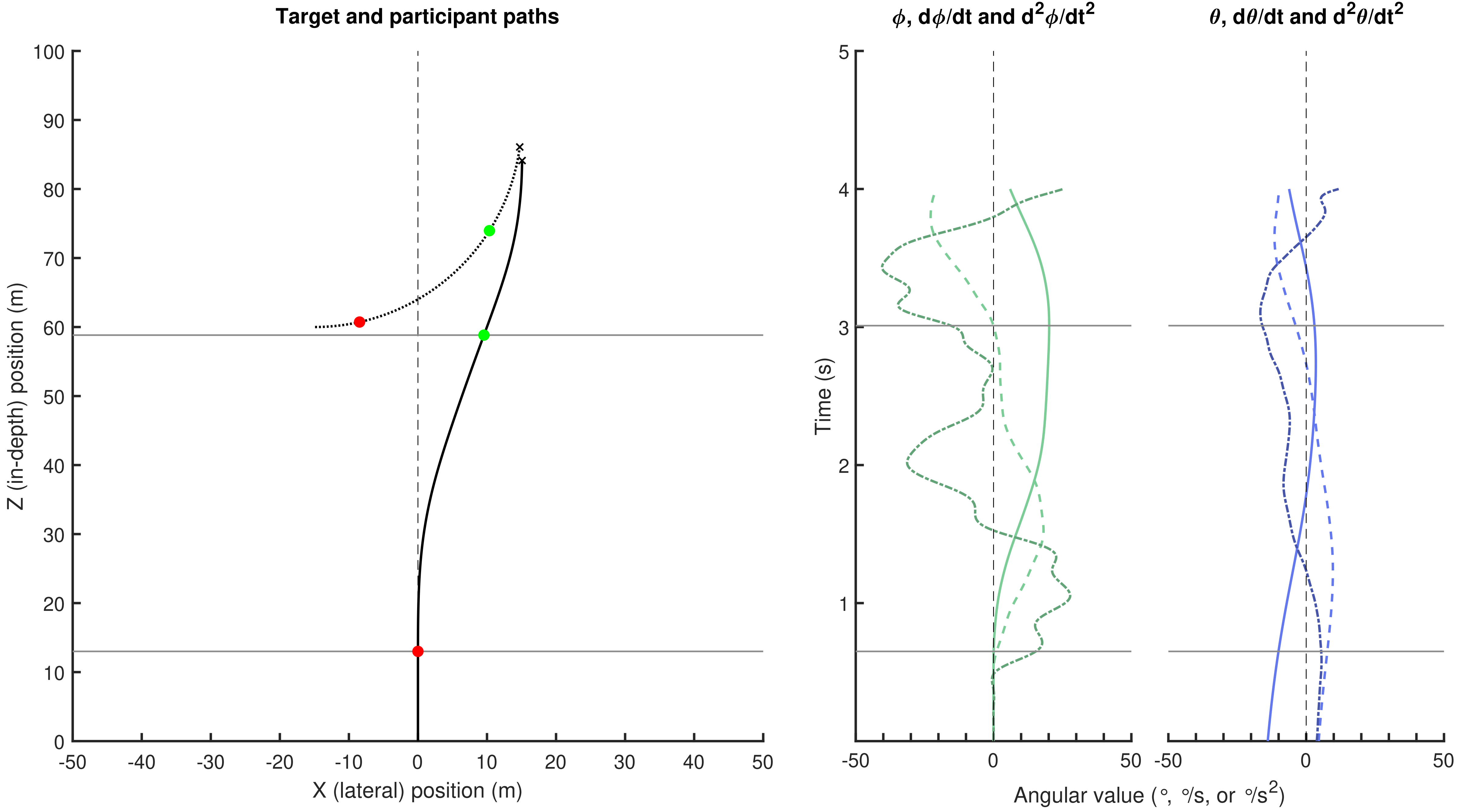

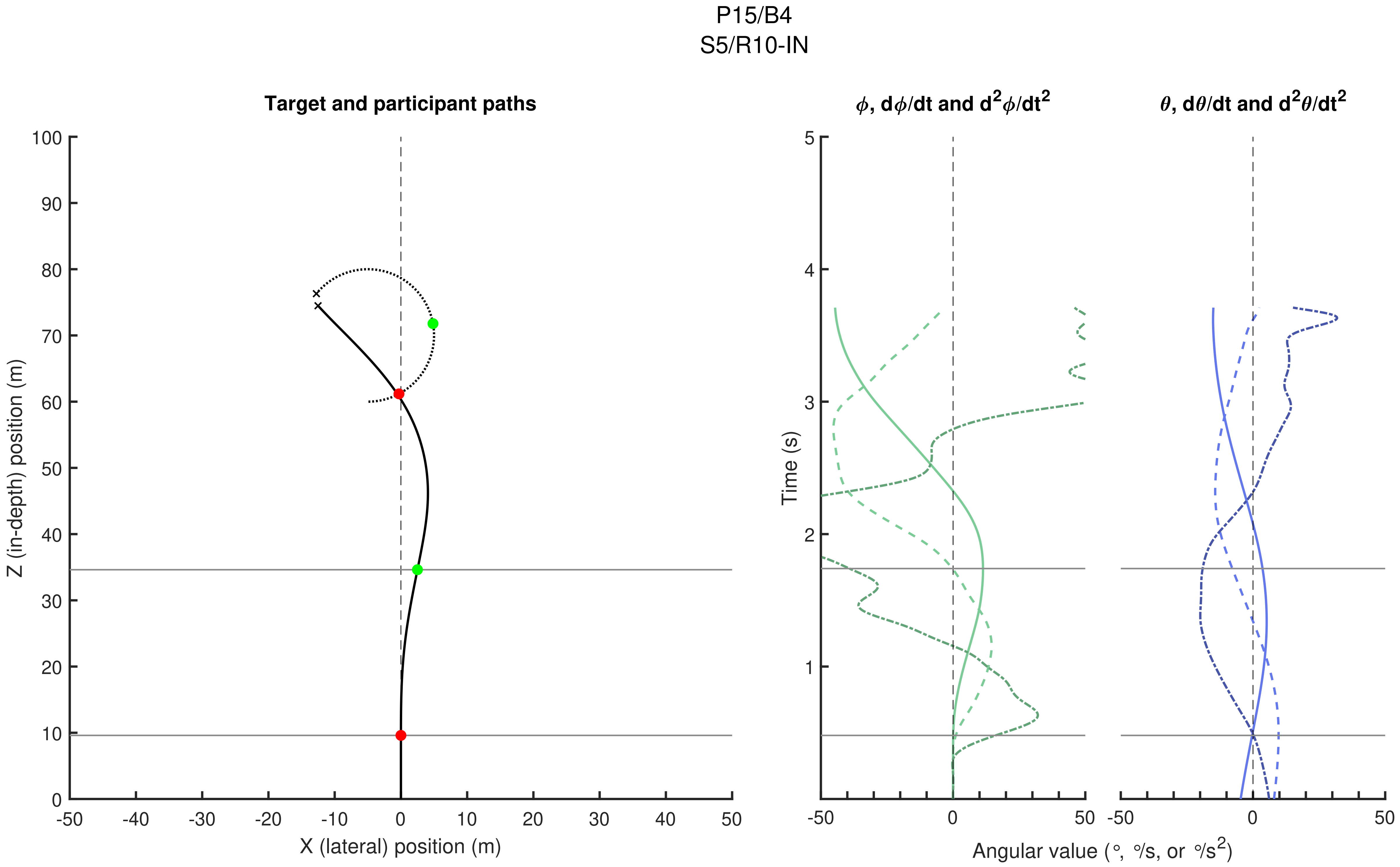

P15/B4
S5/R10-IN
Target and participant paths
φ, dφ/dt and d²φ/dt²
θ, dθ/dt and d²θ/dt²
Z (in-depth) position (m)
X (lateral) position (m)
Time (s)
Angular value (°, °/s, or °/s²)

P15/B4
S5/R10-OUT

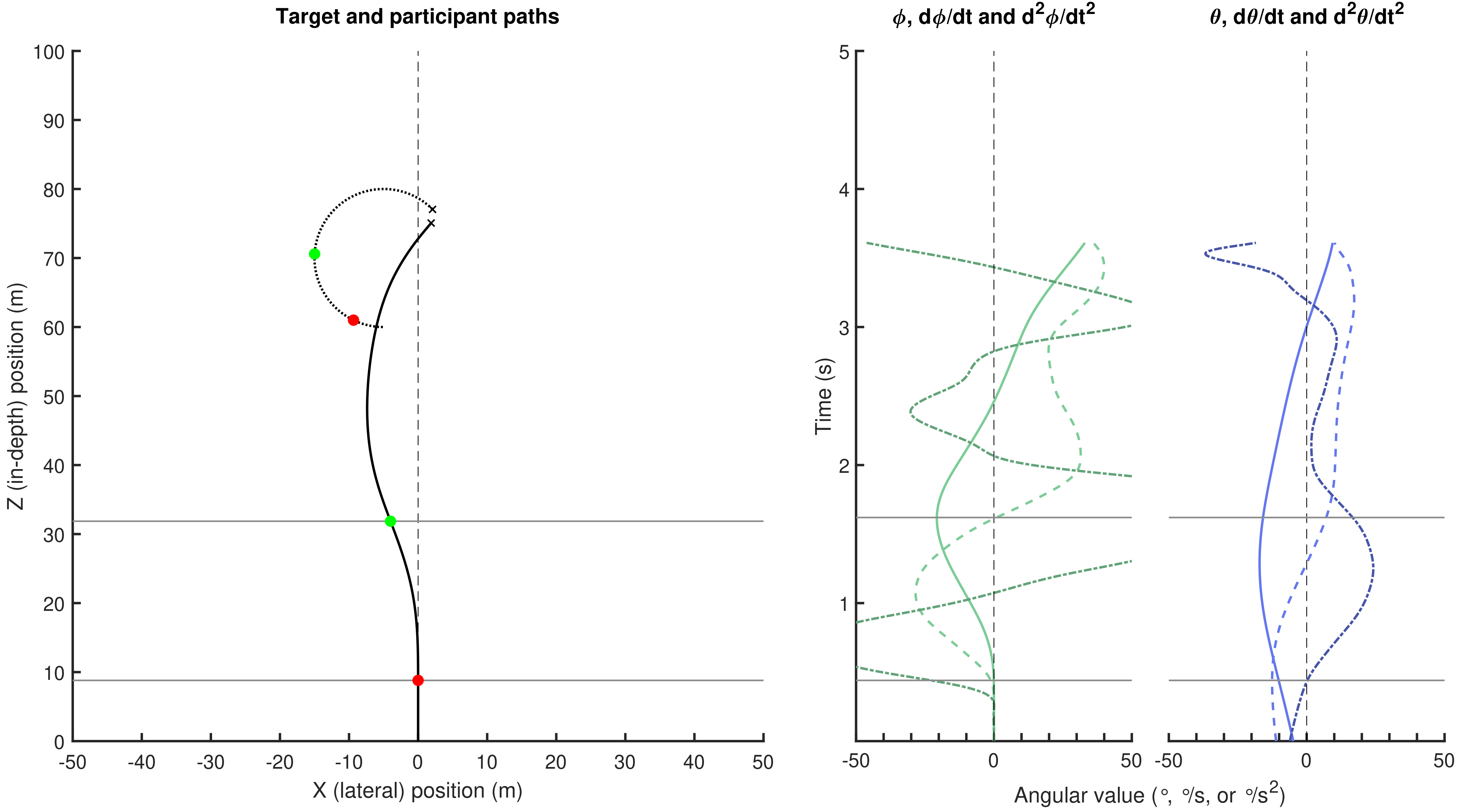

P15/B4
S5/R20-IN

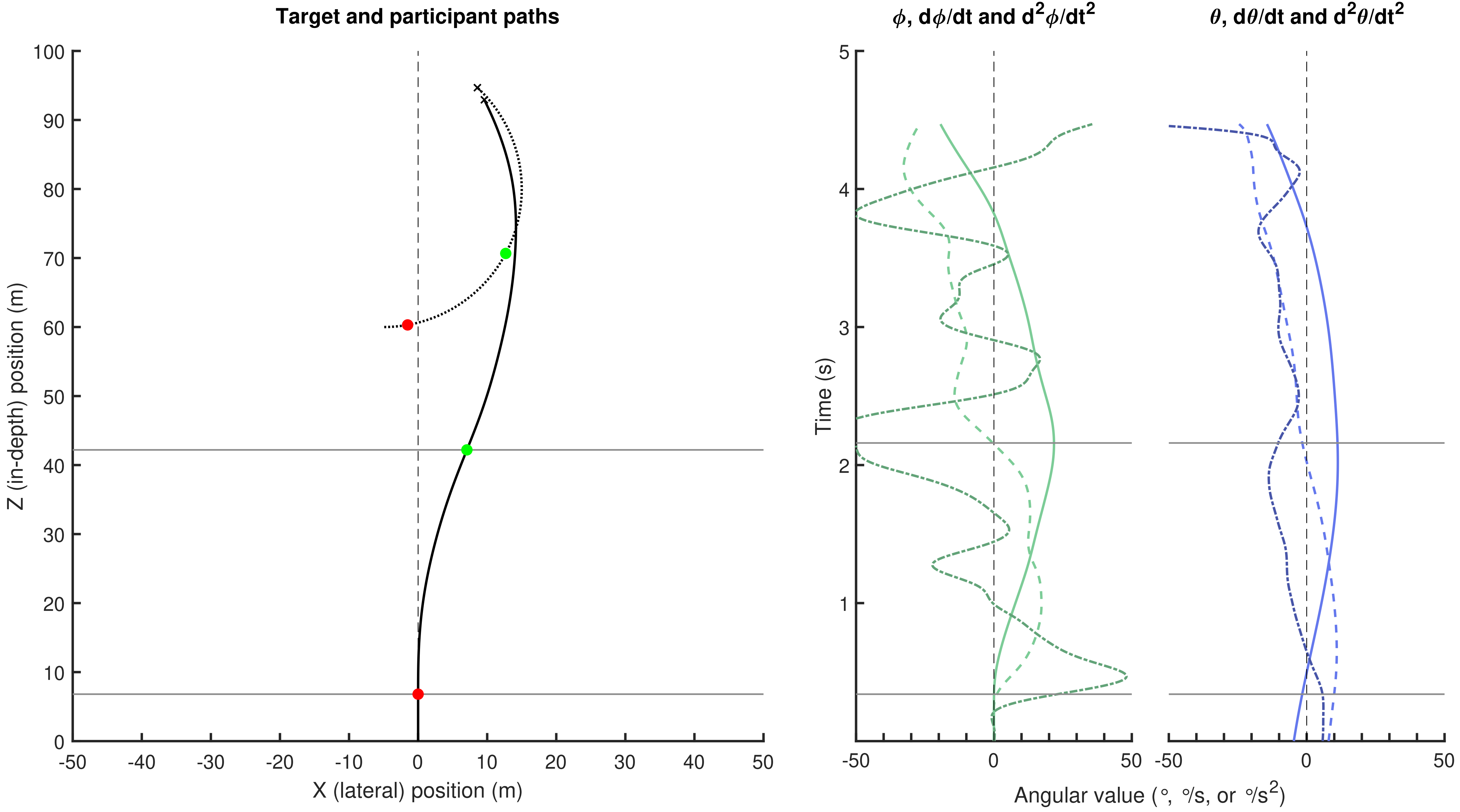

P15/B4
S5/R20-OUT

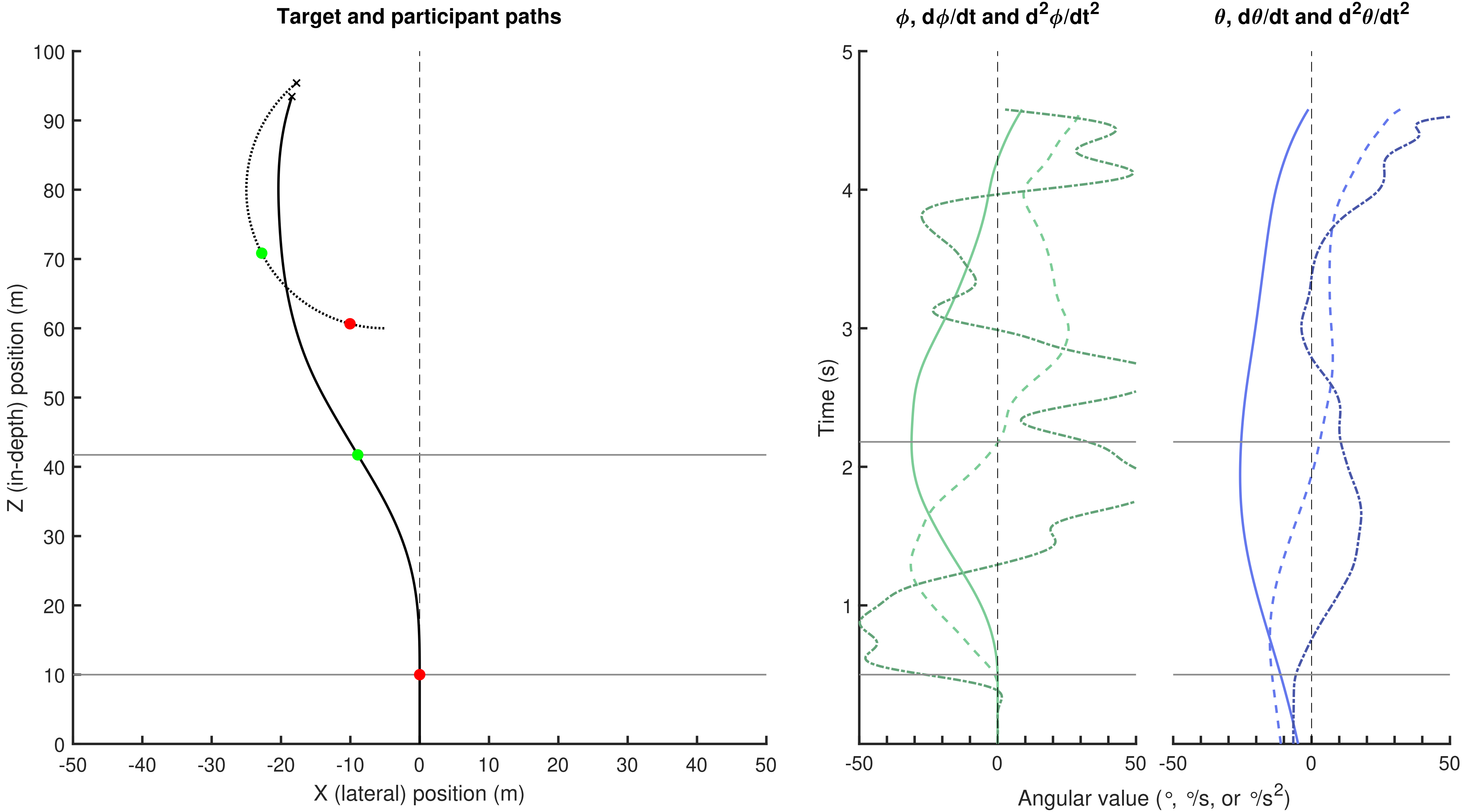

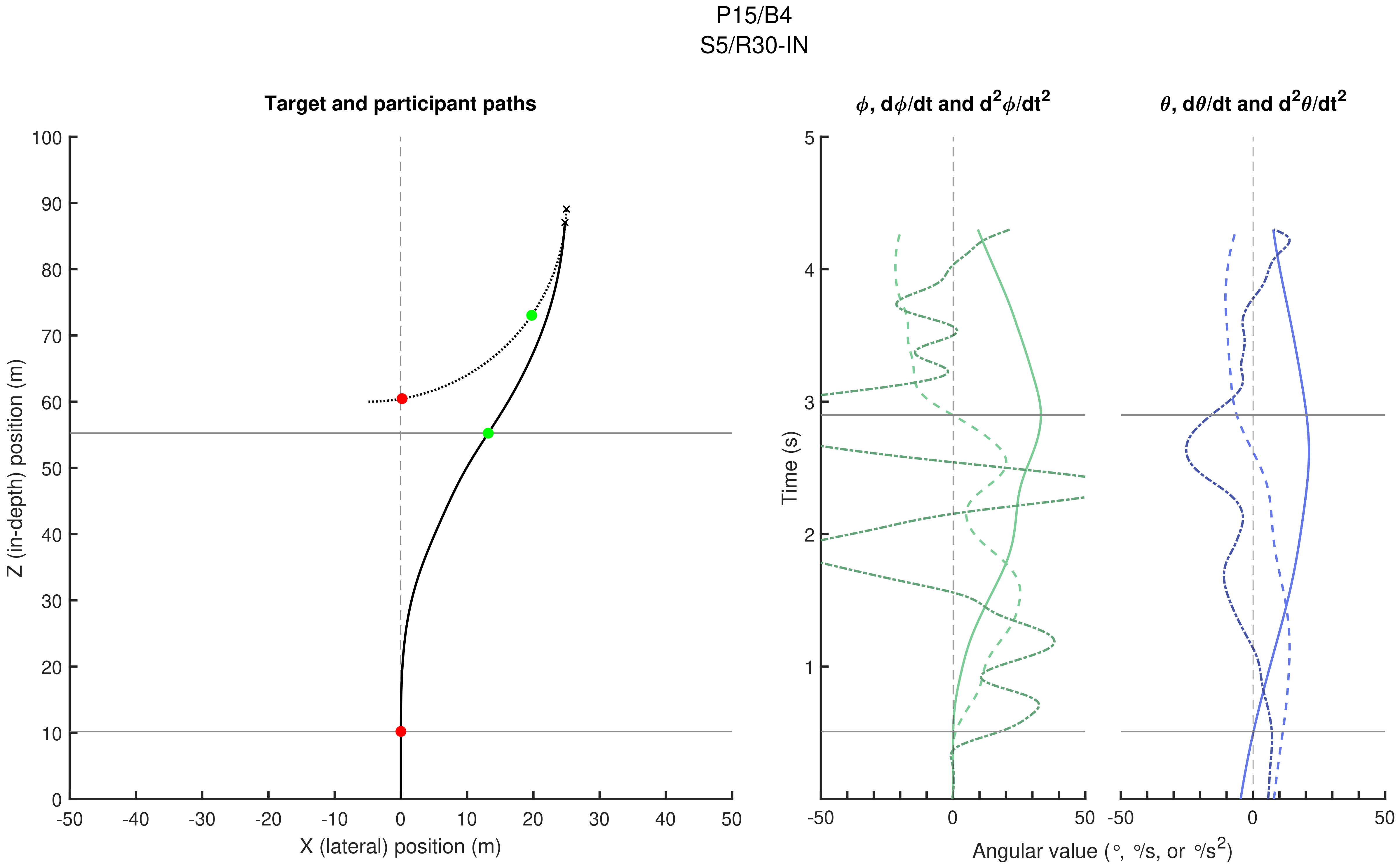
P15/B4
S5/R30-IN
Target and participant paths
Z (in-depth) position (m)
X (lateral) position (m)
100
90
80
70
60
50
40
30
20
10
0
-50
-40
-30
-20
-10
0
10
20
30
40
50
ϕ, dϕ/dt and d$^2\phi$/dt^2
θ, dθ/dt and d$^2\theta$/dt^2
Time (s)
5
4
3
2
1
-50
0
50
-50
0
50
Angular value (°, °/s, or °/s^2)

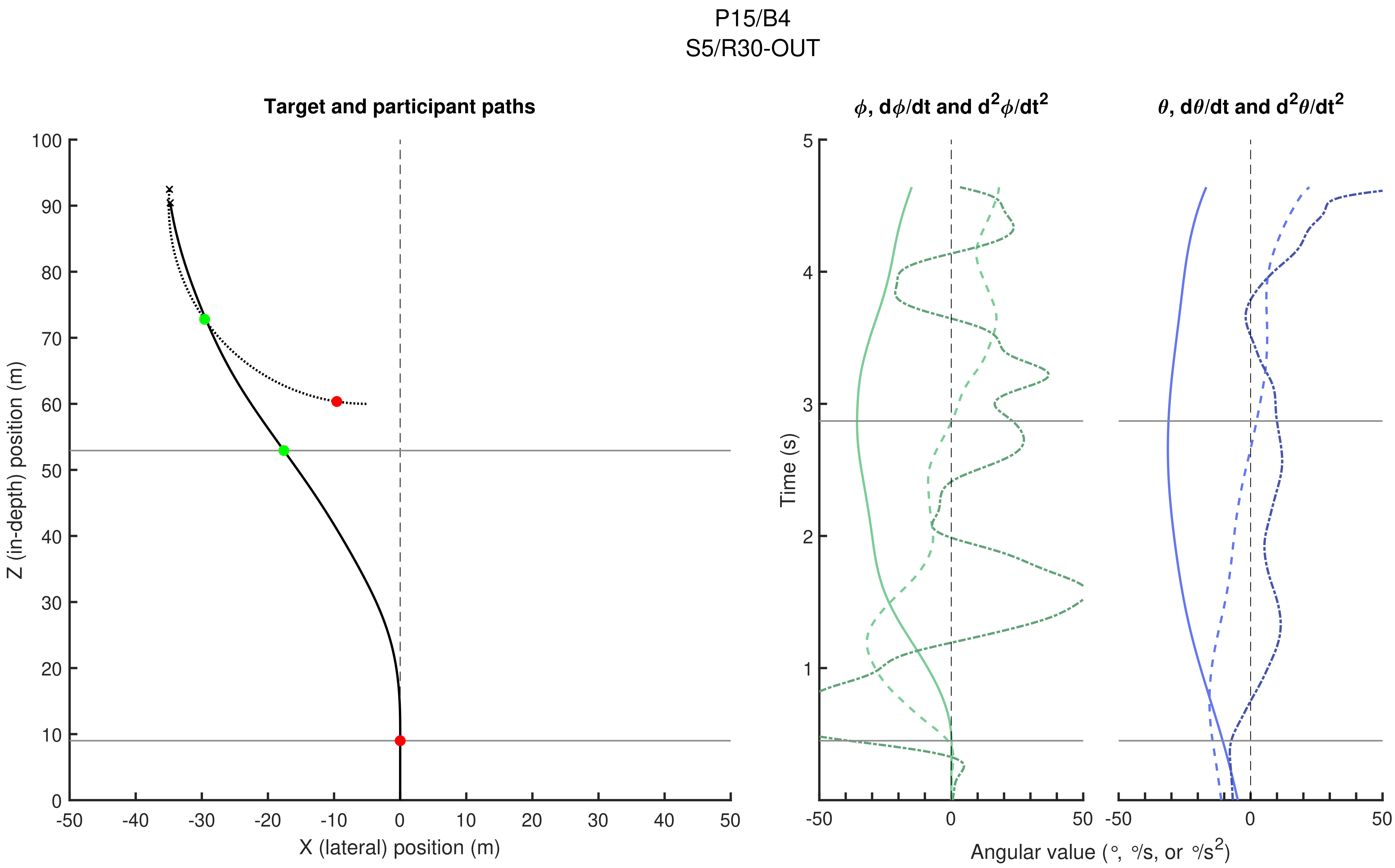
P15/B4
S5/R30-OUT
Target and participant paths
ϕ, dϕ/dt and d$^2\phi$/dt^2
θ, dθ/dt and d$^2\theta$/dt^2
Z (in-depth) position (m)
X (lateral) position (m)
Time (s)
Angular value (°, °/s, or °/s^2)

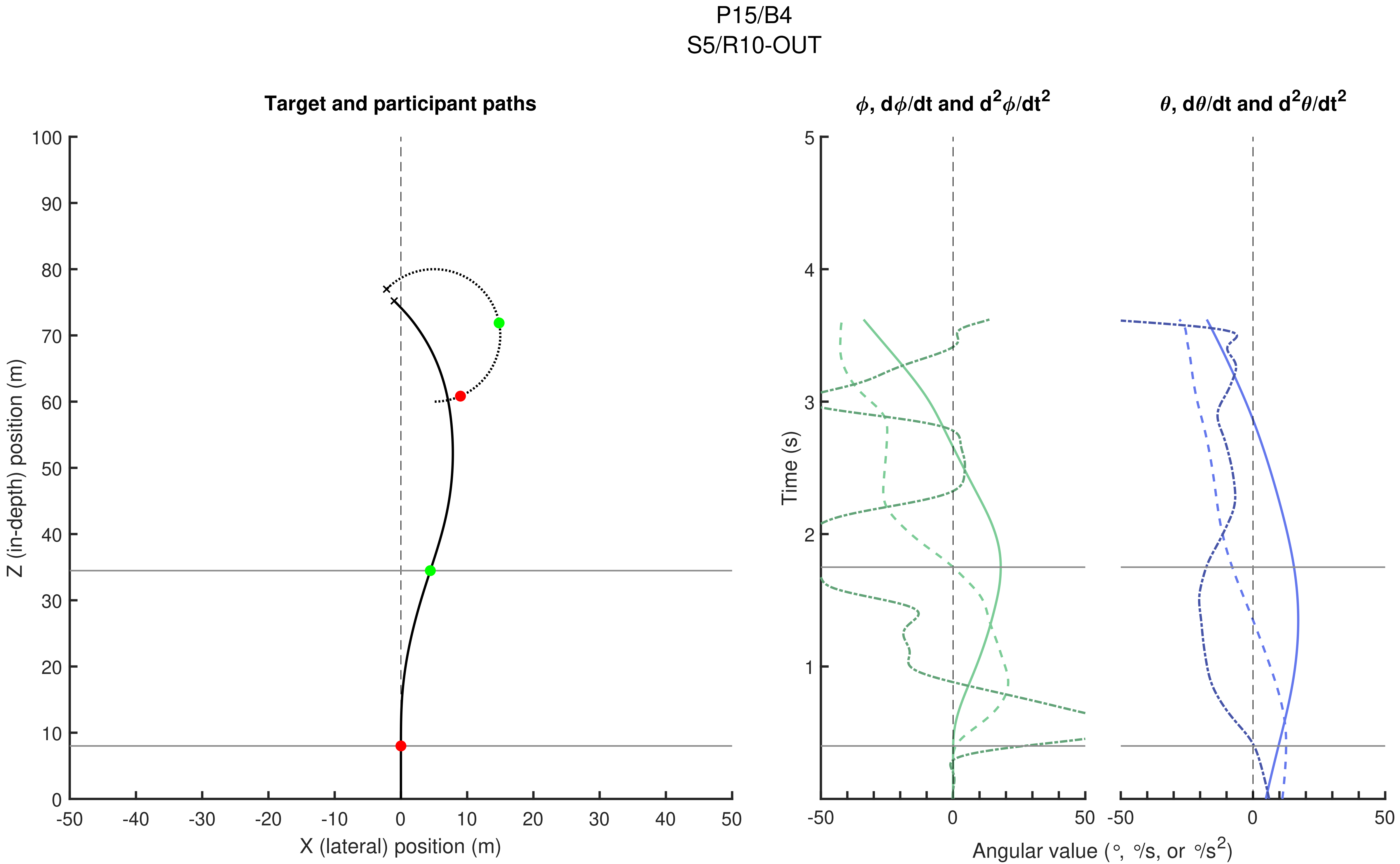
P15/B4
S5/R10-OUT
Target and participant paths
Z (in-depth) position (m)
X (lateral) position (m)
ϕ, dϕ/dt and d$^2\phi$/dt^2
θ, dθ/dt and d$^2\theta$/dt^2
Time (s)
Angular value (°, °/s, or °/s^2)

P15/B4
S5/R10-IN

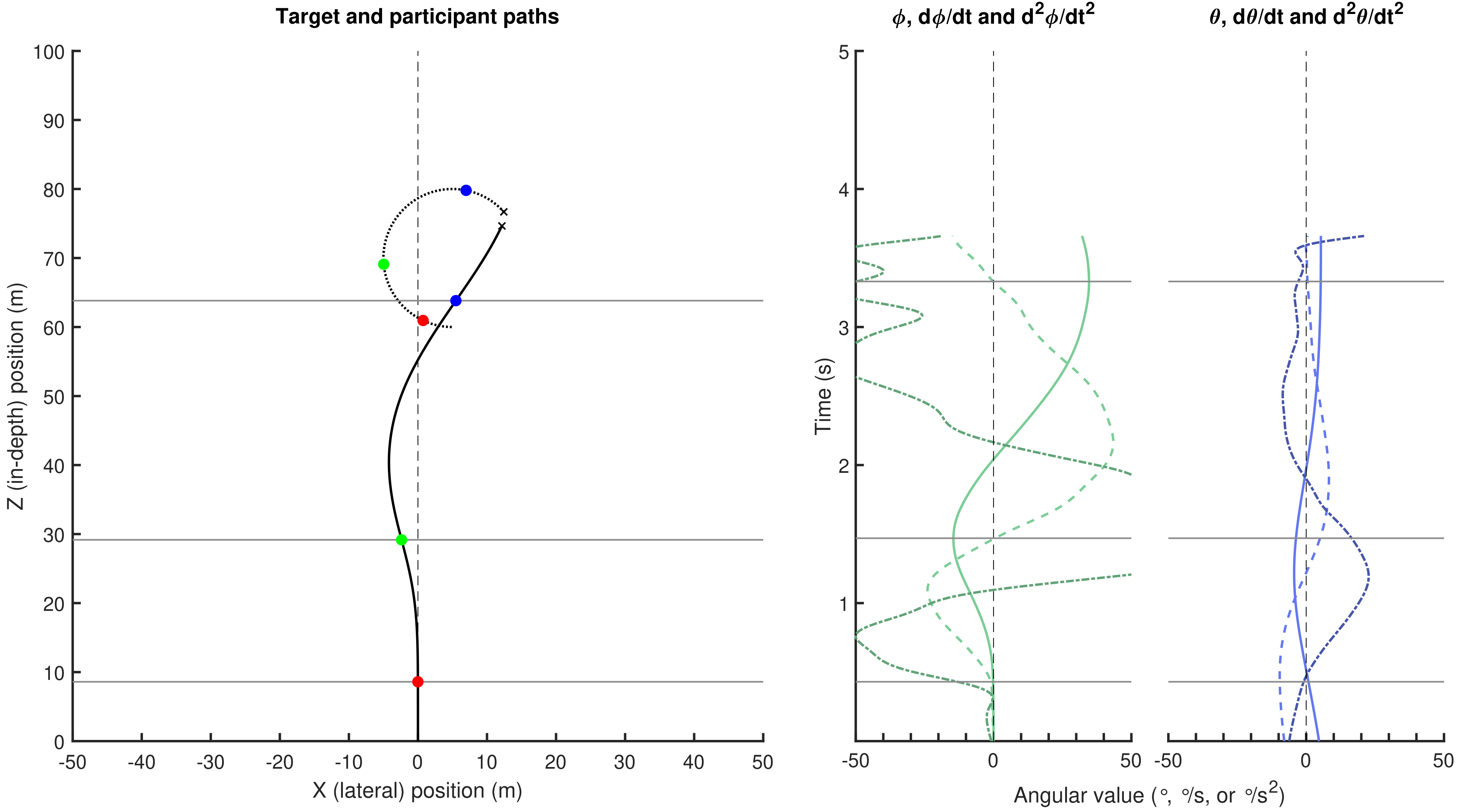

P15/B4
S5/R20-OUT

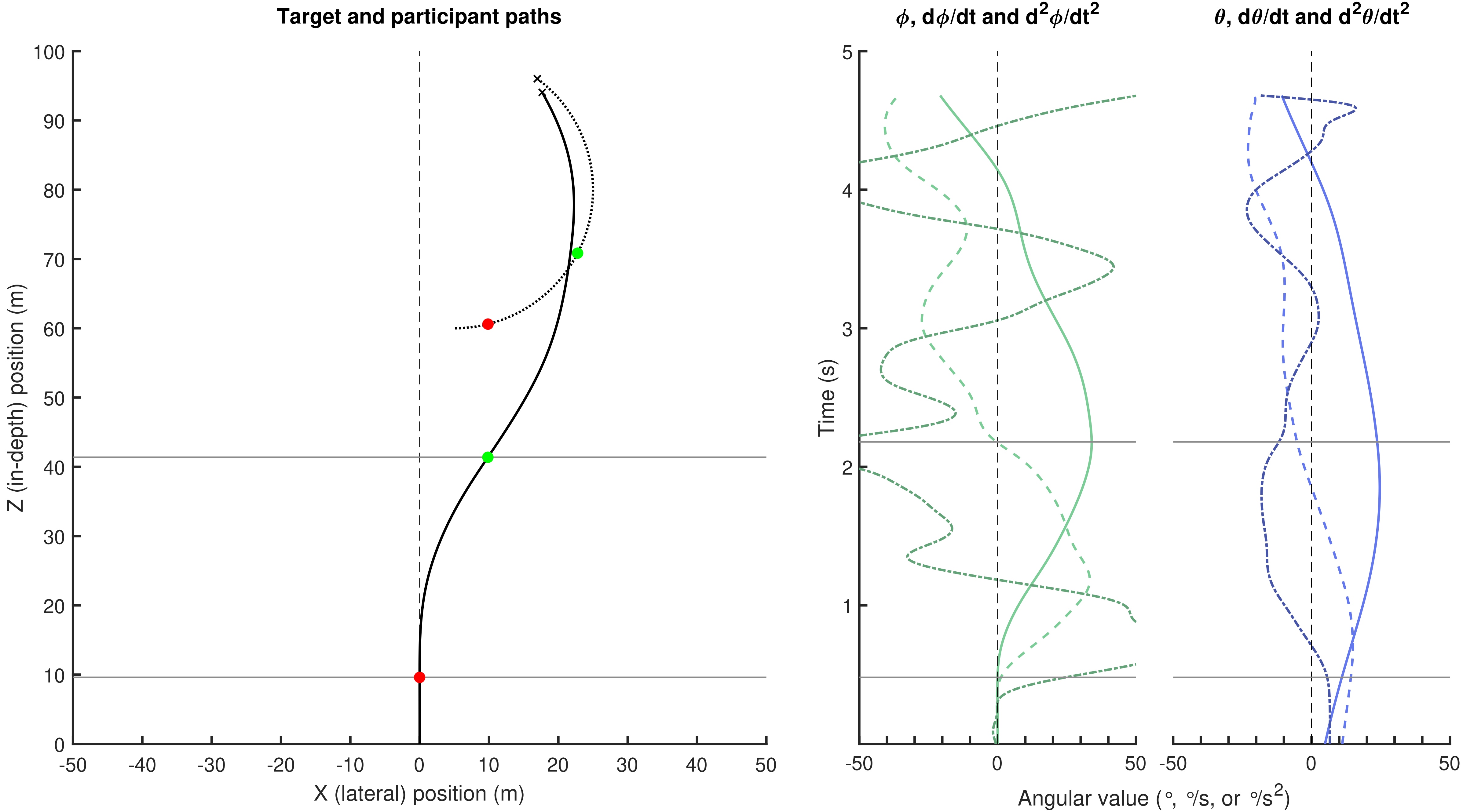

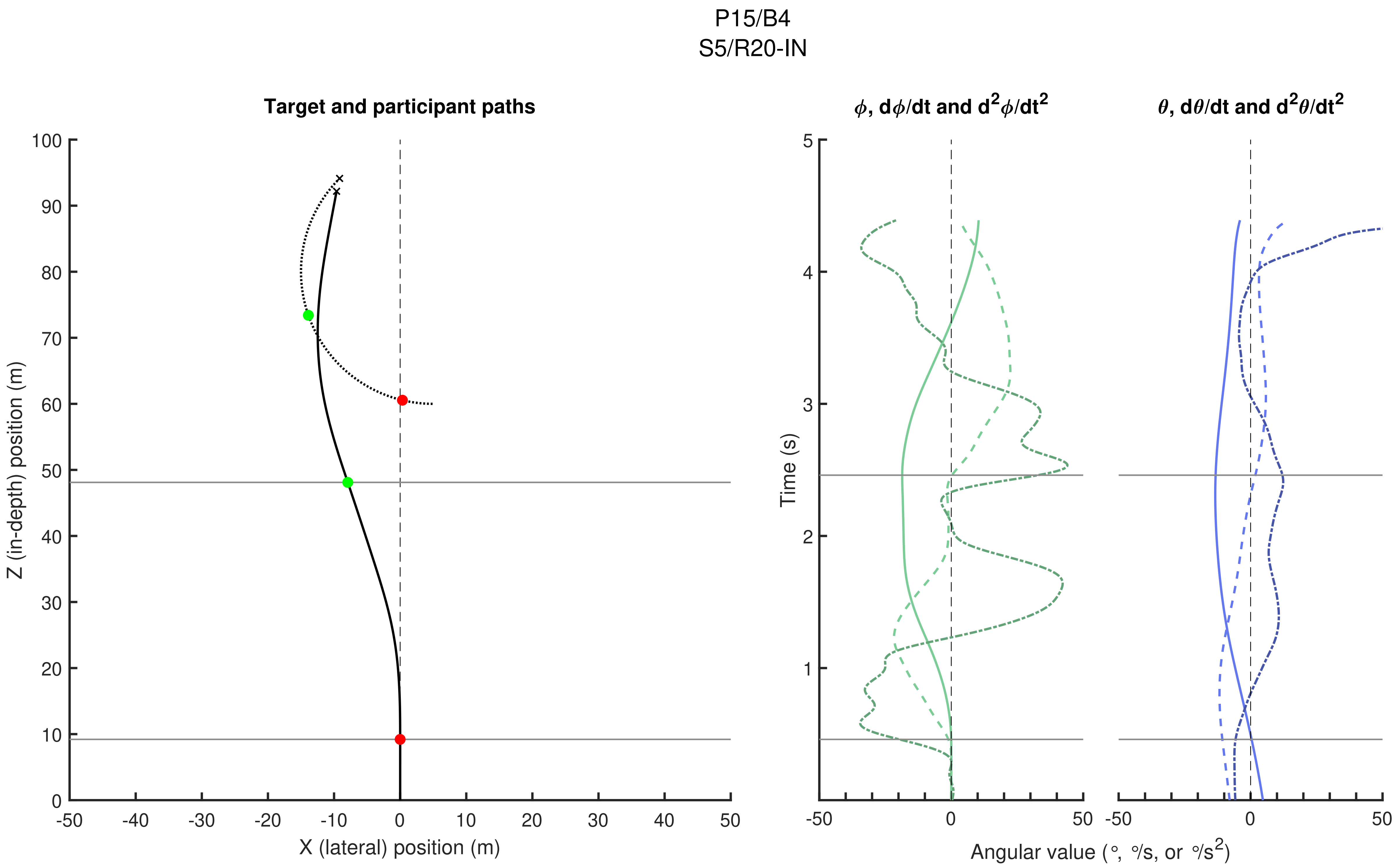
P15/B4
S5/R20-IN
Target and participant paths
Z (in-depth) position (m)
X (lateral) position (m)
ϕ, dϕ/dt and d$^2\phi$/dt^2
θ, dθ/dt and d$^2\theta$/dt^2
Time (s)
Angular value (°, °/s, or °/s^2)

P15/B4
S5/R30-OUT

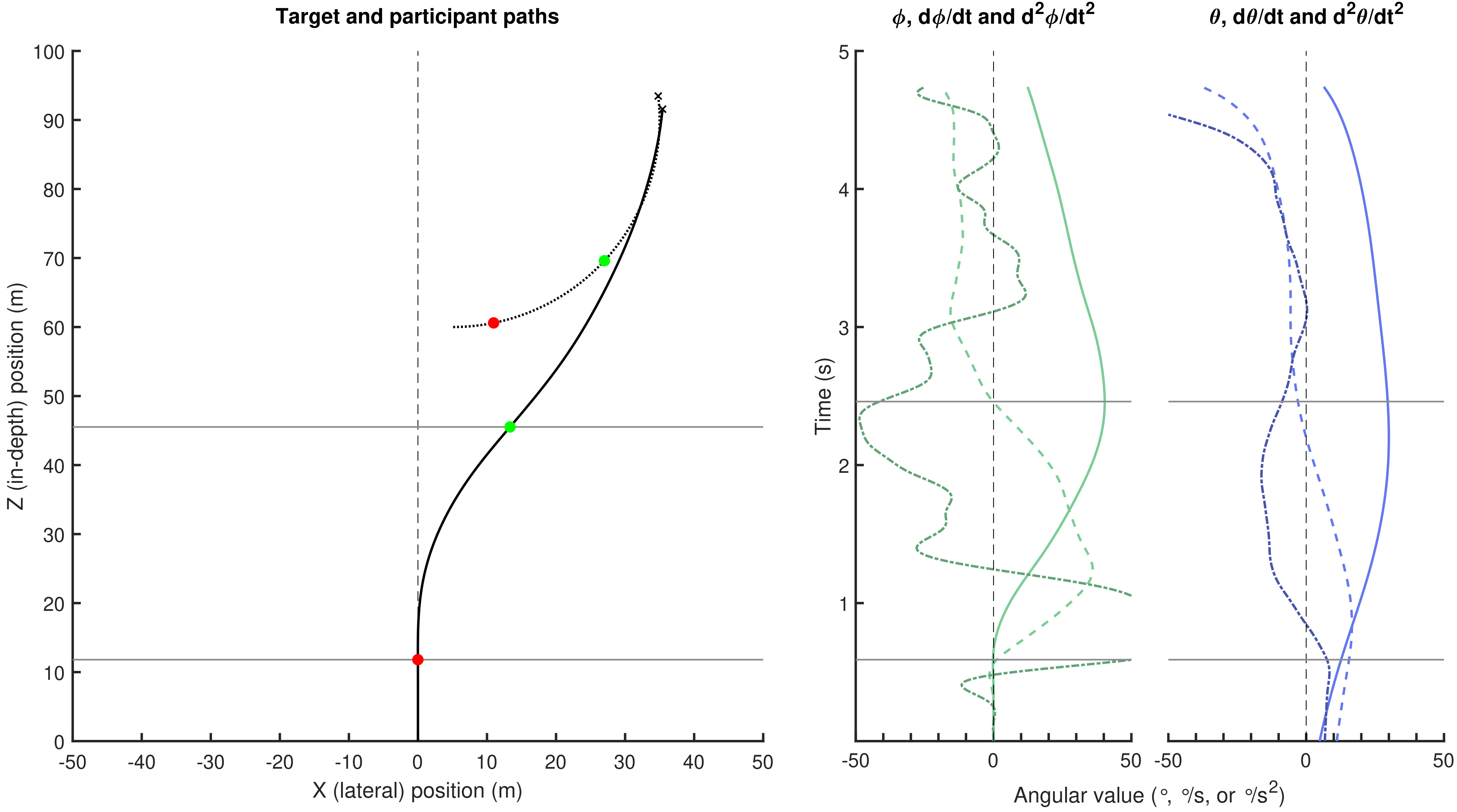

P15/B4
S5/R30-IN

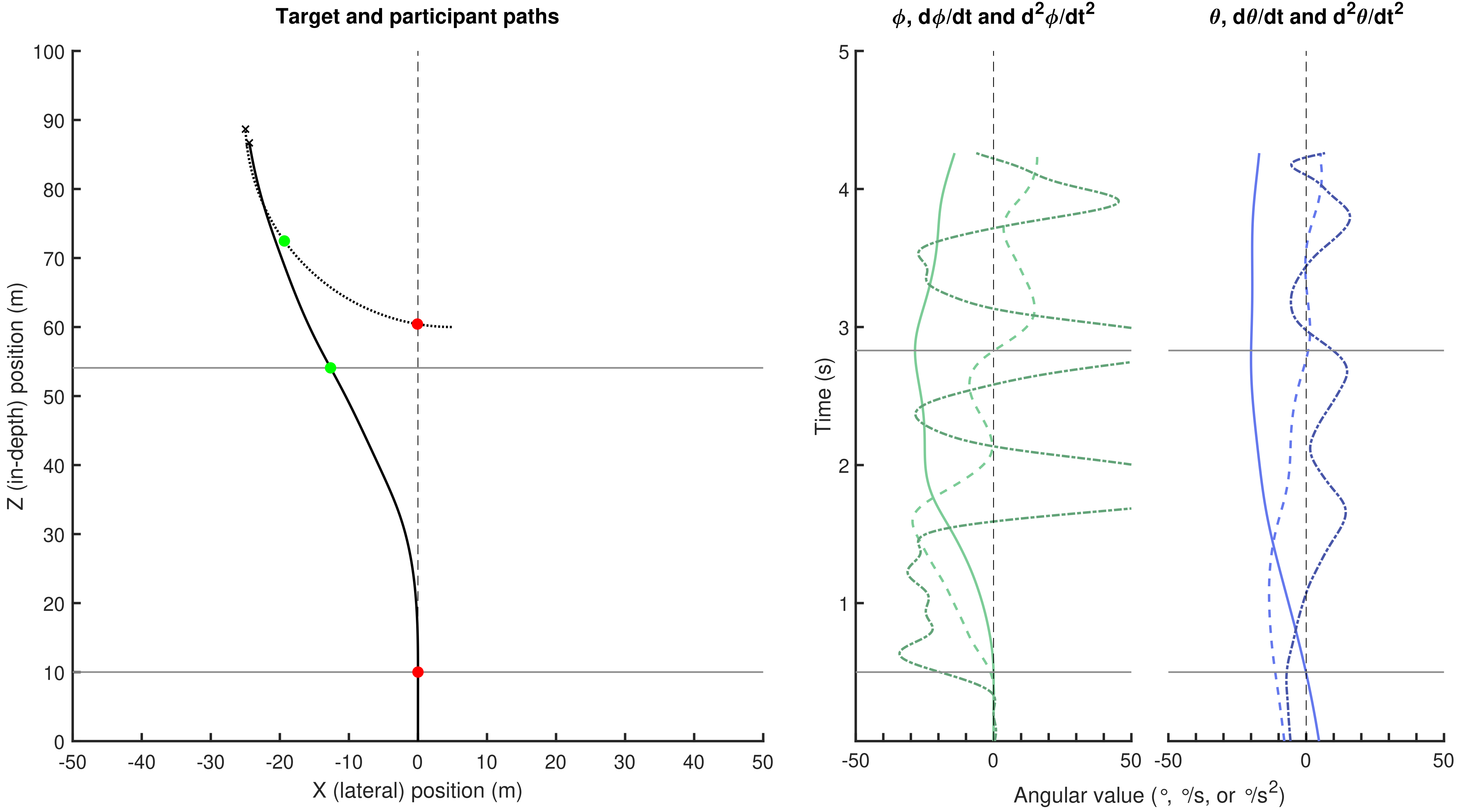

P15/B4
S15/R10-IN

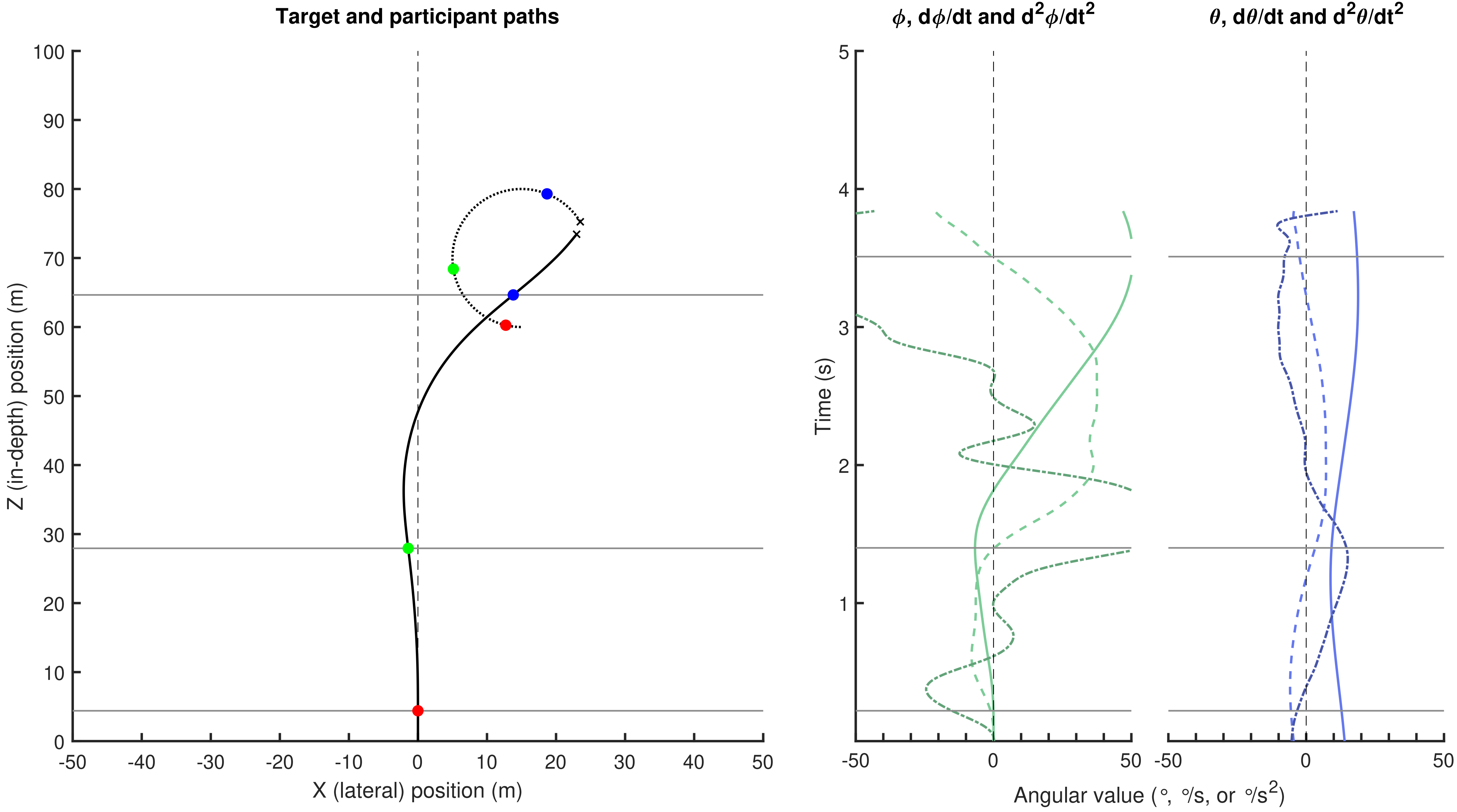

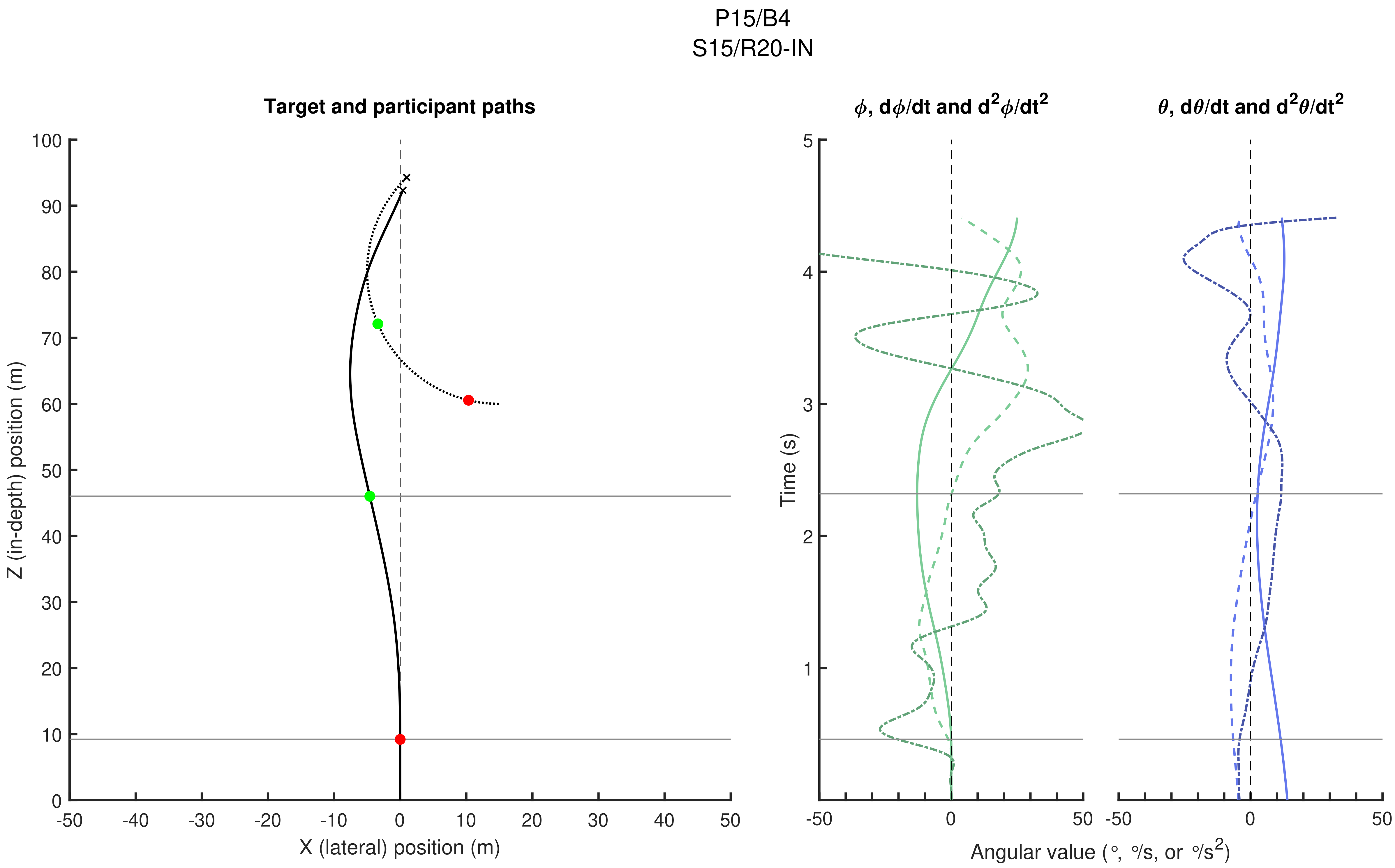
P15/B4
S15/R20-IN
Target and participant paths
Z (in-depth) position (m)
X (lateral) position (m)
0
10
20
30
40
50
60
70
80
90
100
-50
-40
-30
-20
-10
0
10
20
30
40
50
ϕ, dϕ/dt and d$^2\phi$/dt^2
θ, dθ/dt and d$^2\theta$/dt^2
Time (s)
1
2
3
4
5
-50
0
50
-50
0
50
Angular value (°, °/s, or °/s^2)

P15/B4
S15/R30-IN

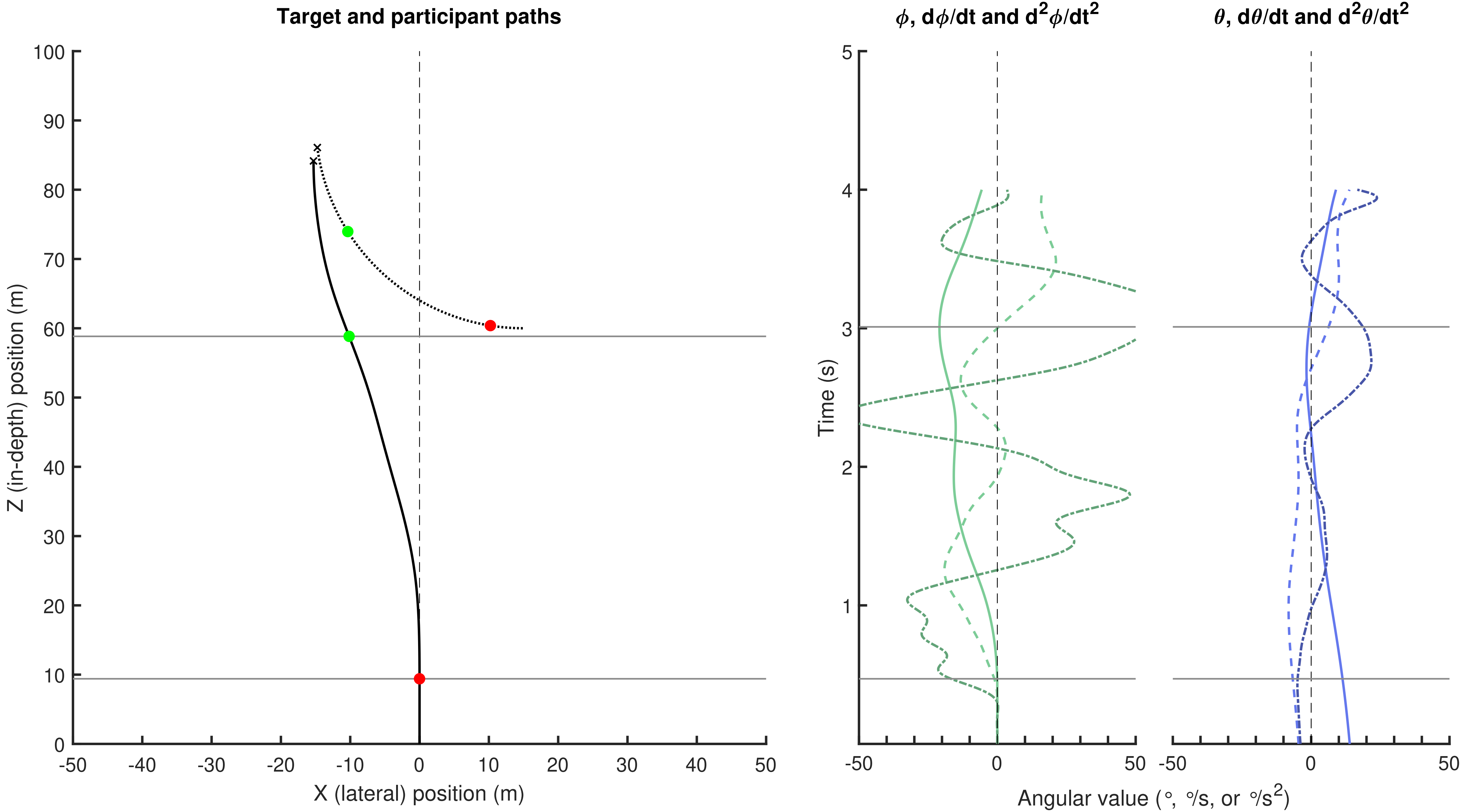

P15/B4
S20/R10-IN

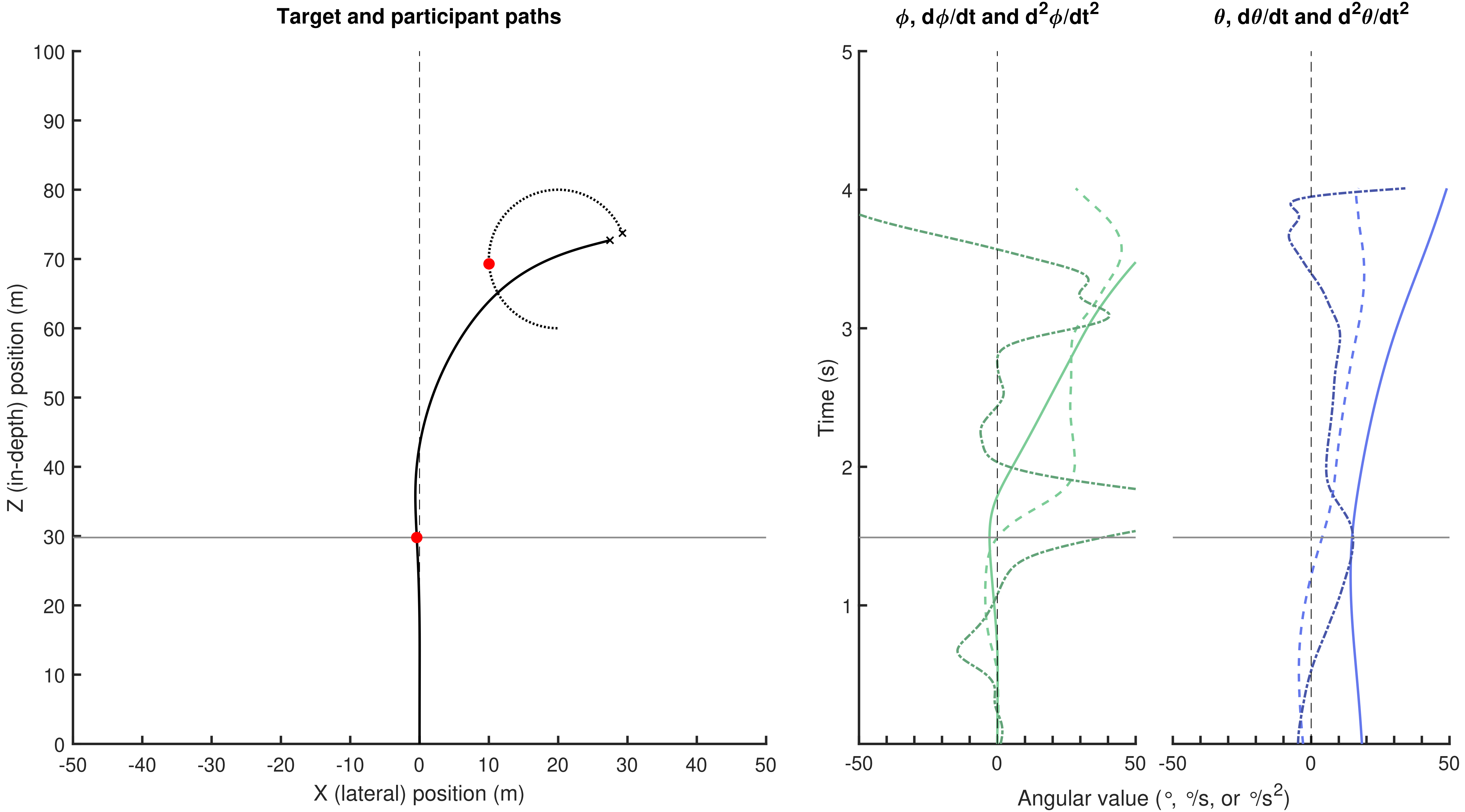

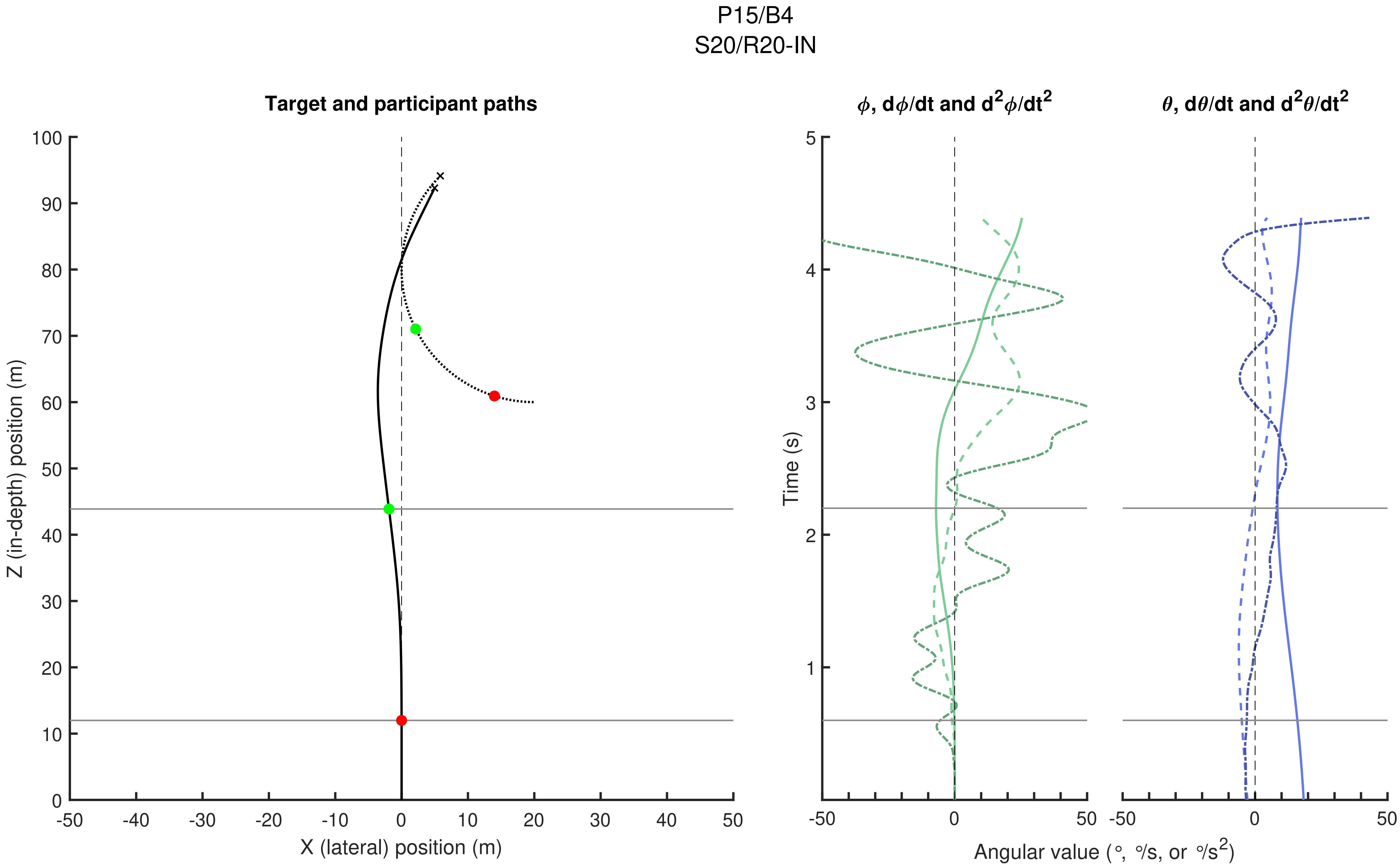
P15/B4
S20/R20-IN
Target and participant paths
ϕ, dϕ/dt and d$^2\phi$/dt^2
θ, dθ/dt and d$^2\theta$/dt^2
Z (in-depth) position (m)
X (lateral) position (m)
Time (s)
Angular value (°, °/s, or °/s^2)

P15/B4
S20/R30-IN

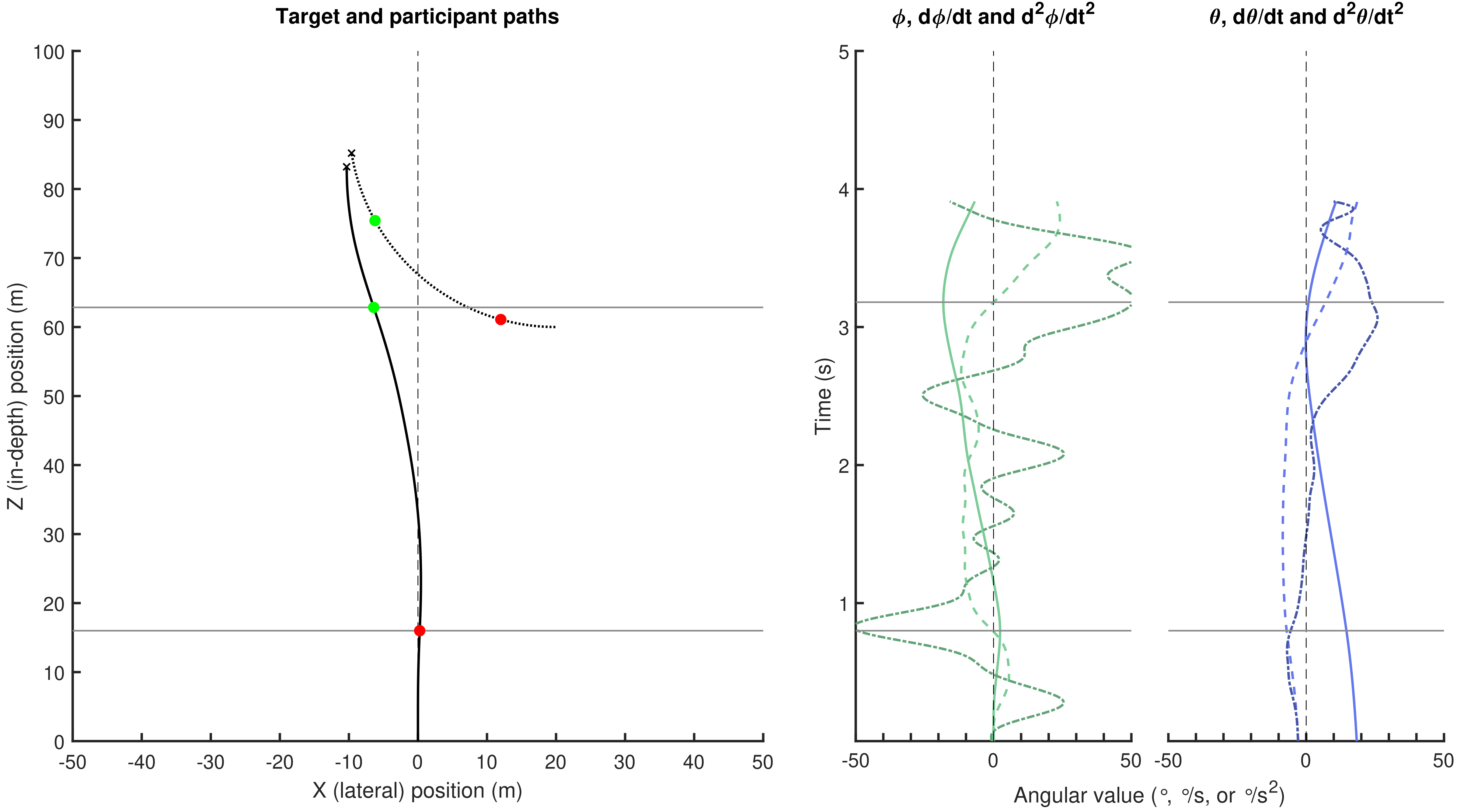

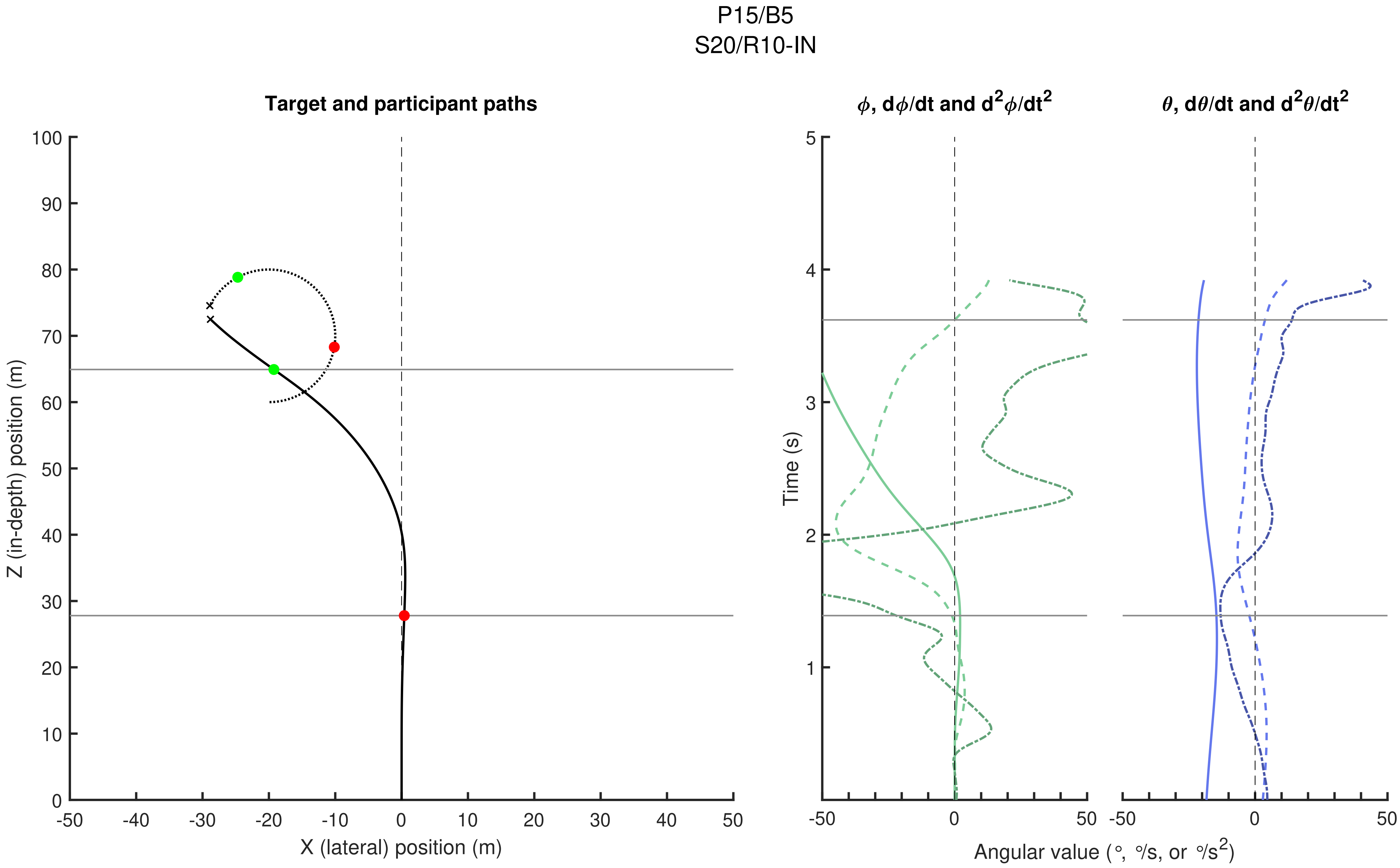
P15/B5
S20/R10-IN
Target and participant paths
ϕ, dϕ/dt and d$^2\phi$/dt^2
θ, dθ/dt and d$^2\theta$/dt^2
Z (in-depth) position (m)
X (lateral) position (m)
Time (s)
Angular value (°, °/s, or °/s^2)
100
90
80
70
60
50
40
30
20
10
0
-50
-40
-30
-20
-10
0
10
20
30
40
50
5
4
3
2
1
-50
0
50
-50
0
50

P15/B5
S20/R20-IN

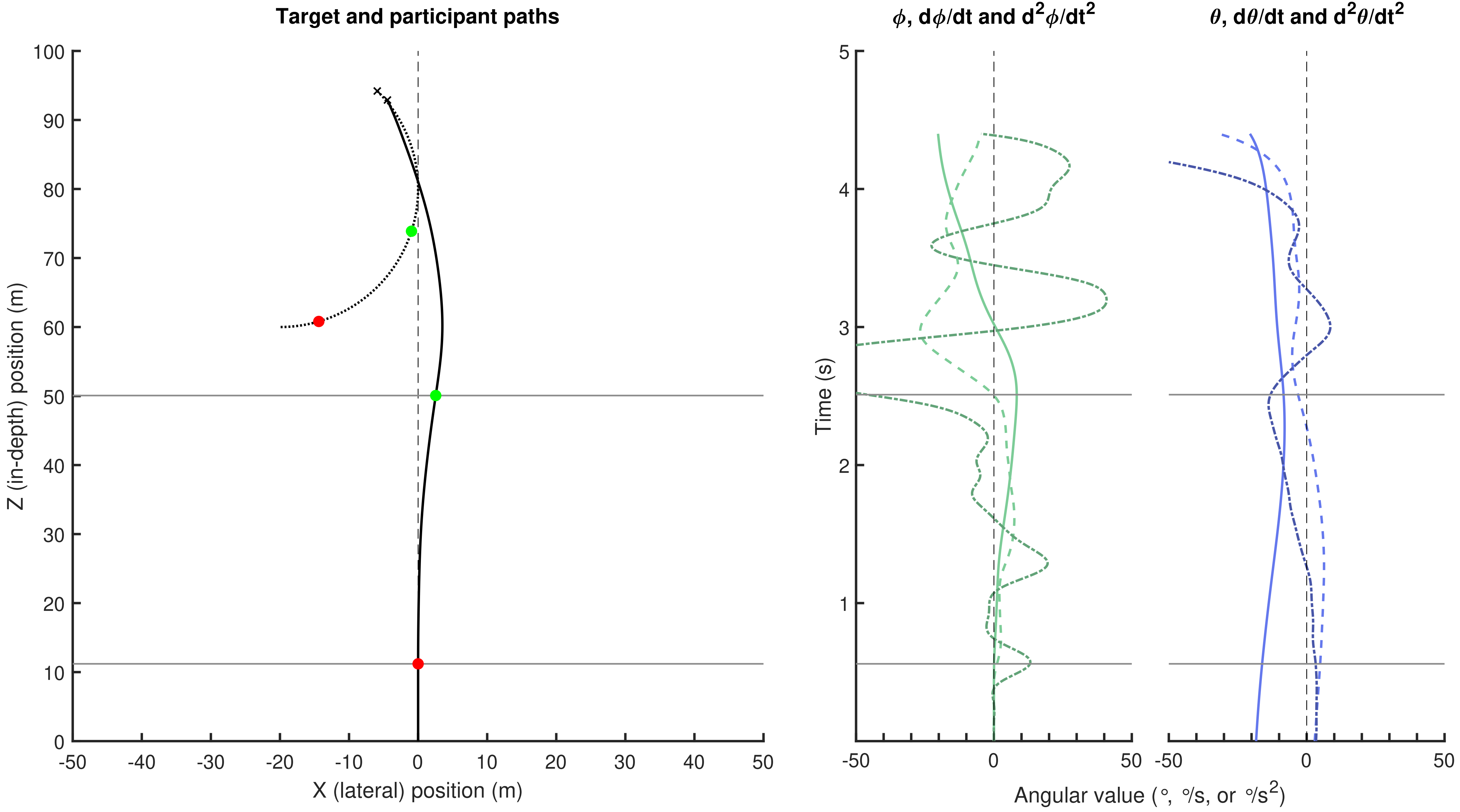

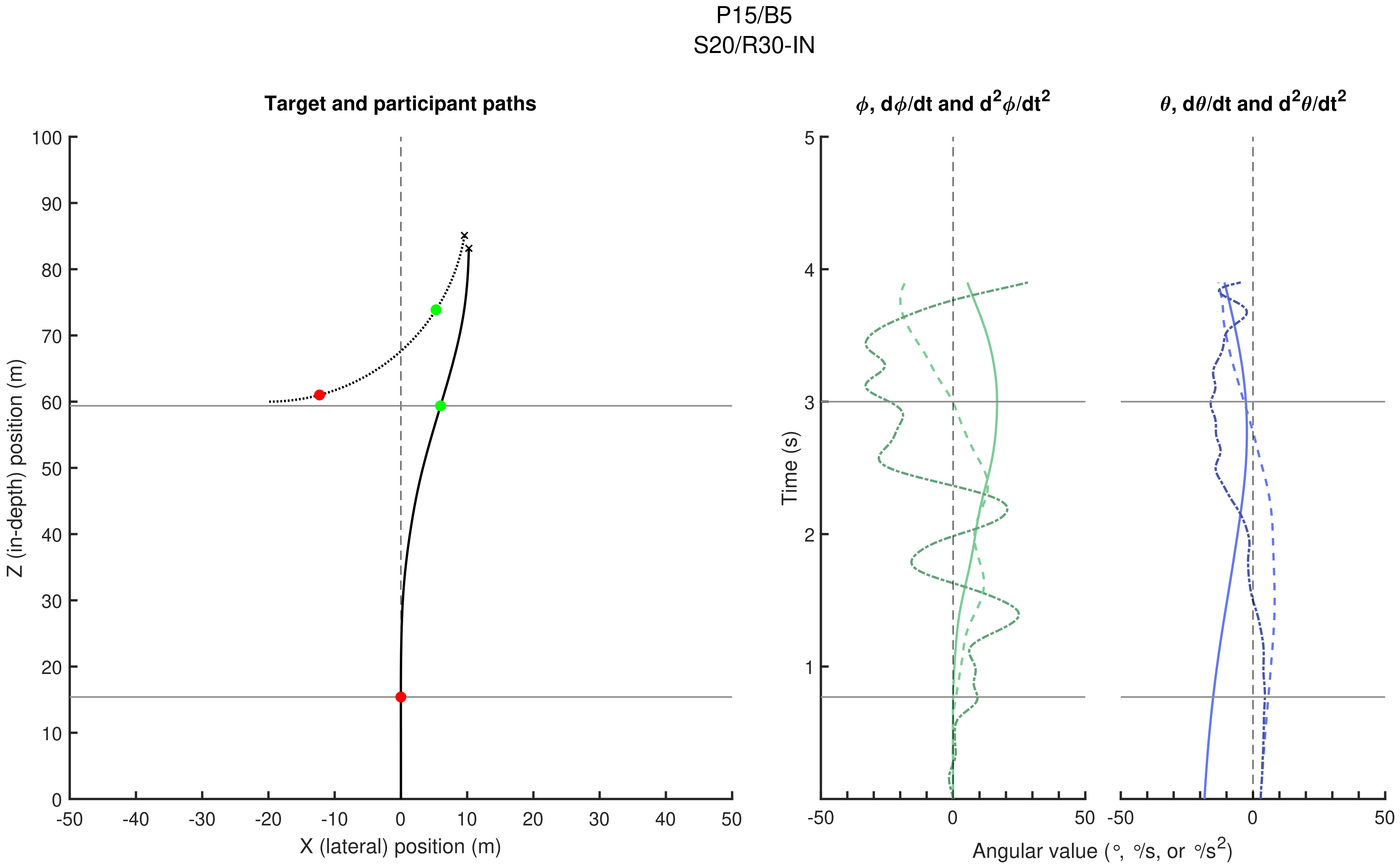
P15/B5
S20/R30-IN
Target and participant paths
Z (in-depth) position (m)
X (lateral) position (m)
100
90
80
70
60
50
40
30
20
10
0
-50
-40
-30
-20
-10
0
10
20
30
40
50
φ, dφ/dt and d²φ/dt²
θ, dθ/dt and d²θ/dt²
Time (s)
5
4
3
2
1
-50
0
50
-50
0
50
Angular value (°, °/s, or °/s²)

P15/B5
S15/R10-IN

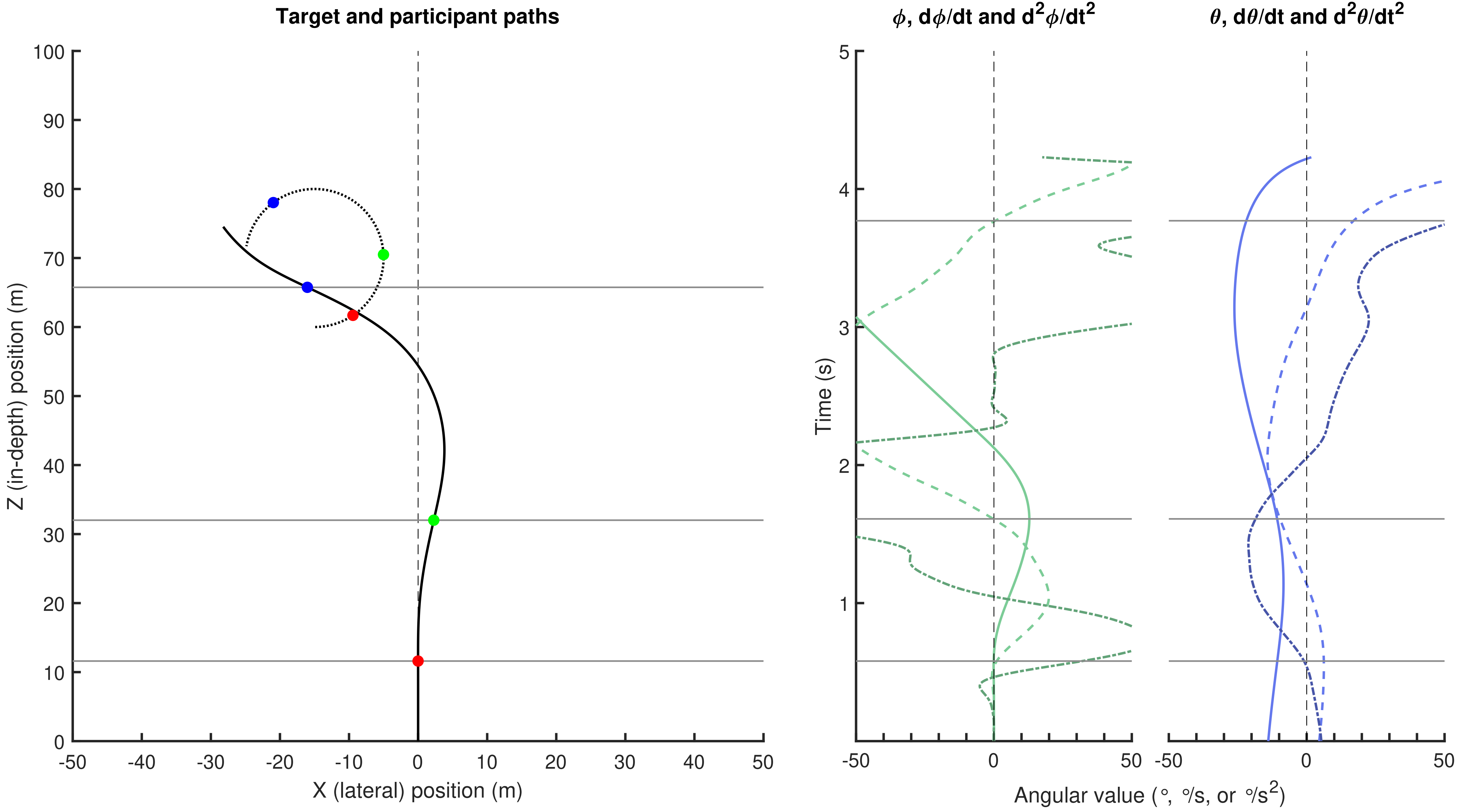

P15/B5
S15/R20-IN

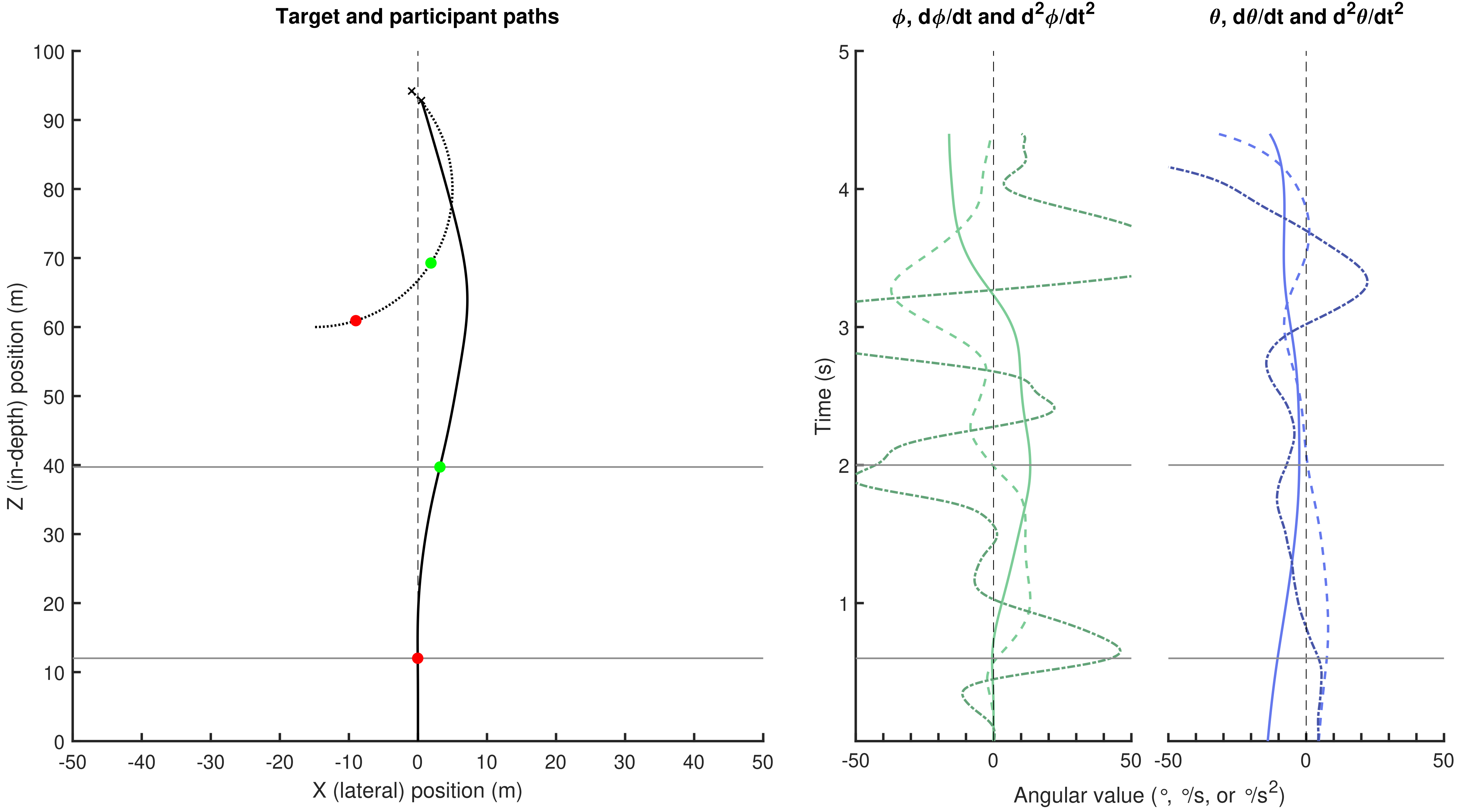

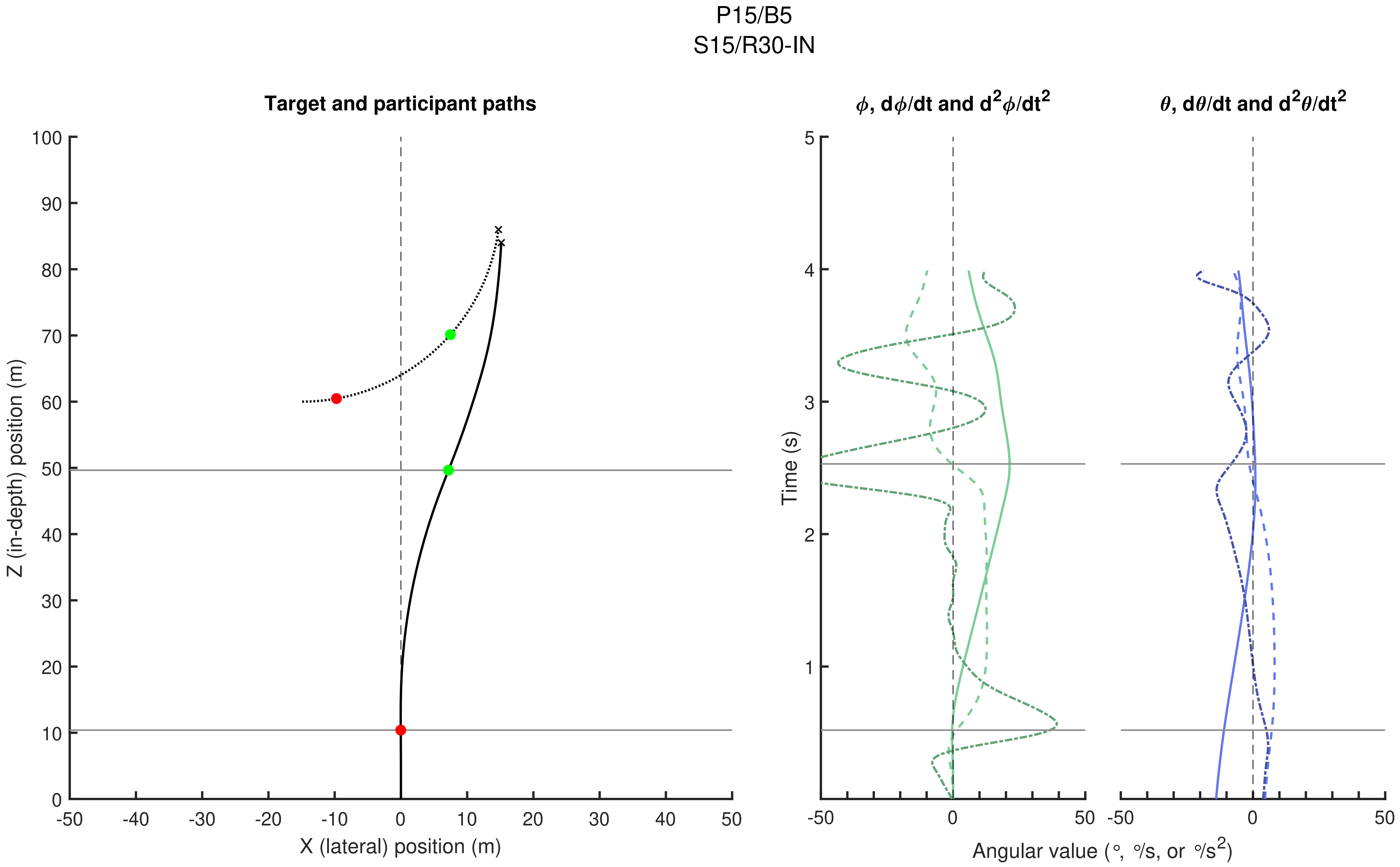

P15/B5
S15/R30-IN
Target and participant paths
Z (in-depth) position (m)
X (lateral) position (m)
ϕ, dϕ/dt and d$^2\phi$/dt^2
θ, dθ/dt and d$^2\theta$/dt^2
Time (s)
Angular value (°, °/s, or °/s^2)

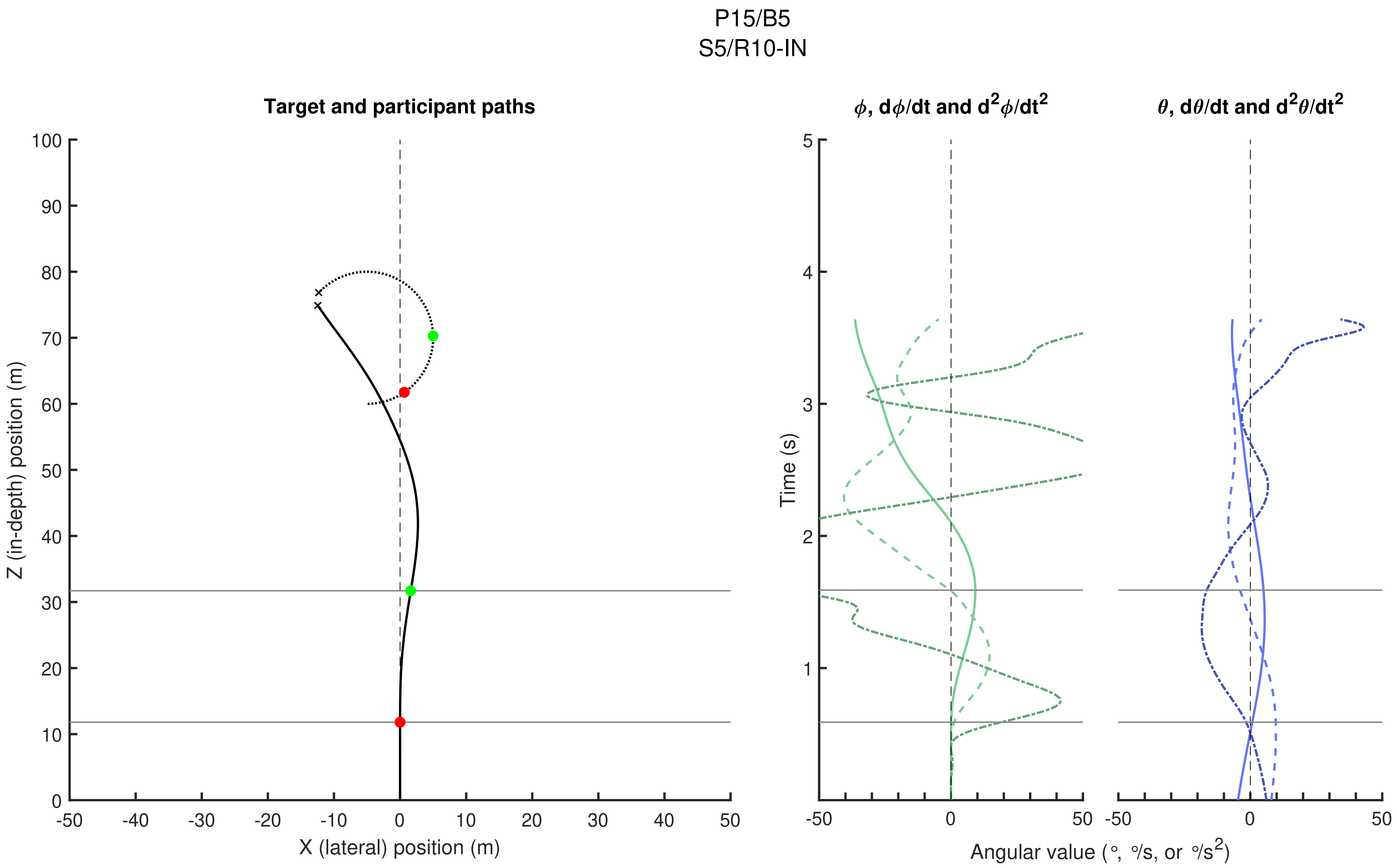
P15/B5
S5/R10-IN
Target and participant paths
Z (in-depth) position (m)
X (lateral) position (m)
100
90
80
70
60
50
40
30
20
10
0
-50
-40
-30
-20
-10
0
10
20
30
40
50
φ, dφ/dt and d²φ/dt²
θ, dθ/dt and d²θ/dt²
Time (s)
5
4
3
2
1
-50
0
50
-50
0
50
Angular value (°, °/s, or °/s²)

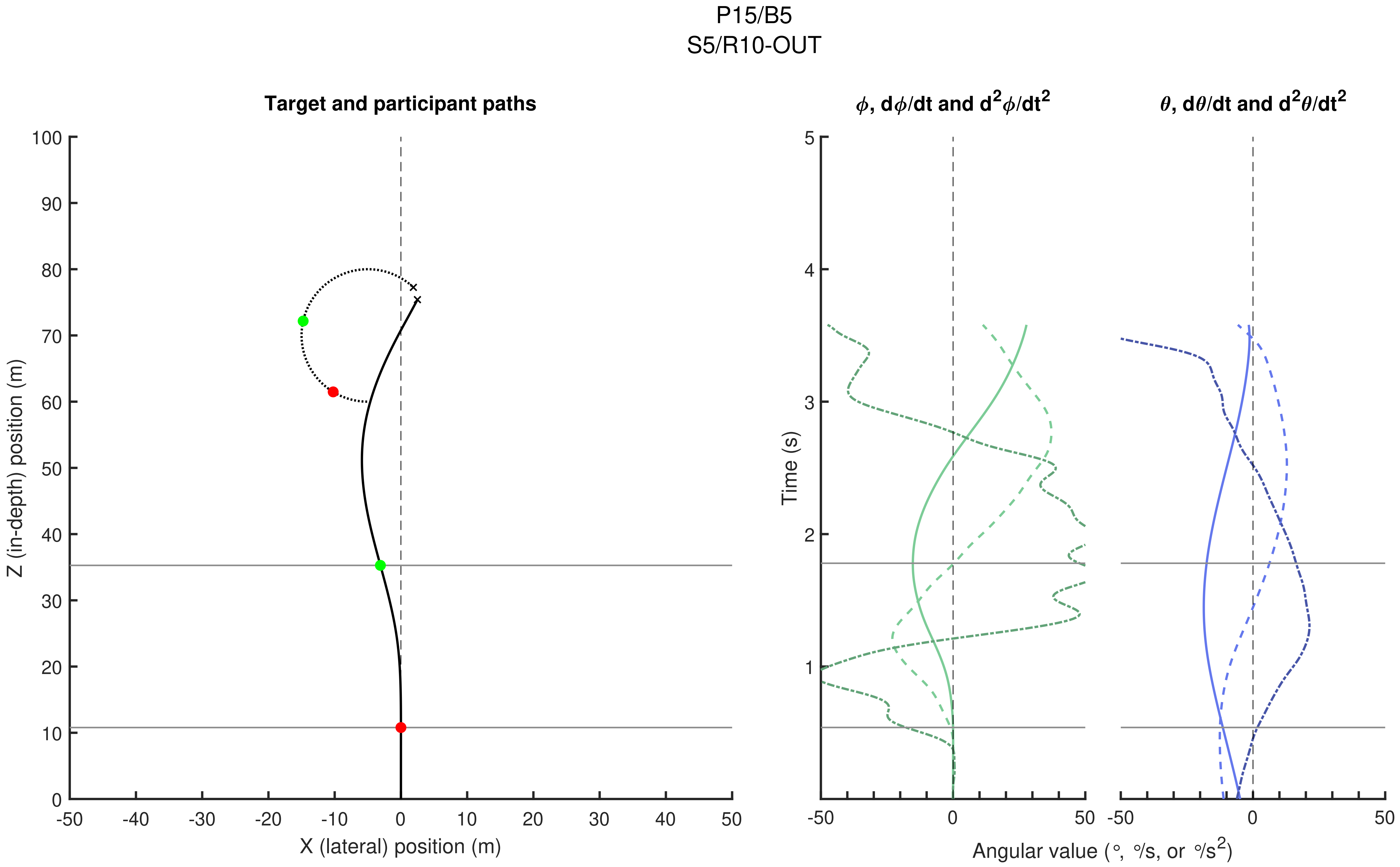

P15/B5
S5/R10-OUT
Target and participant paths
φ, dφ/dt and d²φ/dt²
θ, dθ/dt and d²θ/dt²
Z (in-depth) position (m)
X (lateral) position (m)
Time (s)
Angular value (°, °/s, or °/s²)

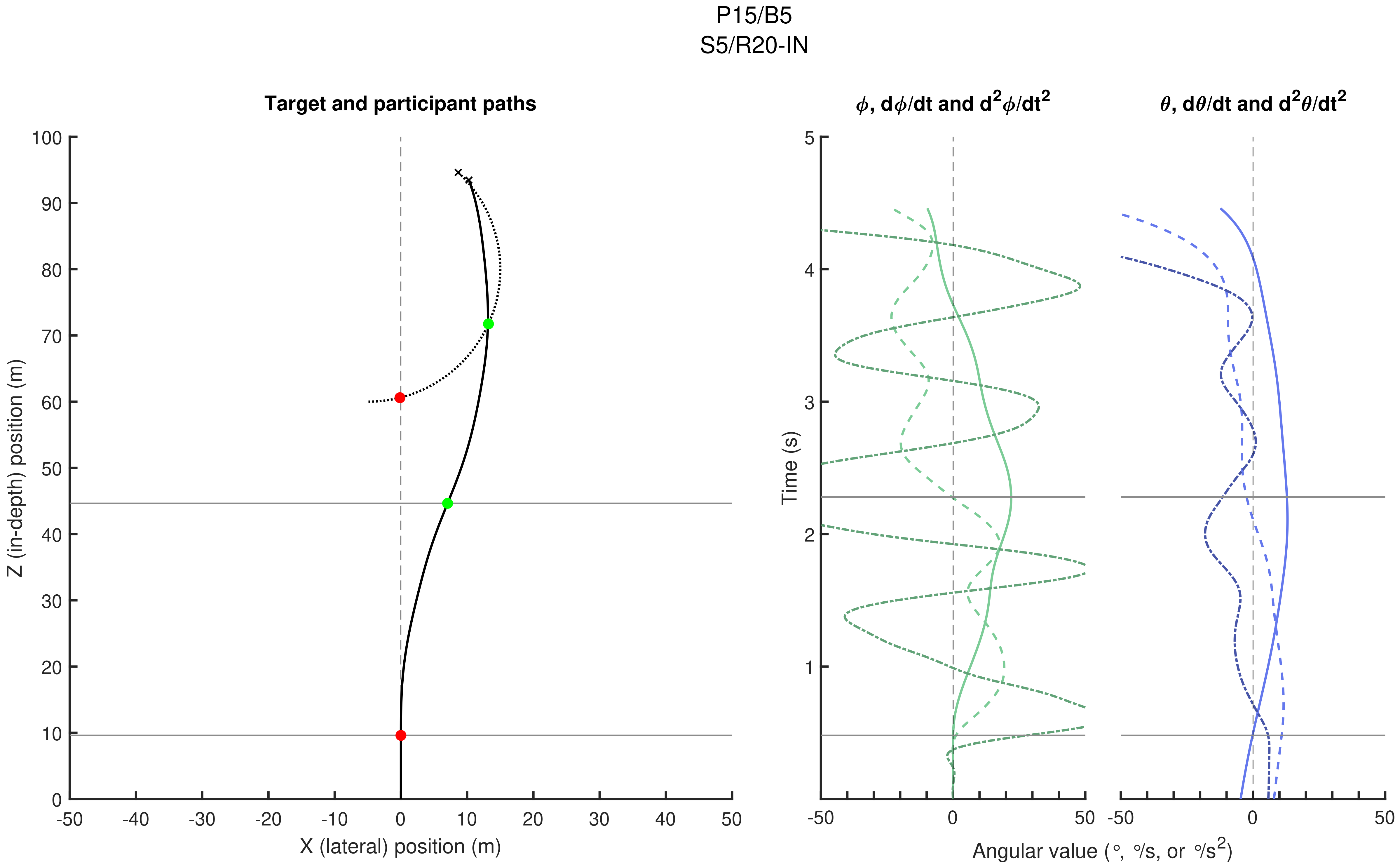

P15/B5
S5/R20-IN
Target and participant paths
Z (in-depth) position (m)
X (lateral) position (m)
ϕ, dϕ/dt and d$^2\phi$/dt^2
θ, dθ/dt and d$^2\theta$/dt^2
Time (s)
Angular value (°, °/s, or °/s^2)

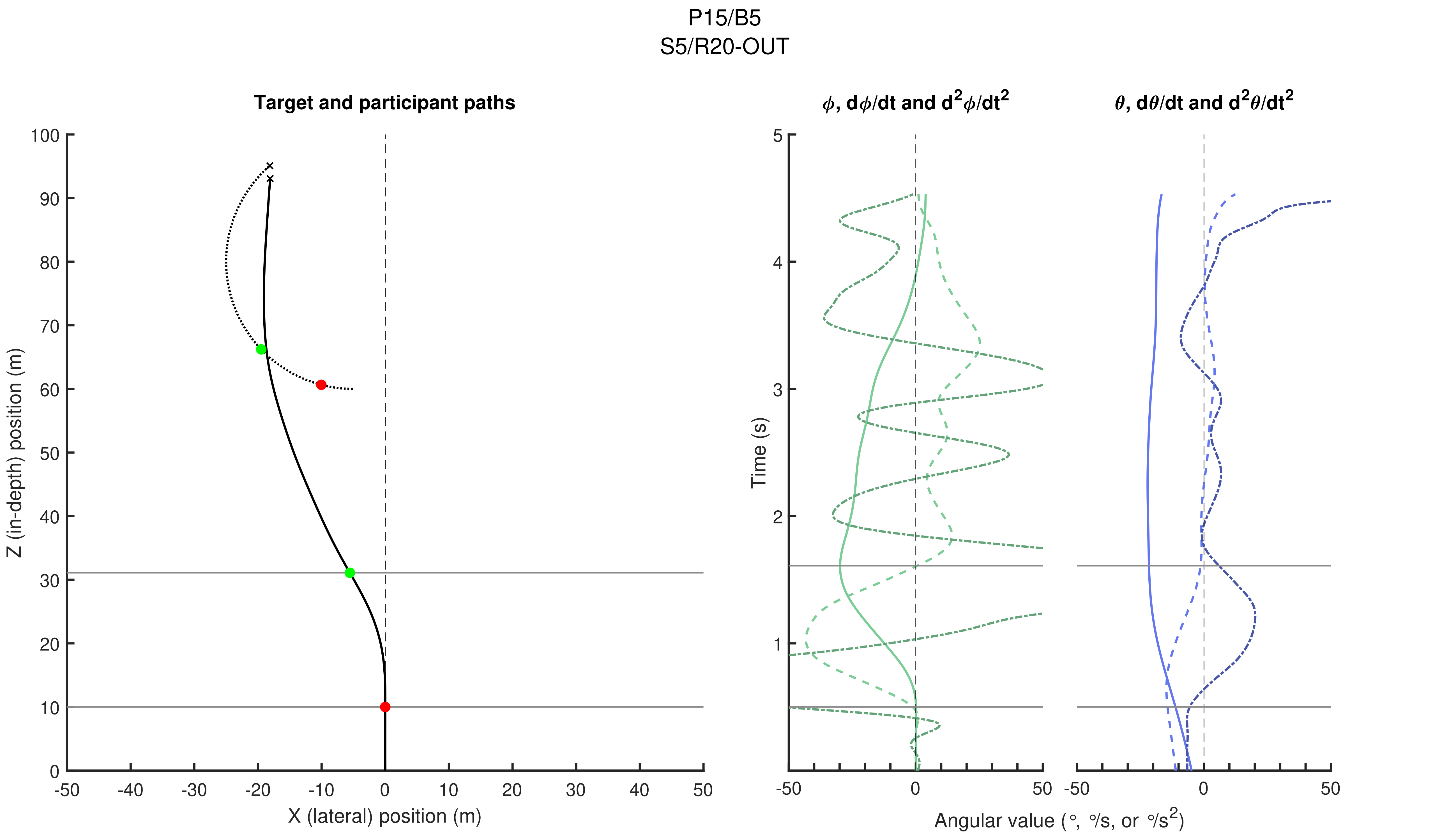
P15/B5
S5/R20-OUT
Target and participant paths
ϕ, dϕ/dt and d$^2\phi$/dt^2
θ, dθ/dt and d$^2\theta$/dt^2
Z (in-depth) position (m)
X (lateral) position (m)
Time (s)
Angular value (°, °/s, or °/s^2)

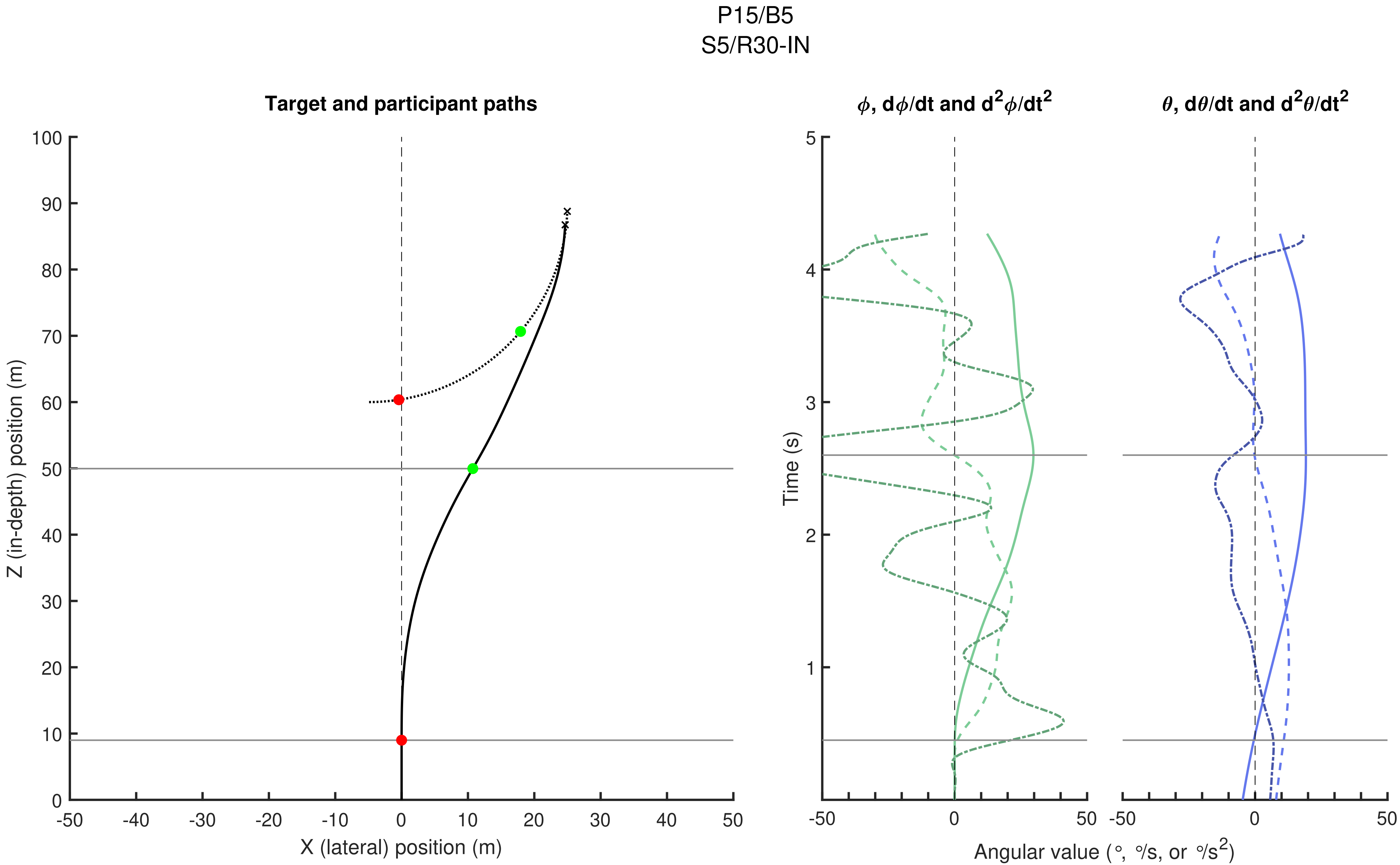
P15/B5
S5/R30-IN
Target and participant paths
Z (in-depth) position (m)
X (lateral) position (m)
100
90
80
70
60
50
40
30
20
10
0
-50
-40
-30
-20
-10
0
10
20
30
40
50
ϕ, dϕ/dt and d$^2\phi$/dt^2
θ, dθ/dt and d$^2\theta$/dt^2
Time (s)
5
4
3
2
1
-50
0
50
-50
0
50
Angular value (°, °/s, or °/s^2)

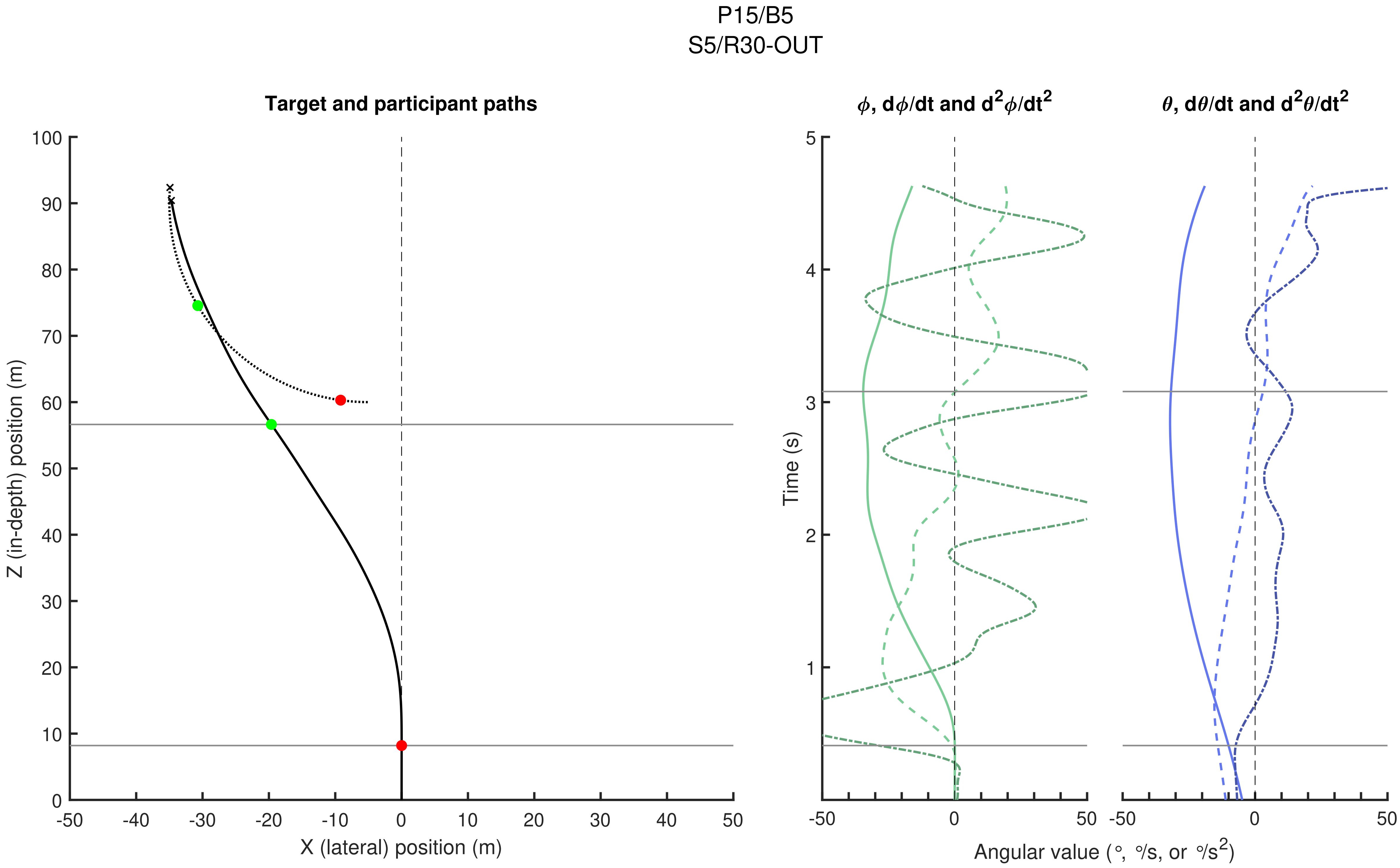
P15/B5
S5/R30-OUT
Target and participant paths
Z (in-depth) position (m)
X (lateral) position (m)
ϕ, dϕ/dt and d$^2\phi$/dt^2
θ, dθ/dt and d$^2\theta$/dt^2
Time (s)
Angular value (°, °/s, or °/s^2)

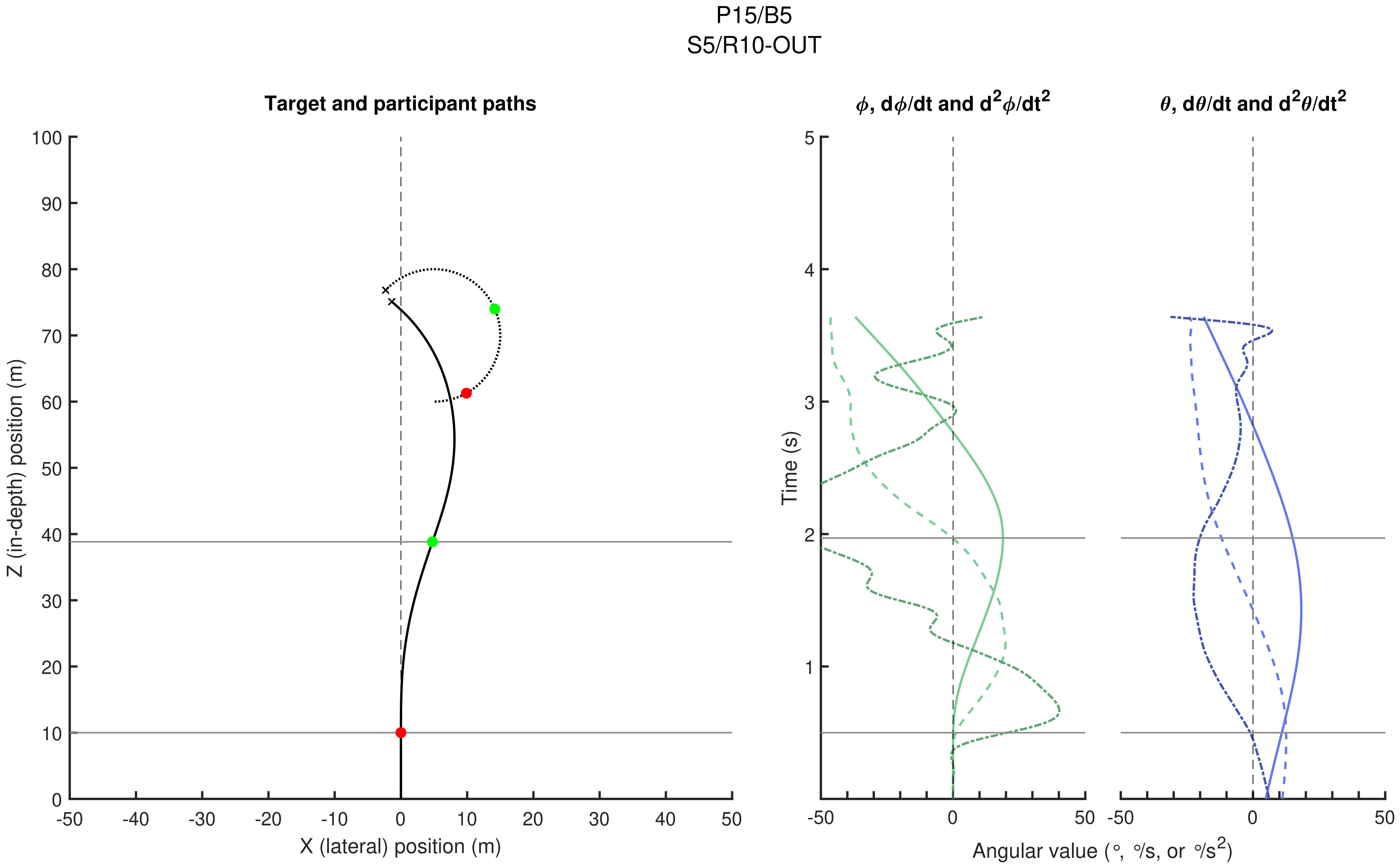

P15/B5
S5/R10-OUT
Target and participant paths
Z (in-depth) position (m)
X (lateral) position (m)
φ, dφ/dt and d²φ/dt²
θ, dθ/dt and d²θ/dt²
Time (s)
Angular value (°, °/s, or °/s²)

P15/B5
S5/R10-IN

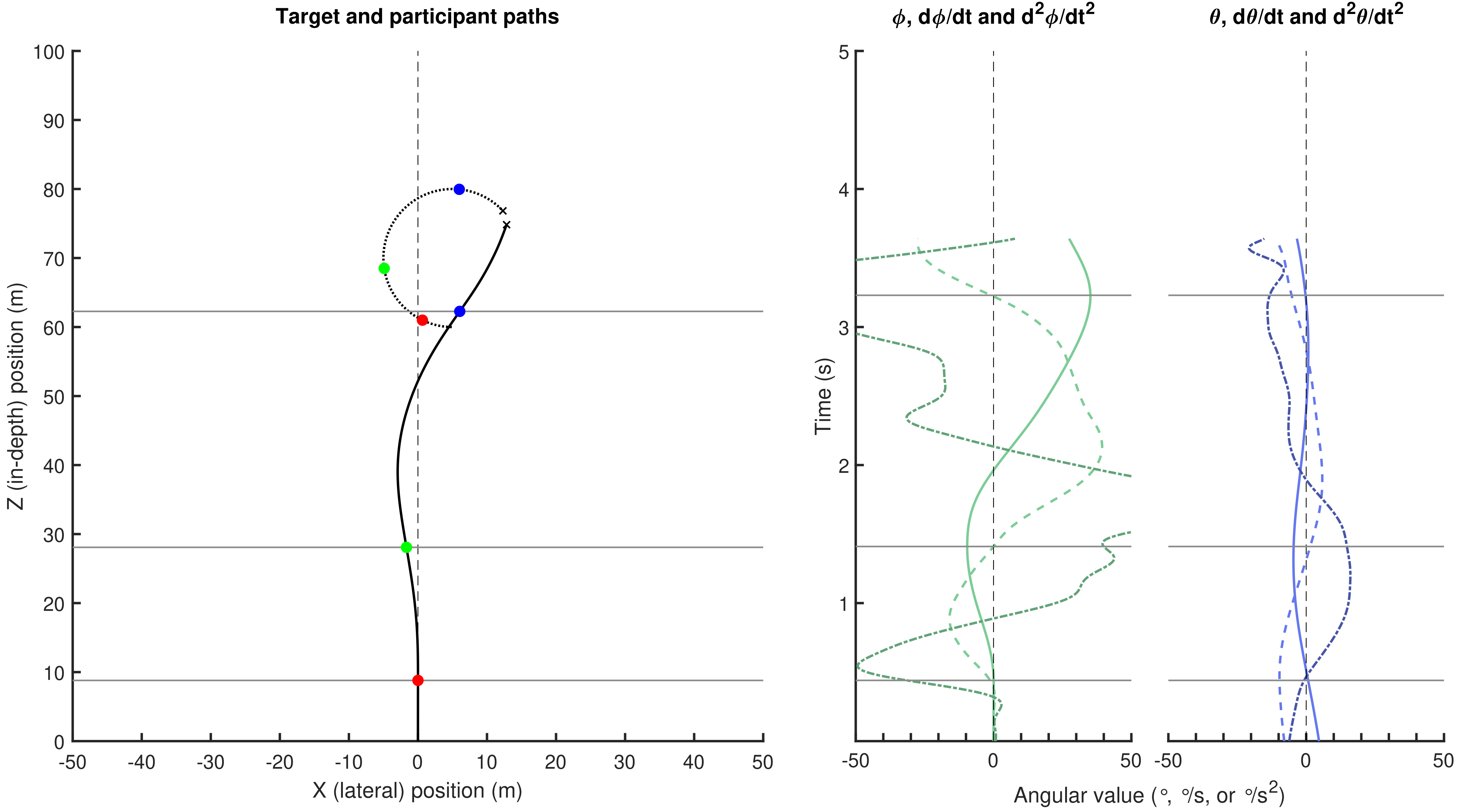

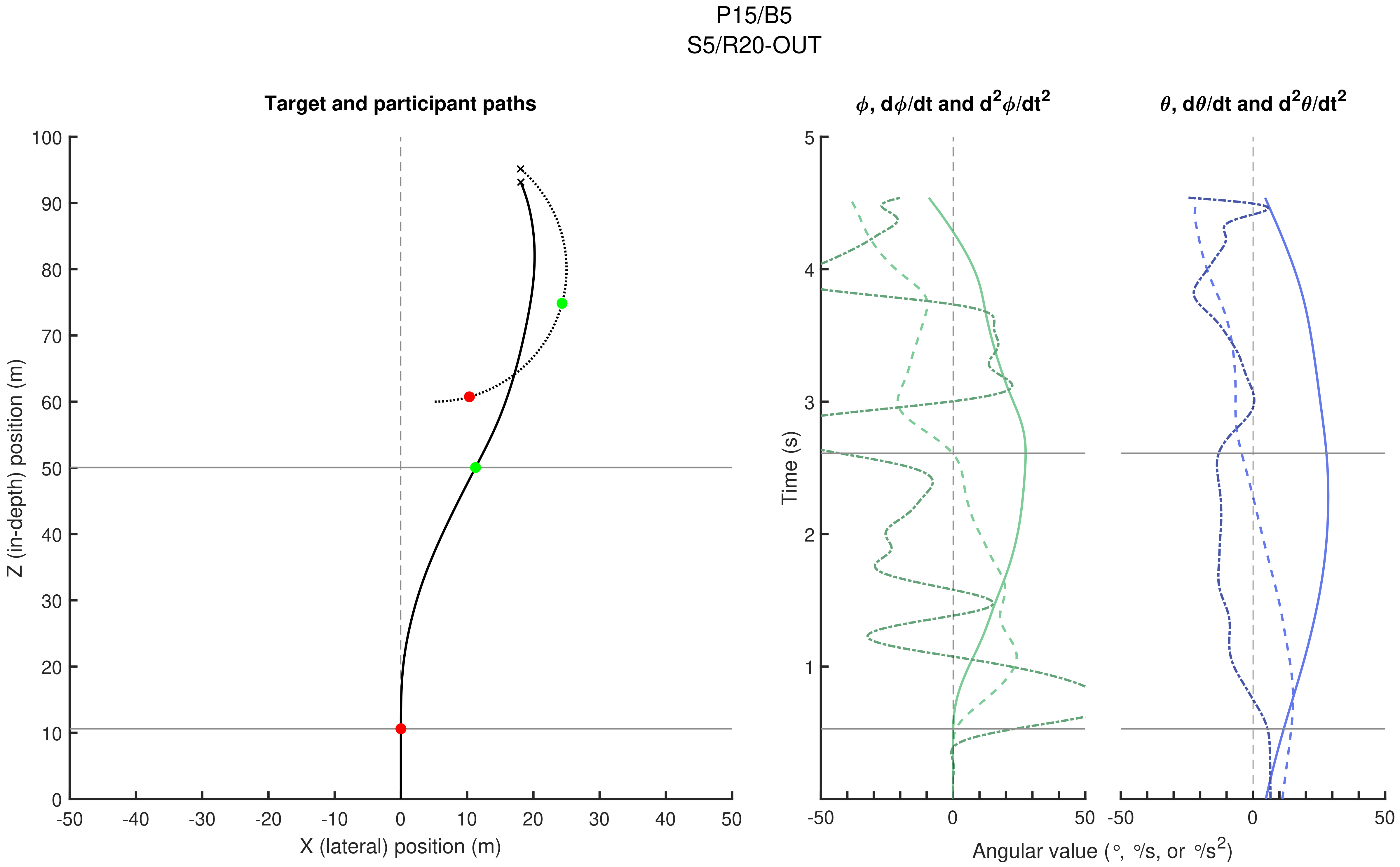
P15/B5
S5/R20-OUT
Target and participant paths
Z (in-depth) position (m)
X (lateral) position (m)
φ, dφ/dt and d²φ/dt²
θ, dθ/dt and d²θ/dt²
Time (s)
Angular value (°, °/s, or °/s²)

P15/B5
S5/R20-IN

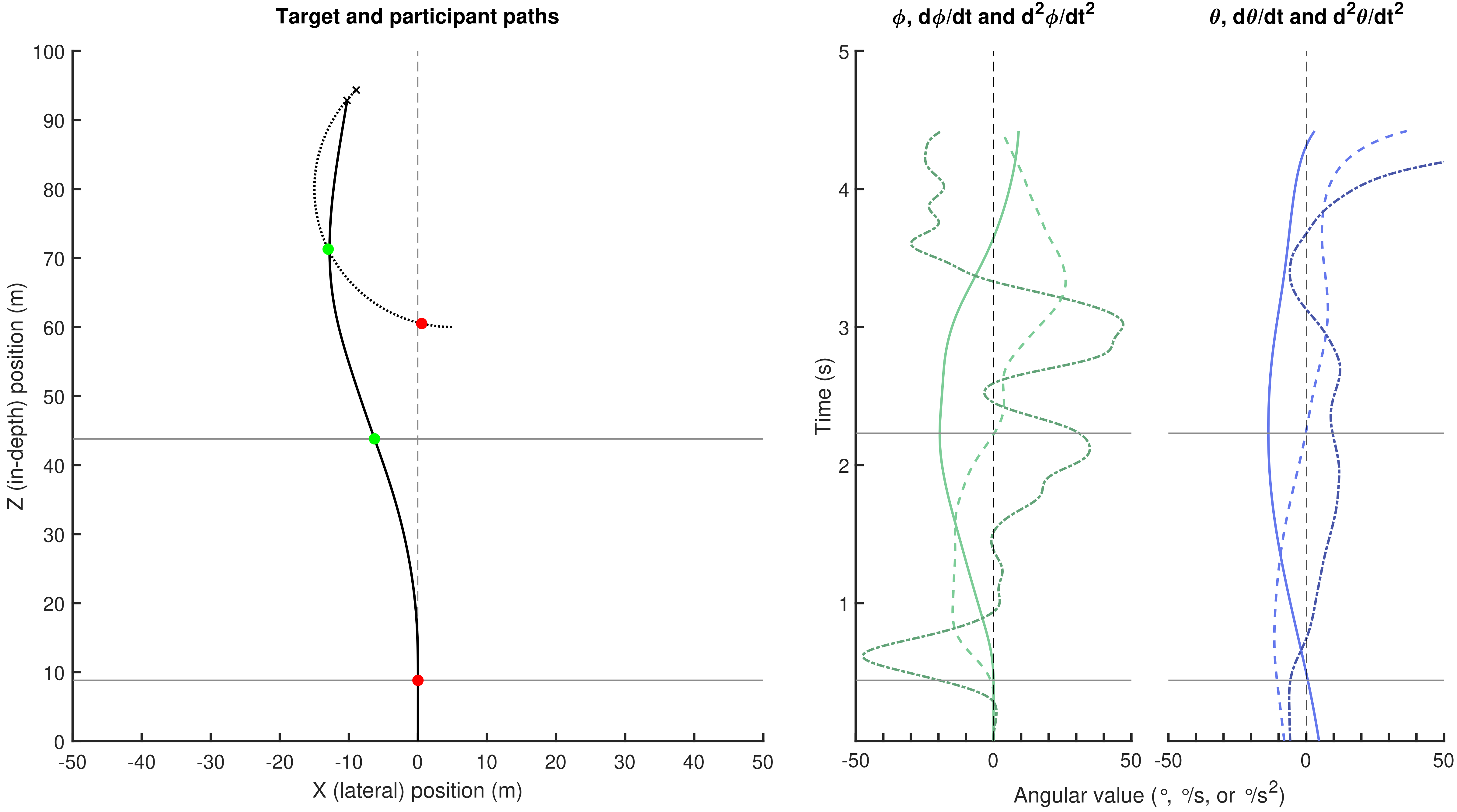

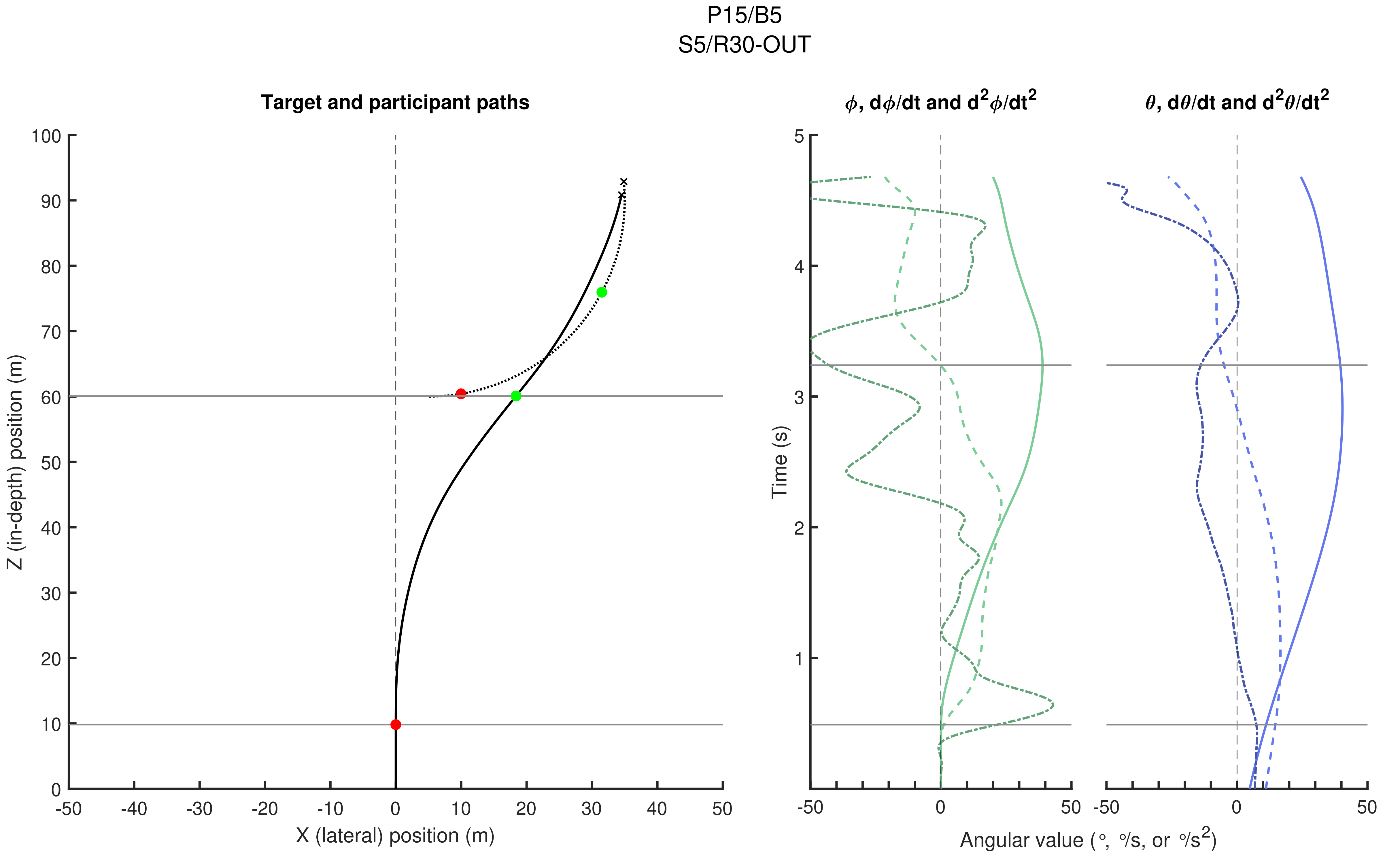

P15/B5
S5/R30-OUT
Target and participant paths
Z (in-depth) position (m)
X (lateral) position (m)
φ, dφ/dt and d²φ/dt²
θ, dθ/dt and d²θ/dt²
Time (s)
Angular value (°, °/s, or °/s²)

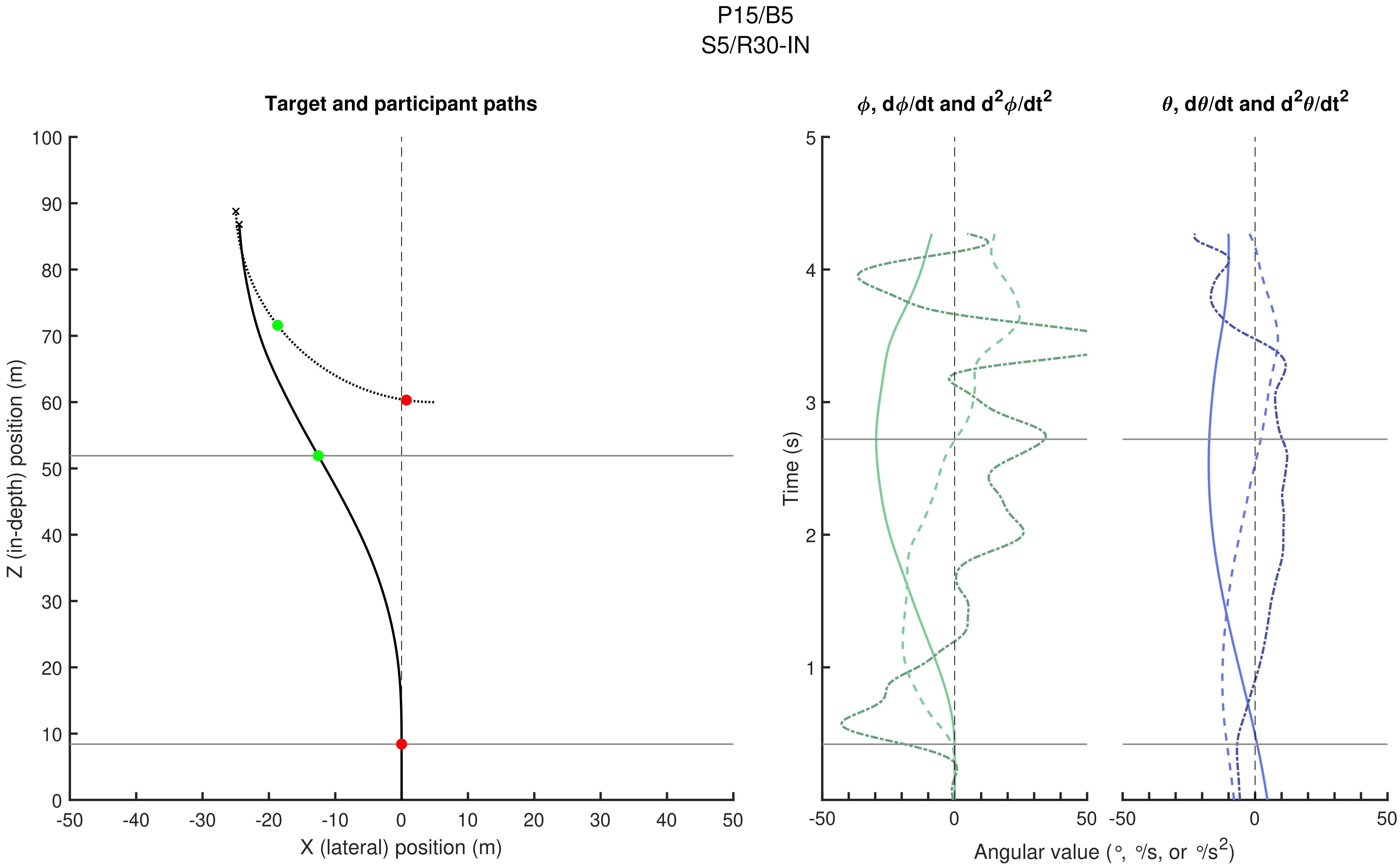
P15/B5
S5/R30-IN
Target and participant paths
Z (in-depth) position (m)
X (lateral) position (m)
ϕ, dϕ/dt and d$^2\phi$/dt^2
θ, dθ/dt and d$^2\theta$/dt^2
Time (s)
Angular value (°, °/s, or °/s^2)

P15/B5
S15/R10-IN

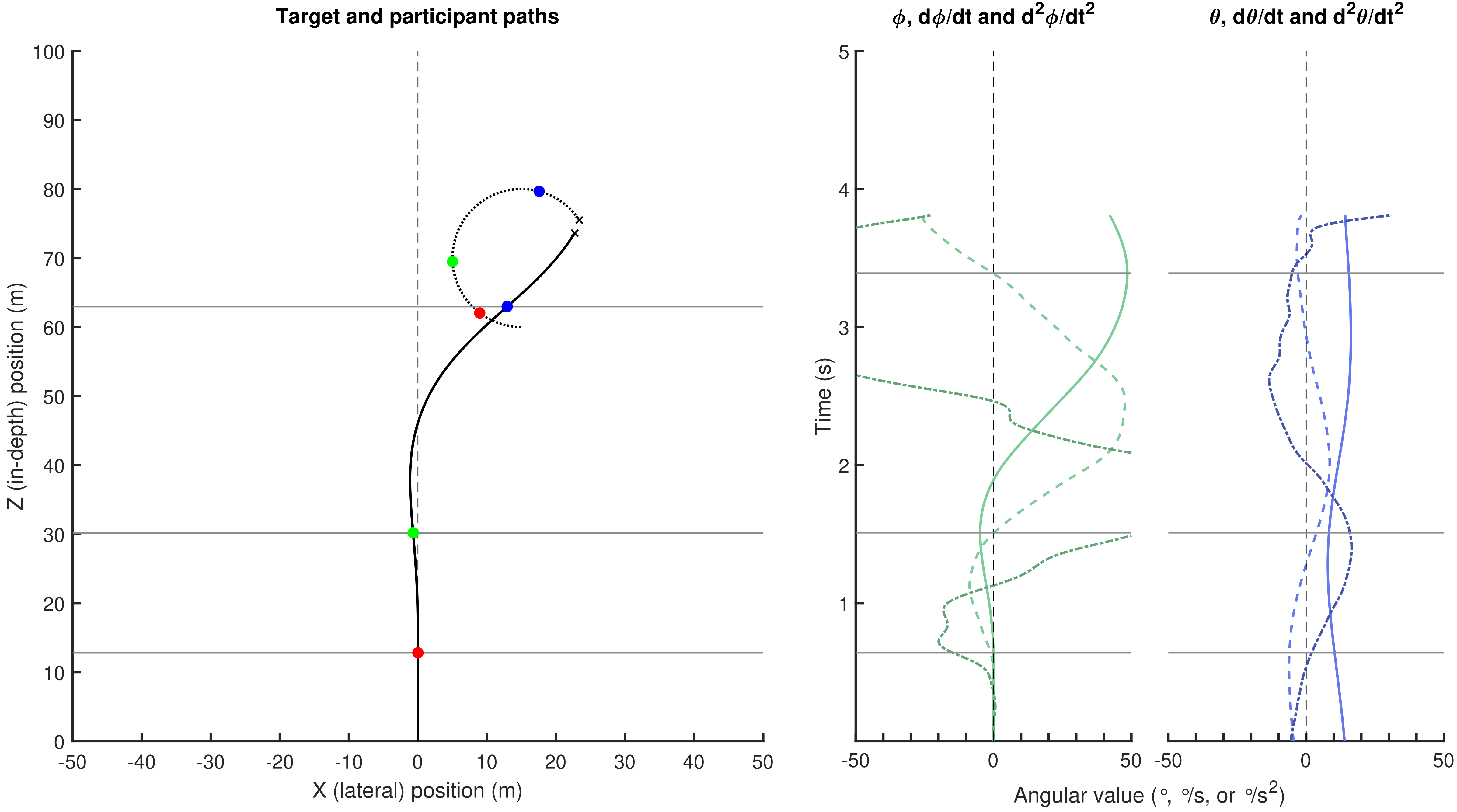

P15/B5
S15/R20-IN

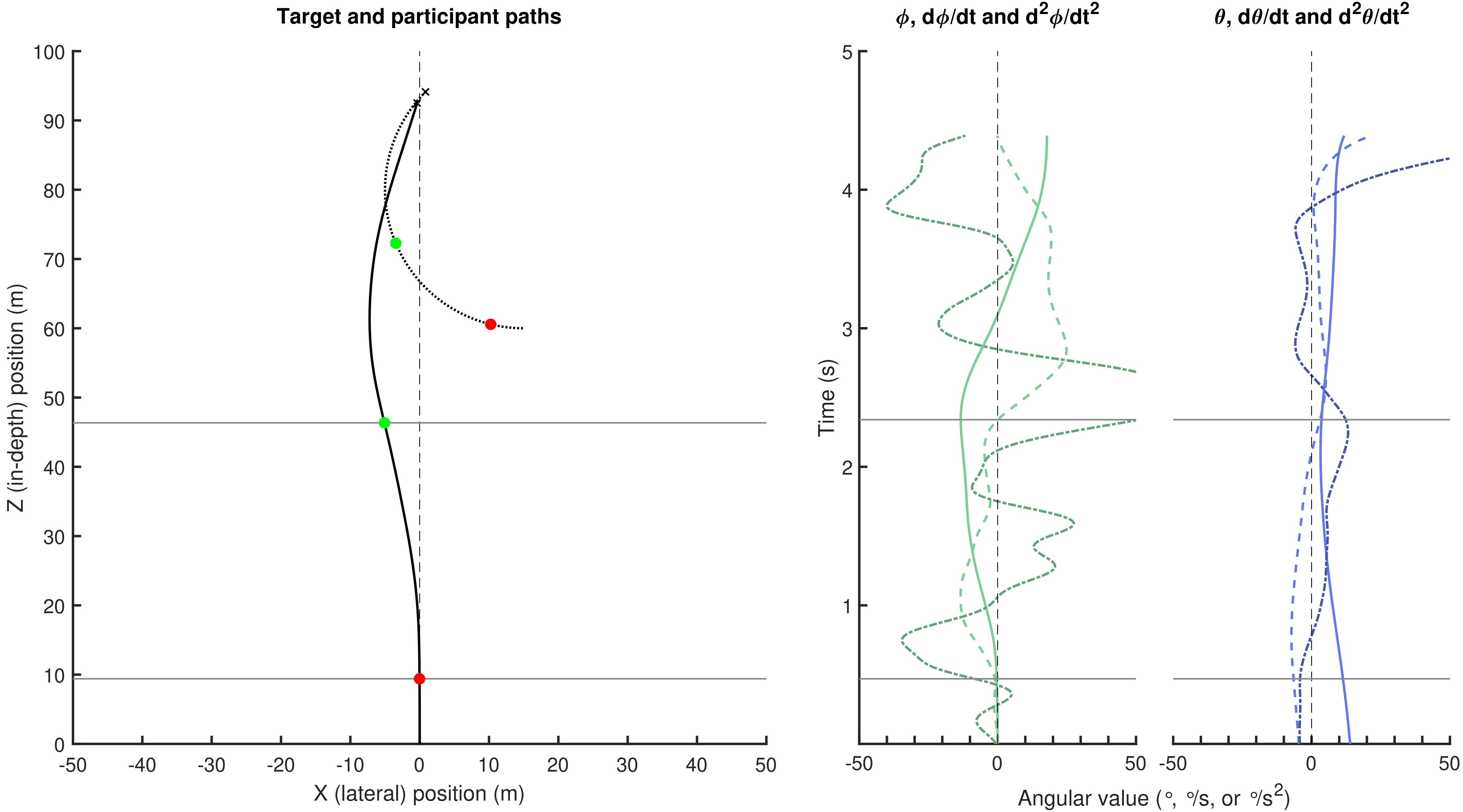

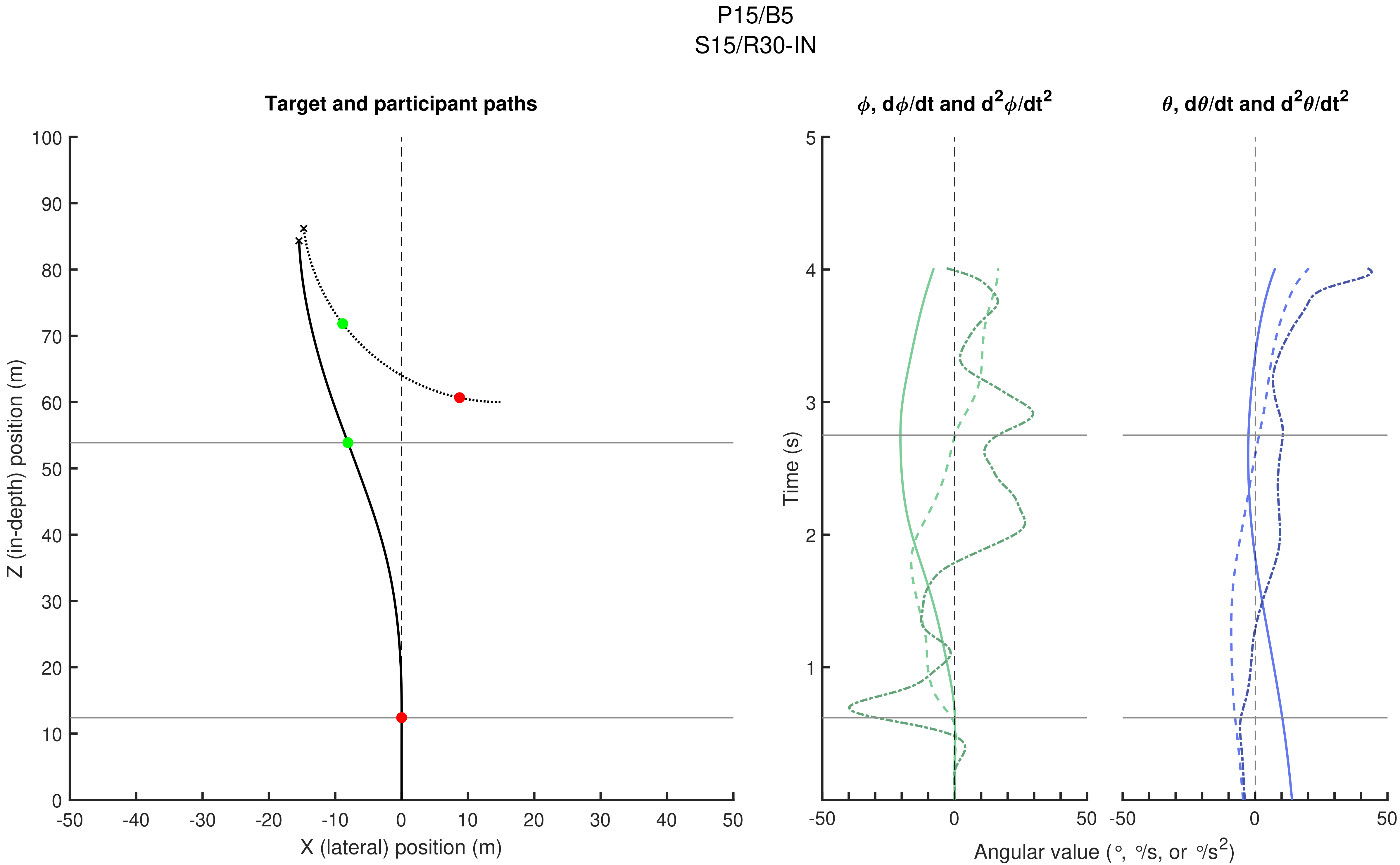
P15/B5
S15/R30-IN
Target and participant paths
Z (in-depth) position (m)
X (lateral) position (m)
ϕ, dϕ/dt and d$^2\phi$/dt^2
θ, dθ/dt and d$^2\theta$/dt^2
Time (s)
Angular value (°, °/s, or °/s^2)

P15/B5
S20/R10-IN

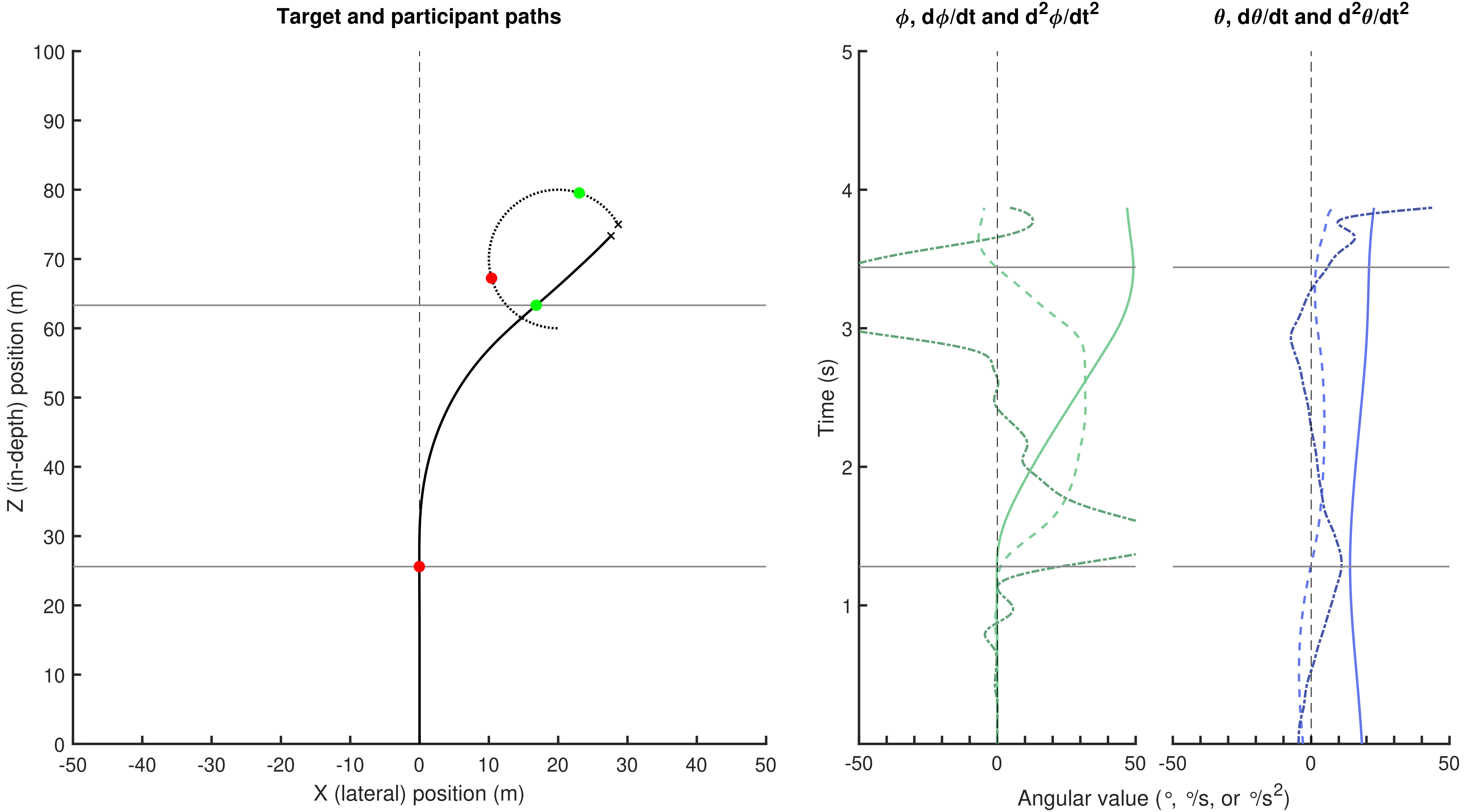

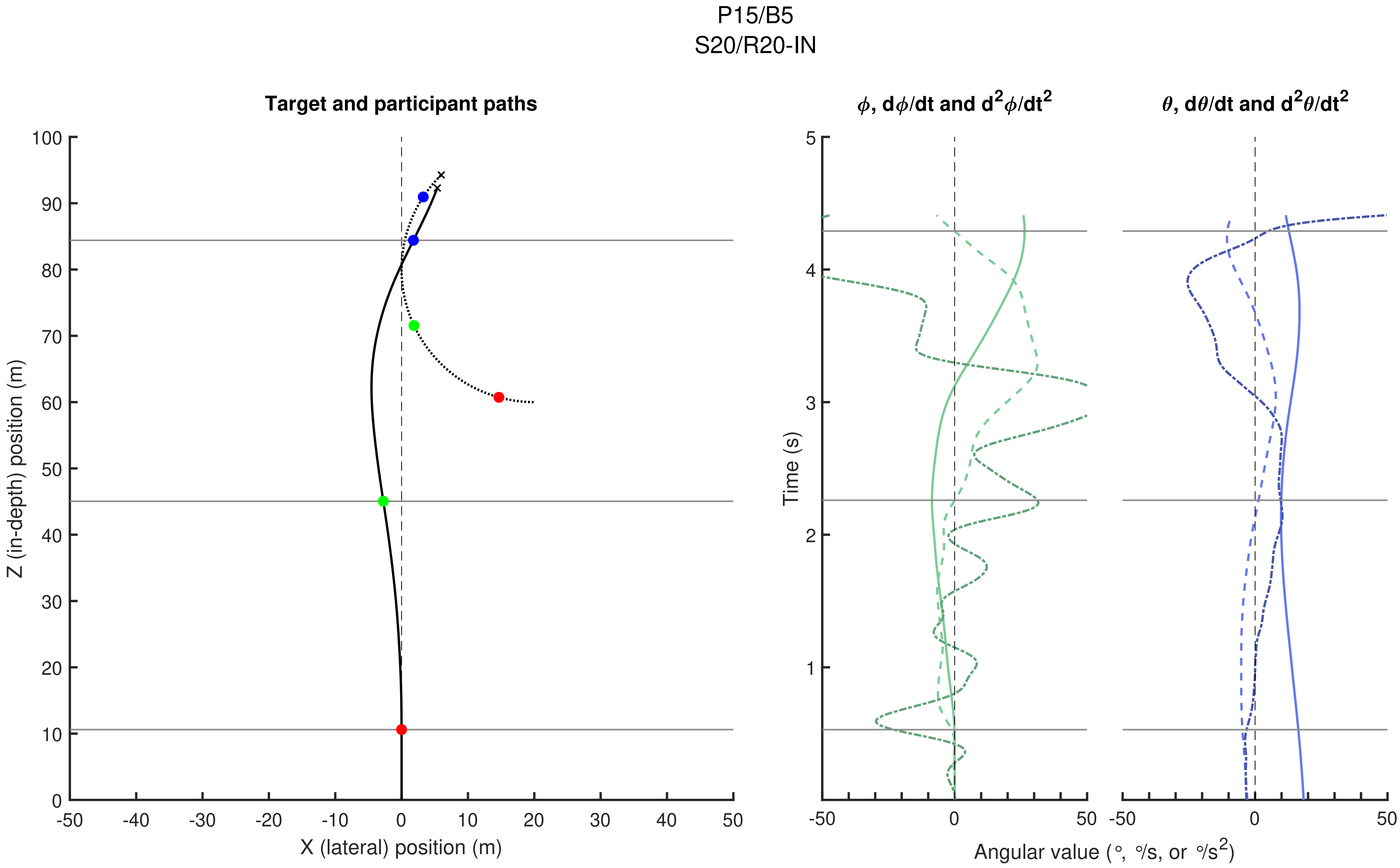
P15/B5
S20/R20-IN
Target and participant paths
Z (in-depth) position (m)
X (lateral) position (m)
ϕ, dϕ/dt and d²ϕ/dt²
θ, dθ/dt and d²θ/dt²
Time (s)
Angular value (°, °/s, or °/s²)

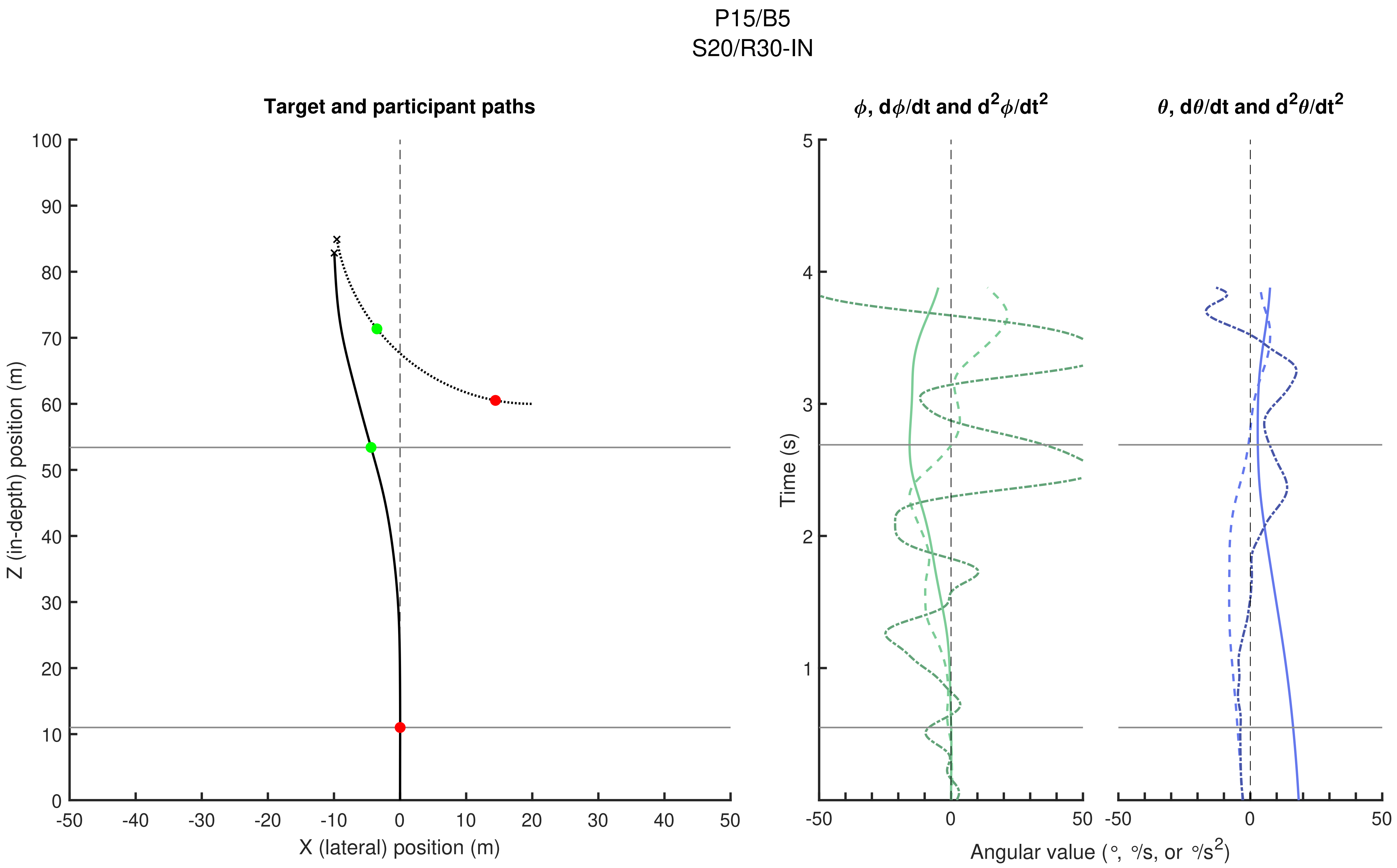
P15/B5
S20/R30-IN
Target and participant paths
Z (in-depth) position (m)
X (lateral) position (m)
100
90
80
70
60
50
40
30
20
10
0
-50
-40
-30
-20
-10
0
10
20
30
40
50
φ, dφ/dt and d²φ/dt²
θ, dθ/dt and d²θ/dt²
Time (s)
5
4
3
2
1
-50
0
50
-50
0
50
Angular value (°, °/s, or °/s²)

P16/B1
S20/R10-IN

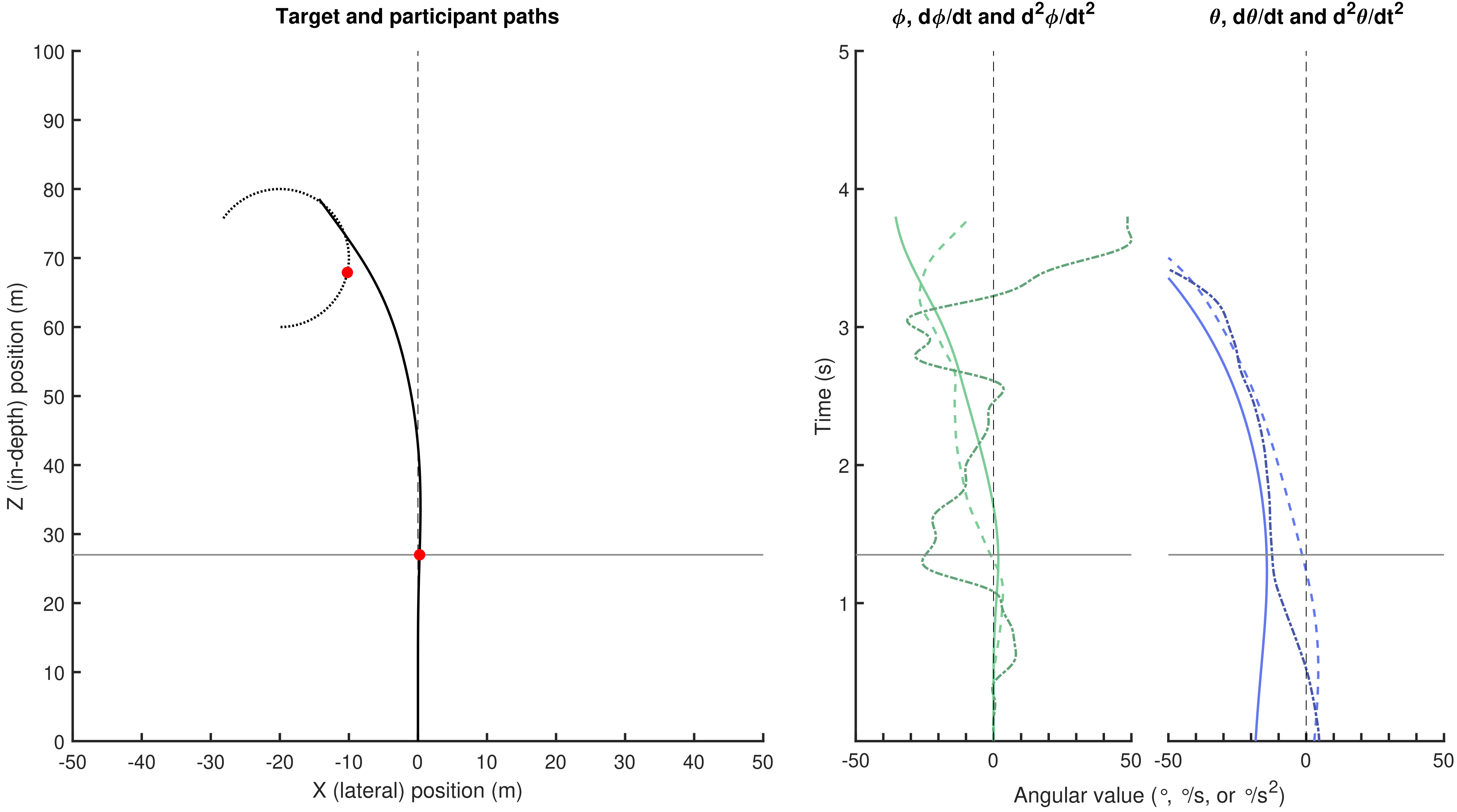

P16/B1
S20/R20-IN

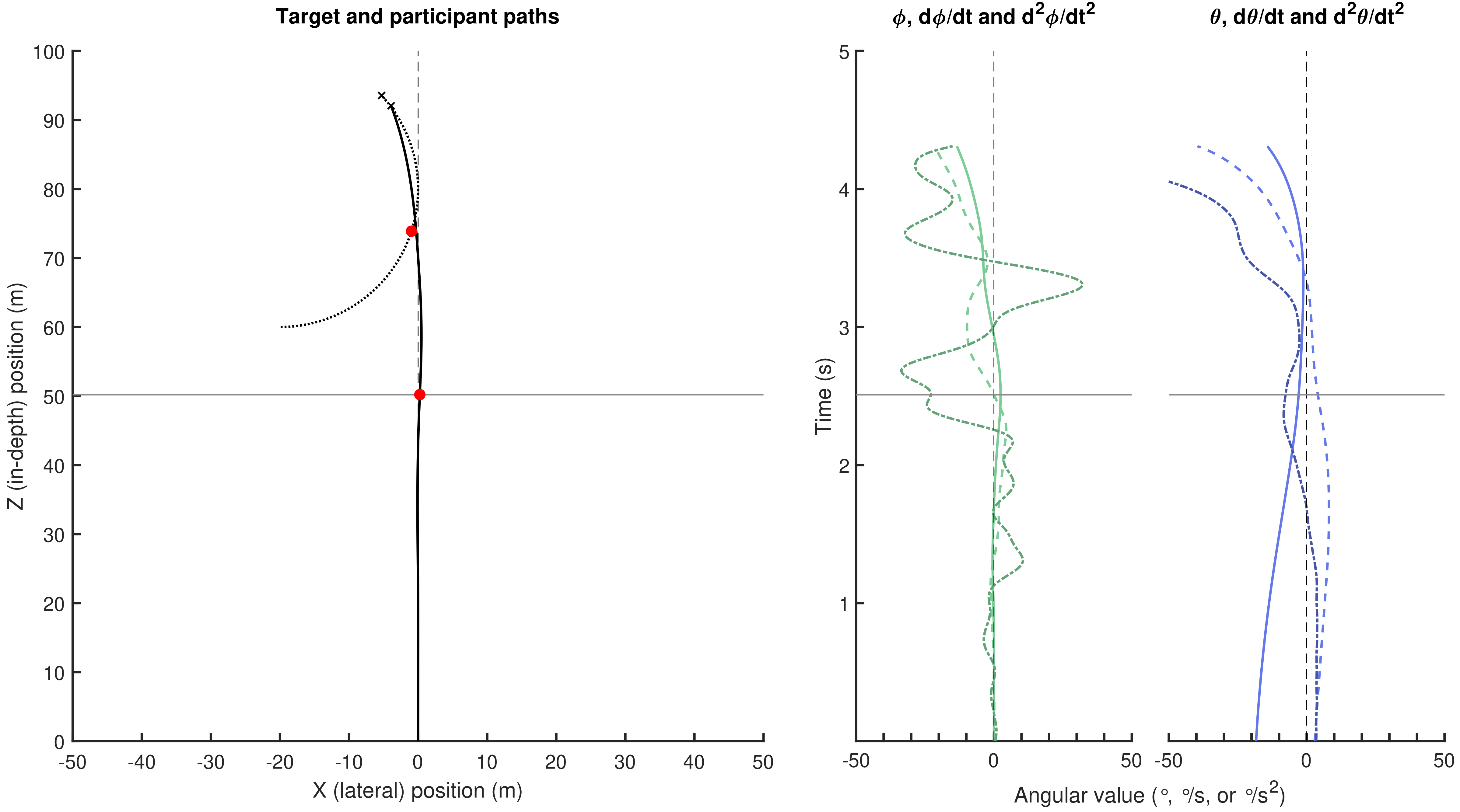

P16/B1
S20/R30-IN

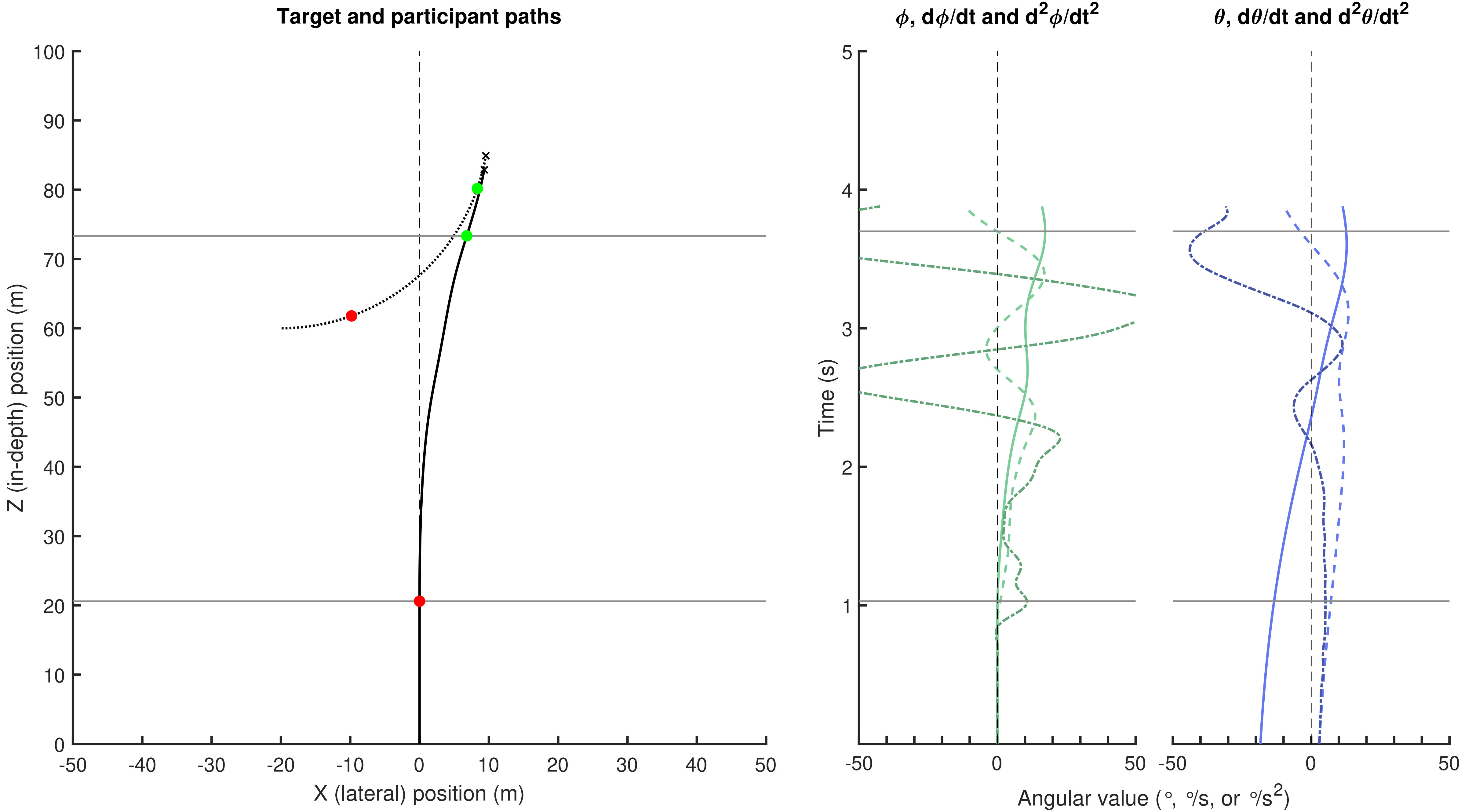

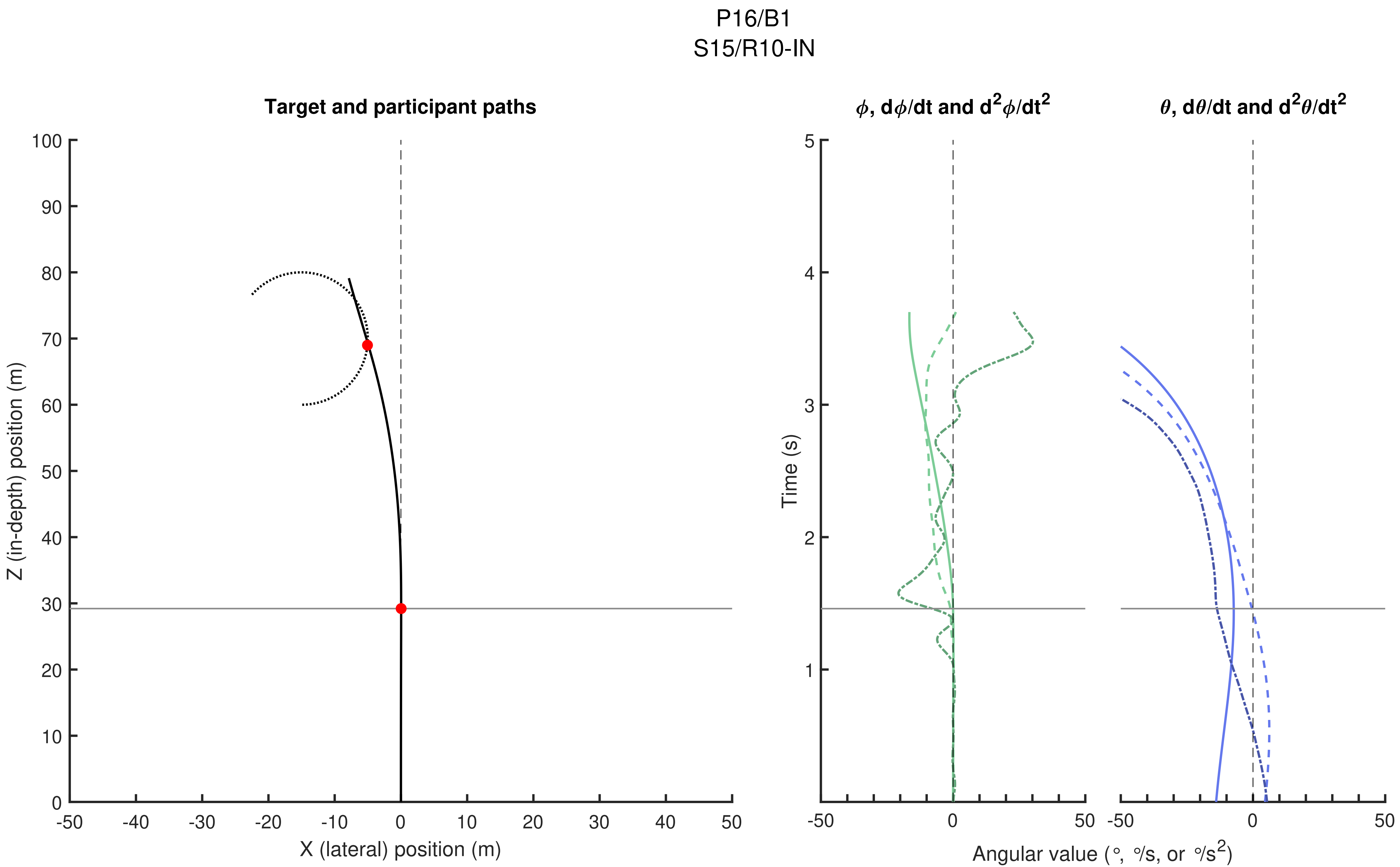
P16/B1
S15/R10-IN
Target and participant paths
Z (in-depth) position (m)
X (lateral) position (m)
100
90
80
70
60
50
40
30
20
10
0
-50
-40
-30
-20
-10
0
10
20
30
40
50
φ, dφ/dt and d²φ/dt²
θ, dθ/dt and d²θ/dt²
Time (s)
5
4
3
2
1
-50
0
50
Angular value (°, °/s, or °/s²)

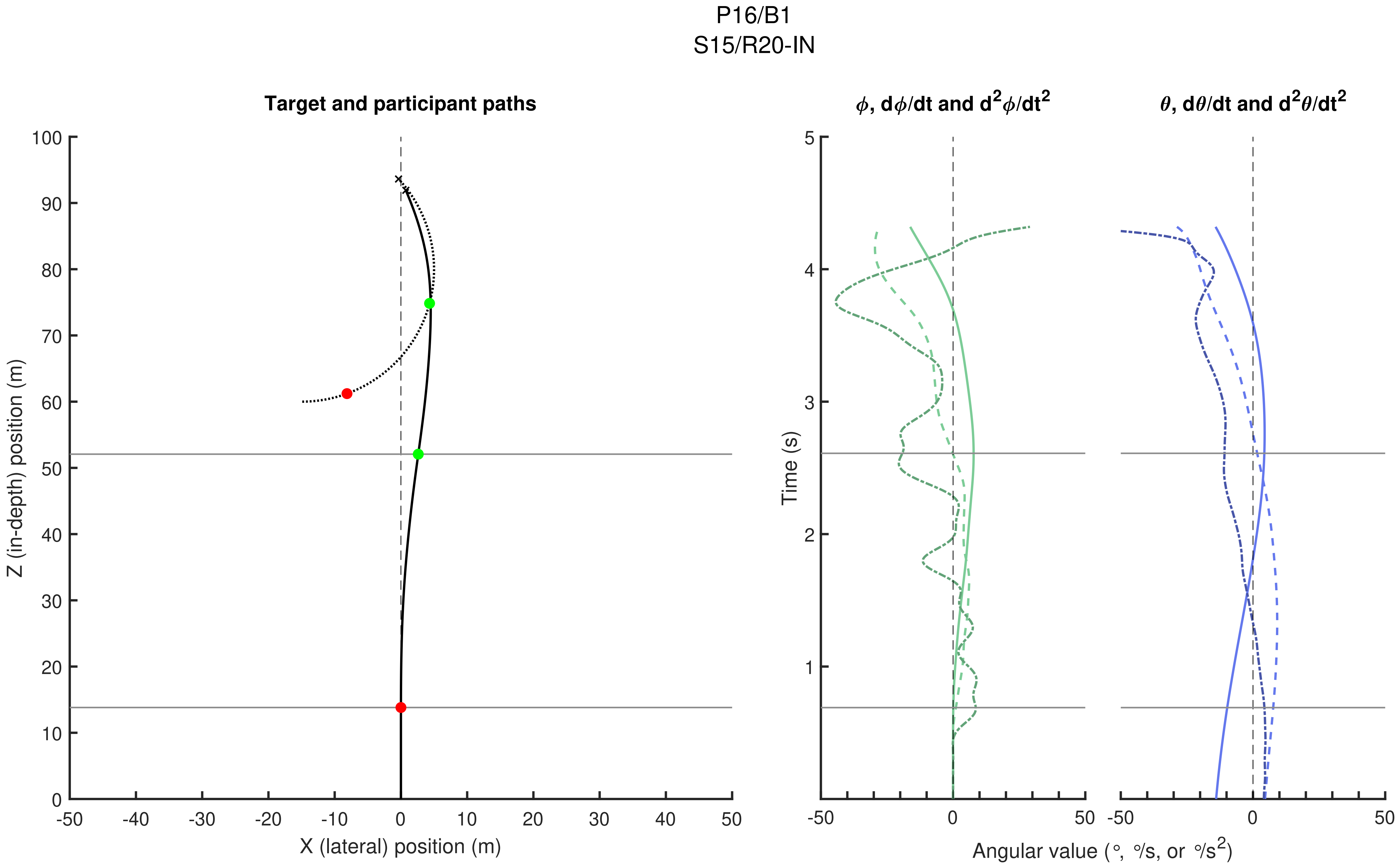
P16/B1
S15/R20-IN
Target and participant paths
Z (in-depth) position (m)
X (lateral) position (m)
ϕ, dϕ/dt and d²ϕ/dt²
θ, dθ/dt and d²θ/dt²
Time (s)
Angular value (°, °/s, or °/s²)

P16/B1
S15/R30-IN

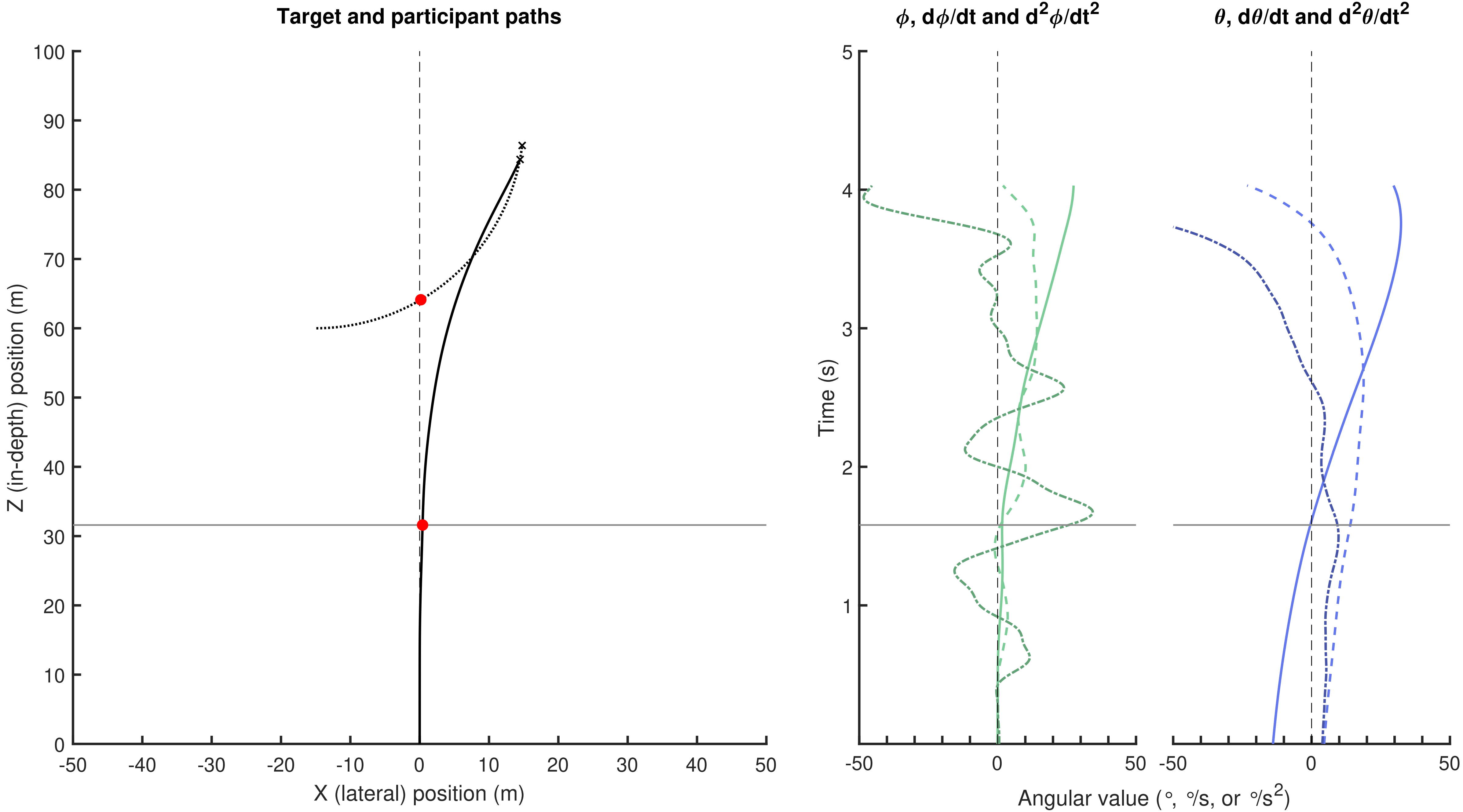

P16/B1
S5/R10-IN

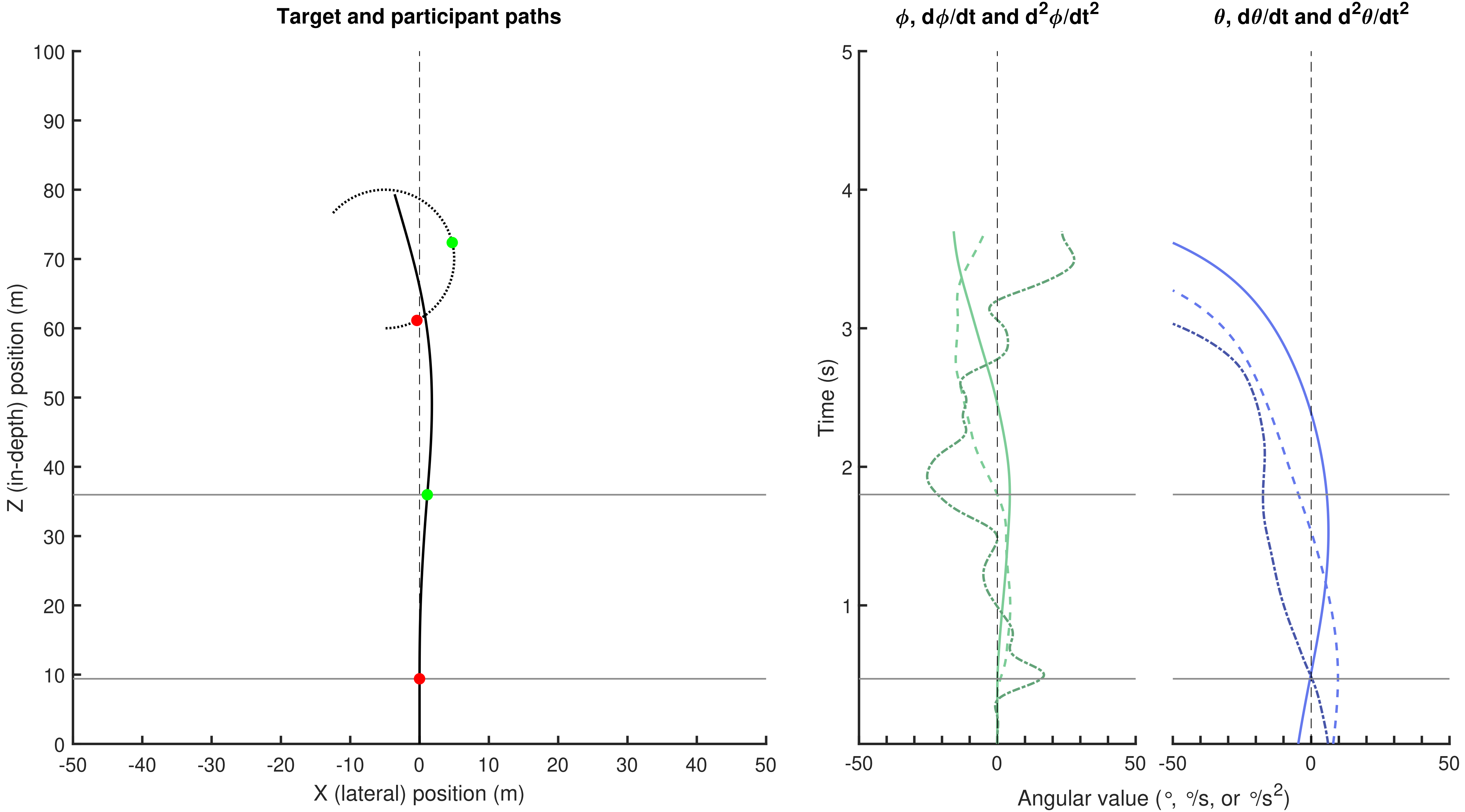

P16/B1
S5/R10-OUT

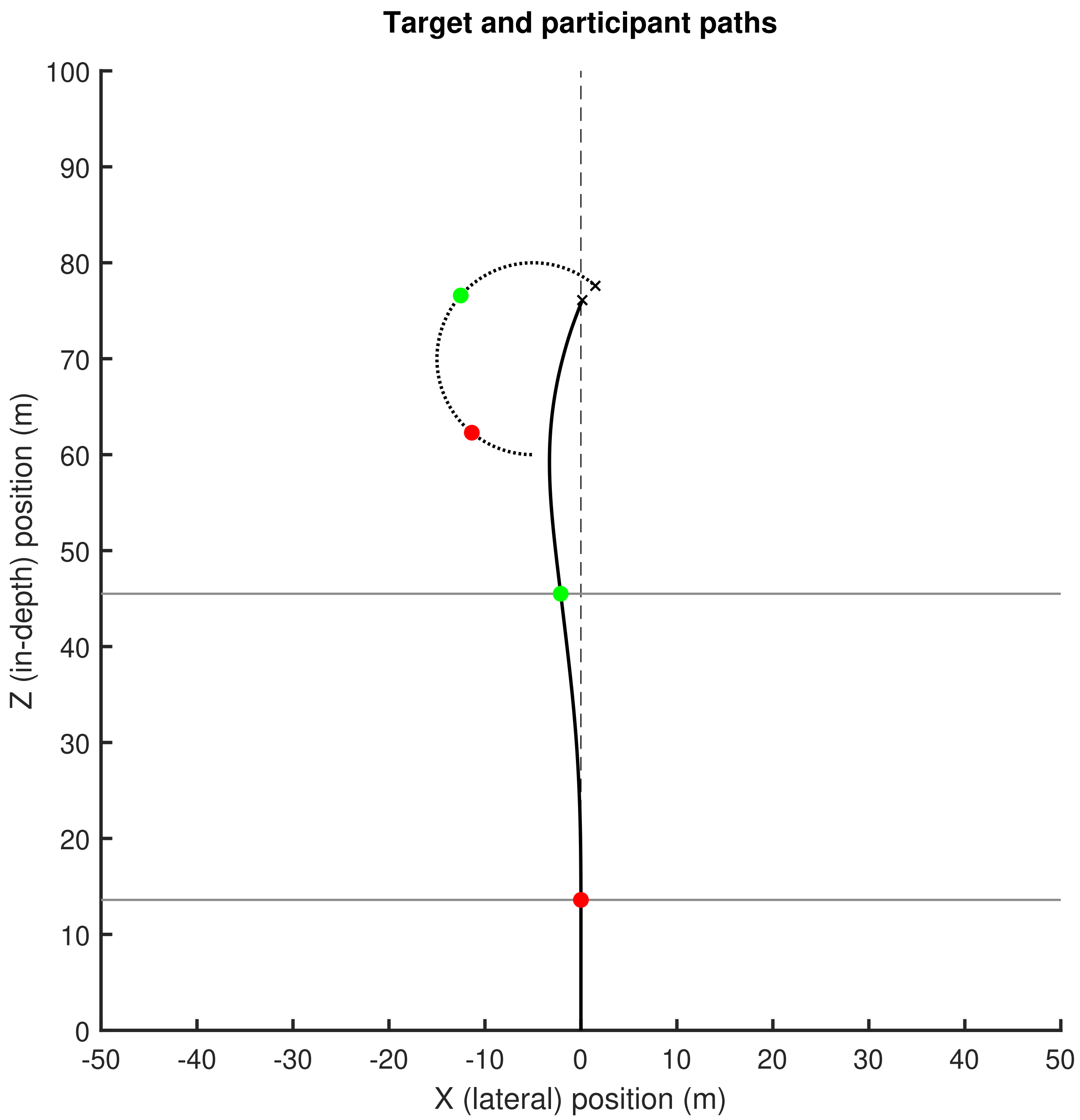

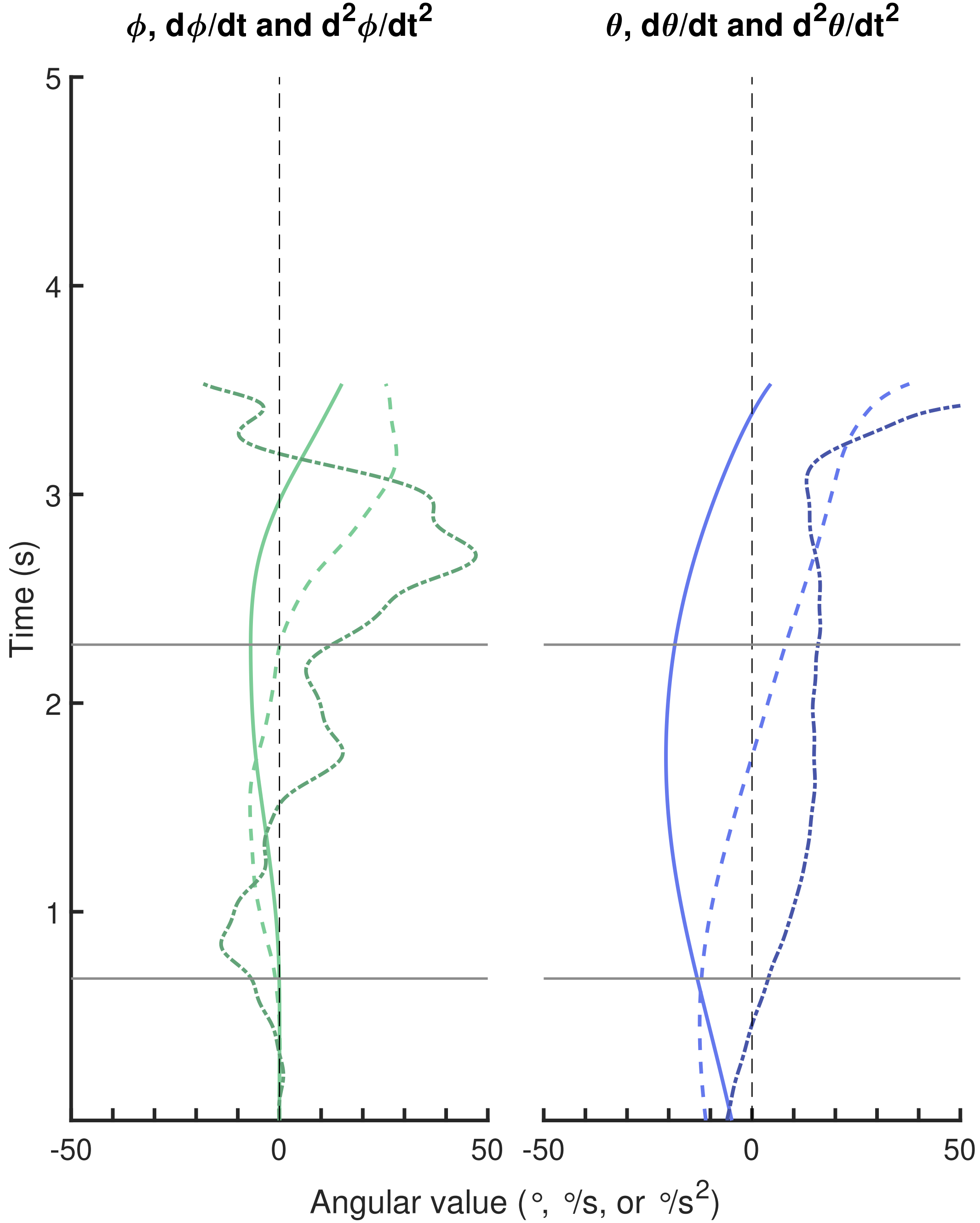

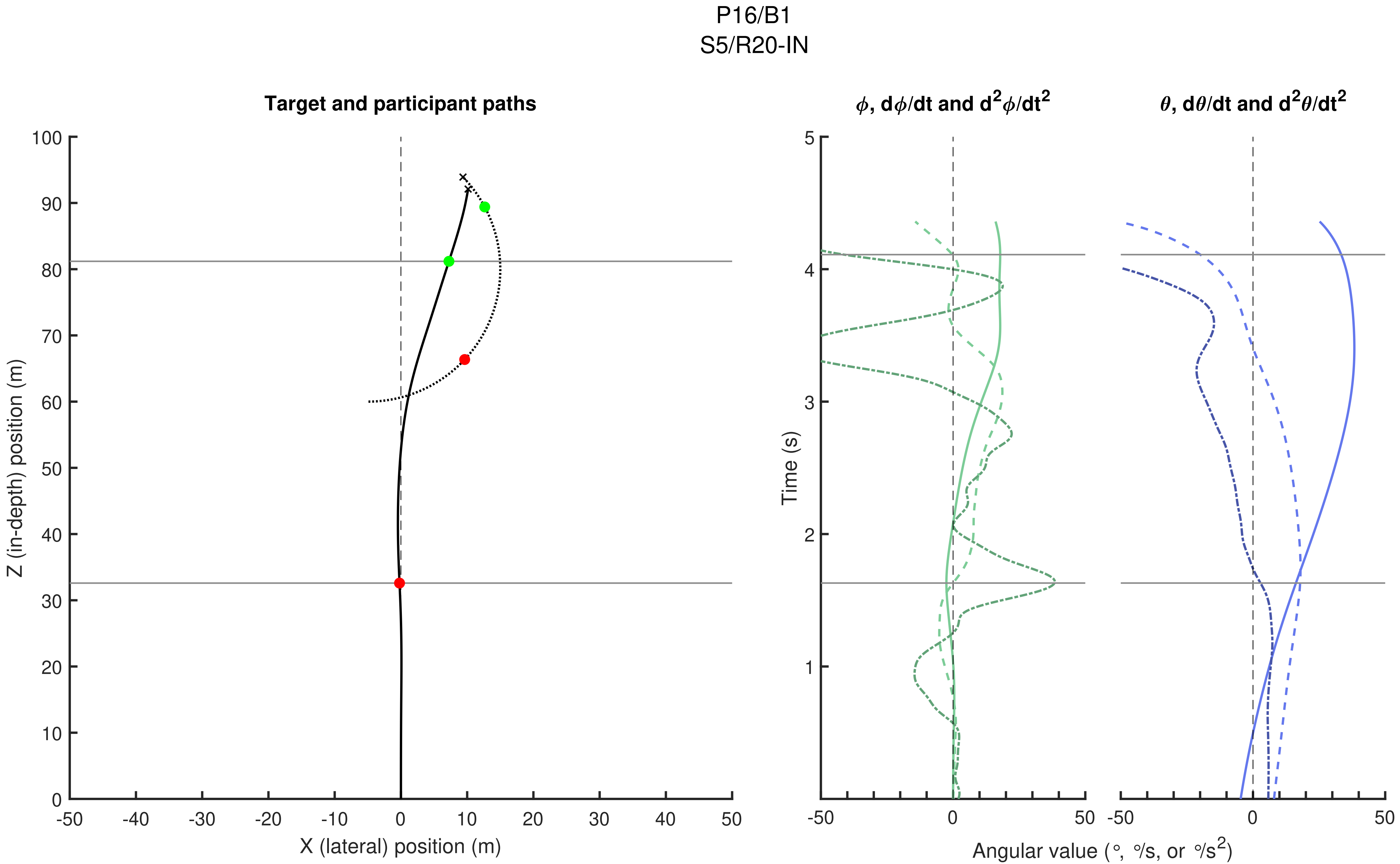
P16/B1
S5/R20-IN
Target and participant paths
Z (in-depth) position (m)
X (lateral) position (m)
φ, dφ/dt and d²φ/dt²
θ, dθ/dt and d²θ/dt²
Time (s)
Angular value (°, °/s, or °/s²)

P16/B1
S5/R20-OUT

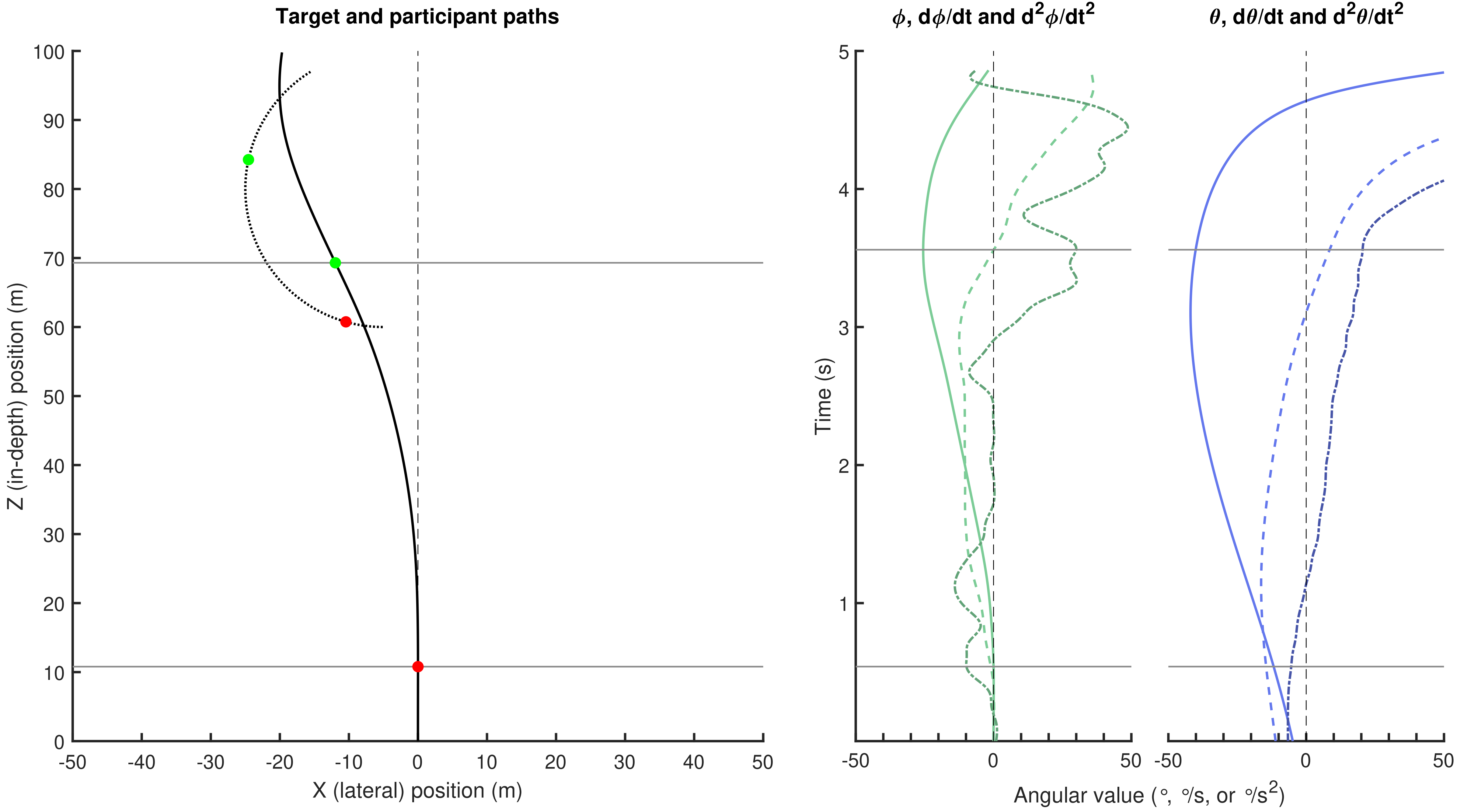

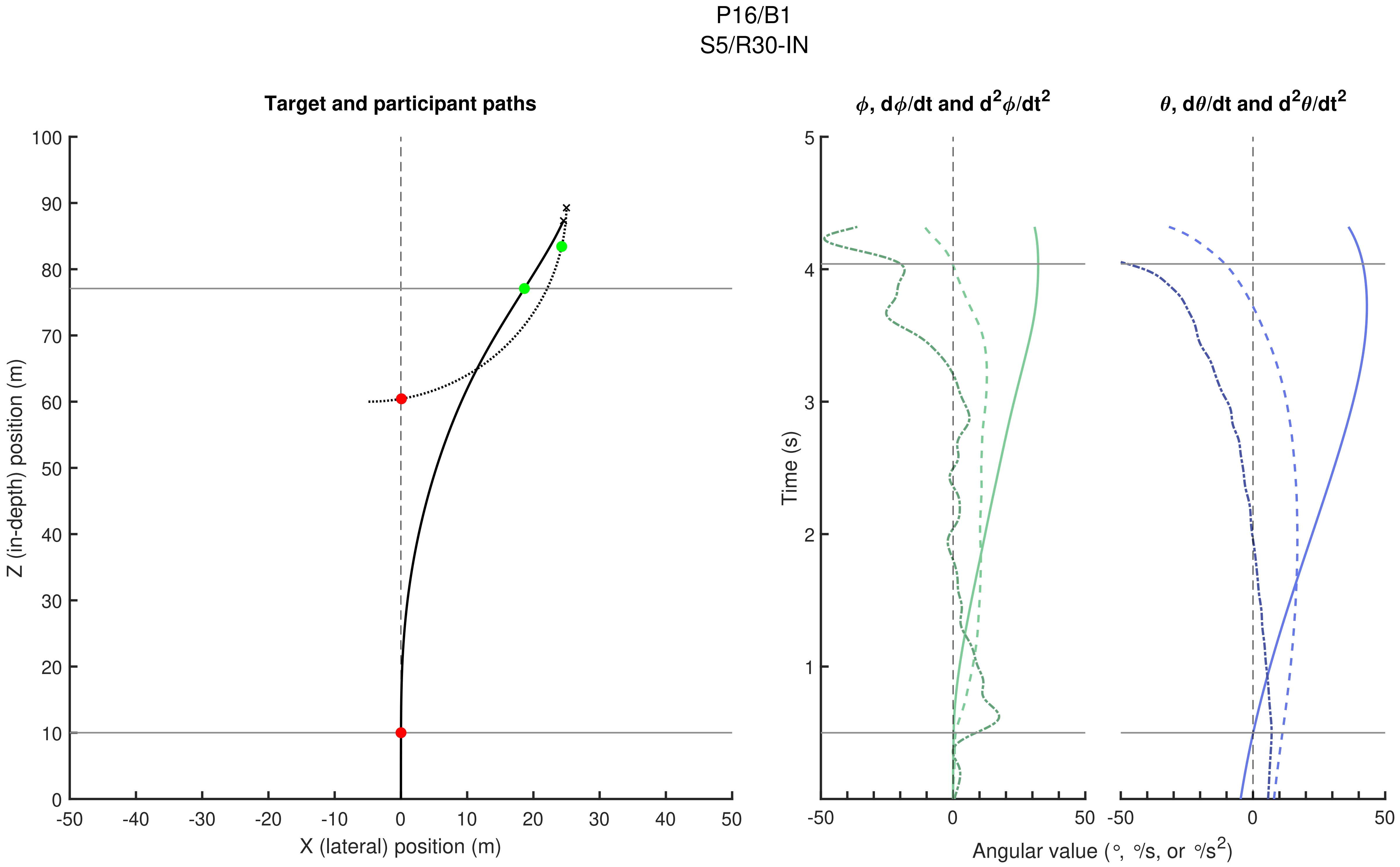
P16/B1
S5/R30-IN
Target and participant paths
Z (in-depth) position (m)
X (lateral) position (m)
φ, dφ/dt and d²φ/dt²
θ, dθ/dt and d²θ/dt²
Time (s)
Angular value (°, °/s, or °/s²)

P16/B1
S5/R30-OUT

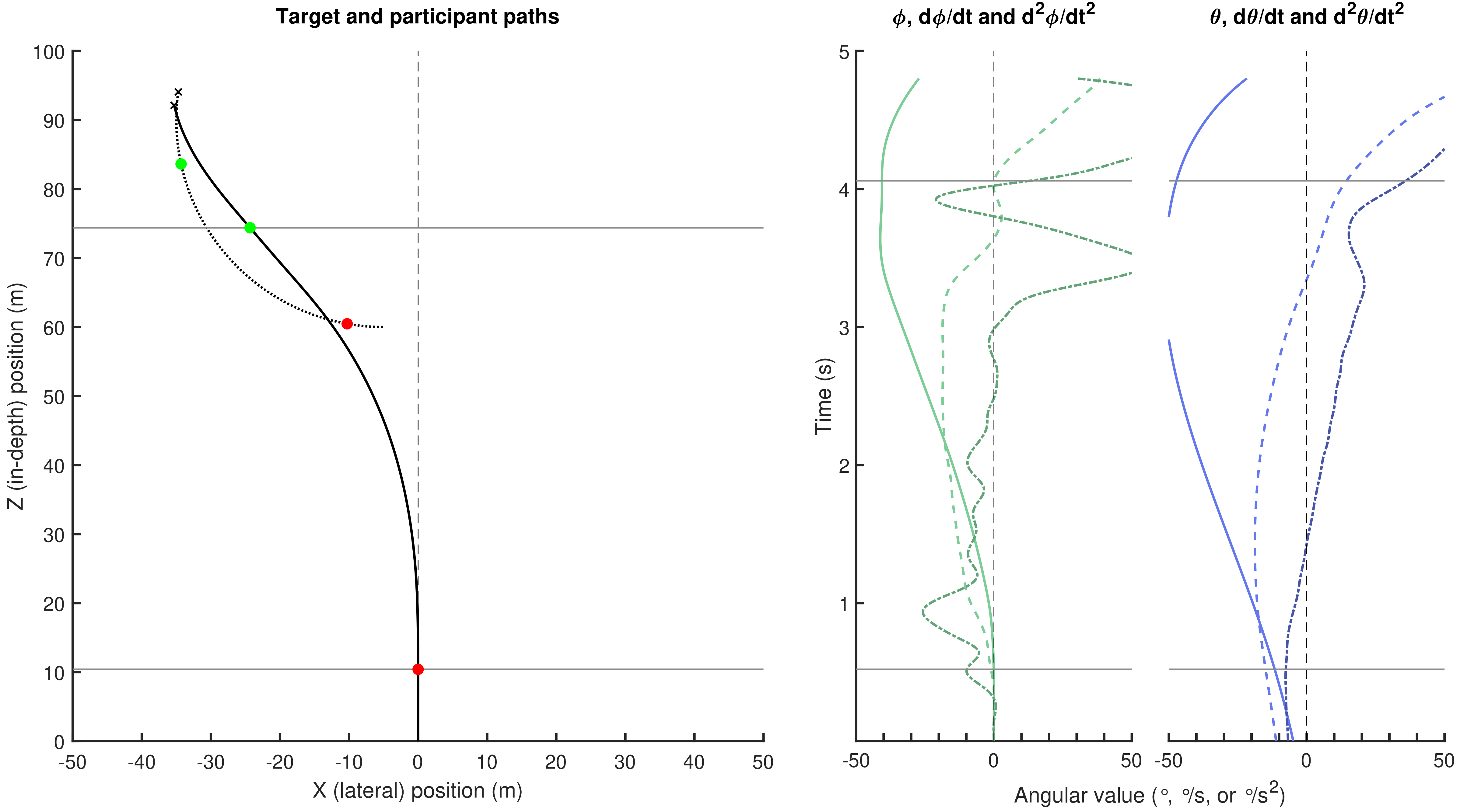

P16/B1
S5/R10-OUT

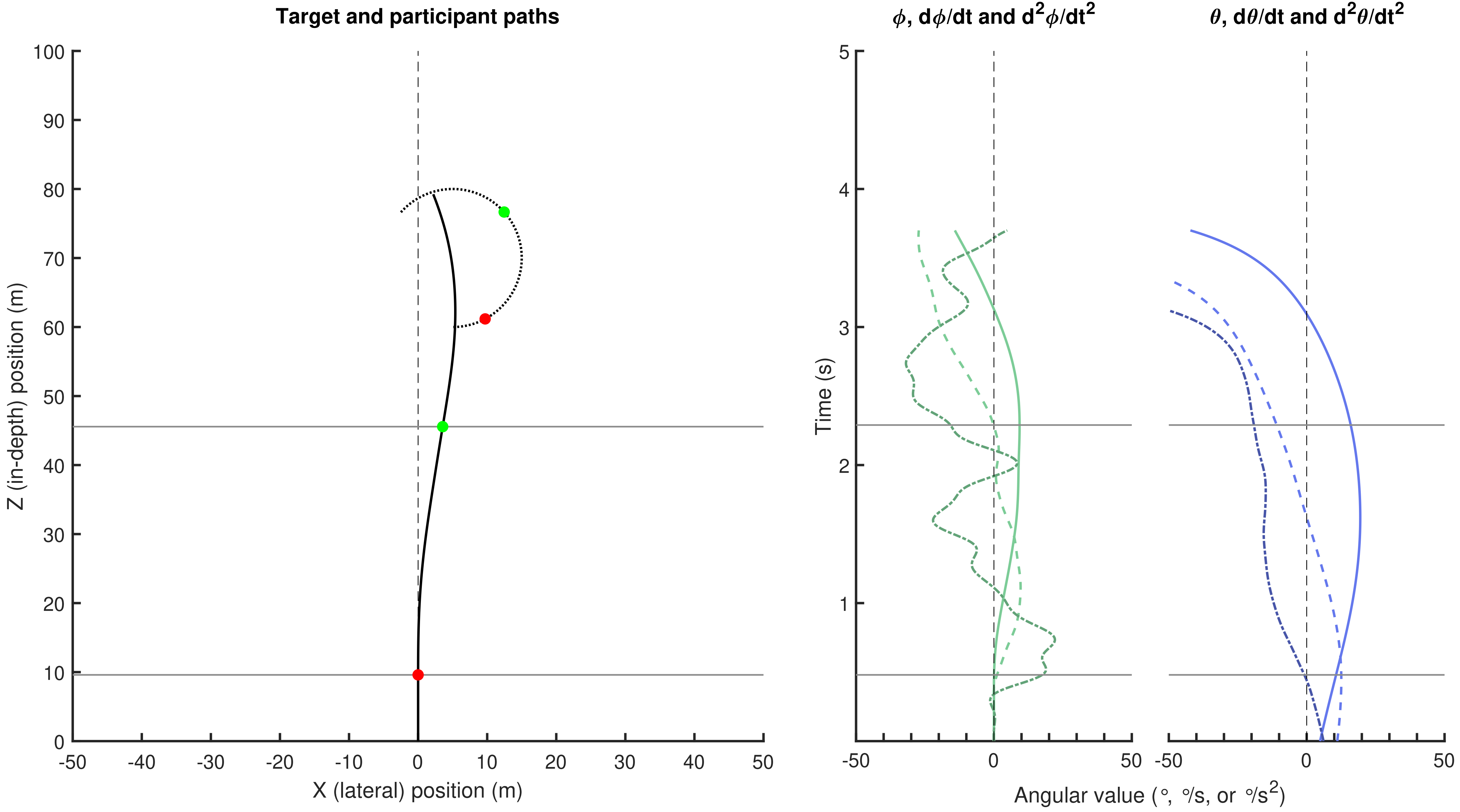

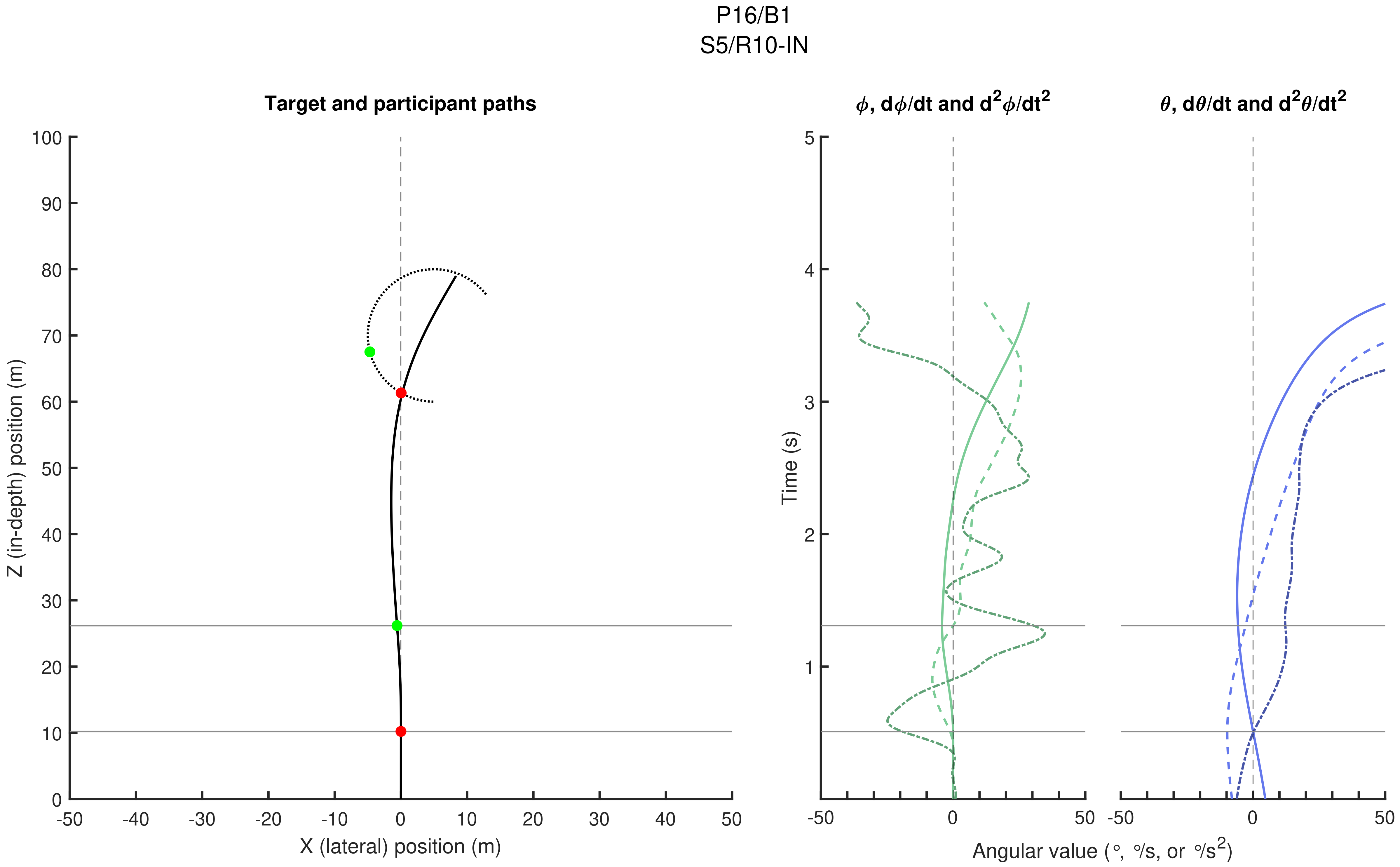

P16/B1
S5/R10-IN
Target and participant paths
Z (in-depth) position (m)
X (lateral) position (m)
100
90
80
70
60
50
40
30
20
10
0
-50
-40
-30
-20
-10
0
10
20
30
40
50
ϕ, dϕ/dt and d$^2\phi$/dt^2
θ, dθ/dt and d$^2\theta$/dt^2
Time (s)
5
4
3
2
1
-50
0
50
-50
0
50
Angular value (°, °/s, or °/s^2)

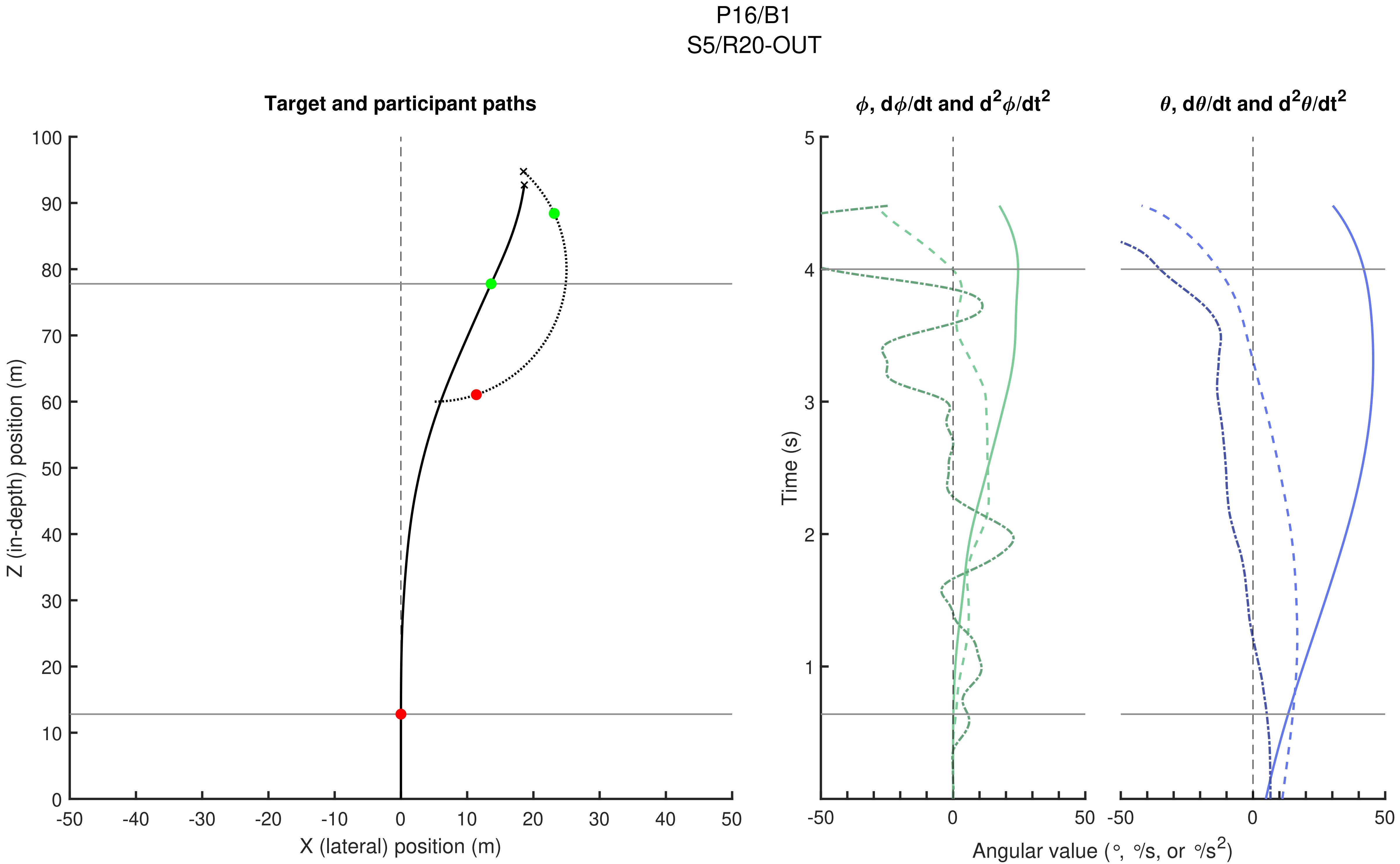

P16/B1
S5/R20-OUT
Target and participant paths
Z (in-depth) position (m)
X (lateral) position (m)
ϕ, dϕ/dt and d$^2\phi$/dt^2
θ, dθ/dt and d$^2\theta$/dt^2
Time (s)
Angular value (°, °/s, or °/s^2)

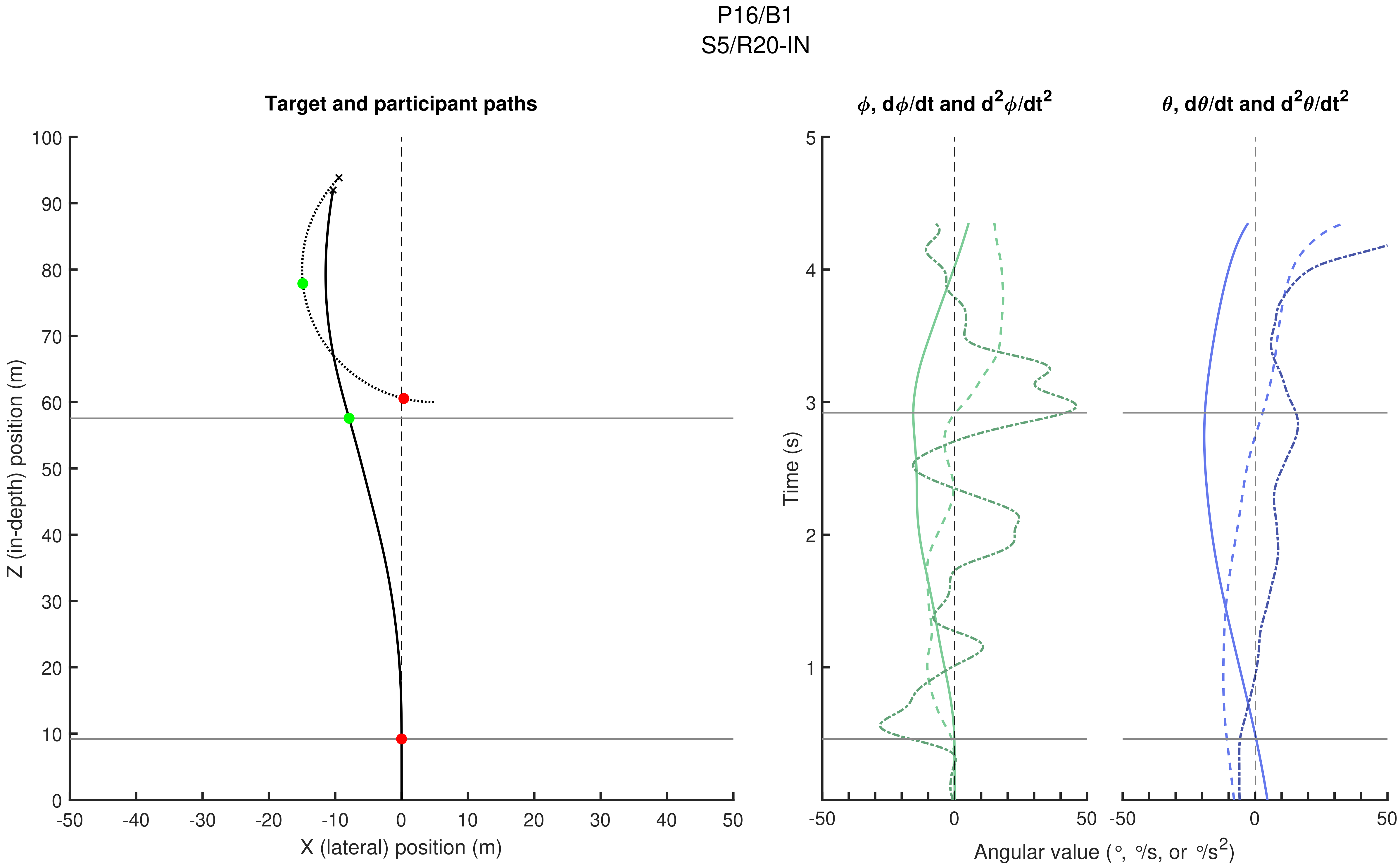

P16/B1
S5/R20-IN
Target and participant paths
Z (in-depth) position (m)
X (lateral) position (m)
ϕ, dϕ/dt and d$^2\phi$/dt^2
θ, dθ/dt and d$^2\theta$/dt^2
Time (s)
Angular value (°, °/s, or °/s^2)

P16/B1
S5/R30-OUT

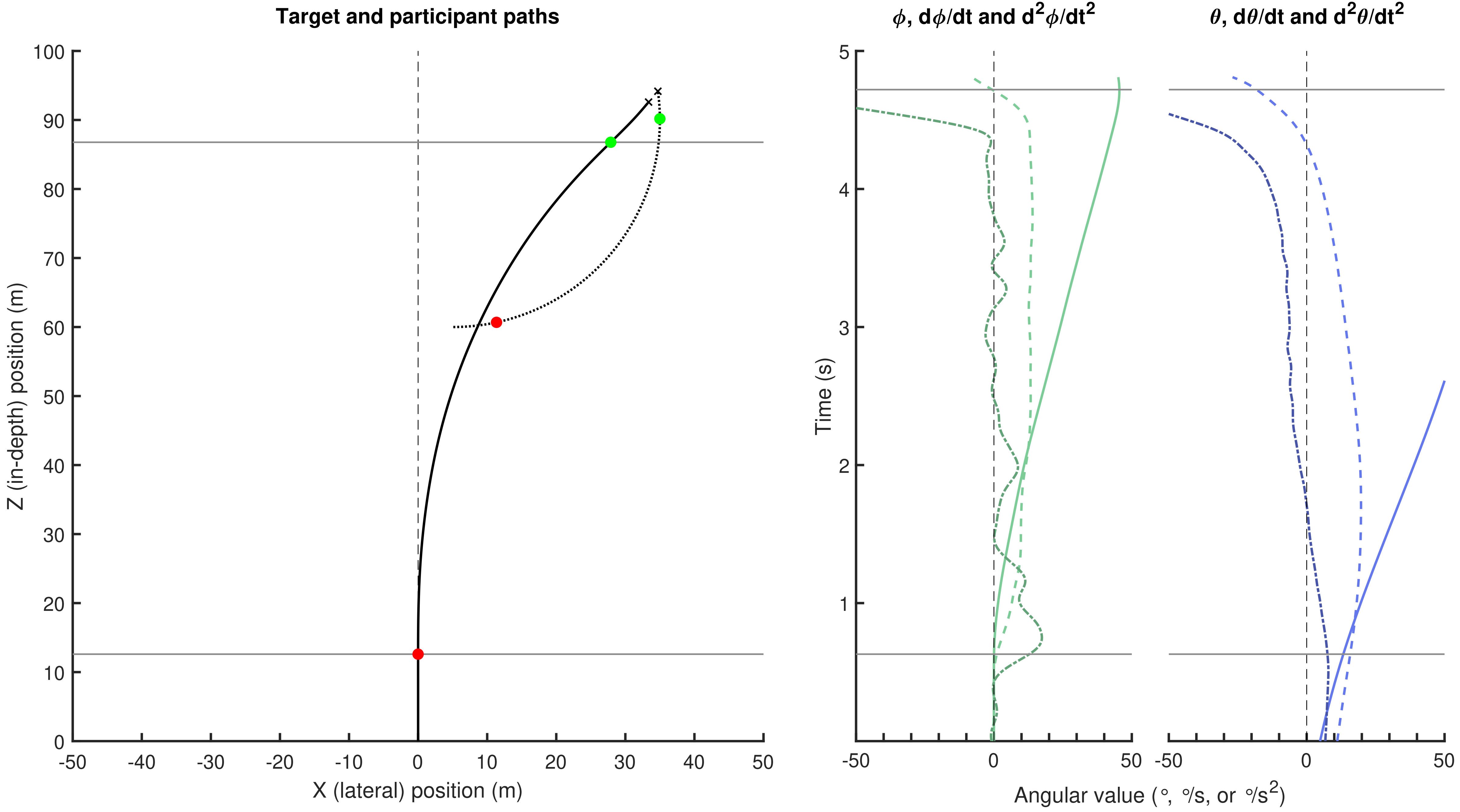

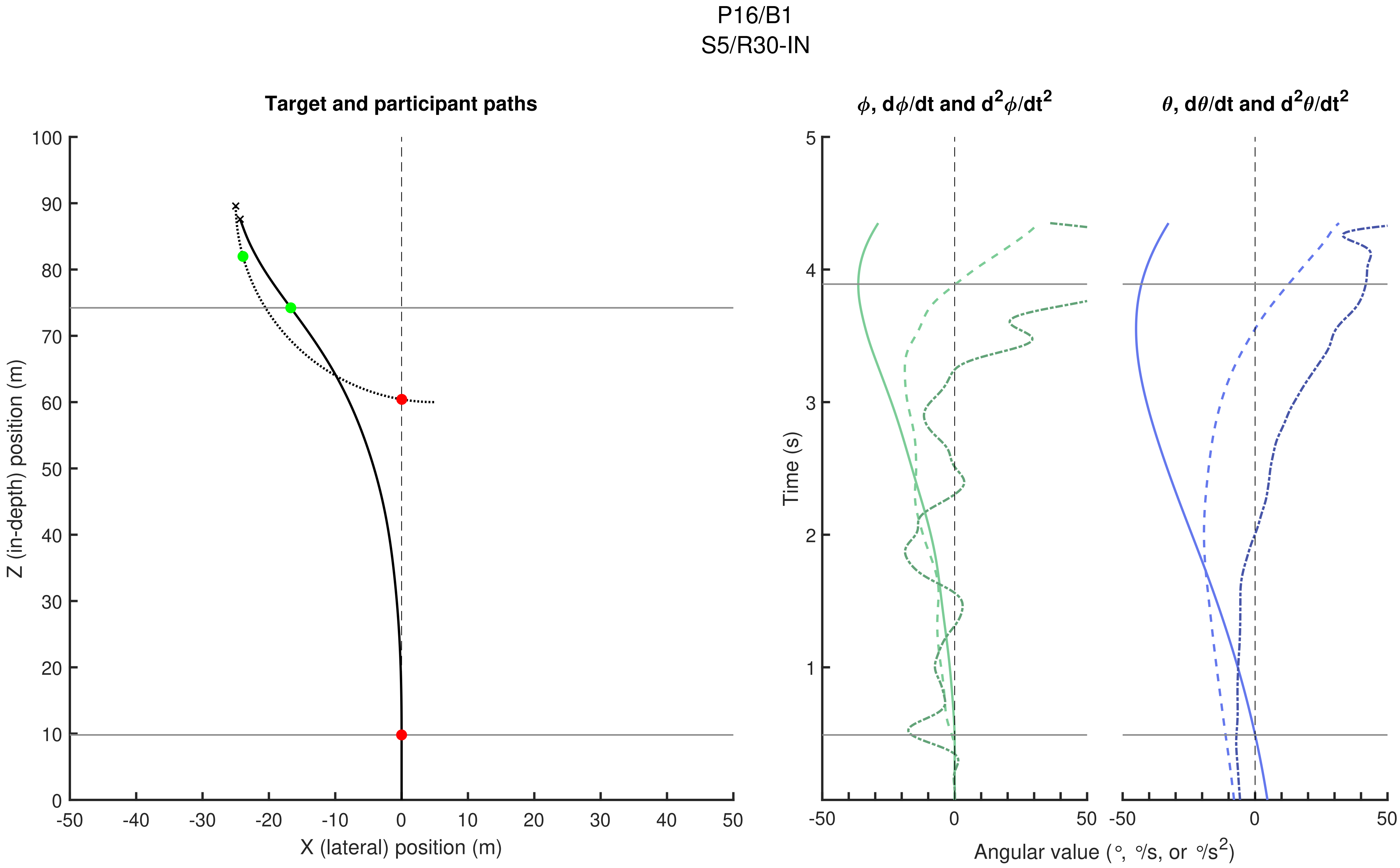

P16/B1
S5/R30-IN
Target and participant paths
Z (in-depth) position (m)
X (lateral) position (m)
100
90
80
70
60
50
40
30
20
10
0
-50
-40
-30
-20
-10
0
10
20
30
40
50
ϕ, dϕ/dt and d$^2\phi$/dt^2
θ, dθ/dt and d$^2\theta$/dt^2
Time (s)
5
4
3
2
1
-50
0
50
-50
0
50
Angular value (°, °/s, or °/s^2)

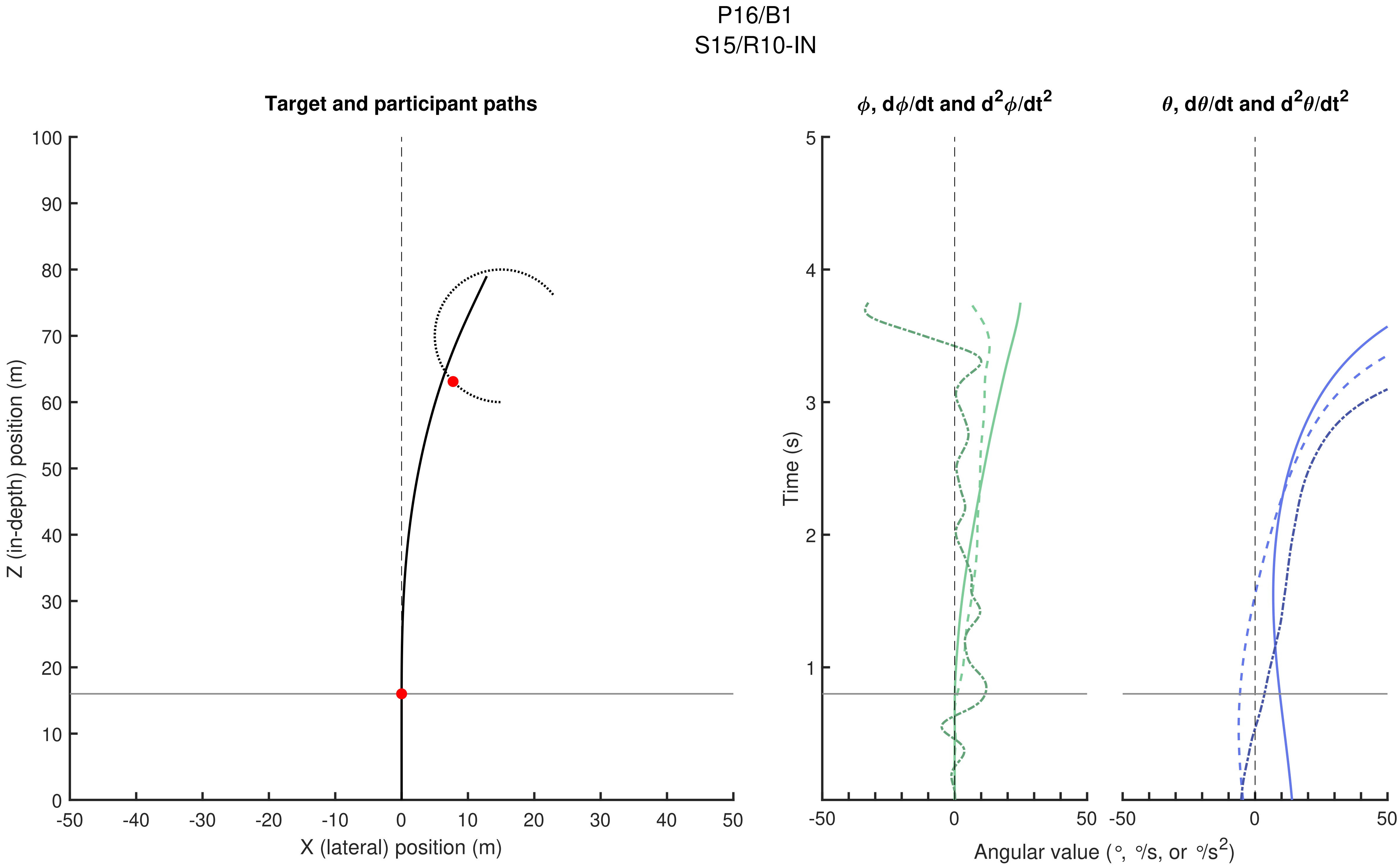
P16/B1
S15/R10-IN
Target and participant paths
Z (in-depth) position (m)
X (lateral) position (m)
100
90
80
70
60
50
40
30
20
10
0
-50
-40
-30
-20
-10
0
10
20
30
40
50
ϕ, dϕ/dt and d$^2\phi$/dt^2
θ, dθ/dt and d$^2\theta$/dt^2
Time (s)
5
4
3
2
1
-50
0
50
-50
0
50
Angular value (°, °/s, or °/s^2)

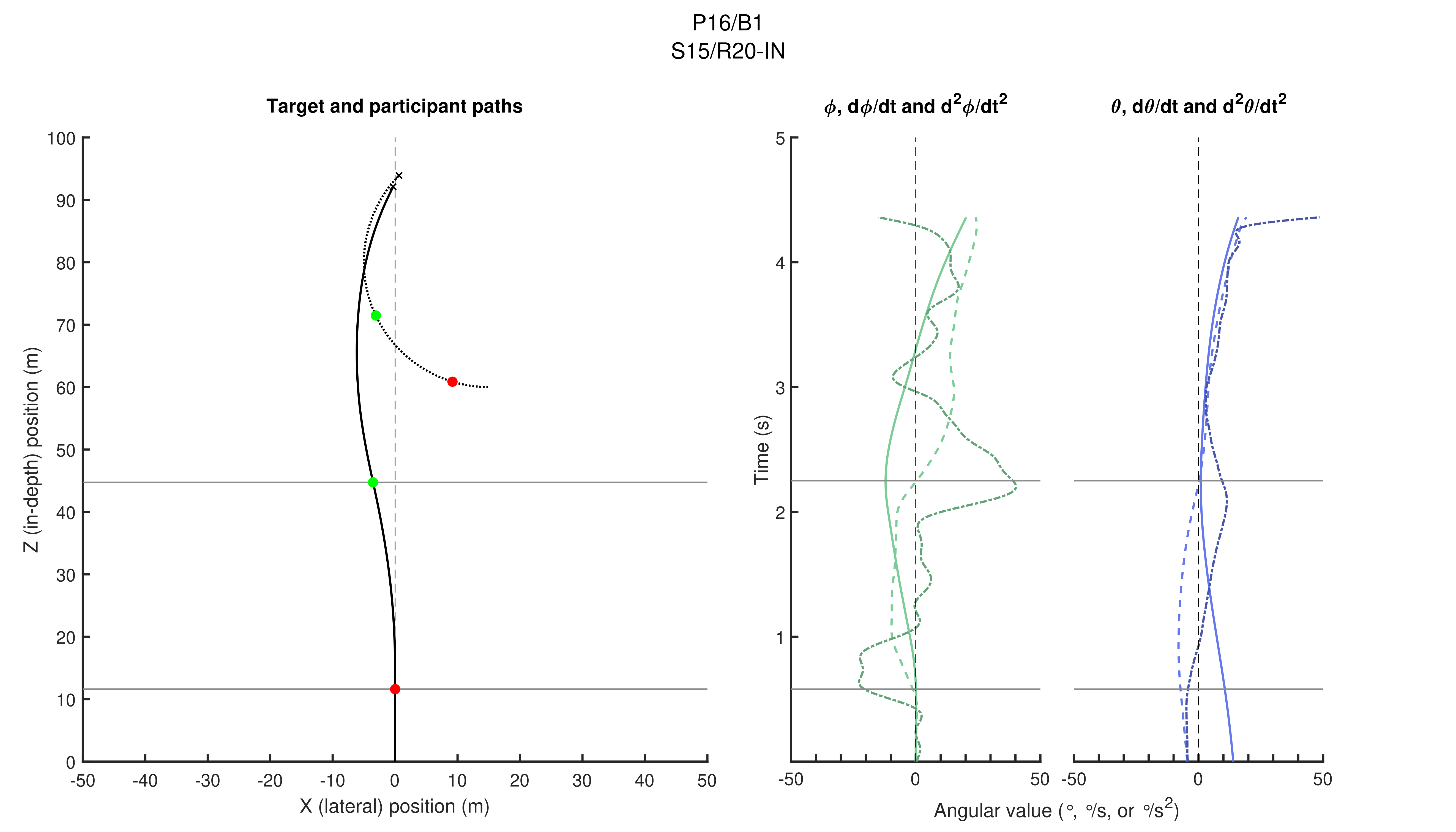

P16/B1
S15/R20-IN
Target and participant paths
Z (in-depth) position (m)
X (lateral) position (m)
100
90
80
70
60
50
40
30
20
10
0
-50
-40
-30
-20
-10
0
10
20
30
40
50
ϕ, dϕ/dt and d²ϕ/dt²
θ, dθ/dt and d²θ/dt²
Time (s)
5
4
3
2
1
-50
0
50
-50
0
50
Angular value (°, °/s, or °/s²)

P16/B1
S15/R30-IN

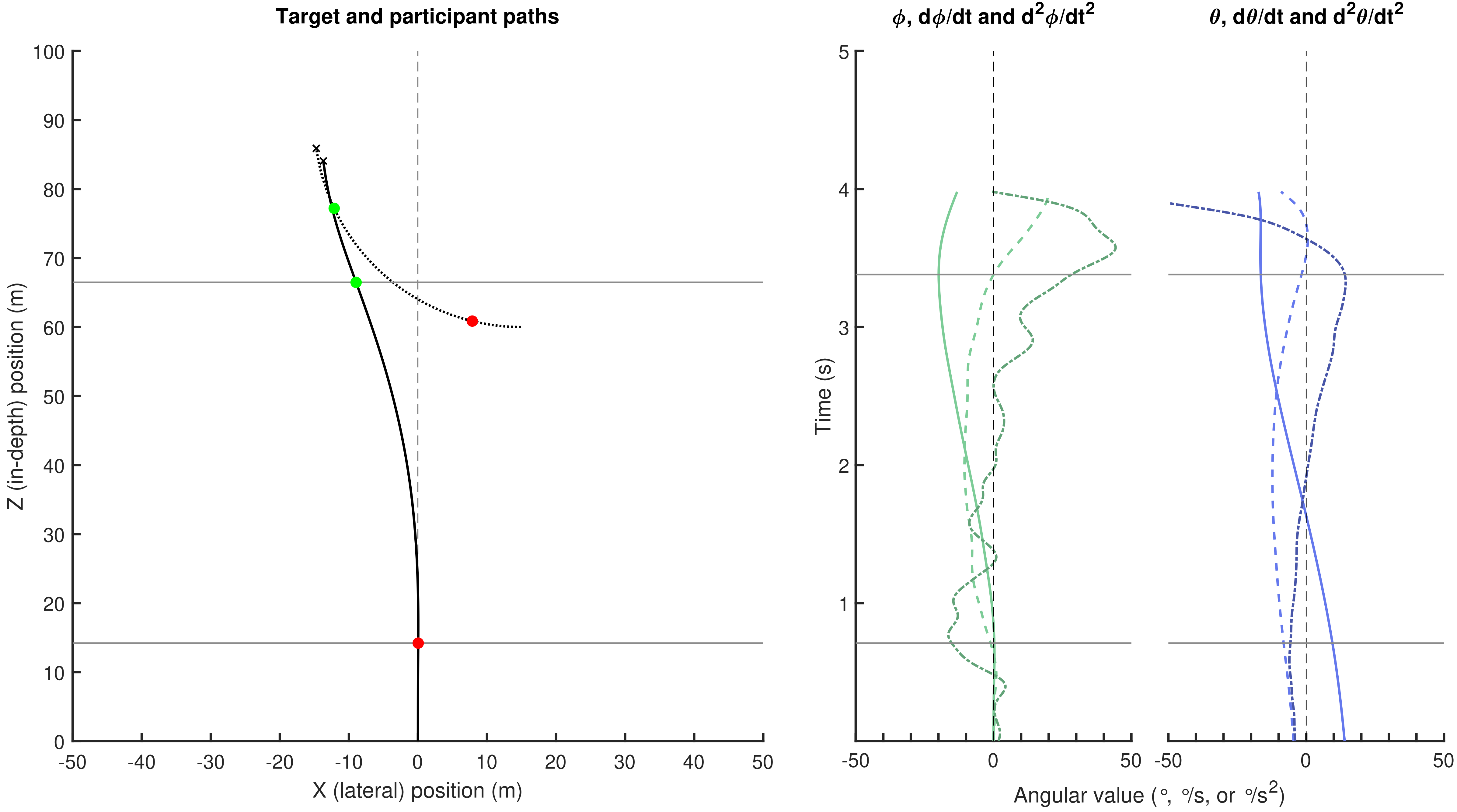

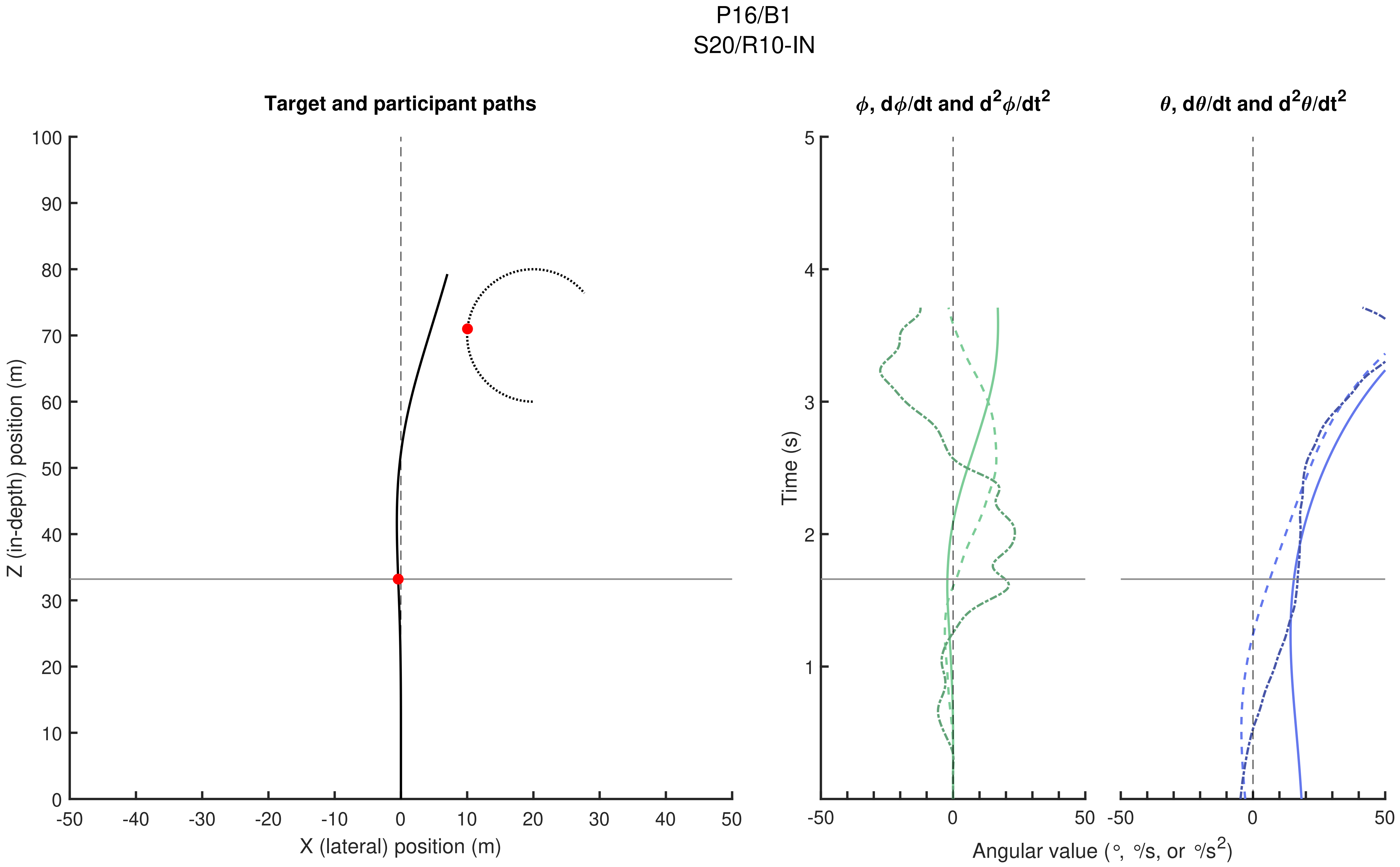
P16/B1
S20/R10-IN
Target and participant paths
ϕ, dϕ/dt and d$^2\phi$/dt^2
θ, dθ/dt and d$^2\theta$/dt^2
Z (in-depth) position (m)
X (lateral) position (m)
Time (s)
Angular value (°, °/s, or °/s^2)
100
90
80
70
60
50
40
30
20
10
0
-50
-40
-30
-20
-10
0
10
20
30
40
50
5
4
3
2
1
-50
0
50
-50
0
50

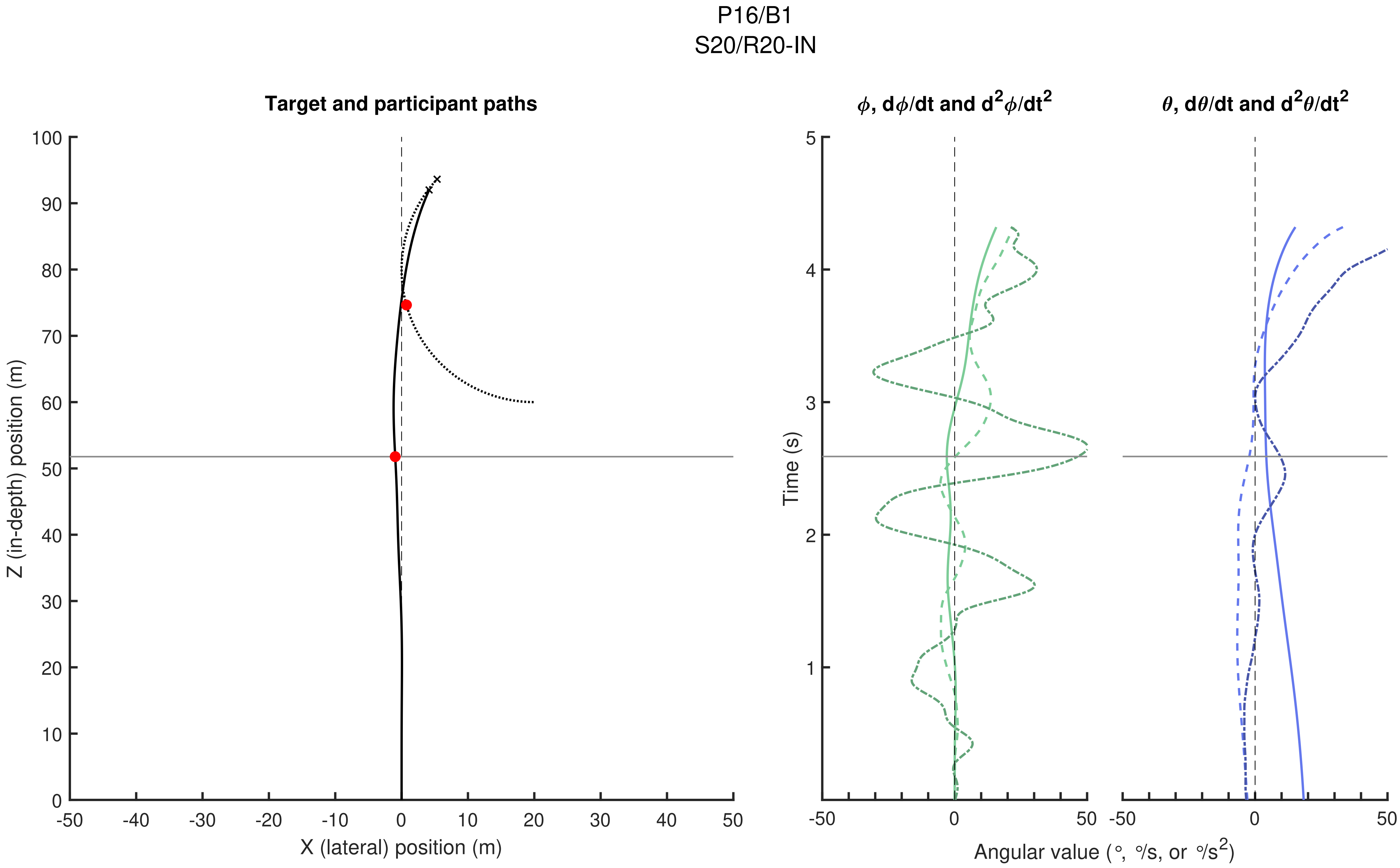
P16/B1
S20/R20-IN
Target and participant paths
Z (in-depth) position (m)
X (lateral) position (m)
100
90
80
70
60
50
40
30
20
10
0
-50
-40
-30
-20
-10
0
10
20
30
40
50
ϕ, dϕ/dt and d$^2\phi$/dt^2
θ, dθ/dt and d$^2\theta$/dt^2
Time (s)
5
4
3
2
1
-50
0
50
-50
0
50
Angular value (°, °/s, or °/s^2)

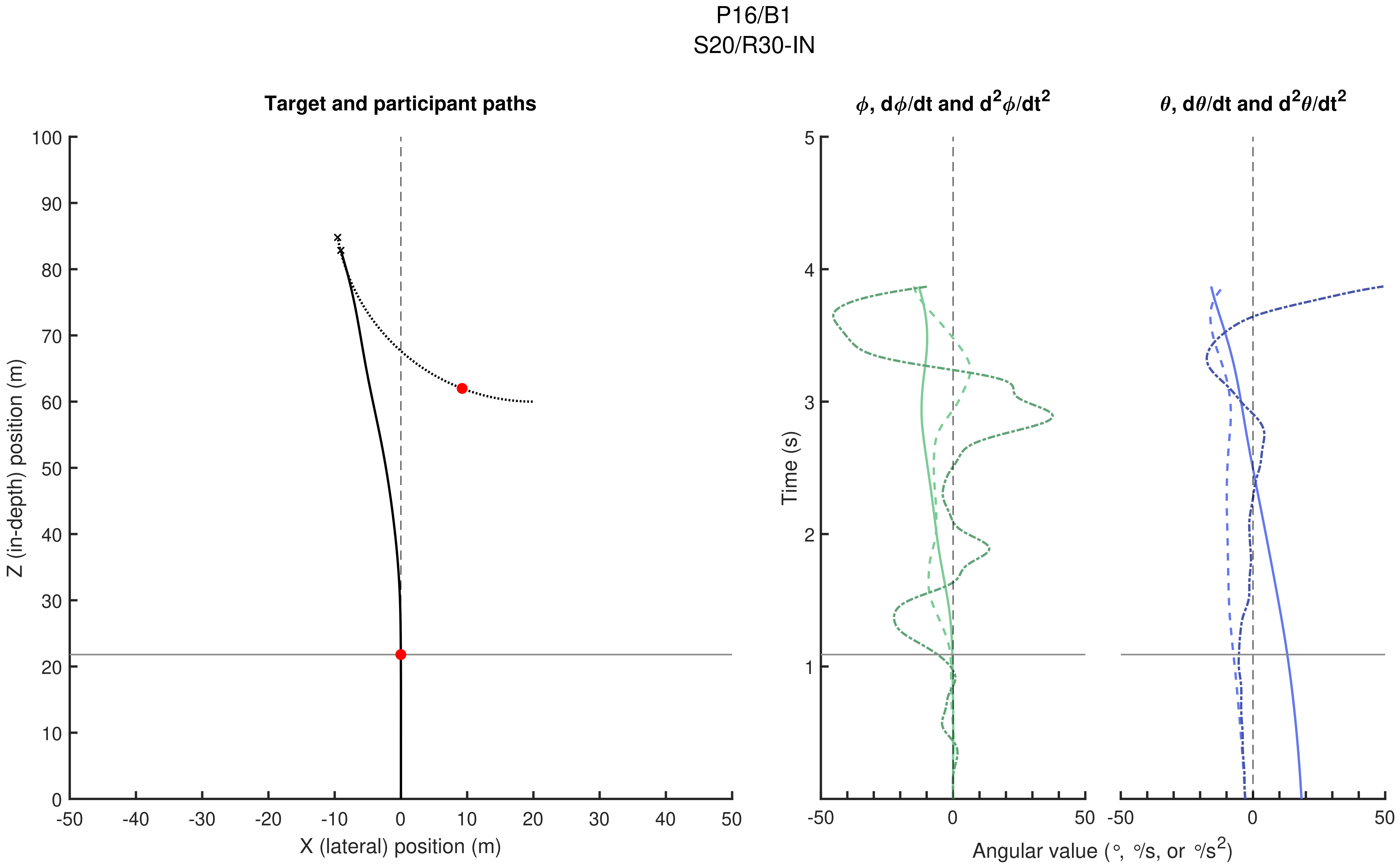
P16/B1
S20/R30-IN
Target and participant paths
Z (in-depth) position (m)
X (lateral) position (m)
0
10
20
30
40
50
60
70
80
90
100
-50
-40
-30
-20
-10
0
10
20
30
40
50
ϕ, dϕ/dt and d$^2\phi$/dt^2
θ, dθ/dt and d$^2\theta$/dt^2
Time (s)
1
2
3
4
5
-50
0
50
-50
0
50
Angular value (°, °/s, or °/s^2)

P16/B2
S20/R10-IN

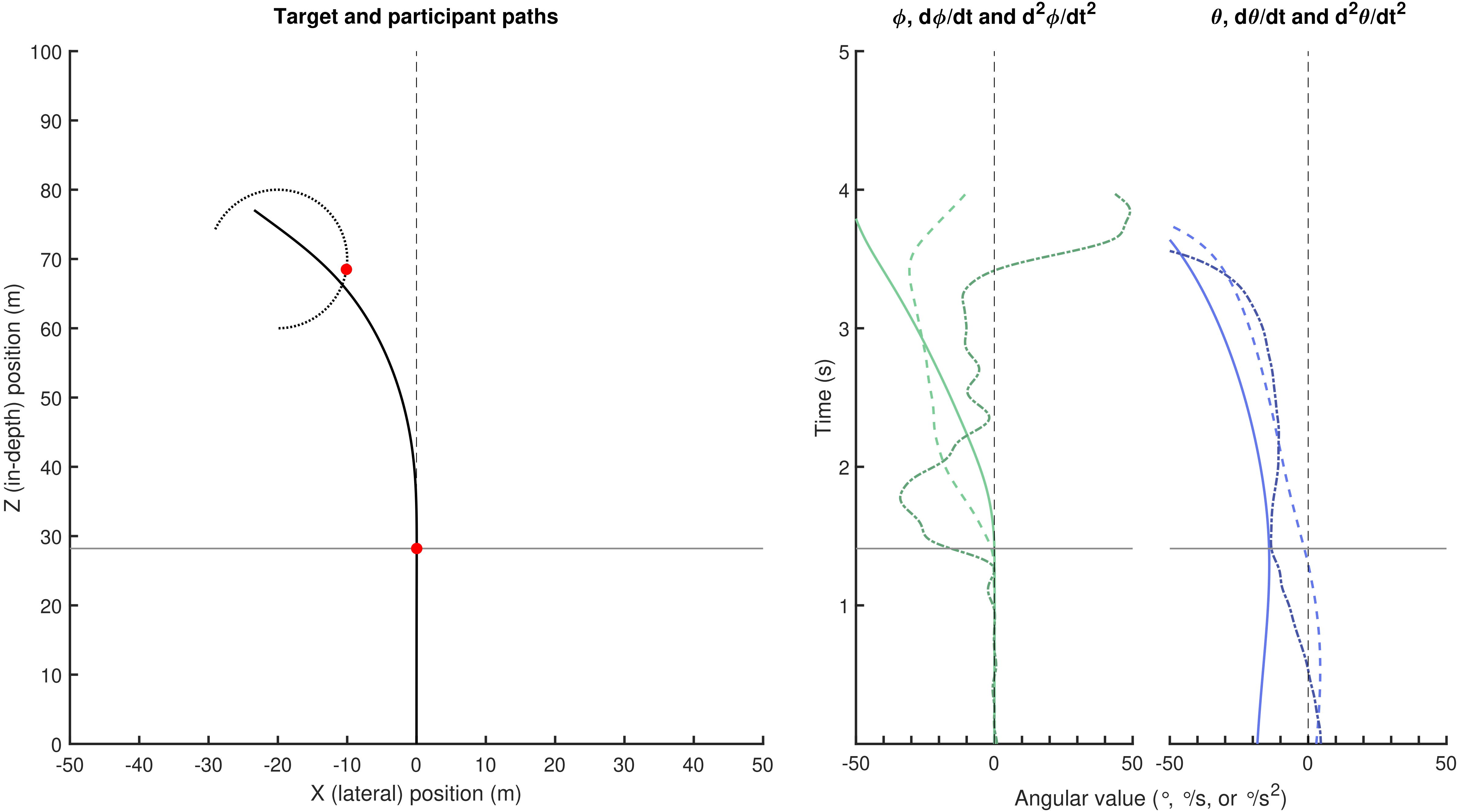

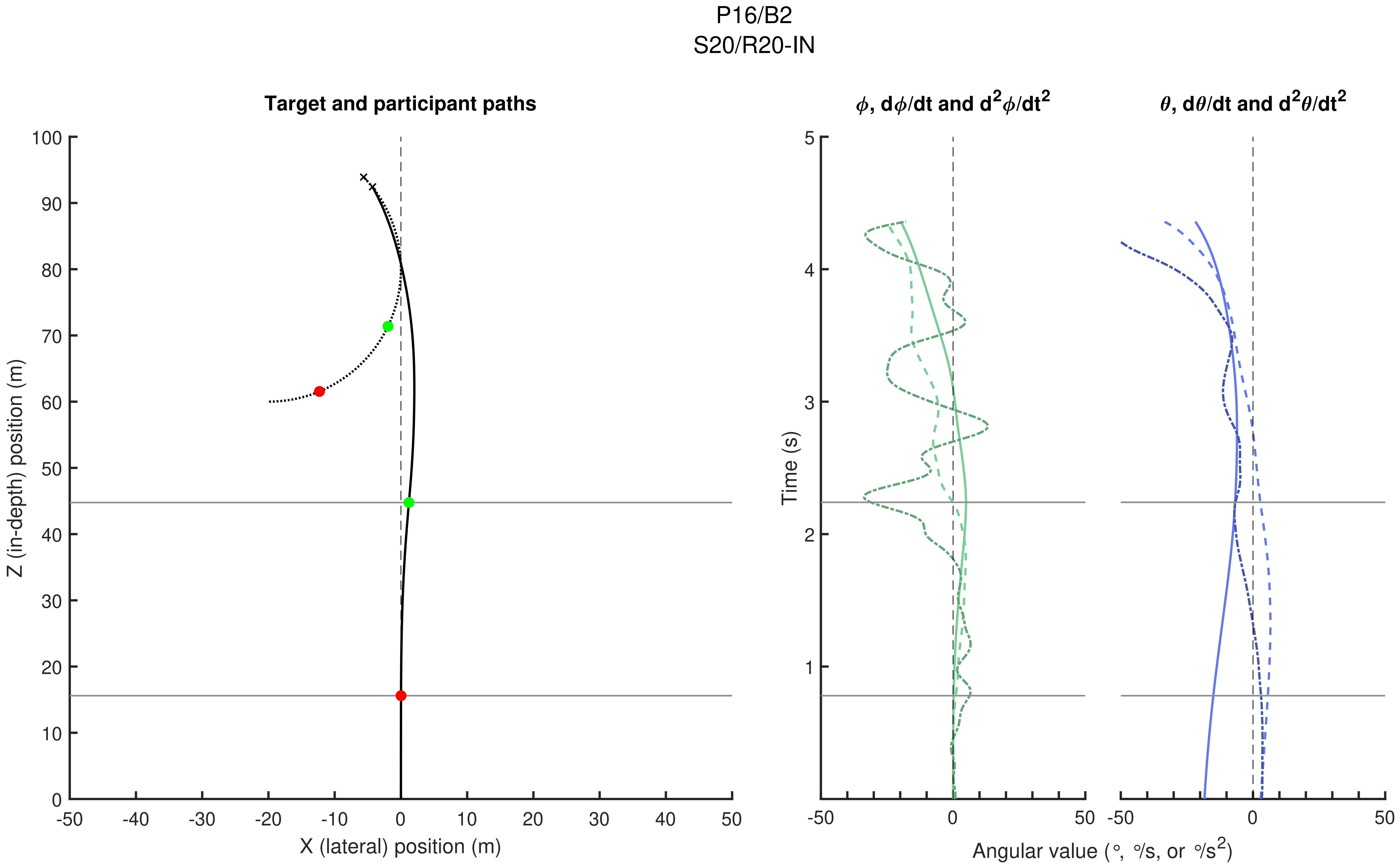

P16/B2
S20/R20-IN
Target and participant paths
ϕ, dϕ/dt and d$^2\phi$/dt^2
θ, dθ/dt and d$^2\theta$/dt^2
Z (in-depth) position (m)
X (lateral) position (m)
Time (s)
Angular value (°, °/s, or °/s^2)
100
90
80
70
60
50
40
30
20
10
0
-50
-40
-30
-20
-10
0
10
20
30
40
50
5
4
3
2
1
-50
0
50
-50
0
50

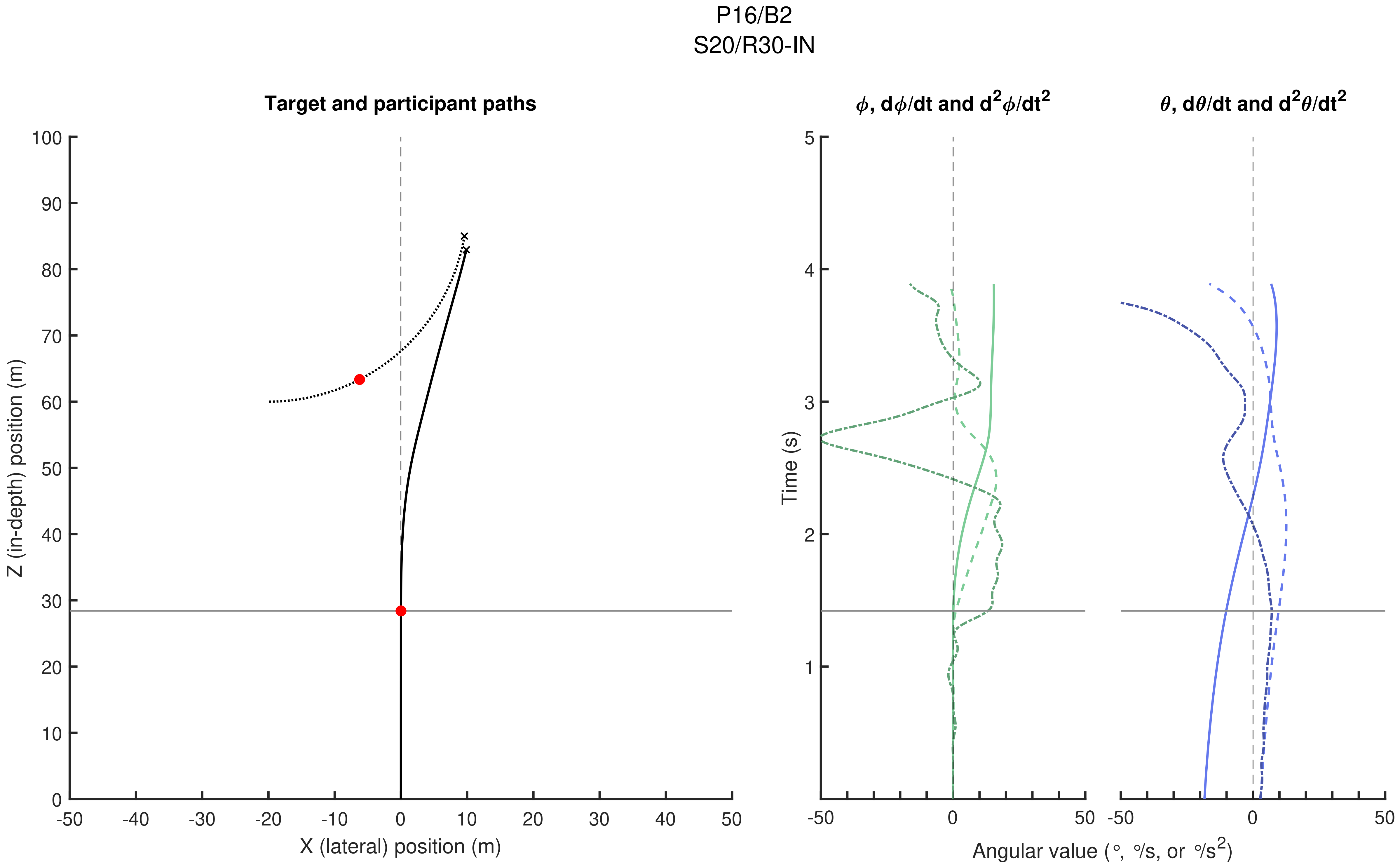

P16/B2
S20/R30-IN
Target and participant paths
Z (in-depth) position (m)
X (lateral) position (m)
0
10
20
30
40
50
60
70
80
90
100
-50
-40
-30
-20
-10
0
10
20
30
40
50
φ, dφ/dt and d²φ/dt²
θ, dθ/dt and d²θ/dt²
Time (s)
1
2
3
4
5
-50
0
50
-50
0
50
Angular value (°, °/s, or °/s²)

P16/B2
S15/R10-IN

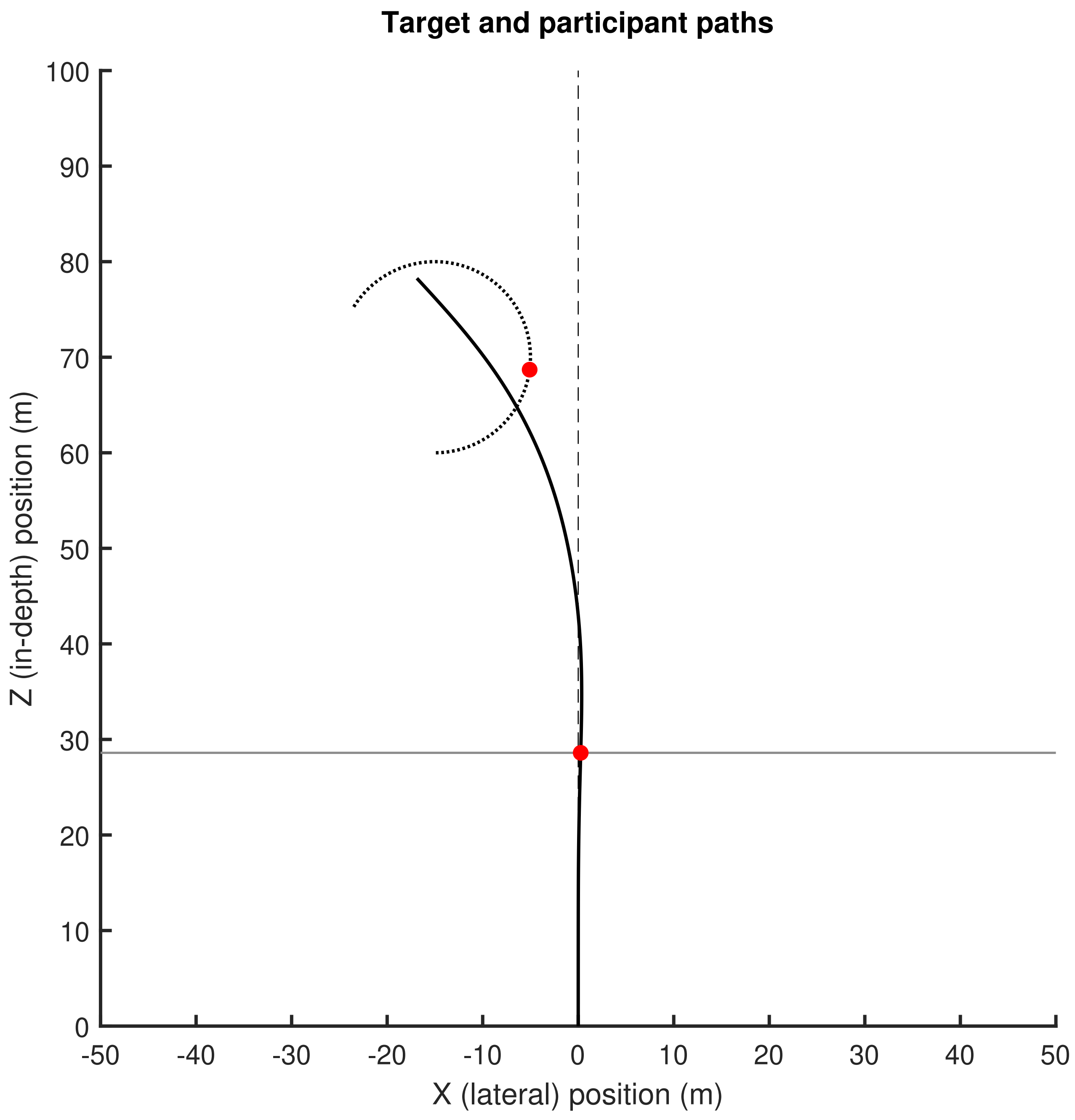

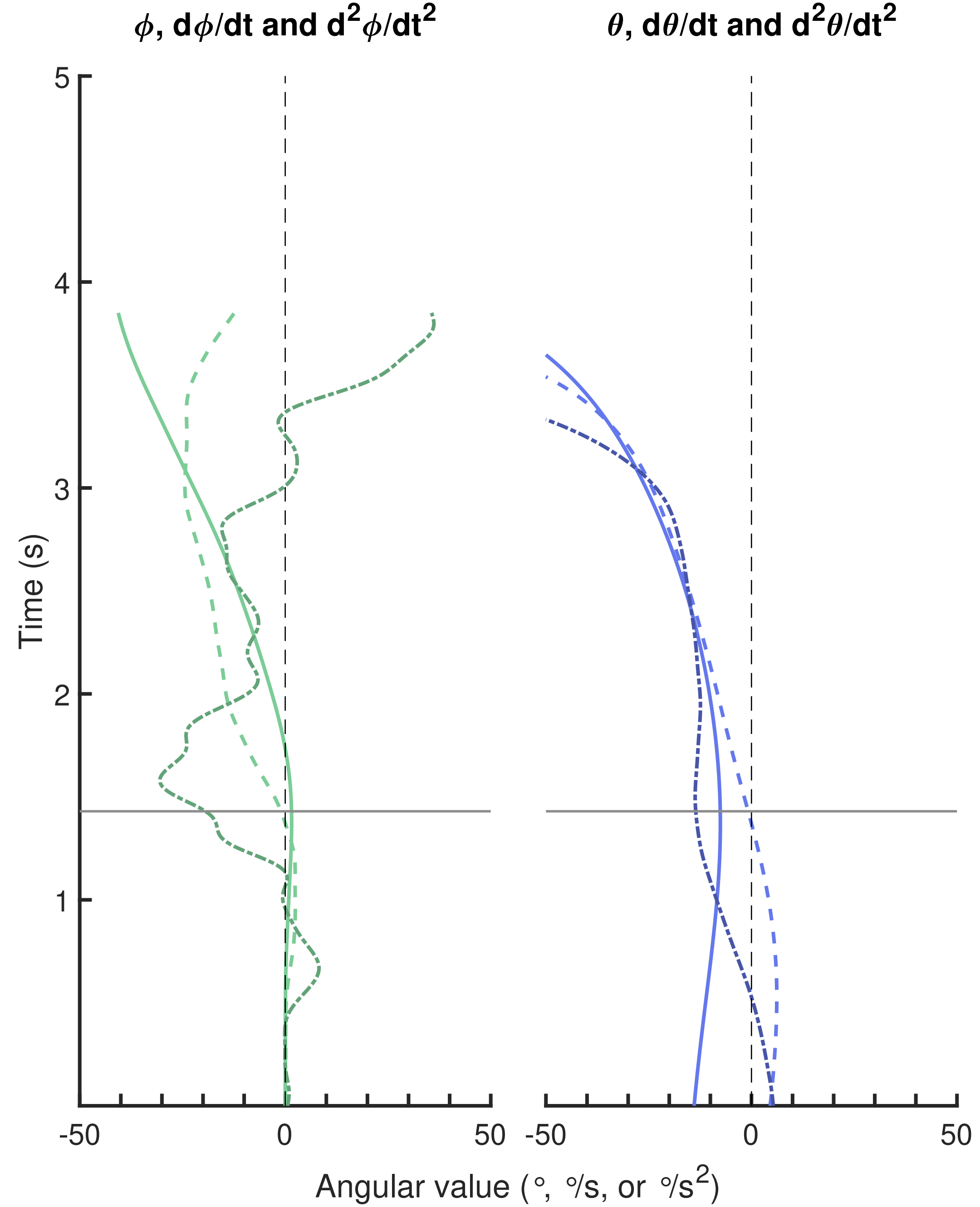

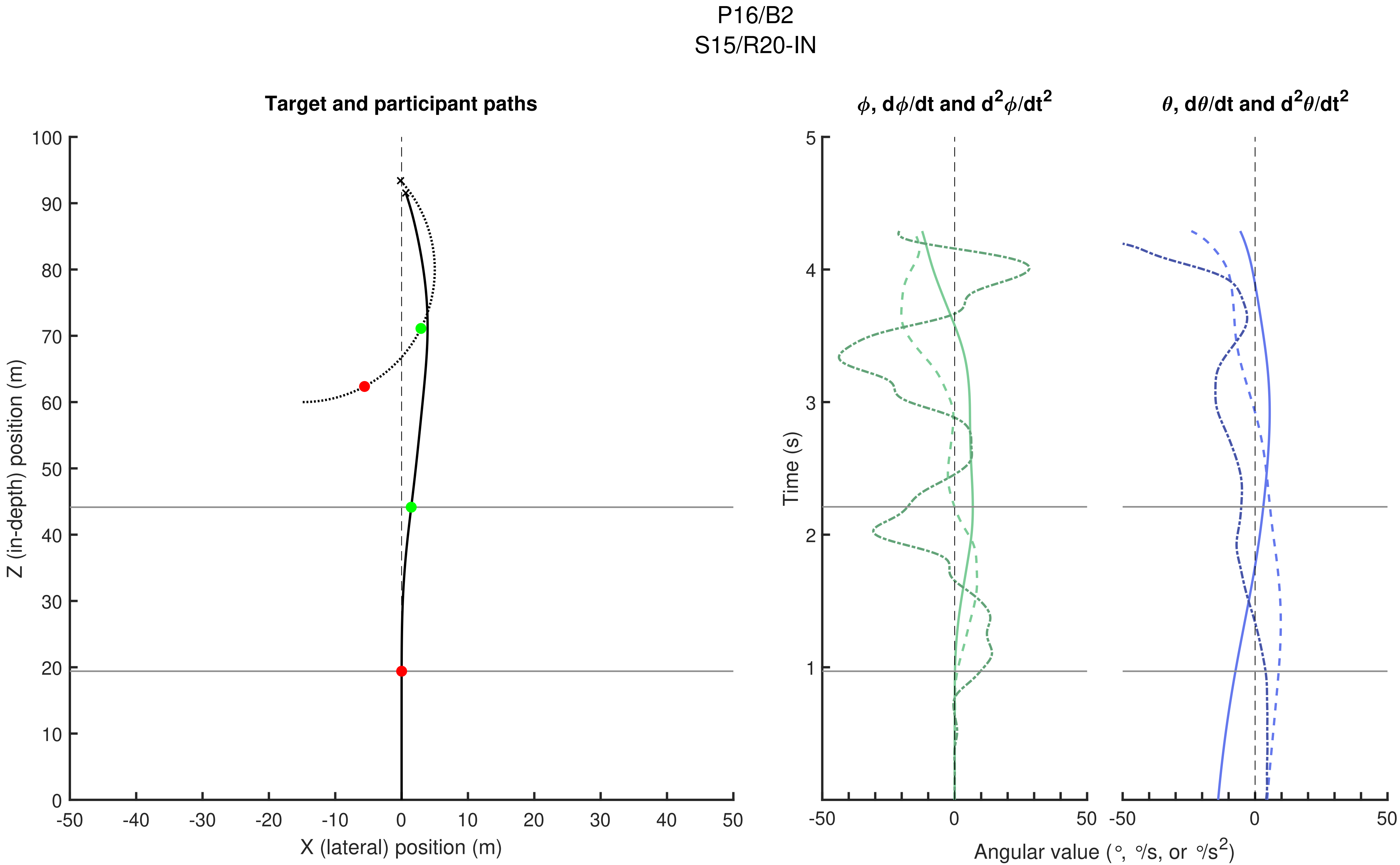
P16/B2
S15/R20-IN
Target and participant paths
ϕ, dϕ/dt and d$^2\phi$/dt^2
θ, dθ/dt and d$^2\theta$/dt^2
Z (in-depth) position (m)
X (lateral) position (m)
Time (s)
Angular value (°, °/s, or °/s^2)

P16/B2
S15/R30-IN

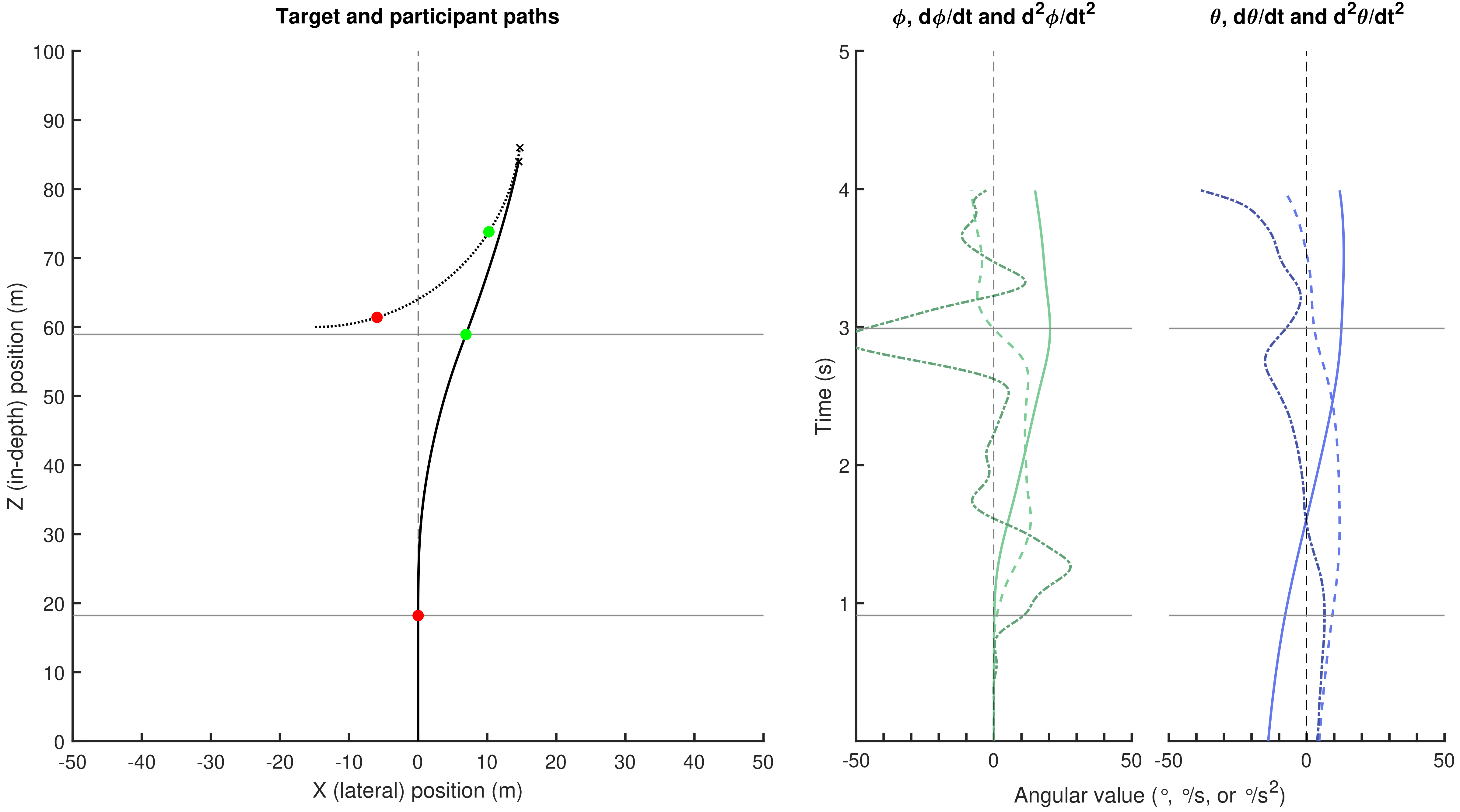

P16/B2
S5/R10-IN

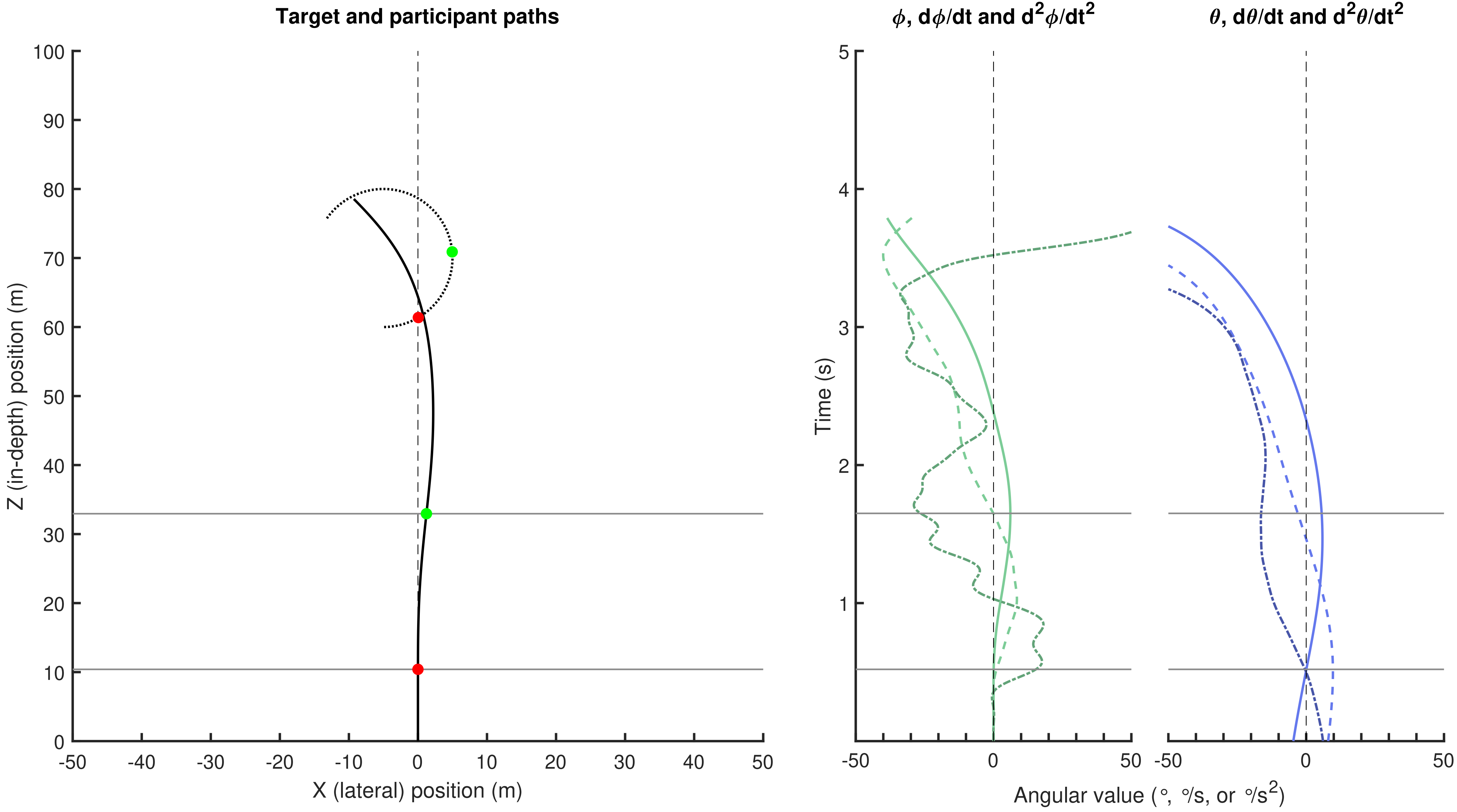

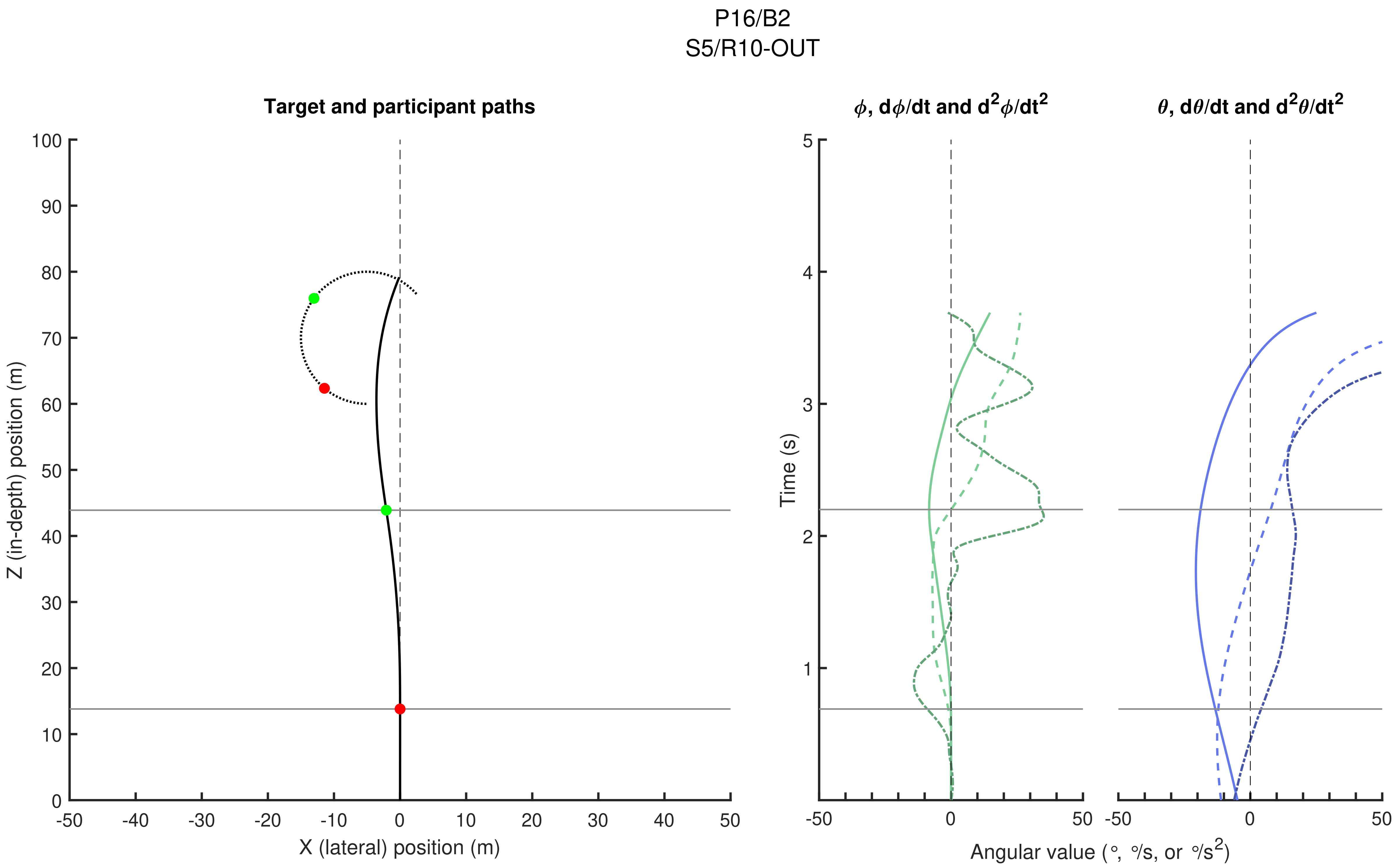
P16/B2
S5/R10-OUT
Target and participant paths
Z (in-depth) position (m)
X (lateral) position (m)
ϕ, dϕ/dt and d²ϕ/dt²
θ, dθ/dt and d²θ/dt²
Time (s)
Angular value (°, °/s, or °/s²)

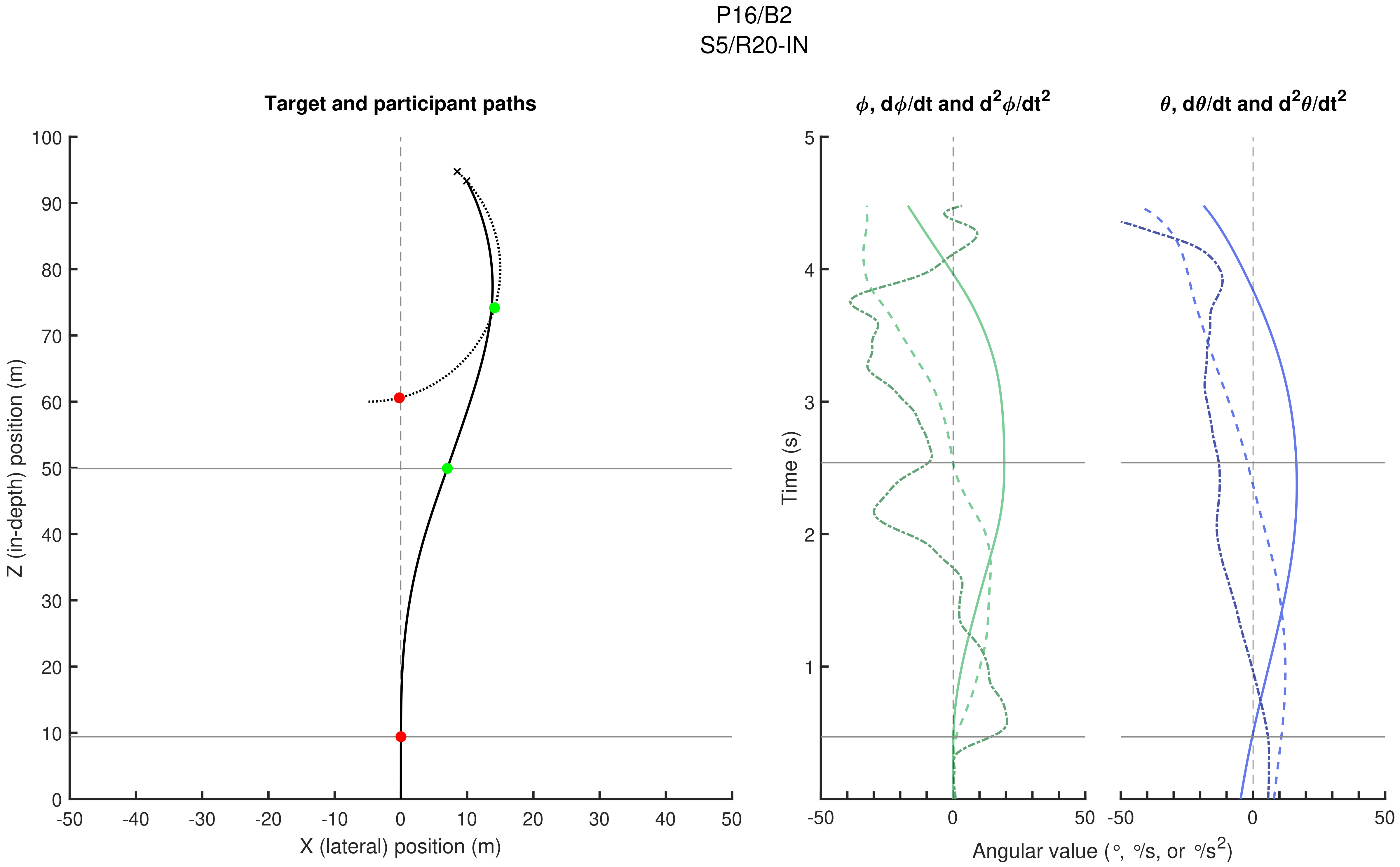

P16/B2
S5/R20-IN
Target and participant paths
Z (in-depth) position (m)
X (lateral) position (m)
100
90
80
70
60
50
40
30
20
10
0
-50
-40
-30
-20
-10
0
10
20
30
40
50
ϕ, dϕ/dt and d²ϕ/dt²
θ, dθ/dt and d²θ/dt²
Time (s)
5
4
3
2
1
-50
0
50
-50
0
50
Angular value (°, °/s, or °/s²)

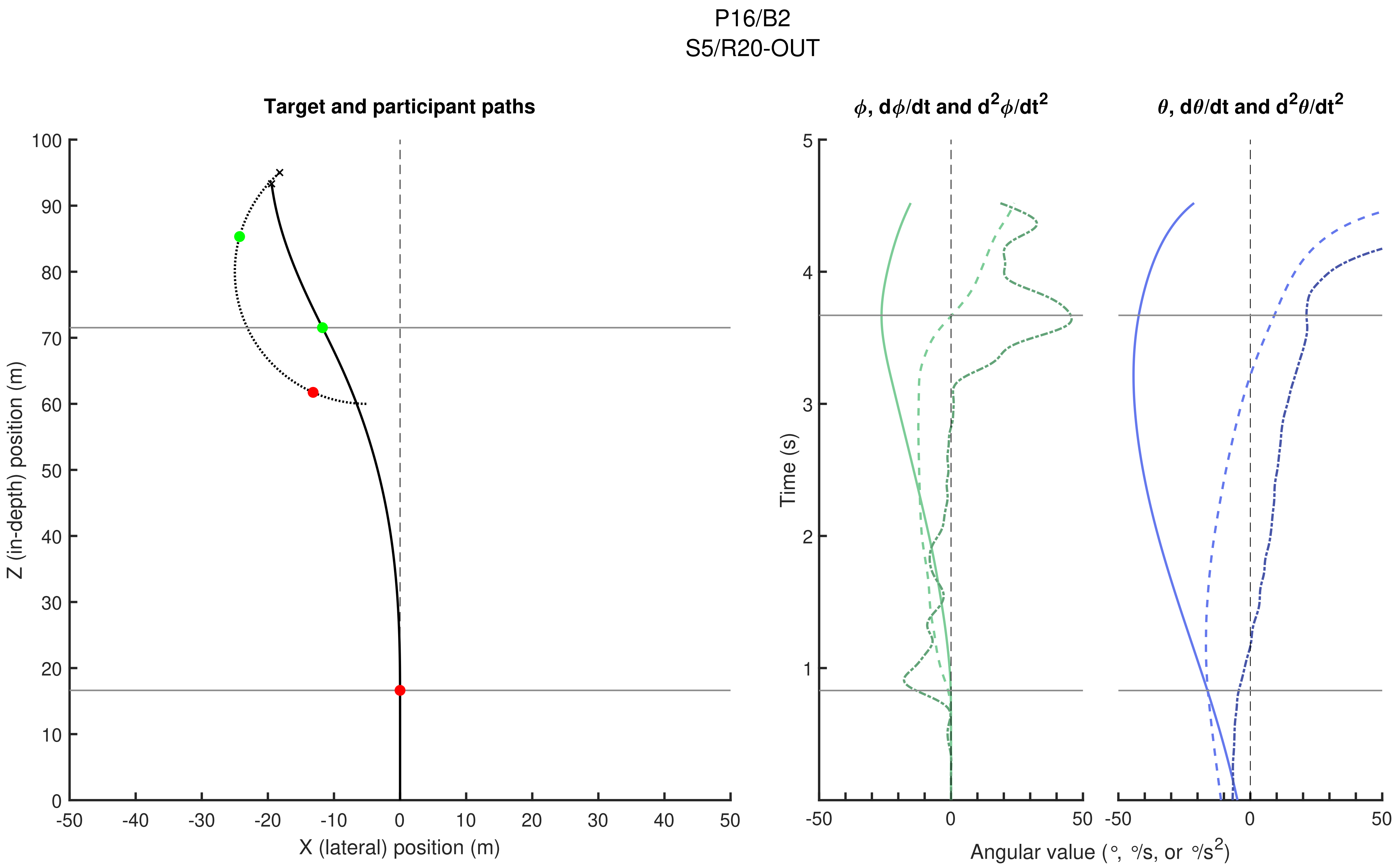
P16/B2
S5/R20-OUT
Target and participant paths
Z (in-depth) position (m)
X (lateral) position (m)
100
90
80
70
60
50
40
30
20
10
0
-50
-40
-30
-20
-10
0
10
20
30
40
50
φ, dφ/dt and d²φ/dt²
θ, dθ/dt and d²θ/dt²
Time (s)
5
4
3
2
1
-50
0
50
-50
0
50
Angular value (°, °/s, or °/s²)

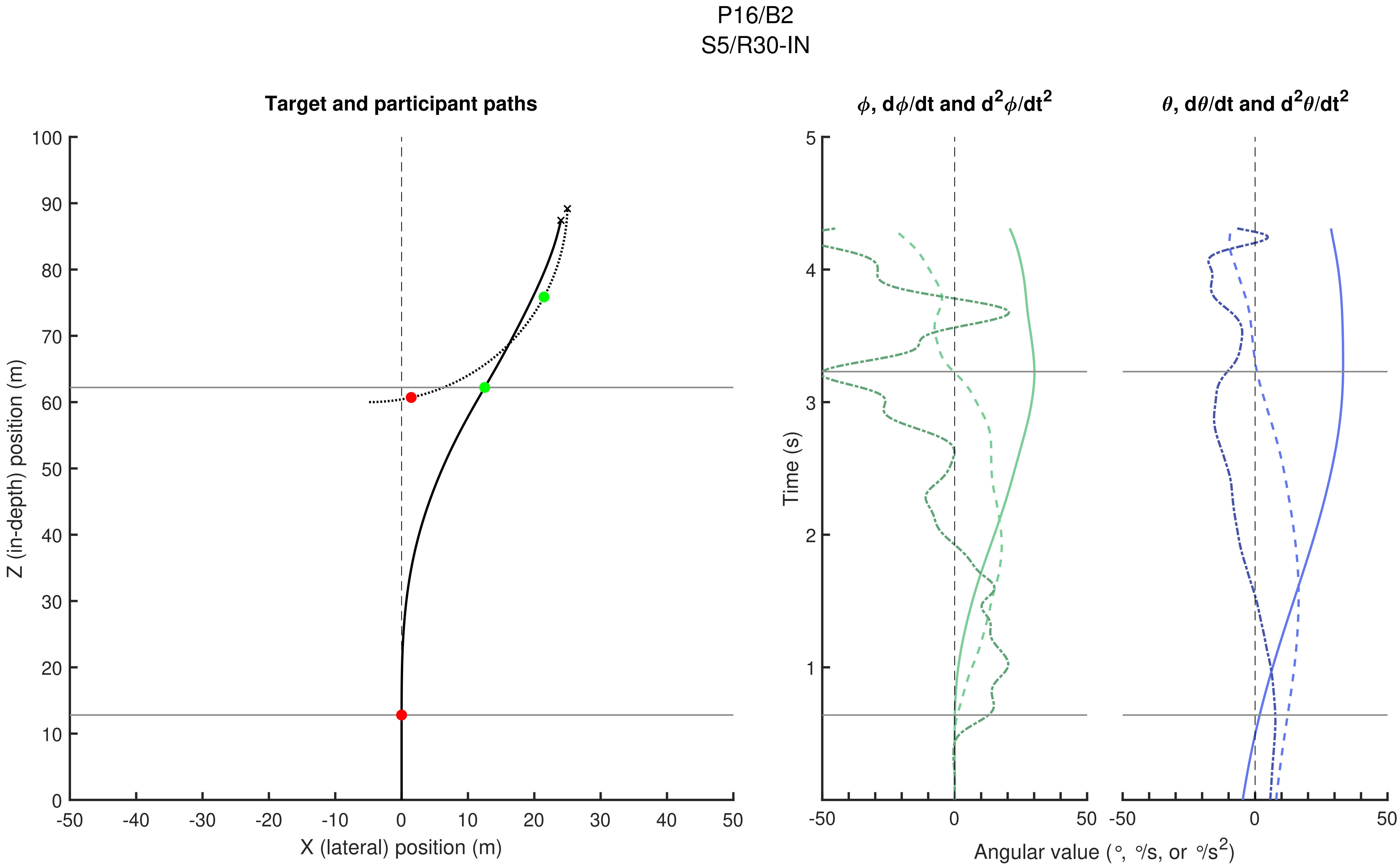

P16/B2
S5/R30-IN
Target and participant paths
Z (in-depth) position (m)
X (lateral) position (m)
100
90
80
70
60
50
40
30
20
10
0
-50
-40
-30
-20
-10
0
10
20
30
40
50
ϕ, dϕ/dt and d$^2\phi$/dt^2
θ, dθ/dt and d$^2\theta$/dt^2
Time (s)
5
4
3
2
1
-50
0
50
-50
0
50
Angular value (°, °/s, or °/s^2)

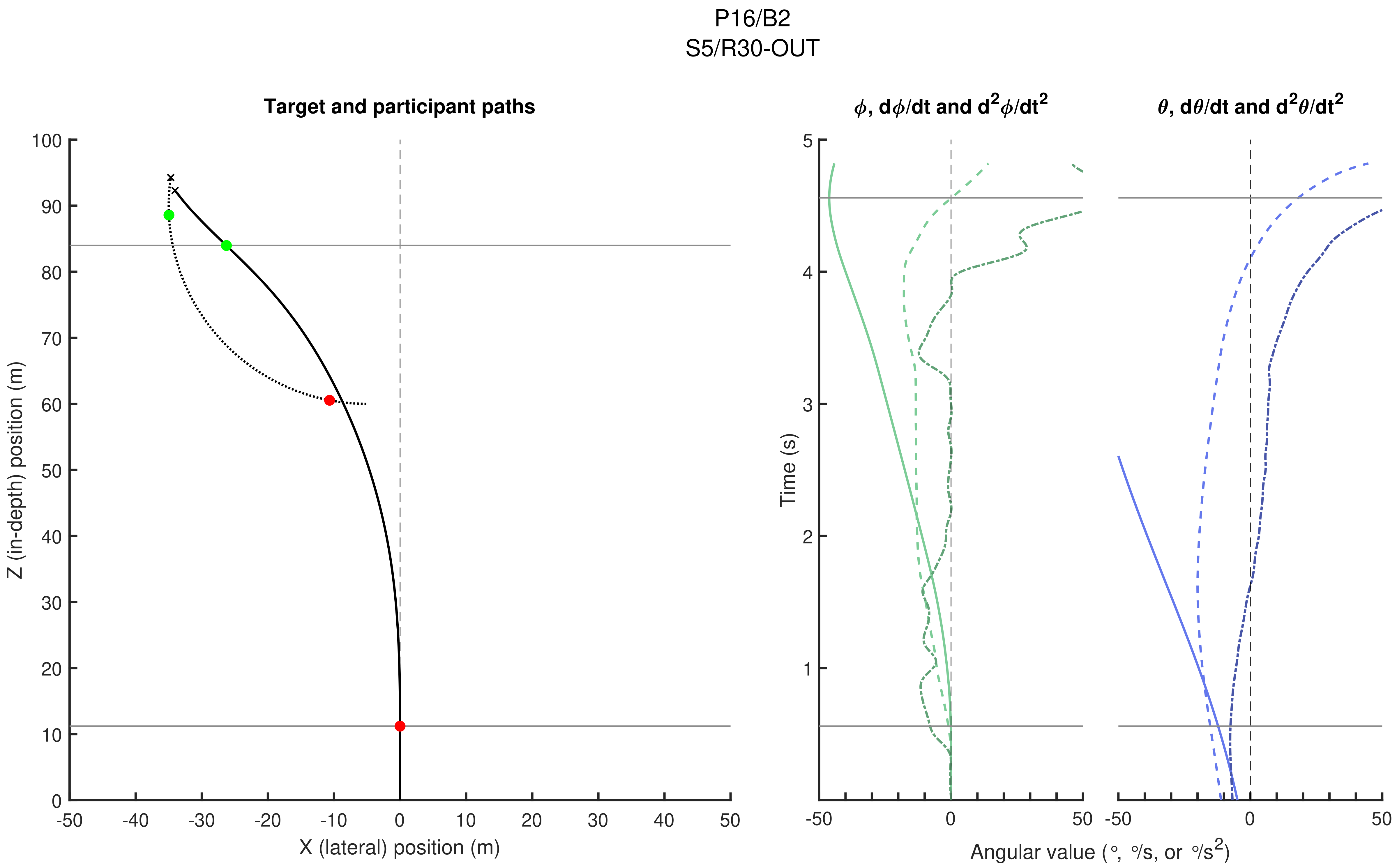
P16/B2
S5/R30-OUT
Target and participant paths
Z (in-depth) position (m)
X (lateral) position (m)
φ, dφ/dt and d²φ/dt²
θ, dθ/dt and d²θ/dt²
Time (s)
Angular value (°, °/s, or °/s²)

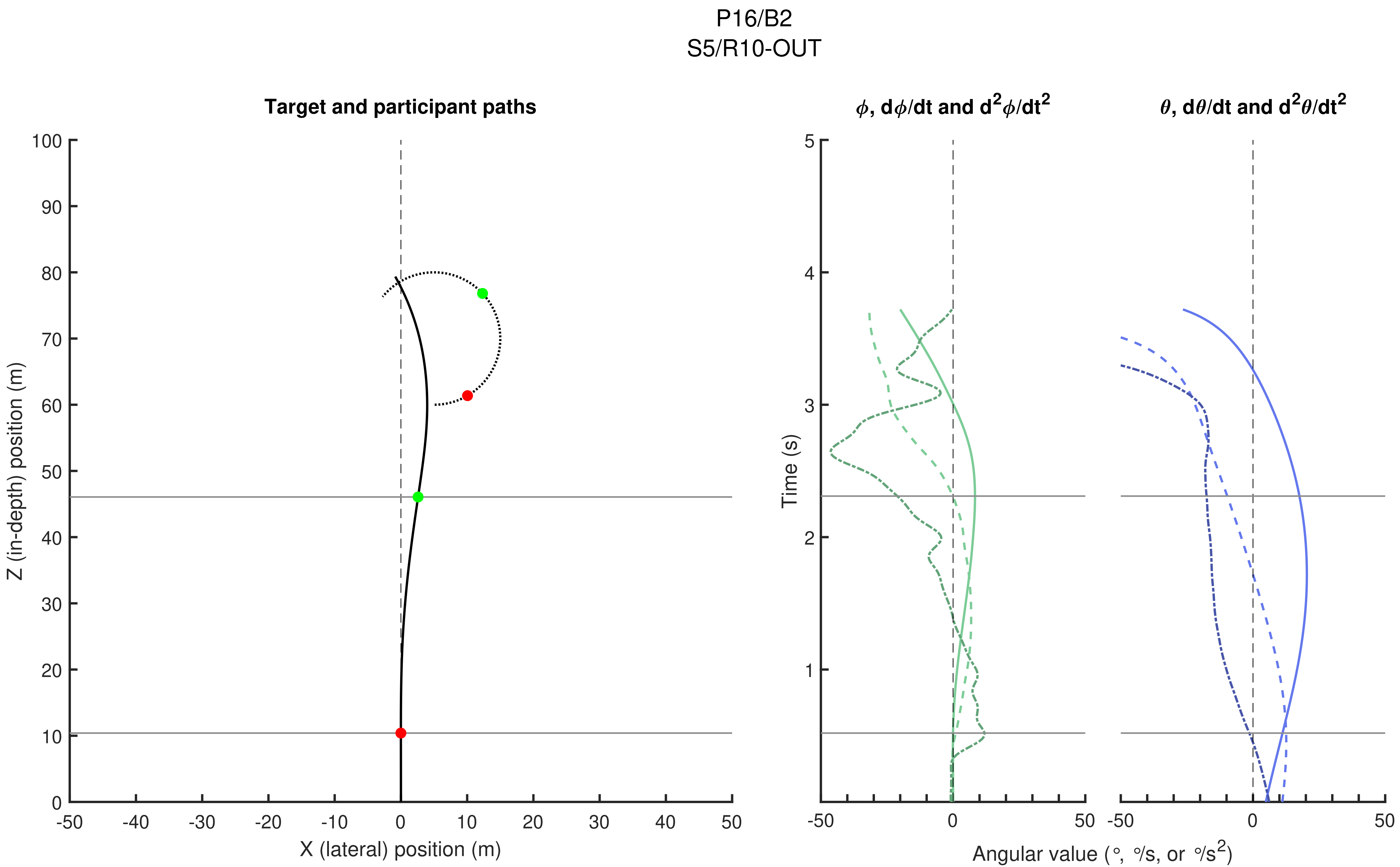
P16/B2
S5/R10-OUT
Target and participant paths
Z (in-depth) position (m)
X (lateral) position (m)
100
90
80
70
60
50
40
30
20
10
0
-50
-40
-30
-20
-10
0
10
20
30
40
50
ϕ, dϕ/dt and d$^2\phi$/dt^2
θ, dθ/dt and d$^2\theta$/dt^2
Time (s)
5
4
3
2
1
-50
0
50
-50
0
50
Angular value (°, °/s, or °/s^2)

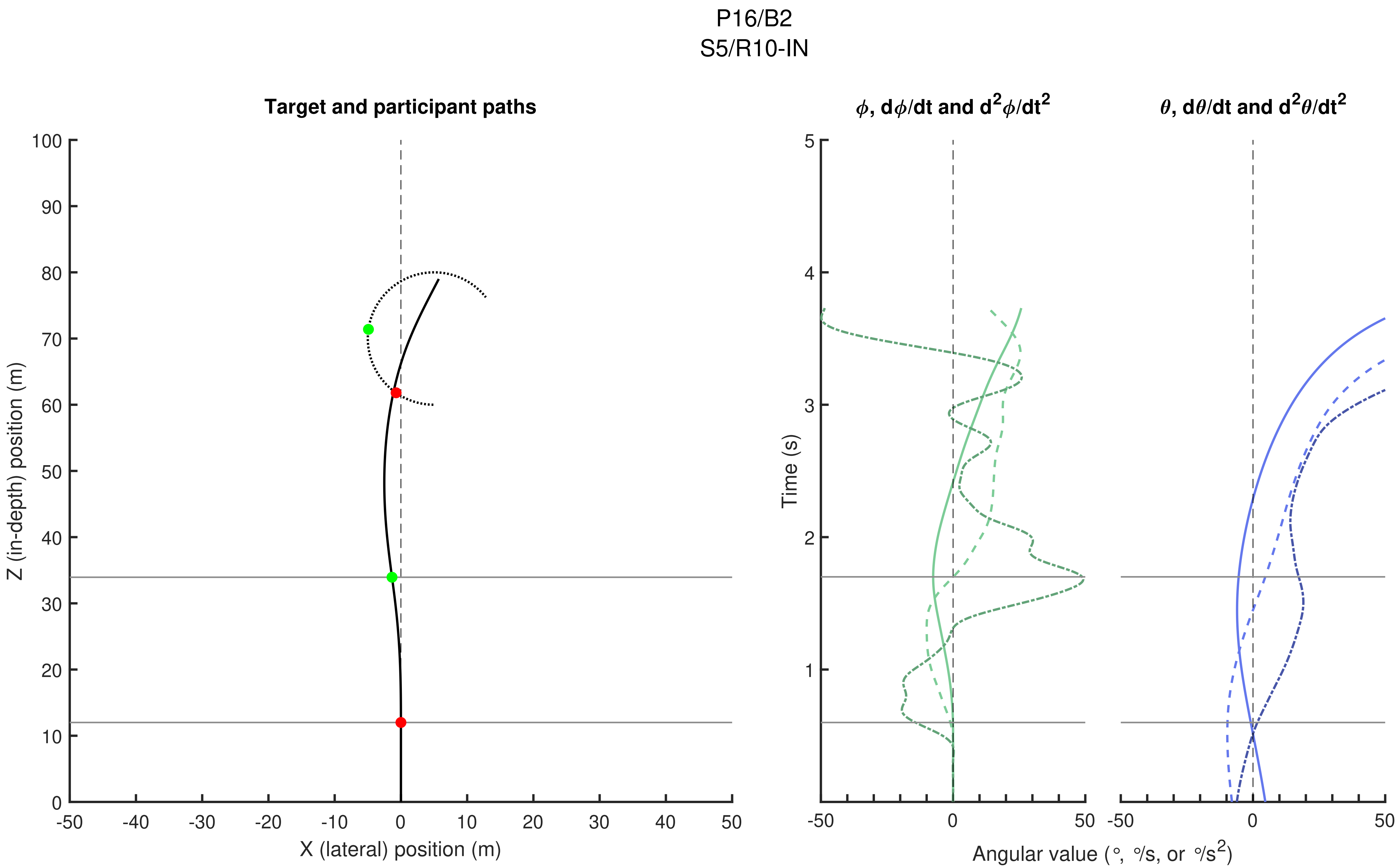
P16/B2
S5/R10-IN
Target and participant paths
Z (in-depth) position (m)
X (lateral) position (m)
100
90
80
70
60
50
40
30
20
10
0
-50
-40
-30
-20
-10
0
10
20
30
40
50
φ, dφ/dt and d²φ/dt²
θ, dθ/dt and d²θ/dt²
Time (s)
5
4
3
2
1
-50
0
50
-50
0
50
Angular value (°, °/s, or °/s²)

P16/B2
S5/R20-OUT

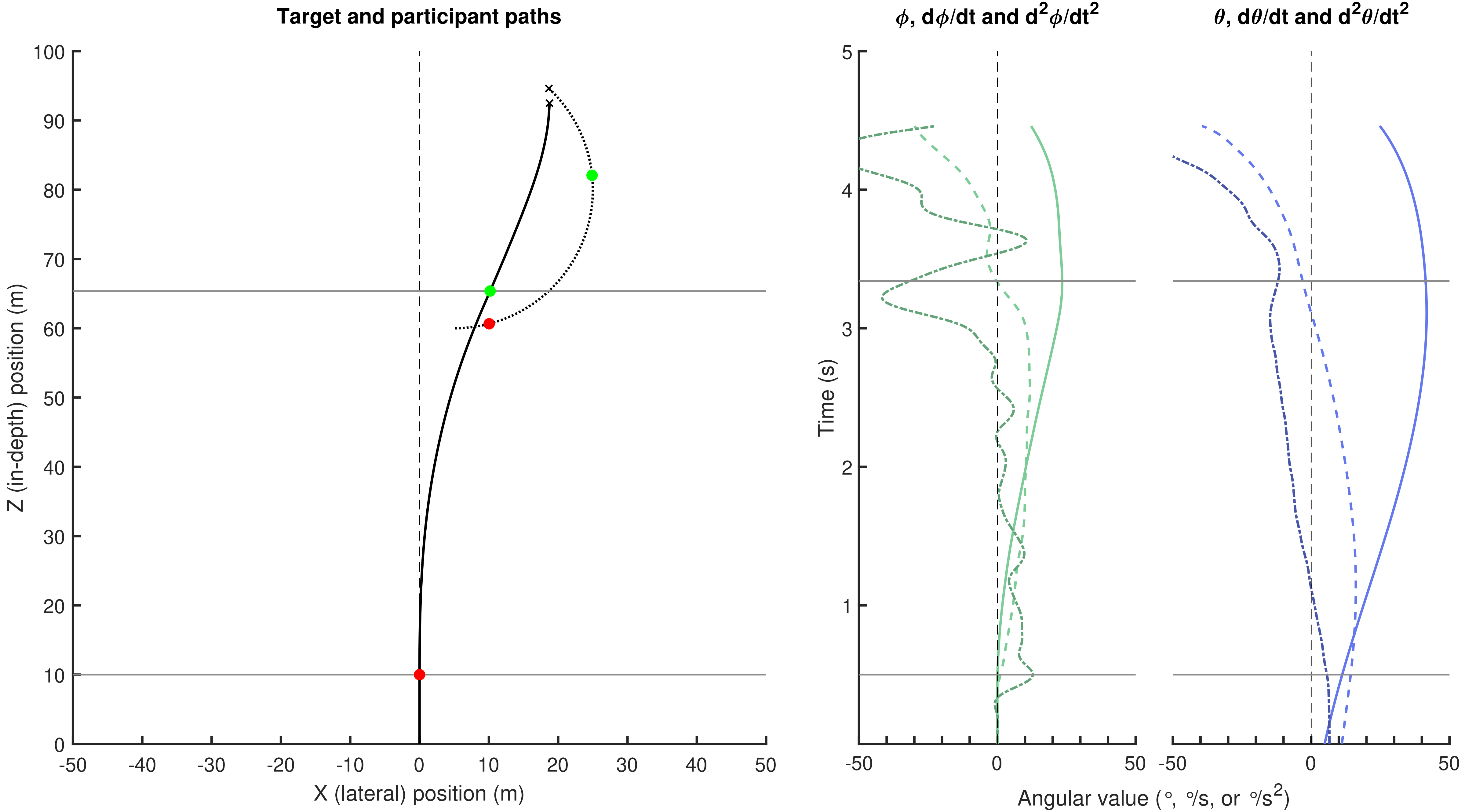

P16/B2
S5/R20-IN

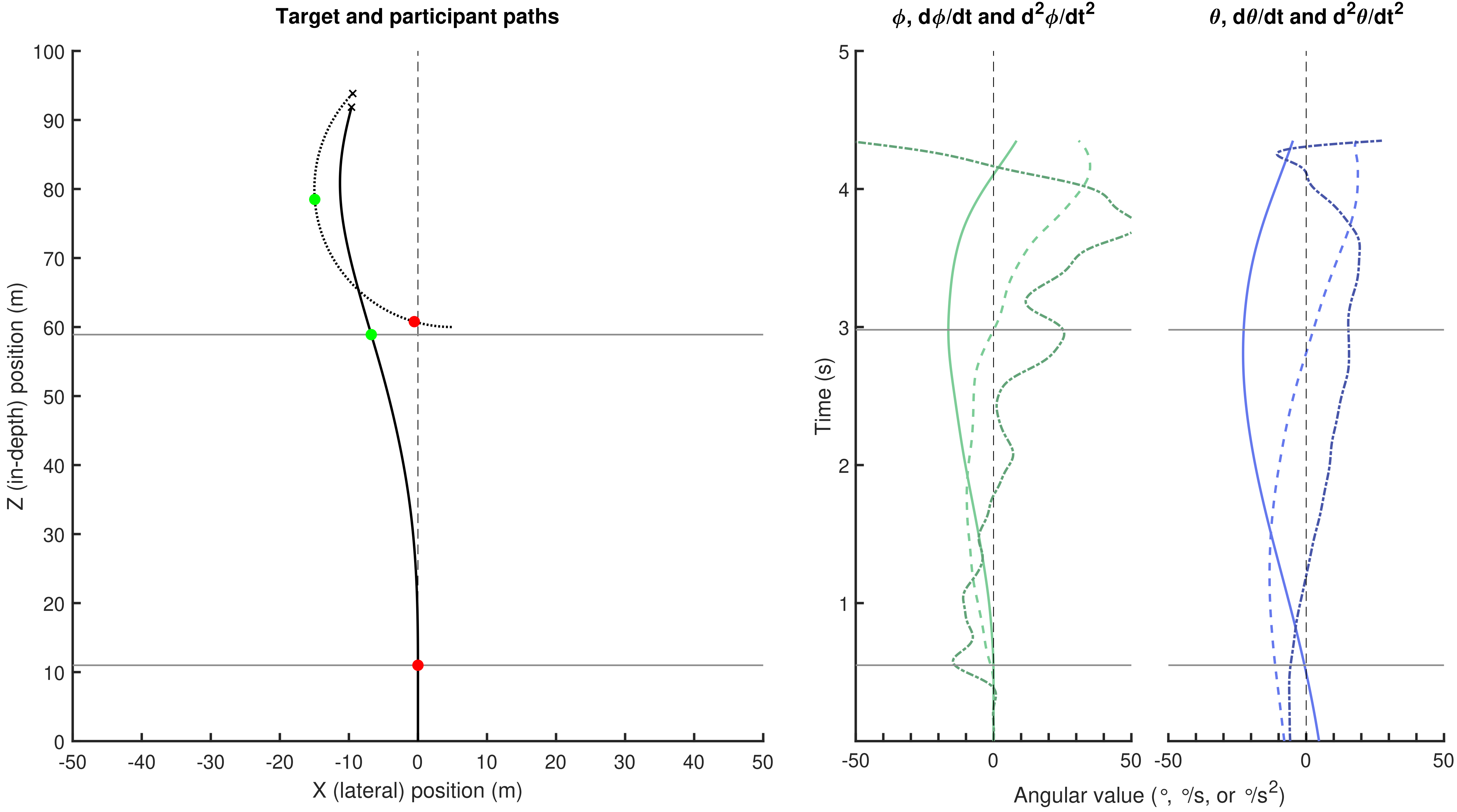

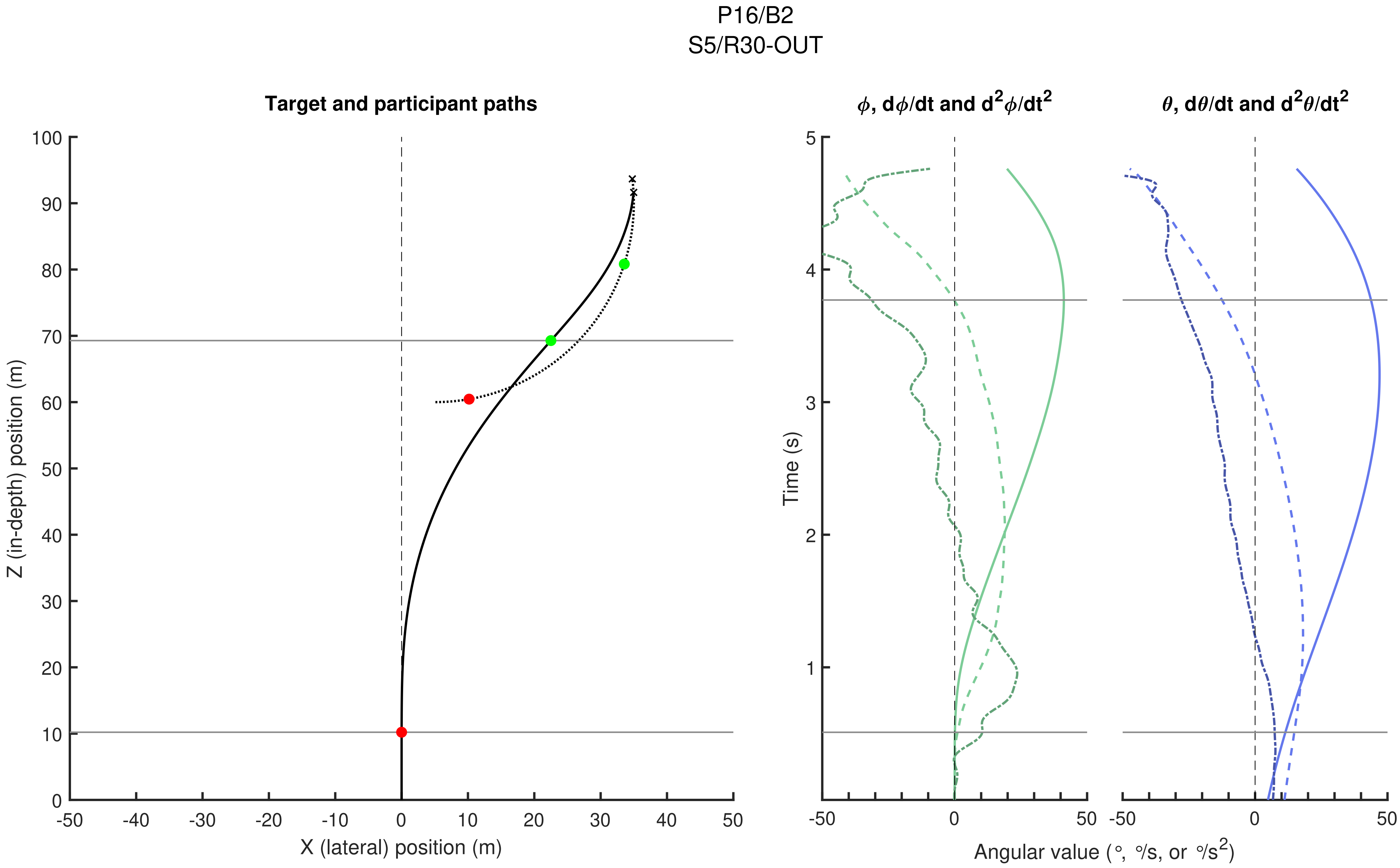

P16/B2
S5/R30-OUT
Target and participant paths
Z (in-depth) position (m)
X (lateral) position (m)
ϕ, dϕ/dt and d$^2\phi$/dt^2
θ, dθ/dt and d$^2\theta$/dt^2
Time (s)
Angular value (°, °/s, or °/s^2)

P16/B2
S5/R30-IN

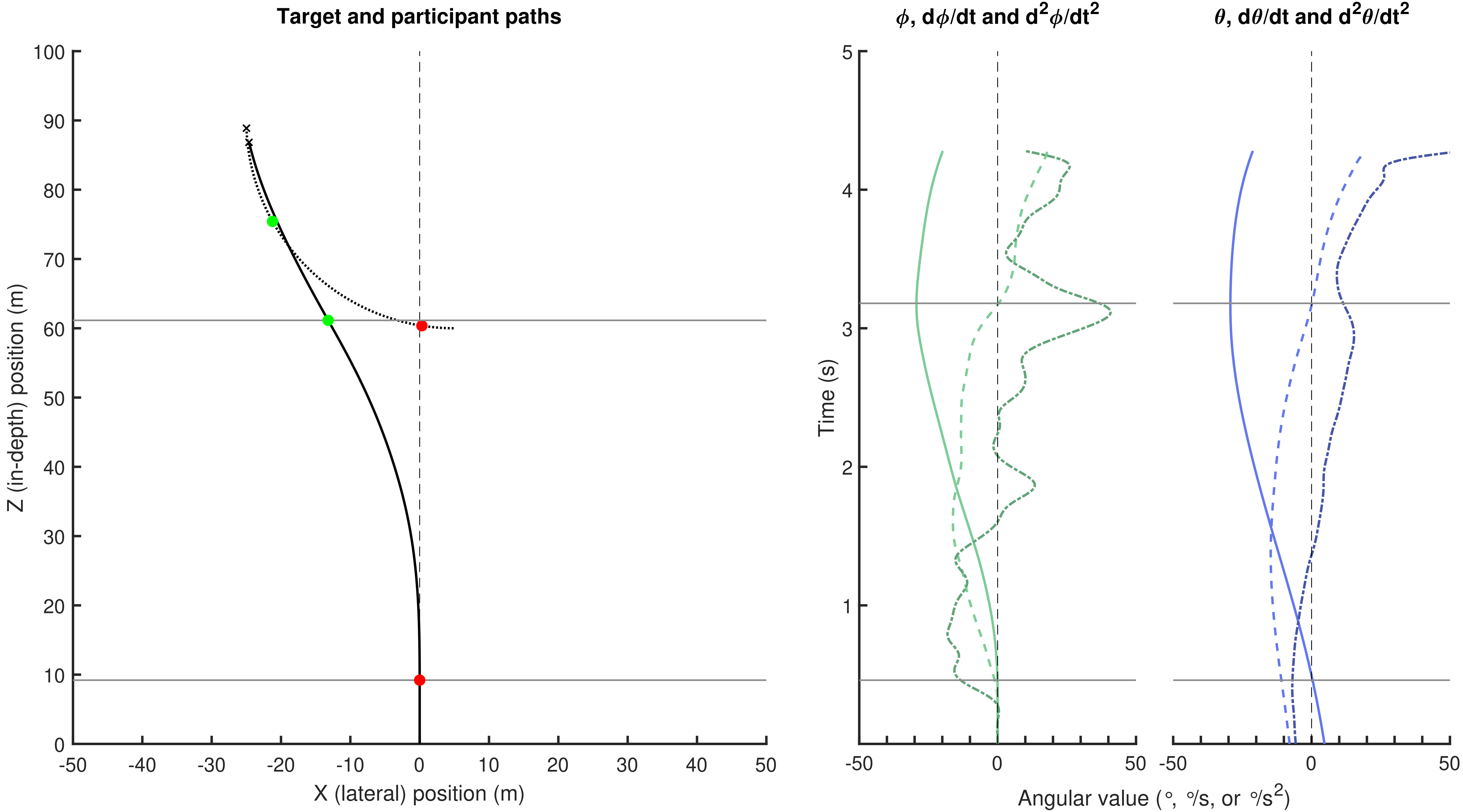

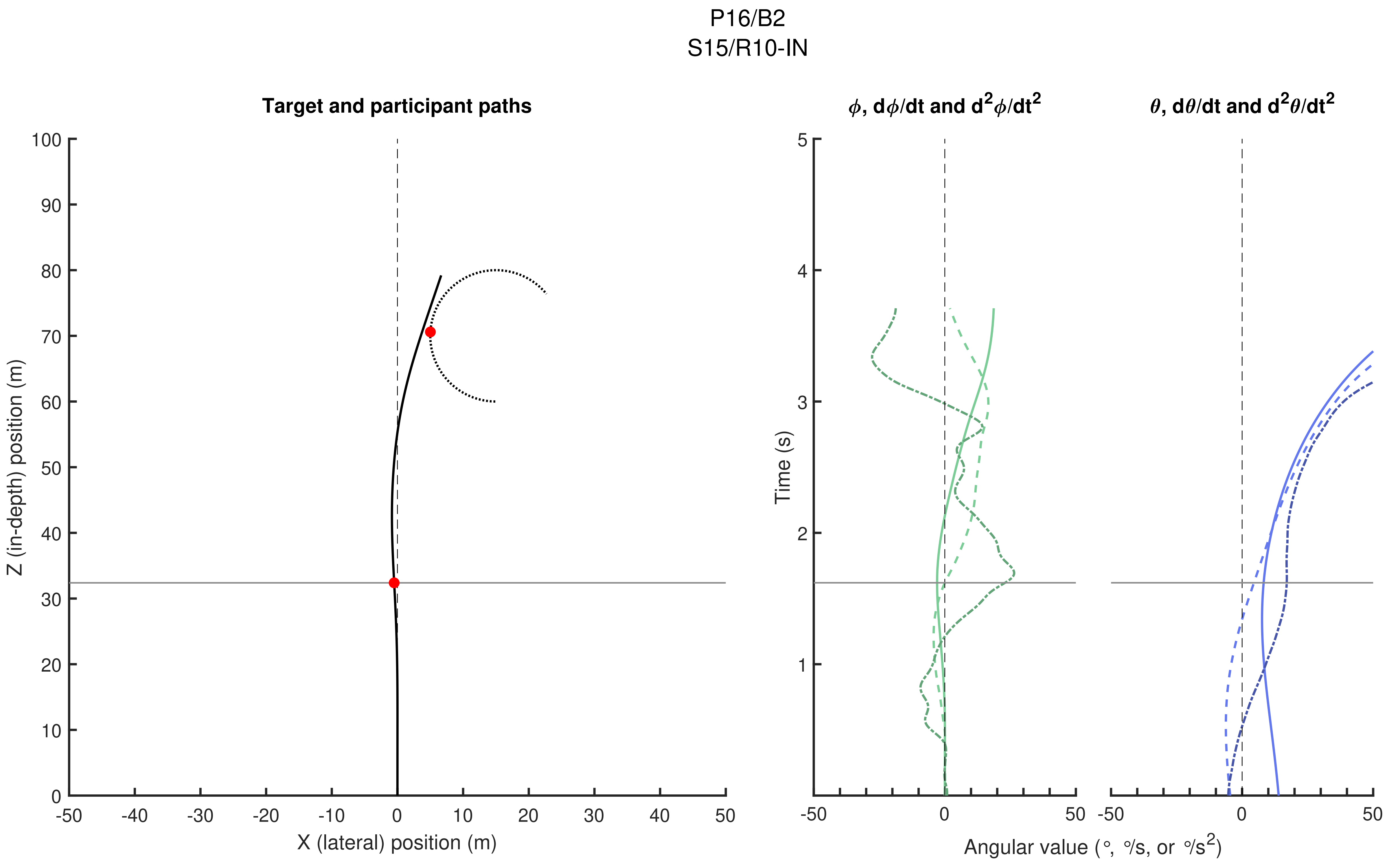

P16/B2
S15/R10-IN
Target and participant paths
Z (in-depth) position (m)
X (lateral) position (m)
100
90
80
70
60
50
40
30
20
10
0
-50
-40
-30
-20
-10
0
10
20
30
40
50
ϕ, dϕ/dt and d$^2\phi$/dt^2
θ, dθ/dt and d$^2\theta$/dt^2
Time (s)
5
4
3
2
1
-50
0
50
-50
0
50
Angular value (°, °/s, or °/s^2)

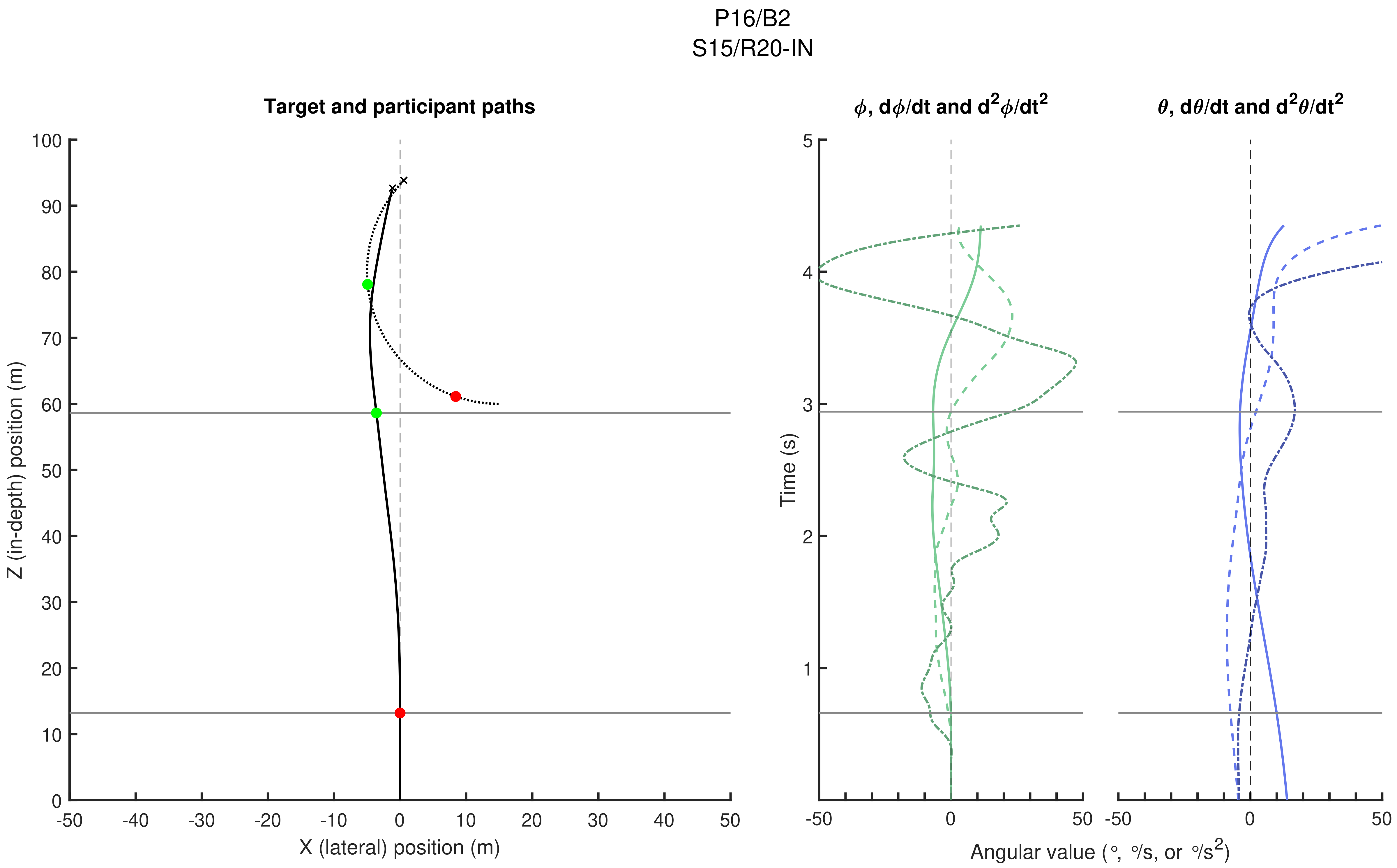
P16/B2
S15/R20-IN
Target and participant paths
Z (in-depth) position (m)
X (lateral) position (m)
ϕ, dϕ/dt and d$^2\phi$/dt^2
θ, dθ/dt and d$^2\theta$/dt^2
Time (s)
Angular value (°, °/s, or °/s^2)

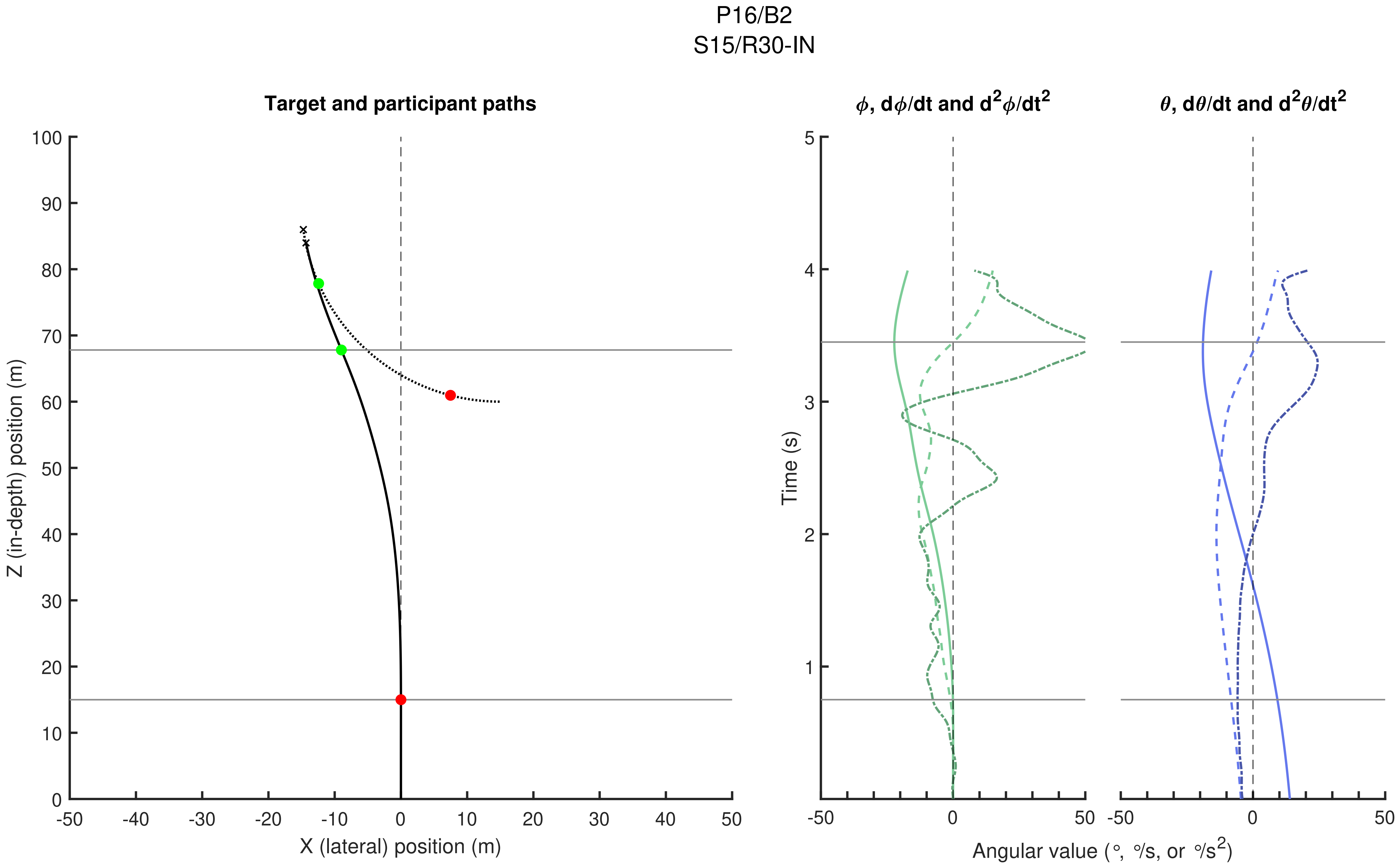

P16/B2
S15/R30-IN
Target and participant paths
Z (in-depth) position (m)
X (lateral) position (m)
ϕ, dϕ/dt and d$^2\phi$/dt^2
θ, dθ/dt and d$^2\theta$/dt^2
Time (s)
Angular value (°, °/s, or °/s^2)

P16/B2
S20/R10-IN

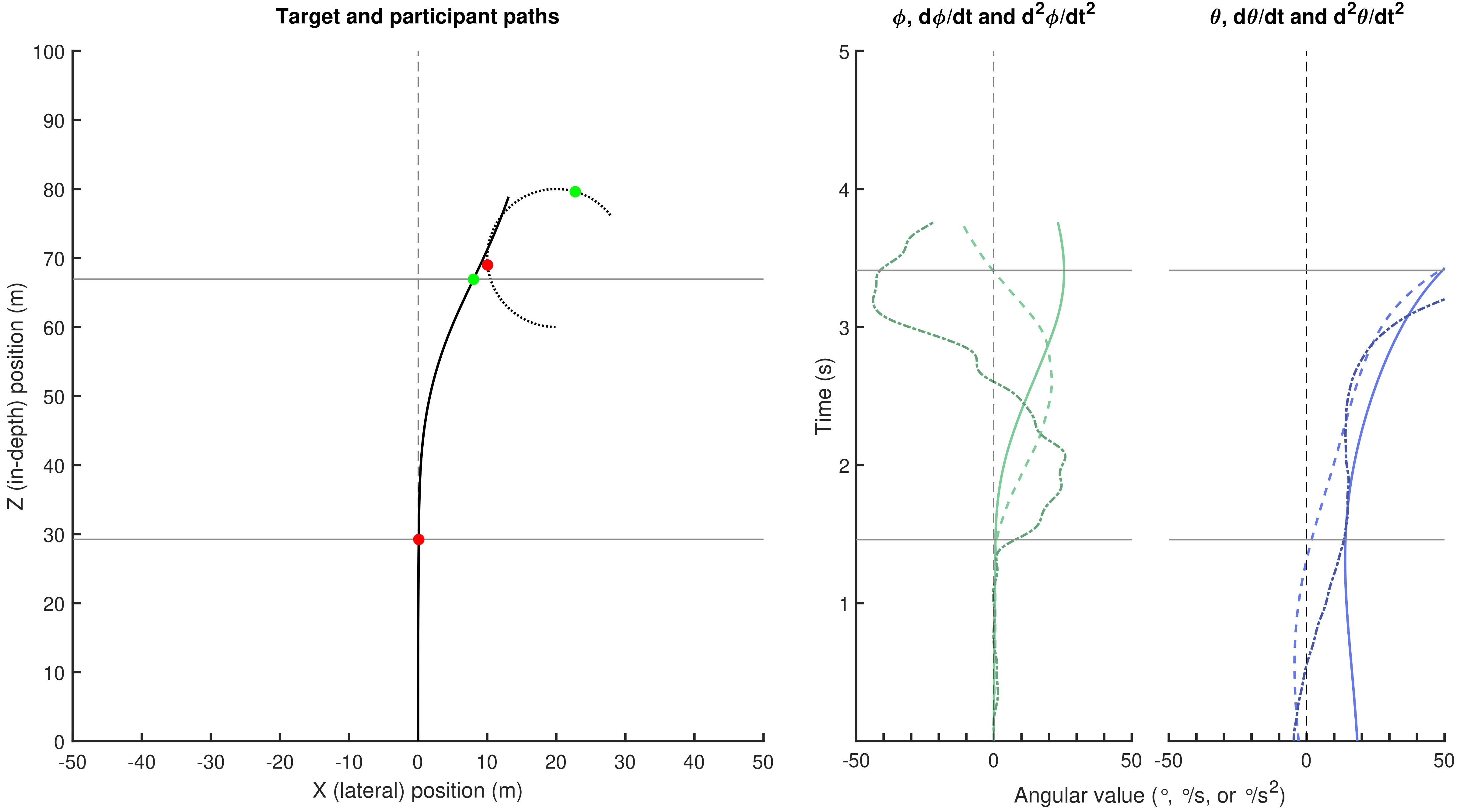

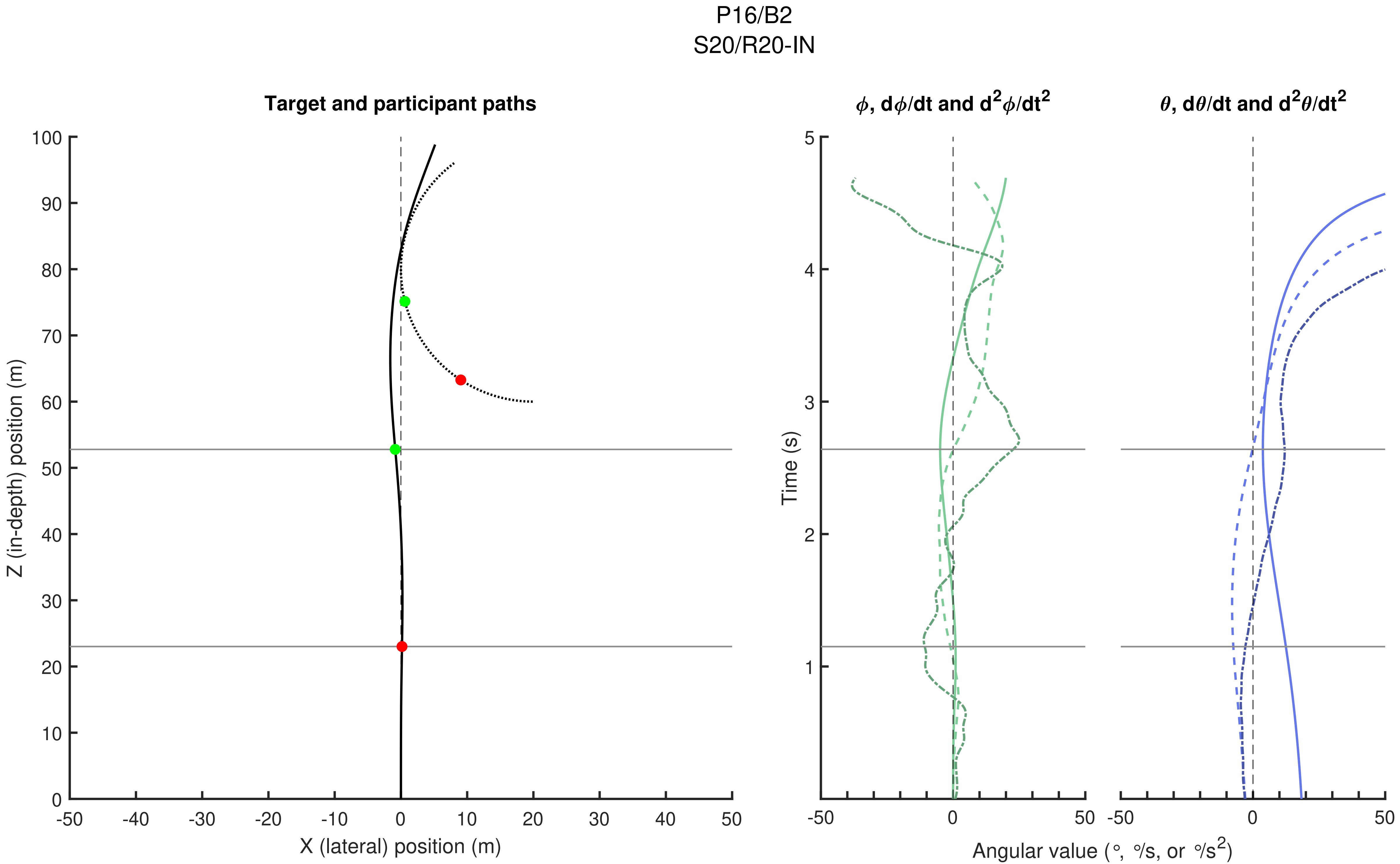
P16/B2
S20/R20-IN
Target and participant paths
Z (in-depth) position (m)
X (lateral) position (m)
ϕ, dϕ/dt and d$^2\phi$/dt^2
θ, dθ/dt and d$^2\theta$/dt^2
Time (s)
Angular value (°, °/s, or °/s^2)

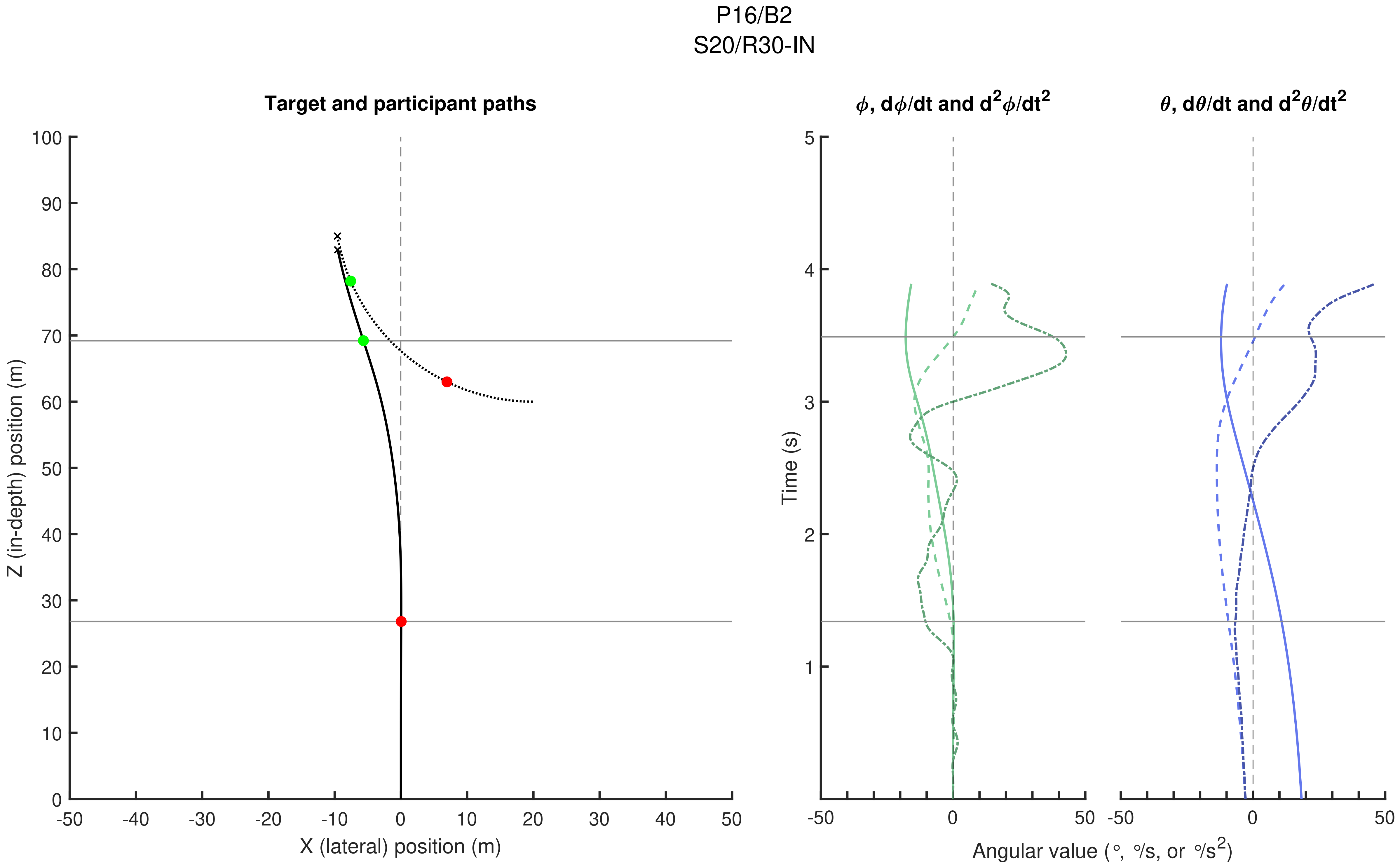

P16/B2
S20/R30-IN
Target and participant paths
Z (in-depth) position (m)
X (lateral) position (m)
ϕ, dϕ/dt and d²ϕ/dt²
θ, dθ/dt and d²θ/dt²
Time (s)
Angular value (°, °/s, or °/s²)

P16/B3
S20/R10-IN

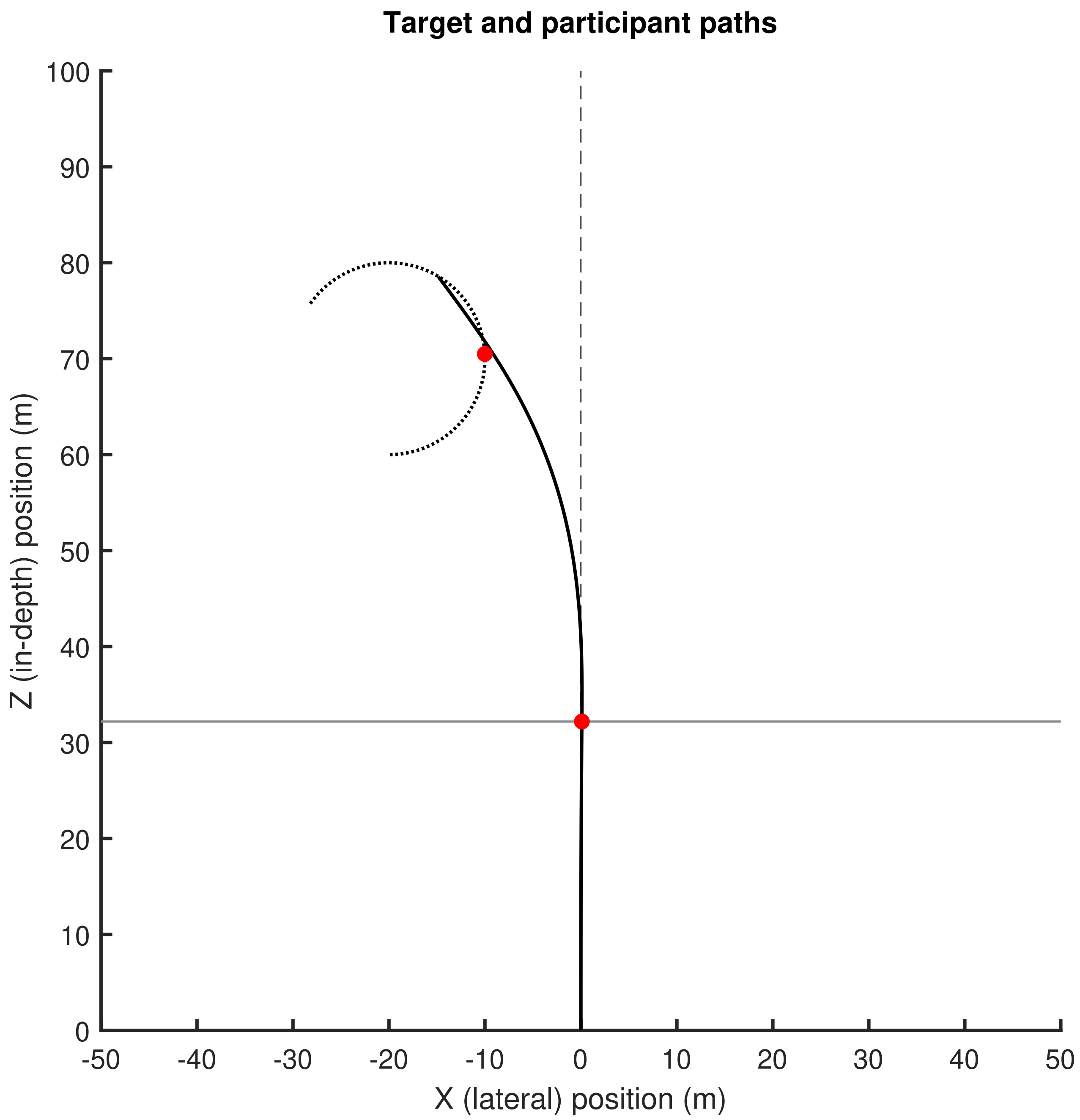

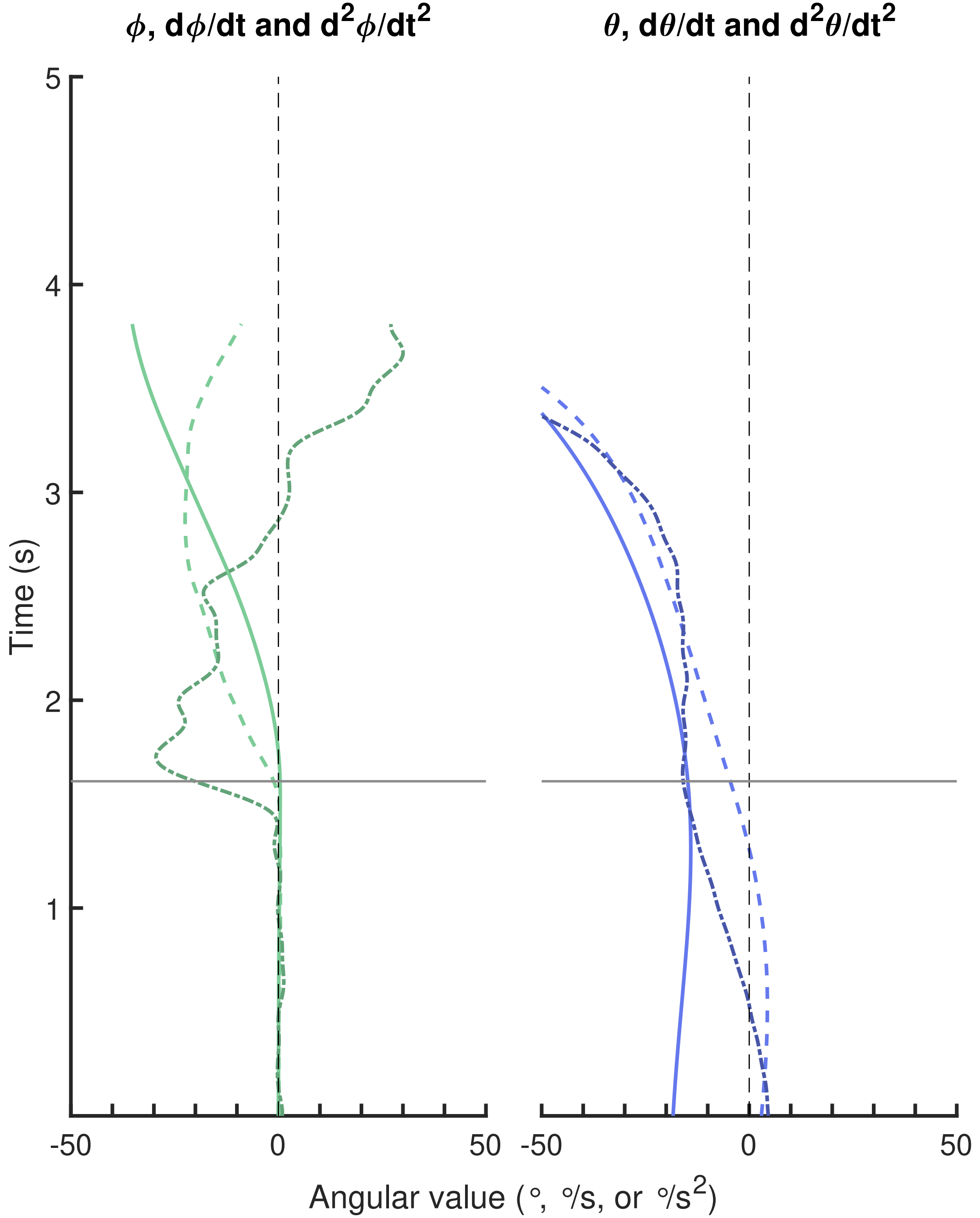

P16/B3
S20/R20-IN

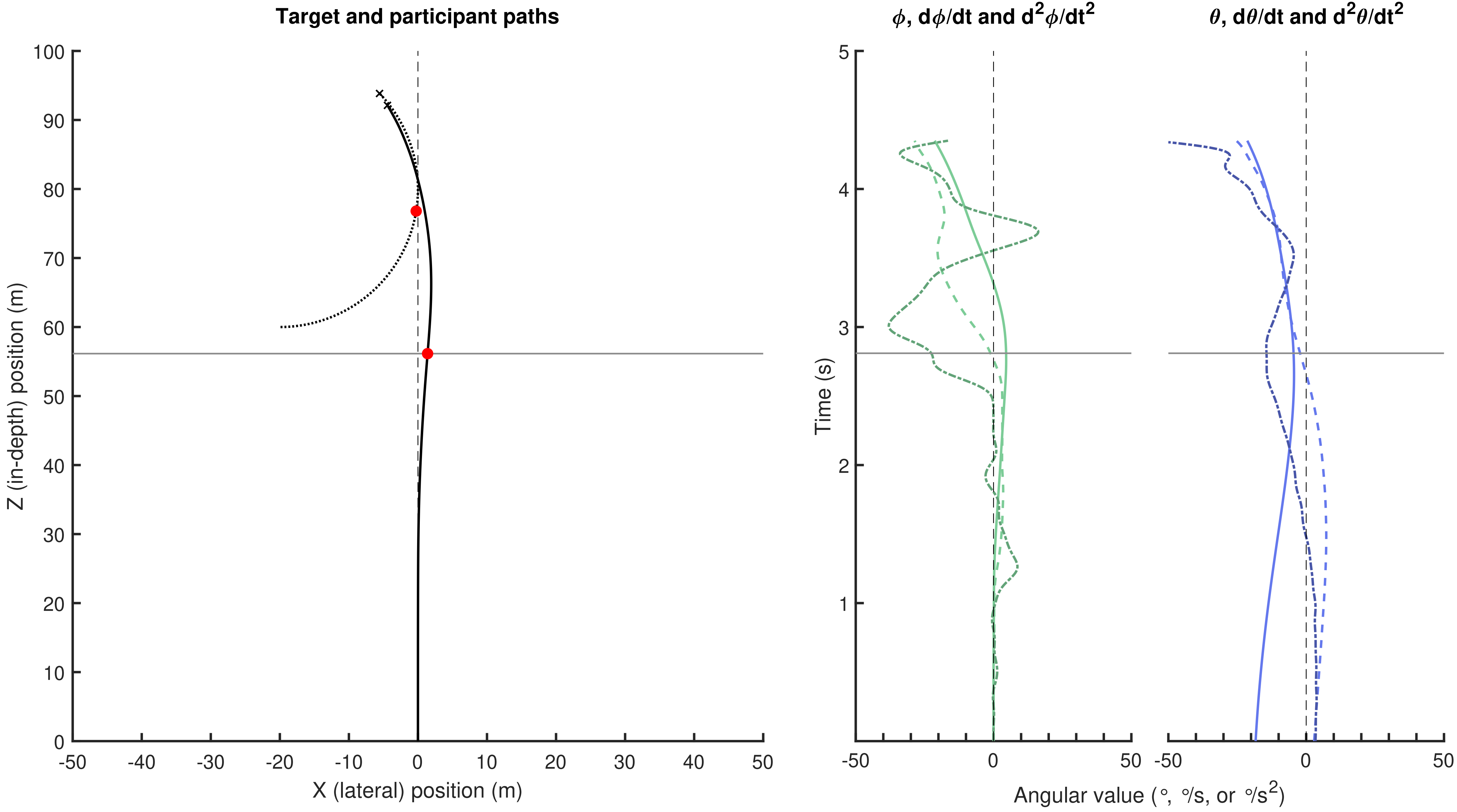

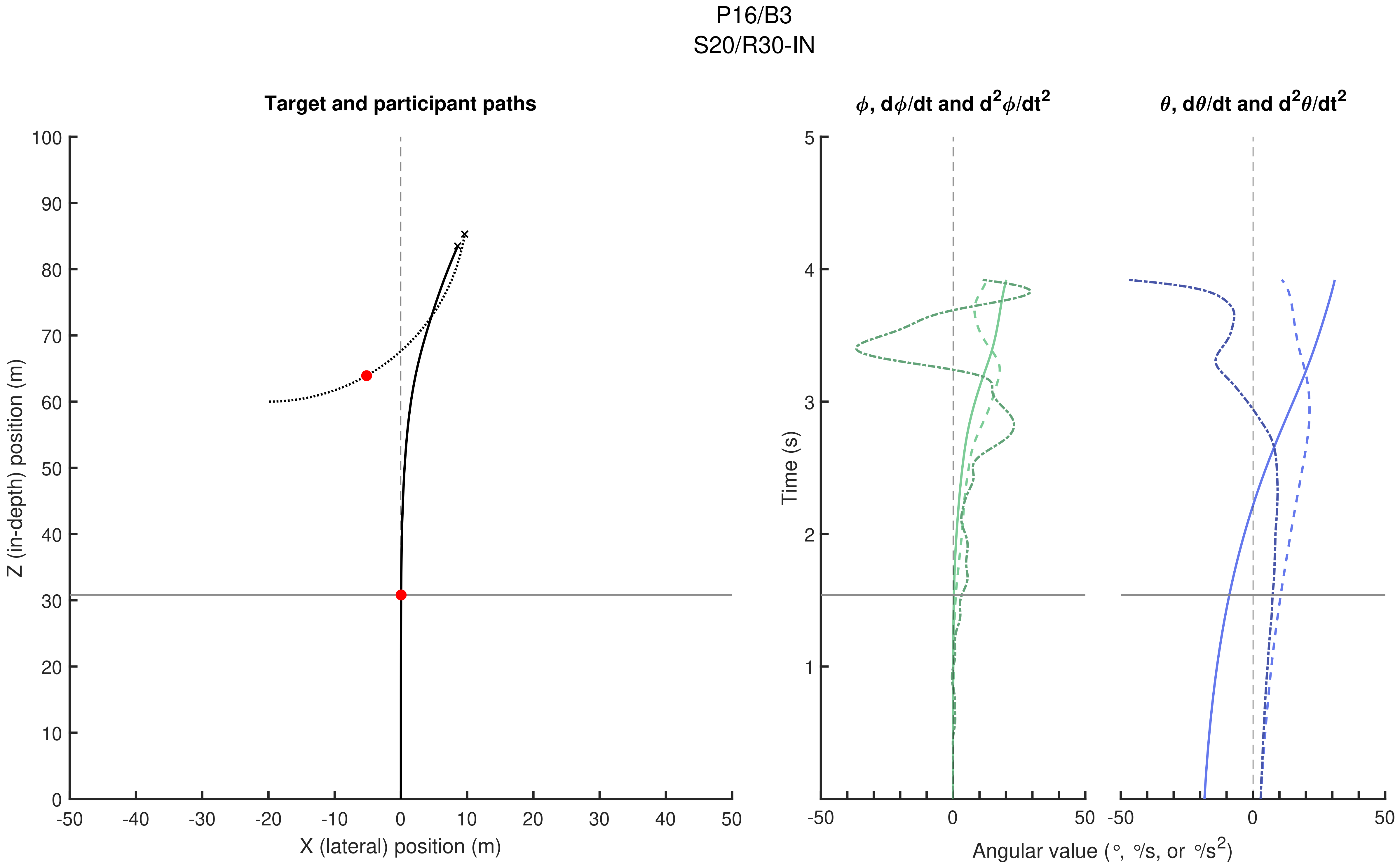
P16/B3
S20/R30-IN
Target and participant paths
Z (in-depth) position (m)
X (lateral) position (m)
100
90
80
70
60
50
40
30
20
10
0
-50
-40
-30
-20
-10
0
10
20
30
40
50
ϕ, dϕ/dt and d$^2\phi$/dt^2
θ, dθ/dt and d$^2\theta$/dt^2
Time (s)
5
4
3
2
1
-50
0
50
-50
0
50
Angular value (°, °/s, or °/s^2)

P16/B3
S15/R10-IN

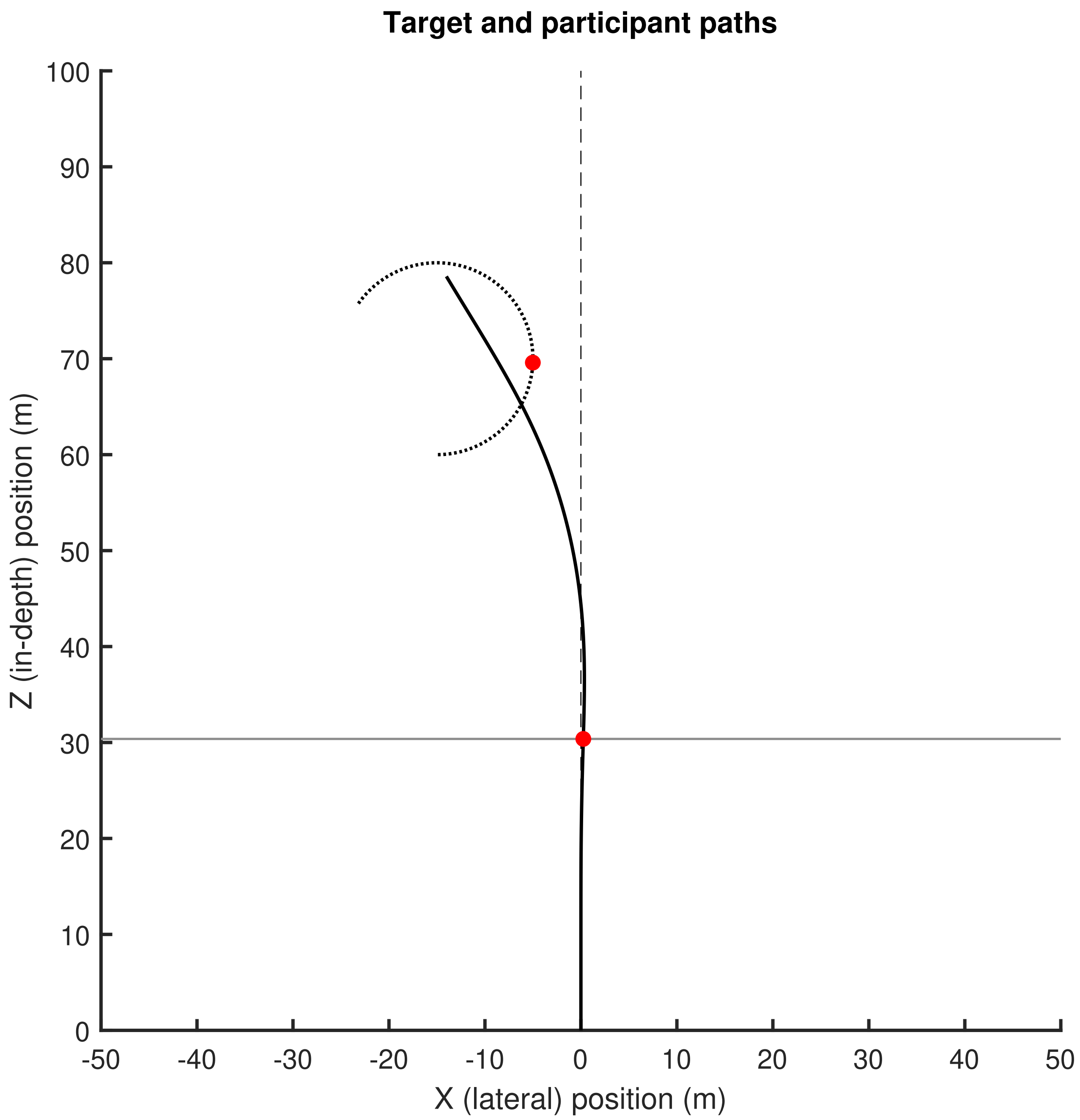

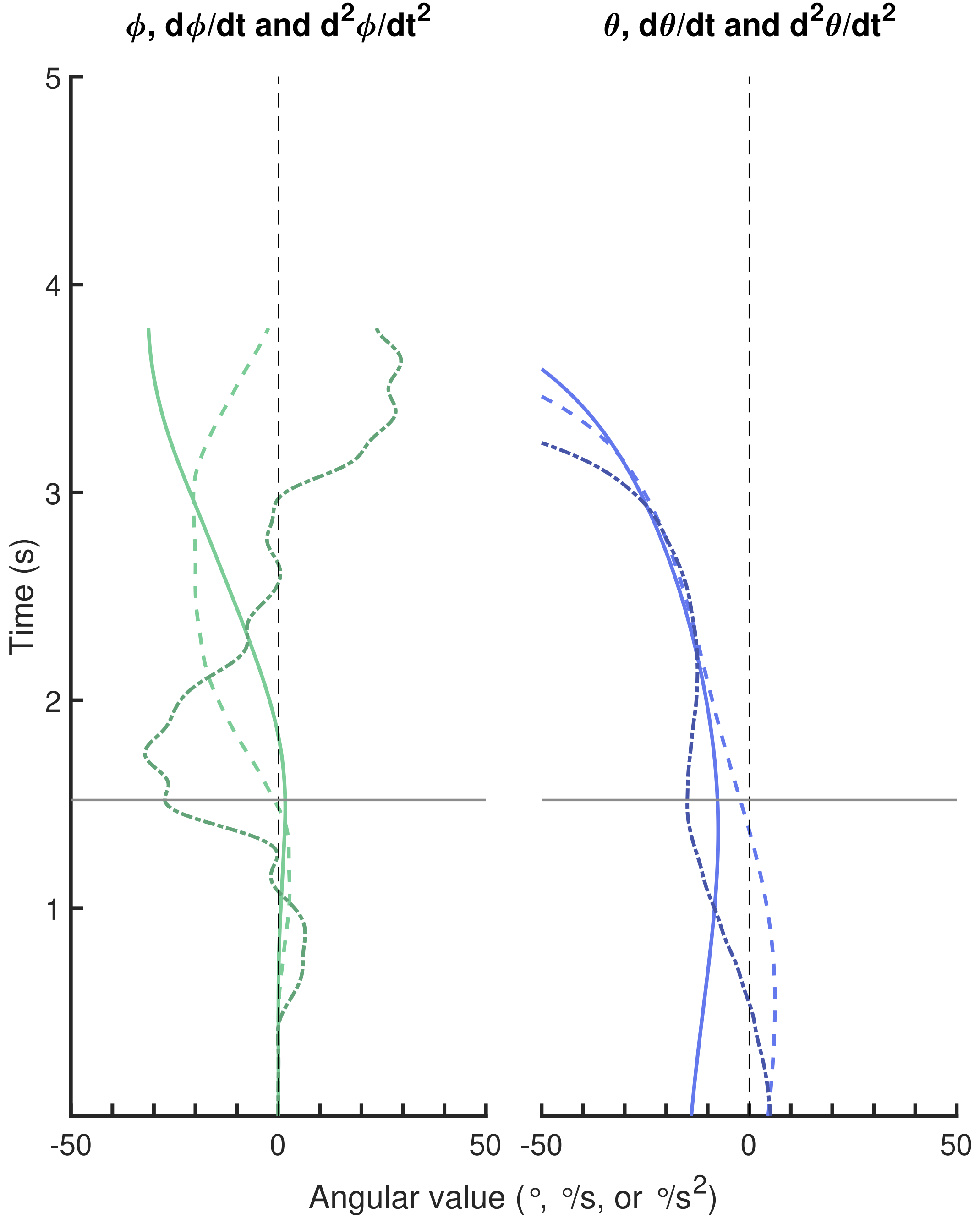

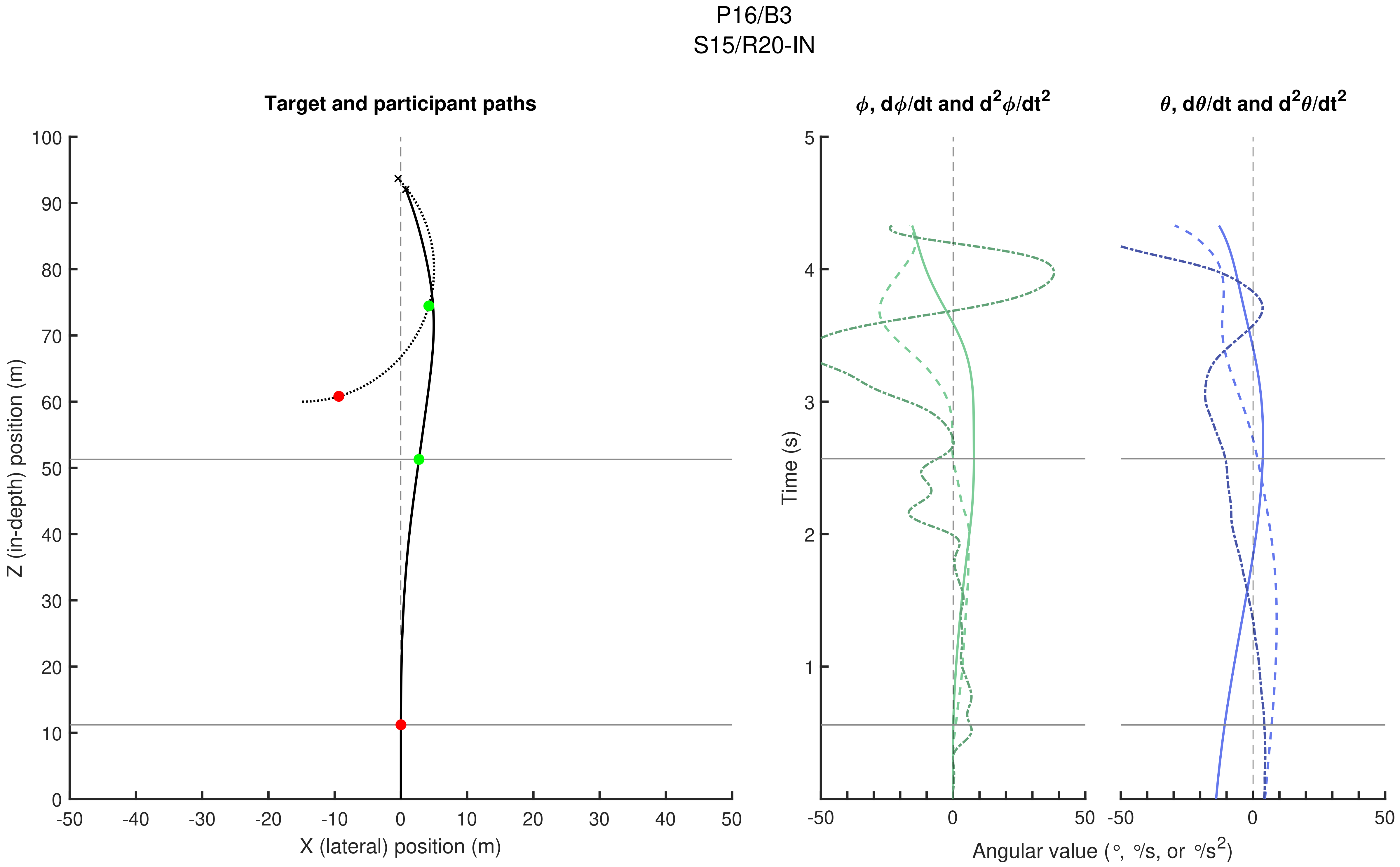

P16/B3
S15/R20-IN
Target and participant paths
Z (in-depth) position (m)
X (lateral) position (m)
0
10
20
30
40
50
60
70
80
90
100
-50
-40
-30
-20
-10
0
10
20
30
40
50
ϕ, dϕ/dt and d$^2\phi$/dt^2
θ, dθ/dt and d$^2\theta$/dt^2
Time (s)
1
2
3
4
5
-50
0
50
-50
0
50
Angular value (°, °/s, or °/s^2)

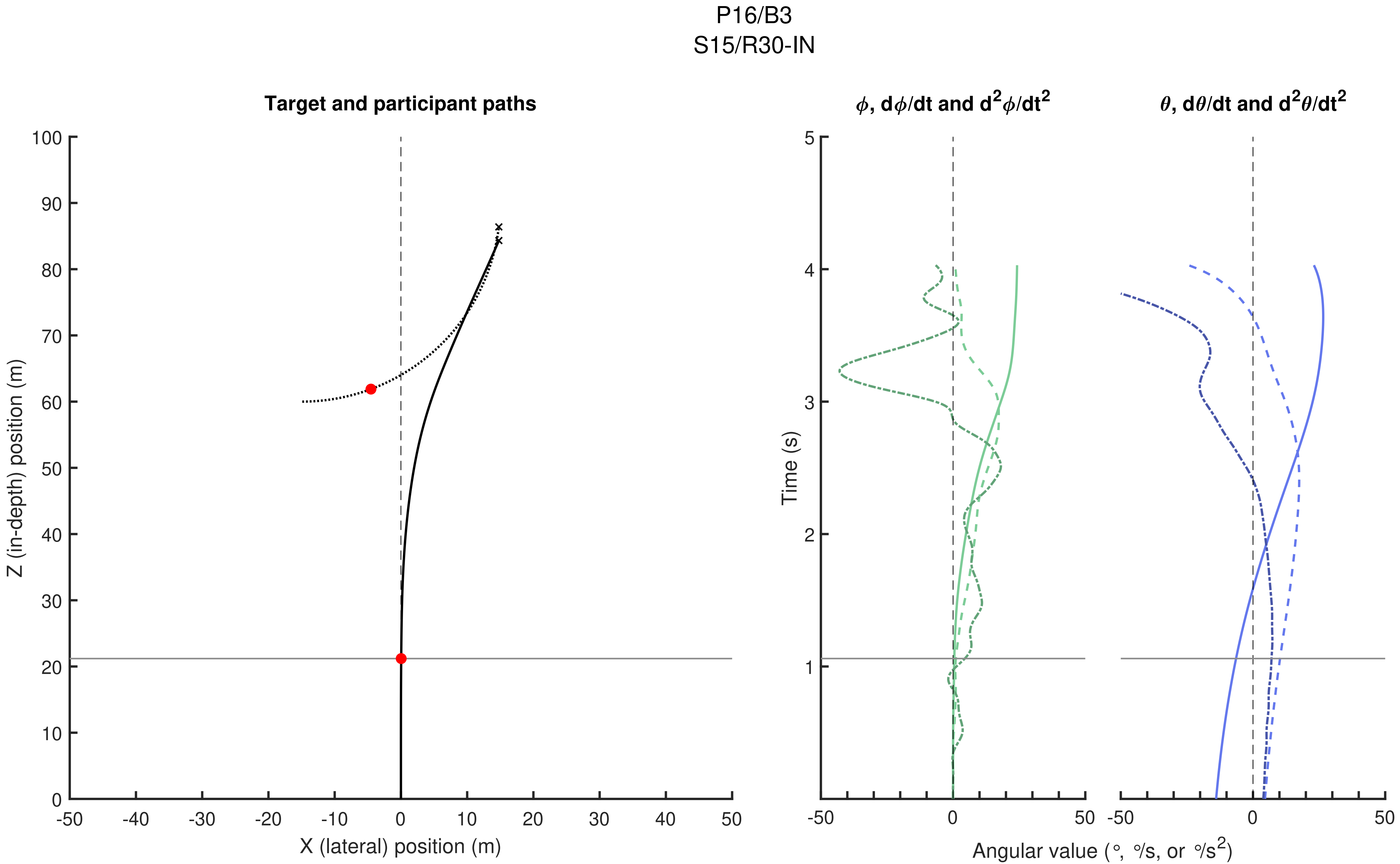
P16/B3
S15/R30-IN
Target and participant paths
Z (in-depth) position (m)
X (lateral) position (m)
100
90
80
70
60
50
40
30
20
10
0
-50
-40
-30
-20
-10
0
10
20
30
40
50
ϕ, dϕ/dt and d$^2\phi$/dt^2
θ, dθ/dt and d$^2\theta$/dt^2
Time (s)
5
4
3
2
1
-50
0
50
-50
0
50
Angular value (°, °/s, or °/s^2)

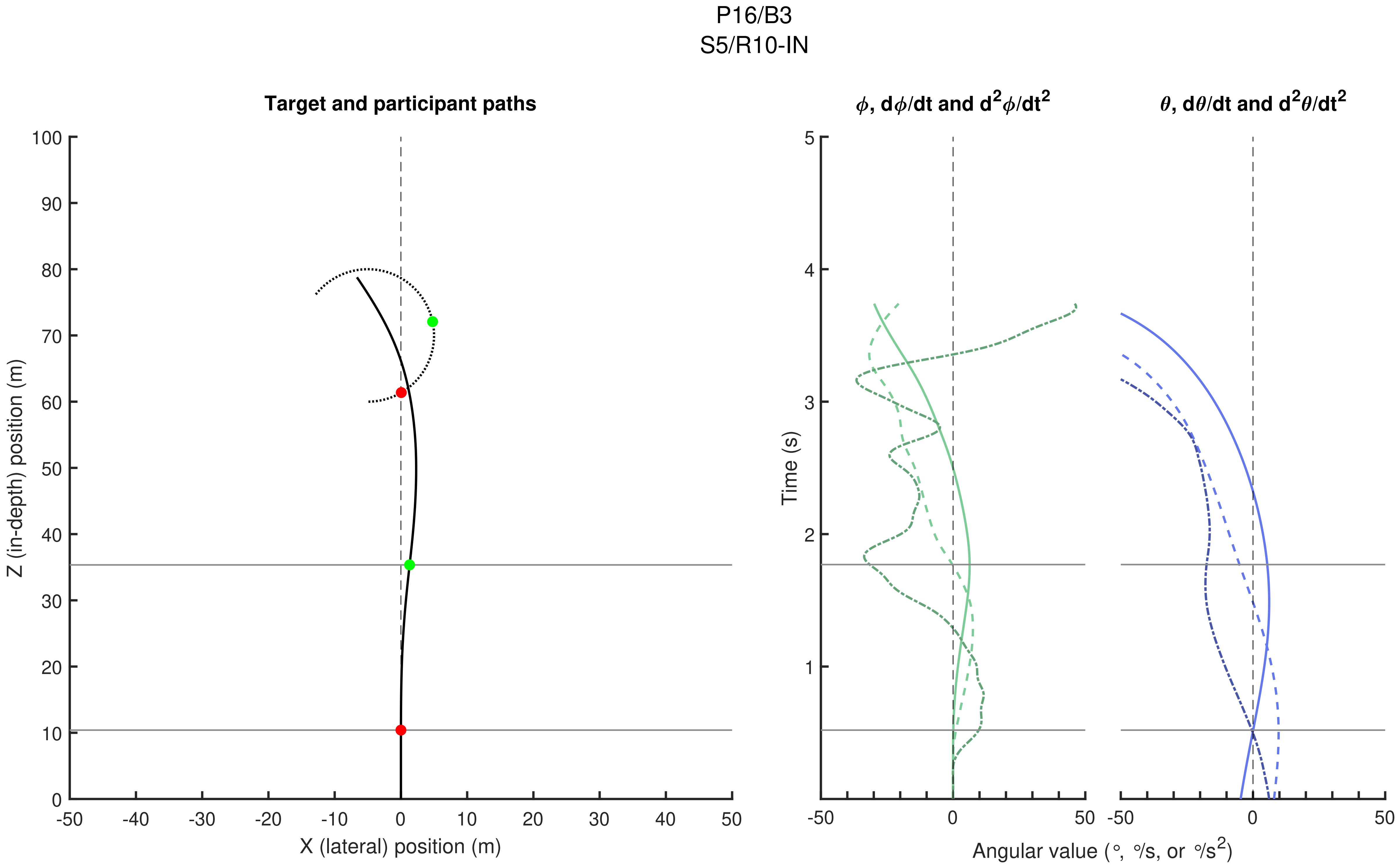
P16/B3
S5/R10-IN
Target and participant paths
Z (in-depth) position (m)
X (lateral) position (m)
ϕ, dϕ/dt and d$^2\phi$/dt^2
θ, dθ/dt and d$^2\theta$/dt^2
Time (s)
Angular value (°, °/s, or °/s^2)

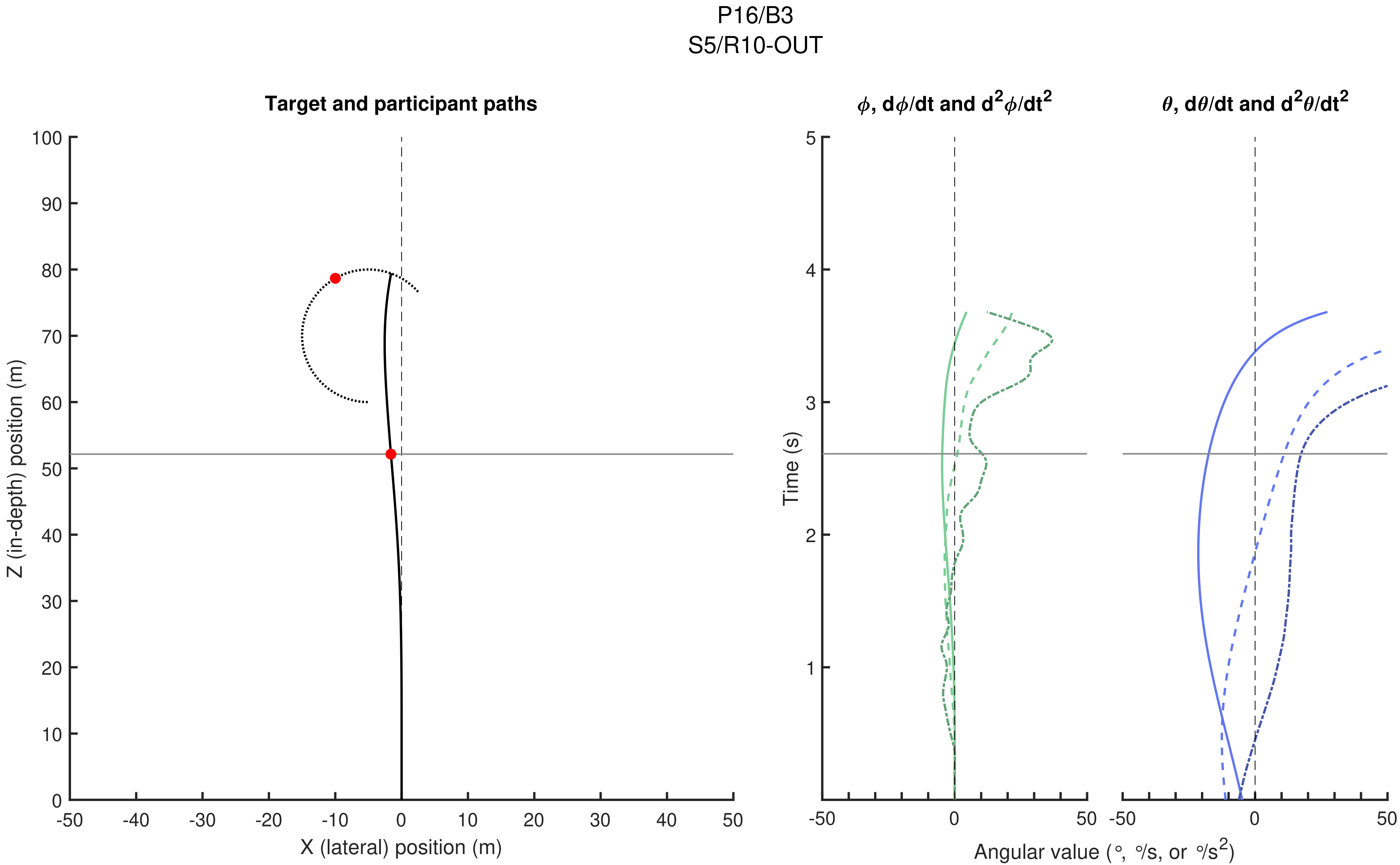

P16/B3
S5/R10-OUT
Target and participant paths
ϕ, dϕ/dt and d$^2\phi$/dt^2
θ, dθ/dt and d$^2\theta$/dt^2
Z (in-depth) position (m)
X (lateral) position (m)
Time (s)
Angular value (°, °/s, or °/s^2)

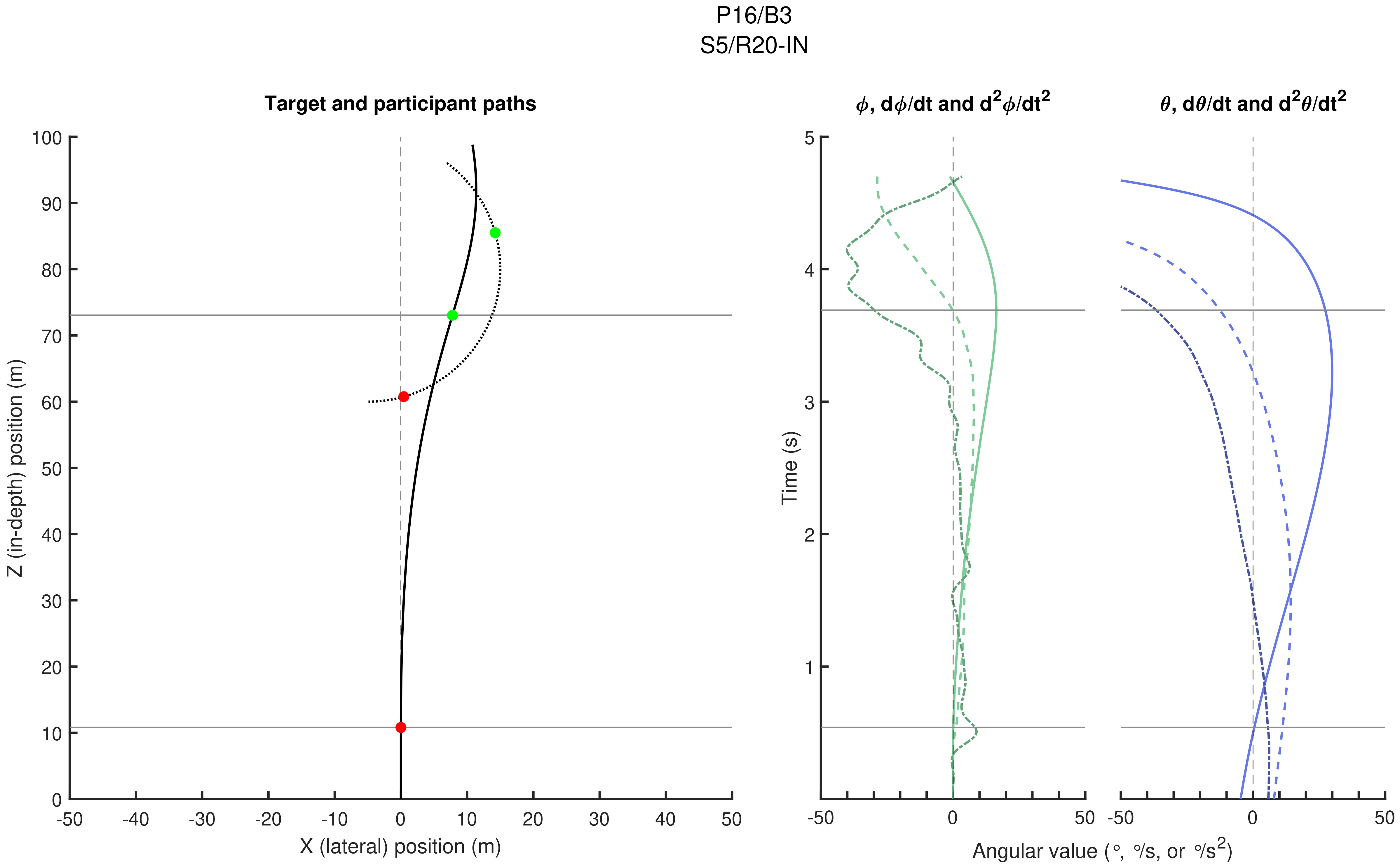

P16/B3
S5/R20-IN
Target and participant paths
Z (in-depth) position (m)
X (lateral) position (m)
ϕ, dϕ/dt and d$^2\phi$/dt^2
θ, dθ/dt and d$^2\theta$/dt^2
Time (s)
Angular value (°, °/s, or °/s^2)

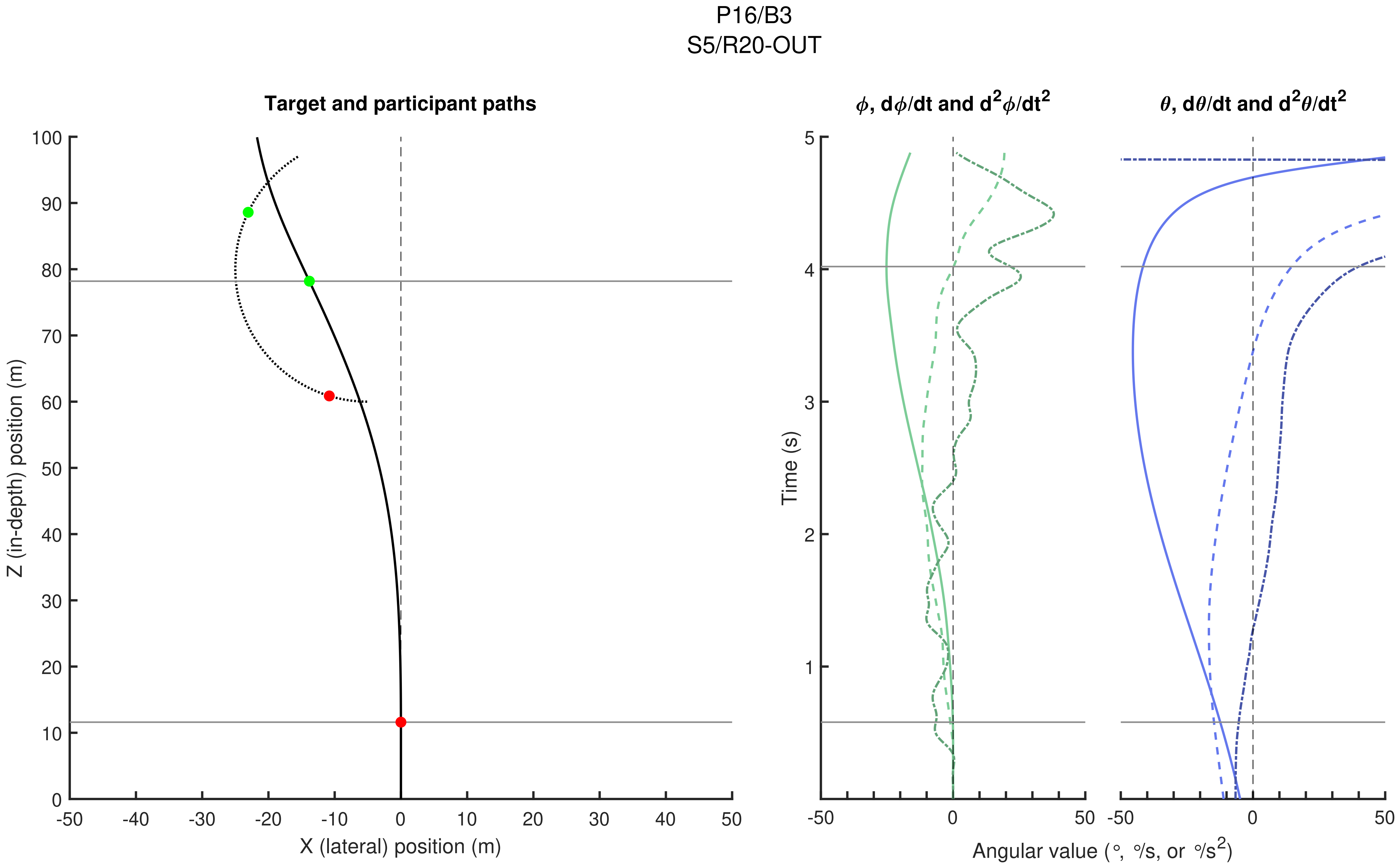
P16/B3
S5/R20-OUT
Target and participant paths
Z (in-depth) position (m)
X (lateral) position (m)
100
90
80
70
60
50
40
30
20
10
0
-50
-40
-30
-20
-10
0
10
20
30
40
50
φ, dφ/dt and d²φ/dt²
θ, dθ/dt and d²θ/dt²
Time (s)
5
4
3
2
1
-50
0
50
-50
0
50
Angular value (°, °/s, or °/s²)

P16/B3
S5/R30-IN

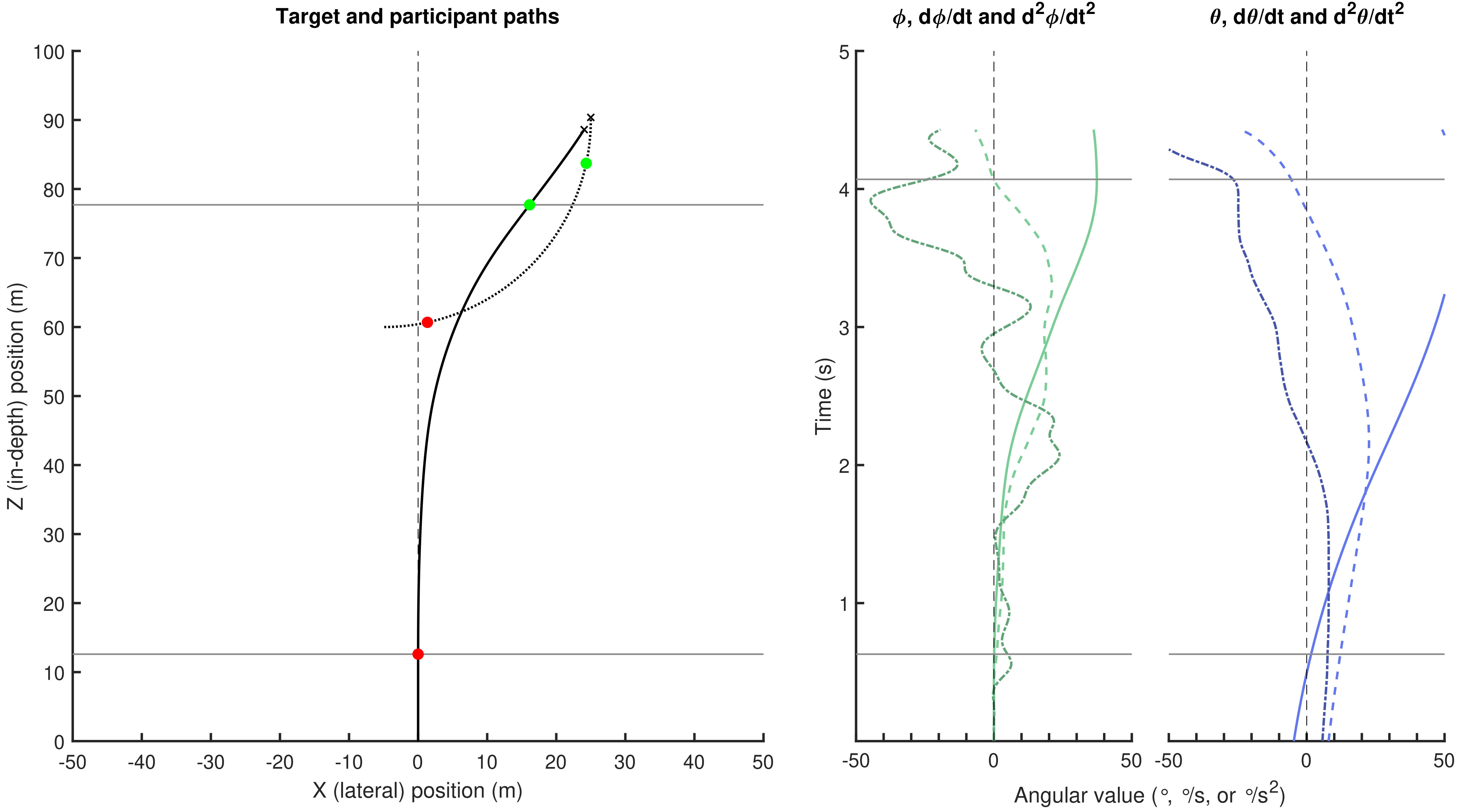

P16/B3
S5/R30-OUT

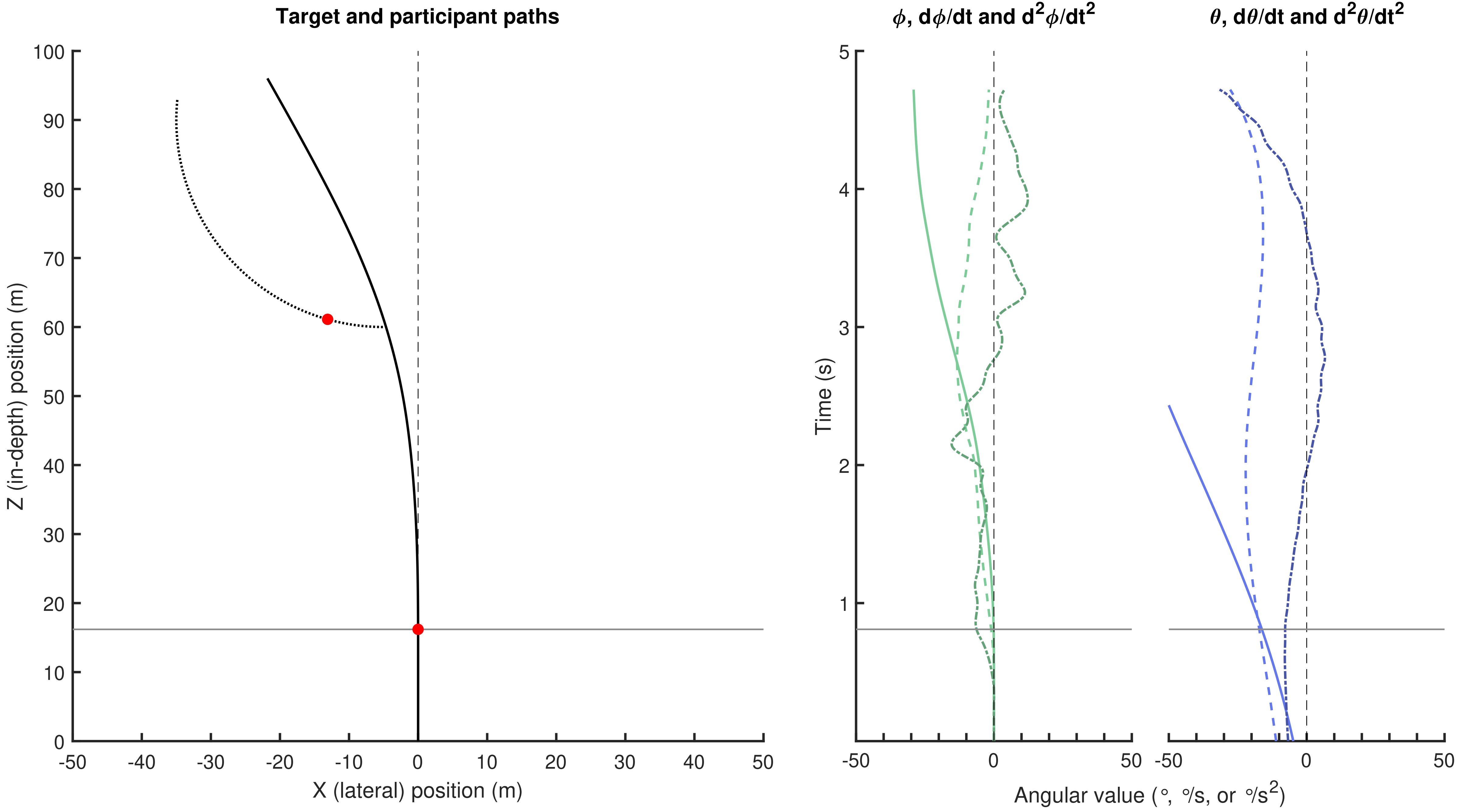

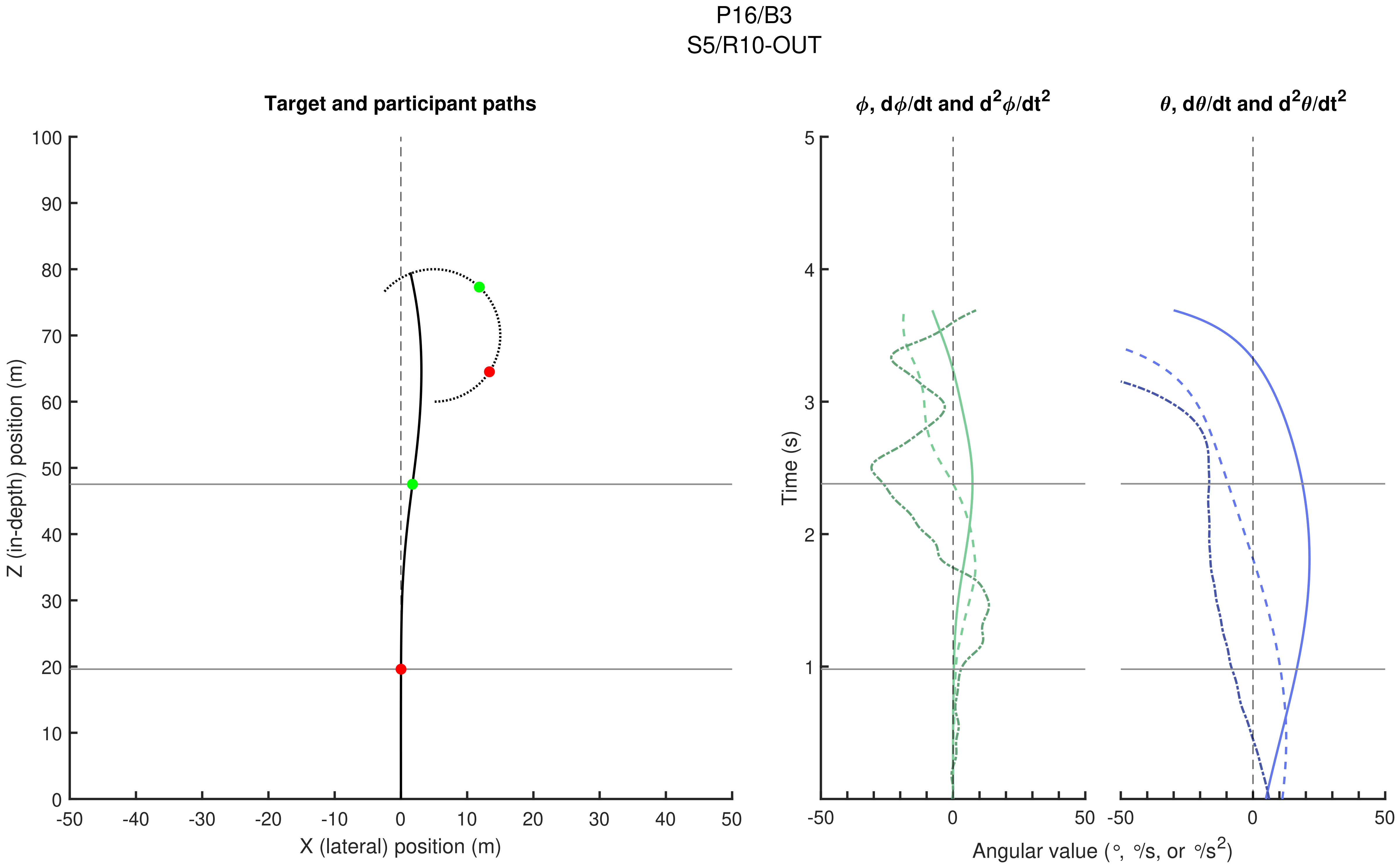

P16/B3
S5/R10-OUT
Target and participant paths
Z (in-depth) position (m)
X (lateral) position (m)
100
90
80
70
60
50
40
30
20
10
0
-50
-40
-30
-20
-10
0
10
20
30
40
50
ϕ, dϕ/dt and d²ϕ/dt²
θ, dθ/dt and d²θ/dt²
Time (s)
5
4
3
2
1
-50
0
50
-50
0
50
Angular value (°, °/s, or °/s²)

P16/B3
S5/R10-IN

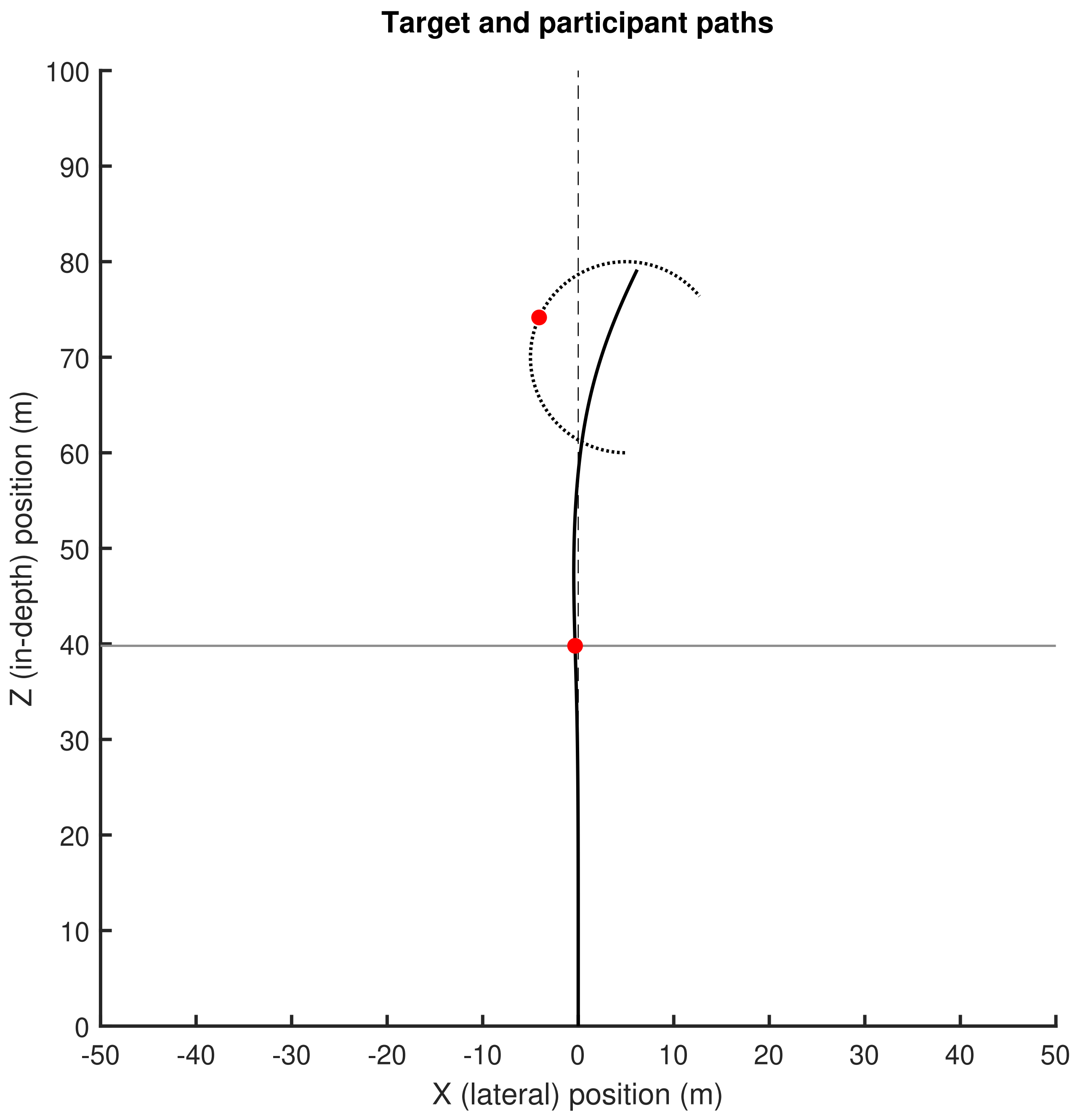

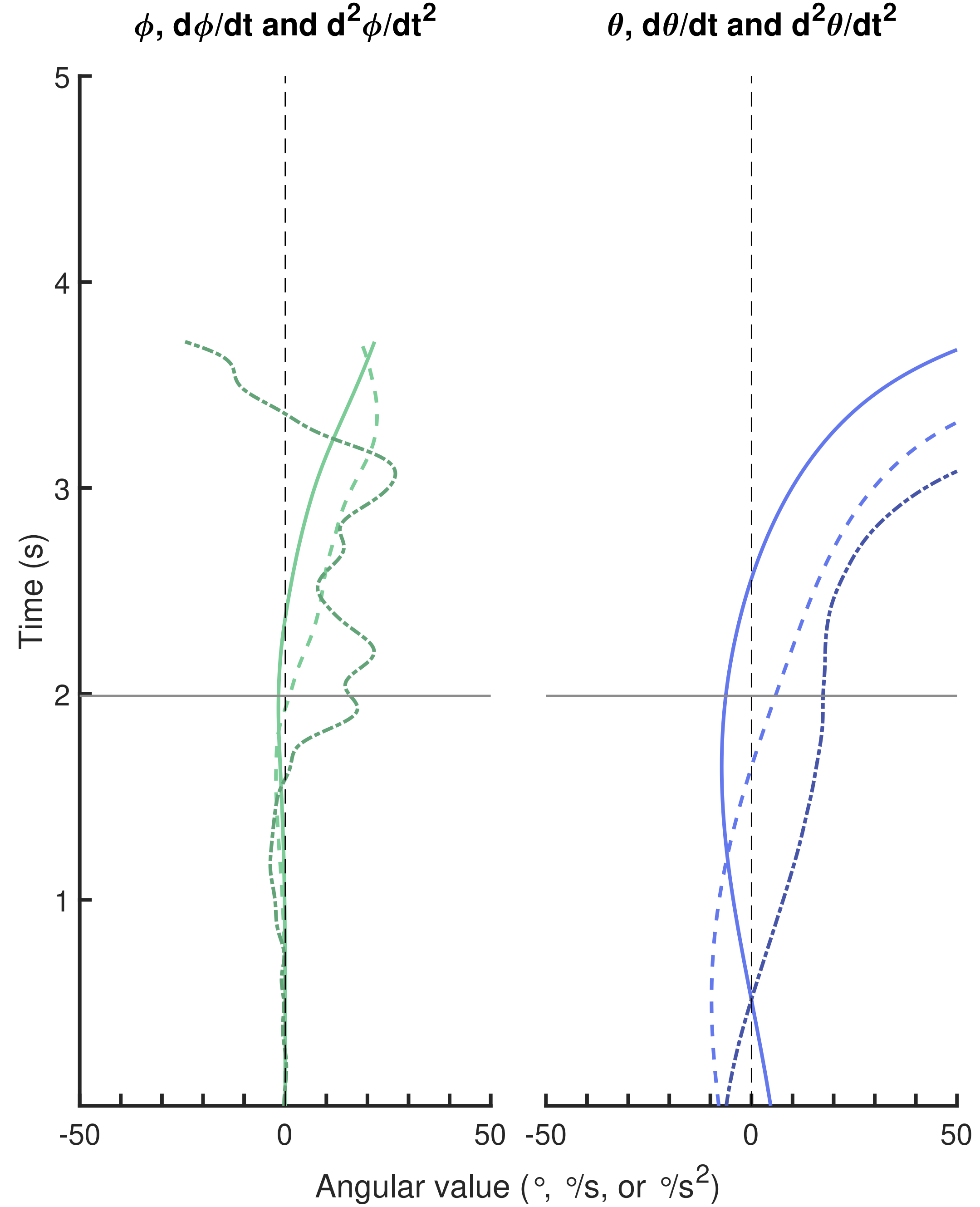

P16/B3
S5/R20-OUT

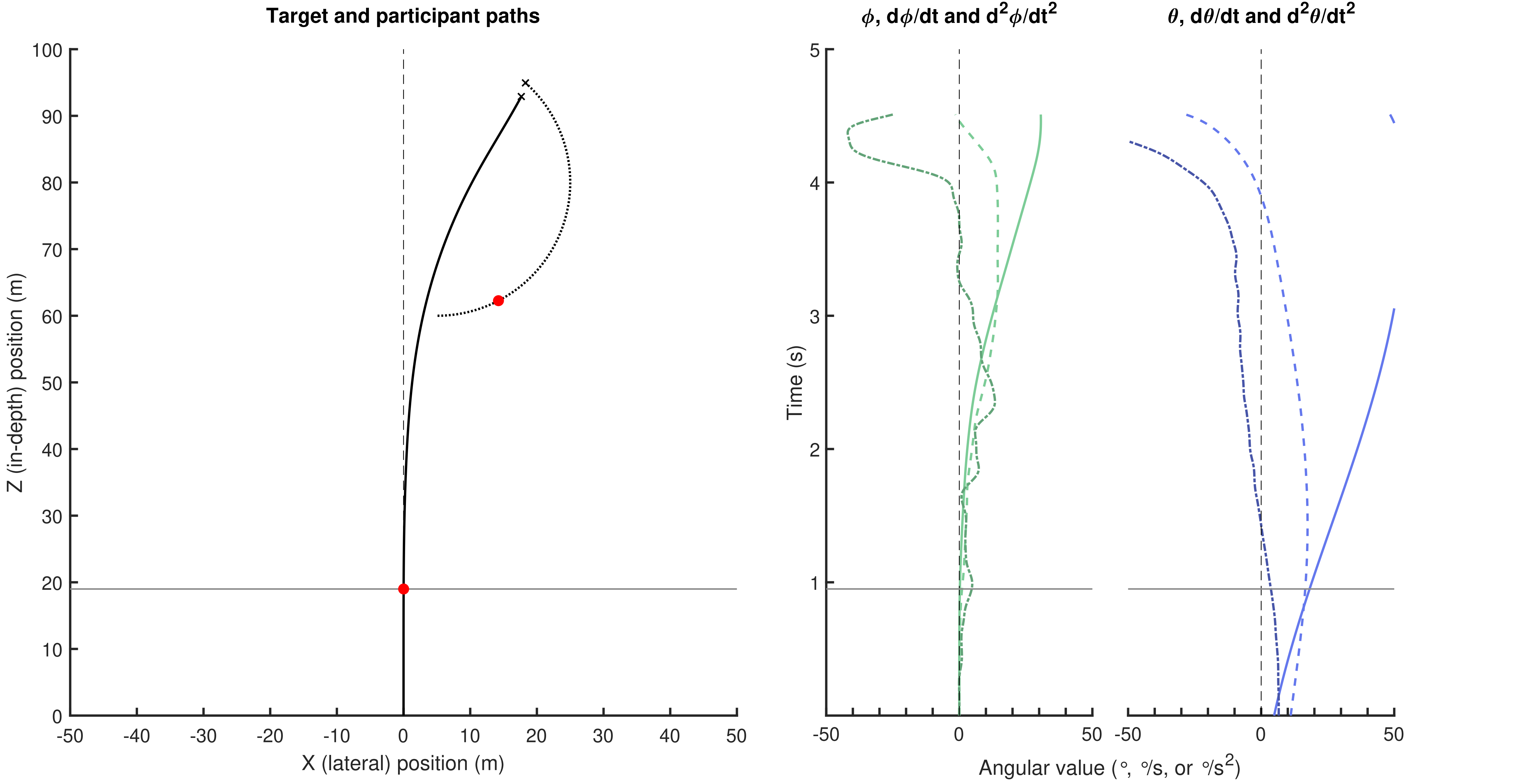

P16/B3
S5/R20-IN

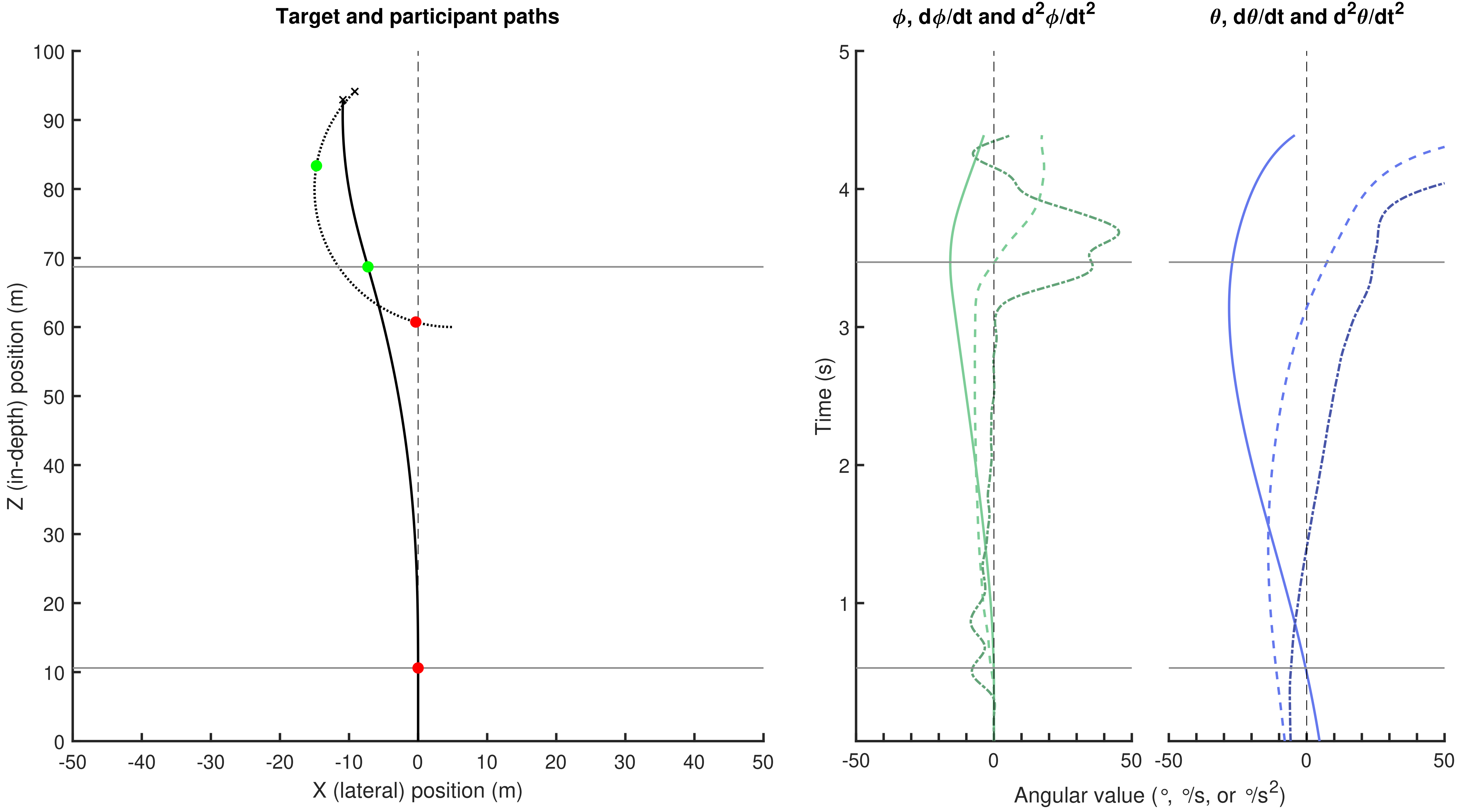

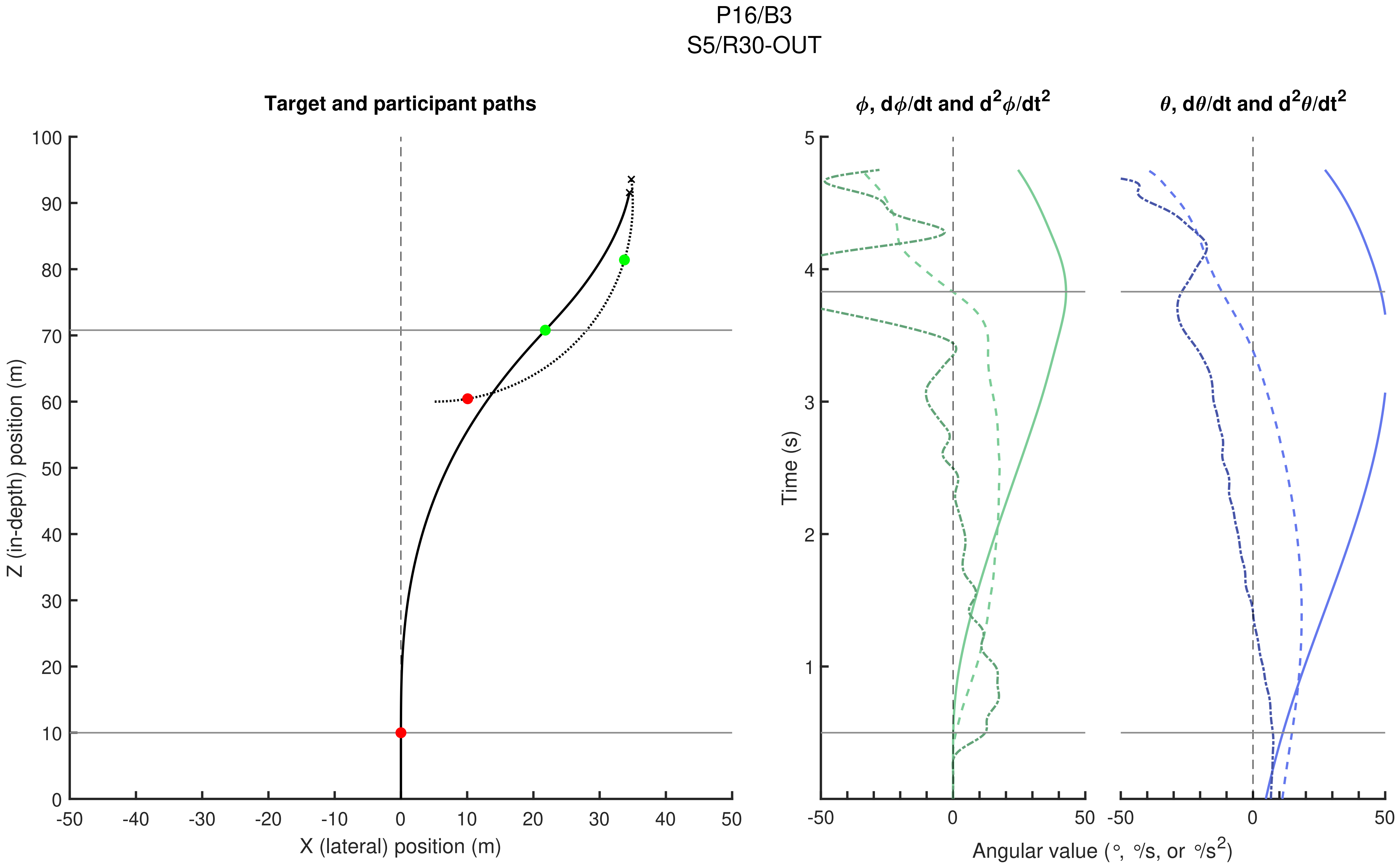
P16/B3
S5/R30-OUT
Target and participant paths
Z (in-depth) position (m)
X (lateral) position (m)
100
90
80
70
60
50
40
30
20
10
0
-50
-40
-30
-20
-10
0
10
20
30
40
50
ϕ, dϕ/dt and d²ϕ/dt²
θ, dθ/dt and d²θ/dt²
Time (s)
5
4
3
2
1
-50
0
50
-50
0
50
Angular value (°, °/s, or °/s²)

P16/B3
S5/R30-IN

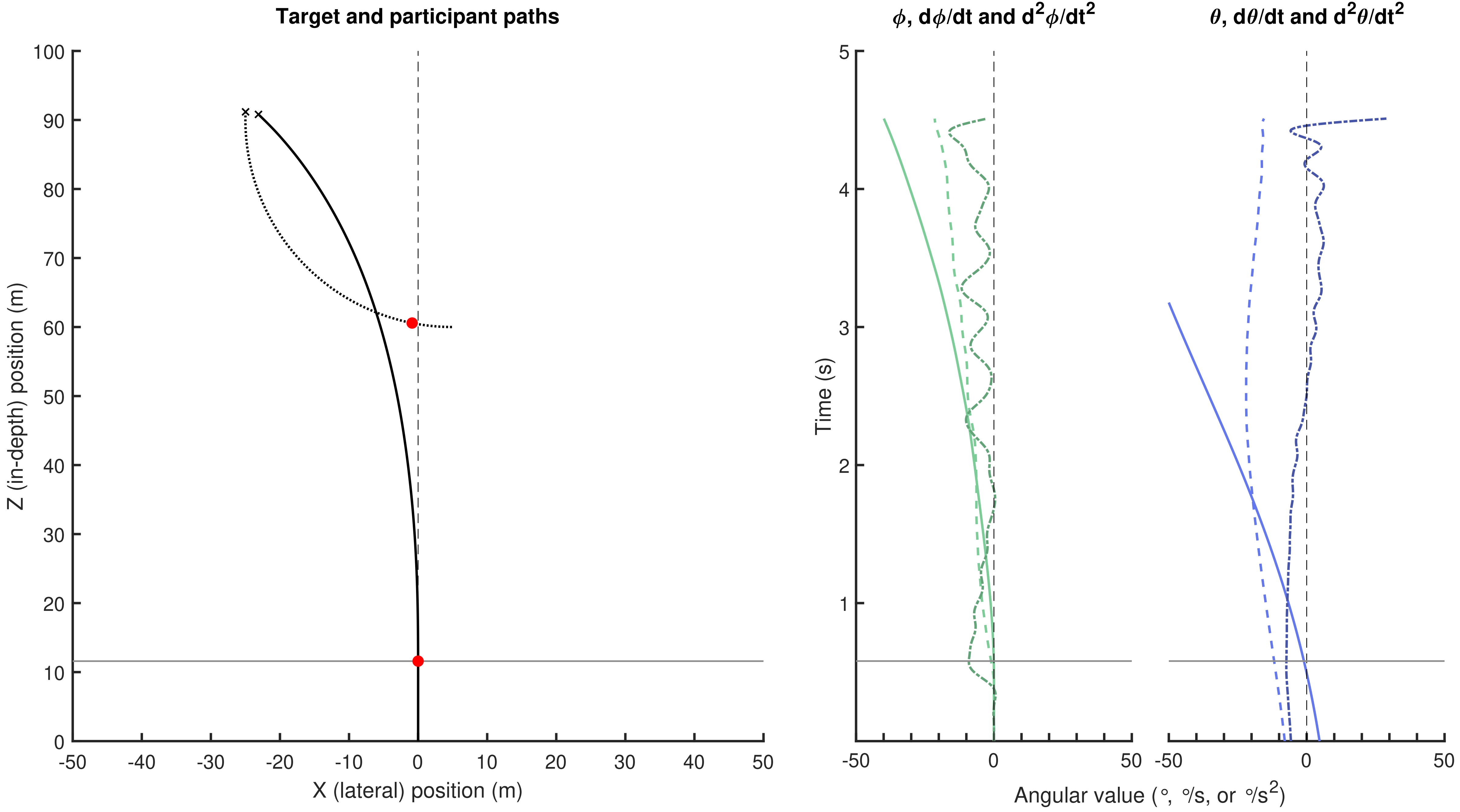

P16/B3
S15/R10-IN

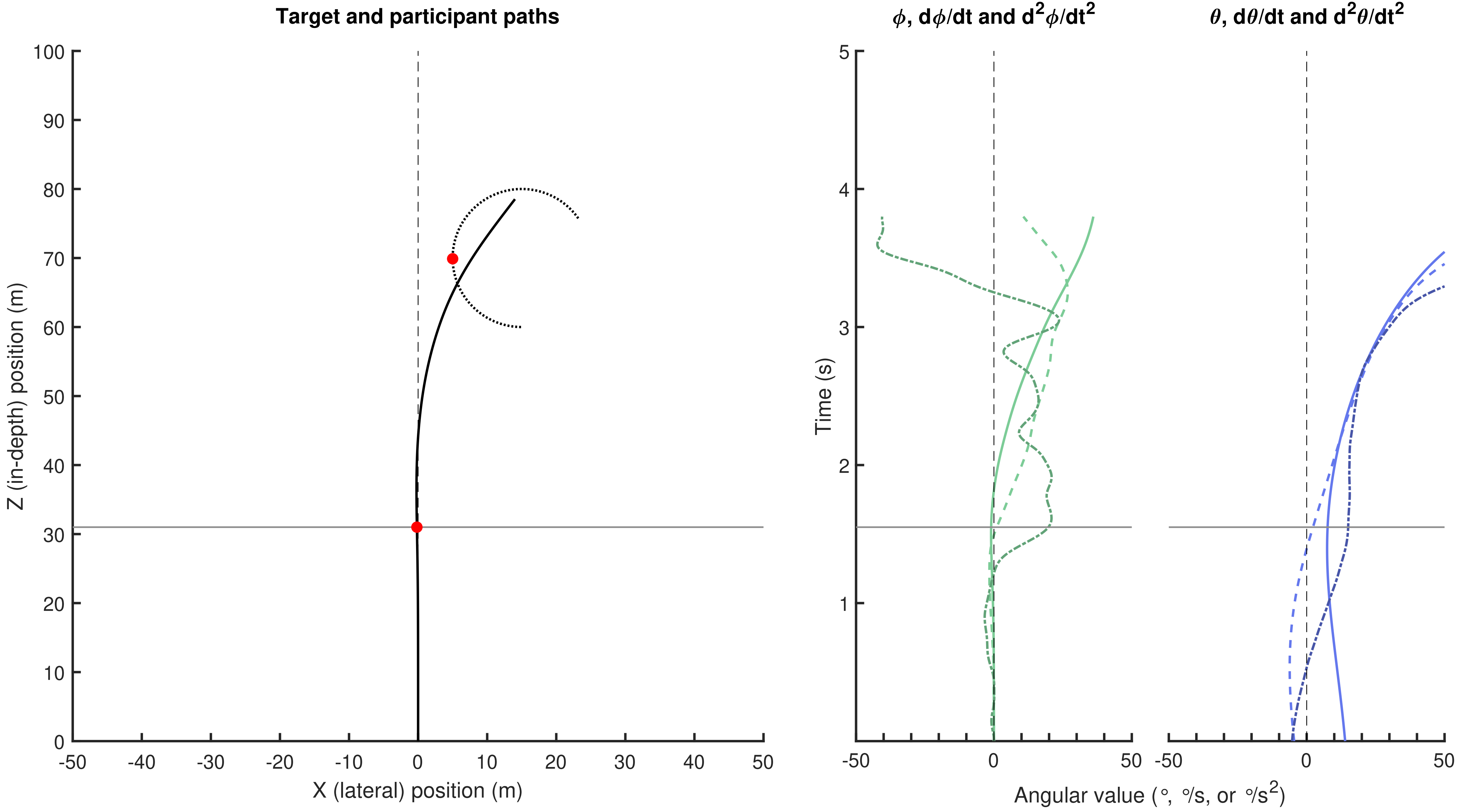

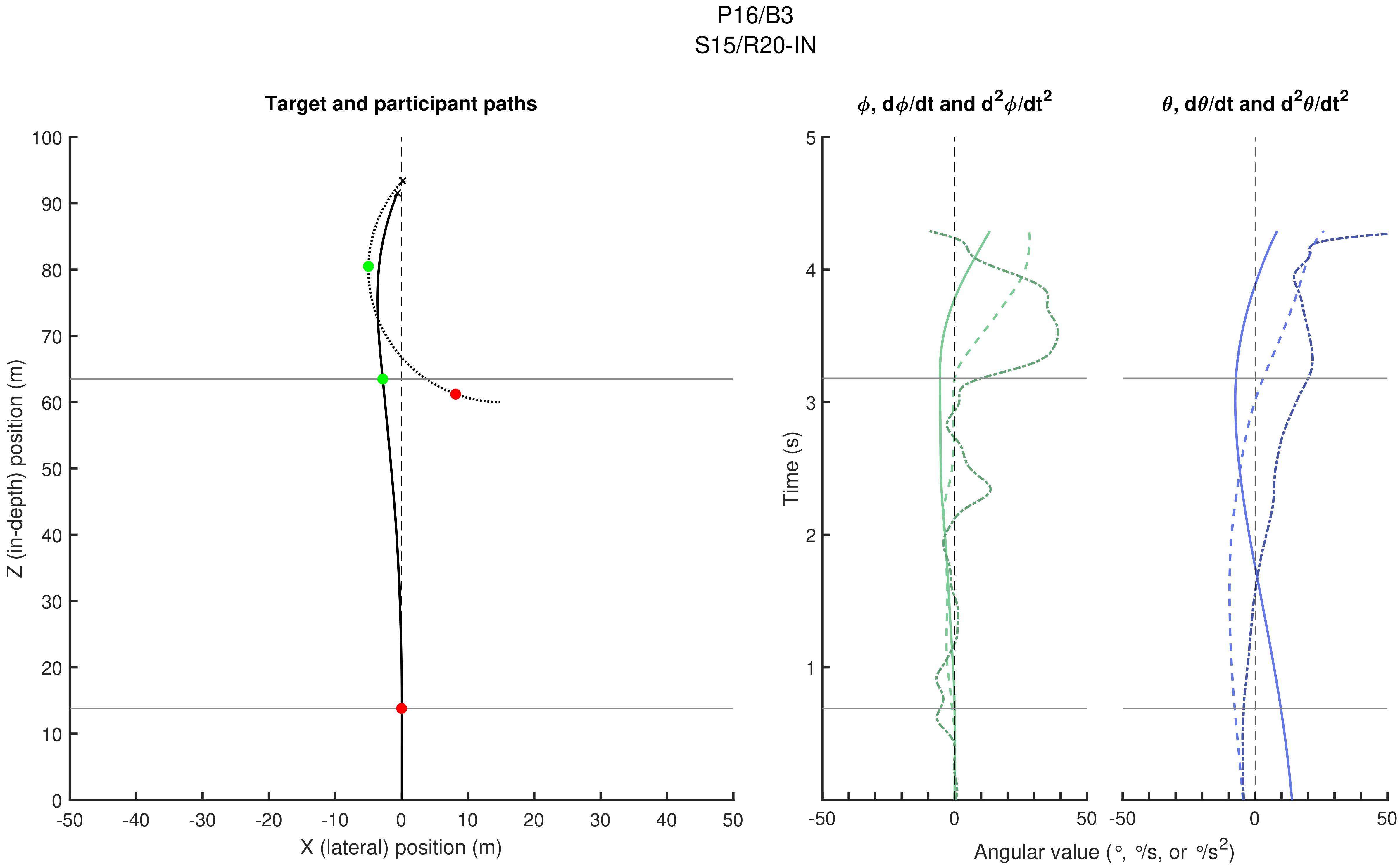
P16/B3
S15/R20-IN
Target and participant paths
Z (in-depth) position (m)
X (lateral) position (m)
100
90
80
70
60
50
40
30
20
10
0
-50
-40
-30
-20
-10
0
10
20
30
40
50
φ, dφ/dt and d²φ/dt²
θ, dθ/dt and d²θ/dt²
Time (s)
5
4
3
2
1
-50
0
50
-50
0
50
Angular value (°, °/s, or °/s²)

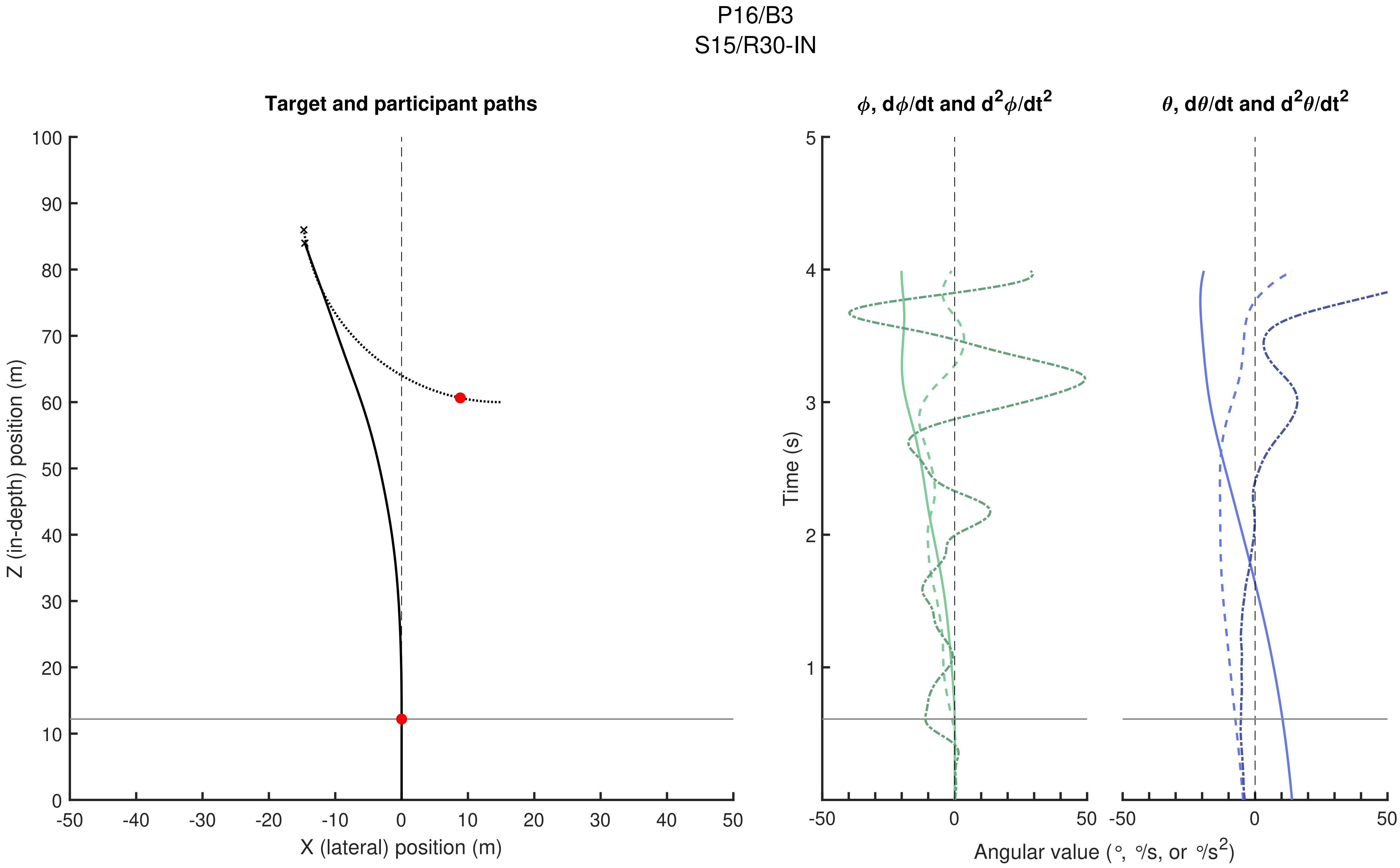
P16/B3
S15/R30-IN
Target and participant paths
Z (in-depth) position (m)
X (lateral) position (m)
ϕ, dϕ/dt and d$^2\phi$/dt^2
θ, dθ/dt and d$^2\theta$/dt^2
Time (s)
Angular value (°, °/s, or °/s^2)

P16/B3
S20/R10-IN

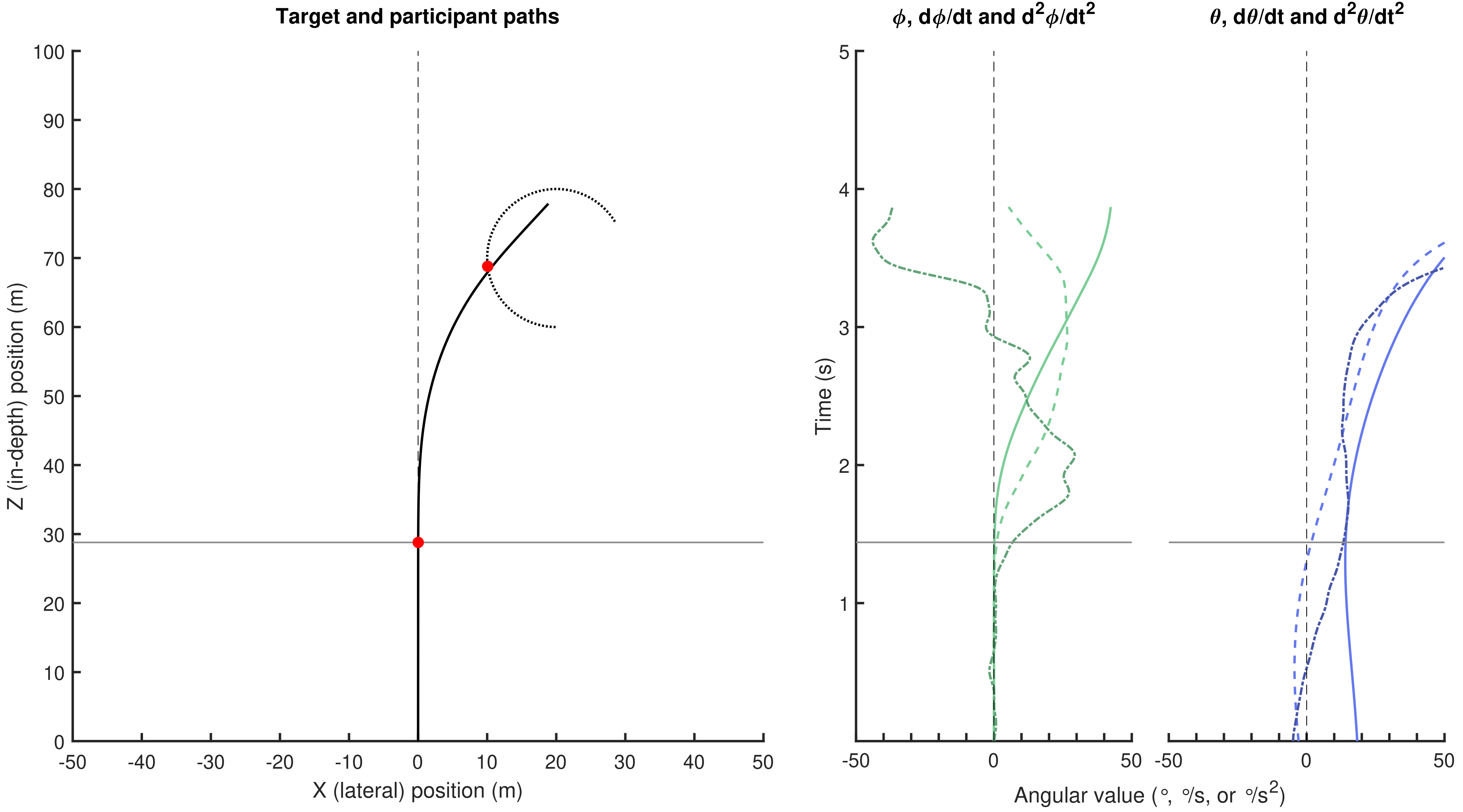

P16/B3
S20/R20-IN

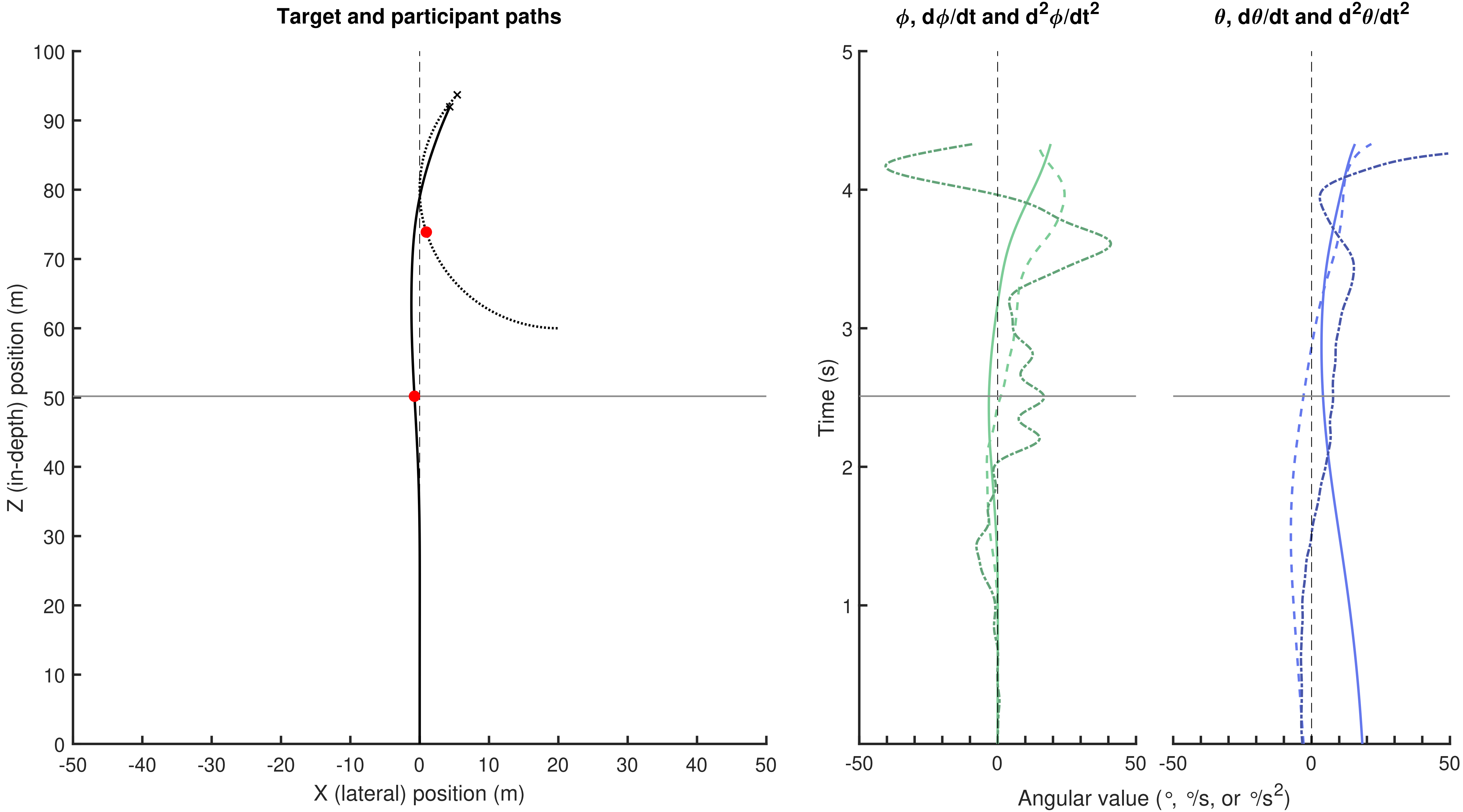

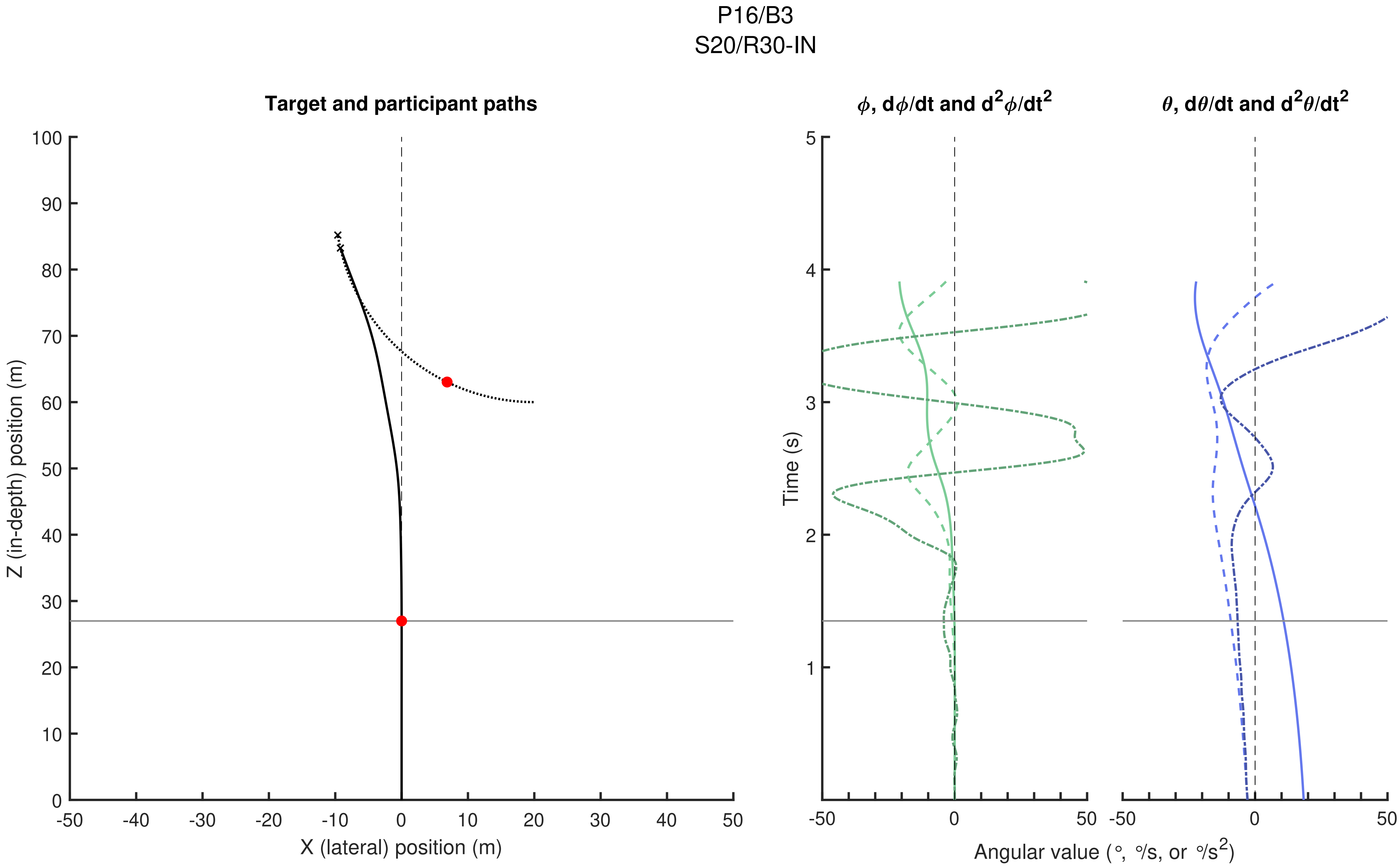
P16/B3
S20/R30-IN
Target and participant paths
Z (in-depth) position (m)
X (lateral) position (m)
100
90
80
70
60
50
40
30
20
10
0
-50
-40
-30
-20
-10
0
10
20
30
40
50
ϕ, dϕ/dt and d²ϕ/dt²
θ, dθ/dt and d²θ/dt²
Time (s)
5
4
3
2
1
-50
0
50
-50
0
50
Angular value (°, °/s, or °/s²)

P16/B4
S20/R10-IN

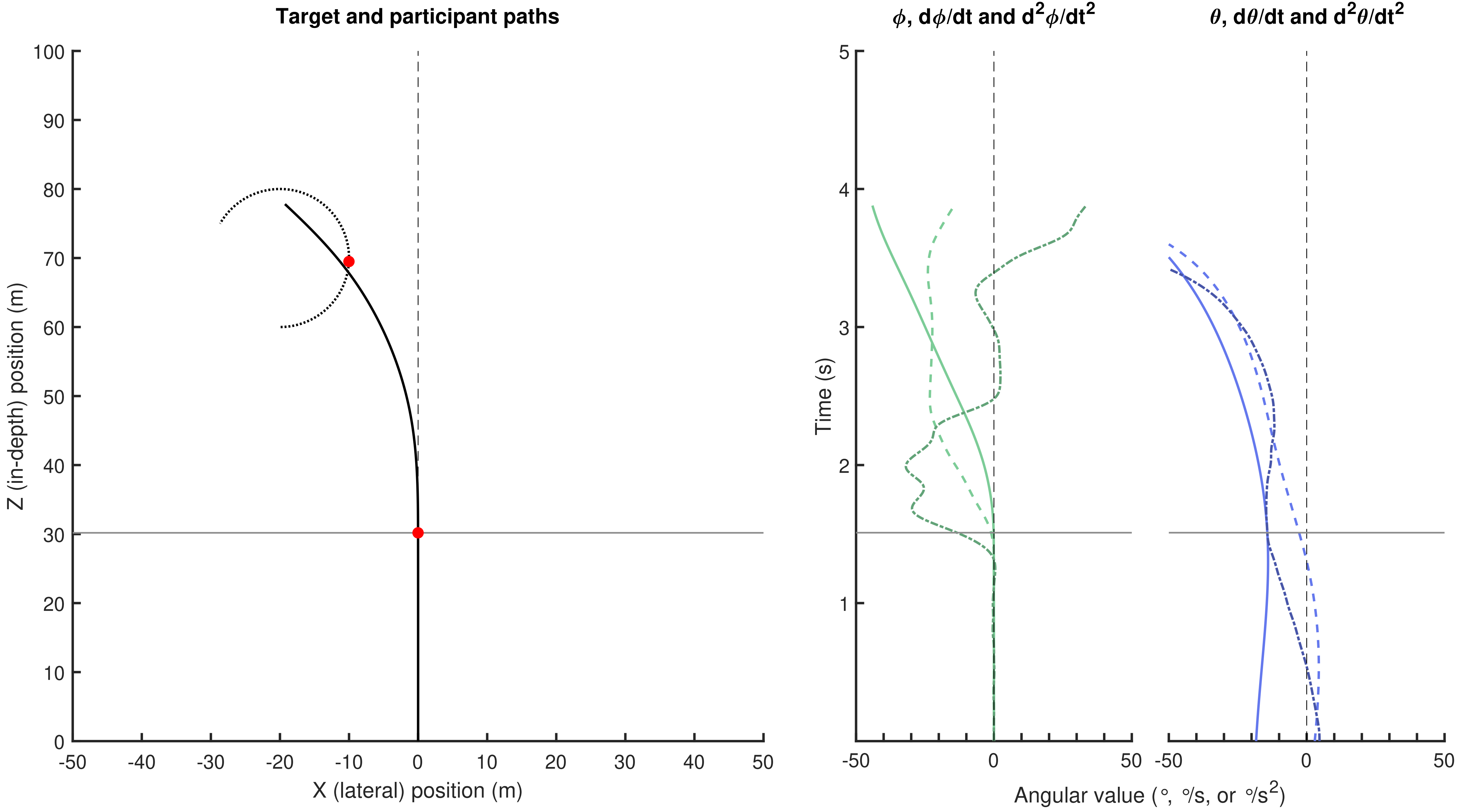

P16/B4
S20/R20-IN

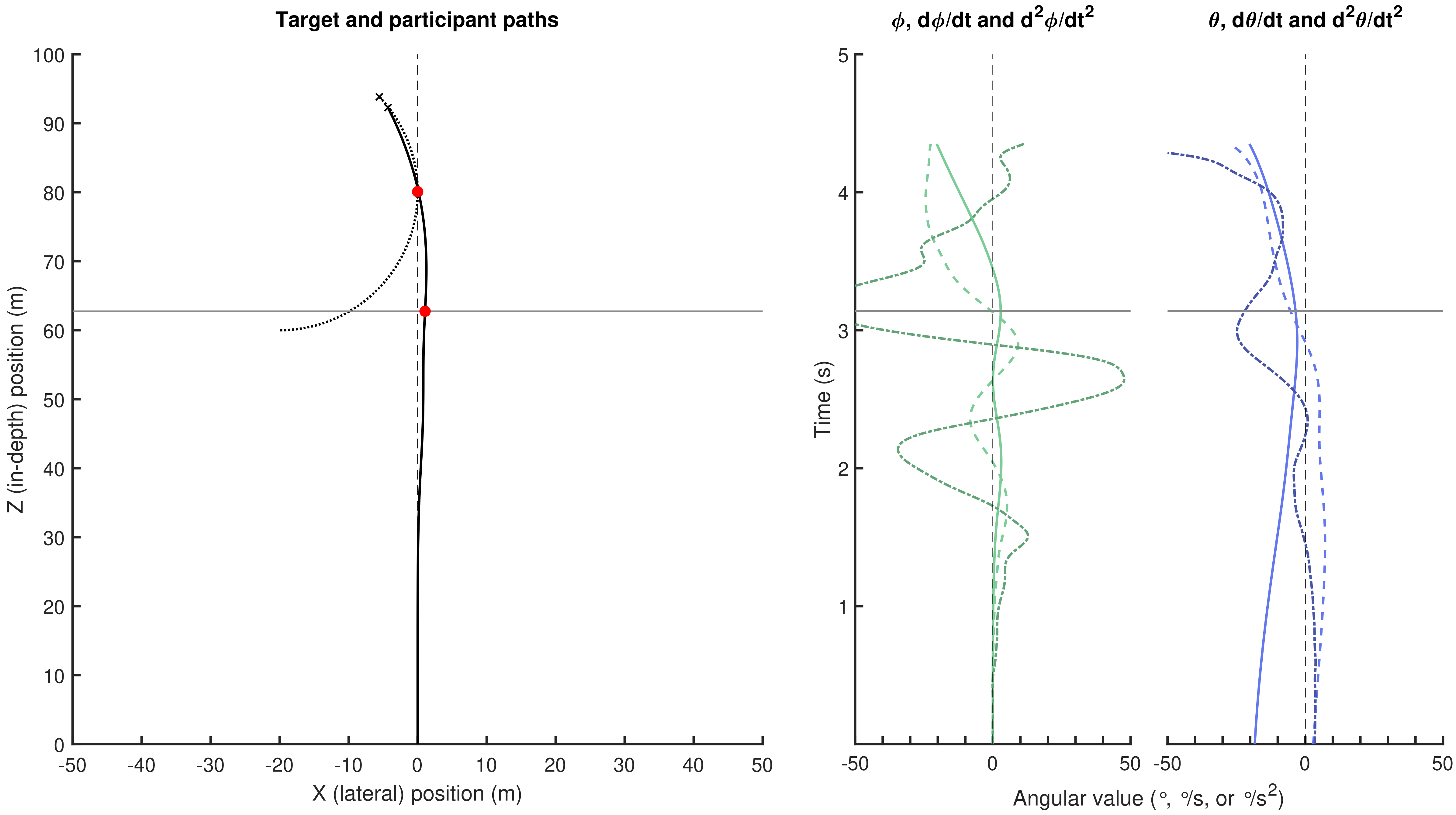

P16/B4
S20/R30-IN

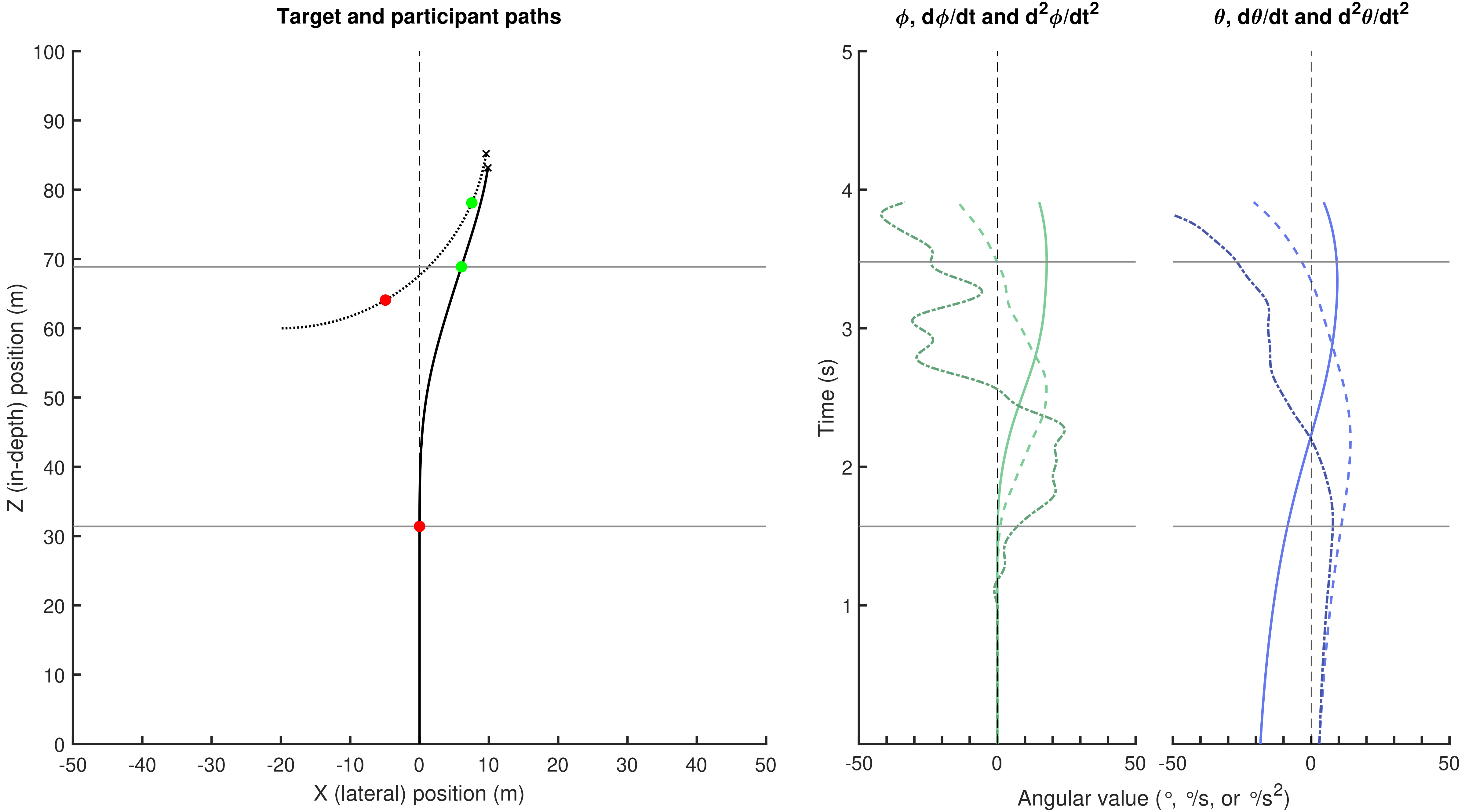

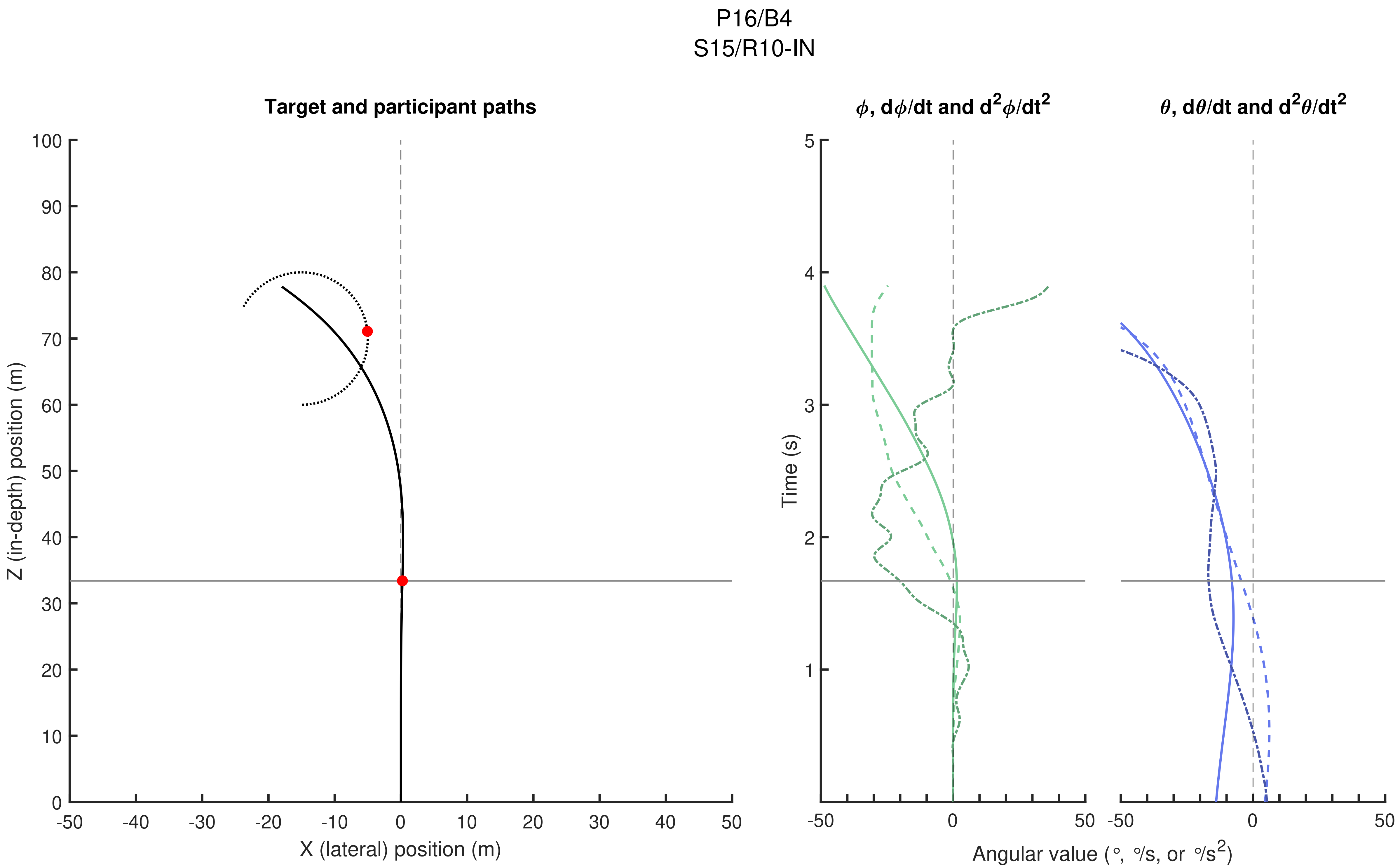
P16/B4
S15/R10-IN
Target and participant paths
Z (in-depth) position (m)
X (lateral) position (m)
ϕ, dϕ/dt and d$^2\phi$/dt^2
θ, dθ/dt and d$^2\theta$/dt^2
Time (s)
Angular value (°, °/s, or °/s^2)

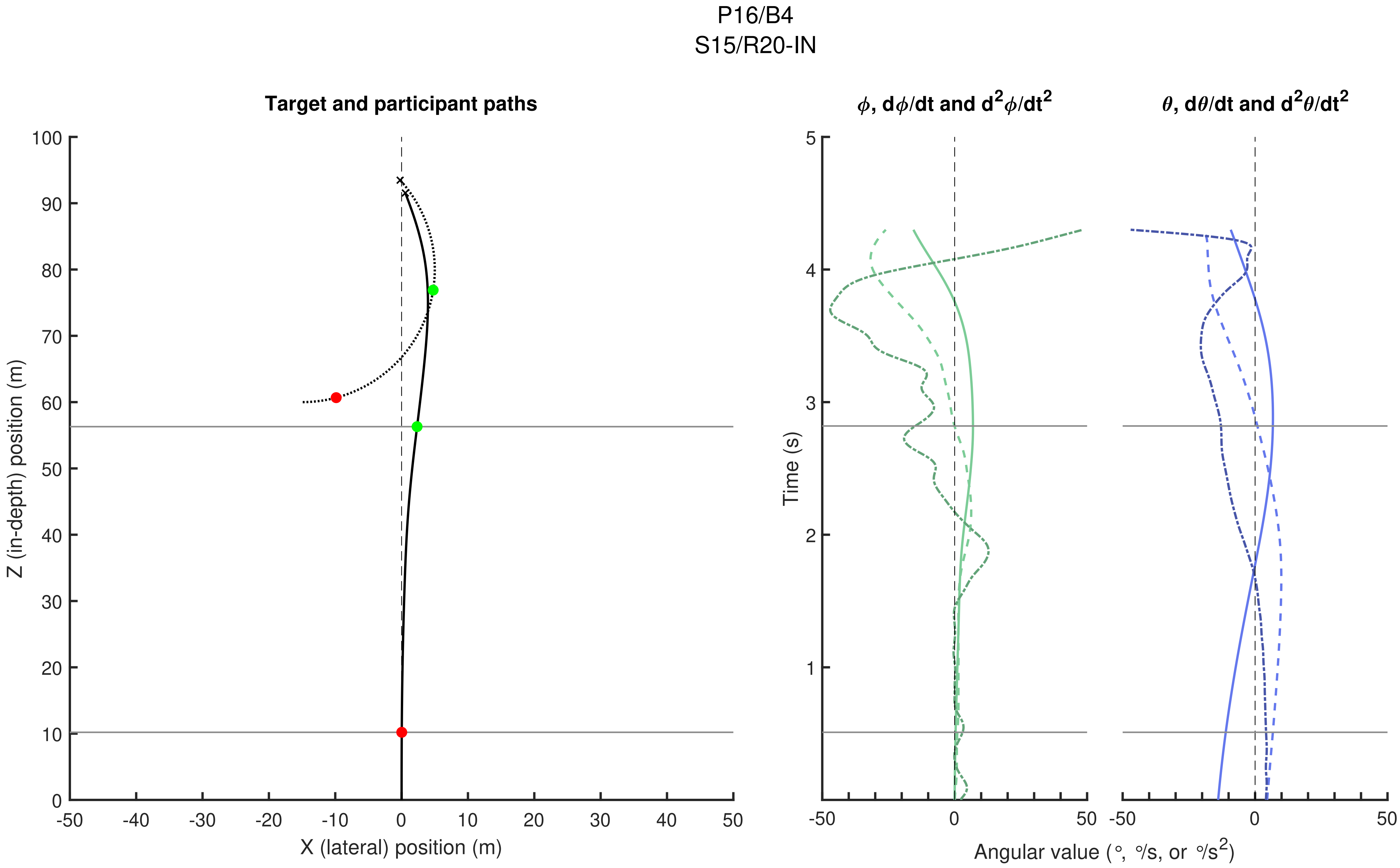

P16/B4
S15/R20-IN
Target and participant paths
Z (in-depth) position (m)
X (lateral) position (m)
ϕ, dϕ/dt and d$^2\phi$/dt^2
θ, dθ/dt and d$^2\theta$/dt^2
Time (s)
Angular value (°, °/s, or °/s^2)

P16/B4
S15/R30-IN

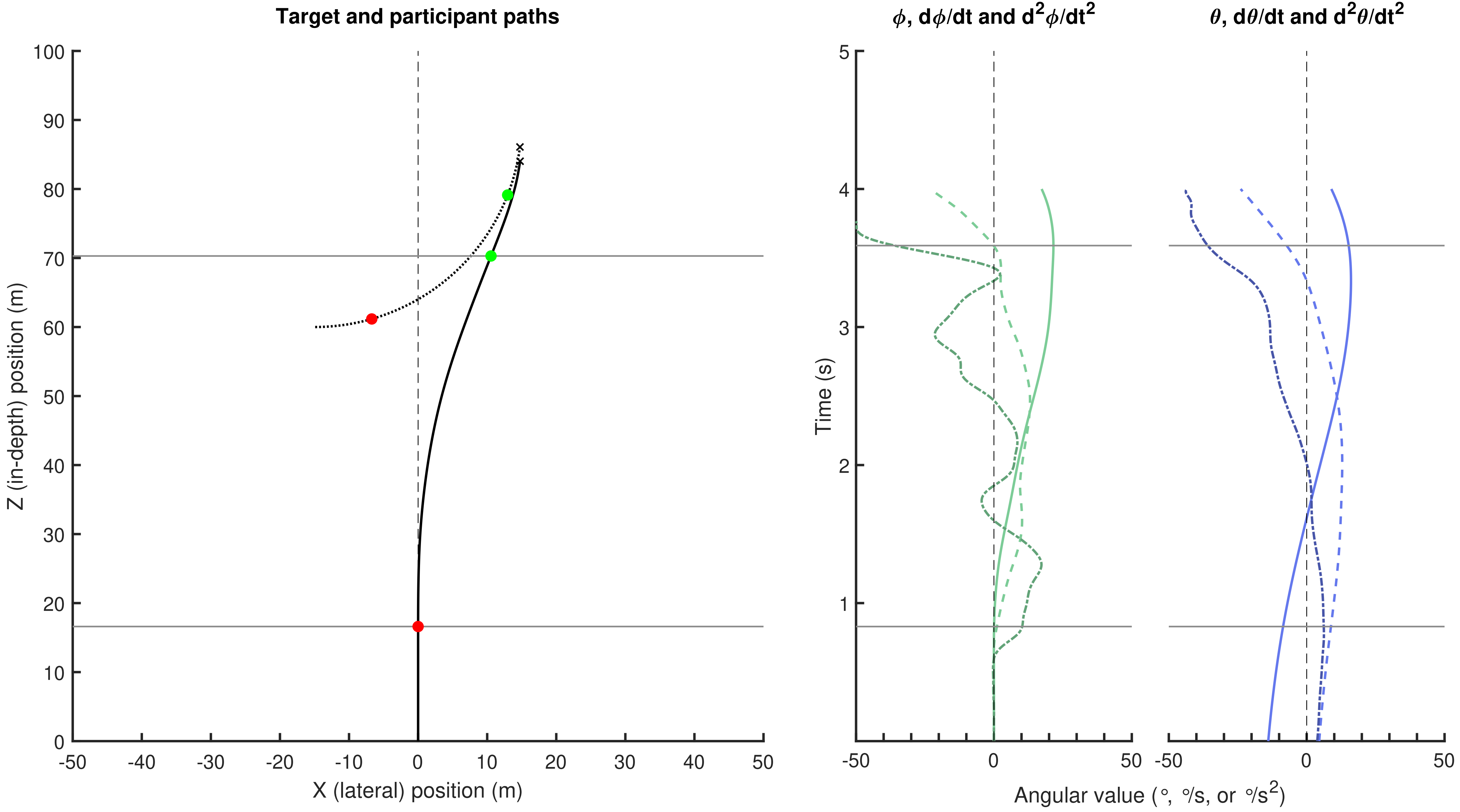

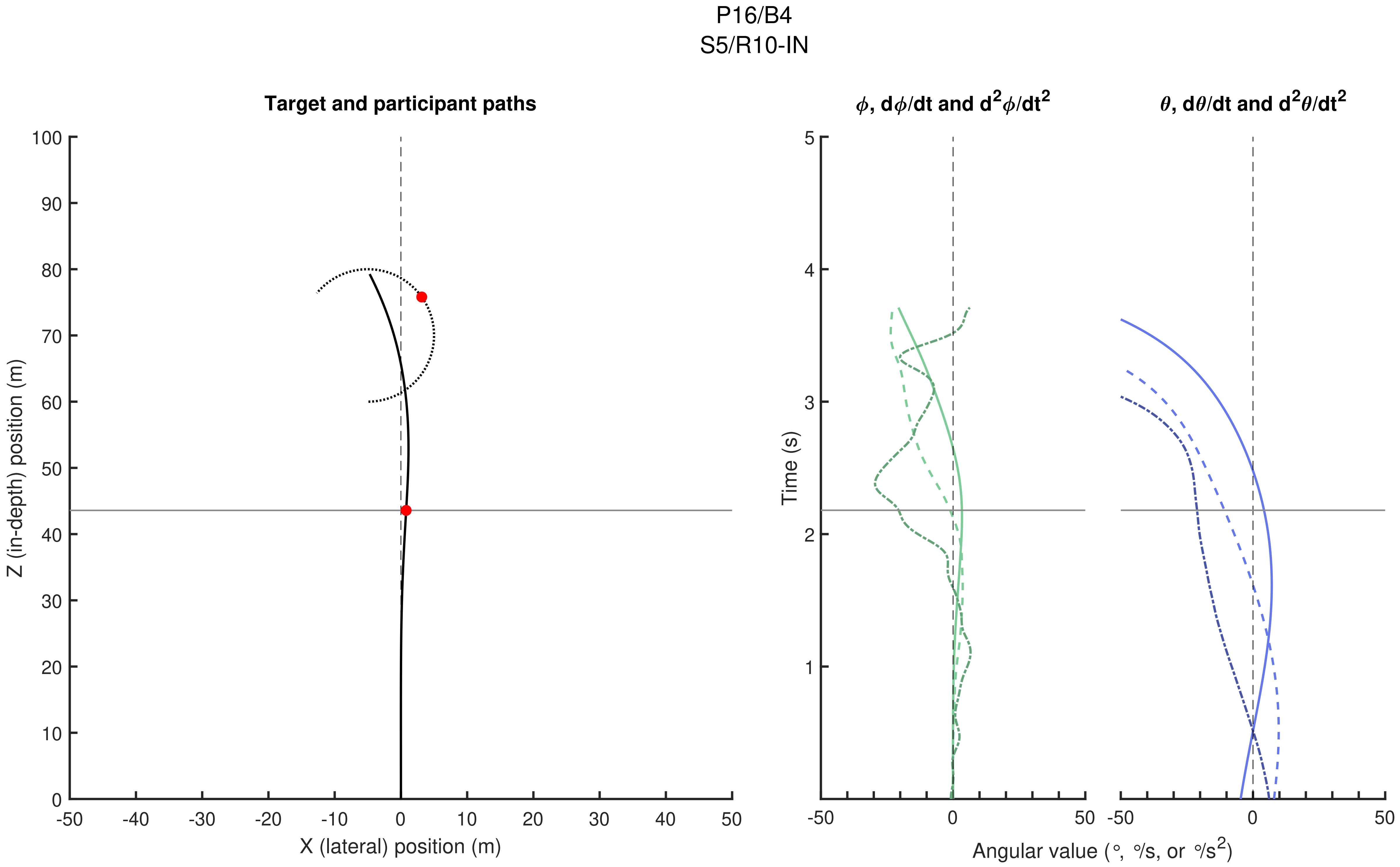
P16/B4
S5/R10-IN
Target and participant paths
ϕ, dϕ/dt and d$^2\phi$/dt^2
θ, dθ/dt and d$^2\theta$/dt^2
Z (in-depth) position (m)
X (lateral) position (m)
Time (s)
Angular value (°, °/s, or °/s^2)
100
90
80
70
60
50
40
30
20
10
0
-50
-40
-30
-20
-10
0
10
20
30
40
50
5
4
3
2
1
-50
0
50
-50
0
50

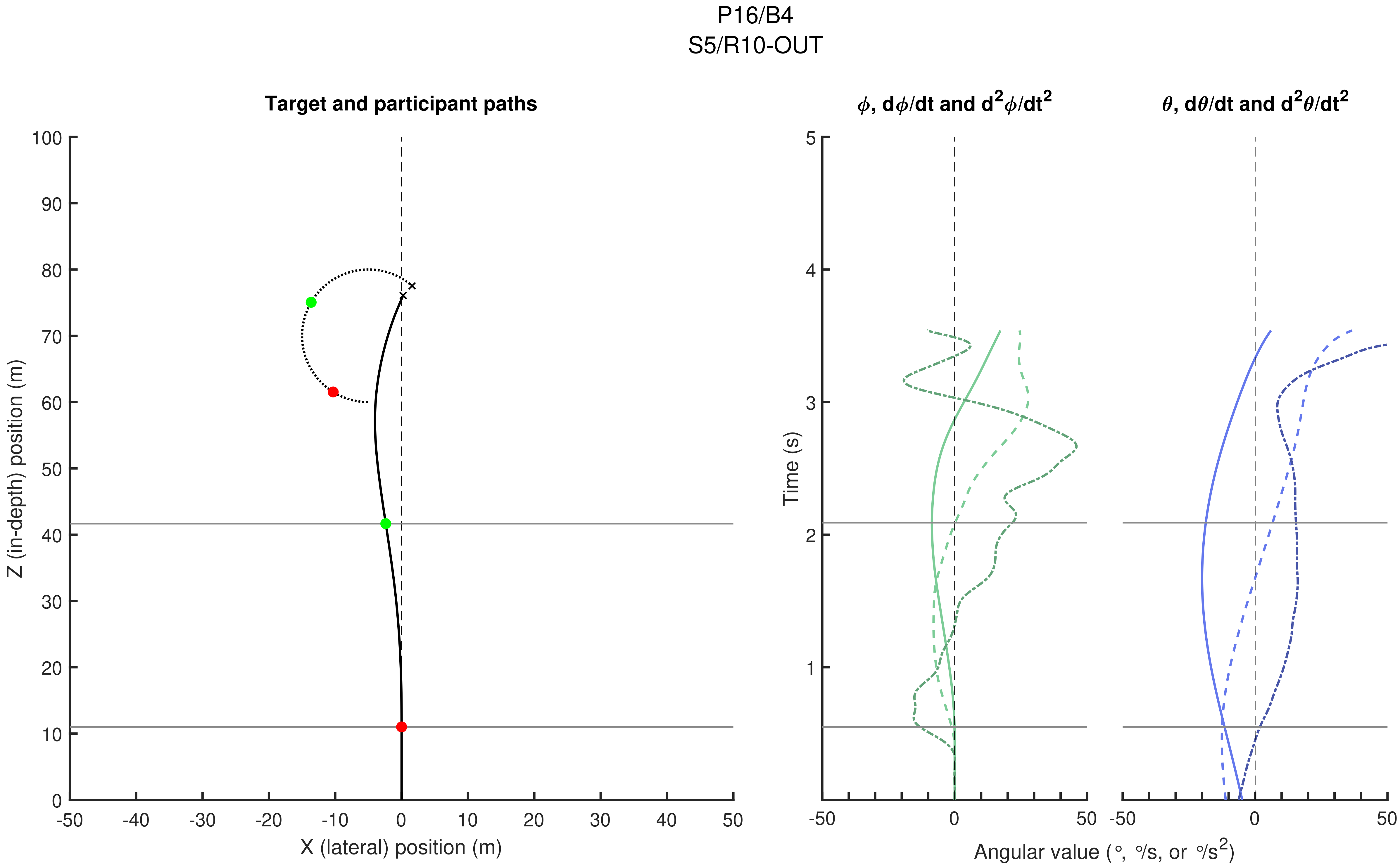

P16/B4
S5/R10-OUT
Target and participant paths
Z (in-depth) position (m)
X (lateral) position (m)
100
90
80
70
60
50
40
30
20
10
0
-50
-40
-30
-20
-10
0
10
20
30
40
50
ϕ, dϕ/dt and d$^2\phi$/dt^2
θ, dθ/dt and d$^2\theta$/dt^2
Time (s)
5
4
3
2
1
-50
0
50
-50
0
50
Angular value (°, °/s, or °/s^2)

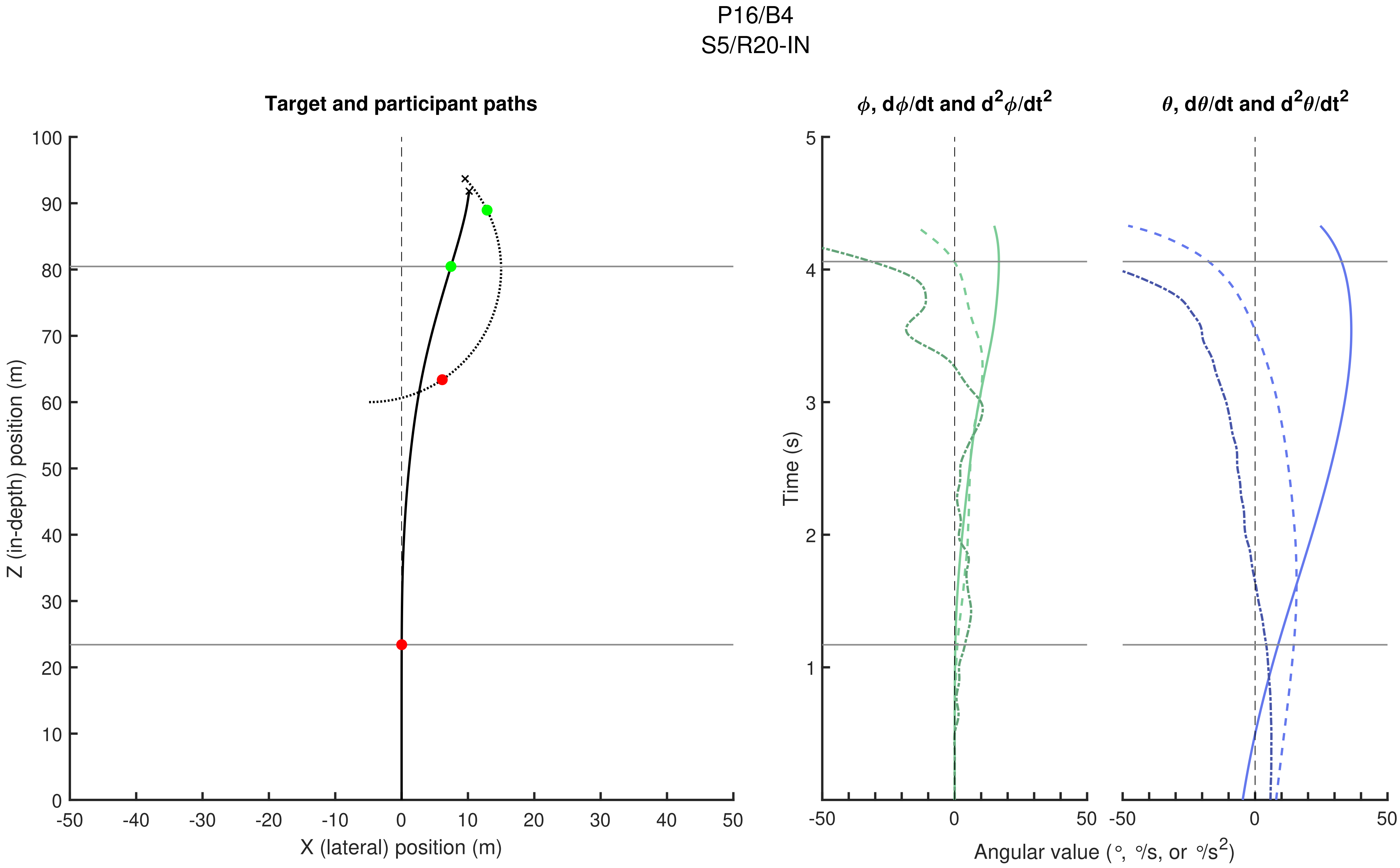
P16/B4
S5/R20-IN
Target and participant paths
Z (in-depth) position (m)
X (lateral) position (m)
100
90
80
70
60
50
40
30
20
10
0
-50
-40
-30
-20
-10
0
10
20
30
40
50
ϕ, dϕ/dt and d²ϕ/dt²
θ, dθ/dt and d²θ/dt²
Time (s)
5
4
3
2
1
-50
0
50
-50
0
50
Angular value (°, °/s, or °/s²)

P16/B4
S5/R20-OUT

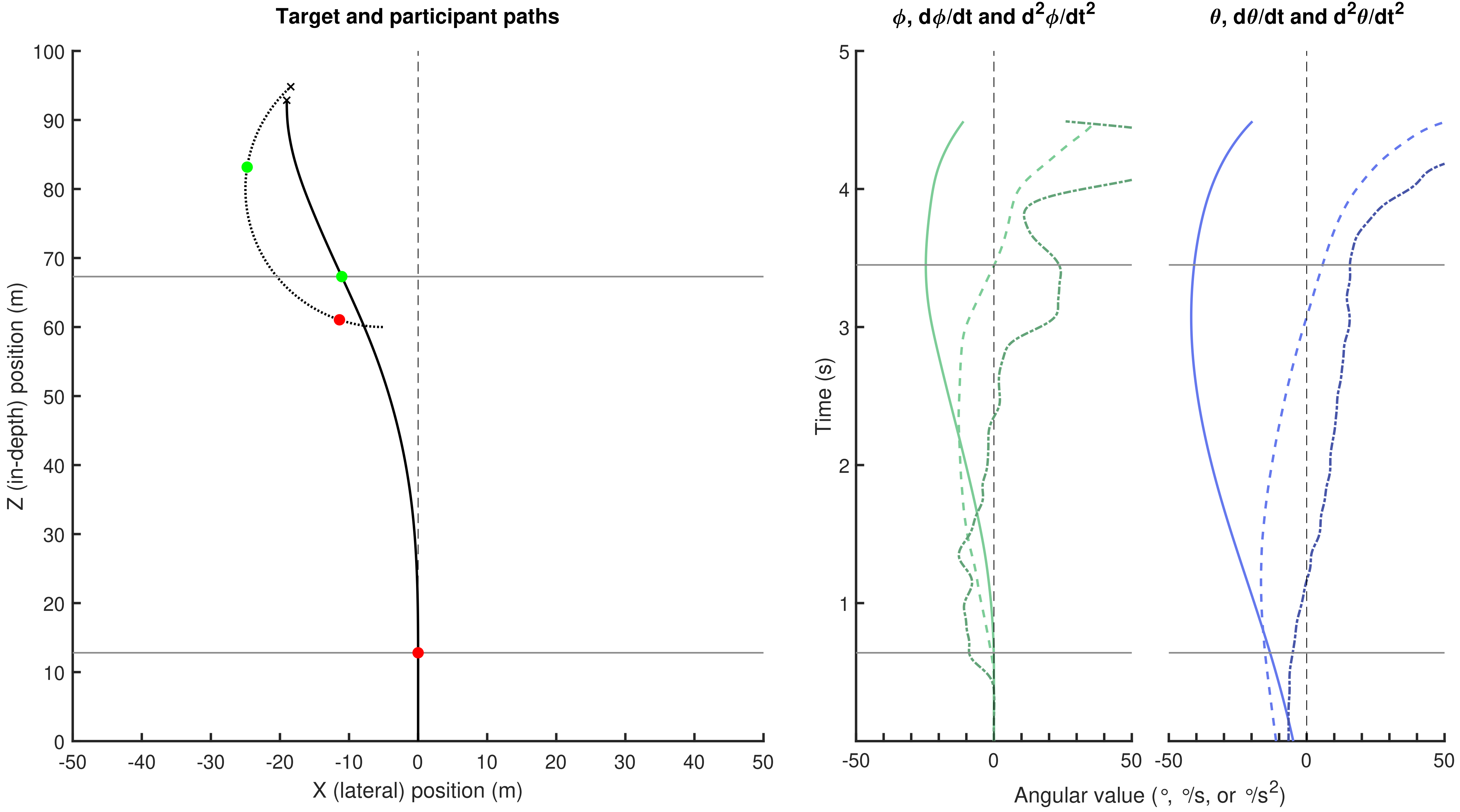

P16/B4
S5/R30-IN

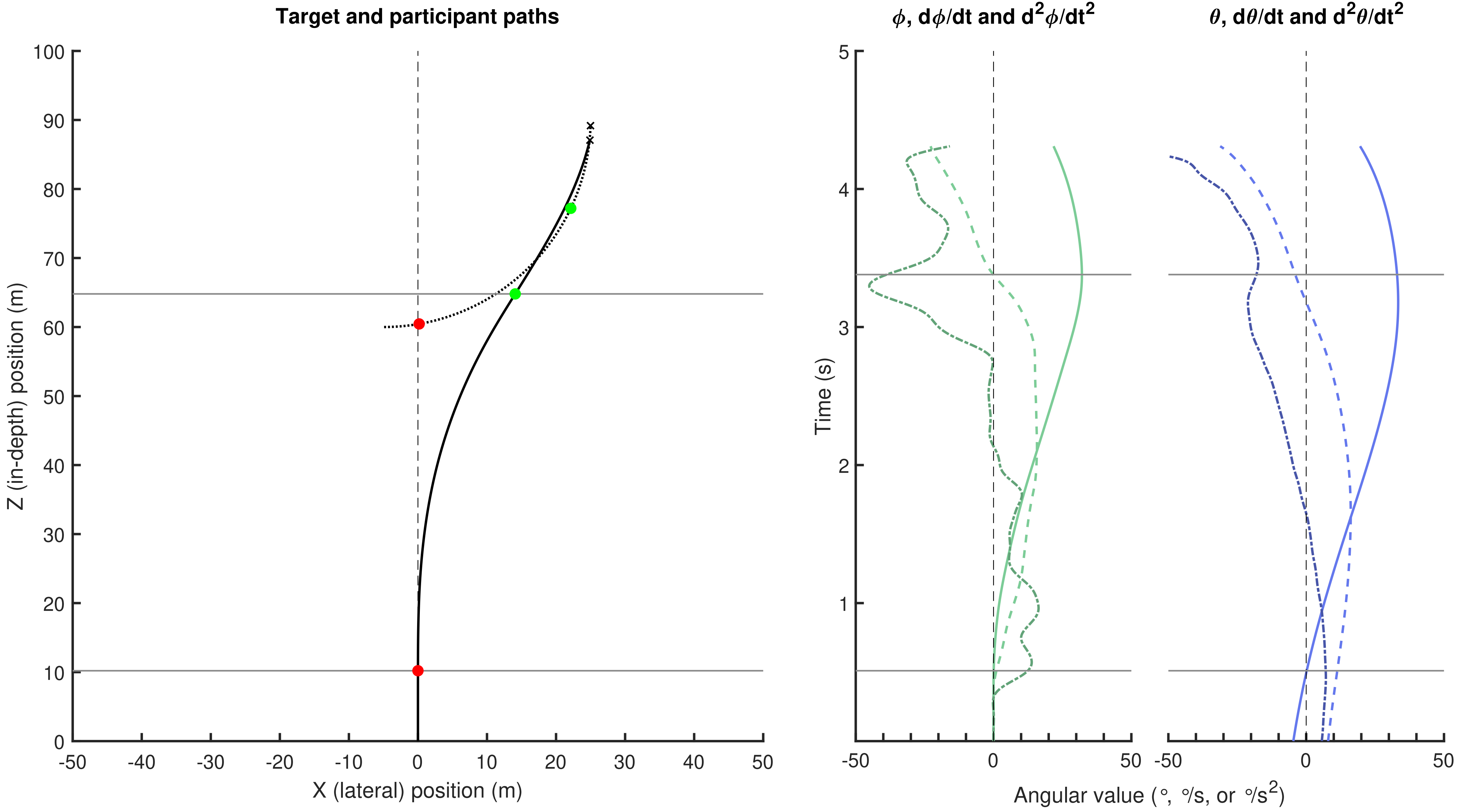

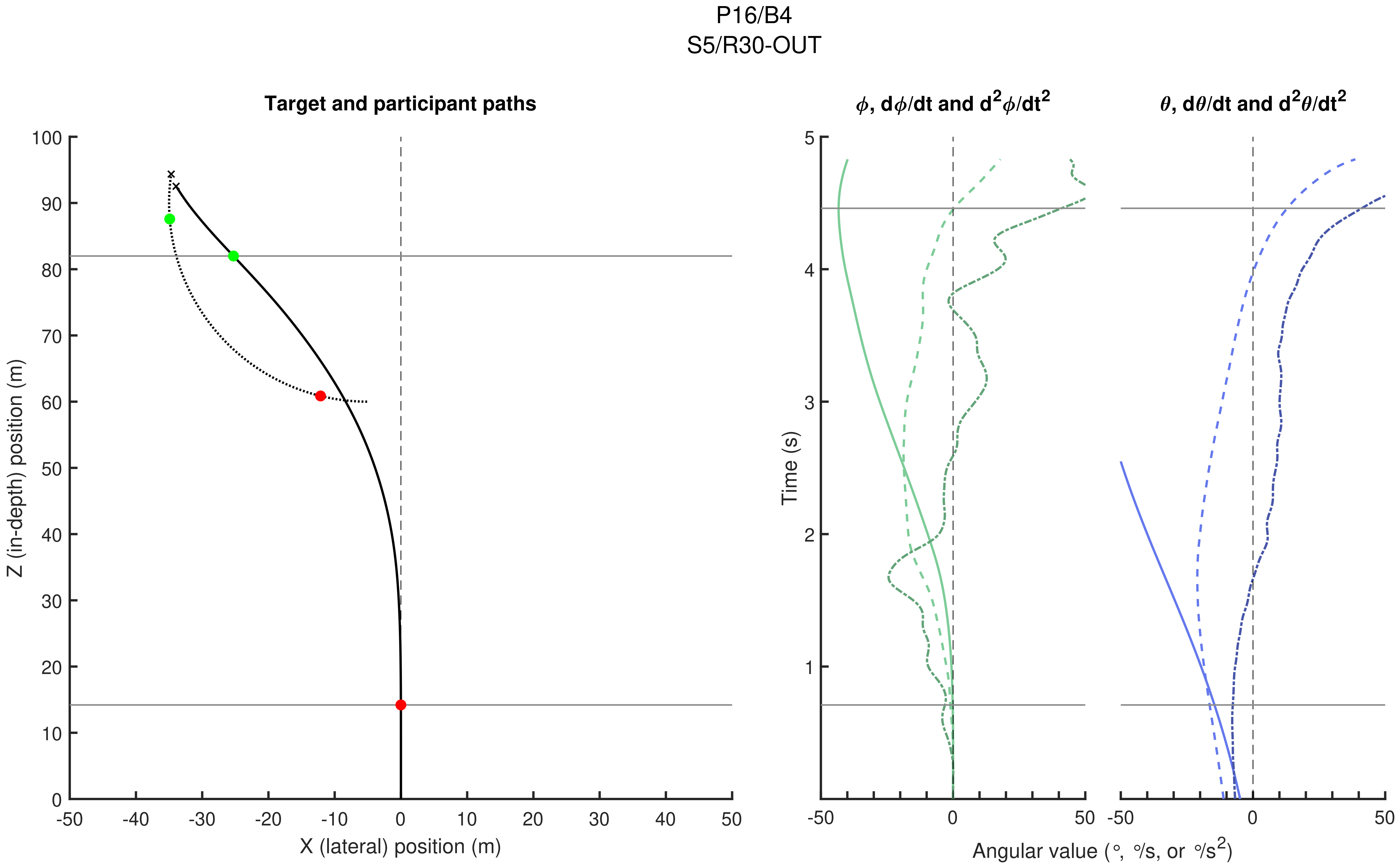
P16/B4
S5/R30-OUT
Target and participant paths
Z (in-depth) position (m)
X (lateral) position (m)
ϕ, dϕ/dt and d²ϕ/dt²
θ, dθ/dt and d²θ/dt²
Time (s)
Angular value (°, °/s, or °/s²)

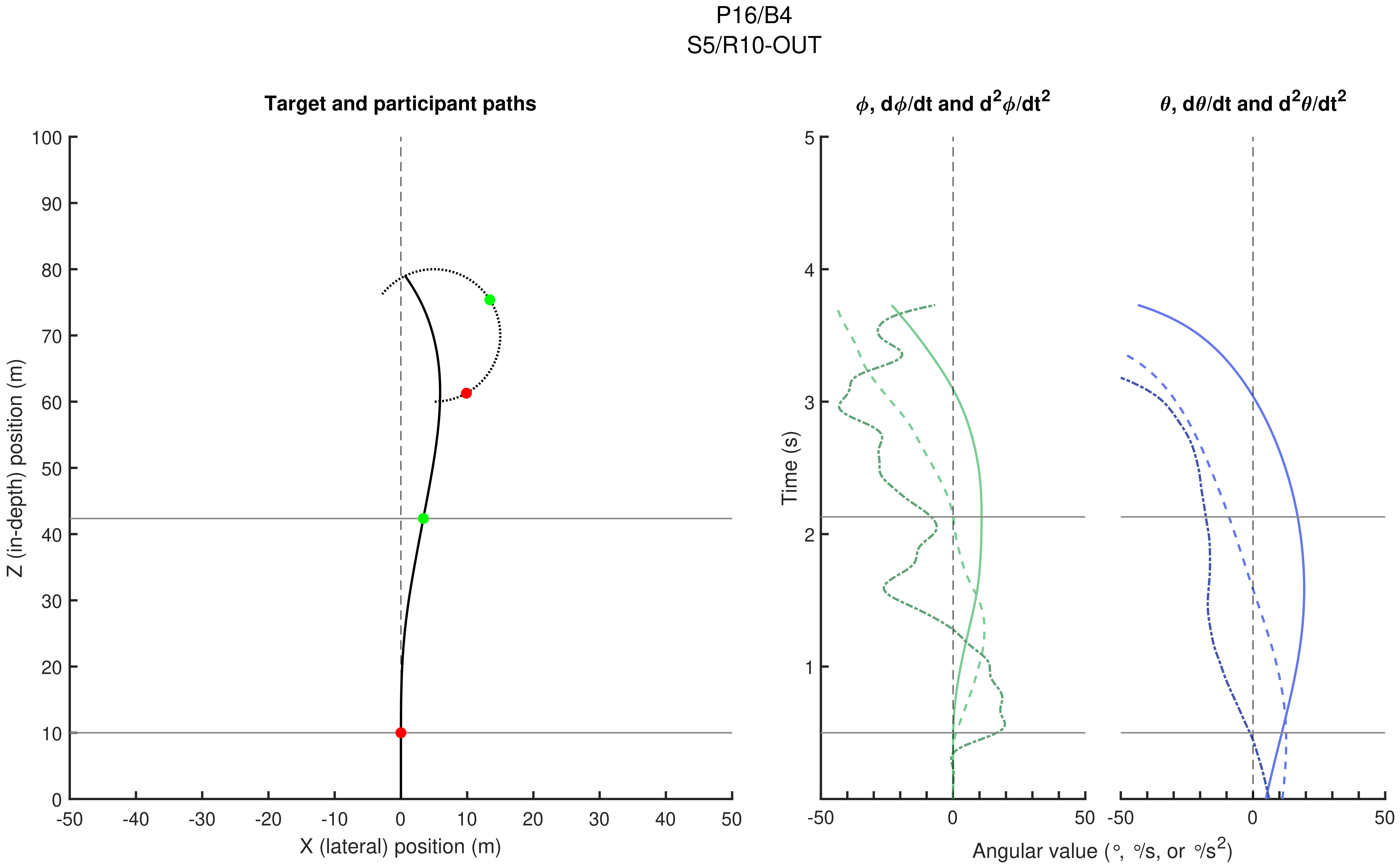

P16/B4
S5/R10-OUT
Target and participant paths
Z (in-depth) position (m)
X (lateral) position (m)
ϕ, dϕ/dt and d$^2\phi$/dt^2
θ, dθ/dt and d$^2\theta$/dt^2
Time (s)
Angular value (°, °/s, or °/s^2)

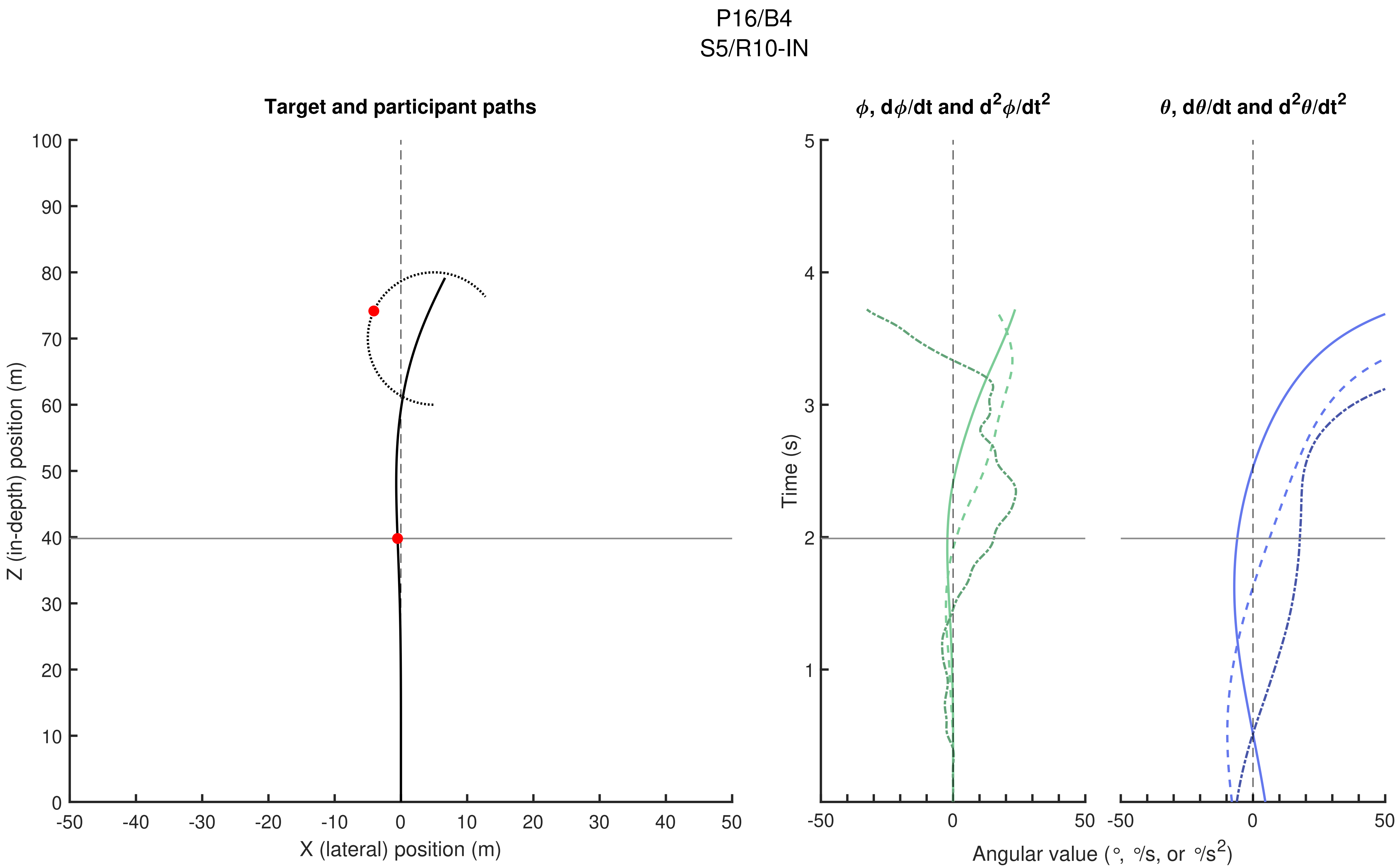
P16/B4
S5/R10-IN
Target and participant paths
Z (in-depth) position (m)
X (lateral) position (m)
ϕ, dϕ/dt and d$^2\phi$/dt^2
θ, dθ/dt and d$^2\theta$/dt^2
Time (s)
Angular value (°, °/s, or °/s^2)

P16/B4
S5/R20-OUT

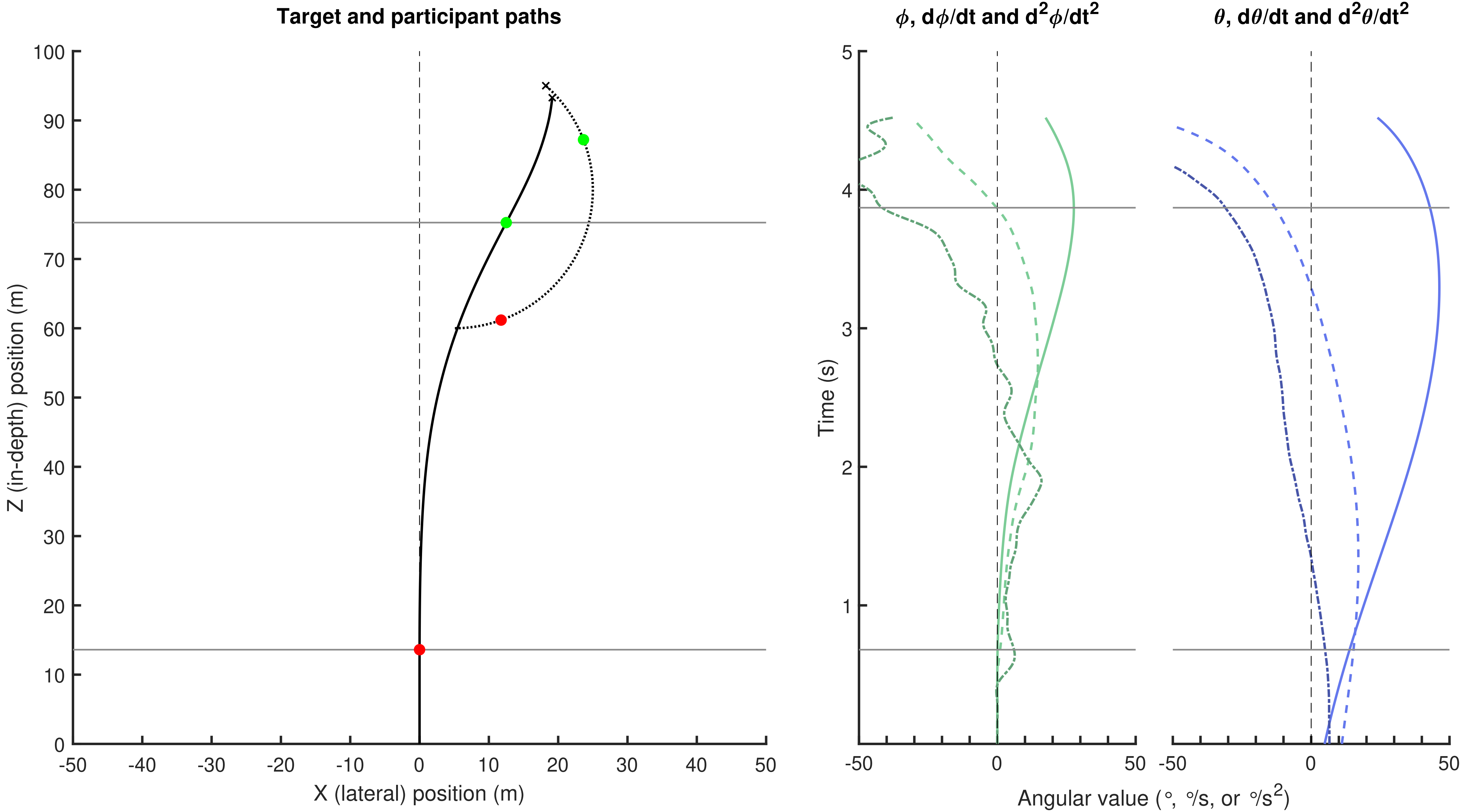

P16/B4
S5/R20-IN

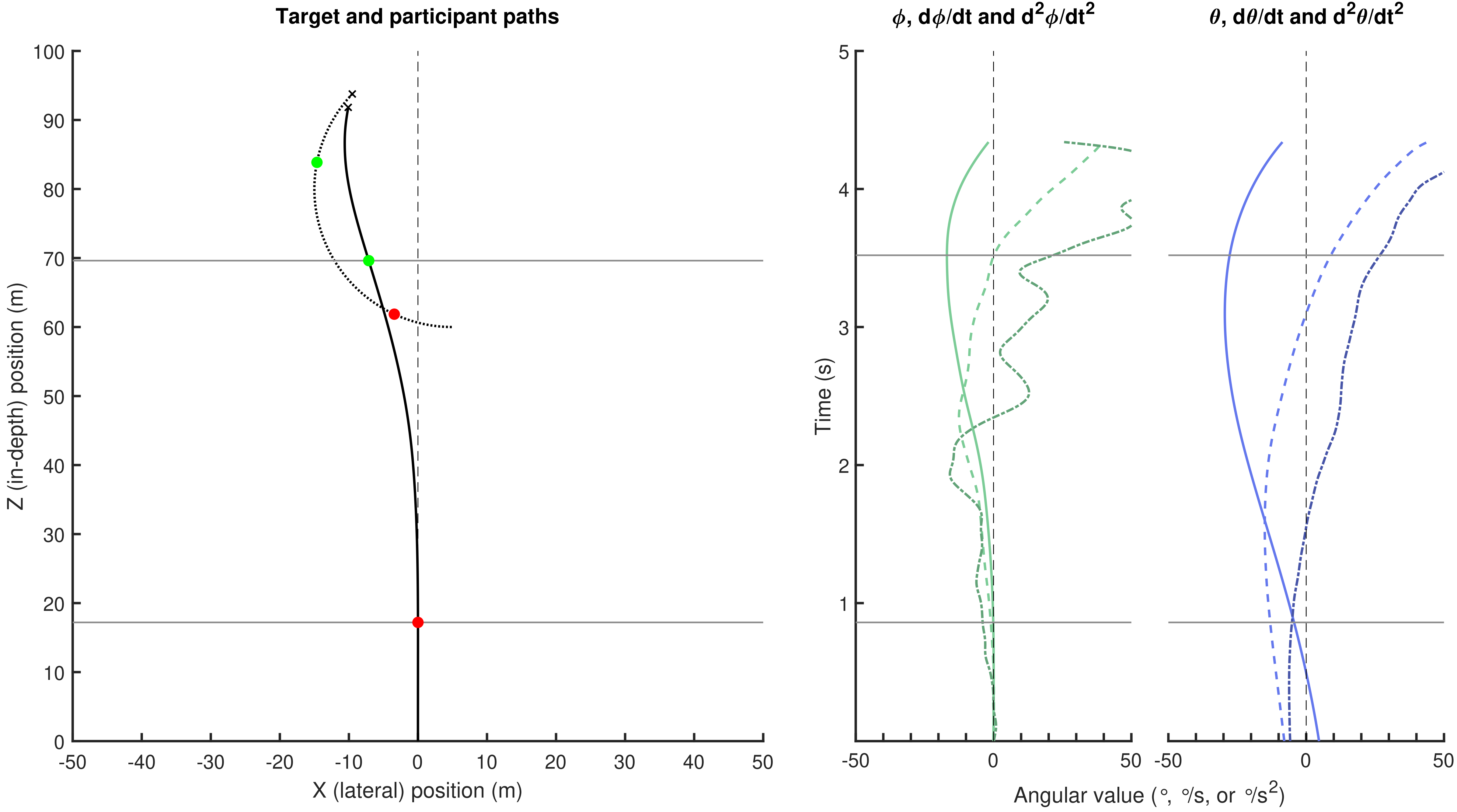

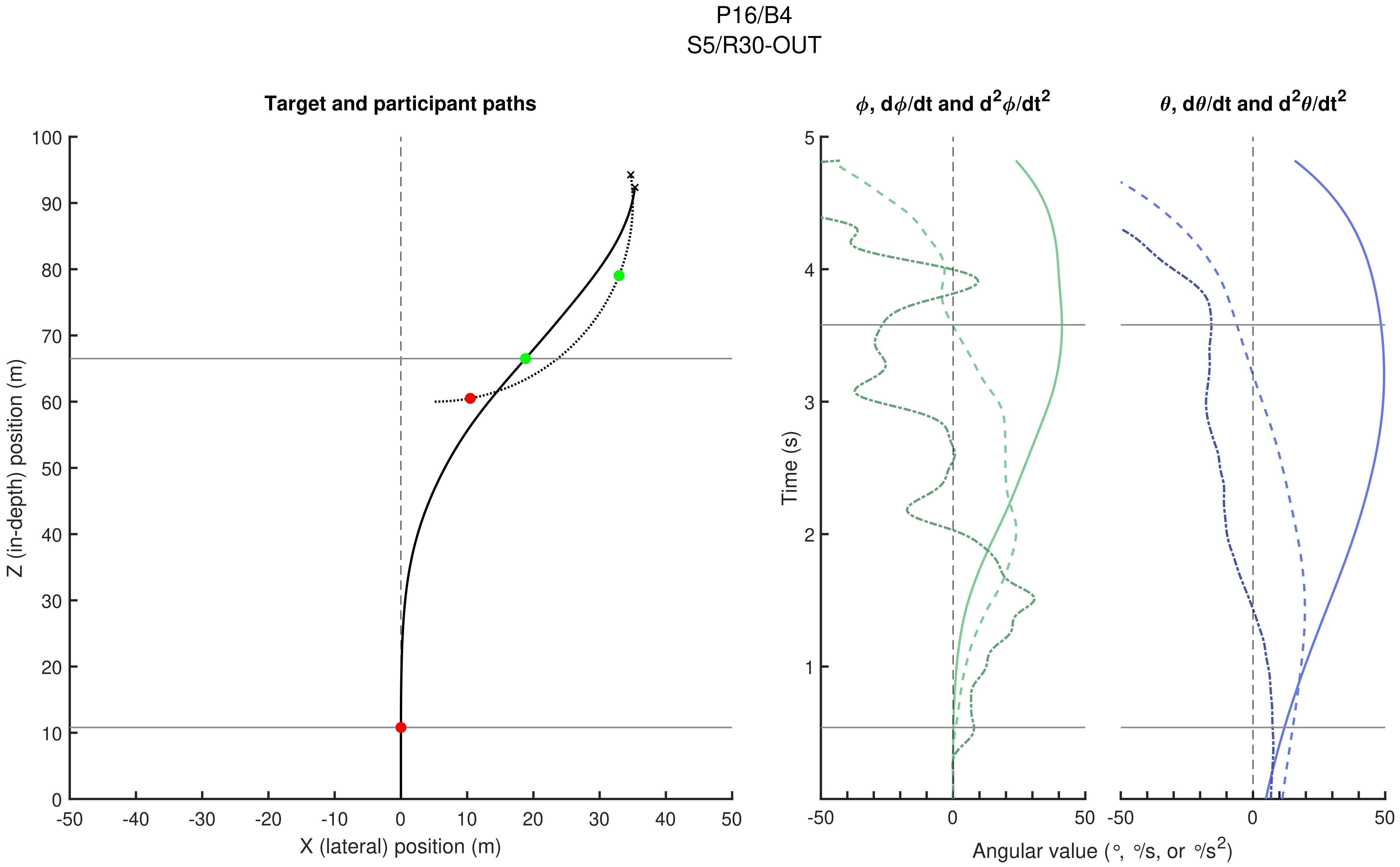

P16/B4
S5/R30-OUT
Target and participant paths
Z (in-depth) position (m)
X (lateral) position (m)
100
90
80
70
60
50
40
30
20
10
0
-50
-40
-30
-20
-10
0
10
20
30
40
50
ϕ, dϕ/dt and d$^2\phi$/dt^2
θ, dθ/dt and d$^2\theta$/dt^2
Time (s)
5
4
3
2
1
-50
0
50
-50
0
50
Angular value (°, °/s, or °/s^2)

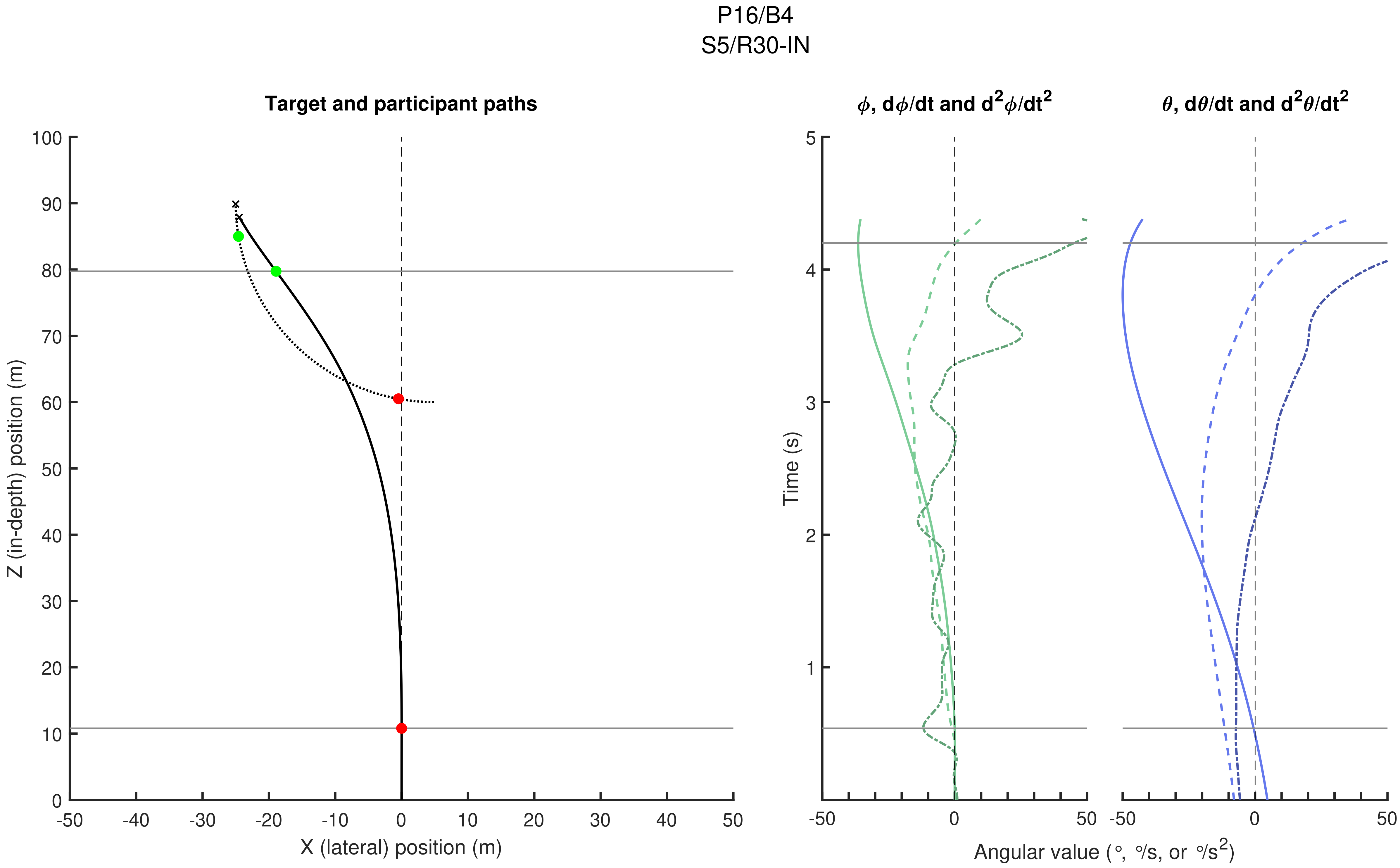

P16/B4
S5/R30-IN
Target and participant paths
Z (in-depth) position (m)
X (lateral) position (m)
100
90
80
70
60
50
40
30
20
10
0
-50
-40
-30
-20
-10
0
10
20
30
40
50
ϕ, dϕ/dt and d2ϕ/dt2
θ, dθ/dt and d2θ/dt2
Time (s)
5
4
3
2
1
-50
0
50
-50
0
50
Angular value (°, °/s, or °/s2)

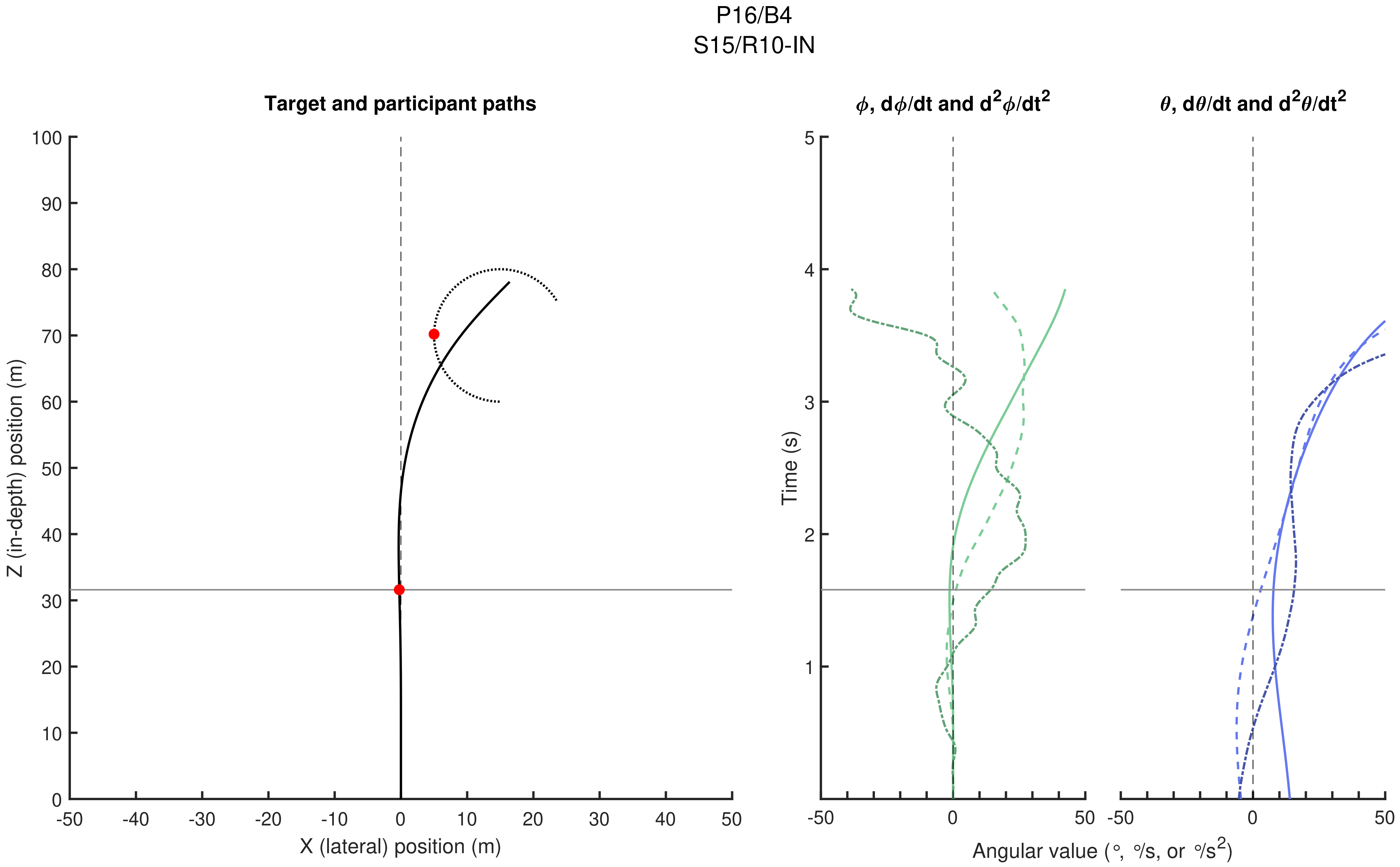

P16/B4
S15/R10-IN
Target and participant paths
Z (in-depth) position (m)
X (lateral) position (m)
ϕ, dϕ/dt and d$^2\phi$/dt^2
θ, dθ/dt and d$^2\theta$/dt^2
Time (s)
Angular value (°, °/s, or °/s^2)

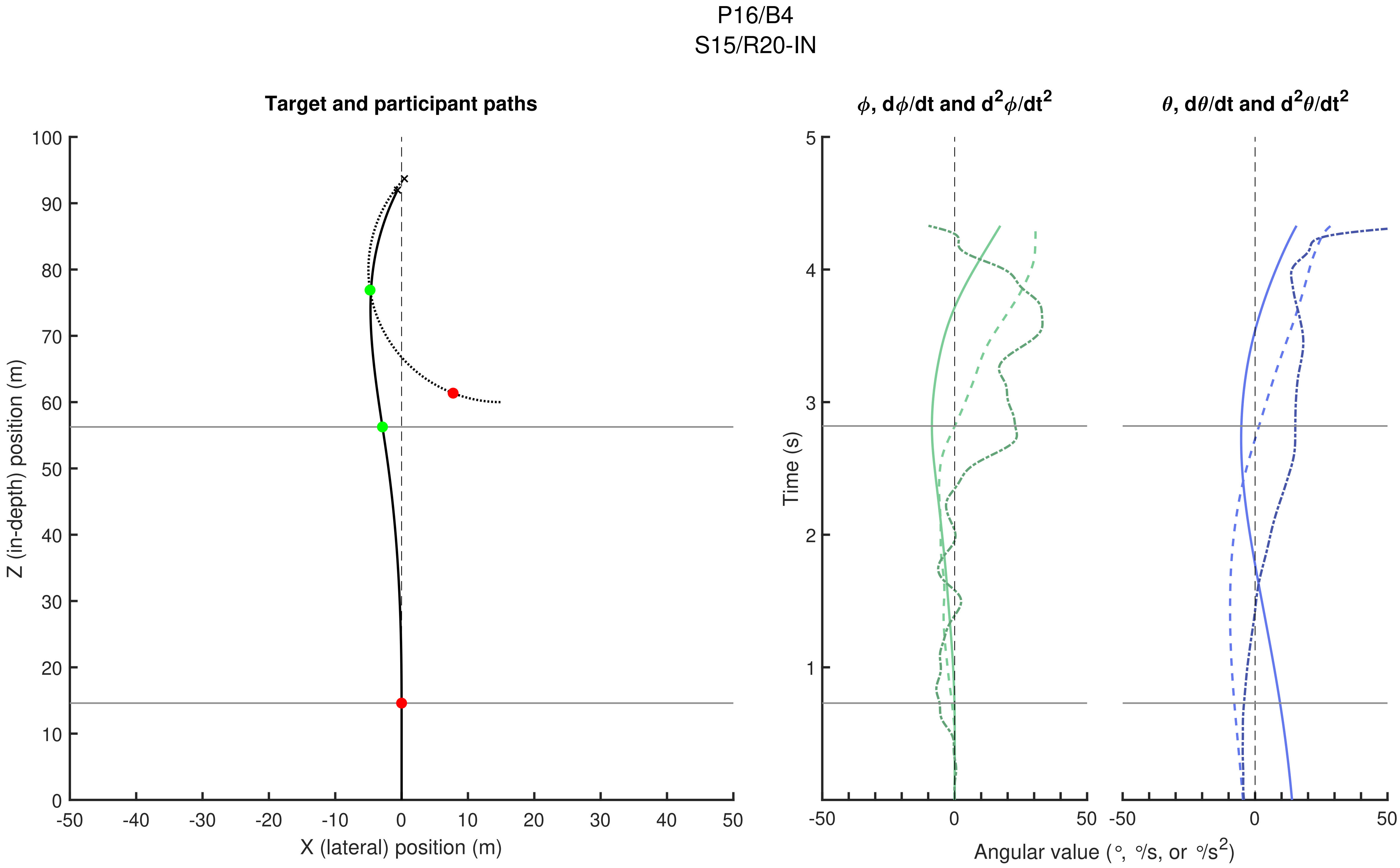
P16/B4
S15/R20-IN
Target and participant paths
Z (in-depth) position (m)
X (lateral) position (m)
ϕ, dϕ/dt and d²ϕ/dt²
θ, dθ/dt and d²θ/dt²
Time (s)
Angular value (°, °/s, or °/s²)

P16/B4
S15/R30-IN

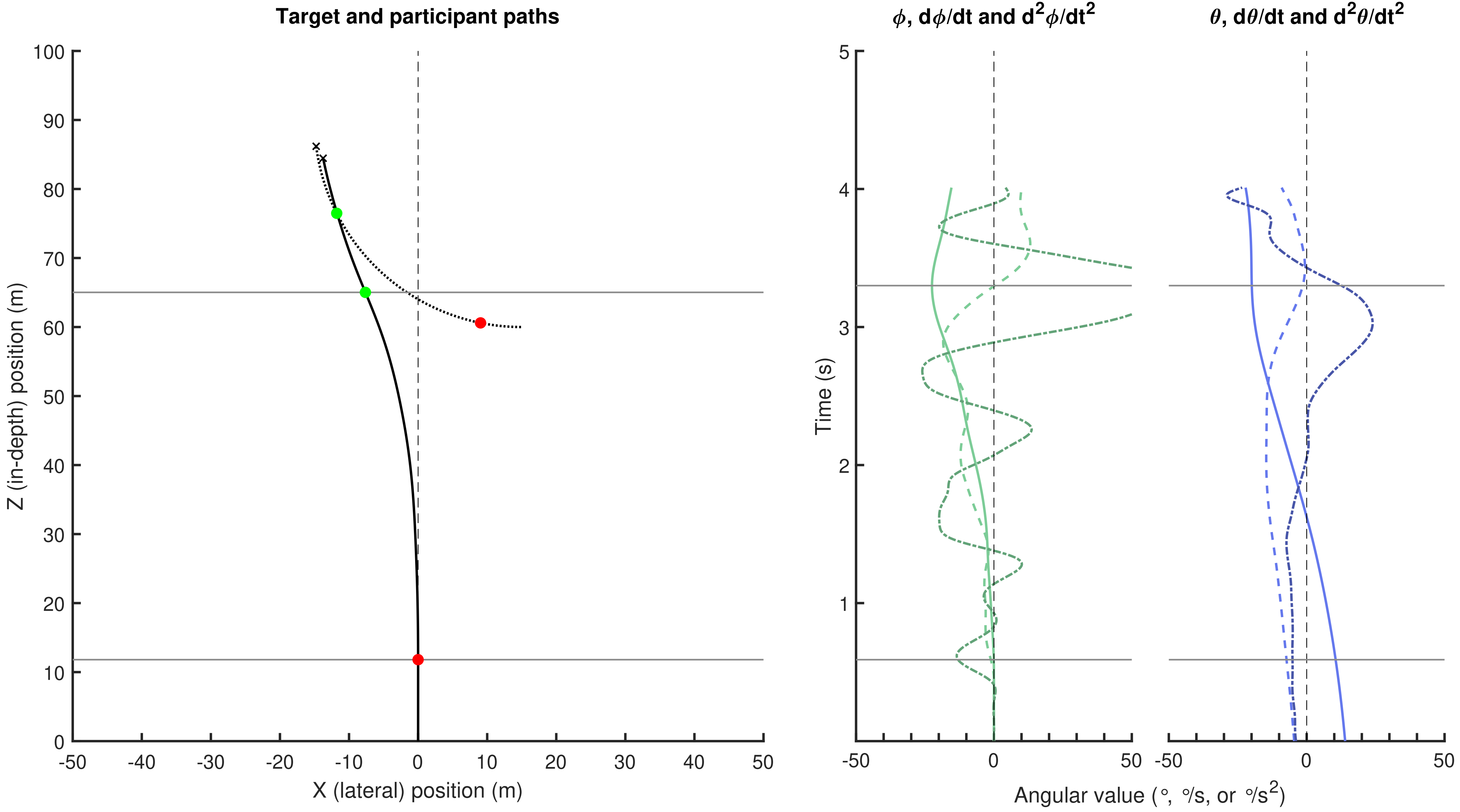

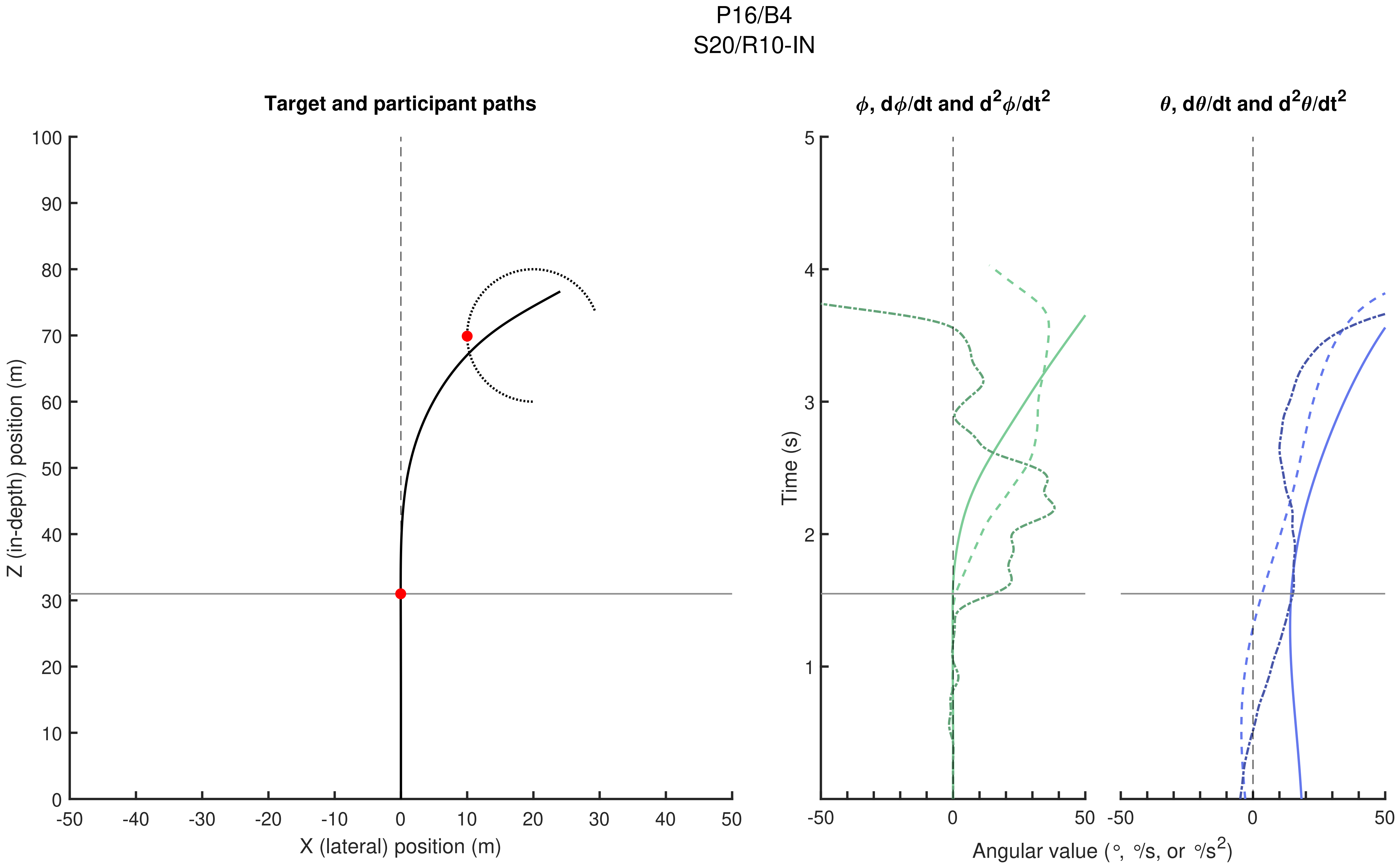
P16/B4
S20/R10-IN
Target and participant paths
ϕ, dϕ/dt and d$^2\phi$/dt^2
θ, dθ/dt and d$^2\theta$/dt^2
Z (in-depth) position (m)
X (lateral) position (m)
Time (s)
Angular value (°, °/s, or °/s^2)

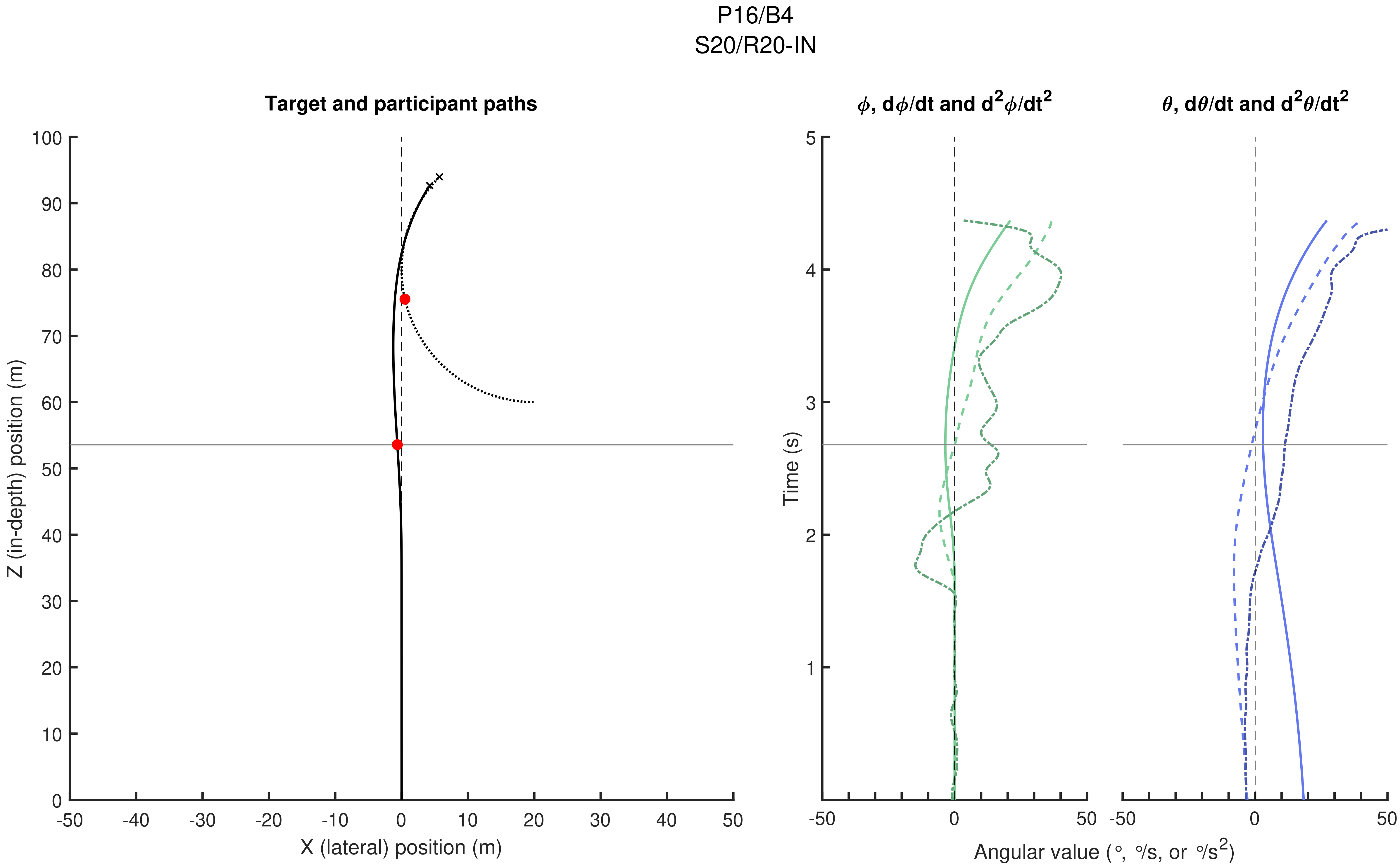
P16/B4
S20/R20-IN
Target and participant paths
Z (in-depth) position (m)
X (lateral) position (m)
100
90
80
70
60
50
40
30
20
10
0
-50
-40
-30
-20
-10
0
10
20
30
40
50
ϕ, dϕ/dt and d²ϕ/dt²
θ, dθ/dt and d²θ/dt²
Time (s)
5
4
3
2
1
-50
0
50
-50
0
50
Angular value (°, °/s, or °/s²)

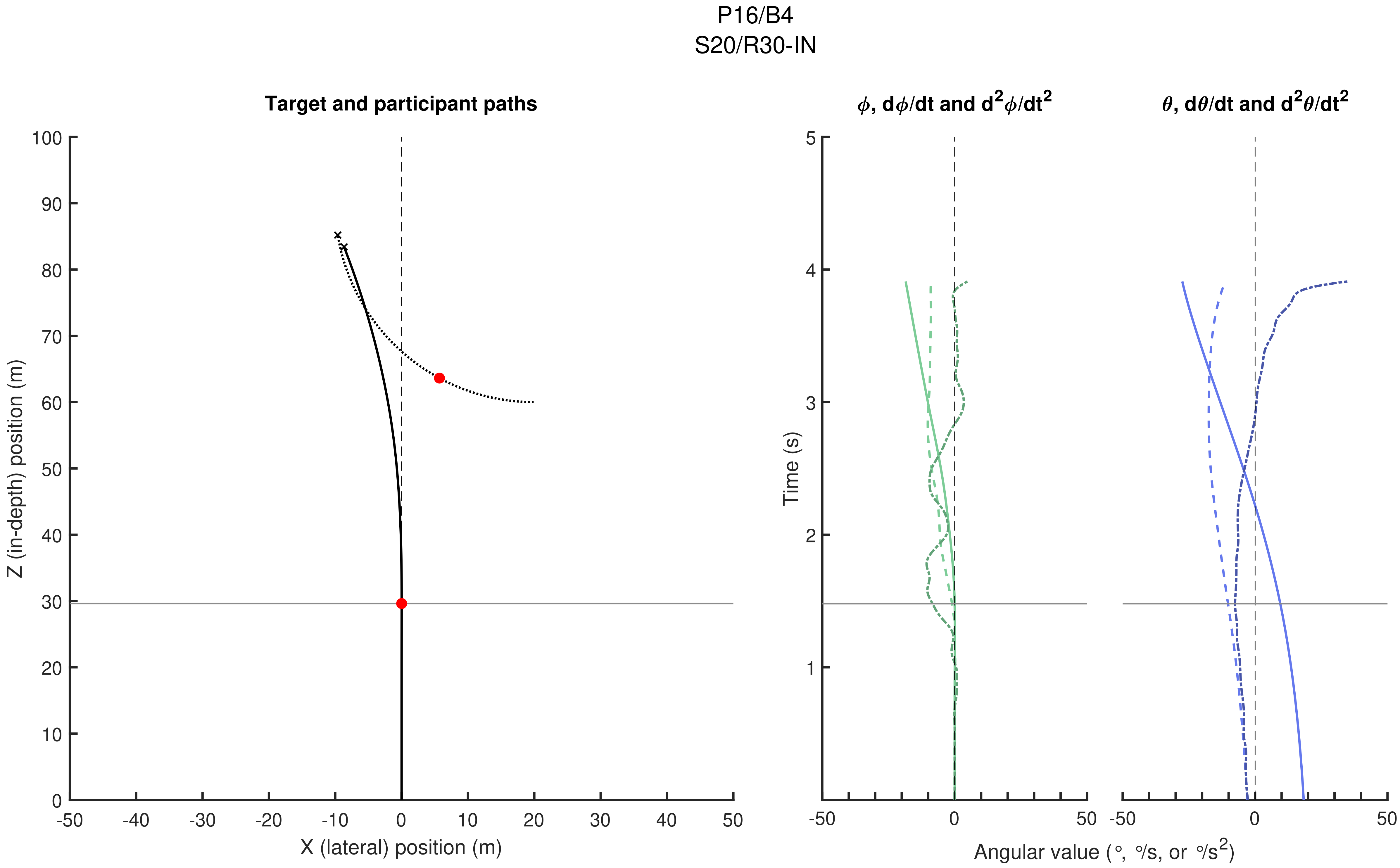
P16/B4
S20/R30-IN
Target and participant paths
Z (in-depth) position (m)
X (lateral) position (m)
φ, dφ/dt and d²φ/dt²
θ, dθ/dt and d²θ/dt²
Time (s)
Angular value (°, °/s, or °/s²)

P16/B5
S20/R10-IN

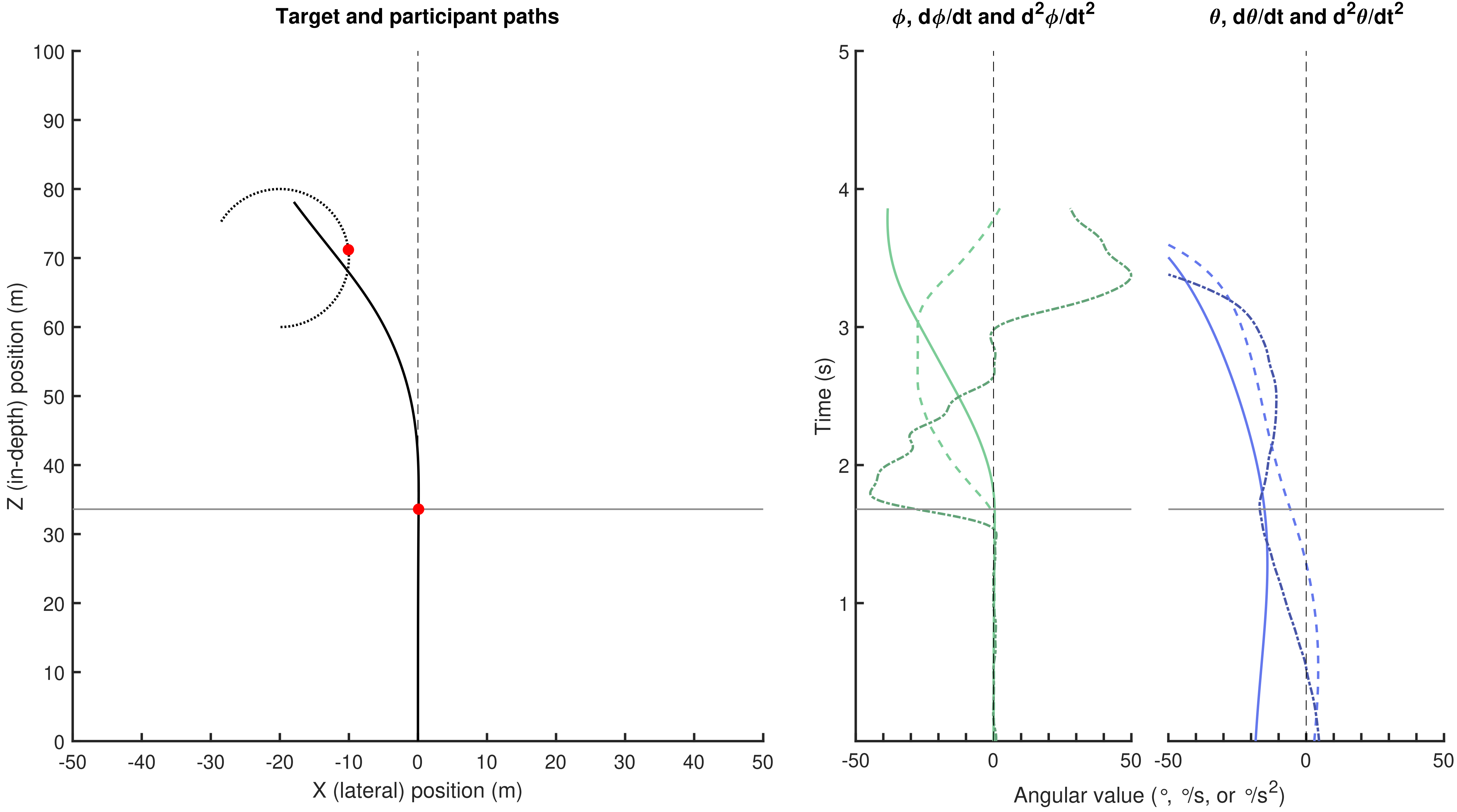

P16/B5
S20/R20-IN

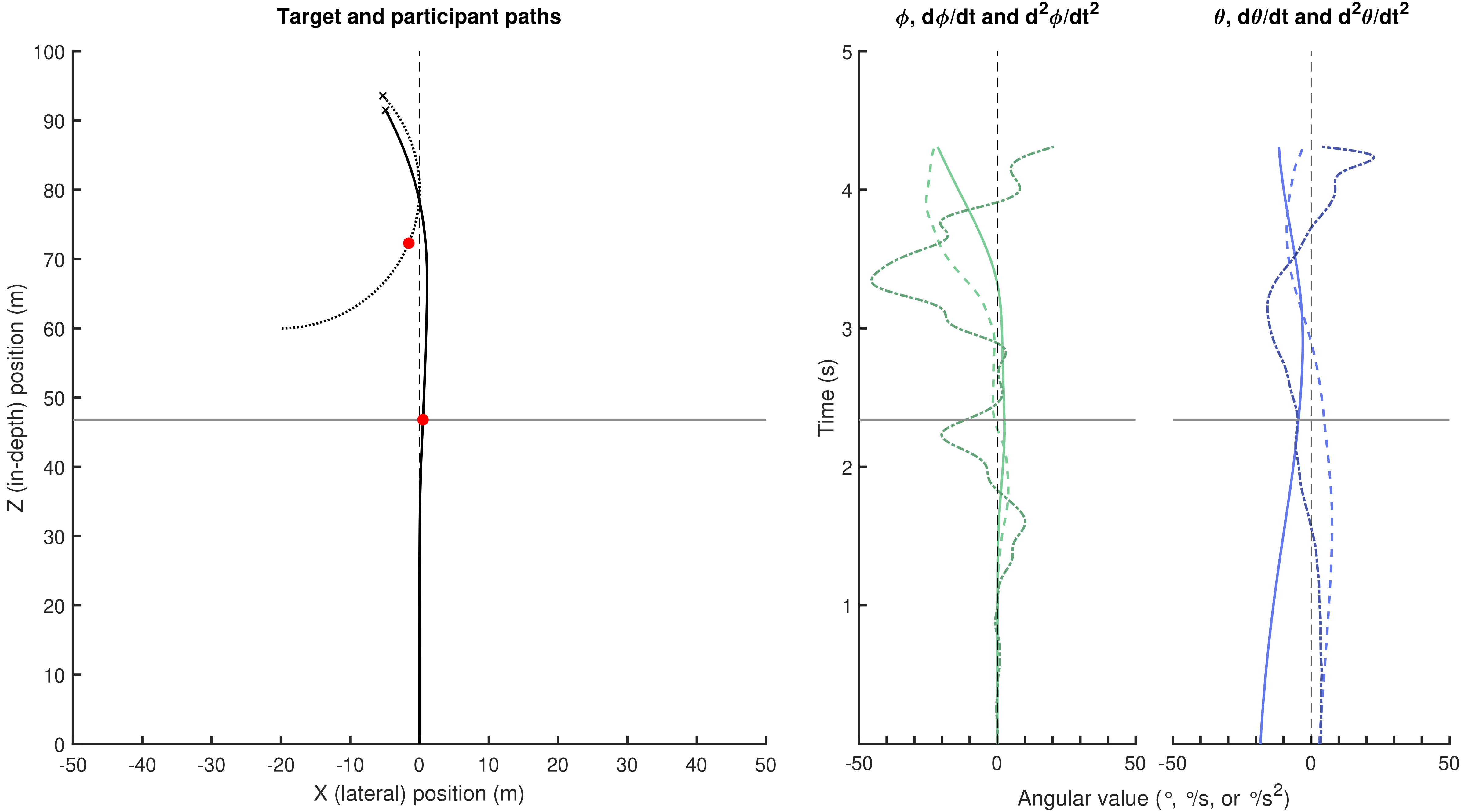

P16/B5
S20/R30-IN

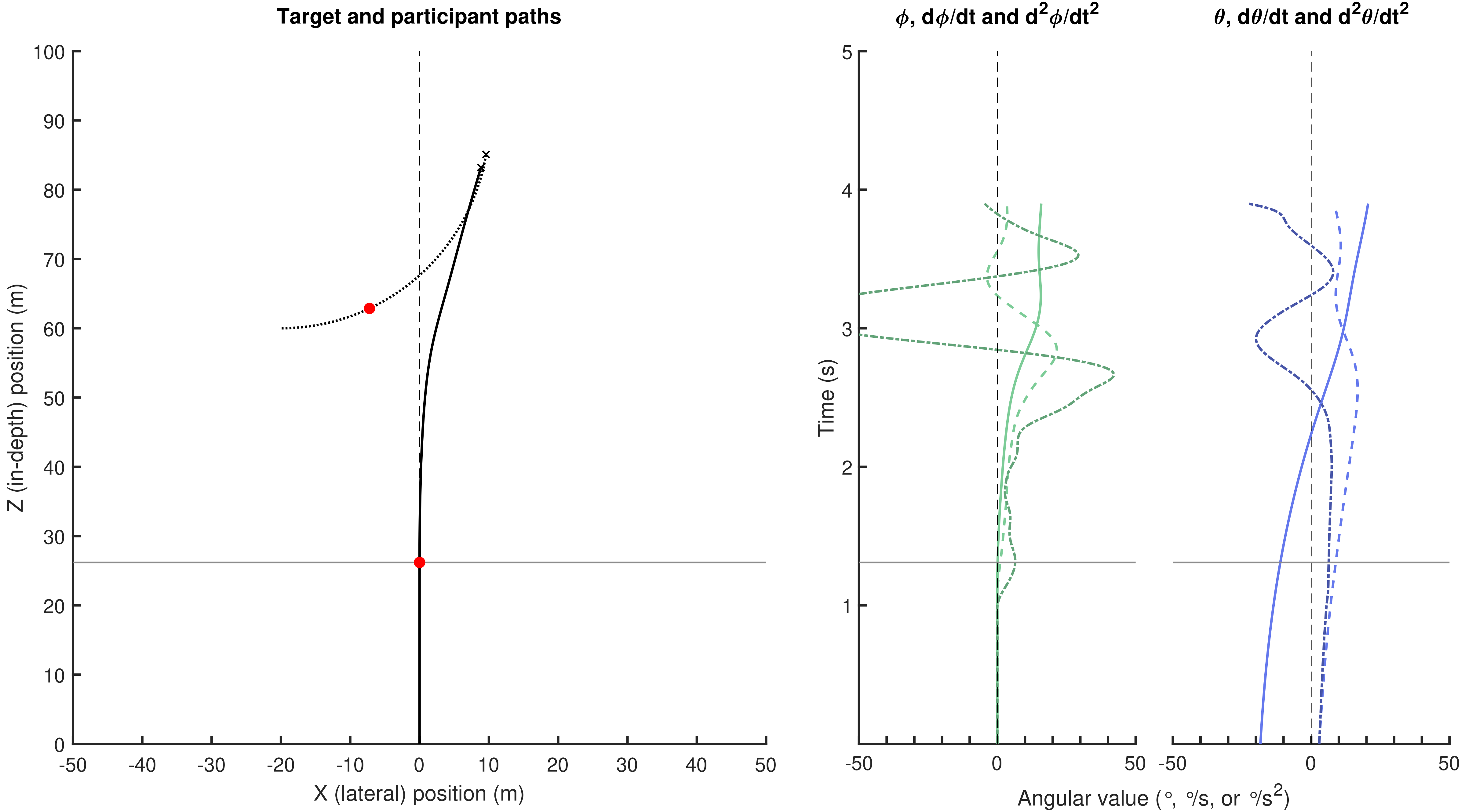

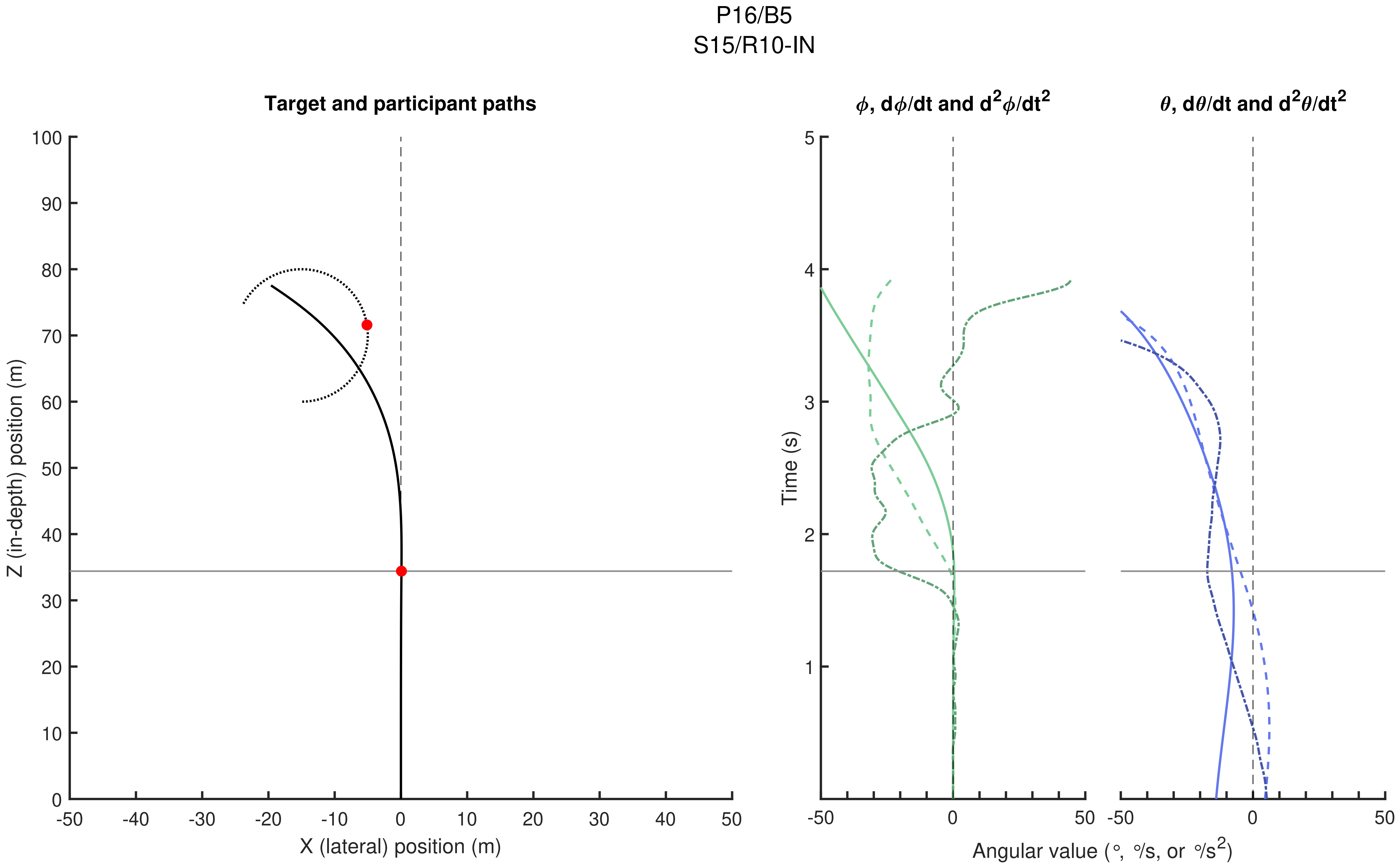
P16/B5
S15/R10-IN
Target and participant paths
ϕ, dϕ/dt and d$^2\phi$/dt^2
θ, dθ/dt and d$^2\theta$/dt^2
Z (in-depth) position (m)
X (lateral) position (m)
Time (s)
Angular value (°, °/s, or °/s^2)
100
90
80
70
60
50
40
30
20
10
0
-50
-40
-30
-20
-10
0
10
20
30
40
50
5
4
3
2
1
-50
0
50
-50
0
50

P16/B5
S15/R20-IN

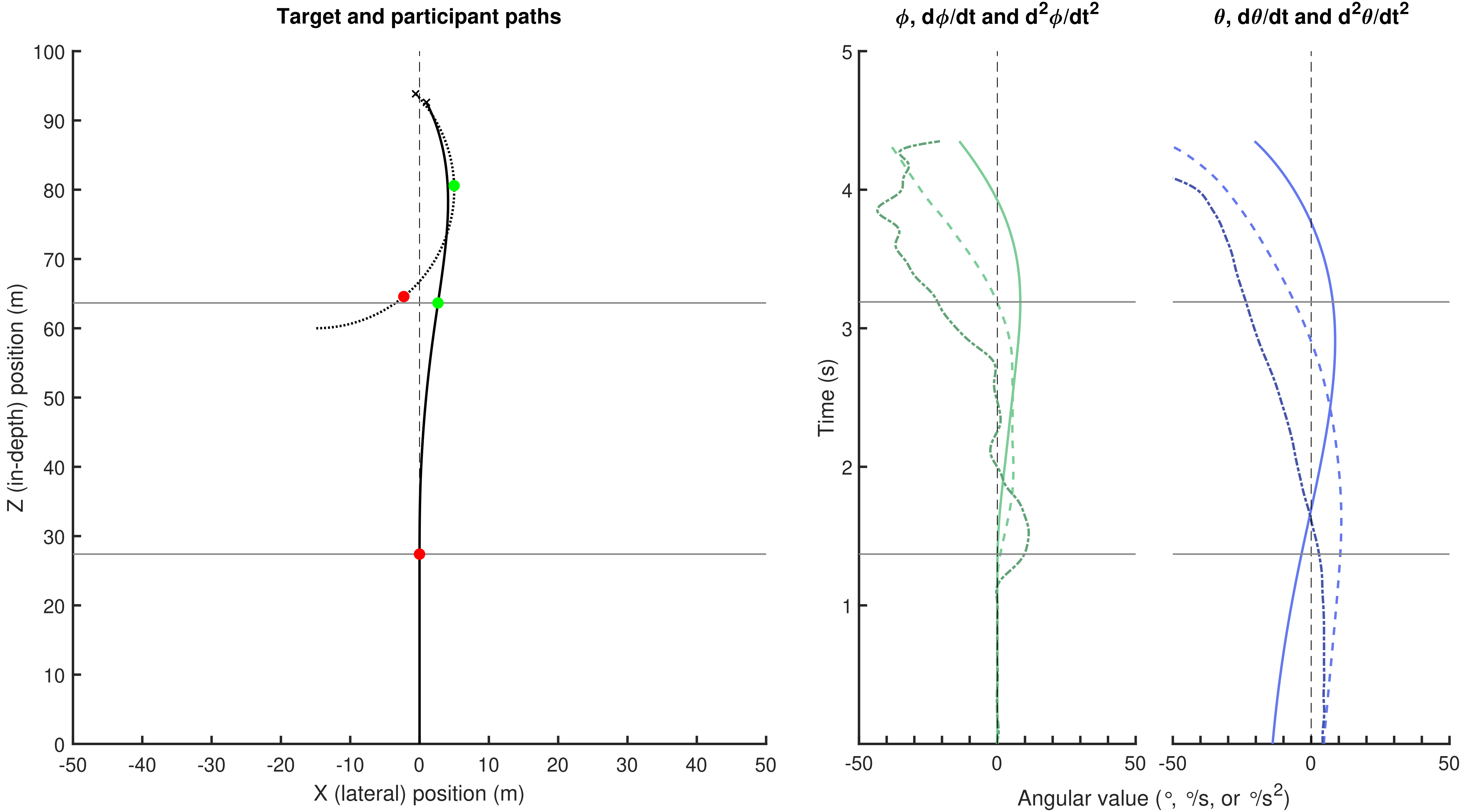

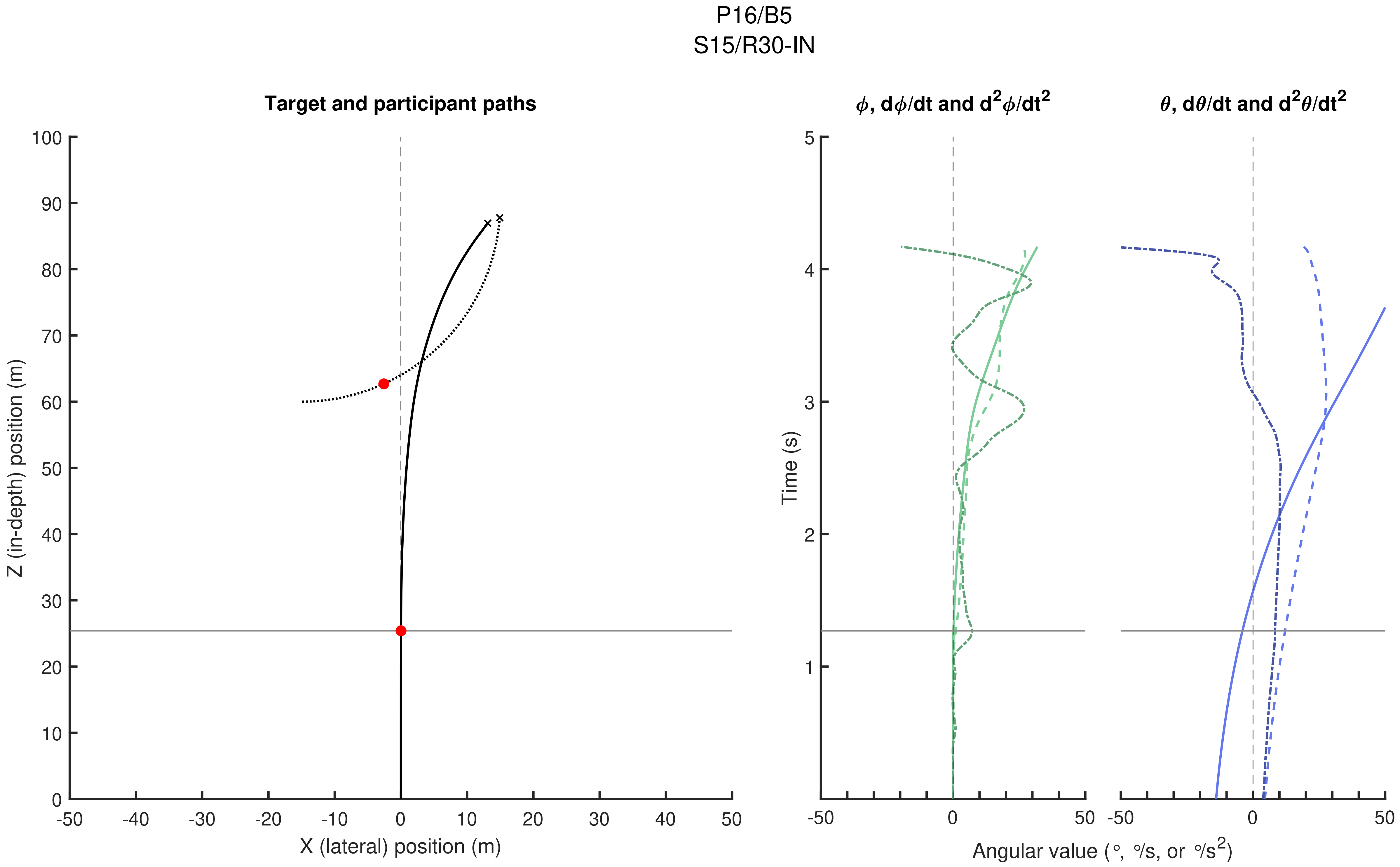

P16/B5
S15/R30-IN
Target and participant paths
ϕ, dϕ/dt and d$^2\phi$/dt^2
θ, dθ/dt and d$^2\theta$/dt^2
Z (in-depth) position (m)
X (lateral) position (m)
Time (s)
Angular value (°, °/s, or °/s^2)

P16/B5
S5/R10-IN

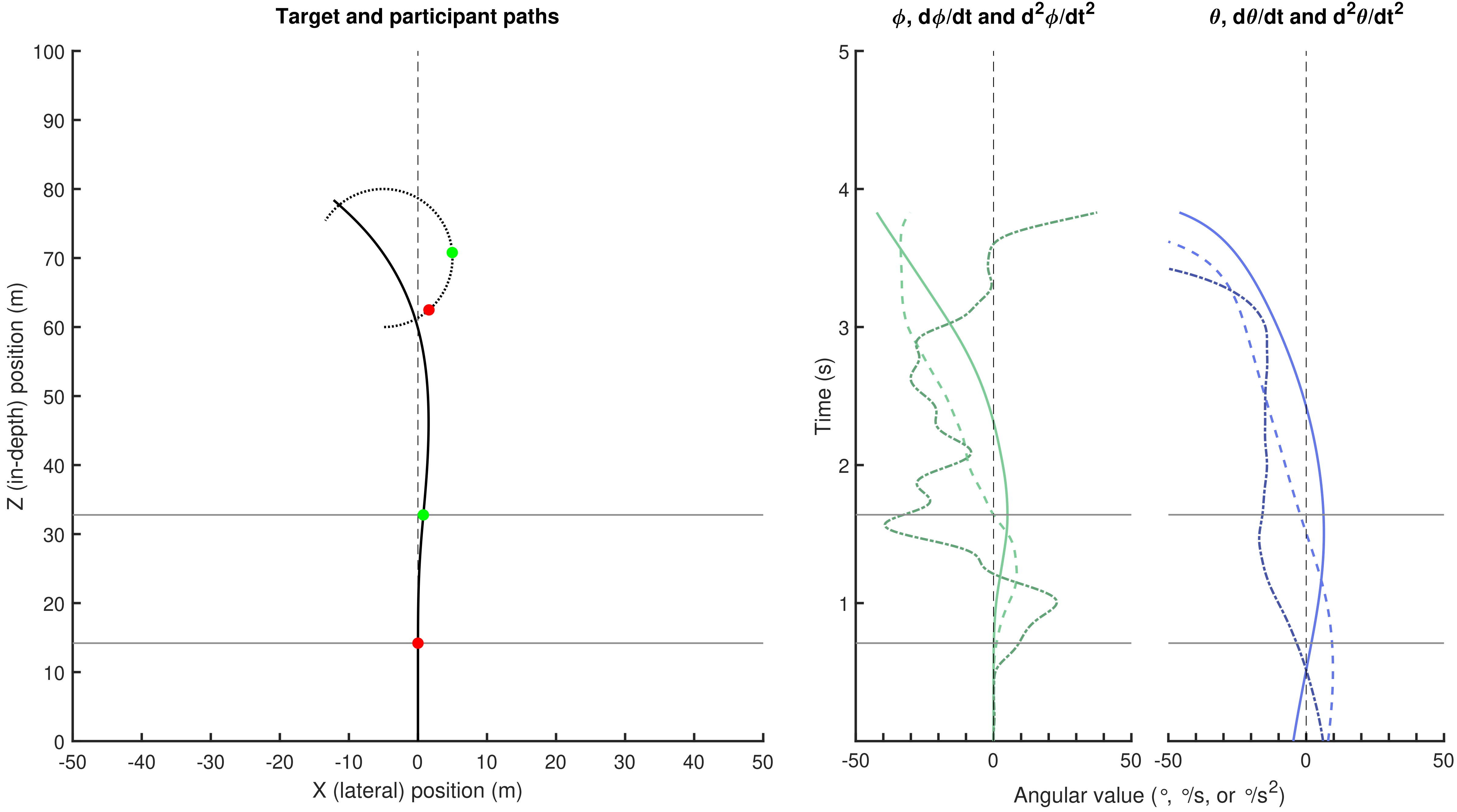

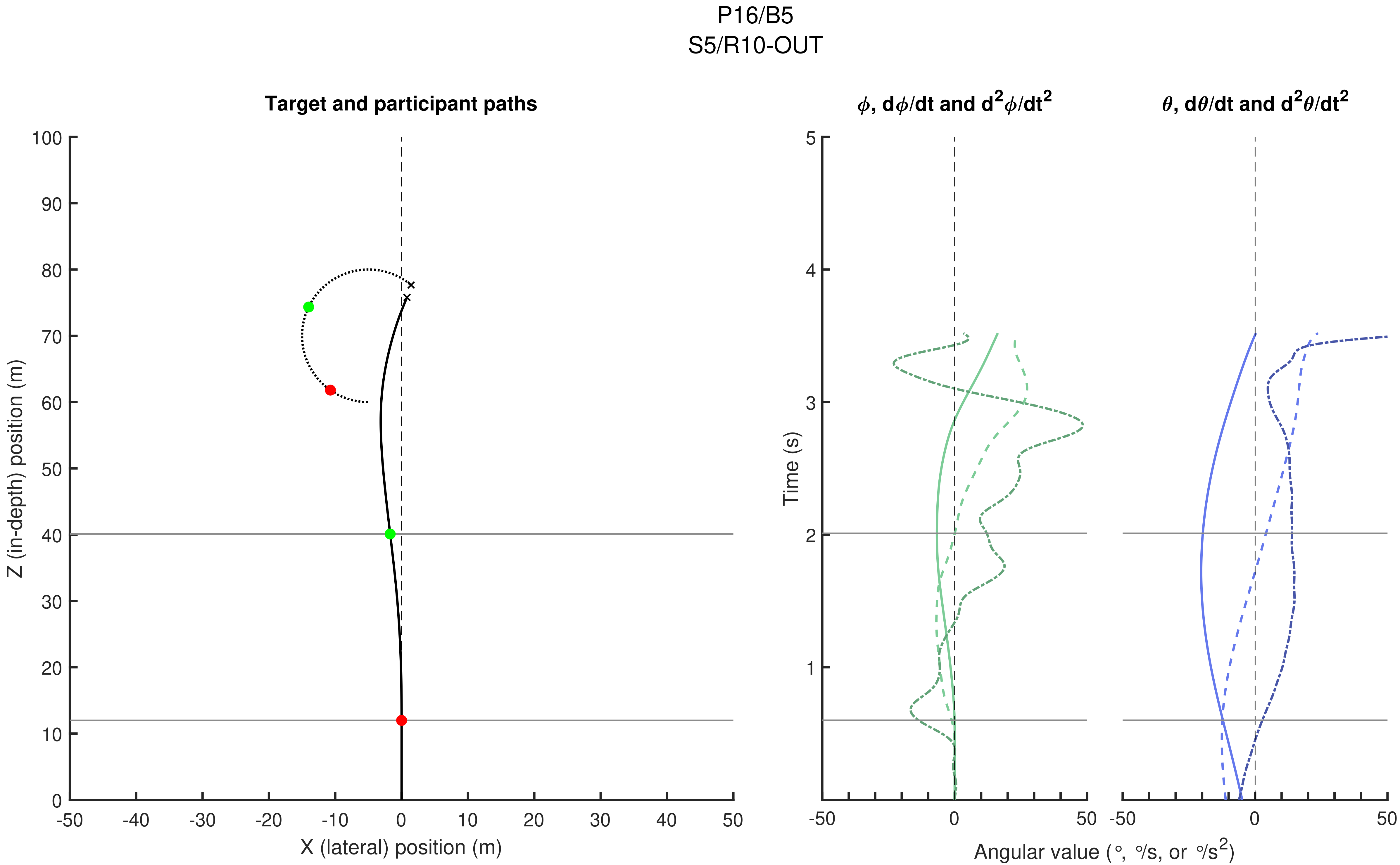

P16/B5
S5/R10-OUT
Target and participant paths
Z (in-depth) position (m)
X (lateral) position (m)
φ, dφ/dt and d²φ/dt²
θ, dθ/dt and d²θ/dt²
Time (s)
Angular value (°, °/s, or °/s²)

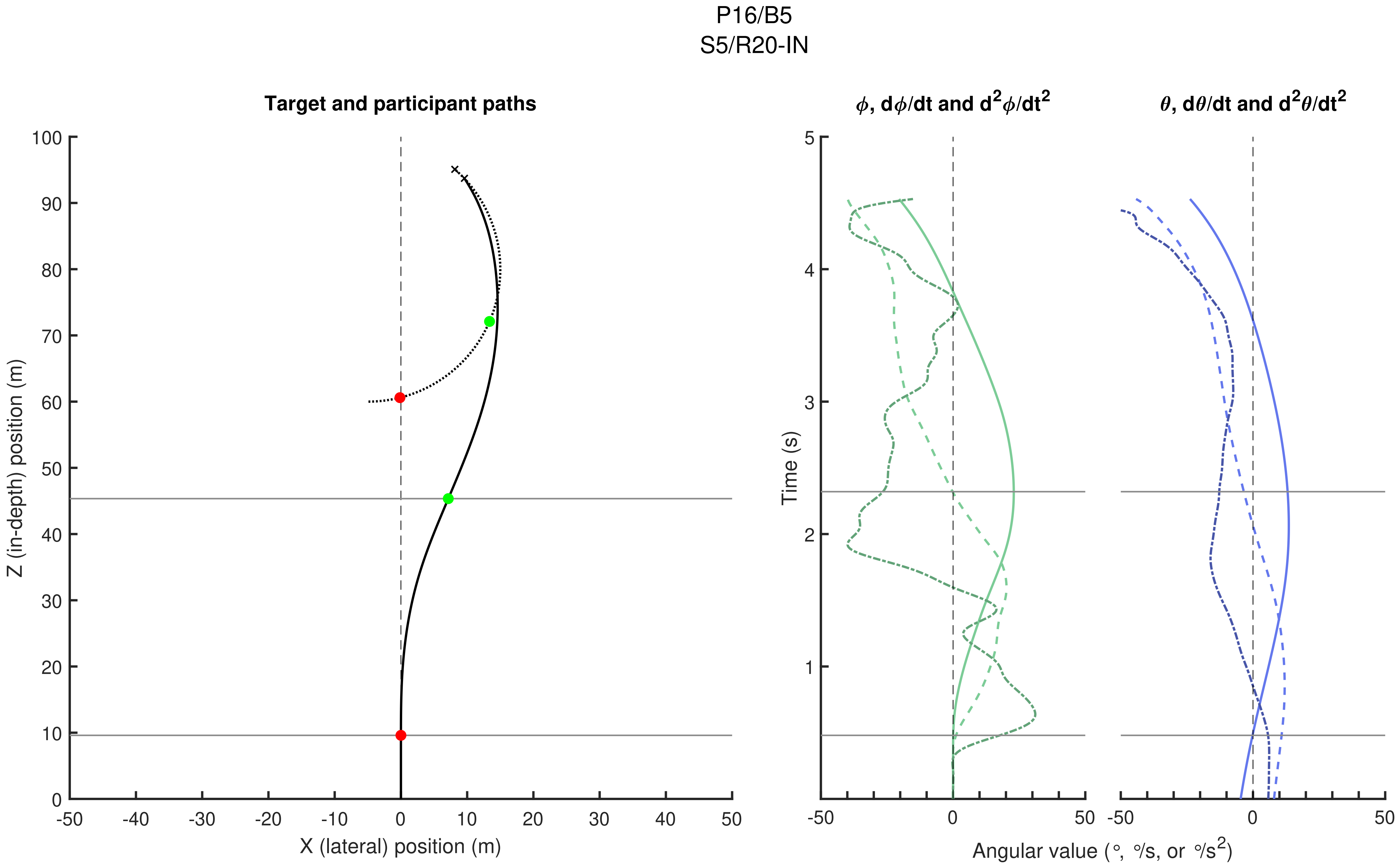

P16/B5
S5/R20-IN
Target and participant paths
Z (in-depth) position (m)
X (lateral) position (m)
φ, dφ/dt and d²φ/dt²
θ, dθ/dt and d²θ/dt²
Time (s)
Angular value (°, °/s, or °/s²)

P16/B5
S5/R20-OUT

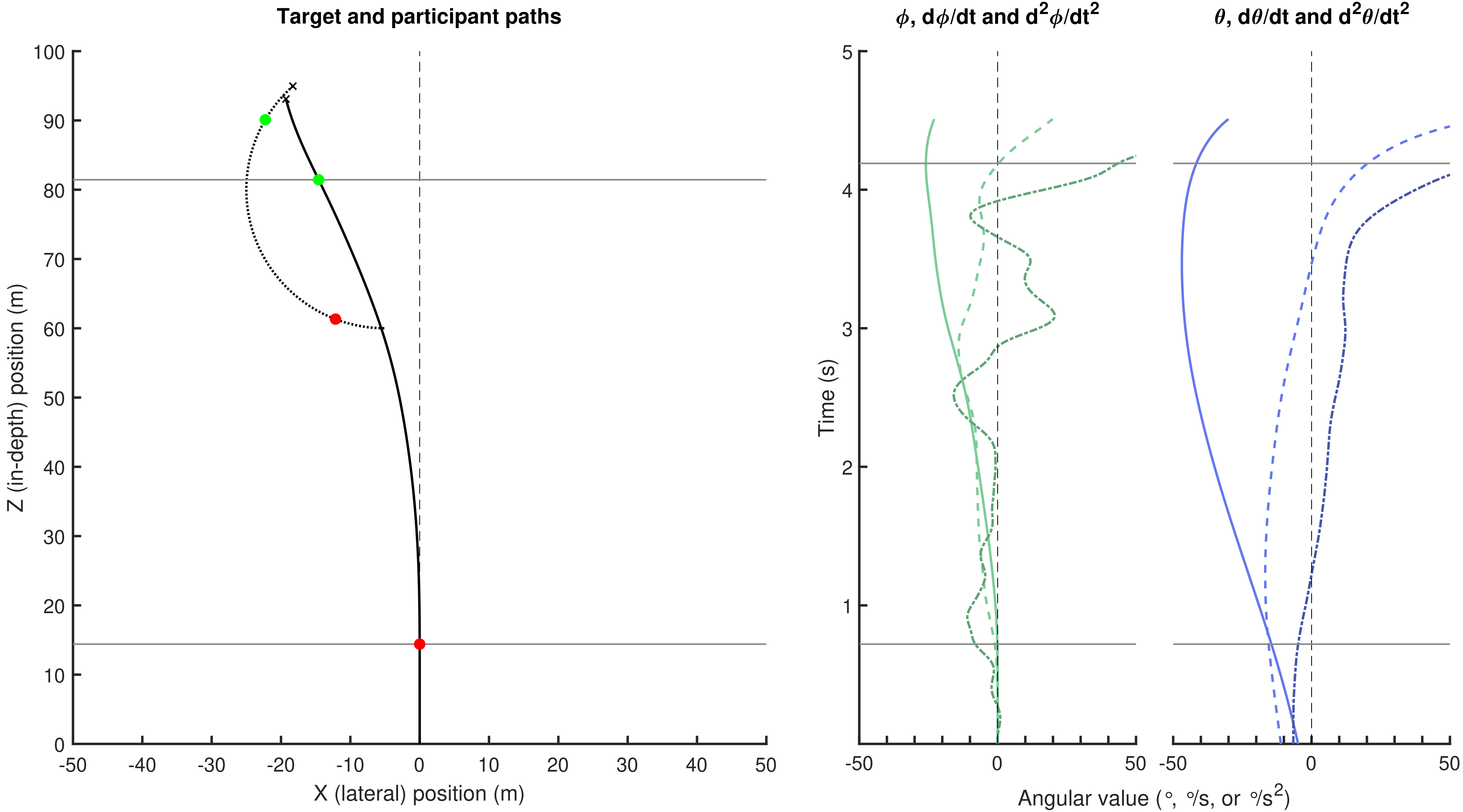

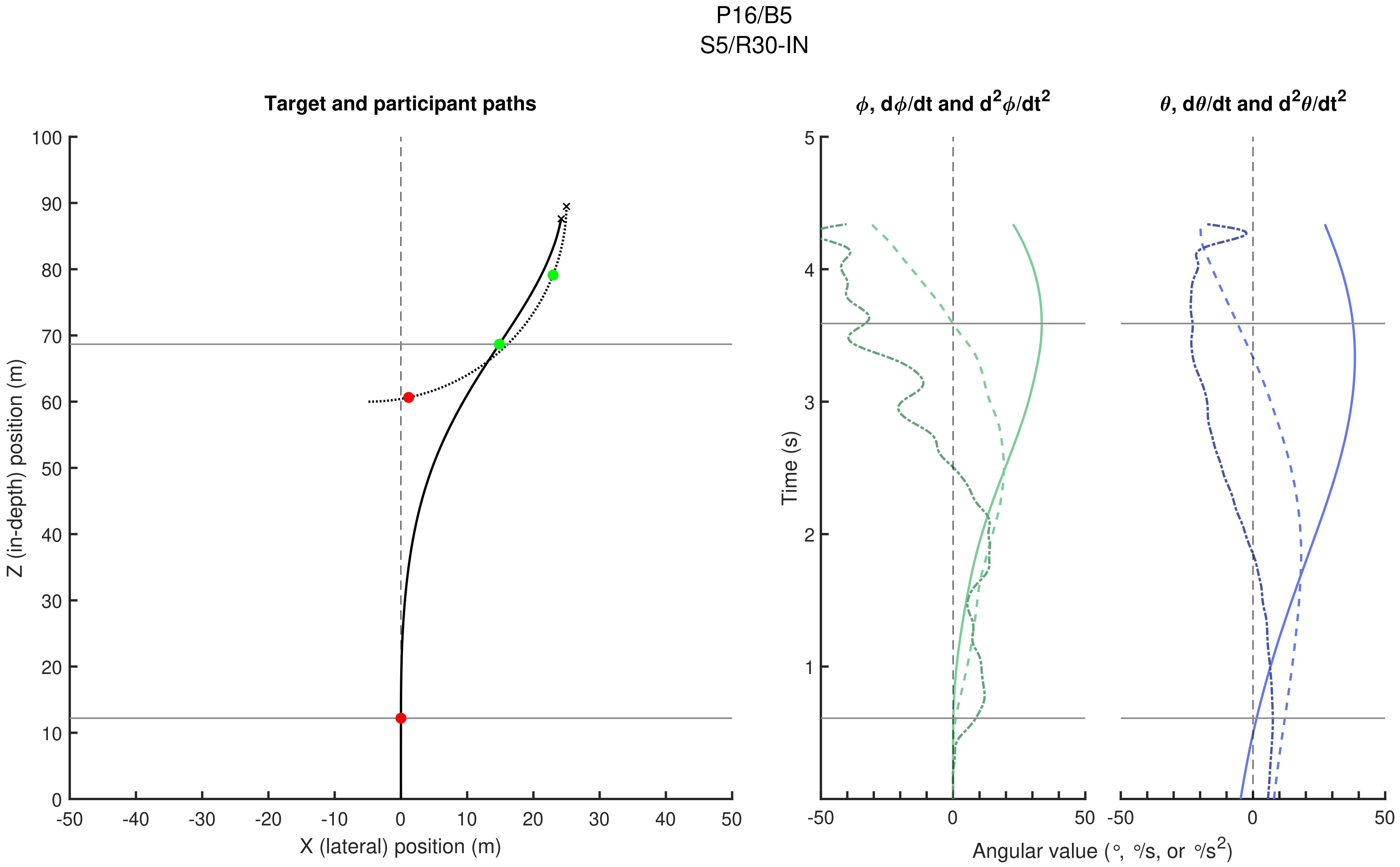

P16/B5
S5/R30-IN
Target and participant paths
Z (in-depth) position (m)
X (lateral) position (m)
ϕ, dϕ/dt and d$^2\phi$/dt^2
θ, dθ/dt and d$^2\theta$/dt^2
Time (s)
Angular value (°, °/s, or °/s^2)

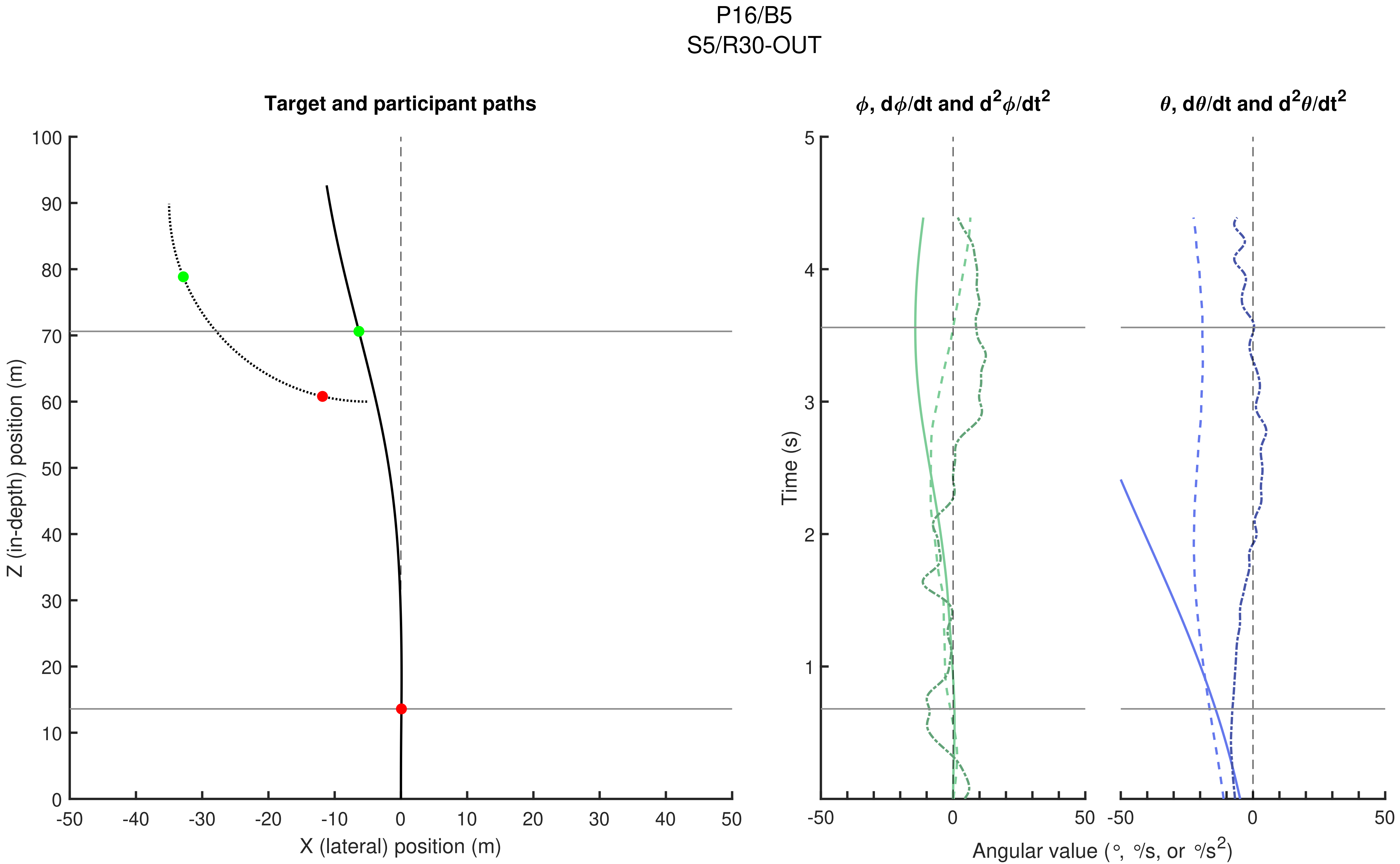
P16/B5
S5/R30-OUT
Target and participant paths
Z (in-depth) position (m)
X (lateral) position (m)
ϕ, dϕ/dt and d$^2\phi$/dt^2
θ, dθ/dt and d$^2\theta$/dt^2
Time (s)
Angular value (°, °/s, or °/s^2)

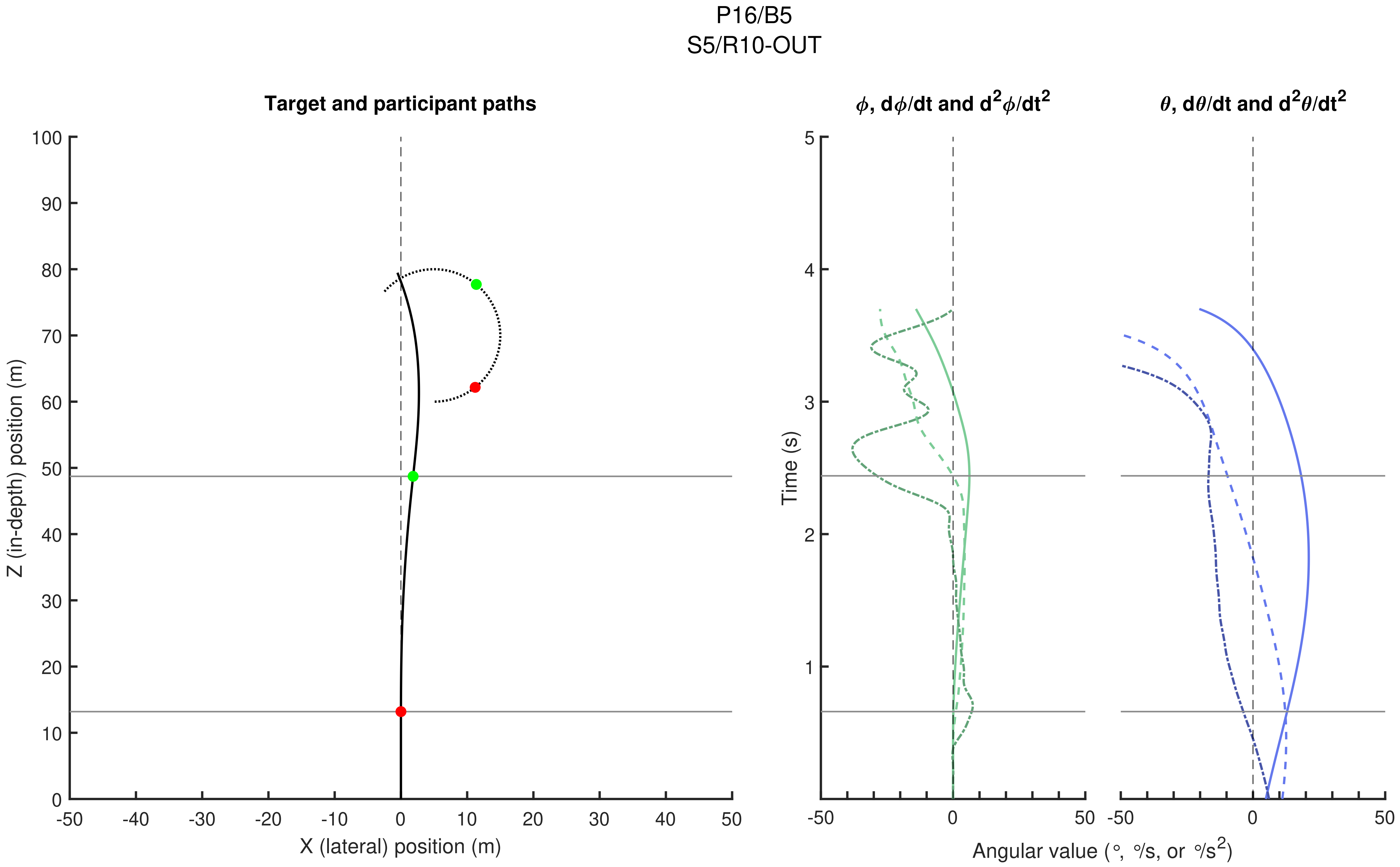

P16/B5
S5/R10-OUT
Target and participant paths
ϕ, dϕ/dt and d$^2\phi$/dt^2
θ, dθ/dt and d$^2\theta$/dt^2
Z (in-depth) position (m)
X (lateral) position (m)
Time (s)
Angular value (°, °/s, or °/s^2)

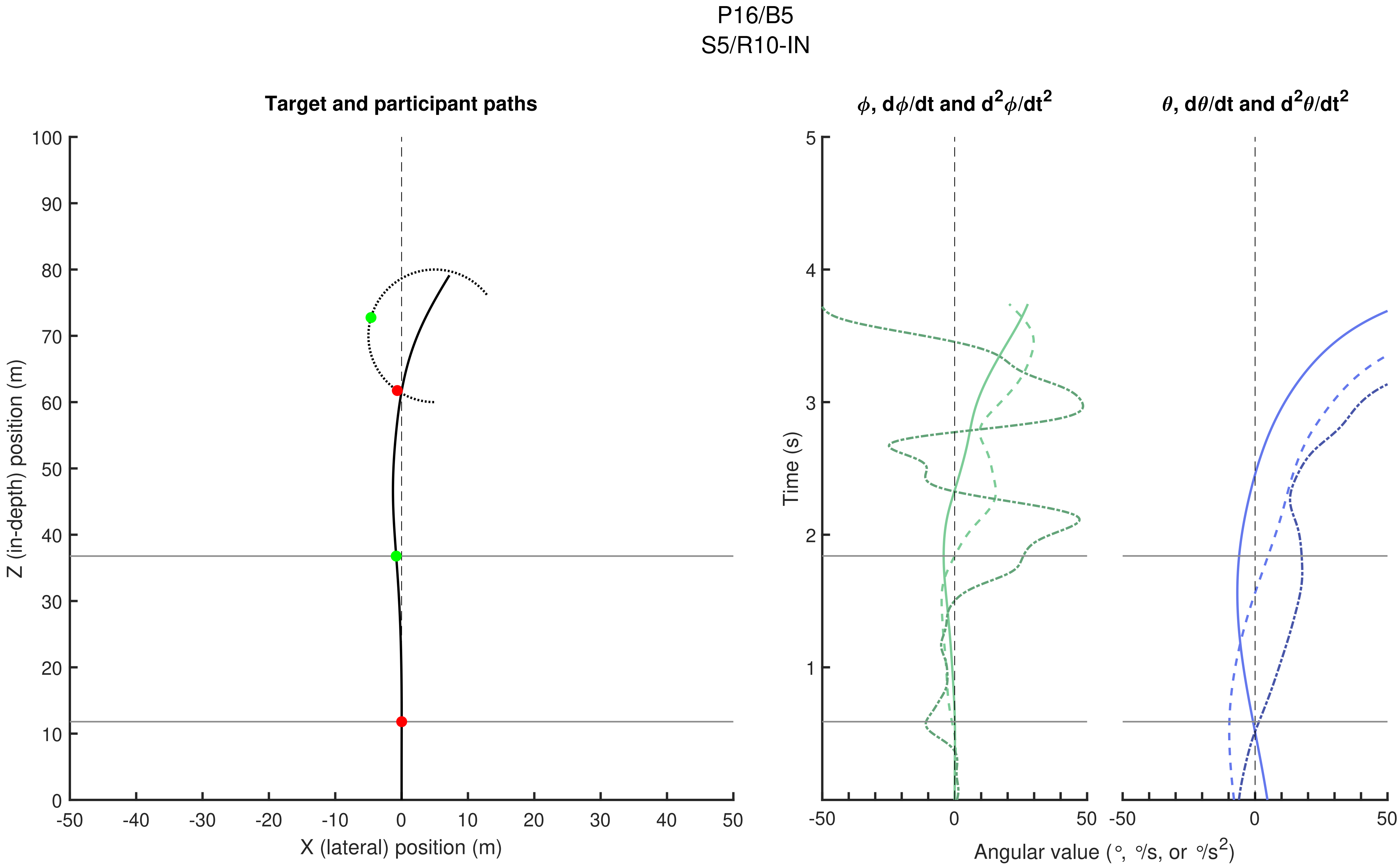

P16/B5
S5/R10-IN
Target and participant paths
Z (in-depth) position (m)
X (lateral) position (m)
ϕ, dϕ/dt and d$^2\phi$/dt^2
θ, dθ/dt and d$^2\theta$/dt^2
Time (s)
Angular value (°, °/s, or °/s^2)

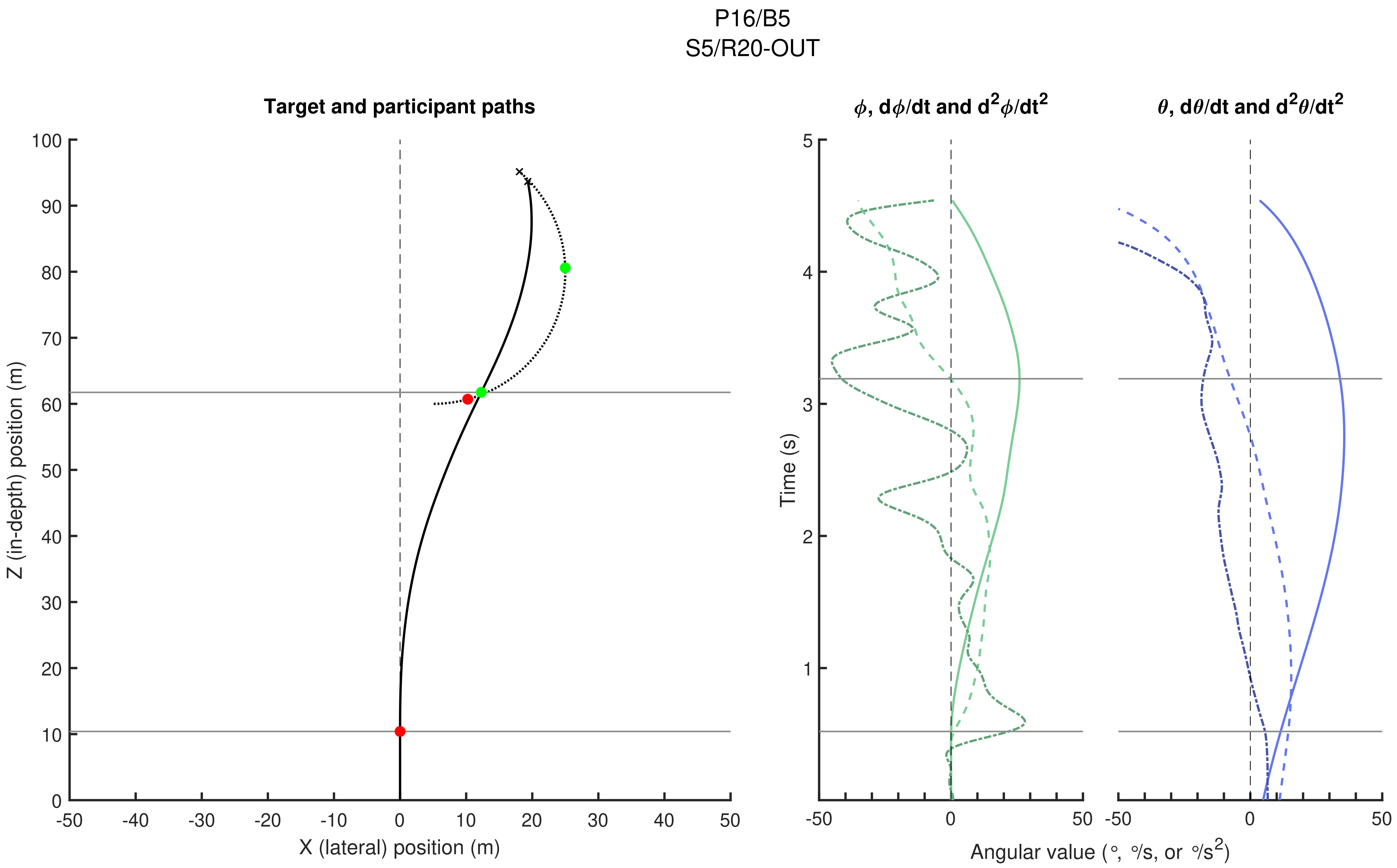

P16/B5
S5/R20-OUT
Target and participant paths
Z (in-depth) position (m)
X (lateral) position (m)
ϕ, dϕ/dt and d$^2\phi$/dt^2
θ, dθ/dt and d$^2\theta$/dt^2
Time (s)
Angular value (°, °/s, or °/s^2)

P16/B5
S5/R20-IN

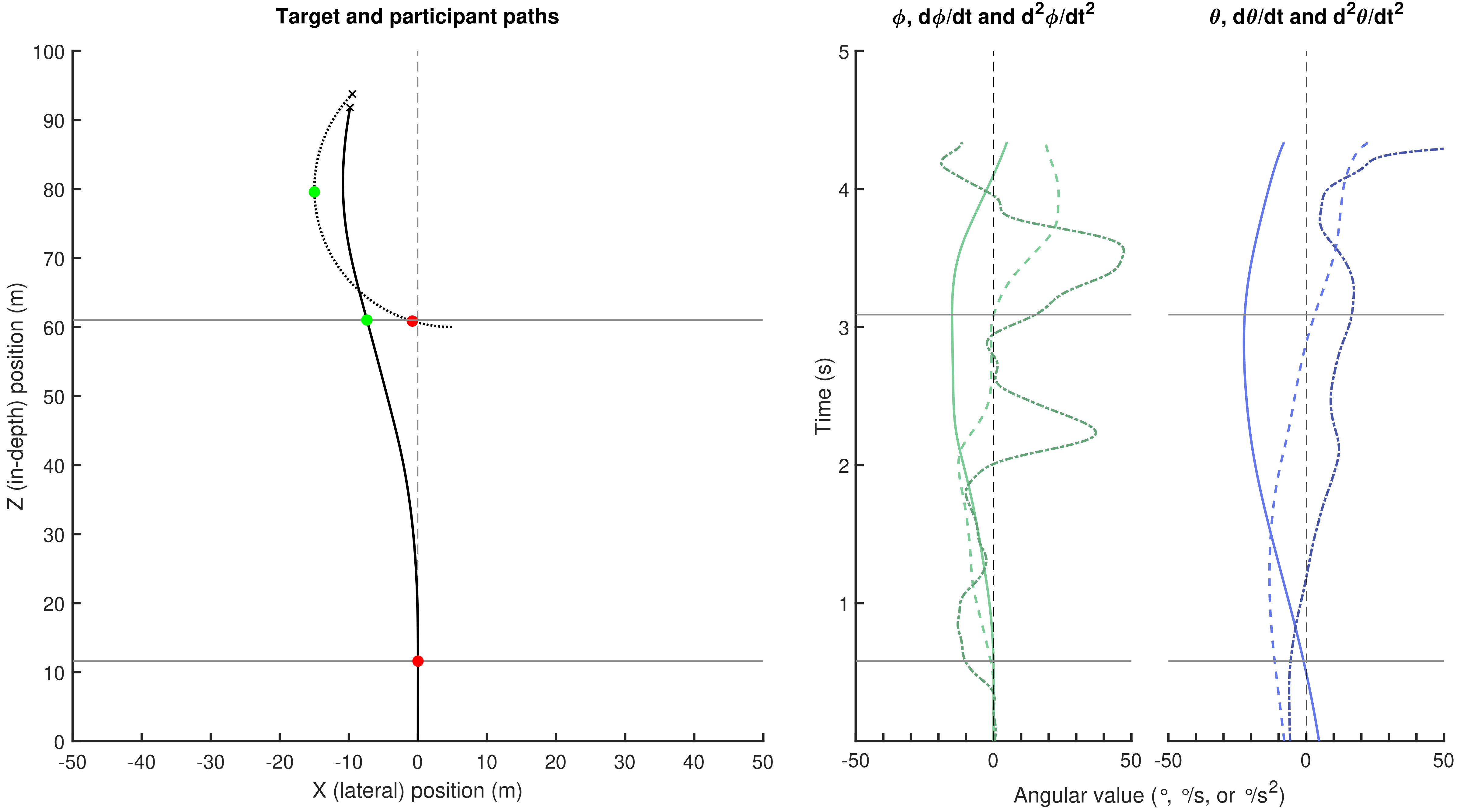

P16/B5
S5/R30-OUT

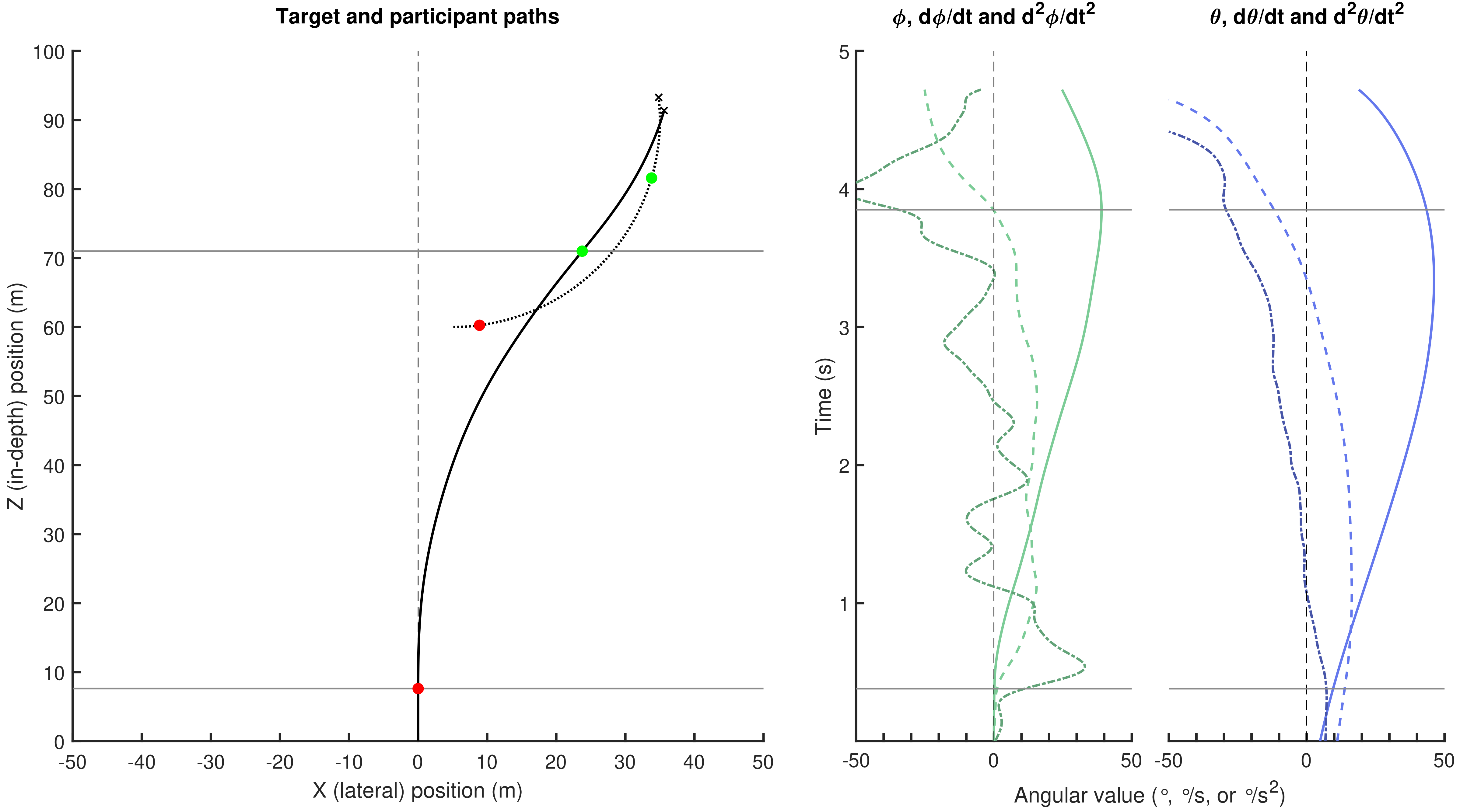

P16/B5
S5/R30-IN

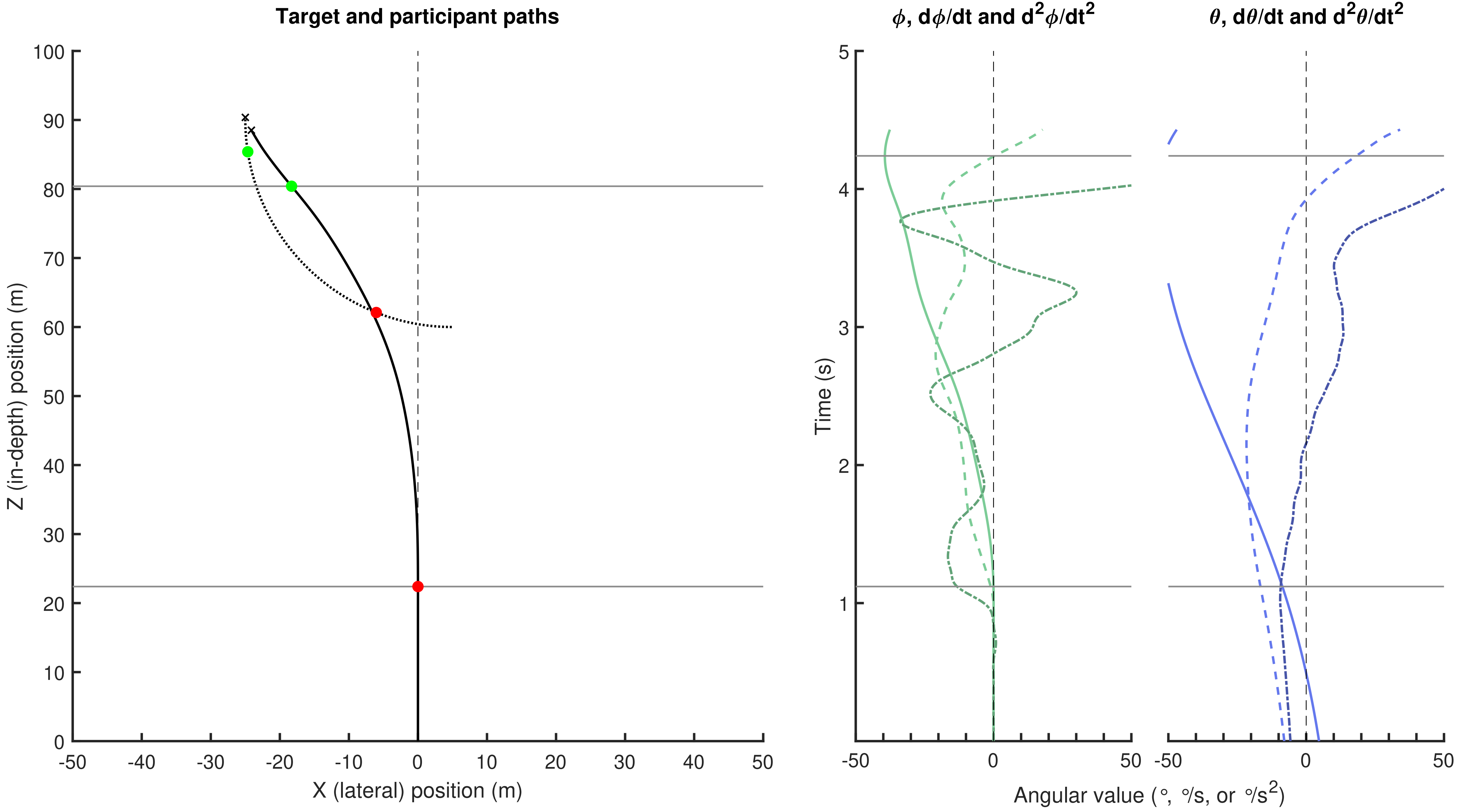

P16/B5
S15/R10-IN

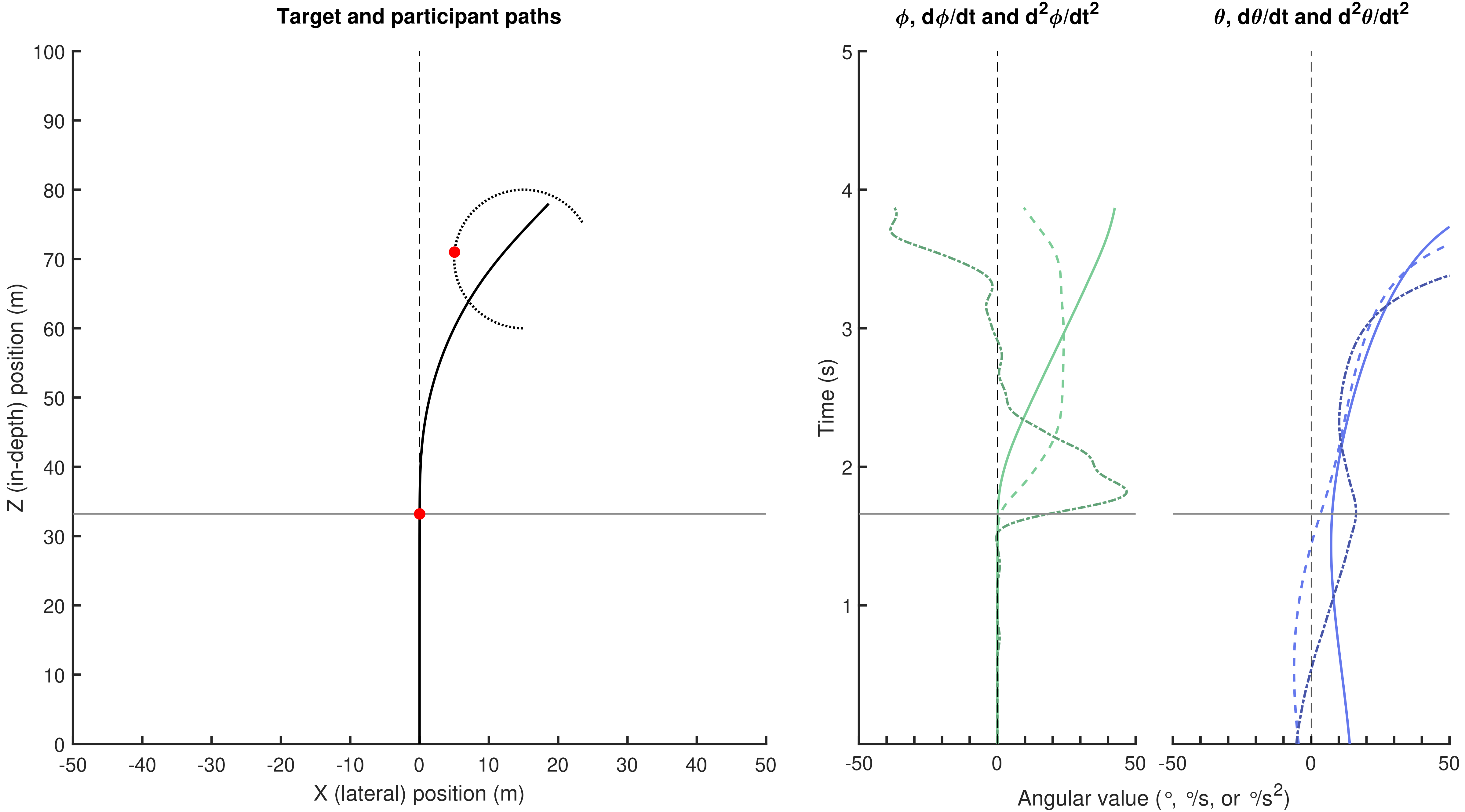

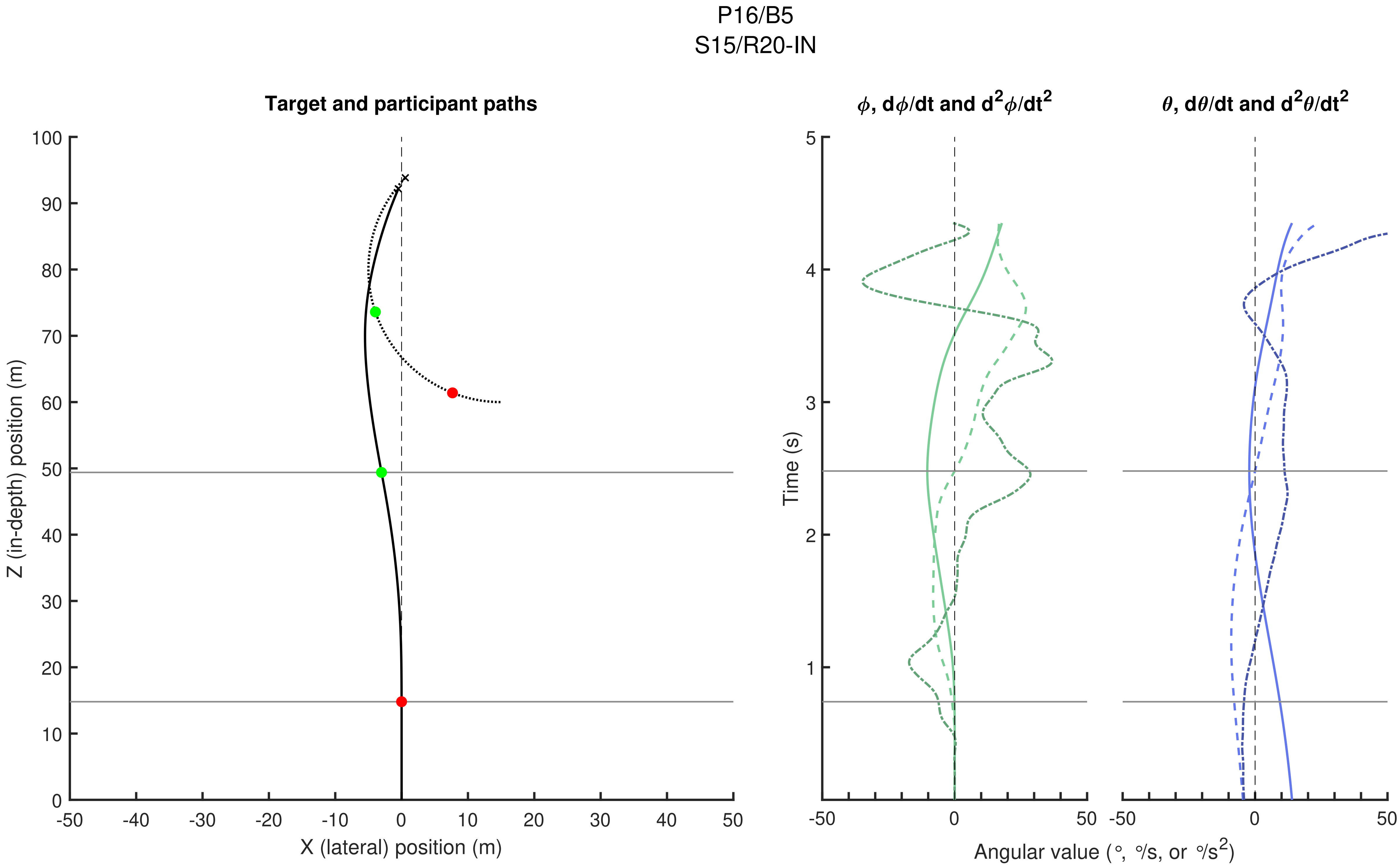
P16/B5
S15/R20-IN
Target and participant paths
Z (in-depth) position (m)
X (lateral) position (m)
ϕ, dϕ/dt and d²ϕ/dt²
θ, dθ/dt and d²θ/dt²
Time (s)
Angular value (°, °/s, or °/s²)

P16/B5
S15/R30-IN

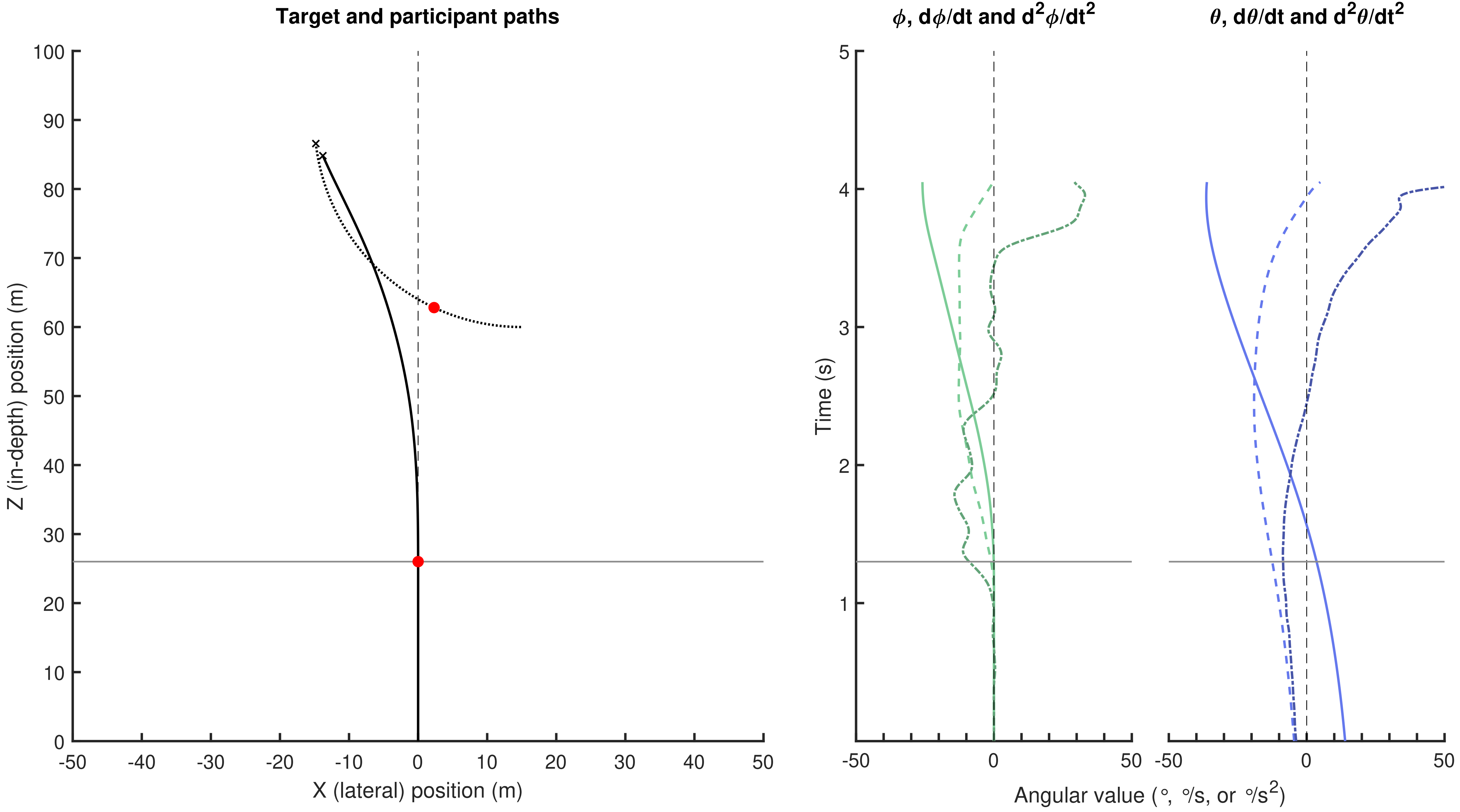

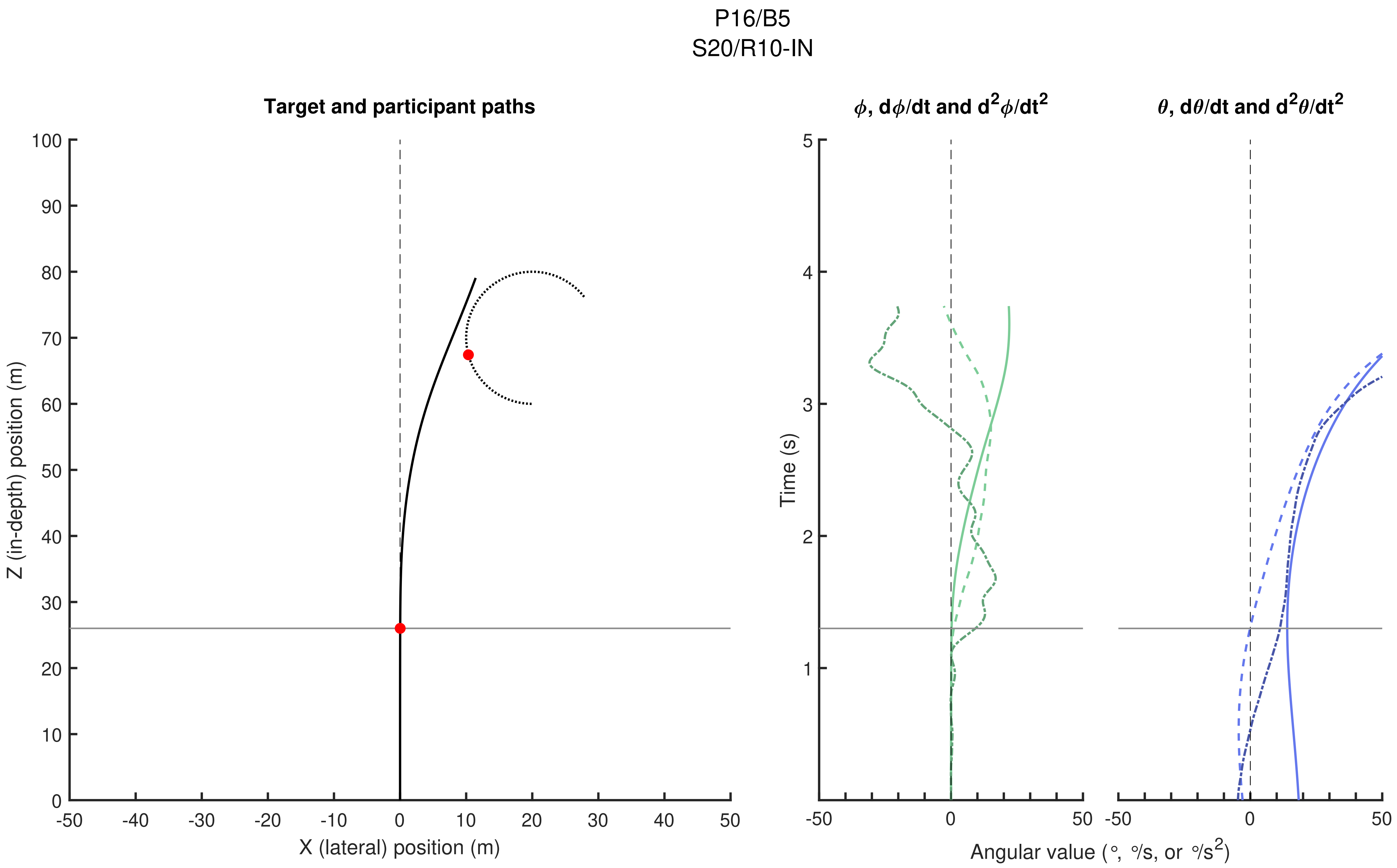
P16/B5
S20/R10-IN
Target and participant paths
Z (in-depth) position (m)
X (lateral) position (m)
ϕ, dϕ/dt and d$^2\phi$/dt^2
θ, dθ/dt and d$^2\theta$/dt^2
Time (s)
Angular value (°, °/s, or °/s^2)

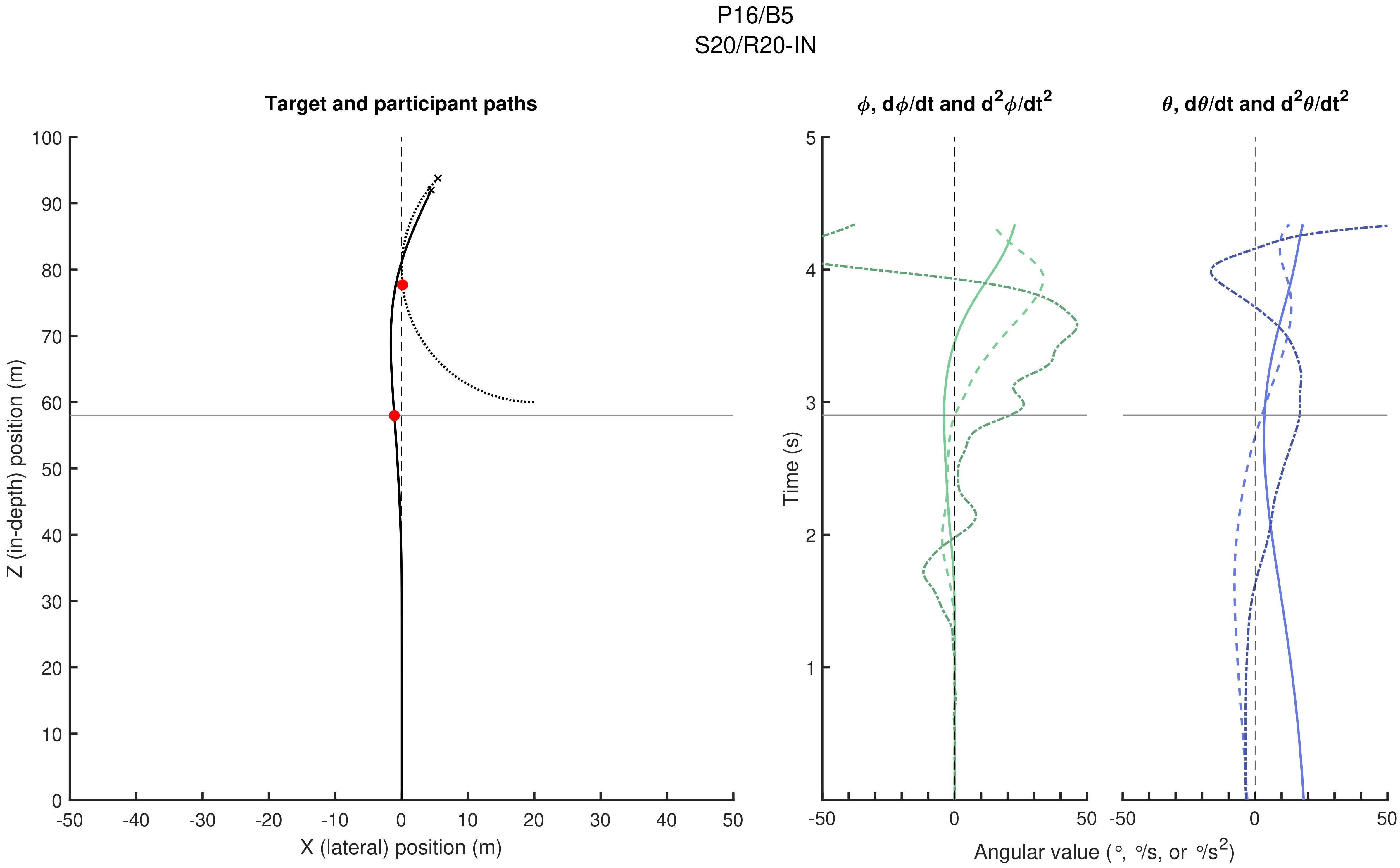
P16/B5
S20/R20-IN
Target and participant paths
Z (in-depth) position (m)
X (lateral) position (m)
100
90
80
70
60
50
40
30
20
10
0
-50
-40
-30
-20
-10
0
10
20
30
40
50
φ, dφ/dt and d²φ/dt²
θ, dθ/dt and d²θ/dt²
Time (s)
5
4
3
2
1
-50
0
50
-50
0
50
Angular value (°, °/s, or °/s²)

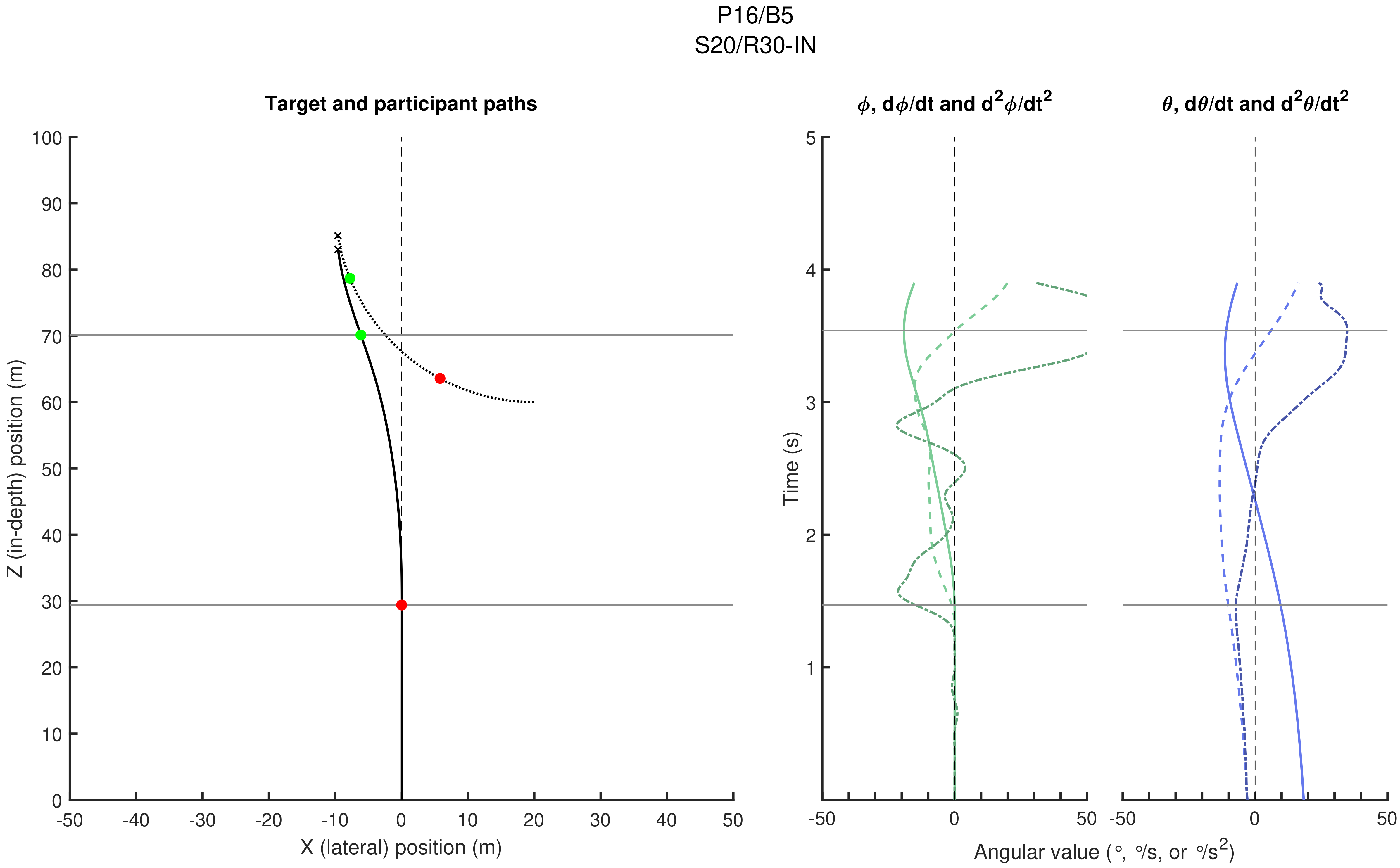

P16/B5
S20/R30-IN
Target and participant paths
Z (in-depth) position (m)
X (lateral) position (m)
ϕ, dϕ/dt and d$^2\phi$/dt^2
θ, dθ/dt and d$^2\theta$/dt^2
Time (s)
Angular value (°, °/s, or °/s^2)

P17/B1
S20/R10-IN

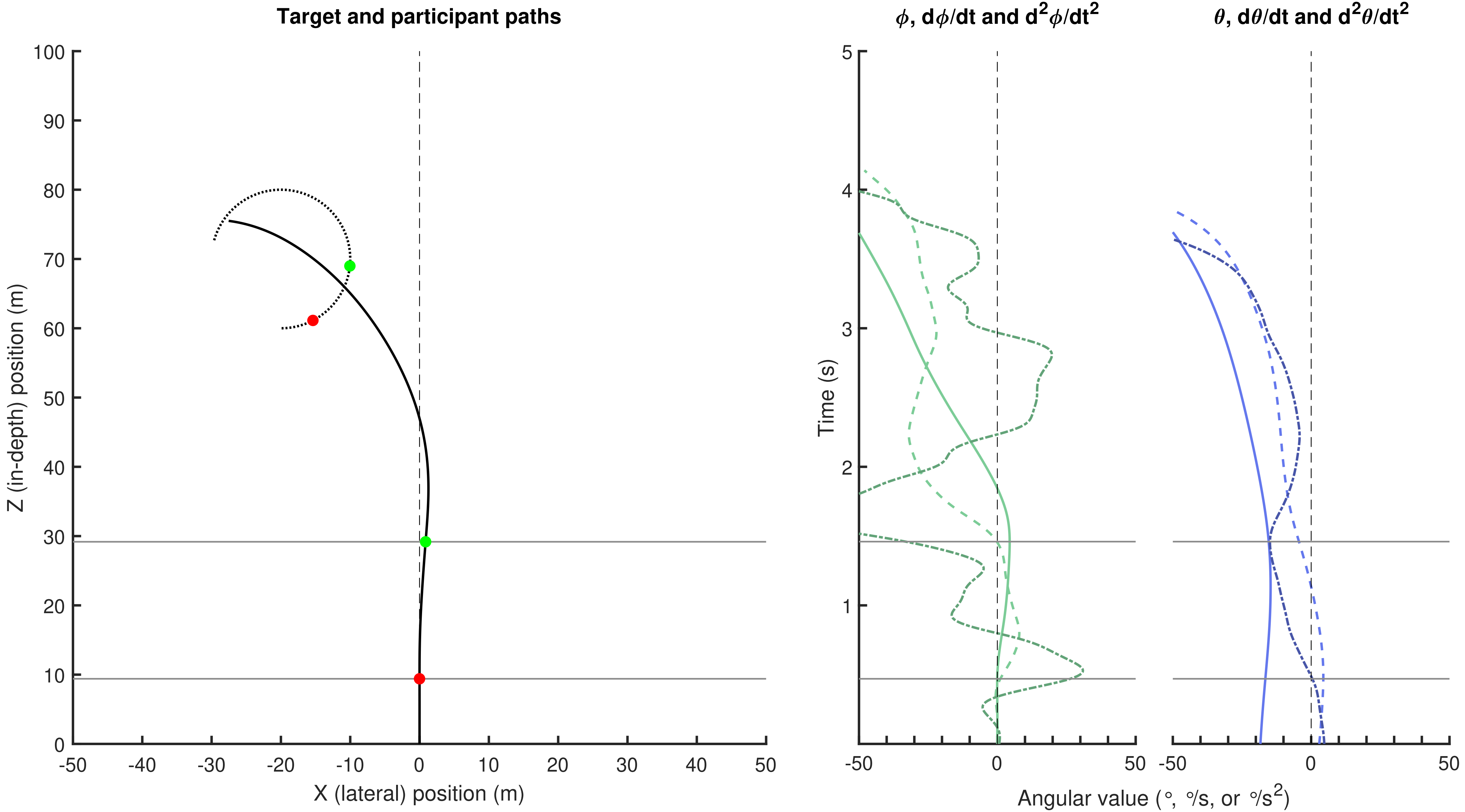

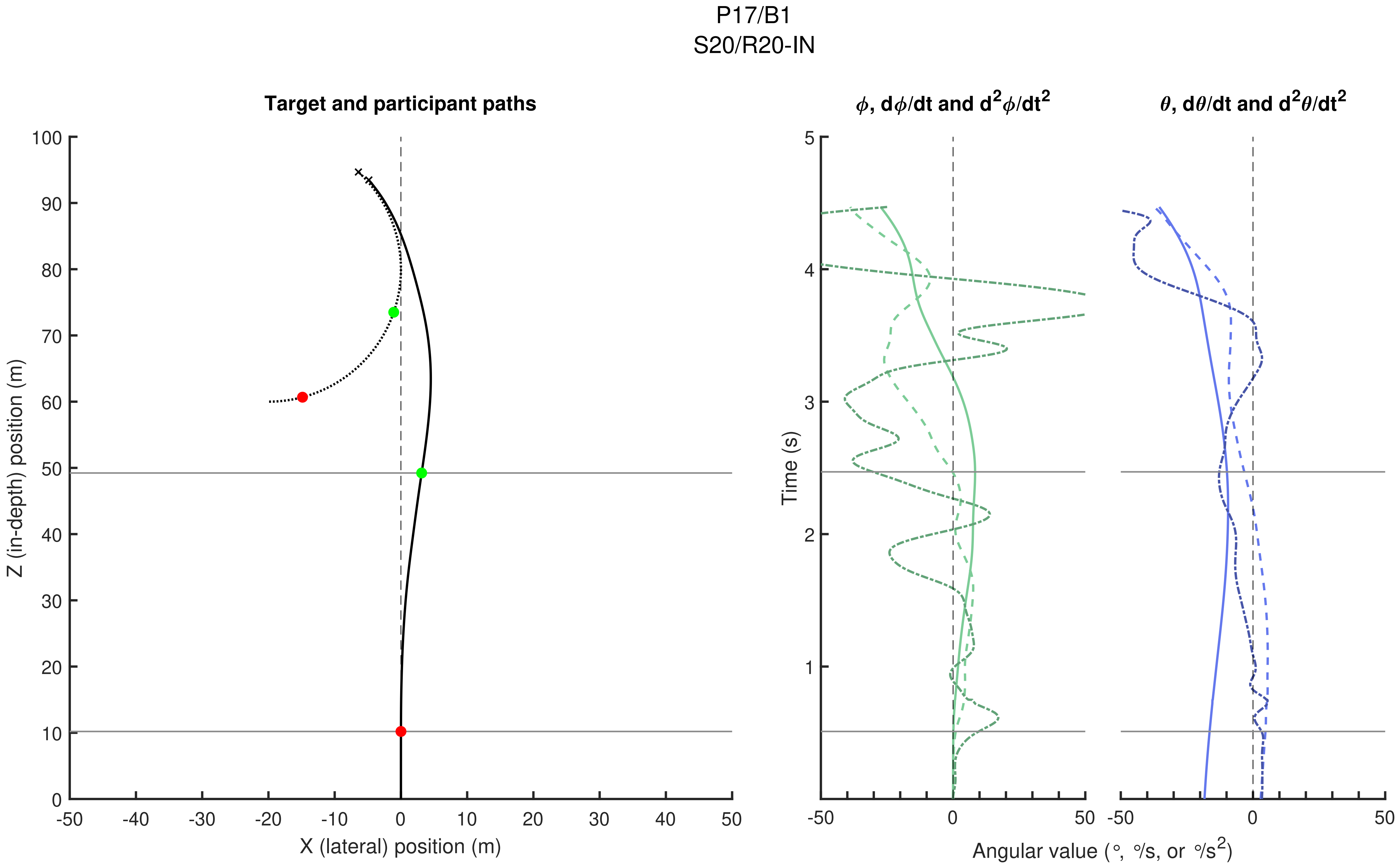
P17/B1
S20/R20-IN
Target and participant paths
ϕ, dϕ/dt and d²ϕ/dt²
θ, dθ/dt and d²θ/dt²
Z (in-depth) position (m)
X (lateral) position (m)
Time (s)
Angular value (°, °/s, or °/s²)
100
90
80
70
60
50
40
30
20
10
0
-50
-40
-30
-20
-10
0
10
20
30
40
50
5
4
3
2
1

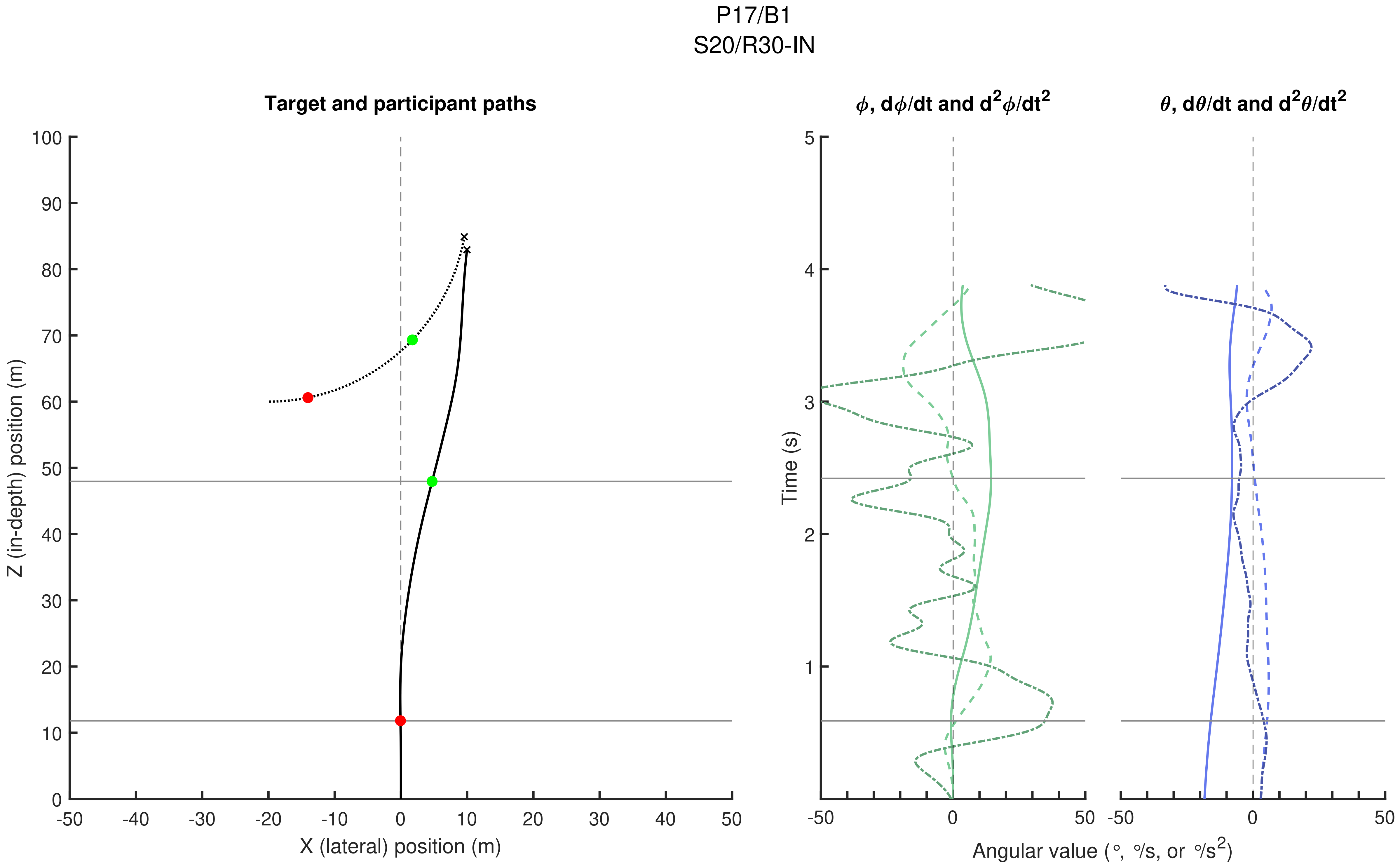

P17/B1
S20/R30-IN
Target and participant paths
Z (in-depth) position (m)
X (lateral) position (m)
ϕ, dϕ/dt and d²ϕ/dt²
θ, dθ/dt and d²θ/dt²
Time (s)
Angular value (°, °/s, or °/s²)

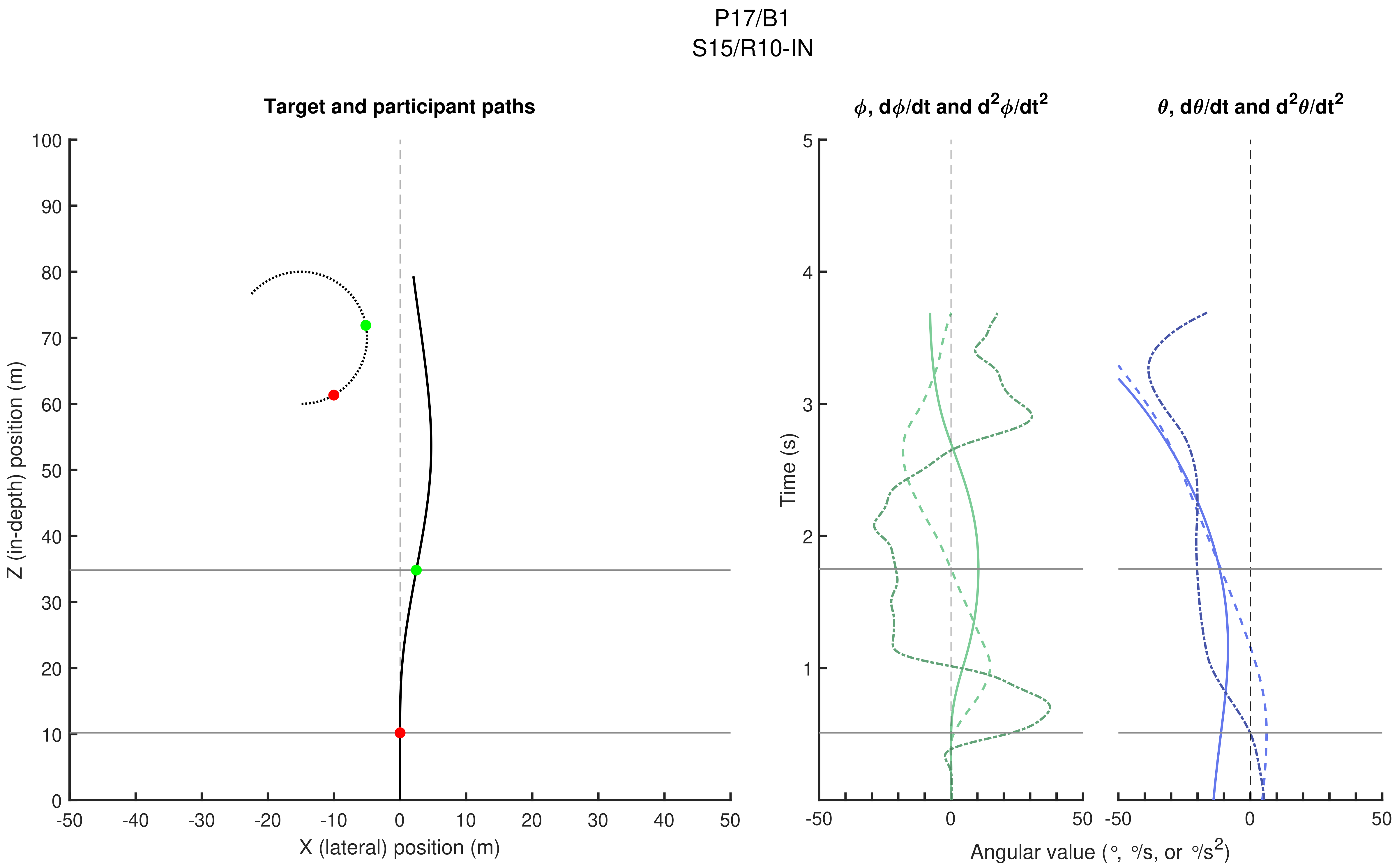
P17/B1
S15/R10-IN
Target and participant paths
Z (in-depth) position (m)
X (lateral) position (m)
ϕ, dϕ/dt and d$^2\phi$/dt^2
θ, dθ/dt and d$^2\theta$/dt^2
Time (s)
Angular value (°, °/s, or °/s^2)

P17/B1
S15/R20-IN

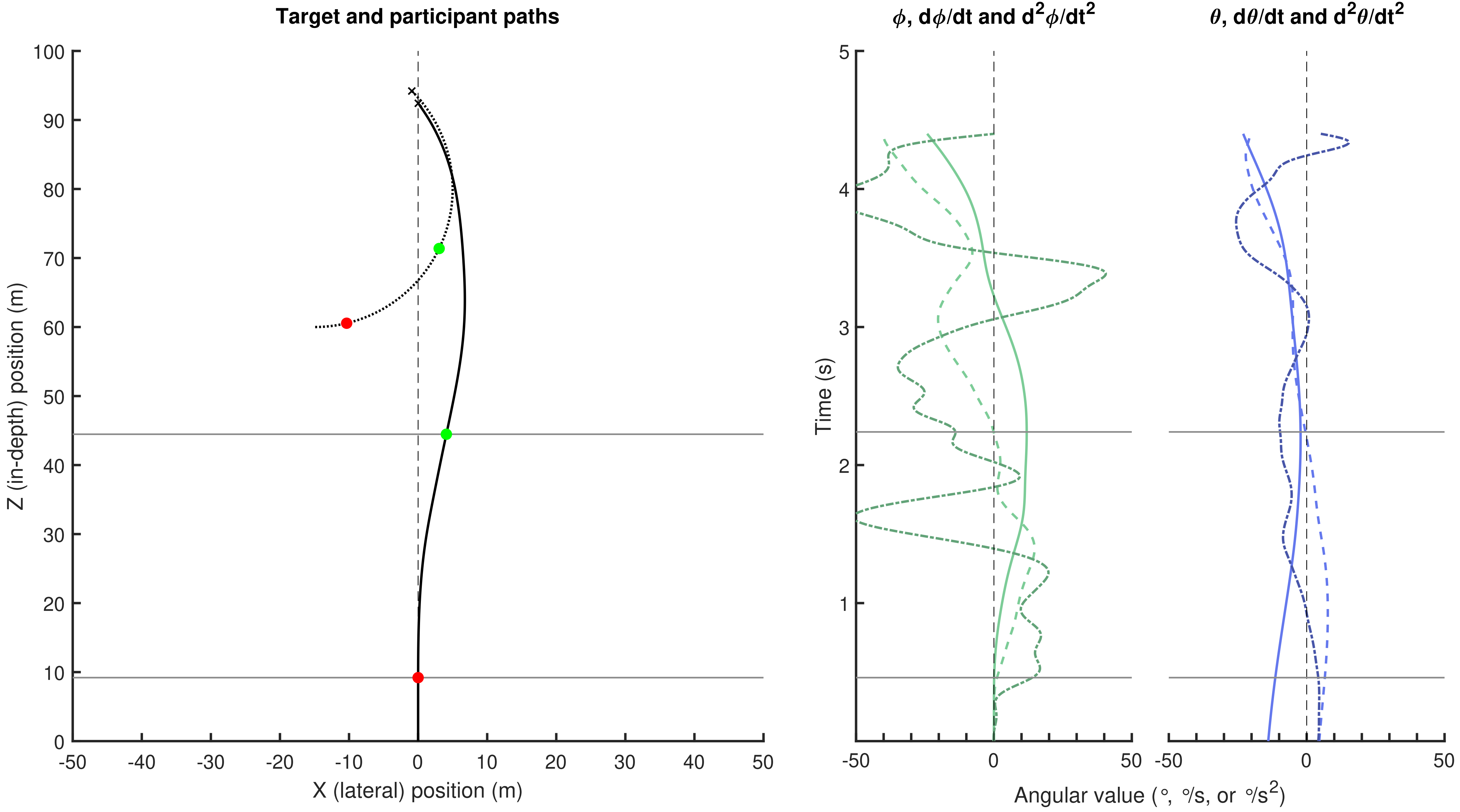

P17/B1
S15/R30-IN

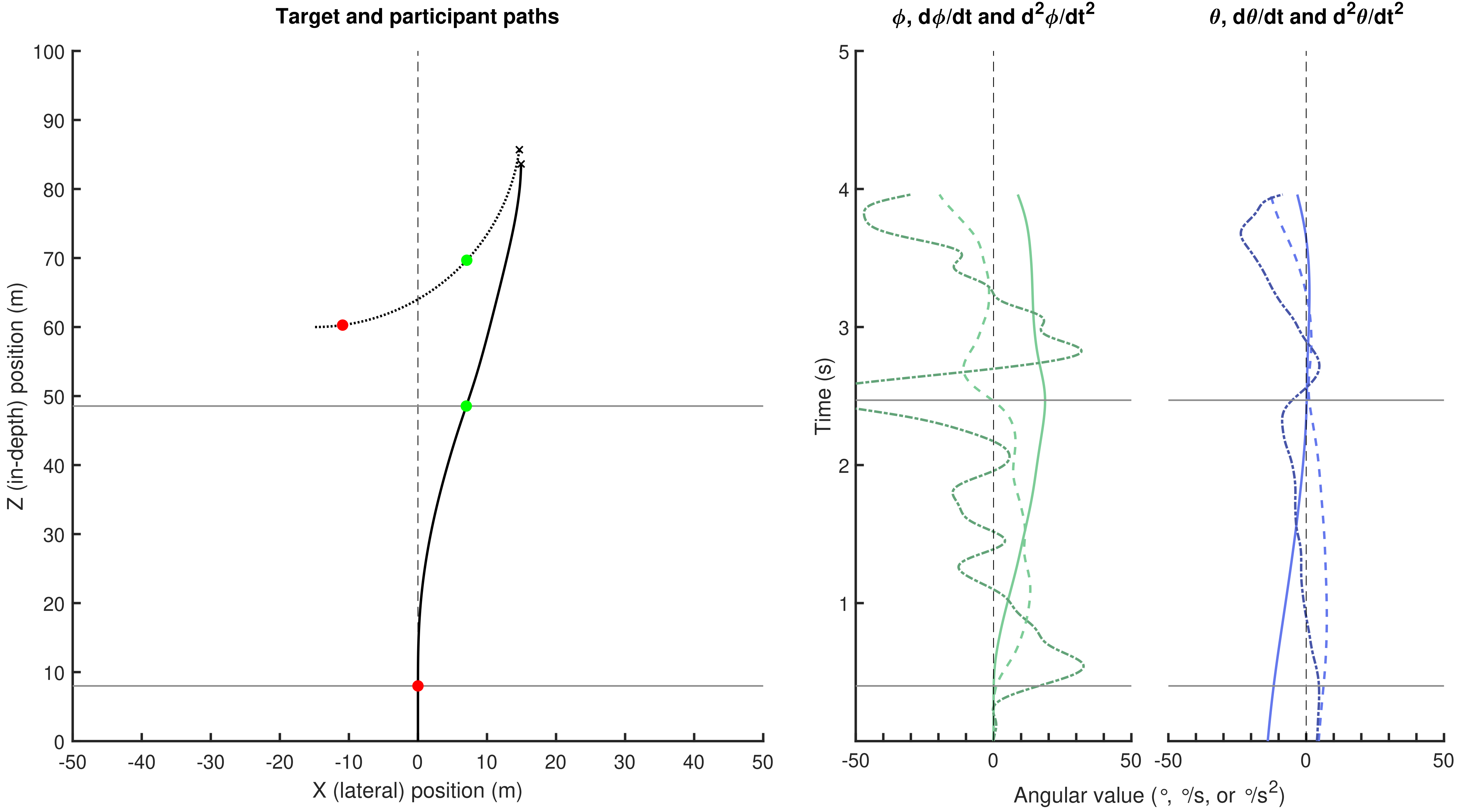

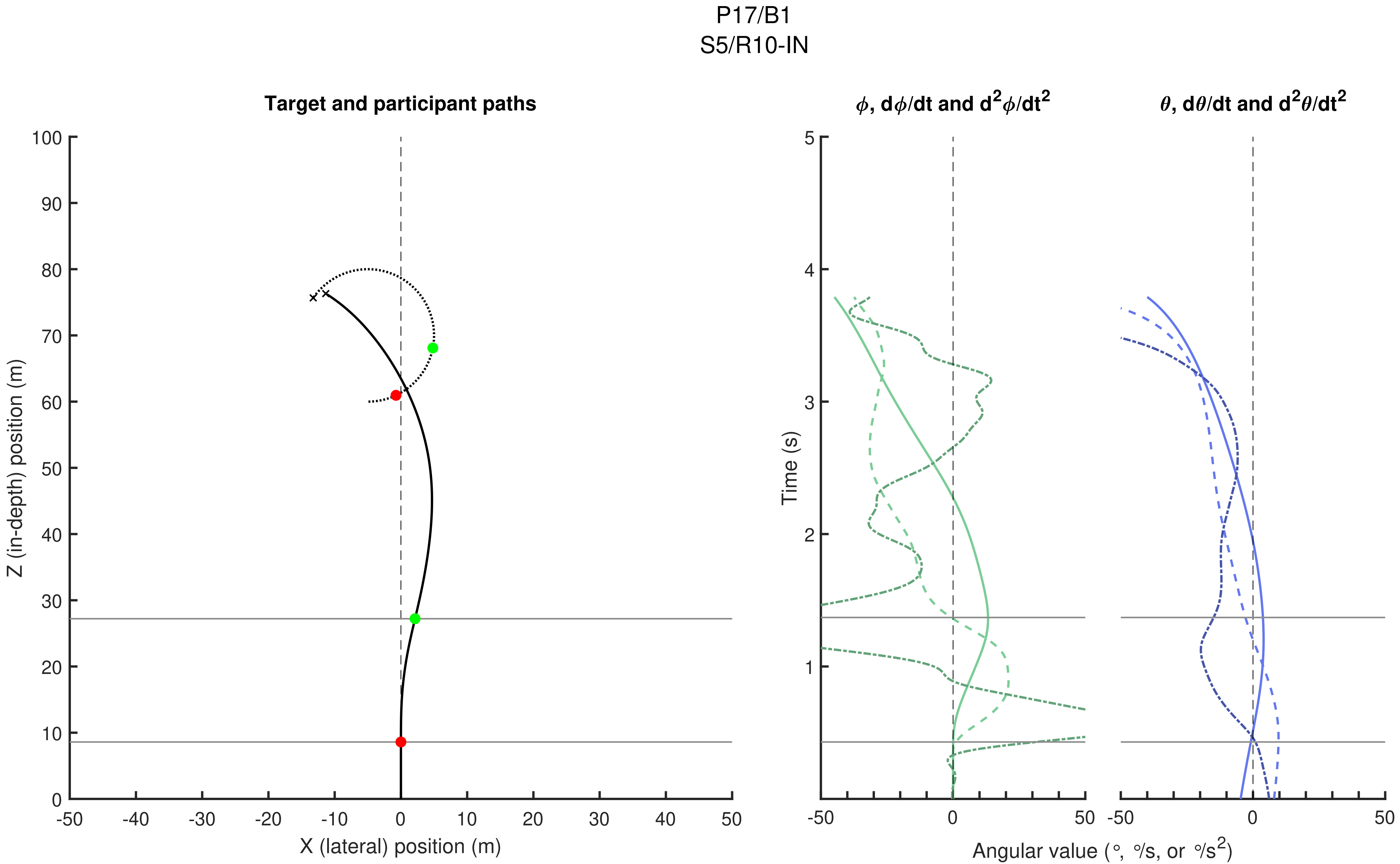
P17/B1
S5/R10-IN
Target and participant paths
ϕ, dϕ/dt and d$^2\phi$/dt^2
θ, dθ/dt and d$^2\theta$/dt^2
Z (in-depth) position (m)
X (lateral) position (m)
Time (s)
Angular value (°, °/s, or °/s^2)
100
90
80
70
60
50
40
30
20
10
0
-50
-40
-30
-20
-10
0
10
20
30
40
50
5
4
3
2
1
-50
0
50
-50
0
50

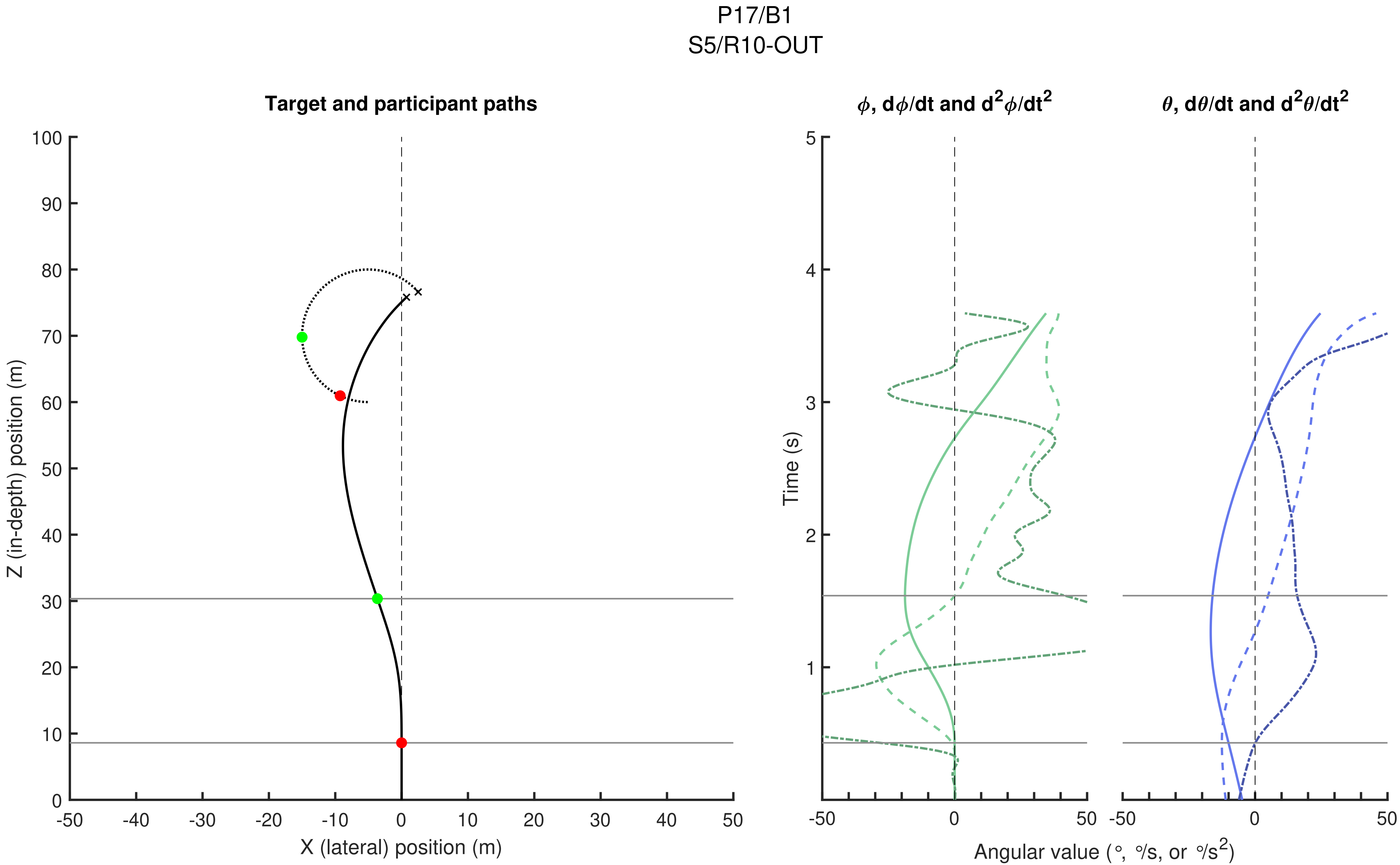
P17/B1
S5/R10-OUT
Target and participant paths
Z (in-depth) position (m)
X (lateral) position (m)
ϕ, dϕ/dt and d²ϕ/dt²
θ, dθ/dt and d²θ/dt²
Time (s)
Angular value (°, °/s, or °/s²)

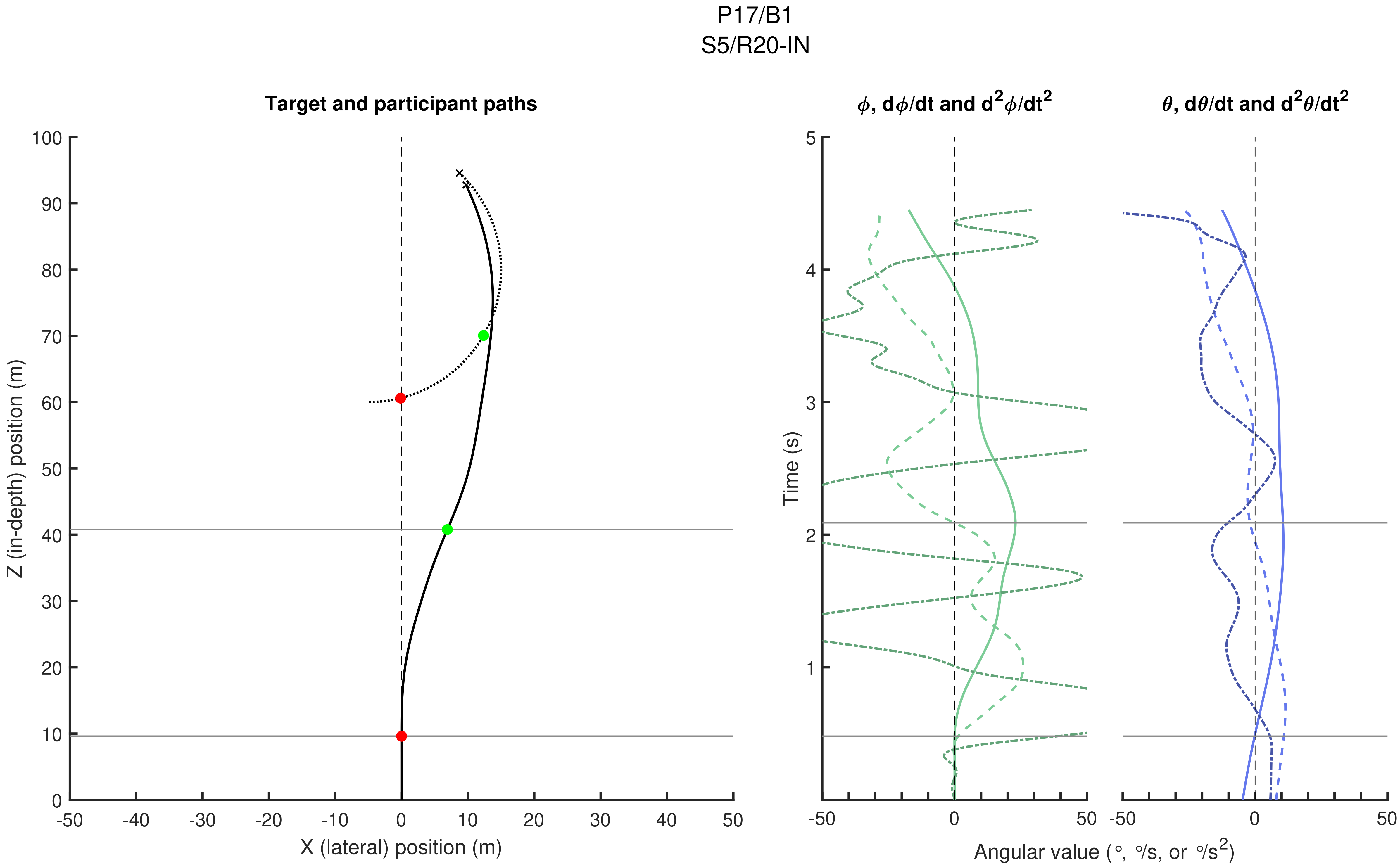

P17/B1
S5/R20-IN
Target and participant paths
Z (in-depth) position (m)
X (lateral) position (m)
ϕ, dϕ/dt and d²ϕ/dt²
θ, dθ/dt and d²θ/dt²
Time (s)
Angular value (°, °/s, or °/s²)

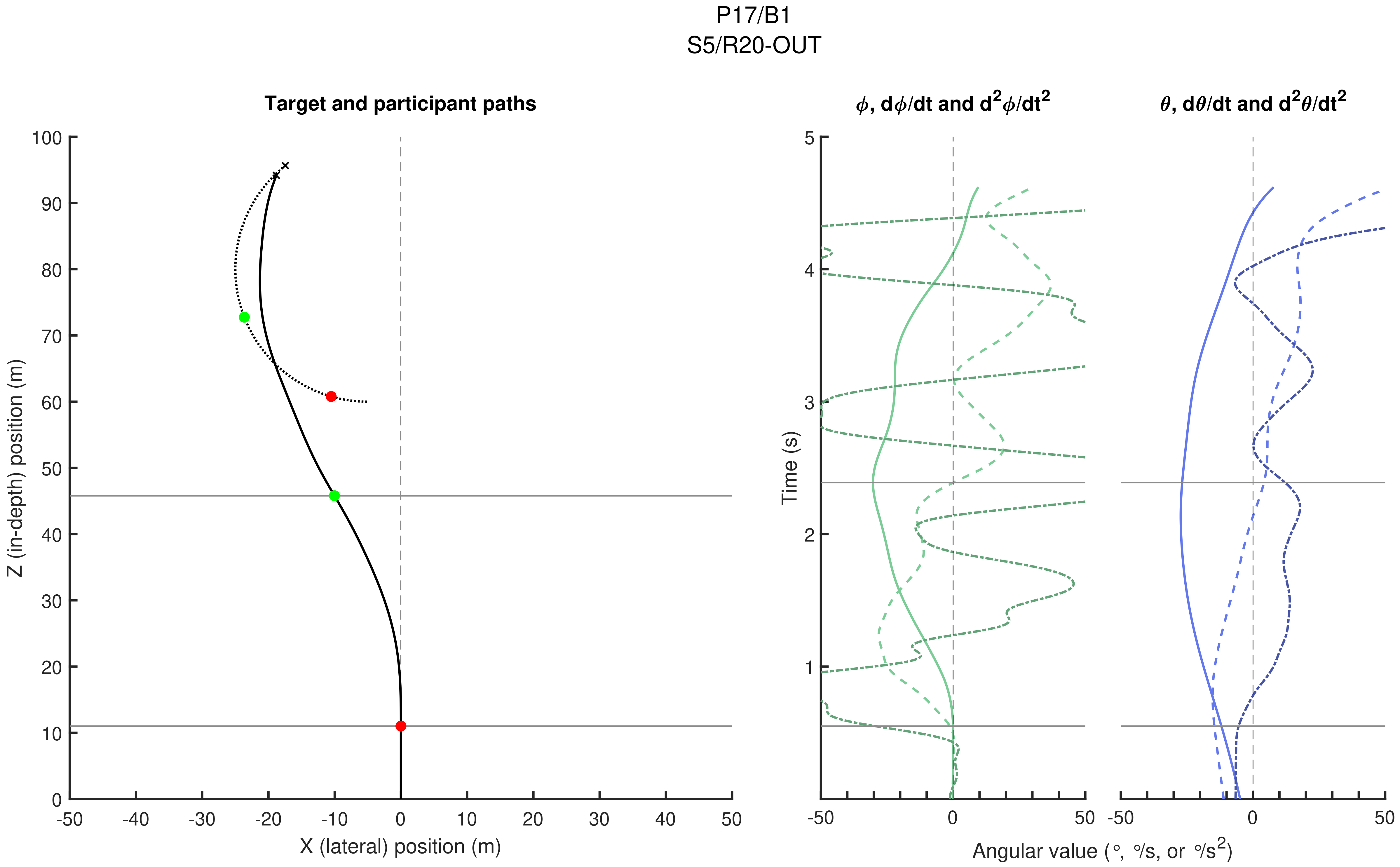

P17/B1
S5/R20-OUT
Target and participant paths
Z (in-depth) position (m)
X (lateral) position (m)
100
90
80
70
60
50
40
30
20
10
0
-50
-40
-30
-20
-10
0
10
20
30
40
50
ϕ, dϕ/dt and d²ϕ/dt²
θ, dθ/dt and d²θ/dt²
Time (s)
5
4
3
2
1
-50
0
50
-50
0
50
Angular value (°, °/s, or °/s²)

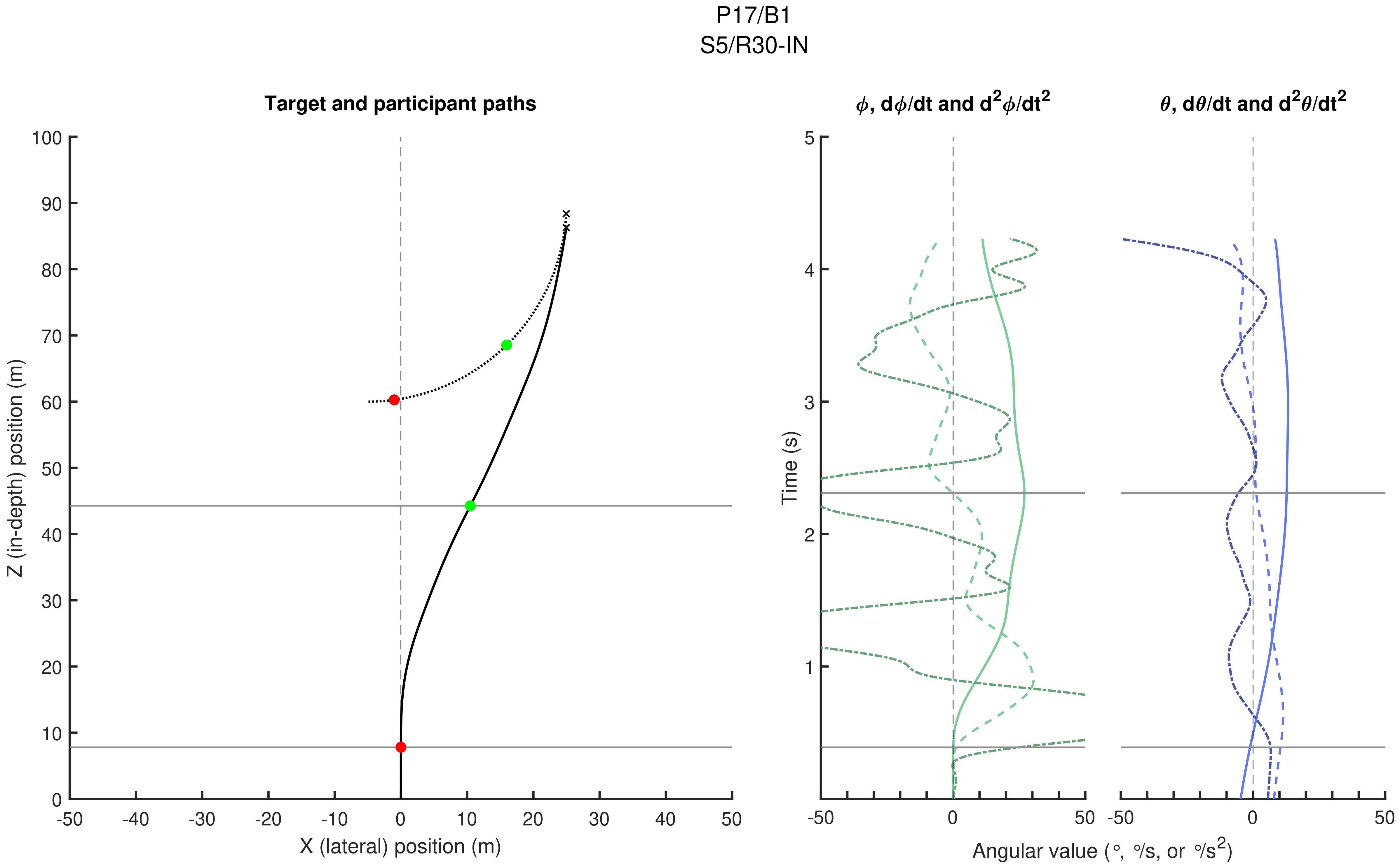
P17/B1
S5/R30-IN
Target and participant paths
Z (in-depth) position (m)
X (lateral) position (m)
φ, dφ/dt and d²φ/dt²
θ, dθ/dt and d²θ/dt²
Time (s)
Angular value (°, °/s, or °/s²)
100
90
80
70
60
50
40
30
20
10
0
-50
-40
-30
-20
-10
0
10
20
30
40
50
5
4
3
2
1
-50
0
50
-50
0
50

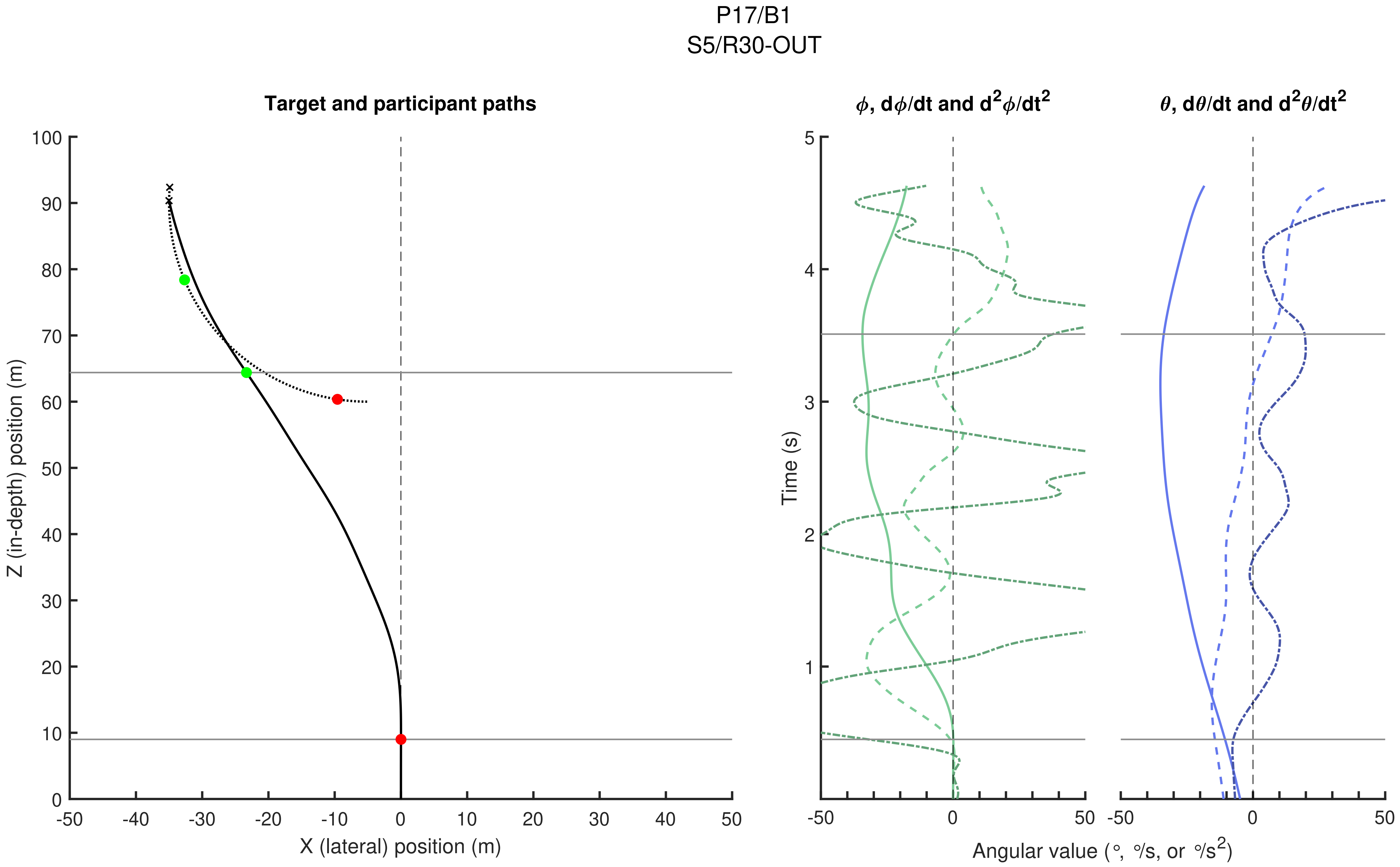
P17/B1
S5/R30-OUT
Target and participant paths
Z (in-depth) position (m)
X (lateral) position (m)
ϕ, dϕ/dt and d²ϕ/dt²
θ, dθ/dt and d²θ/dt²
Time (s)
Angular value (°, °/s, or °/s²)

P17/B1
S5/R10-OUT

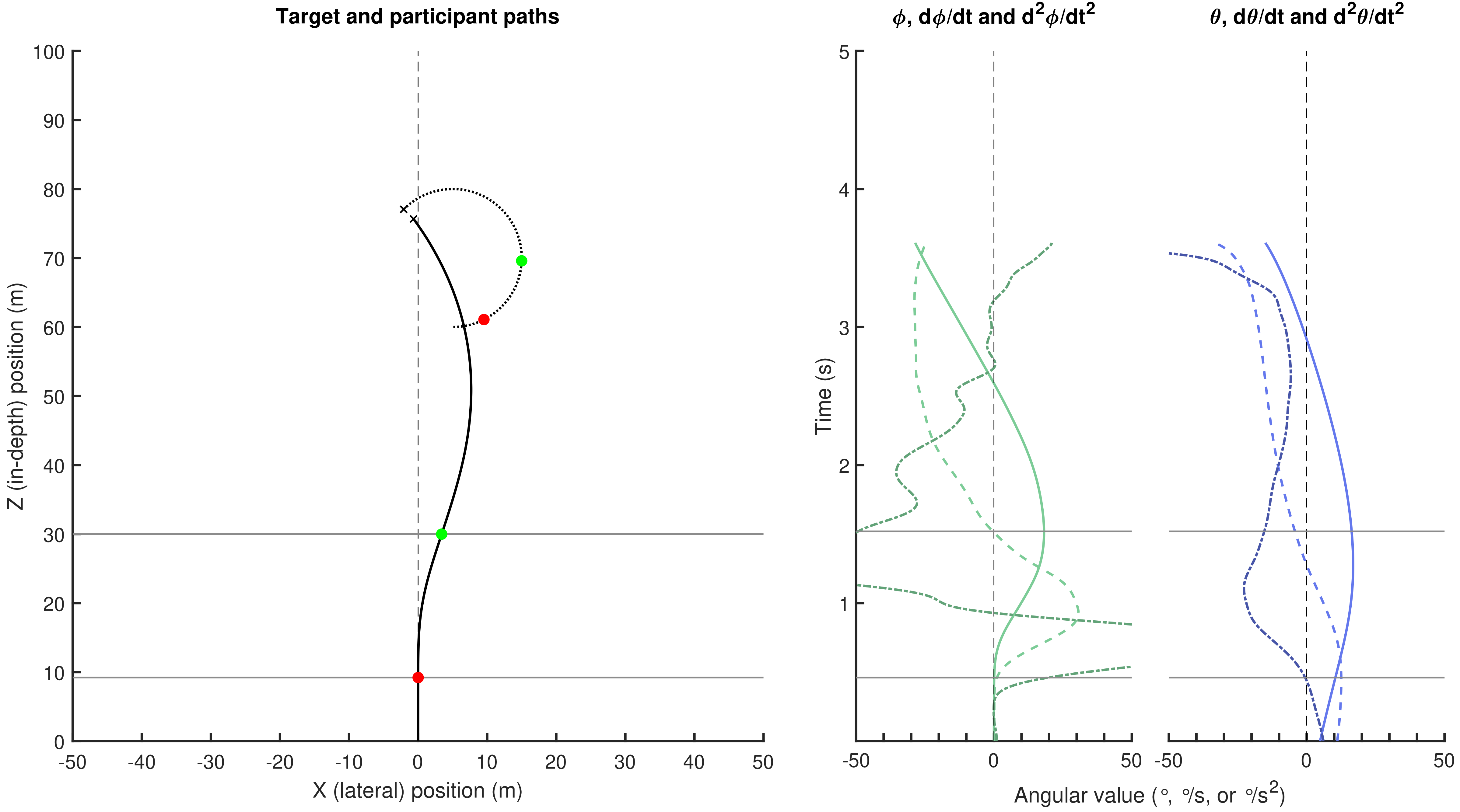

P17/B1
S5/R10-IN

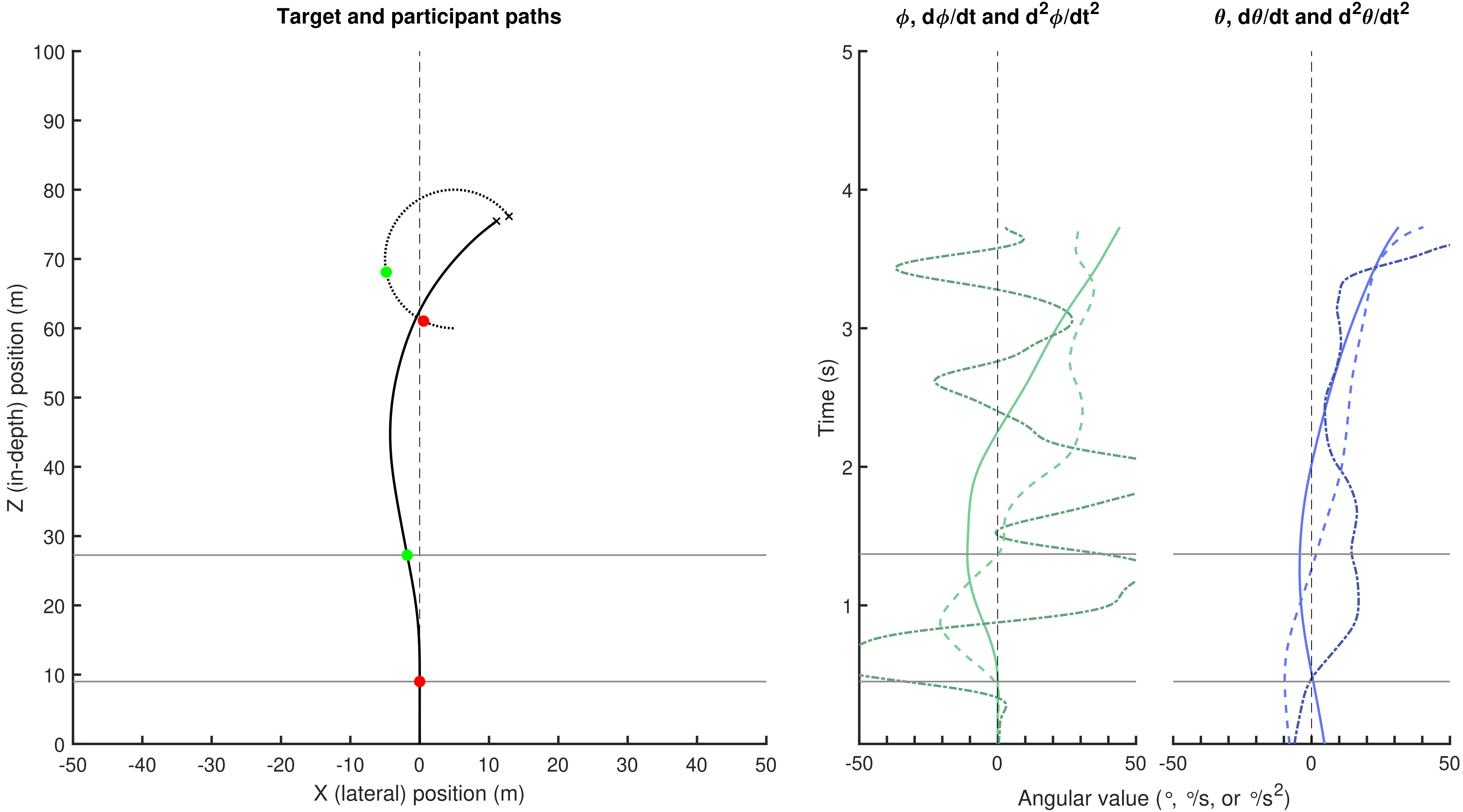

P17/B1
S5/R20-OUT

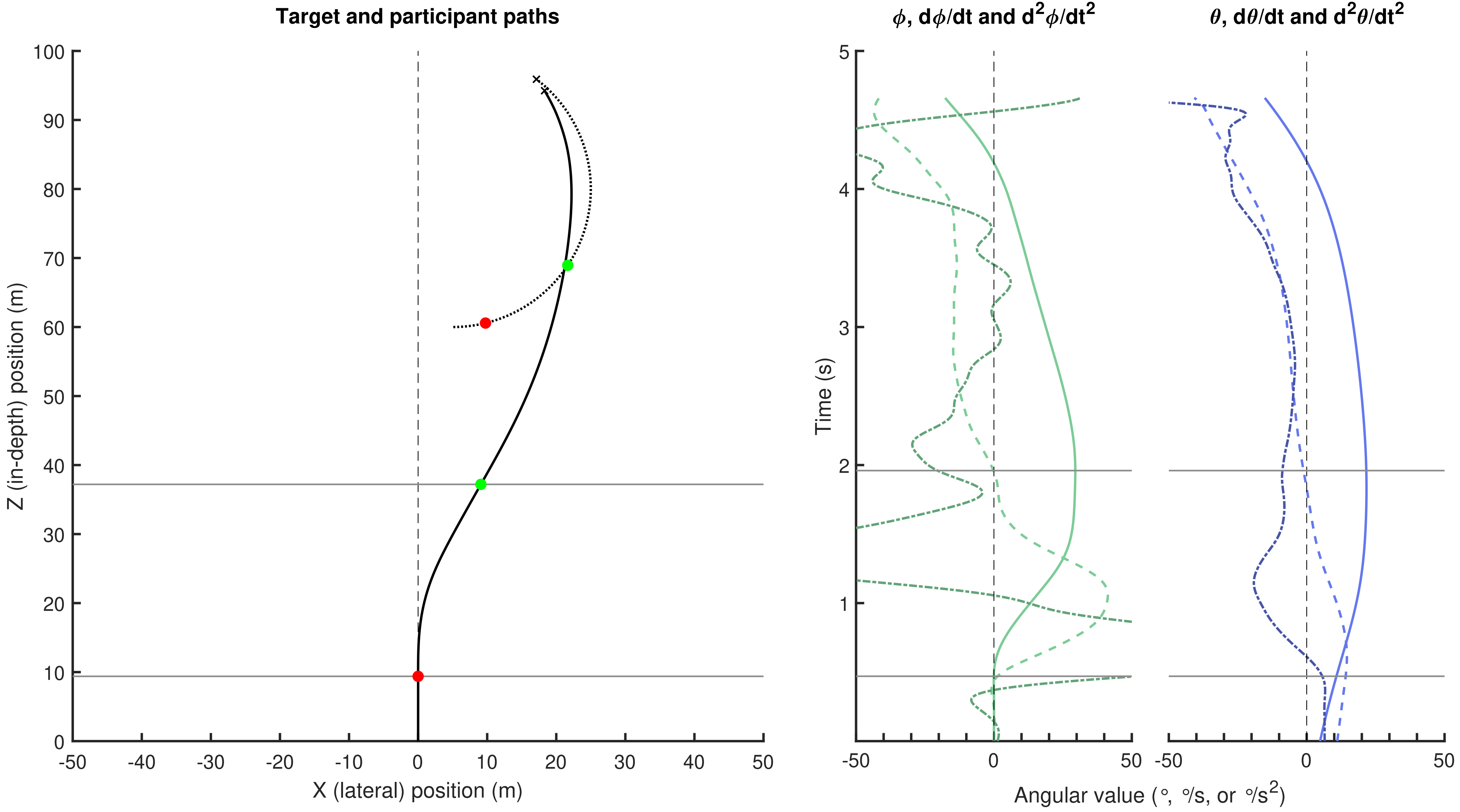

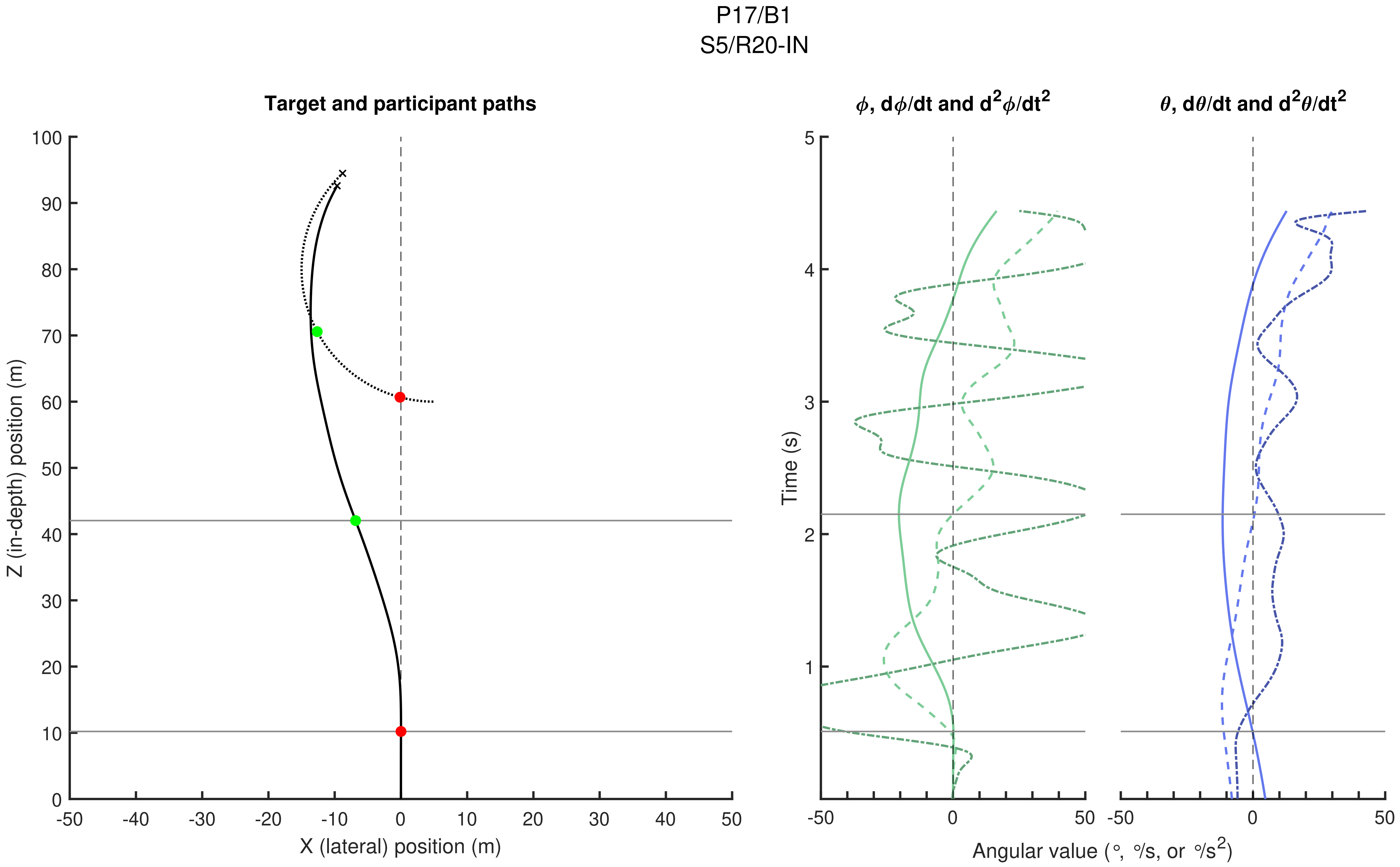
P17/B1
S5/R20-IN
Target and participant paths
Z (in-depth) position (m)
X (lateral) position (m)
ϕ, dϕ/dt and d²ϕ/dt²
θ, dθ/dt and d²θ/dt²
Time (s)
Angular value (°, °/s, or °/s²)

P17/B1
S5/R30-OUT

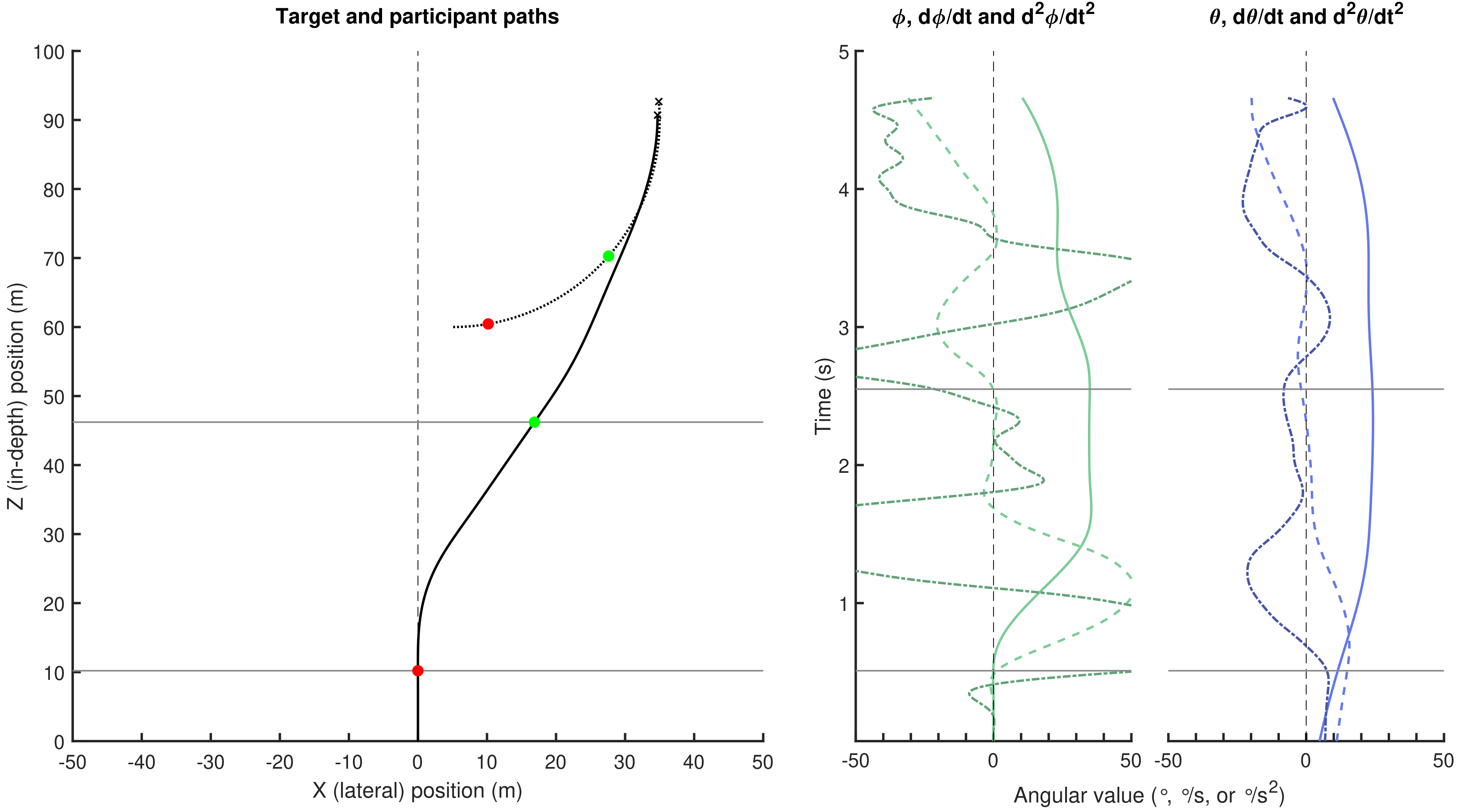

P17/B1
S5/R30-IN

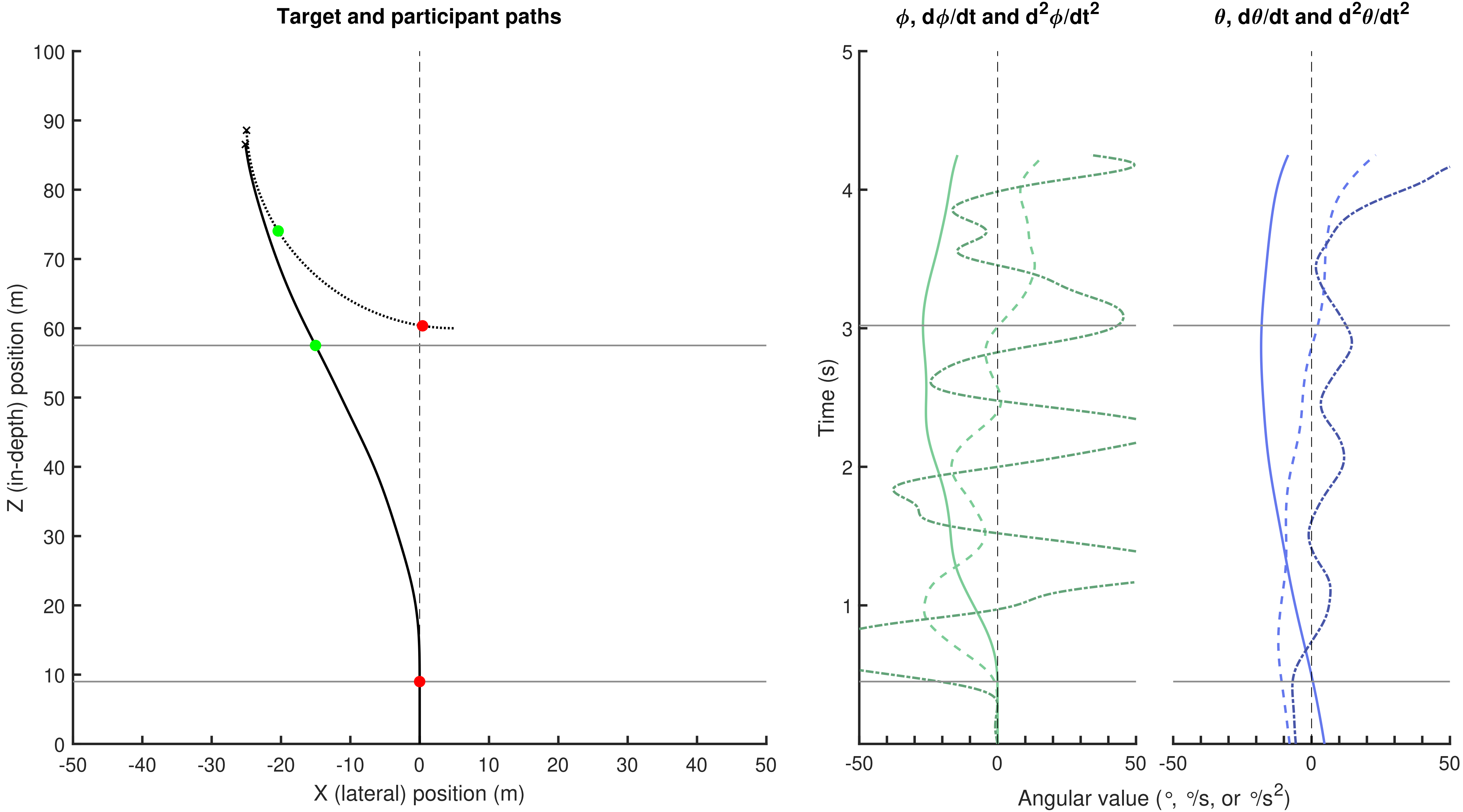

P17/B1
S15/R10-IN

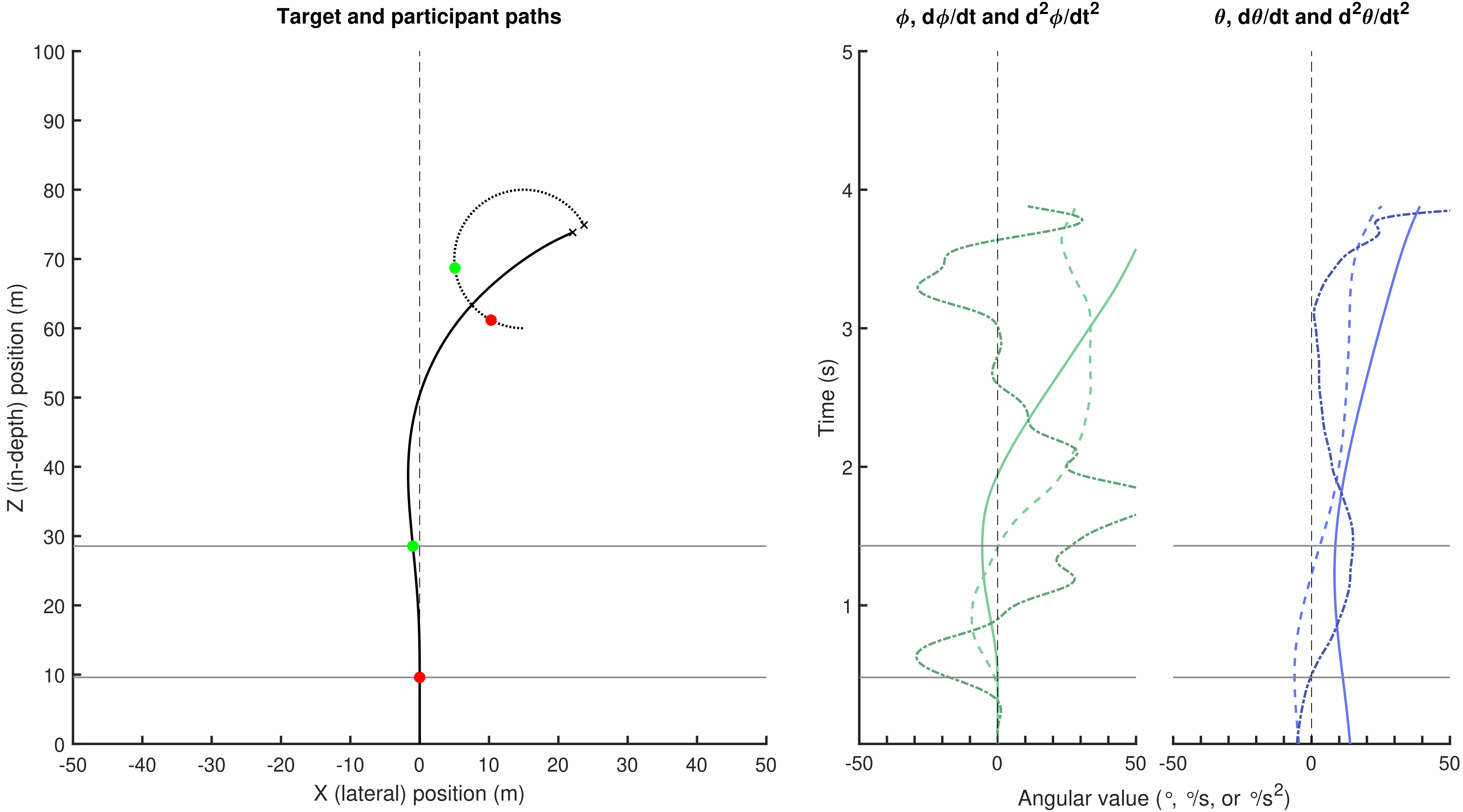

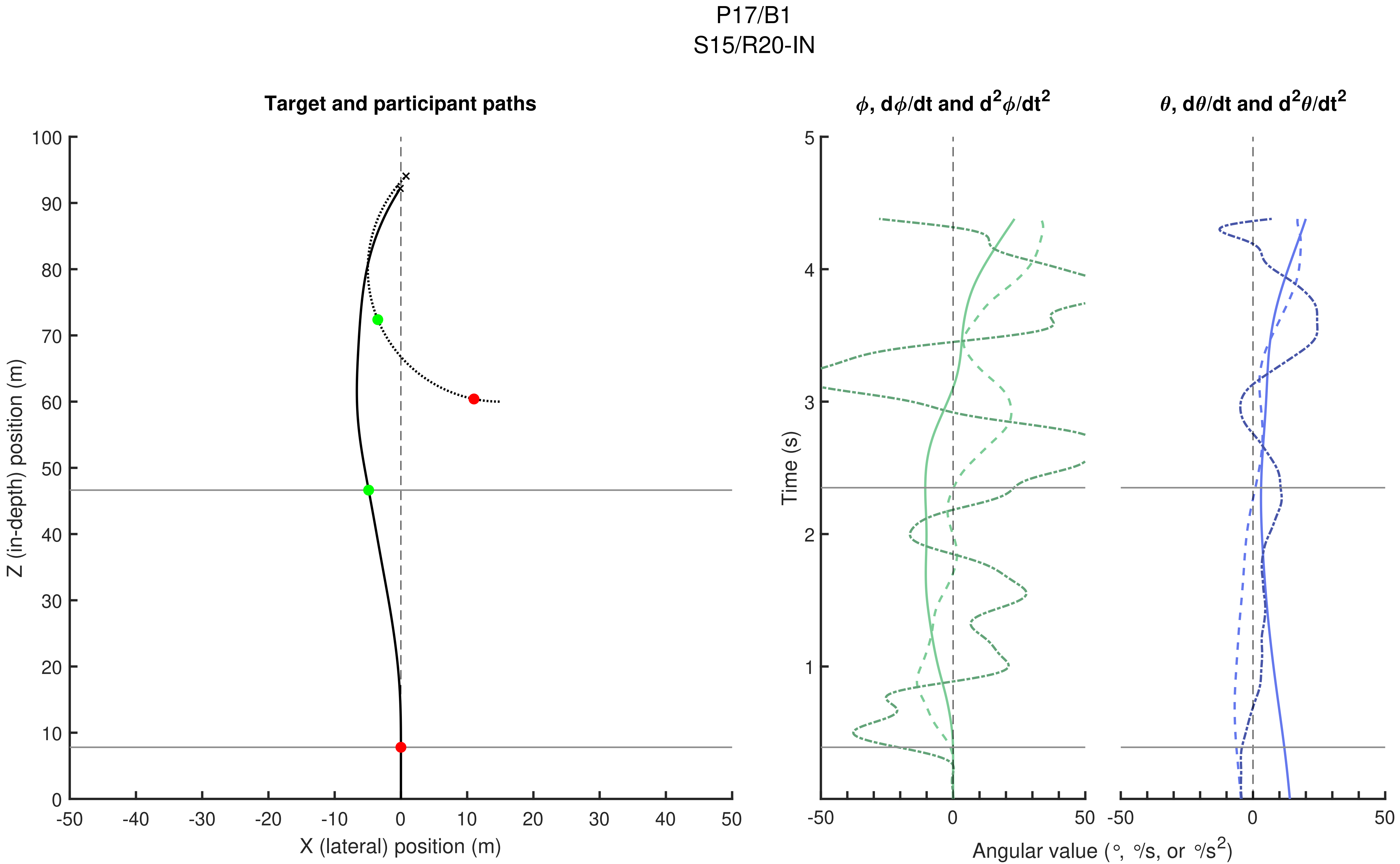
P17/B1
S15/R20-IN
Target and participant paths
Z (in-depth) position (m)
X (lateral) position (m)
0
10
20
30
40
50
60
70
80
90
100
-50
-40
-30
-20
-10
0
10
20
30
40
50
ϕ, dϕ/dt and d²ϕ/dt²
θ, dθ/dt and d²θ/dt²
Time (s)
1
2
3
4
5
-50
0
50
-50
0
50
Angular value (°, °/s, or °/s²)

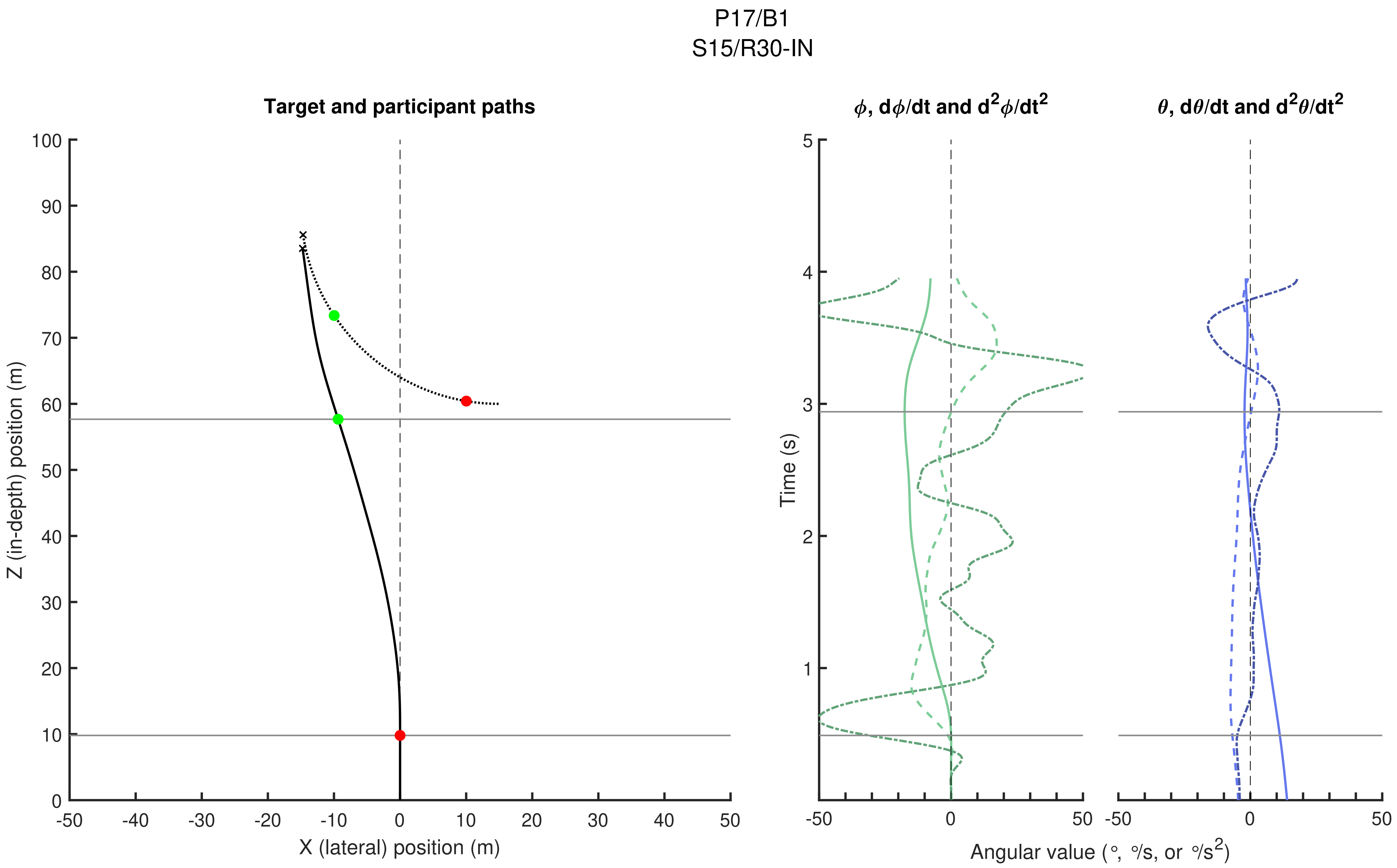
P17/B1
S15/R30-IN
Target and participant paths
Z (in-depth) position (m)
X (lateral) position (m)
φ, dφ/dt and d²φ/dt²
θ, dθ/dt and d²θ/dt²
Time (s)
Angular value (°, °/s, or °/s²)

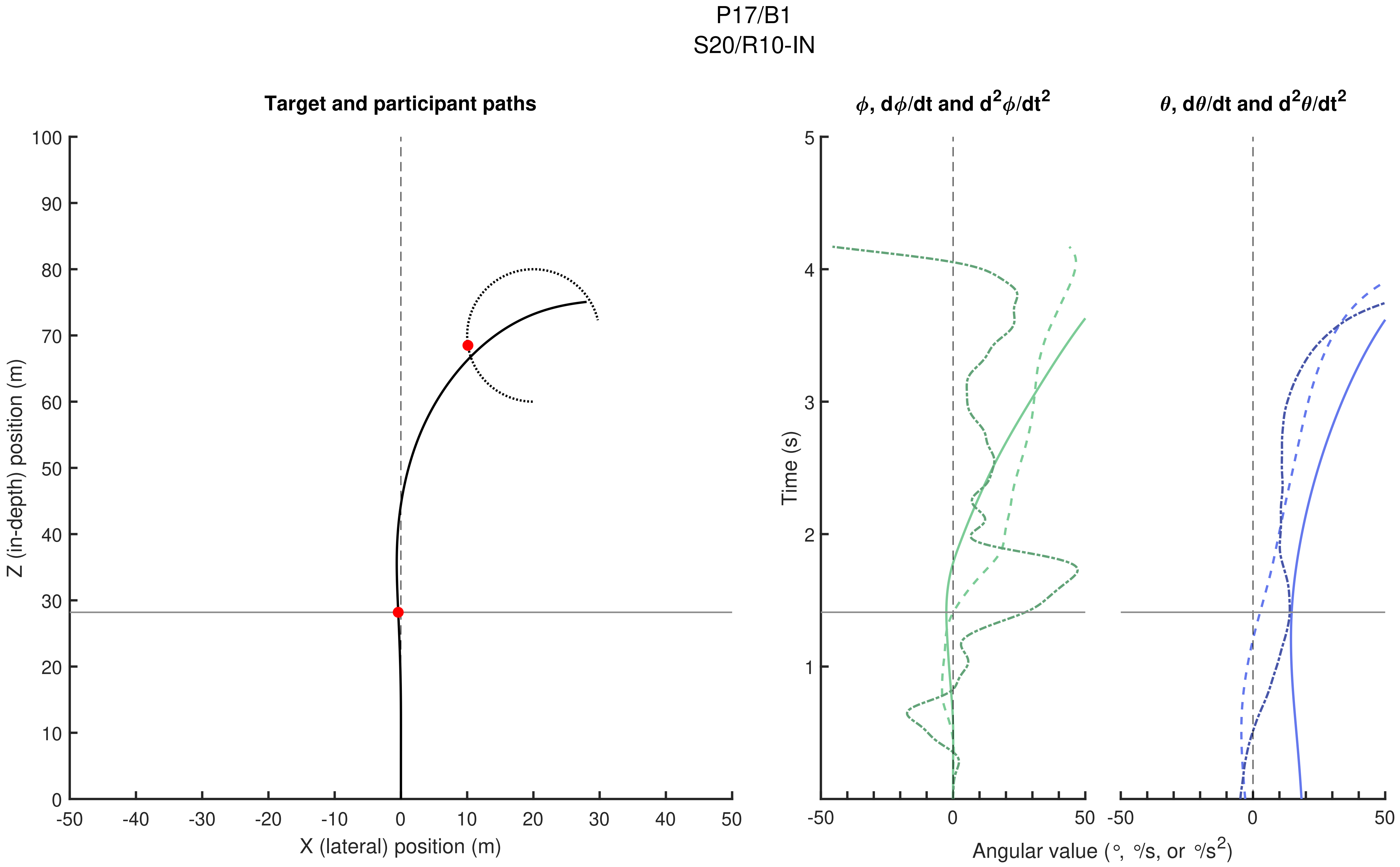

P17/B1
S20/R10-IN
Target and participant paths
ϕ, dϕ/dt and d$^2\phi$/dt^2
θ, dθ/dt and d$^2\theta$/dt^2
Z (in-depth) position (m)
X (lateral) position (m)
Time (s)
Angular value (°, °/s, or °/s^2)
100
90
80
70
60
50
40
30
20
10
0
-50
-40
-30
-20
-10
0
10
20
30
40
50
5
4
3
2
1
-50
0
50
-50
0
50

P17/B1
S20/R20-IN

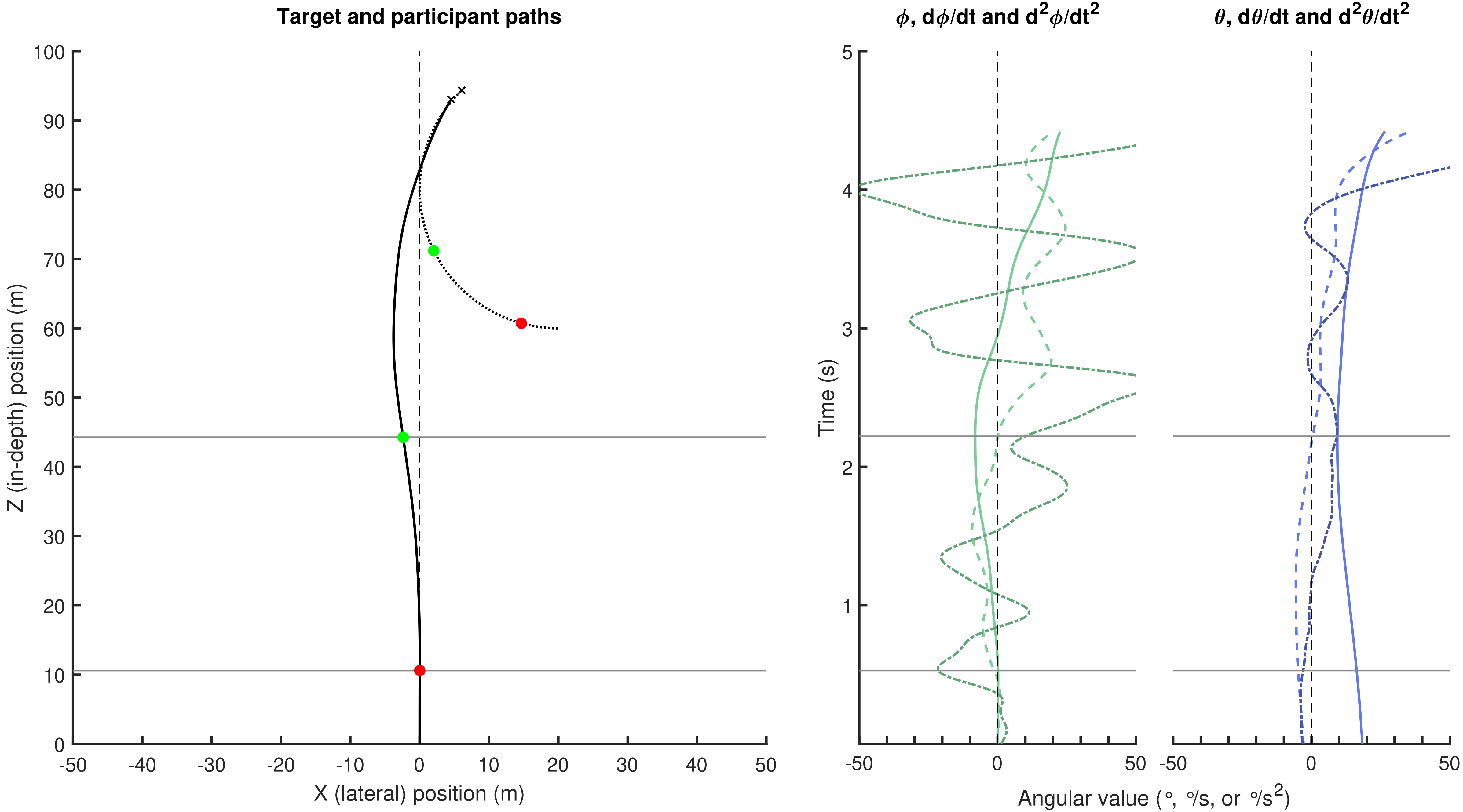

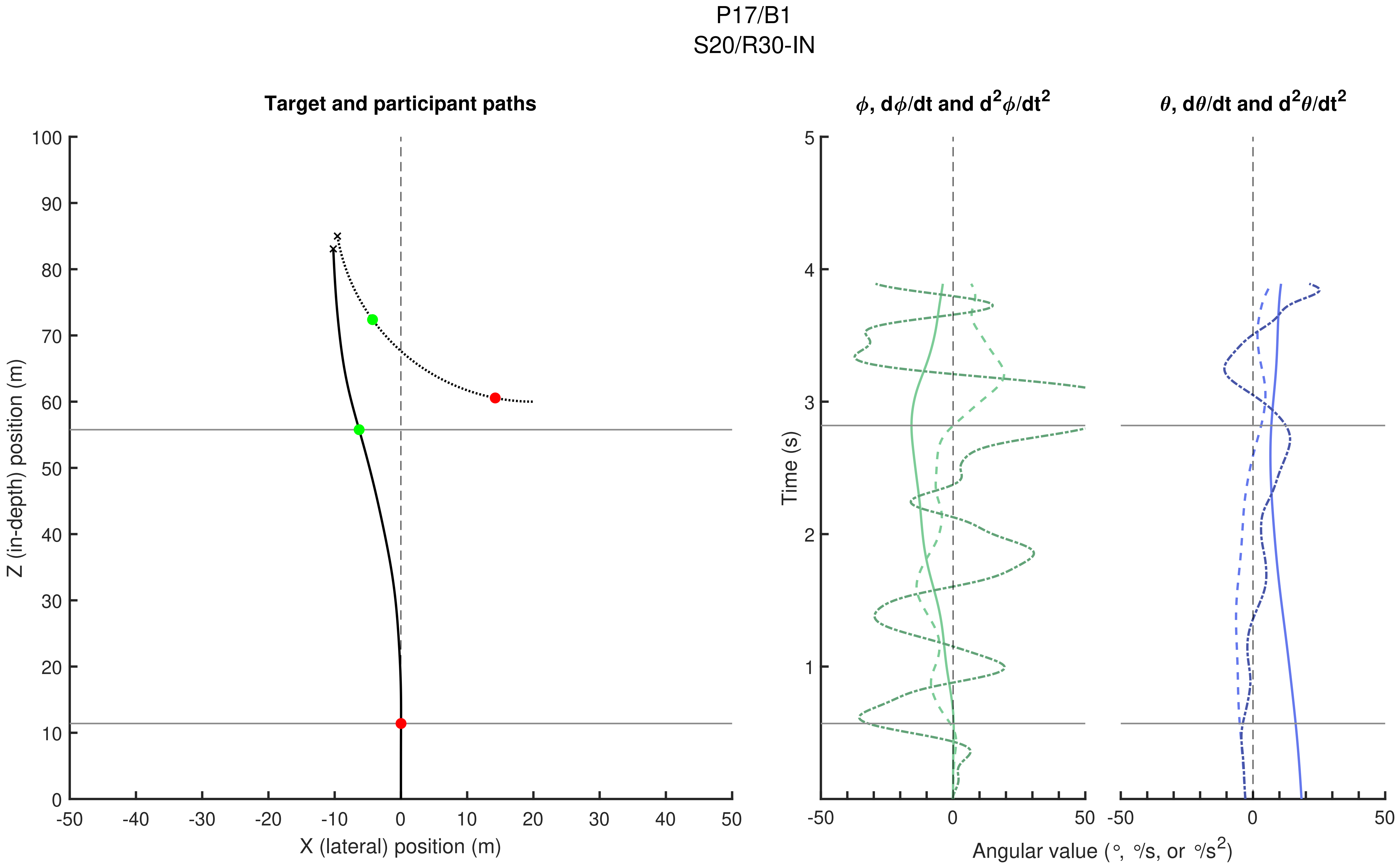
P17/B1
S20/R30-IN
Target and participant paths
ϕ, dϕ/dt and d$^2\phi$/dt^2
θ, dθ/dt and d$^2\theta$/dt^2
Z (in-depth) position (m)
X (lateral) position (m)
Time (s)
Angular value (°, °/s, or °/s^2)
100
90
80
70
60
50
40
30
20
10
0
-50
-40
-30
-20
-10
0
10
20
30
40
50
5
4
3
2
1

P17/B2
S20/R10-IN

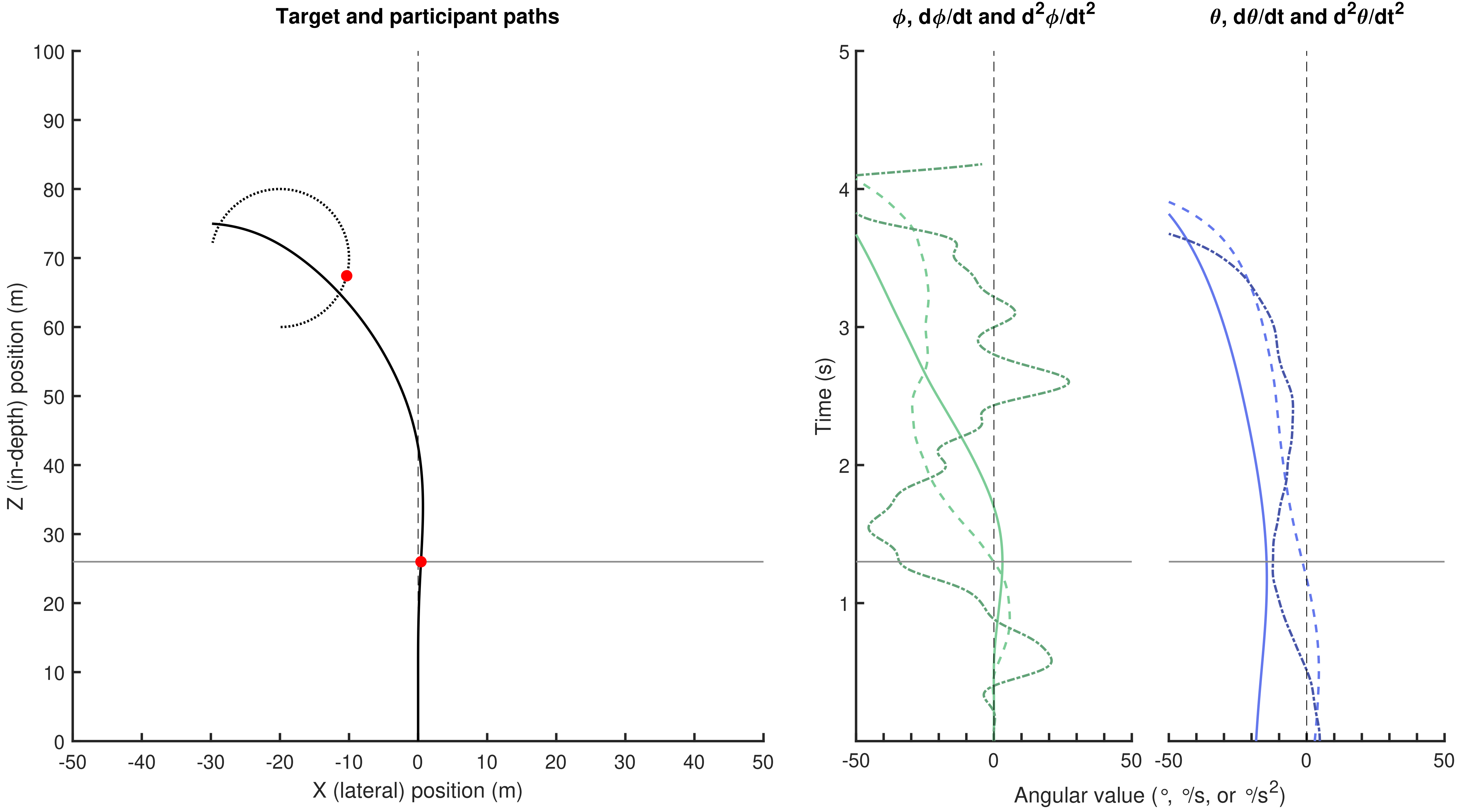

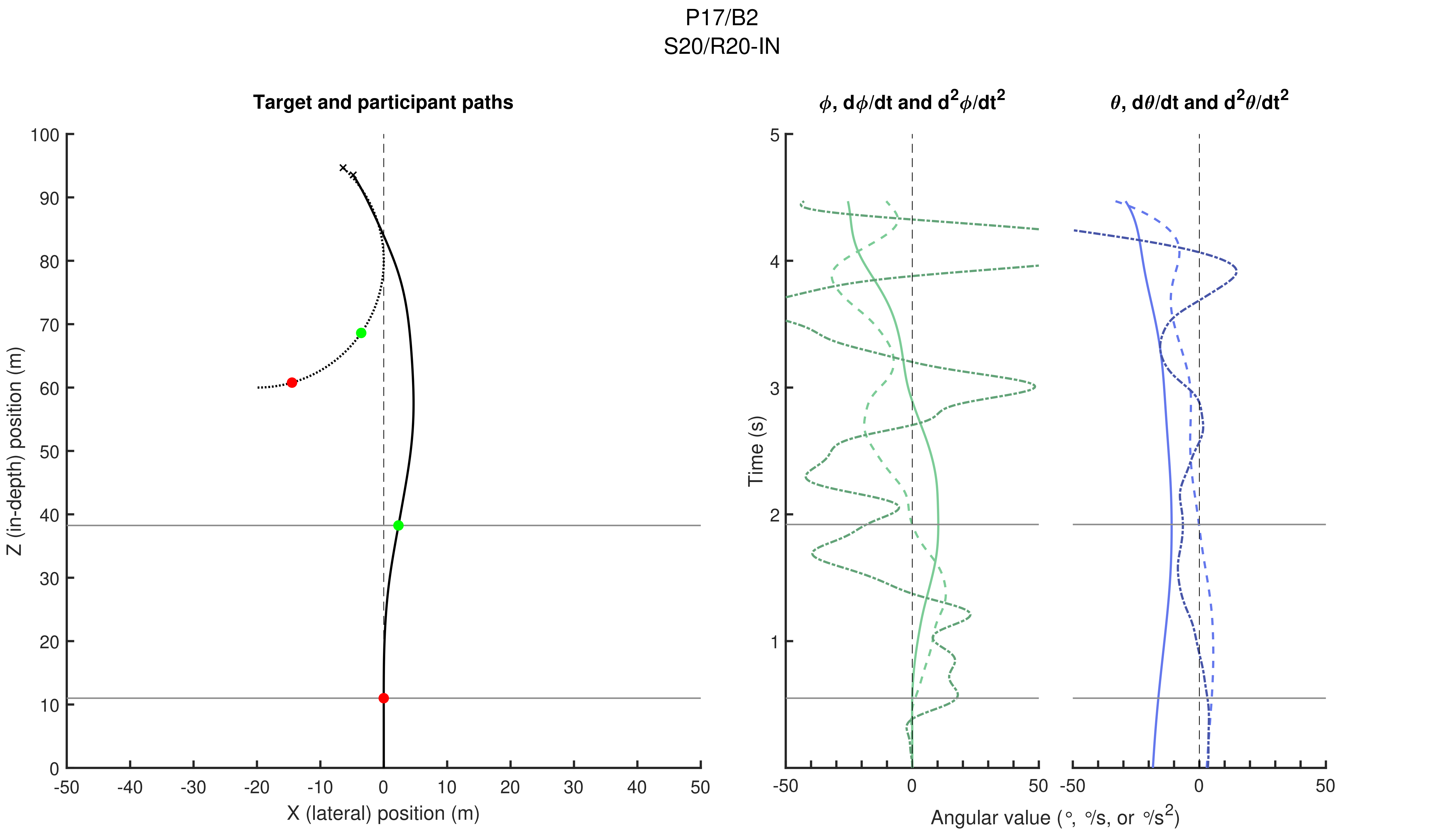

P17/B2
S20/R20-IN
Target and participant paths
Z (in-depth) position (m)
X (lateral) position (m)
ϕ, dϕ/dt and d$^2\phi$/dt^2
θ, dθ/dt and d$^2\theta$/dt^2
Time (s)
Angular value (°, °/s, or °/s^2)

P17/B2
S20/R30-IN

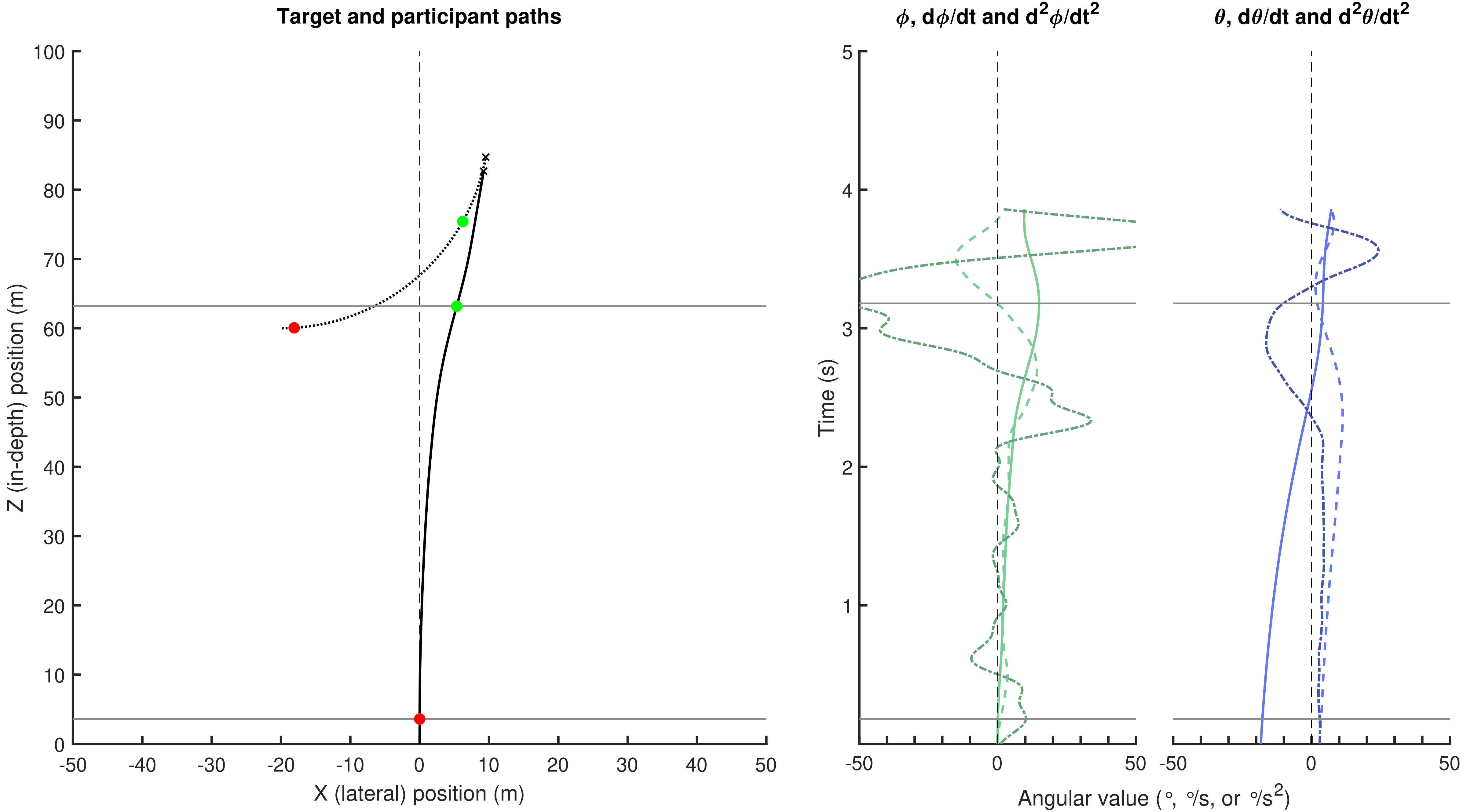

P17/B2
S15/R10-IN

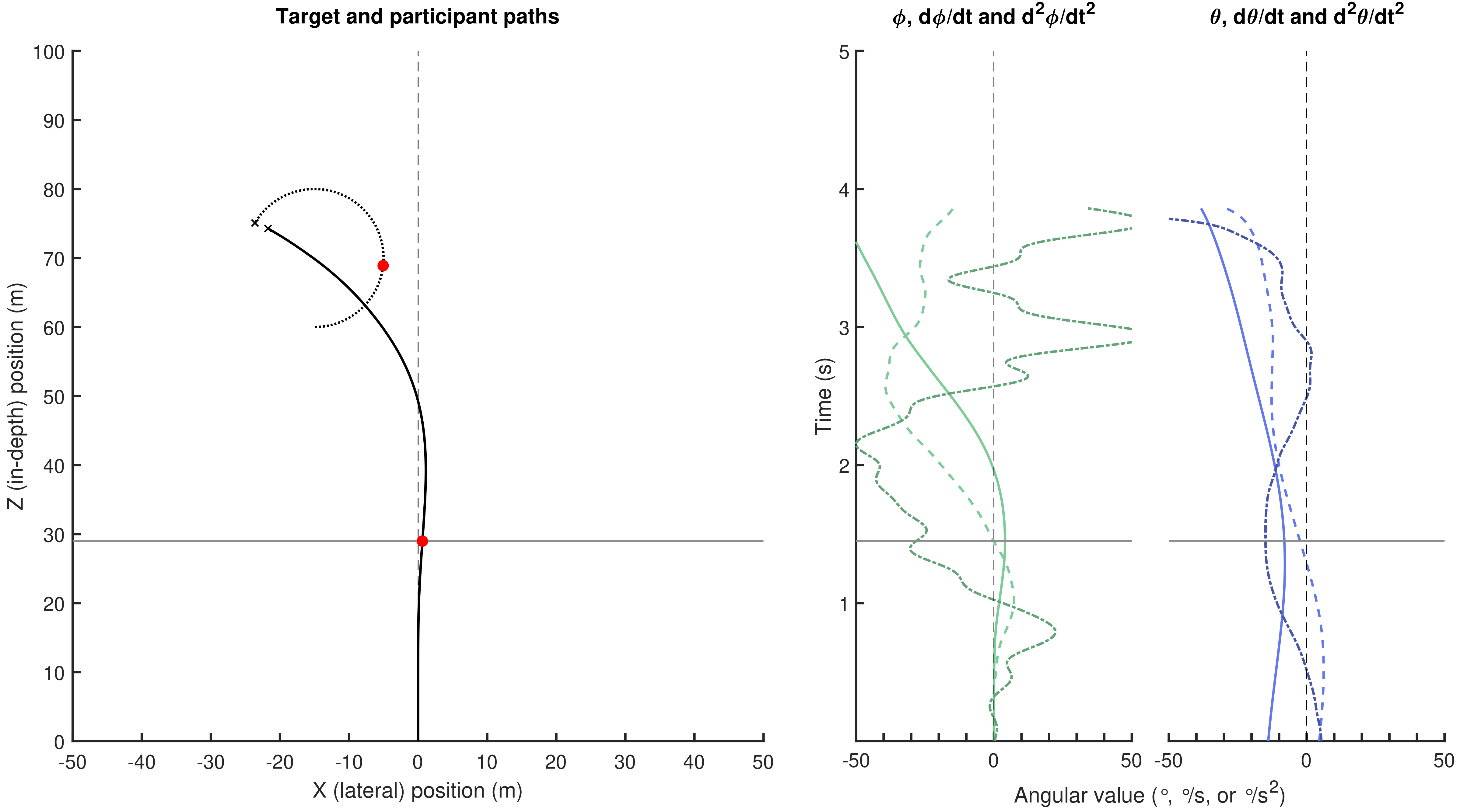

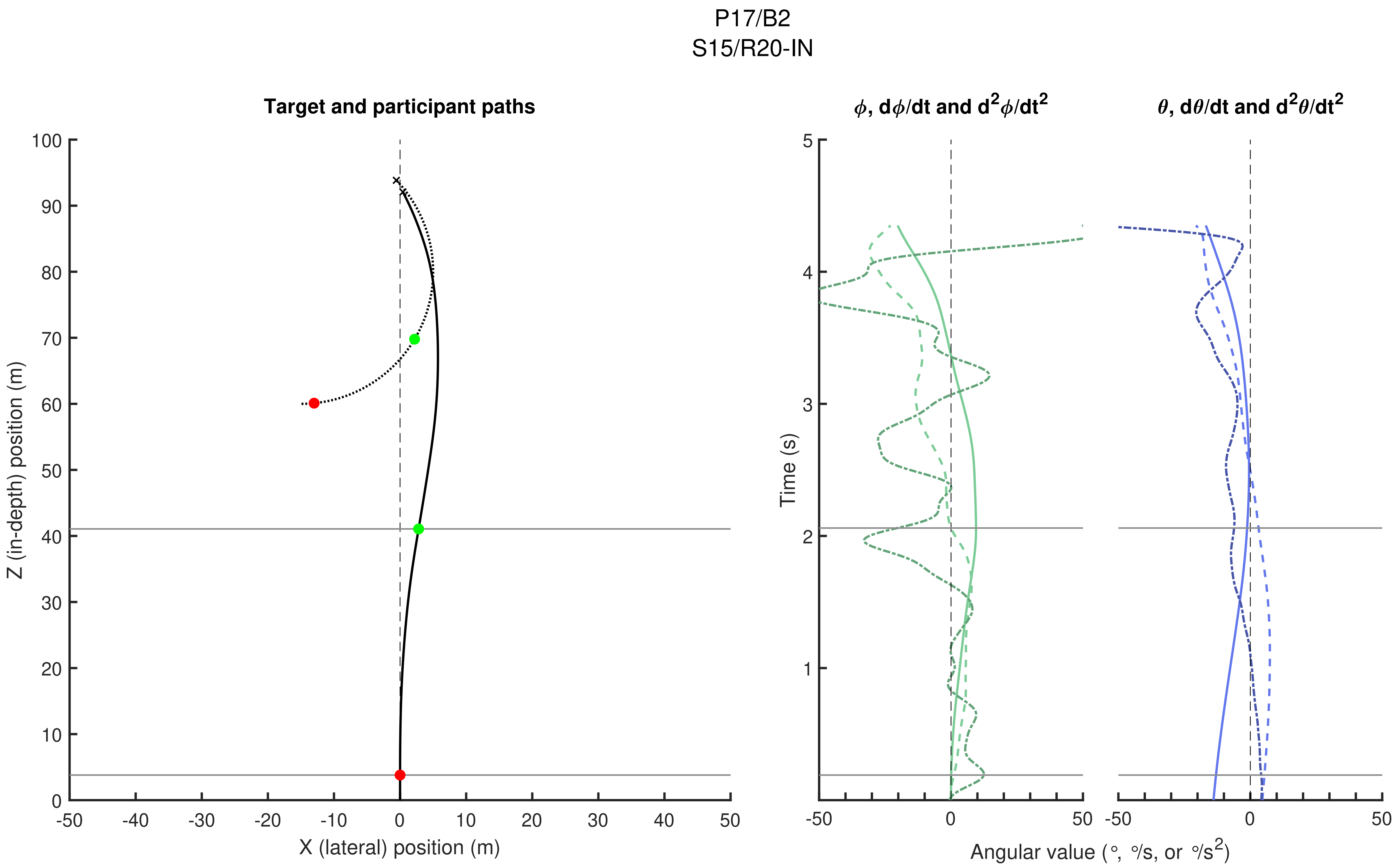
P17/B2
S15/R20-IN
Target and participant paths
Z (in-depth) position (m)
X (lateral) position (m)
φ, dφ/dt and d²φ/dt²
θ, dθ/dt and d²θ/dt²
Time (s)
Angular value (°, °/s, or °/s²)

P17/B2
S15/R30-IN

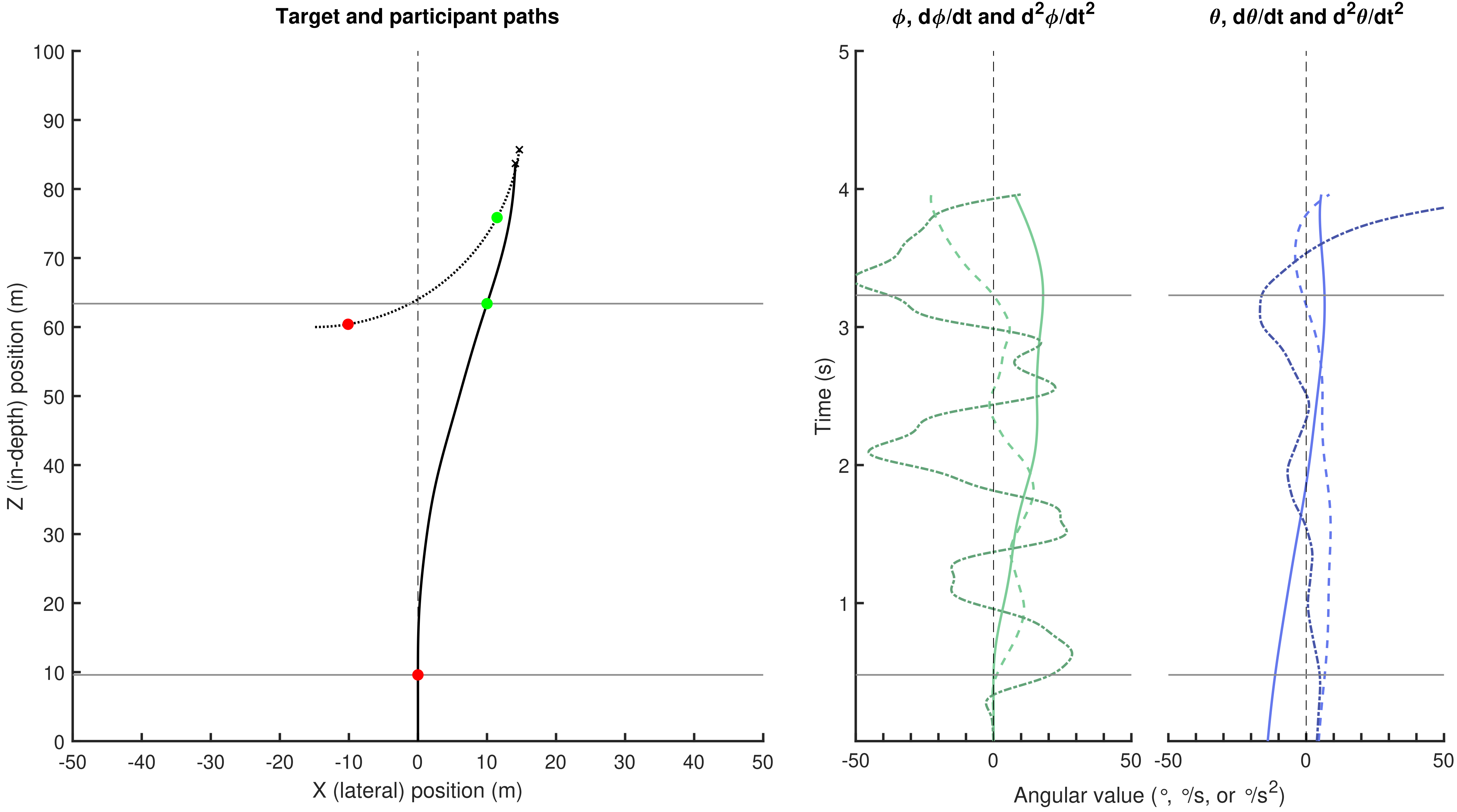

P17/B2
S5/R10-IN

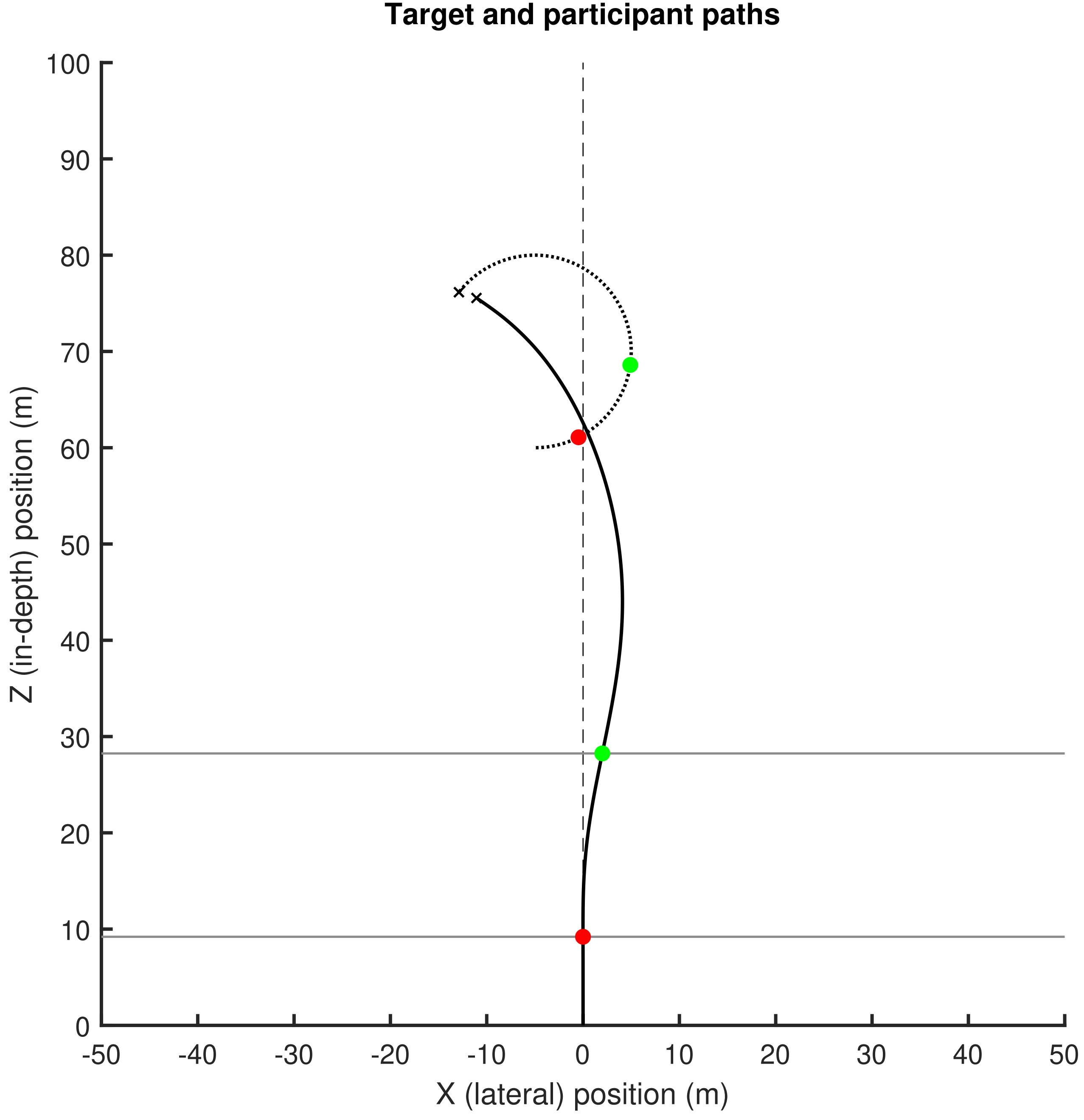

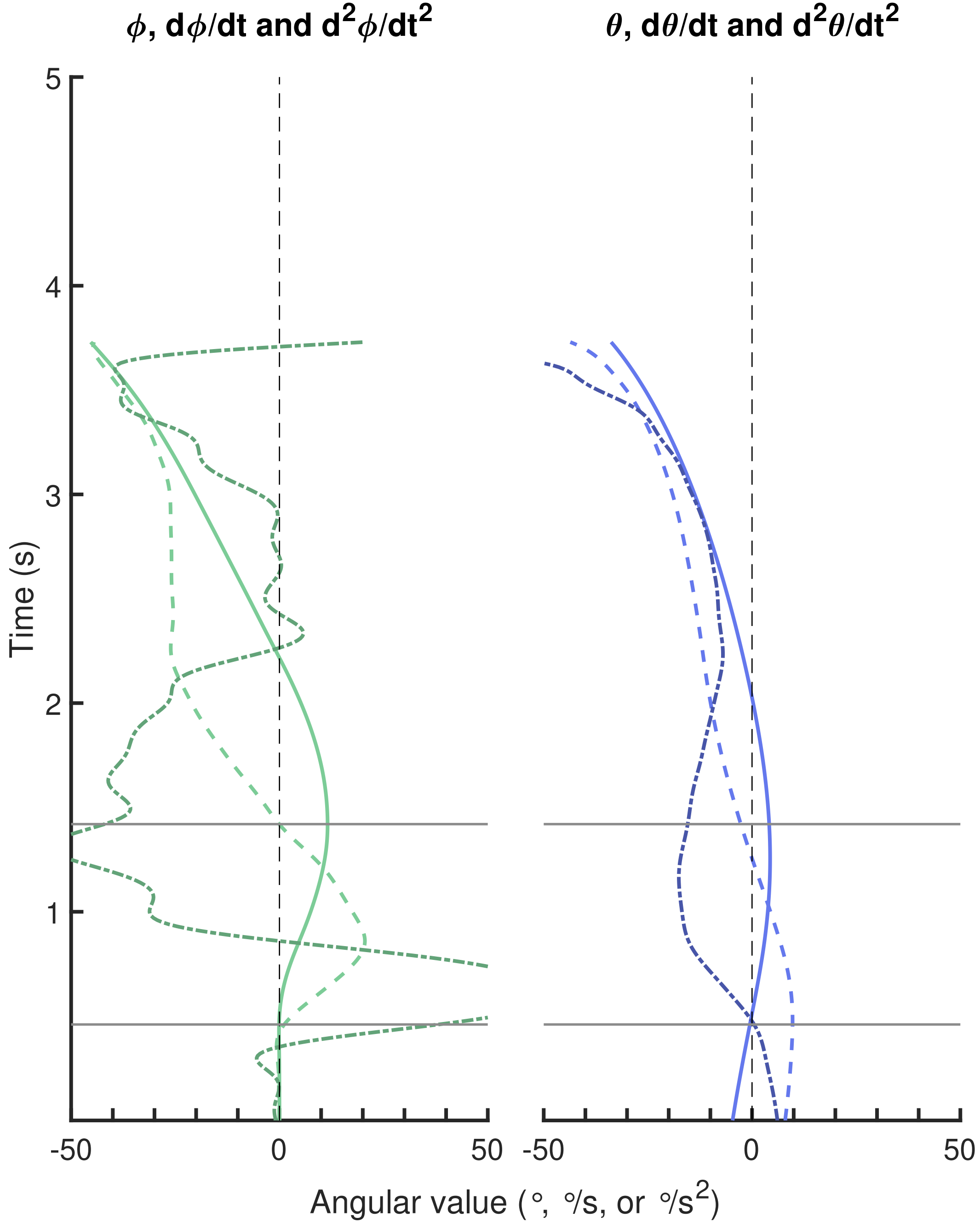

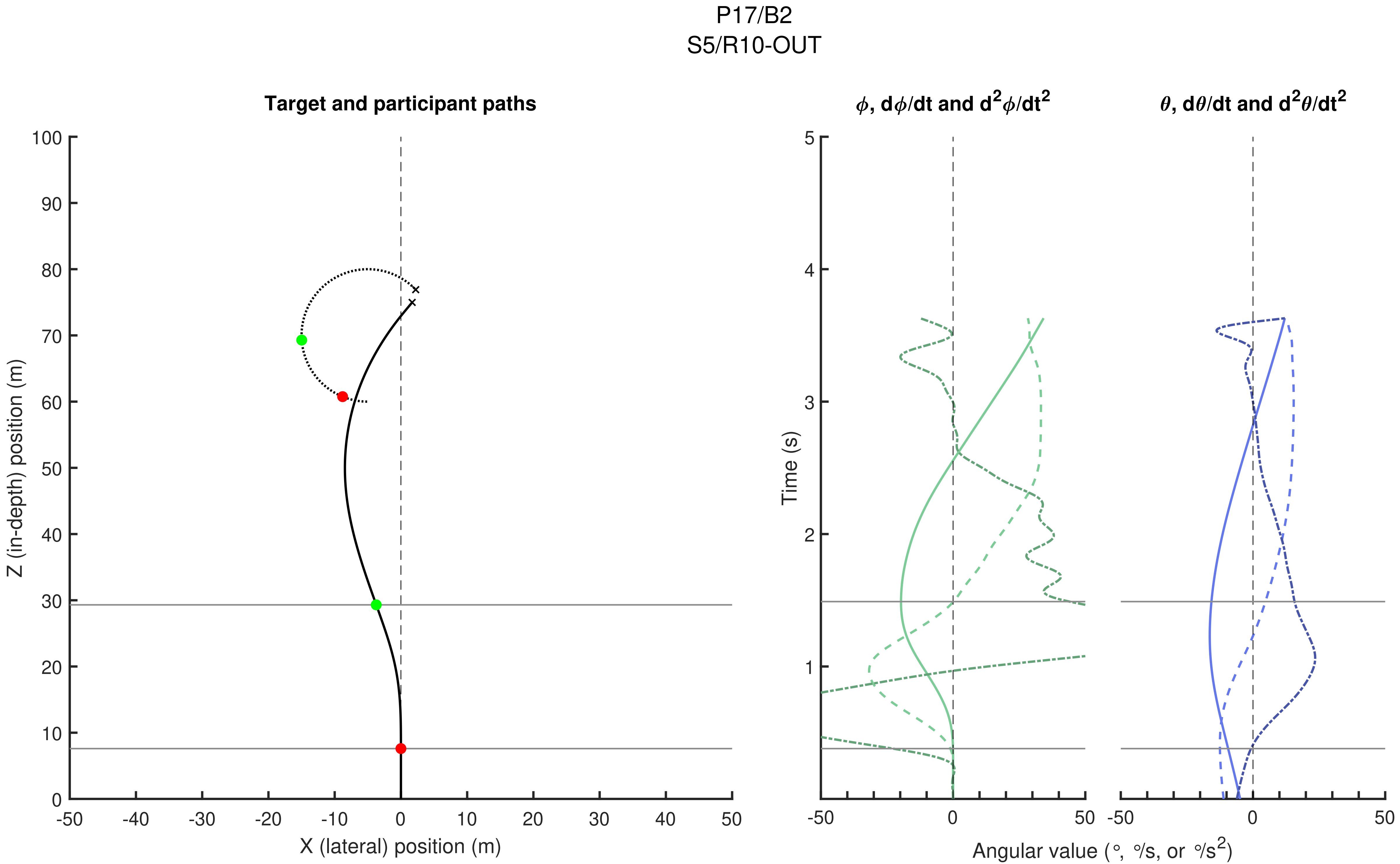

P17/B2
S5/R10-OUT
Target and participant paths
φ, dφ/dt and d²φ/dt²
θ, dθ/dt and d²θ/dt²
Z (in-depth) position (m)
X (lateral) position (m)
Time (s)
Angular value (°, °/s, or °/s²)
100
90
80
70
60
50
40
30
20
10
0
-50
-40
-30
-20
-10
0
10
20
30
40
50
5
4
3
2
1
-50
0
50
-50
0
50

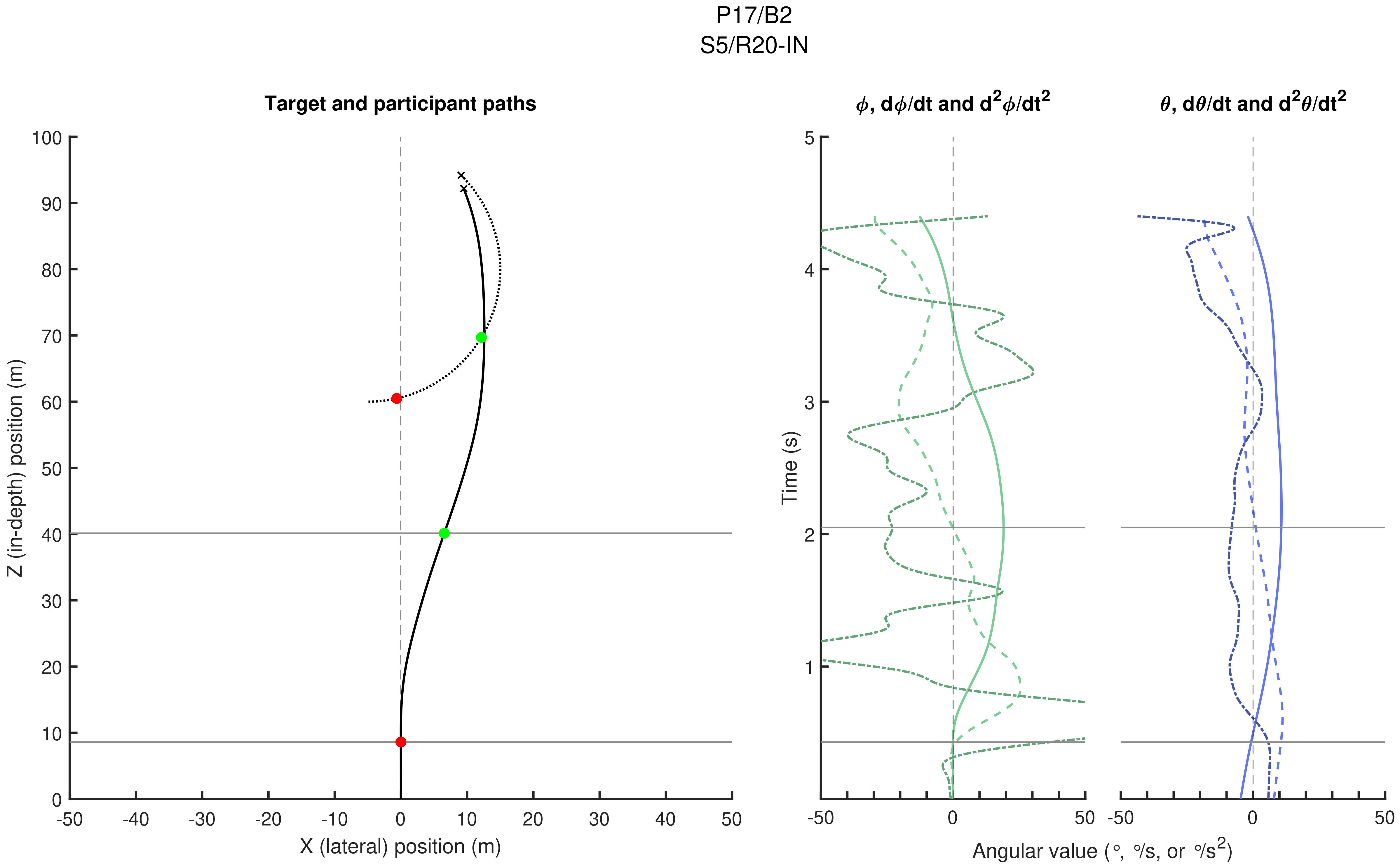
P17/B2
S5/R20-IN
Target and participant paths
Z (in-depth) position (m)
X (lateral) position (m)
100
90
80
70
60
50
40
30
20
10
0
-50
-40
-30
-20
-10
0
10
20
30
40
50
ϕ, dϕ/dt and d²ϕ/dt²
θ, dθ/dt and d²θ/dt²
Time (s)
5
4
3
2
1
-50
0
50
-50
0
50
Angular value (°, °/s, or °/s²)

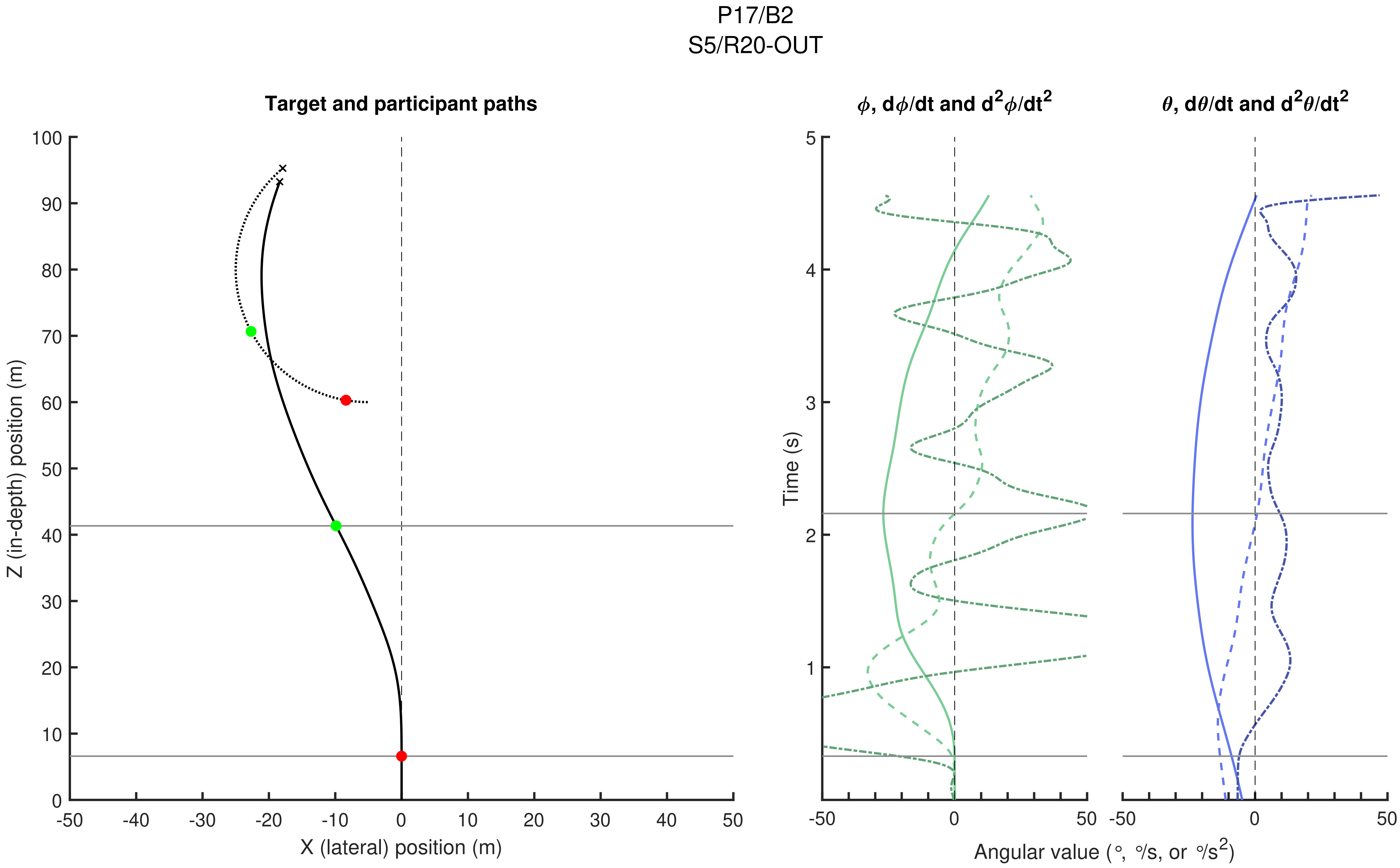

P17/B2
S5/R20-OUT
Target and participant paths
Z (in-depth) position (m)
X (lateral) position (m)
ϕ, dϕ/dt and d²ϕ/dt²
θ, dθ/dt and d²θ/dt²
Time (s)
Angular value (°, °/s, or °/s²)

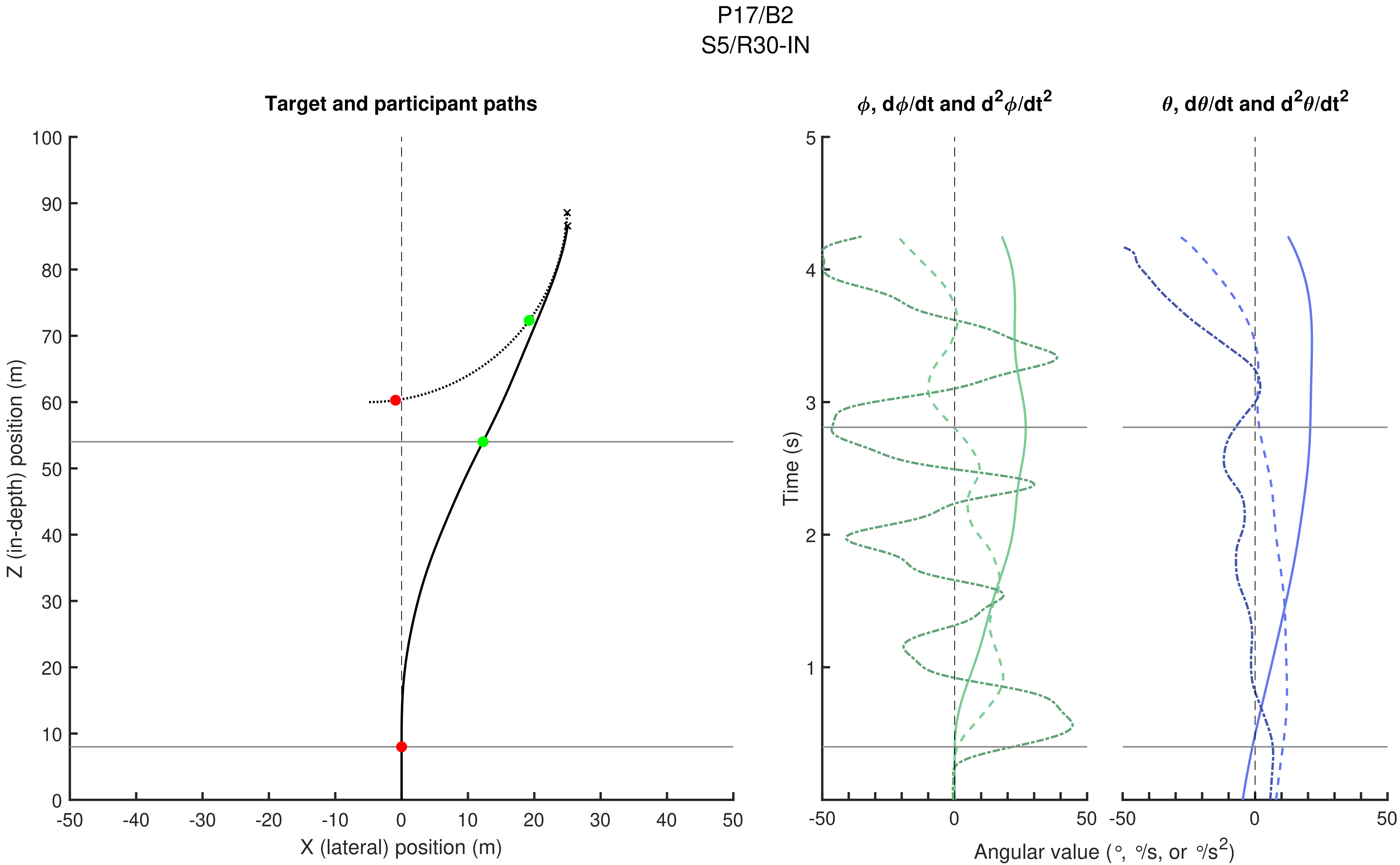

P17/B2
S5/R30-IN
Target and participant paths
ϕ, dϕ/dt and d$^2\phi$/dt^2
θ, dθ/dt and d$^2\theta$/dt^2
Z (in-depth) position (m)
X (lateral) position (m)
Time (s)
Angular value (°, °/s, or °/s^2)

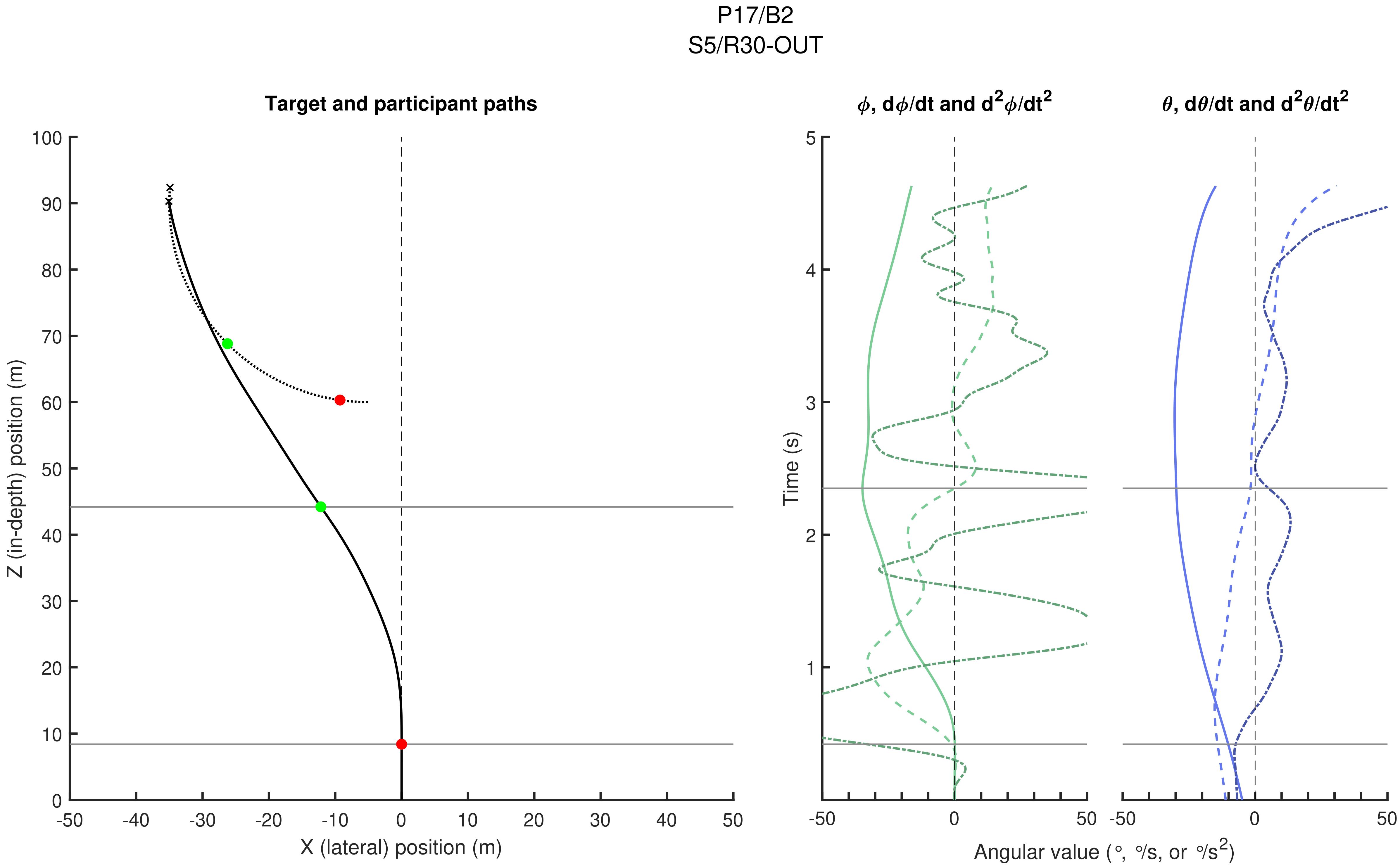

P17/B2
S5/R30-OUT
Target and participant paths
Z (in-depth) position (m)
X (lateral) position (m)
ϕ, dϕ/dt and d$^2\phi$/dt^2
θ, dθ/dt and d$^2\theta$/dt^2
Time (s)
Angular value (°, °/s, or °/s^2)

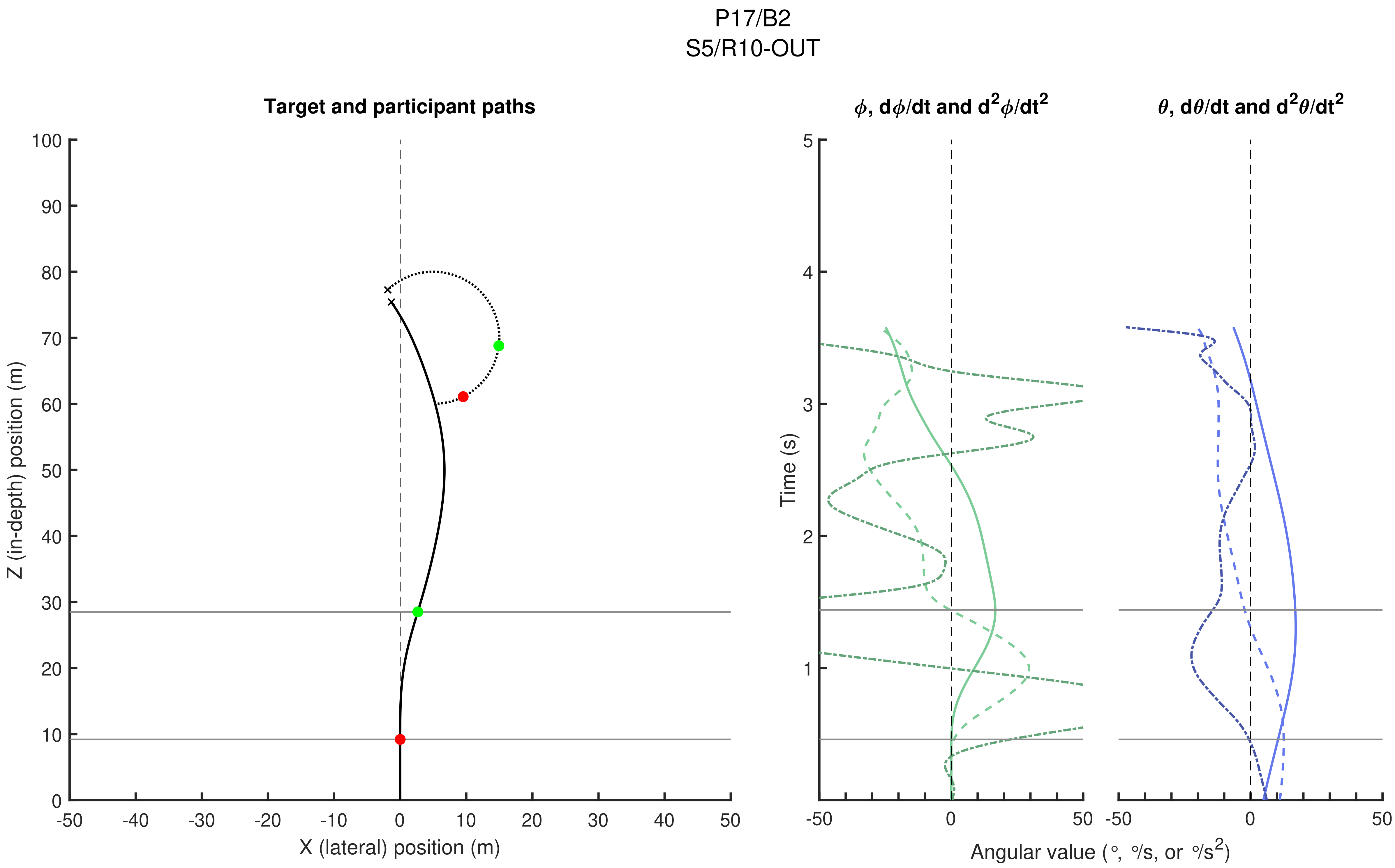

P17/B2
S5/R10-OUT
Target and participant paths
Z (in-depth) position (m)
X (lateral) position (m)
0
10
20
30
40
50
60
70
80
90
100
-50
-40
-30
-20
-10
0
10
20
30
40
50
ϕ, dϕ/dt and d²ϕ/dt²
θ, dθ/dt and d²θ/dt²
Time (s)
1
2
3
4
5
-50
0
50
-50
0
50
Angular value (°, °/s, or °/s²)

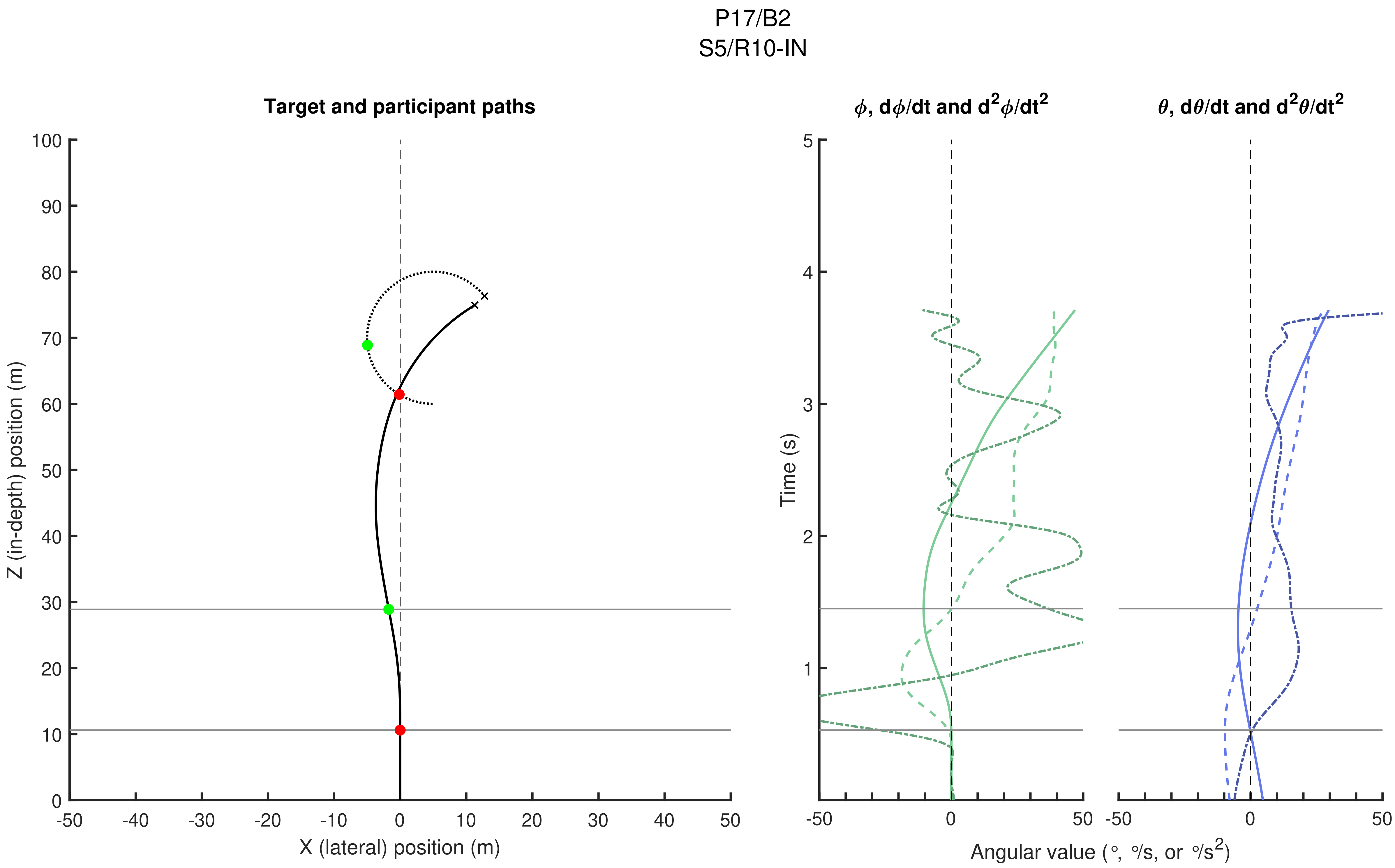
P17/B2
S5/R10-IN
Target and participant paths
Z (in-depth) position (m)
X (lateral) position (m)
ϕ, dϕ/dt and d²ϕ/dt²
θ, dθ/dt and d²θ/dt²
Time (s)
Angular value (°, °/s, or °/s²)

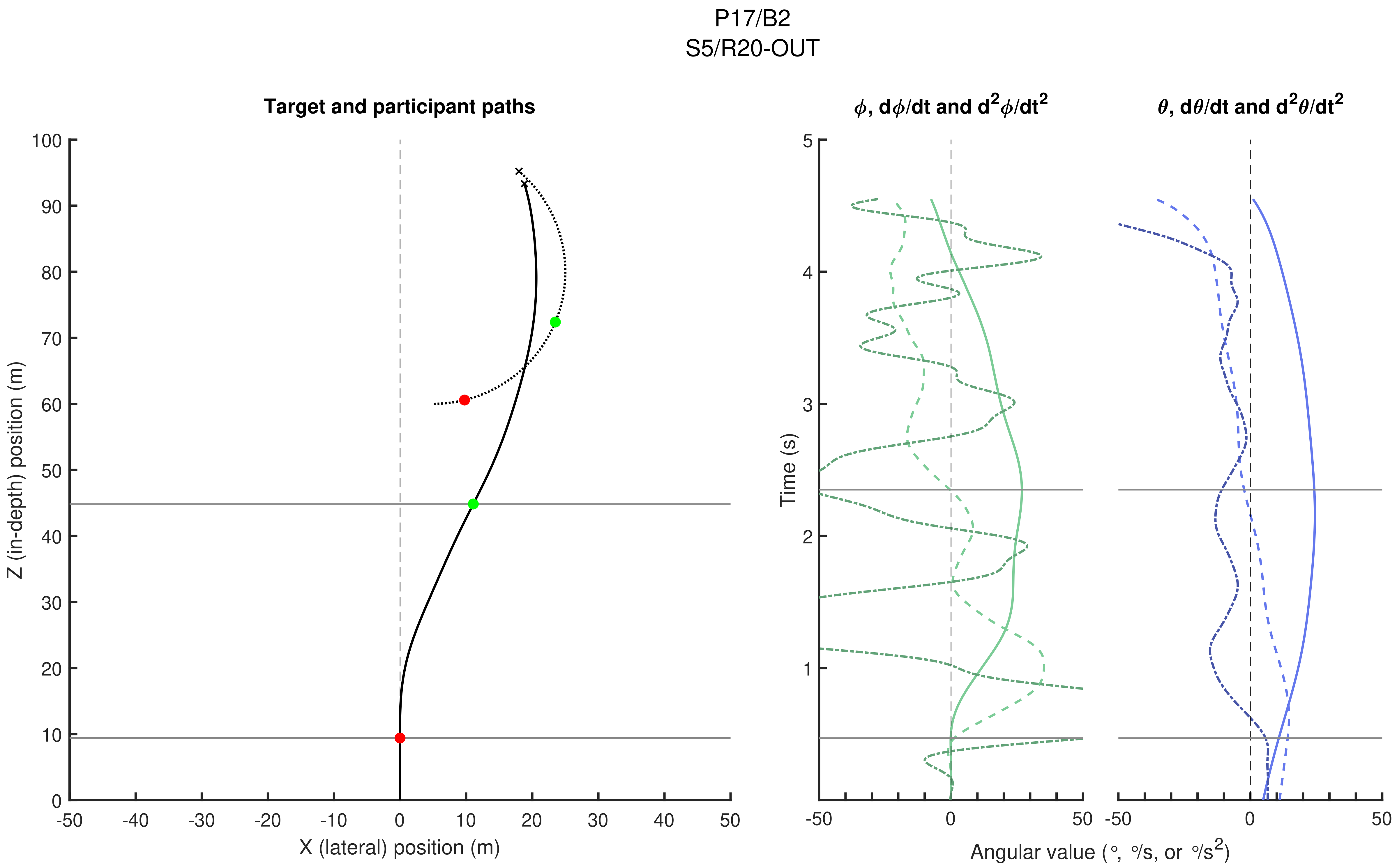

P17/B2
S5/R20-OUT
Target and participant paths
Z (in-depth) position (m)
X (lateral) position (m)
ϕ, dϕ/dt and d$^2\phi$/dt^2
θ, dθ/dt and d$^2\theta$/dt^2
Time (s)
Angular value (°, °/s, or °/s^2)

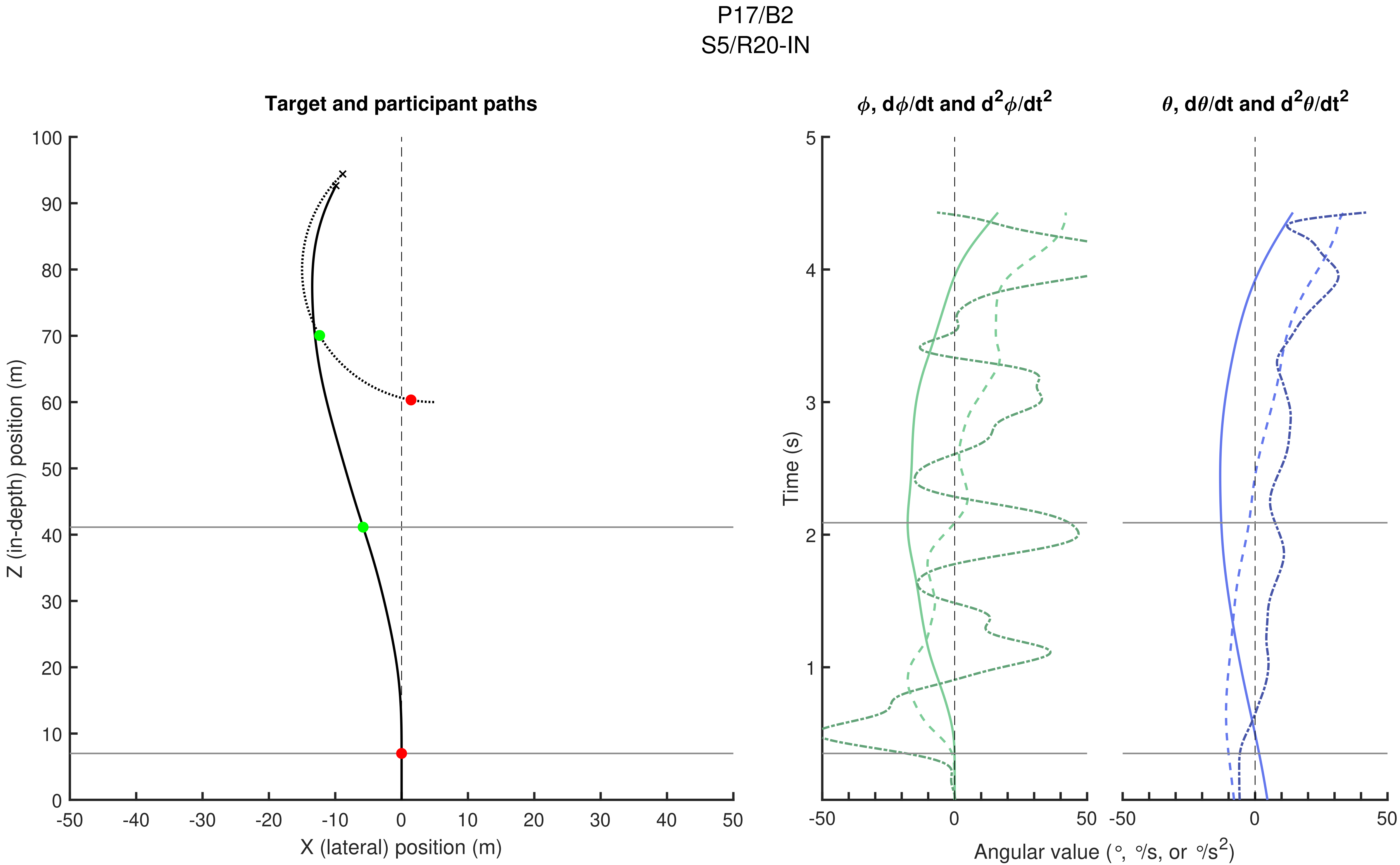

P17/B2
S5/R20-IN
Target and participant paths
Z (in-depth) position (m)
X (lateral) position (m)
ϕ, dϕ/dt and d$^2\phi$/dt^2
θ, dθ/dt and d$^2\theta$/dt^2
Time (s)
Angular value (°, °/s, or °/s^2)

P17/B2
S5/R30-OUT

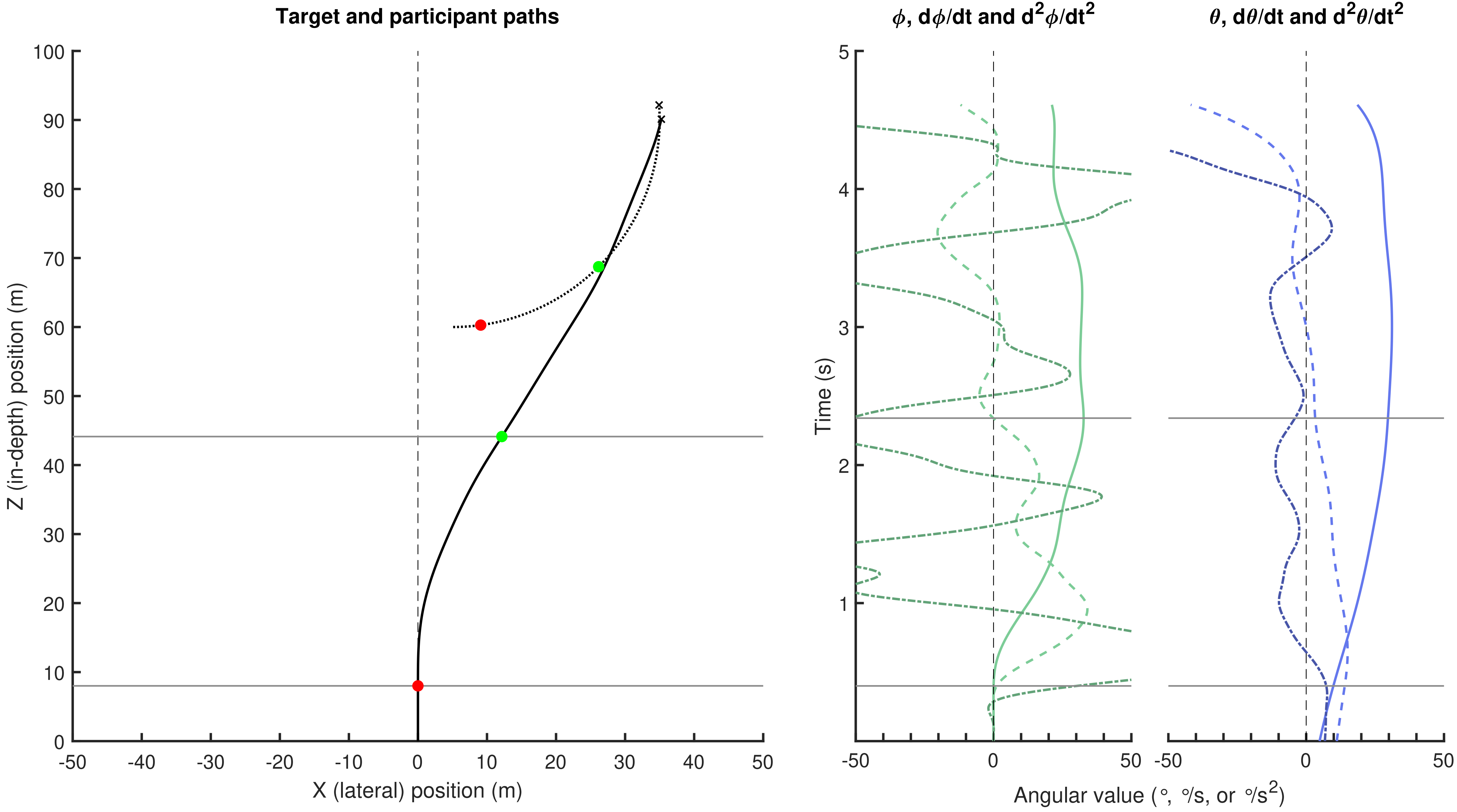

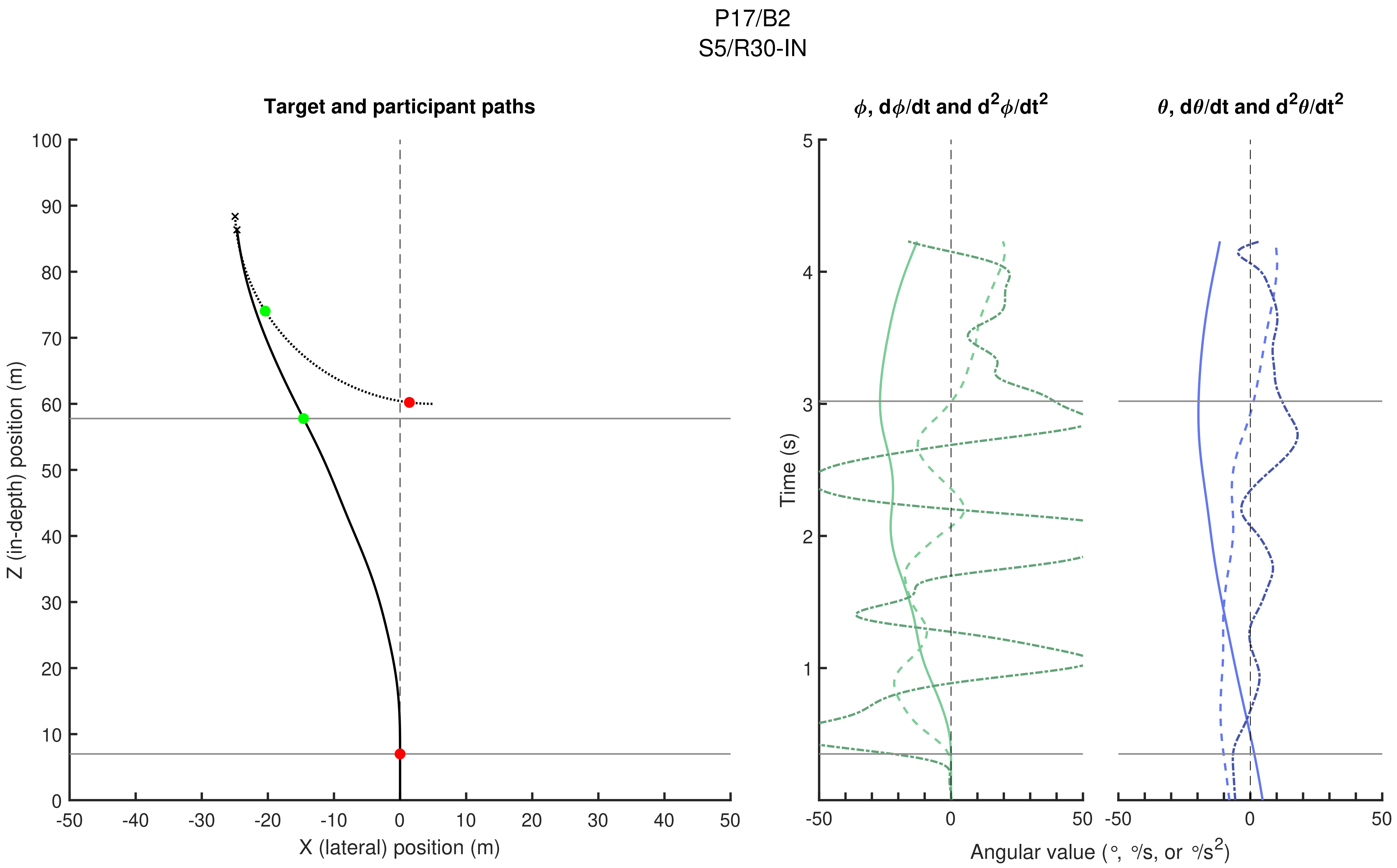
P17/B2
S5/R30-IN
Target and participant paths
Z (in-depth) position (m)
X (lateral) position (m)
φ, dφ/dt and d²φ/dt²
θ, dθ/dt and d²θ/dt²
Time (s)
Angular value (°, °/s, or °/s²)

P17/B2
S15/R10-IN

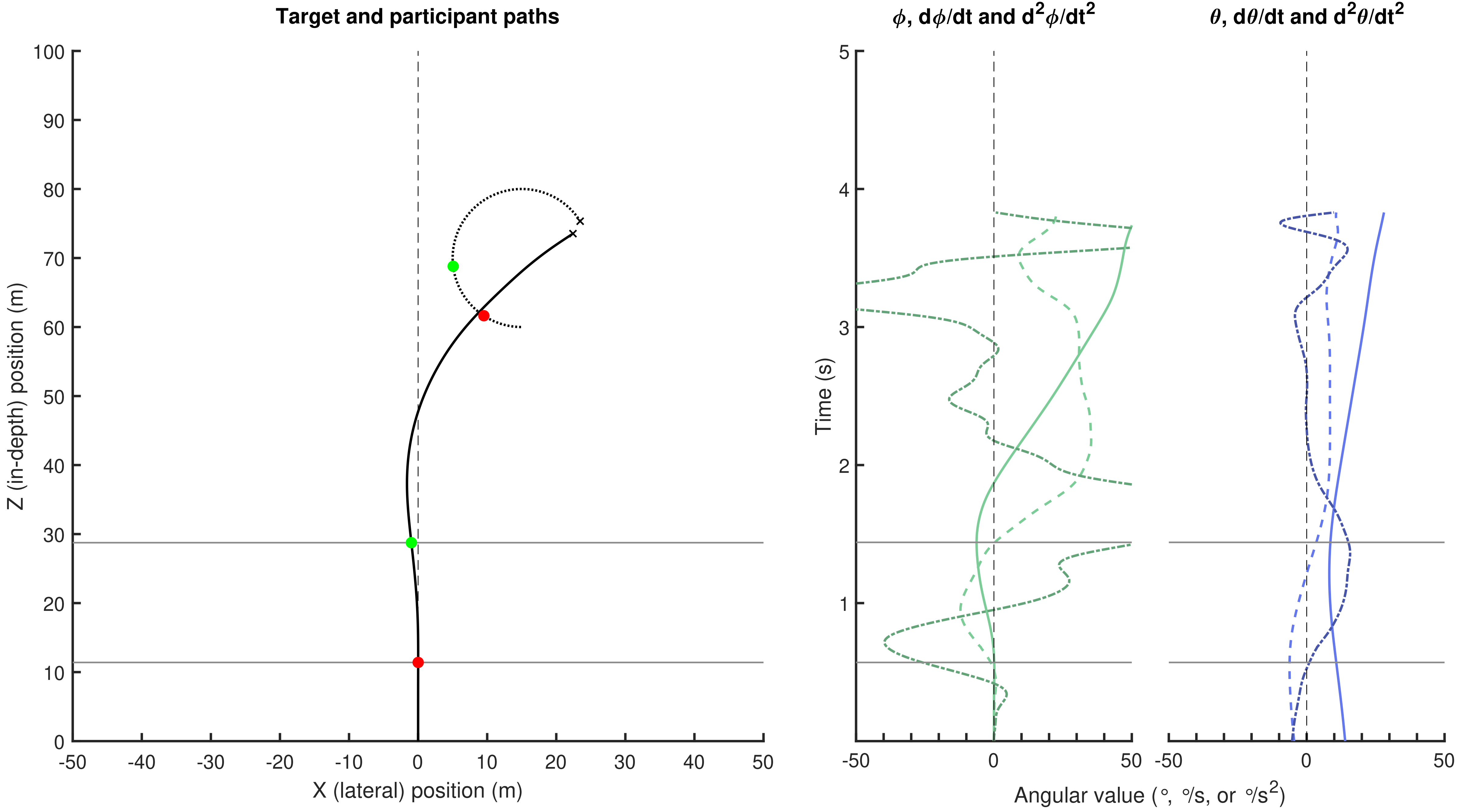

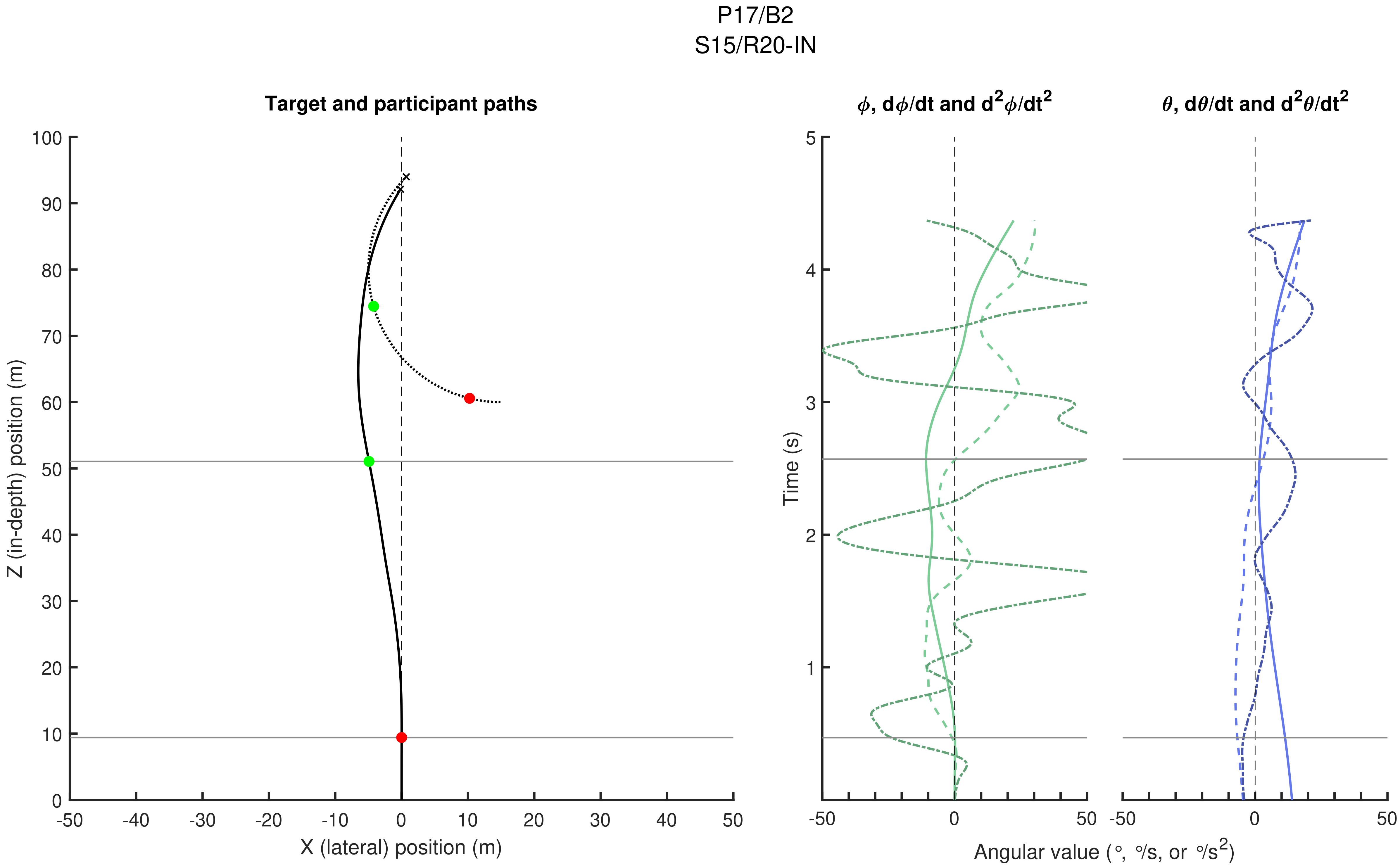
P17/B2
S15/R20-IN
Target and participant paths
Z (in-depth) position (m)
X (lateral) position (m)
0
10
20
30
40
50
60
70
80
90
100
-50
-40
-30
-20
-10
0
10
20
30
40
50
ϕ, dϕ/dt and d²ϕ/dt²
θ, dθ/dt and d²θ/dt²
Time (s)
1
2
3
4
5
-50
0
50
-50
0
50
Angular value (°, °/s, or °/s²)

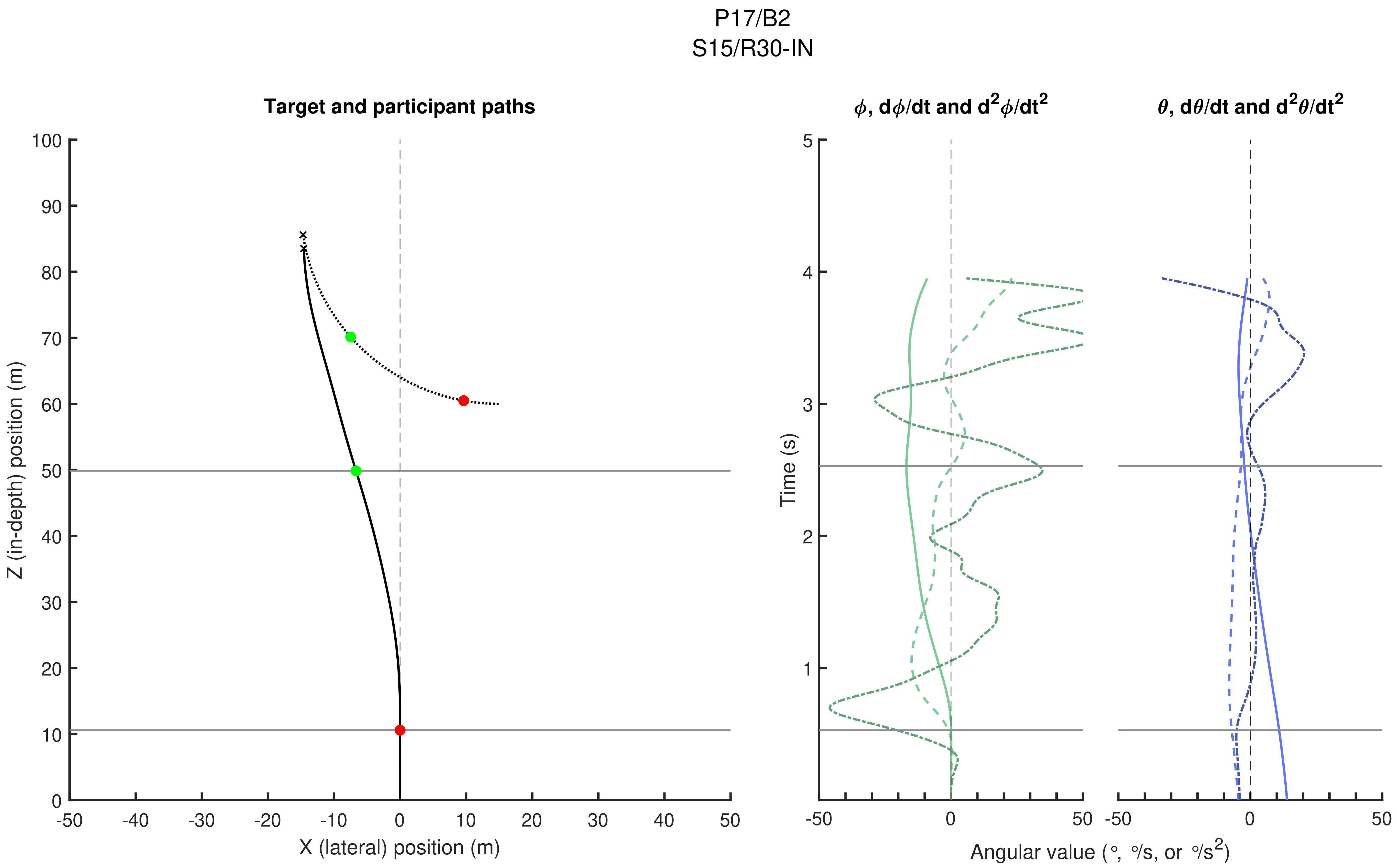
P17/B2
S15/R30-IN
Target and participant paths
ϕ, dϕ/dt and d$^2\phi$/dt^2
θ, dθ/dt and d$^2\theta$/dt^2
Z (in-depth) position (m)
X (lateral) position (m)
Time (s)
Angular value (°, °/s, or °/s^2)
100
90
80
70
60
50
40
30
20
10
0
-50
-40
-30
-20
-10
0
10
20
30
40
50
5
4
3
2
1
-50
0
50
-50
0
50

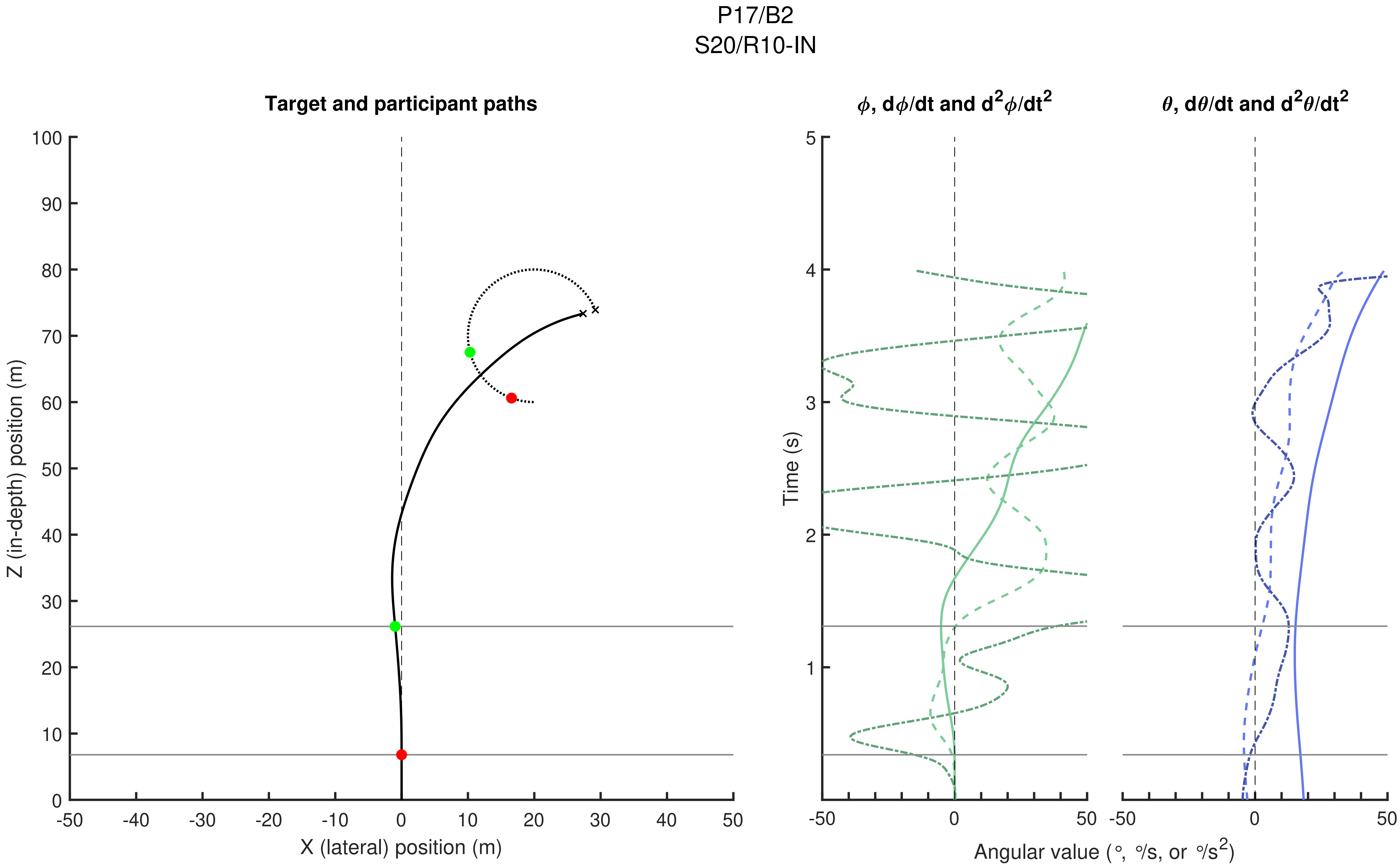
P17/B2
S20/R10-IN
Target and participant paths
Z (in-depth) position (m)
X (lateral) position (m)
100
90
80
70
60
50
40
30
20
10
0
-50
-40
-30
-20
-10
0
10
20
30
40
50
ϕ, dϕ/dt and d²ϕ/dt²
θ, dθ/dt and d²θ/dt²
Time (s)
5
4
3
2
1
-50
0
50
-50
0
50
Angular value (°, °/s, or °/s²)

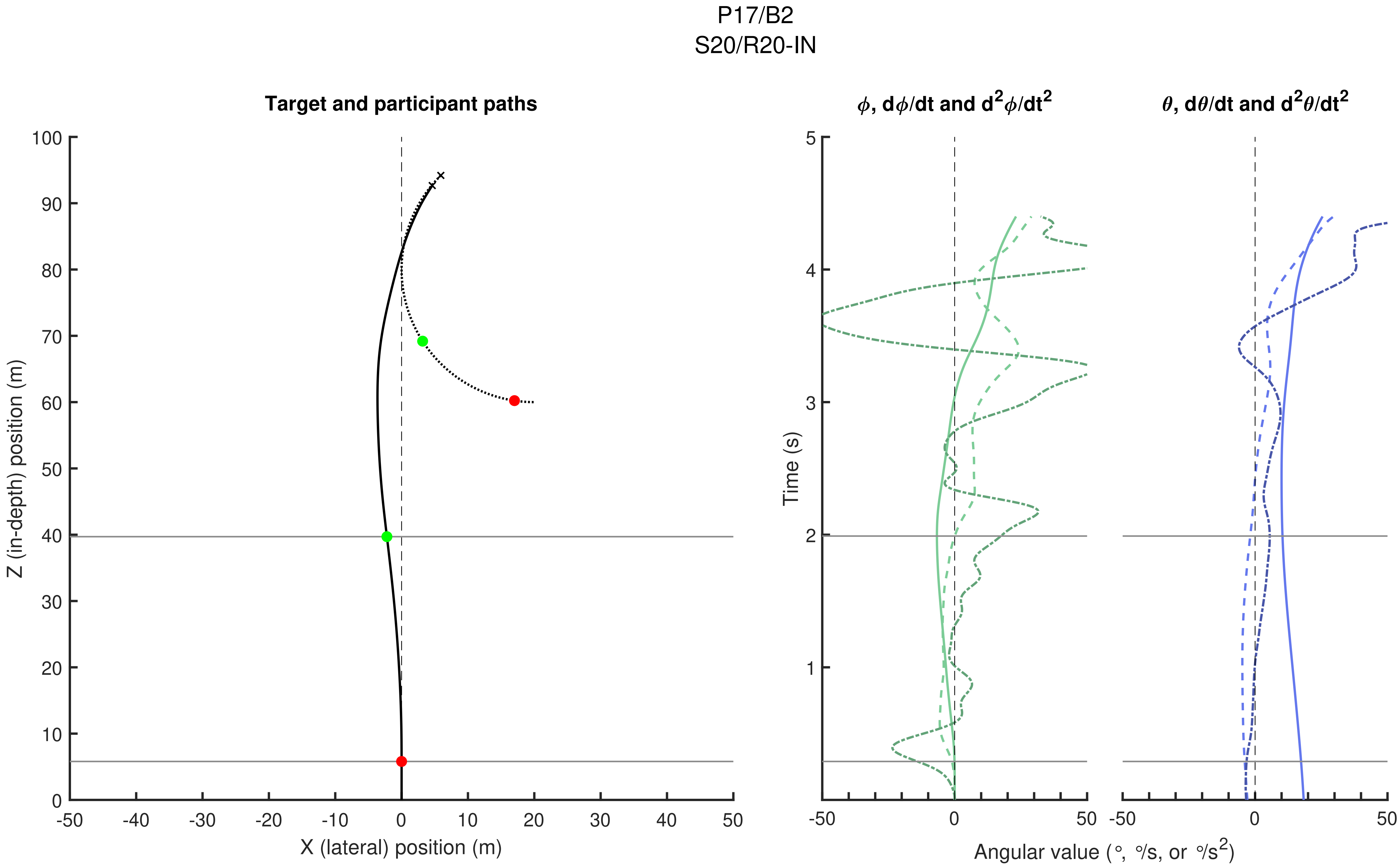

P17/B2
S20/R20-IN
Target and participant paths
ϕ, dϕ/dt and d$^2\phi$/dt^2
θ, dθ/dt and d$^2\theta$/dt^2
Z (in-depth) position (m)
X (lateral) position (m)
Time (s)
Angular value (°, °/s, or °/s^2)
100
90
80
70
60
50
40
30
20
10
0
-50
-40
-30
-20
-10
0
10
20
30
40
50
5
4
3
2
1
-50
0
50
-50
0
50

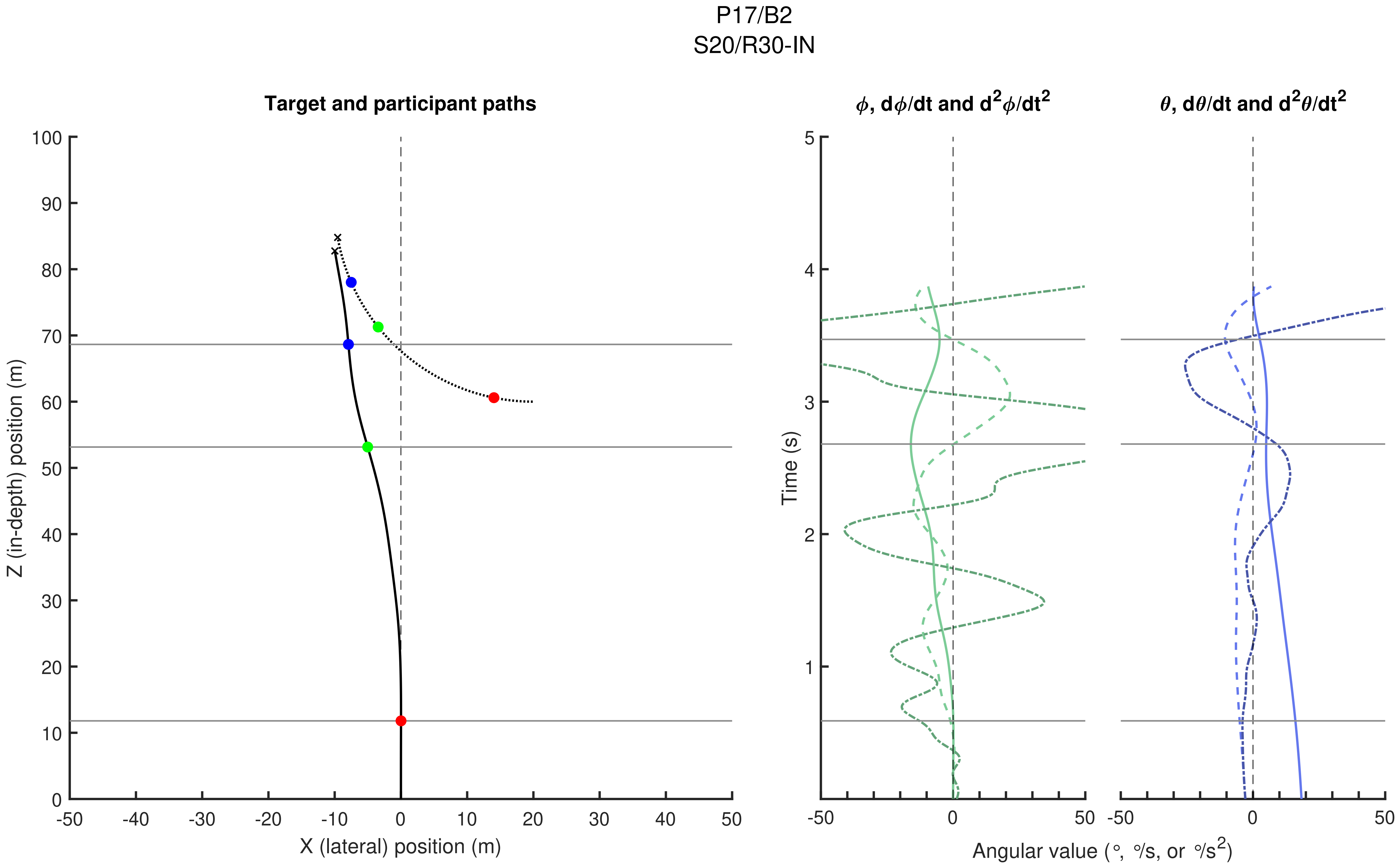

P17/B2
S20/R30-IN
Target and participant paths
ϕ, dϕ/dt and d$^2\phi$/dt^2
θ, dθ/dt and d$^2\theta$/dt^2
Z (in-depth) position (m)
X (lateral) position (m)
Time (s)
Angular value (°, °/s, or °/s^2)

P17/B3
S20/R10-IN

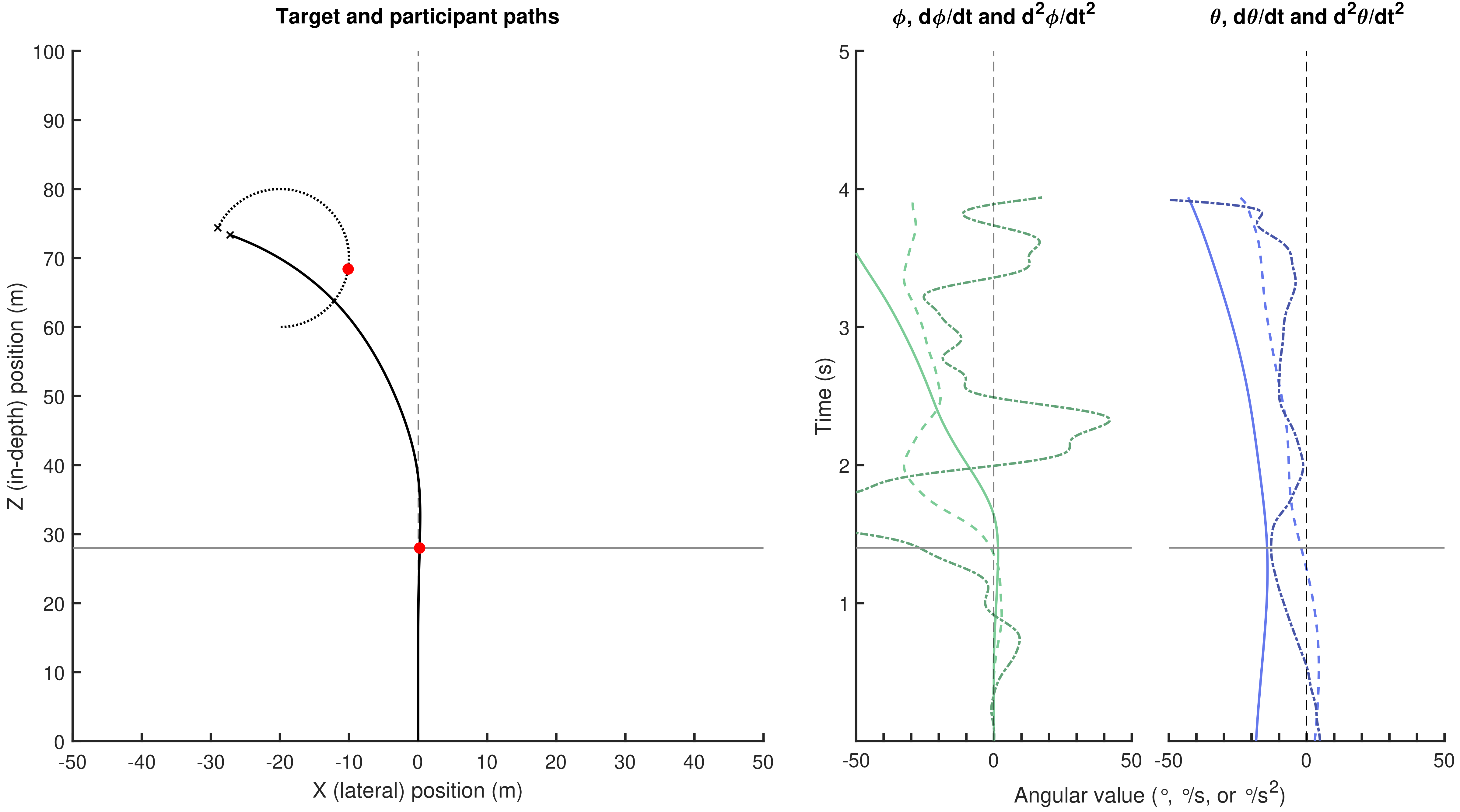

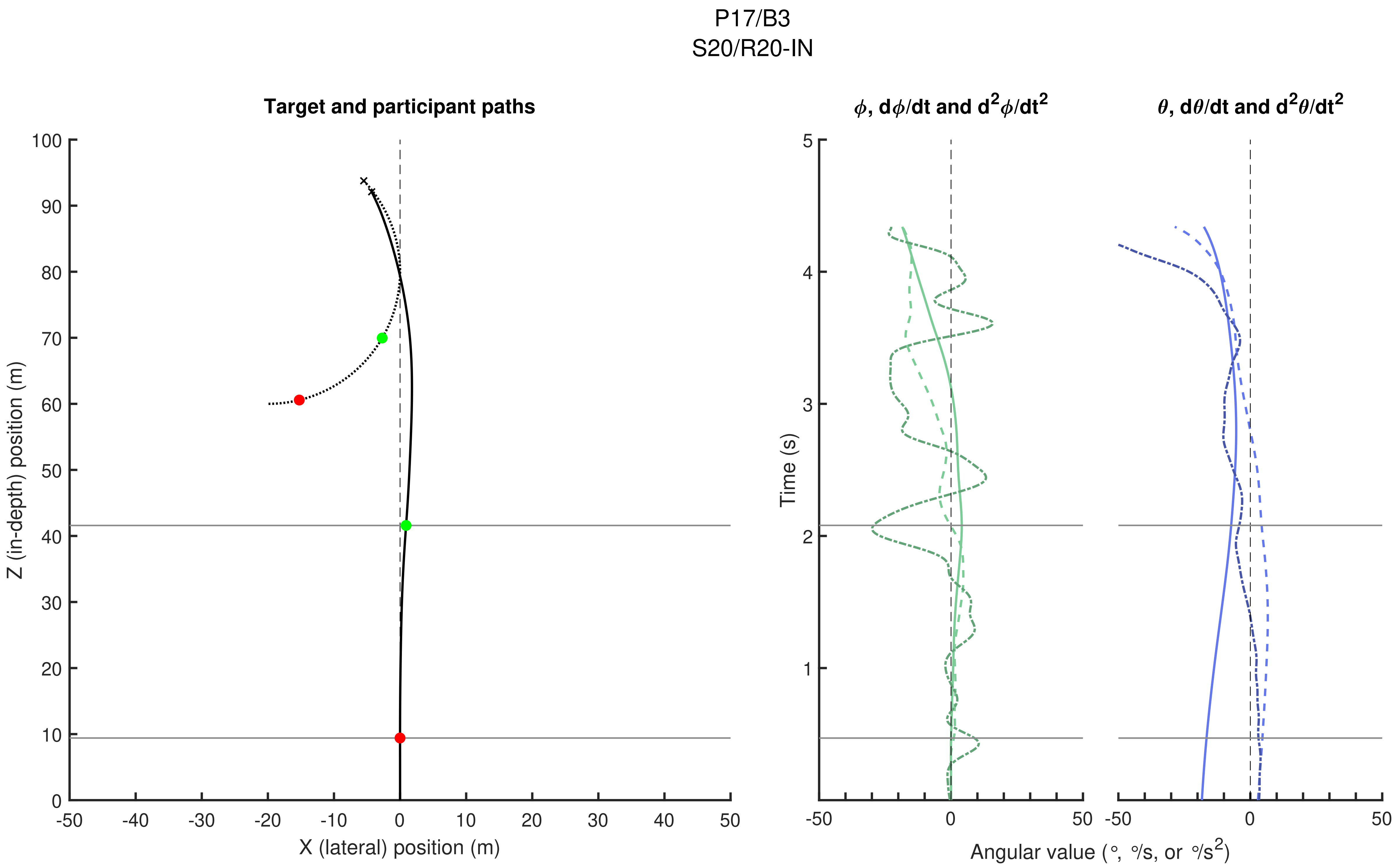
P17/B3
S20/R20-IN
Target and participant paths
ϕ, dϕ/dt and d$^2\phi$/dt^2
θ, dθ/dt and d$^2\theta$/dt^2
Z (in-depth) position (m)
X (lateral) position (m)
Time (s)
Angular value (°, °/s, or °/s^2)

P17/B3
S20/R30-IN

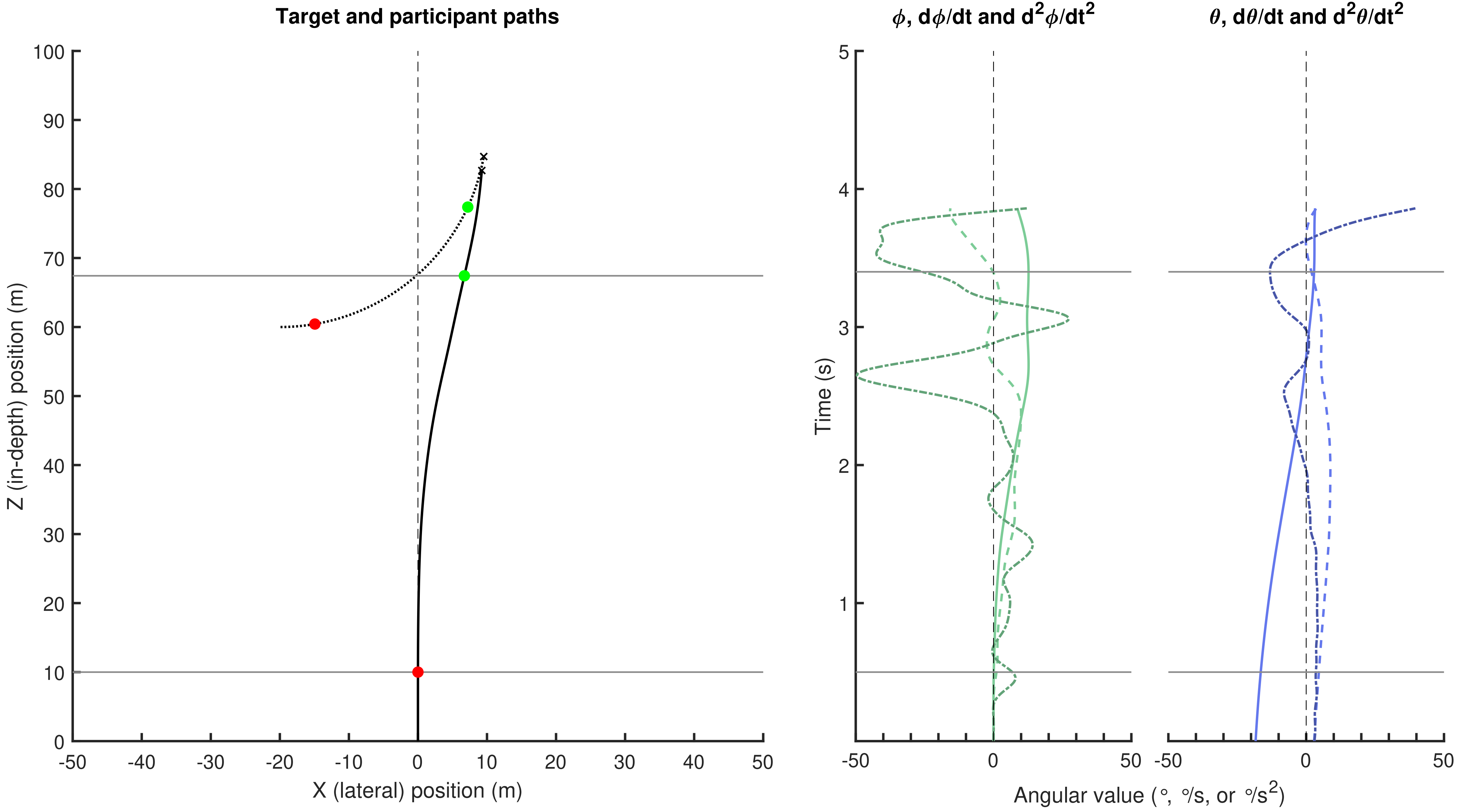

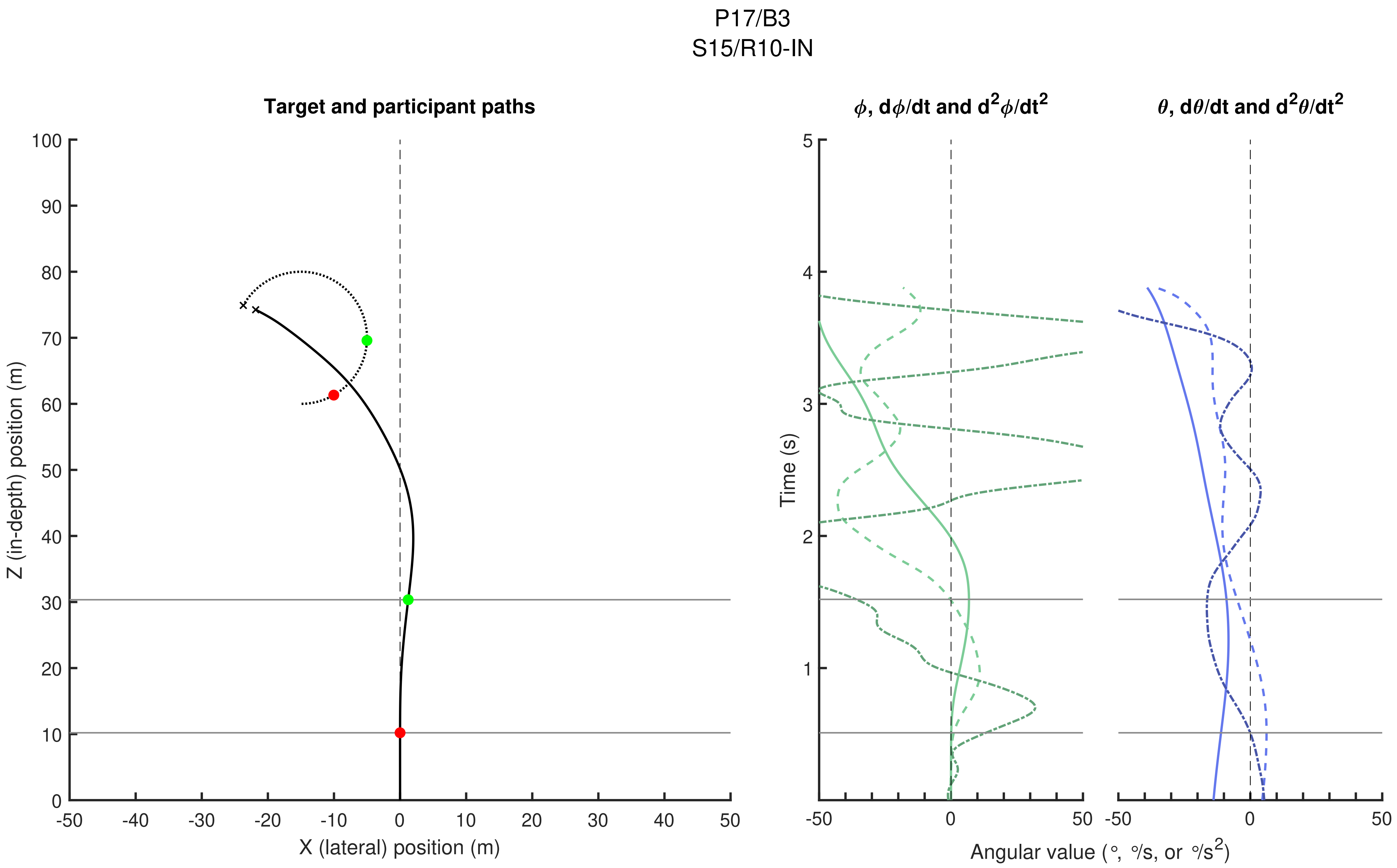
P17/B3
S15/R10-IN
Target and participant paths
Z (in-depth) position (m)
X (lateral) position (m)
ϕ, dϕ/dt and d$^2\phi$/dt^2
θ, dθ/dt and d$^2\theta$/dt^2
Time (s)
Angular value (°, °/s, or °/s^2)

P17/B3
S15/R20-IN

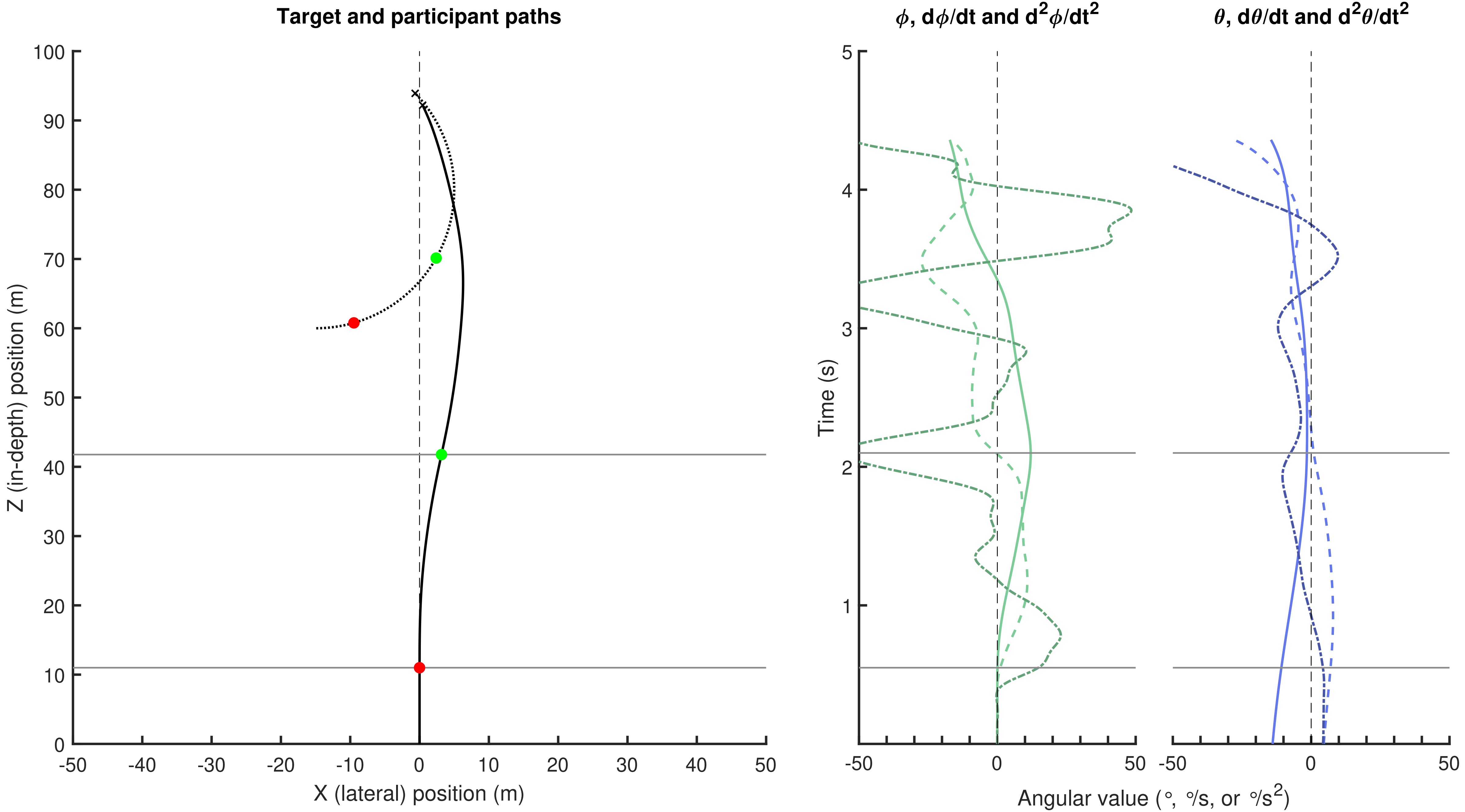

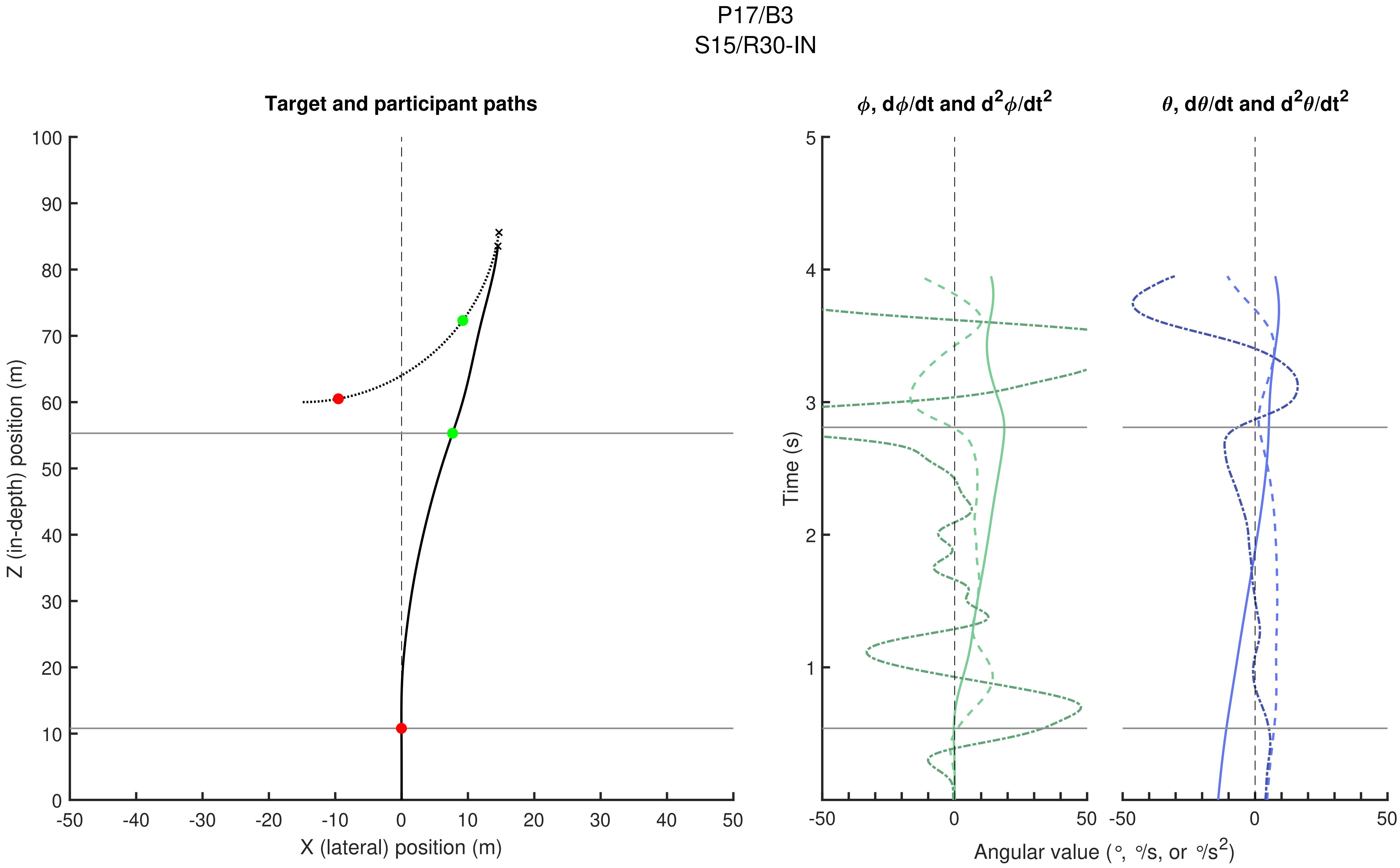

P17/B3
S15/R30-IN
Target and participant paths
Z (in-depth) position (m)
X (lateral) position (m)
ϕ, dϕ/dt and d$^2\phi$/dt^2
θ, dθ/dt and d$^2\theta$/dt^2
Time (s)
Angular value (°, °/s, or °/s^2)

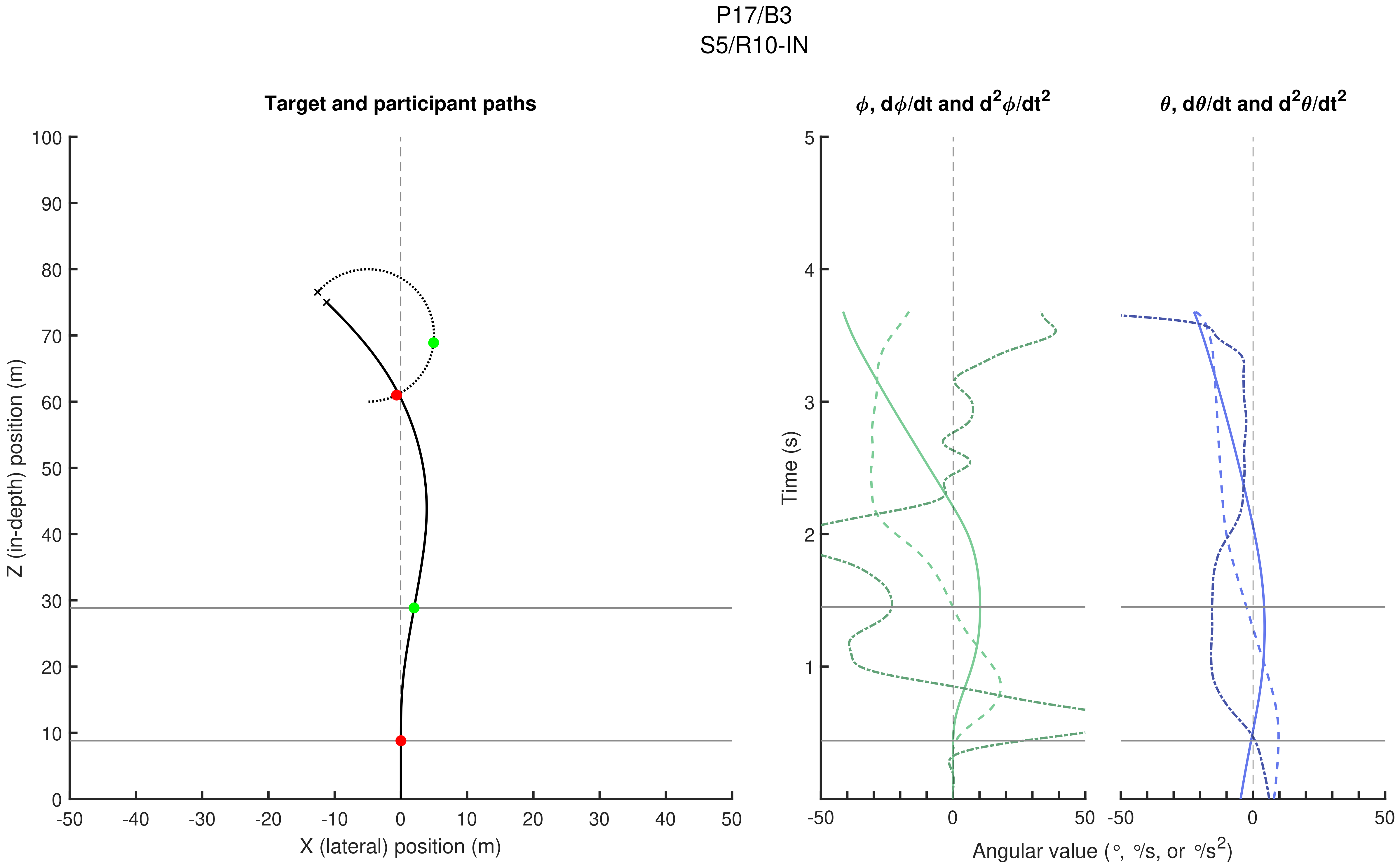

P17/B3
S5/R10-IN
Target and participant paths
Z (in-depth) position (m)
X (lateral) position (m)
ϕ, dϕ/dt and d$^2\phi$/dt^2
θ, dθ/dt and d$^2\theta$/dt^2
Time (s)
Angular value (°, °/s, or °/s^2)

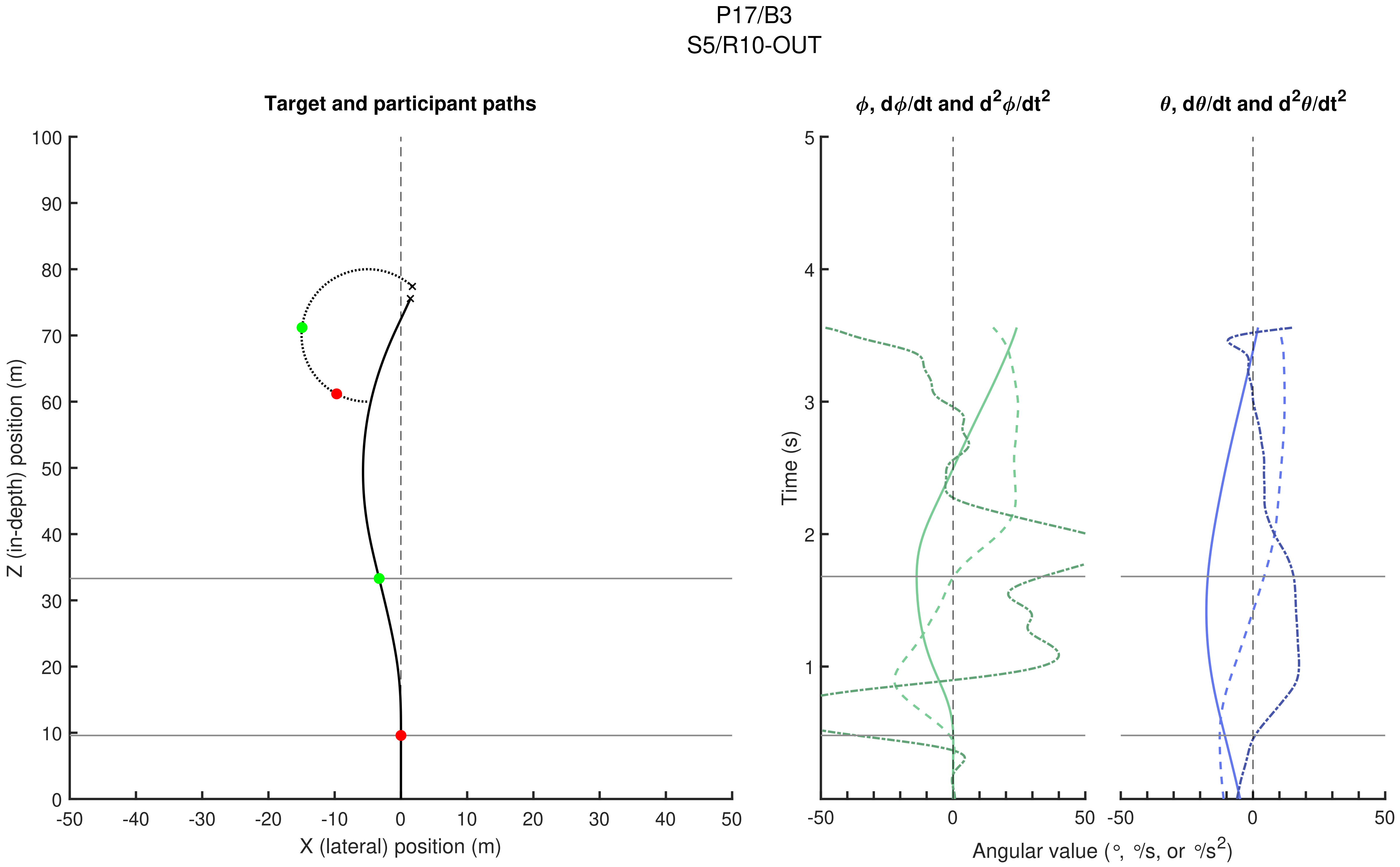

P17/B3
S5/R10-OUT
Target and participant paths
ϕ, dϕ/dt and d$^2\phi$/dt^2
θ, dθ/dt and d$^2\theta$/dt^2
Z (in-depth) position (m)
X (lateral) position (m)
Time (s)
Angular value (°, °/s, or °/s^2)

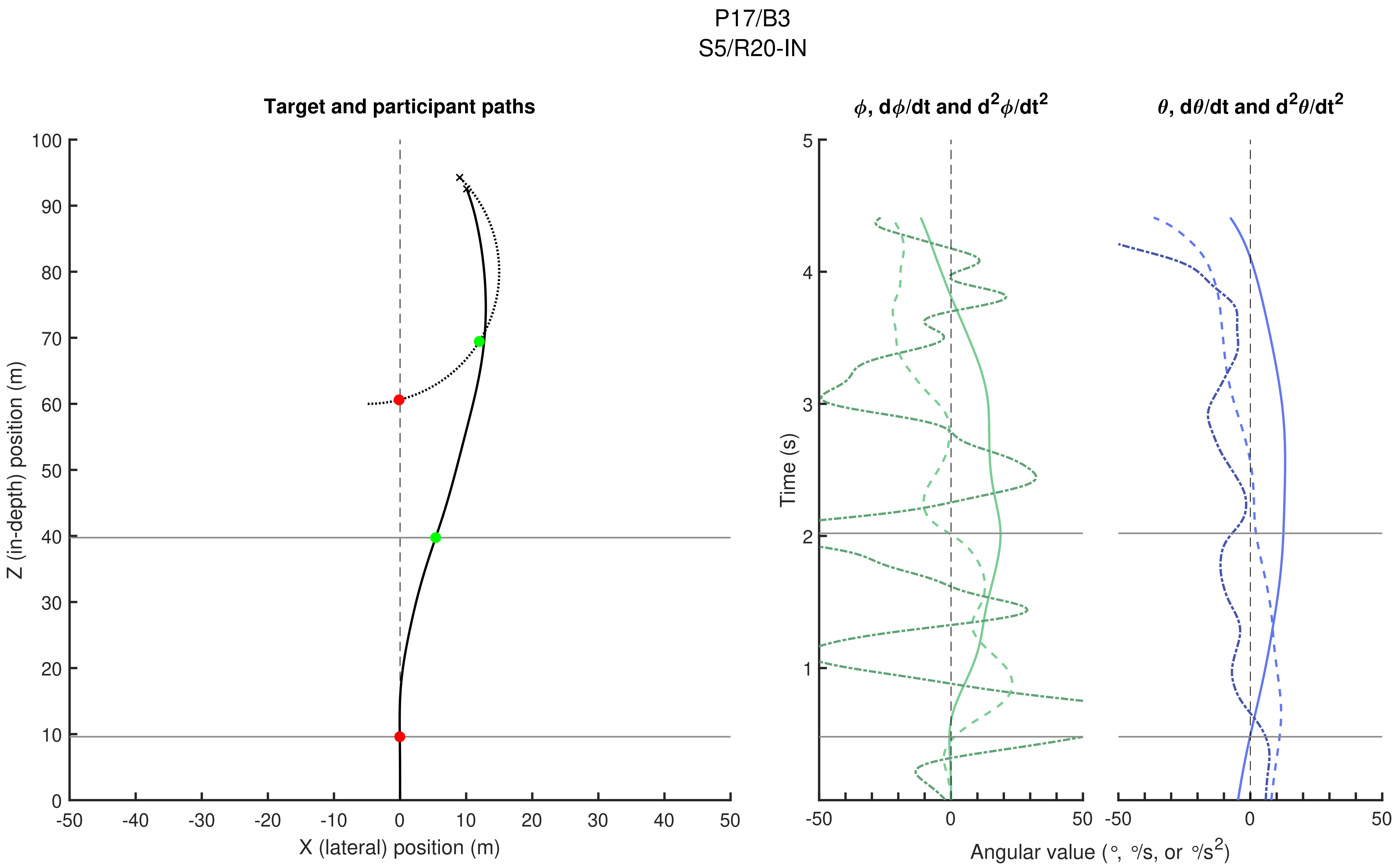
P17/B3
S5/R20-IN
Target and participant paths
Z (in-depth) position (m)
X (lateral) position (m)
ϕ, dϕ/dt and d$^2\phi$/dt^2
θ, dθ/dt and d$^2\theta$/dt^2
Time (s)
Angular value (°, °/s, or °/s^2)

P17/B3
S5/R20-OUT

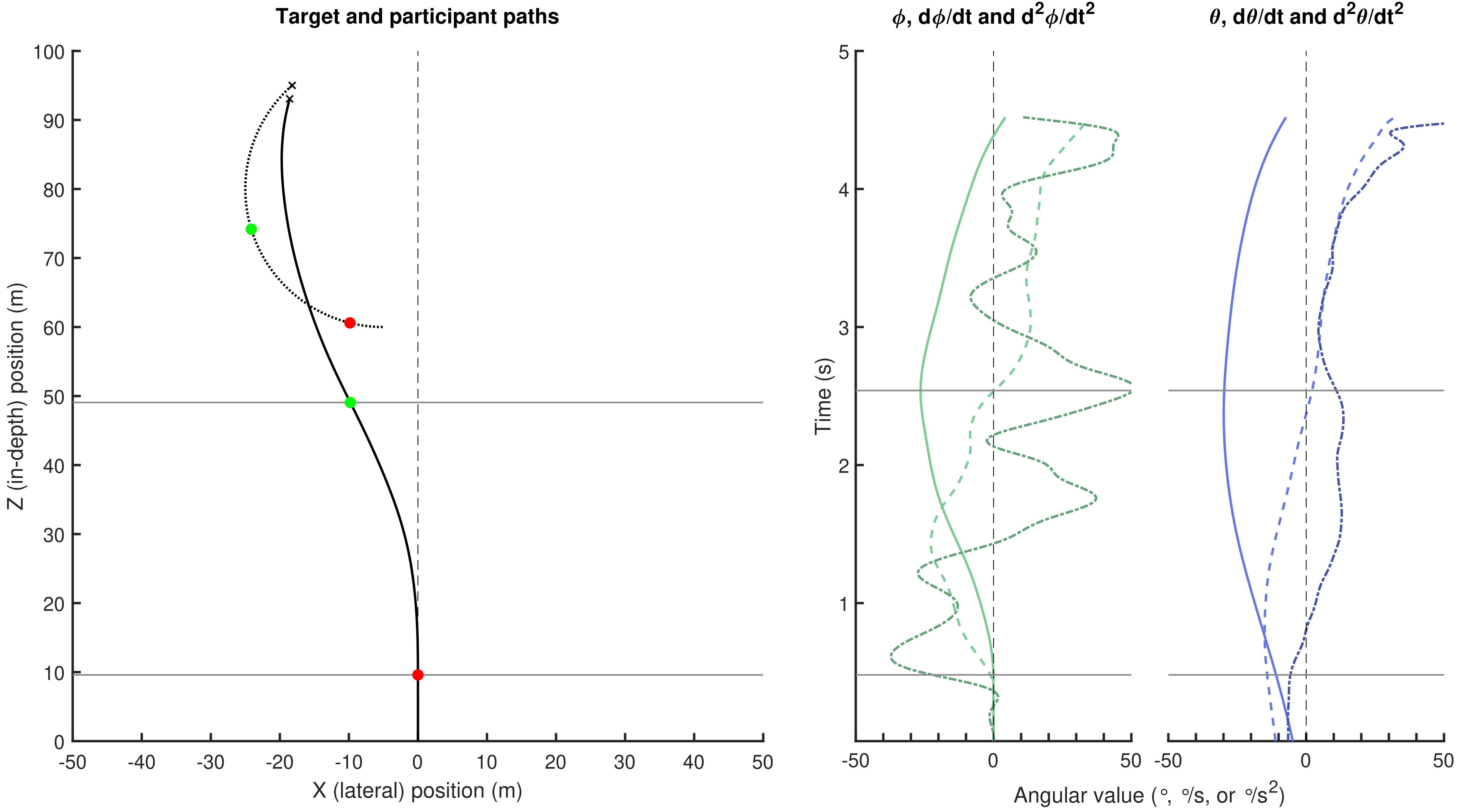

P17/B3
S5/R30-IN

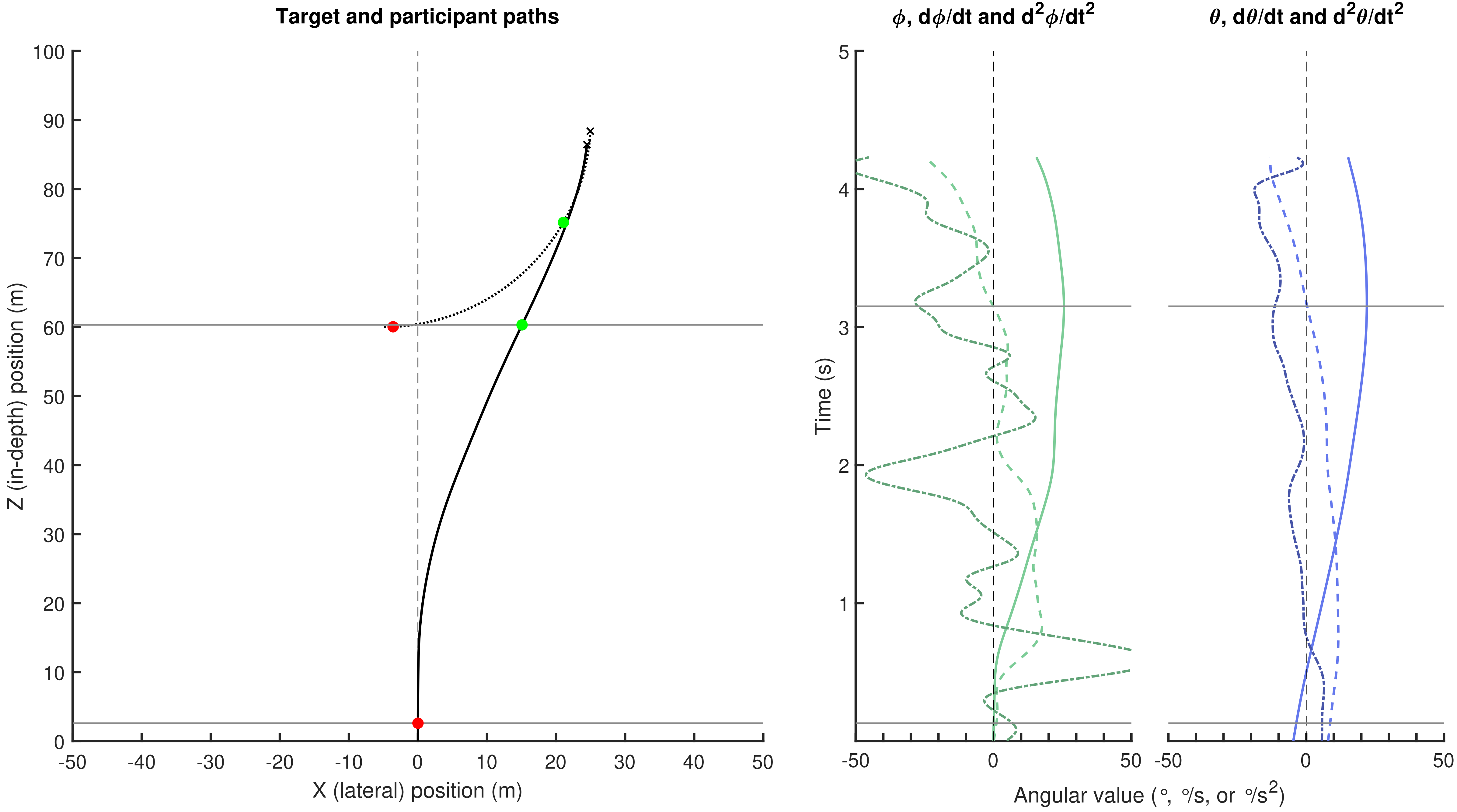

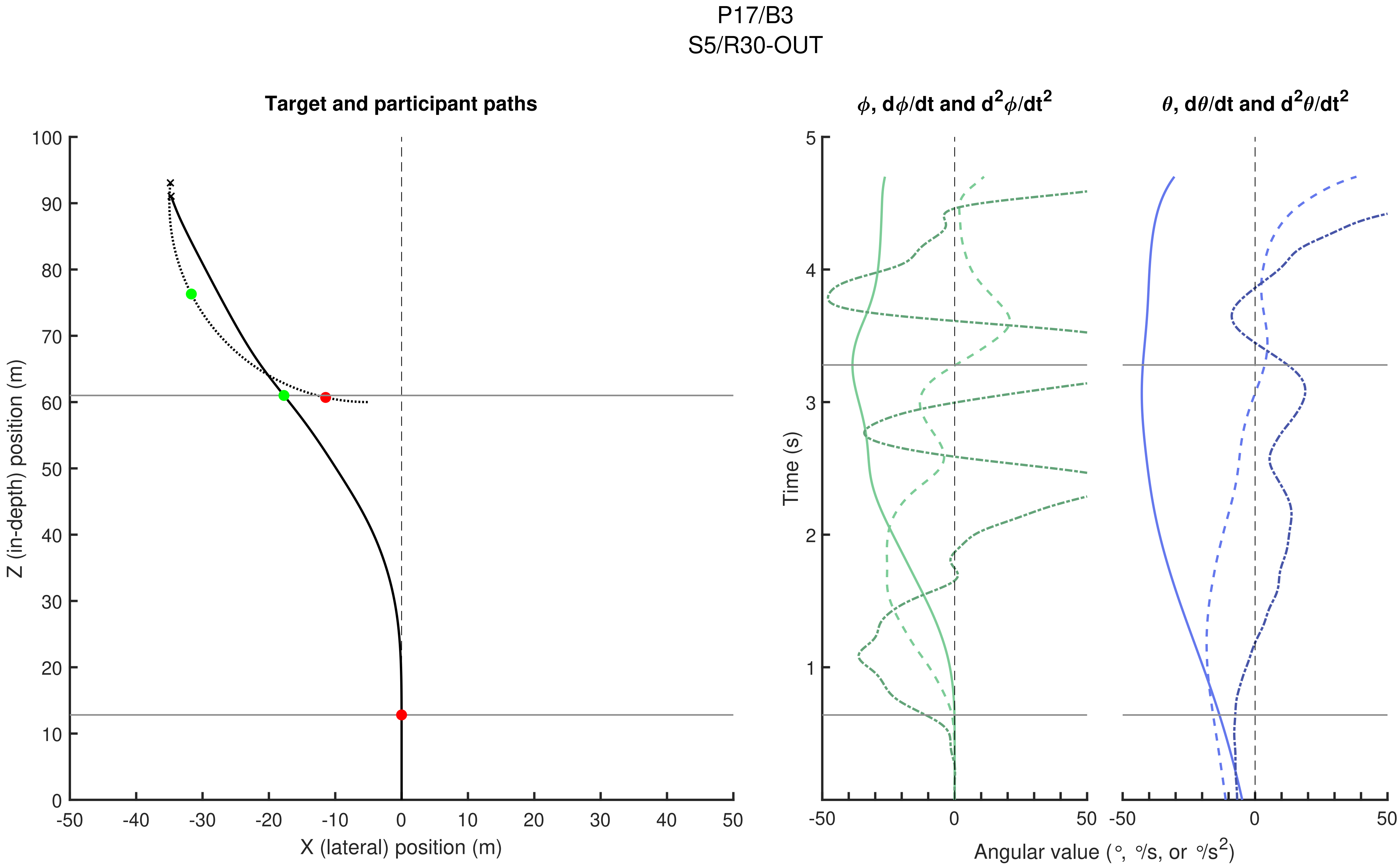
P17/B3
S5/R30-OUT
Target and participant paths
Z (in-depth) position (m)
X (lateral) position (m)
100
90
80
70
60
50
40
30
20
10
0
-50
-40
-30
-20
-10
0
10
20
30
40
50
ϕ, dϕ/dt and d$^2\phi$/dt^2
θ, dθ/dt and d$^2\theta$/dt^2
Time (s)
5
4
3
2
1
-50
0
50
-50
0
50
Angular value (°, °/s, or °/s^2)

P17/B3
S5/R10-OUT

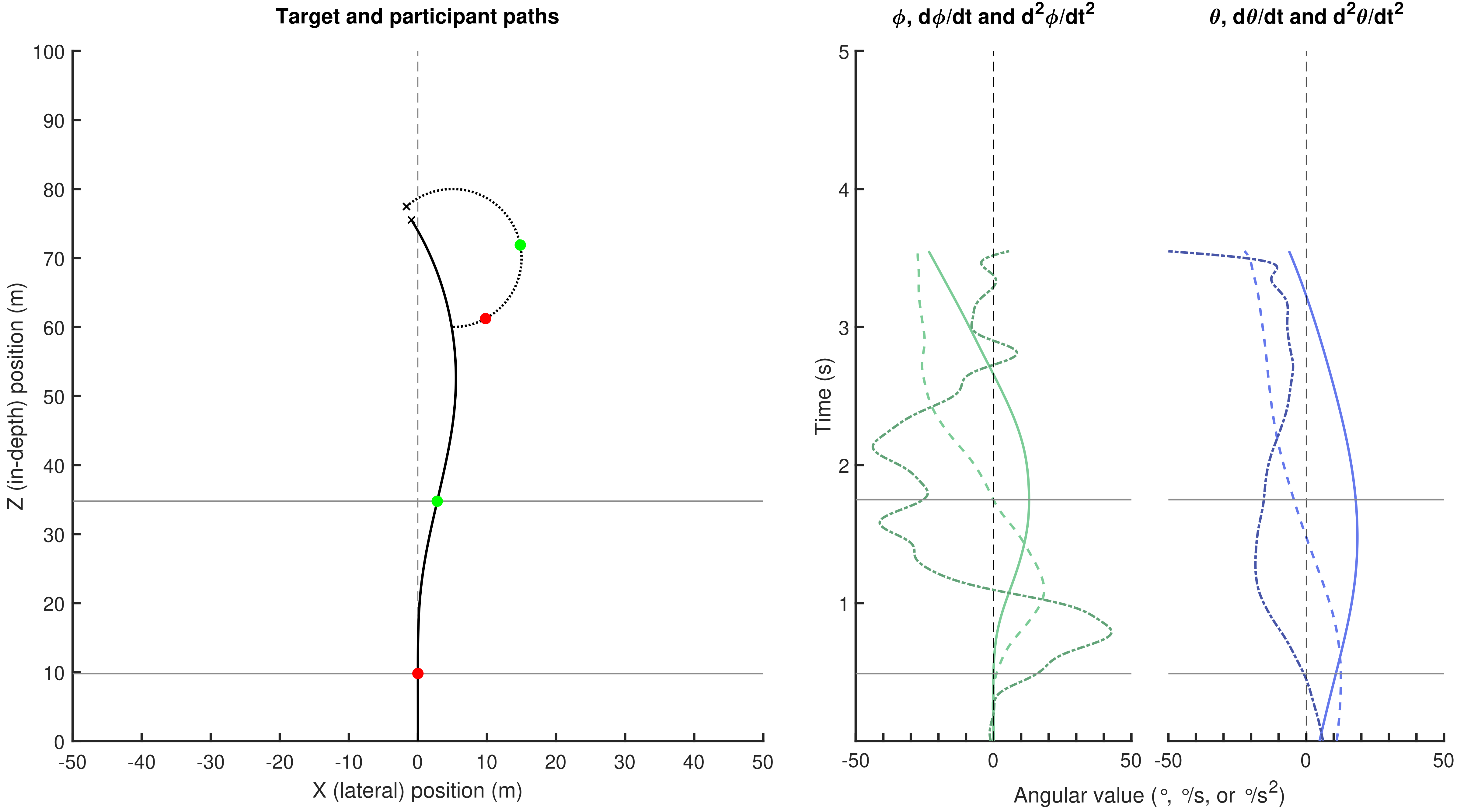

P17/B3
S5/R10-IN

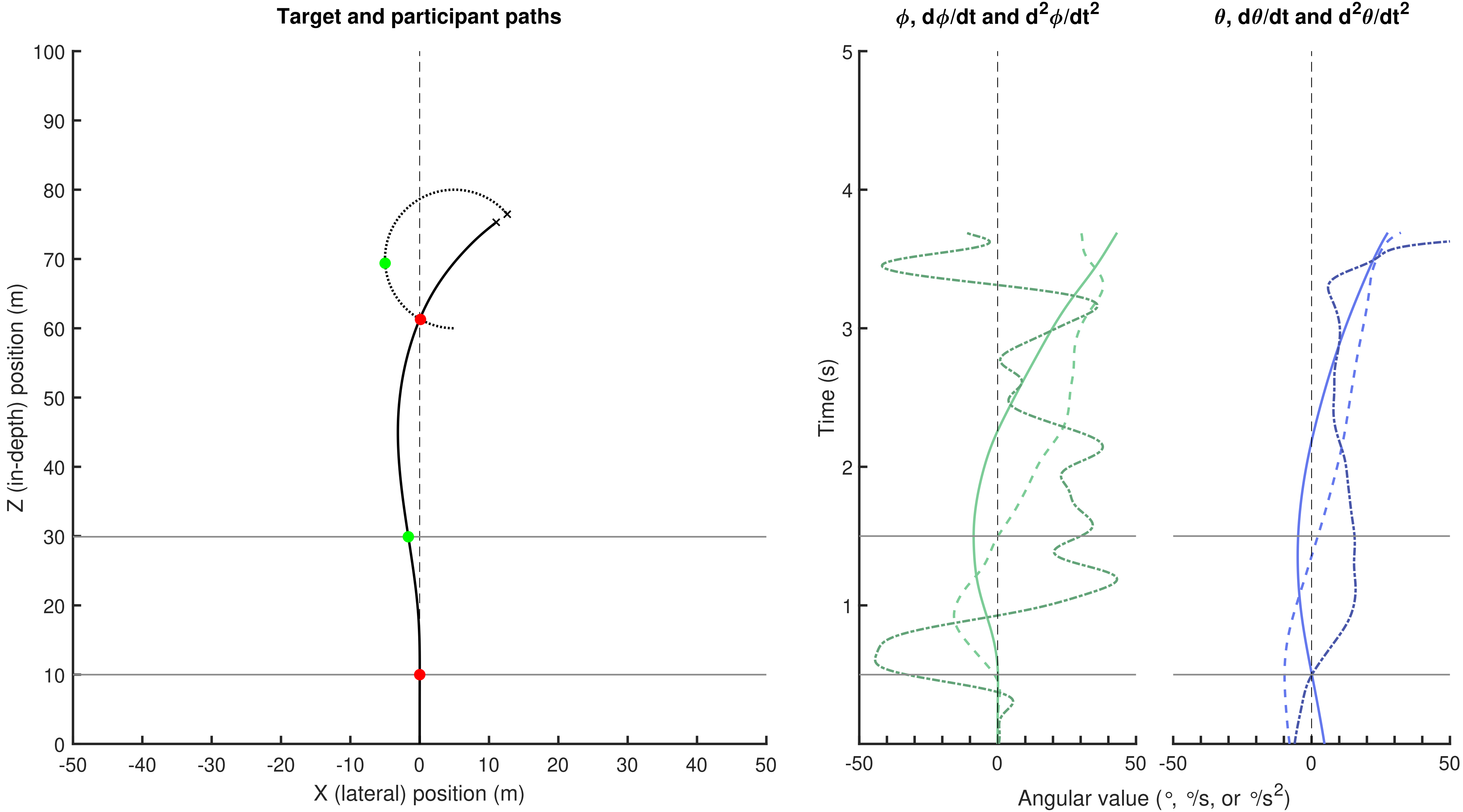

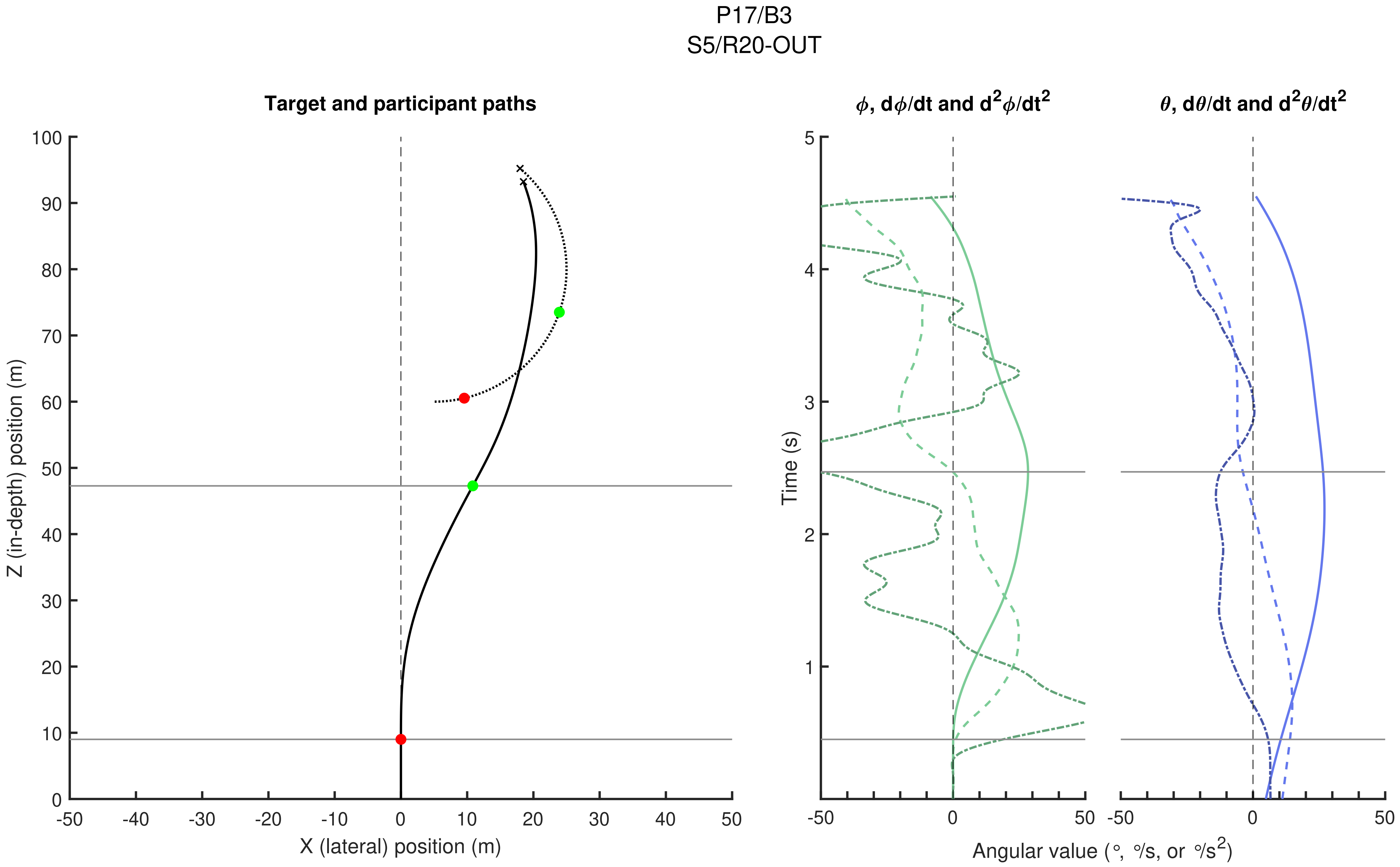
P17/B3
S5/R20-OUT
Target and participant paths
Z (in-depth) position (m)
X (lateral) position (m)
ϕ, dϕ/dt and d²ϕ/dt²
θ, dθ/dt and d²θ/dt²
Time (s)
Angular value (°, °/s, or °/s²)

P17/B3
S5/R20-IN

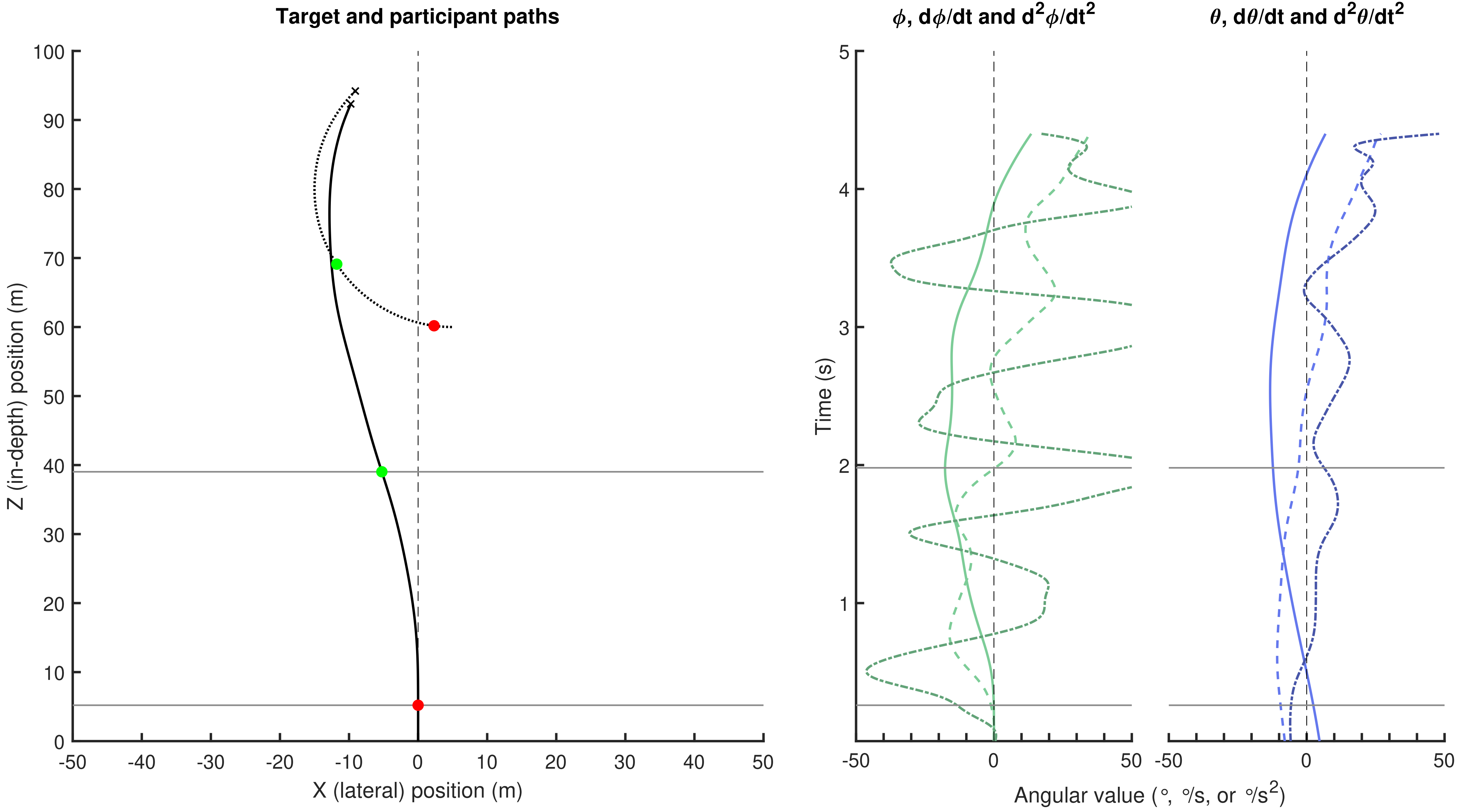

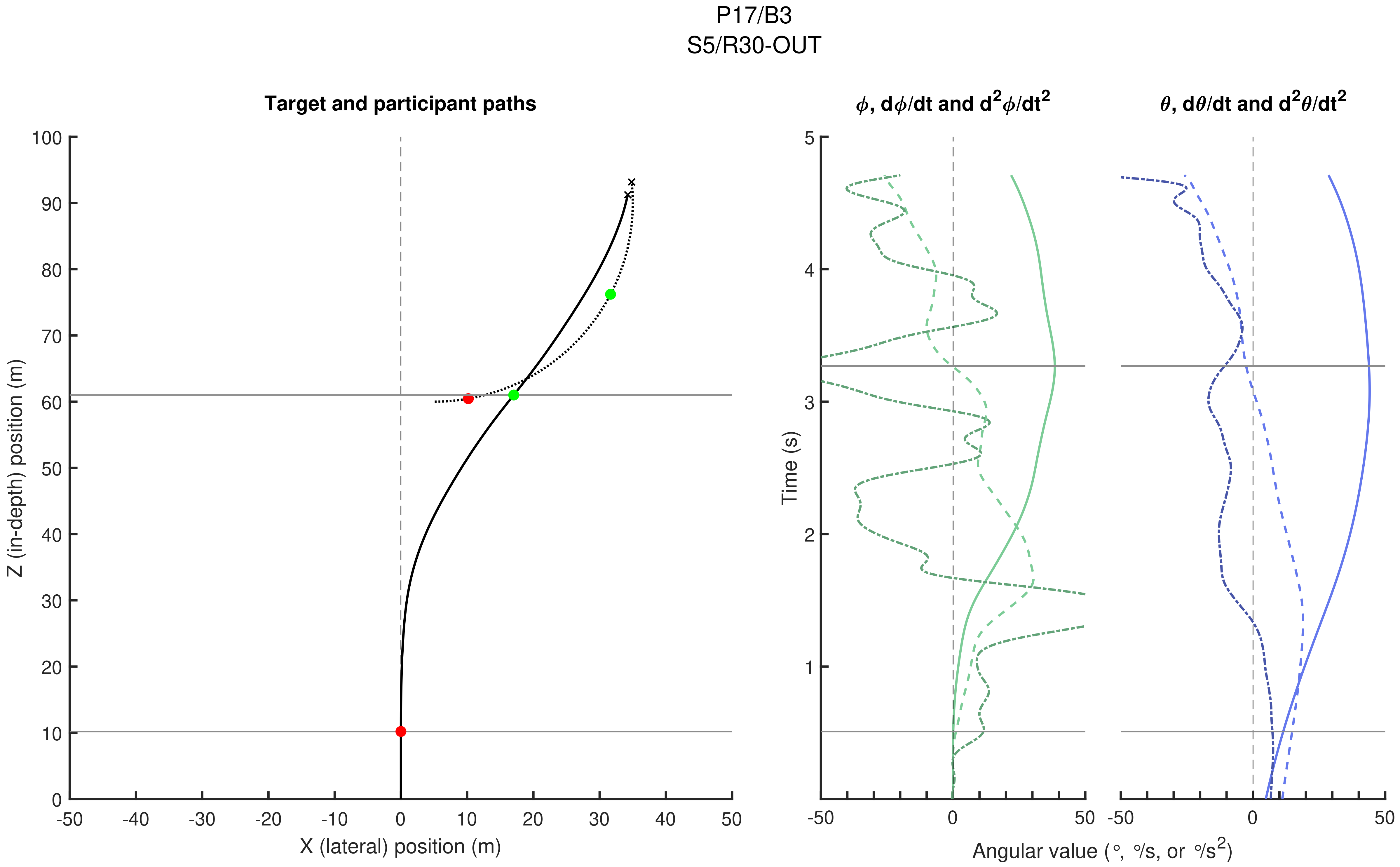

P17/B3
S5/R30-OUT
Target and participant paths
ϕ, dϕ/dt and d$^2\phi$/dt^2
θ, dθ/dt and d$^2\theta$/dt^2
Z (in-depth) position (m)
X (lateral) position (m)
Time (s)
Angular value (°, °/s, or °/s^2)

P17/B3
S5/R30-IN

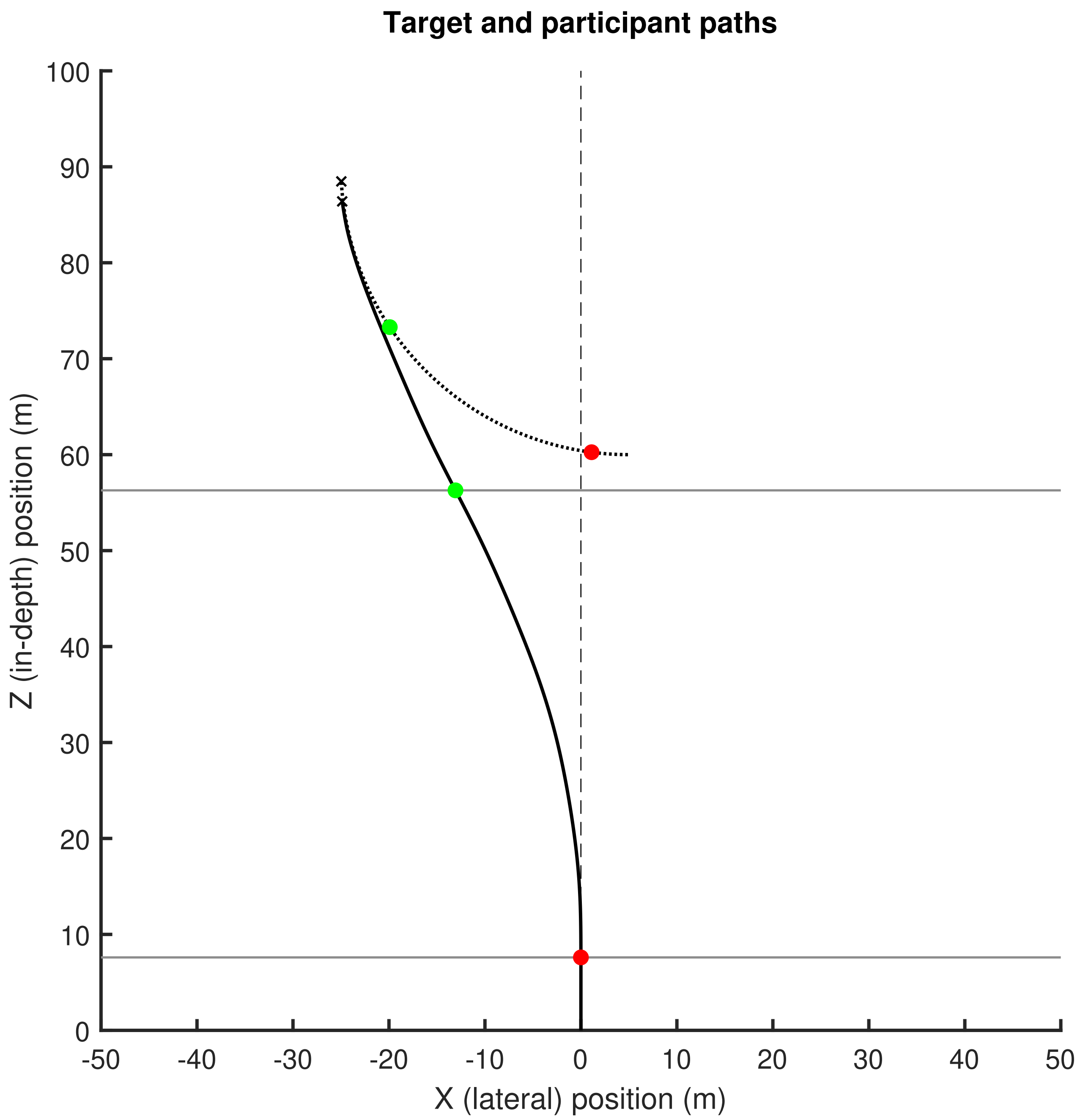

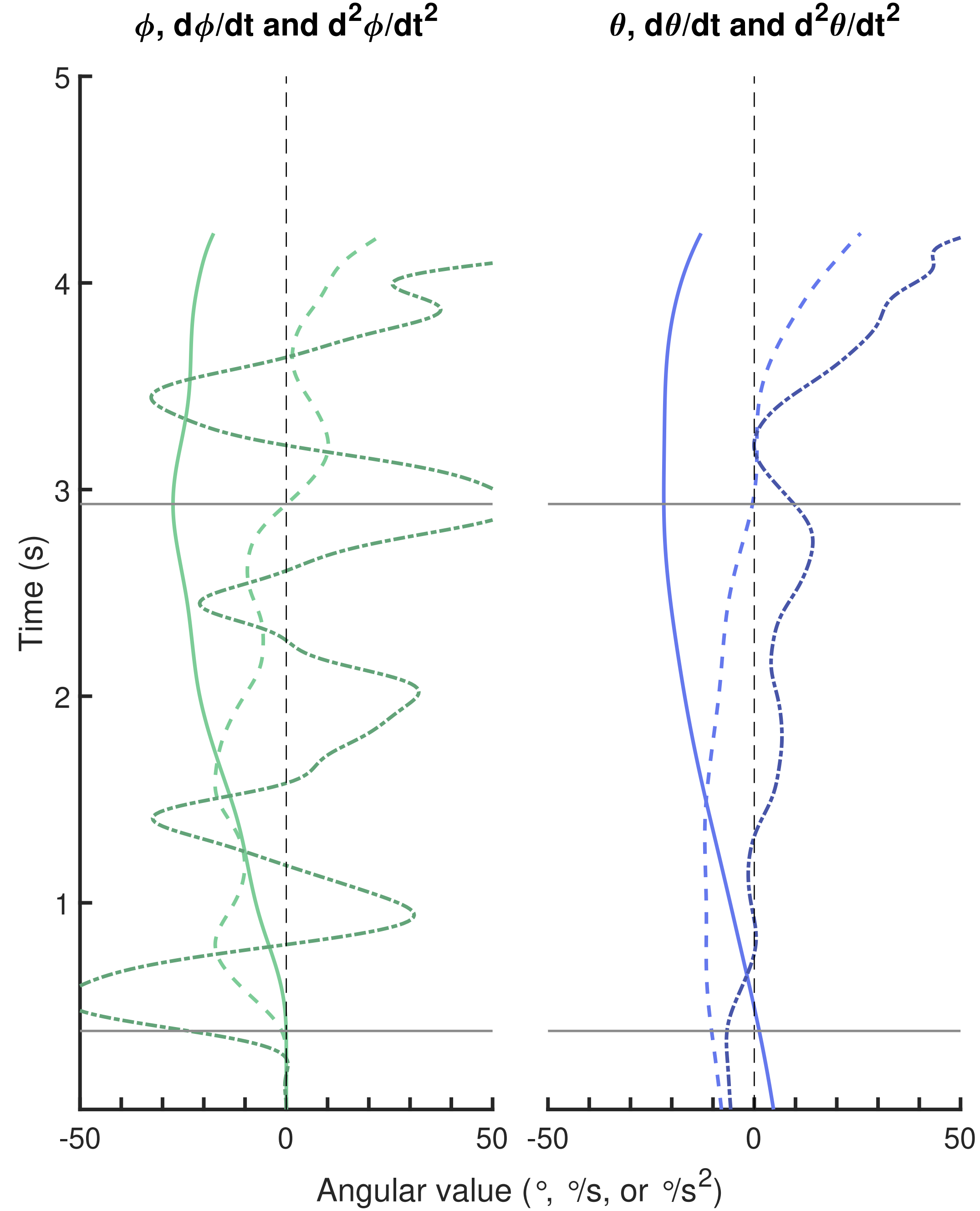

P17/B3
S15/R10-IN

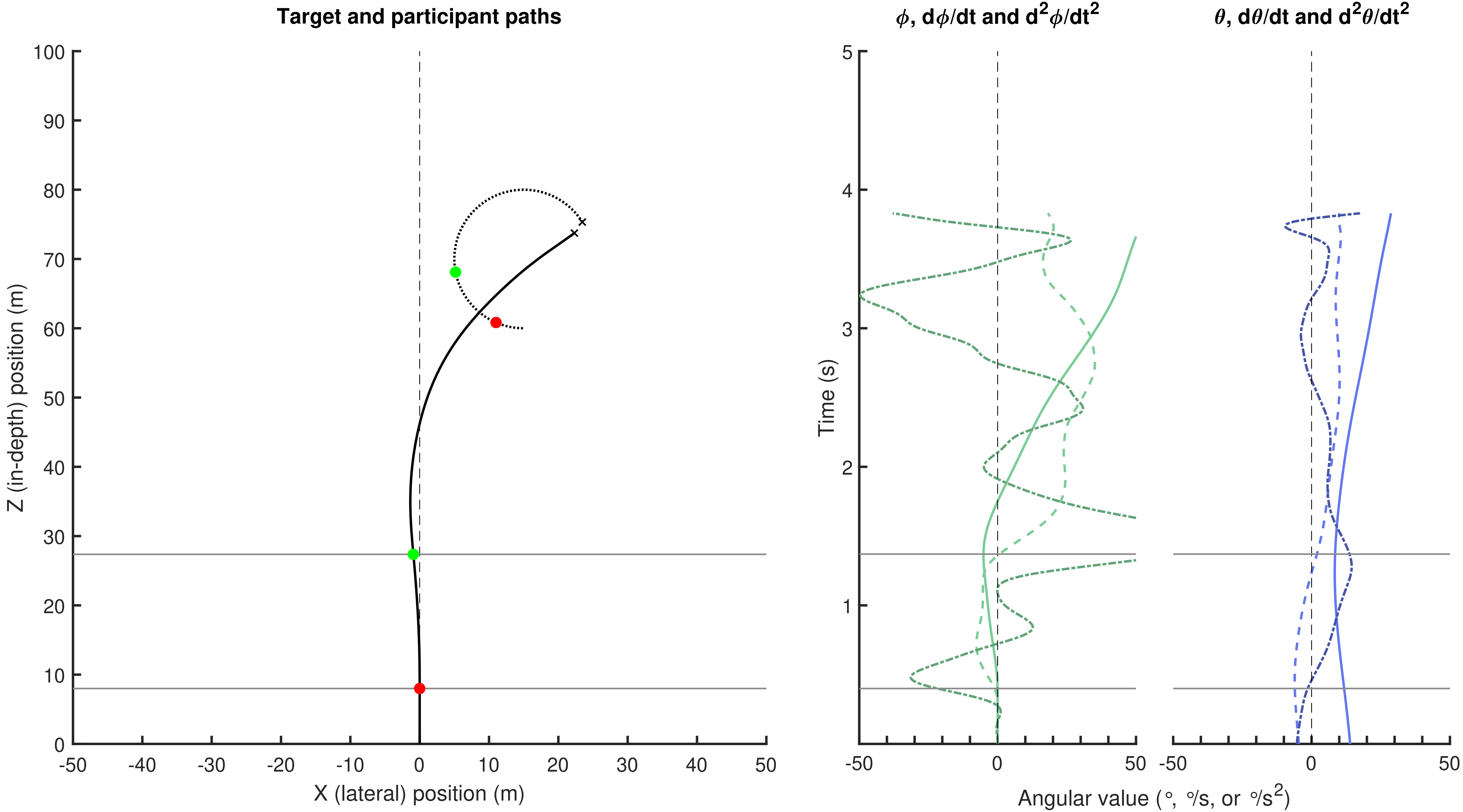

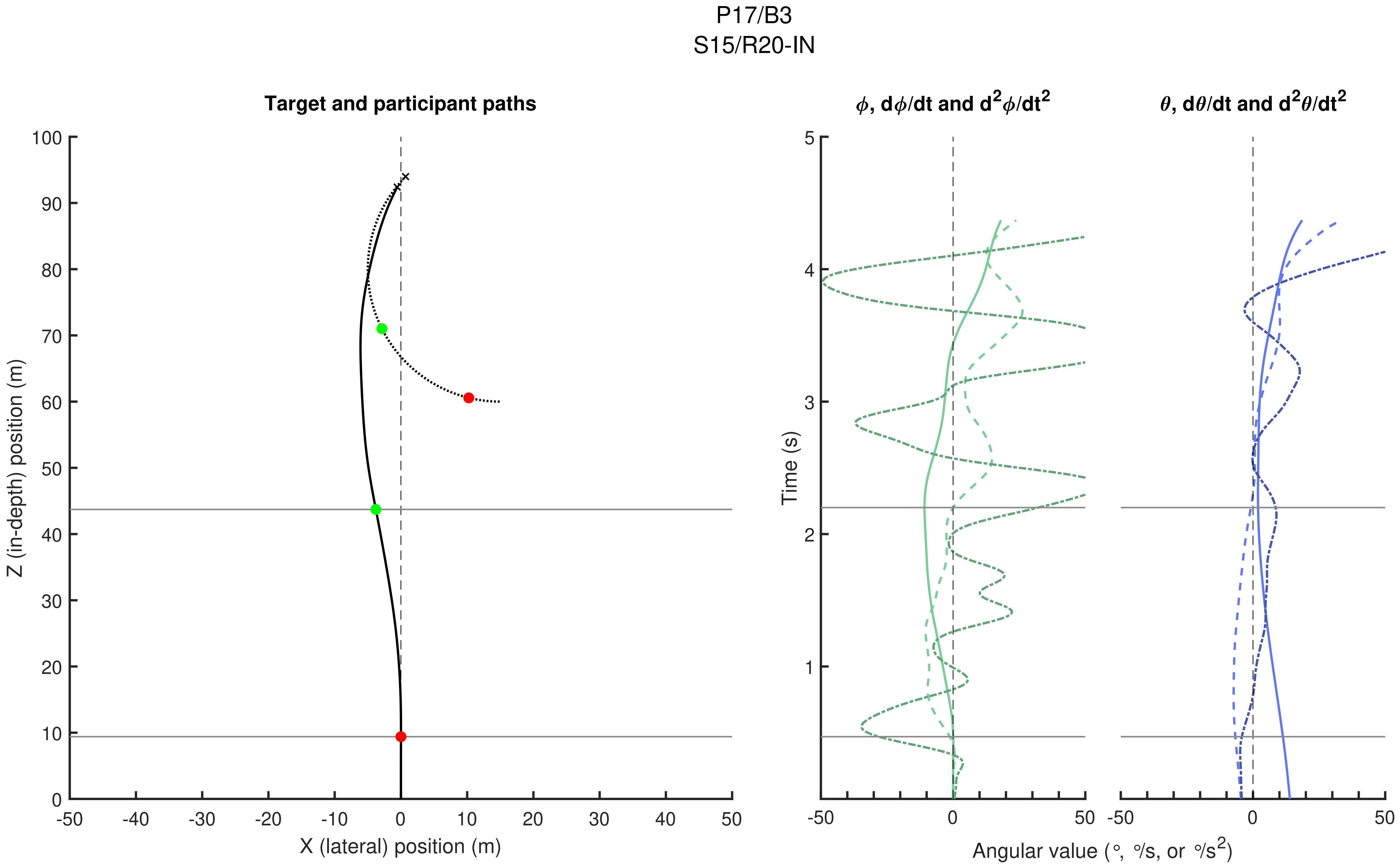
P17/B3
S15/R20-IN
Target and participant paths
Z (in-depth) position (m)
X (lateral) position (m)
ϕ, dϕ/dt and d$^2\phi$/dt^2
θ, dθ/dt and d$^2\theta$/dt^2
Time (s)
Angular value (°, °/s, or °/s^2)

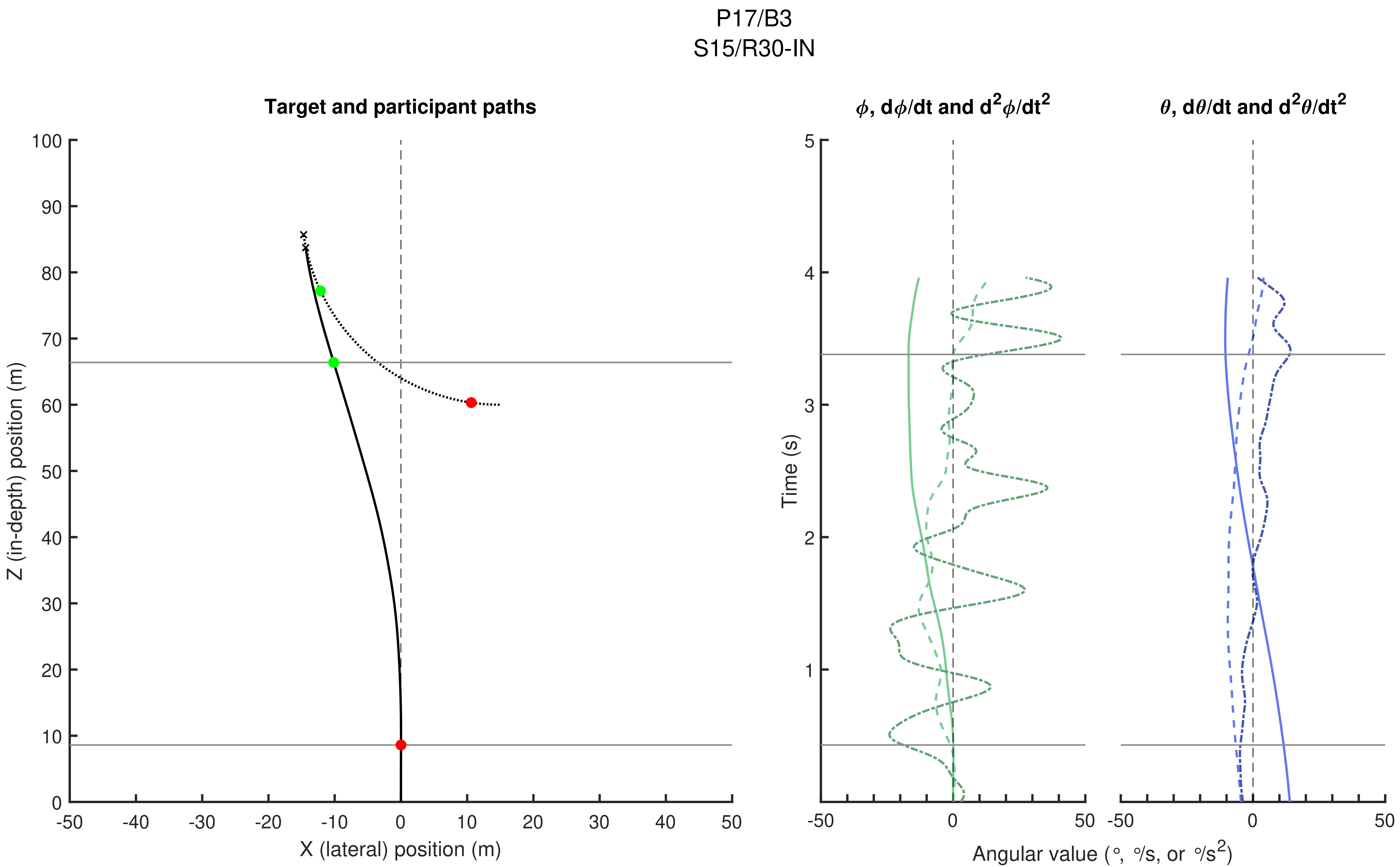

P17/B3
S15/R30-IN
Target and participant paths
Z (in-depth) position (m)
X (lateral) position (m)
φ, dφ/dt and d²φ/dt²
θ, dθ/dt and d²θ/dt²
Time (s)
Angular value (°, °/s, or °/s²)

P17/B3
S20/R10-IN

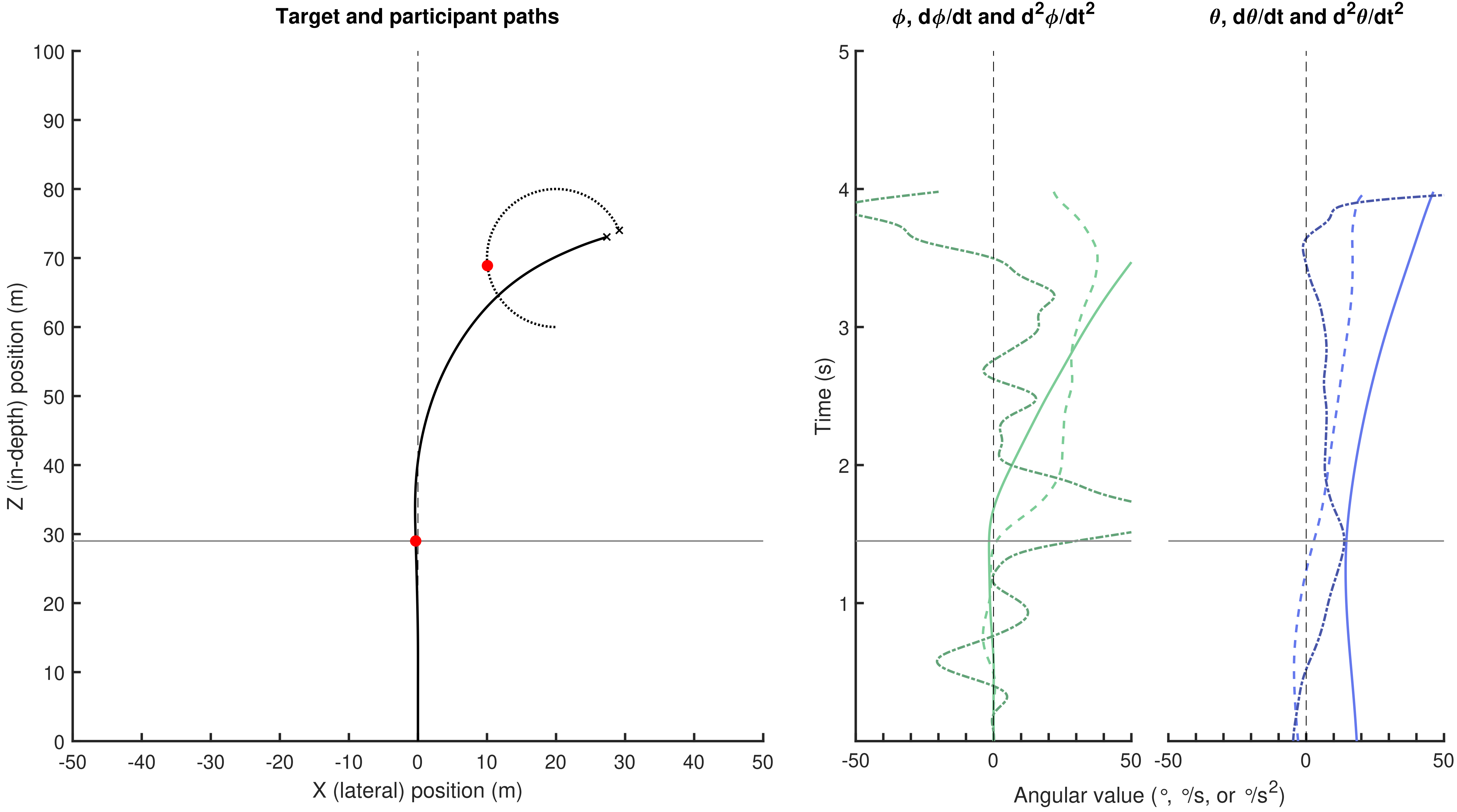

P17/B3
S20/R20-IN

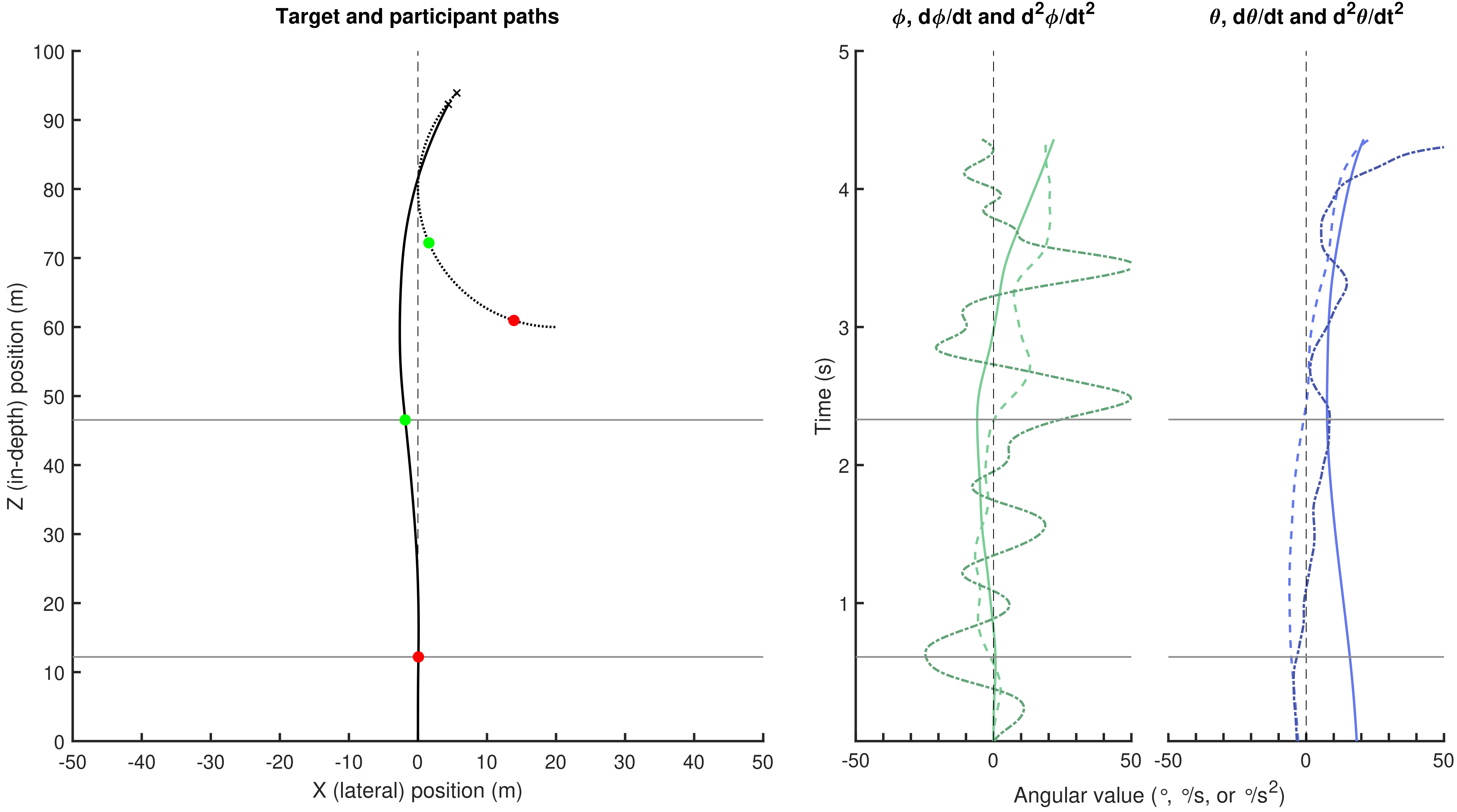

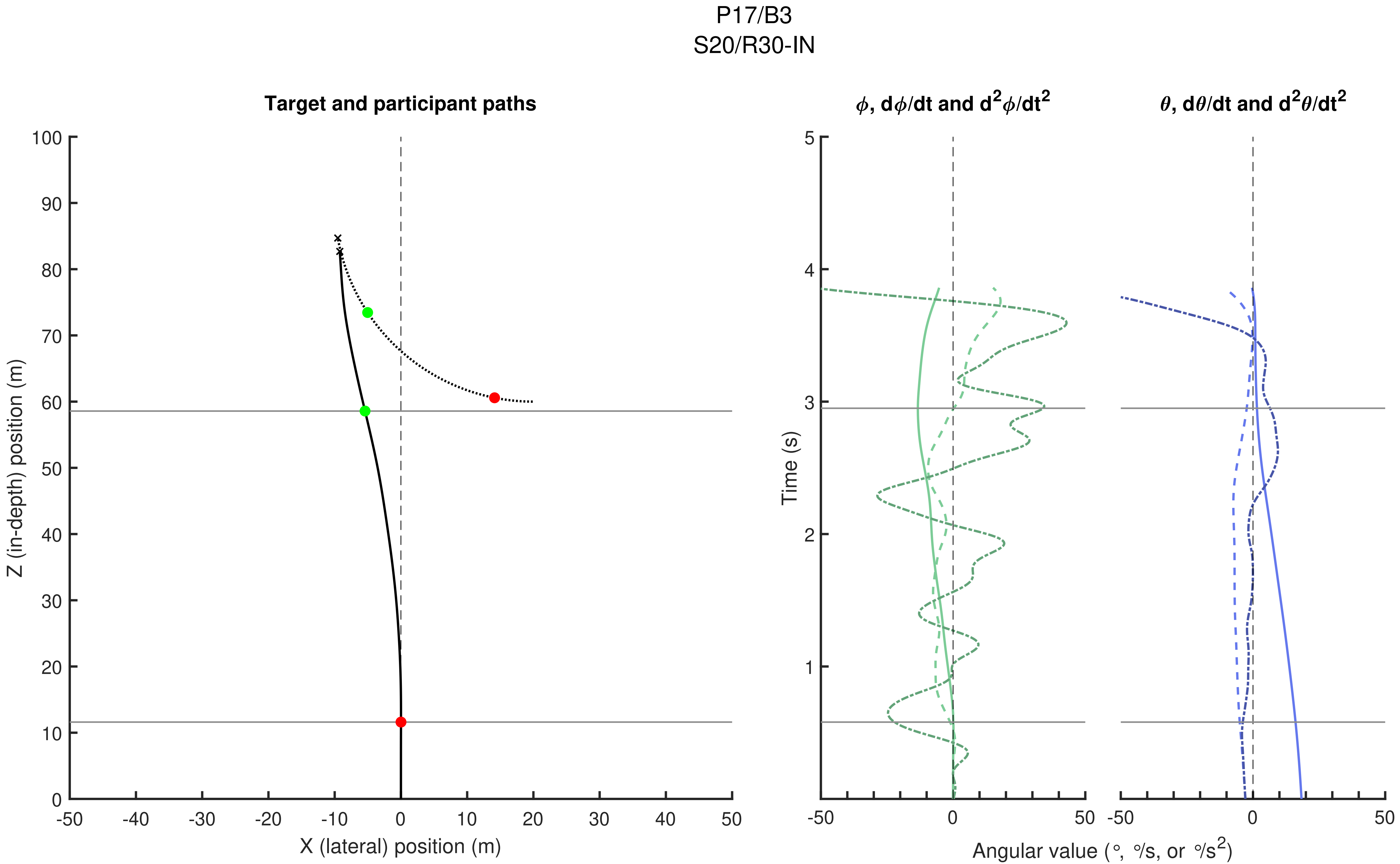

P17/B3
S20/R30-IN
Target and participant paths
Z (in-depth) position (m)
X (lateral) position (m)
100
90
80
70
60
50
40
30
20
10
0
-50
-40
-30
-20
-10
0
10
20
30
40
50
φ, dφ/dt and d²φ/dt²
θ, dθ/dt and d²θ/dt²
Time (s)
5
4
3
2
1
-50
0
50
-50
0
50
Angular value (°, °/s, or °/s²)

P17/B4
S20/R10-IN

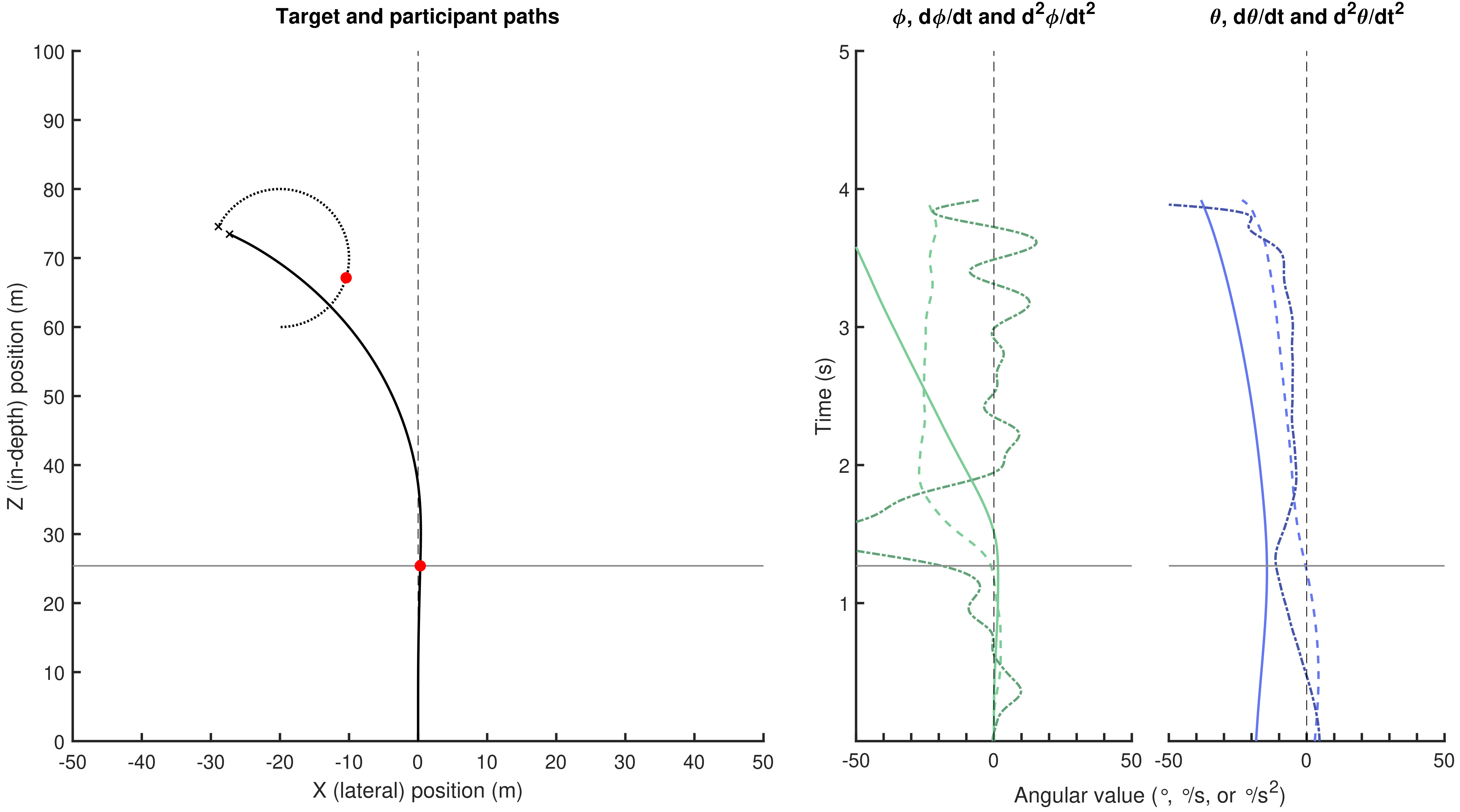

P17/B4
S20/R20-IN

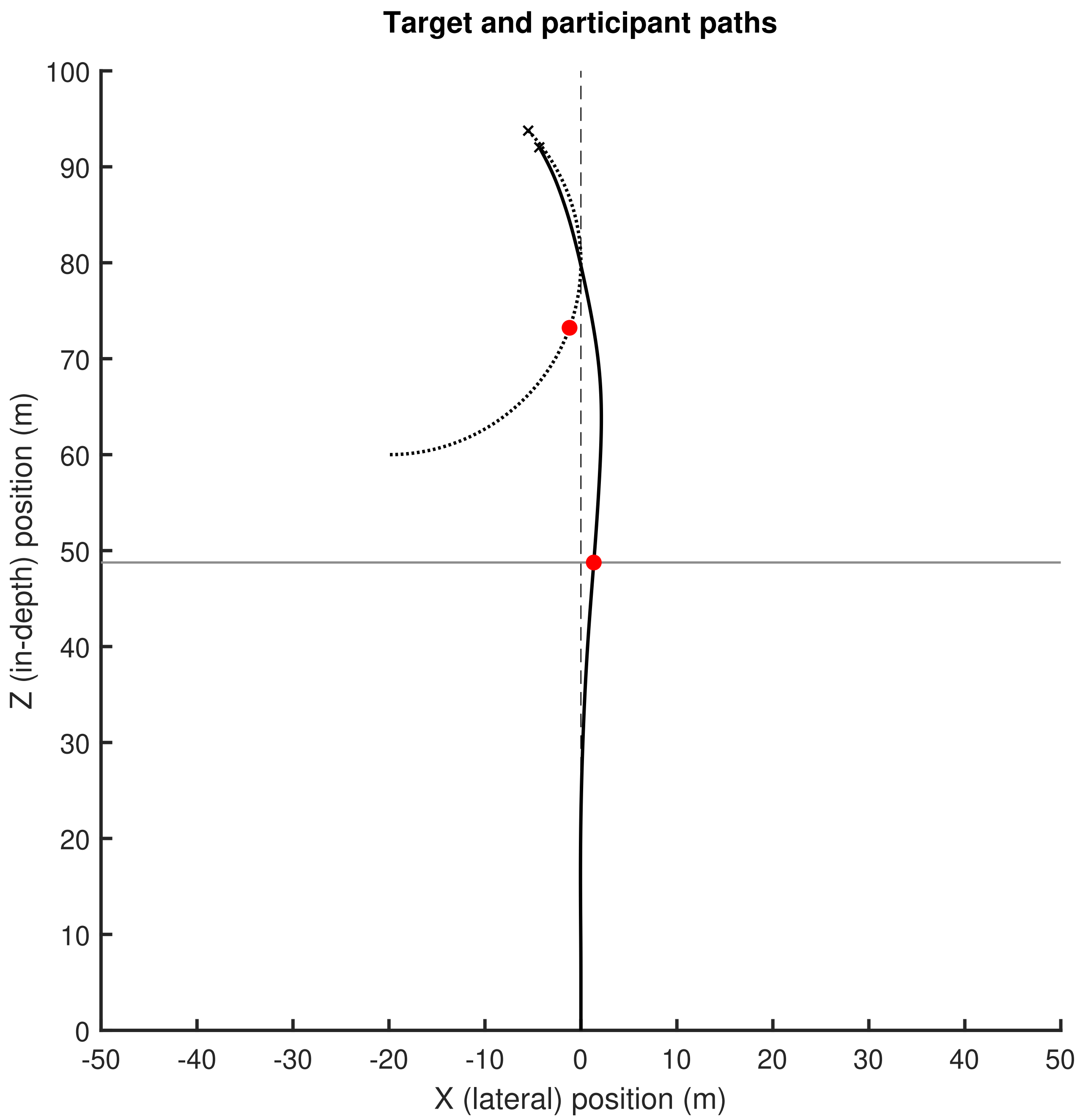

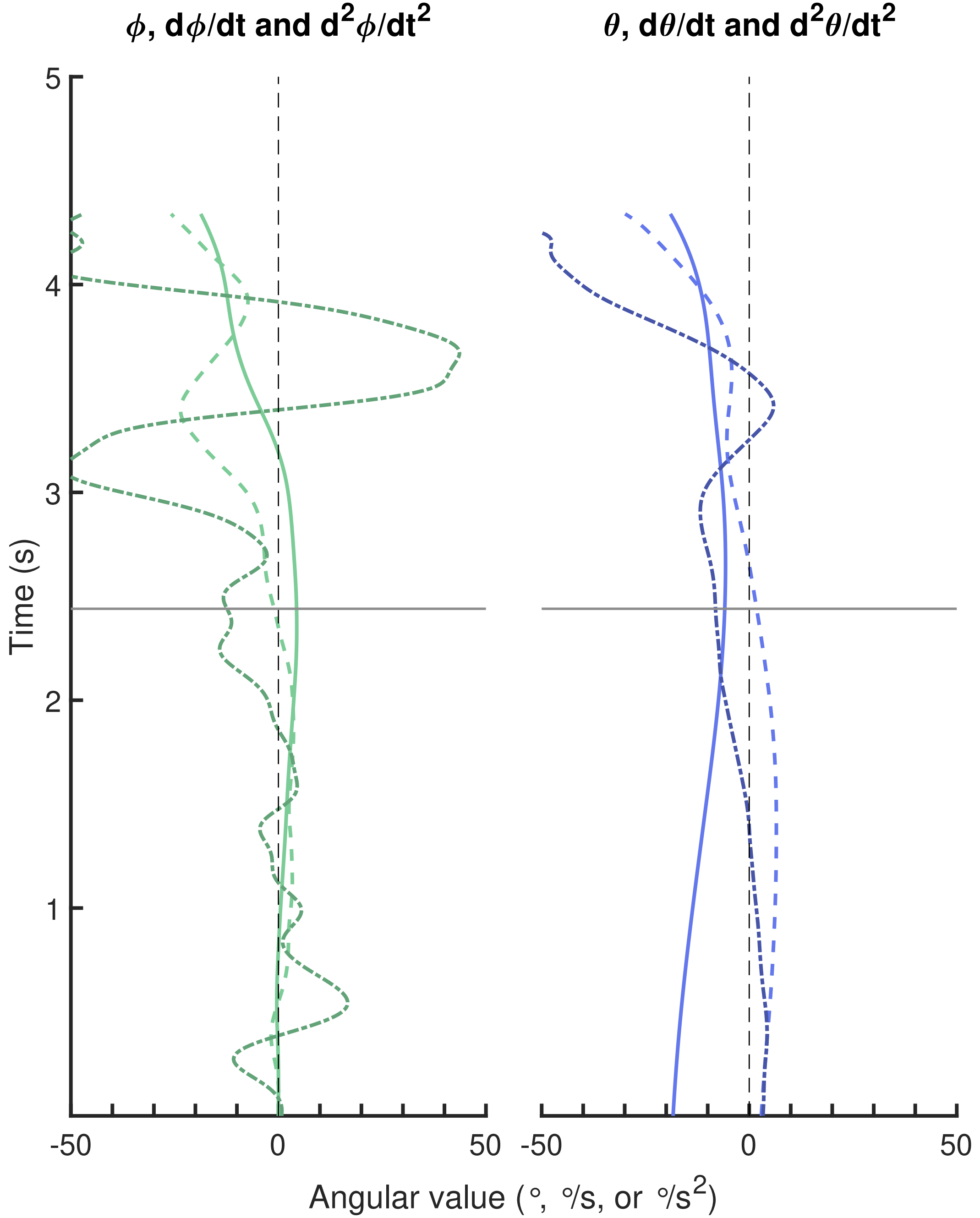

P17/B4
S20/R30-IN

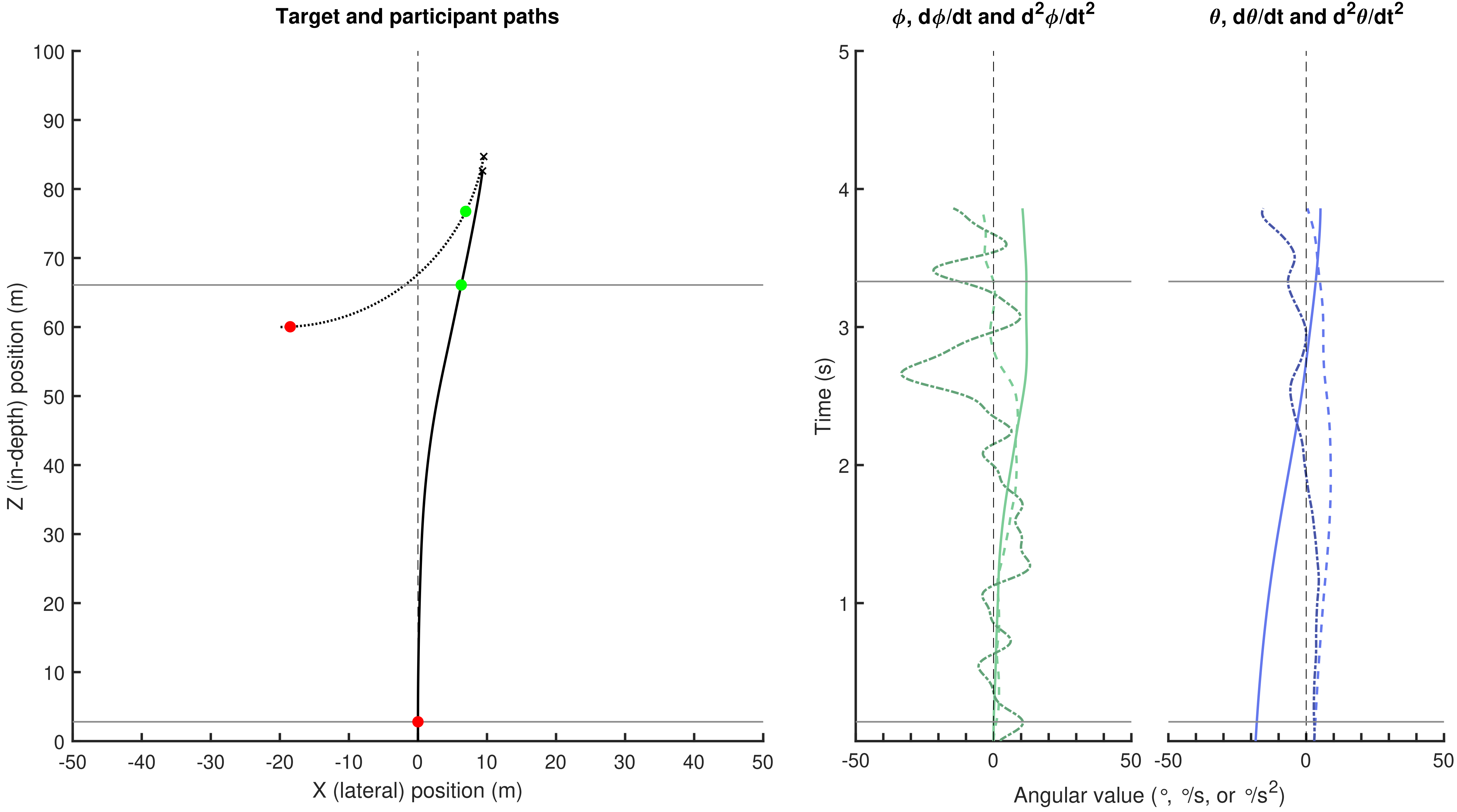

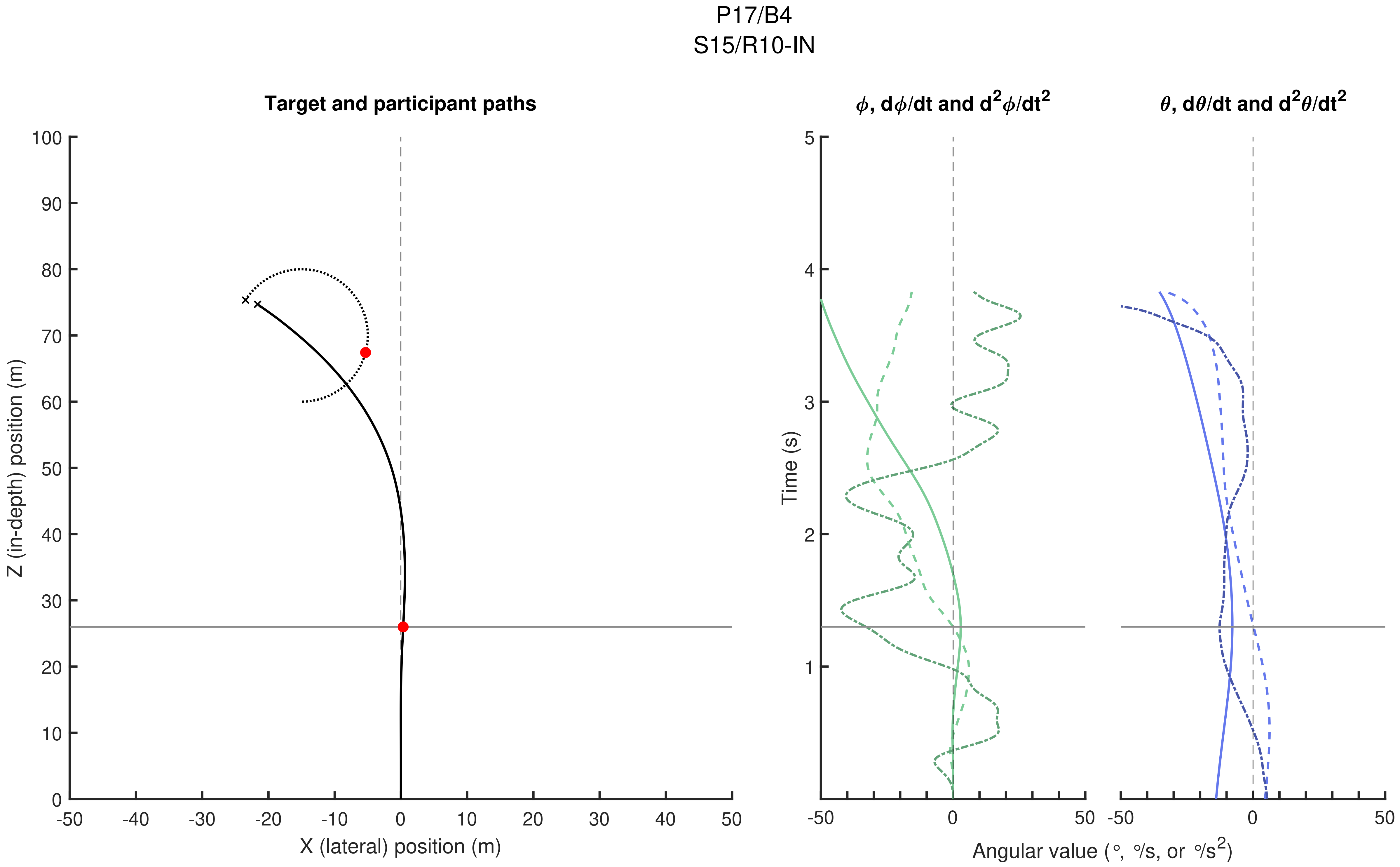
P17/B4
S15/R10-IN
Target and participant paths
Z (in-depth) position (m)
X (lateral) position (m)
ϕ, dϕ/dt and d$^2\phi$/dt^2
θ, dθ/dt and d$^2\theta$/dt^2
Time (s)
Angular value (°, °/s, or °/s^2)

P17/B4
S15/R20-IN

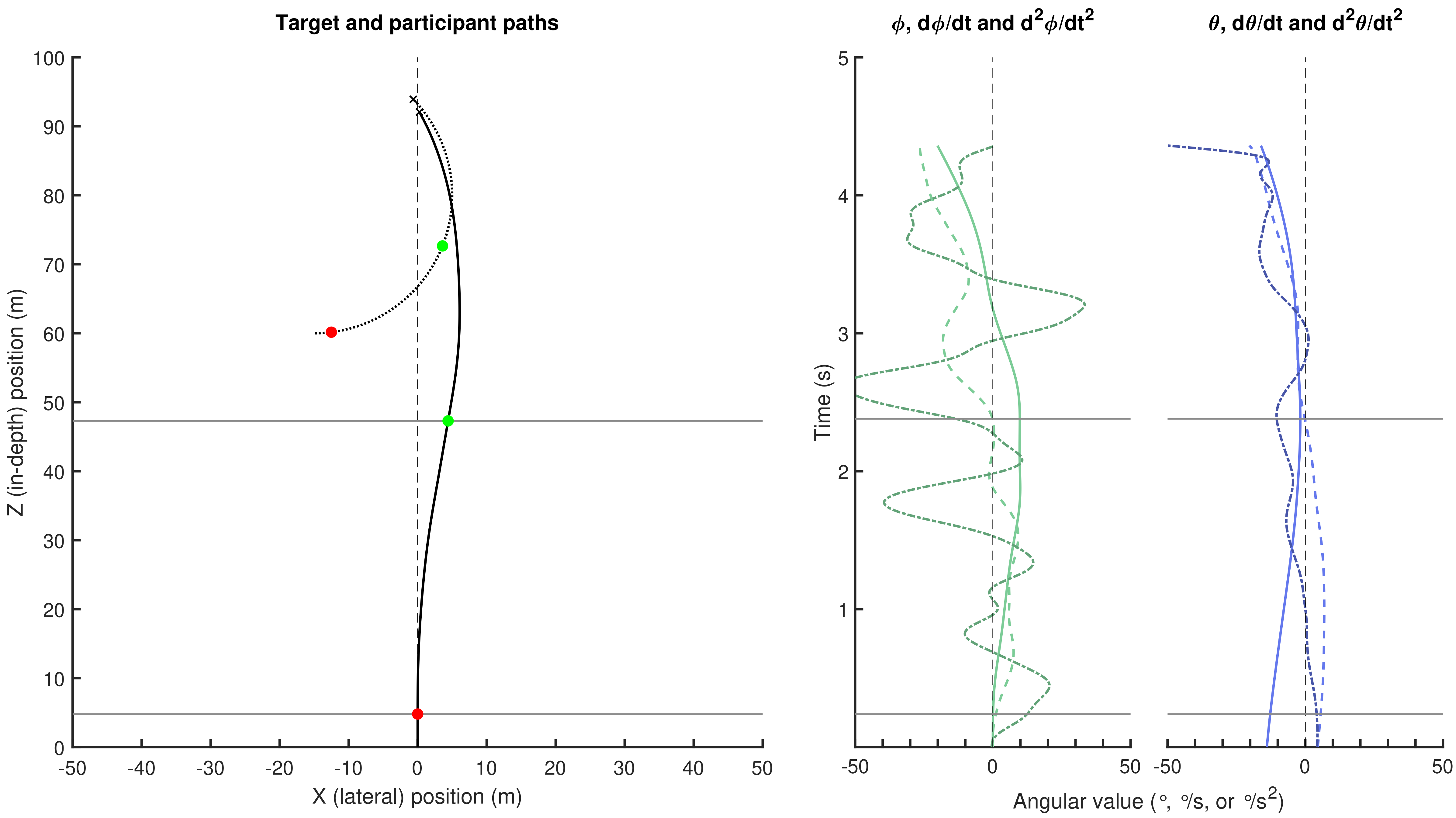

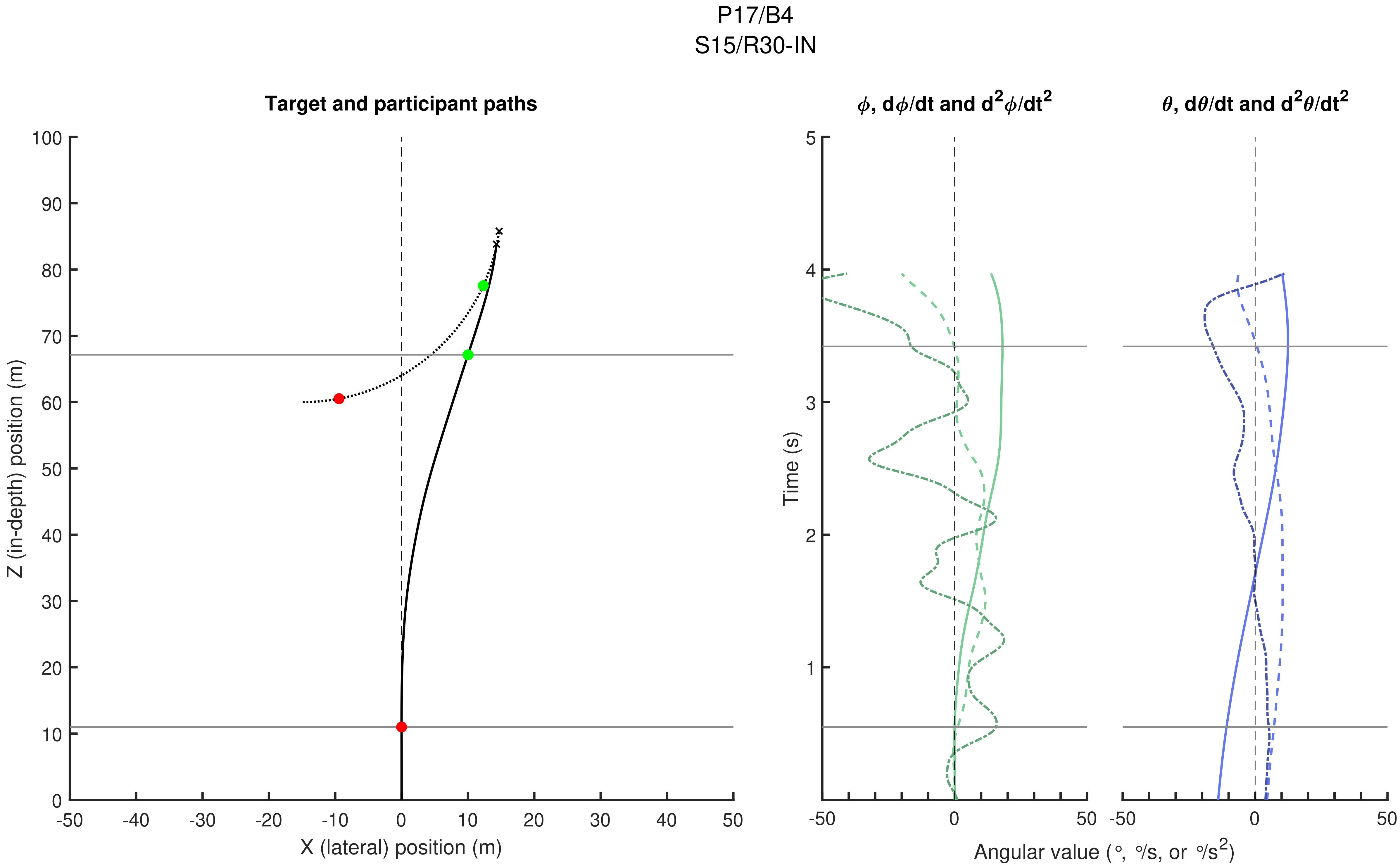

P17/B4
S15/R30-IN
Target and participant paths
Z (in-depth) position (m)
X (lateral) position (m)
-50
-40
-30
-20
-10
0
10
20
30
40
50
100
90
80
70
60
50
40
30
20
10
0
ϕ, dϕ/dt and d$^2\phi$/dt^2
θ, dθ/dt and d$^2\theta$/dt^2
Time (s)
5
4
3
2
1
-50
0
50
-50
0
50
Angular value (°, °/s, or °/s^2)

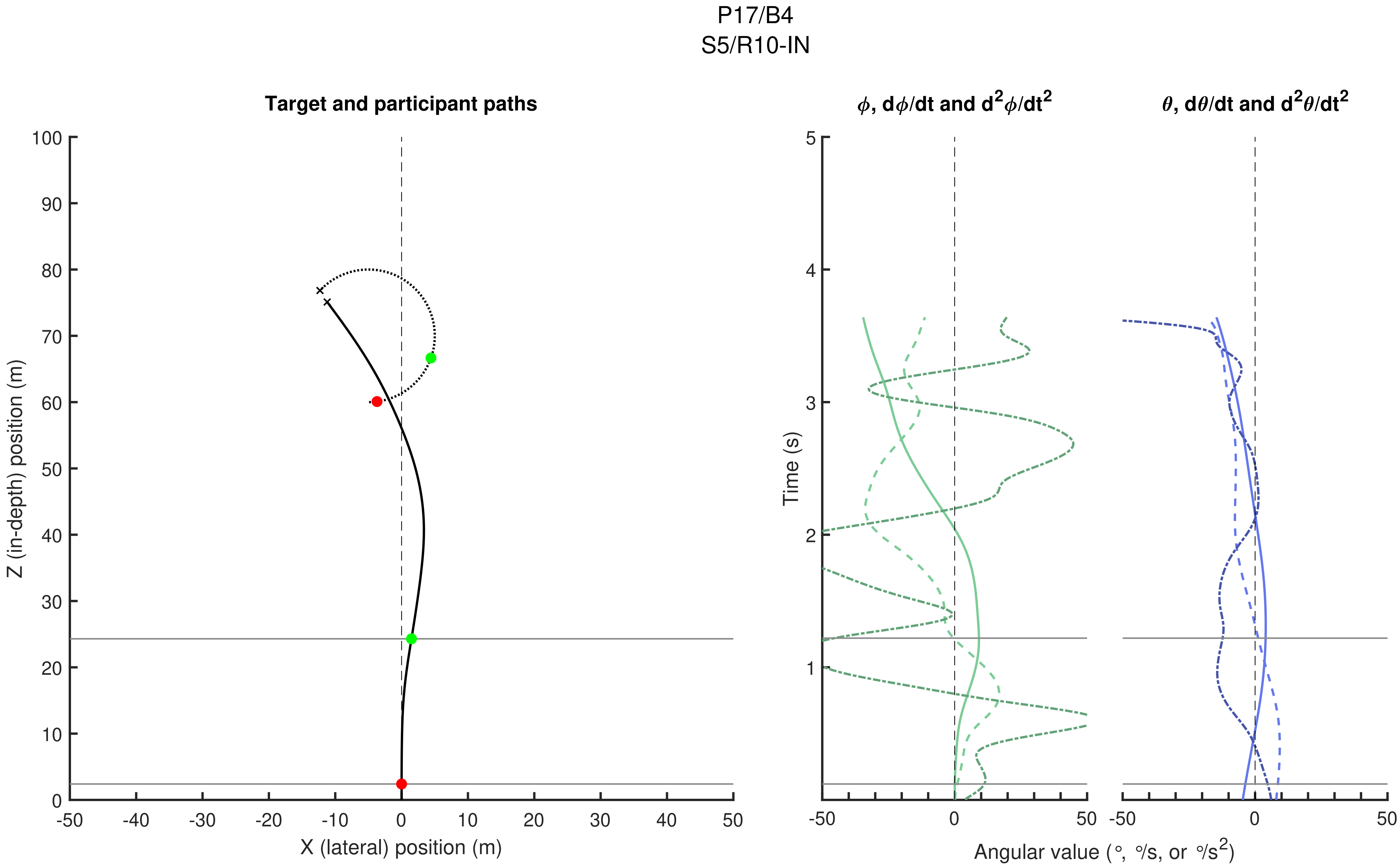
P17/B4
S5/R10-IN
Target and participant paths
Z (in-depth) position (m)
X (lateral) position (m)
100
90
80
70
60
50
40
30
20
10
0
-50
-40
-30
-20
-10
0
10
20
30
40
50
φ, dφ/dt and d²φ/dt²
θ, dθ/dt and d²θ/dt²
Time (s)
5
4
3
2
1
-50
0
50
-50
0
50
Angular value (°, °/s, or °/s²)

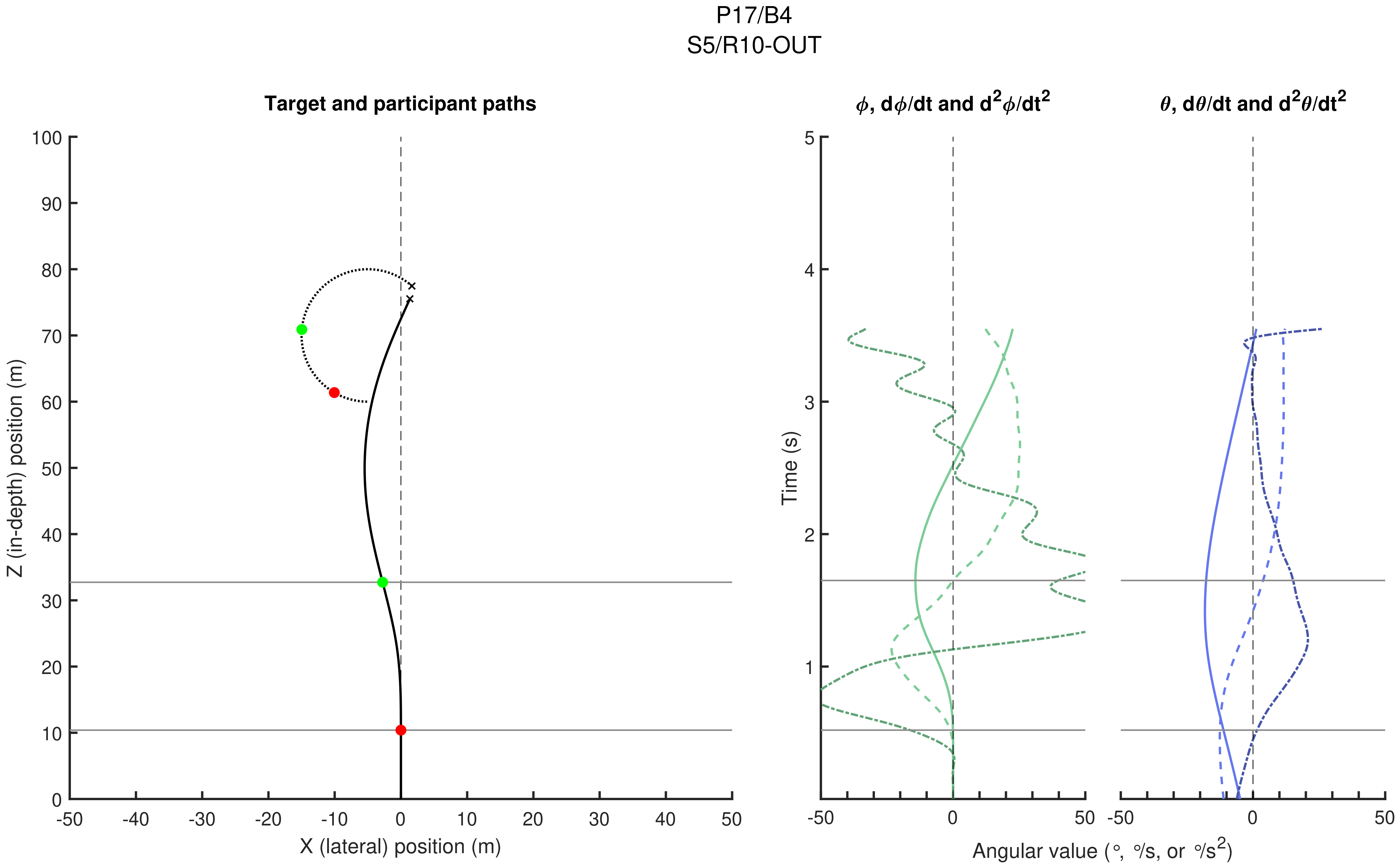
P17/B4
S5/R10-OUT
Target and participant paths
ϕ, dϕ/dt and d$^2\phi$/dt^2
θ, dθ/dt and d$^2\theta$/dt^2
Z (in-depth) position (m)
X (lateral) position (m)
Time (s)
Angular value (°, °/s, or °/s^2)
100
90
80
70
60
50
40
30
20
10
0
-50
-40
-30
-20
-10
0
10
20
30
40
50
5
4
3
2
1
-50
0
50
-50
0
50

P17/B4
S5/R20-IN

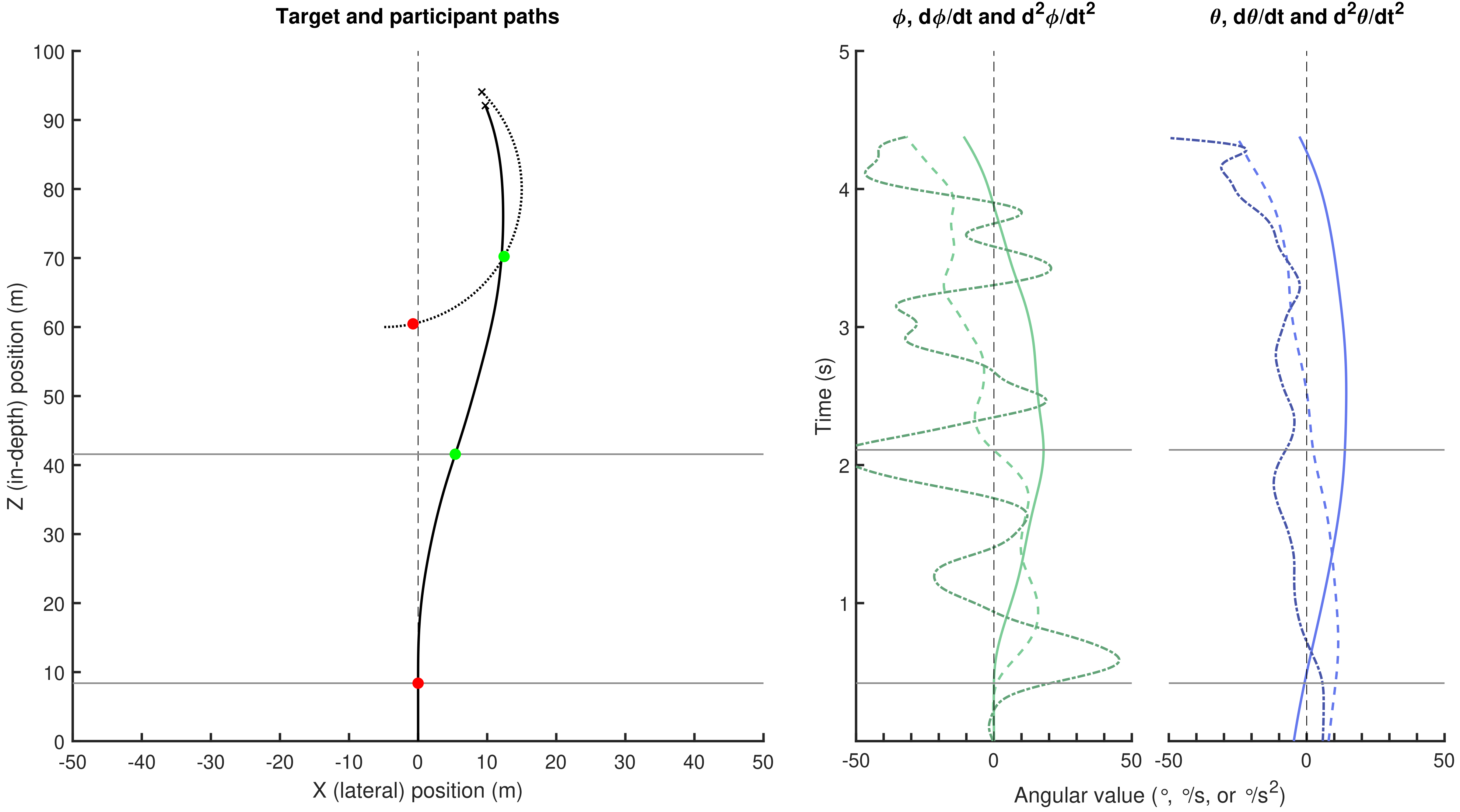

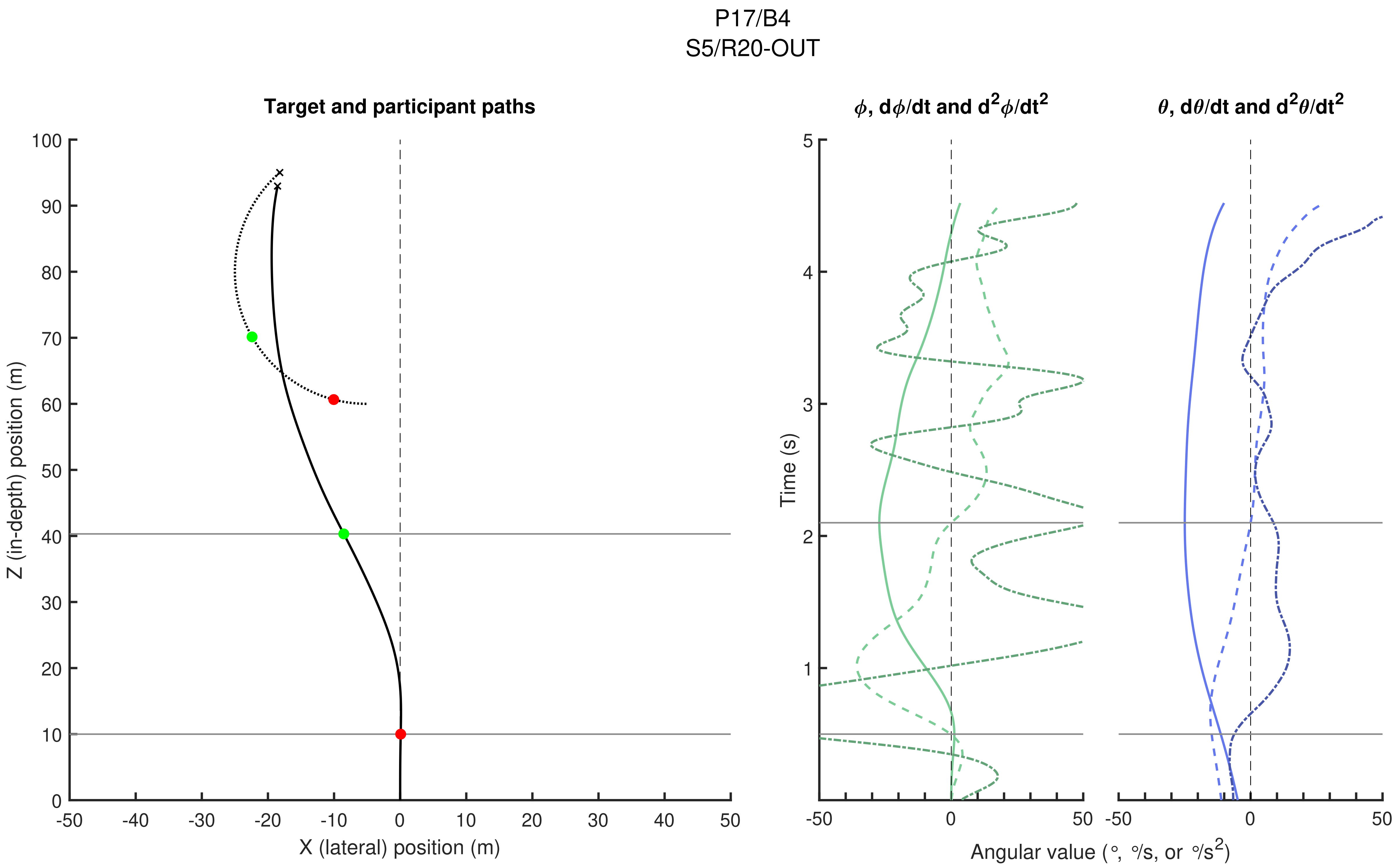
P17/B4
S5/R20-OUT
Target and participant paths
Z (in-depth) position (m)
X (lateral) position (m)
ϕ, dϕ/dt and d$^2\phi$/dt^2
θ, dθ/dt and d$^2\theta$/dt^2
Time (s)
Angular value (°, °/s, or °/s^2)

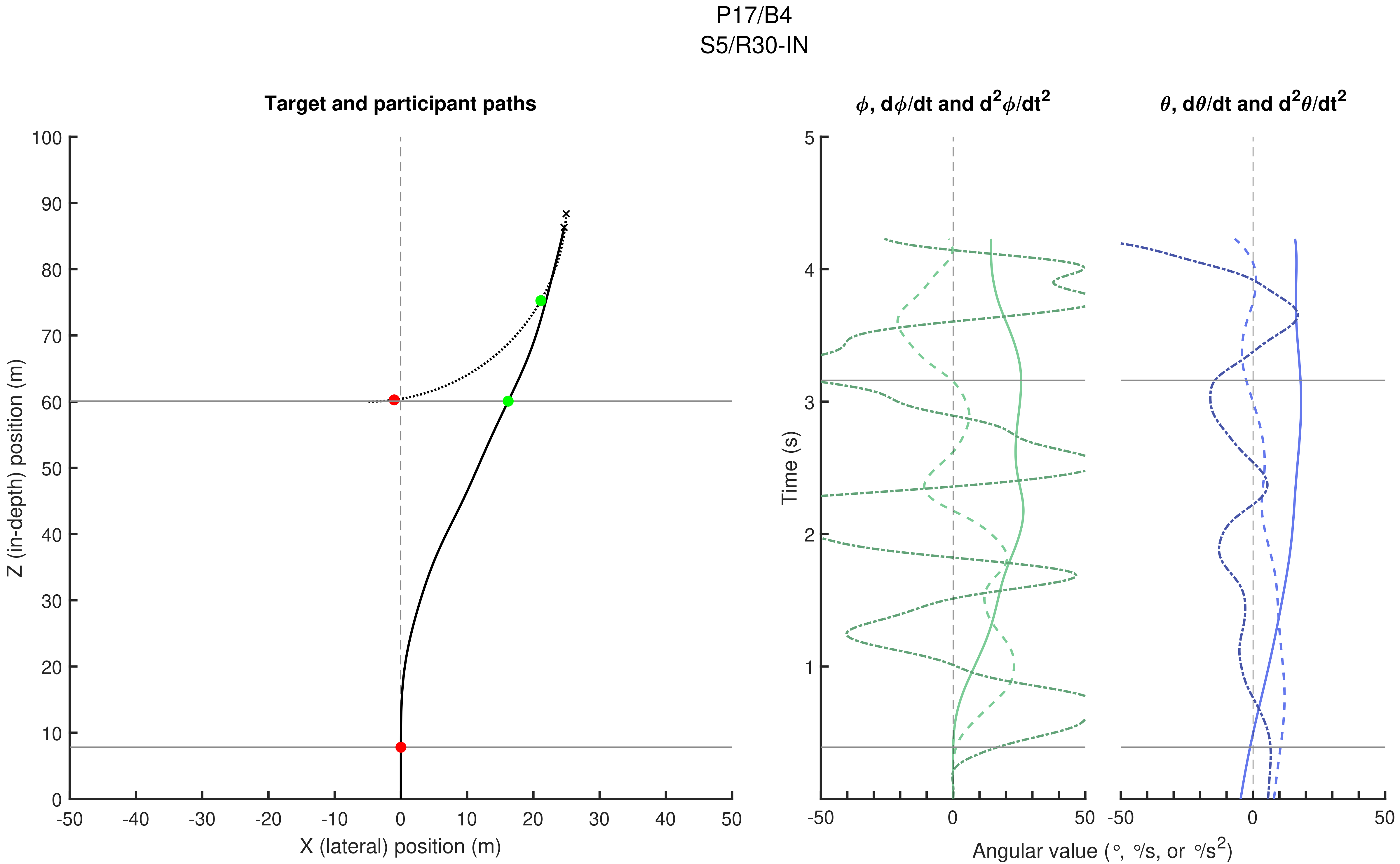
P17/B4
S5/R30-IN
Target and participant paths
Z (in-depth) position (m)
X (lateral) position (m)
100
90
80
70
60
50
40
30
20
10
0
-50
-40
-30
-20
-10
0
10
20
30
40
50
ϕ, dϕ/dt and d²ϕ/dt²
θ, dθ/dt and d²θ/dt²
Time (s)
5
4
3
2
1
-50
0
50
-50
0
50
Angular value (°, °/s, or °/s²)

P17/B4
S5/R30-OUT

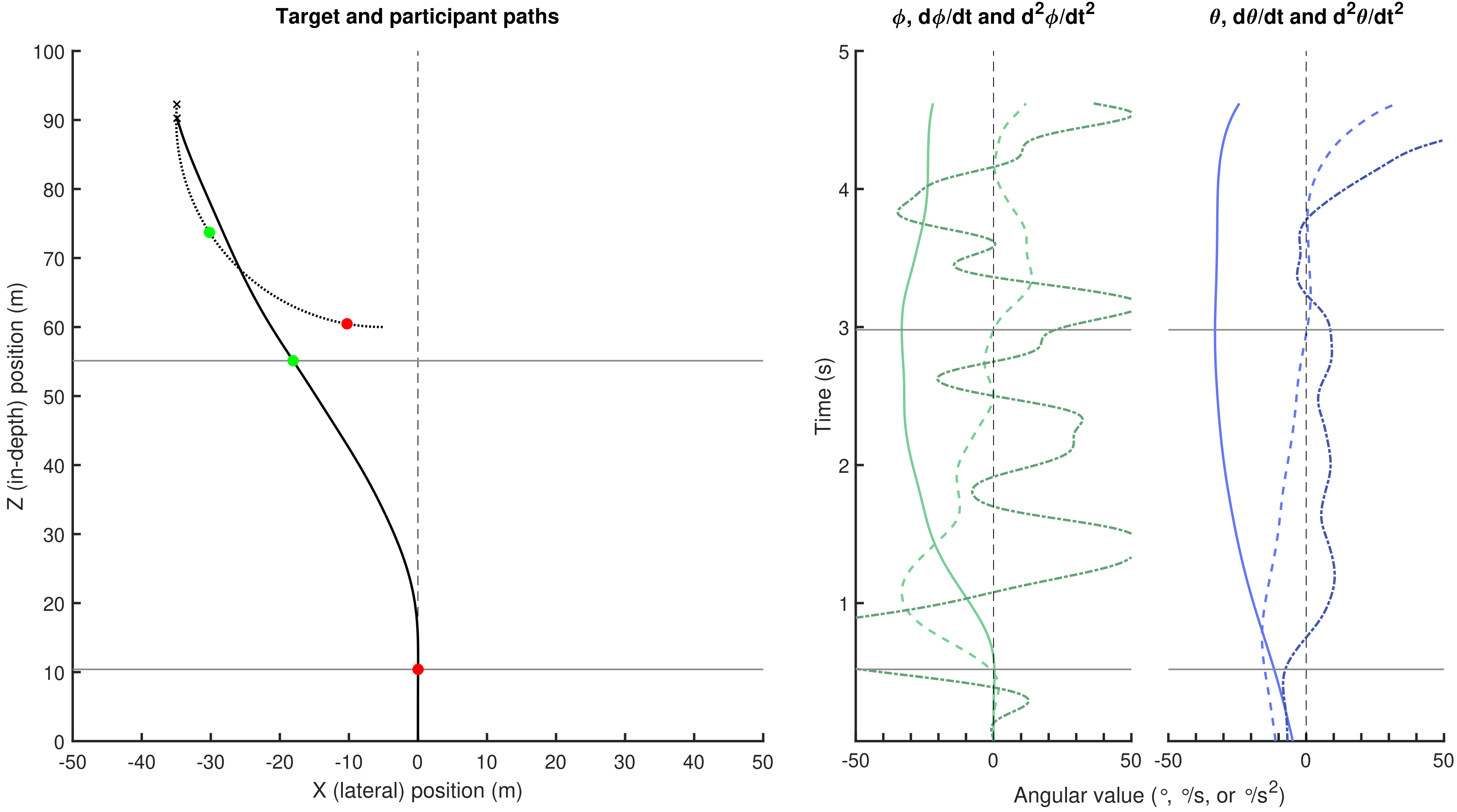

P17/B4
S5/R10-OUT

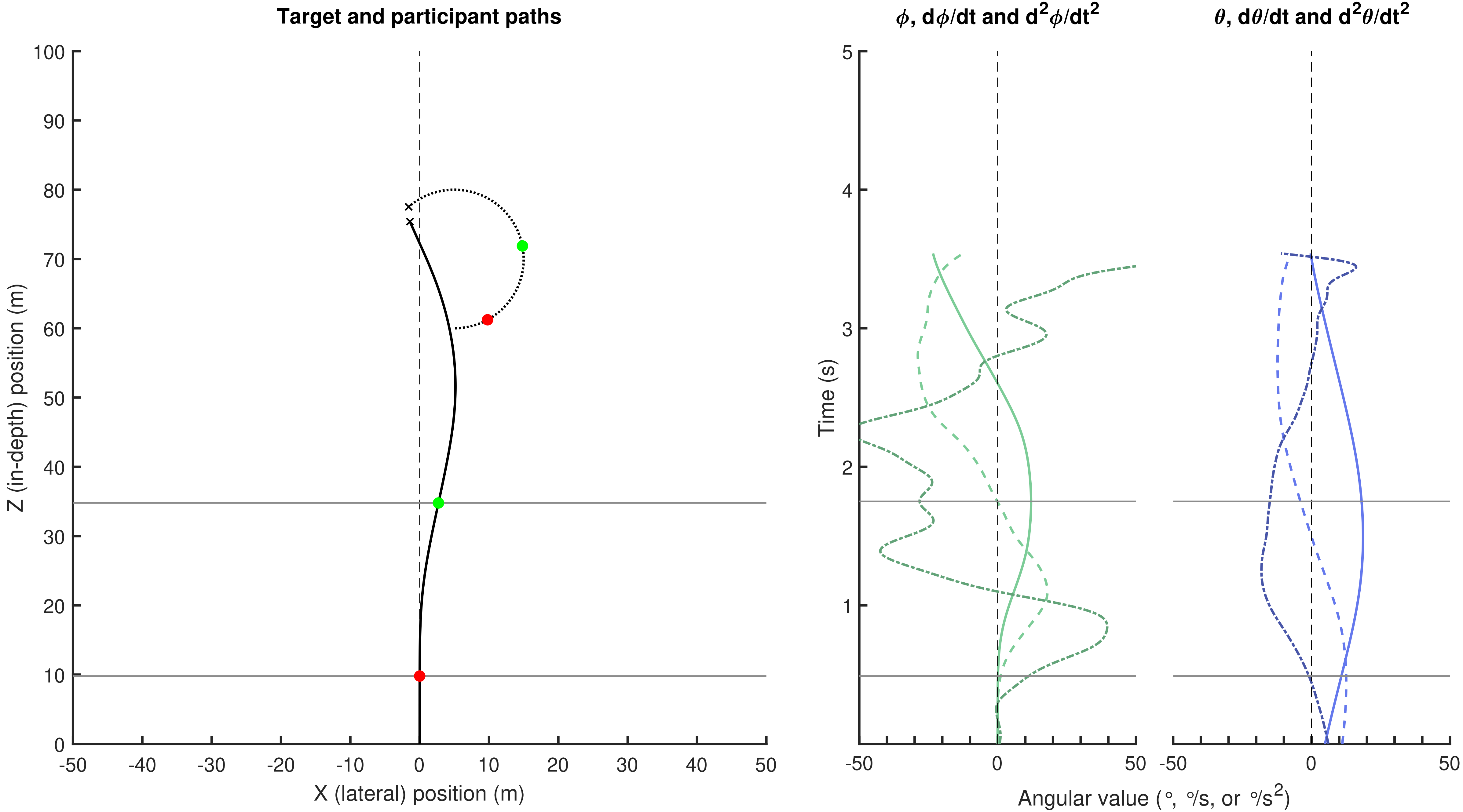

P17/B4
S5/R10-IN

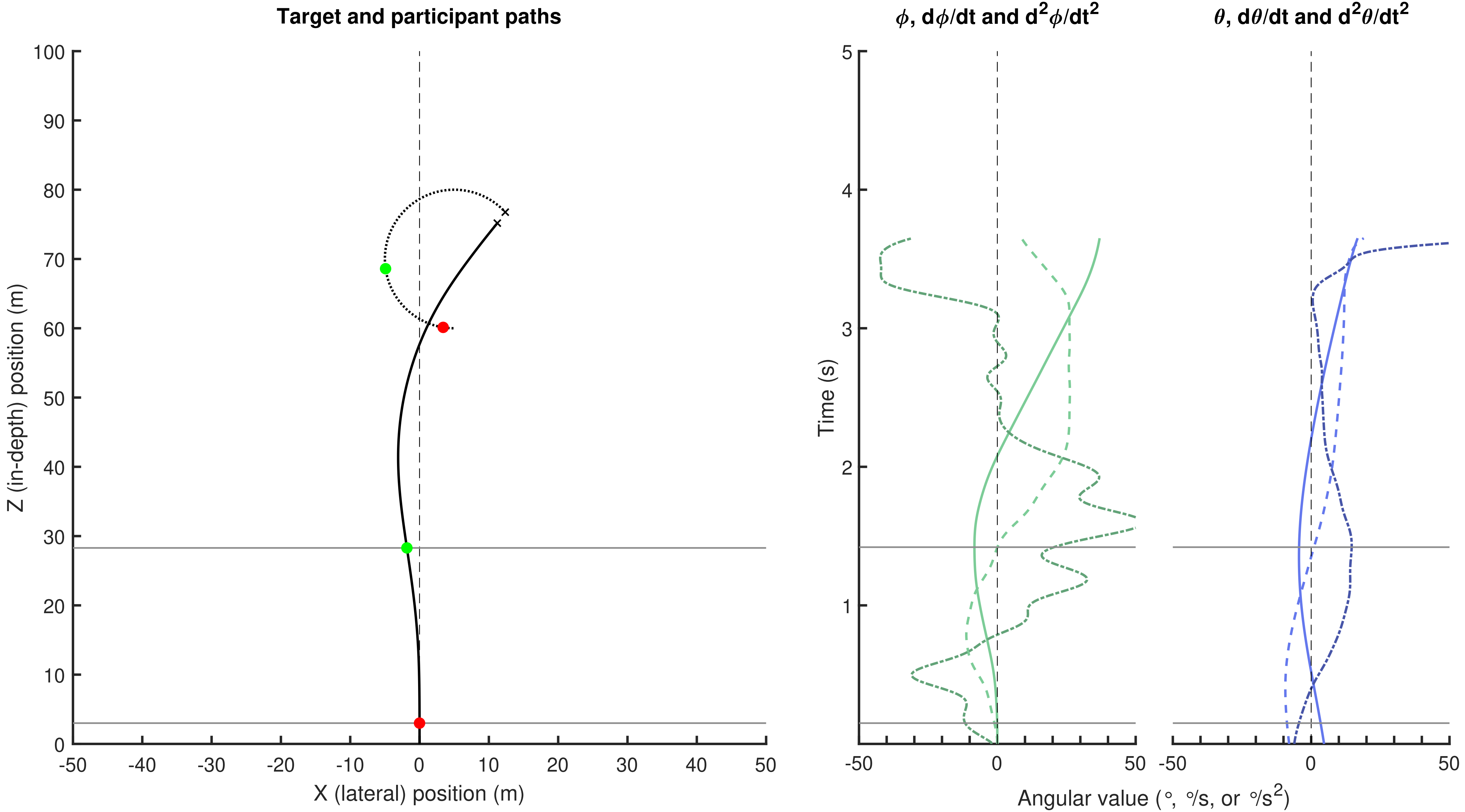

P17/B4
S5/R20-OUT

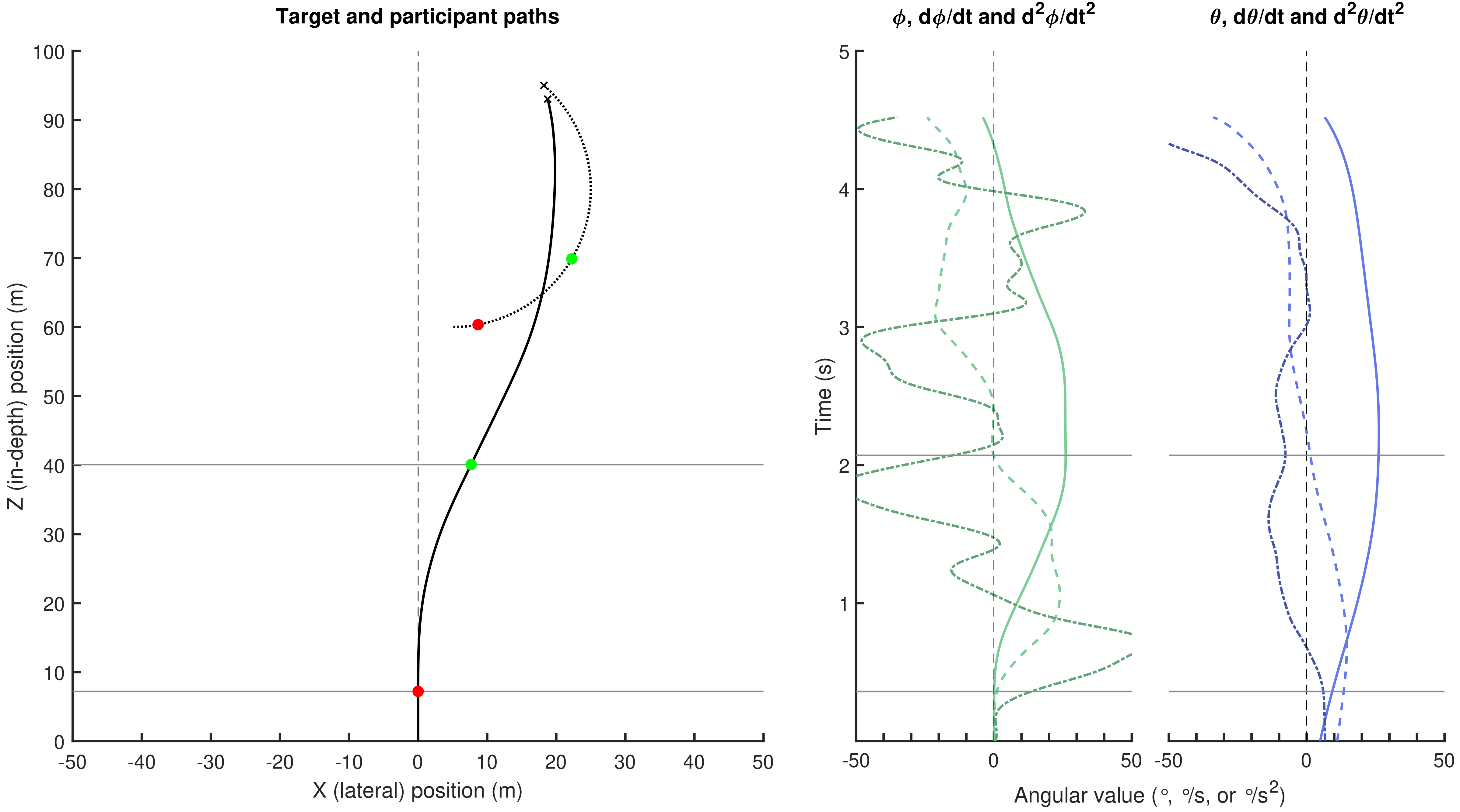

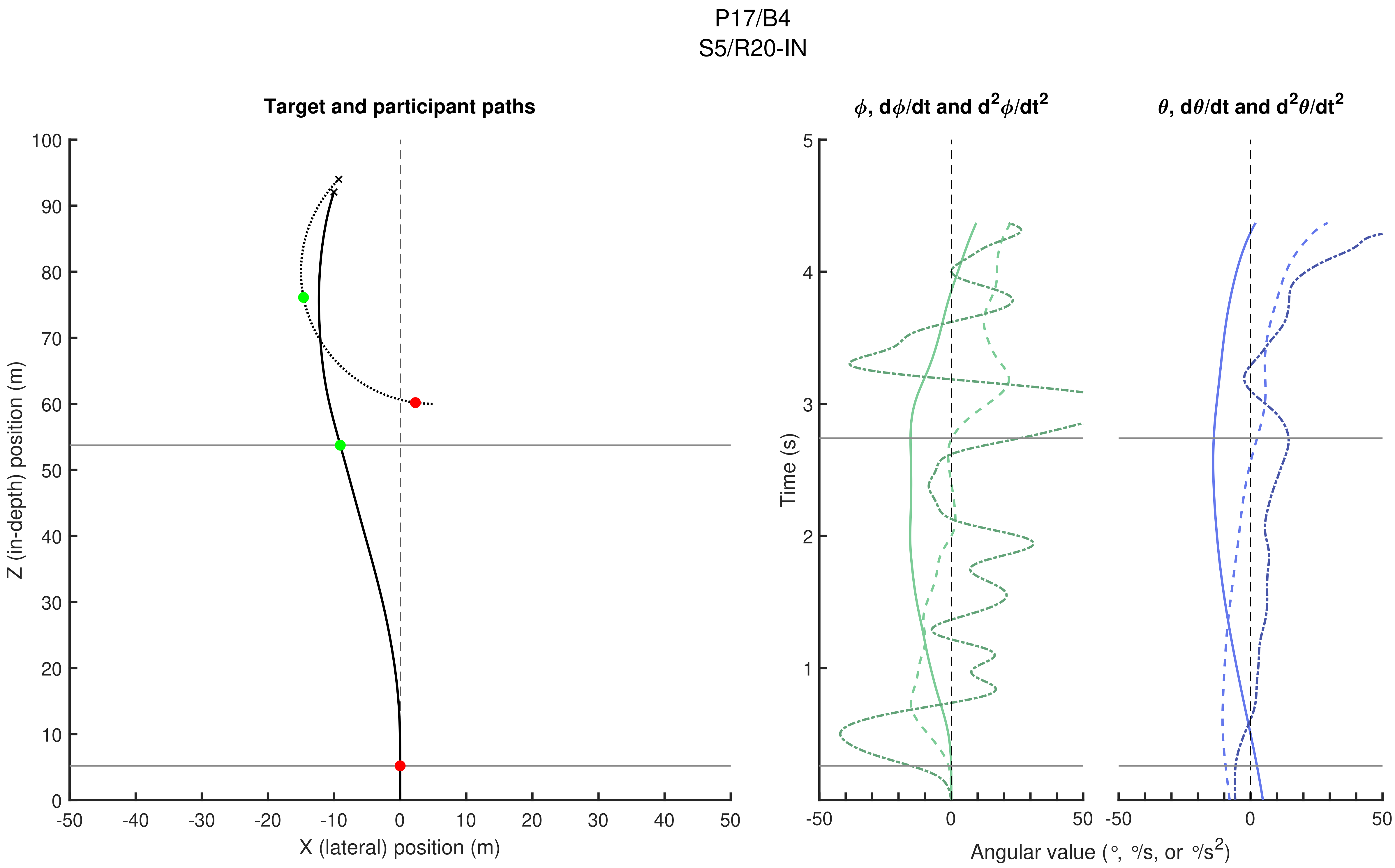
P17/B4
S5/R20-IN
Target and participant paths
Z (in-depth) position (m)
X (lateral) position (m)
φ, dφ/dt and d²φ/dt²
θ, dθ/dt and d²θ/dt²
Time (s)
Angular value (°, °/s, or °/s²)

P17/B4
S5/R30-OUT

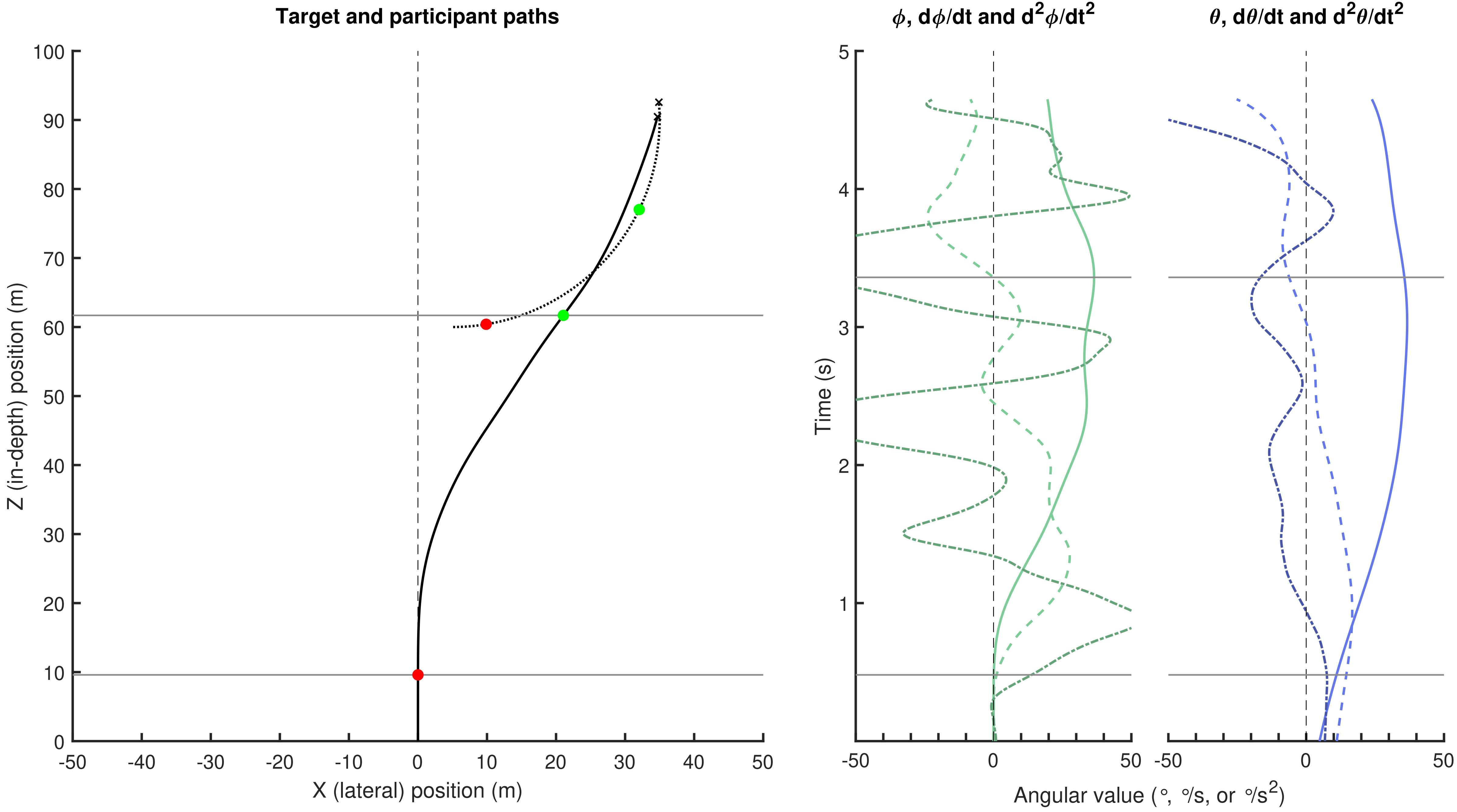

P17/B4
S5/R30-IN

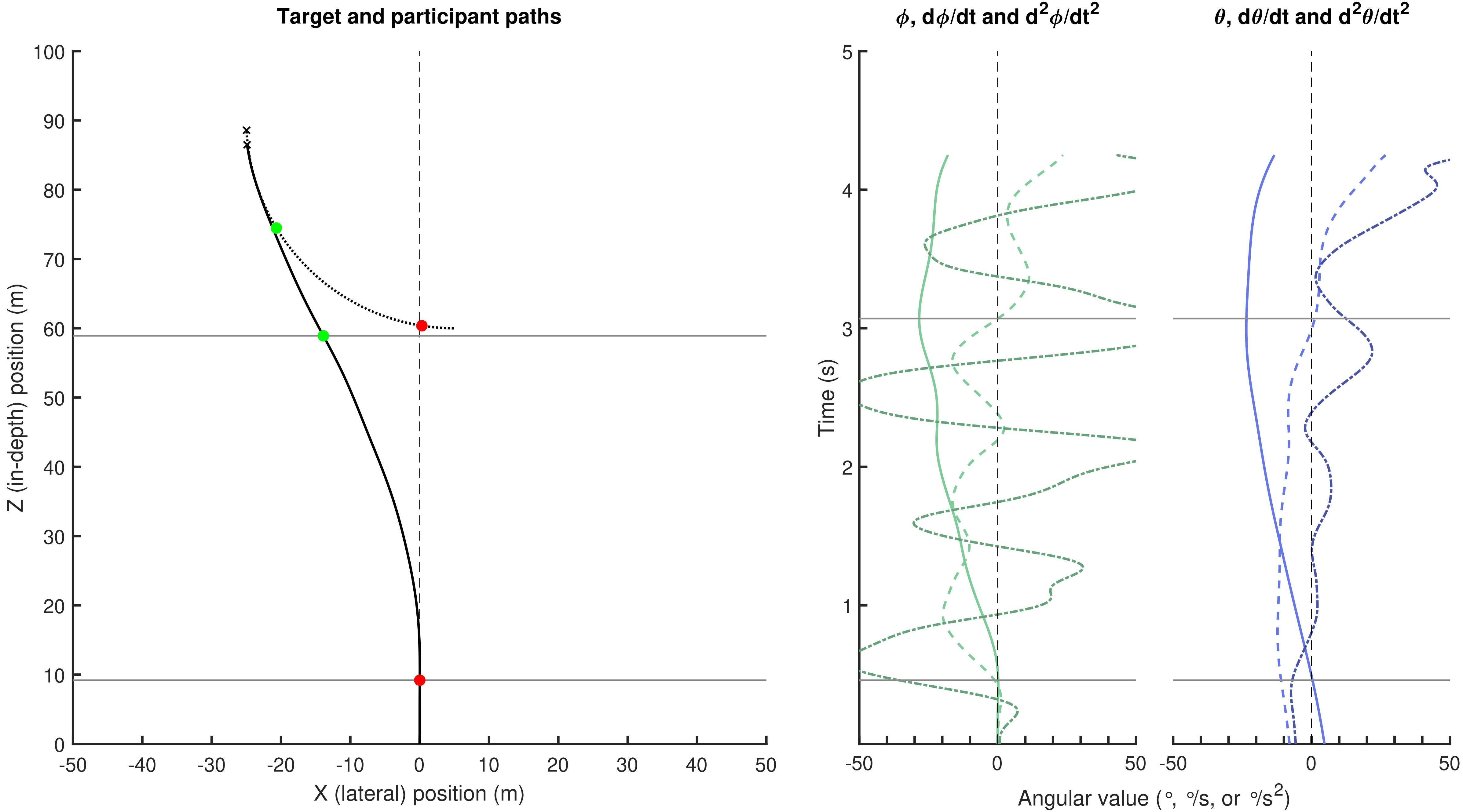

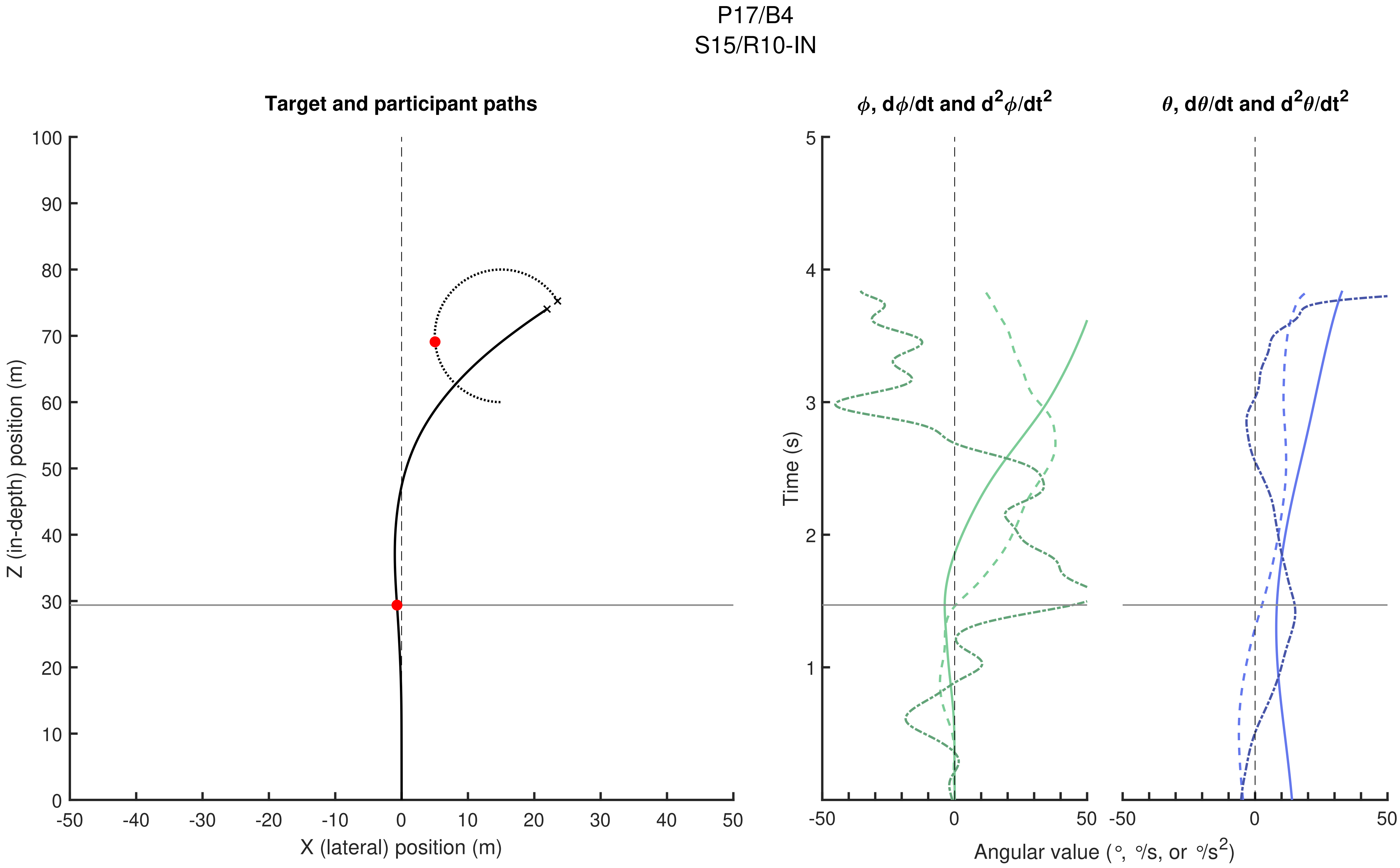
P17/B4
S15/R10-IN
Target and participant paths
Z (in-depth) position (m)
X (lateral) position (m)
100
90
80
70
60
50
40
30
20
10
0
-50
-40
-30
-20
-10
0
10
20
30
40
50
ϕ, dϕ/dt and d²ϕ/dt²
θ, dθ/dt and d²θ/dt²
Time (s)
5
4
3
2
1
-50
0
50
-50
0
50
Angular value (°, °/s, or °/s²)

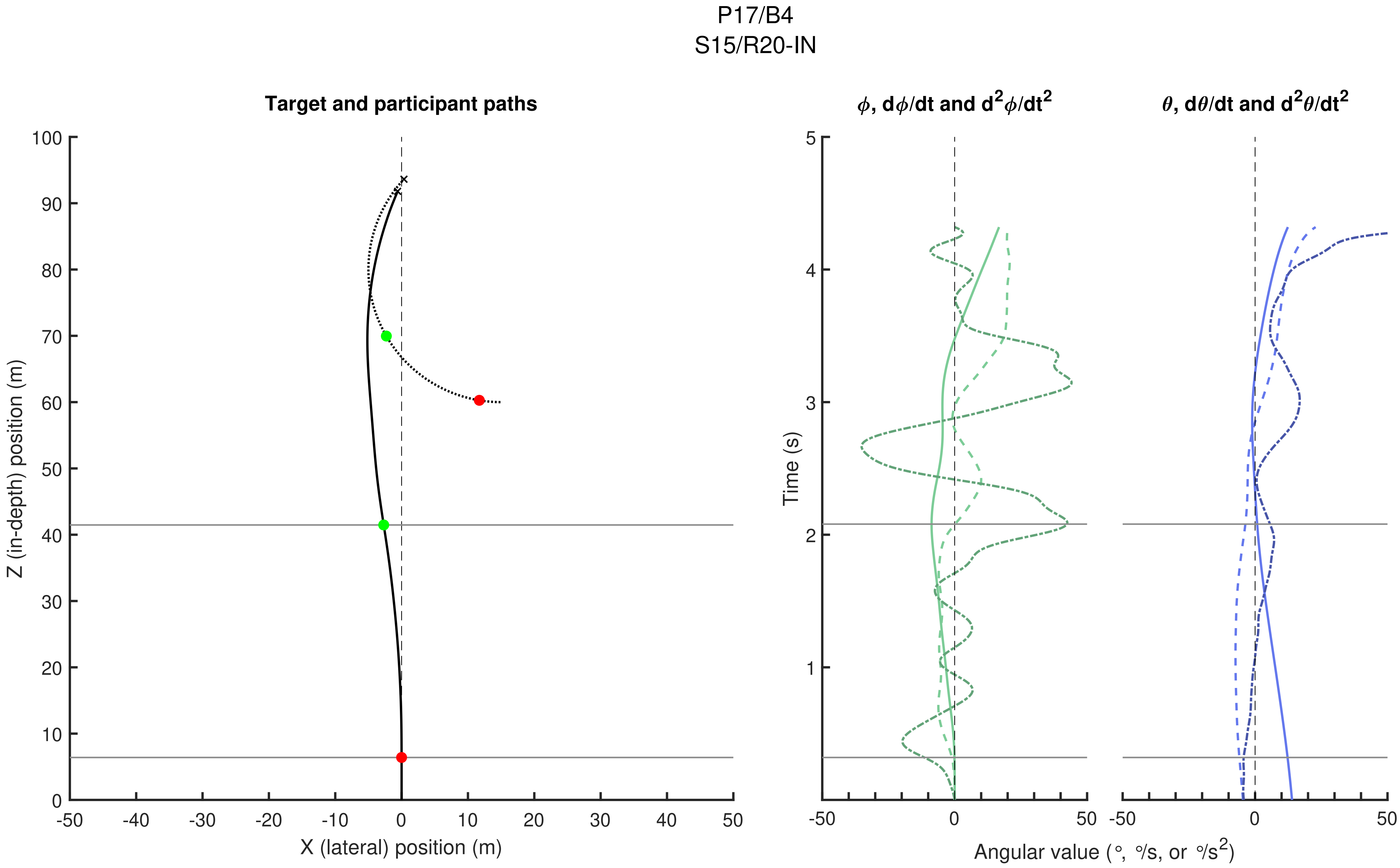
P17/B4
S15/R20-IN
Target and participant paths
Z (in-depth) position (m)
X (lateral) position (m)
100
90
80
70
60
50
40
30
20
10
0
-50
-40
-30
-20
-10
0
10
20
30
40
50
ϕ, dϕ/dt and d$^2\phi$/dt^2
θ, dθ/dt and d$^2\theta$/dt^2
Time (s)
5
4
3
2
1
-50
0
50
-50
0
50
Angular value (°, °/s, or °/s^2)

P17/B4
S15/R30-IN

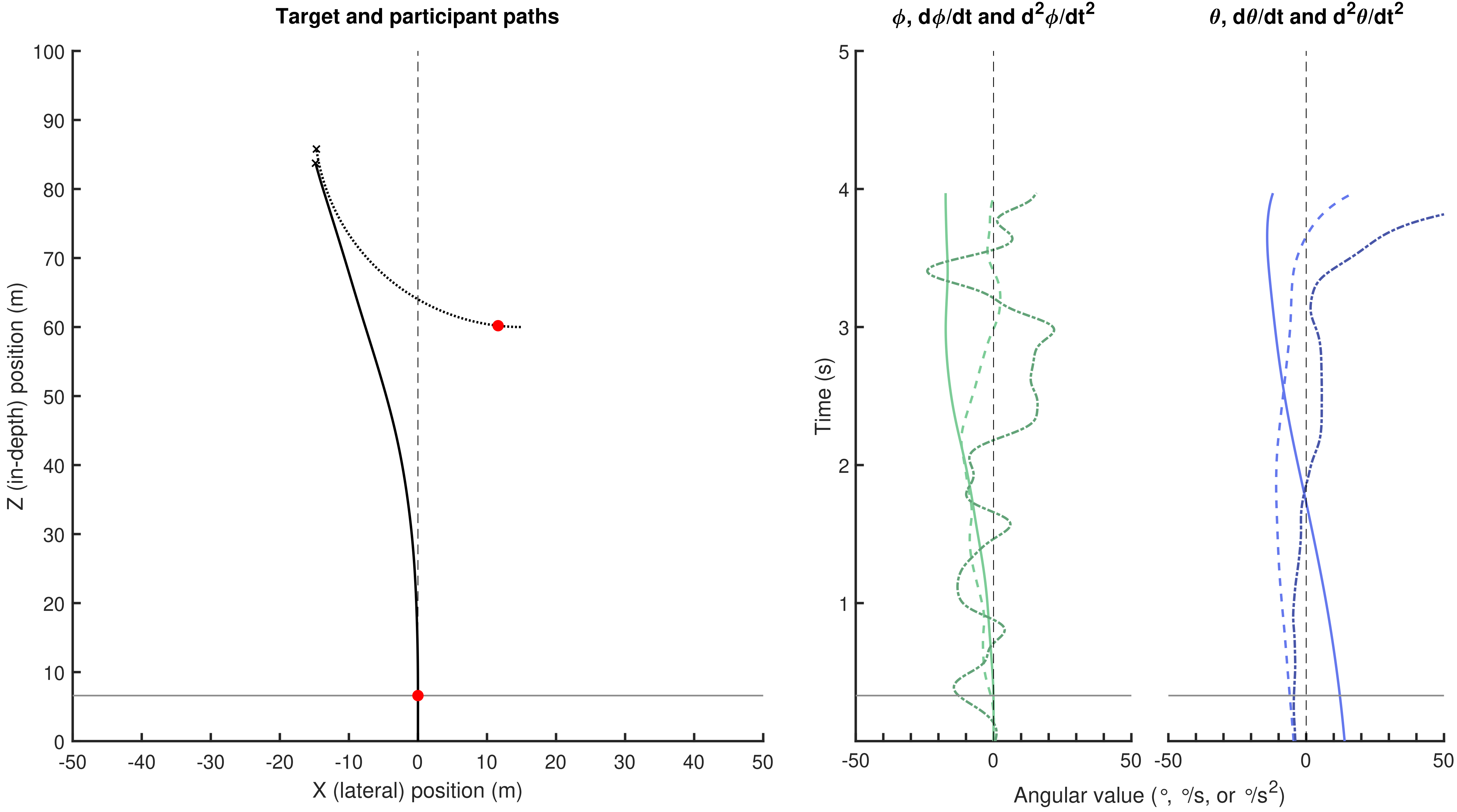

P17/B4
S20/R10-IN

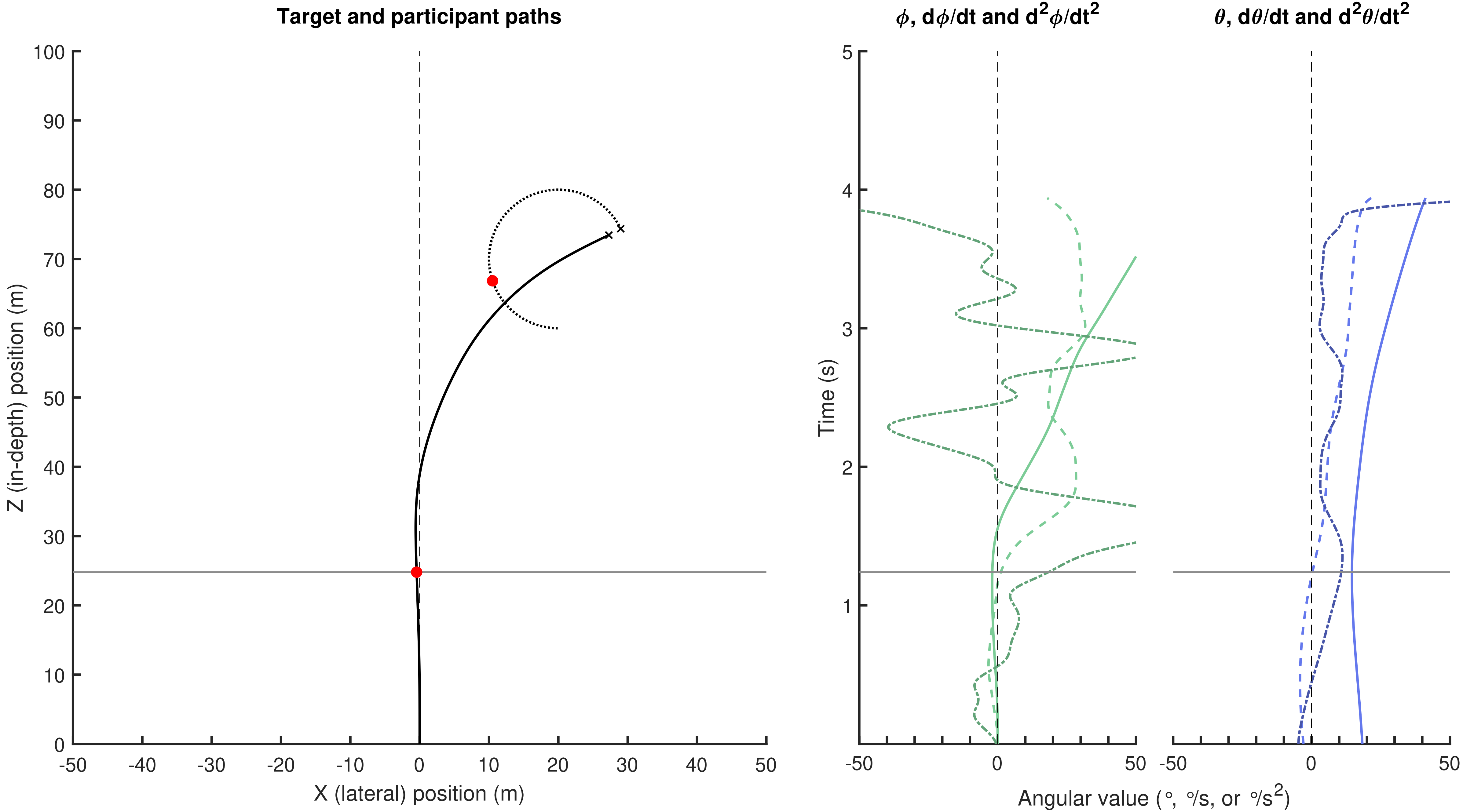

P17/B4
S20/R20-IN

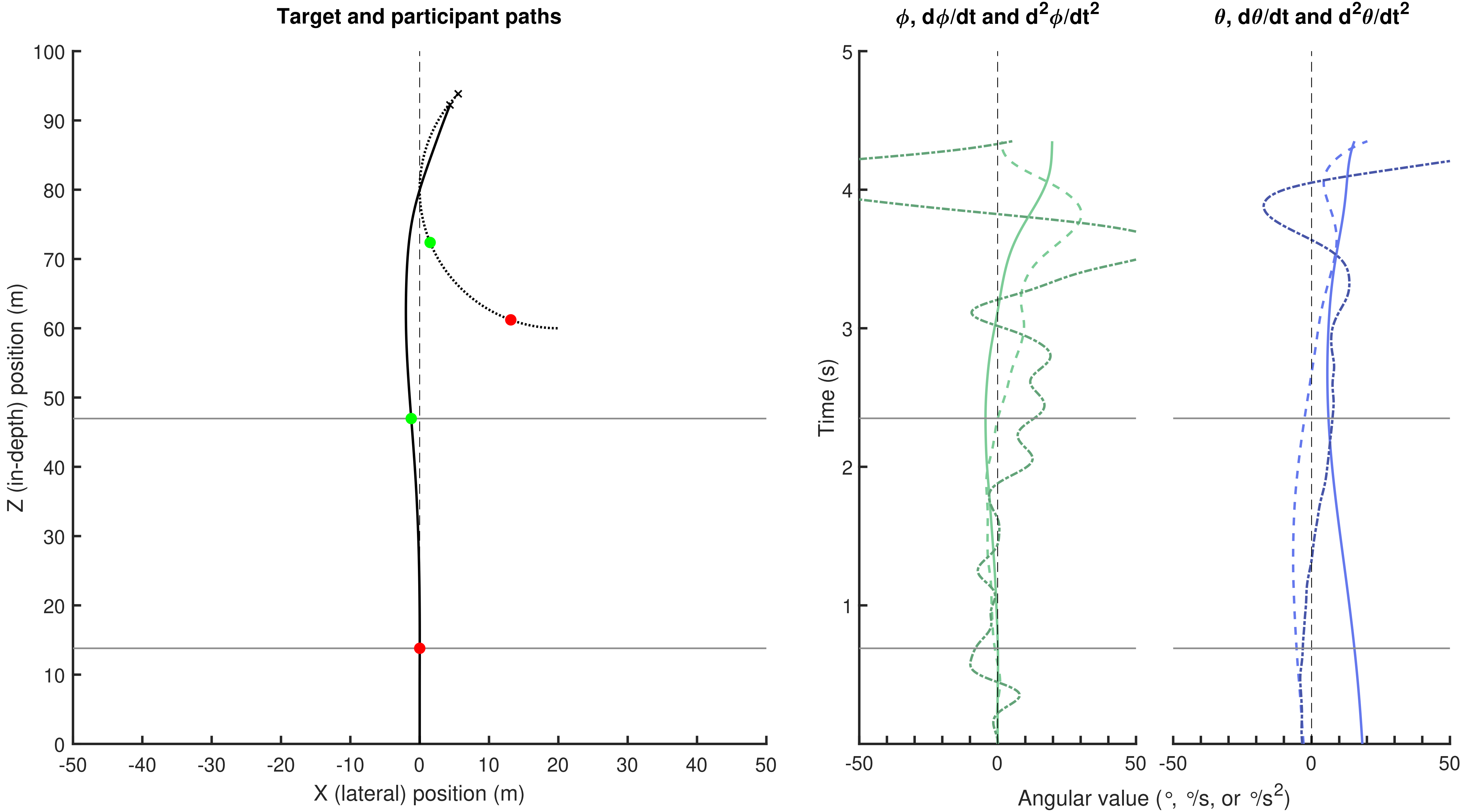

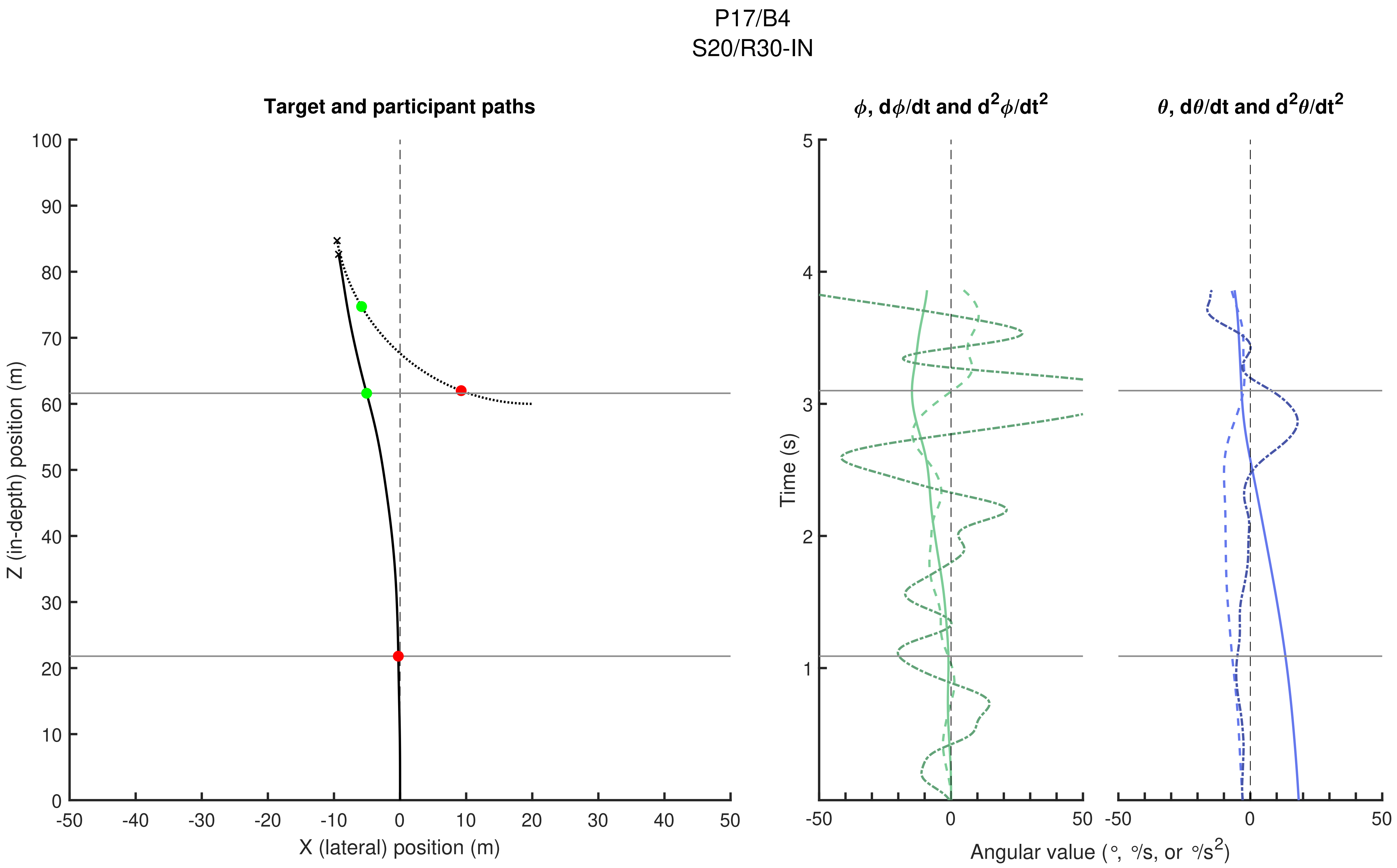
P17/B4
S20/R30-IN
Target and participant paths
Z (in-depth) position (m)
X (lateral) position (m)
ϕ, dϕ/dt and d²ϕ/dt²
θ, dθ/dt and d²θ/dt²
Time (s)
Angular value (°, °/s, or °/s²)

P17/B5
S20/R10-IN

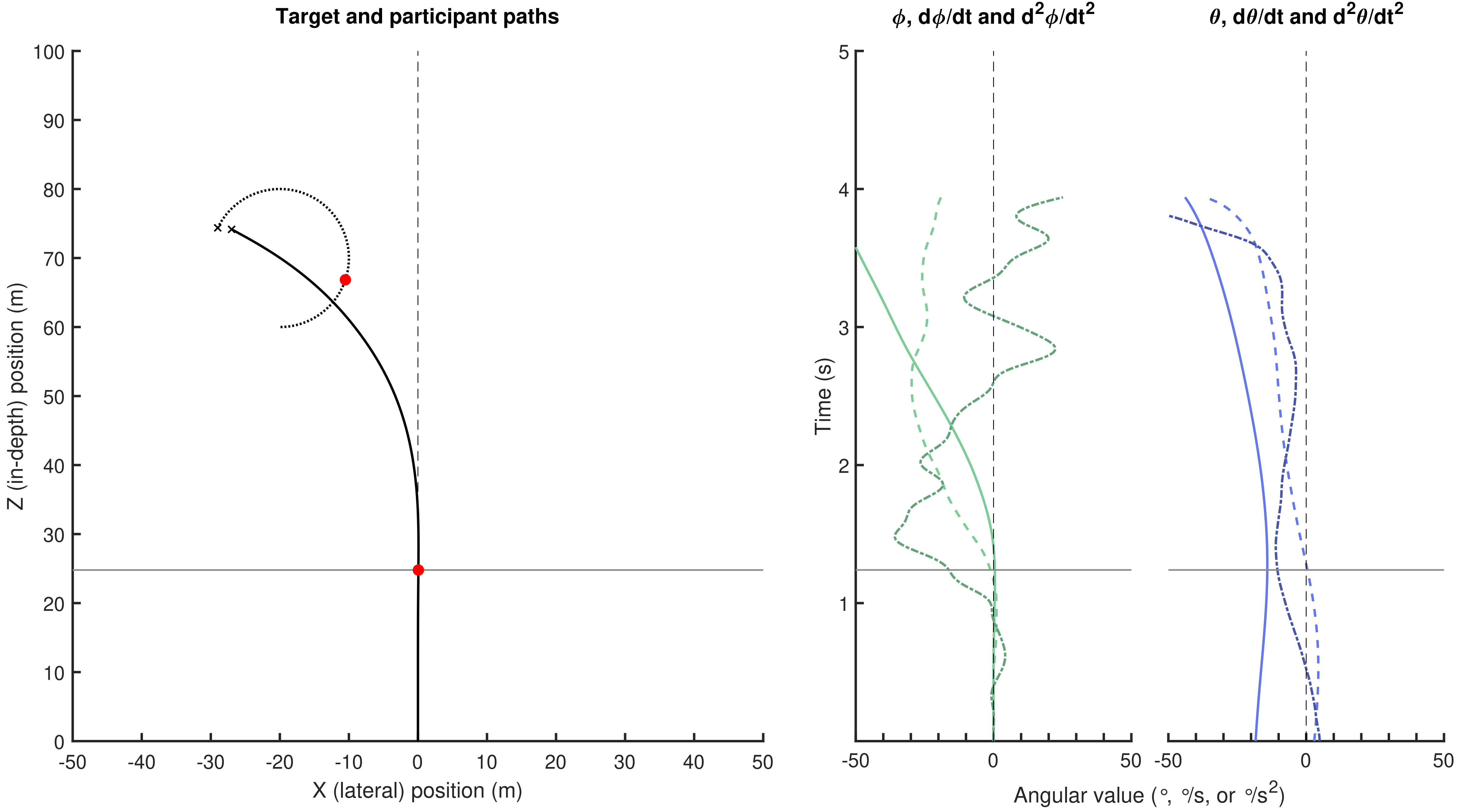

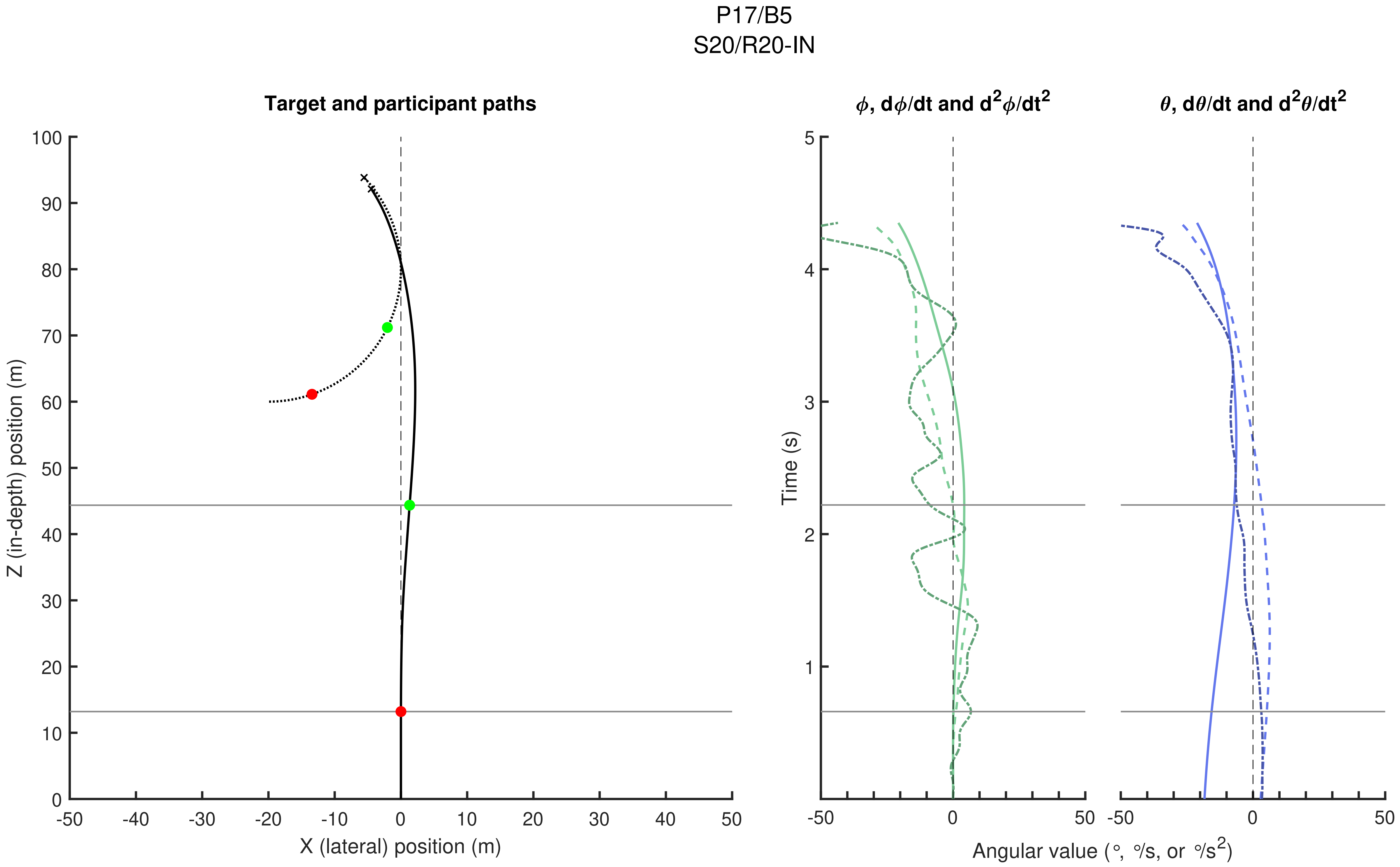

P17/B5
S20/R20-IN
Target and participant paths
Z (in-depth) position (m)
X (lateral) position (m)
ϕ, dϕ/dt and d²ϕ/dt²
θ, dθ/dt and d²θ/dt²
Time (s)
Angular value (°, °/s, or °/s²)

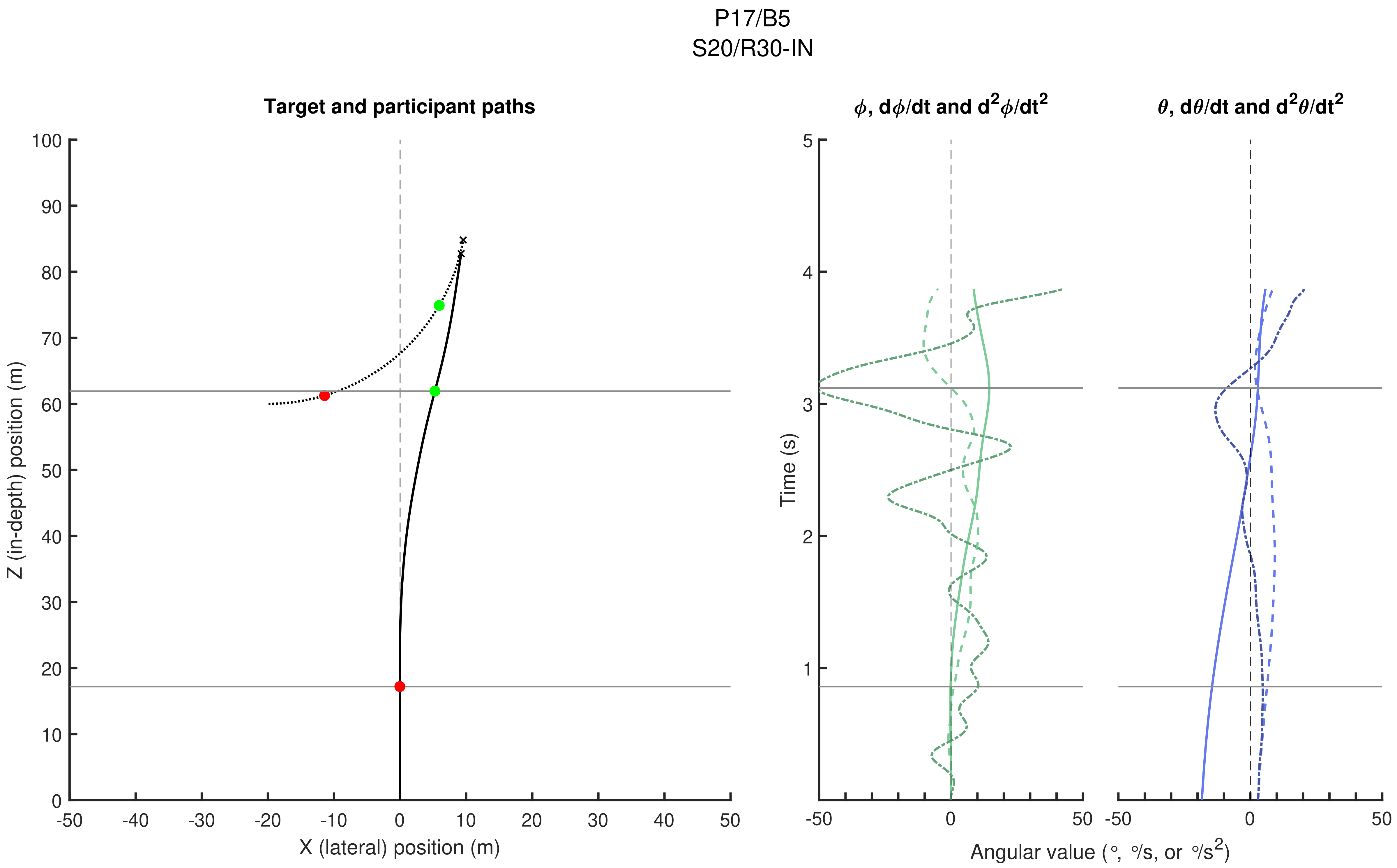
P17/B5
S20/R30-IN
Target and participant paths
ϕ, dϕ/dt and d$^2\phi$/dt^2
θ, dθ/dt and d$^2\theta$/dt^2
Z (in-depth) position (m)
X (lateral) position (m)
Time (s)
Angular value (°, °/s, or °/s^2)

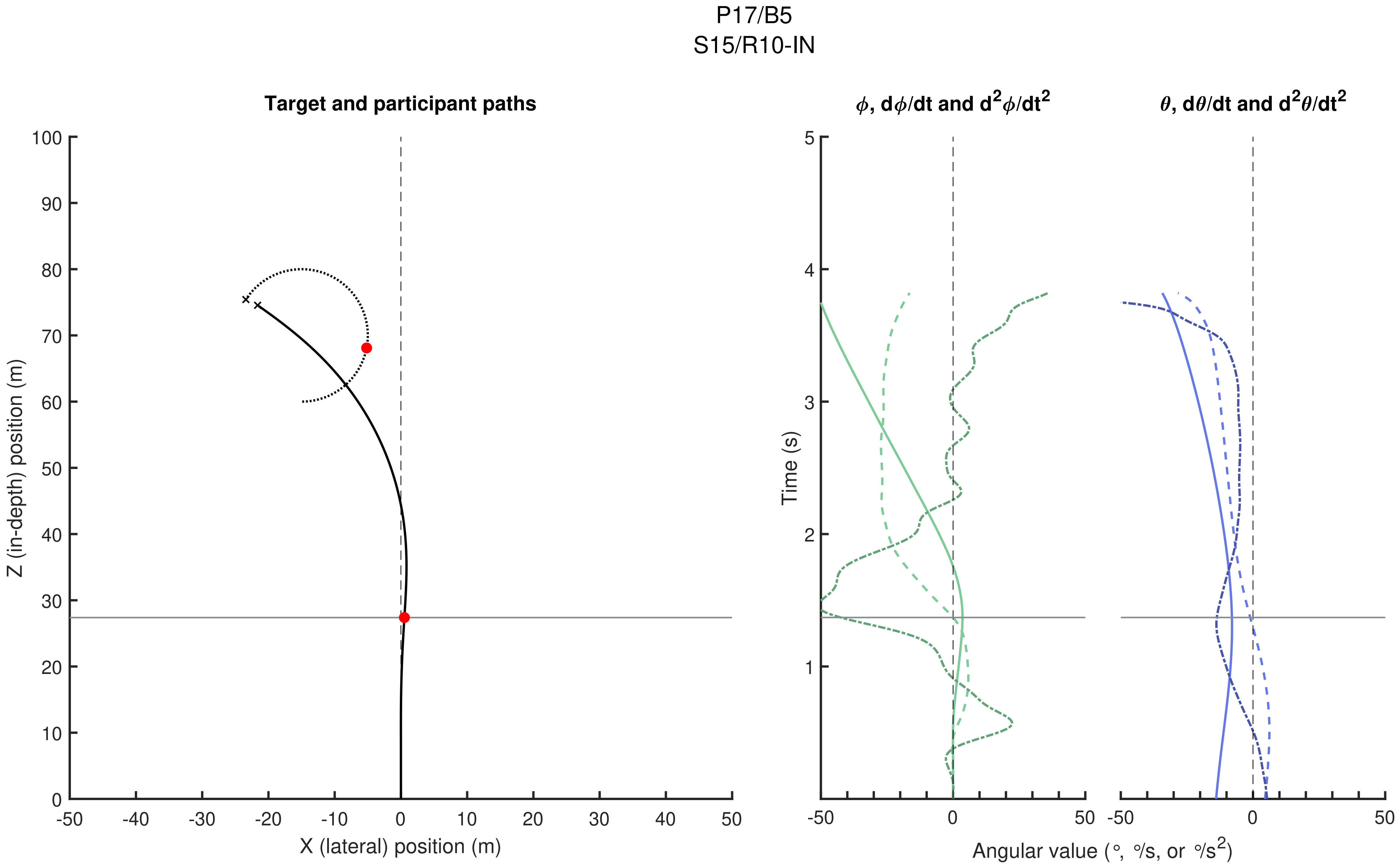
P17/B5
S15/R10-IN
Target and participant paths
Z (in-depth) position (m)
X (lateral) position (m)
φ, dφ/dt and d²φ/dt²
θ, dθ/dt and d²θ/dt²
Time (s)
Angular value (°, °/s, or °/s²)

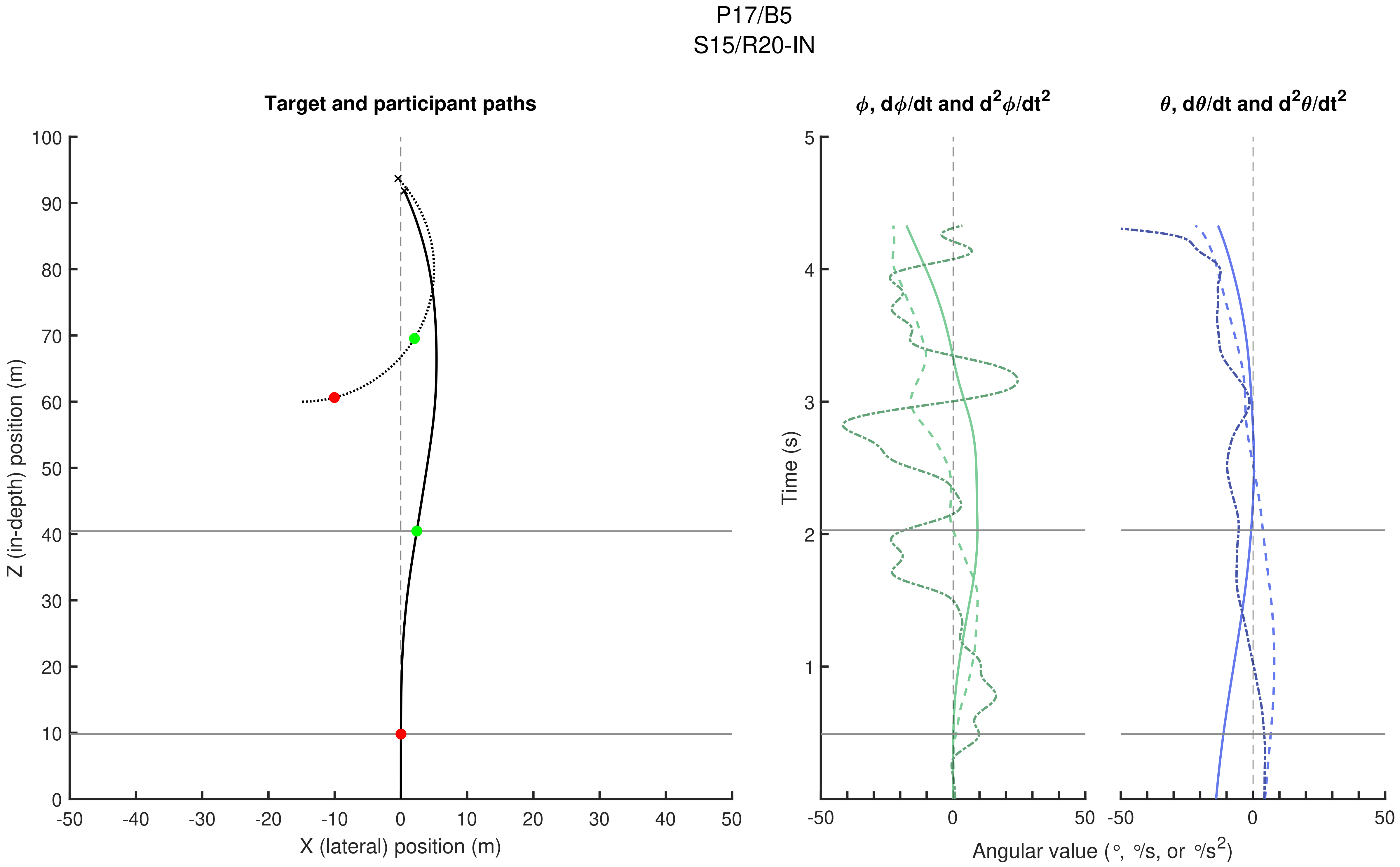

P17/B5
S15/R20-IN
Target and participant paths
Z (in-depth) position (m)
X (lateral) position (m)
ϕ, dϕ/dt and d²ϕ/dt²
θ, dθ/dt and d²θ/dt²
Time (s)
Angular value (°, °/s, or °/s²)

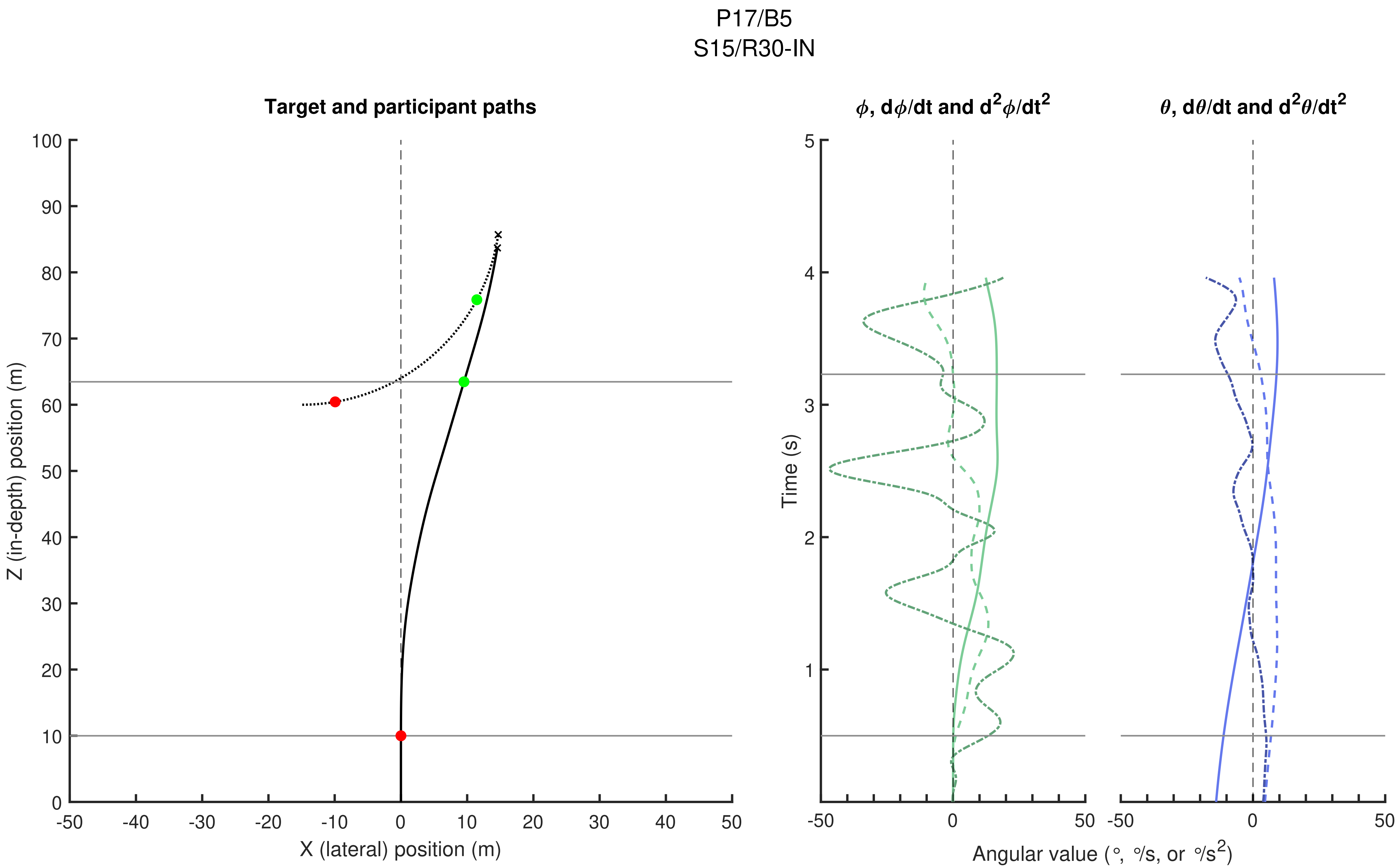
P17/B5
S15/R30-IN
Target and participant paths
Z (in-depth) position (m)
X (lateral) position (m)
ϕ, dϕ/dt and d²ϕ/dt²
θ, dθ/dt and d²θ/dt²
Time (s)
Angular value (°, °/s, or °/s²)

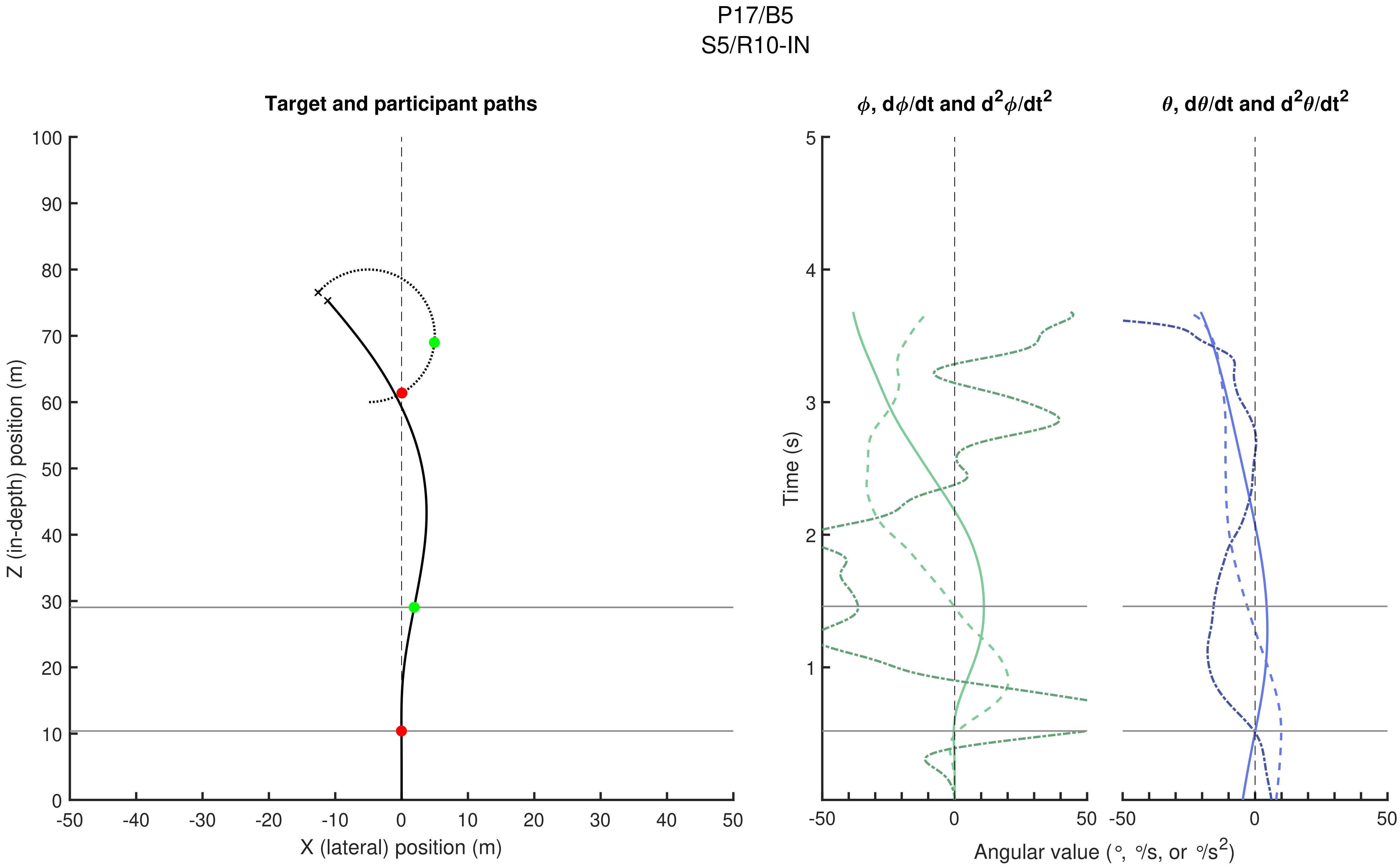

P17/B5
S5/R10-IN
Target and participant paths
Z (in-depth) position (m)
X (lateral) position (m)
ϕ, dϕ/dt and d$^2\phi$/dt^2
θ, dθ/dt and d$^2\theta$/dt^2
Time (s)
Angular value (°, °/s, or °/s^2)

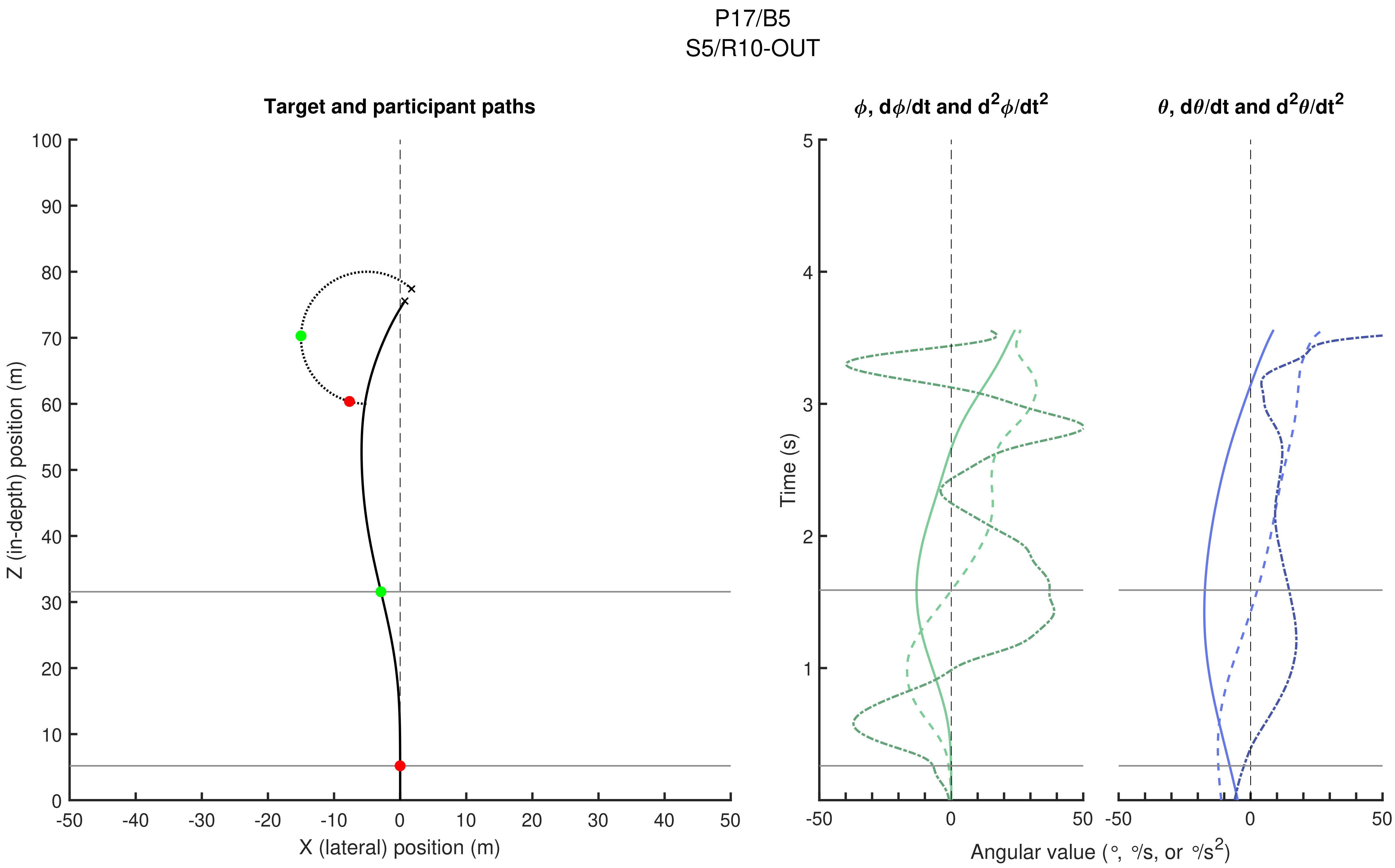
P17/B5
S5/R10-OUT
Target and participant paths
ϕ, dϕ/dt and d$^2\phi$/dt^2
θ, dθ/dt and d$^2\theta$/dt^2
Z (in-depth) position (m)
X (lateral) position (m)
Time (s)
Angular value (°, °/s, or °/s^2)

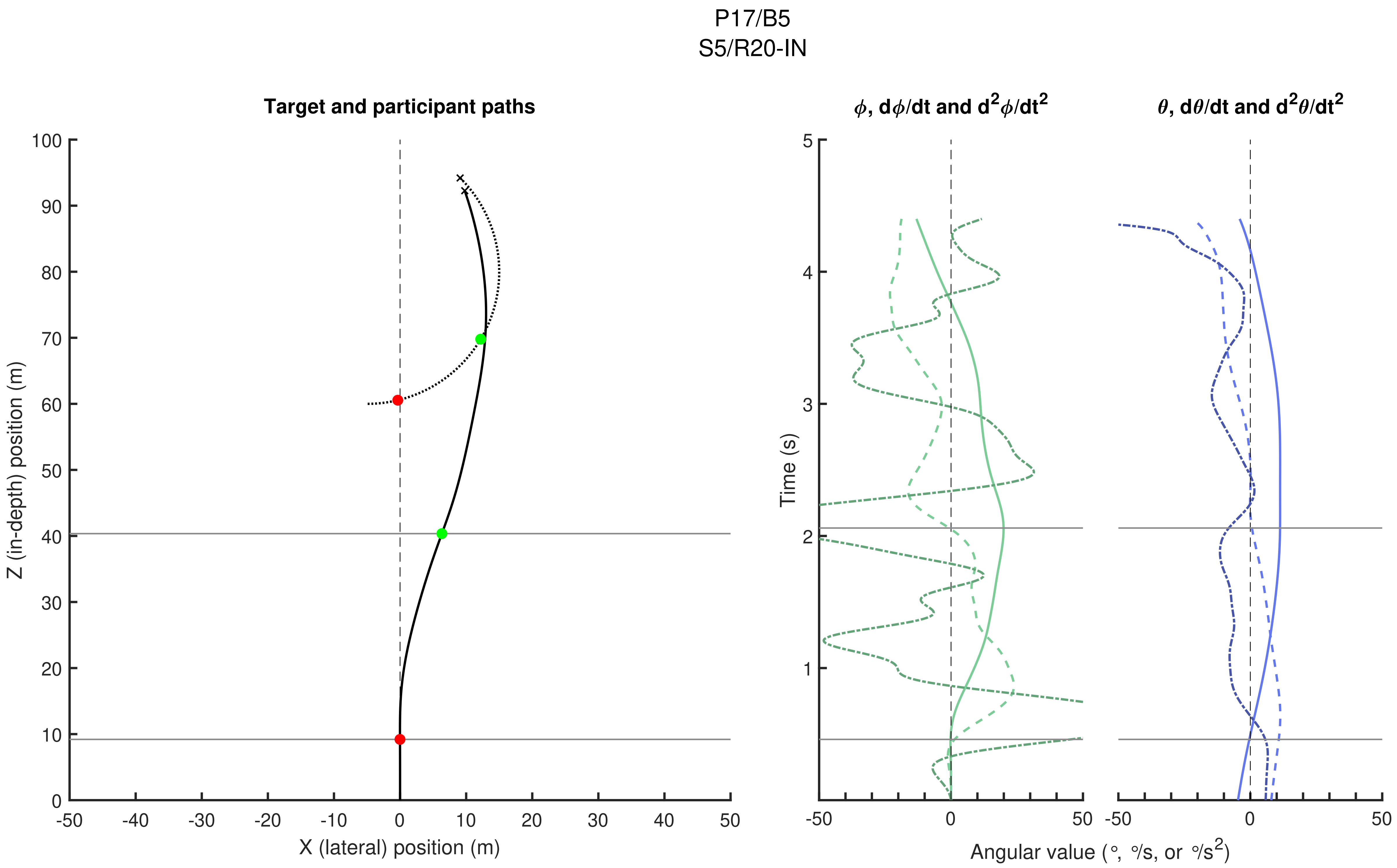
P17/B5
S5/R20-IN
Target and participant paths
Z (in-depth) position (m)
X (lateral) position (m)
100
90
80
70
60
50
40
30
20
10
0
-50
-40
-30
-20
-10
0
10
20
30
40
50
ϕ, dϕ/dt and d$^2\phi$/dt^2
θ, dθ/dt and d$^2\theta$/dt^2
Time (s)
5
4
3
2
1
-50
0
50
-50
0
50
Angular value (°, °/s, or °/s^2)

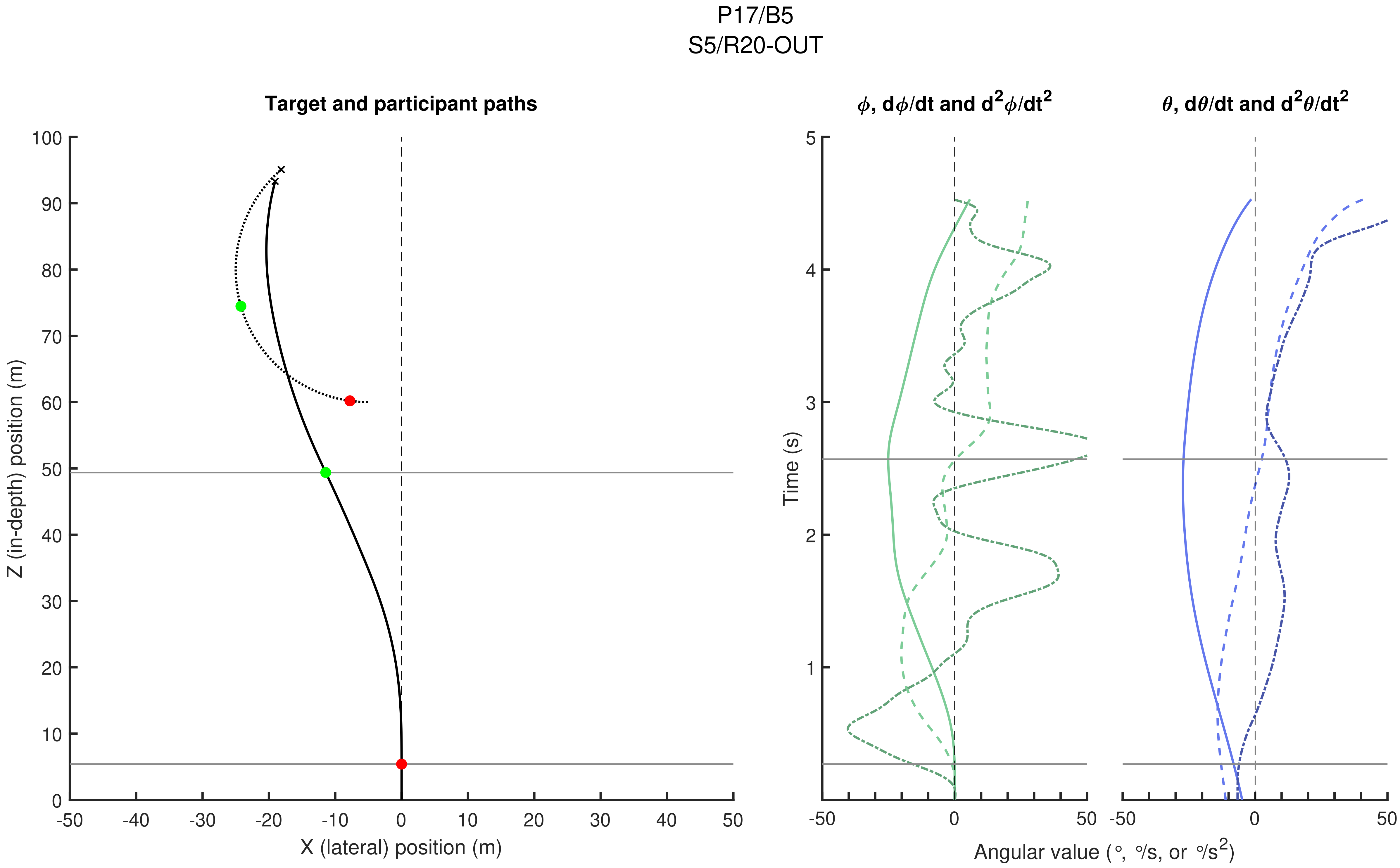

P17/B5
S5/R20-OUT
Target and participant paths
Z (in-depth) position (m)
X (lateral) position (m)
ϕ, dϕ/dt and d$^2\phi$/dt^2
θ, dθ/dt and d$^2\theta$/dt^2
Time (s)
Angular value (°, °/s, or °/s^2)

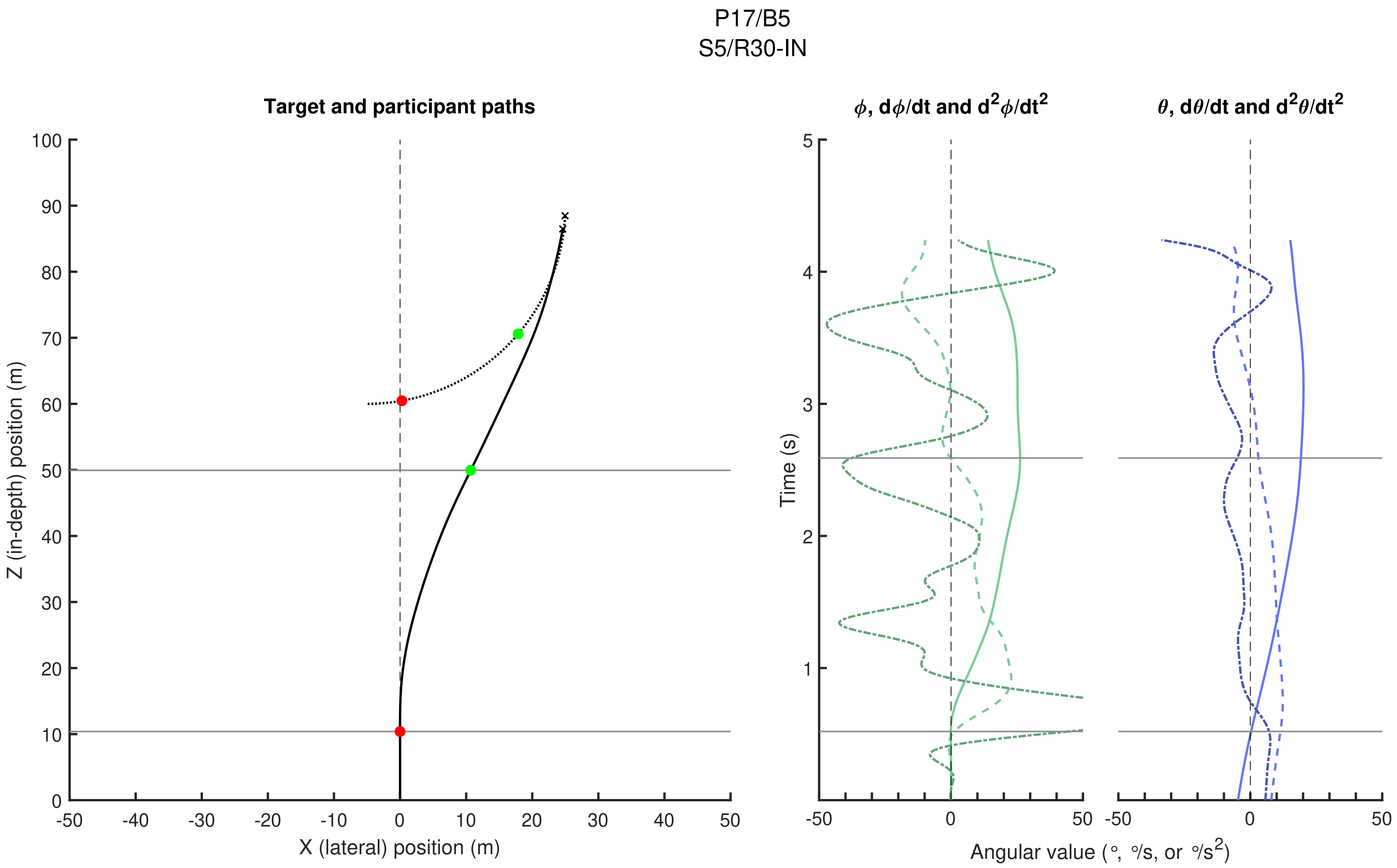
P17/B5
S5/R30-IN
Target and participant paths
Z (in-depth) position (m)
X (lateral) position (m)
100
90
80
70
60
50
40
30
20
10
0
-50
-40
-30
-20
-10
0
10
20
30
40
50
ϕ, dϕ/dt and d$^2\phi$/dt^2
θ, dθ/dt and d$^2\theta$/dt^2
Time (s)
5
4
3
2
1
-50
0
50
-50
0
50
Angular value (°, °/s, or °/s^2)

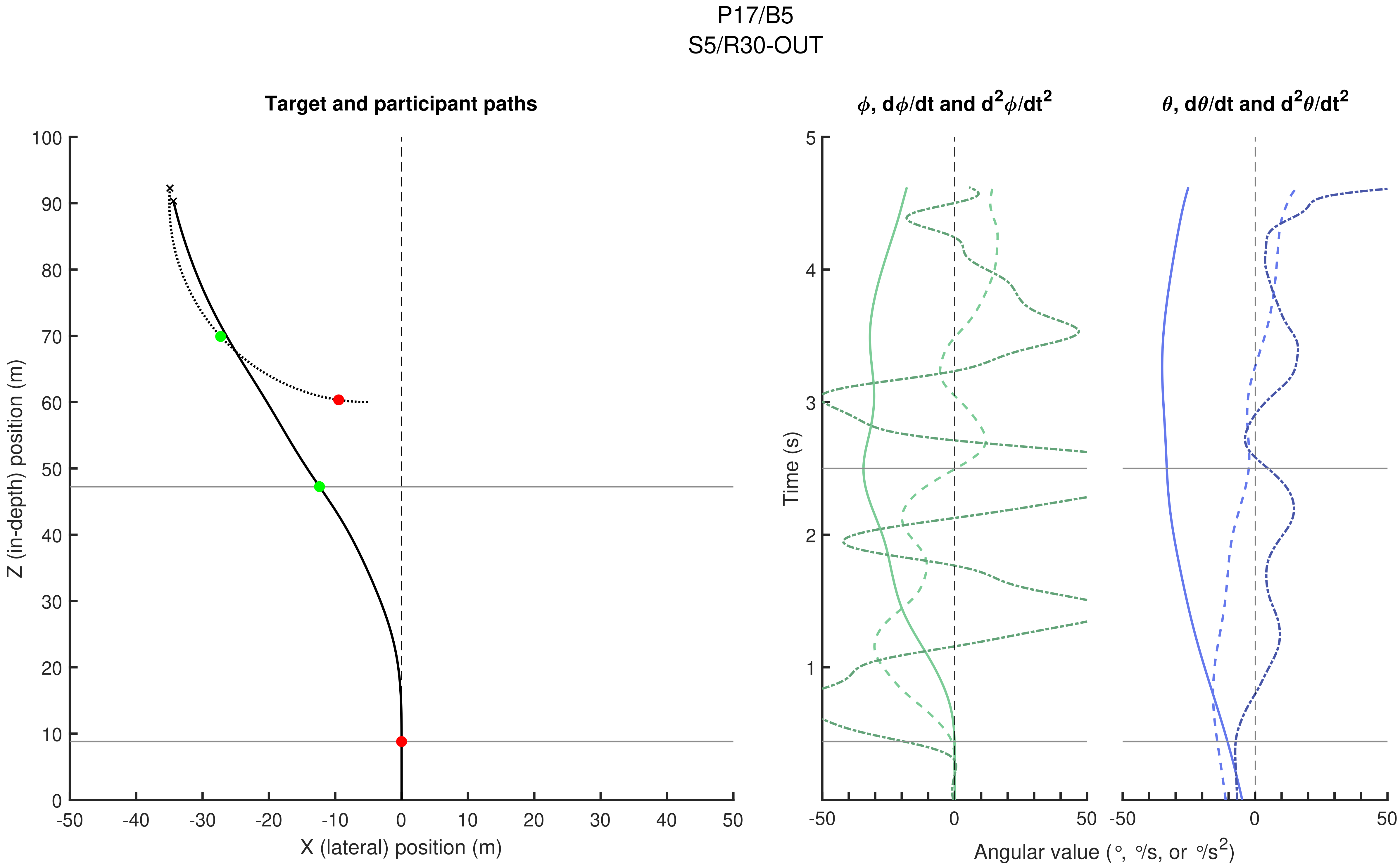
P17/B5
S5/R30-OUT
Target and participant paths
Z (in-depth) position (m)
X (lateral) position (m)
φ, dφ/dt and d²φ/dt²
θ, dθ/dt and d²θ/dt²
Time (s)
Angular value (°, °/s, or °/s²)

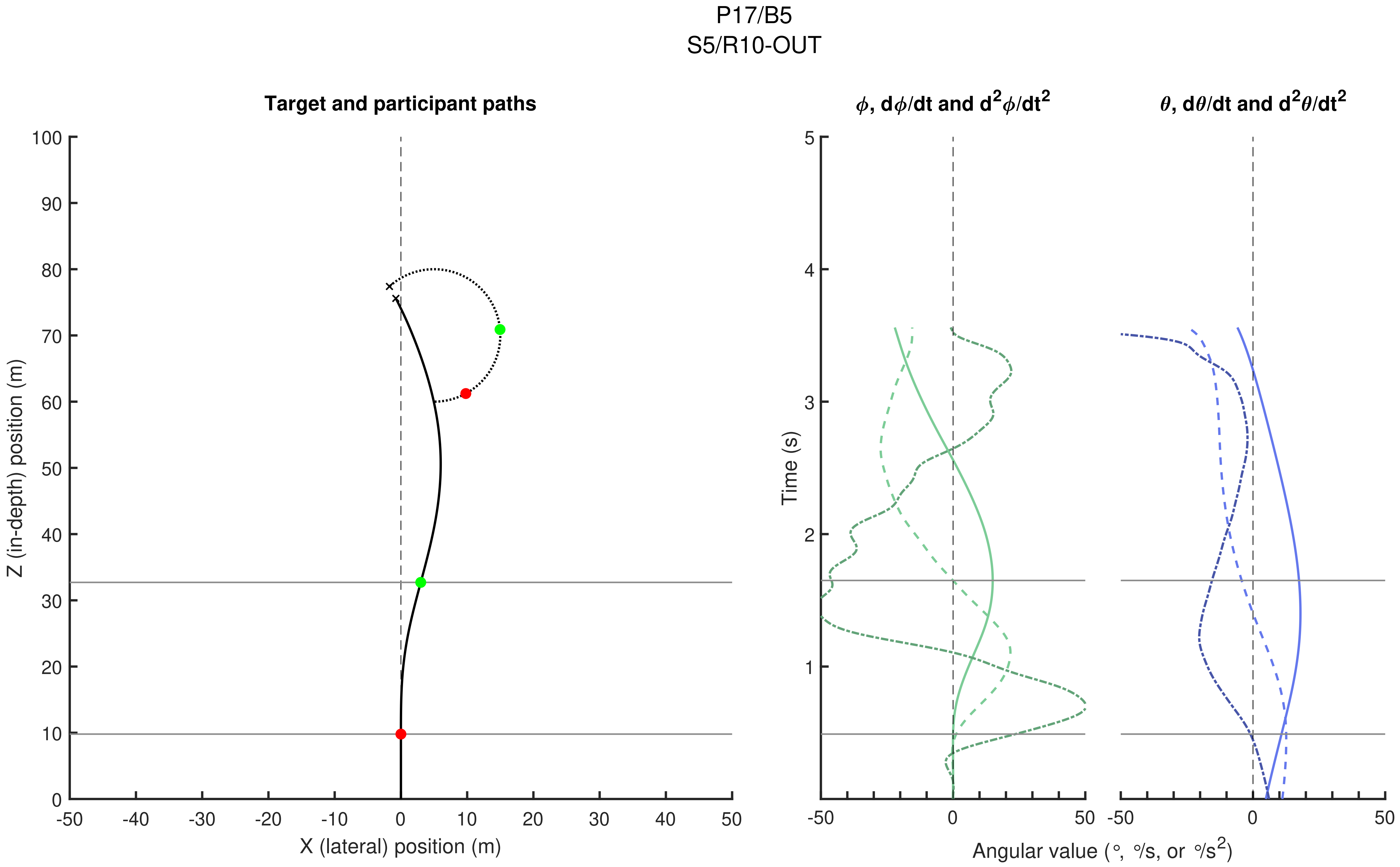

P17/B5
S5/R10-OUT
Target and participant paths
Z (in-depth) position (m)
X (lateral) position (m)
ϕ, dϕ/dt and d²ϕ/dt²
θ, dθ/dt and d²θ/dt²
Time (s)
Angular value (°, °/s, or °/s²)

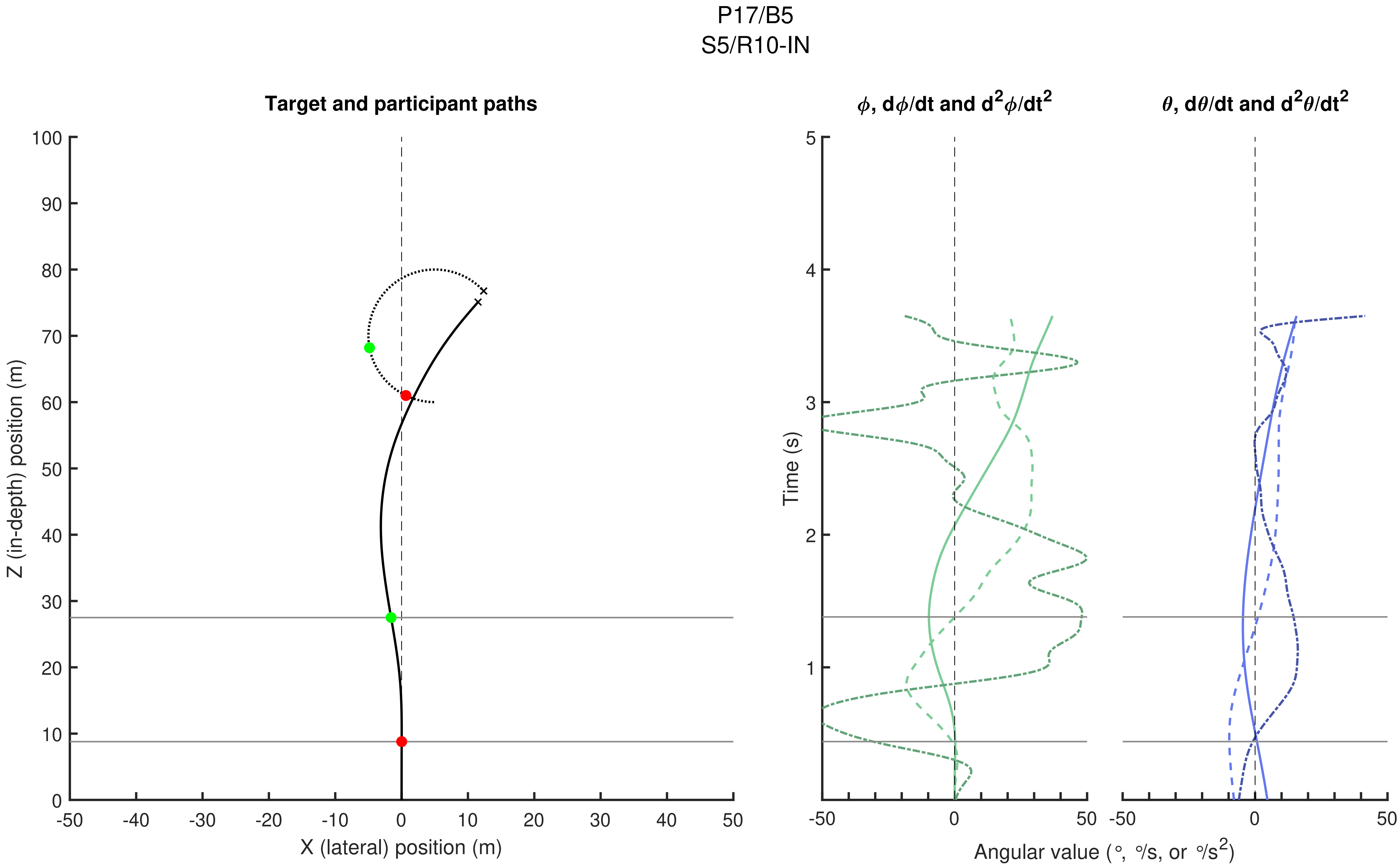

P17/B5
S5/R10-IN
Target and participant paths
Z (in-depth) position (m)
X (lateral) position (m)
φ, dφ/dt and d²φ/dt²
θ, dθ/dt and d²θ/dt²
Time (s)
Angular value (°, °/s, or °/s²)

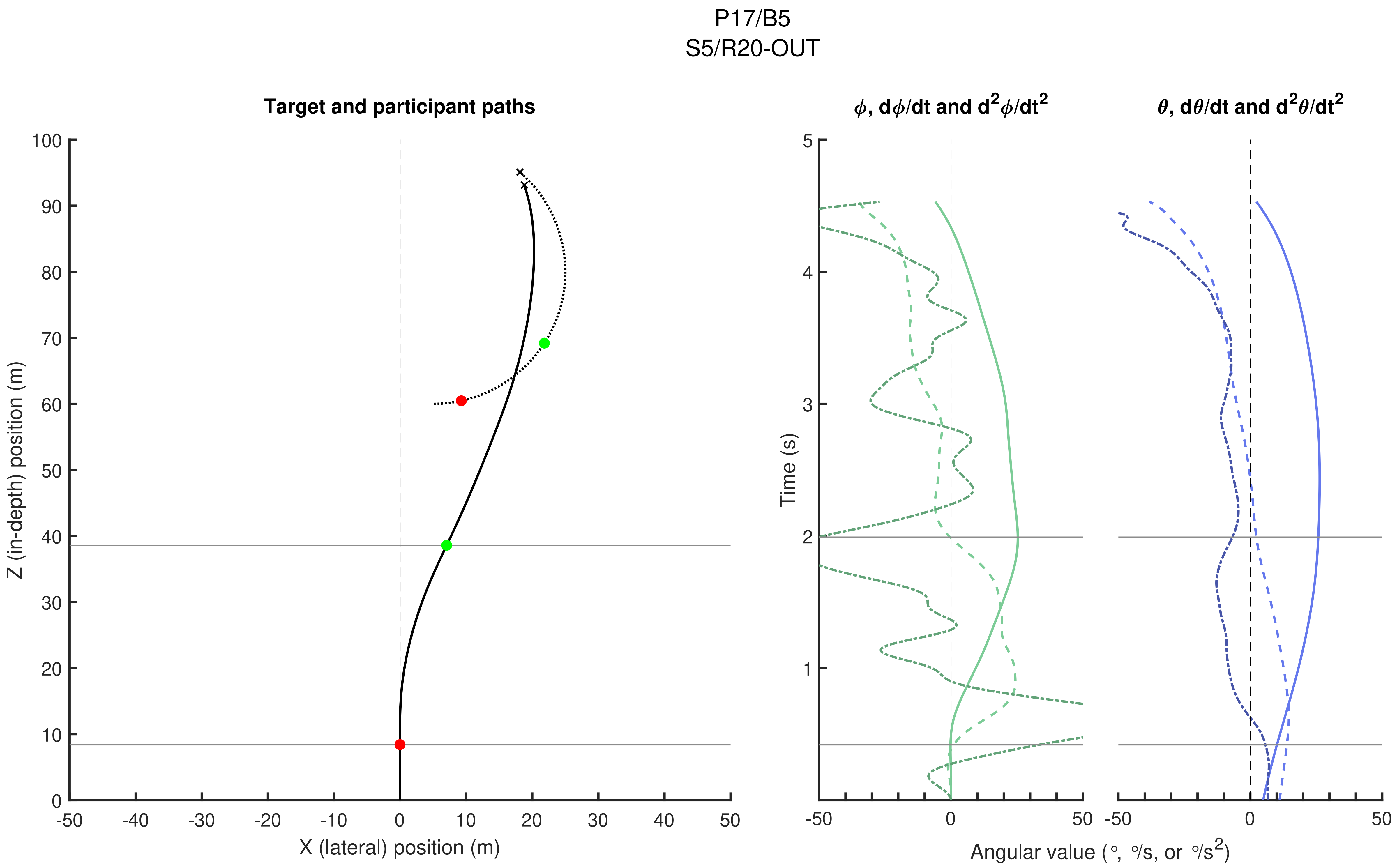
P17/B5
S5/R20-OUT
Target and participant paths
Z (in-depth) position (m)
X (lateral) position (m)
100
90
80
70
60
50
40
30
20
10
0
-50
-40
-30
-20
-10
0
10
20
30
40
50
ϕ, dϕ/dt and d$^2\phi$/dt^2
θ, dθ/dt and d$^2\theta$/dt^2
Time (s)
5
4
3
2
1
-50
0
50
-50
0
50
Angular value (°, °/s, or °/s^2)

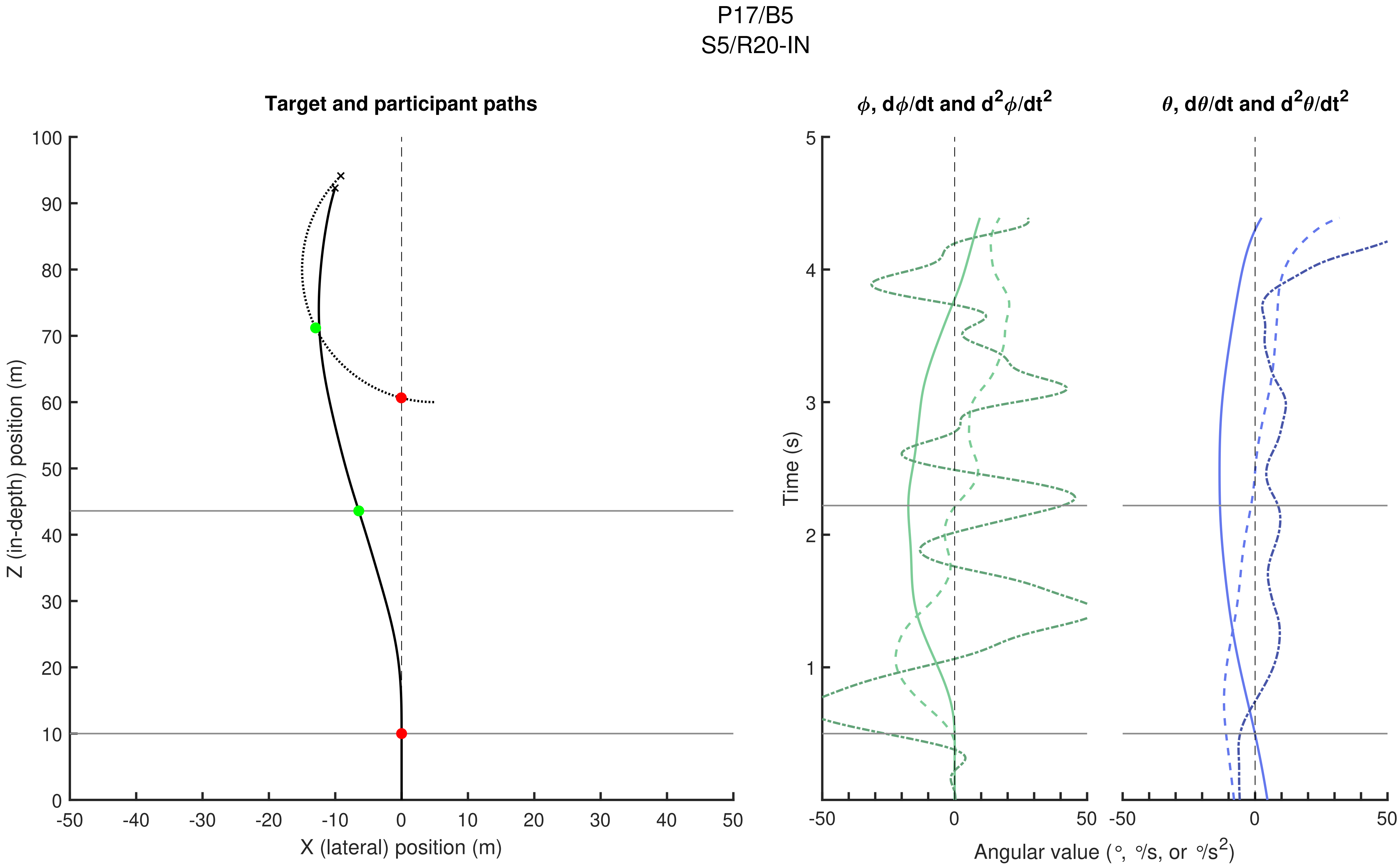

P17/B5
S5/R20-IN
Target and participant paths
Z (in-depth) position (m)
X (lateral) position (m)
ϕ, dϕ/dt and d²ϕ/dt²
θ, dθ/dt and d²θ/dt²
Time (s)
Angular value (°, °/s, or °/s²)

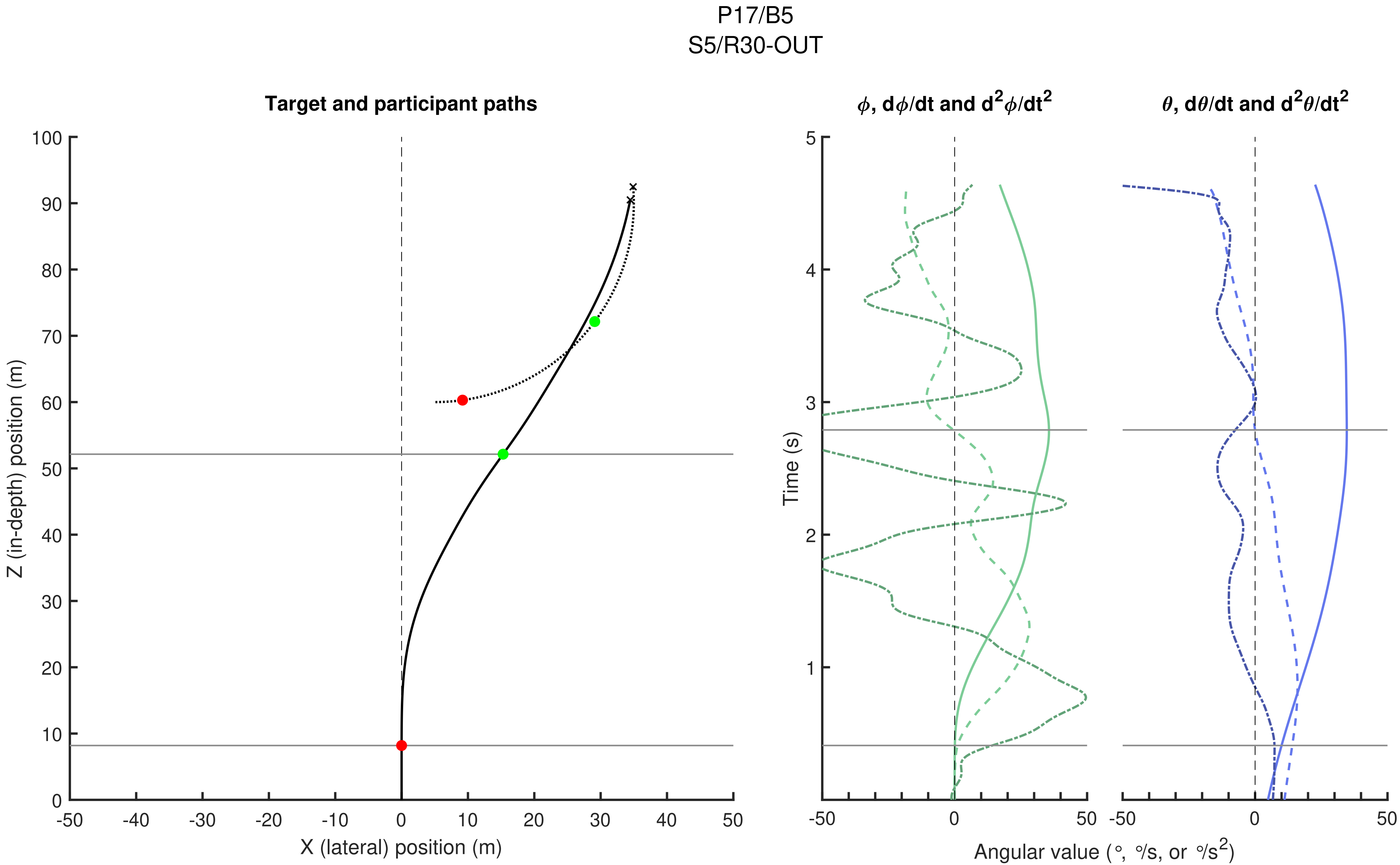
P17/B5
S5/R30-OUT
Target and participant paths
ϕ, dϕ/dt and d$^2\phi$/dt^2
θ, dθ/dt and d$^2\theta$/dt^2
Z (in-depth) position (m)
X (lateral) position (m)
Time (s)
Angular value (°, °/s, or °/s^2)
0
10
20
30
40
50
60
70
80
90
100
-50
-40
-30
-20
-10
0
10
20
30
40
50
1
2
3
4
5
-50
0
50
-50
0
50

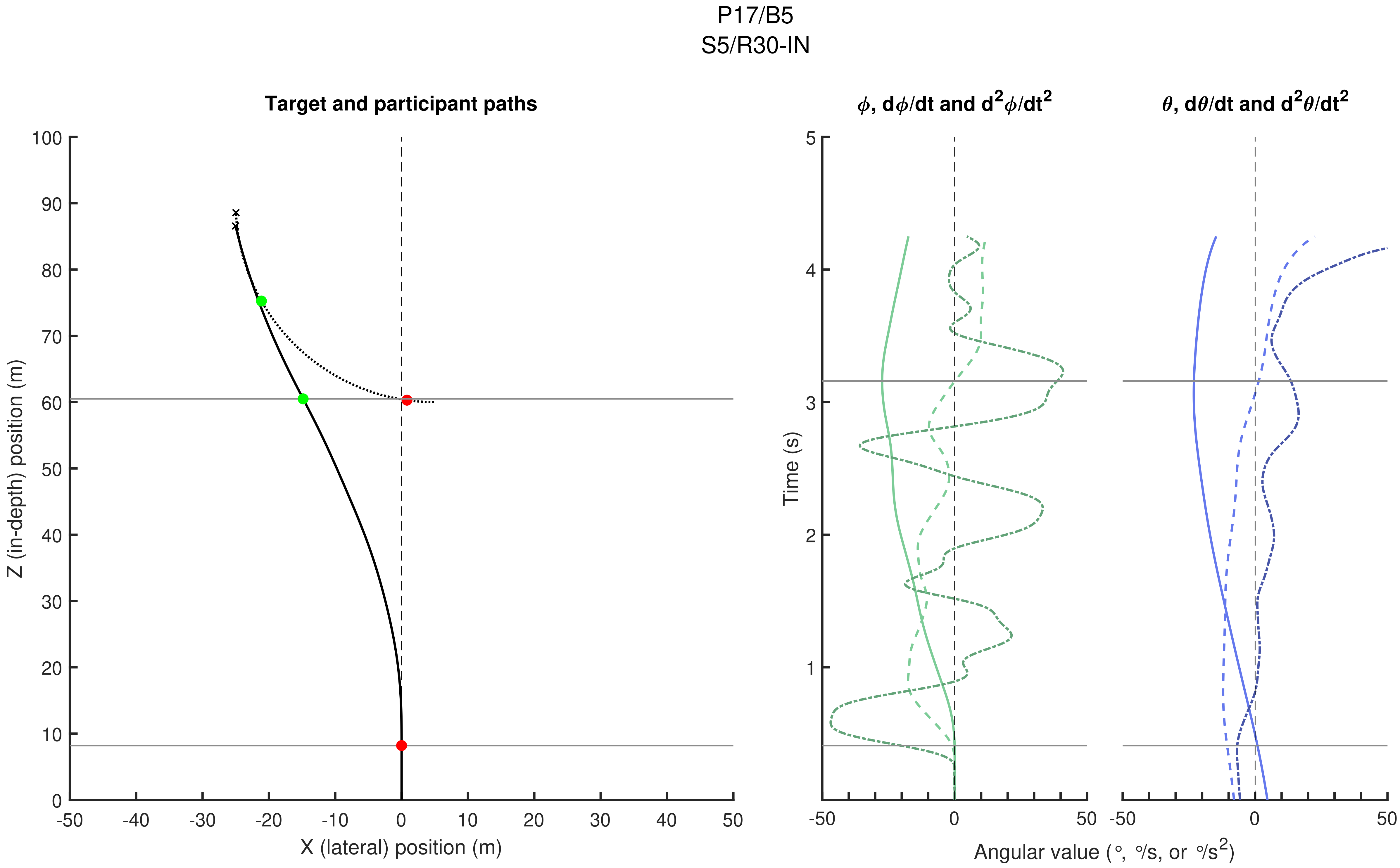
P17/B5
S5/R30-IN
Target and participant paths
Z (in-depth) position (m)
X (lateral) position (m)
100
90
80
70
60
50
40
30
20
10
0
-50
-40
-30
-20
-10
0
10
20
30
40
50
ϕ, dϕ/dt and d2ϕ/dt2
θ, dθ/dt and d2θ/dt2
Time (s)
5
4
3
2
1
-50
0
50
-50
0
50
Angular value (°, °/s, or °/s2)

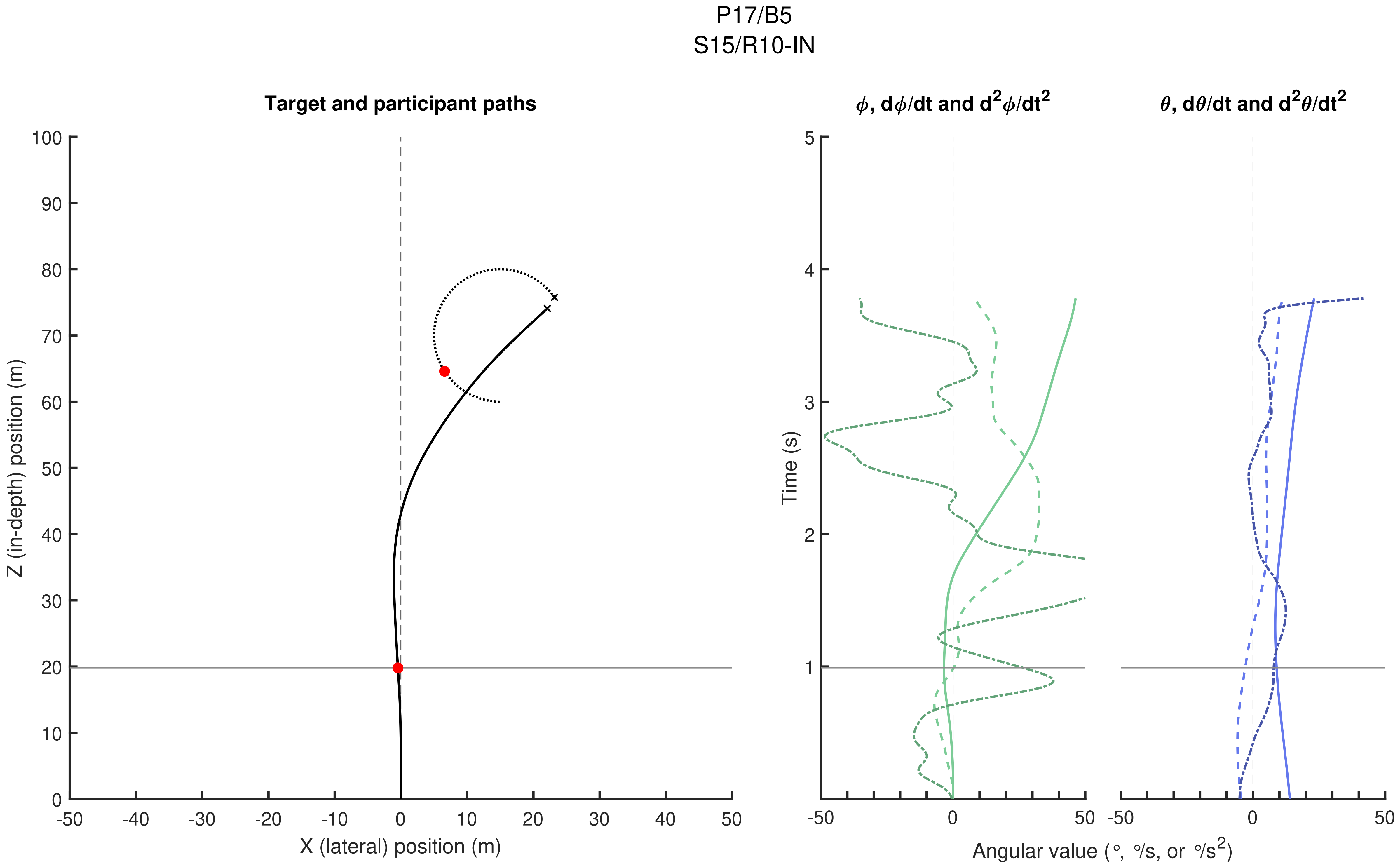
P17/B5
S15/R10-IN
Target and participant paths
Z (in-depth) position (m)
X (lateral) position (m)
100
90
80
70
60
50
40
30
20
10
0
-50
-40
-30
-20
-10
0
10
20
30
40
50
φ, dφ/dt and d²φ/dt²
θ, dθ/dt and d²θ/dt²
Time (s)
5
4
3
2
1
-50
0
50
-50
0
50
Angular value (°, °/s, or °/s²)

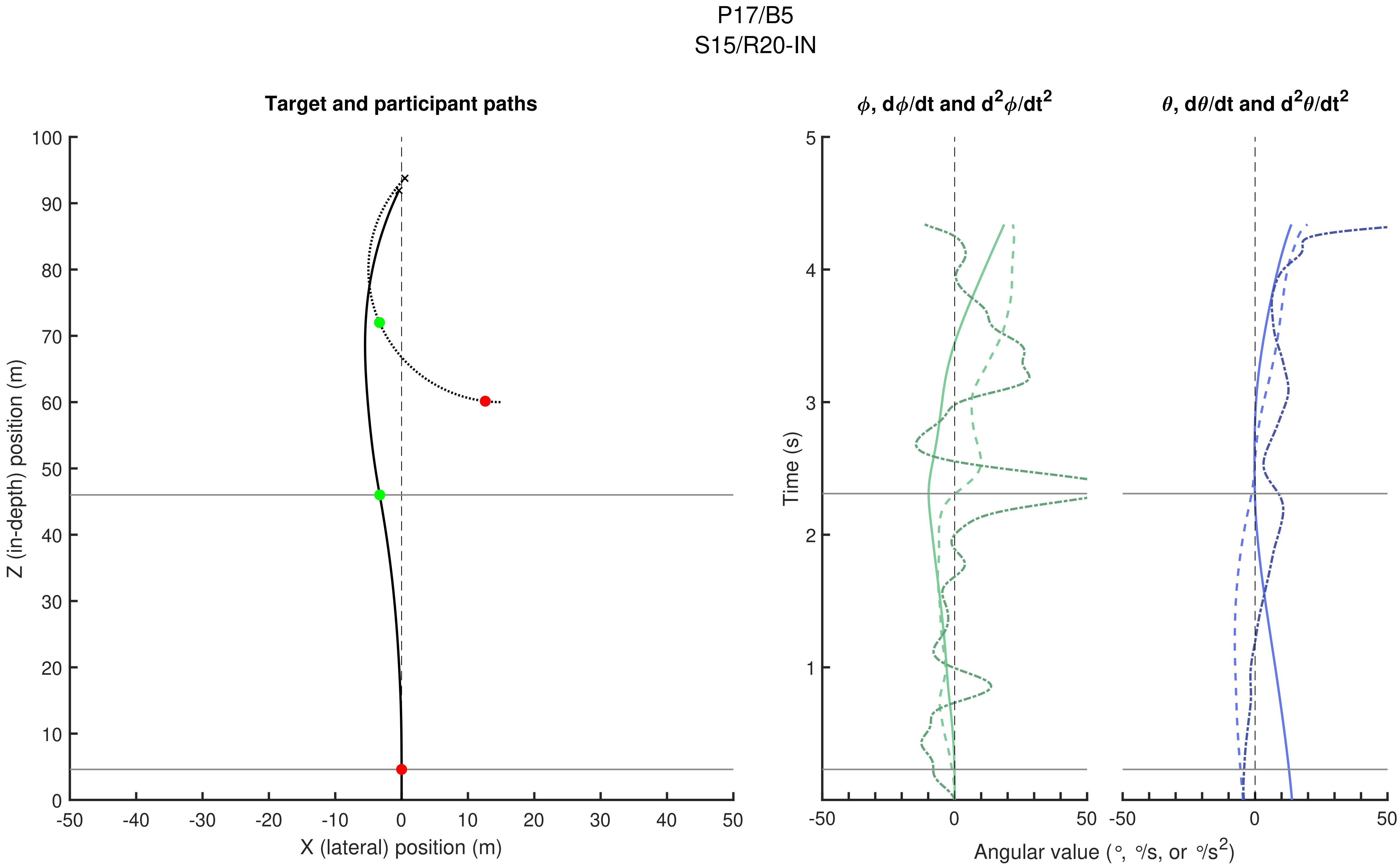
P17/B5
S15/R20-IN
Target and participant paths
Z (in-depth) position (m)
X (lateral) position (m)
100
90
80
70
60
50
40
30
20
10
0
-50
-40
-30
-20
-10
0
10
20
30
40
50
φ, dφ/dt and d²φ/dt²
θ, dθ/dt and d²θ/dt²
Time (s)
5
4
3
2
1
-50
0
50
-50
0
50
Angular value (°, °/s, or °/s²)

P17/B5
S15/R30-IN

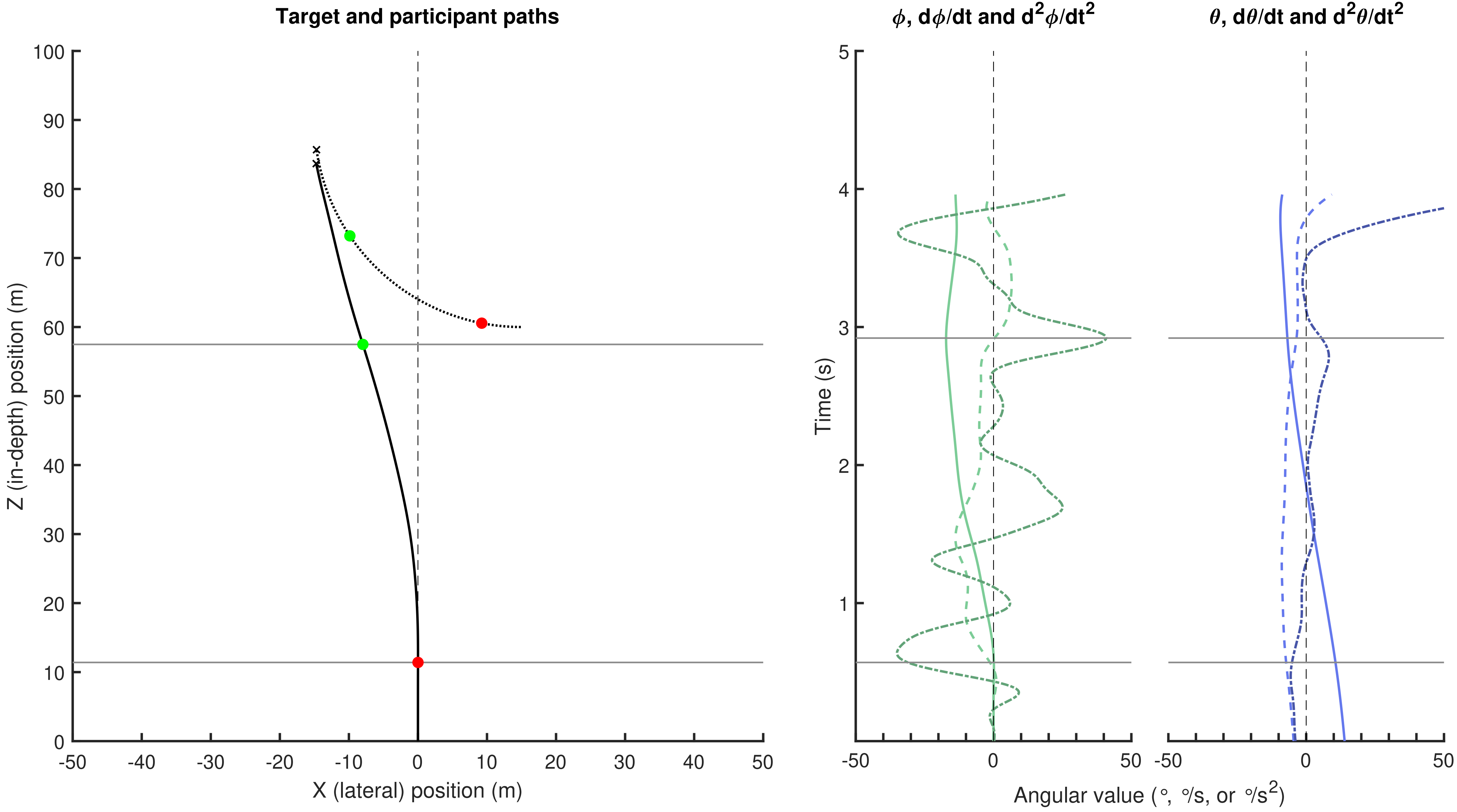

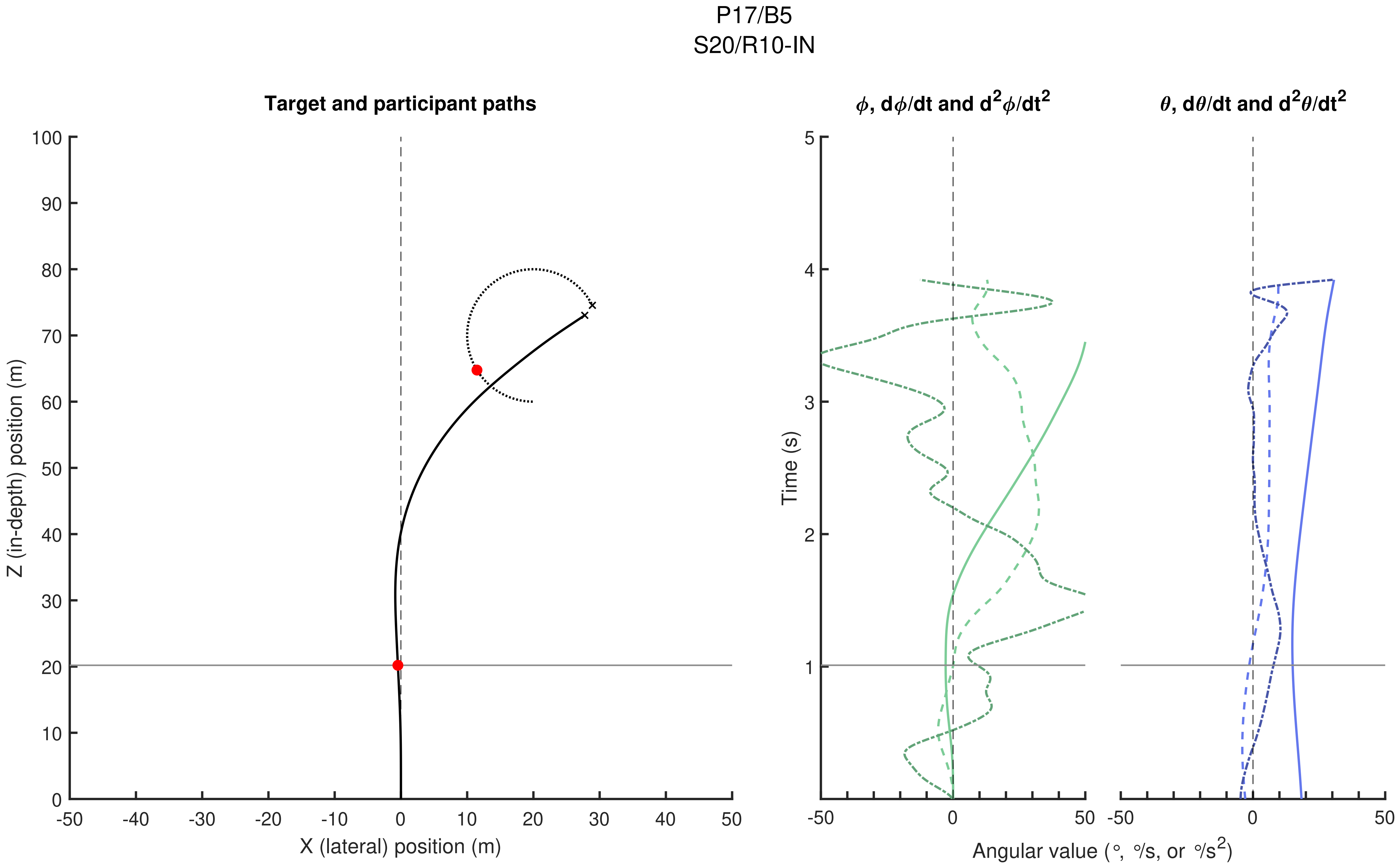

P17/B5
S20/R10-IN
Target and participant paths
ϕ, dϕ/dt and d$^2\phi$/dt^2
θ, dθ/dt and d$^2\theta$/dt^2
Z (in-depth) position (m)
X (lateral) position (m)
Time (s)
Angular value (°, °/s, or °/s^2)

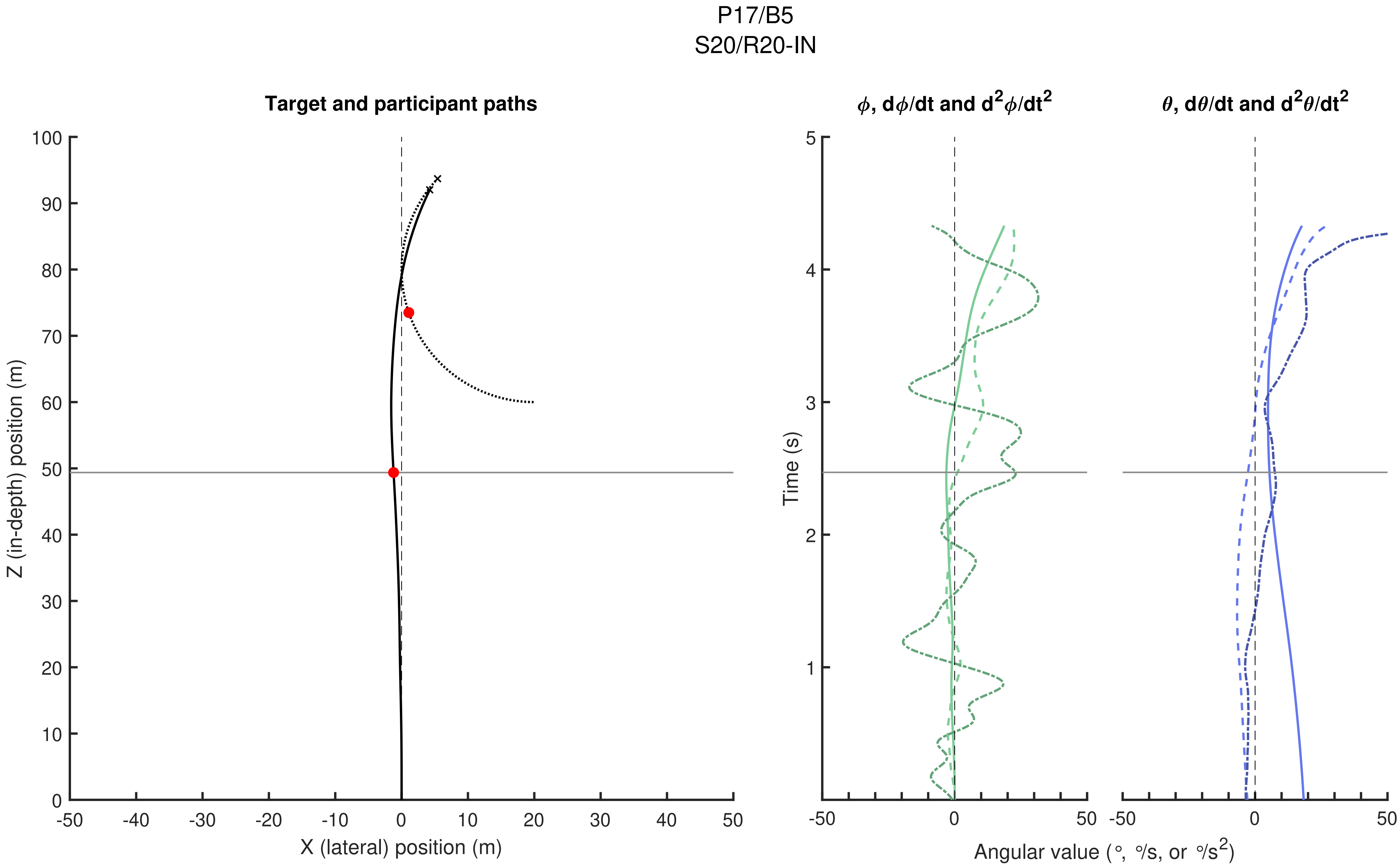

P17/B5
S20/R20-IN
Target and participant paths
Z (in-depth) position (m)
X (lateral) position (m)
100
90
80
70
60
50
40
30
20
10
0
-50
-40
-30
-20
-10
0
10
20
30
40
50
ϕ, dϕ/dt and d$^2\phi$/dt^2
θ, dθ/dt and d$^2\theta$/dt^2
Time (s)
5
4
3
2
1
-50
0
50
-50
0
50
Angular value (°, °/s, or °/s^2)

P17/B5
S20/R30-IN

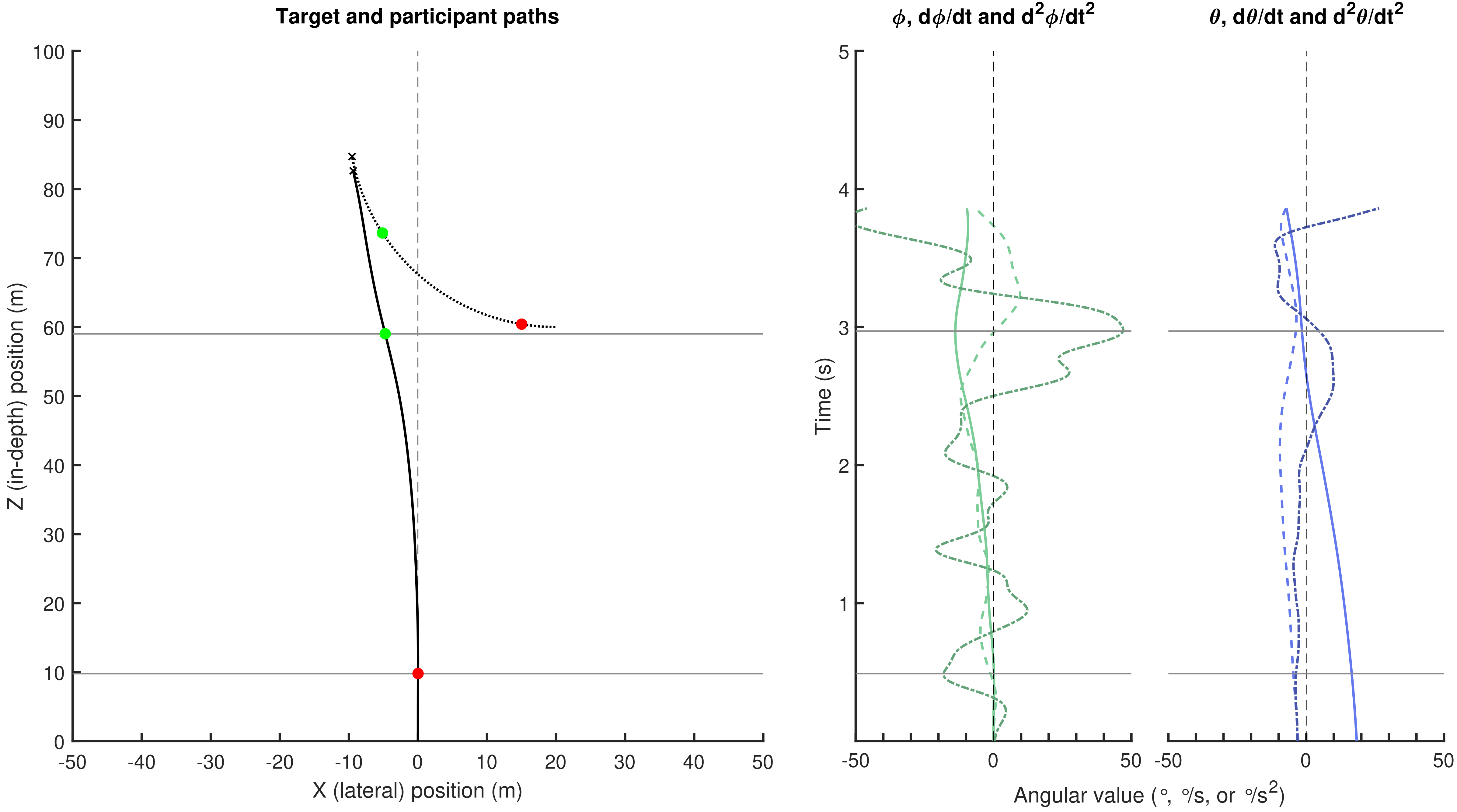

P18/B1
S20/R10-IN

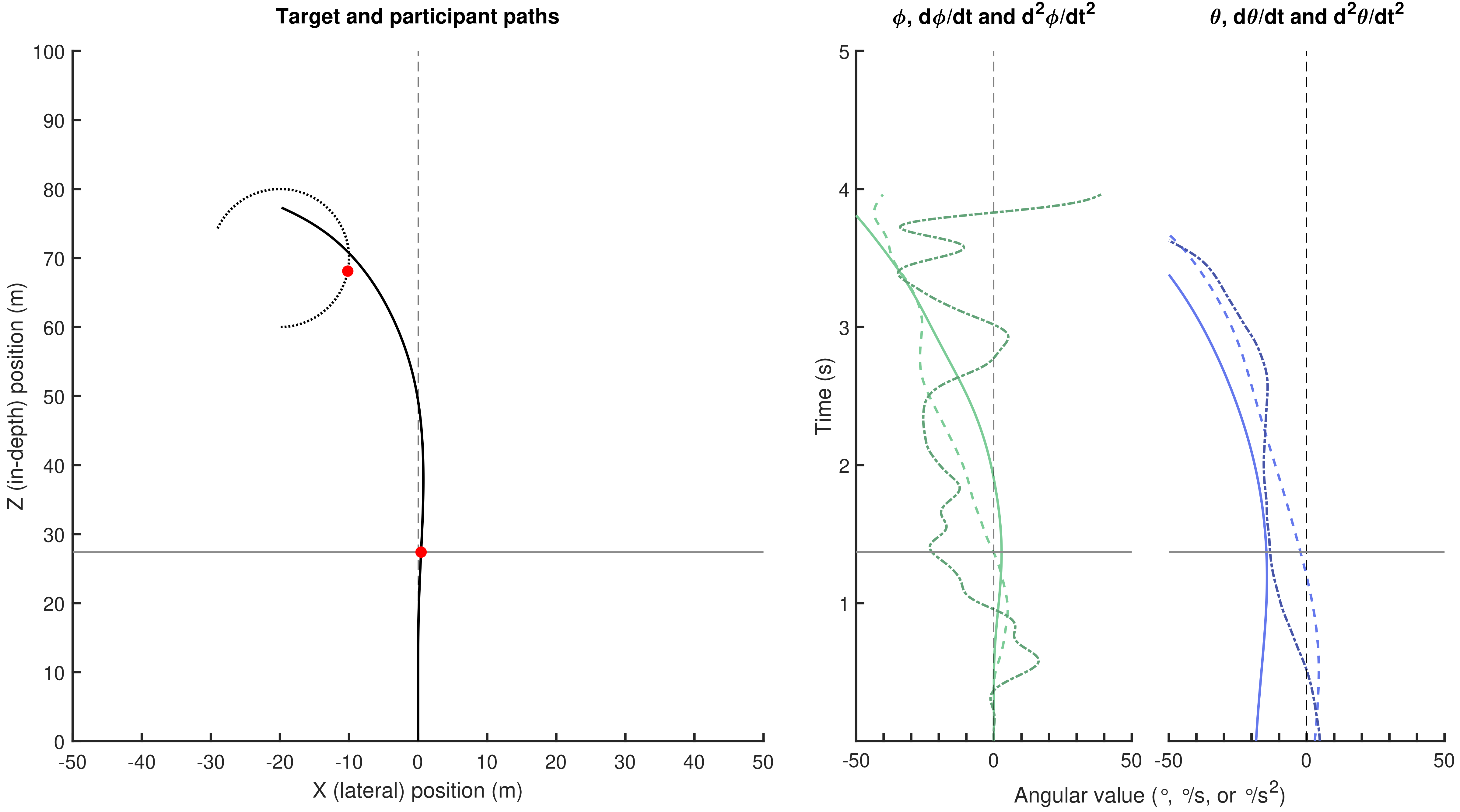

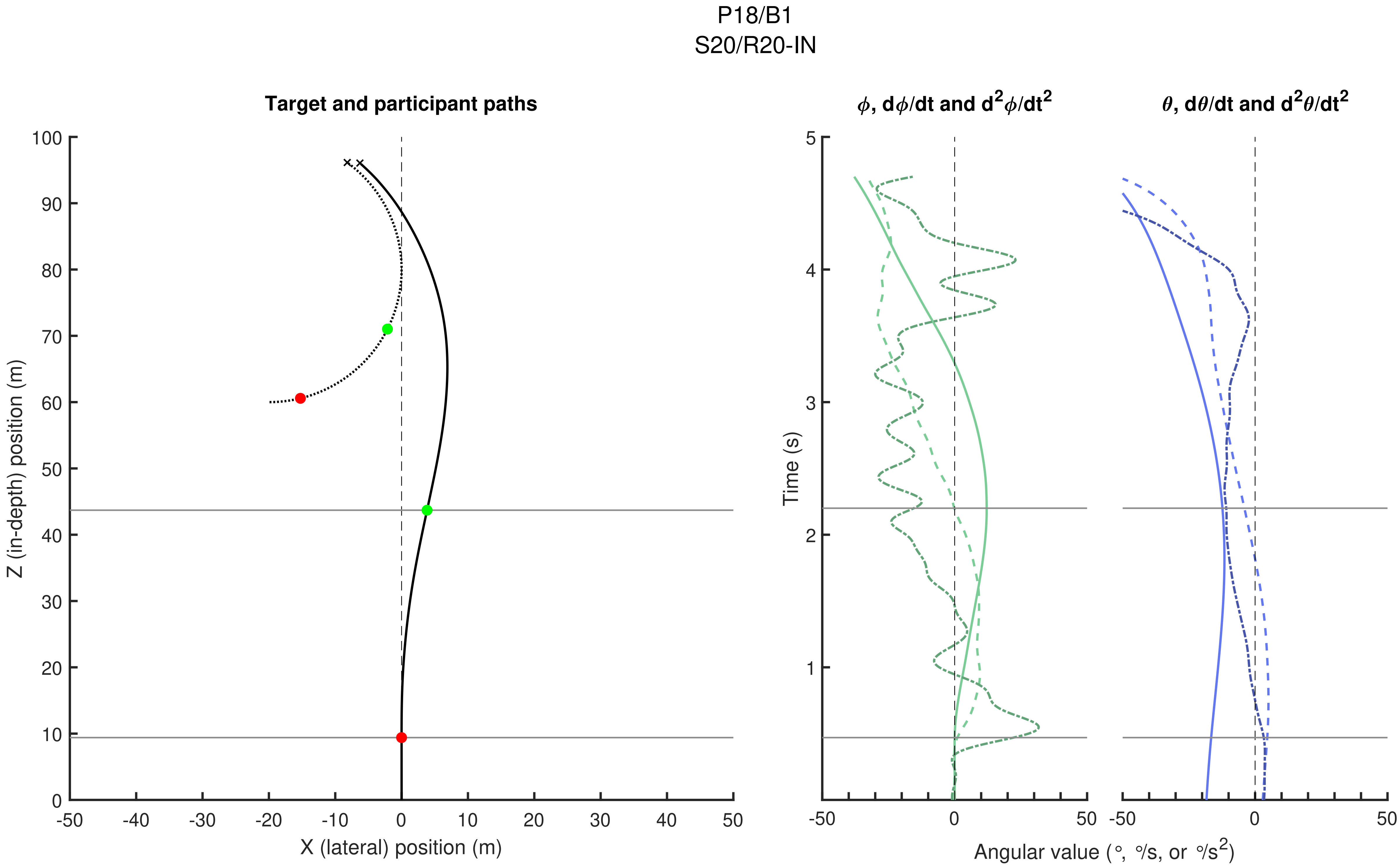
P18/B1
S20/R20-IN
Target and participant paths
Z (in-depth) position (m)
X (lateral) position (m)
ϕ, dϕ/dt and d$^2\phi$/dt^2
θ, dθ/dt and d$^2\theta$/dt^2
Time (s)
Angular value (°, °/s, or °/s^2)

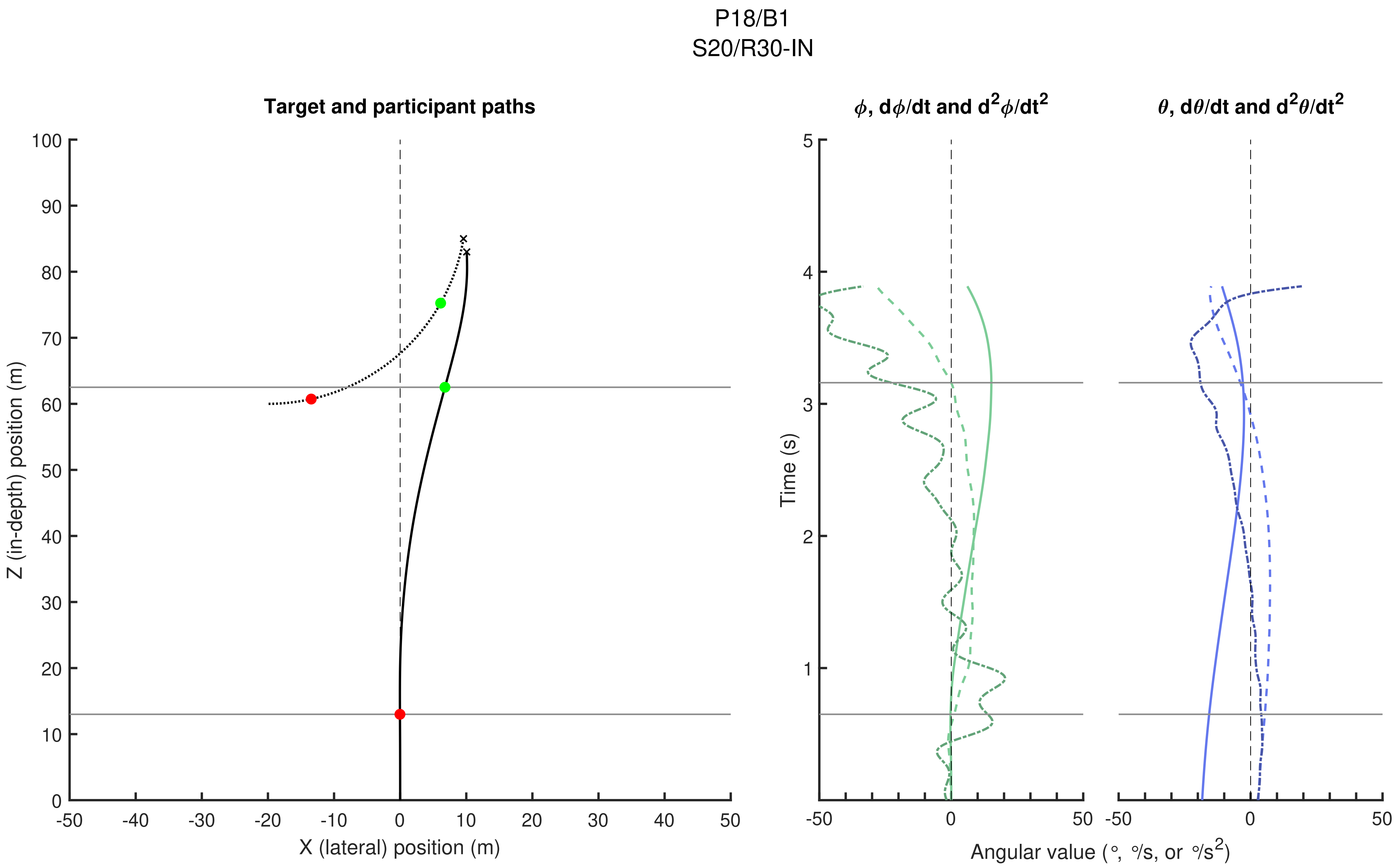

P18/B1
S20/R30-IN
Target and participant paths
Z (in-depth) position (m)
X (lateral) position (m)
100
90
80
70
60
50
40
30
20
10
0
-50
-40
-30
-20
-10
0
10
20
30
40
50
φ, dφ/dt and d²φ/dt²
θ, dθ/dt and d²θ/dt²
Time (s)
5
4
3
2
1
-50
0
50
-50
0
50
Angular value (°, °/s, or °/s²)

P18/B1
S15/R10-IN

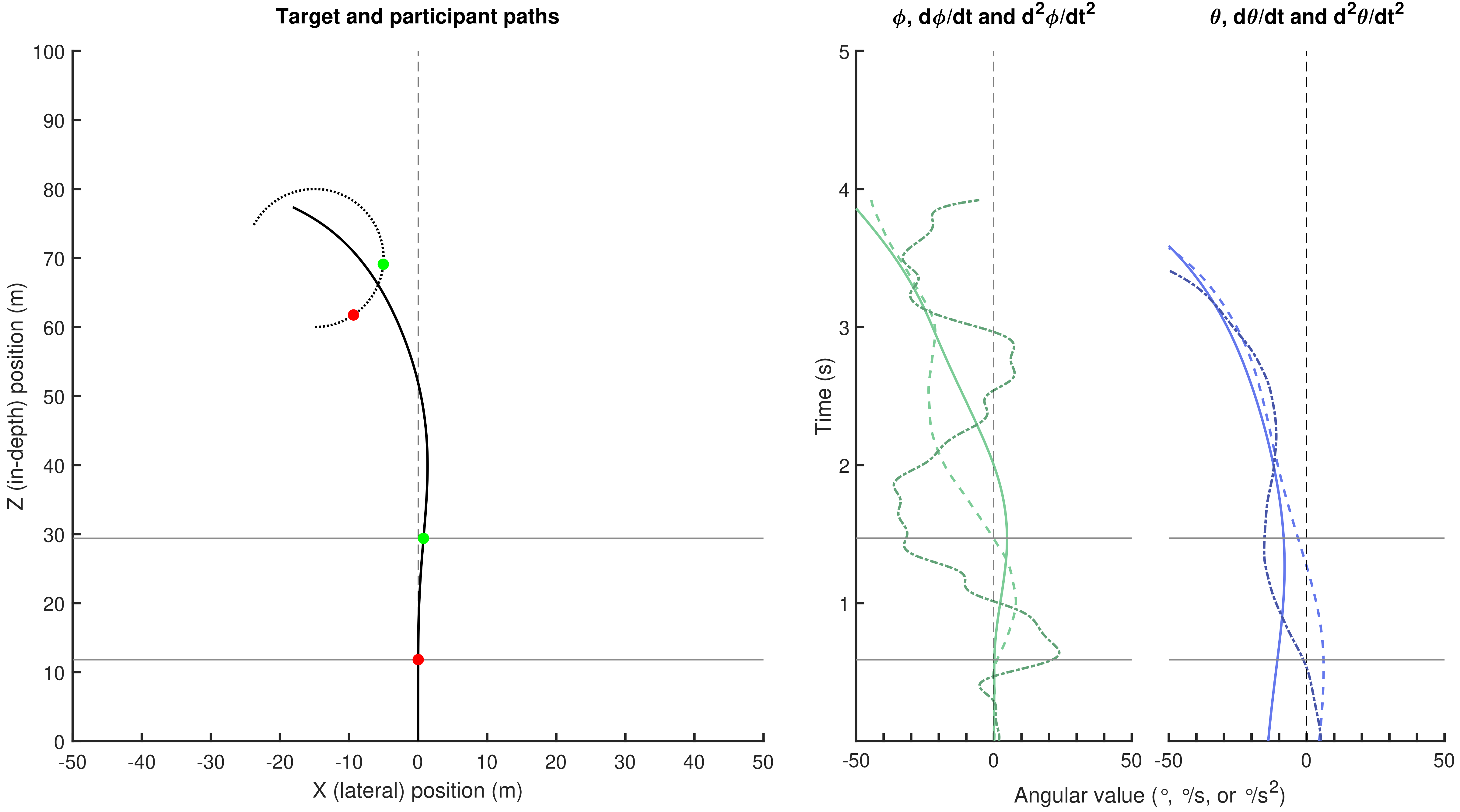

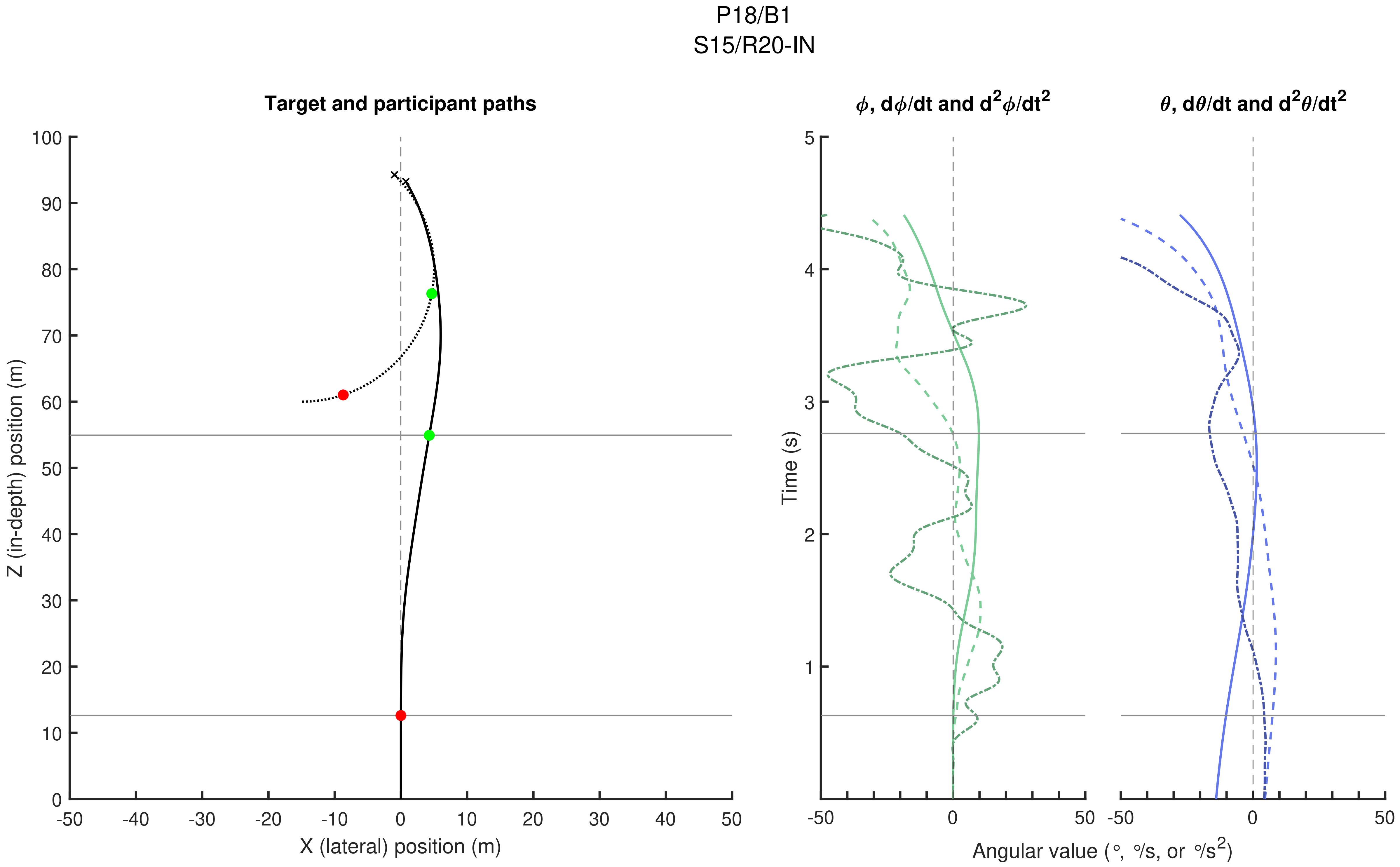
P18/B1
S15/R20-IN
Target and participant paths
Z (in-depth) position (m)
X (lateral) position (m)
ϕ, dϕ/dt and d$^2\phi$/dt^2
θ, dθ/dt and d$^2\theta$/dt^2
Time (s)
Angular value (°, °/s, or °/s^2)
100
90
80
70
60
50
40
30
20
10
0
-50
-40
-30
-20
-10
0
10
20
30
40
50
5
4
3
2
1
-50
0
50
-50
0
50

P18/B1
S15/R30-IN

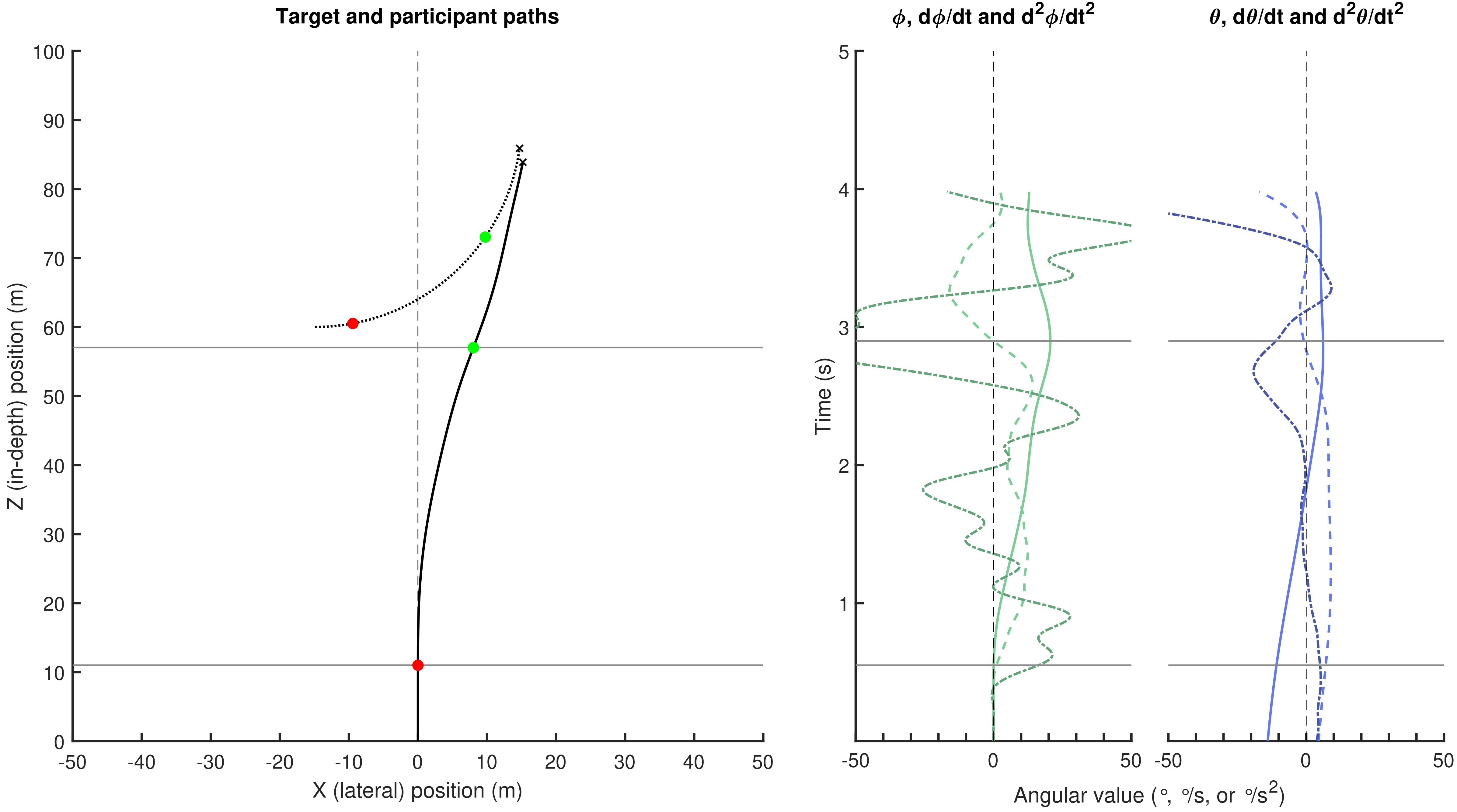

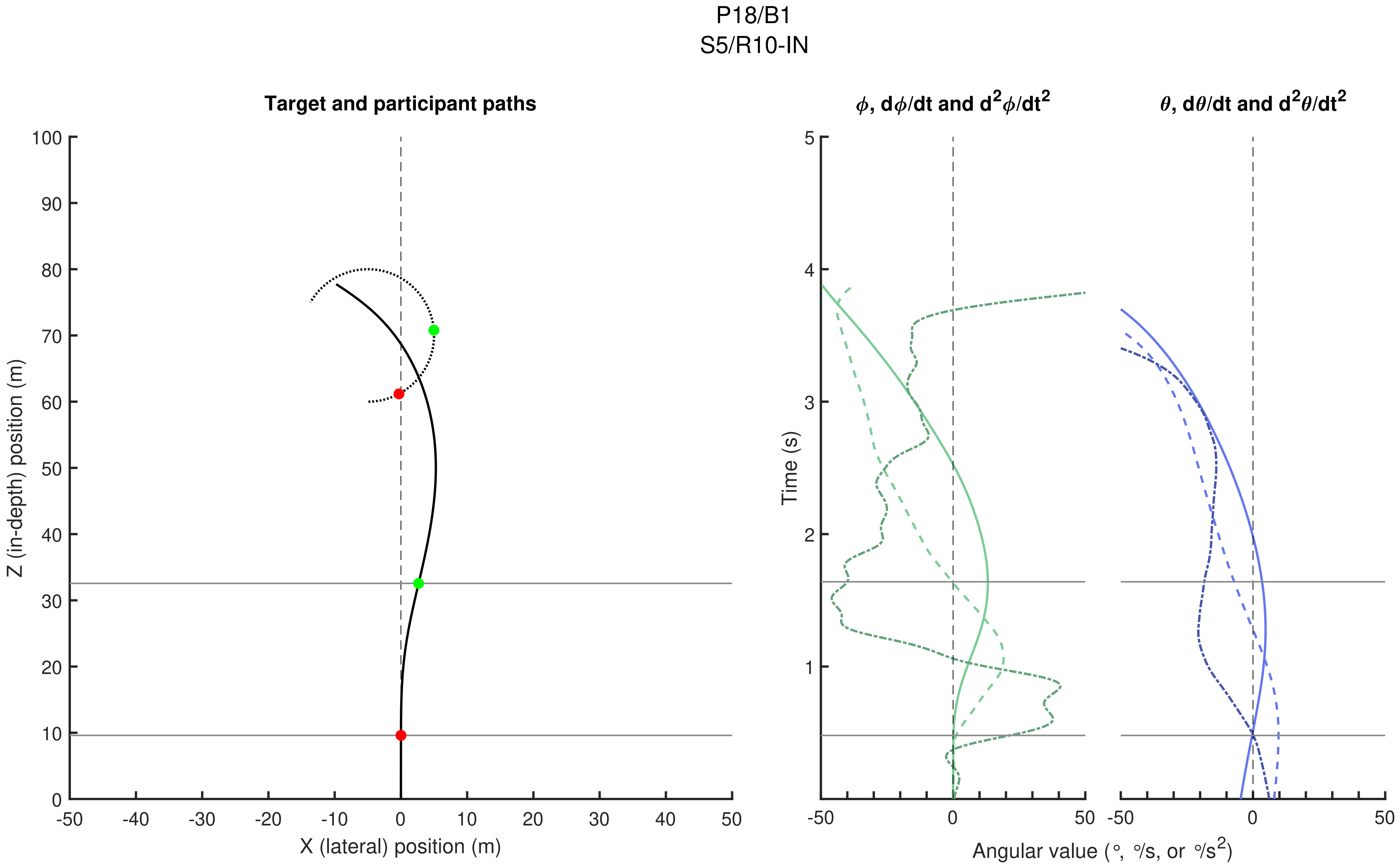

P18/B1
S5/R10-IN
Target and participant paths
Z (in-depth) position (m)
X (lateral) position (m)
100
90
80
70
60
50
40
30
20
10
0
-50
-40
-30
-20
-10
0
10
20
30
40
50
ϕ, dϕ/dt and d$^2\phi$/dt^2
θ, dθ/dt and d$^2\theta$/dt^2
Time (s)
5
4
3
2
1
-50
0
50
-50
0
50
Angular value (°, °/s, or °/s^2)

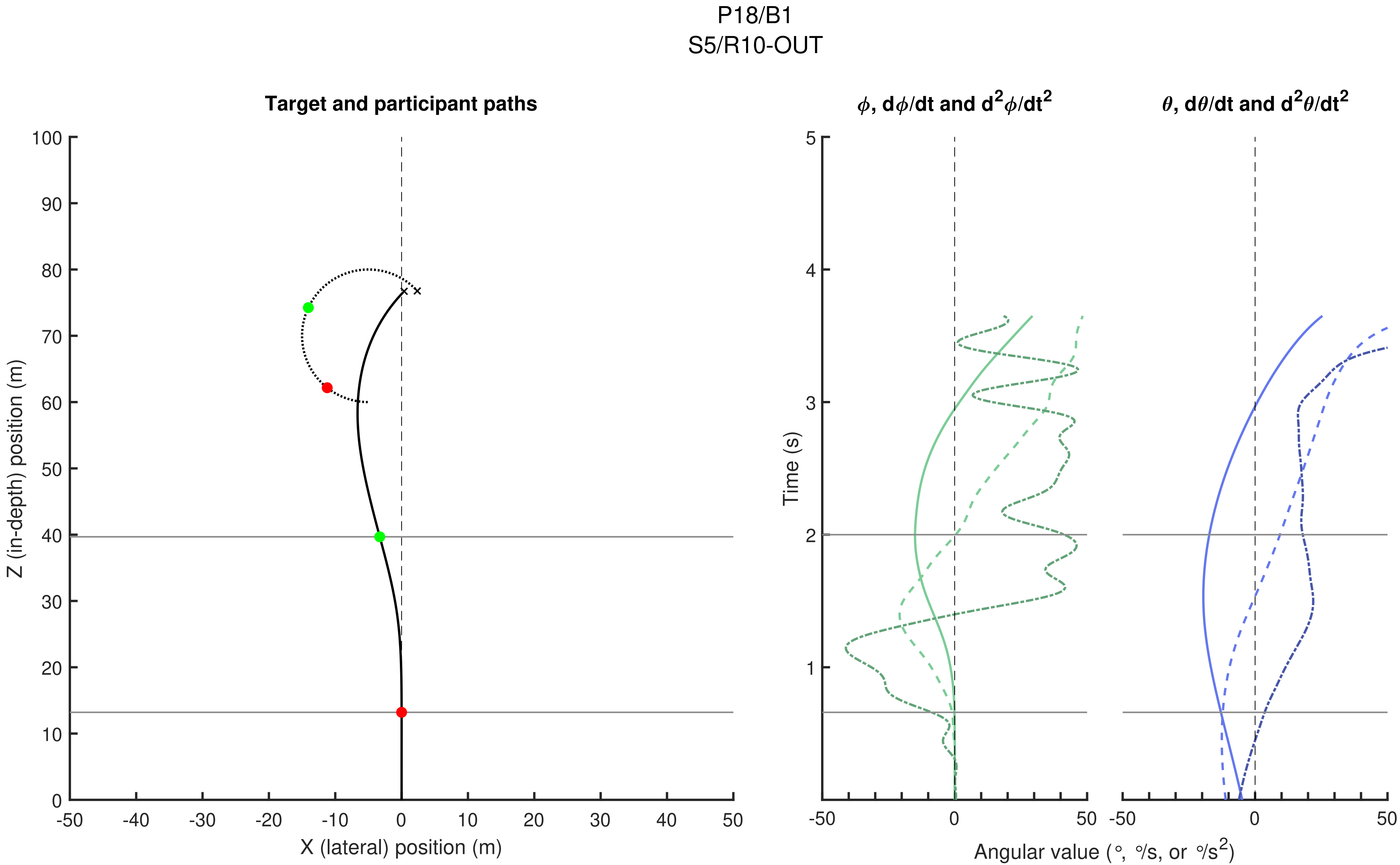
P18/B1
S5/R10-OUT
Target and participant paths
Z (in-depth) position (m)
X (lateral) position (m)
100
90
80
70
60
50
40
30
20
10
0
-50
-40
-30
-20
-10
0
10
20
30
40
50
ϕ, dϕ/dt and d$^2\phi$/dt^2
θ, dθ/dt and d$^2\theta$/dt^2
Time (s)
5
4
3
2
1
-50
0
50
-50
0
50
Angular value (°, °/s, or °/s^2)

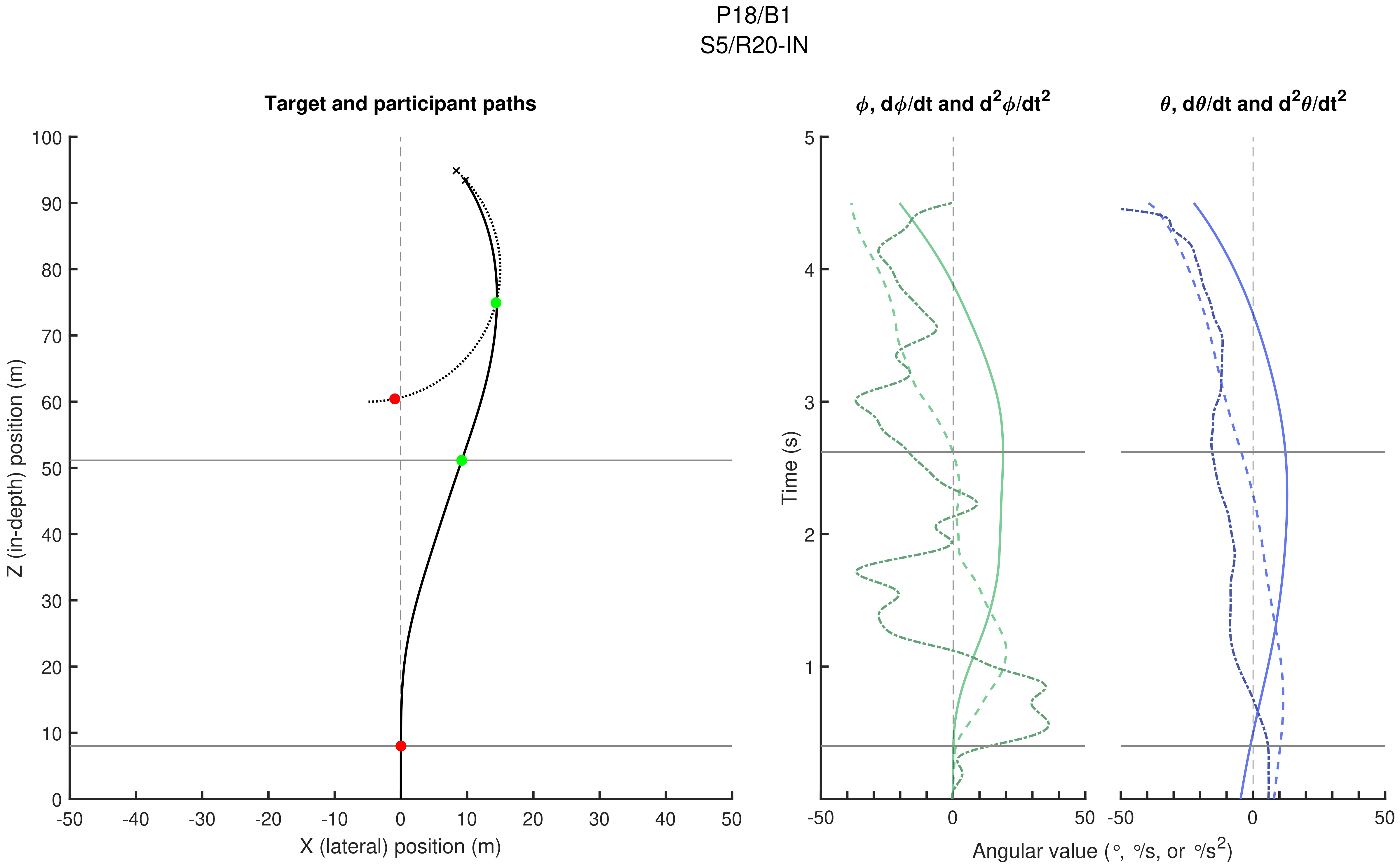

P18/B1
S5/R20-IN
Target and participant paths
Z (in-depth) position (m)
X (lateral) position (m)
ϕ, dϕ/dt and d²ϕ/dt²
θ, dθ/dt and d²θ/dt²
Time (s)
Angular value (°, °/s, or °/s²)

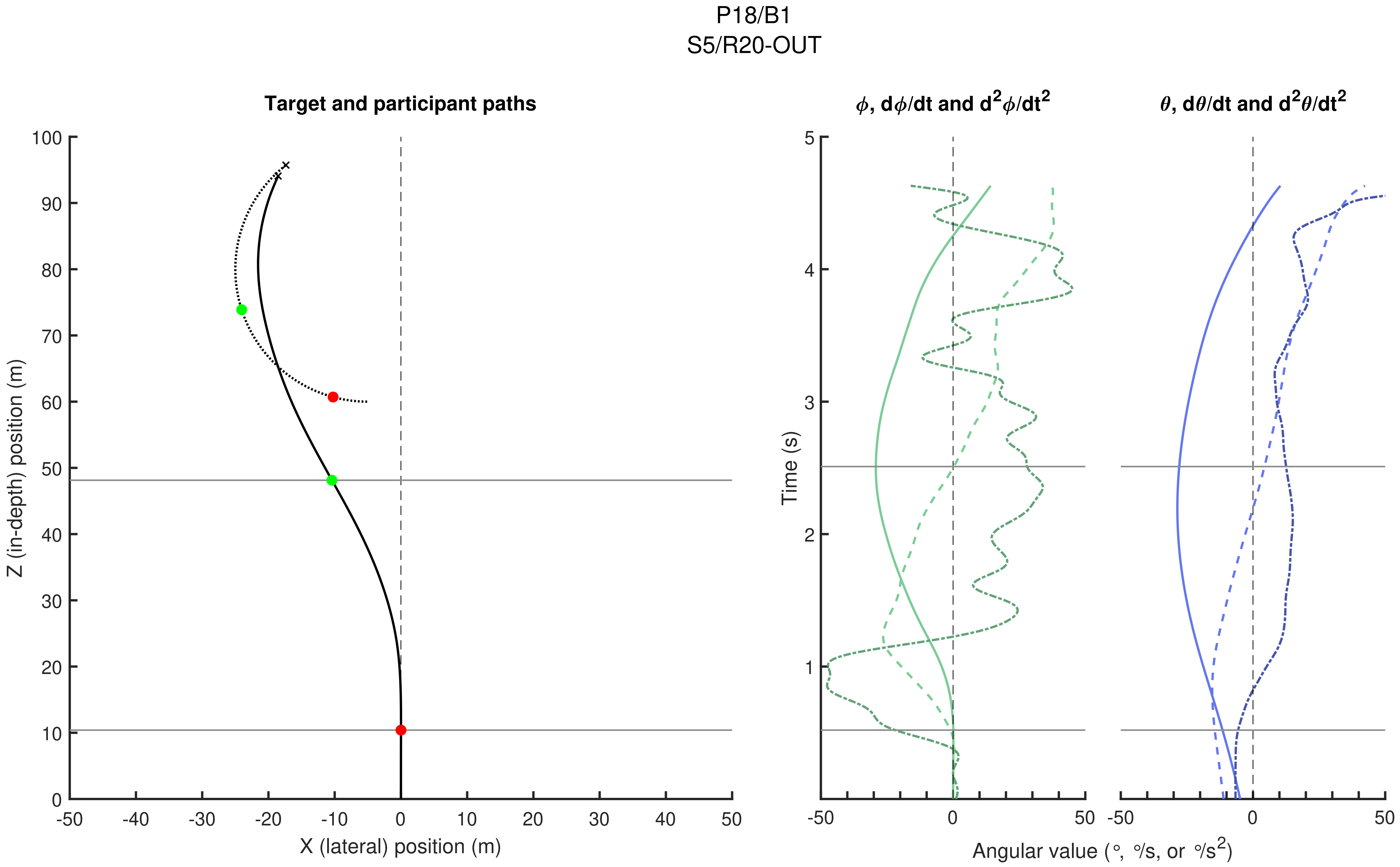
P18/B1
S5/R20-OUT
Target and participant paths
Z (in-depth) position (m)
X (lateral) position (m)
ϕ, dϕ/dt and d²ϕ/dt²
θ, dθ/dt and d²θ/dt²
Time (s)
Angular value (°, °/s, or °/s²)

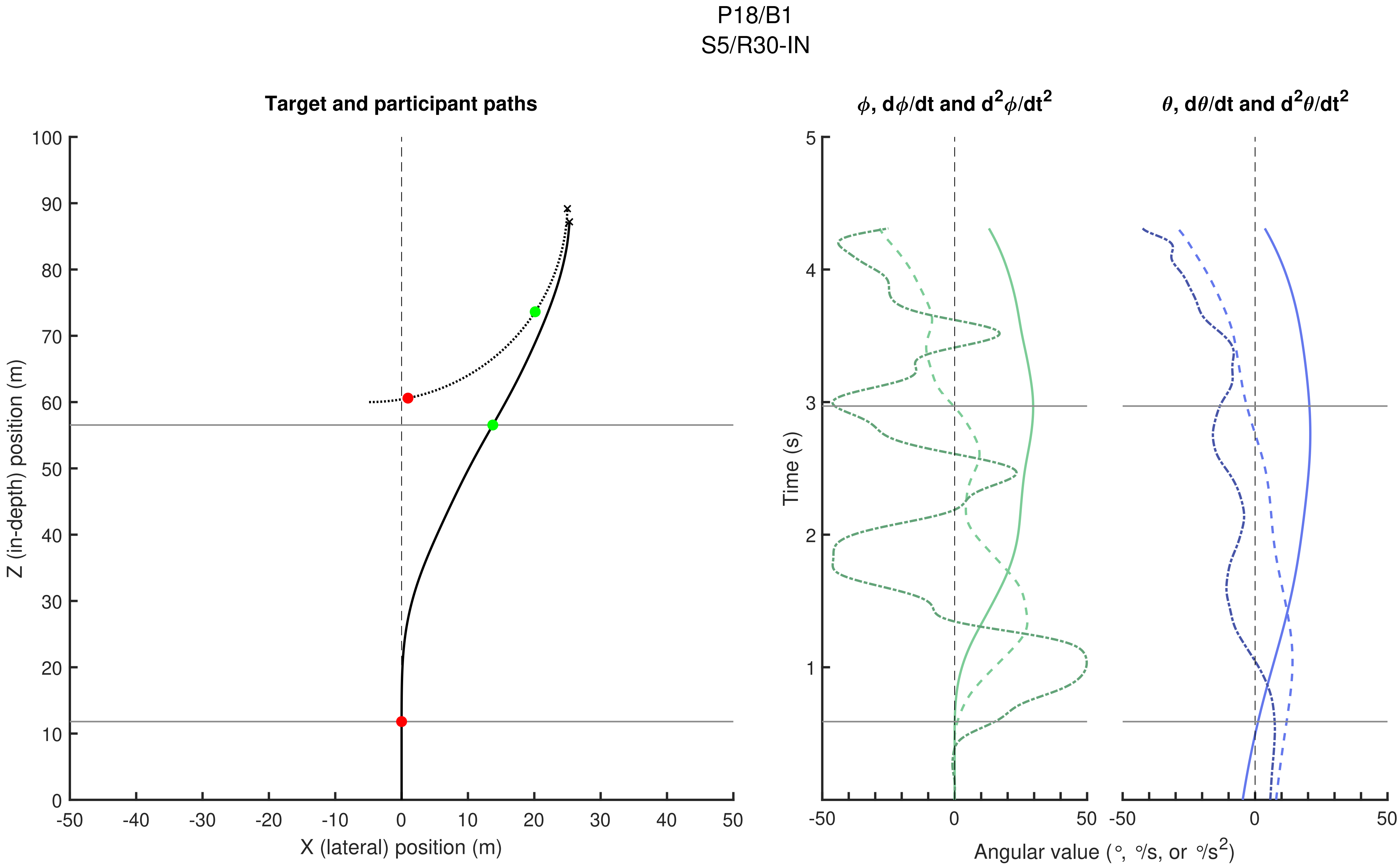

P18/B1
S5/R30-IN
Target and participant paths
Z (in-depth) position (m)
X (lateral) position (m)
100
90
80
70
60
50
40
30
20
10
0
-50
-40
-30
-20
-10
0
10
20
30
40
50
ϕ, dϕ/dt and d²ϕ/dt²
θ, dθ/dt and d²θ/dt²
Time (s)
5
4
3
2
1
-50
0
50
-50
0
50
Angular value (°, °/s, or °/s²)

P18/B1
S5/R30-OUT

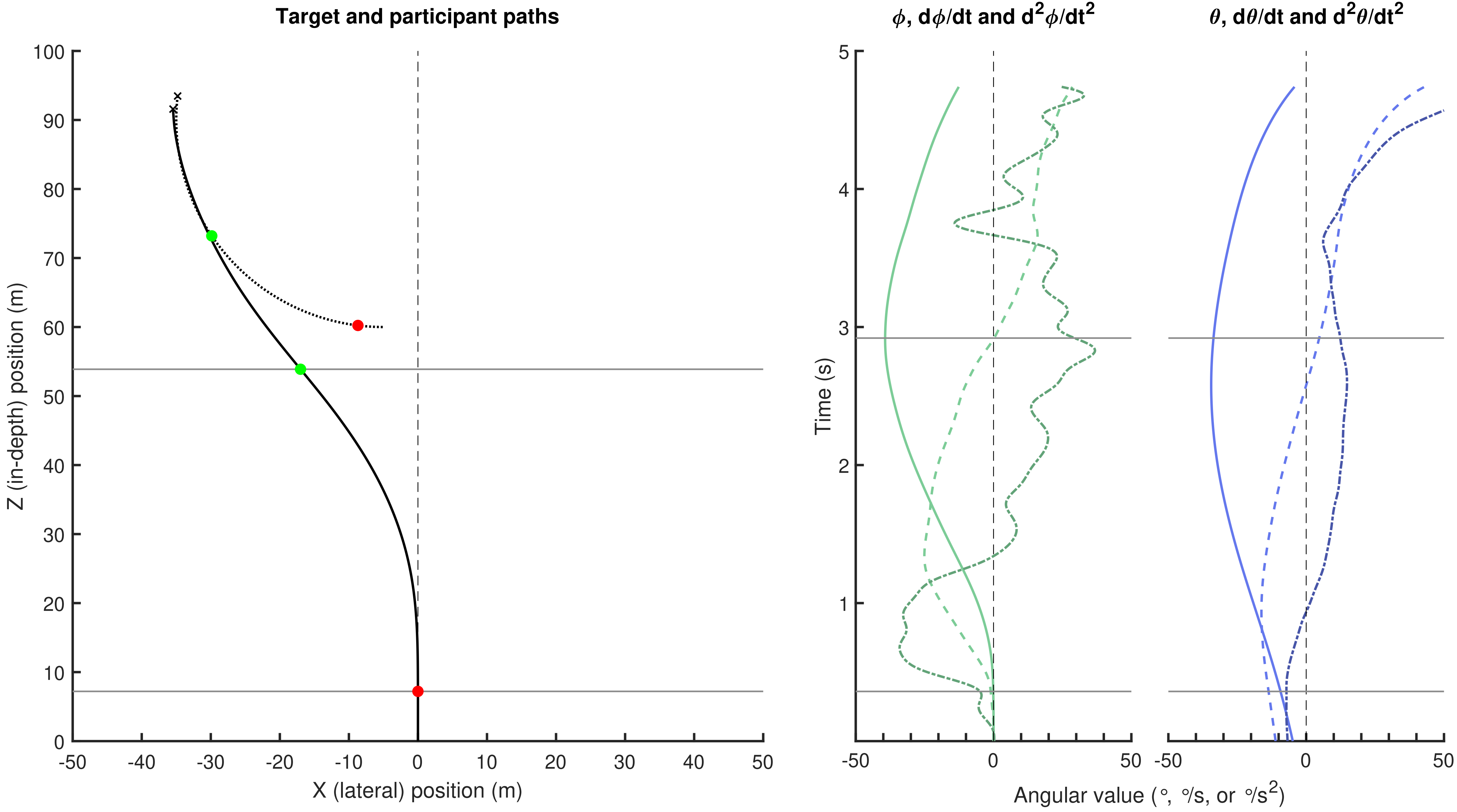

P18/B1
S5/R10-OUT

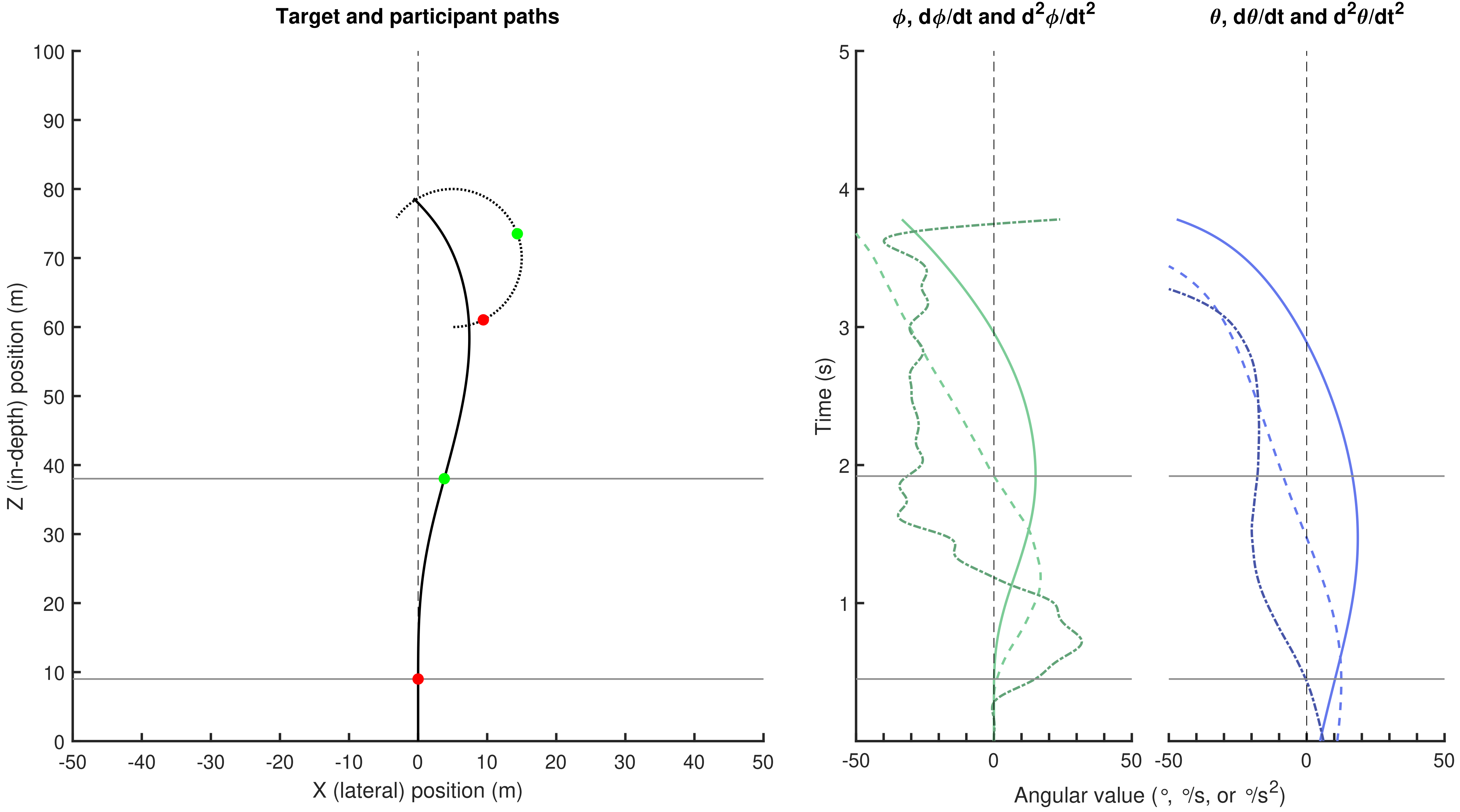

P18/B1
S5/R10-IN

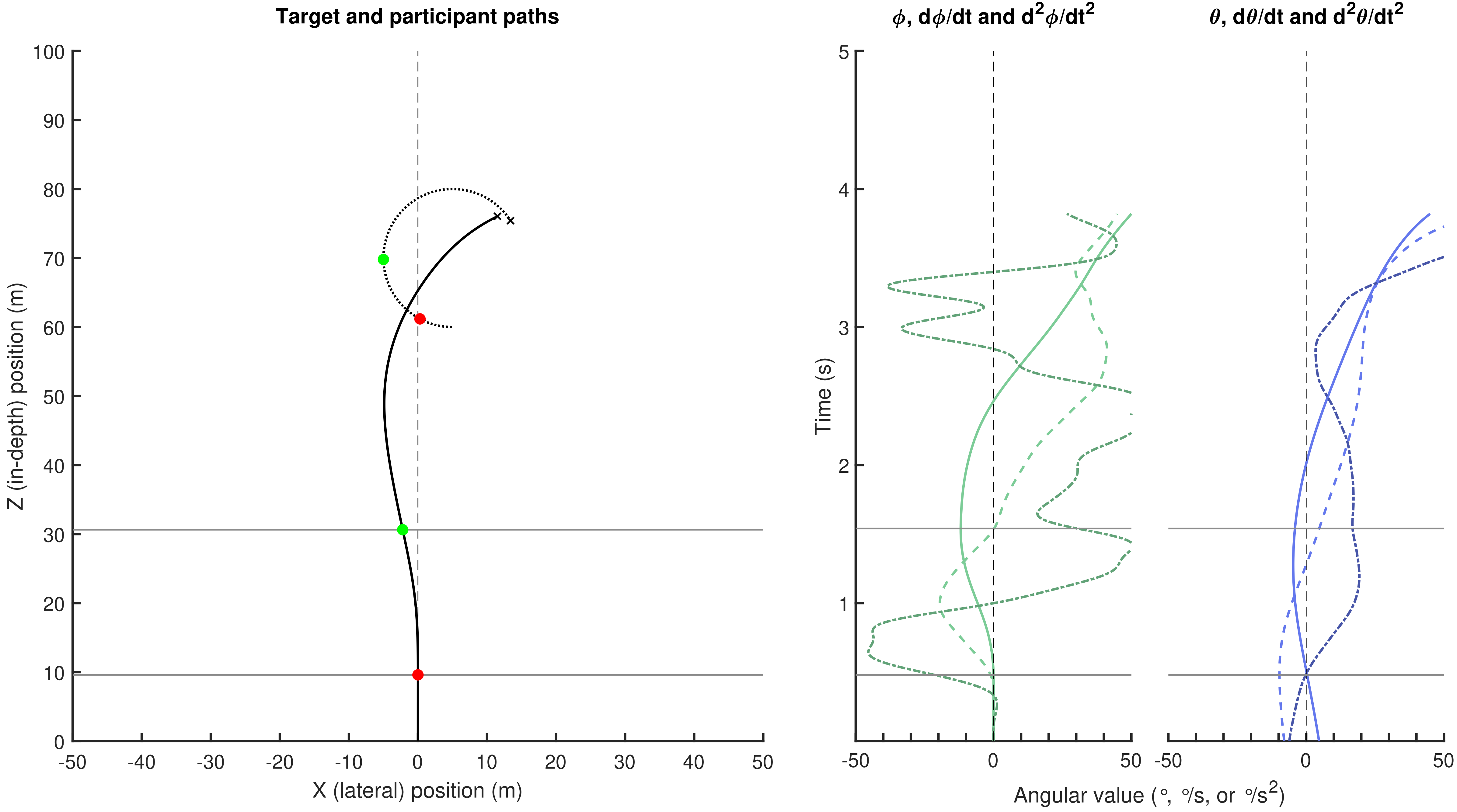

P18/B1
S5/R20-OUT

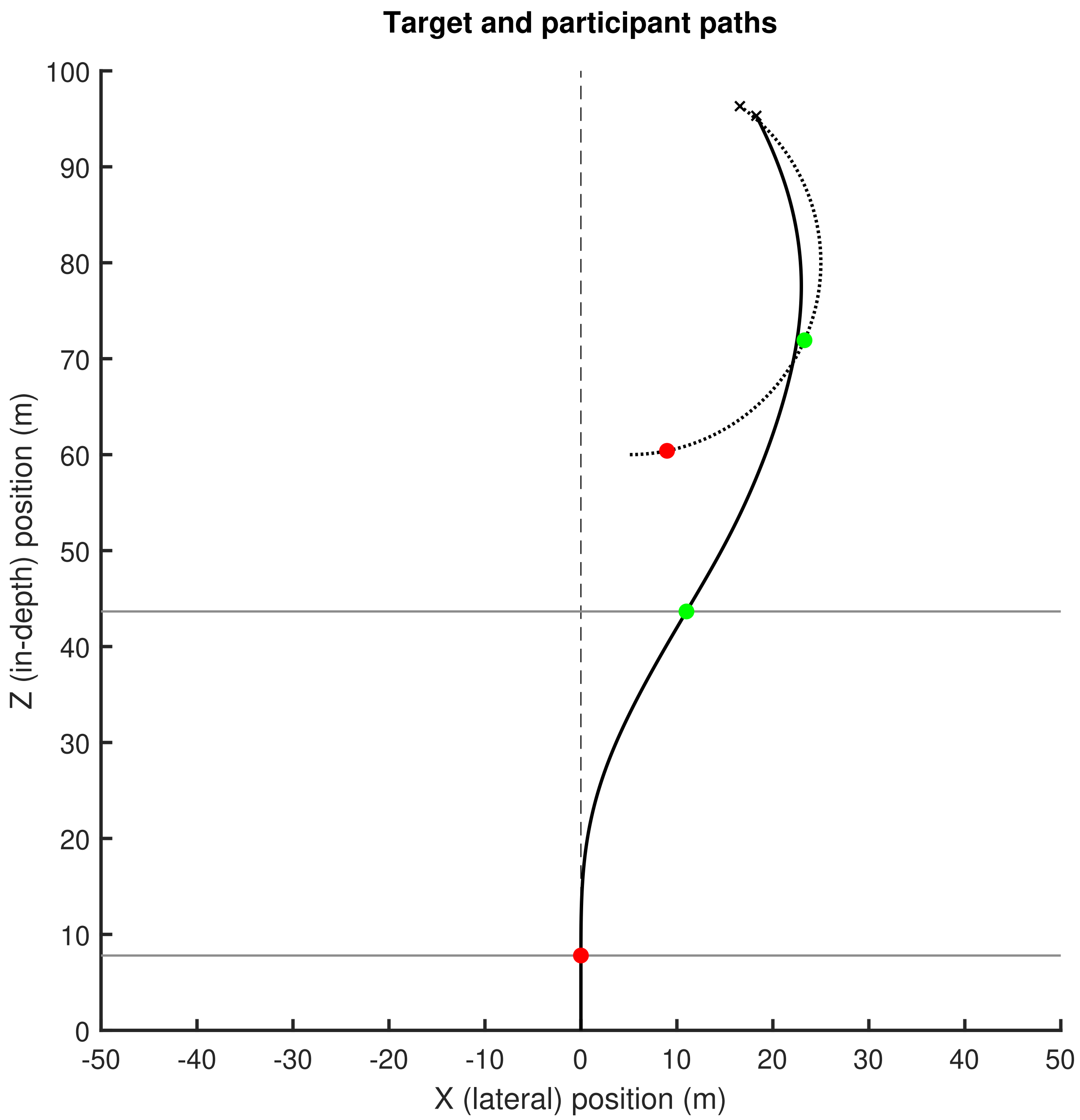

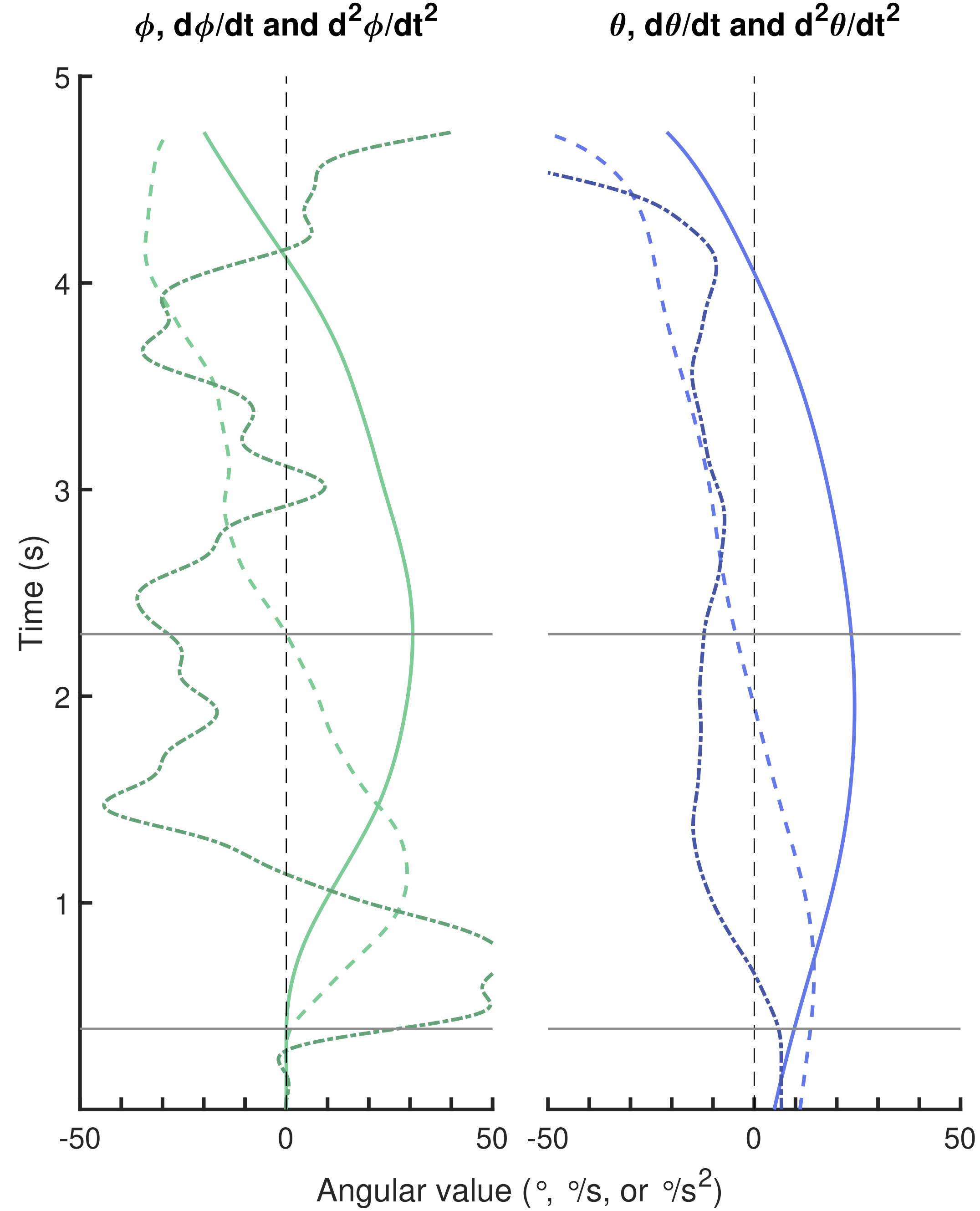

P18/B1
S5/R20-IN

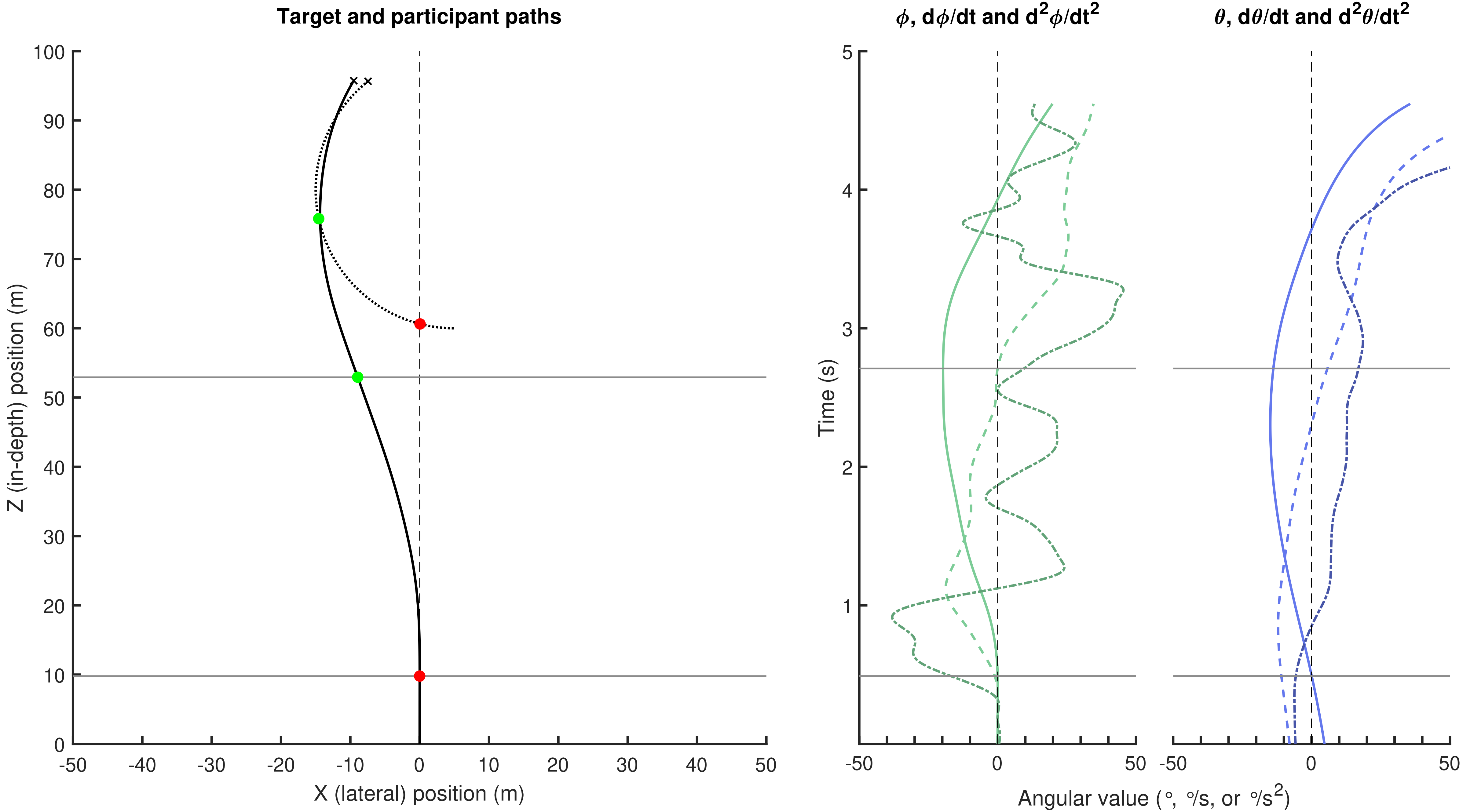

P18/B1
S5/R30-OUT

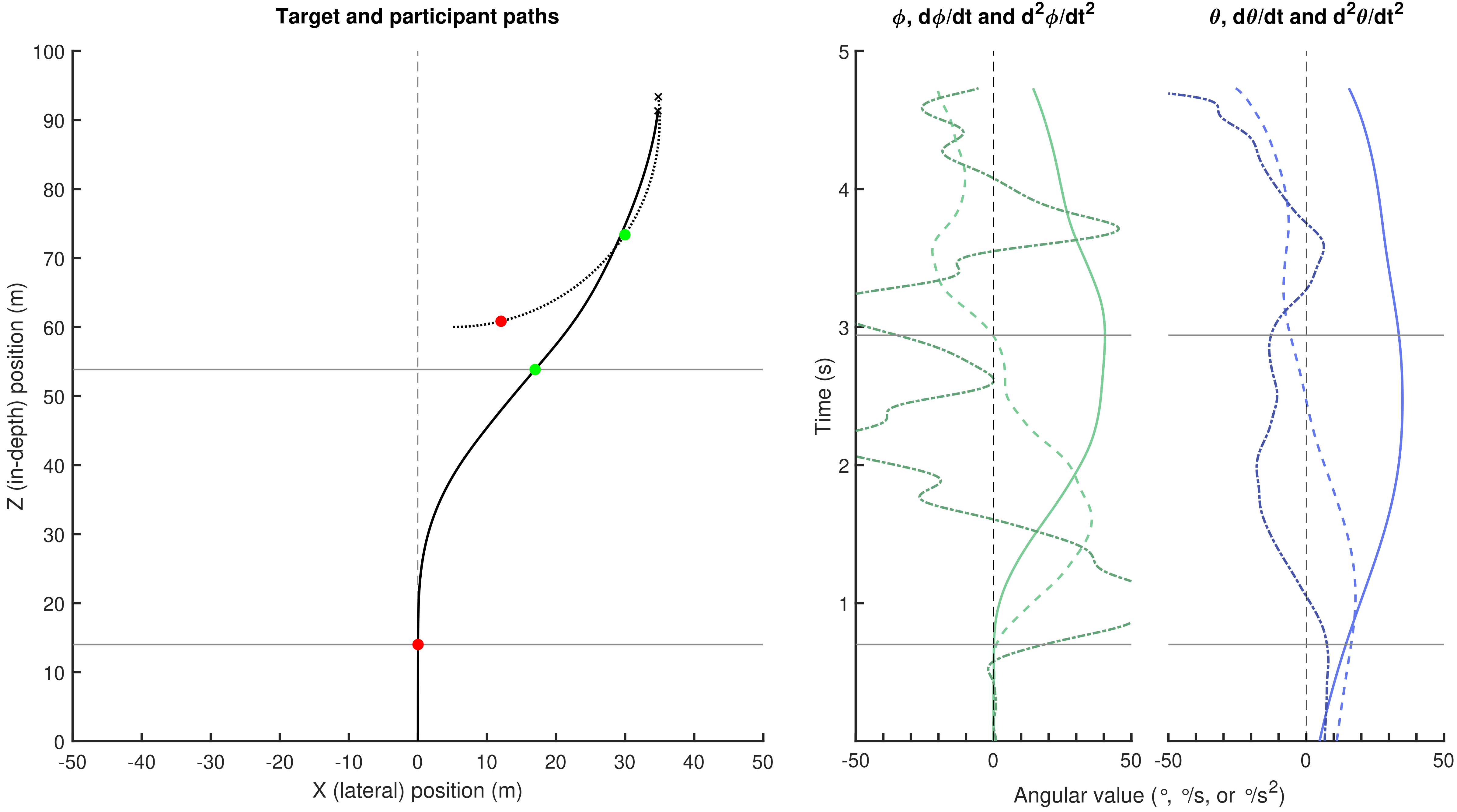

P18/B1
S5/R30-IN

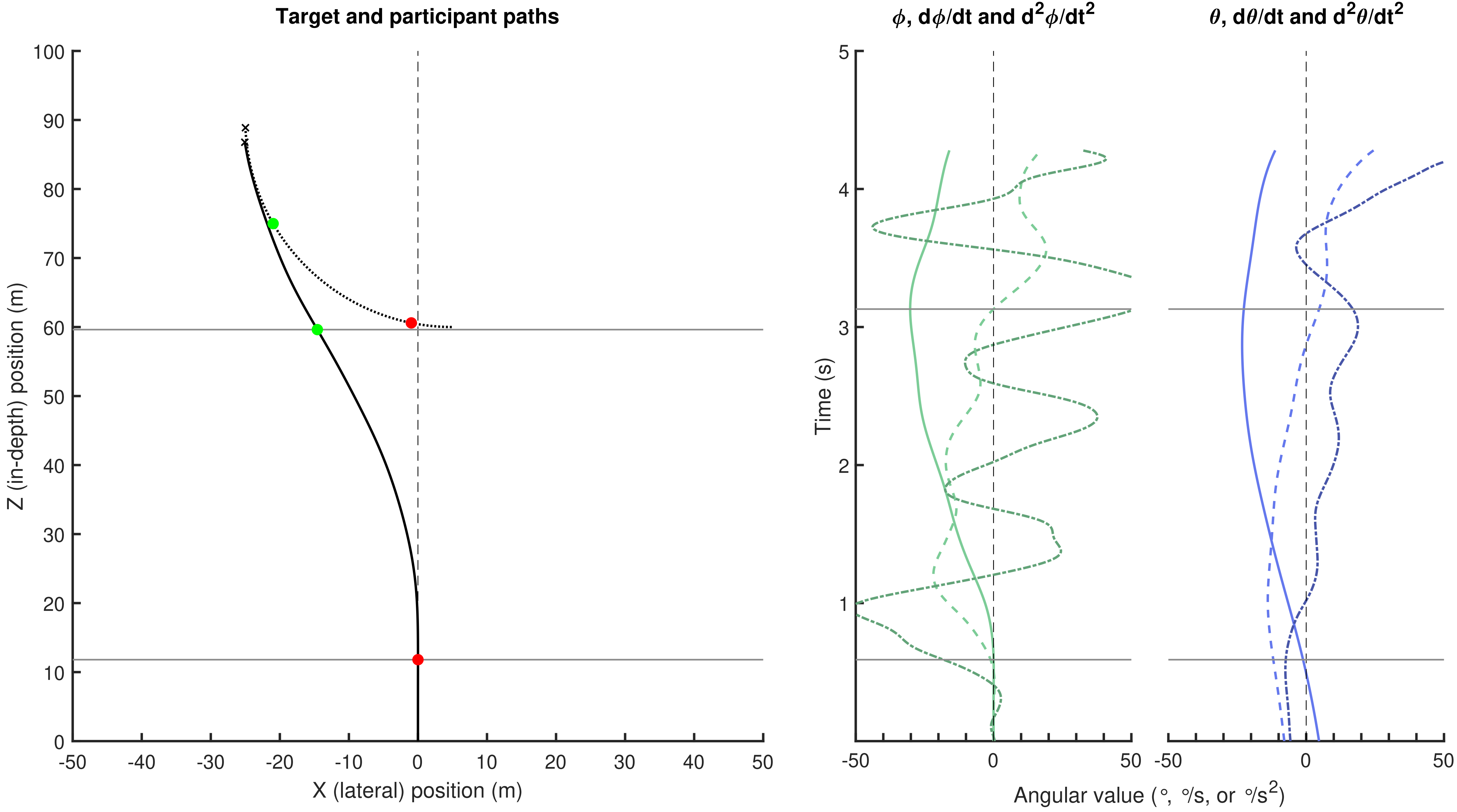

P18/B1
S15/R10-IN

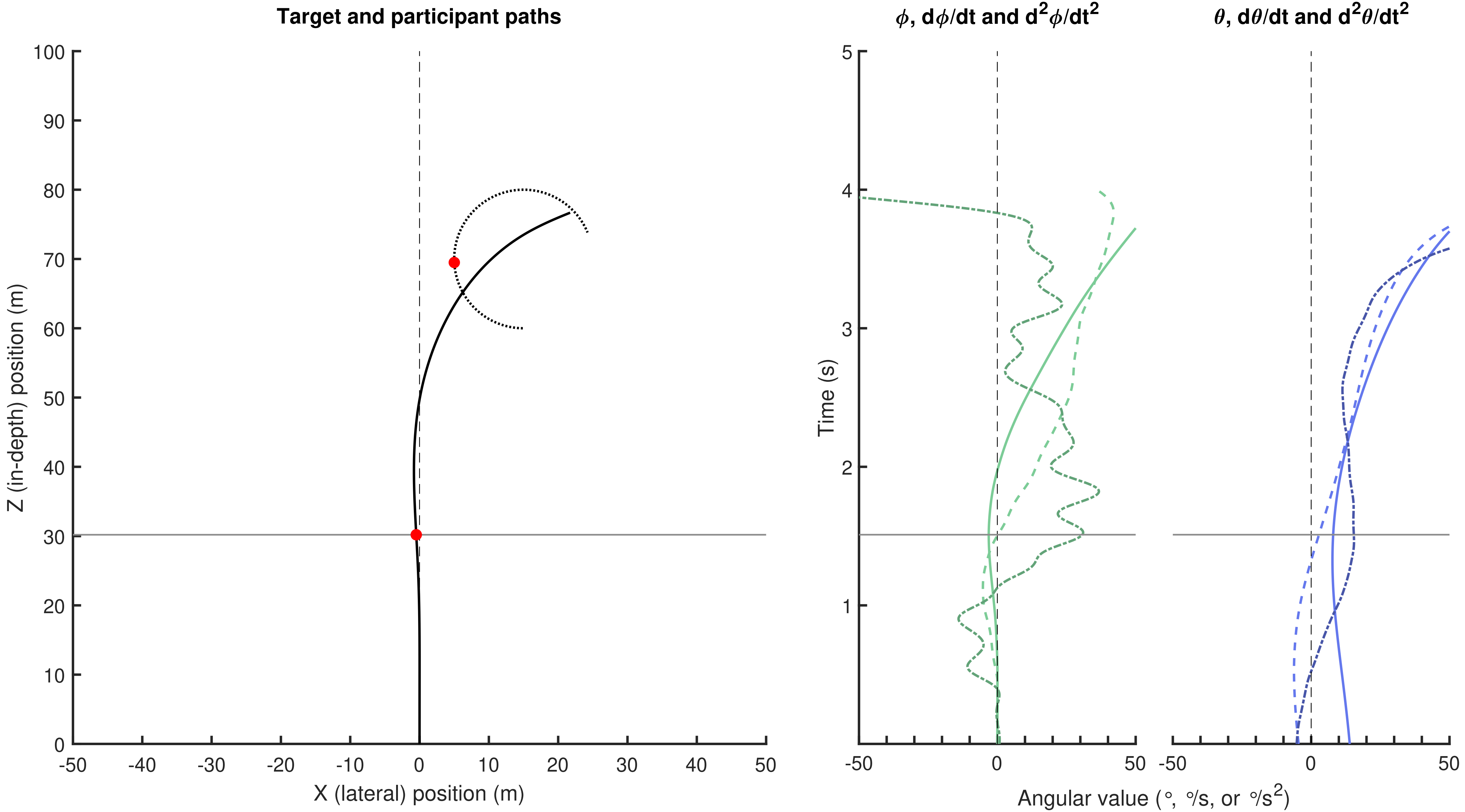

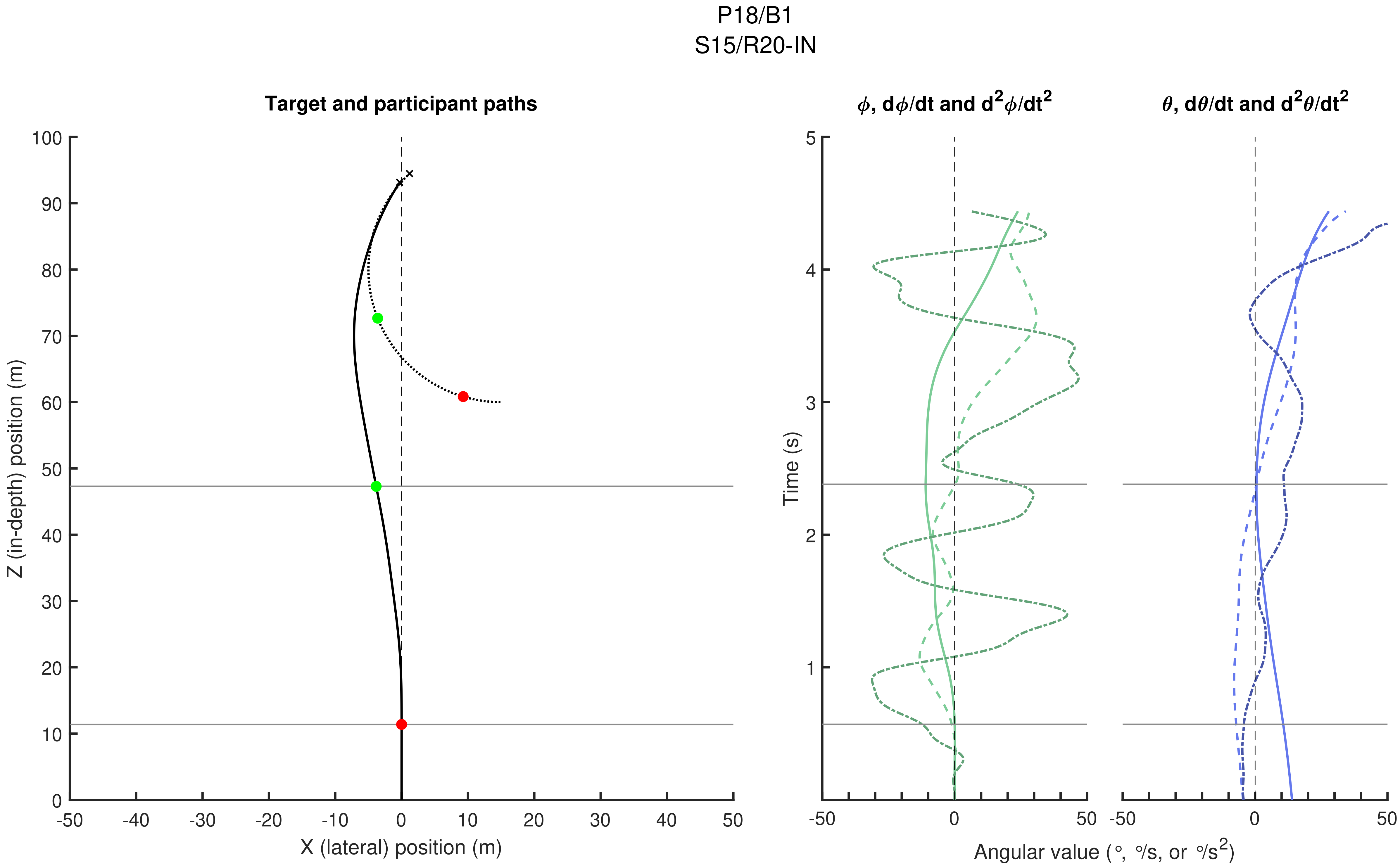
P18/B1
S15/R20-IN
Target and participant paths
Z (in-depth) position (m)
X (lateral) position (m)
ϕ, dϕ/dt and d$^2\phi$/dt^2
θ, dθ/dt and d$^2\theta$/dt^2
Time (s)
Angular value (°, °/s, or °/s^2)

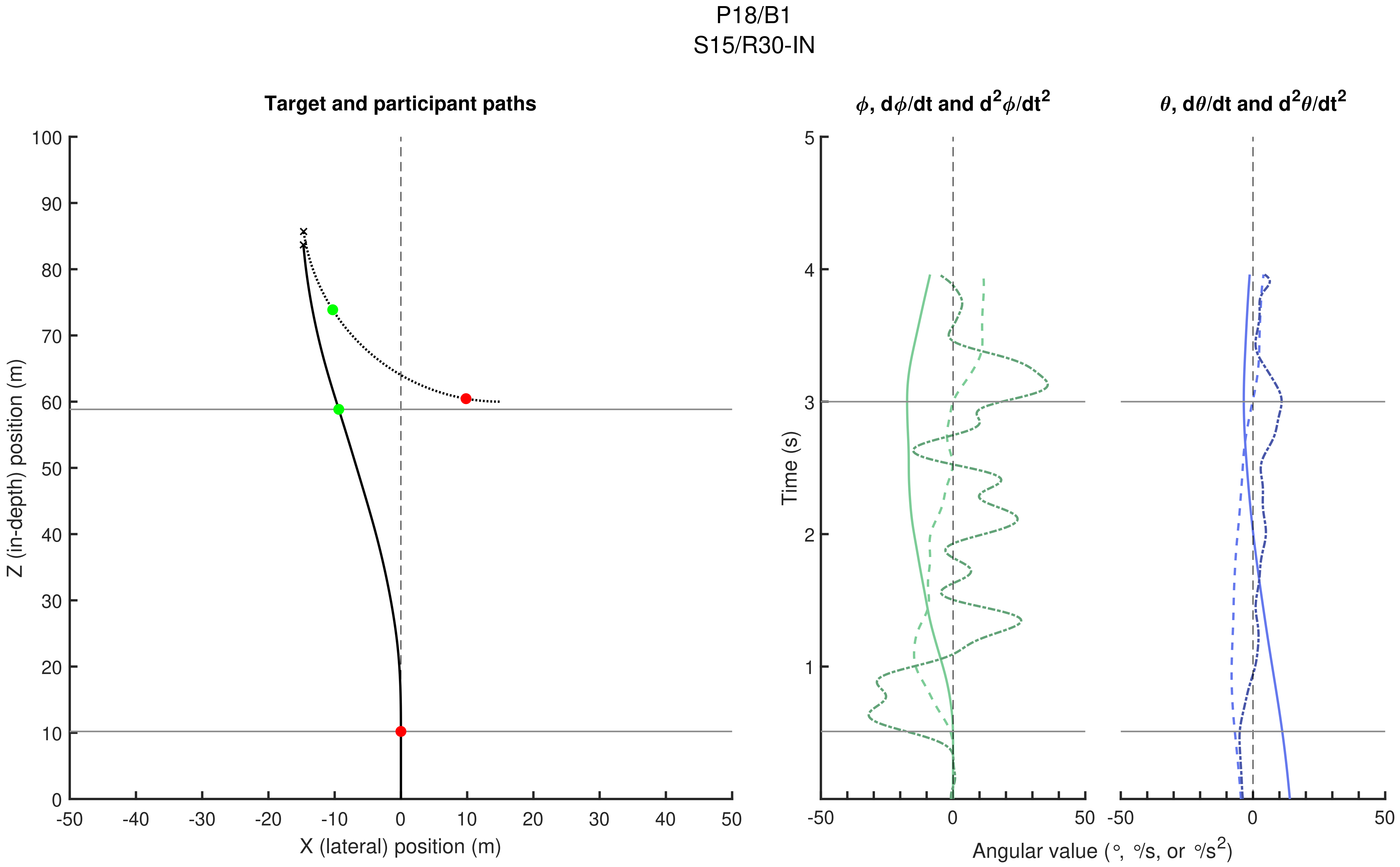
P18/B1
S15/R30-IN
Target and participant paths
Z (in-depth) position (m)
X (lateral) position (m)
ϕ, dϕ/dt and d²ϕ/dt²
θ, dθ/dt and d²θ/dt²
Time (s)
Angular value (°, °/s, or °/s²)

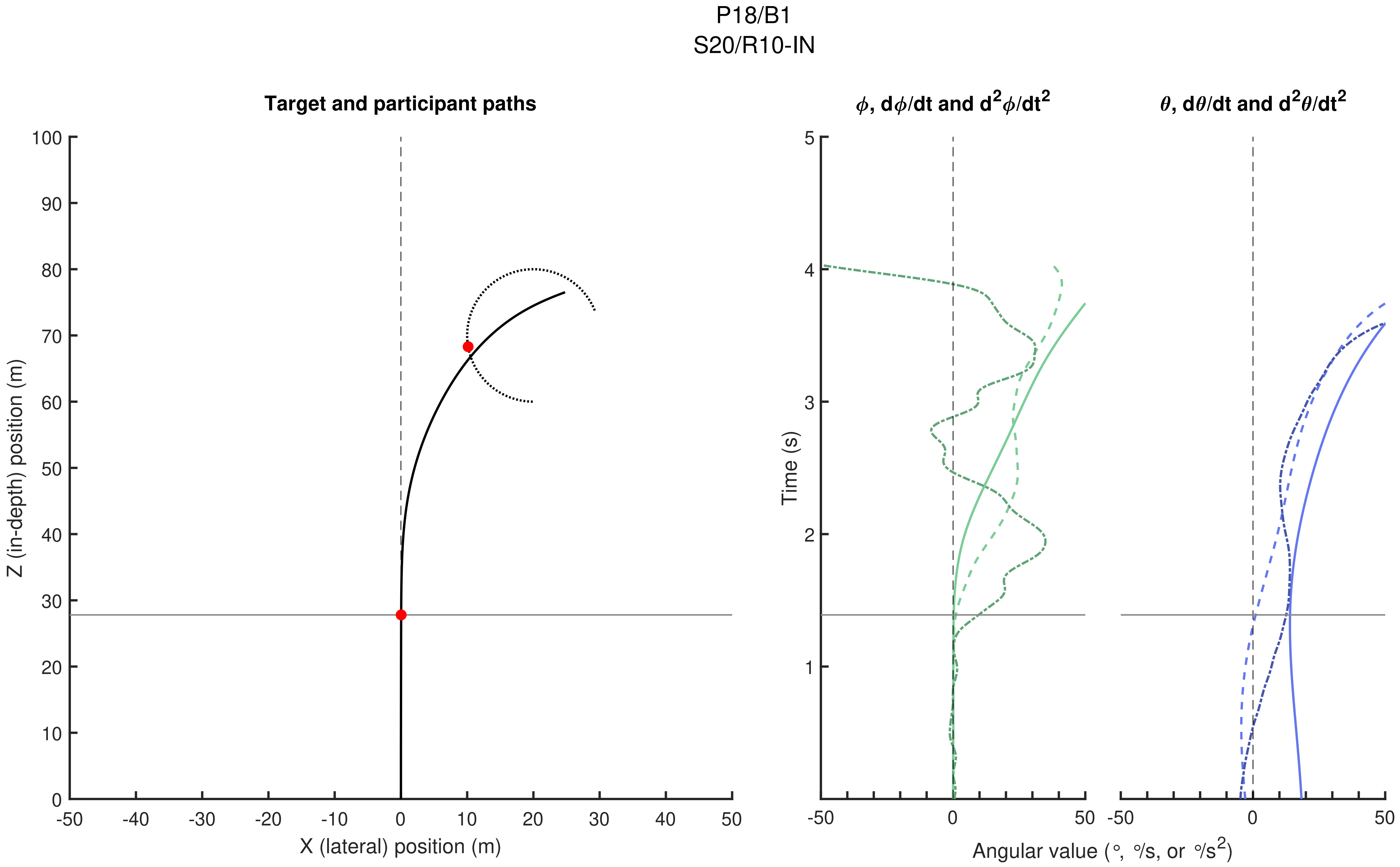

P18/B1
S20/R10-IN
Target and participant paths
Z (in-depth) position (m)
X (lateral) position (m)
ϕ, dϕ/dt and d$^2\phi$/dt^2
θ, dθ/dt and d$^2\theta$/dt^2
Time (s)
Angular value (°, °/s, or °/s^2)

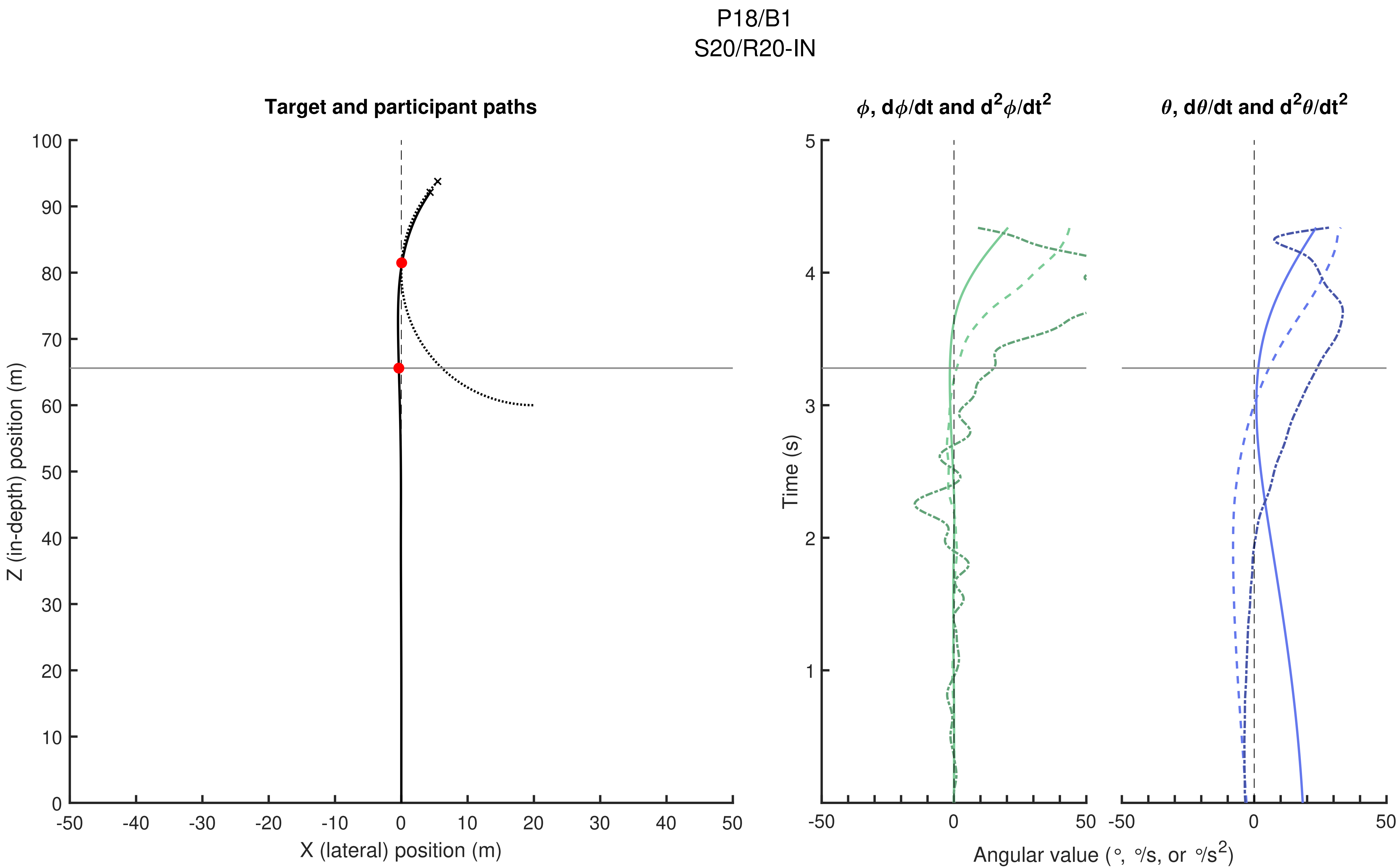
P18/B1
S20/R20-IN
Target and participant paths
Z (in-depth) position (m)
X (lateral) position (m)
φ, dφ/dt and d²φ/dt²
θ, dθ/dt and d²θ/dt²
Time (s)
Angular value (°, °/s, or °/s²)
100
90
80
70
60
50
40
30
20
10
0
-50
-40
-30
-20
-10
0
10
20
30
40
50
5
4
3
2
1

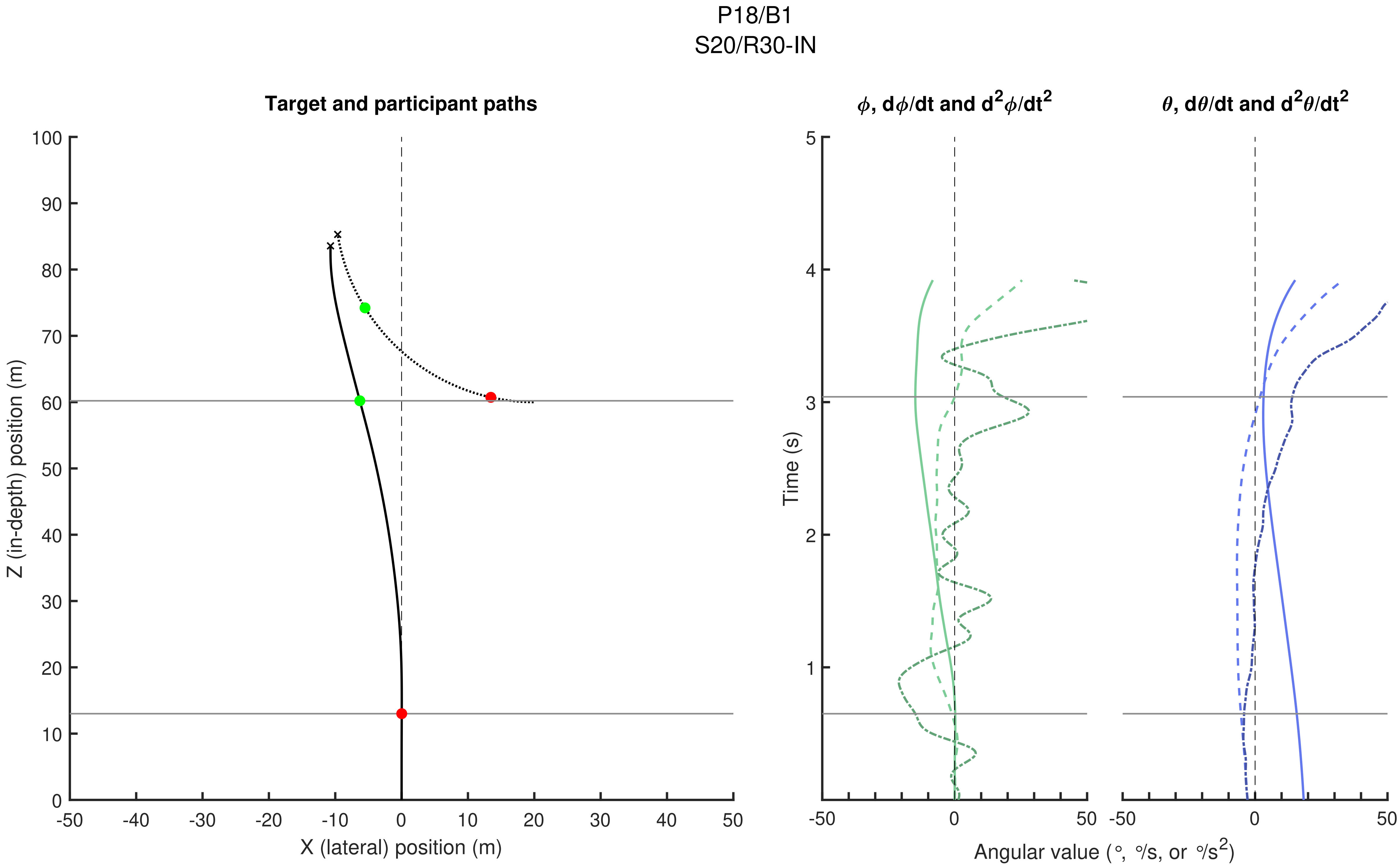

P18/B1
S20/R30-IN
Target and participant paths
Z (in-depth) position (m)
X (lateral) position (m)
ϕ, dϕ/dt and d$^2\phi$/dt^2
θ, dθ/dt and d$^2\theta$/dt^2
Time (s)
Angular value (°, °/s, or °/s^2)

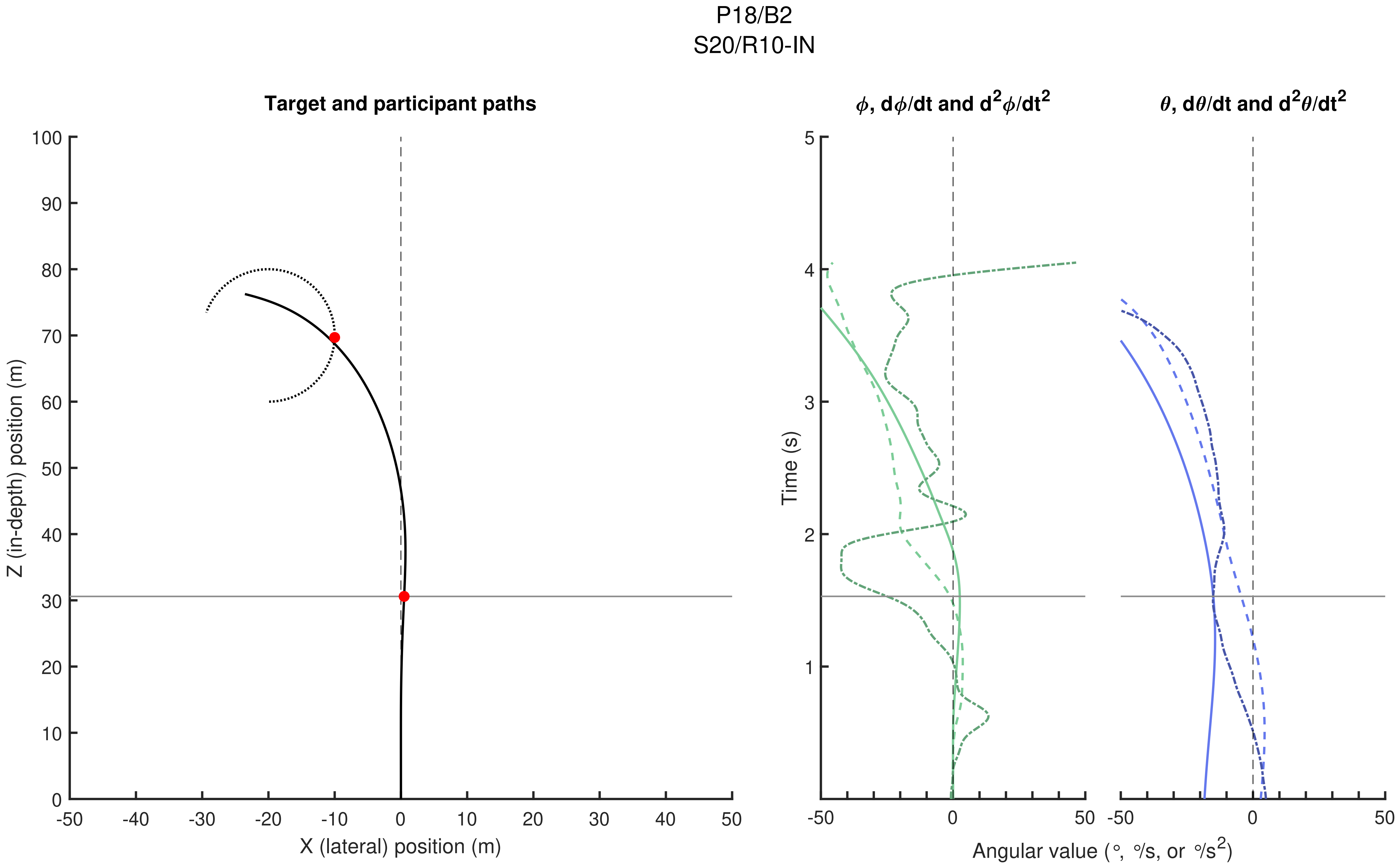

P18/B2
S20/R10-IN
Target and participant paths
Z (in-depth) position (m)
X (lateral) position (m)
100
90
80
70
60
50
40
30
20
10
0
-50
-40
-30
-20
-10
0
10
20
30
40
50
ϕ, dϕ/dt and d$^2\phi$/dt^2
θ, dθ/dt and d$^2\theta$/dt^2
Time (s)
5
4
3
2
1
-50
0
50
-50
0
50
Angular value (°, °/s, or °/s^2)

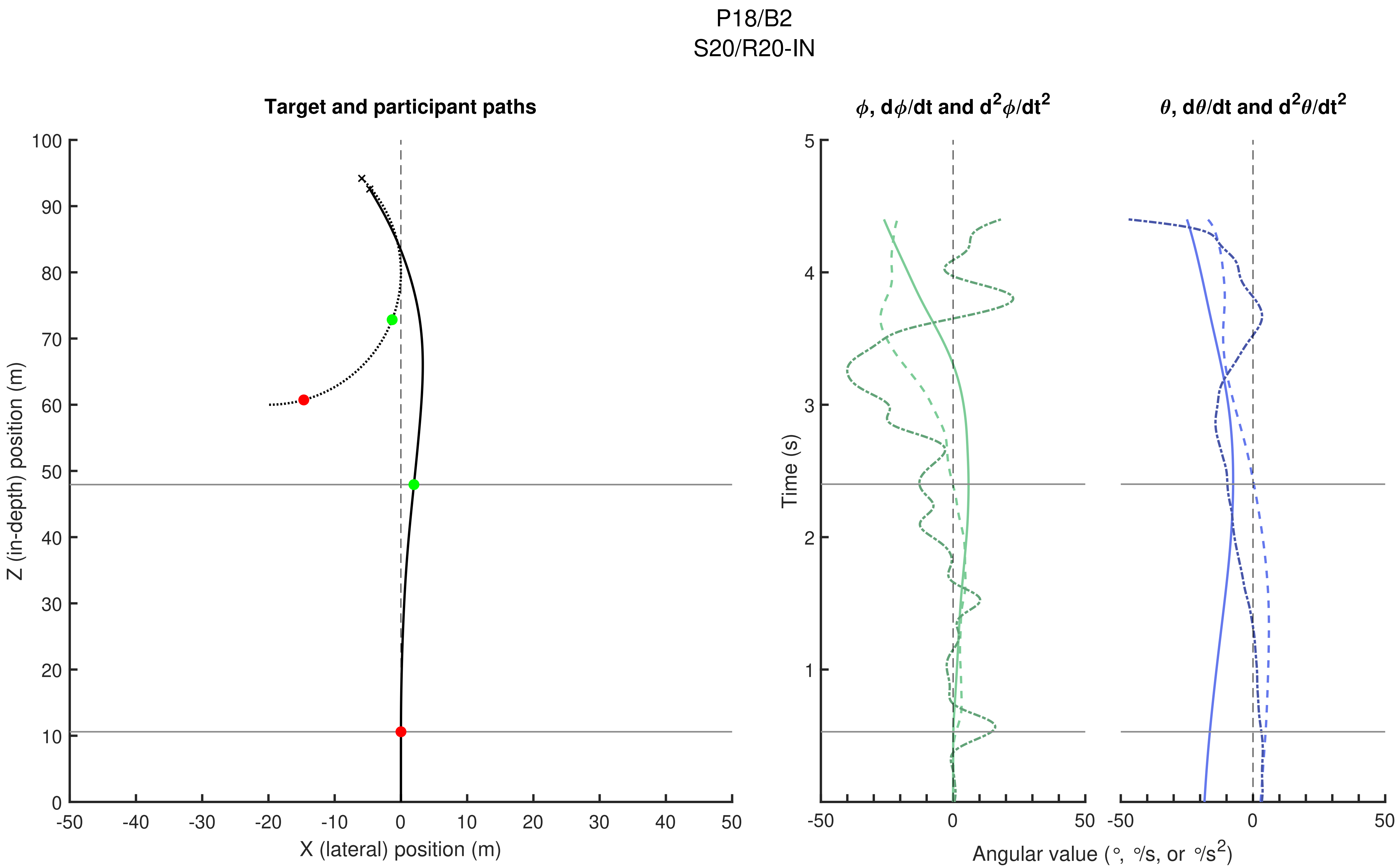
P18/B2
S20/R20-IN
Target and participant paths
ϕ, dϕ/dt and d$^2\phi$/dt^2
θ, dθ/dt and d$^2\theta$/dt^2
Z (in-depth) position (m)
X (lateral) position (m)
Time (s)
Angular value (°, °/s, or °/s^2)

P18/B2
S20/R30-IN

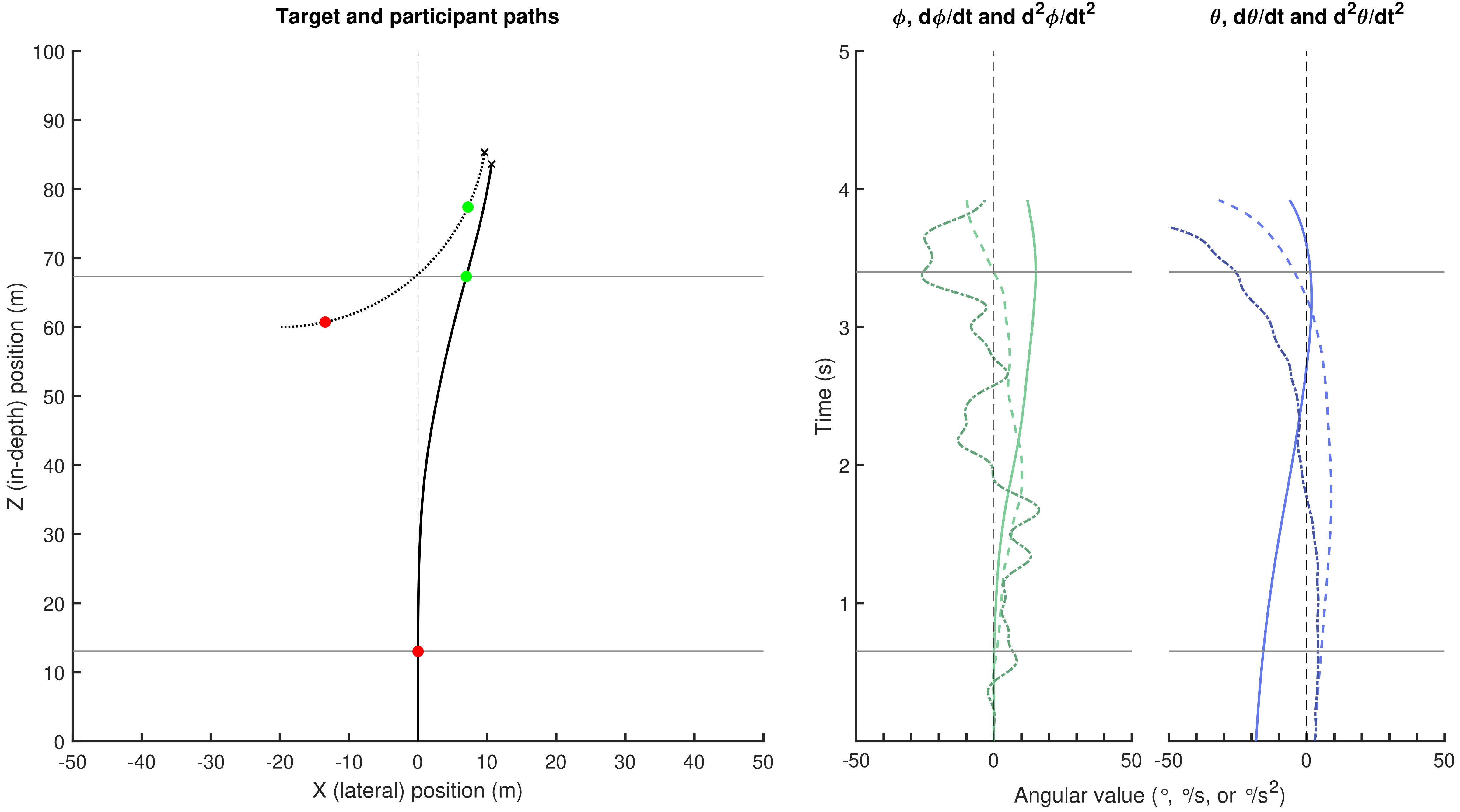

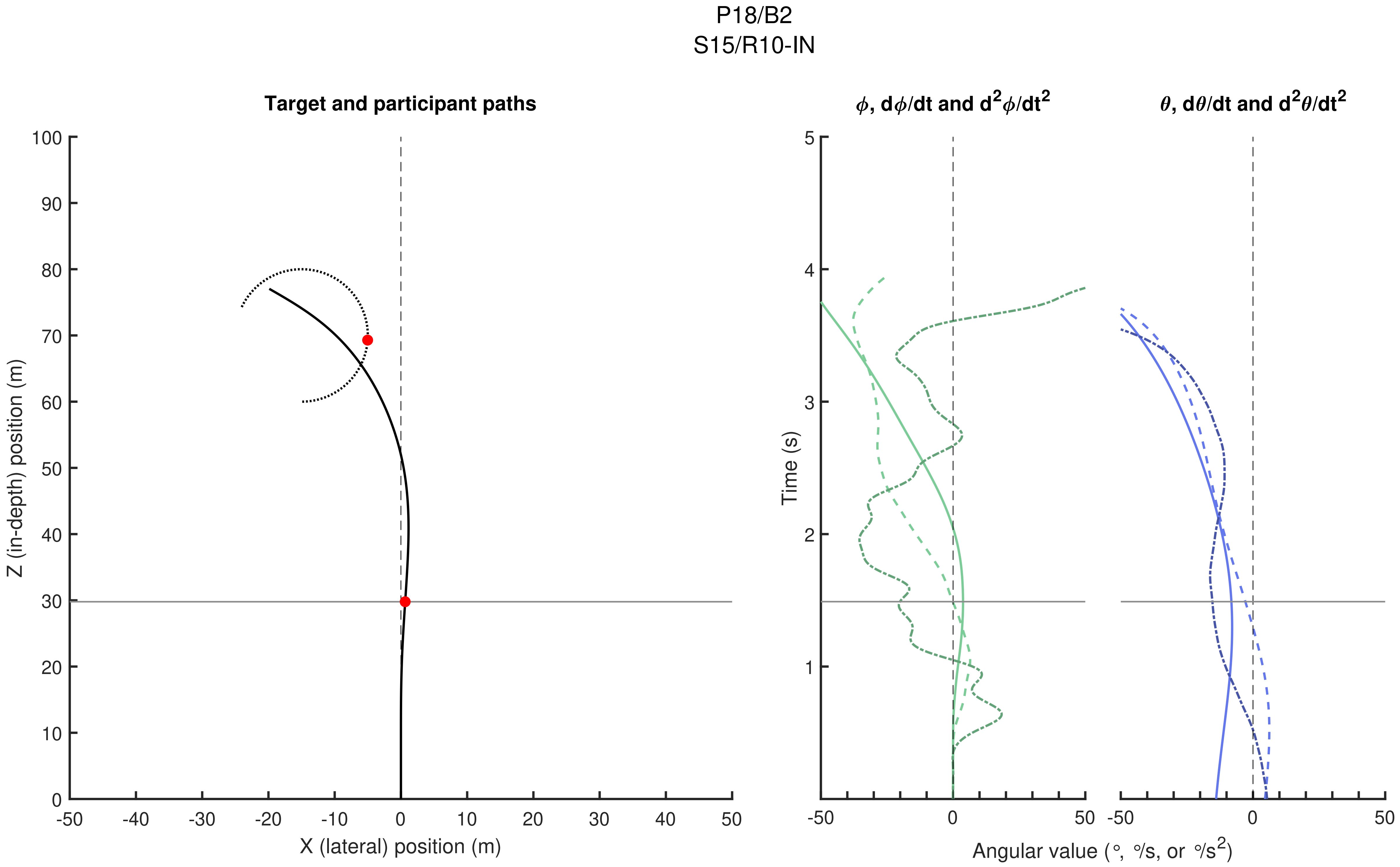

P18/B2
S15/R10-IN
Target and participant paths
Z (in-depth) position (m)
X (lateral) position (m)
ϕ, dϕ/dt and d$^2\phi$/dt^2
θ, dθ/dt and d$^2\theta$/dt^2
Time (s)
Angular value (°, °/s, or °/s^2)

P18/B2
S15/R20-IN

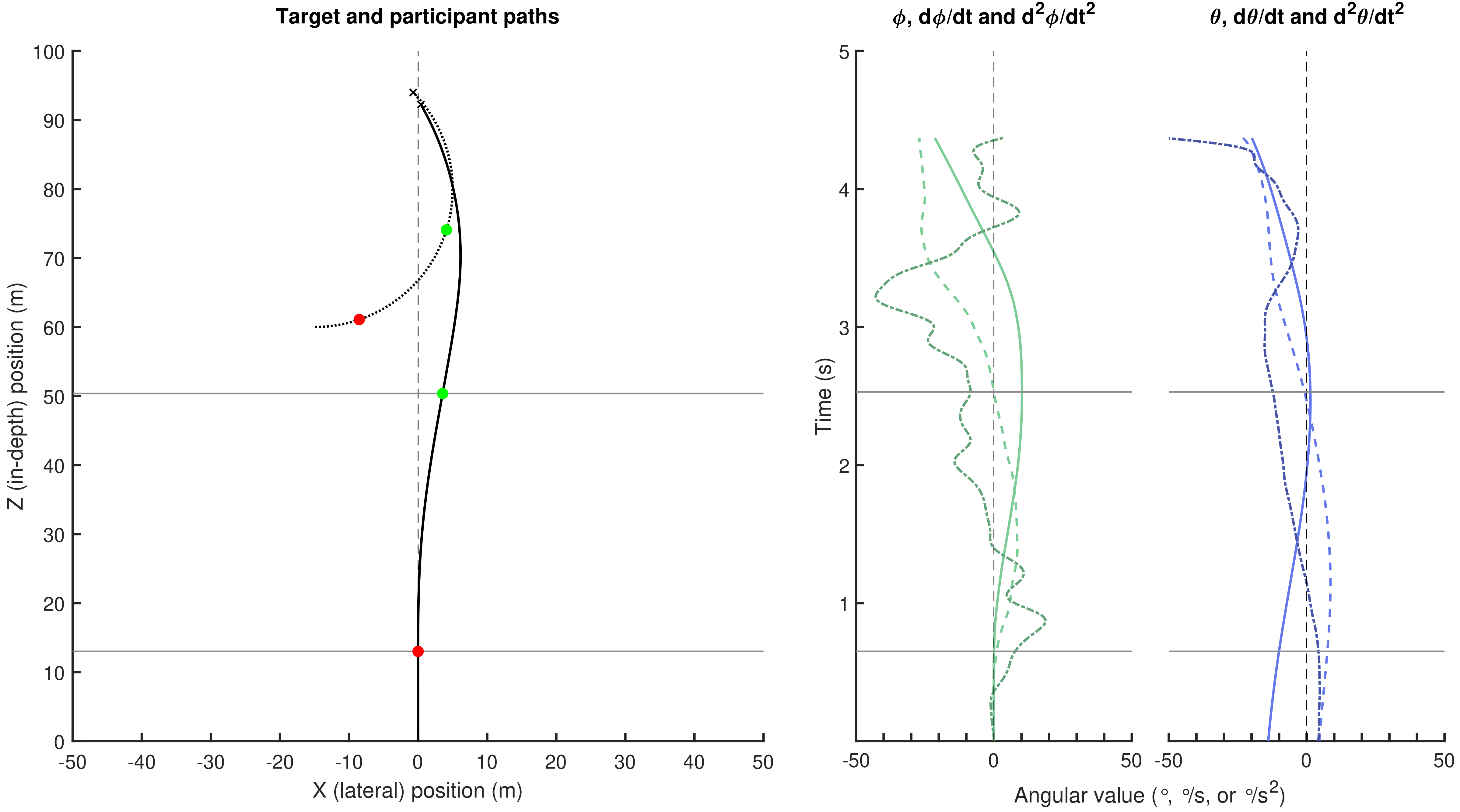

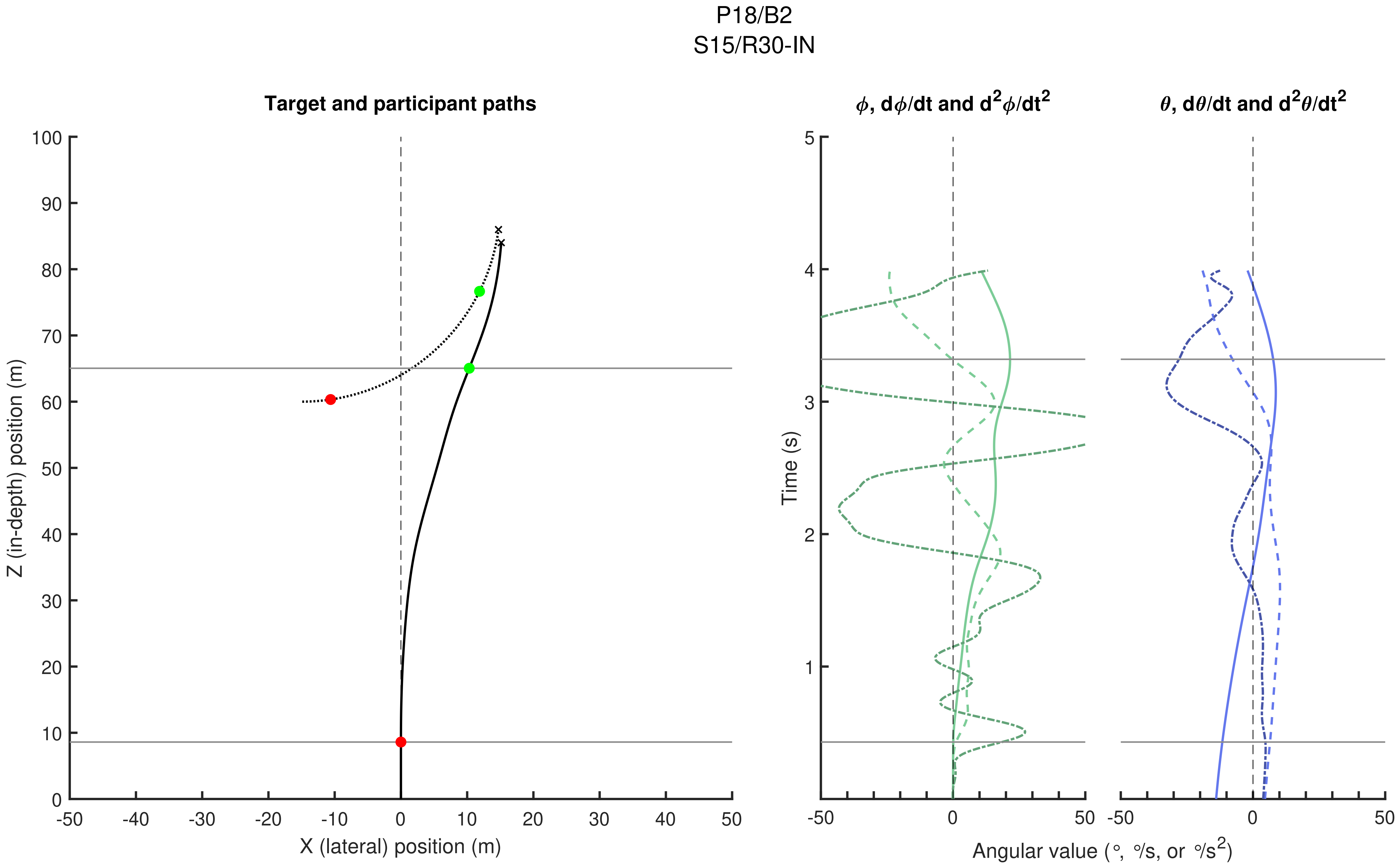

P18/B2
S15/R30-IN
Target and participant paths
Z (in-depth) position (m)
X (lateral) position (m)
φ, dφ/dt and d²φ/dt²
θ, dθ/dt and d²θ/dt²
Time (s)
Angular value (°, °/s, or °/s²)

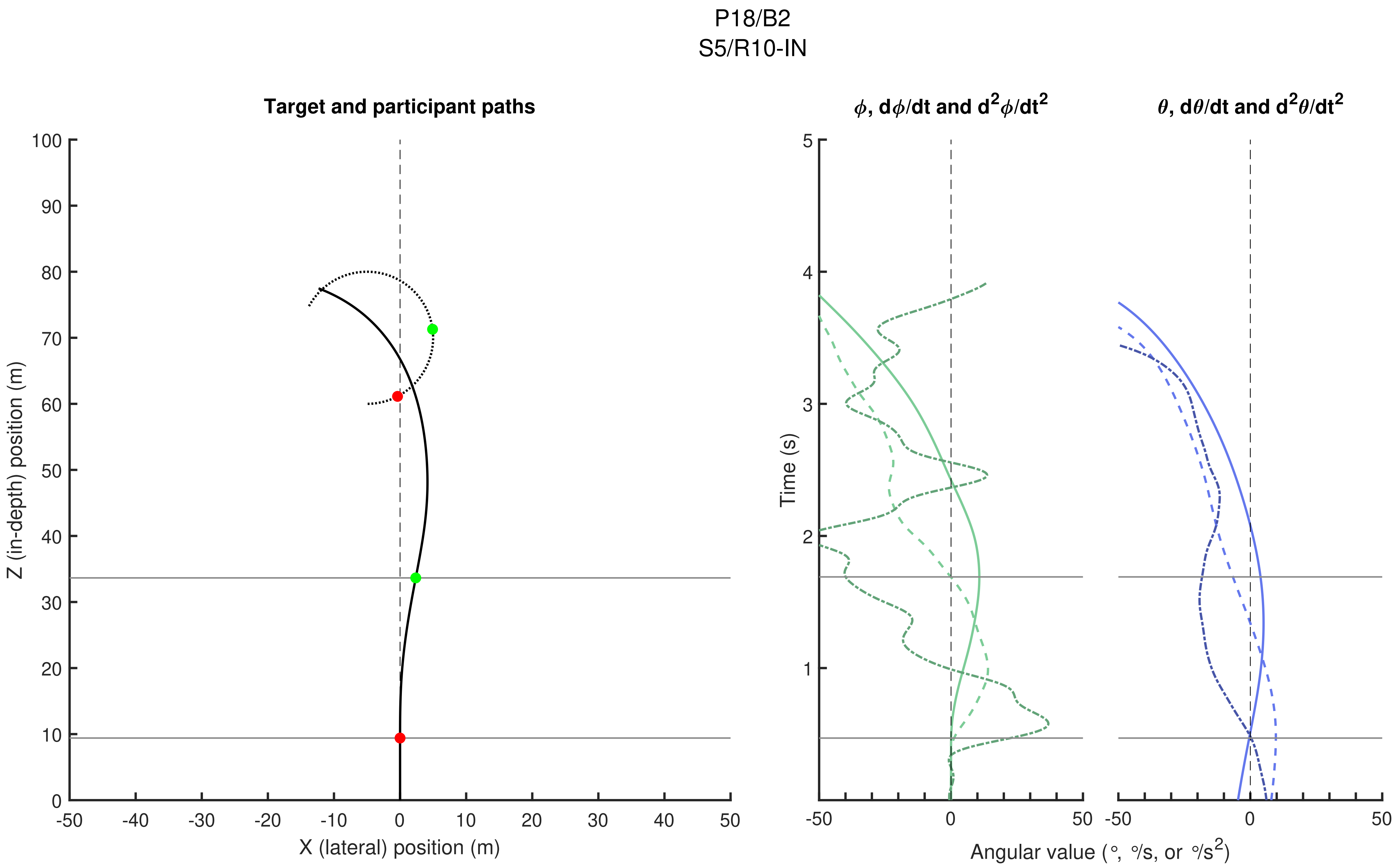
P18/B2
S5/R10-IN
Target and participant paths
Z (in-depth) position (m)
X (lateral) position (m)
ϕ, dϕ/dt and d$^2\phi$/dt^2
θ, dθ/dt and d$^2\theta$/dt^2
Time (s)
Angular value (°, °/s, or °/s^2)

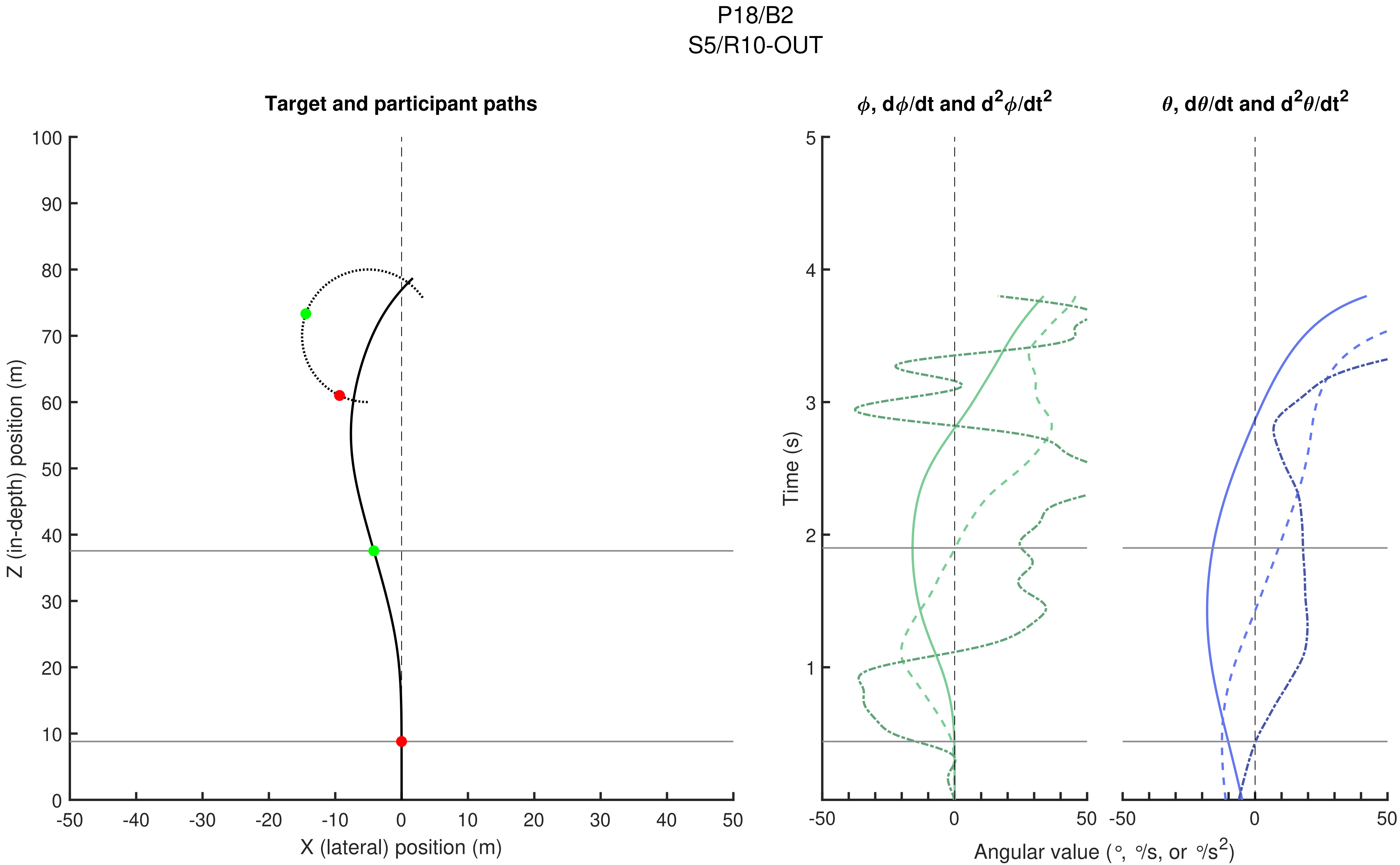

P18/B2
S5/R10-OUT
Target and participant paths
Z (in-depth) position (m)
X (lateral) position (m)
0
10
20
30
40
50
60
70
80
90
100
-50
-40
-30
-20
-10
0
10
20
30
40
50
ϕ, dϕ/dt and d$^2\phi$/dt^2
θ, dθ/dt and d$^2\theta$/dt^2
Time (s)
1
2
3
4
5
-50
0
50
-50
0
50
Angular value (°, °/s, or °/s^2)

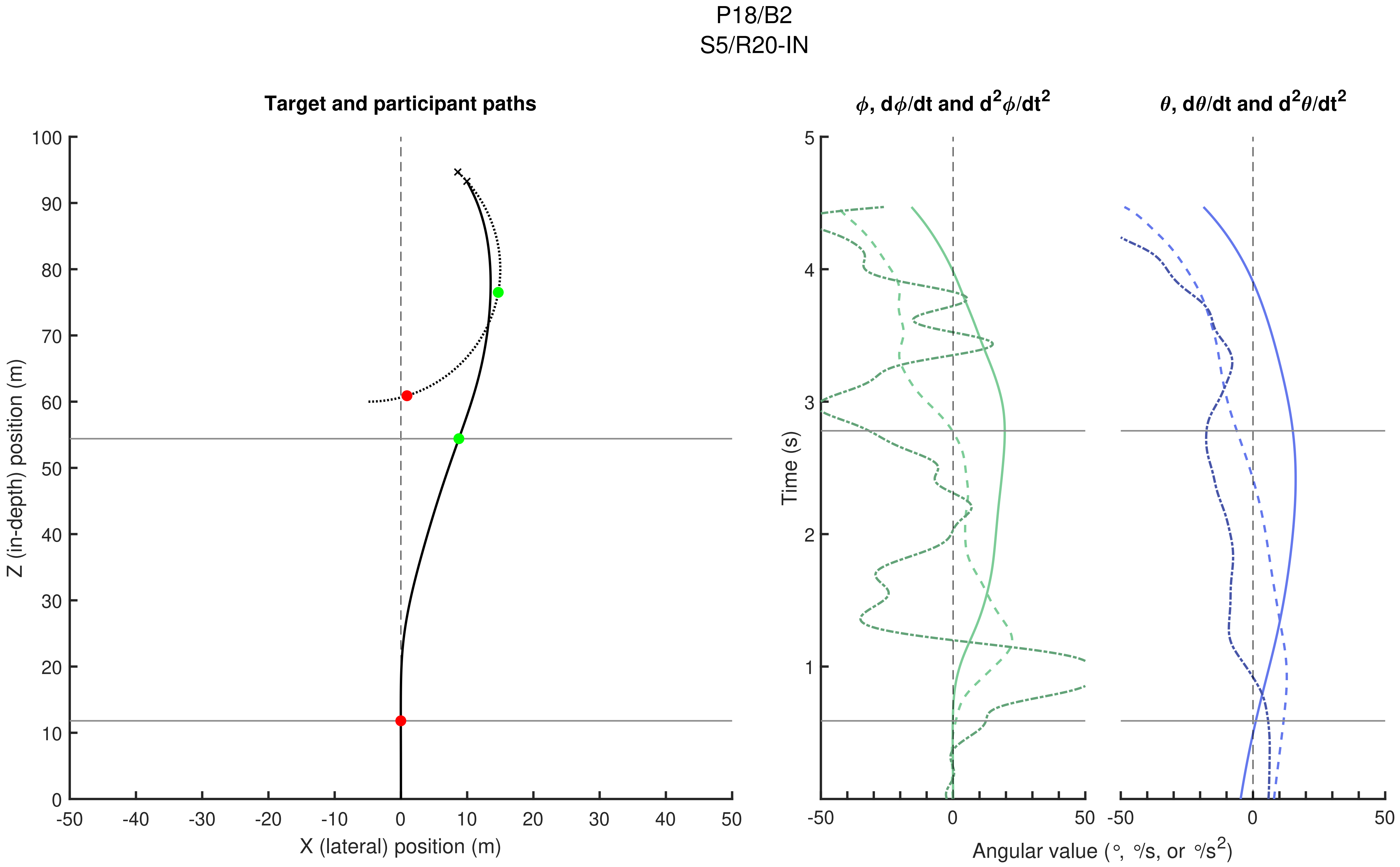
P18/B2
S5/R20-IN
Target and participant paths
Z (in-depth) position (m)
X (lateral) position (m)
100
90
80
70
60
50
40
30
20
10
0
-50
-40
-30
-20
-10
0
10
20
30
40
50
ϕ, dϕ/dt and d²ϕ/dt²
θ, dθ/dt and d²θ/dt²
Time (s)
5
4
3
2
1
-50
0
50
-50
0
50
Angular value (°, °/s, or °/s²)

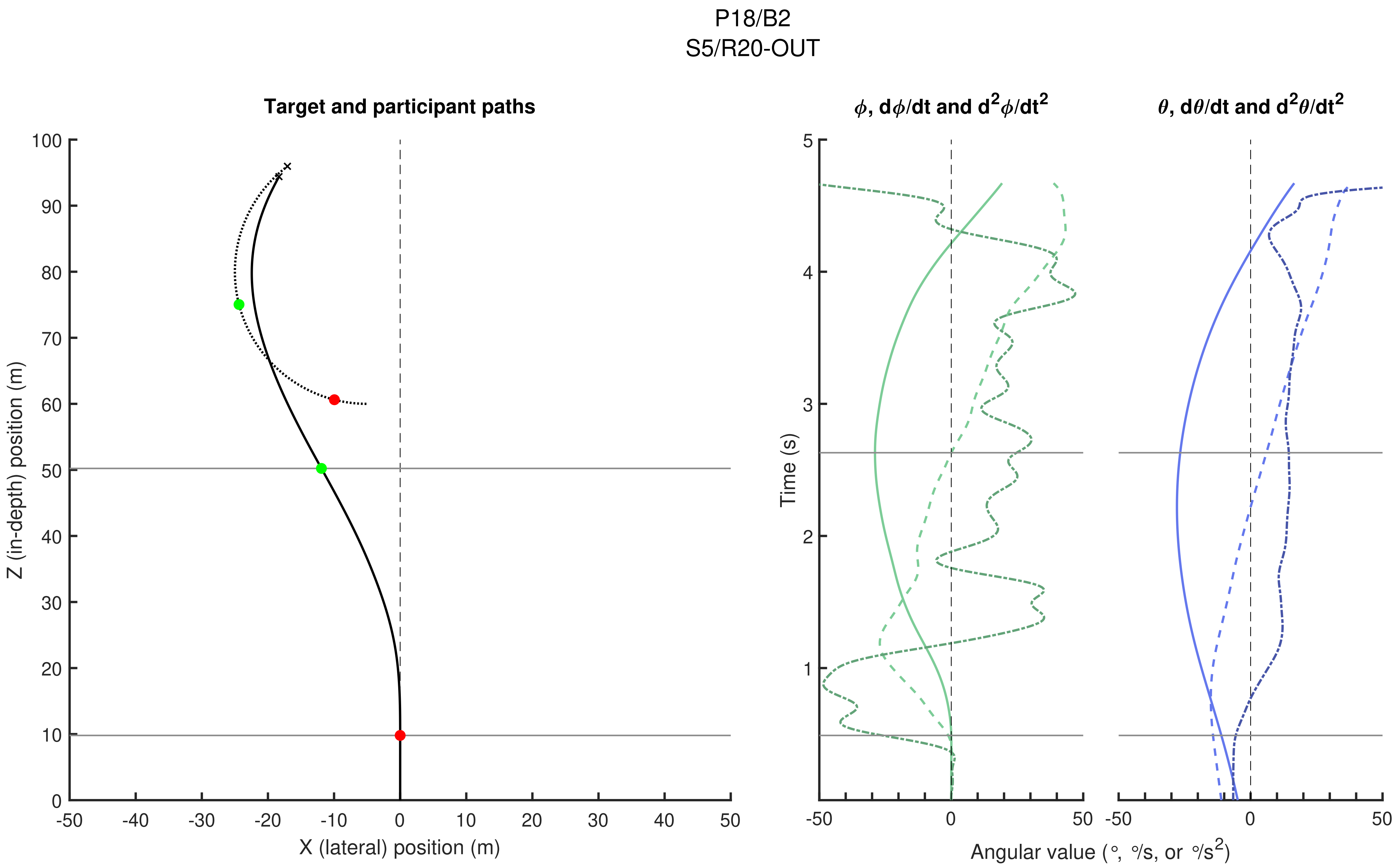

P18/B2
S5/R20-OUT
Target and participant paths
Z (in-depth) position (m)
X (lateral) position (m)
φ, dφ/dt and d²φ/dt²
θ, dθ/dt and d²θ/dt²
Time (s)
Angular value (°, °/s, or °/s²)

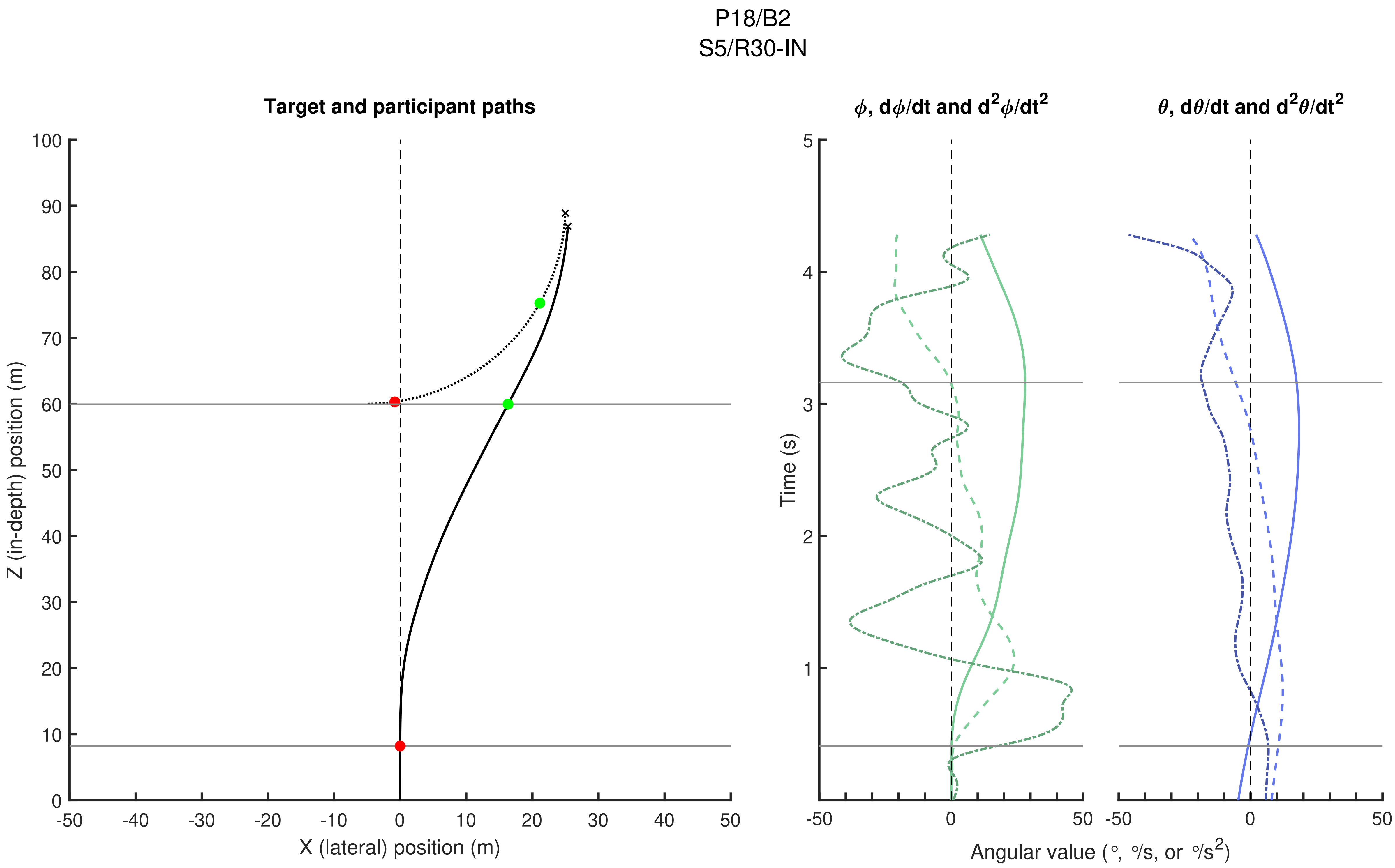
P18/B2
S5/R30-IN
Target and participant paths
Z (in-depth) position (m)
X (lateral) position (m)
0
10
20
30
40
50
60
70
80
90
100
-50
-40
-30
-20
-10
0
10
20
30
40
50
ϕ, dϕ/dt and d$^2\phi$/dt^2
θ, dθ/dt and d$^2\theta$/dt^2
Time (s)
1
2
3
4
5
-50
0
50
-50
0
50
Angular value (°, °/s, or °/s^2)

P18/B2
S5/R30-OUT

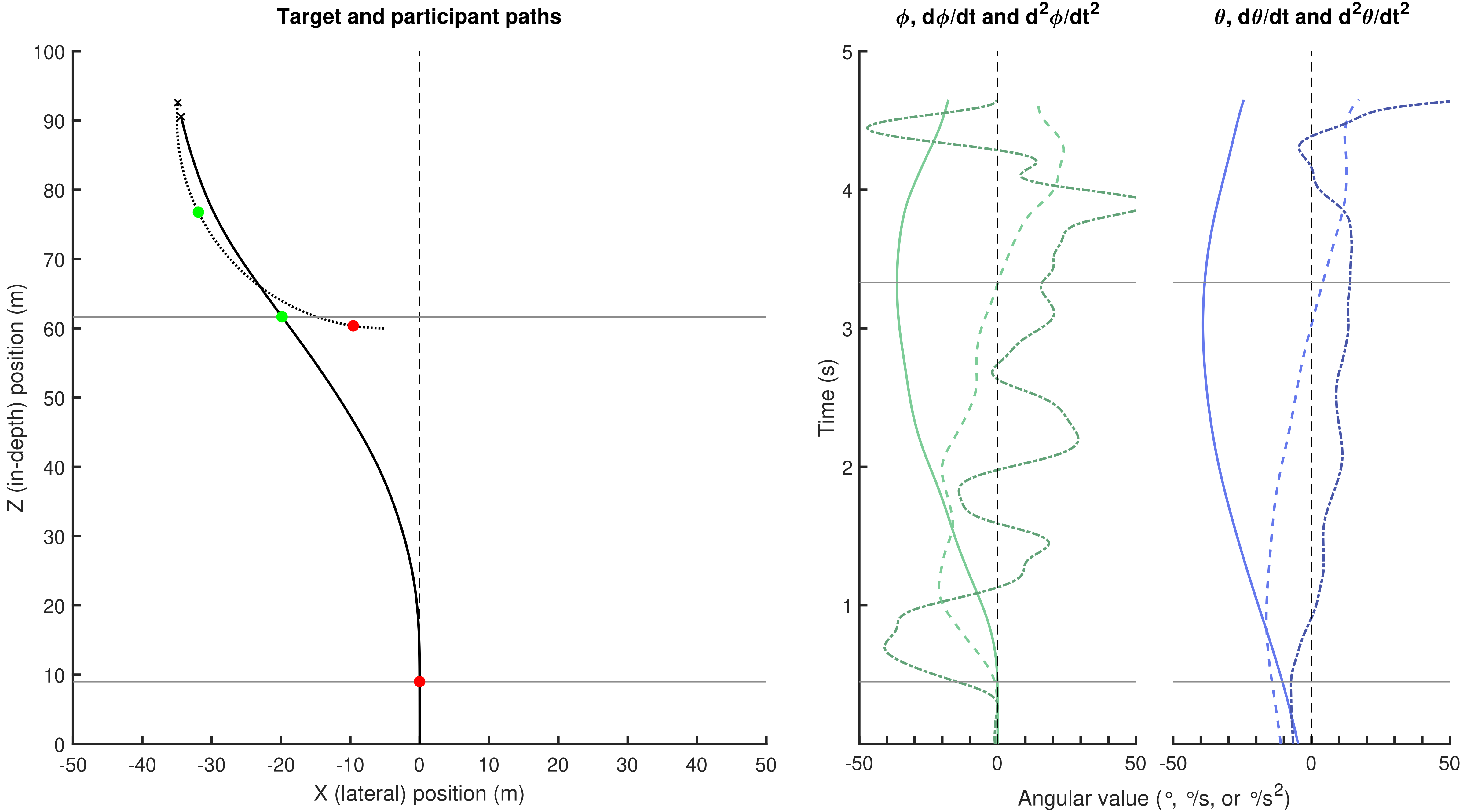

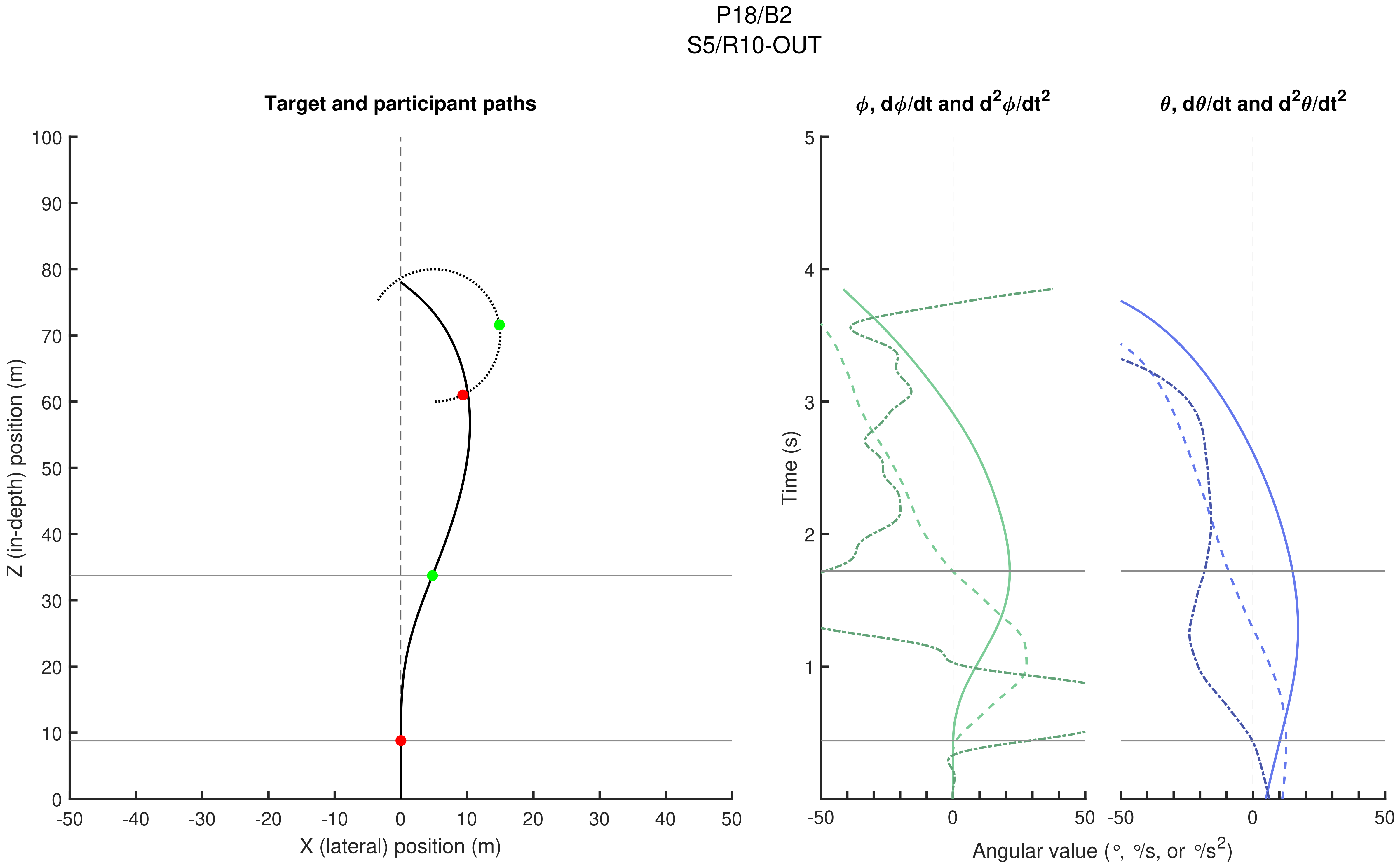

P18/B2
S5/R10-OUT
Target and participant paths
Z (in-depth) position (m)
X (lateral) position (m)
ϕ, dϕ/dt and d²ϕ/dt²
θ, dθ/dt and d²θ/dt²
Time (s)
Angular value (°, °/s, or °/s²)

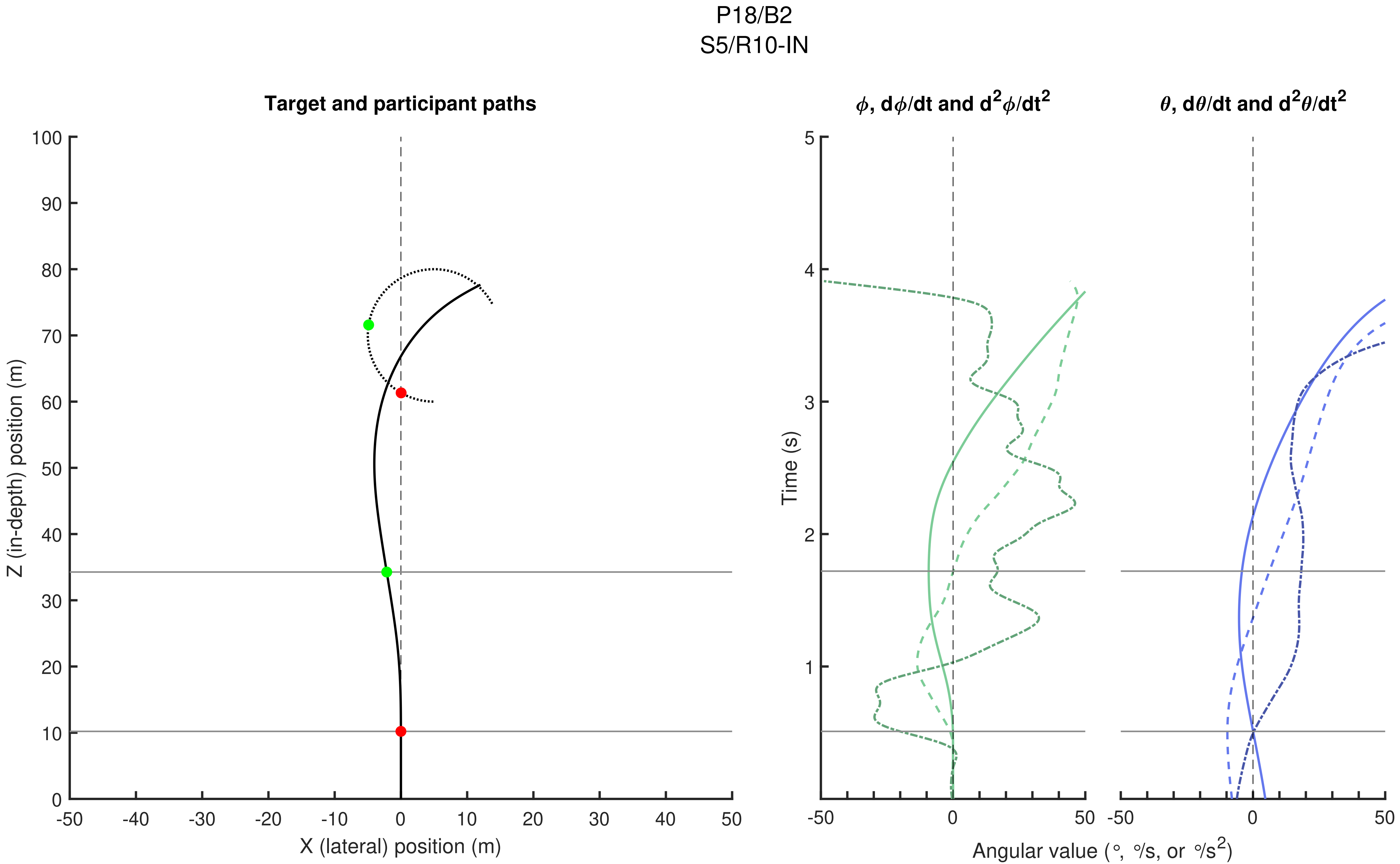
P18/B2
S5/R10-IN
Target and participant paths
Z (in-depth) position (m)
X (lateral) position (m)
100
90
80
70
60
50
40
30
20
10
0
-50
-40
-30
-20
-10
0
10
20
30
40
50
ϕ, dϕ/dt and d$^2\phi$/dt^2
θ, dθ/dt and d$^2\theta$/dt^2
Time (s)
5
4
3
2
1
-50
0
50
-50
0
50
Angular value (°, °/s, or °/s^2)

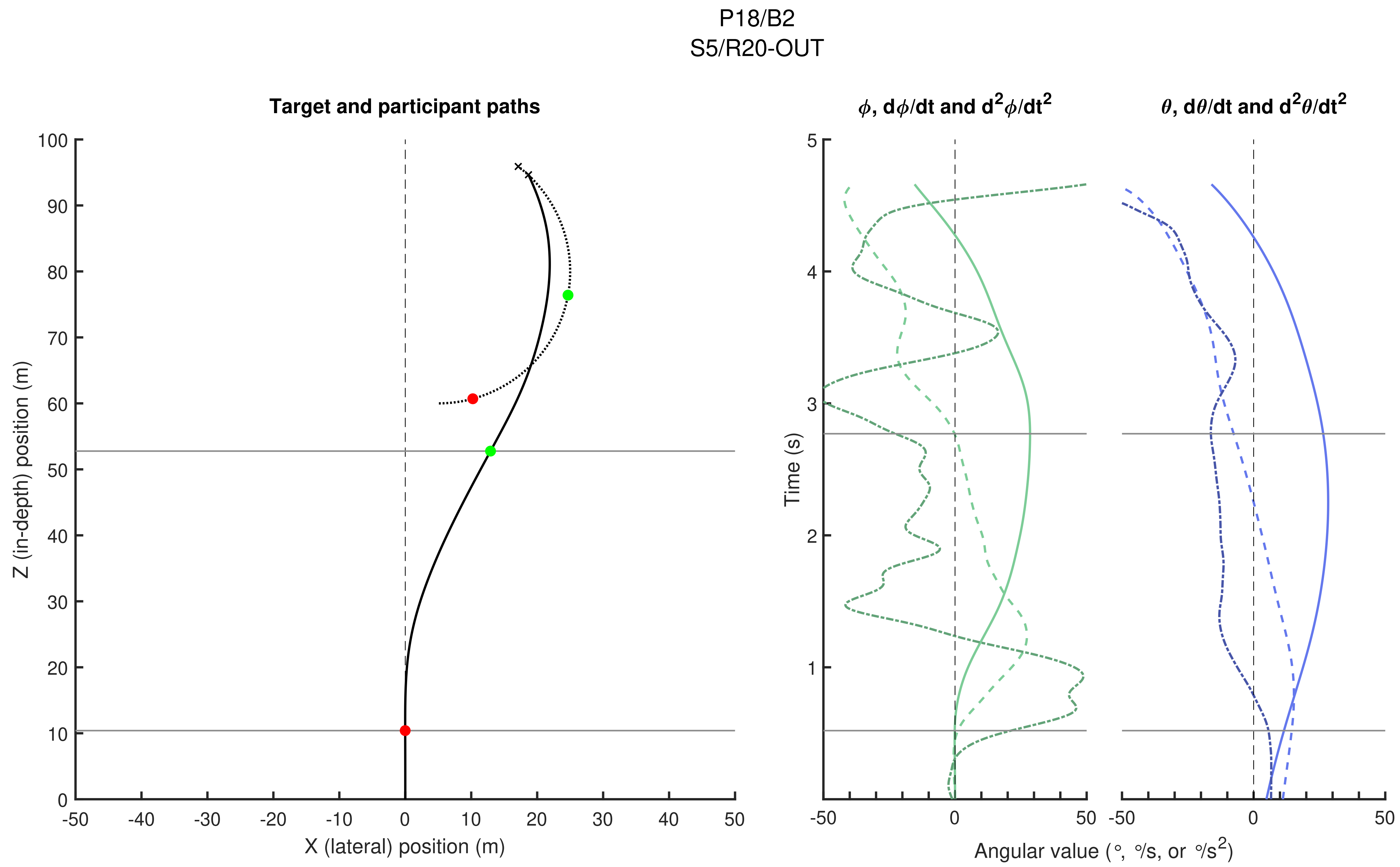

P18/B2
S5/R20-OUT
Target and participant paths
Z (in-depth) position (m)
X (lateral) position (m)
φ, dφ/dt and d²φ/dt²
θ, dθ/dt and d²θ/dt²
Time (s)
Angular value (°, °/s, or °/s²)

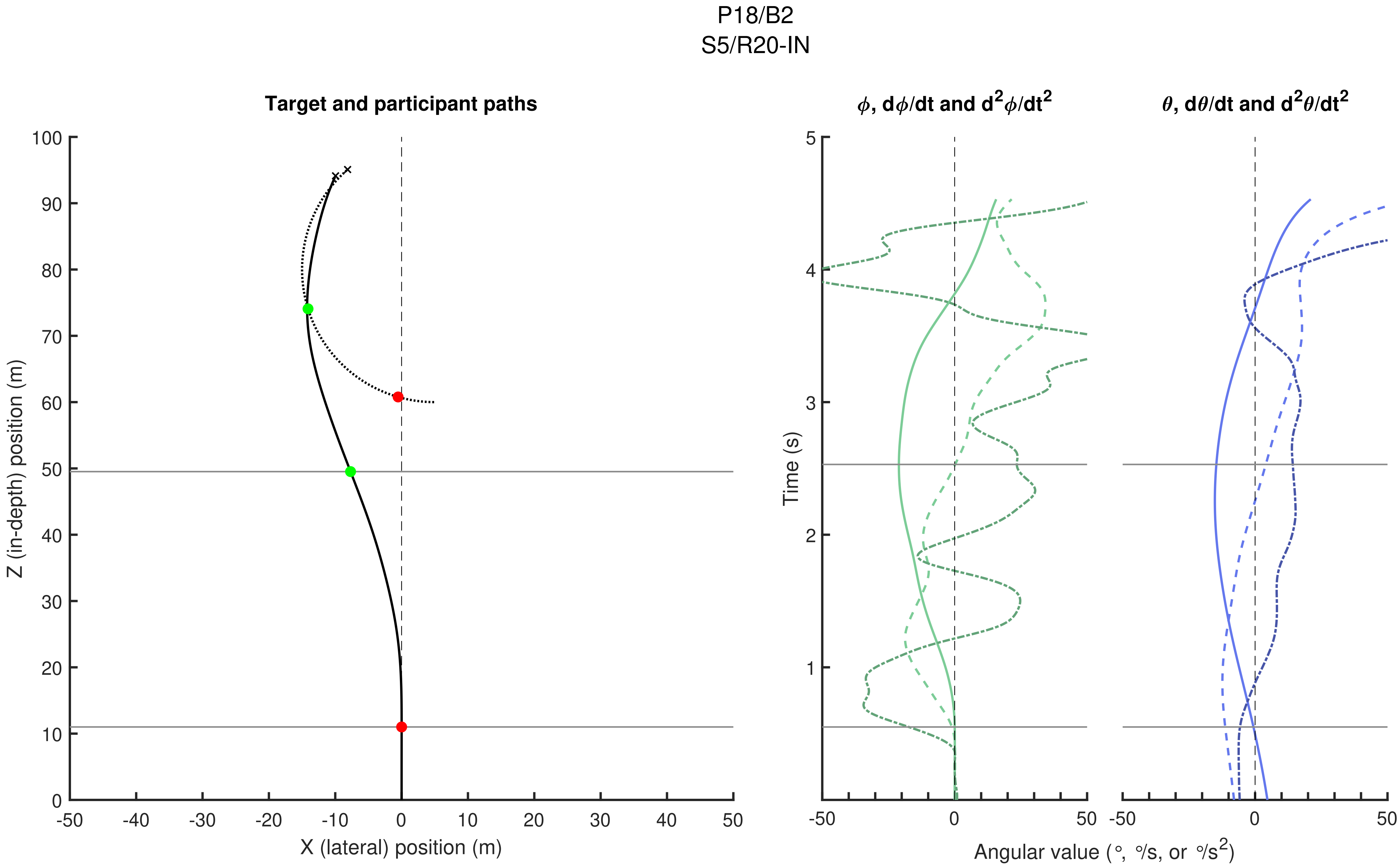
P18/B2
S5/R20-IN
Target and participant paths
Z (in-depth) position (m)
X (lateral) position (m)
100
90
80
70
60
50
40
30
20
10
0
-50
-40
-30
-20
-10
0
10
20
30
40
50
ϕ, dϕ/dt and d²ϕ/dt²
θ, dθ/dt and d²θ/dt²
Time (s)
5
4
3
2
1
-50
0
50
-50
0
50
Angular value (°, °/s, or °/s²)

P18/B2
S5/R30-OUT

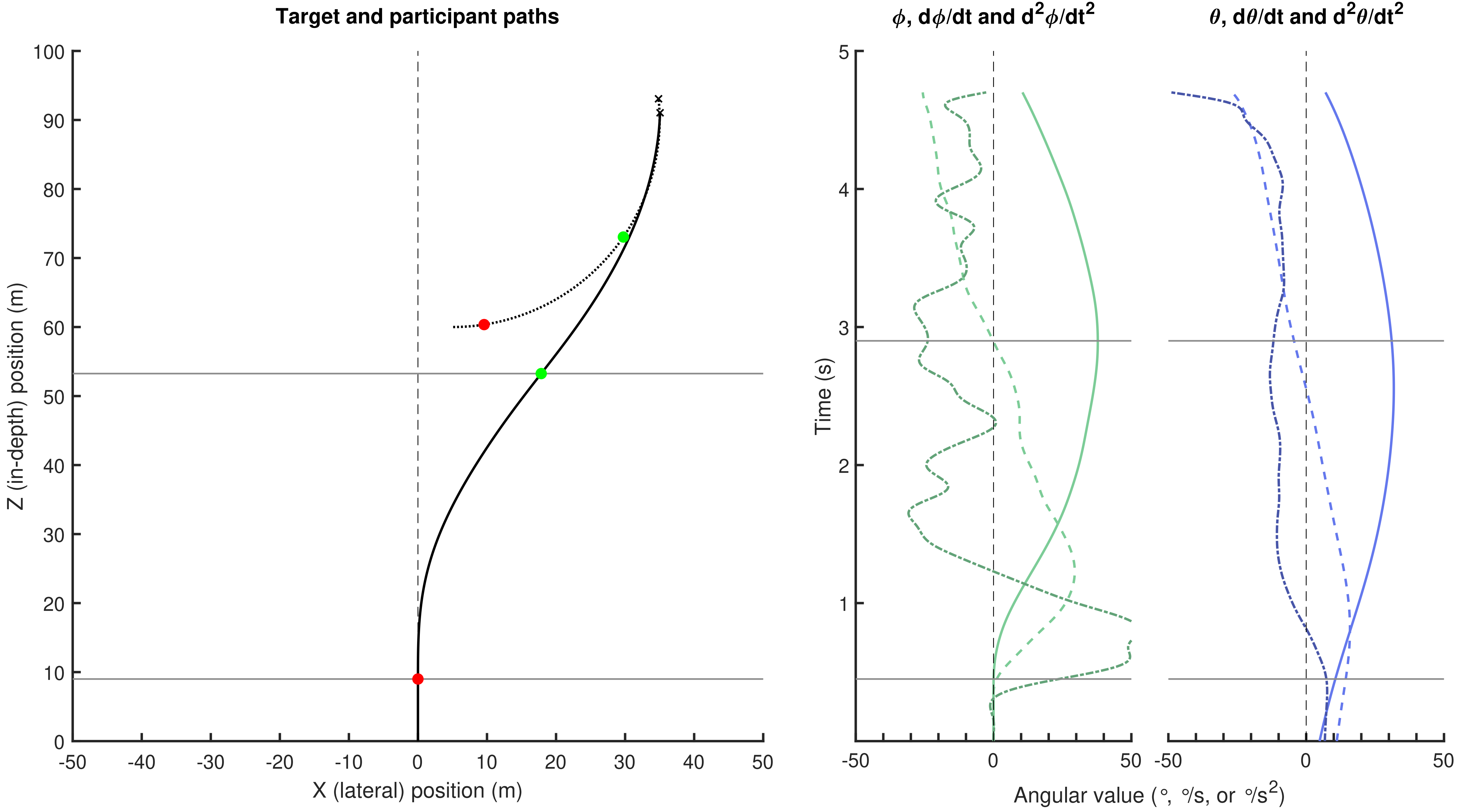

P18/B2
S5/R30-IN

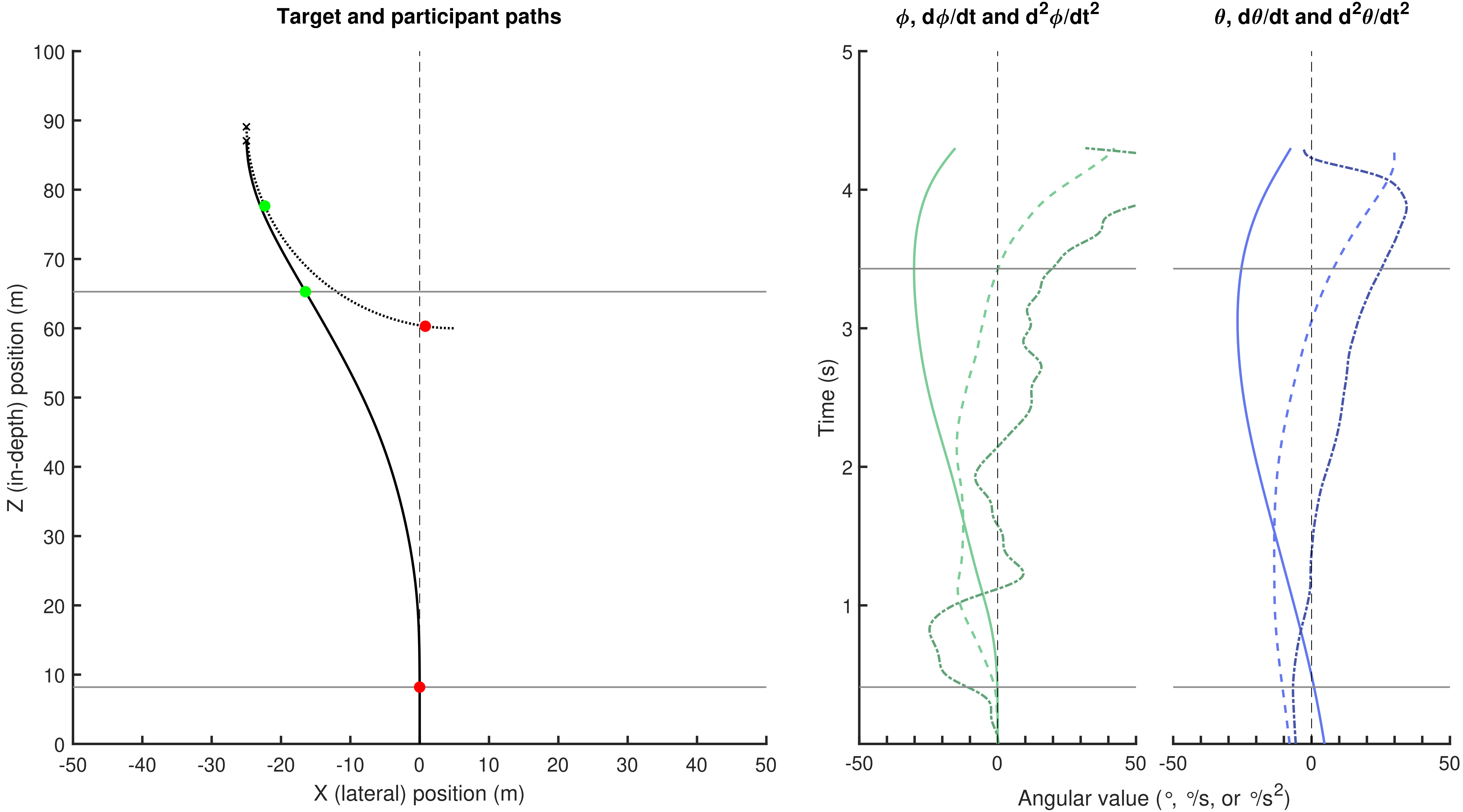

P18/B2
S15/R10-IN

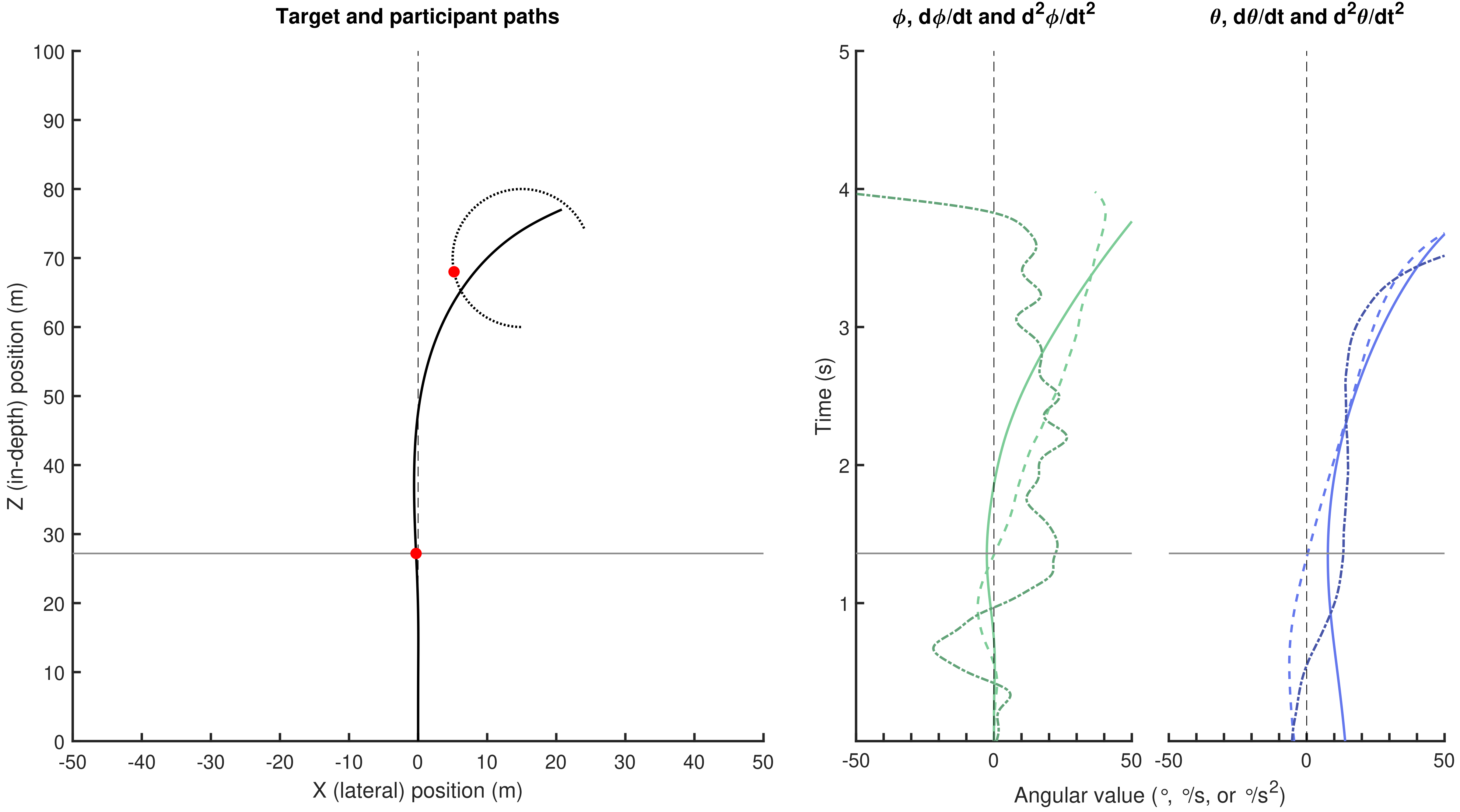

P18/B2
S15/R20-IN

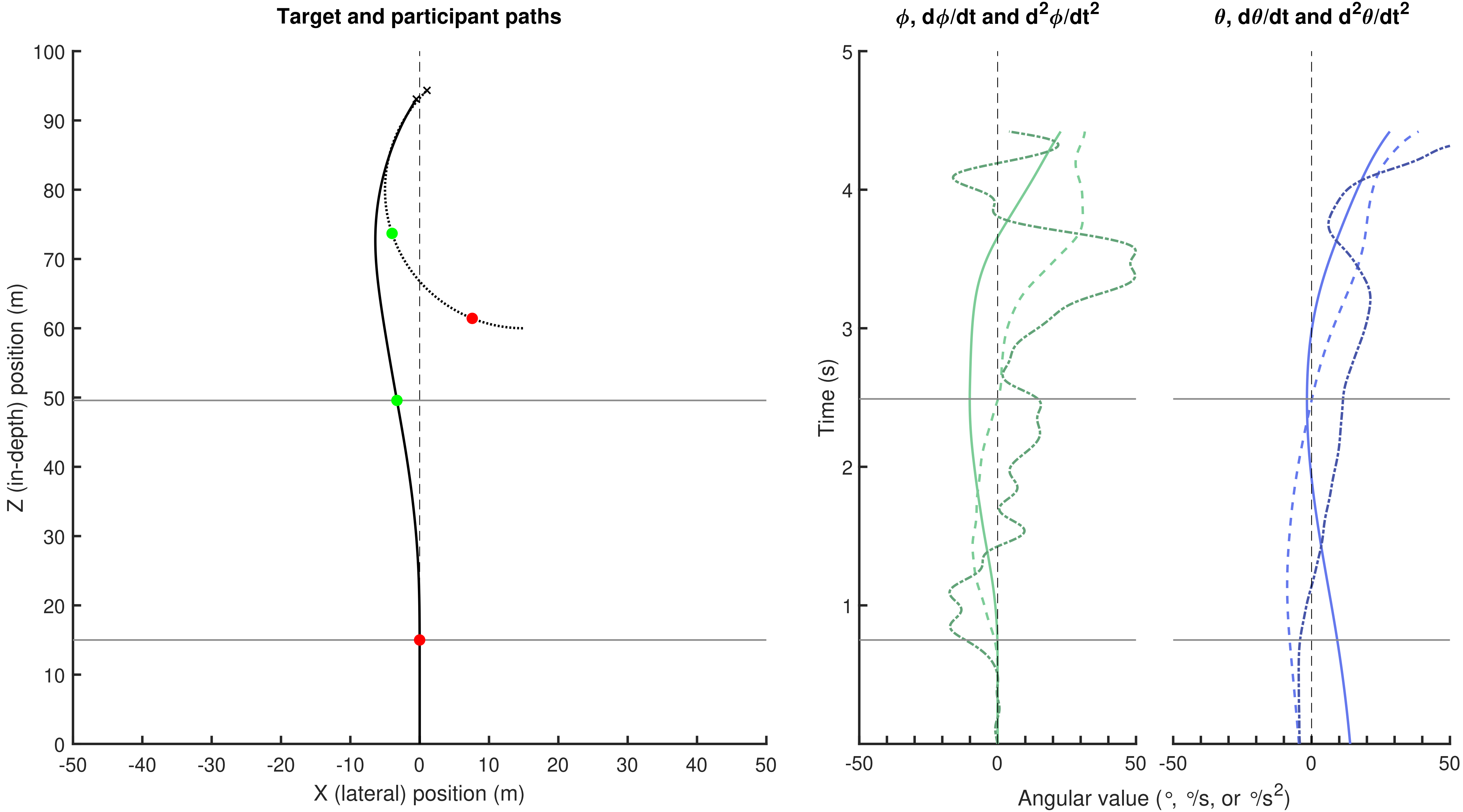

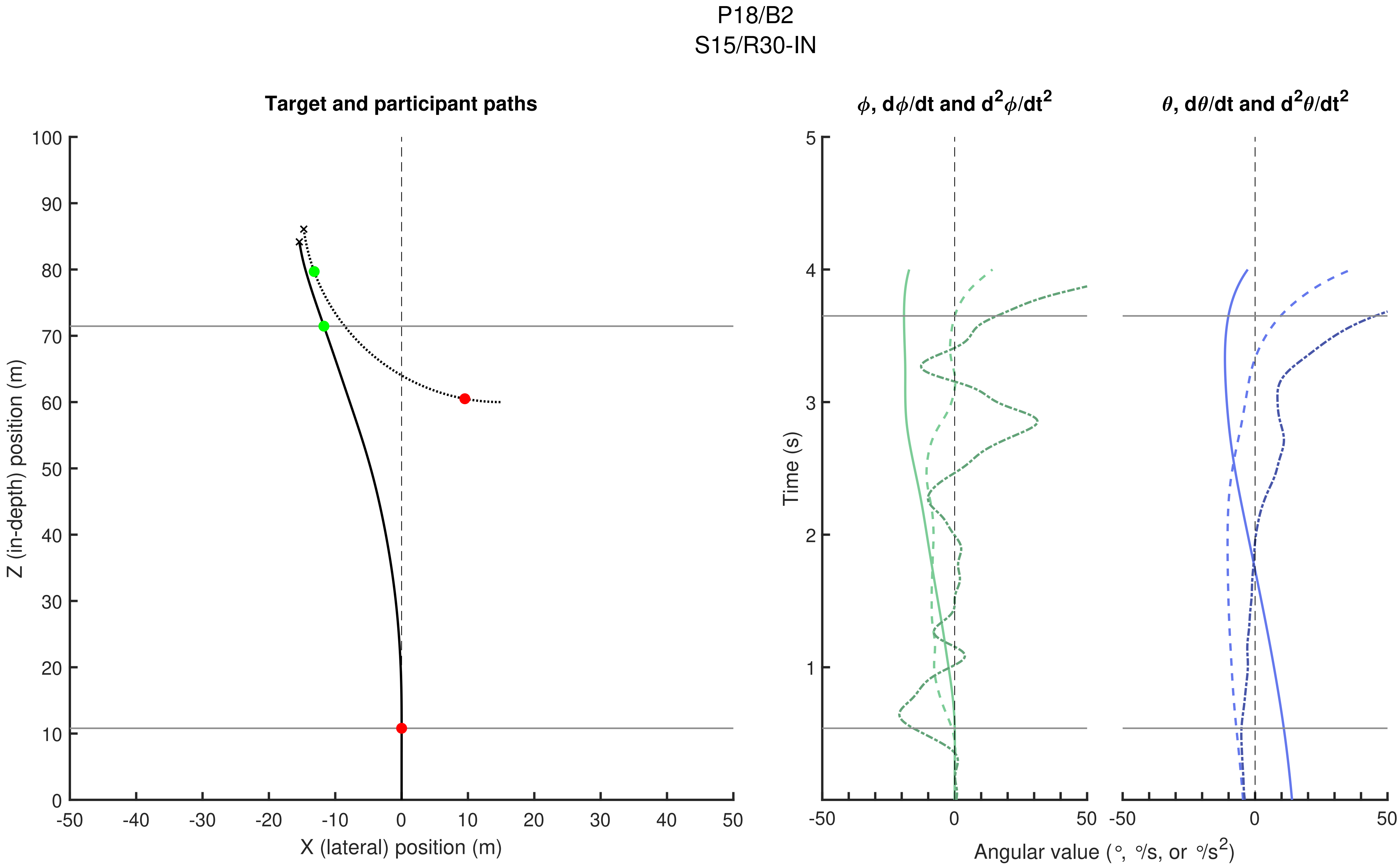
P18/B2
S15/R30-IN
Target and participant paths
Z (in-depth) position (m)
X (lateral) position (m)
100
90
80
70
60
50
40
30
20
10
0
-50
-40
-30
-20
-10
0
10
20
30
40
50
ϕ, dϕ/dt and d$^2\phi$/dt^2
θ, dθ/dt and d$^2\theta$/dt^2
Time (s)
5
4
3
2
1
-50
0
50
-50
0
50
Angular value (°, °/s, or °/s^2)

P18/B2
S20/R10-IN

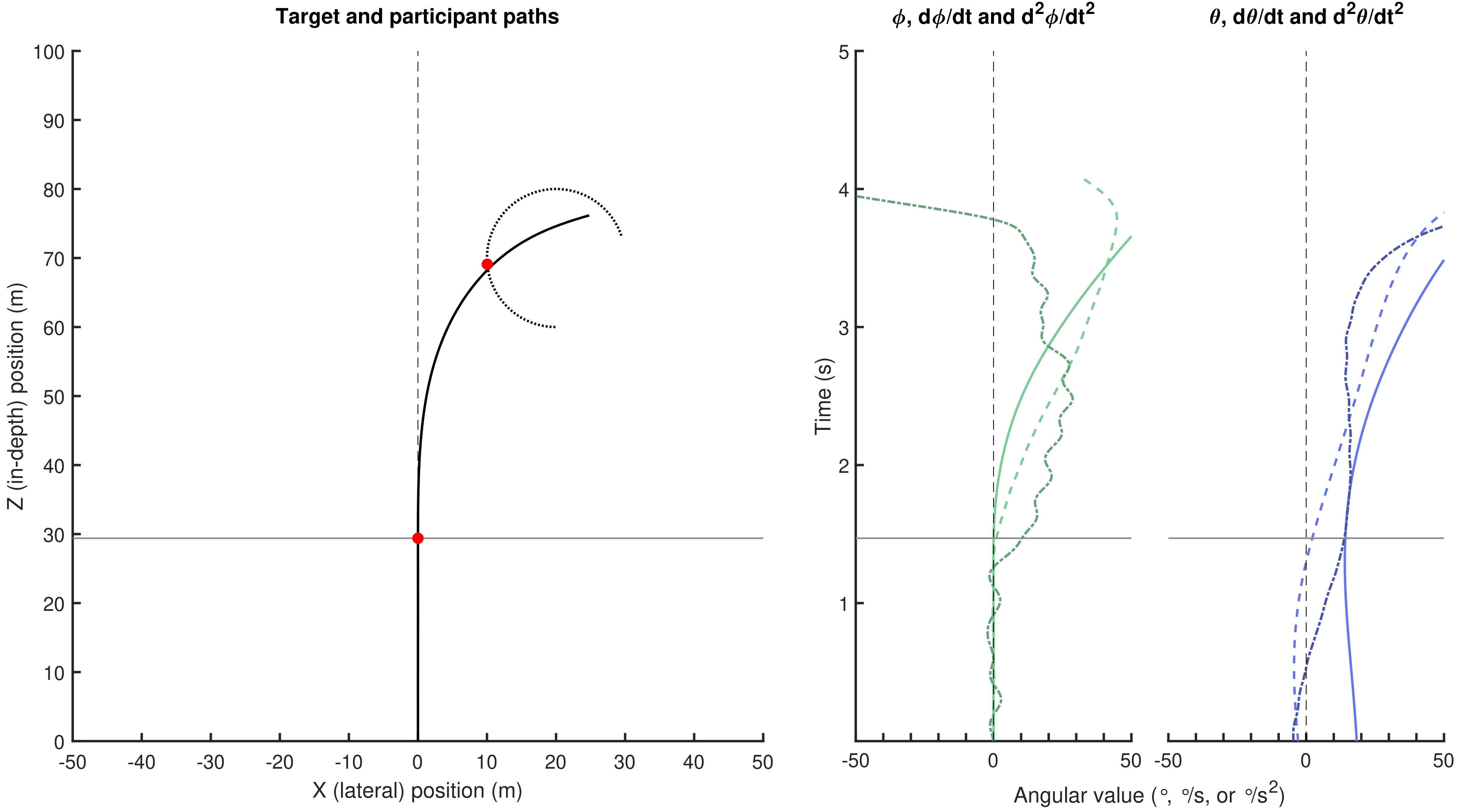

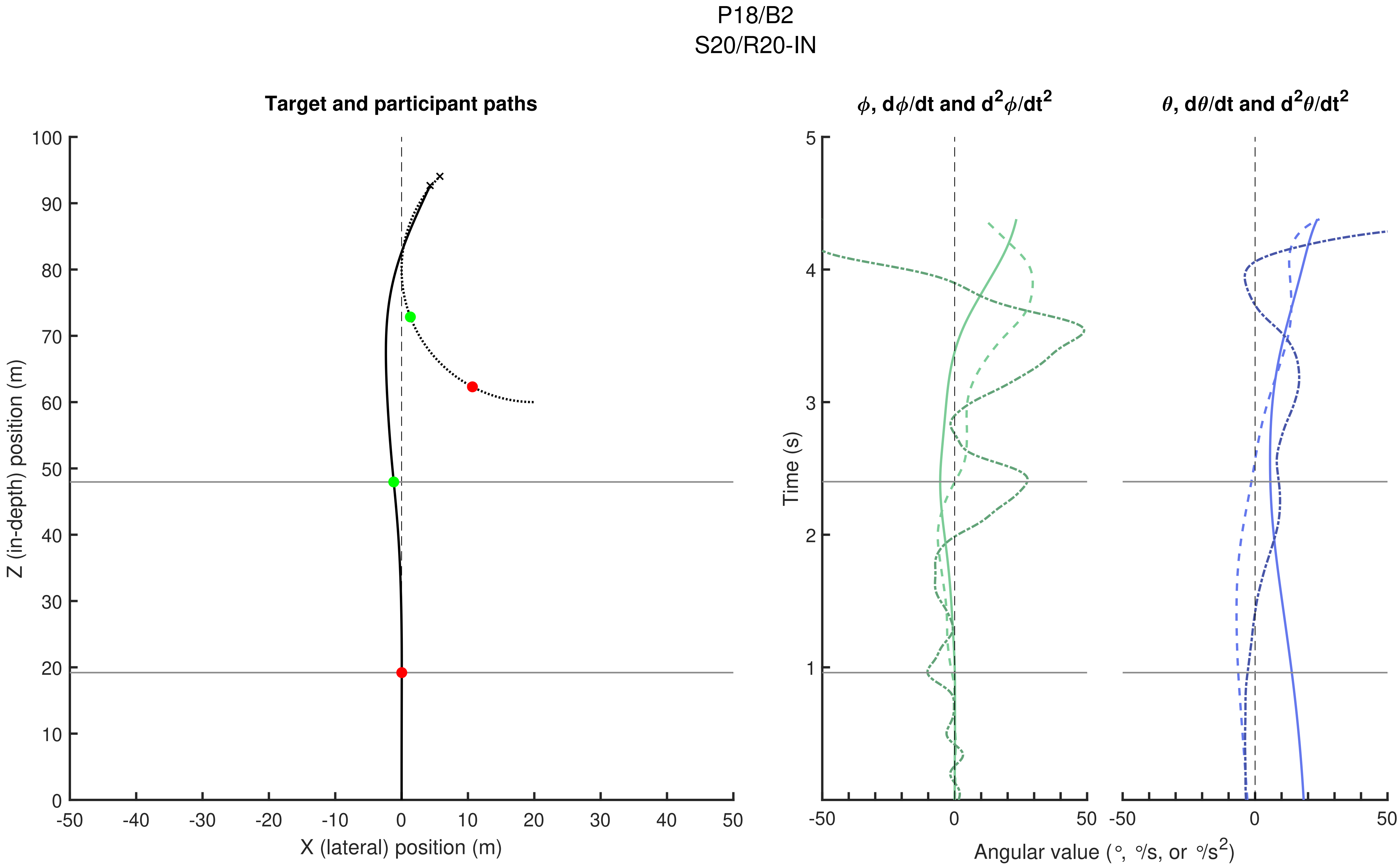

P18/B2
S20/R20-IN
Target and participant paths
Z (in-depth) position (m)
X (lateral) position (m)
ϕ, dϕ/dt and d²ϕ/dt²
θ, dθ/dt and d²θ/dt²
Time (s)
Angular value (°, °/s, or °/s²)

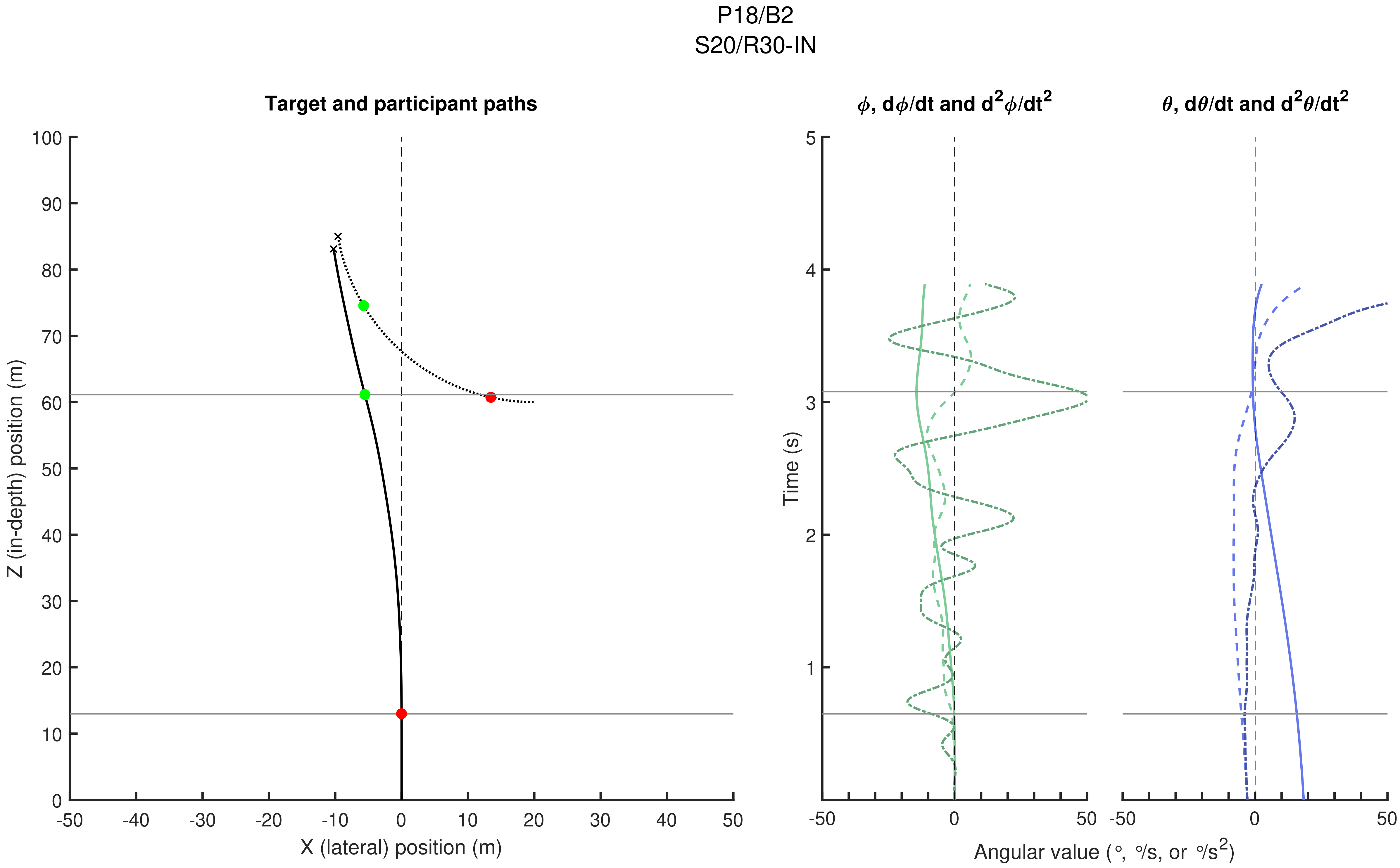

P18/B2
S20/R30-IN
Target and participant paths
Z (in-depth) position (m)
X (lateral) position (m)
ϕ, dϕ/dt and d$^2\phi$/dt^2
θ, dθ/dt and d$^2\theta$/dt^2
Time (s)
Angular value (°, °/s, or °/s^2)

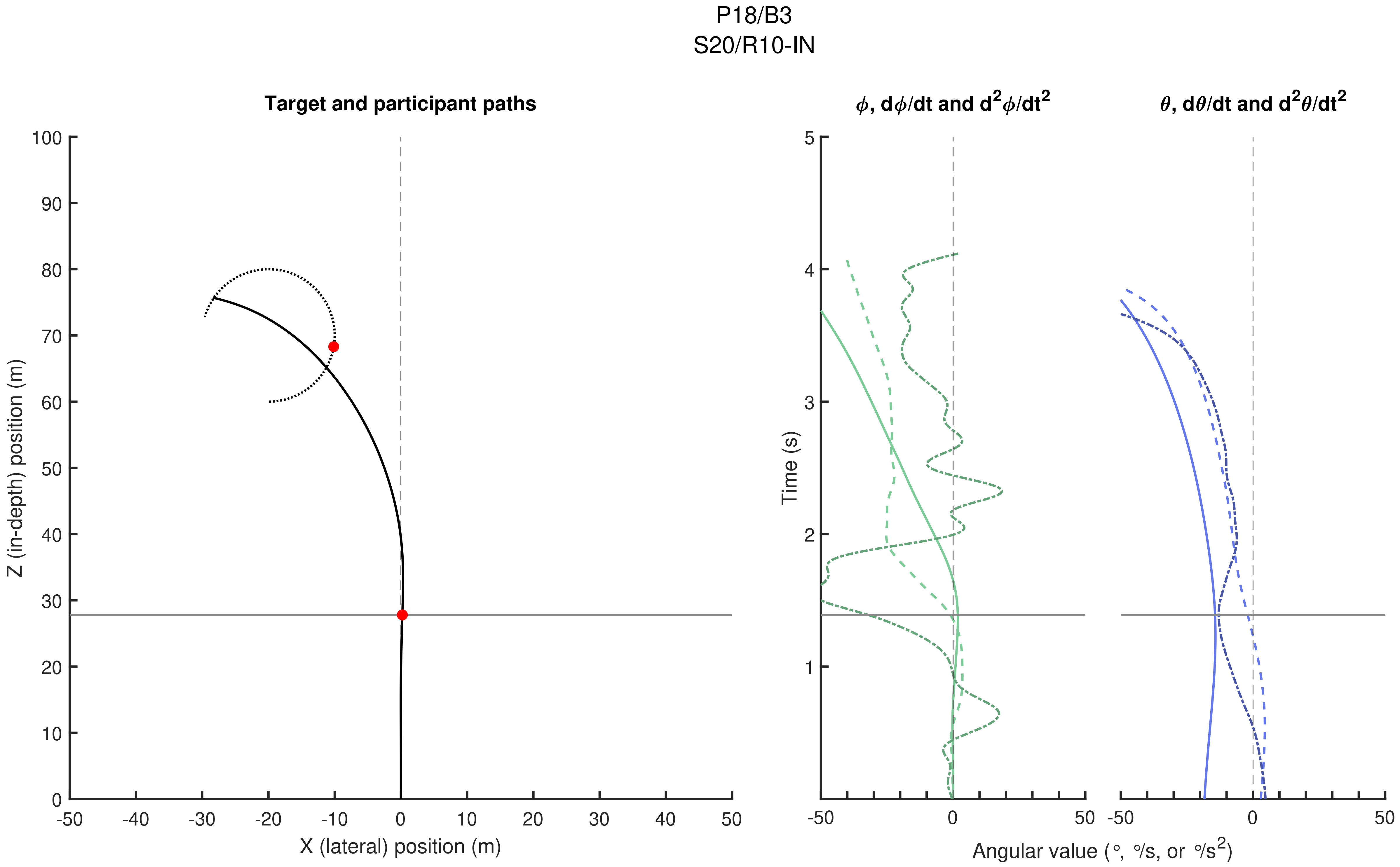
P18/B3
S20/R10-IN
Target and participant paths
Z (in-depth) position (m)
X (lateral) position (m)
φ, dφ/dt and d²φ/dt²
θ, dθ/dt and d²θ/dt²
Time (s)
Angular value (°, °/s, or °/s²)

P18/B3
S20/R20-IN

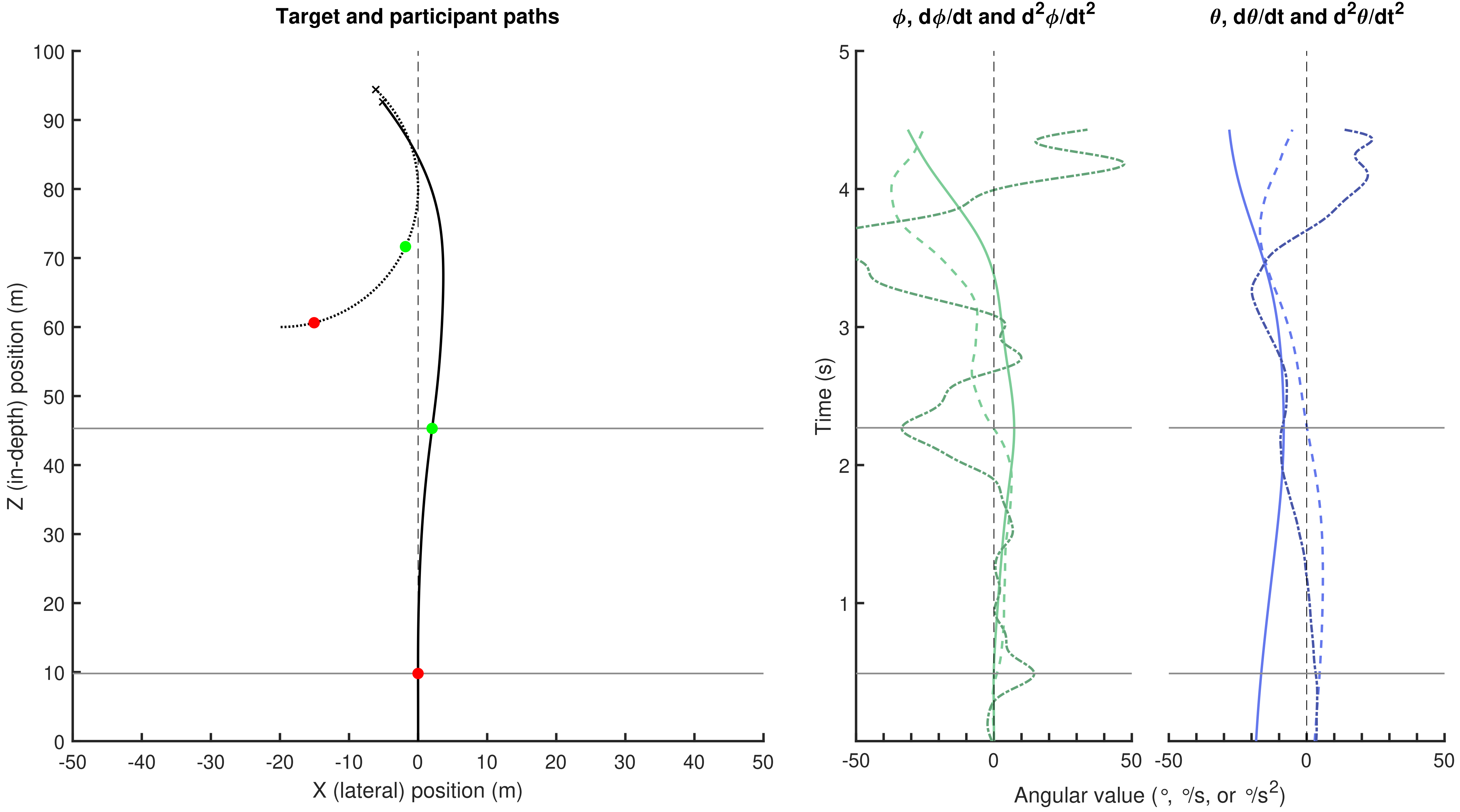

P18/B3
S20/R30-IN

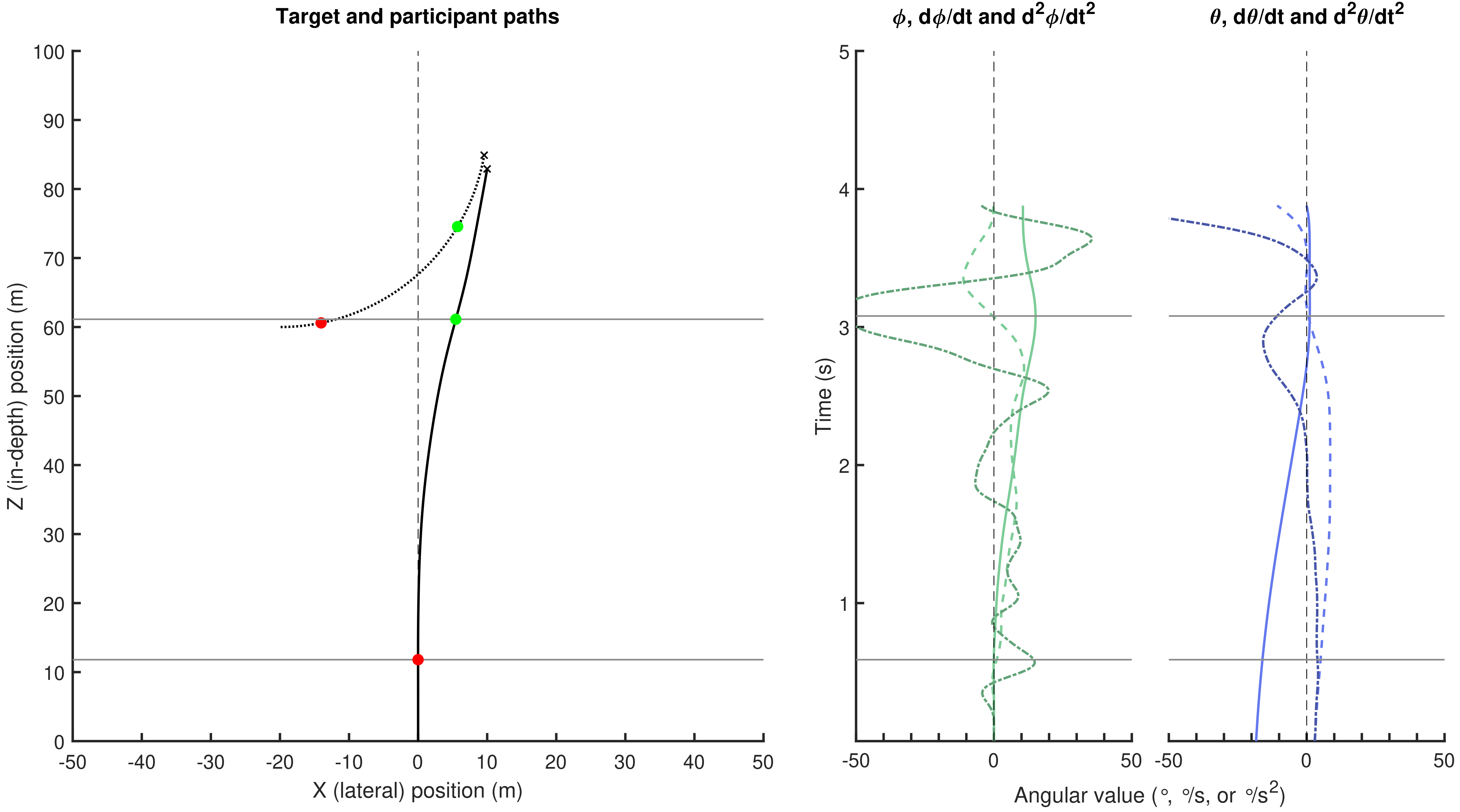

P18/B3
S15/R10-IN

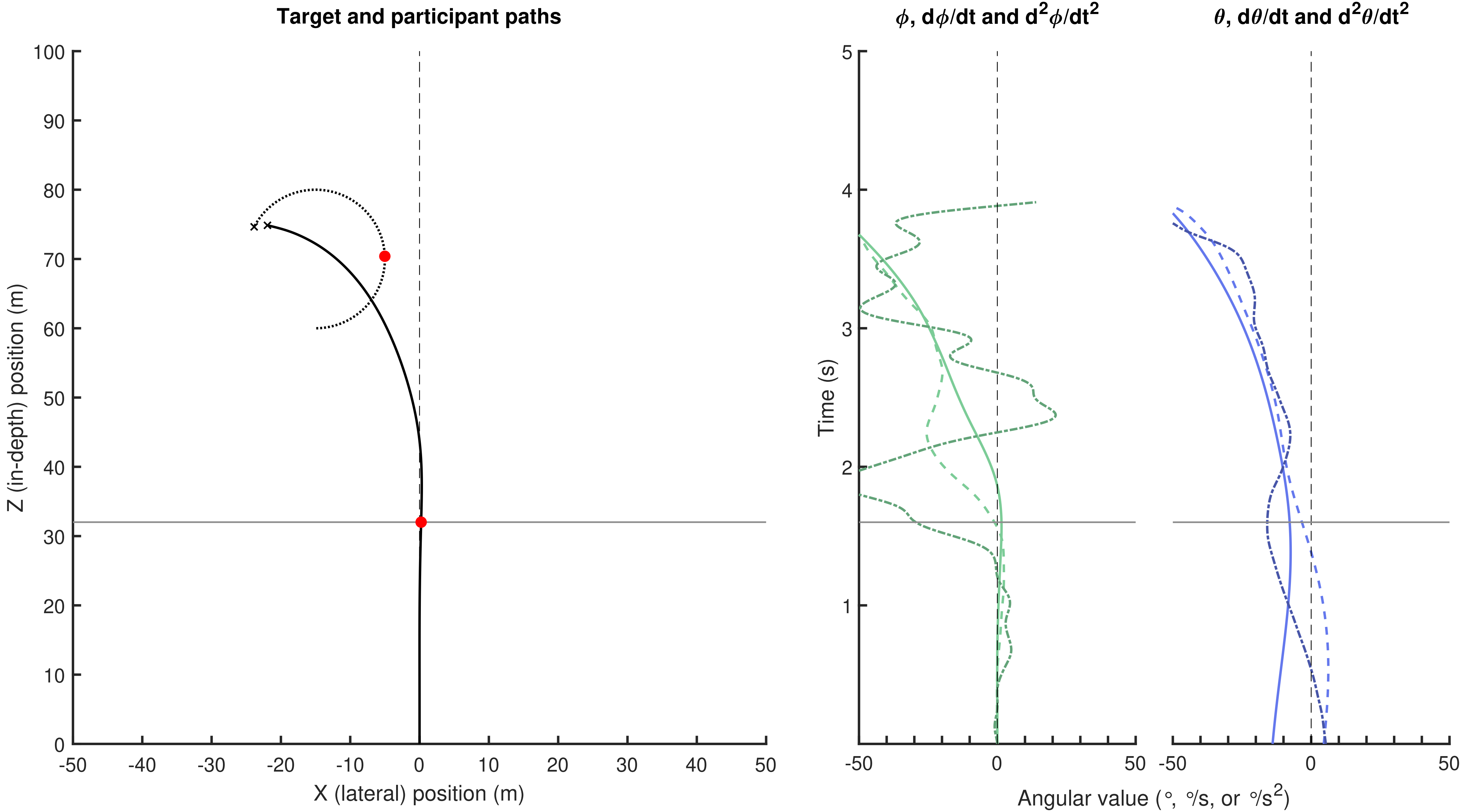

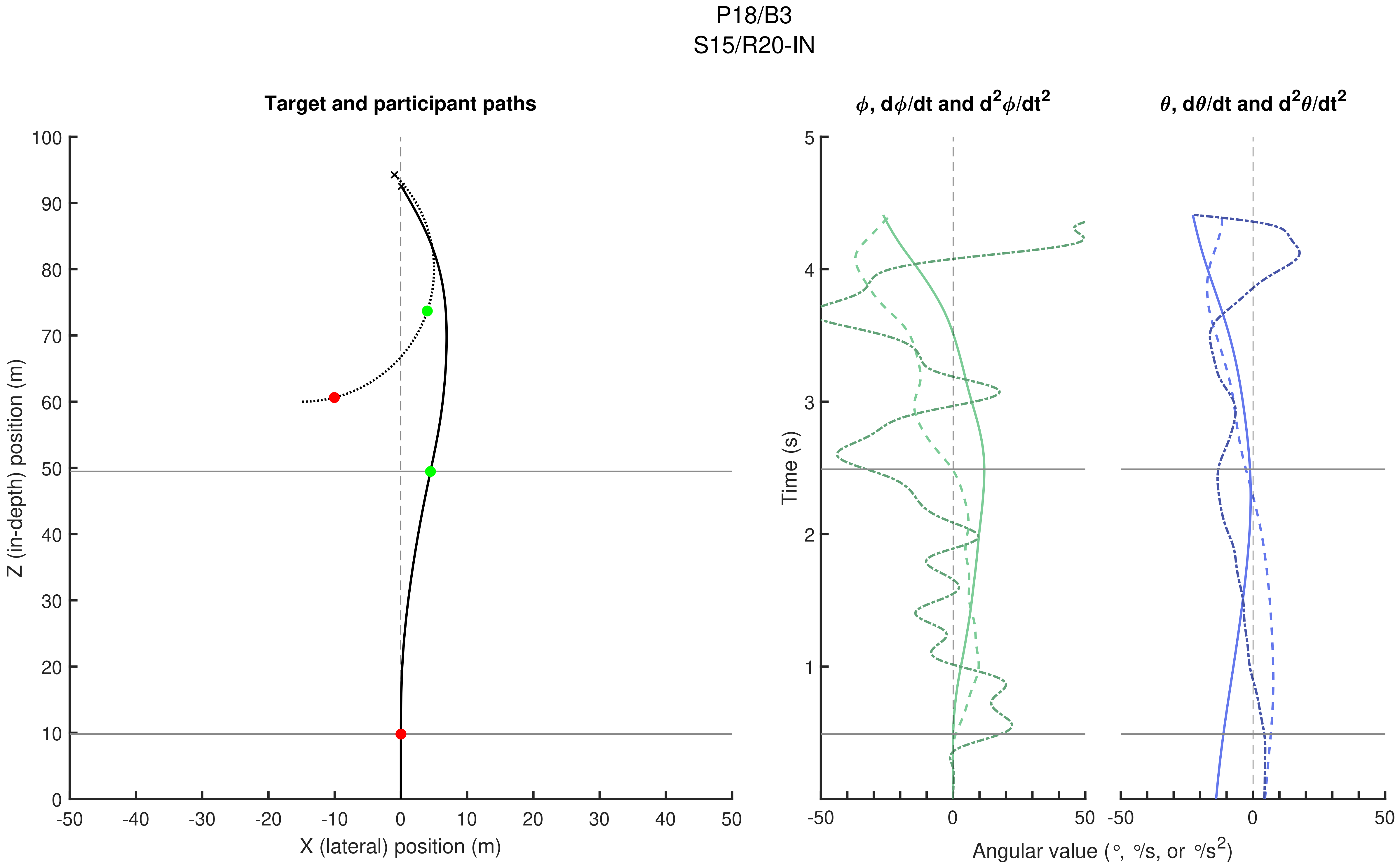

P18/B3
S15/R20-IN
Target and participant paths
Z (in-depth) position (m)
X (lateral) position (m)
ϕ, dϕ/dt and d$^2\phi$/dt^2
θ, dθ/dt and d$^2\theta$/dt^2
Time (s)
Angular value (°, °/s, or °/s^2)

P18/B3
S15/R30-IN

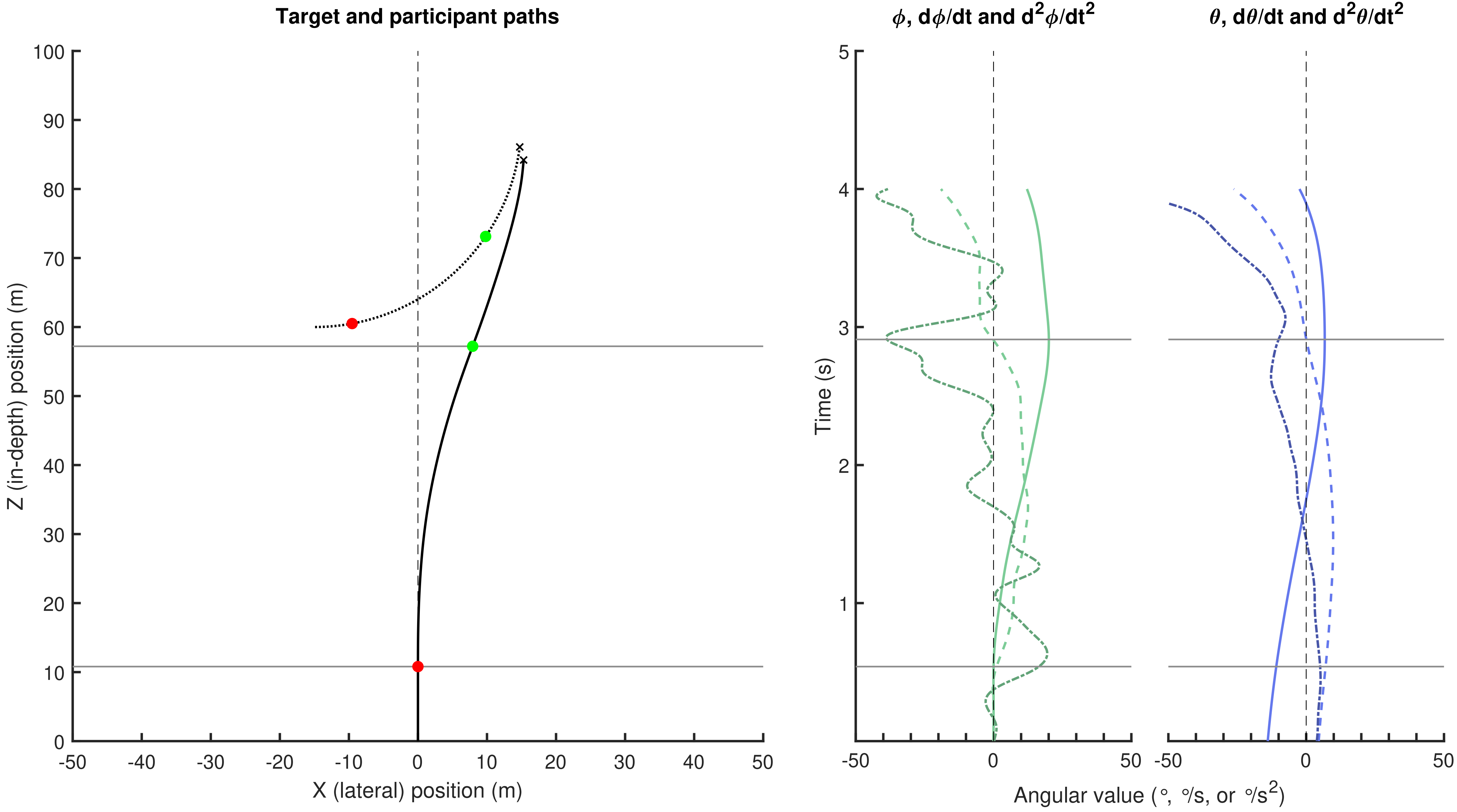

P18/B3
S5/R10-IN

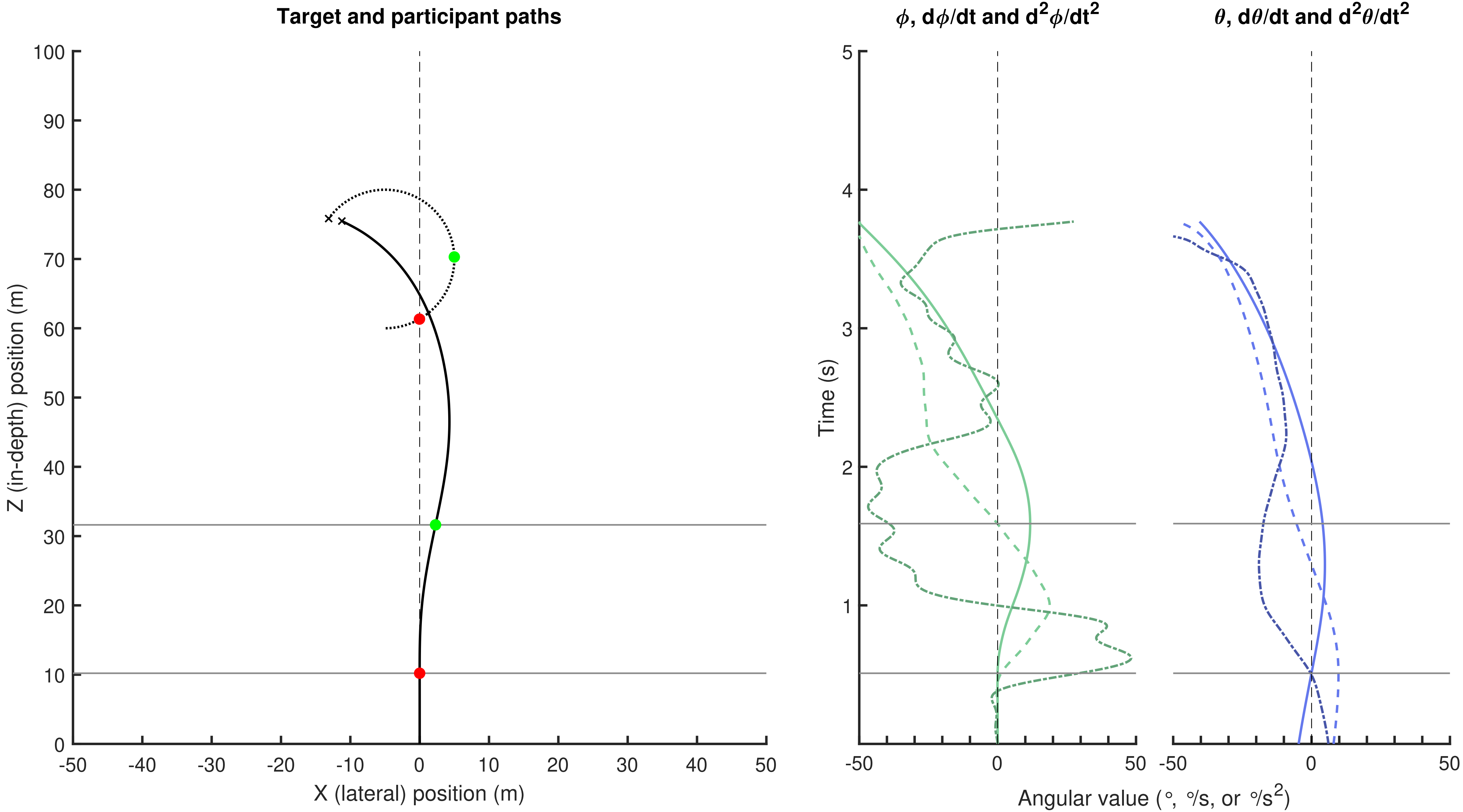

P18/B3
S5/R10-OUT

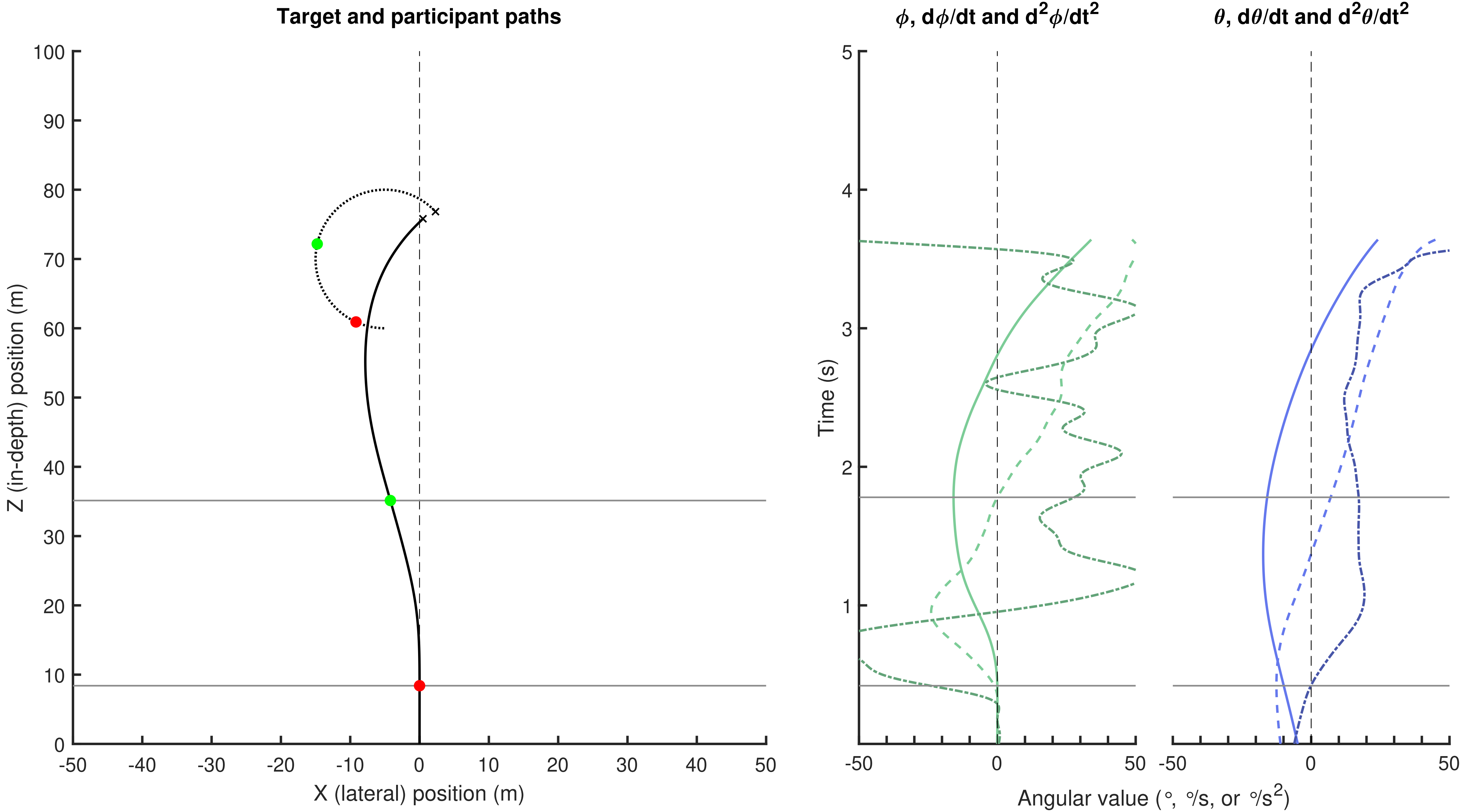

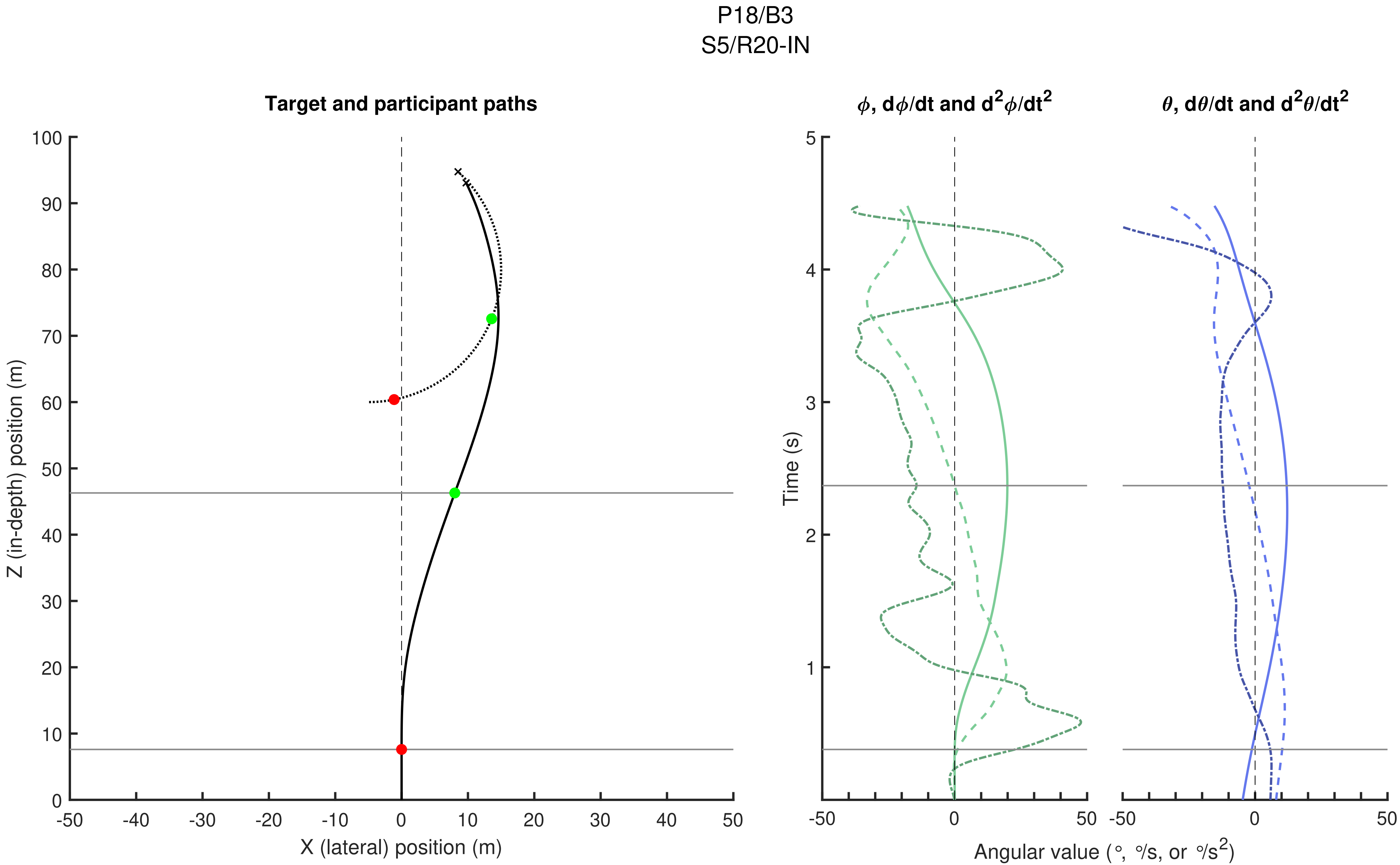
P18/B3
S5/R20-IN
Target and participant paths
Z (in-depth) position (m)
X (lateral) position (m)
100
90
80
70
60
50
40
30
20
10
0
-50
-40
-30
-20
-10
0
10
20
30
40
50
φ, dφ/dt and d²φ/dt²
θ, dθ/dt and d²θ/dt²
Time (s)
5
4
3
2
1
-50
0
50
-50
0
50
Angular value (°, °/s, or °/s²)

P18/B3
S5/R20-OUT

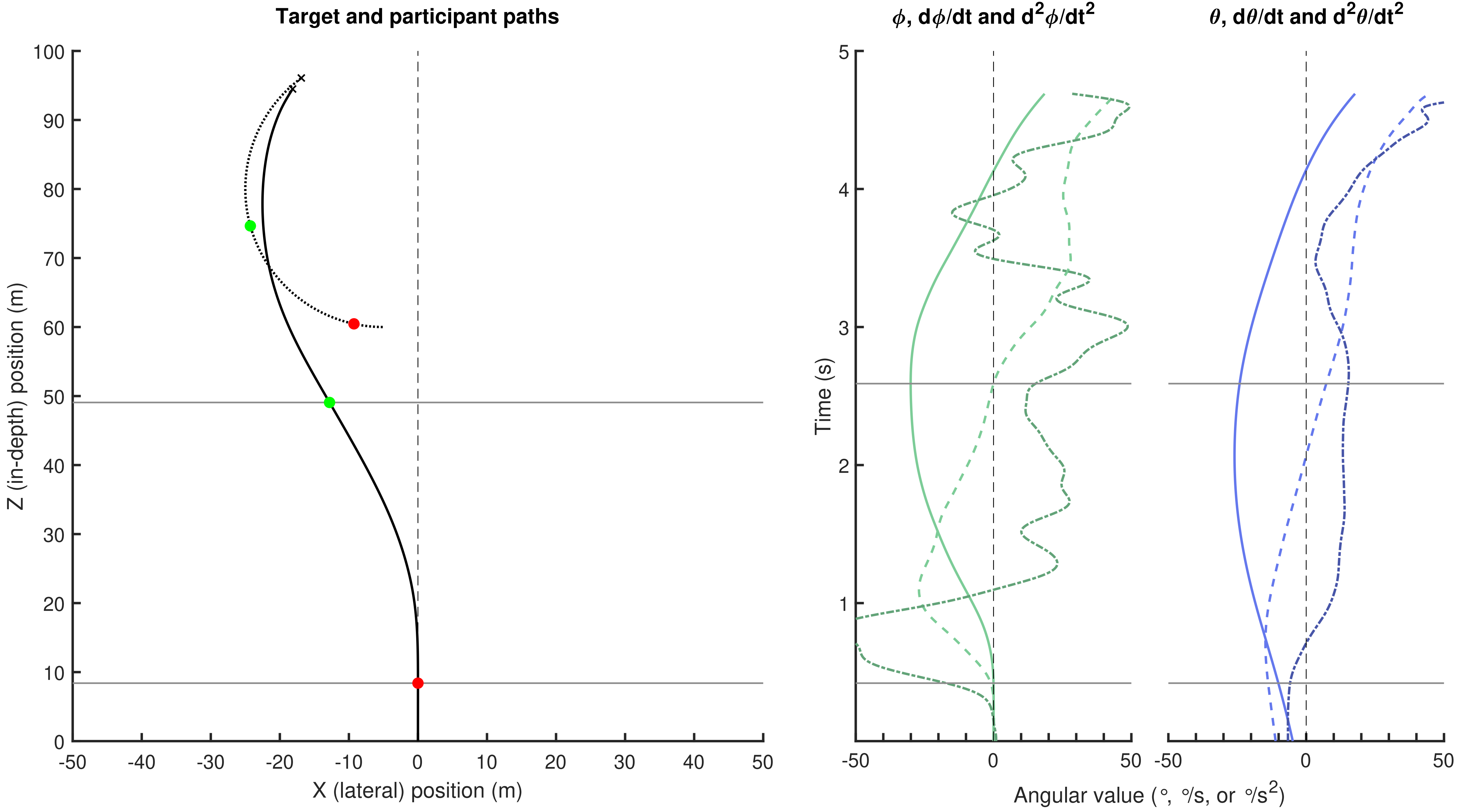

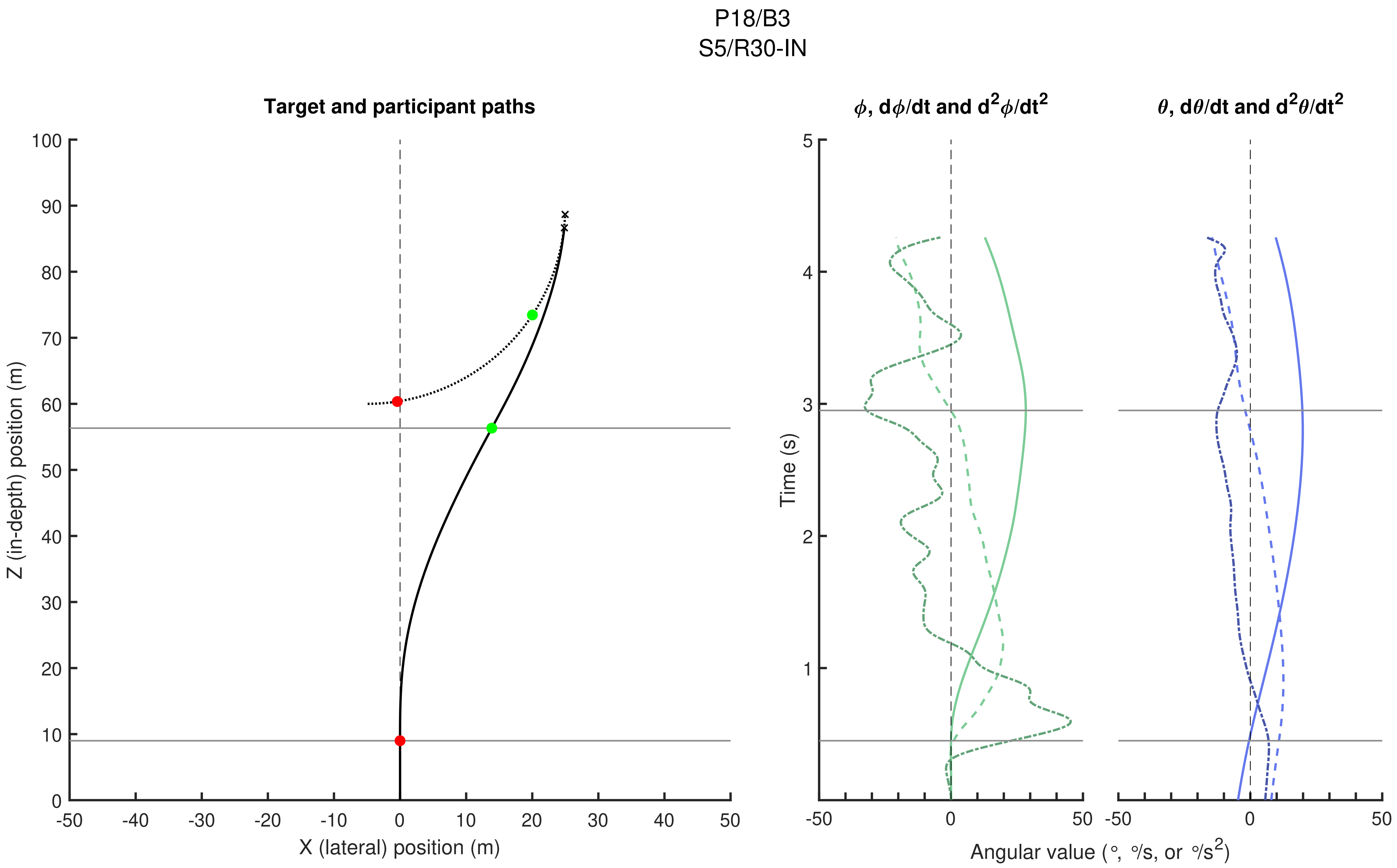
P18/B3
S5/R30-IN
Target and participant paths
Z (in-depth) position (m)
X (lateral) position (m)
φ, dφ/dt and d²φ/dt²
θ, dθ/dt and d²θ/dt²
Time (s)
Angular value (°, °/s, or °/s²)

P18/B3
S5/R30-OUT

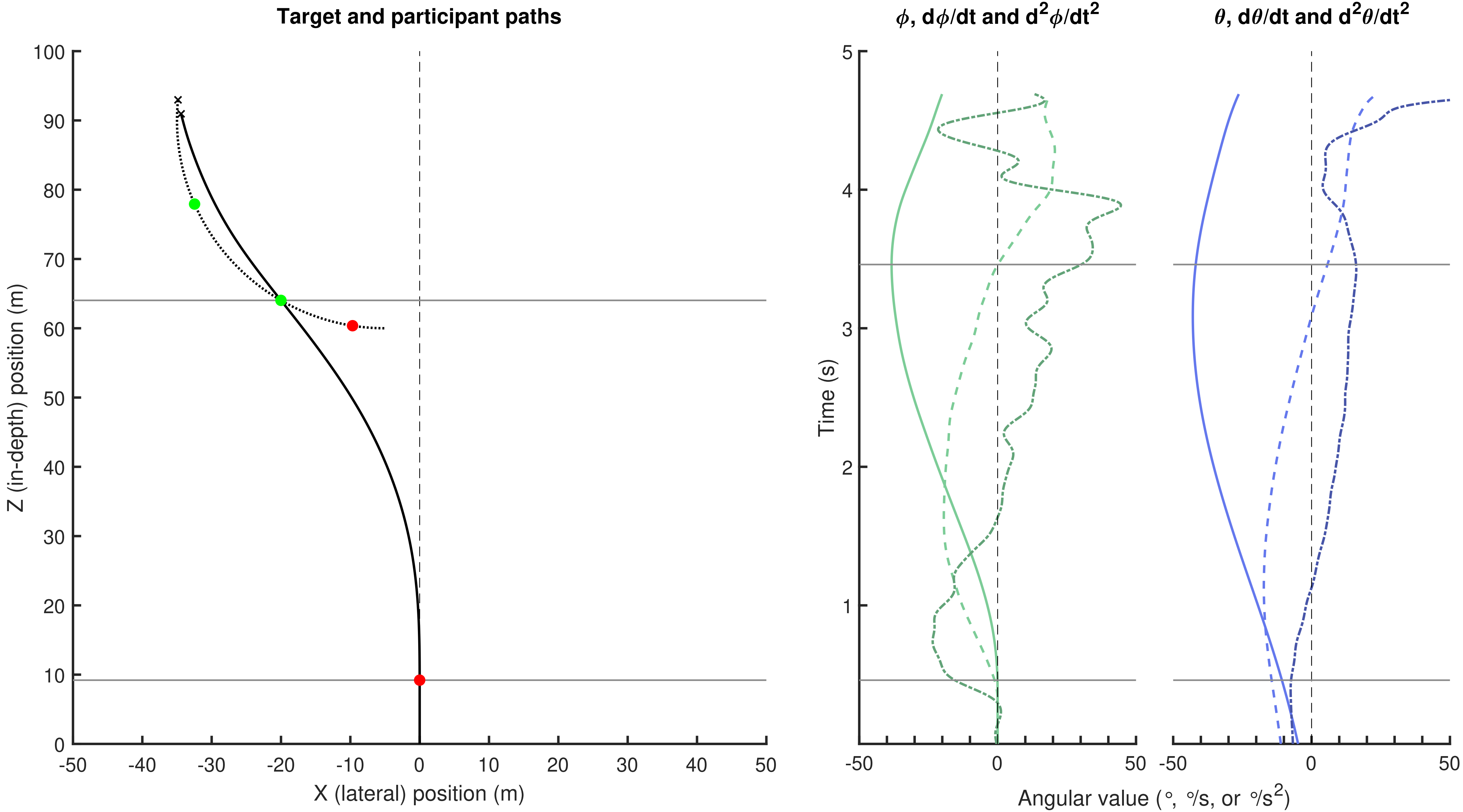

P18/B3
S5/R10-OUT

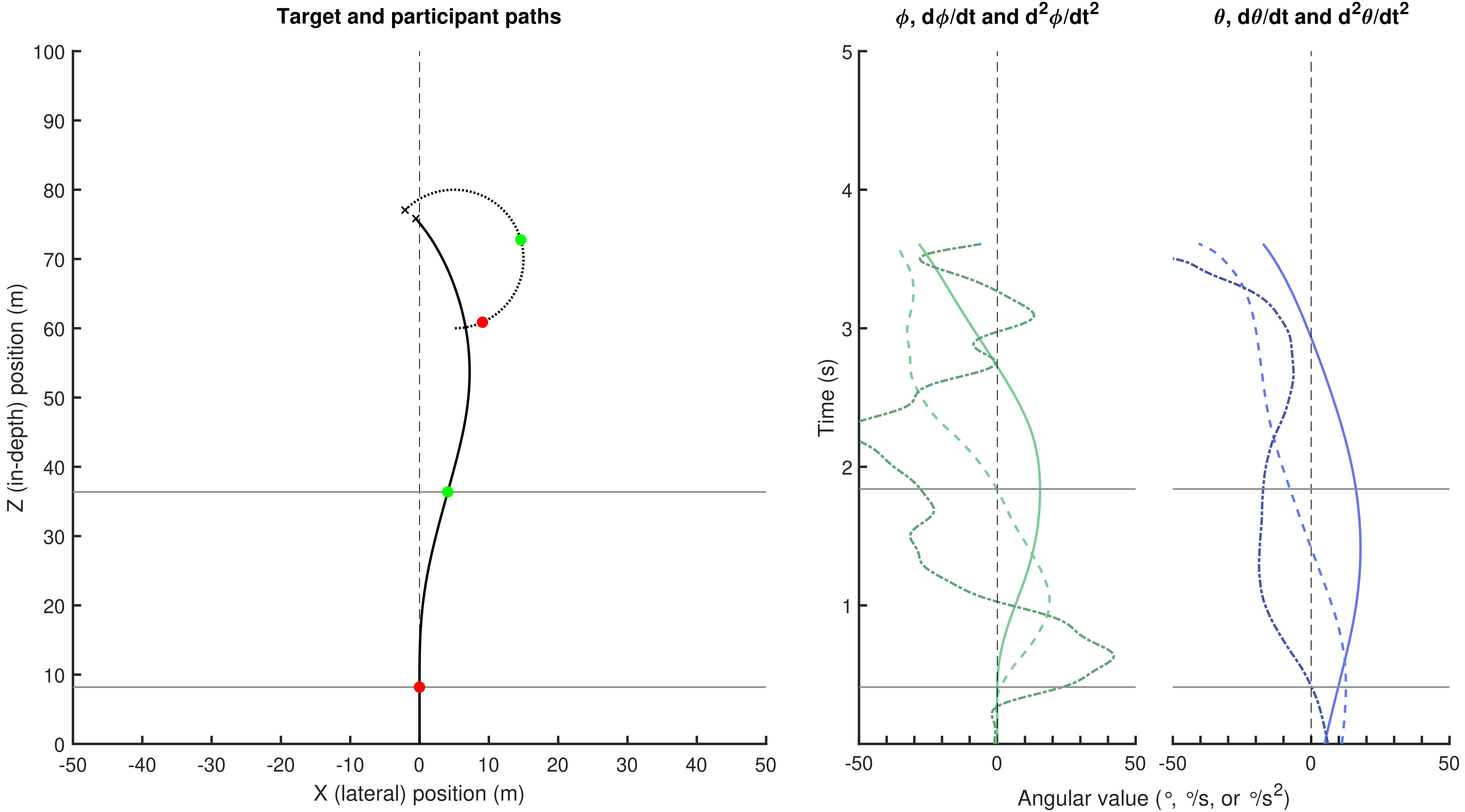

P18/B3
S5/R10-IN

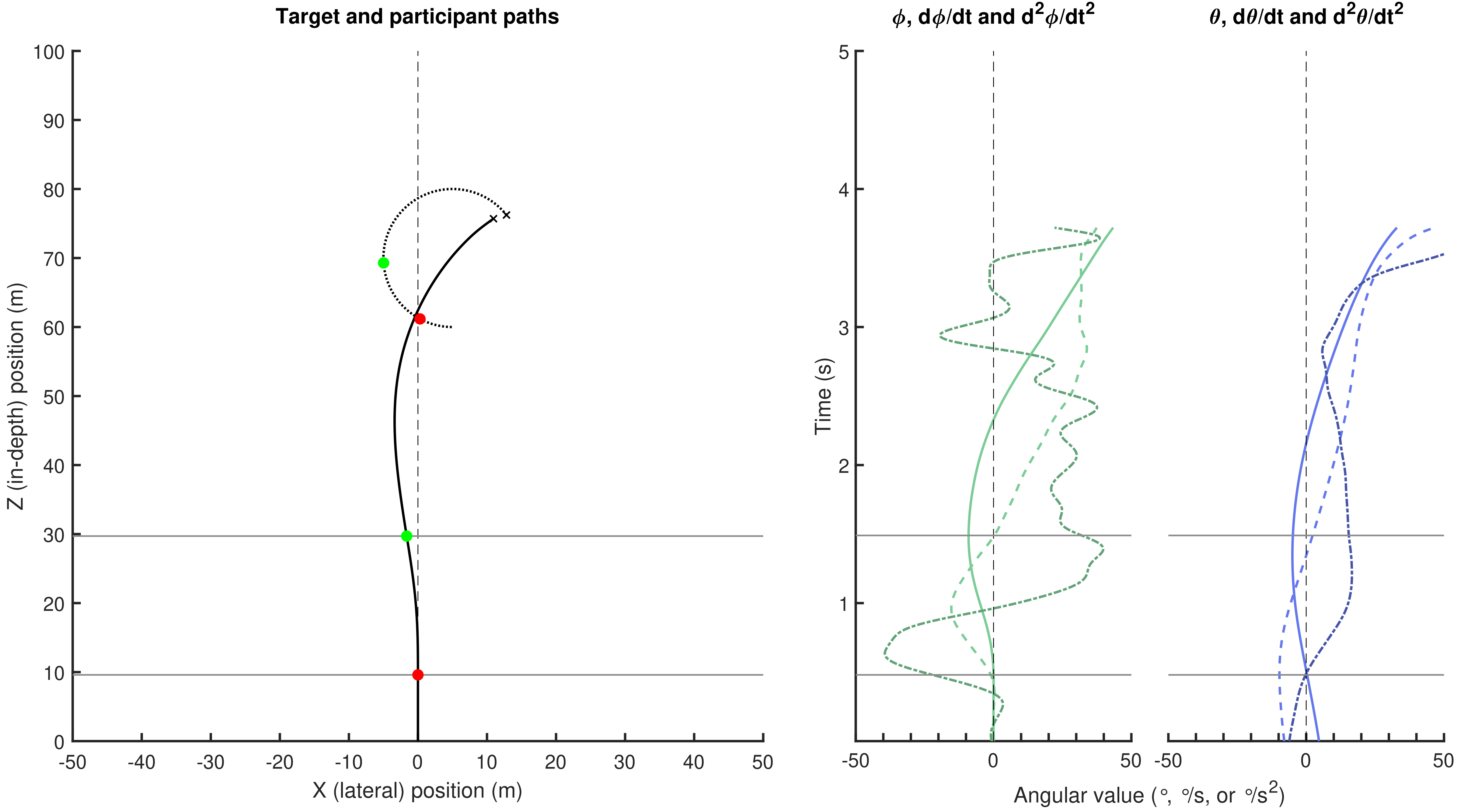

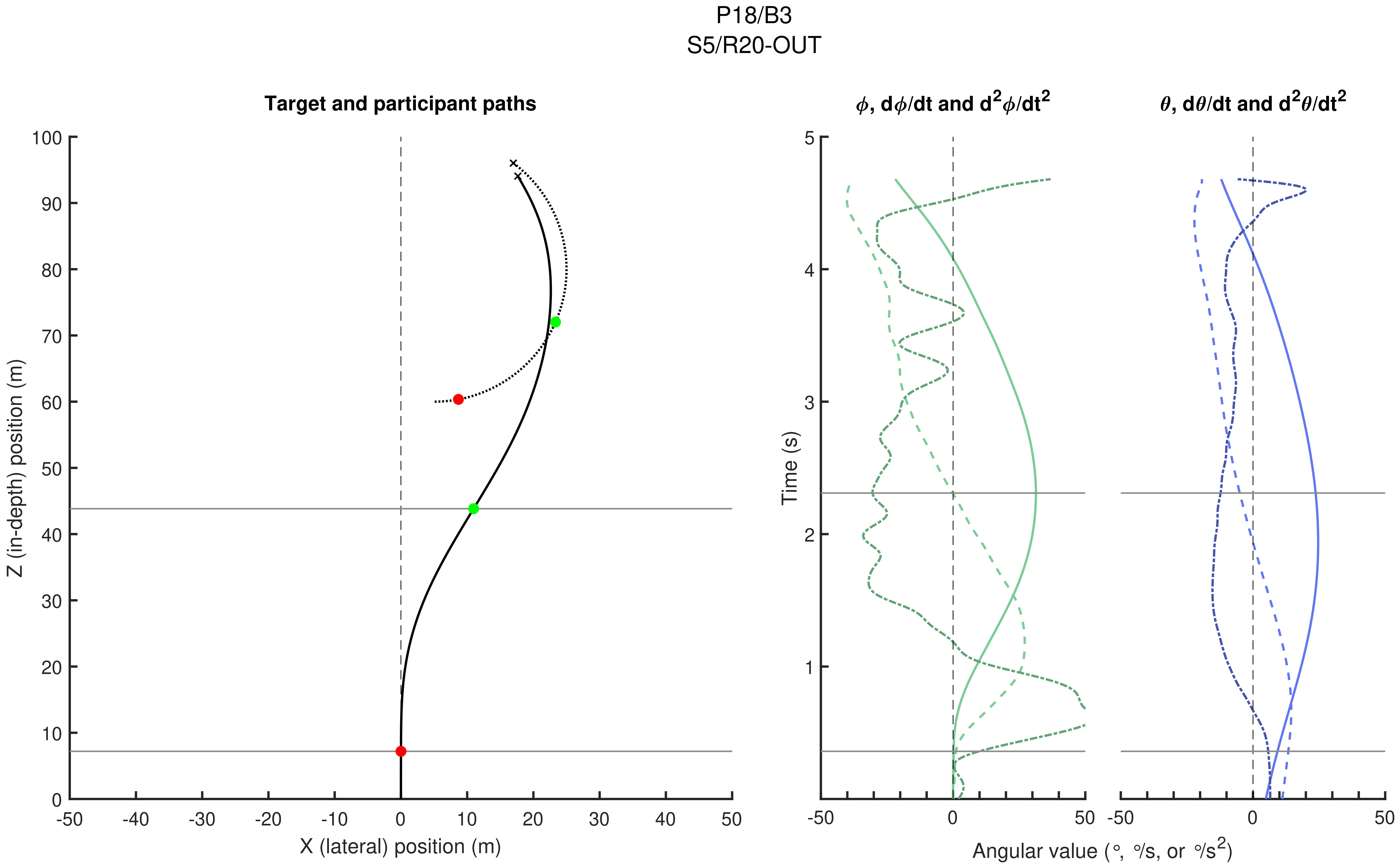

P18/B3
S5/R20-OUT
Target and participant paths
Z (in-depth) position (m)
X (lateral) position (m)
100
90
80
70
60
50
40
30
20
10
0
-50
-40
-30
-20
-10
0
10
20
30
40
50
ϕ, dϕ/dt and d²ϕ/dt²
θ, dθ/dt and d²θ/dt²
Time (s)
5
4
3
2
1
-50
0
50
-50
0
50
Angular value (°, °/s, or °/s²)

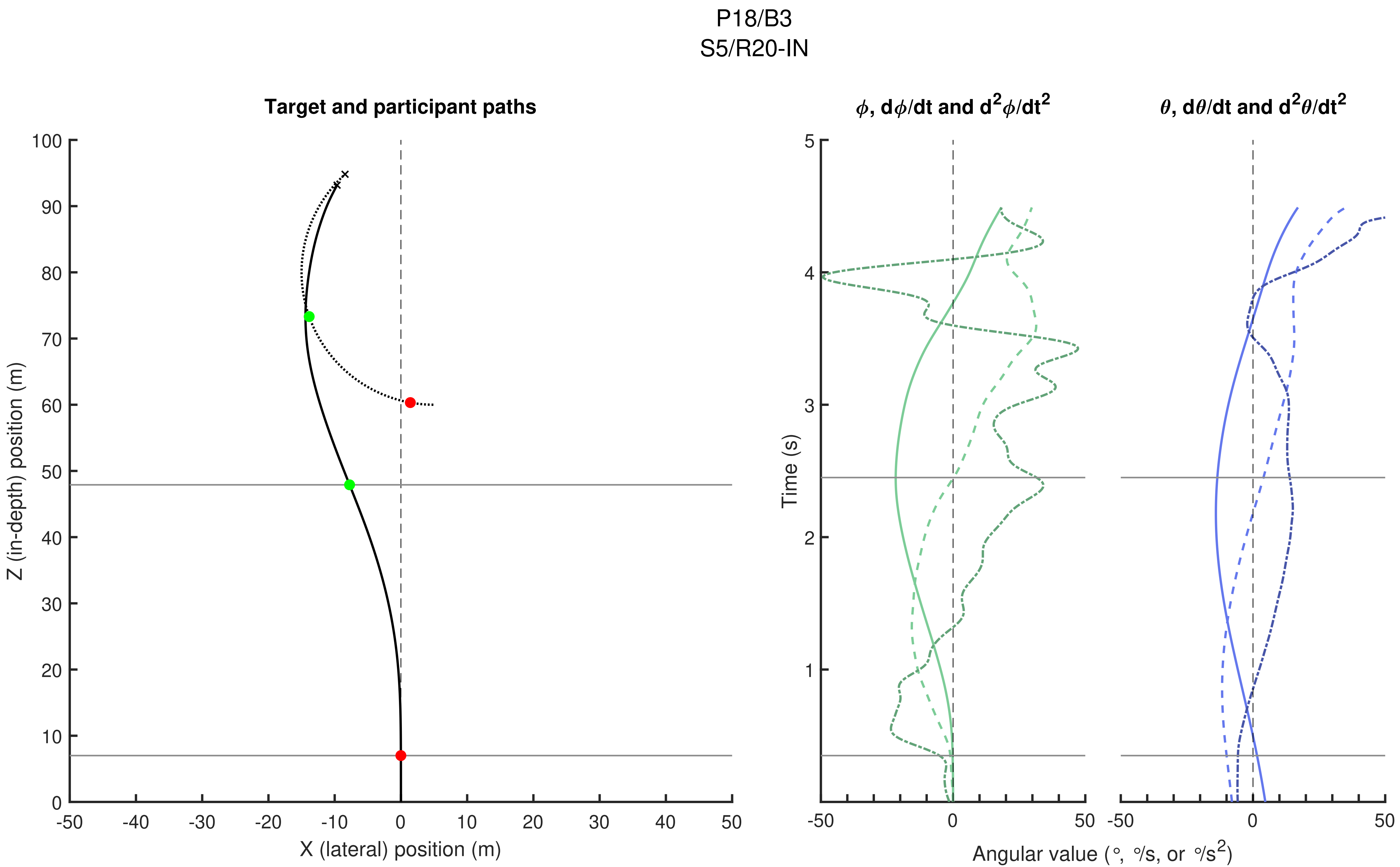

P18/B3
S5/R20-IN
Target and participant paths
Z (in-depth) position (m)
X (lateral) position (m)
ϕ, dϕ/dt and d²ϕ/dt²
θ, dθ/dt and d²θ/dt²
Time (s)
Angular value (°, °/s, or °/s²)

P18/B3
S5/R30-OUT

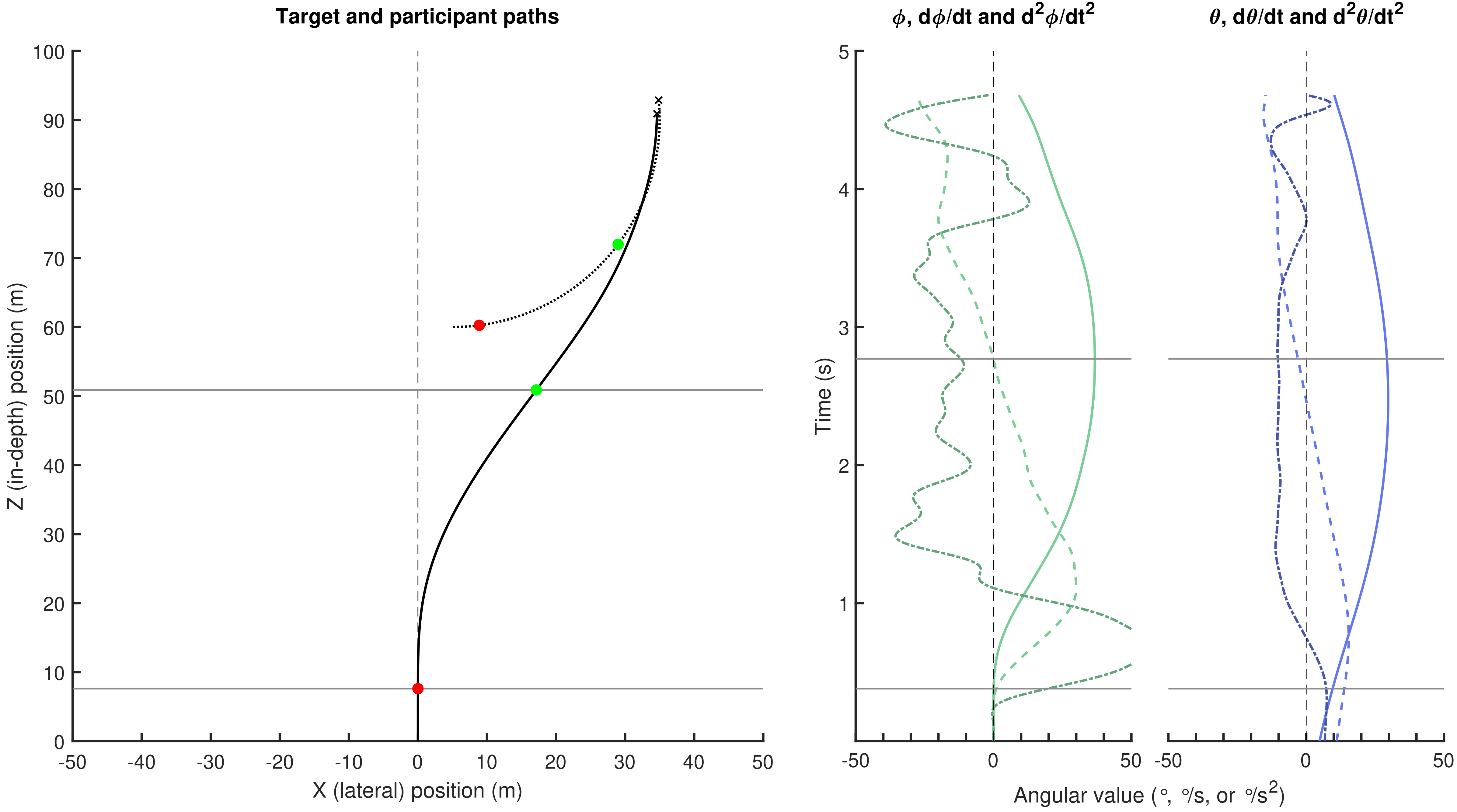

P18/B3
S5/R30-IN

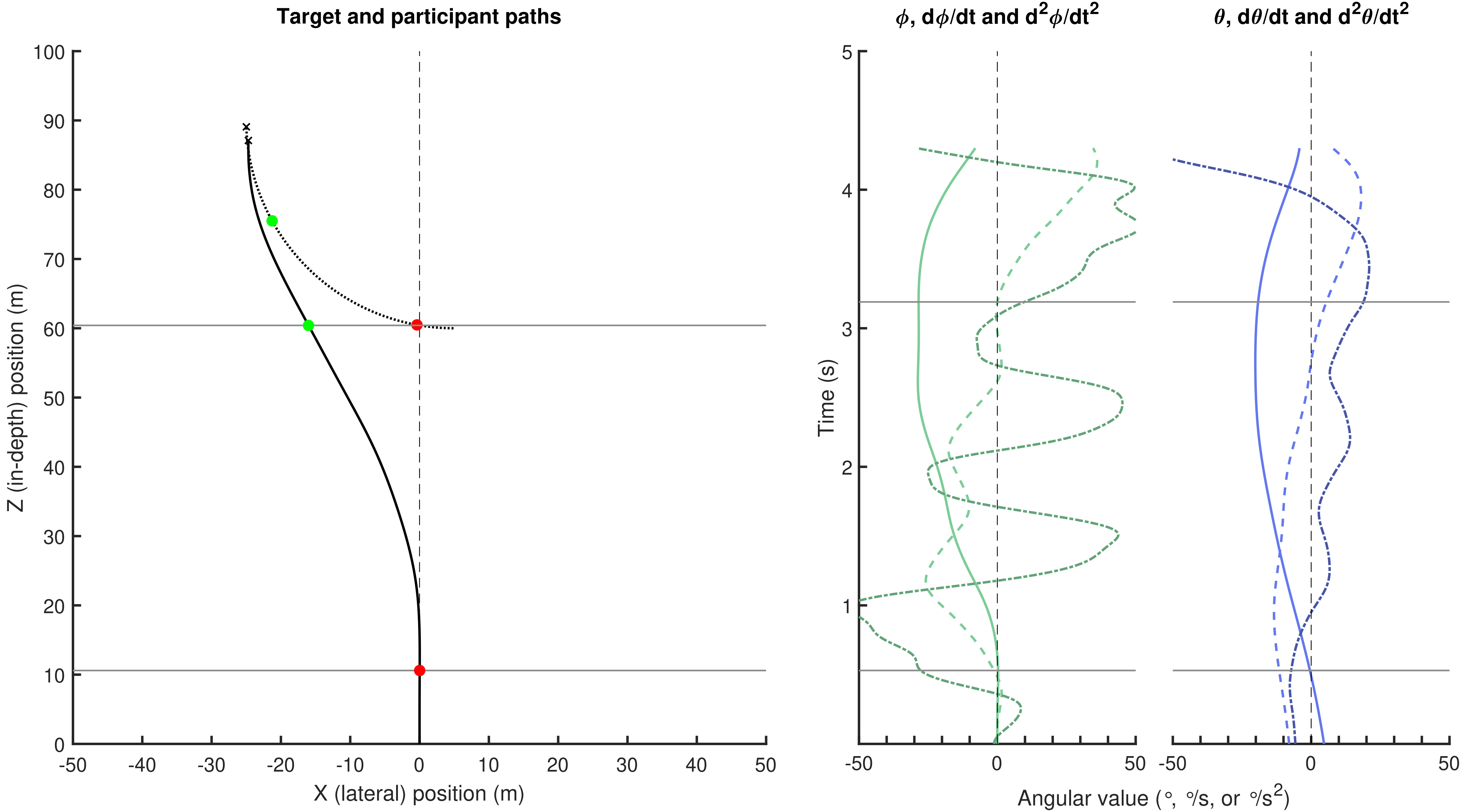

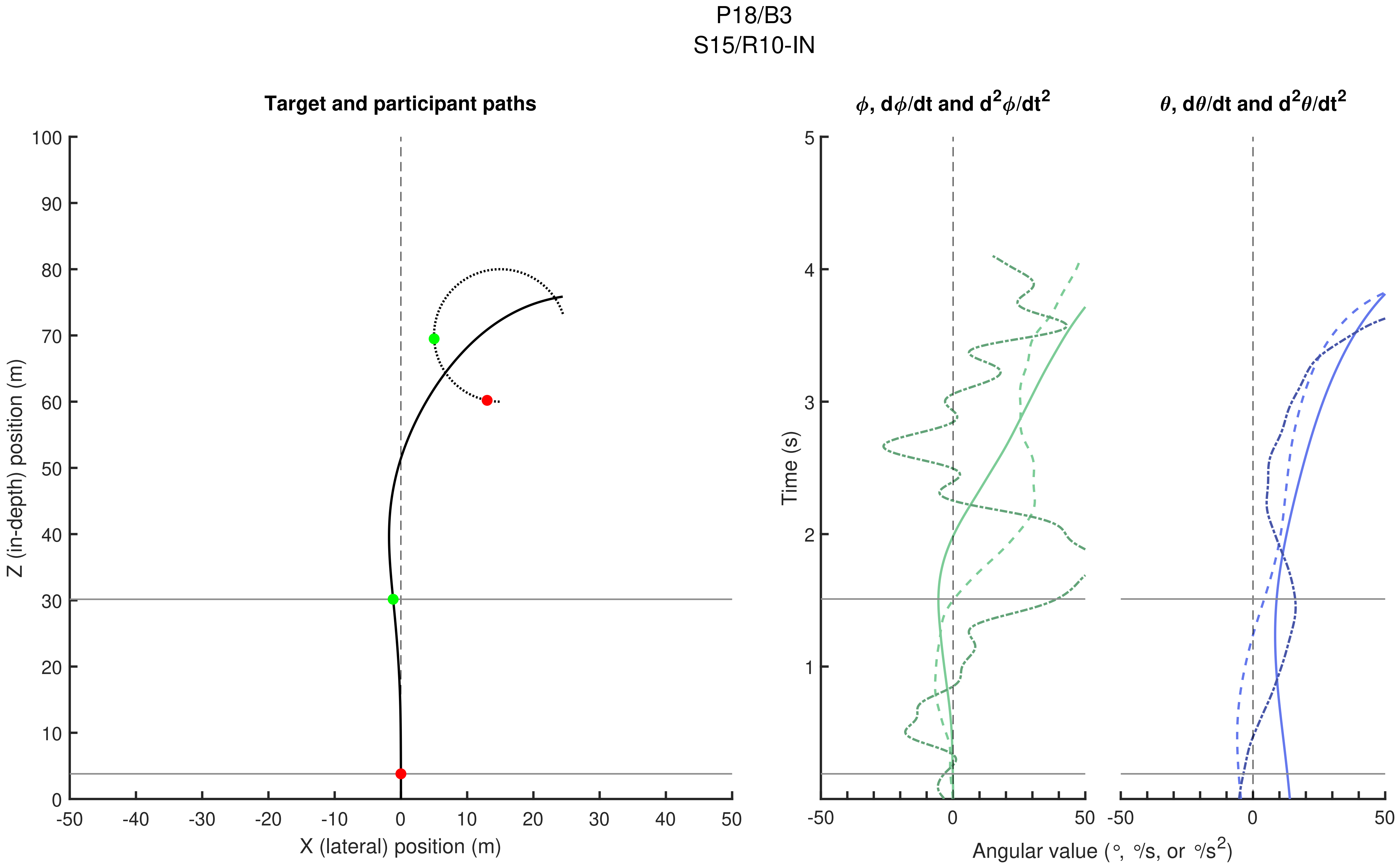

P18/B3
S15/R10-IN
Target and participant paths
Z (in-depth) position (m)
X (lateral) position (m)
100
90
80
70
60
50
40
30
20
10
0
-50
-40
-30
-20
-10
0
10
20
30
40
50
ϕ, dϕ/dt and d$^2\phi$/dt^2
θ, dθ/dt and d$^2\theta$/dt^2
Time (s)
5
4
3
2
1
-50
0
50
-50
0
50
Angular value (°, °/s, or °/s^2)

P18/B3
S15/R20-IN

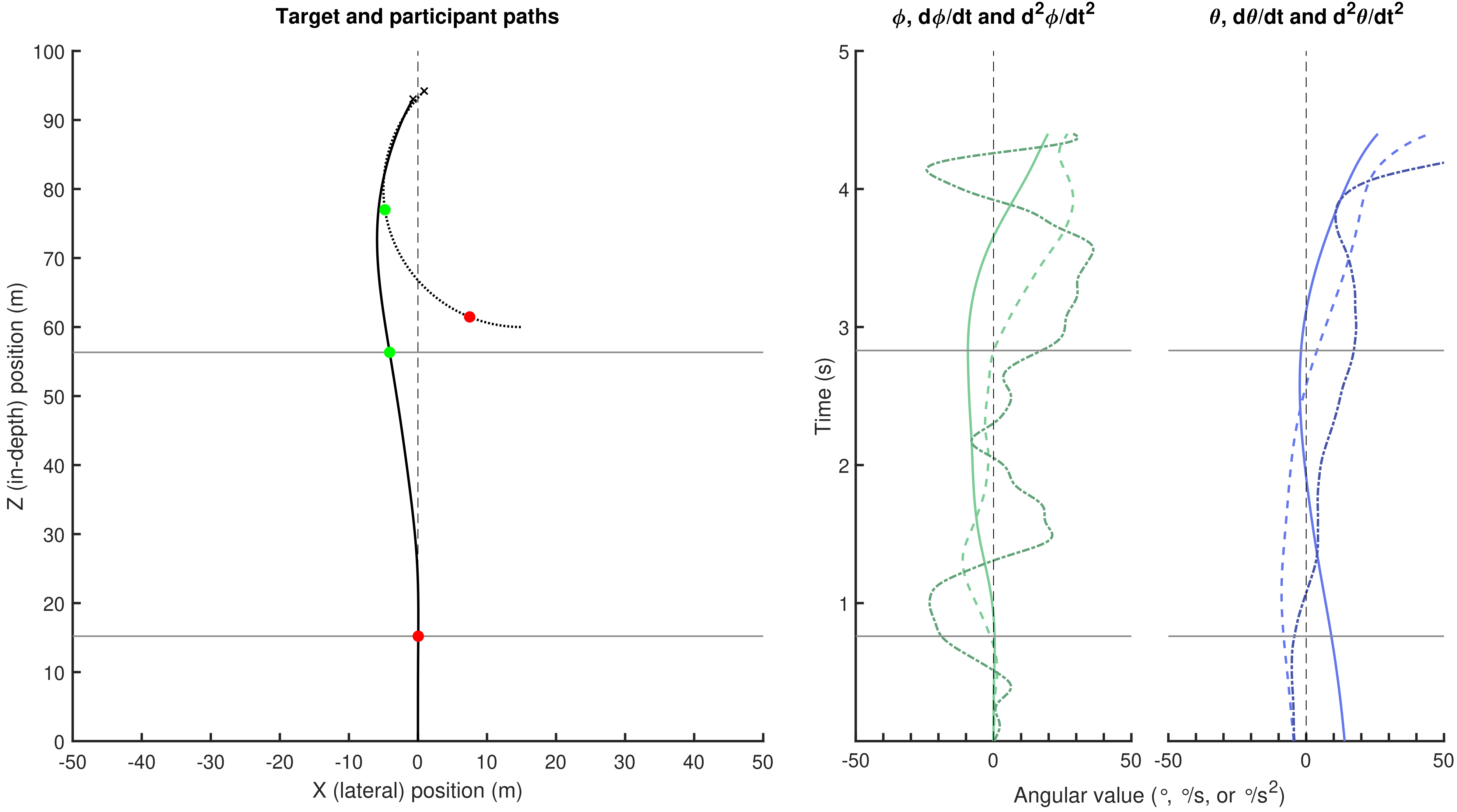

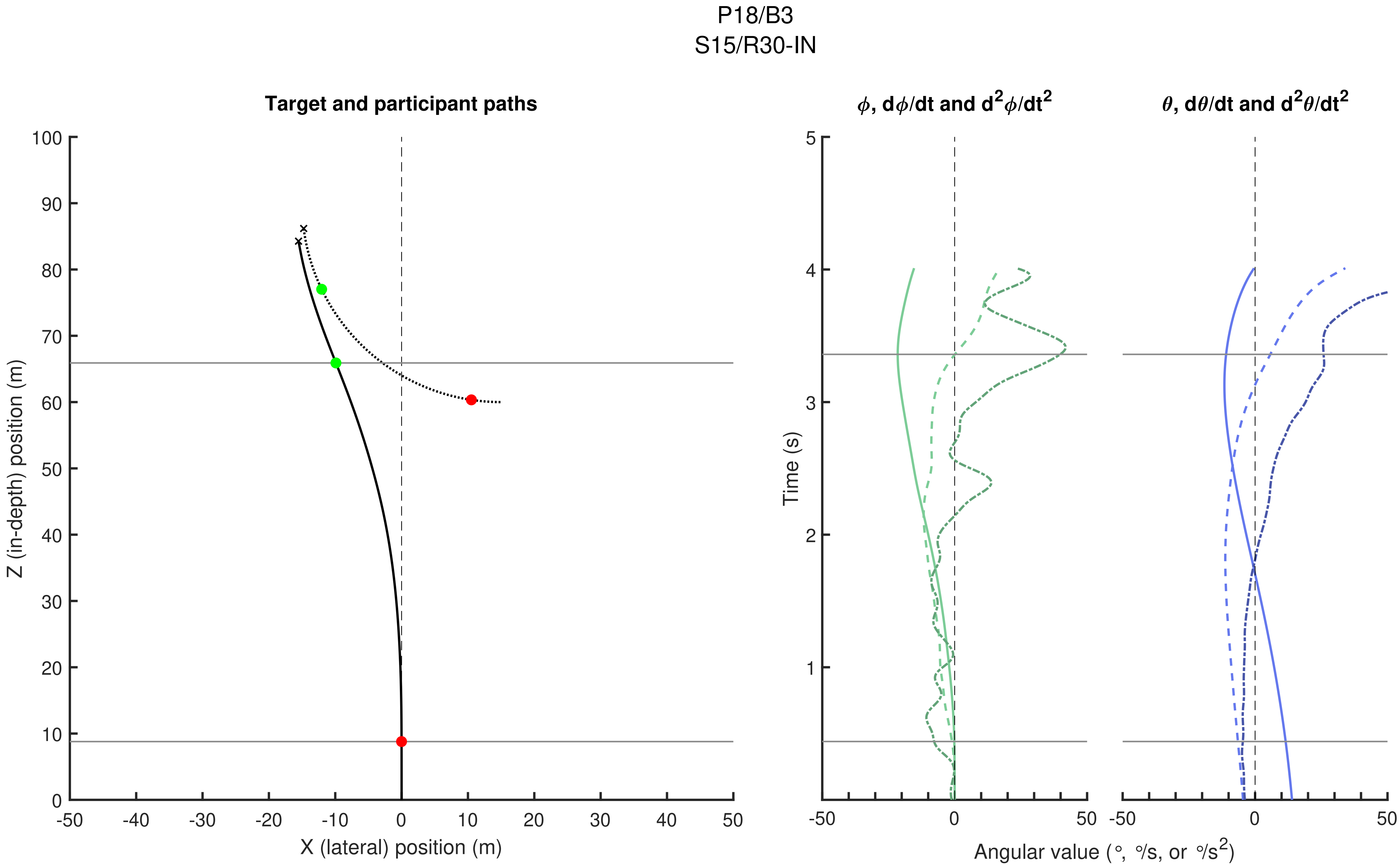
P18/B3
S15/R30-IN
Target and participant paths
Z (in-depth) position (m)
X (lateral) position (m)
0
10
20
30
40
50
60
70
80
90
100
-50
-40
-30
-20
-10
0
10
20
30
40
50
ϕ, dϕ/dt and d$^2\phi$/dt^2
θ, dθ/dt and d$^2\theta$/dt^2
Time (s)
1
2
3
4
5
-50
0
50
-50
0
50
Angular value (°, °/s, or °/s^2)

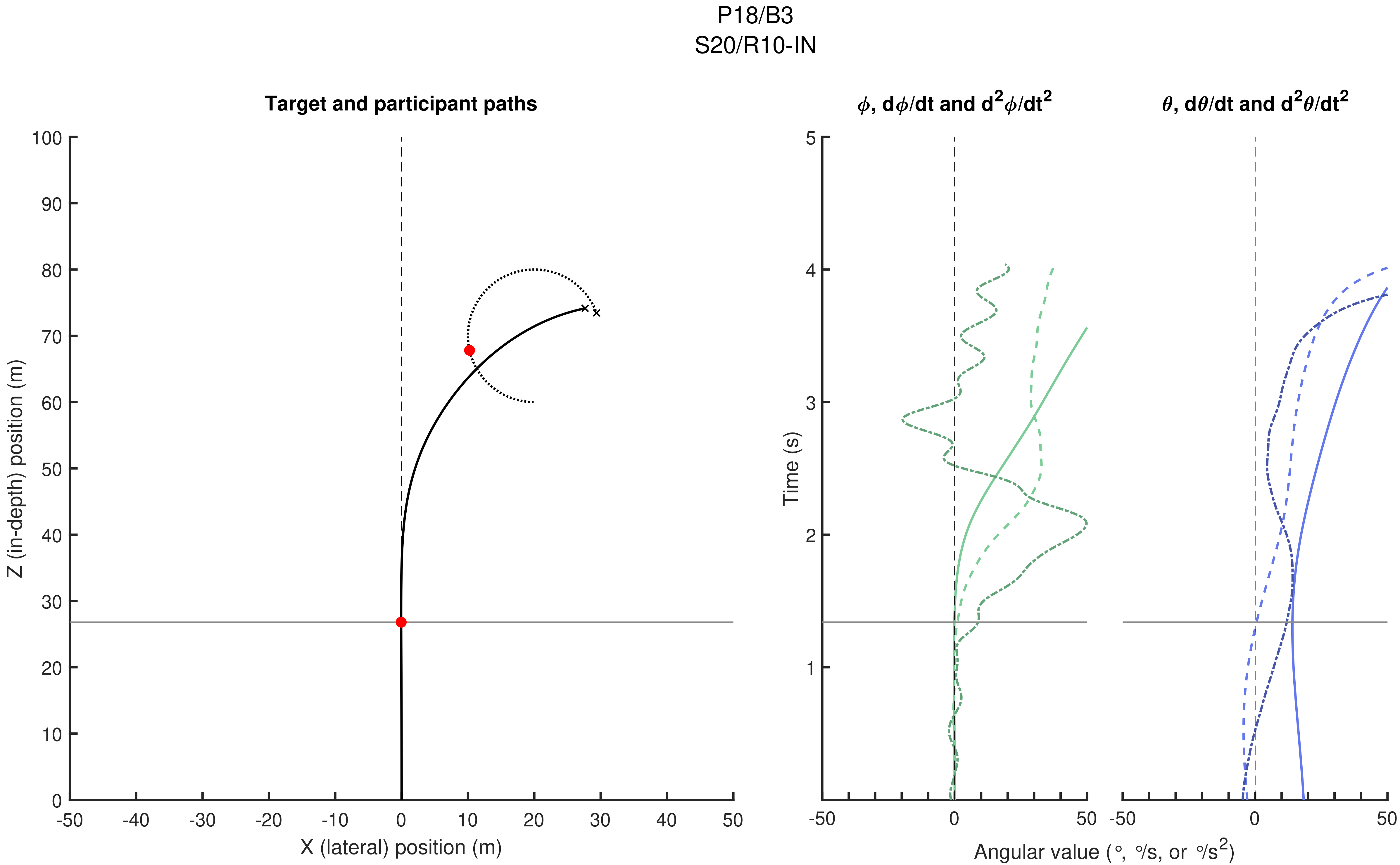
P18/B3
S20/R10-IN
Target and participant paths
ϕ, dϕ/dt and d$^2\phi$/dt^2
θ, dθ/dt and d$^2\theta$/dt^2
Z (in-depth) position (m)
X (lateral) position (m)
Time (s)
Angular value (°, °/s, or °/s^2)
100
90
80
70
60
50
40
30
20
10
0
-50
-40
-30
-20
-10
0
10
20
30
40
50
5
4
3
2
1
-50
0
50
-50
0
50

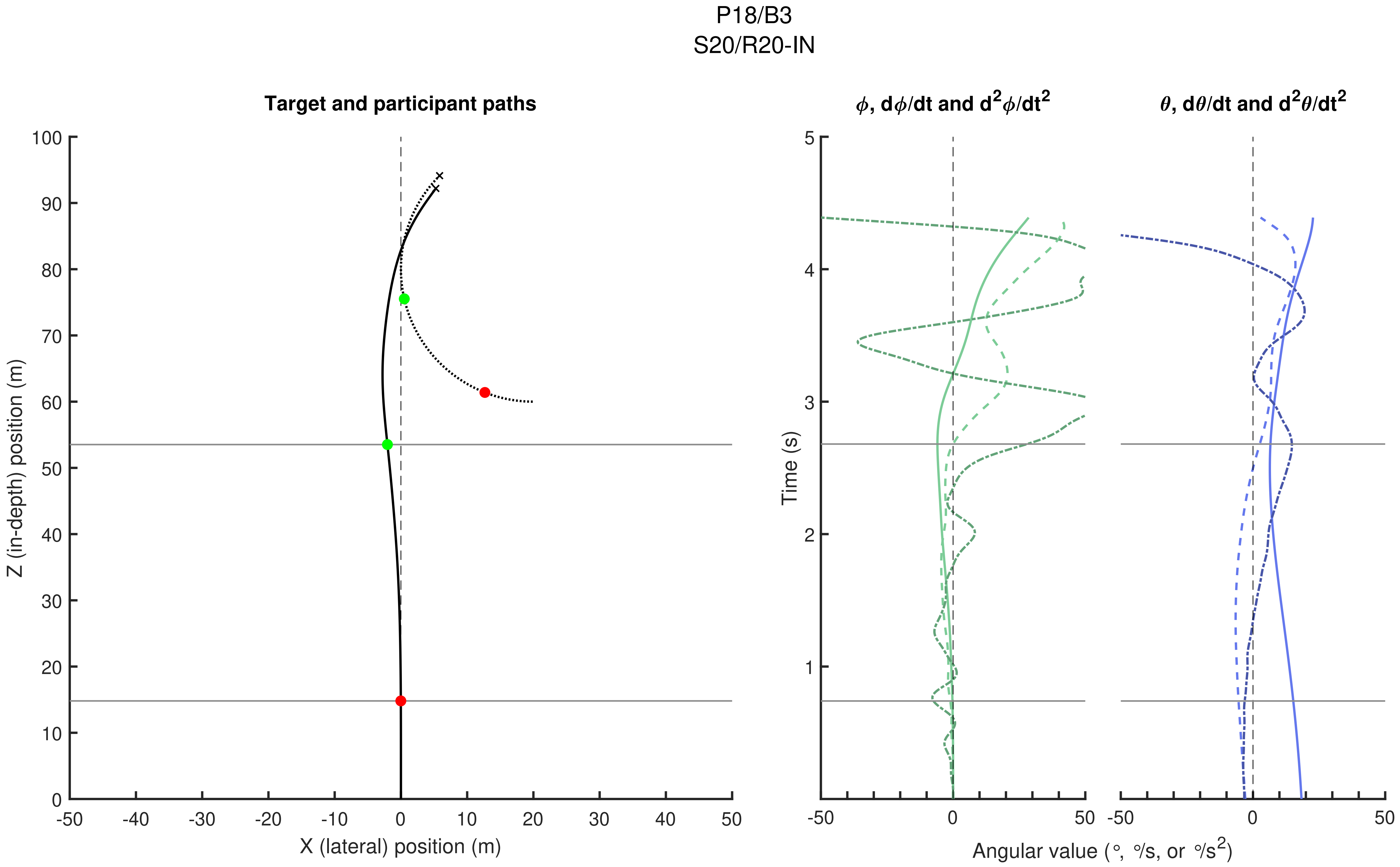
P18/B3
S20/R20-IN
Target and participant paths
Z (in-depth) position (m)
X (lateral) position (m)
φ, dφ/dt and d²φ/dt²
θ, dθ/dt and d²θ/dt²
Time (s)
Angular value (°, °/s, or °/s²)

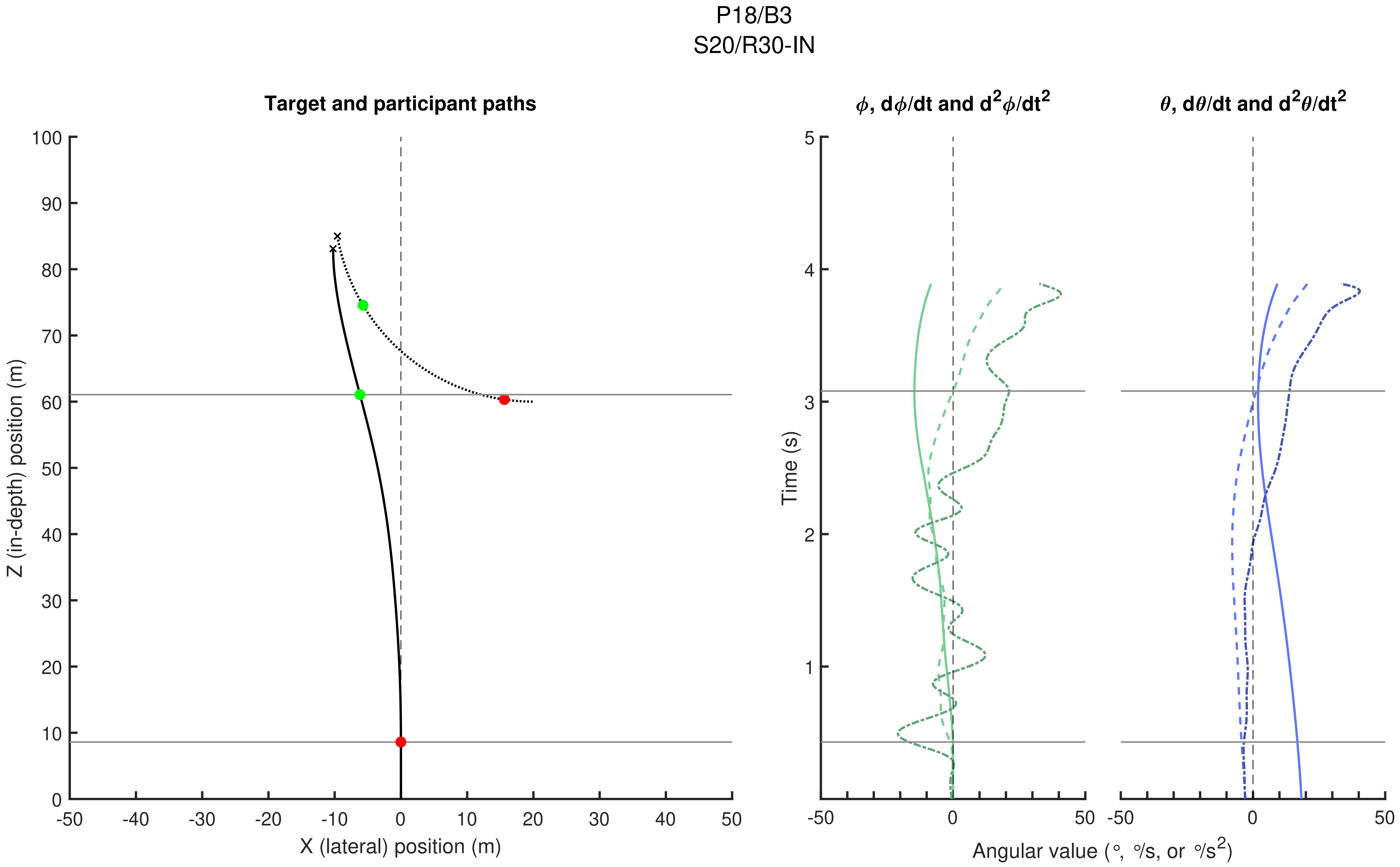
P18/B3
S20/R30-IN
Target and participant paths
Z (in-depth) position (m)
X (lateral) position (m)
ϕ, dϕ/dt and d$^2\phi$/dt^2
θ, dθ/dt and d$^2\theta$/dt^2
Time (s)
Angular value (°, °/s, or °/s^2)
100
90
80
70
60
50
40
30
20
10
0
-50
-40
-30
-20
-10
0
10
20
30
40
50
5
4
3
2
1
-50
0
50
-50
0
50

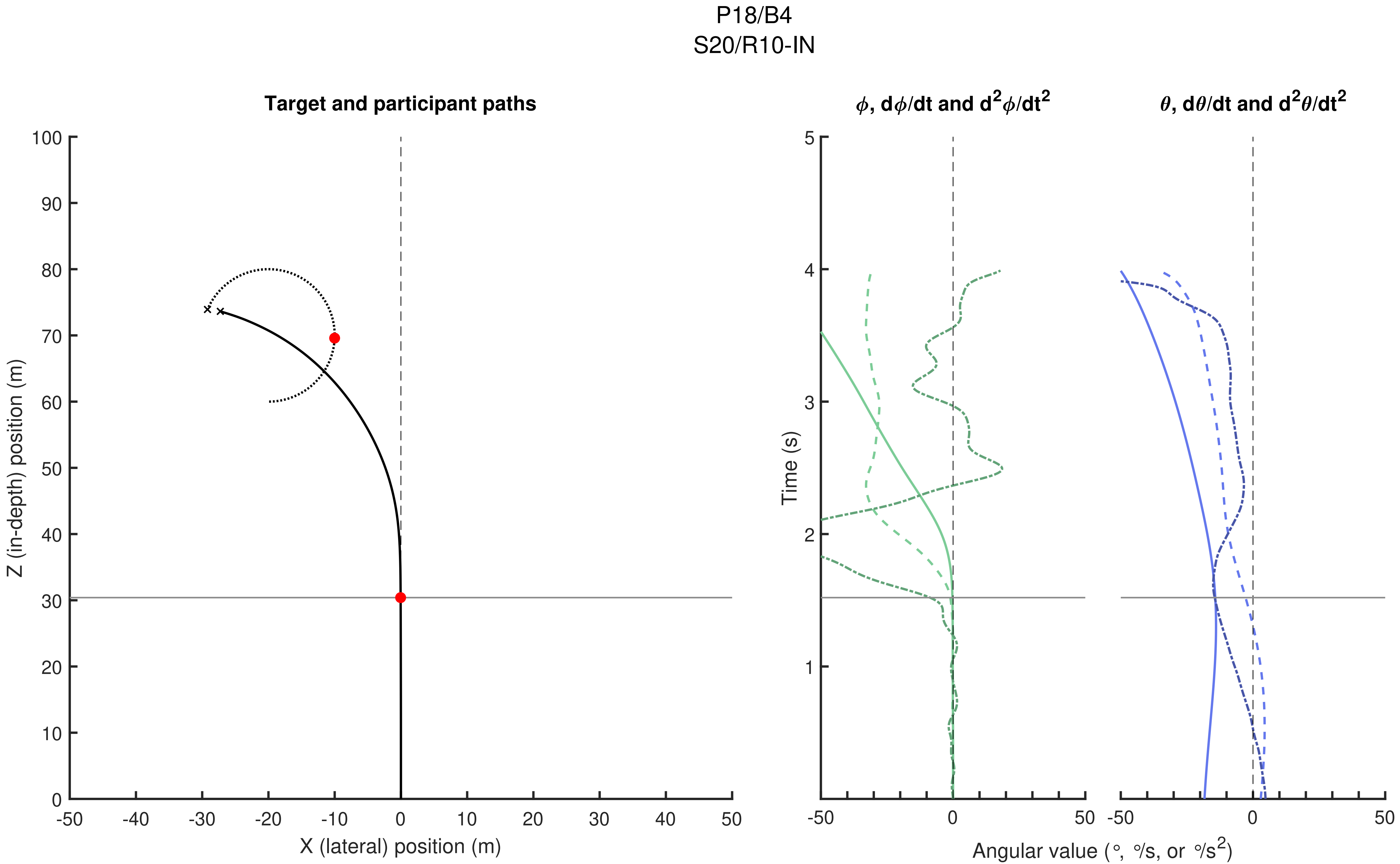
P18/B4
S20/R10-IN
Target and participant paths
Z (in-depth) position (m)
X (lateral) position (m)
φ, dφ/dt and d²φ/dt²
θ, dθ/dt and d²θ/dt²
Time (s)
Angular value (°, °/s, or °/s²)

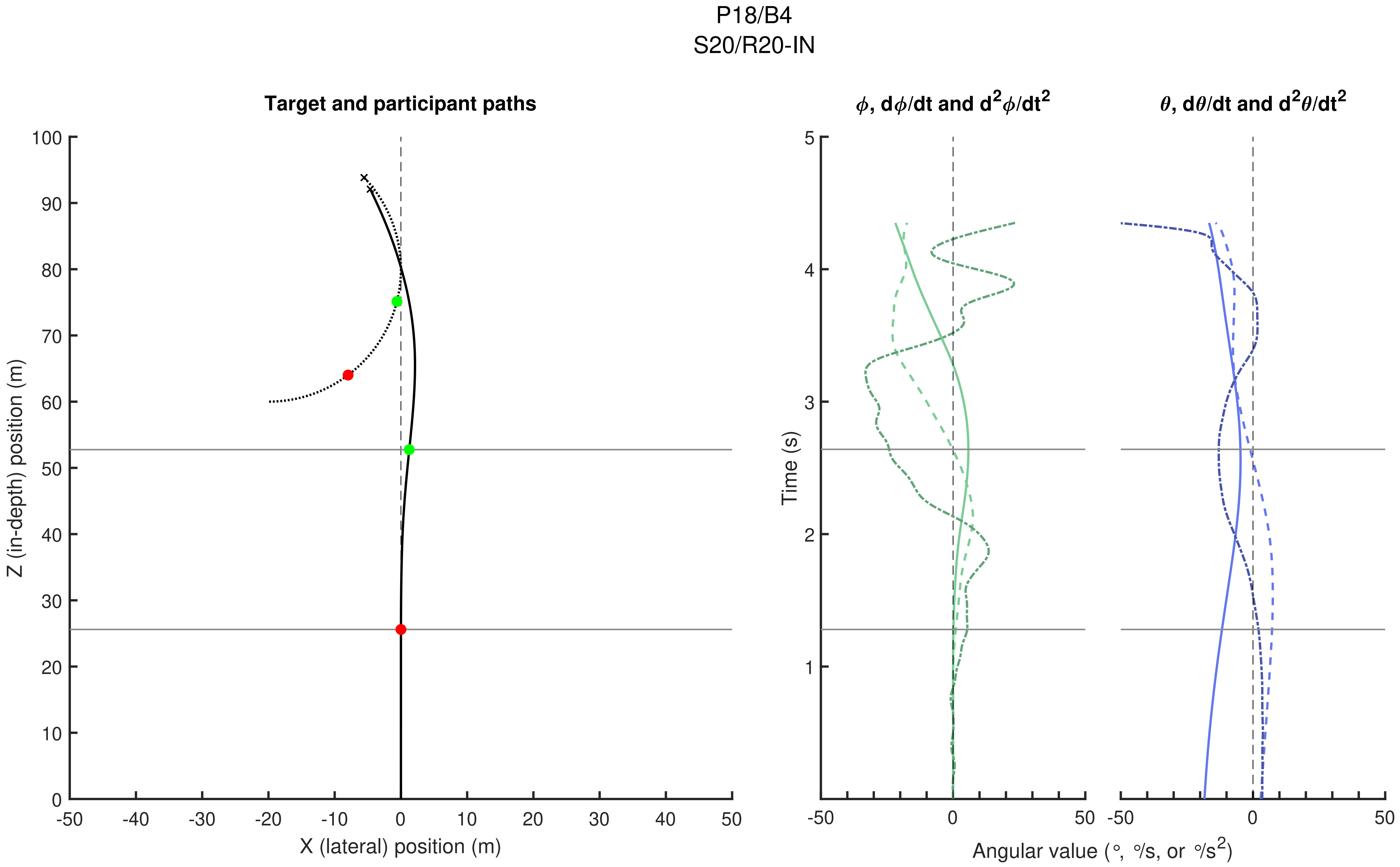

P18/B4
S20/R20-IN
Target and participant paths
Z (in-depth) position (m)
X (lateral) position (m)
ϕ, dϕ/dt and d$^2\phi$/dt^2
θ, dθ/dt and d$^2\theta$/dt^2
Time (s)
Angular value (°, °/s, or °/s^2)

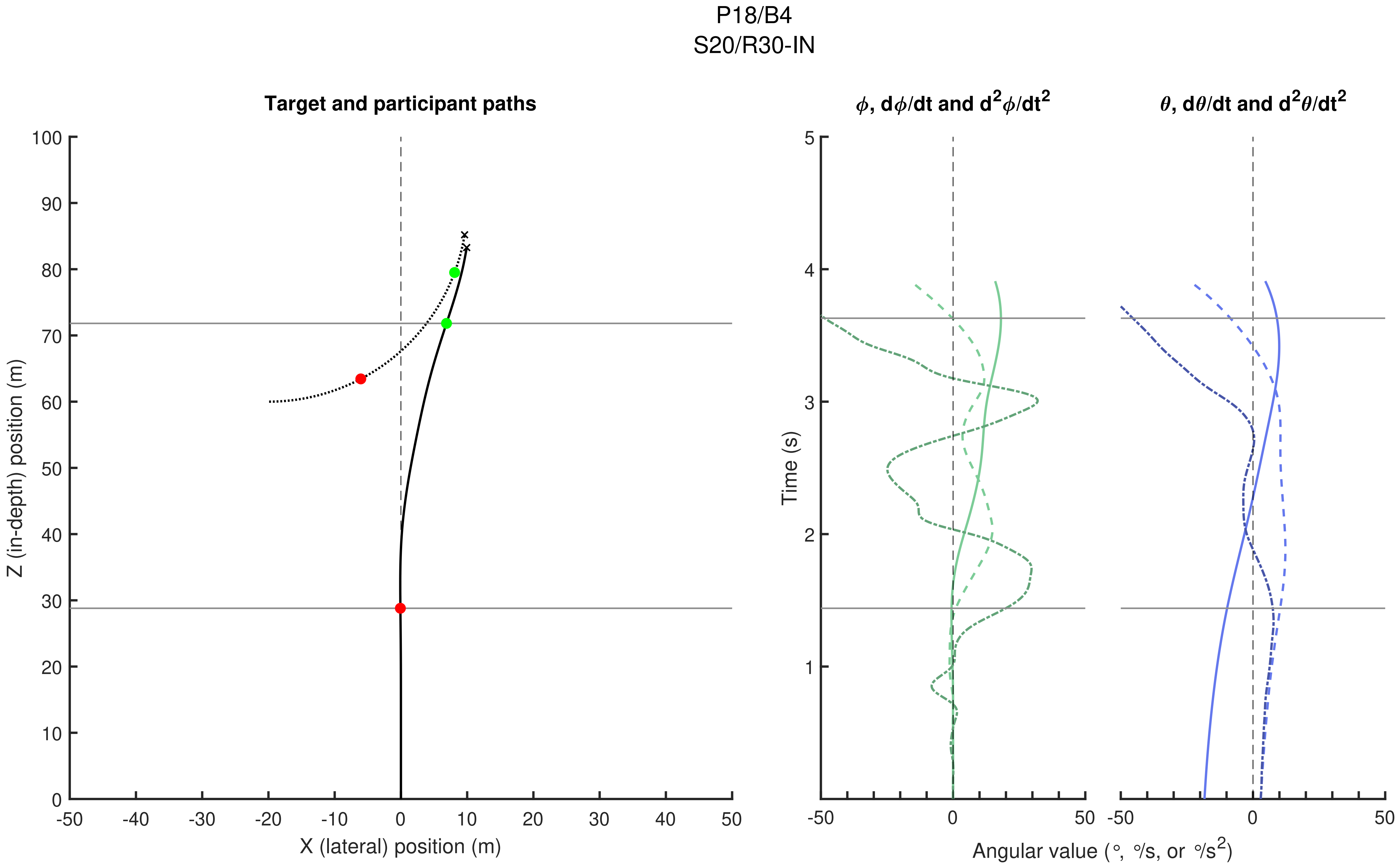
P18/B4
S20/R30-IN
Target and participant paths
ϕ, dϕ/dt and d$^2\phi$/dt^2
θ, dθ/dt and d$^2\theta$/dt^2
Z (in-depth) position (m)
X (lateral) position (m)
Time (s)
Angular value (°, °/s, or °/s^2)
100
90
80
70
60
50
40
30
20
10
0
-50
-40
-30
-20
-10
0
10
20
30
40
50
5
4
3
2
1
-50
0
50
-50
0
50

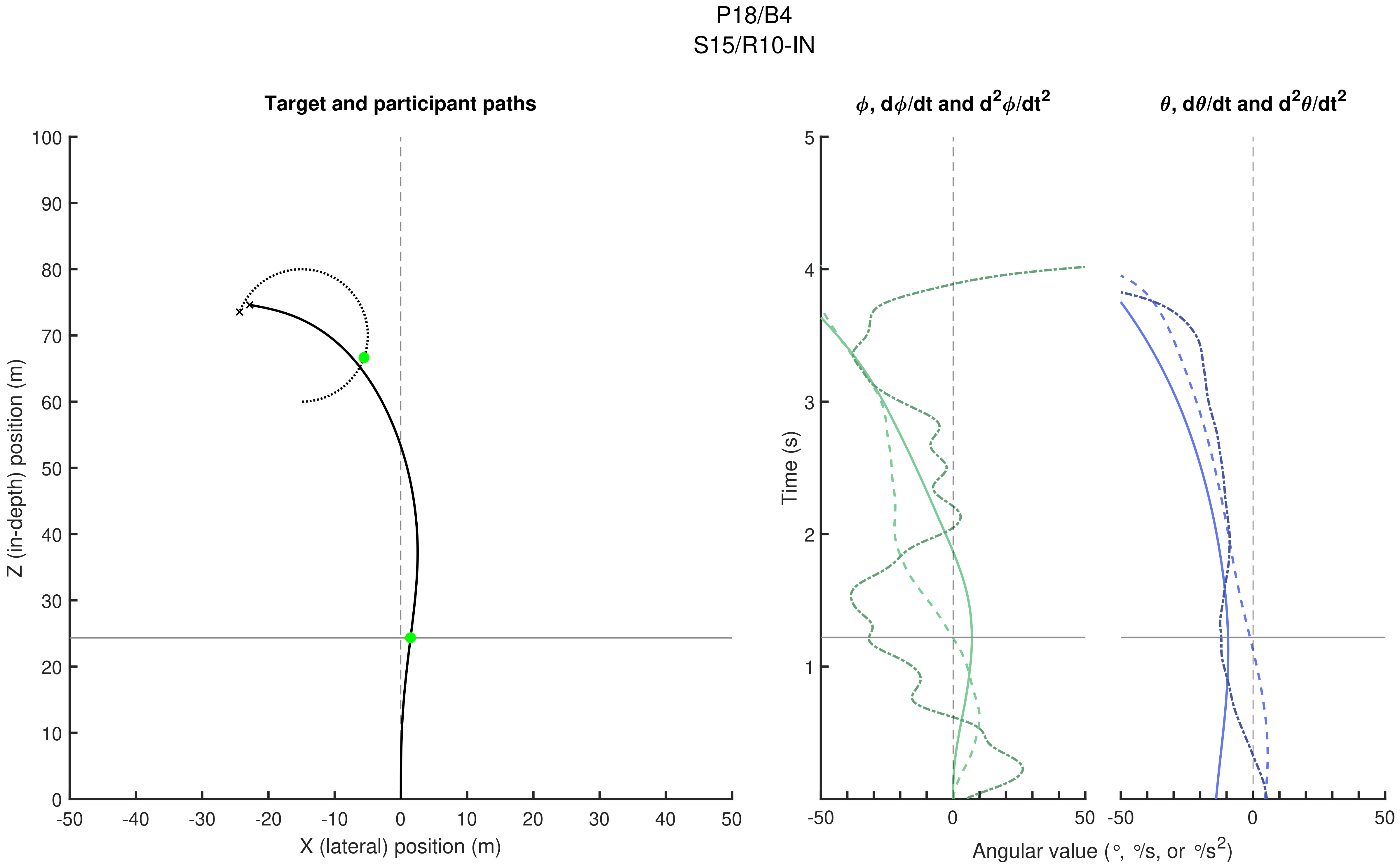
P18/B4
S15/R10-IN
Target and participant paths
Z (in-depth) position (m)
X (lateral) position (m)
φ, dφ/dt and d²φ/dt²
θ, dθ/dt and d²θ/dt²
Time (s)
Angular value (°, °/s, or °/s²)

P18/B4
S15/R20-IN

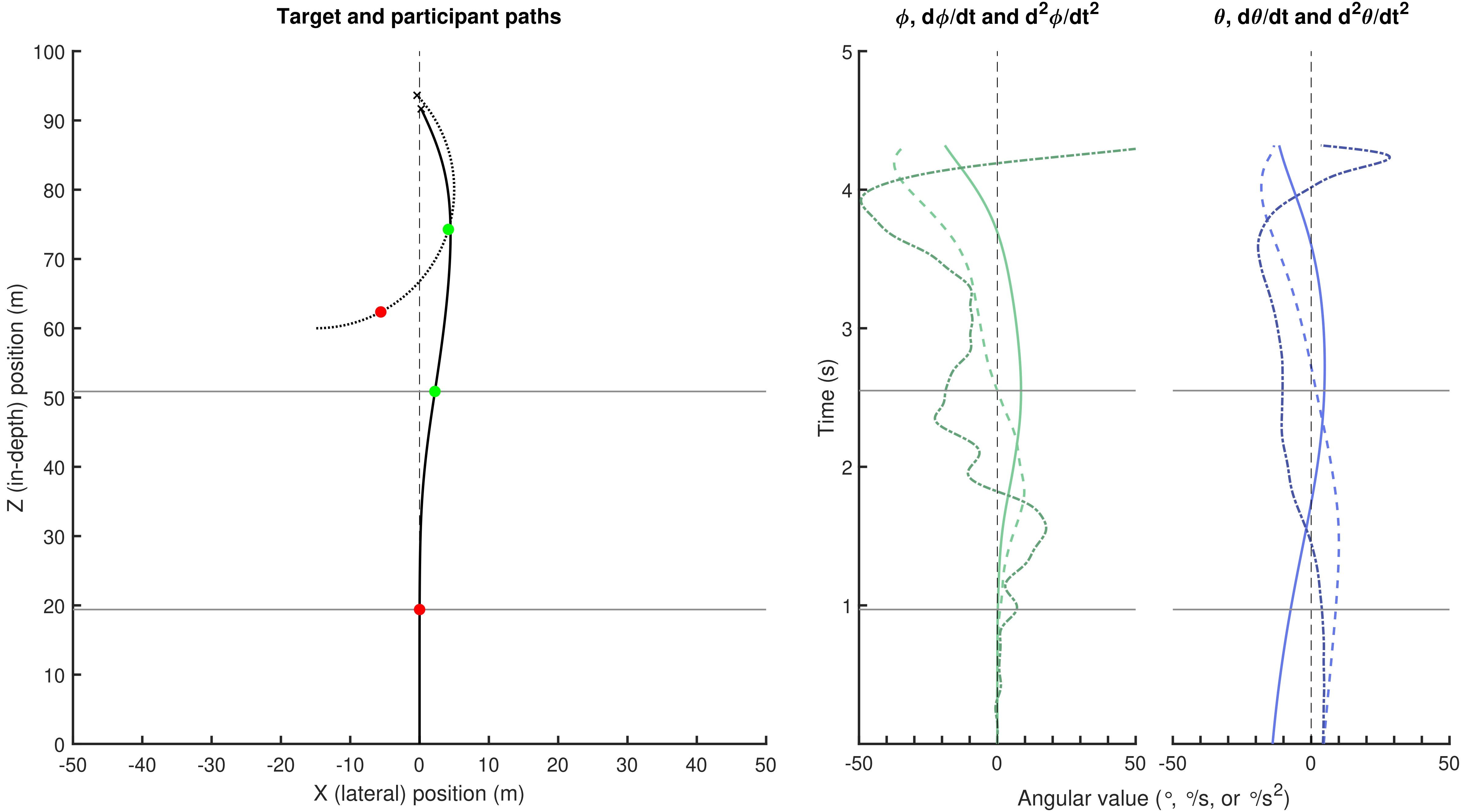

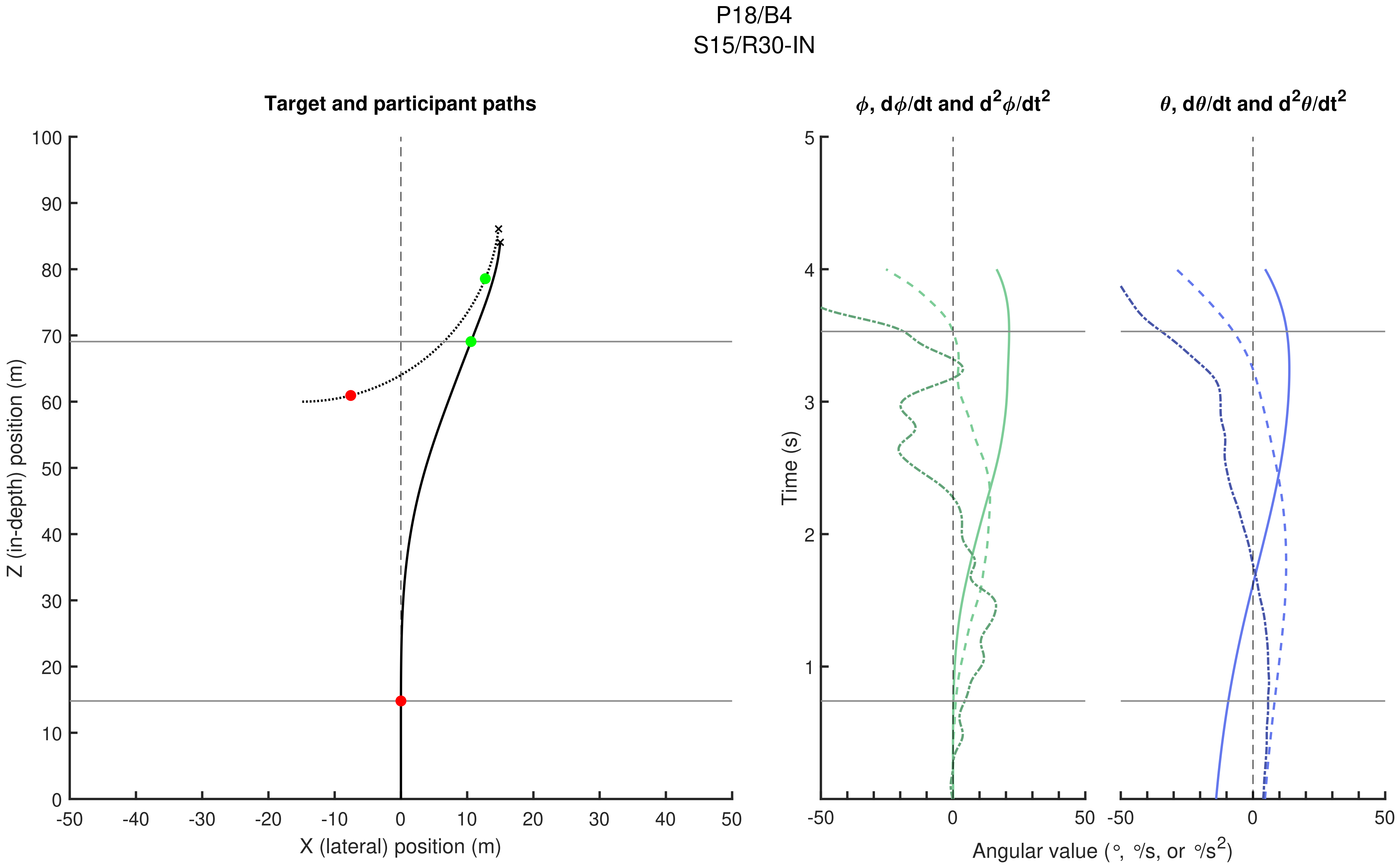
P18/B4
S15/R30-IN
Target and participant paths
Z (in-depth) position (m)
X (lateral) position (m)
ϕ, dϕ/dt and d$^2\phi$/dt^2
θ, dθ/dt and d$^2\theta$/dt^2
Time (s)
Angular value (°, °/s, or °/s^2)

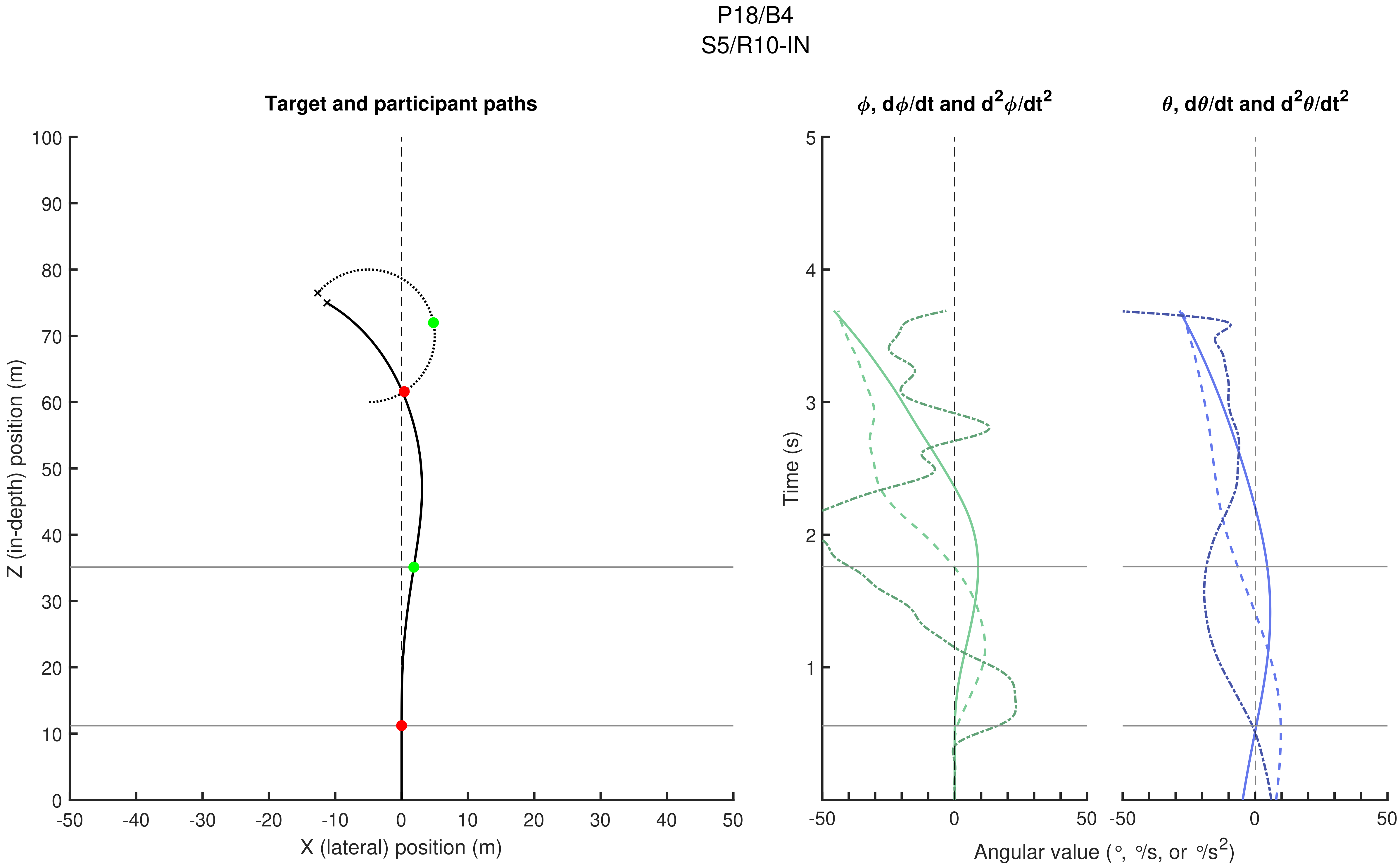
P18/B4
S5/R10-IN
Target and participant paths
φ, dφ/dt and d²φ/dt²
θ, dθ/dt and d²θ/dt²
Z (in-depth) position (m)
X (lateral) position (m)
Time (s)
Angular value (°, °/s, or °/s²)
100
90
80
70
60
50
40
30
20
10
0
-50
-40
-30
-20
-10
0
10
20
30
40
50
5
4
3
2
1
-50
0
50
-50
0
50

P18/B4
S5/R10-OUT

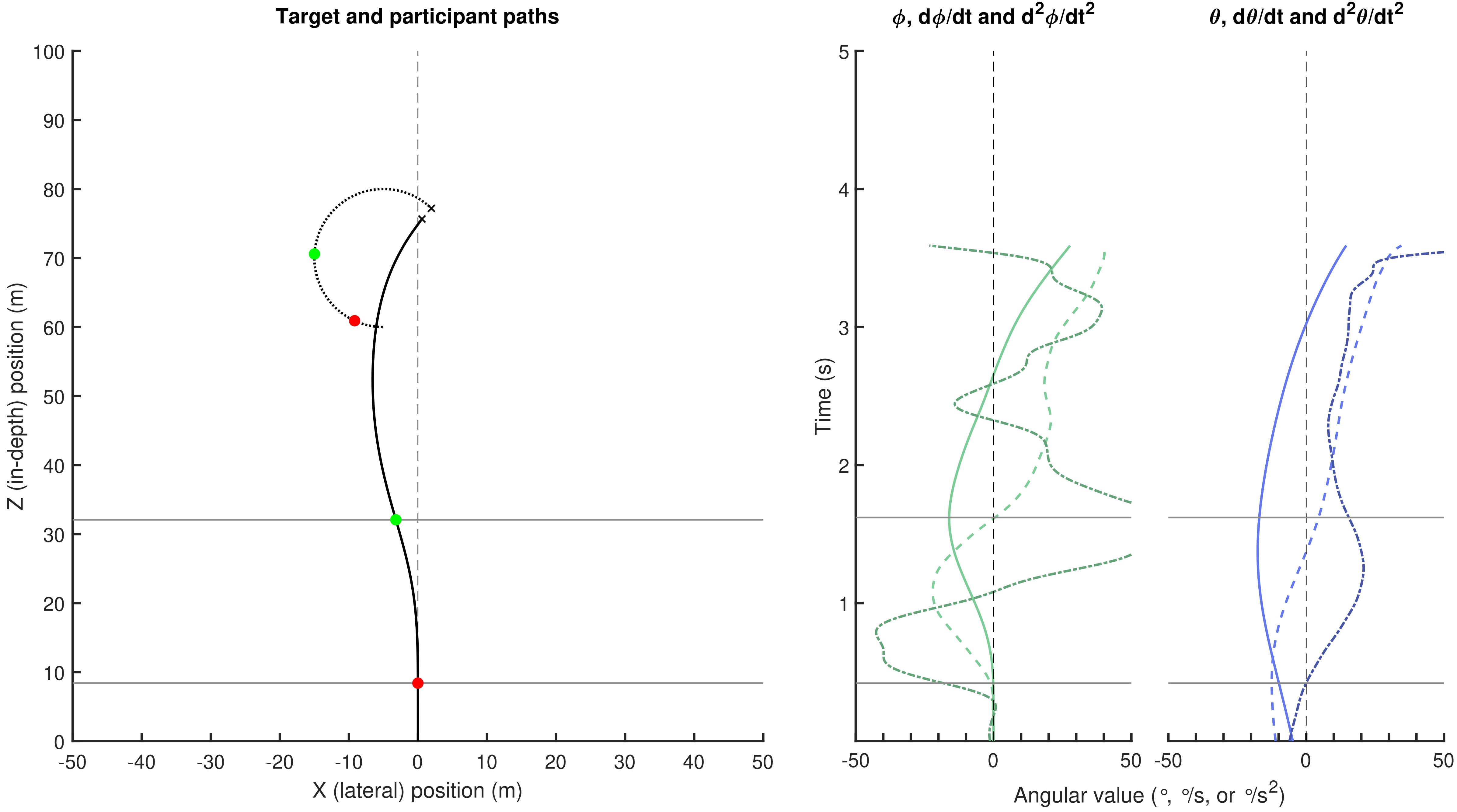

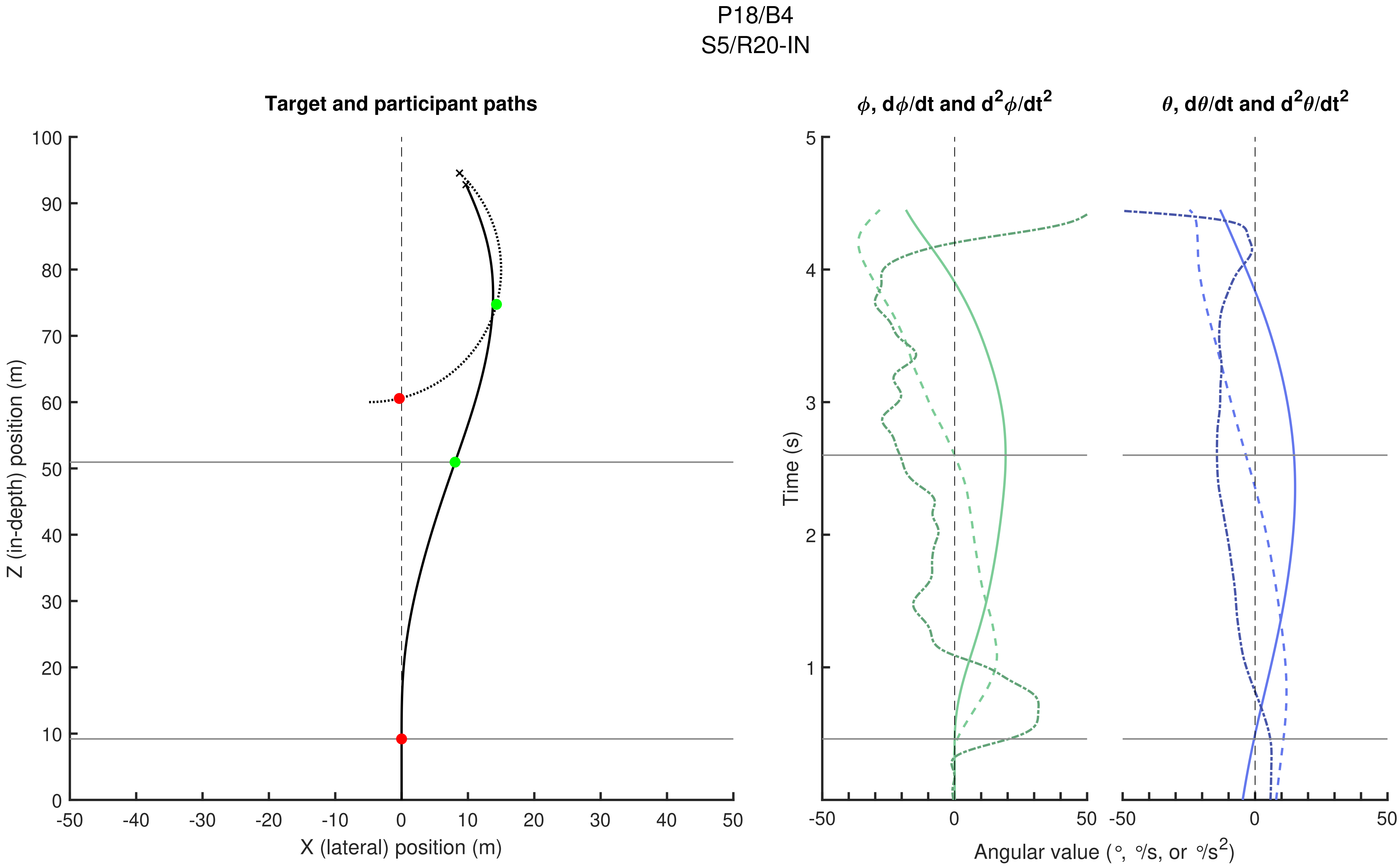
P18/B4
S5/R20-IN
Target and participant paths
Z (in-depth) position (m)
X (lateral) position (m)
ϕ, dϕ/dt and d$^2\phi$/dt^2
θ, dθ/dt and d$^2\theta$/dt^2
Time (s)
Angular value (°, °/s, or °/s^2)

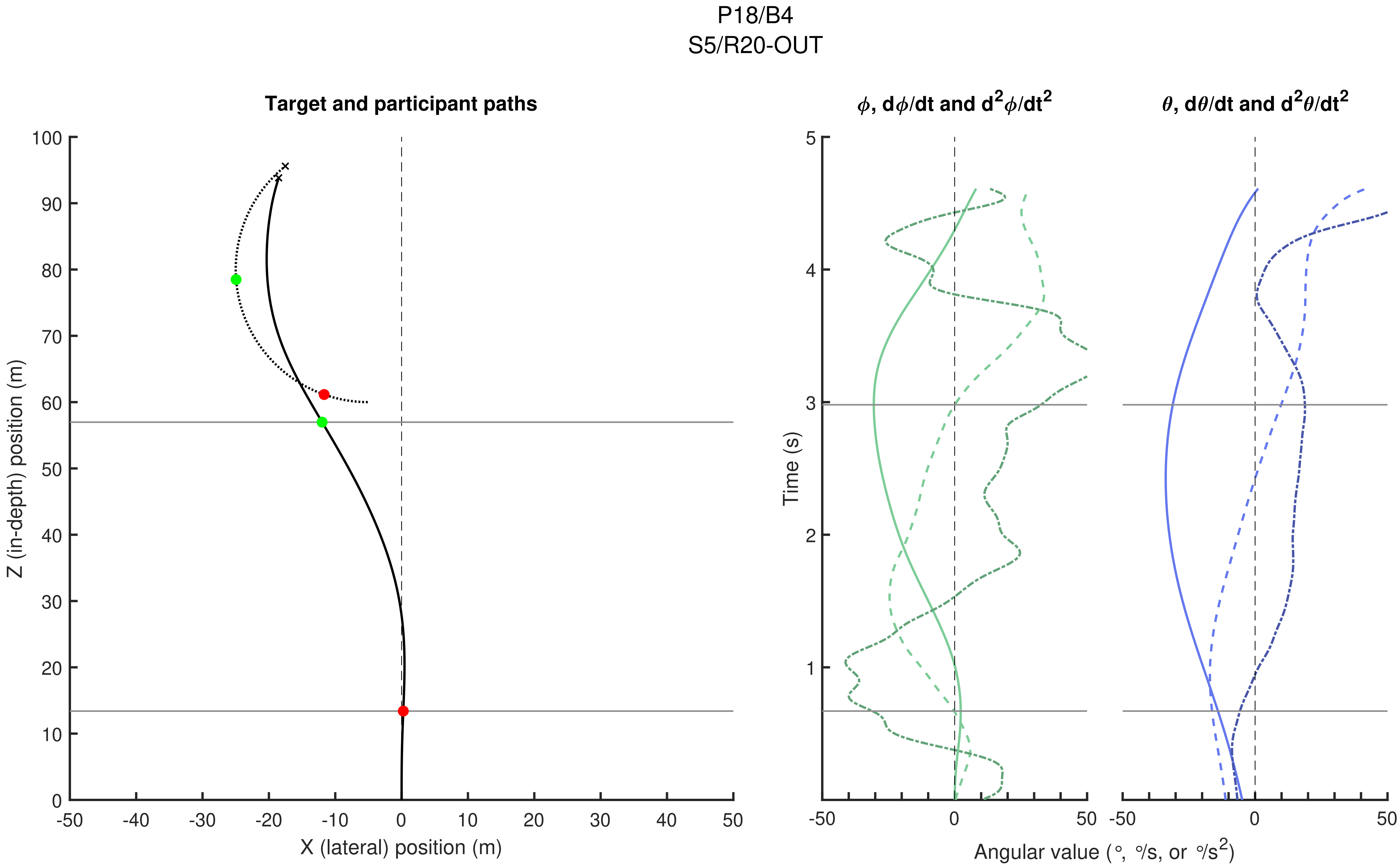
P18/B4
S5/R20-OUT
Target and participant paths
ϕ, dϕ/dt and d$^2\phi$/dt^2
θ, dθ/dt and d$^2\theta$/dt^2
Z (in-depth) position (m)
X (lateral) position (m)
Time (s)
Angular value (°, °/s, or °/s^2)

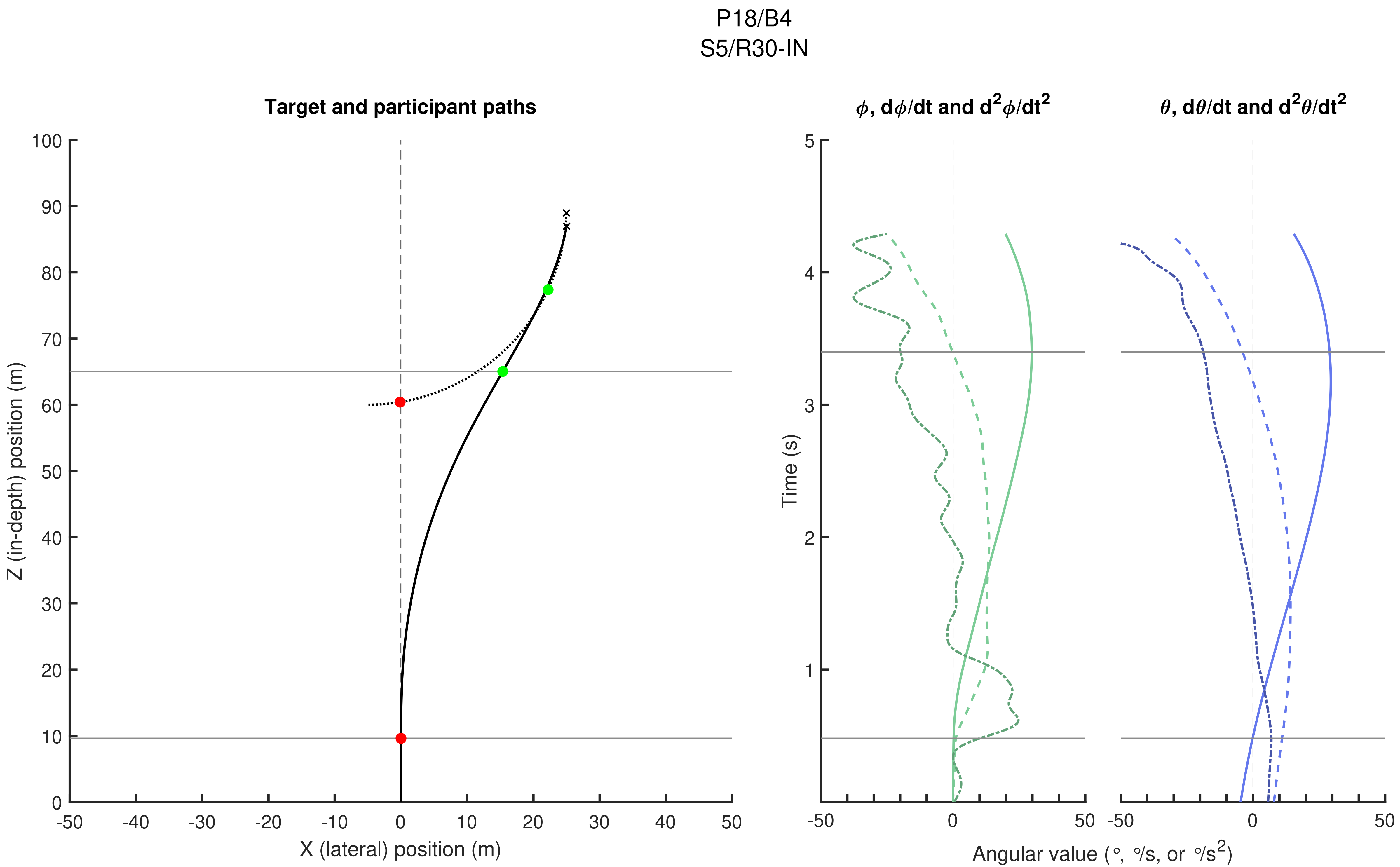
P18/B4
S5/R30-IN
Target and participant paths
Z (in-depth) position (m)
X (lateral) position (m)
100
90
80
70
60
50
40
30
20
10
0
-50
-40
-30
-20
-10
0
10
20
30
40
50
ϕ, dϕ/dt and d²ϕ/dt²
θ, dθ/dt and d²θ/dt²
Time (s)
5
4
3
2
1
-50
0
50
-50
0
50
Angular value (°, °/s, or °/s²)

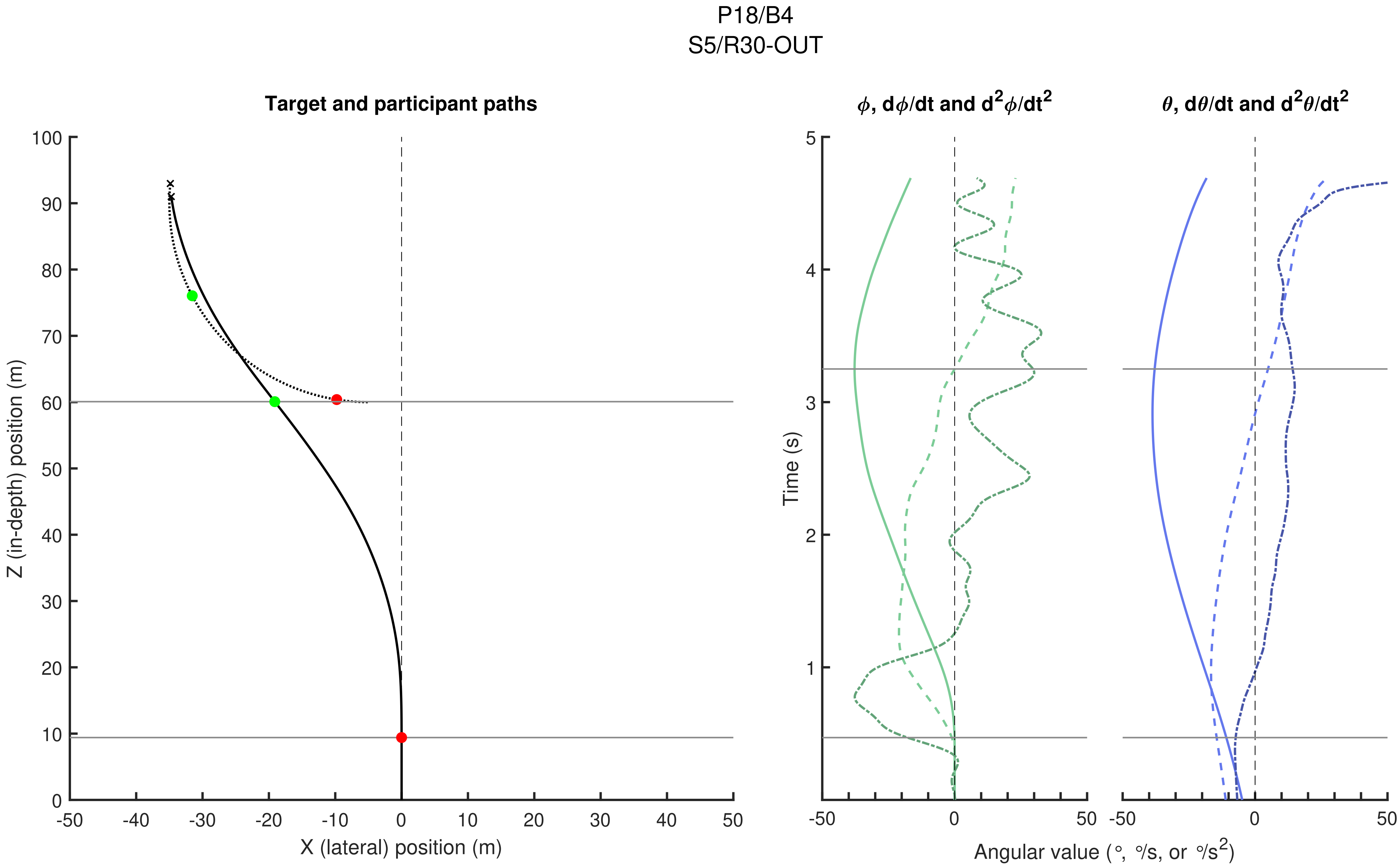
P18/B4
S5/R30-OUT
Target and participant paths
Z (in-depth) position (m)
X (lateral) position (m)
100
90
80
70
60
50
40
30
20
10
0
-50
-40
-30
-20
-10
0
10
20
30
40
50
φ, dφ/dt and d²φ/dt²
θ, dθ/dt and d²θ/dt²
Time (s)
5
4
3
2
1
-50
0
50
-50
0
50
Angular value (°, °/s, or °/s²)

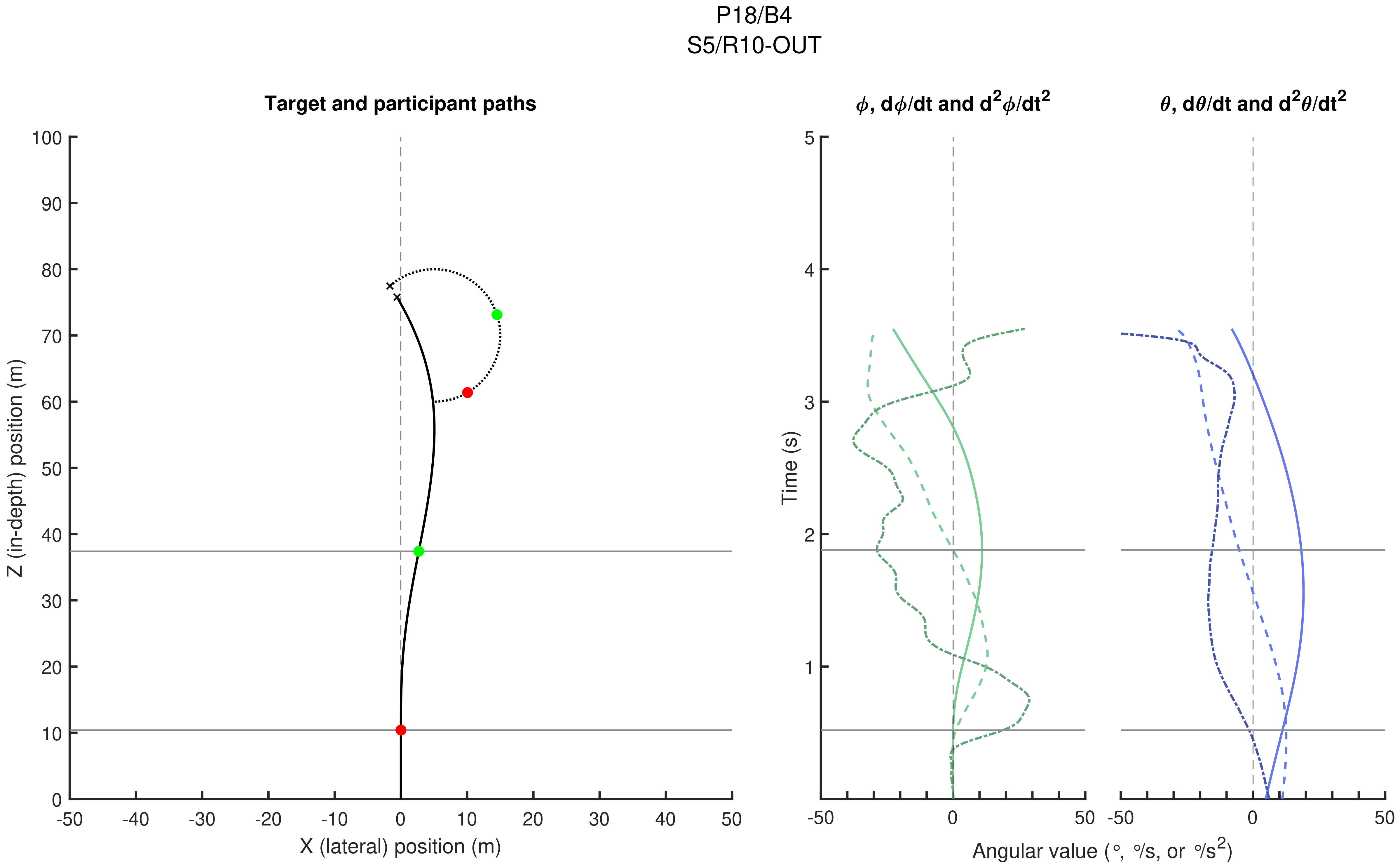
P18/B4
S5/R10-OUT
Target and participant paths
Z (in-depth) position (m)
X (lateral) position (m)
ϕ, dϕ/dt and d²ϕ/dt²
θ, dθ/dt and d²θ/dt²
Time (s)
Angular value (°, °/s, or °/s²)

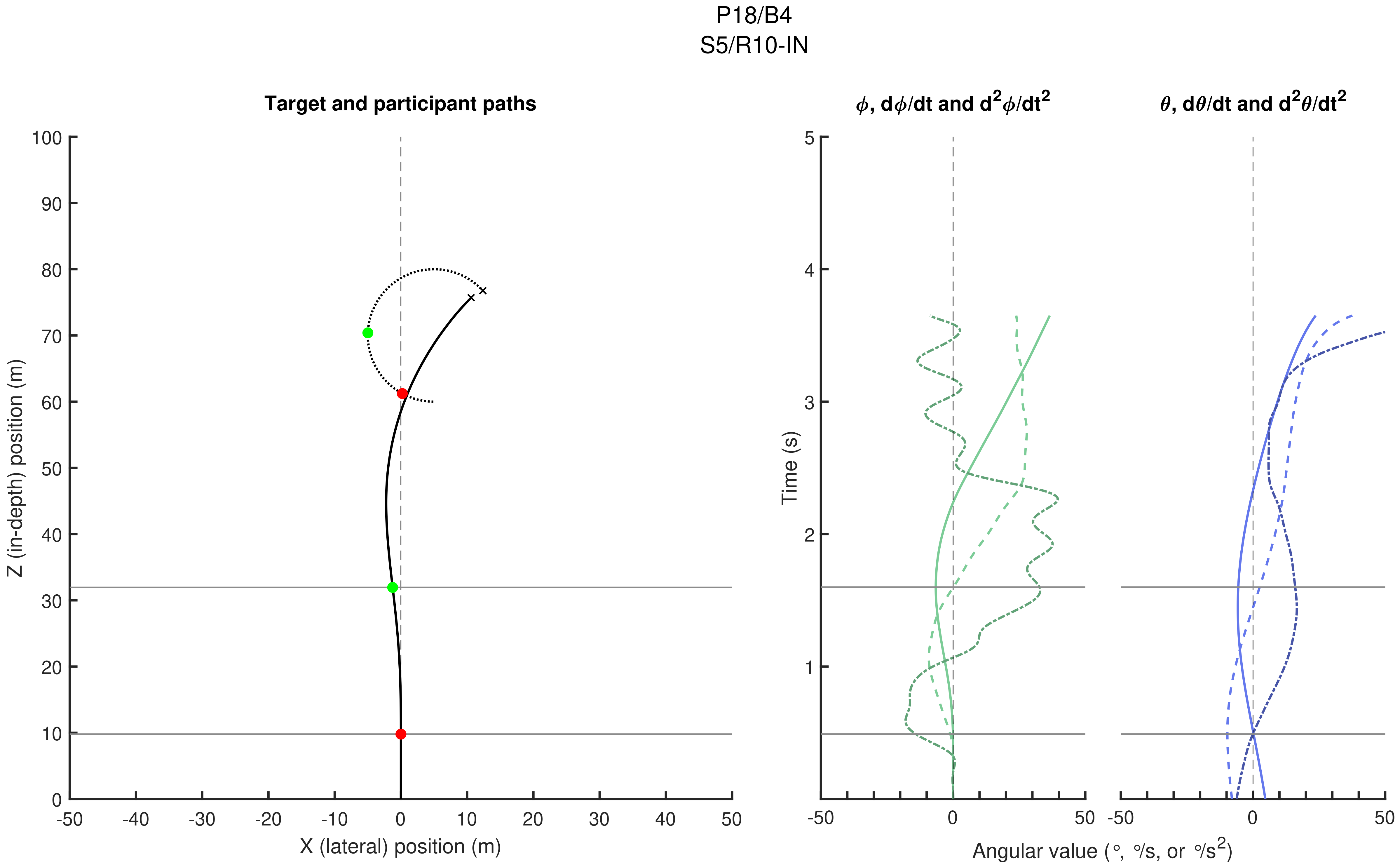
P18/B4
S5/R10-IN
Target and participant paths
Z (in-depth) position (m)
X (lateral) position (m)
100
90
80
70
60
50
40
30
20
10
0
-50
-40
-30
-20
-10
0
10
20
30
40
50
ϕ, dϕ/dt and d²ϕ/dt²
θ, dθ/dt and d²θ/dt²
Time (s)
5
4
3
2
1
-50
0
50
-50
0
50
Angular value (°, °/s, or °/s²)

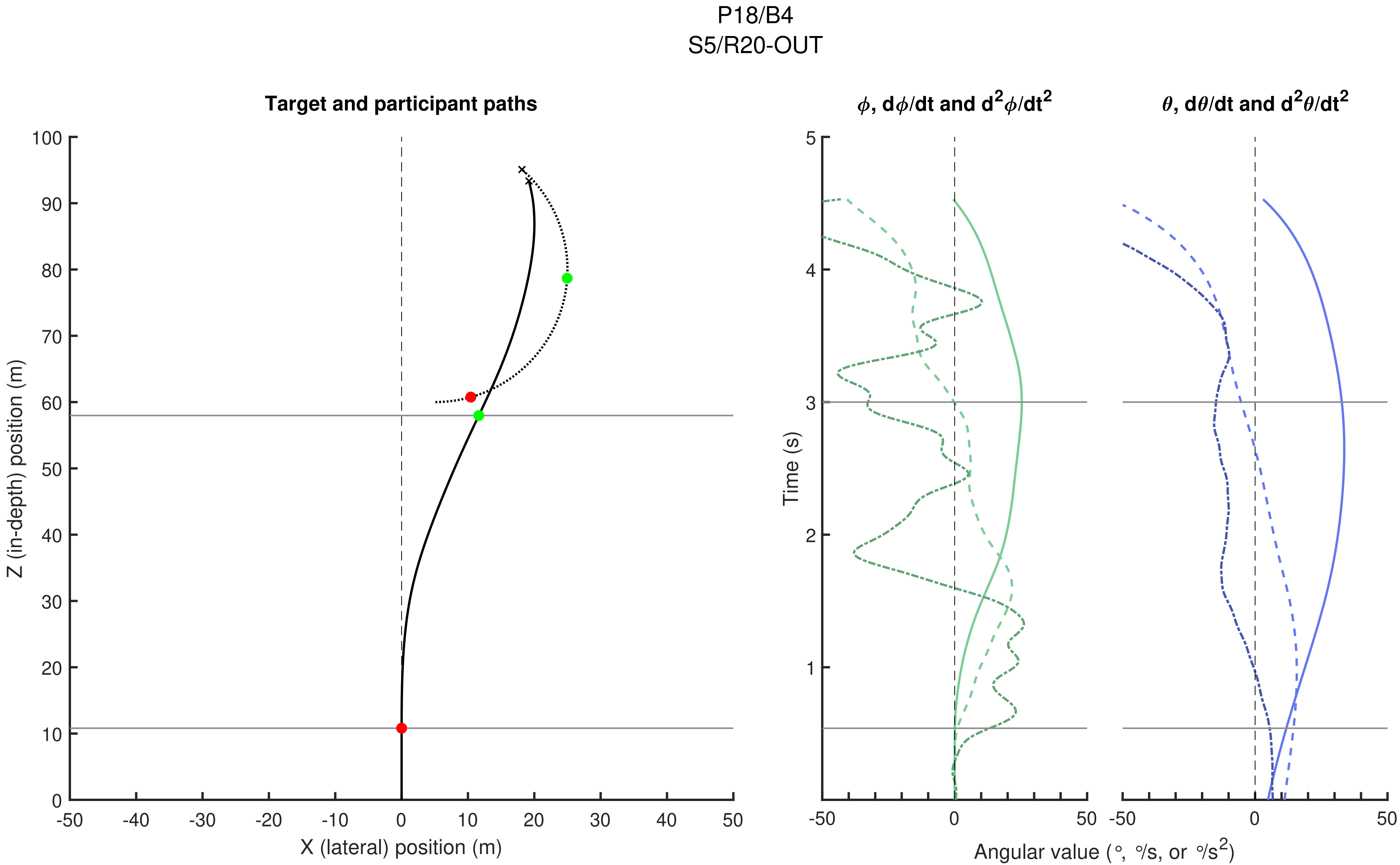
P18/B4
S5/R20-OUT
Target and participant paths
Z (in-depth) position (m)
X (lateral) position (m)
ϕ, dϕ/dt and d²ϕ/dt²
θ, dθ/dt and d²θ/dt²
Time (s)
Angular value (°, °/s, or °/s²)

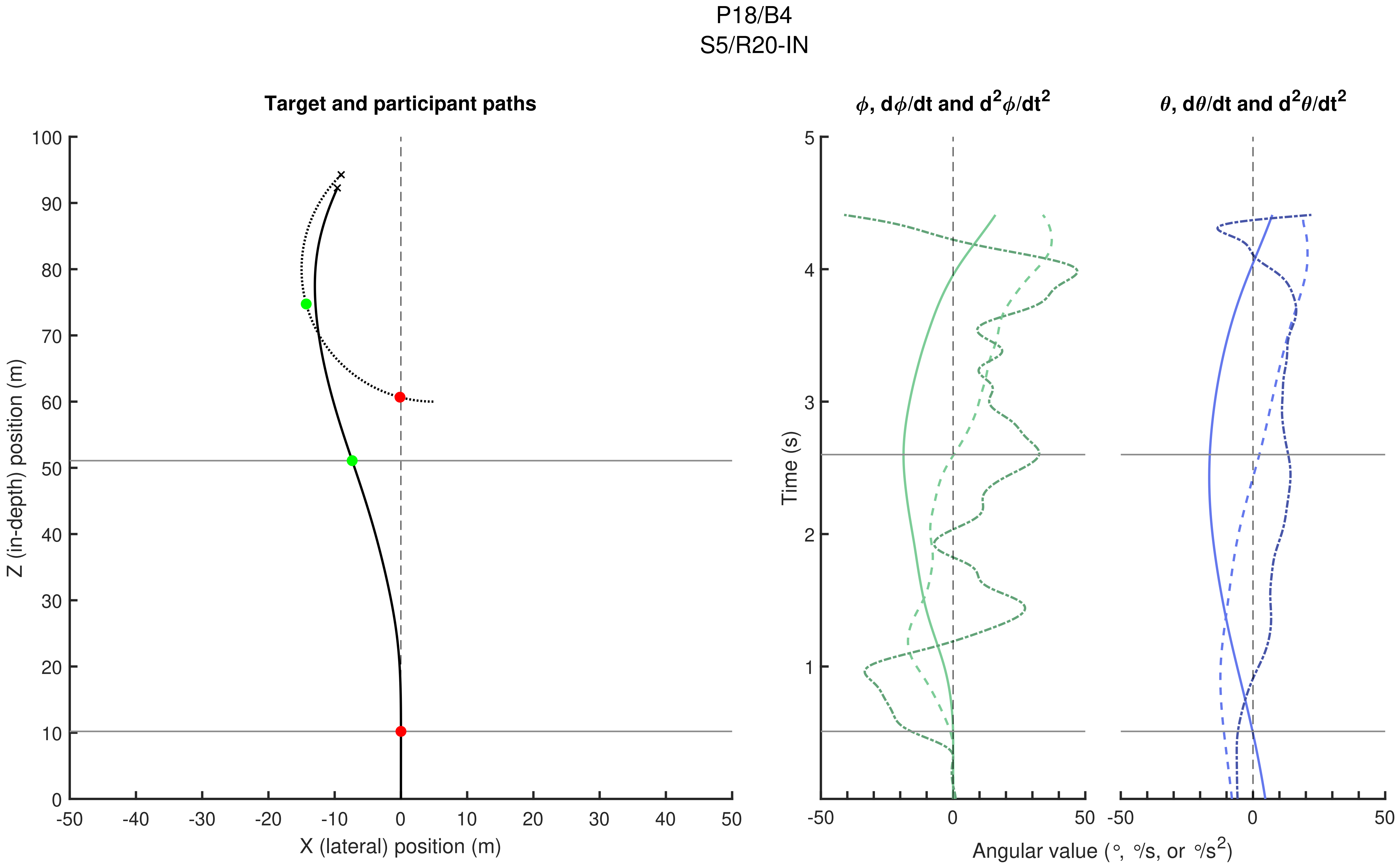
P18/B4
S5/R20-IN
Target and participant paths
Z (in-depth) position (m)
X (lateral) position (m)
ϕ, dϕ/dt and d$^2\phi$/dt^2
θ, dθ/dt and d$^2\theta$/dt^2
Time (s)
Angular value (°, °/s, or °/s^2)

P18/B4
S5/R30-OUT

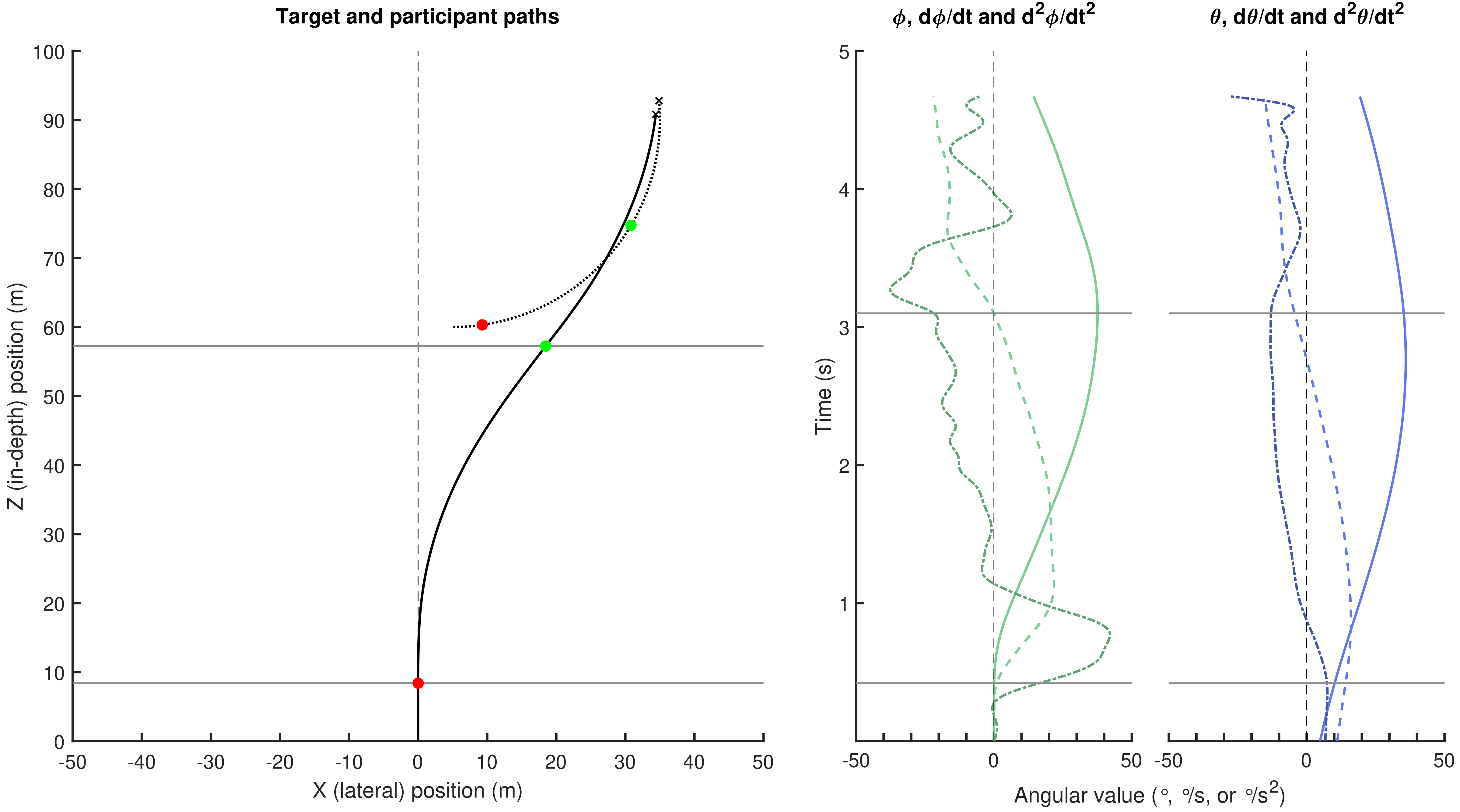

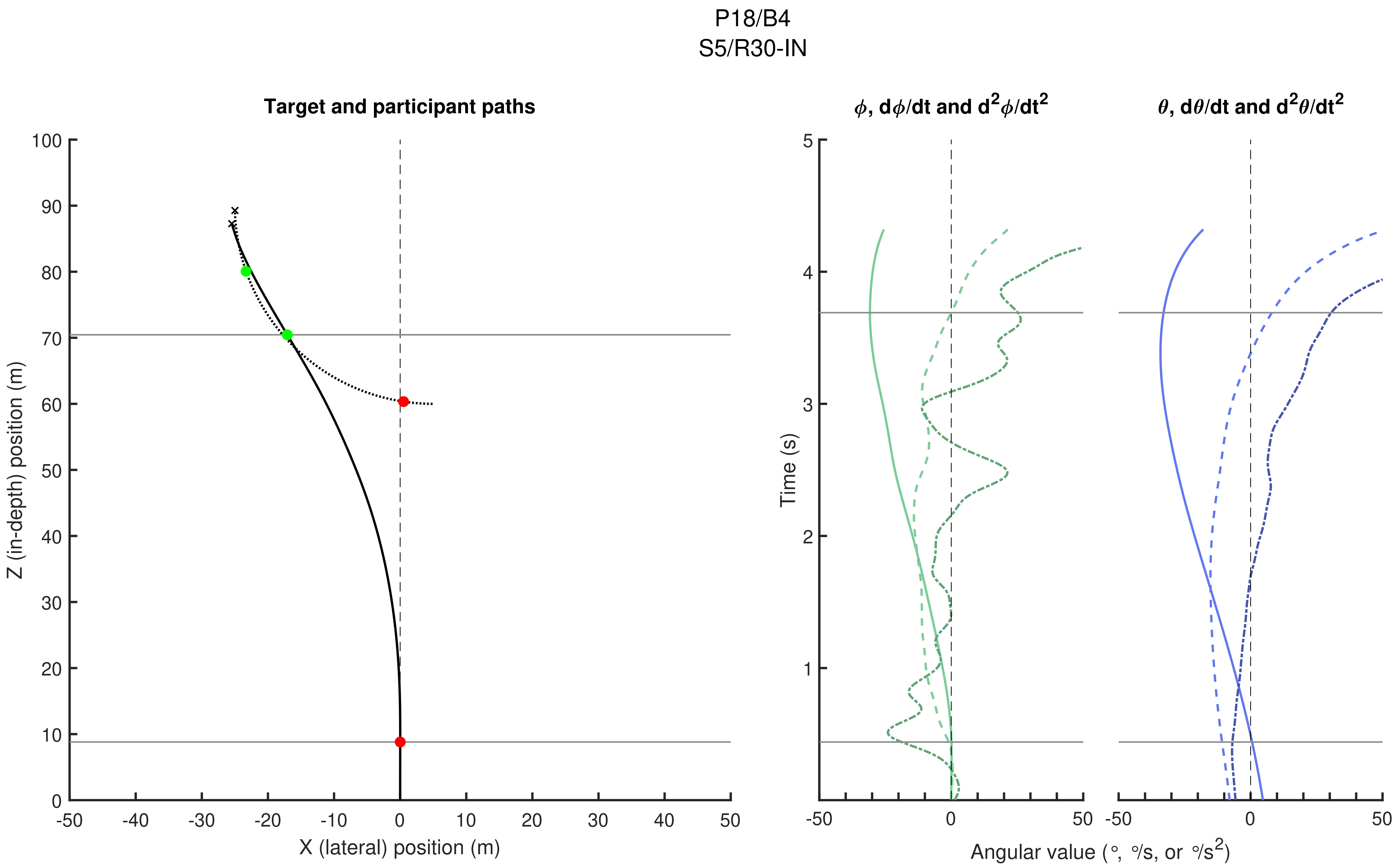
P18/B4
S5/R30-IN
Target and participant paths
φ, dφ/dt and d²φ/dt²
θ, dθ/dt and d²θ/dt²
Z (in-depth) position (m)
X (lateral) position (m)
Time (s)
Angular value (°, °/s, or °/s²)
100
90
80
70
60
50
40
30
20
10
0
-50
-40
-30
-20
-10
0
10
20
30
40
50
5
4
3
2
1
-50
0
50
-50
0
50

P18/B4
S15/R10-IN

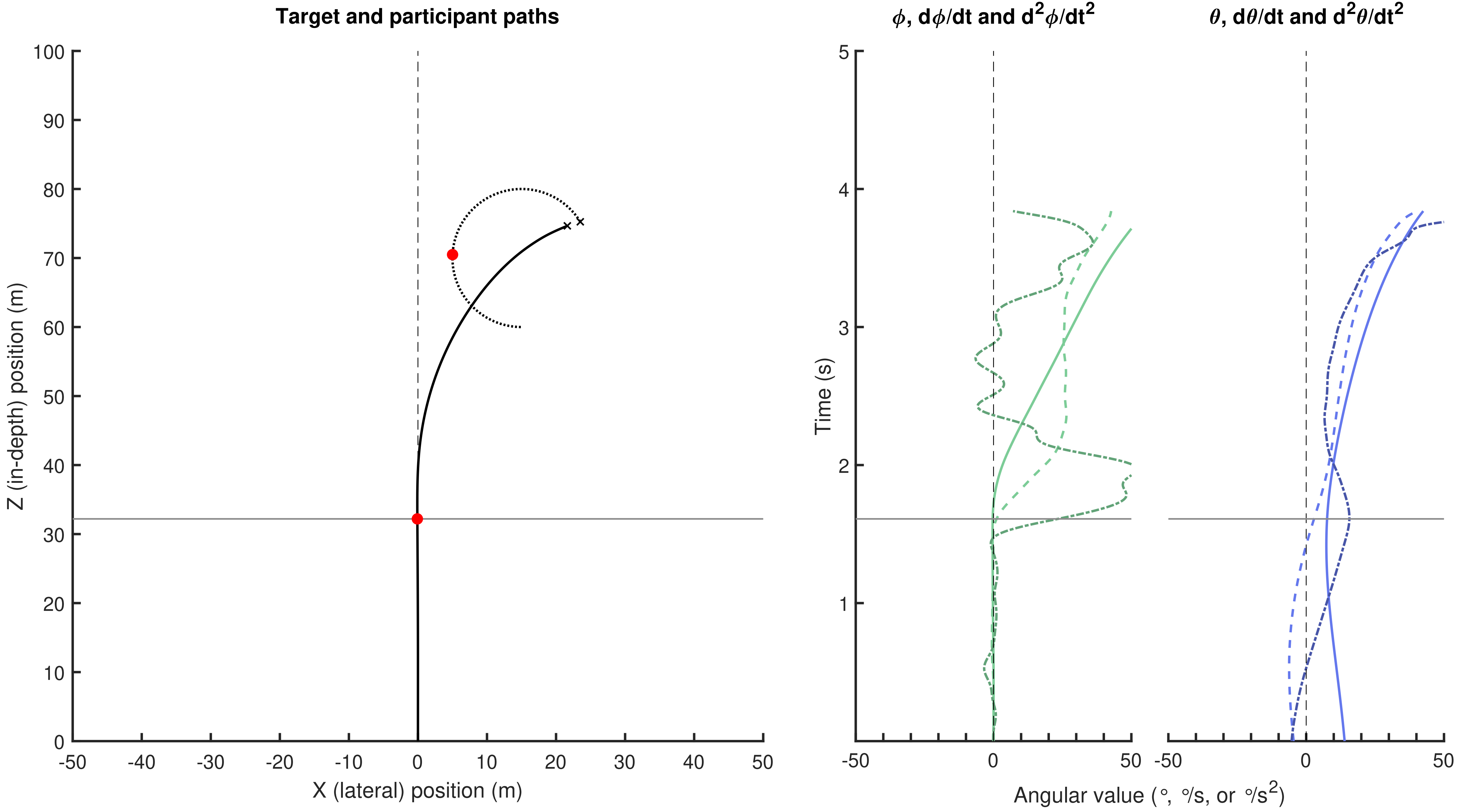

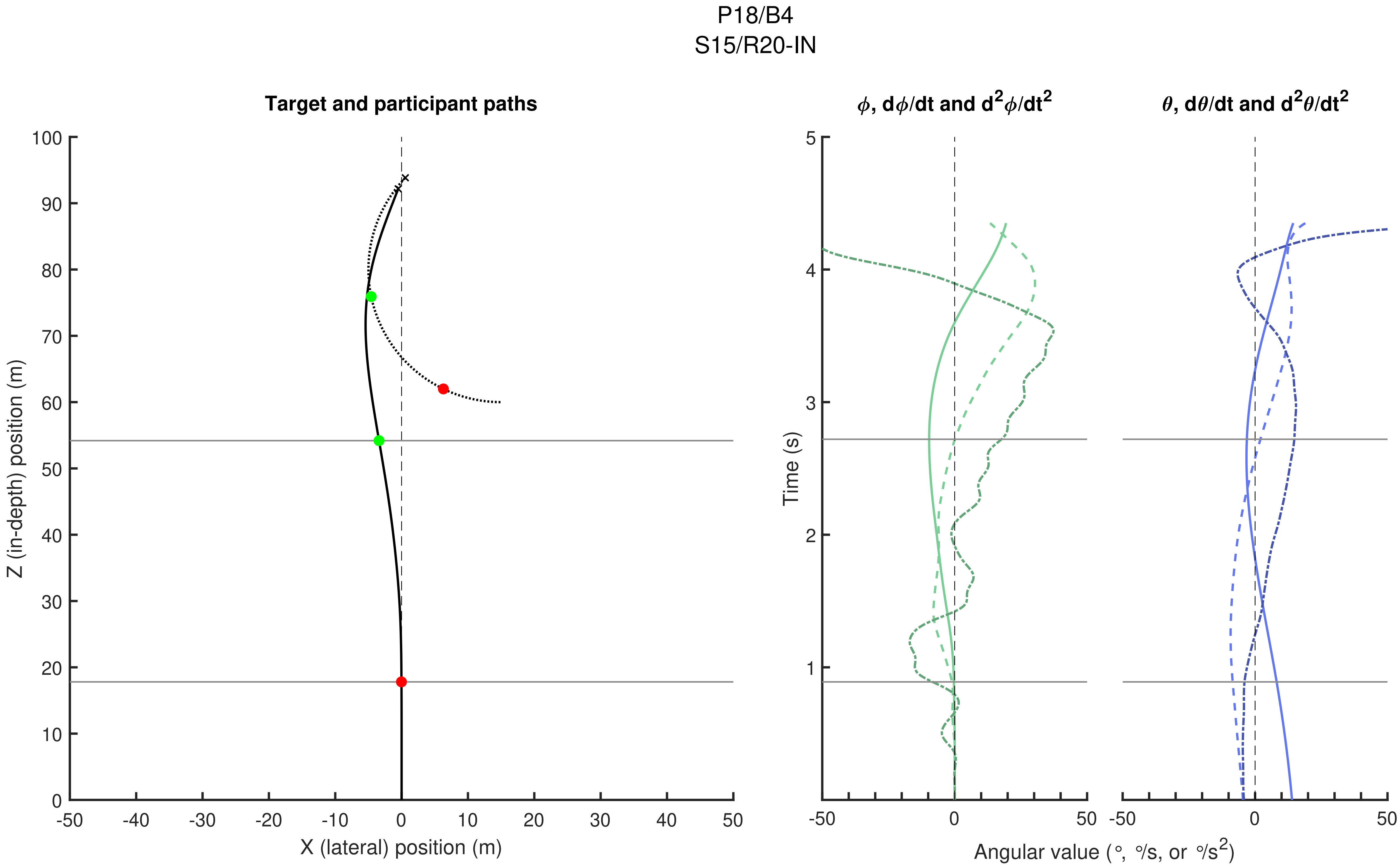
P18/B4
S15/R20-IN
Target and participant paths
Z (in-depth) position (m)
X (lateral) position (m)
ϕ, dϕ/dt and d²ϕ/dt²
θ, dθ/dt and d²θ/dt²
Time (s)
Angular value (°, °/s, or °/s²)

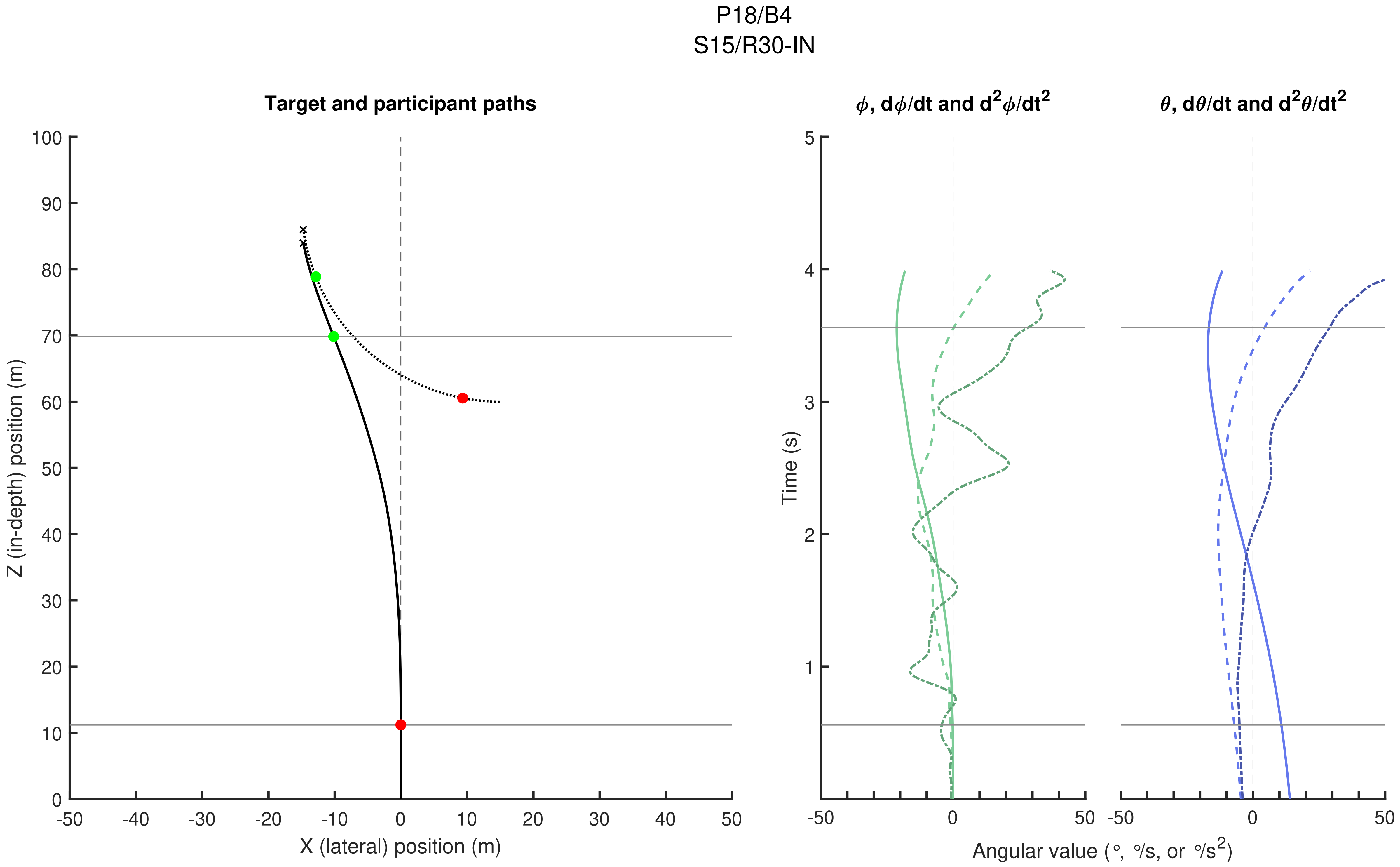
P18/B4
S15/R30-IN
Target and participant paths
ϕ, dϕ/dt and d$^2\phi$/dt^2
θ, dθ/dt and d$^2\theta$/dt^2
Z (in-depth) position (m)
X (lateral) position (m)
Time (s)
Angular value (°, °/s, or °/s^2)
100
90
80
70
60
50
40
30
20
10
0
-50
-40
-30
-20
-10
0
10
20
30
40
50
5
4
3
2
1

P18/B4
S20/R10-IN

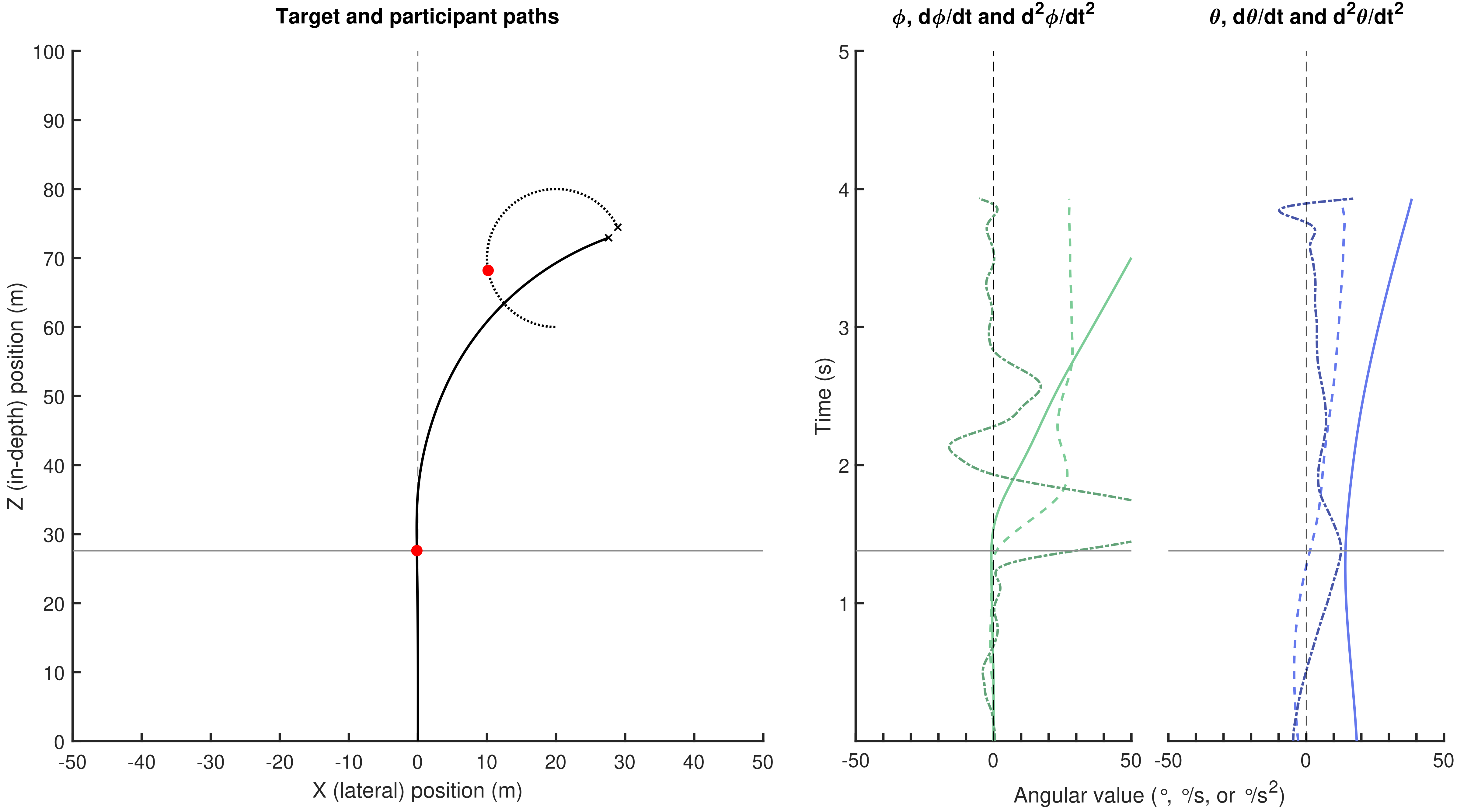

P18/B4
S20/R20-IN

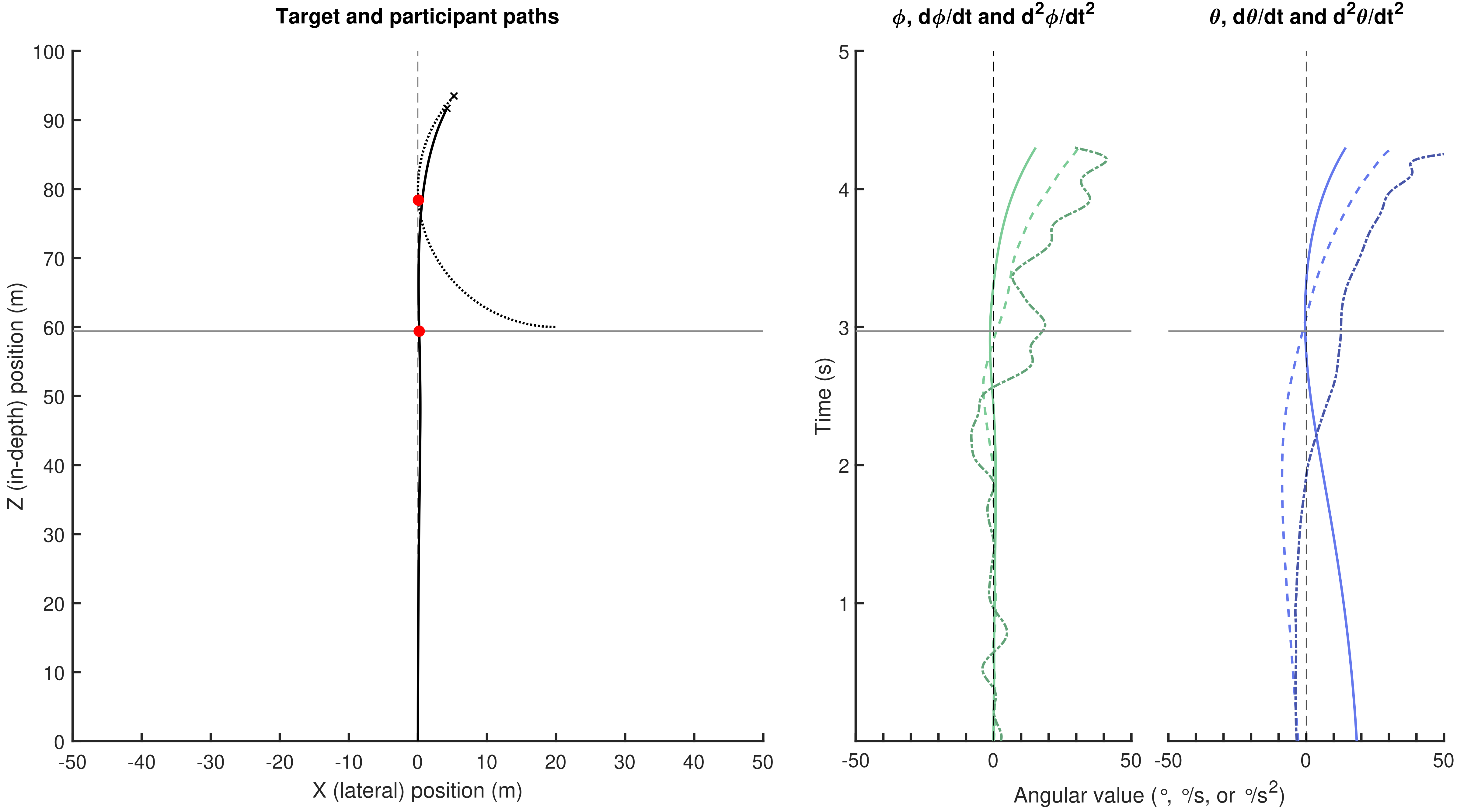

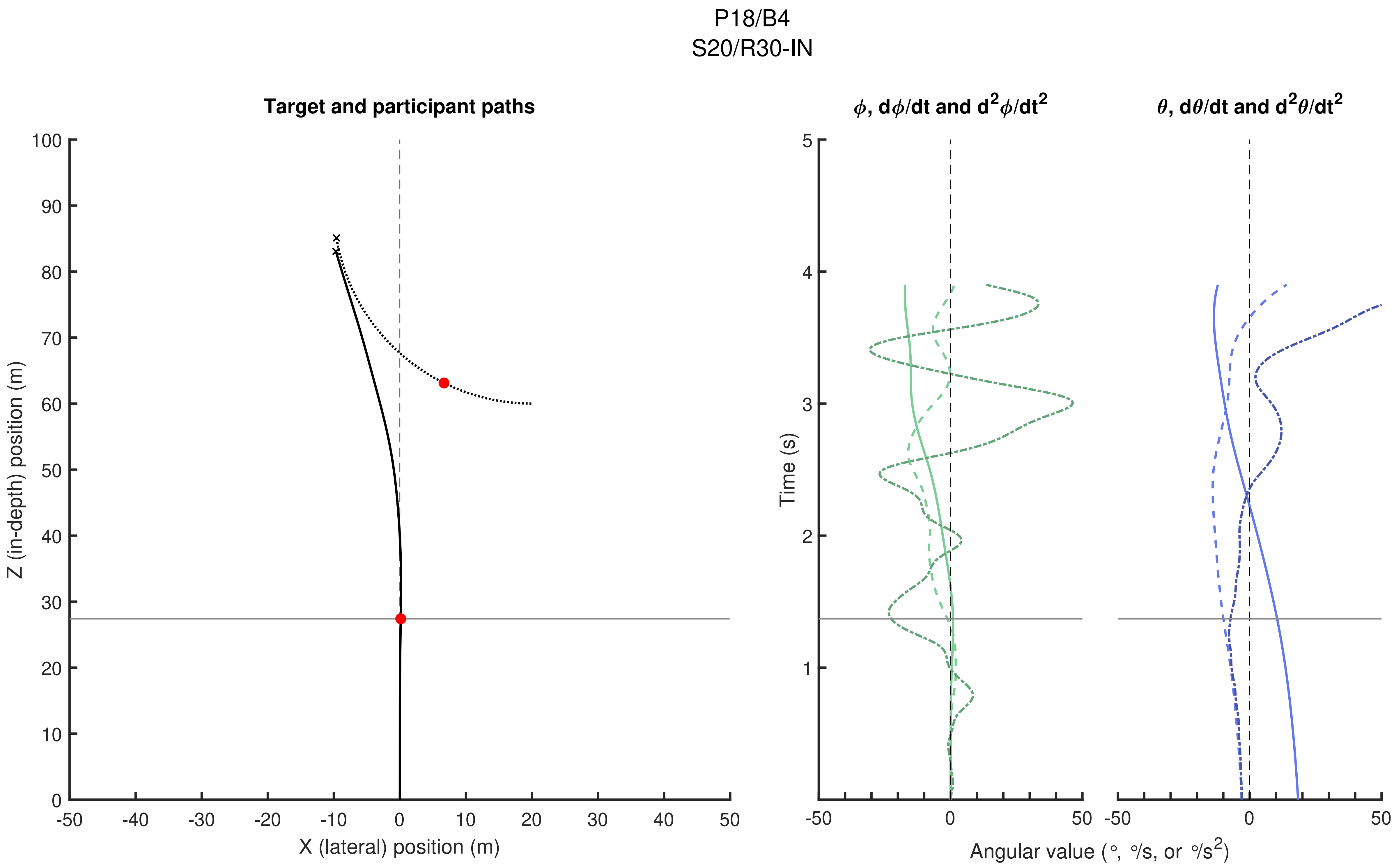

P18/B4
S20/R30-IN
Target and participant paths
ϕ, dϕ/dt and d$^2\phi$/dt^2
θ, dθ/dt and d$^2\theta$/dt^2
Z (in-depth) position (m)
X (lateral) position (m)
Time (s)
Angular value (°, °/s, or °/s^2)
100
90
80
70
60
50
40
30
20
10
0
-50
-40
-30
-20
-10
0
10
20
30
40
50
5
4
3
2
1
-50
0
50
-50
0
50

P18/B5
S20/R10-IN

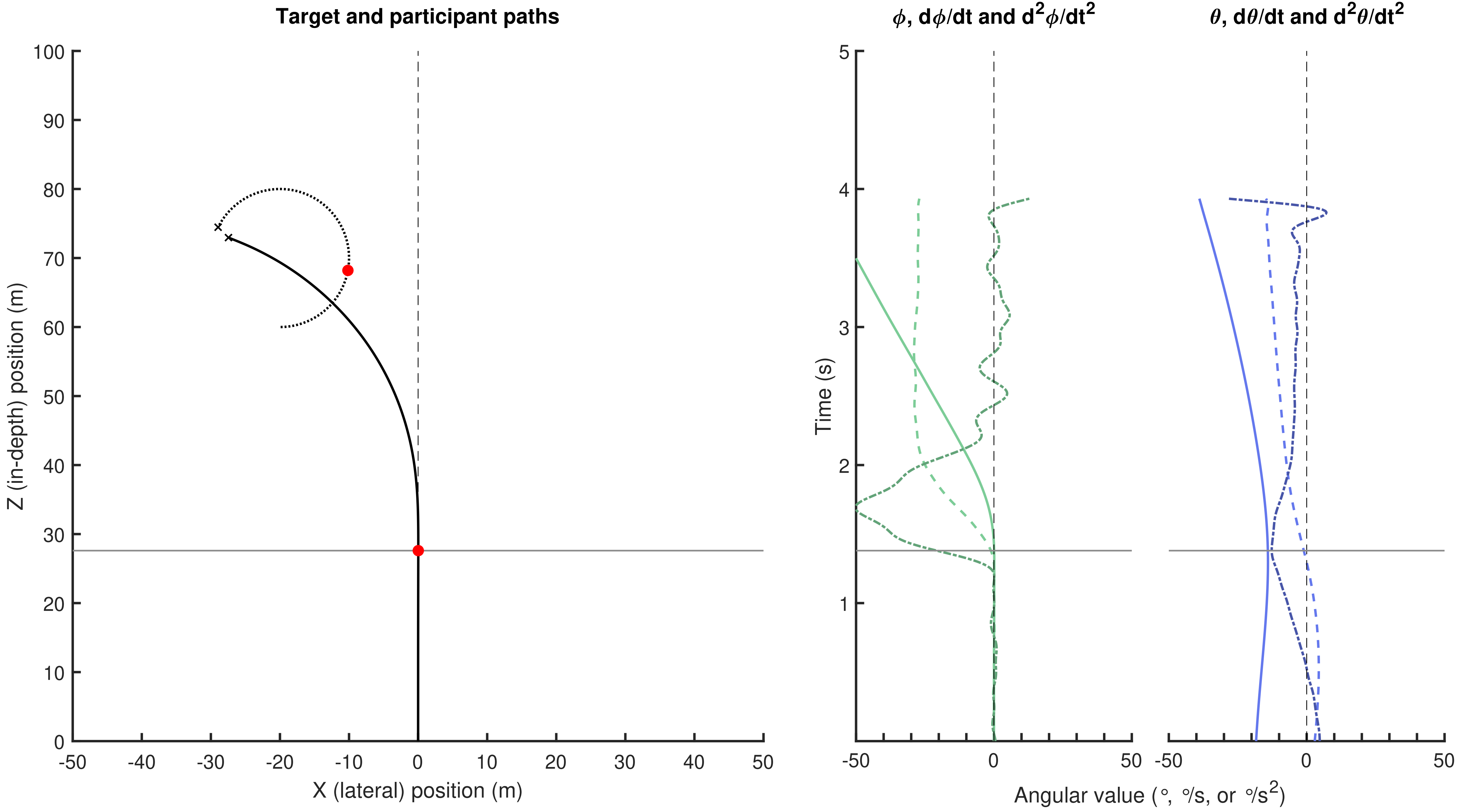

P18/B5
S20/R20-IN

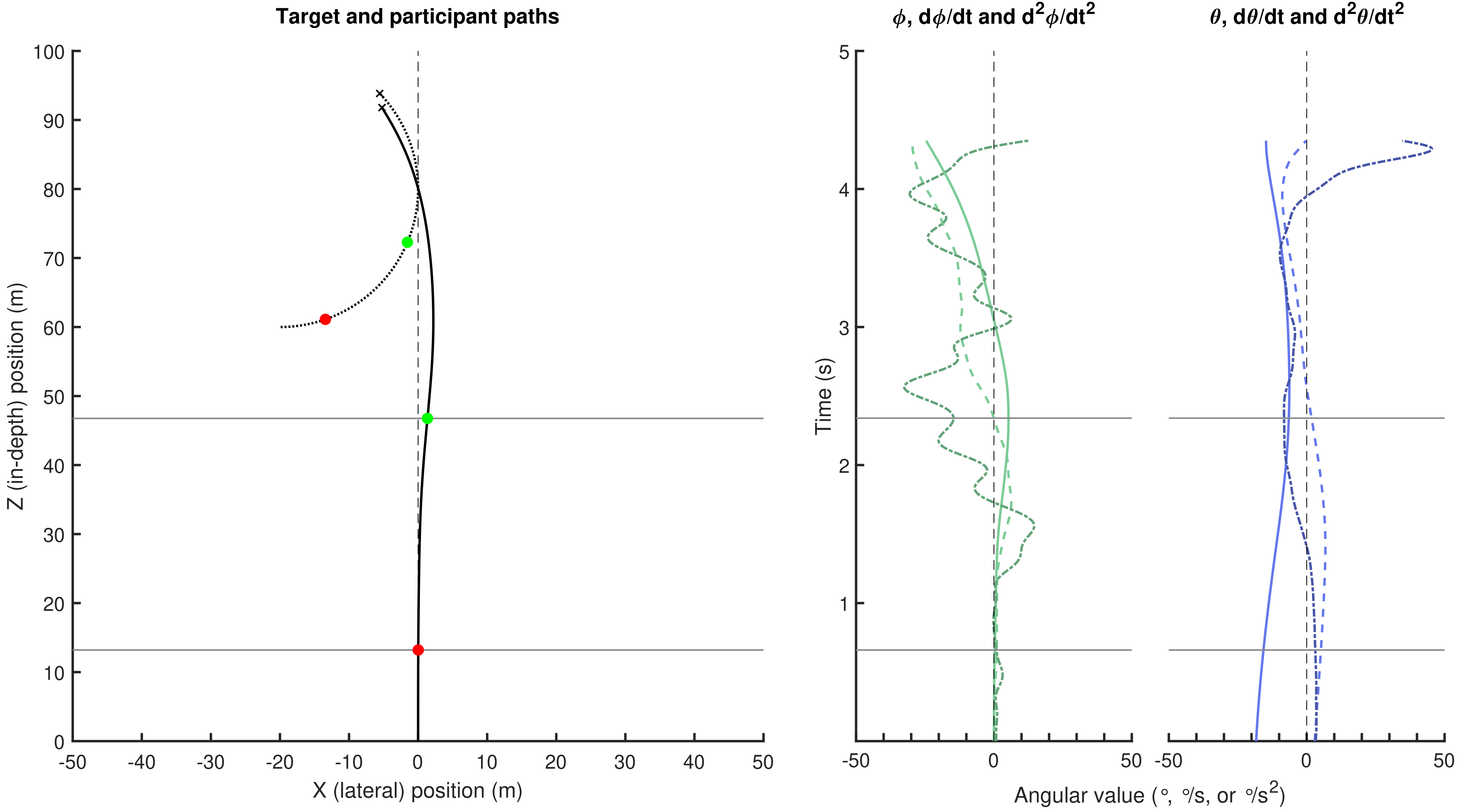

P18/B5
S20/R30-IN

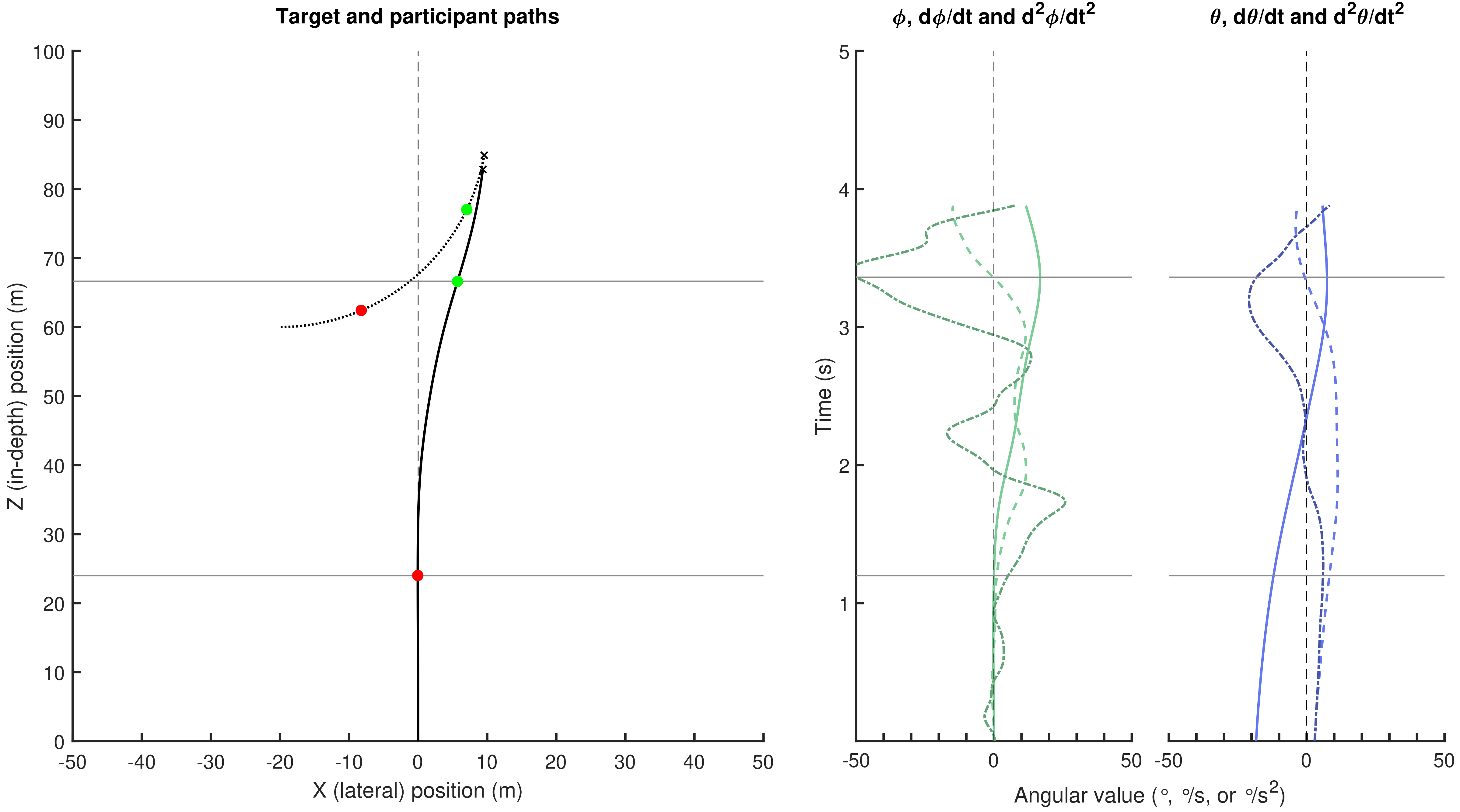

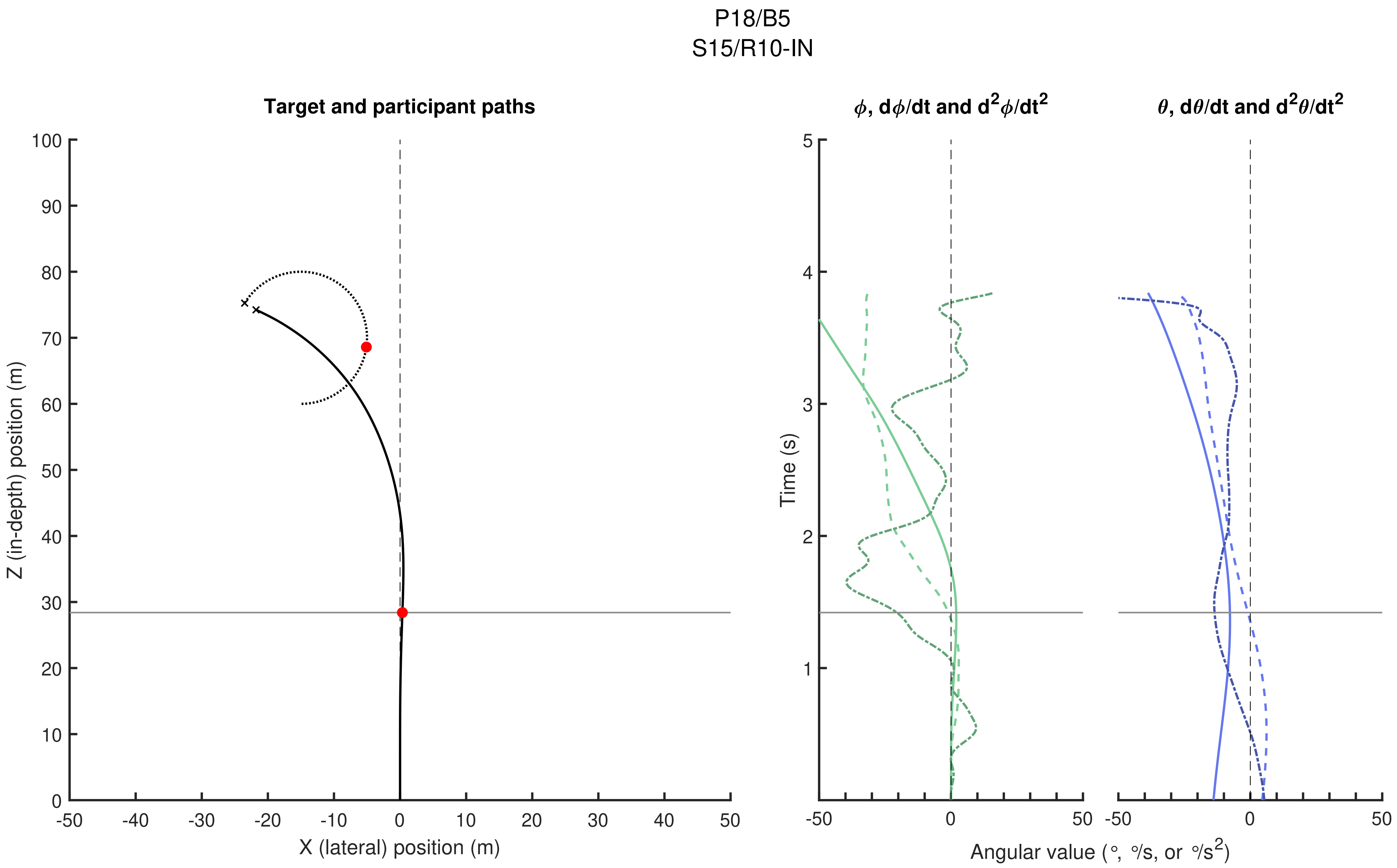
P18/B5
S15/R10-IN
Target and participant paths
Z (in-depth) position (m)
X (lateral) position (m)
0
10
20
30
40
50
60
70
80
90
100
-50
-40
-30
-20
-10
0
10
20
30
40
50
φ, dφ/dt and d²φ/dt²
θ, dθ/dt and d²θ/dt²
Time (s)
1
2
3
4
5
-50
0
50
-50
0
50
Angular value (°, °/s, or °/s²)

P18/B5
S15/R20-IN

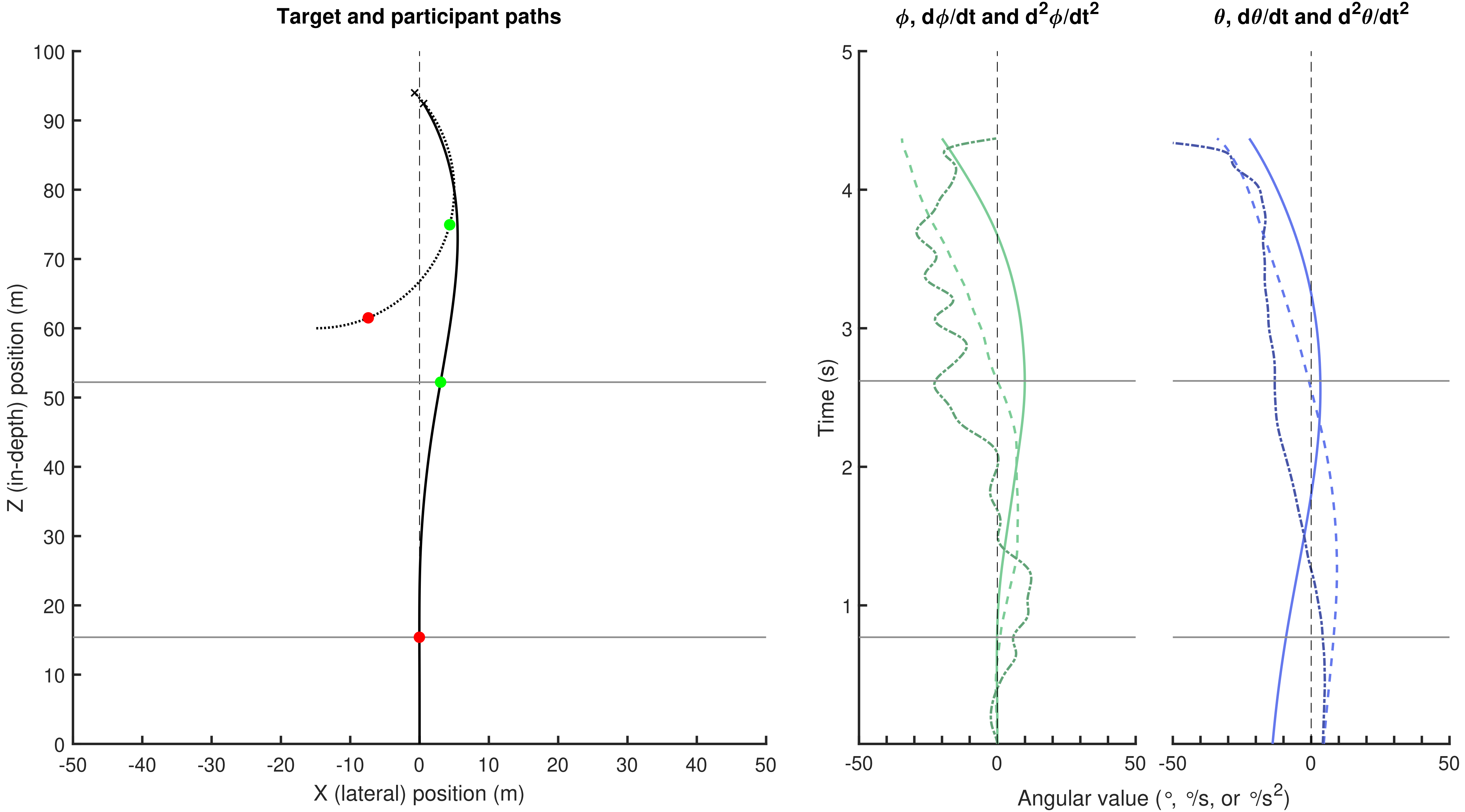

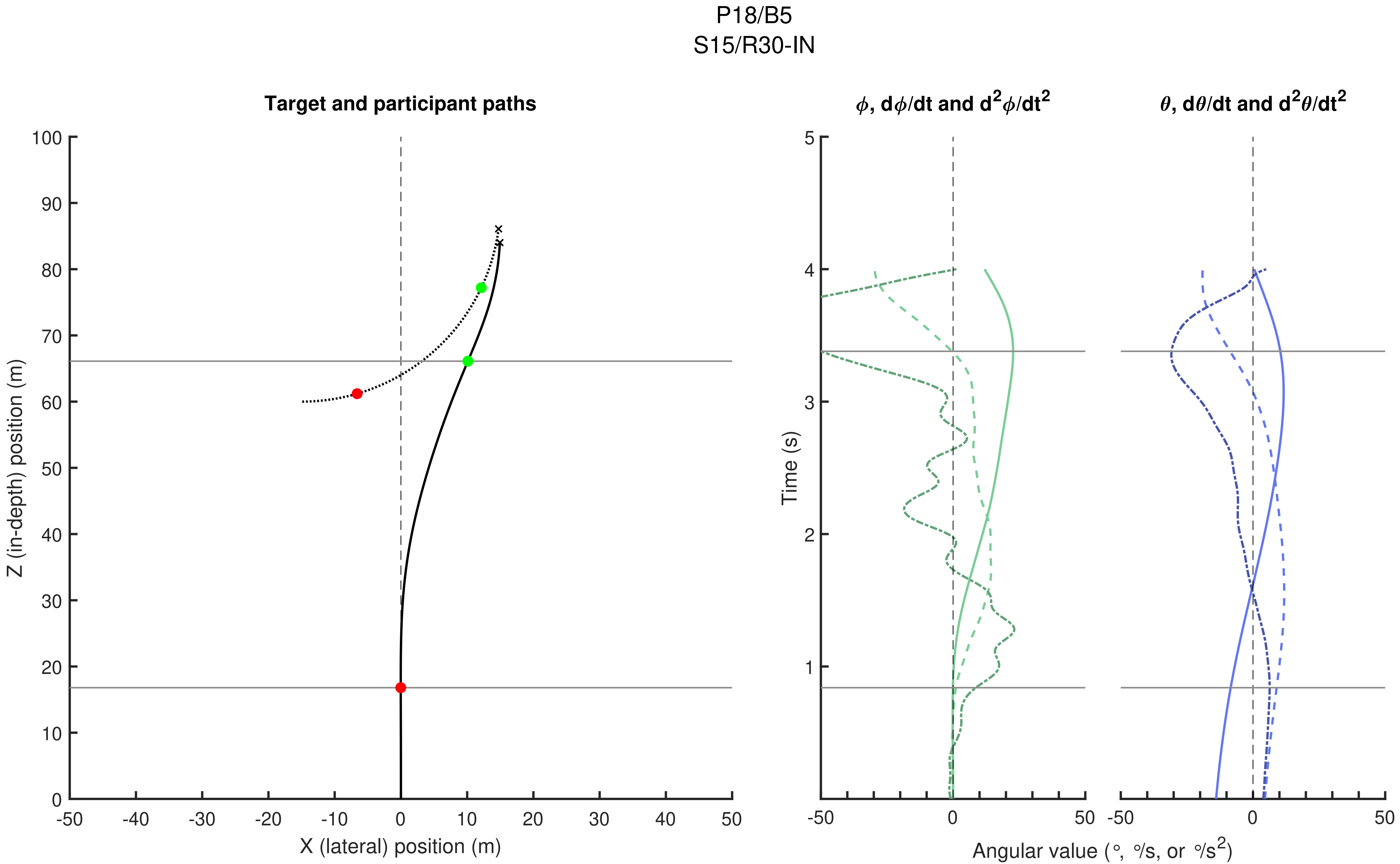

P18/B5
S15/R30-IN
Target and participant paths
Z (in-depth) position (m)
X (lateral) position (m)
100
90
80
70
60
50
40
30
20
10
0
-50
-40
-30
-20
-10
0
10
20
30
40
50
φ, dφ/dt and d²φ/dt²
θ, dθ/dt and d²θ/dt²
Time (s)
5
4
3
2
1
-50
0
50
-50
0
50
Angular value (°, °/s, or °/s²)

P18/B5
S5/R10-IN

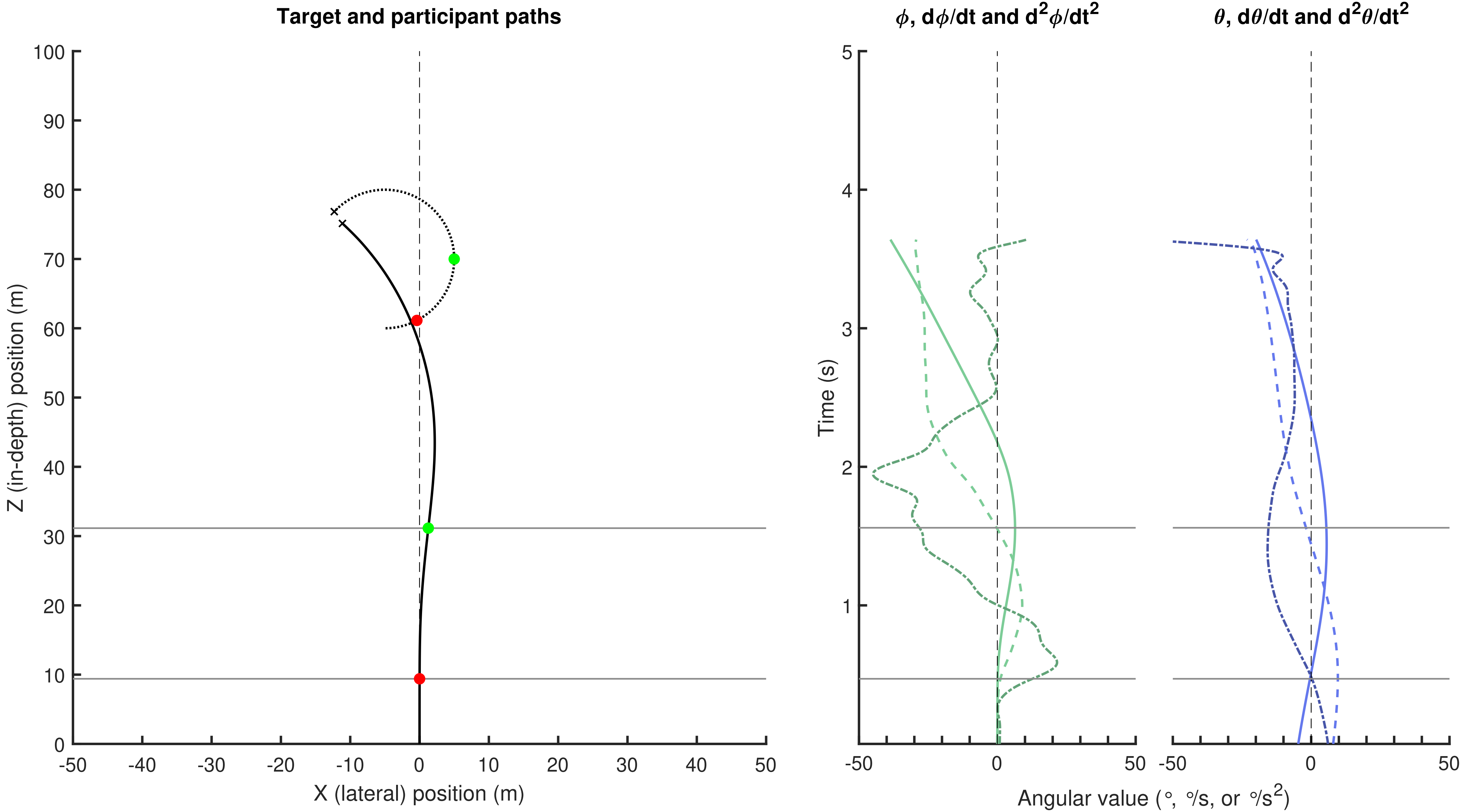

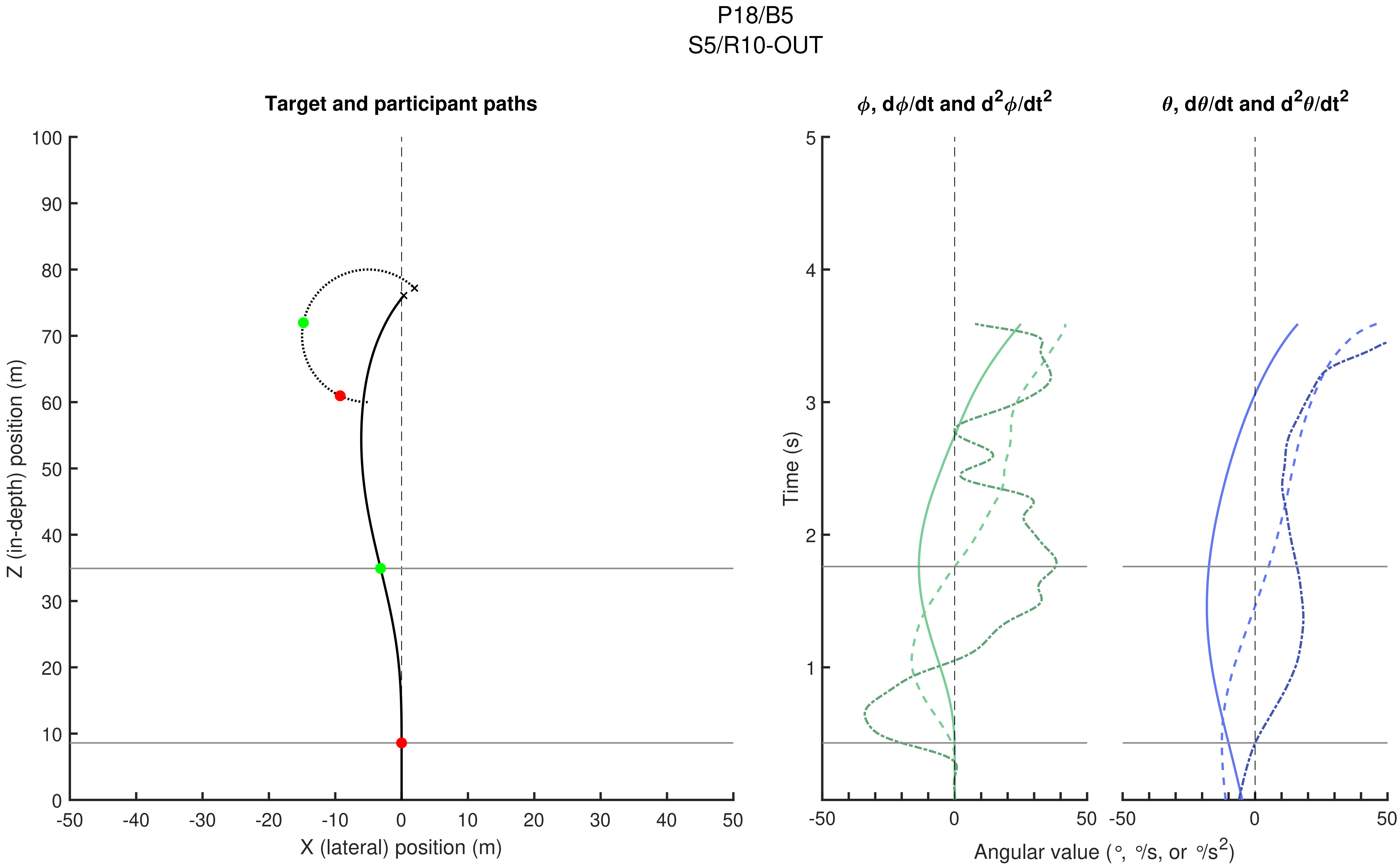

P18/B5
S5/R10-OUT
Target and participant paths
Z (in-depth) position (m)
X (lateral) position (m)
100
90
80
70
60
50
40
30
20
10
0
-50
-40
-30
-20
-10
0
10
20
30
40
50
ϕ, dϕ/dt and d$^2\phi$/dt^2
θ, dθ/dt and d$^2\theta$/dt^2
Time (s)
5
4
3
2
1
-50
0
50
Angular value (°, °/s, or °/s^2)

P18/B5
S5/R20-IN

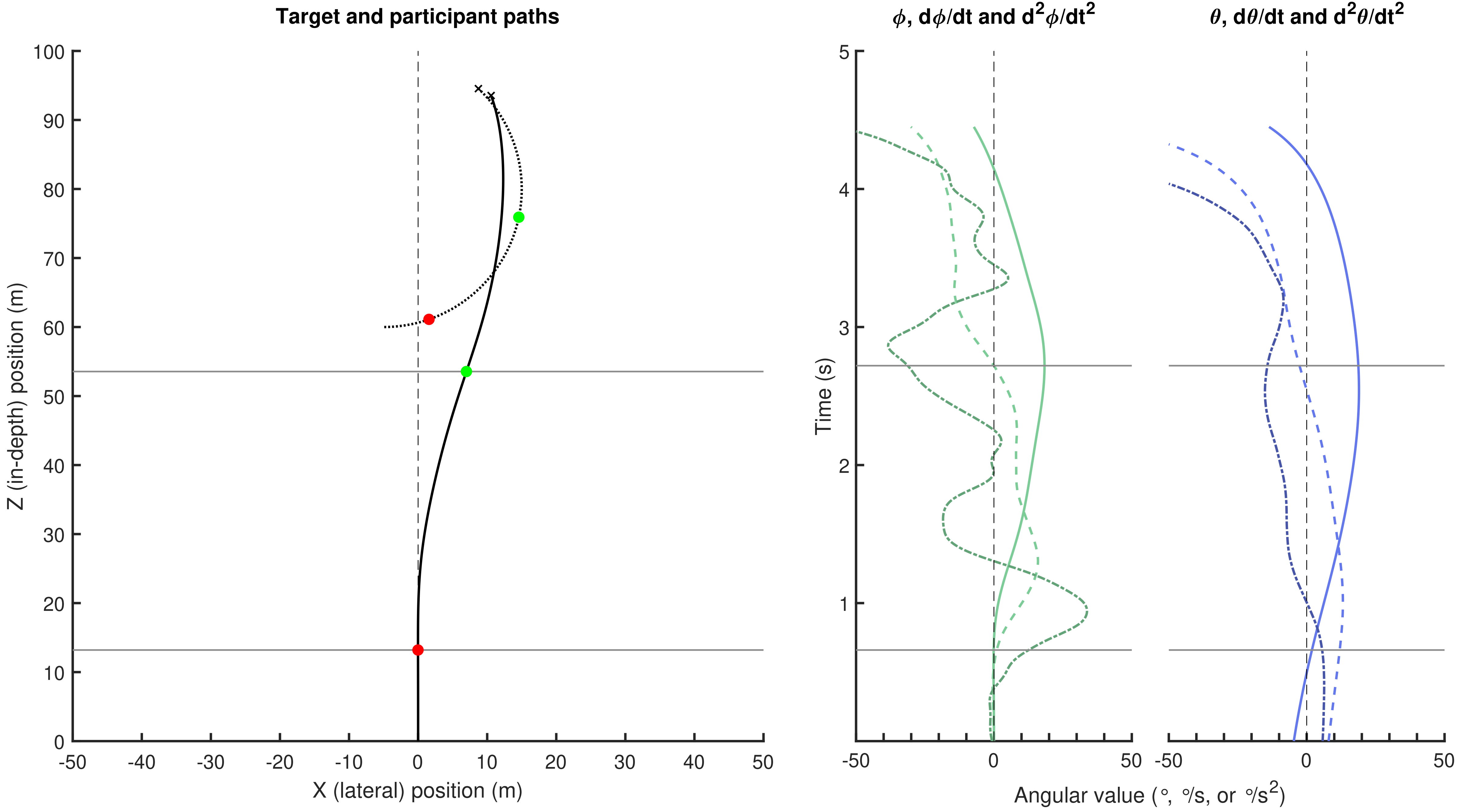

P18/B5
S5/R20-OUT

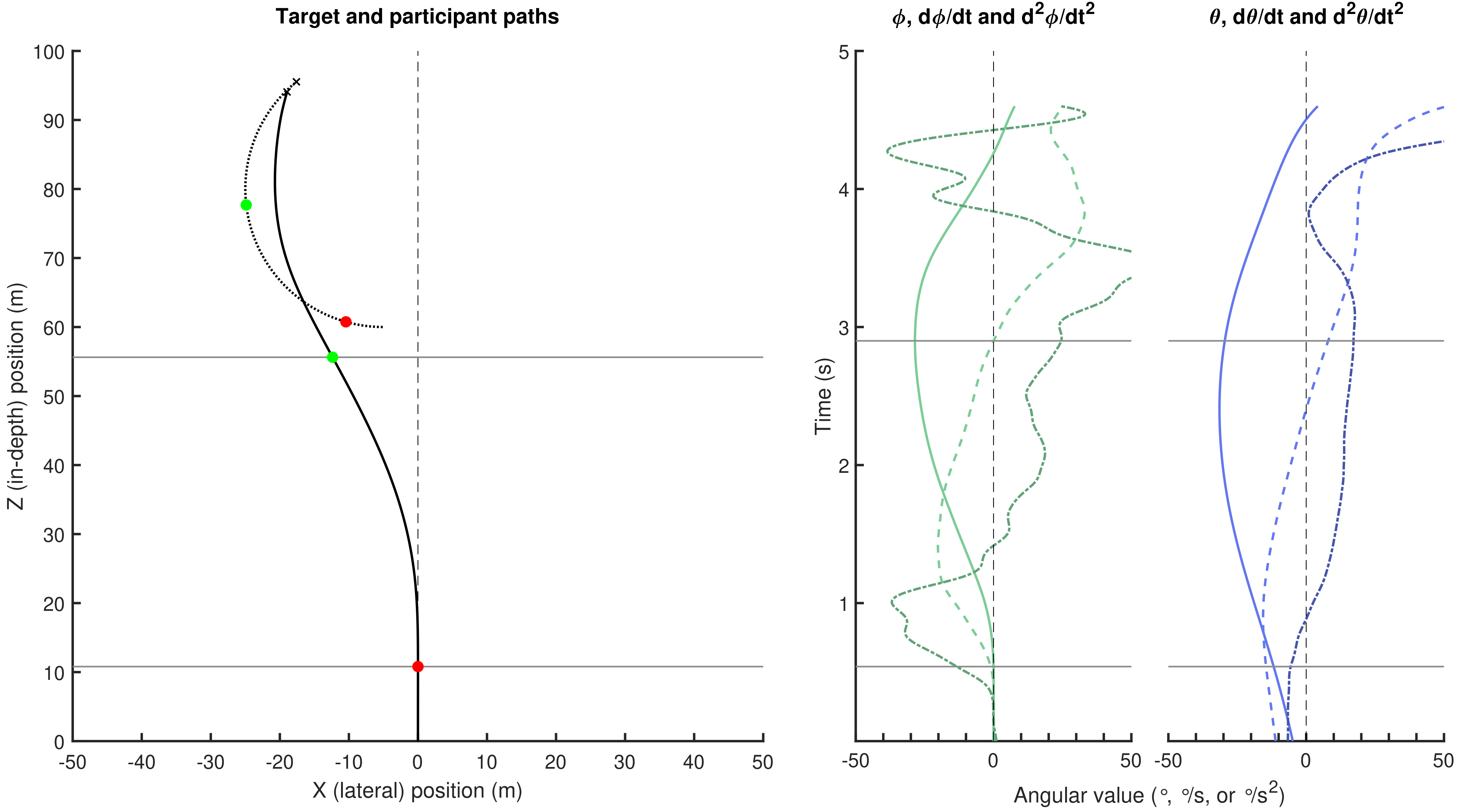

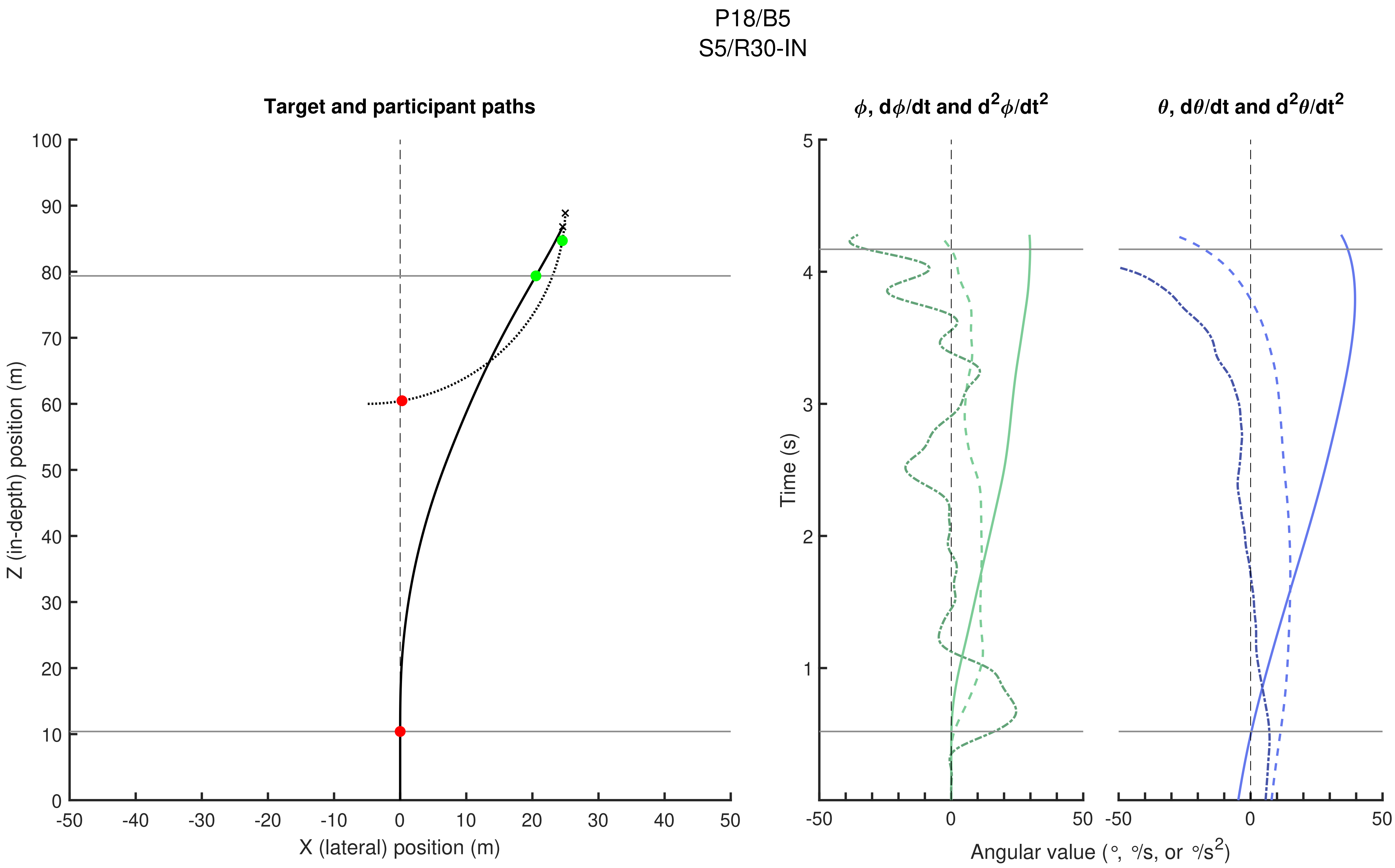
P18/B5
S5/R30-IN
Target and participant paths
Z (in-depth) position (m)
X (lateral) position (m)
100
90
80
70
60
50
40
30
20
10
0
-50
-40
-30
-20
-10
0
10
20
30
40
50
ϕ, dϕ/dt and d$^2\phi$/dt^2
θ, dθ/dt and d$^2\theta$/dt^2
Time (s)
5
4
3
2
1
-50
0
50
-50
0
50
Angular value (°, °/s, or °/s^2)

P18/B5
S5/R30-OUT

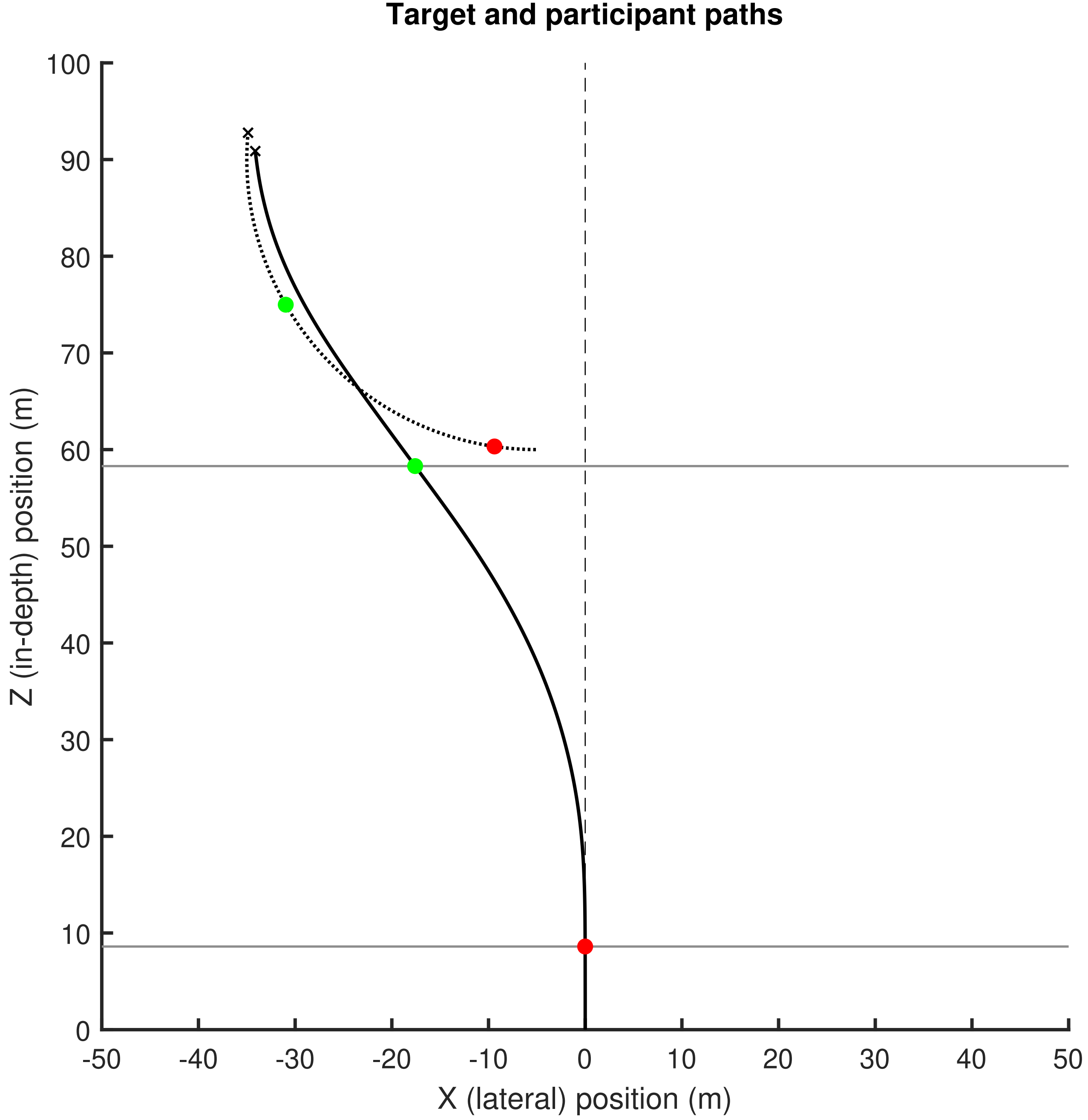

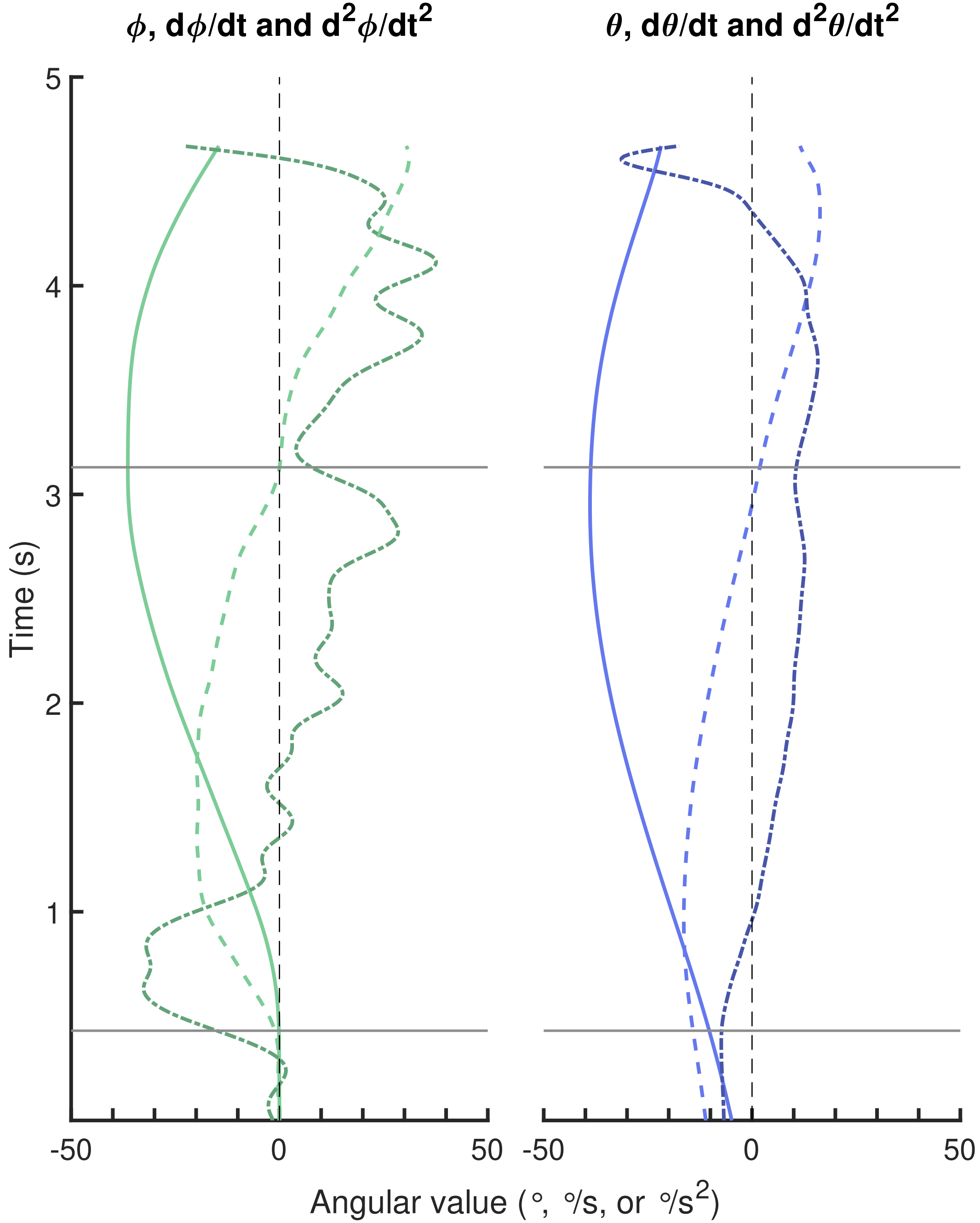

P18/B5
S5/R10-OUT

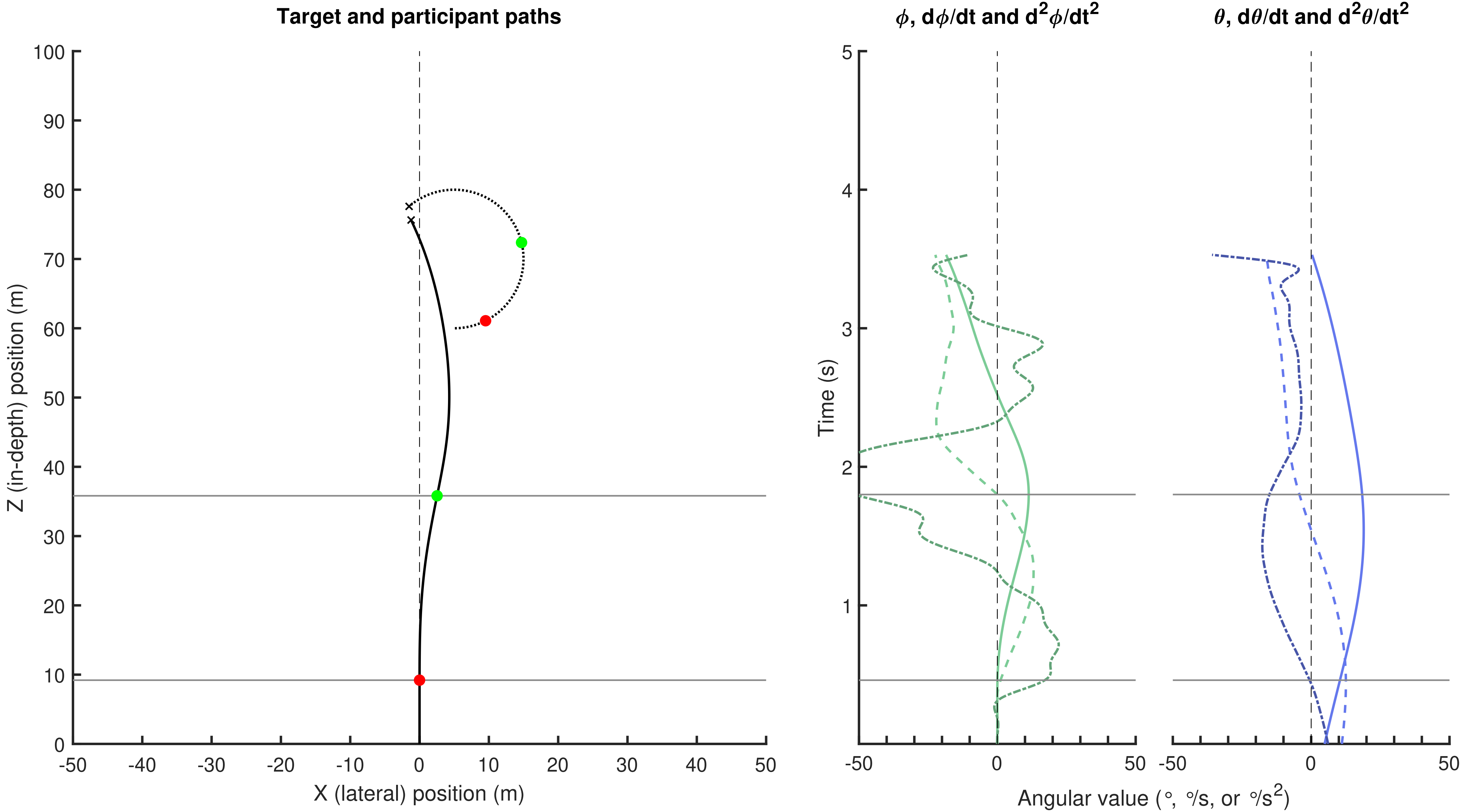

P18/B5
S5/R10-IN

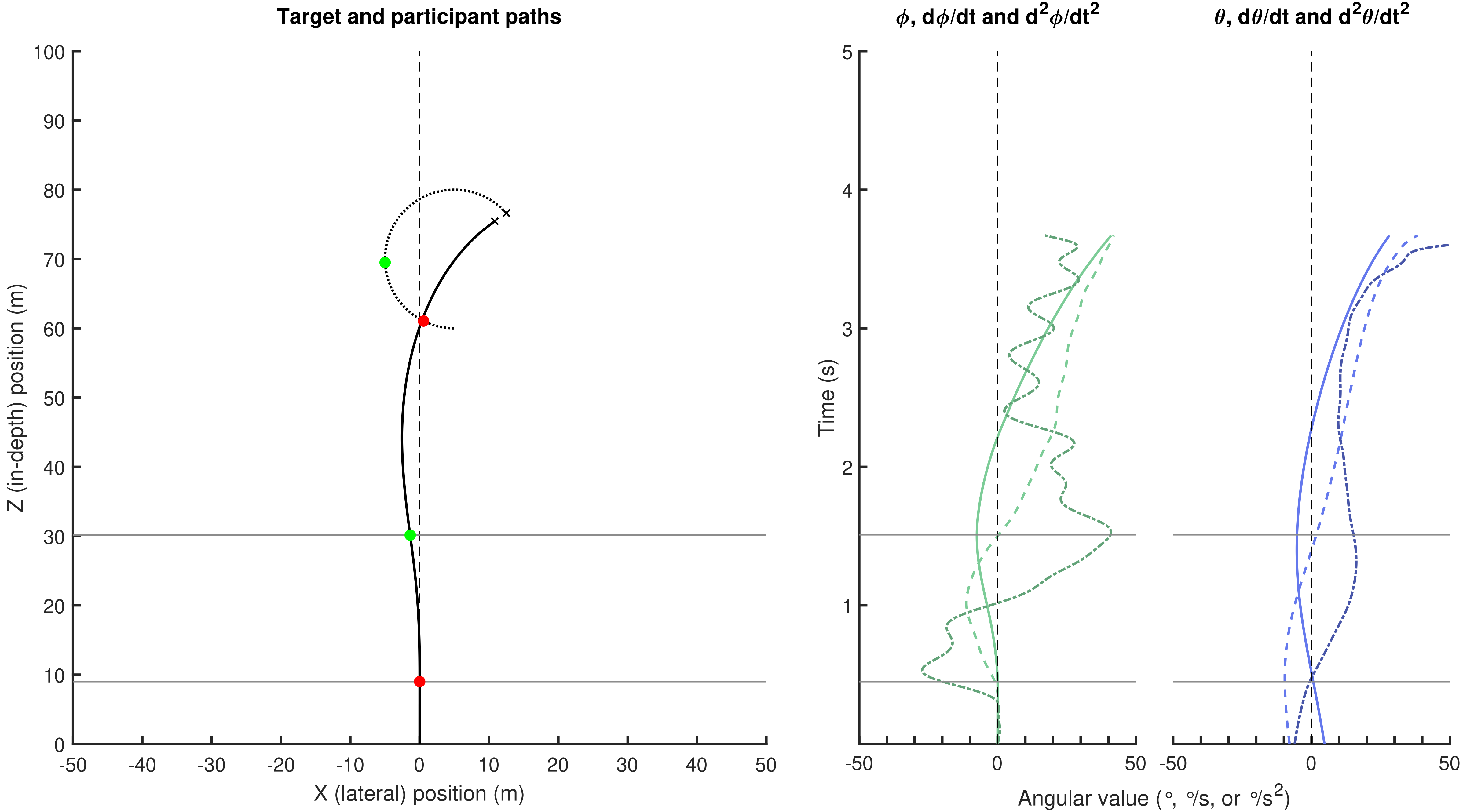

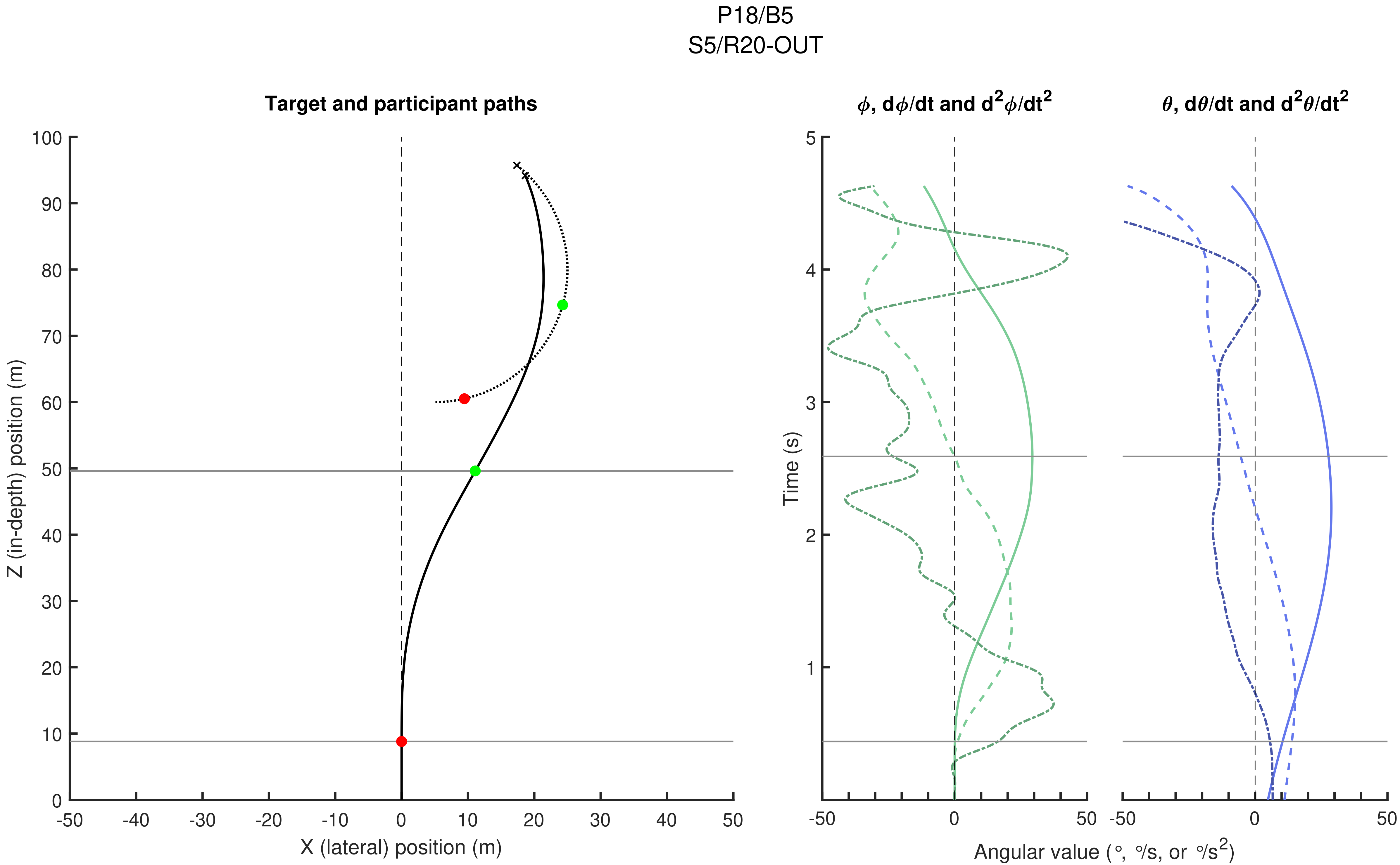
P18/B5
S5/R20-OUT
Target and participant paths
Z (in-depth) position (m)
X (lateral) position (m)
100
90
80
70
60
50
40
30
20
10
0
-50
-40
-30
-20
-10
0
10
20
30
40
50
ϕ, dϕ/dt and d²ϕ/dt²
θ, dθ/dt and d²θ/dt²
Time (s)
5
4
3
2
1
-50
0
50
-50
0
50
Angular value (°, °/s, or °/s²)

P18/B5
S5/R20-IN

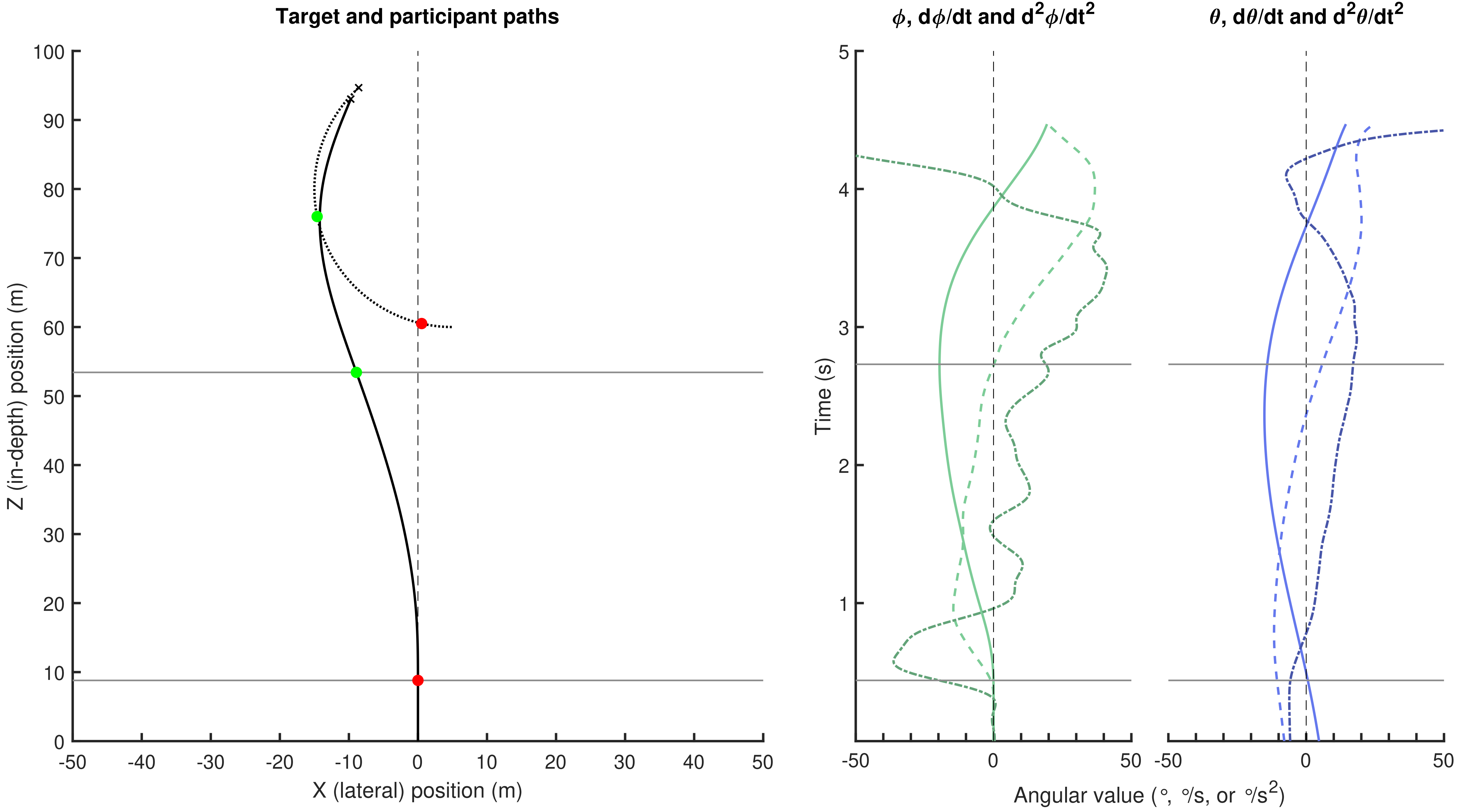

P18/B5
S5/R30-OUT

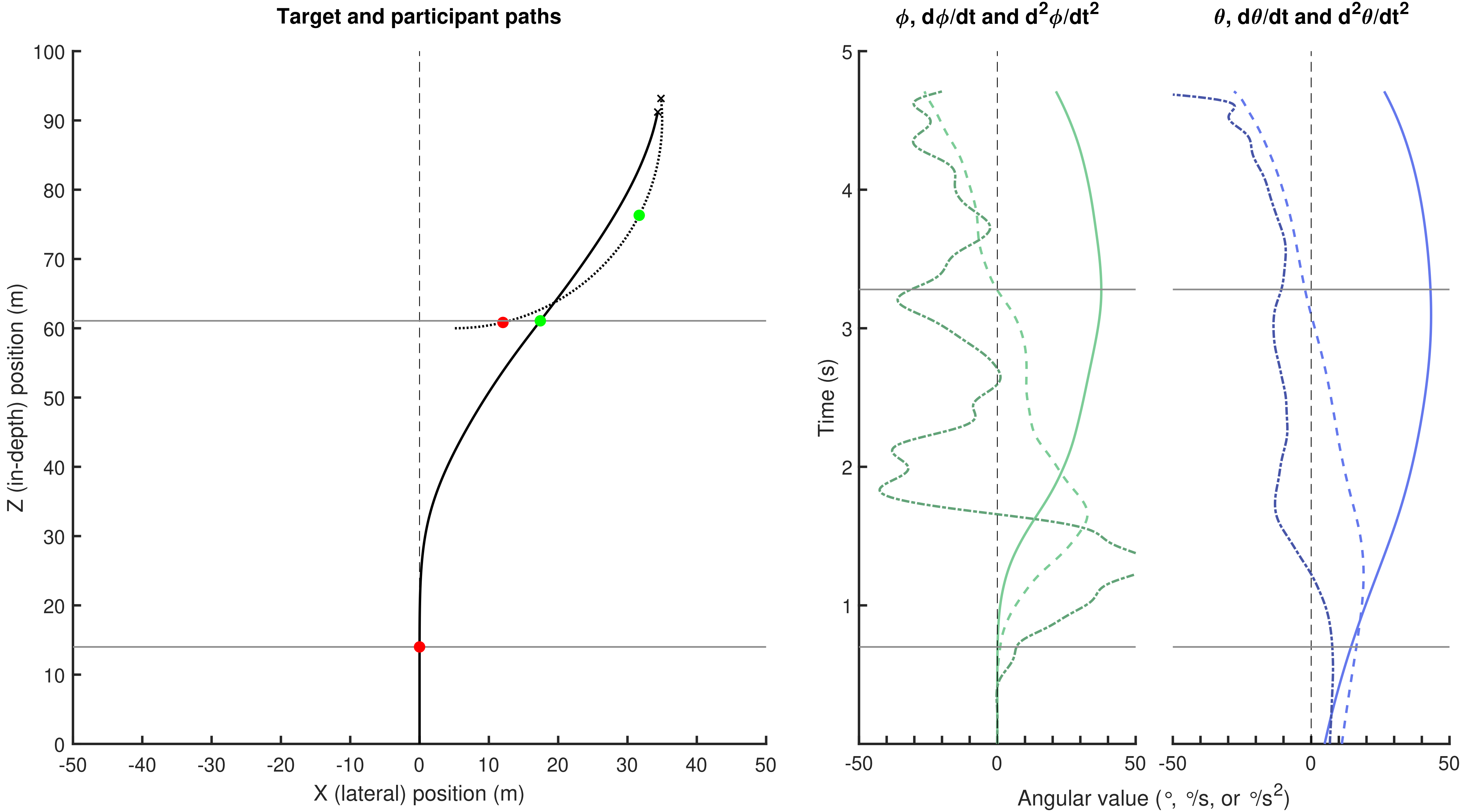

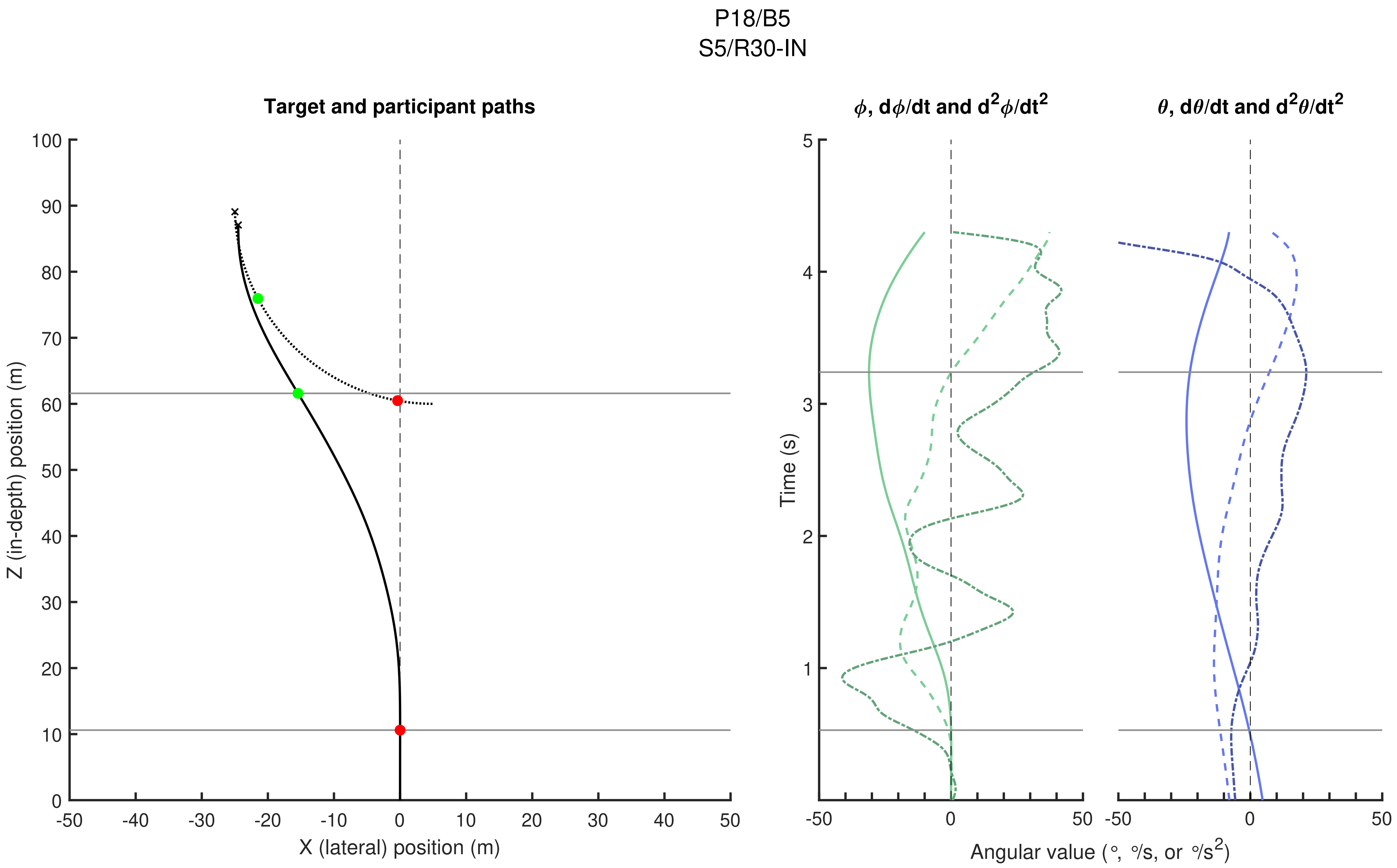
P18/B5
S5/R30-IN
Target and participant paths
Z (in-depth) position (m)
X (lateral) position (m)
ϕ, dϕ/dt and d$^2\phi$/dt^2
θ, dθ/dt and d$^2\theta$/dt^2
Time (s)
Angular value (°, °/s, or °/s^2)
100
90
80
70
60
50
40
30
20
10
0
-50
-40
-30
-20
-10
0
10
20
30
40
50
5
4
3
2
1
-50
0
50
-50
0
50

P18/B5
S15/R10-IN

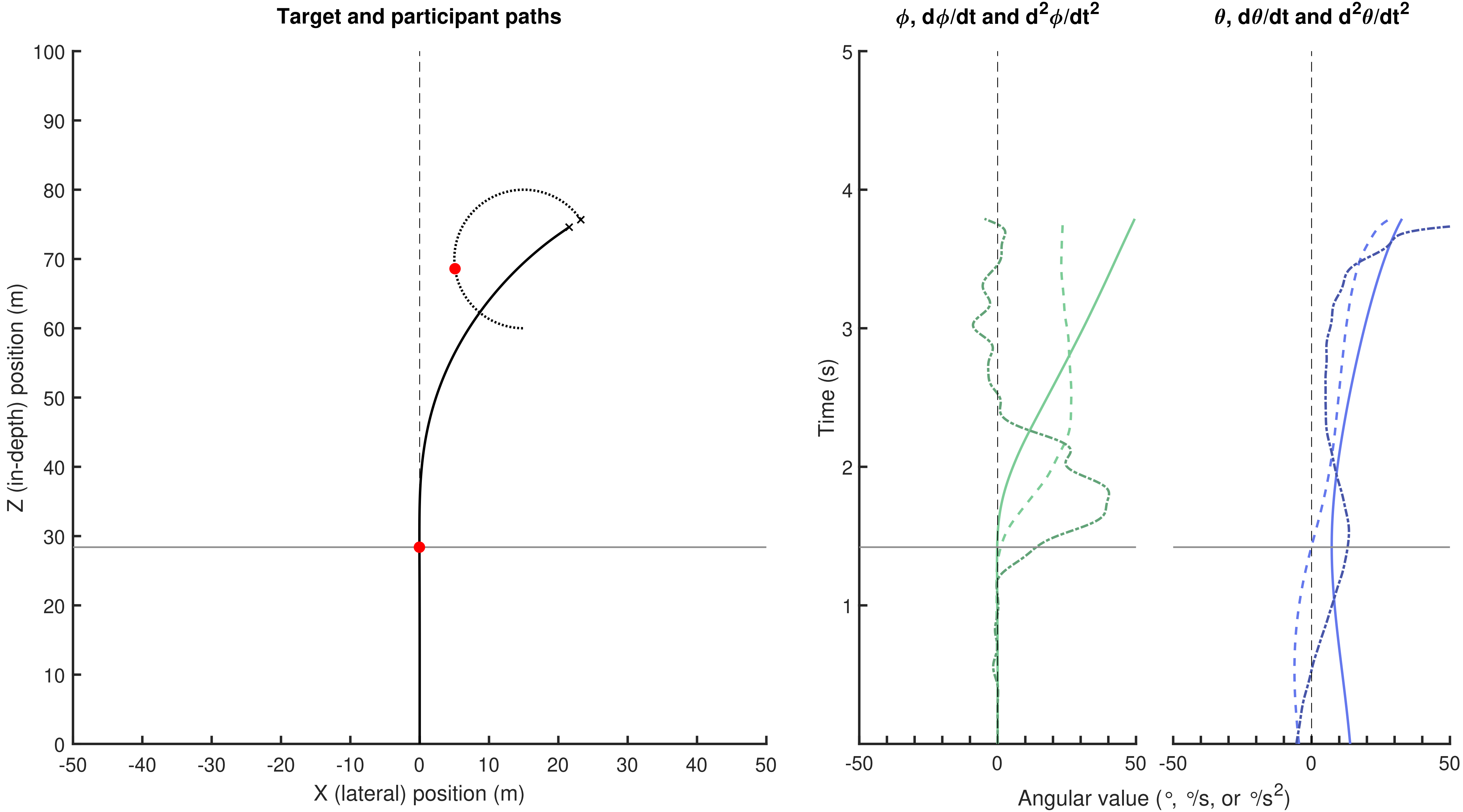

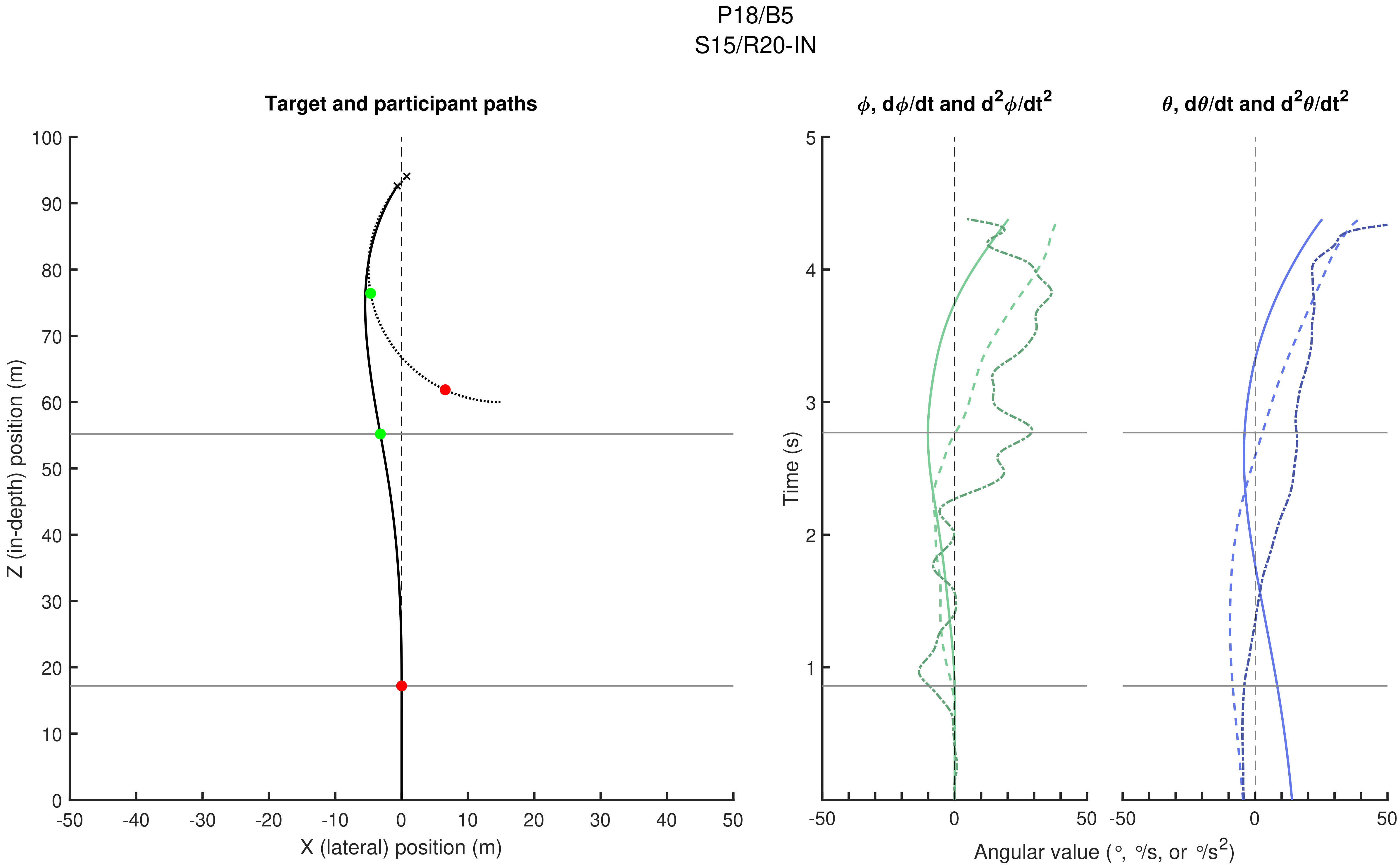
P18/B5
S15/R20-IN
Target and participant paths
ϕ, dϕ/dt and d$^2\phi$/dt^2
θ, dθ/dt and d$^2\theta$/dt^2
Z (in-depth) position (m)
X (lateral) position (m)
Time (s)
Angular value (°, °/s, or °/s^2)

P18/B5
S15/R30-IN

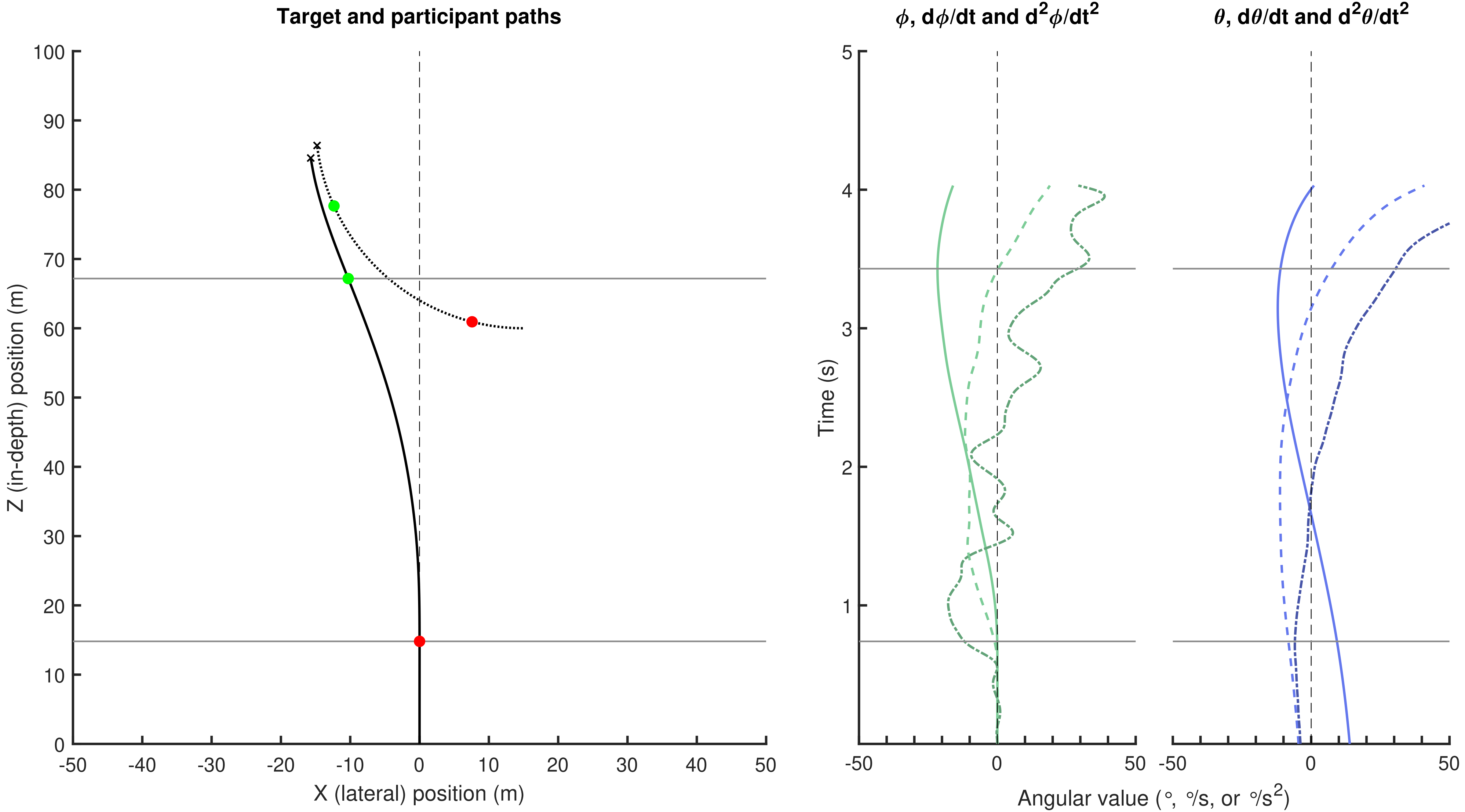

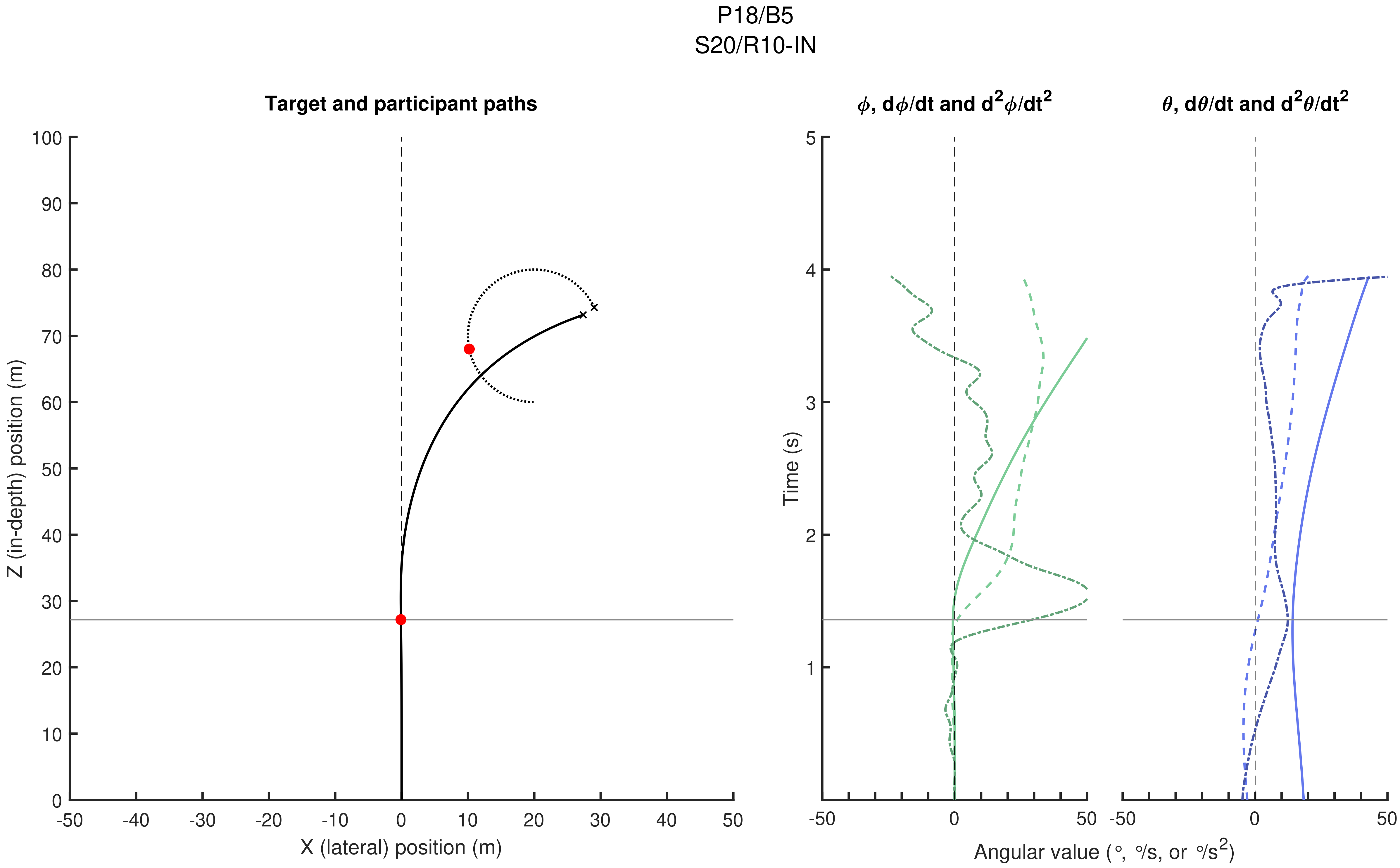
P18/B5
S20/R10-IN
Target and participant paths
Z (in-depth) position (m)
X (lateral) position (m)
ϕ, dϕ/dt and d²ϕ/dt²
θ, dθ/dt and d²θ/dt²
Time (s)
Angular value (°, °/s, or °/s²)

P18/B5
S20/R20-IN

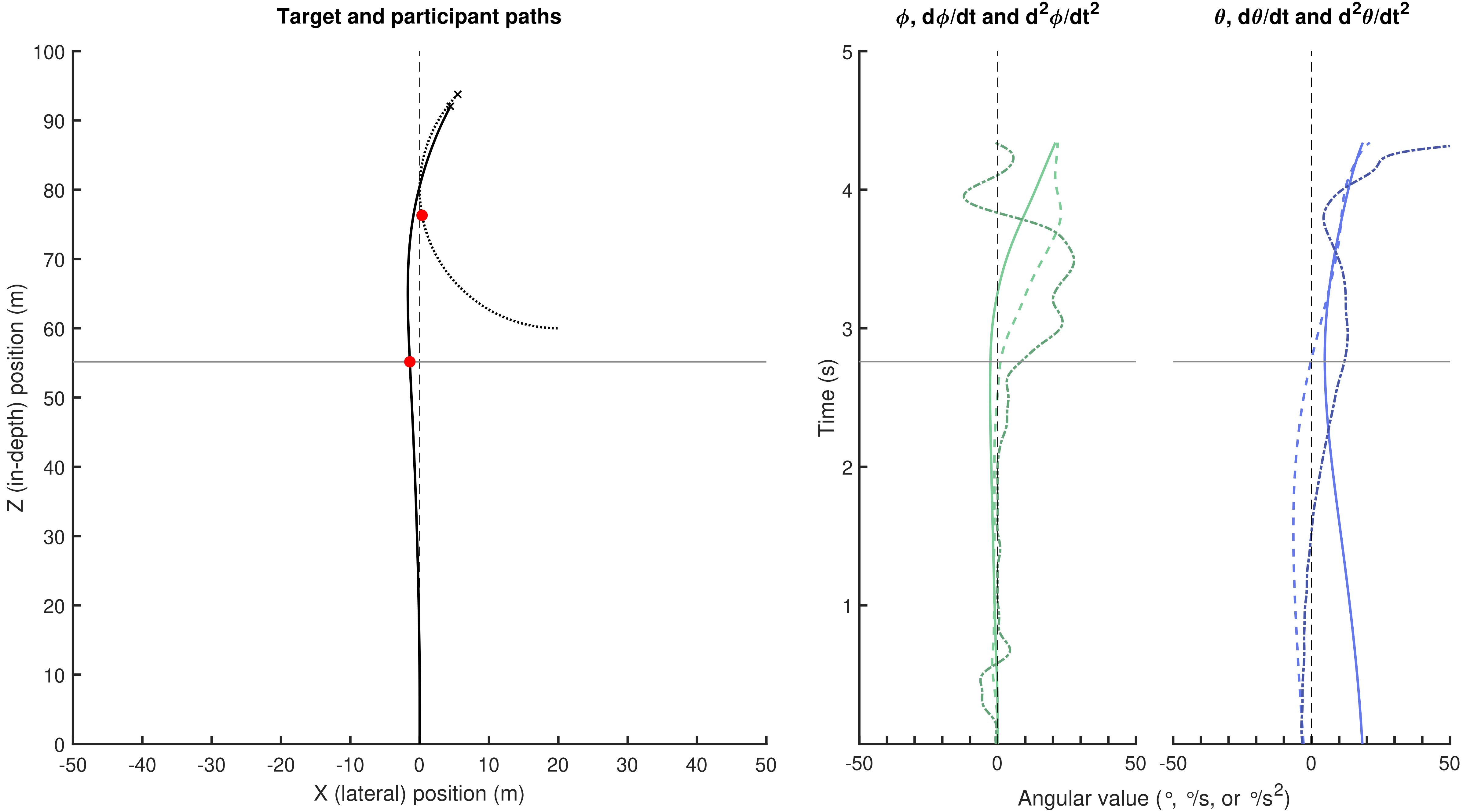

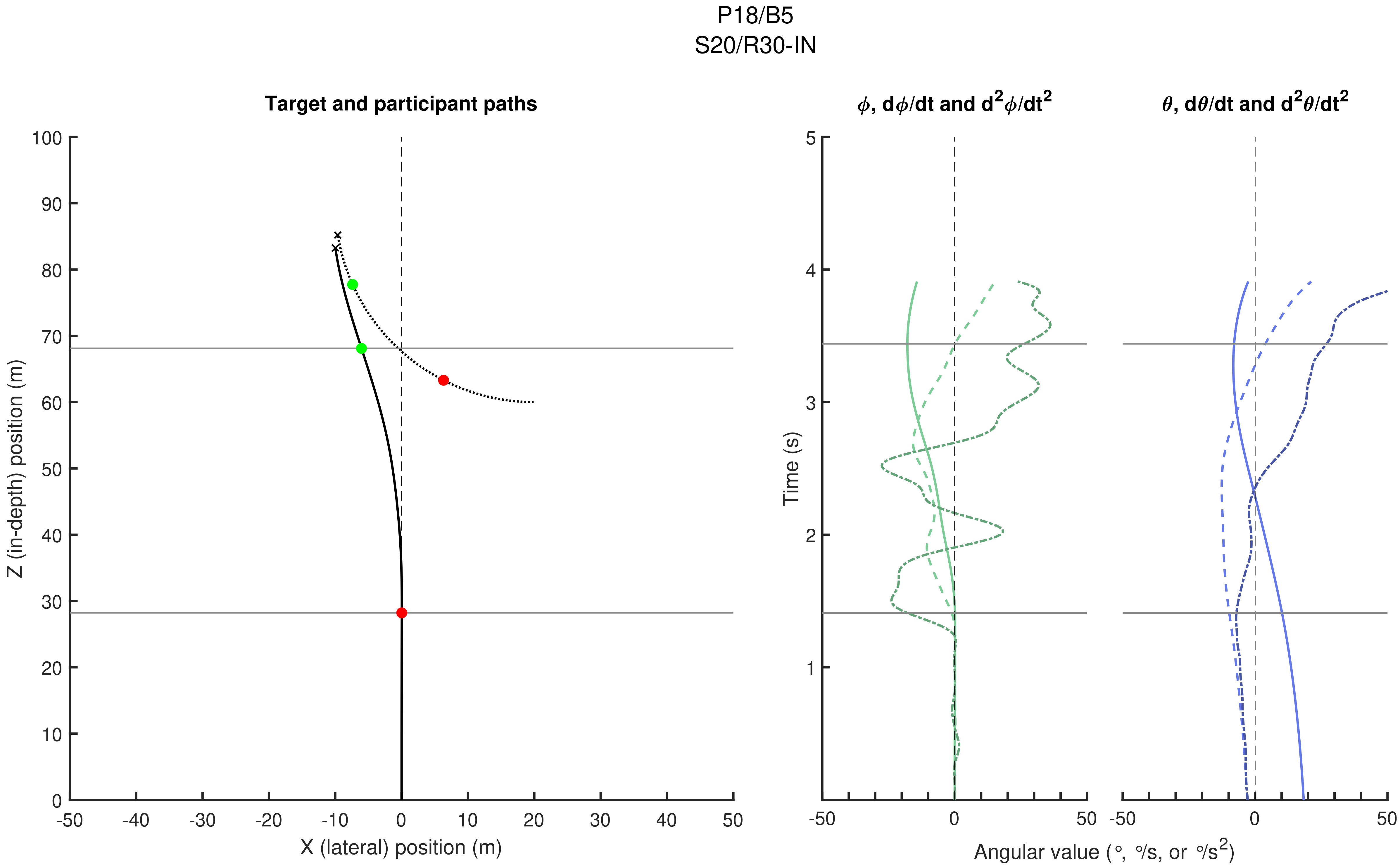
P18/B5
S20/R30-IN
Target and participant paths
Z (in-depth) position (m)
X (lateral) position (m)
ϕ, dϕ/dt and d$^2\phi$/dt^2
θ, dθ/dt and d$^2\theta$/dt^2
Time (s)
Angular value (°, °/s, or °/s^2)

P19/B1
S20/R10-IN

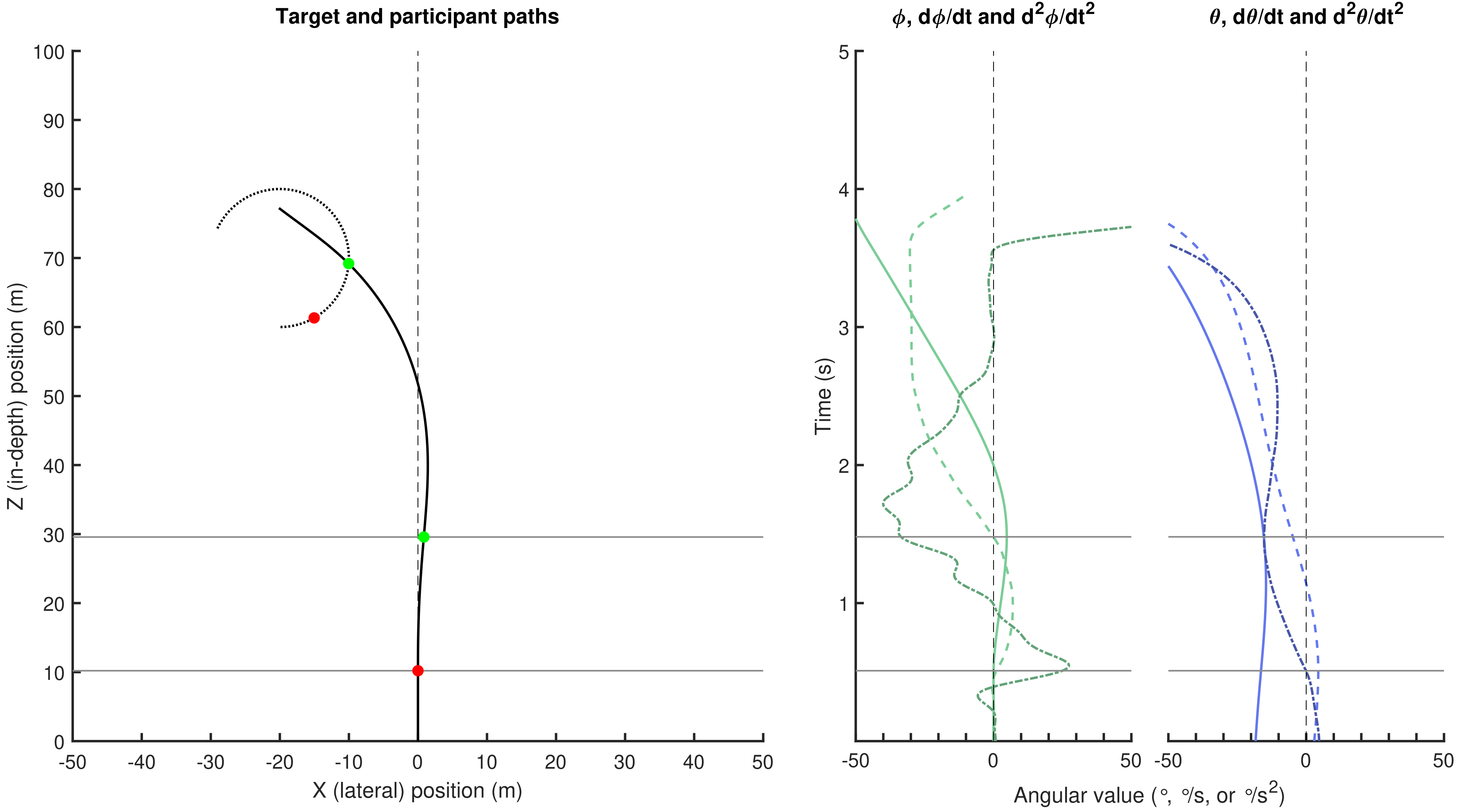

P19/B1
S20/R20-IN

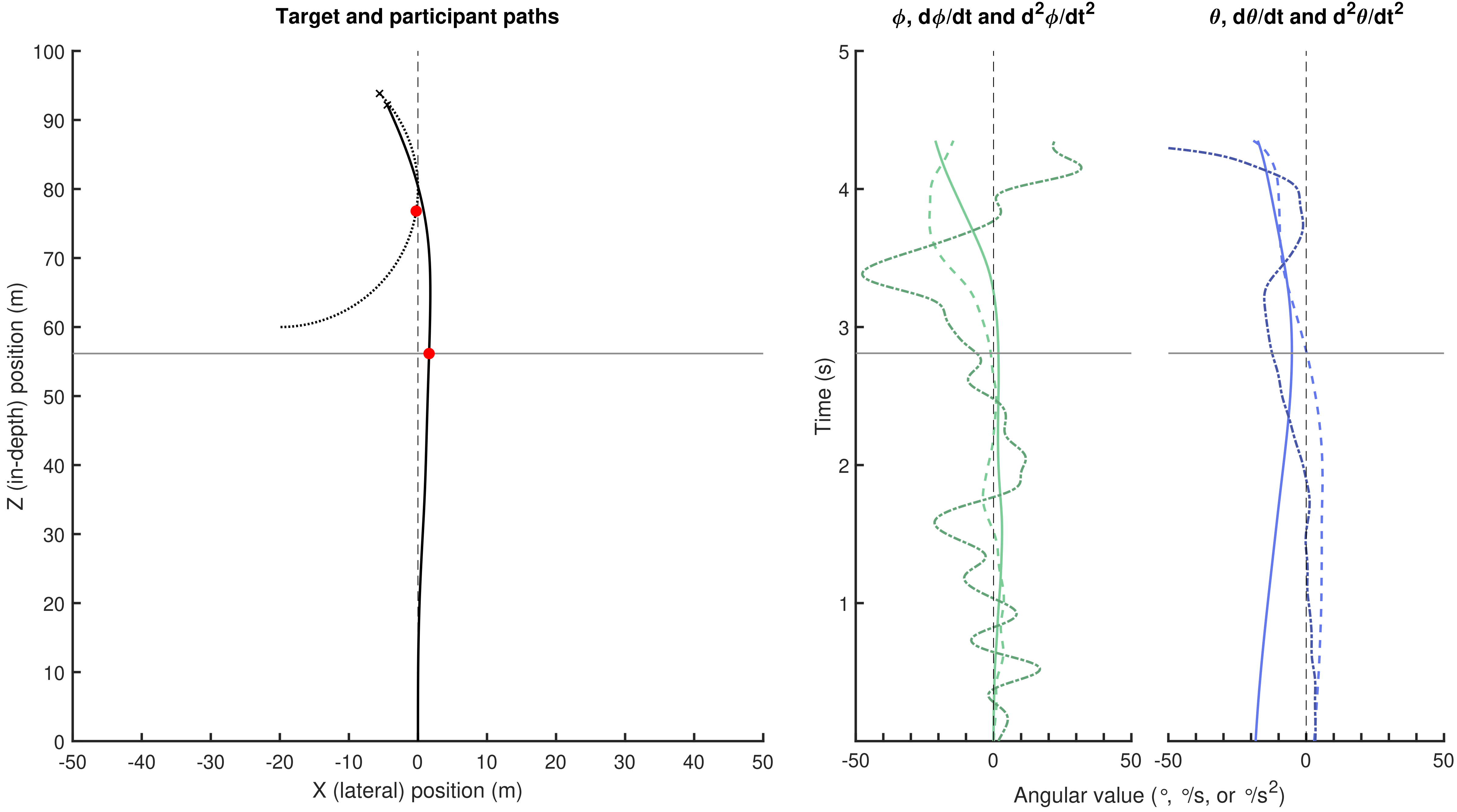

P19/B1
S20/R30-IN

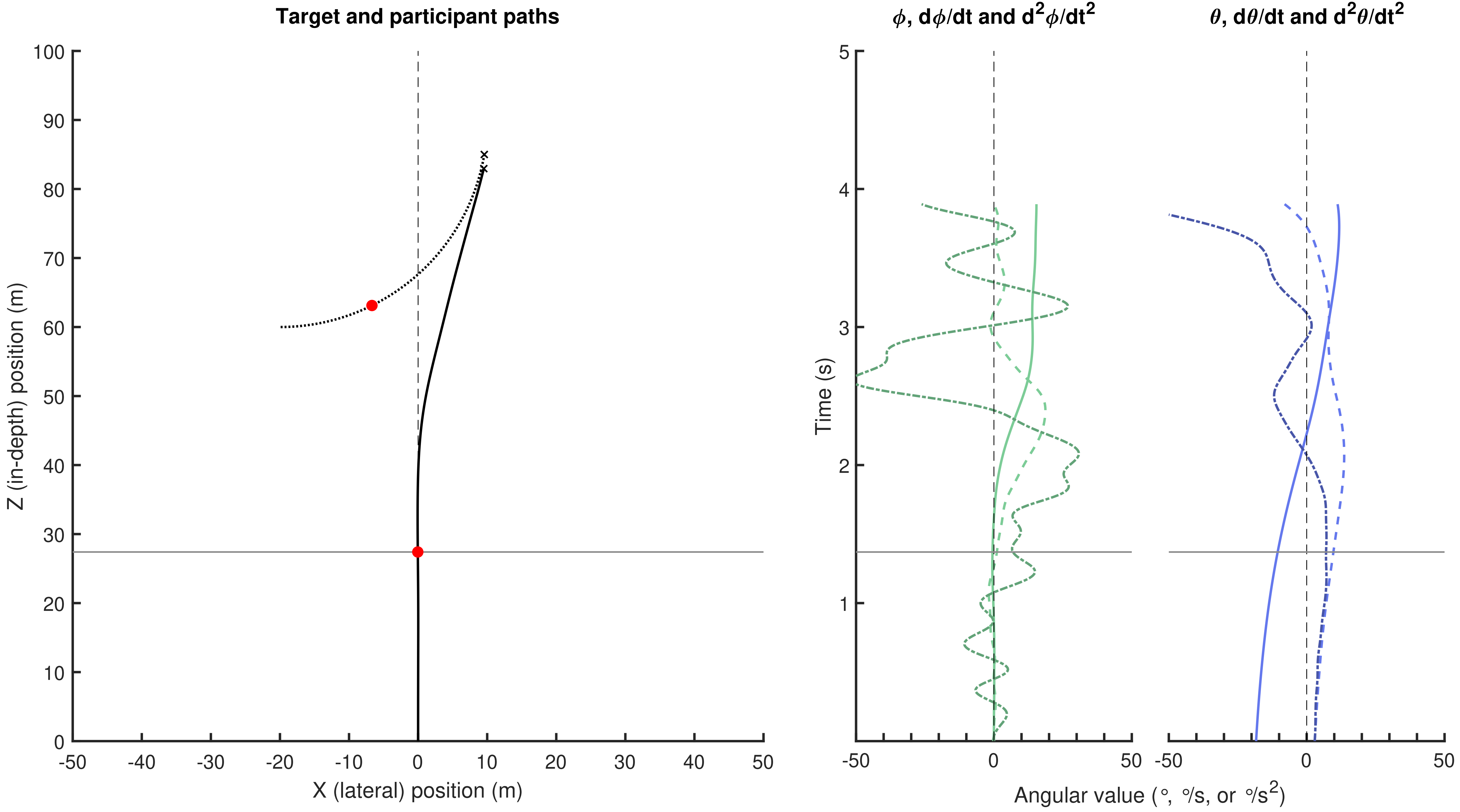

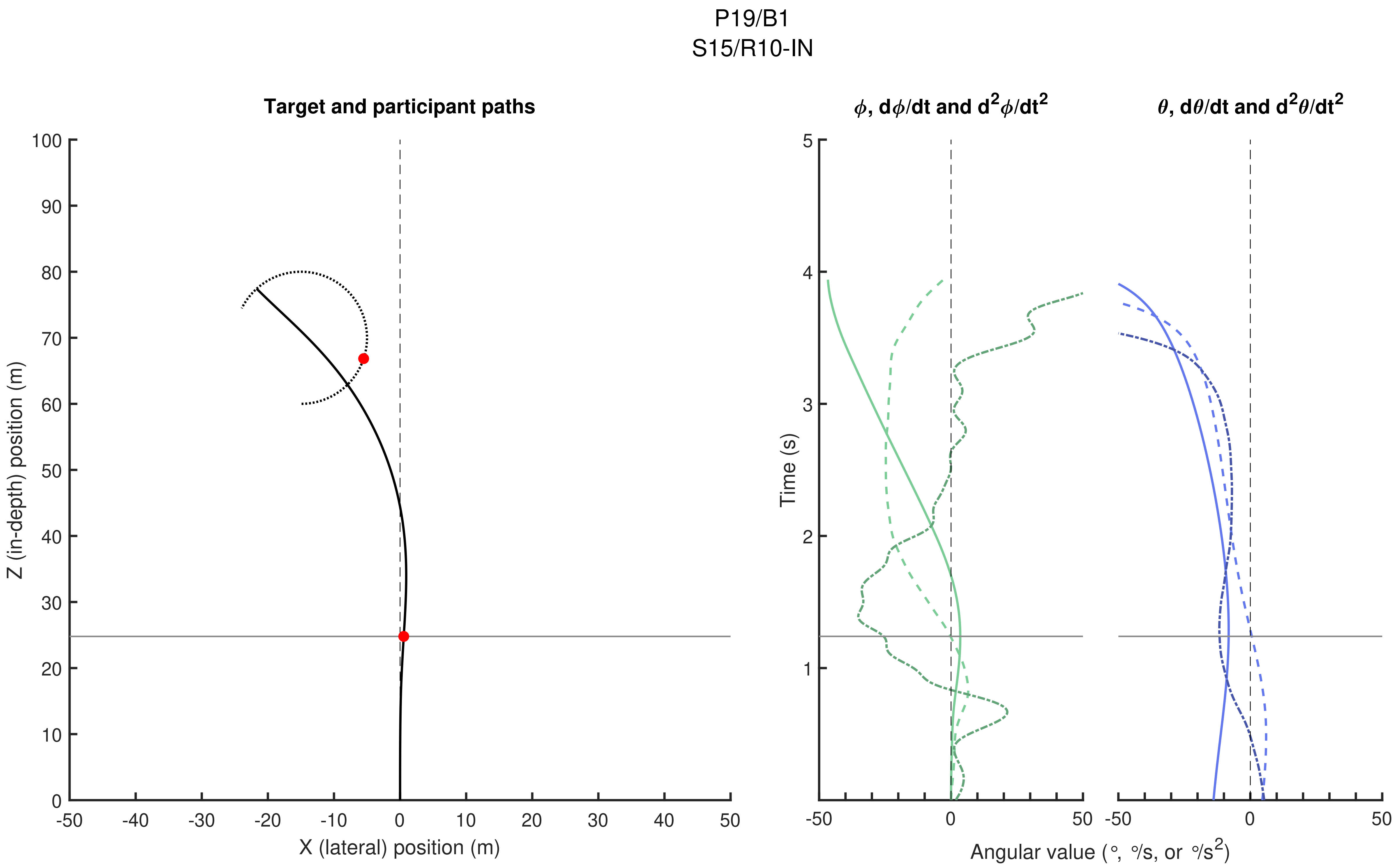
P19/B1
S15/R10-IN
Target and participant paths
ϕ, dϕ/dt and d$^2\phi$/dt^2
θ, dθ/dt and d$^2\theta$/dt^2
Z (in-depth) position (m)
X (lateral) position (m)
Time (s)
Angular value (°, °/s, or °/s^2)

P19/B1
S15/R20-IN

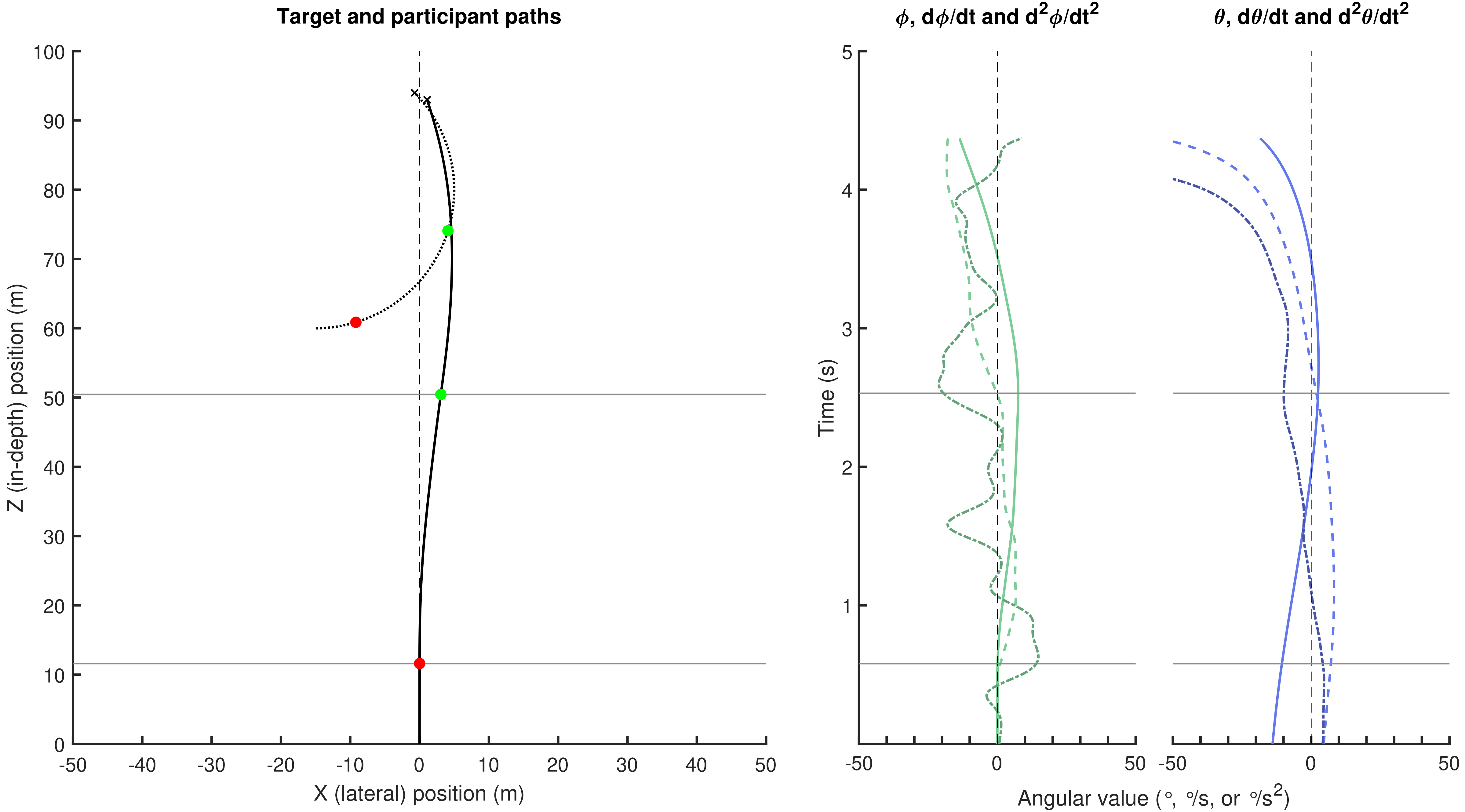

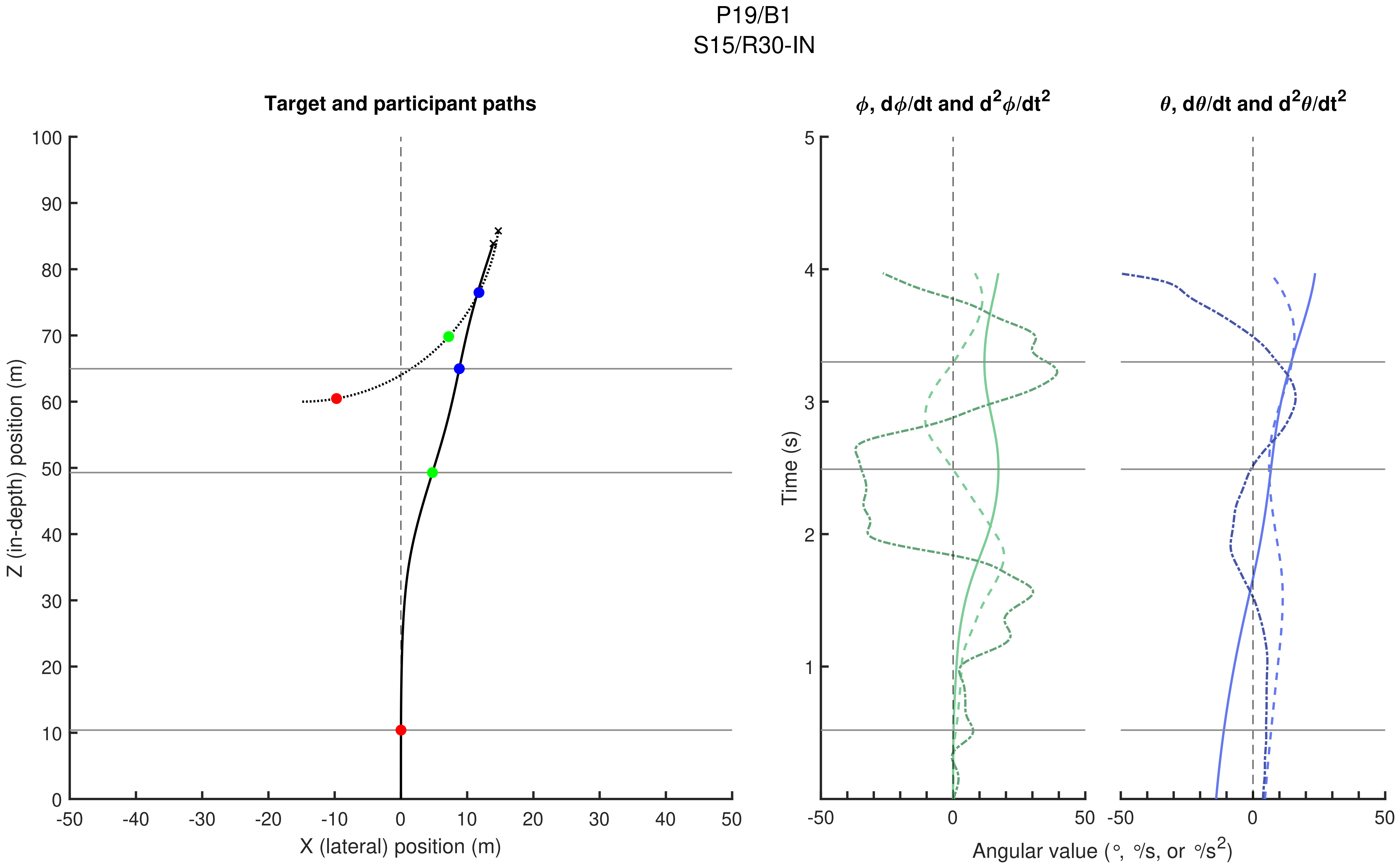

P19/B1
S15/R30-IN
Target and participant paths
Z (in-depth) position (m)
X (lateral) position (m)
ϕ, dϕ/dt and d$^2\phi$/dt^2
θ, dθ/dt and d$^2\theta$/dt^2
Time (s)
Angular value (°, °/s, or °/s^2)

P19/B1
S5/R10-IN

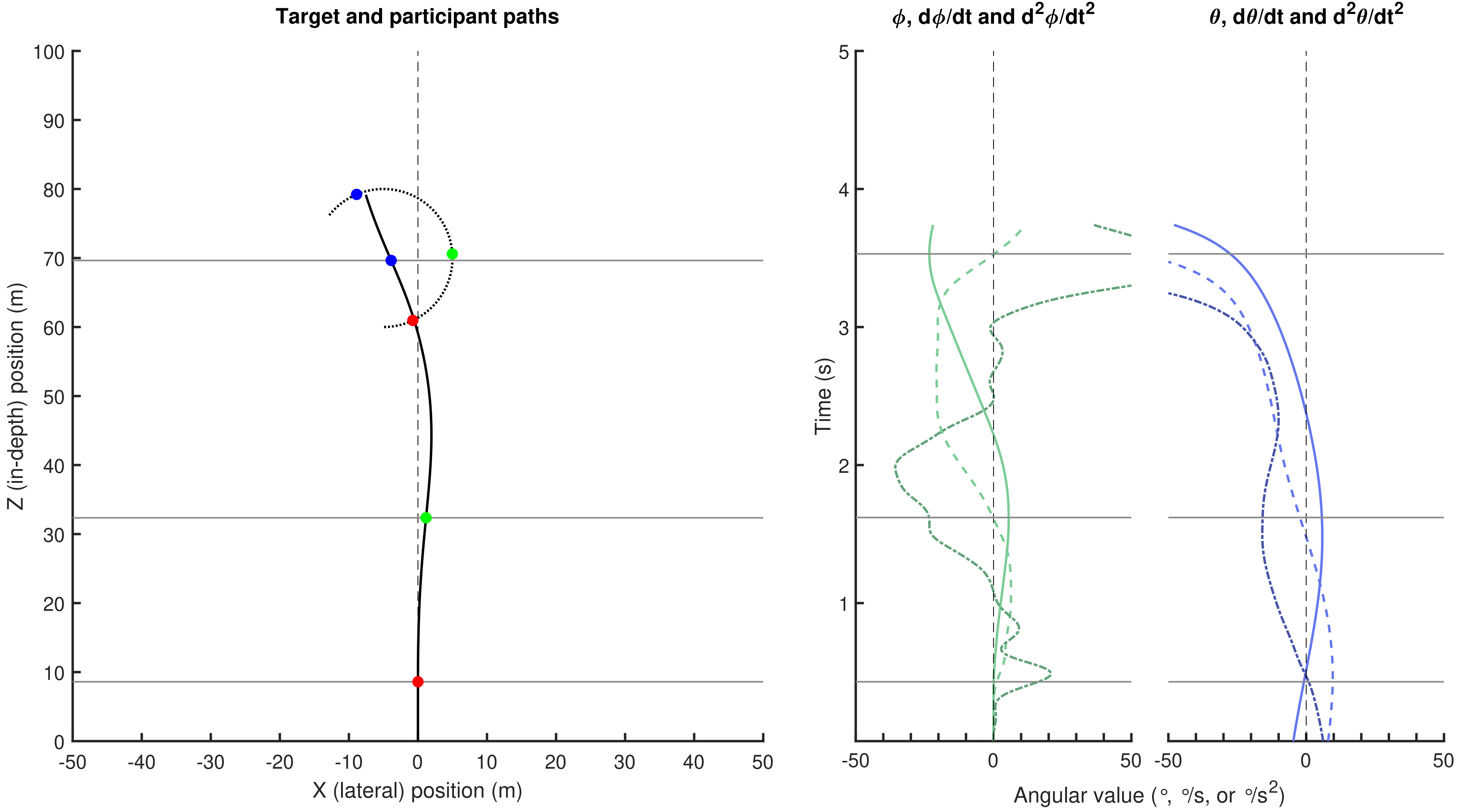

P19/B1
S5/R10-OUT

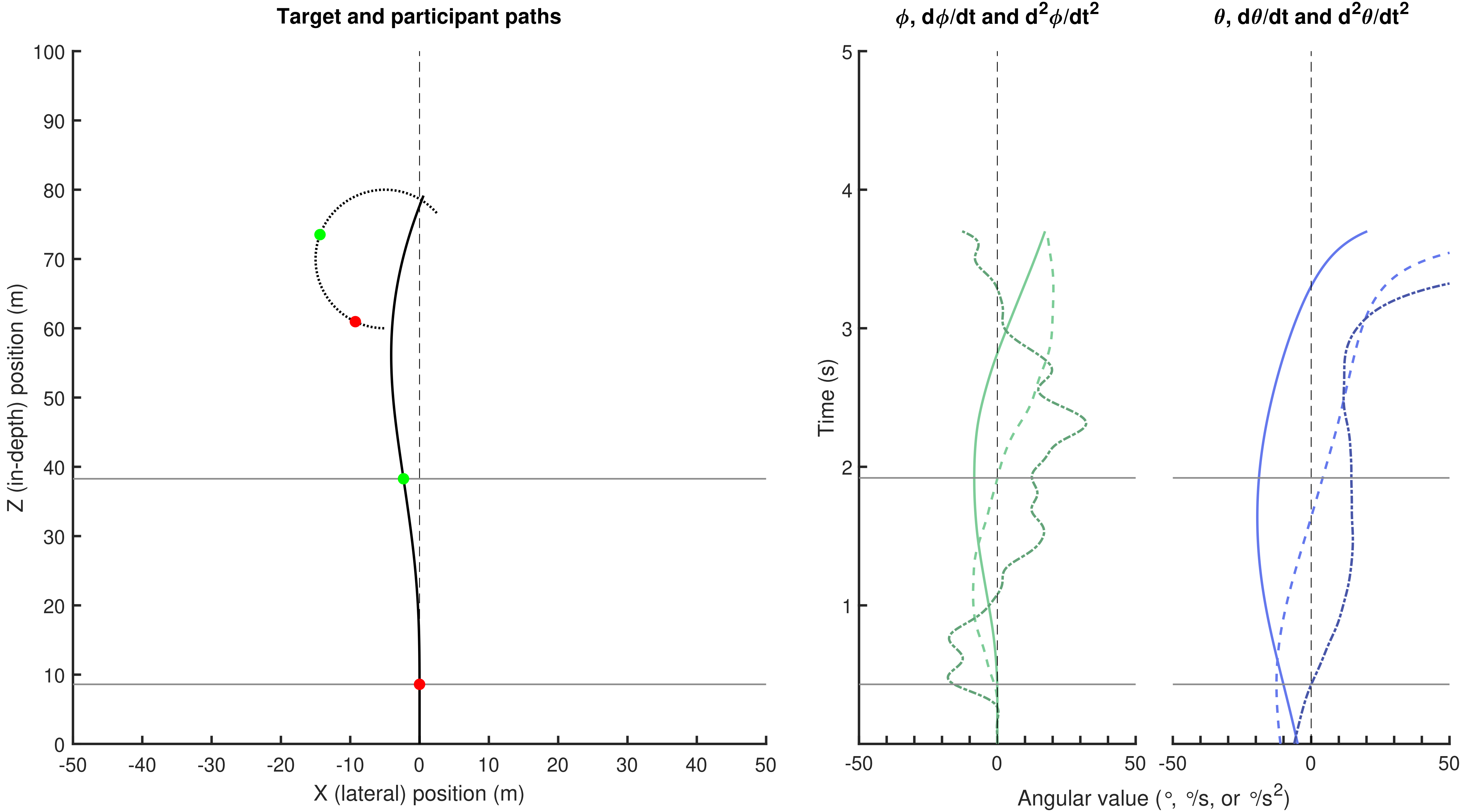

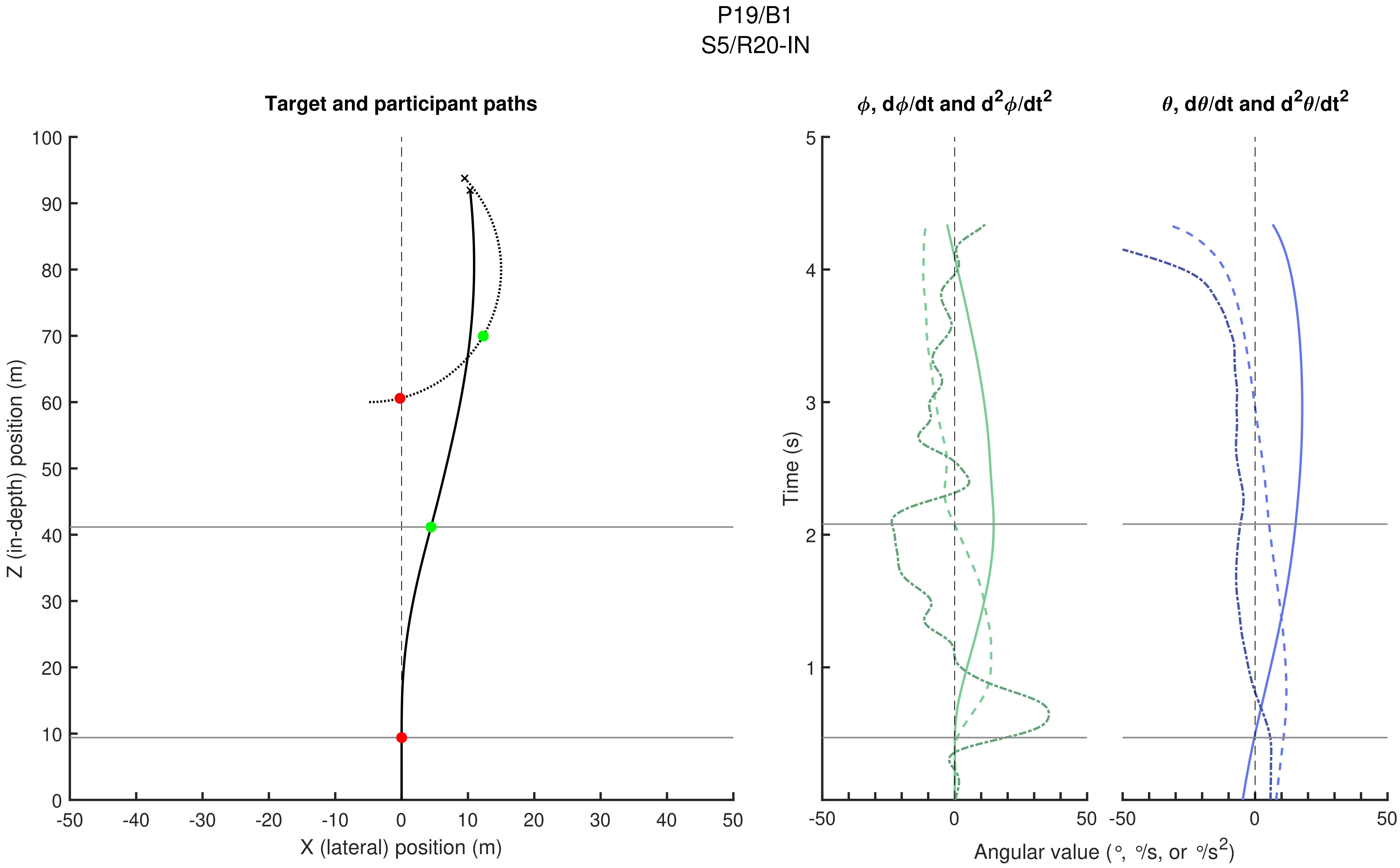
P19/B1
S5/R20-IN
Target and participant paths
Z (in-depth) position (m)
X (lateral) position (m)
ϕ, dϕ/dt and d²ϕ/dt²
θ, dθ/dt and d²θ/dt²
Time (s)
Angular value (°, °/s, or °/s²)

P19/B1
S5/R20-OUT

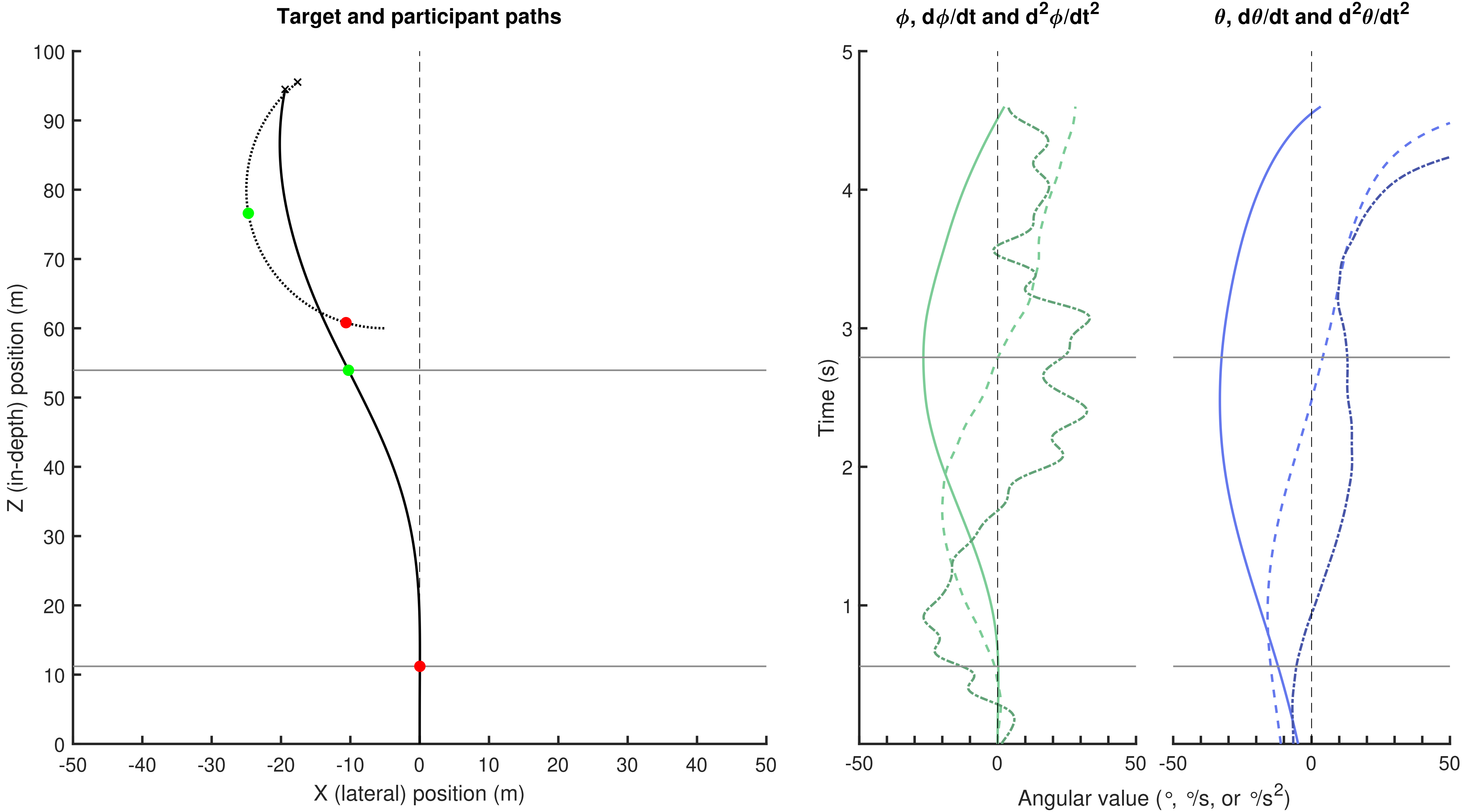

P19/B1
S5/R30-IN

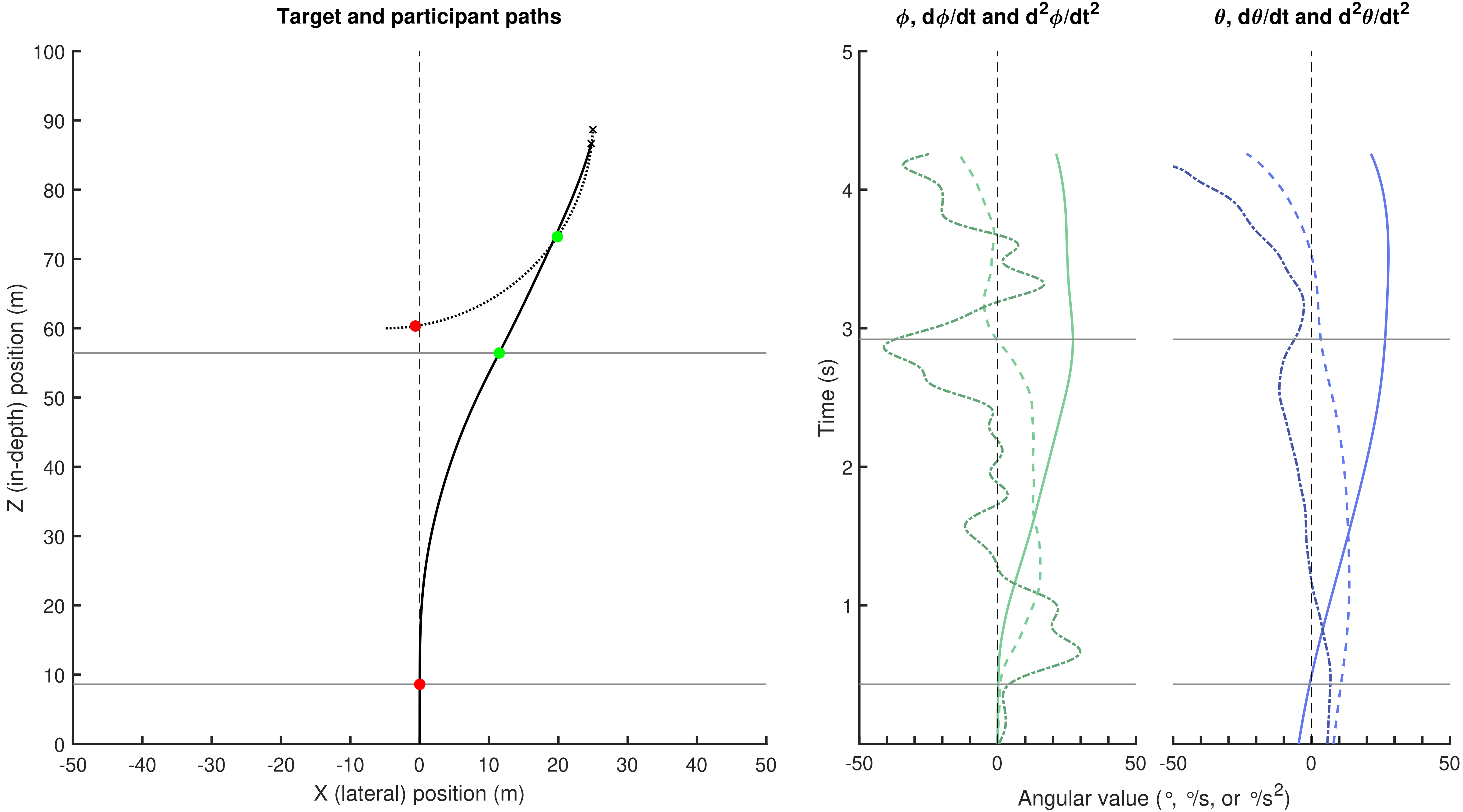

P19/B1
S5/R30-OUT

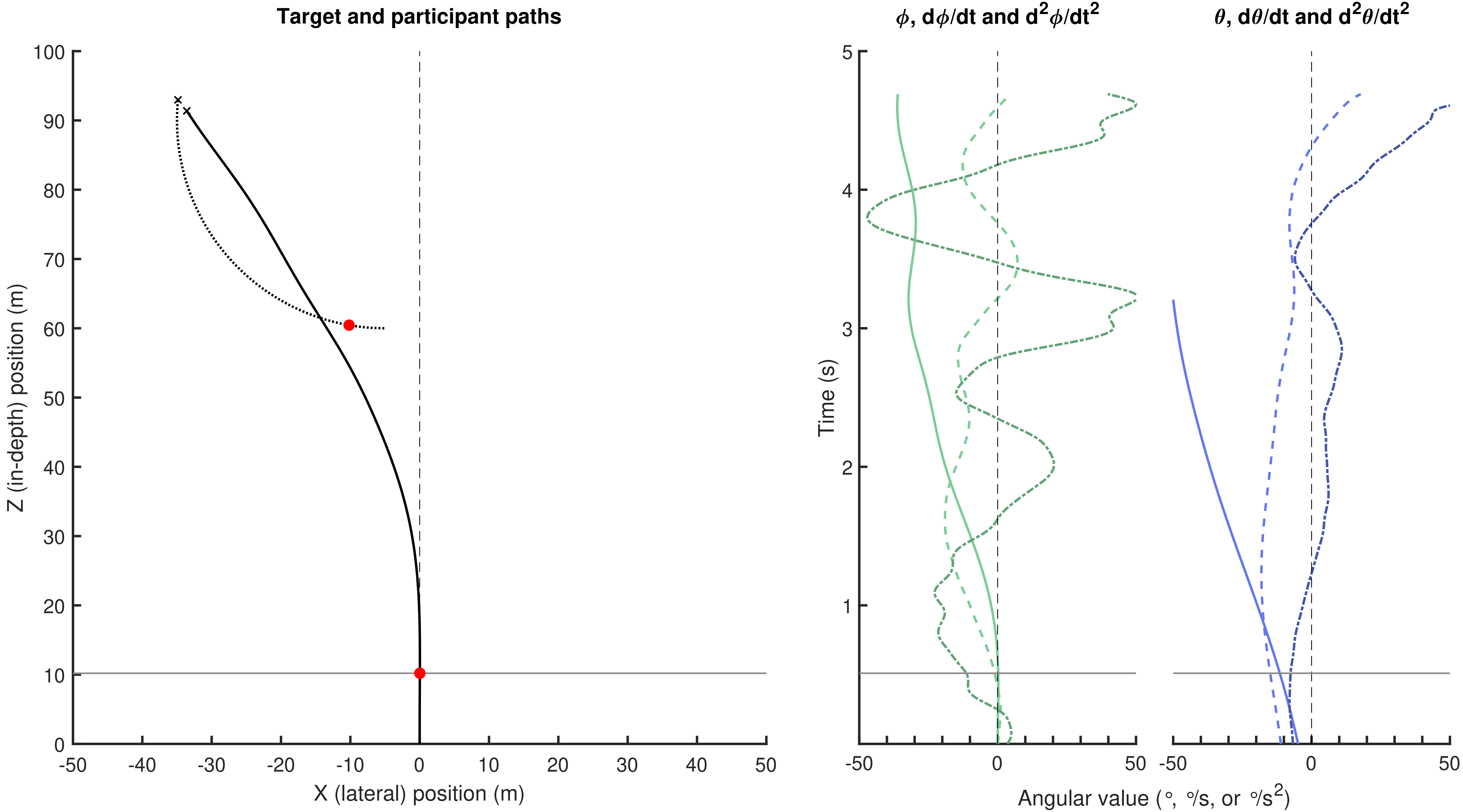

P19/B1
S5/R10-OUT

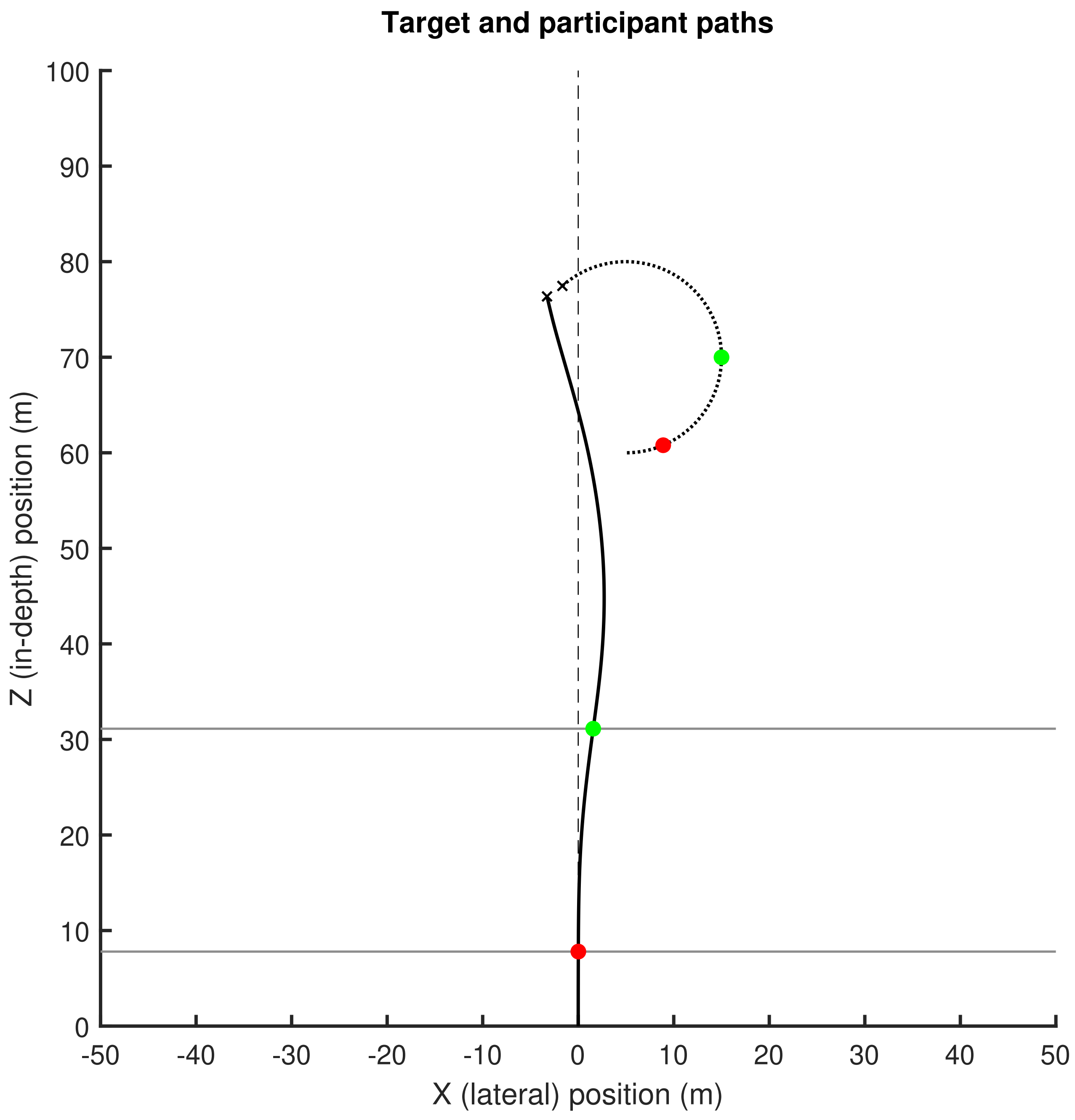

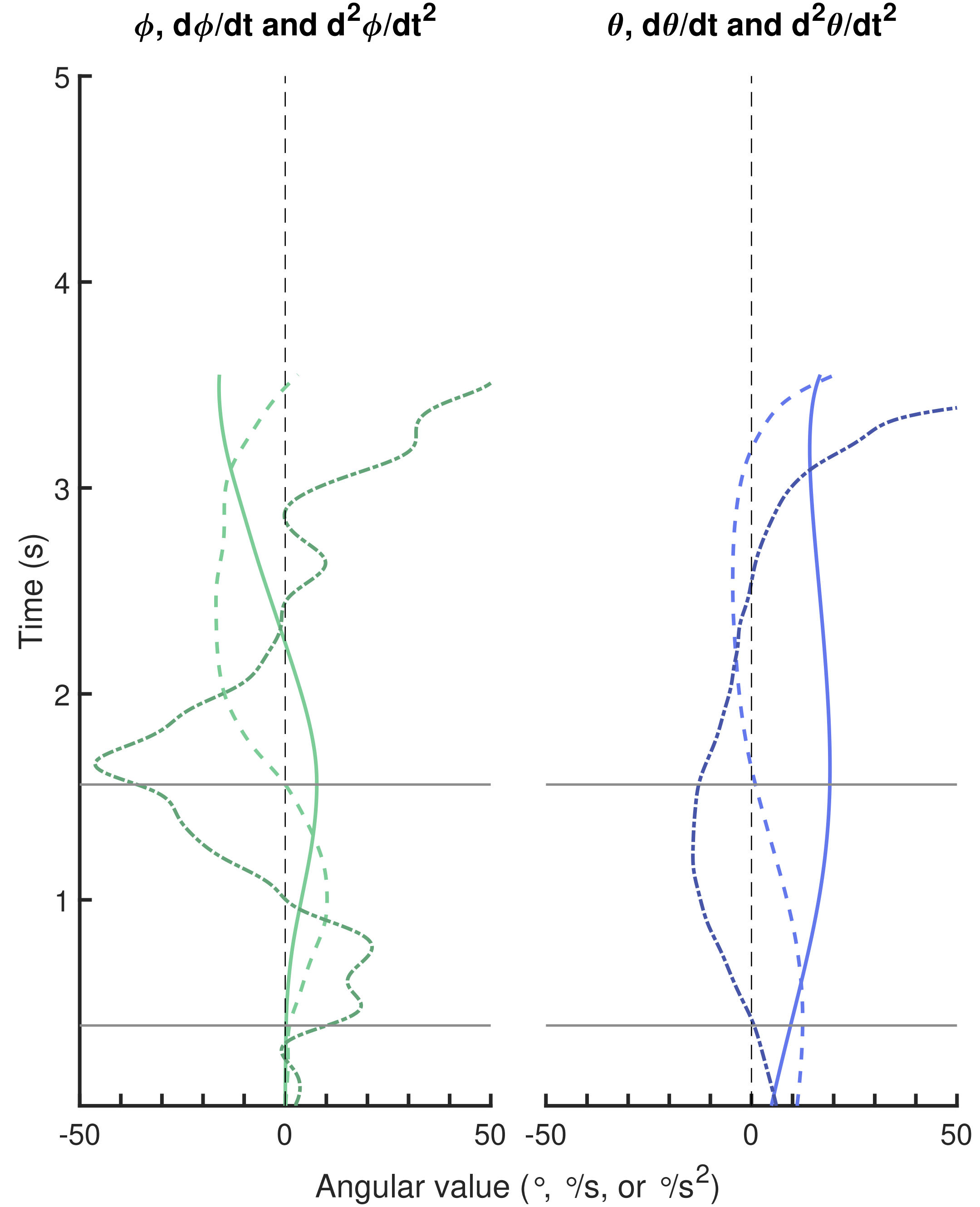

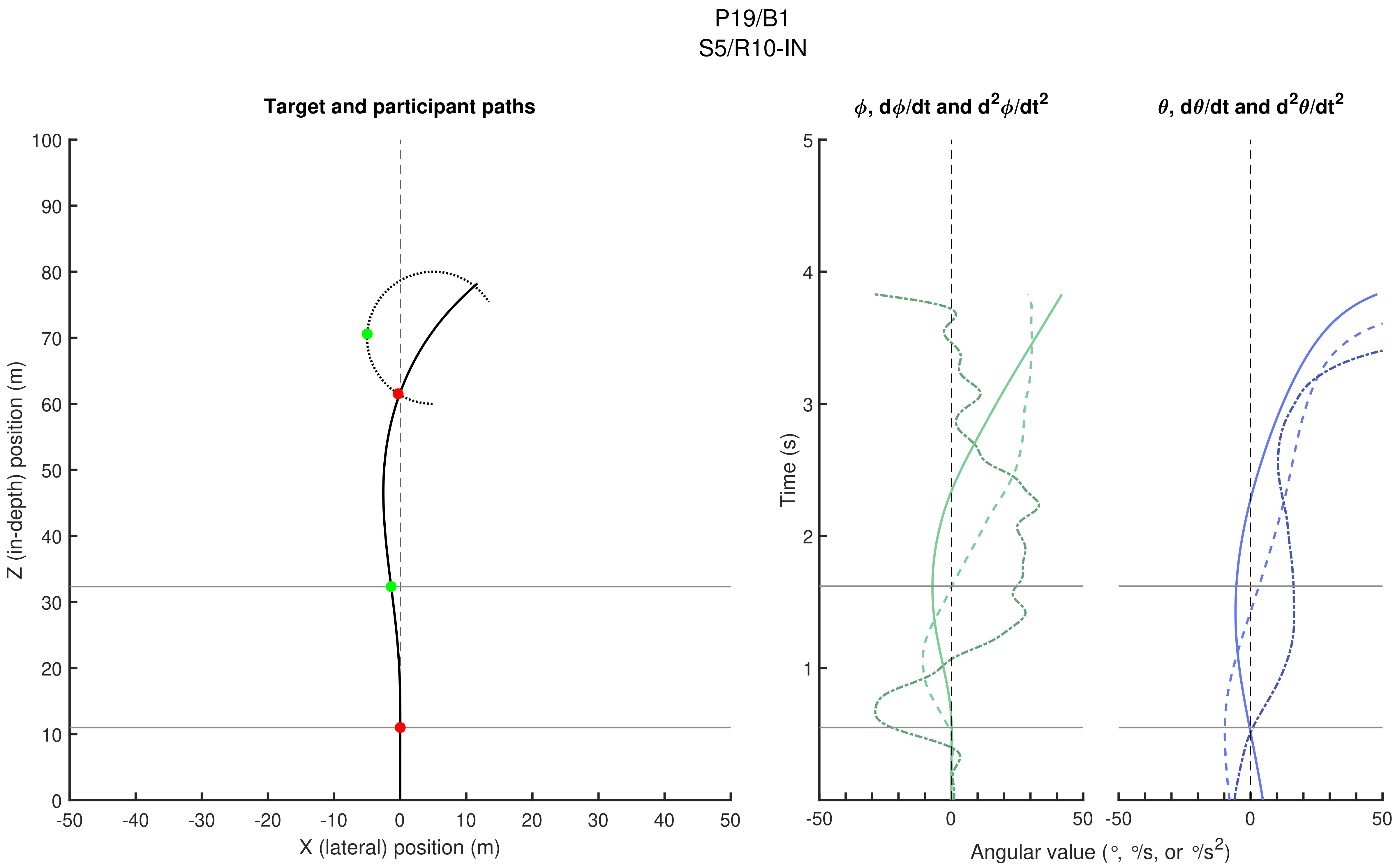

P19/B1
S5/R10-IN
Target and participant paths
Z (in-depth) position (m)
X (lateral) position (m)
φ, dφ/dt and d²φ/dt²
θ, dθ/dt and d²θ/dt²
Time (s)
Angular value (°, °/s, or °/s²)

P19/B1
S5/R20-OUT

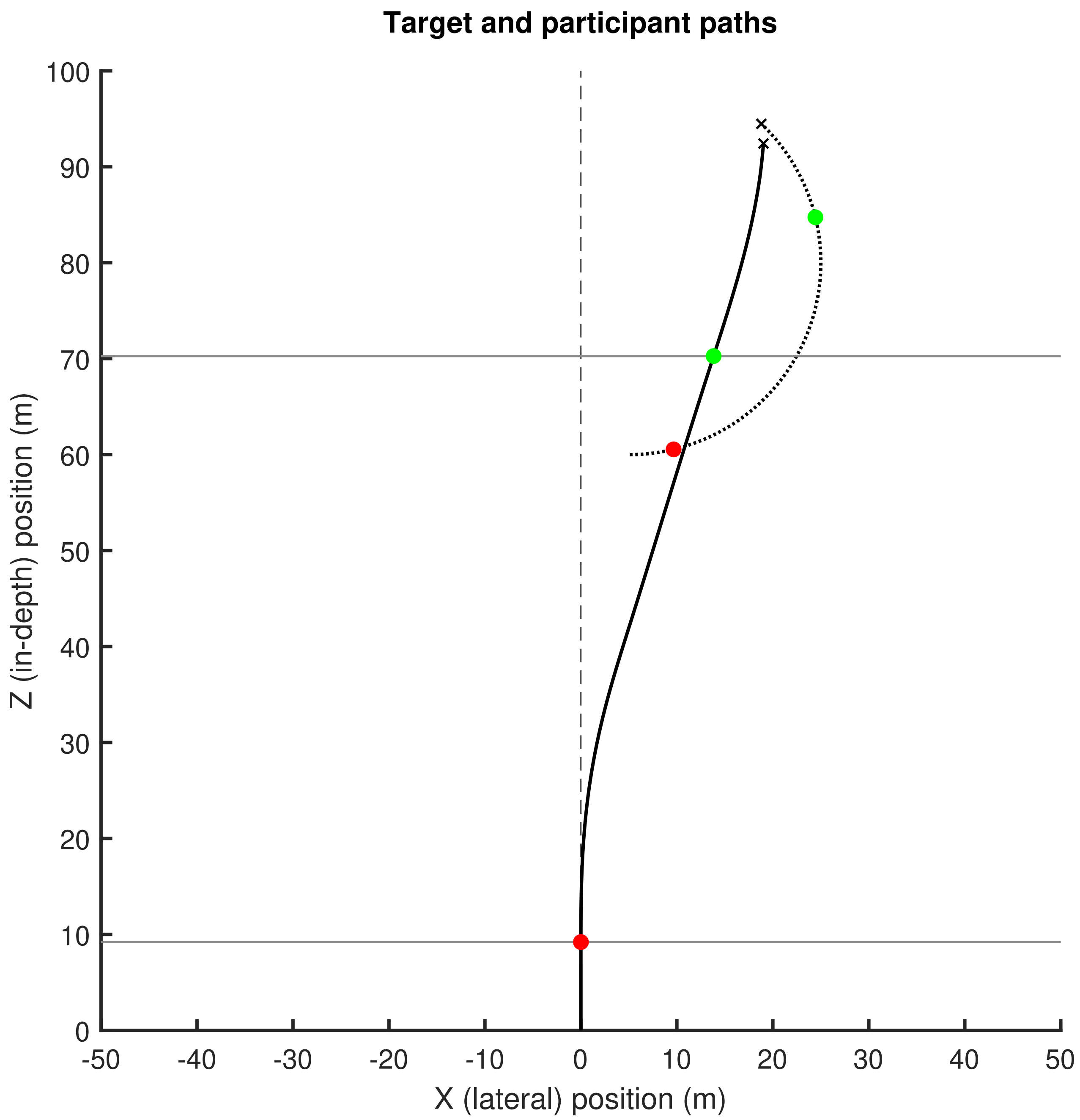

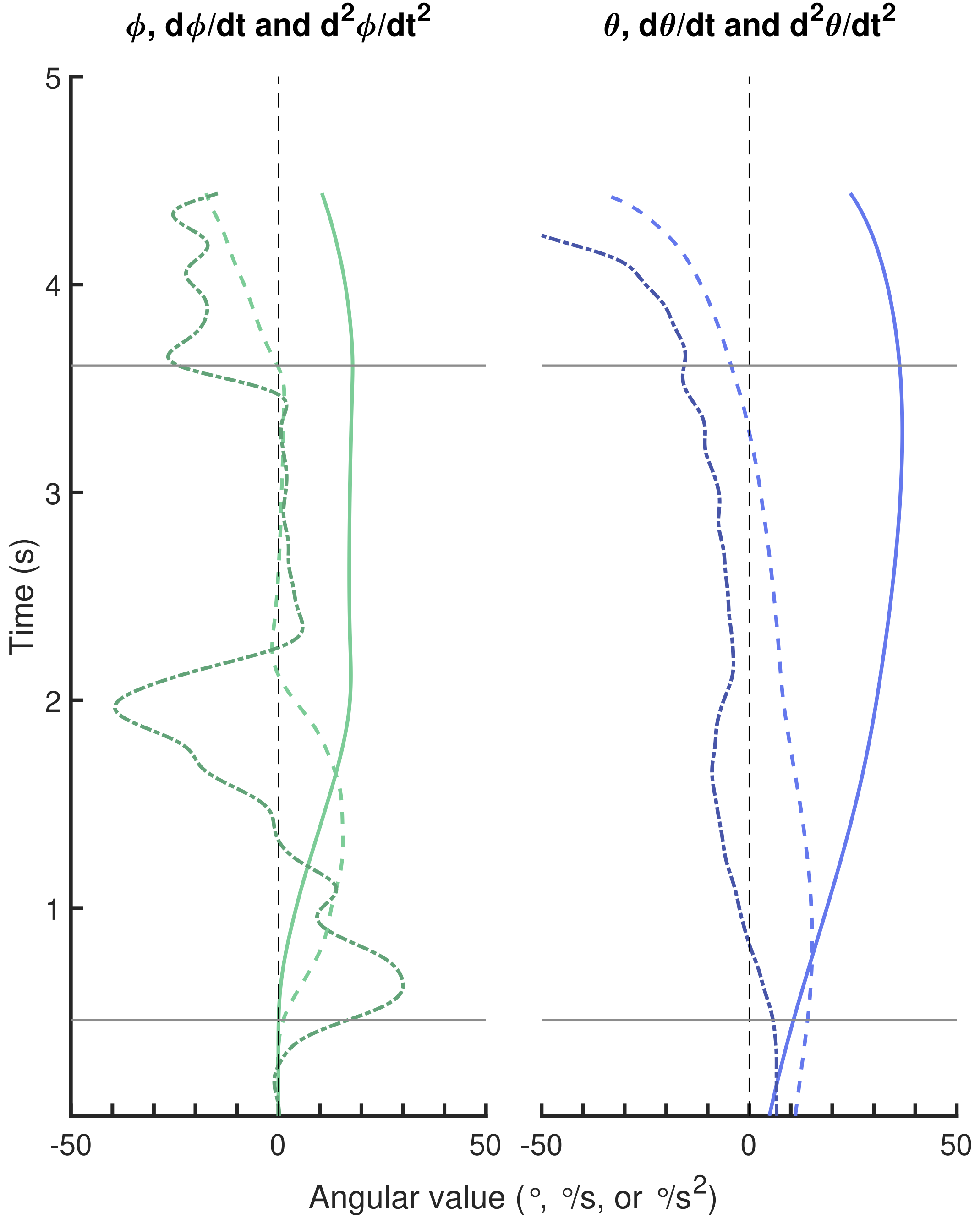

P19/B1
S5/R20-IN

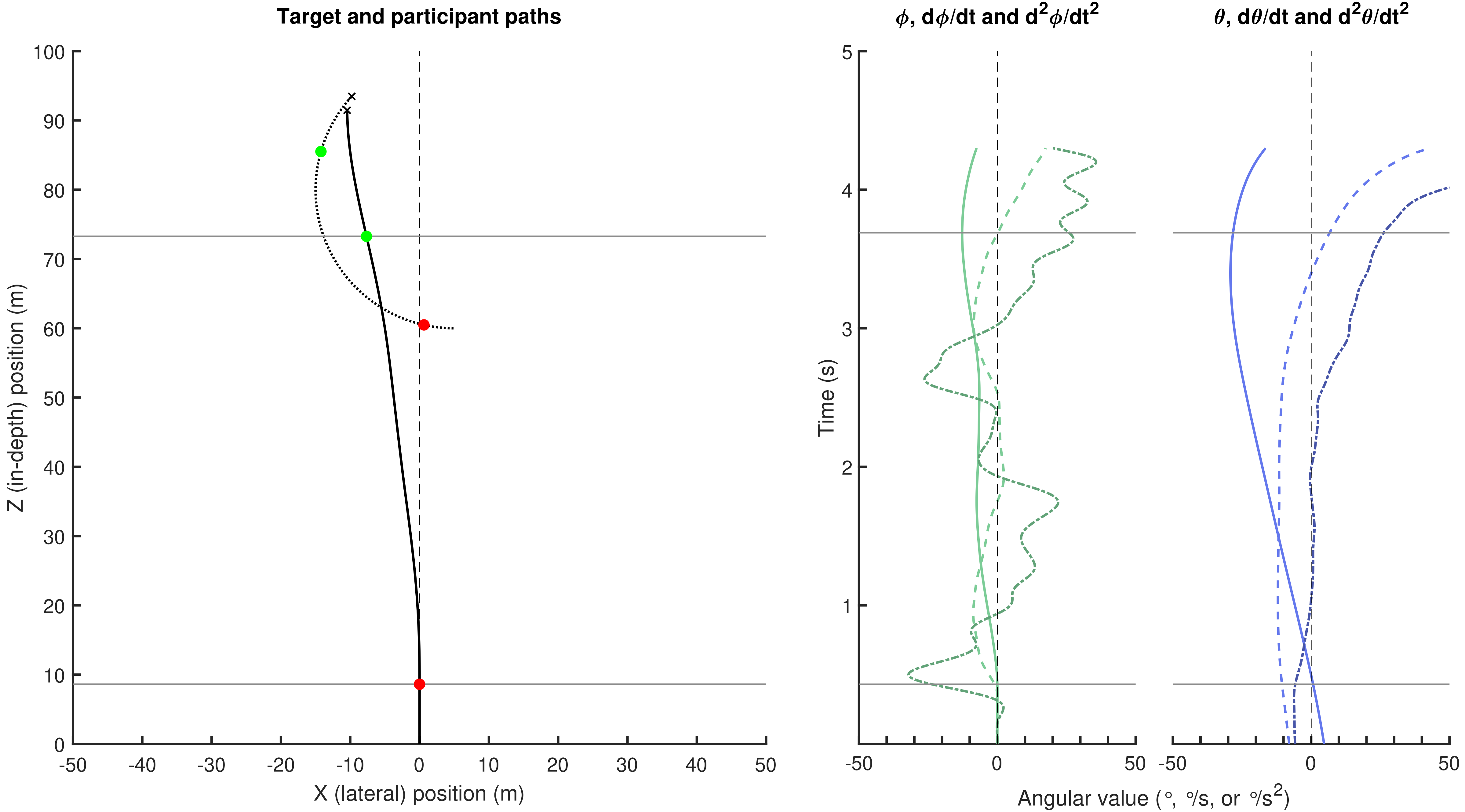

P19/B1
S5/R30-OUT

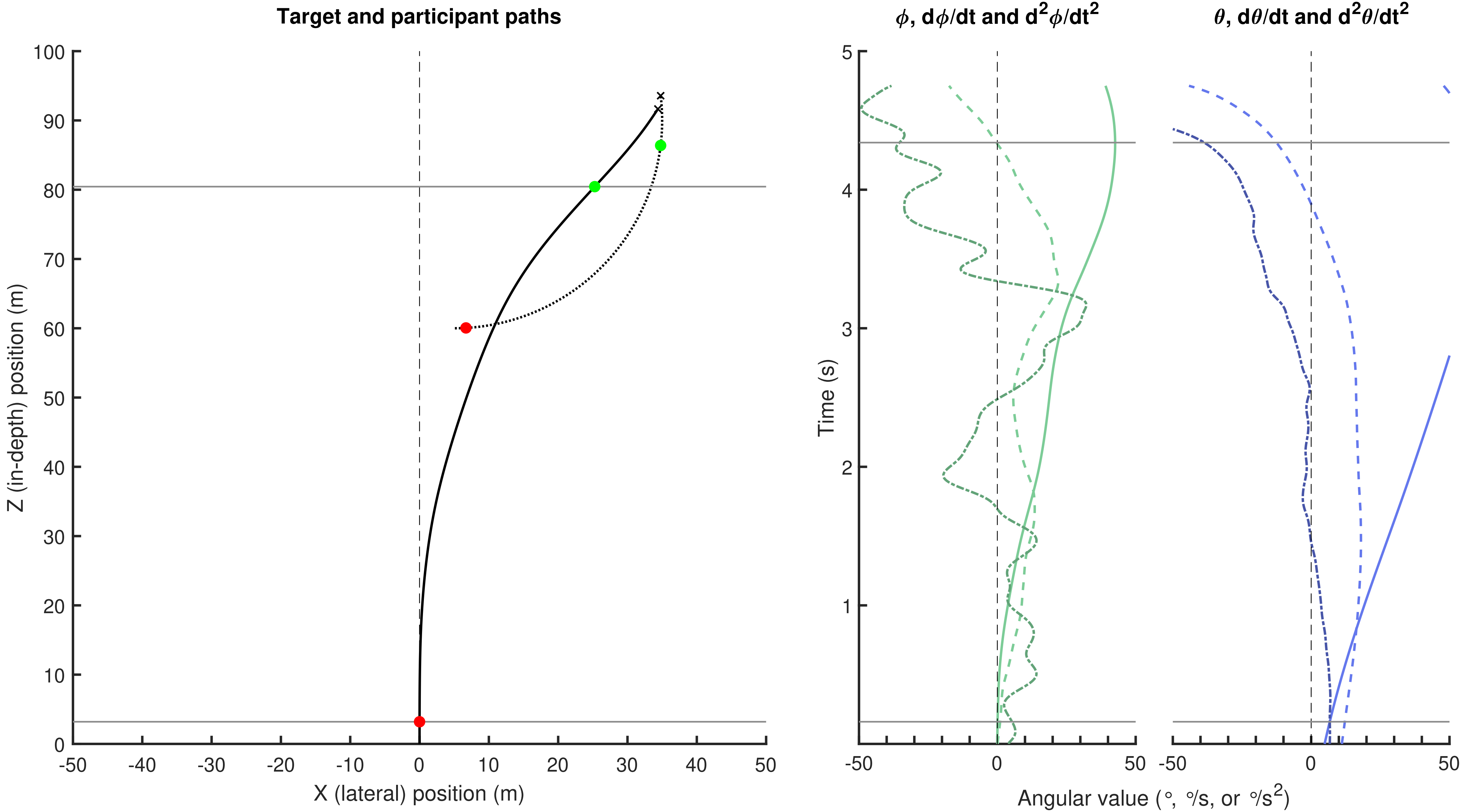

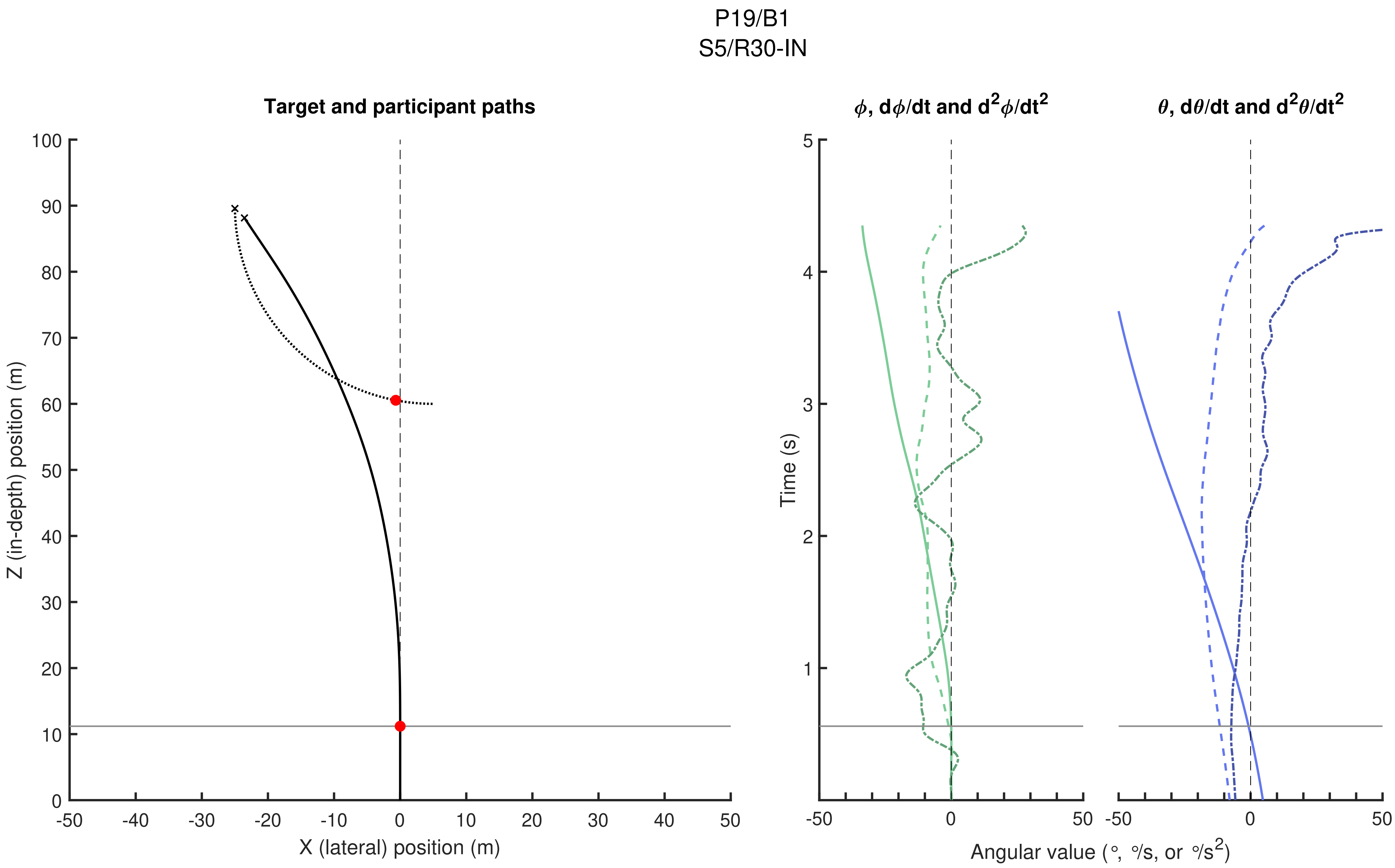

P19/B1
S5/R30-IN
Target and participant paths
Z (in-depth) position (m)
X (lateral) position (m)
ϕ, dϕ/dt and d$^2\phi$/dt^2
θ, dθ/dt and d$^2\theta$/dt^2
Time (s)
Angular value (°, °/s, or °/s^2)

P19/B1
S15/R10-IN

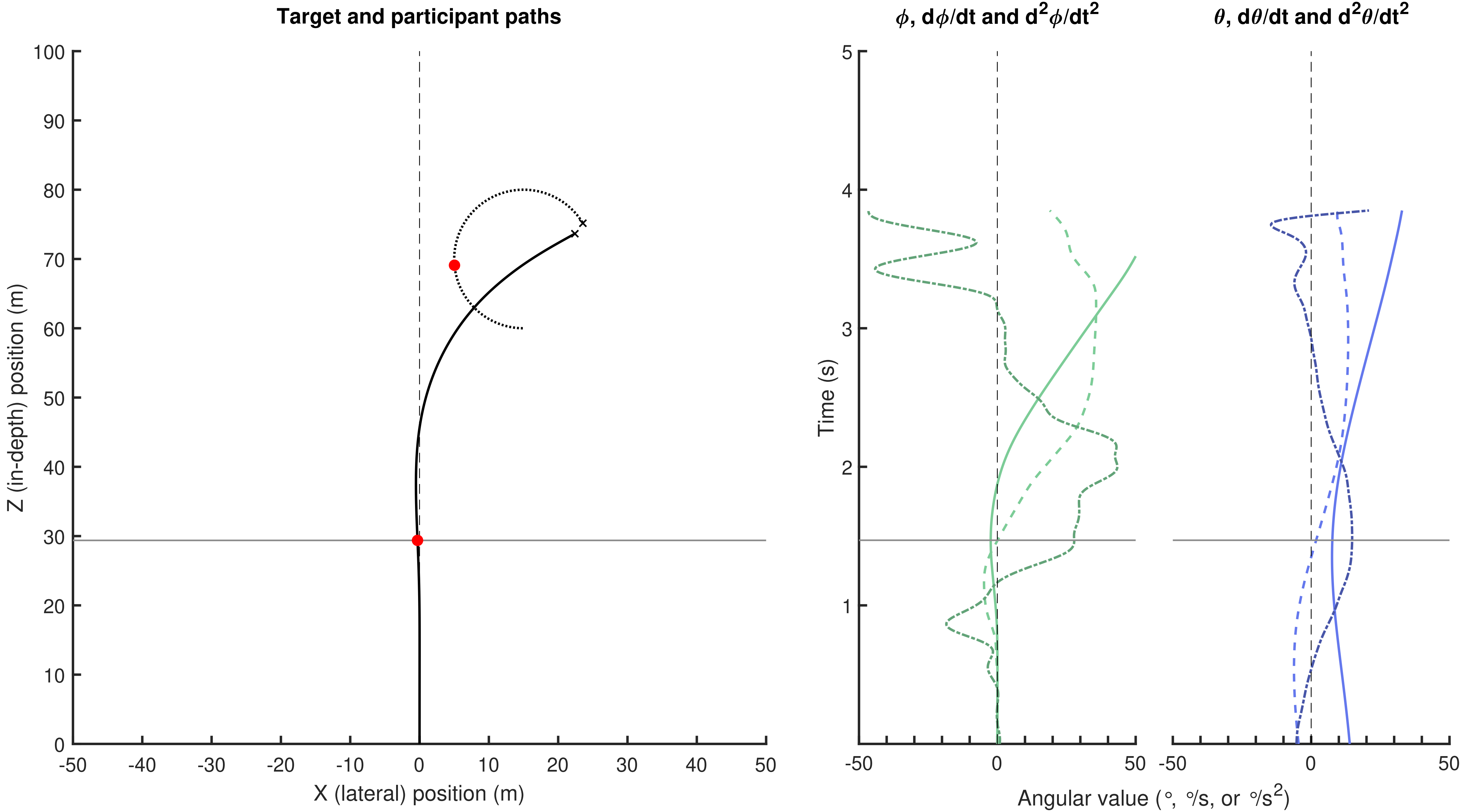

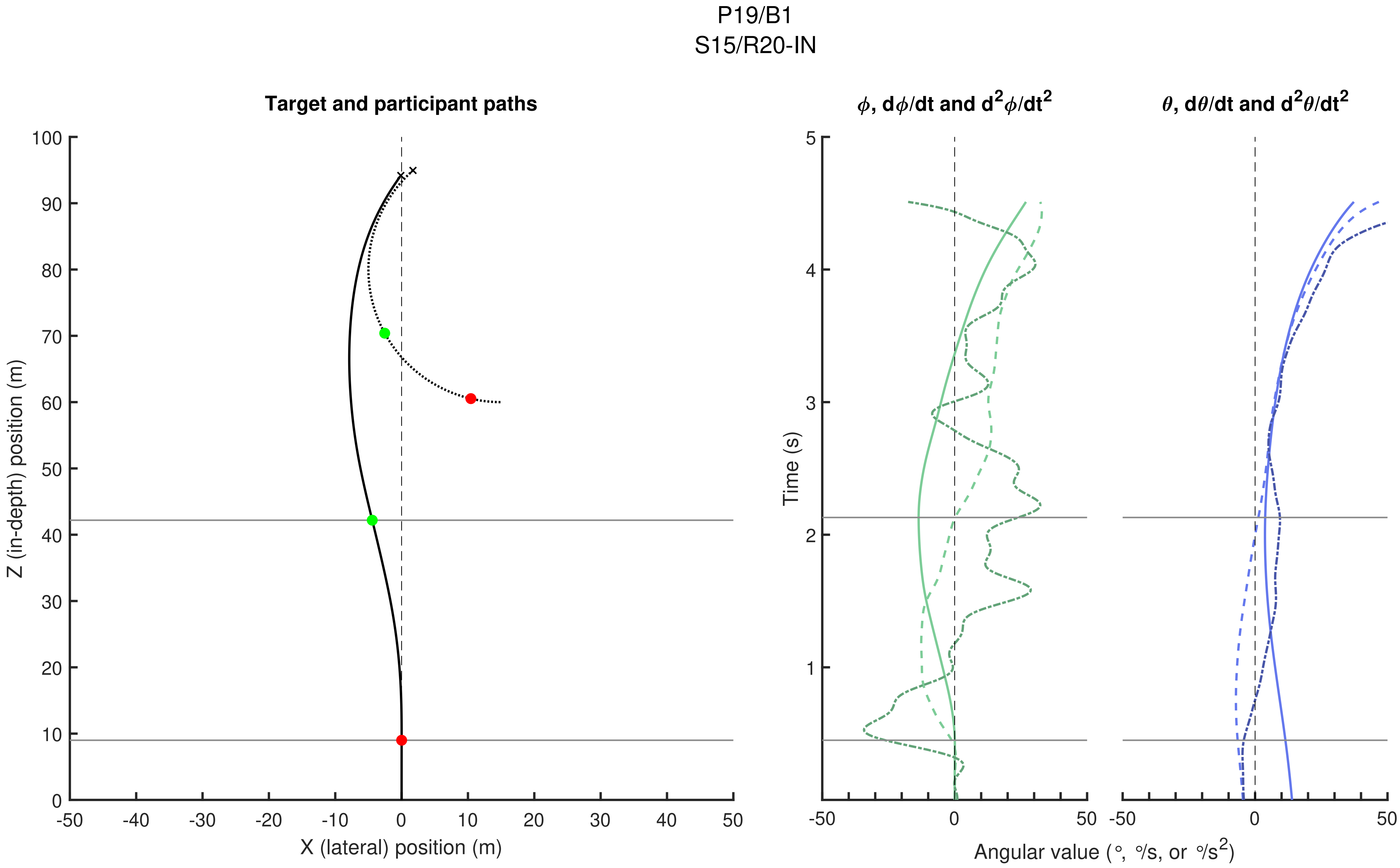
P19/B1
S15/R20-IN
Target and participant paths
Z (in-depth) position (m)
X (lateral) position (m)
φ, dφ/dt and d²φ/dt²
θ, dθ/dt and d²θ/dt²
Time (s)
Angular value (°, °/s, or °/s²)

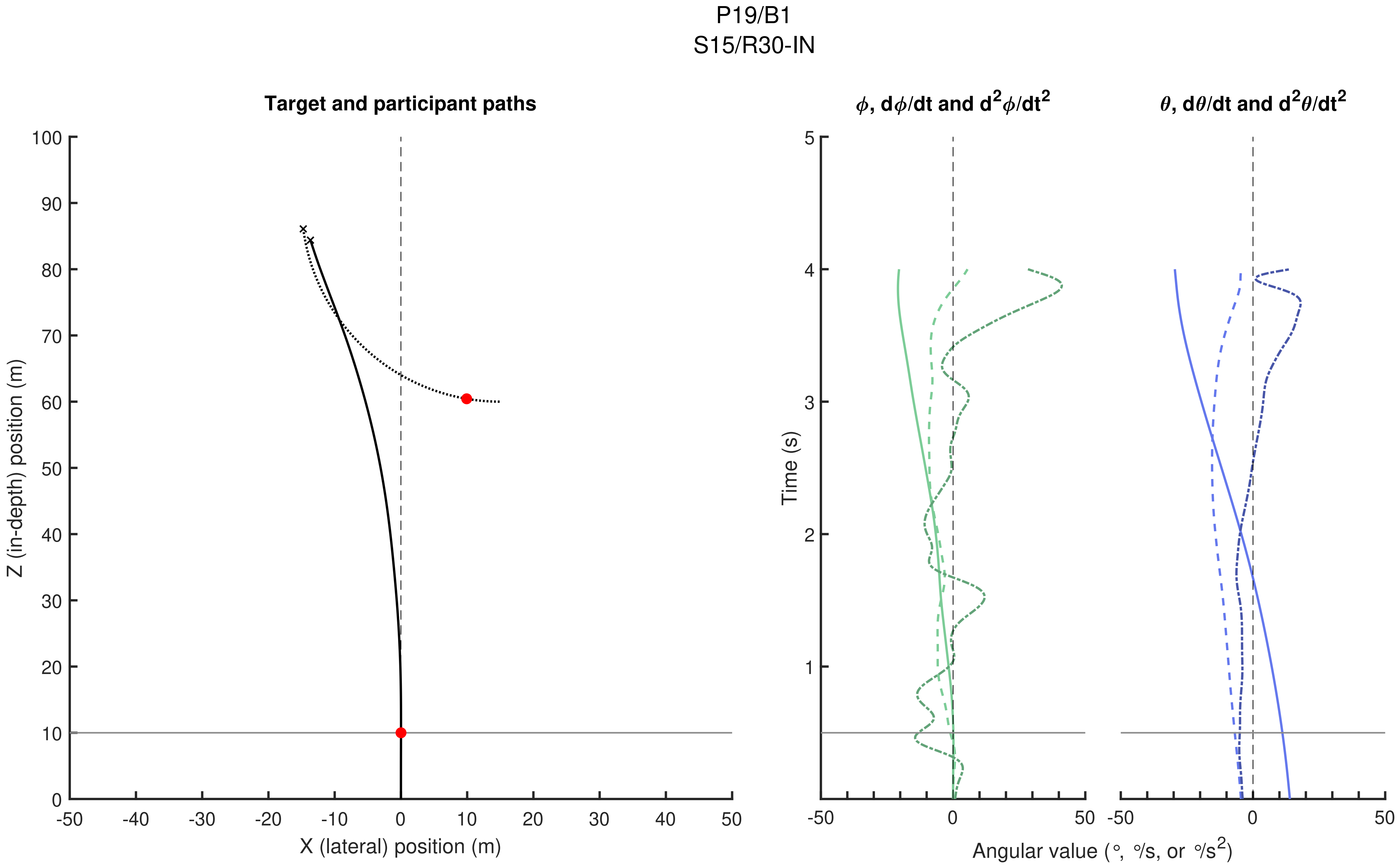

P19/B1
S15/R30-IN
Target and participant paths
Z (in-depth) position (m)
X (lateral) position (m)
ϕ, dϕ/dt and d$^2\phi$/dt^2
θ, dθ/dt and d$^2\theta$/dt^2
Time (s)
Angular value (°, °/s, or °/s^2)

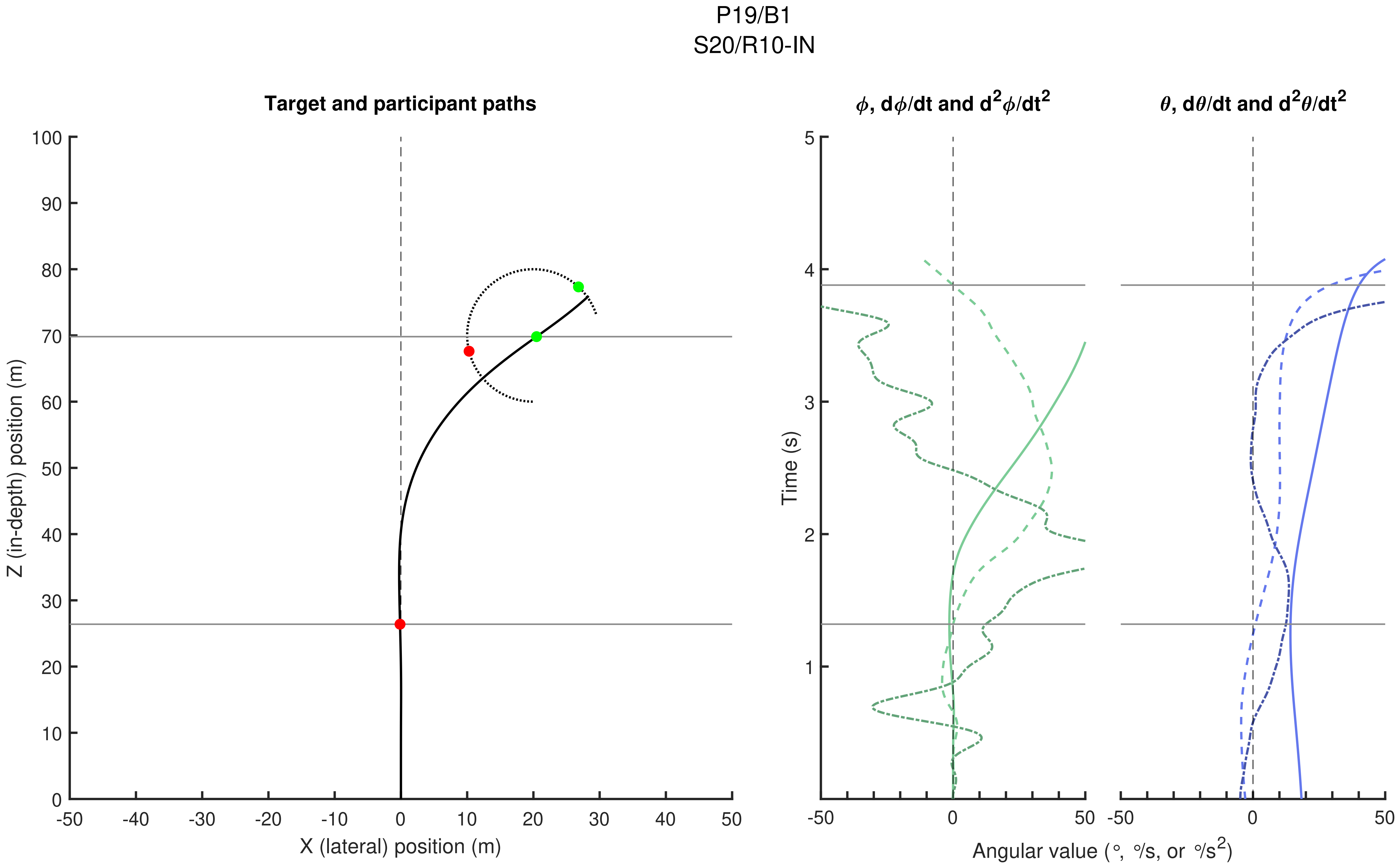

P19/B1
S20/R10-IN
Target and participant paths
Z (in-depth) position (m)
X (lateral) position (m)
ϕ, dϕ/dt and d$^2\phi$/dt^2
θ, dθ/dt and d$^2\theta$/dt^2
Time (s)
Angular value (°, °/s, or °/s^2)

P19/B1
S20/R20-IN

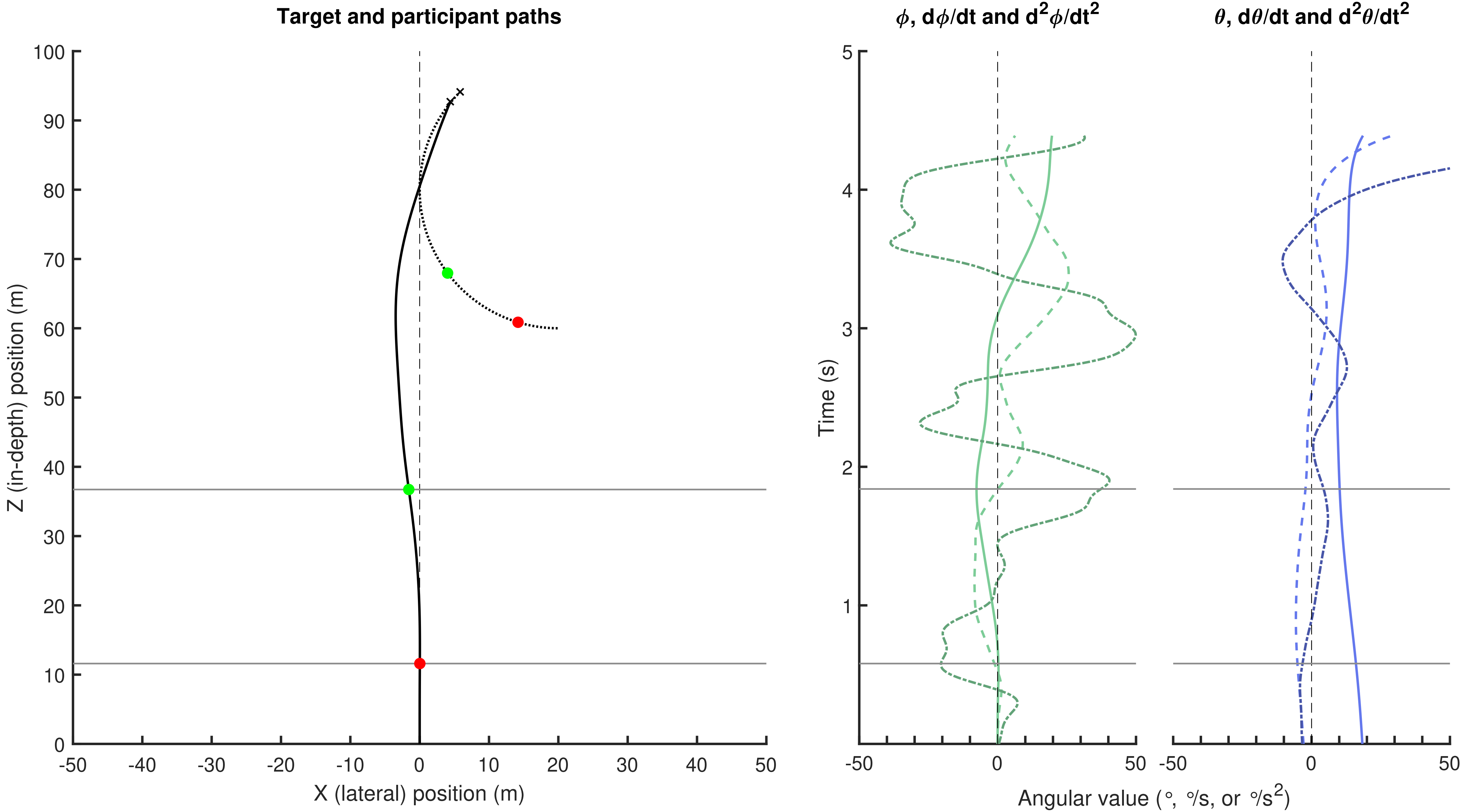

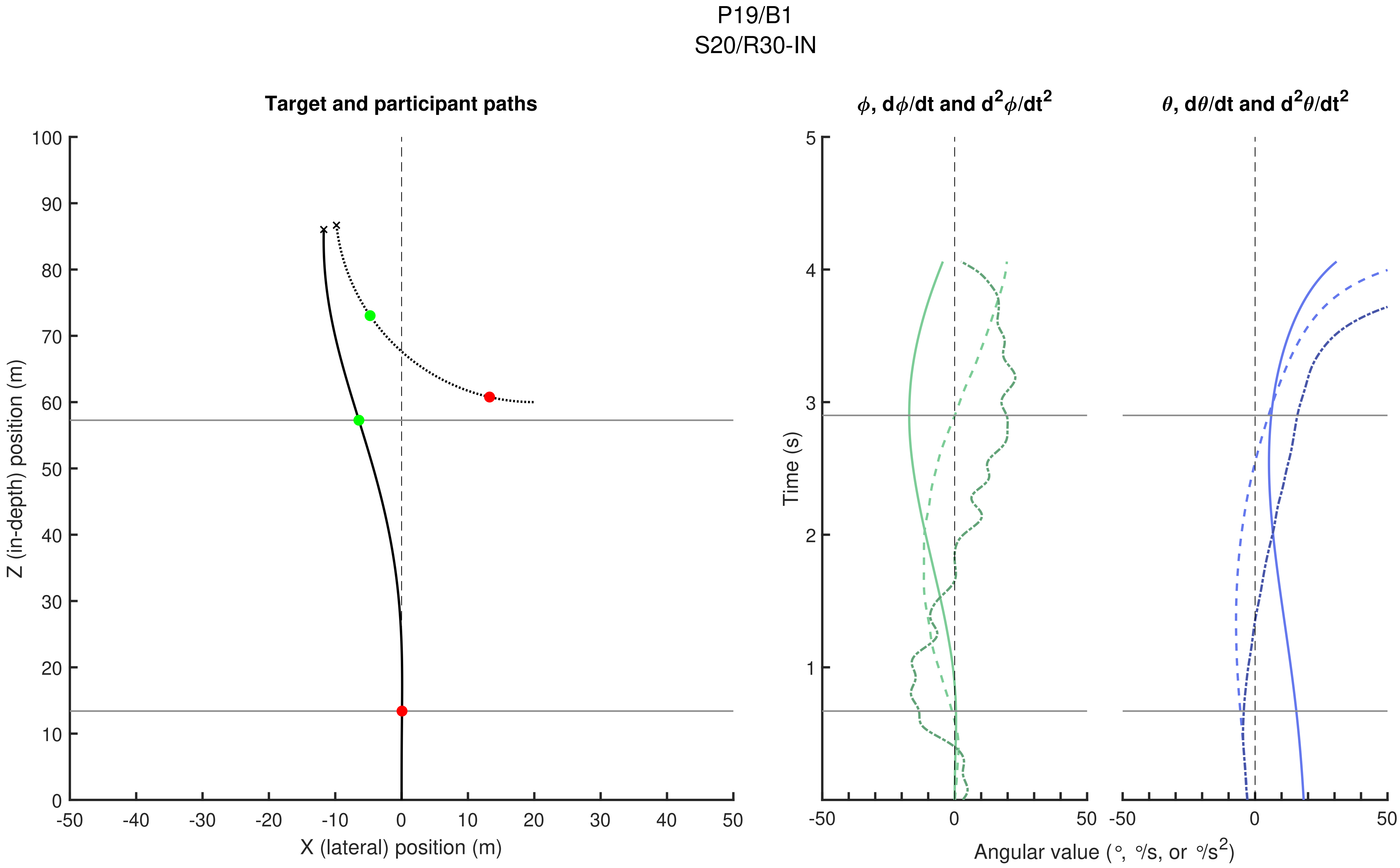

P19/B1
S20/R30-IN
Target and participant paths
Z (in-depth) position (m)
X (lateral) position (m)
ϕ, dϕ/dt and d$^2\phi$/dt^2
θ, dθ/dt and d$^2\theta$/dt^2
Time (s)
Angular value (°, °/s, or °/s^2)

P19/B2
S20/R10-IN

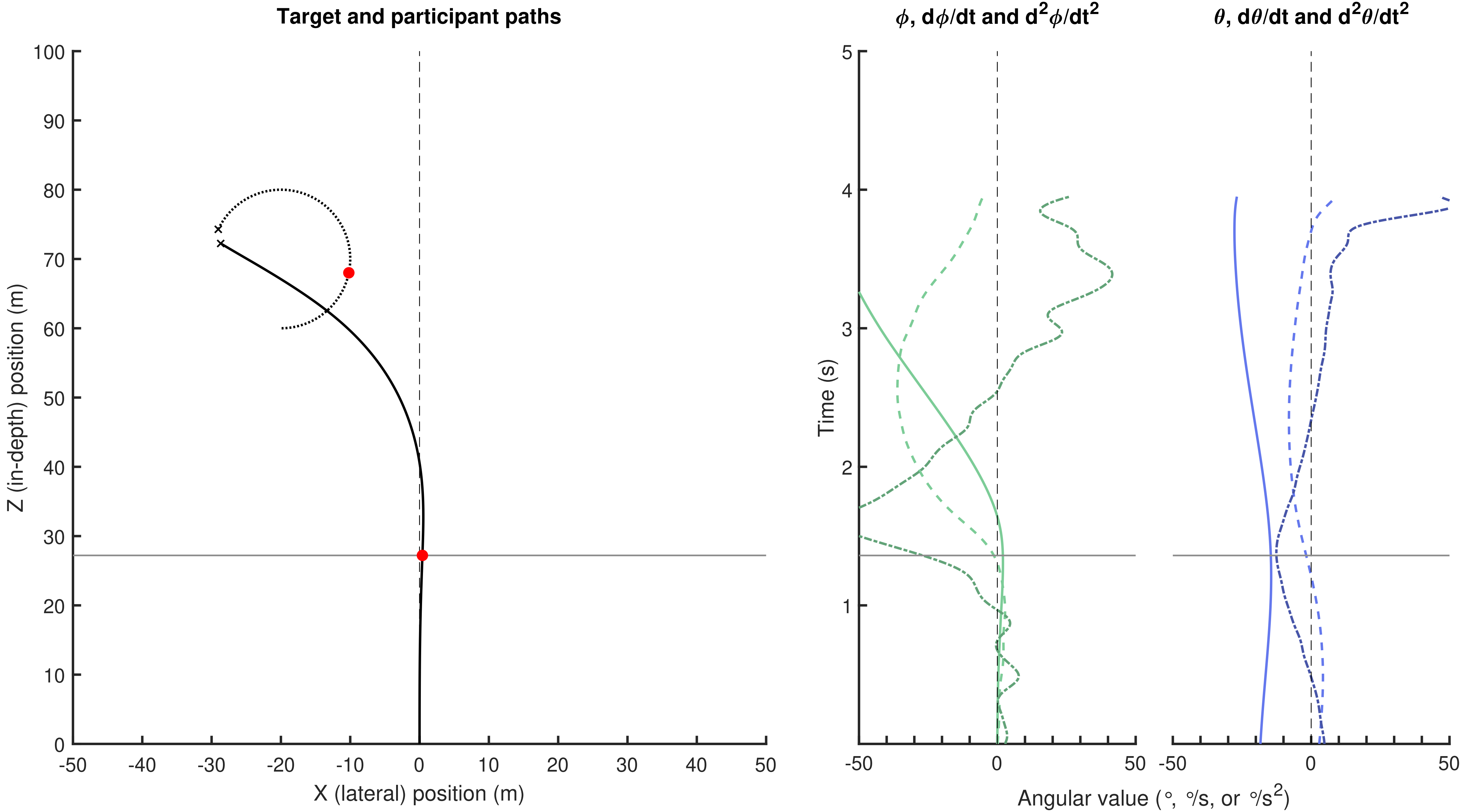

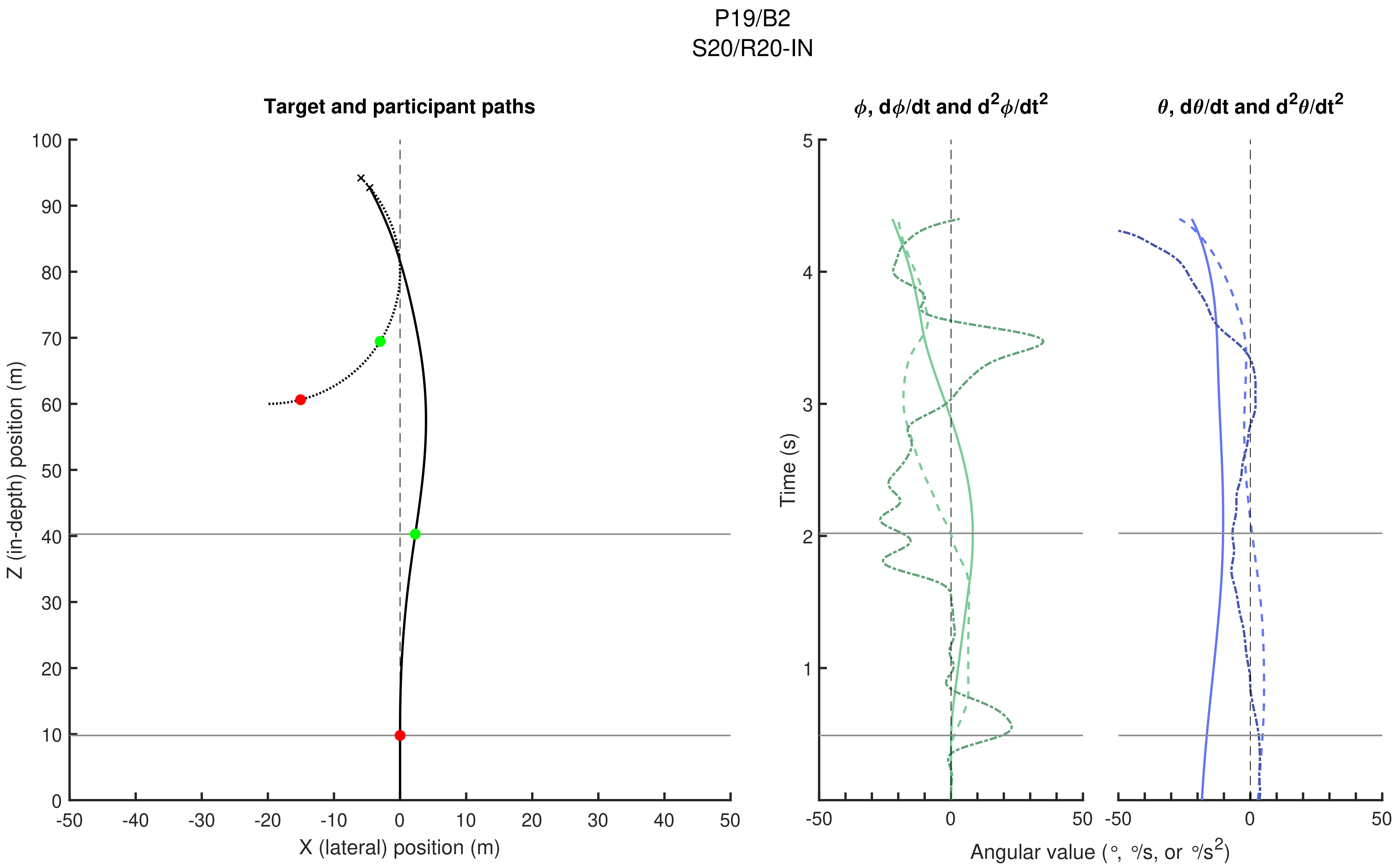
P19/B2
S20/R20-IN
Target and participant paths
Z (in-depth) position (m)
X (lateral) position (m)
ϕ, dϕ/dt and d$^2\phi$/dt^2
θ, dθ/dt and d$^2\theta$/dt^2
Time (s)
Angular value (°, °/s, or °/s^2)

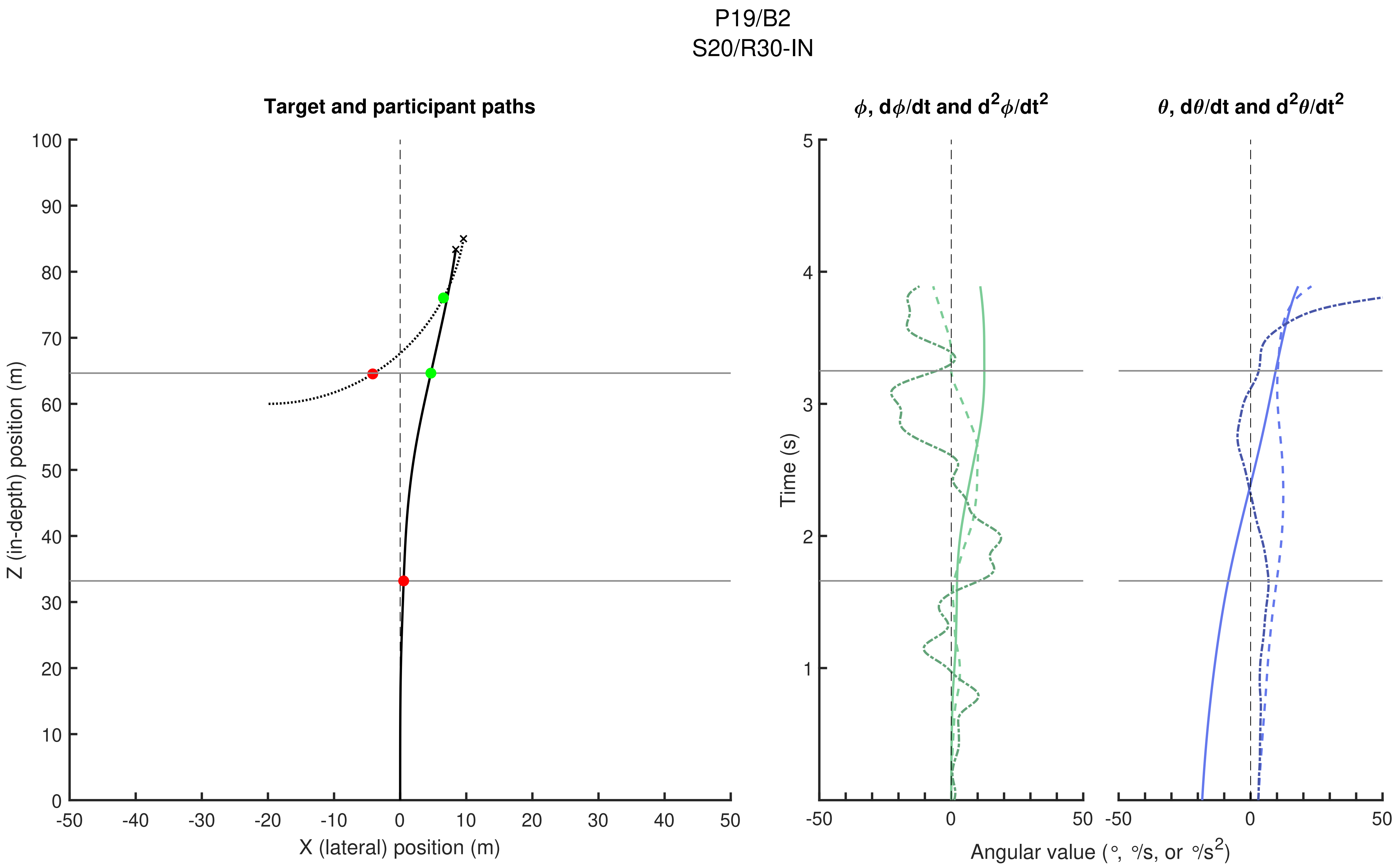

P19/B2
S20/R30-IN
Target and participant paths
Z (in-depth) position (m)
X (lateral) position (m)
ϕ, dϕ/dt and d$^2\phi$/dt^2
θ, dθ/dt and d$^2\theta$/dt^2
Time (s)
Angular value (°, °/s, or °/s^2)

P19/B2
S15/R10-IN

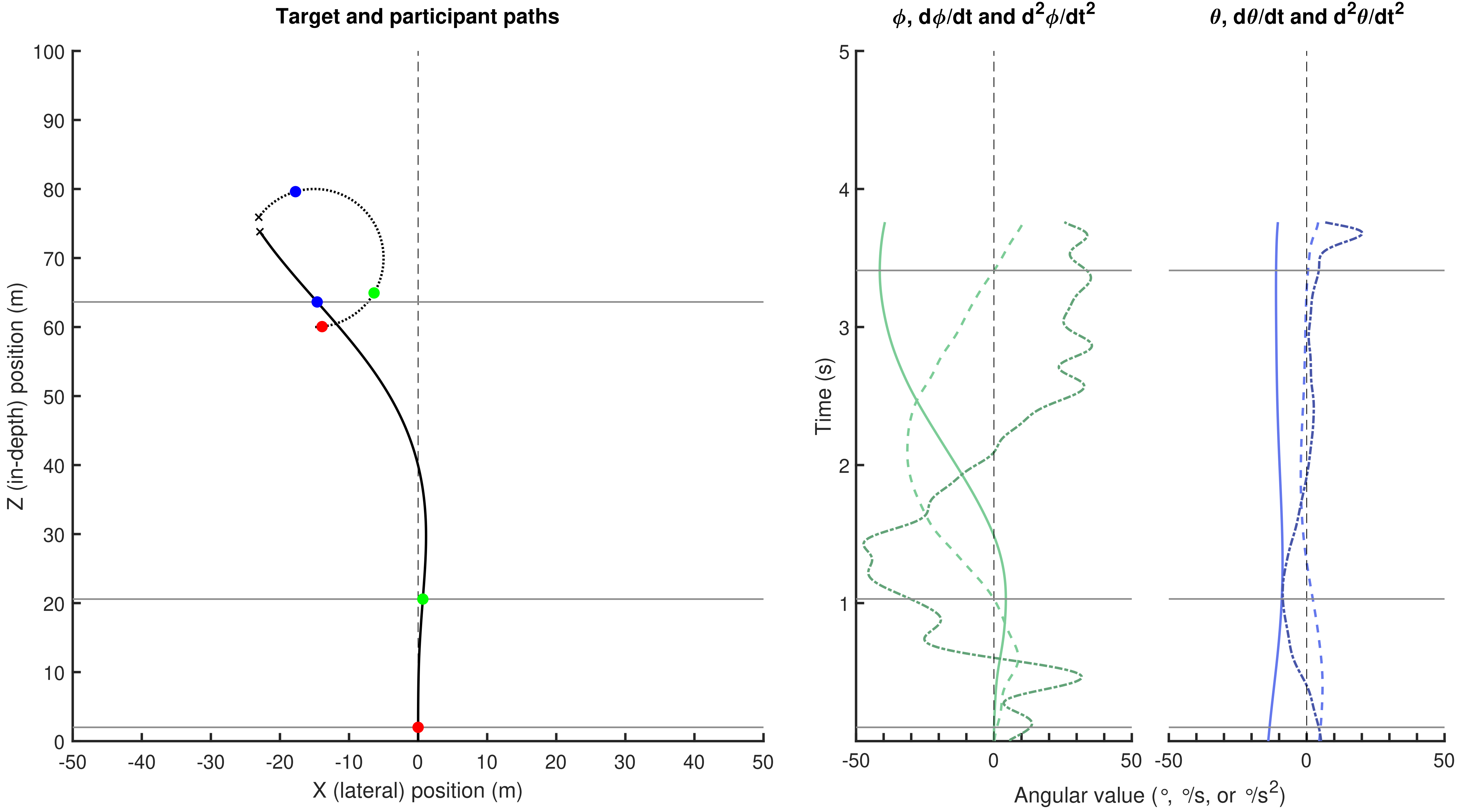

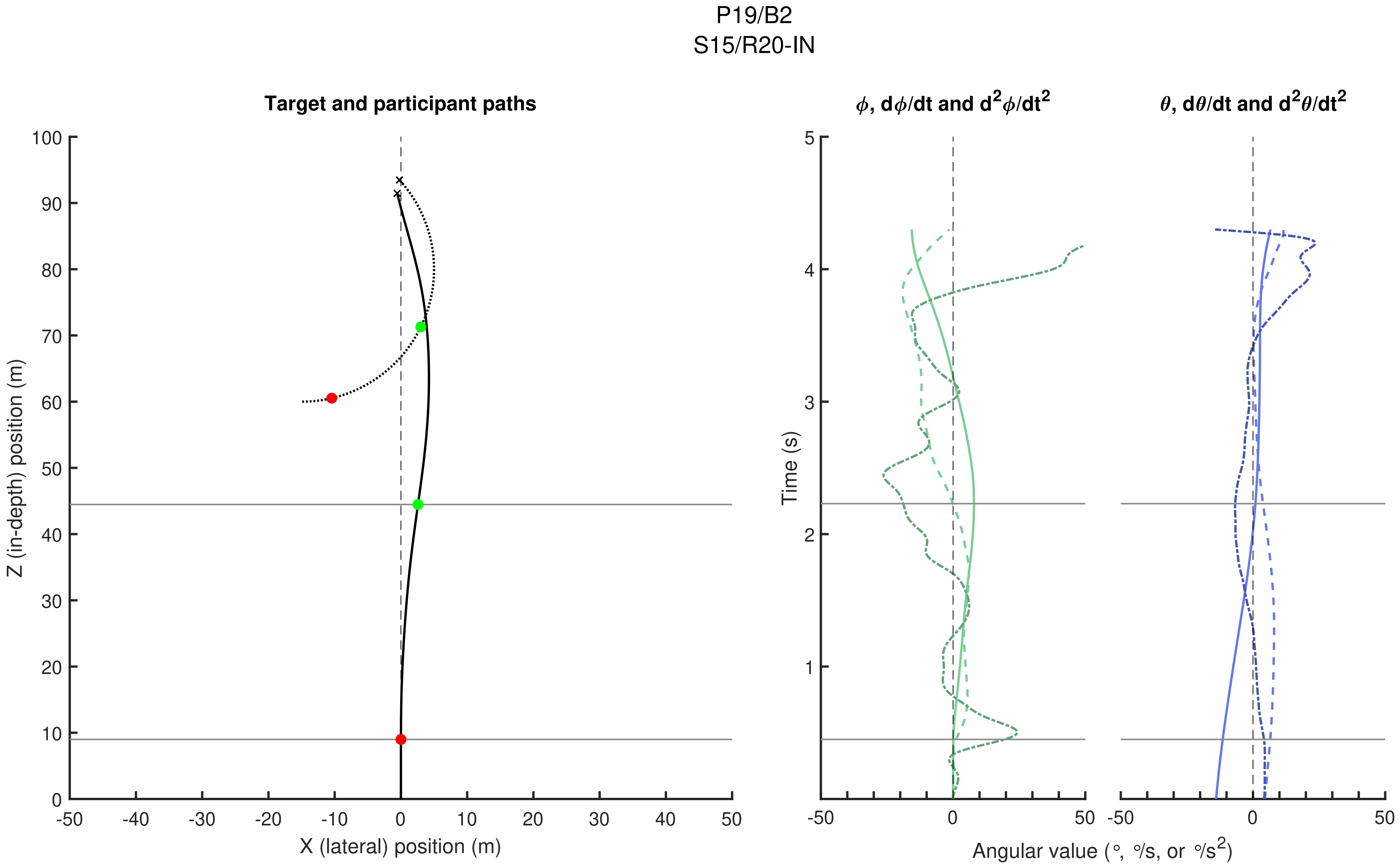
P19/B2
S15/R20-IN
Target and participant paths
Z (in-depth) position (m)
X (lateral) position (m)
100
90
80
70
60
50
40
30
20
10
0
-50
-40
-30
-20
-10
0
10
20
30
40
50
φ, dφ/dt and d²φ/dt²
θ, dθ/dt and d²θ/dt²
Time (s)
5
4
3
2
1
-50
0
50
-50
0
50
Angular value (°, °/s, or °/s²)

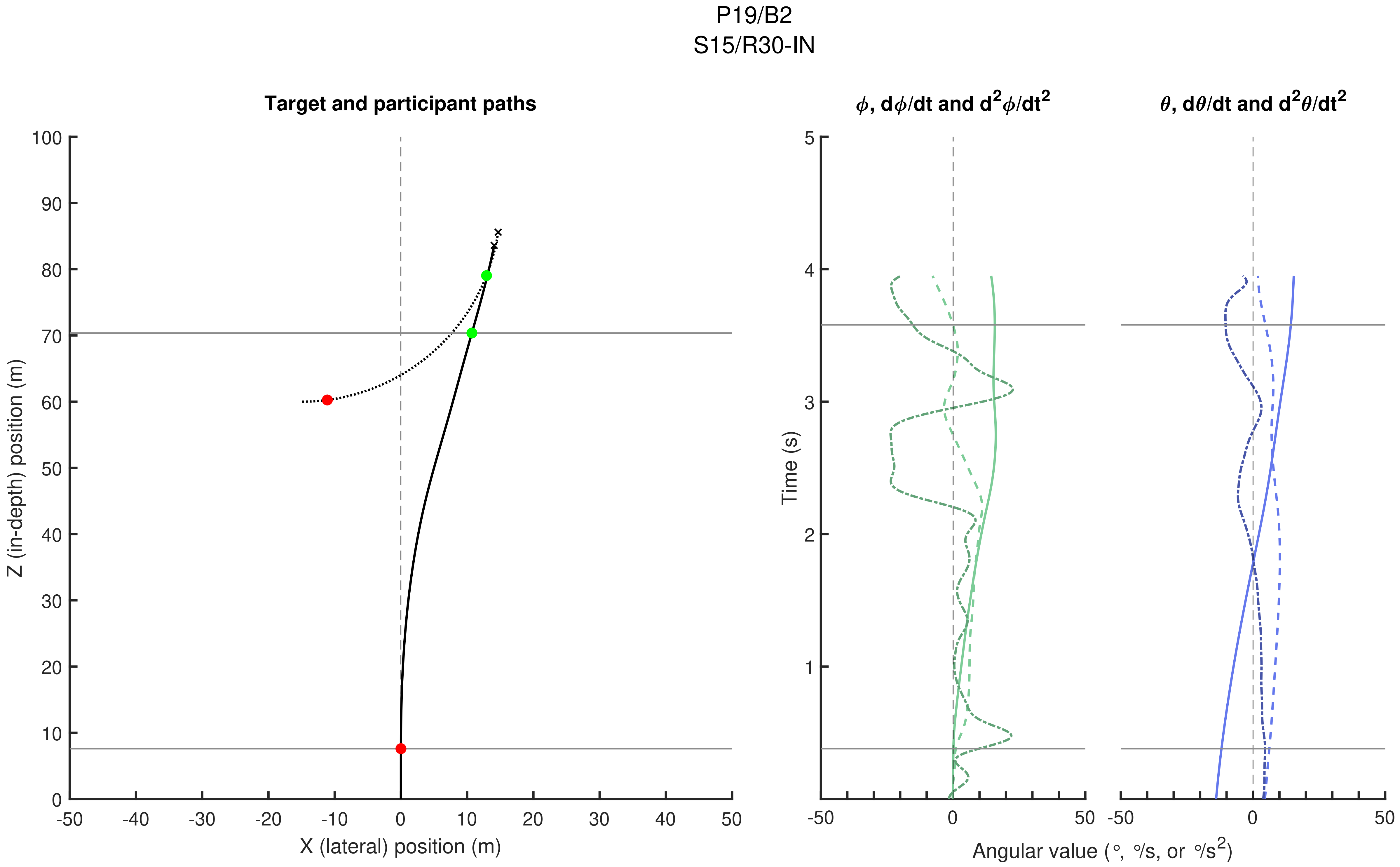
P19/B2
S15/R30-IN
Target and participant paths
Z (in-depth) position (m)
X (lateral) position (m)
φ, dφ/dt and d²φ/dt²
θ, dθ/dt and d²θ/dt²
Time (s)
Angular value (°, °/s, or °/s²)

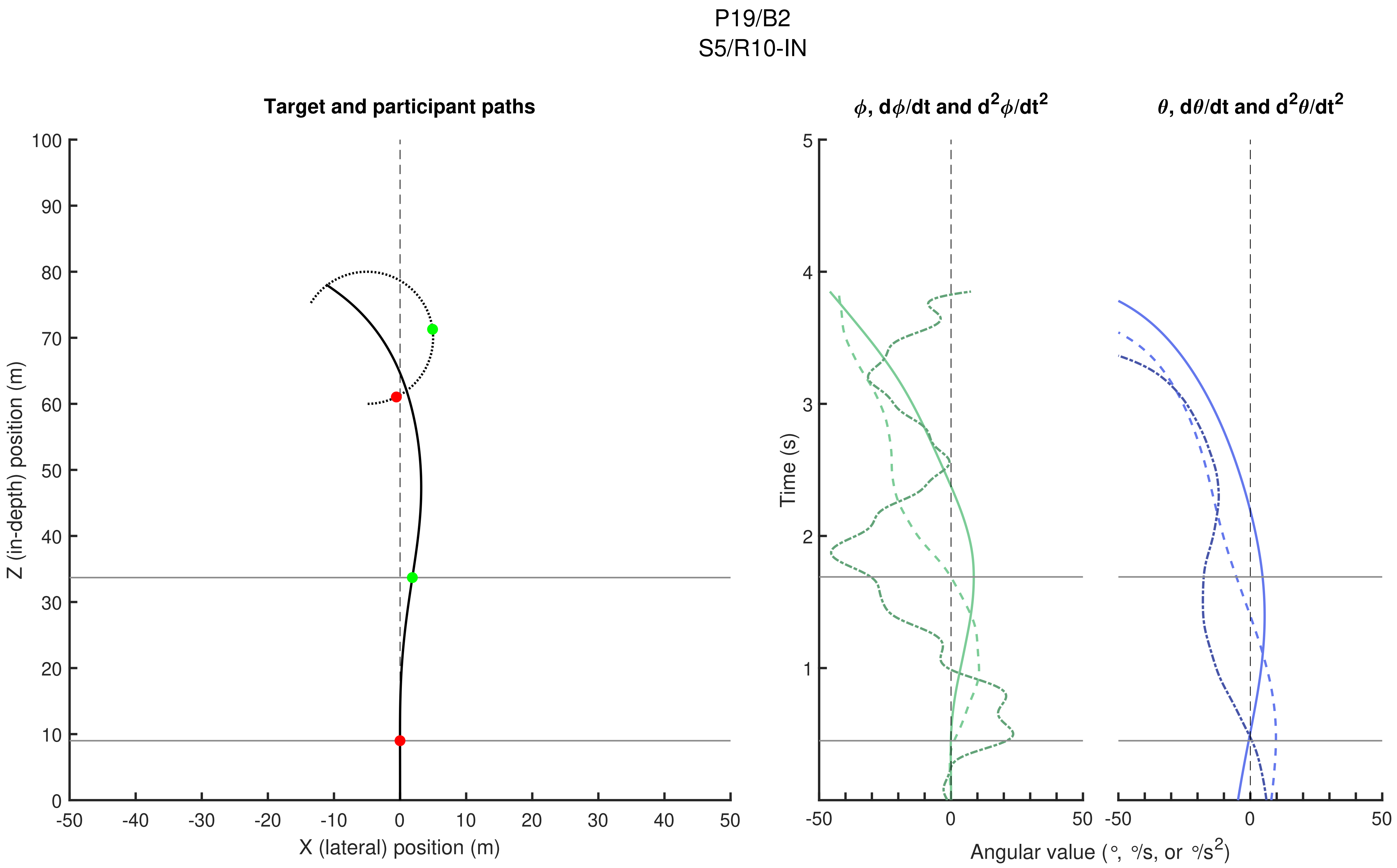
P19/B2
S5/R10-IN
Target and participant paths
Z (in-depth) position (m)
X (lateral) position (m)
0
10
20
30
40
50
60
70
80
90
100
-50
-40
-30
-20
-10
0
10
20
30
40
50
ϕ, dϕ/dt and d²ϕ/dt²
θ, dθ/dt and d²θ/dt²
Time (s)
1
2
3
4
5
-50
0
50
-50
0
50
Angular value (°, °/s, or °/s²)

P19/B2
S5/R10-OUT

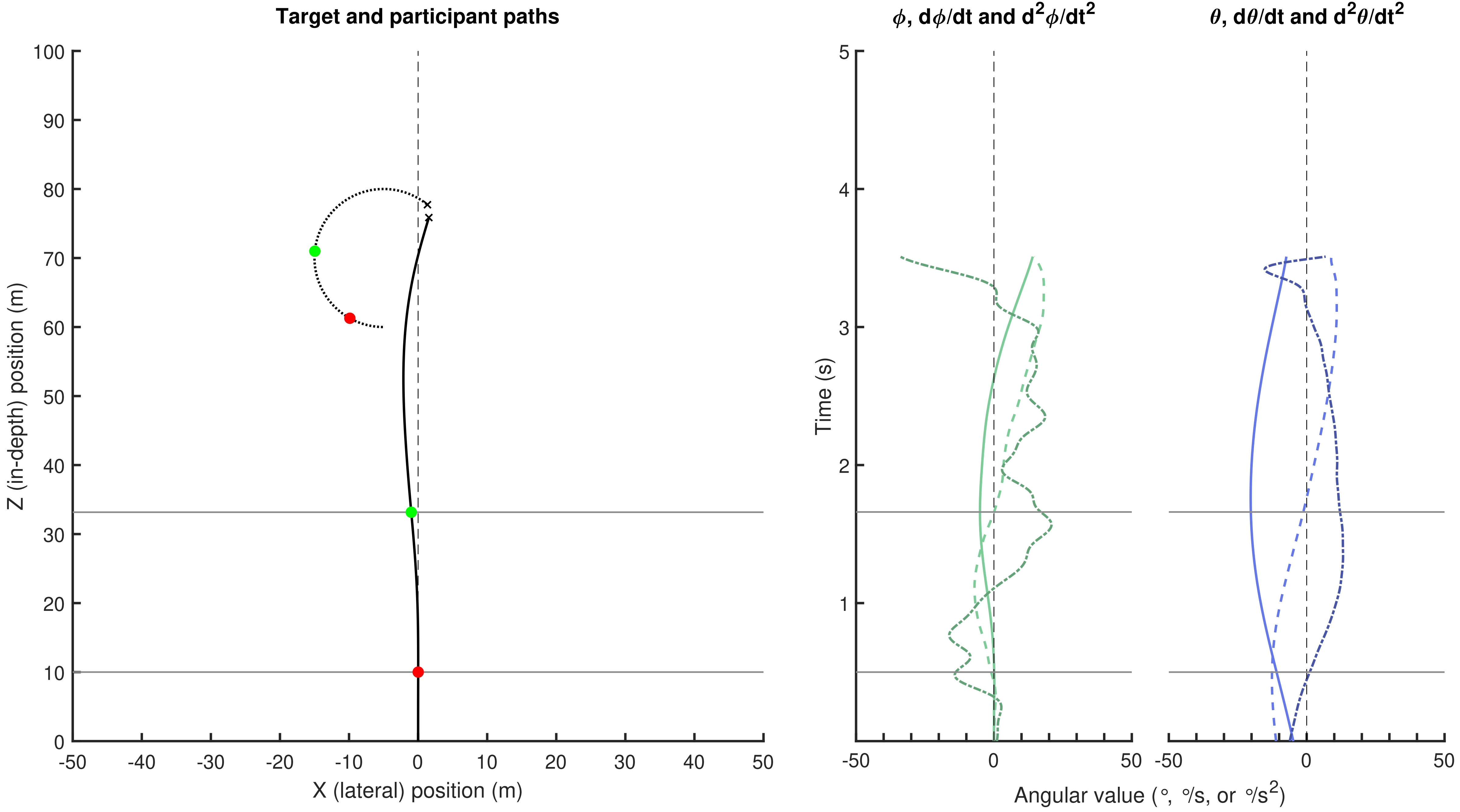

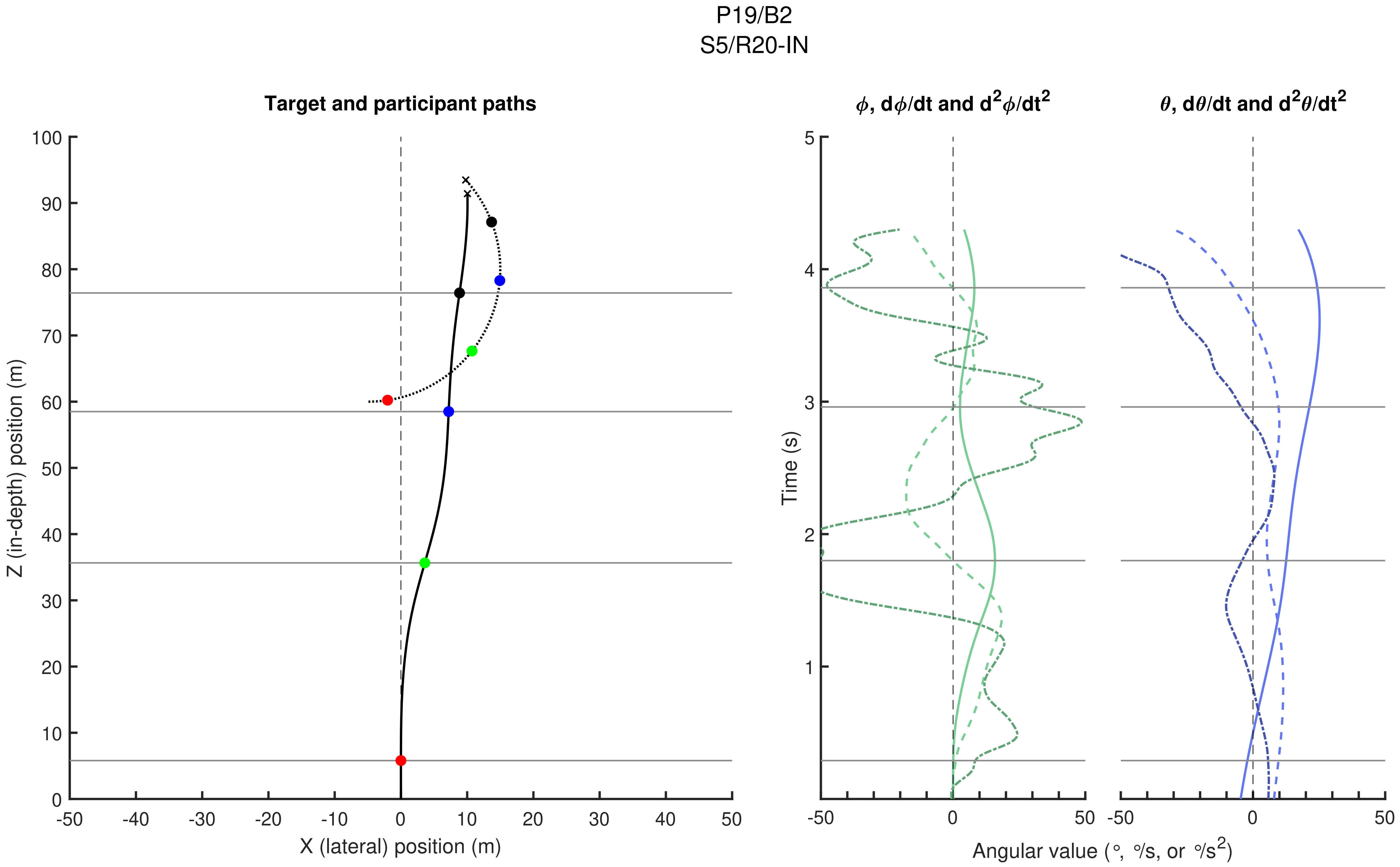

P19/B2
S5/R20-IN
Target and participant paths
Z (in-depth) position (m)
X (lateral) position (m)
φ, dφ/dt and d²φ/dt²
θ, dθ/dt and d²θ/dt²
Time (s)
Angular value (°, °/s, or °/s²)

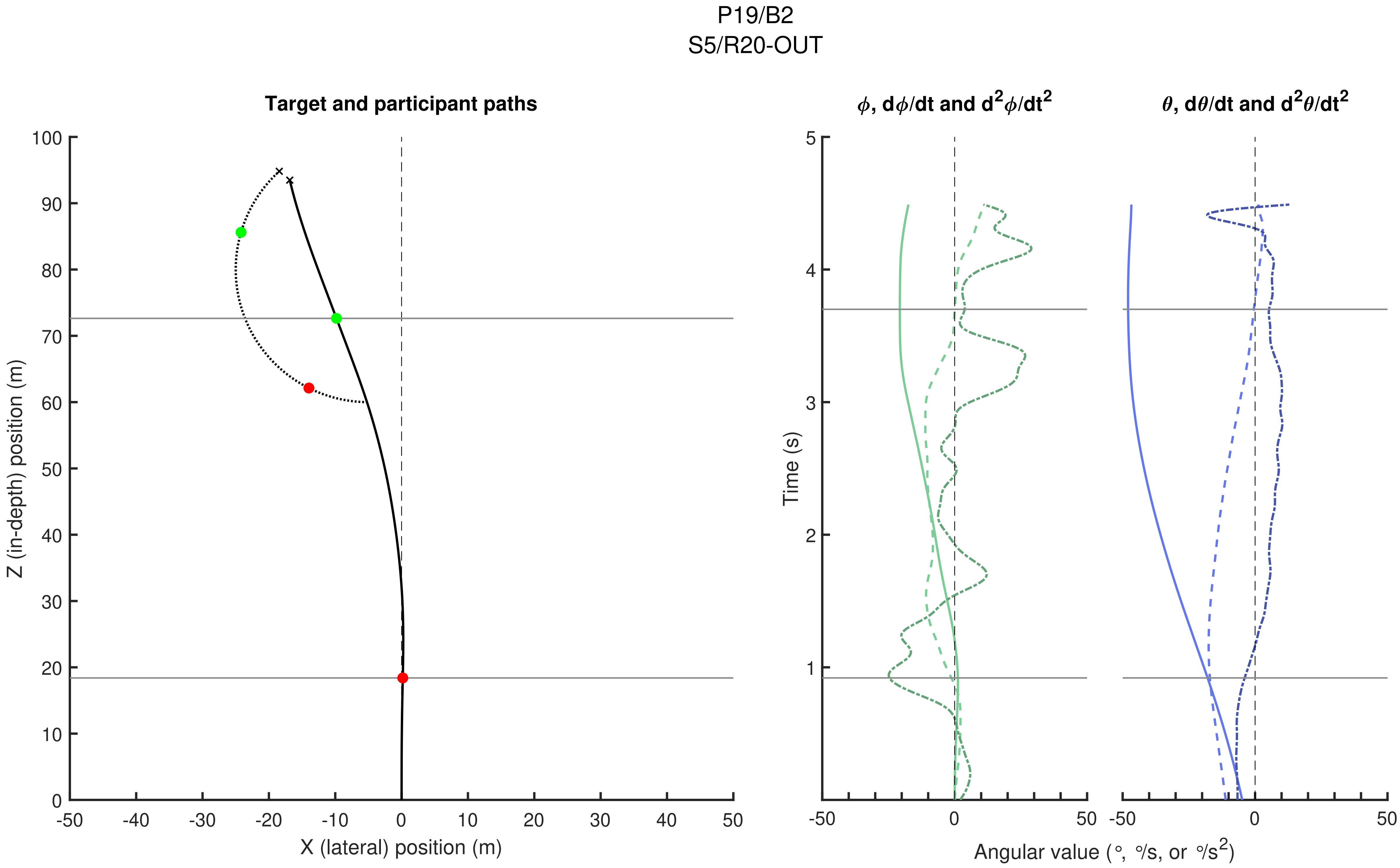

P19/B2
S5/R20-OUT
Target and participant paths
Z (in-depth) position (m)
X (lateral) position (m)
φ, dφ/dt and d²φ/dt²
θ, dθ/dt and d²θ/dt²
Time (s)
Angular value (°, °/s, or °/s²)

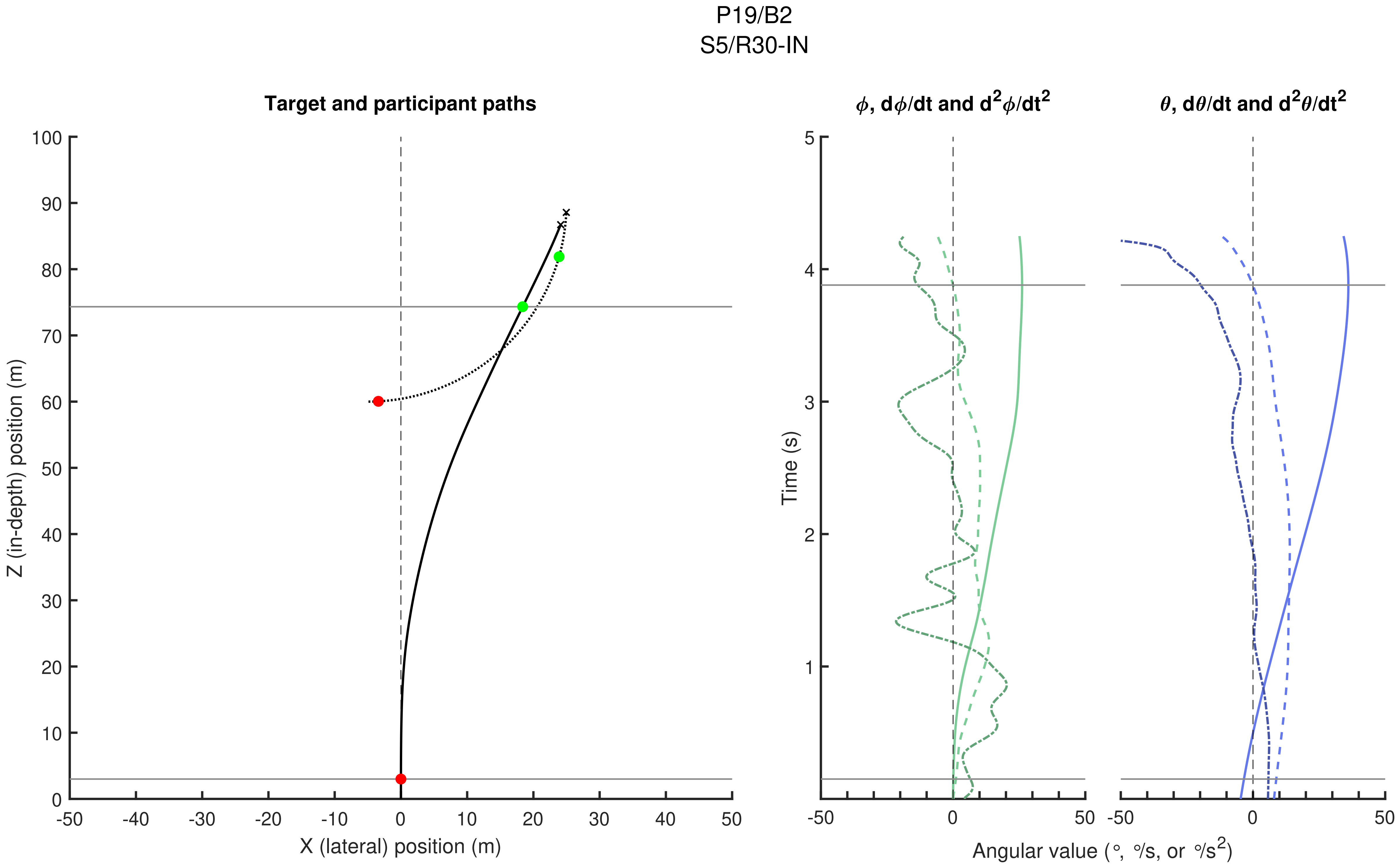
P19/B2
S5/R30-IN
Target and participant paths
ϕ, dϕ/dt and d$^2\phi$/dt^2
θ, dθ/dt and d$^2\theta$/dt^2
Z (in-depth) position (m)
X (lateral) position (m)
Time (s)
Angular value (°, °/s, or °/s^2)

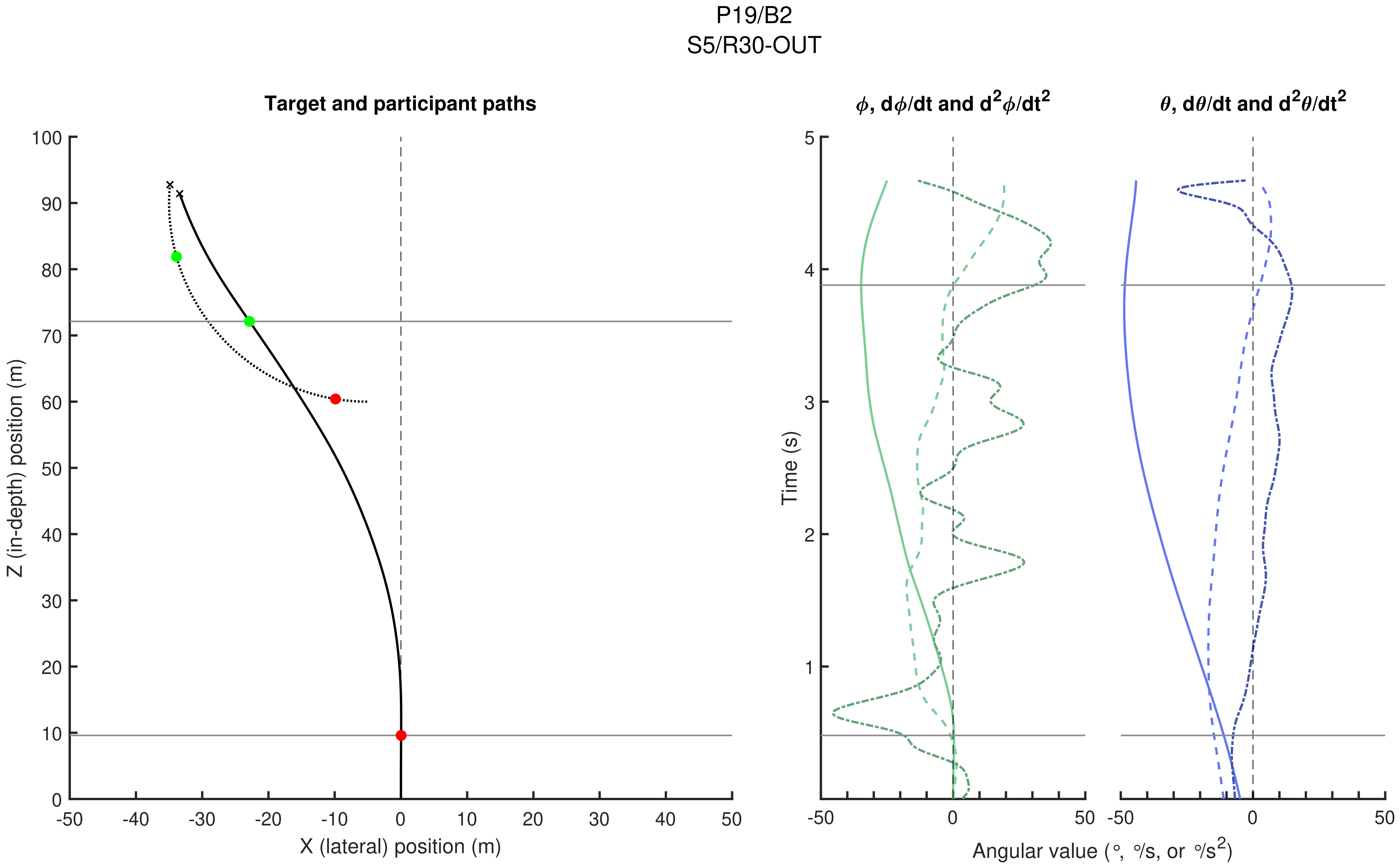
P19/B2
S5/R30-OUT
Target and participant paths
ϕ, dϕ/dt and d$^2\phi$/dt^2
θ, dθ/dt and d$^2\theta$/dt^2
Z (in-depth) position (m)
X (lateral) position (m)
Time (s)
Angular value (°, °/s, or °/s^2)
100
90
80
70
60
50
40
30
20
10
0
-50
-40
-30
-20
-10
0
10
20
30
40
50
5
4
3
2
1
-50
0
50
-50
0
50

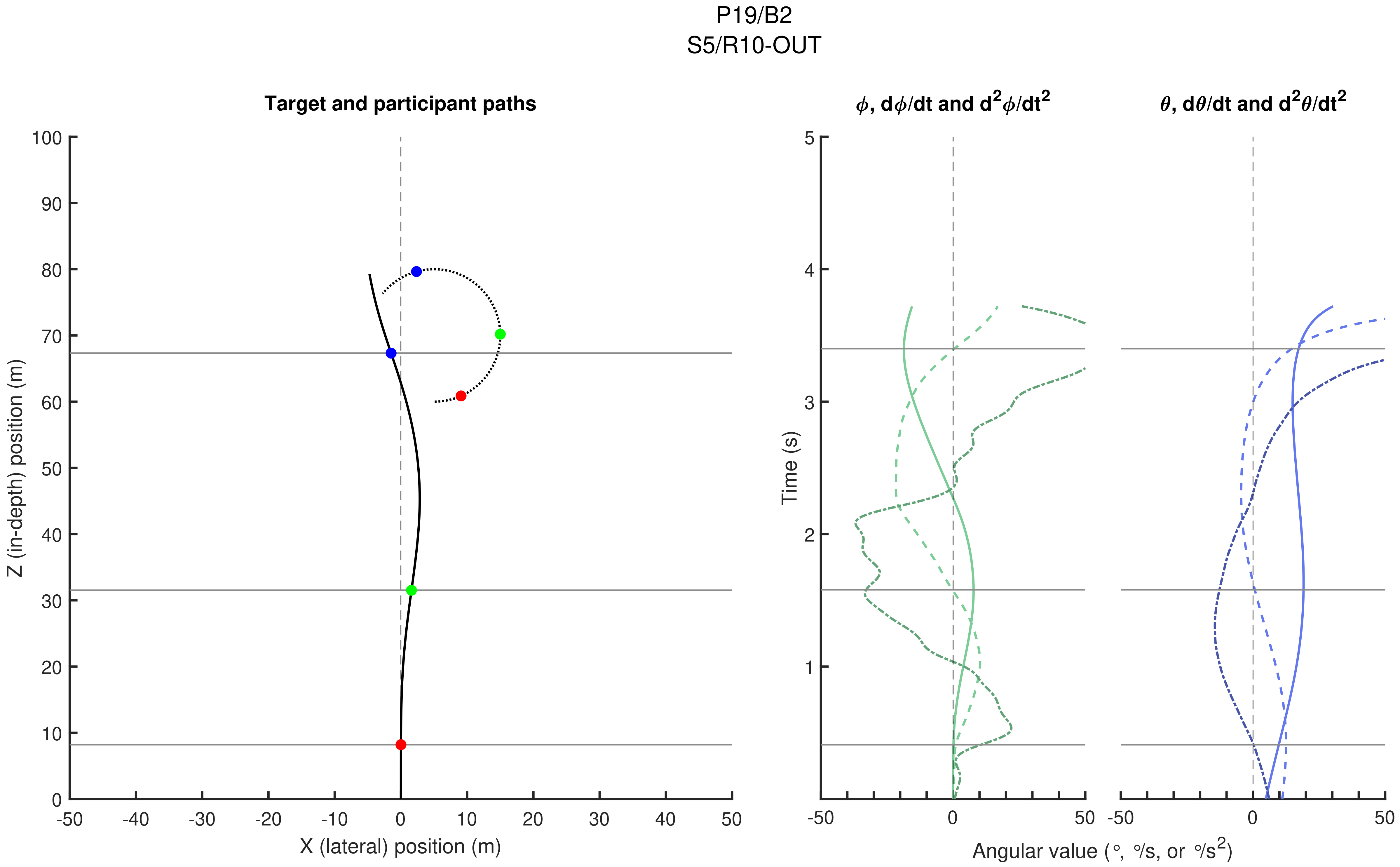
P19/B2
S5/R10-OUT
Target and participant paths
ϕ, dϕ/dt and d²ϕ/dt²
θ, dθ/dt and d²θ/dt²
Z (in-depth) position (m)
X (lateral) position (m)
Time (s)
Angular value (°, °/s, or °/s²)
100
90
80
70
60
50
40
30
20
10
0
-50
-40
-30
-20
-10
0
10
20
30
40
50
5
4
3
2
1
-50
0
50
-50
0
50

P19/B2
S5/R10-IN

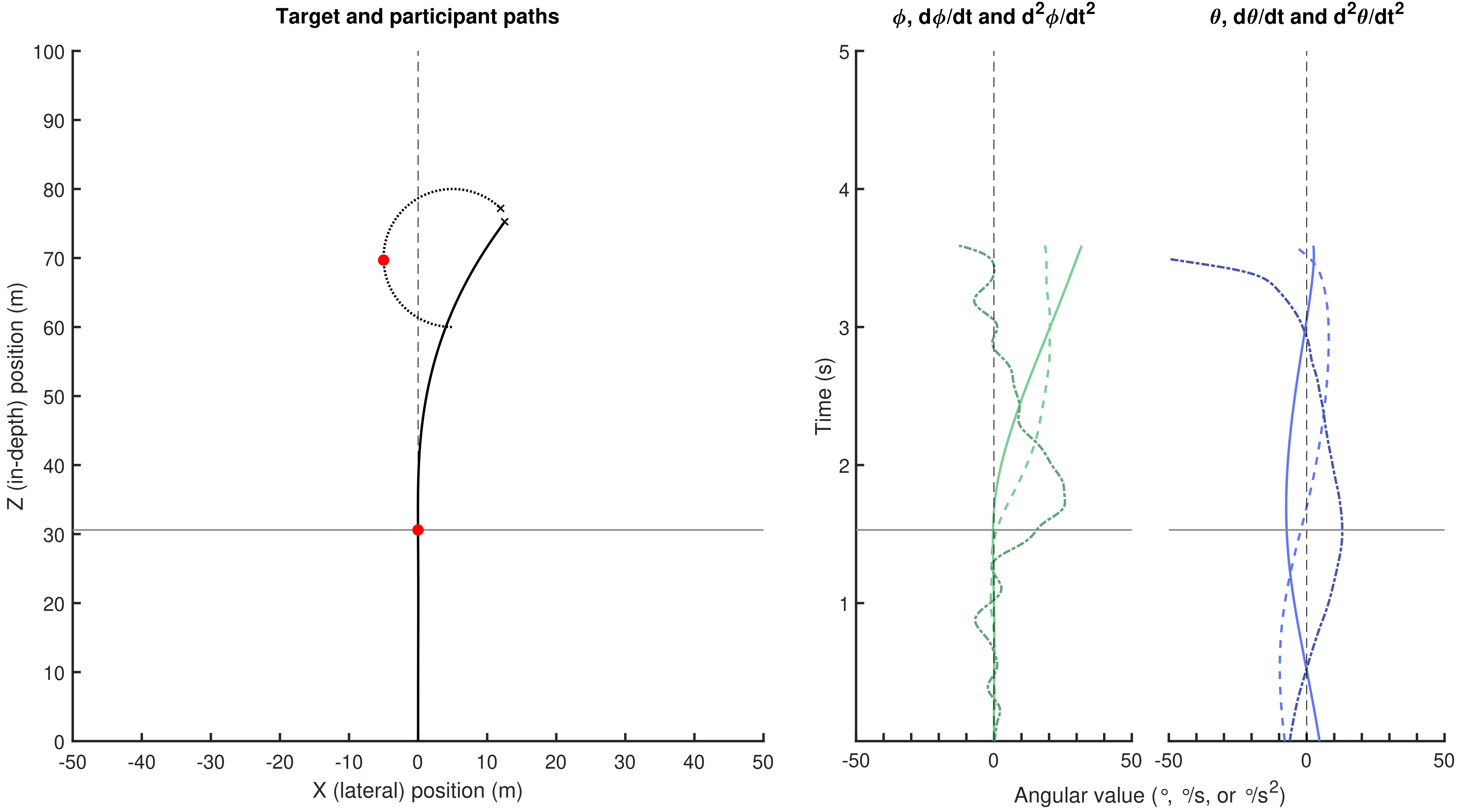

P19/B2
S5/R20-OUT

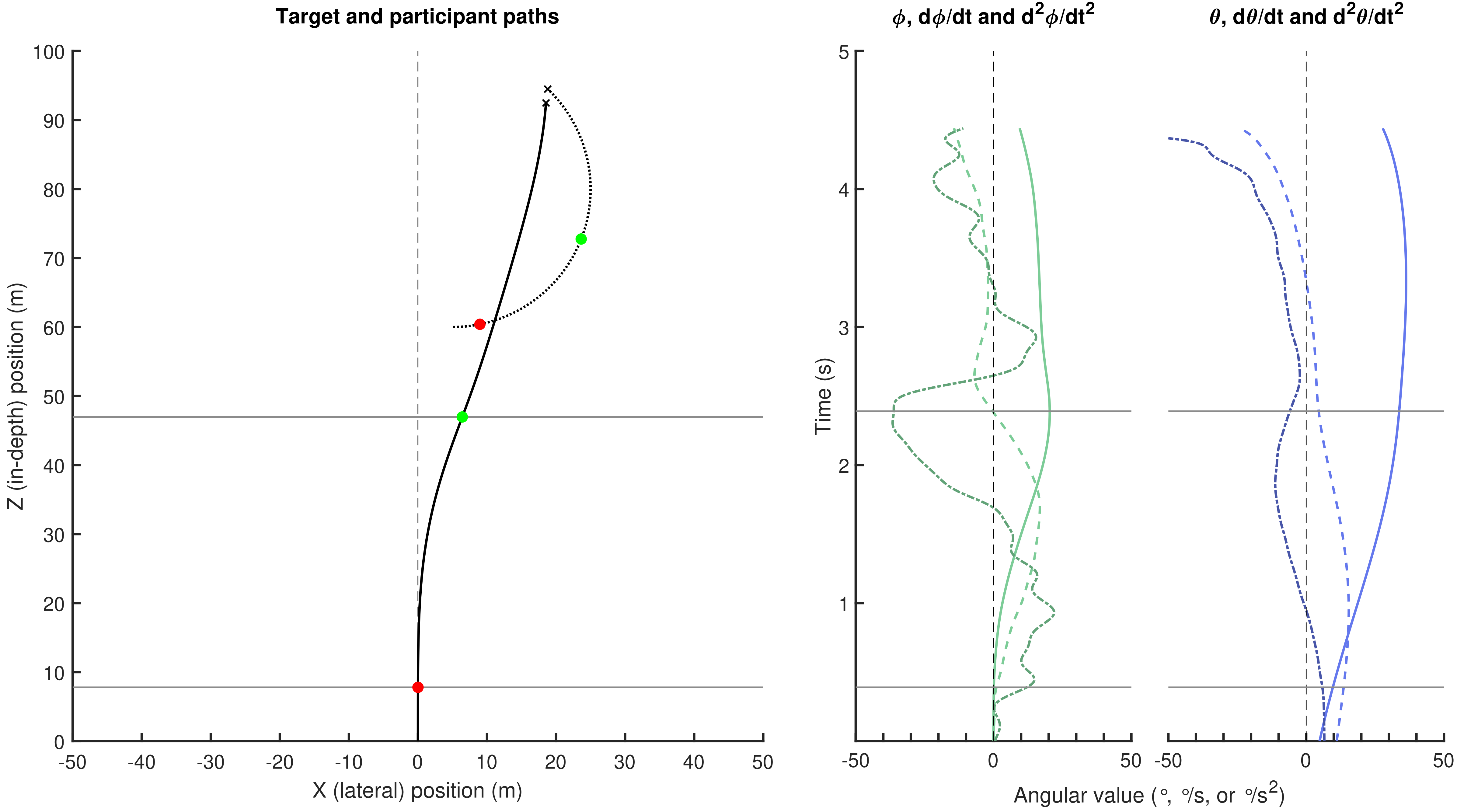

P19/B2
S5/R20-IN

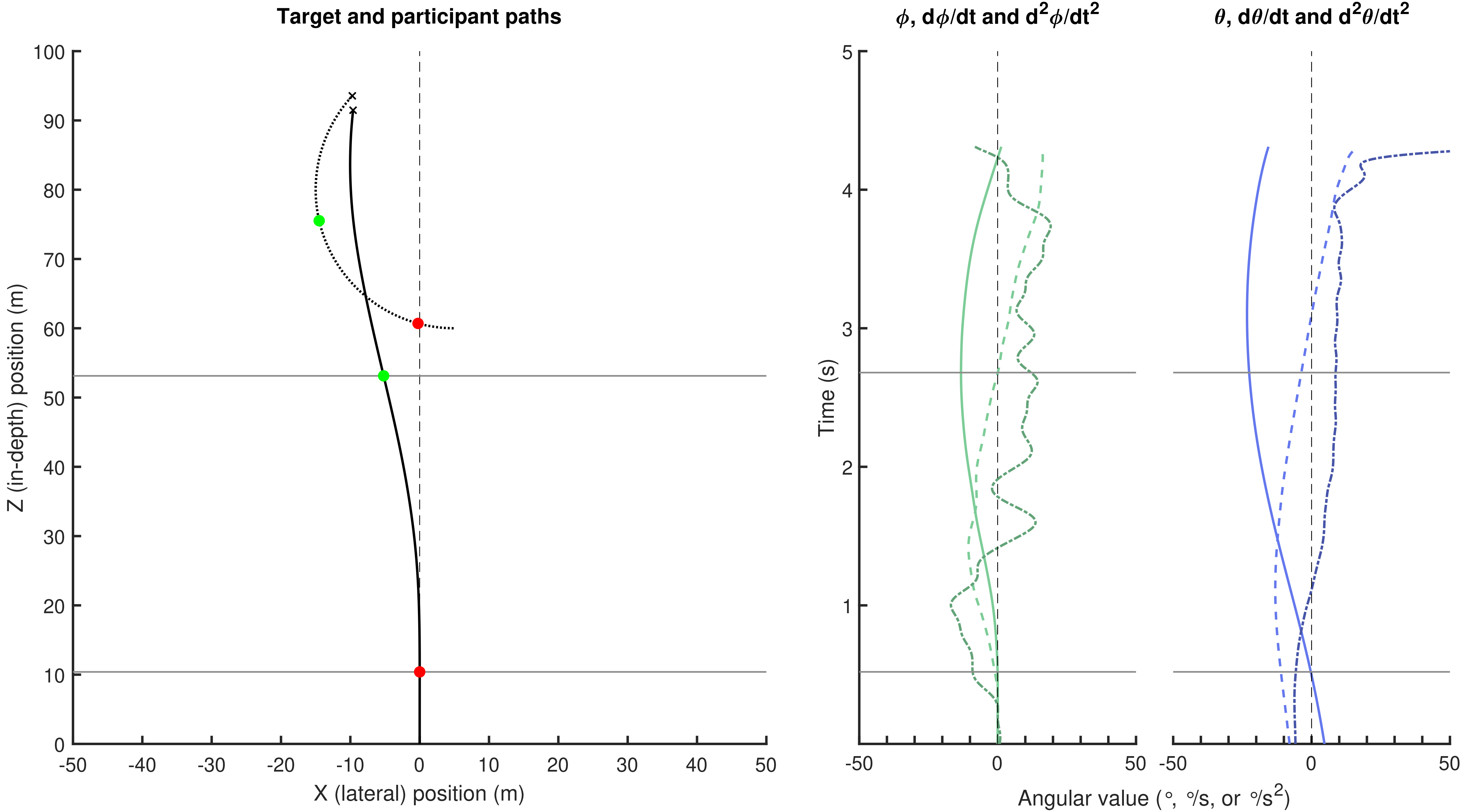

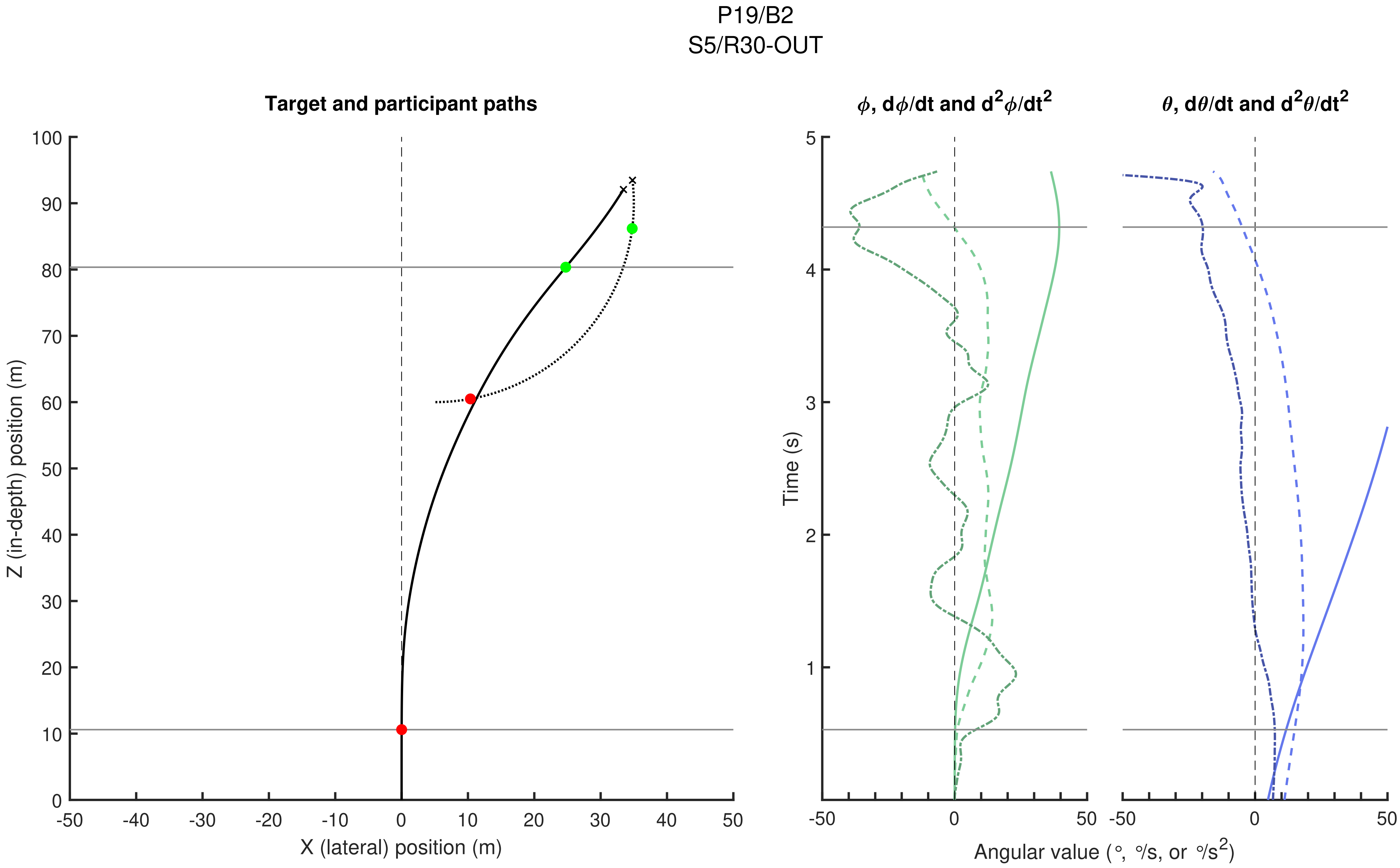
P19/B2
S5/R30-OUT
Target and participant paths
Z (in-depth) position (m)
X (lateral) position (m)
100
90
80
70
60
50
40
30
20
10
0
-50
-40
-30
-20
-10
0
10
20
30
40
50
φ, dφ/dt and d²φ/dt²
θ, dθ/dt and d²θ/dt²
Time (s)
5
4
3
2
1
-50
0
50
-50
0
50
Angular value (°, °/s, or °/s²)

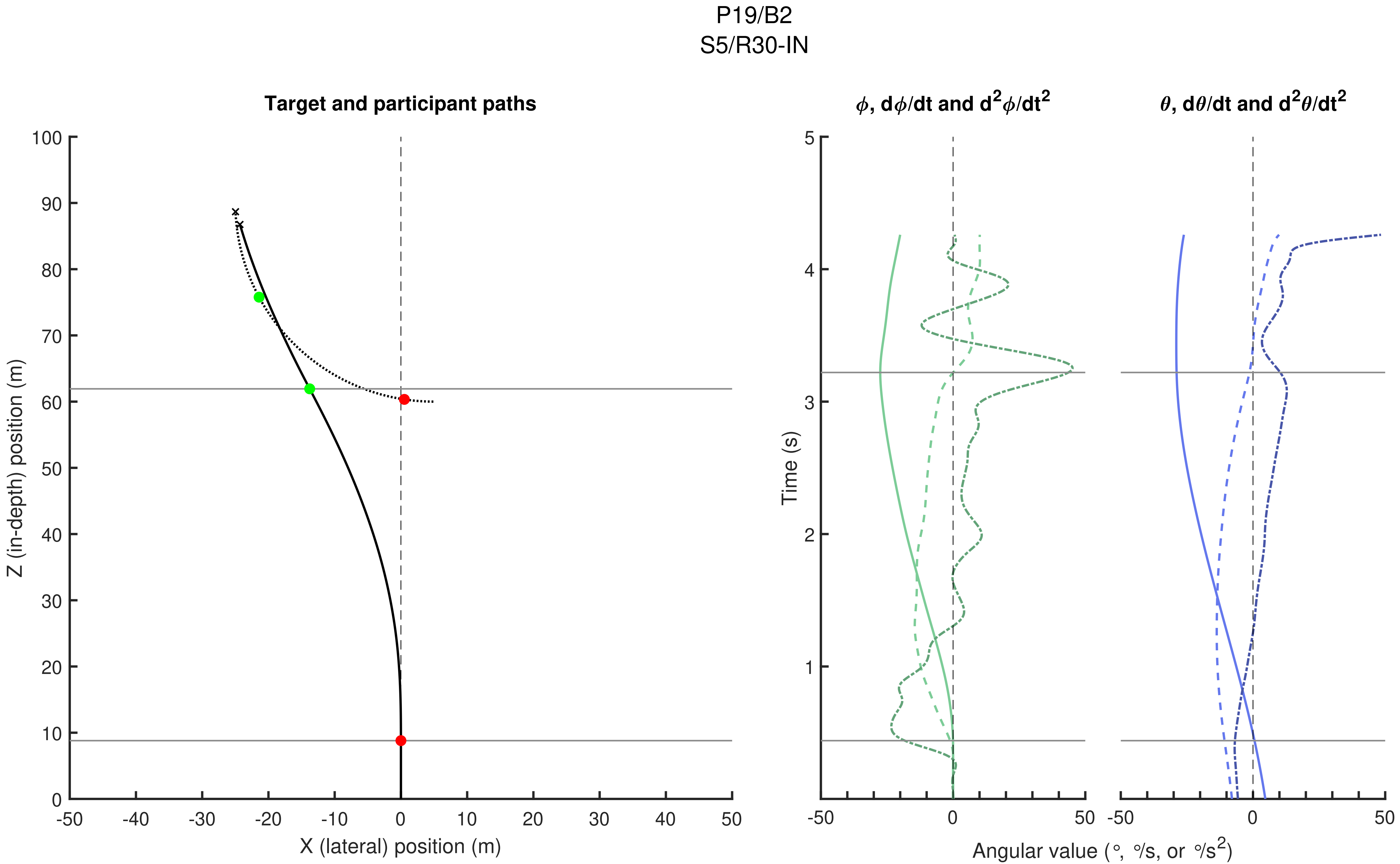

P19/B2
S5/R30-IN
Target and participant paths
Z (in-depth) position (m)
X (lateral) position (m)
ϕ, dϕ/dt and d²ϕ/dt²
θ, dθ/dt and d²θ/dt²
Time (s)
Angular value (°, °/s, or °/s²)

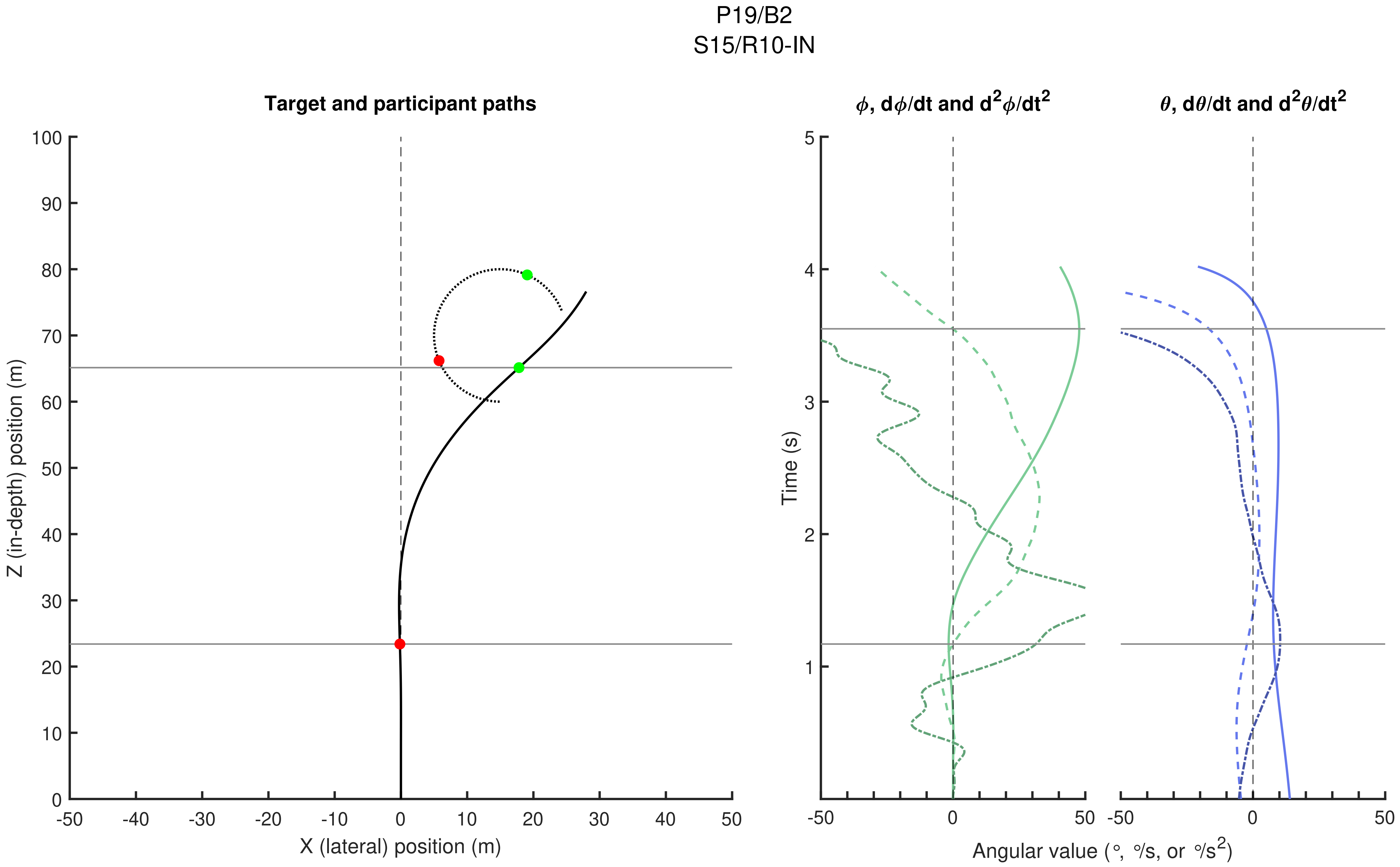

P19/B2
S15/R10-IN
Target and participant paths
Z (in-depth) position (m)
X (lateral) position (m)
100
90
80
70
60
50
40
30
20
10
0
-50
-40
-30
-20
-10
0
10
20
30
40
50
ϕ, dϕ/dt and d$^2\phi$/dt^2
θ, dθ/dt and d$^2\theta$/dt^2
Time (s)
5
4
3
2
1
-50
0
50
-50
0
50
Angular value (°, °/s, or °/s^2)

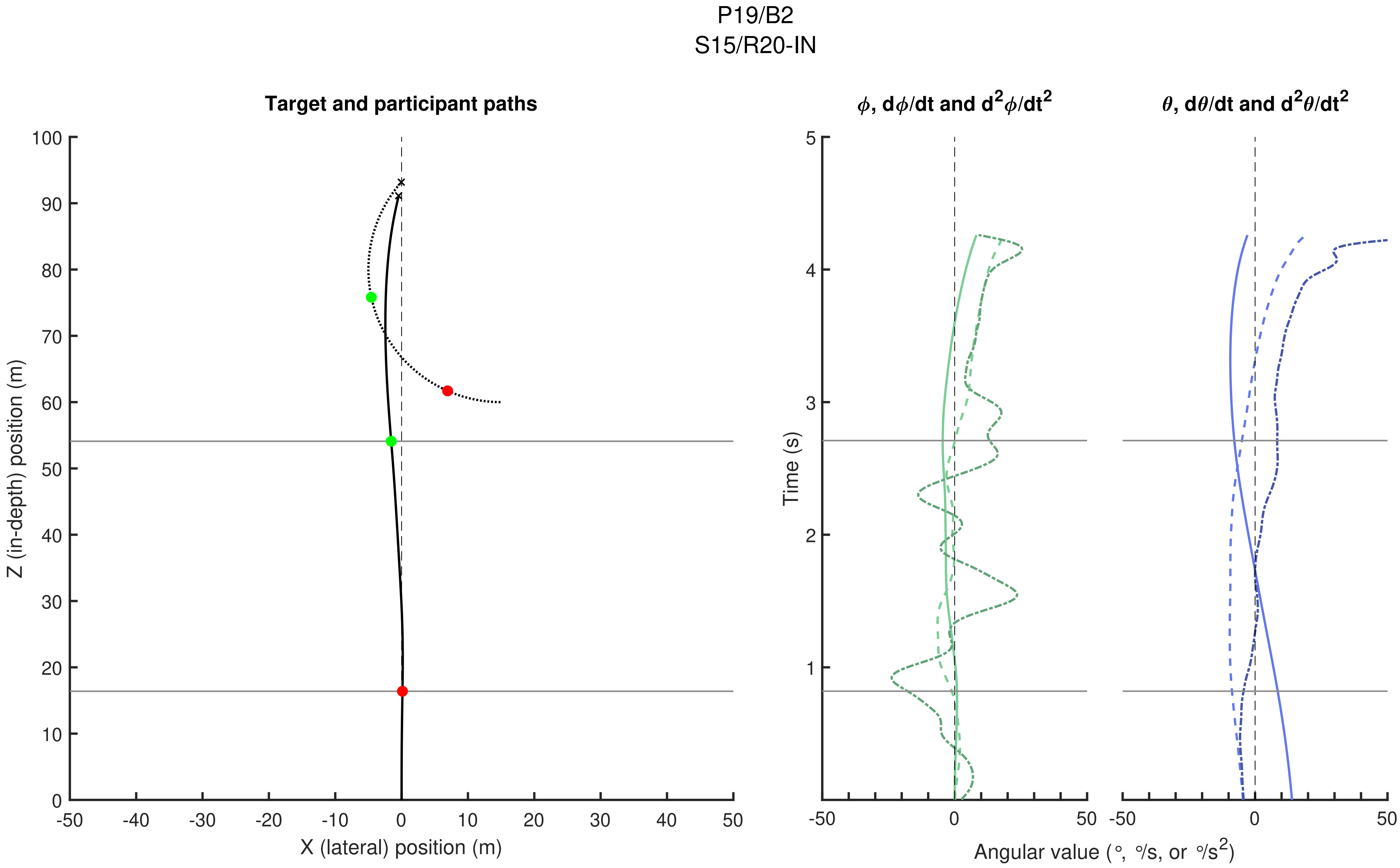
P19/B2
S15/R20-IN
Target and participant paths
Z (in-depth) position (m)
X (lateral) position (m)
ϕ, dϕ/dt and d²ϕ/dt²
θ, dθ/dt and d²θ/dt²
Time (s)
Angular value (°, °/s, or °/s²)

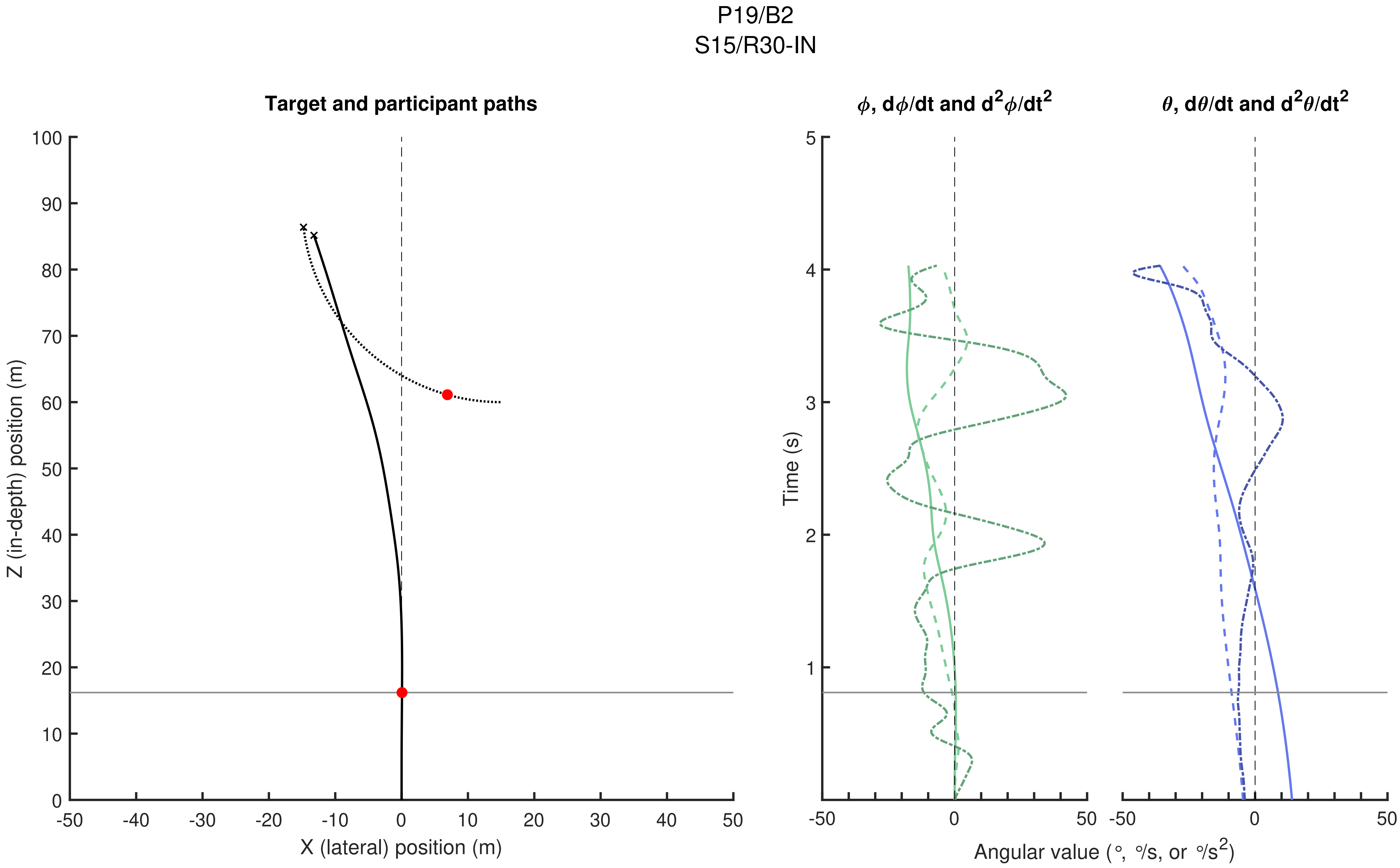

P19/B2
S15/R30-IN
Target and participant paths
Z (in-depth) position (m)
X (lateral) position (m)
ϕ, dϕ/dt and d²ϕ/dt²
θ, dθ/dt and d²θ/dt²
Time (s)
Angular value (°, °/s, or °/s²)

P19/B2
S20/R10-IN

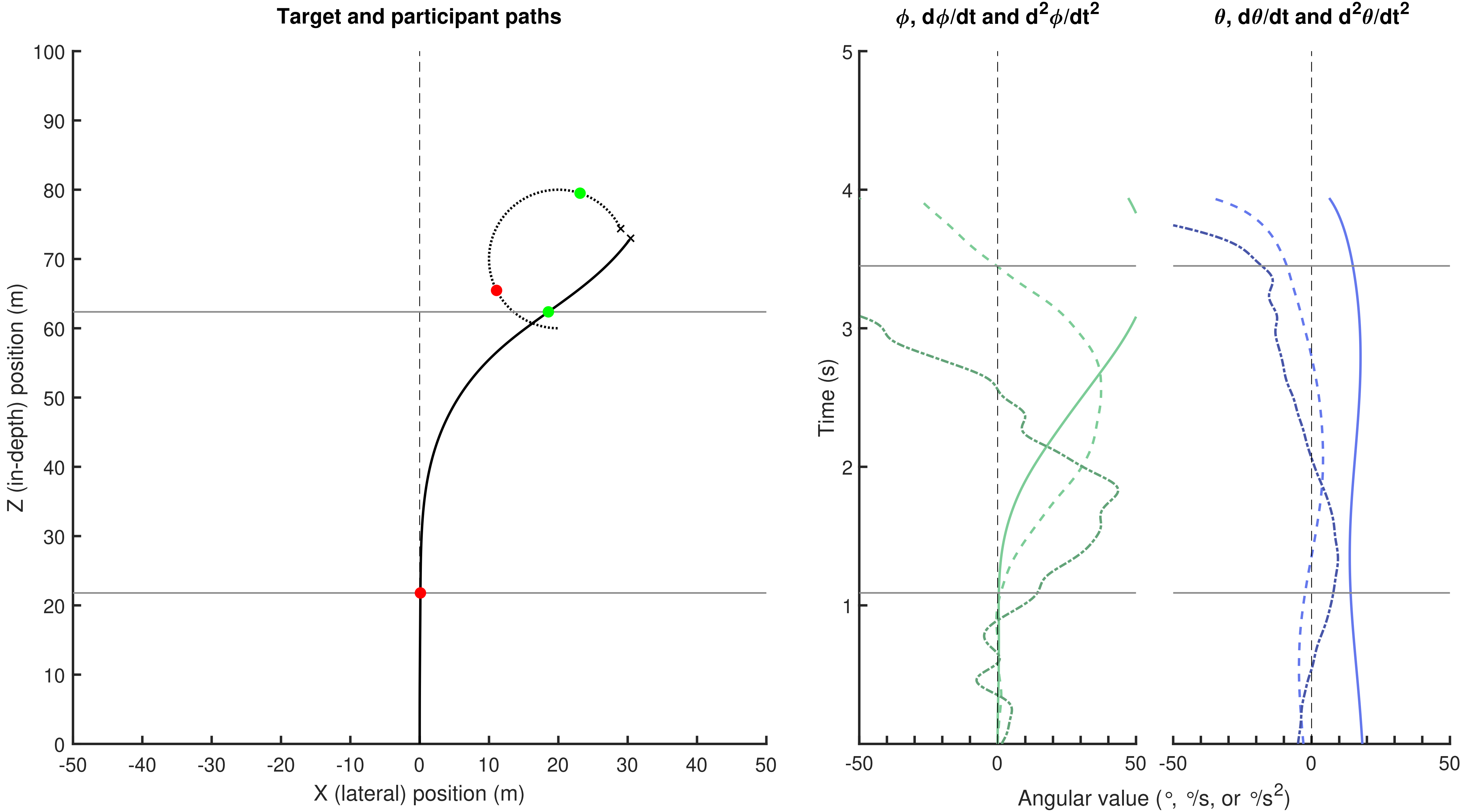

P19/B2
S20/R20-IN

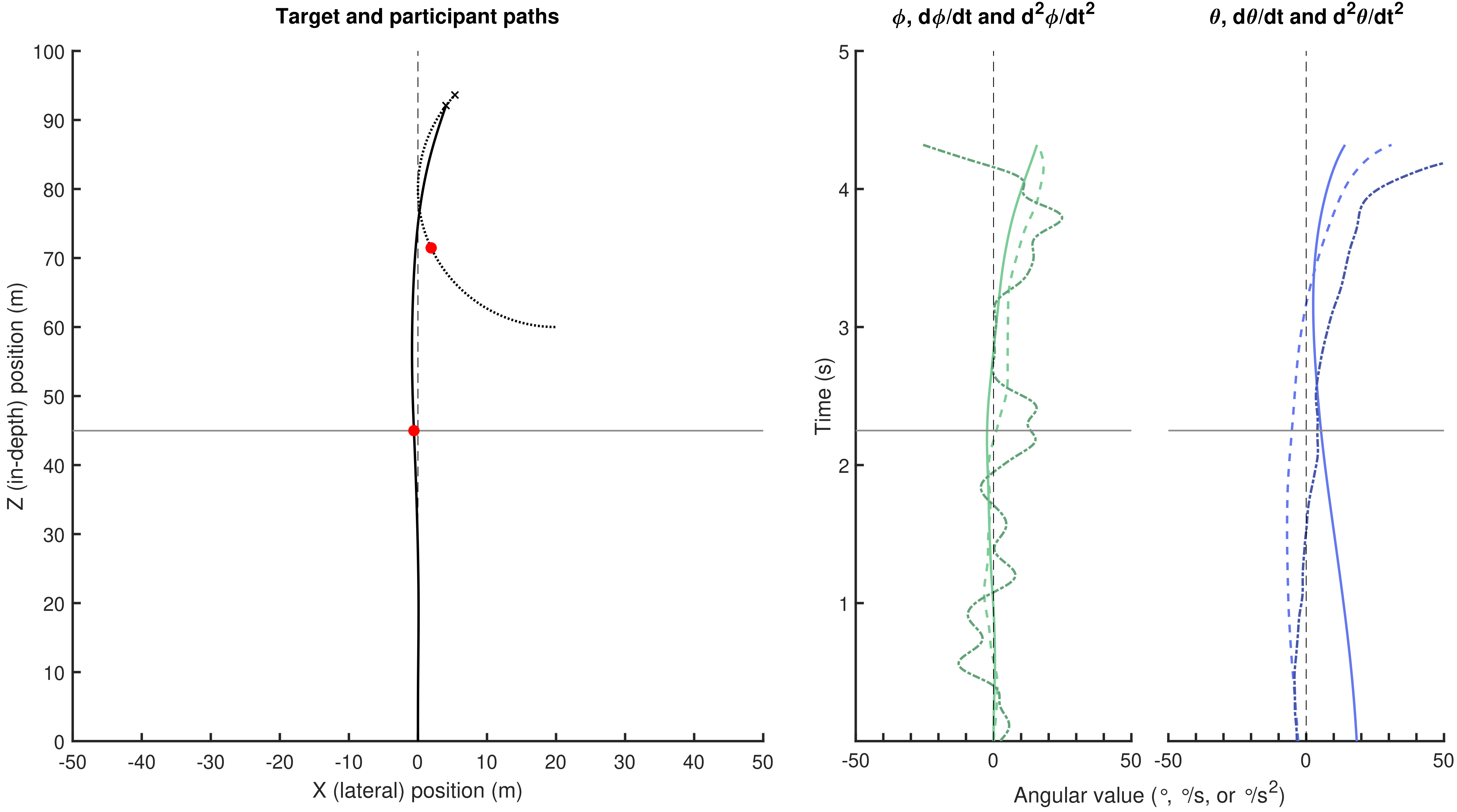

P19/B2
S20/R30-IN

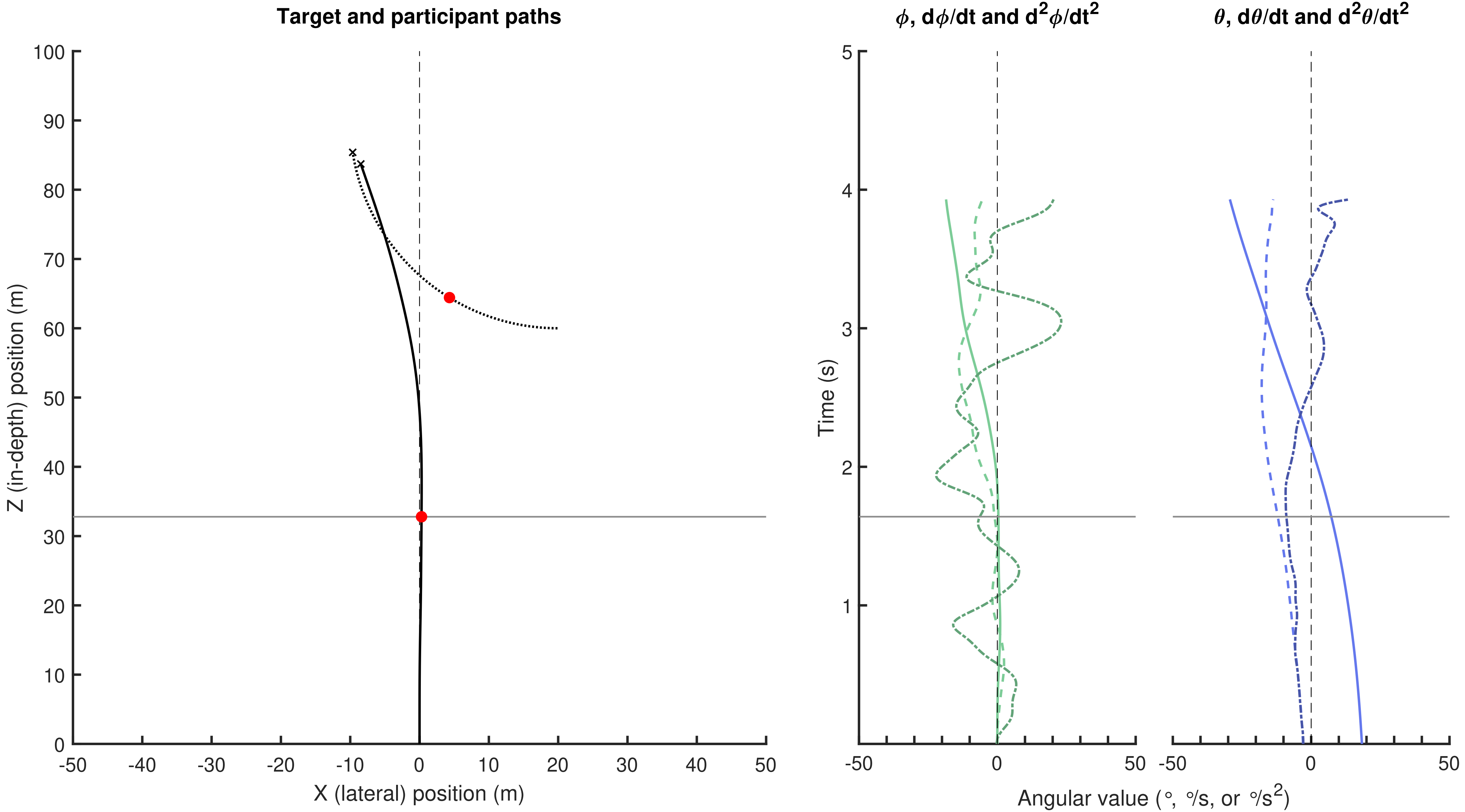

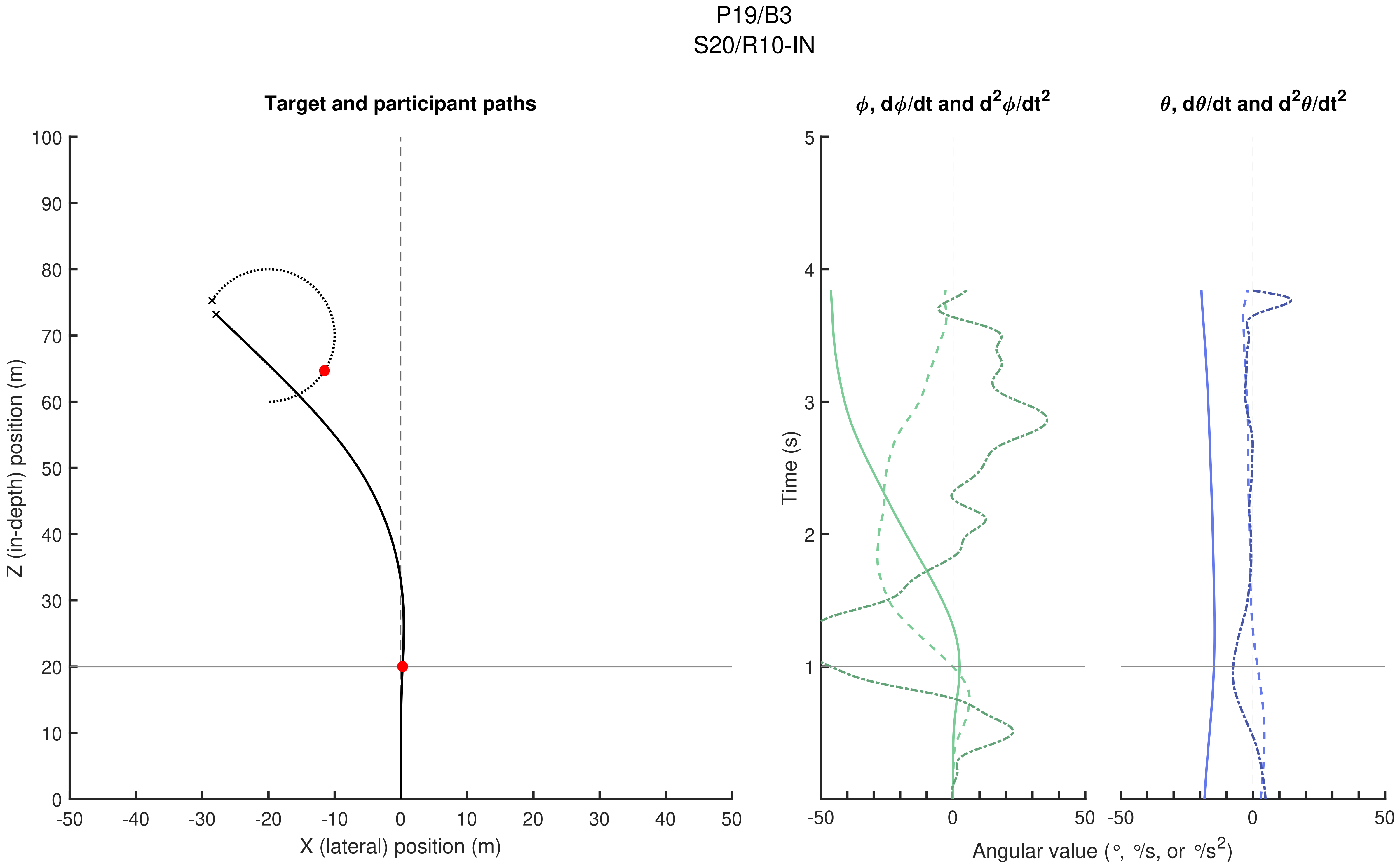

P19/B3
S20/R10-IN
Target and participant paths
Z (in-depth) position (m)
X (lateral) position (m)
100
90
80
70
60
50
40
30
20
10
0
-50
-40
-30
-20
-10
0
10
20
30
40
50
ϕ, dϕ/dt and d²ϕ/dt²
θ, dθ/dt and d²θ/dt²
Time (s)
5
4
3
2
1
-50
0
50
-50
0
50
Angular value (°, °/s, or °/s²)

P19/B3
S20/R20-IN

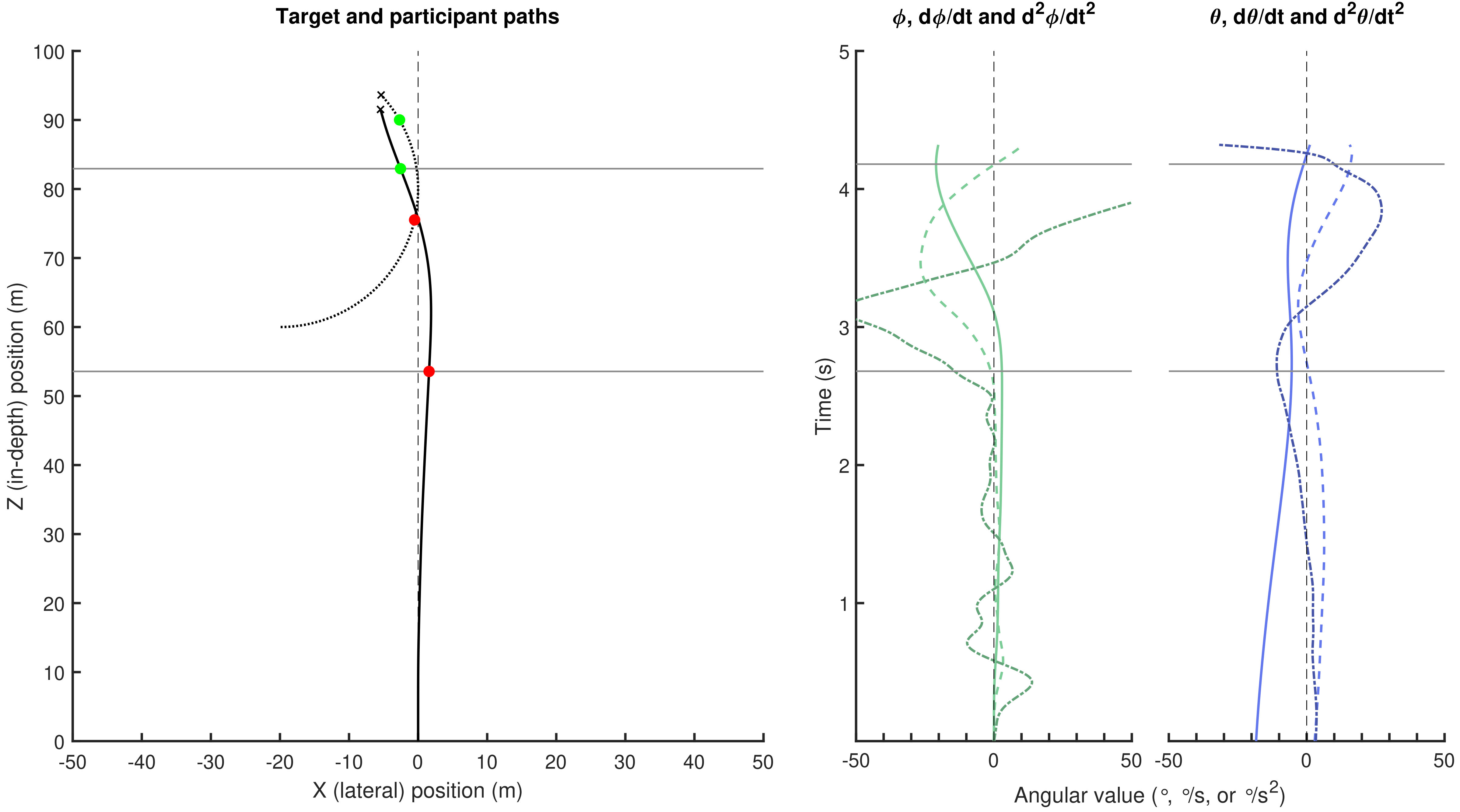

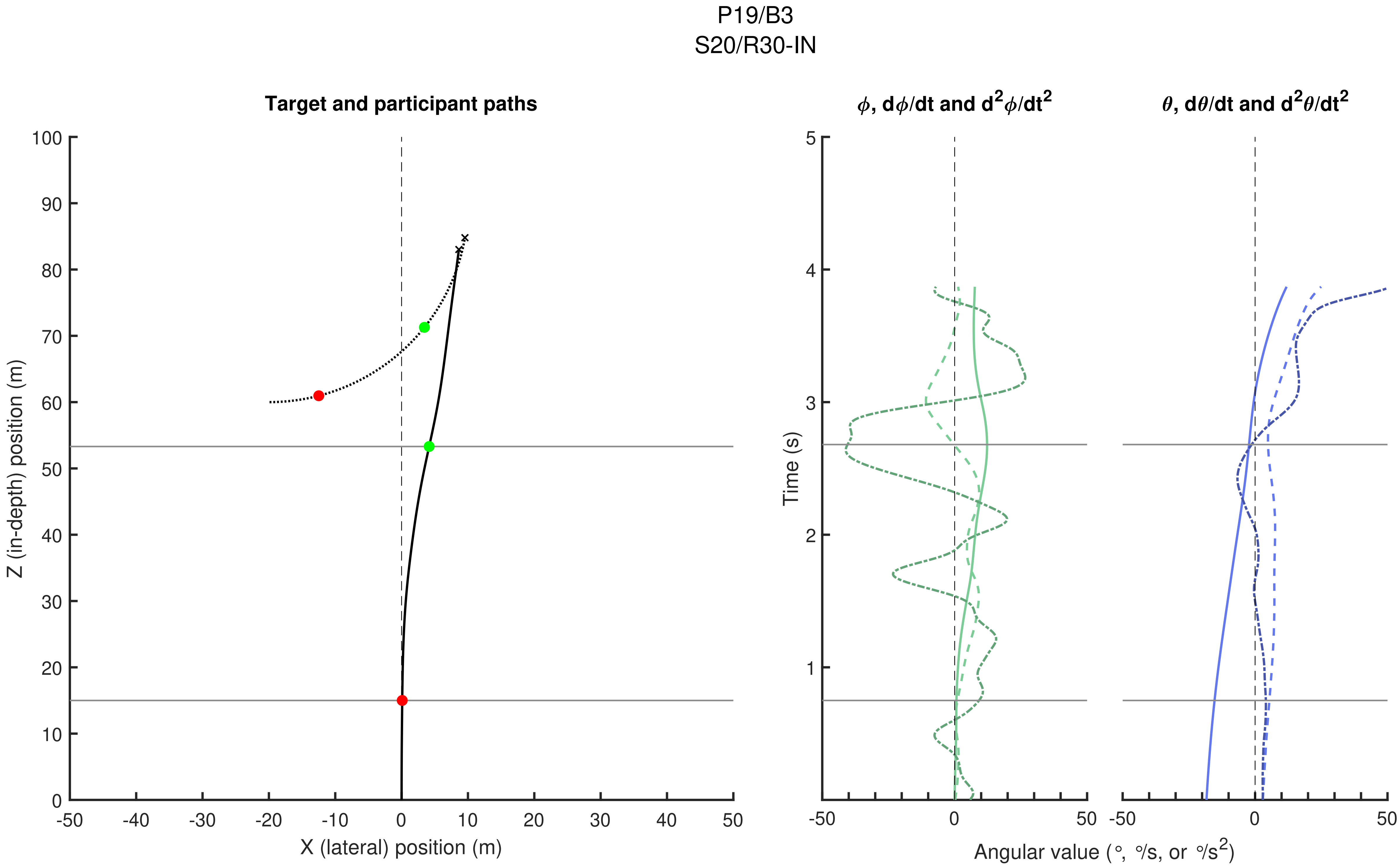

P19/B3
S20/R30-IN
Target and participant paths
ϕ, dϕ/dt and d$^2\phi$/dt^2
θ, dθ/dt and d$^2\theta$/dt^2
Z (in-depth) position (m)
X (lateral) position (m)
Time (s)
Angular value (°, °/s, or °/s^2)

P19/B3
S15/R10-IN

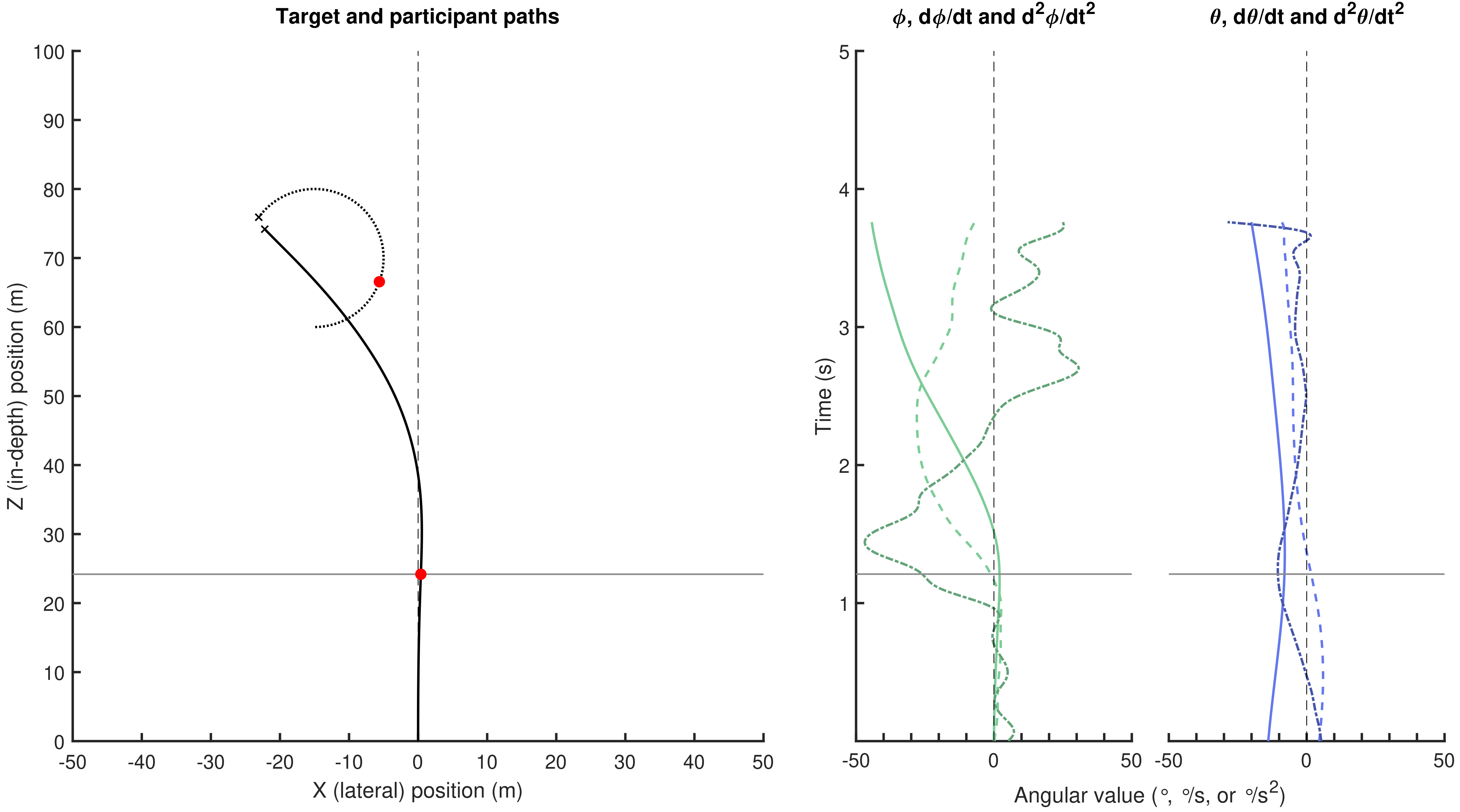

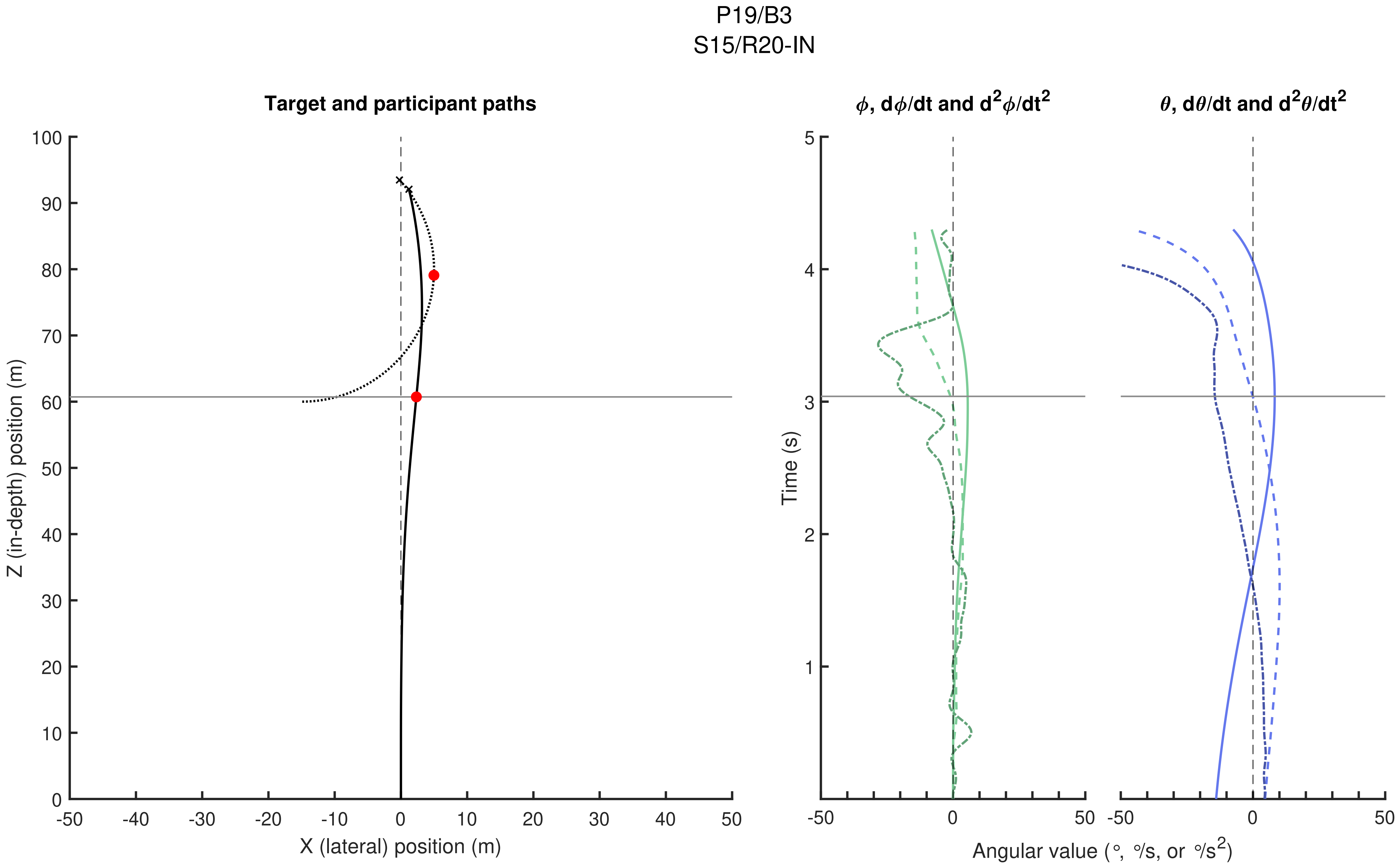

P19/B3
S15/R20-IN
Target and participant paths
Z (in-depth) position (m)
X (lateral) position (m)
100
90
80
70
60
50
40
30
20
10
0
-50
-40
-30
-20
-10
0
10
20
30
40
50
ϕ, dϕ/dt and d²ϕ/dt²
θ, dθ/dt and d²θ/dt²
Time (s)
5
4
3
2
1
-50
0
50
-50
0
50
Angular value (°, °/s, or °/s²)

P19/B3
S15/R30-IN

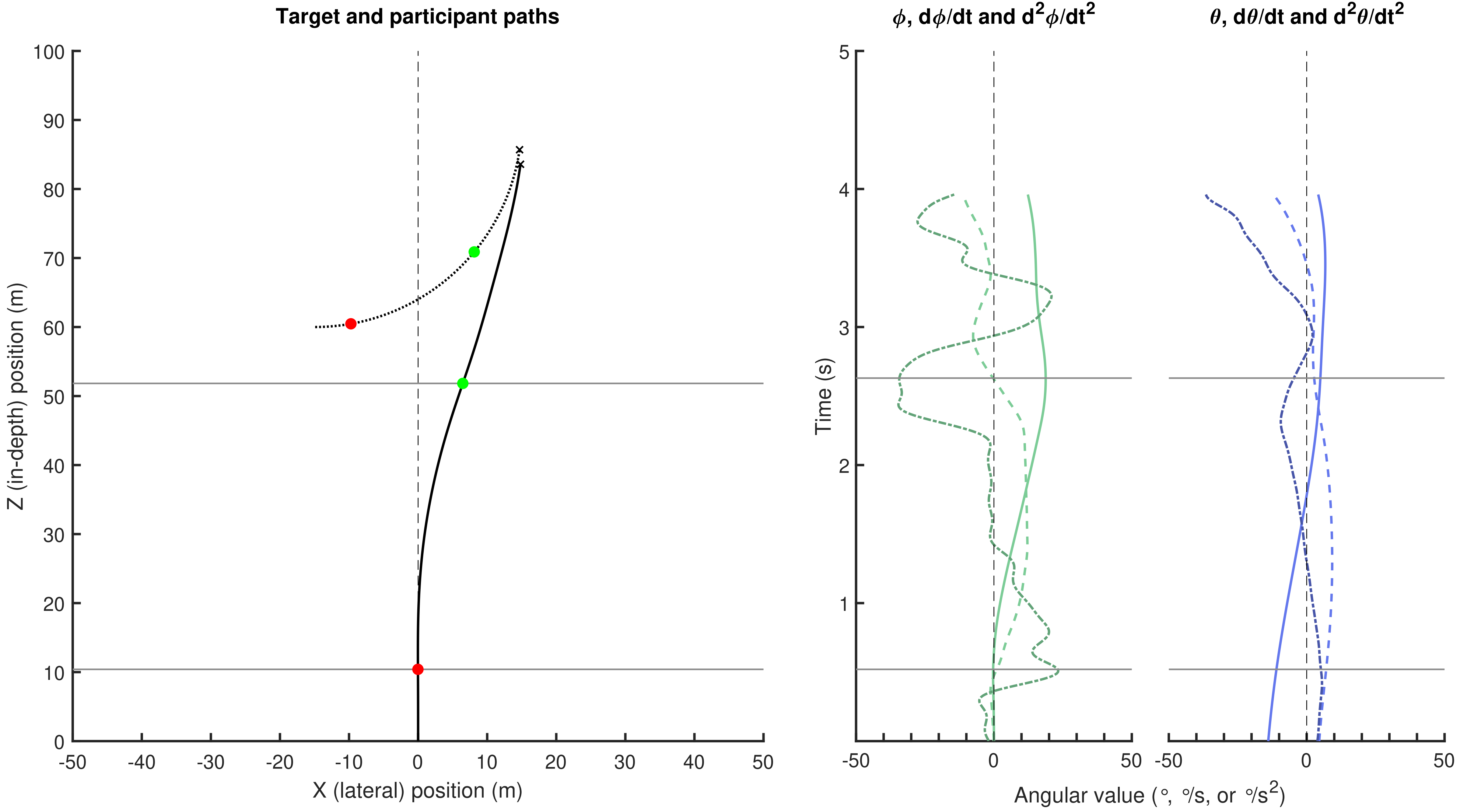

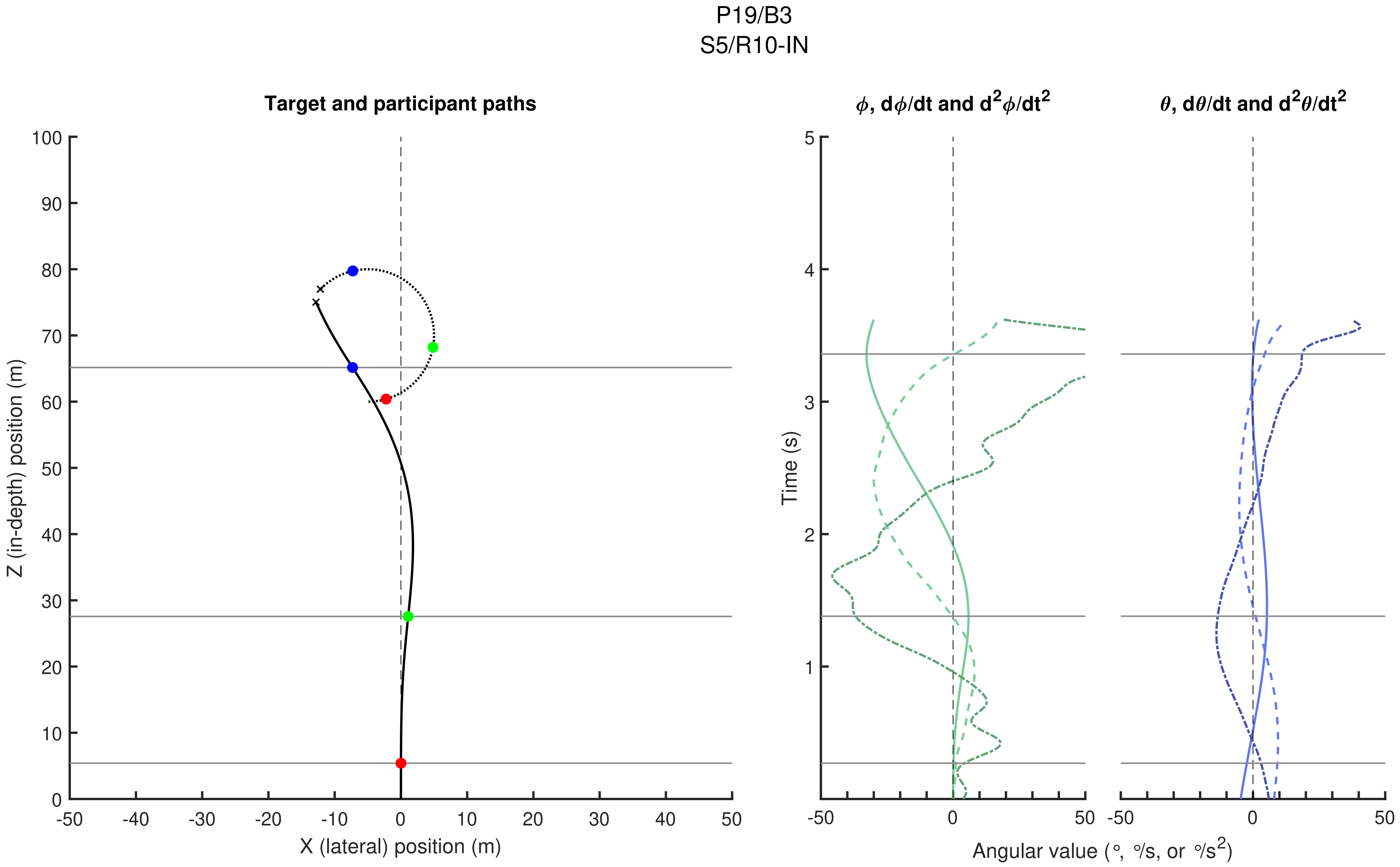
P19/B3
S5/R10-IN
Target and participant paths
Z (in-depth) position (m)
X (lateral) position (m)
φ, dφ/dt and d²φ/dt²
θ, dθ/dt and d²θ/dt²
Time (s)
Angular value (°, °/s, or °/s²)

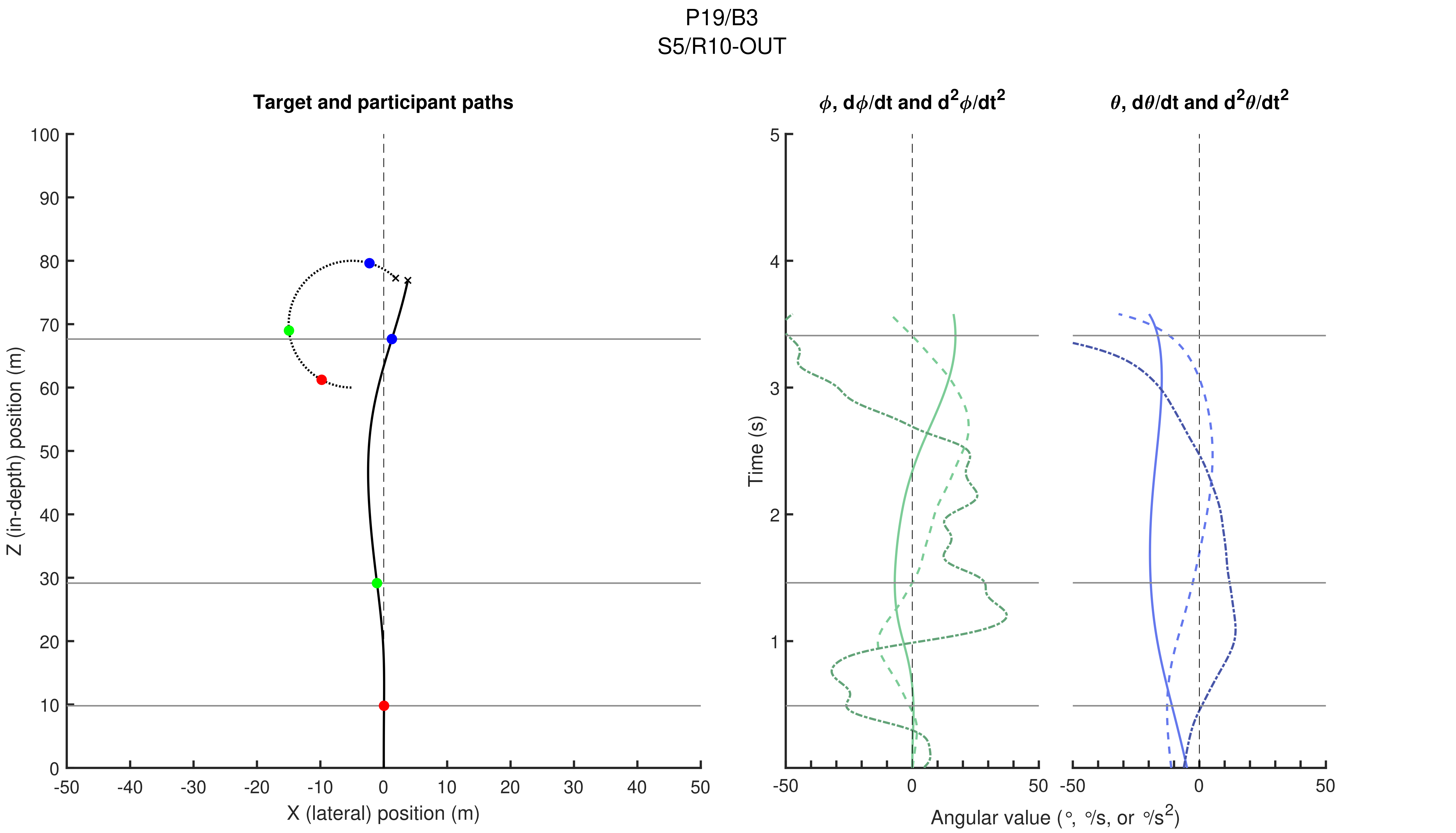

P19/B3
S5/R10-OUT
Target and participant paths
Z (in-depth) position (m)
X (lateral) position (m)
0
10
20
30
40
50
60
70
80
90
100
-50
-40
-30
-20
-10
0
10
20
30
40
50
ϕ, dϕ/dt and d$^2\phi$/dt^2
θ, dθ/dt and d$^2\theta$/dt^2
Time (s)
1
2
3
4
5
-50
0
50
-50
0
50
Angular value (°, °/s, or °/s^2)

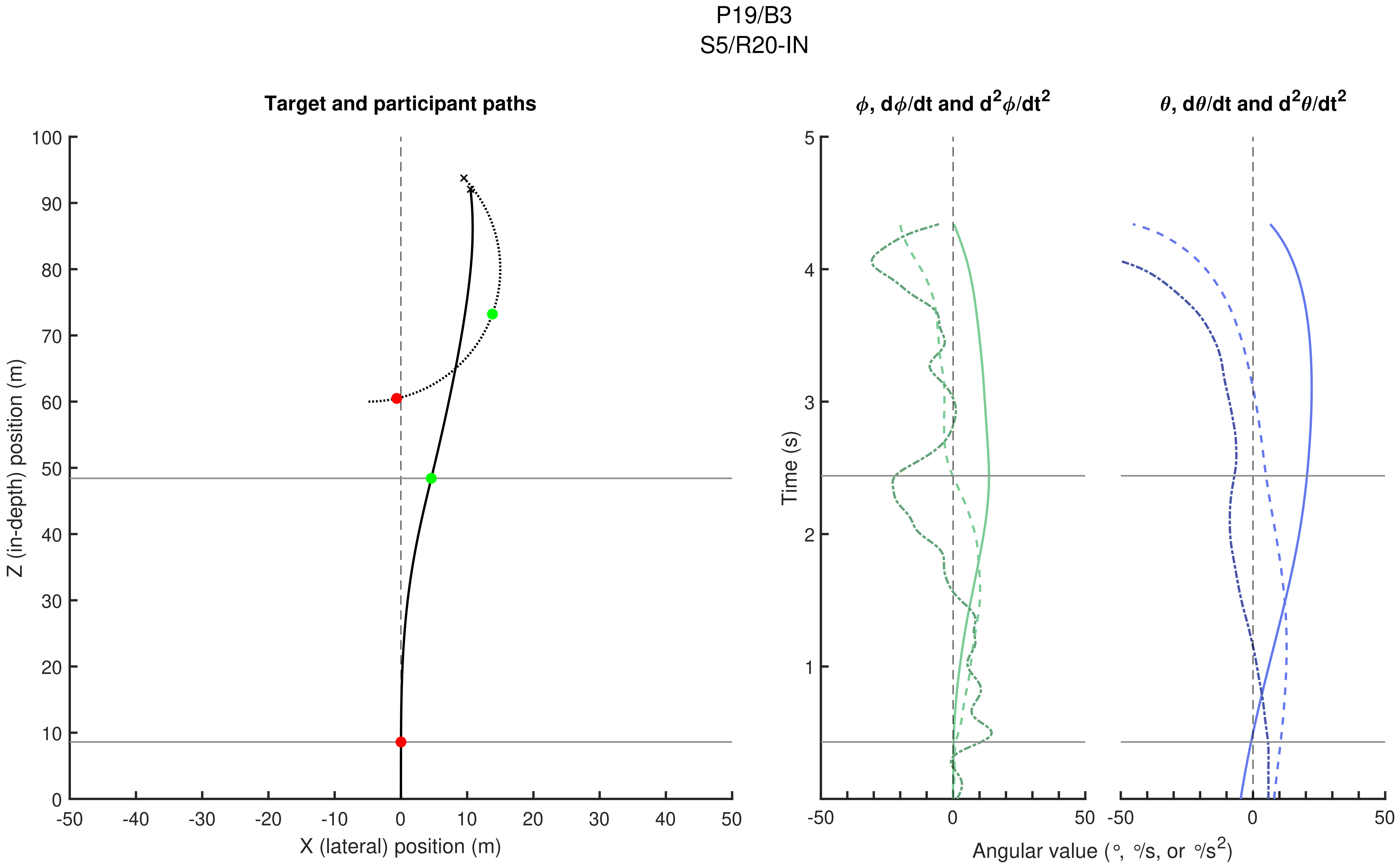

P19/B3
S5/R20-IN
Target and participant paths
ϕ, dϕ/dt and d²ϕ/dt²
θ, dθ/dt and d²θ/dt²
Z (in-depth) position (m)
X (lateral) position (m)
Time (s)
Angular value (°, °/s, or °/s²)
100
90
80
70
60
50
40
30
20
10
0
-50
-40
-30
-20
-10
0
10
20
30
40
50
5
4
3
2
1

P19/B3
S5/R20-OUT

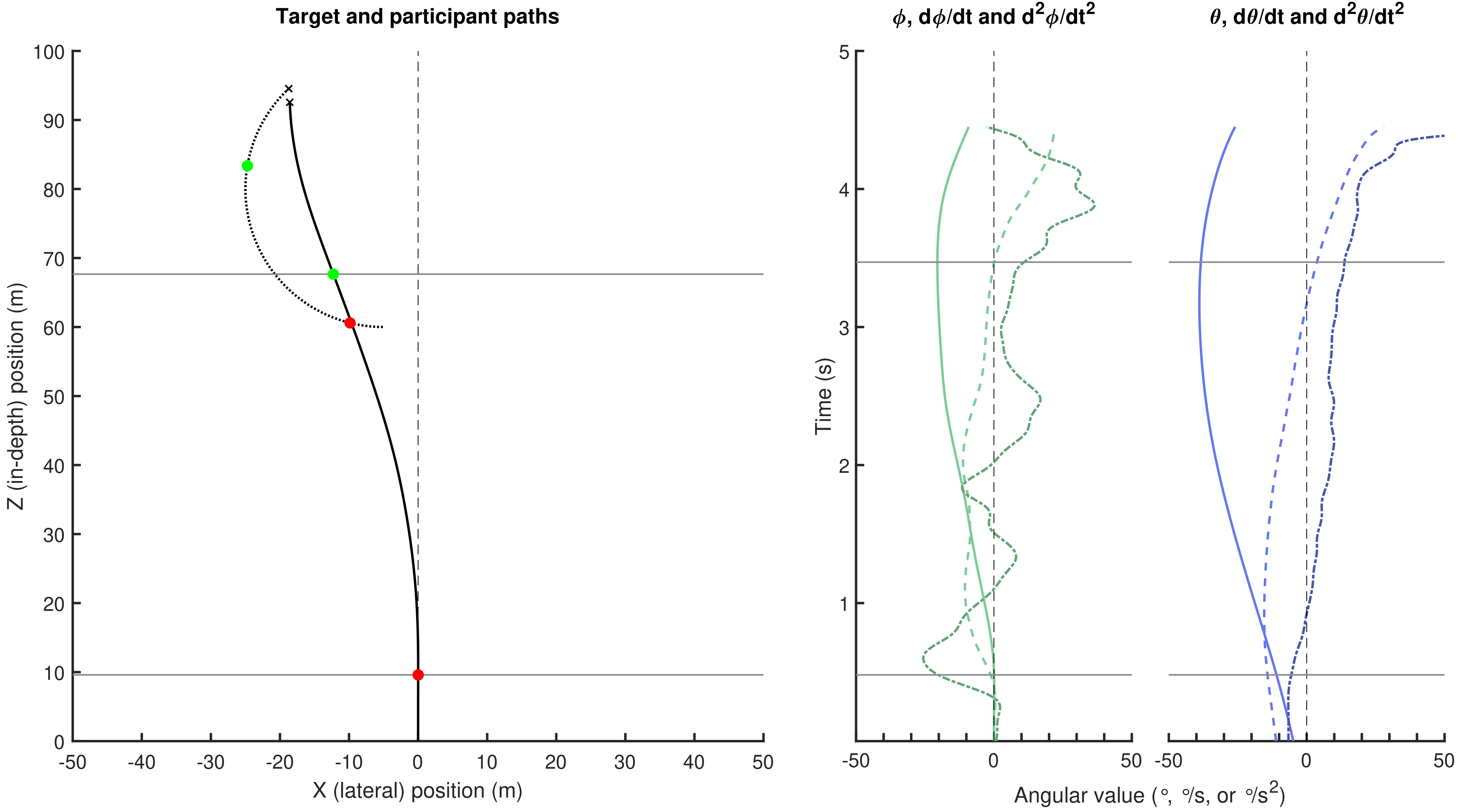

P19/B3
S5/R30-IN

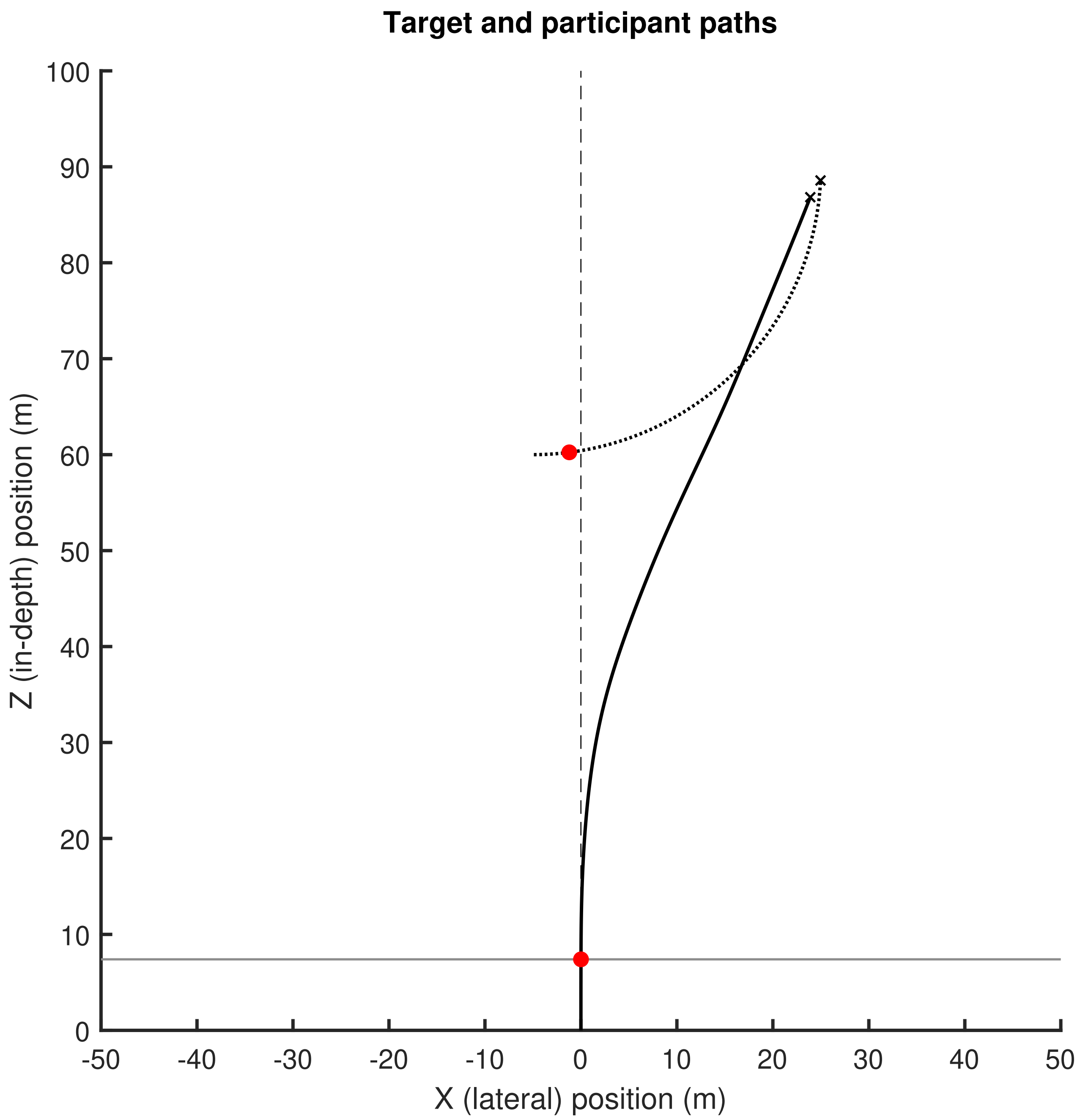

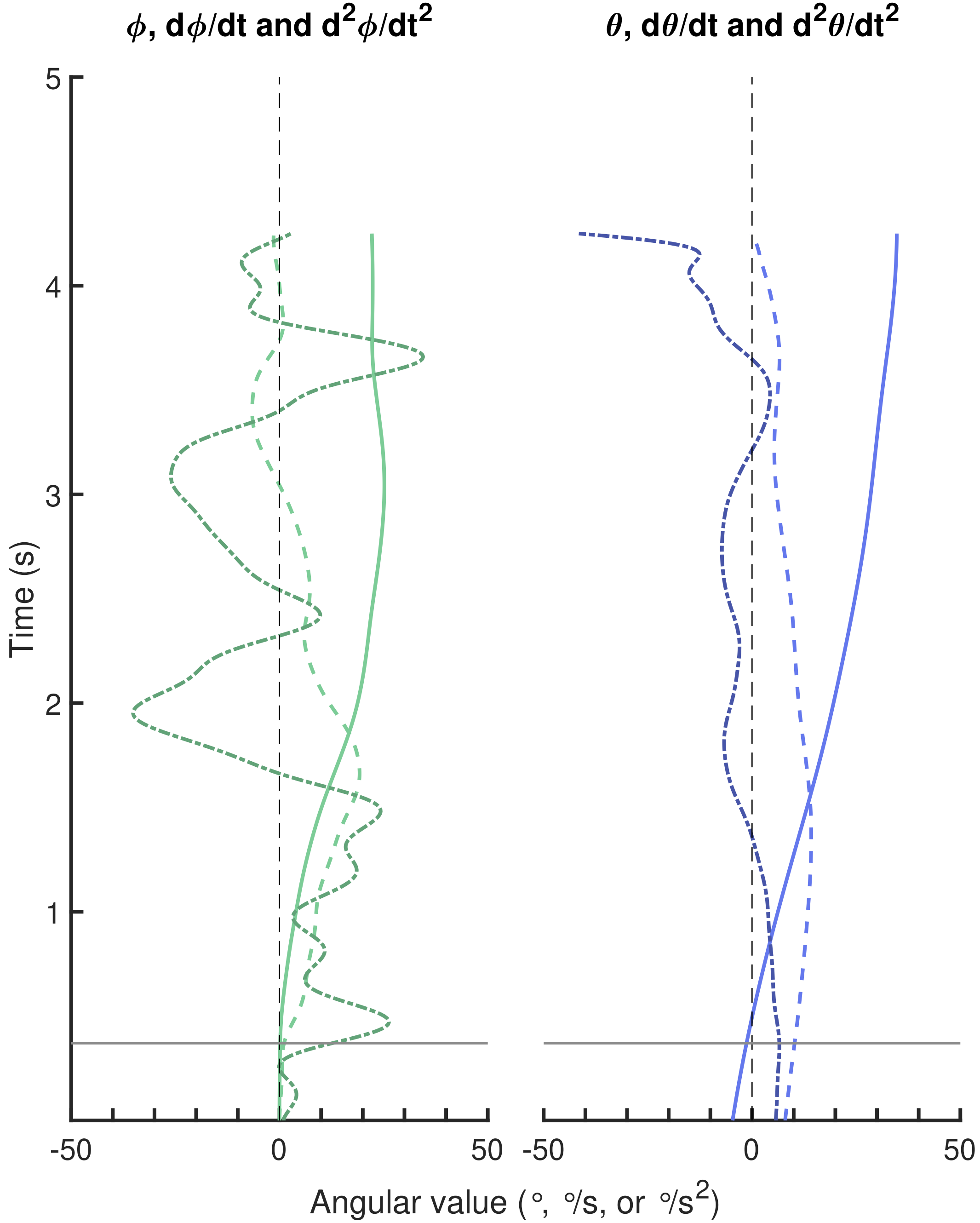

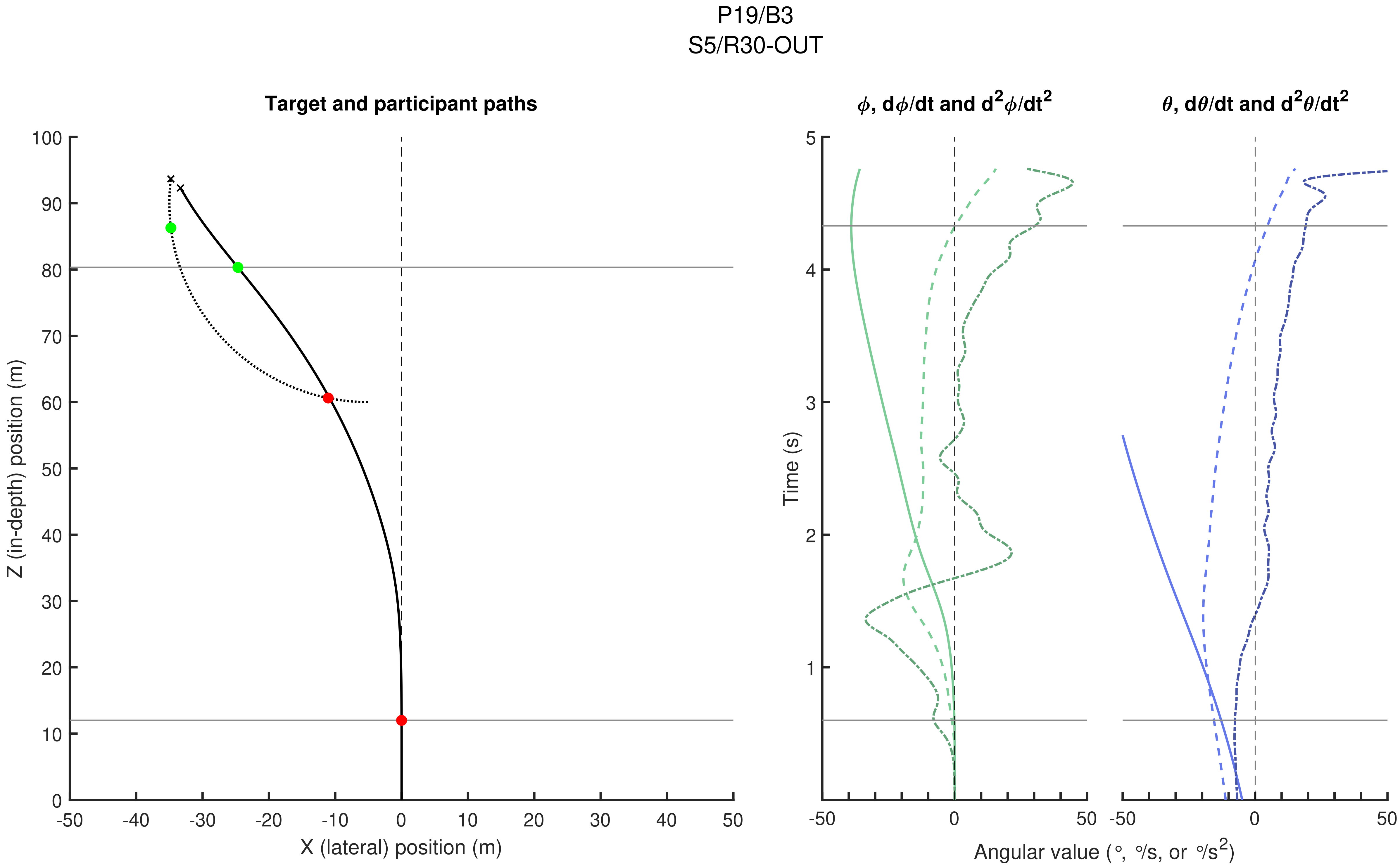

P19/B3
S5/R30-OUT
Target and participant paths
Z (in-depth) position (m)
X (lateral) position (m)
ϕ, dϕ/dt and d²ϕ/dt²
θ, dθ/dt and d²θ/dt²
Time (s)
Angular value (°, °/s, or °/s²)

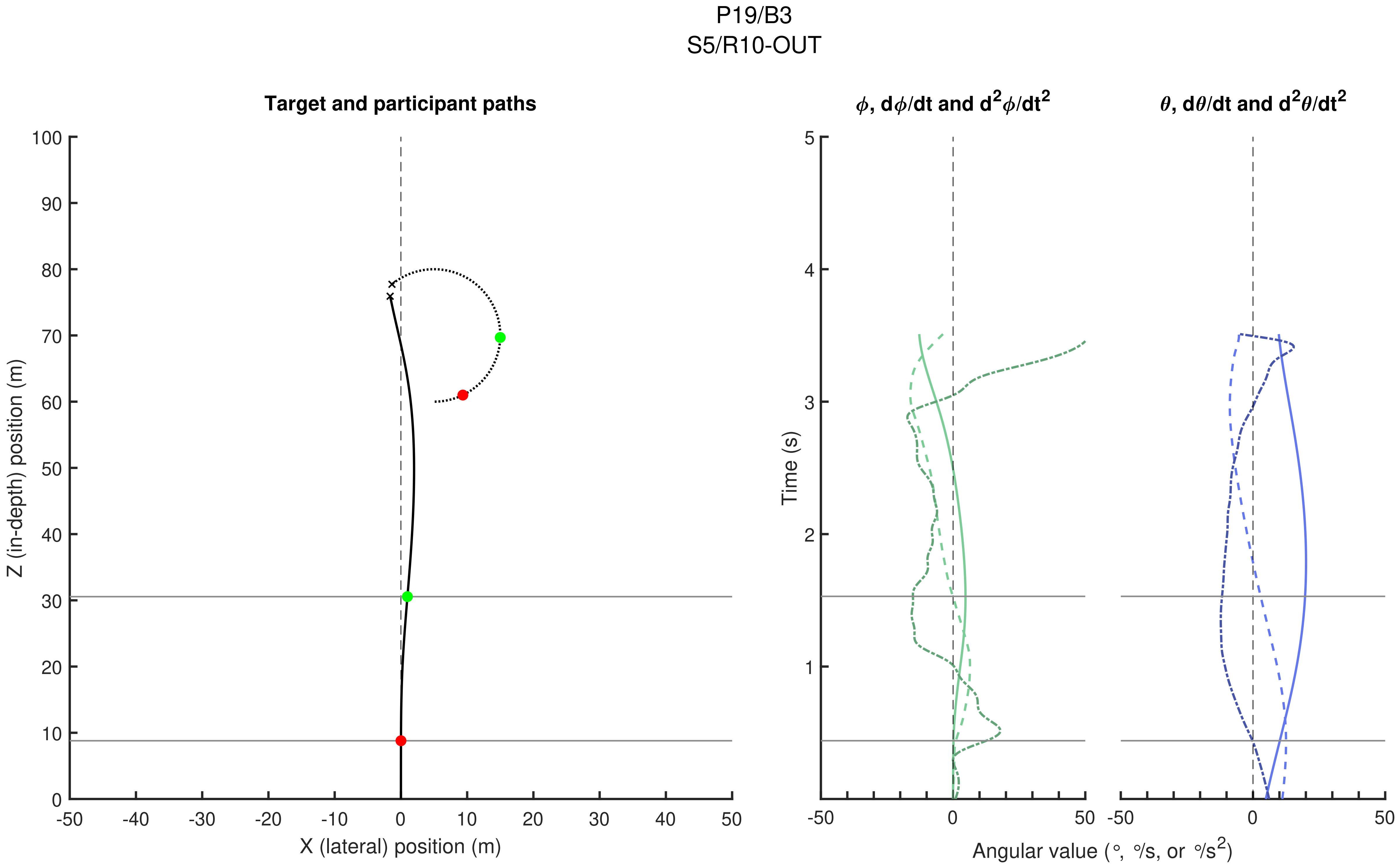
P19/B3
S5/R10-OUT
Target and participant paths
Z (in-depth) position (m)
X (lateral) position (m)
φ, dφ/dt and d²φ/dt²
θ, dθ/dt and d²θ/dt²
Time (s)
Angular value (°, °/s, or °/s²)

P19/B3
S5/R10-IN

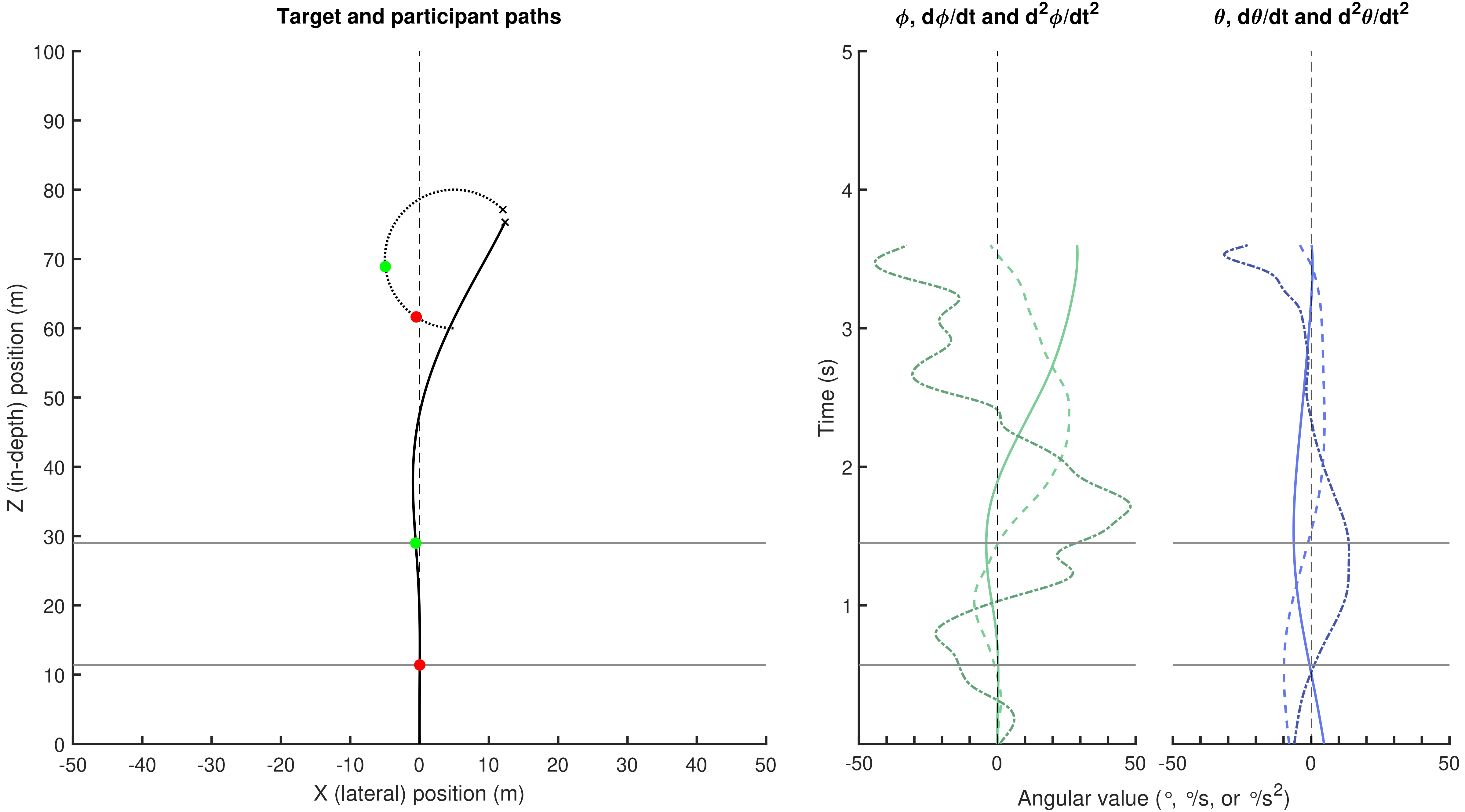

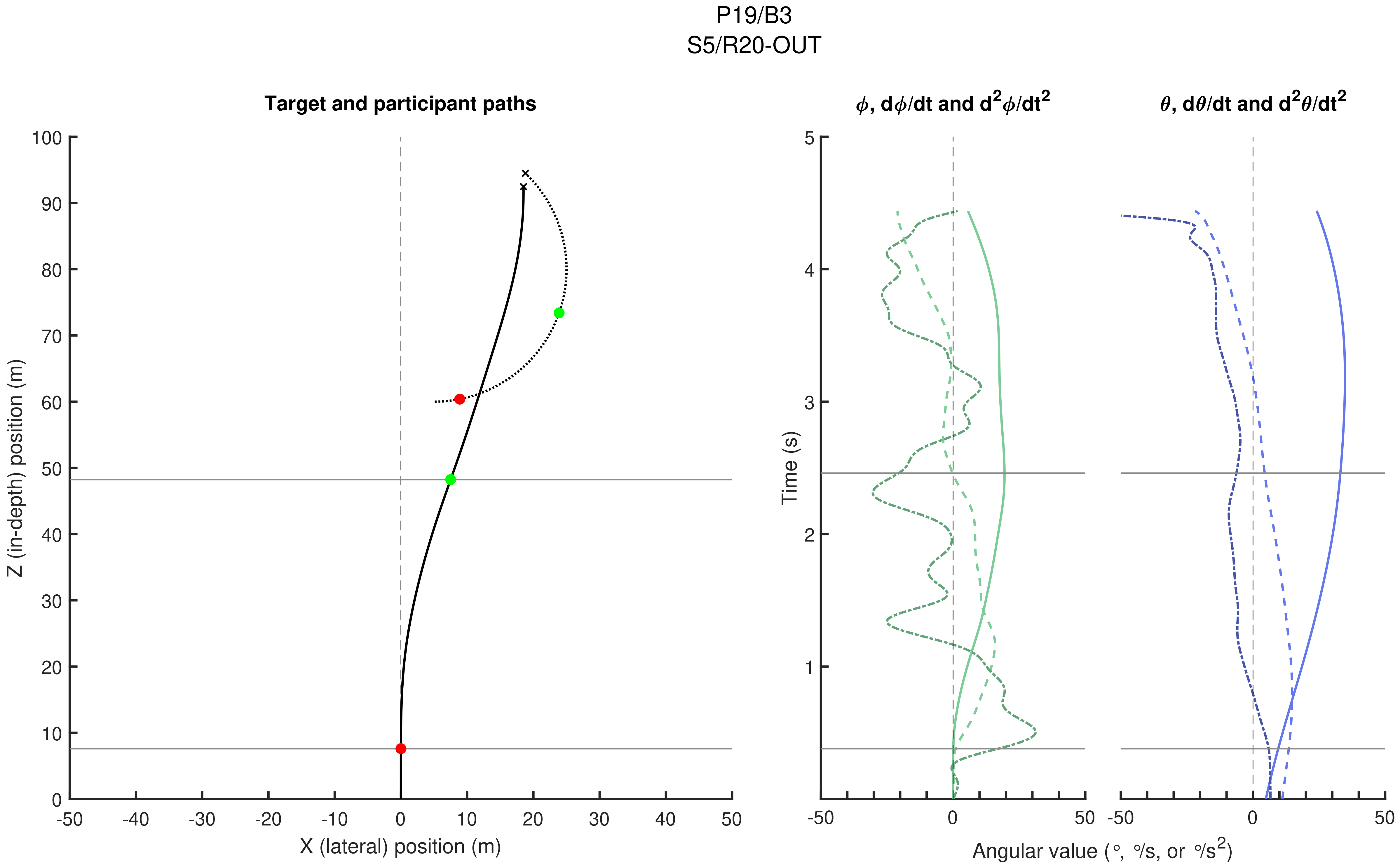
P19/B3
S5/R20-OUT
Target and participant paths
Z (in-depth) position (m)
X (lateral) position (m)
ϕ, dϕ/dt and d²ϕ/dt²
θ, dθ/dt and d²θ/dt²
Time (s)
Angular value (°, °/s, or °/s²)
100
90
80
70
60
50
40
30
20
10
0
-50
-40
-30
-20
-10
0
10
20
30
40
50
5
4
3
2
1
-50
0
50
-50
0
50

P19/B3
S5/R20-IN

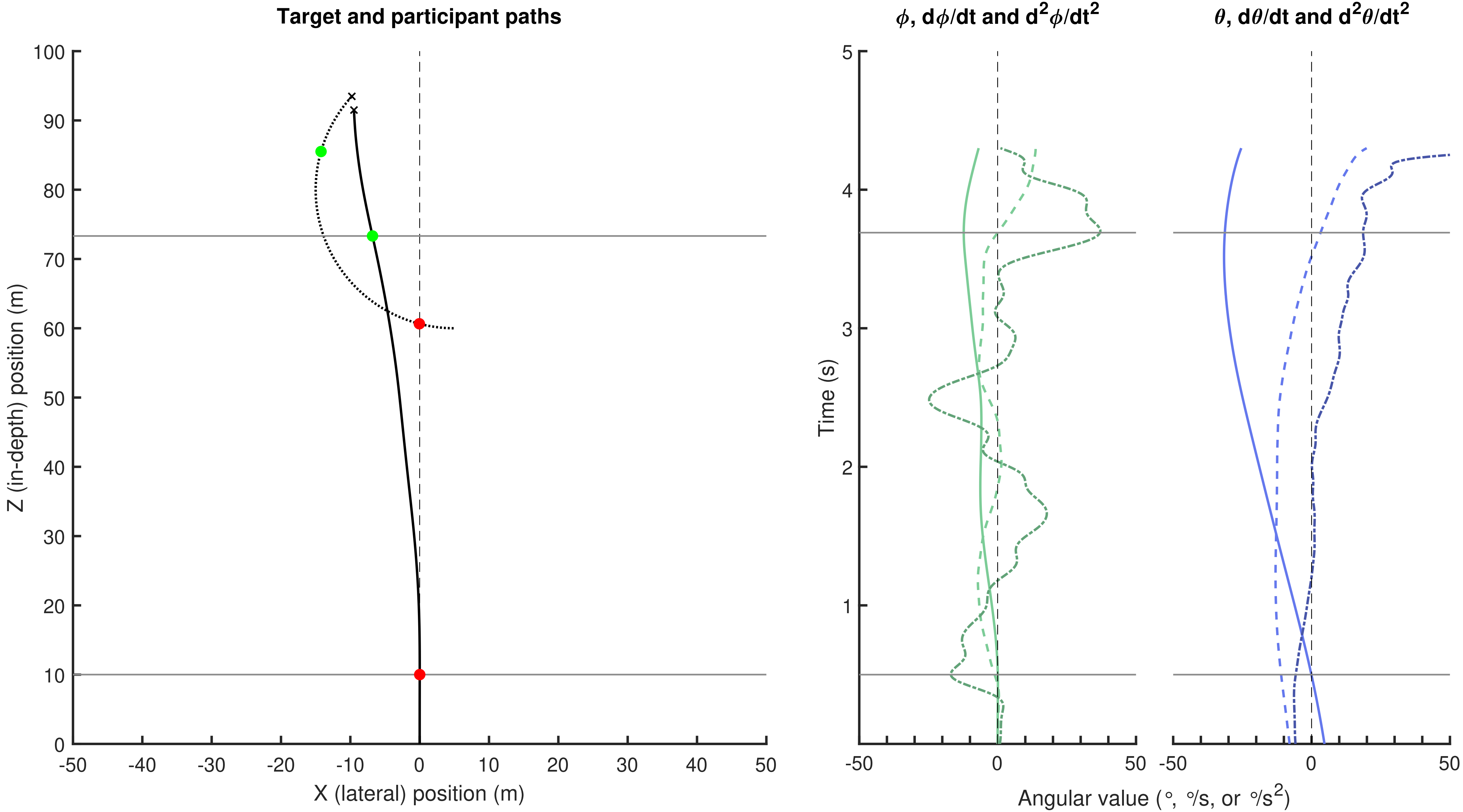

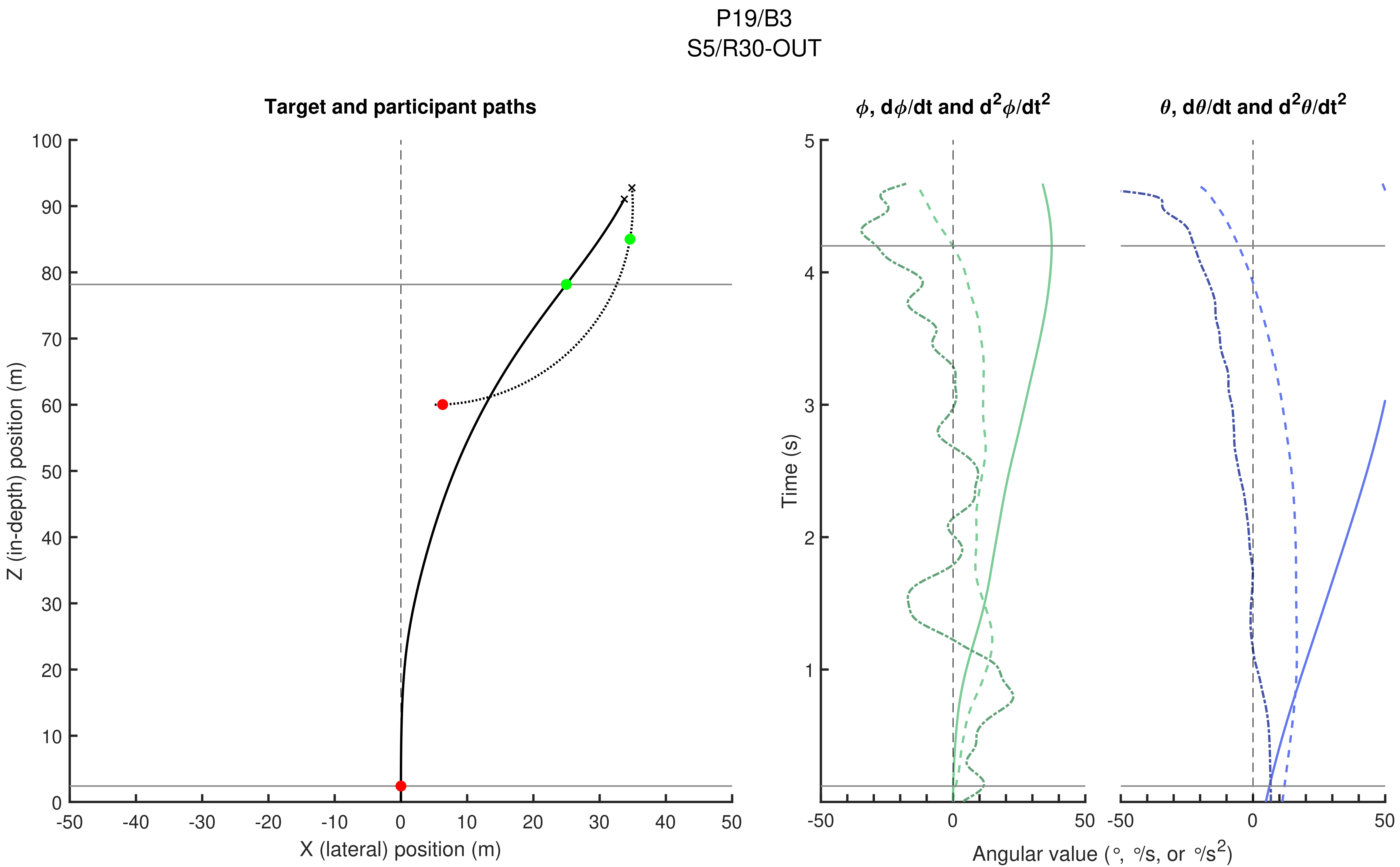

P19/B3
S5/R30-OUT
Target and participant paths
Z (in-depth) position (m)
X (lateral) position (m)
φ, dφ/dt and d²φ/dt²
θ, dθ/dt and d²θ/dt²
Time (s)
Angular value (°, °/s, or °/s²)

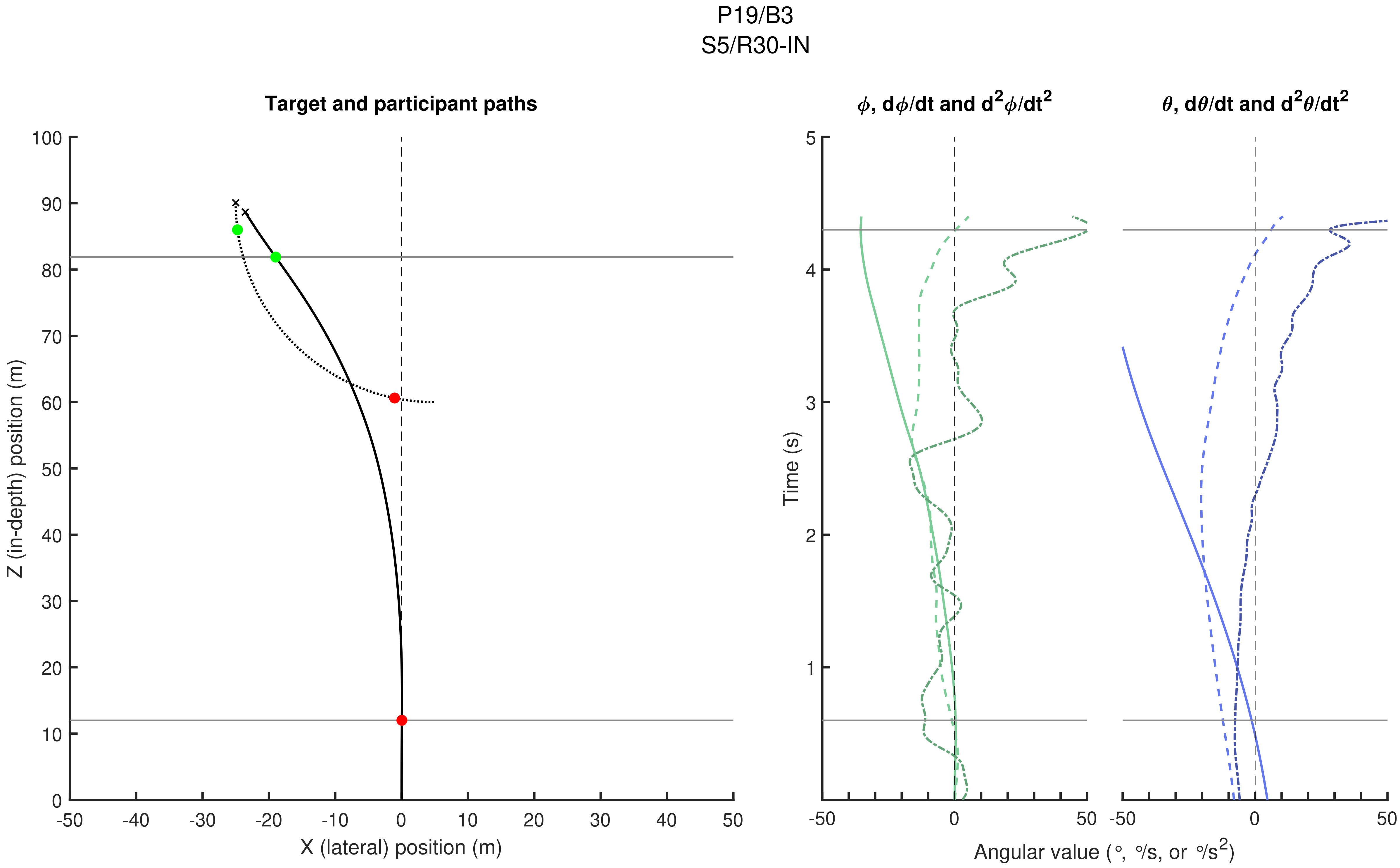
P19/B3
S5/R30-IN
Target and participant paths
Z (in-depth) position (m)
X (lateral) position (m)
ϕ, dϕ/dt and d²ϕ/dt²
θ, dθ/dt and d²θ/dt²
Time (s)
Angular value (°, °/s, or °/s²)

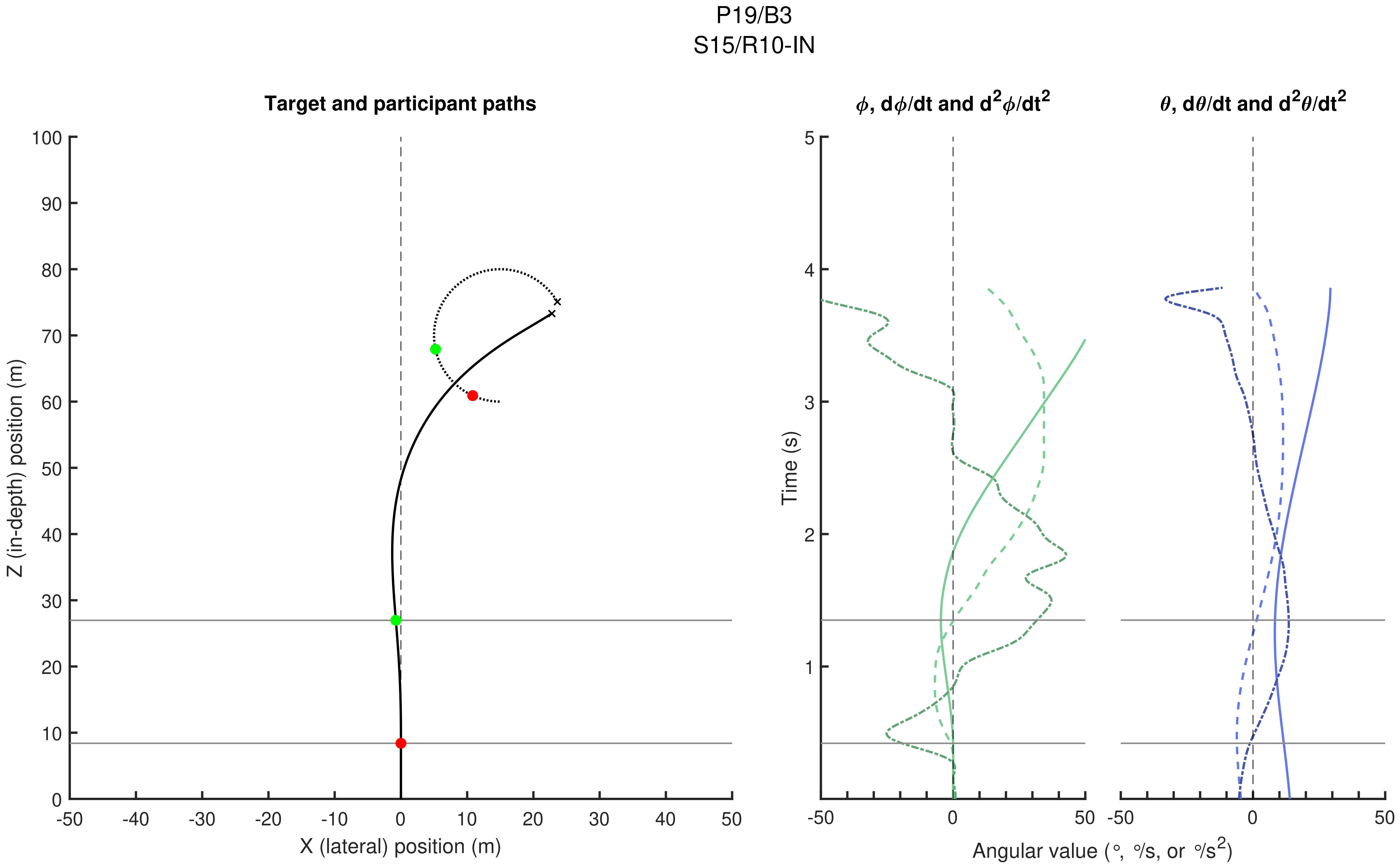
P19/B3
S15/R10-IN
Target and participant paths
ϕ, dϕ/dt and d$^2\phi$/dt^2
θ, dθ/dt and d$^2\theta$/dt^2
Z (in-depth) position (m)
X (lateral) position (m)
Time (s)
Angular value (°, °/s, or °/s^2)

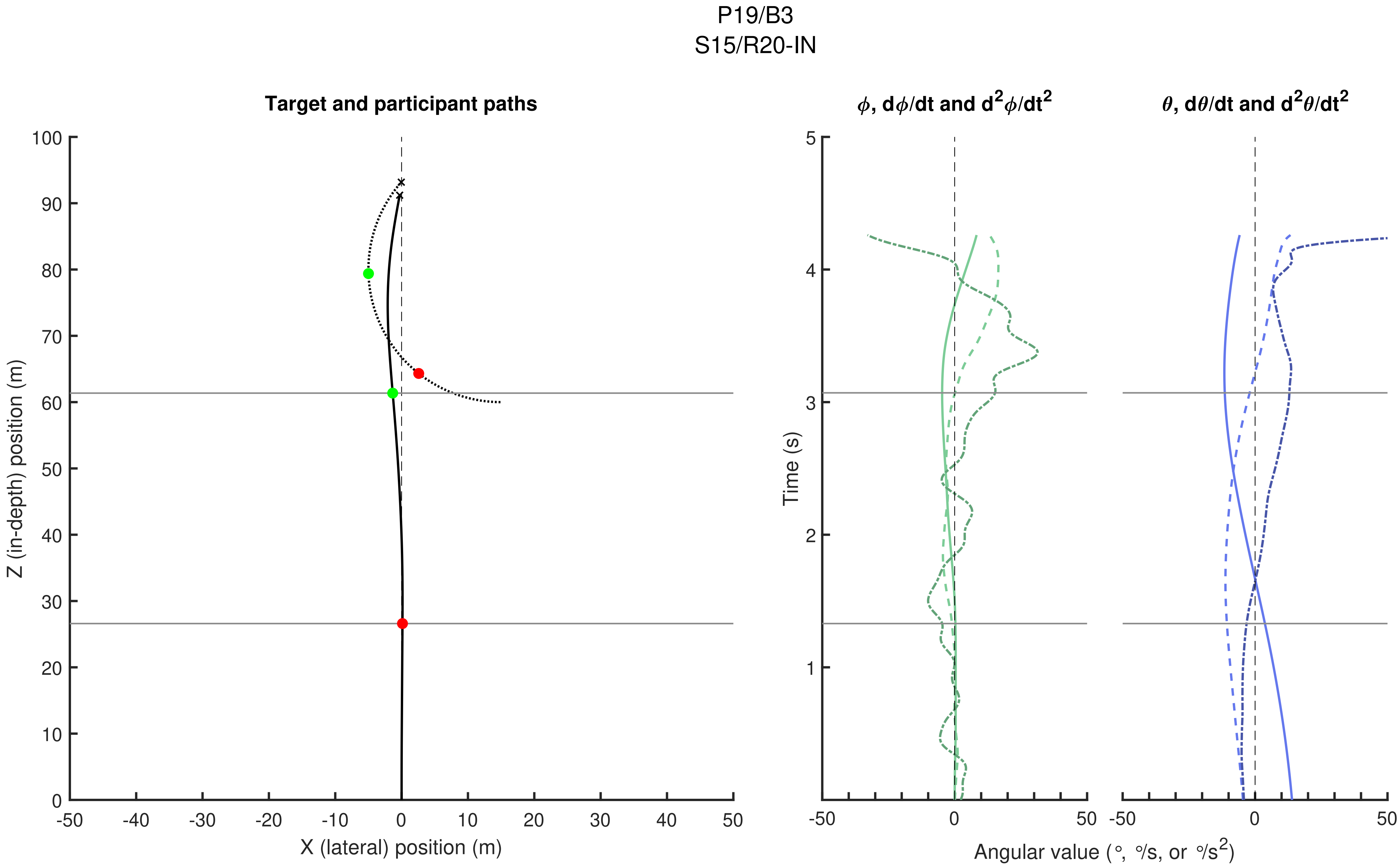
P19/B3
S15/R20-IN
Target and participant paths
Z (in-depth) position (m)
X (lateral) position (m)
φ, dφ/dt and d²φ/dt²
θ, dθ/dt and d²θ/dt²
Time (s)
Angular value (°, °/s, or °/s²)

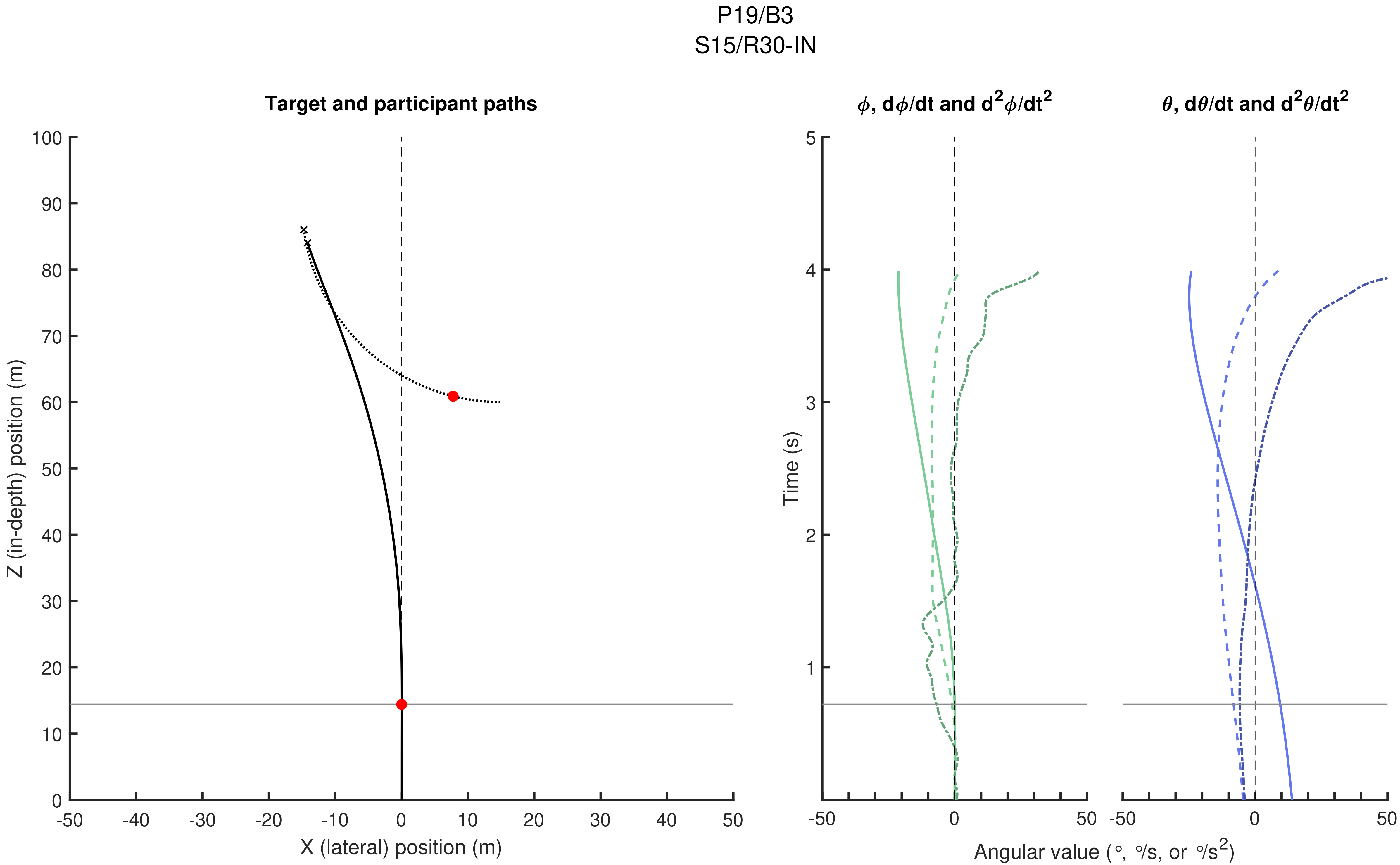
P19/B3
S15/R30-IN
Target and participant paths
Z (in-depth) position (m)
X (lateral) position (m)
100
90
80
70
60
50
40
30
20
10
0
-50
-40
-30
-20
-10
0
10
20
30
40
50
ϕ, dϕ/dt and d$^2\phi$/dt^2
θ, dθ/dt and d$^2\theta$/dt^2
Time (s)
5
4
3
2
1
-50
0
50
-50
0
50
Angular value (°, °/s, or °/s^2)

P19/B3
S20/R10-IN

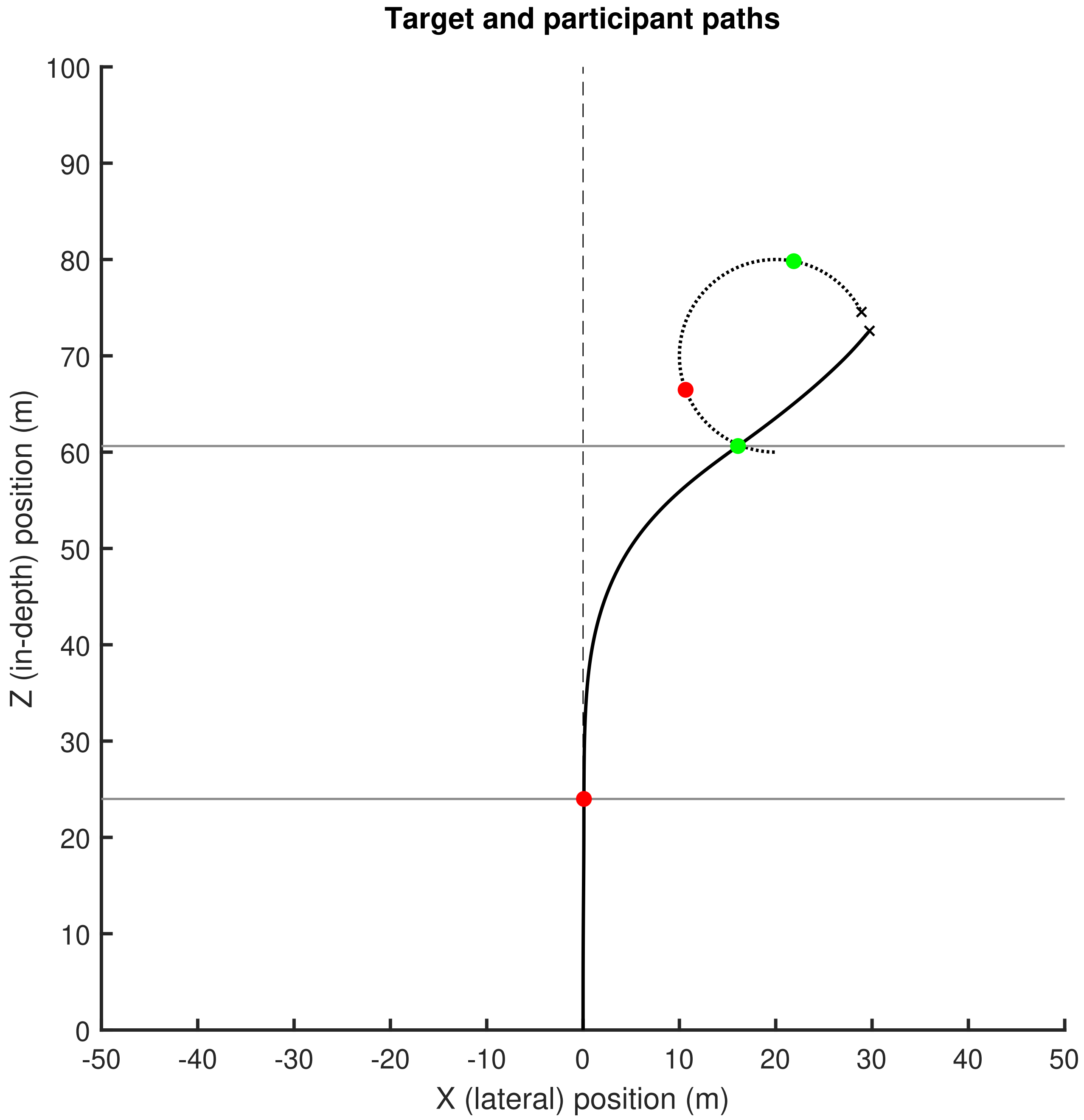

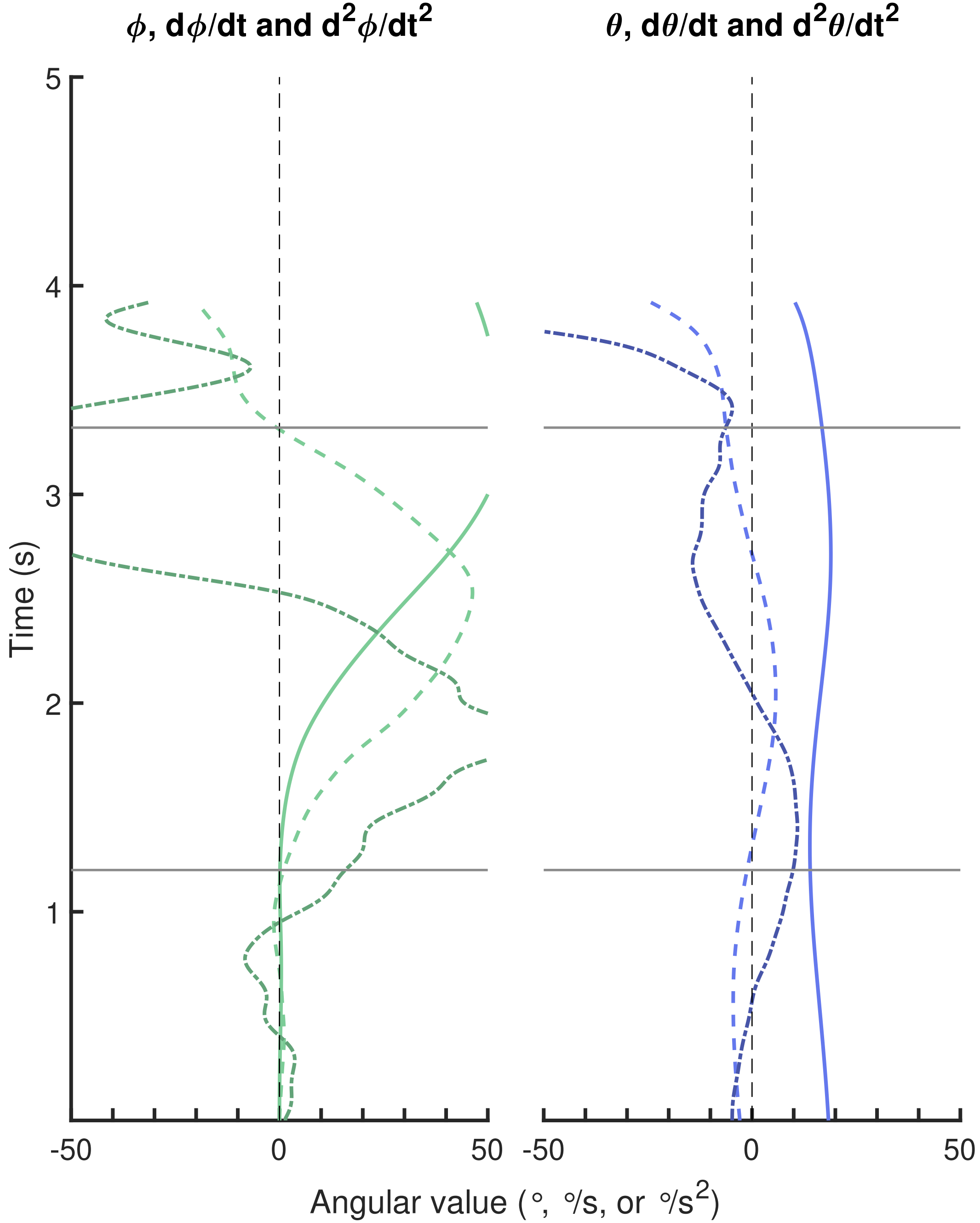

P19/B3
S20/R20-IN

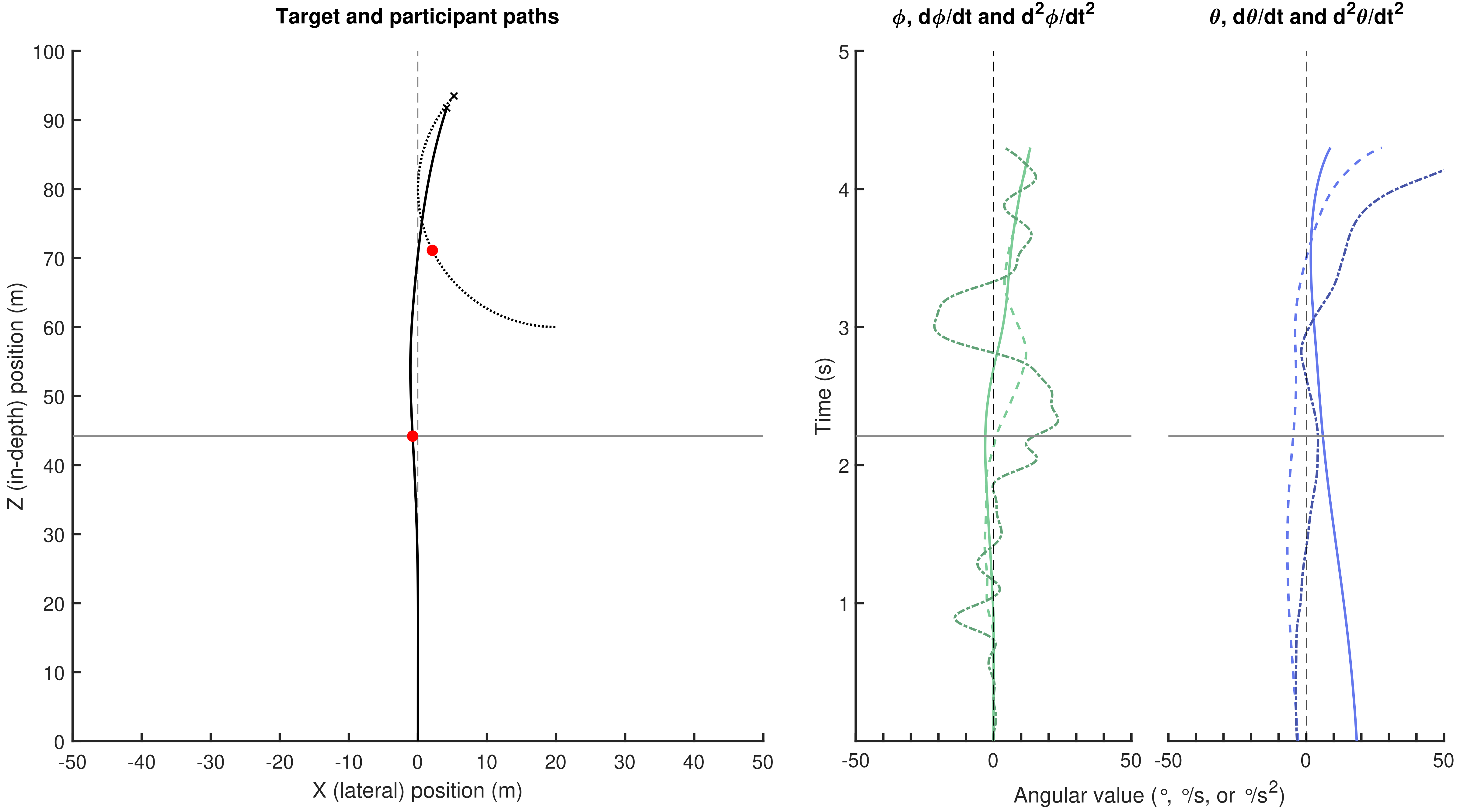

P19/B3
S20/R30-IN

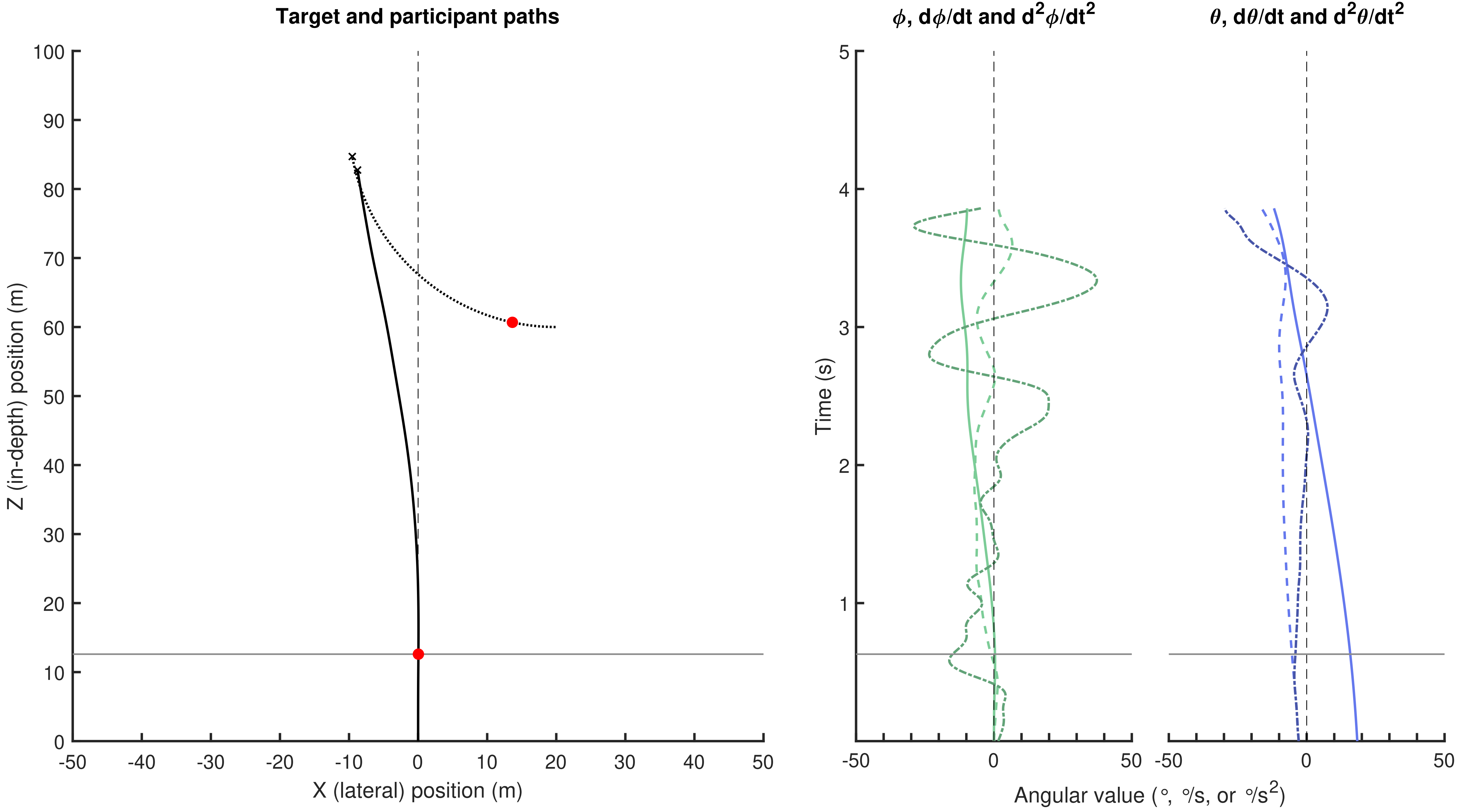

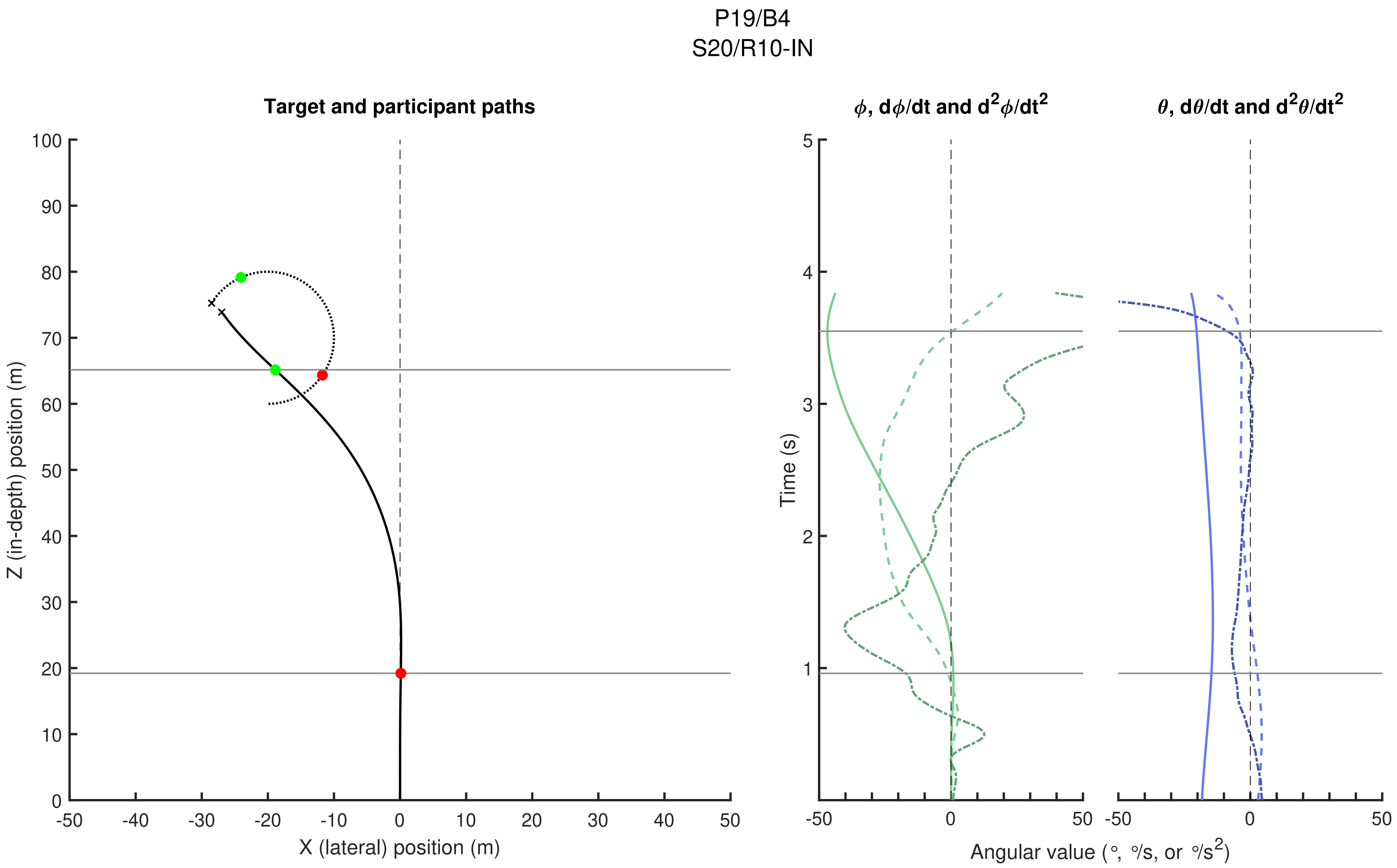
P19/B4
S20/R10-IN
Target and participant paths
Z (in-depth) position (m)
X (lateral) position (m)
ϕ, dϕ/dt and d$^2\phi$/dt^2
θ, dθ/dt and d$^2\theta$/dt^2
Time (s)
Angular value (°, °/s, or °/s^2)

P19/B4
S20/R20-IN

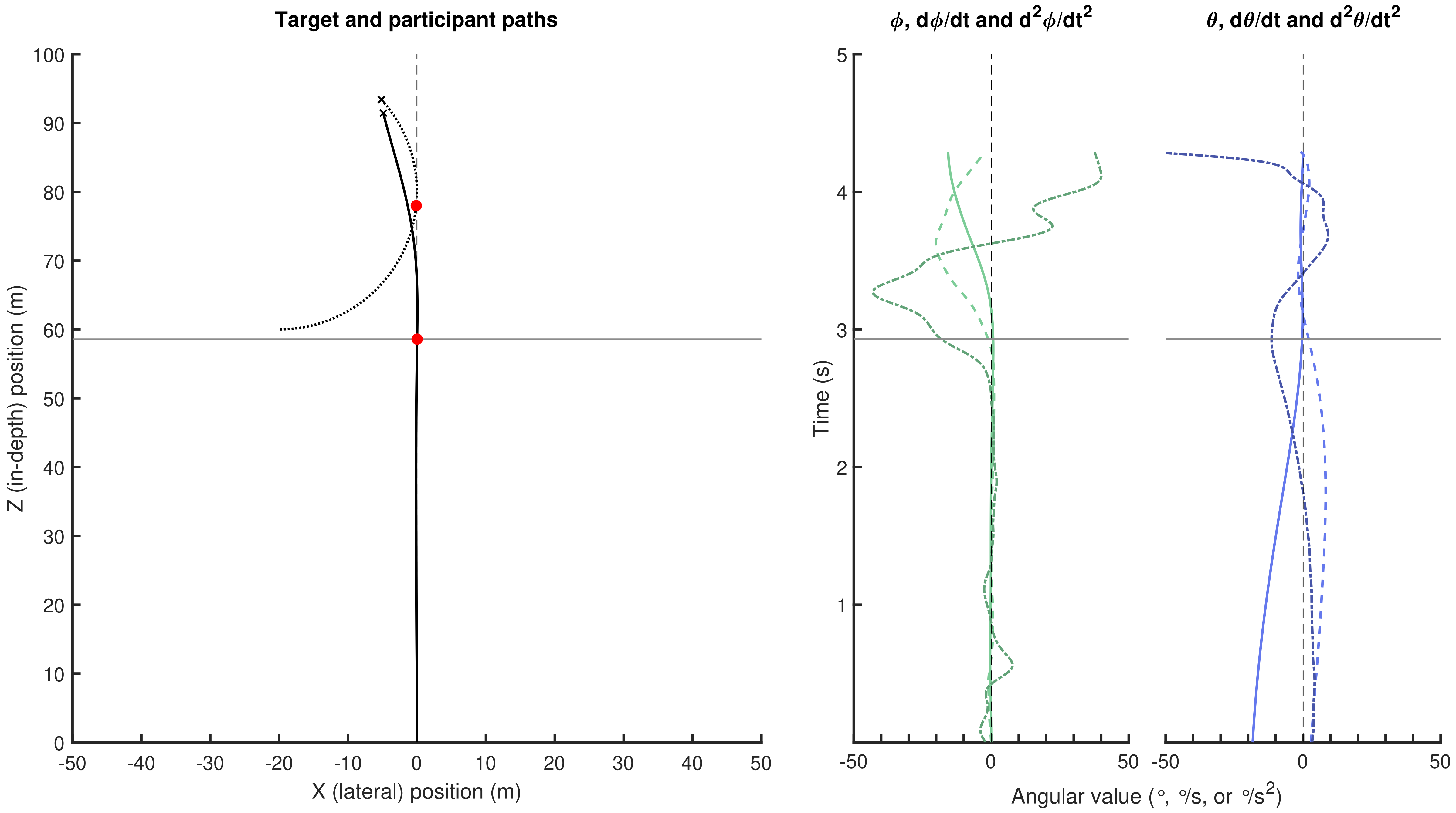

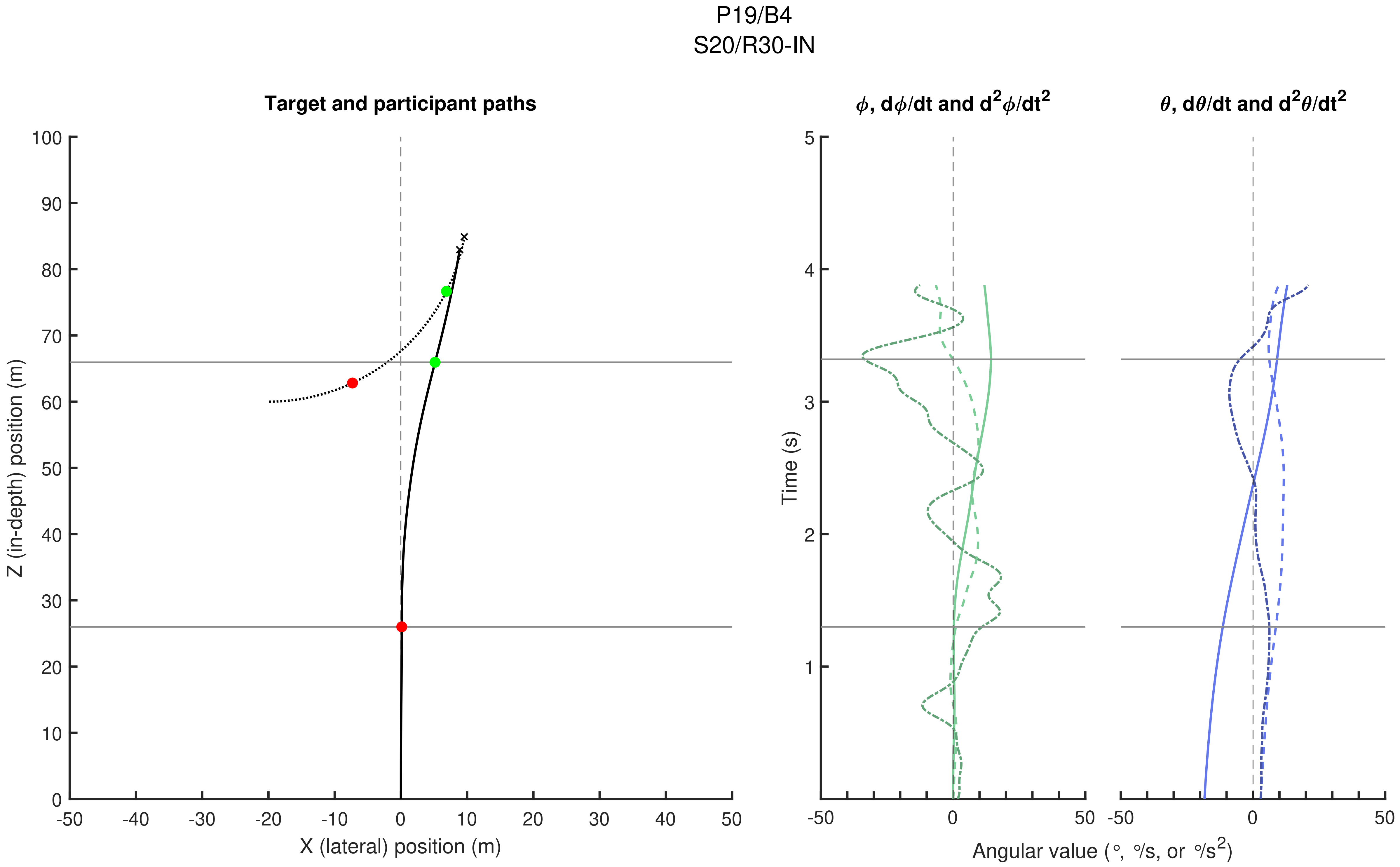
P19/B4
S20/R30-IN
Target and participant paths
Z (in-depth) position (m)
X (lateral) position (m)
ϕ, dϕ/dt and d$^2\phi$/dt^2
θ, dθ/dt and d$^2\theta$/dt^2
Time (s)
Angular value (°, °/s, or °/s^2)

P19/B4
S15/R10-IN

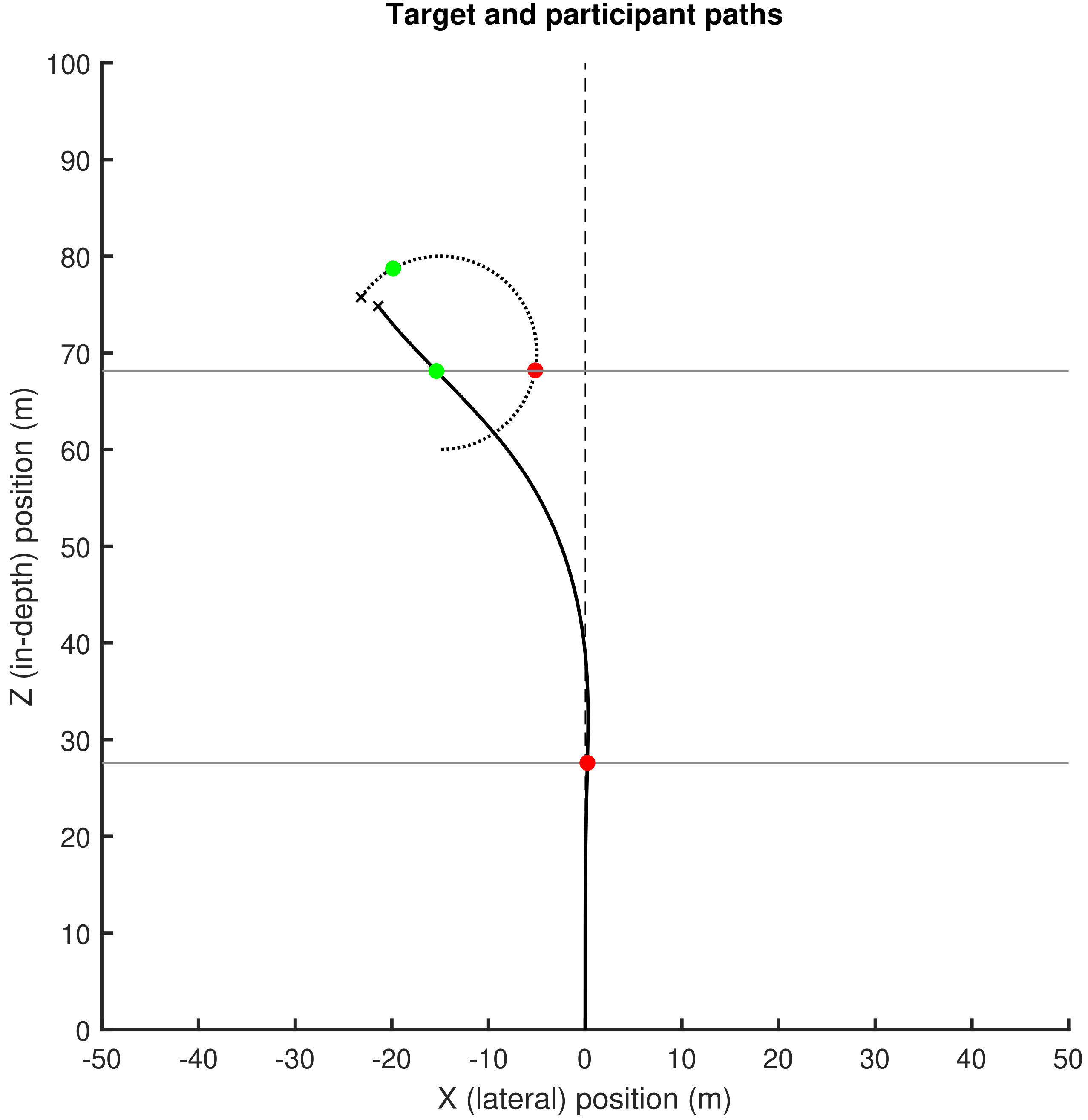

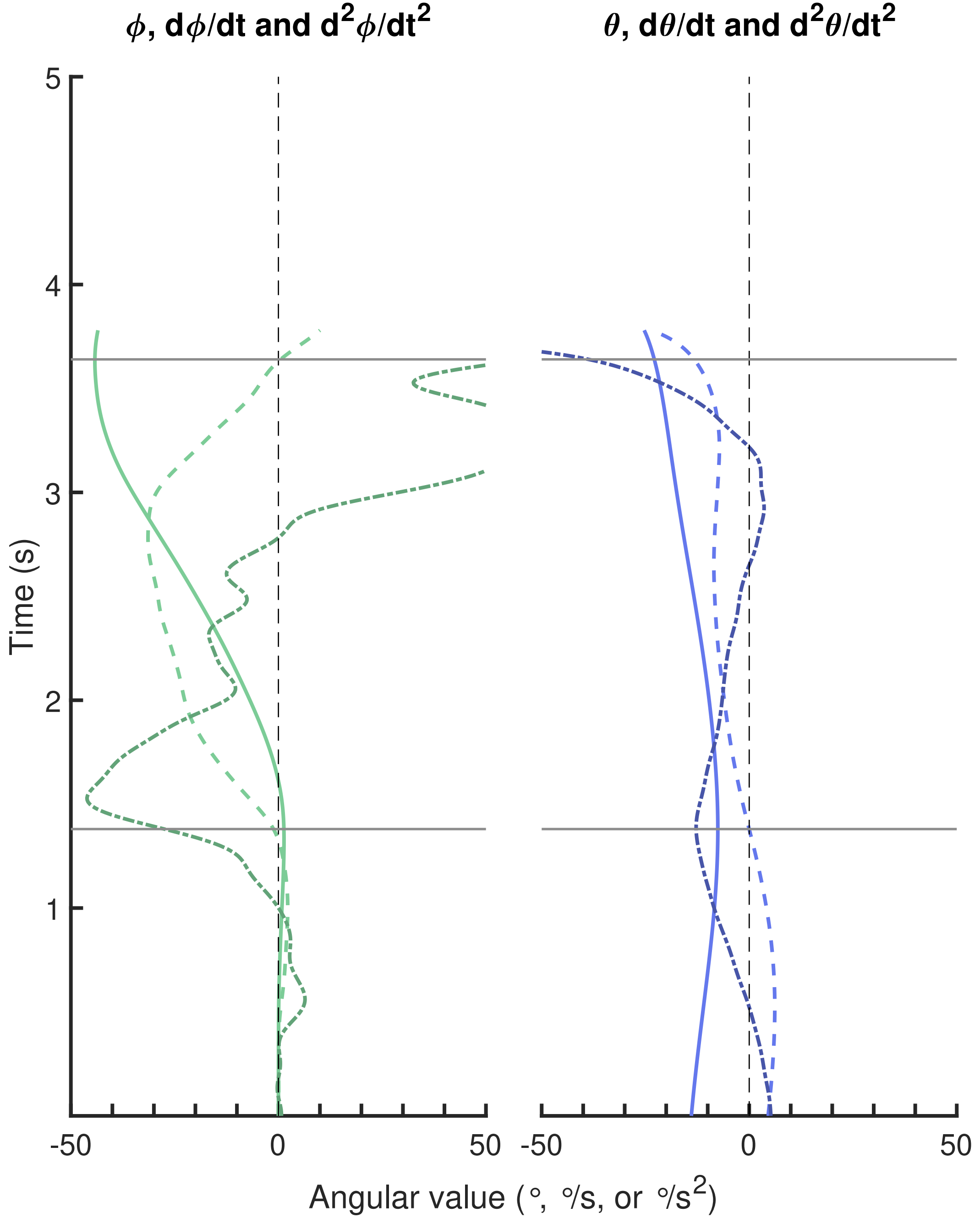

P19/B4
S15/R20-IN

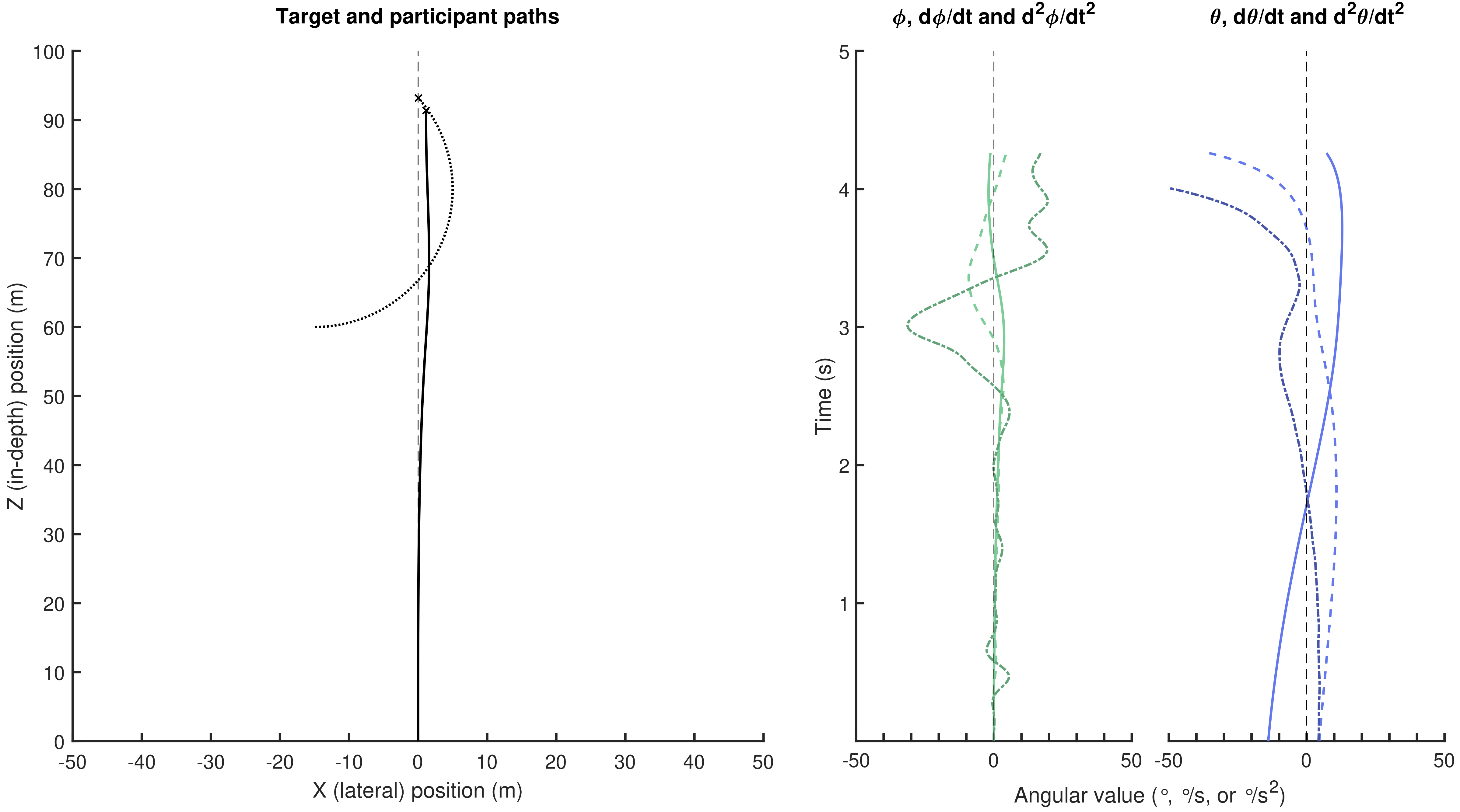

P19/B4
S15/R30-IN

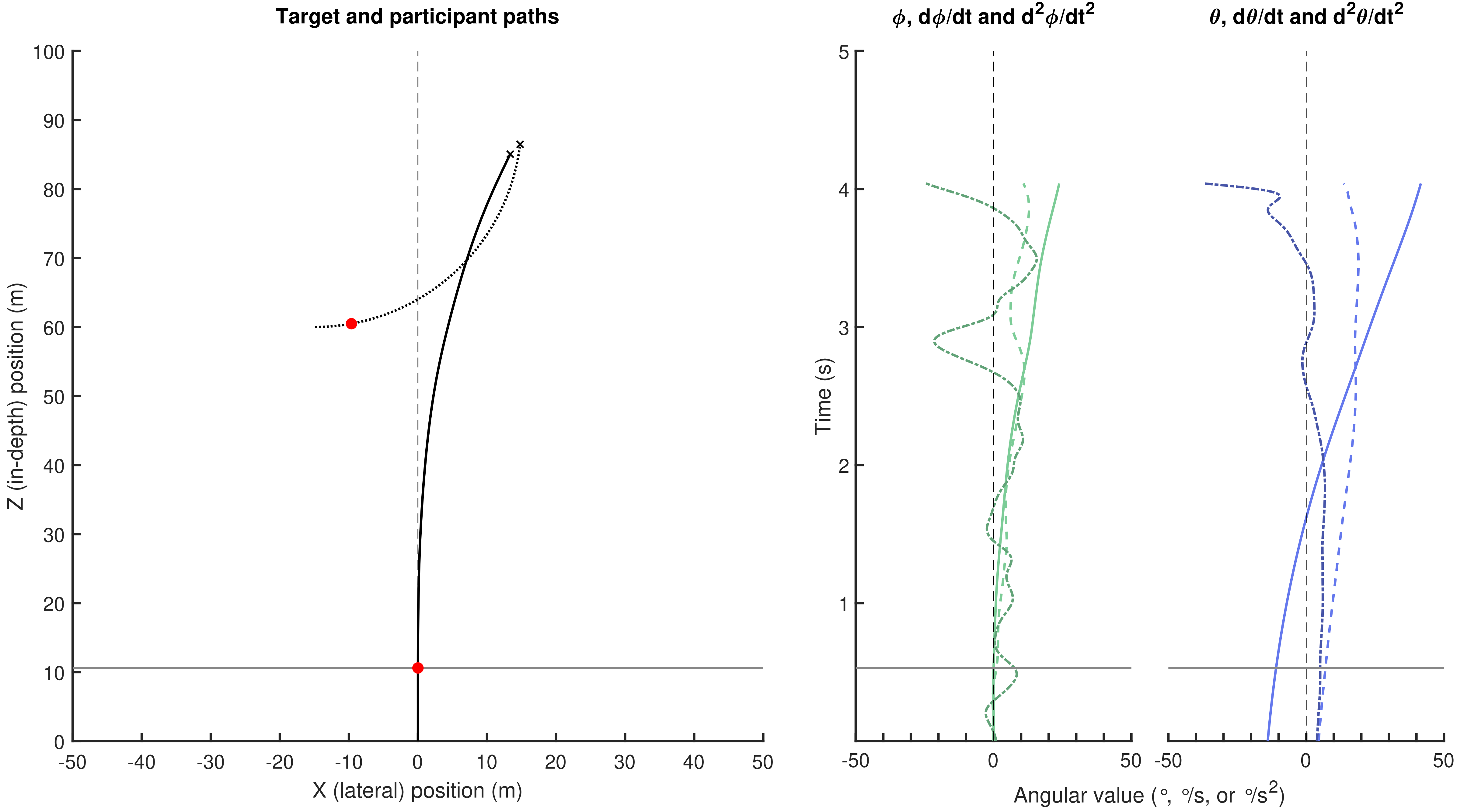

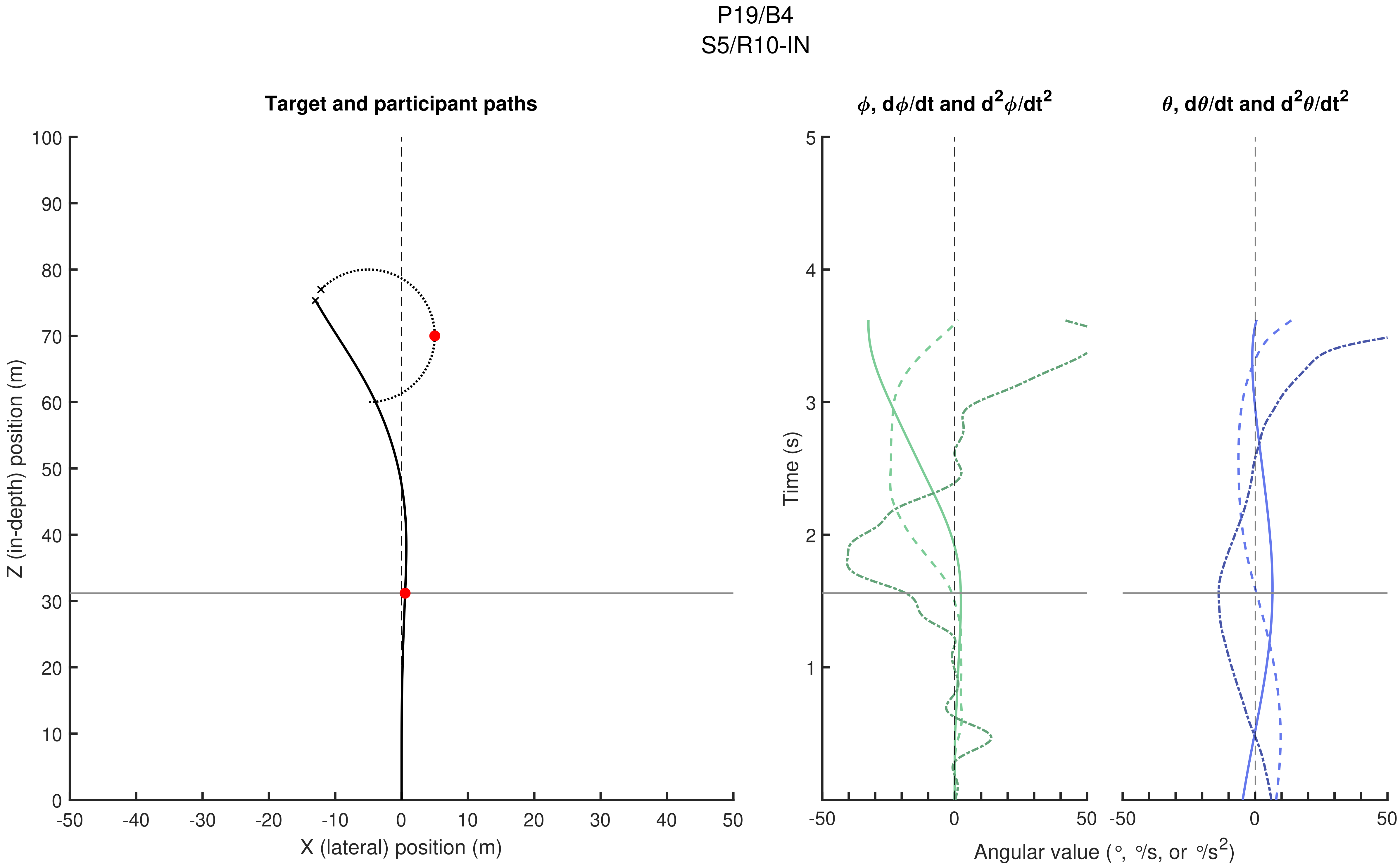
P19/B4
S5/R10-IN
Target and participant paths
ϕ, dϕ/dt and d$^2\phi$/dt^2
θ, dθ/dt and d$^2\theta$/dt^2
Z (in-depth) position (m)
X (lateral) position (m)
Time (s)
Angular value (°, °/s, or °/s^2)
100
90
80
70
60
50
40
30
20
10
0
-50
-40
-30
-20
-10
0
10
20
30
40
50
5
4
3
2
1
-50
0
50
-50
0
50

P19/B4
S5/R10-OUT

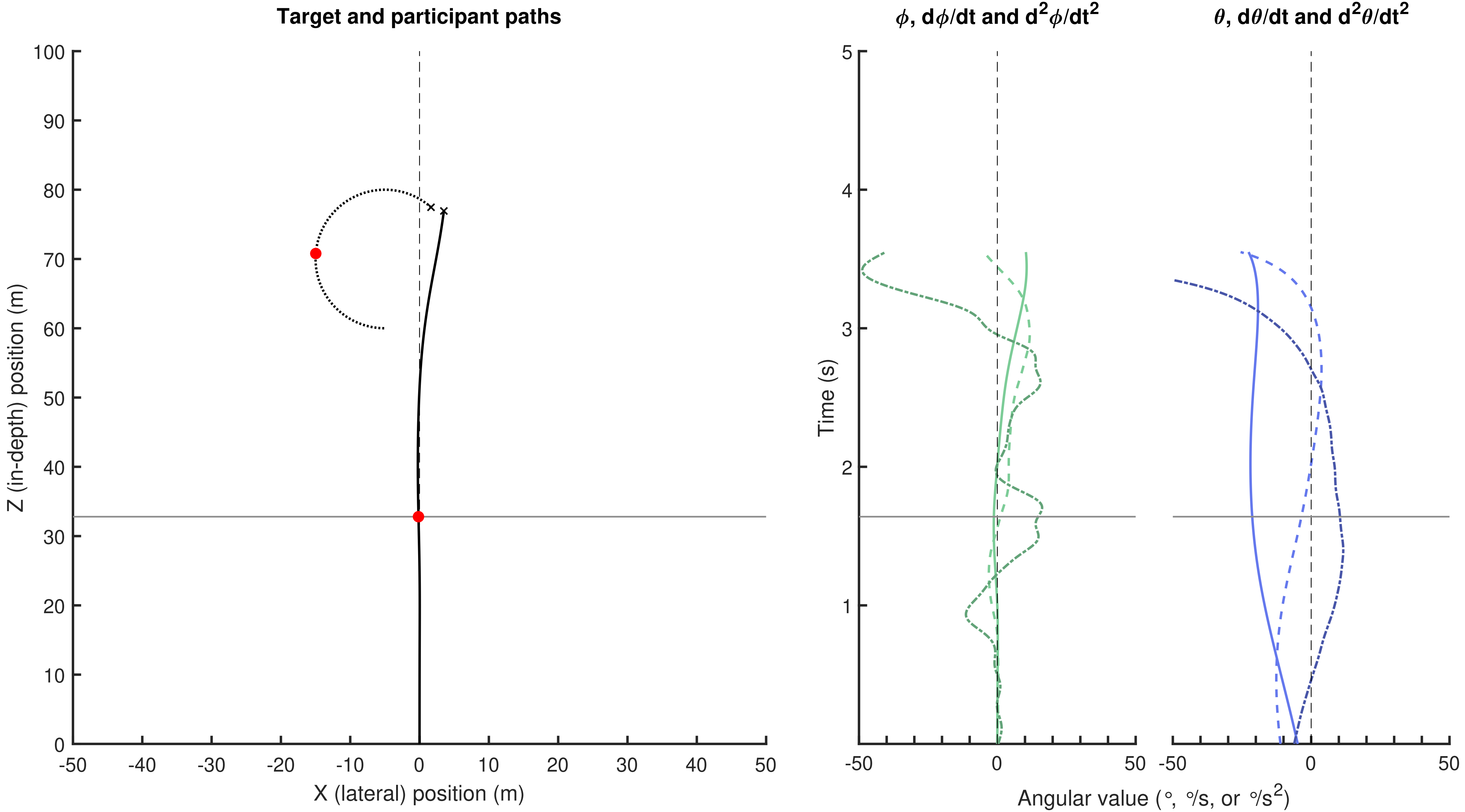

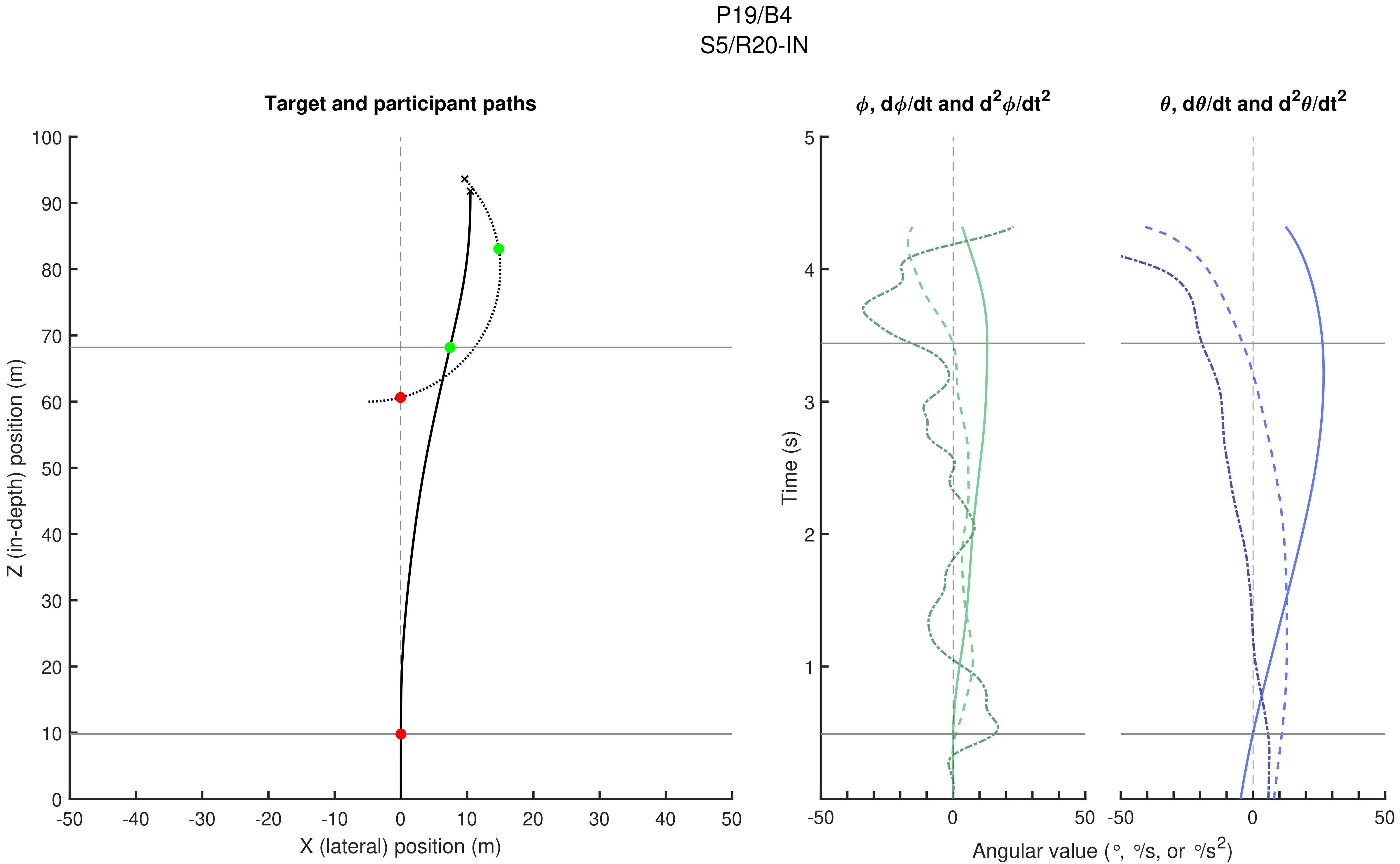
P19/B4
S5/R20-IN
Target and participant paths
Z (in-depth) position (m)
X (lateral) position (m)
ϕ, dϕ/dt and d$^2\phi$/dt^2
θ, dθ/dt and d$^2\theta$/dt^2
Time (s)
Angular value (°, °/s, or °/s^2)

P19/B4
S5/R20-OUT

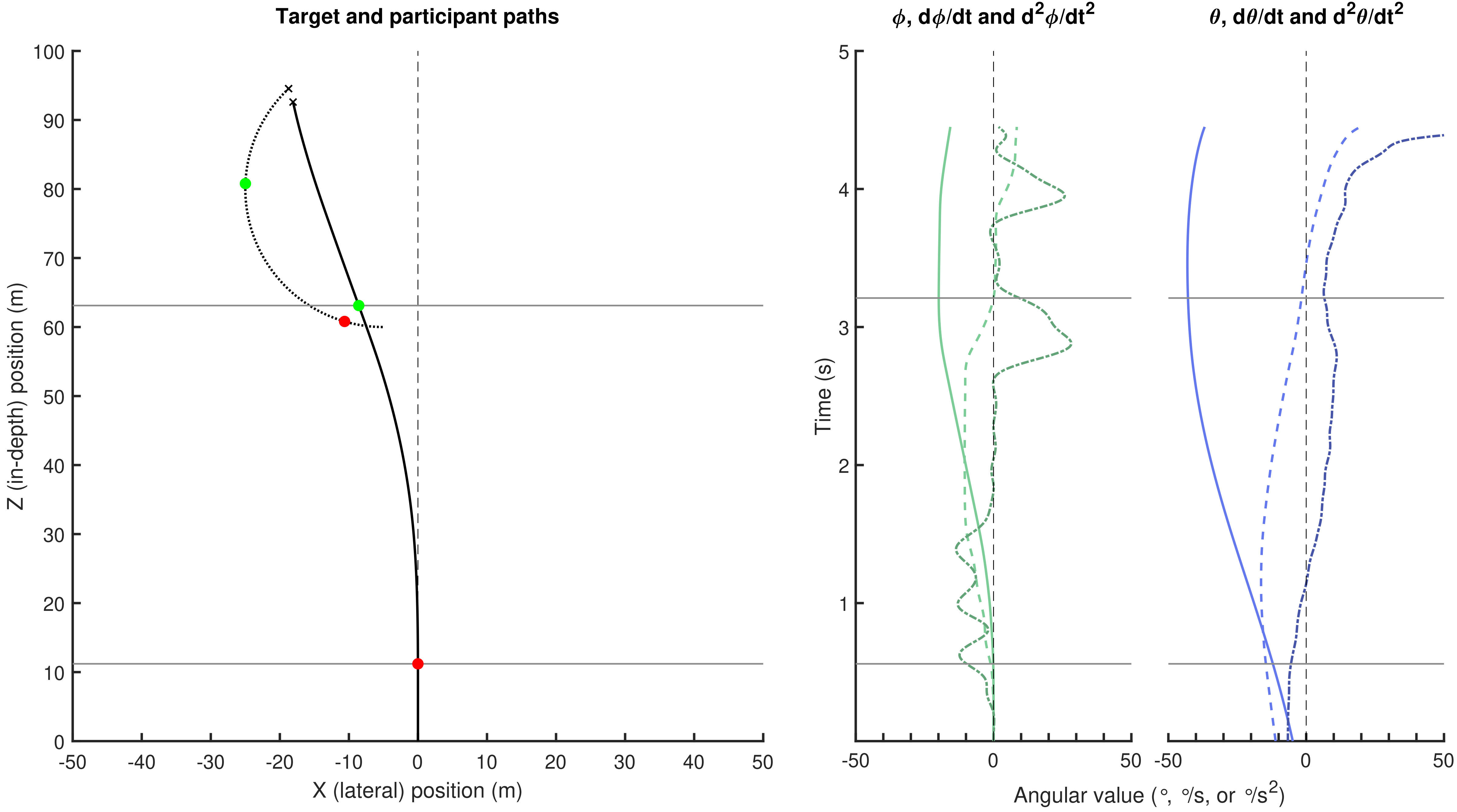

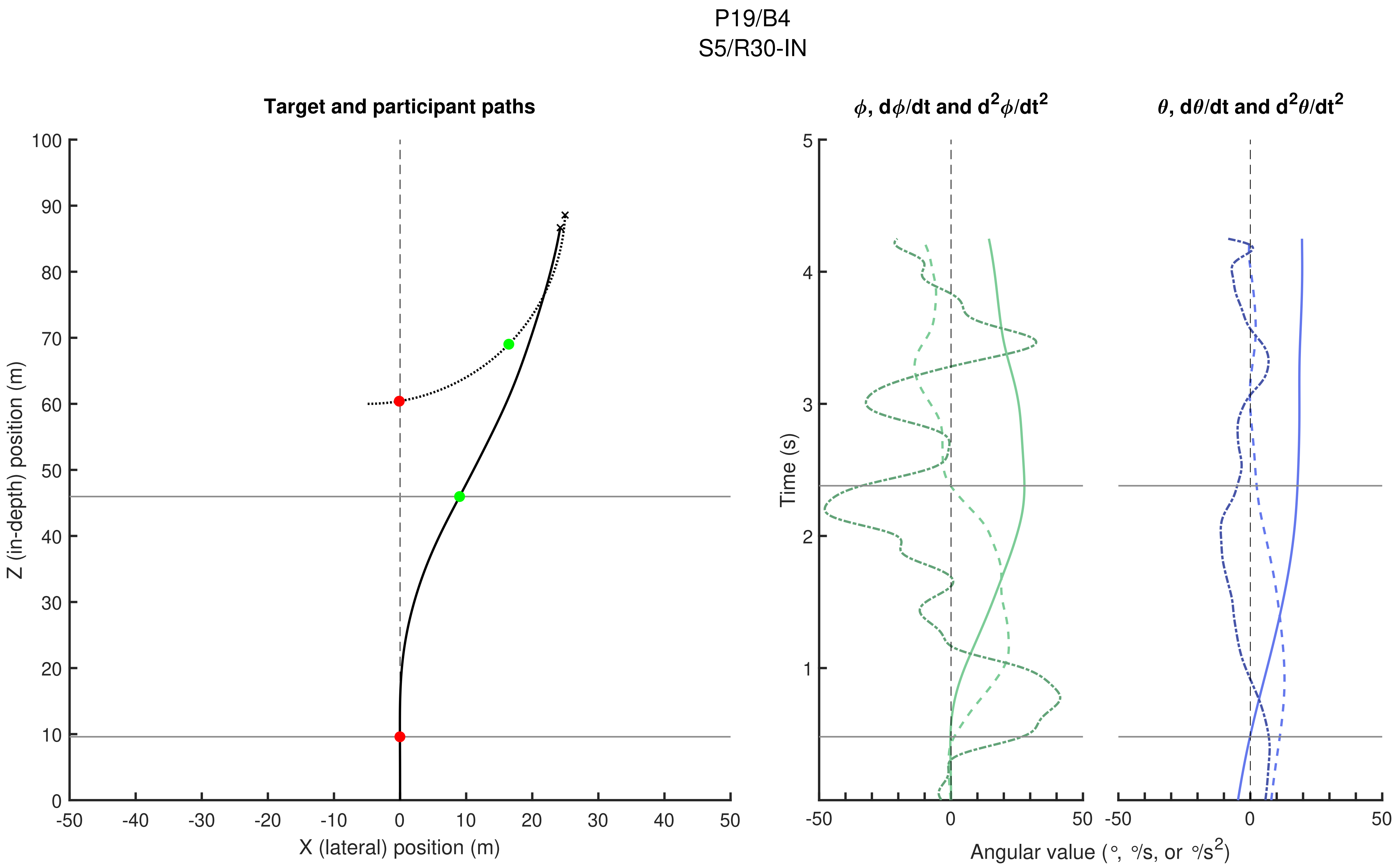

P19/B4
S5/R30-IN
Target and participant paths
Z (in-depth) position (m)
X (lateral) position (m)
0
10
20
30
40
50
60
70
80
90
100
-50
-40
-30
-20
-10
0
10
20
30
40
50
ϕ, dϕ/dt and d²ϕ/dt²
θ, dθ/dt and d²θ/dt²
Time (s)
1
2
3
4
5
-50
0
50
-50
0
50
Angular value (°, °/s, or °/s²)

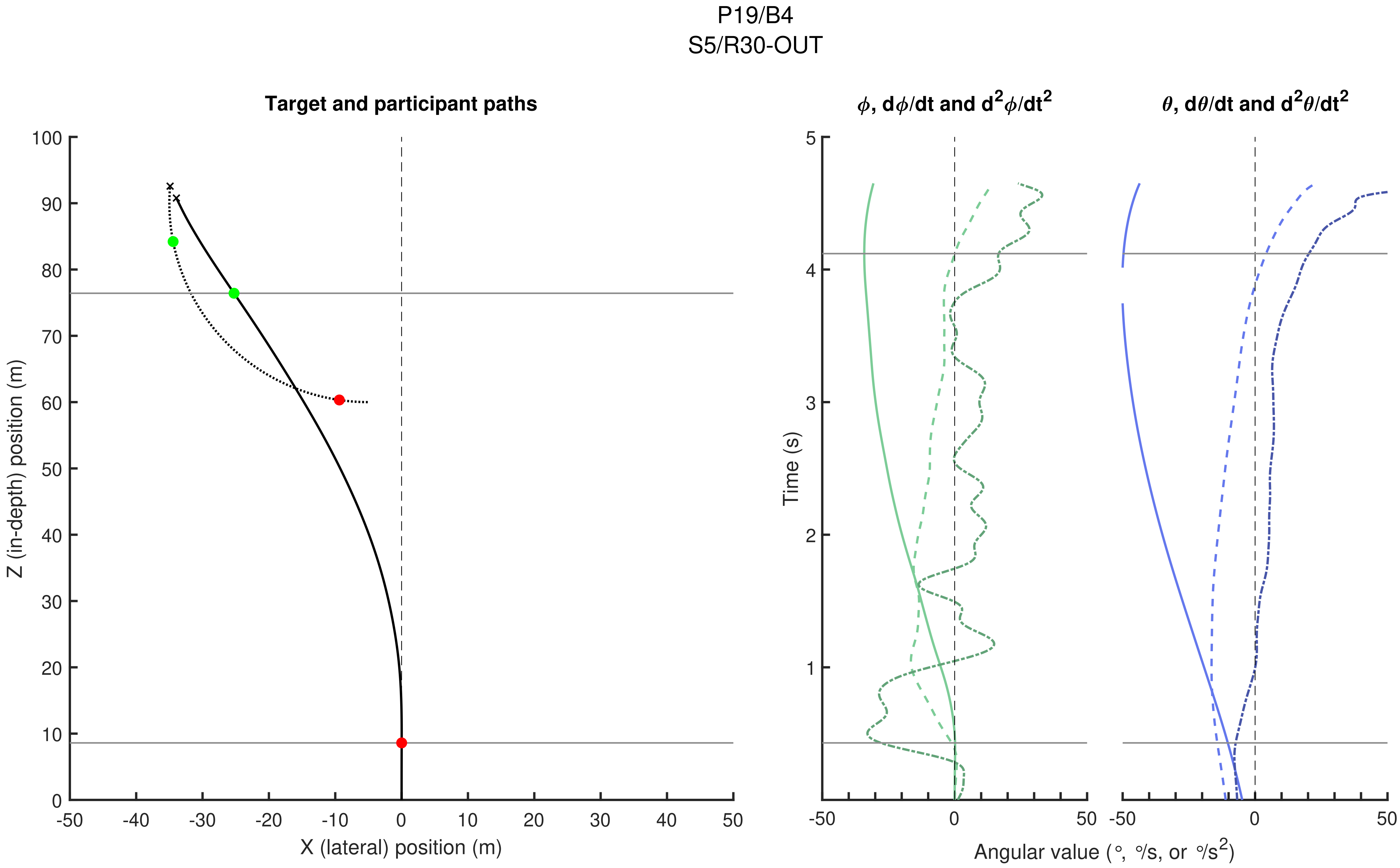
P19/B4
S5/R30-OUT
Target and participant paths
ϕ, dϕ/dt and d$^2\phi$/dt^2
θ, dθ/dt and d$^2\theta$/dt^2
Z (in-depth) position (m)
X (lateral) position (m)
Time (s)
Angular value (°, °/s, or °/s^2)
100
90
80
70
60
50
40
30
20
10
0
-50
-40
-30
-20
-10
0
10
20
30
40
50
5
4
3
2
1
-50
0
50
-50
0
50

P19/B4
S5/R10-OUT

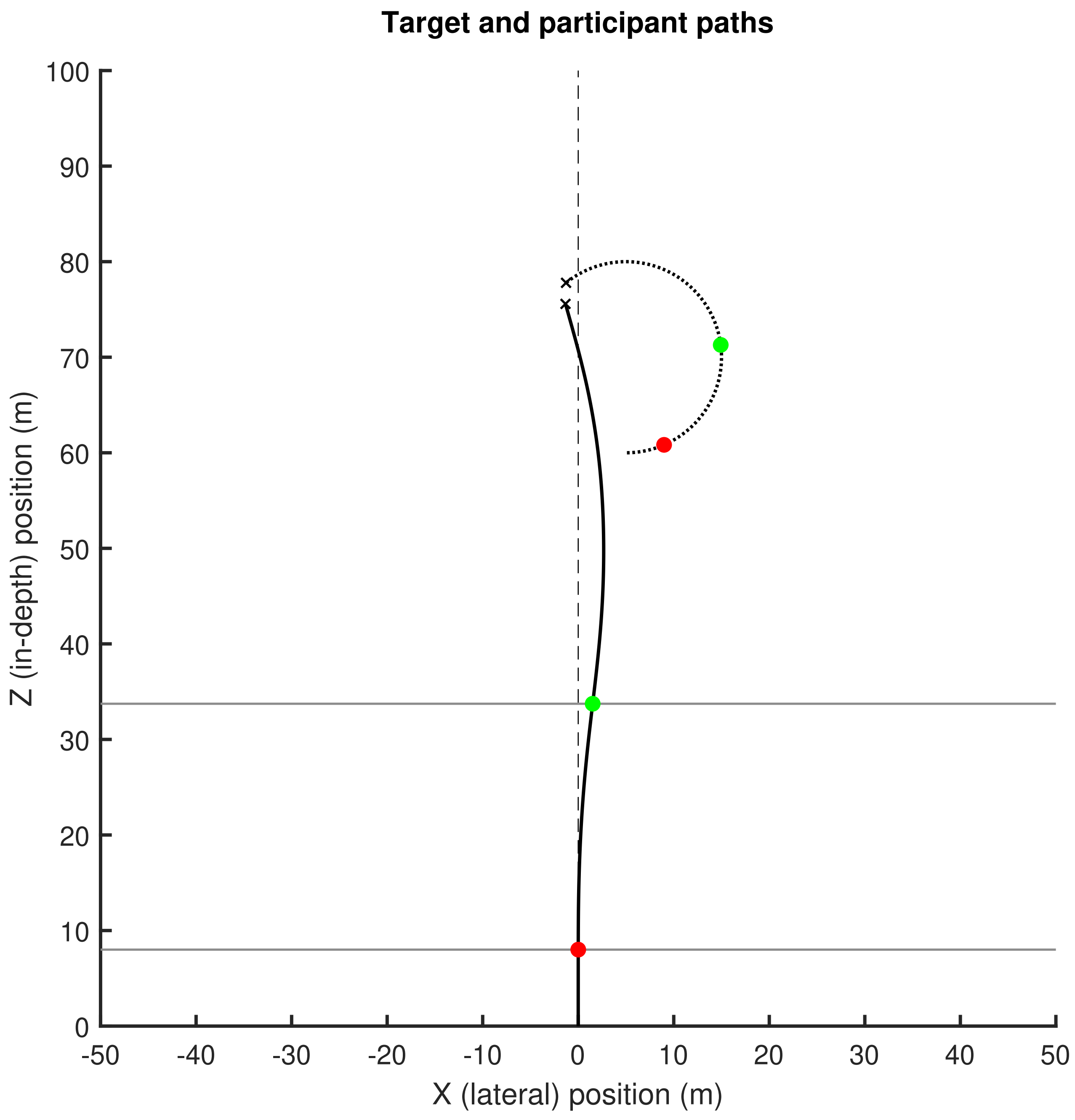

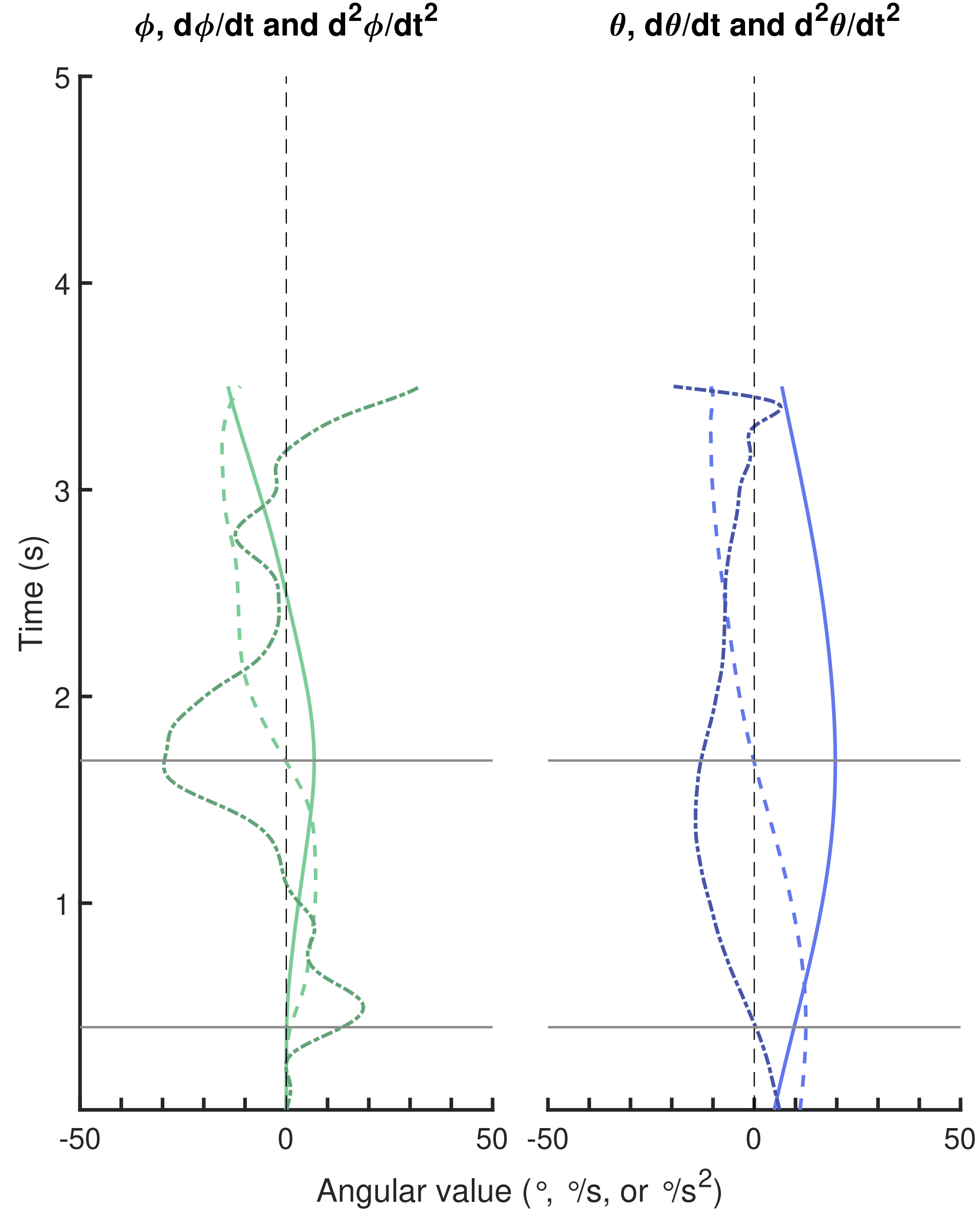

P19/B4
S5/R10-IN

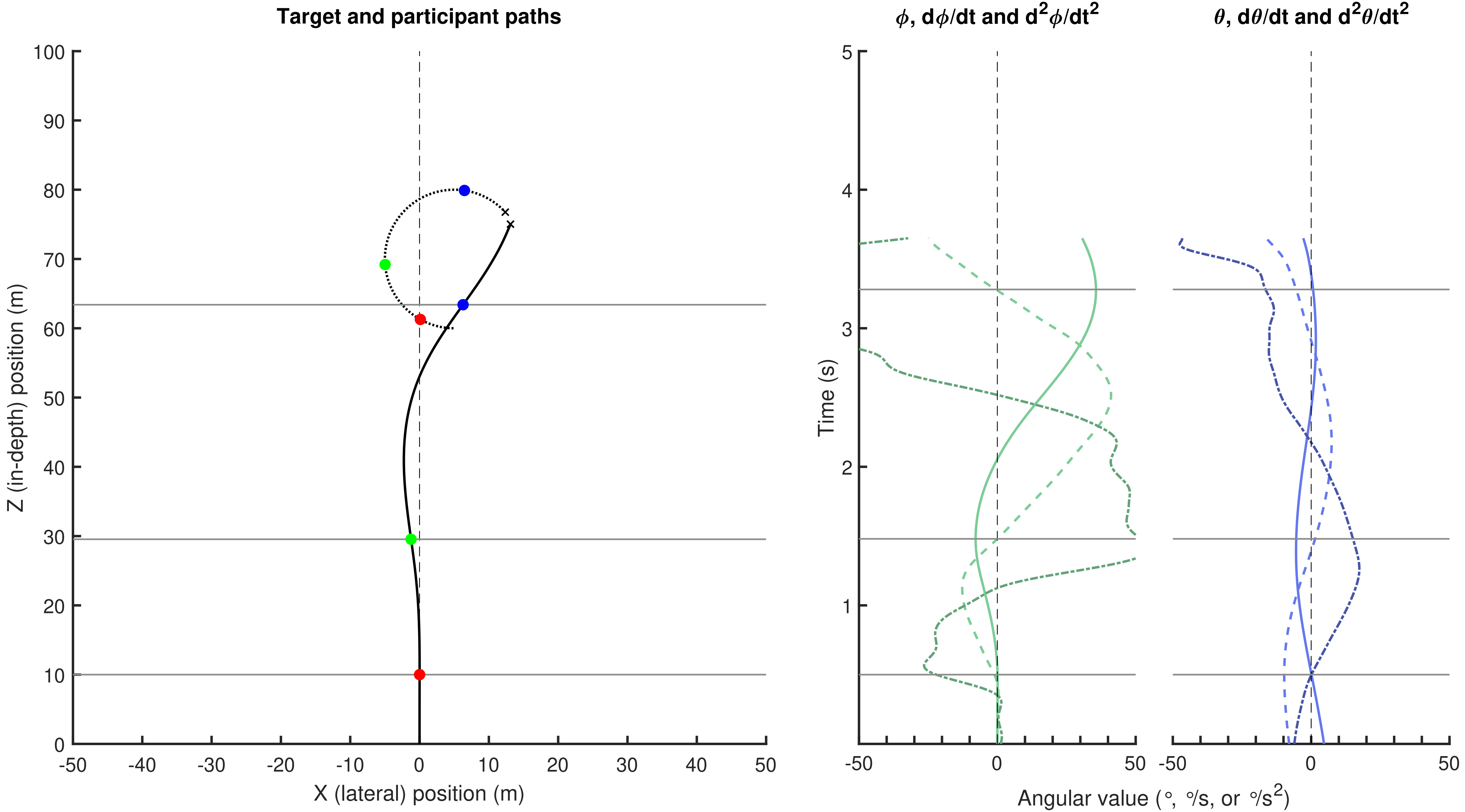

P19/B4
S5/R20-OUT

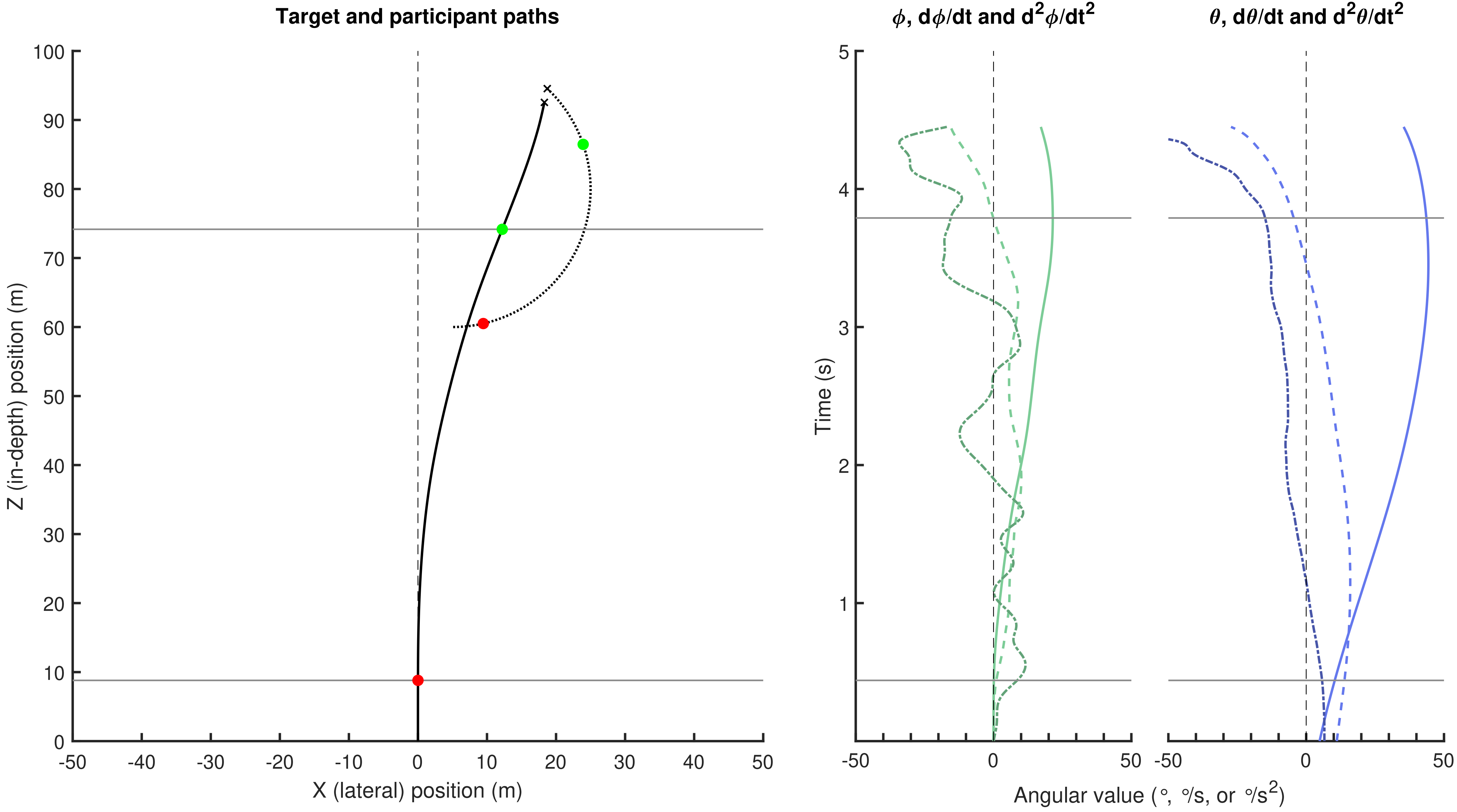

P19/B4
S5/R20-IN

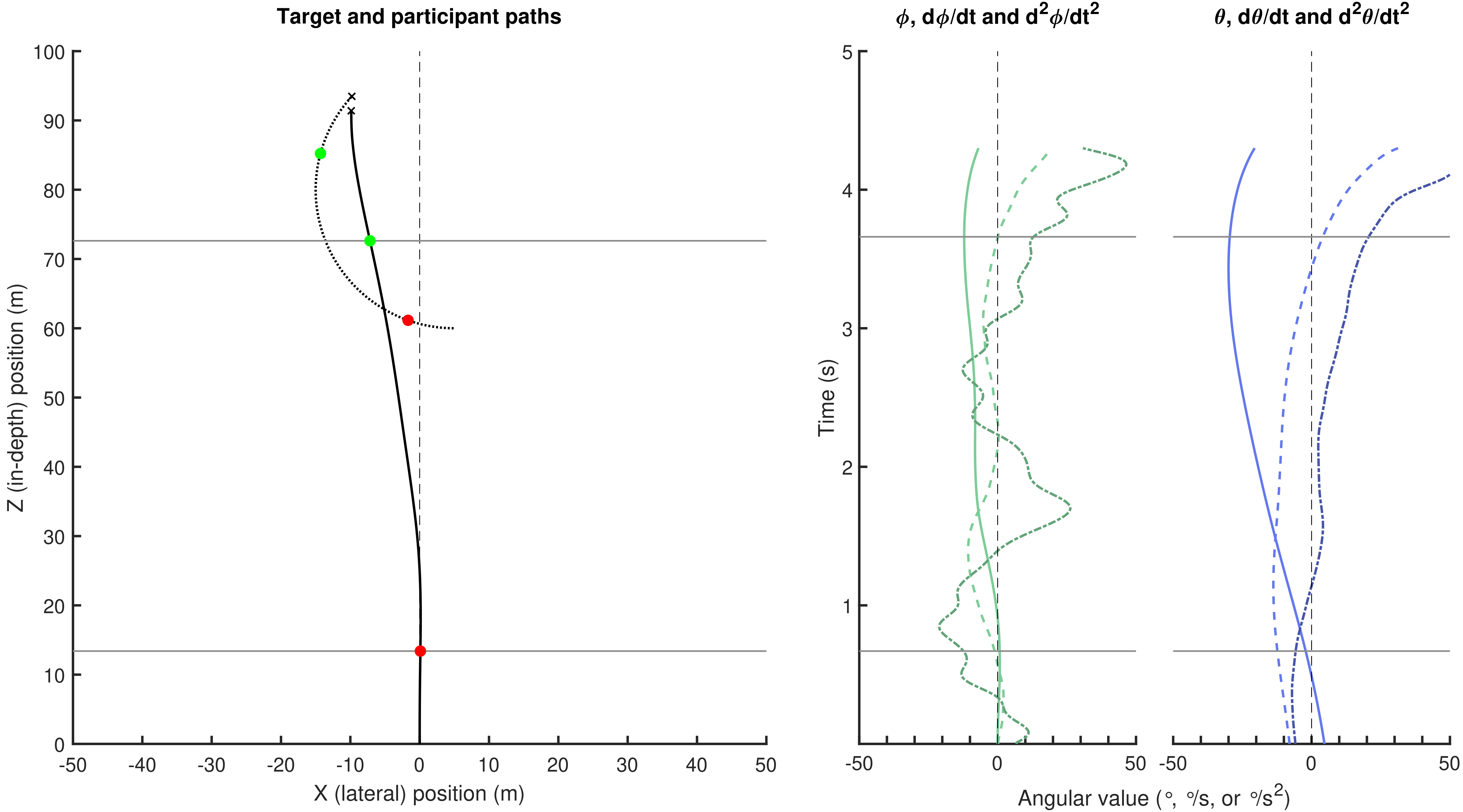

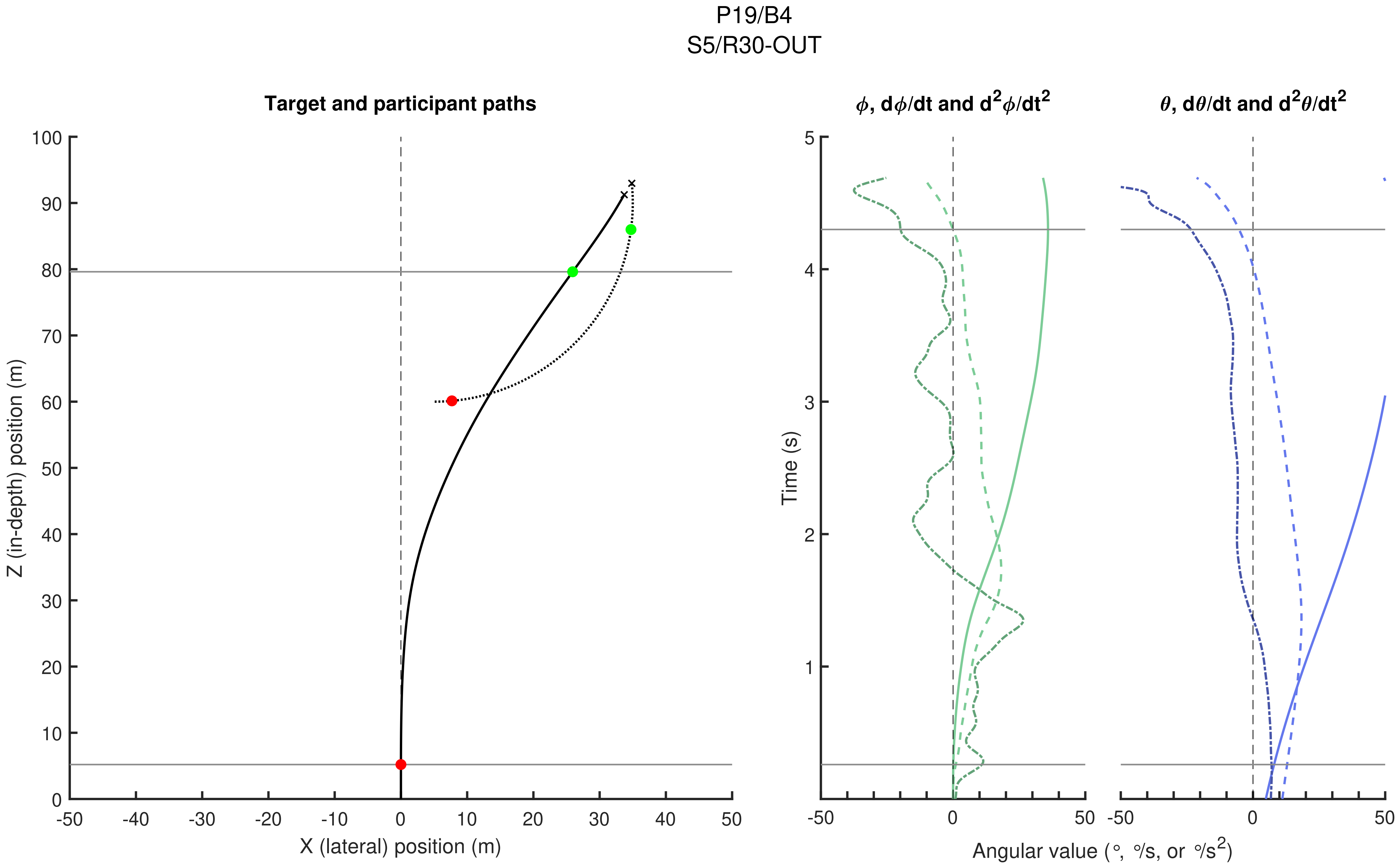

P19/B4
S5/R30-OUT
Target and participant paths
Z (in-depth) position (m)
X (lateral) position (m)
100
90
80
70
60
50
40
30
20
10
0
-50
-40
-30
-20
-10
0
10
20
30
40
50
ϕ, dϕ/dt and d²ϕ/dt²
θ, dθ/dt and d²θ/dt²
Time (s)
5
4
3
2
1
-50
0
50
-50
0
50
Angular value (°, °/s, or °/s²)

P19/B4
S5/R30-IN

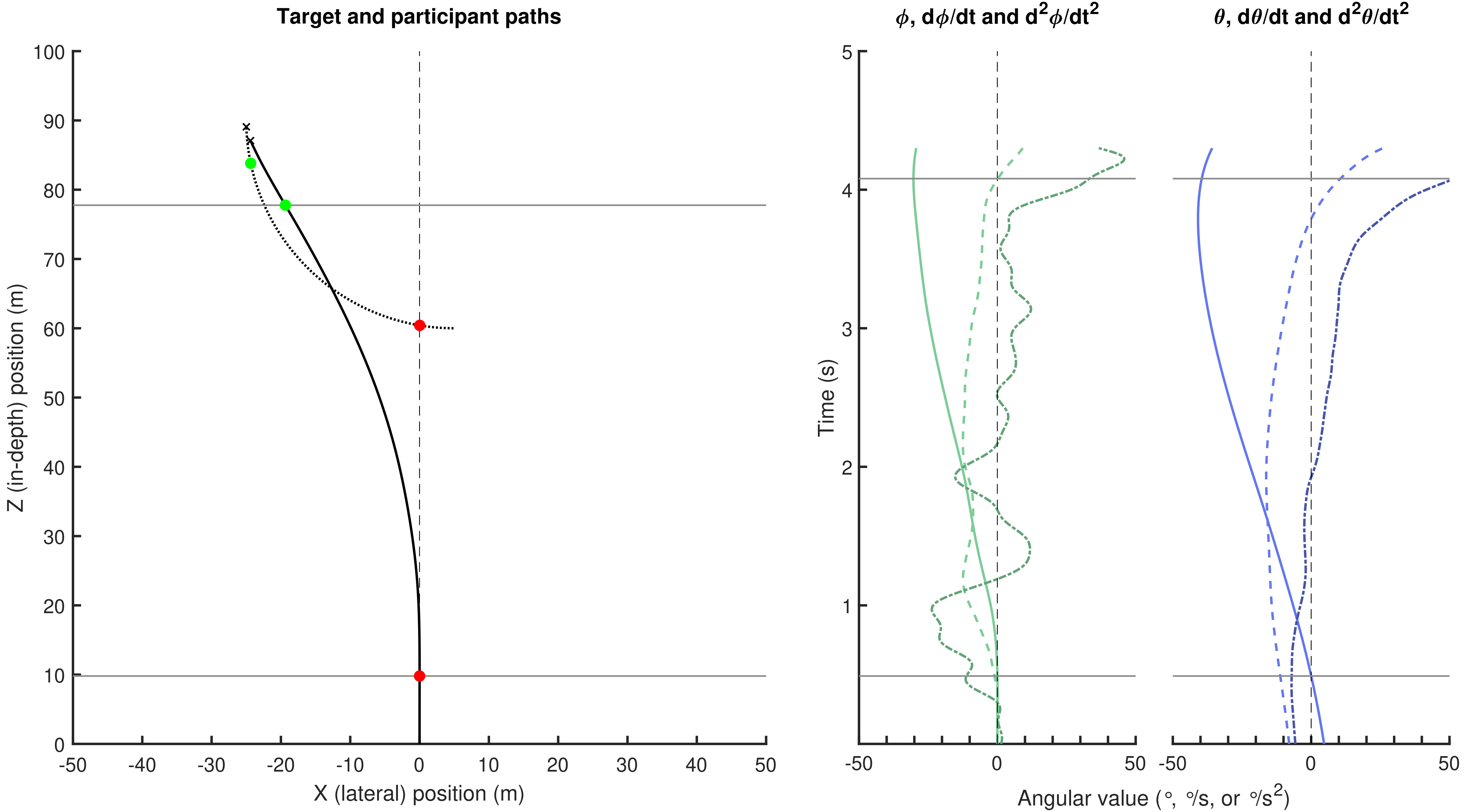

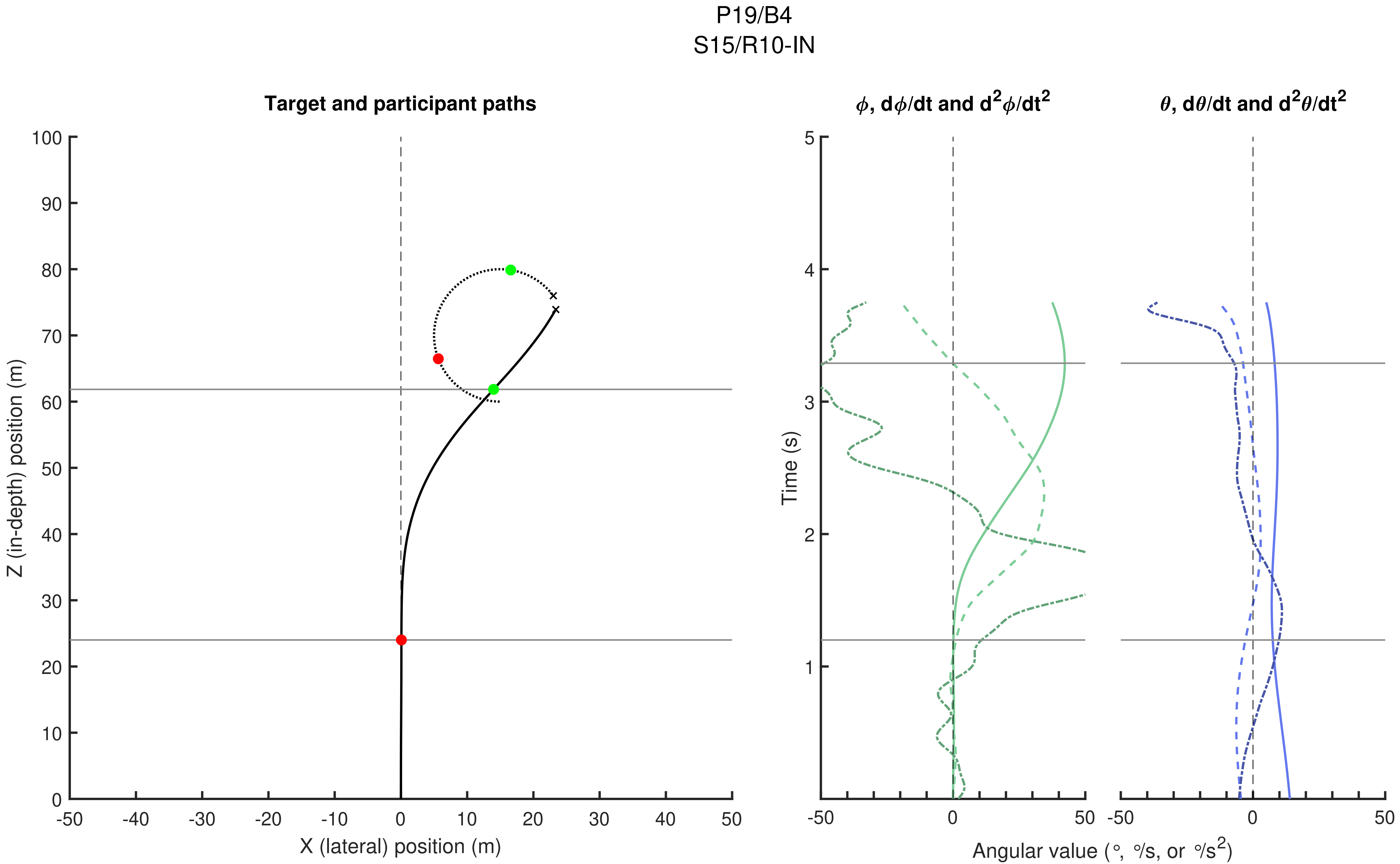
P19/B4
S15/R10-IN
Target and participant paths
Z (in-depth) position (m)
X (lateral) position (m)
100
90
80
70
60
50
40
30
20
10
0
-50
-40
-30
-20
-10
0
10
20
30
40
50
ϕ, dϕ/dt and d²ϕ/dt²
θ, dθ/dt and d²θ/dt²
Time (s)
5
4
3
2
1
-50
0
50
-50
0
50
Angular value (°, °/s, or °/s²)

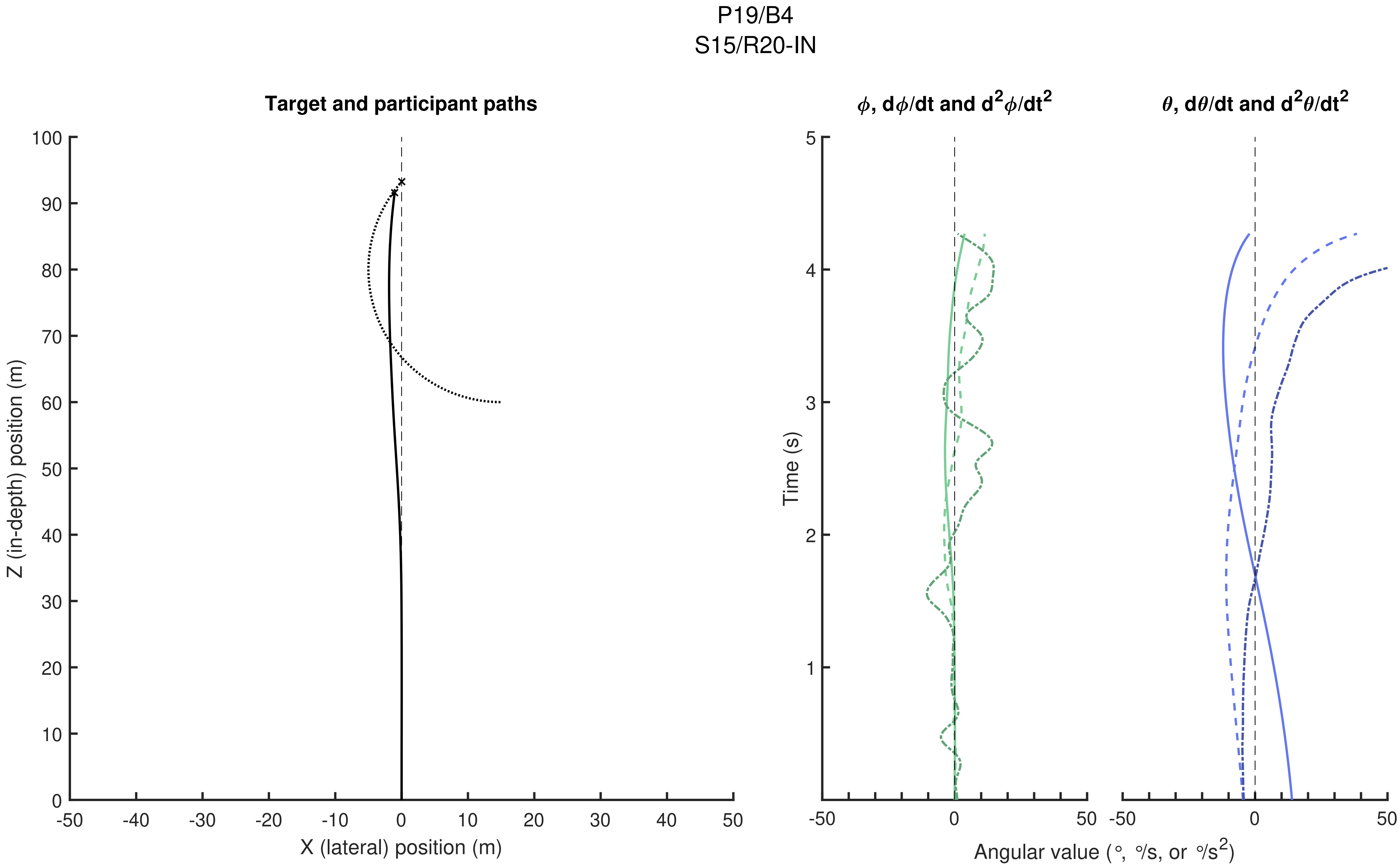
P19/B4
S15/R20-IN
Target and participant paths
Z (in-depth) position (m)
X (lateral) position (m)
ϕ, dϕ/dt and d$^2\phi$/dt^2
θ, dθ/dt and d$^2\theta$/dt^2
Time (s)
Angular value (°, °/s, or °/s^2)

P19/B4
S15/R30-IN

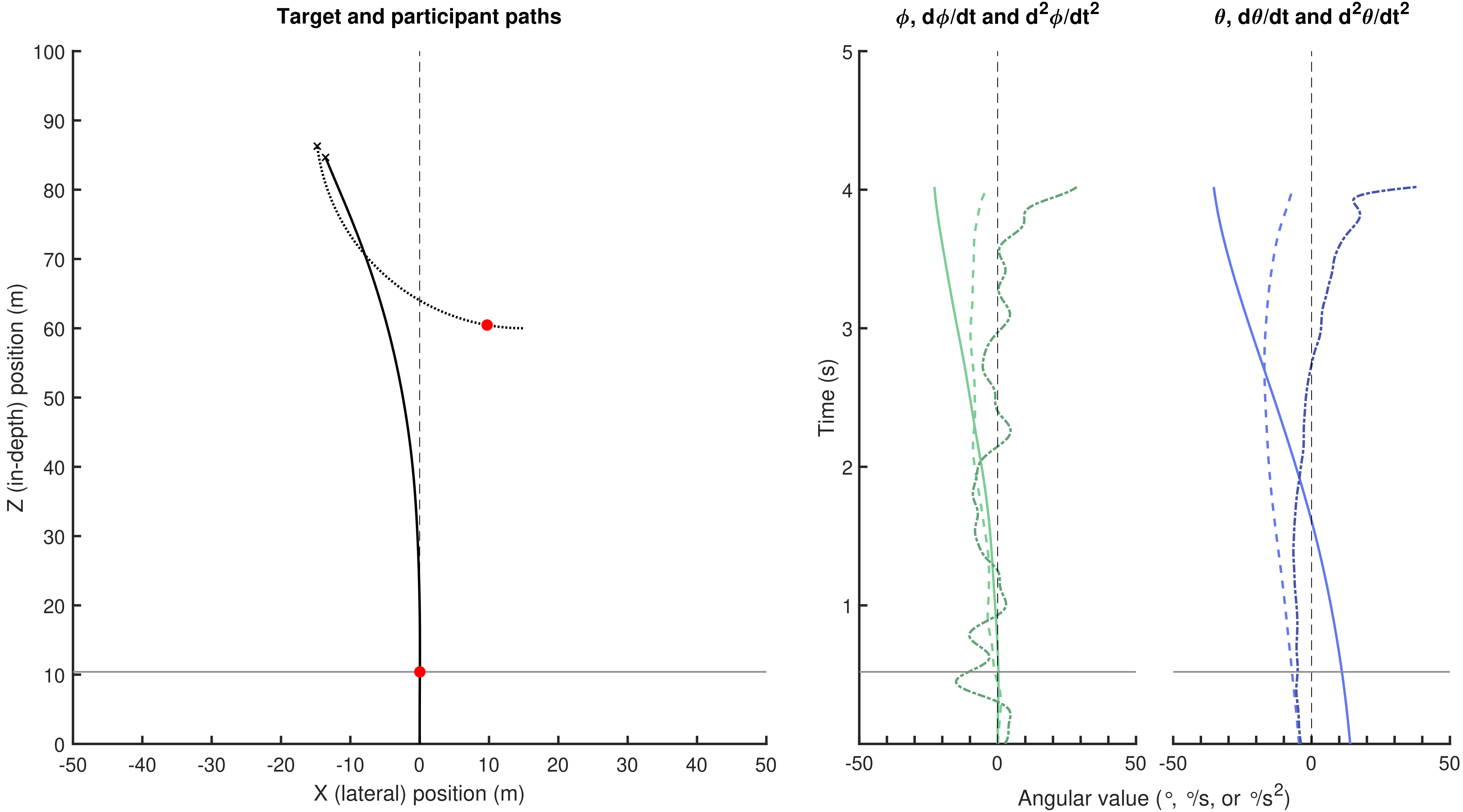

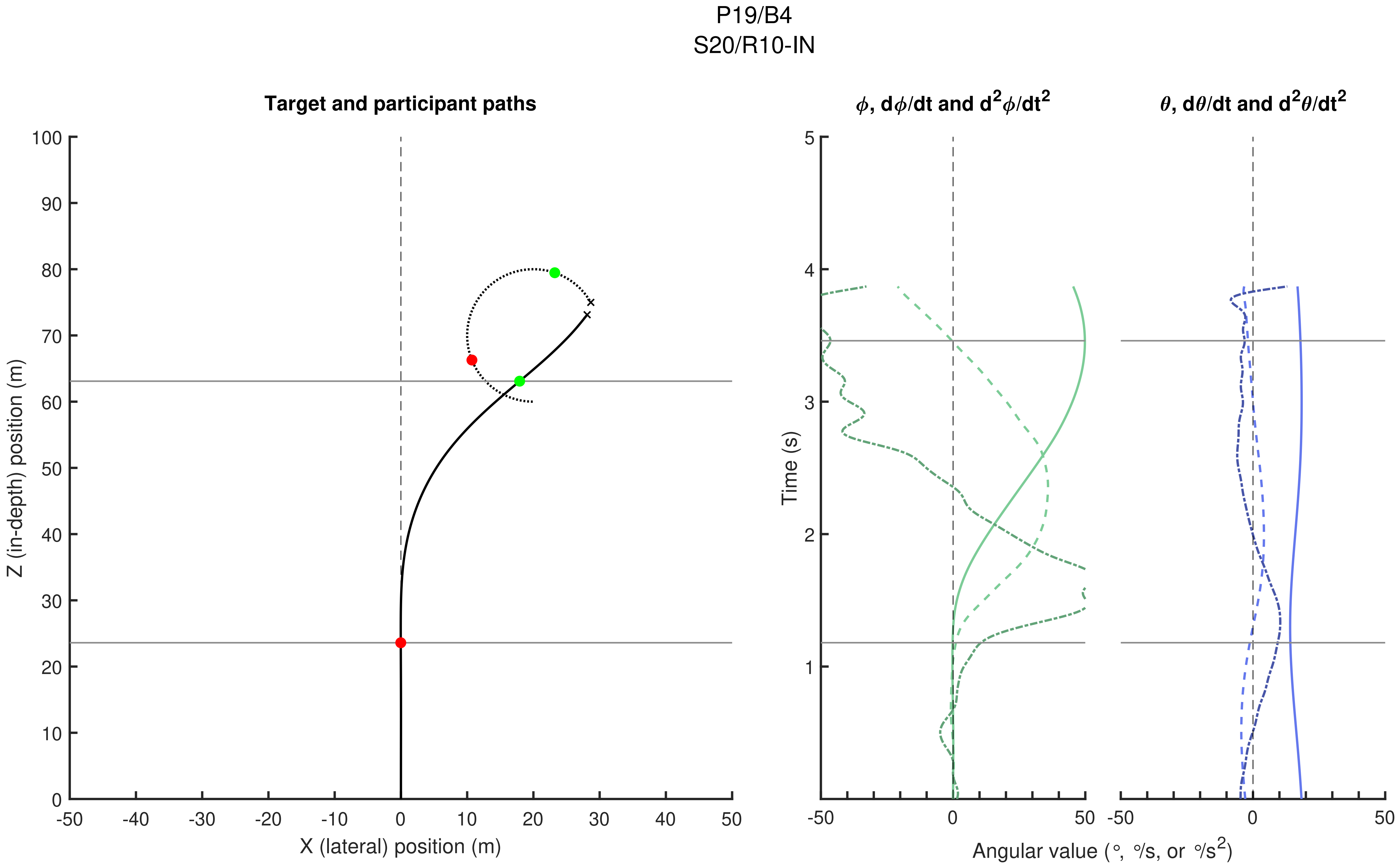

P19/B4
S20/R10-IN
Target and participant paths
Z (in-depth) position (m)
X (lateral) position (m)
100
90
80
70
60
50
40
30
20
10
0
-50
-40
-30
-20
-10
0
10
20
30
40
50
ϕ, dϕ/dt and d²ϕ/dt²
θ, dθ/dt and d²θ/dt²
Time (s)
5
4
3
2
1
-50
0
50
-50
0
50
Angular value (°, °/s, or °/s²)

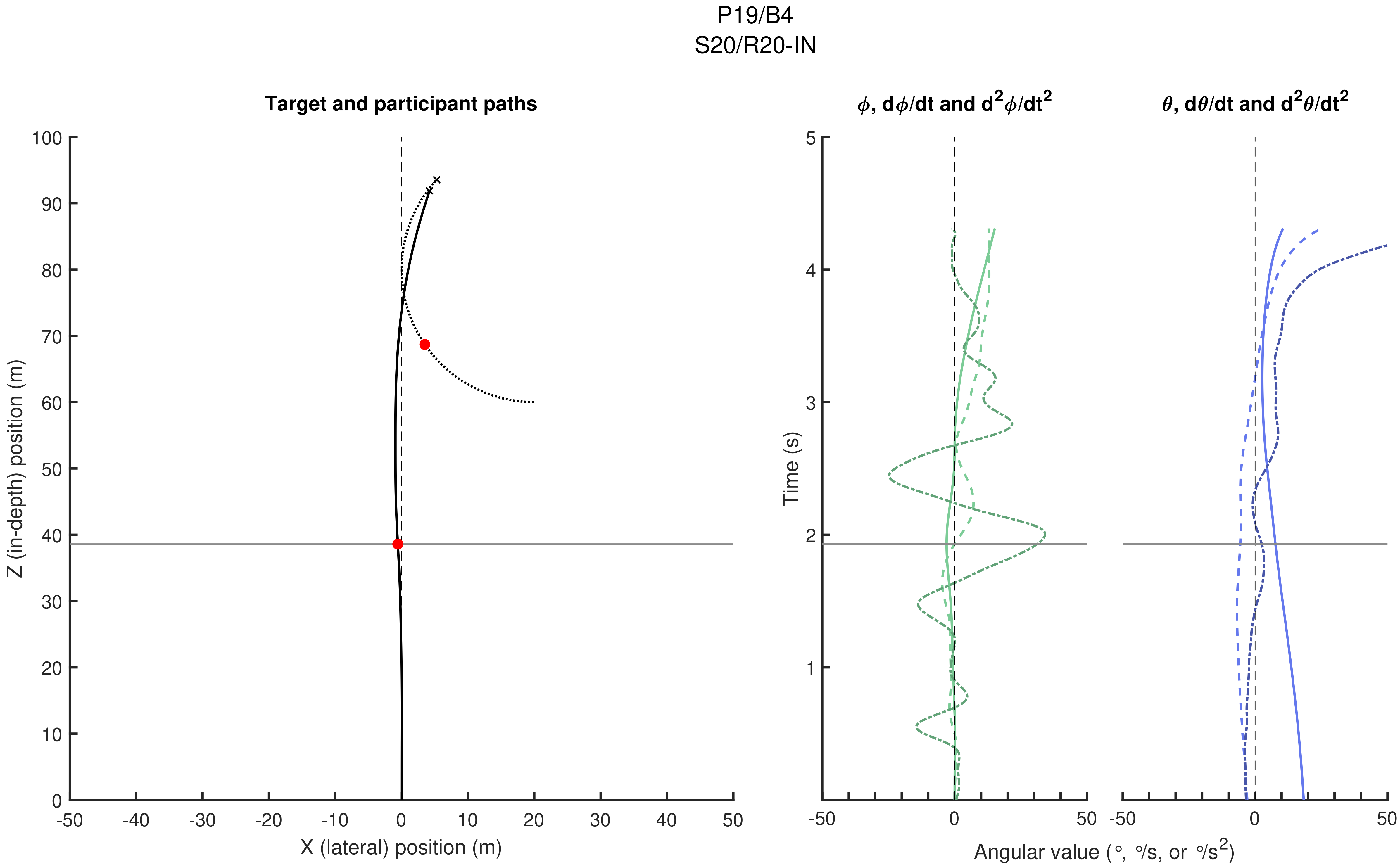

P19/B4
S20/R20-IN
Target and participant paths
Z (in-depth) position (m)
X (lateral) position (m)
100
90
80
70
60
50
40
30
20
10
0
-50
-40
-30
-20
-10
0
10
20
30
40
50
φ, dφ/dt and d²φ/dt²
θ, dθ/dt and d²θ/dt²
Time (s)
5
4
3
2
1
-50
0
50
-50
0
50
Angular value (°, °/s, or °/s²)

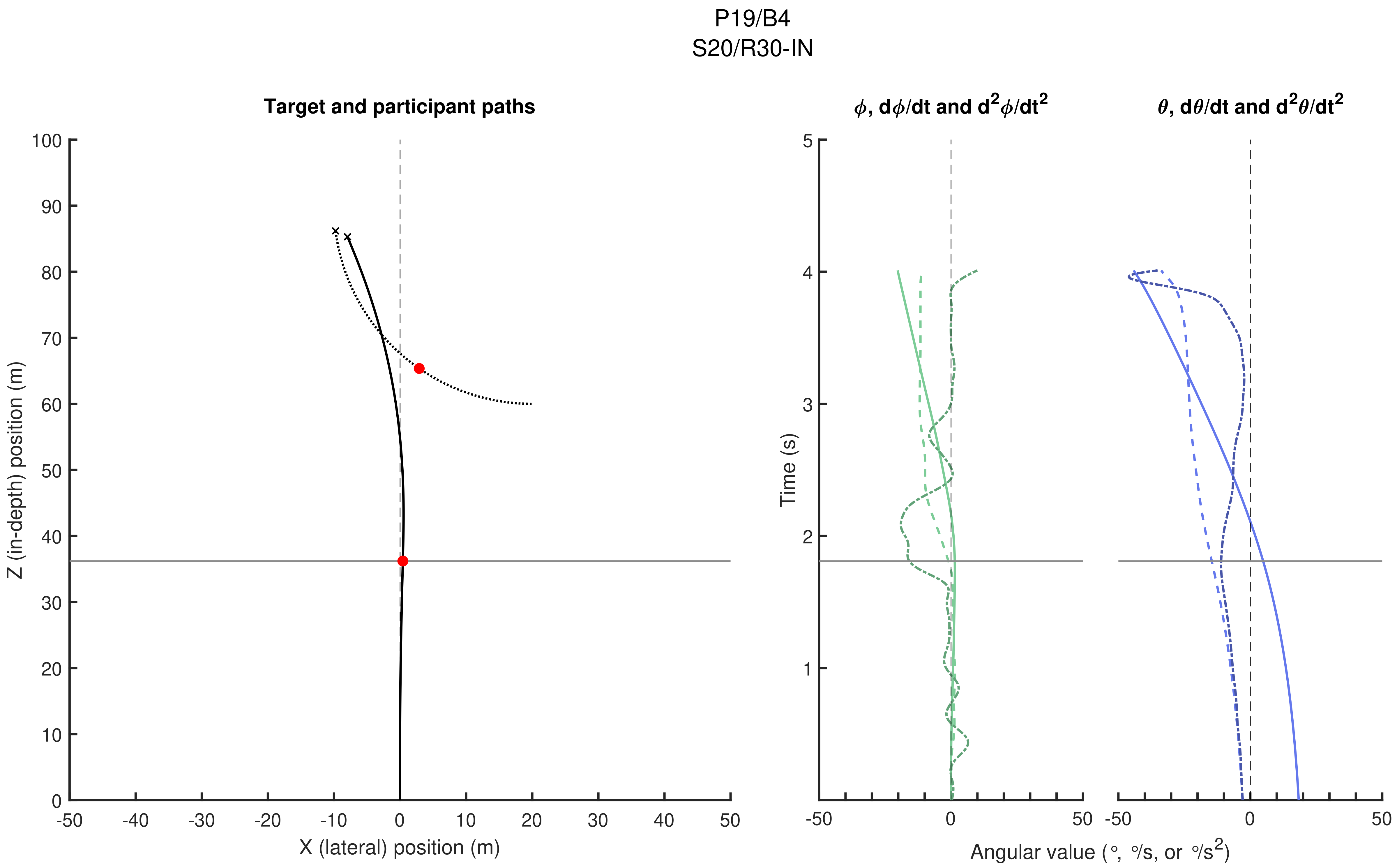
P19/B4
S20/R30-IN
Target and participant paths
Z (in-depth) position (m)
X (lateral) position (m)
ϕ, dϕ/dt and d$^2\phi$/dt^2
θ, dθ/dt and d$^2\theta$/dt^2
Time (s)
Angular value (°, °/s, or °/s^2)

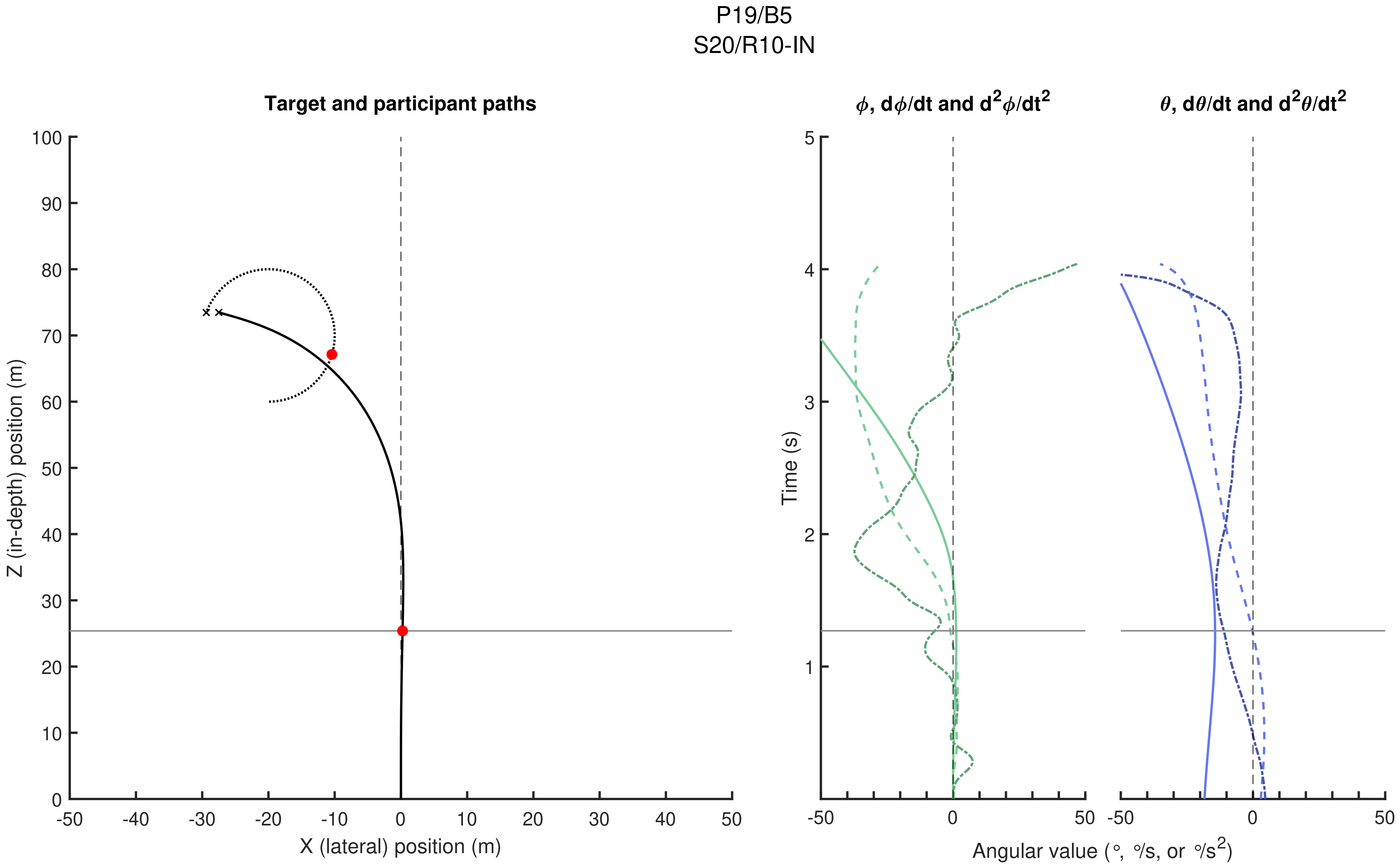

P19/B5
S20/R10-IN
Target and participant paths
ϕ, dϕ/dt and d$^2\phi$/dt^2
θ, dθ/dt and d$^2\theta$/dt^2
100
90
80
70
60
50
40
30
20
10
0
-50
-40
-30
-20
-10
0
10
20
30
40
50
Z (in-depth) position (m)
X (lateral) position (m)
5
4
3
2
1
Time (s)
-50
0
50
-50
0
50
Angular value (°, °/s, or °/s^2)

P19/B5
S20/R20-IN

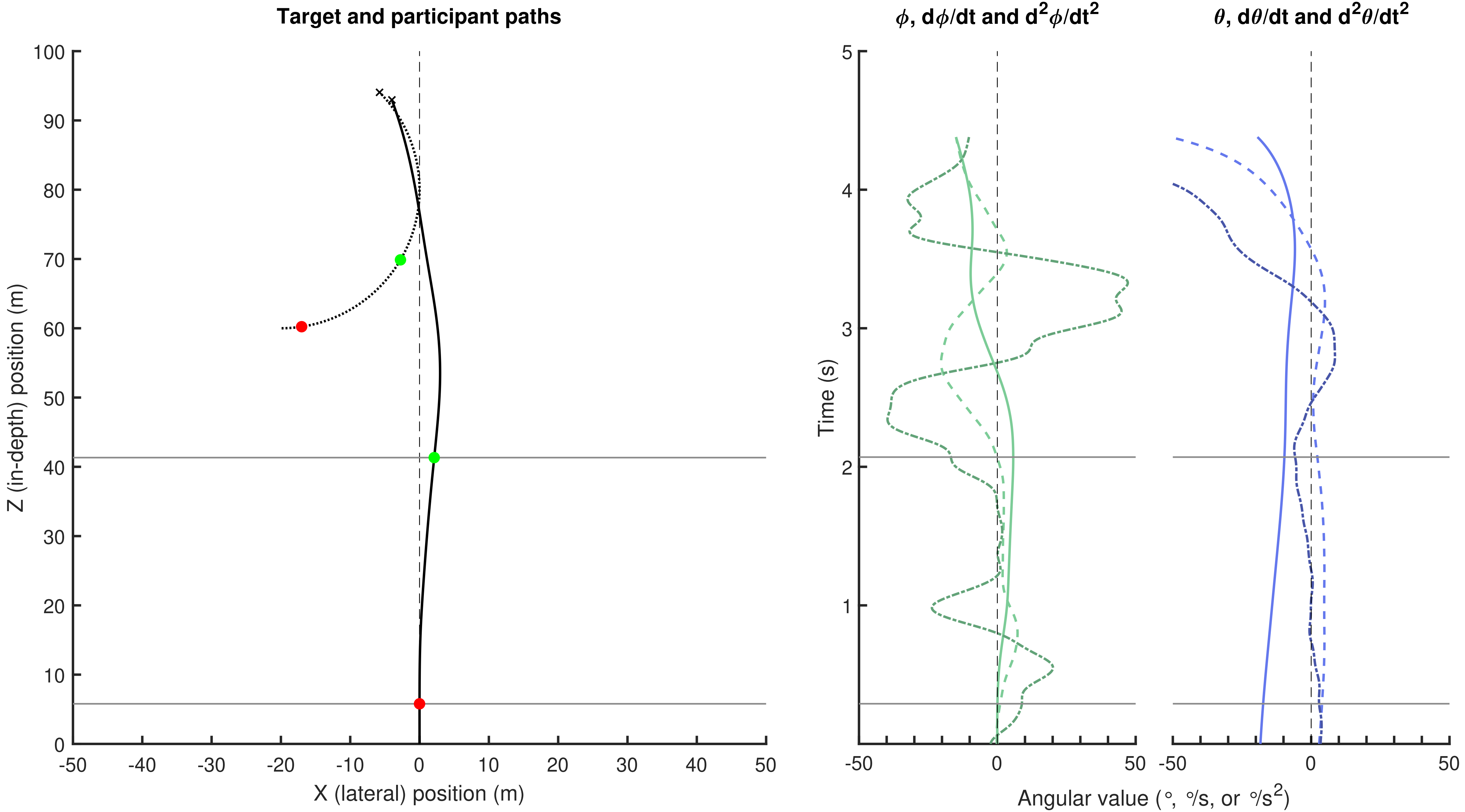

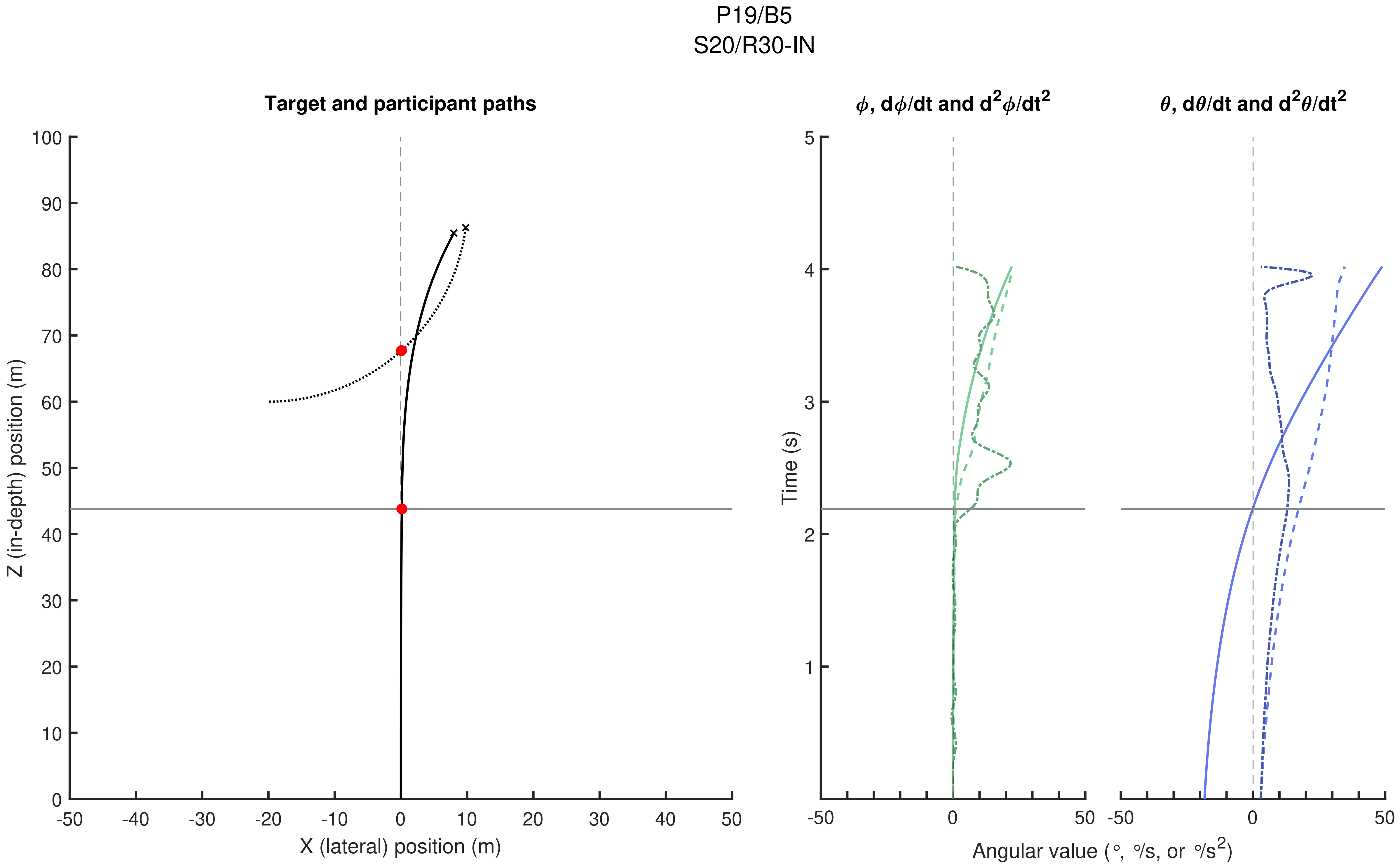
P19/B5
S20/R30-IN
Target and participant paths
Z (in-depth) position (m)
X (lateral) position (m)
φ, dφ/dt and d²φ/dt²
θ, dθ/dt and d²θ/dt²
Time (s)
Angular value (°, °/s, or °/s²)
100
90
80
70
60
50
40
30
20
10
0
-50
-40
-30
-20
-10
0
10
20
30
40
50
5
4
3
2
1
-50
0
50
-50
0
50

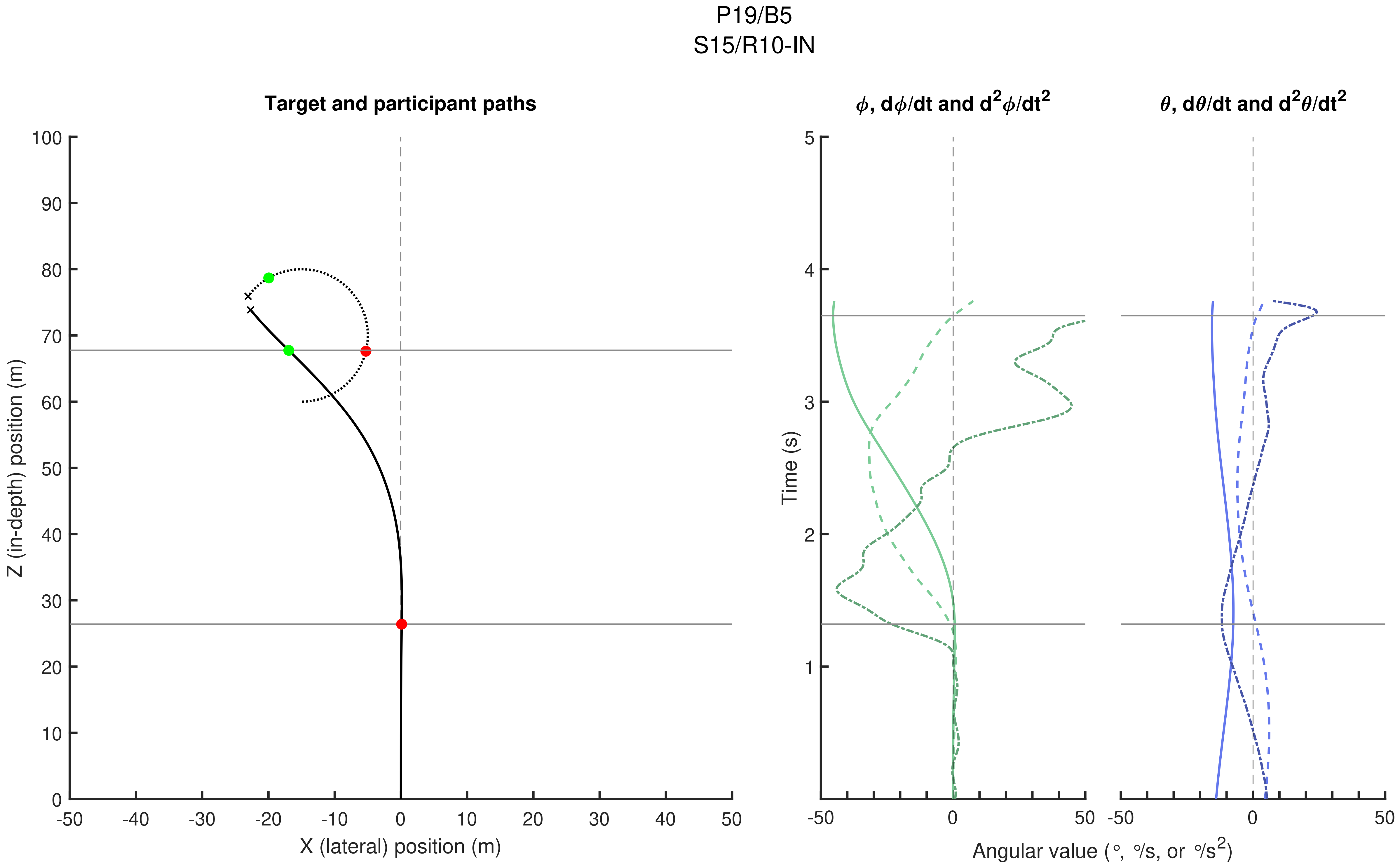
P19/B5
S15/R10-IN
Target and participant paths
Z (in-depth) position (m)
X (lateral) position (m)
ϕ, dϕ/dt and d$^2\phi$/dt^2
θ, dθ/dt and d$^2\theta$/dt^2
Time (s)
Angular value (°, °/s, or °/s^2)

P19/B5
S15/R20-IN

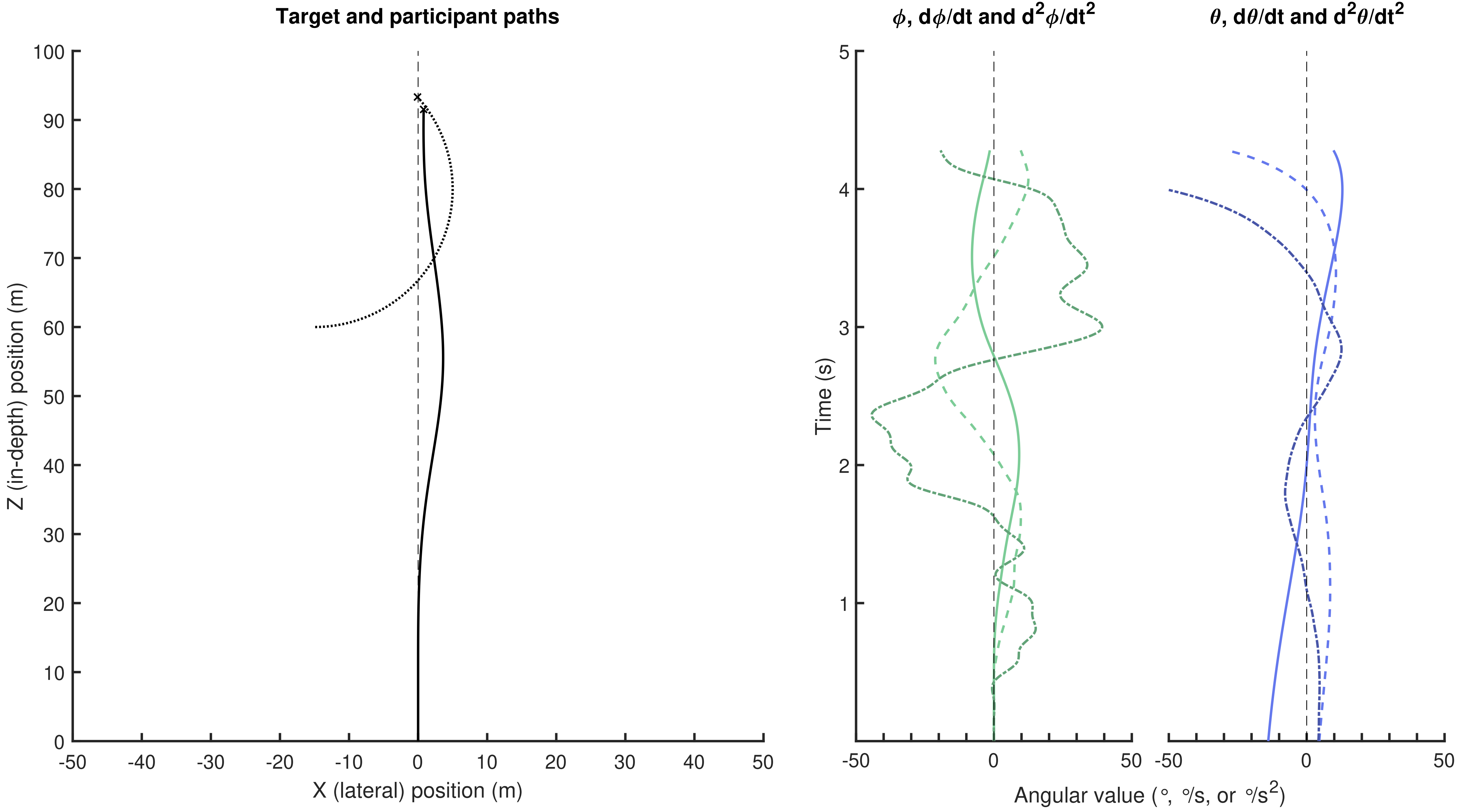

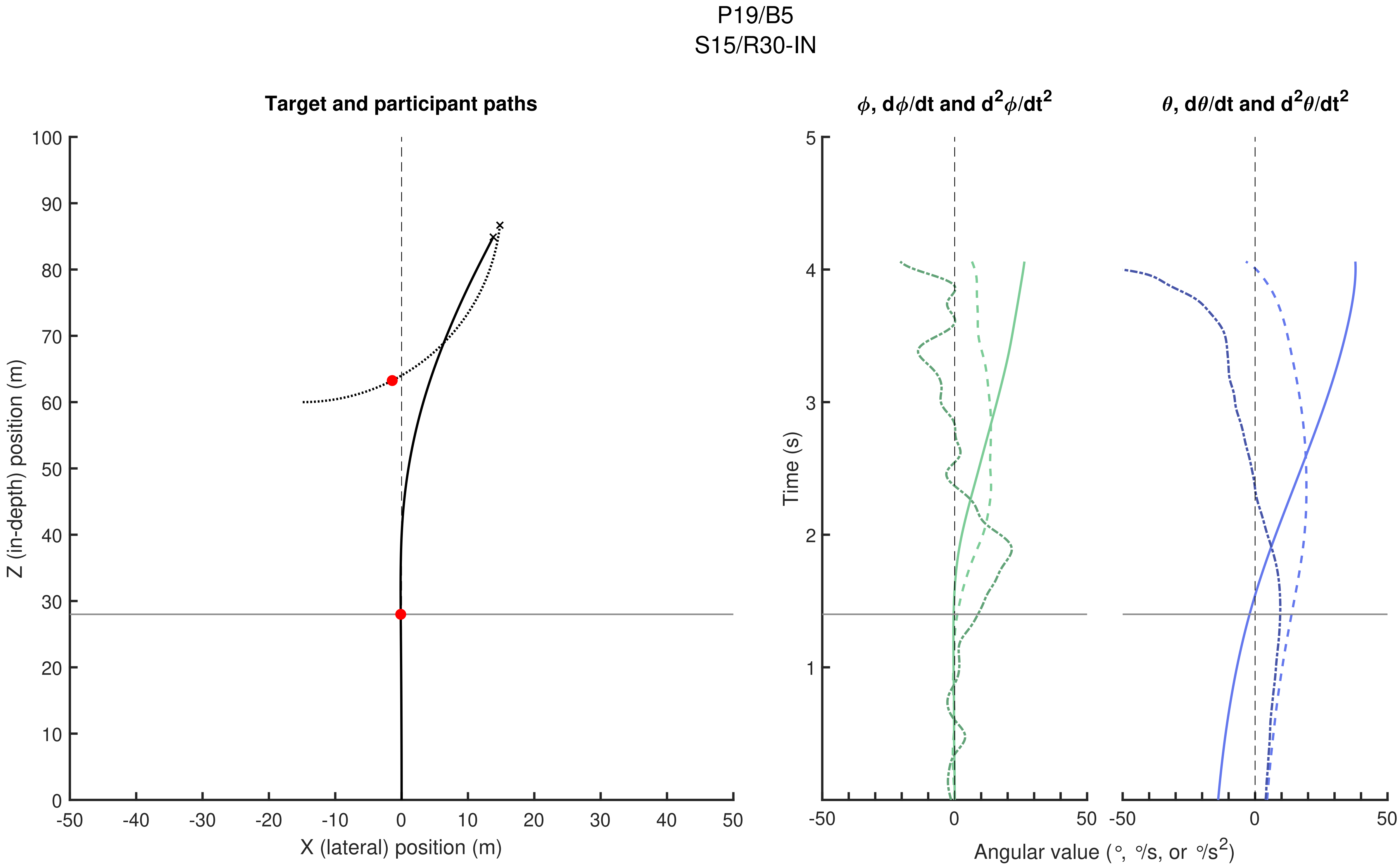
P19/B5
S15/R30-IN
Target and participant paths
ϕ, dϕ/dt and d$^2\phi$/dt^2
θ, dθ/dt and d$^2\theta$/dt^2
Z (in-depth) position (m)
X (lateral) position (m)
Time (s)
Angular value (°, °/s, or °/s^2)
100
90
80
70
60
50
40
30
20
10
0
-50
-40
-30
-20
-10
0
10
20
30
40
50
5
4
3
2
1
-50
0
50
-50
0
50

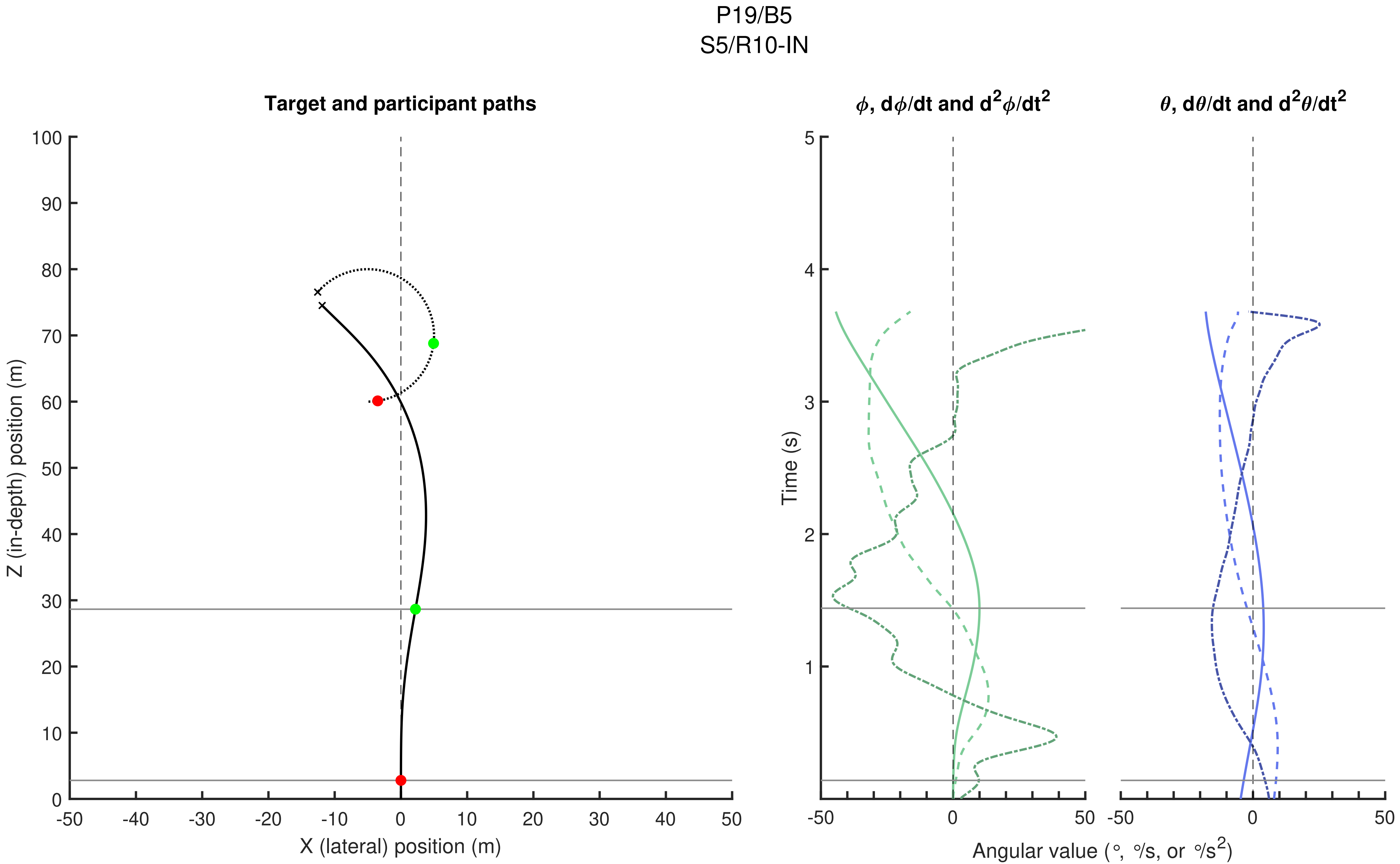
P19/B5
S5/R10-IN
Target and participant paths
Z (in-depth) position (m)
X (lateral) position (m)
100
90
80
70
60
50
40
30
20
10
0
-50
-40
-30
-20
-10
0
10
20
30
40
50
ϕ, dϕ/dt and d$^2\phi$/dt^2
θ, dθ/dt and d$^2\theta$/dt^2
Time (s)
5
4
3
2
1
-50
0
50
-50
0
50
Angular value (°, °/s, or °/s^2)

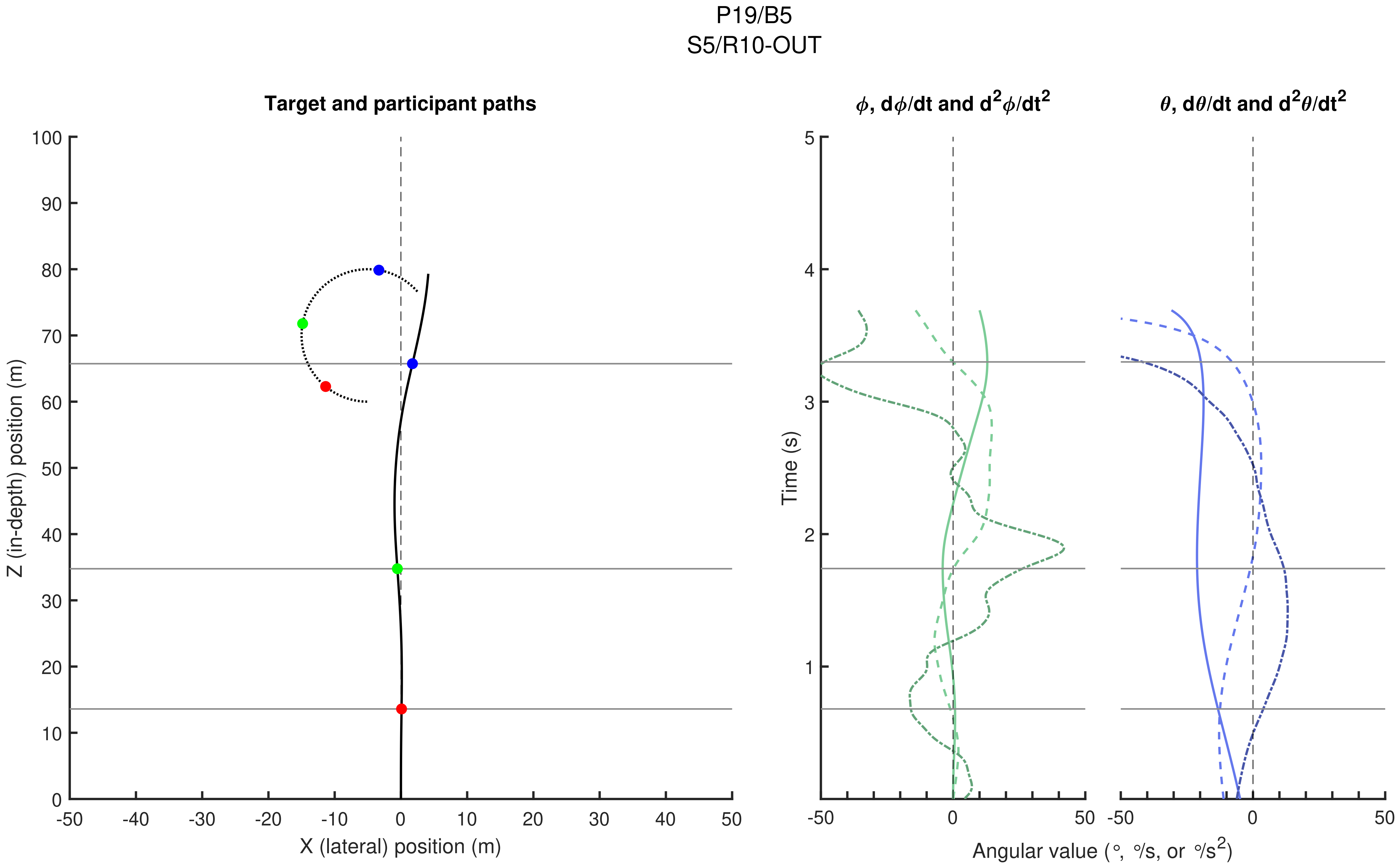
P19/B5
S5/R10-OUT
Target and participant paths
Z (in-depth) position (m)
X (lateral) position (m)
100
90
80
70
60
50
40
30
20
10
0
-50
-40
-30
-20
-10
0
10
20
30
40
50
ϕ, dϕ/dt and d²ϕ/dt²
θ, dθ/dt and d²θ/dt²
Time (s)
5
4
3
2
1
-50
0
50
-50
0
50
Angular value (°, °/s, or °/s²)

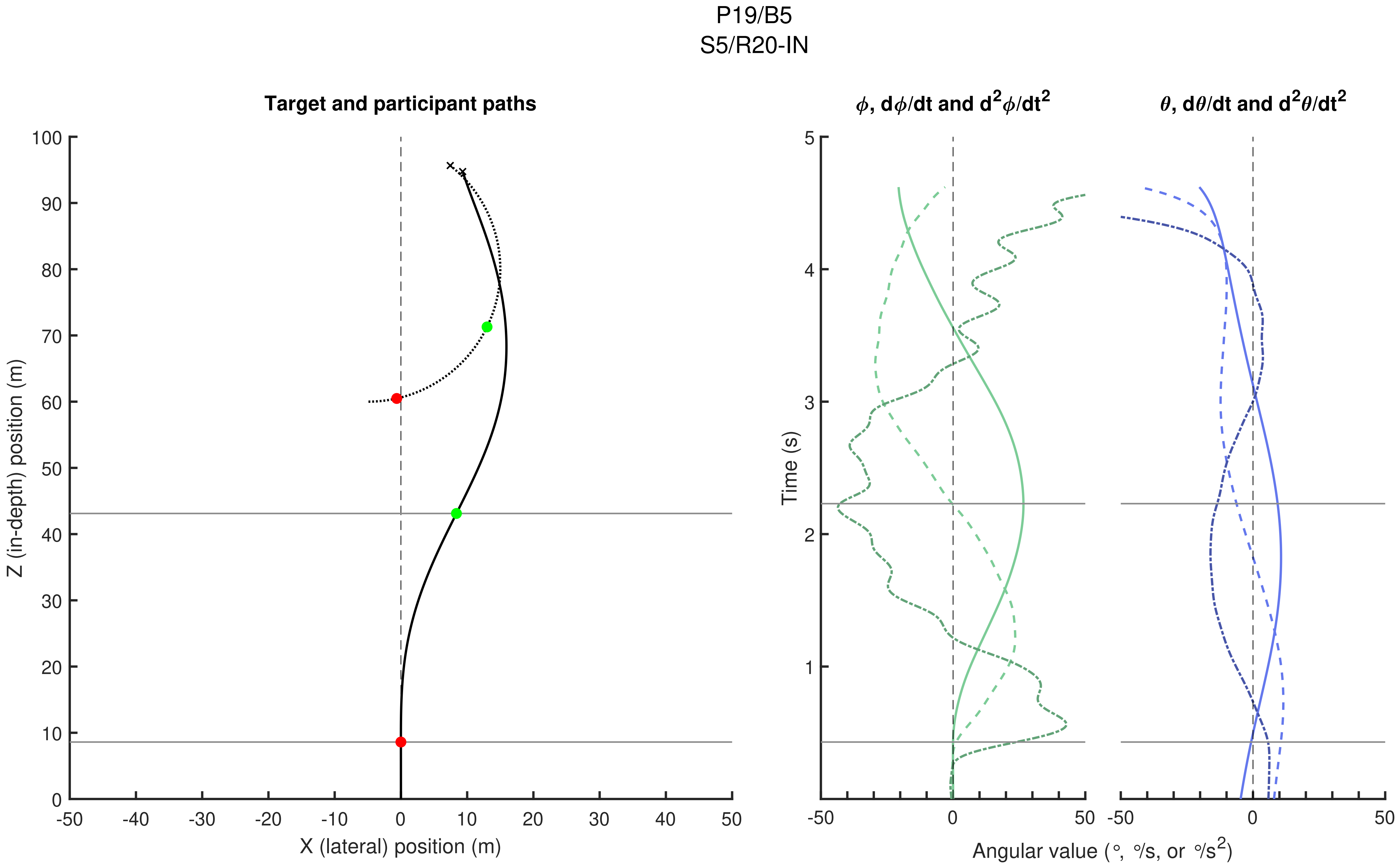

P19/B5
S5/R20-IN
Target and participant paths
Z (in-depth) position (m)
X (lateral) position (m)
ϕ, dϕ/dt and d$^2\phi$/dt^2
θ, dθ/dt and d$^2\theta$/dt^2
Time (s)
Angular value (°, °/s, or °/s^2)

P19/B5
S5/R20-OUT

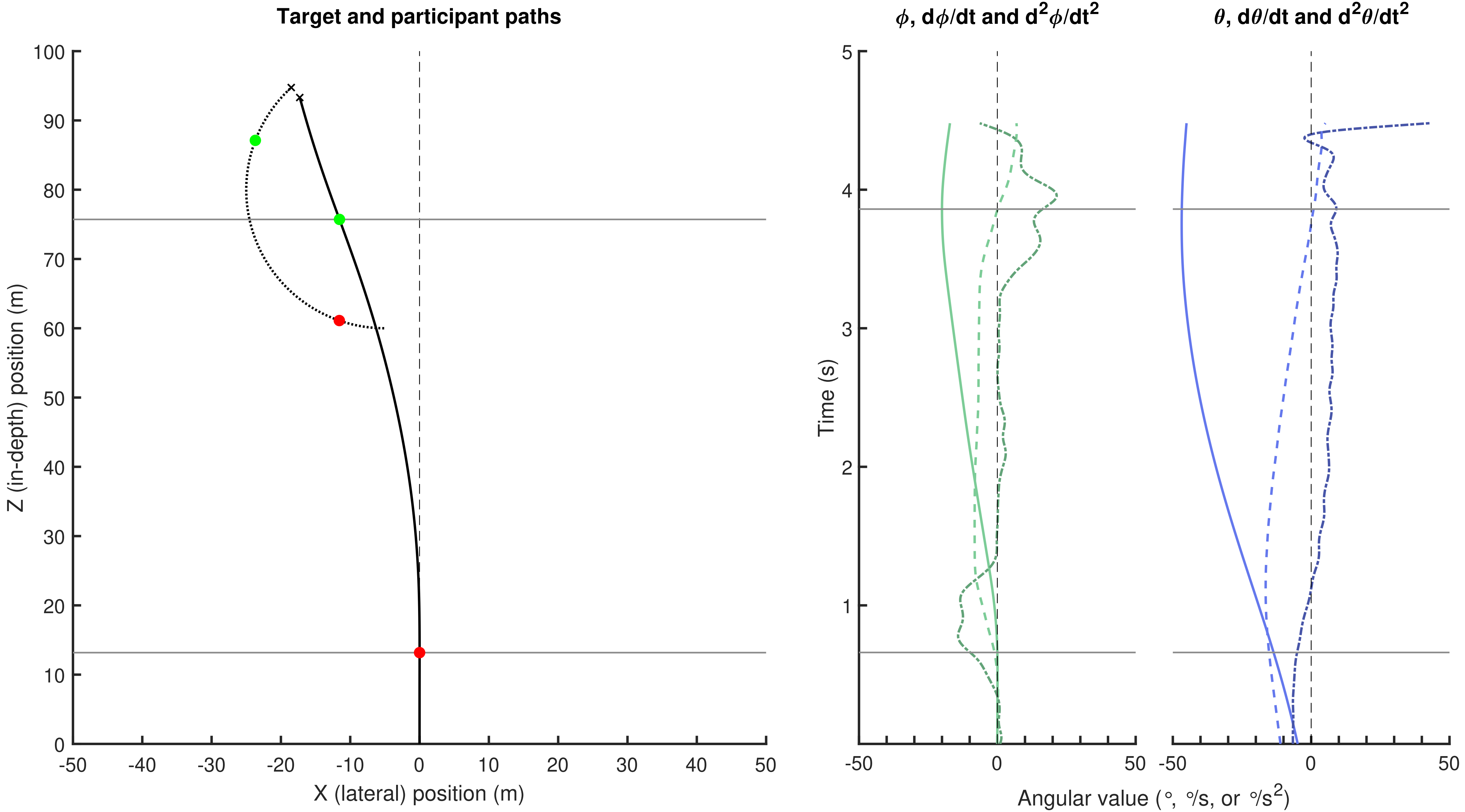

P19/B5
S5/R30-IN

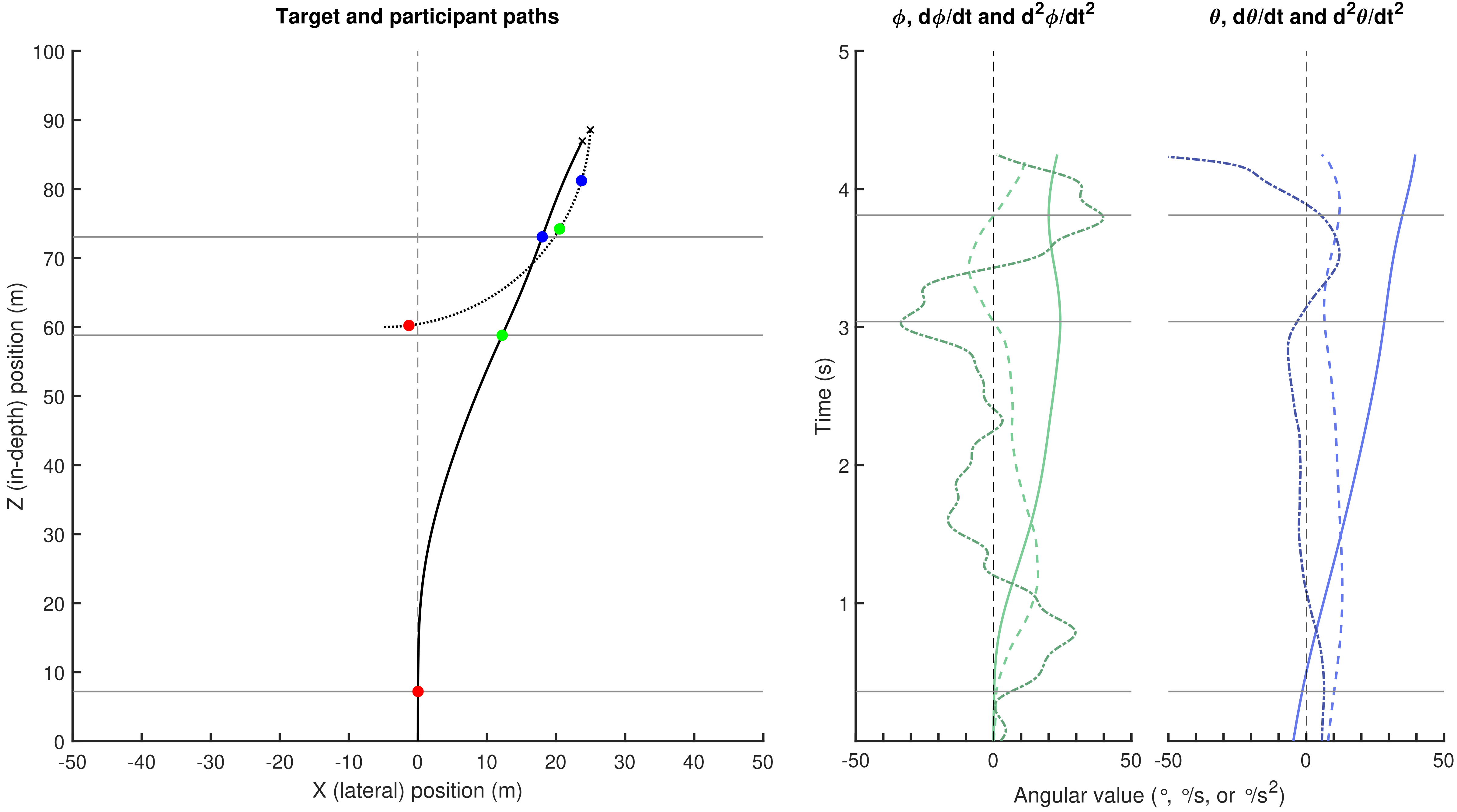

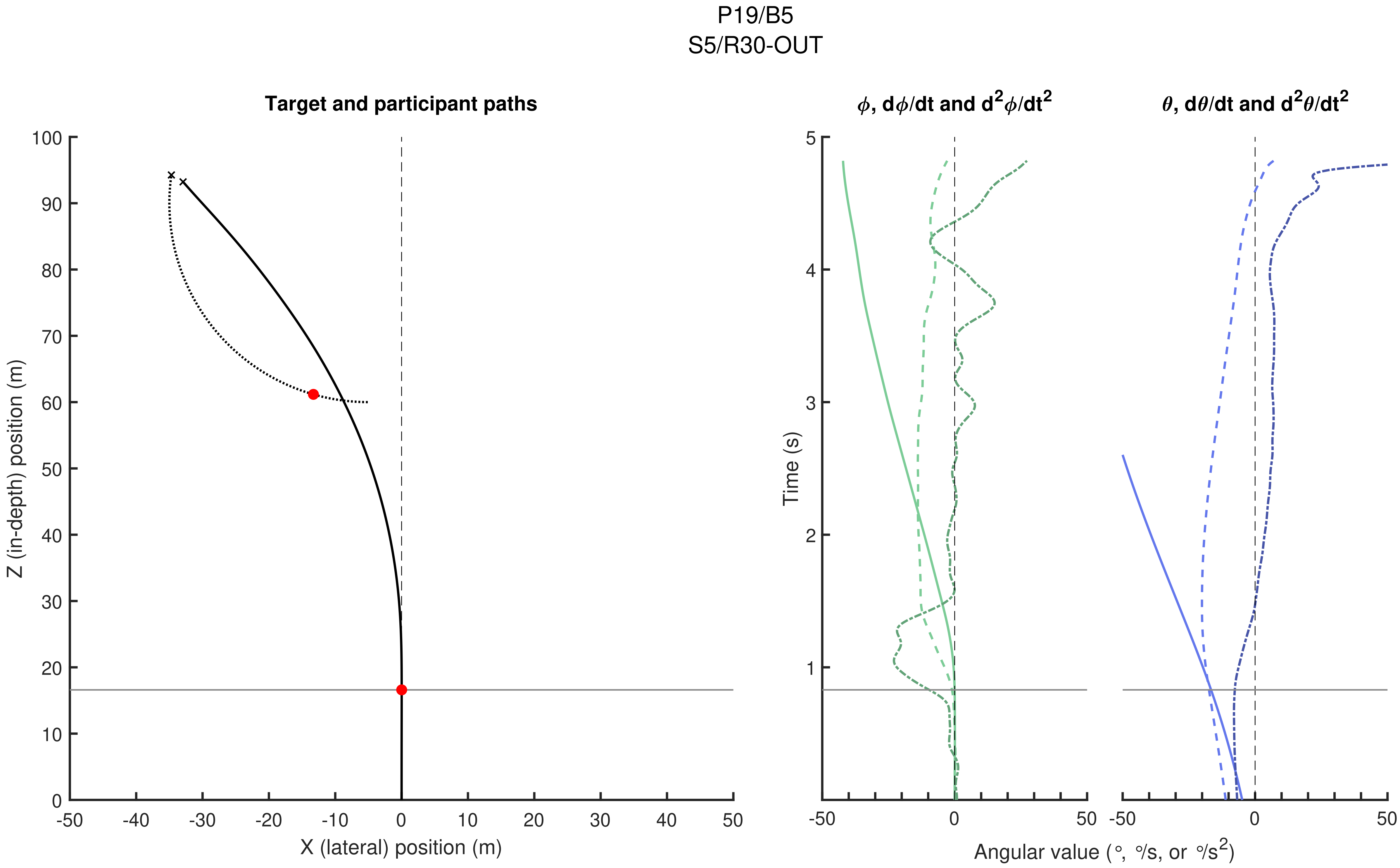

P19/B5
S5/R30-OUT
Target and participant paths
Z (in-depth) position (m)
X (lateral) position (m)
100
90
80
70
60
50
40
30
20
10
0
-50
-40
-30
-20
-10
0
10
20
30
40
50
φ, dφ/dt and d²φ/dt²
θ, dθ/dt and d²θ/dt²
Time (s)
5
4
3
2
1
-50
0
50
Angular value (°, °/s, or °/s²)

P19/B5
S5/R10-OUT

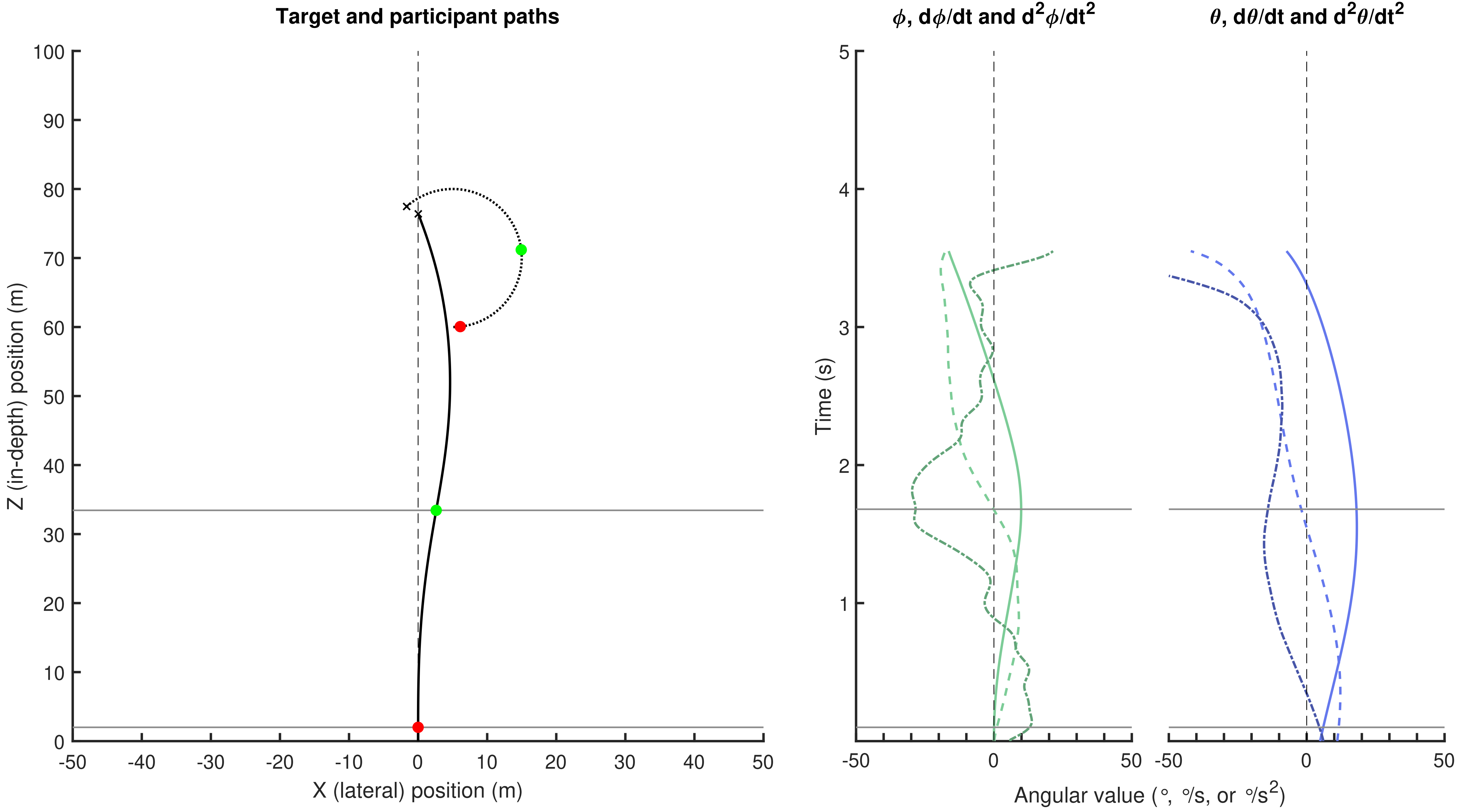

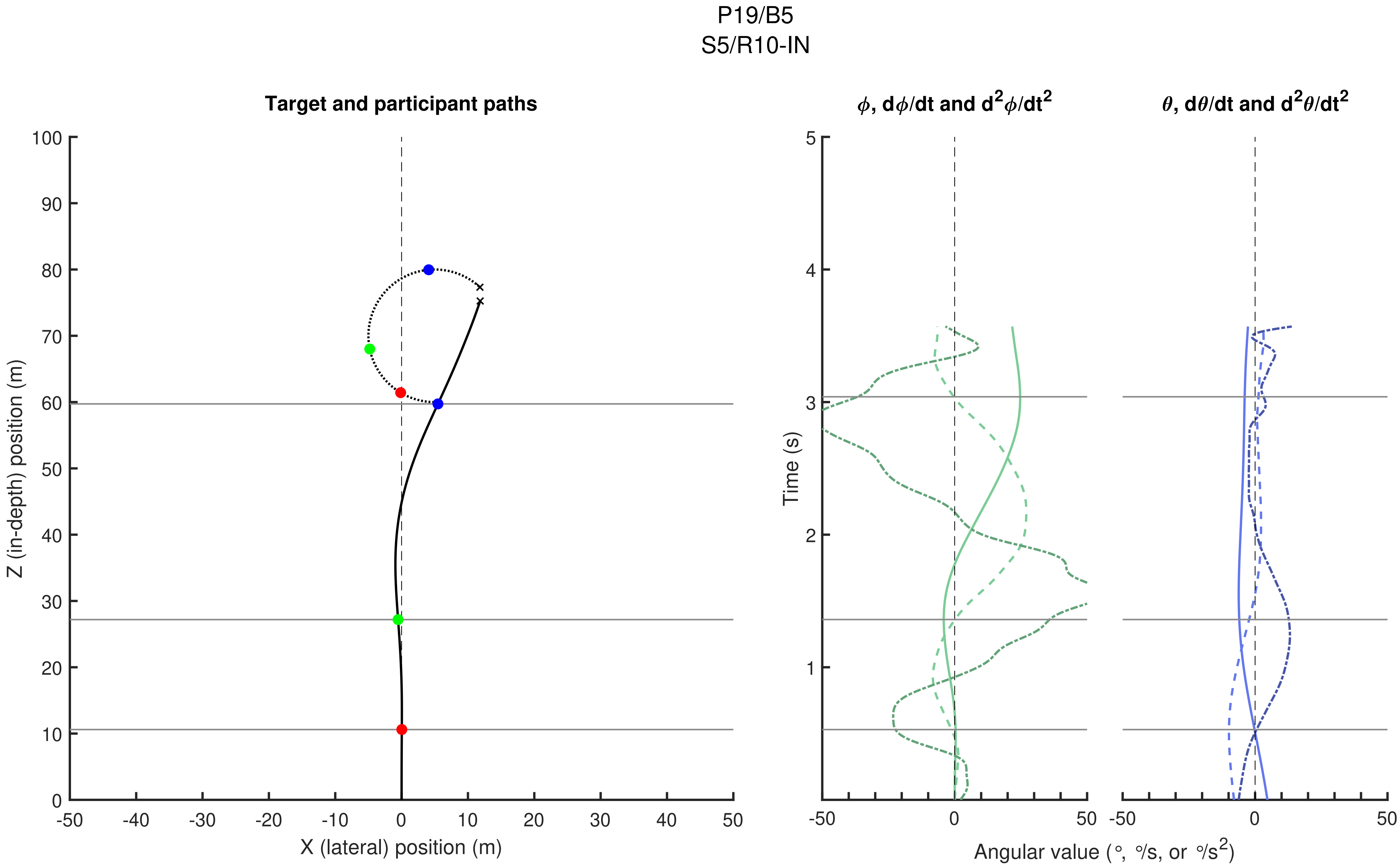
P19/B5
S5/R10-IN
Target and participant paths
Z (in-depth) position (m)
X (lateral) position (m)
φ, dφ/dt and d²φ/dt²
θ, dθ/dt and d²θ/dt²
Time (s)
Angular value (°, °/s, or °/s²)

P19/B5
S5/R20-OUT

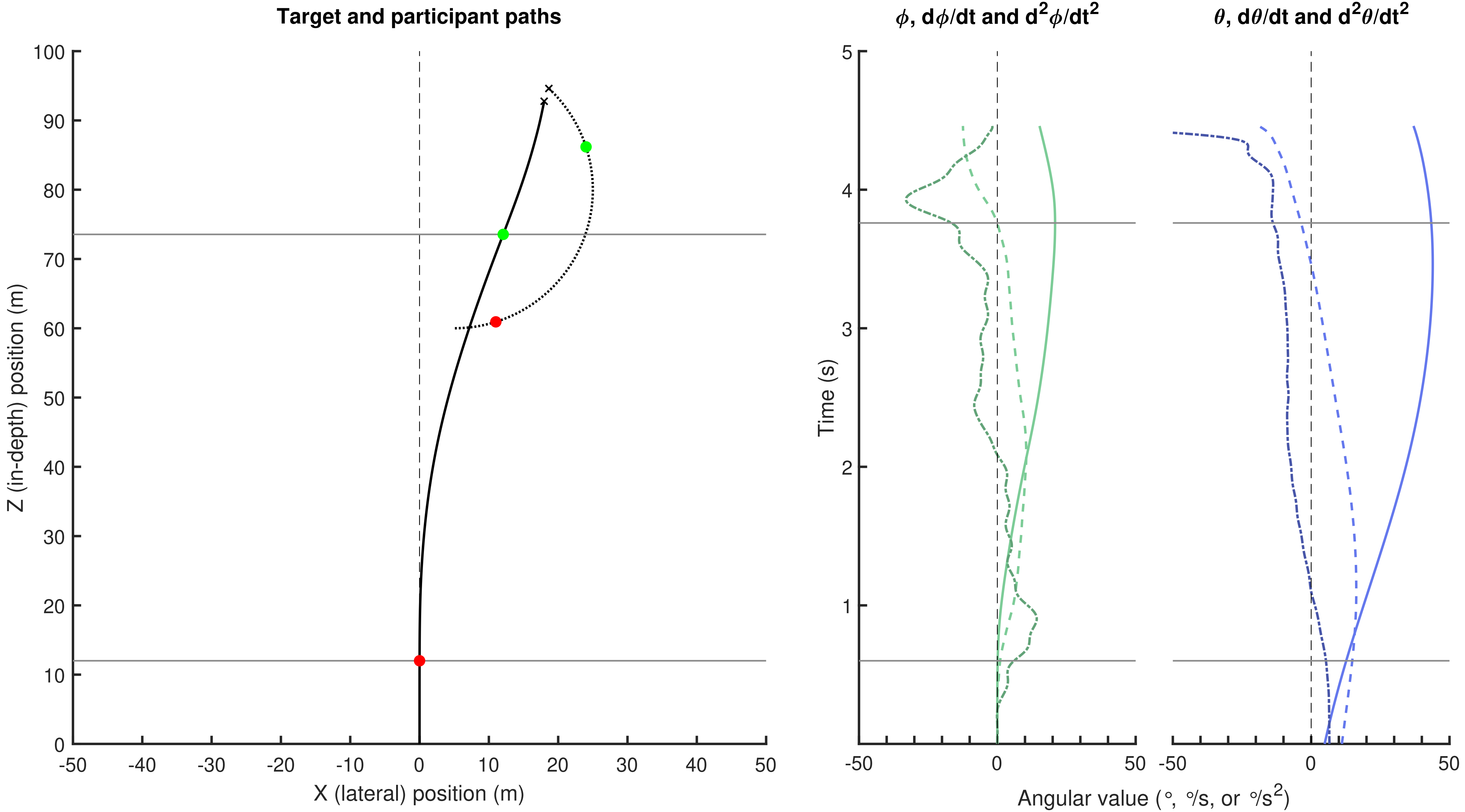

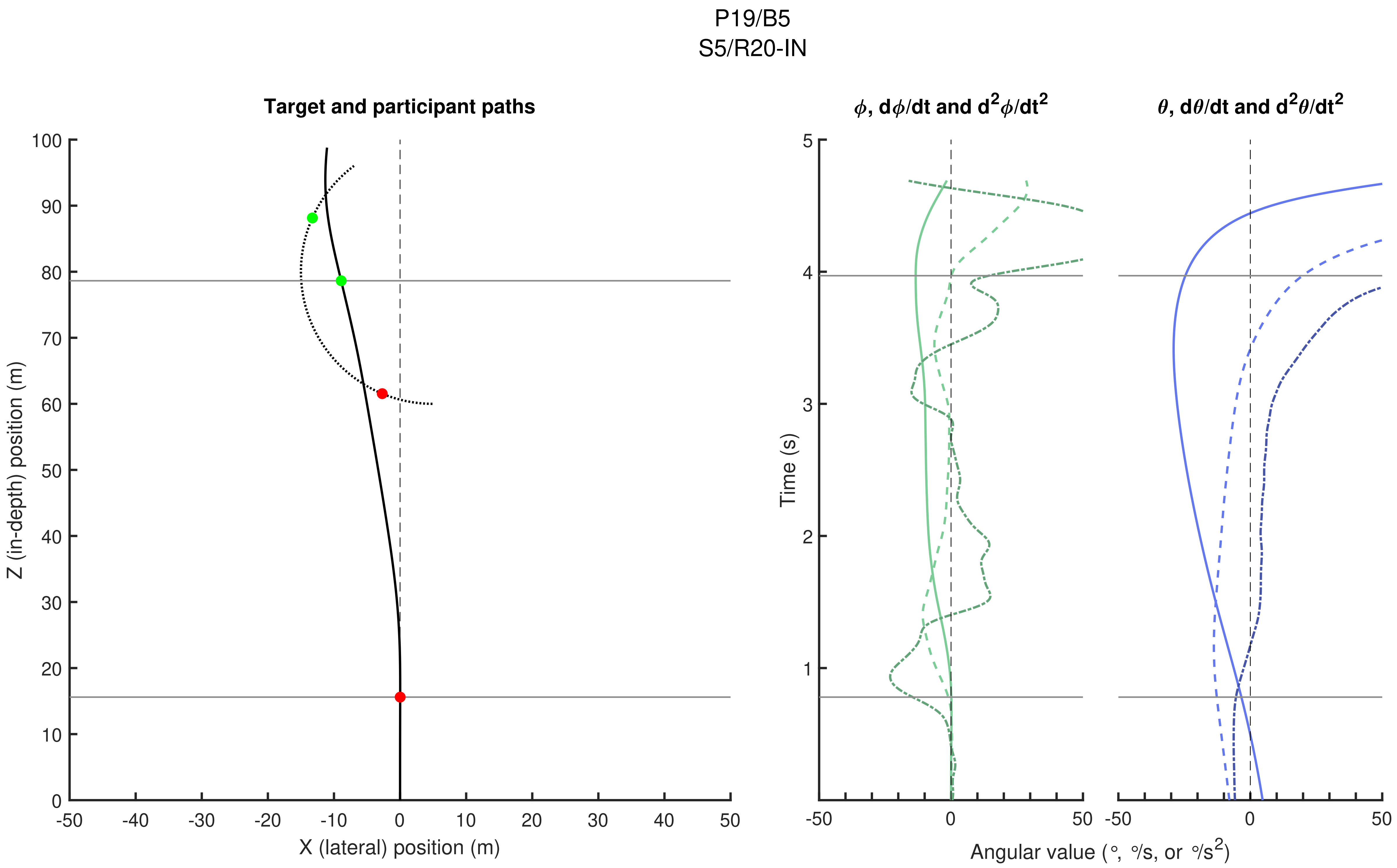

P19/B5
S5/R20-IN
Target and participant paths
ϕ, dϕ/dt and d$^2\phi$/dt^2
θ, dθ/dt and d$^2\theta$/dt^2
Z (in-depth) position (m)
X (lateral) position (m)
Time (s)
Angular value (°, °/s, or °/s^2)

P19/B5
S5/R30-OUT

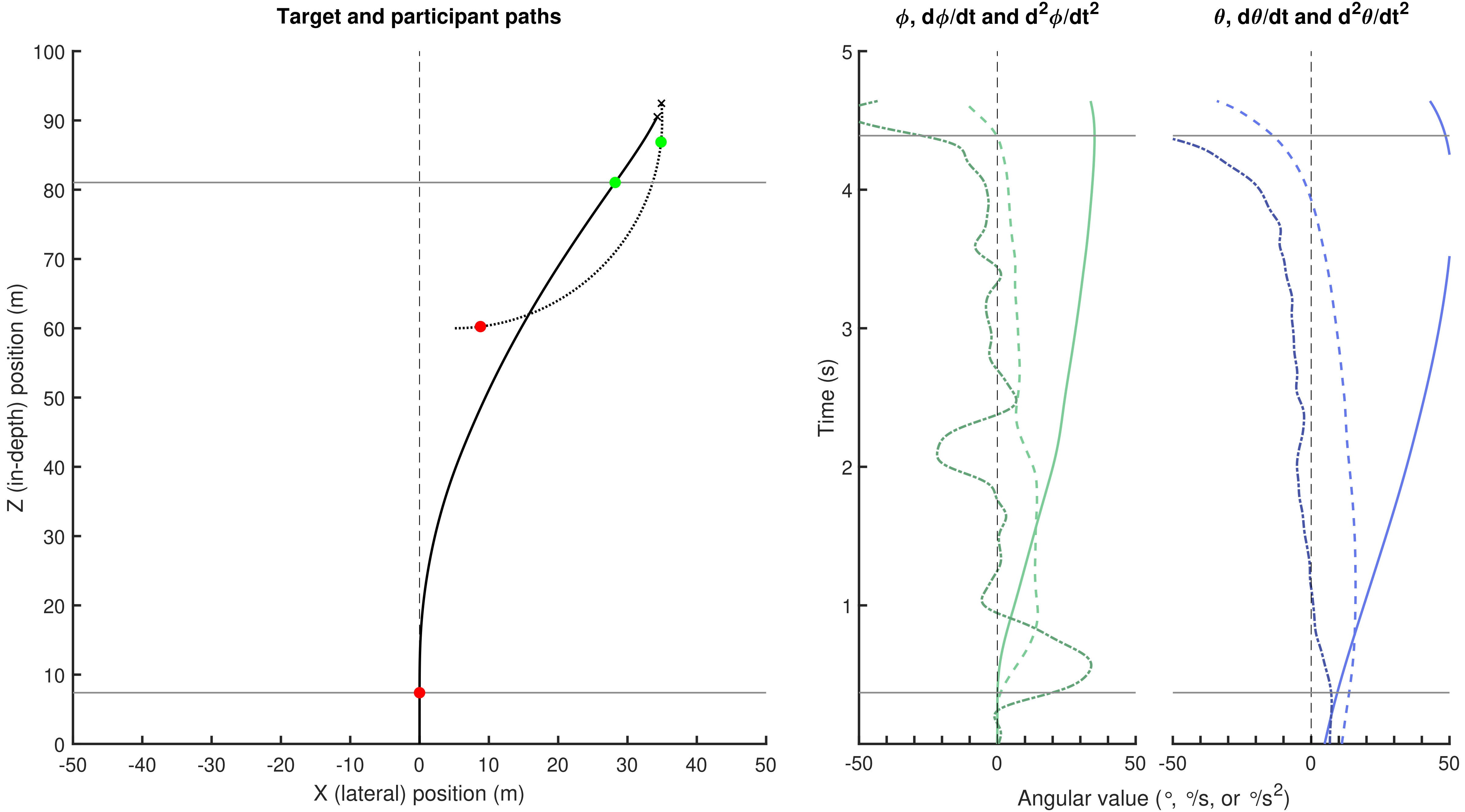

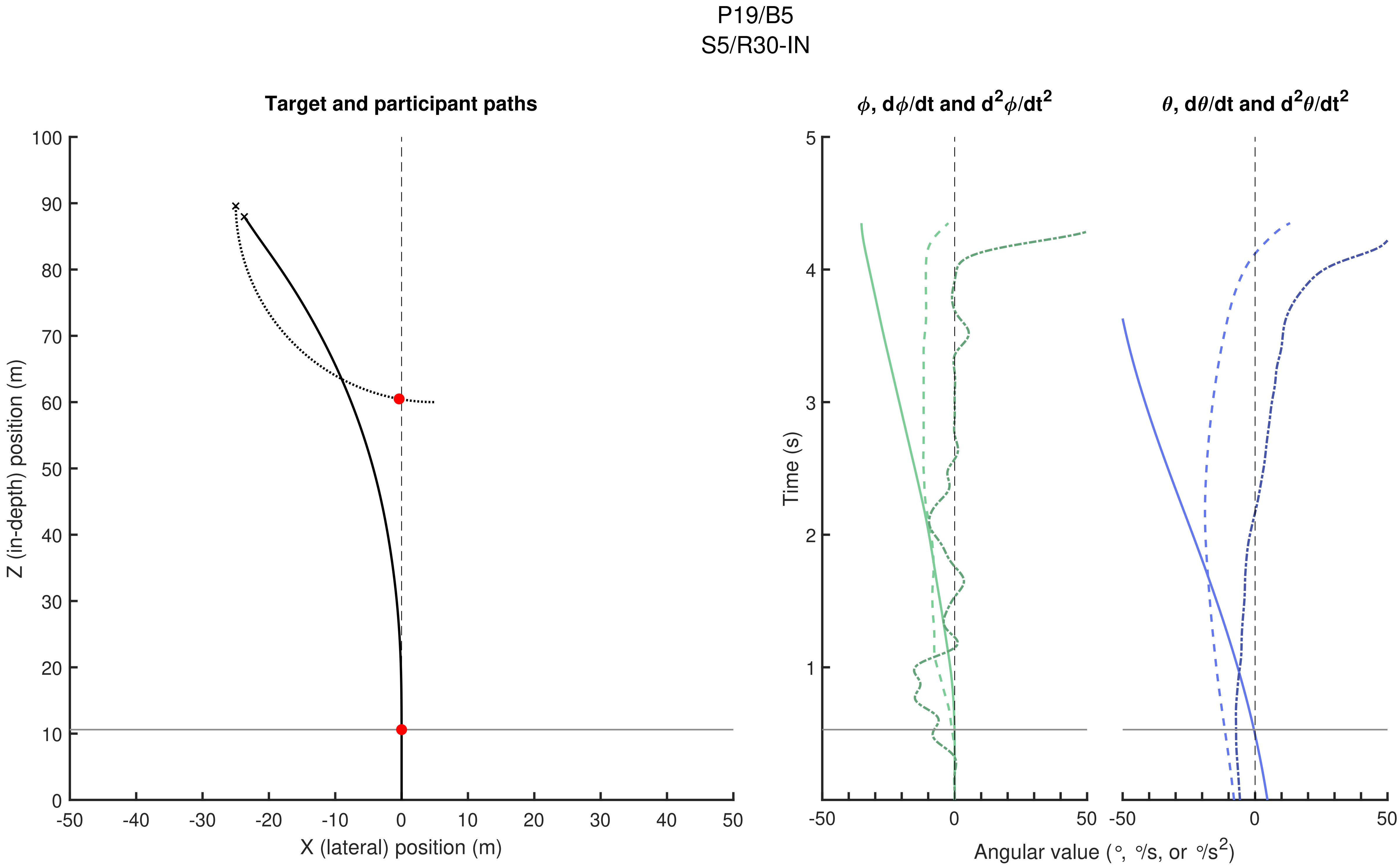

P19/B5
S5/R30-IN
Target and participant paths
ϕ, dϕ/dt and d$^2\phi$/dt^2
θ, dθ/dt and d$^2\theta$/dt^2
Z (in-depth) position (m)
X (lateral) position (m)
Time (s)
Angular value (°, °/s, or °/s^2)

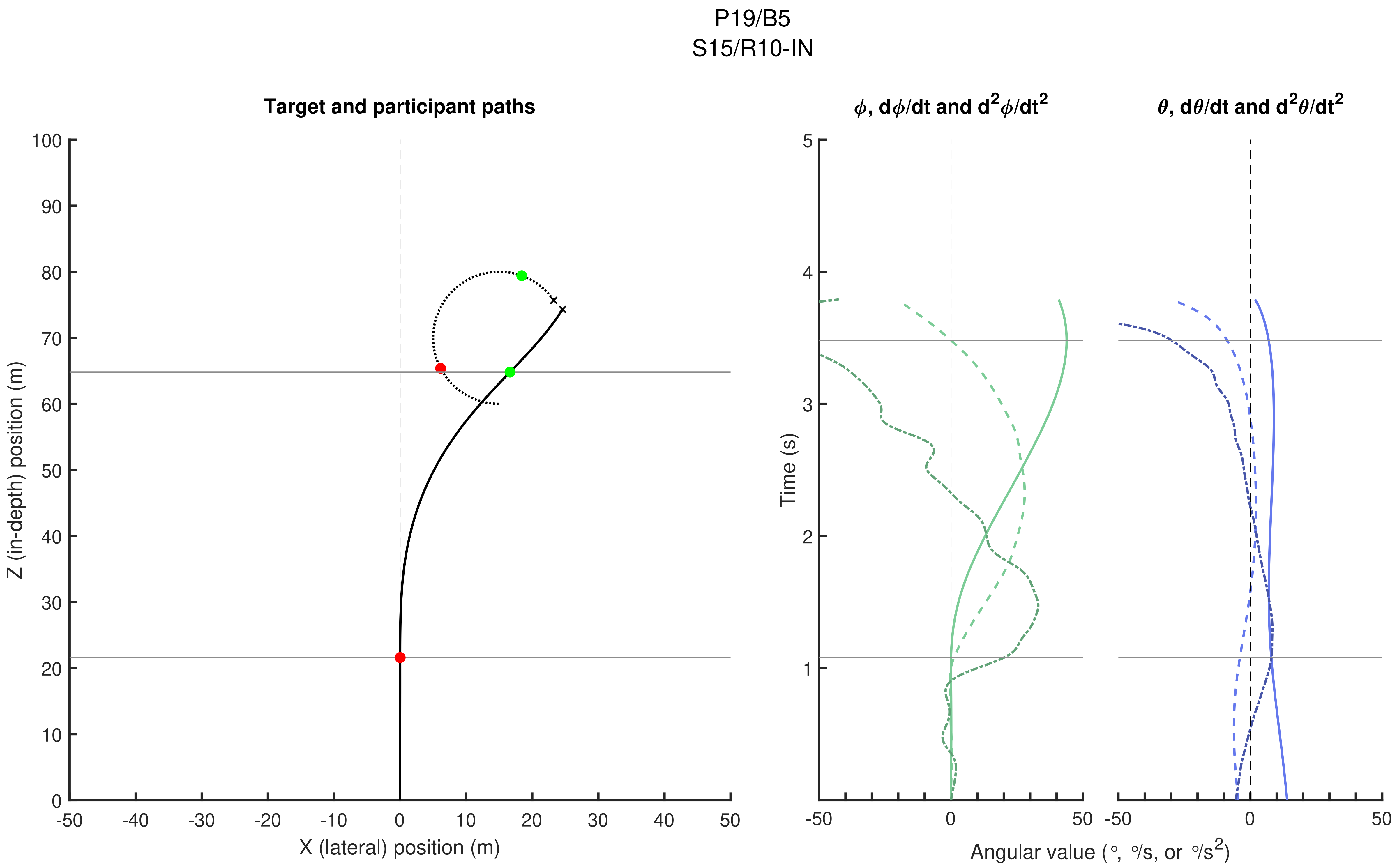

P19/B5
S15/R10-IN
Target and participant paths
Z (in-depth) position (m)
X (lateral) position (m)
ϕ, dϕ/dt and d$^2\phi$/dt^2
θ, dθ/dt and d$^2\theta$/dt^2
Time (s)
Angular value (°, °/s, or °/s^2)

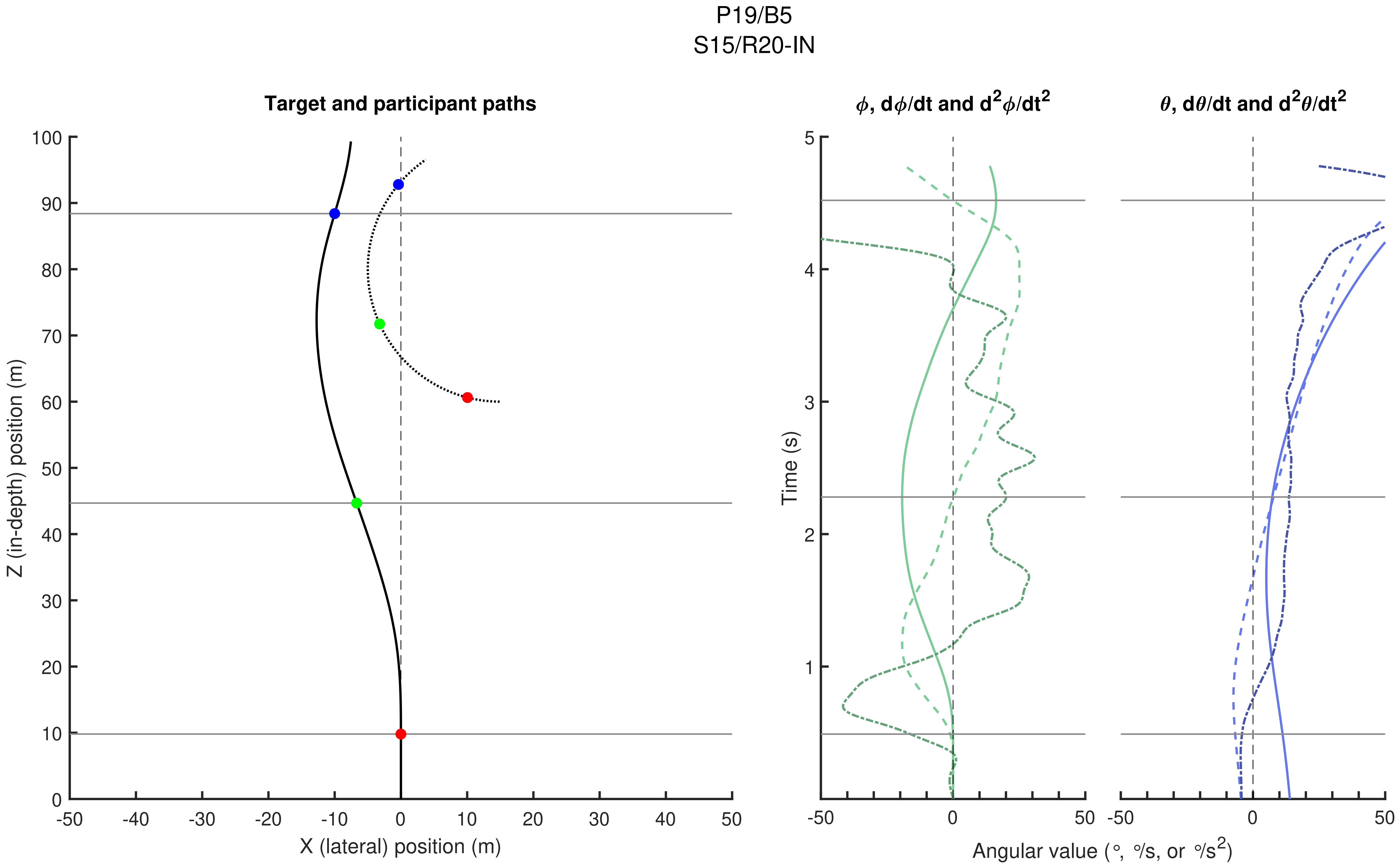
P19/B5
S15/R20-IN
Target and participant paths
Z (in-depth) position (m)
X (lateral) position (m)
ϕ, dϕ/dt and d²ϕ/dt²
θ, dθ/dt and d²θ/dt²
Time (s)
Angular value (°, °/s, or °/s²)

P19/B5
S15/R30-IN

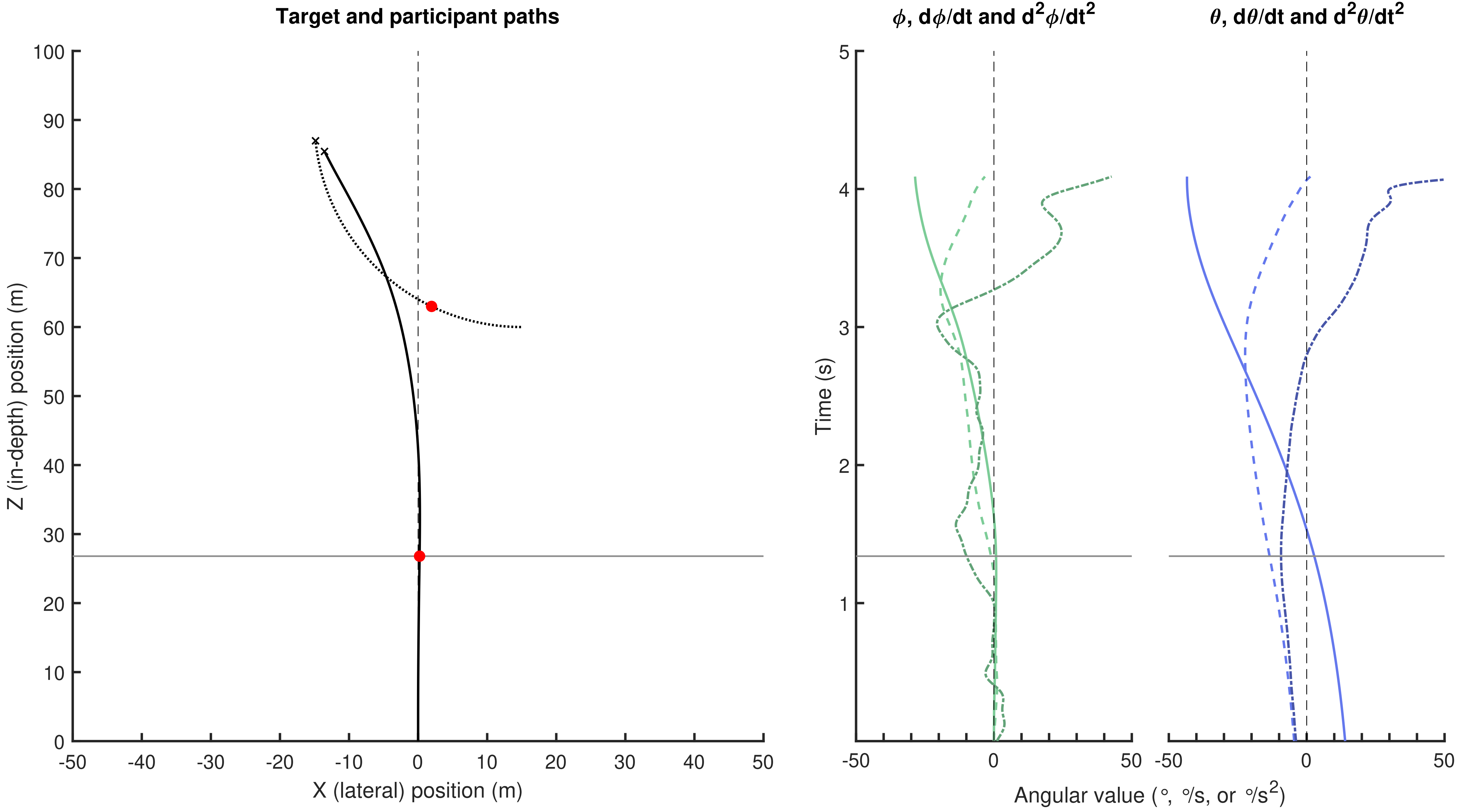

P19/B5
S20/R10-IN

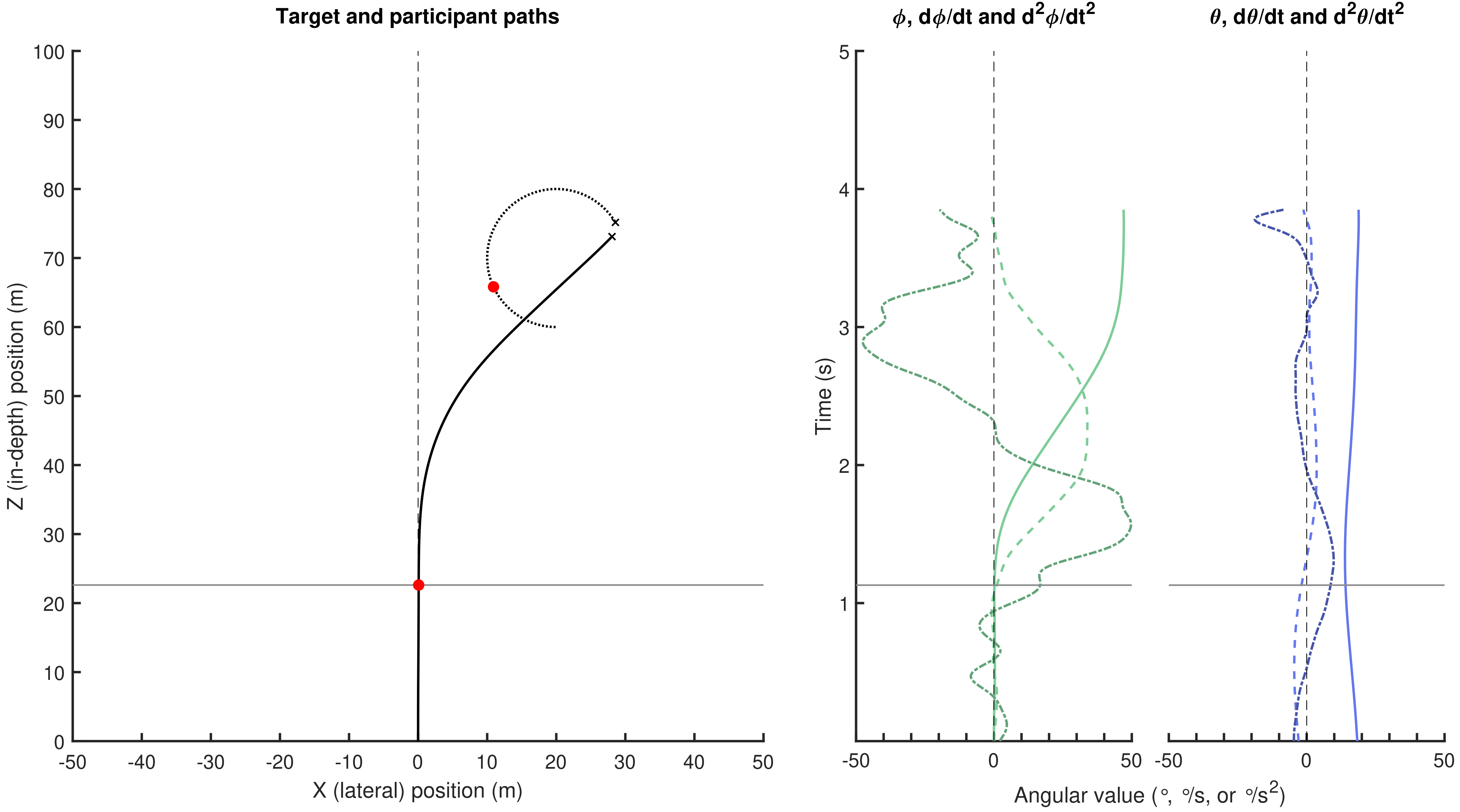

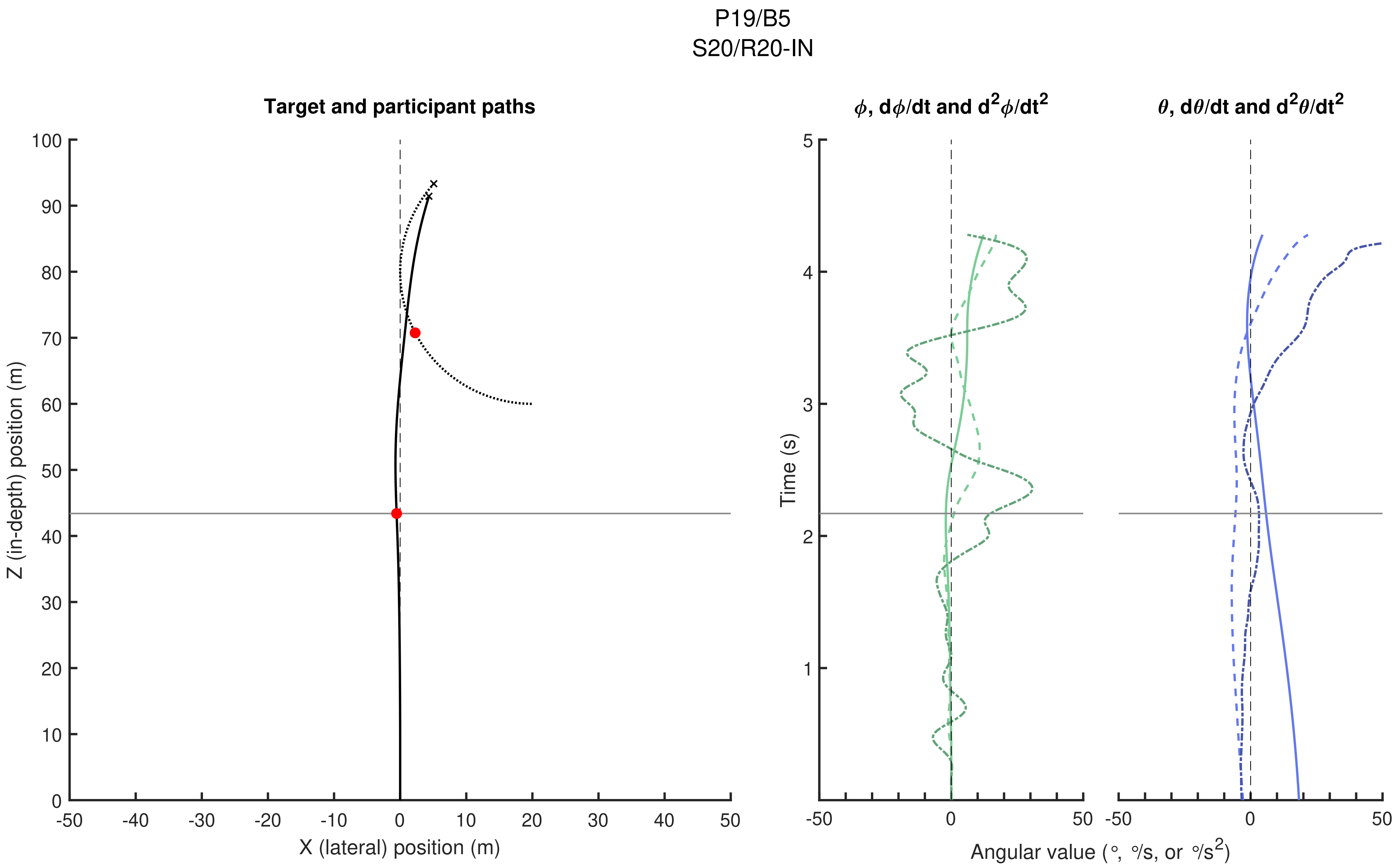
P19/B5
S20/R20-IN
Target and participant paths
Z (in-depth) position (m)
X (lateral) position (m)
ϕ, dϕ/dt and d$^2\phi$/dt^2
θ, dθ/dt and d$^2\theta$/dt^2
Time (s)
Angular value (°, °/s, or °/s^2)

P19/B5
S20/R30-IN

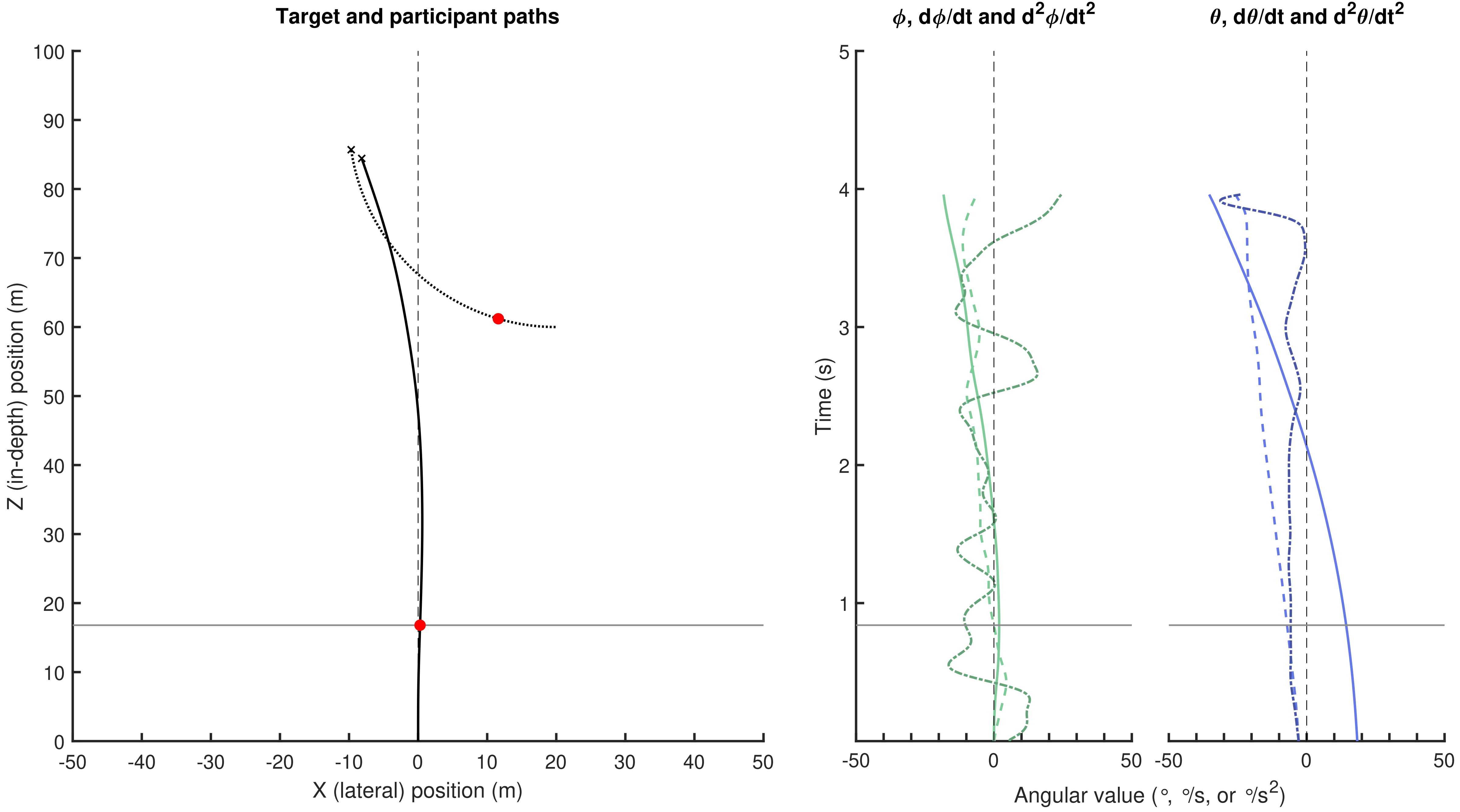

Supplement: Supplement 5 [file jovi-23-3-11_s005.pdf]
